# Data on genome annotation and analysis of earthworm *Eisenia fetida*

**DOI:** 10.1016/j.dib.2018.08.067

**Published:** 2018-08-29

**Authors:** Sayan Paul, Arun Arumugaperumal, Rashmi Rathy, Vasanthakumar Ponesakki, Palavesam Arunachalam, Sudhakar Sivasubramaniam

**Affiliations:** a Department of Biotechnology, Manonmaniam Sundaranar University, Tirunelveli, Tamilnadu 627012, India; b Department of Animal Science, Manonmaniam Sundaranar University, Tirunelveli, Tamilnadu 627012, India

**Keywords:** *Eisenia fetida*, Genome annotation, Orthologous groups, Regeneration

## Abstract

The present article reports the complete draft genome annotation of earthworm *Eisenia fetida*, obtained from the manuscript entitled “Timing and Scope of Genomic Expansion within Annelida: Evidence from Homeoboxes in the Genome of the Earthworm *E. fetida*” (Zwarycz et al., 2015) and provides the data on the repetitive elements, protein coding genes and noncoding RNAs present in the genome dataset of the species. The *E. fetida* protein coding genes were predicted from AUGUSTUS gene prediction and subsequently annotated based on their sequence similarity, Gene Ontology (GO) functional terms, InterPro domains, Clusters of Orthologous Groups (COGs) and KEGG (Kyoto Encyclopedia of Genes and Genomes) pathways information. The genome wide comparison of orthologous clusters and phylogenomic analysis of the core genes were performed to understand the events of genome evolution and genomic diversity between *E. fetida* and its related metazoans. In addition, the genome dataset was screened to identify the crucial stem cell markers, regeneration specific genes and immune-related genes and their functionally enriched GO terms were predicted from Fisher׳s enrichment analysis. The *E. fetida* genome annotation data containing the GFF (general feature format) annotation file, predicted coding gene sequences and translated protein sequences were deposited to the figshare repository under the DOI: https://doi.org/10.6084/m9.figshare.6142322.v1.

**Specifications Table**

**Table:** 

Subject area	*Biology*
More specific subject area	*Bioinformatics (Genomics)*
Type of data	*Table, figure, GFF (general feature format) file*
How data was acquired	*The repeat elements identification and masking were performed by using the tools: TEclass and RepeatMasker. The draft genome annotation was performed by using the AUGUSTUS web server. The GO, KEGG and COG annotation of the E. fetida protein coding genes were carried out by using the BLAST2GO software version 4.1. The prediction and annotation of the noncoding RNAs were performed using the Rfam analysis with Infernal software version 1.1.*
Data format	*Analyzed and annotated*
Experimental factors	*Repeat identification and masking; genome annotation; GO function prediction; orthologous group analysis; KEGG pathway analysis; phylogenomic analysis.*
Experimental features	*Repeat masking, genome annotation and genomic data analysis.*
Data source location	*Whitney Laboratory for Marine Bioscience, University of Florida, Gainesville, USA.*
Data accessibility	*Data are available in this article and at figshare (*https://doi.org/10.6084/m9.figshare.6142322.v1*).*
Related research article	Zwarycz et al. [1].

**Value of the data**

• The annotated genome resource of earthworm *E. fetida* can be effectively utilized by the ecological and regeneration biologists to monitor the key genes, regulating the soil quality and fertility, environmental toxicity and different aspects of annelid regeneration.
• The Riboflavin metabolism and noncoding RNA annotation data can be utilized further to interpret the event of horizontal gene transfer from the gut microbes and endosymbionts to the worm and monitoring their role in regulating the key features like autofluorescence and regeneration of the worm.
• The data related to the genome-wide comparison of the orthologous clusters and phylogenomic analysis of the core genes across the metazoans will be significant to understand the events of genome evolution and genomic diversity both within the annelid lineages (intraphylum) and across the annelids and their neighboring phyla like Echinodermata, Mollusca and Platyhelminthes (interphylum).
• The predicted stem cell markers and immune-related gene datasets and their associated enriched functions can be used further as a valuable resource to interpret the essential genetic, molecular and biochemical pathways associated with the processes like segmental regeneration, organogenesis and innate immune response of the species.

## 1 Data

**Fig. 1:**
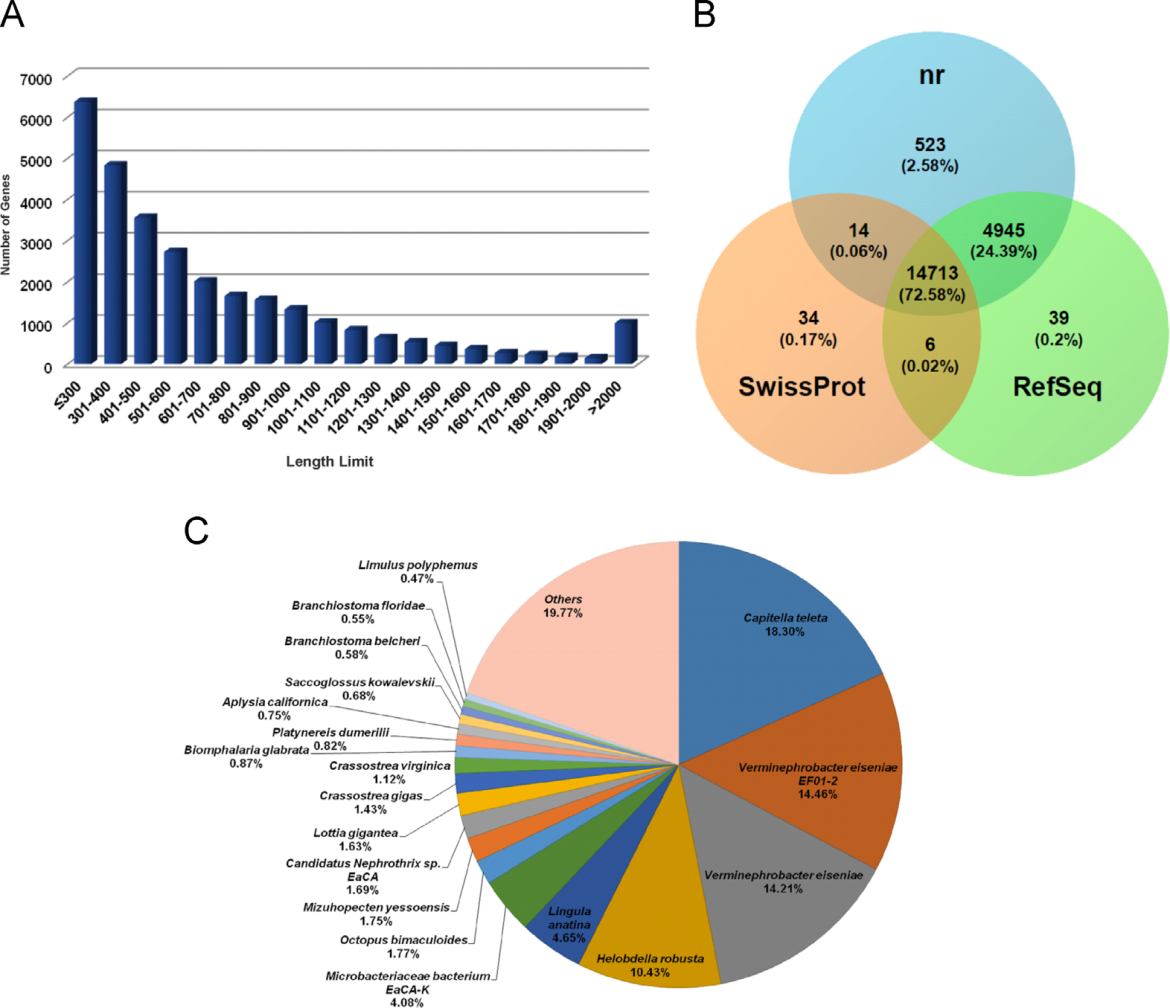
**(A)** Histogram representing the length distribution of the *Eisenia fetida* protein coding genes predicted by AUGUSTUS gene prediction server. **(B)** A three way Venn diagram representing the unique and overlapped coding genes annotated against the public databases nr, SwissProt and RefSeq (BLASTX algorithm; E-value threshold 1E-5). **(C)** Pie chart denoting the top hit species distribution summary of the *Eisenia fetida* genes annotated against nr database with an E-value cut-off of 1E-5.

**Fig. 2:**
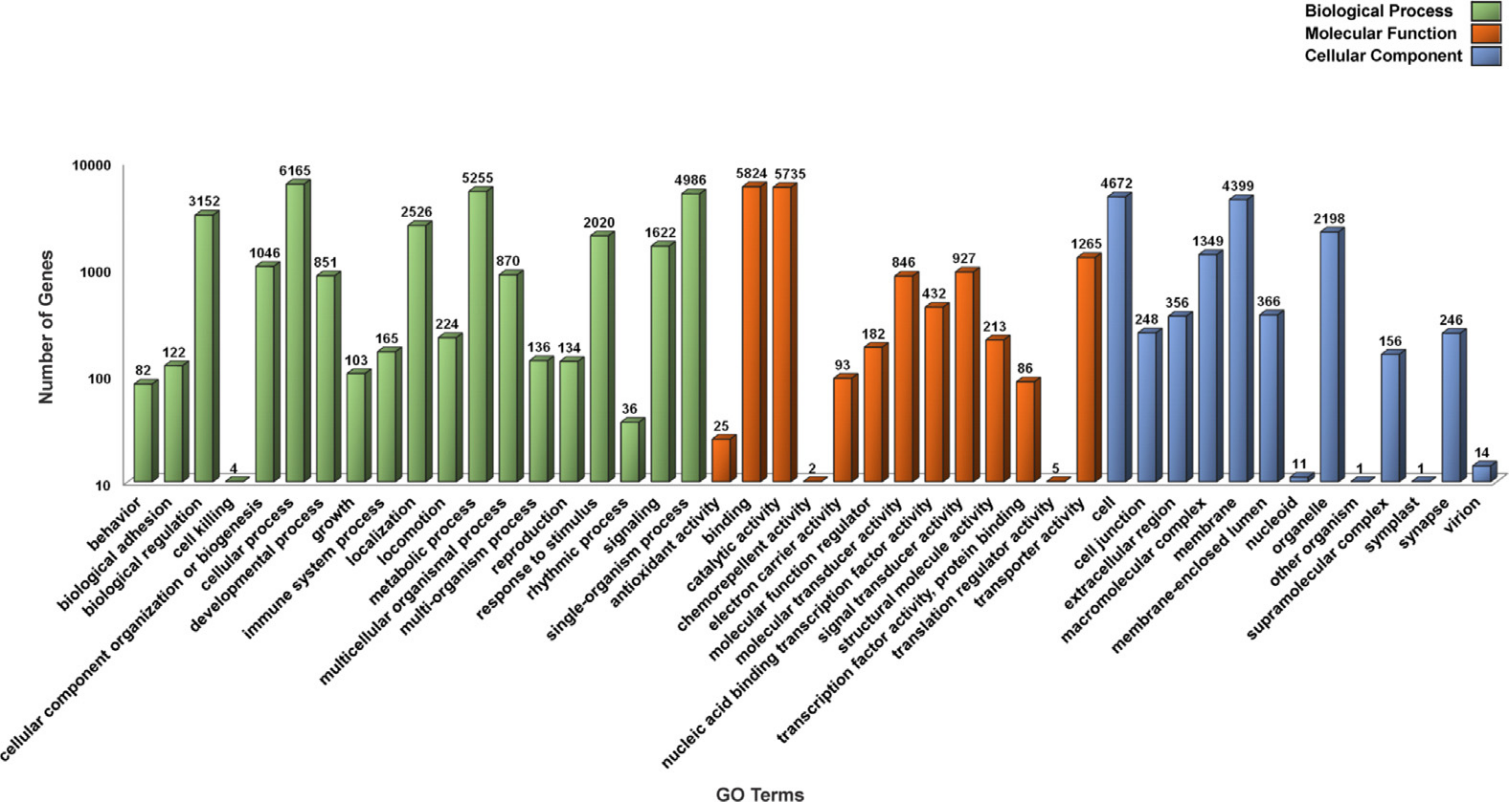
Histogram representing the gene ontology distribution of the annotated *Eisenia fetida* genes. The functionally annotated genes were assigned to three main GO categories: Biological Process (BP), Molecular Function (MF) and Cellular Component (CC).

**Fig. 3:**
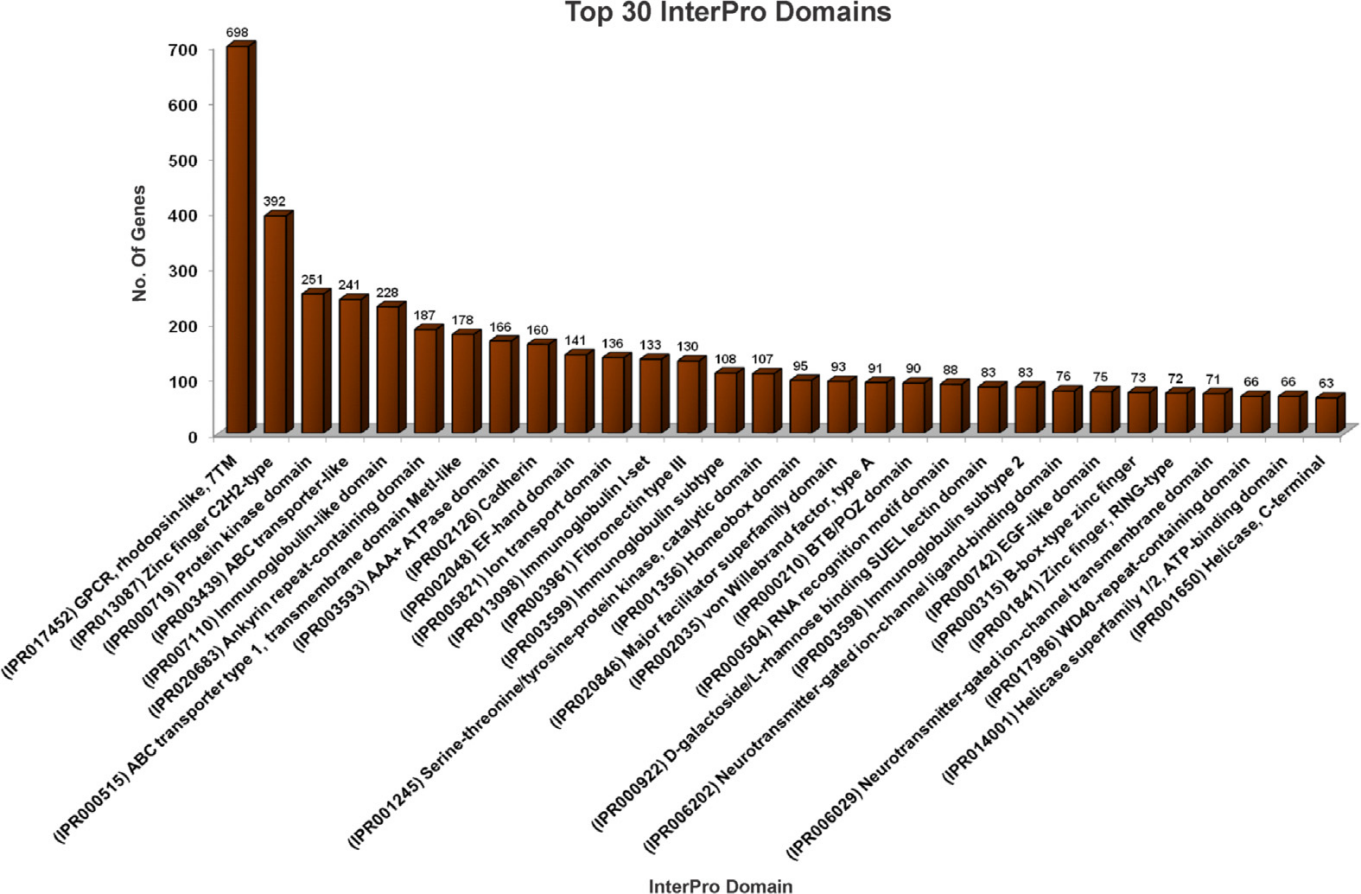
Histogram of top 30 InterPro domains distribution of the *Eisenia fetida* genes obtained from InterProScan annotation using the BLAST2GO software.

**Fig. 4:**
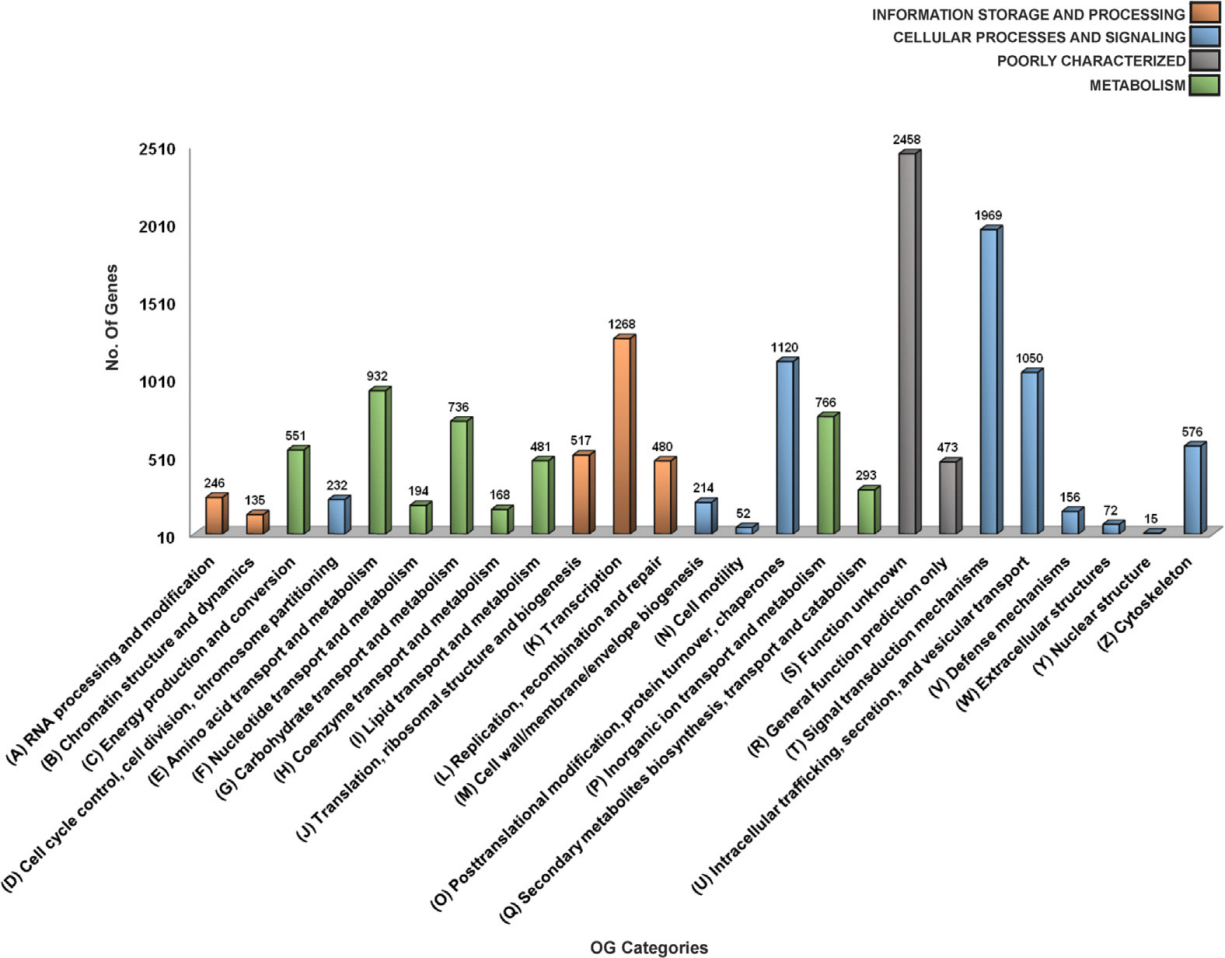
Clusters of Orthologous Groups (COG) distribution of the protein coding genes in *Eisenia fetida* obtained from EggNog database. The genes were annotated and classified into 25 COG functional categories.

**Fig. 5:**
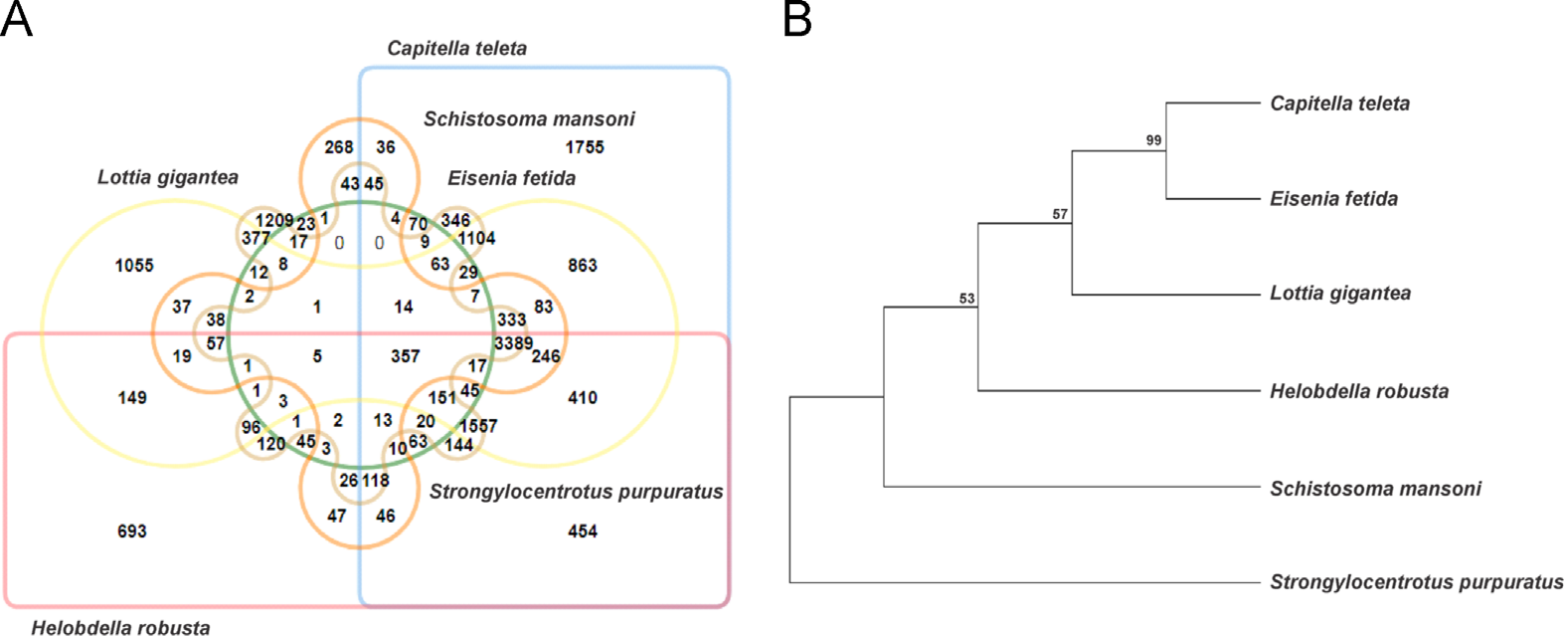
**(A)** Genome-wide comparison and analysis of the orthologous clusters between six different metazoans species: *Eisenia fetida, Capitella teleta*, *Helobdella robusta*, *Lottia gigantea*, *Schistosoma mansoni* and *Strongylocentrotus purpuratus*. **(B)** Phylogenomic tree constructed based on core genes comparison between the genomes of these six metazoans species using the MEGA7 software.

**Fig. 6:**
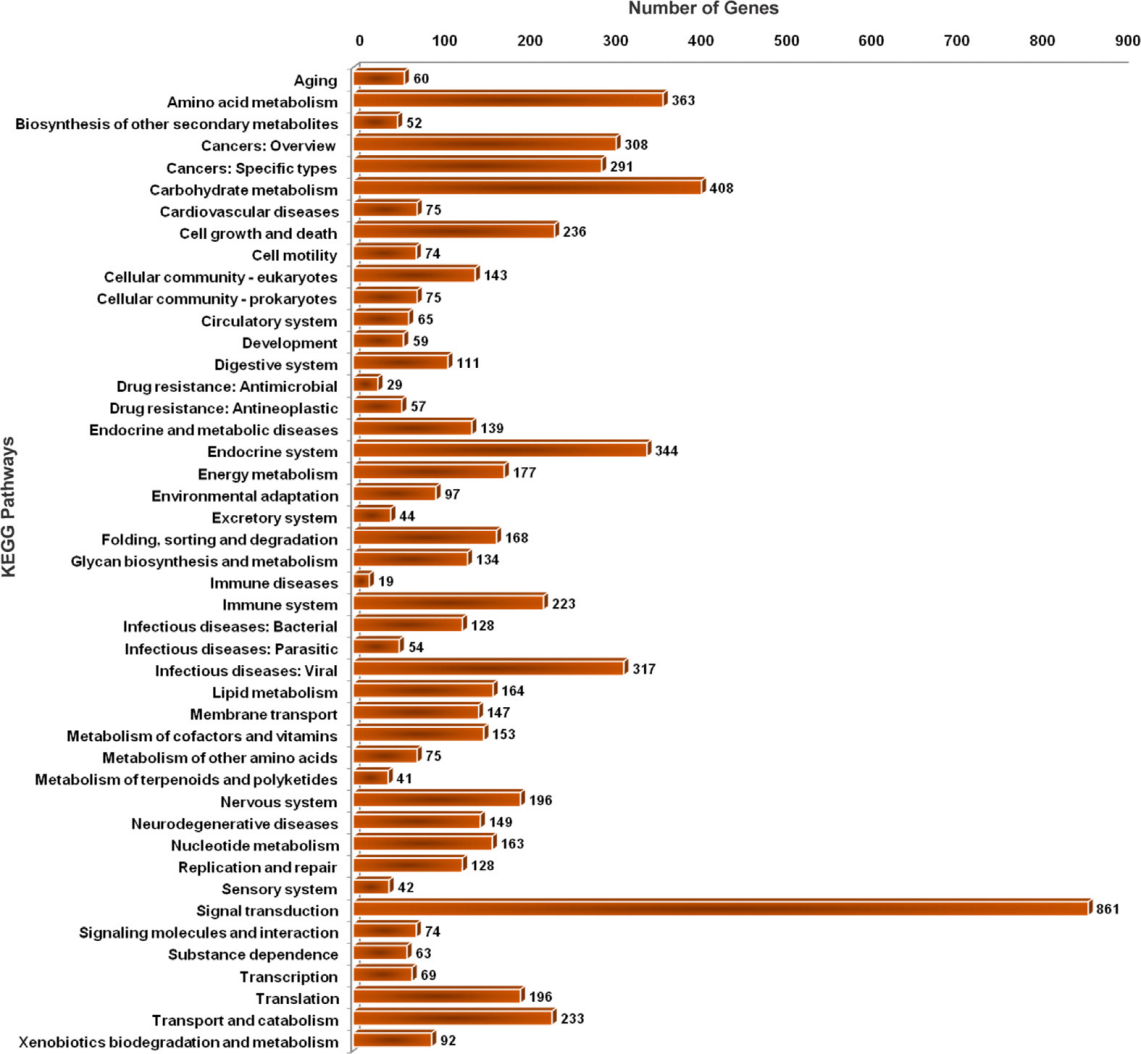
Bar chart representing the distribution of KEGG pathways associated with the genome of earthworm *Eisenia fetida*. The KEGG pathways were assigned by annotating the protein coding genes using the KAAS (KEGG Automatic Annotation Server) web server.

**Fig. 7:**
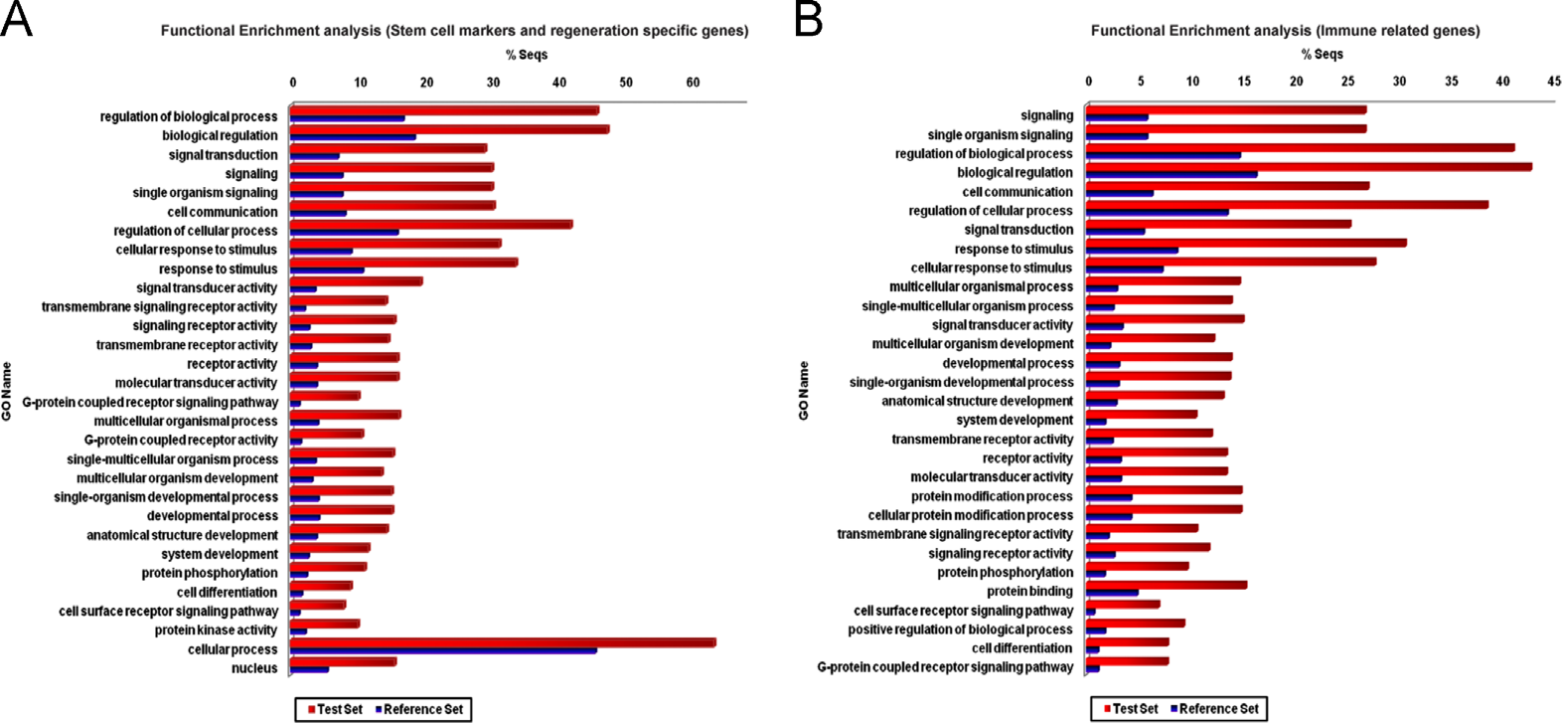
Bar chart representing the top 30 functionally enriched gene ontology terms associated with the predicted (A) stem cell markers and regeneration specific genes and (B) immune-related genes in comparison to the annotated genome dataset of earthworm *Eisenia fetida*.

**Fig. 8:**
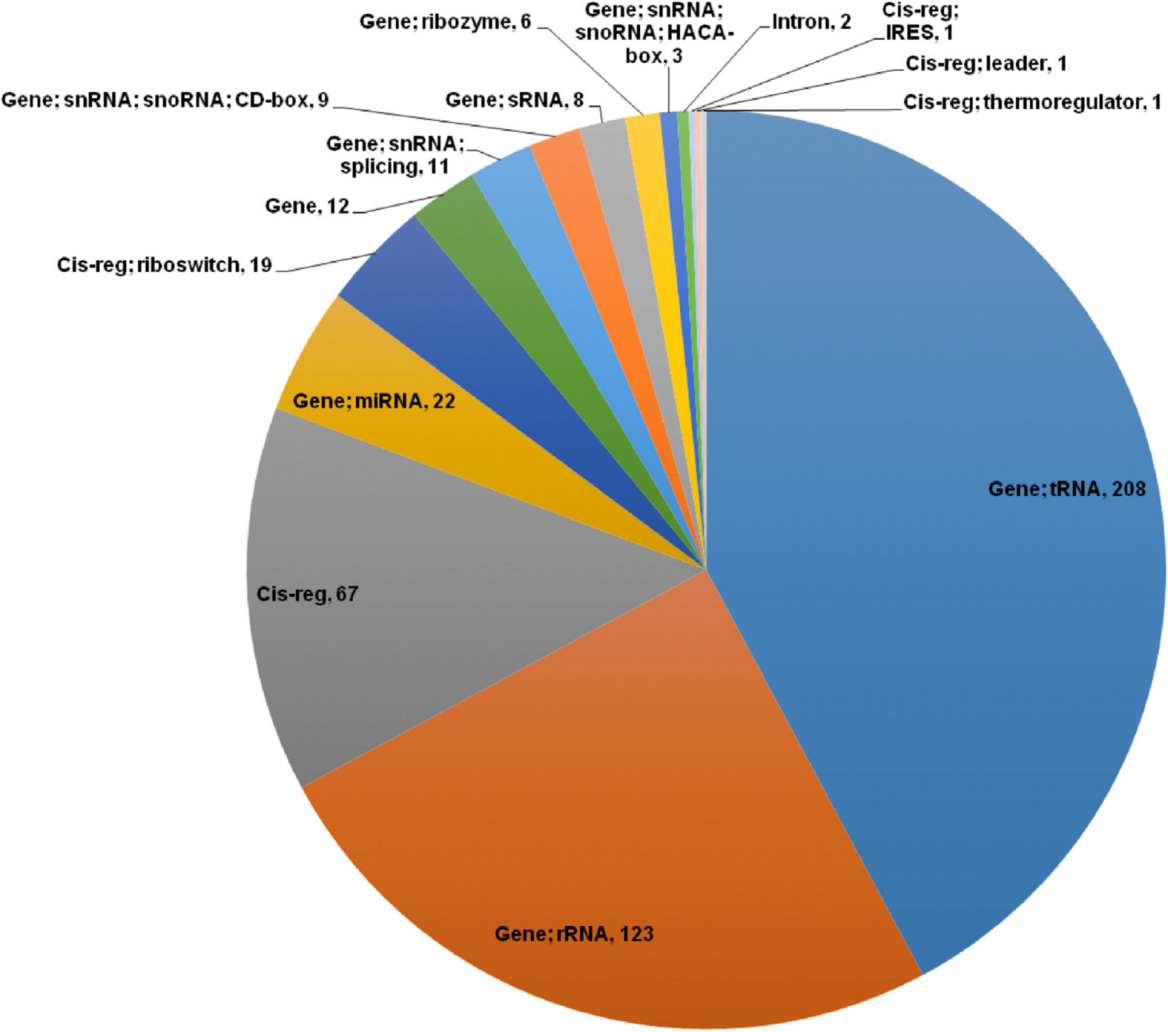
Pie chart summarizing the distribution of the noncoding RNA genes in the genome dataset of earthworm *Eisenia fetida*.

The overall data represent the genome annotation framework of earthworm *Eisenia fetida*. Table 1 denotes the summary statistics of the identified repetitive elements and total number of bases masked in the assembled genome. The genome annotation summary, describing the protein coding genes and their annotation statistics has been documented in Table 2. The length distribution (Fig. 1A), sequence based annotation (Fig. 1A) and BLAST top hit species distribution summary (Fig. 1c) of the predicted protein coding genes are demonstrated in Fig. 1. The list of the functionally annotated genes is documented in table S1. Table S2 reports the list of *E. fetida* genes showing sequence homology to the bacterial genome. The functional gene ontology (GO) terms, top 30 InterPro conserved domains and cluster of orthologous groups (COGs) distributions of the annotated *E. fetida* genes are illustrated in Fig. 2, Fig. 3. and Fig. 4 respectively. Fig. 5 illustrates the genome-wide comparison data of the orthologous clusters (Fig. 5A) and phylogenomic analysis of the core genes (Fig. 5B) between *E. fetida* and its closely related metazoan species. The KEGG pathways annotation data and list of the mapped pathways are given in Fig. 6 and table S3 respectively. Table S4 demonstrates the Riboflavin biosynthesizing enzymes in *E. fetida* having BLAST sequence homology with the bacterial sequences. The stem cell and regeneration specific genes and the immune-related genes identified in the genome dataset are listed in table S5 and S6 respectively. Top 30 functionally enriched GO terms associated with the stem cell markers (Fig. 7A) and immune-related genes (Fig. 7B) are shown in Fig. 7 and lists of all the enriched functions along with FDR corrected P values in both stem cell markers and immune-related genes are demonstrated in table S7 and table S8 respectively. The distribution of the noncoding RNA genes and their annotation details are summarized in Fig. 8 and table S9 respectively.

**Table 1:** Summary of repetitive elements in the assembled genome of earthworm *Eisenia fetida*.

Total Seq length (bp)	1,052,631,503
Total bases masked (bp)	25,336,204
Percentage of bases masked	2.47
Number of Simple repeats	428,154
Number of Low complexity Repeats	34,901
Number of Satellites	11
Number of DNA transposons	506,168
Number of LTRs	365,755
Number of LINEs	204,621
Number of SINEs	86,934

**Table 2:** Genome annotation summary of earthworm *Eisenia fetida*.

**Summary**	**Number**
Number of gene models	29,552
BLAST annotation (nr)	20,274 (68.60%)
InterProScan annotation	22,218 (75.18%)
GO annotation	12,287 (41.58%)
EC annotation	3470 (11.74%)
KEGG annotation	7096 (24.01%)
COG annotation	9143 (30.94%)

## 2 Experimental design, materials and methods

### 2.1 Repeat identification, masking and gene model prediction

The repetitive contents including the tandem repeats and transposable elements were detected by using the repeat identification tools namely TEclass (http://www.bioinformatics.uni-muenster.de/tools/teclass/index.hbi?lang=en) [2] and RepeatMasker (http://www.repeatmasker.org/cgi-bin/WEBRepeatMasker) [3]. The repeat-masked contig sequences were subjected to gene model prediction using the AUGUSTUS web server (http://augustus.gobics.de/submission) [4]. For accurate prediction of gene models, we have performed the evidence-based gene prediction by using the previously assembled nerve cord transcriptome dataset of earthworm, *E. fetida* [5], [6]. The GFF annotation file, predicted coding gene sequences and translated protein sequences were deposited to the figshare public repository (https://doi.org/10.6084/m9.figshare.6142322.v1).

### 2.2 Identification, functional annotation and analysis of E. fetida protein coding genes

The *E. fetida* protein coding genes were annotated by BLAST search against the NCBI nr (non-redundant), RefSeq and Swiss Prot databases using the BLASTx with E-value 1E-05. The BLAST annotation data of the *E. fetida* protein coding genes against these three databases was demonstrated by three-way Venn diagram, plotted by using the Venny 2.1 (http://bioinfogp.cnb.csic.es/tools/venny/) tool [7]. Simultaneously, we have also aligned the *E. fetida* protein coding genes with the bacterial genome sequences available in the NCBI database using the BLASTN search with E-value threshold 1E-5.

The gene sequences with nr BLAST hits were subjected to GO (Gene Ontology) annotation by using the BLAST2GO software version 4.1 (https://www.blast2go.com/) [8], [9]. The data on the conserved domains, protein families and motifs associated with the *E. fetida* genes were extracted from the InterProScan annotation (https://www.ebi.ac.uk/interpro/) using the BLAST2GO [10]. The orthologous groups (COGs) associated with the *E. fetida* genes were annotated and classified using the EggNog tool (http://eggnogdb.embl.de/) of the BLAST2GO [11]. To further analyze the ortholog patterns across the metazoans we have compared the proteome of *E. fetida* against the proteomes of five other closely related metazoan species: *Capitella teleta* (Annelida), *Helobdella robusta* (Annelida), *Lottia gigantea* (Mollusca), *Schistosoma mansoni* (Platyhelminthes) and *Strongylocentrotus purpuratus* (Echinodermata) and clustered them into groups based upon sequence similarity using the OrthoVenn web server (http://www.bioinfogenome.net/OrthoVenn/) [12]. The predicted core gene sequences were concatenated by using the Geneious bioinformatics software version R11 (https://www.geneious.com/) [13] and subjected to phylogenomic analysis by aligning them with multiple sequence alignment with ClustalW program [14]. The phylogenomic reconstruction was performed through maximum likelihood method along with 100 bootstrap replicates using the MEGA software (www.megasoftware.net/) version 7.0 [15].

The cellular and metabolic pathways associated with the genome dataset of *E. fetida* were identified by annotating the protein coding genes against the KEGG online database using KAAS (KEGG Automatic Annotation Server) web annotation tool (http://www.genome.jp/tools/kaas/) [16]. The KEGG pathway annotation data were further screened to identify the crucial Riboflavin biosynthesizing enzymes having significant BLAST sequence homology with the bacterial sequences.

### 2.3 Identification of stem cell and regeneration associated genes and immune responsive genes, functional enrichment analysis and prediction of noncoding RNAs

The stem cell and regeneration specific genes, regulating the anterior and posterior regeneration and organogenesis process of the worm were identified in the genome dataset by comparing the *E. fetida* gene sequences against 3700 reported stem cell markers and regeneration related genes acquired from the sources like human fetal amniocytes [17] and REGene database (regeneration gene database) (http://regene.bioinfo-minzhao.org/) [18]. The annotation was performed through local BLASTX search with E-value cut-off< 1E-5. Simultaneously, the immune-related genes, triggering innate immune response of the species were monitored by annotating the genome dataset against 5919 previously reported innate immune response genes curated from the InnateDB database (http://www.innatedb.com/) [19]. Consequently the enrichment analysis of the functional GO terms related with these identified stem cell and regeneration specific genes (test set) and the immune-related genes (test set) in comparison to the entire annotated genome dataset (reference set) of *E. fetida* was carried out through Fisher׳s exact test using the BLAST2GO software [20]. The FDR (false discovery rate) was controlled by the Benjamini-Hochberg correction method and the FDR corrected P-Values < 0.05 were taken as statistically significant. The ncRNA (noncoding RNA) genes residing in the genome dataset of earthworm *E. fetida* were screened by annotating the draft genome contigs against the Rfam (RNA family) database (http://rfam.xfam.org/) [21] using the cmscan program integrated into the Infernal software version 1.1 [22].
