# Supplementary material for: Data on genome annotation and analysis of earthworm Eisenia fetida
Source: Data Brief. 2018 Aug 29;20:525–34. doi: 10.1016/j.dib.2018.08.067 (PMC6126081; doi:10.1016/j.dib.2018.08.067)
Supplement: Supplementary file 2 — Supplementary material [file mmc2.docx]

Table S1: BLAST2GO functional annotation of AUGUSTUS predicted gene models against nr database

| **SeqName** | **Description** | **Length** | **e-Value** | **Mean similarity (Percentage)** | **GO Names** |
| --- | --- | --- | --- | --- | --- |
| Efet.01.100021.g1.t1 | lambda-crystallin homolog | 897 | 2.94E-71 | 68.95 | F:oxidoreductase activity; F:3-hydroxyacyl-CoA dehydrogenase activity; P:oxidation-reduction process; P:fatty acid metabolic process |
| Efet.01.10003.g732.t1 | tankyrase-1-like | 729 | 6.15E-133 | 85.85 | P:multicellular organism growth; P:regulation of multicellular organism growth; P:negative regulation of telomere maintenance via telomere lengthening; P:protein polyubiquitination; P:positive regulation of telomere capping; P:positive regulation of canonical Wnt signaling pathway; P:telomere maintenance; C:Golgi membrane; C:nuclear chromosome, telomeric region; F:NAD+ ADP-ribosyltransferase activity; C:nuclear envelope; C:pericentriolar material; P:spindle assembly; C:perinuclear region of cytoplasm; F:metal ion binding; P:protein localization to chromosome, telomeric region; P:positive regulation of telomere maintenance via telomerase; C:cytosol; P:Wnt signaling pathway; P:protein auto-ADP-ribosylation; F:enzyme binding |
| Efet.01.100069.g2.t1 | neurobeachin-like isoform X5 | 1320 | 2.67E-108 | 75.45 |  |
| Efet.01.100093.g3.t1 | hypothetical protein CAPTEDRAFT_182197 | 753 | 3.75E-09 | 51 | F:G-protein coupled receptor activity; F:G-protein coupled peptide receptor activity; P:G-protein coupled receptor signaling pathway; P:signal transduction; C:membrane; C:integral component of membrane; F:signal transducer activity |
| Efet.01.100110.g5.t1 | SH2B adapter 1 | 681 | 3.45E-21 | 61.3 | P:regulation of cellular process |
| Efet.01.100128.g6.t1 | galactoside 2-alpha-L-fucosyltransferase 1-like | 672 | 2.08E-19 | 52.85 | P:carbohydrate metabolic process; F:transferase activity, transferring glycosyl groups; P:fucosylation; C:membrane; C:integral component of membrane; F:transferase activity; F:galactoside 2-alpha-L-fucosyltransferase activity |
| Efet.01.100157.g8.t1 | Low-density lipo receptor-related | 714 | 4.61E-22 | 54.5 | P:ion transport; F:calcium ion binding; P:ion transmembrane transport; C:membrane; C:integral component of membrane; F:extracellular ligand-gated ion channel activity; P:transport |
| Efet.01.100171.g10.t1 | GRAM domain-containing 1B-like isoform X2 | 243 | 1.58E-09 | 73.55 |  |
| Efet.01.100179.g11.t1 | DNA-binding SATB2 | 1341 | 1.12E-30 | 89.55 | C:nucleus; F:DNA binding |
| Efet.01.100202.g14.t1 | nucleosome assembly 1-like 1 | 297 | 2.45E-19 | 50 | C:nucleus; P:nucleosome assembly |
| Efet.01.100270.g15.t1 | protocadherin-9 isoform X1 | 537 | 2.37E-27 | 49.7 | F:calcium ion binding; C:membrane; C:integral component of membrane; P:cell adhesion; P:homophilic cell adhesion via plasma membrane adhesion molecules; F:carbohydrate binding; C:plasma membrane |
| Efet.01.100279.g16.t1 | prolactin-releasing peptide receptor isoform X1 | 702 | 8.98E-23 | 47.54 | P:opioid receptor signaling pathway; F:neuropeptide binding; P:G-protein coupled receptor signaling pathway; P:signal transduction; P:G-protein coupled receptor signaling pathway, coupled to cyclic nucleotide second messenger; P:chemical synaptic transmission; C:neuron projection; F:signal transducer activity; F:neuropeptide Y receptor activity; F:G-protein coupled receptor activity; F:opioid receptor activity; C:integral component of plasma membrane; P:neuropeptide signaling pathway; C:membrane; C:integral component of membrane |
| Efet.01.100286.g17.t1 | hypothetical protein CAPTEDRAFT_221462 | 987 | 9.38E-52 | 53.15 | P:signal transduction |
| Efet.01.100296.g18.t1 | enkurin-like | 963 | 2.02E-10 | 68.5 |  |
| Efet.01.10032.g735.t1 | Krueppel-like factor 15 | 750 | 2.34E-37 | 74.85 | F:nucleic acid binding; F:metal ion binding |
| Efet.01.100352.g21.t1 | monocarboxylate transporter 12-like | 1374 | 4.44E-70 | 72 | P:transmembrane transport; C:integral component of membrane |
| Efet.01.100355.g22.t1 | BTB POZ domain-containing 17-like isoform X8 | 258 | 2.02E-15 | 71.75 |  |
| Efet.01.100396.g23.t1 | short transient receptor potential channel 3- | 348 | 4.18E-21 | 67.67 | F:calcium channel activity; C:integral component of membrane; P:calcium ion transmembrane transport |
| Efet.01.100417.g24.t1 | A-kinase anchor 9-like isoform X14 | 1746 | 2.90E-20 | 55 |  |
| Efet.01.100429.g27.t1 | WD repeat and FYVE domain-containing 3-like isoform X1 | 531 | 2.82E-56 | 85.9 | F:metal ion binding |
| Efet.01.100458.g28.t1 | TNF receptor-associated factor | 573 | 4.98E-97 | 83.1 | P:Toll signaling pathway; C:cytoplasmic side of plasma membrane; C:endosome; P:regulation of interferon-beta production; P:apoptotic process; C:cytosol; C:CD40 receptor complex; F:thioesterase binding; F:ligase activity; F:protein kinase binding; P:negative regulation of type I interferon production; F:zinc ion binding; F:protein phosphatase binding; P:negative regulation of NF-kappaB transcription factor activity; P:regulation of proteolysis; F:tumor necrosis factor receptor binding; P:regulation of apoptotic process; F:signal transducer activity; F:ubiquitin-protein transferase activity; P:tumor necrosis factor-mediated signaling pathway; F:ubiquitin protein ligase binding; P:innate immune response; C:mitochondrion; P:TRIF-dependent toll-like receptor signaling pathway; P:protein ubiquitination; P:regulation of defense response to virus |
| Efet.01.100486.g32.t1 | AN1-type zinc finger 3-like | 252 | 1.55E-40 | 80.8 | F:DNA binding; F:zinc ion binding |
| Efet.01.100498.g34.t1 | cell division cycle 123 homolog | 999 | 4.12E-96 | 60.35 | P:regulation of cellular process; P:single-organism cellular process |
| Efet.01.100498.g35.t1 | hypothetical protein CAPTEDRAFT_221354 | 948 | 1.87E-06 | 66 | C:membrane; C:integral component of membrane |
| Efet.01.100499.g36.t1 | gelsolin 2 | 366 | 2.70E-27 | 68.85 | F:actin binding |
| Efet.01.100503.g37.t1 | endophilin-A3-like isoform X2 | 366 | 1.01E-37 | 70.1 | C:cytoplasm; P:endocytosis |
| Efet.01.100504.g38.t1 | G -activated inward rectifier potassium channel 3-like | 642 | 4.09E-53 | 87.45 | P:potassium ion transmembrane transport; C:integral component of membrane; F:inward rectifier potassium channel activity; P:regulation of ion transmembrane transport |
| Efet.01.100504.g39.t1 | G -activated inward rectifier potassium channel 3-like | 768 | 1.18E-46 | 75.85 | P:potassium ion transmembrane transport; C:integral component of membrane; F:inward rectifier potassium channel activity; P:regulation of ion transmembrane transport |
| Efet.01.100540.g40.t1 | inositol 1,4,5-trisphosphate receptor type 2- | 453 | 7.43E-46 | 94.85 | P:inositol phosphate-mediated signaling; C:integral component of membrane; F:inositol 1,4,5-trisphosphate-sensitive calcium-release channel activity; P:calcium ion transmembrane transport; C:endoplasmic reticulum |
| Efet.01.100563.g41.t1 | glycogen debranching enzyme-like | 240 | 2.43E-12 | 60.6 | P:carbohydrate metabolic process; P:glycogen biosynthetic process; F:catalytic activity; P:glycogen catabolic process; F:amylo-alpha-1,6-glucosidase activity; F:4-alpha-glucanotransferase activity |
| Efet.01.100563.g42.t1 | glycogen debranching enzyme-like | 552 | 6.46E-59 | 73.85 | P:glycogen metabolic process; F:glycogen debranching enzyme activity; F:binding; C:intracellular part |
| Efet.01.100588.g43.t1 | coiled-coil domain-containing 180-like | 381 | 1.37E-24 | 69.1 |  |
| Efet.01.10060.g736.t1 | small G signaling modulator 1-like isoform X1 | 291 | 2.21E-18 | 70.75 |  |
| Efet.01.100617.g45.t1 | Tetratricopeptide repeat | 438 | 2.53E-54 | 91.05 | C:axonemal microtubule; C:motile cilium; P:protein polyglutamylation; F:tubulin-glycine ligase activity; P:intraciliary transport; P:cilium assembly; P:opsin transport; P:establishment of planar polarity; C:intraciliary transport particle B; F:transporter activity; P:determination of left/right symmetry |
| Efet.01.100624.g46.t1 | transcription factor Sox-11-like | 1134 | 2.66E-58 | 77 | F:2-alkenal reductase [NAD(P)] activity; P:oxidation-reduction process |
| Efet.01.100646.g48.t1 | cilia- and flagella-associated 44-like isoform X1 | 258 | 3.52E-16 | 77.95 |  |
| Efet.01.100653.g50.t1 | dixin-like isoform X2 | 753 | 3.80E-17 | 49.3 | C:cytosol; P:canonical Wnt signaling pathway; P:Wnt signaling pathway |
| Efet.01.100684.g51.t1 | OTU domain-containing | 660 | 3.76E-15 | 52.88 | P:regulation of transcription, DNA-templated |
| Efet.01.100686.g52.t1 | cytochrome P450 2U1-like | 363 | 1.39E-25 | 57.75 | F:monooxygenase activity |
| Efet.01.100760.g55.t1 | something about silencing 10 | 408 | 2.45E-09 | 54.05 | C:small-subunit processome; P:maturation of SSU-rRNA from tricistronic rRNA transcript (SSU-rRNA, 5.8S rRNA, LSU-rRNA); C:nucleus; P:brain development; C:nucleolus; F:RNA binding |
| Efet.01.100812.g57.t1 | protocadherin gamma-B1-like | 2376 | 2.23E-127 | 53.05 | C:membrane |
| Efet.01.100862.g58.t1 | cyclic GMP-AMP synthase-like | 783 | 4.74E-06 | 42 |  |
| Efet.01.100870.g59.t1 | ras-related Rab-1A | 441 | 1.43E-65 | 77.15 | C:endoplasmic reticulum membrane; F:GTP binding; P:ER to Golgi vesicle-mediated transport; P:small GTPase mediated signal transduction; C:Golgi membrane |
| Efet.01.100874.g60.t1 | coiled-coil domain-containing 97-like | 438 | 7.76E-21 | 63.05 | C:U2 snRNP |
| Efet.01.100874.g61.t1 | coiled-coil domain-containing 97-like | 285 | 2.63E-18 | 71.4 | C:U2 snRNP |
| Efet.01.100889.g62.t1 | protocadherin beta-12 | 2925 | 2.23E-171 | 55.35 | F:calcium ion binding; C:membrane; C:integral component of membrane; P:cell adhesion; P:homophilic cell adhesion via plasma membrane adhesion molecules; C:plasma membrane |
| Efet.01.100925.g66.t1 | nuclear hormone receptor HR96-like isoform X8 | 387 | 6.91E-65 | 83.3 | F:thyroid hormone receptor activity; C:nucleus; F:zinc ion binding; P:transcription, DNA-templated; F:steroid hormone receptor activity; P:regulation of transcription, DNA-templated; F:sequence-specific DNA binding; P:steroid hormone mediated signaling pathway; P:intracellular receptor signaling pathway |
| Efet.01.100931.g67.t1 | neuronal acetylcholine receptor subunit alpha-7-like | 333 | 3.15E-33 | 75.35 | F:ion channel activity; P:ion transmembrane transport; C:synapse; C:membrane part; C:plasma membrane |
| Efet.01.100938.g68.t1 | Dual specificity tyrosine-phosphorylation-regulated kinase 2 | 438 | 4.03E-34 | 69.75 | F:nucleotide binding; F:protein kinase activity; P:phosphorylation |
| Efet.01.100965.g69.t1 | hypothetical protein HELRODRAFT_171217 | 420 | 2.20E-51 | 82 |  |
| Efet.01.101039.g71.t1 | G coupled receptor | 462 | 6.13E-60 | 77.55 | P:G-protein coupled receptor signaling pathway; C:integral component of membrane; F:vasopressin receptor activity |
| Efet.01.101048.g72.t1 | brunelleschi | 732 | 1.17E-36 | 53.75 |  |
| Efet.01.101055.g73.t1 | synaptotagmin-14 2 | 375 | 8.69E-39 | 79.5 | P:calcium ion-regulated exocytosis of neurotransmitter; F:syntaxin binding; P:vesicle fusion; F:calcium ion binding; F:clathrin binding; P:regulation of calcium ion-dependent exocytosis; C:plasma membrane; F:calcium-dependent phospholipid binding |
| Efet.01.101062.g75.t1 | udp-n-acetylglucosamine--peptide n-acetylglucosaminyltransferase | 792 | 2.29E-64 | 53.35 | F:transferase activity, transferring glycosyl groups; C:membrane; C:integral component of membrane; F:transferase activity |
| Efet.01.101109.g76.t1 | transmembrane 196-like | 387 | 6.37E-45 | 73.56 | C:integral component of membrane |
| Efet.01.101188.g77.t1 | ecto-ADP-ribosyltransferase 5 isoform X1 | 786 | 2.23E-14 | 45 | F:NAD+ nucleosidase activity; F:transferase activity, transferring glycosyl groups; P:protein ADP-ribosylation; F:NAD(P)+-protein-arginine ADP-ribosyltransferase activity; C:membrane; F:transferase activity; F:NAD+ ADP-ribosyltransferase activity |
| Efet.01.101192.g78.t1 | pre-mRNA 3 end processing WDR33 isoform X3 | 1305 | 1.18E-36 | 83.5 | C:mRNA cleavage and polyadenylation specificity factor complex; C:nucleolus; C:proteinaceous extracellular matrix; F:RNA binding; P:mRNA cleavage |
| Efet.01.101208.g79.t1 | hypothetical protein HELRODRAFT_191448 | 384 | 5.45E-06 | 72 |  |
| Efet.01.101219.g80.t1 | microtubule-actin cross-linking factor 1-like isoform X6 | 624 | 7.55E-58 | 69.35 | P:cell cycle arrest; C:cytoskeleton; F:actin binding; F:calcium ion binding |
| Efet.01.101223.g81.t1 | serine threonine- kinase DCLK2 isoform X1 | 1575 | 6.66E-35 | 78.95 | F:ATP binding; F:protein serine/threonine kinase activity; P:protein phosphorylation; C:intracellular; P:intracellular signal transduction |
| Efet.01.101259.g82.t1 | TNF receptor-associated factor 3 | 456 | 2.53E-36 | 61 |  |
| Efet.01.101276.g83.t1 | 5-hydroxytryptamine receptor 2A-like | 1017 | 1.08E-50 | 43.1 | F:G-protein coupled receptor activity; P:G-protein coupled receptor signaling pathway; C:membrane; P:negative regulation of biological process; P:multicellular organismal process |
| Efet.01.101318.g85.t1 | Cys-loop ligand-gated ion channel | 420 | 2.95E-09 | 46.13 | P:ion transport; P:ion transmembrane transport; C:membrane; C:integral component of membrane; F:extracellular ligand-gated ion channel activity; P:transport |
| Efet.01.101363.g88.t1 | ankyrin repeat domain-containing 12-like isoform X1 | 792 | 5.23E-34 | 59.25 | C:nucleus; P:skeletal system development; P:head morphogenesis; P:animal organ morphogenesis |
| Efet.01.101392.g90.t1 | cartilage matrix -like | 3411 | 5.68E-42 | 61.7 | C:collagen trimer; F:calcium ion binding |
| Efet.01.101430.g93.t1 | 5-hydroxytryptamine receptor 1A-like | 1416 | 2.90E-153 | 62.4 | F:G-protein coupled receptor activity; P:G-protein coupled receptor signaling pathway; C:membrane |
| Efet.01.101457.g94.t1 | transmembrane 145 | 1272 | 5.86E-38 | 59.35 |  |
| Efet.01.101473.g95.t1 | gamma-1-syntrophin isoform X1 | 285 | 1.22E-25 | 70.4 | F:structural molecule activity |
| Efet.01.101493.g96.t1 | WD repeat-containing 19-like | 297 | 1.33E-37 | 84.15 | C:cilium; C:intraciliary transport particle A; P:intraciliary retrograde transport |
| Efet.01.10153.g739.t1 | FMRFamide-activated amiloride-sensitive sodium channel isoform X1 | 1992 | 0 | 46.4 | P:transport |
| Efet.01.101539.g100.t1 | collagen alpha-1(XXI) chain-like | 492 | 2.03E-13 | 53.65 | F:calcium ion binding; C:collagen trimer |
| Efet.01.101555.g101.t1 | methenyltetrahydrofolate synthase domain-containing isoform X1 | 960 | 1.36E-34 | 78.4 | F:nucleotide binding; F:nucleic acid binding |
| Efet.01.101567.g103.t1 | fibroblast growth factor receptor substrate 2 | 564 | 1.33E-42 | 75.9 | F:insulin receptor binding |
| Efet.01.101590.g104.t1 | dihydroxyacetone phosphate acyltransferase-like | 465 | 1.38E-53 | 68.45 | F:O-acyltransferase activity; P:cellular lipid metabolic process |
| Efet.01.101628.g106.t1 | octopamine receptor beta-2R-like | 594 | 1.85E-50 | 63.85 | F:G-protein coupled receptor activity; P:G-protein coupled receptor signaling pathway; C:integral component of membrane |
| Efet.01.101635.g107.t1 | C-type lectin 37Da | 654 | 6.11E-07 | 58 | F:protein binding |
| Efet.01.10178.g740.t1 | kyphoscoliosis peptidase | 1695 | 0 | 64.6 |  |
| Efet.01.101832.g110.t1 | nuclear receptor subfamily 0 group B member 2-like | 1464 | 7.46E-28 | 55.24 | P:regulation of cellular process; P:cellular process |
| Efet.01.101850.g111.t1 | uncharacterized protein LOC110049576 | 1413 | 3.85E-13 | 51.8 |  |
| Efet.01.10187.g741.t1 | acid trehalase 1 | 264 | 2.94E-19 | 60.95 | P:carbohydrate metabolic process; F:G-protein coupled receptor activity; P:G-protein coupled receptor signaling pathway; F:catalytic activity; C:cell wall; C:membrane; C:integral component of membrane; F:hydrolase activity |
| Efet.01.101873.g113.t1 | hypothetical protein CAPTEDRAFT_65438, partial | 753 | 7.56E-48 | 54 |  |
| Efet.01.101884.g114.t1 | dynamin-1 | 204 | 6.50E-37 | 93.2 | F:GTP binding; F:GTPase activity |
| Efet.01.101887.g115.t1 | cyclic nucleotide-gated cation channel beta-3-like isoform X2 | 348 | 2.35E-19 | 66.3 | P:ion transmembrane transport; C:integral component of membrane; F:intracellular cyclic nucleotide activated cation channel activity |
| Efet.01.10192.g742.t1 | alpha-tocopherol transfer -like | 438 | 2.34E-23 | 50.5 | P:transport; F:transporter activity; C:intracellular; F:hydrolase activity |
| Efet.01.101927.g118.t1 | Glyco 3-alpha-L-fucosyltransferase A | 894 | 1.52E-18 | 51.1 | F:transferase activity, transferring glycosyl groups; F:fucosyltransferase activity; C:Golgi cisterna membrane; P:fucosylation; C:membrane; C:integral component of membrane; P:protein glycosylation; C:Golgi apparatus; F:transferase activity |
| Efet.01.101967.g120.t1 | extended synaptotagmin 2a | 351 | 1.55E-17 | 72.67 | C:membrane |
| Efet.01.102008.g123.t1 | Collagen alpha-3(VI) chain | 573 | 2.29E-24 | 47.6 | F:calcium ion binding; C:collagen trimer |
| Efet.01.102079.g126.t1 | probable ATP-dependent RNA helicase DDX23 | 585 | 1.70E-91 | 77.65 | P:RNA splicing, via transesterification reactions; F:ATP binding; C:extracellular exosome; C:catalytic step 2 spliceosome; C:U5 snRNP; P:RNA secondary structure unwinding; C:nucleolus; F:ATP-dependent RNA helicase activity; F:isomerase activity; F:RNA binding |
| Efet.01.102138.g127.t1 | heparan sulfate glucosamine 3-O-sulfotransferase | 471 | 1.05E-54 | 83.1 | C:integral component of membrane; F:sulfotransferase activity |
| Efet.01.10219.g746.t1 | sulfurtransferase | 405 | 1.51E-76 | 85.9 | F:transferase activity |
| Efet.01.10219.g747.t1 | type III pantothenate kinase | 771 | 5.58E-172 | 87.2 | F:ATP binding; C:cytoplasm; P:coenzyme A biosynthetic process; F:pantothenate kinase activity; P:phosphorylation |
| Efet.01.102244.g129.t1 | tripartite motif-containing 2-like isoform X3 | 2235 | 0 | 52.2 | F:zinc ion binding; F:metal ion binding; C:intracellular |
| Efet.01.102252.g130.t1 | homeobox Mohawk-like | 615 | 7.37E-06 | 79 | F:DNA binding; C:nucleus; P:regulation of transcription, DNA-templated; F:sequence-specific DNA binding |
| Efet.01.102269.g131.t1 | 29-kDa galactose-binding lectin | 225 | 7.45E-13 | 67.7 | F:carbohydrate binding |
| Efet.01.102305.g132.t1 | maternal exuperantia-2-like | 369 | 2.03E-17 | 56.05 | F:nucleic acid binding |
| Efet.01.102419.g137.t1 | hypothetical protein CAPTEDRAFT_217222 | 753 | 1.22E-55 | 56.05 | C:membrane; C:integral component of membrane; F:carbohydrate binding |
| Efet.01.102426.g139.t1 | neuronal acetylcholine receptor subunit alpha-10-like | 657 | 3.51E-108 | 90.35 | C:postsynaptic membrane; P:cation transmembrane transport; C:integral component of membrane; C:cell junction; F:extracellular ligand-gated ion channel activity |
| Efet.01.102454.g140.t1 | arfaptin- | 378 | 7.57E-59 | 86.55 | F:protein domain specific binding; F:identical protein binding; F:cadherin binding involved in cell-cell adhesion; F:phosphatidylinositol-4-phosphate binding; C:cell-cell adherens junction; C:cell cortex; P:ruffle organization; C:ruffle; P:cell-cell adhesion; P:actin cytoskeleton organization; C:trans-Golgi network membrane; F:GTP-dependent protein binding; P:regulation of Arp2/3 complex-mediated actin nucleation |
| Efet.01.102468.g141.t1 | GMP synthase [glutamine-hydrolyzing] | 432 | 9.79E-27 | 73.15 | F:catalytic activity; P:purine nucleotide biosynthetic process |
| Efet.01.102473.g142.t1 | dual specificity tyrosine-phosphorylation-regulated kinase | 705 | 2.60E-111 | 89.7 | P:peptidyl-tyrosine phosphorylation; F:identical protein binding; P:negative regulation of DNA damage response, signal transduction by p53 class mediator; P:positive regulation of protein deacetylation; C:nuclear speck; P:nervous system development; P:protein autophosphorylation; F:protein serine/threonine kinase activity; P:circadian rhythm; F:protein self-association; F:ATP binding; F:protein serine/threonine/tyrosine kinase activity; C:intracellular ribonucleoprotein complex; F:non-membrane spanning protein tyrosine kinase activity; P:regulation of alternative mRNA splicing, via spliceosome; P:negative regulation of mRNA splicing, via spliceosome; F:tau protein binding; P:peptidyl-serine phosphorylation; P:peptidyl-threonine phosphorylation |
| Efet.01.102475.g143.t1 | Ubiquitin carboxyl-terminal hydrolase 42 | 4278 | 1.53E-71 | 58.25 | P:proteolysis; F:hydrolase activity |
| Efet.01.102479.g144.t1 | dynein heavy chain axonemal-like | 798 | 2.45E-129 | 84.1 | F:ATPase activity; F:ATP binding; F:microtubule motor activity; C:dynein complex; P:microtubule-based movement |
| Efet.01.102652.g150.t1 | 60S ribosomal L18 | 273 | 1.24E-47 | 92.35 | F:RNA binding; F:structural constituent of ribosome; C:cytosolic large ribosomal subunit; P:translation |
| Efet.01.102675.g152.t1 | hypothetical protein CAPTEDRAFT_155564 | 546 | 4.28E-07 | 66 | P:peptidyl-tyrosine phosphorylation; F:ATP binding; F:protein tyrosine kinase activity; C:membrane; C:integral component of membrane; F:protein kinase activity; P:protein phosphorylation |
| Efet.01.102678.g153.t1 | testis-specific serine threonine- kinase 4-like | 903 | 1.60E-97 | 74.55 | C:nucleus; F:ATP binding; C:cytoplasm; F:protein serine/threonine kinase activity; P:protein phosphorylation; P:intracellular signal transduction |
| Efet.01.102725.g154.t1 | zinc finger 2 | 1317 | 5.34E-61 | 52.15 | F:nucleic acid binding; F:DNA binding; C:nucleus; F:transcription factor activity, sequence-specific DNA binding; F:metal ion binding; P:transcription, DNA-templated; P:regulation of transcription, DNA-templated |
| Efet.01.102725.g155.t1 | gastrula zinc finger -like | 816 | 4.49E-53 | 54.2 | F:nucleic acid binding; F:DNA binding; C:nucleus; F:transcription factor activity, sequence-specific DNA binding; F:metal ion binding; P:transcription, DNA-templated; P:regulation of transcription, DNA-templated |
| Efet.01.10275.g749.t1 | hypothetical protein CRN43_20910 | 378 | 4.01E-72 | 85 |  |
| Efet.01.102754.g156.t1 | nucleoredoxin-like | 360 | 6.54E-25 | 64.1 |  |
| Efet.01.102758.g157.t1 | FAM69A isoform X2 | 816 | 8.90E-68 | 59 | C:membrane; C:integral component of membrane |
| Efet.01.102773.g158.t1 | WD repeat-containing 3 | 351 | 6.93E-24 | 62.95 | C:preribosome |
| Efet.01.102779.g159.t1 | zinc finger 184-like | 1608 | 1.22E-75 | 56.95 | F:nucleic acid binding; F:metal ion binding |
| Efet.01.102785.g160.t1 | regulator of nonsense transcripts 2-like | 837 | 2.79E-83 | 68.05 | F:RNA binding |
| Efet.01.102824.g162.t1 | F0F1 ATP synthase subunit delta | 588 | 3.16E-94 | 70.7 | C:membrane; C:cell part; P:ATP synthesis coupled proton transport |
| Efet.01.102833.g163.t1 | histamine H2 receptor-like | 1743 | 2.13E-49 | 46.05 | F:G-protein coupled receptor activity; P:G-protein coupled receptor signaling pathway; C:membrane; C:integral component of membrane |
| Efet.01.102846.g164.t1 | dachsous | 1800 | 1.33E-87 | 50.4 | F:calcium ion binding; C:membrane; C:integral component of membrane; P:cell adhesion; P:homophilic cell adhesion via plasma membrane adhesion molecules; C:plasma membrane |
| Efet.01.102855.g165.t1 | hypothetical protein CAPTEDRAFT_221004 | 687 | 5.64E-15 | 50 | C:membrane; C:integral component of membrane |
| Efet.01.10286.g750.t1 | NACHT domain- and WD repeat-containing 1-like isoform X1 | 2913 | 3.52E-92 | 45.05 |  |
| Efet.01.10287.g751.t1 | gill-specific calpain | 225 | 1.95E-18 | 59.55 | F:hydrolase activity |
| Efet.01.102932.g166.t1 | Ankyrin repeat and fibronectin type-III domain-containing 1 | 273 | 2.68E-12 | 65.45 | P:signal transduction |
| Efet.01.102935.g167.t1 | fibroblast growth factor receptor 2-like isoform X3 | 273 | 5.34E-16 | 59.2 | P:peptidyl-tyrosine phosphorylation; P:positive regulation of cell proliferation; P:vacuolar phosphate transport; P:fibroblast growth factor receptor signaling pathway; P:protein autophosphorylation; C:Golgi apparatus; F:heparin binding; P:negative regulation of fibroblast growth factor production; P:positive regulation of proteolysis; C:nucleoplasm; F:nucleotide binding; C:integral component of plasma membrane; F:fibroblast growth factor-activated receptor activity; P:vitamin D3 metabolic process; P:regulation of extracellular matrix disassembly; P:positive regulation of parathyroid hormone secretion; P:cell migration; P:positive regulation of DNA biosynthetic process; P:alveolar secondary septum development; P:phosphate ion homeostasis; C:endoplasmic reticulum; P:negative regulation of gene expression; C:cell-cell junction; P:positive regulation of catalytic activity; P:positive regulation of gene expression; P:glucose homeostasis; P:positive regulation of ERK1 and ERK2 cascade; P:organ induction; F:fibroblast growth factor binding; P:regulation of cholesterol homeostasis; C:transport vesicle; P:regulation of bile acid biosynthetic process |
| Efet.01.10295.g752.t1 | midasin | 1311 | 4.46E-15 | 56.25 | C:intracellular part |
| Efet.01.102958.g168.t1 | Chondroitin sulfate synthase | 1638 | 9.67E-160 | 60.4 | P:multicellular organism development |
| Efet.01.103071.g173.t1 | hypothetical protein CAPTEDRAFT_202513 | 972 | 2.88E-20 | 47 | F:G-protein coupled receptor activity; P:G-protein coupled receptor signaling pathway; C:membrane; C:integral component of membrane |
| Efet.01.103239.g175.t1 | Tektin-1 | 1341 | 1.87E-150 | 70.75 |  |
| Efet.01.103284.g176.t1 | DNA methyltransferase 1-associated 1-like | 249 | 2.53E-26 | 88.9 | P:histone H4 acetylation; F:DNA binding; P:DNA repair; P:chromatin remodeling; C:NuA4 histone acetyltransferase complex; P:histone H2A acetylation; P:negative regulation of transcription, DNA-templated; F:methyltransferase activity; P:methylation |
| Efet.01.103302.g177.t1 | glypican-6 | 351 | 2.86E-47 | 78.95 | F:heparan sulfate proteoglycan binding; C:proteinaceous extracellular matrix; C:plasma membrane; C:anchored component of membrane |
| Efet.01.103327.g178.t1 | neurogenic differentiation factor 6 | 555 | 8.09E-29 | 73.1 | P:camera-type eye development; P:regulation of transcription, DNA-templated; P:inner ear receptor cell development; F:protein dimerization activity; C:intracellular part; P:endocrine pancreas development; P:posterior lateral line neuromast hair cell differentiation |
| Efet.01.103354.g179.t1 | atrial natriuretic peptide receptor 1-like | 228 | 1.41E-28 | 69.8 | F:ATP binding; P:cGMP biosynthetic process; C:integral component of membrane; F:guanylate cyclase activity; F:protein kinase activity; P:protein phosphorylation; C:intracellular; P:intracellular signal transduction |
| Efet.01.10337.g757.t1 | ankyrin repeat domain-containing SOWAHC-like | 345 | 3.18E-24 | 70 |  |
| Efet.01.103444.g181.t1 | serine threonine- phosphatase 6 regulatory subunit 3 isoform X2 | 429 | 4.58E-11 | 73.55 |  |
| Efet.01.103454.g183.t1 | unconventional myosin-VIIa-like | 288 | 1.60E-10 | 62.1 | C:myosin complex; F:ATP binding; F:motor activity |
| Efet.01.103465.g184.t1 | hypothetical protein CAPTEDRAFT_228772 | 1419 | 5.00E-08 | 74.5 | C:membrane; C:integral component of membrane |
| Efet.01.103468.g185.t1 | sterol regulatory element-binding 1 isoform X2 | 1887 | 2.01E-57 | 68.2 | C:membrane |
| Efet.01.103473.g186.t1 | collagen | 333 | 7.20E-08 | 66.4 | F:DNA binding; P:DNA integration |
| Efet.01.103493.g187.t1 | RNA pseudouridylate synthase domain-containing 1 | 393 | 1.26E-42 | 69.7 | P:tRNA pseudouridine synthesis; C:mitochondrion; F:pseudouridine synthase activity; F:deaminase activity |
| Efet.01.103517.g189.t1 | dynein intermediate chain ciliary-like isoform X2 | 522 | 1.05E-57 | 77.85 | C:cytoplasm; P:multicellular organism development; C:cilium; C:dynein complex; C:microtubule; F:motor activity |
| Efet.01.103536.g190.t1 | symplekin-like | 339 | 4.09E-18 | 67.6 |  |
| Efet.01.103538.g191.t1 | 5-hydroxytryptamine receptor 1A-like | 1719 | 6.64E-114 | 58.35 | F:G-protein coupled receptor activity; P:G-protein coupled receptor signaling pathway; C:membrane |
| Efet.01.10360.g758.t1 | titin-like isoform X1 | 591 | 1.09E-28 | 51.2 | F:nucleotide binding; F:ATP binding; F:protein kinase activity; P:phosphorylation; F:transferase activity; P:protein phosphorylation; F:kinase activity |
| Efet.01.103634.g196.t1 | transcriptional repressor p66-beta-like | 771 | 5.06E-37 | 74.3 | P:negative regulation of transcription from RNA polymerase II promoter; F:transcription factor activity, sequence-specific DNA binding; F:zinc ion binding; P:DNA methylation; C:NuRD complex; F:RNA polymerase II regulatory region sequence-specific DNA binding |
| Efet.01.103667.g199.t1 | brefeldin A-inhibited guanine nucleotide-exchange 1-like isoform X2 | 411 | 6.43E-80 | 93.45 | F:ARF guanyl-nucleotide exchange factor activity; P:regulation of ARF protein signal transduction; P:positive regulation of GTPase activity |
| Efet.01.103667.g200.t1 | Brefeldin A-inhibited guanine nucleotide-exchange 1 | 573 | 3.34E-83 | 68.5 | F:ARF guanyl-nucleotide exchange factor activity; P:regulation of ARF protein signal transduction; P:positive regulation of GTPase activity |
| Efet.01.103754.g203.t1 | BTB POZ domain-containing 6-like | 777 | 1.80E-16 | 44 | P:regulation of neurogenesis |
| Efet.01.103764.g205.t1 | serine threonine- kinase 26-like | 249 | 7.57E-06 | 54 |  |
| Efet.01.103837.g208.t1 | hypothetical protein HELRODRAFT_191437 | 477 | 1.37E-06 | 51 |  |
| Efet.01.103839.g209.t1 | hypothetical protein CAPTEDRAFT_222229 | 285 | 1.67E-19 | 70.7 | C:membrane |
| Efet.01.103840.g210.t1 | peregrin isoform X1 | 885 | 1.12E-116 | 80.15 | P:histone H3 acetylation; F:zinc ion binding; P:positive regulation of transcription, DNA-templated; C:membrane; C:MOZ/MORF histone acetyltransferase complex |
| Efet.01.103840.g211.t1 | peregrin-like isoform X1 | 687 | 2.07E-41 | 59 | F:zinc ion binding; F:metal ion binding |
| Efet.01.10385.g760.t1 | ATP-binding cassette sub-family A member 3 | 846 | 4.16E-101 | 71.3 | F:nucleotide binding; P:lipid transport; F:ATPase activity, coupled to transmembrane movement of substances; C:intracellular membrane-bounded organelle; C:membrane |
| Efet.01.103884.g215.t1 | discoidin domain-containing receptor 2-like isoform X1 | 948 | 4.19E-76 | 53.4 | F:ATP binding; P:peptidyl-tyrosine phosphorylation; C:integral component of plasma membrane; F:protein tyrosine kinase activity; F:transmembrane receptor protein tyrosine kinase activity; C:membrane; C:integral component of membrane; F:protein tyrosine kinase collagen receptor activity; P:transmembrane receptor protein tyrosine kinase signaling pathway; F:protein kinase activity; P:collagen-activated tyrosine kinase receptor signaling pathway; P:protein phosphorylation |
| Efet.01.103898.g216.t1 | WD repeat-containing on Y chromosome-like isoform X2 | 447 | 8.64E-22 | 49.6 | F:calcium ion binding; C:membrane; C:integral component of membrane |
| Efet.01.103900.g217.t1 | CD109 antigen-like isoform X1 | 504 | 7.80E-39 | 66.15 | C:extracellular space; F:endopeptidase inhibitor activity; F:RNA binding; P:negative regulation of endopeptidase activity; C:extracellular region |
| Efet.01.10393.g761.t1 | D(2) dopamine receptor isoform X1 | 1155 | 7.35E-21 | 45.85 | F:G-protein coupled receptor activity; P:G-protein coupled receptor signaling pathway; P:signal transduction; C:membrane; C:integral component of membrane; F:signal transducer activity |
| Efet.01.103931.g218.t1 | hypothetical protein CAPTEDRAFT_222159 | 210 | 1.60E-13 | 71.5 | C:membrane |
| Efet.01.103999.g220.t1 | A disintegrin and metallo ase with thrombospondin motifs 20 | 495 | 5.01E-12 | 47.2 | F:zinc ion binding; P:integrin-mediated signaling pathway; P:proteolysis; F:peptidase activity; C:extracellular matrix; F:metalloendopeptidase activity; C:proteinaceous extracellular matrix; F:metallopeptidase activity |
| Efet.01.104013.g221.t1 | Tudor domain-containing 1 | 816 | 1.18E-23 | 48.55 | C:intracellular part |
| Efet.01.10407.g763.t1 | cytokine-inducible SH2-containing -like | 1044 | 3.98E-32 | 56.4 | P:negative regulation of cellular process; P:cellular process |
| Efet.01.104091.g223.t1 | triosephosphate isomerase | 465 | 8.07E-51 | 77.85 | P:gluconeogenesis; P:pentose-phosphate shunt; P:glycolytic process; F:triose-phosphate isomerase activity |
| Efet.01.104125.g224.t1 | microtubule-actin cross-linking factor isoforms 1 2 3 5-like | 729 | 7.02E-18 | 52.86 | P:cell cycle arrest; C:cytoskeleton; F:calcium ion binding |
| Efet.01.104145.g226.t1 | family | 1584 | 0 | 85.6 |  |
| Efet.01.104145.g227.t1 | septation A | 537 | 2.12E-90 | 83 | P:cell division; C:integral component of plasma membrane; P:cell cycle |
| Efet.01.104145.g228.t1 | peptidylprolyl isomerase | 930 | 4.37E-139 | 89.75 | F:peptidyl-prolyl cis-trans isomerase activity; P:protein peptidyl-prolyl isomerization |
| Efet.01.104145.g229.t1 | alpha-methylacyl- racemase | 597 | 2.64E-134 | 75.1 | F:catalytic activity |
| Efet.01.104145.g230.t1 | acyl- synthetase | 1512 | 0 | 77.3 | F:ATP binding; F:catalytic activity; F:metal ion binding; F:cofactor binding |
| Efet.01.104145.g231.t1 | acyl- dehydrogenase | 1146 | 0 | 88.95 | F:acyl-CoA dehydrogenase activity; F:flavin adenine dinucleotide binding; P:oxidation-reduction process |
| Efet.01.104145.g232.t1 | family transcriptional regulator | 585 | 8.91E-94 | 71.25 | F:DNA binding; P:transcription, DNA-templated; P:regulation of transcription, DNA-templated |
| Efet.01.104145.g233.t1 | ABC transporter substrate-binding | 531 | 9.44E-102 | 86.5 | C:outer membrane-bounded periplasmic space |
| Efet.01.104161.g234.t1 | RING finger 37 | 1515 | 8.94E-70 | 45.35 | F:ubiquitin-protein transferase activity; F:zinc ion binding; F:metal ion binding; P:protein ubiquitination |
| Efet.01.104179.g235.t1 | basement membrane-specific heparan sulfate proteoglycan core -like | 336 | 4.13E-11 | 52.5 | F:calcium ion binding |
| Efet.01.104187.g238.t1 | BAI1-associated 3-like isoform X1 | 351 | 1.85E-08 | 61 |  |
| Efet.01.104261.g240.t1 | slit homolog 3 -like isoform X3 | 663 | 1.59E-21 | 53.75 | F:G-protein coupled receptor activity; P:G-protein coupled receptor signaling pathway; P:signal transduction; C:membrane; C:integral component of membrane; F:signal transducer activity; F:protein-hormone receptor activity |
| Efet.01.104282.g241.t1 | beta-lactamase 2 isoform X3 | 672 | 1.26E-60 | 74.25 |  |
| Efet.01.10430.g764.t1 | lysine-specific demethylase 7B-like | 321 | 3.19E-42 | 78.95 | C:nucleus; F:zinc ion binding |
| Efet.01.10432.g765.t1 | probable G- coupled receptor 139 | 1200 | 3.29E-98 | 58.6 | F:G-protein coupled receptor activity; P:G-protein coupled receptor signaling pathway; P:signal transduction; C:membrane; C:integral component of membrane; F:signal transducer activity |
| Efet.01.10434.g766.t1 | glutamine--fructose-6-phosphate aminotransferase [isomerizing] 2-like isoform X5 | 735 | 1.44E-142 | 90.1 | P:carbohydrate metabolic process; F:glutamine-fructose-6-phosphate transaminase (isomerizing) activity; P:fructose 6-phosphate metabolic process; P:UDP-N-acetylglucosamine metabolic process; F:amidophosphoribosyltransferase activity; P:carbohydrate derivative biosynthetic process; F:carbohydrate binding |
| Efet.01.104343.g243.t1 | splicing factor 3A subunit 2-like | 1824 | 1.70E-09 | 45.33 | F:nucleotide binding; F:GTP binding; P:clathrin-dependent endocytosis; F:GTPase activity |
| Efet.01.104372.g244.t1 | ras-related Rab-39B-like | 228 | 1.59E-07 | 64.5 | F:GTP binding; P:small GTPase mediated signal transduction; C:intracellular |
| Efet.01.104377.g245.t1 | hypothetical protein BOX15_Mlig000372g1, partial | 444 | 4.86E-06 | 47 |  |
| Efet.01.104408.g246.t1 | pre-mRNA cleavage complex 2 Pcf11-like isoform X1 | 1242 | 2.33E-08 | 58 |  |
| Efet.01.104408.g247.t1 | Pre-mRNA cleavage complex 2 Pcf11 | 1806 | 1.02E-54 | 55.55 |  |
| Efet.01.104428.g248.t1 | terminal uridylyltransferase 4 isoform X8 | 327 | 7.88E-33 | 67.45 | F:nucleic acid binding; F:zinc ion binding; F:nucleotidyltransferase activity |
| Efet.01.104466.g249.t1 | leucine-rich repeat and fibronectin type III domain-containing 1 | 1236 | 8.05E-122 | 56.5 | C:integral component of membrane |
| Efet.01.104469.g250.t1 | hypothetical protein BRAFLDRAFT_101236 | 381 | 1.08E-06 | 51.17 | F:histamine N-methyltransferase activity; P:methylation |
| Efet.01.104485.g251.t1 | hypothetical protein CAPTEDRAFT_52833, partial | 249 | 2.07E-07 | 68.5 | P:ion transport |
| Efet.01.104514.g252.t1 | Hydrocephalus-inducing -like | 387 | 8.07E-13 | 78 | P:cilium movement |
| Efet.01.104527.g253.t1 | leucine-rich repeat serine threonine- kinase 1-like | 462 | 1.65E-56 | 75.25 | F:purine ribonucleoside triphosphate binding; F:purine ribonucleoside binding; P:phosphorylation; F:kinase activity; F:purine ribonucleotide binding |
| Efet.01.104529.g254.t1 | singed | 930 | 4.96E-134 | 70.6 | F:actin filament binding; C:cytoskeleton; C:cytoplasm; P:actin filament organization; F:protein binding, bridging |
| Efet.01.104578.g255.t1 | neurocalcin homolog | 396 | 4.70E-82 | 94.3 | F:calcium ion binding; F:NAD(P)H oxidase activity; P:oxidation-reduction process |
| Efet.01.104616.g256.t1 | dynein heavy chain axonemal | 393 | 5.58E-51 | 77.45 | F:ATPase activity; F:ATP binding; F:microtubule motor activity; C:dynein complex; P:microtubule-based movement |
| Efet.01.104647.g257.t1 | solute carrier family 28 member 3-like | 534 | 1.85E-47 | 68.2 | F:nucleoside transmembrane transporter activity; C:membrane; C:integral component of membrane; P:nucleoside transmembrane transport; P:transport |
| Efet.01.104647.g258.t1 | solute carrier family 28 member 3-like | 387 | 6.65E-41 | 82.7 | F:nucleoside:sodium symporter activity; C:integral component of plasma membrane; P:nucleoside transmembrane transport |
| Efet.01.104647.g259.t1 | solute carrier family 28 member 3-like | 324 | 2.38E-20 | 71.1 | C:membrane; P:transport |
| Efet.01.104647.g260.t1 | hypothetical protein HELRODRAFT_93209 | 777 | 4.42E-07 | 63 | F:nucleoside transmembrane transporter activity; C:membrane; C:integral component of membrane; P:nucleoside transmembrane transport; P:transport |
| Efet.01.104647.g261.t1 | tripartite motif-containing 2-like | 1131 | 3.35E-19 | 44.05 | F:zinc ion binding; F:metal ion binding; C:intracellular |
| Efet.01.104669.g262.t1 | Integrator complex subunit 3 | 258 | 1.89E-18 | 68.5 | P:DNA repair; P:snRNA processing; F:protein binding; P:mitotic cell cycle checkpoint; P:snRNA transcription from RNA polymerase II promoter; C:SOSS complex; C:integrator complex; P:response to ionizing radiation |
| Efet.01.104751.g263.t1 | Smoothelin 1 | 516 | 1.14E-61 | 83.4 |  |
| Efet.01.10485.g768.t1 | glucokinase | 579 | 7.83E-113 | 83.05 | P:glucose 6-phosphate metabolic process; F:glucokinase activity; P:phosphorylation |
| Efet.01.10485.g769.t1 | carboxymuconolactone decarboxylase family | 474 | 1.76E-85 | 78.6 | F:peroxidase activity; P:cellular oxidant detoxification; P:oxidation-reduction process; F:peroxiredoxin activity |
| Efet.01.104862.g265.t1 | E3 ubiquitin- ligase TRIM71-like | 1134 | 5.91E-13 | 48.6 | F:zinc ion binding; F:metal ion binding; C:intracellular |
| Efet.01.104867.g266.t1 | extracellular serine threonine CG31145 isoform X1 | 252 | 1.80E-12 | 63.95 |  |
| Efet.01.104908.g267.t1 | Neuropeptides capa receptor | 483 | 6.26E-48 | 73 | P:neuropeptide signaling pathway; C:integral component of membrane; F:neuromedin U receptor activity |
| Efet.01.104937.g268.t1 | pikachurin-like isoform X2 | 396 | 4.26E-15 | 63.45 | F:calcium ion binding |
| Efet.01.104942.g269.t1 | 5-dehydro-2-deoxygluconokinase | 1305 | 0 | 87.95 | P:phosphorylation; F:phosphotransferase activity, alcohol group as acceptor; F:kinase activity |
| Efet.01.104942.g270.t1 | 3D-(3,5 4)-trihydroxycyclohexane-1,2-dione acylhydrolase (decyclizing) | 2583 | 0 | 84.35 | F:magnesium ion binding; F:acetolactate synthase activity; F:thiamine pyrophosphate binding; F:hydrolase activity, acting on acid carbon-carbon bonds, in ketonic substances; P:inositol catabolic process |
| Efet.01.104959.g272.t1 | beta-1,3-galactosyltransferase 1-like | 921 | 1.32E-17 | 58.45 | F:transferase activity, transferring glycosyl groups; C:membrane; C:integral component of membrane; P:protein glycosylation; F:galactosyltransferase activity; C:Golgi apparatus; C:Golgi membrane; F:transferase activity |
| Efet.01.104975.g273.t1 | Transcription factor SUM-1 | 1179 | 3.77E-30 | 88 | P:muscle organ development; C:nucleus; F:DNA binding; P:transcription, DNA-templated; P:regulation of transcription, DNA-templated; F:protein dimerization activity |
| Efet.01.105043.g275.t1 | progestin and adipoQ receptor family member 3-like | 249 | 6.00E-10 | 65.33 | C:membrane; C:integral component of membrane |
| Efet.01.105150.g277.t1 | slit homolog 3 -like | 1182 | 1.34E-50 | 56.25 | C:membrane; C:integral component of membrane |
| Efet.01.105168.g278.t1 | hypothetical protein CAPTEDRAFT_227908 | 414 | 4.47E-07 | 58 |  |
| Efet.01.105280.g284.t1 | zinc metallo ase nas-14-like | 912 | 9.59E-58 | 60.45 | F:hydrolase activity |
| Efet.01.105281.g285.t1 | lamin-B2-like | 864 | 4.82E-09 | 52.33 |  |
| Efet.01.105314.g287.t1 | sodium calcium exchanger 1-like isoform X1 | 297 | 2.21E-27 | 81.55 | P:cell communication; P:calcium ion transport; C:integral component of membrane; P:sodium ion transmembrane transport; F:calcium:sodium antiporter activity |
| Efet.01.105316.g288.t1 | hypothetical protein CAPTEDRAFT_216221 | 321 | 9.12E-18 | 63 | C:membrane |
| Efet.01.105341.g289.t1 | pre-mRNA-splicing factor SYF1 | 348 | 1.87E-39 | 81.95 | P:RNA processing |
| Efet.01.105427.g290.t1 | cyclic GMP-AMP synthase-like | 741 | 5.74E-21 | 51 | C:membrane; C:integral component of membrane |
| Efet.01.105459.g291.t1 | TXLR2_EISFE ame: Full=Lysenin-related 2 Short=LRP-2 ame: Full=Fetidin ame: Full=Hemolysin ame: Full=Lysenin-3 ame: Full=efL3 | 1017 | 5.37E-47 | 57.82 | P:cytolysis; P:ion transport; C:membrane; C:integral component of membrane; P:hemolysis in other organism; C:other organism membrane; C:extracellular region; P:defense response to bacterium; P:transport; C:other organism cell membrane |
| Efet.01.105471.g292.t1 | calmodulin isoform 1 | 216 | 1.41E-15 | 75.4 | F:phosphatidylinositol 3-kinase binding; F:protein domain specific binding; C:voltage-gated potassium channel complex; F:calcium-dependent protein binding; F:adenylate cyclase binding; F:ion channel binding; C:growth cone; C:nucleus; F:type 3 metabotropic glutamate receptor binding; P:G2/M transition of mitotic cell cycle; C:cytoplasm; F:calcium ion binding; F:protein N-terminus binding; F:nitric-oxide synthase regulator activity; F:nitric-oxide synthase binding |
| Efet.01.105554.g294.t1 | hypothetical protein HELRODRAFT_191437 | 435 | 7.83E-06 | 48 |  |
| Efet.01.10562.g775.t1 | tyramine receptor tyra-2-like | 1650 | 1.63E-22 | 50.65 | F:G-protein coupled receptor activity; P:insulin secretion; P:G-protein coupled receptor signaling pathway; P:signal transduction; C:membrane; C:integral component of membrane; C:receptor complex; F:phosphatidylcholine binding; F:signal transducer activity |
| Efet.01.105633.g296.t1 | leucine Rich Repeat family | 1797 | 3.66E-130 | 42.6 | F:ubiquitin-protein transferase activity; C:nucleus; P:ubiquitin-dependent protein catabolic process |
| Efet.01.105645.g297.t1 | alpha beta hydrolase domain-containing 17A | 1020 | 4.39E-70 | 60.2 | C:membrane; F:hydrolase activity |
| Efet.01.105706.g299.t1 | Clathrin light | 303 | 8.42E-11 | 63.63 | P:vesicle-mediated transport; C:clathrin coat of trans-Golgi network vesicle; C:clathrin coat of coated pit; F:structural molecule activity; P:intracellular protein transport |
| Efet.01.105711.g300.t1 | gamma-glutamylcyclotransferase isoform X1 | 516 | 1.69E-58 | 64.8 | F:transferase activity, transferring acyl groups; P:cellular process |
| Efet.01.105757.g302.t1 | filamin-interacting FAM101B | 552 | 7.05E-17 | 54.22 |  |
| Efet.01.105787.g303.t1 | thymidylate kinase | 288 | 2.53E-13 | 59.35 | P:nucleotide phosphorylation; F:thymidylate kinase activity; F:ATP binding; P:dTDP biosynthetic process; P:phosphorylation; F:kinase activity |
| Efet.01.105788.g304.t1 | solute carrier family 17 member 9-like | 225 | 5.68E-10 | 69.85 | C:membrane |
| Efet.01.105790.g305.t1 | histone-lysine N-methyltransferase Suv4-20 | 231 | 1.50E-39 | 88.9 | P:histone H4-K20 trimethylation; F:histone methyltransferase activity (H4-K20 specific) |
| Efet.01.105799.g306.t1 | Transaldolase | 408 | 4.08E-44 | 78.75 | P:carbohydrate metabolic process; F:sedoheptulose-7-phosphate:D-glyceraldehyde-3-phosphate glyceronetransferase activity; C:cytoplasm; P:pentose-phosphate shunt |
| Efet.01.105815.g308.t1 | hypothetical protein HELRODRAFT_168985 | 975 | 3.26E-63 | 55.45 |  |
| Efet.01.105815.g309.t1 | ankyrin repeat and death domain-containing 1A isoform X2 | 1194 | 2.84E-10 | 83.45 | P:signal transduction |
| Efet.01.105828.g310.t1 | deglycase DJ-1-like isoform X2 | 384 | 2.53E-28 | 72.2 | P:dopamine metabolic process; P:phosphatidylinositol 3-kinase signaling; P:mitochondrial ATP synthesis coupled electron transport; P:regulation of transcription from RNA polymerase II promoter; C:nucleus; P:regulation of protein kinase B signaling; P:neuron cellular homeostasis; F:glyoxalase III activity; F:transcription coactivator activity; P:methylglyoxal catabolic process to D-lactate via S-lactoyl-glutathione; C:mitochondrion; C:cytosol; P:cellular response to hydrogen peroxide |
| Efet.01.105831.g311.t1 | dynein heavy chain axonemal-like | 918 | 1.31E-163 | 77.9 | F:ATP binding; F:microtubule motor activity; C:dynein complex; P:microtubule-based movement |
| Efet.01.105861.g312.t1 | DNA polymerase | 237 | 7.80E-20 | 69.15 | F:nucleotide binding; F:nucleic acid binding; F:DNA-directed DNA polymerase activity; P:DNA biosynthetic process |
| Efet.01.105872.g313.t1 | calmodulin D | 234 | 2.18E-36 | 88.75 | F:calcium ion binding |
| Efet.01.10588.g777.t1 | probable G- coupled receptor No9 isoform X1 | 1371 | 1.50E-48 | 47.6 | F:G-protein coupled receptor activity; P:G-protein coupled receptor signaling pathway; P:signal transduction; C:membrane; C:integral component of membrane; F:signal transducer activity |
| Efet.01.10589.g778.t1 | acetylcholine receptor subunit beta-like 1 | 465 | 4.08E-24 | 47.95 | C:membrane |
| Efet.01.105929.g314.t1 | radial spoke head 4 homolog A isoform X1 | 222 | 2.83E-19 | 84.8 | P:axoneme assembly; P:cilium movement; C:nucleolus; C:axoneme; C:nucleoplasm |
| Efet.01.105934.g315.t1 | mitoferrin-1 isoform X3 | 675 | 2.55E-59 | 76.65 | C:integral component of membrane; P:transport |
| Efet.01.105973.g316.t1 | transmembrane 177 | 720 | 1.84E-45 | 54.85 | C:membrane; C:integral component of membrane |
| Efet.01.10601.g779.t1 | Solute carrier organic anion transporter family member 5A1 | 621 | 2.75E-54 | 66.5 | C:membrane; P:transport |
| Efet.01.106049.g318.t1 | PREDICTED: uncharacterized protein LOC109483725 | 420 | 2.33E-61 | 75.9 | F:catalytic activity |
| Efet.01.106064.g319.t1 | uncharacterized protein LOC111121124 | 459 | 3.54E-11 | 53.22 | C:membrane; C:integral component of membrane |
| Efet.01.10614.g780.t1 | expanded-like isoform X1 | 1956 | 2.03E-38 | 65.75 | C:cytoskeleton |
| Efet.01.106191.g324.t1 | probable E3 ubiquitin- ligase ARI9- | 420 | 1.90E-06 | 58 |  |
| Efet.01.10627.g781.t1 | diaminopimelate epimerase | 609 | 2.47E-30 | 56.3 | P:lysine biosynthetic process; F:diaminopimelate epimerase activity; C:cytoplasm; P:lysine biosynthetic process via diaminopimelate; P:cellular amino acid biosynthetic process; F:isomerase activity |
| Efet.01.106275.g327.t1 | hypothetical protein CAPTEDRAFT_225909 | 558 | 1.40E-100 | 61.45 | C:integral component of membrane |
| Efet.01.106310.g328.t1 | hypothetical protein | 1470 | 1.97E-07 | 81 |  |
| Efet.01.106326.g331.t1 | DNA repair SWI5 | 297 | 3.33E-14 | 72.25 | P:double-strand break repair via homologous recombination; P:cellular response to ionizing radiation; C:Swi5-Sfr1 complex |
| Efet.01.106418.g337.t1 | phosphatidylinositol 3-kinase catalytic subunit type 3 | 261 | 2.34E-28 | 88.6 | F:ATP binding; C:phosphatidylinositol 3-kinase complex, class III, type I; P:phosphatidylinositol-mediated signaling; P:phosphatidylinositol-3-phosphate biosynthetic process; P:phosphatidylinositol phosphorylation; C:intracellular; F:1-phosphatidylinositol-3-kinase activity |
| Efet.01.106459.g339.t1 | nicotinic acetylcholine receptor alpha5 | 516 | 7.89E-79 | 71.75 | P:ion transmembrane transport; C:integral component of membrane; F:extracellular ligand-gated ion channel activity |
| Efet.01.106464.g340.t1 | cadherin-23 isoform X1 | 345 | 3.25E-12 | 50.63 | F:calcium ion binding; C:membrane; C:integral component of membrane; P:cell adhesion; P:homophilic cell adhesion via plasma membrane adhesion molecules; C:plasma membrane |
| Efet.01.106467.g341.t1 | receptor-type guanylate cyclase gcy-5-like isoform X1 | 576 | 2.98E-54 | 58.9 | F:catalytic activity; P:primary metabolic process; P:organic substance metabolic process; P:phosphate-containing compound metabolic process |
| Efet.01.106547.g344.t1 | furry isoform X1 | 447 | 2.30E-76 | 84.9 | C:site of polarized growth; P:neuron projection development; C:cell cortex; P:cell morphogenesis; C:integral component of membrane; P:actin filament reorganization |
| Efet.01.106592.g345.t1 | membrane progestin receptor gamma | 570 | 2.86E-46 | 64.6 | C:membrane; C:integral component of membrane |
| Efet.01.106615.g346.t1 | hypothetical protein CAPTEDRAFT_186431 | 291 | 8.00E-23 | 57.3 | F:G-protein coupled receptor activity; P:G-protein coupled receptor signaling pathway; C:membrane; C:integral component of membrane |
| Efet.01.106619.g347.t1 | neurotrypsin-like | 270 | 8.03E-11 | 55.15 | F:hydrolase activity |
| Efet.01.10662.g784.t1 | radial spoke head 3 homolog B-like | 402 | 4.60E-14 | 66.5 |  |
| Efet.01.106665.g348.t1 | Wnt-7b-like isoform X4 | 423 | 8.94E-47 | 72.5 | C:membrane; C:extracellular region; P:single-organism process |
| Efet.01.10671.g786.t1 | HEAT repeat-containing 1 | 402 | 3.10E-26 | 64.7 |  |
| Efet.01.106765.g350.t1 | hypothetical protein CAPTEDRAFT_177706 | 333 | 1.05E-12 | 55 | P:transport |
| Efet.01.106847.g353.t1 | RIB43A-like with coiled-coils 2 | 300 | 1.83E-17 | 77.1 |  |
| Efet.01.10686.g787.t1 | ankyrin repeat domain-containing 49 | 687 | 3.12E-65 | 67 | P:positive regulation of transcription, DNA-templated |
| Efet.01.10686.g788.t1 | WD repeat-containing 34-like | 987 | 6.85E-91 | 73.5 |  |
| Efet.01.106873.g354.t1 | carbohydrate sulfotransferase 3-like | 1377 | 1.32E-21 | 49.5 | C:membrane; C:integral component of membrane; F:sulfotransferase activity |
| Efet.01.10688.g789.t1 | hypothetical protein HELRODRAFT_193192 | 309 | 1.83E-06 | 65.5 | F:chitin binding; P:chitin metabolic process; C:extracellular region |
| Efet.01.106880.g355.t1 | hemoglobin subunit mu-like | 651 | 2.73E-49 | 52.9 | F:oxygen binding; F:metal ion binding; P:oxygen transport; F:oxygen transporter activity; P:transport; F:heme binding |
| Efet.01.106921.g356.t1 | zinc finger SNAI2-like | 903 | 4.15E-71 | 81.05 | F:nucleic acid binding; F:metal ion binding |
| Efet.01.106987.g360.t1 | sodium glucose cotransporter 4-like | 567 | 2.31E-24 | 55.95 | P:transmembrane transport; C:membrane; C:integral component of membrane; P:transport; F:transporter activity |
| Efet.01.107008.g362.t1 | PREDICTED: uncharacterized protein LOC106151306 | 534 | 1.62E-09 | 50 |  |
| Efet.01.107010.g363.t1 | helicase with zinc finger domain 2 | 375 | 8.64E-08 | 49.07 | F:nucleotide binding; F:nucleic acid binding; F:5'-flap endonuclease activity; F:ATP binding; F:zinc ion binding; F:metal ion binding; F:single-stranded DNA-dependent ATPase activity; F:helicase activity; P:DNA replication, Okazaki fragment processing |
| Efet.01.107111.g366.t1 | ralBP1-associated Eps domain-containing 1-like isoform X2 | 981 | 1.21E-57 | 64.65 | F:calcium ion binding |
| Efet.01.107118.g367.t1 | serine threonine- kinase PAK 3-like | 273 | 8.40E-37 | 82.65 | F:protein kinase binding; F:identical protein binding; P:negative regulation of cysteine-type endopeptidase activity involved in execution phase of apoptosis; P:protein autophosphorylation; P:signal transduction by protein phosphorylation; P:mitotic cell cycle; P:dendritic spine development; C:nucleoplasm; F:cadherin binding involved in cell-cell adhesion; F:ATP binding; F:signal transducer, downstream of receptor, with serine/threonine kinase activity; C:cytoplasm; C:cell-cell adherens junction; P:axonogenesis; F:protein tyrosine kinase activator activity; P:positive regulation of extrinsic apoptotic signaling pathway; P:peptidyl-serine phosphorylation; P:actin cytoskeleton organization; P:cellular response to organic cyclic compound; P:positive regulation of peptidyl-tyrosine phosphorylation |
| Efet.01.107149.g369.t1 | dual specificity tyrosine-phosphorylation-regulated kinase 2-like | 609 | 2.16E-84 | 69.1 | P:regulation of signal transduction by p53 class mediator; P:smoothened signaling pathway; P:intrinsic apoptotic signaling pathway in response to DNA damage by p53 class mediator; F:protein serine/threonine kinase activity; P:protein phosphorylation; P:positive regulation of glycogen biosynthetic process; C:nucleoplasm; F:magnesium ion binding; F:ATP binding; F:protein tyrosine kinase activity; C:ubiquitin ligase complex; F:ubiquitin binding; C:cytoplasm; F:manganese ion binding; P:negative regulation of NFAT protein import into nucleus |
| Efet.01.107154.g370.t1 | probable domain-containing histone demethylation 2C isoform X4 | 1458 | 6.15E-117 | 58.65 |  |
| Efet.01.107186.g372.t1 | origin recognition complex subunit 1-like | 366 | 2.59E-48 | 78.8 | P:cell division; F:ATP binding; P:DNA replication initiation; F:chromatin binding |
| Efet.01.107198.g373.t1 | von Willebrand factor type EGF and pentraxin domain-containing 1-like | 726 | 3.72E-53 | 46.7 | F:calcium ion binding |
| Efet.01.107245.g374.t1 | PKD domain | 1332 | 1.64E-177 | 45.5 | P:carbohydrate metabolic process; C:membrane; C:integral component of membrane; F:hydrolase activity, hydrolyzing O-glycosyl compounds |
| Efet.01.107292.g376.t1 | helicase domino-like isoform X1 | 861 | 8.77E-69 | 69.7 | F:organic cyclic compound binding; F:heterocyclic compound binding |
| Efet.01.107320.g377.t1 | elongation factor Tu GTP-binding domain-containing 1 | 225 | 2.61E-19 | 92.05 | F:GTP binding; F:translation elongation factor activity; F:GTPase activity; P:translational elongation |
| Efet.01.107322.g378.t1 | myomodulin prepropeptide | 321 | 4.53E-24 | 73.4 | P:neuropeptide signaling pathway; C:extracellular region |
| Efet.01.107327.g379.t1 | disintegrin and metallo ase domain-containing 10-like | 330 | 3.44E-18 | 59.8 | F:zinc ion binding; P:integrin-mediated signaling pathway; P:Notch signaling pathway; P:membrane protein ectodomain proteolysis; P:proteolysis; C:membrane; C:integral component of membrane; F:metalloendopeptidase activity; F:metallopeptidase activity |
| Efet.01.107332.g380.t1 | crumbs homolog 1-like | 996 | 7.53E-101 | 59.45 | C:extracellular region part |
| Efet.01.107350.g381.t1 | mRNA decay activator ZFP36L1-like | 1185 | 6.78E-40 | 74.7 | F:metal ion binding |
| Efet.01.107380.g382.t1 | leucine rich repeat | 1302 | 2.61E-61 | 54.55 |  |
| Efet.01.107380.g383.t1 | serine threonine- kinase roco4 | 2055 | 2.65E-119 | 47.4 |  |
| Efet.01.107404.g387.t1 | propionyl- carboxylase alpha mitochondrial isoform X3 | 354 | 8.85E-15 | 57.4 | F:nucleotide binding; F:ATP binding; F:propionyl-CoA carboxylase activity; F:metal ion binding; F:catalytic activity; C:mitochondrion; F:biotin carboxylase activity; F:ligase activity; F:enzyme binding |
| Efet.01.107507.g389.t1 | transmembrane 104-like | 759 | 6.75E-109 | 74.15 | C:membrane |
| Efet.01.1076.g100.t1 | serine threonine- phosphatase 6 regulatory ankyrin repeat subunit B-like | 744 | 4.01E-24 | 54.55 | P:proteolysis; F:serine-type peptidase activity; F:serine-type endopeptidase activity; P:intracellular signal transduction; C:intracellular; F:serine-type exopeptidase activity |
| Efet.01.1076.g99.t1 | ankyrin-1-like isoform X6 | 1848 | 2.60E-61 | 50.05 | F:ubiquitin-protein transferase activity; P:protein ubiquitination |
| Efet.01.107634.g393.t1 | Pyroglutamylated RFamide peptide receptor | 621 | 3.63E-46 | 59.45 | F:G-protein coupled receptor activity; P:G-protein coupled receptor signaling pathway; C:integral component of membrane |
| Efet.01.107675.g394.t1 | UV excision repair RAD23 homolog | 312 | 5.75E-34 | 72.65 | C:nuclear part; P:nucleotide-excision repair; F:binding |
| Efet.01.107723.g396.t1 | intraflagellar transport 88 homolog isoform X1 | 399 | 6.11E-26 | 78.2 | P:inner ear receptor stereocilium organization; P:spermatid nucleus elongation; P:epidermal stem cell homeostasis; C:outer acrosomal membrane; P:protein localization; P:epidermis development; P:anterior/posterior pattern specification; P:spinal cord dorsal/ventral patterning; P:microtubule-based movement; P:regulation of smoothened signaling pathway; P:embryonic digit morphogenesis; C:motile cilium; C:apical part of cell; P:kidney development; C:kinocilium; P:establishment of localization in cell; P:cardiac muscle cell differentiation; P:cochlea development; P:smoothened signaling pathway; P:non-motile cilium assembly; P:Notch signaling pathway; P:heart formation; P:negative regulation of epithelial cell proliferation; C:photoreceptor connecting cilium; P:telencephalon development; P:neural precursor cell proliferation; P:palate development; P:lung vasculature development; P:regulation of cilium assembly; P:forebrain morphogenesis; P:positive regulation of insulin-like growth factor receptor signaling pathway; C:trans-Golgi network; C:ciliary tip; C:axonemal basal plate; P:endothelial cell morphogenesis; C:ciliary base; P:liver development; P:response to fluid shear stress; P:pancreas development; C:ciliary basal body; P:sperm axoneme assembly; P:regulation of odontogenesis of dentin-containing tooth; P:regulation of fat cell differentiation; P:cardiac septum morphogenesis; P:determination of left/right symmetry; P:regulation of feeding behavior; P:tissue homeostasis; P:eye development; C:centriole; F:kinesin binding; P:regulation of autophagosome assembly; C:intraciliary transport particle B; P:cytoplasmic microtubule organization; P:regulation of protein processing |
| Efet.01.107779.g399.t1 | 5-hydroxytryptamine receptor 2A | 981 | 2.04E-34 | 58.6 | P:signal transduction; C:membrane; F:signal transducer activity |
| Efet.01.107792.g400.t1 | myelin regulatory factor | 282 | 1.87E-25 | 79.9 | F:DNA binding; F:transcription factor activity, sequence-specific DNA binding; C:integral component of membrane; P:regulation of transcription, DNA-templated |
| Efet.01.107793.g401.t1 | NADH dehydrogenase [ubiquinone] flavo mitochondrial | 234 | 1.50E-28 | 85.6 | F:FMN binding; F:NAD binding; F:NADH dehydrogenase (ubiquinone) activity; F:4 iron, 4 sulfur cluster binding; P:aerobic respiration |
| Efet.01.107849.g403.t1 | heterogeneous nuclear ribonucleo K-like isoform X1 | 411 | 5.41E-25 | 74.85 | F:RNA binding; C:intracellular ribonucleoprotein complex; C:viral nucleocapsid |
| Efet.01.107875.g405.t1 | activating signal cointegrator 1 complex subunit 1-like | 702 | 2.11E-27 | 61.8 | F:nucleic acid binding; F:RNA binding |
| Efet.01.107889.g406.t1 | pannexin 4 | 1236 | 0 | 74.75 | C:gap junction; P:ion transport; P:transmembrane transport; C:integral component of membrane; P:cell communication by electrical coupling; C:synapse; F:gap junction channel activity; C:plasma membrane |
| Efet.01.107901.g407.t1 | Zinc transporter 2 | 726 | 1.28E-61 | 65.35 | P:cation transport; P:transmembrane transport; C:membrane; F:cation transmembrane transporter activity |
| Efet.01.107906.g408.t1 | ubiquitin carboxyl-terminal hydrolase 24-like | 288 | 4.32E-28 | 74.35 | P:protein deubiquitination; F:thiol-dependent ubiquitinyl hydrolase activity; P:ubiquitin-dependent protein catabolic process |
| Efet.01.107914.g410.t1 | BTB POZ domain-containing 17-like | 816 | 2.20E-45 | 63.8 |  |
| Efet.01.107920.g411.t1 | galactose-3-O-sulfotransferase 2-like | 1017 | 1.87E-90 | 61.3 | F:galactosylceramide sulfotransferase activity; F:calcium ion binding; C:membrane; C:integral component of membrane; P:glycolipid biosynthetic process; C:Golgi apparatus; F:transferase activity |
| Efet.01.107973.g415.t1 | inosine-5 -monophosphate dehydrogenase 2 isoform X3 | 531 | 3.76E-62 | 65.15 | F:catalytic activity; P:single-organism metabolic process |
| Efet.01.107997.g416.t1 | hypothetical protein CRN43_09195 | 369 | 2.69E-12 | 60 |  |
| Efet.01.108049.g418.t1 | PREDICTED: uncharacterized protein LOC106072951 | 387 | 1.97E-28 | 66.85 |  |
| Efet.01.108059.g419.t1 | tyrosine- phosphatase 10D-like isoform X1 | 348 | 3.93E-34 | 69.45 | P:protein dephosphorylation; F:phosphatase activity |
| Efet.01.10808.g794.t1 | hypothetical protein HELRODRAFT_169584 | 285 | 5.28E-16 | 62.5 | C:membrane; C:integral component of membrane |
| Efet.01.1081.g101.t1 | tiptop-like isoform X5 | 3963 | 3.19E-55 | 58 | P:negative regulation of transcription from RNA polymerase II promoter; F:metal ion binding; P:epidermis morphogenesis; P:regulation of gene expression; P:compound eye development |
| Efet.01.10811.g795.t1 | prolactin-releasing peptide receptor-like | 1026 | 5.13E-83 | 61.5 | F:G-protein coupled receptor activity; P:G-protein coupled receptor signaling pathway; C:membrane |
| Efet.01.108200.g425.t1 | glucose transporter type 1-like | 840 | 1.41E-93 | 56.4 | F:substrate-specific transmembrane transporter activity; P:transmembrane transport; F:transmembrane transporter activity; C:membrane; C:integral component of membrane; P:transport; F:transporter activity |
| Efet.01.108219.g426.t1 | mitogen-activated kinase kinase kinase 10 | 429 | 4.63E-19 | 57.35 | F:ATP binding; F:protein kinase activity; P:phosphorylation; F:protein serine/threonine kinase activity; P:protein phosphorylation; F:kinase activity |
| Efet.01.108232.g427.t1 | BTB POZ domain-containing 6-like | 687 | 4.38E-19 | 63.55 |  |
| Efet.01.108253.g429.t1 | ATP-dependent DNA helicase Q1-like | 459 | 1.14E-08 | 67 | C:nucleus; F:DNA binding; F:ATP binding; C:cytoplasm; F:ATP-dependent 3'-5' DNA helicase activity; P:DNA duplex unwinding; P:double-strand break repair via homologous recombination; F:four-way junction helicase activity; C:chromosome |
| Efet.01.108288.g430.t1 | polycystic kidney disease 1-like 2 | 876 | 4.57E-56 | 74.05 | P:detection of mechanical stimulus; F:calcium channel activity; C:integral component of membrane |
| Efet.01.108288.g431.t1 | polycystic kidney disease 1-like 2 | 1155 | 1.21E-168 | 62.9 | F:calcium ion binding; C:integral component of membrane |
| Efet.01.108288.g432.t1 | ankyrin repeat domain-containing 50-like | 828 | 5.68E-29 | 46.6 | P:intracellular signal transduction; C:intracellular |
| Efet.01.108338.g433.t1 | FMRFamide receptor-like | 1272 | 1.69E-54 | 52.35 | F:G-protein coupled receptor activity; F:G-protein coupled peptide receptor activity; P:G-protein coupled receptor signaling pathway; C:membrane; C:integral component of membrane |
| Efet.01.108371.g434.t1 | hypothetical protein HELRODRAFT_169095 | 738 | 4.90E-18 | 42.67 |  |
| Efet.01.108412.g436.t1 | Calcium-dependent kinase 5 | 693 | 1.14E-39 | 48.35 | F:calcium ion binding; P:phosphorylation; F:kinase activity |
| Efet.01.108412.g437.t1 | Phosphoenolpyruvate carboxykinase [GTP] | 474 | 4.54E-61 | 72.3 | F:phosphoenolpyruvate carboxykinase activity; F:purine nucleotide binding; P:metabolic process |
| Efet.01.108427.g438.t1 | hypothetical protein CAPTEDRAFT_200569 | 804 | 1.93E-50 | 64.33 | C:membrane; C:integral component of membrane |
| Efet.01.108427.g439.t1 | DUF2961 domain-containing | 960 | 3.99E-66 | 48.5 | C:membrane; C:integral component of membrane |
| Efet.01.108447.g442.t1 | unc-93 homolog A | 831 | 4.45E-53 | 55.5 | C:membrane |
| Efet.01.108472.g443.t1 | Ubiquitin carboxyl-terminal hydrolase 35 | 879 | 2.28E-18 | 42.65 | F:thiol-dependent ubiquitin-specific protease activity; P:protein deubiquitination; F:thiol-dependent ubiquitinyl hydrolase activity; F:hydrolase activity; P:ubiquitin-dependent protein catabolic process |
| Efet.01.108486.g445.t1 | protocadherin gamma-B5 | 2499 | 7.61E-96 | 50.4 | F:calcium ion binding; C:membrane; C:integral component of membrane; P:cell adhesion; P:homophilic cell adhesion via plasma membrane adhesion molecules; C:plasma membrane |
| Efet.01.108495.g446.t1 | ankyrin repeat domain-containing 33B-like | 480 | 5.82E-39 | 59.9 |  |
| Efet.01.108496.g447.t1 | hypothetical protein CAPTEDRAFT_191938 | 387 | 9.53E-06 | 53 | C:membrane; C:integral component of membrane |
| Efet.01.1085.g102.t1 | hypothetical protein CAPTEDRAFT_220899 | 714 | 1.75E-22 | 50 | C:membrane; C:integral component of membrane |
| Efet.01.108592.g450.t1 | cytochrome P450 4V2-like | 375 | 4.50E-19 | 76.7 | F:iron ion binding; F:oxidoreductase activity, acting on paired donors, with incorporation or reduction of molecular oxygen; C:integral component of membrane; P:oxidation-reduction process; F:monooxygenase activity; F:heme binding |
| Efet.01.10868.g796.t1 | CREB-binding -like | 2145 | 0 | 82.65 | P:response to estrogen; F:protein C-terminus binding; P:transcription-coupled nucleotide-excision repair; F:RNA polymerase II regulatory region sequence-specific DNA binding; P:intrinsic apoptotic signaling pathway in response to DNA damage by p53 class mediator; P:regulation of transcription from RNA polymerase II promoter in response to hypoxia; P:positive regulation of protein acetylation; P:regulation of anatomical structure morphogenesis; P:fat cell differentiation; P:positive regulation of sequence-specific DNA binding transcription factor activity; P:cellular response to oxygen-containing compound; P:regulation of androgen receptor signaling pathway; F:RNA polymerase II activating transcription factor binding; P:response to glucose; P:positive regulation of cell growth; P:response to retinoic acid; P:N-terminal peptidyl-lysine acetylation; P:cellular response to organonitrogen compound; F:protein kinase binding; F:zinc ion binding; P:positive regulation of gene expression, epigenetic; P:positive regulation of type I interferon production; P:regulation of signal transduction by p53 class mediator; P:anatomical structure formation involved in morphogenesis; P:response to hydrogen peroxide; P:circadian rhythm; F:core promoter proximal region DNA binding; P:tissue development; F:lysine N-acetyltransferase activity, acting on acetyl phosphate as donor; P:Notch signaling pathway; F:androgen receptor binding; P:positive regulation of binding; P:regulation of autophagy; P:myeloid cell differentiation; P:positive regulation by host of viral transcription; P:histone H4 acetylation; P:histone H2B acetylation; P:regulation of cellular response to heat; P:regulation of cell size; P:nervous system development; P:negative regulation of cell death; P:beta-catenin-TCF complex assembly; F:core promoter binding; P:stimulatory C-type lectin receptor signaling pathway; P:negative regulation of transcription from RNA polymerase II promoter; F:transcription factor activity, sequence-specific DNA binding; P:positive regulation of protein transport; P:DNA damage response, signal transduction by p53 class mediator resulting in cell cycle arrest; F:antigen binding; P:positive regulation of organelle organization; P:cellular response to UV; P:response to metal ion; P:cellular response to antibiotic; P:transcription, DNA-templated; P:tube development; P:regulation of proteolysis; P:regulation of tubulin deacetylation; P:positive regulation of transcription from RNA polymerase II promoter involved in unfolded protein response; F:chromatin binding; F:beta-catenin binding; P:cell development; F:damaged DNA binding; C:histone acetyltransferase complex; F:histone acetyltransferase activity; P:cellular response to corticosteroid stimulus; F:transcription coactivator activity; C:cytoplasm; P:response to drug; P:positive regulation of cell development; P:protein stabilization; P:response to dexamethasone |
| Efet.01.108695.g453.t1 | thyrotropin-releasing hormone receptor-like | 726 | 1.25E-25 | 53.15 | P:G-protein coupled receptor signaling pathway; C:membrane; F:signal transducer activity |
| Efet.01.108794.g456.t1 | FMRFamide receptor-like | 477 | 1.65E-43 | 69.8 | F:G-protein coupled receptor activity; P:G-protein coupled receptor signaling pathway; C:integral component of membrane |
| Efet.01.108854.g460.t1 | fork head domain transcription factor slp2-like | 834 | 2.99E-49 | 83.95 | C:nucleus; F:transcription factor activity, sequence-specific DNA binding; P:transcription, DNA-templated; P:regulation of transcription, DNA-templated; F:sequence-specific DNA binding |
| Efet.01.108866.g461.t1 | hypothetical protein HELRODRAFT_188617 | 423 | 1.03E-09 | 54 |  |
| Efet.01.10888.g799.t1 | hypothetical protein CAPTEDRAFT_211244 | 456 | 3.76E-11 | 51.5 |  |
| Efet.01.108910.g462.t1 | dihydrolipoyllysine-residue succinyltransferase component of 2-oxoglutarate dehydrogenase mitochondrial isoform X2 | 501 | 5.18E-94 | 70.15 | F:heat shock protein binding; C:myelin sheath; P:tricarboxylic acid cycle; C:plasma membrane; P:2-oxoglutarate metabolic process; P:lysine catabolic process; C:nucleus; F:dihydrolipoyllysine-residue succinyltransferase activity; P:NADH metabolic process; C:extracellular exosome; C:mitochondrial matrix; P:glyoxylate metabolic process; C:oxoglutarate dehydrogenase complex; F:chaperone binding |
| Efet.01.108959.g465.t1 | isoforms A H I S isoform X5 | 603 | 1.41E-22 | 57.85 | F:protein binding; P:positive regulation of cellular process; P:regulation of transcription, DNA-templated; P:single-organism cellular process; P:negative regulation of cellular process; C:intracellular part |
| Efet.01.10896.g800.t1 | Histone H2B | 357 | 9.52E-11 | 59.05 | C:intracellular organelle |
| Efet.01.108991.g467.t1 | ATPase family AAA domain-containing 1-B-like | 231 | 3.47E-10 | 72.4 | F:nucleotide binding; F:ATP binding; C:membrane; C:integral component of membrane |
| Efet.01.109031.g469.t1 | guanidinobutyrase-like isoform X1 | 243 | 1.40E-21 | 80.9 | F:hydrolase activity, acting on carbon-nitrogen (but not peptide) bonds, in linear amidines; F:metal ion binding |
| Efet.01.109054.g471.t1 | zinc finger MYM-type 1-like | 1167 | 9.65E-129 | 69.5 |  |
| Efet.01.109054.g472.t1 | zinc finger MYM-type 1-like | 579 | 2.12E-15 | 48.3 |  |
| Efet.01.109087.g473.t1 | PRRC2C-like isoform X1 | 2283 | 5.96E-32 | 53.9 |  |
| Efet.01.109096.g474.t1 | kinase DC2 isoform X1 | 210 | 1.63E-22 | 99.85 | F:ATP binding; F:protein serine/threonine kinase activity; P:protein phosphorylation |
| Efet.01.109107.g475.t1 | gastrula zinc finger -like | 1182 | 1.50E-60 | 52.35 | F:nucleic acid binding; F:metal ion binding |
| Efet.01.109181.g478.t1 | ras-related and estrogen-regulated growth inhibitor | 408 | 5.97E-39 | 69.75 | F:nucleotide binding; F:GTP binding; P:small GTPase mediated signal transduction; P:signal transduction; C:membrane; C:integral component of membrane; C:intracellular |
| Efet.01.109186.g479.t1 | hypothetical protein CAPTEDRAFT_197781 | 1626 | 9.66E-162 | 62 | C:membrane; C:integral component of membrane |
| Efet.01.109200.g480.t1 | ankyrin repeat | 1128 | 3.24E-46 | 52.5 | C:membrane; C:integral component of membrane |
| Efet.01.109208.g481.t1 | class B secretin-like G- coupled receptor | 1317 | 3.36E-24 | 41 | F:G-protein coupled receptor activity; F:transmembrane signaling receptor activity; P:immune response; P:G-protein coupled receptor signaling pathway; C:membrane; C:integral component of membrane; F:scavenger receptor activity; P:cell surface receptor signaling pathway; P:receptor-mediated endocytosis; F:polysaccharide binding |
| Efet.01.109208.g482.t1 | G- coupled receptor Mth2-like | 1158 | 1.48E-50 | 56.45 | F:G-protein coupled receptor activity; F:transmembrane signaling receptor activity; P:immune response; P:G-protein coupled receptor signaling pathway; C:membrane; C:integral component of membrane; F:scavenger receptor activity; P:cell surface receptor signaling pathway; P:receptor-mediated endocytosis; F:polysaccharide binding |
| Efet.01.109319.g485.t1 | titin isoform X2 | 633 | 6.77E-16 | 55.2 | F:ATP binding; C:membrane; C:integral component of membrane; F:protein kinase activity; P:protein phosphorylation |
| Efet.01.109332.g486.t1 | dynein heavy chain axonemal-like | 372 | 5.44E-14 | 75.65 | F:nucleoside-triphosphatase activity |
| Efet.01.109361.g487.t1 | Nucleoside diphosphate-linked moiety X motif 22 | 864 | 1.94E-103 | 59.9 | C:cellular_component; F:hydrolase activity |
| Efet.01.109368.g488.t1 | RING finger 17-like | 723 | 2.20E-11 | 55.05 | F:zinc ion binding; F:metal ion binding; C:intracellular |
| Efet.01.10938.g801.t1 | SNF-related serine threonine- kinase-like | 405 | 3.21E-48 | 77.7 | F:magnesium ion binding; C:nucleus; F:ATP binding; C:cytoplasm; P:myeloid cell differentiation; F:protein serine/threonine kinase activity; P:protein phosphorylation; P:intracellular signal transduction |
| Efet.01.109397.g491.t1 | Proactivator polypeptide | 210 | 1.19E-06 | 70.33 | P:lipid metabolic process; C:lysosome; P:sphingolipid metabolic process |
| Efet.01.10942.g802.t1 | ankyrin repeat and MYND domain-containing 1-like isoform X1 | 1620 | 8.16E-38 | 58.1 | F:metal ion binding |
| Efet.01.109420.g493.t1 | nurim homolog | 528 | 2.43E-11 | 53.75 | C:membrane; C:integral component of membrane |
| Efet.01.109444.g494.t1 | fibrinogen 1 | 1251 | 3.76E-35 | 59.3 |  |
| Efet.01.109476.g496.t1 | eukaryotic initiation factor 4A-II-like | 342 | 6.53E-13 | 90.55 | P:negative regulation of RNA-directed 5'-3' RNA polymerase activity; P:translational initiation; P:regulation of translational initiation; F:ATP-dependent RNA helicase activity; F:RNA binding; F:translation initiation factor activity; F:ATP binding; C:perinuclear region of cytoplasm; C:extracellular exosome; F:protein binding; F:double-stranded RNA binding; C:membrane; P:RNA secondary structure unwinding; C:extracellular matrix |
| Efet.01.109479.g497.t1 | Leucine-rich repeat serine threonine- kinase 1 | 717 | 4.32E-30 | 49.15 | F:GTP binding; F:ATP binding; P:small GTPase mediated signal transduction; C:membrane; C:integral component of membrane; F:protein kinase activity; P:phosphorylation; P:protein phosphorylation; F:kinase activity; C:intracellular |
| Efet.01.109485.g500.t1 | Polycystic kidney disease 1-like 2 | 297 | 1.37E-07 | 66.67 | F:calcium ion binding; C:membrane; C:integral component of membrane; F:extracellular matrix structural constituent; C:proteinaceous extracellular matrix |
| Efet.01.10949.g803.t1 | exocyst complex component 4-like isoform X4 | 531 | 1.09E-43 | 68.4 | P:transport |
| Efet.01.109539.g501.t1 | coiled-coil domain-containing 189-like | 282 | 1.04E-27 | 84.4 |  |
| Efet.01.10961.g804.t1 | 1-phosphatidylinositol 4,5-bisphosphate phosphodiesterase delta-4 | 360 | 2.09E-25 | 64.9 | P:lipid metabolic process; F:phosphoric diester hydrolase activity; P:cellular process |
| Efet.01.109634.g502.t1 | Rho GTPase-activating 20 | 234 | 2.22E-15 | 69.1 | P:signal transduction |
| Efet.01.109714.g504.t1 | myocyte-specific enhancer factor 2C-like | 255 | 1.10E-33 | 95.15 | P:positive regulation of cardiac muscle cell proliferation; C:nuclear speck; F:RNA polymerase II core promoter proximal region sequence-specific DNA binding; P:ventricular cardiac muscle cell differentiation; P:cardiac ventricle formation; P:learning or memory; F:RNA polymerase II distal enhancer sequence-specific DNA binding; F:transcriptional activator activity, RNA polymerase II core promoter proximal region sequence-specific binding; P:regulation of germinal center formation; P:skeletal muscle cell differentiation; F:protein heterodimerization activity; P:cellular response to fluid shear stress; P:glomerulus morphogenesis; P:cellular response to parathyroid hormone stimulus; P:apoptotic process; P:humoral immune response; P:regulation of synaptic plasticity; P:platelet formation; P:regulation of AMPA receptor activity; P:heart looping; P:transdifferentiation; P:regulation of NMDA receptor activity; P:positive regulation of macrophage apoptotic process; P:monocyte differentiation; F:activating transcription factor binding; P:positive regulation of alkaline phosphatase activity; P:cardiac muscle hypertrophy in response to stress; P:regulation of synaptic transmission, glutamatergic; P:positive regulation of bone mineralization; P:regulation of synapse assembly; F:miRNA binding; P:excitatory postsynaptic potential; F:histone deacetylase binding; P:neural crest cell differentiation; P:regulation of dendritic spine development; P:sinoatrial valve morphogenesis; P:B cell proliferation; P:regulation of megakaryocyte differentiation; C:protein complex; F:transcription factor activity, RNA polymerase II core promoter sequence-specific; F:core promoter sequence-specific DNA binding; P:positive regulation of cardiac muscle cell differentiation; P:primary heart field specification; P:cellular response to transforming growth factor beta stimulus; P:secondary heart field specification; P:negative regulation of epithelial cell proliferation; P:positive regulation of skeletal muscle cell differentiation; P:endochondral ossification; P:positive regulation of B cell proliferation; P:cellular response to trichostatin A; P:epithelial cell proliferation involved in renal tubule morphogenesis; F:HMG box domain binding; P:regulation of synaptic activity; P:palate development; P:chondrocyte differentiation; P:outflow tract morphogenesis; P:positive regulation of neuron differentiation; P:positive regulation of myoblast differentiation; F:transcriptional activator activity, RNA polymerase II distal enhancer sequence-specific binding; P:positive regulation of osteoblast differentiation; P:positive regulation of transcription from RNA polymerase II promoter; P:positive regulation of protein homodimerization activity; P:negative regulation of neuron apoptotic process; P:negative regulation of transcription from RNA polymerase II promoter; P:MAPK cascade; P:neuron migration; P:smooth muscle cell differentiation; P:blood vessel development; P:cellular response to calcium ion; P:osteoblast differentiation; P:B cell receptor signaling pathway; P:regulation of sarcomere organization; P:regulation of neurotransmitter secretion; P:transcription, DNA-templated; P:cellular response to drug; P:cartilage morphogenesis; F:AT DNA binding; P:muscle cell fate determination; P:cell morphogenesis involved in neuron differentiation; F:chromatin binding; P:nephron tubule epithelial cell differentiation; P:B cell homeostasis; P:embryonic viscerocranium morphogenesis; P:germinal center formation; P:melanocyte differentiation; P:blood vessel remodeling; P:cellular response to lipopolysaccharide; P:positive regulation of behavioral fear response; C:cytoplasm; P:cellular response to retinoic acid; P:positive regulation of cell proliferation in bone marrow; P:negative regulation of ossification |
| Efet.01.109727.g505.t1 | calcineurin-binding cabin-1-like isoform X1 | 1548 | 9.40E-14 | 48.5 | C:intracellular part |
| Efet.01.10974.g805.t1 | valine--tRNA ligase | 207 | 1.01E-17 | 91.9 | F:valine-tRNA ligase activity; F:ATP binding; P:valyl-tRNA aminoacylation; C:cytoplasm; P:regulation of translational fidelity; F:aminoacyl-tRNA editing activity |
| Efet.01.109754.g506.t1 | hypothetical protein CAPTEDRAFT_228772 | 471 | 4.68E-27 | 52.78 | C:membrane; C:integral component of membrane |
| Efet.01.10983.g806.t1 | dual specificity phosphatase 16-like | 501 | 1.88E-07 | 80 | P:inactivation of MAPK activity; F:protein tyrosine phosphatase activity; P:protein dephosphorylation; F:phosphatase activity; P:dephosphorylation; P:peptidyl-tyrosine dephosphorylation; F:MAP kinase tyrosine/serine/threonine phosphatase activity; F:protein tyrosine/serine/threonine phosphatase activity; F:hydrolase activity |
| Efet.01.109887.g509.t1 | KPCD1_HUMAN ame: Full=Serine threonine- kinase D1 ame: Full= kinase C mu type ame: Full= kinase D ame: Full=nPKC-D1 ame: Full=nPKC-mu | 315 | 1.68E-06 | 54.67 | P:protein kinase D signaling; P:negative regulation of endocytosis; P:positive regulation of endothelial cell chemotaxis by VEGF-activated vascular endothelial growth factor receptor signaling pathway; P:protein autophosphorylation; C:Golgi apparatus; P:positive regulation of NF-kappaB transcription factor activity; P:positive regulation of angiogenesis; P:inflammatory response; P:apoptotic process; C:cytosol; P:positive regulation of neuron projection development; P:intracellular signal transduction; P:positive regulation of peptidyl-serine phosphorylation; P:sphingolipid biosynthetic process; P:Ras protein signal transduction; F:protein kinase activity; F:protein serine/threonine kinase activity; P:immune system process; C:plasma membrane; C:nucleus; P:vascular endothelial growth factor receptor signaling pathway; C:cell-cell junction; F:protein binding; F:metal ion binding; P:positive regulation of CREB transcription factor activity; P:peptidyl-serine phosphorylation; P:cell proliferation; P:Golgi organization; P:positive regulation of I-kappaB kinase/NF-kappaB signaling; P:nervous system development; P:negative regulation of cell death; P:positive regulation of transcription factor import into nucleus; P:positive regulation of transcription from RNA polymerase II promoter; P:positive regulation of osteoblast differentiation; P:protein phosphorylation; F:nucleotide binding; F:ATP binding; C:integral component of plasma membrane; C:trans-Golgi network; P:integrin-mediated signaling pathway; P:angiogenesis; P:regulation of keratinocyte proliferation; P:positive regulation of endothelial cell chemotaxis; P:phosphorylation; P:positive regulation of endothelial cell migration; F:identical protein binding; P:regulation of integrin-mediated signaling pathway; P:defense response to Gram-negative bacterium; P:signal transduction; P:positive regulation of blood vessel endothelial cell migration; F:protein kinase C activity; P:positive regulation of histone deacetylase activity; P:regulation of release of sequestered calcium ion into cytosol; P:regulation of protein stability; C:cytoplasm; P:innate immune response; C:cell cortex; C:membrane; P:cell differentiation; P:cellular response to oxidative stress; P:positive regulation of endothelial cell proliferation; P:cellular response to vascular endothelial growth factor stimulus; F:transferase activity; F:kinase activity; P:Golgi vesicle transport |
| Efet.01.10996.g807.t1 | solute carrier family facilitated glucose transporter member 1-like | 396 | 3.11E-36 | 65.8 | F:transmembrane transporter activity; C:membrane; P:transport |
| Efet.01.109963.g510.t1 | dynein heavy chain axonemal-like | 708 | 2.51E-52 | 65.05 | C:intracellular organelle; P:cellular process |
| Efet.01.109981.g511.t1 | axin-1-like | 1068 | 9.21E-29 | 51.15 | C:beta-catenin destruction complex; P:pseudouridine synthesis; F:RNA binding; C:cytoplasm; P:signal transduction; P:RNA modification; F:GTPase activator activity; F:pseudouridine synthase activity; C:plasma membrane; P:positive regulation of GTPase activity; F:receptor signaling complex scaffold activity |
| Efet.01.110045.g512.t1 | dynein regulatory complex subunit 3 | 351 | 5.58E-22 | 52.95 | C:cytoplasm |
| Efet.01.110051.g513.t1 | neuroligin- X-linked-like isoform X2 | 543 | 3.38E-68 | 71.2 | C:integral component of membrane |
| Efet.01.110103.g515.t1 | hypothetical protein CAPTEDRAFT_216867 | 639 | 6.22E-26 | 48.9 | C:membrane; C:integral component of membrane; F:carbohydrate binding |
| Efet.01.110119.g517.t1 | Calcineurin subunit B type 1 | 306 | 1.90E-31 | 97.15 | F:calcium ion binding |
| Efet.01.110123.g518.t1 | innexin unc-9 isoform X6 | 219 | 7.34E-16 | 70.35 | C:membrane; C:cell junction; P:transport |
| Efet.01.110133.g519.t1 | transmembrane 45B-like | 927 | 6.23E-51 | 55.5 | C:membrane; C:integral component of membrane |
| Efet.01.11015.g808.t1 | Zinc finger MYM-type | 1017 | 3.14E-71 | 64.4 | F:nucleic acid binding; F:protein dimerization activity |
| Efet.01.11015.g809.t1 | Zinc finger MYM-type | 504 | 3.13E-49 | 66.6 | F:nucleic acid binding; F:protein dimerization activity |
| Efet.01.110194.g522.t1 | 26S protease regulatory subunit | 204 | 4.63E-31 | 97.1 | C:proteasome regulatory particle, base subcomplex; P:Fc-epsilon receptor signaling pathway; P:transcription from RNA polymerase II promoter; P:regulation of mRNA stability; P:negative regulation of programmed cell death; P:positive regulation of proteasomal protein catabolic process; P:regulation of cellular amino acid metabolic process; C:nucleoplasm; P:stimulatory C-type lectin receptor signaling pathway; F:ATP binding; P:MAPK cascade; P:antigen processing and presentation of exogenous peptide antigen via MHC class I, TAP-dependent; C:extracellular exosome; P:negative regulation of transcription, DNA-templated; C:blood microparticle; C:cytoplasmic vesicle; C:cytosolic proteasome complex; F:thyrotropin-releasing hormone receptor binding; P:Wnt signaling pathway, planar cell polarity pathway; C:nuclear proteasome complex; F:TBP-class protein binding; C:inclusion body; F:proteasome-activating ATPase activity; P:T cell receptor signaling pathway; P:protein polyubiquitination; P:positive regulation of canonical Wnt signaling pathway; P:NIK/NF-kappaB signaling; P:positive regulation of inclusion body assembly; F:peptidase activity; P:positive regulation of RNA polymerase II transcriptional preinitiation complex assembly; P:negative regulation of canonical Wnt signaling pathway; P:tumor necrosis factor-mediated signaling pathway; F:transcription cofactor activity; C:membrane; P:anaphase-promoting complex-dependent catabolic process; P:ER-associated ubiquitin-dependent protein catabolic process; P:positive regulation of ubiquitin-protein ligase activity involved in regulation of mitotic cell cycle transition; P:negative regulation of ubiquitin-protein ligase activity involved in mitotic cell cycle |
| Efet.01.11025.g810.t1 | peroxiredoxin 1 | 255 | 1.56E-45 | 89.2 | F:peroxidase activity; P:cellular oxidant detoxification; P:oxidation-reduction process; F:peroxiredoxin activity |
| Efet.01.110309.g525.t1 | kanadaptin | 1593 | 9.54E-154 | 67.15 | P:RNA phosphodiester bond hydrolysis, endonucleolytic; F:endoribonuclease activity, producing 5'-phosphomonoesters |
| Efet.01.11031.g811.t1 | small G signaling modulator 3 homolog isoform X1 | 465 | 8.06E-78 | 91.5 |  |
| Efet.01.110329.g526.t1 | Polyadenylate-binding 4 | 489 | 1.40E-78 | 88.8 | F:nucleotide binding; C:nucleus; C:cytoplasmic stress granule; F:poly(A) binding; F:poly(C) RNA binding; P:myeloid cell development; F:mRNA binding; P:regulation of mRNA stability; F:poly(U) RNA binding |
| Efet.01.110337.g527.t1 | hypothetical protein CAPTEDRAFT_223662 | 1047 | 3.28E-19 | 47.33 |  |
| Efet.01.110378.g529.t1 | hypothetical protein HELRODRAFT_193047 | 207 | 1.10E-21 | 72.2 | F:calcium ion binding |
| Efet.01.110390.g530.t1 | endoplasmic reticulum mannosyl-oligosaccharide 1,2-alpha-mannosidase-like | 324 | 4.31E-51 | 82.9 | F:calcium ion binding; P:metabolic process; C:integral component of membrane; F:mannosyl-oligosaccharide 1,2-alpha-mannosidase activity |
| Efet.01.110423.g531.t1 | muscle M-line assembly unc-89- | 903 | 9.79E-27 | 51.2 | F:transferase activity; P:regulation of biological process; P:cellular process |
| Efet.01.110471.g532.t1 | tripartite motif-containing 45-like isoform X6 | 1695 | 5.55E-94 | 56.2 | F:zinc ion binding; F:metal ion binding; C:intracellular |
| Efet.01.110486.g533.t1 | inactive serine threonine- kinase | 2355 | 4.62E-09 | 52.25 | F:ATP binding; F:protein kinase activity; P:phosphorylation; P:protein phosphorylation; F:kinase activity |
| Efet.01.11049.g814.t1 | synaptotagmin-4-like isoform X2 | 837 | 2.88E-34 | 57.45 | C:membrane; C:integral component of membrane; P:exocytosis |
| Efet.01.110553.g536.t1 | conserved oligomeric Golgi complex subunit 7-like | 741 | 1.64E-49 | 55.3 | C:Golgi transport complex; P:intracellular protein transport |
| Efet.01.110569.g537.t1 | hippocampus abundant transcript 1 | 309 | 2.28E-08 | 88.1 | P:transmembrane transport; C:integral component of membrane; F:transporter activity |
| Efet.01.110636.g540.t1 | VWFA and cache domain-containing 1 | 786 | 6.13E-22 | 54.05 | C:membrane |
| Efet.01.110726.g542.t1 | long-chain-fatty-acid-- ligase ACSBG2-like | 429 | 2.78E-48 | 74.7 | F:nucleotide binding; F:ligase activity; P:fatty acid metabolic process |
| Efet.01.110733.g543.t1 | FMRFamide receptor-like | 1089 | 4.94E-138 | 56.95 | F:G-protein coupled receptor activity; C:membrane |
| Efet.01.110753.g544.t1 | FMRFamide receptor-like | 852 | 5.37E-38 | 54.75 | F:signal transducer activity |
| Efet.01.110813.g545.t1 | zinc finger GLI4-like | 1905 | 3.23E-17 | 43.55 | F:nucleic acid binding; F:metal ion binding; P:smoothened signaling pathway; P:regulation of transcription, DNA-templated; C:Hedgehog signaling complex |
| Efet.01.110830.g546.t1 | voltage gated hydrogen channel 1 | 204 | 1.18E-19 | 70 | P:ion transmembrane transport; C:integral component of membrane; P:regulation of ion transmembrane transport; F:voltage-gated ion channel activity |
| Efet.01.110832.g547.t1 | serine threonine- kinase PAK 6-like isoform X2 | 780 | 1.94E-10 | 58.15 | F:organic cyclic compound binding; F:heterocyclic compound binding; F:transferase activity |
| Efet.01.110841.g548.t1 | Serine threonine- kinase H1 | 522 | 1.12E-17 | 67.45 | F:kinase activity |
| Efet.01.110869.g550.t1 | leucine-rich repeat and immunoglobulin-like domain-containing nogo receptor-interacting 1 | 1788 | 4.19E-23 | 46.7 | P:Roundabout signaling pathway; F:Roundabout binding; C:extracellular space; P:regulation of axonogenesis; P:axonogenesis; C:membrane; C:integral component of membrane; F:heparin binding; C:proteinaceous extracellular matrix; P:axon extension involved in axon guidance |
| Efet.01.110892.g551.t1 | ribosome-releasing factor mitochondrial-like | 438 | 3.05E-39 | 64.25 | F:organic cyclic compound binding; F:heterocyclic compound binding; C:intracellular; P:translation |
| Efet.01.110949.g552.t1 | atrial natriuretic peptide receptor 1-like | 651 | 3.64E-10 | 50.38 | P:cGMP biosynthetic process; P:G-protein coupled receptor signaling pathway; F:phosphorus-oxygen lyase activity; F:lyase activity; F:guanylate cyclase activity; F:protein kinase activity; P:protein phosphorylation; C:intracellular; F:nucleotide binding; F:G-protein coupled receptor activity; F:ATP binding; F:G-protein coupled GABA receptor activity; C:membrane; C:integral component of membrane; P:intracellular signal transduction; P:cyclic nucleotide biosynthetic process |
| Efet.01.110989.g553.t1 | bark beetle | 3999 | 0 | 56.55 | C:membrane |
| Efet.01.111015.g554.t1 | ADP-dependent glucokinase-like | 315 | 1.73E-31 | 78.35 | P:carbohydrate metabolic process; C:integral component of membrane; F:phosphotransferase activity, alcohol group as acceptor |
| Efet.01.111025.g555.t1 | hypothetical protein LOTGIDRAFT_136055, partial | 318 | 1.49E-12 | 51.75 |  |
| Efet.01.111025.g556.t1 | piggyBac transposable element-derived 4 | 474 | 6.37E-10 | 61 |  |
| Efet.01.111051.g557.t1 | golgin subfamily B member 1-like isoform X2 | 1317 | 2.15E-14 | 72.05 |  |
| Efet.01.111192.g559.t1 | E3 ubiquitin ligase complex SCF subunit sconC | 528 | 3.04E-08 | 58.7 | P:regulation of cellular process; P:protein catabolic process; P:cellular protein metabolic process; C:macromolecular complex; P:single-organism cellular process; P:organelle organization; C:intracellular part |
| Efet.01.111193.g560.t1 | E3 ubiquitin- ligase TRIM71-like | 1209 | 1.43E-10 | 50.5 | F:zinc ion binding; P:signal transduction; P:ion transmembrane transport; F:GABA-A receptor activity; F:extracellular ligand-gated ion channel activity; C:GABA-A receptor complex; C:synapse; P:transport; C:intracellular; C:plasma membrane; P:ion transport; F:metal ion binding; C:membrane; C:integral component of membrane; C:cell junction |
| Efet.01.11121.g816.t1 | deleted in malignant brain tumors 1 -like | 1377 | 7.64E-59 | 44.55 | C:membrane; C:integral component of membrane; F:scavenger receptor activity; P:receptor-mediated endocytosis |
| Efet.01.111220.g561.t1 | CALL_CAEEL ame: Full=Calmodulin | 303 | 7.48E-06 | 73 | F:metal ion binding; P:embryo development ending in birth or egg hatching; F:calcium ion binding |
| Efet.01.111227.g563.t1 | hypothetical protein CAPTEDRAFT_183356, partial | 1326 | 1.81E-06 | 45 | F:nucleotide binding; F:ATP binding; F:protein kinase activity; F:protein serine/threonine kinase activity; P:protein phosphorylation |
| Efet.01.11125.g817.t1 | kelch 20 | 1437 | 4.19E-19 | 47.85 | F:ubiquitin-protein transferase activity; C:Cul3-RING ubiquitin ligase complex; P:protein ubiquitination |
| Efet.01.111274.g564.t1 | activating signal cointegrator 1 complex subunit 2 | 2238 | 2.20E-163 | 55.65 | C:nucleus; P:transcription, DNA-templated; P:regulation of transcription, DNA-templated; C:activating signal cointegrator 1 complex; F:molecular_function |
| Efet.01.111416.g569.t1 | dynein gamma flagellar outer arm-like | 537 | 1.48E-59 | 74.75 | F:ATP binding; F:microtubule motor activity; C:dynein complex; P:microtubule-based movement |
| Efet.01.11143.g818.t1 | lipoma HMGIC fusion partner-like 3 | 423 | 2.58E-33 | 58.65 | C:membrane; C:integral component of membrane |
| Efet.01.111457.g570.t1 | Zinc finger FYVE domain-containing 9 | 201 | 1.24E-09 | 77.8 | F:metal ion binding |
| Efet.01.111457.g571.t1 | zinc finger FYVE domain-containing 16-like isoform X1 | 738 | 4.86E-48 | 52.6 | F:metal ion binding |
| Efet.01.111476.g573.t1 | tudor domain-containing 10 | 306 | 5.08E-07 | 53.25 | F:nucleotide binding |
| Efet.01.111477.g574.t1 | cyclic pyranopterin monophosphate synthase | 546 | 2.18E-59 | 74.75 | P:molybdopterin cofactor biosynthetic process |
| Efet.01.111477.g575.t1 | molybdenum cofactor biosynthesis 1 isoform X2 | 489 | 3.40E-52 | 59.7 | F:binding |
| Efet.01.111501.g577.t1 | ryanodine receptor | 447 | 2.08E-75 | 93.2 | F:ryanodine-sensitive calcium-release channel activity; F:calcium ion binding; C:integral component of membrane; P:release of sequestered calcium ion into cytosol; C:intracellular |
| Efet.01.111502.g578.t1 | tektin-3-like isoform X1 | 318 | 2.65E-46 | 81.75 |  |
| Efet.01.111504.g579.t1 | dynein heavy chain axonemal-like | 678 | 4.09E-112 | 83.7 | F:ATPase activity; F:ATP binding; P:heart development; C:outer dynein arm; F:microtubule motor activity; P:lateral ventricle development; P:flagellated sperm motility; P:outer dynein arm assembly; C:cytoplasm; P:cilium movement; P:determination of left/right symmetry; C:microtubule |
| Efet.01.111579.g581.t1 | Ubiquitin carboxyl-terminal hydrolase 4 | 810 | 1.64E-08 | 61.2 | P:protein deubiquitination; F:zinc ion binding; P:ubiquitin-dependent protein catabolic process; F:thiol-dependent ubiquitinyl hydrolase activity |
| Efet.01.111589.g582.t1 | RNMT-activating mini -like | 423 | 4.77E-13 | 70.6 | C:mRNA cap binding complex; F:RNA binding; P:methylation |
| Efet.01.111627.g583.t1 | muscarinic acetylcholine receptor DM1 | 1902 | 1.65E-86 | 81.85 | P:gastric acid secretion; C:integral component of plasma membrane; P:synaptic transmission, cholinergic; P:phospholipase C-activating G-protein coupled acetylcholine receptor signaling pathway; P:adenylate cyclase-inhibiting G-protein coupled acetylcholine receptor signaling pathway; F:G-protein coupled acetylcholine receptor activity; C:synapse |
| Efet.01.111676.g584.t1 | serine threonine- kinase roco4 | 3192 | 0 | 44.29 |  |
| Efet.01.111728.g588.t1 | serine arginine repetitive matrix 2-like isoform X3 | 1947 | 4.78E-22 | 59.8 | P:protein deubiquitination; F:thiol-dependent ubiquitinyl hydrolase activity |
| Efet.01.111775.g591.t1 | heat shock 70 kDa 12A- | 525 | 4.54E-46 | 78.65 | C:extracellular exosome; C:integral component of membrane |
| Efet.01.111788.g592.t1 | neuropilin and tolloid 2 | 726 | 2.68E-25 | 51.05 | F:zinc ion binding; F:metal ion binding; F:calcium ion binding; C:membrane; P:proteolysis; C:integral component of membrane; F:peptidase activity; F:receptor activity; F:metalloendopeptidase activity; P:receptor-mediated endocytosis; F:metallopeptidase activity; F:hydrolase activity |
| Efet.01.111804.g593.t1 | nuclear factor of activated T-cells 5-like isoform X5 | 441 | 1.56E-58 | 78.9 | C:nucleus; F:transcription factor activity, sequence-specific DNA binding; F:RNA polymerase II core promoter proximal region sequence-specific DNA binding; C:integral component of membrane; P:regulation of transcription, DNA-templated; P:transcription from RNA polymerase II promoter |
| Efet.01.111819.g594.t1 | calcium-transporting atpase sarcoplasmic endoplasmic reticulum type (calcium pump) | 213 | 1.05E-34 | 90.15 | C:integral component of plasma membrane; F:ATP binding; F:metal ion binding; P:calcium ion transmembrane transport; F:calcium-transporting ATPase activity; C:sarcoplasmic reticulum membrane; P:cellular calcium ion homeostasis |
| Efet.01.111832.g595.t1 | 2-oxoglutarate mitochondrial isoform X3 | 459 | 1.49E-74 | 78.25 | F:thiamine pyrophosphate binding; C:mitochondrial membrane; F:protein binding; C:mitochondrial matrix; P:glyoxylate metabolic process; C:oxoglutarate dehydrogenase complex; F:oxoglutarate dehydrogenase (succinyl-transferring) activity; P:nicotinamide nucleotide metabolic process; C:cytosol; P:tricarboxylic acid cycle; P:olfactory bulb development; P:lysine catabolic process |
| Efet.01.111859.g596.t1 | uncharacterized protein LOC110448181 | 1164 | 3.09E-29 | 54.8 |  |
| Efet.01.111860.g597.t1 | dynamin-binding -like | 555 | 2.58E-14 | 61.85 | P:biological regulation |
| Efet.01.111966.g601.t1 | class I SAM-dependent methyltransferase | 1335 | 1.49E-29 | 45.2 | C:membrane; C:integral component of membrane |
| Efet.01.112036.g606.t1 | group XV phospholipase A2-like | 222 | 2.31E-14 | 68.2 | F:O-acyltransferase activity; P:lipid metabolic process |
| Efet.01.112046.g609.t1 | 40S ribosomal S6 | 543 | 3.68E-103 | 94.5 | F:structural constituent of ribosome; C:cytosolic small ribosomal subunit; P:ribosomal small subunit biogenesis; P:rRNA processing; P:translation |
| Efet.01.112053.g610.t1 | fasciclin-2 isoform X1 | 417 | 9.84E-29 | 50.75 | C:membrane; C:integral component of membrane; P:cell adhesion |
| Efet.01.112073.g611.t1 | neuroepithelial cell-transforming gene 1 -like | 387 | 4.67E-26 | 59.9 | P:regulation of Rho protein signal transduction; F:guanyl-nucleotide exchange factor activity; F:Rho guanyl-nucleotide exchange factor activity; P:intracellular signal transduction; C:intracellular; P:positive regulation of GTPase activity |
| Efet.01.112099.g613.t1 | TBC1 domain family member 2B-like | 1434 | 2.85E-97 | 56.2 | F:ligase activity |
| Efet.01.11211.g820.t1 | hypothetical protein CAPTEDRAFT_227345 | 1110 | 3.58E-53 | 49.6 |  |
| Efet.01.112123.g614.t1 | m-phase inducer phosphatase | 636 | 4.95E-17 | 80.2 | F:protein tyrosine phosphatase activity; P:peptidyl-tyrosine dephosphorylation; C:intracellular |
| Efet.01.112159.g615.t1 | nuclear 1 | 324 | 5.52E-08 | 72.06 | F:DNA binding; C:nucleus; F:chromatin binding; P:regulation of transcription from RNA polymerase II promoter |
| Efet.01.112162.g616.t1 | vacuolar sorting-associated 8 homolog | 564 | 2.41E-44 | 62.25 | F:zinc ion binding; P:vesicle-mediated transport; F:metal ion binding; P:intracellular protein transport; C:intracellular |
| Efet.01.112168.g617.t1 | band 7 AGAP004871-like isoform X1 | 465 | 7.16E-22 | 79.75 | C:integral component of membrane |
| Efet.01.112190.g618.t1 | E3 ubiquitin- ligase HUWE1-like isoform X1 | 1236 | 1.57E-93 | 82.05 | F:ubiquitin-protein transferase activity; F:zinc ion binding; C:integral component of membrane; F:ligase activity; P:protein ubiquitination |
| Efet.01.112205.g620.t1 | sacsin-like isoform X1 [Orbicella faveolata] | 873 | 8.04E-15 | 53.33 |  |
| Efet.01.112228.g621.t1 | BTB POZ domain-containing 9-like | 675 | 7.83E-82 | 88.2 | P:serotonin metabolic process; P:circadian sleep/wake cycle, non-REM sleep; P:multicellular organismal iron ion homeostasis; P:regulation of proteolysis; P:regulation of synaptic vesicle endocytosis; P:adult locomotory behavior; P:protein ubiquitination involved in ubiquitin-dependent protein catabolic process; P:long-term memory; P:proteasome-mediated ubiquitin-dependent protein catabolic process; F:ubiquitin protein ligase binding; C:cytoplasm; P:modulation of synaptic transmission; C:SCF ubiquitin ligase complex; P:sensory perception of temperature stimulus |
| Efet.01.112282.g623.t1 | flocculation FLO11-like isoform X1 | 843 | 5.30E-07 | 53.95 | F:nucleotide binding; F:GTP binding; P:small GTPase mediated signal transduction; P:signal transduction; F:GTPase activity; P:negative regulation of voltage-gated calcium channel activity; C:membrane; C:intracellular; C:cytoplasmic side of plasma membrane |
| Efet.01.11233.g824.t1 | coiled-coil domain-containing 146-like | 396 | 2.21E-30 | 68.25 |  |
| Efet.01.11234.g825.t1 | importin-11 | 372 | 8.27E-20 | 61.2 | F:protein binding; F:protein transporter activity; C:intracellular part; P:ribosomal protein import into nucleus |
| Efet.01.112345.g625.t1 | ubiquitin carboxyl-terminal hydrolase 16-like isoform X1 | 312 | 7.19E-15 | 52.8 | P:protein deubiquitination; F:zinc ion binding; F:metal ion binding; P:proteolysis; F:cysteine-type peptidase activity; F:peptidase activity; P:ubiquitin-dependent protein catabolic process; F:thiol-dependent ubiquitinyl hydrolase activity; F:hydrolase activity |
| Efet.01.112376.g627.t1 | titin isoform X2 | 786 | 6.12E-47 | 57 |  |
| Efet.01.112387.g628.t1 | hypothetical protein CAPTEDRAFT_208890 | 807 | 4.31E-62 | 56.3 |  |
| Efet.01.112387.g629.t1 | acetylglutamate kinase-like | 357 | 4.33E-17 | 56.8 | F:transferase activity |
| Efet.01.112431.g630.t1 | hypothetical protein HELRODRAFT_108787 | 267 | 7.88E-07 | 96 | P:transmembrane transport; C:integral component of membrane |
| Efet.01.112440.g631.t1 | rab GTPase-binding effector 1-like | 201 | 2.59E-08 | 66.67 | F:binding |
| Efet.01.112494.g633.t1 | DNA-directed RNA polymerase II subunit RPB1 isoform X9 | 297 | 5.12E-13 | 61.7 |  |
| Efet.01.112585.g637.t1 | zinc finger 585B-like | 1149 | 1.97E-72 | 54.7 | F:nucleic acid binding; F:metal ion binding |
| Efet.01.112692.g639.t1 | hypothetical protein HELRODRAFT_166585 | 420 | 2.14E-24 | 62.1 | C:membrane; C:integral component of membrane; F:carbohydrate binding |
| Efet.01.112709.g640.t1 | nicotinic acetylcholine receptor subunit alpha | 450 | 3.07E-79 | 72.75 | P:ion transmembrane transport; C:integral component of membrane; F:extracellular ligand-gated ion channel activity |
| Efet.01.112717.g641.t1 | phosphatidylinositol 4,5-bisphosphate 3-kinase catalytic subunit delta isoform | 315 | 4.09E-18 | 61.85 | P:phosphorylation; F:transferase activity; P:single-organism process |
| Efet.01.112729.g643.t1 | dynein heavy chain axonemal | 396 | 9.86E-53 | 79.85 | F:ATPase activity; F:ATP binding; F:microtubule motor activity; C:dynein complex; P:microtubule-based movement |
| Efet.01.112740.g644.t1 | COUP transcription factor 1 | 345 | 3.34E-35 | 80.4 | C:nucleus; F:DNA binding; F:RNA polymerase II transcription factor activity, ligand-activated sequence-specific DNA binding; P:transcription, DNA-templated; F:steroid hormone receptor activity; P:regulation of transcription, DNA-templated; P:steroid hormone mediated signaling pathway; P:intracellular receptor signaling pathway |
| Efet.01.112787.g646.t1 | Low-density lipo receptor-related 4 | 459 | 5.59E-56 | 55 | C:integral component of membrane |
| Efet.01.11284.g826.t1 | hypothetical protein CAPTEDRAFT_188333 | 414 | 2.36E-07 | 51 |  |
| Efet.01.112918.g651.t1 | dmX 2 | 408 | 4.35E-14 | 49.85 |  |
| Efet.01.112926.g652.t1 | glutamine--fructose-6-phosphate aminotransferase [isomerizing] 2- | 447 | 4.08E-48 | 96.05 | P:carbohydrate metabolic process; F:glutamine-fructose-6-phosphate transaminase (isomerizing) activity; P:carbohydrate derivative biosynthetic process; F:carbohydrate binding |
| Efet.01.11294.g827.t1 | receptor-type guanylate cyclase daf-11 isoform X2 | 1962 | 2.09E-104 | 56.75 | F:nucleotide binding; P:cGMP biosynthetic process; C:integral component of membrane; F:guanylate cyclase activity; C:intracellular; P:intracellular signal transduction |
| Efet.01.113.g13.t1 | Ankyrin repeat domain-containing 6 | 1200 | 4.95E-07 | 47.11 |  |
| Efet.01.113002.g655.t1 | dual specificity phosphatase 1 | 999 | 6.10E-38 | 47.5 | F:hydrolase activity |
| Efet.01.113039.g656.t1 | E3 ubiquitin- ligase TRIM71-like | 954 | 1.83E-15 | 47.75 |  |
| Efet.01.113046.g657.t1 | activating transcription factor of chaperone isoform X2 | 660 | 2.06E-08 | 66.7 | F:RNA polymerase II distal enhancer sequence-specific DNA binding; C:nucleus; P:pupariation; P:pupation; P:molting cycle, chitin-based cuticle; P:response to starvation; P:triglyceride metabolic process; P:trehalose metabolic process; P:metamorphosis; F:RNA polymerase II transcription coactivator activity; P:positive regulation of transcription from RNA polymerase II promoter; F:RNA polymerase II activating transcription factor binding |
| Efet.01.113110.g659.t1 | Low-density lipo receptor-related 6 | 588 | 1.03E-91 | 82.25 | F:Wnt-activated receptor activity; C:integral component of membrane; P:Wnt signaling pathway involved in dorsal/ventral axis specification; P:canonical Wnt signaling pathway; C:receptor complex; F:Wnt-protein binding; C:plasma membrane |
| Efet.01.113124.g660.t1 | Chloride channel CLIC 1 | 510 | 8.21E-21 | 61.7 | C:cytoplasm; C:intracellular membrane-bounded organelle; C:membrane; P:transport |
| Efet.01.113147.g661.t1 | monocarboxylate transporter 13- | 345 | 6.33E-21 | 78.25 | C:integral component of membrane |
| Efet.01.113149.g663.t1 | hypothetical protein HELRODRAFT_161503 | 411 | 2.66E-08 | 59 | F:carbohydrate binding |
| Efet.01.113197.g667.t1 | hypothetical protein HELRODRAFT_169512 | 441 | 7.82E-06 | 84 |  |
| Efet.01.113202.g668.t1 | hypothetical protein CAPTEDRAFT_196014 | 915 | 3.75E-15 | 46.75 | F:G-protein coupled receptor activity; P:G-protein coupled receptor signaling pathway; P:signal transduction; C:membrane; C:integral component of membrane; F:signal transducer activity |
| Efet.01.11323.g828.t1 | polyubiquitin 8-like | 675 | 2.89E-48 | 53.7 | F:ubiquitin-protein transferase activity; F:ubiquitin protein ligase activity; F:ubiquitin conjugating enzyme binding; C:ubiquitin ligase complex; C:cytoplasm; F:metal ion binding; P:protein polyubiquitination; P:positive regulation of proteasomal ubiquitin-dependent protein catabolic process; P:protein ubiquitination involved in ubiquitin-dependent protein catabolic process; P:protein ubiquitination |
| Efet.01.113232.g670.t1 | enolase-phosphatase E1 isoform X11 | 1749 | 2.79E-100 | 45.05 | F:zinc ion binding; F:metal ion binding |
| Efet.01.113264.g671.t1 | nucleolar RNA helicase 2 | 294 | 4.38E-14 | 87.1 | C:nucleus; F:ATP binding; F:RNA binding; F:helicase activity |
| Efet.01.113291.g673.t1 | Nucleolar pre-ribosomal-associated 1 | 333 | 6.82E-20 | 53.85 | C:nucleolus; F:RNA binding |
| Efet.01.113299.g674.t1 | nuclear factor NF-kappa-B p105 subunit-like | 315 | 6.84E-19 | 63.4 | P:regulation of cellular process |
| Efet.01.113308.g675.t1 | retinol dehydrogenase 12-like | 678 | 1.44E-47 | 64.95 |  |
| Efet.01.113356.g676.t1 | nerve growth factor receptor | 1164 | 2.05E-77 | 70.1 | F:nucleotide binding; F:protein tyrosine kinase activity; F:transmembrane signaling receptor activity; C:membrane; F:lyase activity; P:protein phosphorylation; P:single-organism process |
| Efet.01.113358.g677.t1 | coronin-2B-like isoform X2 | 333 | 1.05E-34 | 77.35 |  |
| Efet.01.113369.g679.t1 | serine threonine- phosphatase 6 regulatory ankyrin repeat subunit A- | 2175 | 5.90E-106 | 52.05 |  |
| Efet.01.113369.g680.t1 | AChain Crystal Structure Of Darpin-darpin Rigid Variant Dd_d12_09_d12 | 939 | 1.43E-30 | 48.4 |  |
| Efet.01.113415.g681.t1 | DGCR14 isoform X2 | 294 | 5.59E-24 | 72.2 | C:catalytic step 2 spliceosome; P:nervous system development; P:RNA processing |
| Efet.01.113449.g682.t1 | mitogen-activated kinase kinase kinase 3 | 300 | 2.27E-12 | 61.6 | F:signal transducer, downstream of receptor, with serine/threonine kinase activity; C:cytoplasm; P:phosphorylation |
| Efet.01.113454.g683.t1 | disrupted in renal carcinoma 2 homolog | 525 | 4.02E-22 | 62.3 | F:ion channel activity; C:membrane; P:transport |
| Efet.01.113474.g684.t1 | serine threonine- kinase 17B-like | 795 | 4.14E-15 | 83.25 | F:ATP binding; P:protein phosphorylation; F:myosin light chain kinase activity |
| Efet.01.11348.g829.t1 | hypothetical protein HELRODRAFT_155469 | 252 | 3.04E-09 | 76 | F:DNA binding; C:nucleus; P:regulation of transcription, DNA-templated |
| Efet.01.1135.g104.t1 | gastrula zinc finger -like | 468 | 1.30E-28 | 54.25 |  |
| Efet.01.11356.g830.t1 | hypothetical protein HELRODRAFT_167085 | 1791 | 5.89E-28 | 51.5 |  |
| Efet.01.113572.g687.t1 | hypothetical protein CAPTEDRAFT_226427 | 675 | 1.01E-29 | 50 | F:calcium ion binding; C:membrane; C:integral component of membrane |
| Efet.01.11367.g831.t1 | WASH complex subunit strumpellin | 258 | 2.20E-29 | 82.3 | C:early endosome; P:protein transport; C:WASH complex |
| Efet.01.113675.g691.t1 | RUN and FYVE domain-containing 2-like isoform X2 | 387 | 3.96E-16 | 80.55 |  |
| Efet.01.1137.g105.t1 | zinc finger MIZ domain-containing 1-like isoform X1 | 726 | 8.98E-09 | 68.4 | F:zinc ion binding |
| Efet.01.113700.g693.t1 | serine arginine repetitive matrix 1-like | 1584 | 1.70E-42 | 59.1 |  |
| Efet.01.113702.g694.t1 | tripartite motif-containing | 444 | 4.10E-12 | 48.05 | P:regulation of cellular process; F:binding; C:intracellular |
| Efet.01.113717.g697.t1 | Follistatin-related 3 | 414 | 9.95E-21 | 68.45 | P:mitochondrial DNA repair; F:single-stranded DNA exodeoxyribonuclease activity; C:mitochondrion |
| Efet.01.113724.g698.t1 | Voltage-dependent calcium channel gamma-1 subunit | 318 | 1.75E-19 | 54.9 | C:membrane; C:integral component of membrane |
| Efet.01.113788.g699.t1 | reticulon 4b isoform X2 | 567 | 2.66E-13 | 60.05 | P:forebrain development; P:motor neuron axon guidance; P:neuromast development; C:membrane; P:camera-type eye development; C:endomembrane system; P:peripheral nervous system development; P:oculomotor nerve development; P:trochlear nerve development; P:axon regeneration; P:optic nerve development; P:embryonic viscerocranium morphogenesis |
| Efet.01.113809.g700.t1 | calcium release-activated calcium channel 1 isoform X2 | 393 | 2.00E-55 | 77.75 | F:store-operated calcium channel activity; P:store-operated calcium entry; C:integral component of membrane |
| Efet.01.113811.g701.t1 | innexin 4 | 582 | 5.08E-94 | 69.55 | C:gap junction; P:ion transport; C:integral component of membrane; P:cell communication by electrical coupling; C:synapse; F:gap junction channel activity; C:plasma membrane |
| Efet.01.113818.g702.t1 | hypothetical protein CAPTEDRAFT_226427 | 315 | 9.32E-24 | 63.92 | F:nucleic acid binding; C:membrane; F:protein dimerization activity |
| Efet.01.113833.g703.t1 | leucine-rich repeats and immunoglobulin-like domains 3 isoform X2 | 888 | 2.16E-69 | 74.1 | C:membrane |
| Efet.01.113870.g705.t1 | Centrosomal of 152 kDa | 1053 | 2.61E-12 | 65.15 | F:protein kinase binding; C:centrosome; C:microtubule organizing center part; P:de novo centriole assembly involved in multi-ciliated epithelial cell differentiation; C:deuterosome; P:centriole replication |
| Efet.01.113895.g707.t1 | cadherin-23-like isoform X1 | 354 | 5.62E-12 | 63.2 | C:membrane; P:cell adhesion |
| Efet.01.113904.g708.t1 | hypothetical protein CAPTEDRAFT_220352 | 585 | 2.02E-28 | 52.26 | C:membrane; C:integral component of membrane |
| Efet.01.113921.g709.t1 | gelsolin 2 | 252 | 1.27E-37 | 84.05 | C:cytoskeleton; P:actin filament severing; F:actin binding; P:actin filament capping; C:cytoplasm; P:multicellular organism development; F:calcium ion binding; P:actin nucleation; P:actin filament polymerization; P:actin filament fragmentation |
| Efet.01.113939.g711.t1 | SPRY domain-containing SOCS box 4-like isoform X1 | 528 | 2.69E-75 | 75.1 | P:intracellular signal transduction |
| Efet.01.113956.g712.t1 | neuroglian-like isoform X1 | 363 | 1.11E-53 | 59.35 | C:integral component of membrane |
| Efet.01.114006.g713.t1 | kinesin KIF2A | 291 | 4.28E-13 | 74.25 | F:ATP binding; F:microtubule motor activity; P:microtubule-based movement; C:microtubule; F:microtubule binding |
| Efet.01.114007.g714.t1 | histone-lysine N-methyltransferase NSD3 isoform X4 | 1368 | 2.39E-28 | 53.85 | F:transferase activity |
| Efet.01.114013.g715.t1 | collagen alpha-1(XII) chain-like | 1275 | 1.59E-122 | 54.4 | F:calcium ion binding |
| Efet.01.114077.g716.t1 | YTH domain-containing family 2-like isoform X2 | 1368 | 2.00E-116 | 68.5 | F:RNA binding; C:membrane; C:integral component of membrane |
| Efet.01.114082.g717.t1 | 5 -3 exoribonuclease 2 homolog | 2910 | 0 | 75.8 | F:binding |
| Efet.01.114158.g720.t1 | protocadherin-11 X-linked-like | 2046 | 8.06E-111 | 54.7 | F:calcium ion binding; C:membrane; C:integral component of membrane; P:cell adhesion; P:homophilic cell adhesion via plasma membrane adhesion molecules; C:plasma membrane |
| Efet.01.114171.g722.t1 | septin-7-like isoform X2 | 468 | 3.85E-32 | 74.25 | F:GTP binding |
| Efet.01.114177.g723.t1 | coiled-coil domain-containing 18 isoform X1 | 3057 | 7.56E-23 | 47.65 |  |
| Efet.01.11418.g832.t1 | FMRFamide receptor-like | 1368 | 1.08E-36 | 51.45 | F:G-protein coupled receptor activity; F:G-protein coupled peptide receptor activity; P:G-protein coupled receptor signaling pathway; P:signal transduction; C:membrane; C:integral component of membrane; F:signal transducer activity |
| Efet.01.114191.g724.t1 | dystonin isoform X1 | 495 | 5.90E-21 | 50.36 | C:cytoskeleton |
| Efet.01.114219.g725.t1 | dynein heavy chain | 489 | 8.66E-51 | 93.7 | F:ATPase activity; F:ATP binding; C:motile cilium; F:microtubule motor activity; C:cytoplasm; P:cell projection organization; C:dynein complex; P:microtubule-based movement; C:microtubule |
| Efet.01.114219.g726.t1 | dynein beta ciliary-like | 264 | 1.36E-31 | 84.6 | F:ATPase activity; F:ATP binding; C:outer dynein arm; F:microtubule motor activity; F:protein binding; C:cytoplasm; P:cilium movement; C:microtubule |
| Efet.01.11422.g833.t1 | TANC2-like isoform X4 | 213 | 1.69E-09 | 66 |  |
| Efet.01.114237.g727.t1 | cartilage matrix | 303 | 2.82E-14 | 68.3 | C:collagen trimer; F:calcium ion binding |
| Efet.01.114243.g728.t1 | tripartite tricarboxylate transporter substrate binding | 942 | 0 | 79 | C:outer membrane-bounded periplasmic space |
| Efet.01.114243.g729.t1 | PIN domain-containing | 402 | 7.15E-94 | 87.1 | F:magnesium ion binding; P:RNA phosphodiester bond hydrolysis; F:ribonuclease activity |
| Efet.01.114260.g731.t1 | importin subunit beta-1 isoform X2 | 1092 | 3.19E-114 | 79.55 | P:intracellular protein transport; F:Ran GTPase binding; C:intracellular |
| Efet.01.114262.g732.t1 | Poly [ADP-ribose] polymerase 14 | 462 | 8.59E-11 | 49.25 | F:nucleotide binding; F:nucleic acid binding |
| Efet.01.114262.g733.t1 | macro domain-containing | 1248 | 3.92E-41 | 58.65 | F:nucleotide binding; F:nucleic acid binding; F:transferase activity, transferring glycosyl groups; F:transferase activity; F:NAD+ ADP-ribosyltransferase activity |
| Efet.01.114272.g735.t1 | voltage-dependent T-type calcium channel subunit alpha-1H-like | 549 | 4.46E-22 | 61.15 | F:ion channel activity; P:ion transport |
| Efet.01.114292.g739.t1 | ubiquitin-conjugating enzyme E2Q 1 | 297 | 4.80E-50 | 80.2 |  |
| Efet.01.114309.g741.t1 | heterogeneous nuclear ribonucleo U 1 | 393 | 4.78E-33 | 77.05 | C:intracellular ribonucleoprotein complex; C:viral nucleocapsid |
| Efet.01.114337.g742.t1 | hypothetical protein CAPTEDRAFT_195059 | 834 | 9.20E-138 | 49.95 | C:intracellular; P:intracellular signal transduction |
| Efet.01.114354.g744.t1 | nuclear pore complex Nup160 | 846 | 4.77E-30 | 53.05 | P:nucleocytoplasmic transport; F:nucleocytoplasmic transporter activity; C:nuclear pore |
| Efet.01.114398.g750.t1 | Adventurous-gliding motility Z | 498 | 1.48E-11 | 59.41 | P:proteolysis |
| Efet.01.114400.g751.t1 | probable G- coupled receptor 139 | 1431 | 2.09E-109 | 54.15 | F:signal transducer activity |
| Efet.01.114416.g752.t1 | Zinc finger MYM-type 4 | 786 | 3.39E-61 | 76.25 | F:zinc ion binding |
| Efet.01.114445.g753.t1 | ileal sodium bile acid cotransporter-like | 801 | 1.49E-23 | 53.95 | C:integral component of membrane; F:organic anion transmembrane transporter activity; C:plasma membrane |
| Efet.01.114476.g755.t1 | membrane-bound O-acyltransferase domain-containing 2-like | 204 | 6.28E-21 | 68.1 | C:integral component of membrane |
| Efet.01.1145.g106.t1 | neuropeptide Y receptor type 5-like | 219 | 4.16E-31 | 81.25 | P:neuropeptide signaling pathway; C:integral component of membrane; F:neuropeptide Y receptor activity |
| Efet.01.114530.g756.t1 | zinc finger | 759 | 2.32E-43 | 75.85 | F:nucleic acid binding |
| Efet.01.114533.g757.t1 | V-type proton ATPase 116 kDa subunit a isoform 1 | 306 | 6.26E-27 | 69.35 | P:proton transport; C:proton-transporting V-type ATPase, V0 domain |
| Efet.01.114562.g758.t1 | protocadherin-9 isoform X3 | 2817 | 3.44E-155 | 54.05 | F:calcium ion binding; C:membrane; C:integral component of membrane; P:cell adhesion; P:homophilic cell adhesion via plasma membrane adhesion molecules; C:plasma membrane |
| Efet.01.114586.g759.t1 | CREBRF homolog isoform X2 | 651 | 7.03E-28 | 60.4 | F:transcription factor activity, sequence-specific DNA binding; C:membrane; C:integral component of membrane; P:regulation of transcription, DNA-templated; F:sequence-specific DNA binding |
| Efet.01.11461.g835.t1 | cold shock domain-containing E1-like | 594 | 2.82E-52 | 68.55 | F:nucleic acid binding |
| Efet.01.114621.g760.t1 | SEC14 1 isoform X2 | 447 | 6.69E-57 | 79.95 | F:molecular function regulator; P:choline transport; P:innate immune response; F:RIG-I binding; C:integral component of membrane; C:Golgi apparatus; P:negative regulation of RIG-I signaling pathway; F:transporter activity |
| Efet.01.114629.g761.t1 | prolow-density lipo receptor-related 1-like isoform X2 | 288 | 8.36E-24 | 62.75 | C:membrane |
| Efet.01.114632.g762.t1 | epidermal growth factor receptor-like isoform X1 | 549 | 1.95E-54 | 66.75 | F:protein kinase activity; P:phosphorylation |
| Efet.01.114634.g764.t1 | mitoferrin-2 isoform X1 | 1083 | 6.38E-44 | 74.05 | C:mitochondrion; C:integral component of membrane; F:ferrous iron transmembrane transporter activity; P:transport |
| Efet.01.114635.g765.t1 | proline synthase co-transcribed bacterial homolog | 693 | 3.83E-82 | 72.8 | F:pyridoxal phosphate binding; C:intracellular; C:membrane-bounded organelle |
| Efet.01.11471.g836.t1 | Potassium sodium hyperpolarization-activated cyclic nucleotide-gated channel 2 | 1122 | 8.22E-28 | 75.75 | P:potassium ion transport; F:ion channel activity; P:ion transmembrane transport; C:integral component of membrane |
| Efet.01.114723.g768.t1 | Periotrophin | 300 | 8.80E-10 | 58.9 | F:chitin binding; P:chitin metabolic process; C:extracellular region |
| Efet.01.114833.g774.t1 | seleno O | 279 | 1.89E-08 | 59 |  |
| Efet.01.114833.g775.t1 | seleno O isoform X2 | 270 | 4.97E-34 | 78.9 |  |
| Efet.01.114845.g776.t1 | endothelin-converting enzyme-like 1 | 798 | 2.36E-24 | 54.6 | C:membrane; P:proteolysis; C:integral component of membrane; F:metalloendopeptidase activity; F:metallopeptidase activity |
| Efet.01.114862.g777.t1 | hypothetical protein CAPTEDRAFT_186249 | 348 | 1.49E-29 | 56.25 | C:membrane; C:integral component of membrane; F:carbohydrate binding |
| Efet.01.114865.g778.t1 | papilin-like isoform X5 | 1029 | 6.82E-54 | 55.65 | F:zinc ion binding; F:peptidase activity; C:extracellular region; F:metalloendopeptidase activity; C:proteinaceous extracellular matrix; F:metallopeptidase activity; F:peptidase inhibitor activity; F:serine-type endopeptidase inhibitor activity; P:proteolysis; C:membrane; C:integral component of membrane; P:negative regulation of endopeptidase activity; C:extracellular matrix; P:negative regulation of peptidase activity |
| Efet.01.114897.g779.t1 | ATP-dependent RNA helicase TDRD12 | 543 | 1.37E-10 | 53.95 | F:organic cyclic compound binding; F:heterocyclic compound binding; C:P granule; C:PET complex; P:single-organism cellular process; P:single organism reproductive process |
| Efet.01.114928.g781.t1 | early growth response 1-B-like | 1182 | 1.28E-57 | 76.65 | F:nucleic acid binding; F:metal ion binding |
| Efet.01.115000.g783.t1 | FMRFamide receptor-like | 960 | 3.50E-51 | 62.05 | P:signal transduction; C:membrane; F:signal transducer activity |
| Efet.01.115066.g784.t1 | eukaryotic translation initiation factor 2-alpha kinase 3 | 237 | 3.86E-23 | 70.1 | P:PERK-mediated unfolded protein response; P:insulin secretion; P:eiF2alpha phosphorylation in response to endoplasmic reticulum stress; P:cellular response to amino acid starvation; P:chondrocyte development; P:protein autophosphorylation; P:regulation of endoplasmic reticulum stress-induced intrinsic apoptotic signaling pathway; P:ER overload response; P:positive regulation of transcription from RNA polymerase I promoter; F:nucleotide binding; P:angiogenesis; P:activation of cysteine-type endopeptidase activity involved in apoptotic process; P:calcium-mediated signaling; P:endocrine pancreas development; P:positive regulation of vascular endothelial growth factor production; P:positive regulation of protein localization to nucleus; F:protein phosphatase binding; F:protein homodimerization activity; P:bone mineralization; P:insulin-like growth factor receptor signaling pathway; P:protein homooligomerization; P:regulation of endoplasmic reticulum stress-induced eIF2 alpha phosphorylation; F:eukaryotic translation initiation factor 2alpha kinase activity; P:endoplasmic reticulum organization; P:cellular response to glucose starvation; C:integral component of endoplasmic reticulum membrane; P:negative regulation of translational initiation in response to stress; P:peptidyl-serine phosphorylation; P:cellular response to cold; P:negative regulation of myelination |
| Efet.01.11508.g839.t1 | ras-related Rab-39B-like | 288 | 9.02E-23 | 62.75 | P:single-organism process |
| Efet.01.115080.g785.t1 | band 7 AGAP004871-like | 252 | 6.10E-35 | 91.65 | C:integral component of membrane |
| Efet.01.115129.g787.t1 | hypothetical protein CAPTEDRAFT_189926 | 702 | 4.24E-11 | 45 | C:membrane; C:integral component of membrane |
| Efet.01.115134.g788.t1 | neuroligin- X-linked-like isoform X2 | 633 | 6.26E-32 | 52.85 | C:membrane; C:integral component of membrane |
| Efet.01.115229.g790.t1 | hypothetical protein HELRODRAFT_169090 | 570 | 3.50E-18 | 50.5 | F:chitin binding; P:chitin metabolic process; C:extracellular region |
| Efet.01.115245.g792.t1 | Syntaxin-binding 5 | 1395 | 6.29E-17 | 67.3 | P:vesicle-mediated transport; C:integral component of membrane |
| Efet.01.115311.g794.t1 | FMRFamide receptor-like | 1161 | 2.01E-123 | 68 | F:G-protein coupled receptor activity; P:signal transduction; C:membrane |
| Efet.01.115317.g795.t1 | homeobox Nkx- | 867 | 7.43E-29 | 72.25 | C:nucleus; F:transcription factor activity, sequence-specific DNA binding; P:transcription, DNA-templated; F:double-stranded DNA binding; P:regulation of transcription, DNA-templated; F:sequence-specific DNA binding; P:ventral spinal cord interneuron differentiation; P:respiratory tube development |
| Efet.01.115358.g796.t1 | USP6 N-terminal isoform X1 | 1047 | 2.40E-14 | 63.9 |  |
| Efet.01.11539.g840.t1 | hypothetical protein HELRODRAFT_190749 | 309 | 3.28E-21 | 69.25 | P:response to stress |
| Efet.01.115390.g797.t1 | glucose dehydrogenase [ quinone] | 555 | 1.45E-71 | 66.6 | F:oxidoreductase activity; C:membrane |
| Efet.01.115447.g799.t1 | von Willebrand factor type EGF and pentraxin domain-containing | 1989 | 3.77E-111 | 47.9 | F:calcium ion binding; F:chromatin binding |
| Efet.01.115447.g800.t1 | von Willebrand factor type EGF and pentraxin domain-containing 1-like | 2586 | 3.45E-165 | 47.35 | F:calcium ion binding |
| Efet.01.115477.g801.t1 | galactoside 2-alpha-L-fucosyltransferase 2-like | 816 | 1.01E-52 | 56.35 | P:carbohydrate metabolic process; F:transferase activity, transferring glycosyl groups; P:fucosylation; C:membrane; C:integral component of membrane; F:transferase activity; F:galactoside 2-alpha-L-fucosyltransferase activity |
| Efet.01.11549.g841.t1 | piggyBac transposable element-derived 4-like | 1986 | 2.76E-106 | 53.7 | F:zinc ion binding; C:intracellular |
| Efet.01.115517.g802.t1 | helicase carboxy-terminal domain | 1746 | 2.11E-26 | 53 | P:protein targeting; F:ATP binding; C:membrane; F:helicase activity; P:intracellular protein transport; P:protein import; C:intracellular |
| Efet.01.115517.g803.t1 | helicase carboxy-terminal domain | 1095 | 3.04E-36 | 67.5 | P:protein targeting; F:ATP binding; C:membrane; F:helicase activity; P:intracellular protein transport; P:protein import; C:intracellular |
| Efet.01.115560.g804.t1 | junctophilin-2 isoform X2 | 540 | 9.93E-63 | 83.7 | F:calcium-release channel activity; P:multicellular organism development; P:positive regulation of ryanodine-sensitive calcium-release channel activity; F:phosphatidylinositol-3,5-bisphosphate binding; C:plasma membrane; F:phosphatidylinositol-3,4,5-trisphosphate binding; C:Z disc; F:phosphatidylinositol-4,5-bisphosphate binding; F:phosphatidylinositol-4-phosphate binding; F:phosphatidylserine binding; F:protein binding; C:integral component of membrane; F:phosphatidylinositol-5-phosphate binding; P:regulation of cardiac muscle tissue development; C:cytosol; F:phosphatidic acid binding; F:phosphatidylinositol-3-phosphate binding; P:release of sequestered calcium ion into cytosol; C:junctional sarcoplasmic reticulum membrane; C:junctional membrane complex |
| Efet.01.115606.g805.t1 | homeobox Nkx- -like | 558 | 7.31E-39 | 66.85 | C:nucleus; P:regulation of cardiac chamber formation; P:heart looping; P:regulation of transcription, DNA-templated; P:pharyngeal arch artery morphogenesis; F:sequence-specific DNA binding; P:cardiac ventricle development; P:cardiac muscle cell differentiation; P:cardiac muscle cell proliferation |
| Efet.01.115608.g806.t1 | tyrosine- kinase Src42A-like | 879 | 0 | 93.55 | F:ATP binding; P:peptidyl-tyrosine phosphorylation; F:non-membrane spanning protein tyrosine kinase activity |
| Efet.01.115611.g807.t1 | galactoside 2-alpha-L-fucosyltransferase 2-like | 816 | 4.82E-53 | 52.15 | P:carbohydrate metabolic process; P:fucosylation; C:membrane; C:integral component of membrane; F:galactoside 2-alpha-L-fucosyltransferase activity |
| Efet.01.11564.g842.t1 | Wnt-5b-like | 321 | 1.63E-44 | 75.4 | P:multicellular organism development; P:Wnt signaling pathway; F:receptor binding; C:proteinaceous extracellular matrix |
| Efet.01.115642.g809.t1 | piccolo-like isoform X1 | 372 | 9.28E-15 | 64.15 | C:membrane; P:intracellular protein transport; F:Rab GTPase binding; C:intracellular |
| Efet.01.115668.g812.t1 | BTB POZ domain-containing 3-like | 1020 | 6.27E-38 | 45.35 | F:isomerase activity |
| Efet.01.115671.g813.t1 | hypothetical protein CAPTEDRAFT_186144 | 1209 | 5.46E-16 | 61 | P:regulation of apoptotic process |
| Efet.01.115691.g814.t1 | PREDICTED: uncharacterized protein LOC108043058 | 249 | 3.88E-06 | 85 |  |
| Efet.01.11571.g843.t1 | crumbs homolog 1-like | 474 | 1.55E-23 | 70 | P:regulation of cellular process; P:cell communication; C:integral component of membrane; F:ion binding; P:phosphorylation; F:kinase activity; P:single-organism developmental process |
| Efet.01.115747.g815.t1 | transmembrane and TPR repeat-containing 3 | 327 | 1.15E-36 | 77.9 | C:integral component of membrane |
| Efet.01.115760.g817.t1 | polycystic kidney disease and receptor for egg jelly-related | 429 | 5.30E-06 | 49 | F:calcium ion binding; C:membrane; C:integral component of membrane |
| Efet.01.11583.g844.t1 | hypothetical protein HELRODRAFT_161716 | 756 | 3.10E-13 | 63 | C:membrane; C:integral component of membrane |
| Efet.01.115834.g821.t1 | KIBRA-like isoform X2 | 1035 | 9.20E-25 | 55.45 |  |
| Efet.01.115844.g823.t1 | melanotransferrin-like isoform X1 | 477 | 1.05E-66 | 60.65 | P:iron ion homeostasis; C:extracellular space; P:ion transport; F:metal ion binding |
| Efet.01.115845.g824.t1 | COUP transcription factor 2 isoform X3 | 369 | 1.05E-54 | 93.95 | C:nucleus; F:DNA binding; F:RNA polymerase II transcription factor activity, ligand-activated sequence-specific DNA binding; P:transcription, DNA-templated; F:steroid hormone receptor activity; P:regulation of transcription, DNA-templated; P:steroid hormone mediated signaling pathway; P:intracellular receptor signaling pathway |
| Efet.01.115880.g827.t1 | lethal(2) giant larvae homolog 1-like | 252 | 5.87E-18 | 83.5 |  |
| Efet.01.115899.g828.t1 | phosphatidylinositol N-acetylglucosaminyltransferase subunit Q | 447 | 6.44E-12 | 83.9 | P:GPI anchor biosynthetic process; C:glycosylphosphatidylinositol-N-acetylglucosaminyltransferase (GPI-GnT) complex; C:integral component of membrane; F:phosphatidylinositol N-acetylglucosaminyltransferase activity |
| Efet.01.11593.g845.t1 | rho guanine nucleotide exchange factor 17-like isoform X1 | 417 | 3.09E-21 | 76.65 | P:regulation of Rho protein signal transduction; F:Rho guanyl-nucleotide exchange factor activity; P:positive regulation of GTPase activity |
| Efet.01.11593.g846.t1 | rho guanine nucleotide exchange factor 17-like isoform X1 | 543 | 1.12E-30 | 71.65 | P:biological regulation |
| Efet.01.115932.g830.t1 | hypothetical protein CAPTEDRAFT_220904 | 729 | 8.85E-17 | 42.95 | C:membrane; C:integral component of membrane; F:carbohydrate binding |
| Efet.01.115981.g831.t1 | GAS2 1 | 1113 | 1.10E-71 | 64 | P:cell cycle arrest; C:cytoplasm; C:stress fiber; P:cellular response to starvation; C:microtubule |
| Efet.01.115982.g832.t1 | Leucine-rich repeats and immunoglobulin-like domains 3 | 339 | 1.04E-10 | 69 | C:membrane |
| Efet.01.115985.g833.t1 | type-1 angiotensin II receptor-like isoform X1 | 540 | 5.93E-39 | 65.5 | F:G-protein coupled receptor activity; P:G-protein coupled receptor signaling pathway; C:integral component of membrane |
| Efet.01.116043.g835.t1 | ABHD17B-like | 624 | 2.72E-102 | 82.6 | F:hydrolase activity |
| Efet.01.11608.g848.t1 | hypothetical protein CAPTEDRAFT_195684 | 372 | 1.44E-31 | 65 |  |
| Efet.01.116082.g836.t1 | Actin-related 2 3 complex subunit 5 | 282 | 2.32E-10 | 70.88 | P:regulation of actin filament polymerization; C:actin cytoskeleton |
| Efet.01.116098.g837.t1 | RAC-gamma serine threonine- kinase-like isoform X1 | 489 | 3.73E-52 | 72.4 | F:protein kinase C binding; C:Golgi apparatus; P:brain morphogenesis; F:protein serine/threonine kinase activity; C:plasma membrane; C:nucleoplasm; P:mitochondrial genome maintenance; P:cellular response to insulin stimulus; F:ATP binding; P:positive regulation of cell size; P:homeostasis of number of cells within a tissue; P:positive regulation of sodium ion transport; P:peptidyl-serine phosphorylation; P:positive regulation of TOR signaling; P:peptidyl-threonine phosphorylation; P:intracellular signal transduction |
| Efet.01.116118.g839.t1 | C3 and PZP-like alpha-2-macroglobulin domain-containing 8 | 423 | 5.98E-21 | 56.45 |  |
| Efet.01.116149.g841.t1 | short transient receptor potential channel 3- | 810 | 1.70E-51 | 63.1 | F:calcium channel activity; C:integral component of membrane; P:metal ion transport; P:single-organism process |
| Efet.01.116175.g842.t1 | signal CUB and EGF-like domain-containing 1 isoform X1 | 426 | 8.71E-55 | 72.4 | F:protein binding; F:calcium ion binding; C:plasma membrane part |
| Efet.01.11618.g849.t1 | nesprin-1-like isoform X3 | 738 | 6.66E-26 | 54.5 | C:nuclear envelope; F:actin filament binding; F:actin binding; C:membrane; C:integral component of membrane; P:cytoskeletal anchoring at nuclear membrane; C:LINC complex |
| Efet.01.116192.g843.t1 | collagen alpha-1(XIV) chain isoform X1 | 327 | 2.64E-20 | 59.05 | C:extracellular space; C:extracellular exosome; F:calcium ion binding; C:extracellular matrix; F:RNA binding |
| Efet.01.116233.g845.t1 | FAM110B-like | 576 | 6.22E-19 | 55.9 |  |
| Efet.01.11624.g850.t1 | allatostatin-A receptor | 855 | 6.74E-50 | 61.5 | P:signal transduction; F:signal transducer activity |
| Efet.01.116244.g846.t1 | JNK1 MAPK8-associated membrane | 390 | 4.20E-46 | 70.95 | C:membrane |
| Efet.01.116268.g849.t1 | zinc finger C2H2 type | 1365 | 8.95E-08 | 72.89 | F:metal ion binding |
| Efet.01.116285.g851.t1 | RNA-binding 1 | 228 | 2.42E-27 | 78.8 | F:nucleotide binding; F:nucleic acid binding |
| Efet.01.11636.g852.t1 | ras-related Rab-40C | 399 | 3.84E-42 | 55 | F:nucleotide binding; F:GTP binding; P:small GTPase mediated signal transduction; P:intracellular signal transduction; C:intracellular |
| Efet.01.11637.g853.t1 | disintegrin and metallo ase domain-containing 10-like | 759 | 3.83E-21 | 49.05 | F:zinc ion binding; P:caveola assembly; P:integrin-mediated signaling pathway; P:Notch signaling pathway; P:membrane protein ectodomain proteolysis; P:proteolysis; C:membrane; C:integral component of membrane; F:metalloendopeptidase activity; F:metallopeptidase activity |
| Efet.01.11637.g854.t1 | disintegrin and metallo ase domain-containing 10-like | 348 | 1.49E-40 | 83.35 | F:zinc ion binding; P:proteolysis; C:integral component of membrane; F:protein kinase activity; P:protein phosphorylation; F:metalloendopeptidase activity |
| Efet.01.116376.g854.t1 | F-actin-methionine sulfoxide oxidase MICAL3-like isoform X1 | 3777 | 2.09E-09 | 64.35 |  |
| Efet.01.11638.g855.t1 | hypothetical protein CAPTEDRAFT_212319 | 5049 | 1.02E-25 | 56.33 | F:metal ion binding |
| Efet.01.116429.g857.t1 | eukaryotic peptide chain release factor | 228 | 1.11E-38 | 79.1 | C:nucleus; P:translational termination; F:protein binding; P:regulation of translational termination; P:nuclear-transcribed mRNA catabolic process, nonsense-mediated decay; F:ribosome binding; C:cytosol; P:protein methylation; F:translation release factor activity, codon specific; F:RNA binding |
| Efet.01.116475.g858.t1 | AT-rich interactive domain-containing 2-like isoform X1 | 516 | 2.48E-22 | 57.2 | F:DNA binding; F:metal ion binding; P:regulation of transcription, DNA-templated |
| Efet.01.116475.g859.t1 | AT-rich interactive domain-containing 2 | 258 | 2.19E-14 | 70.45 | F:binding |
| Efet.01.116489.g860.t1 | mitochondrial ATP synthase subunit d precursor | 333 | 5.10E-46 | 69.4 | C:mitochondrial proton-transporting ATP synthase complex, coupling factor F(o); F:hydrogen ion transmembrane transporter activity; P:ATP synthesis coupled proton transport |
| Efet.01.116557.g862.t1 | Chloride intracellular channel 4 | 636 | 1.95E-102 | 59.3 |  |
| Efet.01.11656.g856.t1 | PR-domain containing | 1608 | 1.59E-76 | 55.3 | F:histone methyltransferase activity (H3-K4 specific); C:nucleus; P:histone lysine methylation; P:positive regulation of reciprocal meiotic recombination; P:spermatogenesis; F:recombination hotspot binding; F:sequence-specific DNA binding; P:positive regulation of transcription from RNA polymerase II promoter |
| Efet.01.116602.g864.t1 | steroid 17-alpha-hydroxylase 17,20 lyase-like isoform X2 | 1695 | 3.55E-85 | 47.85 | F:monooxygenase activity |
| Efet.01.11662.g857.t1 | rho GTPase-activating 190-like isoform X1 | 318 | 1.77E-23 | 67.7 | P:signal transduction |
| Efet.01.116666.g868.t1 | polycystin-1 isoform X1 | 2832 | 1.14E-37 | 44 | P:regulation of cellular process; P:kidney development; P:tube development; C:cell part; P:epithelium development |
| Efet.01.116678.g870.t1 | peripheral-type benzodiazepine receptor-associated 1 isoform X1 | 1272 | 3.33E-35 | 71.95 | P:negative regulation of phosphatase activity; C:membrane |
| Efet.01.116692.g871.t1 | Fasciculation and elongation zeta-2 | 222 | 4.22E-16 | 76.95 |  |
| Efet.01.116704.g872.t1 | calmodulin 1 | 444 | 6.11E-31 | 59.35 | C:extracellular exosome; F:calcium ion binding |
| Efet.01.116755.g874.t1 | atonal homolog 8-like | 828 | 6.08E-44 | 84.45 | F:transcription factor activity, sequence-specific DNA binding; P:cell differentiation; P:regulation of transcription, DNA-templated; F:protein dimerization activity |
| Efet.01.116771.g876.t1 | Serine threonine- kinase | 444 | 3.58E-14 | 57 | F:zinc ion binding; F:metal ion binding; P:phosphorylation; F:transferase activity; F:kinase activity; C:intracellular |
| Efet.01.116847.g877.t1 | BTB POZ domain-containing 7 | 543 | 1.95E-65 | 74.25 | C:membrane |
| Efet.01.11687.g860.t1 | cilia- and flagella-associated 221-like | 744 | 2.41E-50 | 52.6 |  |
| Efet.01.116871.g879.t1 | hypothetical protein HELRODRAFT_186383 | 759 | 2.92E-11 | 82.5 |  |
| Efet.01.116915.g881.t1 | epidermal growth factor receptor substrate 15-like 1 isoform X3 | 2157 | 7.92E-150 | 53.6 | F:calcium ion binding |
| Efet.01.116925.g882.t1 | TBC1 domain family member 2B-like | 597 | 5.52E-29 | 60.6 | P:biological regulation; C:cell part |
| Efet.01.116967.g884.t1 | peroxisomal membrane 11B | 528 | 3.27E-38 | 56.6 | P:peroxisome fission; C:integral component of peroxisomal membrane |
| Efet.01.116993.g885.t1 | glycoside hydrolase subgroup catalytic core | 498 | 1.59E-44 | 58.85 | F:hydrolase activity |
| Efet.01.117068.g887.t1 | hypothetical protein CAPTEDRAFT_220546 | 684 | 4.17E-25 | 49.15 | C:membrane; C:integral component of membrane; F:carbohydrate binding |
| Efet.01.11708.g863.t1 | dynein heavy chain axonemal-like isoform X1 | 783 | 4.97E-128 | 84.45 | F:ATPase activity; F:ATP binding; F:microtubule motor activity; C:cytoplasm; C:axonemal dynein complex; P:cilium or flagellum-dependent cell motility; P:microtubule-based movement; C:microtubule |
| Efet.01.117161.g889.t1 | coiled-coil domain-containing 146-like | 657 | 6.08E-39 | 72.5 |  |
| Efet.01.117178.g891.t1 | innexin unc-9-like isoform X6 | 1083 | 3.09E-138 | 67.85 | C:gap junction; P:ion transport; C:integral component of membrane; C:plasma membrane |
| Efet.01.117183.g892.t1 | nuclear transcription factor Y subunit alpha-like isoform X1 | 420 | 4.61E-40 | 76.35 | C:CCAAT-binding factor complex; F:DNA binding; F:transcription factor activity, sequence-specific DNA binding; P:regulation of transcription, DNA-templated |
| Efet.01.117203.g895.t1 | fasciclin-2 isoform X1 | 468 | 6.09E-29 | 56.85 | C:membrane; C:integral component of membrane; P:cell adhesion |
| Efet.01.117247.g896.t1 | sterile alpha and TIR motif-containing 1-like isoform X10 | 1923 | 2.38E-160 | 64.65 | P:signal transduction |
| Efet.01.117326.g899.t1 | Anopheles gambiae PEST AGAP012554- | 291 | 1.99E-32 | 74.3 | C:integral component of membrane |
| Efet.01.117329.g900.t1 | hypothetical protein HELRODRAFT_82550 | 225 | 1.52E-09 | 85 | P:transmembrane transport; C:integral component of membrane; F:transporter activity |
| Efet.01.117335.g901.t1 | ATP-dependent 6-phosphofructokinase-like isoform X2 | 570 | 1.37E-70 | 60.65 | P:organophosphate metabolic process; P:carbohydrate derivative metabolic process; P:phosphorylation; F:binding; F:transferase activity |
| Efet.01.117410.g902.t1 | mucin-17-like isoform X1 | 4212 | 2.06E-09 | 59.36 |  |
| Efet.01.117435.g904.t1 | GTP-binding nuclear Ran | 258 | 2.46E-50 | 95.3 | F:GTP binding; C:nucleus; P:nucleocytoplasmic transport; P:small GTPase mediated signal transduction; F:GTPase activity; P:intracellular protein transport |
| Efet.01.11750.g866.t1 | acetylcholine receptor subunit alpha-like | 1134 | 6.60E-166 | 69.85 | C:postsynaptic membrane; P:cation transmembrane transport; C:integral component of membrane; C:cell junction; F:extracellular ligand-gated ion channel activity |
| Efet.01.11753.g867.t1 | zinc finger Gfi-1b | 411 | 1.84E-27 | 90.4 | F:nucleic acid binding; F:metal ion binding; P:hemopoiesis; P:regulation of transcription, DNA-templated; P:regulation of histone H3-K4 methylation |
| Efet.01.117532.g908.t1 | hypothetical protein HELRODRAFT_162326 | 780 | 9.06E-17 | 67.5 | F:calcium-activated potassium channel activity; P:potassium ion transmembrane transport; C:integral component of membrane |
| Efet.01.117537.g909.t1 | E3 ubiquitin- ligase RFWD3 | 225 | 2.09E-08 | 53.55 | F:protein binding; P:cellular macromolecule metabolic process; F:catalytic activity; P:regulation of response to stress; P:regulation of cell cycle; P:cellular response to DNA damage stimulus; P:primary metabolic process; C:nucleoplasm |
| Efet.01.117547.g910.t1 | Lysophospholipid acyltransferase | 339 | 2.94E-15 | 57.1 | F:transferase activity, transferring acyl groups; C:membrane; C:integral component of membrane; F:transferase activity |
| Efet.01.117617.g913.t1 | guanine nucleotide-binding G(q) subunit alpha | 507 | 1.09E-89 | 83.95 | F:GTP binding; F:G-protein coupled receptor binding; P:G-protein coupled receptor signaling pathway; F:GTPase activity; F:G-protein beta/gamma-subunit complex binding; F:signal transducer activity |
| Efet.01.117621.g914.t1 | WD repeat-containing 43-like | 735 | 1.32E-21 | 58.7 | P:angiogenesis; C:membrane; C:integral component of membrane; P:ribosome biogenesis; P:hematopoietic stem cell differentiation; P:vasculogenesis; C:intracellular; P:embryonic viscerocranium morphogenesis |
| Efet.01.117688.g915.t1 | hypothetical protein HELRODRAFT_185820 | 219 | 3.31E-10 | 82 | F:iron ion binding; F:oxidoreductase activity; C:integral component of membrane; P:oxidation-reduction process; P:fatty acid biosynthetic process |
| Efet.01.117723.g916.t1 | EH domain-containing 1 | 948 | 2.09E-176 | 88.2 | F:GTP binding; F:calcium ion binding; C:intracellular; P:endocytic recycling |
| Efet.01.117731.g917.t1 | leucine-rich repeat-containing 15-like isoform X7 | 1755 | 7.90E-139 | 44.9 | C:extracellular space; P:axonogenesis; C:membrane; C:integral component of membrane; C:proteinaceous extracellular matrix |
| Efet.01.1178.g107.t1 | sex peptide receptor-like | 1122 | 9.73E-87 | 64.4 | F:G-protein coupled receptor activity; P:G-protein coupled receptor signaling pathway; C:membrane |
| Efet.01.117865.g919.t1 | alpha-actinin isoform X1 | 297 | 2.52E-30 | 75.3 | F:actin filament binding; F:calcium ion binding; P:protein localization to bicellular tight junction; P:bicellular tight junction assembly |
| Efet.01.1179.g108.t1 | zinc finger MIZ domain-containing 1-like isoform X1 | 522 | 1.93E-07 | 75.8 | F:zinc ion binding |
| Efet.01.117912.g920.t1 | slit homolog 3 -like | 1824 | 4.62E-74 | 49.65 | C:membrane; C:integral component of membrane |
| Efet.01.117934.g922.t1 | hypothetical protein CAPTEDRAFT_204754 | 621 | 8.72E-24 | 53.45 | C:membrane; C:integral component of membrane; F:sulfotransferase activity |
| Efet.01.117940.g923.t1 | Calcium-transporting ATPase sarcoplasmic endoplasmic reticulum type | 435 | 4.94E-53 | 72.45 | F:nucleotide binding; P:calcium ion transport; F:ion binding; C:endoplasmic reticulum; C:membrane part; F:hydrolase activity |
| Efet.01.117982.g924.t1 | chromodomain helicase DNA binding | 357 | 2.06E-60 | 81.7 | P:positive regulation by host of viral transcription; C:nucleus; F:DNA binding; F:ATP binding; P:chromatin remodeling; C:cytoplasm; F:methylated histone binding; C:membrane; F:helicase activity |
| Efet.01.117985.g925.t1 | hypothetical protein CAPTEDRAFT_211886 | 657 | 1.14E-47 | 51.8 | C:membrane; C:integral component of membrane; F:carbohydrate binding |
| Efet.01.11799.g871.t1 | Casein kinase I isoform | 336 | 2.34E-30 | 95.2 | P:regulation of cell shape; C:nuclear speck; P:Golgi organization; P:intermediate filament cytoskeleton organization; C:condensed chromosome kinetochore; F:peptide binding; F:magnesium ion binding; P:cell division; F:ATP binding; C:keratin filament; C:intracellular ribonucleoprotein complex; C:mRNA cleavage and polyadenylation specificity factor complex; C:cytosol; P:Wnt signaling pathway; F:glycoprotein binding; P:cell morphogenesis; P:mitotic nuclear division; P:regulation of GTP binding; F:protein serine/threonine kinase activity; C:beta-catenin destruction complex; P:negative regulation of canonical Wnt signaling pathway; C:centrosome; C:membrane; P:phagocytosis; F:phosphoprotein binding; P:peptidyl-serine phosphorylation; P:positive regulation of proteasomal ubiquitin-dependent protein catabolic process |
| Efet.01.118004.g927.t1 | forkhead box K2 | 519 | 1.41E-26 | 64.1 | F:DNA binding; F:transcription factor activity, sequence-specific DNA binding; P:regulation of transcription, DNA-templated |
| Efet.01.118045.g929.t1 | tyrosine- phosphatase Lar-like isoform X1 | 654 | 1.39E-34 | 69.45 | F:protein tyrosine phosphatase activity; P:peptidyl-tyrosine dephosphorylation |
| Efet.01.118059.g931.t1 | Transmembrane 26 | 738 | 9.08E-22 | 50.1 | C:membrane; C:integral component of membrane |
| Efet.01.11807.g872.t1 | transmembrane protease serine 6 | 258 | 4.76E-11 | 60.4 | P:regulation of transcription, DNA-templated; P:negative regulation of biological process; F:hydrolase activity |
| Efet.01.118099.g932.t1 | chondroitin sulfate synthase 1-like | 1059 | 1.36E-103 | 50.95 | C:Golgi cisterna membrane; C:membrane; C:integral component of membrane; F:acetylgalactosaminyltransferase activity |
| Efet.01.118120.g936.t1 | centrosomal of 89 kDa-like | 249 | 6.66E-12 | 67.17 |  |
| Efet.01.118150.g937.t1 | teneurin-m-like isoform X1 | 1338 | 5.01E-146 | 61.05 |  |
| Efet.01.118189.g940.t1 | protocadherin alpha-11-like | 2454 | 6.97E-142 | 51.95 | F:calcium ion binding; C:membrane; C:integral component of membrane; P:cell adhesion; P:homophilic cell adhesion via plasma membrane adhesion molecules; C:plasma membrane |
| Efet.01.118190.g941.t1 | G- coupled receptor 54 | 447 | 7.49E-28 | 67.35 | F:G-protein coupled receptor activity; P:G-protein coupled receptor signaling pathway; C:integral component of membrane |
| Efet.01.118218.g942.t1 | von Willebrand factor D and EGF domain-containing | 936 | 2.75E-53 | 51.25 | C:membrane; C:integral component of membrane |
| Efet.01.11827.g874.t1 | sex peptide receptor-like | 1086 | 8.67E-85 | 65.4 | F:G-protein coupled receptor activity; F:G-protein coupled peptide receptor activity; P:neuropeptide signaling pathway; P:G-protein coupled receptor signaling pathway; C:membrane; C:integral component of membrane |
| Efet.01.118288.g943.t1 | methylmalonyl- mitochondrial | 846 | 6.30E-102 | 66.45 | C:mitochondrion; F:methylmalonyl-CoA mutase activity |
| Efet.01.118295.g945.t1 | calretinin-like | 297 | 2.49E-24 | 63.25 | C:gap junction; C:cytoplasm; F:calcium ion binding |
| Efet.01.118366.g946.t1 | hypothetical protein CAPTEDRAFT_104243, partial | 663 | 6.87E-07 | 80 | C:postsynaptic membrane; P:cation transmembrane transport; C:integral component of membrane; C:cell junction; F:extracellular ligand-gated ion channel activity |
| Efet.01.118393.g947.t1 | DDB1- and CUL4-associated factor 8-like | 891 | 2.38E-11 | 57.17 | C:nucleus; C:cytoplasm |
| Efet.01.118429.g948.t1 | hypothetical protein HELRODRAFT_172268 | 321 | 3.64E-15 | 72 | P:regulation of cellular process; P:cellular process |
| Efet.01.118434.g949.t1 | 60S ribosomal L4 | 852 | 0 | 89.95 | F:structural constituent of ribosome; C:ribosome; P:translation |
| Efet.01.118442.g950.t1 | AChain Quisqualate Bound To The D655a Mutant Of The Ligand Binding Domain Of Glua3 | 777 | 4.78E-64 | 59.2 | C:AMPA glutamate receptor complex; C:postsynaptic density; F:identical protein binding; F:kainate selective glutamate receptor activity; F:excitatory extracellular ligand-gated ion channel activity; C:neuronal cell body; C:protein complex; C:cytoplasmic part; F:AMPA glutamate receptor activity; C:intracellular organelle part; P:establishment of protein localization; C:dendritic shaft; P:protein tetramerization; C:dendritic spine; F:PDZ domain binding; P:response to lithium ion; C:vesicle; C:kainate selective glutamate receptor complex; P:positive regulation of synaptic transmission, glutamatergic; P:ionotropic glutamate receptor signaling pathway; P:receptor internalization; P:regulation of receptor recycling |
| Efet.01.11849.g876.t1 | cholecystokinin receptor type A-like | 1227 | 3.47E-38 | 68.2 | P:signal transduction; C:membrane; F:signal transducer activity |
| Efet.01.118494.g953.t1 | otoferlin isoform X8 | 648 | 1.66E-70 | 70.4 | C:membrane |
| Efet.01.11852.g877.t1 | cytoplasmic polyadenylation element-binding 2 isoform X9 | 348 | 4.53E-18 | 88.7 | F:translation repressor activity, nucleic acid binding; F:translation factor activity, RNA binding; C:synapse; C:neuron projection; F:mRNA 3'-UTR binding; P:translation; F:nucleotide binding; P:cellular response to insulin stimulus; C:nucleus; C:messenger ribonucleoprotein complex; C:cytoplasm; F:ribosome binding; P:cellular response to hypoxia; P:negative regulation of cytoplasmic translation |
| Efet.01.118523.g954.t1 | SWI SNF-related matrix-associated actin-dependent regulator of chromatin subfamily A containing DEAD H box | 312 | 4.30E-29 | 67.15 | F:ATP binding |
| Efet.01.118525.g955.t1 | replication factor C subunit 1-like | 402 | 1.17E-23 | 74.15 | C:nucleus; F:ATP binding; P:DNA repair; F:DNA clamp loader activity; P:DNA replication; C:DNA replication factor C complex |
| Efet.01.118537.g956.t1 | Rho GTPase-activating 100F | 480 | 4.76E-46 | 68.7 | P:signal transduction |
| Efet.01.11855.g878.t1 | cholecystokinin receptor type A-like | 375 | 2.92E-25 | 82.1 | P:G-protein coupled receptor signaling pathway; F:gastrin receptor activity; C:integral component of membrane |
| Efet.01.118560.g957.t1 | vigilin-like | 903 | 1.04E-88 | 66.65 | F:nucleic acid binding |
| Efet.01.118567.g958.t1 | dynein heavy chain axonemal-like | 300 | 6.46E-16 | 75.3 | F:ATPase activity; F:ATP binding; F:microtubule motor activity; C:dynein complex; P:microtubule-based movement |
| Efet.01.118611.g960.t1 | beta-1,4-galactosyltransferase galt-1-like | 885 | 2.26E-46 | 55.5 | C:membrane; C:integral component of membrane |
| Efet.01.11872.g879.t1 | translational GTPase | 357 | 4.54E-58 | 91.15 | F:GTP binding; F:GTPase activity; F:sulfate adenylyltransferase (ATP) activity |
| Efet.01.118788.g967.t1 | transmembrane 189-like | 501 | 2.24E-87 | 83.4 | C:integral component of membrane |
| Efet.01.118792.g968.t1 | kelch 35 | 1038 | 8.56E-23 | 48.2 |  |
| Efet.01.118795.g970.t1 | calcium-activated potassium channel subunit alpha-1-like isoform X2 | 375 | 1.32E-29 | 88.15 | F:calcium-activated potassium channel activity; F:voltage-gated potassium channel activity; P:potassium ion transmembrane transport; C:voltage-gated potassium channel complex |
| Efet.01.118819.g971.t1 | nuclear receptor subfamily 2 group C member 2 isoform X1 | 375 | 2.13E-18 | 80.25 | F:zinc ion binding; P:p38MAPK cascade; P:transcription, DNA-templated; P:cerebellum development; P:spermatogenesis; F:sequence-specific DNA binding; P:positive regulation of myoblast differentiation; P:positive regulation of transcription from RNA polymerase II promoter; P:positive regulation of embryonic development; P:steroid hormone mediated signaling pathway; C:nucleoplasm; P:meiotic cell cycle; F:RNA polymerase II transcription factor activity, ligand-activated sequence-specific DNA binding; F:protein heterodimerization activity; P:positive regulation of behavior; F:steroid hormone receptor activity; P:positive regulation of macromitophagy in response to mitochondrial depolarization; P:intracellular receptor signaling pathway |
| Efet.01.118824.g972.t1 | rho guanine nucleotide exchange factor 11-like isoform X10 | 540 | 1.05E-15 | 77.15 | P:regulation of Rho protein signal transduction; C:cytoplasm; F:metal ion binding; F:Rho guanyl-nucleotide exchange factor activity; P:intracellular signal transduction; P:positive regulation of GTPase activity |
| Efet.01.118853.g974.t1 | cadherin EGF LAG seven-pass G-type receptor 2-like isoform X2 | 252 | 4.34E-11 | 60.65 | F:G-protein coupled receptor activity; F:transmembrane signaling receptor activity; P:G-protein coupled receptor signaling pathway; P:signal transduction; F:calcium ion binding; C:membrane; C:integral component of membrane; P:cell surface receptor signaling pathway; P:cell adhesion; P:homophilic cell adhesion via plasma membrane adhesion molecules; F:signal transducer activity; C:plasma membrane |
| Efet.01.118863.g975.t1 | zinc finger and BTB domain-containing 24-like | 1434 | 3.27E-07 | 50.4 |  |
| Efet.01.118867.g976.t1 | voltage-gated sodium channel para | 369 | 9.04E-29 | 80.25 | P:regulation of postsynaptic membrane potential; F:calcium ion binding; P:regulation of ion transmembrane transport; C:voltage-gated sodium channel complex; P:sodium ion transmembrane transport; F:voltage-gated sodium channel activity |
| Efet.01.118871.g977.t1 | UBX domain-containing 4 | 387 | 1.03E-13 | 57.85 | C:membrane; C:integral component of membrane |
| Efet.01.11889.g880.t1 | hypothetical protein HELRODRAFT_176694 | 723 | 2.88E-73 | 59.6 | C:membrane; C:integral component of membrane |
| Efet.01.118916.g980.t1 | synaptotagmin 7 | 669 | 1.21E-31 | 79.15 | C:presynaptic membrane; P:regulation of glucagon secretion; F:phosphatidylinositol-4,5-bisphosphate binding; P:regulation of insulin secretion; C:early phagosome; C:extracellular exosome; C:terminal bouton; F:calcium ion binding; C:cytosol; P:calcium ion regulated lysosome exocytosis; P:vesicle-mediated cholesterol transport; P:synaptic vesicle recycling; F:syntaxin binding; P:phagosome-lysosome fusion; P:positive regulation of calcium ion-dependent exocytosis; C:lysosomal membrane; F:calcium-dependent phospholipid binding; C:peroxisomal membrane; F:calmodulin binding; P:calcium ion-regulated exocytosis of neurotransmitter; P:plasma membrane repair; P:regulation of bone remodeling; C:integral component of membrane; C:cell junction; C:phagocytic vesicle membrane; C:synaptic vesicle membrane; C:dense core granule; F:clathrin binding; P:regulation of phagocytosis |
| Efet.01.118922.g981.t1 | hypothetical protein CAPTEDRAFT_221374 | 2064 | 1.13E-14 | 51 |  |
| Efet.01.118933.g982.t1 | F-box LRR-repeat 7-like isoform X2 | 1527 | 7.49E-156 | 74.2 |  |
| Efet.01.11897.g882.t1 | TCF LEF | 231 | 4.97E-21 | 88.3 | C:nucleus; P:Wnt signaling pathway; P:regulation of transcription from RNA polymerase II promoter |
| Efet.01.119082.g989.t1 | RNA polymerase-associated RTF1 homolog | 684 | 7.86E-21 | 80.3 | P:histone H3-K4 trimethylation; P:stem cell population maintenance; C:nucleolus; F:single-stranded DNA binding; F:RNA binding; P:transcription elongation from RNA polymerase II promoter; P:negative regulation of transcription from RNA polymerase II promoter; P:positive regulation of transcription elongation from RNA polymerase II promoter; F:protein binding; P:endodermal cell fate commitment; P:blastocyst growth; P:positive regulation of histone H3-K4 methylation; C:Cdc73/Paf1 complex; P:Wnt signaling pathway |
| Efet.01.119092.g990.t1 | ELKS Rab6-interacting CAST family member 1-like isoform X2 | 582 | 7.47E-24 | 57.2 | C:presynaptic active zone |
| Efet.01.11918.g883.t1 | ankyrin-3 isoform X1 | 2076 | 0 | 47.15 | P:intracellular signal transduction; C:intracellular |
| Efet.01.119183.g991.t1 | long-chain-fatty-acid-- ligase ACSBG2-like | 546 | 8.74E-71 | 76.5 | P:metabolic process; F:ligase activity |
| Efet.01.119208.g992.t1 | histamine H2 receptor-like | 537 | 3.88E-14 | 69.95 | C:integral component of plasma membrane; F:G-protein coupled receptor activity; P:peptidyl-tyrosine phosphorylation; F:non-membrane spanning protein tyrosine kinase activity; P:G-protein coupled receptor signaling pathway; P:cell surface receptor signaling pathway |
| Efet.01.119214.g993.t1 | A disintegrin and metallo ase with thrombospondin motifs 2-like | 417 | 6.74E-08 | 54.4 | F:zinc ion binding; P:spermatogenesis; F:peptidase activity; F:metalloendopeptidase activity; P:skin development; C:proteinaceous extracellular matrix; F:metallopeptidase activity; P:integrin-mediated signaling pathway; P:proteolysis; P:protein processing; P:collagen fibril organization; C:extracellular matrix; P:lung development |
| Efet.01.119224.g994.t1 | hypothetical protein | 330 | 1.34E-06 | 67 |  |
| Efet.01.119224.g995.t1 | hypothetical protein HELRODRAFT_188380 | 345 | 1.34E-23 | 75.5 |  |
| Efet.01.119247.g996.t1 | pyrazinamidase nicotinamidase-like | 273 | 1.87E-11 | 84.7 | F:catalytic activity; F:calcium ion binding; P:metabolic process |
| Efet.01.11926.g884.t1 | histone acetyltransferase p300 | 618 | 7.26E-66 | 79.2 | F:histone acetyltransferase activity; F:zinc ion binding; F:transcription coactivator activity; P:regulation of transcription, DNA-templated; P:histone acetylation; C:histone acetyltransferase complex |
| Efet.01.119260.g997.t1 | mediator of RNA polymerase II transcription subunit 12 isoform X4 | 318 | 5.11E-15 | 89.45 | C:mediator complex; F:RNA polymerase II transcription cofactor activity; P:regulation of transcription from RNA polymerase II promoter |
| Efet.01.119272.g998.t1 | neuronal acetylcholine receptor subunit alpha-10 isoform X2 | 462 | 3.08E-40 | 50.95 | F:ion channel activity; P:cation transport; P:ion transmembrane transport; C:membrane part; P:single-organism process; C:plasma membrane; P:cellular process |
| Efet.01.119297.g999.t1 | hypothetical protein HELRODRAFT_159178 | 207 | 5.94E-18 | 67.6 | C:membrane |
| Efet.01.119304.g1000.t1 | heterogeneous nuclear ribonucleo K-like | 213 | 5.72E-22 | 79.4 | C:postsynaptic density; P:response to salt; P:protein sumoylation; F:RNA polymerase II core promoter proximal region sequence-specific DNA binding; P:transcription from RNA polymerase II promoter; P:negative regulation of protein binding; F:mRNA 3'-UTR binding; F:transcriptional activator activity, RNA polymerase II core promoter proximal region sequence-specific binding; C:catalytic step 2 spliceosome; F:pre-mRNA 3'-splice site binding; P:kidney development; P:cellular response to glucose stimulus; C:dendritic spine; C:cytosol; F:heat shock protein binding; F:protein domain specific binding; P:positive regulation of protein localization to cell surface; P:cerebellum development; P:regulation of intrinsic apoptotic signaling pathway in response to DNA damage by p53 class mediator; P:negative regulation of branching morphogenesis of a nerve; P:positive regulation of synaptic transmission; P:peripheral nervous system development; P:positive regulation of dendritic spine development; P:cerebral cortex development; F:lamin binding; C:axon terminus; C:nuclear chromatin; P:regulation of low-density lipoprotein particle clearance; C:protein-DNA complex; P:response to activity; P:positive regulation of dendrite extension; P:positive regulation of long-term synaptic potentiation; P:positive regulation of RNA splicing; C:podosome; C:nucleoplasm; P:mRNA splicing, via spliceosome; P:thymus development; P:negative regulation of transcription from RNA polymerase II promoter; P:cellular response to insulin stimulus; P:cellular response to amino acid stimulus; C:extracellular exosome; C:focal adhesion; P:liver development; P:negative regulation of mRNA splicing, via spliceosome; P:camera-type eye development; P:viral process; P:regulation of lipid transport by positive regulation of transcription from RNA polymerase II promoter; P:positive regulation of receptor-mediated endocytosis; F:C-rich single-stranded DNA binding; P:lung development; P:positive regulation of synapse maturation; P:positive regulation of low-density lipoprotein particle receptor biosynthetic process; F:actinin binding; P:signal transduction; P:cellular response to forskolin; P:hippocampus development; F:transcription factor binding; P:aging; P:acute-phase response; P:ovarian follicle development; P:cellular response to rapamycin; C:cell cortex; P:negative regulation of apoptotic process; C:mitochondrion; C:membrane; F:ribonucleoprotein complex binding; F:mRNA CDS binding; F:ATPase binding |
| Efet.01.119353.g1001.t1 | Ubiquitin-conjugating enzyme E2 | 273 | 4.73E-11 | 60.25 |  |
| Efet.01.119388.g1003.t1 | 5-hydroxytryptamine receptor 1A | 1614 | 9.18E-116 | 55.3 | F:G-protein coupled receptor activity; P:signal transduction; C:membrane |
| Efet.01.11940.g885.t1 | tyrosine- kinase PR2-like isoform X1 | 1743 | 9.68E-13 | 74.05 | F:ATP binding; P:peptidyl-tyrosine phosphorylation; F:protein tyrosine kinase activity |
| Efet.01.119443.g1004.t1 | hypothetical protein CAPTEDRAFT_222382 | 384 | 8.46E-20 | 63.5 |  |
| Efet.01.119475.g1005.t1 | sterile alpha motif domain-containing 9-like | 888 | 3.61E-08 | 43 |  |
| Efet.01.119475.g1006.t1 | sterile alpha motif domain-containing 9-like | 930 | 2.71E-22 | 48.67 |  |
| Efet.01.119477.g1007.t1 | neuroglian-like isoform X1 | 627 | 2.00E-32 | 52.1 | C:membrane; C:integral component of membrane |
| Efet.01.119502.g1008.t1 | glutathione S-transferase | 1101 | 1.48E-83 | 92.65 | F:transferase activity |
| Efet.01.119502.g1009.t1 | adenylosuccinate lyase | 1380 | 0 | 95.95 | F:N6-(1,2-dicarboxyethyl)AMP AMP-lyase (fumarate-forming) activity; P:'de novo' AMP biosynthetic process; P:'de novo' IMP biosynthetic process; F:(S)-2-(5-amino-1-(5-phospho-D-ribosyl)imidazole-4-carboxamido)succinate AMP-lyase (fumarate-forming) activity |
| Efet.01.119547.g1013.t1 | cAMP dependent kinase catalytic subunit | 618 | 1.73E-85 | 77.95 | F:ATP binding; F:protein serine/threonine kinase activity; P:protein phosphorylation |
| Efet.01.119550.g1014.t1 | Nuclear inhibitor of phosphatase 1 | 930 | 5.27E-114 | 69.7 |  |
| Efet.01.119612.g1015.t1 | hypothetical protein CAPTEDRAFT_220098 | 936 | 5.06E-07 | 64.5 | F:metal ion binding |
| Efet.01.119693.g1017.t1 | mitoferrin-2-like isoform X2 | 342 | 9.58E-58 | 81.1 | P:transmembrane transport; C:mitochondrion; C:integral component of membrane |
| Efet.01.119785.g1019.t1 | b(0,+)-type amino acid transporter 1 isoform X1 | 234 | 8.00E-09 | 75.53 | C:integral component of membrane; F:amino acid transmembrane transporter activity; P:amino acid transmembrane transport |
| Efet.01.119791.g1020.t1 | hypothetical protein CRN43_19005, partial | 300 | 1.73E-07 | 75 |  |
| Efet.01.11980.g888.t1 | IST1 homolog | 456 | 6.57E-10 | 72.1 | P:protein transport |
| Efet.01.11981.g889.t1 | DNA-binding inhibitor ID-3 | 345 | 3.87E-10 | 68.5 | C:nucleus; F:protein binding; F:organic cyclic compound binding; F:heterocyclic compound binding; P:negative regulation of transcription, DNA-templated; P:negative regulation of cell differentiation; P:animal organ development; P:cellular process |
| Efet.01.11985.g890.t1 | probable E3 ubiquitin- ligase HERC1 | 897 | 4.09E-98 | 70.7 | P:cerebellar Purkinje cell differentiation; F:catalytic activity; P:neuron projection development; P:negative regulation of autophagy; P:neuromuscular process controlling balance |
| Efet.01.119857.g1024.t1 | tripartite motif-containing 2-like isoform X3 | 954 | 6.65E-78 | 55.65 | F:zinc ion binding; F:metal ion binding; C:intracellular |
| Efet.01.11987.g891.t1 | calponin-2 isoform X3 | 747 | 4.66E-110 | 78.5 | F:protein binding |
| Efet.01.119872.g1025.t1 | hypothetical protein HELRODRAFT_188380 | 342 | 2.07E-08 | 55.75 |  |
| Efet.01.119901.g1026.t1 | RNA-binding 15B | 495 | 4.43E-07 | 53 |  |
| Efet.01.119901.g1027.t1 | RNA-binding 15 | 1137 | 1.85E-55 | 60.95 | F:organic cyclic compound binding; F:heterocyclic compound binding; C:cell part; P:cellular process |
| Efet.01.11992.g892.t1 | hypothetical protein CAPTEDRAFT_202279 | 879 | 2.36E-18 | 59 |  |
| Efet.01.119970.g1029.t1 | neural-cadherin 2 | 513 | 2.61E-56 | 61.95 | F:calcium ion binding; C:integral component of membrane; P:homophilic cell adhesion via plasma membrane adhesion molecules; C:plasma membrane |
| Efet.01.119974.g1030.t1 | rho-associated kinase 2-like isoform X2 | 1131 | 1.18E-35 | 53.2 | F:nucleotide binding; F:ATP binding; F:metal ion binding; F:protein kinase activity; P:phosphorylation; F:transferase activity; F:Rho GTPase binding; F:protein serine/threonine kinase activity; P:protein phosphorylation; F:kinase activity; P:intracellular signal transduction; C:intracellular |
| Efet.01.119982.g1031.t1 | pancreas transcription factor 1 subunit alpha-like | 513 | 1.78E-22 | 68.7 | F:protein dimerization activity |
| Efet.01.119991.g1033.t1 | WD repeat and HMG-box DNA-binding 1-like | 396 | 7.93E-12 | 84 | F:DNA binding |
| Efet.01.120037.g1034.t1 | PREDICTED: uncharacterized protein LOC106162403 | 375 | 3.77E-10 | 58 |  |
| Efet.01.120059.g1036.t1 | gastrula zinc finger xFG20-1-like | 2235 | 1.44E-38 | 63.25 | F:nucleic acid binding; F:metal ion binding |
| Efet.01.120090.g1037.t1 | centromere J-like | 1122 | 1.87E-54 | 84.85 |  |
| Efet.01.120093.g1038.t1 | beta-hexosaminidase subunit beta isoform X2 | 327 | 2.18E-51 | 82.25 | P:carbohydrate metabolic process; P:angiogenesis; F:beta-N-acetylhexosaminidase activity |
| Efet.01.120093.g1039.t1 | beta-hexosaminidase subunit beta-like isoform X2 | 525 | 9.30E-82 | 81 | P:carbohydrate metabolic process; P:angiogenesis; F:beta-N-acetylhexosaminidase activity |
| Efet.01.120102.g1040.t1 | Golgi apparatus 1 | 291 | 2.68E-07 | 54.45 | C:membrane; C:integral component of membrane; C:Golgi membrane |
| Efet.01.120114.g1041.t1 | von Willebrand factor A domain-containing 9 | 582 | 1.61E-45 | 64.65 | C:cytoplasm |
| Efet.01.120120.g1042.t1 | piggyBac transposable element-derived 4-like | 801 | 2.55E-82 | 58.65 |  |
| Efet.01.120161.g1043.t1 | tripartite motif-containing 2-like isoform X5 | 2526 | 3.10E-135 | 54.75 | F:zinc ion binding; F:metal ion binding; C:intracellular |
| Efet.01.120171.g1046.t1 | cap-specific mRNA (nucleoside-2 -O-)-methyltransferase 1 isoform X1 | 372 | 1.30E-25 | 59.2 | P:mRNA metabolic process; F:transferase activity; P:methylation |
| Efet.01.120224.g1048.t1 | T family of potassium channels 7-like | 390 | 1.81E-37 | 73.4 | C:integral component of plasma membrane; P:potassium ion transmembrane transport; P:stabilization of membrane potential; F:potassium ion leak channel activity |
| Efet.01.120233.g1049.t1 | frizzled-2 | 426 | 1.71E-18 | 54.6 | F:transmembrane signaling receptor activity; C:membrane; P:Wnt signaling pathway; P:animal organ morphogenesis |
| Efet.01.12024.g896.t1 | probable cationic amino acid transporter | 234 | 4.34E-12 | 81.7 | P:L-alpha-amino acid transmembrane transport; C:integral component of membrane; F:amino acid transmembrane transporter activity |
| Efet.01.120276.g1051.t1 | dynein heavy chain axonemal-like isoform X5 | 225 | 6.48E-20 | 92.15 | F:microtubule motor activity; C:dynein complex; P:microtubule-based movement |
| Efet.01.120285.g1052.t1 | PREDICTED: uncharacterized protein LOC109075683 | 225 | 5.52E-07 | 56 |  |
| Efet.01.120308.g1054.t1 | receptor-type tyrosine- phosphatase F-like isoform X1 | 324 | 4.69E-08 | 55.2 | F:phosphoprotein phosphatase activity; F:protein tyrosine phosphatase activity; P:protein dephosphorylation; C:membrane; F:phosphatase activity; C:integral component of membrane; P:dephosphorylation; P:peptidyl-tyrosine dephosphorylation; F:hydrolase activity |
| Efet.01.120315.g1055.t1 | neuronal acetylcholine receptor subunit alpha-9-like | 231 | 3.26E-10 | 74 | P:ion transmembrane transport; C:integral component of membrane; F:extracellular ligand-gated ion channel activity |
| Efet.01.12032.g897.t1 | peptidoglycan-recognition LE-like isoform X1 | 1281 | 1.06E-14 | 54.85 | F:zinc ion binding; P:innate immune response; P:peptidoglycan catabolic process; C:membrane; C:integral component of membrane; F:N-acetylmuramoyl-L-alanine amidase activity; P:immune system process; F:hydrolase activity |
| Efet.01.120355.g1059.t1 | peroxidasin homolog | 543 | 1.37E-37 | 46.5 | F:peroxidase activity; P:response to oxidative stress; F:prostaglandin-endoperoxide synthase activity; F:oxidoreductase activity; P:cellular oxidant detoxification; P:oxidation-reduction process; F:heme binding |
| Efet.01.120379.g1061.t1 | oxysterol-binding 1-like isoform X1 | 225 | 3.24E-31 | 86.95 | P:lipid transport |
| Efet.01.120426.g1063.t1 | dopamine D2-like | 819 | 6.57E-09 | 77.5 | F:G-protein coupled receptor activity; P:G-protein coupled receptor signaling pathway; C:integral component of membrane |
| Efet.01.120447.g1064.t1 | IL16_MACMU ame: Full=Pro-interleukin-16 Contains: ame: Full=Interleukin-16 Short=IL-16 ame: Full=Lymphocyte chemoattractant factor Short=LCF | 213 | 7.02E-06 | 58 | P:chemotaxis; C:nucleus; C:extracellular space; C:cytoplasm; P:transcription, DNA-templated; P:regulation of transcription, DNA-templated; C:extracellular region; F:cytokine activity |
| Efet.01.12049.g898.t1 | sarcoplasmic endoplasmic reticulum calcium ATPase 2 isoform X2 | 315 | 7.90E-40 | 92.75 | F:ATP binding; F:metal ion binding; C:integral component of membrane; P:calcium ion transmembrane transport; F:calcium-transporting ATPase activity; C:sarcoplasmic reticulum membrane |
| Efet.01.120490.g1065.t1 | spliceosome-associated | 285 | 3.88E-35 | 88.55 |  |
| Efet.01.12051.g899.t1 | Star-like isoform X1 | 399 | 6.13E-20 | 69.3 | C:membrane; C:integral component of membrane |
| Efet.01.120517.g1067.t1 | cAMP-dependent kinase type II regulatory subunit-like | 477 | 1.91E-47 | 61.65 | F:nucleotide binding; P:regulation of protein phosphorylation |
| Efet.01.120524.g1069.t1 | BTB POZ domain-containing 7 isoform X2 | 774 | 1.90E-68 | 70.9 | C:membrane |
| Efet.01.120535.g1070.t1 | hypothetical protein CAPTEDRAFT_224277 | 609 | 8.86E-41 | 55.4 | C:membrane; P:proteolysis; C:integral component of membrane; F:metallopeptidase activity |
| Efet.01.120585.g1072.t1 | obg-like ATPase | 270 | 2.03E-39 | 94.65 | F:GTP binding; F:ATPase activity; F:ATP binding; C:cytoplasm; F:ribosome binding; F:ribosomal large subunit binding |
| Efet.01.120619.g1073.t1 | FMRFamide receptor-like | 693 | 8.00E-61 | 60 | F:G-protein coupled receptor activity; C:membrane |
| Efet.01.12062.g900.t1 | hypothetical protein CAPTEDRAFT_223037 | 966 | 3.98E-09 | 59 |  |
| Efet.01.120620.g1074.t1 | atrial natriuretic peptide receptor 1-like | 765 | 1.18E-17 | 45.35 | F:G-protein coupled receptor activity; F:G-protein coupled GABA receptor activity; P:G-protein coupled receptor signaling pathway; C:membrane; C:integral component of membrane |
| Efet.01.120627.g1075.t1 | Fanconi anemia group I -like | 1176 | 6.65E-52 | 61 | P:cellular response to DNA damage stimulus |
| Efet.01.120650.g1076.t1 | Guanylate cyclase 32E | 468 | 7.48E-14 | 54.7 | F:catalytic activity; P:cellular process |
| Efet.01.120660.g1077.t1 | gonadotropin-releasing hormone II receptor-like | 684 | 2.43E-46 | 68.9 | C:integral component of plasma membrane; F:G-protein coupled receptor activity; P:G-protein coupled receptor signaling pathway; P:response to peptide; F:peptide binding; P:cellular response to hormone stimulus |
| Efet.01.120667.g1078.t1 | hypothetical protein HELRODRAFT_194205 | 1431 | 6.18E-14 | 51 | C:membrane; C:integral component of membrane; P:regulation of apoptotic process |
| Efet.01.120850.g1081.t1 | T family of potassium channels 18-like | 453 | 1.85E-38 | 63.85 | P:ion transport; C:membrane |
| Efet.01.120850.g1083.t1 | T family of potassium channels 18-like | 480 | 1.13E-44 | 67 | P:ion transport; C:membrane |
| Efet.01.120884.g1084.t1 | neuropeptide F receptor | 1053 | 3.63E-58 | 59.05 | P:signal transduction; F:signal transducer activity |
| Efet.01.120938.g1086.t1 | solute carrier family 13 member 2 | 228 | 5.22E-23 | 83.15 | P:sodium ion transport; P:transmembrane transport; C:integral component of membrane; F:transporter activity |
| Efet.01.120962.g1087.t1 | TGF-beta-activated kinase 1 and MAP3K7-binding 1 | 339 | 4.47E-30 | 71.45 | F:kinase activator activity; P:positive regulation of MAP kinase activity; P:transforming growth factor beta receptor signaling pathway; P:coronary vasculature development; C:protein complex; P:phosphate-containing compound metabolic process; C:nucleoplasm; P:cardiac septum development; P:heart morphogenesis; F:mitogen-activated protein kinase p38 binding; P:activation of MAPKKK activity; C:cytoplasm; F:catalytic activity; P:in utero embryonic development; P:aorta development; F:protein complex binding; P:lung development |
| Efet.01.120962.g1088.t1 | TGF-beta-activated kinase 1 and MAP3K7-binding 1 | 585 | 2.07E-12 | 55.85 | F:catalytic activity; P:cellular protein metabolic process; P:phosphate-containing compound metabolic process |
| Efet.01.120981.g1089.t1 | reticulon-1-A isoform X6 | 201 | 3.04E-24 | 86.5 | C:endoplasmic reticulum membrane; C:integral component of membrane |
| Efet.01.121013.g1090.t1 | cysteine-rich motor neuron 1 -like | 1332 | 3.14E-20 | 41.1 | F:peptidase inhibitor activity; F:serine-type endopeptidase inhibitor activity; P:negative regulation of catalytic activity; F:enzyme inhibitor activity; P:negative regulation of endopeptidase activity; P:negative regulation of peptidase activity |
| Efet.01.121040.g1092.t1 | glypican-5- | 753 | 1.73E-73 | 59.1 | C:membrane |
| Efet.01.121049.g1093.t1 | Allene oxide synthase-lipoxygenase | 624 | 1.64E-68 | 62.45 | F:dioxygenase activity |
| Efet.01.121050.g1094.t1 | Atrial natriuretic peptide receptor | 288 | 3.68E-41 | 72.7 | F:ATP binding; P:cGMP biosynthetic process; C:integral component of membrane; F:guanylate cyclase activity; F:protein kinase activity; P:protein phosphorylation; C:intracellular; P:intracellular signal transduction |
| Efet.01.121052.g1095.t1 | hypothetical protein CAPTEDRAFT_187679, partial | 825 | 4.43E-07 | 53 |  |
| Efet.01.121075.g1098.t1 | inositol hexakisphosphate kinase 1 isoform X2 | 396 | 1.78E-21 | 60.4 | C:nucleus; C:cytoplasm; F:inositol-1,4,5-trisphosphate 3-kinase activity; P:phosphatidylinositol phosphorylation; P:phosphorylation; C:nucleolus; F:kinase activity |
| Efet.01.121148.g1099.t1 | methionine gamma-lyase | 264 | 1.29E-18 | 70.65 | F:catalytic activity |
| Efet.01.121156.g1100.t1 | isoform E | 789 | 7.37E-22 | 46 | P:single-organism behavior; P:neuron recognition |
| Efet.01.121196.g1101.t1 | hemoglobin subunit mu-like | 732 | 4.97E-43 | 54.5 | F:oxygen binding; F:metal ion binding; P:oxygen transport; C:membrane; C:integral component of membrane; F:oxygen transporter activity; P:transport; F:heme binding |
| Efet.01.121204.g1102.t1 | glycogenin-1-like isoform X1 | 804 | 1.52E-08 | 57.67 | F:transferase activity, transferring glycosyl groups; F:transferase activity |
| Efet.01.121270.g1105.t1 | GLIPR1 1 | 498 | 4.05E-11 | 58.45 | P:signal transduction; C:membrane |
| Efet.01.121317.g1107.t1 | hypothetical protein CAPTEDRAFT_227481 | 660 | 3.68E-38 | 67 |  |
| Efet.01.121322.g1108.t1 | alpha-(1,6)-fucosyltransferase-like | 393 | 7.78E-57 | 81.55 | C:Golgi cisterna membrane; P:N-glycan processing; P:transforming growth factor beta receptor signaling pathway; P:receptor metabolic process; P:N-glycan fucosylation; P:oligosaccharide biosynthetic process; P:respiratory gaseous exchange; P:L-fucose catabolic process; P:GDP-L-fucose metabolic process; F:glycoprotein 6-alpha-L-fucosyltransferase activity; P:regulation of cellular response to oxidative stress; P:integrin-mediated signaling pathway; C:extracellular exosome; P:in utero embryonic development; C:integral component of membrane; P:regulation of gene expression; P:protein N-linked glycosylation via asparagine; F:SH3 domain binding; P:cell migration; P:protein glycosylation in Golgi |
| Efet.01.121374.g1110.t1 | sodium calcium exchanger 3-like isoform X1 | 405 | 9.87E-52 | 77.7 | P:cell communication; P:calcium ion transport; C:integral component of membrane; P:sodium ion transmembrane transport; F:calcium:sodium antiporter activity |
| Efet.01.121428.g1112.t1 | serine threonine- kinase NLK isoform X3 | 342 | 4.08E-49 | 81.55 | F:ATP binding; P:MAPK cascade; F:MAP kinase activity; C:intracellular |
| Efet.01.121470.g1113.t1 | hypothetical protein CAPTEDRAFT_203186 | 336 | 1.11E-37 | 64.05 | C:membrane |
| Efet.01.121499.g1115.t1 | von Willebrand factor A domain-containing 8 | 420 | 4.94E-30 | 80.2 | F:ATPase activity; F:ATP binding |
| Efet.01.12151.g904.t1 | DNA replication licensing factor mcm4-A-like | 612 | 8.29E-85 | 72.05 | F:nucleotide binding; P:DNA replication; F:helicase activity |
| Efet.01.121514.g1116.t1 | hypothetical protein CAPTEDRAFT_220082 | 879 | 1.79E-08 | 60 | F:calcium-activated potassium channel activity; P:potassium ion transport; P:potassium ion transmembrane transport; C:membrane; C:integral component of membrane |
| Efet.01.121515.g1117.t1 | M-phase inducer phosphatase-like | 366 | 4.70E-25 | 76.3 | C:centrosome; P:positive regulation of cellular process; F:protein tyrosine phosphatase activity; C:spindle pole; P:single-organism cellular process; P:peptidyl-tyrosine dephosphorylation |
| Efet.01.121530.g1118.t1 | ABC transporter G family member 22-like | 363 | 2.50E-11 | 57.33 | F:nucleotide binding; F:ATPase activity; F:ATP binding; F:ATPase activity, coupled to transmembrane movement of substances; P:transmembrane transport; C:membrane; C:integral component of membrane; C:plasma membrane |
| Efet.01.121546.g1120.t1 | galactoside 2-alpha-L-fucosyltransferase 2-like | 1074 | 2.27E-49 | 52.1 | P:carbohydrate metabolic process; F:transferase activity, transferring glycosyl groups; P:fucosylation; C:membrane; C:integral component of membrane; F:transferase activity; F:galactoside 2-alpha-L-fucosyltransferase activity |
| Efet.01.121554.g1121.t1 | BTB POZ domain-containing KCTD5-like | 267 | 2.69E-18 | 83.6 | P:protein homooligomerization; F:3',5'-cyclic-nucleotide phosphodiesterase activity |
| Efet.01.121554.g1122.t1 | tRNA nucleotidyltransferase poly(A) polymerase family | 303 | 1.43E-41 | 84.05 | F:tRNA adenylyltransferase activity; F:RNA binding; P:RNA processing |
| Efet.01.121571.g1123.t1 | ankyrin repeat | 1026 | 5.55E-07 | 52.33 |  |
| Efet.01.121585.g1125.t1 | hunchback transcription factor | 744 | 1.45E-14 | 68.15 | F:transcription factor activity, sequence-specific DNA binding; F:binding; P:regulation of biological process |
| Efet.01.1216.g110.t1 | dynamin-binding -like | 642 | 5.28E-39 | 54.95 | P:regulation of Rho protein signal transduction; C:cytoplasm; F:guanyl-nucleotide exchange factor activity; F:Rho guanyl-nucleotide exchange factor activity; P:intracellular signal transduction; P:positive regulation of GTPase activity |
| Efet.01.1216.g111.t1 | dynamin-binding isoform X1 | 228 | 6.46E-12 | 67.75 | P:regulation of Rho protein signal transduction; C:cytoplasm; F:Rho guanyl-nucleotide exchange factor activity; P:intracellular signal transduction; P:positive regulation of GTPase activity |
| Efet.01.121615.g1126.t1 | glutamate receptor kainate 2- | 219 | 2.45E-19 | 77.4 | P:ion transport; F:transmembrane signaling receptor activity; F:extracellular ligand-gated ion channel activity; C:synapse; C:membrane part; C:plasma membrane |
| Efet.01.12164.g906.t1 | plexin-A2-like | 603 | 8.47E-48 | 55.5 | F:transmembrane signaling receptor activity; P:cell surface receptor signaling pathway; P:central nervous system neuron differentiation; P:gland development; F:transferase activity; P:cell migration; P:cerebellum morphogenesis |
| Efet.01.121668.g1127.t1 | type 1 collagen alpha | 294 | 3.05E-14 | 81.6 | C:collagen trimer; F:extracellular matrix structural constituent |
| Efet.01.121710.g1128.t1 | proline-rich transmembrane 1-like | 792 | 1.58E-07 | 54.67 | P:response to biotic stimulus; C:membrane; C:integral component of membrane |
| Efet.01.121726.g1129.t1 | proteoglycan 4-like | 462 | 3.14E-15 | 73.33 |  |
| Efet.01.121733.g1130.t1 | hypothetical protein CRN43_00585, partial | 549 | 4.09E-15 | 60.2 |  |
| Efet.01.121746.g1131.t1 | 5-hydroxytryptamine receptor-like | 1296 | 7.36E-103 | 77.7 | C:integral component of plasma membrane; P:G-protein coupled receptor signaling pathway; P:serotonin receptor signaling pathway; F:G-protein coupled serotonin receptor activity |
| Efet.01.12178.g909.t1 | growth hormone secretagogue receptor type 1-like | 1167 | 6.86E-67 | 48.05 | F:G-protein coupled receptor activity; F:G-protein coupled peptide receptor activity; P:G-protein coupled receptor signaling pathway; P:signal transduction; C:membrane; C:integral component of membrane; F:signal transducer activity |
| Efet.01.121798.g1134.t1 | transposase domain-containing | 831 | 2.51E-36 | 58.5 |  |
| Efet.01.121812.g1135.t1 | hypothetical protein CRN43_21760 | 303 | 2.40E-12 | 57.67 |  |
| Efet.01.121829.g1136.t1 | dual specificity phosphatase 6 | 624 | 1.55E-44 | 59.05 | F:phosphoprotein phosphatase activity; P:regulation of signal transduction |
| Efet.01.121846.g1138.t1 | Homeobox-containing 1 | 339 | 1.20E-19 | 66.4 | C:nucleus; F:DNA binding; P:positive regulation of transcription, DNA-templated |
| Efet.01.121904.g1140.t1 | partitioning defective 6 homolog beta-like | 750 | 2.80E-79 | 85.6 |  |
| Efet.01.121917.g1141.t1 | FMRFamide receptor-like | 1215 | 2.28E-131 | 60.1 | F:G-protein coupled receptor activity; C:membrane |
| Efet.01.121926.g1142.t1 | calcium-activated potassium channel subunit alpha-1-like isoform X2 | 321 | 1.58E-42 | 84.5 | F:voltage-gated potassium channel activity; P:potassium ion transmembrane transport; C:voltage-gated potassium channel complex; F:large conductance calcium-activated potassium channel activity |
| Efet.01.121950.g1143.t1 | SLIT-ROBO Rho GTPase-activating 1-like isoform X4 | 312 | 6.06E-41 | 83.4 | C:cytoplasm; P:signal transduction; F:GTPase activator activity; F:Rac GTPase binding; P:negative regulation of cell migration; P:positive regulation of GTPase activity |
| Efet.01.121979.g1145.t1 | cilia- and flagella-associated 58 | 1224 | 0 | 91.55 |  |
| Efet.01.122009.g1146.t1 | L-lactate dehydrogenase | 1158 | 1.81E-133 | 67.05 | F:oxidoreductase activity; P:metabolic process; C:cell part |
| Efet.01.122024.g1148.t1 | transmembrane 132C isoform X1 | 1011 | 1.03E-34 | 68.7 | C:membrane |
| Efet.01.122168.g1152.t1 | nmrA-like family domain-containing 1 | 213 | 2.65E-11 | 68 |  |
| Efet.01.122205.g1153.t1 | unc-79 homolog | 756 | 4.07E-18 | 69.3 |  |
| Efet.01.122241.g1155.t1 | ADP-ribosylation factor GTPase-activating 2-like | 237 | 9.56E-16 | 69.25 | F:GTPase activator activity; P:positive regulation of GTPase activity |
| Efet.01.122286.g1159.t1 | fibrinolytic enzyme | 294 | 4.19E-24 | 77.4 | P:proteolysis; F:serine-type endopeptidase activity; P:phosphorylation; F:kinase activity; P:hemostasis |
| Efet.01.122310.g1160.t1 | serine- kinase ATM-like | 696 | 1.01E-11 | 70.5 | F:nucleotide binding; P:biological regulation; P:chromosome organization; P:phosphorylation; P:DNA metabolic process; P:cellular response to DNA damage stimulus; F:kinase activity |
| Efet.01.122331.g1163.t1 | sodium- and chloride-dependent glycine transporter 1-like | 360 | 2.22E-23 | 63.85 | C:membrane; F:symporter activity; P:transport |
| Efet.01.122381.g1167.t1 | hypothetical protein CAPTEDRAFT_213274 | 594 | 2.98E-18 | 62 | F:insulin receptor binding |
| Efet.01.12240.g914.t1 | FRAS1-related extracellular matrix 1 | 2616 | 3.57E-148 | 51.15 | P:cell adhesion; C:extracellular region |
| Efet.01.122416.g1168.t1 | AChain Crystal Structure Of The Bromodomain Of Human Crebbp In Complex With An Isoxazolyl-benzimidazole Ligand | 477 | 8.09E-26 | 88.35 | P:response to estrogen; C:RNA polymerase II transcription factor complex; F:RNA polymerase II core promoter sequence-specific DNA binding; P:cellular response to nerve growth factor stimulus; F:RNA polymerase II core promoter proximal region sequence-specific DNA binding; P:transcription-coupled nucleotide-excision repair; P:positive regulation of NIK/NF-kappaB signaling; P:intrinsic apoptotic signaling pathway in response to DNA damage by p53 class mediator; P:regulation of androgen receptor signaling pathway; P:regulation of smoothened signaling pathway; P:positive regulation of collagen biosynthetic process; F:protein antigen binding; P:positive regulation of muscle atrophy; P:positive regulation of protein secretion; P:positive regulation of gene expression, epigenetic; P:positive regulation of cell adhesion molecule production; P:protein-DNA complex assembly; P:positive regulation of axon extension; F:androgen receptor binding; P:response to interleukin-1; P:positive regulation of CREB transcription factor activity; P:positive regulation of cell death; F:mitogen-activated protein kinase binding; P:response to tumor necrosis factor; P:cell proliferation; P:positive regulation by host of viral transcription; P:regulation of cellular response to heat; P:stimulatory C-type lectin receptor signaling pathway; P:heart development; P:protein kinase B signaling; P:positive regulation of cell size; P:liver development; F:TFIIB-class transcription factor binding; P:response to cobalt ion; F:glucocorticoid receptor binding; P:regulation of tubulin deacetylation; F:damaged DNA binding; F:histone acetyltransferase activity; C:cytoplasm; P:cellular response to retinoic acid; P:skeletal muscle tissue development; P:positive regulation of protein phosphorylation; P:cellular response to mineralocorticoid stimulus; P:cellular response to dexamethasone stimulus; P:cell-cell adhesion; P:positive regulation of protein binding; P:positive regulation of translation; C:PML body; F:protein C-terminus binding; F:transcriptional activator activity, RNA polymerase II transcription regulatory region sequence-specific binding; P:regulation of transcription from RNA polymerase II promoter in response to hypoxia; P:positive regulation of DNA binding; P:fat cell differentiation; F:RNA polymerase II transcription coactivator activity; P:memory; F:MRF binding; P:transcription initiation from RNA polymerase II promoter; F:RNA polymerase II activating transcription factor binding; F:peroxisome proliferator activated receptor binding; P:embryonic digit morphogenesis; F:transcriptional repressor activity, RNA polymerase II core promoter proximal region sequence-specific binding; F:p53 binding; P:digestive tract development; P:cellular response to glucose stimulus; P:negative regulation of cysteine-type endopeptidase activity involved in apoptotic process; P:N-terminal peptidyl-lysine acetylation; P:platelet formation; F:protein complex binding; F:zinc ion binding; P:B cell differentiation; P:positive regulation of type I interferon production; P:regulation of signal transduction by p53 class mediator; C:condensed chromosome outer kinetochore; P:circadian rhythm; F:signal transducer activity; P:animal organ morphogenesis; F:NF-kappaB binding; F:lysine N-acetyltransferase activity, acting on acetyl phosphate as donor; C:nuclear chromatin; P:Notch signaling pathway; P:somitogenesis; P:regulation of autophagy; P:cellular response to cAMP; C:protein-DNA complex; P:cellular response to trichostatin A; P:positive regulation of histone acetylation; P:histone H4 acetylation; P:histone H3 acetylation; P:positive regulation of protein import into nucleus, translocation; P:histone H2B acetylation; P:positive regulation of glycoprotein biosynthetic process; P:cellular lipid metabolic process; P:positive regulation of proteolysis; P:beta-catenin-TCF complex assembly; P:negative regulation of transcription from RNA polymerase II promoter; P:response to ethanol; P:DNA damage response, signal transduction by p53 class mediator resulting in cell cycle arrest; P:viral process; P:cellular response to UV; P:megakaryocyte development; P:response to fatty acid; P:cellular response to hydrogen peroxide; P:lung development; F:chromatin DNA binding; P:regulation of angiotensin metabolic process; P:positive regulation of sarcomere organization; P:positive regulation of G1/S transition of mitotic cell cycle; P:cellular response to drug; P:positive regulation of transcription from RNA polymerase II promoter involved in unfolded protein response; F:beta-catenin binding; C:histone acetyltransferase complex; P:negative regulation of miRNA metabolic process; F:pre-mRNA intronic binding; F:SMAD binding; P:germ-line stem cell population maintenance; P:cellular response to hepatocyte growth factor stimulus; P:response to calcium ion; C:integral component of membrane; P:protein stabilization |
| Efet.01.122424.g1169.t1 | DNA topoisomerase 2- | 636 | 1.23E-113 | 90.05 | C:nucleoid; P:sister chromatid segregation; F:protein C-terminus binding; P:protein sumoylation; C:DNA topoisomerase complex (ATP-hydrolyzing); F:DNA binding, bending; P:resolution of meiotic recombination intermediates; P:DNA topological change; P:mitotic DNA integrity checkpoint; C:nucleolus; P:DNA unwinding involved in DNA replication; P:axon target recognition; C:nucleoplasm; F:magnesium ion binding; F:ATP binding; P:neuron migration; P:forebrain development; F:protein heterodimerization activity; C:cytosol; F:protein kinase C binding; P:retinal ganglion cell axon guidance; F:histone deacetylase binding; F:chromatin binding; P:mitotic recombination; C:heterochromatin; F:ribonucleoprotein complex binding; P:retina layer formation; F:DNA topoisomerase type II (ATP-hydrolyzing) activity |
| Efet.01.122454.g1170.t1 | polypeptide N-acetylgalactosaminyltransferase 10 | 729 | 1.83E-82 | 57.05 | C:membrane; C:Golgi apparatus; P:O-glycan processing; F:polypeptide N-acetylgalactosaminyltransferase activity |
| Efet.01.122517.g1172.t1 | peripherin-2-like isoform X2 | 360 | 3.05E-18 | 47.85 | C:membrane; C:integral component of membrane |
| Efet.01.122517.g1173.t1 | Photoreceptor outer segment membrane glyco 2 | 780 | 4.45E-20 | 45.05 | C:membrane; C:integral component of membrane; P:visual perception |
| Efet.01.122538.g1175.t1 | U5 small nuclear ribonucleo 200 kDa helicase | 561 | 1.47E-96 | 80.7 | F:ATP binding; F:identical protein binding; C:catalytic step 2 spliceosome; C:membrane; C:U5 snRNP; P:osteoblast differentiation; C:viral nucleocapsid; C:nucleoplasm; F:ATP-dependent RNA helicase activity; F:RNA binding; P:cis assembly of pre-catalytic spliceosome |
| Efet.01.122559.g1176.t1 | tripartite motif-containing 2-like | 2718 | 1.18E-59 | 54.8 | F:zinc ion binding; F:metal ion binding; C:intracellular |
| Efet.01.122617.g1179.t1 | 26S proteasome non-ATPase regulatory subunit 2-like | 222 | 2.73E-14 | 84.75 | C:nucleus; P:proteasome-mediated ubiquitin-dependent protein catabolic process; C:proteasome regulatory particle, base subcomplex; P:regulation of catalytic activity; F:endopeptidase activity; P:regulation of protein catabolic process; C:proteasome storage granule; F:enzyme regulator activity |
| Efet.01.122620.g1180.t1 | Tetratricopeptide repeat 28 | 3708 | 6.71E-123 | 50.2 |  |
| Efet.01.122718.g1182.t1 | serine threonine- kinase WNK3 | 1284 | 5.93E-16 | 86.85 | F:ATP binding; P:regulation of ion homeostasis; P:positive regulation of calcium ion transport; C:cytosol; F:protein serine/threonine kinase activity; P:protein phosphorylation; P:positive regulation of ion transmembrane transporter activity; P:intracellular signal transduction |
| Efet.01.122724.g1183.t1 | ETS translocation variant 1-like | 501 | 2.35E-27 | 85.4 | P:regulation of branching involved in mammary gland duct morphogenesis; P:motor neuron axon guidance; F:RNA polymerase II core promoter proximal region sequence-specific DNA binding; P:transcription from RNA polymerase II promoter; C:nucleolus; P:positive regulation of transcription from RNA polymerase II promoter; P:branching involved in mammary gland duct morphogenesis; P:peripheral nervous system neuron development; C:nucleoplasm; F:transcriptional activator activity, RNA polymerase II core promoter proximal region sequence-specific binding; F:protein binding; P:stem cell differentiation; P:negative regulation of mammary gland epithelial cell proliferation |
| Efet.01.12283.g918.t1 | Cleavage and polyadenylation specificity factor subunit 2 | 1566 | 0 | 64.5 | P:oxidation-reduction process; F:succinate dehydrogenase activity; C:intracellular part |
| Efet.01.122843.g1186.t1 | suppressor of lurcher 1-like | 270 | 2.80E-25 | 71.15 | C:integral component of membrane |
| Efet.01.122900.g1187.t1 | hypothetical protein RF11_15787 | 501 | 3.44E-28 | 61.08 |  |
| Efet.01.122920.g1189.t1 | endoribonuclease Dicer-like | 324 | 9.18E-32 | 66.4 | F:nucleic acid binding; P:nucleic acid phosphodiester bond hydrolysis; P:posttranscriptional gene silencing by RNA; P:ncRNA metabolic process; P:cellular macromolecule catabolic process; P:production of small RNA involved in gene silencing by RNA; P:nucleobase-containing compound catabolic process; F:endoribonuclease activity, producing 5'-phosphomonoesters; C:intracellular part |
| Efet.01.122927.g1190.t1 | synaptotagmin-1-like isoform X2 | 345 | 6.85E-28 | 70.9 | C:exocytic vesicle; P:calcium ion-regulated exocytosis of neurotransmitter; F:syntaxin binding; P:vesicle fusion; F:calcium ion binding; F:clathrin binding; P:regulation of calcium ion-dependent exocytosis; C:plasma membrane; F:calcium-dependent phospholipid binding |
| Efet.01.122965.g1192.t1 | hypothetical protein CAPTEDRAFT_220857 | 252 | 1.92E-07 | 59 |  |
| Efet.01.12297.g919.t1 | Coiled-coil domain-containing | 621 | 8.30E-37 | 58.2 |  |
| Efet.01.122985.g1193.t1 | eukaryotic translation initiation factor 4B-like | 1302 | 1.54E-08 | 66.88 | F:RNA strand-exchange activity; P:cytoplasmic translation; F:RNA strand annealing activity; F:ribosomal small subunit binding; P:translational initiation; P:eukaryotic translation initiation factor 4F complex assembly; C:polysome; F:nucleotide binding; F:nucleic acid binding; F:translation initiation factor activity; P:formation of translation preinitiation complex; F:RNA binding; C:eukaryotic translation initiation factor 4F complex |
| Efet.01.122992.g1194.t1 | sorting nexin-1- | 408 | 1.12E-36 | 65.15 | F:protein homodimerization activity; F:epidermal growth factor receptor binding; F:phosphatidylinositol binding; P:positive regulation of protein catabolic process; F:insulin receptor binding; P:intracellular protein transport; F:leptin receptor binding; C:early endosome membrane; F:cadherin binding involved in cell-cell adhesion; P:vesicle organization; C:cell-cell adherens junction; F:protein heterodimerization activity; C:retromer, tubulation complex; C:intracellular membrane-bounded organelle; P:lamellipodium morphogenesis; F:transferrin receptor binding; C:cytosol; P:early endosome to Golgi transport; C:extrinsic component of membrane; P:receptor internalization; P:negative regulation of transforming growth factor beta receptor signaling pathway |
| Efet.01.123031.g1196.t1 | torsin- | 1209 | 1.75E-45 | 51.55 | C:nuclear envelope; F:ATPase activity; F:ATP binding; C:endoplasmic reticulum lumen; C:extracellular exosome; C:cytoplasm; P:protein homooligomerization; P:chaperone mediated protein folding requiring cofactor; P:nuclear membrane organization; C:endoplasmic reticulum; P:endoplasmic reticulum organization |
| Efet.01.123091.g1197.t1 | Arrestin domain-containing 2 | 651 | 2.91E-07 | 54.4 |  |
| Efet.01.123115.g1199.t1 | receptor-type tyrosine- phosphatase delta-like | 759 | 8.53E-08 | 40 | F:phosphoprotein phosphatase activity; F:protein tyrosine phosphatase activity; C:membrane; P:protein dephosphorylation; F:phosphatase activity; C:integral component of membrane; P:dephosphorylation; P:peptidyl-tyrosine dephosphorylation; F:hydrolase activity |
| Efet.01.123153.g1200.t1 | probable global transcription activator SNF2L2 isoform X1 | 768 | 6.85E-85 | 78.2 | F:ATPase activity; C:SWI/SNF complex; F:ATP binding; P:chromatin remodeling; F:histone binding; P:regulation of transcription, DNA-templated |
| Efet.01.123170.g1201.t1 | aspartyl asparaginyl beta-hydroxylase-like | 210 | 8.50E-36 | 90.2 | P:peptidyl-amino acid modification; C:integral component of membrane |
| Efet.01.123179.g1202.t1 | monocarboxylate transporter 2-like | 525 | 4.64E-21 | 53.75 | P:transmembrane transport; C:membrane; C:integral component of membrane |
| Efet.01.123185.g1203.t1 | S-acyltransferase 24-like | 393 | 2.38E-20 | 64.2 | F:transferase activity |
| Efet.01.123197.g1204.t1 | GTPase-activating and VPS9 domain-containing 1-like | 378 | 2.69E-49 | 80.75 | P:signal transduction; P:regulation of GTPase activity |
| Efet.01.123223.g1205.t1 | hypothetical protein BOX15_Mlig000533g1 | 222 | 2.55E-07 | 58.67 |  |
| Efet.01.123223.g1206.t1 | sidekick-2 isoform X1 | 897 | 8.40E-50 | 56.6 | C:membrane; C:integral component of membrane |
| Efet.01.123250.g1209.t1 | steroid 17-alpha-hydroxylase 17,20 lyase-like | 756 | 1.38E-40 | 53.9 | F:monooxygenase activity |
| Efet.01.123299.g1210.t1 | neuronal acetylcholine receptor subunit alpha-10-like | 318 | 4.48E-29 | 86.75 | C:postsynaptic membrane; P:cation transmembrane transport; C:integral component of membrane; C:cell junction; F:extracellular ligand-gated ion channel activity |
| Efet.01.123351.g1211.t1 | zinc finger 84-like | 1989 | 4.88E-31 | 46.25 | F:nucleic acid binding; F:metal ion binding |
| Efet.01.123373.g1212.t1 | Sarcoplasmic calcium-binding | 372 | 1.63E-53 | 63.05 | F:calcium ion binding |
| Efet.01.123375.g1213.t1 | probable Bax inhibitor 1 | 354 | 2.49E-22 | 65.45 | P:negative regulation of apoptotic process; C:membrane; C:integral component of membrane |
| Efet.01.123398.g1214.t1 | Dynein heavy chain axonemal | 369 | 4.40E-63 | 90.3 | F:ATPase activity; F:ATP binding; F:microtubule motor activity; C:integral component of membrane; C:dynein complex; P:microtubule-based movement |
| Efet.01.123452.g1216.t1 | peroxisomal fatty acid beta-oxidation multifunctional AIM1-like | 699 | 2.15E-74 | 53.45 | F:zinc ion binding; F:oxidoreductase activity; F:3-hydroxyacyl-CoA dehydrogenase activity; F:metal ion binding; P:oxidation-reduction process; P:fatty acid metabolic process |
| Efet.01.12346.g920.t1 | glycine receptor subunit alpha-3-like isoform X1 | 219 | 5.02E-26 | 71.9 | P:ion transmembrane transport; C:integral component of membrane; C:cell junction; F:extracellular ligand-gated ion channel activity; C:synapse; C:plasma membrane |
| Efet.01.123478.g1219.t1 | T family of potassium channels 18-like | 498 | 1.50E-11 | 63.5 | P:potassium ion transmembrane transport; C:integral component of membrane; F:potassium channel activity |
| Efet.01.123532.g1220.t1 | N,N -diacetylchitobiase | 330 | 2.56E-13 | 68.2 | F:hydrolase activity, hydrolyzing O-glycosyl compounds; F:carbohydrate binding |
| Efet.01.123558.g1222.t1 | PREDICTED: uncharacterized protein LOC106176837 | 2013 | 8.68E-14 | 42.25 | F:metal ion binding |
| Efet.01.123569.g1224.t1 | gastrula zinc finger -like isoform X2 | 732 | 8.58E-70 | 67.8 | F:nucleic acid binding; P:transmembrane transport; F:transmembrane transporter activity; F:metal ion binding; C:membrane; C:integral component of membrane; F:methyltransferase activity; F:transferase activity; F:transporter activity; P:methylation |
| Efet.01.123603.g1225.t1 | malate cytoplasmic-like | 273 | 2.09E-40 | 78.4 | P:carbohydrate metabolic process; F:L-malate dehydrogenase activity; P:malate metabolic process; P:tricarboxylic acid cycle |
| Efet.01.12365.g921.t1 | sodium channel para-like isoform X4 | 714 | 3.46E-14 | 96.45 | P:regulation of postsynaptic membrane potential; F:zinc ion binding; F:calcium ion binding; P:proteolysis; P:regulation of ion transmembrane transport; C:voltage-gated sodium channel complex; P:sodium ion transmembrane transport; F:metalloendopeptidase activity; C:proteinaceous extracellular matrix; F:voltage-gated sodium channel activity |
| Efet.01.123652.g1227.t1 | fructose-1,6-bisphosphatase 1-like | 234 | 6.07E-38 | 83.8 | P:carbohydrate metabolic process; F:fructose 1,6-bisphosphate 1-phosphatase activity; P:dephosphorylation |
| Efet.01.12368.g922.t1 | homeobox-containing 1-like | 762 | 2.98E-09 | 76.6 | C:nucleus; F:DNA binding; P:positive regulation of transcription, DNA-templated |
| Efet.01.123704.g1228.t1 | growth hormone secretagogue receptor type 1-like | 990 | 3.75E-71 | 54.7 | F:G-protein coupled receptor activity; F:G-protein coupled peptide receptor activity; P:G-protein coupled receptor signaling pathway; P:signal transduction; C:membrane; C:integral component of membrane; F:signal transducer activity |
| Efet.01.12375.g923.t1 | hypothetical protein CAPTEDRAFT_186654 | 1059 | 2.21E-23 | 45.5 | F:G-protein coupled receptor activity; P:G-protein coupled receptor signaling pathway; C:membrane; C:integral component of membrane |
| Efet.01.123792.g1231.t1 | Rho GTPase-activating 100F | 1332 | 8.04E-42 | 74.35 | P:signal transduction |
| Efet.01.123795.g1232.t1 | phosphatase 1 regulatory subunit 3B isoform X2 | 1113 | 1.33E-33 | 47.95 | P:carbohydrate metabolic process; C:protein phosphatase type 1 complex; P:glycogen metabolic process; P:regulation of catalytic activity; P:regulation of glycogen catabolic process; F:protein phosphatase regulator activity |
| Efet.01.123835.g1233.t1 | collagen alpha-1(XIV) chain-like [Orbicella faveolata] | 414 | 2.50E-17 | 51.22 | F:chitin binding; P:chitin metabolic process; C:extracellular region |
| Efet.01.123863.g1235.t1 | thyrotropin-releasing hormone receptor-like | 876 | 3.87E-15 | 49.7 | F:G-protein coupled receptor activity; P:neuropeptide signaling pathway; P:G-protein coupled receptor signaling pathway; P:signal transduction; C:membrane; C:integral component of membrane; F:signal transducer activity |
| Efet.01.123920.g1238.t1 | hypothetical protein HELRODRAFT_161626 | 834 | 1.13E-69 | 58.15 | F:phosphatidylinositol phospholipase C activity; P:signal transduction; C:membrane; P:lipid metabolic process; C:integral component of membrane; P:intracellular signal transduction; C:intracellular |
| Efet.01.123958.g1239.t1 | solute carrier organic anion transporter family member 2B1-like | 948 | 1.19E-16 | 51.6 | C:membrane; C:integral component of membrane; P:transport; F:transporter activity |
| Efet.01.12399.g924.t1 | short transient receptor potential channel 6 | 696 | 3.40E-79 | 70.5 | F:calcium channel activity; C:integral component of membrane; P:calcium ion transmembrane transport; P:single-organism process |
| Efet.01.124003.g1240.t1 | voltage- gated calcium | 1221 | 1.88E-42 | 62.65 | P:transmembrane transport; F:calcium channel activity; C:membrane; P:calcium ion transport; F:voltage-gated ion channel activity |
| Efet.01.124036.g1241.t1 | all-trans-retinol 13,14-reductase | 402 | 1.18E-49 | 72.9 | F:oxidoreductase activity; C:integral component of membrane; P:oxidation-reduction process; F:binding |
| Efet.01.124056.g1242.t1 | zinc finger 664-like isoform X2 | 330 | 2.59E-16 | 56.15 | F:nucleic acid binding; P:biological_process; F:metal ion binding |
| Efet.01.124091.g1243.t1 | cysteinyl leukotriene receptor 2-like | 1170 | 4.98E-33 | 50.2 | F:G-protein coupled receptor activity; P:G-protein coupled receptor signaling pathway; C:membrane; C:integral component of membrane |
| Efet.01.12410.g925.t1 | pre-mRNA-splicing factor syf2-like | 507 | 4.17E-57 | 84.05 | C:prespliceosome; C:catalytic step 2 spliceosome; C:post-mRNA release spliceosomal complex; C:catalytic step 1 spliceosome; C:Prp19 complex; P:mRNA splicing, via spliceosome |
| Efet.01.124113.g1244.t1 | protocadherin alpha-8 isoform X12 | 738 | 9.40E-41 | 59.75 | F:calcium ion binding; C:membrane; C:integral component of membrane; P:cell adhesion; P:homophilic cell adhesion via plasma membrane adhesion molecules; C:plasma membrane |
| Efet.01.124124.g1245.t1 | Krueppel-like factor 1 | 1470 | 1.24E-51 | 80.85 | F:nucleic acid binding; F:metal ion binding |
| Efet.01.124138.g1246.t1 | hu-li tai shao | 570 | 1.16E-42 | 64.7 |  |
| Efet.01.124194.g1247.t1 | Shroom-like isoform X1 | 351 | 1.22E-09 | 60.5 | F:actin filament binding |
| Efet.01.124196.g1248.t1 | exostosin-like 2 | 255 | 7.50E-09 | 65.9 | P:heparan sulfate proteoglycan biosynthetic process; C:integral component of membrane; P:glycosaminoglycan biosynthetic process; C:Golgi apparatus; F:transferase activity |
| Efet.01.124202.g1249.t1 | nucleolar 4-like isoform X2 | 288 | 4.25E-33 | 95.2 |  |
| Efet.01.124209.g1250.t1 | spondin-2-like | 594 | 1.80E-19 | 61.95 | C:membrane; C:integral component of membrane; F:arylesterase activity; F:hydrolase activity |
| Efet.01.124299.g1252.t1 | elongation factor 1-beta | 903 | 1.51E-69 | 60.9 | F:translation elongation factor activity; P:translational elongation; C:eukaryotic translation elongation factor 1 complex; P:translation |
| Efet.01.124318.g1254.t1 | Two pore potassium channel sup-9 | 645 | 6.99E-63 | 73.65 | P:potassium ion transmembrane transport; C:integral component of membrane; F:potassium channel activity |
| Efet.01.124372.g1257.t1 | uncharacterized protein LOC111108228 isoform X2 | 852 | 3.99E-38 | 51.6 |  |
| Efet.01.124388.g1258.t1 | orexin receptor type 2-like | 303 | 9.72E-33 | 82.3 | C:integral component of plasma membrane; P:peptidyl-tyrosine phosphorylation; P:neuropeptide signaling pathway; F:non-membrane spanning protein tyrosine kinase activity; P:feeding behavior; P:response to peptide; F:peptide binding; P:cellular response to hormone stimulus; F:orexin receptor activity; F:neuropeptide Y receptor activity |
| Efet.01.12448.g926.t1 | beta-1,3-galactosyltransferase 1-like | 1161 | 2.71E-141 | 64.15 | F:transferase activity, transferring glycosyl groups; C:membrane; C:Golgi apparatus |
| Efet.01.124504.g1263.t1 | growth differentiation factor 8-like | 498 | 2.21E-10 | 71.5 | F:growth factor activity; C:extracellular region; P:growth |
| Efet.01.124508.g1264.t1 | calcium-transporting ATPase sarcoplasmic endoplasmic reticulum type-like isoform X4 | 627 | 2.14E-85 | 78.55 | F:ATP binding; F:metal ion binding; C:integral component of membrane; P:calcium ion transmembrane transport; F:calcium-transporting ATPase activity; C:sarcoplasmic reticulum membrane |
| Efet.01.124511.g1265.t1 | calmodulin | 480 | 6.42E-31 | 64.5 | F:metal ion binding; F:calcium ion binding |
| Efet.01.12452.g927.t1 | integrator complex subunit 6-like | 354 | 1.13E-22 | 72.2 |  |
| Efet.01.124546.g1266.t1 | SLIT-ROBO Rho GTPase-activating 1-like isoform X4 | 2091 | 4.95E-68 | 70.6 | P:signal transduction |
| Efet.01.124548.g1267.t1 | dnaJ homolog subfamily A member 1 | 285 | 6.78E-27 | 70.45 | F:protein binding; F:ion binding; C:cytosol |
| Efet.01.124548.g1268.t1 | dnaJ homolog subfamily A member 1 isoform X2 | 768 | 4.01E-116 | 74.05 | F:C3HC4-type RING finger domain binding; P:negative regulation of JUN kinase activity; P:response to unfolded protein; C:cytoplasmic side of endoplasmic reticulum membrane; F:G-protein coupled receptor binding; F:ATP binding; P:response to heat; C:extracellular exosome; F:low-density lipoprotein particle receptor binding; P:regulation of protein transport; P:protein localization to mitochondrion; F:chaperone binding; C:cytosol; P:negative regulation of protein ubiquitination; P:androgen receptor signaling pathway; F:Hsp70 protein binding; P:spermatogenesis; F:unfolded protein binding; P:protein folding; P:toxin transport; F:ubiquitin protein ligase binding; P:flagellated sperm motility; C:ubiquitin ligase complex; F:metal ion binding; P:negative regulation of apoptotic process; P:positive regulation of apoptotic process |
| Efet.01.124579.g1271.t1 | uncharacterized protein LOC111120476 isoform X3 | 822 | 3.27E-16 | 51.3 | C:membrane; C:integral component of membrane |
| Efet.01.124583.g1272.t1 | venom -2 | 222 | 6.12E-13 | 58.2 | C:membrane; C:integral component of membrane |
| Efet.01.124604.g1274.t1 | probable cytochrome P450 CYP44 | 255 | 8.72E-07 | 51 |  |
| Efet.01.124679.g1279.t1 | ankyrin-3-like isoform X13 | 339 | 4.09E-16 | 60.1 | P:signal transduction |
| Efet.01.124738.g1280.t1 | hypothetical protein HELRODRAFT_163366 | 504 | 1.47E-44 | 66.95 | C:integral component of membrane; F:carbohydrate binding |
| Efet.01.12482.g929.t1 | homocysteine-responsive endoplasmic reticulum-resident ubiquitin-like domain member 2 isoform X1 | 822 | 6.93E-16 | 42.05 | P:spermatogenesis; C:membrane; C:integral component of membrane |
| Efet.01.124832.g1284.t1 | Transcription factor AP-2-epsilon | 315 | 1.68E-25 | 100 | C:nucleus; F:transcription factor activity, sequence-specific DNA binding; P:regulation of transcription, DNA-templated |
| Efet.01.124892.g1286.t1 | hypothetical protein CAPTEDRAFT_218786 | 1386 | 3.87E-11 | 47 | P:intracellular signal transduction; C:intracellular |
| Efet.01.124933.g1287.t1 | E3 ubiquitin- ligase HECTD4 | 684 | 3.26E-92 | 79.35 | C:nucleus; F:ubiquitin-protein transferase activity; C:cytoplasm; P:glucose homeostasis; C:integral component of membrane; P:glucose metabolic process; F:ligase activity; P:protein ubiquitination involved in ubiquitin-dependent protein catabolic process |
| Efet.01.124949.g1288.t1 | allatostatin-C neuropeptide precursor | 258 | 3.34E-11 | 82 | P:neuropeptide signaling pathway |
| Efet.01.124954.g1289.t1 | kalirin isoform X5 | 285 | 4.68E-37 | 89.45 | F:ATP binding; P:regulation of Rho protein signal transduction; P:transmembrane receptor protein tyrosine phosphatase signaling pathway; P:positive regulation of apoptotic process; C:cytosol; F:Rho guanyl-nucleotide exchange factor activity; F:protein serine/threonine kinase activity; P:protein phosphorylation; P:intracellular signal transduction; P:positive regulation of GTPase activity; F:enzyme binding |
| Efet.01.124977.g1290.t1 | WD repeat-containing 86-like isoform X1 | 228 | 1.50E-11 | 68.5 |  |
| Efet.01.124977.g1291.t1 | WD repeat-containing 86-like | 564 | 9.43E-70 | 69.65 |  |
| Efet.01.125030.g1.t1 | monocarboxylate transporter 14-like | 732 | 2.09E-26 | 58.95 | P:transmembrane transport; C:membrane; C:integral component of membrane |
| Efet.01.12504.g931.t1 | transmembrane 211-like | 597 | 2.58E-53 | 57.45 | C:membrane; C:integral component of membrane |
| Efet.01.125089.g5.t1 | Blood vessel epicardial substance | 519 | 1.68E-31 | 57.9 | P:regulation of cell shape; C:lateral plasma membrane; P:cell migration involved in heart development; P:hematopoietic progenitor cell differentiation; P:positive regulation of locomotion; P:sinoatrial node cell development; P:regulation of heart rate; C:caveola; C:bicellular tight junction; F:protein binding; P:response to ischemia; P:regulation of membrane potential; P:positive regulation of receptor recycling; C:sarcolemma; P:vesicle docking; P:regulation of GTPase activity; C:integral component of membrane; P:substrate adhesion-dependent cell spreading; C:cell projection membrane; F:cAMP binding; P:regulation of endocytic recycling |
| Efet.01.125093.g6.t1 | glucose-fructose oxidoreductase domain-containing 1-like | 1017 | 3.84E-174 | 71.3 | F:oxidoreductase activity; P:oxidation-reduction process |
| Efet.01.12513.g933.t1 | hypothetical protein CAPTEDRAFT_226427 | 1266 | 3.16E-44 | 44.4 | F:calcium ion binding; C:membrane; C:integral component of membrane |
| Efet.01.12513.g934.t1 | hypothetical protein CAPTEDRAFT_226427 | 594 | 1.95E-30 | 50.67 | F:calcium ion binding; C:membrane; C:integral component of membrane |
| Efet.01.125155.g7.t1 | collagen alpha-1(XII) chain-like | 1098 | 5.88E-55 | 52.4 | C:collagen trimer |
| Efet.01.125188.g9.t1 | DC-STAMP domain-containing 2-like | 801 | 8.10E-30 | 46.86 | C:membrane; C:integral component of membrane |
| Efet.01.125196.g10.t1 | uncharacterized family 31 glucosidase KIAA1161-like | 1845 | 0 | 62.7 | P:metabolic process; F:binding; F:hydrolase activity |
| Efet.01.125217.g12.t1 | patched isoform X2 | 522 | 2.25E-12 | 60.65 | C:membrane |
| Efet.01.125233.g13.t1 | protocadherin-11 X-linked-like | 2313 | 4.28E-124 | 49.95 | F:calcium ion binding; C:membrane; C:integral component of membrane; P:cell adhesion; P:homophilic cell adhesion via plasma membrane adhesion molecules; C:plasma membrane |
| Efet.01.125243.g14.t1 | PDZ and LIM domain 7-like | 207 | 8.68E-08 | 84 |  |
| Efet.01.125255.g15.t1 | dynein beta ciliary-like | 597 | 1.58E-105 | 89.05 | F:ATPase activity; F:ATP binding; C:motile cilium; F:microtubule motor activity; C:cytoplasm; P:cell projection organization; C:dynein complex; P:microtubule-based movement; C:microtubule |
| Efet.01.125278.g16.t1 | PREDICTED: uncharacterized protein LOC109051156 | 807 | 1.26E-07 | 47.6 |  |
| Efet.01.125281.g17.t1 | collagen alpha-3(VI) chain-like isoform X9 | 393 | 6.59E-25 | 58.65 | F:calcium ion binding |
| Efet.01.125292.g18.t1 | ubiquitin carboxyl-terminal hydrolase 34-like | 1353 | 9.31E-25 | 62.7 | P:proteolysis; P:cellular protein metabolic process |
| Efet.01.125339.g19.t1 | laminin subunit gamma-1-like | 468 | 9.53E-42 | 70.25 | P:single-organism process |
| Efet.01.125340.g20.t1 | WD repeat-containing on Y chromosome-like | 414 | 1.32E-36 | 68.4 | F:calcium ion binding |
| Efet.01.125379.g21.t1 | Neuropeptide FF receptor 2 | 645 | 8.66E-22 | 69 | C:integral component of plasma membrane; F:neuropeptide binding; P:neuropeptide signaling pathway; P:G-protein coupled receptor signaling pathway, coupled to cyclic nucleotide second messenger; F:transferase activity; C:neuron projection; F:neuropeptide Y receptor activity |
| Efet.01.12538.g935.t1 | hypothetical protein HELRODRAFT_172421 | 1068 | 0 | 74.15 | F:catalytic activity; P:nucleoside metabolic process |
| Efet.01.125383.g22.t1 | muscleblind 1 isoform X1 | 390 | 1.29E-24 | 68.75 | F:metal ion binding |
| Efet.01.125413.g24.t1 | tripartite motif-containing 2-like | 2013 | 6.95E-114 | 41.25 | F:zinc ion binding; F:metal ion binding; C:intracellular |
| Efet.01.125459.g26.t1 | Appr-1-p processing | 1176 | 1.07E-20 | 58.75 | F:zinc ion binding; F:metal ion binding; P:phosphorylation; F:kinase activity |
| Efet.01.125496.g27.t1 | neuralized isoform X2 | 399 | 1.14E-25 | 68.5 |  |
| Efet.01.125507.g29.t1 | transient receptor potential cation channel subfamily M member 2-like isoform X3 | 321 | 1.53E-10 | 57.4 | P:ion transport; P:transmembrane transport |
| Efet.01.125508.g30.t1 | palmitoyltransferase ZDHHC22 | 1035 | 1.54E-86 | 50.8 | F:transferase activity |
| Efet.01.125550.g33.t1 | pre-mRNA-processing-splicing factor 8 | 441 | 1.15E-83 | 76.1 | F:snRNA binding |
| Efet.01.125554.g34.t1 | hypothetical protein HELRODRAFT_175651 | 939 | 2.10E-07 | 44 |  |
| Efet.01.125565.g35.t1 | NFX1-type zinc finger-containing 1-like | 2040 | 4.12E-20 | 47.75 | C:nucleus; F:transcription factor activity, sequence-specific DNA binding; F:zinc ion binding; F:metal ion binding; C:membrane; C:integral component of membrane; P:regulation of transcription, DNA-templated |
| Efet.01.125602.g37.t1 | prickle 3 isoform X4 | 363 | 4.00E-74 | 62.85 | F:metal ion binding |
| Efet.01.12564.g936.t1 | V-type proton ATPase subunit | 528 | 3.65E-38 | 76.9 | C:vacuolar proton-transporting V-type ATPase, V1 domain; P:ATP hydrolysis coupled proton transport; F:proton-transporting ATPase activity, rotational mechanism |
| Efet.01.12565.g937.t1 | ADP-ribosylation factor-binding GGA1-like | 291 | 9.20E-16 | 66.45 | P:vesicle-mediated transport; C:clathrin adaptor complex; P:intracellular protein transport |
| Efet.01.125671.g39.t1 | ubiquitin- ligase E3A | 864 | 3.09E-150 | 82.5 | F:ubiquitin protein ligase activity; P:positive regulation of phosphatidylinositol 3-kinase signaling; P:protein K48-linked ubiquitination; C:proteasome complex; P:prostate gland growth; P:brain development; P:regulation of circadian rhythm; P:positive regulation of transcription from RNA polymerase II promoter; P:protein ubiquitination involved in ubiquitin-dependent protein catabolic process; P:ovarian follicle development; C:nucleus; F:protein binding; F:transcription coactivator activity; F:metal ion binding; P:viral process; P:positive regulation of protein ubiquitination; P:regulation of protein ubiquitination involved in ubiquitin-dependent protein catabolic process; C:cytosol; P:androgen receptor signaling pathway; P:sperm entry; F:ligase activity; P:protein autoubiquitination |
| Efet.01.125724.g44.t1 | extracellular sulfatase Sulf-1 homolog | 243 | 2.46E-16 | 74.5 | F:hydrolase activity, acting on ester bonds; P:cellular metabolic process |
| Efet.01.125754.g45.t1 | FRAS1-related extracellular matrix 1-like isoform X1 | 813 | 5.59E-50 | 56.7 | P:cell adhesion; C:proteinaceous extracellular matrix |
| Efet.01.12578.g939.t1 | flotillin 1 | 306 | 6.86E-50 | 94.5 |  |
| Efet.01.125784.g48.t1 | 4-(cytidine 5 -diphospho)-2-C-methyl-D-erythritol kinase | 738 | 9.36E-128 | 84.1 | F:ATP binding; P:isopentenyl diphosphate biosynthetic process, methylerythritol 4-phosphate pathway; F:4-(cytidine 5'-diphospho)-2-C-methyl-D-erythritol kinase activity; P:phosphorylation; P:terpenoid biosynthetic process |
| Efet.01.125816.g49.t1 | FTS and Hook-interacting -like isoform X2 | 894 | 1.63E-30 | 50.7 |  |
| Efet.01.125837.g50.t1 | NF-X1-type zinc finger NFXL1-like | 249 | 1.08E-08 | 74.65 | C:nucleus; F:transcriptional repressor activity, RNA polymerase II core promoter proximal region sequence-specific binding; F:zinc ion binding; C:integral component of membrane; F:RNA polymerase II regulatory region sequence-specific DNA binding; P:regulation of transcription, DNA-templated; P:transcription from RNA polymerase II promoter |
| Efet.01.12593.g940.t1 | Tetratricopeptide repeat | 975 | 7.55E-47 | 68.2 |  |
| Efet.01.12593.g941.t1 | toxin-antitoxin system family antitoxin | 531 | 1.77E-47 | 64.25 |  |
| Efet.01.125957.g53.t1 | hypothetical protein CAPTEDRAFT_205146 | 405 | 1.02E-19 | 70.5 | C:membrane |
| Efet.01.125993.g54.t1 | HEAT repeat-containing 5B isoform X1 | 438 | 1.64E-28 | 85.05 |  |
| Efet.01.125993.g55.t1 | HEAT repeat-containing 5B-like isoform X1 | 669 | 1.05E-79 | 60.95 |  |
| Efet.01.126000.g56.t1 | serine threonine- kinase tousled-like 2 isoform X2 | 213 | 5.07E-14 | 98.2 | F:ATP binding; F:protein serine/threonine kinase activity; P:protein phosphorylation |
| Efet.01.126049.g57.t1 | glycine receptor subunit alpha-3-like isoform X1 | 309 | 1.96E-22 | 67.45 | P:ion transport; C:membrane |
| Efet.01.126065.g58.t1 | 5-aminolevulinate mitochondrial | 291 | 7.70E-39 | 74.4 | F:pyridoxal phosphate binding; C:mitochondrial matrix; P:protoporphyrinogen IX biosynthetic process; F:5-aminolevulinate synthase activity |
| Efet.01.126086.g59.t1 | patronin isoform X8 | 1398 | 1.65E-30 | 56.6 | F:calmodulin binding; F:spectrin binding; P:neuron projection development; F:microtubule binding |
| Efet.01.126117.g60.t1 | B9 domain-containing | 504 | 1.78E-70 | 76.45 |  |
| Efet.01.126124.g61.t1 | histone | 387 | 1.68E-67 | 97.9 | F:DNA binding; C:nuclear chromatin; F:protein heterodimerization activity; P:chromatin silencing; C:nucleosome |
| Efet.01.126146.g62.t1 | autocrine proliferation repressor A-like | 339 | 4.99E-15 | 67 | C:membrane; C:integral component of membrane |
| Efet.01.126148.g63.t1 | tumor necrosis factor alpha-induced 3 | 1950 | 2.44E-25 | 48.2 | P:protein deubiquitination |
| Efet.01.126150.g64.t1 | orphan G- coupled receptor 51 | 1272 | 6.54E-67 | 55.85 | F:G-protein coupled receptor activity; F:G-protein coupled peptide receptor activity; P:G-protein coupled receptor signaling pathway; C:membrane; C:integral component of membrane |
| Efet.01.126151.g65.t1 | inner nuclear membrane Man1 | 354 | 1.16E-45 | 79.2 | F:nucleotide binding; P:negative regulation of BMP signaling pathway; F:DNA binding; F:protein binding; C:integral component of nuclear inner membrane; P:negative regulation of activin receptor signaling pathway; P:negative regulation of transforming growth factor beta receptor signaling pathway |
| Efet.01.126172.g68.t1 | hypothetical protein HELRODRAFT_176332 | 828 | 4.36E-08 | 52 |  |
| Efet.01.12620.g942.t1 | Serine threonine- kinase SMG1 | 222 | 4.37E-06 | 62 |  |
| Efet.01.126259.g73.t1 | serine arginine-rich splicing factor 6-like | 294 | 6.94E-17 | 61.47 |  |
| Efet.01.126264.g74.t1 | one cut domain family member 2-like isoform X1 | 975 | 3.93E-61 | 66.2 | C:nucleus; F:DNA binding; P:transcription, DNA-templated; P:regulation of transcription, DNA-templated |
| Efet.01.12627.g943.t1 | tetratricopeptide repeat 30A | 420 | 2.84E-63 | 83.1 |  |
| Efet.01.126276.g75.t1 | hypothetical protein HELRODRAFT_168488 | 1263 | 3.57E-107 | 53.15 | C:membrane; C:integral component of membrane; F:sulfotransferase activity |
| Efet.01.126359.g77.t1 | hypothetical protein HELRODRAFT_192338 | 1167 | 6.09E-11 | 71 | F:nucleic acid binding; F:RNA binding; P:RNA processing |
| Efet.01.12636.g944.t1 | oxysterol-binding 2-like | 942 | 1.01E-81 | 60.65 | P:lipid transport; P:transport |
| Efet.01.12640.g945.t1 | hypothetical protein HELRODRAFT_169358 | 1020 | 1.93E-06 | 66 |  |
| Efet.01.126405.g78.t1 | hypothetical protein HELRODRAFT_192371 | 288 | 7.39E-09 | 72.57 | F:nucleic acid binding; F:metal ion binding |
| Efet.01.12642.g946.t1 | hypothetical protein CAPTEDRAFT_192952 | 294 | 2.31E-16 | 65 |  |
| Efet.01.126432.g81.t1 | dynein heavy chain axonemal-like | 12351 | 0 | 74.85 | F:nucleotide binding; F:nucleoside-triphosphatase activity; C:intracellular; P:cellular process |
| Efet.01.126439.g82.t1 | phytanoyl- hydroxylase-interacting -like isoform X2 | 582 | 5.12E-35 | 57.25 | C:membrane; C:integral component of membrane |
| Efet.01.12644.g947.t1 | 26S proteasome non-ATPase regulatory subunit 10-like | 1224 | 1.18E-52 | 54.45 |  |
| Efet.01.126447.g84.t1 | steroid 17-alpha-hydroxylase 17,20 lyase-like | 762 | 1.01E-59 | 62.2 | F:oxidoreductase activity; F:binding |
| Efet.01.126454.g85.t1 | Zinc finger and BTB domain-containing 10 | 1284 | 5.94E-18 | 64.39 | F:nucleic acid binding; F:metal ion binding |
| Efet.01.126454.g86.t1 | hypothetical protein CAPTEDRAFT_77208, partial | 285 | 1.78E-10 | 93 | F:nucleic acid binding; F:metal ion binding |
| Efet.01.126460.g87.t1 | receptor-type tyrosine- phosphatase O isoform X3 | 603 | 1.84E-20 | 47.8 | F:phosphoprotein phosphatase activity; F:protein tyrosine phosphatase activity; C:membrane; P:protein dephosphorylation; C:integral component of membrane; F:phosphatase activity; P:dephosphorylation; P:peptidyl-tyrosine dephosphorylation; F:hydrolase activity |
| Efet.01.126465.g89.t1 | hypothetical protein CAPTEDRAFT_204171 | 561 | 6.89E-45 | 58.65 |  |
| Efet.01.126469.g90.t1 | twitchin-like isoform X1 | 648 | 5.66E-88 | 57.5 | F:nucleotide binding; P:phosphorylation; F:kinase activity |
| Efet.01.126489.g92.t1 | usherin-like isoform X2 | 744 | 3.50E-75 | 63.05 | C:membrane |
| Efet.01.126489.g93.t1 | usherin-like isoform X2 | 618 | 1.00E-49 | 56.8 | C:membrane; C:integral component of membrane |
| Efet.01.126495.g94.t1 | phosphorylase b kinase gamma catalytic skeletal muscle heart isoform isoform X1 | 213 | 1.59E-21 | 71.7 | F:nucleotide binding; P:cellular macromolecule metabolic process; C:membrane; P:phosphorylation; F:protein serine/threonine kinase activity; P:primary metabolic process |
| Efet.01.1265.g113.t1 | casein kinase I-like | 849 | 1.21E-124 | 79.65 | F:ATP binding; F:protein serine/threonine kinase activity; P:protein phosphorylation |
| Efet.01.126569.g95.t1 | water dikinase | 654 | 3.07E-133 | 91.35 | F:ATP binding; P:phosphorylation; F:kinase activity |
| Efet.01.126609.g98.t1 | pleckstrin homology-like domain family B member 1 isoform X1 | 873 | 1.24E-82 | 70.6 |  |
| Efet.01.126610.g99.t1 | ephrin type-B receptor 1-B-like isoform X4 | 765 | 7.05E-64 | 60.9 | P:phosphorylation; F:kinase activity |
| Efet.01.126673.g103.t1 | zinc finger Y-chromosomal 2-like | 555 | 2.81E-17 | 54.65 | F:nucleic acid binding; F:metal ion binding |
| Efet.01.126712.g104.t1 | PRA1 family 2 | 339 | 1.20E-41 | 80.85 | C:integral component of membrane |
| Efet.01.126743.g105.t1 | complement factor H | 573 | 7.28E-15 | 45.9 |  |
| Efet.01.126778.g106.t1 | growth differentiation factor 11 | 492 | 7.89E-09 | 49.67 |  |
| Efet.01.12683.g951.t1 | ubiquitin conjugation factor E4 B | 693 | 4.27E-112 | 68.3 | F:ubiquitin protein ligase activity; C:intracellular part; P:single-organism process; P:response to stimulus; P:ubiquitin-dependent protein catabolic process; P:protein ubiquitination |
| Efet.01.126884.g111.t1 | potassium voltage-gated channel Shaw-like isoform X1 | 1032 | 4.08E-99 | 68.25 | F:ion channel activity; P:ion transport; C:membrane |
| Efet.01.126895.g112.t1 | zinc finger 236-like isoform X1 | 3444 | 2.02E-38 | 61.7 | F:nucleic acid binding; F:metal ion binding |
| Efet.01.126906.g115.t1 | 1-phosphatidylinositol 3-phosphate 5-kinase isoform X1 | 387 | 3.63E-17 | 57.5 | P:cellular process |
| Efet.01.126965.g117.t1 | prolactin-releasing peptide receptor-like | 1026 | 7.11E-120 | 62.35 | P:signal transduction; F:signal transducer activity |
| Efet.01.126968.g118.t1 | zinc metallo ase | 714 | 6.02E-71 | 59.45 |  |
| Efet.01.126987.g119.t1 | 39S ribosomal mitochondrial | 285 | 4.66E-14 | 65.25 | C:intracellular organelle lumen; C:mitochondrion; C:ribosome; C:intracellular organelle part; P:translation |
| Efet.01.127057.g121.t1 | hypothetical protein HELRODRAFT_188458 | 1011 | 1.36E-83 | 64 |  |
| Efet.01.127082.g122.t1 | ankyrin-3-like isoform X10 | 336 | 8.04E-47 | 75.4 |  |
| Efet.01.127085.g123.t1 | 2 ,3 -cyclic-nucleotide 3 -phosphodiesterase-like | 786 | 2.30E-55 | 61.5 | C:cytoplasm; C:membrane; P:cyclic nucleotide catabolic process; F:2',3'-cyclic-nucleotide 3'-phosphodiesterase activity |
| Efet.01.12709.g952.t1 | malectin-B-like isoform X1 | 291 | 2.23E-09 | 90.14 | C:integral component of membrane |
| Efet.01.127111.g124.t1 | SET and MYND domain-containing 4-like | 2046 | 0 | 51 |  |
| Efet.01.127169.g125.t1 | leucine rich repeat | 1464 | 1.48E-33 | 54.4 |  |
| Efet.01.127202.g127.t1 | AChain Crystal Structure Of Clasp2 Tog Domain (tog3) | 453 | 1.99E-61 | 80.95 |  |
| Efet.01.127209.g129.t1 | kelch 9 | 1677 | 0 | 57.7 | C:integral component of membrane; P:single-organism cellular process |
| Efet.01.127230.g131.t1 | transcription factor AP-2-epsilon-like isoform X1 | 351 | 4.10E-48 | 81.95 | C:nucleus; F:RNA polymerase II regulatory region sequence-specific DNA binding; P:anatomical structure development; P:regulation of cell proliferation; F:RNA polymerase II transcription factor activity, sequence-specific DNA binding; P:regulation of transcription from RNA polymerase II promoter |
| Efet.01.127243.g132.t1 | coiled-coil domain-containing 186-like isoform X1 | 636 | 2.88E-34 | 65.9 |  |
| Efet.01.127299.g134.t1 | endoplasmic reticulum mannosyl-oligosaccharide 1,2-alpha- | 459 | 2.72E-50 | 85.55 | C:extracellular vesicle; P:trimming of terminal mannose on B branch; F:calcium ion binding; C:integral component of membrane; C:endoplasmic reticulum quality control compartment; F:mannosyl-oligosaccharide 1,2-alpha-mannosidase activity; P:ER-associated ubiquitin-dependent protein catabolic process; P:mannose trimming involved in glycoprotein ERAD pathway |
| Efet.01.127336.g135.t1 | wiskott-Aldrich syndrome family member 3- | 291 | 1.38E-27 | 72.8 | C:cytoskeleton; F:actin binding; P:actin cytoskeleton organization |
| Efet.01.127396.g136.t1 | A disintegrin and metallo ase with thrombospondin motifs 7-like | 513 | 2.40E-61 | 66.95 | F:peptidase activity |
| Efet.01.127425.g137.t1 | tetratricopeptide repeat 17-like | 747 | 4.29E-61 | 69.8 |  |
| Efet.01.127471.g139.t1 | ras-related Rab-9A-like | 543 | 5.42E-79 | 75.2 | F:GTP binding; P:small GTPase mediated signal transduction; P:retrograde transport, endosome to Golgi; C:intracellular |
| Efet.01.12748.g954.t1 | endoribonuclease Dicer-like | 405 | 1.02E-54 | 68.6 | P:RNA phosphodiester bond hydrolysis, endonucleolytic; F:RNA binding; F:ribonuclease III activity; P:rRNA catabolic process; P:RNA processing |
| Efet.01.127568.g144.t1 | sodium-dependent glucose transporter 1A-like | 1323 | 4.97E-46 | 58.65 | P:transmembrane transport; C:membrane; C:integral component of membrane; P:carbohydrate transport |
| Efet.01.12763.g955.t1 | beta-N-acetylhexosaminidase | 501 | 1.23E-36 | 65.85 | F:hydrolase activity, hydrolyzing O-glycosyl compounds |
| Efet.01.12768.g956.t1 | beta-1,3-galactosyltransferase 1-like | 1026 | 1.15E-41 | 53.7 | F:transferase activity, transferring glycosyl groups; C:membrane; C:integral component of membrane; P:protein glycosylation; F:galactosyltransferase activity; C:Golgi membrane; C:Golgi apparatus; F:transferase activity |
| Efet.01.127686.g147.t1 | specificity 1 | 807 | 2.44E-13 | 71.25 | F:nucleic acid binding; F:metal ion binding |
| Efet.01.12769.g957.t1 | synaptotagmin 7 | 288 | 8.28E-43 | 79.35 | C:integral component of membrane; P:exocytosis |
| Efet.01.127714.g149.t1 | excitatory amino acid transporter-like isoform X1 | 228 | 1.45E-16 | 74.75 | P:dicarboxylic acid transport; P:transmembrane transport; F:sodium:dicarboxylate symporter activity; C:integral component of membrane; P:larval locomotory behavior |
| Efet.01.12775.g958.t1 | PREDICTED: protein lin-41-like | 822 | 1.28E-08 | 47 |  |
| Efet.01.12775.g960.t1 | dual specificity tyrosine-phosphorylation-regulated kinase 2 | 1524 | 0 | 76.8 | P:regulation of signal transduction by p53 class mediator; P:smoothened signaling pathway; P:intrinsic apoptotic signaling pathway in response to DNA damage by p53 class mediator; F:protein serine/threonine kinase activity; P:protein phosphorylation; P:positive regulation of glycogen biosynthetic process; C:nucleoplasm; F:magnesium ion binding; F:ATP binding; F:protein tyrosine kinase activity; C:ubiquitin ligase complex; F:ubiquitin binding; C:cytoplasm; F:manganese ion binding; P:negative regulation of NFAT protein import into nucleus |
| Efet.01.127766.g152.t1 | Four and a half LIM domains 2 | 588 | 6.06E-85 | 75.25 | F:zinc ion binding; F:protein domain specific binding; C:membrane; C:M band |
| Efet.01.127775.g154.t1 | pelota homolog | 249 | 2.61E-22 | 84.2 | F:retroviral 3' processing activity; P:nucleic acid phosphodiester bond hydrolysis; P:ribosome disassembly; F:T/G mismatch-specific endonuclease activity; F:phosphoric ester hydrolase activity; P:cell cycle; P:translation; P:cell division; P:nuclear-transcribed mRNA catabolic process, non-stop decay; C:nucleus; F:protein binding; C:cytoplasm; F:metal ion binding; P:nonfunctional rRNA decay; P:RNA surveillance; F:ribosome binding; P:nuclear-transcribed mRNA catabolic process, no-go decay |
| Efet.01.127823.g155.t1 | gonadotropin-releasing hormone II receptor-like | 1404 | 1.58E-135 | 65.75 | F:G-protein coupled receptor activity; P:response to organic substance; P:G-protein coupled receptor signaling pathway; P:response to endogenous stimulus; C:integral component of membrane; C:plasma membrane |
| Efet.01.127827.g156.t1 | probable E3 ubiquitin- ligase HECTD4 | 927 | 5.20E-85 | 70.7 | F:catalytic activity |
| Efet.01.127827.g157.t1 | probable E3 ubiquitin- ligase HECTD4 | 2955 | 0 | 56.15 | C:nucleus; F:ubiquitin-protein transferase activity; C:cytoplasm; C:membrane; C:integral component of membrane; P:protein ubiquitination involved in ubiquitin-dependent protein catabolic process; F:ligase activity; P:protein ubiquitination |
| Efet.01.127835.g158.t1 | rod shape-determining | 423 | 9.50E-43 | 67.35 | C:membrane |
| Efet.01.127835.g159.t1 | rod shape-determining | 870 | 0 | 91.7 | P:cell morphogenesis |
| Efet.01.127898.g161.t1 | selenocysteine insertion sequence-binding 2-like isoform X1 | 849 | 2.76E-15 | 63.17 | F:GTP binding; P:small GTPase mediated signal transduction; C:membrane; C:integral component of membrane; C:intracellular |
| Efet.01.127911.g162.t1 | G patch domain and ankyrin repeat-containing 1 | 1872 | 1.57E-12 | 51 |  |
| Efet.01.127939.g163.t1 | kinase C and casein kinase substrate in neurons 1 isoform X1 | 240 | 1.94E-25 | 77.1 | P:phosphorylation; F:kinase activity |
| Efet.01.127944.g164.t1 | histone acetyltransferase KAT8 | 216 | 4.95E-25 | 78.2 | P:transcription, DNA-templated; F:methylated histone binding; P:histone H4-K5 acetylation; F:histone acetyltransferase activity (H4-K16 specific); C:kinetochore; F:transcription factor binding; P:histone H4-K8 acetylation; F:histone acetyltransferase activity (H4-K8 specific); F:histone acetyltransferase activity (H4-K5 specific); P:histone H4-K16 acetylation; F:nucleic acid binding; P:negative regulation of transcription, DNA-templated; C:MSL complex; F:metal ion binding; P:positive regulation of transcription, DNA-templated; P:regulation of autophagy; P:myeloid cell differentiation; C:MLL1 complex; F:enzyme binding |
| Efet.01.127955.g165.t1 | cilia- and flagella-associated 57-like | 432 | 5.27E-62 | 82.8 | C:integral component of membrane |
| Efet.01.127962.g166.t1 | leucine-rich repeat neuronal 1-like | 1665 | 1.04E-50 | 47.2 | P:peptidyl-tyrosine phosphorylation; F:ATP binding; F:protein tyrosine kinase activity; C:membrane; C:integral component of membrane; F:protein kinase activity; P:phosphorylation; P:protein phosphorylation; F:kinase activity |
| Efet.01.127972.g167.t1 | polypeptide N-acetylgalactosaminyltransferase 10 | 276 | 3.21E-24 | 83.25 | F:transferase activity, transferring glycosyl groups; C:integral component of membrane; P:protein glycosylation; C:Golgi membrane; F:carbohydrate binding |
| Efet.01.128014.g168.t1 | transcription elongation regulator 1 | 423 | 1.65E-41 | 92.95 |  |
| Efet.01.128015.g169.t1 | inactive tyrosine- kinase transmembrane receptor ROR1-like | 1410 | 2.95E-111 | 71.85 | F:nucleotide binding; F:protein tyrosine kinase activity; C:membrane; P:protein phosphorylation |
| Efet.01.128095.g172.t1 | tectonin beta-propeller repeat-containing 2-like isoform X1 | 2253 | 6.18E-24 | 56.8 |  |
| Efet.01.128111.g173.t1 | synaptotagmin-12-like | 285 | 1.52E-23 | 69.9 | P:regulation of neurotransmitter secretion; F:protein binding; P:vesicle-mediated transport; C:integral component of membrane; F:ion binding; C:cell part; P:long-term synaptic potentiation; P:single-organism cellular process |
| Efet.01.128190.g175.t1 | synaptogyrin-3-like isoform 2 | 264 | 8.92E-29 | 70.8 | F:catalytic activity; C:integral component of membrane; C:synapse |
| Efet.01.128203.g176.t1 | hypothetical protein HELRODRAFT_178410 | 477 | 1.83E-08 | 70.5 |  |
| Efet.01.128219.g178.t1 | transcription factor hamlet-like | 1086 | 6.32E-53 | 70.4 | F:nucleic acid binding; F:metal ion binding; P:regulation of transcription, DNA-templated |
| Efet.01.128237.g179.t1 | tripartite motif-containing 2-like isoform X5 | 2262 | 0 | 68.1 | F:metal ion binding |
| Efet.01.128245.g180.t1 | laminin subunit beta-1-like | 1155 | 1.71E-46 | 70.15 | F:catalytic activity |
| Efet.01.128250.g181.t1 | hypothetical protein CAPTEDRAFT_214965 | 807 | 1.01E-39 | 49.85 |  |
| Efet.01.128253.g182.t1 | 2-acylglycerol O-acyltransferase 2-A-like | 456 | 3.66E-48 | 68.2 | F:transferase activity |
| Efet.01.128293.g186.t1 | ras-related Rap-2c-like | 540 | 2.14E-90 | 85.95 | F:GTP binding; P:small GTPase mediated signal transduction; C:membrane; C:intracellular |
| Efet.01.128323.g188.t1 | rho GTPase-activating 100F-like isoform X1 | 723 | 1.63E-63 | 72.05 | P:signal transduction |
| Efet.01.128333.g190.t1 | kinesin KIF13A | 210 | 2.64E-30 | 95.95 | F:ATP binding; F:microtubule motor activity; P:plus-end-directed vesicle transport along microtubule; C:integral component of membrane; C:microtubule; F:microtubule binding |
| Efet.01.12835.g966.t1 | tRNA modification GTPase mitochondrial-like | 363 | 1.54E-19 | 60.6 | F:GTP binding; F:nucleotide binding; P:tRNA processing; F:GTPase activity; P:tRNA modification; C:intracellular |
| Efet.01.12835.g967.t1 | calcium calmodulin-dependent kinase kinase 1-like isoform X4 | 426 | 3.93E-24 | 58.45 | F:transferase activity |
| Efet.01.128390.g192.t1 | shaker cognate | 474 | 8.19E-33 | 73.55 | P:potassium ion transport; P:transmembrane transport; C:integral component of membrane; F:voltage-gated ion channel activity; F:potassium channel activity; P:cellular process; F:metallopeptidase activity |
| Efet.01.128428.g193.t1 | hypothetical protein HELRODRAFT_176722 | 1323 | 3.18E-08 | 56 | F:metal ion binding |
| Efet.01.128485.g199.t1 | hypothetical protein CAPTEDRAFT_129833, partial | 297 | 3.17E-08 | 82.5 |  |
| Efet.01.128495.g200.t1 | Krueppel-like factor 5 | 2004 | 9.36E-07 | 90 | F:nucleic acid binding; F:metal ion binding |
| Efet.01.128518.g201.t1 | ribosomal S6 kinase delta-1-like | 816 | 1.58E-21 | 54.25 | F:ATP binding; F:phosphatidylinositol binding; F:protein kinase activity; P:phosphorylation; P:protein phosphorylation; F:kinase activity |
| Efet.01.128524.g202.t1 | neuroligin- X-linked-like | 1002 | 9.80E-13 | 56.4 | C:integral component of plasma membrane; P:synapse organization; F:neurexin family protein binding; C:membrane; C:integral component of membrane; P:cell adhesion; C:synapse |
| Efet.01.12854.g968.t1 | uncharacterized protein LOC111118921 | 1002 | 5.34E-51 | 50.45 | P:signal transduction |
| Efet.01.128550.g203.t1 | hypothetical protein CAPTEDRAFT_195133 | 2661 | 0 | 55.8 | C:membrane |
| Efet.01.128562.g204.t1 | zinc finger CCCH domain-containing 10-like | 1143 | 6.52E-65 | 54.3 | F:metal ion binding |
| Efet.01.12857.g969.t1 | ephrin type-B receptor 1-B-like isoform X4 | 642 | 1.86E-101 | 72.2 | F:nucleotide binding; F:protein tyrosine kinase activity; C:membrane; P:protein phosphorylation; F:hydrolase activity, acting on glycosyl bonds |
| Efet.01.128586.g205.t1 | hypothetical protein CAPTEDRAFT_217943 | 381 | 8.47E-11 | 61.5 | C:membrane; C:integral component of membrane |
| Efet.01.128591.g206.t1 | tudor domain-containing 1 | 1458 | 1.06E-21 | 44.15 |  |
| Efet.01.128654.g207.t1 | hypothetical protein CAPTEDRAFT_192539 | 354 | 1.72E-38 | 63.78 |  |
| Efet.01.128690.g209.t1 | macrophage mannose receptor 1-like isoform X1 | 327 | 2.92E-08 | 60.7 | C:membrane; C:integral component of membrane; F:carbohydrate binding |
| Efet.01.128724.g210.t1 | golgin subfamily A member 3-like | 1017 | 2.17E-17 | 54.71 | C:extrinsic component of Golgi membrane; P:spermatogenesis |
| Efet.01.128756.g212.t1 | hypothetical protein CAPTEDRAFT_223751 | 420 | 6.47E-34 | 72.85 | C:integral component of membrane; F:carbohydrate binding |
| Efet.01.128805.g214.t1 | rho GTPase-activating 32- | 375 | 1.30E-53 | 82.85 | P:signal transduction; F:phosphatidylinositol binding |
| Efet.01.128861.g217.t1 | whirlin-like isoform X1 | 723 | 9.35E-29 | 48.5 | P:sensory perception of sound; P:sensory perception of light stimulus |
| Efet.01.128888.g218.t1 | heterogeneous nuclear ribonucleo Q isoform X3 | 423 | 3.30E-40 | 74.7 | F:organic cyclic compound binding; F:heterocyclic compound binding |
| Efet.01.128955.g221.t1 | exportin-4 isoform X2 | 372 | 1.40E-06 | 59.5 | C:nucleus; C:cytoplasm; P:protein transport; P:transport |
| Efet.01.128967.g222.t1 | lysine-specific demethylase 5A | 504 | 2.50E-08 | 42 |  |
| Efet.01.128981.g223.t1 | ethanolamine-phosphate phospho-lyase isoform X2 | 264 | 3.46E-15 | 77.3 | F:pyridoxal phosphate binding; P:phosphatidylethanolamine biosynthetic process; C:mitochondrial matrix; F:transaminase activity; F:ethanolamine-phosphate phospho-lyase activity |
| Efet.01.128998.g224.t1 | Protocadherin Fat 4 | 1335 | 5.59E-106 | 49.35 | F:calcium ion binding; C:membrane; C:integral component of membrane; P:cell adhesion; P:homophilic cell adhesion via plasma membrane adhesion molecules; C:plasma membrane |
| Efet.01.1290.g115.t1 | mitochondrial substrate carrier family | 396 | 4.91E-39 | 66.4 | F:structural constituent of ribosome; C:membrane; P:transport; P:translation |
| Efet.01.129079.g226.t1 | serine protease 33-like | 399 | 1.75E-12 | 56.55 | F:hydrolase activity |
| Efet.01.129137.g228.t1 | cilia- and flagella-associated 45-like | 609 | 1.48E-19 | 71.55 |  |
| Efet.01.129137.g229.t1 | cilia- and flagella-associated 45-like | 567 | 1.98E-35 | 77.65 | C:nucleoplasm |
| Efet.01.129147.g232.t1 | Calpain-5 | 1752 | 0 | 66.15 | F:peptidase activity |
| Efet.01.12917.g970.t1 | N-acetyllactosaminide beta-1,6-N-acetylglucosaminyl-transferase-like | 978 | 7.97E-29 | 46.4 | F:acetylglucosaminyltransferase activity; C:membrane; C:integral component of membrane |
| Efet.01.129195.g234.t1 | aryl hydrocarbon receptor nuclear translocator | 378 | 4.08E-17 | 71.6 | C:nucleus; C:transcription factor complex; F:DNA binding; F:transcription factor activity, sequence-specific DNA binding; C:cytoplasm; P:transcription, DNA-templated; P:regulation of transcription, DNA-templated; F:protein dimerization activity |
| Efet.01.129222.g235.t1 | M-phase inducer phosphatase isoform X1 | 663 | 4.18E-12 | 63.67 | F:protein tyrosine phosphatase activity; P:protein dephosphorylation; P:peptidyl-tyrosine dephosphorylation; C:intracellular |
| Efet.01.129224.g236.t1 | BTB POZ domain-containing adapter for CUL3-mediated degradation 3 | 453 | 4.25E-76 | 84.9 | P:protein homooligomerization |
| Efet.01.129282.g238.t1 | leucine-rich repeat-containing 24-like | 2220 | 1.16E-48 | 44.8 | C:membrane; C:integral component of membrane |
| Efet.01.129346.g239.t1 | cell division cycle 20 homolog | 207 | 3.52E-13 | 80.45 | P:positive regulation of synapse maturation; F:protein C-terminus binding; C:anaphase-promoting complex; P:regulation of meiotic nuclear division; C:nucleoplasm; P:cell division; F:ubiquitin-protein transferase activator activity; C:cytoplasm; P:positive regulation of synaptic plasticity; P:regulation of catalytic activity; P:anaphase-promoting complex-dependent catabolic process; F:anaphase-promoting complex binding; F:enzyme binding |
| Efet.01.129420.g244.t1 | ankyrin-3-like isoform X9 | 969 | 1.58E-90 | 69.35 | P:signal transduction |
| Efet.01.129444.g245.t1 | Rho-related GTP-binding | 228 | 8.56E-23 | 74.75 | P:regulation of cell shape; P:cytoskeleton organization; P:small GTPase mediated signal transduction; C:Golgi apparatus; P:single-organism cellular process; C:intracellular organelle part; C:plasma membrane; P:positive regulation of protein targeting to mitochondrion; F:GTP binding; F:protein binding; F:metal ion binding; P:regulation of small GTPase mediated signal transduction; C:cytosol; C:cell junction |
| Efet.01.129452.g246.t1 | serine threonine- kinase WNK1-like isoform X7 | 327 | 2.34E-10 | 68.7 | P:regulation of ion homeostasis; C:cytosol; F:protein serine/threonine kinase activity; P:protein phosphorylation; P:positive regulation of ion transmembrane transporter activity; P:intracellular signal transduction |
| Efet.01.12946.g971.t1 | intersectin-1 isoform X3 | 207 | 1.93E-20 | 82.25 | P:regulation of Rho protein signal transduction; F:Rho guanyl-nucleotide exchange factor activity; P:positive regulation of GTPase activity |
| Efet.01.129522.g247.t1 | type III glutamate--ammonia ligase | 432 | 3.70E-42 | 65.45 | P:nitrogen compound metabolic process; F:ligase activity |
| Efet.01.12954.g972.t1 | acetyl-coenzyme A cytoplasmic | 345 | 4.40E-35 | 74.9 | F:ATP binding; F:acetate-CoA ligase activity; F:AMP binding; C:intracellular membrane-bounded organelle; C:membrane; P:acetyl-CoA biosynthetic process from acetate |
| Efet.01.12961.g974.t1 | neuronal acetylcholine receptor subunit alpha-7-like isoform X1 | 984 | 4.16E-23 | 64.75 | C:postsynaptic membrane; P:cation transmembrane transport; C:integral component of membrane; C:cell junction; F:extracellular ligand-gated ion channel activity |
| Efet.01.129634.g249.t1 | endo-beta-1,4-mannanase | 240 | 1.82E-21 | 67 | P:mannan catabolic process; F:mannan endo-1,4-beta-mannosidase activity |
| Efet.01.129692.g250.t1 | UDP-glucuronosyltransferase 2C1-like | 762 | 5.60E-40 | 51.95 | F:transferase activity |
| Efet.01.1297.g117.t1 | dnaJ homolog subfamily C member 11 | 729 | 6.11E-38 | 81.65 |  |
| Efet.01.129732.g252.t1 | rho-associated kinase 2-like isoform X2 | 471 | 1.36E-89 | 96.25 | F:ATP binding; F:metal ion binding; F:protein serine/threonine kinase activity; F:Rho GTPase binding; P:protein phosphorylation; C:intracellular; P:intracellular signal transduction |
| Efet.01.12974.g977.t1 | sialin-like | 624 | 2.94E-36 | 56.85 | P:transmembrane transport; C:membrane; C:integral component of membrane; F:transporter activity |
| Efet.01.129786.g253.t1 | Collagen alpha-4(VI) chain | 1254 | 1.53E-101 | 54.8 | F:calcium ion binding; C:collagen trimer |
| Efet.01.129786.g254.t1 | collagen alpha-3(VI) chain-like isoform X1 | 1812 | 1.17E-164 | 47.1 | F:calcium ion binding |
| Efet.01.129807.g256.t1 | NDRG3-like isoform X1 | 255 | 1.62E-21 | 60.85 |  |
| Efet.01.129821.g257.t1 | innexin 1 | 1239 | 0 | 76.35 | C:gap junction; P:ion transport; P:transmembrane transport; C:integral component of membrane; P:cell communication by electrical coupling; C:synapse; F:gap junction channel activity; C:plasma membrane |
| Efet.01.129827.g258.t1 | zinc finger SNAI2-like | 696 | 2.16E-87 | 86.85 | F:nucleic acid binding; F:metal ion binding |
| Efet.01.12984.g978.t1 | ephrin type-B receptor 1-B-like isoform X4 | 528 | 1.77E-40 | 73.25 | F:ATP binding; P:peptidyl-tyrosine phosphorylation; F:protein tyrosine kinase activity; P:ephrin receptor signaling pathway; C:integral component of membrane |
| Efet.01.129841.g260.t1 | F-box LRR-repeat 12-like | 939 | 1.29E-48 | 53.35 |  |
| Efet.01.129913.g261.t1 | neurogenic locus notch homolog 1 | 234 | 1.63E-23 | 75.6 | P:Notch signaling pathway; P:multicellular organism development; F:calcium ion binding; C:integral component of membrane; P:cell differentiation |
| Efet.01.129961.g264.t1 | Wnt-11b-like | 354 | 1.07E-34 | 68.3 | C:membrane; C:extracellular region; P:single-organism process |
| Efet.01.129965.g265.t1 | hypothetical protein HELRODRAFT_194984 | 606 | 6.41E-06 | 64 | C:membrane; C:integral component of membrane; P:transport; F:transporter activity |
| Efet.01.129972.g266.t1 | hypothetical protein CAPTEDRAFT_191217 | 690 | 6.06E-26 | 53 | P:sodium ion transport; P:ion transport; F:sodium channel activity; C:membrane; C:integral component of membrane; P:sodium ion transmembrane transport; P:transport |
| Efet.01.130001.g267.t1 | hypothetical protein CAPTEDRAFT_151003 | 879 | 5.01E-155 | 69.25 | F:catalytic activity; P:nucleoside metabolic process |
| Efet.01.130010.g268.t1 | sodium glucose cotransporter 4-like isoform X2 | 255 | 2.67E-22 | 71.3 | P:transmembrane transport; C:integral component of membrane; F:transporter activity |
| Efet.01.13005.g979.t1 | Neuronal acetylcholine receptor subunit alpha-10 | 396 | 2.83E-64 | 75.4 | P:synaptic transmission, cholinergic; F:acetylcholine receptor activity; P:neuromuscular synaptic transmission; C:postsynaptic membrane; P:cation transmembrane transport; C:cell junction; F:extracellular ligand-gated ion channel activity; F:acetylcholine binding; C:acetylcholine-gated channel complex; P:response to nicotine |
| Efet.01.130056.g271.t1 | AChain Ligand-binding Domain Of Glua2 (flip) Ionotropic Glutamate Receptor In Complex With An Allosteric Modulator | 597 | 8.08E-28 | 64.65 | C:AMPA glutamate receptor complex; C:postsynaptic density; F:identical protein binding; F:kainate selective glutamate receptor activity; P:positive regulation of synaptic transmission; C:neuronal cell body; C:protein complex; F:AMPA glutamate receptor activity; F:calcium channel regulator activity; P:establishment of protein localization; C:dendritic shaft; P:protein tetramerization; C:dendritic spine; F:PDZ domain binding; P:response to lithium ion; P:receptor internalization; P:regulation of synaptic transmission, glutamatergic; P:regulation of receptor recycling |
| Efet.01.130066.g272.t1 | muscarinic acetylcholine receptor DM1 | 1746 | 5.19E-95 | 82.25 | P:gastric acid secretion; C:integral component of plasma membrane; P:synaptic transmission, cholinergic; P:phospholipase C-activating G-protein coupled acetylcholine receptor signaling pathway; P:adenylate cyclase-inhibiting G-protein coupled acetylcholine receptor signaling pathway; F:G-protein coupled acetylcholine receptor activity; C:synapse |
| Efet.01.130073.g273.t1 | D(3) dopamine receptor isoform X1 | 1290 | 3.11E-90 | 48.3 | F:G-protein coupled receptor activity; P:G-protein coupled receptor signaling pathway; C:integral component of membrane |
| Efet.01.130094.g274.t1 | neuropeptide CCHamide-1 receptor-like | 1032 | 3.85E-98 | 61.8 | F:G-protein coupled receptor activity; P:signal transduction |
| Efet.01.130148.g277.t1 | hypothetical protein HELRODRAFT_178859 | 453 | 5.48E-20 | 61.35 |  |
| Efet.01.130155.g278.t1 | TATA-binding -associated factor 172 | 306 | 1.91E-12 | 72 | F:ATP binding |
| Efet.01.130186.g280.t1 | heme transporter hrg1-B-like | 318 | 7.15E-14 | 58.33 | F:heme transporter activity; C:membrane; C:integral component of membrane; P:heme transport |
| Efet.01.130201.g281.t1 | hypothetical protein HELRODRAFT_193201 | 1872 | 5.13E-165 | 56.8 | F:transferase activity, transferring glycosyl groups; C:membrane; C:integral component of membrane |
| Efet.01.13025.g981.t1 | ankyrin repeat-containing | 1308 | 5.65E-32 | 45.2 |  |
| Efet.01.130273.g282.t1 | Thymidylate | 270 | 9.73E-24 | 71 | P:phosphorylation; F:kinase activity; P:nucleotide metabolic process |
| Efet.01.130301.g284.t1 | E3 SUMO- ligase PIAS2-like | 273 | 8.75E-10 | 85.3 | F:zinc ion binding; F:SUMO transferase activity |
| Efet.01.130331.g285.t1 | sex peptide receptor-like | 1299 | 5.17E-96 | 62.9 | F:G-protein coupled receptor activity; F:G-protein coupled peptide receptor activity; P:G-protein coupled receptor signaling pathway; C:membrane; C:integral component of membrane |
| Efet.01.130345.g286.t1 | cytoplasmic dynein 2 heavy chain 1-like | 1389 | 5.95E-134 | 87.05 | F:ATPase activity; P:Golgi organization; P:spinal cord motor neuron differentiation; P:coronary vasculature development; P:non-motile cilium assembly; C:Golgi apparatus; C:plasma membrane; C:axoneme; P:determination of left/right symmetry; C:microtubule; P:asymmetric protein localization; P:dorsal/ventral pattern formation; F:ATP binding; P:embryonic limb morphogenesis; C:cytoplasmic dynein complex; C:motile cilium; F:microtubule motor activity; C:extracellular exosome; P:forebrain development; C:apical part of cell; P:positive regulation of smoothened signaling pathway; F:dynein light intermediate chain binding; P:protein processing; P:intraciliary retrograde transport |
| Efet.01.130348.g287.t1 | PKD domain-containing | 888 | 1.01E-47 | 58.33 |  |
| Efet.01.130354.g288.t1 | Pre-mRNA-processing-splicing factor 8 | 750 | 3.49E-137 | 91.6 | F:U6 snRNA binding; F:U5 snRNA binding; P:ribonucleoprotein complex assembly; C:spliceosomal complex; C:spliceosomal snRNP complex; P:mRNA splicing, via spliceosome |
| Efet.01.13036.g982.t1 | ELKS Rab6-interacting CAST family member 1 isoform X2 | 528 | 7.98E-15 | 66.1 | C:presynaptic active zone; P:I-kappaB phosphorylation; C:IkappaB kinase complex |
| Efet.01.130368.g290.t1 | mitochondrial import receptor subunit TOM70 | 501 | 5.48E-27 | 57.85 | C:extracellular exosome; C:mitochondrion; C:membrane; C:integral component of membrane |
| Efet.01.130371.g291.t1 | G -activated inward rectifier potassium channel 4-like isoform X1 | 660 | 1.03E-63 | 64.85 | P:potassium ion transport; C:membrane; F:voltage-gated ion channel activity |
| Efet.01.130388.g293.t1 | 60 kDa neurofilament -like | 579 | 2.23E-57 | 73 | F:structural molecule activity; C:intermediate filament |
| Efet.01.1304.g119.t1 | Integrin alpha pat-2 | 423 | 3.23E-17 | 61.95 | C:membrane |
| Efet.01.130402.g294.t1 | band 3 isoform X3 | 459 | 1.87E-19 | 56.7 | C:cytoskeleton; F:actin binding; C:cytoplasm; F:structural molecule activity; F:cytoskeletal protein binding; C:extrinsic component of membrane |
| Efet.01.130466.g296.t1 | death domain-associated 6 | 858 | 9.93E-06 | 58 |  |
| Efet.01.130466.g297.t1 | death domain-associated 6 | 324 | 1.93E-24 | 72.65 |  |
| Efet.01.130536.g298.t1 | disks large 1 tumor suppressor -like isoform X3 | 276 | 1.11E-17 | 90.05 | C:postsynaptic density; F:ionotropic glutamate receptor binding; P:purine ribonucleotide metabolic process; C:voltage-gated potassium channel complex; P:guanosine-containing compound metabolic process; P:nervous system development; P:chemical synaptic transmission; C:juxtaparanode region of axon; F:guanylate kinase activity; P:receptor clustering; C:ionotropic glutamate receptor complex; P:receptor localization to synapse; P:negative regulation of phosphatase activity; C:postsynaptic membrane; P:establishment or maintenance of epithelial cell apical/basal polarity; C:basolateral plasma membrane; F:kinase binding |
| Efet.01.130566.g299.t1 | probable G- coupled receptor 139 | 672 | 2.11E-27 | 50.5 | F:G-protein coupled receptor activity; F:G-protein coupled peptide receptor activity; P:G-protein coupled receptor signaling pathway; P:signal transduction; C:membrane; C:integral component of membrane; F:signal transducer activity |
| Efet.01.130594.g300.t1 | hypothetical protein HELRODRAFT_109499 | 228 | 1.87E-08 | 87 | P:regulation of muscle contraction; C:troponin complex |
| Efet.01.130636.g302.t1 | pericentriolar material 1 isoform X2 | 561 | 2.72E-42 | 67.85 | C:centrosome; P:organelle organization |
| Efet.01.130638.g303.t1 | hypothetical protein CAPTEDRAFT_222036 | 705 | 3.56E-18 | 60.58 | C:membrane; C:integral component of membrane |
| Efet.01.13064.g984.t1 | Small conductance calcium-activated potassium channel | 447 | 2.15E-61 | 85.8 | F:calmodulin binding; P:potassium ion transmembrane transport; C:integral component of membrane; F:small conductance calcium-activated potassium channel activity |
| Efet.01.130643.g304.t1 | misshapen-like kinase 1 isoform X3 | 285 | 5.67E-28 | 88.35 | F:ATP binding; F:signal transducer, downstream of receptor, with serine/threonine kinase activity; C:cytoplasm; P:signal transduction by protein phosphorylation |
| Efet.01.13065.g985.t1 | ankyrin repeat domain-containing 26-like isoform X1 | 495 | 7.64E-16 | 62.4 |  |
| Efet.01.130655.g306.t1 | unnamed protein product | 261 | 9.50E-06 | 53 | C:membrane; C:integral component of membrane; F:semaphorin receptor activity; P:semaphorin-plexin signaling pathway |
| Efet.01.13073.g986.t1 | CYTSA_TAKRU ame: Full=Cytospin-A ame: Full=SPECC1 ame: Full=Sperm antigen with calponin homology and coiled-coil domains 1-like | 1710 | 6.66E-06 | 66 | C:gap junction; P:cell division; C:cytoskeleton; C:cytoplasm; C:spindle; C:cell junction; P:cell cycle |
| Efet.01.13075.g987.t1 | dual specificity kinase TTK-like | 390 | 1.11E-28 | 68.95 | F:nucleotide binding; F:protein kinase activity; P:cellular process |
| Efet.01.130831.g311.t1 | hypothetical protein CAPTEDRAFT_215028 | 459 | 6.19E-30 | 62.95 | P:proteolysis; F:metallopeptidase activity |
| Efet.01.130837.g312.t1 | ubiquinol-cytochrome-c reductase complex assembly factor 1 | 723 | 1.17E-63 | 68.5 |  |
| Efet.01.130911.g315.t1 | plasma membrane calcium-transporting ATPase 2 isoform X1 | 471 | 3.51E-67 | 90.85 | F:ATP binding; F:metal ion binding; C:integral component of membrane; P:calcium ion transmembrane transport; F:calcium-transporting ATPase activity |
| Efet.01.131003.g318.t1 | zinc finger 423-like isoform X1 | 1269 | 6.04E-15 | 67.9 | F:nucleic acid binding; F:metal ion binding |
| Efet.01.131016.g319.t1 | dynein heavy chain domain-containing 1-like | 876 | 1.05E-40 | 47.55 |  |
| Efet.01.131026.g320.t1 | serine threonine- kinase WNK3 | 531 | 1.49E-79 | 69.1 | P:positive regulation of molecular function; F:protein serine/threonine kinase activity; P:protein phosphorylation; P:positive regulation of biological process; P:intracellular signal transduction |
| Efet.01.131113.g324.t1 | serine threonine- kinase N2-like | 363 | 1.30E-13 | 81.55 | F:ATP binding; F:protein serine/threonine kinase activity; P:peptidyl-serine phosphorylation; C:intracellular; P:intracellular signal transduction |
| Efet.01.131122.g326.t1 | UPF0568 C14orf166 homolog | 384 | 5.20E-26 | 64.2 | C:nucleus; F:RNA binding; F:protein binding |
| Efet.01.131135.g327.t1 | prolactin-releasing peptide receptor-like | 1101 | 8.44E-144 | 73.9 | C:integral component of plasma membrane; P:neuropeptide signaling pathway; P:cell surface receptor signaling pathway; F:neuropeptide Y receptor activity |
| Efet.01.1312.g120.t1 | transmembrane 164 | 261 | 1.16E-11 | 73.35 | C:membrane |
| Efet.01.131207.g329.t1 | isoforms A H I | 351 | 9.12E-49 | 77.4 | P:Wnt signaling pathway |
| Efet.01.131221.g330.t1 | dopamine D2-like receptor | 1443 | 4.21E-43 | 70.15 | F:dopamine neurotransmitter receptor activity; P:anesthesia-resistant memory; C:integral component of membrane; P:dopamine receptor signaling pathway |
| Efet.01.131230.g331.t1 | coiled-coil domain-containing 81-like | 579 | 2.02E-39 | 55.8 |  |
| Efet.01.131246.g332.t1 | transitional endoplasmic reticulum ATPase | 1053 | 0 | 93.5 | F:ATP binding; C:integral component of membrane; F:hydrolase activity |
| Efet.01.131248.g333.t1 | uncharacterized protein LOC110460117 isoform X1 | 417 | 1.18E-23 | 55.94 |  |
| Efet.01.13146.g988.t1 | THUMP domain-containing 3 | 297 | 2.01E-14 | 63.35 | F:RNA binding |
| Efet.01.13150.g989.t1 | ubiA prenyltransferase domain-containing 1 homolog | 873 | 1.57E-61 | 55.55 | F:prenyltransferase activity; C:membrane; C:integral component of membrane; F:transferase activity |
| Efet.01.131503.g339.t1 | Suppressor of cytokine signaling 6 | 858 | 1.23E-69 | 86.6 | C:cytoplasm; P:cytokine-mediated signaling pathway; F:protein kinase inhibitor activity; P:negative regulation of protein kinase activity; P:regulation of growth; P:intracellular signal transduction; P:negative regulation of JAK-STAT cascade; P:protein ubiquitination |
| Efet.01.131542.g341.t1 | dynein beta ciliary-like | 744 | 5.80E-107 | 85.2 | F:ATPase activity; F:ATP binding; C:motile cilium; F:microtubule motor activity; C:cytoplasm; P:cell projection organization; C:integral component of membrane; C:dynein complex; P:microtubule-based movement; C:microtubule |
| Efet.01.131542.g342.t1 | dynein heavy chain axonemal | 600 | 2.79E-77 | 78.2 | F:ATPase activity; F:ATP binding; F:microtubule motor activity; C:integral component of membrane; C:dynein complex; P:microtubule-based movement |
| Efet.01.131588.g343.t1 | dual specificity phosphatase 9 | 354 | 9.64E-12 | 82.2 | P:inactivation of MAPK activity; C:nucleus; P:activation of MAPK activity; F:protein binding; F:protein tyrosine phosphatase activity; C:cytosol; P:JNK cascade; P:peptidyl-tyrosine dephosphorylation; F:MAP kinase tyrosine/serine/threonine phosphatase activity |
| Efet.01.131669.g345.t1 | G -activated inward rectifier potassium channel 3-like | 1101 | 3.17E-76 | 70.95 | P:potassium ion transmembrane transport; C:integral component of membrane; F:inward rectifier potassium channel activity; P:regulation of ion transmembrane transport |
| Efet.01.131690.g346.t1 | palmitoleoyl- carboxylesterase NOTUM | 330 | 2.00E-36 | 74.55 | F:hydrolase activity |
| Efet.01.131739.g347.t1 | Activating signal cointegrator 1 complex subunit 3 | 1857 | 0 | 86.9 | P:DNA dealkylation involved in DNA repair; P:cell proliferation; F:ATP binding; F:ATP-dependent 3'-5' DNA helicase activity; P:DNA duplex unwinding; C:membrane; C:Golgi apparatus; F:RNA binding |
| Efet.01.131894.g350.t1 | 1,4-alpha-glucan-branching enzyme | 258 | 3.99E-38 | 85.15 | P:glycogen biosynthetic process; F:1,4-alpha-glucan branching enzyme activity; F:cation binding; F:hydrolase activity, hydrolyzing O-glycosyl compounds |
| Efet.01.131917.g351.t1 | agrin-like isoform X3 | 702 | 2.13E-56 | 63.45 | F:calcium ion binding |
| Efet.01.131945.g353.t1 | Transcription initiation factor TFIID subunit 5 | 972 | 6.05E-84 | 59.8 | C:nucleus; P:cellular macromolecule metabolic process; P:primary metabolic process |
| Efet.01.131948.g354.t1 | PERQ amino acid-rich with GYF domain-containing 2 isoform X3 | 516 | 7.97E-58 | 56.75 |  |
| Efet.01.13208.g990.t1 | hypothetical protein CAPTEDRAFT_38953, partial | 252 | 9.96E-11 | 86 | P:positive regulation of apoptotic process |
| Efet.01.132099.g360.t1 | RNA-binding 26 | 693 | 4.17E-30 | 78.9 | F:nucleotide binding; C:nucleus; F:metal ion binding; P:positive regulation of gene expression; P:negative regulation of mRNA polyadenylation; F:mRNA binding; P:mRNA processing; P:negative regulation of smoothened signaling pathway; P:positive regulation of RNA export from nucleus |
| Efet.01.132146.g361.t1 | metabotropic glutamate receptor isoform X1 | 861 | 5.08E-96 | 71.95 | F:G-protein coupled receptor activity; P:G-protein coupled receptor signaling pathway; C:integral component of membrane; C:plasma membrane |
| Efet.01.132156.g362.t1 | hypothetical protein HELRODRAFT_191922 | 363 | 2.87E-21 | 55.1 | C:membrane; C:integral component of membrane |
| Efet.01.132302.g364.t1 | fructose-bisphosphate aldolase | 426 | 1.52E-62 | 81.7 | P:glycolytic process; F:fructose-bisphosphate aldolase activity |
| Efet.01.132323.g366.t1 | tetratricopeptide repeat 17-like | 285 | 5.49E-14 | 70.75 |  |
| Efet.01.132372.g369.t1 | isocitrate dehydrogenase [NAD] subunit mitochondrial-like | 309 | 6.08E-47 | 87.15 | F:magnesium ion binding; C:nucleus; F:isocitrate dehydrogenase (NAD+) activity; F:NAD binding; C:mitochondrion; P:tricarboxylic acid cycle |
| Efet.01.1324.g121.t1 | LIM and SH3 domain | 843 | 3.31E-17 | 78.6 | F:zinc ion binding |
| Efet.01.13241.g993.t1 | dynein beta ciliary-like | 1080 | 0 | 81.25 | F:ATPase activity; F:ATP binding; F:microtubule motor activity; F:structural constituent of ribosome; C:dynein complex; C:ribosome; P:microtubule-based movement; P:translation |
| Efet.01.132426.g370.t1 | hypothetical protein HELRODRAFT_179908 | 1398 | 5.20E-07 | 60 |  |
| Efet.01.132437.g371.t1 | enoyl- hydratase | 216 | 3.91E-10 | 63.35 | F:catalytic activity; P:metabolic process; F:isomerase activity |
| Efet.01.132446.g373.t1 | discoidin domain-containing receptor 2-like isoform X1 | 1182 | 7.39E-42 | 66.75 | F:ATP binding; P:peptidyl-tyrosine phosphorylation; F:protein tyrosine kinase activity; C:membrane; C:integral component of membrane; F:protein kinase activity; P:protein phosphorylation |
| Efet.01.132451.g375.t1 | scaffold attachment factor B1-like | 1983 | 8.60E-15 | 51.55 | F:nucleotide binding; F:nucleic acid binding |
| Efet.01.132471.g376.t1 | kelch 24 | 651 | 1.49E-09 | 43.2 | F:ubiquitin-protein transferase activity; C:Cul3-RING ubiquitin ligase complex; P:protein ubiquitination |
| Efet.01.132501.g377.t1 | collagen | 651 | 1.52E-15 | 49.05 |  |
| Efet.01.132523.g379.t1 | cilia- and flagella-associated 47-like isoform X3 | 267 | 3.87E-19 | 71.75 |  |
| Efet.01.132533.g381.t1 | hypothetical protein CAPTEDRAFT_221479 | 261 | 2.03E-09 | 62 | F:peptidase inhibitor activity; C:extracellular region; P:negative regulation of peptidase activity |
| Efet.01.132564.g382.t1 | filamin-C isoform X1 | 552 | 1.57E-19 | 40.05 | F:actin filament binding; F:actin binding |
| Efet.01.132636.g384.t1 | hypothetical protein CAPTEDRAFT_189638 | 429 | 9.46E-14 | 62 |  |
| Efet.01.132671.g385.t1 | hypothetical protein CAPTEDRAFT_150374 | 468 | 4.16E-08 | 59 | F:ATP binding; P:peptidyl-tyrosine phosphorylation; F:protein tyrosine kinase activity; C:membrane; C:integral component of membrane; F:protein kinase activity; P:protein phosphorylation |
| Efet.01.132695.g386.t1 | G- coupled receptor 54-like | 429 | 2.82E-10 | 79 | F:G-protein coupled receptor activity; P:G-protein coupled receptor signaling pathway; C:integral component of membrane |
| Efet.01.132704.g387.t1 | Membralin | 276 | 4.71E-08 | 80.3 | C:integral component of membrane |
| Efet.01.1328.g122.t1 | serine threonine- kinase pim-3 | 861 | 5.46E-111 | 72 | F:nucleotide binding; F:protein kinase activity; P:phosphorylation |
| Efet.01.132814.g390.t1 | cadherin-87A isoform X1 | 456 | 1.46E-19 | 59.5 | C:membrane; P:cell-cell adhesion |
| Efet.01.132852.g391.t1 | neuroligin- X-linked-like isoform X1 | 567 | 3.96E-48 | 61.7 | C:membrane; C:integral component of membrane |
| Efet.01.132895.g392.t1 | GRB2-associated-binding 1-like isoform X8 | 231 | 9.33E-12 | 70.63 |  |
| Efet.01.132920.g393.t1 | neuronal acetylcholine receptor subunit alpha-10 isoform X2 | 495 | 2.06E-28 | 48.35 | P:biological regulation; P:ion transport; C:membrane; C:cell part; P:single-organism process; P:cellular process |
| Efet.01.132937.g394.t1 | RIB43A-like with coiled-coils 2 | 336 | 1.72E-38 | 78.35 |  |
| Efet.01.13296.g994.t1 | FLamide receptor 1 | 471 | 1.41E-27 | 56.45 | P:signal transduction; C:membrane; F:signal transducer activity |
| Efet.01.132988.g397.t1 | titin-like | 276 | 1.12E-14 | 58.5 |  |
| Efet.01.133008.g400.t1 | polyadenylate-binding 1 | 738 | 1.73E-123 | 80.55 | F:nucleotide binding; F:RNA binding; C:cytoplasm |
| Efet.01.13303.g995.t1 | TATA box-binding -associated factor RNA polymerase I subunit A-like | 1302 | 5.91E-22 | 51.45 |  |
| Efet.01.133039.g401.t1 | RNA polymerase II subunit A C-terminal domain phosphatase-like | 1695 | 7.01E-45 | 68.5 | F:GTP binding; C:nucleus; F:phosphoprotein phosphatase activity; P:small GTPase mediated signal transduction; P:protein dephosphorylation |
| Efet.01.13306.g996.t1 | calcium-independent phospholipase A2-gamma-like | 849 | 2.32E-07 | 72 |  |
| Efet.01.133068.g403.t1 | hypothetical protein CAPTEDRAFT_86011, partial | 261 | 2.35E-06 | 62.5 |  |
| Efet.01.133136.g408.t1 | hamartin-like isoform X1 | 1023 | 2.09E-10 | 70.1 | P:regulation of actin cytoskeleton organization; C:membrane; P:regulation of GTPase activity; F:GTPase regulator activity; C:TSC1-TSC2 complex; P:regulation of cell cycle; P:negative regulation of TOR signaling; P:negative regulation of insulin receptor signaling pathway |
| Efet.01.133173.g409.t1 | breast cancer anti-estrogen resistance 1 | 1848 | 4.31E-36 | 44.05 |  |
| Efet.01.133179.g410.t1 | DNA-binding RFX2-like isoform X1 | 426 | 1.14E-14 | 76.5 | F:DNA binding; P:regulation of transcription, DNA-templated |
| Efet.01.133298.g411.t1 | allatostatin-A receptor-like | 408 | 2.43E-24 | 54.55 | F:G-protein coupled receptor activity; P:neuropeptide signaling pathway; P:G-protein coupled receptor signaling pathway; P:signal transduction; C:membrane; C:integral component of membrane; F:signal transducer activity; F:neuropeptide Y receptor activity |
| Efet.01.133377.g412.t1 | hypothetical protein HELRODRAFT_70180, partial | 531 | 1.78E-09 | 75 | C:postsynaptic membrane; P:cation transmembrane transport; C:integral component of membrane; C:cell junction; F:extracellular ligand-gated ion channel activity |
| Efet.01.133441.g414.t1 | msx2-interacting -like | 5679 | 4.02E-10 | 54 |  |
| Efet.01.13349.g998.t1 | D(1A) dopamine receptor | 831 | 2.27E-09 | 47 | P:dopamine transport; F:dopamine binding; P:dopamine receptor signaling pathway; P:visual learning; P:striatum development; P:associative learning; P:learning; P:memory; C:endoplasmic reticulum membrane; F:G-protein coupled receptor activity; F:dopamine neurotransmitter receptor activity, coupled via Gs; P:positive regulation of cytosolic calcium ion concentration involved in phospholipase C-activating G-protein coupled signaling pathway; C:cytosol; C:ciliary membrane; P:long-term synaptic potentiation; P:positive regulation of adenylate cyclase activity involved in G-protein coupled receptor signaling pathway; P:positive regulation of synaptic transmission, glutamatergic; P:long term synaptic depression; P:G-protein coupled receptor signaling pathway; P:G-protein coupled receptor signaling pathway, coupled to cyclic nucleotide second messenger; P:positive regulation of cAMP biosynthetic process; P:response to cocaine; P:adenylate cyclase-activating G-protein coupled receptor signaling pathway; P:phospholipase C-activating dopamine receptor signaling pathway; F:signal transducer activity; P:dentate gyrus development; C:plasma membrane; P:mating behavior; C:nucleus; P:protein import into nucleus; P:positive regulation of cell migration; P:operant conditioning; P:adenylate cyclase-activating dopamine receptor signaling pathway; P:astrocyte development; P:habituation; P:cerebral cortex GABAergic interneuron migration; P:feeding behavior; P:vasodilation; P:maternal behavior; P:adult walking behavior; F:dopamine neurotransmitter receptor activity; C:integral component of plasma membrane; P:grooming behavior; P:locomotory behavior; P:synaptic transmission, dopaminergic; P:behavioral response to cocaine; P:sensitization; P:muscle contraction; P:cellular response to catecholamine stimulus; P:transmission of nerve impulse; P:activation of adenylate cyclase activity; P:neuronal action potential; P:signal transduction; C:endoplasmic reticulum; P:glucose import; P:hippocampus development; P:positive regulation of release of sequestered calcium ion into cytosol; P:conditioned taste aversion; P:behavioral fear response; P:response to amphetamine; P:regulation of dopamine metabolic process; P:response to drug; C:membrane; P:temperature homeostasis; C:integral component of membrane; P:peristalsis |
| Efet.01.1335.g123.t1 | limbic system-associated membrane -like isoform X4 | 504 | 1.83E-29 | 69.35 | P:cellular process |
| Efet.01.133540.g417.t1 | Spermatogenesis-associated 18 | 1260 | 3.37E-92 | 69.2 | P:cellular response to stress; C:mitochondrial outer membrane |
| Efet.01.13356.g999.t1 | glutathione-specific gamma-glutamylcyclotransferase 2 | 354 | 1.47E-39 | 72.4 |  |
| Efet.01.133581.g418.t1 | subolesin | 291 | 2.78E-08 | 63.05 |  |
| Efet.01.133622.g419.t1 | matrix metallo ase-17 | 204 | 8.93E-10 | 64 | F:metal ion binding; F:metallopeptidase activity |
| Efet.01.133692.g421.t1 | cubilin isoform X1 | 273 | 5.41E-07 | 52.33 |  |
| Efet.01.133698.g422.t1 | neuronal acetylcholine receptor subunit alpha-7-like | 531 | 1.72E-81 | 68.15 | P:ion transmembrane transport; C:integral component of membrane; F:extracellular ligand-gated ion channel activity; C:synapse; C:plasma membrane |
| Efet.01.133708.g423.t1 | protocadherin-9-like isoform X1 | 888 | 6.56E-41 | 50.85 | F:calcium ion binding; C:membrane; C:integral component of membrane; P:cell adhesion; P:homophilic cell adhesion via plasma membrane adhesion molecules; C:plasma membrane |
| Efet.01.133754.g426.t1 | myocyte-specific enhancer factor 2D | 324 | 6.91E-52 | 92.9 | C:nucleus; F:DNA binding; P:transcription, DNA-templated; P:regulation of transcription, DNA-templated; F:protein dimerization activity |
| Efet.01.13376.g1001.t1 | histone acetyltransferase p300-like | 1464 | 1.33E-82 | 60.4 | F:transcription cofactor activity; C:PML body; F:metal ion binding; F:transferase activity |
| Efet.01.133775.g428.t1 | interferon-induced very large GTPase 1 | 660 | 1.81E-13 | 58.5 | F:GTP binding |
| Efet.01.133791.g430.t1 | PH and SEC7 domain-containing 2-like | 936 | 2.13E-06 | 73 | F:ARF guanyl-nucleotide exchange factor activity; P:regulation of ARF protein signal transduction; F:phospholipid binding; P:positive regulation of GTPase activity |
| Efet.01.133793.g431.t1 | hypothetical protein CAPTEDRAFT_218723 | 2103 | 6.32E-115 | 45.6 | C:membrane; C:integral component of membrane |
| Efet.01.133809.g432.t1 | hypothetical protein CAPTEDRAFT_222036 | 330 | 1.90E-12 | 59.67 |  |
| Efet.01.133812.g433.t1 | specificity 1 | 1029 | 1.58E-08 | 74.25 | F:metal ion binding |
| Efet.01.133899.g437.t1 | SH3 domain-binding glutamic acid-rich 3 | 330 | 7.99E-09 | 62.67 | C:cell; F:protein disulfide oxidoreductase activity; F:electron carrier activity; P:cell redox homeostasis; P:oxidation-reduction process |
| Efet.01.133911.g438.t1 | lactadherin-like isoform X5 | 324 | 2.05E-28 | 74.05 |  |
| Efet.01.133987.g441.t1 | inactive tyrosine- kinase 7-like | 291 | 2.35E-21 | 67 | F:ATP binding; C:membrane; C:integral component of membrane; F:protein kinase activity; P:phosphorylation; P:protein phosphorylation; F:kinase activity |
| Efet.01.134015.g442.t1 | transmembrane 64-like | 807 | 2.02E-48 | 66.95 | C:integral component of membrane |
| Efet.01.13403.g1002.t1 | chloride channel C-like | 300 | 8.05E-24 | 80.05 | P:regulation of anion transmembrane transport; P:ion transmembrane transport; C:integral component of membrane; P:chloride transport; F:voltage-gated chloride channel activity |
| Efet.01.134049.g443.t1 | NADP-dependent malic enzyme-like | 462 | 1.33E-62 | 84.75 | C:nucleus; F:transcription factor activity, sequence-specific DNA binding; F:zinc ion binding; P:malate metabolic process; F:NAD binding; F:malate dehydrogenase (decarboxylating) (NAD+) activity; P:regulation of transcription, DNA-templated; F:sequence-specific DNA binding; P:oxidation-reduction process |
| Efet.01.134061.g445.t1 | serine threonine- phosphatase 2A 65 kDa regulatory subunit A beta isoform | 342 | 1.12E-32 | 80.6 | P:apoptotic process involved in morphogenesis; P:positive regulation of extrinsic apoptotic signaling pathway in absence of ligand; C:extracellular exosome; P:protein complex assembly; C:membrane raft; P:regulation of phosphoprotein phosphatase activity |
| Efet.01.134086.g447.t1 | kelch 8 isoform X1 | 375 | 5.50E-17 | 50.95 | F:nucleic acid binding; F:ubiquitin-protein transferase activity; F:metal ion binding; C:Cul3-RING ubiquitin ligase complex; P:protein ubiquitination involved in ubiquitin-dependent protein catabolic process; C:nucleoplasm; P:protein ubiquitination |
| Efet.01.134099.g448.t1 | hypothetical protein LOTGIDRAFT_153005 | 864 | 7.19E-06 | 47 | C:mediator complex; F:RNA polymerase II transcription cofactor activity; P:regulation of transcription from RNA polymerase II promoter |
| Efet.01.134140.g449.t1 | zinc finger 11-like isoform X2 | 1029 | 3.63E-24 | 60 | F:nucleic acid binding; F:metal ion binding |
| Efet.01.134179.g450.t1 | serine protease 27-like | 576 | 2.76E-40 | 56.8 | F:hydrolase activity |
| Efet.01.134257.g451.t1 | radixin isoform X2 | 780 | 4.02E-29 | 70.1 | P:regulation of cell shape; C:myelin sheath; C:cleavage furrow; P:regulation of actin filament bundle assembly; P:negative regulation of homotypic cell-cell adhesion; P:positive regulation of cellular protein catabolic process; P:regulation of organelle assembly; P:cellular response to thyroid hormone stimulus; C:extracellular exosome; C:focal adhesion; C:cell tip; C:microvillus; P:apical protein localization; P:negative regulation of cell size; C:ruffle; C:stereocilium; C:cortical actin cytoskeleton; C:lamellipodium; F:protein domain specific binding; P:negative regulation of GTPase activity; F:protein homodimerization activity; P:positive regulation of G1/S transition of mitotic cell cycle; P:establishment of protein localization to plasma membrane; P:regulation of ruffle assembly; P:establishment of endothelial barrier; F:RNA binding; P:positive regulation of protein localization to early endosome; F:cadherin binding involved in cell-cell adhesion; C:T-tubule; P:barbed-end actin filament capping; C:extracellular space; F:actin binding; C:cell-cell adherens junction; P:positive regulation of gene expression; P:microvillus assembly; C:filopodium; P:positive regulation of early endosome to late endosome transport; C:midbody; C:extrinsic component of membrane; P:negative regulation of adherens junction organization; C:apical plasma membrane; F:ATPase binding; P:positive regulation of cell migration |
| Efet.01.134295.g452.t1 | EF-hand calcium-binding domain-containing 12-like isoform X1 | 348 | 9.17E-10 | 66.2 |  |
| Efet.01.134336.g454.t1 | bicaudal C homolog 1 | 408 | 1.79E-33 | 73.15 | F:RNA binding; P:multicellular organism development |
| Efet.01.134348.g455.t1 | 20 kDa calcium-binding (Antigen SM20) | 315 | 2.28E-42 | 67.65 | F:calcium ion binding |
| Efet.01.134364.g456.t1 | 60S ribosomal L11-like | 213 | 5.19E-24 | 88.7 | P:protein targeting; C:extracellular exosome; F:structural constituent of ribosome; P:ribosomal large subunit biogenesis; C:membrane; C:cytosolic large ribosomal subunit; P:rRNA processing; C:nucleolus; F:RNA binding; P:translation |
| Efet.01.134415.g459.t1 | hypothetical protein HELRODRAFT_188458 | 900 | 3.78E-85 | 69.5 |  |
| Efet.01.134415.g460.t1 | MULTISPECIES: ankyrin repeat domain-containing | 312 | 5.50E-31 | 58.16 |  |
| Efet.01.134419.g462.t1 | MOG interacting and ectopic P-granules 1-like | 1965 | 9.48E-48 | 46.4 | F:metal ion binding |
| Efet.01.134477.g464.t1 | hypothetical protein HELRODRAFT_96284, partial | 696 | 2.85E-10 | 69.44 | F:nucleic acid binding; F:metal ion binding |
| Efet.01.134484.g465.t1 | polymerase delta-interacting 3 | 864 | 8.52E-13 | 80.15 | F:nucleotide binding; F:nucleic acid binding |
| Efet.01.134498.g467.t1 | ceramide glucosyltransferase-like | 282 | 4.36E-23 | 80.95 | F:transferase activity, transferring glycosyl groups; C:integral component of membrane |
| Efet.01.134510.g468.t1 | collagen alpha-1(XII) chain-like | 498 | 9.54E-39 | 52.4 | F:calcium ion binding |
| Efet.01.134538.g469.t1 | nischarin isoform X2 | 288 | 1.47E-07 | 68.55 | P:Rac protein signal transduction; F:identical protein binding; C:endosome; C:cytoplasm; C:membrane; P:apoptotic process; F:phosphatidylinositol binding; C:cytosol; F:integrin binding; P:actin cytoskeleton organization; C:plasma membrane; P:negative regulation of cell migration |
| Efet.01.13456.g1006.t1 | homeobox Hox-B3-like | 429 | 3.99E-08 | 93.05 | C:nucleus; F:transcription factor activity, sequence-specific DNA binding; P:multicellular organism development; P:regulation of transcription, DNA-templated; F:sequence-specific DNA binding |
| Efet.01.134621.g471.t1 | uncharacterized protein LOC111028731 | 387 | 3.13E-16 | 51.9 | F:zinc ion binding; F:oxidoreductase activity; F:catalytic activity; P:metabolic process; P:oxidation-reduction process; F:transferase activity |
| Efet.01.134718.g474.t1 | microtubule-actin cross-linking factor isoforms 1 2 3 5-like | 1218 | 5.60E-22 | 44.1 |  |
| Efet.01.13472.g1007.t1 | dual serine threonine and tyrosine kinase-like | 450 | 2.77E-49 | 76.55 | P:cellular response to fibroblast growth factor stimulus; P:positive regulation of fibroblast growth factor receptor signaling pathway; F:protein serine/threonine kinase activity; P:protein phosphorylation; F:ATP binding; F:protein tyrosine kinase activity; F:protein serine/threonine/tyrosine kinase activity; P:positive regulation of ERK1 and ERK2 cascade; C:cytosol; C:cell junction; P:positive regulation of kinase activity; C:basolateral plasma membrane; C:apical plasma membrane; P:intracellular signal transduction |
| Efet.01.134763.g476.t1 | hypothetical protein BGI32_03965 | 507 | 5.96E-08 | 60.29 |  |
| Efet.01.134765.g477.t1 | Short transient receptor potential channel 3 | 387 | 2.31E-42 | 80.85 | C:integral component of plasma membrane; F:store-operated calcium channel activity; P:manganese ion transport; P:regulation of cytosolic calcium ion concentration; P:calcium ion transmembrane transport; P:single fertilization |
| Efet.01.134780.g478.t1 | forkhead-associated domain-containing 1-like isoform X1 | 453 | 1.69E-24 | 64.15 |  |
| Efet.01.134820.g479.t1 | neuroblastoma-amplified sequence | 390 | 1.24E-06 | 62.13 |  |
| Efet.01.134844.g481.t1 | vacuolar sorting-associated 13A-like | 486 | 7.60E-45 | 71.05 | P:protein localization to organelle; C:membrane part |
| Efet.01.134855.g484.t1 | photoreceptor-specific nuclear receptor-like | 303 | 2.65E-16 | 73.75 | C:nucleus; F:transcription factor activity, sequence-specific DNA binding; F:zinc ion binding; P:transcription, DNA-templated; F:steroid hormone receptor activity; P:regulation of transcription, DNA-templated; F:sequence-specific DNA binding; P:steroid hormone mediated signaling pathway |
| Efet.01.134877.g485.t1 | neogenin-like isoform X5 | 291 | 2.10E-08 | 79.95 | C:integral component of plasma membrane; P:positive regulation of BMP signaling pathway; P:iron ion homeostasis; P:axon guidance; F:receptor activity |
| Efet.01.135019.g487.t1 | hypothetical protein CAPTEDRAFT_213088 | 1107 | 3.91E-09 | 50 | P:transport |
| Efet.01.135066.g489.t1 | LSM14 homolog A-like isoform X1 | 216 | 8.39E-10 | 69.75 |  |
| Efet.01.135095.g491.t1 | barH-like 1 homeobox | 273 | 1.38E-10 | 81.5 | C:nucleus; P:regulation of transcription, DNA-templated; F:sequence-specific DNA binding |
| Efet.01.135105.g493.t1 | sodium-coupled monocarboxylate transporter 1-like | 201 | 1.61E-10 | 71.95 | C:integral component of plasma membrane; F:symporter activity; P:transport |
| Efet.01.135109.g494.t1 | septin-7-like isoform X5 | 252 | 6.75E-13 | 78.95 | F:GTP binding; C:septin complex |
| Efet.01.135116.g495.t1 | hypothetical protein CAPTEDRAFT_220233 | 561 | 1.77E-13 | 58 | F:RNA binding; P:RNA processing; F:adenosine deaminase activity |
| Efet.01.135136.g496.t1 | hepatocyte growth factor-regulated tyrosine kinase substrate-like | 576 | 1.87E-69 | 78.3 | F:metal ion binding; P:intracellular protein transport; C:intracellular |
| Efet.01.135137.g497.t1 | importin-13 isoform X1 | 207 | 3.45E-11 | 67.8 | F:binding; P:intracellular protein transport; C:intracellular part |
| Efet.01.135150.g499.t1 | transient receptor potential cation channel subfamily M member 1 isoform X4 | 570 | 4.58E-12 | 61.95 | P:transport |
| Efet.01.135161.g500.t1 | roundabout homolog 1-like isoform X1 | 849 | 5.93E-91 | 63.8 | C:membrane |
| Efet.01.135190.g501.t1 | centrosomal of 135 kDa-like isoform X1 | 423 | 4.09E-16 | 80.3 | P:centriole replication; P:centriole-centriole cohesion |
| Efet.01.135195.g502.t1 | thyrotropin-releasing hormone receptor-like | 1542 | 1.97E-24 | 55.25 | F:G-protein coupled receptor activity; F:G-protein coupled peptide receptor activity; P:G-protein coupled receptor signaling pathway; P:signal transduction; C:membrane; C:integral component of membrane; F:signal transducer activity |
| Efet.01.135203.g503.t1 | neuroligin- Y-linked-like | 486 | 1.71E-51 | 67.6 | C:membrane |
| Efet.01.135232.g505.t1 | adenylate kinase isoenzyme 6 | 567 | 7.87E-79 | 83.05 | F:ATPase activity; F:adenylate kinase activity; F:ATP binding; P:nucleotide phosphorylation; C:cytoplasm; C:Cajal body |
| Efet.01.13524.g1010.t1 | neuroblast differentiation-associated AHNAK isoform X1 | 1578 | 3.69E-12 | 40.85 | C:cell part |
| Efet.01.135278.g506.t1 | Laminin subunit alpha | 336 | 1.01E-34 | 52.3 | P:peptidyl-tyrosine phosphorylation; F:transmembrane receptor protein tyrosine kinase activity; P:regulation of embryonic development; P:cell adhesion; P:regulation of cell adhesion; F:transferase activity; P:regulation of cell migration; F:receptor binding |
| Efet.01.135281.g508.t1 | MEP- isoform A | 3360 | 5.22E-16 | 44.95 | C:nucleus; F:binding |
| Efet.01.135314.g509.t1 | zinc finger homeobox 4-like isoform X1 | 5415 | 1.73E-125 | 52.1 | F:binding |
| Efet.01.135355.g510.t1 | Dynein heavy chain axonemal | 288 | 1.55E-47 | 86.75 | F:ATPase activity; F:ATP binding; F:microtubule motor activity; C:dynein complex; P:microtubule-based movement |
| Efet.01.135385.g513.t1 | bifunctional glutamate proline--tRNA ligase-like | 531 | 1.08E-100 | 89.6 | P:protein complex assembly; P:glutamyl-tRNA aminoacylation; P:prolyl-tRNA aminoacylation; C:GAIT complex; F:ATP binding; C:intracellular ribonucleoprotein complex; F:proline-tRNA ligase activity; F:GTPase binding; C:membrane; F:glutamate-tRNA ligase activity; F:RNA stem-loop binding; C:cytosol; C:aminoacyl-tRNA synthetase multienzyme complex; P:cellular response to interferon-gamma; P:negative regulation of translation |
| Efet.01.135395.g514.t1 | 2-oxoglutarate mitochondrial isoform X1 | 489 | 2.74E-29 | 83 | F:thiamine pyrophosphate binding; C:mitochondrial membrane; C:mitochondrial matrix; F:metal ion binding; F:oxoglutarate dehydrogenase (succinyl-transferring) activity; P:glycolytic process; P:tricarboxylic acid cycle |
| Efet.01.13545.g1011.t1 | cation channel sperm-associated 2-like | 279 | 2.14E-10 | 60.8 | P:transmembrane transport; P:ion transmembrane transport; F:cation channel activity; F:voltage-gated ion channel activity; P:transport; C:plasma membrane; F:ion channel activity; F:calcium activated cation channel activity; P:ion transport; P:flagellated sperm motility; C:membrane; P:cation transmembrane transport; P:calcium ion transport; C:integral component of membrane; P:regulation of ion transmembrane transport; P:membrane depolarization during action potential; C:CatSper complex |
| Efet.01.135513.g518.t1 | apoptosis stimulating of | 336 | 2.03E-51 | 84.3 | C:nucleus; F:p53 binding; P:intrinsic apoptotic signaling pathway |
| Efet.01.135528.g519.t1 | piezo-type mechanosensitive ion channel component 2 isoform X3 | 459 | 3.24E-07 | 47.5 |  |
| Efet.01.135531.g520.t1 | hypothetical protein HELRODRAFT_189841 | 1842 | 2.51E-23 | 48.25 | C:membrane; C:integral component of membrane |
| Efet.01.135549.g522.t1 | LRR and PYD domains-containing 14-like isoform X4 | 975 | 7.29E-75 | 58.95 |  |
| Efet.01.135556.g524.t1 | DC-STAMP domain-containing 2-like | 1032 | 1.96E-71 | 50.75 | F:zinc ion binding; C:membrane; C:integral component of membrane |
| Efet.01.135564.g526.t1 | E3 ubiquitin- ligase PDZRN3-like | 1110 | 1.24E-09 | 76.75 | F:ubiquitin-protein transferase activity; F:zinc ion binding; P:protein ubiquitination |
| Efet.01.135624.g527.t1 | FAM136A-like | 294 | 2.22E-37 | 70.2 |  |
| Efet.01.13566.g1012.t1 | glycine receptor subunit alpha-3-like isoform X1 | 207 | 6.24E-20 | 69.05 | P:ion transport; C:membrane |
| Efet.01.135665.g528.t1 | histone acetyltransferase p300-like | 855 | 1.85E-36 | 47.75 | F:histone acetyltransferase activity; C:nucleus; F:transferase activity, transferring acyl groups; F:zinc ion binding; F:transcription cofactor activity; F:transcription coactivator activity; F:metal ion binding; P:regulation of transcription, DNA-templated; P:histone acetylation; F:transferase activity; C:histone acetyltransferase complex |
| Efet.01.135694.g531.t1 | mu-type opioid receptor-like | 936 | 5.83E-60 | 58.45 | F:G-protein coupled receptor activity; P:signal transduction; C:membrane |
| Efet.01.135706.g532.t1 | hypothetical protein CAPTEDRAFT_222036 | 219 | 1.75E-10 | 64 | C:membrane; C:integral component of membrane |
| Efet.01.135715.g533.t1 | allatostatin-A receptor-like | 1176 | 5.43E-85 | 61.7 | F:G-protein coupled receptor activity; P:transmembrane transport; P:G-protein coupled receptor signaling pathway; P:signal transduction; C:membrane; C:integral component of membrane; F:metal ion transmembrane transporter activity; P:metal ion transport; F:signal transducer activity |
| Efet.01.135717.g534.t1 | hypothetical protein CAPTEDRAFT_191673 | 1542 | 8.16E-103 | 54.8 | F:transferase activity, transferring glycosyl groups; C:membrane; C:integral component of membrane; P:Wnt signaling pathway; P:negative regulation of Wnt signaling pathway; F:beta-catenin binding |
| Efet.01.135733.g538.t1 | guanine nucleotide-binding G(I) G(S) G(O) subunit gamma-5 | 201 | 1.60E-23 | 66.65 | C:heterotrimeric G-protein complex; P:G-protein coupled receptor signaling pathway; F:signal transducer activity |
| Efet.01.135754.g540.t1 | Collagen alpha-1(XII) chain | 261 | 1.30E-09 | 60.75 | C:collagen trimer |
| Efet.01.135873.g542.t1 | PREDICTED: uncharacterized protein LOC106159026 | 1695 | 2.66E-35 | 50.67 | C:membrane; C:integral component of membrane; P:regulation of apoptotic process |
| Efet.01.135897.g544.t1 | Four and a half LIM domains | 321 | 2.57E-10 | 54.25 | F:identical protein binding; F:zinc ion binding; F:transcription factor binding; P:response to hormone; P:heart trabecula formation; P:regulation of transcription from RNA polymerase II promoter; P:negative regulation of transcription from RNA polymerase II promoter; C:nucleus; C:focal adhesion; P:negative regulation of transcription, DNA-templated; F:metal ion binding; P:negative regulation of apoptotic process; P:osteoblast differentiation; C:actin cytoskeleton; P:atrial cardiac muscle cell development; P:ventricular cardiac muscle cell development |
| Efet.01.136001.g548.t1 | ran-binding 3-like | 426 | 4.48E-12 | 61.05 | P:intracellular transport; C:intracellular |
| Efet.01.136013.g549.t1 | calcium-activated potassium channel alpha | 441 | 9.10E-36 | 65.5 | F:calcium-activated potassium channel activity; P:potassium ion transport; P:transmembrane transport; C:integral component of membrane |
| Efet.01.13603.g1013.t1 | inositol-3-phosphate synthase 1-A-like | 432 | 2.47E-53 | 74.8 | F:inositol-3-phosphate synthase activity; P:inositol biosynthetic process; P:phospholipid biosynthetic process |
| Efet.01.136066.g552.t1 | acetylcholine receptor subunit beta-like 1 isoform X6 | 546 | 3.79E-59 | 76.2 | C:postsynaptic membrane; P:cation transmembrane transport; C:integral component of membrane; C:cell junction; F:extracellular ligand-gated ion channel activity |
| Efet.01.136082.g553.t1 | Myosin heavy muscle | 234 | 8.86E-08 | 84 | C:myosin complex; F:motor activity |
| Efet.01.136089.g554.t1 | peptidase C19 family | 732 | 2.08E-21 | 45.75 | P:macromolecule metabolic process; F:peptidase activity; P:primary metabolic process; P:cellular process |
| Efet.01.136172.g557.t1 | enhancer of mRNA-decapping 4 isoform X1 | 324 | 5.30E-13 | 65.75 | C:intracellular membrane-bounded organelle |
| Efet.01.13619.g1014.t1 | cytoplasmic dynein 1 heavy chain 1-like | 2313 | 0 | 75.85 | F:ATPase activity; F:ATP binding; F:microtubule motor activity; C:dynein complex; P:microtubule-based movement |
| Efet.01.136195.g558.t1 | ankyrin and armadillo repeat-containing -like | 1794 | 3.95E-115 | 51.25 | C:nucleus; C:cytoplasm |
| Efet.01.136213.g559.t1 | titin-like isoform X2 | 1248 | 7.39E-72 | 50.75 | F:nucleotide binding; P:regulation of Rho protein signal transduction; F:ATP binding; C:membrane; C:integral component of membrane; F:protein kinase activity; F:Rho guanyl-nucleotide exchange factor activity; P:phosphorylation; F:transferase activity; P:protein phosphorylation; F:kinase activity; P:positive regulation of GTPase activity |
| Efet.01.136219.g560.t1 | hypothetical protein CAPTEDRAFT_199057 | 1296 | 7.10E-99 | 53.25 | F:transferase activity, transferring glycosyl groups; C:membrane; C:integral component of membrane |
| Efet.01.136224.g561.t1 | rho GTPase-activating 39-like isoform X3 | 243 | 1.41E-30 | 81.2 | C:cytoskeleton; P:signal transduction |
| Efet.01.13624.g1015.t1 | E3 ubiquitin- ligase MIB2-like isoform X1 | 381 | 4.15E-19 | 52.45 | F:phospholipase A2 activity; F:ubiquitin-protein transferase activity; F:zinc ion binding; C:cytoplasm; F:metal ion binding; F:hydrolase activity; P:protein ubiquitination |
| Efet.01.13630.g1016.t1 | plastin-2 isoform X2 | 249 | 1.53E-20 | 78.05 | F:nicotinate-nucleotide diphosphorylase (carboxylating) activity; F:actin binding; F:calcium ion binding; C:integral component of membrane; P:NAD biosynthetic process |
| Efet.01.136340.g563.t1 | Membrane-associated progesterone receptor component 2 | 303 | 9.14E-08 | 70.1 | F:oxidoreductase activity; C:integral component of membrane |
| Efet.01.13635.g1017.t1 | probable phosphatase CG10417 | 348 | 1.41E-26 | 76.4 | F:protein serine/threonine phosphatase activity; F:metal ion binding; P:protein dephosphorylation |
| Efet.01.136354.g564.t1 | beta-1,3-galactosyltransferase 1-like | 1131 | 1.76E-69 | 57.35 | C:membrane; F:transferase activity |
| Efet.01.136375.g567.t1 | transcription factor jun-D-like | 972 | 2.79E-42 | 52.5 | P:response to organic substance; P:regulation of transcription, DNA-templated; F:binding |
| Efet.01.136390.g568.t1 | 1,4-butanediol diacrylate esterase | 738 | 7.09E-164 | 78.45 |  |
| Efet.01.136421.g571.t1 | hypothetical protein HELRODRAFT_193290 | 396 | 1.62E-33 | 65 | C:membrane |
| Efet.01.136447.g572.t1 | probable G- coupled receptor 139 isoform X1 | 999 | 1.97E-76 | 58.2 | C:membrane |
| Efet.01.136471.g573.t1 | excitatory amino acid transporter-like | 519 | 2.97E-64 | 83.65 | C:presynaptic membrane; P:multicellular organism growth; P:L-glutamate transmembrane transport; P:telencephalon development; P:L-glutamate import; P:response to wounding; P:response to amino acid; P:positive regulation of glucose import; F:high-affinity glutamate transmembrane transporter activity; P:visual behavior; C:cell surface; C:integral component of plasma membrane; P:cellular response to extracellular stimulus; C:dendritic shaft; P:response to drug; C:dendritic spine; F:sodium:dicarboxylate symporter activity; P:multicellular organism aging; C:axolemma; P:D-aspartate import; P:adult behavior; F:glutamate:sodium symporter activity |
| Efet.01.136474.g574.t1 | hypothetical protein BRAFLDRAFT_75947 | 246 | 9.54E-06 | 71 | F:metal ion binding; C:synapse; P:intracellular protein transport; F:Rab GTPase binding; C:intracellular |
| Efet.01.136565.g578.t1 | F-box only 21-like | 219 | 6.21E-21 | 74.6 | F:DNA binding |
| Efet.01.136589.g583.t1 | pikachurin-like isoform X2 | 222 | 9.30E-15 | 68.6 | F:calcium ion binding |
| Efet.01.136650.g586.t1 | hypothetical protein CAPTEDRAFT_152663 | 633 | 1.30E-20 | 74 | P:ion transmembrane transport; C:integral component of membrane; F:extracellular ligand-gated ion channel activity |
| Efet.01.136661.g587.t1 | dystonin isoform X1 | 894 | 4.13E-47 | 52.85 | P:cell cycle arrest; C:cytoskeleton; F:calcium ion binding |
| Efet.01.136685.g588.t1 | GGDEF domain-containing | 1209 | 0 | 78.75 | F:phosphorelay sensor kinase activity; P:phosphorelay signal transduction system; P:signal transduction by protein phosphorylation; C:intracellular |
| Efet.01.136744.g591.t1 | junctophilin-1-like isoform X1 | 1170 | 4.60E-148 | 72.25 |  |
| Efet.01.13681.g1018.t1 | ubiquitin carboxyl-terminal hydrolase CYLD | 402 | 1.55E-45 | 70.35 | P:protein deubiquitination; F:thiol-dependent ubiquitinyl hydrolase activity |
| Efet.01.136846.g594.t1 | zinc finger 474 | 204 | 3.25E-21 | 74.2 |  |
| Efet.01.13690.g1019.t1 | peroxisomal multifunctional enzyme type 2 | 915 | 5.81E-77 | 62 | F:oxidoreductase activity; P:oxidation-reduction process |
| Efet.01.136977.g601.t1 | dopamine D2-like receptor | 450 | 1.60E-16 | 88.85 | C:integral component of plasma membrane; F:dopamine neurotransmitter receptor activity; P:dopamine receptor signaling pathway |
| Efet.01.136987.g602.t1 | alkyldihydroxyacetonephosphate peroxisomal-like | 486 | 1.22E-57 | 62 | F:catalytic activity; C:intracellular membrane-bounded organelle |
| Efet.01.13700.g1020.t1 | fibroblast growth factor receptor substrate 2-like | 858 | 2.93E-33 | 67.9 | F:insulin receptor binding |
| Efet.01.137003.g604.t1 | peroxisomal leader peptide-processing protease-like isoform X2 | 423 | 3.53E-22 | 56.6 | C:peroxisome; F:identical protein binding; P:regulation of fatty acid beta-oxidation; P:proteolysis; C:membrane; F:serine-type endopeptidase activity; P:protein homooligomerization; F:protease binding; P:protein processing |
| Efet.01.137045.g607.t1 | nucleolar complex 3 homolog | 330 | 5.60E-22 | 64.55 | C:nuclear speck; P:fat cell differentiation; C:nucleolus; F:binding |
| Efet.01.137063.g608.t1 | GTP-binding nuclear GSP1 Ran | 207 | 1.27E-21 | 96.9 | F:GTP binding; C:nucleus; P:small GTPase mediated signal transduction; F:GTPase activity; C:cytosol; P:intracellular protein transport; P:negative regulation of G2/M transition of mitotic cell cycle; P:poly(A)+ mRNA export from nucleus |
| Efet.01.137089.g609.t1 | D2-like isoform X3 | 366 | 9.40E-66 | 85.35 |  |
| Efet.01.137155.g612.t1 | interleukin-17 receptor A-like | 819 | 2.03E-06 | 42 |  |
| Efet.01.137181.g613.t1 | atrial natriuretic peptide receptor 1-like | 246 | 9.98E-11 | 60.55 | P:signal transduction; F:phosphorus-oxygen lyase activity; C:membrane; C:cell part; P:cyclic nucleotide biosynthetic process; F:cyclase activity |
| Efet.01.1372.g125.t1 | myosin heavy chain isoform | 438 | 3.49E-50 | 81.9 | C:myosin complex; F:ATP binding; F:actin binding; P:metabolic process; F:amino acid binding; F:motor activity |
| Efet.01.1372.g126.t1 | myosin heavy striated muscle-like isoform X7 | 2133 | 0 | 79.55 | C:myosin complex; F:ATP binding; F:actin binding; P:metabolic process; F:amino acid binding; F:motor activity |
| Efet.01.1372.g127.t1 | myosin head | 642 | 3.83E-44 | 71.45 | F:nucleotide binding; F:anion binding |
| Efet.01.1372.g128.t1 | AChain Rigor-Like Structures Of Muscle Myosins Reveal Key Mechanical Elements In The Transduction Pathways Of This Allosteric Motor | 450 | 1.51E-91 | 89.3 | F:ATP binding; F:calmodulin binding; F:actin binding; C:myosin filament; C:myofibril; F:motor activity |
| Efet.01.137222.g614.t1 | sperm receptor for egg jelly-like | 576 | 2.12E-11 | 49.67 |  |
| Efet.01.137261.g615.t1 | gelsolin 2 | 612 | 4.20E-13 | 65.9 | F:actin binding |
| Efet.01.137274.g617.t1 | E3 ubiquitin- ligase RNF34 isoform X3 | 345 | 1.92E-22 | 70.4 | P:negative regulation of extrinsic apoptotic signaling pathway via death domain receptors; F:zinc ion binding; F:ubiquitin protein ligase activity; P:nucleotide-binding domain, leucine rich repeat containing receptor signaling pathway; P:protein K48-linked ubiquitination; F:phosphatidylinositol phosphate binding; P:negative regulation of cysteine-type endopeptidase activity involved in execution phase of apoptosis; P:negative regulation of signal transduction by p53 class mediator; C:plasma membrane; P:protein ubiquitination involved in ubiquitin-dependent protein catabolic process; C:nucleoplasm; F:p53 binding; P:proteasome-mediated ubiquitin-dependent protein catabolic process; F:ubiquitin protein ligase binding; C:cytosol; F:ligase activity |
| Efet.01.13729.g1021.t1 | Suppressor of cytokine signaling 7 | 348 | 1.17E-37 | 80.25 | C:cytoplasm; P:cytokine-mediated signaling pathway; F:protein kinase inhibitor activity; P:negative regulation of protein kinase activity; P:regulation of growth; P:intracellular signal transduction; P:negative regulation of JAK-STAT cascade |
| Efet.01.137298.g619.t1 | kinase associated domain 1 | 420 | 1.35E-55 | 72.7 | C:microtubule cytoskeleton; P:cytoskeleton organization; F:protein serine/threonine kinase activity; P:protein phosphorylation; C:plasma membrane; F:magnesium ion binding; F:tau-protein kinase activity; F:ATP binding; F:phosphatidylinositol-4,5-bisphosphate binding; F:phosphatidylserine binding; P:neuron migration; C:cytoplasm; F:phosphatidic acid binding; P:intracellular signal transduction |
| Efet.01.137299.g620.t1 | glycogen muscle form-like | 336 | 1.30E-52 | 87.4 | F:glycogen phosphorylase activity; P:carbohydrate metabolic process; F:pyridoxal phosphate binding |
| Efet.01.137301.g621.t1 | endoplasmic reticulum metallopeptidase 1 | 636 | 2.97E-39 | 62.25 | C:membrane; C:integral component of membrane |
| Efet.01.137315.g622.t1 | high affinity cAMP-specific and IBMX-insensitive 3 ,5 -cyclic phosphodiesterase 8B-like isoform X5 | 405 | 2.09E-29 | 78.75 | F:phosphoric diester hydrolase activity |
| Efet.01.137323.g623.t1 | potassium voltage-gated channel Shaw-like isoform X1 | 243 | 2.45E-30 | 81.2 | F:voltage-gated potassium channel activity; P:potassium ion transmembrane transport; F:metal ion binding; C:voltage-gated potassium channel complex; P:regulation of ion transmembrane transport; P:protein homooligomerization |
| Efet.01.137348.g625.t1 | corticotropin-releasing factor-binding -like | 408 | 2.20E-27 | 55.6 | F:corticotropin-releasing hormone binding |
| Efet.01.13736.g1022.t1 | Inositol 1,4,5-trisphosphate receptor type | 690 | 7.00E-76 | 76.3 | P:inositol phosphate-mediated signaling; C:integral component of membrane; F:inositol 1,4,5-trisphosphate-sensitive calcium-release channel activity; P:calcium ion transmembrane transport; C:endoplasmic reticulum |
| Efet.01.13740.g1023.t1 | dynein beta ciliary-like | 1482 | 4.97E-146 | 74.75 | F:ATPase activity; F:ATP binding; F:microtubule motor activity; C:cilium; C:dynein complex; P:microtubule-based movement |
| Efet.01.137401.g629.t1 | aldehyde dehydrogenase family 8 member A1 | 273 | 6.57E-13 | 91.6 | F:aldehyde dehydrogenase (NAD) activity; F:GTP binding; F:GTPase activity; P:liver development; F:retinal dehydrogenase activity; C:integral component of membrane; P:oxidation-reduction process |
| Efet.01.137404.g630.t1 | transcription initiation factor TFIID subunit 3 isoform X1 | 507 | 1.15E-14 | 57.3 | F:translation initiation factor activity; F:zinc ion binding; F:metal ion binding; P:translational initiation |
| Efet.01.137420.g632.t1 | serine-rich adhesin for platelets-like | 5097 | 8.70E-43 | 63.07 | F:SMAD binding |
| Efet.01.137421.g633.t1 | hypothetical protein CAPTEDRAFT_212009 | 615 | 2.54E-32 | 52 |  |
| Efet.01.137425.g634.t1 | transmembrane 169 | 789 | 3.16E-40 | 62.85 | C:membrane; C:integral component of membrane |
| Efet.01.137467.g637.t1 | calcyphosin | 603 | 2.13E-41 | 58.25 | F:calcium ion binding |
| Efet.01.137473.g638.t1 | F-box only 43 | 1179 | 5.58E-07 | 73 |  |
| Efet.01.137515.g640.t1 | kinesin-associated 3-like | 633 | 5.63E-82 | 68.1 | C:membrane; C:integral component of membrane; F:kinesin binding; C:kinesin complex |
| Efet.01.137525.g641.t1 | lysine-specific histone demethylase 1A-like isoform X1 | 525 | 7.15E-39 | 85.7 | C:nucleus; F:DNA binding; F:oxidoreductase activity; F:flavin adenine dinucleotide binding; P:regulation of transcription, DNA-templated; P:histone H3-K4 demethylation; F:methyltransferase activity; P:oxidation-reduction process; P:methylation |
| Efet.01.137568.g642.t1 | limb region 1 | 396 | 9.97E-48 | 79.95 | C:integral component of membrane; P:embryonic digit morphogenesis |
| Efet.01.137584.g644.t1 | axin 1 isoform | 882 | 2.27E-57 | 62.3 | C:beta-catenin destruction complex; P:signal transduction; F:receptor signaling complex scaffold activity |
| Efet.01.137589.g645.t1 | hypothetical protein CAPTEDRAFT_144547 | 1821 | 8.41E-107 | 49.65 | F:calcium ion binding; C:membrane; C:integral component of membrane; P:cell adhesion; P:homophilic cell adhesion via plasma membrane adhesion molecules; C:plasma membrane |
| Efet.01.137618.g646.t1 | Neuropeptide FF receptor 2 | 921 | 1.30E-57 | 63.55 | F:neuropeptide receptor activity; P:G-protein coupled receptor signaling pathway; C:membrane |
| Efet.01.137653.g647.t1 | hypothetical protein CAPTEDRAFT_209319 | 453 | 8.45E-07 | 59 |  |
| Efet.01.137664.g648.t1 | BTB and MATH domain-containing 38-like | 630 | 1.76E-82 | 60.95 |  |
| Efet.01.1377.g129.t1 | sentrin-specific protease 6-like isoform X1 | 555 | 5.71E-39 | 67.1 | P:proteolysis; F:cysteine-type peptidase activity |
| Efet.01.137709.g650.t1 | hypothetical protein CAPTEDRAFT_202676 | 510 | 3.28E-26 | 77.8 | C:integral component of membrane |
| Efet.01.137726.g651.t1 | cytosolic carboxypeptidase 1 | 837 | 3.24E-69 | 63.35 | F:zinc ion binding; F:metallocarboxypeptidase activity; P:proteolysis |
| Efet.01.13773.g1025.t1 | methionine aminopeptidase 2-like | 1005 | 0 | 88.4 | P:protein initiator methionine removal; C:cytoplasm; F:metal ion binding; P:proteolysis; F:metalloaminopeptidase activity |
| Efet.01.137754.g654.t1 | rho GTPase-activating 1-like isoform X4 | 480 | 1.62E-71 | 73.95 | P:signal transduction |
| Efet.01.137780.g655.t1 | solute carrier family 45 member 3-like | 1629 | 1.35E-177 | 65.45 | C:membrane |
| Efet.01.137784.g656.t1 | secretory carrier-associated membrane 2 | 315 | 3.07E-18 | 75.25 | C:intracellular membrane-bounded organelle; P:protein transport; C:integral component of membrane; C:endomembrane system; C:cytoplasmic part; C:intracellular organelle part |
| Efet.01.137788.g657.t1 | lysine-specific demethylase 7B-like | 306 | 5.14E-21 | 63.91 | C:nucleus; F:zinc ion binding; F:metal ion binding |
| Efet.01.137795.g658.t1 | Dol-P-Glc:Glc(2)Man(9) c(2)-PP-Dol alpha-1,2-glucosyltransferase | 639 | 8.82E-21 | 59.3 | C:membrane part; F:transferase activity |
| Efet.01.137812.g662.t1 | midasin-like isoform X2 | 1335 | 6.36E-36 | 48.4 | F:nucleotide binding; F:ATPase activity; C:nucleus; F:ATP binding; P:ribosomal large subunit assembly |
| Efet.01.137812.g663.t1 | midasin-like | 993 | 1.27E-52 | 64.45 | F:nucleotide binding; F:ATPase activity; C:nucleus; F:ATP binding; P:ribosomal large subunit assembly |
| Efet.01.137871.g664.t1 | beta-1,3-galactosyl-O-glycosyl-glyco beta-1,6-N-acetylglucosaminyltransferase 3-like | 1293 | 9.38E-110 | 66.95 | C:membrane |
| Efet.01.137915.g667.t1 | 2-hydroxyacylsphingosine 1-beta-galactosyltransferase-like | 1608 | 3.28E-85 | 54.25 | F:transferase activity |
| Efet.01.137988.g669.t1 | p53-induced death domain-containing 1-like isoform X2 | 723 | 1.52E-07 | 53 | P:signal transduction |
| Efet.01.138007.g672.t1 | transcription cofactor vestigial 4 isoform X2 | 417 | 1.01E-13 | 52.25 | P:regulation of transcription, DNA-templated |
| Efet.01.138016.g673.t1 | Retinal dehydrogenase 1 | 351 | 7.36E-34 | 74 | F:oxidoreductase activity; P:metabolic process |
| Efet.01.138022.g674.t1 | hypothetical protein CAPTEDRAFT_222901, partial | 267 | 3.10E-09 | 64 |  |
| Efet.01.138038.g675.t1 | neuron navigator 2 isoform X6 | 288 | 6.09E-09 | 52 |  |
| Efet.01.138074.g676.t1 | twitchin-like isoform X2 | 339 | 3.07E-35 | 68.4 | F:ATP binding; F:protein kinase activity; P:protein phosphorylation |
| Efet.01.138103.g677.t1 | Wnt-6 | 447 | 2.69E-56 | 68.7 | C:extracellular region; P:single-organism process |
| Efet.01.138154.g678.t1 | hypothetical protein CAPTEDRAFT_213274 | 546 | 1.10E-14 | 61.5 | F:insulin receptor binding |
| Efet.01.138228.g679.t1 | LIM and SH3 domain | 249 | 4.72E-15 | 83.9 | F:ATP binding; F:microtubule motor activity; F:zinc ion binding; F:substrate-specific transmembrane transporter activity; P:transmembrane transport; F:structural constituent of ribosome; C:mitochondrion; C:integral component of membrane; C:ribosome; P:microtubule-based movement; F:microtubule binding; P:translation |
| Efet.01.138239.g680.t1 | transporter SVOPL | 1230 | 2.62E-97 | 59.65 | F:transmembrane transporter activity; C:membrane |
| Efet.01.138281.g681.t1 | cilia- and flagella-associated 36-like | 567 | 1.40E-16 | 70.1 |  |
| Efet.01.138334.g683.t1 | EF-hand domain-containing 1-like | 1089 | 0 | 80.9 | C:motile cilium; F:calcium ion binding |
| Efet.01.138347.g684.t1 | gastrula zinc finger -like | 1968 | 6.82E-47 | 58.25 | F:nucleic acid binding; F:metal ion binding |
| Efet.01.13838.g1027.t1 | chromodomain-helicase-DNA-binding 8-like | 549 | 6.09E-51 | 70.05 | F:DNA binding; C:nucleus; F:ATP binding; F:hydrolase activity, acting on acid anhydrides; F:helicase activity |
| Efet.01.138414.g685.t1 | neuronal acetylcholine receptor subunit alpha-10-like | 324 | 2.10E-20 | 85.3 | C:postsynaptic membrane; P:cation transmembrane transport; C:integral component of membrane; C:cell junction; F:extracellular ligand-gated ion channel activity |
| Efet.01.138427.g686.t1 | LMBR1 domain-containing 2 | 459 | 6.85E-30 | 88.3 | C:integral component of membrane |
| Efet.01.138431.g687.t1 | c-binding -like | 708 | 5.57E-19 | 44.05 | C:membrane; C:integral component of membrane |
| Efet.01.138459.g688.t1 | Receptor-type tyrosine- phosphatase | 594 | 1.08E-08 | 61.7 | F:hydrolase activity |
| Efet.01.138477.g689.t1 | RNA-binding 15B | 2070 | 8.40E-147 | 51.8 | F:nucleotide binding; F:nucleic acid binding |
| Efet.01.138483.g690.t1 | discoidin domain-containing receptor 2-like isoform X1 | 513 | 1.04E-63 | 71.5 | F:transmembrane receptor protein tyrosine kinase activity; C:integral component of membrane; P:protein phosphorylation |
| Efet.01.138508.g691.t1 | spermatogenesis-associated 6 | 204 | 6.23E-16 | 70.95 |  |
| Efet.01.138518.g693.t1 | Pre-mRNA-splicing factor ATP-dependent RNA helicase PRP16 | 612 | 4.86E-10 | 52.6 |  |
| Efet.01.138522.g694.t1 | inositol polyphosphate 5-phosphatase K-like | 522 | 6.86E-43 | 62.6 | F:hydrolase activity; P:phosphatidylinositol dephosphorylation |
| Efet.01.13857.g1028.t1 | zinc finger 774 | 684 | 5.91E-50 | 52.6 | F:nucleic acid binding; F:metal ion binding |
| Efet.01.138649.g697.t1 | FMRFamide receptor-like | 1116 | 2.74E-103 | 61.15 | F:G-protein coupled receptor activity; F:G-protein coupled peptide receptor activity; P:G-protein coupled receptor signaling pathway; C:membrane; C:integral component of membrane |
| Efet.01.138652.g698.t1 | g1 s-specific cyclin-d2 | 405 | 2.48E-10 | 61.8 | C:nucleus; P:cell cycle |
| Efet.01.138671.g699.t1 | pleckstrin homology domain-containing family G member 5-like isoform X1 | 285 | 1.34E-20 | 74.2 | P:regulation of Rho protein signal transduction; C:membrane; F:Rho guanyl-nucleotide exchange factor activity; P:positive regulation of GTPase activity |
| Efet.01.138694.g701.t1 | hypothetical protein CAPTEDRAFT_228538 | 366 | 9.06E-11 | 55 |  |
| Efet.01.138695.g702.t1 | AP-2 complex subunit mu-like | 345 | 9.74E-73 | 97.25 | P:negative regulation of protein localization to plasma membrane; C:secretory granule; F:lipid binding; F:signal sequence binding; P:intracellular protein transport; P:endocytosis; F:ion channel binding; C:plasma membrane; C:extracellular exosome; F:low-density lipoprotein particle receptor binding; C:terminal bouton; C:clathrin-coated pit; C:mitochondrion; C:clathrin adaptor complex |
| Efet.01.138697.g703.t1 | calmodulin 5 | 249 | 6.08E-20 | 67.45 | C:extracellular exosome; F:calcium ion binding |
| Efet.01.138710.g704.t1 | cysteine-rich motor neuron 1 | 717 | 8.46E-10 | 44.56 | F:serine-type endopeptidase inhibitor activity; C:extracellular exosome; P:negative regulation of catalytic activity; F:enzyme inhibitor activity; P:regulation of cell growth; C:membrane; C:integral component of membrane; F:PDZ domain binding; P:negative regulation of endopeptidase activity; C:extracellular region; F:insulin-like growth factor binding |
| Efet.01.138731.g706.t1 | E3 ubiquitin- ligase TRIM71 | 924 | 2.67E-21 | 49.17 | F:zinc ion binding; F:metal ion binding; C:intracellular |
| Efet.01.138762.g708.t1 | immunoglobulin I-set domain | 663 | 3.73E-19 | 51.75 | F:nucleotide binding; P:regulation of Rho protein signal transduction; F:ATP binding; F:protein kinase activity; F:Rho guanyl-nucleotide exchange factor activity; P:phosphorylation; F:transferase activity; P:protein phosphorylation; F:kinase activity; P:positive regulation of GTPase activity |
| Efet.01.138797.g709.t1 | hypothetical protein HELRODRAFT_160537 | 348 | 3.21E-39 | 67.75 | C:membrane; F:carbohydrate binding |
| Efet.01.138816.g710.t1 | N-acetylated-alpha-linked acidic dipeptidase 2-like isoform X5 | 402 | 8.25E-11 | 63.86 | F:carboxypeptidase activity; P:proteolysis; C:membrane; C:integral component of membrane; F:hydrolase activity |
| Efet.01.138845.g711.t1 | Bloom syndrome homolog isoform X1 | 2943 | 0 | 72.75 | F:nucleic acid binding; F:ATP binding; P:DNA repair; F:ATP-dependent 3'-5' DNA helicase activity; P:DNA replication; P:DNA duplex unwinding; P:DNA recombination |
| Efet.01.138851.g712.t1 | partitioning defective 6-like | 762 | 5.07E-93 | 76.8 | P:cell division; C:bicellular tight junction; C:cytoplasm; C:protein complex; C:apical plasma membrane; P:cell cycle |
| Efet.01.138853.g713.t1 | hypothetical protein HELRODRAFT_185255 | 210 | 1.92E-06 | 73 | C:cytoskeleton; F:actin binding; C:cytoplasm; C:extrinsic component of membrane; F:cytoskeletal protein binding |
| Efet.01.138870.g714.t1 | ATP-dependent DNA helicase pif1 | 405 | 9.20E-25 | 59.7 | P:DNA repair; F:ATP binding; F:DNA helicase activity; P:DNA duplex unwinding; P:telomere maintenance; F:helicase activity |
| Efet.01.138902.g715.t1 | DNA polymerase alpha subunit B isoform X4 | 318 | 3.29E-38 | 76.25 | P:DNA metabolic process; P:cellular macromolecule biosynthetic process |
| Efet.01.138915.g716.t1 | mucin-5AC-like isoform X1 | 627 | 2.06E-10 | 78.8 | P:inactivation of MAPK activity; F:protein tyrosine phosphatase activity; P:peptidyl-tyrosine dephosphorylation; F:MAP kinase tyrosine/serine/threonine phosphatase activity |
| Efet.01.138975.g719.t1 | dual specificity kinase CLK2 isoform X2 | 531 | 2.50E-37 | 53.95 | F:kinase activity |
| Efet.01.139002.g720.t1 | Suppressor of cytokine signaling 6 | 1488 | 1.53E-81 | 78.85 | C:cytoplasm; P:cytokine-mediated signaling pathway; F:protein kinase inhibitor activity; P:negative regulation of protein kinase activity; P:regulation of growth; P:intracellular signal transduction; P:negative regulation of JAK-STAT cascade |
| Efet.01.139008.g721.t1 | cilia- and flagella-associated 45-like | 402 | 8.81E-32 | 70.7 |  |
| Efet.01.139023.g723.t1 | fem-1 homolog C-like | 1875 | 0 | 75.65 |  |
| Efet.01.139038.g725.t1 | piggyBac transposable element-derived 4-like | 1071 | 1.80E-74 | 60.3 |  |
| Efet.01.139123.g728.t1 | probable ATP-dependent RNA helicase DHX34 | 3594 | 0 | 71.85 | F:nucleotide binding; F:hydrolase activity |
| Efet.01.13915.g1029.t1 | DNA N6-methyl adenine demethylase-like isoform X1 | 450 | 9.91E-32 | 81.2 | F:nucleotide binding; C:cytoskeleton |
| Efet.01.13915.g1030.t1 | Methylcytosine dioxygenase TET2 | 771 | 5.04E-19 | 80 | F:dioxygenase activity; P:oxidation-reduction process |
| Efet.01.139194.g730.t1 | 5-hydroxytryptamine receptor-like | 1551 | 1.09E-95 | 76.8 | P:cell proliferation; P:regulation of behavior; C:integral component of plasma membrane; P:G-protein coupled receptor signaling pathway; P:regulation of hormone secretion; P:serotonin receptor signaling pathway; P:vasoconstriction; F:G-protein coupled serotonin receptor activity |
| Efet.01.139204.g731.t1 | NADH-cytochrome b5 reductase 2 | 213 | 4.45E-11 | 76.15 | F:cytochrome-b5 reductase activity, acting on NAD(P)H; C:intracellular membrane-bounded organelle; C:membrane; P:oxidation-reduction process |
| Efet.01.139310.g735.t1 | palmitoleoyl- carboxylesterase NOTUM | 324 | 3.18E-39 | 72.3 | F:hydrolase activity |
| Efet.01.139358.g737.t1 | filamin-C isoform X1 | 507 | 9.05E-68 | 64.7 | F:actin filament binding; F:actin binding |
| Efet.01.13936.g1032.t1 | hunchback transcription factor | 3087 | 2.10E-72 | 60.35 | F:nucleic acid binding; C:nucleus; F:transcription factor activity, sequence-specific DNA binding; P:regulation of development, heterochronic; F:metal ion binding; P:transcription, DNA-templated; F:RNA polymerase II transcription factor activity, sequence-specific DNA binding; P:regulation of transcription from RNA polymerase II promoter |
| Efet.01.13938.g1033.t1 | neogenin-like isoform X6 | 330 | 1.02E-33 | 78.25 | C:integral component of plasma membrane; P:positive regulation of BMP signaling pathway; P:iron ion homeostasis; P:axon guidance; F:receptor activity |
| Efet.01.139400.g740.t1 | tyrosine- kinase transmembrane receptor Ror2-like | 1197 | 1.35E-107 | 68.7 | F:protein kinase activity; P:phosphorylation |
| Efet.01.139424.g742.t1 | unc-93 homolog A | 480 | 2.28E-53 | 65.5 | C:integral component of membrane |
| Efet.01.139446.g743.t1 | protocadherin beta-15 isoform X1 | 2430 | 1.24E-129 | 54 | F:calcium ion binding; C:membrane; C:integral component of membrane; P:cell adhesion; P:homophilic cell adhesion via plasma membrane adhesion molecules; C:plasma membrane |
| Efet.01.139469.g745.t1 | hypothetical protein CAPTEDRAFT_199151 | 774 | 3.47E-18 | 58 | F:G-protein coupled receptor activity; F:G-protein coupled peptide receptor activity; P:G-protein coupled receptor signaling pathway; C:membrane; C:integral component of membrane |
| Efet.01.139553.g749.t1 | exportin-7-like isoform X10 | 201 | 4.11E-15 | 85.8 | P:intracellular protein transport; F:Ran GTPase binding; C:intracellular |
| Efet.01.139632.g750.t1 | folliculin-like | 234 | 3.37E-16 | 71.8 | P:negative regulation of phosphate metabolic process; P:regulation of organelle organization; P:negative regulation of intracellular signal transduction; P:positive regulation of signal transduction; C:intracellular part; P:single-organism process; P:negative regulation of nucleobase-containing compound metabolic process; P:cellular process; P:regulation of transcription from RNA polymerase II promoter; P:negative regulation of gene expression; P:negative regulation of cellular biosynthetic process; P:regulation of localization; P:positive regulation of macromolecule metabolic process; P:regulation of protein phosphorylation; P:negative regulation of multicellular organismal process; P:positive regulation of cellular metabolic process; P:regulation of TOR signaling; P:regulation of multicellular organismal development |
| Efet.01.139632.g751.t1 | Folliculin | 222 | 3.70E-08 | 61.7 | P:positive regulation of cellular process; P:regulation of intracellular signal transduction; P:negative regulation of macromolecule metabolic process; P:regulation of protein modification process; P:negative regulation of nucleobase-containing compound metabolic process |
| Efet.01.139671.g753.t1 | serine arginine-rich splicing factor RS31-like isoform X1 | 633 | 8.81E-25 | 63.85 | F:organic cyclic compound binding; F:heterocyclic compound binding |
| Efet.01.139694.g754.t1 | PREDICTED: uncharacterized protein LOC107075466 | 1224 | 7.96E-06 | 45 |  |
| Efet.01.139722.g756.t1 | acyl-coenzyme A amino acid N-acyltransferase 2-like | 225 | 2.11E-10 | 58.55 | P:acyl-CoA metabolic process; F:transferase activity, transferring acyl groups; F:thiolester hydrolase activity; F:transferase activity |
| Efet.01.139786.g757.t1 | tetratricopeptide repeat 30A | 306 | 7.54E-27 | 80.95 | P:intraciliary transport; C:cilium; C:intraciliary transport particle B |
| Efet.01.139844.g758.t1 | ras association domain-containing 1-like isoform X3 | 552 | 1.52E-37 | 63.15 | P:signal transduction; P:intracellular signal transduction; C:intracellular |
| Efet.01.139856.g759.t1 | E3 ubiquitin- ligase UBR2-like | 372 | 3.95E-25 | 69.45 | F:ubiquitin protein ligase activity; C:ubiquitin ligase complex; P:ubiquitin-dependent protein catabolic process via the N-end rule pathway; C:cytoplasm |
| Efet.01.139870.g760.t1 | isoform C | 654 | 1.18E-16 | 48.15 | C:membrane; C:integral component of membrane |
| Efet.01.139876.g761.t1 | transient receptor potential cation channel subfamily M member 3-like | 294 | 7.72E-12 | 68.33 | P:ion transport; P:transmembrane transport; C:membrane |
| Efet.01.139887.g762.t1 | neurogenic locus Notch -like | 1206 | 9.41E-19 | 77 | P:regulation of developmental process; P:Notch signaling pathway; P:multicellular organism development; F:calcium ion binding; C:integral component of membrane; P:cell differentiation |
| Efet.01.13991.g1034.t1 | hypothetical protein HELRODRAFT_183186 | 591 | 1.09E-09 | 89 | C:integral component of membrane |
| Efet.01.139967.g765.t1 | von Willebrand factor D and EGF domain-containing -like | 273 | 7.27E-38 | 56.8 | F:chitin binding; F:calcium ion binding; C:membrane; P:chitin metabolic process; C:integral component of membrane; P:homophilic cell adhesion via plasma membrane adhesion molecules; C:extracellular region |
| Efet.01.139967.g766.t1 | hypothetical protein CAPTEDRAFT_223239 | 237 | 1.06E-32 | 87 |  |
| Efet.01.139973.g767.t1 | S1 RNA-binding domain-containing 1 isoform X1 | 249 | 1.82E-20 | 73.45 | F:nucleic acid binding; P:nucleobase-containing compound metabolic process |
| Efet.01.140014.g769.t1 | cadherin-related tumor suppressor | 951 | 3.47E-48 | 55.6 | F:oxidoreductase activity; F:calcium ion binding; C:membrane; C:integral component of membrane; P:cell adhesion; P:oxidation-reduction process; F:alcohol dehydrogenase (NAD) activity; P:homophilic cell adhesion via plasma membrane adhesion molecules; C:plasma membrane |
| Efet.01.140014.g770.t1 | cadherin-related tumor suppressor | 1686 | 1.29E-71 | 52.3 | F:calcium ion binding; C:membrane; C:integral component of membrane; P:cell adhesion; P:homophilic cell adhesion via plasma membrane adhesion molecules; C:plasma membrane |
| Efet.01.140014.g771.t1 | cadherin-related tumor suppressor | 762 | 1.56E-22 | 55.5 | F:calcium ion binding; C:membrane; C:integral component of membrane; P:cell adhesion; P:homophilic cell adhesion via plasma membrane adhesion molecules; C:plasma membrane |
| Efet.01.140020.g772.t1 | tripartite motif-containing 2-like isoform X1 | 2409 | 1.63E-59 | 54.45 | F:zinc ion binding; F:metal ion binding; C:intracellular |
| Efet.01.140044.g773.t1 | hypothetical protein CAPTEDRAFT_223746 | 237 | 1.53E-15 | 60 |  |
| Efet.01.140057.g774.t1 | Cadherin-23 | 1518 | 9.78E-80 | 48.7 | F:calcium ion binding; C:membrane; C:integral component of membrane; P:cell adhesion; P:homophilic cell adhesion via plasma membrane adhesion molecules; C:plasma membrane |
| Efet.01.140059.g775.t1 | cytochrome c oxidase assembly COX15 homolog | 414 | 2.11E-56 | 75 | F:cytochrome-c oxidase activity; F:oxidoreductase activity, acting on the CH-CH group of donors; C:integral component of membrane; P:heme a biosynthetic process; P:oxidation-reduction process; F:oxidoreductase activity, acting on NAD(P)H, heme protein as acceptor; C:mitochondrial inner membrane; P:respiratory chain complex IV assembly |
| Efet.01.140082.g776.t1 | helicase with zinc finger domain 2-like isoform X1 | 1023 | 4.28E-54 | 54.75 | F:metal ion binding; F:helicase activity |
| Efet.01.140110.g777.t1 | hypothetical protein CAPTEDRAFT_222213 | 591 | 8.46E-15 | 57 |  |
| Efet.01.140145.g779.t1 | hypothetical protein CAPTEDRAFT_213185 | 333 | 1.52E-06 | 51.5 | F:chitin binding; C:membrane; P:chitin metabolic process; C:integral component of membrane; C:extracellular region |
| Efet.01.14015.g1035.t1 | cytosolic carboxypeptidase 6 | 465 | 1.94E-81 | 85.6 | F:tubulin binding; F:zinc ion binding; C:centriole; C:ciliary basal body; F:metallocarboxypeptidase activity; P:proteolysis; P:C-terminal protein deglutamylation; C:cytosol; C:Golgi apparatus; P:protein side chain deglutamylation |
| Efet.01.140171.g780.t1 | cullin-1 | 1410 | 1.82E-31 | 42.45 | F:ubiquitin protein ligase activity; F:ubiquitin protein ligase binding; C:cullin-RING ubiquitin ligase complex; C:SCF ubiquitin ligase complex; P:protein ubiquitination involved in ubiquitin-dependent protein catabolic process; P:ubiquitin-dependent protein catabolic process |
| Efet.01.140171.g781.t1 | cullin-2 | 435 | 5.99E-14 | 56.55 | F:ubiquitin protein ligase binding; C:cullin-RING ubiquitin ligase complex; P:ubiquitin-dependent protein catabolic process |
| Efet.01.140210.g783.t1 | sorting nexin-13-like isoform X3 | 312 | 1.18E-17 | 62.95 | C:membrane; C:integral component of membrane; F:phosphatidylinositol binding |
| Efet.01.140240.g784.t1 | ankyrin repeat | 1611 | 1.10E-37 | 47.25 | P:signal transduction; C:membrane; C:integral component of membrane |
| Efet.01.14025.g1036.t1 | glutamate receptor 3- | 222 | 1.46E-31 | 80.4 | C:postsynaptic membrane; P:ion transmembrane transport; C:integral component of membrane; C:cell junction; P:ionotropic glutamate receptor signaling pathway; F:ionotropic glutamate receptor activity; F:extracellular-glutamate-gated ion channel activity |
| Efet.01.140293.g786.t1 | hypothetical protein CAPTEDRAFT_219540 | 1398 | 2.23E-09 | 57 |  |
| Efet.01.140299.g787.t1 | glycine mitochondrial-like | 336 | 9.12E-42 | 64.45 | F:amidinotransferase activity |
| Efet.01.140300.g788.t1 | multidrug resistance 1-like | 297 | 1.80E-35 | 83.4 | F:ATP binding; F:ATPase activity, coupled to transmembrane movement of substances; P:transmembrane transport; C:integral component of membrane |
| Efet.01.140326.g789.t1 | Histone-lysine N-methyltransferase MLL4 | 903 | 3.58E-78 | 71.2 | F:DNA binding; F:metal ion binding; P:metabolic process; F:transferase activity |
| Efet.01.140364.g790.t1 | 40S ribosomal X isoform | 801 | 5.39E-148 | 90.6 | P:chordate embryonic development; F:structural constituent of ribosome; P:brain development; C:ribosome; F:rRNA binding; P:translation |
| Efet.01.1404.g132.t1 | poly(U)-binding-splicing factor PUF60-like isoform X2 | 291 | 2.51E-39 | 82.05 | F:nucleotide binding; F:RNA binding |
| Efet.01.140413.g791.t1 | collagen alpha-3(VI) chain isoform X1 | 456 | 8.29E-12 | 52.9 | C:extracellular region part |
| Efet.01.140426.g793.t1 | teneurin-m-like isoform X1 | 528 | 1.84E-60 | 56.1 | C:membrane; C:integral component of membrane |
| Efet.01.140426.g794.t1 | teneurin-m-like isoform X8 | 1524 | 0 | 61.65 | F:catalytic activity; C:membrane; C:integral component of membrane |
| Efet.01.14047.g1038.t1 | glycine receptor subunit alpha-3-like isoform X1 | 264 | 1.19E-20 | 81.7 | F:transmitter-gated ion channel activity; P:chloride transmembrane transport; C:external side of plasma membrane; P:protein heterooligomerization; P:regulation of respiratory gaseous exchange by neurological system process; C:perikaryon; C:chloride channel complex; C:integral component of plasma membrane; C:dendrite; P:adult walking behavior; P:cellular response to amino acid stimulus; P:cellular response to zinc ion; P:startle response; C:postsynaptic membrane; P:muscle contraction; F:glycine binding; P:action potential; C:inhibitory synapse; P:synaptic transmission, glycinergic; F:zinc ion binding; P:righting reflex; F:extracellular-glycine-gated chloride channel activity; P:acrosome reaction; P:protein homooligomerization; C:endoplasmic reticulum; P:visual perception; P:cellular response to ethanol; P:neuromuscular process controlling posture; P:neuropeptide signaling pathway; P:negative regulation of transmission of nerve impulse; F:protein binding; F:taurine binding; P:inhibitory postsynaptic potential; P:positive regulation of acrosome reaction; C:cell junction |
| Efet.01.140506.g799.t1 | nuclear hormone receptor HR96-like isoform X8 | 780 | 2.22E-103 | 76.55 | F:thyroid hormone receptor activity; C:nucleus; F:zinc ion binding; P:transcription, DNA-templated; F:steroid hormone receptor activity; P:regulation of transcription, DNA-templated; F:sequence-specific DNA binding; P:steroid hormone mediated signaling pathway; P:intracellular receptor signaling pathway |
| Efet.01.140602.g800.t1 | homeobox prophet of Pit-1-like | 549 | 2.82E-26 | 65.35 | C:nucleus; P:regulation of transcription, DNA-templated; F:sequence-specific DNA binding |
| Efet.01.140618.g802.t1 | Ras-related Rab-33 isoform X2 | 210 | 1.46E-07 | 69.85 | F:GTP binding; P:small GTPase mediated signal transduction; P:antigen processing and presentation; C:intracellular |
| Efet.01.140629.g803.t1 | potassium voltage-gated channel Shaw-like | 249 | 2.97E-13 | 54.55 | P:transport |
| Efet.01.140689.g804.t1 | Polypeptide N-acetylgalactosaminyltransferase 1 | 1488 | 1.70E-138 | 47.45 | C:membrane; P:protein glycosylation; C:cytoplasmic part; F:transferase activity |
| Efet.01.14070.g1039.t1 | ankyrin repeat domain-containing | 1053 | 2.09E-06 | 49.25 |  |
| Efet.01.140705.g805.t1 | chondroitin sulfate N-acetylgalactosaminyltransferase 2-like | 1221 | 9.89E-98 | 65.3 | C:membrane; F:transferase activity |
| Efet.01.140739.g807.t1 | zinc finger 532 | 285 | 2.69E-13 | 63.5 | F:binding |
| Efet.01.140774.g808.t1 | uncharacterized protein LOC110989168 isoform X1 | 324 | 5.68E-16 | 61.55 |  |
| Efet.01.140797.g809.t1 | Excitatory amino acid transporter | 702 | 1.83E-37 | 62.25 | P:dicarboxylic acid transport; P:transmembrane transport; P:single-multicellular organism process; C:membrane; C:cell part; F:symporter activity; F:dicarboxylic acid transmembrane transporter activity; P:acidic amino acid transport; P:response to stimulus; P:single-organism developmental process |
| Efet.01.140823.g810.t1 | 5 -AMP-activated kinase subunit gamma- | 261 | 3.32E-24 | 76.05 | F:protein kinase binding; F:protein kinase activator activity; P:regulation of glycolytic process; F:ADP binding; F:cAMP-dependent protein kinase regulator activity; F:cAMP-dependent protein kinase inhibitor activity; F:phosphorylase kinase regulator activity; F:ATP binding; C:extracellular space; P:glycogen metabolic process; P:activation of protein kinase activity; P:positive regulation of peptidyl-threonine phosphorylation; P:phosphorylation; P:negative regulation of protein serine/threonine kinase activity; F:kinase activity; P:intracellular signal transduction; C:nucleotide-activated protein kinase complex; P:regulation of fatty acid metabolic process |
| Efet.01.140836.g812.t1 | hypothetical protein CAPTEDRAFT_224076 | 3609 | 1.51E-12 | 43 | C:mediator complex; F:RNA polymerase II transcription cofactor activity; P:regulation of transcription from RNA polymerase II promoter |
| Efet.01.140856.g813.t1 | mediator of RNA polymerase II transcription subunit 12 | 804 | 3.55E-95 | 73.35 | C:mediator complex; F:RNA polymerase II transcription cofactor activity; P:regulation of transcription from RNA polymerase II promoter |
| Efet.01.140871.g814.t1 | CBFA2T1-like isoform X6 | 399 | 2.10E-33 | 62.15 | F:transcription factor activity, sequence-specific DNA binding; P:negative regulation of transcription, DNA-templated; F:transcription corepressor activity |
| Efet.01.140892.g815.t1 | U3 small nucleolar RNA-associated 6 homolog | 474 | 6.37E-13 | 42.2 | C:small-subunit processome; P:maturation of SSU-rRNA from tricistronic rRNA transcript (SSU-rRNA, 5.8S rRNA, LSU-rRNA); F:snoRNA binding; P:RNA processing; C:Pwp2p-containing subcomplex of 90S preribosome; C:nucleolus |
| Efet.01.140902.g816.t1 | leucine-rich repeat-containing G- coupled receptor 4-like | 1326 | 5.01E-27 | 48.45 | F:ATP binding; P:peptidyl-tyrosine phosphorylation; F:protein tyrosine kinase activity; C:membrane; C:integral component of membrane; F:protein kinase activity; P:phosphorylation; P:protein phosphorylation; F:kinase activity |
| Efet.01.140907.g818.t1 | AP-3 complex subunit beta-2-like isoform X1 | 276 | 1.71E-09 | 67.45 | C:membrane; P:transport |
| Efet.01.140931.g819.t1 | chondroitin proteoglycan 2-like | 366 | 2.68E-17 | 54.05 | F:chitin binding; P:chitin metabolic process; C:membrane; C:integral component of membrane; C:extracellular region |
| Efet.01.140944.g820.t1 | Catenin beta- | 954 | 9.87E-78 | 61.4 | P:lung-associated mesenchyme development; C:Scrib-APC-beta-catenin complex; F:RNA polymerase II core promoter sequence-specific DNA binding; P:dorsal/ventral axis specification; P:positive regulation of epithelial cell proliferation involved in prostate gland development; P:embryonic axis specification; P:central nervous system vasculogenesis; P:positive regulation of sequence-specific DNA binding transcription factor activity; P:lens morphogenesis in camera-type eye; P:proximal/distal pattern formation; P:anterior/posterior axis specification; P:synaptic vesicle transport; P:positive regulation of skeletal muscle tissue development; F:protein heterodimerization activity; P:in utero embryonic development; P:gastrulation with mouth forming second; P:embryonic brain development; P:cell fate specification; C:lamellipodium; P:regulation of centromeric sister chromatid cohesion; F:protein kinase binding; F:protein phosphatase binding; P:cell morphogenesis involved in differentiation; P:chemical synaptic transmission; P:negative regulation of mesenchymal to epithelial transition involved in metanephros morphogenesis; P:positive regulation of chromatin-mediated maintenance of transcription; P:response to hormone; P:canonical Wnt signaling pathway involved in positive regulation of cardiac outflow tract cell proliferation; F:ion channel binding; P:regulation of nephron tubule epithelial cell differentiation; P:lung cell differentiation; C:Z disc; P:renal outer medulla development; P:renal inner medulla development; P:metanephros morphogenesis; P:regulation of protein localization to cell surface; P:endodermal cell fate commitment; P:male genitalia development; C:fascia adherens; P:mesenchymal cell proliferation involved in lung development; P:cellular response to growth factor stimulus; P:hair follicle morphogenesis; P:adherens junction assembly; P:chromatin-mediated maintenance of transcription; P:layer formation in cerebral cortex; P:regulation of calcium ion import; P:negative regulation of cell proliferation; P:fungiform papilla formation; P:positive regulation of I-kappaB kinase/NF-kappaB signaling; P:hair follicle placode formation; P:canonical Wnt signaling pathway involved in positive regulation of epithelial to mesenchymal transition; P:positive regulation of osteoblast differentiation; P:positive regulation of transcription from RNA polymerase II promoter; P:convergent extension; P:canonical Wnt signaling pathway involved in negative regulation of apoptotic process; P:positive regulation of neuron apoptotic process; P:dorsal root ganglion development; P:thymus development; P:regulation of smooth muscle cell proliferation; F:transcription factor activity, sequence-specific DNA binding; P:neuron migration; C:extracellular exosome; P:cardiac vascular smooth muscle cell differentiation; P:negative regulation of osteoclast differentiation; P:negative regulation of protein sumoylation; P:positive regulation of determination of dorsal identity; P:single organismal cell-cell adhesion; P:T cell differentiation in thymus; P:lung induction; P:cranial skeletal system development; P:positive regulation of MAPK cascade; P:glial cell fate determination; C:beta-catenin destruction complex; P:regulation of centriole-centriole cohesion; F:transcription coactivator activity; P:branching involved in ureteric bud morphogenesis; P:nephron tubule formation; C:catenin complex; P:positive regulation of DNA-templated transcription, initiation; P:negative regulation of apoptotic signaling pathway; P:protein localization to cell surface; P:trachea formation; P:epithelial tube branching involved in lung morphogenesis; C:lateral plasma membrane; F:protein C-terminus binding; P:negative regulation of oxidative stress-induced neuron death; P:primary metabolic process; P:positive regulation of endothelial cell differentiation; P:negative regulation of oligodendrocyte differentiation; F:RNA polymerase II activating transcription factor binding; P:embryonic digit morphogenesis; F:alpha-catenin binding; C:apical part of cell; P:hindbrain development; P:bone resorption; F:cadherin binding; C:cytosol; C:beta-catenin-TCF7L2 complex; P:regulation of histone H3-K4 methylation; P:embryonic forelimb morphogenesis; P:embryonic hindlimb morphogenesis; P:genitalia morphogenesis; P:synapse organization; P:positive regulation of neuroblast proliferation; P:positive regulation of mesenchymal cell proliferation; P:positive regulation of heparan sulfate proteoglycan biosynthetic process; P:midbrain dopaminergic neuron differentiation; P:regulation of secondary heart field cardioblast proliferation; P:negative regulation of mitotic cell cycle, embryonic; P:positive regulation of telomerase activity; P:cellular response to indole-3-methanol; C:protein-DNA complex; P:response to estradiol; C:nuclear euchromatin; F:estrogen receptor binding; C:basolateral plasma membrane; C:flotillin complex; P:oviduct development; P:regulation of euchromatin binding; C:Wnt signalosome; P:protein complex assembly; P:endothelial tube morphogenesis; P:cranial ganglion development; P:positive regulation of fibroblast growth factor receptor signaling pathway; P:odontogenesis of dentin-containing tooth; P:ectoderm development; P:positive regulation of core promoter binding; P:embryonic foregut morphogenesis; P:cardiac muscle tissue development; P:epithelial cell differentiation involved in prostate gland development; F:euchromatin binding; P:negative regulation of transcription from RNA polymerase II promoter; P:neural plate development; C:bicellular tight junction; P:cellular macromolecule metabolic process; C:focal adhesion; P:branching involved in blood vessel morphogenesis; P:pancreas development; P:cell-matrix adhesion; P:embryonic skeletal limb joint morphogenesis; P:regulation of T cell proliferation; P:sympathetic ganglion development; P:cell maturation; P:embryonic heart tube development; P:osteoclast differentiation; P:renal vesicle formation; C:microvillus membrane; C:centrosome; C:perinuclear region of cytoplasm; F:repressing transcription factor binding; P:oocyte development; C:cell cortex; P:response to drug; P:positive regulation of telomere maintenance via telomerase; P:regulation of myelination; P:negative regulation of chondrocyte differentiation; F:I-SMAD binding |
| Efet.01.140965.g822.t1 | zinc finger 226 isoform X1 | 1749 | 6.83E-51 | 48.15 | F:nucleic acid binding; F:metal ion binding; P:regulation of transcription, DNA-templated; C:intracellular |
| Efet.01.140965.g823.t1 | kelch 7 | 522 | 1.10E-07 | 52.33 | F:ubiquitin-protein transferase activity; C:Cul3-RING ubiquitin ligase complex; P:protein ubiquitination involved in ubiquitin-dependent protein catabolic process |
| Efet.01.140993.g826.t1 | Homeobox Nkx- | 1128 | 3.92E-29 | 90.25 | C:nucleus; P:multicellular organism development; C:integral component of membrane; P:regulation of transcription, DNA-templated; F:sequence-specific DNA binding |
| Efet.01.141017.g828.t1 | 1-phosphatidylinositol 4,5-bisphosphate phosphodiesterase gamma-1 | 543 | 4.90E-31 | 62.8 | P:lipid metabolic process; P:cellular process; F:hydrolase activity |
| Efet.01.141098.g830.t1 | ankyrin repeat domain-containing 32 | 1401 | 6.01E-09 | 48 |  |
| Efet.01.141099.g831.t1 | UPF0046 -like | 594 | 2.12E-89 | 60.15 | F:hydrolase activity |
| Efet.01.14110.g1041.t1 | gastrula zinc finger -like isoform X1 | 1524 | 1.31E-65 | 50.9 | F:nucleic acid binding; F:DNA binding; F:metal ion binding |
| Efet.01.141110.g833.t1 | phosphoenolpyruvate carboxykinase [GTP]-like | 756 | 2.83E-125 | 78.85 | F:GTP binding; P:gluconeogenesis; F:phosphoenolpyruvate carboxykinase (GTP) activity; C:mitochondrion; P:phosphorylation; F:kinase activity |
| Efet.01.14115.g1042.t1 | neuromacin propeptide | 249 | 8.39E-22 | 52.67 | C:extracellular region; P:defense response |
| Efet.01.141154.g834.t1 | microtubule-associated 1B-like isoform X2 | 654 | 2.25E-53 | 52.25 | P:microtubule cytoskeleton organization; F:microtubule binding; C:microtubule |
| Efet.01.141160.g835.t1 | PREDICTED: uncharacterized protein LOC106156997 | 597 | 3.37E-10 | 45 |  |
| Efet.01.141182.g837.t1 | SKI family transcriptional corepressor 1 homolog-B-like | 1185 | 1.47E-119 | 88.25 | P:negative regulation of transcription from RNA polymerase II promoter; P:negative regulation of BMP signaling pathway; C:nucleus; F:DNA binding; F:SMAD binding; C:cytoplasm; F:transcription corepressor activity; P:negative regulation of transforming growth factor beta receptor signaling pathway |
| Efet.01.141193.g838.t1 | serine threonine- kinase Nek8-like | 243 | 1.32E-23 | 81.8 | F:ATP binding; F:protein serine/threonine kinase activity; P:protein phosphorylation; P:animal organ morphogenesis |
| Efet.01.141207.g841.t1 | NAD(P)-binding [Auricularia subglabra TFB-10046 SS5] | 357 | 7.99E-12 | 76.1 |  |
| Efet.01.141269.g842.t1 | fructose-2,6-bisphosphatase TIGAR | 1011 | 9.03E-11 | 58.9 | F:catalytic activity; P:metabolic process; F:phosphatase activity; C:cytosol; P:dephosphorylation |
| Efet.01.141269.g843.t1 | ADP-dependent glucokinase-like | 465 | 1.14E-54 | 74.2 | C:membrane |
| Efet.01.14128.g1044.t1 | innexin unc-9-like isoform X2 | 1023 | 1.47E-84 | 57.2 | C:membrane |
| Efet.01.141290.g844.t1 | myosin light chain smooth muscle isoform X1 | 1311 | 9.02E-12 | 56.2 | P:regulation of response to stimulus; P:regulation of cellular process; C:cytoskeletal part; P:skeletal muscle tissue development; C:actin cytoskeleton; F:binding; P:skeletal myofibril assembly; F:kinase activity; P:cardiac muscle tissue development; C:sarcomere |
| Efet.01.14131.g1045.t1 | NDNF-like isoform X2 | 858 | 5.25E-19 | 45.25 | P:single-organism cellular process |
| Efet.01.141364.g846.t1 | golgin 4 subfamily a | 255 | 6.14E-09 | 74.3 | C:extracellular exosome; P:protein targeting to Golgi; C:cytoplasm; C:intracellular membrane-bounded organelle; F:GTPase binding; P:Golgi to plasma membrane protein transport; P:positive regulation of axon extension; C:Golgi apparatus; C:nucleoplasm |
| Efet.01.141367.g847.t1 | transcription elongation regulator 1-like | 291 | 8.61E-31 | 81.25 |  |
| Efet.01.141383.g849.t1 | twitchin-like isoform X1 | 507 | 4.98E-59 | 66.05 | F:nucleotide binding; P:phosphorylation; F:kinase activity |
| Efet.01.14139.g1046.t1 | Down syndrome cell adhesion molecule homolog | 1233 | 1.60E-75 | 50.5 | P:nervous system development |
| Efet.01.141468.g852.t1 | diacyglycerol O-acyltransferase Rv1760 isoform X2 | 1092 | 3.01E-27 | 48.95 | F:transferase activity, transferring acyl groups; C:membrane; C:integral component of membrane; P:glycerolipid biosynthetic process; F:diacylglycerol O-acyltransferase activity; F:transferase activity |
| Efet.01.141476.g853.t1 | hypothetical protein CAPTEDRAFT_212404 | 573 | 4.88E-11 | 60.4 | C:membrane; C:integral component of membrane |
| Efet.01.141479.g854.t1 | dystonin isoform X1 | 2424 | 1.35E-155 | 49.8 | C:cytoskeleton |
| Efet.01.141480.g855.t1 | dystonin isoform X1 | 1566 | 5.05E-96 | 49.45 | C:cytoskeleton |
| Efet.01.141489.g856.t1 | serine threonine- phosphatase 2A regulatory subunit B subunit | 669 | 4.19E-115 | 88.85 | F:calcium ion binding |
| Efet.01.14155.g1048.t1 | nuclear hormone receptor HR96 isoform X11 | 327 | 4.82E-43 | 92.1 |  |
| Efet.01.141550.g858.t1 | dynein heavy chain axonemal- | 1083 | 0 | 73.7 | F:nucleotide binding; F:ATPase activity; C:inner dynein arm; F:microtubule motor activity; C:cytoplasm; F:ion binding; P:cilium or flagellum-dependent cell motility; P:cilium movement; P:inner dynein arm assembly |
| Efet.01.141582.g859.t1 | attractin 1 isoform X2 | 402 | 2.66E-27 | 60.55 | P:synaptic target recognition |
| Efet.01.141662.g860.t1 | tyrosine- kinase SYK-like | 744 | 6.88E-130 | 79.3 | F:ATP binding; F:non-membrane spanning protein tyrosine kinase activity; P:innate immune response; P:cell differentiation; C:extrinsic component of cytoplasmic side of plasma membrane; P:transmembrane receptor protein tyrosine kinase signaling pathway; P:peptidyl-tyrosine autophosphorylation; F:receptor binding; P:regulation of cell proliferation |
| Efet.01.141671.g861.t1 | centrosome-associated 350-like isoform X2 | 2751 | 1.51E-10 | 56.2 | C:centrosome; P:microtubule anchoring; F:microtubule binding |
| Efet.01.141680.g862.t1 | DNA-binding SATB2 | 780 | 1.07E-18 | 66.2 | F:DNA binding |
| Efet.01.14170.g1049.t1 | zinc finger GLI4-like | 651 | 1.35E-32 | 54.2 | F:binding; P:single-organism process |
| Efet.01.141710.g865.t1 | glyco 3-alpha-L-fucosyltransferase A | 1077 | 9.82E-35 | 51.85 | F:transferase activity, transferring glycosyl groups; F:fucosyltransferase activity; C:Golgi cisterna membrane; P:fucosylation; C:membrane; C:integral component of membrane; P:protein glycosylation; C:Golgi apparatus; F:transferase activity |
| Efet.01.141777.g866.t1 | hypothetical protein HELRODRAFT_190830 | 210 | 1.26E-20 | 74 |  |
| Efet.01.141809.g869.t1 | hypothetical protein CAPTEDRAFT_224889 | 852 | 1.39E-08 | 83 |  |
| Efet.01.141818.g871.t1 | homeobox cut-like 1 isoform X2 | 642 | 2.00E-64 | 76.15 | P:negative regulation of transcription from RNA polymerase II promoter; F:protein tyrosine kinase activity; P:cellular macromolecule metabolic process; F:RNA polymerase II regulatory region sequence-specific DNA binding; C:cytosol; C:Golgi apparatus; P:primary metabolic process; P:positive regulation of dendrite morphogenesis; C:nucleoplasm |
| Efet.01.141875.g874.t1 | PREDICTED: uncharacterized protein LOC105321710 isoform X1 | 759 | 1.57E-19 | 41.4 |  |
| Efet.01.141878.g875.t1 | dynein heavy chain axonemal-like | 666 | 2.28E-93 | 71.3 | F:ATPase activity; F:ATP binding; F:microtubule motor activity; C:dynein complex; P:microtubule-based movement |
| Efet.01.141878.g876.t1 | dynein heavy chain axonemal-like | 213 | 1.65E-20 | 54.05 | F:nucleotide binding; F:ATPase activity; F:ATP binding; F:microtubule motor activity; F:actin binding; C:dynein complex; P:phosphorylation; P:microtubule-based movement; F:kinase activity; C:intracellular |
| Efet.01.141878.g877.t1 | dynein heavy chain axonemal-like isoform X1 | 828 | 4.45E-105 | 75.05 | F:nucleotide binding; F:nucleoside-triphosphatase activity; C:intracellular; P:cellular process |
| Efet.01.141938.g879.t1 | mitochondrial-processing peptidase subunit beta | 336 | 8.45E-37 | 88.2 | F:zinc ion binding; C:mitochondrial matrix; C:mitochondrial respiratory chain complex III; P:protein processing; P:mitochondrial electron transport, ubiquinol to cytochrome c; P:aerobic respiration; F:metalloendopeptidase activity |
| Efet.01.14198.g1050.t1 | bromodomain-containing 2 isoform X2 | 348 | 1.15E-12 | 60.56 | F:transferase activity |
| Efet.01.142004.g880.t1 | protocadherin-11 X-linked- | 513 | 1.03E-24 | 60.7 | C:extracellular exosome; P:negative regulation of phosphatase activity; C:integral component of membrane; C:plasma membrane |
| Efet.01.142004.g881.t1 | protocadherin-9-like isoform X2 | 2124 | 2.89E-97 | 51.4 | F:calcium ion binding; C:membrane; C:integral component of membrane; P:cell adhesion; P:homophilic cell adhesion via plasma membrane adhesion molecules; C:plasma membrane |
| Efet.01.142124.g883.t1 | novel [Xenopus tropicalis] | 1296 | 1.08E-64 | 50.6 |  |
| Efet.01.142126.g884.t1 | 3-methyl-2-oxobutanoate dehydrogenase [lipoamide] mitochondrial-like | 375 | 2.00E-48 | 70.6 | F:transferase activity, transferring phosphorus-containing groups |
| Efet.01.14215.g1051.t1 | cAMP-specific 3 ,5 -cyclic phosphodiesterase 4D | 531 | 3.62E-47 | 61.1 | F:phosphoric diester hydrolase activity |
| Efet.01.142155.g885.t1 | inositol-trisphosphate 3-kinase B | 261 | 3.75E-21 | 80.7 | F:inositol-1,4,5-trisphosphate 3-kinase activity; F:metal ion binding; C:integral component of membrane; P:phosphorylation |
| Efet.01.142171.g886.t1 | stabilizer of axonemal microtubules 2-like | 609 | 4.92E-27 | 44.2 | F:microtubule binding |
| Efet.01.142179.g887.t1 | serine threonine- kinase PAK 2-like | 501 | 1.51E-15 | 62.15 | P:positive regulation of JUN kinase activity; P:negative regulation of cell proliferation involved in contact inhibition; P:Fc-epsilon receptor signaling pathway; P:neuron projection morphogenesis; P:protein autophosphorylation; C:Golgi apparatus; P:Fc-gamma receptor signaling pathway involved in phagocytosis; P:branching morphogenesis of an epithelial tube; C:neuron projection; P:stimulatory C-type lectin receptor signaling pathway; P:MAPK cascade; P:wound healing; C:ruffle; C:cytosol; C:invadopodium; P:actin cytoskeleton reorganization; P:T cell receptor signaling pathway; P:positive regulation of peptidyl-serine phosphorylation; P:T cell costimulation; C:intracellular non-membrane-bounded organelle; C:protein complex; F:protein serine/threonine kinase activity; C:intracellular organelle part; C:plasma membrane; C:cell-cell junction; F:collagen binding; P:positive regulation of stress fiber assembly; C:supramolecular fiber; P:ephrin receptor signaling pathway; P:positive regulation of intracellular estrogen receptor signaling pathway; P:positive regulation of cell migration |
| Efet.01.142185.g888.t1 | transcription factor AP-2-epsilon-like isoform X3 | 339 | 1.12E-25 | 75.5 | C:nucleus; F:transcription factor activity, sequence-specific DNA binding; P:regulation of transcription, DNA-templated |
| Efet.01.142242.g890.t1 | kelch 17 | 2106 | 9.22E-155 | 59.4 | F:ubiquitin-protein transferase activity; C:Cul3-RING ubiquitin ligase complex; P:protein ubiquitination involved in ubiquitin-dependent protein catabolic process; P:protein ubiquitination |
| Efet.01.142275.g891.t1 | Catenin alpha-2 | 771 | 8.34E-102 | 76.65 | F:actin filament binding; F:cadherin binding; P:cell adhesion |
| Efet.01.142313.g892.t1 | protocadherin alpha-3-like | 1320 | 6.61E-114 | 58.05 | C:membrane |
| Efet.01.142314.g893.t1 | hypothetical protein CAPTEDRAFT_198078 | 255 | 6.90E-06 | 58 | F:electron carrier activity; C:membrane; F:NADH dehydrogenase (ubiquinone) activity; P:oxidation-reduction process |
| Efet.01.142316.g894.t1 | Zinc transporter ZIP1 | 408 | 1.90E-19 | 65.15 | F:zinc ion transmembrane transporter activity; P:zinc II ion transmembrane transport; C:plasma membrane |
| Efet.01.142329.g895.t1 | E3 SUMO- ligase 2-like | 672 | 3.87E-20 | 38.15 | P:biological_process; F:catalytic activity; C:mitochondrion; P:positive regulation of apoptotic process; C:membrane; C:integral component of membrane; C:cellular_component; F:molecular_function |
| Efet.01.142346.g896.t1 | USP6 N-terminal | 1458 | 4.99E-14 | 57.25 |  |
| Efet.01.14235.g1052.t1 | tyrosinase tyr-3 | 375 | 3.71E-13 | 57.65 | F:metal ion binding; F:oxidoreductase activity; P:metabolic process; P:oxidation-reduction process |
| Efet.01.142355.g897.t1 | grpE mitochondrial | 462 | 1.53E-67 | 84.45 | F:protein homodimerization activity; C:mitochondrial matrix; F:chaperone binding; P:regulation of catalytic activity; P:protein folding; F:adenyl-nucleotide exchange factor activity |
| Efet.01.142360.g898.t1 | Disks large 1 tumor suppressor | 336 | 1.47E-37 | 78 |  |
| Efet.01.142382.g899.t1 | dynein heavy chain | 447 | 1.73E-50 | 93.15 | C:inner dynein arm; F:ATP binding; F:microtubule motor activity; C:cytoplasm; P:cilium or flagellum-dependent cell motility; P:microtubule-based movement; C:microtubule |
| Efet.01.142382.g900.t1 | dynein heavy chain axonemal-like | 303 | 1.14E-51 | 94.05 | F:ATPase activity; F:ATP binding; F:microtubule motor activity; C:dynein complex; P:microtubule-based movement |
| Efet.01.142415.g901.t1 | titin-like isoform X2 | 5718 | 1.02E-23 | 66.7 | C:membrane; C:integral component of membrane |
| Efet.01.142449.g903.t1 | glypican-5-like isoform X1 | 828 | 3.15E-18 | 46.1 | F:heparan sulfate proteoglycan binding; C:membrane; C:proteinaceous extracellular matrix |
| Efet.01.142468.g905.t1 | DNA helicase INO80 isoform X1 | 855 | 3.00E-31 | 57.4 | P:cellular process |
| Efet.01.142486.g906.t1 | mRNA-capping enzyme | 849 | 3.92E-68 | 55.75 | F:phosphatase activity; P:RNA processing; P:dephosphorylation |
| Efet.01.1425.g134.t1 | ribonuclease E G | 651 | 2.50E-87 | 94.25 | F:RNA binding; P:RNA phosphodiester bond hydrolysis; F:ribonuclease activity; P:RNA processing |
| Efet.01.142520.g908.t1 | hypothetical protein CAPTEDRAFT_227900 | 837 | 1.35E-45 | 57.89 |  |
| Efet.01.142555.g909.t1 | titin homolog isoform X6 | 570 | 1.88E-07 | 75 |  |
| Efet.01.142566.g910.t1 | Dynein heavy chain axonemal | 1062 | 5.59E-107 | 61.95 | C:cell part; C:organelle; P:microtubule-based movement |
| Efet.01.142586.g911.t1 | T-complex 11 1 | 246 | 1.70E-21 | 73.35 | C:integral component of membrane |
| Efet.01.142614.g913.t1 | C-ets-2 isoform X3 | 240 | 5.20E-39 | 84.55 | P:transcription, DNA-templated; F:RNA polymerase II core promoter proximal region sequence-specific DNA binding; P:mesoderm development; P:positive regulation of transcription from RNA polymerase II promoter; C:plasma membrane; C:nucleoplasm; P:negative regulation of transcription from RNA polymerase II promoter; F:transcriptional repressor activity, RNA polymerase II core promoter proximal region sequence-specific binding; P:skeletal system development; C:cytoplasm; P:ectodermal cell fate commitment; F:glucocorticoid receptor binding; P:primitive streak formation |
| Efet.01.142616.g914.t1 | dedicator of cytokinesis 11-like | 270 | 1.19E-24 | 87.6 | P:small GTPase mediated signal transduction; F:guanyl-nucleotide exchange factor activity; C:intracellular; P:positive regulation of GTPase activity |
| Efet.01.1427.g135.t1 | SUN domain-containing ossification factor-like | 357 | 1.10E-10 | 61.5 | C:membrane; C:integral component of membrane |
| Efet.01.142769.g916.t1 | 2-phospho-D-glycerate hydrolase, partial | 375 | 1.33E-38 | 73 | F:catalytic activity |
| Efet.01.142807.g918.t1 | anoctamin-8-like isoform X2 | 585 | 1.24E-27 | 74.8 | C:membrane |
| Efet.01.142807.g919.t1 | anoctamin-8-like isoform X1 | 909 | 8.10E-88 | 60.95 | C:membrane; C:integral component of membrane |
| Efet.01.14281.g1055.t1 | proteasome subunit beta type-5-like | 357 | 1.61E-47 | 81.95 | C:nucleus; C:cytoplasm; C:proteasome core complex; F:threonine-type endopeptidase activity; P:proteolysis involved in cellular protein catabolic process |
| Efet.01.142966.g923.t1 | hu-li tai shao isoform X5 | 549 | 1.22E-13 | 56.25 |  |
| Efet.01.143099.g926.t1 | kalirin-like isoform X11 | 417 | 6.18E-43 | 79.1 | P:regulation of cellular process; F:guanyl-nucleotide exchange factor activity; P:cellular process |
| Efet.01.143134.g927.t1 | probable ATP-dependent RNA helicase DDX41 | 600 | 4.64E-88 | 86.5 | F:nucleic acid binding; F:ATP binding; F:zinc ion binding; C:catalytic step 2 spliceosome; P:RNA secondary structure unwinding; F:ATP-dependent RNA helicase activity |
| Efet.01.143151.g928.t1 | alpha-1,3-mannosyl-glyco 4-beta-N-acetylglucosaminyltransferase C-like | 945 | 9.31E-19 | 46.4 | P:carbohydrate metabolic process; F:transferase activity, transferring hexosyl groups; C:membrane; C:integral component of membrane |
| Efet.01.143159.g929.t1 | neural-cadherin-like | 345 | 2.11E-54 | 57.1 | F:calcium ion binding; C:integral component of membrane; P:homophilic cell adhesion via plasma membrane adhesion molecules; C:plasma membrane |
| Efet.01.143181.g930.t1 | Dynein heavy chain | 339 | 5.93E-46 | 81.8 | F:ATP binding; F:microtubule motor activity; F:calcium ion binding; C:dynein complex; P:microtubule-based movement |
| Efet.01.143191.g932.t1 | Transposon Ty3-G Gag-Pol poly | 600 | 7.36E-10 | 60.9 |  |
| Efet.01.143193.g934.t1 | spectrin beta chain-like isoform X1 | 375 | 5.86E-47 | 84.6 | C:postsynaptic density; P:Golgi to plasma membrane protein transport; C:nucleolus; C:extracellular exosome; F:ankyrin binding; F:GTPase binding; P:mitotic cytokinesis; P:protein targeting to plasma membrane; C:M band; F:protein complex binding; P:common-partner SMAD protein phosphorylation; P:positive regulation of interleukin-2 secretion; P:SMAD protein import into nucleus; P:membrane assembly; P:positive regulation of protein localization to plasma membrane; F:structural constituent of cytoskeleton; C:protein complex; F:phospholipid binding; F:RNA binding; F:cadherin binding involved in cell-cell adhesion; F:calmodulin binding; F:actin binding; P:actin filament capping; C:cell-cell adherens junction; C:axolemma; C:spectrin; C:cuticular plate |
| Efet.01.143193.g935.t1 | spectrin beta non-erythrocytic 1- | 417 | 8.17E-67 | 89.65 | C:postsynaptic density; P:Golgi to plasma membrane protein transport; C:nucleolus; C:extracellular exosome; F:ankyrin binding; F:GTPase binding; P:mitotic cytokinesis; P:protein targeting to plasma membrane; C:M band; F:protein complex binding; P:common-partner SMAD protein phosphorylation; P:positive regulation of interleukin-2 secretion; P:SMAD protein import into nucleus; P:membrane assembly; P:positive regulation of protein localization to plasma membrane; F:structural constituent of cytoskeleton; C:protein complex; F:phospholipid binding; F:RNA binding; F:cadherin binding involved in cell-cell adhesion; F:calmodulin binding; F:actin binding; P:actin filament capping; C:cell-cell adherens junction; C:axolemma; C:spectrin; C:cuticular plate |
| Efet.01.1432.g136.t1 | otopetrin-2-like isoform X2 | 831 | 3.01E-53 | 57.45 | C:membrane; C:integral component of membrane |
| Efet.01.143203.g936.t1 | GRB2-associated and regulator of MAPK 2 | 1161 | 1.04E-17 | 60.75 |  |
| Efet.01.143327.g946.t1 | microspherule 1 | 231 | 3.74E-15 | 64 | C:nuclear lumen |
| Efet.01.143406.g947.t1 | Histone-lysine N-methyltransferase | 669 | 1.76E-53 | 65.25 | F:transferase activity |
| Efet.01.143468.g949.t1 | homeobox SIX6 | 687 | 1.87E-84 | 82.25 | C:nucleus; F:DNA binding; P:neural retina development; P:cell proliferation involved in compound eye morphogenesis; P:brain development; F:RNA polymerase II transcription factor activity, sequence-specific DNA binding; P:regulation of transcription from RNA polymerase II promoter |
| Efet.01.143622.g953.t1 | neurotrophin receptor B x -alpha | 411 | 4.95E-07 | 46.43 | P:peptidyl-tyrosine phosphorylation; P:transmembrane receptor protein tyrosine kinase signaling pathway; F:protein kinase activity; P:protein phosphorylation; F:nucleotide binding; C:integral component of plasma membrane; F:ATP binding; F:protein tyrosine kinase activity; F:transmembrane receptor protein tyrosine kinase activity; F:calcium ion binding; C:membrane; C:integral component of membrane; P:basement membrane organization; P:fin morphogenesis; P:fin development; P:phosphorylation; F:transferase activity; F:kinase activity |
| Efet.01.143655.g955.t1 | muscarinic acetylcholine receptor DM1 | 2394 | 8.82E-84 | 83.2 | P:gastric acid secretion; C:integral component of plasma membrane; P:synaptic transmission, cholinergic; P:phospholipase C-activating G-protein coupled acetylcholine receptor signaling pathway; P:adenylate cyclase-inhibiting G-protein coupled acetylcholine receptor signaling pathway; F:G-protein coupled acetylcholine receptor activity; C:synapse |
| Efet.01.143656.g956.t1 | hypothetical protein CAPTEDRAFT_199444 | 537 | 1.53E-41 | 65 |  |
| Efet.01.143711.g957.t1 | neurogenic locus delta | 261 | 4.56E-15 | 56.65 | P:Notch signaling pathway; P:multicellular organism development; P:cell communication; F:calcium ion binding; C:membrane; C:integral component of membrane |
| Efet.01.143720.g959.t1 | CBFA2T2 isoform X2 | 393 | 6.37E-15 | 85.25 | P:negative regulation of cell proliferation; F:RNA polymerase II regulatory region sequence-specific DNA binding; P:chondrocyte differentiation; P:transcription from RNA polymerase II promoter; P:negative regulation of glycolytic process; C:Golgi membrane; F:transcriptional activator activity, RNA polymerase II transcription regulatory region sequence-specific binding; C:nucleolus; P:positive regulation of transcription from RNA polymerase II promoter; P:positive regulation of angiogenesis; P:peripheral nervous system neuron development; C:nucleoplasm; F:core promoter binding; F:ATP binding; P:response to hypoxia; P:negative regulation of transcription, DNA-templated; F:protein heterodimerization activity; P:ossification; F:calcium ion binding; P:positive regulation of interleukin-2 production; P:generation of precursor metabolites and energy; F:protein homodimerization activity; P:negative regulation of fat cell differentiation; F:transcription factor binding; P:regulation of aerobic respiration; P:positive regulation of granulocyte differentiation; F:transcription corepressor activity; C:mitochondrion; C:nuclear matrix; P:hematopoietic stem cell proliferation; P:granulocyte differentiation; P:positive regulation of proteasomal ubiquitin-dependent protein catabolic process; P:negative regulation of granulocyte differentiation |
| Efet.01.14375.g1057.t1 | zinc finger CCCH domain-containing 11A | 429 | 1.16E-23 | 76.45 | F:metal ion binding |
| Efet.01.143758.g961.t1 | neuronal acetylcholine receptor subunit alpha-7-like | 315 | 5.13E-32 | 75.3 | C:postsynaptic membrane; P:cation transmembrane transport; C:integral component of membrane; C:cell junction; F:extracellular ligand-gated ion channel activity |
| Efet.01.143784.g962.t1 | serine-rich adhesin for platelets-like isoform X6 | 2514 | 2.56E-09 | 45 |  |
| Efet.01.143791.g963.t1 | hypothetical protein CAPTEDRAFT_165192 | 369 | 1.28E-12 | 60 | C:membrane |
| Efet.01.143799.g964.t1 | homeobox Nkx- -like | 1050 | 2.30E-53 | 84.3 | C:nucleus; P:regulation of transcription, DNA-templated; F:sequence-specific DNA binding |
| Efet.01.1438.g137.t1 | Cullin-4A | 246 | 2.05E-28 | 62.95 | F:ubiquitin protein ligase binding; F:calcium ion binding; C:integral component of membrane; F:serine-type endopeptidase activity; C:extracellular region; C:Cul4-RING E3 ubiquitin ligase complex; P:ubiquitin-dependent protein catabolic process |
| Efet.01.14385.g1058.t1 | midasin isoform X1 | 741 | 1.77E-61 | 69.4 |  |
| Efet.01.143861.g967.t1 | outer membrane | 1365 | 0 | 56.9 |  |
| Efet.01.143861.g968.t1 | response regulator | 627 | 3.45E-128 | 95 | F:RNA binding; P:phosphorelay signal transduction system; C:intracellular |
| Efet.01.143861.g969.t1 | nitrate transporter | 1233 | 0 | 94 |  |
| Efet.01.143861.g970.t1 | nitrate ABC permease | 357 | 5.59E-61 | 98.9 | F:nitrate transmembrane transporter activity; C:integral component of membrane; P:nitrate transport; C:plasma membrane |
| Efet.01.143887.g971.t1 | serine threonine- kinase MRCK alpha-like isoform X3 | 318 | 1.40E-32 | 74.1 | F:ATP binding; F:metal ion binding; F:protein serine/threonine kinase activity; P:protein phosphorylation; C:intracellular; P:intracellular signal transduction |
| Efet.01.143912.g972.t1 | sodium channel para isoform X1 | 1092 | 7.22E-114 | 60.65 | F:ion channel activity; P:ion transport; P:transmembrane transport; C:membrane |
| Efet.01.144008.g976.t1 | neuropilin and tolloid | 414 | 4.43E-30 | 73.05 | P:neuromuscular junction development; P:hatching behavior; P:postsynaptic membrane organization; P:neuromuscular synaptic transmission; P:flight; P:postsynaptic density organization; P:lateral inhibition; P:glutamate receptor clustering; P:regulation of cell cycle; C:ionotropic glutamate receptor complex; P:regulation of BMP signaling pathway; C:muscle cell postsynaptic density |
| Efet.01.144034.g977.t1 | DCN1 3 | 966 | 6.07E-92 | 70.55 |  |
| Efet.01.144077.g979.t1 | limbic system-associated membrane -like isoform X1 | 291 | 3.67E-23 | 65.95 | P:peptidyl-tyrosine phosphorylation; F:transmembrane receptor protein tyrosine kinase activity; P:transmembrane receptor protein tyrosine kinase signaling pathway |
| Efet.01.144093.g980.t1 | coiled-coil domain-containing 166-like | 360 | 1.30E-20 | 73.95 |  |
| Efet.01.144126.g981.t1 | hypothetical protein CAPTEDRAFT_210002 | 657 | 7.43E-17 | 54 | F:calcium ion binding; C:membrane; P:homophilic cell adhesion via plasma membrane adhesion molecules |
| Efet.01.14416.g1059.t1 | hypothetical protein HELRODRAFT_188253 | 741 | 4.65E-10 | 49 |  |
| Efet.01.144204.g983.t1 | C2 calcium-dependent domain-containing 6-like isoform X1 | 1053 | 3.43E-15 | 40.25 |  |
| Efet.01.144207.g984.t1 | transcription factor Sp9-like | 1383 | 4.30E-79 | 68.1 | F:nucleic acid binding; F:metal ion binding |
| Efet.01.144230.g985.t1 | hypothetical protein HELRODRAFT_191395 | 384 | 3.97E-24 | 63.5 | F:zinc ion binding; F:actin binding; P:cytoskeleton organization; F:metal ion binding |
| Efet.01.144382.g989.t1 | dexamethasone-induced Ras-related 1 isoform X2 | 297 | 1.48E-42 | 87.75 | F:GTP binding; C:sarcoplasmic reticulum; P:small GTPase mediated signal transduction; C:membrane |
| Efet.01.144392.g990.t1 | leucine-rich repeat neuronal 3-like | 888 | 2.30E-13 | 49.17 | C:membrane; C:integral component of membrane |
| Efet.01.144396.g991.t1 | hypothetical protein CAPTEDRAFT_217222 | 504 | 7.87E-13 | 48.95 | C:membrane; C:integral component of membrane; F:carbohydrate binding |
| Efet.01.144443.g992.t1 | hypothetical protein CAPTEDRAFT_4034 | 342 | 4.32E-14 | 62 | C:membrane; C:integral component of membrane |
| Efet.01.144470.g993.t1 | hypothetical protein HELRODRAFT_191982 | 705 | 1.15E-10 | 42.67 | F:transferase activity, transferring glycosyl groups; C:membrane; C:integral component of membrane; P:Wnt signaling pathway; P:negative regulation of Wnt signaling pathway; F:beta-catenin binding |
| Efet.01.14452.g1063.t1 | ubiquitin carboxyl-terminal hydrolase BAP1 | 303 | 1.70E-34 | 85.6 | F:thiol-dependent ubiquitin-specific protease activity; P:Notch signaling pathway; C:cytoplasm; P:regulation of cell growth; C:PR-DUB complex; F:chromatin binding; P:regulation of cell cycle; P:monoubiquitinated histone H2A deubiquitination; P:ubiquitin-dependent protein catabolic process; P:protein K48-linked deubiquitination |
| Efet.01.144520.g995.t1 | hypothetical protein CAPTEDRAFT_219724 | 993 | 2.70E-36 | 55.3 | C:membrane |
| Efet.01.144530.g996.t1 | cilia- and flagella-associated 69-like | 1329 | 1.75E-125 | 69.35 |  |
| Efet.01.144556.g998.t1 | G2 mitotic-specific cyclin-B2 | 375 | 1.03E-28 | 68.45 | P:single-organism cellular process |
| Efet.01.144561.g999.t1 | zinc finger | 2106 | 8.37E-67 | 75.6 | F:nucleic acid binding; F:metal ion binding |
| Efet.01.144599.g1000.t1 | PREDICTED: uncharacterized protein LOC106162403 | 432 | 7.51E-17 | 56.5 |  |
| Efet.01.144661.g1001.t1 | Retinol dehydrogenase 3-like | 363 | 1.30E-27 | 66.65 | F:oxidoreductase activity; P:anatomical structure morphogenesis; P:animal organ development; P:single-organism metabolic process |
| Efet.01.144689.g1002.t1 | echinoderm microtubule-associated -like 6 | 297 | 7.69E-20 | 74.15 | C:extracellular exosome; F:catalytic activity |
| Efet.01.144794.g1006.t1 | short transient receptor potential channel 6-like | 429 | 4.40E-58 | 72.1 | F:calcium channel activity; C:integral component of membrane; P:calcium ion transmembrane transport |
| Efet.01.144802.g1007.t1 | dynein heavy chain axonemal-like | 684 | 2.10E-40 | 60.6 | F:nucleotide binding; F:ATP binding; F:microtubule motor activity; C:dynein complex; P:microtubule-based movement |
| Efet.01.144817.g1008.t1 | zinc finger 33B-like | 1398 | 1.99E-31 | 69.75 | F:nucleic acid binding; F:metal ion binding |
| Efet.01.144856.g1009.t1 | transient receptor potential cation channel subfamily V member 5-like isoform X1 | 309 | 1.13E-12 | 53.15 |  |
| Efet.01.144857.g1010.t1 | polybromo-1-like isoform X3 | 294 | 2.24E-18 | 47.95 |  |
| Efet.01.144919.g1011.t1 | TBC1 domain family member 25 | 345 | 2.93E-19 | 73.4 | P:regulation of vesicle fusion; P:regulation of autophagosome maturation; P:activation of GTPase activity; F:GTPase activator activity; C:endomembrane system; P:intracellular protein transport; F:Rab GTPase binding; C:autophagosome |
| Efet.01.14494.g1065.t1 | amine oxidase [flavin-containing] B | 1539 | 0 | 68.15 | C:mitochondrion; C:membrane |
| Efet.01.144957.g1012.t1 | catenin delta-2-like isoform X1 | 714 | 1.65E-70 | 69.9 | P:single-organism process |
| Efet.01.144972.g1013.t1 | twitchin-like isoform X1 | 453 | 9.72E-62 | 70.5 | F:ATP binding; F:protein kinase activity; P:protein phosphorylation |
| Efet.01.145017.g1014.t1 | still isoform SIF type 1-like isoform X1 | 1701 | 1.20E-12 | 78.4 | P:regulation of Rho protein signal transduction; F:signal transducer activity, downstream of receptor; F:Rho guanyl-nucleotide exchange factor activity; F:phospholipid binding; C:intracellular; P:intracellular signal transduction; P:positive regulation of GTPase activity |
| Efet.01.145026.g1016.t1 | uncharacterized protein LOC110459495 | 477 | 4.34E-42 | 71.25 | C:membrane |
| Efet.01.145056.g1018.t1 | dual specificity phosphatase 1-like | 507 | 3.60E-14 | 61.6 | F:phosphoprotein phosphatase activity; P:dephosphorylation |
| Efet.01.14508.g1066.t1 | CCA tRNA nucleotidyltransferase mitochondrial-like | 393 | 2.11E-29 | 58.05 | F:nucleotidyltransferase activity; F:RNA binding; P:RNA processing; F:transferase activity |
| Efet.01.145090.g1019.t1 | cyclic GMP-AMP synthase-like | 936 | 5.17E-16 | 47.65 |  |
| Efet.01.145141.g1021.t1 | amine oxidoreductase | 915 | 4.71E-32 | 43.55 | F:oxidoreductase activity; C:membrane; C:integral component of membrane; P:oxidation-reduction process; F:monooxygenase activity; P:defense response to bacterium |
| Efet.01.145232.g1024.t1 | sodium channel | 228 | 1.53E-13 | 63.6 | P:regulation of postsynaptic membrane potential; P:regulation of ion transmembrane transport; C:voltage-gated sodium channel complex; P:sodium ion transmembrane transport; F:voltage-gated sodium channel activity |
| Efet.01.145250.g1025.t1 | dynein heavy chain axonemal-like isoform X1 | 306 | 1.71E-38 | 79.4 | F:ATPase activity; F:ATP binding; F:microtubule motor activity; C:dynein complex; P:microtubule-based movement |
| Efet.01.145279.g1027.t1 | A-kinase anchor mitochondrial-like | 1050 | 7.56E-29 | 65.45 | C:mitochondrion; P:protein localization |
| Efet.01.145288.g1029.t1 | AChain The Structure Of The Axh Domain Of Ataxin- | 1050 | 5.46E-25 | 77.3 | C:nuclear inclusion body; F:identical protein binding; P:nuclear export; F:poly(G) binding; P:negative regulation of transcription, DNA-templated; C:cytoplasm; C:nuclear RNA export factor complex; F:protein C-terminus binding; C:nuclear matrix; P:RNA processing; F:poly(U) RNA binding; F:protein self-association |
| Efet.01.1453.g138.t1 | acid-sensing ion channel 1-like isoform X4 | 441 | 3.83E-21 | 78.8 | F:sodium channel activity; C:integral component of membrane; P:sodium ion transmembrane transport |
| Efet.01.145321.g1030.t1 | adenosine deaminase CECR1-like | 549 | 5.11E-45 | 55.25 | C:extracellular space; F:deaminase activity |
| Efet.01.145329.g1031.t1 | interferon-induced helicase C domain-containing 1-like | 1977 | 1.74E-13 | 43.45 | F:nucleic acid binding; F:DNA binding; F:ATP binding; C:nucleus; F:zinc ion binding; F:metal ion binding; F:hydrolase activity |
| Efet.01.145361.g1032.t1 | acid-sensing ion channel 1-like | 588 | 5.49E-09 | 53.8 | P:sodium ion transport; P:ion transport; F:sodium channel activity; C:membrane; C:integral component of membrane; P:sodium ion transmembrane transport; P:transport |
| Efet.01.145373.g1033.t1 | ABHD17B-like | 651 | 1.52E-117 | 86.75 | F:hydrolase activity |
| Efet.01.145380.g1034.t1 | peptidoglycan recognition 1 | 375 | 1.69E-41 | 71.85 | F:zinc ion binding; P:peptidoglycan catabolic process; P:innate immune response; C:membrane; F:N-acetylmuramoyl-L-alanine amidase activity |
| Efet.01.14541.g1069.t1 | TPR repeat-containing DDB_G0287407-like isoform X1 | 561 | 1.79E-26 | 51.35 | F:ATP binding |
| Efet.01.145487.g1035.t1 | hypothetical protein CAPTEDRAFT_219851 | 363 | 9.29E-09 | 57 |  |
| Efet.01.145532.g1039.t1 | PREDICTED: protein cramped-like | 330 | 3.96E-06 | 56 |  |
| Efet.01.145588.g1040.t1 | bromodomain adjacent to zinc finger domain 2B-like isoform X5 | 627 | 1.57E-50 | 73.75 | C:nucleus; F:DNA binding; F:zinc ion binding |
| Efet.01.145589.g1041.t1 | RNA polymerase II-associated factor 1 homolog | 351 | 3.16E-58 | 86.25 | P:histone modification; C:Cdc73/Paf1 complex; P:transcription elongation from RNA polymerase II promoter |
| Efet.01.145617.g1042.t1 | androglobin isoform X5 | 726 | 2.27E-81 | 73.7 | F:binding |
| Efet.01.145642.g1043.t1 | ras-specific guanine nucleotide-releasing factor 2-like | 450 | 3.45E-50 | 67.15 | P:biological regulation |
| Efet.01.145755.g1046.t1 | PREDICTED: uncharacterized protein LOC101864447 | 441 | 5.95E-37 | 60.6 | C:membrane; C:integral component of membrane |
| Efet.01.145792.g1047.t1 | 60S ribosomal L27a | 273 | 6.05E-50 | 86.45 | F:structural constituent of ribosome; C:cytosolic large ribosomal subunit; P:translation |
| Efet.01.145838.g1048.t1 | transmembrane 229B | 555 | 2.04E-78 | 80.35 | C:integral component of membrane |
| Efet.01.145886.g1049.t1 | Cubilin | 594 | 1.18E-44 | 52.45 | F:calcium ion binding; C:membrane; C:integral component of membrane; F:receptor activity; P:receptor-mediated endocytosis |
| Efet.01.145887.g1050.t1 | manganese-dependent ADP-ribose CDP-alcohol diphosphatase-like | 216 | 8.09E-17 | 69.3 | F:hydrolase activity |
| Efet.01.145963.g1051.t1 | argonaute-2-like isoform X1 | 447 | 8.42E-79 | 94.9 | F:nucleic acid binding |
| Efet.01.146017.g1054.t1 | sex peptide receptor | 870 | 1.76E-55 | 64.2 | F:G-protein coupled receptor activity; F:G-protein coupled peptide receptor activity; P:G-protein coupled receptor signaling pathway; C:membrane; C:integral component of membrane |
| Efet.01.146035.g1055.t1 | probable G- coupled receptor 139 isoform X1 | 969 | 2.68E-94 | 61.95 | F:G-protein coupled receptor activity; P:G-protein coupled receptor signaling pathway; C:membrane; C:integral component of membrane |
| Efet.01.146043.g1056.t1 | Proteasome subunit beta type-3 | 411 | 1.66E-11 | 55 | C:nucleus; F:calcium ion binding; C:proteasome core complex; C:membrane; C:integral component of membrane; F:endopeptidase activity; F:threonine-type endopeptidase activity; C:proteasome complex; F:extracellular matrix structural constituent; P:proteolysis involved in cellular protein catabolic process; C:proteinaceous extracellular matrix |
| Efet.01.146100.g1057.t1 | hypothetical protein CAPTEDRAFT_225483 | 312 | 7.97E-17 | 58.35 | C:membrane; C:integral component of membrane; F:carbohydrate binding |
| Efet.01.146103.g1058.t1 | Beta-1,4-N-acetylgalactosaminyltransferase bre-4 | 216 | 2.02E-19 | 69.35 | P:carbohydrate metabolic process; F:transferase activity, transferring glycosyl groups |
| Efet.01.146173.g1061.t1 | hypothetical protein CAPTEDRAFT_187274 | 1473 | 0 | 58.47 | C:integral component of membrane |
| Efet.01.14618.g1071.t1 | TRAF3-interacting 1-like isoform X2 | 2493 | 3.58E-08 | 77.64 | F:microtubule binding |
| Efet.01.146213.g1062.t1 | afadin-like isoform X1 | 1377 | 3.10E-06 | 63.75 |  |
| Efet.01.146232.g1063.t1 | histone-lysine N-methyltransferase ASH1L-like isoform X10 | 561 | 4.97E-64 | 63.05 | F:binding; F:transferase activity |
| Efet.01.146249.g1065.t1 | barH-like 1 homeobox | 381 | 1.62E-30 | 71.3 | C:nucleus; P:regulation of transcription, DNA-templated; F:sequence-specific DNA binding |
| Efet.01.146252.g1067.t1 | tripartite motif-containing 45 | 1857 | 1.29E-32 | 46.25 | F:zinc ion binding; F:metal ion binding; F:ligase activity; C:intracellular |
| Efet.01.146254.g1068.t1 | lymphocyte expansion molecule-like | 312 | 8.13E-22 | 64.6 |  |
| Efet.01.146303.g1069.t1 | Down syndrome cell adhesion molecule Dscam2 | 450 | 2.19E-31 | 60.75 | C:membrane; C:integral component of membrane |
| Efet.01.146349.g1071.t1 | hypothetical protein CAPTEDRAFT_123191 | 483 | 4.32E-38 | 50 | P:lipid metabolic process; F:phosphoric diester hydrolase activity |
| Efet.01.146352.g1072.t1 | extracellular sulfatase Sulf-1 homolog | 573 | 4.29E-08 | 63.64 |  |
| Efet.01.146363.g1073.t1 | hypothetical protein HELRODRAFT_161512 | 600 | 8.91E-06 | 55 |  |
| Efet.01.146415.g1075.t1 | dystonin isoform X1 | 534 | 1.18E-43 | 55.45 | P:cell cycle arrest; C:cytoskeleton; F:calcium ion binding |
| Efet.01.146454.g1076.t1 | hypothetical protein CAPTEDRAFT_199538 | 828 | 2.29E-92 | 61.15 | P:single-organism process |
| Efet.01.146456.g1077.t1 | 1-phosphatidylinositol 4,5-bisphosphate phosphodiesterase epsilon-1 | 324 | 1.09E-30 | 79.45 | F:phosphoric diester hydrolase activity; P:intracellular signal transduction |
| Efet.01.146547.g1082.t1 | E3 ubiquitin- ligase NEDD4- | 201 | 3.32E-32 | 86.15 | F:ubiquitin protein ligase activity; P:response to salt stress; P:positive regulation of dendrite extension; P:cellular sodium ion homeostasis; P:regulation of membrane repolarization; P:regulation of membrane depolarization; C:nucleoplasm; P:negative regulation of transcription from RNA polymerase II promoter; P:sodium ion transport; C:extracellular exosome; C:cytosol; P:response to metal ion; P:regulation of bicellular tight junction assembly; P:negative regulation of sodium ion transmembrane transporter activity; P:positive regulation of caveolin-mediated endocytosis; F:ligase activity; P:water homeostasis; P:protein K48-linked ubiquitination; P:ion transmembrane transport; P:ventricular cardiac muscle cell action potential; P:negative regulation of systemic arterial blood pressure; P:positive regulation of protein catabolic process; P:negative regulation of potassium ion transmembrane transporter activity; F:ion channel binding; P:protein monoubiquitination; C:plasma membrane; P:excretion; P:protein ubiquitination involved in ubiquitin-dependent protein catabolic process; P:proteasome-mediated ubiquitin-dependent protein catabolic process; P:negative regulation of protein localization to cell surface; F:potassium channel inhibitor activity; P:positive regulation of sodium ion transport; P:viral life cycle; F:sodium channel inhibitor activity; P:positive regulation of cation channel activity |
| Efet.01.14658.g1073.t1 | PREDICTED: uncharacterized protein LOC106177189 | 1470 | 3.14E-13 | 62.4 | F:catalytic activity; P:metabolic process; C:membrane; C:integral component of membrane |
| Efet.01.146595.g1083.t1 | actin-related 2 3 complex subunit 5-like | 453 | 1.03E-100 | 75.8 | P:Arp2/3 complex-mediated actin nucleation; C:Arp2/3 protein complex |
| Efet.01.146595.g1084.t1 | transmembrane and coiled-coil domains 2 | 438 | 3.03E-18 | 82 | C:membrane |
| Efet.01.146621.g1085.t1 | calcipressin-3 isoform X2 | 339 | 2.74E-13 | 65.4 | P:regulation of cellular process; C:intracellular |
| Efet.01.146631.g1086.t1 | histone acetyltransferase KAT6B isoform X1 | 816 | 2.29E-29 | 61.9 | C:intracellular organelle; F:binding; F:transferase activity; P:chromatin organization |
| Efet.01.146634.g1087.t1 | transmembrane 186 | 561 | 4.50E-38 | 63.55 | C:mitochondrion; C:membrane; C:integral component of membrane |
| Efet.01.146716.g1089.t1 | Growth differentiation factor 8 | 324 | 3.40E-40 | 75.5 | C:extracellular space; F:growth factor activity; P:regulation of MAPK cascade; F:transforming growth factor beta receptor binding; P:SMAD protein signal transduction; P:regulation of apoptotic process; P:cell development; F:cytokine activity; P:positive regulation of pathway-restricted SMAD protein phosphorylation |
| Efet.01.146794.g1093.t1 | Cadherin-related tumor suppressor | 2058 | 1.27E-34 | 55.45 | F:calcium ion binding; C:membrane; C:integral component of membrane; P:cell adhesion; P:homophilic cell adhesion via plasma membrane adhesion molecules; C:plasma membrane |
| Efet.01.146812.g1094.t1 | hypothetical protein CAPTEDRAFT_220899 | 579 | 4.22E-47 | 61.9 | C:membrane; C:integral component of membrane |
| Efet.01.146813.g1095.t1 | hypothetical protein HELRODRAFT_180534 | 477 | 3.00E-16 | 58 |  |
| Efet.01.146861.g1096.t1 | ileal sodium bile acid cotransporter-like | 771 | 6.29E-12 | 56.2 | C:membrane; C:integral component of membrane |
| Efet.01.146892.g1097.t1 | histone-lysine N-methyltransferase 2C-like | 1578 | 3.27E-166 | 64.65 | F:metal ion binding; F:methyltransferase activity; P:methylation |
| Efet.01.14695.g1074.t1 | twitchin-like isoform X2 | 360 | 2.57E-28 | 65.5 | F:ATP binding; F:protein kinase activity; P:protein phosphorylation |
| Efet.01.146962.g1098.t1 | adenylate kinase 9-like | 390 | 7.97E-43 | 78.05 | P:cellular metabolic process |
| Efet.01.146998.g1100.t1 | gastrula zinc finger -like isoform X1 | 1311 | 9.03E-103 | 63.15 |  |
| Efet.01.147028.g1101.t1 | MOG interacting and ectopic P-granules 1-like | 1713 | 3.04E-33 | 44.1 | F:metal ion binding |
| Efet.01.147059.g1102.t1 | bone morphogenetic 4-like | 873 | 1.34E-46 | 49.9 | F:growth factor activity; C:extracellular region; P:growth |
| Efet.01.147115.g1104.t1 | hypothetical protein CAPTEDRAFT_199134 | 1617 | 1.33E-105 | 45.67 | P:transport |
| Efet.01.147138.g1105.t1 | hypothetical protein CAPTEDRAFT_222036 | 267 | 7.11E-22 | 64.56 | C:membrane; C:integral component of membrane |
| Efet.01.147229.g1108.t1 | laminin subunit beta-1- | 630 | 3.32E-80 | 77.35 | F:glycosphingolipid binding; P:neuronal-glial interaction involved in cerebral cortex radial glia guided migration; P:neuron projection development; P:negative regulation of cell adhesion; C:laminin-8 complex; F:integrin binding; P:odontogenesis; C:laminin-10 complex; F:extracellular matrix structural constituent; P:embryo implantation; P:learning or memory; C:nucleus; C:extracellular space; C:perinuclear region of cytoplasm; C:extracellular exosome; C:laminin-1 complex; C:laminin-2 complex; P:substrate adhesion-dependent cell spreading; F:enzyme binding; P:positive regulation of cell migration |
| Efet.01.147235.g1109.t1 | hypothetical protein CAPTEDRAFT_209611, partial | 987 | 1.18E-11 | 53 |  |
| Efet.01.147262.g1110.t1 | ubiquitin carboxyl-terminal hydrolase MINDY-1-like isoform X1 | 564 | 3.02E-18 | 77.9 |  |
| Efet.01.147347.g1111.t1 | dolichyl-diphosphooligosaccharide-- glycosyltransferase subunit STT3B | 369 | 3.23E-59 | 77.25 | C:integral component of membrane; P:protein glycosylation; F:oligosaccharyl transferase activity |
| Efet.01.1474.g139.t1 | plasma serine protease inhibitor | 453 | 1.47E-16 | 63.35 | C:intracellular organelle; C:extracellular space; F:serine-type endopeptidase inhibitor activity; C:membrane; C:serine protease inhibitor complex; C:vesicle; F:binding |
| Efet.01.1474.g140.t1 | leukocyte elastase inhibitor-like isoform X1 | 459 | 1.06E-23 | 51.9 | F:peptidase inhibitor activity; C:extracellular space; F:serine-type endopeptidase inhibitor activity; P:proteolysis; P:negative regulation of endopeptidase activity; F:peptidase activity; P:negative regulation of peptidase activity |
| Efet.01.147429.g1113.t1 | 5-oxoprolinase | 228 | 7.49E-26 | 91.5 | F:hydrolase activity |
| Efet.01.147445.g1114.t1 | acyl- dehydrogenase | 516 | 3.14E-86 | 93 | F:acyl-CoA dehydrogenase activity; F:flavin adenine dinucleotide binding; P:oxidation-reduction process |
| Efet.01.147445.g1115.t1 | family transcriptional regulator | 597 | 3.63E-109 | 80.3 | F:DNA binding; P:transcription, DNA-templated; P:regulation of transcription, DNA-templated |
| Efet.01.147445.g1116.t1 | 23S rRNA (adenine(2030)-N(6))-methyltransferase | 882 | 0 | 89.25 | F:RNA binding; P:rRNA base methylation; F:23S rRNA (adenine(2030)-N(6))-methyltransferase activity |
| Efet.01.147479.g1117.t1 | protocadherin gamma-B1 | 636 | 2.09E-27 | 55 | F:calcium ion binding; C:membrane; C:integral component of membrane; P:cell adhesion; P:homophilic cell adhesion via plasma membrane adhesion molecules; C:plasma membrane |
| Efet.01.147479.g1118.t1 | protocadherin beta-12 | 2055 | 9.62E-58 | 49.1 | F:calcium ion binding; C:membrane; C:integral component of membrane; P:cell adhesion; P:homophilic cell adhesion via plasma membrane adhesion molecules; C:plasma membrane |
| Efet.01.14748.g1077.t1 | guanine nucleotide-binding -like 3 | 714 | 1.47E-69 | 73.7 | F:GTPase activity; P:ribosome biogenesis; C:nucleolus |
| Efet.01.14748.g1078.t1 | Small integral membrane 15 | 411 | 1.50E-17 | 76.33 | C:integral component of membrane |
| Efet.01.1475.g141.t1 | E3 ubiquitin- ligase NEURL1B | 312 | 7.86E-27 | 77.1 | F:zinc ion binding |
| Efet.01.147525.g1119.t1 | teneurin-m-like isoform X1 | 843 | 3.01E-67 | 57.4 | P:self proteolysis; C:membrane; C:integral component of membrane |
| Efet.01.147535.g1120.t1 | PREDICTED: uncharacterized protein LOC100208603 | 228 | 1.88E-06 | 68 |  |
| Efet.01.147577.g1123.t1 | sarcoplasmic calcium-binding isoform X1 | 531 | 1.99E-26 | 53.05 | F:metal ion binding; F:calcium ion binding |
| Efet.01.147578.g1124.t1 | ribosomal- -alanine N-acetyltransferase | 375 | 4.74E-06 | 56 | F:acetyltransferase activity; P:N-terminal protein amino acid acetylation; F:transferase activity; F:N-acetyltransferase activity |
| Efet.01.147608.g1125.t1 | single-strand selective monofunctional uracil DNA glycosylase-like | 342 | 8.40E-35 | 70.45 | F:uracil DNA N-glycosylase activity; F:oxidized pyrimidine nucleobase lesion DNA N-glycosylase activity; C:nuclear lumen |
| Efet.01.14763.g1080.t1 | receptor-type guanylate cyclase gcy-5-like isoform X1 | 807 | 2.56E-54 | 42.75 | P:peptidyl-tyrosine phosphorylation; P:cGMP biosynthetic process; P:G-protein coupled receptor signaling pathway; F:lyase activity; F:guanylate cyclase activity; F:protein kinase activity; P:protein phosphorylation; F:nucleotide binding; F:ATP binding; F:protein tyrosine kinase activity; F:G-protein coupled GABA receptor activity; C:membrane; C:integral component of membrane |
| Efet.01.14766.g1081.t1 | sodium channel | 792 | 5.64E-113 | 78.05 | P:regulation of postsynaptic membrane potential; F:calcium ion binding; P:regulation of ion transmembrane transport; C:voltage-gated sodium channel complex; P:sodium ion transmembrane transport; F:voltage-gated sodium channel activity |
| Efet.01.147732.g1130.t1 | ATP-dependent RNA helicase DHX29 | 357 | 4.38E-06 | 71 | F:nucleotide binding; F:nucleic acid binding; F:ATP binding; F:ATP-dependent helicase activity; F:helicase activity; F:hydrolase activity |
| Efet.01.147743.g1131.t1 | arf-GAP with ANK repeat and PH domain-containing 1- | 390 | 2.46E-56 | 81.7 | F:GTP binding; P:small GTPase mediated signal transduction; C:membrane; F:GTPase activator activity; F:phospholipid binding; C:intracellular; P:positive regulation of GTPase activity |
| Efet.01.147776.g1132.t1 | Ankyrin repeat and fibronectin type-III domain-containing 1 | 738 | 5.16E-77 | 77.05 | P:signal transduction |
| Efet.01.14778.g1082.t1 | erythroid differentiation-related factor 1-like | 474 | 1.06E-27 | 57.35 |  |
| Efet.01.14779.g1083.t1 | PREDICTED: uncharacterized protein LOC102808008 | 372 | 5.68E-10 | 56.95 |  |
| Efet.01.14779.g1084.t1 | Peptidyl-prolyl cis-trans isomerase B | 531 | 5.07E-47 | 57.6 | P:regulation of biological quality; C:endoplasmic reticulum chaperone complex; F:binding; P:positive regulation of biological process |
| Efet.01.147804.g1134.t1 | voltage-dependent anion-selective channel 2-like | 216 | 2.02E-12 | 69.8 | C:mitochondrion; P:transport; P:regulation of anion transport |
| Efet.01.147809.g1135.t1 | protocadherin gamma-A11-like | 948 | 3.80E-45 | 52.7 | F:calcium ion binding; C:membrane; C:integral component of membrane; P:cell adhesion; P:homophilic cell adhesion via plasma membrane adhesion molecules; C:plasma membrane |
| Efet.01.147884.g1137.t1 | cGMP-dependent isozyme 2 forms cD4 T1 T3A T3B-like isoform X1 | 312 | 4.35E-09 | 60.86 | F:ATP binding; F:cGMP-dependent protein kinase activity; F:protein kinase activity; P:phosphorylation; F:protein serine/threonine kinase activity; P:protein phosphorylation; F:kinase activity |
| Efet.01.14789.g1085.t1 | serine arginine-rich splicing factor 2 | 594 | 1.04E-24 | 88.55 | F:protein kinase C binding; F:pre-mRNA binding; C:PML body; C:nuclear speck; C:spliceosomal complex; C:interchromatin granule; P:termination of RNA polymerase II transcription; P:pathogenesis; P:negative regulation of nucleic acid-templated transcription; F:RNA binding; P:mRNA splicing, via spliceosome; F:nucleotide binding; P:mRNA export from nucleus; C:extracellular exosome; F:transcription corepressor activity; P:regulation of alternative mRNA splicing, via spliceosome; C:cell wall; C:membrane; P:mRNA 3'-end processing; F:IgG binding |
| Efet.01.147933.g1138.t1 | inorganic phosphate cotransporter isoform X1 | 234 | 2.41E-06 | 77 |  |
| Efet.01.147969.g1139.t1 | diphthine methyltransferase | 528 | 4.47E-21 | 52 | P:peptidyl-diphthamide biosynthetic process from peptidyl-histidine |
| Efet.01.147980.g1140.t1 | hypothetical protein | 219 | 6.13E-07 | 77.93 | P:biological_process; C:cellular_component; F:molecular_function |
| Efet.01.147989.g1141.t1 | bromo adjacent homology (BAH) domain-containing | 1110 | 4.36E-55 | 63.05 | F:binding; P:chromatin organization; C:intracellular organelle part |
| Efet.01.148005.g1143.t1 | cyclic nucleotide-binding domain-containing 2-like | 210 | 9.43E-08 | 64.8 |  |
| Efet.01.14806.g1087.t1 | hypothetical protein CAPTEDRAFT_200225 | 783 | 1.62E-32 | 55.8 | C:integral component of membrane; F:carbohydrate binding |
| Efet.01.148128.g1147.t1 | hypothetical protein CAPTEDRAFT_221857 | 486 | 8.28E-19 | 56 |  |
| Efet.01.148148.g1148.t1 | Neuropeptide FF receptor 2 | 378 | 1.46E-44 | 70.1 | P:neuropeptide signaling pathway; C:integral component of membrane; F:neuropeptide Y receptor activity |
| Efet.01.148158.g1149.t1 | beta-1,3-galactosyl-O-glycosyl-glyco beta-1,6-N-acetylglucosaminyltransferase 4-like | 1053 | 3.36E-87 | 66.85 | C:membrane |
| Efet.01.148177.g1150.t1 | wings apart homolog | 390 | 4.37E-24 | 67.55 |  |
| Efet.01.14825.g1090.t1 | serine threonine- kinase SIK2-like | 1446 | 7.50E-30 | 57.2 | F:kinase activity |
| Efet.01.148267.g1152.t1 | transmembrane protease serine 9 | 348 | 1.91E-15 | 53.9 | F:hydrolase activity |
| Efet.01.148280.g1153.t1 | zinc finger 226 | 1701 | 2.54E-94 | 57.4 | F:nucleic acid binding; F:transcription factor activity, sequence-specific DNA binding; F:metal ion binding; P:regulation of transcription, DNA-templated; C:intracellular |
| Efet.01.1483.g142.t1 | Ubiquitin carboxyl-terminal hydrolase 4 | 393 | 3.03E-16 | 75.2 | P:protein deubiquitination; F:thiol-dependent ubiquitinyl hydrolase activity; P:ubiquitin-dependent protein catabolic process |
| Efet.01.148332.g1154.t1 | E3 ubiquitin- ligase CBL-B | 495 | 2.94E-83 | 88.8 | F:phosphotyrosine binding; C:nucleus; F:ubiquitin-protein transferase activity; F:zinc ion binding; P:regulation of signaling; F:calcium ion binding; P:cell surface receptor signaling pathway; F:signal transducer activity; F:ligase activity; P:protein ubiquitination |
| Efet.01.148364.g1157.t1 | malonyl- mitochondrial-like | 237 | 1.01E-23 | 74.7 | C:mitochondrion; P:cellular metabolic process; P:organic substance metabolic process |
| Efet.01.148442.g1158.t1 | malignant fibrous histiocytoma-amplified sequence 1 homolog isoform X1 | 2679 | 0 | 53.15 | F:GTP binding; P:small GTPase mediated signal transduction; C:intracellular |
| Efet.01.14845.g1091.t1 | imidazoleglycerol-phosphate dehydratase | 342 | 2.36E-49 | 93.45 | F:imidazoleglycerol-phosphate dehydratase activity; C:cytoplasm; P:histidine biosynthetic process |
| Efet.01.148480.g1160.t1 | SWI SNF complex subunit SMARCC1-like | 285 | 2.47E-23 | 77.8 | C:SWI/SNF complex; F:DNA binding; F:catalytic activity; F:metal ion binding; P:ATP-dependent chromatin remodeling; P:regulation of transcription from RNA polymerase II promoter |
| Efet.01.148529.g1161.t1 | zinc finger weckle-like | 264 | 9.64E-07 | 58.89 | F:nucleic acid binding; F:metal ion binding |
| Efet.01.148541.g1162.t1 | zinc finger 845-like isoform X2 | 1011 | 1.14E-29 | 48.83 | F:nucleic acid binding; F:metal ion binding |
| Efet.01.148544.g1163.t1 | Major egg antigen (p40) | 987 | 7.79E-17 | 47.5 | F:DNA binding; P:DNA replication; C:nuclear origin of replication recognition complex |
| Efet.01.148596.g1165.t1 | RING finger 121-like | 201 | 1.43E-14 | 84.55 | C:endoplasmic reticulum membrane; F:zinc ion binding; F:ubiquitin protein ligase activity; P:endoplasmic reticulum unfolded protein response; C:integral component of membrane; C:Golgi membrane; P:ER-associated ubiquitin-dependent protein catabolic process; P:protein ubiquitination |
| Efet.01.148771.g1169.t1 | tyrosine- phosphatase Lar isoform X1 | 549 | 1.16E-28 | 68.25 | F:phosphatase activity; P:dephosphorylation |
| Efet.01.148789.g1170.t1 | IQ motif and SEC7 domain-containing 1 isoform X2 | 1653 | 2.10E-27 | 81.9 | F:ARF guanyl-nucleotide exchange factor activity; P:regulation of ARF protein signal transduction; P:positive regulation of GTPase activity |
| Efet.01.148818.g1174.t1 | probable serine threonine- kinase DDB_G0282963 isoform X2 | 582 | 1.40E-13 | 51.43 | F:protein dimerization activity |
| Efet.01.148845.g1175.t1 | zinc transporter ZIP1-like | 1083 | 3.46E-67 | 64 | C:membrane; P:transport |
| Efet.01.14885.g1092.t1 | neurogenic locus notch | 1578 | 7.56E-149 | 62.55 | C:membrane; C:integral component of membrane |
| Efet.01.148859.g1176.t1 | titin-like isoform X1 | 606 | 6.29E-35 | 58.35 | P:myofibril assembly; P:chordate embryonic development; P:heart morphogenesis; P:muscle contraction; F:structural molecule activity; P:cardiac muscle cell development; C:I band; C:A band; F:cytoskeletal protein binding; C:intracellular organelle part; P:regulation of biological process |
| Efet.01.148879.g1177.t1 | methylenetetrahydrofolate reductase isoform X2 | 615 | 7.48E-64 | 57.15 | P:small molecule metabolic process; P:single-organism cellular process; P:cellular metabolic process |
| Efet.01.148885.g1178.t1 | histidine--tRNA cytoplasmic isoform X2 | 492 | 2.93E-54 | 83.35 | F:histidine-tRNA ligase activity; F:ATP binding; P:histidyl-tRNA aminoacylation; C:cytoplasm |
| Efet.01.148946.g1181.t1 | hypothetical protein CRN43_14440 | 309 | 2.17E-25 | 65.17 |  |
| Efet.01.14895.g1094.t1 | Plasma alpha-L-fucosidase | 600 | 4.54E-61 | 73.95 | F:alpha-L-fucosidase activity; P:fucose metabolic process |
| Efet.01.148956.g1182.t1 | neurobeachin isoform X3 | 270 | 6.85E-07 | 84.45 |  |
| Efet.01.149.g16.t1 | alpha beta hydrolase domain-containing 17B | 738 | 9.75E-121 | 81.05 | F:hydrolase activity |
| Efet.01.149020.g1183.t1 | Zinc finger with UFM1-specific peptidase domain | 870 | 6.96E-09 | 47.8 | F:metal ion binding |
| Efet.01.14911.g1095.t1 | SH2 domain-containing adapter B-like isoform X4 | 309 | 1.56E-25 | 82.85 |  |
| Efet.01.149138.g1188.t1 | rab s geranylgeranyltransferase component A 2-like | 381 | 5.08E-48 | 81.9 | F:GDP-dissociation inhibitor activity; C:Rab-protein geranylgeranyltransferase complex; F:oxidoreductase activity; P:small GTPase mediated signal transduction; P:regulation of catalytic activity; P:oxidation-reduction process; P:protein geranylgeranylation; F:transferase activity; P:intracellular protein transport |
| Efet.01.149163.g1190.t1 | NSFL1 cofactor p47 | 270 | 2.09E-08 | 66.4 | P:membrane fusion; P:Golgi organization; C:Golgi apparatus; F:lipid binding; C:chromosome; F:phospholipid binding; C:Golgi stack; C:plasma membrane; C:nucleoplasm; C:nucleus; P:proteasome-mediated ubiquitin-dependent protein catabolic process; F:protein binding; C:VCP-NSFL1C complex; F:ubiquitin binding; C:intermediate filament cytoskeleton; C:cytoplasm; C:cytosol; P:nuclear envelope reassembly; F:ATPase binding |
| Efet.01.14921.g1096.t1 | low-density lipo receptor-related 6 | 357 | 2.68E-42 | 76.65 | F:Wnt-activated receptor activity; P:negative regulation of smooth muscle cell apoptotic process; C:Golgi apparatus; C:neuronal cell body; C:synapse; P:positive regulation of sequence-specific DNA binding transcription factor activity; P:thalamus development; P:face morphogenesis; C:caveola; F:frizzled binding; F:coreceptor activity involved in canonical Wnt signaling pathway; F:Wnt-protein binding; F:apolipoprotein binding; P:embryonic pattern specification; F:low-density lipoprotein receptor activity; P:positive regulation of canonical Wnt signaling pathway; P:chemical synaptic transmission; C:receptor complex; P:cerebral cortex development; P:pericardium morphogenesis; P:cerebellum morphogenesis; C:early endosome membrane; C:Wnt-Frizzled-LRP5/6 complex; P:neural crest formation; P:protein localization to plasma membrane; C:Wnt signalosome; P:receptor-mediated endocytosis involved in cholesterol transport; P:external genitalia morphogenesis; P:palate development; P:Wnt signaling pathway involved in dorsal/ventral axis specification; P:positive regulation of cell cycle; P:odontogenesis of dentin-containing tooth; P:canonical Wnt signaling pathway involved in neural crest cell differentiation; P:Wnt signaling pathway involved in midbrain dopaminergic neuron differentiation; C:extracellular region; P:positive regulation of transcription from RNA polymerase II promoter; P:convergent extension; P:neural tube closure; P:canonical Wnt signaling pathway involved in regulation of cell proliferation; P:cellular response to cholesterol; P:positive regulation of Wnt signaling pathway involved in dorsal/ventral axis specification; F:protein homodimerization activity; P:axis elongation involved in somitogenesis; P:Wnt signaling pathway involved in somitogenesis; P:midbrain-hindbrain boundary development; P:beta-catenin destruction complex disassembly; P:embryonic retina morphogenesis in camera-type eye; C:cell surface; P:trachea cartilage morphogenesis; P:negative regulation of canonical Wnt signaling pathway; F:kinase inhibitor activity; P:negative regulation of protein serine/threonine kinase activity; F:toxin transporter activity; P:primitive streak formation |
| Efet.01.149246.g1192.t1 | hypothetical protein CAPTEDRAFT_225776 | 654 | 9.19E-37 | 51.33 | C:membrane; C:integral component of membrane |
| Efet.01.149246.g1193.t1 | signal recognition particle receptor subunit alpha homolog | 783 | 3.20E-160 | 91.9 | F:GTP binding; P:SRP-dependent cotranslational protein targeting to membrane; F:GTPase activity; C:signal recognition particle receptor complex; F:signal recognition particle binding |
| Efet.01.149279.g1196.t1 | Collagen alpha-4(VI) chain | 918 | 1.22E-58 | 52.55 | F:calcium ion binding; C:collagen trimer |
| Efet.01.149294.g1198.t1 | innexin unc-9-like isoform X2 | 564 | 1.05E-63 | 66.95 | C:gap junction; P:ion transport; C:integral component of membrane; P:cell communication by electrical coupling; C:synapse; F:gap junction channel activity; C:plasma membrane |
| Efet.01.149345.g1202.t1 | FMRFamide receptor-like | 1383 | 2.68E-82 | 61.5 | F:G-protein coupled receptor activity; F:G-protein coupled peptide receptor activity; P:G-protein coupled receptor signaling pathway; C:membrane; C:integral component of membrane |
| Efet.01.14940.g1098.t1 | O-linked-mannose beta-1,4-N-acetylglucosaminyltransferase 2-like isoform X2 | 330 | 2.63E-32 | 74.64 | F:transferase activity, transferring glycosyl groups; C:membrane |
| Efet.01.149441.g1203.t1 | UDP- c:betaGal beta-1,3-N-acetylglucosaminyltransferase LOC100288842 | 471 | 6.48E-33 | 55.15 | F:transferase activity, transferring glycosyl groups; C:membrane; C:integral component of membrane; P:protein glycosylation; F:galactosyltransferase activity; C:Golgi apparatus; C:Golgi membrane; F:transferase activity |
| Efet.01.149454.g1204.t1 | tubulin alpha-1C chain- | 420 | 2.85E-47 | 97.75 | F:GTP binding; C:myelin sheath; C:extracellular exosome; F:ubiquitin protein ligase binding; F:double-stranded RNA binding; F:GTPase activity; P:cellular response to interleukin-4; F:structural constituent of cytoskeleton; C:cytoplasmic microtubule; P:microtubule cytoskeleton organization |
| Efet.01.149454.g1205.t1 | tubulin alpha chain | 447 | 4.48E-86 | 95.4 | F:GTP binding; C:nucleus; C:cytoplasmic ribonucleoprotein granule; F:protein domain specific binding; C:myelin sheath; C:extracellular exosome; F:GTPase activity; C:recycling endosome; F:structural constituent of cytoskeleton; C:cytoplasmic microtubule; P:microtubule-based process |
| Efet.01.149464.g1206.t1 | glyceraldehyde-3-phosphate dehydrogenase | 516 | 1.62E-92 | 85.4 | F:NADP binding; F:NAD binding; P:glycolytic process; P:oxidation-reduction process; P:glucose metabolic process; F:glyceraldehyde-3-phosphate dehydrogenase (NAD+) (phosphorylating) activity |
| Efet.01.1496.g143.t1 | cadherin-23 isoform X4 | 564 | 8.34E-15 | 52.65 | P:L-phenylalanine biosynthetic process; F:calcium ion binding; C:membrane; C:integral component of membrane; P:cell adhesion; P:homophilic cell adhesion via plasma membrane adhesion molecules; C:plasma membrane; F:prephenate dehydratase activity |
| Efet.01.149634.g1211.t1 | MSHA biogenesis | 273 | 2.86E-10 | 56.05 | C:membrane; C:integral component of membrane |
| Efet.01.149684.g1212.t1 | glyco 3-alpha-L-fucosyltransferase A-like | 996 | 1.20E-46 | 56.15 | F:transferase activity, transferring glycosyl groups; F:fucosyltransferase activity; C:Golgi cisterna membrane; P:fucosylation; C:membrane; C:integral component of membrane; P:protein glycosylation; C:Golgi apparatus; F:transferase activity |
| Efet.01.149756.g1216.t1 | long-chain-fatty-acid-- ligase ACSBG2-like | 417 | 2.75E-37 | 64.65 | F:catalytic activity; P:metabolic process; F:ligase activity |
| Efet.01.149786.g1217.t1 | pyroglutamylated RFamide peptide receptor isoform X1 | 1203 | 7.17E-37 | 62.75 | F:G-protein coupled receptor activity; P:G-protein coupled receptor signaling pathway; C:membrane |
| Efet.01.149819.g1218.t1 | sodium-coupled monocarboxylate transporter 1-like isoform X1 | 396 | 3.09E-30 | 65.05 | C:membrane; P:transport |
| Efet.01.149830.g1219.t1 | BTB POZ domain-containing kctd15-like | 252 | 1.20E-16 | 93.4 | P:protein homooligomerization |
| Efet.01.149893.g1222.t1 | vacuolar sorting-associated 52 homolog | 453 | 1.20E-62 | 82.85 |  |
| Efet.01.149916.g1225.t1 | tartrate-resistant acid phosphatase type 5-like | 261 | 6.35E-20 | 59.1 | P:dephosphorylation; F:acid phosphatase activity; F:hydrolase activity |
| Efet.01.14995.g1101.t1 | dachshund homolog 1-like | 354 | 7.10E-63 | 88.9 | C:nucleus; C:transcription factor complex; P:RNA polymerase II transcriptional preinitiation complex assembly; F:transcription factor activity, RNA polymerase II core promoter sequence-specific binding involved in preinitiation complex assembly; P:multicellular organism development; P:regulation of transcription, DNA-templated |
| Efet.01.150032.g1.t1 | runt-related transcription factor 1-like isoform X2 | 378 | 2.13E-46 | 80.3 | C:nucleus; F:DNA binding; F:ATP binding; F:transcription factor activity, sequence-specific DNA binding; P:regulation of transcription, DNA-templated |
| Efet.01.150063.g3.t1 | EF-hand domain-containing D1 | 381 | 9.79E-34 | 76.5 | F:calcium ion binding; C:mitochondrion |
| Efet.01.150082.g4.t1 | condensin-2 complex subunit G2 | 765 | 1.68E-40 | 52.75 | C:nucleus; P:inner cell mass cell proliferation; C:condensin complex; F:bHLH transcription factor binding |
| Efet.01.150117.g7.t1 | WD repeat-containing 20-like | 282 | 2.85E-26 | 70.6 |  |
| Efet.01.150164.g8.t1 | Potassium voltage-gated channel eag | 420 | 4.14E-52 | 84.5 | F:phosphorelay sensor kinase activity; F:voltage-gated potassium channel activity; P:potassium ion transmembrane transport; P:phosphorelay signal transduction system; C:integral component of membrane; P:signal transduction by protein phosphorylation; C:intracellular |
| Efet.01.15020.g1102.t1 | solute carrier family 35 member E1 | 444 | 8.20E-60 | 71.8 | C:integral component of membrane; P:carbohydrate transmembrane transport |
| Efet.01.150234.g9.t1 | acetylcholinesterase-like isoform X3 | 1425 | 2.43E-133 | 57.15 | C:membrane; C:integral component of membrane; F:cholinesterase activity; F:hydrolase activity |
| Efet.01.150236.g10.t1 | arrestin domain-containing 4-like | 1143 | 3.13E-92 | 56.15 |  |
| Efet.01.150236.g11.t1 | cell division cycle 7-related kinase-like | 261 | 1.29E-09 | 81.09 | P:regulation of cell shape; C:nucleus; F:ATP binding; P:DNA replication initiation; C:cytoplasm; P:double-strand break repair via break-induced replication; C:membrane; P:phagocytosis; F:protein serine/threonine kinase activity; P:peptidyl-serine phosphorylation |
| Efet.01.150286.g14.t1 | dystroglycan isoform X1 | 786 | 1.83E-32 | 53.1 | F:protein binding; P:regulation of cellular process; C:membrane; P:cellular component organization; P:animal organ development; P:muscle structure development; P:cell development; P:tissue development; C:cell periphery |
| Efet.01.150304.g16.t1 | dynein heavy chain axonemal-like | 1644 | 0 | 89.15 | F:microtubule motor activity; F:calcium ion binding; C:dynein complex; P:microtubule-based movement |
| Efet.01.150310.g17.t1 | myosin heavy chain isoform | 612 | 7.57E-49 | 84.85 | C:myosin complex; F:ATP binding; F:actin binding; P:metabolic process; F:amino acid binding; F:motor activity |
| Efet.01.150392.g21.t1 | WD repeat-containing on Y chromosome-like | 1095 | 2.08E-60 | 52.9 | F:calcium ion binding |
| Efet.01.150398.g22.t1 | hypothetical protein HELRODRAFT_175590 | 363 | 7.91E-23 | 67.67 | F:zinc ion binding; F:metal ion binding; F:carbonate dehydratase activity; P:one-carbon metabolic process |
| Efet.01.15043.g1103.t1 | CDP-diacylglycerol--glycerol-3-phosphate 3- mitochondrial | 816 | 2.91E-78 | 64.95 | F:transferase activity |
| Efet.01.150442.g23.t1 | teneurin-m isoform X4 | 300 | 3.50E-18 | 62.5 | F:protein binding; P:retinal ganglion cell axon guidance; C:membrane; P:cell adhesion; P:dendrite guidance; C:cell projection |
| Efet.01.150464.g24.t1 | synaptotagmin-7-like isoform X4 | 501 | 1.62E-31 | 79.8 | C:presynaptic membrane; P:regulation of glucagon secretion; F:phosphatidylinositol-4,5-bisphosphate binding; P:regulation of insulin secretion; C:early phagosome; C:extracellular exosome; C:terminal bouton; F:calcium ion binding; C:cytosol; P:calcium ion regulated lysosome exocytosis; P:vesicle-mediated cholesterol transport; P:synaptic vesicle recycling; F:syntaxin binding; P:phagosome-lysosome fusion; P:positive regulation of calcium ion-dependent exocytosis; C:lysosomal membrane; F:calcium-dependent phospholipid binding; C:peroxisomal membrane; F:calmodulin binding; P:calcium ion-regulated exocytosis of neurotransmitter; P:plasma membrane repair; P:regulation of bone remodeling; C:integral component of membrane; C:cell junction; C:phagocytic vesicle membrane; C:synaptic vesicle membrane; C:dense core granule; F:clathrin binding; P:regulation of phagocytosis |
| Efet.01.150499.g25.t1 | hypothetical protein HELRODRAFT_173674 | 564 | 2.51E-06 | 58 |  |
| Efet.01.150508.g27.t1 | ankyrin repeat domain-containing 63 | 285 | 4.60E-22 | 71.65 |  |
| Efet.01.150541.g31.t1 | contactin-associated -like 2 | 408 | 3.25E-18 | 49.8 | F:calcium ion binding; C:membrane; F:scavenger receptor activity; P:receptor-mediated endocytosis |
| Efet.01.150583.g32.t1 | FMRFamide receptor-like | 1257 | 9.74E-113 | 55.15 | F:G-protein coupled receptor activity; P:signal transduction; C:membrane |
| Efet.01.150601.g34.t1 | GTP-binding GEM | 219 | 3.26E-14 | 69.94 | F:GTP binding; P:small GTPase mediated signal transduction; C:membrane; C:intracellular |
| Efet.01.150634.g35.t1 | dachsous | 1248 | 4.05E-111 | 60.7 | C:membrane |
| Efet.01.150845.g41.t1 | F-box LRR-repeat 16-like | 1293 | 2.41E-85 | 69.35 |  |
| Efet.01.150866.g42.t1 | potassium voltage-gated channel subfamily A member 1-like isoform X1 | 1467 | 4.65E-105 | 69.7 | F:voltage-gated potassium channel activity; P:potassium ion transmembrane transport; C:voltage-gated potassium channel complex; P:regulation of ion transmembrane transport; P:protein homooligomerization |
| Efet.01.150884.g44.t1 | connectin-like | 1137 | 1.46E-39 | 52.35 | C:extracellular exosome; C:blood microparticle |
| Efet.01.150888.g45.t1 | transcriptional-regulating factor 1 isoform X1 | 2586 | 5.52E-11 | 86.75 | C:nucleus; F:DNA binding; F:metal ion binding |
| Efet.01.150917.g47.t1 | FMRFamide receptor | 1455 | 2.80E-32 | 52.65 | F:G-protein coupled receptor activity; P:G-protein coupled receptor signaling pathway; P:signal transduction; C:membrane; C:integral component of membrane; F:signal transducer activity |
| Efet.01.150928.g49.t1 | zinc finger 513 isoform X2 | 948 | 1.29E-73 | 59 | F:nucleic acid binding; F:metal ion binding |
| Efet.01.150930.g50.t1 | acetylornithine deacetylase | 1215 | 0 | 77.95 | F:hydrolase activity, acting on carbon-nitrogen (but not peptide) bonds, in linear amidines; P:proteolysis; F:metallopeptidase activity |
| Efet.01.150930.g51.t1 | family transcriptional regulator | 861 | 1.03E-162 | 76.8 | F:DNA binding; F:transcription factor activity, sequence-specific DNA binding; P:transcription, DNA-templated; P:regulation of transcription, DNA-templated |
| Efet.01.150930.g52.t1 | sodium-independent anion transporter | 1743 | 0 | 87.45 | F:secondary active sulfate transmembrane transporter activity; C:integral component of membrane; P:sulfate transmembrane transport |
| Efet.01.150930.g53.t1 | dihydroxy-acid dehydratase | 372 | 2.05E-57 | 92.95 | F:dihydroxy-acid dehydratase activity; F:L-arabinonate dehydratase activity; P:metabolic process |
| Efet.01.150930.g54.t1 | dihydroxy-acid dehydratase | 1089 | 0 | 90.95 | F:dihydroxy-acid dehydratase activity; F:L-arabinonate dehydratase activity; P:metabolic process |
| Efet.01.150944.g55.t1 | LIN-24 (Twenty-four) Like | 903 | 6.48E-82 | 55.35 |  |
| Efet.01.15097.g1105.t1 | SET and MYND domain-containing 4-like | 837 | 5.66E-55 | 54.85 |  |
| Efet.01.15097.g1106.t1 | SET and MYND domain-containing 4-like | 666 | 2.51E-14 | 60.5 |  |
| Efet.01.151001.g57.t1 | transmembrane 260-like | 258 | 6.21E-27 | 62.5 | C:membrane |
| Efet.01.151043.g59.t1 | somatostatin receptor type 2-like | 1377 | 4.88E-92 | 60.8 | F:G-protein coupled receptor activity; P:G-protein coupled receptor signaling pathway; C:membrane |
| Efet.01.1511.g144.t1 | FAM78A isoform X2 | 585 | 3.35E-86 | 65.05 |  |
| Efet.01.1511.g145.t1 | hypothetical protein HELRODRAFT_75099 | 522 | 3.06E-07 | 93 |  |
| Efet.01.15111.g1108.t1 | Y-box factor homolog | 345 | 1.56E-20 | 90.8 | P:male gonad development; P:transcription, DNA-templated; P:spermatogenesis; F:RNA polymerase II regulatory region sequence-specific DNA binding; P:positive regulation of organ growth; P:fertilization; F:transcriptional repressor activity, RNA polymerase II transcription regulatory region sequence-specific binding; P:negative regulation of intrinsic apoptotic signaling pathway in response to osmotic stress; F:single-stranded DNA binding; F:mRNA 3'-UTR binding; P:negative regulation of necroptotic process; C:polysome; P:negative regulation of transcription from RNA polymerase II promoter; C:nucleus; C:cytoplasm; P:negative regulation of skeletal muscle tissue development; P:cellular hyperosmotic response; P:cellular response to tumor necrosis factor |
| Efet.01.151149.g60.t1 | acetyl- carboxylase 1 isoform X6 | 231 | 1.41E-40 | 93.3 | F:ATP binding; F:acetyl-CoA carboxylase activity; F:metal ion binding; F:biotin carboxylase activity; P:fatty acid biosynthetic process |
| Efet.01.151176.g61.t1 | neuroligin- X-linked-like isoform X1 | 279 | 1.22E-16 | 89.4 | F:neurexin family protein binding; C:integral component of membrane |
| Efet.01.151180.g62.t1 | SPRY domain-containing SOCS box 1-like | 822 | 3.33E-130 | 80.4 | C:intracellular; P:intracellular signal transduction |
| Efet.01.151196.g63.t1 | microtubule-associated futsch | 2823 | 2.27E-99 | 53.9 | P:microtubule cytoskeleton organization; F:microtubule binding; C:microtubule |
| Efet.01.151234.g64.t1 | addiction module family | 435 | 1.30E-73 | 82.9 |  |
| Efet.01.151234.g65.t1 | ATP-dependent helicase | 720 | 2.87E-161 | 56.45 | F:DNA binding; F:ATP binding; P:DNA duplex unwinding; F:ATP-dependent DNA helicase activity |
| Efet.01.151234.g66.t1 | type IV secretion system | 384 | 1.05E-79 | 79.5 | C:integral component of membrane |
| Efet.01.151234.g67.t1 | disulfide isomerase | 255 | 2.68E-12 | 64.3 | C:cell; P:cell redox homeostasis |
| Efet.01.151234.g68.t1 | hypothetical protein | 1185 | 0 | 62.63 | C:integral component of membrane |
| Efet.01.151234.g69.t1 | hypothetical protein | 1119 | 0 | 77.5 |  |
| Efet.01.151234.g70.t1 | type IV pilin | 495 | 9.04E-101 | 61.1 | C:integral component of membrane |
| Efet.01.151234.g71.t1 | conjugal transfer | 2094 | 0 | 73.4 | P:protein secretion |
| Efet.01.151234.g72.t1 | -like C-terminal domain | 1305 | 0 | 90.7 |  |
| Efet.01.151234.g73.t1 | ATP-dependent helicase | 942 | 2.25E-166 | 60 | F:DNA binding; F:ATP binding; P:DNA duplex unwinding; F:ATP-dependent DNA helicase activity |
| Efet.01.151234.g74.t1 | DUF2442 domain-containing | 501 | 2.57E-88 | 77.45 | F:sequence-specific DNA binding |
| Efet.01.151234.g75.t1 | DUF4113 domain-containing | 267 | 4.20E-53 | 67.45 |  |
| Efet.01.151234.g76.t1 | aspartate aminotransferase family | 1476 | 0 | 86.2 | F:pyridoxal phosphate binding; F:transaminase activity |
| Efet.01.151234.g77.t1 | aldehyde dehydrogenase | 1269 | 0 | 73.35 | P:oxidation-reduction process; F:oxidoreductase activity, acting on the aldehyde or oxo group of donors, NAD or NADP as acceptor |
| Efet.01.151234.g78.t1 | BMC domain-containing | 324 | 1.39E-59 | 81.85 |  |
| Efet.01.151237.g79.t1 | FRAS1-related extracellular matrix 1 | 627 | 1.00E-38 | 63.25 | P:cell-matrix adhesion; C:basement membrane; P:cell communication; C:integral component of membrane; P:craniofacial suture morphogenesis |
| Efet.01.151237.g80.t1 | FRAS1-related extracellular matrix 1-like | 873 | 1.56E-50 | 52.7 | P:cell-matrix adhesion; C:basement membrane; P:cell communication; F:calcium ion binding; C:membrane; C:integral component of membrane; P:homophilic cell adhesion via plasma membrane adhesion molecules |
| Efet.01.15124.g1110.t1 | Calcium calmodulin-dependent kinase type II alpha chain | 276 | 1.64E-16 | 72.95 | F:nucleotide binding; P:phosphorylation; F:protein serine/threonine kinase activity |
| Efet.01.151251.g81.t1 | hypothetical protein HELRODRAFT_192854 | 432 | 7.37E-14 | 51 | F:metal ion binding |
| Efet.01.151299.g84.t1 | probable G- coupled receptor 139 | 1200 | 5.74E-55 | 57.3 | F:G-protein coupled receptor activity; P:G-protein coupled receptor signaling pathway; P:signal transduction; C:membrane; C:integral component of membrane; F:signal transducer activity |
| Efet.01.151322.g85.t1 | peptidyl-prolyl cis-trans isomerase CWC27 homolog | 1359 | 4.56E-133 | 61.85 | C:catalytic step 2 spliceosome; P:mRNA splicing, via spliceosome |
| Efet.01.151411.g87.t1 | growth differentiation factor 3 | 1155 | 6.16E-25 | 69.95 | F:growth factor activity; P:regulation of MAPK cascade; P:regulation of epiboly involved in gastrulation with mouth forming second; F:transforming growth factor beta receptor binding; P:SMAD protein signal transduction; P:regulation of nodal signaling pathway involved in determination of left/right asymmetry; P:regulation of apoptotic process; P:neuron development; F:cytokine activity; P:growth; C:extracellular space; P:BMP signaling pathway; P:heart looping; P:positive regulation of pathway-restricted SMAD protein phosphorylation |
| Efet.01.151414.g88.t1 | hypothetical protein A3Q56_04706, partial | 417 | 8.65E-07 | 54 |  |
| Efet.01.15149.g1111.t1 | Zonadhesin | 2682 | 3.03E-12 | 41.5 |  |
| Efet.01.151563.g92.t1 | aquaporin -like | 417 | 3.70E-11 | 75.8 | C:integral component of plasma membrane; P:water transport; F:glycerol channel activity; P:cellular water homeostasis; P:glycerol transport; P:ion transmembrane transport; F:water channel activity |
| Efet.01.151599.g94.t1 | EH domain-containing 1 | 231 | 2.20E-36 | 93.2 | F:GTP binding; F:calcium ion binding; C:intracellular; P:endocytic recycling |
| Efet.01.151599.g95.t1 | Receptor Mediated Endocytosis | 621 | 3.46E-107 | 88.75 | F:GTP binding; F:ATPase activity; P:lipid tube assembly involved in organelle fission; F:protein domain specific binding; F:calcium ion binding; P:embryo development ending in birth or egg hatching; P:positive regulation of necrotic cell death; C:recycling endosome; P:meiotic nuclear division; P:endocytic recycling |
| Efet.01.151644.g96.t1 | hypothetical protein CAPTEDRAFT_187422 | 2235 | 8.09E-10 | 42.33 | C:membrane; C:integral component of membrane |
| Efet.01.151652.g97.t1 | endoplasmic reticulum mannosyl-oligosaccharide 1,2-alpha-mannosidase | 243 | 1.36E-21 | 78.95 | F:calcium ion binding; P:metabolic process; C:integral component of membrane; F:mannosyl-oligosaccharide 1,2-alpha-mannosidase activity |
| Efet.01.151661.g98.t1 | acetyl- carboxylase carboxyltransferase subunit | 915 | 0 | 80.75 | F:propionyl-CoA carboxylase activity; F:methylcrotonoyl-CoA carboxylase activity; F:transferase activity |
| Efet.01.151661.g99.t1 | acyl- dehydrogenase | 1158 | 0 | 91.65 | F:isovaleryl-CoA dehydrogenase activity; F:flavin adenine dinucleotide binding; P:oxidation-reduction process |
| Efet.01.151662.g100.t1 | potassium voltage-gated channel Shab-like | 558 | 5.00E-25 | 86.2 | F:voltage-gated potassium channel activity; P:potassium ion transmembrane transport; C:voltage-gated potassium channel complex; P:regulation of ion transmembrane transport; P:protein homooligomerization |
| Efet.01.151663.g101.t1 | heparan sulfate glucosamine 3-O-sulfotransferase 6-like | 609 | 2.49E-23 | 78.55 | C:integral component of membrane; F:[heparan sulfate]-glucosamine 3-sulfotransferase 1 activity |
| Efet.01.151664.g102.t1 | sacsin-like | 519 | 1.06E-43 | 68.8 |  |
| Efet.01.151710.g104.t1 | F-box LRR-repeat 14 | 1242 | 3.14E-169 | 78.5 | F:ubiquitin-protein transferase activity; C:SCF ubiquitin ligase complex; P:protein ubiquitination |
| Efet.01.151736.g105.t1 | hypothetical protein LOTGIDRAFT_160410 | 432 | 4.74E-07 | 50.5 |  |
| Efet.01.15175.g1113.t1 | kinase C theta type | 246 | 1.01E-17 | 86.15 | F:ATP binding; F:metal ion binding; P:protein phosphorylation; C:intracellular; P:intracellular signal transduction; F:protein kinase C activity |
| Efet.01.15180.g1114.t1 | DNA-binding D-ETS-3-like | 510 | 1.49E-26 | 83.75 | F:RNA polymerase II distal enhancer sequence-specific DNA binding; C:nucleus; F:transcriptional activator activity, RNA polymerase II core promoter proximal region sequence-specific binding; F:protein binding; F:RNA polymerase II core promoter proximal region sequence-specific DNA binding; P:megakaryocyte development; P:transcription from RNA polymerase II promoter; P:blood circulation; F:chromatin binding; P:positive regulation of transcription from RNA polymerase II promoter; P:hemostasis; P:animal organ morphogenesis |
| Efet.01.15181.g1115.t1 | FMRFamide receptor-like | 759 | 3.39E-65 | 70.6 | F:G-protein coupled receptor activity; P:G-protein coupled receptor signaling pathway; C:integral component of membrane |
| Efet.01.151815.g108.t1 | guanylate cyclase 32E | 228 | 2.72E-31 | 83.1 | F:ATP binding; P:cGMP biosynthetic process; C:integral component of membrane; F:guanylate cyclase activity; F:protein kinase activity; P:protein phosphorylation; C:intracellular; P:intracellular signal transduction |
| Efet.01.151826.g109.t1 | Down syndrome cell adhesion molecule Dscam2 | 555 | 1.45E-36 | 47.65 |  |
| Efet.01.15184.g1116.t1 | cleavage stimulation factor subunit 3-like | 282 | 6.28E-33 | 87.85 | C:nucleus; P:RNA 3'-end processing; F:mRNA binding; P:mRNA processing; P:mRNA cleavage |
| Efet.01.151853.g110.t1 | hypothetical protein HELRODRAFT_189210 | 504 | 3.20E-29 | 49.58 | F:oxidoreductase activity; P:metabolic process; C:membrane; C:integral component of membrane; P:oxidation-reduction process |
| Efet.01.151863.g111.t1 | guanylyl cyclase GC-E-like | 1038 | 3.84E-45 | 46.3 | F:phosphorus-oxygen lyase activity; F:protein kinase activity; F:phosphate ion binding; P:protein phosphorylation; P:transport; C:intracellular; F:ATP binding; P:phosphate ion transport; C:membrane; C:integral component of membrane; P:intracellular signal transduction; P:phosphate ion transmembrane transport; P:cyclic nucleotide biosynthetic process; C:ATP-binding cassette (ABC) transporter complex |
| Efet.01.151865.g112.t1 | FMRFamide receptor | 1203 | 7.37E-91 | 55.9 | F:signal transducer activity |
| Efet.01.151917.g114.t1 | hypothetical protein HELRODRAFT_160645 | 456 | 9.78E-06 | 54 |  |
| Efet.01.151958.g116.t1 | SMG7-like isoform X1 | 573 | 1.62E-16 | 58.25 |  |
| Efet.01.15198.g1117.t1 | NADP-dependent malic enzyme-like | 309 | 7.97E-44 | 82.25 | P:malate metabolic process; F:metal ion binding; F:NAD binding; F:malate dehydrogenase (decarboxylating) (NAD+) activity; P:oxidation-reduction process |
| Efet.01.152042.g120.t1 | calmodulin isoform X2 | 219 | 6.82E-25 | 83.45 | P:regulation of release of sequestered calcium ion into cytosol by sarcoplasmic reticulum; P:positive regulation of cyclic-nucleotide phosphodiesterase activity; C:calcium channel complex; C:extracellular exosome; P:positive regulation of cyclic nucleotide metabolic process; F:protein phosphatase activator activity; F:calcium ion binding; F:phospholipase binding; P:regulation of cytokinesis; F:titin binding; F:thioesterase binding; F:N-terminal myristoylation domain binding; F:protein kinase binding; F:protein domain specific binding; C:spindle pole; P:positive regulation of ryanodine-sensitive calcium-release channel activity; F:ion channel binding; P:substantia nigra development; C:nucleus; C:spindle microtubule; P:regulation of heart rate; P:detection of calcium ion; C:centrosome; P:positive regulation of phosphoprotein phosphatase activity; P:regulation of cardiac muscle contraction; C:sarcomere |
| Efet.01.152059.g121.t1 | Hepatocyte nuclear factor 4- | 978 | 1.10E-69 | 84.1 | C:nucleus; F:zinc ion binding; F:RNA polymerase II transcription factor activity, ligand-activated sequence-specific DNA binding; P:transcription, DNA-templated; F:steroid hormone receptor activity; P:regulation of transcription, DNA-templated; F:sequence-specific DNA binding; P:steroid hormone mediated signaling pathway; P:intracellular receptor signaling pathway |
| Efet.01.152133.g122.t1 | Alpha-N-acetylgalactosaminidase | 411 | 1.28E-19 | 63.9 | F:hydrolase activity, hydrolyzing O-glycosyl compounds; P:organic substance metabolic process |
| Efet.01.152178.g125.t1 | hypothetical protein CAPTEDRAFT_180887 | 543 | 7.81E-50 | 83.35 | C:integral component of membrane |
| Efet.01.15219.g1118.t1 | pleckstrin homology domain-containing family G member 7-like | 345 | 9.74E-12 | 81.44 | P:regulation of Rho protein signal transduction; F:Rho guanyl-nucleotide exchange factor activity; P:positive regulation of GTPase activity |
| Efet.01.152194.g128.t1 | hypothetical protein HELRODRAFT_183289 | 1719 | 2.22E-08 | 50 | C:membrane; C:integral component of membrane |
| Efet.01.152211.g129.t1 | hypothetical protein CAPTEDRAFT_193290 | 1620 | 4.51E-23 | 55.67 |  |
| Efet.01.152236.g130.t1 | beta-2 adrenergic receptor-like | 1047 | 8.86E-112 | 64.35 | C:integral component of membrane; P:dopamine receptor signaling pathway; F:G-protein coupled amine receptor activity |
| Efet.01.15226.g1120.t1 | dopamine D2-like receptor | 2493 | 1.24E-25 | 68.4 | C:integral component of plasma membrane; F:dopamine neurotransmitter receptor activity; P:dopamine receptor signaling pathway |
| Efet.01.15227.g1121.t1 | hypothetical protein CAPTEDRAFT_194969 | 201 | 2.56E-11 | 60.85 | C:membrane; C:integral component of membrane; F:carbohydrate binding |
| Efet.01.152294.g132.t1 | ubiquinone biosynthesis O- mitochondrial-like | 603 | 1.74E-50 | 70.3 | F:3-demethylubiquinone-n 3-O-methyltransferase activity; P:metabolic process; F:S-adenosylmethionine-dependent methyltransferase activity |
| Efet.01.152379.g135.t1 | potassium voltage-gated channel eag-like | 264 | 4.57E-25 | 76.65 | F:phosphorelay sensor kinase activity; C:integral component of plasma membrane; F:voltage-gated potassium channel activity; P:regulation of membrane potential; P:potassium ion transmembrane transport; P:phosphorelay signal transduction system; P:signal transduction by protein phosphorylation; C:intracellular |
| Efet.01.152432.g136.t1 | 60S ribosomal L24 | 333 | 3.91E-59 | 85.8 | C:ribosome |
| Efet.01.152439.g137.t1 | LARGE xylosyl- and glucuronyltransferase 1-like isoform X1 | 3159 | 1.49E-70 | 48.4 | F:transferase activity, transferring glycosyl groups; C:intracellular membrane-bounded organelle; P:metabolic process; F:binding |
| Efet.01.152440.g138.t1 | histone-lysine N-methyltransferase SETDB1-B-like isoform X1 | 702 | 9.42E-66 | 73.35 | C:intracellular organelle; F:methyltransferase activity; F:binding; P:methylation |
| Efet.01.152511.g139.t1 | contactin | 399 | 2.44E-32 | 66.45 | C:membrane |
| Efet.01.152524.g140.t1 | LETM1 and EF-hand domain-containing | 843 | 1.56E-90 | 74.25 | F:calcium ion binding; F:ribosome binding; C:integral component of membrane |
| Efet.01.152535.g142.t1 | adenylate kinase 7-like | 750 | 6.66E-48 | 69.5 | F:ATP binding; P:phosphorylation; F:nucleobase-containing compound kinase activity; P:nucleobase-containing compound metabolic process |
| Efet.01.152657.g144.t1 | 5 exonuclease Apollo | 1998 | 2.48E-75 | 59.55 | P:DNA repair; F:exonuclease activity |
| Efet.01.152661.g145.t1 | collagen alpha-1(XXII) chain | 246 | 3.04E-23 | 64.25 | C:extracellular region part; F:binding |
| Efet.01.152705.g146.t1 | otoferlin isoform X8 | 822 | 8.24E-63 | 60.85 | C:intracellular organelle; C:membrane; P:response to auditory stimulus; C:endomembrane system; C:cytoplasmic part; C:synapse; P:neuromuscular process controlling balance; C:membrane-bounded organelle |
| Efet.01.152715.g148.t1 | deoxyhypusine hydroxylase | 417 | 3.44E-58 | 80.75 | F:metal ion binding; F:deoxyhypusine monooxygenase activity; P:oxidation-reduction process; P:peptidyl-lysine modification to peptidyl-hypusine |
| Efet.01.152766.g150.t1 | serine arginine-rich splicing factor 7-like isoform X1 | 267 | 2.00E-20 | 82.3 | F:nucleotide binding; F:nucleic acid binding; F:zinc ion binding |
| Efet.01.152768.g151.t1 | vesicle-fusing ATPase 1-like | 525 | 3.31E-95 | 76.8 | F:ATPase activity; F:ATP binding; P:Golgi vesicle docking; P:Golgi to plasma membrane protein transport; P:intra-Golgi vesicle-mediated transport; C:Golgi stack |
| Efet.01.152908.g155.t1 | dimmed-like | 831 | 8.55E-38 | 75.5 | F:protein dimerization activity |
| Efet.01.15292.g1125.t1 | outer dense fiber 2 isoform X2 | 465 | 2.62E-17 | 57.25 |  |
| Efet.01.152924.g156.t1 | NEC1_MOUSE ame: Full=Neuroendocrine convertase 1 Short=NEC 1 ame: Full=Furin homolog ame: Full=PC3 ame: Full=Prohormone convertase 1 ame: Full=Propeptide-processing protease ame: Full=Pro convertase 1 Short=PC1 Flags: Precursor | 354 | 7.00E-06 | 80 | C:extracellular space; P:peptide biosynthetic process; P:proteolysis; F:serine-type peptidase activity; F:serine-type endopeptidase activity; F:endopeptidase activity; C:cytoplasmic vesicle; P:protein processing; F:peptidase activity; C:transport vesicle; P:peptide hormone processing; F:hydrolase activity |
| Efet.01.152930.g157.t1 | F0F1 ATP synthase subunit beta | 570 | 3.06E-130 | 99.6 | C:proton-transporting ATP synthase complex, catalytic core F(1); F:ATP binding; P:ATP hydrolysis coupled proton transport; C:plasma membrane; F:proton-transporting ATP synthase activity, rotational mechanism; P:plasma membrane ATP synthesis coupled proton transport |
| Efet.01.152944.g158.t1 | pikachurin-like | 267 | 2.02E-29 | 78.6 | F:calcium ion binding; C:membrane |
| Efet.01.152957.g159.t1 | hypothetical protein CAPTEDRAFT_215767 | 570 | 6.54E-48 | 68 |  |
| Efet.01.152966.g160.t1 | Bardet-Biedl syndrome 5 homolog | 258 | 1.83E-27 | 78.9 | C:BBSome; C:ciliary basal body; P:intracellular transport; F:phosphatidylinositol-3-phosphate binding |
| Efet.01.152984.g161.t1 | contactin | 522 | 8.62E-24 | 56.2 | C:membrane; C:integral component of membrane |
| Efet.01.152987.g162.t1 | bicaudal D-like isoform X2 | 939 | 8.16E-24 | 59.4 | C:Golgi apparatus; P:transport |
| Efet.01.152987.g163.t1 | bicaudal D-like isoform X1 | 279 | 8.76E-22 | 71.6 | C:Golgi apparatus; P:transport |
| Efet.01.152999.g164.t1 | tripartite tricarboxylate transporter substrate binding | 642 | 2.09E-140 | 71.3 | C:outer membrane-bounded periplasmic space |
| Efet.01.153003.g165.t1 | Ankyrin repeat | 1119 | 9.78E-39 | 50 | P:transport |
| Efet.01.153064.g167.t1 | gliomedin-like isoform X2 | 750 | 2.36E-93 | 58.55 | P:regulation of cellular process |
| Efet.01.153118.g169.t1 | cAMP-specific 3 ,5 -cyclic phosphodiesterase 4C-like isoform X9 | 519 | 1.92E-53 | 72.65 | F:phosphoric diester hydrolase activity |
| Efet.01.15315.g1127.t1 | thyrotropin-releasing hormone receptor-like | 1353 | 2.63E-69 | 56.5 | F:G-protein coupled receptor activity; P:G-protein coupled receptor signaling pathway; P:signal transduction; C:membrane; C:integral component of membrane; F:signal transducer activity |
| Efet.01.153212.g170.t1 | outer membrane | 459 | 4.61E-50 | 66.11 |  |
| Efet.01.153261.g174.t1 | polypeptide N-acetylgalactosaminyltransferase 1-like | 1983 | 0 | 62.15 | C:membrane |
| Efet.01.15329.g1128.t1 | tetratricopeptide repeat 14-like isoform X2 | 327 | 1.80E-20 | 71.1 | F:nucleic acid binding |
| Efet.01.153308.g179.t1 | mediator of RNA polymerase II transcription subunit 13-like | 1380 | 6.60E-125 | 59.8 | C:mediator complex; F:RNA polymerase II transcription cofactor activity; P:regulation of transcription from RNA polymerase II promoter |
| Efet.01.153393.g180.t1 | FMRFamide receptor-like | 369 | 1.08E-23 | 69.35 | F:G-protein coupled peptide receptor activity; P:G-protein coupled receptor signaling pathway; C:integral component of membrane |
| Efet.01.153393.g181.t1 | FMRFamide receptor-like | 627 | 8.97E-43 | 66 | F:G-protein coupled receptor activity; C:membrane |
| Efet.01.153394.g182.t1 | Zinc finger SWIM domain-containing | 435 | 8.11E-43 | 78.8 | C:extracellular space; F:zinc ion binding |
| Efet.01.153418.g184.t1 | forkhead box A | 534 | 9.88E-39 | 85.95 | F:transcription factor activity, sequence-specific DNA binding; F:protein domain specific binding; P:regulation of transcription, DNA-templated; F:sequence-specific DNA binding; F:transcription factor binding |
| Efet.01.153445.g185.t1 | kelch 20 | 381 | 1.57E-51 | 75.9 | F:ubiquitin-protein transferase activity; C:trans-Golgi network; P:proteasome-mediated ubiquitin-dependent protein catabolic process; C:PML body; P:negative regulation of apoptotic process; P:protein K33-linked ubiquitination; C:Cul3-RING ubiquitin ligase complex; F:interferon-gamma binding; P:response to interferon-alpha; P:Golgi to endosome transport; P:protein ubiquitination involved in ubiquitin-dependent protein catabolic process |
| Efet.01.153447.g186.t1 | inosine-uridine preferring nucleoside hydrolase-like | 483 | 2.29E-28 | 63.9 | F:hydrolase activity |
| Efet.01.15345.g1130.t1 | dystonin-like isoform X11 | 7332 | 9.19E-103 | 51.7 | P:cell cycle arrest; C:cytoskeleton; F:calcium ion binding |
| Efet.01.153538.g188.t1 | acetyl- carboxylase-like | 600 | 8.31E-106 | 88.95 | F:ATP binding; F:acetyl-CoA carboxylase activity; F:metal ion binding; F:biotin carboxylase activity; P:fatty acid biosynthetic process |
| Efet.01.153538.g189.t1 | acetyl- carboxylase 2 isoform X5 | 708 | 9.35E-95 | 66.6 | F:ATP binding; F:acetyl-CoA carboxylase activity; F:metal ion binding; F:biotin carboxylase activity; P:fatty acid biosynthetic process |
| Efet.01.153578.g191.t1 | rho GTPase-activating 190 isoform X1 | 300 | 1.42E-28 | 72.35 | P:signal transduction |
| Efet.01.153611.g192.t1 | leukocyte elastase inhibitor | 432 | 5.44E-23 | 62.8 | F:peptidase inhibitor activity; C:extracellular region part |
| Efet.01.153611.g193.t1 | serpin family | 243 | 1.90E-16 | 70.95 | C:extracellular space |
| Efet.01.153618.g195.t1 | hypothetical protein HELRODRAFT_170716 | 453 | 1.68E-13 | 81.5 | C:integral component of membrane |
| Efet.01.153657.g198.t1 | translocon-associated subunit beta-like | 345 | 3.42E-26 | 61.8 | C:integral component of membrane; C:endoplasmic reticulum |
| Efet.01.15367.g1131.t1 | hypothetical protein CAPTEDRAFT_227790 | 633 | 3.59E-14 | 65 |  |
| Efet.01.15369.g1132.t1 | zinc finger 845-like | 495 | 6.88E-07 | 42.26 | F:nucleic acid binding; F:metal ion binding |
| Efet.01.153765.g199.t1 | TKL kinase | 2829 | 1.39E-51 | 50.15 | F:ATP binding; P:peptidyl-tyrosine phosphorylation; F:protein tyrosine kinase activity; C:membrane; C:integral component of membrane; F:protein kinase activity; P:phosphorylation; P:protein phosphorylation; F:kinase activity |
| Efet.01.153934.g201.t1 | ADP-ribose mitochondrial | 318 | 8.47E-36 | 77.7 | F:hydrolase activity |
| Efet.01.153967.g202.t1 | calcium-dependent cysteine | 345 | 2.87E-50 | 76.15 | F:transcription factor activity, sequence-specific DNA binding; C:cytoplasm; F:metal ion binding; P:proteolysis; F:calcium-dependent cysteine-type endopeptidase activity |
| Efet.01.154084.g204.t1 | uncharacterized aminotransferase -like | 276 | 2.12E-13 | 59.75 | F:catalytic activity |
| Efet.01.15412.g1133.t1 | predicted protein | 1584 | 4.39E-11 | 54.78 | C:membrane; C:integral component of membrane |
| Efet.01.154124.g205.t1 | sarcoplasmic calcium-binding | 270 | 1.10E-17 | 62.5 | F:calcium ion binding |
| Efet.01.154148.g208.t1 | E3 ubiquitin- ligase RNF213- | 663 | 1.42E-07 | 58 |  |
| Efet.01.154210.g211.t1 | potassium voltage-gated channel Shaw-like isoform X5 | 561 | 6.55E-45 | 89.5 | F:voltage-gated potassium channel activity; P:potassium ion transmembrane transport; F:metal ion binding; C:voltage-gated potassium channel complex; P:regulation of ion transmembrane transport; P:protein homooligomerization |
| Efet.01.154252.g214.t1 | hypothetical protein CAPTEDRAFT_191992 | 1518 | 6.16E-31 | 46.5 | C:membrane; C:integral component of membrane |
| Efet.01.154297.g215.t1 | Histone-lysine N-methyltransferase MLL5 | 822 | 1.62E-30 | 81.05 | F:zinc ion binding; F:metal ion binding; F:methyltransferase activity; F:transferase activity; P:methylation |
| Efet.01.154321.g216.t1 | hypothetical protein CAPTEDRAFT_220544 | 2976 | 3.14E-10 | 45 | P:signal transduction; F:GTPase activator activity; F:phospholipid binding; P:positive regulation of GTPase activity |
| Efet.01.154353.g218.t1 | TELO2-interacting 2-like | 342 | 4.86E-12 | 63.6 | C:membrane; C:integral component of membrane |
| Efet.01.154386.g220.t1 | SUMO-conjugating enzyme UBC9-B | 303 | 3.41E-67 | 96.55 | C:nucleus; F:ATP binding; F:ubiquitin protein ligase activity; F:ubiquitin protein ligase binding; C:cytoplasm; P:protein sumoylation; F:SUMO transferase activity |
| Efet.01.15443.g1134.t1 | soluble guanylate cyclase 88E isoform X1 | 318 | 2.43E-10 | 71.62 | P:single-organism process; P:cellular process |
| Efet.01.154433.g222.t1 | tripartite motif-containing 2-like isoform X3 | 1503 | 9.93E-124 | 57.15 | F:zinc ion binding; F:metal ion binding; C:intracellular |
| Efet.01.154440.g226.t1 | RUN domain-containing 3A-like | 291 | 2.75E-13 | 66.55 |  |
| Efet.01.154453.g227.t1 | histone-lysine N-methyltransferase SETD1 | 1410 | 1.30E-17 | 52.15 |  |
| Efet.01.154453.g228.t1 | SET domain-containing | 348 | 3.01E-67 | 87.15 | F:histone methyltransferase activity (H3-K4 specific); P:stem cell population maintenance; P:transcription, DNA-templated; P:regulation of hematopoietic stem cell differentiation; C:nuclear speck; P:regulation of chromatin organization; F:beta-catenin binding; F:transcription factor binding; P:regulation of erythrocyte differentiation; C:Set1C/COMPASS complex; F:nucleotide binding; F:euchromatin binding; F:RNA binding; P:positive regulation of stem cell proliferation; P:positive regulation of neural precursor cell proliferation; C:nuclear euchromatin; P:chromatin-mediated maintenance of transcription; P:histone H3-K4 methylation |
| Efet.01.15446.g1135.t1 | KLF2-like transcription factor | 480 | 4.00E-48 | 87.55 | F:nucleic acid binding; F:metal ion binding |
| Efet.01.154538.g230.t1 | hypothetical protein CAPTEDRAFT_191180 | 1215 | 4.71E-08 | 57 |  |
| Efet.01.154553.g231.t1 | twist-related 2 | 393 | 5.51E-08 | 83.55 | C:nucleus; C:cytoplasm; F:protein dimerization activity; P:negative regulation of osteoblast differentiation |
| Efet.01.154584.g233.t1 | poly [ADP-ribose] polymerase 14-like | 900 | 4.81E-24 | 44.25 |  |
| Efet.01.15460.g1138.t1 | gamma-aminobutyric acid receptor subunit delta-like isoform X2 | 321 | 3.79E-53 | 76.2 | P:ion transmembrane transport; C:integral component of membrane; C:cell junction; F:extracellular ligand-gated ion channel activity; C:synapse; C:plasma membrane |
| Efet.01.154615.g235.t1 | transmembrane INAFM2 | 384 | 1.18E-08 | 85.5 | C:integral component of membrane |
| Efet.01.154627.g236.t1 | pannexin 4 | 705 | 2.23E-46 | 79.55 | C:gap junction; P:ion transport; P:transmembrane transport; C:integral component of membrane; P:cell communication by electrical coupling; C:synapse; F:gap junction channel activity; C:plasma membrane |
| Efet.01.154664.g241.t1 | Dynactin subunit 1 | 276 | 5.48E-14 | 74.6 | C:dynactin complex; P:transport along microtubule |
| Efet.01.154690.g242.t1 | F-box only 16-like | 639 | 3.63E-20 | 54.8 |  |
| Efet.01.154752.g245.t1 | Voltage-dependent calcium channel subunit alpha-2 delta-2 | 627 | 1.56E-17 | 41.67 | C:membrane; C:integral component of membrane |
| Efet.01.15477.g1141.t1 | cyclic nucleotide-gated cation channel alpha-3-like isoform X1 | 408 | 7.99E-37 | 72.05 | P:ion transport; P:transmembrane transport; C:membrane |
| Efet.01.154784.g247.t1 | T family of potassium channels 18-like | 1095 | 6.02E-31 | 64.6 | P:ion transport; C:membrane |
| Efet.01.154796.g249.t1 | leucine-rich repeat-containing 24-like | 1716 | 4.34E-62 | 50.65 | C:membrane; C:integral component of membrane |
| Efet.01.154828.g251.t1 | sugar phosphate isomerase epimerase | 744 | 1.02E-167 | 72.4 | F:isomerase activity |
| Efet.01.154831.g252.t1 | briggsae CBR-LNTL-1 | 804 | 2.54E-88 | 56 |  |
| Efet.01.154917.g253.t1 | hypothetical protein CAPTEDRAFT_211886 | 930 | 2.51E-66 | 55.5 | C:membrane; C:integral component of membrane; F:carbohydrate binding |
| Efet.01.154962.g255.t1 | iroquois-class homeodomain IRX-6-like | 237 | 1.77E-11 | 62.91 | F:DNA binding; C:nucleus; P:regulation of transcription, DNA-templated; F:sequence-specific DNA binding |
| Efet.01.154995.g256.t1 | Serine threonine- kinase pim-1 | 480 | 3.93E-43 | 73.35 | F:nucleotide binding; P:regulation of macromolecule metabolic process; C:cytoplasm; P:negative regulation of apoptotic process; F:ion binding; P:protein autophosphorylation; F:protein serine/threonine kinase activity; P:single-organism process; P:regulation of primary metabolic process; P:regulation of mitotic cell cycle; P:regulation of cellular metabolic process; P:regulation of molecular function |
| Efet.01.154996.g257.t1 | serine threonine- kinase pim-1-like | 657 | 2.51E-95 | 78.75 | F:ATP binding; P:camera-type eye morphogenesis; P:negative regulation of apoptotic process; F:protein serine/threonine kinase activity; P:protein phosphorylation; P:optokinetic behavior |
| Efet.01.1550.g148.t1 | intraflagellar transport 46 homolog isoform X2 | 447 | 8.98E-58 | 81.65 | P:intraciliary transport; C:cilium |
| Efet.01.155025.g258.t1 | TAF5-like RNA polymerase II p300 CBP-associated factor-associated factor 65 kDa subunit 5L | 459 | 2.97E-17 | 68.87 | C:intracellular membrane-bounded organelle |
| Efet.01.155056.g259.t1 | disrupted in renal carcinoma 2-like | 519 | 3.64E-22 | 63.3 | C:membrane |
| Efet.01.155064.g260.t1 | unc-93 homolog A | 354 | 5.74E-18 | 58.5 | C:membrane; C:integral component of membrane |
| Efet.01.1551.g149.t1 | neuronal acetylcholine receptor subunit alpha-7 | 558 | 2.12E-45 | 82.25 | C:postsynaptic membrane; P:cation transmembrane transport; C:integral component of membrane; C:cell junction; F:extracellular ligand-gated ion channel activity |
| Efet.01.155110.g262.t1 | BTB POZ domain-containing KCTD16 | 762 | 8.19E-62 | 66.05 | P:protein homooligomerization |
| Efet.01.155130.g264.t1 | lateral signaling target 2 homolog | 300 | 9.36E-47 | 86.75 | F:metal ion binding |
| Efet.01.155144.g265.t1 | flagellin | 1653 | 1.08E-180 | 64.05 | C:bacterial-type flagellum filament; P:bacterial-type flagellum organization; F:structural molecule activity; P:bacterial-type flagellum-dependent cell motility; C:extracellular region |
| Efet.01.155169.g266.t1 | somatostatin receptor type 2-like | 1200 | 1.46E-98 | 64.1 | F:G-protein coupled peptide receptor activity; P:G-protein coupled receptor signaling pathway; C:membrane |
| Efet.01.155178.g267.t1 | ATP-dependent RNA helicase DDX3X-like isoform X2 | 453 | 1.51E-89 | 92.15 | F:poly(A) binding; C:nuclear speck; F:ribosomal small subunit binding; P:chromosome segregation; P:mature ribosome assembly; P:extrinsic apoptotic signaling pathway via death domain receptors; P:positive regulation of cell growth; F:CTPase activity; P:positive regulation of chemokine (C-C motif) ligand 5 production; P:negative regulation of cell growth; F:GTPase activity; P:positive regulation of interferon-beta production; P:negative regulation of cysteine-type endopeptidase activity involved in apoptotic process; P:Wnt signaling pathway; P:negative regulation of intrinsic apoptotic signaling pathway; P:negative regulation of translation; P:stress granule assembly; P:translational initiation; P:positive regulation of cysteine-type endopeptidase activity involved in apoptotic process; P:protein localization to cytoplasmic stress granule; C:cell-cell adherens junction; P:cellular response to arsenic-containing substance; P:RNA secondary structure unwinding; F:eukaryotic initiation factor 4E binding; P:positive regulation of translational initiation; C:cytosolic small ribosomal subunit; P:intrinsic apoptotic signaling pathway; F:ATP-dependent DNA helicase activity; P:response to virus; P:positive regulation of transcription from RNA polymerase II promoter; C:eukaryotic translation initiation factor 3 complex; F:ATP-dependent RNA helicase activity; F:ATP binding; C:extracellular exosome; P:cellular response to osmotic stress; F:mRNA 5'-UTR binding; P:DNA duplex unwinding; F:RNA stem-loop binding; P:viral process; P:negative regulation of protein complex assembly; P:positive regulation of viral genome replication; C:cytoplasmic stress granule; P:positive regulation of G1/S transition of mitotic cell cycle; P:transcription, DNA-templated; F:transcription factor binding; C:mitochondrial outer membrane; F:cadherin binding involved in cell-cell adhesion; F:DNA binding; P:innate immune response; P:cell-cell adhesion |
| Efet.01.155246.g269.t1 | Rab GDP dissociation inhibitor beta | 201 | 1.22E-18 | 90.1 | F:Rab GDP-dissociation inhibitor activity; F:oxidoreductase activity; P:small GTPase mediated signal transduction; P:protein transport; P:regulation of catalytic activity; P:oxidation-reduction process; C:intracellular |
| Efet.01.155399.g274.t1 | ATP-dependent RNA helicase DDX51 | 462 | 3.39E-16 | 61.7 | F:organic cyclic compound binding; F:heterocyclic compound binding |
| Efet.01.155461.g275.t1 | allatostatin-A receptor-like | 474 | 1.75E-21 | 57.15 | F:G-protein coupled receptor activity; P:transmembrane transport; P:G-protein coupled receptor signaling pathway; P:signal transduction; C:membrane; C:integral component of membrane; F:metal ion transmembrane transporter activity; P:metal ion transport; F:signal transducer activity |
| Efet.01.155461.g276.t1 | allatostatin-A receptor-like isoform X1 | 750 | 3.32E-36 | 55.65 | F:G-protein coupled receptor activity; P:G-protein coupled receptor signaling pathway; P:signal transduction; C:membrane; C:integral component of membrane; F:signal transducer activity |
| Efet.01.155465.g277.t1 | LIM homeobox Lhx9- | 390 | 8.01E-21 | 71.1 | F:DNA binding; F:metal ion binding |
| Efet.01.155472.g278.t1 | headcase | 903 | 2.87E-53 | 70.7 |  |
| Efet.01.155474.g279.t1 | leucine-rich repeat neuronal 2-like | 1050 | 2.67E-38 | 53.5 | C:membrane; C:integral component of membrane |
| Efet.01.155508.g281.t1 | hypothetical protein CAPTEDRAFT_223749 | 432 | 1.06E-20 | 58.36 | C:membrane; C:integral component of membrane |
| Efet.01.155556.g282.t1 | hypothetical protein CAPTEDRAFT_222884 | 258 | 2.05E-18 | 59.15 | C:membrane; C:integral component of membrane; F:carbohydrate binding |
| Efet.01.155565.g283.t1 | histone demethylase UTY isoform X3 | 612 | 3.27E-45 | 76.75 |  |
| Efet.01.155603.g284.t1 | sterol regulatory element-binding cleavage-activating | 549 | 4.17E-11 | 75.1 | C:endoplasmic reticulum membrane; P:SREBP signaling pathway; F:cholesterol binding; C:integral component of membrane; P:cholesterol metabolic process; C:Golgi membrane |
| Efet.01.155614.g286.t1 | neuronal acetylcholine receptor subunit alpha-10-like isoform X1 | 546 | 1.34E-24 | 65.7 | P:ion transport; C:integral component of membrane |
| Efet.01.155650.g288.t1 | eukaryotic translation initiation factor 3 subunit J | 525 | 1.61E-11 | 67.13 | C:translation preinitiation complex; P:translational initiation |
| Efet.01.155679.g289.t1 | amiloride-sensitive amine oxidase [copper-containing]-like | 852 | 1.81E-36 | 53.1 | P:amine metabolic process; F:quinone binding; F:copper ion binding; F:oxidoreductase activity; F:metal ion binding; F:primary amine oxidase activity; C:membrane; C:integral component of membrane; P:oxidation-reduction process |
| Efet.01.155711.g290.t1 | tctex1 domain-containing 4 | 237 | 1.51E-17 | 68.5 |  |
| Efet.01.155713.g292.t1 | beta-1,4-galactosyltransferase galt-1-like | 1035 | 7.68E-77 | 46.3 | C:membrane; C:integral component of membrane |
| Efet.01.155731.g293.t1 | ubiquitin-conjugating enzyme E2 J2-like | 345 | 3.06E-20 | 56.7 | F:ubiquitin protein ligase activity; F:ubiquitin protein ligase binding; P:protein polyubiquitination; C:membrane; C:integral component of membrane; C:endoplasmic reticulum; P:ER-associated ubiquitin-dependent protein catabolic process |
| Efet.01.155734.g294.t1 | Serine threonine- kinase WNK2 | 4377 | 4.59E-08 | 64 | C:cytoplasm; F:protein kinase activity; P:negative regulation of cellular process; P:protein phosphorylation |
| Efet.01.155760.g295.t1 | anoctamin-1-like isoform X5 | 363 | 1.75E-12 | 76.38 | C:integral component of membrane; F:protein dimerization activity |
| Efet.01.155767.g296.t1 | major egg antigen-like | 1122 | 3.36E-129 | 59.65 |  |
| Efet.01.155790.g297.t1 | hornerin-like isoform X1 | 2544 | 2.15E-30 | 76 | C:nucleus; F:DNA binding; P:regulation of transcription, DNA-templated |
| Efet.01.155818.g300.t1 | uncharacterized protein LOC111088095 | 1143 | 9.19E-12 | 47 |  |
| Efet.01.155841.g301.t1 | rab3 GTPase-activating catalytic subunit | 276 | 4.70E-19 | 72.7 | C:intracellular organelle; P:regulation of biological quality; F:molecular function regulator; P:head development; P:positive regulation of cellular process; C:cytoplasm; P:regulation of GTPase activity; P:modulation of synaptic transmission; P:animal organ development; P:cellular process; C:membrane-bounded organelle |
| Efet.01.155842.g302.t1 | R-spondin-2 isoform X1 | 225 | 5.73E-09 | 67.6 |  |
| Efet.01.155879.g304.t1 | hypothetical protein CAPTEDRAFT_226205 | 231 | 6.55E-08 | 66 | F:nucleotide binding; F:ATP binding; F:protein kinase activity; P:protein phosphorylation |
| Efet.01.155881.g305.t1 | diacylglycerol kinase gamma isoform X5 | 246 | 2.67E-18 | 68.15 |  |
| Efet.01.155910.g307.t1 | probable E3 ubiquitin- ligase HERC1 | 1065 | 9.30E-14 | 51.65 | F:ubiquitin-protein transferase activity; P:cerebellar Purkinje cell differentiation; P:neuron projection development; P:negative regulation of autophagy; P:neuromuscular process controlling balance; F:ligase activity; P:protein ubiquitination |
| Efet.01.155922.g308.t1 | guanine nucleotide-binding G(t) subunit alpha-3 | 261 | 6.15E-19 | 78.45 | P:adenylate cyclase-inhibiting G-protein coupled receptor signaling pathway; P:sensory perception of umami taste; P:detection of visible light; P:sensory perception of sweet taste; P:response to nicotine; P:protein folding; P:visual perception; F:signal transducer activity; P:phototransduction; C:axoneme; F:GTP binding; F:G-protein coupled receptor binding; P:eye development; C:heterotrimeric G-protein complex; F:metal ion binding; C:acrosomal vesicle; C:photoreceptor cell cilium; F:GTPase activity; P:sensory perception of bitter taste; F:G-protein beta/gamma-subunit complex binding; C:apical plasma membrane |
| Efet.01.155968.g309.t1 | tyramine receptor 1-like | 1713 | 5.71E-81 | 69.1 | F:G-protein coupled receptor activity; P:G-protein coupled receptor signaling pathway; C:integral component of membrane |
| Efet.01.156006.g310.t1 | von Willebrand factor A domain-containing 8 | 372 | 4.05E-60 | 88.8 | F:ATPase activity; F:ATP binding; C:mitochondrion; C:extracellular region |
| Efet.01.156040.g311.t1 | WD repeat-containing 53-like | 1032 | 3.89E-111 | 57.05 |  |
| Efet.01.156104.g313.t1 | tubulin-specific chaperone E-like | 1050 | 2.29E-88 | 60.6 | P:single-multicellular organism process; P:cellular process; P:single-organism developmental process |
| Efet.01.15612.g1151.t1 | polycystin-1-like isoform X2 | 2442 | 1.31E-71 | 45.2 | P:regulation of cellular process; P:kidney development; P:tube development; C:cell part; P:epithelium development |
| Efet.01.156122.g316.t1 | hypothetical protein CGI_10016314 | 258 | 3.10E-09 | 71.44 |  |
| Efet.01.156196.g318.t1 | insulin-like growth factor-binding complex acid labile subunit | 1470 | 1.28E-50 | 43.7 | P:peptidyl-tyrosine phosphorylation; F:ATP binding; F:protein tyrosine kinase activity; P:signal transduction; C:membrane; C:integral component of membrane; F:protein kinase activity; P:phosphorylation; P:protein phosphorylation; F:kinase activity |
| Efet.01.15623.g1152.t1 | synapse differentiation-inducing gene 1-like | 870 | 6.09E-13 | 53.75 | P:response to biotic stimulus; C:membrane; C:integral component of membrane |
| Efet.01.156237.g322.t1 | negative elongation factor D-like | 522 | 3.56E-16 | 62.35 |  |
| Efet.01.156258.g323.t1 | baculoviral IAP repeat-containing 6-like isoform X4 | 300 | 4.80E-15 | 73.6 | F:ubiquitin-protein transferase activity; C:cytoplasm; P:cellular protein modification process; P:negative regulation of cellular process; P:placenta development; C:intracellular organelle part; P:regulation of signal transduction |
| Efet.01.156308.g326.t1 | PREDICTED: T-complex-associated testis-expressed protein 1-like | 849 | 2.87E-140 | 78.05 |  |
| Efet.01.156318.g327.t1 | follistatin-related 5 isoform X2 | 225 | 3.42E-14 | 64.9 |  |
| Efet.01.156339.g330.t1 | interferon-induced very large GTPase 1 | 1353 | 3.28E-33 | 46.33 | F:GTP binding |
| Efet.01.156339.g331.t1 | interferon-induced very large GTPase 1-like | 1860 | 4.01E-116 | 52.55 | F:GTP binding |
| Efet.01.156360.g332.t1 | glutaminase kidney mitochondrial-like isoform X5 | 900 | 2.47E-126 | 74.55 | F:glutaminase activity; P:glutamate biosynthetic process; C:mitochondrial matrix; P:glutamine catabolic process |
| Efet.01.156414.g335.t1 | neuroligin- X-linked-like isoform X1 | 786 | 2.16E-16 | 51.15 | C:integral component of membrane |
| Efet.01.156415.g336.t1 | calsyntenin-1-like isoform X7 | 324 | 1.01E-09 | 70.05 | F:X11-like protein binding; C:extracellular exosome; P:regulation of cellular process; C:intracellular membrane-bounded organelle; F:kinesin binding; C:endomembrane system; P:cell adhesion; C:cytoplasmic part; C:membrane part; F:beta-amyloid binding |
| Efet.01.156418.g337.t1 | Chitin synthase 1 | 333 | 1.42E-27 | 75.5 | F:transferase activity, transferring hexosyl groups; C:integral component of membrane |
| Efet.01.156506.g340.t1 | substance-P receptor-like | 939 | 3.05E-30 | 44.55 | C:gap junction; F:G-protein coupled receptor activity; P:G-protein coupled receptor signaling pathway; P:signal transduction; C:membrane; C:integral component of membrane; F:signal transducer activity |
| Efet.01.156546.g342.t1 | 6-phosphofructo-2-kinase fructose-2,6-bisphosphatase-like isoform X4 | 1248 | 0 | 80.45 | F:ATP binding; C:integral component of membrane; P:fructose 2,6-bisphosphate metabolic process; P:fructose metabolic process; P:carbohydrate phosphorylation; F:6-phosphofructo-2-kinase activity |
| Efet.01.156547.g343.t1 | hypothetical protein HELRODRAFT_192228 | 246 | 4.85E-21 | 67.5 | F:metal ion binding |
| Efet.01.156618.g345.t1 | probable ATP-dependent RNA helicase DDX43 | 2325 | 0 | 68.75 | F:organic cyclic compound binding; F:heterocyclic compound binding |
| Efet.01.15666.g1153.t1 | hypothetical protein CAPTEDRAFT_197976 | 201 | 3.81E-07 | 64 |  |
| Efet.01.156672.g349.t1 | Insulin gene enhancer ISL- | 405 | 2.43E-39 | 89.45 | F:ligand-dependent nuclear receptor binding; P:trigeminal nerve development; P:ventricular cardiac muscle tissue morphogenesis; F:RNA polymerase II core promoter proximal region sequence-specific DNA binding; P:positive regulation of DNA binding; F:RNA polymerase II transcription coactivator activity; P:positive regulation of angiogenesis; F:RNA polymerase II activating transcription factor binding; F:bHLH transcription factor binding; P:endocardial cushion morphogenesis; P:sensory system development; F:transcriptional activator activity, RNA polymerase II core promoter proximal region sequence-specific binding; P:positive regulation of granulocyte macrophage colony-stimulating factor production; P:positive regulation of interferon-gamma production; P:positive regulation of tumor necrosis factor production; P:positive regulation of granulocyte colony-stimulating factor production; P:positive regulation of vascular endothelial growth factor production; F:zinc ion binding; P:negative regulation of inflammatory response; P:cardiac cell fate determination; P:pharyngeal system development; P:regulation of secondary heart field cardioblast proliferation; F:enhancer sequence-specific DNA binding; P:pituitary gland development; P:cardiac right ventricle morphogenesis; P:secondary heart field specification; P:neural crest cell migration; F:estrogen receptor binding; P:positive regulation of interleukin-6 production; P:positive regulation of histone acetylation; P:positive regulation of tyrosine phosphorylation of Stat3 protein; P:positive regulation of cell proliferation; P:innervation; P:negative regulation of neuron differentiation; P:negative regulation of protein homodimerization activity; P:positive regulation of transcription from RNA polymerase II promoter; P:peripheral nervous system neuron axonogenesis; C:nucleoplasm; P:negative regulation of neuron apoptotic process; P:negative regulation of transcription from RNA polymerase II promoter; P:outflow tract septum morphogenesis; P:positive regulation of insulin secretion; P:pancreas development; P:retinal ganglion cell axon guidance; P:visceral motor neuron differentiation; F:chromatin binding; P:cardiac muscle cell myoblast differentiation; P:spinal cord motor neuron cell fate specification; P:atrial septum morphogenesis; P:negative regulation of canonical Wnt signaling pathway; P:positive regulation of interleukin-12 production; P:positive regulation of interleukin-1 alpha production; P:negative regulation of intracellular estrogen receptor signaling pathway; P:positive regulation of interleukin-1 beta production |
| Efet.01.156737.g352.t1 | matrix metallo ase-21-like | 942 | 7.27E-69 | 52 | F:metal ion binding; F:peptidase activity |
| Efet.01.156748.g353.t1 | phosphatase 1 regulatory subunit 16A isoform X1 | 303 | 5.99E-16 | 57.4 |  |
| Efet.01.156767.g355.t1 | transcriptional repressor p66-beta isoform X2 | 1572 | 1.60E-52 | 73.15 | F:transcription factor activity, sequence-specific DNA binding; F:zinc ion binding; P:regulation of transcription, DNA-templated; F:sequence-specific DNA binding |
| Efet.01.156770.g356.t1 | homeodomain transcription factor 2 isoform X1 | 900 | 2.42E-42 | 88.45 | F:DNA binding; C:integral component of membrane |
| Efet.01.156857.g360.t1 | pancreas transcription factor 1 subunit alpha-like | 1128 | 2.22E-12 | 65.8 | C:membrane; F:binding; P:pancreas development |
| Efet.01.15688.g1154.t1 | zinc finger 570 isoform X4 | 1071 | 3.20E-55 | 56.65 | F:binding |
| Efet.01.156916.g361.t1 | testis-specific serine threonine- kinase 1-like | 288 | 5.75E-21 | 75.9 | F:ATP binding; F:protein serine/threonine kinase activity; P:protein phosphorylation |
| Efet.01.156935.g363.t1 | probable ATP-dependent RNA helicase DDX27 | 294 | 2.82E-09 | 67.2 | F:nucleotide binding; F:hydrolase activity |
| Efet.01.156944.g364.t1 | dual specificity phosphatase 10 isoform X3 | 849 | 9.47E-69 | 57.95 | P:protein dephosphorylation; F:protein tyrosine/serine/threonine phosphatase activity |
| Efet.01.156956.g365.t1 | activated CDC42 kinase 1 isoform X3 | 1251 | 8.05E-22 | 46 |  |
| Efet.01.156964.g366.t1 | vacuolar sorting-associated 26B-like | 552 | 3.48E-79 | 76.05 | C:endosome; C:retromer complex; C:cytosol; F:protein transporter activity; P:intracellular protein transport; P:retrograde transport, endosome to Golgi |
| Efet.01.157022.g367.t1 | twitchin | 498 | 1.76E-55 | 70.55 | F:ATP binding; F:protein kinase activity; P:protein phosphorylation |
| Efet.01.157035.g368.t1 | TBC1 domain family member 15-like isoform X2 | 564 | 2.10E-36 | 57.3 | P:regulation of vesicle fusion; P:activation of GTPase activity; F:GTPase activator activity; C:endomembrane system; P:intracellular protein transport; F:Rab GTPase binding; C:intracellular |
| Efet.01.157094.g371.t1 | hypothetical protein CAPTEDRAFT_199133 | 393 | 1.32E-18 | 55 | P:ion transport; P:ion transmembrane transport; C:membrane; C:integral component of membrane; F:extracellular ligand-gated ion channel activity; P:transport |
| Efet.01.157121.g373.t1 | substance-K receptor-like | 840 | 8.56E-55 | 61.7 | P:signal transduction; F:signal transducer activity |
| Efet.01.157126.g374.t1 | atonal homolog 8-like | 672 | 1.76E-50 | 89.15 | F:transcription factor activity, sequence-specific DNA binding; P:cell differentiation; P:regulation of transcription, DNA-templated; F:protein dimerization activity |
| Efet.01.157129.g375.t1 | procollagen-lysine,2-oxoglutarate 5-dioxygenase 3 | 489 | 1.50E-35 | 74.75 | P:chordate embryonic development; P:system development; F:oxidoreductase activity, acting on paired donors, with incorporation or reduction of molecular oxygen; P:tube development; F:ion binding; P:extracellular matrix organization; P:anatomical structure morphogenesis; P:epithelium development; C:membrane-bounded organelle |
| Efet.01.157186.g377.t1 | hypothetical protein HELRODRAFT_188889 | 261 | 5.40E-21 | 86.25 |  |
| Efet.01.1571945.g4.t1 | tetratricopeptide repeat 17-like | 228 | 3.84E-15 | 84.5 | C:integral component of membrane |
| Efet.01.157224.g381.t1 | E3 ubiquitin- ligase UBR5 | 534 | 4.12E-55 | 78.6 | F:ubiquitin-protein transferase activity; F:zinc ion binding; F:RNA binding; F:ubiquitin binding; F:ligase activity; P:protein ubiquitination |
| Efet.01.157252.g382.t1 | tyrosine- kinase Fer-like isoform X1 | 600 | 1.10E-44 | 61.25 | F:kinase activity |
| Efet.01.15726.g1157.t1 | cholecystokinin receptor type A-like | 735 | 1.43E-14 | 60.75 | P:signal transduction; C:membrane; F:signal transducer activity |
| Efet.01.15726.g1158.t1 | muscarinic acetylcholine receptor M2-like | 474 | 1.13E-34 | 67.95 | P:signal transduction; C:membrane; F:signal transducer activity |
| Efet.01.157331.g383.t1 | cysteine serine-rich nuclear 2-like | 777 | 4.06E-70 | 71.85 |  |
| Efet.01.157336.g386.t1 | lon protease mitochondrial | 795 | 4.13E-137 | 86.55 | P:oxidation-dependent protein catabolic process; F:serine-type endopeptidase activity; P:protein homooligomerization; F:sequence-specific DNA binding; F:ATP-dependent peptidase activity; F:ADP binding; P:mitochondrion organization; C:nucleoplasm; C:mitochondrial nucleoid; F:single-stranded DNA binding; F:ATP binding; F:mitochondrial light strand promoter anti-sense binding; F:DNA polymerase binding; P:response to hypoxia; P:misfolded or incompletely synthesized protein catabolic process; F:G-quadruplex DNA binding; F:mitochondrial heavy strand promoter sense binding; F:mitochondrial heavy strand promoter anti-sense binding; C:membrane; F:single-stranded RNA binding; F:mitochondrial light strand promoter sense binding; P:chaperone-mediated protein complex assembly; P:cellular response to oxidative stress |
| Efet.01.157338.g387.t1 | alpha amylase | 213 | 2.25E-20 | 67.45 | F:hydrolase activity |
| Efet.01.157363.g388.t1 | WD repeat-containing 97-like | 366 | 5.78E-32 | 63.95 |  |
| Efet.01.15738.g1159.t1 | glycine-rich domain-containing 2-like [Orbicella faveolata] | 1080 | 6.79E-87 | 62.35 |  |
| Efet.01.15739.g1160.t1 | LYR motif-containing 5 | 243 | 1.24E-26 | 84.85 | C:mitochondrion |
| Efet.01.1573918.g5.t1 | fumarate hydratase | 207 | 2.29E-28 | 91.25 | P:generation of precursor metabolites and energy; F:fumarate hydratase activity |
| Efet.01.157392.g390.t1 | deformed epidermal autoregulatory factor 1 homolog isoform X1 | 237 | 2.08E-10 | 64.89 | F:DNA binding; F:oxidoreductase activity; P:oxidation-reduction process |
| Efet.01.157394.g391.t1 | eukaryotic translation initiation factor 2 subunit | 258 | 6.87E-33 | 95.2 | C:multi-eIF complex; C:cytoplasmic stress granule; P:cellular response to amino acid starvation; P:protein autophosphorylation; P:positive regulation of defense response to virus by host; C:ribosome; C:eukaryotic translation initiation factor 2B complex; C:eukaryotic 48S preinitiation complex; P:translational initiation; P:xenophagy; F:RNA binding; C:nucleus; F:translation initiation factor activity; C:extracellular exosome; F:protein binding; F:ribosome binding; C:membrane; P:cellular response to UV; P:positive regulation of macromitophagy in response to mitochondrial depolarization; P:response to manganese-induced endoplasmic reticulum stress; P:negative regulation of translational initiation in response to stress |
| Efet.01.157405.g392.t1 | Midasin | 1167 | 3.63E-135 | 66.2 | F:nucleotide binding; F:ATPase activity; F:ATP binding; C:nucleus; P:ribosomal large subunit assembly |
| Efet.01.15747.g1162.t1 | ankyrin repeat and EF-hand domain-containing 1 | 387 | 4.05E-17 | 68.8 | F:calcium ion binding |
| Efet.01.157478.g394.t1 | zinc finger SWIM domain-containing 1-like | 1461 | 6.32E-51 | 45.45 |  |
| Efet.01.157478.g395.t1 | probable D-lactate mitochondrial | 318 | 6.45E-49 | 86.05 |  |
| Efet.01.157496.g398.t1 | adenylate kinase 9-like | 270 | 4.96E-17 | 72.95 | C:membrane; P:cellular metabolic process |
| Efet.01.157549.g401.t1 | chondroitin sulfate proteoglycan 4-like | 366 | 4.33E-09 | 55.2 | C:integral component of plasma membrane; C:membrane; C:integral component of membrane; P:cell migration |
| Efet.01.157607.g403.t1 | hypothetical protein HELRODRAFT_188674 | 483 | 2.11E-22 | 48 | C:membrane; C:integral component of membrane |
| Efet.01.157607.g404.t1 | hypothetical protein HELRODRAFT_188674 | 504 | 2.01E-26 | 46.55 | C:membrane; C:integral component of membrane |
| Efet.01.157687.g406.t1 | 5-hydroxytryptamine receptor 1A-like | 744 | 6.51E-40 | 65.2 | F:G-protein coupled receptor activity; P:G-protein coupled receptor signaling pathway; C:integral component of membrane |
| Efet.01.15772.g1163.t1 | CUGBP Elav-like family member 4 | 246 | 9.52E-21 | 95 | F:nucleotide binding; F:nucleic acid binding |
| Efet.01.157735.g408.t1 | hypothetical protein CAPTEDRAFT_228532 | 1320 | 1.82E-17 | 49 | F:ATP binding; F:protein kinase activity; P:protein phosphorylation |
| Efet.01.157783.g409.t1 | nucleoporin SEH1 isoform X2 | 489 | 1.44E-71 | 72.45 | C:nuclear pore outer ring; P:nuclear pore organization; P:macromolecule localization; C:kinetochore; P:transport; P:response to stress; P:attachment of mitotic spindle microtubules to kinetochore |
| Efet.01.157857.g413.t1 | cytidylate kinase | 708 | 8.19E-112 | 69.05 | F:nucleotide binding; P:phosphorylation; F:kinase activity; P:nucleotide metabolic process |
| Efet.01.157889.g416.t1 | ATP-dependent RNA helicase DDX55 | 249 | 1.17E-23 | 79.2 | F:ATP binding; F:RNA binding; P:RNA secondary structure unwinding; F:ATP-dependent RNA helicase activity |
| Efet.01.157949.g419.t1 | hypothetical protein CAPTEDRAFT_219019 | 276 | 6.84E-28 | 68.9 | C:integral component of membrane; F:carbohydrate binding |
| Efet.01.157951.g420.t1 | hypothetical protein HELRODRAFT_164068 | 1734 | 4.80E-12 | 53.5 |  |
| Efet.01.157976.g422.t1 | hypothetical protein CAPTEDRAFT_204443 | 912 | 5.55E-25 | 50 |  |
| Efet.01.158006.g424.t1 | netrin receptor UNC5B | 576 | 1.18E-36 | 62.75 | P:regulation of cellular process |
| Efet.01.158036.g425.t1 | homeobox abdominal-B | 798 | 7.90E-09 | 80.05 | C:nucleus; P:regulation of transcription, DNA-templated; F:sequence-specific DNA binding |
| Efet.01.15804.g1165.t1 | NC domain | 1296 | 8.73E-16 | 50.45 | P:RNA phosphodiester bond hydrolysis, endonucleolytic; F:nucleic acid binding; F:RNA-DNA hybrid ribonuclease activity; C:membrane; C:integral component of membrane |
| Efet.01.158046.g426.t1 | tripartite motif-containing 2-like isoform X3 | 1692 | 0 | 50.05 | F:zinc ion binding; C:intracellular |
| Efet.01.158093.g428.t1 | chromodomain-helicase-DNA-binding 7 isoform X1 | 300 | 7.16E-28 | 83.2 | C:nucleus; F:ATP binding; F:hydrolase activity, acting on acid anhydrides |
| Efet.01.158098.g429.t1 | ribosome biogenesis BMS1 homolog | 423 | 1.90E-24 | 80.75 | C:nucleus; F:organic cyclic compound binding; F:heterocyclic compound binding; C:cytoplasm; C:intracellular non-membrane-bounded organelle; P:ribosome biogenesis; C:intracellular organelle part; P:cellular process |
| Efet.01.158109.g430.t1 | hypothetical protein CAPTEDRAFT_195876 | 549 | 1.74E-30 | 62.7 | F:oxidoreductase activity; C:membrane; C:integral component of membrane; P:oxidation-reduction process |
| Efet.01.158179.g431.t1 | Ras-like GTP-binding | 720 | 1.68E-75 | 69.1 | P:wound healing, spreading of cells; P:skeletal muscle satellite cell migration |
| Efet.01.158236.g432.t1 | bromodomain testis-specific | 333 | 2.12E-12 | 52.4 | F:histone acetyltransferase activity; F:transferase activity, transferring acyl groups; P:histone acetylation; F:transferase activity |
| Efet.01.158239.g433.t1 | allatostatin-A receptor-like isoform X2 | 1098 | 5.13E-82 | 58.8 | F:G-protein coupled receptor activity; P:G-protein coupled receptor signaling pathway; C:membrane |
| Efet.01.158248.g434.t1 | specificity 1 | 1119 | 9.47E-15 | 75.05 | F:nucleic acid binding; F:metal ion binding |
| Efet.01.15829.g1167.t1 | beta-1,3-galactosyltransferase 1-like isoform X1 | 1191 | 3.64E-90 | 56.65 | C:membrane; F:transferase activity |
| Efet.01.158293.g435.t1 | hypothetical protein HELRODRAFT_179552 | 579 | 4.61E-18 | 71 | F:calcium ion binding; C:membrane; P:homophilic cell adhesion via plasma membrane adhesion molecules |
| Efet.01.1583077.g6.t1 | homeobox MSX-2 | 210 | 6.52E-29 | 86.55 | C:nucleus; P:regulation of transcription, DNA-templated; F:sequence-specific DNA binding |
| Efet.01.158375.g438.t1 | hypothetical protein CAPTEDRAFT_189487 | 2277 | 0 | 59.1 | C:membrane |
| Efet.01.158375.g439.t1 | of unassigned function | 834 | 1.98E-135 | 60.95 | C:integral component of membrane |
| Efet.01.158399.g440.t1 | tolloid 1 | 1629 | 2.95E-54 | 62.25 | P:multicellular organism development |
| Efet.01.15840.g1168.t1 | FRAS1-related extracellular matrix 1 | 1557 | 1.91E-88 | 52.9 | P:cell-matrix adhesion; C:basement membrane; P:cell communication; F:calcium ion binding; C:membrane; C:integral component of membrane; P:homophilic cell adhesion via plasma membrane adhesion molecules |
| Efet.01.158441.g441.t1 | splicing factor 3B subunit 3 isoform X2 | 714 | 1.35E-131 | 90.05 | F:nucleic acid binding; C:nucleus |
| Efet.01.158445.g442.t1 | SPRY domain-containing SOCS box 3-like | 540 | 1.55E-13 | 72.65 | P:intracellular signal transduction; C:intracellular |
| Efet.01.158454.g443.t1 | pleckstrin homology domain-containing family G member 2 isoform X2 | 291 | 1.56E-10 | 58.8 | P:regulation of Rho protein signal transduction; C:membrane; C:integral component of membrane; F:Rho guanyl-nucleotide exchange factor activity; P:positive regulation of GTPase activity |
| Efet.01.158458.g444.t1 | chromodomain-helicase-DNA-binding Mi-2 homolog isoform X1 | 1416 | 7.41E-63 | 58.6 | P:histone H4 acetylation; P:regulation of transcription involved in cell fate commitment; F:histone deacetylase binding; F:heterocyclic compound binding; P:negative regulation of cell proliferation; P:spermatogenesis, exchange of chromosomal proteins; P:positive regulation of signal transduction by p53 class mediator; F:H3K27me3 modified histone binding; P:cerebral cortex neuron differentiation; C:heterochromatin; F:organic cyclic compound binding; P:regulation of cell differentiation; C:NuRD complex; F:hydrolase activity |
| Efet.01.15847.g1169.t1 | E1A-binding p400 | 1146 | 9.21E-33 | 72.85 | F:ATP binding |
| Efet.01.158481.g446.t1 | hypothetical protein CAPTEDRAFT_204754 | 1029 | 3.49E-62 | 55.95 | C:membrane; C:integral component of membrane; F:sulfotransferase activity |
| Efet.01.158539.g447.t1 | homeobox orthopedia-like | 513 | 4.12E-10 | 52.1 | F:DNA binding |
| Efet.01.158725.g456.t1 | ARI4B_MOUSE ame: Full=AT-rich interactive domain-containing 4B Short=ARID domain-containing 4B ame: Full=180 kDa Sin3-associated polypeptide Short=Sin3-associated polypeptide p180 ame: Full=Histone deacetylase complex subunit SAP180 | 546 | 9.45E-06 | 58 | F:DNA binding; C:nucleus; P:histone H3-K9 trimethylation; P:regulation of gene expression by genetic imprinting; F:protein binding; P:establishment of Sertoli cell barrier; P:transcription, DNA-templated; P:spermatogenesis; F:transcription regulatory region DNA binding; P:regulation of transcription, DNA-templated; P:histone H4-K20 trimethylation; P:positive regulation of transcription from RNA polymerase II promoter |
| Efet.01.1587334.g8.t1 | 1,4-dihydroxy-2-naphthoyl- synthase | 210 | 7.39E-42 | 99.7 | F:1,4-dihydroxy-2-naphthoyl-CoA synthase activity; P:menaquinone biosynthetic process |
| Efet.01.158739.g458.t1 | tripartite motif-containing 3-like | 1452 | 6.21E-25 | 45.55 | F:zinc ion binding; F:metal ion binding; C:intracellular |
| Efet.01.158783.g459.t1 | sex peptide receptor-like | 417 | 1.48E-46 | 69.95 | F:G-protein coupled peptide receptor activity; P:G-protein coupled receptor signaling pathway; C:integral component of membrane |
| Efet.01.158801.g461.t1 | twitchin-like isoform X3 | 351 | 1.29E-17 | 58.95 | F:nucleotide binding; F:ATP binding; F:protein kinase activity; P:phosphorylation; F:transferase activity; P:protein phosphorylation; F:kinase activity |
| Efet.01.158801.g462.t1 | titin-like isoform X1 | 1899 | 6.11E-125 | 52 |  |
| Efet.01.158816.g463.t1 | PREDICTED: uncharacterized protein LOC105339366 | 669 | 1.53E-21 | 58.2 | C:membrane; C:integral component of membrane |
| Efet.01.158840.g465.t1 | hypothetical protein LOTGIDRAFT_148200, partial | 1116 | 5.00E-07 | 45 |  |
| Efet.01.1589751.g10.t1 | hypothetical protein HELRODRAFT_192078 | 252 | 7.03E-20 | 68.5 | C:integral component of membrane |
| Efet.01.158986.g469.t1 | ras-related Rab-30 | 270 | 4.84E-09 | 57 | F:GTP binding; P:small GTPase mediated signal transduction; C:intracellular |
| Efet.01.158998.g470.t1 | hypothetical protein BRAFLDRAFT_63830 | 630 | 9.75E-09 | 49.33 | F:zinc ion binding; P:signal transduction; P:ion transmembrane transport; F:GABA-A receptor activity; F:extracellular ligand-gated ion channel activity; C:GABA-A receptor complex; C:synapse; P:transport; C:intracellular; C:plasma membrane; P:ion transport; F:metal ion binding; C:membrane; C:integral component of membrane; C:cell junction |
| Efet.01.159011.g471.t1 | Transposon Ty3-G Gag-Pol poly | 696 | 1.29E-47 | 57.65 |  |
| Efet.01.159022.g472.t1 | sprouty homolog 2-like | 789 | 7.12E-30 | 54.45 | P:regulation of fibroblast growth factor receptor signaling pathway; P:negative regulation of signal transduction; P:animal organ development |
| Efet.01.159030.g474.t1 | tripartite motif-containing 3-like | 891 | 1.35E-23 | 48.43 |  |
| Efet.01.1590408.g11.t1 | kinetochore-associated 1-like | 201 | 1.10E-19 | 67.4 | C:cytoskeleton; C:intracellular organelle part |
| Efet.01.159041.g476.t1 | synapsin-like isoform X1 | 312 | 1.77E-12 | 54.64 | F:ATP binding; F:metal ion binding; F:catalytic activity; C:synaptic vesicle; P:neurotransmitter secretion |
| Efet.01.159116.g479.t1 | transient receptor potential cation channel subfamily V member 5-like isoform X8 | 222 | 4.29E-11 | 61.35 | P:ion transport; P:transmembrane transport; C:membrane |
| Efet.01.1591239.g13.t1 | uncharacterized protein LOC111330178 | 225 | 3.31E-10 | 60.67 |  |
| Efet.01.15914.g1173.t1 | phospholipase DDHD1-like | 828 | 5.71E-43 | 58.7 | F:metal ion binding |
| Efet.01.159207.g480.t1 | chondroitin sulfate proteoglycan 4-like | 1716 | 5.56E-46 | 50.65 | C:integral component of plasma membrane; C:membrane; C:integral component of membrane; P:cell migration |
| Efet.01.159251.g481.t1 | hypothetical protein CAPTEDRAFT_195695 | 762 | 1.71E-16 | 52 |  |
| Efet.01.159287.g484.t1 | potassium sodium hyperpolarization-activated cyclic nucleotide-gated channel 4 | 240 | 1.06E-40 | 92.65 | C:integral component of plasma membrane; F:voltage-gated potassium channel activity; P:regulation of membrane potential; P:potassium ion transmembrane transport |
| Efet.01.1593054.g15.t1 | hypothetical protein CRN43_12040 | 201 | 7.37E-42 | 83.67 |  |
| Efet.01.1593073.g16.t1 | pikachurin-like | 222 | 1.29E-29 | 79.55 | F:calcium ion binding; C:integral component of membrane; P:oxidation-reduction process |
| Efet.01.159407.g487.t1 | hypothetical protein HELRODRAFT_191437 | 738 | 2.24E-17 | 44 |  |
| Efet.01.1594087.g17.t1 | cytoplasmic FMR1-interacting -like isoform X1 | 222 | 9.63E-38 | 85.4 | P:retinal ganglion cell axon guidance; P:retina layer formation |
| Efet.01.159430.g488.t1 | rho GTPase-activating 100F-like | 819 | 7.34E-36 | 80.85 | P:signal transduction |
| Efet.01.159453.g490.t1 | Inositol phosphoceramide mannosyltransferase 3 | 768 | 1.29E-11 | 54.35 | C:membrane; C:integral component of membrane |
| Efet.01.159463.g491.t1 | kelch 24 | 861 | 3.94E-20 | 44.9 |  |
| Efet.01.159466.g492.t1 | homeobox SIX6 | 645 | 8.44E-105 | 90.55 | C:nucleus; F:DNA binding; P:eye development; P:transcription from RNA polymerase II promoter; F:transcriptional activator activity, RNA polymerase II distal enhancer sequence-specific binding; P:brain development; P:visual perception; P:positive regulation of transcription from RNA polymerase II promoter; P:animal organ morphogenesis |
| Efet.01.1594716.g18.t1 | DNA-directed RNA polymerase II subunit RPB2 | 228 | 2.88E-39 | 93.85 | P:7-methylguanosine mRNA capping; P:transcription-coupled nucleotide-excision repair; P:fibroblast growth factor receptor signaling pathway; F:chromatin binding; P:snRNA transcription from RNA polymerase II promoter; C:nuclear chromosome, telomeric region; P:transcription initiation from RNA polymerase II promoter; C:DNA-directed RNA polymerase II, core complex; F:RNA binding; P:mRNA splicing, via spliceosome; P:transcription elongation from RNA polymerase II promoter; F:DNA binding; F:RNA polymerase II activity; F:protein binding; F:ribonucleoside binding; F:metal ion binding; C:membrane; P:somatic stem cell population maintenance; P:gene silencing by RNA; P:positive regulation of viral transcription |
| Efet.01.159489.g495.t1 | pyroglutamylated RFamide peptide receptor-like | 453 | 4.82E-32 | 65.6 | F:G-protein coupled receptor activity; P:G-protein coupled receptor signaling pathway; C:integral component of membrane |
| Efet.01.159492.g496.t1 | probable phospholipid-transporting ATPase IM isoform X2 | 399 | 2.51E-40 | 88.45 | F:magnesium ion binding; F:ATP binding; P:phospholipid translocation; P:Golgi organization; C:integral component of membrane; C:Golgi apparatus; F:phospholipid-translocating ATPase activity; C:plasma membrane |
| Efet.01.159512.g497.t1 | integrator complex subunit 5-like | 420 | 7.19E-33 | 67.2 |  |
| Efet.01.159556.g499.t1 | pglyrp1 isoform X1 [Xenopus tropicalis] | 882 | 2.64E-58 | 57 |  |
| Efet.01.159578.g500.t1 | bromodomain-containing 3-like isoform X4 | 255 | 3.06E-09 | 90.3 |  |
| Efet.01.159614.g501.t1 | Dynein heavy chain axonemal | 219 | 1.02E-33 | 90 | F:ATPase activity; F:ATP binding; F:microtubule motor activity; C:dynein complex; P:microtubule-based movement |
| Efet.01.159620.g502.t1 | pikachurin-like | 279 | 3.96E-23 | 70.75 | F:calcium ion binding; C:membrane; C:integral component of membrane; P:oxidation-reduction process |
| Efet.01.159641.g504.t1 | serine threonine- phosphatase 6 regulatory ankyrin repeat subunit B- | 2331 | 4.06E-104 | 51.5 | F:ubiquitin-protein transferase activity; P:protein ubiquitination |
| Efet.01.159648.g505.t1 | dystonin isoform X5 | 3519 | 0 | 61.05 | C:postsynaptic density; C:microtubule cytoskeleton; F:protein homodimerization activity; C:neurofilament cytoskeleton; C:cytoplasmic side of plasma membrane; F:microtubule binding; C:nuclear envelope; C:endoplasmic reticulum membrane; C:Z disc; C:focal adhesion; C:perinuclear endoplasmic reticulum; C:sarcolemma; P:axonogenesis; C:stress fiber; C:H zone; C:intercalated disc; P:regulation of microtubule polymerization or depolymerization; C:hemidesmosome; P:cytoplasmic microtubule organization; P:retrograde axonal transport |
| Efet.01.1596852.g20.t1 | DUF2961 domain-containing | 213 | 8.57E-43 | 87.6 |  |
| Efet.01.159707.g507.t1 | fat-like cadherin-related tumor suppressor homolog isoform X1 | 423 | 7.00E-22 | 56.3 | F:calcium ion binding; C:membrane; C:integral component of membrane; P:cell adhesion; P:homophilic cell adhesion via plasma membrane adhesion molecules; C:plasma membrane |
| Efet.01.159727.g508.t1 | equilibrative nucleoside transporter 4 | 546 | 1.59E-37 | 61.6 | P:transport |
| Efet.01.1597384.g21.t1 | metal ABC transporter permease | 207 | 3.41E-20 | 82.6 | F:ATP binding; F:ATPase activity, coupled to transmembrane movement of substances; P:transmembrane transport; C:integral component of membrane |
| Efet.01.1597529.g22.t1 | 4-hydroxy-3-methylbut-2-en-1-yl diphosphate synthase | 243 | 4.79E-51 | 88.6 | F:iron ion binding; P:isopentenyl diphosphate biosynthetic process, methylerythritol 4-phosphate pathway; P:oxidation-reduction process; F:4 iron, 4 sulfur cluster binding; F:4-hydroxy-3-methylbut-2-en-1-yl diphosphate synthase activity; P:terpenoid biosynthetic process |
| Efet.01.159847.g514.t1 | endonuclease exonuclease phosphatase family domain-containing 1-like | 795 | 1.19E-53 | 54.8 | P:DNA repair; F:DNA binding; P:nucleic acid phosphodiester bond hydrolysis; F:exonuclease activity; F:endonuclease activity |
| Efet.01.1598616.g24.t1 | kinesin KIF17 | 234 | 6.11E-27 | 78.6 | F:ATPase activity; F:ATP binding; F:microtubule motor activity; C:kinesin complex; P:microtubule-based movement; C:microtubule; F:microtubule binding |
| Efet.01.159900.g518.t1 | gamma-glutamylcyclotransferase isoform X1 | 633 | 1.53E-42 | 55.6 | F:transferase activity |
| Efet.01.159902.g519.t1 | E3 ubiquitin- ligase NEURL1-like | 567 | 5.12E-17 | 75.1 | F:metal ion binding |
| Efet.01.159951.g520.t1 | serine threonine- kinase MAK isoform X4 | 492 | 2.08E-79 | 73.5 | C:photoreceptor connecting cilium; P:protein autophosphorylation; F:cyclin-dependent protein serine/threonine kinase activity; C:axoneme; C:photoreceptor outer segment; P:negative regulation of non-motile cilium assembly; C:nucleus; F:ATP binding; C:centrosome; C:motile cilium; C:ciliary tip; F:protein binding; C:mitotic spindle; F:transcription coactivator activity; P:photoreceptor cell maintenance; C:midbody; P:regulation of cell cycle; C:photoreceptor inner segment |
| Efet.01.15999.g1178.t1 | potassium voltage-gated channel Shaw-like isoform X1 | 237 | 1.71E-22 | 91.25 | F:voltage-gated potassium channel activity; P:potassium ion transmembrane transport; F:metal ion binding; C:voltage-gated potassium channel complex; P:regulation of ion transmembrane transport; P:protein homooligomerization |
| Efet.01.16.g1.t1 | polycystic kidney disease 1-like 2 | 894 | 1.99E-36 | 53.65 | P:detection of mechanical stimulus; F:zinc ion binding; F:metal ion binding; F:calcium ion binding; F:calcium channel activity; C:membrane; C:integral component of membrane; P:calcium ion transmembrane transport; C:cytosol; P:retrograde transport, endosome to Golgi |
| Efet.01.160017.g521.t1 | PDZ domain-containing GIPC1-like | 705 | 2.27E-111 | 80.35 |  |
| Efet.01.16002.g1179.t1 | U11 U12 small nuclear ribonucleo 48 kDa | 453 | 3.32E-26 | 61.25 | C:intracellular ribonucleoprotein complex; C:viral nucleocapsid |
| Efet.01.1600498.g1.t1 | kinesin KIF21A isoform X3 | 207 | 1.56E-14 | 79.55 | F:microtubule motor activity; P:microtubule-based movement |
| Efet.01.160072.g524.t1 | 60S ribosomal export NMD3 | 444 | 1.72E-19 | 64.8 | C:nucleus; F:binding |
| Efet.01.160082.g525.t1 | Telomeric repeat-binding factor 1 | 1287 | 4.19E-20 | 42.6 | C:intracellular non-membrane-bounded organelle |
| Efet.01.1600837.g2.t1 | gonadotropin-releasing hormone receptor | 207 | 1.30E-18 | 66.75 | P:cellular response to ethanol; C:integral component of plasma membrane; P:neuropeptide signaling pathway; P:behavioral response to ethanol; P:acetaldehyde metabolic process; F:corazonin receptor activity |
| Efet.01.160091.g527.t1 | dispatched homolog 1 | 2952 | 4.34E-179 | 53.65 | P:embryo development; P:regulation of cellular process; P:nervous system development; P:cell differentiation; P:muscle structure development; P:regionalization; P:response to stimulus; P:animal organ morphogenesis |
| Efet.01.160106.g528.t1 | outer dense fiber 3-like | 312 | 8.51E-13 | 64 | C:cytoplasm |
| Efet.01.16012.g1180.t1 | Pre-mRNA cleavage complex 2 | 537 | 3.19E-25 | 61.7 | P:mRNA metabolic process; P:gene expression |
| Efet.01.160125.g530.t1 | hypothetical protein CAPTEDRAFT_187913 | 435 | 9.86E-20 | 53.33 | C:membrane; C:integral component of membrane |
| Efet.01.160140.g531.t1 | potassium channel subfamily K member 1-like | 402 | 3.88E-41 | 74.7 | P:potassium ion transmembrane transport; C:integral component of membrane; F:potassium channel activity |
| Efet.01.160206.g532.t1 | Round spermatid basic 1 | 375 | 6.13E-29 | 67.05 | C:nucleus |
| Efet.01.160243.g536.t1 | uncharacterized protein LOC110395878 | 897 | 2.31E-54 | 59 |  |
| Efet.01.16026.g1182.t1 | phenylalanine--tRNA mitochondrial isoform X2 | 390 | 9.46E-53 | 71.85 | F:magnesium ion binding; F:ATP binding; C:cytoplasm; P:tRNA processing; F:phenylalanine-tRNA ligase activity; P:phenylalanyl-tRNA aminoacylation; F:tRNA binding |
| Efet.01.160304.g541.t1 | dynein heavy chain domain-containing 1-like | 885 | 3.51E-29 | 54.8 | F:nucleotide binding; F:ATPase activity; F:ATP binding; F:microtubule motor activity; C:motile cilium; C:axonemal dynein complex; C:dynein complex; P:cilium movement; P:microtubule-based movement; P:transport |
| Efet.01.160307.g542.t1 | chromodomain-helicase-DNA-binding 1 isoform X2 | 318 | 1.44E-09 | 61.55 | P:positive regulation by host of viral transcription; C:nucleus; F:organic cyclic compound binding; F:heterocyclic compound binding; C:cytoplasm; F:methylated histone binding; F:ATP-dependent helicase activity; P:regulation of transcription, DNA-templated |
| Efet.01.160333.g545.t1 | tolloid 1 | 300 | 1.91E-12 | 57.85 | C:membrane; C:integral component of membrane |
| Efet.01.16036.g1183.t1 | phosphatidylserine decarboxylase mitochondrial isoform X4 | 492 | 5.14E-27 | 61.55 | F:lyase activity |
| Efet.01.1603813.g4.t1 | methyl-accepting chemotaxis | 237 | 1.01E-20 | 85.45 | P:chemotaxis; F:transmembrane signaling receptor activity; P:signal transduction; C:integral component of membrane; P:phosphorylation; F:kinase activity |
| Efet.01.1603858.g5.t1 | histone acetyltransferase KAT6B-like | 243 | 3.76E-34 | 87.2 | C:nucleus; F:DNA binding; F:histone acetyltransferase activity; F:zinc ion binding; P:regulation of transcription, DNA-templated; P:histone acetylation; C:nucleosome; P:nucleosome assembly |
| Efet.01.1603872.g6.t1 | trafficking kinesin-binding 1 | 255 | 5.50E-21 | 76.2 |  |
| Efet.01.1604238.g8.t1 | tektin-4 | 243 | 3.42E-25 | 78.85 |  |
| Efet.01.1604645.g9.t1 | glutamate receptor kainate 3-like | 207 | 7.28E-23 | 68.75 | C:postsynaptic membrane; P:ion transmembrane transport; C:integral component of membrane; C:cell junction; P:ionotropic glutamate receptor signaling pathway; F:ionotropic glutamate receptor activity; F:extracellular-glutamate-gated ion channel activity |
| Efet.01.16048.g1184.t1 | transcription elongation factor B polypeptide 3 | 1209 | 3.76E-26 | 56.85 | F:nucleic acid binding; P:cellular nitrogen compound biosynthetic process; P:gene expression; P:primary metabolic process; P:cellular macromolecule biosynthetic process |
| Efet.01.160487.g549.t1 | probable G- coupled receptor 83 | 477 | 7.40E-31 | 66.85 | C:integral component of plasma membrane; F:G-protein coupled receptor activity; P:G-protein coupled receptor signaling pathway; P:cell surface receptor signaling pathway |
| Efet.01.160516.g551.t1 | hypothetical protein CAPTEDRAFT_187411 | 3156 | 8.72E-68 | 64.09 | P:detection of mechanical stimulus; F:G-protein coupled receptor activity; F:transmembrane signaling receptor activity; P:G-protein coupled receptor signaling pathway; F:calcium channel activity; C:membrane; C:integral component of membrane; P:calcium ion transmembrane transport; P:cell surface receptor signaling pathway |
| Efet.01.160557.g552.t1 | paxillin isoform X5 | 228 | 3.67E-11 | 80.75 | P:cell-matrix adhesion; F:zinc ion binding; C:cytoskeleton |
| Efet.01.160610.g553.t1 | gamma dynein heavy | 348 | 1.03E-53 | 95.45 | F:ATPase activity; F:ATP binding; F:microtubule motor activity; C:cytoplasm; C:axonemal dynein complex; P:cilium or flagellum-dependent cell motility; C:sperm principal piece; P:microtubule-based movement; C:microtubule |
| Efet.01.1606549.g12.t1 | dynein regulatory complex subunit 3-like | 246 | 2.66E-09 | 66.7 | C:cytoplasm |
| Efet.01.160657.g554.t1 | E3 ubiquitin- ligase | 300 | 2.16E-09 | 58 | F:zinc ion binding; F:metal ion binding |
| Efet.01.160672.g555.t1 | Leucine-rich repeat-containing 14 | 708 | 3.30E-56 | 55.41 |  |
| Efet.01.16069.g1186.t1 | cytochrome P450 2B4-like | 294 | 1.95E-13 | 70.8 | F:iron ion binding; F:oxidoreductase activity, acting on paired donors, with incorporation or reduction of molecular oxygen; C:integral component of membrane; P:oxidation-reduction process; F:monooxygenase activity; F:heme binding |
| Efet.01.1607092.g14.t1 | helicase SKI2W-like | 240 | 2.52E-16 | 66.65 | F:nucleic acid binding; F:helicase activity |
| Efet.01.160734.g556.t1 | alpha-glucosidase isozyme I | 231 | 2.01E-15 | 71.2 | C:membrane |
| Efet.01.160741.g558.t1 | hypothetical protein CAPTEDRAFT_226930 | 765 | 1.76E-11 | 65 |  |
| Efet.01.160746.g559.t1 | hemicentin-1 precursor | 222 | 6.51E-10 | 63.65 | P:fin morphogenesis; P:basement membrane organization |
| Efet.01.1607541.g15.t1 | Innexin unc-7 | 285 | 9.26E-29 | 71.55 | C:gap junction; P:ion transport; C:integral component of membrane |
| Efet.01.160755.g560.t1 | helicase with zinc finger domain 2-like isoform X1 | 1113 | 3.09E-35 | 49.65 | F:metal ion binding; F:helicase activity |
| Efet.01.160768.g562.t1 | AT-rich interactive domain-containing 3A-like | 315 | 1.07E-18 | 75.8 | F:DNA binding |
| Efet.01.160779.g563.t1 | uncharacterized protein LOC111134311 isoform X2 | 2160 | 1.20E-98 | 49.45 |  |
| Efet.01.160824.g565.t1 | cAMP-dependent kinase type II regulatory subunit | 1110 | 8.76E-179 | 72.55 | P:regulation of protein kinase activity; F:cAMP binding; P:phosphorylation; F:cAMP-dependent protein kinase regulator activity; F:kinase activity; C:cAMP-dependent protein kinase complex |
| Efet.01.160831.g566.t1 | stum homolog | 465 | 3.27E-22 | 71.33 | C:integral component of membrane |
| Efet.01.1608399.g16.t1 | cilia- and flagella-associated 157-like | 279 | 2.46E-20 | 73.1 | C:membrane |
| Efet.01.160883.g569.t1 | cAMP-specific 3 ,5 -cyclic phosphodiesterase 7B isoform X1 | 201 | 3.06E-09 | 69.6 | F:hydrolase activity |
| Efet.01.160917.g570.t1 | lifeguard 3-like | 297 | 3.30E-10 | 73.9 | C:integral component of membrane |
| Efet.01.1609179.g17.t1 | peptidylprolyl isomerase | 219 | 2.93E-41 | 75.8 | C:membrane; P:cellular process; F:isomerase activity |
| Efet.01.160922.g571.t1 | otopetrin-2-like isoform X1 | 1512 | 5.68E-10 | 50.82 | C:membrane; C:integral component of membrane |
| Efet.01.160923.g572.t1 | PREDICTED: ankyrin-3-like | 330 | 2.87E-34 | 83 | P:signal transduction |
| Efet.01.1609271.g18.t1 | bifunctional 3 -phosphoadenosine 5 -phosphosulfate synthase-like | 201 | 7.54E-30 | 84.05 | F:ATP binding; F:adenylylsulfate kinase activity; F:sulfate adenylyltransferase (ATP) activity; P:phosphorylation; P:sulfate assimilation |
| Efet.01.160929.g573.t1 | hypothetical protein CAPTEDRAFT_186186 | 1245 | 4.14E-12 | 52.5 |  |
| Efet.01.160959.g574.t1 | dynein heavy chain axonemal-like isoform X2 | 1473 | 4.67E-167 | 62.75 | C:cilium; C:dynein complex; P:single-organism cellular process; F:binding; P:microtubule-based process |
| Efet.01.16098.g1187.t1 | hypothetical protein CAPTEDRAFT_204443 | 909 | 1.89E-15 | 43 |  |
| Efet.01.1610020.g22.t1 | cysteine ABC transporter substrate-binding | 228 | 5.44E-48 | 67 | C:membrane; P:ionotropic glutamate receptor signaling pathway; F:ionotropic glutamate receptor activity; P:transport |
| Efet.01.16102.g1188.t1 | oxysterol-binding -related 1 | 354 | 4.89E-10 | 66.7 |  |
| Efet.01.161059.g576.t1 | RB1-inducible coiled-coil 1-like isoform X1 | 1365 | 6.35E-27 | 45 | P:signal transduction |
| Efet.01.16107.g1189.t1 | FAD-dependent oxidoreductase domain-containing 2-like | 765 | 3.05E-49 | 66.6 | F:oxidoreductase activity; P:single-organism metabolic process |
| Efet.01.161084.g578.t1 | microtubule-actin cross-linking factor 1 isoform X3 | 255 | 2.84E-16 | 69.7 | C:cytoskeleton |
| Efet.01.1611.g152.t1 | FGFR1 oncogene partner 2 homolog | 333 | 7.42E-13 | 63.35 | C:cytoplasm |
| Efet.01.16116.g1190.t1 | neogenin-like isoform X2 | 408 | 6.88E-09 | 50.8 | P:iron ion homeostasis; P:positive regulation of BMP signaling pathway; C:integral component of plasma membrane; P:nervous system development; P:axon guidance; F:receptor activity |
| Efet.01.1611785.g24.t1 | unc-112-related -like | 264 | 1.05E-40 | 86.7 | C:cytoskeleton |
| Efet.01.16119.g1191.t1 | thrombospondin type-1 domain-containing 7A | 297 | 2.27E-08 | 50.85 | C:extracellular exosome; C:membrane; C:integral component of membrane |
| Efet.01.1612062.g25.t1 | Wnt-4-like | 243 | 1.75E-25 | 76.2 | P:regulation of reproductive process; P:gamete generation; P:negative regulation of developmental process; P:regulation of anatomical structure morphogenesis; P:branching morphogenesis of an epithelial tube; P:positive regulation of developmental process; P:positive regulation of steroid hormone biosynthetic process; C:proteinaceous extracellular matrix; P:nephron epithelium morphogenesis; P:mesonephric tubule development; F:frizzled binding; P:negative regulation of signal transduction; P:regulation of cell differentiation; P:positive regulation of cellular component biogenesis; P:regulation of canonical Wnt signaling pathway; P:positive regulation of organelle organization; P:negative regulation of multicellular organismal process; P:mammary gland epithelium development; P:positive regulation of macromolecule biosynthetic process; P:negative regulation of steroid biosynthetic process; P:neuron differentiation; P:T cell differentiation in thymus; P:adenohypophysis development; C:cell part; P:regulation of transcription, DNA-templated; P:hormone metabolic process; P:positive regulation of ossification; P:epithelial cell differentiation; P:negative regulation of gene expression; P:epithelial tube formation; C:extracellular space; P:negative regulation of cellular biosynthetic process; P:gonad development; P:cell fate commitment; P:metanephric nephron development; P:regulation of ketone biosynthetic process; P:regulation of cell adhesion; P:non-canonical Wnt signaling pathway; P:regulation of multicellular organismal development |
| Efet.01.1612352.g26.t1 | solute carrier family 40 member 1-like | 264 | 3.47E-11 | 68.6 | C:membrane |
| Efet.01.1612459.g27.t1 | NADH dehydrogenase [ubiquinone] flavo mitochondrial-like | 231 | 1.79E-22 | 89.7 | F:FMN binding; F:NAD binding; F:NADH dehydrogenase (ubiquinone) activity; F:4 iron, 4 sulfur cluster binding; P:aerobic respiration |
| Efet.01.161247.g580.t1 | poly(ADP-ribose) polymerase pme-5-like | 489 | 1.73E-40 | 64.25 | P:DNA ligation involved in DNA repair; F:transferase activity, transferring glycosyl groups; F:DNA ligase (ATP) activity; P:lagging strand elongation; C:cytoplasm; P:cellular protein metabolic process |
| Efet.01.161270.g581.t1 | neurofascin-like isoform X1 | 426 | 5.23E-19 | 55.1 | F:rhodopsin kinase activity; C:membrane; C:integral component of membrane; F:transferase activity; P:protein phosphorylation |
| Efet.01.1612933.g28.t1 | medium-chain specific acyl- mitochondrial | 201 | 4.39E-07 | 90.75 | P:post-embryonic development; P:response to starvation; P:regulation of gluconeogenesis; P:response to cold; P:glycogen biosynthetic process; P:fatty acid beta-oxidation using acyl-CoA dehydrogenase; F:medium-chain-acyl-CoA dehydrogenase activity; F:flavin adenine dinucleotide binding; P:liver development; P:carnitine metabolic process, CoA-linked; C:mitochondrion; P:medium-chain fatty acid metabolic process; P:cardiac muscle cell differentiation |
| Efet.01.161348.g584.t1 | 3-hydroxyisobutyrate mitochondrial-like | 375 | 1.70E-66 | 89.45 | F:phosphogluconate dehydrogenase (decarboxylating) activity; F:NAD binding; C:mitochondrion; P:valine catabolic process; P:oxidation-reduction process; F:3-hydroxyisobutyrate dehydrogenase activity |
| Efet.01.161350.g585.t1 | hypothetical protein HELRODRAFT_168859 | 201 | 4.30E-10 | 58.5 | C:membrane; C:integral component of membrane |
| Efet.01.161363.g587.t1 | DNA repair and recombination RAD26 | 2274 | 1.09E-52 | 44.21 | C:membrane; C:integral component of membrane |
| Efet.01.161368.g588.t1 | metal transporter CNNM4-like | 1398 | 2.31E-141 | 71.9 | C:integral component of membrane |
| Efet.01.161370.g589.t1 | type I inositol 1,4,5-trisphosphate 5-phosphatase-like isoform X1 | 867 | 7.42E-14 | 51.17 |  |
| Efet.01.161403.g590.t1 | hypothetical protein CAPTEDRAFT_218836 | 276 | 3.18E-11 | 56 | C:membrane; C:integral component of membrane |
| Efet.01.161437.g592.t1 | vacuolar sorting-associated 11 homolog | 465 | 5.74E-30 | 88.2 | P:regulation of SNARE complex assembly; P:endosome organization; C:CORVET complex; P:positive regulation of cellular protein catabolic process; C:late endosome; P:regulation of organelle assembly; P:intracellular protein transport; F:nucleotide binding; C:early endosome; P:autophagy; C:AP-3 adaptor complex; P:lysosome organization; F:zinc ion binding; F:protein domain specific binding; F:syntaxin binding; P:endosome to lysosome transport; C:actin filament; P:toxin transport; C:lysosomal membrane; P:positive regulation of protein targeting to mitochondrion; C:endocytic vesicle; P:regulation of protein stability; P:vesicle docking involved in exocytosis; P:positive regulation of early endosome to late endosome transport; P:endosomal vesicle fusion; F:protein binding, bridging; C:HOPS complex |
| Efet.01.161439.g593.t1 | glutamine synthetase | 699 | 2.81E-28 | 56.5 | P:nitrogen compound metabolic process; F:catalytic activity; P:glutamine biosynthetic process; F:ligase activity; F:glutamate-ammonia ligase activity |
| Efet.01.161456.g594.t1 | glycylpeptide N-tetradecanoyltransferase | 537 | 1.22E-81 | 80.05 | F:glycylpeptide N-tetradecanoyltransferase activity; P:N-terminal protein myristoylation |
| Efet.01.161479.g595.t1 | cyclic nucleotide-gated cation channel subunit A | 294 | 6.02E-33 | 83.95 | C:integral component of plasma membrane; F:voltage-gated potassium channel activity; P:regulation of membrane potential; P:potassium ion transmembrane transport; F:cGMP-dependent protein kinase activity; F:intracellular cAMP activated cation channel activity; P:protein phosphorylation; F:intracellular cGMP activated cation channel activity; F:cGMP binding |
| Efet.01.1614985.g30.t1 | eIF-2-alpha kinase activator GCN1 | 273 | 1.53E-16 | 73.05 | P:regulation of kinase activity; P:regulation of cellular protein metabolic process; F:binding |
| Efet.01.16152.g1192.t1 | hypothetical protein HELRODRAFT_168350 | 306 | 1.27E-12 | 61 |  |
| Efet.01.1615240.g31.t1 | crooked neck 1 | 297 | 1.44E-39 | 83.1 | C:prespliceosome; C:catalytic step 2 spliceosome; C:post-mRNA release spliceosomal complex; C:precatalytic spliceosome; C:catalytic step 1 spliceosome; P:positive regulation of macromitophagy in response to mitochondrial depolarization; C:Prp19 complex; P:positive regulation of defense response to virus by host; P:xenophagy; P:spliceosomal complex assembly; F:RNA binding |
| Efet.01.161542.g597.t1 | glutamyl aminopeptidase-like | 342 | 8.76E-20 | 57.3 | F:zinc ion binding; P:proteolysis; C:membrane; C:integral component of membrane; F:aminopeptidase activity; F:metallopeptidase activity |
| Efet.01.1615663.g32.t1 | suppressor of lurcher 1-like | 282 | 2.31E-26 | 66.4 | C:membrane |
| Efet.01.161592.g600.t1 | ras-related Rab-33A isoform X1 | 291 | 4.31E-21 | 79.8 | F:GTP binding; P:small GTPase mediated signal transduction; P:regulation of autophagosome assembly; P:antigen processing and presentation; C:intracellular |
| Efet.01.1616067.g35.t1 | receptor-type tyrosine- phosphatase F | 231 | 1.40E-39 | 94 | P:establishment of endothelial intestinal barrier; P:negative regulation of cell proliferation; P:regulation of axon regeneration; F:heparin binding; C:neuronal cell body; P:homophilic cell adhesion via plasma membrane adhesion molecules; F:insulin receptor binding; P:negative regulation of epidermal growth factor receptor signaling pathway; P:positive regulation of neuron apoptotic process; P:spinal cord development; C:growth cone; P:negative regulation of cytokine-mediated signaling pathway; P:negative regulation of neurotrophin TRK receptor signaling pathway; C:endosome; C:extracellular exosome; C:intracellular membrane-bounded organelle; P:cell migration; P:peptidyl-tyrosine dephosphorylation; P:positive regulation of dendrite morphogenesis; P:cerebellum development; C:excitatory synapse; F:chondroitin sulfate proteoglycan binding; P:negative regulation of receptor binding; P:hippocampus development; F:transmembrane receptor protein tyrosine phosphatase activity; P:cerebral cortex development; F:phosphate ion binding; P:negative regulation of insulin receptor signaling pathway; C:plasma membrane; P:corpus callosum development; C:integral component of membrane; P:regulation of synapse structure or activity; P:neuron projection regeneration; F:receptor tyrosine kinase binding; P:negative regulation of cell projection organization |
| Efet.01.1616258.g36.t1 | voltage-dependent calcium channel gamma-5 subunit-like | 222 | 1.30E-10 | 63.75 | P:calcium ion transport; C:membrane; C:integral component of membrane |
| Efet.01.161631.g602.t1 | cytochrome b5 reductase 4 isoform X1 | 345 | 9.91E-17 | 80.9 | P:generation of precursor metabolites and energy; P:insulin secretion; P:response to antibiotic; C:endoplasmic reticulum; P:cell development; F:heme binding; F:cytochrome-b5 reductase activity, acting on NAD(P)H; C:perinuclear region of cytoplasm; P:superoxide metabolic process; F:metal ion binding; F:NAD(P)H oxidase activity; P:glucose homeostasis; P:oxidation-reduction process |
| Efet.01.161632.g603.t1 | golgin subfamily A member 4-like isoform X2 | 651 | 4.52E-26 | 55.35 |  |
| Efet.01.16168.g1194.t1 | neuroligin- Y-linked-like isoform X4 | 207 | 5.11E-21 | 86.05 | F:neurexin family protein binding; C:integral component of membrane |
| Efet.01.161682.g605.t1 | CAMK DAPK DAPK variant | 1017 | 8.28E-55 | 50.5 | F:nucleotide binding; F:ATP binding; P:signal transduction; F:protein kinase activity; P:phosphorylation; P:protein phosphorylation; F:kinase activity |
| Efet.01.161682.g606.t1 | leucine-rich repeat SHOC-2-like | 888 | 2.81E-69 | 65.75 |  |
| Efet.01.161702.g607.t1 | rho GTPase-activating 32-like isoform X1 | 210 | 2.51E-12 | 79.95 | P:small GTPase mediated signal transduction; C:cell cortex; F:GTPase activator activity; C:actin cytoskeleton; F:phosphatidylinositol binding |
| Efet.01.16172.g1195.t1 | germinal-center associated nuclear | 414 | 1.45E-57 | 77.95 | F:nucleotide binding; C:nuclear membrane; C:cytosol |
| Efet.01.161730.g608.t1 | TAR DNA-binding 43-like | 288 | 5.39E-31 | 80.65 | F:nucleotide binding; F:nucleic acid binding |
| Efet.01.161735.g609.t1 | baculoviral IAP repeat-containing 6-like isoform X2 | 423 | 4.39E-23 | 62.7 | F:ubiquitin-protein transferase activity; P:apoptotic process; P:regulation of cytokinesis; P:protein ubiquitination |
| Efet.01.161735.g610.t1 | baculoviral IAP repeat-containing 6-like | 489 | 3.51E-36 | 58.95 | C:intracellular organelle; P:regulation of cellular process; P:single-organism cellular process |
| Efet.01.1617567.g40.t1 | hypothetical protein HELRODRAFT_125965, partial | 315 | 1.63E-09 | 78 |  |
| Efet.01.1617647.g41.t1 | DNA mismatch repair Mlh1-like | 210 | 3.90E-07 | 89.15 | F:ATP binding; P:mismatch repair; F:mismatched DNA binding |
| Efet.01.1617917.g43.t1 | ESF1 homolog | 216 | 2.65E-22 | 77.6 | C:membrane |
| Efet.01.1618204.g46.t1 | arrestin domain-containing 3-like | 318 | 2.30E-28 | 83.95 | P:signal transduction |
| Efet.01.1618262.g47.t1 | hypothetical protein BRAFLDRAFT_58690 | 219 | 9.40E-06 | 54 | F:iron ion binding; F:metal ion binding; F:oxidoreductase activity, acting on paired donors, with incorporation or reduction of molecular oxygen; F:oxidoreductase activity; F:steroid hydroxylase activity; P:oxidation-reduction process; F:monooxygenase activity; F:heme binding |
| Efet.01.161827.g612.t1 | serine threonine- phosphatase 6 regulatory ankyrin repeat subunit B- | 1734 | 1.02E-86 | 53.4 | P:signal transduction |
| Efet.01.16184.g1196.t1 | myosin heavy chain cardiac muscle specific isoform | 1623 | 1.03E-107 | 68.85 | F:nucleotide binding; F:anion binding |
| Efet.01.161840.g613.t1 | phosphatidylinositol 4,5-bisphosphate 3-kinase catalytic subunit alpha isoform-like | 519 | 1.48E-60 | 77.95 | C:phosphatidylinositol 3-kinase complex; P:phosphatidylinositol-mediated signaling; P:phosphatidylinositol-3-phosphate biosynthetic process; P:phosphatidylinositol phosphorylation; F:phosphatidylinositol-4,5-bisphosphate 3-kinase activity; F:1-phosphatidylinositol-4-phosphate 3-kinase activity; C:intracellular; C:plasma membrane; F:1-phosphatidylinositol-3-kinase activity |
| Efet.01.161841.g614.t1 | kelch 5 isoform X1 | 936 | 8.15E-25 | 54.2 | F:ubiquitin-protein transferase activity; C:Cul3-RING ubiquitin ligase complex; P:protein ubiquitination |
| Efet.01.1618969.g49.t1 | transmembrane 2-like | 204 | 6.38E-29 | 78.75 | C:integral component of membrane |
| Efet.01.1619110.g51.t1 | hypothetical protein CAPTEDRAFT_209261 | 261 | 7.94E-15 | 62 | C:membrane; C:integral component of membrane |
| Efet.01.1619149.g53.t1 | suppressor of lurcher 1-like | 201 | 1.08E-20 | 80.9 | C:integral component of membrane |
| Efet.01.161918.g617.t1 | tetrameric potassium-selective cyclic nucleotide gated channel | 474 | 1.69E-06 | 70.33 | P:potassium ion transport; F:ion channel activity; F:voltage-gated potassium channel activity; P:ion transport; P:transmembrane transport; P:potassium ion transmembrane transport; P:ion transmembrane transport; C:membrane; C:integral component of membrane; P:transport |
| Efet.01.1619239.g54.t1 | methylmalonate-semialdehyde dehydrogenase ( acylating) | 243 | 3.91E-34 | 87.15 | F:methylmalonate-semialdehyde dehydrogenase (acylating) activity; P:oxidation-reduction process |
| Efet.01.161938.g618.t1 | RNA-directed DNA polymerase homolog | 465 | 7.30E-13 | 77.9 | F:nucleic acid binding; F:transcription factor activity, sequence-specific DNA binding; F:zinc ion binding; F:aspartic-type endopeptidase activity; P:proteolysis; P:DNA integration; P:regulation of transcription, DNA-templated |
| Efet.01.161958.g619.t1 | homeobox | 375 | 7.41E-13 | 87 | C:nucleus; P:regulation of transcription, DNA-templated; F:sequence-specific DNA binding |
| Efet.01.16198.g1197.t1 | TNF receptor-associated factor 6-like isoform X2 | 957 | 2.04E-56 | 71.45 | F:ubiquitin-protein transferase activity; F:zinc ion binding; F:metal ion binding; P:signal transduction; P:regulation of apoptotic process; P:protein ubiquitination |
| Efet.01.161981.g620.t1 | solute carrier family 15 member 4-like | 264 | 2.72E-11 | 68.55 | P:oligopeptide transport; C:integral component of membrane; F:transporter activity |
| Efet.01.161993.g622.t1 | amiloride-sensitive sodium channel subunit alpha | 786 | 9.77E-51 | 52.65 | P:sodium ion transport; C:membrane |
| Efet.01.161993.g623.t1 | FMRFamide-activated amiloride-sensitive sodium channel isoform X1 | 1017 | 1.89E-102 | 48.05 | P:ion transport |
| Efet.01.1619965.g55.t1 | arrestin domain-containing 17 | 306 | 7.03E-24 | 63.25 |  |
| Efet.01.1620228.g56.t1 | ketol-acid reductoisomerase | 264 | 4.92E-43 | 86.85 | P:isoleucine biosynthetic process; P:valine biosynthetic process; P:oxidation-reduction process; F:ketol-acid reductoisomerase activity; F:isomerase activity |
| Efet.01.1620234.g57.t1 | ATP-binding cassette sub-family A member 3-like | 270 | 2.60E-25 | 71.65 | F:nucleotide binding; P:lipid transport; F:ATPase activity, coupled to transmembrane movement of substances; C:intracellular membrane-bounded organelle; C:membrane |
| Efet.01.162043.g624.t1 | CD109 antigen | 255 | 1.81E-35 | 80.3 | C:extracellular space; F:endopeptidase inhibitor activity; P:negative regulation of endopeptidase activity |
| Efet.01.1620676.g58.t1 | EF-hand domain-containing D2-like | 207 | 1.10E-16 | 69.45 | F:calcium ion binding |
| Efet.01.162097.g626.t1 | hypothetical protein CAPTEDRAFT_187428 | 1182 | 8.62E-18 | 50.83 |  |
| Efet.01.162109.g628.t1 | guanine nucleotide exchange factor DBS-like | 369 | 5.19E-30 | 75.55 | P:regulation of Rho protein signal transduction; F:Rho guanyl-nucleotide exchange factor activity; C:intracellular; P:intracellular signal transduction; P:positive regulation of GTPase activity |
| Efet.01.162123.g629.t1 | T-box transcription factor TBX1 | 378 | 2.17E-39 | 84.65 | P:cell proliferation; P:soft palate development; P:negative regulation of mesenchymal cell apoptotic process; P:coronary artery morphogenesis; P:mesoderm development; P:anterior/posterior pattern specification; P:outer ear morphogenesis; P:muscle cell fate commitment; P:middle ear morphogenesis; F:sequence-specific DNA binding; P:tongue morphogenesis; P:positive regulation of transcription from RNA polymerase II promoter; P:face morphogenesis; P:thymus development; P:mesenchymal cell apoptotic process; P:outflow tract septum morphogenesis; P:sensory perception of sound; F:transcription factor activity, sequence-specific DNA binding; P:retinoic acid receptor signaling pathway; P:enamel mineralization; P:angiogenesis; P:negative regulation of cell differentiation; P:lymph vessel development; P:cell fate specification; P:positive regulation of epithelial cell proliferation; P:social behavior; F:protein homodimerization activity; P:transcription, DNA-templated; P:cochlea morphogenesis; P:cellular response to fibroblast growth factor stimulus; P:muscle tissue morphogenesis; P:vagus nerve morphogenesis; P:positive regulation of mesenchymal cell proliferation; P:positive regulation of MAPK cascade; P:pharyngeal system development; P:determination of left/right symmetry; P:parathyroid gland development; P:embryonic viscerocranium morphogenesis; P:epithelial cell differentiation; C:nucleus; P:aorta morphogenesis; P:thyroid gland development; P:neural crest cell migration; P:semicircular canal morphogenesis; P:cellular response to retinoic acid; P:regulation of organ morphogenesis; P:positive regulation of tongue muscle cell differentiation |
| Efet.01.1621643.g63.t1 | dynein heavy chain axonemal-like | 234 | 9.62E-37 | 87.1 | F:ATPase activity; F:ATP binding; F:microtubule motor activity; C:dynein complex; P:microtubule-based movement |
| Efet.01.162174.g630.t1 | tyrosine- kinase ZAP- | 402 | 1.67E-27 | 77.05 | F:ATP binding; F:non-membrane spanning protein tyrosine kinase activity; P:innate immune response; P:cell differentiation; C:extrinsic component of cytoplasmic side of plasma membrane; P:transmembrane receptor protein tyrosine kinase signaling pathway; P:peptidyl-tyrosine autophosphorylation; F:receptor binding; P:regulation of cell proliferation |
| Efet.01.162228.g631.t1 | testis-specific serine threonine- kinase 1 | 462 | 2.69E-78 | 73.75 | F:ATP binding; F:protein serine/threonine kinase activity; P:protein phosphorylation |
| Efet.01.1622333.g64.t1 | tumor suppressor candidate | 231 | 4.32E-19 | 89.1 | C:integral component of membrane |
| Efet.01.1622408.g65.t1 | Collagen alpha-3(VI) chain | 249 | 2.12E-11 | 61.75 | C:collagen trimer; F:calcium ion binding |
| Efet.01.162293.g632.t1 | hypothetical protein CAPTEDRAFT_225957 | 249 | 3.28E-20 | 67 | C:membrane |
| Efet.01.16232.g1198.t1 | hypothetical protein CAPTEDRAFT_219720 | 231 | 1.53E-15 | 58.71 | C:membrane; C:integral component of membrane; F:carbohydrate binding |
| Efet.01.162340.g634.t1 | DNA topoisomerase 3-beta-1 isoform X1 | 1437 | 0 | 67.1 | C:intracellular organelle; F:DNA binding; F:DNA topoisomerase type I activity; P:DNA topological change |
| Efet.01.1624194.g69.t1 | hypothetical protein CRN43_05520, partial | 255 | 5.26E-44 | 92 |  |
| Efet.01.162431.g635.t1 | phospholipase A-2-activating | 597 | 1.94E-43 | 66.95 | C:extracellular exosome; F:protein binding; P:signal transduction; P:phospholipid metabolic process; F:phospholipase A2 activator activity |
| Efet.01.1624319.g70.t1 | probable G- coupled receptor 139 | 327 | 1.31E-22 | 66.8 | P:signal transduction; C:membrane; F:signal transducer activity |
| Efet.01.162446.g636.t1 | polycystic kidney disease 1-like 2 | 1542 | 1.70E-28 | 47.05 | C:membrane; C:integral component of membrane |
| Efet.01.1624973.g72.t1 | WD and tetratricopeptide repeats 1-like isoform X1 | 240 | 1.12E-22 | 81.4 | P:transmembrane transport; C:integral component of membrane |
| Efet.01.1625073.g73.t1 | cholecystokinin receptor type A-like | 246 | 1.86E-11 | 87.2 | F:rhodopsin kinase activity; P:G-protein coupled receptor signaling pathway; F:gastrin receptor activity; C:integral component of membrane; P:protein phosphorylation |
| Efet.01.1625268.g75.t1 | UDP-galactose translocator | 210 | 2.25E-20 | 75.45 | C:membrane; P:single-organism transport |
| Efet.01.1625714.g77.t1 | dynein heavy chain axonemal | 240 | 2.88E-37 | 82.9 |  |
| Efet.01.1625906.g78.t1 | neuronal acetylcholine receptor subunit alpha-5-like isoform X1 | 213 | 5.25E-25 | 81.2 | C:postsynaptic membrane; P:cation transmembrane transport; C:integral component of membrane; C:cell junction; F:extracellular ligand-gated ion channel activity |
| Efet.01.162597.g639.t1 | long-chain-fatty-acid-- ligase ACSBG2 isoform X1 | 525 | 3.75E-39 | 81.35 | P:metabolic process; F:ligase activity |
| Efet.01.1626162.g80.t1 | tyrosine phosphatase receptor | 234 | 4.85E-30 | 72.7 | F:protein binding; P:regulation of cellular process; F:protein tyrosine phosphatase activity; C:membrane; P:negative regulation of biological process; P:regulation of multicellular organismal process; P:peptidyl-tyrosine dephosphorylation; P:single-organism process; P:response to stimulus; P:multicellular organismal process |
| Efet.01.162659.g641.t1 | clavaminate synthase At3g21360 | 1017 | 1.77E-121 | 66.85 | C:nucleus; F:oxidoreductase activity; P:oxidation-reduction process |
| Efet.01.162667.g643.t1 | Tyrosine- kinase transmembrane receptor | 318 | 2.74E-09 | 55.85 | F:transferase activity |
| Efet.01.16268.g1202.t1 | peptidyl-prolyl cis-trans isomerase CYP63-like isoform X1 | 264 | 4.64E-13 | 73.05 | C:integral component of membrane |
| Efet.01.1626886.g81.t1 | gamma-aminobutyric acid receptor subunit gamma-3 | 318 | 6.84E-31 | 76.75 | P:ion transmembrane transport; C:integral component of membrane; F:extracellular ligand-gated ion channel activity |
| Efet.01.1627090.g82.t1 | Cadherin- isoform A | 264 | 1.67E-25 | 66.9 | P:calcium-dependent cell-cell adhesion via plasma membrane cell adhesion molecules; F:protein homodimerization activity; P:ommatidial rotation; P:retinal ganglion cell axon guidance; P:R7 cell development; P:homophilic cell adhesion via plasma membrane adhesion molecules; C:plasma membrane; P:axon target recognition; P:axonal fasciculation; P:axon extension involved in axon guidance; C:dendrite; P:negative regulation of dendrite morphogenesis; C:cell-cell junction; P:R8 cell development; P:regulation of axon extension involved in axon guidance; F:calcium ion binding; F:cadherin binding; P:cell-cell adhesion mediated by cadherin; C:integral component of membrane; P:sensory perception of pain; C:axon |
| Efet.01.1627232.g83.t1 | adhesion G -coupled receptor L4-like | 258 | 4.50E-22 | 54.5 | P:signal transduction; F:signal transducer activity |
| Efet.01.162740.g644.t1 | Dynein beta flagellar outer arm | 615 | 4.33E-66 | 73 | F:microtubule motor activity; C:motile cilium; P:microtubule-based movement |
| Efet.01.16275.g1203.t1 | RNA-binding Raly | 378 | 1.86E-31 | 72.15 | F:nucleotide binding; C:nucleus; F:metal ion binding; F:RNA binding |
| Efet.01.162760.g645.t1 | 60 kDa neurofilament -like | 507 | 1.47E-39 | 76.15 | F:structural molecule activity; C:intermediate filament |
| Efet.01.162777.g647.t1 | cAMP-responsive element-binding -like 2 | 225 | 1.07E-21 | 83.85 | F:transcription factor activity, sequence-specific DNA binding; P:regulation of transcription, DNA-templated; F:sequence-specific DNA binding |
| Efet.01.162807.g648.t1 | pikachurin-like isoform X2 | 276 | 1.39E-23 | 67.85 | F:calcium ion binding |
| Efet.01.1628109.g85.t1 | carboxypeptidase B-like | 240 | 1.70E-18 | 69.5 | F:carboxypeptidase activity; F:ion binding |
| Efet.01.162818.g649.t1 | Wnt-11b-2-like | 225 | 2.39E-26 | 77.95 | P:multicellular organism development; C:integral component of membrane; P:Wnt signaling pathway; F:receptor binding; C:proteinaceous extracellular matrix |
| Efet.01.162851.g653.t1 | essential MCU mitochondrial-like | 339 | 7.63E-16 | 67.85 |  |
| Efet.01.1628545.g87.t1 | Erythronolide modules 1 and 2 | 300 | 3.33E-58 | 66.15 | F:catalytic activity; P:metabolic process; F:transferase activity |
| Efet.01.162857.g654.t1 | Zinc metallo ase | 306 | 1.71E-20 | 80 | P:proteolysis; F:metallopeptidase activity |
| Efet.01.1628885.g89.t1 | Fibronectin type III domain-containing 1 | 222 | 5.57E-29 | 76.65 | C:membrane |
| Efet.01.162890.g656.t1 | receptor-type guanylate cyclase gcy-4-like isoform X1 | 948 | 3.91E-41 | 54.7 | P:phosphorylation; P:primary metabolic process; F:kinase activity; P:organic substance metabolic process |
| Efet.01.1628922.g90.t1 | hypothetical protein CAPTEDRAFT_227592 | 273 | 1.50E-31 | 86 |  |
| Efet.01.162919.g657.t1 | steroid receptor seven- isoforms B C | 249 | 3.79E-38 | 74.95 | F:DNA binding; F:transcription factor activity, sequence-specific DNA binding; F:metal ion binding; P:signal transduction; F:signal transducer activity |
| Efet.01.162939.g658.t1 | tRNA (N6-isopentenyl adenosine(37)-C2)-methylthiotransferase | 999 | 7.96E-146 | 79.8 | C:cytoplasm; F:iron ion binding; F:4 iron, 4 sulfur cluster binding; F:transferase activity; P:tRNA modification |
| Efet.01.1629497.g95.t1 | bicaudal D isoform X2 | 267 | 6.00E-09 | 72.1 | C:Golgi apparatus; P:transport |
| Efet.01.1629507.g96.t1 | kinase C epsilon type | 213 | 1.63E-22 | 88.9 | F:ATP binding; F:metal ion binding; P:peptidyl-serine phosphorylation; C:intracellular; P:intracellular signal transduction; F:protein kinase C activity |
| Efet.01.1629704.g97.t1 | hypothetical protein B5766_02165 | 213 | 1.57E-42 | 85.88 |  |
| Efet.01.1629803.g99.t1 | gamma-glutamyltranspeptidase 1-like | 216 | 6.29E-19 | 75.45 | P:glutathione metabolic process; F:gamma-glutamyltransferase activity |
| Efet.01.162983.g659.t1 | hypothetical protein CAPTEDRAFT_216524 | 1497 | 1.51E-26 | 72 | C:membrane |
| Efet.01.1629875.g100.t1 | gametogenetin-binding 2 isoform X4 | 288 | 1.79E-06 | 71.25 |  |
| Efet.01.1629985.g101.t1 | phosphatidylinositol 4-kinase beta | 231 | 2.99E-25 | 76.7 | P:phosphatidylinositol-mediated signaling; P:phosphatidylinositol phosphorylation; F:kinase activity; C:intracellular |
| Efet.01.1630309.g103.t1 | inorganic pyrophosphatase | 252 | 3.53E-54 | 95.35 | F:magnesium ion binding; F:inorganic diphosphatase activity; C:cytoplasm; P:phosphate-containing compound metabolic process |
| Efet.01.1630470.g105.t1 | phospho-sugar mutase | 234 | 5.97E-48 | 81.45 | F:magnesium ion binding; P:carbohydrate metabolic process; F:intramolecular transferase activity, phosphotransferases |
| Efet.01.163053.g666.t1 | ribonucleo PTB-binding 1-like isoform X1 | 432 | 1.26E-32 | 57.6 | F:nucleotide binding; F:nucleic acid binding; C:nucleus; C:intracellular ribonucleoprotein complex; C:virion; C:viral nucleocapsid; P:mRNA splicing, via spliceosome |
| Efet.01.1630598.g106.t1 | neuronal acetylcholine receptor subunit alpha-10-like | 234 | 1.08E-14 | 75.4 | C:postsynaptic membrane; P:cation transmembrane transport; C:integral component of membrane; C:cell junction; F:extracellular ligand-gated ion channel activity |
| Efet.01.1630802.g107.t1 | DNA glycosylase | 297 | 7.02E-52 | 83.3 | F:hydrolase activity, hydrolyzing N-glycosyl compounds; F:zinc ion binding; P:base-excision repair; P:nucleotide-excision repair; F:DNA-(apurinic or apyrimidinic site) lyase activity; F:damaged DNA binding |
| Efet.01.1630916.g108.t1 | hypothetical protein HELRODRAFT_188726 | 309 | 1.33E-08 | 65 |  |
| Efet.01.1630939.g109.t1 | lamin dm0-like isoform x1 | 288 | 2.90E-30 | 80.35 | C:nuclear part; F:structural molecule activity; C:intermediate filament |
| Efet.01.1631066.g111.t1 | gastrula zinc finger -like | 369 | 3.01E-36 | 64.6 | F:nucleic acid binding; F:metal ion binding |
| Efet.01.16311.g1205.t1 | tyrosine- kinase Btk29A isoform X2 | 3684 | 1.17E-141 | 54.7 | F:protein kinase activity; P:phosphorylation |
| Efet.01.1631129.g112.t1 | split ends-like | 282 | 2.43E-16 | 65.9 | F:DNA binding; C:nucleus; P:regulation of transcription, DNA-templated |
| Efet.01.163117.g668.t1 | Serine threonine- kinase B-raf | 654 | 3.18E-17 | 56.25 | F:transferase activity; P:cellular process |
| Efet.01.163121.g669.t1 | LIM domain only 3-like isoform X1 | 207 | 9.92E-16 | 84.7 | F:zinc ion binding |
| Efet.01.163130.g670.t1 | dynein heavy chain axonemal-like | 567 | 6.68E-38 | 60.8 | F:nucleotide binding; F:ATP binding; F:microtubule motor activity; C:dynein complex; P:microtubule-based movement |
| Efet.01.1631465.g113.t1 | paramyosin-like isoform X1 | 270 | 2.25E-23 | 72.25 | C:myosin complex; F:motor activity |
| Efet.01.163163.g671.t1 | sugar ABC transporter substrate-binding | 573 | 1.08E-56 | 92.3 |  |
| Efet.01.16318.g1206.t1 | E3 ubiquitin- ligase Bre1 | 807 | 3.74E-35 | 80.45 | F:zinc ion binding; F:ligase activity |
| Efet.01.1632087.g116.t1 | Location of vulva defective 1 | 324 | 7.14E-34 | 71.65 | C:membrane |
| Efet.01.1632125.g117.t1 | G -coupled receptor kinase b | 207 | 1.81E-07 | 60 | F:transferase activity |
| Efet.01.1632146.g118.t1 | thyrotropin-releasing hormone receptor-like | 303 | 3.76E-35 | 80.25 | F:thyrotropin-releasing hormone receptor activity; P:G-protein coupled receptor signaling pathway; C:integral component of membrane |
| Efet.01.1632263.g119.t1 | serine threonine- kinase D3 | 270 | 2.61E-24 | 84.55 | F:ATP binding; F:metal ion binding; P:protein phosphorylation; C:intracellular; P:intracellular signal transduction; F:protein kinase C activity |
| Efet.01.1632425.g121.t1 | serine threonine- phosphatase 6 regulatory ankyrin repeat subunit B-like | 336 | 2.77E-06 | 58 |  |
| Efet.01.1632489.g122.t1 | cell division | 285 | 4.83E-33 | 73.85 | P:cell division; C:cytoplasm; P:cell cycle |
| Efet.01.1632518.g123.t1 | branched-chain amino acid ABC transporter substrate-binding | 273 | 1.57E-57 | 88.8 | P:amino acid transport |
| Efet.01.1632648.g125.t1 | double-strand-break repair rad21 homolog isoform X1 | 243 | 9.45E-20 | 87.1 | C:nuclear chromosome |
| Efet.01.1632798.g127.t1 | adenosine 3 -phospho 5 -phosphosulfate transporter 2 isoform X1 | 333 | 7.53E-07 | 56 |  |
| Efet.01.1633180.g129.t1 | family | 342 | 1.88E-30 | 69.35 | C:membrane |
| Efet.01.163319.g675.t1 | iroquois-class homeodomain IRX-6-like | 288 | 4.51E-16 | 85.45 | C:nucleus; P:regulation of transcription, DNA-templated; F:sequence-specific DNA binding |
| Efet.01.1633238.g130.t1 | hypothetical protein CAPTEDRAFT_223749 | 228 | 1.08E-07 | 58 | C:membrane; C:integral component of membrane |
| Efet.01.1633285.g131.t1 | BDNF NT-3 growth factors receptor- | 216 | 7.27E-23 | 78 | F:ATP binding; P:peptidyl-tyrosine phosphorylation; F:protein tyrosine kinase activity; C:integral component of membrane |
| Efet.01.1633463.g132.t1 | lysis | 219 | 1.73E-16 | 75.95 |  |
| Efet.01.1633521.g133.t1 | Laminin subunit alpha | 324 | 1.71E-13 | 58.6 | P:regulation of embryonic development; P:cell adhesion; P:regulation of cell adhesion; P:regulation of cell migration; F:receptor binding |
| Efet.01.1633564.g135.t1 | hypothetical protein CAPTEDRAFT_200949, partial | 222 | 3.14E-10 | 77 | F:endopeptidase inhibitor activity; P:negative regulation of endopeptidase activity |
| Efet.01.1633572.g136.t1 | Phorbol ester diacylglycerol-binding unc- | 318 | 2.24E-17 | 80.4 | F:G-protein coupled peptide receptor activity; F:RNA binding; P:G-protein coupled receptor signaling pathway; F:metal ion binding; C:integral component of membrane; F:diacylglycerol binding; P:chemical synaptic transmission; P:RNA processing; F:adenosine deaminase activity; C:intracellular; P:intracellular signal transduction |
| Efet.01.1633599.g137.t1 | transcription termination factor Rho | 243 | 8.44E-39 | 92.1 | F:ATP binding |
| Efet.01.1633846.g140.t1 | Multidrug efflux pump subunit | 267 | 4.02E-31 | 74.45 | C:membrane |
| Efet.01.1633975.g141.t1 | disintegrin and metallo ase domain-containing 12-like isoform X2 | 234 | 1.74E-10 | 66.8 | F:hydrolase activity |
| Efet.01.163428.g679.t1 | UPF0415 C7orf25 homolog | 1014 | 1.63E-54 | 63.5 | P:biological_process; C:cellular_component; F:molecular_function |
| Efet.01.163432.g680.t1 | tigger transposable element-derived 2-like | 660 | 1.51E-15 | 63.1 | F:nucleic acid binding |
| Efet.01.1634322.g145.t1 | hypothetical protein CAPTEDRAFT_225002 | 225 | 1.33E-11 | 73 | C:membrane |
| Efet.01.1634377.g146.t1 | turtle homolog A | 306 | 3.33E-33 | 65.2 | C:inhibitory synapse; C:dendrite; P:regulation of synapse organization; F:protein binding involved in cell-cell adhesion; C:membrane; C:integral component of membrane; P:homophilic cell adhesion via plasma membrane adhesion molecules; C:axon; P:dendrite development |
| Efet.01.1634468.g147.t1 | Kinesin heavy chain | 225 | 1.19E-20 | 79.2 | F:ATP binding; F:microtubule motor activity; P:microtubule-based movement; C:microtubule; F:microtubule binding |
| Efet.01.1634560.g149.t1 | hypothetical protein CAPTEDRAFT_227455 | 234 | 2.64E-13 | 56 | C:membrane; P:proteolysis; C:integral component of membrane; F:serine-type peptidase activity |
| Efet.01.1634637.g150.t1 | dnaJ homolog subfamily C member 1 | 246 | 2.53E-08 | 86.07 | F:DNA binding |
| Efet.01.1634649.g151.t1 | Oxysterol-binding -related 6 | 294 | 1.39E-11 | 73.95 | P:lipid transport; P:transport |
| Efet.01.1634715.g153.t1 | dynamin-1-like isoform X1 | 231 | 7.90E-23 | 89.5 | F:GTP binding; P:clathrin-dependent endocytosis; F:GTPase activity |
| Efet.01.1634772.g155.t1 | Kinesin-like | 234 | 1.16E-28 | 86.7 | F:ATPase activity; F:ATP binding; F:microtubule motor activity; C:kinesin complex; P:microtubule-based movement; C:microtubule; F:microtubule binding |
| Efet.01.1634867.g156.t1 | recombinase family | 273 | 7.16E-56 | 93.7 | F:DNA binding; F:recombinase activity; P:DNA recombination |
| Efet.01.1634986.g157.t1 | methionine--tRNA cytoplasmic isoform X2 | 207 | 9.38E-28 | 90.8 | F:ferrous iron binding; P:methionyl-tRNA aminoacylation; P:rRNA transcription; C:nucleolus; F:tRNA binding; F:ATP binding; C:extracellular exosome; F:methionine-tRNA ligase activity; F:oxidoreductase activity; C:mitochondrion; C:membrane; C:cytosol; P:oxidation-reduction process |
| Efet.01.163499.g683.t1 | myrosinase 1-like | 258 | 5.45E-14 | 68.8 | F:beta-glucosidase activity; P:glycosyl compound metabolic process |
| Efet.01.1635148.g158.t1 | 60S ribosomal L26 | 303 | 1.23E-28 | 94.1 | F:structural constituent of ribosome; C:large ribosomal subunit; P:translation |
| Efet.01.1635206.g159.t1 | zinc finger 678-like | 375 | 9.68E-27 | 72.3 | F:nucleic acid binding; F:metal ion binding |
| Efet.01.163521.g685.t1 | Guanine nucleotide-binding subunit gamma-1 | 213 | 1.09E-39 | 74.85 | C:heterotrimeric G-protein complex; P:G-protein coupled receptor signaling pathway; F:signal transducer activity |
| Efet.01.1635333.g160.t1 | target of rapamycin | 279 | 8.77E-17 | 80.75 | F:ATP binding; F:protein serine/threonine kinase activity; P:protein phosphorylation; F:macromolecular complex binding |
| Efet.01.1635455.g161.t1 | fibulin-1-like isoform X3 | 294 | 4.27E-31 | 59.75 | P:DNA repair; C:extracellular region; F:binding |
| Efet.01.163558.g687.t1 | kinase c delta type | 291 | 7.40E-25 | 55.5 | F:nucleotide binding; F:protein binding; C:membrane; F:ion binding; P:cellular response to stimulus; P:single-organism cellular process; F:protein serine/threonine kinase activity; P:protein phosphorylation; P:positive regulation of signal transduction; C:intracellular part |
| Efet.01.1635604.g162.t1 | CUGBP Elav-like family member | 300 | 8.63E-25 | 91.6 | F:nucleotide binding; C:nucleus; P:RNA splicing; F:RNA binding; P:flagellated sperm motility; C:cytoplasm; P:regulation of alternative mRNA splicing, via spliceosome; P:positive regulation of mRNA splicing, via spliceosome; P:spermatogenesis; P:mRNA processing |
| Efet.01.1635692.g163.t1 | epidermal growth factor receptor-like isoform X2 | 270 | 4.09E-27 | 91.75 | F:ATP binding; P:peptidyl-tyrosine phosphorylation; F:transmembrane receptor protein tyrosine kinase activity; C:integral component of membrane; P:transmembrane receptor protein tyrosine kinase signaling pathway |
| Efet.01.1635737.g164.t1 | MFS transporter | 270 | 7.28E-43 | 95.75 | P:transmembrane transport; F:transmembrane transporter activity; C:integral component of membrane |
| Efet.01.1635837.g166.t1 | proline racemase | 222 | 8.12E-42 | 82.65 | F:proline racemase activity |
| Efet.01.1635872.g167.t1 | neuronal acetylcholine receptor subunit alpha-10-like isoform X1 | 300 | 3.15E-07 | 69.5 | F:ion channel activity; P:ion transport; C:postsynaptic membrane; P:ion transmembrane transport; C:membrane; P:cation transmembrane transport; C:integral component of membrane; F:extracellular ligand-gated ion channel activity; C:cell junction; C:synapse; P:transport; C:plasma membrane |
| Efet.01.1635923.g168.t1 | hypothetical protein CAPTEDRAFT_219563 | 309 | 3.03E-10 | 54 | C:membrane; C:integral component of membrane |
| Efet.01.1635950.g169.t1 | suppressor of lurcher 1-like | 210 | 1.89E-07 | 67.25 | C:membrane; C:integral component of membrane |
| Efet.01.1635971.g170.t1 | chromodomain-helicase-DNA-binding 8-like isoform X3 | 306 | 4.15E-23 | 82.95 | C:nucleus; F:DNA binding; F:ATP binding; F:helicase activity |
| Efet.01.1636371.g175.t1 | hypothetical protein CAPTEDRAFT_192952 | 324 | 1.15E-16 | 75 |  |
| Efet.01.1636384.g176.t1 | forkhead box O-like | 408 | 1.76E-35 | 63 | F:DNA binding; C:nucleus; F:transcription factor activity, sequence-specific DNA binding; P:transcription, DNA-templated; P:regulation of transcription, DNA-templated; F:sequence-specific DNA binding |
| Efet.01.1636455.g177.t1 | T-box transcription factor TBX20-like isoform X2 | 204 | 3.74E-10 | 61.35 | F:DNA binding; C:nucleus; F:transcription factor activity, sequence-specific DNA binding; P:transcription, DNA-templated; P:regulation of transcription, DNA-templated |
| Efet.01.1636636.g178.t1 | 40S ribosomal S9 | 273 | 1.34E-40 | 90.55 | F:structural constituent of ribosome; C:small ribosomal subunit; F:rRNA binding; P:translation |
| Efet.01.1636673.g179.t1 | magnesium transporter | 240 | 7.08E-46 | 92.5 |  |
| Efet.01.1636724.g180.t1 | isoleucine--tRNA cytoplasmic | 234 | 1.85E-36 | 94.85 | P:isoleucyl-tRNA aminoacylation; P:regulation of sprouting angiogenesis; F:ATP binding; F:isoleucine-tRNA ligase activity; P:regulation of translational fidelity; F:aminoacyl-tRNA editing activity; C:cytosol; F:tRNA binding |
| Efet.01.1636785.g181.t1 | ABC transporter permease | 291 | 2.79E-53 | 89.15 | C:integral component of membrane; P:transport; C:plasma membrane; F:transporter activity |
| Efet.01.1637086.g184.t1 | DNA-directed RNA polymerase subunit omega | 279 | 9.06E-31 | 90 | F:DNA binding; F:DNA-directed 5'-3' RNA polymerase activity; P:transcription, DNA-templated |
| Efet.01.163725.g691.t1 | Potassium voltage-gated channel subfamily A member 2 | 1056 | 1.38E-108 | 77.7 | P:potassium ion transmembrane transport; C:voltage-gated potassium channel complex; F:delayed rectifier potassium channel activity; P:regulation of ion transmembrane transport; P:protein homooligomerization |
| Efet.01.163781.g693.t1 | extended synaptotagmin-2-like | 246 | 3.16E-18 | 73.05 | C:membrane |
| Efet.01.163808.g695.t1 | Negative elongation factor D | 246 | 1.17E-22 | 83.7 | C:nucleus; P:negative regulation of transcription, DNA-templated |
| Efet.01.163811.g696.t1 | membrane-associated guanylate WW and PDZ domain-containing 2 | 285 | 1.39E-15 | 73.9 | C:postsynaptic density; C:slit diaphragm; P:negative regulation of cell proliferation; C:late endosome; P:protein heterooligomerization; C:dendrite; P:planar cell polarity pathway involved in axis elongation; C:bicellular tight junction; P:mitotic cell cycle arrest; P:negative regulation of activin receptor signaling pathway; P:positive regulation of neuron projection development; F:beta-1 adrenergic receptor binding; F:phosphatase binding; P:positive regulation of receptor internalization; P:SMAD protein signal transduction; C:protein complex; P:receptor clustering; C:plasma membrane; P:nerve growth factor signaling pathway; C:nucleus; C:perinuclear region of cytoplasm; P:positive regulation of phosphoprotein phosphatase activity; F:SMAD binding; P:glomerular visceral epithelial cell development; F:type II activin receptor binding; P:negative regulation of cell migration; F:receptor signaling complex scaffold activity; P:negative regulation of protein kinase B signaling |
| Efet.01.1638137.g190.t1 | myosin-VIIa isoform X1 | 219 | 2.54E-26 | 80.25 | C:myosin complex; F:ATP binding; F:actin binding; F:motor activity |
| Efet.01.1638252.g192.t1 | SET-like isoform X2 | 318 | 1.45E-53 | 88.75 | C:nucleus; P:nucleosome assembly |
| Efet.01.1638266.g193.t1 | family lipo | 297 | 1.05E-23 | 59.15 |  |
| Efet.01.1638395.g194.t1 | carbonate dehydratase | 312 | 7.75E-52 | 73.7 | F:lyase activity |
| Efet.01.1638608.g195.t1 | calmodulin 3 | 312 | 1.06E-17 | 70.2 | C:extracellular exosome; F:metal ion binding |
| Efet.01.1638668.g196.t1 | carbamoyl-phosphate synthase large subunit | 222 | 1.35E-35 | 94.1 | F:ATP binding; F:metal ion binding; P:nitrogen compound metabolic process; F:carbamoyl-phosphate synthase (glutamine-hydrolyzing) activity |
| Efet.01.16387.g1210.t1 | gonadotropin-releasing hormone receptor | 399 | 5.85E-27 | 70.6 | F:G-protein coupled receptor activity; P:G-protein coupled receptor signaling pathway; C:integral component of membrane |
| Efet.01.1638719.g197.t1 | hypothetical protein HELRODRAFT_163003 | 249 | 3.05E-18 | 63.42 | F:nucleic acid binding; F:protein dimerization activity |
| Efet.01.1638728.g198.t1 | thymidylate synthase | 237 | 3.85E-49 | 88.6 | F:thymidylate synthase activity; C:cytoplasm; P:dTMP biosynthetic process; P:dTTP biosynthetic process; P:methylation |
| Efet.01.1638731.g199.t1 | ABC transporter ATP-binding | 363 | 1.35E-72 | 83.6 | F:ATPase activity; F:ATP binding |
| Efet.01.1638827.g200.t1 | ABC transporter permease | 255 | 2.58E-52 | 89.3 |  |
| Efet.01.1638858.g201.t1 | dynein light chain cytoplasmic | 222 | 1.02E-47 | 100 | C:motile cilium; C:dynein complex; P:microtubule-based movement; F:ATPase activity, coupled |
| Efet.01.1638959.g202.t1 | Anhydro-N-acetylmuramic acid kinase | 351 | 5.66E-42 | 72.8 | P:carbohydrate derivative metabolic process; F:transferase activity, transferring phosphorus-containing groups |
| Efet.01.163908.g702.t1 | kinase C and casein kinase substrate in neurons 1-like isoform X3 | 330 | 6.08E-16 | 69.25 | P:phosphorylation; F:kinase activity |
| Efet.01.163912.g703.t1 | probable G- coupled receptor | 1503 | 1.97E-49 | 58.4 | F:G-protein coupled receptor activity; F:G-protein coupled peptide receptor activity; P:G-protein coupled receptor signaling pathway; C:membrane; C:integral component of membrane |
| Efet.01.1639240.g204.t1 | sarcoplasmic calcium-binding -like | 300 | 9.96E-22 | 57.78 | F:metal ion binding; F:calcium ion binding |
| Efet.01.1639252.g205.t1 | disintegrin and metallo ase domain-containing 10-like | 216 | 4.43E-07 | 53 |  |
| Efet.01.163929.g704.t1 | phosphate carrier mitochondrial-like | 273 | 5.04E-46 | 90.95 | P:transmembrane transport; C:integral component of membrane |
| Efet.01.16393.g1211.t1 | para-nitrobenzyl esterase-like | 555 | 2.29E-07 | 46 | C:membrane; C:integral component of membrane; F:hydrolase activity |
| Efet.01.1639369.g206.t1 | septate junction | 270 | 2.00E-13 | 57.6 | C:membrane; C:integral component of membrane; P:regulation of apoptotic process |
| Efet.01.1639375.g207.t1 | sugar ABC transporter permease | 225 | 6.59E-43 | 96.5 | C:integral component of membrane; P:transport; C:plasma membrane |
| Efet.01.163945.g705.t1 | neurabin-1-like isoform X2 | 492 | 3.25E-19 | 79.5 |  |
| Efet.01.1639525.g208.t1 | UDP-N-acetylmuramoyl-tripeptide--D-alanyl-D-alanine ligase | 396 | 3.09E-90 | 68.6 | F:nucleotide binding; P:cell wall organization or biogenesis; P:cellular component organization or biogenesis; P:biosynthetic process; P:single-organism cellular process; F:ligase activity |
| Efet.01.1639631.g210.t1 | bromodomain and WD repeat-containing 3 | 222 | 8.64E-11 | 77.9 |  |
| Efet.01.1639754.g211.t1 | G3E family | 210 | 1.10E-28 | 78.25 |  |
| Efet.01.1639813.g213.t1 | AAA family ATPase | 267 | 3.13E-24 | 84.75 |  |
| Efet.01.163991.g706.t1 | dynein heavy chain axonemal-like | 1119 | 8.67E-102 | 79.35 | F:microtubule motor activity; F:calcium ion binding; C:dynein complex; P:microtubule-based movement |
| Efet.01.1639979.g217.t1 | transcriptional regulator | 261 | 1.11E-30 | 71.95 | F:DNA binding |
| Efet.01.163999.g708.t1 | apoptosis-stimulating of p53 1 isoform X3 | 2085 | 1.00E-38 | 80.4 |  |
| Efet.01.1640158.g218.t1 | hypothetical protein HELRODRAFT_162389 | 318 | 1.37E-39 | 75 |  |
| Efet.01.1640270.g219.t1 | phosphonate ABC transporter ATP-binding | 249 | 3.15E-48 | 87.7 | F:ATP binding; P:organic phosphonate transport; P:organophosphate ester transport; F:ATPase-coupled organic phosphonate transmembrane transporter activity; C:ATP-binding cassette (ABC) transporter complex |
| Efet.01.1640504.g221.t1 | ATPase | 201 | 8.94E-22 | 75.55 |  |
| Efet.01.164062.g711.t1 | muscle calcium channel subunit alpha-1 isoform X12 | 546 | 1.18E-82 | 85.9 | C:voltage-gated calcium channel complex; P:regulation of ion transmembrane transport; P:calcium ion transmembrane transport; F:voltage-gated calcium channel activity |
| Efet.01.1640710.g224.t1 | neuronal calcium sensor 2 | 387 | 4.79E-50 | 67.25 | F:calcium ion binding |
| Efet.01.1640726.g225.t1 | molybdopterin oxidoreductase | 327 | 2.38E-41 | 73.75 | F:oxidoreductase activity; P:oxidation-reduction process |
| Efet.01.164073.g712.t1 | ADP-ribosylation factor GTPase-activating 2-like isoform X2 | 387 | 5.86E-24 | 66.65 | F:GTPase activator activity; P:positive regulation of GTPase activity |
| Efet.01.1640802.g226.t1 | probable cationic amino acid transporter | 333 | 2.28E-18 | 68.7 | C:membrane |
| Efet.01.1640932.g227.t1 | hypothetical protein CAPTEDRAFT_219222 | 405 | 5.11E-06 | 54 | C:membrane; C:integral component of membrane |
| Efet.01.1641038.g228.t1 | Ankyrin repeat and FYVE domain-containing 1 | 243 | 3.06E-21 | 71.4 | C:endosome; P:vesicle-mediated transport; P:cellular localization; C:membrane; F:ion binding |
| Efet.01.1641110.g229.t1 | potential E3 ubiquitin- ligase ariadne-2-like | 258 | 9.54E-11 | 74.64 | C:nucleus; F:zinc ion binding; F:ubiquitin conjugating enzyme binding; F:ubiquitin protein ligase activity; C:ubiquitin ligase complex; C:cytoplasm; P:protein polyubiquitination; P:positive regulation of proteasomal ubiquitin-dependent protein catabolic process; P:protein ubiquitination involved in ubiquitin-dependent protein catabolic process |
| Efet.01.1641193.g231.t1 | dynein heavy chain axonemal-like isoform X2 | 258 | 3.35E-26 | 81.1 | F:ATPase activity; F:ATP binding; F:microtubule motor activity; C:dynein complex; P:microtubule-based movement |
| Efet.01.164135.g713.t1 | phosphatidylinositol 3-kinase regulatory subunit alpha isoform X3 | 1527 | 4.74E-90 | 56.9 | C:phosphatidylinositol 3-kinase complex; F:phosphatidylinositol 3-kinase regulator activity; F:metal ion binding; P:signal transduction; P:regulation of phosphatidylinositol 3-kinase activity; P:phosphorylation; F:kinase activity; P:intracellular signal transduction; C:intracellular |
| Efet.01.1641399.g235.t1 | charged multivesicular body 4b | 240 | 1.17E-11 | 80.45 | P:vacuolar transport; C:intracellular |
| Efet.01.1641483.g236.t1 | Trimeric intracellular cation channel type B-A | 261 | 2.71E-07 | 67.05 | P:monovalent inorganic cation transport; F:cation channel activity; C:membrane; P:cation transmembrane transport; C:integral component of membrane |
| Efet.01.1641603.g237.t1 | family transcriptional regulator | 363 | 3.94E-62 | 69.95 | F:DNA binding; F:transcription factor activity, sequence-specific DNA binding; P:transcription, DNA-templated; P:regulation of transcription, DNA-templated |
| Efet.01.1641636.g238.t1 | DNA topoisomerase IV subunit A | 237 | 2.01E-44 | 81.45 | F:DNA binding; F:ATP binding; C:chromosome; F:DNA topoisomerase type II (ATP-hydrolyzing) activity; P:DNA topological change |
| Efet.01.164166.g714.t1 | F-box LRR-repeat 5-like | 372 | 9.49E-32 | 73.1 | P:iron ion homeostasis; C:perinuclear region of cytoplasm; F:iron ion binding; P:positive regulation of cellular protein catabolic process; P:SCF-dependent proteasomal ubiquitin-dependent protein catabolic process; C:SCF ubiquitin ligase complex; P:protein ubiquitination |
| Efet.01.1641752.g240.t1 | phosphoserine transaminase | 324 | 1.44E-57 | 84.55 | F:pyridoxal phosphate binding; P:pyridoxine biosynthetic process; C:cytoplasm; F:O-phospho-L-serine:2-oxoglutarate aminotransferase activity; P:L-serine biosynthetic process |
| Efet.01.1641790.g241.t1 | RIB43A-like with coiled-coils 2 | 240 | 1.61E-31 | 86.3 |  |
| Efet.01.16418.g1213.t1 | dimethyladenosine transferase mitochondrial | 750 | 8.63E-119 | 78.4 | F:DNA binding; F:RNA binding; C:mitochondrial matrix; P:transcription, DNA-templated; P:rRNA methylation; P:regulation of transcription, DNA-templated; F:rRNA (adenine-N6,N6-)-dimethyltransferase activity |
| Efet.01.164183.g715.t1 | RNA polymerase I-specific transcription initiation factor RRN3 | 1662 | 2.21E-109 | 53.9 | P:cellular process |
| Efet.01.164184.g716.t1 | neuronal calcium sensor 2 | 357 | 3.36E-47 | 68.5 | F:calcium ion binding |
| Efet.01.1641975.g244.t1 | MFS transporter | 435 | 7.32E-62 | 72.6 | P:transmembrane transport; C:integral component of membrane |
| Efet.01.1642001.g245.t1 | E3 ubiquitin- ligase HERC2-like isoform X3 | 258 | 2.47E-06 | 63 | F:ubiquitin-protein transferase activity; C:nucleus; F:zinc ion binding; C:cytoplasm; F:metal ion binding; P:protein ubiquitination involved in ubiquitin-dependent protein catabolic process; P:protein ubiquitination |
| Efet.01.1642005.g246.t1 | hypothetical protein B5766_06910 | 231 | 8.80E-44 | 98 |  |
| Efet.01.1642157.g247.t1 | helix-turn-helix transcriptional regulator | 234 | 1.83E-23 | 69.4 | F:DNA binding |
| Efet.01.1642204.g249.t1 | plexin-A2-like isoform X1 | 276 | 5.87E-17 | 65 | P:regulation of locomotion; C:integral component of membrane; P:axon guidance; P:regulation of cellular component movement; P:semaphorin-plexin signaling pathway |
| Efet.01.1642265.g251.t1 | synaptotagmin-7-like isoform X2 | 276 | 6.75E-24 | 73.45 | C:membrane; P:exocytosis |
| Efet.01.1642306.g252.t1 | DUF2147 domain-containing | 378 | 1.02E-84 | 89.85 |  |
| Efet.01.164231.g720.t1 | enoyl- delta isomerase peroxisomal-like | 369 | 6.11E-23 | 61.1 | F:catalytic activity; P:metabolic process; C:membrane; C:integral component of membrane |
| Efet.01.1642338.g254.t1 | potassium voltage-gated channel Shab | 213 | 5.45E-15 | 82.85 | P:potassium ion transmembrane transport; C:voltage-gated potassium channel complex; F:delayed rectifier potassium channel activity; P:regulation of ion transmembrane transport; P:protein homooligomerization |
| Efet.01.1642340.g255.t1 | tRNA (adenine(58)-N(1))-methyltransferase catalytic subunit TRMT61A-like | 333 | 2.06E-46 | 81.45 | C:nucleus; C:tRNA (m1A) methyltransferase complex; F:tRNA (adenine-N1-)-methyltransferase activity; P:tRNA methylation |
| Efet.01.1642449.g258.t1 | hypothetical protein HELRODRAFT_170257 | 219 | 7.07E-08 | 65.5 | C:membrane |
| Efet.01.1642510.g260.t1 | UPF0182 family | 246 | 3.36E-48 | 89.35 | C:integral component of membrane; C:plasma membrane |
| Efet.01.164265.g721.t1 | peroxidasin homolog isoform X2 | 852 | 6.60E-117 | 71.35 | F:peroxidase activity; P:response to oxidative stress; C:extracellular space; C:extracellular exosome; P:extracellular matrix organization; P:cellular oxidant detoxification; C:endoplasmic reticulum; P:oxidation-reduction process; C:extracellular matrix; F:extracellular matrix structural constituent; F:heme binding; P:hydrogen peroxide catabolic process |
| Efet.01.1642716.g263.t1 | hypothetical protein B5766_10745 | 276 | 2.18E-56 | 100 |  |
| Efet.01.1642751.g264.t1 | hypothetical protein B5766_09700 | 246 | 1.90E-50 | 63.7 | F:hydrolase activity |
| Efet.01.16428.g1214.t1 | neo-calmodulin-like isoform X4 | 297 | 6.28E-20 | 81 | F:calcium ion binding |
| Efet.01.1642880.g267.t1 | 3-deoxy-D-manno-octulosonic acid transferase | 435 | 2.34E-101 | 66.6 | F:transferase activity |
| Efet.01.1642882.g268.t1 | methyltransferase NSUN7 | 285 | 2.72E-18 | 63.6 | F:methyltransferase activity; F:transferase activity; P:methylation |
| Efet.01.164289.g722.t1 | coiled-coil domain-containing 102A | 672 | 2.62E-16 | 74.45 | C:myosin complex; F:motor activity |
| Efet.01.1643010.g269.t1 | acid trehalase 1 | 270 | 3.69E-30 | 67.8 | F:G-protein coupled receptor activity; P:carbohydrate metabolic process; P:G-protein coupled receptor signaling pathway; F:catalytic activity; C:integral component of membrane |
| Efet.01.1643086.g270.t1 | tripartite tricarboxylate transporter substrate binding | 366 | 1.66E-75 | 87.45 | C:outer membrane-bounded periplasmic space |
| Efet.01.1643152.g272.t1 | centrosomal of 290 kDa-like | 270 | 5.41E-07 | 72.38 | C:nucleus; C:centrosome; P:cilium assembly |
| Efet.01.1643236.g274.t1 | ATP-dependent DNA helicase | 363 | 1.28E-68 | 96.75 | F:DNA binding; F:ATP binding; P:DNA duplex unwinding; F:ATP-dependent DNA helicase activity; P:nucleobase-containing compound metabolic process |
| Efet.01.1643253.g275.t1 | GH3 auxin-responsive promoter | 333 | 8.00E-53 | 83.15 |  |
| Efet.01.1643329.g276.t1 | bcl-2 1 | 309 | 1.61E-24 | 60.15 | P:regulation of cellular process |
| Efet.01.1643383.g277.t1 | Sodium potassium-transporting ATPase subunit alpha-4 | 261 | 1.37E-38 | 86.7 | P:sodium ion export from cell; P:ATP hydrolysis coupled proton transport; F:sodium:potassium-exchanging ATPase activity; F:alpha-D-galactofuranose transporter activity; P:cellular sodium ion homeostasis; P:brain morphogenesis; C:integral component of plasma membrane; F:ATP binding; P:cellular potassium ion homeostasis; P:histone lysine methylation; P:otolith development; P:regulation of cellular pH; P:bract development; P:cardiocyte differentiation; P:spermatogenesis; P:embryonic body morphogenesis; P:fertilization; P:midbrain-hindbrain boundary development; P:embryonic heart tube morphogenesis; P:determination of left/right symmetry; P:cell migration involved in heart formation; P:ventricular system development; C:nucleus; P:flagellated sperm motility; P:regulation of membrane potential; F:metal ion binding; P:otic vesicle formation; F:histone-lysine N-methyltransferase activity; P:establishment or maintenance of transmembrane electrochemical gradient; P:extracellular matrix organization; P:potassium ion import |
| Efet.01.164342.g725.t1 | anoctamin-5-like isoform X1 | 729 | 1.43E-69 | 56.45 | C:membrane; C:integral component of membrane; F:protein dimerization activity |
| Efet.01.1643494.g280.t1 | Coatomer subunit | 345 | 4.25E-64 | 96.3 | P:retrograde vesicle-mediated transport, Golgi to ER; P:somite development; F:structural molecule activity; C:Golgi membrane; P:intra-Golgi vesicle-mediated transport; P:intracellular protein transport; C:extracellular space; P:ER to Golgi vesicle-mediated transport; C:extracellular exosome; P:notochord development; F:N-acetyltransferase activity; P:pancreatic juice secretion; C:COPI vesicle coat |
| Efet.01.1643521.g281.t1 | seleno T2-like | 318 | 4.93E-21 | 69.9 | C:membrane |
| Efet.01.1643610.g282.t1 | neural-cadherin 2 | 312 | 1.38E-34 | 64.55 | C:membrane; P:cell adhesion |
| Efet.01.1643636.g283.t1 | glycine receptor subunit alpha-3-like isoform X1 | 348 | 3.37E-28 | 71.15 | P:ion transmembrane transport; C:integral component of membrane; C:cell junction; F:extracellular ligand-gated ion channel activity; C:synapse; C:plasma membrane |
| Efet.01.164364.g727.t1 | AUGMIN subunit 2-like isoform X2 | 243 | 3.52E-08 | 76.44 | P:spindle assembly; P:centrosome organization; C:HAUS complex; P:microtubule organizing center organization |
| Efet.01.1643783.g284.t1 | DUF4191 domain-containing | 243 | 3.11E-38 | 85.25 | C:integral component of membrane |
| Efet.01.1643789.g285.t1 | ubiquinol-cytochrome c reductase cytochrome b subunit | 231 | 1.10E-43 | 93.6 | F:oxidoreductase activity; F:electron carrier activity; P:respiratory electron transport chain; C:integral component of membrane |
| Efet.01.1643836.g287.t1 | arsenate reductase | 258 | 1.24E-52 | 85.95 |  |
| Efet.01.164386.g728.t1 | adenosine deaminase CECR1 | 357 | 1.50E-16 | 60.2 | P:nucleobase-containing small molecule metabolic process; P:purine-containing compound metabolic process |
| Efet.01.164391.g729.t1 | angel homolog 2 isoform X3 | 795 | 1.53E-56 | 54.6 | P:3'-UTR-mediated mRNA stabilization; C:cytoplasm; C:Cajal body; P:negative regulation of mitotic cell cycle; F:mRNA 3'-UTR binding |
| Efet.01.1643972.g288.t1 | fructose-bisphosphatase class II | 270 | 3.48E-39 | 90.6 | P:glycerol metabolic process; P:gluconeogenesis; F:metal ion binding; F:fructose 1,6-bisphosphate 1-phosphatase activity; P:dephosphorylation |
| Efet.01.1643975.g289.t1 | glyco 3-alpha-L-fucosyltransferase A-like | 372 | 1.05E-22 | 63.3 | C:membrane; F:transferase activity |
| Efet.01.164398.g731.t1 | zinc transporter 1-like | 351 | 8.00E-27 | 60.85 | P:response to zinc ion; P:regulation of sequestering of zinc ion; P:cation transmembrane transport; F:zinc ion transmembrane transporter activity; C:plasma membrane |
| Efet.01.1644061.g290.t1 | Ras-related Rab-39B | 231 | 2.05E-35 | 88.15 | F:GTP binding; P:small GTPase mediated signal transduction; C:intracellular |
| Efet.01.1644091.g291.t1 | methyltransferase 24 | 291 | 2.74E-14 | 57.4 | C:membrane; C:integral component of membrane |
| Efet.01.1644117.g292.t1 | SEC14 1 isoform X2 | 318 | 8.59E-51 | 91.1 | C:integral component of membrane; P:transport; C:intracellular; F:transporter activity |
| Efet.01.1644166.g294.t1 | hypothetical protein BSL78_06940 | 342 | 3.87E-10 | 54.85 | C:membrane; C:integral component of membrane |
| Efet.01.164424.g732.t1 | sodium channel type 4 subunit alpha B-like isoform X1 | 528 | 7.98E-14 | 65 | P:biological regulation; P:sodium ion transport; P:ion transmembrane transport; F:sodium channel activity; C:integral component of membrane; F:voltage-gated ion channel activity |
| Efet.01.1644242.g296.t1 | uncharacterized protein LOC110048656 | 300 | 1.59E-09 | 55.2 | P:carbohydrate metabolic process; F:carbohydrate binding; F:hydrolase activity, hydrolyzing O-glycosyl compounds; F:beta-N-acetylhexosaminidase activity; F:polysaccharide binding |
| Efet.01.1644272.g297.t1 | NFX1-type zinc finger-containing 1-like | 414 | 7.14E-33 | 63.5 | C:nucleus; F:transcription factor activity, sequence-specific DNA binding; F:zinc ion binding; P:regulation of transcription, DNA-templated |
| Efet.01.1644458.g298.t1 | hypothetical protein CAPTEDRAFT_224968 | 318 | 6.21E-12 | 60 |  |
| Efet.01.1644477.g299.t1 | poly(U)-specific endoribonuclease-like | 447 | 4.40E-18 | 69.8 | P:nucleic acid phosphodiester bond hydrolysis; F:RNA binding; F:endoribonuclease activity |
| Efet.01.1644488.g300.t1 | sodium potassium adenosine | 249 | 5.84E-40 | 93.3 | C:integral component of plasma membrane; F:ATP binding; P:cellular potassium ion homeostasis; P:sodium ion export from cell; P:ATP hydrolysis coupled proton transport; F:sodium:potassium-exchanging ATPase activity; F:metal ion binding; C:intracellular membrane-bounded organelle; P:establishment or maintenance of transmembrane electrochemical gradient; P:cellular sodium ion homeostasis; P:potassium ion import |
| Efet.01.1644550.g302.t1 | soluble guanylate cyclase 88E-like isoform X1 | 336 | 9.87E-32 | 70 | P:single-organism process; P:cellular process |
| Efet.01.1644584.g303.t1 | E3 ubiquitin- ligase RNF123-like | 279 | 5.37E-26 | 69.65 | F:metal ion binding |
| Efet.01.1644605.g304.t1 | CNPV223 ankyrin repeat | 252 | 1.31E-08 | 61 | P:fungal-type cell wall organization; F:transcription factor activity, sequence-specific DNA binding; C:TORC2 complex; F:aspartic-type endopeptidase activity; C:cytoplasm; P:regulation of cell growth; P:proteolysis; P:regulation of transcription, DNA-templated; F:sequence-specific DNA binding; P:establishment or maintenance of actin cytoskeleton polarity |
| Efet.01.1644608.g305.t1 | IS630 family transposase | 201 | 1.43E-16 | 71.4 | F:nucleic acid binding |
| Efet.01.164462.g733.t1 | Homeobox Nkx- | 1194 | 3.27E-39 | 95.3 | C:nucleus; P:regulation of transcription, DNA-templated; F:sequence-specific DNA binding |
| Efet.01.1644650.g307.t1 | hypothetical protein CRN43_17300 | 327 | 1.10E-19 | 74 |  |
| Efet.01.1644717.g308.t1 | hsp90 co-chaperone Cdc37 | 255 | 2.82E-22 | 88.05 | F:protein kinase binding |
| Efet.01.164472.g735.t1 | multidrug resistance-associated 1-like | 435 | 1.80E-25 | 69.9 | F:nucleotide binding; F:ATPase activity; F:ATP binding; F:ATPase activity, coupled to transmembrane movement of substances; P:transmembrane transport; C:membrane; C:integral component of membrane; P:transport; F:transporter activity |
| Efet.01.1644723.g309.t1 | ADP-ribosylglycohydrolase family | 384 | 2.13E-83 | 65.5 |  |
| Efet.01.1644749.g310.t1 | ras family member 10B | 312 | 8.01E-22 | 68 | F:nucleotide binding; P:signal transduction |
| Efet.01.1644771.g311.t1 | ABC transporter ATP-binding | 414 | 2.60E-74 | 81 | F:ATPase activity; F:ATP binding |
| Efet.01.1644773.g312.t1 | response regulator | 201 | 4.74E-35 | 90.4 | F:DNA binding; P:phosphorelay signal transduction system; C:intracellular |
| Efet.01.1644783.g313.t1 | hypothetical protein CAPTEDRAFT_222036 | 276 | 3.46E-15 | 58.18 | C:membrane; C:integral component of membrane |
| Efet.01.1644818.g314.t1 | collagen alpha-1(VII) chain | 468 | 4.59E-32 | 55.05 | F:calcium ion binding; C:membrane; F:scavenger receptor activity; P:receptor-mediated endocytosis |
| Efet.01.1644859.g315.t1 | sidekick isoform X2 | 231 | 8.54E-11 | 57.9 |  |
| Efet.01.164496.g736.t1 | hypothetical protein CAPTEDRAFT_195684 | 486 | 6.16E-24 | 56 |  |
| Efet.01.1645055.g316.t1 | solute carrier family 25 member 45-like isoform X1 | 249 | 5.21E-19 | 67.55 | C:membrane |
| Efet.01.164514.g737.t1 | coiled-coil domain-containing 170-like | 1143 | 5.89E-54 | 57.55 |  |
| Efet.01.1645249.g319.t1 | tripartite motif-containing 2-like | 354 | 4.76E-26 | 60.67 | F:zinc ion binding; F:metal ion binding; C:intracellular |
| Efet.01.1645250.g320.t1 | glycine receptor subunit alpha-3-like isoform X1 | 228 | 1.77E-22 | 68.7 | P:ion transport; C:membrane |
| Efet.01.1645270.g321.t1 | coiled-coil domain-containing 65-like | 384 | 9.66E-29 | 87.4 |  |
| Efet.01.1645469.g323.t1 | titin isoform X2 | 252 | 6.50E-32 | 74.8 |  |
| Efet.01.164554.g740.t1 | Cyclin-dependent kinase 9 | 930 | 1.89E-153 | 89.15 | P:cell division; F:ATP binding; F:protein serine/threonine kinase activity; P:protein phosphorylation |
| Efet.01.1645560.g325.t1 | family transporter | 231 | 1.70E-44 | 78.2 | C:integral component of plasma membrane; P:transport; F:transporter activity |
| Efet.01.1645600.g326.t1 | hypothetical protein CAPTEDRAFT_223749 | 321 | 5.80E-24 | 54.93 | C:membrane; C:integral component of membrane |
| Efet.01.1645605.g327.t1 | polypeptide N-acetylgalactosaminyltransferase 6 | 444 | 5.24E-26 | 59.95 | F:transferase activity, transferring glycosyl groups; C:membrane; C:integral component of membrane; P:protein glycosylation; C:Golgi membrane; C:Golgi apparatus; F:transferase activity; F:carbohydrate binding |
| Efet.01.164567.g741.t1 | calcium channel voltage-dependent alpha2 delta subunit 1 | 594 | 3.82E-55 | 64.8 | F:binding |
| Efet.01.164571.g742.t1 | receptor tyrosine phosphatase | 204 | 9.88E-19 | 73.14 | F:protein tyrosine phosphatase activity; C:integral component of membrane; P:peptidyl-tyrosine dephosphorylation |
| Efet.01.1645757.g330.t1 | calcium calmodulin-dependent 3 ,5 -cyclic nucleotide phosphodiesterase 1A isoform X8 | 249 | 4.26E-20 | 80.65 | F:metal ion binding; P:signal transduction; C:membrane; F:3',5'-cyclic-nucleotide phosphodiesterase activity |
| Efet.01.1645765.g332.t1 | homeobox unc-4 homolog | 234 | 2.74E-12 | 99.8 | C:nucleus; P:regulation of transcription, DNA-templated; F:sequence-specific DNA binding |
| Efet.01.1645788.g333.t1 | probable G- coupled receptor 139 | 246 | 5.19E-23 | 65.15 | P:signal transduction; C:membrane; F:signal transducer activity |
| Efet.01.1645818.g334.t1 | leucine carboxyl methyltransferase | 267 | 9.20E-14 | 71.5 | P:C-terminal protein methylation; C:cytosol; F:protein C-terminal leucine carboxyl O-methyltransferase activity |
| Efet.01.164589.g744.t1 | hypothetical protein HELRODRAFT_172085 | 465 | 4.36E-18 | 60.33 | P:signal transduction |
| Efet.01.1645908.g335.t1 | phospho-N-acetylmuramoyl-pentapeptide-transferase | 495 | 7.39E-99 | 97.55 | P:regulation of cell shape; P:cell division; F:UDP-N-acetylmuramoyl-L-alanyl-D-glutamyl-meso-2,6-diaminopimelyl-D-alanyl-D-alanine:undecaprenyl-phosphate transferase activity; P:peptidoglycan biosynthetic process; C:integral component of membrane; P:cell wall organization; C:plasma membrane; P:cell cycle; F:phospho-N-acetylmuramoyl-pentapeptide-transferase activity |
| Efet.01.1645911.g336.t1 | Fe-S oxidoreductase | 297 | 6.43E-52 | 82.2 | C:integral component of membrane; F:iron-sulfur cluster binding |
| Efet.01.1645954.g337.t1 | cyclic nucleotide-gated cation channel alpha-3-like | 288 | 1.81E-52 | 90.95 |  |
| Efet.01.164603.g745.t1 | kinesin KIF21A isoform X3 | 276 | 1.17E-21 | 49.65 | F:nucleotide binding; F:ATP binding; F:microtubule motor activity; P:microtubule-based movement; F:microtubule binding |
| Efet.01.1646036.g338.t1 | hypothetical protein CRN43_14830 | 411 | 6.41E-34 | 64.35 |  |
| Efet.01.1646040.g339.t1 | sidekick-2-like isoform X3 | 330 | 5.60E-11 | 55.73 | C:membrane; C:integral component of membrane |
| Efet.01.1646133.g341.t1 | cytoplasmic dynein 1 heavy chain 1-like | 225 | 1.55E-34 | 94.1 | F:ATPase activity; F:ATP binding; F:microtubule motor activity; C:dynein complex; P:microtubule-based movement |
| Efet.01.1646205.g342.t1 | cholesterol 25-hydroxylase member 2 | 309 | 4.78E-27 | 62.7 | F:iron ion binding; F:oxidoreductase activity; C:membrane; C:integral component of membrane; P:oxidation-reduction process; P:fatty acid biosynthetic process |
| Efet.01.164621.g746.t1 | neutral alpha-glucosidase AB-like | 663 | 6.36E-67 | 65.85 | P:metabolic process; F:binding; F:hydrolase activity, acting on glycosyl bonds |
| Efet.01.1646325.g346.t1 | homeobox Hox-A1-like | 300 | 6.87E-58 | 86.45 | C:nucleus; P:regulation of transcription, DNA-templated; F:sequence-specific DNA binding |
| Efet.01.1646516.g349.t1 | pfkB carbohydrate kinase family | 330 | 9.76E-63 | 69.4 | F:transferase activity |
| Efet.01.16466.g1215.t1 | homeobox prospero | 417 | 4.26E-23 | 96.9 | F:DNA binding; C:integral component of membrane |
| Efet.01.1646605.g351.t1 | beta keto-acyl synthase | 375 | 2.73E-74 | 73.7 | F:catalytic activity; P:metabolic process; F:transferase activity |
| Efet.01.1646664.g352.t1 | armadillo segment polarity isoform X3 | 243 | 1.24E-23 | 67.55 | C:intracellular part; P:single-organism process; P:cellular process |
| Efet.01.1646673.g353.t1 | pyridine nucleotide-disulfide oxidoreductase | 231 | 4.26E-26 | 80 | F:oxidoreductase activity; P:oxidation-reduction process |
| Efet.01.1646823.g357.t1 | synaptotagmin 4 | 333 | 2.98E-37 | 71.35 | C:membrane |
| Efet.01.1646835.g358.t1 | transcriptional family | 435 | 6.15E-94 | 88.95 | F:DNA binding; F:transcription factor activity, sequence-specific DNA binding; F:pyridoxal phosphate binding; F:L-phenylalanine:2-oxoglutarate aminotransferase activity; P:transcription, DNA-templated; P:regulation of transcription, DNA-templated; F:L-aspartate:2-oxoglutarate aminotransferase activity |
| Efet.01.1646855.g359.t1 | Transcriptional regulator | 219 | 5.49E-42 | 71.5 | F:carbohydrate binding |
| Efet.01.1646861.g360.t1 | Clavaminate synthase | 279 | 8.41E-12 | 57.1 | F:dioxygenase activity; F:oxidoreductase activity; P:oxidation-reduction process |
| Efet.01.1646869.g361.t1 | low-density lipo receptor-related 8 isoform X2 | 216 | 8.96E-16 | 67.1 | F:apolipoprotein binding; F:low-density lipoprotein receptor activity; P:regulation of transcription, DNA-templated; C:receptor complex; P:cell surface receptor signaling pathway; P:endocytosis; P:positive regulation of dendrite development; P:pallium development; P:positive regulation of molecular function; C:caveola; F:transmembrane signaling receptor activity; F:very-low-density lipoprotein particle receptor activity; P:retinoid metabolic process; P:cellular response to organic substance; C:neuron part; C:integral component of membrane; P:positive regulation of peptidyl-tyrosine phosphorylation |
| Efet.01.1646877.g362.t1 | SURF1 family | 375 | 1.10E-78 | 79.1 | C:integral component of membrane |
| Efet.01.1646950.g364.t1 | ABC transporter permease | 315 | 6.03E-47 | 85.55 | F:strictosidine synthase activity; C:integral component of membrane; P:carbohydrate transport; P:biosynthetic process; C:plasma membrane; F:transporter activity |
| Efet.01.1646951.g365.t1 | potassium transporter | 309 | 7.50E-54 | 94.1 | P:potassium ion transport; F:solute:proton antiporter activity; C:integral component of membrane; P:hydrogen ion transmembrane transport; C:plasma membrane |
| Efet.01.1647088.g366.t1 | proline dehydrogenase mitochondrial isoform X1 | 378 | 2.71E-14 | 70.15 | P:single-organism metabolic process |
| Efet.01.1647127.g367.t1 | hypothetical protein CAPTEDRAFT_222159 | 243 | 1.54E-14 | 60.57 | C:membrane; C:integral component of membrane |
| Efet.01.1647142.g368.t1 | potassium sodium hyperpolarization-activated cyclic nucleotide-gated channel 4 | 330 | 4.65E-11 | 76.05 | F:voltage-gated potassium channel activity; P:potassium ion transmembrane transport; C:integral component of membrane |
| Efet.01.1647161.g369.t1 | probable 39S ribosomal mitochondrial | 315 | 6.08E-30 | 59.3 | C:intracellular organelle; C:intracellular ribonucleoprotein complex; C:cytoplasmic part |
| Efet.01.1647181.g370.t1 | zinc finger SWIM domain-containing 8-like | 315 | 2.29E-30 | 91.9 | F:zinc ion binding |
| Efet.01.1647190.g371.t1 | DUF1501 domain-containing | 291 | 1.39E-52 | 91.1 | F:catalytic activity; P:metabolic process |
| Efet.01.1647192.g372.t1 | 3-oxoadipyl- thiolase | 276 | 1.48E-55 | 96.25 | F:acetyl-CoA C-acyltransferase activity; P:3,4-dihydroxybenzoate catabolic process; F:3-oxoadipyl-CoA thiolase activity |
| Efet.01.1647246.g373.t1 | phosphatidylinositol 5-phosphate 4-kinase type-2 beta isoform X1 | 276 | 3.35E-06 | 54.59 | F:phosphatidylinositol phosphate kinase activity; P:phosphatidylinositol phosphorylation; P:phosphatidylinositol metabolic process; P:phosphorylation; F:kinase activity |
| Efet.01.1647264.g374.t1 | PIN domain | 471 | 2.08E-111 | 73.4 |  |
| Efet.01.1647439.g378.t1 | sacsin-like | 372 | 4.21E-19 | 58.35 |  |
| Efet.01.164747.g749.t1 | PR domain zinc finger 14-like | 222 | 2.27E-21 | 79.95 | F:nucleic acid binding; F:metal ion binding |
| Efet.01.164747.g750.t1 | PR domain zinc finger 14-like | 348 | 7.96E-44 | 84.85 | F:nucleic acid binding; C:nucleus; F:metal ion binding; P:axonogenesis |
| Efet.01.1647493.g379.t1 | hypothetical protein CAPTEDRAFT_192565 | 351 | 8.56E-51 | 69.67 |  |
| Efet.01.1647571.g381.t1 | acetoacetyl- synthetase | 303 | 1.64E-53 | 89.5 | F:acetoacetate-CoA ligase activity; P:lipid metabolic process |
| Efet.01.1647694.g383.t1 | ankyrin repeat domain-containing 46-like | 354 | 2.98E-12 | 55.5 | C:membrane; C:integral component of membrane |
| Efet.01.1647703.g384.t1 | endoplasmic reticulum mannosyl-oligosaccharide 1,2-alpha-mannosidase | 354 | 1.33E-30 | 74.2 | C:membrane; F:hydrolase activity, acting on glycosyl bonds |
| Efet.01.1647785.g386.t1 | nuclear hormone receptor HR96 isoform X11 | 243 | 1.88E-19 | 91 |  |
| Efet.01.164792.g752.t1 | glyco 3-alpha-L-fucosyltransferase A isoform X1 | 366 | 1.21E-42 | 71.6 | P:glycosylation; F:transferase activity, transferring glycosyl groups; C:membrane; C:Golgi apparatus |
| Efet.01.1647925.g389.t1 | helicase-like transcription factor | 222 | 7.56E-18 | 84.05 | F:nucleic acid binding; F:ATPase activity; C:nucleus; F:ATP binding; F:zinc ion binding; F:ubiquitin protein ligase activity; P:protein polyubiquitination; F:helicase activity; P:postreplication repair; P:regulation of transcription from RNA polymerase II promoter |
| Efet.01.1648008.g390.t1 | beta-1,3-galactosyl-O-glycosyl-glyco beta-1,6-N-acetylglucosaminyltransferase 3-like | 387 | 9.27E-51 | 71.3 | F:acetylglucosaminyltransferase activity; C:membrane |
| Efet.01.1648029.g391.t1 | FMRF-amide neuropeptide | 396 | 8.56E-33 | 60.64 | P:neuropeptide signaling pathway; C:extracellular region |
| Efet.01.1648054.g396.t1 | hypothetical protein HELRODRAFT_159014 | 321 | 6.29E-16 | 59.64 | F:ion binding |
| Efet.01.164807.g753.t1 | synaptotagmin 7d | 390 | 7.00E-39 | 83.9 | C:presynaptic membrane; C:neuronal cell body; P:regulation of glucagon secretion; C:dendrite; F:phosphatidylinositol-4,5-bisphosphate binding; P:regulation of insulin secretion; C:early phagosome; F:phosphatidylserine binding; C:extracellular exosome; C:terminal bouton; F:calcium ion binding; C:cytosol; P:short-term synaptic potentiation; P:calcium ion regulated lysosome exocytosis; P:vesicle-mediated cholesterol transport; P:regulation of dopamine secretion; P:synaptic vesicle recycling; F:syntaxin binding; P:phagosome-lysosome fusion; C:lysosomal membrane; F:calcium-dependent phospholipid binding; C:peroxisomal membrane; F:calmodulin binding; P:calcium ion-regulated exocytosis of neurotransmitter; P:plasma membrane repair; P:regulation of bone remodeling; C:integral component of membrane; C:cell junction; C:phagocytic vesicle membrane; C:synaptic vesicle membrane; F:clathrin binding; P:regulation of calcium ion-dependent exocytosis; P:regulation of phagocytosis |
| Efet.01.1648084.g397.t1 | trafficking particle complex subunit 1 | 438 | 6.54E-76 | 84.65 | C:TRAPP complex; P:ER to Golgi vesicle-mediated transport; C:cytoplasm; F:Rab guanyl-nucleotide exchange factor activity; P:positive regulation of GTPase activity |
| Efet.01.1648100.g398.t1 | methionine synthase | 414 | 3.88E-59 | 80.2 | P:methionine biosynthetic process; P:pteridine-containing compound metabolic process; F:methionine synthase activity; F:zinc ion binding; F:cobalamin binding; C:intracellular; F:S-adenosylmethionine-homocysteine S-methyltransferase activity; P:methylation |
| Efet.01.1648211.g400.t1 | aquaporin-9 | 210 | 4.65E-22 | 79.8 | P:inorganic anion transport; F:glycerol transmembrane transporter activity; P:water transport; P:glycerol transport; F:water transmembrane transporter activity; P:urea transmembrane transport; C:integral component of membrane; F:arsenite transmembrane transporter activity; F:urea transmembrane transporter activity |
| Efet.01.1648222.g401.t1 | phosphoenolpyruvate carboxylase | 453 | 5.40E-98 | 69.5 | F:catalytic activity |
| Efet.01.1648258.g402.t1 | prenyltransferase family | 495 | 2.03E-111 | 56.09 | C:membrane; C:integral component of membrane |
| Efet.01.164833.g754.t1 | alpha-2A adrenergic receptor-like | 969 | 1.28E-98 | 74.8 | C:integral component of plasma membrane; F:adrenergic receptor activity; P:adenylate cyclase-activating adrenergic receptor signaling pathway; P:cell-cell signaling; F:transferase activity |
| Efet.01.1648381.g406.t1 | ATP-binding | 510 | 8.01E-103 | 64.7 | F:ATP binding |
| Efet.01.1648452.g407.t1 | hypothetical protein B5766_11380 | 321 | 1.39E-56 | 71.67 |  |
| Efet.01.1648475.g408.t1 | dynein heavy chain axonemal-like | 246 | 3.57E-33 | 84.3 | F:ATPase activity; F:ATP binding; F:microtubule motor activity; C:dynein complex; P:microtubule-based movement |
| Efet.01.1648483.g409.t1 | tetratricopeptide repeat 25 isoform X2 | 333 | 6.24E-06 | 63 |  |
| Efet.01.1648513.g410.t1 | phosphatase 1 regulatory subunit 32-like | 309 | 3.69E-25 | 74.5 |  |
| Efet.01.1648606.g412.t1 | hypothetical protein HELRODRAFT_190829 | 357 | 4.83E-16 | 65.5 | F:metal ion binding |
| Efet.01.1648656.g414.t1 | isotocin receptor-like | 264 | 1.09E-11 | 78.05 | C:integral component of plasma membrane; P:G-protein coupled receptor signaling pathway; F:vasopressin receptor activity; P:oviposition; P:response to peptide; F:peptide binding; P:cellular response to hormone stimulus; F:protein-hormone receptor activity |
| Efet.01.1648752.g415.t1 | homoserine kinase | 213 | 3.00E-40 | 91.3 | F:homoserine kinase activity; F:ATP binding; C:cytoplasm; P:threonine biosynthetic process; P:phosphorylation |
| Efet.01.1648811.g417.t1 | zinc finger and BTB domain-containing 9 | 420 | 1.45E-15 | 55.45 | F:nucleic acid binding; F:metal ion binding |
| Efet.01.1648839.g418.t1 | twitchin isoform X8 | 288 | 3.03E-10 | 56.1 | F:nucleotide binding; F:ATP binding; F:protein kinase activity; P:phosphorylation; F:transferase activity; P:protein phosphorylation; F:kinase activity |
| Efet.01.1648887.g419.t1 | DNA replication licensing factor mcm4-A-like | 372 | 6.92E-58 | 86.5 | C:nucleus; F:DNA binding; F:ATP binding; P:DNA replication initiation; F:DNA helicase activity; P:DNA duplex unwinding; C:MCM complex |
| Efet.01.1648930.g421.t1 | alpha-amylase 2-like | 219 | 3.38E-27 | 78.8 | F:alpha-amylase activity; P:carbohydrate metabolic process; F:cation binding |
| Efet.01.1648952.g422.t1 | goliath isoform X1 | 267 | 2.63E-26 | 76.3 | F:zinc ion binding; C:integral component of membrane; F:ligase activity |
| Efet.01.1648991.g423.t1 | family transcriptional regulator | 228 | 1.07E-42 | 75.7 | F:DNA binding; F:transcription factor activity, sequence-specific DNA binding; P:transcription, DNA-templated; P:regulation of transcription, DNA-templated |
| Efet.01.1648997.g424.t1 | aspartate aminotransferase family | 504 | 9.94E-106 | 76.5 | F:transaminase activity |
| Efet.01.1649042.g425.t1 | D-arabino 3-hexulose 6-phosphate aldehyde lyase | 363 | 3.42E-77 | 68.75 | F:catalytic activity |
| Efet.01.1649088.g426.t1 | hypothetical protein CAPTEDRAFT_223170 | 456 | 3.95E-20 | 81 | C:integral component of membrane |
| Efet.01.1649099.g427.t1 | peptidase S8 | 318 | 4.57E-51 | 64.4 | F:hydrolase activity |
| Efet.01.1649133.g428.t1 | MFS transporter | 402 | 8.79E-74 | 62.1 | P:transmembrane transport; C:membrane; C:integral component of membrane |
| Efet.01.164922.g757.t1 | spastin isoform X1 | 489 | 2.35E-27 | 54.45 | F:nucleotide binding; F:ATP binding; C:membrane; C:integral component of membrane |
| Efet.01.1649227.g430.t1 | fructose-bisphosphate | 297 | 5.48E-54 | 79.4 | P:glycolytic process; F:fructose-bisphosphate aldolase activity |
| Efet.01.1649255.g431.t1 | fibrinogen 1 | 330 | 3.46E-41 | 71.9 | P:system development |
| Efet.01.1649327.g433.t1 | serine O-acetyltransferase | 270 | 2.95E-58 | 81.75 | C:cytoplasm; F:serine O-acetyltransferase activity; P:cysteine biosynthetic process from serine |
| Efet.01.1649379.g434.t1 | dystonin-like isoform X1 | 300 | 3.38E-23 | 68.3 | P:cell cycle arrest; C:cytoskeleton; F:calcium ion binding |
| Efet.01.1649392.g435.t1 | TNF receptor-associated factor 2-like | 309 | 1.79E-54 | 89.05 | F:ubiquitin-protein transferase activity; F:zinc ion binding; P:signal transduction; P:regulation of apoptotic process; P:protein ubiquitination |
| Efet.01.1649484.g438.t1 | glycoside hydrolase family | 318 | 3.89E-41 | 57.65 | P:carbohydrate metabolic process; F:hydrolase activity, hydrolyzing O-glycosyl compounds; F:hydrolase activity |
| Efet.01.1649509.g439.t1 | soluble pyridine nucleotide transhydrogenase | 405 | 5.18E-70 | 85.85 | C:cytoplasm; F:flavin adenine dinucleotide binding; F:NAD(P)+ transhydrogenase (B-specific) activity; P:hydrogen ion transmembrane transport; P:cell redox homeostasis; P:oxidation-reduction process |
| Efet.01.1649513.g440.t1 | allatostatin-A receptor-like | 519 | 9.52E-23 | 72.15 | F:G-protein coupled receptor activity; P:G-protein coupled receptor signaling pathway; C:integral component of membrane |
| Efet.01.1649523.g442.t1 | hypothetical protein | 264 | 1.71E-41 | 89.5 |  |
| Efet.01.1649552.g444.t1 | hypothetical protein | 477 | 1.46E-20 | 63.25 |  |
| Efet.01.1649555.g445.t1 | family nutrient uptake outer membrane | 330 | 1.64E-69 | 71.95 |  |
| Efet.01.1649609.g448.t1 | N-acetylmannosamine-6-phosphate 2-epimerase | 309 | 7.38E-18 | 59.35 | P:organic substance metabolic process; F:isomerase activity |
| Efet.01.1649692.g450.t1 | beta-1-syntrophin-like | 381 | 6.93E-14 | 55.64 | P:synapse organization; C:dystrophin-associated glycoprotein complex; F:structural molecule activity |
| Efet.01.1649724.g452.t1 | FAR-RED ELONGATED HYPOCOTYL 3-like | 360 | 4.42E-21 | 56.39 | F:zinc ion binding; F:metal ion binding |
| Efet.01.1649875.g453.t1 | neo-calmodulin-like isoform X4 | 207 | 1.31E-21 | 82.55 | F:calcium ion binding |
| Efet.01.1649902.g455.t1 | fibrinogen C domain-containing 1-like | 399 | 4.85E-19 | 56.2 |  |
| Efet.01.1649921.g456.t1 | Wnt-4 isoform 2 precursor | 489 | 1.09E-55 | 69.2 | C:extracellular space; F:frizzled binding; P:nephrostome development; P:neuron differentiation; P:embryonic pattern specification; P:glomus development; P:cell fate commitment; P:Wnt signaling pathway; P:pronephric nephron tubule development |
| Efet.01.1649994.g457.t1 | hypothetical protein CAPTEDRAFT_136979 | 543 | 2.86E-65 | 51.15 |  |
| Efet.01.1650013.g459.t1 | FMRFamide receptor-like | 525 | 1.87E-37 | 63.15 | F:G-protein coupled receptor activity; P:signal transduction; C:membrane |
| Efet.01.1650034.g460.t1 | Kyphoscoliosis peptidase | 393 | 1.40E-14 | 57.85 |  |
| Efet.01.1650052.g461.t1 | hypothetical protein CAPTEDRAFT_102797, partial | 465 | 4.53E-68 | 68.85 | C:integral component of membrane |
| Efet.01.1650074.g462.t1 | family nutrient uptake outer membrane | 375 | 4.58E-39 | 66.55 |  |
| Efet.01.1650088.g463.t1 | Zinc finger FYVE domain-containing | 321 | 2.33E-28 | 76.5 | F:metal ion binding |
| Efet.01.16501.g1217.t1 | glucose dehydrogenase [ quinone] | 819 | 1.88E-134 | 62.45 | F:oxidoreductase activity, acting on CH-OH group of donors; F:flavin adenine dinucleotide binding; C:integral component of membrane; P:oxidation-reduction process |
| Efet.01.1650143.g465.t1 | EH domain-containing 3 isoform X2 | 456 | 1.58E-59 | 78.65 | F:GTP binding; F:calcium ion binding; C:intracellular; P:endocytic recycling |
| Efet.01.1650173.g466.t1 | rho GTPase-activating 45-like isoform X3 | 216 | 1.09E-06 | 58.43 |  |
| Efet.01.1650250.g467.t1 | hypothetical protein CAPTEDRAFT_187300 | 312 | 3.81E-12 | 53.92 | C:membrane; C:integral component of membrane; F:carbohydrate binding |
| Efet.01.1650306.g468.t1 | tau-tubulin kinase homolog Asator isoform X7 | 384 | 1.04E-46 | 75.85 | F:ATP binding; F:protein kinase activity; P:protein phosphorylation |
| Efet.01.1650343.g469.t1 | recombinase | 279 | 1.36E-44 | 85.6 | F:DNA binding; P:nucleic acid phosphodiester bond hydrolysis; F:nuclease activity |
| Efet.01.1650365.g470.t1 | tripartite tricarboxylate transporter substrate binding | 408 | 2.75E-71 | 75.15 | C:outer membrane-bounded periplasmic space |
| Efet.01.1650384.g472.t1 | pantothenate kinase 3-like | 438 | 1.43E-56 | 73.2 | P:regulation of bile acid metabolic process; P:spermatid development; F:pantothenate kinase activity; F:ATP binding; P:mitochondrion morphogenesis; P:regulation of mitochondrial membrane potential; C:mitochondrion; P:coenzyme A biosynthetic process; C:cytosol; P:phosphorylation; P:aerobic respiration; P:regulation of triglyceride metabolic process; P:regulation of fatty acid metabolic process |
| Efet.01.1650422.g473.t1 | hypothetical protein CRN43_00975 | 378 | 3.13E-85 | 69.3 |  |
| Efet.01.1650446.g474.t1 | hypothetical protein HELRODRAFT_180234 | 441 | 7.07E-06 | 60 | P:sodium ion transport; P:ion transport; F:sodium channel activity; C:membrane; C:integral component of membrane; P:sodium ion transmembrane transport; P:transport |
| Efet.01.1650507.g475.t1 | glycogen liver form- | 255 | 2.76E-27 | 80.3 | F:glycogen phosphorylase activity; F:pyridoxal phosphate binding; F:AMP binding; F:protein homodimerization activity; F:drug binding; F:purine nucleobase binding; F:bile acid binding; P:glycogen catabolic process; C:plasma membrane; P:5-phosphoribose 1-diphosphate biosynthetic process; F:ATP binding; C:extracellular exosome; C:cytoplasm; P:necroptotic process; P:glucose homeostasis; F:carbohydrate binding; F:vitamin binding |
| Efet.01.165051.g760.t1 | PREDICTED: uncharacterized protein C18orf8-like | 378 | 7.53E-12 | 73.75 |  |
| Efet.01.1650523.g477.t1 | fibrinogen related domain variant | 312 | 7.81E-12 | 76.3 | C:integral component of membrane |
| Efet.01.1650560.g478.t1 | DUF898 domain-containing | 387 | 1.25E-61 | 54.55 | C:integral component of membrane |
| Efet.01.1650564.g479.t1 | cilia- and flagella-associated 44-like | 408 | 8.53E-26 | 80.2 |  |
| Efet.01.1650599.g480.t1 | hypothetical protein CAPTEDRAFT_209714 | 243 | 7.31E-22 | 66.95 | C:membrane |
| Efet.01.1650606.g481.t1 | type II secretion system F | 480 | 9.01E-100 | 95.2 | C:integral component of membrane |
| Efet.01.1650614.g483.t1 | hypothetical protein CAPTEDRAFT_200338 | 330 | 2.82E-11 | 79 |  |
| Efet.01.1650683.g484.t1 | delta-1-pyrroline-5-carboxylate synthase | 258 | 3.94E-20 | 84.25 | F:glutamate 5-kinase activity; C:mitochondrion; F:glutamate-5-semialdehyde dehydrogenase activity; P:proline biosynthetic process; P:oxidation-reduction process; P:phosphorylation |
| Efet.01.1650758.g486.t1 | myosin-VIIa-like | 270 | 8.33E-44 | 88.15 | C:myosin complex; F:ATP binding; F:actin binding; C:integral component of membrane; F:motor activity |
| Efet.01.1650866.g488.t1 | formamidopyrimidine-DNA glycosylase | 276 | 7.77E-59 | 81.95 | F:zinc ion binding; P:base-excision repair; P:nucleotide-excision repair; F:damaged DNA binding; F:oxidized purine nucleobase lesion DNA N-glycosylase activity |
| Efet.01.1650919.g489.t1 | tigger transposable element-derived 6 | 372 | 8.75E-20 | 62.2 | F:nucleic acid binding; F:DNA binding |
| Efet.01.1650954.g491.t1 | restriction endonuclease subunit R | 372 | 2.00E-60 | 74.8 | F:DNA binding; F:ATP binding; F:deoxyribonuclease activity; P:DNA metabolic process |
| Efet.01.1650983.g493.t1 | hypothetical protein HELRODRAFT_171217 | 378 | 1.21E-20 | 57 |  |
| Efet.01.1651011.g494.t1 | tRNA preQ1(34) S-adenosylmethionine ribosyltransferase-isomerase | 258 | 4.05E-35 | 85.9 | P:queuosine biosynthetic process; C:cytoplasm; F:transferase activity; F:isomerase activity |
| Efet.01.1651032.g495.t1 | von Willebrand factor A domain-containing 8 | 339 | 4.41E-34 | 80.4 | F:ATPase activity; F:ATP binding; C:mitochondrion |
| Efet.01.1651061.g497.t1 | hypothetical protein Veis_0715 | 336 | 1.16E-26 | 97 |  |
| Efet.01.1651071.g498.t1 | dynein heavy chain axonemal | 348 | 6.56E-41 | 81.15 | F:microtubule motor activity; F:calcium ion binding; C:dynein complex; P:microtubule-based movement |
| Efet.01.165110.g761.t1 | short transient receptor potential channel 3-like | 366 | 1.41E-18 | 70.21 | F:calcium channel activity; C:integral component of membrane; P:calcium ion transmembrane transport |
| Efet.01.1651153.g500.t1 | polyketide synthase | 279 | 3.84E-53 | 58.53 | F:transferase activity |
| Efet.01.1651186.g501.t1 | ATP-binding cassette sub-family G member 1-like isoform X1 | 279 | 8.79E-34 | 81.1 | F:ATPase activity; F:ATP binding; F:zinc ion binding; C:integral component of membrane |
| Efet.01.1651240.g503.t1 | cyclic nucleotide-gated cation channel alpha-3-like | 249 | 3.13E-12 | 68.38 | F:voltage-gated potassium channel activity; P:potassium ion transmembrane transport; C:integral component of membrane |
| Efet.01.16513.g1218.t1 | steroid hormone receptor ERR1 isoform X2 | 339 | 2.57E-08 | 63.25 | P:regulation of cellular process; F:organic cyclic compound binding; P:single-organism process; P:cellular process |
| Efet.01.1651300.g504.t1 | coiled-coil domain-containing 170-like | 273 | 6.37E-21 | 68.75 |  |
| Efet.01.1651340.g506.t1 | orotidine 5 -phosphate decarboxylase | 414 | 4.12E-74 | 95.1 | P:'de novo' pyrimidine nucleobase biosynthetic process; F:orotidine-5'-phosphate decarboxylase activity; P:'de novo' UMP biosynthetic process |
| Efet.01.1651431.g507.t1 | globin | 279 | 6.44E-62 | 89.55 | F:oxygen binding; P:oxygen transport; F:heme binding |
| Efet.01.165146.g762.t1 | peptidyl-prolyl cis-trans isomerase FKBP62-like | 807 | 5.72E-29 | 50.7 | C:endoplasmic reticulum membrane; F:peptidyl-prolyl cis-trans isomerase activity; F:FK506 binding; P:vasculature development; P:chaperone-mediated protein folding; P:protein peptidyl-prolyl isomerization |
| Efet.01.1651564.g509.t1 | hypothetical protein HELRODRAFT_159340 | 276 | 3.85E-27 | 65.05 | C:membrane; F:binding |
| Efet.01.1651567.g510.t1 | DNA polymerase epsilon catalytic subunit A-like | 474 | 1.96E-33 | 72.8 | F:nucleic acid binding; C:nucleus; F:nucleotidyltransferase activity; P:DNA metabolic process; P:cellular macromolecule biosynthetic process |
| Efet.01.1651608.g511.t1 | Growth arrest-specific 8 | 222 | 8.91E-14 | 86.6 | P:cell motility; C:motile cilium |
| Efet.01.1651610.g512.t1 | translation initiation factor eIF-2B subunit epsilon-like | 519 | 9.74E-48 | 70.65 | P:biosynthetic process; F:transferase activity |
| Efet.01.1651621.g513.t1 | DNA-binding response regulator | 336 | 3.74E-73 | 91.8 | F:DNA binding; P:transcription, DNA-templated; P:phosphorelay signal transduction system; P:regulation of transcription, DNA-templated; C:intracellular |
| Efet.01.1651659.g514.t1 | midasin isoform X2 | 243 | 5.63E-22 | 87.7 | F:ATPase activity; C:nucleus; F:ATP binding; P:ribosomal large subunit assembly |
| Efet.01.1651662.g515.t1 | MFS transporter | 273 | 5.18E-17 | 82.25 |  |
| Efet.01.1651663.g516.t1 | VOC family | 477 | 7.09E-63 | 63.25 | F:transferase activity |
| Efet.01.1651667.g517.t1 | FMRFamide receptor-like | 402 | 1.99E-20 | 73 | F:G-protein coupled receptor activity; P:G-protein coupled receptor signaling pathway; C:integral component of membrane |
| Efet.01.1651673.g518.t1 | glycine cleavage system H | 369 | 2.89E-80 | 85.05 | P:glycine decarboxylation via glycine cleavage system; C:glycine cleavage complex |
| Efet.01.1651695.g521.t1 | kinase C delta type isoform X2 | 300 | 1.26E-11 | 70.55 | P:negative regulation of platelet aggregation; P:negative regulation of glial cell apoptotic process; P:negative regulation of filopodium assembly; P:negative regulation of protein binding; P:cell chemotaxis; F:calcium-independent protein kinase C activity; P:termination of signal transduction; C:cytosol; P:histone phosphorylation; P:positive regulation of glucosylceramide catabolic process; P:positive regulation of sphingomyelin catabolic process; F:protein kinase binding; P:cellular response to angiotensin; P:positive regulation of protein import into nucleus; P:positive regulation of response to DNA damage stimulus; P:negative regulation of inflammatory response; P:intrinsic apoptotic signaling pathway in response to oxidative stress; P:defense response to bacterium; P:negative regulation of insulin receptor signaling pathway; C:plasma membrane; P:cellular component disassembly involved in execution phase of apoptosis; F:insulin receptor substrate binding; P:cellular response to hydroperoxide; P:positive regulation of protein dephosphorylation; P:regulation of receptor activity; P:positive regulation of endodeoxyribonuclease activity; P:peptidyl-serine phosphorylation; P:peptidyl-threonine phosphorylation; P:neutrophil activation; P:negative regulation of MAP kinase activity; P:cellular senescence; F:ion binding; F:enzyme activator activity; P:regulation of mRNA stability; P:Fc-gamma receptor signaling pathway involved in phagocytosis; C:nucleoplasm; P:stimulatory C-type lectin receptor signaling pathway; F:nucleotide binding; C:extracellular exosome; P:activation of protein kinase activity; P:cellular response to hydrogen peroxide; P:platelet activation; P:negative regulation of peptidyl-tyrosine phosphorylation; P:interferon-gamma-mediated signaling pathway; P:positive regulation of phospholipid scramblase activity; P:activation of phospholipase C activity; C:endoplasmic reticulum; P:negative regulation of actin filament polymerization; P:cytokine production; P:protein stabilization; P:positive regulation of ceramide biosynthetic process; F:TIR domain binding; P:positive regulation of superoxide anion generation |
| Efet.01.1651700.g522.t1 | T-box transcription factor | 303 | 1.14E-58 | 95.9 | C:nucleus; F:DNA binding; F:transcription factor activity, sequence-specific DNA binding; P:transcription, DNA-templated; P:regulation of transcription, DNA-templated |
| Efet.01.1651721.g523.t1 | hypothetical protein HELRODRAFT_170343 | 246 | 6.82E-11 | 56 |  |
| Efet.01.1651765.g525.t1 | Mannose-6-phosphate class I | 363 | 2.59E-78 | 68.65 | P:phosphorylation; F:kinase activity |
| Efet.01.1651862.g527.t1 | NADH dehydrogenase ubiquinone 1 beta subcomplex subunit 6 | 435 | 2.03E-38 | 60.15 | C:membrane; C:integral component of membrane |
| Efet.01.1651873.g528.t1 | MFS transporter | 207 | 2.01E-31 | 81.5 | P:transmembrane transport; C:integral component of membrane |
| Efet.01.1651903.g532.t1 | hypothetical protein B5766_04950 | 228 | 4.10E-12 | 100 |  |
| Efet.01.1651908.g533.t1 | Leucine-rich repeat and IQ domain-containing 3 | 384 | 4.33E-16 | 61.35 |  |
| Efet.01.165193.g763.t1 | ATP-dependent RNA helicase FAL1 | 339 | 3.61E-47 | 79.15 | C:FAL1-SGD1 complex; F:translation initiation factor activity; F:ATP binding; P:maturation of SSU-rRNA from tricistronic rRNA transcript (SSU-rRNA, 5.8S rRNA, LSU-rRNA); P:multicellular organism development; P:regulation of gene expression; P:RNA secondary structure unwinding; C:cytosol; C:nucleolus; P:translational initiation; F:ATP-dependent RNA helicase activity |
| Efet.01.1651931.g535.t1 | phosphatase | 204 | 2.99E-17 | 92.6 | F:protein serine/threonine phosphatase activity; P:protein dephosphorylation; C:integral component of membrane |
| Efet.01.1651955.g536.t1 | RNA-binding | 261 | 9.93E-58 | 80.55 | F:nucleotide binding; F:nucleic acid binding |
| Efet.01.1651974.g537.t1 | cell filamentation Fic | 393 | 3.59E-26 | 75.05 |  |
| Efet.01.165204.g765.t1 | Alpha- sarcomeric | 336 | 1.53E-53 | 87.45 | P:vesicle transport along actin filament; P:protein localization to bicellular tight junction; P:protein transport; F:RNA polymerase II regulatory region sequence-specific DNA binding; P:negative regulation of cellular component movement; P:positive regulation of NIK/NF-kappaB signaling; P:regulation of nucleic acid-templated transcription; C:neuron projection; F:retinoic acid receptor binding; P:retinoic acid receptor signaling pathway; C:extracellular exosome; C:focal adhesion; C:intracellular ribonucleoprotein complex; F:calcium ion binding; C:stress fiber; P:peroxisome proliferator activated receptor signaling pathway; C:cortical cytoskeleton; F:chromatin DNA binding; F:protein homodimerization activity; P:negative regulation of substrate adhesion-dependent cell spreading; P:regulation of apoptotic process; C:protein complex; F:ion channel binding; F:nucleoside binding; F:RNA binding; P:positive regulation of pinocytosis; F:actin filament binding; C:nucleus; C:Z disc; C:extracellular space; C:cell-cell junction; C:perinuclear region of cytoplasm; F:protein N-terminus binding; F:ligand-dependent nuclear receptor transcription coactivator activity; P:bicellular tight junction assembly; C:pseudopodium; P:actin filament bundle assembly; P:positive regulation of cell migration |
| Efet.01.1652040.g538.t1 | toxin | 525 | 1.22E-83 | 72.6 | F:ATP binding; P:phosphorylation; F:kinase activity |
| Efet.01.1652059.g540.t1 | hypothetical protein CAPTEDRAFT_228779 | 372 | 4.75E-13 | 60.43 |  |
| Efet.01.1652076.g542.t1 | hexokinase-2-like isoform X2 | 318 | 5.32E-10 | 74.9 | F:nucleotide binding; P:cellular glucose homeostasis; P:glycolytic process; F:glucokinase activity; C:cytosol; F:mannokinase activity; F:fructokinase activity |
| Efet.01.1652115.g543.t1 | cell cycle control 50A isoform X2 | 282 | 3.67E-21 | 67.2 | C:membrane |
| Efet.01.1652141.g546.t1 | ABC transporter substrate-binding | 300 | 2.30E-60 | 66.6 | P:thiamine biosynthetic process |
| Efet.01.1652197.g548.t1 | GS homeobox 2 | 357 | 1.36E-33 | 83.7 | P:olfactory bulb interneuron differentiation; P:spinal cord association neuron differentiation; P:telencephalon regionalization; P:subpallium neuron fate commitment; P:regulation of transcription, DNA-templated; P:forebrain dorsal/ventral pattern formation; F:sequence-specific DNA binding; P:regulation of respiratory gaseous exchange by neurological system process; P:forebrain morphogenesis; P:positive regulation of Notch signaling pathway; P:hindbrain morphogenesis; P:positive regulation of oligodendrocyte differentiation; C:nucleus; P:neuron fate specification; P:regulation of cell migration |
| Efet.01.1652245.g549.t1 | family transcriptional regulator | 372 | 6.85E-72 | 82.45 | F:DNA binding; F:transcription factor activity, sequence-specific DNA binding; P:transcription, DNA-templated; P:regulation of transcription, DNA-templated |
| Efet.01.1652260.g550.t1 | TIGR02099 family | 480 | 6.25E-75 | 80.8 | C:integral component of membrane |
| Efet.01.1652285.g551.t1 | growth differentiation factor 11 | 369 | 1.38E-15 | 53.3 | F:growth factor activity; C:extracellular region; P:growth |
| Efet.01.1652286.g552.t1 | 28S ribosomal mitochondrial | 282 | 2.90E-23 | 68.95 | C:mitochondrial small ribosomal subunit |
| Efet.01.165229.g766.t1 | steroid hormone receptor ERR2-like isoform X1 | 594 | 2.98E-63 | 67.5 | C:nucleus; F:transcription factor activity, sequence-specific DNA binding; F:zinc ion binding; P:transcription, DNA-templated; F:steroid hormone receptor activity; P:regulation of transcription, DNA-templated; F:sequence-specific DNA binding; P:steroid hormone mediated signaling pathway |
| Efet.01.1652308.g553.t1 | mannose-1-phosphate guanylyltransferase | 429 | 8.07E-57 | 70.05 | F:transferase activity |
| Efet.01.1652319.g554.t1 | hypothetical protein CAPTEDRAFT_217222 | 216 | 6.21E-15 | 65.3 | C:membrane; C:integral component of membrane; F:carbohydrate binding |
| Efet.01.1652388.g555.t1 | probable calcium-binding CML23 | 225 | 2.47E-28 | 64.35 | F:calcium ion binding |
| Efet.01.1652420.g556.t1 | hypothetical protein CAPTEDRAFT_127120 | 411 | 1.47E-16 | 54 | C:membrane; C:integral component of membrane |
| Efet.01.1652431.g557.t1 | Hydrocephalus-inducing -like | 486 | 2.50E-60 | 81.1 | P:cilium movement |
| Efet.01.1652469.g559.t1 | transmembrane 211-like | 552 | 5.11E-16 | 49.75 | C:membrane; C:integral component of membrane |
| Efet.01.1652512.g561.t1 | basic proline-rich -like isoform X5 | 243 | 6.44E-19 | 72.9 | F:transferase activity, transferring glycosyl groups |
| Efet.01.1652518.g562.t1 | Pre-mRNA-processing-splicing factor 8 | 273 | 3.20E-50 | 94.35 | C:nuclear speck; C:U5 snRNP; F:RNA binding; P:spliceosomal tri-snRNP complex assembly; F:second spliceosomal transesterification activity; F:U6 snRNA binding; F:U5 snRNA binding; F:pre-mRNA intronic binding; C:catalytic step 2 spliceosome; P:cellular response to lipopolysaccharide; F:K63-linked polyubiquitin binding; F:U1 snRNA binding; C:membrane; P:cellular response to tumor necrosis factor; F:U2 snRNA binding |
| Efet.01.1652532.g564.t1 | titin-like isoform X1 | 420 | 1.67E-52 | 65.15 |  |
| Efet.01.1652570.g566.t1 | DUF3372 domain-containing | 444 | 3.53E-45 | 71.55 | F:catalytic activity |
| Efet.01.1652577.g567.t1 | actin-related 2-A- | 234 | 4.67E-37 | 94.55 | F:ATP binding; P:Arp2/3 complex-mediated actin nucleation; C:Arp2/3 protein complex |
| Efet.01.1652585.g568.t1 | ABC transporter [Burkholderia territorii] | 414 | 3.26E-91 | 58.5 | F:ATPase activity; F:ATP binding; P:transmembrane transport; C:ATP-binding cassette (ABC) transporter complex; F:hydrolase activity, acting on acid anhydrides, catalyzing transmembrane movement of substances |
| Efet.01.1652593.g569.t1 | low-density lipo receptor-related 6-like | 204 | 1.91E-30 | 84.25 | F:Wnt-activated receptor activity; C:integral component of membrane; P:Wnt signaling pathway involved in dorsal/ventral axis specification; P:canonical Wnt signaling pathway; C:receptor complex; F:Wnt-protein binding; C:plasma membrane |
| Efet.01.1652623.g570.t1 | UDP-N-acetylmuramate--L-alanine ligase | 273 | 1.57E-50 | 84.4 | P:regulation of cell shape; P:cell division; F:ATP binding; C:cytoplasm; P:peptidoglycan biosynthetic process; F:UDP-N-acetylmuramate-L-alanine ligase activity; P:cell wall organization; P:cell cycle |
| Efet.01.1652640.g571.t1 | hypothetical protein CRN43_13925 | 333 | 1.16E-73 | 98 |  |
| Efet.01.1652651.g572.t1 | 4-hydroxybenzoyl- thioesterase | 213 | 1.26E-42 | 79.1 |  |
| Efet.01.1652652.g573.t1 | family transcriptional regulator | 336 | 4.89E-71 | 93.4 | C:outer membrane-bounded periplasmic space |
| Efet.01.1652684.g574.t1 | otopetrin-3-like isoform X3 | 471 | 1.71E-14 | 70.3 | C:membrane |
| Efet.01.1652696.g575.t1 | IS110 family | 528 | 4.77E-123 | 92.5 | F:DNA binding; F:transposase activity; P:transposition, DNA-mediated |
| Efet.01.1652709.g576.t1 | hypothetical protein HELRODRAFT_165596 | 201 | 3.38E-09 | 75.5 | C:membrane |
| Efet.01.1652720.g578.t1 | glutamate 5-kinase | 423 | 1.83E-65 | 83.8 | F:ATP binding; F:glutamate 5-kinase activity; C:cytoplasm; P:phosphorylation; P:L-proline biosynthetic process |
| Efet.01.1652727.g579.t1 | family transcriptional regulator | 405 | 6.52E-73 | 67.65 | F:DNA binding; P:transcription, DNA-templated; P:regulation of transcription, DNA-templated |
| Efet.01.1652728.g580.t1 | zinc and double PHD fingers family 2-like [Xenopus tropicalis] | 291 | 1.05E-22 | 79.6 | F:zinc ion binding |
| Efet.01.1652764.g581.t1 | monofunctional biosynthetic peptidoglycan transglycosylase | 498 | 6.12E-119 | 82.8 | P:regulation of cell shape; C:peptidoglycan-based cell wall; P:peptidoglycan biosynthetic process; C:integral component of membrane; P:cell wall organization; F:transferase activity, transferring pentosyl groups; C:plasma membrane |
| Efet.01.1652856.g586.t1 | 5-hydroxytryptamine receptor 1A-like | 441 | 6.22E-44 | 69.85 | C:integral component of plasma membrane; F:G-protein coupled acetylcholine receptor activity; P:G-protein coupled acetylcholine receptor signaling pathway |
| Efet.01.1652885.g587.t1 | phospho-N-acetylmuramoyl-pentapeptide-transferase | 306 | 2.04E-43 | 95.95 | P:regulation of cell shape; P:cell division; F:UDP-N-acetylmuramoyl-L-alanyl-D-glutamyl-meso-2,6-diaminopimelyl-D-alanyl-D-alanine:undecaprenyl-phosphate transferase activity; P:peptidoglycan biosynthetic process; C:integral component of membrane; P:cell wall organization; C:plasma membrane; P:cell cycle; F:phospho-N-acetylmuramoyl-pentapeptide-transferase activity |
| Efet.01.1652886.g588.t1 | PREDICTED: uncharacterized protein LOC106152298 | 351 | 1.35E-51 | 73.65 | C:integral component of membrane |
| Efet.01.1652891.g589.t1 | gfo Idh family oxidoreductase | 360 | 7.17E-79 | 64.45 | F:oxidoreductase activity; P:oxidation-reduction process |
| Efet.01.1652905.g590.t1 | synapsin-like isoform X2 | 306 | 1.54E-10 | 51.4 | F:ATP binding; F:catalytic activity; F:metal ion binding; C:synaptic vesicle; P:neurotransmitter secretion |
| Efet.01.1652915.g591.t1 | UPF0669 C6orf120 homolog | 495 | 4.80E-45 | 64.25 | C:extracellular region |
| Efet.01.1652933.g592.t1 | ABC transporter substrate-binding | 522 | 3.13E-120 | 54.15 | P:transmembrane transport; C:ATP-binding cassette (ABC) transporter complex |
| Efet.01.1652994.g594.t1 | piggyBac transposable element-derived 2 | 570 | 3.12E-30 | 60.05 |  |
| Efet.01.1653029.g596.t1 | hypothetical protein CAPTEDRAFT_219353 | 387 | 7.63E-16 | 58 |  |
| Efet.01.1653071.g597.t1 | TNF receptor-associated factor 5-like | 297 | 2.46E-17 | 58.2 | F:ubiquitin-protein transferase activity; F:zinc ion binding; F:metal ion binding; P:protein ubiquitination |
| Efet.01.1653164.g599.t1 | phosphopantothenate--cysteine ligase | 243 | 6.26E-10 | 73.4 | F:ligase activity |
| Efet.01.1653198.g600.t1 | hypothetical protein CAPTEDRAFT_208916 | 246 | 1.41E-12 | 64 | P:ion transmembrane transport; C:membrane; F:extracellular ligand-gated ion channel activity; P:transport |
| Efet.01.1653216.g601.t1 | nephrocystin-4 isoform X3 | 471 | 4.75E-64 | 76.05 | P:negative regulation of canonical Wnt signaling pathway; C:cytoskeleton; P:system development; C:ciliary transition zone; P:single-organism cellular process |
| Efet.01.1653231.g602.t1 | tripartite tricarboxylate transporter substrate binding | 501 | 2.17E-34 | 77.9 | C:outer membrane-bounded periplasmic space |
| Efet.01.1653234.g603.t1 | circularly permutated Ras 1-like | 222 | 3.18E-19 | 74.65 | F:zinc ion binding; P:ER to Golgi vesicle-mediated transport; P:intracellular protein transport; C:COPII vesicle coat |
| Efet.01.1653250.g605.t1 | AChain Crystal Structure Of Engineered Northeast Structural Genomics Consortium Target | 447 | 5.58E-24 | 62.05 | F:catalytic activity; P:nucleoside metabolic process; C:cellular_component |
| Efet.01.1653270.g606.t1 | serine-rich adhesin for platelets-like | 462 | 8.75E-39 | 63.7 | F:zinc ion binding; F:metal ion binding; C:intracellular |
| Efet.01.1653348.g608.t1 | hypothetical protein | 366 | 1.87E-35 | 68.2 |  |
| Efet.01.1653352.g609.t1 | chromodomain-helicase-DNA-binding 7 | 282 | 1.44E-36 | 86.05 | C:nucleus; F:ATP binding; F:hydrolase activity, acting on acid anhydrides |
| Efet.01.1653365.g610.t1 | ABC transporter permease | 447 | 1.35E-55 | 98 | C:integral component of membrane; P:transport; C:plasma membrane |
| Efet.01.1653369.g611.t1 | dmX 2 isoform X3 | 399 | 3.65E-26 | 75.8 |  |
| Efet.01.1653386.g612.t1 | lanC 3 | 555 | 1.17E-38 | 57.95 | F:catalytic activity |
| Efet.01.1653412.g614.t1 | non-ribosomal peptide synthetase | 576 | 6.42E-118 | 79.5 | F:catalytic activity; P:metabolic process; F:phosphopantetheine binding |
| Efet.01.1653414.g615.t1 | surfeit locus 4 homolog | 369 | 1.18E-44 | 83 | C:integral component of membrane |
| Efet.01.165344.g767.t1 | guanine nucleotide-binding G(q) subunit alpha | 240 | 9.27E-47 | 98.6 | F:GTP binding; F:G-protein coupled receptor binding; P:phototransduction, visible light; P:entrainment of circadian clock; F:metal ion binding; F:GTPase activity; C:membrane; P:G-protein coupled acetylcholine receptor signaling pathway; F:G-protein beta/gamma-subunit complex binding; F:signal transducer activity; C:photoreceptor outer segment |
| Efet.01.165346.g768.t1 | hypothetical protein CAPTEDRAFT_200225 | 504 | 8.27E-11 | 46.29 | F:pyridoxal phosphate binding; P:tryptophan catabolic process; F:kynureninase activity; C:cytoplasm; F:catalytic activity; C:membrane; C:integral component of membrane; P:NAD biosynthetic process; F:carbohydrate binding |
| Efet.01.1653483.g618.t1 | Zinc finger 771 | 225 | 3.12E-11 | 67.1 |  |
| Efet.01.1653487.g619.t1 | sugar ABC transporter permease | 480 | 1.54E-97 | 91.95 | C:integral component of membrane; P:transport; C:plasma membrane |
| Efet.01.1653493.g620.t1 | soluble guanylate cyclase 89Db-like | 288 | 2.34E-25 | 67.3 | P:signal transduction; F:phosphorus-oxygen lyase activity; C:intracellular; P:cyclic nucleotide biosynthetic process; F:cyclase activity |
| Efet.01.1653519.g621.t1 | serine protease | 249 | 2.38E-48 | 74.8 | P:proteolysis; C:integral component of membrane; F:peptidase activity |
| Efet.01.1653531.g622.t1 | sex peptide receptor-like | 270 | 7.87E-36 | 73.95 | F:G-protein coupled peptide receptor activity; P:G-protein coupled receptor signaling pathway; C:integral component of membrane |
| Efet.01.1653536.g623.t1 | peptidylprolyl isomerase | 624 | 7.76E-151 | 75.85 | P:cellular process; F:isomerase activity |
| Efet.01.1653542.g624.t1 | synaptic vesicle membrane VAT-1 homolog-like | 228 | 5.11E-11 | 69.3 | F:zinc ion binding; F:oxidoreductase activity; P:oxidation-reduction process |
| Efet.01.1653575.g625.t1 | MULTISPECIES: DUF2469 domain-containing | 324 | 1.31E-67 | 98.5 |  |
| Efet.01.1653579.g626.t1 | site-specific DNA-methyltransferase | 225 | 6.76E-45 | 100 | F:nucleic acid binding; F:methyltransferase activity; P:methylation |
| Efet.01.1653592.g628.t1 | phosphatase 1B-like | 579 | 4.65E-97 | 80.9 | F:magnesium ion binding; F:protein serine/threonine phosphatase activity; C:cytoskeleton; P:protein dephosphorylation; F:manganese ion binding |
| Efet.01.1653602.g630.t1 | mediator of RNA polymerase II transcription subunit 21-like | 429 | 9.08E-60 | 80.8 | C:mediator complex; F:RNA polymerase II transcription cofactor activity; P:regulation of transcription from RNA polymerase II promoter |
| Efet.01.1653621.g631.t1 | GH3 auxin-responsive promoter | 636 | 1.86E-140 | 84.4 |  |
| Efet.01.1653625.g632.t1 | LIX1 | 360 | 4.53E-66 | 88.65 | P:autophagosome maturation; C:cytoplasm |
| Efet.01.1653631.g633.t1 | vacuolar fusion MON1 homolog A-like | 360 | 6.94E-24 | 70.75 | P:cellular iron ion homeostasis; P:vesicle-mediated transport; F:guanyl-nucleotide exchange factor activity; C:endomembrane system; P:protein secretion |
| Efet.01.1653712.g634.t1 | cartilage matrix -like isoform X1 | 279 | 2.52E-09 | 54.7 | F:calcium ion binding; C:collagen trimer; C:membrane; C:integral component of membrane; C:proteinaceous extracellular matrix |
| Efet.01.1653719.g635.t1 | PAC2 family | 285 | 3.81E-43 | 80.95 | F:ligase activity |
| Efet.01.1653749.g636.t1 | hypothetical protein CAPTEDRAFT_220098 | 351 | 8.42E-13 | 72.57 | F:binding |
| Efet.01.1653783.g637.t1 | A disintegrin and metallo ase with thrombospondin motifs 6-like | 333 | 1.61E-12 | 54.65 | P:circulatory system development |
| Efet.01.1653796.g638.t1 | Wnt-6 | 387 | 6.89E-39 | 65.45 | P:multicellular organism development; C:membrane; C:integral component of membrane; P:Wnt signaling pathway; C:extracellular region; F:receptor binding; C:proteinaceous extracellular matrix |
| Efet.01.1653800.g639.t1 | metal-sulfur cluster assembly factor | 333 | 2.29E-73 | 92.85 |  |
| Efet.01.1653821.g640.t1 | 16S rRNA methyltransferase | 441 | 1.23E-86 | 72 | F:transferase activity |
| Efet.01.1653830.g641.t1 | potassium sodium hyperpolarization-activated cyclic nucleotide-gated channel 2-like | 642 | 1.03E-30 | 69.55 | P:potassium ion transport; F:ion channel activity; P:ion transmembrane transport; C:membrane |
| Efet.01.1653899.g642.t1 | ankyrin 2,3 | 396 | 6.08E-23 | 71.2 | F:hydrolase activity |
| Efet.01.16539.g1220.t1 | KN motif and ankyrin repeat domain-containing 1-like isoform X1 | 1260 | 5.88E-14 | 69.4 |  |
| Efet.01.1653926.g644.t1 | innexin 1 | 471 | 2.53E-77 | 82.8 | C:gap junction; P:ion transport; P:transmembrane transport; C:integral component of membrane; P:cell communication by electrical coupling; C:synapse; F:gap junction channel activity; C:plasma membrane |
| Efet.01.1653934.g646.t1 | tRNA (guanine(26)-N(2))-dimethyltransferase | 534 | 3.94E-72 | 70.8 | P:tRNA processing; F:methyltransferase activity; F:binding; P:methylation |
| Efet.01.1653988.g650.t1 | succinyl-diaminopimelate desuccinylase | 366 | 8.77E-79 | 83.2 | P:lysine biosynthetic process via diaminopimelate; F:succinyl-diaminopimelate desuccinylase activity |
| Efet.01.1653994.g651.t1 | family transcriptional regulator | 309 | 2.40E-65 | 76.6 | F:DNA binding; F:transcription factor activity, sequence-specific DNA binding; P:transcription, DNA-templated; P:regulation of transcription, DNA-templated |
| Efet.01.16540.g1221.t1 | zinc finger | 729 | 8.18E-55 | 85.75 | C:nucleus; F:DNA binding; F:metal ion binding; P:transcription, DNA-templated; P:regulation of transcription, DNA-templated |
| Efet.01.1654014.g654.t1 | family toxin | 312 | 1.34E-34 | 75.8 |  |
| Efet.01.1654027.g655.t1 | glutamate receptor kainate 2-like | 621 | 2.78E-104 | 88.1 | C:postsynaptic membrane; P:ion transmembrane transport; C:integral component of membrane; C:cell junction; P:ionotropic glutamate receptor signaling pathway; F:ionotropic glutamate receptor activity; F:extracellular-glutamate-gated ion channel activity |
| Efet.01.1654033.g656.t1 | hypothetical protein CAPTEDRAFT_225483 | 240 | 4.89E-12 | 55.6 | C:membrane; C:integral component of membrane; F:carbohydrate binding |
| Efet.01.1654050.g657.t1 | cytidine deaminase | 360 | 2.18E-82 | 93.9 | F:zinc ion binding; P:cytidine deamination; F:cytidine deaminase activity |
| Efet.01.165407.g770.t1 | golgin subfamily B member 1-like | 1872 | 3.80E-06 | 50 |  |
| Efet.01.1654073.g658.t1 | lipo | 285 | 2.96E-58 | 74.25 | C:outer membrane-bounded periplasmic space |
| Efet.01.1654088.g659.t1 | FMRFamide receptor-like | 522 | 1.25E-33 | 55.45 | P:signal transduction; C:membrane; F:signal transducer activity |
| Efet.01.1654121.g660.t1 | hypothetical protein CRN43_15250 | 384 | 3.48E-65 | 64.85 |  |
| Efet.01.165414.g771.t1 | retinol dehydrogenase 7-like isoform X2 | 396 | 2.16E-12 | 48.7 | F:oxidoreductase activity; C:membrane; C:integral component of membrane; P:oxidation-reduction process |
| Efet.01.1654151.g664.t1 | sulfite reductase | 456 | 6.43E-98 | 93.4 | F:sulfite reductase (ferredoxin) activity; F:metal ion binding; P:oxidation-reduction process; F:4 iron, 4 sulfur cluster binding; F:heme binding |
| Efet.01.1654152.g665.t1 | homoserine dehydrogenase | 456 | 2.11E-41 | 66.35 |  |
| Efet.01.1654159.g666.t1 | hypothetical protein B5766_12745 | 429 | 2.49E-73 | 70.9 |  |
| Efet.01.1654162.g667.t1 | ABC transporter ATP-binding | 468 | 4.78E-110 | 71.7 | F:nucleotide binding |
| Efet.01.1654190.g668.t1 | alpha-L-fucosidase | 264 | 1.74E-54 | 85.8 | F:alpha-L-fucosidase activity; P:fucose metabolic process |
| Efet.01.1654205.g670.t1 | acyl carrier | 249 | 1.47E-48 | 92.25 | C:cytoplasm; P:fatty acid biosynthetic process; F:ACP phosphopantetheine attachment site binding involved in fatty acid biosynthetic process |
| Efet.01.1654222.g671.t1 | family nutrient uptake outer membrane | 549 | 9.98E-130 | 83.4 |  |
| Efet.01.1654225.g672.t1 | glycogen starch alpha-glucan phosphorylase | 540 | 2.22E-66 | 76.75 | F:glycogen phosphorylase activity; F:pyridoxal phosphate binding; C:cytoplasm; P:glycogen catabolic process |
| Efet.01.1654229.g673.t1 | ABC transporter permease | 579 | 2.40E-116 | 70.75 | C:membrane |
| Efet.01.1654259.g676.t1 | hypothetical protein HELRODRAFT_145549, partial | 354 | 5.82E-06 | 63 | C:membrane; C:integral component of membrane |
| Efet.01.1654264.g677.t1 | sodium bicarbonate transporter 11 isoform X1 | 213 | 1.77E-09 | 76.75 | C:integral component of plasma membrane; P:regulation of intracellular pH; P:sodium ion transmembrane transport; F:inorganic anion exchanger activity; P:borate transmembrane transport |
| Efet.01.1654282.g678.t1 | growth arrest and DNA damage-inducible s-interacting 1-like | 429 | 1.53E-22 | 61.6 | C:nucleus; P:cell cycle |
| Efet.01.1654284.g679.t1 | D-ala-D-ala transporter subunit | 405 | 1.17E-58 | 70.15 | C:membrane |
| Efet.01.1654295.g680.t1 | bifunctional glutamine-synthetase adenylyltransferase deadenyltransferase | 405 | 6.68E-82 | 74.85 | F:nucleotide binding; F:nucleotidyltransferase activity |
| Efet.01.1654343.g683.t1 | hydrogenase maturation protease | 483 | 2.23E-61 | 75.9 | P:positive regulation of catalytic activity; P:proteolysis; F:enzyme activator activity; F:peptidase activity |
| Efet.01.1654352.g684.t1 | 5-hydroxytryptamine receptor 2C isoform X2 | 393 | 2.13E-22 | 78.3 | P:cGMP biosynthetic process; F:drug binding; P:regulation of appetite; P:dopamine receptor signaling pathway; P:feeding behavior; P:regulation of corticotropin-releasing hormone secretion; P:positive regulation of fat cell differentiation; P:behavioral fear response; F:serotonin binding; C:integral component of plasma membrane; F:Gq/11-coupled serotonin receptor activity; F:dopamine neurotransmitter receptor activity; P:phospholipase C-activating serotonin receptor signaling pathway; P:locomotory behavior; P:response to drug; P:positive regulation of cytosolic calcium ion concentration involved in phospholipase C-activating G-protein coupled signaling pathway; P:positive regulation of ERK1 and ERK2 cascade; F:1-(4-iodo-2,5-dimethoxyphenyl)propan-2-amine binding; C:cytosol; P:positive regulation of phosphatidylinositol biosynthetic process; P:release of sequestered calcium ion into cytosol; P:regulation of neurological system process |
| Efet.01.1654378.g685.t1 | NADH dehydrogenase | 492 | 3.73E-68 | 86.35 | F:NADH dehydrogenase (quinone) activity; F:quinone binding; F:NAD binding; P:oxidation-reduction process; P:transport; C:plasma membrane |
| Efet.01.1654382.g686.t1 | group XV phospholipase A2-like | 357 | 7.10E-14 | 68.5 | F:O-acyltransferase activity; P:lipid metabolic process |
| Efet.01.1654414.g688.t1 | turtle 3 | 390 | 1.32E-27 | 58.9 | C:membrane; C:integral component of membrane |
| Efet.01.1654430.g689.t1 | hypothetical protein CRN43_14440 | 345 | 3.85E-10 | 62.5 |  |
| Efet.01.1654473.g692.t1 | -tyrosine sulfotransferase 1-like | 597 | 4.30E-95 | 75 | F:protein-tyrosine sulfotransferase activity; P:peptidyl-tyrosine sulfation |
| Efet.01.1654483.g694.t1 | metal tolerance 9-like | 294 | 1.74E-38 | 79.75 | P:cation transmembrane transport; C:integral component of membrane; F:cation transmembrane transporter activity |
| Efet.01.1654503.g695.t1 | hypothetical protein CAPTEDRAFT_223621 | 639 | 9.97E-15 | 61 | P:regulation of apoptotic process |
| Efet.01.1654506.g696.t1 | prephenate dehydratase | 291 | 1.36E-62 | 91.75 | F:chorismate mutase activity; P:L-phenylalanine biosynthetic process; C:cytoplasm; F:amino acid binding; F:prephenate dehydratase activity |
| Efet.01.1654540.g697.t1 | steroid hormone receptor ERR2-like isoform X1 | 273 | 2.27E-21 | 68.3 | F:DNA binding; P:regulation of cellular process; F:metal ion binding; P:cellular process |
| Efet.01.1654548.g698.t1 | biotin-independent malonate decarboxylase subunit beta | 378 | 6.47E-72 | 83.4 | F:methylmalonyl-CoA carboxytransferase activity; F:propionyl-CoA carboxylase activity; F:acetyl-CoA carboxylase activity; P:cellular carbohydrate metabolic process; P:fatty acid biosynthetic process; C:acetyl-CoA carboxylase complex; F:carboxy-lyase activity |
| Efet.01.1654625.g700.t1 | paramyosin | 396 | 2.23E-24 | 77 | C:myosin complex; C:cytoplasm |
| Efet.01.1654661.g701.t1 | NAD-dependent succinate-semialdehyde dehydrogenase | 606 | 1.25E-97 | 71.4 | P:oxidation-reduction process; F:oxidoreductase activity, acting on the aldehyde or oxo group of donors, NAD or NADP as acceptor |
| Efet.01.1654681.g704.t1 | collagen alpha-4(VI) chain- | 477 | 1.19E-30 | 57.45 | F:calcium ion binding; C:membrane; C:integral component of membrane; C:proteinaceous extracellular matrix |
| Efet.01.1654704.g705.t1 | SprT-like family protein | 429 | 1.47E-100 | 63.45 |  |
| Efet.01.1654715.g706.t1 | eukaryotic elongation factor 2 kinase-like isoform X1 | 513 | 4.47E-10 | 61.38 | F:ATP binding; F:protein serine/threonine kinase activity; P:protein phosphorylation |
| Efet.01.1654736.g707.t1 | leukocyte elastase inhibitor-like | 558 | 3.69E-37 | 59.85 | C:extracellular space; F:serine-type endopeptidase inhibitor activity; P:negative regulation of endopeptidase activity |
| Efet.01.1654746.g708.t1 | glycogenin-2 isoform X1 | 366 | 1.67E-53 | 81.65 | P:glycogen biosynthetic process; C:extracellular exosome; F:protein binding; F:metal ion binding; C:membrane; C:cytosol; F:glycogenin glucosyltransferase activity; C:lysosomal lumen; P:glycogen catabolic process |
| Efet.01.165477.g773.t1 | homeobox PKNOX2-like | 525 | 3.78E-30 | 72.15 | P:retinoic acid receptor signaling pathway; P:pharyngeal system development |
| Efet.01.1654783.g711.t1 | stomatin 2 | 609 | 2.50E-138 | 95.65 | C:membrane |
| Efet.01.1654805.g714.t1 | hypothetical protein CAPTEDRAFT_222159 | 318 | 1.14E-29 | 62.86 | C:membrane |
| Efet.01.1654813.g715.t1 | ATP-binding cassette sub-family A member 3 | 390 | 3.28E-46 | 73.3 | F:ATPase activity; F:ATP binding; C:integral component of membrane; P:transport; F:transporter activity |
| Efet.01.1654833.g716.t1 | hypothetical protein B5766_04805 | 333 | 1.93E-53 | 64.9 | C:membrane |
| Efet.01.1654846.g717.t1 | FAD-dependent oxidoreductase | 486 | 8.28E-76 | 93.55 | F:oxidoreductase activity; C:integral component of membrane; P:oxidation-reduction process |
| Efet.01.1654847.g718.t1 | DNA polymerase subunit gamma-1 | 294 | 7.06E-30 | 90.9 | F:DNA binding; F:DNA-directed DNA polymerase activity; C:extracellular exosome; P:DNA replication; P:DNA biosynthetic process; F:protease binding; C:gamma DNA polymerase complex; C:protein complex; F:chromatin binding; P:base-excision repair, gap-filling; C:mitochondrial nucleoid |
| Efet.01.1654880.g721.t1 | helix-turn-helix domain-containing | 270 | 7.47E-57 | 60.4 |  |
| Efet.01.1654936.g723.t1 | adenosine receptor A2b-like | 237 | 1.06E-20 | 64.45 | F:G-protein coupled receptor activity; P:G-protein coupled receptor signaling pathway; C:membrane |
| Efet.01.1654947.g724.t1 | gfo Idh family oxidoreductase | 588 | 2.32E-111 | 87.3 | F:inositol 2-dehydrogenase activity; P:oxidation-reduction process |
| Efet.01.1654997.g725.t1 | sugar kinase | 513 | 9.62E-115 | 65.35 | P:phosphorylation; F:transferase activity; F:kinase activity |
| Efet.01.1654998.g726.t1 | family transcriptional regulator | 486 | 2.21E-109 | 97.45 | F:DNA binding; P:carbohydrate metabolic process; F:transcription factor activity, sequence-specific DNA binding; P:regulation of transcription, DNA-templated; F:carbohydrate binding |
| Efet.01.1655.g153.t1 | sodium channel | 552 | 3.45E-51 | 72.6 | P:regulation of postsynaptic membrane potential; P:regulation of ion transmembrane transport; C:voltage-gated sodium channel complex; P:sodium ion transmembrane transport; F:voltage-gated sodium channel activity |
| Efet.01.1655019.g729.t1 | neuronal acetylcholine receptor subunit alpha-10-like | 393 | 3.73E-24 | 69.45 | F:ion channel activity; P:synaptic transmission, cholinergic; F:acetylcholine receptor activity; P:neuromuscular synaptic transmission; P:signal transduction; P:cation transmembrane transport; F:acetylcholine binding; C:acetylcholine-gated channel complex; C:synapse; P:response to nicotine |
| Efet.01.1655035.g731.t1 | DUF4065 domain-containing | 402 | 6.75E-89 | 77.5 |  |
| Efet.01.1655036.g732.t1 | thymidine kinase | 387 | 3.09E-88 | 84.1 | F:ATP binding; P:DNA biosynthetic process; P:deoxyribonucleoside monophosphate biosynthetic process; P:phosphorylation; F:thymidine kinase activity |
| Efet.01.1655038.g733.t1 | sugar ABC transporter substrate-binding | 516 | 1.40E-101 | 70.95 | P:transport; F:transporter activity |
| Efet.01.1655063.g734.t1 | hypothetical protein CAPTEDRAFT_19685 | 321 | 3.46E-17 | 66.25 | F:N-acetyltransferase activity |
| Efet.01.165507.g774.t1 | calcium-transporting ATPase sarcoplasmic endoplasmic reticulum type isoform X1 | 1002 | 1.87E-103 | 75.7 | F:ATP binding; F:metal ion binding; C:integral component of membrane; P:calcium ion transmembrane transport; F:calcium-transporting ATPase activity; C:sarcoplasmic reticulum membrane |
| Efet.01.1655073.g736.t1 | malonate decarboxylase subunit alpha | 309 | 1.25E-52 | 90.7 | P:metabolic process; F:CoA-transferase activity |
| Efet.01.1655088.g737.t1 | perilipin-2-like isoform X1 | 381 | 2.81E-11 | 66.33 |  |
| Efet.01.1655123.g738.t1 | E3 SUMO- ligase KIAA1586-like | 489 | 1.03E-28 | 63.2 | F:nucleic acid binding |
| Efet.01.1655129.g739.t1 | dihydropteroate synthase | 450 | 4.08E-102 | 75.25 | F:metal ion binding; P:folic acid biosynthetic process; P:tetrahydrofolate biosynthetic process; F:dihydropteroate synthase activity |
| Efet.01.1655157.g740.t1 | helicase | 384 | 1.61E-83 | 82.05 | F:DNA binding; F:ATP binding; F:helicase activity |
| Efet.01.1655187.g741.t1 | ribonuclease HII | 366 | 2.59E-84 | 79 | P:RNA phosphodiester bond hydrolysis, endonucleolytic; F:RNA-DNA hybrid ribonuclease activity; F:RNA binding; C:cytoplasm; F:manganese ion binding; P:RNA catabolic process |
| Efet.01.1655191.g742.t1 | low-density lipo receptor-related 6 | 591 | 2.26E-45 | 64.2 | P:neural tube development; C:intracellular organelle; P:single-organism cellular localization; F:signaling receptor activity; P:axis specification; P:regulation of transcription, DNA-templated; C:cytoplasmic part; P:receptor-mediated endocytosis; P:regulation of molecular function; C:membrane-bounded organelle; C:plasma membrane part; P:animal organ morphogenesis; F:protein binding; P:forebrain development; P:positive regulation of cellular process; P:somitogenesis; P:morphogenesis of an epithelium; P:embryonic morphogenesis; P:negative regulation of protein phosphorylation; P:mesenchymal cell differentiation; P:Wnt signaling pathway; C:macromolecular complex; P:macromolecule localization |
| Efet.01.1655217.g743.t1 | Uncharacterized conserved DUF4415 family | 381 | 6.56E-71 | 86.55 |  |
| Efet.01.1655218.g744.t1 | CDP-alcohol phosphatidyltransferase | 606 | 4.15E-70 | 54.94 | C:membrane; F:phosphotransferase activity, for other substituted phosphate groups |
| Efet.01.1655255.g745.t1 | Innexin unc-9 | 213 | 4.51E-13 | 69.53 | C:membrane; C:cell junction; P:transport |
| Efet.01.1655262.g746.t1 | dehydrogenase | 285 | 2.74E-38 | 93.85 | F:zinc ion binding; F:S-(hydroxymethyl)glutathione dehydrogenase activity; P:oxidation-reduction process |
| Efet.01.1655266.g747.t1 | hypothetical protein CAPTEDRAFT_219500 | 348 | 1.41E-08 | 50.8 | C:membrane; C:integral component of membrane |
| Efet.01.1655278.g748.t1 | MFS transporter | 393 | 6.05E-55 | 92.2 | P:transmembrane transport; C:integral component of membrane; F:transporter activity |
| Efet.01.1655294.g750.t1 | tripartite motif-containing 2-like | 441 | 1.36E-39 | 58.15 | F:zinc ion binding; F:metal ion binding; C:intracellular |
| Efet.01.1655299.g751.t1 | TBC1 domain family member 9 | 270 | 1.15E-09 | 67.95 | P:regulation of vesicle fusion; P:activation of GTPase activity; F:calcium ion binding; F:GTPase activator activity; C:endomembrane system; P:intracellular protein transport; F:Rab GTPase binding; C:intracellular |
| Efet.01.1655335.g753.t1 | bark beetle-like isoform X1 | 348 | 2.00E-39 | 58.3 | C:membrane; F:scavenger receptor activity; P:receptor-mediated endocytosis |
| Efet.01.1655369.g754.t1 | family [Chryseolinea serpens] | 345 | 1.50E-69 | 63.45 |  |
| Efet.01.1655371.g755.t1 | DNA-binding response regulator | 324 | 4.13E-72 | 80.9 | F:DNA binding; P:transcription, DNA-templated; P:phosphorelay signal transduction system; P:regulation of transcription, DNA-templated; C:intracellular |
| Efet.01.1655416.g757.t1 | tyrosine--tRNA ligase | 507 | 1.17E-109 | 74.15 | F:nucleotide binding; P:tRNA aminoacylation for protein translation; F:aminoacyl-tRNA ligase activity |
| Efet.01.1655418.g758.t1 | family transcriptional regulator | 633 | 6.54E-114 | 70.55 |  |
| Efet.01.1655466.g761.t1 | diacylglycerol kinase epsilon-like | 684 | 5.43E-79 | 61.75 | F:nucleotide binding; P:signal transduction; F:kinase activity |
| Efet.01.1655507.g764.t1 | hypothetical protein HELRODRAFT_77866 | 228 | 2.38E-07 | 71 | C:membrane; P:transport |
| Efet.01.1655513.g765.t1 | nucleotidyltransferase domain-containing | 378 | 1.79E-32 | 66.4 | F:nucleotidyltransferase activity; F:transferase activity |
| Efet.01.165553.g777.t1 | histone-lysine N-methyltransferase NSD2 isoform X1 | 1164 | 8.35E-26 | 61.5 | P:regulation of nucleobase-containing compound metabolic process; P:regulation of macromolecule metabolic process; P:histone lysine methylation; P:animal organ development; F:binding; F:transferase activity |
| Efet.01.1655550.g767.t1 | atrial natriuretic peptide receptor 3-like | 315 | 1.43E-36 | 65.8 | C:membrane |
| Efet.01.1655572.g768.t1 | ABC transporter ATP-binding | 246 | 1.64E-48 | 89.2 | F:ATPase activity; F:ATP binding |
| Efet.01.1655581.g769.t1 | phenylacetate- oxygenase subunit | 342 | 4.28E-75 | 92.85 | P:phenylacetate catabolic process |
| Efet.01.1655588.g770.t1 | ABC transporter ATP-binding | 294 | 2.09E-59 | 93.4 | F:ATP binding; F:taurine-transporting ATPase activity; P:amine transport |
| Efet.01.1655607.g771.t1 | RNA polymerase-binding | 315 | 6.40E-58 | 89 | F:bacterial-type RNA polymerase core enzyme binding; P:transcription, DNA-templated; P:positive regulation of transcription, DNA-templated |
| Efet.01.1655654.g775.t1 | NFX1-type zinc finger-containing 1 | 648 | 1.26E-16 | 50.35 | C:nucleus; F:transcription factor activity, sequence-specific DNA binding; F:zinc ion binding; P:regulation of transcription, DNA-templated; F:RNA binding |
| Efet.01.1655655.g776.t1 | neuropeptide SIFamide receptor-like | 366 | 1.36E-11 | 72.3 | P:neuropeptide signaling pathway; C:integral component of membrane; F:neuropeptide Y receptor activity |
| Efet.01.1655662.g777.t1 | non-ribosomal peptide synthetase | 570 | 1.93E-108 | 85 | F:catalytic activity; P:metabolic process; F:phosphopantetheine binding |
| Efet.01.1655672.g779.t1 | family transcriptional regulator | 432 | 2.09E-100 | 68.9 | P:regulation of DNA repair |
| Efet.01.1655691.g780.t1 | matrix metallo ase-2-like | 333 | 9.07E-12 | 58.3 | F:metal ion binding; F:metallopeptidase activity |
| Efet.01.1655703.g781.t1 | hypothetical protein KP79_PYT00168 | 387 | 1.29E-09 | 50.85 | C:trans-Golgi network; C:endosome; C:membrane; C:integral component of membrane; C:Golgi apparatus |
| Efet.01.1655707.g782.t1 | B-box type zinc finger ncl-1-like | 561 | 3.40E-38 | 64.5 | F:zinc ion binding; F:metal ion binding; C:intracellular |
| Efet.01.1655711.g783.t1 | neuropeptide Y receptor-like | 438 | 6.01E-37 | 68.4 | F:neuropeptide receptor activity; P:G-protein coupled receptor signaling pathway; C:membrane |
| Efet.01.1655718.g785.t1 | neuromedin-U receptor 2-like | 528 | 1.86E-61 | 69.5 | P:neuropeptide signaling pathway; C:integral component of membrane; F:neuromedin U receptor activity |
| Efet.01.1655723.g786.t1 | Pre-mRNA-processing-splicing factor 8 | 408 | 1.40E-68 | 97.25 | C:U5 snRNP; F:protein kinase activity; P:protein phosphorylation; F:RNA binding; P:spliceosomal tri-snRNP complex assembly; F:second spliceosomal transesterification activity; F:ATP binding; F:U6 snRNA binding; F:U5 snRNA binding; F:pre-mRNA intronic binding; C:catalytic step 2 spliceosome; P:cellular response to lipopolysaccharide; F:K63-linked polyubiquitin binding; F:U1 snRNA binding; C:membrane; P:cellular response to tumor necrosis factor; F:U2 snRNA binding |
| Efet.01.1655741.g787.t1 | hypothetical protein CAPTEDRAFT_216220 | 333 | 2.32E-10 | 57 | F:G-protein coupled receptor activity; P:G-protein coupled receptor signaling pathway; C:membrane; C:integral component of membrane |
| Efet.01.1655753.g788.t1 | epsin-2-like isoform X2 | 582 | 1.78E-83 | 87.35 | C:clathrin coat of endocytic vesicle; P:endocytosis |
| Efet.01.1655754.g789.t1 | protocadherin-18-like isoform X1 | 315 | 1.13E-20 | 58.3 | C:membrane |
| Efet.01.1655777.g791.t1 | tRNA pseudouridine(55) synthase | 492 | 1.05E-114 | 75.4 | P:pseudouridine synthesis; P:tRNA processing; F:isomerase activity |
| Efet.01.16558.g1222.t1 | cyclic nucleotide-gated channel rod photoreceptor subunit alpha-like | 1299 | 9.60E-75 | 69.8 | P:potassium ion transport; F:ion channel activity; P:ion transmembrane transport; C:membrane |
| Efet.01.1655808.g792.t1 | neurogenic locus Notch -like | 417 | 1.47E-47 | 72.65 | P:cell cycle arrest; P:negative regulation of cell proliferation; P:positive regulation of Ras protein signal transduction; C:extracellular region; C:cell surface; P:animal organ morphogenesis; C:nucleus; C:integral component of plasma membrane; P:heart development; P:marginal zone B cell differentiation; P:Notch signaling pathway; C:cytosol; P:cell growth |
| Efet.01.1655873.g794.t1 | kelch repeat | 342 | 4.18E-12 | 56.05 | F:ubiquitin-protein transferase activity; F:actin binding; C:cytoplasm; C:actin cytoskeleton; C:Cul3-RING ubiquitin ligase complex; P:protein ubiquitination |
| Efet.01.1655878.g795.t1 | hypothetical protein CAPTEDRAFT_220266 | 552 | 7.53E-32 | 61 |  |
| Efet.01.1655883.g796.t1 | GGDEF domain-containing | 282 | 9.08E-42 | 94.6 | F:phosphorelay sensor kinase activity; P:phosphorelay signal transduction system; C:integral component of membrane; P:signal transduction by protein phosphorylation; C:intracellular |
| Efet.01.1655885.g797.t1 | sex peptide receptor-like | 513 | 5.15E-51 | 73.95 | F:G-protein coupled peptide receptor activity; P:G-protein coupled receptor signaling pathway; C:integral component of membrane |
| Efet.01.1655890.g798.t1 | GTP-binding 1-like | 390 | 2.36E-36 | 73.5 | F:GTP binding; F:GTPase activity |
| Efet.01.1655891.g799.t1 | histidinol phosphatase | 441 | 4.66E-96 | 80.65 | P:phosphatidylinositol phosphorylation; P:dephosphorylation; F:histidinol-phosphatase activity |
| Efet.01.1655895.g800.t1 | Mitogen-activated kinase kinase kinase | 417 | 2.53E-16 | 55.9 | P:system development; P:response to wounding; P:cellular response to stimulus; P:regulation of neutrophil chemotaxis; F:protein kinase activity; P:phosphorylation; P:single-organism cellular process; P:positive regulation of biological process; P:immune system process |
| Efet.01.1655926.g802.t1 | ATP-dependent helicase | 498 | 5.60E-79 | 79.75 | F:DNA binding; F:ATP binding; P:DNA duplex unwinding; F:ATP-dependent DNA helicase activity |
| Efet.01.1655934.g804.t1 | efflux RND transporter periplasmic adaptor subunit | 321 | 1.79E-25 | 77.85 | P:transmembrane transport; C:integral component of membrane |
| Efet.01.1655942.g805.t1 | segregation condensation A | 501 | 1.16E-112 | 84.55 |  |
| Efet.01.1655956.g807.t1 | tripartite tricarboxylate transporter substrate binding | 438 | 1.64E-94 | 64.45 | C:outer membrane-bounded periplasmic space |
| Efet.01.1655957.g808.t1 | phosphopyruvate hydratase | 558 | 1.53E-97 | 86.55 | F:magnesium ion binding; F:phosphopyruvate hydratase activity; P:glycolytic process; C:extracellular region; C:phosphopyruvate hydratase complex; C:cell surface |
| Efet.01.1655969.g809.t1 | transient-receptor-potential | 273 | 1.75E-26 | 84.2 | F:calcium channel activity; C:integral component of membrane; P:calcium ion transmembrane transport |
| Efet.01.1655978.g810.t1 | acriflavin resistance | 540 | 1.31E-80 | 89.2 | C:integral component of membrane; P:transport; F:transporter activity |
| Efet.01.1655997.g811.t1 | dual specificity tyrosine-phosphorylation-regulated kinase | 270 | 1.52E-47 | 94.85 | P:peptidyl-tyrosine phosphorylation; F:identical protein binding; P:negative regulation of DNA damage response, signal transduction by p53 class mediator; P:positive regulation of protein deacetylation; C:nuclear speck; P:nervous system development; P:protein autophosphorylation; F:protein serine/threonine kinase activity; P:circadian rhythm; F:protein self-association; F:ATP binding; F:protein serine/threonine/tyrosine kinase activity; C:intracellular ribonucleoprotein complex; F:non-membrane spanning protein tyrosine kinase activity; P:regulation of alternative mRNA splicing, via spliceosome; P:negative regulation of mRNA splicing, via spliceosome; F:tau protein binding; P:peptidyl-serine phosphorylation; P:peptidyl-threonine phosphorylation |
| Efet.01.16560.g1223.t1 | probable ATP-dependent RNA helicase DHX58 | 2079 | 1.29E-64 | 60.2 | F:DNA binding; F:ATP binding; F:hydrolase activity, acting on acid anhydrides; P:regulation of apoptotic process; F:hydrolase activity |
| Efet.01.16560.g1224.t1 | interferon-induced helicase C domain-containing 1 | 483 | 1.66E-09 | 47.93 | F:DNA binding; F:ATP binding; F:hydrolase activity, acting on acid anhydrides; F:helicase activity; F:hydrolase activity |
| Efet.01.1656000.g813.t1 | beta-N-acetylglucosaminidase | 552 | 4.99E-112 | 56.7 |  |
| Efet.01.1656005.g814.t1 | Uncharacterized membrane Bax inhibitor family | 288 | 9.89E-39 | 88.9 | C:integral component of membrane |
| Efet.01.1656043.g817.t1 | nuclease HARBI1 | 507 | 1.09E-37 | 68.25 |  |
| Efet.01.1656069.g818.t1 | cytochrome P450 | 573 | 3.61E-129 | 53.45 | F:iron ion binding; F:oxidoreductase activity, acting on paired donors, with incorporation or reduction of molecular oxygen; P:oxidation-reduction process; F:heme binding |
| Efet.01.1656075.g819.t1 | serine protease | 573 | 4.33E-122 | 66.9 | F:peptidase activity |
| Efet.01.1656078.g820.t1 | neurobeachin-like isoform X3 | 684 | 3.27E-10 | 52.1 |  |
| Efet.01.1656084.g821.t1 | 1-acyl-sn-glycerol-3-phosphate acyltransferase delta-like | 282 | 3.08E-11 | 63 | F:transferase activity, transferring acyl groups; P:metabolic process; C:membrane; C:integral component of membrane |
| Efet.01.1656108.g826.t1 | cytochrome P450 26A1 | 690 | 5.29E-13 | 69.2 | F:oxidoreductase activity; F:binding |
| Efet.01.1656111.g827.t1 | family transcriptional regulator | 219 | 3.20E-41 | 88.85 | F:DNA binding; F:transcription factor activity, sequence-specific DNA binding; P:transcription, DNA-templated; P:regulation of transcription, DNA-templated |
| Efet.01.1656111.g828.t1 | aldehyde dehydrogenase | 261 | 2.27E-53 | 91.14 | P:oxidation-reduction process; F:oxidoreductase activity, acting on the aldehyde or oxo group of donors, NAD or NADP as acceptor |
| Efet.01.1656116.g829.t1 | glycine dehydrogenase (aminomethyl-transferring) | 630 | 2.01E-146 | 85.4 | P:glycine decarboxylation via glycine cleavage system; F:glycine dehydrogenase (decarboxylating) activity; P:oxidation-reduction process |
| Efet.01.1656127.g830.t1 | retinol retinaldehyde reductase | 297 | 3.12E-19 | 72.05 | F:oxidoreductase activity; C:integral component of membrane; P:oxidation-reduction process |
| Efet.01.1656175.g831.t1 | short-chain dehydrogenase | 654 | 4.83E-123 | 81 | F:oxidoreductase activity; P:oxidation-reduction process |
| Efet.01.1656178.g832.t1 | hypothetical protein HELRODRAFT_119038, partial | 408 | 4.33E-13 | 71.67 | P:biological regulation; P:sodium ion transport; P:ion transmembrane transport; F:sodium channel activity; C:integral component of membrane; F:voltage-gated ion channel activity |
| Efet.01.165618.g778.t1 | hypothetical protein HELRODRAFT_173659 | 429 | 3.57E-11 | 76.67 |  |
| Efet.01.1656182.g833.t1 | OHCU decarboxylase | 570 | 1.76E-102 | 87.8 | P:allantoin biosynthetic process; F:hydrolase activity, acting on carbon-nitrogen (but not peptide) bonds, in linear amidines; F:lyase activity; F:N-carbamoyl-L-amino-acid hydrolase activity |
| Efet.01.1656187.g834.t1 | hypothetical protein OCBIM_22004104mg | 273 | 1.03E-15 | 61.9 |  |
| Efet.01.1656193.g836.t1 | ABC transporter permease | 651 | 1.21E-134 | 90.9 | F:strictosidine synthase activity; C:integral component of membrane; P:biosynthetic process; P:transport; C:plasma membrane; F:transporter activity |
| Efet.01.165620.g779.t1 | innexin 4 | 870 | 2.22E-73 | 69.05 | C:gap junction; P:ion transport; C:integral component of membrane; P:cell communication by electrical coupling; C:synapse; F:gap junction channel activity; C:plasma membrane |
| Efet.01.1656205.g837.t1 | non-ribosomal peptide synthetase | 471 | 4.15E-89 | 83.1 | F:catalytic activity; P:metabolic process; F:phosphopantetheine binding |
| Efet.01.1656217.g838.t1 | mannose-6-phosphate isomerase | 528 | 2.36E-99 | 69.6 |  |
| Efet.01.1656219.g839.t1 | cysteine hydrolase | 387 | 5.59E-87 | 92.6 | P:metabolic process; F:hydrolase activity |
| Efet.01.1656231.g840.t1 | RNA polymerase subunit sigma | 306 | 8.06E-56 | 87.4 | F:DNA binding; F:sigma factor activity; F:transcription factor activity, sequence-specific DNA binding; P:regulation of transcription, DNA-templated; P:DNA-templated transcription, initiation |
| Efet.01.1656247.g842.t1 | carbohydrate ABC transporter permease | 513 | 7.76E-106 | 83.7 | C:integral component of membrane; P:transport; C:plasma membrane |
| Efet.01.1656264.g844.t1 | RNA polymerase sigma-70 factor | 555 | 5.15E-135 | 64.45 | F:DNA binding; F:sigma factor activity; F:transcription factor activity, sequence-specific DNA binding; P:transcription, DNA-templated; P:regulation of transcription, DNA-templated; P:DNA-templated transcription, initiation |
| Efet.01.1656266.g845.t1 | flagellar basal body | 378 | 8.64E-78 | 77.65 | P:chemotaxis; C:integral component of membrane; P:bacterial-type flagellum-dependent cell motility; C:bacterial-type flagellum basal body |
| Efet.01.1656276.g846.t1 | hypothetical protein B5766_03700 | 405 | 2.98E-85 | 73 |  |
| Efet.01.1656279.g847.t1 | ABC transporter permease | 696 | 4.14E-157 | 95.45 | C:integral component of membrane; P:transport; C:plasma membrane |
| Efet.01.1656280.g848.t1 | melanotransferrin-like isoform X1 | 288 | 9.69E-17 | 63.85 | P:iron ion homeostasis; C:extracellular space; P:ion transport; F:metal ion binding |
| Efet.01.1656291.g850.t1 | NADH dehydrogenase [ubiquinone] 1 alpha subcomplex subunit mitochondrial | 591 | 4.12E-53 | 59.75 |  |
| Efet.01.1656309.g852.t1 | Cap-specific mRNA (nucleoside-2 -O-)-methyltransferase 1 | 438 | 2.23E-54 | 72.05 | P:mRNA metabolic process; F:methyltransferase activity; P:methylation |
| Efet.01.1656345.g854.t1 | phosphoglucosamine mutase | 558 | 8.61E-108 | 84.7 | F:magnesium ion binding; P:carbohydrate metabolic process; F:phosphoglucosamine mutase activity |
| Efet.01.1656349.g856.t1 | glyco endo-alpha-1,2-mannosidase | 585 | 3.80E-72 | 72.55 |  |
| Efet.01.1656378.g857.t1 | carbohydrate ABC transporter substrate-binding CUT1 family | 504 | 6.27E-58 | 66.25 | P:transport; F:transporter activity |
| Efet.01.1656379.g858.t1 | dystonin isoform X1 | 561 | 9.25E-36 | 57.1 | P:cell cycle arrest; C:cytoskeleton; F:calcium ion binding |
| Efet.01.1656388.g859.t1 | urease subunit gamma | 303 | 1.60E-64 | 98.75 | P:urea catabolic process; C:cytoplasm; F:nickel cation binding; F:urease activity |
| Efet.01.1656396.g860.t1 | transcriptional regulator | 444 | 8.16E-100 | 79.65 | F:DNA binding; F:transcription factor activity, sequence-specific DNA binding; P:transcription, DNA-templated; P:regulation of transcription, DNA-templated; C:intracellular |
| Efet.01.1656422.g863.t1 | Calcineurin B homologous 1 | 243 | 3.23E-17 | 86.95 | F:calcium ion binding |
| Efet.01.1656424.g864.t1 | eukaryotic initiation factor 4A-I | 456 | 1.28E-41 | 95.6 | F:translation initiation factor activity; F:ATP binding; C:cytosol; F:helicase activity; P:translational initiation |
| Efet.01.1656428.g865.t1 | arabinose isomerase | 450 | 2.92E-94 | 93.3 | F:L-fucose isomerase activity; C:cytoplasm; F:metal ion binding; F:L-arabinose isomerase activity; P:fucose metabolic process; P:L-arabinose catabolic process to xylulose 5-phosphate |
| Efet.01.1656440.g868.t1 | hypothetical protein MIC448_750005 | 237 | 1.81E-09 | 84.5 |  |
| Efet.01.1656442.g869.t1 | hypothetical protein HELRODRAFT_194765 | 339 | 2.49E-37 | 69.4 | C:membrane; C:integral component of membrane |
| Efet.01.1656465.g871.t1 | spermidine putrescine ABC transporter permease | 393 | 1.04E-78 | 62.85 | C:integral component of membrane; P:transport; C:plasma membrane |
| Efet.01.1656470.g872.t1 | innexin 1 | 420 | 5.65E-46 | 68.75 | C:membrane; P:cell communication by electrical coupling; C:cell junction; C:synapse; F:gap junction channel activity; P:transport |
| Efet.01.1656473.g873.t1 | ATP synthase F0 subunit C | 273 | 1.26E-23 | 95.1 | F:hydrogen ion transmembrane transporter activity; P:ATP hydrolysis coupled proton transport; C:proton-transporting ATP synthase complex, coupling factor F(o); C:integral component of membrane; C:plasma membrane; P:ATP synthesis coupled proton transport; F:hydrolase activity |
| Efet.01.1656497.g875.t1 | restriction endonuclease subunit R | 462 | 8.69E-91 | 79.8 | F:DNA binding; F:ATP binding; P:nucleic acid phosphodiester bond hydrolysis; F:endonuclease activity |
| Efet.01.1656508.g877.t1 | kelch-like ECH-associated 1 | 579 | 1.49E-77 | 70.75 | C:Cul3-RING ubiquitin ligase complex; P:protein ubiquitination |
| Efet.01.1656526.g878.t1 | ABC transporter substrate-binding | 492 | 2.69E-98 | 78.9 | P:amino acid transport |
| Efet.01.1656529.g879.t1 | hypothetical protein CAPTEDRAFT_217495 | 234 | 1.51E-07 | 62.25 | C:membrane; C:integral component of membrane; F:carbohydrate binding |
| Efet.01.1656537.g880.t1 | FAD-dependent oxidoreductase | 432 | 4.66E-76 | 75.35 | F:FAD binding; C:integral component of membrane; P:oxidation-reduction process; F:monooxygenase activity |
| Efet.01.1656547.g881.t1 | E3 ubiquitin- ligase CBL-B | 513 | 9.92E-70 | 81.1 | F:phosphotyrosine binding; C:nucleus; F:ubiquitin-protein transferase activity; F:zinc ion binding; P:regulation of signaling; F:calcium ion binding; P:cell surface receptor signaling pathway; F:signal transducer activity; F:ligase activity; P:protein ubiquitination |
| Efet.01.1656555.g882.t1 | Neuroglian | 330 | 1.87E-14 | 54.72 | F:growth factor activity; C:Nem1-Spo7 phosphatase complex; C:membrane; C:integral component of membrane; P:positive regulation of protein dephosphorylation; C:extracellular region; P:growth |
| Efet.01.1656574.g885.t1 | bone morphogenetic 4 | 420 | 2.01E-14 | 49.2 | P:epithelial tube branching involved in lung morphogenesis; P:positive regulation of transport; P:regulation of morphogenesis of a branching structure; P:forebrain development; P:ossification; P:BMP signaling pathway; P:regulation of epithelial cell proliferation; P:positive regulation of nucleobase-containing compound metabolic process; P:positive regulation of transmembrane receptor protein serine/threonine kinase signaling pathway; P:regulation of apoptotic process; P:response to organic cyclic compound; P:negative regulation of kidney development; P:response to hormone; P:cell differentiation involved in kidney development; P:sensory organ development; P:growth; P:positive regulation of ossification; P:epithelial cell differentiation; P:negative regulation of gene expression; P:positive regulation of molecular function; P:positive regulation of epithelial cell differentiation; P:positive regulation of gene expression; P:mesodermal cell differentiation; P:myeloid cell differentiation; P:positive regulation of cell death; P:cell proliferation; P:regulation of cellular localization; P:chordate embryonic development; P:regulation of cartilage development; P:regulation of MAPK cascade; P:negative regulation of cell proliferation; P:positive regulation of cell proliferation; P:negative regulation of cell death; C:extracellular region; P:regulation of animal organ formation; P:regulation of muscle cell differentiation; P:heart development; P:angiogenesis; P:negative regulation of cell differentiation; P:response to lipid; P:positive regulation of macromolecule biosynthetic process; P:nephron epithelium development; P:negative regulation of morphogenesis of an epithelium; P:cell development; F:receptor binding; P:regionalization; P:skeletal system morphogenesis; P:positive regulation of cellular biosynthetic process; P:metanephros development; P:ureteric bud development; P:positive regulation of protein phosphorylation; P:positive regulation of cell development; P:developmental induction; P:negative regulation of cellular metabolic process |
| Efet.01.1656581.g887.t1 | Small conductance calcium-activated potassium channel | 360 | 9.49E-20 | 73.05 | F:calmodulin binding; P:potassium ion transmembrane transport; C:integral component of membrane; F:small conductance calcium-activated potassium channel activity; C:neuronal cell body; C:plasma membrane |
| Efet.01.1656600.g889.t1 | receptor-interacting serine threonine- kinase 1 | 258 | 2.11E-11 | 61 | P:positive regulation of phosphorylation; P:protein complex assembly; P:regulation of intracellular signal transduction; P:positive regulation of programmed cell death; P:cellular protein metabolic process; C:cell part; P:regulation of apoptotic process; P:positive regulation of signal transduction; F:protein binding; P:positive regulation of macromolecule metabolic process; P:cellular response to stimulus; P:phosphorylation; P:programmed cell death; P:regulation of primary metabolic process |
| Efet.01.1656606.g890.t1 | flagellar biosynthetic | 768 | 1.04E-163 | 92.55 | P:protein targeting; P:bacterial-type flagellum assembly; C:integral component of membrane; C:plasma membrane; C:bacterial-type flagellum basal body |
| Efet.01.1656622.g893.t1 | family pseudouridine synthase | 492 | 1.23E-115 | 86.8 | P:pseudouridine synthesis; F:RNA binding; F:pseudouridine synthase activity |
| Efet.01.1656626.g894.t1 | solute carrier family 12 | 231 | 2.60E-34 | 75.65 | F:potassium:chloride symporter activity; P:chloride transmembrane transport; C:integral component of membrane |
| Efet.01.1656637.g896.t1 | retinol retinaldehyde reductase | 297 | 2.60E-12 | 66.2 | C:membrane |
| Efet.01.1656647.g897.t1 | glucosamine-6-phosphate deaminase | 648 | 1.65E-154 | 68.25 | P:carbohydrate metabolic process; P:N-acetylglucosamine metabolic process; F:glucosamine-6-phosphate deaminase activity |
| Efet.01.1656660.g898.t1 | zinc finger FYVE domain-containing 1-like | 480 | 4.84E-74 | 84.4 | F:phosphatidylinositol-3,4,5-trisphosphate binding; F:zinc ion binding; C:perinuclear region of cytoplasm; P:negative regulation of phosphatase activity; C:pre-autophagosomal structure; C:ER-mitochondrion membrane contact site; C:endoplasmic reticulum; C:extrinsic component of omegasome membrane; F:phosphatidylinositol-3,4-bisphosphate binding; C:Golgi stack; C:autophagosome; F:1-phosphatidylinositol binding |
| Efet.01.1656667.g899.t1 | peptidoglycan binding domain-containing | 495 | 1.47E-99 | 54.2 | C:membrane; C:integral component of membrane |
| Efet.01.1656675.g900.t1 | hypothetical protein CAPTEDRAFT_118770, partial | 231 | 6.40E-07 | 65 | C:nuclear envelope; F:actin filament binding; F:actin binding; P:cytoskeletal anchoring at nuclear membrane; C:LINC complex |
| Efet.01.1656677.g901.t1 | actin beta gamma 1 | 567 | 1.62E-95 | 83.45 | F:ATP binding |
| Efet.01.1656719.g904.t1 | family | 537 | 1.34E-39 | 63.2 | C:membrane; C:integral component of membrane |
| Efet.01.1656720.g905.t1 | hypothetical protein | 393 | 7.88E-73 | 78.65 |  |
| Efet.01.1656720.g906.t1 | ATP-dependent Clp protease ATP-binding subunit | 375 | 5.19E-70 | 68.35 | F:ATP binding |
| Efet.01.1656721.g907.t1 | DNA primase | 534 | 6.96E-18 | 55.25 | C:membrane; C:integral component of membrane |
| Efet.01.1656733.g908.t1 | hypothetical protein | 303 | 1.79E-19 | 75.8 |  |
| Efet.01.1656766.g910.t1 | NAD(P) FAD-dependent oxidoreductase | 642 | 6.79E-150 | 70.9 | F:oxidoreductase activity; P:oxidation-reduction process |
| Efet.01.1656769.g911.t1 | ATPase | 219 | 5.20E-38 | 100 | F:phosphorelay sensor kinase activity; P:phosphorelay signal transduction system; C:integral component of membrane; P:signal transduction by protein phosphorylation; C:intracellular |
| Efet.01.1656772.g913.t1 | glutamate--cysteine ligase | 456 | 7.38E-55 | 73.2 | F:nucleotide binding; F:ligase activity, forming carbon-nitrogen bonds |
| Efet.01.1656780.g914.t1 | peptidyl-prolyl cis-trans isomerase [Fabibacter pacificus] | 594 | 3.12E-70 | 71.05 |  |
| Efet.01.1656809.g915.t1 | trehalose-phosphatase | 738 | 9.11E-172 | 81.45 | F:metal ion binding; F:trehalose-phosphatase activity; P:trehalose biosynthetic process; P:dephosphorylation |
| Efet.01.1656814.g916.t1 | sphingosine-1-phosphate phosphatase 1-like | 789 | 2.70E-53 | 57.3 | C:integral component of membrane |
| Efet.01.1656827.g917.t1 | tachykinin-like peptides receptor 99D | 495 | 5.80E-37 | 78.85 | P:neuropeptide signaling pathway; C:integral component of membrane; P:tachykinin receptor signaling pathway; C:plasma membrane; F:tachykinin receptor activity; F:neuropeptide Y receptor activity |
| Efet.01.1656869.g919.t1 | integrator complex subunit 2 isoform X2 | 306 | 9.51E-37 | 76.05 | P:snRNA processing; C:integrator complex |
| Efet.01.1656872.g920.t1 | urocanate hydratase | 753 | 1.19E-157 | 93.75 | C:cytoplasm; F:urocanate hydratase activity; P:histidine catabolic process to glutamate and formate; P:histidine catabolic process to glutamate and formamide |
| Efet.01.1656988.g925.t1 | sugar isomerase | 318 | 4.04E-32 | 79 | P:carbohydrate metabolic process; F:carbohydrate binding |
| Efet.01.1656989.g926.t1 | glutamate receptor NMDA 2B isoform X3 | 606 | 2.05E-08 | 45.7 | C:membrane |
| Efet.01.1657001.g929.t1 | phosphatase 1L isoform X2 | 423 | 2.24E-71 | 88.25 | C:endoplasmic reticulum membrane; F:protein serine/threonine phosphatase activity; P:MAPK cascade; C:extracellular exosome; F:metal ion binding; P:protein dephosphorylation; C:integral component of membrane; P:transmembrane receptor protein serine/threonine kinase signaling pathway; P:sphingolipid biosynthetic process |
| Efet.01.1657003.g930.t1 | NADP-dependent oxidoreductase | 699 | 4.58E-105 | 76.95 | F:zinc ion binding; F:oxidoreductase activity; P:oxidation-reduction process |
| Efet.01.1657015.g931.t1 | DUF4131 domain-containing | 249 | 2.55E-21 | 75.6 | C:integral component of membrane |
| Efet.01.1657017.g933.t1 | chitin synthase | 492 | 1.62E-27 | 82.7 | F:transferase activity, transferring hexosyl groups; C:integral component of membrane |
| Efet.01.1657020.g934.t1 | Outer membrane beta-barrel domain-containing | 228 | 1.38E-46 | 67.6 | C:integral component of membrane |
| Efet.01.1657029.g935.t1 | 3-isopropylmalate dehydratase large subunit | 765 | 1.04E-143 | 82.6 | F:metal ion binding; P:leucine biosynthetic process; F:4 iron, 4 sulfur cluster binding; F:3-isopropylmalate dehydratase activity; F:isomerase activity |
| Efet.01.1657052.g936.t1 | potassium voltage-gated channel subfamily H member 7-like isoform X3 | 447 | 1.54E-52 | 79.8 | F:voltage-gated potassium channel activity; P:potassium ion transmembrane transport; C:integral component of membrane |
| Efet.01.1657070.g938.t1 | TIGR03759 family integrating conjugative element | 534 | 1.63E-83 | 86.9 | C:cell; F:protein disulfide oxidoreductase activity; F:electron carrier activity; P:cell redox homeostasis; P:oxidation-reduction process |
| Efet.01.1657080.g939.t1 | acyl- dehydrogenase | 564 | 4.35E-107 | 84.7 |  |
| Efet.01.1657095.g942.t1 | zinc finger basonuclin-2-like isoform X1 | 300 | 1.93E-27 | 77.25 | F:nucleic acid binding; F:metal ion binding |
| Efet.01.1657106.g943.t1 | SH3 domain-containing 19-like | 285 | 1.78E-20 | 77.8 |  |
| Efet.01.1657118.g944.t1 | hypothetical protein CAPTEDRAFT_195684 | 570 | 2.54E-48 | 58.29 | C:membrane; C:integral component of membrane |
| Efet.01.1657123.g945.t1 | Zn-dependent minimal metalloprotease (MMP)-like domain | 426 | 8.96E-59 | 87.3 |  |
| Efet.01.1657160.g947.t1 | septum formation initiator | 474 | 5.45E-88 | 74.35 | C:integral component of membrane |
| Efet.01.1657170.g948.t1 | 30S ribosomal S15 | 267 | 4.27E-44 | 98.15 | F:structural constituent of ribosome; C:ribosome; F:rRNA binding; P:translation |
| Efet.01.1657181.g951.t1 | tricalbin-3 isoform X6 | 348 | 2.54E-16 | 68.4 | C:membrane |
| Efet.01.1657182.g952.t1 | polyketide cyclase | 459 | 9.47E-107 | 73.25 |  |
| Efet.01.1657186.g953.t1 | conserved hypothetical protein | 453 | 9.14E-84 | 92.15 |  |
| Efet.01.1657188.g954.t1 | hypothetical protein HELRODRAFT_191437 | 618 | 2.13E-23 | 50.5 |  |
| Efet.01.1657200.g955.t1 | chromosomal replication initiator | 438 | 3.49E-76 | 80.95 | F:DNA replication origin binding; F:ATP binding; P:DNA replication initiation; C:cytoplasm; P:regulation of DNA replication |
| Efet.01.1657216.g958.t1 | arginyl-tRNA-- transferase 1 isoform X1 | 411 | 2.37E-10 | 61.55 | F:transferase activity, transferring acyl groups |
| Efet.01.1657234.g959.t1 | aminoglycoside phosphotransferase family | 708 | 4.42E-153 | 60.44 | F:transferase activity |
| Efet.01.1657240.g960.t1 | FMRFamide receptor | 717 | 1.42E-86 | 58.65 | F:G-protein coupled receptor activity; P:signal transduction; C:membrane |
| Efet.01.1657249.g963.t1 | family transcriptional regulator | 435 | 5.85E-88 | 80.9 | F:DNA binding; F:transcription factor activity, sequence-specific DNA binding; P:transcription, DNA-templated; P:regulation of transcription, DNA-templated |
| Efet.01.1657281.g964.t1 | MULTISPECIES: hypothetical protein | 276 | 3.83E-19 | 68.89 |  |
| Efet.01.1657281.g965.t1 | FABP family | 282 | 6.01E-47 | 81.55 |  |
| Efet.01.1657283.g966.t1 | ammonia channel | 555 | 7.20E-125 | 95.3 | F:ammonium transmembrane transporter activity; P:ammonium transmembrane transport; C:integral component of membrane |
| Efet.01.1657287.g967.t1 | uncharacterized protein K02A2.6-like | 339 | 3.05E-27 | 61.3 |  |
| Efet.01.1657288.g968.t1 | tRNA methyltransferase 10 homolog B-like | 384 | 8.24E-34 | 67.95 | F:methyltransferase activity; F:transferase activity; P:methylation |
| Efet.01.1657319.g970.t1 | hypothetical protein HELRODRAFT_185352 | 429 | 1.50E-09 | 58.25 | C:membrane; C:integral component of membrane; P:transport; F:transporter activity |
| Efet.01.1657332.g971.t1 | ubiquinol-cytochrome C reductase | 588 | 8.90E-142 | 89.7 | F:oxidoreductase activity, acting on diphenols and related substances as donors; F:metal ion binding; C:integral component of membrane; P:oxidation-reduction process; F:2 iron, 2 sulfur cluster binding |
| Efet.01.1657335.g972.t1 | pyruvate kinase | 498 | 5.73E-112 | 93.45 | F:magnesium ion binding; F:pyruvate kinase activity; F:potassium ion binding; P:glycolytic process; F:kinase activity |
| Efet.01.1657336.g973.t1 | dynein gamma flagellar outer arm-like | 552 | 4.76E-27 | 55.5 | F:nucleotide binding; F:ATP binding; F:microtubule motor activity; C:dynein complex; P:microtubule-based movement |
| Efet.01.1657353.g975.t1 | hypothetical protein B5766_11670 | 498 | 3.53E-116 | 74.6 |  |
| Efet.01.1657366.g976.t1 | GPI transamidase component PIG-T | 333 | 3.28E-36 | 68.35 | P:attachment of GPI anchor to protein; C:GPI-anchor transamidase complex |
| Efet.01.1657368.g977.t1 | sugar ABC transporter ATP-binding | 678 | 9.10E-110 | 77.05 | F:ATPase activity; F:ATP binding |
| Efet.01.1657372.g978.t1 | M-phase inducer phosphatase-like | 465 | 5.04E-06 | 55 |  |
| Efet.01.1657387.g979.t1 | sugar phosphate isomerase epimerase | 690 | 5.50E-142 | 89.1 | P:nucleic acid phosphodiester bond hydrolysis; F:endonuclease activity; F:isomerase activity |
| Efet.01.1657388.g980.t1 | acetyl- acetyltransferase | 318 | 2.36E-67 | 96.7 | F:acetyl-CoA C-acetyltransferase activity; P:metabolic process |
| Efet.01.1657411.g982.t1 | hypothetical protein B5766_09480 | 309 | 3.03E-15 | 99 |  |
| Efet.01.1657429.g984.t1 | zinc finger 239 | 432 | 1.66E-26 | 56 | F:nucleic acid binding; F:metal ion binding |
| Efet.01.1657445.g985.t1 | Synaptic vesicular amine transporter | 282 | 3.69E-15 | 75.85 | P:transmembrane transport; C:integral component of membrane |
| Efet.01.1657446.g986.t1 | tripartite tricarboxylate transporter substrate binding | 606 | 1.21E-100 | 76.4 | C:outer membrane-bounded periplasmic space |
| Efet.01.1657455.g988.t1 | UDP-N-acetylmuramoyl-L-alanine--D-glutamate ligase | 504 | 1.19E-101 | 84.55 | F:tetrahydrofolylpolyglutamate synthase activity; P:regulation of cell shape; P:cell division; F:ATP binding; C:cytoplasm; P:peptidoglycan biosynthetic process; F:UDP-N-acetylmuramoylalanine-D-glutamate ligase activity; P:cell wall organization; P:tetrahydrofolylpolyglutamate biosynthetic process; P:cell cycle |
| Efet.01.1657461.g990.t1 | modification methylase 7I | 453 | 2.46E-66 | 66.05 | F:DNA binding; F:methyltransferase activity; P:DNA modification; P:methylation |
| Efet.01.1657480.g992.t1 | hypothetical protein HELRODRAFT_191437 | 624 | 4.07E-08 | 54 |  |
| Efet.01.1657492.g994.t1 | peptide ABC transporter substrate-binding | 819 | 0 | 67.7 | P:transmembrane transport; C:ATP-binding cassette (ABC) transporter complex |
| Efet.01.1657493.g995.t1 | serine incorporator 1-like | 342 | 2.10E-25 | 72.65 | C:membrane; P:cellular lipid metabolic process; P:organonitrogen compound metabolic process |
| Efet.01.1657518.g997.t1 | hypothetical protein CRN43_15250 | 378 | 3.92E-42 | 67.89 |  |
| Efet.01.1657526.g998.t1 | ABC transporter permease | 558 | 6.49E-80 | 85.6 | C:integral component of membrane; P:transport; C:plasma membrane; F:transporter activity |
| Efet.01.1657527.g999.t1 | cell division | 456 | 2.70E-85 | 89.05 | P:cell division; F:DNA binding; F:ATP binding; C:integral component of membrane |
| Efet.01.1657533.g1000.t1 | glycoside hydrolase family 127 | 627 | 3.63E-144 | 79.1 | F:catalytic activity |
| Efet.01.1657535.g1001.t1 | general transcription factor IIE subunit 1-like | 558 | 1.09E-50 | 73.4 | C:intracellular part |
| Efet.01.1657541.g1002.t1 | excinuclease ABC subunit | 576 | 1.05E-96 | 91.15 | F:ATPase activity; F:DNA binding; F:ATP binding; P:nucleic acid phosphodiester bond hydrolysis; F:excinuclease ABC activity; C:excinuclease repair complex; C:cytoplasm; F:metal ion binding; P:nucleotide-excision repair |
| Efet.01.1657542.g1003.t1 | branched-chain-amino-acid aminotransferase 2 | 234 | 2.62E-42 | 71.4 | F:catalytic activity; P:metabolic process |
| Efet.01.1657544.g1004.t1 | guanylate-binding 1-like | 348 | 1.47E-11 | 61.25 | F:GTP binding; F:nucleotide binding; F:GTPase activity |
| Efet.01.1657553.g1006.t1 | T-complex 1 subunit epsilon-like | 246 | 7.26E-37 | 89.2 | F:ATP binding; C:cytoplasm; F:unfolded protein binding; P:protein folding |
| Efet.01.1657565.g1008.t1 | tripartite tricarboxylate transporter substrate binding | 651 | 8.88E-74 | 72.55 | C:outer membrane-bounded periplasmic space |
| Efet.01.1657570.g1009.t1 | aldo keto reductase | 588 | 3.58E-112 | 74.8 |  |
| Efet.01.1657574.g1010.t1 | aminobutyraldehyde dehydrogenase | 204 | 1.45E-40 | 94.2 | F:1-pyrroline dehydrogenase activity; F:aminobutyraldehyde dehydrogenase activity; P:oxidation-reduction process |
| Efet.01.1657589.g1012.t1 | ABC transporter ATP-binding | 810 | 0 | 69.6 | F:nucleotide binding; F:ATPase activity; P:single-organism transport; C:plasma membrane; F:hydrolase activity, acting on acid anhydrides, catalyzing transmembrane movement of substances; P:organic substance transport |
| Efet.01.1657590.g1013.t1 | inorganic pyrophosphatase | 486 | 2.37E-90 | 93.35 | F:magnesium ion binding; F:inorganic diphosphatase activity; C:cytoplasm; P:phosphate-containing compound metabolic process |
| Efet.01.1657603.g1014.t1 | VWA domain-containing | 336 | 3.63E-51 | 63.25 | C:membrane; C:integral component of membrane |
| Efet.01.1657616.g1016.t1 | fluoride efflux transporter | 381 | 6.00E-43 | 68.55 | C:membrane; P:transport |
| Efet.01.1657620.g1017.t1 | succinate dehydrogenase subunit | 534 | 5.99E-122 | 66.45 | F:hydrolase activity, acting on ester bonds; F:metal ion binding; P:metabolic process |
| Efet.01.1657625.g1018.t1 | sensor histidine kinase | 564 | 8.83E-128 | 83.5 | F:phosphorelay sensor kinase activity; F:ATP binding; P:phosphorelay signal transduction system; C:integral component of membrane; P:signal transduction by protein phosphorylation; C:intracellular |
| Efet.01.1657633.g1019.t1 | 4-hydroxy-tetrahydrodipicolinate reductase | 558 | 1.33E-98 | 78.35 | F:NADP binding; F:oxidoreductase activity, acting on CH or CH2 groups, NAD or NADP as acceptor; C:cytoplasm; F:NAD binding; P:lysine biosynthetic process via diaminopimelate; F:4-hydroxy-tetrahydrodipicolinate reductase; P:oxidation-reduction process; P:diaminopimelate biosynthetic process |
| Efet.01.1657637.g1020.t1 | zinc C2H2 | 255 | 1.40E-25 | 84.3 | C:nucleus; F:metal ion binding; P:regulation of transcription, DNA-templated; F:sequence-specific DNA binding |
| Efet.01.1657652.g1023.t1 | methylmalonate-semialdehyde dehydrogenase ( acylating) | 678 | 7.66E-151 | 86.7 | F:methylmalonate-semialdehyde dehydrogenase (acylating) activity; P:oxidation-reduction process |
| Efet.01.1657672.g1024.t1 | FAA hydrolase family | 708 | 2.51E-126 | 75.15 | F:5-carboxymethyl-2-hydroxymuconate delta-isomerase activity; P:metabolic process |
| Efet.01.1657687.g1025.t1 | homoserine dehydrogenase | 558 | 6.35E-84 | 89.15 | P:methionine biosynthetic process; P:isoleucine biosynthetic process; F:NADP binding; P:threonine biosynthetic process; P:oxidation-reduction process; F:amino acid binding; F:homoserine dehydrogenase activity |
| Efet.01.1657691.g1026.t1 | elongation factor-like GTPase 1 | 252 | 7.59E-26 | 72.5 | F:nucleotide binding; F:GTPase activity; P:mature ribosome assembly; C:intracellular; P:translation |
| Efet.01.1657703.g1027.t1 | Carboxypeptidase regulatory-like domain-containing protein | 726 | 1.04E-63 | 57.7 | C:membrane; F:receptor activity; P:transport |
| Efet.01.1657708.g1028.t1 | acid sphingomyelinase-like phosphodiesterase 3b | 432 | 4.97E-39 | 64.6 | C:extracellular space; F:sphingomyelin phosphodiesterase activity; C:membrane; C:integral component of membrane; P:sphingomyelin catabolic process; F:hydrolase activity |
| Efet.01.1657728.g1029.t1 | sensor histidine kinase | 741 | 9.02E-133 | 80.4 | F:phosphorelay sensor kinase activity; F:ATP binding; P:phosphorelay signal transduction system; C:integral component of membrane; P:signal transduction by protein phosphorylation; C:intracellular |
| Efet.01.1657733.g1030.t1 | undecaprenyldiphospho-muramoylpentapeptide beta-N-acetylglucosaminyltransferase | 738 | 2.63E-105 | 86.85 | P:regulation of cell shape; P:cell division; P:carbohydrate metabolic process; F:UDP-N-acetyl-D-glucosamine:N-acetylmuramoyl-L-alanyl-D-glutamyl-meso-2,6-diaminopimelyl-D-alanyl-D-alanine-diphosphoundecaprenol 4-beta-N-acetylglucosaminlytransferase activity; P:peptidoglycan biosynthetic process; P:cell wall organization; C:plasma membrane; F:undecaprenyldiphospho-muramoylpentapeptide beta-N-acetylglucosaminyltransferase activity; P:cell cycle; P:lipid glycosylation |
| Efet.01.1657737.g1031.t1 | SPRY domain-containing 3-like | 597 | 1.48E-06 | 44 |  |
| Efet.01.1657744.g1032.t1 | APC family permease | 285 | 1.54E-52 | 87.35 | F:DNA binding; C:integral component of membrane; F:amino acid transmembrane transporter activity; P:amino acid transmembrane transport |
| Efet.01.1657750.g1033.t1 | aspartate aminotransferase | 210 | 4.19E-40 | 87.85 | F:pyridoxal phosphate binding; F:L-phenylalanine:2-oxoglutarate aminotransferase activity; P:biosynthetic process; F:L-aspartate:2-oxoglutarate aminotransferase activity |
| Efet.01.1657769.g1035.t1 | sphingosine-1-phosphate phosphatase 2-like | 504 | 8.50E-52 | 58.15 | C:membrane; C:integral component of membrane |
| Efet.01.1657781.g1036.t1 | ABC transporter substrate-binding | 450 | 3.86E-82 | 69.45 | P:transmembrane transport; C:ATP-binding cassette (ABC) transporter complex |
| Efet.01.1657789.g1037.t1 | Leucine-rich repeat and fibronectin type III domain-containing 1 | 249 | 1.40E-20 | 66.69 | C:membrane |
| Efet.01.1657797.g1038.t1 | DNA-binding response regulator | 525 | 8.35E-52 | 85.45 | F:DNA binding; P:transcription, DNA-templated; P:phosphorelay signal transduction system; P:regulation of transcription, DNA-templated; C:intracellular |
| Efet.01.1657802.g1039.t1 | conjugal transfer | 690 | 2.05E-12 | 46.15 | F:hydrolase activity |
| Efet.01.1657811.g1040.t1 | Bcl-2 1 | 495 | 1.45E-41 | 59.25 | P:regulation of cellular process; C:membrane |
| Efet.01.1657814.g1041.t1 | Uncharacterised | 255 | 1.24E-39 | 74 |  |
| Efet.01.1657814.g1042.t1 | toxin | 399 | 2.76E-87 | 80.2 | F:ATP binding; P:phosphorylation; F:kinase activity |
| Efet.01.1657819.g1043.t1 | DNA-binding response regulator | 549 | 3.81E-88 | 81.4 | F:DNA binding; P:transcription, DNA-templated; P:phosphorelay signal transduction system; P:regulation of transcription, DNA-templated; C:intracellular |
| Efet.01.1657830.g1044.t1 | hypothetical protein [Diaphorobacter polyhydroxybutyrativorans] | 345 | 8.85E-45 | 62 |  |
| Efet.01.165784.g783.t1 | la-related 4-like isoform X2 | 297 | 1.34E-22 | 68 | F:nucleotide binding; F:nucleic acid binding |
| Efet.01.1657851.g1046.t1 | shikimate dehydrogenase | 513 | 1.00E-43 | 65.7 | F:catalytic activity |
| Efet.01.1657854.g1047.t1 | 60S ribosomal L13a | 360 | 2.03E-30 | 83.6 | F:structural constituent of ribosome; F:mRNA binding; C:cytosolic large ribosomal subunit; P:translation |
| Efet.01.1657871.g1048.t1 | FMRFamide receptor-like | 795 | 2.01E-89 | 57.3 | F:G-protein coupled receptor activity; C:membrane |
| Efet.01.1657876.g1049.t1 | threonine serine dehydratase | 969 | 2.86E-171 | 85.55 | F:L-threonine ammonia-lyase activity; F:isomerase activity |
| Efet.01.1657884.g1050.t1 | sex peptide receptor-like | 357 | 3.47E-32 | 72.05 | F:G-protein coupled receptor activity; C:membrane |
| Efet.01.1657885.g1051.t1 | threonylcarbamoyl-AMP synthase | 321 | 1.58E-61 | 78.15 | F:nucleotide binding; F:double-stranded RNA binding |
| Efet.01.1657890.g1052.t1 | neuralized isoform X2 | 489 | 2.04E-77 | 78.25 | F:metal ion binding |
| Efet.01.1657891.g1053.t1 | creatininase | 396 | 4.58E-79 | 86.3 | P:creatinine catabolic process; F:creatininase activity; P:creatine biosynthetic process |
| Efet.01.1657891.g1054.t1 | sulfite exporter family | 306 | 7.18E-63 | 92.25 | C:integral component of membrane; C:plasma membrane |
| Efet.01.1657901.g1055.t1 | N-acetyltransferase | 453 | 1.92E-105 | 88.1 | F:N-acetyltransferase activity |
| Efet.01.1657906.g1057.t1 | chromosome segregation SMC | 888 | 7.93E-120 | 77.85 | F:DNA binding; F:ATP binding; P:sister chromatid cohesion; C:cytoplasm; P:DNA replication; P:chromosome condensation; C:chromosome |
| Efet.01.1657907.g1058.t1 | ABC transporter substrate-binding | 462 | 3.34E-102 | 95.75 | P:amino acid transport |
| Efet.01.1657909.g1059.t1 | monocarboxylate transporter 12 | 384 | 1.96E-22 | 63.95 | P:transmembrane transport; C:integral component of membrane |
| Efet.01.1657920.g1061.t1 | 30S ribosomal S11 | 399 | 2.15E-65 | 99.45 | F:structural constituent of ribosome; C:ribosome; F:rRNA binding; P:translation |
| Efet.01.1657921.g1062.t1 | chorismate synthase | 630 | 1.33E-128 | 94 | P:aromatic amino acid family biosynthetic process; F:chorismate synthase activity; P:chorismate biosynthetic process |
| Efet.01.1657931.g1063.t1 | aminoglycoside phosphotransferase | 792 | 2.02E-139 | 70.55 | F:transferase activity |
| Efet.01.1657942.g1065.t1 | 1-deoxy-D-xylulose-5-phosphate synthase | 621 | 7.62E-97 | 82.2 | F:magnesium ion binding; F:thiamine pyrophosphate binding; F:1-deoxy-D-xylulose-5-phosphate synthase activity; P:terpenoid biosynthetic process; P:thiamine biosynthetic process; P:1-deoxy-D-xylulose 5-phosphate biosynthetic process |
| Efet.01.1657947.g1066.t1 | DUF2189 domain-containing | 651 | 1.49E-110 | 80.65 | C:integral component of membrane |
| Efet.01.1657948.g1067.t1 | inner centromere A-like isoform X1 | 960 | 1.05E-12 | 46.5 | F:DNA binding; F:oxidoreductase activity; P:oxidation-reduction process |
| Efet.01.1657951.g1068.t1 | rhomboid family intramembrane serine protease | 870 | 0 | 74.4 | C:membrane |
| Efet.01.1657953.g1069.t1 | Outer membrane | 858 | 7.26E-130 | 56.05 | P:transport; F:transporter activity |
| Efet.01.1657963.g1070.t1 | carboxymuconolactone decarboxylase family | 369 | 7.66E-42 | 87.65 | F:peroxidase activity; C:integral component of membrane; P:cellular oxidant detoxification; P:oxidation-reduction process; P:transport; C:plasma membrane; F:peroxiredoxin activity |
| Efet.01.1657969.g1071.t1 | mandelate racemase | 909 | 0 | 65 | P:cellular amino acid catabolic process; F:catalytic activity; P:metabolic process |
| Efet.01.1657970.g1072.t1 | tRNA (adenosine(37)-N6)-threonylcarbamoyltransferase complex transferase subunit | 690 | 1.54E-111 | 83.85 | C:cytoplasm; F:iron ion binding; P:proteolysis; P:tRNA threonylcarbamoyladenosine modification; P:phosphorylation; F:metalloendopeptidase activity; F:kinase activity; F:N(6)-L-threonylcarbamoyladenine synthase |
| Efet.01.1657974.g1073.t1 | hypothetical protein CRN43_19745, partial | 222 | 1.93E-06 | 64 |  |
| Efet.01.1657977.g1074.t1 | elongation factor P | 516 | 6.81E-123 | 97.05 | F:translation elongation factor activity; C:cytoplasm; P:translational elongation |
| Efet.01.1657978.g1075.t1 | cysteine hydrolase | 723 | 1.01E-159 | 65.75 | F:catalytic activity; P:metabolic process; F:hydrolase activity |
| Efet.01.1657992.g1076.t1 | dihydroorotate dehydrogenase | 702 | 1.78E-98 | 75.6 | P:transmembrane transport; C:integral component of membrane; F:metal ion transmembrane transporter activity; P:metal ion transport |
| Efet.01.1657993.g1077.t1 | specificity 1 | 864 | 2.81E-12 | 69.6 | F:metal ion binding |
| Efet.01.1658007.g1079.t1 | acyl- dehydrogenase | 825 | 0 | 93.95 | F:acyl-CoA dehydrogenase activity; F:flavin adenine dinucleotide binding; P:oxidation-reduction process |
| Efet.01.1658008.g1080.t1 | ribonuclease E G | 450 | 1.20E-93 | 99.05 | F:magnesium ion binding; P:RNA phosphodiester bond hydrolysis, endonucleolytic; F:zinc ion binding; F:RNA binding; C:cytoplasm; P:tRNA processing; F:ribonuclease E activity; P:rRNA processing; P:mRNA catabolic process; C:cytoplasmic side of plasma membrane; F:endoribonuclease activity |
| Efet.01.1658019.g1081.t1 | peregrin-like isoform X1 | 642 | 6.65E-38 | 58.75 | F:zinc ion binding; F:metal ion binding |
| Efet.01.1658020.g1082.t1 | relaxase (plasmid) | 360 | 1.25E-30 | 81.06 |  |
| Efet.01.1658029.g1083.t1 | repulsive guidance molecule A-like | 729 | 2.53E-38 | 53.7 | C:membrane; C:integral component of membrane |
| Efet.01.1658030.g1084.t1 | ABC transporter permease | 849 | 1.06E-169 | 74.35 | C:integral component of membrane; P:transport; C:plasma membrane |
| Efet.01.1658033.g1085.t1 | GGDEF domain-containing | 801 | 2.42E-158 | 83.45 | F:phosphorelay sensor kinase activity; P:phosphorelay signal transduction system; P:signal transduction by protein phosphorylation; C:intracellular |
| Efet.01.1658036.g1086.t1 | acyl- dehydrogenase | 579 | 3.55E-128 | 89.9 | F:acyl-CoA dehydrogenase activity; F:flavin adenine dinucleotide binding; P:oxidation-reduction process |
| Efet.01.1658037.g1087.t1 | titin-like isoform X2 | 375 | 1.09E-22 | 56.45 | F:nucleotide binding; F:ATP binding; F:protein kinase activity; P:phosphorylation; F:transferase activity; P:protein phosphorylation; F:kinase activity |
| Efet.01.1658050.g1089.t1 | KCNA5_RABIT ame: Full=Potassium voltage-gated channel subfamily A member 5 ame: Full=Voltage-gated potassium channel subunit | 258 | 3.23E-06 | 67 | P:regulation of potassium ion transport; F:outward rectifier potassium channel activity; C:Golgi apparatus; P:negative regulation of potassium ion transport; C:potassium channel complex; F:voltage-gated potassium channel activity involved in bundle of His cell action potential repolarization; F:protein kinase binding; P:potassium ion transmembrane transport; F:voltage-gated potassium channel activity involved in atrial cardiac muscle cell action potential repolarization; C:voltage-gated potassium channel complex; P:protein homooligomerization; F:voltage-gated ion channel activity; P:transport; C:plasma membrane; C:Z disc; F:protein binding; P:Notch signaling pathway; P:regulation of membrane potential; F:voltage-gated potassium channel activity involved in SA node cell action potential repolarization; F:potassium channel inhibitor activity; P:membrane hyperpolarization; C:intercalated disc; P:positive regulation of myoblast proliferation; P:membrane repolarization during bundle of His cell action potential; P:regulation of heart rate by cardiac conduction; P:protein oligomerization; P:membrane repolarization during SA node cell action potential; P:atrial cardiac muscle cell action potential; P:potassium ion homeostasis; F:delayed rectifier potassium channel activity; F:alpha-actinin binding; P:potassium ion transport; C:integral component of plasma membrane; F:voltage-gated potassium channel activity; P:ion transport; P:regulation of ion transmembrane transport; P:potassium ion export; P:membrane repolarization during atrial cardiac muscle cell action potential; P:regulation of vasoconstriction; P:transmembrane transport; P:positive regulation of G1/S transition of mitotic cell cycle; P:regulation of atrial cardiac muscle cell membrane repolarization; P:negative regulation of cytosolic calcium ion concentration; C:endoplasmic reticulum; F:potassium channel activity; F:receptor binding; C:intracellular canaliculus; F:scaffold protein binding; F:ion channel activity; C:perinuclear region of cytoplasm; C:membrane raft; C:membrane; C:integral component of membrane |
| Efet.01.1658055.g1090.t1 | aldehyde dehydrogenase | 315 | 9.71E-62 | 93.95 | F:oxidoreductase activity; F:electron carrier activity; P:oxidation-reduction process; F:heme binding |
| Efet.01.1658060.g1091.t1 | ankyrin repeat and KH domain-containing 1-like | 729 | 3.11E-08 | 59 |  |
| Efet.01.1658062.g1092.t1 | sugar ABC transporter permease | 822 | 1.47E-168 | 77 | C:integral component of membrane; P:transport; C:plasma membrane |
| Efet.01.1658067.g1093.t1 | family transcriptional regulator | 267 | 7.30E-58 | 69.67 | F:DNA binding; F:transcription factor activity, sequence-specific DNA binding; P:transcription, DNA-templated; P:regulation of transcription, DNA-templated; F:sequence-specific DNA binding |
| Efet.01.1658069.g1094.t1 | hypothetical protein, partial | 843 | 6.27E-64 | 64.8 |  |
| Efet.01.1658077.g1095.t1 | phospholipid carrier-dependent glycosyltransferase | 594 | 1.22E-111 | 93.9 | F:mannosyltransferase activity; P:protein O-linked glycosylation; P:mannosylation; C:integral component of membrane |
| Efet.01.1658078.g1096.t1 | ABC transporter substrate-binding | 588 | 6.06E-125 | 64.05 | P:transmembrane transport; C:ATP-binding cassette (ABC) transporter complex |
| Efet.01.1658080.g1097.t1 | ABC transporter substrate-binding | 966 | 0 | 75.65 | C:outer membrane-bounded periplasmic space |
| Efet.01.1658086.g1100.t1 | ABC transporter substrate-binding | 873 | 8.32E-152 | 74.65 |  |
| Efet.01.1658091.g1102.t1 | G -activated inward rectifier potassium channel 3-like | 666 | 8.43E-40 | 67.7 | P:potassium ion transport; C:membrane; F:voltage-gated ion channel activity |
| Efet.01.1658095.g1103.t1 | tryptophan--tRNA ligase | 720 | 6.27E-165 | 85.35 | F:ATP binding; C:cytoplasm; P:tryptophanyl-tRNA aminoacylation; F:tryptophan-tRNA ligase activity |
| Efet.01.1658096.g1104.t1 | pyridine nucleotide-disulfide oxidoreductase | 621 | 1.05E-128 | 83.4 | F:oxidoreductase activity; P:oxidation-reduction process |
| Efet.01.1658105.g1105.t1 | adenylosuccinate synthase | 612 | 4.92E-136 | 91.15 | F:magnesium ion binding; F:GTP binding; P:'de novo' AMP biosynthetic process; F:adenylosuccinate synthase activity; C:cytoplasm |
| Efet.01.1658111.g1106.t1 | DUF4199 domain-containing | 369 | 3.64E-71 | 57.5 | C:membrane; C:integral component of membrane |
| Efet.01.1658112.g1107.t1 | innexin 4 | 567 | 3.48E-70 | 65.85 | C:membrane; P:cell communication by electrical coupling; C:cell junction; C:synapse; F:gap junction channel activity; P:transport |
| Efet.01.1658126.g1110.t1 | arrestin domain-containing 3-like | 402 | 1.10E-09 | 72.56 |  |
| Efet.01.1658141.g1111.t1 | tripartite motif-containing 2-like | 501 | 7.43E-08 | 49.2 | F:zinc ion binding; F:metal ion binding; C:intracellular |
| Efet.01.1658144.g1112.t1 | Monocarboxylate transporter 4 | 840 | 1.46E-22 | 68 | P:signal transduction |
| Efet.01.1658150.g1113.t1 | Fe-S | 444 | 1.41E-99 | 83.2 |  |
| Efet.01.1658150.g1114.t1 | hydrolase | 423 | 3.39E-91 | 89.4 | F:hydrolase activity, acting on carbon-nitrogen (but not peptide) bonds; F:transferase activity, transferring acyl groups; P:nitrogen compound metabolic process |
| Efet.01.1658157.g1116.t1 | ribosome maturation factor | 396 | 6.41E-78 | 63.15 | C:cytoplasm; P:ribosomal small subunit biogenesis; P:ribosome biogenesis |
| Efet.01.1658158.g1117.t1 | fer3 | 537 | 1.38E-21 | 78.55 | F:protein dimerization activity |
| Efet.01.1658175.g1119.t1 | RNA degradosome polyphosphate kinase | 801 | 2.49E-149 | 81.65 | F:ATP binding; C:polyphosphate kinase complex; P:phosphorylation; F:polyphosphate kinase activity; P:polyphosphate biosynthetic process |
| Efet.01.1658179.g1120.t1 | spermidine putrescine ABC transporter substrate-binding | 831 | 0 | 87.9 |  |
| Efet.01.1658184.g1122.t1 | RNA polymerase subunit sigma | 864 | 0 | 96.6 | F:DNA binding; F:sigma factor activity; F:transcription factor activity, sequence-specific DNA binding; P:regulation of transcription, DNA-templated; P:DNA-templated transcription, initiation; P:sporulation resulting in formation of a cellular spore |
| Efet.01.1658186.g1123.t1 | Brefeldin A-inhibited guanine nucleotide-exchange 3 | 936 | 5.05E-21 | 67.65 | F:ARF guanyl-nucleotide exchange factor activity; P:regulation of ARF protein signal transduction; P:positive regulation of GTPase activity |
| Efet.01.1658203.g1124.t1 | glutamate receptor 2-like isoform X2 | 276 | 4.21E-23 | 69.4 | C:presynaptic membrane; C:postsynaptic density; F:kainate selective glutamate receptor activity; C:perikaryon; F:extracellular-glutamate-gated ion channel activity; C:growth cone; P:ion transport; C:terminal bouton; C:postsynaptic membrane; C:dendritic shaft; P:protein tetramerization; C:dendritic spine; F:PDZ domain binding; P:ionotropic glutamate receptor signaling pathway; P:regulation of synaptic transmission, glutamatergic; C:AMPA glutamate receptor complex; F:protein kinase binding; F:identical protein binding; P:positive regulation of synaptic transmission; P:chemical synaptic transmission; C:endoplasmic reticulum; C:protein complex; F:AMPA glutamate receptor activity; C:cell surface; P:establishment of protein localization; C:dendrite cytoplasm; C:synaptic vesicle membrane; F:cytoskeletal protein binding; P:receptor internalization; P:regulation of receptor recycling |
| Efet.01.1658204.g1125.t1 | serine threonine- kinase WNK3- | 705 | 1.36E-15 | 93.05 | F:ATP binding; P:regulation of ion homeostasis; P:positive regulation of calcium ion transport; C:cytosol; F:protein serine/threonine kinase activity; P:protein phosphorylation; P:positive regulation of ion transmembrane transporter activity; P:intracellular signal transduction |
| Efet.01.1658206.g1126.t1 | hypothetical protein CRN43_06545 | 924 | 0 | 53.17 |  |
| Efet.01.1658209.g1129.t1 | efflux RND transporter periplasmic adaptor subunit | 684 | 1.40E-89 | 89.75 | P:transmembrane transport; C:integral component of membrane |
| Efet.01.1658215.g1131.t1 | DUF4172 domain-containing | 522 | 3.97E-93 | 86.25 | P:cell division |
| Efet.01.1658216.g1132.t1 | DNA-binding transcriptional regulator | 486 | 1.04E-92 | 70.1 | F:carbohydrate binding |
| Efet.01.1658216.g1133.t1 | ABC transporter substrate-binding | 240 | 2.14E-45 | 84.6 | C:integral component of membrane; P:transport; F:transporter activity |
| Efet.01.1658222.g1134.t1 | pyrimidine-specific ribonucleoside hydrolase | 297 | 8.48E-64 | 76.1 | F:hydrolase activity, hydrolyzing N-glycosyl compounds; P:metabolic process |
| Efet.01.1658238.g1136.t1 | ABC transporter permease | 657 | 6.67E-77 | 59.85 | C:membrane; C:integral component of membrane; P:transport; C:plasma membrane; F:transporter activity |
| Efet.01.1658248.g1138.t1 | sugar ABC transporter substrate-binding | 636 | 9.68E-146 | 68.4 |  |
| Efet.01.1658249.g1139.t1 | ABC transporter ATP-binding | 633 | 5.57E-140 | 82.3 | F:ATPase activity; F:ATP binding |
| Efet.01.1658253.g1140.t1 | chromosome segregation SMC | 831 | 1.68E-173 | 93.85 | F:DNA binding; F:ATP binding; P:sister chromatid cohesion; C:cytoplasm; P:DNA replication; P:chromosome condensation; C:chromosome |
| Efet.01.1658262.g1142.t1 | glycoside hydrolase family 92 | 705 | 5.97E-125 | 83.45 | P:carbohydrate metabolic process; F:carbohydrate binding; F:hydrolase activity |
| Efet.01.1658265.g1143.t1 | thyrotropin-releasing hormone receptor-like | 714 | 3.04E-26 | 52.5 | P:signal transduction; C:membrane; F:signal transducer activity |
| Efet.01.1658267.g1144.t1 | Steroid receptor seven- isoform A | 339 | 3.59E-55 | 95.2 | C:nucleus; F:DNA binding; F:RNA polymerase II transcription factor activity, ligand-activated sequence-specific DNA binding; P:transcription, DNA-templated; F:steroid hormone receptor activity; P:regulation of transcription, DNA-templated; P:steroid hormone mediated signaling pathway; P:intracellular receptor signaling pathway |
| Efet.01.1658268.g1145.t1 | ester cyclase | 348 | 9.23E-41 | 90.95 |  |
| Efet.01.1658272.g1146.t1 | hypothetical protein HELRODRAFT_172421 | 714 | 1.63E-111 | 73.1 | F:catalytic activity; P:nucleoside metabolic process |
| Efet.01.1658273.g1147.t1 | isoprenyl transferase | 288 | 1.04E-50 | 94.55 | F:magnesium ion binding; F:prenyltransferase activity |
| Efet.01.1658277.g1148.t1 | hypothetical protein B5766_10095 | 663 | 8.10E-147 | 52.2 | C:membrane; C:integral component of membrane |
| Efet.01.1658278.g1149.t1 | hydrocephalus-inducing -like | 468 | 1.46E-19 | 83.65 | P:cilium movement |
| Efet.01.1658296.g1151.t1 | eukaryotic initiation factor 4A-III | 225 | 1.15E-33 | 90.8 | C:exon-exon junction complex; P:RNA splicing; P:positive regulation of translation; F:poly(A) binding; C:nuclear speck; P:embryonic cranial skeleton morphogenesis; P:rRNA processing; P:mRNA processing; P:translational initiation; F:ATP-dependent RNA helicase activity; F:translation initiation factor activity; F:ATP binding; C:catalytic step 2 spliceosome; P:nuclear-transcribed mRNA catabolic process, nonsense-mediated decay; P:regulation of alternative mRNA splicing, via spliceosome; C:membrane; P:RNA secondary structure unwinding; P:mRNA transport |
| Efet.01.1658305.g1152.t1 | DUF2141 domain-containing | 435 | 4.37E-76 | 56.6 |  |
| Efet.01.1658305.g1153.t1 | ABC transporter ATP-binding | 324 | 1.85E-33 | 74.05 | F:ATPase activity; F:ATP binding |
| Efet.01.1658315.g1154.t1 | transcriptional regulator | 756 | 0 | 95.6 | F:DNA binding; P:transcription, DNA-templated; P:regulation of transcription, DNA-templated |
| Efet.01.1658339.g1157.t1 | phosphoglycolate phosphatase | 579 | 3.62E-136 | 85.9 | P:glycolate biosynthetic process; P:carbohydrate metabolic process; F:phosphoglycolate phosphatase activity; F:metal ion binding; P:dephosphorylation |
| Efet.01.1658349.g1159.t1 | uncharacterized protein LOC111133750 | 501 | 4.57E-08 | 51 |  |
| Efet.01.1658350.g1160.t1 | polymerase | 363 | 3.47E-78 | 82.05 | C:integral component of membrane |
| Efet.01.1658350.g1161.t1 | acyltransferase | 516 | 4.78E-110 | 66.95 | F:transferase activity, transferring acyl groups other than amino-acyl groups; C:integral component of membrane |
| Efet.01.1658351.g1162.t1 | ABC transporter permease | 972 | 1.12E-169 | 73.35 | C:integral component of membrane; P:transport; C:plasma membrane |
| Efet.01.1658364.g1163.t1 | TonB-dependent siderophore receptor | 909 | 0 | 90.15 | F:iron ion binding; P:siderophore transport; C:cell outer membrane; F:receptor activity |
| Efet.01.1658367.g1164.t1 | Collagen alpha-2(IV) chain | 441 | 3.00E-43 | 88.85 | C:collagen trimer; F:extracellular matrix structural constituent; C:proteinaceous extracellular matrix |
| Efet.01.1658370.g1165.t1 | acetyl- carboxylase biotin carboxylase subunit | 489 | 9.80E-93 | 80.9 | F:ATP binding; F:acetyl-CoA carboxylase activity; F:metal ion binding; F:biotin carboxylase activity |
| Efet.01.1658372.g1166.t1 | mannosyl phosphorylinositol ceramide synthase CSH1-like | 636 | 8.32E-51 | 53.35 | C:membrane; C:integral component of membrane |
| Efet.01.1658379.g1167.t1 | 4 -phosphopantetheinyl transferase | 363 | 2.62E-26 | 62.4 | F:magnesium ion binding; F:metal ion binding; F:transferase activity; P:fatty acid biosynthetic process; F:holo-[acyl-carrier-protein] synthase activity |
| Efet.01.1658387.g1168.t1 | TIR-like domain-containing | 447 | 2.64E-73 | 69.45 |  |
| Efet.01.1658391.g1169.t1 | uncharacterized protein LOC110061142 | 567 | 1.20E-06 | 48 |  |
| Efet.01.1658394.g1170.t1 | glycoside hydrolase family 2 | 348 | 3.50E-28 | 63 | F:hydrolase activity |
| Efet.01.1658398.g1171.t1 | 2-isopropylmalate synthase | 711 | 3.81E-160 | 94.95 | F:2-isopropylmalate synthase activity; P:leucine biosynthetic process |
| Efet.01.1658401.g1172.t1 | Wobble nucleotide-excising tRNase | 801 | 3.40E-111 | 67.4 |  |
| Efet.01.1658403.g1173.t1 | glycosyltransferase family 4 | 804 | 2.29E-179 | 85.5 | F:transferase activity |
| Efet.01.1658406.g1174.t1 | hypothetical protein CRN43_05335, partial | 786 | 1.97E-153 | 53.95 | C:membrane; C:integral component of membrane |
| Efet.01.1658413.g1175.t1 | IS4 family | 510 | 1.15E-96 | 75 |  |
| Efet.01.1658415.g1176.t1 | family transcriptional regulator | 531 | 1.49E-66 | 70.65 | F:DNA binding |
| Efet.01.1658428.g1179.t1 | ABC transporter substrate-binding | 609 | 1.09E-94 | 58 | P:transport; F:transporter activity |
| Efet.01.1658436.g1182.t1 | insulinase family | 732 | 3.76E-90 | 78.95 | F:metal ion binding; P:proteolysis; F:metalloendopeptidase activity |
| Efet.01.1658439.g1184.t1 | 6-bladed beta-propeller | 888 | 9.16E-09 | 47.35 | F:zinc ion binding; F:metal ion binding; C:intracellular |
| Efet.01.1658449.g1185.t1 | ABC transporter ATP-binding | 885 | 4.00E-161 | 94.35 | F:ATPase activity; F:ATP binding |
| Efet.01.1658452.g1186.t1 | NAD(P)-dependent glycerol-3-phosphate dehydrogenase | 777 | 7.53E-172 | 86.3 | P:carbohydrate metabolic process; P:glycerol-3-phosphate biosynthetic process; C:glycerol-3-phosphate dehydrogenase complex; F:NAD binding; P:glycerol-3-phosphate catabolic process; P:phospholipid biosynthetic process; F:glycerol-3-phosphate dehydrogenase [NADP+] activity; F:glycerol-3-phosphate dehydrogenase [NAD(P)+] activity; P:oxidation-reduction process; P:glycerophospholipid metabolic process; F:glycerol-3-phosphate dehydrogenase [NAD+] activity |
| Efet.01.1658456.g1187.t1 | ABC transporter permease | 822 | 1.28E-108 | 83.35 | C:integral component of membrane; C:plasma membrane |
| Efet.01.1658468.g1189.t1 | acetyl- C-acyltransferase | 537 | 3.07E-80 | 77.6 | F:acetyl-CoA C-acetyltransferase activity; P:metabolic process |
| Efet.01.1658479.g1190.t1 | branched-chain amino acid ABC transporter substrate-binding | 291 | 6.84E-58 | 96.55 |  |
| Efet.01.1658479.g1191.t1 | aromatic-ring-hydroxylating dioxygenase subunit beta | 465 | 2.01E-85 | 89.25 | F:dioxygenase activity; P:cellular aromatic compound metabolic process; P:oxidation-reduction process |
| Efet.01.1658480.g1192.t1 | 1-deoxy-D-xylulose-5-phosphate reductoisomerase | 777 | 2.22E-165 | 93.45 | F:1-deoxy-D-xylulose-5-phosphate reductoisomerase activity; F:metal ion binding; F:NADPH binding; P:isopentenyl diphosphate biosynthetic process, methylerythritol 4-phosphate pathway; P:oxidation-reduction process; P:terpenoid biosynthetic process; F:isomerase activity |
| Efet.01.1658481.g1193.t1 | Mitotic spindle assembly checkpoint MAD2B | 624 | 2.71E-34 | 58 |  |
| Efet.01.1658488.g1194.t1 | netrin-1-like isoform X3 | 927 | 1.27E-114 | 76.3 | C:membrane |
| Efet.01.1658489.g1195.t1 | tartrate-resistant acid phosphatase type 5 | 300 | 6.40E-26 | 76.7 | P:response to bacterium; P:response to organic substance; F:iron ion binding; P:single-multicellular organism process; C:integral component of membrane; P:negative regulation of cytokine production; F:acid phosphatase activity; C:membrane-bounded organelle; P:negative regulation of reactive oxygen species metabolic process |
| Efet.01.1658493.g1196.t1 | carbohydrate sulfotransferase 1-like | 897 | 1.50E-65 | 47.85 | C:membrane; C:integral component of membrane; F:sulfotransferase activity |
| Efet.01.1658500.g1198.t1 | Zeta toxin | 963 | 9.29E-74 | 59.25 | F:ATP binding; P:phosphorylation; F:kinase activity |
| Efet.01.1658501.g1199.t1 | chromosome segregation SMC | 690 | 3.98E-148 | 75.35 | F:ATP binding |
| Efet.01.1658505.g1200.t1 | metabotropic glutamate receptor-like | 972 | 3.33E-27 | 53.8 | F:G-protein coupled receptor activity; P:G-protein coupled receptor signaling pathway; P:signal transduction; C:membrane; C:integral component of membrane; F:signal transducer activity |
| Efet.01.1658506.g1201.t1 | BTB POZ domain-containing 2 | 288 | 6.05E-10 | 54.25 |  |
| Efet.01.165851.g787.t1 | myosin heavy non-muscle- | 801 | 2.87E-18 | 65.65 |  |
| Efet.01.1658511.g1202.t1 | sugar ABC transporter substrate-binding | 702 | 8.01E-124 | 79.65 |  |
| Efet.01.1658514.g1203.t1 | poly(3-hydroxyalkanoate) granule-associated | 372 | 2.25E-47 | 68.67 |  |
| Efet.01.1658521.g1205.t1 | glycine C-acetyltransferase | 735 | 2.21E-150 | 83.05 | F:pyridoxal phosphate binding; P:L-threonine catabolic process to glycine; F:glycine C-acetyltransferase activity; P:biosynthetic process; F:ligase activity |
| Efet.01.1658522.g1206.t1 | gremlin-2 | 714 | 1.18E-21 | 54.15 | P:negative regulation of BMP signaling pathway; F:BMP binding; P:determination of dorsal identity; C:extracellular space; P:regulation of cytokine activity; F:protein homodimerization activity; P:sequestering of BMP from receptor via BMP binding; P:cytokine-mediated signaling pathway; P:embryonic body morphogenesis; F:heparin binding; C:extracellular region |
| Efet.01.1658526.g1208.t1 | ATP-dependent Clp protease proteolytic subunit | 690 | 1.26E-161 | 81.9 | C:cytoplasm; P:proteolysis; F:serine-type endopeptidase activity |
| Efet.01.1658533.g1210.t1 | innexin unc-9-like isoform X2 | 765 | 2.86E-103 | 72.25 | C:gap junction; P:ion transport; C:integral component of membrane; C:plasma membrane |
| Efet.01.1658536.g1211.t1 | bromodomain-containing 2 isoform X2 | 399 | 6.94E-13 | 77.15 | P:macromolecule metabolic process; F:lysine-acetylated histone binding; F:chromatin binding; C:intracellular part; P:nucleosome assembly; P:regulation of transcription from RNA polymerase II promoter |
| Efet.01.1658542.g1212.t1 | 50S ribosomal L31 | 255 | 9.88E-56 | 97.15 | F:structural constituent of ribosome; C:ribosome; P:translation |
| Efet.01.1658542.g1213.t1 | cell envelope integrity | 312 | 1.73E-50 | 94.2 | C:integral component of membrane; P:transport; F:transporter activity |
| Efet.01.1658553.g1215.t1 | pyridoxamine 5 -phosphate oxidase | 429 | 9.39E-71 | 93.75 | F:pyridoxamine-phosphate oxidase activity; P:pyridoxal phosphate biosynthetic process; P:pyridoxine biosynthetic process; F:FMN binding; P:oxidation-reduction process |
| Efet.01.1658555.g1216.t1 | E3 ubiquitin- ligase TRIM56-like | 972 | 1.58E-12 | 45.65 | F:zinc ion binding; F:RNA binding; F:metal ion binding; C:intracellular |
| Efet.01.1658570.g1218.t1 | helix-turn-helix transcriptional regulator | 687 | 2.86E-147 | 65.8 | F:DNA binding |
| Efet.01.1658583.g1220.t1 | Fic family | 813 | 0 | 67.75 | P:cell division |
| Efet.01.1658584.g1221.t1 | FAD-binding oxidoreductase | 312 | 2.06E-35 | 83.1 | F:oxidoreductase activity, acting on CH-OH group of donors; F:flavin adenine dinucleotide binding; F:alkylglycerone-phosphate synthase activity; P:lipid biosynthetic process; P:oxidation-reduction process |
| Efet.01.1658588.g1223.t1 | FMRFamide-activated amiloride-sensitive sodium channel isoform X1 | 1062 | 3.23E-151 | 51.65 | F:sodium channel activity; C:integral component of membrane; P:sodium ion transmembrane transport |
| Efet.01.1658591.g1224.t1 | para-aminobenzoate synthase | 474 | 2.57E-81 | 81.05 | P:glutamine metabolic process; F:transferase activity |
| Efet.01.1658605.g1226.t1 | SGNH GDSL hydrolase family | 279 | 2.53E-62 | 63.3 |  |
| Efet.01.1658606.g1227.t1 | zinc finger 239-like | 852 | 1.32E-72 | 61.95 | F:nucleic acid binding; F:metal ion binding |
| Efet.01.1658611.g1228.t1 | TBC1 domain family member 25 | 798 | 6.71E-108 | 79.55 | P:regulation of vesicle fusion; P:regulation of autophagosome maturation; P:activation of GTPase activity; F:GTPase activator activity; C:endomembrane system; F:sulfotransferase activity; P:intracellular protein transport; F:Rab GTPase binding; C:autophagosome |
| Efet.01.1658613.g1229.t1 | phosphosulfolactate synthase | 735 | 6.10E-146 | 89.85 | F:phosphosulfolactate synthase activity |
| Efet.01.1658616.g1231.t1 | polymerase | 1050 | 0 | 60.3 | C:integral component of membrane |
| Efet.01.1658617.g1232.t1 | somatostatin receptor type 2 | 1089 | 1.16E-65 | 50 | F:G-protein coupled receptor activity; P:G-protein coupled receptor signaling pathway; C:membrane |
| Efet.01.1658622.g1233.t1 | Metal transporter | 927 | 4.90E-136 | 84.3 | C:integral component of membrane |
| Efet.01.1658626.g1234.t1 | elongation factor Tu | 918 | 1.71E-178 | 98.1 | F:GTP binding; F:translation elongation factor activity; C:cytoplasm; F:GTPase activity; P:translational elongation |
| Efet.01.1658627.g1235.t1 | PREDICTED: cadherin-13 | 282 | 4.38E-06 | 50 |  |
| Efet.01.1658628.g1236.t1 | 30S ribosomal S3 | 741 | 1.23E-165 | 94.95 | F:structural constituent of ribosome; F:mRNA binding; C:small ribosomal subunit; F:rRNA binding; P:translation |
| Efet.01.1658633.g1237.t1 | hypothetical protein CRN43_17300 | 396 | 4.50E-26 | 66 |  |
| Efet.01.1658652.g1238.t1 | Type I phosphodiesterase nucleotide pyrophosphatase | 858 | 0 | 68.5 | F:catalytic activity; P:metabolic process |
| Efet.01.1658654.g1239.t1 | sugar ABC transporter permease | 525 | 1.00E-104 | 70.2 | C:membrane |
| Efet.01.1658657.g1240.t1 | sugar ABC transporter ATP-binding | 822 | 0 | 71.6 | F:nucleotide binding; F:ATPase activity; P:carbohydrate transport |
| Efet.01.1658658.g1241.t1 | c-binding -like | 924 | 1.16E-40 | 55.8 | C:membrane; C:integral component of membrane |
| Efet.01.1658659.g1242.t1 | malonate decarboxylase subunit alpha | 825 | 1.82E-143 | 90 | P:metabolic process; F:CoA-transferase activity |
| Efet.01.1658666.g1243.t1 | hypothetical protein A3F76_05545 | 381 | 1.09E-35 | 76 |  |
| Efet.01.1658668.g1244.t1 | MFS transporter | 1026 | 0 | 93.2 | P:transmembrane transport; F:transmembrane transporter activity; C:integral component of membrane |
| Efet.01.1658669.g1245.t1 | hypothetical protein B5766_02895 | 630 | 5.56E-16 | 89 |  |
| Efet.01.1658675.g1246.t1 | ABC transporter ATP-binding | 438 | 2.11E-71 | 52 | F:nucleotide binding; F:ATPase activity; F:ATP binding |
| Efet.01.1658676.g1247.t1 | DUF2304 domain-containing | 384 | 1.29E-54 | 82.9 | C:integral component of membrane |
| Efet.01.1658677.g1248.t1 | 2-amino-4-hydroxy-6-hydroxymethyldihydropteridine diphosphokinase | 498 | 7.42E-102 | 73.3 | F:transferase activity, transferring phosphorus-containing groups; P:cellular metabolic process |
| Efet.01.1658679.g1249.t1 | acetyl- acetyltransferase | 972 | 0 | 89.8 | F:acetyl-CoA C-acetyltransferase activity; P:metabolic process |
| Efet.01.1658685.g1250.t1 | peptide-methionine (S)-S-oxide reductase | 561 | 4.22E-135 | 89.25 | P:protein repair; P:response to oxidative stress; P:cellular protein modification process; F:peptide-methionine (S)-S-oxide reductase activity; P:oxidation-reduction process |
| Efet.01.1658692.g1251.t1 | Carboxypeptidase regulatory-like domain-containing protein | 828 | 1.70E-180 | 55.4 | C:membrane; F:receptor activity; F:carbohydrate binding; P:transport |
| Efet.01.1658696.g1252.t1 | protocadherin Fat 4-like | 300 | 1.03E-12 | 62.1 | C:membrane; P:cell adhesion |
| Efet.01.1658706.g1254.t1 | hypothetical protein CAPTEDRAFT_195499 | 1092 | 1.61E-29 | 49 | F:G-protein coupled receptor activity; P:G-protein coupled receptor signaling pathway; C:membrane; C:integral component of membrane |
| Efet.01.1658718.g1255.t1 | lipoyl synthase | 963 | 0 | 94.2 | C:cytoplasm; F:metal ion binding; F:lipoate synthase activity; F:4 iron, 4 sulfur cluster binding; P:lipoate biosynthetic process; P:protein lipoylation |
| Efet.01.1658720.g1256.t1 | phosphohydrolase | 555 | 1.23E-77 | 86.35 | F:hydrolase activity |
| Efet.01.1658720.g1257.t1 | biotin synthase | 471 | 2.91E-97 | 89 |  |
| Efet.01.1658725.g1258.t1 | sugar ABC transporter permease | 357 | 2.27E-64 | 94.05 | C:integral component of membrane; P:transport; C:plasma membrane |
| Efet.01.1658725.g1259.t1 | sugar ABC transporter permease | 303 | 2.48E-34 | 90.2 | C:integral component of membrane; P:transport; C:plasma membrane |
| Efet.01.1658727.g1260.t1 | family transcriptional regulator | 705 | 1.28E-156 | 89.1 | F:DNA binding; P:negative regulation of transcription, DNA-templated; P:transcription, DNA-templated |
| Efet.01.1658742.g1262.t1 | carbohydrate kinase | 591 | 1.75E-33 | 82.3 | F:ATPase activity; F:ATP binding; P:carbohydrate metabolic process; P:transmembrane transport; P:phosphorylation; F:phosphotransferase activity, alcohol group as acceptor; F:kinase activity; C:ATP-binding cassette (ABC) transporter complex; F:hydrolase activity, acting on acid anhydrides, catalyzing transmembrane movement of substances |
| Efet.01.1658745.g1263.t1 | hypothetical protein B7Z23_14020, partial | 819 | 2.10E-114 | 79.55 |  |
| Efet.01.1658764.g1266.t1 | extracellular solute-binding | 507 | 1.05E-94 | 63.31 | C:membrane; C:integral component of membrane; P:transport; F:transporter activity |
| Efet.01.1658764.g1267.t1 | mandelate racemase muconate lactonizing enzyme family | 459 | 1.76E-101 | 73.55 | F:metal ion binding; F:catalytic activity; P:metabolic process |
| Efet.01.1658765.g1268.t1 | metal-dependent hydrolase | 687 | 8.59E-102 | 86.95 | F:ATPase activity; F:ATP binding; C:integral component of membrane; P:transport; C:plasma membrane; F:transporter activity |
| Efet.01.1658772.g1269.t1 | ADP-ribosylation factor 6 | 546 | 3.61E-93 | 81.95 | P:intermembrane transport; P:small GTPase mediated signal transduction; P:regulation of Notch signaling pathway; P:brain development; P:fat cell differentiation; P:visual perception; P:determination of left/right symmetry; P:gastrulation; P:regulation of smoothened signaling pathway; F:GTP binding; C:extracellular exosome; P:protein localization to non-motile cilium; C:membrane; P:Kupffer's vesicle development; C:cilium; P:retina layer formation; C:cytosol; P:Wnt signaling pathway; P:pectoral fin development; P:melanosome transport |
| Efet.01.1658777.g1271.t1 | replicative DNA helicase | 375 | 5.49E-80 | 94.5 | F:DNA binding; F:ATP binding; F:DNA helicase activity; P:DNA duplex unwinding; C:primosome complex; P:DNA replication, synthesis of RNA primer |
| Efet.01.1658778.g1272.t1 | FAD-binding | 528 | 5.07E-123 | 80.7 | F:D-arabinono-1,4-lactone oxidase activity; F:flavin adenine dinucleotide binding; C:membrane; P:oxidation-reduction process |
| Efet.01.1658780.g1273.t1 | hypothetical protein CRN43_06535 | 663 | 2.92E-159 | 83 |  |
| Efet.01.1658784.g1275.t1 | hypothetical protein B5766_09495 | 366 | 1.77E-33 | 98 |  |
| Efet.01.1658788.g1276.t1 | ankyrin domain | 621 | 2.04E-21 | 56.4 | F:ATP binding; C:membrane; C:integral component of membrane; F:protein kinase activity; P:protein phosphorylation |
| Efet.01.1658789.g1277.t1 | Kinesin KIF2A | 255 | 1.67E-33 | 87.45 | F:ATP binding; F:microtubule motor activity; P:microtubule-based movement; C:microtubule; F:microtubule binding |
| Efet.01.1658795.g1278.t1 | unconventional myosin-Vc | 402 | 4.81E-12 | 63.6 | F:binding |
| Efet.01.165880.g789.t1 | menin | 207 | 9.87E-08 | 77.8 | C:nucleus |
| Efet.01.1658804.g1279.t1 | family transcriptional regulator | 417 | 2.39E-91 | 89.55 | F:DNA binding; F:transcription factor activity, sequence-specific DNA binding; P:transcription, DNA-templated; P:regulation of transcription, DNA-templated |
| Efet.01.1658806.g1280.t1 | acetylcholine receptor subunit beta-like 1 | 1020 | 4.08E-98 | 64.35 | F:transmembrane receptor activity; P:ion transmembrane transport; C:integral component of membrane; P:chemical synaptic transmission; P:response to stimulus; C:plasma membrane part |
| Efet.01.1658807.g1281.t1 | tubulin polyglutamylase TTLL6-like isoform X1 | 246 | 5.75E-07 | 70.86 | F:ATP binding; P:cellular protein modification process |
| Efet.01.1658810.g1282.t1 | Potassium voltage-gated channel subfamily A member 2 | 1221 | 0 | 79.8 | P:potassium ion transmembrane transport; C:voltage-gated potassium channel complex; F:delayed rectifier potassium channel activity; P:regulation of ion transmembrane transport; P:protein homooligomerization |
| Efet.01.1658815.g1284.t1 | pfkB carbohydrate kinase family | 531 | 2.53E-115 | 61.55 | P:phosphorylation; F:transferase activity; F:kinase activity |
| Efet.01.1658815.g1285.t1 | aldolase [Rhodococcus hoagii] | 297 | 7.59E-62 | 68.4 | F:L-fuculose-phosphate aldolase activity; F:lyase activity |
| Efet.01.1658819.g1286.t1 | TonB-dependent receptor | 969 | 0 | 77.25 | C:cell outer membrane; F:receptor activity; P:transport |
| Efet.01.1658820.g1287.t1 | early growth response | 237 | 1.99E-08 | 57 |  |
| Efet.01.1658821.g1288.t1 | Transglutaminase-like superfamily | 942 | 0 | 71 |  |
| Efet.01.1658830.g1289.t1 | proton channel family | 582 | 1.08E-122 | 72 | C:membrane; P:transport |
| Efet.01.1658836.g1291.t1 | type-2 restriction enzyme 7I | 672 | 6.08E-58 | 58.2 | F:DNA binding; P:nucleic acid phosphodiester bond hydrolysis; F:endonuclease activity; F:Type II site-specific deoxyribonuclease activity; P:DNA restriction-modification system; F:hydrolase activity |
| Efet.01.1658837.g1292.t1 | endopeptidase La | 324 | 4.16E-64 | 86.05 | F:ATP binding; P:misfolded or incompletely synthesized protein catabolic process; C:cytoplasm; F:metal ion binding; F:serine-type endopeptidase activity; P:cellular response to stress; F:sequence-specific DNA binding; F:ATP-dependent peptidase activity |
| Efet.01.1658839.g1293.t1 | ABC transporter permease | 624 | 1.85E-110 | 69.75 | C:membrane |
| Efet.01.1658840.g1294.t1 | hypothetical protein B5766_08000 | 888 | 0 | 57.8 | F:zinc ion binding; F:metal ion binding; P:catabolic process |
| Efet.01.1658843.g1295.t1 | fibrinogen C domain-containing 1-A-like | 630 | 1.31E-30 | 57.35 | F:carbohydrate binding |
| Efet.01.1658858.g1296.t1 | microcin ABC transporter permease | 387 | 4.24E-55 | 99.7 | C:integral component of membrane; P:transport; C:plasma membrane |
| Efet.01.1658858.g1297.t1 | peptide ABC transporter permease | 711 | 2.95E-96 | 92.15 | C:integral component of membrane; P:transport; C:plasma membrane |
| Efet.01.1658859.g1298.t1 | pyruvate carboxylase | 975 | 2.25E-179 | 94.2 | F:ATP binding; P:gluconeogenesis; F:pyruvate carboxylase activity; F:metal ion binding; F:biotin binding; F:biotin carboxylase activity; P:pyruvate metabolic process |
| Efet.01.1658861.g1299.t1 | 3-oxoacyl-[acyl-carrier- ] reductase | 744 | 5.09E-141 | 88 | F:3-oxoacyl-[acyl-carrier-protein] reductase (NADPH) activity; F:NAD binding; P:oxidation-reduction process; P:fatty acid biosynthetic process |
| Efet.01.1658863.g1300.t1 | iron dicitrate transport regulator | 621 | 1.19E-138 | 63.65 | C:membrane; C:integral component of membrane |
| Efet.01.1658864.g1301.t1 | ABC transporter | 375 | 5.38E-82 | 94.85 | F:ATPase activity; F:ATP binding |
| Efet.01.1658864.g1302.t1 | 23S rRNA (guanosine(2251)-2 -O)-methyltransferase | 747 | 7.51E-178 | 94.55 | P:rRNA 2'-O-methylation; F:RNA binding; C:cytoplasm; F:rRNA (guanosine-2'-O-)-methyltransferase activity |
| Efet.01.1658865.g1303.t1 | cell division | 813 | 2.51E-145 | 81.75 | P:regulation of cell shape; P:FtsZ-dependent cytokinesis; C:integral component of plasma membrane; C:cell division site; P:peptidoglycan biosynthetic process; P:lipid-linked peptidoglycan transport; F:lipid-linked peptidoglycan transporter activity; P:cell wall organization |
| Efet.01.1658872.g1306.t1 | sugar kinase | 873 | 0 | 62.6 | P:phosphorylation; F:transferase activity; F:kinase activity |
| Efet.01.1658878.g1307.t1 | aldolase | 741 | 1.85E-156 | 75.5 | F:catalytic activity; F:metal ion binding |
| Efet.01.1658878.g1308.t1 | virulence factor MVIN family | 303 | 1.38E-15 | 88 | C:integral component of membrane |
| Efet.01.1658880.g1310.t1 | creatininase | 447 | 6.43E-101 | 52.4 | F:creatininase activity; F:hydrolase activity |
| Efet.01.1658880.g1311.t1 | family | 384 | 9.97E-89 | 68.6 |  |
| Efet.01.1658882.g1312.t1 | sensor histidine kinase | 522 | 5.13E-89 | 62.75 | F:phosphorelay sensor kinase activity; P:phosphorelay signal transduction system; C:membrane; C:integral component of membrane; F:protein dimerization activity; P:phosphorylation; F:transferase activity; P:signal transduction by protein phosphorylation; F:kinase activity; C:intracellular |
| Efet.01.1658883.g1313.t1 | glutamine--fructose-6-phosphate transaminase (isomerizing) | 1089 | 0 | 92.15 | P:carbohydrate metabolic process; C:cytoplasm; F:glutamine-fructose-6-phosphate transaminase (isomerizing) activity; P:glutamine metabolic process; P:carbohydrate derivative biosynthetic process; F:carbohydrate binding |
| Efet.01.1658886.g1314.t1 | IS256 family transposase | 900 | 0 | 91.1 | F:DNA binding; F:transposase activity; P:transposition, DNA-mediated |
| Efet.01.1658890.g1315.t1 | acyl- dehydrogenase | 927 | 0 | 88.1 | F:acyl-CoA dehydrogenase activity; F:flavin adenine dinucleotide binding; P:oxidation-reduction process |
| Efet.01.1658891.g1316.t1 | hypothetical protein B5766_03955 | 360 | 4.41E-62 | 79.55 |  |
| Efet.01.1658897.g1318.t1 | oligopeptide transport system ATP-binding | 324 | 3.82E-71 | 81.75 | F:ATPase activity; F:ATP binding; P:peptide transport |
| Efet.01.1658900.g1319.t1 | hypothetical protein CAPTEDRAFT_99765 | 690 | 4.17E-12 | 76 | F:ion channel activity; P:ion transmembrane transport; C:synapse; C:membrane part; C:plasma membrane |
| Efet.01.1658901.g1320.t1 | carnitine dehydratase | 834 | 4.57E-107 | 84.9 | F:pyridoxal phosphate binding; F:transaminase activity; P:oxidation-reduction process; F:oxidoreductase activity, acting on the aldehyde or oxo group of donors, NAD or NADP as acceptor |
| Efet.01.1658916.g1323.t1 | acyl-ACP--UDP-N-acetylglucosamine O-acyltransferase | 585 | 3.67E-108 | 81.95 | C:cytoplasm; F:acyl-[acyl-carrier-protein]-UDP-N-acetylglucosamine O-acyltransferase activity; P:lipid A biosynthetic process |
| Efet.01.1658917.g1324.t1 | TIGR02099 family | 1092 | 0 | 75.1 | C:membrane |
| Efet.01.1658918.g1325.t1 | glutamine synthetase | 1104 | 0 | 95.8 | C:cytoplasm; P:glutamine biosynthetic process; P:nitrogen fixation; F:glutamate-ammonia ligase activity |
| Efet.01.1658922.g1326.t1 | general secretion pathway | 681 | 2.26E-92 | 74.7 | C:integral component of membrane; P:protein secretion; C:plasma membrane |
| Efet.01.1658933.g1327.t1 | outer membrane lipo -sorting | 702 | 2.14E-153 | 63.15 |  |
| Efet.01.1658935.g1328.t1 | peptidase | 741 | 9.60E-49 | 80.2 | C:integral component of membrane |
| Efet.01.1658942.g1329.t1 | Indolepyruvate ferredoxin oxidoreductase | 891 | 2.75E-161 | 85.05 | F:thiamine pyrophosphate binding; F:indolepyruvate ferredoxin oxidoreductase activity; P:oxidation-reduction process |
| Efet.01.1658952.g1330.t1 | serine threonine- kinase | 936 | 0 | 85.85 | F:ATP binding; C:integral component of membrane; F:protein serine/threonine kinase activity; P:protein phosphorylation |
| Efet.01.1658954.g1331.t1 | non-ribosomal peptide synthetase | 1023 | 1.82E-55 | 56.85 | F:catalytic activity |
| Efet.01.1658956.g1332.t1 | carbon monoxide dehydrogenase | 609 | 3.21E-107 | 87.8 | C:integral component of membrane |
| Efet.01.1658956.g1333.t1 | VWA domain-containing | 363 | 1.01E-56 | 91.5 |  |
| Efet.01.1658958.g1334.t1 | ABC transporter ATP-binding | 756 | 3.95E-180 | 91.95 | F:ATPase activity; F:ATP binding; P:peptide transport |
| Efet.01.1658958.g1335.t1 | ABC transporter ATP-binding | 405 | 9.82E-90 | 83.2 | F:ATPase activity; F:ATP binding; P:peptide transport |
| Efet.01.1658959.g1336.t1 | hypothetical protein CAPTEDRAFT_109895 | 435 | 6.35E-46 | 64.8 | C:membrane; C:integral component of membrane; C:outer membrane-bounded periplasmic space |
| Efet.01.165896.g791.t1 | nucleolar gtp-binding 1 | 522 | 4.09E-59 | 65.55 | F:GTP binding; C:nucleus; P:ferrous iron transport; C:integral component of membrane; P:ribosome biogenesis; P:ferrous iron transmembrane transport; F:ferrous iron transmembrane transporter activity; C:nucleolus; F:hydrolase activity |
| Efet.01.1658963.g1337.t1 | 50S ribosomal L28 | 360 | 1.88E-49 | 98.85 | F:structural constituent of ribosome; C:ribosome; P:translation |
| Efet.01.1658963.g1338.t1 | 30S ribosomal S14 | 306 | 2.85E-65 | 94.7 | F:structural constituent of ribosome; C:ribosome; F:rRNA binding; P:translation |
| Efet.01.1658966.g1339.t1 | helicase SNF2 | 993 | 0 | 83.25 | F:DNA binding; P:nucleic acid phosphodiester bond hydrolysis; F:endonuclease activity; P:DNA restriction-modification system |
| Efet.01.1658967.g1340.t1 | tudor domain-containing 5-like | 681 | 6.07E-25 | 48.95 |  |
| Efet.01.1658970.g1343.t1 | hypothetical protein CRN43_14440 | 516 | 6.81E-16 | 53 |  |
| Efet.01.1658974.g1344.t1 | G1 S-specific cyclin-D2-like | 414 | 8.69E-18 | 62.25 | C:nucleus; P:cell cycle |
| Efet.01.1658975.g1345.t1 | hairy enhancer-of-split related with YRPW motif 1-like | 546 | 3.07E-22 | 50.55 | P:regulation of transcription, DNA-templated |
| Efet.01.1658980.g1347.t1 | Protein-disulfide isomerase | 936 | 4.10E-173 | 66.3 | C:membrane; C:integral component of membrane |
| Efet.01.1658981.g1348.t1 | proton-coupled folate transporter-like | 1260 | 5.27E-69 | 54.6 | P:transmembrane transport; C:membrane; C:integral component of membrane; F:transporter activity |
| Efet.01.1658989.g1349.t1 | hydrocephalus-inducing -like | 855 | 7.34E-06 | 55 |  |
| Efet.01.1658990.g1350.t1 | gastrula zinc finger -like | 1203 | 1.02E-09 | 43.85 | F:nucleic acid binding; F:zinc ion binding; F:metal ion binding |
| Efet.01.1658992.g1351.t1 | UPF0365 family | 981 | 0 | 89.85 | C:integral component of membrane; C:plasma membrane |
| Efet.01.1658998.g1353.t1 | calreticulin precursor | 351 | 8.01E-45 | 85.45 | F:calcium ion binding; C:endoplasmic reticulum; F:unfolded protein binding; P:protein folding |
| Efet.01.1659001.g1354.t1 | family transcriptional regulator | 531 | 1.50E-122 | 78.7 | F:DNA binding; F:transcription factor activity, sequence-specific DNA binding; P:transcription, DNA-templated; P:regulation of transcription, DNA-templated |
| Efet.01.1659001.g1355.t1 | gfo Idh family oxidoreductase | 462 | 5.96E-91 | 72 | F:phthalate 4,5-cis-dihydrodiol dehydrogenase activity; P:oxidation-reduction process |
| Efet.01.1659007.g1356.t1 | DUF3488 domain-containing | 858 | 1.18E-133 | 84.6 | C:integral component of membrane |
| Efet.01.1659010.g1357.t1 | glycogen synthase | 609 | 3.42E-145 | 91.8 | F:starch synthase activity; F:alpha-1,4-glucan synthase activity |
| Efet.01.1659012.g1358.t1 | dihydropyrimidinase | 984 | 0 | 94.1 | C:cytoplasm; F:dihydropyrimidinase activity |
| Efet.01.1659013.g1359.t1 | chromosome segregation SMC | 453 | 3.77E-69 | 86.85 | F:DNA binding; F:ATP binding; P:sister chromatid cohesion; C:cytoplasm; P:DNA replication; P:chromosome condensation; C:chromosome |
| Efet.01.1659014.g1360.t1 | exostosin-2 | 591 | 1.44E-06 | 47.45 | P:heparan sulfate proteoglycan biosynthetic process, polysaccharide chain biosynthetic process; P:heparan sulfate proteoglycan biosynthetic process; F:acetylglucosaminyltransferase activity; F:glucuronosyl-N-acetylglucosaminyl-proteoglycan 4-alpha-N-acetylglucosaminyltransferase activity; F:N-acetylglucosaminyl-proteoglycan 4-beta-glucuronosyltransferase activity; F:glucuronosyltransferase activity; P:glycosaminoglycan biosynthetic process; P:smoothened signaling pathway; P:N-acetylglucosamine metabolic process; C:endoplasmic reticulum; C:Golgi apparatus; P:decapentaplegic signaling pathway; P:chondroitin sulfate biosynthetic process; F:protein binding; C:membrane; C:integral component of membrane; P:Wnt signaling pathway; P:heparin biosynthetic process |
| Efet.01.1659015.g1361.t1 | TSP1_XENLA ame: Full=Thrombospondin-1 Flags: Precursor | 1083 | 3.47E-06 | 55 | P:negative regulation of angiogenesis; F:extracellular matrix binding; C:extracellular space; P:inflammatory response; C:sarcoplasmic reticulum; F:calcium ion binding; P:cell adhesion; C:endoplasmic reticulum; F:heparin binding; C:extracellular region; P:response to unfolded protein |
| Efet.01.1659017.g1363.t1 | magnesium transporter | 1164 | 0 | 82.25 | F:metal ion binding; C:integral component of membrane; F:magnesium ion transmembrane transporter activity; P:magnesium ion transmembrane transport; C:plasma membrane |
| Efet.01.1659019.g1364.t1 | 30S ribosomal S5 | 219 | 3.72E-31 | 96.3 | F:structural constituent of ribosome; C:small ribosomal subunit; F:rRNA binding; P:translation |
| Efet.01.1659019.g1365.t1 | 50S ribosomal L15 | 432 | 2.50E-82 | 94.65 | F:structural constituent of ribosome; C:large ribosomal subunit; F:rRNA binding; P:translation |
| Efet.01.1659026.g1367.t1 | aldehyde dehydrogenase | 1008 | 0 | 86.4 | P:oxidation-reduction process; F:oxidoreductase activity, acting on the aldehyde or oxo group of donors, NAD or NADP as acceptor |
| Efet.01.1659028.g1368.t1 | Lsr2 family | 453 | 5.22E-63 | 62.8 |  |
| Efet.01.1659030.g1369.t1 | sugar ABC transporter permease | 360 | 1.00E-62 | 81.4 | C:integral component of membrane; P:transport; C:plasma membrane |
| Efet.01.1659031.g1370.t1 | flagellar motor switch | 1257 | 0 | 96.3 | P:bacterial-type flagellum-dependent cell motility; C:bacterial-type flagellum basal body; F:motor activity |
| Efet.01.1659032.g1371.t1 | leucine-rich repeat and immunoglobulin-like domain-containing nogo receptor-interacting 2 isoform X2 | 825 | 1.31E-21 | 49.35 | F:catalytic activity |
| Efet.01.1659033.g1372.t1 | short-chain dehydrogenase | 462 | 8.27E-107 | 88.9 | F:3-oxoacyl-[acyl-carrier-protein] reductase (NADPH) activity; P:oxidation-reduction process |
| Efet.01.1659033.g1373.t1 | glycerol dehydrogenase | 744 | 9.21E-144 | 78.35 | F:metal ion binding; P:oxidation-reduction process; F:glycerol dehydrogenase [NAD+] activity |
| Efet.01.1659037.g1375.t1 | alcohol dehydrogenase | 1035 | 0 | 82.6 | F:zinc ion binding; F:oxidoreductase activity; P:oxidation-reduction process |
| Efet.01.1659039.g1376.t1 | FH1 FH2 domain-containing 3-like | 780 | 1.21E-10 | 61.33 |  |
| Efet.01.1659041.g1377.t1 | DNA-binding | 969 | 1.29E-112 | 74.2 | C:membrane |
| Efet.01.1659042.g1378.t1 | Sporulation and spore germination | 684 | 5.25E-150 | 61.9 |  |
| Efet.01.1659044.g1379.t1 | copper transporter | 1242 | 0 | 83.35 | C:integral component of membrane |
| Efet.01.1659046.g1380.t1 | transcriptional family | 1182 | 0 | 92.8 | F:DNA binding; F:transcription factor activity, sequence-specific DNA binding; F:pyridoxal phosphate binding; F:L-phenylalanine:2-oxoglutarate aminotransferase activity; P:transcription, DNA-templated; P:regulation of transcription, DNA-templated; F:L-aspartate:2-oxoglutarate aminotransferase activity |
| Efet.01.1659047.g1381.t1 | thiol peroxidase | 240 | 3.78E-25 | 73.3 | F:peroxidase activity; P:single-organism process; P:cellular process |
| Efet.01.1659048.g1382.t1 | hypothetical protein B5766_02665 | 741 | 6.59E-164 | 67.67 |  |
| Efet.01.165905.g792.t1 | tubulin alpha-1 chain-like | 747 | 2.98E-176 | 99 | F:GTP binding; C:nucleus; C:myelin sheath; C:extracellular exosome; F:ubiquitin protein ligase binding; F:double-stranded RNA binding; F:GTPase activity; P:cellular response to interleukin-4; F:structural constituent of cytoskeleton; C:cytoplasmic microtubule; P:microtubule-based process |
| Efet.01.1659051.g1383.t1 | cyanophycin synthetase | 858 | 5.66E-143 | 83.1 | F:ATP binding; F:metal ion binding; P:macromolecule biosynthetic process; F:ligase activity |
| Efet.01.1659053.g1384.t1 | flagellar motor | 1302 | 0 | 53.5 | C:membrane; C:integral component of membrane; C:cell outer membrane |
| Efet.01.1659054.g1385.t1 | family transcriptional regulator | 1383 | 0 | 50.6 | F:DNA binding |
| Efet.01.1659057.g1386.t1 | ABC transporter ATP-binding | 279 | 9.14E-57 | 79.5 | F:ATPase activity; F:ATP binding |
| Efet.01.1659057.g1387.t1 | ABC transporter ATP-binding | 651 | 2.46E-109 | 72.8 | F:ATPase activity; F:ATP binding; F:substrate-specific transmembrane transporter activity; P:transport |
| Efet.01.1659061.g1389.t1 | Chaperone of endosialidase [Chryseobacterium arachidis] | 411 | 1.92E-17 | 59.6 |  |
| Efet.01.1659071.g1390.t1 | hypothetical protein CAPTEDRAFT_227646 | 966 | 7.62E-06 | 41 | F:metal ion binding |
| Efet.01.1659077.g1391.t1 | oxidoreductase | 1257 | 0 | 88.9 | P:aromatic compound catabolic process; F:iron ion binding; P:oxidation-reduction process; F:3-phenylpropionate dioxygenase activity; F:2 iron, 2 sulfur cluster binding |
| Efet.01.1659078.g1392.t1 | hypothetical protein CAPTEDRAFT_197038 | 996 | 1.17E-49 | 56 |  |
| Efet.01.1659079.g1393.t1 | hypothetical protein HELRODRAFT_167174 | 636 | 2.67E-33 | 51.5 |  |
| Efet.01.1659083.g1394.t1 | T9SS C-terminal target domain-containing | 615 | 1.04E-79 | 53.75 |  |
| Efet.01.1659085.g1395.t1 | DNA recombination | 1107 | 0 | 95.55 | C:integral component of membrane |
| Efet.01.1659086.g1396.t1 | [Naegleria gruberi] | 1176 | 1.83E-44 | 59.35 | F:organic cyclic compound binding; F:heterocyclic compound binding |
| Efet.01.1659087.g1397.t1 | acetolactate synthase small subunit | 900 | 3.46E-107 | 94.85 | F:acetolactate synthase activity; F:amino acid binding; P:branched-chain amino acid biosynthetic process |
| Efet.01.1659088.g1398.t1 | neuropeptide F receptor-like | 1338 | 9.64E-143 | 66.65 | F:G-protein coupled receptor activity; P:G-protein coupled receptor signaling pathway; C:membrane |
| Efet.01.1659089.g1399.t1 | dynein beta ciliary-like | 1224 | 0 | 83.55 | F:ATPase activity; F:ATP binding; F:microtubule motor activity; C:integral component of membrane; C:dynein complex; P:microtubule-based movement |
| Efet.01.165909.g794.t1 | PREDICTED: uncharacterized protein LOC107186184 | 285 | 6.88E-07 | 54 |  |
| Efet.01.1659092.g1400.t1 | oxidoreductase | 1317 | 0 | 77.65 | F:oxidoreductase activity, acting on the CH-OH group of donors, NAD or NADP as acceptor; F:NAD binding; P:oxidation-reduction process |
| Efet.01.1659093.g1401.t1 | ribosome silencing factor | 318 | 2.60E-48 | 82.25 | C:cytoplasm; P:mature ribosome assembly; P:negative regulation of translation; P:negative regulation of ribosome biogenesis |
| Efet.01.1659093.g1402.t1 | ATP-dependent zinc metalloprotease | 744 | 6.24E-72 | 72.5 | F:nucleotide binding; P:protein metabolic process; F:metal ion binding; C:membrane; F:metallopeptidase activity |
| Efet.01.1659098.g1404.t1 | beta-1,3-galactosyltransferase 1-like | 1188 | 4.89E-86 | 58.8 | F:transferase activity, transferring glycosyl groups; C:membrane; C:Golgi apparatus |
| Efet.01.1659102.g1405.t1 | C4-dicarboxylate ABC transporter | 1332 | 0 | 86.25 | C:integral component of membrane |
| Efet.01.1659107.g1406.t1 | oxygen-independent coproporphyrinogen III oxidase | 663 | 4.84E-151 | 88.3 | P:porphyrin-containing compound biosynthetic process; C:cytoplasm; F:coproporphyrinogen oxidase activity; P:oxidation-reduction process; F:iron-sulfur cluster binding |
| Efet.01.1659111.g1407.t1 | hypothetical protein SAMN05216567_12589 | 1257 | 5.25E-120 | 56.85 |  |
| Efet.01.1659114.g1409.t1 | lysozyme | 714 | 1.31E-58 | 88.65 | P:cell wall macromolecule catabolic process; P:cytolysis; P:peptidoglycan catabolic process; P:defense response to bacterium; F:lysozyme activity |
| Efet.01.1659114.g1410.t1 | 3-isopropylmalate dehydratase large subunit | 459 | 1.96E-83 | 81 | F:metal ion binding; P:leucine biosynthetic process; F:4 iron, 4 sulfur cluster binding; F:3-isopropylmalate dehydratase activity; F:isomerase activity |
| Efet.01.1659117.g1412.t1 | urea ABC transporter permease subunit | 336 | 6.26E-54 | 96.2 | C:integral component of membrane; P:transport; C:plasma membrane; F:transporter activity |
| Efet.01.1659117.g1413.t1 | urea ABC transporter permease subunit | 738 | 3.69E-132 | 95.15 | C:integral component of membrane; P:transport; C:plasma membrane; F:transporter activity |
| Efet.01.1659124.g1415.t1 | NAD(P)-dependent oxidoreductase | 759 | 1.10E-137 | 69.5 |  |
| Efet.01.1659128.g1416.t1 | Xaa-Pro aminopeptidase | 1386 | 0 | 61.95 | F:hydrolase activity |
| Efet.01.1659131.g1417.t1 | replication region DNA-binding N-term | 528 | 5.73E-25 | 84 |  |
| Efet.01.1659131.g1418.t1 | FMN-binding negative transcriptional regulator | 645 | 1.97E-151 | 91.05 | F:FMN binding; F:oxidoreductase activity; P:oxidation-reduction process |
| Efet.01.1659132.g1419.t1 | branched-chain amino acid ABC transporter permease | 861 | 1.18E-155 | 73.8 | C:integral component of membrane; P:transport; C:plasma membrane; F:transporter activity |
| Efet.01.1659132.g1420.t1 | ABC transporter substrate-binding | 507 | 1.27E-117 | 75.9 | P:amino acid transport |
| Efet.01.1659133.g1421.t1 | transcriptional regulator | 768 | 0 | 76.95 | F:DNA binding; P:transcription, DNA-templated; P:regulation of transcription, DNA-templated |
| Efet.01.1659133.g1422.t1 | Mannose-6-phosphate cupin superfamily [Paraburkholderia susongensis] | 705 | 3.09E-145 | 74.4 |  |
| Efet.01.1659137.g1423.t1 | ABC transporter ATP-binding | 306 | 4.43E-62 | 83.35 | F:ATPase activity; F:ATP binding |
| Efet.01.1659137.g1424.t1 | family transcriptional regulator | 834 | 1.37E-165 | 75.3 | F:DNA binding; F:transcription factor activity, sequence-specific DNA binding; P:transcription, DNA-templated; P:regulation of transcription, DNA-templated |
| Efet.01.1659138.g1425.t1 | ABC transporter substrate-binding | 783 | 0 | 60 |  |
| Efet.01.1659142.g1427.t1 | sugar phosphate isomerase epimerase | 693 | 1.04E-149 | 88.05 | P:nucleic acid phosphodiester bond hydrolysis; F:endonuclease activity; F:isomerase activity |
| Efet.01.1659142.g1428.t1 | DUF1993 domain-containing | 630 | 1.48E-119 | 93.35 |  |
| Efet.01.1659146.g1429.t1 | hypothetical protein | 336 | 1.02E-12 | 63 |  |
| Efet.01.1659146.g1430.t1 | hypothetical protein A3F76_05545 | 291 | 1.86E-23 | 79 |  |
| Efet.01.1659151.g1432.t1 | hypothetical protein | 339 | 1.10E-12 | 57.2 |  |
| Efet.01.1659153.g1433.t1 | hypothetical protein B5766_06260 | 1185 | 0 | 56.4 | C:membrane; C:integral component of membrane |
| Efet.01.1659154.g1434.t1 | ABC transporter | 1299 | 0 | 95.9 | F:ATP binding; F:ATPase activity, coupled to transmembrane movement of substances; P:transmembrane transport; C:integral component of membrane |
| Efet.01.1659155.g1435.t1 | phosphoglucosamine mutase | 606 | 1.21E-129 | 83.5 | F:magnesium ion binding; P:carbohydrate metabolic process; F:phosphoglucomutase activity; F:phosphoglucosamine mutase activity |
| Efet.01.1659157.g1436.t1 | peptide ABC transporter ATP-binding | 999 | 0 | 78.1 | F:ATPase activity; F:ATP binding; P:peptide transport |
| Efet.01.1659160.g1437.t1 | hypothetical protein CAPTEDRAFT_222921 | 1467 | 6.56E-88 | 41.63 | P:lipid transport; F:lipid transporter activity |
| Efet.01.1659161.g1438.t1 | N-acetyltransferase | 1023 | 5.99E-80 | 88.7 | F:N-acetyltransferase activity |
| Efet.01.1659166.g1440.t1 | insulinase family | 1248 | 0 | 85.65 | F:metal ion binding; P:proteolysis; F:metalloendopeptidase activity |
| Efet.01.1659168.g1441.t1 | chloride channel | 486 | 4.63E-59 | 74.5 | F:ion channel activity; P:ion transport; P:transmembrane transport; C:membrane |
| Efet.01.1659170.g1442.t1 | nicotinate-nucleotide adenylyltransferase | 582 | 8.27E-140 | 79.3 | F:ATP binding; F:nicotinate-nucleotide adenylyltransferase activity; P:NAD biosynthetic process |
| Efet.01.1659172.g1443.t1 | galanin receptor type 1-like | 1053 | 1.58E-25 | 49.4 | F:G-protein coupled receptor activity; C:integral component of plasma membrane; P:G-protein coupled receptor signaling pathway; P:signal transduction; C:membrane; C:integral component of membrane; P:cell surface receptor signaling pathway; F:signal transducer activity |
| Efet.01.1659177.g1445.t1 | xylose isomerase | 828 | 0 | 65.7 | F:isomerase activity |
| Efet.01.1659180.g1446.t1 | nitrate reductase | 1299 | 0 | 89.05 | F:electron carrier activity; F:molybdenum ion binding; P:oxidation-reduction process; F:nitrate reductase (NADH) activity; F:iron-sulfur cluster binding |
| Efet.01.1659182.g1447.t1 | non-canonical purine NTP diphosphatase | 1275 | 1.30E-130 | 75.15 | F:pyrophosphatase activity; F:binding; P:nucleotide metabolic process |
| Efet.01.1659183.g1448.t1 | sugar ABC transporter ATP-binding | 267 | 1.19E-51 | 69.7 | F:ATPase activity; F:ATP binding |
| Efet.01.1659186.g1449.t1 | POU class transcription factor 1 isoform X3 | 432 | 3.94E-27 | 82.4 | F:transcription factor activity, sequence-specific DNA binding; P:negative regulation of transcription, DNA-templated; P:transcription, DNA-templated; C:endoplasmic reticulum; F:sequence-specific DNA binding; C:nucleoplasm |
| Efet.01.1659188.g1450.t1 | rod shape-determining | 870 | 6.32E-101 | 87.75 | P:regulation of cell shape; P:cell division; C:integral component of membrane; P:cell cycle; F:hydrolase activity |
| Efet.01.1659189.g1451.t1 | cyclohexanecarboxylate- ligase | 1224 | 0 | 79.95 | P:metabolic process; F:ligase activity |
| Efet.01.1659190.g1452.t1 | hypothetical protein HELRODRAFT_171923 | 513 | 4.13E-11 | 48.25 | F:chitin binding; F:calcium ion binding; C:membrane; P:chitin metabolic process; C:integral component of membrane; C:extracellular region |
| Efet.01.1659191.g1453.t1 | ABC transporter permease | 1092 | 6.61E-100 | 61.5 | C:membrane |
| Efet.01.1659192.g1454.t1 | neurotrypsin-like | 378 | 1.79E-27 | 71.35 | C:membrane; F:scavenger receptor activity; P:receptor-mediated endocytosis |
| Efet.01.1659193.g1455.t1 | family transcriptional regulator | 726 | 2.64E-159 | 80.3 | F:DNA binding; P:transcription, DNA-templated; P:regulation of transcription, DNA-templated |
| Efet.01.1659194.g1456.t1 | DUF1640 domain-containing | 366 | 2.00E-51 | 63.95 | C:integral component of membrane |
| Efet.01.16592.g1225.t1 | discoidin domain-containing receptor 2 isoform X1 | 390 | 1.80E-42 | 60.8 | C:integral component of membrane; F:transferase activity |
| Efet.01.1659201.g1457.t1 | alpha beta hydrolase | 438 | 2.99E-85 | 68.7 | F:peroxidase activity; F:oxidoreductase activity; P:cellular oxidant detoxification; P:oxidation-reduction process |
| Efet.01.1659201.g1458.t1 | carbohydrate ABC transporter permease | 897 | 1.07E-179 | 95.9 | C:integral component of membrane; P:transport; C:plasma membrane |
| Efet.01.1659203.g1459.t1 | bark beetle-like isoform X1 | 1341 | 1.22E-168 | 60.45 | C:integral component of membrane; F:scavenger receptor activity; P:receptor-mediated endocytosis |
| Efet.01.1659205.g1460.t1 | formate dehydrogenase | 1533 | 0 | 96.2 | F:oxidoreductase activity; F:molybdenum ion binding; P:oxidation-reduction process; F:iron-sulfur cluster binding |
| Efet.01.1659209.g1461.t1 | family phosphatase | 1014 | 0 | 88.8 |  |
| Efet.01.1659210.g1462.t1 | alpha beta hydrolase | 903 | 0 | 68.65 | C:integral component of membrane; F:hydrolase activity |
| Efet.01.1659214.g1465.t1 | mobilization | 1023 | 0 | 76.3 | F:transferase activity |
| Efet.01.1659216.g1466.t1 | DNA-binding response regulator | 546 | 1.41E-126 | 81.35 | F:DNA binding; P:transcription, DNA-templated; P:phosphorelay signal transduction system; P:regulation of transcription, DNA-templated; C:intracellular |
| Efet.01.1659217.g1467.t1 | DUF4433 domain-containing | 399 | 2.86E-22 | 60.6 |  |
| Efet.01.1659217.g1468.t1 | IS110 family transposase ISBcen8 | 801 | 2.00E-147 | 89.3 | F:DNA binding; F:transposase activity; P:transposition, DNA-mediated |
| Efet.01.1659219.g1469.t1 | taurine catabolism dioxygenase | 864 | 1.74E-100 | 76.35 | F:oxidoreductase activity; P:oxidation-reduction process |
| Efet.01.1659219.g1470.t1 | Sua5 family | 414 | 5.48E-71 | 57.5 | F:double-stranded RNA binding |
| Efet.01.1659226.g1471.t1 | proline dehydrogenase | 690 | 3.46E-88 | 73.95 | P:single-organism metabolic process |
| Efet.01.1659227.g1472.t1 | 8-amino-7-oxononanoate synthase | 1005 | 0 | 85.2 | F:pyridoxal phosphate binding; F:glycine C-acetyltransferase activity; P:biosynthetic process; F:ligase activity |
| Efet.01.1659228.g1473.t1 | CDP-6-deoxy-delta-3,4-glucoseen reductase | 1053 | 0 | 91.55 | F:oxidoreductase activity; F:metal ion binding; F:electron carrier activity; P:oxidation-reduction process; F:2 iron, 2 sulfur cluster binding |
| Efet.01.1659229.g1474.t1 | chromate transporter | 639 | 2.42E-80 | 87.35 | C:integral component of membrane; F:chromate transmembrane transporter activity; P:chromate transport |
| Efet.01.1659231.g1475.t1 | Kyphoscoliosis peptidase | 1326 | 1.02E-166 | 60.8 | F:metal ion binding |
| Efet.01.1659233.g1476.t1 | DUF2191 domain-containing | 207 | 5.79E-34 | 91.3 |  |
| Efet.01.1659233.g1477.t1 | endonuclease | 786 | 1.68E-105 | 77.3 | F:catalytic activity |
| Efet.01.1659235.g1478.t1 | hypothetical protein HELRODRAFT_183479 | 972 | 3.87E-08 | 86.5 | F:nucleic acid binding; F:metal ion binding |
| Efet.01.1659236.g1479.t1 | long-chain fatty acid-- ligase | 1326 | 0 | 95 | P:metabolic process; F:ligase activity |
| Efet.01.1659237.g1480.t1 | CBS domain-containing | 483 | 1.62E-102 | 94.75 |  |
| Efet.01.1659237.g1481.t1 | 2-dehydro-3-deoxygalactonokinase | 726 | 4.58E-156 | 81.5 | P:D-galactonate catabolic process; F:2-dehydro-3-deoxygalactonokinase activity; P:carbohydrate phosphorylation |
| Efet.01.1659239.g1482.t1 | ABC transporter permease | 804 | 2.46E-155 | 79.8 | C:integral component of membrane; P:transport; C:plasma membrane |
| Efet.01.1659239.g1483.t1 | ABC transporter permease | 735 | 1.05E-137 | 83.3 | C:integral component of membrane; P:transport; C:plasma membrane |
| Efet.01.1659240.g1484.t1 | MFS transporter | 705 | 8.43E-144 | 78.85 | P:transmembrane transport; C:integral component of membrane |
| Efet.01.1659242.g1485.t1 | potassium transporter | 1365 | 0 | 86.9 | P:potassium ion transport; F:solute:proton antiporter activity; C:integral component of membrane; P:hydrogen ion transmembrane transport; C:plasma membrane |
| Efet.01.1659243.g1486.t1 | multiple coagulation factor deficiency 2 homolog | 366 | 1.91E-27 | 65.2 | F:calcium ion binding; C:membrane; C:integral component of membrane |
| Efet.01.1659246.g1487.t1 | L-threonine 3-dehydrogenase | 984 | 0 | 86.8 | F:zinc ion binding; P:L-threonine catabolic process to glycine; C:cytoplasm; P:oxidation-reduction process; F:L-threonine 3-dehydrogenase activity |
| Efet.01.1659248.g1488.t1 | DNA helicase | 1254 | 0 | 93.15 | F:nucleic acid binding; F:ATP binding; P:DNA repair; F:ATP-dependent 3'-5' DNA helicase activity; P:DNA replication; P:DNA duplex unwinding; P:DNA recombination; P:SOS response; C:intracellular |
| Efet.01.1659255.g1489.t1 | hypothetical protein | 654 | 2.55E-60 | 65.05 |  |
| Efet.01.1659255.g1490.t1 | integrating conjugative element pfgi-1 | 414 | 3.21E-18 | 71.75 |  |
| Efet.01.1659263.g1491.t1 | beta-glycosidase | 1170 | 1.27E-154 | 73.85 | P:carbohydrate metabolic process; F:hydrolase activity, hydrolyzing O-glycosyl compounds |
| Efet.01.1659264.g1492.t1 | tripartite tricarboxylate transporter substrate binding | 330 | 1.58E-58 | 69.2 | C:outer membrane-bounded periplasmic space |
| Efet.01.1659264.g1493.t1 | CoA-binding domain protein | 1089 | 9.26E-163 | 82.25 | F:ATP binding; F:metal ion binding; F:cofactor binding; F:transferase activity; F:ligase activity |
| Efet.01.1659265.g1494.t1 | family transcriptional regulator | 507 | 9.70E-120 | 74.75 | F:DNA binding; P:transcription, DNA-templated; P:regulation of transcription, DNA-templated |
| Efet.01.1659265.g1495.t1 | tetratricopeptide repeat | 585 | 6.85E-61 | 65.55 |  |
| Efet.01.1659268.g1497.t1 | EF-hand domain-containing 1-like | 438 | 5.36E-24 | 59.8 | F:calcium ion binding |
| Efet.01.1659269.g1498.t1 | hypothetical protein CRN43_01970 | 1389 | 0 | 58.75 |  |
| Efet.01.1659273.g1500.t1 | L-fuculose kinase | 651 | 9.70E-85 | 55.11 | P:carbohydrate metabolic process; P:phosphorylation; F:phosphotransferase activity, alcohol group as acceptor; F:kinase activity |
| Efet.01.1659273.g1501.t1 | fatty acid desaturase | 624 | 2.69E-109 | 76.45 | P:sodium ion transport; F:metal ion binding; P:lipid metabolic process; C:integral component of membrane; P:oxidation-reduction process; F:2 iron, 2 sulfur cluster binding; F:oxidoreductase activity, acting on NAD(P)H, quinone or similar compound as acceptor |
| Efet.01.1659275.g1502.t1 | GDSL family lipase | 729 | 6.26E-89 | 83.05 | F:lysophospholipase activity |
| Efet.01.1659275.g1503.t1 | family transcriptional regulator | 645 | 1.61E-120 | 75.25 | F:DNA binding; P:transcription, DNA-templated; P:regulation of transcription, DNA-templated |
| Efet.01.1659277.g1505.t1 | dihydroxy-acid dehydratase | 897 | 0 | 95 | F:dihydroxy-acid dehydratase activity; P:metabolic process |
| Efet.01.1659277.g1506.t1 | 3-hydroxyisobutyrate dehydrogenase | 363 | 1.75E-76 | 94.2 | F:phosphogluconate dehydrogenase (decarboxylating) activity; F:NAD binding; P:valine catabolic process; P:oxidation-reduction process; F:3-hydroxyisobutyrate dehydrogenase activity |
| Efet.01.1659279.g1508.t1 | RNA-dependent RNA polymerase 1 | 846 | 1.51E-103 | 70.7 | F:RNA-directed 5'-3' RNA polymerase activity; P:transcription, RNA-templated |
| Efet.01.1659281.g1509.t1 | sodium channel para- | 813 | 4.88E-99 | 72.95 | P:regulation of postsynaptic membrane potential; P:regulation of ion transmembrane transport; C:voltage-gated sodium channel complex; P:sodium ion transmembrane transport; F:voltage-gated sodium channel activity |
| Efet.01.1659283.g1510.t1 | DNA-directed RNA polymerase subunit beta | 549 | 3.53E-124 | 77.1 | F:DNA-directed 5'-3' RNA polymerase activity; P:RNA biosynthetic process |
| Efet.01.1659285.g1512.t1 | hypothetical protein B5766_12210 | 357 | 5.59E-10 | 95 |  |
| Efet.01.1659286.g1513.t1 | cyanophycin synthetase | 1122 | 0 | 94.05 | F:ATP binding; F:metal ion binding; P:macromolecule biosynthetic process; F:ligase activity |
| Efet.01.1659287.g1514.t1 | aldo keto reductase | 837 | 0 | 83.55 | F:oxidoreductase activity; P:oxidation-reduction process |
| Efet.01.1659288.g1515.t1 | hypothetical protein B5766_02430 | 1767 | 0 | 66.25 | P:carbohydrate metabolic process |
| Efet.01.1659289.g1516.t1 | 3-keto-5-aminohexanoate cleavage | 654 | 3.17E-155 | 91.4 | P:L-lysine catabolic process to acetate; F:transferase activity |
| Efet.01.1659289.g1517.t1 | peptidase M48 | 408 | 5.27E-26 | 54.15 | C:membrane; F:metallopeptidase activity |
| Efet.01.1659290.g1518.t1 | cell division | 501 | 1.63E-90 | 91.45 | P:FtsZ-dependent cytokinesis; C:integral component of plasma membrane; C:cell division site |
| Efet.01.1659290.g1519.t1 | D-alanine--D-alanine ligase | 996 | 0 | 82.95 | F:magnesium ion binding; P:regulation of cell shape; F:ATP binding; F:D-alanine-D-alanine ligase activity; C:cytoplasm; P:peptidoglycan biosynthetic process; F:manganese ion binding; P:cell wall organization |
| Efet.01.1659292.g1520.t1 | AAA family ATPase | 795 | 0 | 91.55 | F:ATP binding |
| Efet.01.1659292.g1521.t1 | type VI secretion | 762 | 4.49E-90 | 90.65 | C:integral component of membrane; P:unidirectional conjugation |
| Efet.01.1659293.g1522.t1 | transferase | 1101 | 0 | 83.55 | F:formyl-CoA transferase activity; F:succinyl-CoA:(R)-benzylsuccinate CoA-transferase activity |
| Efet.01.1659293.g1523.t1 | synthetase | 297 | 1.17E-56 | 71.2 | P:metabolic process; F:CoA-transferase activity |
| Efet.01.1659295.g1525.t1 | sugar ABC transporter permease | 927 | 9.79E-168 | 71.35 | C:integral component of membrane; P:transport; C:plasma membrane |
| Efet.01.1659297.g1526.t1 | Transcriptional i Type IV TA system | 471 | 3.11E-104 | 80.55 |  |
| Efet.01.1659297.g1527.t1 | nucleotidyl transferase ii ii toxin family | 975 | 0 | 73.65 |  |
| Efet.01.1659299.g1529.t1 | apolipo L3-like | 1314 | 1.63E-11 | 51 |  |
| Efet.01.1659300.g1530.t1 | NAD-dependent DNA ligase | 909 | 6.11E-175 | 88.5 | F:dioxygenase activity; F:DNA binding; P:DNA repair; F:DNA ligase (NAD+) activity; F:metal ion binding; P:DNA replication; P:DNA ligation; P:oxidation-reduction process |
| Efet.01.1659300.g1531.t1 | cell division | 603 | 1.79E-137 | 93 | P:cell division; C:integral component of membrane |
| Efet.01.1659305.g1534.t1 | fibronectin type III domain-containing | 1359 | 0 | 70 | P:self proteolysis |
| Efet.01.1659307.g1535.t1 | citrate synthase | 783 | 9.88E-180 | 86.7 | F:citrate (Si)-synthase activity; C:cytoplasm; P:tricarboxylic acid cycle |
| Efet.01.1659308.g1536.t1 | teneurin-m-like isoform X1 | 1611 | 7.39E-177 | 55.65 | P:self proteolysis |
| Efet.01.1659309.g1537.t1 | sulfite oxidase-like | 1575 | 0 | 67.8 | F:metal ion binding; P:single-organism metabolic process |
| Efet.01.165931.g796.t1 | UDP-galactose UDP-glucose transporter 7 isoform X5 | 750 | 5.10E-89 | 65 | C:integral component of membrane |
| Efet.01.1659311.g1538.t1 | ABC transporter permease | 777 | 3.91E-121 | 91.05 | C:integral component of membrane; P:transport; C:plasma membrane; F:transporter activity |
| Efet.01.1659311.g1539.t1 | ABC transporter permease | 699 | 2.39E-65 | 94 | C:integral component of membrane; P:transport; C:plasma membrane; F:transporter activity |
| Efet.01.1659313.g1540.t1 | TonB-dependent receptor | 726 | 1.94E-164 | 65.75 | C:membrane; F:receptor activity; P:transport |
| Efet.01.1659314.g1541.t1 | myo-inosose-2 dehydratase | 255 | 1.53E-48 | 82.25 | F:isomerase activity |
| Efet.01.1659314.g1542.t1 | xylose isomerase | 744 | 1.34E-147 | 85.85 | F:isomerase activity |
| Efet.01.1659314.g1543.t1 | glycerol-3-phosphate dehydrogenase | 765 | 4.04E-160 | 90.5 | P:carbohydrate metabolic process; P:glycerol-3-phosphate biosynthetic process; C:glycerol-3-phosphate dehydrogenase complex; F:NAD binding; P:glycerol-3-phosphate catabolic process; P:phospholipid biosynthetic process; F:glycerol-3-phosphate dehydrogenase [NADP+] activity; F:glycerol-3-phosphate dehydrogenase [NAD(P)+] activity; P:oxidation-reduction process; P:glycerophospholipid metabolic process; F:glycerol-3-phosphate dehydrogenase [NAD+] activity |
| Efet.01.1659315.g1544.t1 | exoribonuclease R | 642 | 5.65E-140 | 62.8 |  |
| Efet.01.1659315.g1545.t1 | peptidoglycan-binding domain-containing | 813 | 5.50E-171 | 57.35 |  |
| Efet.01.1659316.g1546.t1 | lactaldehyde reductase | 432 | 2.86E-77 | 58.95 | F:oxidoreductase activity; F:metal ion binding; P:oxidation-reduction process |
| Efet.01.1659316.g1547.t1 | aldehyde dehydrogenase | 1470 | 0 | 76.65 | F:betaine-aldehyde dehydrogenase activity; P:oxidation-reduction process |
| Efet.01.1659317.g1548.t1 | 1-acyl-sn-glycerol-3-phosphate acyltransferase | 1251 | 0 | 54.45 | F:1-acylglycerol-3-phosphate O-acyltransferase activity; F:transferase activity, transferring acyl groups; P:metabolic process; F:transferase activity |
| Efet.01.1659318.g1549.t1 | family transcriptional regulator | 348 | 3.13E-75 | 88.05 | F:DNA binding; F:transcription factor activity, sequence-specific DNA binding; P:transcription, DNA-templated; P:regulation of transcription, DNA-templated |
| Efet.01.1659318.g1550.t1 | acyl- dehydrogenase | 810 | 0 | 94.6 | F:acyl-CoA dehydrogenase activity; F:flavin adenine dinucleotide binding; P:oxidation-reduction process |
| Efet.01.1659320.g1551.t1 | alpha-adducin-like isoform X1 | 405 | 8.60E-39 | 71.25 |  |
| Efet.01.1659325.g1552.t1 | monovalent cation H+ antiporter subunit A | 1407 | 0 | 88.3 | F:NADH dehydrogenase (quinone) activity; C:integral component of membrane; P:oxidation-reduction process |
| Efet.01.1659325.g1553.t1 | Na+ H+ antiporter subunit C | 288 | 4.09E-62 | 91.2 | C:integral component of membrane; F:oxidoreductase activity, acting on NAD(P)H; P:ATP synthesis coupled electron transport |
| Efet.01.1659326.g1554.t1 | nucleolar 58-like | 516 | 3.68E-48 | 77.75 | C:box C/D snoRNP complex; C:small-subunit processome; P:rRNA modification; F:snoRNA binding |
| Efet.01.1659327.g1555.t1 | asteroid homolog 1 | 960 | 4.27E-16 | 41.5 |  |
| Efet.01.1659328.g1556.t1 | ABC transporter permease | 1689 | 0 | 92.1 | C:integral component of membrane; P:transport; C:plasma membrane; F:transporter activity |
| Efet.01.1659330.g1557.t1 | enoyl- hydratase isomerase family | 1335 | 0 | 70.35 | F:hydrolase activity |
| Efet.01.1659332.g1558.t1 | hypothetical protein CAPTEDRAFT_199057 | 1575 | 1.56E-89 | 50.25 | F:transferase activity, transferring glycosyl groups; C:membrane; C:integral component of membrane |
| Efet.01.1659333.g1559.t1 | hypothetical protein CAPTEDRAFT_168270 | 1479 | 1.62E-139 | 53.85 |  |
| Efet.01.1659334.g1560.t1 | family transcriptional regulator | 981 | 0 | 95.6 | F:DNA binding; P:carbohydrate metabolic process; F:transcription factor activity, sequence-specific DNA binding; P:regulation of transcription, DNA-templated; F:carbohydrate binding |
| Efet.01.1659334.g1561.t1 | isoaspartyl peptidase L-asparaginase | 450 | 1.98E-98 | 96.5 | P:proteolysis; F:beta-aspartyl-peptidase activity |
| Efet.01.1659335.g1562.t1 | ATP-dependent chaperone | 1680 | 0 | 94.25 |  |
| Efet.01.1659337.g1564.t1 | acetylcholinesterase-like | 1578 | 0 | 67.95 | C:extracellular space; C:membrane; F:carboxylic ester hydrolase activity |
| Efet.01.1659338.g1565.t1 | methylmalonyl Co-A mutase-associated GTPase | 537 | 3.38E-86 | 91.5 | F:GTP binding; F:GTPase activity |
| Efet.01.1659338.g1566.t1 | methylmalonyl- mutase | 1374 | 0 | 91.8 | F:metal ion binding; F:cobalamin binding; P:metabolic process; F:methylmalonyl-CoA mutase activity |
| Efet.01.1659339.g1567.t1 | VWA domain-containing | 924 | 1.35E-175 | 56.4 | C:membrane; C:integral component of membrane |
| Efet.01.1659339.g1568.t1 | [Methylomonas denitrificans] | 456 | 1.46E-87 | 62.6 |  |
| Efet.01.1659340.g1569.t1 | GH3 auxin-responsive promoter | 834 | 0 | 86.65 |  |
| Efet.01.1659341.g1570.t1 | Methyltransferase type 12 | 399 | 1.98E-87 | 83.45 | F:methyltransferase activity; P:methylation |
| Efet.01.1659341.g1571.t1 | restriction endonuclease | 840 | 4.35E-131 | 95.3 | P:nucleic acid phosphodiester bond hydrolysis; F:endonuclease activity |
| Efet.01.1659342.g1572.t1 | chromosome segregation SMC | 1095 | 4.54E-179 | 86.3 | F:DNA binding; F:ATP binding; P:sister chromatid cohesion; C:cytoplasm; P:DNA replication; P:chromosome condensation; C:chromosome |
| Efet.01.1659342.g1573.t1 | signal recognition particle-docking | 891 | 0 | 90.05 | F:GTP binding; P:cell division; P:SRP-dependent cotranslational protein targeting to membrane; C:cytoplasm; F:GTPase activity; C:intrinsic component of plasma membrane |
| Efet.01.1659343.g1574.t1 | death domain-containing 1-like isoform X1 | 618 | 1.17E-14 | 49.95 | P:signal transduction |
| Efet.01.1659345.g1575.t1 | DNA topoisomerase III | 1227 | 0 | 90.25 | F:DNA binding; F:DNA topoisomerase type I activity; P:DNA topological change |
| Efet.01.1659346.g1576.t1 | 16S rRNA (cytidine(1402)-2 -O)-methyltransferase | 849 | 1.40E-111 | 89.35 | C:cytoplasm; F:rRNA (cytosine-2'-O-)-methyltransferase activity; P:enzyme-directed rRNA 2'-O-methylation |
| Efet.01.1659346.g1577.t1 | family | 435 | 5.10E-57 | 84.9 | F:nucleic acid binding; P:nucleic acid phosphodiester bond hydrolysis; F:nuclease activity |
| Efet.01.1659346.g1578.t1 | phosphoheptose isomerase | 585 | 1.14E-115 | 87.4 | P:carbohydrate metabolic process; F:carbohydrate binding; F:isomerase activity |
| Efet.01.1659347.g1579.t1 | alpha beta hydrolase | 648 | 3.89E-140 | 64.85 | F:hydrolase activity |
| Efet.01.1659347.g1580.t1 | short chain dehydrogenase | 693 | 2.47E-128 | 65 | F:oxidoreductase activity; P:oxidation-reduction process |
| Efet.01.1659347.g1581.t1 | tripartite tricarboxylate transporter substrate binding | 444 | 5.02E-101 | 62.05 | C:outer membrane-bounded periplasmic space |
| Efet.01.1659348.g1582.t1 | hypothetical protein | 402 | 1.59E-68 | 89.45 |  |
| Efet.01.1659348.g1583.t1 | BAX inhibitor (BI)-1 family | 696 | 7.57E-148 | 95.45 | C:integral component of membrane |
| Efet.01.1659348.g1584.t1 | heme-binding | 408 | 4.19E-64 | 94.7 |  |
| Efet.01.1659349.g1585.t1 | urea ABC transporter substrate-binding | 1350 | 0 | 97.05 |  |
| Efet.01.1659349.g1586.t1 | urea ABC transporter permease subunit | 429 | 2.26E-44 | 86.45 | C:integral component of membrane; P:transport; C:plasma membrane; F:transporter activity |
| Efet.01.1659351.g1587.t1 | DNA alkylation repair | 771 | 9.77E-64 | 62.35 |  |
| Efet.01.1659351.g1588.t1 | hypothetical protein | 603 | 1.48E-35 | 67.85 |  |
| Efet.01.1659352.g1589.t1 | cobalamin adenosyltransferase | 294 | 6.52E-49 | 90.6 | F:transferase activity |
| Efet.01.1659352.g1590.t1 | catechol 2,3-dioxygenase | 906 | 0 | 94.65 | P:aromatic compound catabolic process; F:ferrous iron binding; P:oxidation-reduction process; F:catechol 2,3-dioxygenase activity |
| Efet.01.1659352.g1591.t1 | ferredoxin | 363 | 4.00E-62 | 80.65 | F:metal ion binding; F:electron carrier activity; F:2 iron, 2 sulfur cluster binding |
| Efet.01.1659354.g1592.t1 | DUF1854 domain-containing | 522 | 6.82E-112 | 86.05 |  |
| Efet.01.1659354.g1593.t1 | virulence factor | 279 | 1.35E-60 | 91.4 |  |
| Efet.01.1659354.g1594.t1 | hypothetical protein | 438 | 1.92E-71 | 68.1 |  |
| Efet.01.1659356.g1596.t1 | cytidylate kinase | 804 | 7.52E-130 | 88 | F:cytidylate kinase activity; F:ATP binding; P:nucleotide phosphorylation; P:aromatic amino acid family biosynthetic process; F:3-phosphoshikimate 1-carboxyvinyltransferase activity; C:cytoplasm; P:chorismate biosynthetic process |
| Efet.01.1659356.g1597.t1 | prephenate dehydrogenase | 900 | 0 | 92.25 | P:tyrosine biosynthetic process; P:oxidation-reduction process; F:prephenate dehydrogenase (NAD+) activity; F:prephenate dehydrogenase (NADP+) activity |
| Efet.01.1659357.g1598.t1 | lytic transglycosylase | 1767 | 0 | 66.4 | P:peptidoglycan metabolic process; C:membrane; F:lyase activity; F:lytic transglycosylase activity |
| Efet.01.1659360.g1599.t1 | alpha beta hydrolase | 420 | 2.95E-84 | 66.95 | F:hydrolase activity |
| Efet.01.1659360.g1600.t1 | long-chain fatty acid-- ligase | 1620 | 0 | 79.1 | P:long-chain fatty acid metabolic process; F:long-chain fatty acid-CoA ligase activity |
| Efet.01.1659362.g1602.t1 | ABC transporter permease | 528 | 7.87E-74 | 75.5 | C:integral component of membrane; P:transport; C:plasma membrane; F:transporter activity |
| Efet.01.1659362.g1603.t1 | ABC transporter permease | 1044 | 7.31E-149 | 75.45 | C:integral component of membrane; P:transport; C:plasma membrane; F:transporter activity |
| Efet.01.1659364.g1605.t1 | serine threonine- kinase RIO1 | 204 | 1.30E-23 | 84.2 | F:ATP binding; F:protein serine/threonine kinase activity; P:protein phosphorylation |
| Efet.01.1659365.g1606.t1 | elongation factor Ts | 711 | 5.98E-148 | 89 | F:translation elongation factor activity; C:cytoplasm; P:translational elongation |
| Efet.01.1659365.g1607.t1 | 30S ribosomal S2 | 741 | 4.15E-180 | 96.1 | F:structural constituent of ribosome; C:small ribosomal subunit; P:translation |
| Efet.01.1659367.g1608.t1 | carbamoyl-phosphate synthase large subunit | 1434 | 0 | 82.35 | F:ATP binding; F:metal ion binding; F:biotin carboxylase activity |
| Efet.01.1659369.g1610.t1 | L-fuculose-phosphate aldolase | 702 | 4.94E-158 | 73.55 | F:L-fuculose-phosphate aldolase activity |
| Efet.01.1659370.g1611.t1 | argininosuccinate synthase | 1272 | 0 | 95.7 | F:ATP binding; F:protein homodimerization activity; P:arginine biosynthetic process; C:cytoplasm; F:argininosuccinate synthase activity |
| Efet.01.1659370.g1612.t1 | family transcriptional regulator | 405 | 6.50E-76 | 82.2 | F:DNA binding; F:transcription factor activity, sequence-specific DNA binding; P:transcription, DNA-templated; P:regulation of transcription, DNA-templated |
| Efet.01.1659371.g1613.t1 | transcriptional regulator | 417 | 1.80E-81 | 75.3 | F:DNA binding; F:transcription factor activity, sequence-specific DNA binding; P:transcription, DNA-templated; P:regulation of transcription, DNA-templated; C:intracellular |
| Efet.01.1659371.g1614.t1 | sugar ABC transporter substrate-binding | 909 | 4.18E-173 | 83.85 |  |
| Efet.01.1659372.g1615.t1 | cell filamentation Fic | 1404 | 0 | 82.75 | P:cell division |
| Efet.01.1659373.g1616.t1 | pyrroline-5-carboxylate reductase | 849 | 3.28E-171 | 87.75 | F:pyrroline-5-carboxylate reductase activity; C:cytoplasm; P:oxidation-reduction process; P:L-proline biosynthetic process |
| Efet.01.1659373.g1617.t1 | glycerol kinase | 975 | 0 | 88.6 | F:ATP binding; P:glycerol-3-phosphate metabolic process; F:glycerol kinase activity; P:glycerol catabolic process; P:phosphorylation |
| Efet.01.1659374.g1618.t1 | peptide ABC transporter permease | 516 | 2.81E-103 | 82.95 | P:transmembrane transport; C:ATP-binding cassette (ABC) transporter complex |
| Efet.01.1659374.g1619.t1 | ABC transporter permease | 936 | 0 | 89 | C:integral component of membrane; P:transport; C:plasma membrane |
| Efet.01.1659375.g1620.t1 | hemin ABC transporter substrate-binding | 873 | 0 | 81.1 |  |
| Efet.01.1659375.g1621.t1 | hemin-degrading factor | 915 | 0 | 75.65 | P:iron ion transport |
| Efet.01.1659376.g1622.t1 | integration host factor subunit alpha | 351 | 4.38E-71 | 95.35 | P:regulation of translation; F:DNA binding; P:transcription, DNA-templated; P:DNA recombination; P:regulation of transcription, DNA-templated |
| Efet.01.1659376.g1623.t1 | family transcriptional regulator | 423 | 1.64E-98 | 83 | F:DNA binding; P:regulation of transcription, DNA-templated |
| Efet.01.1659376.g1624.t1 | hypothetical protein | 264 | 1.74E-24 | 91 |  |
| Efet.01.1659377.g1625.t1 | tripartite tricarboxylate transporter substrate binding | 1086 | 0 | 89.8 | C:outer membrane-bounded periplasmic space |
| Efet.01.1659377.g1626.t1 | acyl- dehydrogenase | 666 | 1.51E-136 | 94.25 | F:acyl-CoA dehydrogenase activity; F:flavin adenine dinucleotide binding; F:butyryl-CoA dehydrogenase activity; P:oxidation-reduction process |
| Efet.01.1659378.g1627.t1 | acyl :acetate 3-ketoacid transferase | 1821 | 2.80E-148 | 76.5 | F:acetate CoA-transferase activity; P:ketone body catabolic process |
| Efet.01.1659380.g1628.t1 | ABC transporter ATP-binding | 2013 | 0 | 80.75 | F:ATPase activity; F:ATP binding; P:peptide transport |
| Efet.01.1659382.g1629.t1 | T family of potassium channels 18-like | 1596 | 1.52E-36 | 67.55 | P:ion transport; C:membrane |
| Efet.01.1659383.g1630.t1 | sugar kinase | 957 | 1.24E-167 | 70.25 | P:phosphorylation; F:kinase activity |
| Efet.01.1659383.g1631.t1 | ABC transporter permease | 951 | 0 | 75.8 | C:membrane |
| Efet.01.1659386.g1632.t1 | meiosis arrest female 1 homolog isoform X1 | 1464 | 1.45E-73 | 62.6 | F:binding |
| Efet.01.1659387.g1633.t1 | hypothetical protein CAPTEDRAFT_135953 | 303 | 4.43E-06 | 52 | F:hydrolase activity |
| Efet.01.1659388.g1634.t1 | BMP family ABC transporter substrate-binding | 1155 | 0 | 93.75 | C:plasma membrane |
| Efet.01.1659388.g1635.t1 | ABC transporter permease | 939 | 2.86E-150 | 94.1 | C:integral component of membrane; P:transport; C:plasma membrane; F:transporter activity |
| Efet.01.1659389.g1636.t1 | amino acid ABC transporter ATP-binding | 294 | 1.64E-61 | 95.3 | F:ATP binding; P:amino acid transmembrane transport; F:amino acid-transporting ATPase activity |
| Efet.01.1659389.g1637.t1 | amino acid ABC transporter permease | 1230 | 1.81E-140 | 92.45 | C:integral component of membrane; P:transport; C:plasma membrane; F:transporter activity |
| Efet.01.1659389.g1638.t1 | L-idonate 5-dehydrogenase | 483 | 1.02E-98 | 87.3 | F:zinc ion binding; F:L-idonate 5-dehydrogenase activity; P:oxidation-reduction process |
| Efet.01.1659390.g1639.t1 | SH3 domain-containing | 1215 | 4.52E-10 | 52 | C:membrane; C:integral component of membrane |
| Efet.01.1659391.g1640.t1 | fibronectin type III domain-containing | 1413 | 2.27E-144 | 61.85 | F:carbohydrate binding |
| Efet.01.1659392.g1641.t1 | flagellar motor stator | 546 | 5.39E-114 | 86.3 | P:protein transport; C:integral component of membrane; F:protein transporter activity |
| Efet.01.1659392.g1642.t1 | glucose-1-phosphate cytidylyltransferase | 771 | 9.63E-174 | 85.9 | F:glucose-1-phosphate cytidylyltransferase activity; P:biosynthetic process |
| Efet.01.1659393.g1643.t1 | outer membrane lipo -sorting | 1455 | 2.50E-164 | 70.1 |  |
| Efet.01.1659394.g1644.t1 | Acyl- dehydrogenase | 666 | 8.21E-142 | 74.85 | F:oxidoreductase activity, acting on the CH-CH group of donors; P:metabolic process |
| Efet.01.1659394.g1645.t1 | dihydroflavonol-4-reductase | 822 | 2.81E-143 | 61 | F:coenzyme binding; F:catalytic activity; F:isomerase activity |
| Efet.01.1659394.g1646.t1 | beta-ketoacyl-[acyl-carrier- ] synthase family | 558 | 2.18E-112 | 72.75 | F:transferase activity |
| Efet.01.1659395.g1647.t1 | hypothetical protein | 297 | 3.68E-52 | 67.17 | C:integral component of membrane |
| Efet.01.1659395.g1648.t1 | methylmalonyl- carboxyltransferase | 1533 | 0 | 96.2 | F:transferase activity; F:ligase activity |
| Efet.01.1659395.g1649.t1 | methylmalonyl Co-A mutase-associated GTPase | 222 | 1.72E-27 | 84 | F:phosphorelay sensor kinase activity; F:GTP binding; F:GTPase activity; P:phosphorelay signal transduction system; P:signal transduction by protein phosphorylation; C:intracellular |
| Efet.01.1659396.g1650.t1 | gluconate 2-dehydrogenase gamma chain | 594 | 9.56E-144 | 84.55 |  |
| Efet.01.1659396.g1651.t1 | GMC family oxidoreductase | 1698 | 0 | 86.4 | F:oxidoreductase activity, acting on CH-OH group of donors; F:flavin adenine dinucleotide binding; P:oxidation-reduction process |
| Efet.01.1659397.g1652.t1 | ornithine cyclodeaminase | 258 | 8.24E-41 | 80.6 | F:ornithine cyclodeaminase activity |
| Efet.01.1659397.g1653.t1 | dCTP deaminase | 573 | 1.77E-139 | 97.2 | P:dUTP biosynthetic process; F:dCTP deaminase activity; P:pyrimidine ribonucleotide biosynthetic process; P:dUMP biosynthetic process |
| Efet.01.1659397.g1654.t1 | guanine deaminase | 1092 | 0 | 85.85 | F:zinc ion binding; F:guanine deaminase activity; P:guanine catabolic process |
| Efet.01.1659399.g1655.t1 | peptidase S11 | 477 | 9.63E-83 | 91.35 | F:serine-type D-Ala-D-Ala carboxypeptidase activity; P:proteolysis; C:integral component of membrane |
| Efet.01.1659399.g1656.t1 | 2-isopropylmalate synthase | 1473 | 0 | 95 | F:2-isopropylmalate synthase activity; P:leucine biosynthetic process |
| Efet.01.1659400.g1657.t1 | peptide ABC transporter substrate-binding | 1557 | 0 | 86.05 | P:transmembrane transport; C:ATP-binding cassette (ABC) transporter complex |
| Efet.01.1659400.g1658.t1 | ABC transporter permease | 288 | 6.33E-58 | 82.4 | C:integral component of membrane; P:transport; C:plasma membrane |
| Efet.01.1659401.g1659.t1 | family muropeptide MFS transporter | 1353 | 0 | 87.8 | P:transmembrane transport; C:integral component of membrane; F:acetyl-CoA transporter activity; P:acetyl-CoA transport |
| Efet.01.1659401.g1660.t1 | DNA-binding response regulator | 756 | 2.24E-160 | 91.7 | F:DNA binding; P:transcription, DNA-templated; P:phosphorelay signal transduction system; P:regulation of transcription, DNA-templated; C:intracellular |
| Efet.01.1659402.g1661.t1 | branched-chain amino acid ABC transporter permease | 1398 | 1.80E-167 | 81.3 | C:integral component of membrane; P:transport; C:plasma membrane; F:transporter activity |
| Efet.01.1659402.g1662.t1 | branched-chain amino acid ABC transporter permease | 933 | 3.21E-168 | 75.2 | C:integral component of membrane; P:transport; C:plasma membrane; F:transporter activity |
| Efet.01.1659403.g1663.t1 | DUF2339 domain-containing | 1995 | 0 | 87.5 | C:integral component of membrane |
| Efet.01.1659404.g1664.t1 | helicase domain (plasmid) | 1554 | 1.69E-65 | 50.7 | F:binding; P:DNA metabolic process |
| Efet.01.1659408.g1665.t1 | tRNA uridine-5-carboxymethylaminomethyl(34) synthesis GTPase | 813 | 9.13E-161 | 93.55 | F:GTP binding; C:cytoplasm; F:metal ion binding; F:GTPase activity; P:tRNA modification |
| Efet.01.1659408.g1666.t1 | membrane insertase | 1347 | 0 | 95.05 | P:protein transport; C:integral component of membrane; C:plasma membrane; P:protein insertion into membrane |
| Efet.01.1659409.g1667.t1 | Enoyl- hydratase | 804 | 2.37E-169 | 66.25 | P:metabolic process; F:enoyl-CoA hydratase activity |
| Efet.01.1659409.g1668.t1 | acyl- dehydrogenase | 1167 | 0 | 89.1 | F:isovaleryl-CoA dehydrogenase activity; F:flavin adenine dinucleotide binding; P:oxidation-reduction process |
| Efet.01.1659410.g1669.t1 | carbohydrate ABC transporter permease | 486 | 1.88E-105 | 75.95 | C:membrane |
| Efet.01.1659410.g1670.t1 | sugar isomerase domain-containing | 690 | 1.28E-163 | 67.9 | P:carbohydrate metabolic process; F:carbohydrate binding; F:isomerase activity |
| Efet.01.1659410.g1671.t1 | xylulokinase | 693 | 1.74E-150 | 54.2 | P:xylulose metabolic process; P:carbohydrate metabolic process; F:xylulokinase activity; P:phosphorylation; F:phosphotransferase activity, alcohol group as acceptor; F:transferase activity; P:carbohydrate phosphorylation; F:kinase activity |
| Efet.01.1659411.g1672.t1 | cyanophycin synthetase | 1755 | 0 | 93.85 | F:ATP binding; F:metal ion binding; P:macromolecule biosynthetic process; F:ligase activity |
| Efet.01.1659411.g1673.t1 | creA family | 504 | 4.70E-108 | 93.75 |  |
| Efet.01.1659413.g1674.t1 | adenosine deaminase | 1890 | 0 | 92.15 | F:adenine deaminase activity; F:metal ion binding; F:adenosine deaminase activity |
| Efet.01.1659414.g1675.t1 | Mechanosensitive ion channel | 1227 | 0 | 87.7 | P:transmembrane transport; C:integral component of membrane |
| Efet.01.1659414.g1676.t1 | gfo Idh family oxidoreductase | 954 | 0 | 92.35 | F:oxidoreductase activity; P:oxidation-reduction process |
| Efet.01.1659416.g1677.t1 | sugar phosphate isomerase epimerase | 870 | 0 | 83.25 | F:isomerase activity |
| Efet.01.1659416.g1678.t1 | nucleoside nucleotide kinase family | 465 | 7.92E-88 | 79.15 | F:ATP binding; P:phosphorylation; F:kinase activity |
| Efet.01.1659416.g1679.t1 | ABC transporter substrate-binding | 852 | 3.08E-153 | 88.9 | P:carbohydrate transport |
| Efet.01.1659417.g1680.t1 | mitogen-activated kinase kinase kinase 11-like | 489 | 1.43E-22 | 55.9 | F:nucleotide binding; F:ATP binding; F:protein kinase activity; P:phosphorylation; P:protein phosphorylation; F:kinase activity |
| Efet.01.1659418.g1681.t1 | amino acid dehydrogenase | 1347 | 0 | 91.65 | F:D-amino-acid dehydrogenase activity; P:oxidation-reduction process |
| Efet.01.1659418.g1682.t1 | transcriptional regulator | 918 | 2.53E-170 | 87.35 | F:DNA binding; F:transcription factor activity, sequence-specific DNA binding; P:transcription, DNA-templated; P:regulation of transcription, DNA-templated |
| Efet.01.1659421.g1683.t1 | isochorismatase | 303 | 2.07E-63 | 83.9 | P:metabolic process; F:hydrolase activity |
| Efet.01.1659421.g1684.t1 | aminopeptidase P family | 1215 | 0 | 94.3 | P:proteolysis; F:aminopeptidase activity |
| Efet.01.1659422.g1685.t1 | transglutaminase domain | 2448 | 0 | 66.55 | C:integral component of membrane |
| Efet.01.1659423.g1686.t1 | DNA polymerase III subunit delta | 1254 | 0 | 83.4 | F:DNA binding; P:DNA replication |
| Efet.01.1659425.g1687.t1 | hypothetical protein Veis_2545 | 1131 | 0 | 53.7 |  |
| Efet.01.1659425.g1688.t1 | ACP S-malonyltransferase | 465 | 3.69E-88 | 71.8 | F:[acyl-carrier-protein] S-malonyltransferase activity; P:metabolic process |
| Efet.01.1659425.g1689.t1 | hypothetical protein | 573 | 3.91E-114 | 97 | C:integral component of membrane |
| Efet.01.1659427.g1690.t1 | phenylpropionate dioxygenase | 318 | 7.59E-71 | 96.6 | F:2-chlorobenzoate 1,2-dioxygenase activity; P:cellular aromatic compound metabolic process; P:oxidation-reduction process |
| Efet.01.1659427.g1691.t1 | oxidoreductase | 1479 | 0 | 95 | P:aromatic compound catabolic process; F:iron ion binding; F:oxidoreductase activity, acting on paired donors, with incorporation or reduction of molecular oxygen, NAD(P)H as one donor, and incorporation of two atoms of oxygen into one donor; P:oxidation-reduction process; F:2 iron, 2 sulfur cluster binding |
| Efet.01.1659428.g1692.t1 | polyphosphate--nucleotide phosphotransferase | 894 | 0 | 82.75 | F:phosphotransferase activity, phosphate group as acceptor; P:polyphosphate metabolic process |
| Efet.01.1659428.g1693.t1 | glutamate dehydrogenase | 1302 | 0 | 92.4 | F:oxidoreductase activity, acting on the CH-NH2 group of donors, NAD or NADP as acceptor; P:cellular amino acid metabolic process; P:oxidation-reduction process |
| Efet.01.1659429.g1694.t1 | ribosomal RNA small subunit methyltransferase I | 798 | 3.85E-136 | 83.35 | F:methyltransferase activity; P:methylation |
| Efet.01.1659429.g1695.t1 | cysteine synthase | 1203 | 0 | 92.6 |  |
| Efet.01.1659430.g1696.t1 | carbohydrate ABC transporter substrate-binding CUT1 family (TC -) | 561 | 6.33E-116 | 84.7 | P:transport; F:transporter activity |
| Efet.01.1659430.g1697.t1 | carbohydrate ABC transporter permease | 888 | 0 | 66.95 | C:membrane |
| Efet.01.1659431.g1698.t1 | DUF2339 domain-containing | 1446 | 0 | 76.85 | C:integral component of membrane |
| Efet.01.1659431.g1699.t1 | DUF3999 domain-containing | 1179 | 0 | 80.15 | C:integral component of membrane |
| Efet.01.1659432.g1700.t1 | flagellar biosynthetic | 669 | 1.02E-112 | 80.85 | C:integral component of membrane; P:protein secretion |
| Efet.01.1659432.g1701.t1 | flagellar biosynthesis | 1959 | 0 | 94.9 | P:bacterial-type flagellum assembly; C:integral component of membrane; P:protein secretion |
| Efet.01.1659433.g1702.t1 | peptidase S11 | 408 | 1.08E-92 | 91.35 | F:serine-type D-Ala-D-Ala carboxypeptidase activity; P:proteolysis; C:integral component of membrane |
| Efet.01.1659433.g1703.t1 | family transcriptional regulator | 861 | 0 | 92.25 | F:DNA binding; P:transcription, DNA-templated; P:regulation of transcription, DNA-templated |
| Efet.01.1659433.g1704.t1 | Arginyltransferase | 582 | 7.40E-134 | 89.95 | C:cytoplasm; F:arginyltransferase activity; P:protein arginylation |
| Efet.01.1659433.g1705.t1 | leucyl phenylalanyl-tRNA-- transferase | 573 | 6.87E-130 | 83.25 | F:leucyltransferase activity; C:cytoplasm; P:protein catabolic process |
| Efet.01.1659434.g1706.t1 | relaxase | 2067 | 8.91E-06 | 44 |  |
| Efet.01.1659435.g1707.t1 | flagellar biosynthesis | 924 | 0 | 85.7 | F:GTP binding; P:SRP-dependent cotranslational protein targeting to membrane; F:GTPase activity; P:bacterial-type flagellum organization; C:intracellular |
| Efet.01.1659435.g1708.t1 | RNA polymerase sigma factor | 645 | 4.87E-151 | 92.7 | P:transcription initiation from bacterial-type RNA polymerase promoter; F:DNA binding; F:sigma factor activity; F:transcription factor activity, sequence-specific DNA binding; F:DNA-directed 5'-3' RNA polymerase activity; C:cytoplasm; P:regulation of transcription, DNA-templated; P:sporulation resulting in formation of a cellular spore |
| Efet.01.1659435.g1709.t1 | glycerate kinase | 1155 | 0 | 85 | F:hydroxypyruvate reductase activity; P:oxidation-reduction process; P:phosphorylation; F:kinase activity |
| Efet.01.1659436.g1710.t1 | RNA helicase | 2949 | 0 | 74.2 | F:ATP binding |
| Efet.01.1659437.g1711.t1 | 2-methylcitrate dehydratase | 1458 | 0 | 89.75 | P:propionate catabolic process, 2-methylcitrate cycle; F:2-methylcitrate dehydratase activity; F:2 iron, 2 sulfur cluster binding |
| Efet.01.1659437.g1712.t1 | methylisocitrate lyase | 807 | 4.00E-156 | 92.3 | F:magnesium ion binding; P:propionate catabolic process, 2-methylcitrate cycle; F:methylisocitrate lyase activity |
| Efet.01.1659438.g1713.t1 | dihydrolipoyllysine-residue succinyltransferase | 678 | 1.64E-164 | 97.4 | F:dihydrolipoyllysine-residue succinyltransferase activity; C:oxoglutarate dehydrogenase complex; P:tricarboxylic acid cycle; P:L-lysine catabolic process to acetyl-CoA via saccharopine |
| Efet.01.1659438.g1714.t1 | dihydrolipoyl dehydrogenase | 1179 | 0 | 90.15 | C:cell; F:flavin adenine dinucleotide binding; P:glycolytic process; P:cell redox homeostasis; P:oxidation-reduction process; F:dihydrolipoyl dehydrogenase activity |
| Efet.01.1659438.g1715.t1 | cell division | 576 | 3.46E-121 | 92 | F:ATP binding |
| Efet.01.1659439.g1716.t1 | ABC transporter permease | 519 | 5.36E-78 | 83.2 | C:integral component of membrane; P:transport; C:plasma membrane |
| Efet.01.1659439.g1717.t1 | ABC transporter substrate-binding | 1716 | 0 | 77.45 |  |
| Efet.01.1659440.g1718.t1 | cation diffusion facilitator family transporter | 939 | 5.38E-159 | 83.75 | P:cation transmembrane transport; C:integral component of membrane; F:cation transmembrane transporter activity; C:plasma membrane |
| Efet.01.1659440.g1719.t1 | isovaleryl- dehydrogenase | 1191 | 0 | 93.85 | F:acyl-CoA dehydrogenase activity; F:flavin adenine dinucleotide binding; P:oxidation-reduction process |
| Efet.01.1659440.g1720.t1 | carbonate dehydratase | 618 | 4.92E-133 | 93.9 | F:zinc ion binding; F:carbonate dehydratase activity; P:carbon utilization |
| Efet.01.1659441.g1721.t1 | chromosome partition Smc [Duganella phyllosphaerae] | 1788 | 0 | 75.75 | C:membrane |
| Efet.01.1659441.g1722.t1 | cell envelope biogenesis | 354 | 1.51E-52 | 77.15 | C:membrane |
| Efet.01.1659441.g1723.t1 | cell envelope biogenesis | 378 | 7.70E-37 | 74.65 | C:membrane |
| Efet.01.1659442.g1724.t1 | DNA-binding response regulator | 222 | 6.09E-27 | 95.05 | F:DNA binding; P:transcription, DNA-templated; P:phosphorelay signal transduction system; P:regulation of transcription, DNA-templated; C:intracellular |
| Efet.01.1659442.g1725.t1 | hybrid sensor histidine kinase response regulator | 2526 | 0 | 82.75 | F:phosphorelay sensor kinase activity; P:phosphorelay signal transduction system; C:integral component of membrane; P:signal transduction by protein phosphorylation; C:intracellular |
| Efet.01.1659443.g1726.t1 | glycerol kinase | 1455 | 0 | 72.9 | P:carbohydrate metabolic process; C:cytoplasm; P:phosphorylation; F:phosphotransferase activity, alcohol group as acceptor; F:kinase activity |
| Efet.01.1659443.g1727.t1 | transcriptional regulator | 888 | 3.49E-172 | 72.8 | F:DNA binding; F:transcription factor activity, sequence-specific DNA binding; P:transcription, DNA-templated; P:regulation of transcription, DNA-templated; C:intracellular |
| Efet.01.1659443.g1728.t1 | carbamoyl-phosphate synthase large subunit | 450 | 1.30E-83 | 92.3 | F:ATP binding; F:propionyl-CoA carboxylase activity; F:metal ion binding; F:biotin carboxylase activity |
| Efet.01.1659444.g1729.t1 | Transposon Ty3-G Gag-Pol poly | 2271 | 0 | 62.1 | F:binding |
| Efet.01.1659445.g1730.t1 | multidrug transporter | 3186 | 0 | 91.2 | C:integral component of membrane; P:transport; F:transporter activity |
| Efet.01.1659446.g1731.t1 | long-chain fatty acid-- ligase | 1527 | 0 | 64.45 | P:metabolic process; F:ligase activity |
| Efet.01.1659446.g1732.t1 | nuclear transport factor 2 family | 513 | 1.05E-107 | 82.25 |  |
| Efet.01.1659446.g1733.t1 | enoyl- hydratase | 813 | 0 | 85.6 | P:metabolic process; F:enoyl-CoA hydratase activity; F:isomerase activity |
| Efet.01.1659447.g1734.t1 | nitrate reductase | 1065 | 0 | 88.3 | P:transmembrane transport; C:integral component of membrane; F:transporter activity |
| Efet.01.1659447.g1735.t1 | microcin ABC transporter ATP-binding | 1695 | 0 | 84.95 | F:ATPase activity; F:ATP binding; P:peptide transport |
| Efet.01.1659448.g1736.t1 | 2-pyrone-4,6-dicarboxylate hydrolase | 918 | 0 | 91.7 | F:hydrolase activity |
| Efet.01.1659448.g1737.t1 | 4-oxalomesaconate hydratase | 1038 | 0 | 92.35 | F:hydrolase activity |
| Efet.01.1659448.g1738.t1 | molybdenum ABC transporter substrate-binding | 693 | 8.75E-136 | 83.45 |  |
| Efet.01.1659449.g1739.t1 | mammalian cell entry | 444 | 4.60E-83 | 89.55 | C:integral component of membrane |
| Efet.01.1659449.g1740.t1 | ABC transporter permease | 744 | 5.71E-128 | 81 | P:transport; C:ATP-binding cassette (ABC) transporter complex |
| Efet.01.1659449.g1741.t1 | histidine kinase | 1158 | 0 | 73.55 | P:phosphorylation; F:kinase activity |
| Efet.01.1659450.g1742.t1 | ABC transporter ATP-binding | 717 | 3.75E-135 | 73.25 | F:ATPase activity; F:ATP binding |
| Efet.01.1659450.g1743.t1 | ABC transporter permease | 2193 | 0 | 69.1 | C:membrane |
| Efet.01.1659451.g1744.t1 | chemotaxis response regulator -glutamate methylesterase | 528 | 1.92E-123 | 93.05 | P:chemotaxis; C:cytoplasm; P:phosphorelay signal transduction system; F:protein-glutamate methylesterase activity; F:phosphorelay response regulator activity |
| Efet.01.1659451.g1745.t1 | ABC transporter ATP-binding | 1920 | 0 | 86.65 | F:ATPase activity; F:DNA binding; F:ATP binding |
| Efet.01.1659451.g1746.t1 | poly granule associated | 552 | 4.75E-80 | 91.2 |  |
| Efet.01.1659452.g1747.t1 | DUF192 domain-containing | 270 | 4.05E-60 | 96 |  |
| Efet.01.1659452.g1748.t1 | superoxide dismutase | 582 | 4.02E-121 | 95.5 | F:metal ion binding; P:removal of superoxide radicals; P:oxidation-reduction process; F:superoxide dismutase activity |
| Efet.01.1659452.g1749.t1 | exodeoxyribonuclease VII large subunit | 1140 | 0 | 88.05 | F:nucleic acid binding; P:DNA catabolic process; P:nucleic acid phosphodiester bond hydrolysis; C:cytoplasm; C:exodeoxyribonuclease VII complex; F:exodeoxyribonuclease VII activity |
| Efet.01.1659453.g1750.t1 | type IV pilus biogenesis stability | 774 | 5.83E-172 | 88 |  |
| Efet.01.1659453.g1751.t1 | 23S rRNA (adenine(2503)-C(2))-methyltransferase | 1041 | 0 | 92.6 | F:rRNA (adenine-C2-)-methyltransferase activity; C:cytoplasm; F:metal ion binding; P:rRNA base methylation; F:tRNA (adenine-C2-)-methyltransferase activity; F:4 iron, 4 sulfur cluster binding; F:tRNA binding; F:rRNA binding; P:tRNA methylation |
| Efet.01.1659453.g1752.t1 | nucleoside-diphosphate kinase | 462 | 7.86E-98 | 95.4 | F:ATP binding; P:nucleic acid phosphodiester bond hydrolysis; P:UTP biosynthetic process; C:cytoplasm; F:metal ion binding; P:CTP biosynthetic process; F:endonuclease activity; P:GTP biosynthetic process; P:nucleoside diphosphate phosphorylation; F:nucleoside diphosphate kinase activity |
| Efet.01.1659454.g1753.t1 | cell division | 1230 | 0 | 87.15 | F:GTP binding; P:FtsZ-dependent cytokinesis; P:protein polymerization; C:cell division site; C:cytoplasm; F:GTPase activity |
| Efet.01.1659454.g1754.t1 | cell division | 1233 | 0 | 94.1 | P:regulation of cell shape; P:FtsZ-dependent cytokinesis; C:cell division site; C:cytoplasmic side of plasma membrane |
| Efet.01.1659455.g1755.t1 | DNA topoisomerase III | 1188 | 0 | 92.15 | F:DNA binding; F:DNA topoisomerase type I activity; P:DNA topological change |
| Efet.01.1659455.g1756.t1 | alpha-hydroxy-acid oxidizing | 1245 | 0 | 96.15 | F:FMN binding; F:L-lactate dehydrogenase (cytochrome) activity; P:oxidation-reduction process |
| Efet.01.1659455.g1757.t1 | acetoacetate-- ligase | 717 | 1.41E-162 | 93.05 | F:acetoacetate-CoA ligase activity; P:lipid metabolic process |
| Efet.01.1659456.g1758.t1 | -like domain-containing [Paraburkholderia caballeronis] | 447 | 5.84E-94 | 66.2 |  |
| Efet.01.1659456.g1759.t1 | 2-pyrone-4,6-dicarboxylate hydrolase | 909 | 0 | 78.65 | F:hydrolase activity |
| Efet.01.1659456.g1760.t1 | 23S rRNA (uracil(1939)-C(5))-methyltransferase | 927 | 0 | 88.35 | F:rRNA (uridine-C5-)-methyltransferase activity; F:RNA binding; F:iron ion binding; P:rRNA methylation; F:4 iron, 4 sulfur cluster binding |
| Efet.01.1659457.g1761.t1 | hypothetical protein Veis_1316 | 456 | 2.43E-46 | 62.75 | F:catalytic activity; P:metabolic process |
| Efet.01.1659457.g1762.t1 | beta-ketoacyl-ACP synthase II | 1026 | 0 | 84.15 | F:3-oxoacyl-[acyl-carrier-protein] synthase activity; P:metabolic process |
| Efet.01.1659457.g1763.t1 | 3-oxoacyl-ACP reductase | 588 | 3.81E-93 | 90.1 | F:3-oxoacyl-[acyl-carrier-protein] reductase (NADPH) activity; P:oxidation-reduction process |
| Efet.01.1659457.g1764.t1 | dehydratase | 408 | 1.85E-49 | 65.85 |  |
| Efet.01.1659458.g1765.t1 | family cyclic di-GMP-binding | 489 | 2.28E-110 | 89.35 |  |
| Efet.01.1659458.g1766.t1 | beta-glucosidase | 2373 | 0 | 74.65 | F:nucleotide binding; C:membrane; F:hydrolase activity |
| Efet.01.1659460.g1767.t1 | transcriptional family | 858 | 0 | 80 | F:peroxidase activity; F:DNA binding; F:transcription factor activity, sequence-specific DNA binding; P:transcription, DNA-templated; P:cellular oxidant detoxification; P:regulation of transcription, DNA-templated |
| Efet.01.1659460.g1768.t1 | sugar ABC transporter substrate-binding | 939 | 0 | 80.3 |  |
| Efet.01.1659460.g1769.t1 | sugar ABC transporter ATP-binding | 750 | 1.32E-163 | 93.45 | F:ATPase activity; F:ATP binding |
| Efet.01.1659462.g1770.t1 | nitrate transport permease nrtB | 969 | 2.19E-157 | 87.05 | C:integral component of membrane; P:transport; C:plasma membrane |
| Efet.01.1659462.g1771.t1 | ABC transporter ATP-binding | 696 | 6.21E-165 | 86.05 | F:ATPase activity; F:ATP binding |
| Efet.01.1659462.g1772.t1 | urea carboxylase | 819 | 0 | 79.15 |  |
| Efet.01.1659462.g1773.t1 | urea carboxylase | 699 | 1.44E-170 | 85.4 |  |
| Efet.01.1659463.g1774.t1 | NAD(P) FAD-dependent oxidoreductase | 1077 | 0 | 80.65 | F:FAD binding; F:oxidoreductase activity; P:oxidation-reduction process |
| Efet.01.1659463.g1775.t1 | beta-ketoacyl-[acyl-carrier- ] synthase II | 906 | 1.87E-129 | 73.2 | F:catalytic activity |
| Efet.01.1659465.g1776.t1 | negative regulator of -dependent stress response | 462 | 2.31E-62 | 86.1 | C:integral component of membrane |
| Efet.01.1659465.g1777.t1 | histidine--tRNA ligase | 1266 | 0 | 90.1 | F:histidine-tRNA ligase activity; F:ATP binding; P:histidyl-tRNA aminoacylation; C:cytoplasm |
| Efet.01.1659465.g1778.t1 | 4-hydroxy-3-methylbut-2-en-1-yl diphosphate synthase | 1149 | 0 | 94.7 | F:iron ion binding; P:isopentenyl diphosphate biosynthetic process, methylerythritol 4-phosphate pathway; P:oxidation-reduction process; F:4 iron, 4 sulfur cluster binding; F:4-hydroxy-3-methylbut-2-en-1-yl diphosphate synthase activity; P:terpenoid biosynthetic process |
| Efet.01.1659466.g1779.t1 | 50S ribosomal L6 | 534 | 3.72E-91 | 95.35 | F:structural constituent of ribosome; C:ribosome; F:rRNA binding; P:translation |
| Efet.01.1659466.g1780.t1 | 30S ribosomal S8 | 396 | 2.00E-88 | 97.6 | F:structural constituent of ribosome; C:ribosome; F:rRNA binding; P:translation |
| Efet.01.1659466.g1781.t1 | 50S ribosomal L5 | 465 | 3.56E-107 | 100 | F:structural constituent of ribosome; C:ribosome; F:tRNA binding; F:rRNA binding; P:translation |
| Efet.01.1659466.g1782.t1 | 50S ribosomal L14 | 606 | 1.12E-109 | 97.8 | F:structural constituent of ribosome; C:large ribosomal subunit; F:rRNA binding; P:translation |
| Efet.01.1659466.g1783.t1 | FAD-dependent oxidoreductase | 219 | 1.46E-25 | 86.95 | P:glycerol-3-phosphate metabolic process; C:glycerol-3-phosphate dehydrogenase complex; P:oxidation-reduction process; F:glycerol-3-phosphate dehydrogenase activity |
| Efet.01.1659467.g1784.t1 | Twin-arginine translocation pathway signal | 972 | 0 | 93.3 | C:outer membrane-bounded periplasmic space |
| Efet.01.1659467.g1785.t1 | transferase | 1155 | 0 | 81.65 | F:catalytic activity |
| Efet.01.1659470.g1788.t1 | OTU domain-containing | 2829 | 1.05E-74 | 45.65 |  |
| Efet.01.1659471.g1789.t1 | branched-chain amino acid ABC transporter permease | 555 | 8.18E-93 | 90.2 | C:integral component of membrane; P:transport; C:plasma membrane; F:transporter activity |
| Efet.01.1659471.g1790.t1 | ABC transporter ATP-binding | 612 | 7.80E-112 | 84.8 | F:ATPase activity; F:ATP binding |
| Efet.01.1659471.g1791.t1 | ABC transporter ATP-binding | 618 | 6.10E-145 | 92.6 | F:ATPase activity; F:ATP binding |
| Efet.01.1659471.g1792.t1 | tripartite tricarboxylate transporter substrate binding | 957 | 0 | 93.3 | C:outer membrane-bounded periplasmic space |
| Efet.01.1659471.g1793.t1 | 3-isopropylmalate dehydratase large subunit | 258 | 1.10E-52 | 97.9 | P:leucine biosynthetic process; F:4 iron, 4 sulfur cluster binding; F:3-isopropylmalate dehydratase activity; F:dimethylmaleate hydratase activity |
| Efet.01.1659472.g1794.t1 | Transposon Ty3-G Gag-Pol poly | 3540 | 0 | 58.55 | F:binding |
| Efet.01.1659473.g1795.t1 | hypothetical protein | 705 | 1.13E-137 | 80.95 |  |
| Efet.01.1659473.g1796.t1 | hypothetical protein | 450 | 4.90E-60 | 78.85 |  |
| Efet.01.1659473.g1797.t1 | type II toxin-antitoxin system family toxin | 1233 | 0 | 87 |  |
| Efet.01.1659473.g1798.t1 | XRE family transcriptional regulator | 237 | 6.57E-48 | 90.85 | F:sequence-specific DNA binding |
| Efet.01.1659475.g1799.t1 | hypothetical protein | 1845 | 0 | 89.8 |  |
| Efet.01.1659475.g1800.t1 | helicase | 1392 | 0 | 88 | F:helicase activity |
| Efet.01.1659476.g1801.t1 | type II toxin-antitoxin system mRNA interferase family | 204 | 7.09E-41 | 87.2 | F:DNA binding; F:transcription factor activity, sequence-specific DNA binding; P:regulation of transcription, DNA-templated |
| Efet.01.1659476.g1802.t1 | antitoxin | 261 | 4.81E-55 | 88 | F:toxic substance binding; P:single-species biofilm formation; F:bacterial-type RNA polymerase core promoter proximal region sequence-specific DNA binding |
| Efet.01.1659476.g1803.t1 | DUF2442 domain-containing | 204 | 6.31E-24 | 71.62 |  |
| Efet.01.1659476.g1804.t1 | xanthine dehydrogenase molybdopterin binding subunit | 1401 | 0 | 85.5 | F:xanthine dehydrogenase activity; F:molybdenum ion binding; P:oxidation-reduction process |
| Efet.01.1659477.g1805.t1 | phosphoglycerate mutase | 657 | 1.79E-123 | 75.85 |  |
| Efet.01.1659477.g1806.t1 | single-stranded-DNA-specific exonuclease | 1404 | 0 | 91.05 | F:nucleic acid binding; F:5'-3' exonuclease activity; P:DNA repair; P:nucleic acid phosphodiester bond hydrolysis; P:DNA recombination |
| Efet.01.1659477.g1807.t1 | IS66 family transposase | 339 | 1.45E-22 | 70.75 |  |
| Efet.01.1659477.g1808.t1 | dihydrodipicolinate synthase | 612 | 6.75E-113 | 87.05 | P:metabolic process; F:4-hydroxy-tetrahydrodipicolinate synthase |
| Efet.01.1659478.g1809.t1 | peptidylprolyl isomerase | 465 | 3.99E-105 | 87.35 | F:peptidyl-prolyl cis-trans isomerase activity; P:protein peptidyl-prolyl isomerization; P:protein folding |
| Efet.01.1659478.g1810.t1 | 4-hydroxy-3-methylbut-2-enyl diphosphate reductase | 810 | 0 | 92.65 | P:dimethylallyl diphosphate biosynthetic process; F:metal ion binding; P:isopentenyl diphosphate biosynthetic process, methylerythritol 4-phosphate pathway; F:3 iron, 4 sulfur cluster binding; P:oxidation-reduction process; F:4-hydroxy-3-methylbut-2-en-1-yl diphosphate reductase activity; P:terpenoid biosynthetic process |
| Efet.01.1659478.g1811.t1 | phosphatidylserine decarboxylase | 990 | 0 | 90.75 | P:phosphatidylethanolamine biosynthetic process; F:phosphatidylserine decarboxylase activity; C:plasma membrane |
| Efet.01.1659479.g1812.t1 | Short-chain dehydrogenase | 837 | 2.15E-180 | 90.9 | F:cholate 7-alpha-dehydrogenase activity; P:oxidation-reduction process |
| Efet.01.1659479.g1813.t1 | family transcriptional regulator | 708 | 2.92E-144 | 88.1 | F:DNA binding; F:transcription factor activity, sequence-specific DNA binding; P:transcription, DNA-templated; P:regulation of transcription, DNA-templated |
| Efet.01.1659479.g1814.t1 | taurine dioxygenase | 840 | 0 | 89.55 | F:taurine dioxygenase activity; P:oxidation-reduction process |
| Efet.01.1659479.g1815.t1 | class II aldolase | 675 | 6.81E-148 | 91.5 | F:lyase activity |
| Efet.01.1659479.g1816.t1 | ABC transporter permease | 471 | 4.32E-84 | 92.35 | C:integral component of membrane; P:transport; C:plasma membrane |
| Efet.01.1659480.g1817.t1 | ABC transporter permease | 810 | 6.52E-169 | 85.2 | C:integral component of membrane; P:transport; C:plasma membrane; F:transporter activity |
| Efet.01.1659480.g1818.t1 | deoxyribose-phosphate aldolase | 960 | 0 | 83.1 | F:deoxyribose-phosphate aldolase activity; C:cytoplasm; P:deoxyribonucleotide catabolic process; P:deoxyribose phosphate catabolic process |
| Efet.01.1659480.g1819.t1 | aldehyde dehydrogenase | 1782 | 0 | 75.5 | F:aldehyde dehydrogenase (NAD) activity; P:oxidation-reduction process |
| Efet.01.1659481.g1820.t1 | hypothetical protein [Diaphorobacter polyhydroxybutyrativorans] | 258 | 3.10E-47 | 75.9 |  |
| Efet.01.1659481.g1821.t1 | ABC transporter permease | 885 | 1.25E-91 | 68.6 | C:membrane |
| Efet.01.1659481.g1822.t1 | ABC transporter ATP-binding | 966 | 4.31E-45 | 69.3 | F:ATPase activity; F:ATP binding |
| Efet.01.1659481.g1823.t1 | ABC transporter ATP-binding | 588 | 4.87E-74 | 73.4 | F:nucleotide binding |
| Efet.01.1659482.g1824.t1 | PIN domain-containing | 429 | 5.24E-65 | 94.3 | F:magnesium ion binding; P:RNA phosphodiester bond hydrolysis; F:ribonuclease activity |
| Efet.01.1659482.g1825.t1 | Ynd [Pseudomonas pseudoalcaligenes KF707 = NBRC 110670] | 420 | 9.36E-76 | 83.45 |  |
| Efet.01.1659482.g1826.t1 | Uncharacterised | 1074 | 0 | 78.05 |  |
| Efet.01.1659483.g1827.t1 | UDP-N-acetylglucosamine diphosphorylase glucosamine-1-phosphate N-acetyltransferase | 1383 | 0 | 85.4 | F:magnesium ion binding; P:regulation of cell shape; F:UDP-N-acetylglucosamine diphosphorylase activity; C:cytoplasm; P:cell morphogenesis; P:peptidoglycan biosynthetic process; F:glucosamine-1-phosphate N-acetyltransferase activity; P:lipopolysaccharide biosynthetic process; P:lipid A biosynthetic process; P:cell wall organization; P:UDP-N-acetylglucosamine biosynthetic process |
| Efet.01.1659483.g1828.t1 | pilus assembly | 2190 | 0 | 64.8 | C:membrane; C:integral component of membrane |
| Efet.01.1659484.g1829.t1 | transcriptional regulator | 846 | 0 | 93.9 | F:DNA binding; P:carbohydrate metabolic process; F:transcription factor activity, sequence-specific DNA binding; P:regulation of transcription, DNA-templated; F:carbohydrate binding |
| Efet.01.1659484.g1830.t1 | transaldolase | 951 | 0 | 94.75 | P:carbohydrate metabolic process; F:sedoheptulose-7-phosphate:D-glyceraldehyde-3-phosphate glyceronetransferase activity; C:cytoplasm; P:pentose-phosphate shunt |
| Efet.01.1659484.g1831.t1 | sugar ABC transporter substrate-binding | 516 | 2.96E-92 | 72.95 | P:transport; F:transporter activity |
| Efet.01.1659484.g1832.t1 | porin | 1125 | 0 | 88.1 | C:membrane; P:transport |
| Efet.01.1659485.g1833.t1 | long-chain fatty acid-- ligase | 1404 | 0 | 74.3 | P:metabolic process; F:ligase activity |
| Efet.01.1659485.g1834.t1 | nitronate monooxygenase | 951 | 0 | 69.35 | F:dioxygenase activity; F:nitronate monooxygenase activity; P:oxidation-reduction process |
| Efet.01.1659485.g1835.t1 | short-chain dehydrogenase | 813 | 0 | 82.15 | F:3-oxoacyl-[acyl-carrier-protein] reductase (NADPH) activity; P:oxidation-reduction process |
| Efet.01.1659485.g1836.t1 | crotonase | 699 | 3.94E-148 | 81.55 | P:metabolic process; F:enoyl-CoA hydratase activity |
| Efet.01.1659486.g1837.t1 | glutathione ABC transporter substrate-binding | 1641 | 0 | 94.85 | P:transmembrane transport; C:ATP-binding cassette (ABC) transporter complex |
| Efet.01.1659486.g1838.t1 | glutathione ABC transporter permease | 921 | 0 | 98.5 | C:integral component of membrane; P:transport; C:plasma membrane |
| Efet.01.1659486.g1839.t1 | glutathione ABC transporter permease | 699 | 2.14E-146 | 97.55 | C:integral component of membrane; P:transport; C:plasma membrane |
| Efet.01.1659487.g1840.t1 | sugar ABC transporter substrate-binding | 1314 | 0 | 83 | P:carbohydrate transport; F:transporter activity |
| Efet.01.1659487.g1841.t1 | sugar kinase | 936 | 0 | 87.7 | P:carbohydrate phosphorylation; F:fructokinase activity |
| Efet.01.1659487.g1842.t1 | ABC transporter permease | 1365 | 1.85E-159 | 90.9 | F:ATP binding; C:integral component of membrane; P:monosaccharide transport; F:monosaccharide-transporting ATPase activity; C:plasma membrane |
| Efet.01.1659488.g1843.t1 | IS4 family | 663 | 2.03E-124 | 89 | C:integral component of membrane |
| Efet.01.1659488.g1844.t1 | transposase | 450 | 1.26E-18 | 72.75 | F:nucleic acid binding |
| Efet.01.1659488.g1845.t1 | TniQ transposition protein | 297 | 1.26E-09 | 58.05 |  |
| Efet.01.1659488.g1846.t1 | transposase | 819 | 3.97E-131 | 83.1 |  |
| Efet.01.1659488.g1847.t1 | transposase | 1002 | 3.59E-137 | 72.5 | F:nucleic acid binding; P:DNA integration |
| Efet.01.1659489.g1848.t1 | glutathione S-transferase | 690 | 2.76E-166 | 89.2 | F:transferase activity |
| Efet.01.1659489.g1849.t1 | MFS transporter | 987 | 0 | 63.4 | P:transmembrane transport; C:integral component of membrane |
| Efet.01.1659489.g1850.t1 | 5-formyltetrahydrofolate cyclo-ligase | 483 | 1.17E-98 | 94.3 | F:ATP binding; F:metal ion binding; F:5-formyltetrahydrofolate cyclo-ligase activity |
| Efet.01.1659489.g1851.t1 | lytic transglycosylase | 948 | 0 | 84.7 | C:periplasmic space; F:hydrolase activity, hydrolyzing O-glycosyl compounds |
| Efet.01.1659490.g1852.t1 | TonB-dependent receptor | 1905 | 0 | 79.75 | C:cell outer membrane; F:receptor activity; P:transport |
| Efet.01.1659490.g1853.t1 | family transcriptional regulator | 969 | 0 | 73.5 | F:transcription factor activity, sequence-specific DNA binding; P:transcription, DNA-templated; P:regulation of transcription, DNA-templated; F:sequence-specific DNA binding |
| Efet.01.1659490.g1854.t1 | hypothetical protein | 585 | 5.20E-97 | 72.2 |  |
| Efet.01.1659491.g1855.t1 | Rare lipo B | 213 | 4.69E-40 | 89.9 | P:Gram-negative-bacterium-type cell outer membrane assembly; C:cell outer membrane |
| Efet.01.1659491.g1856.t1 | leucine--tRNA ligase | 2718 | 0 | 89.3 | F:ATP binding; P:leucyl-tRNA aminoacylation; F:leucine-tRNA ligase activity; C:cytoplasm; P:regulation of translational fidelity; F:aminoacyl-tRNA editing activity |
| Efet.01.1659491.g1857.t1 | 4-hydroxy-tetrahydrodipicolinate reductase | 561 | 1.84E-117 | 93.95 | F:NADP binding; F:oxidoreductase activity, acting on CH or CH2 groups, NAD or NADP as acceptor; C:cytoplasm; F:NAD binding; P:lysine biosynthetic process via diaminopimelate; F:4-hydroxy-tetrahydrodipicolinate reductase; P:oxidation-reduction process; P:diaminopimelate biosynthetic process |
| Efet.01.1659492.g1858.t1 | 2-dehydro-3-deoxyglucarate aldolase | 612 | 5.82E-131 | 79.4 | F:2-dehydro-3-deoxyglucarate aldolase activity; F:metal ion binding; P:glucarate catabolic process |
| Efet.01.1659492.g1859.t1 | tripartite tricarboxylate transporter family receptor | 1044 | 0 | 76.3 | C:membrane; C:outer membrane-bounded periplasmic space |
| Efet.01.1659492.g1860.t1 | tagatose-6-phosphate kinase | 1734 | 0 | 65.6 | F:transferase activity; P:galactitol metabolic process |
| Efet.01.1659493.g1861.t1 | family | 612 | 4.71E-92 | 79 | F:lyase activity |
| Efet.01.1659493.g1862.t1 | acyl- dehydrogenase | 1155 | 0 | 87.25 | F:acyl-CoA dehydrogenase activity; F:flavin adenine dinucleotide binding; F:butyryl-CoA dehydrogenase activity; P:oxidation-reduction process |
| Efet.01.1659493.g1863.t1 | tripartite tricarboxylate transporter substrate binding | 963 | 0 | 78.65 | C:outer membrane-bounded periplasmic space |
| Efet.01.1659493.g1864.t1 | 6-carboxyhexanoate-- ligase | 576 | 1.45E-82 | 88.6 | F:ATP binding; F:cofactor binding; F:ligase activity |
| Efet.01.1659494.g1865.t1 | tRNA 2-thiocytidine(32) synthetase | 405 | 1.47E-92 | 89.35 | F:ATP binding; C:cytoplasm; P:tRNA processing |
| Efet.01.1659494.g1866.t1 | phosphoglycerate mutase | 1146 | 1.92E-127 | 90.55 | F:isomerase activity |
| Efet.01.1659494.g1867.t1 | rRNA methyltransferase | 1173 | 1.08E-156 | 89.75 | F:RNA methyltransferase activity; P:RNA methylation; F:RNA binding; P:RNA processing |
| Efet.01.1659494.g1868.t1 | lipid-A-disaccharide synthase | 1158 | 0 | 89.4 | F:lipid-A-disaccharide synthase activity; P:lipid A biosynthetic process |
| Efet.01.1659495.g1869.t1 | sugar ABC transporter permease | 480 | 1.36E-26 | 75.1 | P:carbohydrate metabolic process; C:integral component of membrane; F:beta-galactosidase activity |
| Efet.01.1659495.g1870.t1 | alpha-N-arabinofuranosidase | 1521 | 0 | 85.05 | P:L-arabinose metabolic process; F:alpha-L-arabinofuranosidase activity |
| Efet.01.1659495.g1871.t1 | ABC transporter substrate-binding | 1329 | 0 | 75.35 | P:transport; F:transporter activity |
| Efet.01.1659495.g1872.t1 | carbohydrate ABC transporter permease | 429 | 7.56E-97 | 93.75 | C:integral component of plasma membrane; C:ATP-binding cassette (ABC) transporter complex, substrate-binding subunit-containing; P:glycerol-3-phosphate transport; P:glycerophosphodiester transport |
| Efet.01.1659496.g1873.t1 | family transcriptional regulator | 375 | 3.26E-78 | 54.95 | F:DNA binding; F:transcription factor activity, sequence-specific DNA binding; P:transcription, DNA-templated; P:regulation of transcription, DNA-templated |
| Efet.01.1659496.g1874.t1 | family transcriptional regulator | 732 | 4.67E-171 | 75 | F:DNA binding; F:transcription factor activity, sequence-specific DNA binding; P:transcription, DNA-templated; P:regulation of transcription, DNA-templated |
| Efet.01.1659496.g1875.t1 | 3-carboxy-cis,cis-muconate cycloisomerase | 1356 | 0 | 95.2 | F:3-carboxy-cis,cis-muconate cycloisomerase activity; P:3,4-dihydroxybenzoate catabolic process; F:lyase activity |
| Efet.01.1659496.g1876.t1 | tripartite tricarboxylate transporter substrate binding | 987 | 0 | 92.75 | C:outer membrane-bounded periplasmic space; F:isomerase activity |
| Efet.01.1659496.g1877.t1 | class II fumarate hydratase | 375 | 2.36E-65 | 91.9 | P:tricarboxylic acid cycle; F:fumarate hydratase activity; P:fumarate metabolic process; C:tricarboxylic acid cycle enzyme complex |
| Efet.01.1659497.g1878.t1 | lipid A export permease ATP-binding | 1551 | 0 | 88.45 | F:ATP binding; P:lipid transport; F:lipid-transporting ATPase activity; P:transmembrane transport; C:ATP-binding cassette (ABC) transporter complex |
| Efet.01.1659497.g1879.t1 | hypothetical protein | 642 | 2.22E-107 | 90.35 |  |
| Efet.01.1659497.g1880.t1 | DNAase | 798 | 0 | 93.75 | F:endodeoxyribonuclease activity, producing 5'-phosphomonoesters; P:DNA metabolic process |
| Efet.01.1659497.g1881.t1 | pilus assembly | 288 | 9.84E-63 | 94.3 | F:cyclic-di-GMP binding |
| Efet.01.1659497.g1882.t1 | DNA polymerase III subunit delta | 879 | 0 | 92.95 | F:DNA-directed DNA polymerase activity; P:DNA biosynthetic process |
| Efet.01.1659498.g1883.t1 | acyl- synthetase | 1035 | 0 | 69.3 | P:metabolic process; F:ligase activity |
| Efet.01.1659498.g1884.t1 | tripartite tricarboxylate transporter substrate binding | 330 | 6.64E-61 | 67.95 | C:outer membrane-bounded periplasmic space |
| Efet.01.1659498.g1885.t1 | family transcriptional regulator | 1125 | 0 | 68.7 | F:DNA binding; F:transcription factor activity, sequence-specific DNA binding; P:transcription, DNA-templated; P:regulation of transcription, DNA-templated |
| Efet.01.1659498.g1886.t1 | NAD(P)-dependent oxidoreductase | 1113 | 6.48E-174 | 83.45 | F:oxidoreductase activity; P:oxidation-reduction process |
| Efet.01.1659499.g1887.t1 | amino acid transporter | 330 | 2.52E-54 | 84.15 | F:ATP binding; P:amino acid transmembrane transport; F:amino acid-transporting ATPase activity |
| Efet.01.1659499.g1888.t1 | amino acid ABC transporter permease | 612 | 4.43E-143 | 80.35 | C:integral component of membrane; P:transport; C:plasma membrane; F:transporter activity |
| Efet.01.1659499.g1889.t1 | ABC transporter | 705 | 1.78E-140 | 77.15 | C:integral component of membrane; P:transport; C:plasma membrane; F:transporter activity |
| Efet.01.1659499.g1890.t1 | ABC transporter substrate-binding | 999 | 0 | 70.5 | P:transport; F:transporter activity |
| Efet.01.1659499.g1891.t1 | agmatinase | 894 | 0 | 90.5 | F:metal ion binding; F:agmatinase activity |
| Efet.01.1659500.g1892.t1 | sugar ABC transporter ATP-binding | 1530 | 0 | 76.9 | F:ATPase activity; F:ATP binding; P:carbohydrate transport |
| Efet.01.1659500.g1893.t1 | sugar ABC transporter substrate-binding | 963 | 0 | 84.7 | P:carbohydrate transport |
| Efet.01.1659500.g1894.t1 | family transcriptional regulator | 1542 | 0 | 75.15 | F:DNA binding; F:transcription factor activity, sequence-specific DNA binding; P:transcription, DNA-templated; P:regulation of transcription, DNA-templated |
| Efet.01.1659501.g1895.t1 | ABC transporter ATP-binding | 1191 | 4.33E-159 | 89.25 | F:ATP binding; P:amino acid transmembrane transport; F:amino acid-transporting ATPase activity |
| Efet.01.1659501.g1896.t1 | type I restriction endonuclease subunit R | 3006 | 0 | 89.85 | F:DNA binding; F:ATP binding; P:nucleic acid phosphodiester bond hydrolysis; F:Type I site-specific deoxyribonuclease activity; F:helicase activity; P:DNA restriction-modification system |
| Efet.01.1659502.g1897.t1 | SIR2-like domain | 3873 | 0 | 74.45 |  |
| Efet.01.1659503.g1898.t1 | hypothetical protein | 3297 | 0 | 58.35 |  |
| Efet.01.1659503.g1899.t1 | hypothetical protein [Diaphorobacter polyhydroxybutyrativorans] | 399 | 4.60E-76 | 87.75 |  |
| Efet.01.1659504.g1900.t1 | 3-oxoadipate -transferase | 654 | 3.51E-128 | 83.5 | P:metabolic process; F:3-oxoadipate CoA-transferase activity |
| Efet.01.1659504.g1901.t1 | 3-oxoadipate -transferase | 468 | 1.75E-94 | 88.15 | P:metabolic process; F:3-oxoadipate CoA-transferase activity |
| Efet.01.1659504.g1902.t1 | thiolase | 1173 | 0 | 88.15 | F:acetate-CoA ligase activity; F:transferase activity, transferring acyl groups other than amino-acyl groups; P:metabolic process |
| Efet.01.1659504.g1903.t1 | 3-hydroxyacyl- dehydrogenase | 1200 | 0 | 81.3 | P:fatty acid beta-oxidation; F:3-hydroxyacyl-CoA dehydrogenase activity; F:dodecenoyl-CoA delta-isomerase activity; F:enoyl-CoA hydratase activity |
| Efet.01.1659505.g1904.t1 | phosphonoacetaldehyde dehydrogenase | 1140 | 0 | 93 | P:oxidation-reduction process; F:oxidoreductase activity, acting on the aldehyde or oxo group of donors, NAD or NADP as acceptor |
| Efet.01.1659505.g1905.t1 | phosphohydrolase | 375 | 5.49E-83 | 81.3 | F:hydrolase activity |
| Efet.01.1659505.g1906.t1 | phosphonate utilization associated membrane | 750 | 4.82E-138 | 68.95 | C:integral component of membrane |
| Efet.01.1659505.g1907.t1 | DUF1177 domain-containing | 942 | 0 | 89.3 |  |
| Efet.01.1659505.g1908.t1 | OPT oligopeptide transporter | 714 | 3.87E-131 | 87.1 | P:transmembrane transport; C:integral component of membrane |
| Efet.01.1659506.g1909.t1 | methylenetetrahydrofolate reductase [NAD(P)H] | 618 | 1.52E-146 | 95.35 | P:methionine biosynthetic process; C:cytosol; P:tetrahydrofolate interconversion; P:oxidation-reduction process; F:methylenetetrahydrofolate reductase (NAD(P)H) activity |
| Efet.01.1659506.g1910.t1 | family RNA methyltransferase | 756 | 2.18E-146 | 70.1 | F:RNA binding; F:methyltransferase activity; P:methylation |
| Efet.01.1659506.g1911.t1 | adenosylhomocysteinase | 1422 | 0 | 93.7 | C:cytoplasm; F:adenosylhomocysteinase activity; P:one-carbon metabolic process |
| Efet.01.1659506.g1912.t1 | peptide chain release factor 3 | 975 | 0 | 93.5 | F:GTP binding; P:translational termination; P:regulation of translational termination; C:cytoplasm; F:GTPase activity; F:translation release factor activity, codon specific |
| Efet.01.1659507.g1913.t1 | 3-isopropylmalate dehydratase large subunit | 618 | 5.27E-121 | 89.75 | P:leucine biosynthetic process; F:4 iron, 4 sulfur cluster binding; F:3-isopropylmalate dehydratase activity |
| Efet.01.1659507.g1914.t1 | 3-isopropylmalate dehydratase | 441 | 2.67E-87 | 87.15 | P:metabolic process; F:3-isopropylmalate dehydratase activity |
| Efet.01.1659507.g1915.t1 | hydantoinase oxoprolinase family | 3576 | 0 | 93.85 | F:5-oxoprolinase (ATP-hydrolyzing) activity; F:ligase activity |
| Efet.01.1659508.g1916.t1 | family transcriptional regulator | 624 | 2.08E-108 | 83.9 | F:DNA binding; F:transcription factor activity, sequence-specific DNA binding; P:transcription, DNA-templated; P:regulation of transcription, DNA-templated |
| Efet.01.1659508.g1917.t1 | transcriptional regulator | 705 | 5.85E-91 | 62.25 | F:DNA binding; P:carbohydrate metabolic process; F:transcription factor activity, sequence-specific DNA binding; P:regulation of transcription, DNA-templated; F:carbohydrate binding |
| Efet.01.1659508.g1918.t1 | hydroxyquinol 1,2-dioxygenase | 1533 | 0 | 86.7 | F:ferric iron binding; F:catechol 1,2-dioxygenase activity; P:oxidation-reduction process; P:catechol-containing compound metabolic process |
| Efet.01.1659508.g1919.t1 | Transposase | 1236 | 0 | 85.3 |  |
| Efet.01.1659509.g1920.t1 | ABC transporter permease | 408 | 9.19E-68 | 91.85 | C:integral component of membrane; P:transport; C:plasma membrane; F:transporter activity |
| Efet.01.1659509.g1921.t1 | phosphotriesterase-related | 981 | 0 | 88.2 | F:hydrolase activity, acting on ester bonds; F:zinc ion binding; P:catabolic process |
| Efet.01.1659509.g1922.t1 | cytoplasmic | 1464 | 4.51E-165 | 88.15 | F:ribokinase activity; P:D-ribose metabolic process; P:carbohydrate phosphorylation |
| Efet.01.1659509.g1923.t1 | family membrane complex biogenesis | 711 | 6.46E-126 | 88.95 | F:catalytic activity; P:metabolic process |
| Efet.01.1659509.g1924.t1 | lytic transglycosylase | 405 | 4.94E-87 | 92.95 | C:periplasmic space; F:hydrolase activity, hydrolyzing O-glycosyl compounds |
| Efet.01.1659510.g1925.t1 | nucleotidyl transferase ii ii toxin family | 522 | 4.15E-93 | 73.35 |  |
| Efet.01.1659510.g1926.t1 | transcriptional XRE family | 465 | 2.40E-105 | 67.05 | F:sequence-specific DNA binding |
| Efet.01.1659510.g1927.t1 | peptidase M20 | 939 | 9.31E-132 | 84.5 | P:proteolysis; F:metallopeptidase activity |
| Efet.01.1659510.g1928.t1 | FAD-dependent oxidoreductase | 2220 | 0 | 87.7 | F:oxidoreductase activity; F:electron carrier activity; P:oxidation-reduction process; F:iron-sulfur cluster binding |
| Efet.01.1659511.g1929.t1 | hypothetical protein [Diaphorobacter polyhydroxybutyrativorans] | 231 | 3.67E-06 | 73 |  |
| Efet.01.1659511.g1930.t1 | hypothetical protein | 423 | 3.08E-64 | 76.5 |  |
| Efet.01.1659511.g1931.t1 | ATP-dependent DNA helicase | 1254 | 0 | 67.15 | F:nucleic acid binding; F:ATP binding; F:ATP-dependent helicase activity; P:nucleobase-containing compound metabolic process |
| Efet.01.1659511.g1932.t1 | DUF1817 domain-containing | 513 | 1.15E-67 | 55.05 |  |
| Efet.01.1659511.g1933.t1 | MULTISPECIES: hypothetical protein | 627 | 5.05E-49 | 60.9 |  |
| Efet.01.1659511.g1934.t1 | type I restriction endonuclease subunit M | 852 | 6.97E-115 | 70.95 | P:nucleic acid metabolic process; F:methyltransferase activity; P:methylation |
| Efet.01.1659512.g1935.t1 | two-component sensor histidine kinase | 1344 | 0 | 80.3 | F:phosphorelay sensor kinase activity; F:ATP binding; P:phosphorelay signal transduction system; C:integral component of membrane; P:signal transduction by protein phosphorylation; C:intracellular; P:peptidyl-histidine phosphorylation |
| Efet.01.1659512.g1936.t1 | DNA-binding response regulator | 663 | 1.04E-153 | 93.35 | F:DNA binding; P:transcription, DNA-templated; P:phosphorelay signal transduction system; P:regulation of transcription, DNA-templated; C:intracellular |
| Efet.01.1659512.g1937.t1 | acriflavine resistance B | 2427 | 0 | 91.15 | C:integral component of membrane; P:transport; F:transporter activity |
| Efet.01.1659513.g1938.t1 | osmotically inducible C | 402 | 1.30E-78 | 94.75 |  |
| Efet.01.1659513.g1939.t1 | DUF3579 domain-containing | 369 | 3.84E-68 | 87.55 |  |
| Efet.01.1659513.g1940.t1 | pyruvate decarboxylase | 1647 | 0 | 78.8 | F:magnesium ion binding; F:acetolactate synthase activity; F:thiamine pyrophosphate binding |
| Efet.01.1659513.g1941.t1 | family transcriptional regulator | 939 | 2.30E-125 | 76.5 | F:DNA binding; F:transcription factor activity, sequence-specific DNA binding; P:transcription, DNA-templated; P:regulation of transcription, DNA-templated |
| Efet.01.1659513.g1942.t1 | tripartite tricarboxylate transporter substrate binding | 1002 | 0 | 71.1 | C:outer membrane-bounded periplasmic space |
| Efet.01.1659513.g1943.t1 | family transcriptional regulator | 345 | 3.76E-71 | 74.3 | F:DNA binding; F:transcription factor activity, sequence-specific DNA binding; P:transcription, DNA-templated; P:regulation of transcription, DNA-templated |
| Efet.01.1659514.g1944.t1 | gamma-glutamyltransferase | 942 | 0 | 91.65 | P:glutathione metabolic process; F:gamma-glutamyltransferase activity |
| Efet.01.1659514.g1945.t1 | NADP-dependent isocitrate dehydrogenase | 2238 | 0 | 94.8 | F:metal ion binding; F:isocitrate dehydrogenase (NADP+) activity; P:tricarboxylic acid cycle |
| Efet.01.1659514.g1946.t1 | FAD-binding oxidoreductase | 1761 | 0 | 93.2 | F:oxidoreductase activity, acting on CH-OH group of donors; F:flavin adenine dinucleotide binding; P:oxidation-reduction process |
| Efet.01.1659514.g1947.t1 | transporter | 309 | 7.63E-65 | 89.1 |  |
| Efet.01.1659515.g1948.t1 | ABC transporter ATP-binding | 1497 | 0 | 79.55 | F:ATPase activity; F:ATP binding; P:peptide transport |
| Efet.01.1659515.g1949.t1 | ABC transporter permease | 579 | 1.69E-95 | 89.6 | C:integral component of membrane; P:transport; C:plasma membrane |
| Efet.01.1659515.g1950.t1 | ABC transporter permease | 891 | 4.03E-164 | 80.95 | C:integral component of membrane; P:transport; C:plasma membrane |
| Efet.01.1659515.g1951.t1 | ABC transporter substrate-binding | 1584 | 0 | 85.95 | P:transmembrane transport; C:ATP-binding cassette (ABC) transporter complex |
| Efet.01.1659515.g1952.t1 | D-amino acid dehydrogenase | 276 | 5.43E-56 | 75 | C:membrane; F:D-amino-acid dehydrogenase activity; P:oxidation-reduction process |
| Efet.01.1659516.g1953.t1 | Uncharacterised | 615 | 2.49E-123 | 86.25 |  |
| Efet.01.1659516.g1954.t1 | IS5 IS1182 family transposase | 684 | 3.04E-13 | 67.35 | F:DNA binding; F:transposase activity; P:transposition, DNA-mediated |
| Efet.01.1659516.g1955.t1 | aldolase | 648 | 8.18E-103 | 74.6 | F:metal ion binding; P:lipid metabolic process; F:phosphoric diester hydrolase activity |
| Efet.01.1659516.g1956.t1 | tripartite tricarboxylate transporter substrate binding | 990 | 0 | 71.7 | C:outer membrane-bounded periplasmic space |
| Efet.01.1659516.g1957.t1 | fumarate hydratase | 555 | 7.79E-120 | 80.35 | F:hydro-lyase activity |
| Efet.01.1659516.g1958.t1 | fumarate hydratase | 873 | 0 | 89.2 | F:fumarate hydratase activity |
| Efet.01.1659516.g1959.t1 | sugar transferase | 405 | 2.20E-91 | 80.15 | C:membrane; F:transferase activity |
| Efet.01.1659517.g1960.t1 | enoyl- hydratase | 858 | 0 | 73.65 | P:metabolic process; F:isomerase activity |
| Efet.01.1659517.g1961.t1 | transferase | 1173 | 0 | 64.9 | F:catalytic activity |
| Efet.01.1659517.g1962.t1 | transcriptional regulator | 729 | 3.23E-138 | 71.3 | F:DNA binding; F:transcription factor activity, sequence-specific DNA binding; P:transcription, DNA-templated; P:regulation of transcription, DNA-templated |
| Efet.01.1659517.g1963.t1 | tripartite tricarboxylate transporter substrate binding | 876 | 0 | 76.85 | C:outer membrane-bounded periplasmic space |
| Efet.01.1659517.g1964.t1 | DUF1446 domain-containing | 1827 | 0 | 78.25 | F:lyase activity |
| Efet.01.1659518.g1965.t1 | MULTISPECIES: hypothetical protein | 429 | 1.50E-37 | 68.33 |  |
| Efet.01.1659518.g1966.t1 | hypothetical protein | 270 | 8.60E-17 | 69 |  |
| Efet.01.1659518.g1967.t1 | hypothetical protein [Paraburkholderia phytofirmans] | 381 | 5.72E-29 | 59.33 |  |
| Efet.01.1659518.g1968.t1 | type II toxin-antitoxin system family toxin | 762 | 1.13E-139 | 79.14 |  |
| Efet.01.1659518.g1969.t1 | nucleotidyl transferase ii ii toxin family | 528 | 1.31E-97 | 71.8 |  |
| Efet.01.1659518.g1970.t1 | Uncharacterised [Kingella negevensis] | 372 | 8.34E-86 | 67.55 |  |
| Efet.01.1659518.g1971.t1 | hypothetical protein | 576 | 6.49E-93 | 65.65 |  |
| Efet.01.1659518.g1972.t1 | hypothetical protein Veis_1422 | 462 | 1.76E-99 | 88 |  |
| Efet.01.1659518.g1973.t1 | hypothetical protein | 477 | 9.02E-94 | 67.05 | C:membrane; C:integral component of membrane |
| Efet.01.1659519.g1974.t1 | uroporphyrinogen-III C-methyltransferase | 666 | 1.31E-105 | 87 | P:porphyrin-containing compound biosynthetic process; P:oxidation-reduction process; F:uroporphyrin-III C-methyltransferase activity; P:methylation |
| Efet.01.1659519.g1975.t1 | DNA-binding | 924 | 3.08E-165 | 91.95 | F:transferase activity, transferring glycosyl groups; P:metabolic process |
| Efet.01.1659519.g1976.t1 | DUF3800 domain-containing | 294 | 4.70E-39 | 68.75 |  |
| Efet.01.1659519.g1977.t1 | twin-arginine translocation pathway signal | 1329 | 0 | 90.9 | F:sulfuric ester hydrolase activity; P:metabolic process |
| Efet.01.1659519.g1978.t1 | ABC transporter substrate-binding | 1038 | 0 | 91.95 | C:outer membrane-bounded periplasmic space |
| Efet.01.1659519.g1979.t1 | methyl-accepting chemotaxis | 1287 | 0 | 75.65 | P:chemotaxis; P:signal transduction; C:integral component of membrane; F:signal transducer activity |
| Efet.01.1659520.g1980.t1 | heme biosynthesis | 1305 | 1.23E-166 | 83.15 | C:integral component of membrane; P:heme metabolic process |
| Efet.01.1659520.g1981.t1 | biotin carboxylase | 4122 | 5.53E-179 | 69.2 | F:ATP binding; F:catalytic activity; F:metal ion binding |
| Efet.01.1659521.g1982.t1 | DNA repair | 363 | 3.56E-12 | 75.25 |  |
| Efet.01.1659521.g1983.t1 | helicase | 2196 | 0 | 81.6 | F:DNA binding; F:ATP binding; F:ATP-dependent helicase activity; P:nucleobase-containing compound metabolic process |
| Efet.01.1659521.g1984.t1 | Uncharacterised | 1212 | 0 | 79.2 |  |
| Efet.01.1659521.g1985.t1 | DUF3732 domain-containing | 1689 | 0 | 88.15 |  |
| Efet.01.1659521.g1986.t1 | DNA repair | 480 | 3.57E-88 | 90.8 | P:nucleic acid phosphodiester bond hydrolysis; F:exonuclease activity |
| Efet.01.1659522.g1987.t1 | family disulfide reductase | 597 | 4.95E-107 | 90.2 | F:oxidoreductase activity; C:integral component of membrane; P:oxidation-reduction process |
| Efet.01.1659522.g1988.t1 | cytochrome c | 591 | 1.88E-75 | 90.55 | F:iron ion binding; F:electron carrier activity; C:periplasmic space; P:electron transport chain; F:heme binding |
| Efet.01.1659522.g1989.t1 | cytochrome b | 570 | 3.02E-58 | 73.65 | F:electron carrier activity; P:respiratory electron transport chain; C:integral component of membrane |
| Efet.01.1659522.g1990.t1 | GCN5 family acetyltransferase | 444 | 1.22E-100 | 91.05 | F:N-acetyltransferase activity |
| Efet.01.1659522.g1991.t1 | peroxiredoxin | 507 | 8.21E-120 | 92.75 | F:oxidoreductase activity; P:oxidation-reduction process |
| Efet.01.1659522.g1992.t1 | 30S ribosomal S17 | 270 | 8.25E-57 | 96.65 | F:structural constituent of ribosome; C:ribosome; F:rRNA binding; P:translation |
| Efet.01.1659522.g1993.t1 | 30S ribosomal S3 | 1122 | 1.55E-133 | 95.05 | F:structural constituent of ribosome; F:mRNA binding; C:small ribosomal subunit; F:rRNA binding; P:translation |
| Efet.01.1659522.g1994.t1 | 50S ribosomal L22 | 333 | 1.80E-73 | 93.95 | F:structural constituent of ribosome; C:large ribosomal subunit; F:rRNA binding; P:translation |
| Efet.01.1659522.g1995.t1 | 50S ribosomal L2 | 825 | 0 | 97.6 | F:structural constituent of ribosome; C:large ribosomal subunit; F:transferase activity; F:rRNA binding; P:translation |
| Efet.01.1659522.g1996.t1 | 50S ribosomal L4 | 348 | 1.05E-62 | 96.85 | F:structural constituent of ribosome; C:ribosome; F:rRNA binding; P:translation |
| Efet.01.1659522.g1997.t1 | 50S ribosomal L3 | 675 | 2.88E-160 | 97.1 | F:structural constituent of ribosome; C:ribosome; F:rRNA binding; P:translation |
| Efet.01.1659522.g1998.t1 | 30S ribosomal S10 | 312 | 5.03E-67 | 99.4 | F:structural constituent of ribosome; C:ribosome; F:tRNA binding; P:translation |
| Efet.01.1659523.g1999.t1 | phosphate transport system regulatory | 600 | 6.41E-84 | 65.45 | P:biological regulation; P:transport |
| Efet.01.1659523.g2000.t1 | FAD-binding oxidoreductase | 1332 | 0 | 88.35 | F:D-amino-acid dehydrogenase activity; F:sarcosine oxidase activity; P:oxidation-reduction process |
| Efet.01.1659523.g2001.t1 | XRE family transcriptional regulator | 627 | 3.08E-151 | 85 | F:sequence-specific DNA binding |
| Efet.01.1659523.g2002.t1 | type III glutamate--ammonia ligase | 1293 | 0 | 90.05 | P:glutamine biosynthetic process; F:glutamate-ammonia ligase activity |
| Efet.01.1659523.g2003.t1 | amidophosphoribosyltransferase | 846 | 0 | 78.7 | P:glutamine metabolic process; P:oxidation-reduction process; F:amidophosphoribosyltransferase activity; F:glutamate synthase (NADPH) activity |
| Efet.01.1659523.g2004.t1 | FMN-binding glutamate synthase family | 1746 | 0 | 94.6 | P:glutamate biosynthetic process; P:oxidation-reduction process; F:glutamate synthase (NADPH) activity; F:transferase activity |
| Efet.01.1659523.g2005.t1 | Uncharacterised [Mycobacterium abscessus abscessus] | 1050 | 0 | 85.2 | F:transferase activity |
| Efet.01.1659523.g2006.t1 | family | 546 | 2.66E-107 | 88.6 | C:integral component of membrane |
| Efet.01.1659524.g2007.t1 | transferase | 969 | 0 | 86.95 | F:formyl-CoA transferase activity |
| Efet.01.1659524.g2008.t1 | tripartite tricarboxylate transporter substrate binding | 816 | 0 | 84 | C:outer membrane-bounded periplasmic space |
| Efet.01.1659524.g2009.t1 | enoyl- hydratase isomerase family | 762 | 0 | 80.3 | P:metabolic process; F:enoyl-CoA hydratase activity; F:isomerase activity |
| Efet.01.1659524.g2010.t1 | family transcriptional regulator | 234 | 1.32E-28 | 68.18 | F:DNA binding; F:transcription factor activity, sequence-specific DNA binding; P:transcription, DNA-templated; P:regulation of transcription, DNA-templated |
| Efet.01.1659524.g2011.t1 | D-alanyl-D-alanine carboxypeptidase | 1209 | 0 | 93.85 | F:serine-type D-Ala-D-Ala carboxypeptidase activity; P:proteolysis |
| Efet.01.1659524.g2012.t1 | potassium transporter Kup | 3189 | 0 | 96.5 | F:potassium ion transmembrane transporter activity; P:potassium ion transmembrane transport; C:integral component of membrane; C:plasma membrane |
| Efet.01.1659524.g2013.t1 | MFS transporter | 1260 | 0 | 74.45 | P:transmembrane transport; F:transmembrane transporter activity; C:integral component of membrane |
| Efet.01.1659525.g2014.t1 | hypothetical protein | 291 | 1.68E-31 | 82.7 |  |
| Efet.01.1659525.g2015.t1 | DUF3422 domain-containing | 1293 | 0 | 90.55 | C:integral component of membrane |
| Efet.01.1659525.g2016.t1 | tripartite tricarboxylate transporter permease | 1374 | 0 | 94 | C:integral component of membrane |
| Efet.01.1659525.g2017.t1 | tripartite tricarboxylate transporter family | 543 | 7.87E-123 | 84.1 | C:integral component of membrane |
| Efet.01.1659525.g2018.t1 | type II secretion system E | 1761 | 0 | 95.3 | F:ATP binding; P:transport |
| Efet.01.1659525.g2019.t1 | spermidine synthase | 660 | 1.55E-137 | 89.25 |  |
| Efet.01.1659525.g2020.t1 | DNA-deoxyinosine glycosylase | 552 | 1.31E-126 | 76.25 |  |
| Efet.01.1659525.g2021.t1 | S-formylglutathione hydrolase | 771 | 5.04E-172 | 89.2 | F:S-formylglutathione hydrolase activity; P:formaldehyde catabolic process; F:carboxylic ester hydrolase activity |
| Efet.01.1659525.g2022.t1 | S-(hydroxymethyl)glutathione synthase | 576 | 3.46E-128 | 86.5 | F:zinc ion binding; P:formaldehyde catabolic process; F:S-(hydroxymethyl)glutathione synthase activity |
| Efet.01.1659525.g2023.t1 | S-(hydroxymethyl)glutathione dehydrogenase | 1116 | 0 | 93.1 | F:zinc ion binding; F:S-(hydroxymethyl)glutathione dehydrogenase activity; P:ethanol oxidation |
| Efet.01.1659526.g2024.t1 | membrane implicated in regulation of membrane protease activity | 279 | 9.00E-61 | 84.75 | P:proteolysis; C:integral component of membrane; F:peptidase activity |
| Efet.01.1659526.g2025.t1 | lysine decarboxylase | 2319 | 0 | 96.75 | F:lysine decarboxylase activity; C:cytoplasm; P:cellular amino acid metabolic process |
| Efet.01.1659526.g2026.t1 | DNA ligase | 1014 | 0 | 90.4 | P:DNA ligation involved in DNA repair; F:ATP binding; F:DNA ligase (ATP) activity; P:DNA recombination |
| Efet.01.1659526.g2027.t1 | tripartite tricarboxylate transporter substrate binding | 966 | 0 | 68.65 | C:outer membrane-bounded periplasmic space |
| Efet.01.1659526.g2028.t1 | CoA-binding protein | 2136 | 0 | 64.7 | F:cofactor binding |
| Efet.01.1659526.g2029.t1 | enoyl- hydratase isomerase family | 738 | 2.81E-180 | 72.5 | P:metabolic process; F:isomerase activity |
| Efet.01.1659526.g2030.t1 | tripartite tricarboxylate transporter substrate binding | 972 | 0 | 65.95 | C:outer membrane-bounded periplasmic space |
| Efet.01.1659526.g2031.t1 | serine threonine kinase | 1419 | 0 | 92.15 | F:ATP binding; F:protein serine/threonine kinase activity; P:protein phosphorylation; P:response to stress |
| Efet.01.1659526.g2032.t1 | metallophosphoesterase | 756 | 2.54E-168 | 83.95 |  |
| Efet.01.1659527.g2033.t1 | translocation | 1266 | 0 | 94.3 | C:periplasmic space; P:protein import |
| Efet.01.1659527.g2034.t1 | peptidoglycan-associated lipo | 549 | 3.80E-112 | 82.45 | C:integral component of membrane; C:cell outer membrane |
| Efet.01.1659527.g2035.t1 | tol-pal system | 666 | 1.71E-155 | 92.2 | P:protein trimerization |
| Efet.01.1659527.g2036.t1 | tRNA threonylcarbamoyladenosine dehydratase | 597 | 6.61E-112 | 71.3 | F:small protein activating enzyme activity |
| Efet.01.1659527.g2037.t1 | type I restriction modification DNA specificity domain | 1683 | 0 | 67.95 | F:DNA binding; P:DNA modification |
| Efet.01.1659527.g2038.t1 | DNA methyltransferase | 1458 | 0 | 89.35 | F:DNA binding; F:N-methyltransferase activity; P:DNA methylation on adenine; F:site-specific DNA-methyltransferase (adenine-specific) activity |
| Efet.01.1659527.g2039.t1 | restriction endonuclease | 2259 | 0 | 73.45 | F:DNA binding; F:ATP binding; F:endonuclease activity; P:DNA modification |
| Efet.01.1659527.g2040.t1 | recombination-associated | 786 | 8.44E-168 | 90.75 | P:nucleic acid phosphodiester bond hydrolysis; F:exonuclease activity; P:DNA recombination |
| Efet.01.166008.g797.t1 | structural maintenance of chromosomes 4-like | 498 | 1.59E-55 | 63.95 | C:nucleus; F:ATP binding; P:chromosome organization; C:chromosome |
| Efet.01.166035.g798.t1 | hypothetical protein CAPTEDRAFT_227181 | 336 | 8.70E-07 | 63 | F:DNA binding; C:nucleus; P:regulation of transcription, DNA-templated; F:sequence-specific DNA binding |
| Efet.01.166054.g799.t1 | PGAP2-interacting | 492 | 1.62E-47 | 83.35 | C:integral component of membrane |
| Efet.01.166066.g801.t1 | dynactin subunit 1-like isoform X4 | 273 | 1.58E-08 | 64.5 | C:dynactin complex; P:transport along microtubule; F:dynein complex binding |
| Efet.01.166172.g805.t1 | sesquipedalian-1-like | 708 | 1.27E-32 | 58.8 |  |
| Efet.01.16623.g1227.t1 | alpha-1,6-mannosyl-glyco 2-beta-N-acetylglucosaminyltransferase | 591 | 1.22E-23 | 71.55 | C:integral component of membrane; F:alpha-1,6-mannosylglycoprotein 2-beta-N-acetylglucosaminyltransferase activity; P:oligosaccharide biosynthetic process; C:Golgi stack |
| Efet.01.166243.g807.t1 | ATP-dependent RNA helicase DHX29 isoform X2 | 861 | 4.87E-102 | 61.45 | F:nucleotide binding; F:nucleic acid binding; F:ATP binding; F:ATP-dependent helicase activity; F:helicase activity; F:hydrolase activity |
| Efet.01.166326.g809.t1 | atrial natriuretic peptide receptor 3-like | 465 | 3.03E-08 | 48.5 | F:G-protein coupled receptor activity; F:G-protein coupled GABA receptor activity; P:G-protein coupled receptor signaling pathway; C:membrane; C:integral component of membrane |
| Efet.01.166383.g812.t1 | FAM214A-like isoform X2 | 417 | 5.87E-27 | 56.95 |  |
| Efet.01.166417.g814.t1 | glutamate receptor | 207 | 7.46E-19 | 70.65 | P:ion transport; F:transmembrane signaling receptor activity; F:extracellular ligand-gated ion channel activity; C:synapse; C:membrane part; C:plasma membrane |
| Efet.01.16642.g1228.t1 | pyrokinin-1 receptor- | 540 | 4.78E-49 | 80 | F:rhodopsin kinase activity; P:neuropeptide signaling pathway; C:integral component of membrane; F:neuromedin U receptor activity; P:protein phosphorylation |
| Efet.01.16645.g1230.t1 | neuron navigator 2 isoform X2 | 399 | 1.72E-33 | 70 | P:cranial nerve development; C:proteinaceous extracellular matrix; P:sensory perception |
| Efet.01.166453.g816.t1 | ral guanine nucleotide dissociation stimulator-like 1 isoform X2 | 267 | 5.61E-31 | 78.5 | P:small GTPase mediated signal transduction; C:membrane; F:guanyl-nucleotide exchange factor activity; C:intracellular; P:positive regulation of GTPase activity |
| Efet.01.16646.g1231.t1 | Serine threonine- phosphatase 4 regulatory subunit | 1689 | 4.41E-78 | 80.75 | P:signal transduction; P:regulation of catalytic activity; C:protein phosphatase 4 complex |
| Efet.01.166489.g817.t1 | ATP-dependent DNA helicase Q1-like | 267 | 5.90E-34 | 85.65 | F:nucleic acid binding; F:ATP binding; P:DNA recombination; F:ATP-dependent helicase activity |
| Efet.01.166531.g818.t1 | peptidyl-alpha-hydroxyglycine alpha-amidating lyase 1 isoform X1 | 795 | 5.18E-49 | 49.75 | F:catalytic activity |
| Efet.01.166543.g819.t1 | E3 ubiquitin- ligase UBR3 | 567 | 1.42E-11 | 56.35 | F:zinc ion binding; F:ligase activity |
| Efet.01.16655.g1232.t1 | MCM domain-containing 2 | 252 | 1.01E-06 | 62.2 | F:DNA binding; F:ATP binding; P:DNA replication |
| Efet.01.166601.g821.t1 | transformation transcription domain-associated -like isoform X3 | 402 | 9.13E-23 | 82.05 | F:transmembrane transporter activity; C:membrane; P:histone acetylation; P:phosphorylation; F:kinase activity; C:histone acetyltransferase complex |
| Efet.01.166612.g822.t1 | hypothetical protein CAPTEDRAFT_221826 | 438 | 8.95E-14 | 51.5 | C:membrane; C:integral component of membrane; F:carbohydrate binding |
| Efet.01.166641.g825.t1 | Hook homolog 3-like isoform X1 | 711 | 2.03E-29 | 61.15 | C:cytoskeleton; C:cytoplasm; P:protein transport; P:transport; C:microtubule |
| Efet.01.166697.g827.t1 | poly [ADP-ribose] polymerase 14-like | 684 | 2.92E-10 | 49.6 |  |
| Efet.01.16676.g1233.t1 | switch-associated 70-like isoform X2 | 459 | 1.88E-07 | 59.67 |  |
| Efet.01.166779.g828.t1 | RING finger 17-like isoform X2 | 5220 | 4.81E-117 | 44.55 | F:zinc ion binding; F:metal ion binding; C:intracellular |
| Efet.01.166830.g831.t1 | receptor-type tyrosine- phosphatase F- | 1416 | 1.22E-131 | 83.2 | F:protein tyrosine phosphatase activity; C:integral component of membrane; P:peptidyl-tyrosine dephosphorylation |
| Efet.01.16684.g1234.t1 | hypothetical protein HELRODRAFT_161577 | 1194 | 2.67E-06 | 74 | F:calcium ion binding |
| Efet.01.166891.g834.t1 | C-terminal-binding 1 isoform X4 | 660 | 2.96E-48 | 64.45 | P:negative regulation of transcription from RNA polymerase II promoter; P:white fat cell differentiation; C:nucleus; C:transcription factor complex; P:negative regulation of histone acetylation; F:transcription factor activity, sequence-specific DNA binding; F:identical protein binding; P:cellular macromolecule metabolic process; F:transcription corepressor activity; C:transcriptional repressor complex; F:transcription factor binding; P:primary metabolic process |
| Efet.01.166921.g837.t1 | N-acetylglucosamine-1-phosphotransferase subunits alpha beta | 1629 | 0 | 68.5 | P:lysosome organization; F:UDP-N-acetylglucosamine-lysosomal-enzyme N-acetylglucosaminephosphotransferase activity; F:protein binding; F:metal ion binding; C:Golgi membrane; P:N-glycan processing to lysosome; P:carbohydrate phosphorylation |
| Efet.01.166921.g838.t1 | N-acetylglucosamine-1-phosphotransferase subunits alpha beta-like | 1038 | 1.40E-111 | 72.3 | P:lysosome organization; F:UDP-N-acetylglucosamine-lysosomal-enzyme N-acetylglucosaminephosphotransferase activity; F:protein binding; F:metal ion binding; C:Golgi membrane; P:secretion of lysosomal enzymes; P:N-glycan processing to lysosome; P:carbohydrate phosphorylation |
| Efet.01.166936.g839.t1 | nachr subunit | 249 | 1.62E-26 | 80.55 | P:synaptic transmission, cholinergic; F:acetylcholine receptor activity; P:neuromuscular synaptic transmission; C:postsynaptic membrane; P:signal transduction; P:cation transmembrane transport; C:cell junction; F:extracellular ligand-gated ion channel activity; F:acetylcholine binding; C:acetylcholine-gated channel complex; P:response to nicotine |
| Efet.01.167007.g840.t1 | Dynein heavy chain axonemal | 771 | 4.11E-129 | 88.95 | F:ATPase activity; F:ATP binding; F:microtubule motor activity; C:dynein complex; P:microtubule-based movement |
| Efet.01.167019.g841.t1 | tropomyosin | 264 | 3.19E-35 | 73.8 |  |
| Efet.01.167044.g842.t1 | cholecystokinin receptor-like | 759 | 4.37E-31 | 56.05 | P:G-protein coupled receptor signaling pathway; C:membrane; F:signal transducer activity |
| Efet.01.167076.g843.t1 | anoctamin-1-like isoform X10 | 276 | 1.14E-14 | 71.05 | C:membrane |
| Efet.01.167098.g844.t1 | unc-112-related -like | 384 | 1.30E-33 | 52.2 | C:cytoskeleton |
| Efet.01.167112.g845.t1 | transmembrane 183-like | 453 | 2.86E-15 | 50.91 |  |
| Efet.01.167139.g847.t1 | RAS and EF-hand domain-containing | 213 | 1.34E-16 | 77.5 | F:GTP binding; P:small GTPase mediated signal transduction; F:calcium ion binding; C:intracellular |
| Efet.01.167143.g848.t1 | long-chain-fatty-acid-- ligase ACSBG2 isoform X2 | 240 | 1.04E-11 | 65.2 | F:catalytic activity; P:metabolic process |
| Efet.01.167162.g849.t1 | actin-binding | 1281 | 4.06E-131 | 66.05 | F:ubiquitin-protein transferase activity; F:actin binding; C:Cul3-RING ubiquitin ligase complex; P:protein ubiquitination |
| Efet.01.167192.g850.t1 | serine arginine-rich splicing factor 7-like | 267 | 2.72E-30 | 87.7 | F:nucleotide binding; F:nucleic acid binding; F:zinc ion binding |
| Efet.01.16725.g1236.t1 | proton-coupled folate transporter-like | 1197 | 1.84E-68 | 46.45 | P:transmembrane transport; C:membrane; C:integral component of membrane |
| Efet.01.167274.g854.t1 | cytochrome P450 family | 366 | 6.99E-26 | 70.25 | F:oxidoreductase activity; F:metal ion binding |
| Efet.01.167297.g856.t1 | hypothetical protein HELRODRAFT_159834 | 618 | 1.28E-08 | 51 | C:membrane; C:integral component of membrane |
| Efet.01.167334.g857.t1 | mitogen-activated kinase | 267 | 1.63E-33 | 87.25 | F:ATP binding; P:MAPK cascade; F:MAP kinase activity; C:intracellular |
| Efet.01.16736.g1238.t1 | diacylglycerol kinase eta isoform X2 | 519 | 3.04E-17 | 68.6 | F:transferase activity; P:cellular process |
| Efet.01.167362.g858.t1 | domain-containing histone demethylation 2c | 273 | 2.69E-36 | 82.95 |  |
| Efet.01.167388.g859.t1 | serine threonine- phosphatase 2A catalytic subunit alpha isoform- | 348 | 3.91E-35 | 99.2 | F:phosphoprotein phosphatase activity; P:protein dephosphorylation |
| Efet.01.167398.g860.t1 | sodium channel type 8 subunit alpha-like isoform X1 | 291 | 2.64E-34 | 69.4 | P:regulation of postsynaptic membrane potential; F:calcium ion binding; P:regulation of ion transmembrane transport; C:voltage-gated sodium channel complex; P:sodium ion transmembrane transport; F:voltage-gated sodium channel activity |
| Efet.01.167398.g861.t1 | hypothetical protein HELRODRAFT_109965 | 345 | 3.85E-18 | 97 | P:regulation of postsynaptic membrane potential; P:regulation of ion transmembrane transport; C:voltage-gated sodium channel complex; P:sodium ion transmembrane transport; F:voltage-gated sodium channel activity |
| Efet.01.167421.g862.t1 | sex peptide | 339 | 3.65E-10 | 50.65 | F:G-protein coupled receptor activity; F:G-protein coupled peptide receptor activity; P:neuropeptide signaling pathway; P:regulation of post-mating oviposition; P:G-protein coupled receptor signaling pathway; C:membrane; C:integral component of membrane; P:negative regulation of female receptivity, post-mating; C:plasma membrane |
| Efet.01.167421.g863.t1 | sex peptide receptor-like | 939 | 6.26E-72 | 62.5 | F:G-protein coupled receptor activity; F:G-protein coupled peptide receptor activity; P:G-protein coupled receptor signaling pathway; C:membrane; C:integral component of membrane |
| Efet.01.167446.g865.t1 | glycogen-binding subunit 76A-like | 984 | 1.01E-36 | 53 |  |
| Efet.01.167467.g868.t1 | PR domain zinc finger 10-like | 1350 | 6.63E-130 | 59.35 | F:nucleic acid binding; C:nucleus; F:metal ion binding; P:ubiquitin-dependent catabolism of misfolded proteins by nucleus-associated proteasome; C:membrane; C:integral component of membrane; P:proteasome localization |
| Efet.01.167518.g869.t1 | tweety-like isoform X1 | 246 | 4.00E-20 | 57.55 | P:ion transport; C:membrane |
| Efet.01.167554.g870.t1 | cytokine-inducible SH2-containing -like | 576 | 1.60E-43 | 60.85 | P:negative regulation of cellular process |
| Efet.01.167584.g872.t1 | TNF receptor-associated factor 2-like | 432 | 2.56E-40 | 72.65 | P:regulation of cellular process; F:metal ion binding; P:cellular process |
| Efet.01.167591.g873.t1 | Transmembrane 26 | 561 | 1.90E-39 | 58.7 | C:membrane; C:integral component of membrane |
| Efet.01.16762.g1239.t1 | Ubiquitin carboxyl-terminal hydrolase 5 | 366 | 4.41E-42 | 74.15 | F:metal ion binding; P:proteolysis; P:cellular protein metabolic process; F:thiol-dependent ubiquitinyl hydrolase activity |
| Efet.01.167644.g875.t1 | ankyrin-2-like isoform X19 | 219 | 3.75E-25 | 85 | P:signal transduction |
| Efet.01.167737.g878.t1 | hypothetical protein HELRODRAFT_160914 | 234 | 1.82E-21 | 76 | P:biological regulation |
| Efet.01.16779.g1240.t1 | tankyrase isoform X3 | 1452 | 4.22E-37 | 48.1 | F:transferase activity, transferring glycosyl groups; F:structural constituent of ribosome; P:protein ADP-ribosylation; F:transferase activity; C:ribosome; C:intracellular; P:translation; F:NAD+ ADP-ribosyltransferase activity |
| Efet.01.167816.g882.t1 | 26S protease regulatory subunit 7 | 411 | 2.34E-45 | 90.9 | C:proteasome regulatory particle, base subcomplex; C:P-body; F:peptidase activity; P:positive regulation of proteasomal protein catabolic process; P:positive regulation of RNA polymerase II transcriptional preinitiation complex assembly; F:ATP binding; C:membrane; P:osteoblast differentiation; C:cytosolic proteasome complex; C:nuclear proteasome complex; F:TBP-class protein binding; P:ER-associated ubiquitin-dependent protein catabolic process; F:proteasome-activating ATPase activity |
| Efet.01.167886.g883.t1 | von Willebrand factor D and EGF domain-containing | 525 | 3.92E-28 | 51.25 | C:membrane; C:integral component of membrane |
| Efet.01.167886.g884.t1 | hypothetical protein HELRODRAFT_182201 | 636 | 1.87E-57 | 54.8 |  |
| Efet.01.167886.g885.t1 | laminin subunit alpha | 312 | 8.38E-07 | 46.93 | F:metal ion binding; C:integral component of membrane; P:chemical synaptic transmission |
| Efet.01.167936.g886.t1 | DNA replication ATP-dependent helicase nuclease DNA2 isoform X2 | 324 | 2.77E-13 | 58.05 | F:5'-flap endonuclease activity; F:single-stranded DNA-dependent ATPase activity; F:helicase activity; P:DNA replication, Okazaki fragment processing |
| Efet.01.167939.g888.t1 | THO complex subunit 1 | 645 | 2.92E-27 | 56.4 | P:signal transduction |
| Efet.01.16795.g1242.t1 | retinol retinaldehyde reductase | 693 | 3.74E-57 | 68.2 | F:oxidoreductase activity; P:oxidation-reduction process |
| Efet.01.168.g17.t1 | Nucleoside diphosphate kinase 7 | 741 | 2.56E-82 | 60.25 | F:transferase activity; P:nucleotide metabolic process |
| Efet.01.16800.g1243.t1 | beta-1,3-galactosyl-O-glycosyl-glyco beta-1,6-N-acetylglucosaminyltransferase-like | 342 | 2.39E-35 | 66.2 | F:acetylglucosaminyltransferase activity; C:membrane |
| Efet.01.168046.g890.t1 | probable G- coupled receptor 139 isoform X1 | 750 | 1.20E-50 | 68.15 | F:G-protein coupled receptor activity; P:G-protein coupled receptor signaling pathway; C:integral component of membrane |
| Efet.01.168063.g891.t1 | dopamine receptor 1-like | 771 | 2.95E-24 | 53.55 | P:cellular process |
| Efet.01.168087.g893.t1 | cullin-5 isoform X4 | 396 | 4.38E-35 | 79.75 | P:cell cycle arrest; P:cell proliferation; F:ubiquitin protein ligase activity; F:calcium channel activity; P:negative regulation of cell proliferation; P:intrinsic apoptotic signaling pathway; F:receptor activity; P:protein ubiquitination involved in ubiquitin-dependent protein catabolic process; F:ubiquitin protein ligase binding; P:G1/S transition of mitotic cell cycle; P:viral process; P:calcium ion transmembrane transport; C:cytosol; P:ERBB2 signaling pathway; C:Cul5-RING ubiquitin ligase complex |
| Efet.01.168088.g894.t1 | SCY1 2 isoform X1 | 465 | 3.49E-37 | 71.45 | F:ATP binding; F:protein kinase activity; P:protein phosphorylation |
| Efet.01.168130.g895.t1 | low-density lipo receptor-related 1 | 609 | 9.85E-86 | 74.95 | F:transferase activity, transferring glycosyl groups; C:membrane |
| Efet.01.168137.g896.t1 | TPA_inf: Pax-beta paired domain | 1527 | 2.60E-08 | 80.13 | C:nucleus; F:DNA binding; P:transcription, DNA-templated; P:multicellular organism development; P:regulation of transcription, DNA-templated |
| Efet.01.168152.g898.t1 | photoreceptor outer segment membrane glyco 2-like | 654 | 4.35E-37 | 60.2 | C:membrane |
| Efet.01.168163.g899.t1 | E3 ubiquitin- ligase UBR4 | 312 | 1.83E-63 | 96.7 | F:zinc ion binding; F:ligase activity |
| Efet.01.168176.g900.t1 | ankyrin repeat domain-containing 13D isoform X1 | 666 | 7.80E-71 | 77.5 |  |
| Efet.01.168210.g901.t1 | nucleolar GTP-binding 1 | 549 | 6.47E-35 | 54.05 | C:intracellular membrane-bounded organelle; P:negative regulation of cellular process |
| Efet.01.16831.g1244.t1 | aldo keto reductase | 210 | 1.75E-10 | 73.3 | F:oxidoreductase activity; P:oxidation-reduction process |
| Efet.01.168338.g902.t1 | multidrug resistance-associated 1-like isoform X1 | 501 | 6.77E-77 | 83 | F:ATP binding; F:ATPase activity, coupled to transmembrane movement of substances; P:transmembrane transport; C:integral component of membrane |
| Efet.01.168341.g903.t1 | glucan 1,6-alpha-glucosidase | 342 | 8.49E-15 | 57.5 | P:carbohydrate metabolic process; F:catalytic activity; C:membrane; C:integral component of membrane |
| Efet.01.168346.g904.t1 | dystroglycan-like | 921 | 1.02E-34 | 61.65 | C:membrane |
| Efet.01.168346.g905.t1 | dystroglycan | 1353 | 2.50E-50 | 52.4 | P:regulation of biological quality; F:protein binding; P:system development; P:cellular component organization; P:regulation of embryonic development; P:regulation of anatomical structure morphogenesis; P:single-organism cellular process; C:extracellular region; C:plasma membrane part; P:regulation of cellular component organization |
| Efet.01.16840.g1245.t1 | neurexin-1-like isoform X1 | 630 | 1.06E-32 | 58.5 | C:presynaptic membrane; F:metal ion binding; C:integral component of membrane; P:chemical synaptic transmission |
| Efet.01.168402.g908.t1 | muscarinic acetylcholine receptor M3 isoform X2 | 771 | 1.19E-16 | 55.25 | P:system process; P:G-protein coupled receptor signaling pathway; C:membrane |
| Efet.01.168408.g909.t1 | neuroendocrine convertase 1-like isoform X2 | 306 | 1.01E-18 | 80.6 | P:proteolysis; C:integral component of membrane; F:serine-type endopeptidase activity |
| Efet.01.168410.g910.t1 | collagen alpha-3(VI) chain-like isoform X9 | 453 | 1.33E-25 | 69.8 |  |
| Efet.01.168410.g911.t1 | collagen alpha-3(VI) chain-like isoform X3 | 348 | 2.30E-13 | 56.5 | F:calcium ion binding |
| Efet.01.168503.g912.t1 | solute carrier family 12 member 6 isoform X5 | 237 | 3.18E-29 | 88.3 | F:potassium:chloride symporter activity; F:protein kinase binding; P:chloride transmembrane transport; F:dipeptidase activity; F:potassium ion symporter activity; F:metalloexopeptidase activity; P:chemical synaptic transmission; F:dipeptidyl-peptidase activity; C:integral component of plasma membrane; P:rubidium ion transport; F:rubidium ion transmembrane transporter activity; F:metal ion binding; P:angiogenesis; P:proteolysis; P:potassium ion import; P:cellular hypotonic salinity response; C:basolateral plasma membrane; C:anchored component of membrane |
| Efet.01.168562.g915.t1 | GAS2 1 | 318 | 1.36E-13 | 72.1 | P:cell cycle arrest |
| Efet.01.168567.g916.t1 | Dual specificity | 552 | 6.05E-66 | 59.75 | F:protein tyrosine phosphatase activity; P:peptidyl-tyrosine dephosphorylation; F:protein tyrosine/serine/threonine phosphatase activity |
| Efet.01.168661.g919.t1 | hypothetical protein HELRODRAFT_189125 | 948 | 2.39E-47 | 57 | C:membrane; C:integral component of membrane |
| Efet.01.168665.g920.t1 | galanin receptor type 1-like | 801 | 1.16E-30 | 52.8 | F:G-protein coupled receptor activity; C:integral component of plasma membrane; P:G-protein coupled receptor signaling pathway; P:signal transduction; C:membrane; C:integral component of membrane; P:cell surface receptor signaling pathway; F:signal transducer activity |
| Efet.01.168675.g921.t1 | transcription factor SOX-11-like | 1152 | 6.65E-50 | 85.1 |  |
| Efet.01.168697.g923.t1 | leucine-rich repeat and fibronectin type III domain-containing 1 | 1527 | 3.23E-118 | 59.4 | C:membrane |
| Efet.01.16871.g1246.t1 | ubiquitin-like modifier-activating enzyme 1 | 207 | 6.69E-23 | 76.55 | F:ATP binding; P:cellular protein modification process; F:small protein activating enzyme activity |
| Efet.01.168711.g925.t1 | voltage-dependent L-type calcium channel subunit beta-1 isoform X1 | 636 | 8.04E-13 | 88.6 | P:biological regulation; P:single-multicellular organism process; C:voltage-gated calcium channel complex; P:calcium ion transmembrane transport; P:single-organism cellular process; F:voltage-gated calcium channel activity |
| Efet.01.168726.g926.t1 | probable cytosolic oligopeptidase A | 795 | 1.60E-42 | 69.1 | P:peptide metabolic process; F:metalloendopeptidase activity |
| Efet.01.168798.g927.t1 | outer dense fiber 2-like isoform X3 | 471 | 4.92E-36 | 69.8 |  |
| Efet.01.168837.g928.t1 | solute carrier family 28 member 3-like | 351 | 1.37E-24 | 74.6 | F:nucleoside:sodium symporter activity; P:nucleobase transport; P:purine-containing compound transmembrane transport; C:integral component of membrane; P:nucleoside transmembrane transport |
| Efet.01.168919.g930.t1 | brain tumor -like | 489 | 6.06E-102 | 93.95 | F:zinc ion binding; C:intracellular |
| Efet.01.168924.g932.t1 | SCY1 2 | 219 | 3.93E-16 | 75.35 | F:ATP binding; F:protein kinase activity; P:protein phosphorylation |
| Efet.01.169.g18.t1 | titin-like isoform X1 | 318 | 3.60E-40 | 70.9 |  |
| Efet.01.169.g19.t1 | titin-like isoform X1 | 1725 | 9.42E-109 | 57.1 |  |
| Efet.01.169028.g935.t1 | GDH 6PGL endoplasmic bifunctional | 1671 | 3.72E-131 | 53.4 | P:metabolic process |
| Efet.01.169044.g937.t1 | cadherin EGF LAG seven-pass G-type receptor 2-like isoform X1 | 495 | 2.39E-20 | 54.5 | F:G-protein coupled receptor activity; F:transmembrane signaling receptor activity; P:G-protein coupled receptor signaling pathway; P:signal transduction; F:calcium ion binding; C:membrane; C:integral component of membrane; P:cell surface receptor signaling pathway; P:cell adhesion; P:homophilic cell adhesion via plasma membrane adhesion molecules; F:signal transducer activity; C:plasma membrane |
| Efet.01.169089.g939.t1 | diacylglycerol kinase eta isoform X1 | 243 | 1.36E-14 | 71.75 | F:ATP binding; P:response to oxidative stress; F:diacylglycerol kinase activity; P:phosphorylation; P:protein kinase C-activating G-protein coupled receptor signaling pathway; C:intracellular; C:plasma membrane; P:intracellular signal transduction; P:diacylglycerol metabolic process |
| Efet.01.169097.g940.t1 | potassium voltage-gated channel subfamily A member 1 isoform X1 | 1407 | 1.00E-170 | 82.35 | F:voltage-gated potassium channel activity; P:potassium ion transmembrane transport; C:voltage-gated potassium channel complex; P:regulation of ion transmembrane transport; P:protein homooligomerization |
| Efet.01.169097.g941.t1 | hypothetical protein CRN43_17300 | 318 | 3.79E-17 | 87 |  |
| Efet.01.169129.g942.t1 | hypothetical protein HELRODRAFT_185802 | 819 | 7.91E-126 | 70.75 | F:actin filament binding; C:cytoskeleton; C:cytoplasm; P:actin filament organization; F:protein binding, bridging |
| Efet.01.16924.g1248.t1 | galactosylgalactosylxylosyl 3-beta-glucuronosyltransferase I | 603 | 1.29E-52 | 70.7 | P:carbohydrate metabolic process; P:chondroitin sulfate proteoglycan biosynthetic process; C:membrane; F:galactosylgalactosylxylosylprotein 3-beta-glucuronosyltransferase activity; P:chondroitin sulfate metabolic process |
| Efet.01.169263.g945.t1 | Calcium-dependent kinase | 465 | 9.58E-68 | 59.6 | F:calcium ion binding; F:protein kinase activity; P:phosphorylation |
| Efet.01.16927.g1249.t1 | spermine oxidase-like | 957 | 3.09E-116 | 59.8 | F:oxidoreductase activity; P:oxidation-reduction process |
| Efet.01.169272.g946.t1 | F-box DNA helicase 1-like | 588 | 3.15E-28 | 61.05 | F:organic cyclic compound binding; F:heterocyclic compound binding; F:hydrolase activity |
| Efet.01.169305.g947.t1 | epidermal growth factor receptor-like isoform X2 | 222 | 2.09E-29 | 87.7 | F:ATP binding; P:peptidyl-tyrosine phosphorylation; F:transmembrane receptor protein tyrosine kinase activity; C:integral component of membrane; P:transmembrane receptor protein tyrosine kinase signaling pathway |
| Efet.01.169384.g948.t1 | early growth response 2- | 1467 | 2.31E-56 | 80.9 | F:nucleic acid binding; F:metal ion binding |
| Efet.01.169387.g949.t1 | proline--tRNA ligase | 549 | 1.24E-88 | 77.6 | F:ATP binding; C:cytoplasm; F:proline-tRNA ligase activity; F:glutamate-tRNA ligase activity; P:glutamyl-tRNA aminoacylation; P:prolyl-tRNA aminoacylation |
| Efet.01.169391.g951.t1 | phosphoenolpyruvate carboxykinase [GTP]-like | 2037 | 0 | 81.95 | F:GTP binding; P:gluconeogenesis; F:phosphoenolpyruvate carboxykinase (GTP) activity; C:mitochondrion; P:phosphorylation; F:kinase activity |
| Efet.01.169393.g952.t1 | arachidonate 5-lipoxygenase | 222 | 9.27E-20 | 65.95 | F:dioxygenase activity; F:metal ion binding; P:single-organism metabolic process |
| Efet.01.169395.g953.t1 | serine threonine- phosphatase 2A activator-like | 411 | 1.20E-23 | 81.4 | F:peptidyl-prolyl cis-trans isomerase activity; C:cytoplasm; P:positive regulation of catalytic activity; F:phosphatase activator activity; P:protein peptidyl-prolyl isomerization |
| Efet.01.16951.g1250.t1 | guanine nucleotide-binding subunit beta | 366 | 8.12E-79 | 97.25 | F:calmodulin binding; C:cytoplasm; P:signal transduction; F:signal transducer activity |
| Efet.01.169527.g956.t1 | stAR-related lipid transfer 13 isoform X5 | 1335 | 1.01E-06 | 53.57 | P:endothelial tube lumen extension; P:signal transduction; F:lipid binding; P:endothelial cell migration; P:negative regulation of cell migration involved in sprouting angiogenesis |
| Efet.01.169624.g958.t1 | unc-13 homolog B-like isoform X11 | 204 | 9.09E-30 | 83.4 | F:metal ion binding; F:diacylglycerol binding; P:chemical synaptic transmission; C:intracellular; P:intracellular signal transduction |
| Efet.01.169632.g959.t1 | titin-like isoform X3 | 780 | 1.45E-14 | 64.21 |  |
| Efet.01.169635.g961.t1 | autophagy-related 101 | 300 | 6.19E-35 | 81.75 | P:autophagosome assembly; C:pre-autophagosomal structure |
| Efet.01.169635.g962.t1 | transmembrane 132E-like | 939 | 6.00E-53 | 53.95 | C:membrane; C:integral component of membrane |
| Efet.01.169719.g965.t1 | aromatic-L-amino-acid decarboxylase isoform X5 | 261 | 4.95E-26 | 83.05 | F:pyridoxal phosphate binding; P:cellular amino acid metabolic process; F:carboxy-lyase activity |
| Efet.01.169800.g968.t1 | chloride intracellular channel 4 | 378 | 5.04E-61 | 56.45 |  |
| Efet.01.16985.g1252.t1 | TRPM8 channel-associated factor homolog | 720 | 4.91E-60 | 58.4 | C:membrane; C:integral component of membrane |
| Efet.01.169880.g971.t1 | sex peptide receptor-like | 834 | 1.12E-67 | 53.15 | F:G-protein coupled receptor activity; F:G-protein coupled peptide receptor activity; P:G-protein coupled receptor signaling pathway; C:membrane; C:integral component of membrane |
| Efet.01.169883.g972.t1 | atrial natriuretic peptide receptor 1-like | 231 | 1.62E-18 | 74.5 | C:guanylate cyclase complex, soluble; F:nucleotide binding; P:cGMP biosynthetic process; P:signal transduction; F:guanylate cyclase activity; F:transferase activity; C:plasma membrane; F:adenylate cyclase activity |
| Efet.01.169940.g973.t1 | follistatin-related 1 | 378 | 5.74E-13 | 59.35 | C:extracellular space; C:extracellular exosome; F:calcium ion binding; P:proteolysis; P:response to starvation; F:peptidase activity |
| Efet.01.169949.g974.t1 | palmitoyltransferase PFA3-like | 762 | 1.10E-68 | 59.45 | F:transferase activity |
| Efet.01.169976.g975.t1 | dynein heavy chain axonemal | 249 | 6.88E-08 | 66.7 | P:movement of cell or subcellular component; P:multicellular organism development; C:dynein complex; C:axoneme; P:microtubule-based process |
| Efet.01.169987.g976.t1 | transportin-3 | 294 | 5.90E-14 | 72.95 | P:intracellular protein transport; F:Ran GTPase binding; C:intracellular |
| Efet.01.170002.g977.t1 | discoidin domain-containing receptor 2-like isoform X1 | 1146 | 1.99E-71 | 58.95 | C:membrane; F:protein kinase activity; P:cellular process |
| Efet.01.170094.g980.t1 | retinol dehydrogenase 12-like | 429 | 7.85E-36 | 54.2 | C:membrane; C:integral component of membrane |
| Efet.01.170165.g981.t1 | transient receptor potential cation channel subfamily M member 2-like | 879 | 1.29E-58 | 67.1 | F:ion channel activity; P:ion transmembrane transport; C:integral component of membrane; F:hydrolase activity |
| Efet.01.170165.g982.t1 | ADP-ribose mitochondrial isoform X2 | 579 | 1.82E-14 | 48.25 | P:ion transport; F:pyrophosphatase activity; C:membrane-bounded organelle |
| Efet.01.17021.g1255.t1 | Glutamate synthase [NADH] amyloplastic | 1206 | 8.82E-136 | 80.05 | P:glutamate biosynthetic process; F:FMN binding; F:iron ion binding; F:glutamate synthase (NADH) activity; F:flavin adenine dinucleotide binding; P:RNA processing; P:oxidation-reduction process; F:iron-sulfur cluster binding |
| Efet.01.170245.g986.t1 | vacuolar sorting-associated 33B-like | 882 | 4.18E-38 | 55.75 | C:intracellular organelle; P:vesicle-mediated transport; P:cellular component organization; C:cytoplasmic part; P:single-organism cellular process; C:membrane-bounded organelle |
| Efet.01.170251.g987.t1 | gastrula zinc finger -like | 1248 | 3.09E-70 | 56.5 | F:nucleic acid binding; F:metal ion binding |
| Efet.01.170253.g988.t1 | T-complex 1 subunit gamma | 258 | 6.10E-40 | 87.9 | F:ATP binding; C:cytoplasm; F:unfolded protein binding; P:protein folding |
| Efet.01.170274.g989.t1 | glycosyltransferase family 57 | 498 | 7.67E-40 | 76.15 | C:endoplasmic reticulum membrane; P:oligosaccharide-lipid intermediate biosynthetic process; F:dolichyl pyrophosphate Man9GlcNAc2 alpha-1,3-glucosyltransferase activity; F:dolichyl pyrophosphate Glc1Man9GlcNAc2 alpha-1,3-glucosyltransferase activity; C:integral component of membrane; P:protein N-linked glycosylation |
| Efet.01.170284.g990.t1 | trithorax group osa-like isoform X7 | 1806 | 1.71E-118 | 59.5 | F:DNA binding; C:BAF-type complex; P:chromatin remodeling |
| Efet.01.170352.g991.t1 | Glutamate decarboxylase 1 | 285 | 2.02E-24 | 71.3 | F:pyridoxal phosphate binding; C:clathrin-sculpted gamma-aminobutyric acid transport vesicle membrane; F:protein binding; P:protein-pyridoxal-5-phosphate linkage; F:glutamate decarboxylase activity; P:glutamate decarboxylation to succinate; P:cellular biosynthetic process; C:plasma membrane; P:neurotransmitter secretion |
| Efet.01.170391.g992.t1 | FACT complex subunit SSRP1 | 204 | 8.83E-09 | 62.41 | F:DNA binding; C:nucleus |
| Efet.01.170391.g993.t1 | FACT complex subunit Ssrp1-like | 243 | 4.77E-18 | 69.2 | F:DNA binding; C:nucleus |
| Efet.01.170452.g995.t1 | CREBRF homolog | 612 | 1.55E-71 | 61.5 | F:transcription factor activity, sequence-specific DNA binding; C:membrane; C:integral component of membrane; P:regulation of transcription, DNA-templated; F:sequence-specific DNA binding |
| Efet.01.17048.g1257.t1 | zinc finger 253-like isoform X1 | 621 | 7.64E-16 | 49.15 | F:nucleic acid binding; C:nucleus; F:zinc ion binding; F:metal ion binding |
| Efet.01.170567.g999.t1 | Ankyrin repeat and FYVE domain-containing | 669 | 5.08E-06 | 58 | F:metal ion binding |
| Efet.01.170631.g1002.t1 | voltage-gated sodium channel | 468 | 9.88E-11 | 60.2 | F:ion channel activity; P:ion transport; P:transmembrane transport; C:membrane |
| Efet.01.170658.g1003.t1 | malate dehydrogenase 1B | 561 | 5.61E-25 | 52.9 | F:catalytic activity |
| Efet.01.170695.g1004.t1 | KN motif and ankyrin repeat domain-containing 3 | 276 | 9.41E-28 | 81.85 | F:NADH dehydrogenase (ubiquinone) activity; P:ATP synthesis coupled electron transport; C:mitochondrial inner membrane |
| Efet.01.170740.g1005.t1 | UDP-glucuronosyltransferase 2C1-like | 435 | 2.00E-31 | 58.4 | F:transferase activity, transferring hexosyl groups; P:metabolic process; C:integral component of membrane |
| Efet.01.170794.g1007.t1 | cartilage-associated -like | 669 | 8.03E-24 | 45.6 | F:L-ascorbic acid binding; F:iron ion binding; F:oxidoreductase activity, acting on paired donors, with incorporation or reduction of molecular oxygen; F:oxidoreductase activity; P:oxidation-reduction process |
| Efet.01.170811.g1008.t1 | hypothetical protein CAPTEDRAFT_224948 | 387 | 7.57E-08 | 79 |  |
| Efet.01.170965.g1010.t1 | glutamate receptor kainate 2-like isoform X1 | 240 | 9.35E-19 | 74.15 | P:ion transport; F:transmembrane signaling receptor activity; F:extracellular ligand-gated ion channel activity; C:synapse; C:membrane part; C:plasma membrane |
| Efet.01.170966.g1011.t1 | protocadherin-9-like isoform X2 | 2787 | 1.53E-152 | 49.45 | F:calcium ion binding; C:membrane; C:integral component of membrane; P:cell adhesion; P:homophilic cell adhesion via plasma membrane adhesion molecules; C:plasma membrane |
| Efet.01.170997.g1013.t1 | small subunit processome component 20 homolog | 915 | 6.11E-39 | 55.6 | P:transport |
| Efet.01.17103.g1258.t1 | glutathione ABC transporter ATP-binding | 537 | 2.47E-98 | 90.45 | F:ATPase activity; F:ATP binding; P:peptide transport |
| Efet.01.17103.g1259.t1 | glutathione ABC transporter ATP-binding | 675 | 6.00E-93 | 97.4 | F:ATPase activity; F:ATP binding; P:peptide transport |
| Efet.01.171047.g1014.t1 | Krueppel-like factor 5 | 444 | 9.19E-31 | 88.7 | F:nucleic acid binding; F:metal ion binding |
| Efet.01.171120.g1017.t1 | tetraspanin 74f | 294 | 1.55E-23 | 59.3 | C:integral component of membrane |
| Efet.01.171145.g1019.t1 | actin-related 2 3 complex subunit 1A-like | 234 | 7.02E-15 | 69.8 | F:actin filament binding; P:Arp2/3 complex-mediated actin nucleation; C:cytoplasmic part; C:Arp2/3 protein complex |
| Efet.01.171146.g1020.t1 | lysosomal alpha-glucosidase-like | 420 | 2.04E-10 | 64.65 | P:metabolic process; F:hydrolase activity, acting on glycosyl bonds |
| Efet.01.171147.g1021.t1 | glutamate synthase [NADPH] isoform X1 | 345 | 2.92E-46 | 84.35 | P:glutamate biosynthetic process; F:FMN binding; F:iron ion binding; F:glutamate synthase (NADH) activity; F:flavin adenine dinucleotide binding; P:oxidation-reduction process; F:iron-sulfur cluster binding |
| Efet.01.17117.g1261.t1 | probable G- coupled receptor 139 | 1509 | 2.45E-120 | 62.35 | F:G-protein coupled receptor activity; P:G-protein coupled receptor signaling pathway; P:signal transduction; C:membrane; C:integral component of membrane; F:signal transducer activity |
| Efet.01.171183.g1024.t1 | sonic hedgehog | 1110 | 2.03E-94 | 65.2 | P:chordate embryonic development; P:tissue morphogenesis; P:neuron differentiation; P:cell development; P:epithelium development; C:extracellular region; P:regionalization; P:regulation of signal transduction; P:embryonic organ morphogenesis; P:heart development; P:eye development; P:forebrain development; F:metal ion binding; P:gliogenesis; P:muscle cell differentiation; P:tube morphogenesis; P:cell fate specification |
| Efet.01.171232.g1025.t1 | pro-interleukin-16 isoform X2 | 375 | 4.36E-22 | 64 | C:cytoplasm; C:intracellular membrane-bounded organelle |
| Efet.01.171243.g1026.t1 | NACHT domain- and WD repeat-containing 1-like isoform X1 | 3648 | 1.96E-54 | 47.55 | F:nucleotide binding; F:ATP binding |
| Efet.01.17128.g1262.t1 | Vacuolar sorting-associated 54 | 555 | 1.00E-38 | 62.5 | C:GARP complex; C:perinuclear region of cytoplasm; F:protein binding; P:lysosomal transport; P:retrograde transport, endosome to Golgi; P:single-organism process; C:trans-Golgi network membrane |
| Efet.01.171370.g1028.t1 | bromodomain adjacent to zinc finger domain 1A isoform X1 | 2385 | 1.71E-74 | 44.45 | F:zinc ion binding; F:metal ion binding; P:DNA-dependent DNA replication; C:CHRAC |
| Efet.01.17138.g1263.t1 | synaptotagmin- | 582 | 5.83E-49 | 67.3 | C:presynaptic membrane; C:synaptic vesicle; C:neuronal cell body; P:regulation of glucagon secretion; C:dendrite; F:phosphatidylinositol-4,5-bisphosphate binding; P:regulation of insulin secretion; C:early phagosome; F:phosphatidylserine binding; C:extracellular exosome; C:terminal bouton; F:calcium ion binding; P:short-term synaptic potentiation; P:calcium ion regulated lysosome exocytosis; P:vesicle-mediated cholesterol transport; P:regulation of dopamine secretion; P:synaptic vesicle recycling; F:syntaxin binding; P:phagosome-lysosome fusion; C:lysosome; F:calcium-dependent phospholipid binding; C:peroxisome; F:calmodulin binding; P:calcium ion-regulated exocytosis of neurotransmitter; C:transport vesicle membrane; P:plasma membrane repair; P:regulation of bone remodeling; F:clathrin binding; P:regulation of calcium ion-dependent exocytosis; P:regulation of phagocytosis |
| Efet.01.17140.g1264.t1 | neuromedin-U receptor 2-like isoform X2 | 375 | 9.73E-19 | 72.55 | F:G-protein coupled receptor activity; P:G-protein coupled receptor signaling pathway; C:membrane |
| Efet.01.171403.g1030.t1 | DNA repair RAD51 homolog 4 | 1014 | 6.12E-65 | 55.3 | F:nucleotide binding; P:DNA repair; F:DNA binding; F:ATP binding; F:DNA-dependent ATPase activity |
| Efet.01.171406.g1031.t1 | hypothetical protein HELRODRAFT_81691, partial | 462 | 7.33E-29 | 59.25 |  |
| Efet.01.171437.g1032.t1 | DUF3483 domain-containing | 651 | 1.15E-133 | 77.75 | C:integral component of membrane; F:iron-sulfur cluster binding |
| Efet.01.171446.g1033.t1 | Superoxide dismutase | 423 | 9.64E-42 | 67.5 | P:superoxide metabolic process; F:oxidoreductase activity |
| Efet.01.1715.g154.t1 | DNA-directed RNA polymerase II subunit RPB3 | 231 | 3.14E-28 | 82.65 | F:DNA-directed 5'-3' RNA polymerase activity; P:transcription, DNA-templated; F:protein dimerization activity |
| Efet.01.171552.g1036.t1 | small conductance calcium-activated potassium channel SK splice variant | 420 | 2.22E-42 | 84.3 | F:calmodulin binding; P:potassium ion transmembrane transport; F:photoreceptor activity; C:integral component of membrane; F:small conductance calcium-activated potassium channel activity; P:regulation of membrane potential in photoreceptor cell |
| Efet.01.171582.g1037.t1 | plasma membrane calcium-transporting ATPase 2 isoform X1 | 1194 | 0 | 89.85 | F:ATP binding; F:metal ion binding; C:integral component of membrane; P:calcium ion transmembrane transport; F:calcium-transporting ATPase activity |
| Efet.01.171632.g1042.t1 | alpha-tocopherol transfer -like | 429 | 2.89E-17 | 50.45 | P:transport; F:transporter activity; C:intracellular |
| Efet.01.171647.g1043.t1 | FMRFamide receptor | 1164 | 5.31E-52 | 50.6 | F:G-protein coupled receptor activity; P:G-protein coupled receptor signaling pathway; P:signal transduction; C:membrane; C:integral component of membrane; F:signal transducer activity |
| Efet.01.17167.g1265.t1 | homer homolog 2-like isoform X8 | 783 | 2.83E-20 | 64.5 | F:G-protein coupled glutamate receptor binding |
| Efet.01.171686.g1044.t1 | solute carrier family 25 member 47 | 501 | 1.90E-41 | 63.9 | C:mitochondrion; C:membrane; P:transport |
| Efet.01.171711.g1045.t1 | cartilage matrix -like isoform X2 | 657 | 1.22E-16 | 54.9 | F:calcium ion binding; C:collagen trimer; C:membrane; C:integral component of membrane; C:proteinaceous extracellular matrix |
| Efet.01.171729.g1046.t1 | potassium voltage-gated channel Shaw-like isoform X1 | 444 | 1.23E-64 | 68.7 | F:ion channel activity; P:ion transport; C:membrane |
| Efet.01.171738.g1047.t1 | cGMP-dependent kinase 1-like isoform X7 | 453 | 3.00E-06 | 62.83 |  |
| Efet.01.17176.g1267.t1 | DNA ligase 1-like isoform X8 | 891 | 3.69E-12 | 65.45 |  |
| Efet.01.171778.g1048.t1 | sarcoplasmic calcium-binding -like | 417 | 3.80E-16 | 58.05 | F:calcium ion binding |
| Efet.01.171826.g1050.t1 | probable indole-3-acetic acid-amido synthetase isoform X1 | 891 | 7.14E-70 | 54.35 | C:membrane; C:integral component of membrane |
| Efet.01.171839.g1051.t1 | plexin-a4 isoform x3 | 969 | 8.43E-76 | 53.55 | P:signal transduction; C:membrane; P:axon guidance |
| Efet.01.171865.g1052.t1 | oxysterol-binding 1 isoform X3 | 351 | 1.47E-34 | 88.95 | P:lipid transport |
| Efet.01.171920.g1053.t1 | B2 bradykinin receptor-like | 639 | 2.01E-09 | 50.55 | F:G-protein coupled receptor activity; P:G-protein coupled receptor signaling pathway; P:signal transduction; F:photoreceptor activity; C:membrane; C:integral component of membrane; P:protein-chromophore linkage; P:visual perception; F:signal transducer activity; P:phototransduction; P:response to stimulus |
| Efet.01.171964.g1054.t1 | hypothetical protein CAPTEDRAFT_193482 | 543 | 8.67E-12 | 59 |  |
| Efet.01.172042.g1056.t1 | E3 SUMO- ligase PIAS2 isoform X6 | 528 | 6.25E-30 | 68.2 | F:zinc ion binding; F:SUMO transferase activity |
| Efet.01.172060.g1058.t1 | double-strand-break repair rad21 homolog | 207 | 3.05E-20 | 68.1 | C:nuclear meiotic cohesin complex; P:atrioventricular valve morphogenesis; P:mitotic sister chromatid cohesion; P:rRNA transcription; P:blood circulation; F:sequence-specific DNA binding; F:chromatin binding; P:ventriculo bulbo valve morphogenesis; P:pharyngeal system development; P:double-strand break repair; P:regulation of transcription from RNA polymerase II promoter; P:translation; P:embryonic viscerocranium morphogenesis; P:positive regulation of cysteine-type endopeptidase activity involved in apoptotic process; P:enteric nervous system development; P:heart looping |
| Efet.01.172152.g1060.t1 | agrin-like isoform X3 | 222 | 5.60E-07 | 67.42 | F:calcium ion binding |
| Efet.01.172246.g1062.t1 | cullin-5-like isoform X1 | 351 | 6.99E-56 | 93.25 | C:nucleus; F:ubiquitin protein ligase activity; F:ubiquitin protein ligase binding; C:cytoplasm; F:protein heterodimerization activity; P:regulation of cytosolic calcium ion concentration; P:cerebral cortex radially oriented cell migration; P:radial glia guided migration of Purkinje cell; C:plasma membrane; C:Cul5-RING ubiquitin ligase complex; P:protein ubiquitination involved in ubiquitin-dependent protein catabolic process |
| Efet.01.172277.g1063.t1 | SH3 domain-containing kinase-binding 1-like isoform X1 | 318 | 1.40E-24 | 80.3 |  |
| Efet.01.172280.g1064.t1 | atrial natriuretic peptide receptor 1-like isoform X1 | 474 | 1.30E-38 | 62.45 | F:lyase activity; P:primary metabolic process; P:single-organism process; P:organic substance metabolic process; P:phosphate-containing compound metabolic process |
| Efet.01.172324.g1065.t1 | leucine-rich repeat lrrA-like | 2745 | 2.42E-179 | 60.05 |  |
| Efet.01.17235.g1272.t1 | exostosin-2 | 534 | 2.13E-70 | 74.15 | P:heparan sulfate proteoglycan biosynthetic process; P:single-multicellular organism process; P:cellular polysaccharide biosynthetic process; F:glucuronosyltransferase activity; C:integral component of membrane; P:glycosaminoglycan biosynthetic process; C:endoplasmic reticulum; F:protein dimerization activity; P:single-organism developmental process |
| Efet.01.172431.g1068.t1 | diacyglycerol O-acyltransferase Rv1760 isoform X2 | 852 | 1.04E-18 | 57.8 | F:transferase activity |
| Efet.01.172503.g1070.t1 | leucine-rich repeat and fibronectin type III domain-containing 1 | 1638 | 1.14E-139 | 55.8 | C:membrane |
| Efet.01.172508.g1071.t1 | ankyrin repeat | 843 | 8.74E-10 | 59 |  |
| Efet.01.172529.g1073.t1 | regulator of G- signaling 22 | 285 | 7.27E-09 | 61 |  |
| Efet.01.172552.g1074.t1 | gonadotropin-releasing hormone receptor-like isoform X2 | 552 | 2.11E-52 | 69.15 | F:G-protein coupled receptor activity; P:G-protein coupled receptor signaling pathway; P:signal transduction; C:membrane; C:integral component of membrane; F:signal transducer activity |
| Efet.01.172576.g1075.t1 | UDP-N-acetylglucosamine--dolichyl-phosphate N-acetylglucosaminephosphotransferase | 354 | 1.83E-33 | 77.1 | C:endoplasmic reticulum membrane; F:UDP-N-acetylglucosamine-dolichyl-phosphate N-acetylglucosaminephosphotransferase activity; F:UDP-N-acetylglucosamine-lysosomal-enzyme N-acetylglucosaminephosphotransferase activity; P:dolichol biosynthetic process; C:integral component of membrane; P:UDP-N-acetylglucosamine metabolic process; P:protein N-linked glycosylation; F:phospho-N-acetylmuramoyl-pentapeptide-transferase activity |
| Efet.01.172588.g1076.t1 | myosin light chain smooth muscle isoform X7 | 1071 | 3.24E-18 | 63.1 | P:bleb assembly; P:positive regulation of wound healing; C:cleavage furrow; P:positive regulation of calcium ion transport; P:positive regulation of defense response to virus by host; P:protein phosphorylation; P:xenophagy; F:myosin light chain kinase activity; P:aorta smooth muscle tissue morphogenesis; C:extracellular exosome; F:protein binding; P:smooth muscle contraction; P:cellular hypotonic response; C:stress fiber; C:cytosol; P:positive regulation of macromitophagy in response to mitochondrial depolarization; C:lamellipodium; P:positive regulation of cell migration |
| Efet.01.172629.g1079.t1 | enoyl- hydratase | 1005 | 1.96E-149 | 75.85 | P:metabolic process; F:lyase activity |
| Efet.01.172645.g1080.t1 | centriolin-like isoform X1 | 531 | 4.25E-36 | 64.8 | P:cell division; P:mitotic cell cycle |
| Efet.01.172665.g1082.t1 | leucine-rich repeat-containing 34-like isoform X1 | 804 | 7.25E-83 | 61.15 |  |
| Efet.01.17273.g1274.t1 | fas apoptotic inhibitory molecule 1 | 282 | 4.35E-26 | 79.4 | P:negative regulation of apoptotic process |
| Efet.01.172818.g1086.t1 | 4-aminobutyrate mitochondrial | 369 | 3.51E-33 | 66.9 | F:transferase activity |
| Efet.01.172827.g1087.t1 | 1-phosphatidylinositol 3-phosphate 5-kinase-like isoform X1 | 465 | 4.75E-41 | 68.85 | F:ATP binding; F:1-phosphatidylinositol-3-phosphate 5-kinase activity; F:phosphatidylinositol phosphate kinase activity; F:metal ion binding; P:phosphatidylinositol phosphorylation; P:phosphatidylinositol metabolic process; P:phosphorylation; F:transferase activity; F:kinase activity; P:intracellular signal transduction; C:intracellular |
| Efet.01.172860.g1089.t1 | potassium channel subfamily K member 1-like isoform X2 | 240 | 3.73E-07 | 70.67 |  |
| Efet.01.172896.g1090.t1 | synaptotagmin-7-like isoform X4 | 558 | 1.44E-87 | 75.5 | C:membrane; P:exocytosis |
| Efet.01.172932.g1092.t1 | catenin beta-like | 690 | 1.16E-66 | 66.85 | C:cytoskeleton; C:cytoplasm; P:signal transduction; C:membrane; C:integral component of membrane; P:cell adhesion; P:adherens junction assembly; F:signal transducer activity |
| Efet.01.172960.g1093.t1 | acid phosphatase type 7 | 279 | 2.44E-46 | 80.95 | F:metal ion binding; P:dephosphorylation; F:acid phosphatase activity |
| Efet.01.173015.g1097.t1 | polycystin-1-like isoform X2 | 744 | 7.46E-26 | 44.5 | F:calcium ion binding; C:membrane; C:integral component of membrane |
| Efet.01.173036.g1099.t1 | ADAMTS 1 isoform X1 | 795 | 1.84E-33 | 48.1 | F:zinc ion binding; P:integrin-mediated signaling pathway; P:proteolysis; F:peptidase activity; C:proteinaceous extracellular matrix; F:metallopeptidase activity |
| Efet.01.173113.g1101.t1 | glutathione S-transferase omega-1 | 306 | 8.96E-29 | 73.5 | P:positive regulation of homeostatic process; P:positive regulation of cellular process; C:cytoplasm; F:oxidoreductase activity; P:glutathione metabolic process; F:glutathione transferase activity; P:single-organism metabolic process; P:regulation of ryanodine-sensitive calcium-release channel activity; P:cellular response to chemical stimulus |
| Efet.01.173114.g1102.t1 | multiple C2 and transmembrane domain-containing 1-like | 330 | 3.11E-29 | 66.15 | F:calcium ion binding; C:membrane; C:integral component of membrane |
| Efet.01.173173.g1104.t1 | vacuolar sorting-associated 11 homolog | 369 | 3.12E-34 | 58.1 | F:binding; P:transport; P:cellular process |
| Efet.01.173182.g1105.t1 | alpha-2A adrenergic receptor-like | 1704 | 2.77E-70 | 74.3 | C:integral component of plasma membrane; P:peptidyl-tyrosine phosphorylation; F:non-membrane spanning protein tyrosine kinase activity; F:adrenergic receptor activity; P:adenylate cyclase-activating adrenergic receptor signaling pathway; P:regulation of muscle contraction; P:cell-cell signaling; P:regulation of heart contraction; P:regulation of blood vessel diameter; P:positive regulation of blood circulation; P:positive regulation of blood pressure by epinephrine-norepinephrine |
| Efet.01.173221.g1106.t1 | sodium-dependent glucose transporter 1-like | 1653 | 2.34E-11 | 47.71 | P:transmembrane transport; C:membrane; C:integral component of membrane |
| Efet.01.173257.g1108.t1 | Coiled-coil domain-containing 132 | 324 | 3.62E-12 | 58.2 | C:perinuclear region of cytoplasm; C:extracellular exosome; C:membrane; C:EARP complex; F:SNARE binding; P:retrograde transport, endosome to Golgi; P:endocytic recycling |
| Efet.01.173336.g1109.t1 | SWI SNF-related matrix-associated actin-dependent regulator of chromatin subfamily A containing DEAD H box 1-like | 291 | 3.77E-46 | 89.8 | F:ATP binding; F:protein complex scaffold; P:proteasome assembly |
| Efet.01.173358.g1110.t1 | zinc finger 33B-like | 432 | 1.21E-37 | 78.65 | F:nucleic acid binding; F:metal ion binding |
| Efet.01.173363.g1111.t1 | aspartate kinase | 1116 | 0 | 88.2 | F:aspartate kinase activity; P:lysine biosynthetic process via diaminopimelate; P:proline biosynthetic process; P:phosphorylation; F:amino acid binding |
| Efet.01.173363.g1112.t1 | integrase family | 243 | 6.15E-42 | 81.1 | F:DNA binding; P:DNA recombination; P:DNA integration |
| Efet.01.17339.g1278.t1 | sodium channel type 11 subunit alpha | 951 | 5.78E-20 | 77 | P:sodium ion transport; C:extracellular exosome; F:protein binding; P:ion transmembrane transport; P:regulation of ion transmembrane transport; C:voltage-gated sodium channel complex; C:C-fiber; P:membrane depolarization during action potential; F:voltage-gated sodium channel activity; P:regulation of sensory perception of pain; P:neuronal action potential |
| Efet.01.173411.g1113.t1 | Kyphoscoliosis peptidase | 1164 | 1.50E-87 | 47.5 |  |
| Efet.01.173443.g1115.t1 | Polypeptide N-acetylgalactosaminyltransferase | 426 | 1.26E-37 | 70.85 | C:membrane; C:integral component of membrane; F:transferase activity |
| Efet.01.173457.g1116.t1 | semaphorin-1A isoform X2 | 771 | 1.10E-101 | 54.3 | P:multicellular organism development; P:cellular process |
| Efet.01.173460.g1117.t1 | neuropeptides capa receptor-like | 288 | 2.29E-13 | 68.95 | F:G-protein coupled receptor activity; P:G-protein coupled receptor signaling pathway; C:membrane |
| Efet.01.173512.g1118.t1 | liprin-beta-1-like isoform X1 | 297 | 3.86E-30 | 74.05 |  |
| Efet.01.173550.g1119.t1 | polypeptide N-acetylgalactosaminyltransferase 13-like isoform X1 | 1860 | 1.99E-117 | 58.25 | F:transferase activity, transferring glycosyl groups; C:membrane; C:integral component of membrane; P:protein glycosylation; C:Golgi membrane; C:Golgi apparatus; F:transferase activity; F:carbohydrate binding |
| Efet.01.173701.g1123.t1 | vacuolar sorting-associated 13A-like | 537 | 8.77E-22 | 65.2 | C:membrane; C:integral component of membrane |
| Efet.01.173773.g1125.t1 | neuroligin- X-linked-like isoform X1 | 708 | 8.41E-47 | 56.65 | C:membrane; C:integral component of membrane |
| Efet.01.173813.g1127.t1 | Abhydrolase domain-containing | 306 | 2.50E-36 | 71.75 | F:hydrolase activity |
| Efet.01.173855.g1128.t1 | Wnt-11 precursor | 354 | 3.37E-18 | 62.8 | P:multicellular organism development; C:membrane; C:integral component of membrane; P:Wnt signaling pathway; C:extracellular region; F:receptor binding; C:proteinaceous extracellular matrix |
| Efet.01.173893.g1130.t1 | phosphatase 1D | 339 | 4.33E-21 | 72.15 | F:phosphoprotein phosphatase activity; F:cation binding |
| Efet.01.173930.g1131.t1 | hypothetical protein HELRODRAFT_189103 | 552 | 5.48E-11 | 50 |  |
| Efet.01.173939.g1132.t1 | germ cell-less -like 1 | 573 | 8.45E-32 | 61.15 | C:nuclear envelope; F:protein binding; C:nuclear matrix; P:regulation of transcription, DNA-templated; P:single-organism process; P:multicellular organismal process |
| Efet.01.173954.g1133.t1 | regeneration-upregulated 2 | 288 | 9.39E-12 | 70.5 | C:cytoskeleton |
| Efet.01.173958.g1134.t1 | acidic mammalian chitinase-like | 783 | 2.01E-70 | 74.95 | F:hydrolase activity, hydrolyzing O-glycosyl compounds; P:organic substance metabolic process |
| Efet.01.173963.g1136.t1 | multidrug resistance 1 | 201 | 4.86E-07 | 67.45 | F:nucleotide binding; F:ATPase activity; F:ATP binding; F:transferase activity, transferring glycosyl groups; F:ATPase activity, coupled to transmembrane movement of substances; P:transmembrane transport; C:membrane; C:integral component of membrane; P:transport |
| Efet.01.17400.g1281.t1 | synaptopodin 2 | 1347 | 1.39E-15 | 72.2 |  |
| Efet.01.17400.g1282.t1 | Synaptopodin 2 | 903 | 8.05E-42 | 46.85 |  |
| Efet.01.174021.g1137.t1 | Glioma pathogenesis-related 1 | 495 | 3.25E-21 | 53.1 | P:signal transduction; C:membrane |
| Efet.01.174166.g1138.t1 | aminopeptidase N | 354 | 3.10E-20 | 64.1 | F:zinc ion binding; P:proteolysis; C:membrane; C:integral component of membrane; F:aminopeptidase activity; F:metallopeptidase activity |
| Efet.01.174197.g1139.t1 | adhesive plaque matrix -like | 1953 | 4.36E-23 | 46 |  |
| Efet.01.174245.g1141.t1 | hypothetical protein CAPTEDRAFT_198413 | 519 | 6.50E-08 | 60 |  |
| Efet.01.174265.g1143.t1 | trunk-like isoform X3 | 891 | 9.42E-17 | 57.94 |  |
| Efet.01.17427.g1283.t1 | gem-associated 5-like | 639 | 4.25E-09 | 51.33 |  |
| Efet.01.174270.g1144.t1 | serine threonine- kinase tousled-like 2 isoform X1 | 483 | 4.61E-40 | 83.65 | F:ATP binding; F:protein serine/threonine kinase activity; P:protein phosphorylation |
| Efet.01.174458.g1152.t1 | retinoblastoma 1 | 255 | 3.51E-12 | 70.95 | C:nucleus; C:transcription factor complex; P:regulation of cell cycle; P:cell cycle; P:regulation of transcription from RNA polymerase II promoter |
| Efet.01.174460.g1153.t1 | hypothetical protein CAPTEDRAFT_220152 | 3120 | 5.48E-21 | 44 |  |
| Efet.01.174484.g1154.t1 | family methyltransferase | 546 | 9.06E-83 | 75.1 | F:methyltransferase activity; P:methylation |
| Efet.01.174549.g1155.t1 | ephrin type-B receptor 1-B isoform X6 | 282 | 1.83E-08 | 60.33 | P:phosphorylation; F:kinase activity |
| Efet.01.17457.g1284.t1 | methionine--tRNA cytoplasmic-like | 270 | 3.89E-36 | 91 | F:ATP binding; C:extracellular exosome; F:methionine-tRNA ligase activity; C:membrane; P:methionyl-tRNA aminoacylation; C:cytosol |
| Efet.01.174585.g1156.t1 | GTP-binding Rhes | 684 | 1.41E-51 | 55.7 | F:GTP binding; F:nucleotide binding; P:small GTPase mediated signal transduction; P:signal transduction; C:membrane; C:intracellular |
| Efet.01.174633.g1158.t1 | RNA-directed DNA polymerase from mobile element jockey-like | 375 | 4.63E-08 | 51.43 | F:RNA-directed DNA polymerase activity; P:RNA-dependent DNA biosynthetic process |
| Efet.01.174672.g1159.t1 | GTP-binding 2-like | 594 | 1.09E-66 | 73.15 | F:GTP binding; F:protein binding; F:translation elongation factor activity; C:intracellular membrane-bounded organelle; F:GTPase activity; P:translational elongation |
| Efet.01.174695.g1160.t1 | adenylate cyclase type 10-like | 1638 | 8.48E-122 | 56.3 | F:phosphorus-oxygen lyase activity; P:intracellular signal transduction; P:cyclic nucleotide biosynthetic process; C:intracellular |
| Efet.01.174699.g1161.t1 | sodium potassium ATPase alpha | 288 | 2.74E-52 | 90.9 | P:potassium ion transport; F:ATP binding; P:sodium ion transport; F:sodium:potassium-exchanging ATPase activity; F:metal ion binding; P:ATP hydrolysis coupled transmembrane transport; C:integral component of membrane; P:establishment or maintenance of transmembrane electrochemical gradient |
| Efet.01.174699.g1162.t1 | sodium potassium-transporting ATPase subunit alpha isoform X1 | 1341 | 0 | 91.7 | P:potassium ion transport; F:ATP binding; P:sodium ion transport; F:sodium:potassium-exchanging ATPase activity; F:metal ion binding; P:ATP hydrolysis coupled transmembrane transport; C:integral component of membrane; P:establishment or maintenance of transmembrane electrochemical gradient |
| Efet.01.17470.g1286.t1 | zinc finger RNA-binding -like isoform X3 | 297 | 1.48E-09 | 69.85 |  |
| Efet.01.17477.g1287.t1 | titin-like isoform X1 | 1233 | 2.74E-94 | 59.55 | F:ATP binding; F:protein kinase activity; P:protein phosphorylation |
| Efet.01.174815.g1165.t1 | E3 ubiquitin- ligase HECTD1 isoform X1 | 690 | 1.42E-82 | 77.7 | F:catalytic activity |
| Efet.01.174826.g1166.t1 | hypothetical protein CAPTEDRAFT_209261 | 348 | 7.62E-23 | 62.75 | C:membrane |
| Efet.01.174840.g1167.t1 | phytanoyl- hydroxylase-interacting -like | 687 | 2.90E-43 | 56.55 |  |
| Efet.01.17490.g1288.t1 | hypothetical protein CAPTEDRAFT_225483 | 282 | 3.92E-25 | 68.85 | P:cellular aromatic compound metabolic process; P:small molecule metabolic process; P:heterocycle metabolic process; P:organic cyclic compound metabolic process; F:catalytic activity; C:membrane; P:organonitrogen compound metabolic process; P:cellular nitrogen compound metabolic process; P:single-organism cellular process; F:binding; P:primary metabolic process |
| Efet.01.174915.g1168.t1 | BTB POZ domain-containing 7 isoform X1 | 828 | 6.60E-32 | 43.05 |  |
| Efet.01.174939.g1169.t1 | vesicular glutamate transporter | 240 | 3.24E-11 | 83.95 | P:transmembrane transport; C:integral component of membrane |
| Efet.01.17494.g1289.t1 | hypothetical protein HELRODRAFT_168783 | 366 | 3.87E-15 | 72 | F:binding |
| Efet.01.17494.g1290.t1 | Zinc finger | 1092 | 2.96E-35 | 47.25 | F:nucleic acid binding; F:metal ion binding |
| Efet.01.174955.g1170.t1 | oxysterol-binding -related 8-like | 459 | 7.14E-30 | 66.8 | P:lipid transport; C:integral component of membrane |
| Efet.01.175019.g1172.t1 | ubiquitin carboxyl-terminal hydrolase 24-like | 258 | 5.59E-24 | 72.15 | P:protein deubiquitination; F:thiol-dependent ubiquitinyl hydrolase activity; P:ubiquitin-dependent protein catabolic process |
| Efet.01.175039.g1173.t1 | 26S proteasome non-ATPase regulatory subunit | 459 | 6.26E-27 | 60.25 | C:proteasome accessory complex; P:proteasome assembly |
| Efet.01.175082.g1174.t1 | probable ATP-dependent RNA helicase DDX46 | 1284 | 0 | 86.9 | F:nucleic acid binding; F:ATP binding; F:helicase activity |
| Efet.01.175115.g1177.t1 | ATS16_HUMAN ame: Full=A disintegrin and metallo ase with thrombospondin motifs 16 Short=ADAM-TS 16 Short=ADAM-TS16 Short=ADAMTS-16 Flags: Precursor | 321 | 6.06E-07 | 49 | F:zinc ion binding; P:regulation of systemic arterial blood pressure; P:regulation of cilium assembly; F:peptidase activity; C:extracellular region; F:metalloendopeptidase activity; C:proteinaceous extracellular matrix; F:metallopeptidase activity; F:metal ion binding; P:branching involved in ureteric bud morphogenesis; P:proteolysis; C:extracellular matrix; F:hydrolase activity |
| Efet.01.175127.g1178.t1 | innexin 1 | 1005 | 1.38E-95 | 71 | C:membrane; P:cell communication by electrical coupling; C:cell junction; C:synapse; F:gap junction channel activity; P:transport |
| Efet.01.175172.g1179.t1 | D-glucuronyl C5-epimerase | 1092 | 1.51E-142 | 72.3 | P:sulfur compound biosynthetic process; F:racemase and epimerase activity, acting on carbohydrates and derivatives; C:membrane; P:glycosaminoglycan biosynthetic process; P:single-organism biosynthetic process; P:oxoacid metabolic process |
| Efet.01.175237.g1182.t1 | protein FAM161A-like | 870 | 4.98E-09 | 57.67 |  |
| Efet.01.175385.g1186.t1 | 39S ribosomal mitochondrial | 333 | 9.33E-28 | 75.15 | C:ribosome |
| Efet.01.175466.g1187.t1 | kinesin KIF20B isoform X1 | 447 | 1.98E-38 | 73.5 | F:ATP binding; F:microtubule motor activity; P:cytokinesis; C:Golgi apparatus; P:microtubule-based movement; C:microtubule; F:microtubule binding |
| Efet.01.175474.g1188.t1 | leukotriene A-4 hydrolase isoform X2 | 522 | 7.57E-35 | 67.65 | F:zinc ion binding; P:leukotriene biosynthetic process; P:peptide catabolic process; P:proteolysis; F:metalloaminopeptidase activity; F:peptide binding; C:plasma membrane; F:epoxide hydrolase activity |
| Efet.01.175532.g1192.t1 | transcription factor AP-4-like | 723 | 2.80E-20 | 55.15 | F:protein dimerization activity |
| Efet.01.175585.g1194.t1 | exportin-7-like isoform X2 | 537 | 1.49E-55 | 73.7 | P:intracellular protein transport; F:Ran GTPase binding; C:intracellular |
| Efet.01.17559.g1291.t1 | FH2 domain-containing 1-like | 2163 | 4.27E-11 | 71.6 | F:actin binding; P:cellular component organization |
| Efet.01.1756.g157.t1 | sterol O-acyltransferase 1-like isoform X2 | 555 | 3.74E-57 | 61.3 | F:transferase activity, transferring acyl groups; C:membrane |
| Efet.01.17560.g1292.t1 | arfaptin-1 isoform X1 | 705 | 3.84E-29 | 57.7 | F:protein domain specific binding |
| Efet.01.175621.g1195.t1 | F-box WD repeat-containing 1A-like isoform X1 | 330 | 3.81E-53 | 86 | P:SCF-dependent proteasomal ubiquitin-dependent protein catabolic process; F:protein dimerization activity; F:ligase activity |
| Efet.01.175628.g1196.t1 | epsin-2-like isoform X1 | 639 | 6.52E-103 | 94.05 | C:clathrin coat of endocytic vesicle; P:endocytosis |
| Efet.01.175657.g1197.t1 | sodium channel para isoform X13 | 321 | 2.45E-25 | 67.7 | F:ion channel activity; P:ion transport; P:transmembrane transport; C:membrane |
| Efet.01.175692.g1198.t1 | S-phase kinase-associated 1 | 384 | 1.14E-83 | 95.65 | P:phosphorylation; F:kinase activity; F:ligase activity; P:ubiquitin-dependent protein catabolic process |
| Efet.01.175693.g1199.t1 | LOW QUALITY PROTEIN: uncharacterized protein C19orf47-like | 423 | 1.46E-32 | 63.95 |  |
| Efet.01.17573.g1294.t1 | transcriptional enhancer factor TEF-1-like isoform X1 | 960 | 5.97E-26 | 47.25 | F:transcription factor activity, sequence-specific DNA binding; P:positive regulation of macromolecule metabolic process; F:transcription regulatory region DNA binding; P:regulation of transcription, DNA-templated; P:embryonic organ development; P:positive regulation of cellular metabolic process; C:intracellular part; P:tissue development; P:cellular process |
| Efet.01.17576.g1295.t1 | transmembrane 184B isoform X1 | 579 | 3.86E-49 | 59.3 | C:membrane; C:integral component of membrane |
| Efet.01.175771.g1201.t1 | protocadherin Fat 4 | 441 | 5.15E-55 | 61.15 | F:calcium ion binding; C:integral component of membrane; P:animal organ development; P:homophilic cell adhesion via plasma membrane adhesion molecules; C:plasma membrane; P:cellular process |
| Efet.01.175783.g1202.t1 | paramyosin-like isoform X3 | 429 | 2.48E-38 | 64.35 | C:myosin complex; F:motor activity |
| Efet.01.175832.g1203.t1 | metal tolerance 9-like | 315 | 5.02E-37 | 83.75 | P:response to zinc ion; P:regulation of sequestering of zinc ion; C:integral component of membrane; F:zinc ion transmembrane transporter activity; P:zinc II ion transmembrane transport |
| Efet.01.175874.g1204.t1 | dynein heavy chain axonemal-like isoform X2 | 453 | 3.17E-43 | 52.85 | F:microtubule motor activity; C:dynein complex; P:microtubule-based movement |
| Efet.01.175913.g1207.t1 | hypothetical protein HELRODRAFT_191437 | 900 | 9.71E-10 | 47 |  |
| Efet.01.175928.g1208.t1 | serine threonine- kinase D3-like isoform X5 | 537 | 1.02E-41 | 61.4 | F:kinase activity |
| Efet.01.17597.g1297.t1 | zinc finger 665-like | 1281 | 2.13E-85 | 57.65 | F:nucleic acid binding; F:metal ion binding |
| Efet.01.176.g20.t1 | SNF-related serine threonine- kinase-like | 372 | 5.79E-32 | 70.4 | F:nucleotide binding; C:nucleus; C:cytoplasm; F:protein serine/threonine kinase activity; P:protein phosphorylation; P:intracellular signal transduction |
| Efet.01.176072.g1210.t1 | hypothetical protein CAPTEDRAFT_205572 | 879 | 7.57E-68 | 61.5 | C:membrane; C:integral component of membrane; P:cell adhesion; P:tissue regeneration |
| Efet.01.176130.g1211.t1 | glutamate receptor 2 | 282 | 4.72E-07 | 69.5 | P:ion transport; F:transmembrane signaling receptor activity; F:extracellular ligand-gated ion channel activity; C:synapse; C:membrane part; C:plasma membrane |
| Efet.01.176160.g1215.t1 | histone-lysine N- H3 lysine-79 specific | 408 | 4.12E-06 | 63 | P:histone H3-K79 methylation; C:nucleus; C:membrane; F:histone methyltransferase activity (H3-K79 specific); C:integral component of membrane; F:histone-lysine N-methyltransferase activity; F:methyltransferase activity; P:regulation of cell cycle; F:transferase activity; P:methylation |
| Efet.01.176165.g1216.t1 | homeobox vab-7-like | 618 | 6.05E-12 | 57.83 | F:DNA binding; C:nucleus; P:regulation of transcription, DNA-templated; F:sequence-specific DNA binding |
| Efet.01.176249.g1220.t1 | vacuolar sorting-associated 13A-like | 483 | 3.83E-30 | 61.25 |  |
| Efet.01.176278.g1222.t1 | Titin | 1401 | 1.53E-107 | 49.6 | F:nucleotide binding; P:regulation of Rho protein signal transduction; F:ATP binding; C:membrane; C:integral component of membrane; F:protein kinase activity; F:Rho guanyl-nucleotide exchange factor activity; P:phosphorylation; F:transferase activity; P:protein phosphorylation; F:kinase activity; P:positive regulation of GTPase activity |
| Efet.01.176279.g1223.t1 | ABHD4 isoform X2 | 444 | 1.46E-51 | 70.8 | F:carboxylic ester hydrolase activity |
| Efet.01.176384.g1228.t1 | arylsulfatase B | 297 | 7.95E-13 | 55.25 | F:catalytic activity; F:sulfuric ester hydrolase activity; P:metabolic process |
| Efet.01.176388.g1229.t1 | peroxidasin homolog | 696 | 2.16E-48 | 54.85 | F:peroxidase activity; P:response to oxidative stress; C:extracellular space; C:extracellular exosome; P:extracellular matrix organization; P:cellular oxidant detoxification; P:oxidation-reduction process; C:endoplasmic reticulum; C:extracellular matrix; F:extracellular matrix structural constituent; F:heme binding; P:hydrogen peroxide catabolic process |
| Efet.01.17644.g1300.t1 | potassium channel subfamily K member 1-like isoform X2 | 744 | 2.92E-33 | 52.85 | C:integral component of plasma membrane; P:ion transport; P:potassium ion transmembrane transport; C:membrane; C:integral component of membrane; F:potassium channel activity; P:stabilization of membrane potential; P:transport; F:potassium ion leak channel activity |
| Efet.01.176441.g1230.t1 | sperm receptor for egg jelly-like | 504 | 2.01E-14 | 47.13 |  |
| Efet.01.176446.g1231.t1 | monocarboxylate transporter 13-like | 1152 | 7.36E-69 | 43.6 | C:integral component of plasma membrane; P:transmembrane transport; C:membrane; C:integral component of membrane; P:plasma membrane lactate transport; F:lactate transmembrane transporter activity |
| Efet.01.176511.g1232.t1 | peptidase inhibitor 16 | 258 | 2.94E-15 | 61.7 | C:extracellular exosome; C:extracellular region |
| Efet.01.176529.g1233.t1 | splicing factor U2AF 35 kDa subunit | 294 | 7.22E-32 | 68.7 | F:organic cyclic compound binding; F:heterocyclic compound binding |
| Efet.01.176534.g1234.t1 | universal stress A | 345 | 4.00E-14 | 77.75 | P:response to stress |
| Efet.01.17668.g1302.t1 | vacuolar sorting-associated 13C | 240 | 3.59E-18 | 76.55 |  |
| Efet.01.176741.g1238.t1 | zinc finger 420 isoform X5 | 1791 | 8.39E-101 | 55.3 | F:nucleic acid binding; F:metal ion binding; P:regulation of transcription, DNA-templated; C:intracellular |
| Efet.01.176760.g1239.t1 | family transcriptional regulator | 501 | 8.64E-113 | 70.1 | F:DNA binding; P:transcription, DNA-templated; P:regulation of transcription, DNA-templated |
| Efet.01.176760.g1240.t1 | transferase | 1197 | 0 | 82.25 | F:bile-acid-CoA hydrolase activity; F:formyl-CoA transferase activity; F:isomerase activity |
| Efet.01.176760.g1241.t1 | acyl- dehydrogenase | 1149 | 0 | 78.55 | F:acyl-CoA dehydrogenase activity; F:flavin adenine dinucleotide binding; P:oxidation-reduction process |
| Efet.01.176760.g1242.t1 | tripartite tricarboxylate transporter substrate binding | 969 | 0 | 67.3 | C:outer membrane-bounded periplasmic space |
| Efet.01.176760.g1243.t1 | tripartite tricarboxylate transporter substrate binding | 684 | 1.59E-117 | 71.5 | C:outer membrane-bounded periplasmic space |
| Efet.01.176760.g1244.t1 | acyl- dehydrogenase | 1659 | 0 | 84.75 | F:acyl-CoA dehydrogenase activity; F:zinc ion binding; F:flavin adenine dinucleotide binding; F:butyryl-CoA dehydrogenase activity; P:oxidation-reduction process |
| Efet.01.176760.g1245.t1 | sulfite exporter family | 819 | 3.60E-123 | 70.35 | C:integral component of membrane; C:plasma membrane |
| Efet.01.176760.g1246.t1 | tripartite tricarboxylate transporter substrate binding | 1002 | 0 | 78.15 | C:outer membrane-bounded periplasmic space |
| Efet.01.176760.g1247.t1 | family transcriptional regulator | 888 | 0 | 84.9 | F:DNA binding; F:transcription factor activity, sequence-specific DNA binding; P:transcription, DNA-templated; P:regulation of transcription, DNA-templated |
| Efet.01.176760.g1248.t1 | succinate dehydrogenase fumarate reductase flavo subunit | 3138 | 0 | 87.5 | P:oxidation-reduction process; F:succinate dehydrogenase activity |
| Efet.01.176760.g1249.t1 | succinate dehydrogenase | 297 | 2.18E-40 | 81.7 | F:oxidoreductase activity, acting on the CH-CH group of donors; C:integral component of membrane; P:oxidation-reduction process |
| Efet.01.176760.g1250.t1 | succinate dehydrogenase fumarate reductase iron-sulfur subunit | 438 | 4.10E-103 | 93.75 | F:succinate dehydrogenase (ubiquinone) activity; F:metal ion binding; F:electron carrier activity; F:3 iron, 4 sulfur cluster binding; P:tricarboxylic acid cycle; F:4 iron, 4 sulfur cluster binding; F:2 iron, 2 sulfur cluster binding |
| Efet.01.176795.g1251.t1 | Glycine oxidase | 825 | 1.14E-124 | 78.95 | F:D-amino-acid dehydrogenase activity; P:oxidation-reduction process; F:glycine oxidase activity |
| Efet.01.176795.g1252.t1 | family transcriptional regulator | 591 | 4.07E-133 | 82.95 | F:DNA binding; P:transcription, DNA-templated; P:regulation of transcription, DNA-templated |
| Efet.01.176795.g1253.t1 | Uncharacterised | 1017 | 1.54E-161 | 65.55 |  |
| Efet.01.176795.g1254.t1 | family transcriptional regulator | 984 | 0 | 61.1 | F:transcription factor activity, sequence-specific DNA binding; P:transcription, DNA-templated; P:regulation of transcription, DNA-templated; F:sequence-specific DNA binding |
| Efet.01.176795.g1255.t1 | ribosomal subunit interface | 1236 | 0 | 87.9 | P:aromatic compound catabolic process; F:iron ion binding; F:oxidoreductase activity, acting on paired donors, with incorporation or reduction of molecular oxygen, NAD(P)H as one donor, and incorporation of two atoms of oxygen into one donor; P:oxidation-reduction process; F:2 iron, 2 sulfur cluster binding |
| Efet.01.176795.g1256.t1 | Isochorismatase family yecD | 786 | 7.11E-121 | 77.3 | P:metabolic process; F:hydrolase activity |
| Efet.01.176795.g1257.t1 | NAD(P)-dependent oxidoreductase | 636 | 3.95E-109 | 79 | F:oxidoreductase activity; P:oxidation-reduction process |
| Efet.01.176806.g1258.t1 | kinesin KIF23 | 459 | 1.89E-51 | 73.1 | F:ATP binding; F:microtubule motor activity; P:plus-end-directed vesicle transport along microtubule; P:actomyosin contractile ring assembly; C:microtubule; F:microtubule binding |
| Efet.01.176840.g1259.t1 | programmed cell death 4-like | 408 | 1.20E-21 | 71.5 |  |
| Efet.01.176877.g1260.t1 | calcitonin gene-related peptide type 1 receptor-like | 474 | 9.05E-20 | 45.05 | F:G-protein coupled receptor activity; F:transmembrane signaling receptor activity; P:G-protein coupled receptor signaling pathway; P:signal transduction; F:calcitonin receptor activity; C:membrane; C:integral component of membrane; P:cell surface receptor signaling pathway; F:signal transducer activity |
| Efet.01.176888.g1261.t1 | dynein intermediate chain axonemal isoform X1 | 204 | 2.40E-12 | 68.25 | C:outer dynein arm; F:microtubule motor activity; F:protein binding; P:outer dynein arm assembly; P:cilium movement; C:sperm flagellum; P:determination of left/right symmetry |
| Efet.01.176903.g1262.t1 | cold shock domain-containing C2-like | 492 | 5.80E-38 | 71.6 | F:nucleic acid binding |
| Efet.01.176913.g1263.t1 | hypothetical protein CAPTEDRAFT_212909 | 765 | 9.40E-29 | 73.5 |  |
| Efet.01.176914.g1264.t1 | hypothetical protein CAPTEDRAFT_212909 | 741 | 7.51E-29 | 73.5 |  |
| Efet.01.176923.g1265.t1 | methylmalonyl- mitochondrial-like | 534 | 1.36E-67 | 81.3 | F:metal ion binding; F:cobalamin binding; C:mitochondrion; P:metabolic process; F:methylmalonyl-CoA mutase activity |
| Efet.01.176942.g1266.t1 | Forkhead box B1 | 870 | 7.31E-63 | 61.4 | F:DNA binding |
| Efet.01.177024.g1267.t1 | hypothetical protein CAPTEDRAFT_219883 | 288 | 3.52E-20 | 75.15 | F:ubiquitin-protein transferase activity; F:zinc ion binding; P:protein ubiquitination |
| Efet.01.17707.g1305.t1 | interferon regulatory factor 2-binding -like | 2001 | 2.44E-87 | 71.8 | P:regulation of transcription from RNA polymerase II promoter |
| Efet.01.177133.g1274.t1 | T family of potassium channels 7-like | 660 | 2.85E-40 | 74 | P:potassium ion transmembrane transport; C:integral component of membrane; F:potassium channel activity |
| Efet.01.177191.g1276.t1 | Chaoptin | 1026 | 1.69E-11 | 48.95 | F:G-protein coupled receptor activity; P:G-protein coupled receptor signaling pathway; P:signal transduction; C:membrane; C:integral component of membrane; P:adenylate cyclase-modulating G-protein coupled receptor signaling pathway; C:plasma membrane |
| Efet.01.177234.g1279.t1 | monoglycylase TTLL8 isoform X14 | 273 | 1.56E-23 | 75.9 | P:cellular protein modification process |
| Efet.01.177285.g1283.t1 | neuronal acetylcholine receptor subunit alpha-7-like isoform X1 | 210 | 9.63E-32 | 85.65 | C:postsynaptic membrane; F:calcium ion binding; P:cation transmembrane transport; C:integral component of membrane; C:cell junction; F:extracellular ligand-gated ion channel activity |
| Efet.01.177294.g1284.t1 | muscarinic acetylcholine receptor M1-like | 2199 | 3.30E-115 | 71.35 | C:integral component of plasma membrane; F:G-protein coupled acetylcholine receptor activity; P:G-protein coupled acetylcholine receptor signaling pathway |
| Efet.01.177344.g1286.t1 | von Willebrand factor type EGF and pentraxin domain-containing 1-like | 279 | 4.72E-15 | 58.75 | C:collagen trimer; F:calcium ion binding |
| Efet.01.177375.g1287.t1 | family | 600 | 1.21E-13 | 64.4 | C:membrane; C:integral component of membrane; F:transferase activity |
| Efet.01.177423.g1288.t1 | neuropeptide SIFamide receptor | 498 | 6.29E-24 | 70.3 | P:neuropeptide signaling pathway; C:integral component of membrane; F:neuropeptide Y receptor activity |
| Efet.01.177429.g1289.t1 | CDK5 regulatory subunit-associated 3-like | 411 | 3.02E-35 | 67.55 | P:regulation of cell cycle |
| Efet.01.177438.g1290.t1 | probable phosphorylase b kinase regulatory subunit alpha | 801 | 2.66E-122 | 82.7 | F:calmodulin binding; P:glycogen metabolic process; F:catalytic activity |
| Efet.01.17744.g1309.t1 | 1-phosphatidylinositol 4,5-bisphosphate phosphodiesterase classes I and II isoform X1 | 408 | 2.86E-57 | 93 | F:phosphatidylinositol phospholipase C activity; F:calcium ion binding; P:lipid catabolic process; F:signal transducer activity; C:intracellular; P:intracellular signal transduction |
| Efet.01.177474.g1291.t1 | G- coupled receptor 54-like | 1185 | 1.26E-73 | 59.95 | F:G-protein coupled receptor activity; P:G-protein coupled receptor signaling pathway; C:integral component of membrane |
| Efet.01.177493.g1292.t1 | hypothetical protein CAPTEDRAFT_228278 | 363 | 4.93E-55 | 67.6 |  |
| Efet.01.17754.g1310.t1 | eukaryotic translation initiation factor 4 gamma 2-like | 594 | 1.16E-47 | 67.7 | F:RNA binding |
| Efet.01.177543.g1293.t1 | bactericidal permeability increasing | 384 | 1.32E-09 | 53.4 | C:extracellular space; F:lipid binding |
| Efet.01.177614.g1294.t1 | FMRFamide receptor-like | 1020 | 7.57E-104 | 57 | F:G-protein coupled receptor activity; F:G-protein coupled peptide receptor activity; P:G-protein coupled receptor signaling pathway; C:membrane; C:integral component of membrane |
| Efet.01.177621.g1295.t1 | hypothetical protein CAPTEDRAFT_187679, partial | 837 | 6.61E-10 | 82 |  |
| Efet.01.177666.g1296.t1 | NAD kinase | 510 | 5.12E-77 | 92.8 | F:NAD+ kinase activity; F:ATP binding; C:cytoplasm; F:metal ion binding; P:NAD metabolic process; P:phosphorylation; P:NADP biosynthetic process |
| Efet.01.177689.g1297.t1 | spectrin beta chain-like isoform X1 | 516 | 2.75E-71 | 85.3 | F:actin binding; F:structural constituent of cytoskeleton; F:phospholipid binding; C:spectrin |
| Efet.01.177769.g1300.t1 | glycogen-binding subunit 76A-like | 534 | 2.46E-26 | 62.05 |  |
| Efet.01.177775.g1302.t1 | AChain Structural Determinants For Improved Thermal Stability Of Designed Ankyrin Repeat s With A Redesigned C- Capping | 744 | 4.39E-28 | 55.5 | F:ATP binding; F:protein kinase activity; P:phosphorylation; P:protein phosphorylation; F:kinase activity |
| Efet.01.177805.g1304.t1 | myosin regulatory light chain smooth muscle minor isoform | 384 | 1.92E-57 | 82.15 | F:calcium ion binding |
| Efet.01.17785.g1312.t1 | nucleolar pre-ribosomal-associated 1-like | 366 | 4.51E-12 | 62.33 |  |
| Efet.01.177893.g1307.t1 | 5-hydroxytryptamine receptor 2C-like | 612 | 1.73E-16 | 69.35 | P:G-protein coupled receptor signaling pathway; C:integral component of membrane; P:serotonin receptor signaling pathway; F:G-protein coupled serotonin receptor activity |
| Efet.01.177907.g1308.t1 | dynein assembly factor axonemal-like | 981 | 2.54E-111 | 71.7 |  |
| Efet.01.177912.g1309.t1 | general transcription factor IIF subunit 1-like | 207 | 3.75E-15 | 86.4 | P:positive regulation of transcription elongation from RNA polymerase II promoter; C:nucleus; F:DNA binding; F:catalytic activity; P:transcription initiation from RNA polymerase II promoter |
| Efet.01.178.g21.t1 | Probable ATP-dependent RNA helicase pitchoune | 246 | 2.10E-30 | 80.55 | F:ATP binding; F:RNA binding; P:RNA secondary structure unwinding; F:ATP-dependent RNA helicase activity |
| Efet.01.178010.g1313.t1 | rho guanine nucleotide exchange factor 12-like | 414 | 3.12E-19 | 64.5 | P:biological regulation |
| Efet.01.178091.g1314.t1 | glycine receptor subunit alphaZ1-like isoform X1 | 345 | 5.92E-24 | 61.05 | C:membrane; P:transport |
| Efet.01.178125.g1315.t1 | transcription factor HES-1-like isoform X2 | 909 | 2.66E-15 | 59.65 | F:DNA binding; C:nucleus; P:transcription, DNA-templated; P:regulation of transcription, DNA-templated; F:protein dimerization activity |
| Efet.01.178149.g1316.t1 | NADH dehydrogenase [ubiquinone] iron-sulfur 5 | 351 | 1.21E-29 | 52.55 |  |
| Efet.01.178168.g1317.t1 | 4,5-DOPA dioxygenase extradiol-like | 273 | 3.16E-15 | 57.56 | P:cellular aromatic compound metabolic process; F:oxidoreductase activity, acting on single donors with incorporation of molecular oxygen; F:zinc ion binding; F:oxidoreductase activity; F:metal ion binding; F:ferrous iron binding; P:oxidation-reduction process |
| Efet.01.178171.g1318.t1 | hypothetical protein HELRODRAFT_190915 | 309 | 4.55E-07 | 68 | C:cytoskeleton |
| Efet.01.178192.g1319.t1 | short transient receptor potential channel 6 | 672 | 7.88E-15 | 64.45 | P:transport |
| Efet.01.178213.g1320.t1 | sex peptide receptor-like | 1125 | 1.85E-132 | 65.4 | F:G-protein coupled peptide receptor activity; P:G-protein coupled receptor signaling pathway; C:integral component of membrane |
| Efet.01.178337.g1326.t1 | CCR4-NOT transcription complex subunit 1-like | 513 | 1.02E-44 | 76.75 | P:gene silencing by miRNA; C:P-body; P:positive regulation of nuclear-transcribed mRNA poly(A) tail shortening; F:retinoic acid receptor binding; P:nuclear-transcribed mRNA poly(A) tail shortening; F:RNA binding; C:peroxisomal membrane; F:poly(A)-specific ribonuclease activity; P:negative regulation of transcription from RNA polymerase II promoter; F:protein complex scaffold; C:extracellular space; P:DNA damage response, signal transduction by p53 class mediator resulting in cell cycle arrest; P:positive regulation of cytoplasmic mRNA processing body assembly; P:negative regulation of retinoic acid receptor signaling pathway; F:armadillo repeat domain binding; P:regulation of stem cell population maintenance; C:cytosol; P:negative regulation of intracellular estrogen receptor signaling pathway; F:estrogen receptor binding; C:CCR4-NOT core complex |
| Efet.01.178385.g1327.t1 | GTP-binding Rhes-like | 300 | 6.34E-32 | 89.25 | F:GTP binding; P:small GTPase mediated signal transduction; C:membrane; C:intracellular |
| Efet.01.178394.g1328.t1 | roundabout homolog 2-like | 414 | 6.22E-39 | 70.8 | P:single-organism process |
| Efet.01.178399.g1329.t1 | potassium voltage-gated channel subfamily KQT member 4-like isoform X1 | 621 | 1.88E-13 | 73.65 | F:voltage-gated potassium channel activity; P:potassium ion transmembrane transport; C:voltage-gated potassium channel complex |
| Efet.01.178419.g1330.t1 | TNF receptor-associated factor 2 isoform X2 | 252 | 3.62E-11 | 67.25 | P:positive regulation of molecular function; C:cytoplasm; F:metal ion binding; P:signal transduction; P:positive regulation of protein phosphorylation; C:protein complex; P:positive regulation of intracellular signal transduction; C:membrane part; F:enzyme binding; P:protein ubiquitination |
| Efet.01.178434.g1331.t1 | inverted formin-2-like isoform X1 | 1395 | 1.65E-28 | 65.55 | F:protein binding; P:cellular component organization |
| Efet.01.178477.g1332.t1 | sperm receptor for egg jelly-like | 840 | 1.28E-10 | 45 |  |
| Efet.01.178576.g1335.t1 | potassium voltage-gated channel subfamily H member 6 isoform X2 | 1062 | 2.25E-129 | 81.65 | C:integral component of plasma membrane; F:voltage-gated potassium channel activity; P:regulation of membrane potential; P:potassium ion transmembrane transport; P:regulation of ion transmembrane transport |
| Efet.01.178646.g1337.t1 | neuron navigator 2- | 846 | 7.74E-68 | 81.45 | P:sensory perception of sound; C:interstitial matrix; P:locomotory behavior; P:sensory perception of smell; F:heparin binding; P:glossopharyngeal nerve development; P:regulation of systemic arterial blood pressure by baroreceptor feedback; P:vagus nerve development; C:nucleoplasm; P:optic nerve development |
| Efet.01.178670.g1338.t1 | RISC-loading complex subunit tarbp2-like | 246 | 3.41E-20 | 72.25 | F:RNA binding; P:gene silencing by miRNA; P:production of small RNA involved in gene silencing by RNA |
| Efet.01.178681.g1339.t1 | sodium hydrogen exchanger 3-like isoform X3 | 396 | 4.83E-06 | 54.43 |  |
| Efet.01.178702.g1341.t1 | _reg-like domain-containing | 951 | 0 | 46.75 |  |
| Efet.01.178851.g1344.t1 | unc-93 homolog A-like | 1209 | 4.45E-72 | 60.7 | C:membrane |
| Efet.01.17892.g1318.t1 | Rho GTPase-activating 20 | 771 | 5.92E-72 | 71.25 | P:signal transduction |
| Efet.01.178996.g1348.t1 | glycerol-3-phosphate acyltransferase 3-like | 546 | 1.85E-87 | 68.1 | C:endoplasmic reticulum membrane; C:integral component of membrane; P:triglyceride biosynthetic process; F:glycerol-3-phosphate O-acyltransferase activity; P:regulation of TOR signaling |
| Efet.01.17903.g1319.t1 | major vault -like | 675 | 5.21E-118 | 82.7 |  |
| Efet.01.179044.g1350.t1 | dopey-1-like isoform X1 | 477 | 5.80E-35 | 65 |  |
| Efet.01.179044.g1351.t1 | dopey-1-like isoform X1 | 1761 | 2.60E-41 | 72.15 |  |
| Efet.01.179047.g1352.t1 | ectopic P granules 5 homolog | 360 | 1.79E-18 | 62.95 | P:autophagy |
| Efet.01.179116.g1353.t1 | Phosphatidylinositol-4-phosphate 5-kinase type-1 alpha | 216 | 1.97E-31 | 87.5 | F:phosphatidylinositol phosphate kinase activity; P:phosphatidylinositol phosphorylation |
| Efet.01.179256.g1356.t1 | calcium uniporter mitochondrial isoform X2 | 282 | 5.51E-19 | 74.65 | C:calcium channel complex; P:regulation of cellular process; F:transmembrane transporter activity; P:chemical homeostasis; C:mitochondrial membrane part; P:single-organism process; C:mitochondrial inner membrane; P:mitochondrial calcium ion transport; P:cellular process |
| Efet.01.179258.g1357.t1 | neuralized-like isoform X1 | 480 | 1.74E-17 | 75.3 | F:zinc ion binding |
| Efet.01.179398.g1358.t1 | dystroglycan-like | 1854 | 2.39E-48 | 45.2 | F:calcium ion binding; C:membrane; C:integral component of membrane; C:dystrophin-associated glycoprotein complex; P:cytoskeletal anchoring at plasma membrane |
| Efet.01.179410.g1359.t1 | high affinity cAMP-specific and IBMX-insensitive 3 ,5 -cyclic phosphodiesterase 8B-like isoform X5 | 222 | 8.62E-16 | 82.1 | F:metal ion binding; P:signal transduction; F:3',5'-cyclic-nucleotide phosphodiesterase activity |
| Efet.01.179434.g1361.t1 | axin-1-like | 1008 | 1.39E-61 | 56.1 | C:beta-catenin destruction complex; P:pseudouridine synthesis; F:RNA binding; P:signal transduction; P:RNA modification; F:pseudouridine synthase activity; F:receptor signaling complex scaffold activity |
| Efet.01.179440.g1362.t1 | calmodulin 5 | 225 | 4.60E-09 | 77 | C:extracellular exosome; F:calcium ion binding |
| Efet.01.179448.g1363.t1 | proto-oncogene tyrosine- kinase ROS isoform X3 | 630 | 4.64E-07 | 48.81 | C:membrane; C:integral component of membrane |
| Efet.01.179480.g1364.t1 | peptidyl-prolyl cis-trans isomerase FKBP2 | 282 | 4.41E-47 | 91.75 | C:endoplasmic reticulum membrane; F:peptidyl-prolyl cis-trans isomerase activity; F:FK506 binding; P:chaperone-mediated protein folding; P:protein peptidyl-prolyl isomerization |
| Efet.01.179484.g1365.t1 | hypothetical protein CAPTEDRAFT_222844 | 1629 | 1.45E-19 | 44 | F:metal ion binding |
| Efet.01.179494.g1366.t1 | dynein heavy chain axonemal | 348 | 1.90E-47 | 90.35 | F:ATPase activity; F:ATP binding; F:microtubule motor activity; C:dynein complex; P:microtubule-based movement |
| Efet.01.179495.g1367.t1 | dopamine D2-like receptor | 285 | 3.18E-33 | 79.1 | C:integral component of plasma membrane; F:dopamine neurotransmitter receptor activity; P:dopamine receptor signaling pathway |
| Efet.01.179505.g1369.t1 | major facilitator superfamily domain-containing 4A-like isoform X1 | 282 | 2.37E-18 | 66.55 | C:membrane |
| Efet.01.179511.g1370.t1 | interleukin enhancer-binding factor 2 | 327 | 2.63E-25 | 71.35 | F:RNA binding; C:nuclear lumen |
| Efet.01.17952.g1321.t1 | RNA-binding 12 | 1884 | 5.45E-40 | 70.05 | F:nucleotide binding; F:nucleic acid binding |
| Efet.01.179539.g1371.t1 | uncharacterized protein KIAA1109 isoform X11 | 1119 | 4.56E-76 | 65 |  |
| Efet.01.179549.g1373.t1 | neuronal acetylcholine receptor subunit alpha-2-like | 1161 | 1.21E-129 | 68.85 | C:postsynaptic membrane; P:cation transmembrane transport; C:integral component of membrane; C:cell junction; F:extracellular ligand-gated ion channel activity |
| Efet.01.179556.g1375.t1 | pyrokinin-1 receptor-like isoform X2 | 480 | 5.39E-54 | 73.75 | P:neuropeptide signaling pathway; C:integral component of membrane; F:neuromedin U receptor activity |
| Efet.01.179564.g1376.t1 | receptor-type tyrosine- phosphatase F isoform X1 | 375 | 3.08E-19 | 63.5 |  |
| Efet.01.179580.g1377.t1 | Heat shock 70 kDa | 1005 | 1.36E-47 | 88.1 |  |
| Efet.01.179606.g1378.t1 | potassium voltage-gated channel Shal-like | 906 | 1.46E-131 | 81.1 | P:potassium ion transmembrane transport; C:voltage-gated potassium channel complex; F:delayed rectifier potassium channel activity; F:A-type (transient outward) potassium channel activity; P:regulation of ion transmembrane transport; P:protein homooligomerization |
| Efet.01.17962.g1324.t1 | regulator of G- signaling | 225 | 6.63E-37 | 95.7 | C:heterotrimeric G-protein complex; P:G-protein coupled receptor signaling pathway; F:signal transducer activity; P:intracellular signal transduction |
| Efet.01.179622.g1380.t1 | ecotropic viral integration site 5 ortholog-like isoform X1 | 399 | 5.69E-20 | 65.15 | F:zinc ion binding; F:metal ion binding; F:GTPase activator activity; P:regulation of GTPase activity; P:positive regulation of GTPase activity; C:intracellular |
| Efet.01.17966.g1325.t1 | GTP-binding 1 | 279 | 8.57E-43 | 93.55 | F:GTP binding; F:GTPase activity |
| Efet.01.179693.g1382.t1 | transmembrane 145 | 744 | 1.78E-30 | 59 |  |
| Efet.01.179721.g1384.t1 | tyrosine- kinase Srms | 216 | 4.30E-13 | 63.2 | F:protein tyrosine kinase activity; F:protein binding; P:negative regulation of signal transduction; C:cytosol; P:peptidyl-tyrosine autophosphorylation; P:single-organism process |
| Efet.01.179724.g1385.t1 | transient receptor potential cation channel subfamily M member 2-like isoform X1 | 390 | 2.24E-35 | 64.85 | P:transport |
| Efet.01.179730.g1386.t1 | hypothetical protein HELRODRAFT_194738 | 1716 | 8.12E-07 | 73 | F:nucleotide binding |
| Efet.01.179781.g1389.t1 | SWI SNF-related matrix-associated actin-dependent regulator of chromatin subfamily A member 5 | 243 | 2.63E-11 | 84.15 | F:ATPase activity; F:DNA binding; F:ATP binding; P:retina development in camera-type eye; F:nucleosome binding; P:ATP-dependent chromatin remodeling; P:cardiac ventricle development; C:NURF complex |
| Efet.01.179794.g1390.t1 | DNA replication licensing factor mcm5 | 288 | 3.55E-12 | 75.9 | F:DNA replication origin binding; F:ATP binding; P:DNA replication initiation; F:DNA helicase activity; P:DNA duplex unwinding; F:heme oxygenase (decyclizing) activity; C:integral component of membrane; F:chromatin binding; C:MCM complex; P:heme oxidation; C:nuclear chromosome, telomeric region; C:nucleoplasm |
| Efet.01.179797.g1391.t1 | SPRY domain-containing SOCS box 3 | 867 | 1.22E-94 | 72 | P:intracellular signal transduction; C:intracellular |
| Efet.01.179862.g1392.t1 | dual oxidase 2-like isoform X1 | 201 | 1.27E-16 | 67.8 | F:oxidoreductase activity; C:membrane; F:binding; P:response to stimulus |
| Efet.01.179914.g1394.t1 | hypothetical protein CAPTEDRAFT_217222 | 234 | 5.14E-22 | 69.2 | C:membrane; F:carbohydrate binding |
| Efet.01.17999.g1326.t1 | E3 SUMO- ligase 2 | 1683 | 5.88E-38 | 66.55 | F:catalytic activity; F:metal ion binding |
| Efet.01.179995.g1395.t1 | neuropeptide CCHamide-1 receptor-like | 1014 | 1.16E-78 | 59.25 | F:allatostatin receptor activity; C:integral component of plasma membrane; P:neuropeptide signaling pathway; F:neuropeptide receptor activity |
| Efet.01.1800.g160.t1 | 60S ribosomal L5 | 381 | 1.02E-71 | 87.85 | F:structural constituent of ribosome; F:5S rRNA binding; C:cytosolic large ribosomal subunit; P:ribosomal large subunit assembly; P:translation |
| Efet.01.18001.g1327.t1 | beta-hexosaminidase subunit beta isoform X3 | 921 | 5.29E-122 | 57.5 | P:metabolic process; F:hydrolase activity, hydrolyzing O-glycosyl compounds |
| Efet.01.180013.g1396.t1 | RNA-directed DNA polymerase from mobile element jockey- | 267 | 7.95E-07 | 54 |  |
| Efet.01.180017.g1397.t1 | phosphofurin acidic cluster sorting 2-like isoform X10 | 564 | 1.03E-43 | 76 |  |
| Efet.01.180028.g1398.t1 | kinesin unc-104 isoform X1 | 474 | 1.88E-42 | 74.95 | F:ATP binding; F:microtubule motor activity; P:microtubule-based movement; F:microtubule binding |
| Efet.01.180054.g1399.t1 | hypothetical protein CAPTEDRAFT_228921 | 753 | 1.70E-54 | 52.8 | C:membrane; C:integral component of membrane; F:carbohydrate binding |
| Efet.01.180054.g1401.t1 | hypothetical protein CAPTEDRAFT_225872 | 402 | 2.19E-22 | 75 | C:membrane |
| Efet.01.180060.g1402.t1 | C3 and PZP-like alpha-2-macroglobulin domain-containing 8 | 768 | 3.10E-32 | 45.95 | C:membrane; C:integral component of membrane; F:methyltransferase activity; F:transferase activity; P:methylation |
| Efet.01.1801.g161.t1 | PREDICTED: uncharacterized protein LOC106152277 | 1503 | 4.76E-114 | 56.25 |  |
| Efet.01.18011.g1328.t1 | enkurin | 213 | 1.07E-13 | 77.7 | C:sperm part; C:organelle |
| Efet.01.180113.g1405.t1 | chromodomain-helicase-DNA-binding 1- | 846 | 2.00E-131 | 77.15 | F:core promoter sequence-specific DNA binding; C:nucleus; F:ATP binding; F:ATP-dependent helicase activity; P:regulation of transcription, DNA-templated |
| Efet.01.18024.g1330.t1 | cement 3B variant 3 | 1176 | 4.33E-95 | 56.6 | P:intracellular signal transduction; C:intracellular |
| Efet.01.180297.g1408.t1 | hypothetical protein CAPTEDRAFT_198762 | 420 | 2.69E-15 | 56.5 | F:calcium-activated potassium channel activity; P:potassium ion transport; P:potassium ion transmembrane transport; C:membrane; C:integral component of membrane |
| Efet.01.180300.g1409.t1 | inositol monophosphatase 1 | 435 | 1.08E-55 | 72.1 | F:magnesium ion binding; F:identical protein binding; F:inositol monophosphate 1-phosphatase activity; P:inositol metabolic process; C:cytoplasm; P:signal transduction; P:phosphatidylinositol metabolic process; P:inositol phosphate dephosphorylation |
| Efet.01.180341.g1413.t1 | espinas isoform X2 | 1590 | 3.11E-15 | 63.95 | P:biological_process; F:zinc ion binding; F:metal ion binding; C:cellular_component; F:molecular_function |
| Efet.01.180342.g1414.t1 | spectrin beta chain-like isoform X1 | 420 | 4.37E-53 | 88.35 | F:actin binding; F:structural constituent of cytoskeleton; F:phospholipid binding; C:spectrin |
| Efet.01.180371.g1416.t1 | cytoplasmic dynein 2 heavy chain 1-like | 471 | 3.46E-72 | 82.5 | F:ATPase activity; F:ATP binding; F:microtubule motor activity; C:dynein complex; P:microtubule-based movement |
| Efet.01.180403.g1417.t1 | potassium voltage-gated channel subfamily H member 7-like isoform X3 | 582 | 7.08E-41 | 76.85 | F:voltage-gated potassium channel activity; P:potassium ion transmembrane transport; C:integral component of membrane |
| Efet.01.18041.g1331.t1 | pyridoxal phosphate phosphatase PHOSPHO2-like | 714 | 3.19E-91 | 64.9 | P:metabolic process |
| Efet.01.180427.g1421.t1 | microtubule-actin cross-linking factor 1 isoform X8 | 804 | 6.67E-29 | 49.15 |  |
| Efet.01.180442.g1422.t1 | flagellar motor | 501 | 6.28E-85 | 76.4 | C:integral component of membrane; C:cell outer membrane |
| Efet.01.180448.g1423.t1 | alpha-2A adrenergic receptor | 573 | 1.49E-34 | 61.7 | F:G-protein coupled receptor activity; P:G-protein coupled receptor signaling pathway; P:signal transduction; C:membrane; C:integral component of membrane; F:signal transducer activity |
| Efet.01.180515.g1425.t1 | nesprin-1 isoform X3 | 924 | 5.15E-15 | 47.75 |  |
| Efet.01.18058.g1332.t1 | sister chromatid cohesion PDS5 homolog A-like | 267 | 1.67E-26 | 80.05 | F:binding; F:hydrolase activity |
| Efet.01.180597.g1427.t1 | AF101063_1intermediate filament filarin | 258 | 4.77E-15 | 52.75 | F:structural molecule activity; C:intermediate filament |
| Efet.01.180648.g1429.t1 | 3 beta-hydroxysteroid dehydrogenase Delta 5-- | 816 | 3.88E-83 | 59.65 | F:oxidoreductase activity |
| Efet.01.180649.g1430.t1 | tyrosine- phosphatase Lar-like isoform X1 | 288 | 1.81E-20 | 62 | F:phosphatase activity; P:dephosphorylation |
| Efet.01.180666.g1431.t1 | multiple epidermal growth factor-like domains 6 | 285 | 5.80E-11 | 67.7 |  |
| Efet.01.180681.g1432.t1 | inter-alpha-trypsin inhibitor heavy chain H2 | 270 | 3.42E-13 | 68.8 | F:serine-type endopeptidase inhibitor activity; P:negative regulation of endopeptidase activity; P:hyaluronan metabolic process |
| Efet.01.18070.g1334.t1 | innexin unc-9-like isoform X2 | 1140 | 0 | 68.3 | C:gap junction; P:ion transport; C:integral component of membrane; C:plasma membrane |
| Efet.01.180729.g1434.t1 | PREDICTED: uncharacterized protein LOC106151497 isoform X1 | 3090 | 9.73E-41 | 46.25 |  |
| Efet.01.180742.g1435.t1 | cystatin precursor | 237 | 1.63E-09 | 58.25 | F:peptidase inhibitor activity; P:negative regulation of proteolysis; C:extracellular region |
| Efet.01.180749.g1436.t1 | titin-like isoform X5 | 351 | 3.91E-41 | 57.45 | F:ATP binding; P:regulation of Rho protein signal transduction; C:integral component of membrane; F:protein kinase activity; F:Rho guanyl-nucleotide exchange factor activity; P:protein phosphorylation; P:positive regulation of GTPase activity |
| Efet.01.180806.g1438.t1 | Solute carrier family 25 member 36-A | 459 | 7.25E-27 | 88.4 | C:mitochondrial membrane; P:transmembrane transport; P:mitochondrial transport; C:integral component of membrane |
| Efet.01.180832.g1439.t1 | transmembrane 120A | 1059 | 2.51E-155 | 74.1 | C:integral component of membrane |
| Efet.01.180873.g1440.t1 | E3 ubiquitin- ligase TRIM71-like | 1137 | 5.30E-30 | 46.9 | F:zinc ion binding; F:metal ion binding; C:intracellular |
| Efet.01.180874.g1441.t1 | thrombospondin type-1 domain-containing 7A-like isoform X1 | 651 | 1.20E-42 | 46.95 | C:membrane; C:integral component of membrane |
| Efet.01.180880.g1443.t1 | 1-phosphatidylinositol 4,5-bisphosphate phosphodiesterase epsilon-1 isoform X4 | 1053 | 2.34E-58 | 54.35 | F:phosphatidylinositol phospholipase C activity; P:small GTPase mediated signal transduction; P:signal transduction; F:guanyl-nucleotide exchange factor activity; P:lipid metabolic process; F:phosphoric diester hydrolase activity; F:signal transducer activity; P:intracellular signal transduction; P:positive regulation of GTPase activity; C:intracellular |
| Efet.01.18092.g1335.t1 | probable G- coupled receptor 139 | 1419 | 1.56E-81 | 59.75 | F:G-protein coupled receptor activity; P:G-protein coupled receptor signaling pathway; P:signal transduction; C:membrane; C:integral component of membrane; F:signal transducer activity |
| Efet.01.181001.g1448.t1 | disulfide-isomerase A4 | 273 | 7.75E-14 | 67.25 | F:protein disulfide isomerase activity; C:endoplasmic reticulum; P:cell redox homeostasis |
| Efet.01.181045.g1450.t1 | cyclin-dependent kinase 13-like | 342 | 3.13E-36 | 82.5 | P:cell division; F:ATP binding; F:protein kinase activity; P:protein phosphorylation |
| Efet.01.18105.g1337.t1 | prelamin-A C-like | 591 | 2.96E-10 | 52 |  |
| Efet.01.181067.g1453.t1 | glutamate receptor 1-like | 306 | 4.29E-18 | 64.85 | P:ion transport; F:transmembrane signaling receptor activity; F:extracellular ligand-gated ion channel activity; C:synapse; C:membrane part; C:plasma membrane |
| Efet.01.181083.g1454.t1 | PREDICTED: uncharacterized protein C1orf112 homolog | 246 | 9.41E-06 | 64 |  |
| Efet.01.181098.g1455.t1 | tudor and KH domain-containing -like | 312 | 3.48E-20 | 67.8 | F:nucleic acid binding; C:membrane |
| Efet.01.181099.g1456.t1 | zinc finger 665-like | 1164 | 1.67E-15 | 38.45 | F:nucleic acid binding; C:nucleus; F:zinc ion binding; F:metal ion binding |
| Efet.01.181114.g1457.t1 | B-cell lymphoma leukemia 11A-like | 342 | 5.63E-10 | 73.95 | C:nucleus; F:transcription factor activity, sequence-specific DNA binding; P:signal transduction; P:multicellular organism development; F:transcription regulatory region DNA binding; P:regulation of transcription, DNA-templated; P:transcription from RNA polymerase II promoter; F:sequence-specific DNA binding |
| Efet.01.18112.g1338.t1 | triple functional domain -like isoform X3 | 393 | 6.45E-49 | 80.5 | F:ATP binding; P:regulation of Rho protein signal transduction; F:Rho guanyl-nucleotide exchange factor activity; F:protein serine/threonine kinase activity; P:protein phosphorylation; C:intracellular; P:intracellular signal transduction; P:positive regulation of GTPase activity |
| Efet.01.181120.g1458.t1 | hypothetical protein CAPTEDRAFT_206678 | 1740 | 4.12E-103 | 42.93 | P:intracellular signal transduction; C:intracellular |
| Efet.01.18115.g1339.t1 | sarcoplasmic endoplasmic reticulum calcium ATPase 2-like | 219 | 4.88E-19 | 89.15 | F:ATP binding; F:metal ion binding; C:integral component of membrane; P:calcium ion transmembrane transport; F:calcium-transporting ATPase activity; C:sarcoplasmic reticulum membrane |
| Efet.01.181188.g1463.t1 | dual specificity tyrosine-phosphorylation-regulated kinase 1A- | 438 | 2.58E-51 | 83 | P:peptidyl-tyrosine phosphorylation; F:identical protein binding; P:negative regulation of DNA damage response, signal transduction by p53 class mediator; P:positive regulation of protein deacetylation; C:nuclear speck; P:nervous system development; P:protein autophosphorylation; F:protein serine/threonine kinase activity; P:circadian rhythm; F:protein self-association; F:ATP binding; F:protein serine/threonine/tyrosine kinase activity; C:intracellular ribonucleoprotein complex; F:non-membrane spanning protein tyrosine kinase activity; P:regulation of alternative mRNA splicing, via spliceosome; P:negative regulation of mRNA splicing, via spliceosome; F:tau protein binding; P:peptidyl-serine phosphorylation; P:peptidyl-threonine phosphorylation |
| Efet.01.181193.g1464.t1 | potassium voltage-gated channel Shal | 1188 | 1.07E-177 | 79.9 | P:potassium ion transmembrane transport; C:voltage-gated potassium channel complex; F:delayed rectifier potassium channel activity; F:A-type (transient outward) potassium channel activity; P:regulation of ion transmembrane transport; P:protein homooligomerization |
| Efet.01.181257.g1465.t1 | neuromedin-U receptor 2-like | 882 | 4.64E-89 | 67.95 | P:neuropeptide signaling pathway; C:integral component of membrane; F:neuromedin U receptor activity |
| Efet.01.181289.g1469.t1 | ATP-dependent RNA helicase SUV3 mitochondrial | 249 | 3.72E-12 | 78.85 | C:nucleus; F:DNA binding; F:ATP binding; F:DNA helicase activity; C:mitochondrial matrix; P:DNA duplex unwinding |
| Efet.01.181324.g1472.t1 | 26S protease regulatory subunit 4 | 666 | 2.25E-135 | 96.55 | F:ATP binding; C:cytoplasm; P:protein catabolic process; P:proteolysis; C:proteasome complex; F:peptidase activity |
| Efet.01.181328.g1473.t1 | E3 ubiquitin- ligase rnf146 | 1401 | 1.67E-31 | 77.6 | F:zinc ion binding; F:ligase activity; P:protein ubiquitination |
| Efet.01.181348.g1474.t1 | gastrin cholecystokinin type B receptor-like | 957 | 1.49E-54 | 58.7 | P:signal transduction; F:signal transducer activity |
| Efet.01.18135.g1342.t1 | FERM and PDZ domain-containing 1 | 366 | 4.17E-15 | 60.1 | C:cytoskeleton; P:signal transduction |
| Efet.01.181455.g1475.t1 | leucine-rich repeat-containing 47-like | 1041 | 1.36E-91 | 61.9 | F:RNA binding; F:phenylalanine-tRNA ligase activity |
| Efet.01.181469.g1477.t1 | hypothetical protein HELRODRAFT_161883 | 210 | 6.97E-13 | 86 | C:integral component of membrane |
| Efet.01.181516.g1479.t1 | Eukaryotic translation initiation factor 3 subunit A | 237 | 4.09E-18 | 73.65 | F:translation initiation factor activity; P:formation of translation preinitiation complex; C:eukaryotic 43S preinitiation complex; C:eukaryotic 48S preinitiation complex; P:regulation of translational initiation; C:eukaryotic translation initiation factor 3 complex |
| Efet.01.181521.g1480.t1 | hypothetical protein CAPTEDRAFT_204443 | 1107 | 1.30E-26 | 52.5 |  |
| Efet.01.181544.g1481.t1 | 5-hydroxytryptamine receptor-like | 1476 | 7.00E-98 | 54.35 | P:signal transduction; C:membrane; F:signal transducer activity |
| Efet.01.181620.g1484.t1 | polycystic kidney disease and receptor for egg jelly-related | 561 | 1.63E-06 | 45 | C:membrane; C:integral component of membrane |
| Efet.01.181656.g1485.t1 | phenylserine dehydratase-like | 342 | 3.09E-26 | 65.7 | F:pyridoxal phosphate binding; P:cellular amino acid metabolic process |
| Efet.01.181701.g1489.t1 | TNF receptor-associated factor 6 | 414 | 4.49E-08 | 63.9 | F:ubiquitin-protein transferase activity; F:zinc ion binding; P:signal transduction; F:metal ion binding; P:regulation of apoptotic process; P:protein ubiquitination |
| Efet.01.181718.g1490.t1 | E3 ubiquitin- ligase RNF213 | 249 | 2.70E-11 | 87.65 | F:ATPase activity; F:ubiquitin-protein transferase activity; F:ATP binding; F:zinc ion binding; P:protein ubiquitination |
| Efet.01.181767.g1491.t1 | beta-1,3-galactosyltransferase 1-like isoform X2 | 1176 | 8.00E-48 | 56.85 | F:transferase activity, transferring glycosyl groups; C:membrane; C:integral component of membrane; P:protein glycosylation; F:galactosyltransferase activity; C:Golgi apparatus; C:Golgi membrane; F:transferase activity |
| Efet.01.181810.g1492.t1 | polyubiquitin 8-like | 654 | 4.31E-48 | 57.6 | P:protein ubiquitination |
| Efet.01.181834.g1493.t1 | gamma-aminobutyric acid receptor subunit beta | 495 | 1.43E-45 | 88.1 | P:signal transduction; P:ion transmembrane transport; C:integral component of membrane; F:GABA-A receptor activity; C:cell junction; F:extracellular ligand-gated ion channel activity; C:synapse; C:plasma membrane |
| Efet.01.181838.g1494.t1 | regulator of G- signaling 3- | 204 | 3.72E-16 | 74.95 | C:nucleus; C:cytoplasm; F:GTPase activator activity; C:plasma membrane |
| Efet.01.181839.g1495.t1 | Lysine-specific demethylase 3B | 1737 | 1.36E-120 | 59.65 | F:catalytic activity |
| Efet.01.181964.g1499.t1 | double-stranded RNA-specific editase 1 | 1554 | 2.37E-11 | 46.8 | P:hypoglossal nerve morphogenesis; P:multicellular organism growth; P:neuromuscular synaptic transmission; P:adenosine to inosine editing; P:innervation; P:facial nerve morphogenesis; P:muscle tissue morphogenesis; P:spinal cord ventral commissure morphogenesis; P:negative regulation of cellular process; P:motor behavior; C:nucleus; P:neuromuscular process controlling posture; F:RNA binding; P:motor neuron apoptotic process; F:hydrolase activity |
| Efet.01.181985.g1500.t1 | dual specificity phosphatase 3 | 237 | 8.49E-14 | 68.8 | F:protein kinase binding; P:negative regulation of JNK cascade; P:positive regulation of mitotic cell cycle; C:immunological synapse; C:nucleoplasm; F:protein tyrosine/serine/threonine phosphatase activity; P:inactivation of MAPK activity; C:extracellular exosome; F:MAP kinase phosphatase activity; P:negative regulation of T cell receptor signaling pathway; F:protein tyrosine phosphatase activity; P:negative regulation of ERK1 and ERK2 cascade; C:cytosol; P:negative regulation of T cell activation; P:peptidyl-tyrosine dephosphorylation |
| Efet.01.181989.g1501.t1 | sel-1 homolog 1-like | 318 | 1.20E-36 | 77.2 | C:Hrd1p ubiquitin ligase ERAD-L complex; F:protein binding; C:integral component of membrane; P:triglyceride metabolic process; P:retrograde protein transport, ER to cytosol; P:protein secretion; C:Derlin-1 retrotranslocation complex |
| Efet.01.182026.g1502.t1 | ribosome biogenesis NSA2 homolog | 261 | 2.84E-32 | 80 |  |
| Efet.01.18203.g1344.t1 | glomulin-like isoform X1 | 996 | 5.44E-21 | 45.33 |  |
| Efet.01.18203.g1345.t1 | glomulin isoform X1 | 729 | 2.53E-20 | 51.15 |  |
| Efet.01.182039.g1503.t1 | inositol hexakisphosphate and diphosphoinositol-pentakisphosphate kinase 2 isoform X1 | 228 | 1.32E-33 | 85.85 | P:inositol phosphate metabolic process; F:inositol heptakisphosphate kinase activity; F:inositol-1,3,4,5,6-pentakisphosphate kinase activity; P:inositol metabolic process; F:inositol hexakisphosphate 5-kinase activity; F:acid phosphatase activity; F:ATP binding; F:diphosphoinositol-pentakisphosphate kinase activity; C:cytosol; P:phosphorylation; F:inositol hexakisphosphate 3-kinase activity; P:dephosphorylation; F:inositol hexakisphosphate 1-kinase activity |
| Efet.01.18206.g1346.t1 | zinc finger Gfi-1b-like | 1614 | 3.98E-67 | 56.65 | F:binding |
| Efet.01.18208.g1347.t1 | clustered mitochondria homolog | 1008 | 1.10E-121 | 91.3 | F:RNA binding; C:cytoplasm; P:intracellular distribution of mitochondria |
| Efet.01.182112.g1504.t1 | trithorax group osa-like isoform X7 | 1944 | 6.47E-80 | 61.7 | F:DNA binding; C:BAF-type complex; P:chromatin remodeling |
| Efet.01.182140.g1505.t1 | Peptidyl-prolyl cis-trans isomerase B | 411 | 1.91E-40 | 77.7 | F:peptidyl-prolyl cis-trans isomerase activity; P:protein peptidyl-prolyl isomerization; P:protein folding |
| Efet.01.182165.g1507.t1 | macoilin-like isoform X1 | 387 | 9.00E-27 | 60.95 | C:nuclear membrane; C:synapse; P:brain development; C:axon; C:neuron projection terminus |
| Efet.01.182168.g1508.t1 | sodium channel type 4 subunit alpha A-like isoform X1 | 264 | 2.07E-21 | 71.55 | P:biological regulation; P:sodium ion transport; P:ion transmembrane transport; F:sodium channel activity; C:integral component of membrane; F:voltage-gated ion channel activity |
| Efet.01.18219.g1349.t1 | hypothetical protein HELRODRAFT_189210 | 945 | 1.08E-138 | 56.6 | C:membrane |
| Efet.01.182257.g1509.t1 | E3 ubiquitin- ligase PDZRN3-like | 549 | 1.77E-20 | 64.1 |  |
| Efet.01.182269.g1510.t1 | PAXX isoform X2 | 366 | 2.32E-12 | 56.58 | P:double-strand break repair via nonhomologous end joining |
| Efet.01.18228.g1350.t1 | epidermal growth factor receptor-like isoform X1 | 222 | 8.54E-24 | 85.6 | F:ATP binding; P:peptidyl-tyrosine phosphorylation; F:transmembrane receptor protein tyrosine kinase activity; C:integral component of membrane; P:transmembrane receptor protein tyrosine kinase signaling pathway |
| Efet.01.182338.g1513.t1 | ubiquitin-like modifier-activating enzyme 1 | 504 | 9.73E-96 | 89.65 | P:cellular response to DNA damage stimulus; C:lysosomal membrane; F:RNA binding; C:heterochromatin; C:nucleus; F:ubiquitin-protein transferase activity; F:ATP binding; C:rough endoplasmic reticulum membrane; C:extracellular exosome; F:protein binding; P:axonogenesis; C:mitochondrion; C:cytosol; F:ubiquitin activating enzyme activity; C:endosome membrane; C:desmosome; P:modification-dependent protein catabolic process; P:protein ubiquitination |
| Efet.01.182350.g1514.t1 | DNA topoisomerase 1 | 426 | 6.29E-73 | 91.9 | P:embryonic cleavage; P:DNA replication; C:nuclear chromosome; C:P-body; F:chromatin binding; P:DNA topological change; C:nucleolus; C:perikaryon; C:nucleoplasm; F:RNA binding; F:core promoter sequence-specific DNA binding; P:chromatin remodeling; P:circadian regulation of gene expression; P:response to drug; F:DNA topoisomerase type I activity; F:DNA topoisomerase type II (ATP-hydrolyzing) activity |
| Efet.01.182447.g1519.t1 | AChain Structure Of A Glutamate Receptor Ligand Binding Core (glur2) Complexed With Kainate | 969 | 6.70E-73 | 49.75 | P:regulation of biological quality; P:regulation of macromolecule metabolic process; P:chemical synaptic transmission; P:cell surface receptor signaling pathway; F:ligand-gated cation channel activity; C:ionotropic glutamate receptor complex; C:organelle; F:ionotropic glutamate receptor activity; P:transport; P:establishment of protein localization; C:dendrite; C:postsynapse; F:protein binding; C:cell projection part; C:cation channel complex; P:regulation of synaptic transmission, glutamatergic; P:regulation of cellular metabolic process |
| Efet.01.182447.g1520.t1 | Transport and Golgi organization 11 | 396 | 4.52E-18 | 50.7 | C:membrane |
| Efet.01.182493.g1522.t1 | probable E3 ubiquitin- ligase MID2 isoform X1 | 1350 | 1.44E-93 | 53.85 | P:regulation of cellular process; F:binding |
| Efet.01.182563.g1527.t1 | filamin-C isoform X1 | 327 | 2.82E-24 | 74.75 | F:actin filament binding |
| Efet.01.182572.g1528.t1 | microtubule-actin cross-linking factor 1 isoform X35 | 306 | 2.44E-10 | 56.2 | C:cytoskeleton |
| Efet.01.182591.g1529.t1 | glycogen brain form- | 393 | 8.87E-58 | 86.85 | F:glycogen phosphorylase activity; F:pyridoxal phosphate binding; F:AMP binding; F:drug binding; F:purine nucleobase binding; F:bile acid binding; P:glycogen catabolic process; C:plasma membrane; F:ATP binding; C:extracellular exosome; C:cytoplasm; P:necroptotic process; P:glucose homeostasis; F:vitamin binding |
| Efet.01.182634.g1531.t1 | transient receptor potential cation channel subfamily M member 5-like | 417 | 4.53E-21 | 60.1 | P:transport |
| Efet.01.182660.g1532.t1 | TNF receptor-associated factor 6-like | 330 | 1.18E-15 | 78.5 | F:DNA binding; F:ubiquitin-protein transferase activity; P:DNA repair; F:zinc ion binding; P:immune response; P:signal transduction; P:regulation of apoptotic process; P:protein ubiquitination |
| Efet.01.182737.g1537.t1 | spectrin alpha non-erythrocytic 1-like isoform X1 | 261 | 2.31E-16 | 86.6 | F:actin binding; F:phospholipid binding |
| Efet.01.182758.g1539.t1 | transmembrane 151B-like | 1143 | 7.98E-69 | 57.7 | C:membrane; C:integral component of membrane |
| Efet.01.182782.g1540.t1 | Anaphase-promoting complex subunit 13 | 240 | 1.26E-11 | 80.5 |  |
| Efet.01.182793.g1541.t1 | SEC14 domain and spectrin repeat-containing 1 | 438 | 8.33E-33 | 59.55 |  |
| Efet.01.182852.g1542.t1 | FAM46C-like | 1272 | 0 | 79.7 |  |
| Efet.01.1829.g162.t1 | guanine nucleotide-binding subunit alpha-12 | 468 | 7.90E-22 | 77.85 | F:GTP binding; P:embryo development; F:G-protein coupled receptor binding; C:heterotrimeric G-protein complex; P:small GTPase mediated signal transduction; F:GTPase activity; P:adenylate cyclase-modulating G-protein coupled receptor signaling pathway; F:G-protein beta/gamma-subunit complex binding; F:signal transducer activity |
| Efet.01.182937.g1545.t1 | ribonuclease P subunit p38 | 708 | 1.90E-22 | 54.35 | P:RNA phosphodiester bond hydrolysis, endonucleolytic; F:ribonuclease P activity; C:nucleolus; C:nucleolar ribonuclease P complex |
| Efet.01.18300.g1351.t1 | E3 ubiquitin- ligase mind- | 588 | 2.32E-26 | 74.4 | C:membrane |
| Efet.01.183000.g1547.t1 | probable imidazolonepropionase | 270 | 2.18E-30 | 76.85 | F:hydrolase activity, acting on carbon-nitrogen (but not peptide) bonds, in cyclic amides; C:cytoplasm; P:histidine catabolic process to glutamate and formamide |
| Efet.01.183029.g1548.t1 | MChain Lumbricus Erythrocruorin At Resolution | 375 | 2.26E-19 | 52.57 |  |
| Efet.01.183058.g1550.t1 | rho GTPase-activating 7-like isoform X1 | 411 | 1.16E-36 | 71.4 | P:signal transduction; F:lipid binding |
| Efet.01.18312.g1352.t1 | cilia- and flagella-associated 54- | 627 | 9.58E-08 | 42.86 |  |
| Efet.01.183135.g1552.t1 | hypothetical protein B9Z55_019763 | 300 | 7.94E-06 | 52 |  |
| Efet.01.183260.g1557.t1 | G- coupled receptor 84 | 1194 | 1.17E-17 | 42.7 | C:integral component of plasma membrane; F:G-protein coupled receptor activity; F:G-protein coupled peptide receptor activity; P:neuropeptide signaling pathway; P:G-protein coupled receptor signaling pathway; C:membrane; C:integral component of membrane; P:response to lipopolysaccharide; C:receptor complex; P:phospholipase C-activating G-protein coupled receptor signaling pathway |
| Efet.01.183265.g1558.t1 | sorbitol dehydrogenase-like | 717 | 1.62E-83 | 80.9 | F:zinc ion binding; F:oxidoreductase activity; P:oxidation-reduction process |
| Efet.01.183274.g1559.t1 | hypothetical protein CAPTEDRAFT_193210 | 636 | 3.58E-48 | 62.67 | C:integral component of membrane |
| Efet.01.18332.g1353.t1 | Dynein heavy chain axonemal | 579 | 5.54E-84 | 89.65 | F:ATPase activity; F:ATP binding; F:microtubule motor activity; C:dynein complex; P:microtubule-based movement |
| Efet.01.18333.g1354.t1 | microtubule-actin cross-linking factor isoforms 1 2 3 5-like | 6831 | 0 | 51.85 | P:cell cycle arrest; C:cytoskeleton; F:calcium ion binding |
| Efet.01.18333.g1355.t1 | microtubule-actin cross-linking factor isoforms 1 2 3 5-like | 888 | 2.12E-69 | 46.5 | P:cell cycle arrest; C:cytoskeleton; F:actin binding; F:calcium ion binding |
| Efet.01.18333.g1356.t1 | microtubule-actin cross-linking factor 1-like isoform X13 | 2832 | 7.11E-179 | 48.45 | P:cell cycle arrest; C:cytoskeleton; F:calcium ion binding |
| Efet.01.18333.g1357.t1 | dystonin isoform X1 | 3099 | 2.67E-120 | 46.6 | C:cytoskeleton |
| Efet.01.18333.g1358.t1 | microtubule-actin cross-linking factor 1-like isoform X10 | 2457 | 1.60E-118 | 47.85 | C:cytoskeleton |
| Efet.01.183367.g1563.t1 | schwannomin interacting | 279 | 1.46E-35 | 81.6 |  |
| Efet.01.183407.g1564.t1 | 60 kDa neurofilament -like | 474 | 3.80E-37 | 71.9 | F:structural molecule activity; C:intermediate filament |
| Efet.01.183459.g1568.t1 | potassium channel subfamily K member 4-like | 822 | 9.79E-62 | 58 | P:ion transport; P:potassium ion transmembrane transport; C:membrane; C:integral component of membrane; F:potassium channel activity; P:transport |
| Efet.01.183507.g1571.t1 | E3 ubiquitin- ligase RGLG4-like | 750 | 7.16E-67 | 68.3 | C:membrane; C:integral component of membrane |
| Efet.01.183512.g1572.t1 | signal recognition particle receptor subunit alpha homolog | 345 | 1.65E-23 | 76.95 | F:GTP binding; P:SRP-dependent cotranslational protein targeting to membrane; F:GTPase activity; C:signal recognition particle receptor complex; F:signal recognition particle binding |
| Efet.01.183541.g1574.t1 | homeobox CDX-1 | 612 | 1.61E-22 | 91.1 | F:RNA polymerase II distal enhancer sequence-specific DNA binding; C:nucleus; P:anterior/posterior axis specification; P:regulation of somitogenesis; P:cell differentiation; P:bone morphogenesis; P:transcription from RNA polymerase II promoter; F:transcriptional activator activity, RNA polymerase II distal enhancer sequence-specific binding; P:positive regulation of transcription from RNA polymerase II promoter |
| Efet.01.183566.g1577.t1 | hypothetical protein CAPTEDRAFT_223621 | 726 | 6.56E-13 | 54 | P:regulation of apoptotic process |
| Efet.01.183596.g1578.t1 | inactive tyrosine- kinase 7-like | 315 | 4.73E-28 | 71.25 | P:cellular macromolecule metabolic process; F:organic cyclic compound binding; F:heterocyclic compound binding; F:transferase activity, transferring phosphorus-containing groups; P:primary metabolic process |
| Efet.01.18361.g1360.t1 | slit-like isoform X2 | 1068 | 6.80E-57 | 49 | C:extracellular region |
| Efet.01.183639.g1579.t1 | sprouty homolog 4 | 741 | 2.84E-14 | 55.25 | P:multicellular organism development; P:negative regulation of ERK1 and ERK2 cascade; C:membrane; P:regulation of signal transduction |
| Efet.01.183662.g1580.t1 | haloacid dehalogenase type II | 855 | 2.59E-158 | 78.65 | F:hydrolase activity, acting on acid halide bonds, in C-halide compounds; P:metabolic process |
| Efet.01.183691.g1581.t1 | EH domain-containing 3 | 648 | 2.88E-105 | 82.8 | F:GTP binding; F:calcium ion binding; C:intracellular; P:endocytic recycling |
| Efet.01.183717.g1582.t1 | LOW QUALITY PROTEIN: uncharacterized protein LOC111123909 | 399 | 9.26E-17 | 63.92 |  |
| Efet.01.18374.g1362.t1 | E3 ubiquitin- ligase TRIM9-like isoform X1 | 354 | 3.60E-36 | 74.55 | F:zinc ion binding; C:intracellular |
| Efet.01.183752.g1583.t1 | adiponectin receptor -like isoform X1 | 252 | 3.91E-09 | 72.85 | C:membrane |
| Efet.01.183787.g1584.t1 | hypothetical protein CAPTEDRAFT_112095, partial | 732 | 1.68E-31 | 60.5 |  |
| Efet.01.183799.g1587.t1 | S-phase kinase-associated 1 | 435 | 6.20E-32 | 76 | C:cytosol; P:SCF-dependent proteasomal ubiquitin-dependent protein catabolic process; C:SCF ubiquitin ligase complex; C:Cul7-RING ubiquitin ligase complex |
| Efet.01.183852.g1588.t1 | alpha beta hydrolase domain-containing 14B | 255 | 8.54E-15 | 70.3 | C:cytoplasm; C:membrane; F:hydrolase activity |
| Efet.01.183867.g1590.t1 | retinol dehydrogenase 14-like | 858 | 1.69E-108 | 75.25 | F:oxidoreductase activity; C:membrane; P:single-organism metabolic process |
| Efet.01.183869.g1591.t1 | GAS2 2 | 1107 | 1.07E-41 | 57.6 | P:cell cycle arrest |
| Efet.01.183929.g1594.t1 | Transmembrane 104 | 453 | 1.14E-19 | 53.4 | C:membrane; C:integral component of membrane |
| Efet.01.183939.g1595.t1 | neuronal acetylcholine receptor subunit alpha-7-like | 339 | 5.56E-50 | 75.7 | C:postsynaptic membrane; P:cation transmembrane transport; C:integral component of membrane; C:cell junction; F:extracellular ligand-gated ion channel activity |
| Efet.01.18397.g1363.t1 | atrial natriuretic peptide receptor 1-like | 357 | 5.57E-10 | 74.67 | F:ATP binding; P:cGMP biosynthetic process; C:integral component of membrane; F:guanylate cyclase activity; F:protein kinase activity; P:protein phosphorylation; C:intracellular; P:intracellular signal transduction |
| Efet.01.183981.g1596.t1 | E3 ubiquitin- ligase arih1-like | 261 | 3.12E-44 | 93.1 | F:zinc ion binding; F:ubiquitin conjugating enzyme binding; F:ubiquitin protein ligase activity; C:ubiquitin ligase complex; C:cytoplasm; P:protein polyubiquitination; P:positive regulation of proteasomal ubiquitin-dependent protein catabolic process; F:ligase activity; P:protein ubiquitination involved in ubiquitin-dependent protein catabolic process |
| Efet.01.18399.g1364.t1 | filamin-A-like isoform X1 | 390 | 1.01E-29 | 68.5 | F:actin binding |
| Efet.01.184031.g1597.t1 | heat shock 70 kDa 12A- | 447 | 2.05E-34 | 62.25 | P:sprouting angiogenesis; P:blood vessel morphogenesis |
| Efet.01.184056.g1598.t1 | U4 U6 small nuclear ribonucleo Prp3 | 348 | 2.70E-28 | 67.9 | P:metabolic process; P:cellular process; P:response to stimulus |
| Efet.01.184112.g1599.t1 | PREDICTED: uncharacterized protein C14orf80 homolog | 771 | 2.97E-27 | 50.08 |  |
| Efet.01.184134.g1601.t1 | small subunit processome component 20 | 204 | 3.07E-06 | 62.67 |  |
| Efet.01.18416.g1366.t1 | hypothetical protein CAPTEDRAFT_219563 | 474 | 1.09E-10 | 54 | C:membrane; C:integral component of membrane |
| Efet.01.184241.g1603.t1 | sprouty- EVH1 domain-containing 1-like | 261 | 4.58E-13 | 65.8 | F:phosphatase binding; F:protein kinase binding; F:protein serine/threonine kinase inhibitor activity; P:positive regulation of DNA damage response, signal transduction by p53 class mediator; P:regulation of protein deacetylation; F:stem cell factor receptor binding; P:fibroblast growth factor receptor signaling pathway; C:plasma membrane; C:nucleoplasm; P:inactivation of MAPK activity; P:MAPK cascade; P:negative regulation of phosphatase activity; P:negative regulation of peptidyl-threonine phosphorylation; C:cytosol; C:cytoplasmic vesicle |
| Efet.01.18433.g1367.t1 | slit homolog 3 -like | 2436 | 5.43E-132 | 51.95 | C:membrane; C:integral component of membrane |
| Efet.01.184366.g1605.t1 | exostosin-1 | 306 | 2.67E-26 | 65.25 | F:transferase activity, transferring glycosyl groups |
| Efet.01.184386.g1606.t1 | translocon-associated subunit gamma-like | 423 | 2.22E-64 | 89.2 | P:SRP-dependent cotranslational protein targeting to membrane; C:integral component of membrane; C:intracellular |
| Efet.01.184390.g1607.t1 | meteorin | 366 | 1.88E-10 | 61.5 | C:extracellular space; P:positive regulation of axonogenesis; P:glial cell differentiation |
| Efet.01.184432.g1608.t1 | neurexin-1a-like isoform X1 | 807 | 1.22E-56 | 55.5 | C:presynaptic membrane; F:metal ion binding; F:calcium ion binding; C:membrane; C:integral component of membrane; P:chemical synaptic transmission |
| Efet.01.184465.g1609.t1 | 5-oxoprolinase isoform X2 | 339 | 4.68E-26 | 66.55 | F:catalytic activity; F:hydrolase activity |
| Efet.01.184491.g1611.t1 | fer3 | 234 | 1.13E-11 | 80.75 | P:negative regulation of transcription from RNA polymerase II promoter; C:nucleus; F:RNA polymerase II regulatory region sequence-specific DNA binding; F:protein dimerization activity; F:transcriptional repressor activity, RNA polymerase II transcription regulatory region sequence-specific binding; P:cell development; P:floor plate development; P:regulation of neurogenesis |
| Efet.01.18451.g1368.t1 | structural maintenance of chromosomes 1A-like | 636 | 2.59E-119 | 90.5 | P:DNA repair; P:stem cell population maintenance; P:mitotic cell cycle checkpoint; F:mediator complex binding; P:mitotic sister chromatid cohesion; P:signal transduction in response to DNA damage; C:kinetochore; F:chromatin binding; P:meiotic nuclear division; C:nucleoplasm; F:RNA binding; P:cell division; F:ATP binding; C:cytoplasm; F:protein heterodimerization activity; C:meiotic cohesin complex; P:negative regulation of DNA endoreduplication |
| Efet.01.184533.g1612.t1 | staphylococcal nuclease domain-containing 1-like | 819 | 6.24E-92 | 64.6 | P:regulation of macromolecule metabolic process; P:regulation of cellular process; P:single-organism process; P:cellular process |
| Efet.01.184536.g1613.t1 | vang 2 | 483 | 2.17E-47 | 73.6 | C:intracellular organelle; P:cochlea development; P:dichotomous subdivision of terminal units involved in lung branching; C:plasma membrane region; P:pattern specification process; P:axon guidance; P:inner ear receptor cell development; P:positive regulation of Wnt signaling pathway, calcium modulating pathway; P:convergent extension involved in gastrulation; P:ventricular septum morphogenesis; P:convergent extension involved in organogenesis; P:axis elongation; P:embryonic organ morphogenesis; P:localization; P:neural tube closure; P:kidney development; P:establishment of planar polarity; C:integral component of membrane; P:Wnt signaling pathway, planar cell polarity pathway |
| Efet.01.184545.g1616.t1 | thyrotropin-releasing hormone receptor-like | 597 | 3.77E-19 | 60.15 | F:G-protein coupled receptor activity; P:G-protein coupled receptor signaling pathway; P:signal transduction; C:membrane; C:integral component of membrane; F:signal transducer activity |
| Efet.01.18459.g1369.t1 | short transient receptor potential channel 3-like | 732 | 1.54E-82 | 72.7 | F:calcium channel activity; C:integral component of membrane; P:calcium ion transmembrane transport |
| Efet.01.184616.g1620.t1 | voltage-dependent calcium channel type A subunit alpha-1-like | 864 | 4.74E-15 | 51.95 | F:ion channel activity; P:ion transport; P:transmembrane transport; C:membrane |
| Efet.01.184649.g1621.t1 | MAATS1 isoform X1 | 249 | 1.24E-20 | 80.45 |  |
| Efet.01.184674.g1622.t1 | homeobox Nkx- | 363 | 2.42E-20 | 86.85 | C:nucleus; P:regulation of transcription, DNA-templated; F:sequence-specific DNA binding |
| Efet.01.184713.g1624.t1 | receptor-type guanylate cyclase Gyc76C-like | 591 | 2.34E-35 | 68.8 | F:nucleotide binding; P:signal transduction; F:phosphorus-oxygen lyase activity; C:membrane; C:intracellular; P:cyclic nucleotide biosynthetic process; F:cyclase activity |
| Efet.01.184744.g1625.t1 | filamin-A isoform X1 | 276 | 3.50E-31 | 80.15 | F:actin filament binding; C:membrane |
| Efet.01.184747.g1626.t1 | neurensin-1-like | 360 | 3.18E-16 | 66.17 | C:membrane |
| Efet.01.184792.g1627.t1 | N-alpha-acetyltransferase auxiliary subunit-like | 393 | 8.99E-55 | 82.05 | F:transferase activity |
| Efet.01.184871.g1629.t1 | calumenin-A-like isoform X1 | 363 | 5.51E-24 | 72 | C:endoplasmic reticulum lumen; F:protein binding; C:sarcoplasmic reticulum; F:calcium ion binding; C:membrane; C:Golgi apparatus; P:response to stimulus |
| Efet.01.184884.g1630.t1 | cAMP-dependent kinase regulatory subunit | 534 | 1.95E-39 | 67.15 | P:phosphorylation; F:kinase activity |
| Efet.01.18489.g1370.t1 | flightless-1 homolog isoform X2 | 231 | 2.06E-16 | 76.65 | P:actin filament severing; F:actin binding; C:intracellular organelle part; P:actin cytoskeleton organization |
| Efet.01.184970.g1633.t1 | hypothetical protein CAPTEDRAFT_223700 | 357 | 1.19E-11 | 60 | C:membrane; C:integral component of membrane |
| Efet.01.185039.g1635.t1 | dual specificity kinase CLK2 | 234 | 6.41E-27 | 80.05 | F:ATP binding; P:peptidyl-tyrosine phosphorylation; F:protein tyrosine kinase activity; P:response to retinoic acid; P:regulation of RNA splicing; P:negative regulation of gluconeogenesis; P:protein autophosphorylation; F:protein serine/threonine kinase activity; C:nucleoplasm; P:response to ionizing radiation |
| Efet.01.185080.g1636.t1 | Metabotropic glutamate receptor | 345 | 3.36E-35 | 67 | F:G-protein coupled receptor activity; P:G-protein coupled receptor signaling pathway; C:integral component of membrane; C:plasma membrane |
| Efet.01.18515.g1372.t1 | leucine-rich repeat-containing 14-like | 216 | 3.86E-10 | 60.5 |  |
| Efet.01.185157.g1639.t1 | ubiquitin-conjugating enzyme E2-24 kDa | 267 | 1.87E-49 | 95.05 | F:ubiquitin-protein transferase activity; F:ATP binding; F:SUMO activating enzyme activity; P:lipid storage; C:cytoplasm; P:protein sumoylation; P:protein polyubiquitination; P:humoral immune response; P:positive regulation of MyD88-dependent toll-like receptor signaling pathway; F:ubiquitin activating enzyme binding; P:positive regulation of NF-kappaB transcription factor activity; C:SUMO activating enzyme complex |
| Efet.01.185271.g1642.t1 | transcription factor AP-1 | 948 | 1.83E-44 | 52.2 | P:positive regulation of RNA biosynthetic process; C:nucleus; P:negative regulation of gene expression; P:response to organic substance; F:protein binding; P:system development; P:positive regulation of gene expression; F:transcription regulatory region DNA binding; P:cellular response to stimulus; P:regulation of transcription, DNA-templated; P:regulation of cell proliferation; P:negative regulation of RNA biosynthetic process |
| Efet.01.185277.g1643.t1 | dynein heavy chain axonemal | 2604 | 0 | 75.4 | F:nucleotide binding; F:nucleoside-triphosphatase activity |
| Efet.01.185285.g1644.t1 | hypothetical protein CAPTEDRAFT_222884 | 459 | 1.31E-25 | 51.2 | C:membrane; C:integral component of membrane; F:carbohydrate binding |
| Efet.01.185297.g1646.t1 | Neurogenic locus Notch | 306 | 5.41E-12 | 66.25 | F:calcium ion binding; C:membrane; C:integral component of membrane |
| Efet.01.185380.g1650.t1 | cartilage matrix -like | 582 | 1.01E-23 | 54.15 | C:collagen trimer; C:membrane; C:integral component of membrane |
| Efet.01.185388.g1651.t1 | plectin-like isoform X4 | 378 | 1.36E-27 | 66.7 | C:cytoskeleton |
| Efet.01.185388.g1652.t1 | microtubule-actin cross-linking factor isoforms 1 2 3 5-like | 243 | 7.11E-11 | 63.42 | C:cytoskeleton |
| Efet.01.185401.g1653.t1 | fibroblast growth factor receptor-like 1 | 633 | 3.40E-07 | 47.75 | C:membrane; C:integral component of membrane |
| Efet.01.18545.g1374.t1 | E3 ubiquitin- ligase Topors-like | 2772 | 2.72E-31 | 47.25 | F:nucleic acid binding; F:zinc ion binding; F:metal ion binding; F:ligase activity |
| Efet.01.18548.g1375.t1 | hypothetical protein HELRODRAFT_162084 | 3276 | 8.56E-14 | 44 |  |
| Efet.01.185526.g1656.t1 | adenosine 3 -phospho 5 -phosphosulfate transporter 1-like | 1281 | 1.90E-136 | 69.95 | P:system development; C:membrane; P:single-organism cellular process; P:transport |
| Efet.01.18559.g1376.t1 | transmembrane 242-like isoform X2 | 249 | 9.10E-22 | 65.7 | C:membrane; C:integral component of membrane |
| Efet.01.185603.g1658.t1 | serpin family | 210 | 1.08E-16 | 72.6 | C:membrane |
| Efet.01.185609.g1660.t1 | hypothetical protein HELRODRAFT_175680 | 441 | 2.59E-21 | 68 |  |
| Efet.01.185615.g1661.t1 | hypothetical protein CAPTEDRAFT_226670 | 303 | 2.59E-06 | 51 | F:nucleotide binding; F:ATP binding; F:microtubule motor activity; P:plus-end-directed vesicle transport along microtubule; P:actomyosin contractile ring assembly; P:cytokinesis; P:microtubule-based movement; F:microtubule binding; C:microtubule |
| Efet.01.185627.g1662.t1 | spermatogenesis-associated 20 | 723 | 2.41E-90 | 72.15 | F:catalytic activity |
| Efet.01.185666.g1664.t1 | transcriptional-regulating factor 1-like isoform X3 | 1386 | 6.87E-07 | 63 |  |
| Efet.01.185693.g1665.t1 | 1-phosphatidylinositol-4,5-bisphosphate phosphodiesterase classes I and | 399 | 3.74E-12 | 70 | P:signal transduction; P:lipid metabolic process; F:phosphoric diester hydrolase activity |
| Efet.01.185695.g1666.t1 | disks large homolog 5 | 327 | 1.17E-08 | 85.65 | C:apical part of cell; F:protein tyrosine phosphatase activity; P:lateral inhibition; P:border follicle cell migration; P:antimicrobial humoral response; P:peptidyl-tyrosine dephosphorylation |
| Efet.01.185727.g1669.t1 | hemoglobin subunit | 297 | 2.96E-41 | 64.25 | C:hemoglobin complex; F:oxygen binding; F:iron ion binding; P:oxygen transport; C:extracellular region; F:oxygen transporter activity; F:heme binding |
| Efet.01.185812.g1672.t1 | Neurogenic locus Notch | 288 | 1.22E-21 | 77.3 | P:regulation of developmental process; P:Notch signaling pathway; P:multicellular organism development; F:calcium ion binding; C:integral component of membrane; P:cell differentiation |
| Efet.01.185916.g1677.t1 | zinc finger E-box-binding homeobox 2-like | 1650 | 1.30E-24 | 74.69 | F:nucleic acid binding; F:metal ion binding |
| Efet.01.185932.g1678.t1 | zinc finger and BTB domain-containing 41 | 1392 | 2.48E-37 | 56.9 | F:nucleic acid binding; F:metal ion binding |
| Efet.01.185941.g1680.t1 | pre-mRNA-splicing factor CWC22 homolog | 618 | 1.01E-34 | 91.1 | F:RNA binding |
| Efet.01.185951.g1681.t1 | gluconolaconase | 990 | 0 | 74 | F:gluconolactonase activity |
| Efet.01.185951.g1682.t1 | creatininase | 819 | 0 | 87.45 | F:hydrolase activity |
| Efet.01.185951.g1683.t1 | ABC transporter ATP-binding | 909 | 1.10E-172 | 80.7 | F:ATPase activity; F:ATP binding |
| Efet.01.185951.g1684.t1 | family transport system permease | 774 | 1.19E-136 | 97.15 | F:ATP binding; C:integral component of membrane; P:transport; C:plasma membrane |
| Efet.01.185951.g1685.t1 | FAD-binding oxidoreductase | 888 | 1.32E-170 | 75 | F:oxidoreductase activity, acting on CH-OH group of donors; F:flavin adenine dinucleotide binding; P:oxidation-reduction process |
| Efet.01.185951.g1686.t1 | ABC transporter substrate-binding | 1056 | 0 | 94.25 | P:thiamine biosynthetic process |
| Efet.01.185952.g1687.t1 | hypothetical protein CAPTEDRAFT_221832 | 213 | 9.70E-15 | 72 | C:membrane |
| Efet.01.18596.g1378.t1 | lactadherin-like isoform X5 | 495 | 6.85E-54 | 71.2 |  |
| Efet.01.185967.g1688.t1 | transcription factor 15-like | 726 | 3.52E-26 | 81.15 | P:embryo development; P:regulation of cartilage development; P:tendon formation; P:transcription, DNA-templated; F:transcription regulatory region DNA binding; F:sequence-specific DNA binding; F:protein dimerization activity; P:negative regulation of cellular process; P:positive regulation of developmental process; C:intracellular part; P:heart valve morphogenesis; P:regulation of transcription from RNA polymerase II promoter; P:bone development; P:positive regulation of transcription, DNA-templated; P:cellular response to BMP stimulus; P:positive regulation of multicellular organismal process; P:cell differentiation |
| Efet.01.18598.g1379.t1 | sex peptide receptor-like | 300 | 3.50E-32 | 75.45 | F:G-protein coupled peptide receptor activity; P:G-protein coupled receptor signaling pathway; C:integral component of membrane |
| Efet.01.18598.g1380.t1 | sex peptide receptor-like | 891 | 2.88E-69 | 62.75 | F:G-protein coupled peptide receptor activity; P:G-protein coupled receptor signaling pathway; C:integral component of membrane |
| Efet.01.186014.g1689.t1 | reelin-like | 429 | 4.46E-33 | 60.75 |  |
| Efet.01.186059.g1692.t1 | hypothetical protein CAPTEDRAFT_213446 | 840 | 1.07E-54 | 49.05 | C:membrane; C:integral component of membrane |
| Efet.01.1861.g165.t1 | Reticulocyte-binding 2-like a | 336 | 2.15E-18 | 60.53 |  |
| Efet.01.186206.g1696.t1 | hypothetical protein CAPTEDRAFT_220197 | 702 | 2.18E-83 | 71 |  |
| Efet.01.186217.g1697.t1 | zinc finger OZF-like isoform X3 | 435 | 1.61E-24 | 74.1 | F:nucleic acid binding; F:metal ion binding |
| Efet.01.186218.g1698.t1 | coiled-coil domain-containing 158-like | 1089 | 7.88E-26 | 56.5 |  |
| Efet.01.18622.g1381.t1 | chondroitin sulfate synthase 3 | 1080 | 1.36E-67 | 55.6 | C:Golgi cisterna membrane; C:membrane; C:integral component of membrane; F:acetylgalactosaminyltransferase activity |
| Efet.01.186255.g1699.t1 | Tyrosine- kinase | 285 | 4.47E-25 | 71.6 | F:protein kinase activity; P:protein phosphorylation |
| Efet.01.186327.g1702.t1 | centaurin-gamma-1A isoform X2 | 201 | 9.63E-31 | 90.1 | F:GTP binding; P:small GTPase mediated signal transduction; C:membrane; F:GTPase activator activity; C:intracellular; P:positive regulation of GTPase activity |
| Efet.01.18637.g1383.t1 | calcium-activated potassium channel slowpoke isoform X16 | 261 | 1.30E-17 | 74.15 | P:potassium ion transport; P:transmembrane transport; F:gated channel activity; C:integral component of membrane; F:potassium channel activity |
| Efet.01.18637.g1384.t1 | calcium-activated potassium channel slowpoke-like isoform X4 | 681 | 5.69E-105 | 68.5 | P:potassium ion transport; P:transmembrane transport; F:gated channel activity; C:integral component of membrane; F:potassium channel activity |
| Efet.01.186462.g1703.t1 | dopey-1 homolog isoform X1 | 840 | 2.65E-105 | 78.1 |  |
| Efet.01.186526.g1704.t1 | hypothetical protein CAPTEDRAFT_223820 | 282 | 2.12E-20 | 64 |  |
| Efet.01.186595.g1706.t1 | spermine oxidase-like | 1014 | 5.22E-56 | 50.1 | P:polyamine metabolic process |
| Efet.01.186608.g1707.t1 | frizzled 9 10 | 1521 | 3.48E-98 | 82.45 | F:G-protein coupled receptor activity; F:Wnt-activated receptor activity; P:G-protein coupled receptor signaling pathway; P:multicellular organism development; C:integral component of membrane; P:canonical Wnt signaling pathway; F:Wnt-protein binding; C:plasma membrane; P:non-canonical Wnt signaling pathway |
| Efet.01.186756.g1710.t1 | cilia- and flagella-associated 157 | 525 | 1.79E-09 | 61.33 |  |
| Efet.01.186761.g1711.t1 | guanylyl cyclase GC-E-like | 1656 | 2.08E-82 | 51.25 | F:phosphorus-oxygen lyase activity; F:protein kinase activity; F:phosphate ion binding; P:protein phosphorylation; P:transport; C:intracellular; F:ATP binding; P:phosphate ion transport; C:membrane; C:integral component of membrane; P:intracellular signal transduction; P:phosphate ion transmembrane transport; P:cyclic nucleotide biosynthetic process; C:ATP-binding cassette (ABC) transporter complex |
| Efet.01.186787.g1712.t1 | pleckstrin homology domain-containing family G member 4B-like isoform X5 | 648 | 5.05E-70 | 73.45 | P:biological regulation |
| Efet.01.186810.g1714.t1 | helicase ARIP4-like isoform X1 | 246 | 7.25E-38 | 77.85 | F:ATP binding |
| Efet.01.186854.g1716.t1 | dual specificity phosphatase 26-like | 1908 | 3.14E-61 | 63.75 | F:protein tyrosine phosphatase activity; P:peptidyl-tyrosine dephosphorylation; F:protein tyrosine/serine/threonine phosphatase activity |
| Efet.01.186881.g1718.t1 | ETS isoform P2 D | 327 | 1.42E-12 | 64.55 | C:nucleus; F:DNA binding; P:cell differentiation; F:RNA polymerase II transcription factor activity, sequence-specific DNA binding; P:regulation of transcription from RNA polymerase II promoter |
| Efet.01.186887.g1719.t1 | ARF GTPase-activating GIT2-like isoform X1 | 234 | 9.07E-26 | 78.55 | F:GTPase activator activity; P:positive regulation of GTPase activity |
| Efet.01.186906.g1721.t1 | zinc finger 239-like | 825 | 4.75E-81 | 68.4 | F:binding |
| Efet.01.186939.g1722.t1 | FAM50 homolog | 498 | 5.81E-107 | 91.4 | C:nucleus |
| Efet.01.186942.g1723.t1 | histone-lysine N-methyltransferase ASH1L-like | 618 | 2.17E-24 | 76.35 | C:nucleus; F:DNA binding; F:zinc ion binding; P:histone lysine methylation; F:histone-lysine N-methyltransferase activity; F:chromatin binding |
| Efet.01.186953.g1724.t1 | lethal(2) giant larvae homolog 1-like | 528 | 3.12E-26 | 68.65 |  |
| Efet.01.186957.g1726.t1 | cyclic nucleotide-gated cation channel alpha-3-like | 363 | 7.14E-54 | 87.75 | C:integral component of plasma membrane; F:voltage-gated potassium channel activity; P:regulation of membrane potential; P:potassium ion transmembrane transport; F:cGMP-dependent protein kinase activity; P:protein phosphorylation |
| Efet.01.186957.g1727.t1 | cyclic-nucleotide-gated cation | 480 | 3.95E-41 | 88.7 | C:integral component of plasma membrane; F:voltage-gated potassium channel activity; P:regulation of membrane potential; P:potassium ion transmembrane transport; F:cGMP-dependent protein kinase activity; P:protein phosphorylation |
| Efet.01.186994.g1730.t1 | FMRFamide receptor-like | 1287 | 1.49E-129 | 56.8 | F:G-protein coupled receptor activity; C:membrane |
| Efet.01.187044.g1733.t1 | dynamin-binding -like | 1248 | 5.38E-46 | 51.9 | P:regulation of Rho protein signal transduction; C:cytoplasm; F:guanyl-nucleotide exchange factor activity; F:Rho guanyl-nucleotide exchange factor activity; P:intracellular signal transduction; P:positive regulation of GTPase activity |
| Efet.01.187047.g1734.t1 | NEDD1 isoform X1 | 207 | 6.41E-26 | 83.2 |  |
| Efet.01.18708.g1386.t1 | Interleukin enhancer-binding factor 2-like | 789 | 5.17E-145 | 86.15 | F:DNA binding; F:ATP binding; P:immune response; C:intracellular ribonucleoprotein complex; F:double-stranded RNA binding; P:transcription, DNA-templated; P:positive regulation of transcription, DNA-templated; C:membrane; C:nucleolus; F:transferase activity; C:nucleoplasm; F:RNA binding |
| Efet.01.187083.g1736.t1 | fibroblast growth factor receptor 2-like isoform X1 | 684 | 8.17E-19 | 65.35 | P:negative regulation of astrocyte differentiation; P:response to axon injury; P:positive regulation of tyrosine phosphorylation of STAT protein; P:lens morphogenesis in camera-type eye; P:central nervous system myelination; P:forebrain development; P:apoptotic process; P:negative regulation of developmental growth; P:positive regulation of protein ubiquitination; P:epithelial cell fate commitment; P:positive regulation of bone mineralization; P:inner ear receptor cell differentiation; P:p38MAPK cascade; P:cochlea development; P:positive regulation of canonical Wnt signaling pathway; C:lysosome; F:protein binding; P:morphogenesis of an epithelium; P:positive regulation of catalytic activity; P:negative regulation of epithelial cell proliferation; P:cartilage development; P:axonogenesis involved in innervation; P:bone trabecula morphogenesis; P:bone morphogenesis; P:positive regulation of neuron apoptotic process; C:cytoplasmic side of plasma membrane; F:nucleotide binding; P:negative regulation of transcription from RNA polymerase II promoter; F:fibroblast growth factor-activated receptor activity; P:positive regulation of MAPKKK cascade by fibroblast growth factor receptor signaling pathway; P:regulation of osteoclast differentiation; P:somatic stem cell population maintenance; C:cytoplasmic vesicle; P:regulation of collagen metabolic process; P:lens fiber cell development; P:post-anal tail morphogenesis; P:alveolar secondary septum development; P:negative regulation of mitotic nuclear division; P:bone mineralization; P:positive regulation of cell differentiation; P:digestive tract morphogenesis; P:negative regulation of smoothened signaling pathway; P:substantia nigra development; P:ERK1 and ERK2 cascade; C:perinuclear region of cytoplasm; P:regulation of bone remodeling; C:integral component of membrane; P:positive regulation of cell proliferation in bone marrow; C:endomembrane system; P:positive regulation of endothelial cell proliferation; P:bone maturation |
| Efet.01.187085.g1737.t1 | turtle isoform X3 | 348 | 8.15E-15 | 56.1 | C:membrane; C:integral component of membrane |
| Efet.01.187196.g1739.t1 | hypothetical protein HELRODRAFT_175680 | 813 | 3.02E-15 | 59.5 | P:regulation of apoptotic process |
| Efet.01.1872.g167.t1 | SCP_HEDDI ame: Full=Sarcoplasmic calcium-binding Short=SCP | 213 | 1.36E-23 | 70.2 | F:calcium ion binding |
| Efet.01.18720.g1389.t1 | DNA polymerase theta-like isoform X1 | 1491 | 6.15E-76 | 66.6 | F:DNA-directed DNA polymerase activity; F:organic cyclic compound binding; F:heterocyclic compound binding; P:negative regulation of double-strand break repair via homologous recombination; P:double-strand break repair via alternative nonhomologous end joining; P:somatic hypermutation of immunoglobulin genes; P:cellular macromolecule biosynthetic process |
| Efet.01.187247.g1740.t1 | Segment polarity dishevelled DVL-3 | 1311 | 1.35E-19 | 59.45 | P:Wnt signaling pathway; P:intracellular signal transduction; C:intracellular |
| Efet.01.187271.g1741.t1 | hypothetical protein HELRODRAFT_187853 | 264 | 1.44E-06 | 69.43 | F:nucleotide binding; F:nucleic acid binding; F:ATP binding |
| Efet.01.187290.g1742.t1 | dynein heavy chain axonemal-like isoform X1 | 1014 | 6.78E-96 | 75.5 | F:nucleotide binding; P:movement of cell or subcellular component; C:dynein complex; F:nucleoside-triphosphatase activity |
| Efet.01.187303.g1743.t1 | hypothetical protein CAPTEDRAFT_222159 | 720 | 3.96E-23 | 59.67 | C:membrane; C:integral component of membrane |
| Efet.01.187322.g1744.t1 | cell death specification 2-like | 291 | 4.59E-11 | 67.46 | F:transcription factor activity, sequence-specific DNA binding; P:regulation of transcription, DNA-templated; F:sequence-specific DNA binding |
| Efet.01.187337.g1745.t1 | ubiquitin carboxyl-terminal hydrolase 25-like isoform X2 | 231 | 9.63E-28 | 80.25 | P:protein deubiquitination; F:thiol-dependent ubiquitinyl hydrolase activity; P:ubiquitin-dependent protein catabolic process |
| Efet.01.18734.g1391.t1 | sodium channel alpha-subunit SCAP1 | 207 | 1.45E-15 | 89.6 | P:regulation of postsynaptic membrane potential; P:regulation of ion transmembrane transport; C:voltage-gated sodium channel complex; P:sodium ion transmembrane transport; F:voltage-gated sodium channel activity |
| Efet.01.187355.g1747.t1 | titin-like isoform X2 | 690 | 4.16E-45 | 60.2 |  |
| Efet.01.187366.g1748.t1 | potassium voltage-gated channel subfamily KQT member 1 isoform X3 | 225 | 4.85E-07 | 61.4 | P:potassium ion transport; F:ion channel activity; F:voltage-gated potassium channel activity; P:ion transport; P:potassium ion transmembrane transport; P:transmembrane transport; C:voltage-gated potassium channel complex; P:ion transmembrane transport; C:membrane; C:integral component of membrane; P:transport |
| Efet.01.187432.g1751.t1 | heme oxygenase | 726 | 1.14E-62 | 58.25 | F:oxidoreductase activity; C:membrane; P:oxidation-reduction process |
| Efet.01.187433.g1752.t1 | RIMS-binding 2 | 513 | 5.10E-12 | 72 | P:negative regulation of phosphatase activity |
| Efet.01.187447.g1753.t1 | hypothetical protein HELRODRAFT_179428 | 894 | 4.88E-06 | 49 | F:zinc ion binding; F:metal ion binding; C:intracellular |
| Efet.01.187450.g1754.t1 | SRPBCC domain-containing | 528 | 9.02E-54 | 82.5 |  |
| Efet.01.187462.g1755.t1 | ZDHHC-type palmitoyltransferase 6 isoform X2 | 450 | 7.37E-24 | 53.95 | F:transferase activity, transferring acyl groups; F:protein-cysteine S-palmitoyltransferase activity; F:zinc ion binding; F:metal ion binding; C:membrane; C:integral component of membrane; F:transferase activity |
| Efet.01.187500.g1757.t1 | G- coupled receptor 158 | 906 | 3.08E-164 | 71.65 | C:integral component of membrane |
| Efet.01.187507.g1758.t1 | neuronal acetylcholine receptor subunit alpha-7-like | 306 | 2.60E-10 | 62.25 | P:synaptic transmission, cholinergic; F:acetylcholine receptor activity; P:neuromuscular synaptic transmission; P:cation transmembrane transport; F:acetylcholine binding; C:acetylcholine-gated channel complex; P:response to nicotine |
| Efet.01.187521.g1759.t1 | dynein heavy chain axonemal-like | 465 | 5.30E-79 | 84.35 | F:ATPase activity; F:ATP binding; F:microtubule motor activity; C:dynein complex; P:microtubule-based movement |
| Efet.01.187521.g1760.t1 | dynein heavy chain axonemal | 249 | 1.55E-42 | 84.9 | F:ATPase activity; F:ATP binding; P:heart development; C:outer dynein arm; F:microtubule motor activity; P:lateral ventricle development; P:flagellated sperm motility; P:outer dynein arm assembly; C:integral component of membrane; P:cilium movement; P:determination of left/right symmetry |
| Efet.01.187525.g1761.t1 | neuropeptide Y receptor-like | 939 | 6.89E-96 | 70.7 | P:neuropeptide signaling pathway; C:integral component of membrane; F:neuropeptide Y receptor activity |
| Efet.01.187527.g1762.t1 | BTB POZ domain-containing KCTD6-like | 645 | 1.08E-32 | 69.55 | F:identical protein binding; F:ankyrin binding |
| Efet.01.187552.g1763.t1 | mitogen-activated kinase 7-like | 954 | 8.25E-135 | 69.35 | F:nucleotide binding; F:protein serine/threonine kinase activity; P:protein phosphorylation |
| Efet.01.187616.g1765.t1 | hypothetical protein CAPTEDRAFT_226428 | 477 | 9.03E-46 | 62.25 | F:nucleic acid binding; F:protein dimerization activity |
| Efet.01.187618.g1766.t1 | inactive rhomboid 1-like | 480 | 5.66E-23 | 65.7 | P:proteolysis; C:integral component of membrane; F:serine-type endopeptidase activity |
| Efet.01.187627.g1767.t1 | neuroglian-like isoform X2 | 312 | 6.09E-40 | 66.4 | C:integral component of membrane |
| Efet.01.18764.g1392.t1 | E3 ubiquitin- ligase Mdm2 isoform X2 | 885 | 2.03E-27 | 41.85 | P:heart development; P:regulation of macromolecule metabolic process; P:negative regulation of cellular process; F:binding; P:cellular process |
| Efet.01.187700.g1768.t1 | transient-receptor-potential | 879 | 2.50E-102 | 78.95 | F:calcium channel activity; C:integral component of membrane; P:calcium ion transmembrane transport |
| Efet.01.187709.g1770.t1 | embryonic stem cell-specific 5-hydroxymethylcytosine-binding | 519 | 5.60E-22 | 69.7 | F:DNA binding; P:proteolysis; F:peptidase activity; F:hydrolase activity |
| Efet.01.18772.g1393.t1 | prefoldin subunit 5 | 264 | 6.30E-26 | 74.5 | F:ATP binding; C:prefoldin complex; F:unfolded protein binding; F:protein serine/threonine kinase activity; P:protein folding; P:protein phosphorylation |
| Efet.01.187731.g1771.t1 | probable helicase with zinc finger domain | 864 | 3.72E-61 | 60.2 | F:binding |
| Efet.01.187760.g1773.t1 | serine threonine- phosphatase 5 | 309 | 9.17E-61 | 92.6 | C:nucleus; F:phosphoprotein phosphatase activity; C:cytoplasm; P:protein dephosphorylation |
| Efet.01.187822.g1775.t1 | dynein heavy chain axonemal | 918 | 3.42E-117 | 62.05 | F:nucleotide binding; F:ATPase activity; F:ATP binding; F:microtubule motor activity; C:dynein complex; P:microtubule-based movement |
| Efet.01.187847.g1776.t1 | N6-adenosine-methyltransferase 70 kDa subunit | 1707 | 0 | 71.8 | C:nucleus; F:mRNA (2'-O-methyladenosine-N6-)-methyltransferase activity; P:mRNA methylation |
| Efet.01.187884.g1777.t1 | ras-related Rab-5B-like | 210 | 7.45E-28 | 89.9 | F:GTP binding; C:endocytic vesicle; C:early endosome; P:small GTPase mediated signal transduction; P:regulation of endocytosis; C:plasma membrane |
| Efet.01.187899.g1778.t1 | exonuclease mut-7 homolog isoform X1 | 369 | 1.16E-18 | 73.35 | F:exonuclease activity; P:nucleobase-containing compound metabolic process |
| Efet.01.187902.g1779.t1 | solute carrier organic anion transporter family member 3A1 isoform X3 | 837 | 5.62E-36 | 48.2 | P:ion transport; C:membrane; C:integral component of membrane; P:transport; F:transporter activity; C:plasma membrane |
| Efet.01.187916.g1780.t1 | mediator of RNA polymerase II transcription subunit 1-like | 630 | 4.50E-07 | 50.4 | F:insulin receptor binding |
| Efet.01.18793.g1394.t1 | copper-transporting ATPase 1-like isoform X3 | 816 | 9.50E-89 | 75.35 | P:cation transport; F:metal ion binding; C:membrane; F:hydrolase activity |
| Efet.01.187959.g1783.t1 | RNA-binding fusilli | 732 | 5.94E-107 | 76.25 | F:nucleotide binding; F:nucleic acid binding |
| Efet.01.187965.g1785.t1 | ATP-binding cassette sub-family C member 8 | 720 | 4.84E-74 | 62.45 | P:potassium ion transport; F:ATP binding; F:sulfonylurea receptor activity; F:ATPase activity, coupled to transmembrane movement of substances; P:transmembrane transport; P:signal transduction; C:integral component of membrane |
| Efet.01.188005.g1787.t1 | glutamate receptor NMDA 3A-like | 633 | 2.12E-16 | 49.6 | P:ion transmembrane transport; C:synapse; F:receptor activity; F:ionotropic glutamate receptor activity; F:extracellular-glutamate-gated ion channel activity; P:transport; C:plasma membrane; F:ion channel activity; P:ion transport; C:postsynaptic membrane; C:membrane; C:integral component of membrane; C:cell junction; P:ionotropic glutamate receptor signaling pathway |
| Efet.01.188016.g1788.t1 | RAC-gamma serine threonine- kinase-like isoform X1 | 258 | 6.48E-15 | 88.2 | P:response to food; P:cellular response to decreased oxygen levels; P:cellular response to nerve growth factor stimulus; P:positive regulation of fat cell differentiation; P:glucose metabolic process; P:positive regulation of sequence-specific DNA binding transcription factor activity; P:positive regulation of glycogen biosynthetic process; P:cellular response to prostaglandin E stimulus; F:phosphatidylinositol-3,4,5-trisphosphate binding; P:inflammatory response; P:positive regulation of cell growth; P:negative regulation of fatty acid beta-oxidation; P:positive regulation of nitric-oxide synthase activity; P:negative regulation of cysteine-type endopeptidase activity involved in apoptotic process; P:protein import into nucleus, translocation; C:cytosol; P:peripheral nervous system myelin maintenance; P:regulation of neuron projection development; P:positive regulation of peptidyl-serine phosphorylation; P:positive regulation of protein localization to nucleus; F:protein kinase binding; P:insulin-like growth factor receptor signaling pathway; C:protein complex; F:protein serine/threonine kinase activity; C:plasma membrane; C:nucleus; P:activation-induced cell death of T cells; P:negative regulation of gene expression; P:cellular response to granulocyte macrophage colony-stimulating factor stimulus; C:cell-cell junction; P:labyrinthine layer blood vessel development; P:negative regulation of autophagy; P:negative regulation of plasma membrane long-chain fatty acid transport; P:glucose homeostasis; P:cellular response to epidermal growth factor stimulus; C:vesicle; P:lipopolysaccharide-mediated signaling pathway; P:maintenance of protein location in mitochondrion; P:establishment of protein localization to mitochondrion; P:response to insulin-like growth factor stimulus; P:glucose transport; P:positive regulation of endodeoxyribonuclease activity; P:peptidyl-serine phosphorylation; P:positive regulation of proteasomal ubiquitin-dependent protein catabolic process; P:peptidyl-threonine phosphorylation; F:14-3-3 protein binding; P:protein ubiquitination; P:positive regulation of nitric oxide biosynthetic process; P:insulin receptor signaling pathway; F:phosphatidylinositol-3,4-bisphosphate binding; P:positive regulation of transcription from RNA polymerase II promoter; P:negative regulation of protein kinase activity; P:execution phase of apoptosis; P:maternal placenta development; P:positive regulation of cyclin-dependent protein serine/threonine kinase activity involved in G1/S transition of mitotic cell cycle; P:glycogen cell differentiation involved in embryonic placenta development; F:ATP binding; P:response to UV-A; P:protein kinase B signaling; F:protein serine/threonine/tyrosine kinase activity; P:apoptotic mitochondrial changes; P:striated muscle cell differentiation; P:osteoblast differentiation; P:response to fluid shear stress; P:germ cell development; F:identical protein binding; P:negative regulation of release of cytochrome c from mitochondria; C:ciliary basal body; P:positive regulation of establishment of protein localization to plasma membrane; P:protein catabolic process; P:positive regulation of glucose import; P:response to growth hormone; P:positive regulation of blood vessel endothelial cell migration; P:positive regulation of lipid biosynthetic process; P:glycogen metabolic process; C:mitochondrion; C:spindle; P:positive regulation of sodium ion transport; P:regulation of myelination; P:positive regulation of endothelial cell proliferation; P:cellular response to vascular endothelial growth factor stimulus; F:nitric-oxide synthase regulator activity; P:positive regulation of fibroblast migration |
| Efet.01.188067.g1789.t1 | RING finger 17-like isoform X1 | 729 | 2.17E-36 | 54.6 |  |
| Efet.01.188078.g1791.t1 | spectrin alpha non-erythrocytic 1-like isoform X1 | 717 | 3.11E-69 | 70.4 | F:binding |
| Efet.01.18810.g1395.t1 | hypothetical protein LOTGIDRAFT_175217 | 1473 | 1.14E-08 | 69 | F:receptor binding |
| Efet.01.188110.g1792.t1 | cytoskeletal protein 4.1 | 324 | 1.85E-06 | 70.67 | C:cytoskeleton; F:actin binding; C:cytoplasm; F:structural molecule activity; F:cytoskeletal protein binding; C:extrinsic component of membrane |
| Efet.01.188217.g1799.t1 | Pleckstrin domain-containing family A member 7 | 525 | 1.60E-40 | 73 |  |
| Efet.01.188256.g1800.t1 | Short transient receptor potential channel 3 | 822 | 7.59E-76 | 79.85 | C:integral component of plasma membrane; F:store-operated calcium channel activity; P:manganese ion transport; P:regulation of cytosolic calcium ion concentration; P:calcium ion transmembrane transport; P:single fertilization |
| Efet.01.188280.g1801.t1 | mitochondrial cardiolipin hydrolase-like | 369 | 3.13E-48 | 75.45 | F:protein homodimerization activity; F:cardiolipin synthase activity; P:spermatid development; P:mitochondrial fusion; P:DNA methylation involved in gamete generation; P:P granule organization; P:piRNA metabolic process; C:mitochondrial outer membrane; P:meiotic nuclear division; P:cardiolipin biosynthetic process; F:cardiolipin hydrolase activity |
| Efet.01.188316.g1803.t1 | eIF-2-alpha kinase activator GCN1-like | 285 | 1.23E-08 | 64.89 | P:regulation of cellular metabolic process |
| Efet.01.188354.g1804.t1 | cell death abnormality 1-like | 1365 | 3.05E-61 | 46.65 | F:calcium ion binding |
| Efet.01.18836.g1397.t1 | dynein heavy chain axonemal-like isoform X1 | 687 | 2.52E-60 | 65.75 | F:nucleotide binding; F:ATPase activity; F:ATP binding; F:microtubule motor activity; C:motile cilium; C:dynein complex; P:microtubule-based movement |
| Efet.01.18836.g1398.t1 | Dynein heavy chain axonemal | 237 | 1.11E-23 | 51.1 | F:nucleotide binding; F:ATPase activity; F:ATP binding; F:microtubule motor activity; C:motile cilium; C:dynein complex; P:microtubule-based movement |
| Efet.01.188391.g1805.t1 | hypothetical protein CAPTEDRAFT_73789, partial | 1230 | 2.88E-23 | 58 | C:integral component of plasma membrane; P:riboflavin transport; C:membrane; C:integral component of membrane; F:riboflavin transporter activity |
| Efet.01.188396.g1806.t1 | pre-mRNA-splicing factor CWC22 homolog | 678 | 2.83E-64 | 74.8 | F:RNA binding; C:catalytic step 2 spliceosome; C:U2-type catalytic step 1 spliceosome; P:mRNA splicing, via spliceosome |
| Efet.01.188445.g1808.t1 | kinase C and casein kinase substrate in neurons 1 isoform X2 | 306 | 3.04E-12 | 69.3 | C:endosome; P:regulation of endocytosis |
| Efet.01.188445.g1809.t1 | kinase C and casein kinase II substrate 3 isoform X1 | 426 | 7.50E-45 | 70.1 | C:endosome; P:regulation of endocytosis |
| Efet.01.18848.g1399.t1 | teneurin-m-like isoform X2 | 1005 | 1.69E-130 | 68.45 | F:catalytic activity; C:membrane; C:integral component of membrane |
| Efet.01.188497.g1810.t1 | parafibromin-like | 216 | 2.74E-11 | 83 | P:histone modification; C:Cdc73/Paf1 complex; P:transcription elongation from RNA polymerase II promoter |
| Efet.01.188519.g1811.t1 | filamin-C-like isoform X1 | 309 | 4.71E-26 | 80.9 |  |
| Efet.01.188578.g1813.t1 | baculoviral IAP repeat-containing 6-like | 435 | 3.19E-39 | 62.29 | F:nucleotide binding; F:ubiquitin-protein transferase activity; F:ATP binding; C:cytoplasm; F:chromatin binding; P:regulation of signal transduction; P:protein ubiquitination |
| Efet.01.188605.g1815.t1 | neural-cadherin 2 | 528 | 1.30E-53 | 68.5 | F:calcium ion binding; C:integral component of membrane; P:homophilic cell adhesion via plasma membrane adhesion molecules; C:plasma membrane |
| Efet.01.188605.g1816.t1 | neural-cadherin-like isoform X2 | 714 | 5.90E-46 | 59.7 | C:membrane |
| Efet.01.188614.g1817.t1 | unconventional myosin-XVIIIa-like isoform X1 | 525 | 2.34E-17 | 48.77 | P:signal transduction |
| Efet.01.188657.g1820.t1 | sodium leak channel non-selective | 357 | 1.46E-32 | 85.85 | C:voltage-gated calcium channel complex; P:regulation of ion transmembrane transport; P:calcium ion transmembrane transport; F:voltage-gated calcium channel activity |
| Efet.01.188662.g1821.t1 | hypothetical protein CAPTEDRAFT_218741 | 438 | 1.72E-18 | 54.8 | F:calcium ion binding |
| Efet.01.188710.g1824.t1 | beta-1,3-galactosyl-O-glycosyl-glyco beta-1,6-N-acetylglucosaminyltransferase 3-like | 921 | 5.53E-103 | 64.95 | F:acetylglucosaminyltransferase activity; C:membrane; C:integral component of membrane |
| Efet.01.188723.g1825.t1 | FMRFamide-activated amiloride-sensitive sodium channel isoform X1 | 1569 | 2.21E-96 | 49.75 | P:sodium ion transport; P:ion transport; F:sodium channel activity; C:membrane; C:integral component of membrane; P:sodium ion transmembrane transport; P:transport |
| Efet.01.188875.g1830.t1 | proline-rich extensin EPR1 isoform X1 | 2229 | 7.75E-90 | 45.55 |  |
| Efet.01.188880.g1831.t1 | dual specificity tyrosine-phosphorylation-regulated kinase 2 isoform X1 | 660 | 4.75E-90 | 82.1 | F:ATP binding; F:protein serine/threonine kinase activity; P:protein phosphorylation |
| Efet.01.188911.g1832.t1 | PREDICTED: uncharacterized protein LOC106068559 isoform X1 | 1167 | 1.43E-07 | 53 |  |
| Efet.01.188981.g1834.t1 | splicing factor 3B subunit 3 | 1848 | 0 | 86.6 | C:nucleus; F:RNA binding; P:mRNA splicing, via spliceosome |
| Efet.01.189.g23.t1 | Chromodomain-helicase-DNA-binding 1 | 1263 | 6.26E-35 | 67.75 | F:DNA binding |
| Efet.01.189006.g1836.t1 | protocadherin-9-like isoform X1 | 888 | 6.56E-41 | 50.85 | F:calcium ion binding; C:membrane; C:integral component of membrane; P:cell adhesion; P:homophilic cell adhesion via plasma membrane adhesion molecules; C:plasma membrane |
| Efet.01.189021.g1837.t1 | microtubule-actin cross-linking factor isoforms 1 2 3 5-like | 837 | 3.11E-73 | 62.2 | C:cytoskeleton |
| Efet.01.189027.g1838.t1 | troponin T-like isoform X5 | 522 | 4.38E-40 | 72.35 | P:regulation of muscle contraction; C:troponin complex |
| Efet.01.189049.g1839.t1 | glutamyl aminopeptidase-like | 588 | 9.35E-14 | 52.15 | F:zinc ion binding; C:membrane; P:proteolysis; C:integral component of membrane; F:aminopeptidase activity; F:metallopeptidase activity |
| Efet.01.18913.g1403.t1 | presenilin-1-like isoform X1 | 708 | 1.07E-12 | 75.35 | P:choline transport; P:negative regulation of epidermal growth factor-activated receptor activity; C:smooth endoplasmic reticulum; P:post-embryonic development; P:protein transport; P:protein glycosylation; C:rough endoplasmic reticulum; C:Golgi membrane; C:kinetochore; C:neuronal cell body; P:T cell activation involved in immune response; P:Cajal-Retzius cell differentiation; P:skin morphogenesis; P:memory; P:hematopoietic progenitor cell differentiation; P:cerebral cortex cell migration; C:endoplasmic reticulum membrane; C:growth cone; P:negative regulation of ubiquitin-protein transferase activity; P:activation of MAPKK activity; P:smooth endoplasmic reticulum calcium ion homeostasis; P:epithelial cell proliferation; P:mitochondrial transport; P:regulation of protein binding; P:positive regulation of transcription, DNA-templated; C:dendritic shaft; F:cadherin binding; P:neural retina development; P:regulation of synaptic plasticity; C:gamma-secretase complex; P:heart looping; C:neuromuscular junction; P:cell fate specification; P:regulation of synaptic transmission, glutamatergic; C:axon; P:intracellular signal transduction; P:neuron apoptotic process; P:dorsal/ventral neural tube patterning; F:aspartic-type endopeptidase activity; P:Notch receptor processing; P:regulation of resting membrane potential; P:positive regulation of dendritic spine development; C:nuclear outer membrane; P:cellular response to DNA damage stimulus; C:lysosomal membrane; P:synaptic vesicle targeting; P:Notch signaling pathway; P:somitogenesis; C:cell junction; P:protein processing; P:positive regulation of proteasomal ubiquitin-dependent protein catabolic process; P:positive regulation of MAP kinase activity; P:brain morphogenesis; P:negative regulation of neuron apoptotic process; P:thymus development; P:autophagosome assembly; P:negative regulation of transcription from RNA polymerase II promoter; C:integral component of plasma membrane; P:response to oxidative stress; P:embryonic limb morphogenesis; C:presynapse; P:neuron migration; P:negative regulation of axonogenesis; P:blood vessel development; P:positive regulation of receptor recycling; P:myeloid dendritic cell differentiation; F:PDZ domain binding; P:calcium ion transmembrane transport; C:cytoplasmic vesicle; P:canonical Wnt signaling pathway; C:aggresome; P:T cell receptor signaling pathway; P:beta-amyloid formation; P:negative regulation of protein ubiquitination involved in ubiquitin-dependent protein catabolic process; C:ciliary rootlet; P:single organismal cell-cell adhesion; F:calcium channel activity; F:beta-catenin binding; P:neuron development; P:skeletal system morphogenesis; C:mitochondrial inner membrane; C:cell surface; C:centrosome; P:membrane protein ectodomain proteolysis; C:membrane raft; P:positive regulation of apoptotic process; P:L-glutamate transport; P:negative regulation of apoptotic signaling pathway; P:positive regulation of coagulation |
| Efet.01.189133.g1841.t1 | circularly permutated Ras 1-like | 312 | 5.84E-13 | 70 |  |
| Efet.01.189150.g1842.t1 | coatomer subunit gamma-2-like | 345 | 1.39E-51 | 80.5 | C:endoplasmic reticulum membrane; P:ER to Golgi vesicle-mediated transport; P:retrograde vesicle-mediated transport, Golgi to ER; F:structural molecule activity; C:cytosol; C:Golgi membrane; P:intra-Golgi vesicle-mediated transport; C:transport vesicle; P:intracellular protein transport; C:COPI vesicle coat |
| Efet.01.18916.g1404.t1 | zinc finger OZF-like | 900 | 1.66E-23 | 50.7 | F:nucleic acid binding; C:nucleus; F:zinc ion binding; F:metal ion binding |
| Efet.01.189228.g1844.t1 | cullin-5-like isoform X2 | 258 | 1.97E-27 | 81.25 | F:ubiquitin protein ligase activity; F:ubiquitin protein ligase binding; C:Cul5-RING ubiquitin ligase complex; P:protein ubiquitination involved in ubiquitin-dependent protein catabolic process |
| Efet.01.189298.g1845.t1 | immediate early response gene 5 | 762 | 1.27E-08 | 73.27 |  |
| Efet.01.189318.g1847.t1 | 5-hydroxytryptamine receptor 2B | 948 | 1.15E-105 | 52.75 | F:G-protein coupled receptor activity; P:G-protein coupled receptor signaling pathway; C:integral component of membrane; C:cell part; C:synapse; P:multicellular organismal process |
| Efet.01.189358.g1848.t1 | Leucine-rich repeat neuronal 1 | 1692 | 2.89E-53 | 48.65 | P:axonogenesis; C:membrane; C:integral component of membrane |
| Efet.01.189358.g1849.t1 | leucine-rich repeat neuronal 1-like | 363 | 4.78E-06 | 56 | C:membrane; C:integral component of membrane |
| Efet.01.189383.g1850.t1 | polycystic kidney disease 2-like 1 | 555 | 1.67E-22 | 92.95 | P:detection of mechanical stimulus; F:calcium channel activity; F:calcium ion binding; C:integral component of membrane; P:calcium ion transmembrane transport |
| Efet.01.18939.g1405.t1 | serine threonine- kinase PLK1-like isoform X2 | 276 | 9.18E-15 | 76.85 | F:ATP binding; F:protein serine/threonine kinase activity; P:protein phosphorylation |
| Efet.01.18939.g1406.t1 | serine threonine- kinase PLK1-like | 741 | 1.09E-84 | 72.2 | P:mitotic spindle assembly checkpoint; P:cell proliferation; P:mitotic nuclear envelope disassembly; C:nucleolus; C:nucleoplasm; F:nucleotide binding; P:negative regulation of transcription from RNA polymerase II promoter; P:regulation of cell cycle G2/M phase transition; P:G2/M transition of mitotic cell cycle; P:regulation of protein binding; P:mitotic cytokinesis; P:meiotic chromosome segregation; C:cytosol; P:G2 DNA damage checkpoint; F:anaphase-promoting complex binding; P:positive regulation of protein localization to nucleus; F:protein kinase binding; P:sister chromatid cohesion; P:regulation of mitotic spindle assembly; C:spindle pole; C:condensed nuclear chromosome outer kinetochore; F:protein serine/threonine kinase activity; P:negative regulation of cyclin-dependent protein serine/threonine kinase activity; P:protein ubiquitination involved in ubiquitin-dependent protein catabolic process; F:microtubule binding; P:protein destabilization; P:establishment of protein localization; C:spindle microtubule; C:centrosome; C:spindle midzone; P:microtubule bundle formation; P:centrosome organization; P:protein localization to chromatin; P:negative regulation of apoptotic process; P:mitotic sister chromatid segregation; P:anaphase-promoting complex-dependent catabolic process; P:positive regulation of peptidyl-threonine phosphorylation; P:regulation of ubiquitin-protein ligase activity involved in mitotic cell cycle; C:midbody; P:peptidyl-serine phosphorylation; P:positive regulation of proteasomal ubiquitin-dependent protein catabolic process; P:positive regulation of ubiquitin-protein ligase activity involved in regulation of mitotic cell cycle transition |
| Efet.01.189434.g1852.t1 | Eka-cGMP gated channel alpha 1 | 606 | 8.29E-26 | 56.05 | P:ion transport |
| Efet.01.189515.g1853.t1 | PR domain zinc finger 10-like | 3204 | 7.45E-08 | 51.95 | F:nucleic acid binding; F:metal ion binding |
| Efet.01.189536.g1855.t1 | hypothetical protein CAPTEDRAFT_193290 | 924 | 7.72E-18 | 45 |  |
| Efet.01.189547.g1856.t1 | acid phosphatase | 789 | 1.25E-76 | 51.55 | P:dephosphorylation; F:acid phosphatase activity; F:hydrolase activity |
| Efet.01.189547.g1857.t1 | rho GTPase-activating 39-like | 540 | 1.49E-74 | 80.75 | C:nucleus; C:cytoskeleton; P:signal transduction; P:regulation of small GTPase mediated signal transduction; F:GTPase activator activity; C:integral component of membrane; C:cytosol; P:positive regulation of GTPase activity |
| Efet.01.189576.g1858.t1 | epidermal growth factor receptor kinase substrate 8 2 isoform X1 | 243 | 8.37E-13 | 65.55 |  |
| Efet.01.189606.g1859.t1 | hemerythrin | 228 | 1.39E-15 | 78.95 |  |
| Efet.01.189659.g1860.t1 | cilia- and flagella-associated 57-like | 564 | 2.49E-48 | 83.05 |  |
| Efet.01.189666.g1861.t1 | serine threonine- kinase SIK3 isoform X1 | 258 | 9.27E-18 | 66.4 | P:phosphorylation; F:kinase activity |
| Efet.01.189783.g1863.t1 | neurocalcin homolog | 453 | 5.28E-33 | 59.15 | P:regulation of cellular process; C:membrane; C:intracellular part |
| Efet.01.189841.g1864.t1 | AN1-type zinc finger 5-like | 528 | 4.48E-13 | 48.95 | F:DNA binding; F:zinc ion binding; F:metal ion binding |
| Efet.01.189875.g1865.t1 | substance-K receptor-like | 432 | 1.78E-27 | 84 | F:leucokinin receptor activity; P:neuropeptide signaling pathway; C:integral component of membrane; F:neuropeptide Y receptor activity; P:hormone-mediated signaling pathway |
| Efet.01.189875.g1866.t1 | neuropeptide Y receptor type 1-like | 438 | 2.46E-31 | 52.6 | P:signal transduction; F:signal transducer activity |
| Efet.01.189944.g1868.t1 | group 2 family | 456 | 6.69E-34 | 71 | F:transferase activity, transferring glycosyl groups; C:integral component of membrane; P:protein glycosylation; C:Golgi membrane; F:carbohydrate binding |
| Efet.01.189949.g1869.t1 | serine threonine- phosphatase 6 regulatory ankyrin repeat subunit B-like | 366 | 1.13E-13 | 56.35 | F:catalytic activity; C:membrane; C:integral component of membrane; P:nucleoside metabolic process; F:ADP binding; P:intracellular signal transduction; C:intracellular |
| Efet.01.189967.g1870.t1 | serine threonine- phosphatase 6 regulatory ankyrin repeat subunit A-like | 642 | 5.45E-30 | 45.8 | C:membrane; C:integral component of membrane; P:intracellular signal transduction; C:intracellular |
| Efet.01.190020.g1872.t1 | wiskott-Aldrich syndrome family member 3 isoform X1 | 342 | 1.17E-36 | 74.95 | P:regulation of developmental process; C:cytoskeleton; P:regulation of cellular process; F:actin binding; P:actin cytoskeleton organization |
| Efet.01.19004.g1407.t1 | NADP-dependent malic enzyme-like | 234 | 1.44E-26 | 77.8 | P:malate metabolic process; F:metal ion binding; F:NAD binding; F:malate dehydrogenase (decarboxylating) (NAD+) activity; P:oxidation-reduction process |
| Efet.01.190094.g1876.t1 | rho guanine nucleotide exchange factor 11-like isoform X2 | 315 | 4.16E-28 | 82.3 | P:regulation of Rho protein signal transduction; C:cytoplasm; F:metal ion binding; F:Rho guanyl-nucleotide exchange factor activity; P:intracellular signal transduction; P:positive regulation of GTPase activity |
| Efet.01.190154.g1877.t1 | Smarce1 variant | 231 | 2.92E-24 | 95.6 | C:SWI/SNF complex; P:covalent chromatin modification; F:ligand-dependent nuclear receptor binding; F:RNA polymerase II core promoter proximal region sequence-specific DNA binding; F:nucleosomal DNA binding; P:neurogenesis; P:nucleosome disassembly; C:nucleoplasm; F:RNA polymerase II distal enhancer sequence-specific DNA binding; C:nuclear chromatin; F:RNA binding; P:negative regulation of transcription, DNA-templated; C:npBAF complex; C:transcriptional repressor complex; P:ATP-dependent chromatin remodeling; F:protein N-terminus binding; C:nBAF complex; F:N-acetyltransferase activity |
| Efet.01.190176.g1878.t1 | homeobox HMX1 | 915 | 2.26E-30 | 95.5 | C:nucleus; P:regulation of transcription, DNA-templated; F:sequence-specific DNA binding; P:retinal cone cell development |
| Efet.01.190280.g1881.t1 | sodium channel type 3 subunit alpha isoform X1 | 354 | 2.18E-12 | 50.2 |  |
| Efet.01.190289.g1882.t1 | probable G- coupled receptor | 1875 | 7.10E-20 | 51.4 | F:G-protein coupled receptor activity; P:G-protein coupled receptor signaling pathway; P:signal transduction; C:membrane; C:integral component of membrane; F:signal transducer activity |
| Efet.01.190308.g1883.t1 | hypothetical protein CAPTEDRAFT_205745 | 708 | 1.61E-13 | 70 | F:chitin binding; P:chitin metabolic process; C:extracellular region |
| Efet.01.190336.g1884.t1 | zinc finger castor homolog 1-like | 429 | 2.99E-63 | 78.3 | F:metal ion binding |
| Efet.01.19034.g1410.t1 | V-type proton ATPase subunit H-like | 453 | 1.39E-65 | 88.6 | C:vacuolar proton-transporting V-type ATPase, V1 domain; P:ATP hydrolysis coupled proton transport; F:proton-transporting ATPase activity, rotational mechanism |
| Efet.01.190369.g1886.t1 | neural-cadherin isoform X3 | 771 | 4.42E-109 | 56.8 | F:calcium ion binding; C:integral component of membrane; P:homophilic cell adhesion via plasma membrane adhesion molecules; C:plasma membrane |
| Efet.01.190380.g1887.t1 | N-alpha-acetyltransferase auxiliary subunit-like | 336 | 1.31E-48 | 84.55 | C:nucleus; C:transcription factor complex; C:NatA complex; P:positive regulation of transcription, DNA-templated; F:ribosome binding; P:N-terminal peptidyl-methionine acetylation; P:negative regulation of apoptotic process; C:membrane; P:protein stabilization; F:peptide alpha-N-acetyltransferase activity; F:RNA binding |
| Efet.01.19041.g1411.t1 | ABC transporter ATP-binding | 411 | 1.14E-75 | 59.45 | F:nucleotide binding |
| Efet.01.190449.g1889.t1 | dedicator of cytokinesis 7 isoform X1 | 252 | 1.59E-17 | 85.85 | P:small GTPase mediated signal transduction; F:guanyl-nucleotide exchange factor activity; C:integral component of membrane; C:intracellular; P:positive regulation of GTPase activity |
| Efet.01.190460.g1890.t1 | ras-related Rab-18-B-like | 240 | 5.95E-06 | 50 | F:GTP binding; P:small GTPase mediated signal transduction; C:intracellular |
| Efet.01.190530.g1892.t1 | beta-1,4-galactosyltransferase galt-1-like | 1362 | 4.51E-11 | 50.5 | C:membrane; C:integral component of membrane |
| Efet.01.190530.g1893.t1 | beta-1,4-galactosyltransferase galt-1-like | 426 | 5.86E-16 | 55.4 | C:membrane; C:integral component of membrane |
| Efet.01.190538.g1895.t1 | sex peptide receptor-like | 381 | 3.83E-21 | 67.05 | F:G-protein coupled receptor activity; C:membrane |
| Efet.01.19055.g1414.t1 | transcription factor Sp9-like isoform X2 | 462 | 1.33E-54 | 77.5 | P:embryonic limb morphogenesis; F:metal ion binding; F:sequence-specific DNA binding |
| Efet.01.19056.g1415.t1 | serine threonine- kinase N2-like | 516 | 1.94E-16 | 73.7 | F:nucleotide binding; F:protein kinase activity; P:phosphorylation |
| Efet.01.190560.g1896.t1 | protocadherin beta-3-like isoform X1 | 468 | 9.89E-22 | 55.25 | P:anatomical structure morphogenesis; P:animal organ development; P:cellular process |
| Efet.01.19058.g1416.t1 | glutaredoxin-related mitochondrial | 444 | 2.99E-64 | 82.3 | F:protein disulfide oxidoreductase activity; C:mitochondrial matrix; F:metal ion binding; F:electron carrier activity; P:hemopoiesis; P:cell redox homeostasis; P:oxidation-reduction process; F:2 iron, 2 sulfur cluster binding; P:iron-sulfur cluster assembly |
| Efet.01.19062.g1417.t1 | mesocentin isoform X1 | 411 | 2.75E-14 | 66.75 | C:cytoskeleton; F:actin binding; C:cytoplasm; F:structural molecule activity; C:extrinsic component of membrane |
| Efet.01.190657.g1898.t1 | O-linked-mannose beta-1,2-N-acetylglucosaminyltransferase 1-like isoform X3 | 315 | 3.02E-25 | 57.3 | F:acetylglucosaminyltransferase activity; C:membrane; C:integral component of membrane; P:protein glycosylation |
| Efet.01.190683.g1899.t1 | Adenylate cyclase type 1 | 432 | 3.45E-14 | 61.86 | F:nucleotide binding; P:cAMP biosynthetic process; F:phosphorus-oxygen lyase activity; C:membrane; F:lyase activity; C:integral component of membrane; P:intracellular signal transduction; F:adenylate cyclase activity; C:plasma membrane; P:cyclic nucleotide biosynthetic process; C:intracellular |
| Efet.01.190689.g1900.t1 | B-cell lymphoma leukemia 11A-like | 2517 | 1.04E-37 | 83.1 | F:nucleic acid binding; F:metal ion binding |
| Efet.01.19076.g1418.t1 | Chitin synthase 6 | 717 | 9.00E-55 | 66 | F:transferase activity, transferring hexosyl groups; C:membrane; C:integral component of membrane |
| Efet.01.190792.g1901.t1 | IS256 family transposase | 411 | 9.50E-33 | 91.3 | F:DNA binding; F:transposase activity; P:transposition, DNA-mediated |
| Efet.01.190792.g1902.t1 | IS256 family transposase | 315 | 1.08E-53 | 83.9 | F:DNA binding; F:transposase activity; P:transposition, DNA-mediated |
| Efet.01.190823.g1904.t1 | LIM homeobox Lhx3-like | 474 | 8.04E-60 | 83.5 | C:nucleus; F:zinc ion binding; P:regulation of transcription, DNA-templated; F:sequence-specific DNA binding |
| Efet.01.190823.g1905.t1 | hypothetical protein CAPTEDRAFT_228137 | 384 | 1.32E-10 | 81 | C:nucleus; F:zinc ion binding; P:regulation of transcription, DNA-templated; F:sequence-specific DNA binding |
| Efet.01.190824.g1906.t1 | reversion-inducing cysteine-rich | 1071 | 1.40E-69 | 53.65 | F:nucleic acid binding; F:transposase activity; P:transposition, DNA-mediated |
| Efet.01.190873.g1910.t1 | neural-cadherin isoform X3 | 384 | 7.90E-49 | 57.9 | F:calcium ion binding; C:integral component of membrane; P:homophilic cell adhesion via plasma membrane adhesion molecules; C:plasma membrane |
| Efet.01.190874.g1911.t1 | titin isoform X1 | 351 | 3.98E-43 | 61.85 |  |
| Efet.01.190934.g1912.t1 | Acetylcholine receptor subunit beta-like 1 | 975 | 2.74E-87 | 58.95 | P:ion transport; C:membrane |
| Efet.01.190936.g1913.t1 | dynein heavy chain axonemal-like | 855 | 8.66E-141 | 86.95 | F:ATPase activity; F:ATP binding; F:microtubule motor activity; C:axonemal dynein complex; P:cilium movement |
| Efet.01.190959.g1914.t1 | hepatocyte growth factor receptor-like | 528 | 1.47E-62 | 75.7 | F:ATP binding; P:peptidyl-tyrosine phosphorylation; F:protein tyrosine kinase activity; C:integral component of membrane |
| Efet.01.190959.g1915.t1 | hypothetical protein HELRODRAFT_162326 | 480 | 1.43E-06 | 83 | F:calcium-activated potassium channel activity; P:potassium ion transport; P:potassium ion transmembrane transport; C:membrane; C:integral component of membrane |
| Efet.01.190962.g1916.t1 | leucine-rich repeat and fibronectin type III domain-containing 1 | 1089 | 1.69E-119 | 57.65 | C:membrane |
| Efet.01.19105.g1420.t1 | acetylcholine receptor subunit alpha-like isoform X2 | 1098 | 1.54E-85 | 65.7 | C:postsynaptic membrane; P:cation transmembrane transport; C:integral component of membrane; C:cell junction; F:extracellular ligand-gated ion channel activity |
| Efet.01.19107.g1422.t1 | protocadherin gamma-B1-like | 2628 | 1.90E-80 | 53 | F:calcium ion binding; C:membrane; C:integral component of membrane; P:cell adhesion; P:homophilic cell adhesion via plasma membrane adhesion molecules; C:plasma membrane |
| Efet.01.191125.g1919.t1 | hypothetical protein CAPTEDRAFT_218728 | 282 | 3.75E-12 | 59.2 | C:membrane; C:integral component of membrane; F:carbohydrate binding |
| Efet.01.191128.g1920.t1 | neuron navigator 2 isoform X2 | 213 | 3.37E-09 | 65.65 | F:nucleotide binding; F:ATP binding |
| Efet.01.191181.g1923.t1 | vacuolar sorting-associated 13A-like | 204 | 5.01E-07 | 62.43 |  |
| Efet.01.191185.g1924.t1 | E3 ubiquitin- ligase TRIM33-like | 975 | 6.23E-19 | 44.8 | F:zinc ion binding; F:metal ion binding; C:intracellular |
| Efet.01.191246.g1926.t1 | S-adenosylmethionine synthase | 423 | 7.29E-69 | 88.55 | F:ATP binding; F:metal ion binding; C:cytosol; P:S-adenosylmethionine biosynthetic process; F:methionine adenosyltransferase activity; P:one-carbon metabolic process |
| Efet.01.191256.g1927.t1 | glyoxylate reductase hydroxypyruvate reductase-like | 498 | 6.63E-68 | 76.9 | P:metabolic process |
| Efet.01.191263.g1928.t1 | hypothetical protein HELRODRAFT_175014 | 1098 | 9.33E-16 | 83 | F:metal ion binding |
| Efet.01.191288.g1930.t1 | Surfeit locus 4 homolog | 483 | 1.31E-74 | 84.65 | C:integral component of membrane |
| Efet.01.191359.g1931.t1 | MICOS complex subunit MIC60-like | 627 | 7.57E-49 | 69.4 | C:integral component of membrane; C:mitochondrial inner membrane |
| Efet.01.191362.g1932.t1 | cyclin-G-associated kinase-like | 204 | 6.91E-25 | 82.35 | F:ATP binding; P:protein dephosphorylation; F:protein kinase activity; P:protein phosphorylation; F:protein tyrosine/serine/threonine phosphatase activity |
| Efet.01.191402.g1934.t1 | phospholipid-transporting ATPase ID isoform X4 | 213 | 8.61E-23 | 89.4 | F:magnesium ion binding; F:ATP binding; P:phospholipid translocation; C:integral component of membrane; F:phospholipid-translocating ATPase activity |
| Efet.01.191461.g1935.t1 | calcium-activated potassium channel slowpoke-like isoform X4 | 357 | 2.48E-42 | 91.7 | F:calcium-activated potassium channel activity; F:voltage-gated potassium channel activity; P:potassium ion transmembrane transport; C:voltage-gated potassium channel complex |
| Efet.01.191488.g1937.t1 | PAXIP1-associated glutamate-rich 1 | 261 | 1.28E-08 | 58.6 |  |
| Efet.01.191556.g1939.t1 | glycine receptor subunit alpha-4-like | 372 | 2.77E-27 | 62.05 | P:monovalent inorganic cation transport; P:chloride transmembrane transport; C:neuronal cell body; C:synapse; C:neuron projection; P:chemical synaptic transmission, postsynaptic; P:cellular response to amino acid stimulus; P:cellular response to zinc ion; P:startle response; C:intracellular membrane-bounded organelle; P:muscle contraction; F:glycine binding; P:metal ion transport; F:zinc ion binding; F:extracellular-glycine-gated chloride channel activity; C:glycine-gated chloride channel complex; P:protein homooligomerization; F:glycine-gated chloride ion channel activity; P:cellular response to ethanol; P:neuropeptide signaling pathway; P:negative regulation of transmission of nerve impulse; F:protein binding; F:taurine binding; P:regulation of membrane potential; P:positive regulation of acrosome reaction |
| Efet.01.19163.g1423.t1 | small conductance calcium-activated potassium channel -like | 1344 | 4.37E-97 | 58.1 | P:potassium ion transport; F:photoreceptor activity; C:integral component of membrane; F:small conductance calcium-activated potassium channel activity; P:regulation of membrane potential in photoreceptor cell |
| Efet.01.191636.g1942.t1 | slit homolog 3 -like | 2418 | 4.16E-139 | 52.6 | C:membrane; C:integral component of membrane |
| Efet.01.191678.g1945.t1 | cathepsin B-like | 282 | 2.13E-38 | 76.9 | P:proteolysis; P:regulation of catalytic activity; F:cysteine-type endopeptidase activity |
| Efet.01.191763.g1946.t1 | Dipeptidyl peptidase 1 | 312 | 1.97E-20 | 76.35 | P:proteolysis; F:cysteine-type peptidase activity |
| Efet.01.191763.g1947.t1 | ADP-ribosylation factor 6 | 459 | 4.35E-34 | 63.2 | F:GTP binding; F:nucleotide binding; P:small GTPase mediated signal transduction; C:intracellular |
| Efet.01.191793.g1949.t1 | phosphoenolpyruvate carboxykinase (GTP) | 465 | 4.40E-79 | 85.85 | F:GTP binding; P:gluconeogenesis; F:phosphoenolpyruvate carboxykinase (GTP) activity; C:cytoplasm; F:manganese ion binding; P:phosphorylation; F:kinase activity |
| Efet.01.1918.g169.t1 | variable lymphocyte receptor B | 858 | 2.81E-20 | 54.6 | F:phosphoprotein phosphatase activity; P:axonogenesis; C:membrane; P:protein dephosphorylation; C:integral component of membrane; F:hydrolase activity |
| Efet.01.191806.g1950.t1 | adenosine receptor A2b | 342 | 4.27E-26 | 63.6 | F:G-protein coupled receptor activity; P:G-protein coupled receptor signaling pathway; C:membrane |
| Efet.01.191884.g1953.t1 | 14-3-3 zeta isoform X1 | 435 | 8.85E-78 | 88.25 | F:protein domain specific binding |
| Efet.01.191906.g1955.t1 | potassium voltage-gated channel subfamily B member 2-like isoform X5 | 381 | 1.30E-39 | 95.65 | F:voltage-gated potassium channel activity; P:potassium ion transmembrane transport; C:voltage-gated potassium channel complex; P:regulation of ion transmembrane transport; P:protein homooligomerization |
| Efet.01.191963.g1959.t1 | hepatocyte growth factor receptor-like | 690 | 2.73E-45 | 53.2 | P:neuron projection guidance; P:regulation of cellular process; C:membrane; C:cell part |
| Efet.01.191976.g1960.t1 | nuclear hormone receptor HR96-like isoform X3 | 570 | 3.11E-79 | 79.75 | F:thyroid hormone receptor activity; C:nucleus; F:zinc ion binding; P:transcription, DNA-templated; F:steroid hormone receptor activity; P:regulation of transcription, DNA-templated; F:sequence-specific DNA binding; P:steroid hormone mediated signaling pathway; P:intracellular receptor signaling pathway |
| Efet.01.191998.g1962.t1 | myosin heavy striated muscle-like isoform X5 | 390 | 2.32E-46 | 81.7 | C:myosin complex; F:ATP binding; F:actin binding; F:motor activity |
| Efet.01.1920.g170.t1 | kelch 20 | 321 | 7.35E-49 | 66.8 | C:nucleus; F:protein binding; C:Golgi apparatus; C:intracellular organelle part; P:transport; P:ubiquitin-dependent protein catabolic process; P:protein ubiquitination |
| Efet.01.192018.g1963.t1 | hypothetical protein CAPTEDRAFT_199259 | 228 | 2.22E-16 | 67.95 | C:membrane; C:integral component of membrane |
| Efet.01.192031.g1964.t1 | calcitonin gene-related peptide type 1 receptor-like | 279 | 2.67E-14 | 73.6 | P:G-protein coupled receptor signaling pathway; F:calcitonin receptor activity; C:integral component of membrane; P:cell surface receptor signaling pathway |
| Efet.01.192052.g1965.t1 | hypothetical protein CAPTEDRAFT_201190 | 372 | 9.47E-10 | 55 |  |
| Efet.01.19207.g1425.t1 | vacuolar sorting-associated 13D | 240 | 1.48E-13 | 67.4 |  |
| Efet.01.192165.g1967.t1 | secretin receptor-like | 357 | 2.94E-34 | 74.05 | F:G-protein coupled receptor activity; P:signal transduction; C:membrane |
| Efet.01.192196.g1969.t1 | Src tyrosine kinase 2 | 216 | 8.37E-19 | 86.25 | F:ATP binding; P:peptidyl-tyrosine phosphorylation; F:non-membrane spanning protein tyrosine kinase activity; C:cytoplasm; P:innate immune response |
| Efet.01.192282.g1974.t1 | POU class transcription factor 4 | 1077 | 8.11E-46 | 84.45 | P:sensory perception of sound; C:nucleus; F:transcription factor activity, sequence-specific DNA binding; P:negative regulation of mesenchymal cell apoptotic process; P:forebrain neuron differentiation; P:cochlea morphogenesis; F:RNA polymerase II regulatory region sequence-specific DNA binding; F:AT DNA binding; P:transcription from RNA polymerase II promoter; P:regulation of transcription from RNA polymerase II promoter |
| Efet.01.19230.g1427.t1 | neural-cadherin-like isoform X1 | 615 | 1.09E-34 | 53.1 | P:cell adhesion; F:alcohol dehydrogenase (NAD) activity; P:homophilic cell adhesion via plasma membrane adhesion molecules; C:plasma membrane; F:protein tyrosine phosphatase activity; F:oxidoreductase activity; F:calcium ion binding; C:membrane; C:integral component of membrane; F:phosphatase activity; P:oxidation-reduction process; P:dephosphorylation; P:peptidyl-tyrosine dephosphorylation; F:hydrolase activity |
| Efet.01.192375.g1976.t1 | hypothetical protein CAPTEDRAFT_229087 | 543 | 2.76E-12 | 52 | C:membrane; C:integral component of membrane; F:carbohydrate binding |
| Efet.01.192379.g1977.t1 | tubulin--tyrosine ligase 12 | 576 | 7.30E-61 | 61.8 | P:cellular protein modification process; F:ligase activity |
| Efet.01.192431.g1979.t1 | glycogen liver form | 270 | 5.90E-39 | 92.5 | F:glycogen phosphorylase activity; P:carbohydrate metabolic process; F:pyridoxal phosphate binding |
| Efet.01.19245.g1428.t1 | ectonucleoside triphosphate diphosphohydrolase 4 isoform X1 | 396 | 1.57E-14 | 57.5 | F:uridine-diphosphatase activity; C:intracellular membrane-bounded organelle; C:integral component of Golgi membrane; C:membrane; C:integral component of membrane; P:UDP catabolic process; F:hydrolase activity |
| Efet.01.192561.g1983.t1 | alpha-mannosidase 2-like | 549 | 6.40E-89 | 79.4 | F:hydrolase activity, hydrolyzing N-glycosyl compounds; P:protein deglycosylation; F:zinc ion binding; P:N-glycan processing; C:integral component of membrane; P:mannose metabolic process; C:Golgi membrane; F:mannosyl-oligosaccharide 1,3-1,6-alpha-mannosidase activity; F:carbohydrate binding |
| Efet.01.192629.g1984.t1 | monocarboxylate transporter 3 | 1137 | 8.91E-17 | 43.6 | P:transmembrane transport; C:membrane; C:integral component of membrane |
| Efet.01.192659.g1986.t1 | kelch repeat and BTB domain-containing 7 | 465 | 1.37E-16 | 53.1 | P:germinal center formation; F:ubiquitin-protein transferase activity; C:Cul3-RING ubiquitin ligase complex; P:protein ubiquitination; P:B cell receptor signaling pathway |
| Efet.01.192672.g1988.t1 | rRNA methyltransferase mitochondrial | 351 | 7.16E-35 | 78.4 | F:rRNA (uridine-2'-O-)-methyltransferase activity; C:mitochondrion; P:enzyme-directed rRNA 2'-O-methylation |
| Efet.01.192672.g1989.t1 | rRNA methyltransferase mitochondrial | 399 | 2.59E-37 | 69.5 | F:transferase activity; P:methylation |
| Efet.01.192694.g1991.t1 | Zinc finger and BTB domain-containing 40 | 3822 | 8.36E-47 | 51.45 | F:metal ion binding |
| Efet.01.192793.g1993.t1 | FMRFamide receptor-like | 1200 | 6.67E-102 | 55.25 | F:G-protein coupled receptor activity; P:signal transduction; C:membrane |
| Efet.01.192795.g1994.t1 | PREDICTED: uncharacterized protein LOC108740099 isoform X2 | 246 | 7.72E-06 | 66 |  |
| Efet.01.192821.g1995.t1 | Receptor-type tyrosine- phosphatase S | 384 | 1.19E-52 | 89.05 | P:establishment of endothelial intestinal barrier; C:extracellular exosome; F:protein tyrosine phosphatase activity; P:cerebellum development; C:integral component of membrane; P:hippocampus development; P:cerebral cortex development; P:peptidyl-tyrosine dephosphorylation; P:spinal cord development; P:corpus callosum development |
| Efet.01.1929.g171.t1 | BTB POZ domain-containing KCTD7 | 489 | 5.21E-28 | 51.65 | P:protein homooligomerization |
| Efet.01.192995.g1997.t1 | golgi-specific 1 brefeldin a-resistance guanine nucleotide exchange | 498 | 1.74E-10 | 82.69 | F:ATP binding; F:ARF guanyl-nucleotide exchange factor activity; F:protein kinase activity; P:regulation of ARF protein signal transduction; P:protein phosphorylation; P:positive regulation of GTPase activity |
| Efet.01.193011.g1998.t1 | rho GTPase-activating 24 isoform X1 | 489 | 4.08E-26 | 64.25 | F:protein binding; P:regulation of small GTPase mediated signal transduction; F:GTPase activator activity; C:cytosol; C:cell junction; P:single-organism developmental process |
| Efet.01.193022.g1999.t1 | hypothetical protein CAPTEDRAFT_224991 | 528 | 1.69E-12 | 45.33 | C:membrane; C:integral component of membrane; F:carbohydrate binding |
| Efet.01.193027.g2000.t1 | transformation transcription domain-associated -like | 966 | 2.76E-89 | 55.95 | C:nucleus; P:histone acetylation; P:phosphorylation; F:kinase activity; C:histone acetyltransferase complex |
| Efet.01.19309.g1429.t1 | Histone-lysine N-methyltransferase | 411 | 4.55E-13 | 85.5 | F:zinc ion binding; C:integral component of membrane; F:methyltransferase activity; P:methylation |
| Efet.01.1931.g172.t1 | Cullin-associated NEDD8-dissociated 1 | 708 | 1.16E-50 | 76.65 | C:extracellular exosome; C:intracellular ribonucleoprotein complex; P:negative regulation of catalytic activity; C:cullin-RING ubiquitin ligase complex; C:membrane; P:cell differentiation; C:Golgi apparatus; F:TBP-class protein binding; P:SCF complex assembly; C:nucleoplasm; P:protein ubiquitination; P:positive regulation of RNA polymerase II transcriptional preinitiation complex assembly |
| Efet.01.193113.g2001.t1 | fibrinolytic enzyme | 462 | 3.17E-50 | 66.5 | F:hydrolase activity |
| Efet.01.19314.g1430.t1 | Proteasome subunit alpha type- | 552 | 5.08E-79 | 80.6 | C:nucleus; C:cytoplasm; C:proteasome core complex, alpha-subunit complex; F:threonine-type endopeptidase activity; P:ubiquitin-dependent protein catabolic process |
| Efet.01.193176.g2004.t1 | Neuroglian | 324 | 1.09E-08 | 58.25 | F:growth factor activity; C:Nem1-Spo7 phosphatase complex; C:membrane; C:integral component of membrane; P:positive regulation of protein dephosphorylation; C:extracellular region; P:growth |
| Efet.01.193204.g2005.t1 | von Willebrand factor type EGF and pentraxin domain-containing 1-like | 282 | 6.24E-14 | 70.35 | F:calcium ion binding |
| Efet.01.19332.g1431.t1 | titin isoform X1 | 1068 | 1.44E-49 | 73.65 |  |
| Efet.01.193330.g2009.t1 | hornerin-like isoform X1 | 522 | 4.26E-35 | 81.5 | C:nucleus; F:DNA binding; P:regulation of transcription, DNA-templated |
| Efet.01.193344.g2010.t1 | Regulation of nuclear pre-mRNA domain-containing 2 | 414 | 1.36E-18 | 73 | C:DNA-directed RNA polymerase II, holoenzyme |
| Efet.01.19335.g1432.t1 | calcineurin-binding cabin-1-like | 405 | 1.88E-10 | 51.32 | P:DNA replication-independent nucleosome assembly |
| Efet.01.193366.g2011.t1 | ankyrin repeat | 519 | 2.00E-11 | 57.45 | F:aspartic-type endopeptidase activity; C:membrane; P:proteolysis; C:integral component of membrane |
| Efet.01.193392.g2012.t1 | MSL1_HUMAN ame: Full=Male-specific lethal 1 homolog Short=MSL-1 ame: Full=Male-specific lethal 1-like 1 Short=MSL1-like 1 ame: Full=Male-specific lethal-1 homolog 1 | 306 | 6.76E-07 | 82 | C:MSL complex; F:chromatin binding; P:histone H4-K16 acetylation |
| Efet.01.193439.g2014.t1 | roundabout homolog 2-like | 474 | 1.63E-24 | 61.8 | C:membrane; C:integral component of membrane |
| Efet.01.193456.g2015.t1 | U4 U6 small nuclear ribonucleo | 258 | 2.04E-06 | 59.33 | C:intracellular ribonucleoprotein complex; C:precatalytic spliceosome; C:U4atac snRNP; C:U4/U6 x U5 tri-snRNP complex; C:spliceosomal tri-snRNP complex; C:viral nucleocapsid; P:mRNA splicing, via spliceosome; P:spliceosomal tri-snRNP complex assembly; C:U4 snRNP |
| Efet.01.193473.g2017.t1 | BTB POZ domain-containing kctd15-like | 648 | 1.30E-85 | 74.1 | P:protein homooligomerization |
| Efet.01.193490.g2018.t1 | sorting nexin-25-like | 336 | 5.34E-08 | 64.33 | C:membrane; C:integral component of membrane; F:phosphatidylinositol binding |
| Efet.01.193515.g2019.t1 | hypothetical protein CAPTEDRAFT_34881, partial | 1377 | 1.26E-15 | 44 | F:metal ion binding |
| Efet.01.193521.g2020.t1 | probable palmitoyltransferase ZDHHC24 | 837 | 6.23E-57 | 51.45 | F:transferase activity, transferring acyl groups; F:zinc ion binding; F:protein-cysteine S-palmitoyltransferase activity; F:metal ion binding; C:membrane; C:integral component of membrane; F:transferase activity |
| Efet.01.193525.g2021.t1 | Zinc transporter | 312 | 1.95E-37 | 88.05 | C:nucleus; F:ligand-dependent nuclear receptor binding; C:cytoskeleton; P:cation transmembrane transport; C:integral component of membrane; F:cation transmembrane transporter activity; F:ligand-dependent nuclear receptor transcription coactivator activity; F:chromatin binding; P:positive regulation of transcription from RNA polymerase II promoter |
| Efet.01.19353.g1433.t1 | oxysterols receptor LXR-alpha isoform X1 | 219 | 5.48E-14 | 64.35 | P:positive regulation of cellular protein metabolic process; C:RNA polymerase II transcription factor complex; P:response to progesterone; F:sequence-specific DNA binding; P:regulation of circadian rhythm; P:positive regulation of transcription from RNA polymerase II promoter; P:negative regulation of immune system process; P:steroid hormone mediated signaling pathway; P:transcription initiation from RNA polymerase II promoter; C:nucleoplasm; P:positive regulation of fatty acid biosynthetic process; P:sterol homeostasis; P:negative regulation of secretion; P:negative regulation of transcription, DNA-templated; F:RNA polymerase II transcription factor activity, ligand-activated sequence-specific DNA binding; P:positive regulation of cholesterol efflux; F:steroid hormone receptor activity; P:positive regulation of receptor biosynthetic process; P:positive regulation of lipoprotein lipase activity; P:intracellular receptor signaling pathway; P:negative regulation of lipid transport; F:cholesterol binding; P:positive regulation of toll-like receptor 4 signaling pathway; P:apoptotic cell clearance; C:receptor complex; P:negative regulation of pinocytosis; C:nuclear chromatin; F:protein binding; F:transcription coactivator activity; P:cellular response to lipopolysaccharide; F:metal ion binding; P:positive regulation of triglyceride biosynthetic process; P:positive regulation of cholesterol homeostasis; P:negative regulation of macrophage derived foam cell differentiation; P:negative regulation of cholesterol storage; F:sterol response element binding; P:negative regulation of defense response |
| Efet.01.193608.g2023.t1 | gastrula zinc finger -like | 501 | 7.17E-53 | 70.9 | F:binding |
| Efet.01.193609.g2024.t1 | hypothetical protein HELRODRAFT_166528 | 4494 | 2.87E-17 | 52 |  |
| Efet.01.193609.g2025.t1 | hypothetical protein HELRODRAFT_166528 | 969 | 1.00E-09 | 60 |  |
| Efet.01.193627.g2027.t1 | double-strand-break repair rad21 homolog | 378 | 1.17E-64 | 85.4 | C:nuclear chromosome |
| Efet.01.193633.g2028.t1 | zinc transporter ZIP1-like | 945 | 4.07E-57 | 54.75 | P:transmembrane transport; C:membrane; C:integral component of membrane; F:metal ion transmembrane transporter activity; P:metal ion transport |
| Efet.01.193637.g2029.t1 | neuropeptide Y receptor-like | 627 | 1.46E-73 | 72.8 | P:neuropeptide signaling pathway; C:integral component of membrane; F:neuropeptide Y receptor activity |
| Efet.01.193642.g2030.t1 | hypothetical protein CAPTEDRAFT_227481 | 612 | 7.11E-12 | 54 |  |
| Efet.01.193724.g2031.t1 | YIPF5 isoform X2 | 273 | 1.94E-06 | 85 | C:membrane; C:integral component of membrane |
| Efet.01.193753.g2032.t1 | protocadherin-19-like isoform X4 | 2217 | 1.04E-97 | 46.6 | P:nervous system development |
| Efet.01.193761.g2033.t1 | epidermal growth factor receptor kinase substrate 8 2 isoform X1 | 288 | 2.74E-17 | 61.25 |  |
| Efet.01.193769.g2034.t1 | MAM and LDL-receptor class A domain-containing 1-like | 492 | 2.24E-16 | 54.5 | C:membrane; C:integral component of membrane |
| Efet.01.193777.g2035.t1 | kelch-like ECH-associated 1 | 411 | 5.13E-47 | 78.05 | C:Cul3-RING ubiquitin ligase complex; P:protein ubiquitination |
| Efet.01.193784.g2036.t1 | hypothetical protein HELRODRAFT_174471 | 588 | 2.47E-32 | 57.05 |  |
| Efet.01.193790.g2037.t1 | ubiquitin ligase | 405 | 6.88E-15 | 62.05 | F:ligase activity |
| Efet.01.193812.g2038.t1 | hypothetical protein CAPTEDRAFT_198743 | 225 | 9.44E-15 | 81.5 | C:integral component of membrane |
| Efet.01.193835.g2039.t1 | dynein beta ciliary-like | 2292 | 0 | 84 | F:ATPase activity; F:ATP binding; C:motile cilium; F:microtubule motor activity; C:cytoplasm; P:cell projection organization; C:dynein complex; P:microtubule-based movement; C:axoneme; C:microtubule |
| Efet.01.193835.g2040.t1 | dynein beta ciliary-like | 399 | 2.81E-70 | 87.2 | F:ATPase activity; F:ATP binding; F:microtubule motor activity; F:structural constituent of ribosome; C:integral component of membrane; C:dynein complex; C:ribosome; P:microtubule-based movement; P:translation |
| Efet.01.193841.g2042.t1 | polycystic kidney disease 1-like 2 | 927 | 1.20E-10 | 44.8 | P:regulation of biological quality; P:tube development; F:cation channel activity; P:anatomical structure morphogenesis; C:membrane part; P:kidney epithelium development; C:intracellular organelle part; P:cellular process; F:protein binding; P:localization; P:metanephros development; P:positive regulation of cellular process; C:cytoplasm; P:response to stimulus |
| Efet.01.193849.g2043.t1 | dmX 2 isoform X11 | 900 | 8.79E-47 | 50 |  |
| Efet.01.193858.g2044.t1 | atrial natriuretic peptide receptor 1-like | 327 | 1.71E-30 | 70.8 | F:nucleotide binding; F:phosphorus-oxygen lyase activity; C:integral component of membrane; P:cyclic nucleotide biosynthetic process |
| Efet.01.193924.g2046.t1 | probable E3 ubiquitin- ligase RNF217 | 981 | 3.09E-26 | 58.85 | F:catalytic activity; P:protein ubiquitination |
| Efet.01.193944.g2047.t1 | myomodulin receptor 2 | 570 | 1.00E-24 | 49.4 | F:G-protein coupled receptor activity; F:G-protein coupled peptide receptor activity; P:G-protein coupled receptor signaling pathway; P:signal transduction; C:membrane; C:integral component of membrane; F:signal transducer activity |
| Efet.01.193944.g2048.t1 | myomodulin receptor 2 | 687 | 1.75E-17 | 55.63 | F:signal transducer activity |
| Efet.01.193974.g2050.t1 | BBSome-interacting 1 | 393 | 1.93E-20 | 86.5 | C:BBSome; P:cilium assembly; P:receptor localization to non-motile cilium; C:cytoplasm; P:Kupffer's vesicle development; P:melanosome transport; P:eating behavior |
| Efet.01.194053.g2051.t1 | Heparan sulfate glucosamine 3-O-sulfotransferase | 390 | 6.89E-46 | 76 | F:transferase activity |
| Efet.01.194061.g2052.t1 | acetyl-coenzyme A synthetase 2- mitochondrial | 567 | 8.06E-75 | 90.5 | F:ATP binding; F:acetate-CoA ligase activity; F:AMP binding; C:mitochondrial matrix; P:acetyl-CoA biosynthetic process from acetate |
| Efet.01.19407.g1436.t1 | tachykinin-like peptides receptor 99D | 483 | 1.18E-51 | 73.55 | C:integral component of membrane; P:tachykinin receptor signaling pathway; C:plasma membrane; F:tachykinin receptor activity |
| Efet.01.19413.g1437.t1 | fibroblast growth factor receptor-like 1 | 450 | 9.74E-28 | 55.7 | C:membrane; C:integral component of membrane |
| Efet.01.194197.g2057.t1 | pyruvate kinase PKLR | 231 | 2.27E-17 | 76.2 | F:magnesium ion binding; F:pyruvate kinase activity; F:potassium ion binding; P:glycolytic process; F:kinase activity |
| Efet.01.19421.g1438.t1 | meckelin isoform X2 | 372 | 9.35E-28 | 59.35 | P:negative regulation of centrosome duplication; C:membrane; C:integral component of membrane; C:MKS complex |
| Efet.01.194248.g2058.t1 | glutathione gamma-glutamylcysteinyltransferase 1-like | 1551 | 0 | 66.7 | P:phytochelatin biosynthetic process; F:glutathione gamma-glutamylcysteinyltransferase activity; F:metal ion binding; P:response to metal ion |
| Efet.01.194264.g2059.t1 | laminin epi- | 390 | 3.52E-10 | 57.25 | C:membrane; C:integral component of membrane |
| Efet.01.194286.g2060.t1 | kalirin-like isoform X11 | 597 | 2.30E-66 | 72.4 | P:regulation of cellular process; F:guanyl-nucleotide exchange factor activity; F:protein kinase activity; P:cellular process |
| Efet.01.194314.g2063.t1 | trissin receptor-like | 375 | 5.01E-21 | 58.7 | P:signal transduction; F:signal transducer activity |
| Efet.01.194314.g2064.t1 | trissin receptor-like | 1251 | 2.23E-12 | 60.5 | P:signal transduction; F:signal transducer activity |
| Efet.01.194380.g2066.t1 | ankyrin repeat domain-containing | 1668 | 0 | 46.8 | P:intracellular signal transduction; C:intracellular |
| Efet.01.1944.g174.t1 | plastin-2 isoform X1 | 399 | 2.50E-51 | 76.15 | C:membrane; F:binding |
| Efet.01.194411.g2067.t1 | 60 kDa neurofilament -like | 609 | 1.17E-46 | 65.75 | F:structural molecule activity; C:intermediate filament |
| Efet.01.194464.g2068.t1 | chromodomain-helicase-DNA-binding 2 | 915 | 7.55E-123 | 58.85 | F:organic cyclic compound binding; F:heterocyclic compound binding |
| Efet.01.194465.g2069.t1 | adhesion G -coupled receptor A3 | 1695 | 5.33E-16 | 45 | F:G-protein coupled receptor activity; F:transmembrane signaling receptor activity; P:G-protein coupled receptor signaling pathway; P:signal transduction; C:membrane; C:integral component of membrane; P:cell surface receptor signaling pathway; F:signal transducer activity |
| Efet.01.19449.g1440.t1 | charged multivesicular body 5 | 372 | 5.88E-28 | 81.5 | P:vacuolar transport; C:intracellular |
| Efet.01.1945.g175.t1 | PREDICTED: dystrophin-like, partial | 291 | 3.65E-06 | 62 |  |
| Efet.01.194568.g2070.t1 | ankyrin repeat domain-containing 50 | 864 | 9.02E-21 | 46.4 | P:intracellular signal transduction; C:intracellular |
| Efet.01.194568.g2071.t1 | Ankyrin repeat domain-containing 1 | 381 | 1.14E-32 | 59.4 | F:methyltransferase activity; F:transferase activity; P:intracellular signal transduction; C:intracellular; P:methylation |
| Efet.01.194571.g2072.t1 | hypothetical protein | 519 | 9.07E-81 | 58.6 |  |
| Efet.01.194579.g2073.t1 | uncharacterized protein LOC106466797 | 273 | 1.24E-10 | 58.5 | F:calcium ion binding; C:membrane; C:integral component of membrane; P:cell adhesion; P:homophilic cell adhesion via plasma membrane adhesion molecules; C:plasma membrane |
| Efet.01.19462.g1441.t1 | tankyrase | 624 | 1.36E-50 | 63.65 | F:transferase activity |
| Efet.01.194631.g2075.t1 | MULTISPECIES: alpha beta hydrolase | 471 | 6.99E-10 | 63.2 | F:hydrolase activity |
| Efet.01.19464.g1442.t1 | protogenin-like isoform X3 | 378 | 1.91E-17 | 58.19 | C:membrane; C:integral component of membrane |
| Efet.01.194660.g2076.t1 | cAMP-dependent kinase catalytic subunit | 312 | 8.16E-15 | 86.35 | F:ATP binding; P:positive regulation of oocyte maturation; P:nematode larval development; F:cAMP-dependent protein kinase activity; P:oviposition; P:protein phosphorylation; C:cAMP-dependent protein kinase complex |
| Efet.01.194666.g2077.t1 | zinc finger 950 isoform X1 | 834 | 3.18E-28 | 47.2 | P:anatomical structure morphogenesis; P:animal organ development; P:cellular process |
| Efet.01.194729.g2081.t1 | protocadherin-like wing polarity stan | 396 | 4.97E-34 | 75.65 | F:transmembrane signaling receptor activity; P:signal transduction; C:membrane |
| Efet.01.194729.g2082.t1 | protocadherin-like wing polarity stan | 3327 | 0 | 63.35 | F:transmembrane signaling receptor activity; P:signal transduction; C:membrane; P:cell adhesion |
| Efet.01.194731.g2083.t1 | regulator of G- signaling 20-like | 438 | 2.10E-08 | 85.75 | C:postsynaptic density; F:protein kinase binding; F:GTPase activator activity; P:Wnt signaling pathway involved in somitogenesis; P:regulation of transcription, DNA-templated; P:dorsal/ventral axis specification; C:cytoplasmic microtubule; F:beta-catenin binding; C:plasma membrane; P:positive regulation of GTPase activity; C:beta-catenin destruction complex; P:negative regulation of canonical Wnt signaling pathway; P:cell death; C:cell cortex; F:armadillo repeat domain binding; P:cell differentiation; P:regulation of catenin import into nucleus; P:cellular response to organic cyclic compound |
| Efet.01.194757.g2084.t1 | chromodomain-helicase-DNA-binding 1-like | 489 | 6.20E-64 | 89.7 | F:core promoter sequence-specific DNA binding; C:nucleus; F:ATP binding; F:ATP-dependent helicase activity; P:regulation of transcription, DNA-templated |
| Efet.01.194817.g2086.t1 | ubiquitin carboxyl-terminal hydrolase 21 isoform X1 | 204 | 1.18E-21 | 84.65 | P:protein deubiquitination; P:central nervous system morphogenesis; F:thiol-dependent ubiquitinyl hydrolase activity; P:ubiquitin-dependent protein catabolic process |
| Efet.01.19488.g1443.t1 | suppressor of cytokine signaling 2-like | 1194 | 1.83E-21 | 51.45 | P:regulation of cellular process |
| Efet.01.194970.g2089.t1 | chromobox homolog 1-like | 363 | 5.96E-16 | 88.3 | C:nucleus |
| Efet.01.194991.g2090.t1 | beta-centractin | 441 | 2.21E-69 | 92.8 | F:ATP binding; C:dynactin complex |
| Efet.01.195060.g2092.t1 | E3 ubiquitin- ligase HUWE1-like | 597 | 1.72E-40 | 65 | F:ubiquitin-protein transferase activity; C:membrane; C:integral component of membrane; F:ligase activity; P:protein ubiquitination |
| Efet.01.195060.g2093.t1 | E3 ubiquitin- ligase HUWE1 isoform X2 | 1104 | 8.52E-113 | 69.65 | C:membrane; C:integral component of membrane |
| Efet.01.195070.g2094.t1 | delta 1 | 765 | 1.40E-51 | 61.05 | P:cell communication; C:membrane; P:single-organism process |
| Efet.01.19509.g1445.t1 | unc-93 homolog A-like | 261 | 3.76E-08 | 70 | C:membrane; C:integral component of membrane |
| Efet.01.195111.g2097.t1 | G- coupled receptor GRL101-like isoform X1 | 312 | 3.87E-28 | 70.85 | F:G-protein coupled receptor activity; P:signal transduction; C:membrane |
| Efet.01.195116.g2098.t1 | hypothetical protein FRACYDRAFT_155556, partial | 243 | 1.80E-06 | 60 |  |
| Efet.01.195183.g2099.t1 | gastrula zinc finger -like | 1011 | 2.08E-53 | 52.8 |  |
| Efet.01.195211.g2100.t1 | RUS1 family C16orf58 homolog | 357 | 1.28E-61 | 87.35 | C:integral component of membrane |
| Efet.01.195276.g2103.t1 | neuronal acetylcholine receptor subunit alpha-10-like | 684 | 1.87E-10 | 75.11 | F:ion channel activity; P:synaptic transmission, cholinergic; F:acetylcholine receptor activity; P:neuromuscular synaptic transmission; P:signal transduction; P:cation transmembrane transport; F:acetylcholine binding; C:acetylcholine-gated channel complex; C:synapse; P:response to nicotine |
| Efet.01.195333.g2105.t1 | growth hormone secretagogue receptor type 1-like isoform X4 | 333 | 9.18E-19 | 71.15 | C:membrane |
| Efet.01.195382.g2107.t1 | otoferlin-like isoform X1 | 303 | 6.85E-11 | 68.15 | C:integral component of membrane |
| Efet.01.195505.g2110.t1 | amyloid beta A4 -like | 282 | 1.68E-10 | 70.25 | C:membrane; F:ion binding |
| Efet.01.195511.g2111.t1 | baculoviral IAP repeat-containing 6-like | 522 | 4.17E-49 | 65.1 | F:ubiquitin-protein transferase activity; C:cytoplasm; P:regulation of signal transduction; P:protein ubiquitination |
| Efet.01.195577.g2114.t1 | hypothetical protein CAPTEDRAFT_201969 | 1308 | 7.19E-09 | 55 | C:membrane; C:integral component of membrane |
| Efet.01.19558.g1446.t1 | leucine-rich repeat serine threonine- kinase 1-like isoform X1 | 975 | 2.88E-11 | 52.6 |  |
| Efet.01.19560.g1447.t1 | alkylated DNA repair alkB homolog 8 | 216 | 4.52E-10 | 65.35 | F:catalytic activity; P:metabolic process |
| Efet.01.19560.g1448.t1 | L-rhamnonate dehydratase | 933 | 8.94E-175 | 84 | P:cellular amino acid catabolic process; F:catalytic activity |
| Efet.01.19562.g1449.t1 | voltage-gated sodium | 375 | 2.60E-45 | 84.75 | F:voltage-gated potassium channel activity; P:regulation of postsynaptic membrane potential; P:potassium ion transmembrane transport; C:voltage-gated potassium channel complex; F:calcium ion binding; P:regulation of ion transmembrane transport; C:voltage-gated sodium channel complex; P:sodium ion transmembrane transport; F:voltage-gated sodium channel activity |
| Efet.01.195713.g2117.t1 | dysferlin-like isoform X2 | 480 | 2.10E-66 | 61.6 | C:membrane; C:integral component of membrane |
| Efet.01.195732.g2118.t1 | probable G- coupled receptor 139 isoform X1 | 876 | 2.25E-71 | 66.7 | C:membrane |
| Efet.01.195756.g2120.t1 | serine threonine- kinase DCLK1-like | 639 | 6.73E-52 | 69.55 | F:kinase activity; P:cellular process |
| Efet.01.195800.g2121.t1 | neuropeptide SIFamide receptor | 309 | 2.54E-24 | 71.65 | P:neuropeptide signaling pathway; C:integral component of membrane; F:neuropeptide Y receptor activity |
| Efet.01.19585.g1450.t1 | Neuronal PAS domain-containing 4 | 426 | 1.08E-36 | 58.7 | C:nucleus; P:transcription, DNA-templated; P:regulation of transcription, DNA-templated; F:protein dimerization activity; P:positive regulation of transcription from RNA polymerase II promoter; F:RNA polymerase II transcription factor activity, sequence-specific DNA binding |
| Efet.01.19585.g1451.t1 | neuronal PAS domain-containing 4 | 921 | 6.87E-18 | 63.33 | C:nucleus; P:transcription, DNA-templated; P:regulation of transcription, DNA-templated; F:protein dimerization activity |
| Efet.01.195866.g2122.t1 | Fibroblast growth factor receptor | 438 | 1.19E-23 | 53.55 | F:ATP binding; C:membrane; C:integral component of membrane; F:protein kinase activity; P:protein phosphorylation |
| Efet.01.195876.g2123.t1 | neogenin-like isoform X1 | 435 | 2.55E-47 | 66.05 | P:biological regulation; C:integral component of membrane; P:nervous system development |
| Efet.01.195918.g2124.t1 | pikachurin-like | 435 | 7.31E-41 | 68.8 | F:calcium ion binding; C:membrane; C:integral component of membrane |
| Efet.01.195919.g2125.t1 | electrogenic sodium bicarbonate cotransporter 1-like | 771 | 1.27E-67 | 54.65 | P:ion transport |
| Efet.01.195950.g2126.t1 | Neuropeptide FF receptor 2 | 429 | 1.68E-28 | 53.71 | F:G-protein coupled receptor activity; P:neuropeptide signaling pathway; P:G-protein coupled receptor signaling pathway; P:signal transduction; C:membrane; C:integral component of membrane; F:signal transducer activity; F:neuropeptide Y receptor activity |
| Efet.01.19597.g1452.t1 | titin-like isoform X6 | 201 | 7.86E-14 | 63.83 |  |
| Efet.01.196051.g2129.t1 | FAM43A-like | 603 | 6.87E-14 | 61.05 | C:membrane; C:integral component of membrane |
| Efet.01.196066.g2130.t1 | NADH dehydrogenase [ubiquinone] flavo mitochondrial | 369 | 1.60E-71 | 96.45 | F:FMN binding; F:NAD binding; F:NADH dehydrogenase (ubiquinone) activity; P:oxidation-reduction process; F:4 iron, 4 sulfur cluster binding |
| Efet.01.19609.g1453.t1 | neuroglian-like isoform X2 | 207 | 2.83E-14 | 67.85 | C:integral component of membrane |
| Efet.01.196095.g2131.t1 | peregrin-like isoform X1 | 798 | 7.22E-102 | 70.1 | F:zinc ion binding; F:metal ion binding; C:membrane; C:integral component of membrane |
| Efet.01.196112.g2134.t1 | hypothetical protein LOTGIDRAFT_236810 | 498 | 2.23E-07 | 84 |  |
| Efet.01.196137.g2136.t1 | SZT2 | 1479 | 7.11E-34 | 50.45 | C:peroxisome; F:galactosylceramide sulfotransferase activity; C:integral component of membrane; P:glycolipid biosynthetic process; C:Golgi apparatus |
| Efet.01.196174.g2138.t1 | hypothetical protein CAPTEDRAFT_225890 | 621 | 5.33E-38 | 60 |  |
| Efet.01.196208.g2142.t1 | hypothetical protein CAPTEDRAFT_209261 | 540 | 1.03E-34 | 63 | C:membrane; C:integral component of membrane |
| Efet.01.196216.g2143.t1 | FMRFamide-activated amiloride-sensitive sodium channel isoform X1 | 2118 | 3.90E-180 | 45.95 | P:transport |
| Efet.01.19622.g1454.t1 | T family of potassium channels 18-like | 975 | 5.62E-26 | 53.45 | P:ion transport; P:potassium ion transmembrane transport; C:membrane; C:integral component of membrane; F:potassium channel activity; P:transport |
| Efet.01.196234.g2144.t1 | CLP1 homolog | 258 | 3.51E-20 | 60.25 | C:nucleus; F:polynucleotide 5'-hydroxyl-kinase activity; P:mRNA processing |
| Efet.01.19626.g1455.t1 | LRR and PYD domains-containing 5 | 2103 | 3.67E-67 | 53.4 |  |
| Efet.01.196343.g2146.t1 | E3 ubiquitin- ligase TRIM56-like | 951 | 6.50E-15 | 58.75 | F:zinc ion binding; F:metal ion binding; C:intracellular |
| Efet.01.196353.g2147.t1 | AP-2 complex subunit sigma | 315 | 1.72E-36 | 91.2 | C:membrane coat; P:vesicle-mediated transport; F:protein transporter activity; P:intracellular protein transport |
| Efet.01.19637.g1456.t1 | 5-hydroxytryptamine receptor 2C-like | 2169 | 7.24E-42 | 72.1 | P:G-protein coupled receptor signaling pathway; C:integral component of membrane; P:serotonin receptor signaling pathway; F:G-protein coupled serotonin receptor activity |
| Efet.01.196477.g2150.t1 | Adenylate cyclase type 9 | 1230 | 1.55E-168 | 71.45 | F:nucleotide binding; F:phosphorus-oxygen lyase activity; C:integral component of membrane; P:adenylate cyclase-modulating G-protein coupled receptor signaling pathway; C:intracellular; P:cyclic nucleotide biosynthetic process; P:intracellular signal transduction |
| Efet.01.196489.g2151.t1 | nucleolin-like isoform X2 | 309 | 7.57E-23 | 69.15 | F:nucleic acid binding |
| Efet.01.196576.g2153.t1 | gastrula zinc finger - | 336 | 1.61E-20 | 57.35 | F:nucleic acid binding; F:metal ion binding |
| Efet.01.196604.g2154.t1 | hypothetical protein CAPTEDRAFT_223037 | 1020 | 1.67E-13 | 53.43 |  |
| Efet.01.196619.g2156.t1 | uncharacterized protein LOC109922783 | 801 | 9.58E-11 | 48.5 |  |
| Efet.01.196628.g2157.t1 | synapsin | 297 | 1.59E-09 | 86.4 | F:ATP binding; F:catalytic activity; F:metal ion binding; C:synaptic vesicle; P:neurotransmitter secretion |
| Efet.01.196678.g2159.t1 | RNA-directed DNA methylation 3-like | 2217 | 1.91E-25 | 41.25 | P:neuropeptide signaling pathway |
| Efet.01.196864.g2165.t1 | adenosine kinase 2 | 1302 | 1.20E-104 | 62.4 | F:transferase activity, transferring phosphorus-containing groups; P:cellular metabolic process |
| Efet.01.19688.g1458.t1 | BTB POZ domain-containing KCTD6-like | 1527 | 1.35E-31 | 74.9 | P:protein homooligomerization |
| Efet.01.19688.g1459.t1 | coiled-coil domain-containing 47-like | 288 | 2.65E-19 | 82.3 | F:calcium ion binding; C:integral component of membrane; C:endoplasmic reticulum |
| Efet.01.196900.g2166.t1 | NADP-dependent malic mitochondrial | 273 | 4.77E-11 | 78.25 | P:malate metabolic process; F:metal ion binding; F:NAD binding; C:mitochondrion; F:malate dehydrogenase (decarboxylating) (NAD+) activity; P:oxidation-reduction process; F:malate dehydrogenase (decarboxylating) (NADP+) activity; P:pyruvate metabolic process |
| Efet.01.196922.g2168.t1 | endoplasmic reticulum aminopeptidase 1-like | 729 | 4.20E-38 | 57.75 | F:hydrolase activity |
| Efet.01.196959.g2169.t1 | Neuronal acetylcholine receptor subunit alpha- | 474 | 6.63E-38 | 67.2 | F:transmembrane receptor activity; P:ion transmembrane transport; C:integral component of membrane; P:chemical synaptic transmission; P:response to stimulus; C:plasma membrane part |
| Efet.01.197001.g2170.t1 | glyco -N-acetylgalactosamine 3-beta-galactosyltransferase 1-like | 825 | 2.95E-65 | 70.95 | F:transferase activity, transferring glycosyl groups; C:membrane |
| Efet.01.197029.g2174.t1 | hypothetical protein | 4644 | 0 | 54.85 |  |
| Efet.01.197029.g2175.t1 | fibronectin type 3 domain-containing | 1860 | 2.97E-161 | 53.5 |  |
| Efet.01.197029.g2176.t1 | T9SS C-terminal target domain-containing | 2685 | 0 | 50.9 |  |
| Efet.01.197029.g2177.t1 | hypothetical protein | 1641 | 0 | 66.4 |  |
| Efet.01.19706.g1460.t1 | Serine threonine- kinase | 507 | 1.15E-55 | 77.5 | F:ATP binding; P:MAPK cascade; F:protein binding; P:peptidyl-amino acid modification; F:MAP kinase activity; P:regulation of signal transduction; C:intracellular |
| Efet.01.19707.g1461.t1 | hypothetical protein CAPTEDRAFT_216691 | 672 | 2.22E-72 | 74.5 | C:integral component of membrane |
| Efet.01.19707.g1462.t1 | hypothetical protein CAPTEDRAFT_216691 | 1494 | 5.31E-172 | 62 | C:membrane; C:integral component of membrane |
| Efet.01.197076.g2180.t1 | midasin isoform X2 | 507 | 1.02E-29 | 61.35 | F:ATPase activity; C:nucleus; F:ATP binding; P:ribosomal large subunit assembly |
| Efet.01.197116.g2181.t1 | Nuclear receptor subfamily 0 group B member 1 | 1872 | 3.87E-20 | 58.3 | F:DNA binding; C:nucleus; P:transcription, DNA-templated; P:regulation of transcription, DNA-templated; F:steroid hormone receptor activity; P:steroid hormone mediated signaling pathway |
| Efet.01.197128.g2183.t1 | ethanolamine kinase 1 | 243 | 8.98E-10 | 74.65 | C:membrane; C:intracellular part; P:phosphate-containing compound metabolic process |
| Efet.01.19713.g1463.t1 | glycosylated lysosomal membrane -like | 267 | 2.30E-10 | 61.85 | C:membrane; C:integral component of membrane |
| Efet.01.197167.g2184.t1 | Wnt-7b isoform X2 | 417 | 2.18E-20 | 68.7 | C:membrane; C:extracellular region; P:single-organism process |
| Efet.01.197205.g2185.t1 | conserved oligomeric Golgi complex subunit 3-like isoform X1 | 282 | 2.27E-21 | 68.8 | C:cis-Golgi network; F:metal ion binding; C:membrane; P:intracellular protein transport |
| Efet.01.197245.g2186.t1 | cofilin | 306 | 2.00E-27 | 67.4 | C:intracellular part |
| Efet.01.197250.g2187.t1 | hillarin isoform X1 | 573 | 5.87E-52 | 58.95 |  |
| Efet.01.197283.g2188.t1 | heat shock 70 kDa 12A | 753 | 2.55E-92 | 60.75 | C:extracellular exosome |
| Efet.01.197366.g2191.t1 | hypothetical protein HELRODRAFT_188009 | 594 | 8.15E-47 | 59 | C:membrane; C:integral component of membrane |
| Efet.01.197449.g2193.t1 | glutamyl-tRNA(Gln) amidotransferase subunit mitochondrial | 507 | 4.71E-38 | 73.4 | C:glutamyl-tRNA(Gln) amidotransferase complex; P:glutaminyl-tRNAGln biosynthesis via transamidation; C:mitochondrion; P:mitochondrial translation; F:glutaminyl-tRNA synthase (glutamine-hydrolyzing) activity |
| Efet.01.197467.g2195.t1 | hypothetical protein CAPTEDRAFT_223573 | 249 | 1.14E-06 | 77.67 | F:calcium ion binding; C:membrane; F:receptor activity; P:receptor-mediated endocytosis |
| Efet.01.197471.g2197.t1 | hypothetical protein HELRODRAFT_160353 | 1752 | 1.12E-06 | 72 |  |
| Efet.01.197487.g2198.t1 | AP-2 complex subunit alpha | 630 | 2.45E-111 | 92.55 | F:protein kinase binding; P:regulation of hematopoietic stem cell differentiation; C:clathrin-coated pit; C:secretory granule; C:clathrin adaptor complex; F:protein transporter activity; F:lipid binding; F:protein complex binding; P:intracellular protein transport; P:endocytosis; C:plasma membrane |
| Efet.01.197569.g2200.t1 | zinc finger 667-like | 1377 | 1.29E-94 | 62.05 | F:binding |
| Efet.01.197610.g2201.t1 | SPFH Band 7 PHB domain | 225 | 1.14E-37 | 95.2 | C:integral component of membrane |
| Efet.01.197649.g2202.t1 | transmembrane 268-like isoform X2 | 633 | 6.40E-33 | 51.25 | P:signal transduction; C:membrane; C:integral component of membrane |
| Efet.01.197677.g2203.t1 | cytochrome c oxidase assembly COX19 | 243 | 8.36E-18 | 76.8 |  |
| Efet.01.197703.g2205.t1 | hypothetical protein HELRODRAFT_81275, partial | 420 | 1.45E-26 | 58.8 |  |
| Efet.01.197727.g2206.t1 | ubiquitin-conjugating enzyme E2-22 kDa | 360 | 3.64E-22 | 86.65 | F:ATP binding |
| Efet.01.197751.g2207.t1 | alpha-actinin isoform X1 | 285 | 6.24E-29 | 79.85 | F:actin filament binding; P:actin crosslink formation; F:calcium ion binding; P:actin filament bundle assembly |
| Efet.01.197764.g2208.t1 | mab-21-like 3 | 1266 | 2.82E-13 | 44.4 |  |
| Efet.01.197765.g2209.t1 | hypothetical protein CAPTEDRAFT_220792 | 1029 | 3.85E-12 | 56.33 |  |
| Efet.01.197807.g2211.t1 | muscarinic acetylcholine receptor M1-like | 2004 | 1.67E-36 | 75.6 | C:integral component of plasma membrane; F:G-protein coupled acetylcholine receptor activity; P:G-protein coupled acetylcholine receptor signaling pathway |
| Efet.01.197835.g2213.t1 | metabotropic glutamate receptor 3-like | 429 | 5.43E-38 | 73.65 | P:signal transduction; C:membrane; F:signal transducer activity |
| Efet.01.197849.g2214.t1 | hypothetical protein CAPTEDRAFT_224889 | 525 | 1.20E-51 | 49.18 |  |
| Efet.01.19789.g1467.t1 | glypican-6-like | 432 | 3.75E-40 | 75.8 | F:heparan sulfate proteoglycan binding; C:proteinaceous extracellular matrix; C:plasma membrane; C:anchored component of membrane |
| Efet.01.197895.g2215.t1 | FAD-dependent oxidoreductase domain-containing 1-like | 309 | 1.55E-40 | 77.65 | F:nucleic acid binding; P:RNA phosphodiester bond hydrolysis; F:oxidoreductase activity; F:endoribonuclease activity, producing 5'-phosphomonoesters; P:oxidation-reduction process |
| Efet.01.197946.g2216.t1 | DNA polymerase eta | 366 | 5.59E-09 | 79.65 | P:DNA repair; F:damaged DNA binding |
| Efet.01.198156.g2221.t1 | solute carrier family 25 member 40-like | 411 | 3.78E-36 | 67.05 | F:structural constituent of ribosome; C:membrane; P:transport; P:translation |
| Efet.01.19816.g1469.t1 | leucine-rich repeats and immunoglobulin-like domains 3 | 285 | 4.56E-27 | 78.85 | F:phosphoprotein phosphatase activity; C:extracellular space; P:protein dephosphorylation; C:integral component of membrane; P:otolith morphogenesis |
| Efet.01.198217.g2223.t1 | TNF receptor-associated factor 4-like | 297 | 1.77E-18 | 59.1 | F:ubiquitin-protein transferase activity; F:zinc ion binding; F:metal ion binding; P:protein ubiquitination |
| Efet.01.198217.g2224.t1 | major egg antigen-like | 1134 | 2.40E-81 | 50.15 |  |
| Efet.01.198222.g2225.t1 | hypothetical protein HELRODRAFT_195043 | 210 | 9.80E-06 | 68 | C:membrane; C:integral component of membrane |
| Efet.01.198283.g2226.t1 | dual specificity phosphatase 7-like | 453 | 2.21E-11 | 56.8 | F:phosphoprotein phosphatase activity; P:regulation of cellular process |
| Efet.01.198330.g2227.t1 | T family of potassium channels 18-like | 1293 | 2.77E-41 | 68.5 | P:ion transport; C:membrane |
| Efet.01.198386.g2229.t1 | transcription factor 21 | 417 | 1.11E-25 | 72.45 | P:palate development; P:Sertoli cell differentiation; P:lung vasculature development; P:spleen development; P:embryonic digestive tract morphogenesis; P:sex determination; P:gland development; P:positive regulation of transcription from RNA polymerase II promoter; F:bHLH transcription factor binding; P:lung morphogenesis; P:negative regulation of androgen receptor signaling pathway; P:negative regulation of transcription from RNA polymerase II promoter; F:transcriptional repressor activity, RNA polymerase II core promoter proximal region sequence-specific binding; P:lung alveolus development; F:transcriptional activator activity, RNA polymerase II core promoter proximal region sequence-specific binding; P:metanephric mesenchymal cell differentiation; F:E-box binding; P:transcription, DNA-templated; F:protein dimerization activity; P:diaphragm development; P:bronchiole development; C:nucleus; F:androgen receptor binding; P:branching involved in ureteric bud morphogenesis; P:branchiomeric skeletal muscle development; P:metanephric glomerular capillary formation |
| Efet.01.198391.g2230.t1 | E3 ubiquitin- ligase pub2 | 1989 | 2.13E-14 | 51.17 |  |
| Efet.01.19840.g1470.t1 | frizzled-4-like | 2031 | 5.69E-134 | 60.85 | F:protein binding; P:vasculature development; P:cell-cell signaling; P:cell surface receptor signaling pathway; P:anatomical structure morphogenesis; P:animal organ development |
| Efet.01.198432.g2231.t1 | ras-related Rab-30-like | 417 | 4.76E-12 | 52.3 | F:GTP binding; P:autophagosome assembly; F:sphingomyelin phosphodiesterase activity; P:small GTPase mediated signal transduction; C:endosome membrane; C:autophagosome; C:intracellular; F:hydrolase activity |
| Efet.01.198432.g2232.t1 | 26S proteasome non-ATPase regulatory subunit | 414 | 6.59E-45 | 92.1 | C:proteasome complex |
| Efet.01.19846.g1473.t1 | amyloid beta A4 -like isoform X1 | 480 | 9.60E-41 | 66.65 | C:membrane; F:ion binding |
| Efet.01.198506.g2233.t1 | DNA damage response | 282 | 2.61E-06 | 54.5 | C:membrane; C:integral component of membrane |
| Efet.01.198561.g2236.t1 | nuclear receptor 2DBD-gamma | 1167 | 1.43E-14 | 81.5 | C:nucleus; F:transcription factor activity, sequence-specific DNA binding; F:zinc ion binding; P:transcription, DNA-templated; P:regulation of transcription, DNA-templated; F:sequence-specific DNA binding |
| Efet.01.198577.g2237.t1 | JNK-interacting 3 isoform X1 | 453 | 8.33E-69 | 80.4 | C:integral component of membrane |
| Efet.01.198602.g2238.t1 | retinaldehyde-binding 1-like | 423 | 4.03E-10 | 52.35 | P:optokinetic behavior; P:transport; P:response to light stimulus; C:intracellular; F:transporter activity |
| Efet.01.198608.g2239.t1 | innexin 4 | 570 | 1.11E-78 | 64.8 | C:membrane; P:cell communication by electrical coupling; C:cell junction; C:synapse; F:gap junction channel activity; P:transport |
| Efet.01.198684.g2240.t1 | hypothetical protein HELRODRAFT_189751 | 276 | 1.05E-08 | 79 |  |
| Efet.01.198772.g2244.t1 | trafficking particle complex subunit 6B | 396 | 3.35E-62 | 88.2 |  |
| Efet.01.198780.g2246.t1 | hypothetical protein HELRODRAFT_159908 | 825 | 4.40E-26 | 57.5 |  |
| Efet.01.19880.g1476.t1 | PREDICTED: uncharacterized protein LOC106171662 | 930 | 6.21E-33 | 49.7 |  |
| Efet.01.19880.g1477.t1 | hypothetical protein CAPTEDRAFT_228736 | 753 | 5.89E-09 | 56.17 |  |
| Efet.01.198828.g2247.t1 | GTPase-activating and VPS9 domain-containing 1-like | 225 | 3.63E-36 | 89.55 | P:signal transduction; F:guanyl-nucleotide exchange factor activity; F:GTPase activator activity; P:endocytosis; F:cadherin binding involved in cell-cell adhesion; C:endosome; P:regulation of protein transport; C:cell-cell adherens junction; C:membrane; P:regulation of GTPase activity; C:cytosol; F:GTPase activating protein binding; P:cell-cell adhesion |
| Efet.01.198835.g2248.t1 | ribonuclease P subunit p25 | 330 | 5.69E-29 | 72.1 | F:transmembrane transporter activity; F:organic cyclic compound binding; F:heterocyclic compound binding; F:oxidoreductase activity; C:membrane |
| Efet.01.198882.g2249.t1 | Radial spoke head 4 | 573 | 2.22E-40 | 63.4 | C:intracellular organelle |
| Efet.01.198883.g2250.t1 | lysine-specific demethylase 2A-like | 201 | 1.50E-13 | 85.7 | F:DNA binding; F:zinc ion binding; F:methyltransferase activity; P:methylation |
| Efet.01.198926.g2254.t1 | targeting for Xklp2 homolog | 408 | 7.66E-21 | 63.7 | C:cytoskeleton |
| Efet.01.198985.g2256.t1 | 1-phosphatidylinositol 4,5-bisphosphate phosphodiesterase epsilon-1-like | 216 | 7.57E-08 | 76.5 | P:biological regulation |
| Efet.01.198985.g2257.t1 | 1-phosphatidylinositol 4,5-bisphosphate phosphodiesterase epsilon-1-like | 987 | 5.12E-52 | 52.1 | P:single-organism process |
| Efet.01.199001.g2258.t1 | solute carrier family 12 member 4 isoform X1 | 267 | 6.78E-19 | 76.9 | F:potassium:chloride symporter activity; F:protein kinase binding; P:potassium ion transmembrane transport; P:chloride transmembrane transport; C:integral component of membrane; F:potassium ion symporter activity; P:chemical synaptic transmission; C:lysosomal membrane |
| Efet.01.199044.g2259.t1 | hypothetical protein CAPTEDRAFT_201544 | 774 | 1.72E-25 | 46.85 | F:chitin binding; C:membrane; P:chitin metabolic process; C:integral component of membrane; C:extracellular region |
| Efet.01.199072.g2261.t1 | tRNA pseudouridine synthase Pus10 | 1623 | 3.43E-173 | 62.15 | P:pseudouridine synthesis; F:RNA binding; P:RNA modification; F:pseudouridine synthase activity |
| Efet.01.199090.g2262.t1 | proliferating cell nuclear antigen-like | 537 | 5.76E-83 | 91.95 | C:nucleus; F:DNA binding; F:DNA polymerase processivity factor activity; P:DNA replication; P:regulation of DNA replication |
| Efet.01.199268.g2269.t1 | scabrous-like | 693 | 1.83E-84 | 63.9 | F:serine-type endopeptidase inhibitor activity; C:membrane; C:integral component of membrane; P:negative regulation of endopeptidase activity |
| Efet.01.199281.g2270.t1 | hepatocyte nuclear factor 4-beta-like isoform X1 | 693 | 5.42E-45 | 74.4 | C:nucleus; F:transcription factor activity, sequence-specific DNA binding; F:zinc ion binding; P:transcription, DNA-templated; F:steroid hormone receptor activity; P:regulation of transcription, DNA-templated; F:sequence-specific DNA binding; P:steroid hormone mediated signaling pathway |
| Efet.01.199312.g2271.t1 | FAM13A-like isoform X1 | 426 | 1.08E-17 | 71.65 | P:single-organism process |
| Efet.01.199318.g2272.t1 | AP-4 complex subunit mu-1 | 303 | 1.39E-28 | 72.55 | C:trans-Golgi network; C:vesicle; P:intracellular protein transport; P:Golgi to endosome transport |
| Efet.01.199326.g2273.t1 | GON-4 | 426 | 3.68E-19 | 53.45 | F:DNA binding; P:B cell differentiation; P:negative regulation of transcription, DNA-templated; F:transcription corepressor activity; P:regulation of transcription, DNA-templated |
| Efet.01.199386.g2276.t1 | pentatricopeptide repeat-containing mitochondrial | 288 | 5.13E-13 | 73.8 | C:mitochondrial matrix; P:tRNA 3'-end processing; F:tRNA binding |
| Efet.01.199402.g2278.t1 | sterol regulatory element-binding 1-like | 327 | 5.03E-16 | 60.2 | C:membrane; C:integral component of membrane; F:protein dimerization activity |
| Efet.01.199441.g2280.t1 | pecanex 1 isoform X2 | 654 | 1.46E-16 | 49.9 | C:membrane; C:integral component of membrane |
| Efet.01.199447.g2281.t1 | allatostatin-A receptor-like | 1167 | 2.57E-34 | 72.2 | F:G-protein coupled receptor activity; P:G-protein coupled receptor signaling pathway; C:integral component of membrane |
| Efet.01.199457.g2282.t1 | Atrial natriuretic peptide receptor 3 | 639 | 1.78E-38 | 58.5 | C:membrane; C:integral component of membrane |
| Efet.01.199459.g2283.t1 | poly [ADP-ribose] polymerase 12-like | 690 | 3.77E-33 | 78.9 | F:metal ion binding; F:NAD+ ADP-ribosyltransferase activity |
| Efet.01.199526.g2284.t1 | DNA-directed RNA polymerase III subunit RPC1-like | 387 | 1.43E-45 | 82.45 | F:RNA polymerase III activity; F:DNA binding; P:transcription from RNA polymerase III promoter; P:regulation of mitophagy; P:positive regulation of interferon-beta production; P:innate immune response; C:membrane; F:chromatin binding; C:DNA-directed RNA polymerase III complex; P:positive regulation of protein targeting to mitochondrion |
| Efet.01.199541.g2286.t1 | hypothetical protein CAPTEDRAFT_222159 | 540 | 1.39E-42 | 51.86 | C:membrane; C:integral component of membrane |
| Efet.01.199545.g2287.t1 | polymorphic outer membrane | 1545 | 1.36E-13 | 45 | F:calcium ion binding; C:membrane |
| Efet.01.199689.g2290.t1 | hypothetical protein CAPTEDRAFT_219019 | 291 | 8.75E-15 | 54.43 | C:membrane; C:integral component of membrane; F:carbohydrate binding |
| Efet.01.199714.g2292.t1 | PREDICTED: protein TBRG4 | 501 | 3.25E-06 | 66 |  |
| Efet.01.199733.g2294.t1 | enkurin | 612 | 7.32E-88 | 74.85 |  |
| Efet.01.19974.g1479.t1 | actin-related 2 isoform X3 | 540 | 1.37E-117 | 96.95 | P:establishment or maintenance of cell polarity; P:asymmetric cell division; F:structural constituent of cytoskeleton; C:Arp2/3 protein complex; F:actin filament binding; P:spindle localization; F:ATP binding; C:extracellular exosome; C:focal adhesion; C:membrane; P:cytosolic transport; P:Arp2/3 complex-mediated actin nucleation; C:cytosol; P:meiotic chromosome movement towards spindle pole; P:meiotic cytokinesis; C:actin cap |
| Efet.01.199879.g2295.t1 | cyclin B | 939 | 1.91E-107 | 69.6 | P:single-organism cellular process |
| Efet.01.199898.g2296.t1 | hypothetical protein CAPTEDRAFT_227790 | 1071 | 9.14E-25 | 63 |  |
| Efet.01.19994.g1480.t1 | ATP-dependent RNA helicase DDX54 | 603 | 3.07E-93 | 81.2 | C:nucleus; F:ATP binding; F:RNA binding; F:ATP-dependent RNA helicase activity |
| Efet.01.199950.g2297.t1 | hypothetical protein HELRODRAFT_169796 | 285 | 2.73E-34 | 68.67 |  |
| Efet.01.199974.g2299.t1 | retinoic acid receptor gamma isoform X3 | 1449 | 7.53E-12 | 51.46 | F:binding |
| Efet.01.200.g27.t1 | roundabout homolog 1-like isoform X1 | 504 | 1.92E-48 | 63.35 | C:membrane; C:integral component of membrane |
| Efet.01.200013.g1.t1 | outer dense fiber 3-like | 306 | 4.89E-32 | 67.45 |  |
| Efet.01.200035.g2.t1 | hypothetical protein CAPTEDRAFT_228965 | 831 | 9.60E-07 | 67 | C:membrane; C:integral component of membrane |
| Efet.01.200062.g5.t1 | hepatocyte growth factor receptor | 294 | 1.44E-09 | 56.45 | P:regulation of cellular process; F:kinase activity; P:single-organism process; P:cellular process |
| Efet.01.200078.g6.t1 | achaete-scute homolog 1b-like | 615 | 2.12E-13 | 58.3 | F:transcription factor activity, sequence-specific DNA binding; C:RNA polymerase II transcription factor complex; F:RNA polymerase II regulatory region sequence-specific DNA binding; P:regulation of neurogenesis; P:regulation of transcription from RNA polymerase II promoter |
| Efet.01.200094.g7.t1 | hypothetical protein HELRODRAFT_159178 | 267 | 4.06E-34 | 71.65 | C:integral component of membrane; F:carbohydrate binding |
| Efet.01.200145.g9.t1 | G- coupled receptor 158 | 390 | 6.04E-42 | 64.35 | C:integral component of membrane |
| Efet.01.200208.g10.t1 | forkhead box O6-like | 411 | 4.05E-39 | 66.95 | C:nucleus; F:transcription factor activity, sequence-specific DNA binding; P:transcription, DNA-templated; P:regulation of transcription, DNA-templated; F:sequence-specific DNA binding |
| Efet.01.200215.g12.t1 | E3 ubiquitin- ligase HERC2-like isoform X1 | 321 | 1.49E-29 | 58 | F:ubiquitin-protein transferase activity; F:ligase activity; P:protein ubiquitination |
| Efet.01.20022.g1483.t1 | eukaryotic translation initiation factor 5-like | 720 | 1.81E-57 | 78.8 | F:nucleotide binding; F:cadherin binding involved in cell-cell adhesion; F:translation initiation factor activity; P:formation of translation preinitiation complex; F:eukaryotic initiation factor eIF2 binding; P:activation of GTPase activity; C:cytoplasm; C:cell-cell adherens junction; P:regulation of translational initiation; P:cell-cell adhesion; C:plasma membrane; F:RNA binding |
| Efet.01.200227.g14.t1 | malectin-B-like isoform X1 | 582 | 2.95E-74 | 78.8 | C:integral component of membrane |
| Efet.01.200233.g15.t1 | 39S ribosomal mitochondrial | 306 | 2.02E-40 | 79.35 | F:structural constituent of ribosome; C:integral component of membrane; C:ribosome; P:translation |
| Efet.01.200238.g16.t1 | NLR family CARD domain-containing 3-like | 2325 | 9.43E-55 | 50.9 | F:ATP binding; F:zinc ion binding; F:protein kinase activity; P:phosphorylation; P:protein phosphorylation; F:kinase activity |
| Efet.01.200243.g17.t1 | serine threonine- kinase PAK | 429 | 4.10E-83 | 85.75 | P:positive regulation of JUN kinase activity; P:negative regulation of cell proliferation involved in contact inhibition; P:Fc-epsilon receptor signaling pathway; P:protein autophosphorylation; C:Golgi apparatus; P:Fc-gamma receptor signaling pathway involved in phagocytosis; P:branching morphogenesis of an epithelial tube; P:exocytosis; P:mitotic cell cycle; P:stimulatory C-type lectin receptor signaling pathway; P:cellular response to insulin stimulus; C:dendrite; F:ATP binding; P:MAPK cascade; P:wound healing; P:response to hypoxia; C:focal adhesion; P:axonogenesis; P:apoptotic process; C:cytosol; C:ruffle membrane; C:axon; C:invadopodium; P:actin cytoskeleton reorganization; P:T cell receptor signaling pathway; P:positive regulation of peptidyl-serine phosphorylation; F:protein kinase binding; P:T cell costimulation; C:Z disc; C:nuclear membrane; F:signal transducer, downstream of receptor, with serine/threonine kinase activity; F:collagen binding; P:positive regulation of stress fiber assembly; P:ephrin receptor signaling pathway; P:positive regulation of intracellular estrogen receptor signaling pathway; C:intercalated disc; C:filamentous actin; P:positive regulation of cell migration |
| Efet.01.200253.g18.t1 | glucose-regulated 94 | 291 | 3.20E-48 | 91.2 | F:ATP binding; F:unfolded protein binding; P:protein folding; P:response to stress |
| Efet.01.200269.g20.t1 | leucine-rich repeat and fibronectin type III domain-containing 1 | 384 | 2.02E-20 | 64 | C:membrane |
| Efet.01.200345.g21.t1 | rho GTPase-activating 39-like isoform X5 | 264 | 4.03E-28 | 78.65 | C:cytoskeleton; P:signal transduction; C:integral component of membrane |
| Efet.01.200355.g22.t1 | dolichyl-diphosphooligosaccharide-- glycosyltransferase subunit STT3A | 315 | 1.74E-65 | 93.85 | C:oligosaccharyltransferase complex; F:dolichyl-diphosphooligosaccharide-protein glycotransferase activity; C:integral component of membrane; P:protein N-linked glycosylation via asparagine; P:co-translational protein modification; P:post-translational protein modification |
| Efet.01.200486.g23.t1 | transcription factor AP-4-like | 828 | 1.05E-18 | 58.25 | C:nucleus; F:transcription factor activity, sequence-specific DNA binding; F:protein dimerization activity; P:regulation of transcription from RNA polymerase II promoter |
| Efet.01.200517.g24.t1 | sperm-associated antigen 16 -like | 282 | 8.15E-30 | 85.55 |  |
| Efet.01.200528.g27.t1 | cytochrome b-c1 complex subunit mitochondrial | 294 | 1.57E-30 | 62.1 | F:catalytic activity; F:metal ion binding |
| Efet.01.200548.g29.t1 | muscle-specific 20-like isoform X3 | 615 | 1.00E-89 | 71.1 | F:calmodulin binding; F:actin binding; P:actomyosin structure organization |
| Efet.01.200630.g30.t1 | dynein regulatory complex subunit 7-like | 627 | 1.66E-22 | 59.7 | C:nucleus |
| Efet.01.200646.g31.t1 | monocarboxylate transporter 2-like | 369 | 1.15E-48 | 76.17 | P:transmembrane transport; C:integral component of membrane |
| Efet.01.200688.g35.t1 | peflin-like | 285 | 1.38E-32 | 77.5 | C:cytoplasm; F:calcium ion binding; P:proteolysis; F:calcium-dependent cysteine-type endopeptidase activity |
| Efet.01.20071.g1485.t1 | beta-1,3-galactosyl-O-glycosyl-glyco beta-1,6-N-acetylglucosaminyltransferase 3-like | 309 | 1.58E-35 | 69.55 | F:acetylglucosaminyltransferase activity; C:membrane |
| Efet.01.200726.g39.t1 | somatostatin receptor type 2 | 747 | 1.75E-28 | 60.5 | F:neuropeptide receptor activity; P:G-protein coupled receptor signaling pathway, coupled to cyclic nucleotide second messenger; C:integral component of membrane; F:binding; C:plasma membrane |
| Efet.01.200761.g40.t1 | udp-n-acetylglucosamine--peptide n-acetylglucosaminyltransferase | 582 | 1.09E-54 | 60 | F:transferase activity, transferring glycosyl groups; C:membrane; C:integral component of membrane; F:transferase activity |
| Efet.01.20078.g1487.t1 | Dedicator of cytokinesis | 528 | 1.19E-33 | 62.8 | P:small GTPase mediated signal transduction; F:guanyl-nucleotide exchange factor activity; C:intracellular; P:positive regulation of GTPase activity |
| Efet.01.200850.g42.t1 | cilia- and flagella-associated 65-like isoform X1 | 351 | 2.91E-16 | 65.95 |  |
| Efet.01.200918.g46.t1 | glutamate receptor kainate 2 isoform X1 | 276 | 1.45E-36 | 82.1 | C:presynaptic membrane; C:postsynaptic density; F:kainate selective glutamate receptor activity; P:adenylate cyclase-inhibiting G-protein coupled glutamate receptor signaling pathway; C:neuronal cell body; P:intracellular protein transport; F:extracellular-glutamate-gated ion channel activity; P:negative regulation of neuron apoptotic process; C:postsynaptic membrane; P:regulation of long-term neuronal synaptic plasticity; C:kainate selective glutamate receptor complex; P:negative regulation of synaptic transmission, glutamatergic; P:ionotropic glutamate receptor signaling pathway; P:neuronal action potential; P:excitatory postsynaptic potential; P:ion transmembrane transport; F:5S rRNA binding; P:positive regulation of synaptic transmission; C:ribosome; F:adenylate cyclase inhibiting G-protein coupled glutamate receptor activity; P:behavioral fear response; P:translation; P:cellular calcium ion homeostasis; F:structural constituent of ribosome; P:inhibitory postsynaptic potential; C:cell junction; P:regulation of short-term neuronal synaptic plasticity; P:synaptic transmission, glutamatergic |
| Efet.01.200931.g49.t1 | afadin-like isoform X1 | 372 | 2.67E-29 | 72.05 |  |
| Efet.01.200938.g50.t1 | acetyl-coenzyme A synthetase 2- mitochondrial | 372 | 2.97E-23 | 65.65 | F:nucleotide binding; F:catalytic activity |
| Efet.01.201018.g52.t1 | hypothetical protein CAPTEDRAFT_193182 | 2268 | 4.82E-103 | 50.5 | C:membrane; C:integral component of membrane |
| Efet.01.20103.g1488.t1 | family transcriptional regulator | 498 | 4.34E-84 | 81.15 | F:DNA binding; F:transcription factor activity, sequence-specific DNA binding; P:transcription, DNA-templated; P:regulation of transcription, DNA-templated |
| Efet.01.201055.g53.t1 | Retrovirus-related Pol poly from transposon | 282 | 1.58E-12 | 62.95 |  |
| Efet.01.20108.g1489.t1 | zinc finger 595-like | 354 | 1.48E-08 | 59.9 | F:nucleic acid binding; F:metal ion binding |
| Efet.01.201104.g54.t1 | hypothetical protein CAPTEDRAFT_220549 | 312 | 2.44E-06 | 62 |  |
| Efet.01.201155.g56.t1 | hypothetical protein HELRODRAFT_175217 | 339 | 4.27E-07 | 69 | C:membrane; C:integral component of membrane |
| Efet.01.201157.g57.t1 | synaptotagmin-7-like isoform X4 | 330 | 7.66E-35 | 72.25 | P:calcium ion-regulated exocytosis of neurotransmitter; F:syntaxin binding; P:vesicle fusion; F:calcium ion binding; F:clathrin binding; P:regulation of calcium ion-dependent exocytosis; C:plasma membrane; F:calcium-dependent phospholipid binding |
| Efet.01.201192.g60.t1 | protocadherin-12 isoform X1 | 705 | 4.41E-43 | 63.55 | C:membrane |
| Efet.01.2012.g178.t1 | low density lipo receptor adapter 1-like | 864 | 6.64E-50 | 61.25 |  |
| Efet.01.201206.g61.t1 | hypothetical protein CAPTEDRAFT_221789 | 1134 | 7.69E-43 | 47.15 | C:membrane; C:integral component of membrane |
| Efet.01.201207.g62.t1 | UPF0602 C4orf47 homolog | 222 | 1.11E-18 | 71.05 | C:centrosome |
| Efet.01.201271.g65.t1 | kelch 5 isoform X3 | 546 | 1.99E-114 | 91.9 | F:ubiquitin-protein transferase activity; C:cytoskeleton; F:actin binding; C:cytoplasm; C:Cul3-RING ubiquitin ligase complex; P:protein ubiquitination |
| Efet.01.201280.g66.t1 | histone deacetylase 4 isoform X2 | 576 | 2.81E-21 | 61.3 | C:intracellular organelle; P:single-multicellular organism process; P:anatomical structure development; P:regulation of transcription, DNA-templated; P:protein deacetylation; F:transcription factor binding; P:negative regulation of cellular process; P:chromatin organization; P:single-organism developmental process; F:DNA binding; P:regulation of cell differentiation; P:positive regulation of cellular process; P:regulation of multicellular organismal process; F:hydrolase activity |
| Efet.01.201400.g67.t1 | potassium sodium hyperpolarization-activated cyclic nucleotide-gated channel 3-like isoform X2 | 798 | 6.35E-32 | 55.5 | P:ion transport |
| Efet.01.201410.g68.t1 | TNF receptor-associated factor 2-like | 576 | 1.71E-32 | 75.95 | F:ubiquitin-protein transferase activity; F:zinc ion binding; P:regulation of protein phosphorylation; P:signal transduction; P:regulation of intracellular signal transduction; P:regulation of apoptotic process; P:protein ubiquitination |
| Efet.01.20143.g1490.t1 | triose-phosphate isomerase | 654 | 6.59E-143 | 86.5 | P:gluconeogenesis; C:cytoplasm; P:pentose-phosphate shunt; P:glycolytic process; F:triose-phosphate isomerase activity |
| Efet.01.201441.g69.t1 | spermatogenesis-associated 5 1 | 639 | 7.25E-39 | 61.55 | F:nucleotide binding; F:ATP binding |
| Efet.01.201452.g70.t1 | BTB POZ domain-containing 6-like | 1086 | 2.26E-35 | 49.05 | P:proteasome-mediated ubiquitin-dependent protein catabolic process; F:ubiquitin protein ligase binding; C:cytoplasm; P:regulation of proteolysis; C:cytosol; P:neurogenesis; C:SCF ubiquitin ligase complex; P:protein ubiquitination involved in ubiquitin-dependent protein catabolic process |
| Efet.01.20147.g1491.t1 | potassium voltage-gated channel Shaw-like isoform X1 | 549 | 7.21E-49 | 76.8 | P:potassium ion transport; P:transmembrane transport; F:delayed rectifier potassium channel activity; C:integral component of membrane; P:cellular process; F:hydrolase activity |
| Efet.01.201471.g72.t1 | Major facilitator superfamily domain-containing 3 | 1038 | 8.13E-60 | 64.25 | C:membrane |
| Efet.01.201591.g77.t1 | nuclear receptor subfamily 2 group E member 1 | 528 | 6.66E-32 | 55.6 | F:binding |
| Efet.01.201615.g79.t1 | hypothetical protein CRN43_19315 | 375 | 9.80E-41 | 67.75 |  |
| Efet.01.201643.g80.t1 | hypothetical protein CAPTEDRAFT_220800 | 366 | 1.72E-08 | 59 | P:regulation of apoptotic process |
| Efet.01.201672.g82.t1 | kelch diablo | 963 | 1.10E-107 | 62.75 | F:ubiquitin-protein transferase activity; C:membrane; C:integral component of membrane; C:Cul3-RING ubiquitin ligase complex; P:protein ubiquitination |
| Efet.01.201672.g83.t1 | kelch diablo | 567 | 4.71E-91 | 58 | F:ATP binding; F:ubiquitin-protein transferase activity; C:membrane; C:integral component of membrane; F:protein kinase activity; P:phosphorylation; C:Cul3-RING ubiquitin ligase complex; P:protein phosphorylation; F:kinase activity; P:protein ubiquitination; F:hydrolase activity |
| Efet.01.201732.g85.t1 | retrovirus-related Pol poly from transposon | 648 | 3.14E-60 | 55.7 | F:nucleic acid binding; F:RNA-directed DNA polymerase activity; F:transcription factor activity, sequence-specific DNA binding; F:zinc ion binding; P:DNA integration; P:regulation of transcription, DNA-templated; P:RNA-dependent DNA biosynthetic process |
| Efet.01.201747.g86.t1 | fat-like cadherin-related tumor suppressor homolog isoform X4 | 777 | 2.24E-67 | 61.65 | C:membrane |
| Efet.01.201760.g87.t1 | poly(U)-specific endoribonuclease-B-like | 309 | 2.08E-24 | 73.65 | F:RNA binding; F:endoribonuclease activity |
| Efet.01.201782.g89.t1 | hepatocyte nuclear factor | 849 | 6.06E-32 | 84.9 | P:B cell differentiation; P:cilium assembly; P:endoderm development; P:transcription from RNA polymerase II promoter; P:spleen development; P:epithelial cell development; P:anatomical structure morphogenesis; P:glucose metabolic process; P:positive regulation of transcription from RNA polymerase II promoter; C:nucleus; F:DNA binding; F:transcriptional activator activity, RNA polymerase II core promoter proximal region sequence-specific binding; P:regulation of cell-matrix adhesion; P:Notch signaling pathway; P:liver development; P:cell fate commitment; P:endocrine pancreas development; P:negative regulation of transforming growth factor beta receptor signaling pathway; P:positive regulation of cell migration |
| Efet.01.201842.g92.t1 | probable G- coupled receptor 139 isoform X1 | 1134 | 3.04E-77 | 61.1 | F:G-protein coupled receptor activity; P:G-protein coupled receptor signaling pathway; C:membrane; C:integral component of membrane |
| Efet.01.201860.g95.t1 | Ras-related Rab-43 | 216 | 1.88E-28 | 85.35 | F:GTP binding; P:small GTPase mediated signal transduction; C:intracellular |
| Efet.01.201898.g96.t1 | Down syndrome critical region 3-like | 231 | 1.15E-11 | 86.3 | C:endosome; F:protein transporter activity; P:intracellular protein transport |
| Efet.01.201905.g97.t1 | neuromedin-U receptor 2-like | 1002 | 1.86E-89 | 69.85 | F:G-protein coupled receptor activity; P:G-protein coupled receptor signaling pathway; C:membrane |
| Efet.01.20195.g1493.t1 | hypothetical protein HELRODRAFT_161680 | 714 | 7.15E-16 | 49.75 | F:chitin binding; F:calcium ion binding; P:chitin metabolic process; C:extracellular region |
| Efet.01.201980.g99.t1 | disulfide-isomerase TMX3 | 273 | 2.65E-12 | 57.3 | C:cell; C:membrane; C:integral component of membrane; P:cell redox homeostasis |
| Efet.01.202003.g100.t1 | TIS11 isoform X2 | 786 | 3.05E-33 | 80.85 | P:regulation of macromolecule metabolic process; F:RNA binding; P:regulation of cellular process; F:metal ion binding; P:hemopoiesis; P:mRNA catabolic process; C:intracellular part |
| Efet.01.202029.g102.t1 | tropomyosin-2-like isoform X4 | 783 | 1.18E-08 | 61.05 |  |
| Efet.01.20203.g1494.t1 | hypothetical protein HELRODRAFT_166807 | 1755 | 7.01E-27 | 60 |  |
| Efet.01.202050.g104.t1 | RD3-like | 468 | 5.18E-10 | 67.5 |  |
| Efet.01.202218.g107.t1 | hypothetical protein HELRODRAFT_191395 | 324 | 3.07E-07 | 53 | F:zinc ion binding; F:actin binding; P:cytoskeleton organization; F:metal ion binding |
| Efet.01.202250.g109.t1 | vinexin-like isoform X1 | 951 | 2.25E-24 | 52.45 | P:insulin receptor signaling pathway; P:actin filament organization |
| Efet.01.202263.g110.t1 | delta(24)-sterol reductase | 267 | 3.35E-38 | 88.75 | P:amyloid precursor protein catabolic process; P:membrane organization; F:oxidoreductase activity, acting on CH-OH group of donors; F:peptide antigen binding; P:cholesterol biosynthetic process; P:protein localization; C:endoplasmic reticulum; F:delta24(24-1) sterol reductase activity; P:skin development; P:tissue development; C:nucleus; P:response to oxidative stress; F:flavin adenine dinucleotide binding; P:male genitalia development; P:plasminogen activation; P:negative regulation of cysteine-type endopeptidase activity involved in apoptotic process; C:integral component of membrane; P:oxidation-reduction process; F:enzyme binding |
| Efet.01.202316.g112.t1 | ornithine mitochondrial | 633 | 1.61E-102 | 85.95 | F:pyridoxal phosphate binding; F:transaminase activity |
| Efet.01.202433.g113.t1 | follicular epithelium yolk subunit | 714 | 5.36E-42 | 52 |  |
| Efet.01.202437.g114.t1 | cAMP-dependent kinase regulatory subunit | 282 | 4.04E-29 | 70.4 | P:phosphorylation; F:kinase activity |
| Efet.01.20247.g1495.t1 | neuronal acetylcholine receptor subunit alpha-10-like | 222 | 6.46E-42 | 72.5 | C:postsynaptic membrane; P:cation transmembrane transport; C:integral component of membrane; C:cell junction; F:extracellular ligand-gated ion channel activity |
| Efet.01.202489.g115.t1 | U3 small nucleolar ribonucleo IMP3-like | 387 | 4.82E-52 | 76.6 | C:small-subunit processome; F:snoRNA binding; P:positive regulation of translational fidelity; F:structural constituent of ribosome; C:Mpp10 complex; C:small ribosomal subunit; P:rRNA processing; F:rRNA binding |
| Efet.01.202501.g118.t1 | coiled-coil domain-containing 89-like | 264 | 2.70E-18 | 69.35 |  |
| Efet.01.202517.g119.t1 | collagen alpha-1(I) chain-like | 885 | 1.58E-46 | 52.25 | C:collagen trimer; C:membrane; C:integral component of membrane; F:extracellular matrix structural constituent; C:proteinaceous extracellular matrix |
| Efet.01.202550.g122.t1 | usherin-like | 552 | 8.17E-57 | 69.45 | C:membrane |
| Efet.01.202586.g123.t1 | cell number regulator 3-like | 471 | 2.91E-49 | 57.95 | C:membrane |
| Efet.01.20264.g1496.t1 | hypothetical protein HELRODRAFT_162456 | 969 | 5.07E-06 | 68 | C:membrane; C:integral component of membrane |
| Efet.01.202644.g124.t1 | neogenin-like isoform X1 | 270 | 7.07E-26 | 60.45 | P:biological regulation; C:integral component of membrane; P:nervous system development |
| Efet.01.202706.g126.t1 | filamin-A-like isoform X3 | 258 | 9.73E-18 | 66.25 | F:actin filament binding |
| Efet.01.202742.g129.t1 | hypothetical protein CAPTEDRAFT_186654 | 504 | 1.52E-17 | 59 | F:G-protein coupled receptor activity; P:G-protein coupled receptor signaling pathway; C:membrane; C:integral component of membrane |
| Efet.01.202756.g130.t1 | 1-phosphatidylinositol 4,5-bisphosphate phosphodiesterase epsilon-1-like isoform X6 | 906 | 1.13E-53 | 48 | F:phosphatidylinositol phospholipase C activity; P:small GTPase mediated signal transduction; P:signal transduction; F:guanyl-nucleotide exchange factor activity; P:lipid metabolic process; F:phosphoric diester hydrolase activity; F:signal transducer activity; P:intracellular signal transduction; P:positive regulation of GTPase activity; C:intracellular |
| Efet.01.20280.g1498.t1 | kinase domain | 687 | 4.46E-67 | 89.7 | F:ATP binding; F:G-protein coupled receptor kinase activity; P:signal transduction; P:protein phosphorylation |
| Efet.01.202820.g133.t1 | hyperpolarizaion-activated cyclic nucleotide-gated cation channel | 504 | 2.10E-100 | 90.8 | F:voltage-gated potassium channel activity; P:potassium ion transmembrane transport; C:integral component of membrane |
| Efet.01.202820.g134.t1 | Potassium sodium hyperpolarization-activated cyclic nucleotide-gated channel | 414 | 1.23E-48 | 88.45 | F:voltage-gated potassium channel activity; P:potassium ion transmembrane transport; C:integral component of membrane |
| Efet.01.202903.g136.t1 | Poly(ADP-ribose) polymerase pme-5 | 357 | 1.29E-13 | 68.3 | F:transferase activity |
| Efet.01.202970.g139.t1 | hypothetical protein CAPTEDRAFT_204696 | 504 | 4.26E-27 | 56.5 | C:membrane; C:integral component of membrane; F:carbohydrate binding |
| Efet.01.202977.g140.t1 | gamma-aminobutyric acid receptor subunit beta-like | 432 | 1.07E-39 | 62.9 | C:membrane; P:transport |
| Efet.01.203005.g143.t1 | mixed-lineage leukemia | 453 | 1.07E-84 | 88.05 | P:exploration behavior; P:protein complex assembly; P:embryonic hemopoiesis; P:negative regulation of cell proliferation; P:post-embryonic development; P:anterior/posterior pattern specification; P:transcription from RNA polymerase II promoter; P:spleen development; F:unmethylated CpG binding; P:visual learning; P:positive regulation of transcription from RNA polymerase II promoter; P:histone H4-K16 acetylation; F:transcription factor activity, sequence-specific DNA binding; P:circadian regulation of gene expression; P:regulation of histone H3-K9 acetylation; P:apoptotic process; C:MLL1 complex; F:histone methyltransferase activity (H3-K4 specific); P:histone H3-K4 dimethylation; P:histone H3-K4 trimethylation; F:zinc ion binding; F:protein homodimerization activity; P:positive regulation of cellular response to drug; F:AT DNA binding; P:regulation of histone H3-K27 acetylation; F:chromatin binding; P:definitive hemopoiesis; F:core promoter sequence-specific DNA binding; P:DNA methylation; C:cytoplasm; P:homeostasis of number of cells within a tissue; P:regulation of histone H3-K14 acetylation; P:positive regulation of histone H3-K4 methylation; F:lysine-acetylated histone binding; P:positive regulation of transporter activity; P:peptidyl-lysine monomethylation; P:membrane depolarization; P:regulation of short-term neuronal synaptic plasticity; P:response to potassium ion |
| Efet.01.203017.g144.t1 | SAM-dependent methyltransferase | 375 | 1.35E-38 | 72.7 | F:O-methyltransferase activity |
| Efet.01.203040.g146.t1 | galanin receptor type 1 | 456 | 2.18E-09 | 55.3 | F:G-protein coupled receptor activity; P:G-protein coupled receptor signaling pathway; C:membrane |
| Efet.01.203049.g147.t1 | dynein heavy chain axonemal-like | 741 | 2.20E-135 | 84.7 | F:ATPase activity; F:ATP binding; F:microtubule motor activity; C:dynein complex; P:microtubule-based movement |
| Efet.01.203066.g148.t1 | beta-1,3-galactosyl-O-glycosyl-glyco beta-1,6-N-acetylglucosaminyltransferase-like | 666 | 2.41E-69 | 72 | F:acetylglucosaminyltransferase activity; C:membrane |
| Efet.01.203082.g149.t1 | FMRFamide receptor-like | 1203 | 6.04E-71 | 48.25 | F:G-protein coupled receptor activity; F:G-protein coupled peptide receptor activity; P:G-protein coupled receptor signaling pathway; P:signal transduction; C:membrane; C:integral component of membrane; F:signal transducer activity |
| Efet.01.20312.g1499.t1 | ATP-dependent DNA helicase Q5 isoform X3 | 699 | 9.20E-80 | 75.5 | P:negative regulation of transcription elongation from RNA polymerase II promoter; P:DNA replication; P:double-strand break repair via homologous recombination; F:four-way junction helicase activity; P:mitotic nuclear division; C:chromosome; F:RNA polymerase II core binding; F:nucleotide binding; F:DNA binding; C:cytoplasm; F:ATP-dependent 3'-5' DNA helicase activity; C:DNA-directed RNA polymerase II, holoenzyme; P:chromosome separation |
| Efet.01.203125.g150.t1 | smoothelin 1 isoform X11 | 279 | 8.37E-36 | 86.45 |  |
| Efet.01.203233.g153.t1 | hypothetical protein HELRODRAFT_180820 | 939 | 8.03E-12 | 70 |  |
| Efet.01.203265.g154.t1 | E3 ubiquitin- ligase TRIM71 | 879 | 2.80E-16 | 46.5 | F:ligase activity |
| Efet.01.203301.g156.t1 | serine threonine- kinase PLK1 | 570 | 5.79E-70 | 65.35 | F:nucleotide binding; F:protein kinase activity; P:phosphorylation |
| Efet.01.203373.g158.t1 | E3 ubiquitin- ligase PDZRN3-like | 1413 | 2.30E-62 | 53.05 |  |
| Efet.01.203477.g160.t1 | chymotrypsinogen B | 669 | 1.14E-78 | 66.8 | F:serine-type peptidase activity |
| Efet.01.203545.g162.t1 | heterogeneous nuclear ribonucleo Q isoform X7 | 1137 | 6.91E-97 | 73.1 | P:posttranscriptional regulation of gene expression; C:nuclear part; F:RNA binding; C:intracellular ribonucleoprotein complex; C:cytoplasm; C:protein complex; P:cellular process |
| Efet.01.20361.g1500.t1 | E3 ubiquitin- ligase SHPRH-like | 1587 | 1.93E-14 | 58.5 | F:metal ion binding |
| Efet.01.203625.g164.t1 | sex peptide receptor-like | 969 | 2.38E-102 | 58.4 | F:G-protein coupled receptor activity; F:G-protein coupled peptide receptor activity; P:G-protein coupled receptor signaling pathway; C:membrane; C:integral component of membrane |
| Efet.01.203642.g165.t1 | neuroblast differentiation-associated AHNAK isoform X1 | 1962 | 4.66E-19 | 41.95 |  |
| Efet.01.203719.g168.t1 | Collagen triple helix repeat-containing | 705 | 4.40E-12 | 63 | C:membrane |
| Efet.01.20383.g1501.t1 | pleckstrin homology-like domain family B member 1 isoform X1 | 525 | 7.44E-62 | 79.75 |  |
| Efet.01.203834.g172.t1 | xyloside xylosyltransferase 1 isoform X2 | 621 | 1.10E-102 | 77.65 | F:transferase activity |
| Efet.01.203932.g173.t1 | tamozhennic | 1356 | 1.60E-09 | 55.85 | F:zinc ion binding; F:metal ion binding; P:multicellular organism development; P:protein transport |
| Efet.01.203950.g174.t1 | 1-phosphatidylinositol 4,5-bisphosphate phosphodiesterase gamma-1 | 894 | 1.18E-90 | 58.4 | P:lipid metabolic process; P:cellular process; F:hydrolase activity |
| Efet.01.203959.g175.t1 | E3 ubiquitin- ligase RNF213-like | 1236 | 6.64E-14 | 46.75 | F:nucleotide binding; F:ATP binding |
| Efet.01.203997.g176.t1 | Janus kinase and microtubule-interacting | 663 | 1.74E-18 | 64.8 | F:catalytic activity |
| Efet.01.204044.g177.t1 | Dynein heavy chain axonemal | 228 | 1.12E-35 | 84.75 | F:ATPase activity; F:ATP binding; F:microtubule motor activity; C:dynein complex; P:microtubule-based movement |
| Efet.01.204094.g181.t1 | spectrin beta chain-like isoform X1 | 879 | 1.56E-136 | 86.35 | C:postsynaptic density; P:Golgi to plasma membrane protein transport; C:nucleolus; C:extracellular exosome; F:ankyrin binding; F:GTPase binding; P:mitotic cytokinesis; P:protein targeting to plasma membrane; C:M band; F:protein complex binding; P:common-partner SMAD protein phosphorylation; P:positive regulation of interleukin-2 secretion; P:SMAD protein import into nucleus; P:membrane assembly; P:positive regulation of protein localization to plasma membrane; F:structural constituent of cytoskeleton; C:protein complex; F:phospholipid binding; F:RNA binding; F:cadherin binding involved in cell-cell adhesion; F:calmodulin binding; F:actin binding; P:actin filament capping; C:cell-cell adherens junction; C:axolemma; C:spectrin; C:cuticular plate |
| Efet.01.204119.g182.t1 | CCR4-NOT transcription complex subunit 1 | 414 | 1.34E-86 | 93.9 | P:RNA phosphodiester bond hydrolysis, exonucleolytic; P:transcription, DNA-templated; P:gene silencing by miRNA; C:P-body; P:positive regulation of nuclear-transcribed mRNA poly(A) tail shortening; F:retinoic acid receptor binding; P:nuclear-transcribed mRNA poly(A) tail shortening; F:RNA binding; C:peroxisomal membrane; F:poly(A)-specific ribonuclease activity; P:negative regulation of transcription from RNA polymerase II promoter; C:nucleus; F:protein complex scaffold; C:extracellular space; P:DNA damage response, signal transduction by p53 class mediator resulting in cell cycle arrest; P:positive regulation of cytoplasmic mRNA processing body assembly; P:negative regulation of retinoic acid receptor signaling pathway; F:armadillo repeat domain binding; P:regulation of stem cell population maintenance; C:cytosol; P:negative regulation of intracellular estrogen receptor signaling pathway; F:estrogen receptor binding; C:CCR4-NOT core complex; P:trophectodermal cell differentiation |
| Efet.01.204180.g183.t1 | TNF receptor-associated factor 2-like | 585 | 1.66E-29 | 67.5 | F:ubiquitin-protein transferase activity; F:zinc ion binding; P:signal transduction; F:metal ion binding; P:regulation of apoptotic process; P:protein ubiquitination |
| Efet.01.204214.g184.t1 | glycine receptor subunit alpha-3-like isoform X1 | 474 | 6.75E-55 | 68.1 | P:ion transport; C:membrane |
| Efet.01.204297.g185.t1 | ankyrin repeat and sterile alpha motif domain-containing 1B isoform X9 | 576 | 3.56E-23 | 63.5 | C:postsynapse; C:membrane; F:ephrin receptor binding; C:organelle; C:intracellular part |
| Efet.01.204317.g188.t1 | ras-related Rab-39B-like | 393 | 5.34E-49 | 70.7 | C:intracellular |
| Efet.01.204323.g189.t1 | sex peptide receptor-like | 1242 | 2.34E-132 | 65.75 | F:G-protein coupled receptor activity; F:G-protein coupled peptide receptor activity; P:G-protein coupled receptor signaling pathway; C:membrane; C:integral component of membrane |
| Efet.01.204398.g191.t1 | Centrosomal of 89 kDa | 687 | 1.33E-24 | 55.15 | C:membrane; C:integral component of membrane |
| Efet.01.20441.g1505.t1 | guanine nucleotide-binding subunit alpha-12-like | 606 | 3.69E-66 | 79.6 | F:GTP binding; C:heterotrimeric G-protein complex; C:brush border membrane; F:GTPase activity; P:adenylate cyclase-modulating G-protein coupled receptor signaling pathway; F:G-protein beta/gamma-subunit complex binding; P:Rho protein signal transduction; F:signal transducer activity; F:D5 dopamine receptor binding |
| Efet.01.204418.g192.t1 | cleavage and polyadenylation specificity factor subunit 3-like | 645 | 9.55E-81 | 74.15 | F:hydrolase activity |
| Efet.01.204442.g193.t1 | SET | 645 | 5.82E-114 | 84.25 | P:negative regulation of histone acetylation; P:DNA replication; C:endoplasmic reticulum; P:regulation of mRNA stability; C:protein complex; P:nucleosome disassembly; P:nucleosome assembly; C:nucleoplasm; P:negative regulation of neuron apoptotic process; F:DNA binding; F:protein phosphatase inhibitor activity; P:nucleocytoplasmic transport; C:perinuclear region of cytoplasm; P:negative regulation of catalytic activity; P:negative regulation of transcription, DNA-templated; F:histone binding; P:viral process; C:cytosol; P:regulation of phosphoprotein phosphatase activity |
| Efet.01.204481.g194.t1 | neurexin-2 isoform X20 | 636 | 7.54E-40 | 55.15 | C:membrane; P:single-organism cellular process |
| Efet.01.204541.g197.t1 | ankyrin repeat | 1455 | 4.00E-10 | 52.9 |  |
| Efet.01.204564.g198.t1 | 2-oxoglutarate mitochondrial isoform X5 | 771 | 1.42E-136 | 82.95 | F:thiamine pyrophosphate binding; F:oxoglutarate dehydrogenase (succinyl-transferring) activity; P:tricarboxylic acid cycle |
| Efet.01.20461.g1506.t1 | FAM161A isoform X2 | 1467 | 4.98E-24 | 58.25 | C:organelle; C:cell projection; C:intracellular part |
| Efet.01.204613.g199.t1 | PA14 domain-containing | 864 | 6.28E-29 | 48.5 |  |
| Efet.01.204620.g200.t1 | von Willebrand factor type EGF and pentraxin domain-containing 1-like | 630 | 1.23E-38 | 79.95 | F:calcium ion binding; C:integral component of membrane |
| Efet.01.204628.g201.t1 | probable ATP-dependent RNA helicase | 435 | 3.35E-65 | 85.8 | F:nucleic acid binding; F:ATP binding; P:positive regulation of transcription, DNA-templated; P:RNA secondary structure unwinding; F:ATP-dependent RNA helicase activity |
| Efet.01.204652.g202.t1 | helicase domino-like isoform X5 | 315 | 5.64E-07 | 57.55 | F:ATP binding; F:helicase activity |
| Efet.01.204681.g204.t1 | uncharacterized transporter slc- -like isoform X2 | 471 | 1.86E-14 | 57.15 | C:membrane; P:transport |
| Efet.01.204772.g205.t1 | alpha-N-acetylgalactosaminidase-like isoform X1 | 375 | 6.75E-35 | 68.5 | C:membrane; P:metabolic process; F:hydrolase activity, hydrolyzing O-glycosyl compounds |
| Efet.01.204807.g206.t1 | KIF1-binding homolog | 1707 | 0 | 61.95 |  |
| Efet.01.204838.g208.t1 | transient-receptor-potential | 762 | 4.03E-80 | 83.7 | F:calcium channel activity; C:integral component of membrane; P:calcium ion transmembrane transport |
| Efet.01.204865.g209.t1 | transcription factor SOX-11-like | 1053 | 7.29E-43 | 69.6 |  |
| Efet.01.204878.g210.t1 | hornerin-like isoform X1 | 1596 | 9.73E-20 | 66.15 | F:DNA binding; C:nucleus; P:regulation of transcription, DNA-templated |
| Efet.01.204928.g212.t1 | sodium channel type 4 subunit alpha B-like isoform X1 | 456 | 2.68E-29 | 70.1 | P:regulation of postsynaptic membrane potential; P:regulation of ion transmembrane transport; C:voltage-gated sodium channel complex; P:sodium ion transmembrane transport; F:voltage-gated sodium channel activity |
| Efet.01.204947.g214.t1 | EF-hand calcium-binding domain-containing 14-like isoform X1 | 234 | 7.19E-13 | 70.14 | C:membrane |
| Efet.01.204956.g215.t1 | Sperm-associated antigen | 399 | 2.56E-20 | 59.25 | C:microtubule cytoskeleton; C:cytoplasm; C:nucleoplasm |
| Efet.01.205009.g216.t1 | hypothetical protein CAPTEDRAFT_221070 | 345 | 1.79E-18 | 55.22 | C:membrane; C:integral component of membrane |
| Efet.01.205050.g217.t1 | hypothetical protein CAPTEDRAFT_144547 | 2787 | 0 | 55.25 | F:calcium ion binding; C:membrane; C:integral component of membrane; P:cell adhesion; P:homophilic cell adhesion via plasma membrane adhesion molecules; C:plasma membrane |
| Efet.01.20512.g1511.t1 | polypeptide N-acetylgalactosaminyltransferase 10 | 528 | 3.92E-40 | 57.25 | F:transferase activity, transferring glycosyl groups; C:integral component of membrane; P:protein glycosylation; C:Golgi membrane; F:carbohydrate binding |
| Efet.01.205168.g220.t1 | chitin synthase | 804 | 4.26E-90 | 61 | F:transferase activity, transferring hexosyl groups; C:integral component of membrane |
| Efet.01.205168.g221.t1 | chitin synthase | 630 | 1.58E-70 | 74.35 | C:myosin complex; F:ATP binding; F:transferase activity, transferring hexosyl groups; C:integral component of membrane; F:motor activity |
| Efet.01.205226.g224.t1 | hypothetical protein CAPTEDRAFT_202568 | 864 | 3.88E-08 | 43.5 | P:sodium ion transport; P:ion transport; F:sodium channel activity; C:membrane; C:integral component of membrane; P:sodium ion transmembrane transport; P:transport |
| Efet.01.205237.g225.t1 | E3 ubiquitin- ligase HUWE1-like | 1548 | 0 | 84.8 | C:nucleus; F:ubiquitin-protein transferase activity; F:zinc ion binding; C:cytoplasm; C:integral component of membrane; F:ligase activity; P:protein ubiquitination involved in ubiquitin-dependent protein catabolic process |
| Efet.01.205270.g228.t1 | insulin-like growth factor-binding complex acid labile subunit isoform X3 | 882 | 3.46E-86 | 51.1 | C:extracellular exosome; C:blood microparticle; C:membrane; C:integral component of membrane |
| Efet.01.205371.g229.t1 | argonaute- | 312 | 1.23E-49 | 87.8 | F:nucleic acid binding; F:metal ion binding |
| Efet.01.205386.g230.t1 | CTP synthase 1 | 225 | 3.79E-08 | 85.9 | F:ATP binding; P:'de novo' CTP biosynthetic process; P:glutamine metabolic process; F:CTP synthase activity |
| Efet.01.205443.g232.t1 | hypothetical protein CAPTEDRAFT_222036 | 360 | 1.69E-21 | 56.17 | C:membrane; C:integral component of membrane |
| Efet.01.205447.g234.t1 | hypothetical protein CAPTEDRAFT_201143 | 1062 | 9.21E-17 | 59 | P:regulation of apoptotic process |
| Efet.01.205455.g235.t1 | 5 -AMP-activated kinase catalytic subunit alpha- | 324 | 2.76E-44 | 93.1 | P:cell cycle arrest; F:protein C-terminus binding; P:cold acclimation; P:protein heterooligomerization; P:glucose metabolic process; P:cellular response to prostaglandin E stimulus; P:activation of MAPK activity; P:positive regulation of skeletal muscle tissue development; C:cytosol; P:Wnt signaling pathway; P:regulation of peptidyl-serine phosphorylation; P:cellular response to hypoxia; P:response to gamma radiation; P:negative regulation of TOR signaling; P:cellular response to organonitrogen compound; P:intracellular signal transduction; P:negative regulation of lipid catabolic process; P:cholesterol biosynthetic process; P:regulation of signal transduction by p53 class mediator; P:cellular response to glucose starvation; F:tau-protein kinase activity; P:cellular response to ethanol; P:positive regulation of autophagy; P:fatty acid oxidation; F:metal ion binding; F:[hydroxymethylglutaryl-CoA reductase (NADPH)] kinase activity; P:positive regulation of gene expression; P:glucose homeostasis; F:[acetyl-CoA carboxylase] kinase activity; P:response to activity; C:apical plasma membrane; C:nucleotide-activated protein kinase complex; P:negative regulation of glucose import in response to insulin stimulus; P:response to camptothecin; P:positive regulation of cell proliferation; P:response to UV; P:positive regulation of cholesterol biosynthetic process; P:regulation of vesicle-mediated transport; P:regulation of circadian rhythm; P:negative regulation of glucosylceramide biosynthetic process; P:positive regulation of glycolytic process; C:nucleoplasm; F:ATP binding; F:AMP-activated protein kinase activity; P:cellular response to hydrogen peroxide; F:kinase binding; P:macroautophagy; P:transcription, DNA-templated; F:histone serine kinase activity; P:fatty acid homeostasis; F:cAMP-dependent protein kinase activity; P:regulation of transcription, DNA-templated; F:chromatin binding; P:fatty acid biosynthetic process; P:rhythmic process; P:negative regulation of apoptotic process; P:regulation of energy homeostasis; P:histone-serine phosphorylation; P:response to caffeine |
| Efet.01.205467.g237.t1 | polyubiquitin 8-like | 222 | 5.66E-12 | 68.7 | F:ubiquitin conjugating enzyme binding; F:ubiquitin protein ligase activity; C:ubiquitin ligase complex; C:cytoplasm; P:protein polyubiquitination; P:positive regulation of proteasomal ubiquitin-dependent protein catabolic process; P:protein ubiquitination involved in ubiquitin-dependent protein catabolic process |
| Efet.01.205468.g239.t1 | potassium voltage-gated channel Shal-like | 1200 | 4.35E-160 | 80.7 | P:potassium ion transmembrane transport; C:voltage-gated potassium channel complex; F:delayed rectifier potassium channel activity; F:A-type (transient outward) potassium channel activity; P:regulation of ion transmembrane transport; P:protein homooligomerization |
| Efet.01.205525.g240.t1 | SON isoform X1 | 519 | 2.03E-13 | 55.55 | F:nucleic acid binding |
| Efet.01.205528.g241.t1 | lon protease homolog peroxisomal-like | 432 | 1.17E-49 | 82.55 | F:ATP binding; P:misfolded or incompletely synthesized protein catabolic process; F:serine-type endopeptidase activity; F:sequence-specific DNA binding; F:ATP-dependent peptidase activity |
| Efet.01.205536.g242.t1 | hypothetical protein CAPTEDRAFT_223749 | 255 | 1.11E-21 | 62.81 | C:membrane; C:integral component of membrane |
| Efet.01.205537.g243.t1 | hypothetical protein CAPTEDRAFT_223749 | 255 | 1.11E-21 | 62.81 | C:membrane; C:integral component of membrane |
| Efet.01.205552.g244.t1 | PAN domain | 696 | 2.57E-36 | 51.35 | F:metal ion binding; F:oxidoreductase activity; P:metabolic process; P:oxidation-reduction process |
| Efet.01.205553.g245.t1 | FMRFamide receptor | 918 | 1.22E-62 | 59.6 | C:membrane |
| Efet.01.20556.g1513.t1 | kinase C iota type | 618 | 3.47E-36 | 54.65 |  |
| Efet.01.205743.g249.t1 | heat shock 70 kDa 12A- | 516 | 2.94E-53 | 64.9 |  |
| Efet.01.205760.g250.t1 | 1-phosphatidylinositol 4,5-bisphosphate phosphodiesterase gamma-1-like | 345 | 4.58E-30 | 67.7 | P:angiogenesis; P:signal transduction; P:lipid catabolic process; P:regulation of heart contraction; P:fin regeneration; P:artery morphogenesis; P:hematopoietic stem cell differentiation; P:vasculogenesis; F:phosphoric diester hydrolase activity; P:granulocyte differentiation; P:regulation of angioblast cell migration involved in selective angioblast sprouting |
| Efet.01.205764.g251.t1 | Transcription factor | 471 | 4.45E-29 | 73.1 | C:nucleus; P:transcription, DNA-templated; F:transcriptional activator activity, RNA polymerase II transcription regulatory region sequence-specific binding; F:sequence-specific DNA binding; P:regulation of transcription from RNA polymerase II promoter |
| Efet.01.205792.g253.t1 | elongation of very long chain fatty acids 7-like | 261 | 1.13E-18 | 64.3 | C:membrane; P:fatty acid metabolic process |
| Efet.01.20585.g1514.t1 | pentatricopeptide repeat-containing mitochondrial-like isoform X1 | 318 | 5.06E-27 | 69.35 |  |
| Efet.01.205875.g254.t1 | insulin-like receptor | 255 | 3.70E-22 | 63.55 | F:nucleotide binding; F:protein tyrosine kinase activity; C:membrane; P:protein phosphorylation |
| Efet.01.205895.g255.t1 | metabotropic glutamate receptor 8-like | 288 | 6.10E-36 | 82.3 | F:G-protein coupled receptor activity; P:G-protein coupled receptor signaling pathway; C:integral component of membrane; C:plasma membrane |
| Efet.01.205904.g256.t1 | 5-hydroxytryptamine receptor 1A-like | 972 | 6.88E-92 | 74.85 | C:integral component of plasma membrane; F:adrenergic receptor activity; P:adrenergic receptor signaling pathway; F:G-protein coupled acetylcholine receptor activity; P:G-protein coupled acetylcholine receptor signaling pathway |
| Efet.01.205927.g258.t1 | Gypsy retrotransposon integrase 1 | 453 | 1.05E-13 | 53.15 | F:nucleic acid binding; P:DNA integration |
| Efet.01.20598.g1515.t1 | gastrula zinc finger -like | 1698 | 7.25E-19 | 39.6 | F:nucleic acid binding; F:metal ion binding |
| Efet.01.205990.g259.t1 | 60 kDa neurofilament -like | 381 | 3.84E-43 | 81.5 | F:structural molecule activity; C:intermediate filament |
| Efet.01.206016.g260.t1 | notchless homolog 1-like | 462 | 1.51E-26 | 57.7 | C:nucleus; C:nucleolus |
| Efet.01.206021.g261.t1 | hypothetical protein CAPTEDRAFT_185469 | 903 | 7.96E-14 | 50 | P:regulation of apoptotic process |
| Efet.01.206089.g263.t1 | 40S ribosomal S3a | 240 | 3.34E-17 | 80.45 | C:extracellular exosome; C:focal adhesion; F:structural constituent of ribosome; C:cytosolic small ribosomal subunit; P:negative regulation of apoptotic process; C:nucleolus; F:RNA binding; P:translation |
| Efet.01.206117.g265.t1 | zinc finger Xfin-like | 6186 | 4.58E-76 | 44.5 | F:nucleic acid binding; F:metal ion binding |
| Efet.01.206120.g266.t1 | elongation of very long chain fatty acids 6-like | 891 | 1.06E-123 | 72.15 | C:membrane; P:fatty acid metabolic process |
| Efet.01.206171.g267.t1 | turtle homolog B- | 288 | 6.48E-35 | 61.95 | C:membrane; C:integral component of membrane |
| Efet.01.20621.g1517.t1 | zinc finger Xfin-like | 5742 | 5.34E-59 | 48.7 | F:nucleic acid binding; F:metal ion binding |
| Efet.01.206216.g269.t1 | acid-sensing ion channel 1-like | 696 | 9.03E-17 | 58.5 | P:ion transport |
| Efet.01.206239.g270.t1 | fibrillin-1 | 369 | 3.02E-26 | 62.7 | C:extracellular matrix |
| Efet.01.20625.g1518.t1 | hypothetical protein CAPTEDRAFT_228965 | 969 | 1.12E-07 | 70 | C:membrane; C:integral component of membrane |
| Efet.01.20626.g1519.t1 | acetylcholine receptor subunit alpha-like isoform X1 | 1203 | 5.32E-132 | 60.85 | P:synaptic transmission, cholinergic; F:acetylcholine receptor activity; P:neuromuscular synaptic transmission; P:cation transmembrane transport; F:acetylcholine binding; C:acetylcholine-gated channel complex; P:response to nicotine |
| Efet.01.20628.g1520.t1 | antimicrobial-like peptide PP-1 | 270 | 1.20E-14 | 59 | P:cytolysis; P:regulation of muscle contraction; P:hemolysis in other organism; P:killing of cells of other organism; C:extracellular region; P:defense response to fungus; C:troponin complex; P:defense response to bacterium |
| Efet.01.206297.g272.t1 | CTP synthase | 732 | 1.93E-95 | 67.8 | P:pyrimidine nucleotide biosynthetic process; F:CTP synthase activity |
| Efet.01.206300.g273.t1 | innexin unc-9-like isoform X1 | 624 | 3.45E-107 | 76.1 | C:gap junction; P:ion transport; C:integral component of membrane; P:cell communication by electrical coupling; C:synapse; F:gap junction channel activity; C:plasma membrane |
| Efet.01.206311.g274.t1 | mucin 17 | 459 | 6.55E-09 | 69.2 |  |
| Efet.01.206322.g275.t1 | Potassium channel subfamily K member 9 | 489 | 1.55E-38 | 79.7 | C:integral component of plasma membrane; P:potassium ion transmembrane transport; P:stabilization of membrane potential; F:potassium ion leak channel activity |
| Efet.01.206361.g277.t1 | Growth hormone secretagogue receptor type 1 | 687 | 1.75E-27 | 50.65 | F:G-protein coupled receptor activity; P:G-protein coupled receptor signaling pathway; P:signal transduction; C:membrane; C:integral component of membrane; F:signal transducer activity |
| Efet.01.206372.g278.t1 | neuronal acetylcholine receptor subunit alpha-9-like | 543 | 5.92E-21 | 46.25 | F:ion channel activity; P:ion transport; C:postsynaptic membrane; P:ion transmembrane transport; C:membrane; P:cation transmembrane transport; C:integral component of membrane; C:cell junction; F:extracellular ligand-gated ion channel activity; C:synapse; P:transport; C:plasma membrane |
| Efet.01.206375.g279.t1 | transcription factor | 726 | 2.67E-27 | 85.3 | P:coronary artery morphogenesis; P:visceral serous pericardium development; P:palate development; F:RNA polymerase II regulatory region sequence-specific DNA binding; P:transcription from RNA polymerase II promoter; P:odontogenesis of dentin-containing tooth; P:cardiac neural crest cell migration involved in outflow tract morphogenesis; P:tongue development; P:apoptotic process involved in heart morphogenesis; P:peripheral nervous system neuron development; P:adult heart development; P:embryonic digit morphogenesis; P:negative regulation of osteoblast differentiation; P:thymus development; P:sympathetic nervous system development; C:transcription factor complex; F:transcriptional activator activity, RNA polymerase II core promoter proximal region sequence-specific binding; P:negative regulation of DNA binding; P:positive regulation of transcription regulatory region DNA binding; F:protein heterodimerization activity; P:angiogenesis; P:in utero embryonic development; P:suckling behavior; P:heart looping; P:positive regulation of cardiac muscle hypertrophy; P:regulation of tissue remodeling; F:activating transcription factor binding; F:E-box binding; F:protein homodimerization activity; P:cartilage morphogenesis; F:AT DNA binding; P:negative regulation of sequence-specific DNA binding transcription factor activity; P:regulation of secondary heart field cardioblast proliferation; P:cell proliferation involved in outflow tract morphogenesis; P:positive regulation of p38MAPK cascade; P:negative regulation of gene expression; C:nuclear chromatin; F:transcription coactivator activity; P:cardiac right ventricle formation; P:positive regulation of ERK1 and ERK2 cascade; P:positive regulation of transcription from RNA polymerase II promoter involved in norepinephrine biosynthetic process; P:positive regulation of semaphorin-plexin signaling pathway involved in outflow tract morphogenesis; P:negative regulation of cardiac muscle cell apoptotic process; P:mesenchymal cell proliferation |
| Efet.01.20638.g1521.t1 | protocadherin-9-like isoform X1 | 2664 | 0 | 55.3 | F:calcium ion binding; C:membrane; C:integral component of membrane; P:cell adhesion; P:homophilic cell adhesion via plasma membrane adhesion molecules; C:plasma membrane |
| Efet.01.206399.g280.t1 | tripartite motif-containing 2-like | 1296 | 1.93E-26 | 47.25 | F:zinc ion binding; F:metal ion binding; C:intracellular |
| Efet.01.20643.g1522.t1 | zinc finger 616-like | 330 | 7.32E-09 | 52.55 | F:nucleic acid binding; C:nucleus; F:zinc ion binding; F:metal ion binding |
| Efet.01.20643.g1523.t1 | zinc finger 408-like | 261 | 3.20E-11 | 52.95 | F:nucleic acid binding; C:nucleus; F:zinc ion binding; F:metal ion binding |
| Efet.01.20643.g1524.t1 | zinc finger and BTB domain-containing 37-like | 231 | 6.41E-07 | 55.3 | F:nucleic acid binding; F:metal ion binding |
| Efet.01.206446.g281.t1 | proton-coupled amino acid transporter CG1139 isoform X4 | 402 | 1.71E-24 | 68.8 | C:membrane; C:integral component of membrane |
| Efet.01.206470.g282.t1 | oocyte zinc finger 6-like | 2022 | 2.32E-84 | 44.15 | F:nucleic acid binding; F:metal ion binding |
| Efet.01.2065.g179.t1 | zinc finger castor homolog 1-like | 2163 | 3.54E-108 | 63.05 | F:metal ion binding |
| Efet.01.206516.g283.t1 | bactericidal permeability increasing | 855 | 5.01E-29 | 48.2 | C:extracellular space; F:lipid binding |
| Efet.01.206518.g284.t1 | succinate dehydrogenase [ubiquinone] iron-sulfur mitochondrial | 267 | 5.11E-49 | 95.15 | F:succinate dehydrogenase (ubiquinone) activity; F:metal ion binding; F:electron carrier activity; F:3 iron, 4 sulfur cluster binding; P:tricarboxylic acid cycle; F:4 iron, 4 sulfur cluster binding; F:2 iron, 2 sulfur cluster binding; C:mitochondrial inner membrane |
| Efet.01.206543.g285.t1 | Na(+) H(+) exchange regulatory cofactor NHE-RF1 | 366 | 4.11E-12 | 74 |  |
| Efet.01.206557.g287.t1 | degenerin unc-105 | 744 | 9.32E-21 | 58.5 | P:ion transport |
| Efet.01.206609.g288.t1 | hypothetical protein HELRODRAFT_175037 | 294 | 1.32E-34 | 66.35 | F:binding |
| Efet.01.206641.g290.t1 | hypothetical protein CAPTEDRAFT_220857 | 450 | 3.78E-11 | 53.2 | C:membrane; C:integral component of membrane |
| Efet.01.206659.g292.t1 | delta(24)-sterol reductase | 363 | 1.04E-50 | 79.3 | P:cell cycle arrest; P:protein localization; C:Golgi apparatus; P:skin development; C:endoplasmic reticulum membrane; P:response to oxidative stress; P:plasminogen activation; P:negative regulation of cysteine-type endopeptidase activity involved in apoptotic process; P:apoptotic process; F:delta24-sterol reductase activity; P:amyloid precursor protein catabolic process; P:membrane organization; F:oxidoreductase activity, acting on CH-OH group of donors; P:cholesterol biosynthetic process via lathosterol; F:peptide antigen binding; P:regulation of neuron death; F:delta24(24-1) sterol reductase activity; P:tissue development; C:nucleus; F:flavin adenine dinucleotide binding; P:male genitalia development; C:integral component of membrane; P:cholesterol biosynthetic process via desmosterol; P:oxidation-reduction process; F:enzyme binding |
| Efet.01.20668.g1526.t1 | aquaporin -like | 582 | 8.87E-73 | 73.75 | C:membrane |
| Efet.01.206687.g293.t1 | hypothetical protein CAPTEDRAFT_221134 | 312 | 7.68E-09 | 64 |  |
| Efet.01.206695.g294.t1 | neuropeptide CCHamide-1 receptor-like | 393 | 1.02E-17 | 69.95 | F:G-protein coupled receptor activity; P:signal transduction |
| Efet.01.206695.g295.t1 | neuropeptide CCHamide-1 receptor-like | 726 | 1.58E-65 | 68.5 | F:G-protein coupled peptide receptor activity; P:G-protein coupled receptor signaling pathway; C:membrane |
| Efet.01.206715.g296.t1 | DNA replication complex GINS PSF3 | 447 | 1.92E-51 | 69.2 | C:nucleus |
| Efet.01.20673.g1527.t1 | hypothetical protein CAPTEDRAFT_220857 | 519 | 1.39E-15 | 53.22 | C:membrane; C:integral component of membrane |
| Efet.01.206739.g297.t1 | C3 and PZP-like alpha-2-macroglobulin domain-containing 8 | 414 | 9.11E-21 | 52.75 | F:methyltransferase activity; F:transferase activity; P:methylation |
| Efet.01.206769.g298.t1 | Propionyl- carboxylase alpha mitochondrial | 543 | 1.38E-53 | 68.15 | F:ATP binding; F:propionyl-CoA carboxylase activity; F:metal ion binding; C:mitochondrion; F:biotin carboxylase activity; F:enzyme binding |
| Efet.01.206818.g300.t1 | guanylate cyclase 2G | 2250 | 2.23E-70 | 47.1 | F:ATP binding; F:phosphorus-oxygen lyase activity; C:membrane; C:integral component of membrane; F:protein kinase activity; F:phosphate ion binding; P:protein phosphorylation; P:intracellular signal transduction; P:phosphate ion transmembrane transport; P:cyclic nucleotide biosynthetic process; C:intracellular; C:ATP-binding cassette (ABC) transporter complex |
| Efet.01.206827.g301.t1 | tRNA pseudouridine(38 39) synthase | 492 | 3.20E-08 | 76.4 | C:nucleus; F:RNA binding; F:oxidosqualene cyclase activity; P:tRNA pseudouridine synthesis; C:cytoplasm; F:pseudouridine synthase activity; P:mRNA pseudouridine synthesis |
| Efet.01.20683.g1529.t1 | Transmembrane 151B | 477 | 2.89E-28 | 51.4 | C:membrane; C:integral component of membrane; F:scavenger receptor activity; P:receptor-mediated endocytosis |
| Efet.01.206847.g303.t1 | atrial natriuretic peptide receptor 1-like | 225 | 1.00E-09 | 62.45 | P:cGMP biosynthetic process; F:phosphorus-oxygen lyase activity; F:lyase activity; F:guanylate cyclase activity; F:protein kinase activity; P:protein phosphorylation; C:intracellular; F:nucleotide binding; F:ATP binding; C:membrane; C:integral component of membrane; P:intracellular signal transduction; P:cyclic nucleotide biosynthetic process |
| Efet.01.206867.g304.t1 | hypothetical protein CAPTEDRAFT_202797 | 1290 | 9.02E-46 | 68 |  |
| Efet.01.20690.g1530.t1 | probable ATP-dependent RNA helicase DDX46 isoform X2 | 417 | 1.59E-39 | 76.3 | F:nucleic acid binding; F:ATP binding; F:helicase activity |
| Efet.01.206916.g306.t1 | zinc finger SWIM domain-containing 6-like | 396 | 1.09E-24 | 65.05 | F:zinc ion binding |
| Efet.01.20693.g1531.t1 | 1-phosphatidylinositol 4,5-bisphosphate phosphodiesterase delta-1 isoform X1 | 258 | 2.06E-16 | 67.9 | P:regulation of cellular process; F:phospholipid binding; F:phosphoric diester hydrolase activity; P:single-organism process; C:intracellular |
| Efet.01.206930.g307.t1 | vascular endothelial growth factor A-like isoform X2 | 468 | 2.62E-15 | 56.5 | F:nucleic acid binding; F:growth factor activity; C:membrane; F:protein dimerization activity |
| Efet.01.207039.g312.t1 | zinc finger 653 | 2340 | 2.67E-11 | 62.6 | F:ubiquitin-protein transferase activity; P:activation of NF-kappaB-inducing kinase activity; P:protein K63-linked ubiquitination; F:binding |
| Efet.01.207064.g314.t1 | hypothetical protein CAPTEDRAFT_186854 | 735 | 4.54E-58 | 52.2 | C:nuclear envelope; C:cytosol; P:transcription factor import into nucleus; F:protein transporter activity; P:intracellular protein transport; F:Ran GTPase binding |
| Efet.01.2071.g181.t1 | hypothetical protein CAPTEDRAFT_159800 | 360 | 4.46E-14 | 49.33 |  |
| Efet.01.2071.g182.t1 | teneurin-m-like isoform X1 | 2010 | 7.03E-132 | 54.7 | P:self proteolysis |
| Efet.01.207101.g316.t1 | CAP-Gly domain-containing linker 1 isoform X2 | 375 | 2.01E-08 | 64.6 |  |
| Efet.01.207156.g320.t1 | tetrameric potassium-selective cyclic nucleotide gated channel | 1323 | 1.67E-91 | 65.7 | F:ion channel activity; P:ion transport; P:transmembrane transport; C:integral component of membrane |
| Efet.01.207233.g322.t1 | potassium channel subfamily K member 10 | 267 | 7.48E-08 | 54 | P:ion transport; P:potassium ion transmembrane transport; C:membrane; C:integral component of membrane; F:potassium channel activity; P:transport |
| Efet.01.207325.g325.t1 | 5-hydroxytryptamine receptor 1A-like | 852 | 8.70E-50 | 53.45 | P:G-protein coupled receptor signaling pathway; C:integral component of membrane; C:plasma membrane; F:G-protein coupled amine receptor activity |
| Efet.01.207326.g326.t1 | sel-1 homolog 1-like | 1260 | 2.30E-17 | 90.1 | C:integral component of membrane |
| Efet.01.207347.g327.t1 | hypothetical protein CAPTEDRAFT_210402 | 1791 | 2.84E-37 | 54.85 | F:catalytic activity; P:nucleoside metabolic process |
| Efet.01.207381.g330.t1 | unc-93 homolog A-like | 321 | 1.53E-18 | 70.5 | C:integral component of membrane |
| Efet.01.207408.g331.t1 | kinesin KIF26A isoform X1 | 366 | 1.46E-36 | 75.7 | F:nucleotide binding; F:ATPase activity; P:enteric nervous system development; F:microtubule motor activity; P:negative regulation of signal transduction; P:cytoskeleton-dependent intracellular transport; P:protein localization; P:axon guidance; C:kinesin complex; P:regulation of cell growth by extracellular stimulus; P:microtubule-based movement; F:microtubule binding |
| Efet.01.20745.g1533.t1 | ankyrin repeat domain-containing 17- | 780 | 5.85E-64 | 89.9 | P:positive regulation of G1/S transition of mitotic cell cycle; P:endoderm development; P:positive regulation of I-kappaB kinase/NF-kappaB signaling; P:positive regulation of MDA-5 signaling pathway; P:negative regulation of smooth muscle cell differentiation; P:regulation of DNA replication; F:chromatin binding; P:positive regulation of RIG-I signaling pathway; C:chromatin; P:defense response to bacterium; F:RNA binding; C:nucleus; F:protein binding; C:cytoplasm; P:blood vessel maturation; P:innate immune response; C:membrane; P:viral process |
| Efet.01.20745.g1534.t1 | ankyrin repeat domain-containing 17-like isoform X2 | 1803 | 2.89E-93 | 73.5 | F:nucleic acid binding |
| Efet.01.20745.g1535.t1 | ankyrin repeat domain-containing 17-like isoform X2 | 969 | 1.98E-10 | 45.5 | F:nucleic acid binding; F:RNA binding |
| Efet.01.207474.g334.t1 | probable phospholipid-transporting ATPase IM isoform X1 | 417 | 1.36E-78 | 93.7 | F:magnesium ion binding; F:ATP binding; P:phospholipid translocation; P:Golgi organization; C:integral component of membrane; C:Golgi apparatus; F:phospholipid-translocating ATPase activity; C:plasma membrane |
| Efet.01.207488.g337.t1 | transmembrane 132C-like | 654 | 3.14E-20 | 44.2 | C:membrane; C:integral component of membrane |
| Efet.01.20750.g1536.t1 | PREDICTED: uncharacterized protein C3orf38 homolog | 780 | 8.25E-50 | 52.75 |  |
| Efet.01.207507.g338.t1 | chromodomain-helicase-DNA-binding 4-like isoform X8 | 729 | 5.95E-23 | 69.9 | F:nucleic acid binding; C:nucleus; F:metal ion binding; F:hydrolase activity, acting on acid anhydrides, in phosphorus-containing anhydrides; P:microtubule cytoskeleton organization |
| Efet.01.207527.g340.t1 | Calcium-binding NCS-1 | 387 | 4.84E-31 | 68.6 | P:regulation of cellular process; C:cytoplasm; F:metal ion binding |
| Efet.01.207533.g341.t1 | G patch domain-containing 2-like | 708 | 2.24E-27 | 59.2 | F:nucleic acid binding |
| Efet.01.20754.g1537.t1 | sodium glucose cotransporter 5 isoform X2 | 387 | 7.61E-32 | 52.25 | C:integral component of plasma membrane; C:extracellular exosome; P:transmembrane transport; C:membrane; C:integral component of membrane; F:symporter activity; P:transport; F:transporter activity |
| Efet.01.207640.g343.t1 | hypothetical protein CTR53_20720, partial | 780 | 8.50E-171 | 62.67 |  |
| Efet.01.207640.g344.t1 | RHS repeat-associated core domain-containing | 2040 | 1.40E-171 | 47 | P:self proteolysis |
| Efet.01.207643.g345.t1 | serine arginine repetitive matrix 2-like isoform X1 | 729 | 1.61E-06 | 69 |  |
| Efet.01.207665.g347.t1 | twitchin | 288 | 1.07E-08 | 56.05 | F:nucleotide binding; F:ATP binding; F:protein kinase activity; P:phosphorylation; F:transferase activity; P:protein phosphorylation; F:kinase activity |
| Efet.01.207683.g348.t1 | prohormone-3 neuropeptide precursor | 387 | 2.75E-31 | 61.5 | P:neuropeptide signaling pathway |
| Efet.01.207713.g349.t1 | chromodomain-helicase-DNA-binding 5 | 201 | 6.32E-27 | 53.95 | F:binding |
| Efet.01.207750.g350.t1 | cullin-3-like isoform X4 | 360 | 3.48E-61 | 93.8 | P:cell cycle arrest; P:embryonic cleavage; F:ubiquitin protein ligase activity; P:negative regulation of Rho protein signal transduction; P:positive regulation of cell proliferation; P:cell projection organization; P:positive regulation of mitotic metaphase/anaphase transition; C:Golgi membrane; P:intrinsic apoptotic signaling pathway; P:stress fiber assembly; C:nucleoplasm; P:negative regulation of transcription from RNA polymerase II promoter; P:MAPK cascade; P:integrin-mediated signaling pathway; C:extracellular exosome; F:protein heterodimerization activity; C:polar microtubule; P:G1/S transition of mitotic cell cycle; C:cytosol; P:Wnt signaling pathway; P:stem cell division; P:cell migration; P:positive regulation of cytokinesis; F:protein homodimerization activity; P:protein polyubiquitination; P:fibroblast apoptotic process; P:protein monoubiquitination; P:protein ubiquitination involved in ubiquitin-dependent protein catabolic process; P:gastrulation; P:COPII vesicle coating; F:POZ domain binding; P:negative regulation of canonical Wnt signaling pathway; P:proteasome-mediated ubiquitin-dependent protein catabolic process; F:ubiquitin protein ligase binding; P:trophectodermal cellular morphogenesis; P:liver morphogenesis; F:cyclin binding; C:Cul3-RING ubiquitin ligase complex; P:mitotic metaphase plate congression |
| Efet.01.207760.g351.t1 | hypothetical protein HELRODRAFT_176694 | 795 | 2.17E-75 | 61.45 | C:membrane |
| Efet.01.207762.g352.t1 | nischarin-like isoform X2 | 2733 | 1.11E-47 | 53.65 | F:phosphatidylinositol binding |
| Efet.01.207773.g353.t1 | zinc finger 525-like | 1128 | 1.17E-06 | 56 |  |
| Efet.01.207785.g354.t1 | FAD-binding oxidoreductase | 435 | 4.05E-65 | 75.4 | F:oxidoreductase activity; C:integral component of membrane; P:oxidation-reduction process |
| Efet.01.207785.g355.t1 | spermidine putrescine ABC transporter substrate-binding | 1035 | 0 | 91.15 |  |
| Efet.01.207785.g356.t1 | ABC transporter ATP-binding | 1023 | 0 | 81.6 | F:ATPase activity; F:ATP binding; P:peptide transport |
| Efet.01.207785.g357.t1 | ABC transporter ATP-binding | 342 | 2.37E-36 | 88.35 | F:ATPase activity; F:ATP binding; P:peptide transport |
| Efet.01.207798.g358.t1 | GIGYF family CG11148-like isoform X4 | 324 | 6.66E-13 | 69.75 |  |
| Efet.01.207916.g363.t1 | mahjong isoform X1 | 312 | 1.45E-27 | 79.45 | P:protein ubiquitination |
| Efet.01.207917.g364.t1 | titin-like isoform X10 | 4002 | 5.68E-20 | 55.69 |  |
| Efet.01.208001.g365.t1 | dynein heavy chain axonemal-like | 459 | 3.41E-29 | 57.4 | F:nucleotide binding; F:ATPase activity; F:ATP binding; F:microtubule motor activity; C:axonemal dynein complex; C:dynein complex; P:cilium movement; P:microtubule-based movement |
| Efet.01.208001.g366.t1 | dynein heavy chain axonemal | 1164 | 1.04E-164 | 78.8 | F:ATPase activity; F:ATP binding; F:microtubule motor activity; C:dynein complex; P:microtubule-based movement |
| Efet.01.20804.g1540.t1 | beta-adrenergic receptor kinase 2-like | 294 | 1.32E-35 | 77.6 | F:ATP binding; F:G-protein coupled receptor kinase activity; P:signal transduction; P:protein phosphorylation |
| Efet.01.20807.g1541.t1 | fantom-like isoform X1 | 507 | 7.36E-41 | 74.05 | P:chemotaxis; C:ciliary transition zone |
| Efet.01.208096.g368.t1 | Shroom3 isoform X1 | 1194 | 2.83E-29 | 69.55 | C:adherens junction; C:cytoskeleton; F:actin binding; P:cellular pigment accumulation; P:cell morphogenesis; C:apical junction complex; P:apical protein localization; P:pattern specification process; C:apical plasma membrane |
| Efet.01.208105.g369.t1 | kinesin KIF23 isoform X1 | 645 | 2.40E-15 | 56.44 | P:single-organism cellular process; F:binding |
| Efet.01.208113.g370.t1 | M23 family peptidase [Pseudoxanthomonas wuyuanensis] | 1386 | 2.46E-140 | 48.95 |  |
| Efet.01.208133.g371.t1 | DUF5103 domain-containing | 657 | 1.27E-145 | 60.4 |  |
| Efet.01.208149.g372.t1 | hypothetical protein CRN43_10290, partial | 525 | 8.37E-18 | 58 |  |
| Efet.01.2082.g183.t1 | rho GTPase-activating 23 | 1476 | 2.14E-21 | 78.35 | C:extracellular exosome; P:signal transduction; F:GTPase activator activity; F:phospholipid binding; P:positive regulation of GTPase activity |
| Efet.01.208210.g374.t1 | filamin-C isoform X1 | 594 | 4.70E-93 | 82 | F:actin filament binding |
| Efet.01.208232.g376.t1 | neuropeptide F receptor-like | 831 | 1.94E-66 | 65.2 | F:G-protein coupled receptor activity; P:G-protein coupled receptor signaling pathway; C:membrane |
| Efet.01.208269.g379.t1 | serine threonine- phosphatase 6 regulatory ankyrin repeat subunit B-like | 3315 | 0 | 55.9 |  |
| Efet.01.208334.g381.t1 | poly(U)-binding-splicing factor PUF60-like isoform X2 | 429 | 3.94E-49 | 81.7 | F:nucleotide binding; F:RNA binding |
| Efet.01.208353.g382.t1 | collagen type IV alpha-3-binding -like | 441 | 9.84E-68 | 77.35 | F:lipid binding |
| Efet.01.208384.g383.t1 | calpain-A-like isoform X7 | 282 | 1.47E-22 | 69.3 | C:extracellular exosome; C:focal adhesion; C:cytoplasm; P:proteolysis; F:calcium-dependent cysteine-type endopeptidase activity; C:cell surface |
| Efet.01.208388.g384.t1 | solute carrier family 35 member E3-like isoform X2 | 552 | 4.67E-58 | 79.4 | C:integral component of membrane |
| Efet.01.208415.g386.t1 | synaptotagmin-1-like isoform X1 | 306 | 7.86E-46 | 97.6 | P:calcium ion-regulated exocytosis of neurotransmitter; F:syntaxin binding; P:vesicle fusion; P:synaptic vesicle endocytosis; F:calcium ion binding; C:cytosol; C:cell junction; C:integral component of synaptic vesicle membrane; F:clathrin binding; P:negative regulation of calcium ion-dependent exocytosis; C:plasma membrane; F:calcium-dependent phospholipid binding |
| Efet.01.208499.g388.t1 | EF-hand domain-containing family member C2 | 321 | 1.60E-13 | 57.8 | F:calcium ion binding |
| Efet.01.20854.g1545.t1 | phosphatidylinositol 4-kinase type 2-beta | 276 | 1.06E-08 | 79.55 | P:phosphorylation; P:pectoral fin development; F:kinase activity |
| Efet.01.208544.g389.t1 | ribonuclease P subunit p20 | 444 | 1.03E-45 | 74.1 | P:RNA phosphodiester bond hydrolysis |
| Efet.01.208546.g390.t1 | laminin subunit beta-1-like | 921 | 1.44E-108 | 69.5 | P:somite development; P:basement membrane assembly; P:muscle attachment |
| Efet.01.208547.g391.t1 | stomatin 1 | 213 | 4.61E-10 | 66.3 | C:membrane; C:integral component of membrane |
| Efet.01.208563.g393.t1 | Ras-related Rab-18-B | 237 | 5.68E-33 | 80.65 | F:GTP binding; P:chordate embryonic development; P:eye development; C:extracellular exosome; P:small GTPase mediated signal transduction; C:membrane; C:endoplasmic reticulum tubular network; F:GDP binding; P:lipid particle organization; P:brain development; P:endoplasmic reticulum tubular network organization; P:transport |
| Efet.01.208608.g394.t1 | UDP-N-acetylglucosamine--peptide N-acetylglucosaminyltransferase 110 kDa subunit isoform X2 | 714 | 8.77E-97 | 72.6 | C:integral component of membrane; F:transferase activity |
| Efet.01.208617.g395.t1 | coronin-6 isoform X1 | 438 | 1.64E-22 | 74.8 |  |
| Efet.01.20868.g1546.t1 | CASP isoform X2 | 405 | 1.23E-31 | 70.4 | C:integral component of membrane |
| Efet.01.208690.g396.t1 | pumilio homolog 2-like isoform X1 | 276 | 2.61E-34 | 77.65 | F:RNA binding |
| Efet.01.208723.g397.t1 | hypothetical protein HELRODRAFT_181170 | 597 | 8.05E-10 | 88 | C:postsynaptic membrane; P:cation transmembrane transport; C:integral component of membrane; C:cell junction; F:extracellular ligand-gated ion channel activity |
| Efet.01.208838.g398.t1 | hypothetical protein CAPTEDRAFT_222036 | 381 | 3.01E-10 | 55 |  |
| Efet.01.208865.g401.t1 | diaphanous-related formin | 327 | 1.12E-17 | 81.5 | F:actin binding; P:actin filament organization; F:Rho GTPase binding; P:cell cycle |
| Efet.01.208898.g402.t1 | uncharacterized protein LOC110453123 | 2580 | 7.39E-06 | 54 |  |
| Efet.01.208946.g403.t1 | thyrotropin-releasing hormone receptor-like | 300 | 9.41E-11 | 61.5 | F:G-protein coupled receptor activity; P:G-protein coupled receptor signaling pathway; P:signal transduction; C:membrane; C:integral component of membrane; F:signal transducer activity |
| Efet.01.208946.g404.t1 | thyrotropin-releasing hormone receptor | 1014 | 4.54E-56 | 60.05 | P:G-protein coupled receptor signaling pathway; C:membrane; F:signal transducer activity |
| Efet.01.208970.g405.t1 | arg8-vasotocin receptor-like | 639 | 8.89E-42 | 68.05 | P:response to organic substance; P:signal transduction; P:response to endogenous stimulus; C:integral component of membrane; F:signal transducer activity |
| Efet.01.209014.g408.t1 | hypothetical protein BHC48_06095 | 3180 | 3.68E-07 | 65 |  |
| Efet.01.209030.g409.t1 | Pantothenate kinase | 651 | 7.45E-14 | 54.35 | F:ATP binding; P:coenzyme A biosynthetic process; F:pantothenate kinase activity; P:phosphorylation; F:kinase activity |
| Efet.01.20905.g1548.t1 | NAD(P)H dehydrogenase [quinone] 1 | 435 | 9.14E-09 | 68.9 | P:response to oxidative stress; F:identical protein binding; C:extracellular exosome; F:NAD(P)H dehydrogenase (quinone) activity; P:negative regulation of catalytic activity; C:cytoplasm; P:oxidation-reduction process; C:nucleoplasm; F:RNA binding |
| Efet.01.209094.g411.t1 | Casein kinase I isoform alpha | 951 | 1.06E-117 | 70.9 | F:nucleotide binding; F:protein kinase activity; P:phosphorylation |
| Efet.01.209105.g413.t1 | SWI SNF complex subunit SMARCC2-like | 651 | 4.22E-68 | 86.8 | F:DNA binding; C:BAF-type complex; P:chromatin remodeling; P:regulation of transcription from RNA polymerase II promoter |
| Efet.01.209215.g414.t1 | hypothetical protein CAPTEDRAFT_227990 | 2148 | 7.25E-41 | 52.5 |  |
| Efet.01.209218.g416.t1 | zinc finger 585A-like isoform X1 | 1386 | 4.25E-47 | 43.1 | F:nucleic acid binding; P:biological_process; F:metal ion binding |
| Efet.01.209265.g418.t1 | rho GTPase-activating 21-like isoform X4 | 774 | 9.24E-63 | 59.85 | P:signal transduction; F:GTPase activator activity; F:phospholipid binding; P:positive regulation of GTPase activity |
| Efet.01.20930.g1549.t1 | hypothetical protein HELRODRAFT_180676 | 2892 | 5.37E-142 | 48.8 | C:membrane; C:integral component of membrane |
| Efet.01.209323.g419.t1 | potassium voltage-gated channel Shaker-like | 1038 | 1.58E-128 | 78.4 | P:potassium ion transmembrane transport; C:voltage-gated potassium channel complex; F:delayed rectifier potassium channel activity; P:regulation of ion transmembrane transport; P:protein homooligomerization |
| Efet.01.209327.g420.t1 | hypothetical protein CAPTEDRAFT_209671 | 297 | 1.08E-11 | 64.5 | C:membrane |
| Efet.01.209372.g421.t1 | zinc finger OZF-like isoform X1 | 894 | 7.54E-70 | 63.1 | F:nucleic acid binding; F:metal ion binding |
| Efet.01.209373.g422.t1 | hypothetical protein CAPTEDRAFT_82130, partial | 1320 | 1.47E-08 | 47 | P:transmembrane transport; C:membrane; C:integral component of membrane |
| Efet.01.2094.g184.t1 | transmembrane 132E-like | 537 | 6.75E-30 | 56.55 | C:membrane |
| Efet.01.209427.g425.t1 | Netrin receptor DCC | 225 | 8.16E-21 | 78.45 | C:integral component of plasma membrane; P:positive regulation of BMP signaling pathway; P:iron ion homeostasis; F:netrin receptor activity; P:apoptotic process; P:axon guidance; P:netrin-activated signaling pathway |
| Efet.01.209460.g427.t1 | Forkhead box G1 | 987 | 2.31E-41 | 65.4 | F:transcription factor activity, sequence-specific DNA binding; P:regulation of transcription, DNA-templated; F:sequence-specific DNA binding |
| Efet.01.209531.g429.t1 | hypothetical protein CAPTEDRAFT_207355 | 228 | 6.11E-06 | 62 |  |
| Efet.01.209606.g431.t1 | retrovirus-related Pol poly from transposon | 414 | 1.24E-42 | 70.5 |  |
| Efet.01.209668.g433.t1 | delta 1 | 843 | 1.67E-22 | 62.7 | P:cell communication; C:membrane; P:single-organism developmental process |
| Efet.01.209679.g434.t1 | alpha-adducin-like isoform X1 | 387 | 1.13E-40 | 83 |  |
| Efet.01.209727.g435.t1 | major facilitator superfamily domain-containing 4A-like isoform X2 | 351 | 1.85E-30 | 74.45 | P:transmembrane transport; C:integral component of membrane |
| Efet.01.20973.g1551.t1 | chondroitin sulfate N-acetylgalactosaminyltransferase 2-like | 1281 | 1.11E-108 | 57.55 | F:transferase activity, transferring glycosyl groups; C:Golgi cisterna membrane; C:membrane; C:integral component of membrane; F:acetylgalactosaminyltransferase activity; F:transferase activity |
| Efet.01.209765.g437.t1 | leucine-rich repeat-containing G- coupled receptor 4-like | 2037 | 1.94E-37 | 45.35 | C:membrane; C:integral component of membrane |
| Efet.01.209822.g439.t1 | hypothetical protein CAPTEDRAFT_201014 | 1596 | 8.10E-86 | 43.15 | C:membrane; C:integral component of membrane |
| Efet.01.209855.g441.t1 | nucleolar 6 isoform X2 | 429 | 1.24E-20 | 45.45 | C:nuclear lumen; C:intracellular non-membrane-bounded organelle |
| Efet.01.209929.g443.t1 | collagen alpha-6(VI) chain isoform X2 | 687 | 7.04E-18 | 50.25 | F:calcium ion binding |
| Efet.01.209985.g444.t1 | cystathionine beta-synthase isoform X3 | 222 | 3.99E-08 | 100 | F:pyridoxal phosphate binding; C:cytoplasm; P:cysteine biosynthetic process via cystathionine; F:cystathionine beta-synthase activity; F:cysteine synthase activity; P:cysteine biosynthetic process from serine |
| Efet.01.210024.g447.t1 | integrin alpha-1-like | 558 | 3.07E-09 | 56.6 | F:calcium ion binding; C:membrane; C:integral component of membrane; C:proteinaceous extracellular matrix |
| Efet.01.210030.g448.t1 | calmodulin isoform X1 | 297 | 4.33E-19 | 71.9 | P:regulation of release of sequestered calcium ion into cytosol; P:positive regulation of hydrolase activity; C:supramolecular fiber; P:response to calcium ion; C:spindle; P:regulation of heart contraction; C:vesicle; P:positive regulation of protein dephosphorylation; F:enzyme binding |
| Efet.01.21004.g1552.t1 | kinesin KIF17 | 3768 | 0 | 82.9 | F:ATP binding; P:cell projection organization; C:kinesin complex; P:neurogenesis; F:ATP-dependent microtubule motor activity, plus-end-directed; P:microtubule-based movement; C:microtubule; F:microtubule binding |
| Efet.01.210134.g450.t1 | unconventional myosin-IXa-like isoform X10 | 573 | 9.22E-20 | 90.4 | C:myosin complex; F:ATP binding; F:metal ion binding; P:intracellular signal transduction; F:motor activity |
| Efet.01.210140.g451.t1 | Potassium sodium hyperpolarization-activated cyclic nucleotide-gated channel 4 | 528 | 7.16E-76 | 91.4 | F:voltage-gated potassium channel activity; P:potassium ion transmembrane transport; C:integral component of membrane |
| Efet.01.210186.g454.t1 | dnaJ homolog subfamily C member 28 | 588 | 1.48E-39 | 65.8 | P:cellular component organization; P:Golgi vesicle transport |
| Efet.01.210277.g455.t1 | carbohydrate sulfotransferase 3-like | 942 | 2.80E-77 | 47.4 | C:membrane; C:integral component of membrane; F:sulfotransferase activity |
| Efet.01.210314.g456.t1 | cubilin | 729 | 7.61E-27 | 50.2 | C:cytoplasm; C:membrane-bounded organelle |
| Efet.01.210359.g458.t1 | kelch 5 isoform X3 | 1503 | 3.17E-11 | 51.25 | F:ubiquitin-protein transferase activity; C:Cul3-RING ubiquitin ligase complex; P:protein ubiquitination involved in ubiquitin-dependent protein catabolic process; P:protein ubiquitination |
| Efet.01.21037.g1553.t1 | hypothetical protein CAPTEDRAFT_226177 | 381 | 2.42E-09 | 46 | C:membrane; C:integral component of membrane; F:carbohydrate binding |
| Efet.01.210398.g460.t1 | filamin-A isoform X6 | 345 | 2.12E-32 | 71.2 | F:actin filament binding |
| Efet.01.210405.g461.t1 | cadherin-related tumor suppressor | 1311 | 5.11E-63 | 51.7 | F:calcium ion binding; C:membrane; C:integral component of membrane; P:cell adhesion; P:homophilic cell adhesion via plasma membrane adhesion molecules; C:plasma membrane |
| Efet.01.2105.g185.t1 | phenylalanine--tRNA ligase alpha subunit | 537 | 1.73E-91 | 79.75 | F:ATP binding; C:cytoplasm; F:phenylalanine-tRNA ligase activity; P:phenylalanyl-tRNA aminoacylation; F:tRNA binding |
| Efet.01.210557.g465.t1 | neurexin-3 isoform X2 | 522 | 1.34E-23 | 62.65 | P:olfactory learning |
| Efet.01.210575.g467.t1 | peregrin-like isoform X12 | 390 | 5.42E-20 | 73.3 | F:metal ion binding; F:transferase activity |
| Efet.01.2106.g186.t1 | probable G- coupled receptor 139 | 1215 | 1.47E-76 | 51.1 | F:G-protein coupled receptor activity; P:G-protein coupled receptor signaling pathway; P:signal transduction; C:membrane; C:integral component of membrane; F:signal transducer activity |
| Efet.01.210609.g469.t1 | leucine-rich repeat and fibronectin type III domain-containing 1 | 1587 | 5.09E-139 | 53.4 | C:membrane; C:integral component of membrane |
| Efet.01.21069.g1555.t1 | KAT8 regulatory NSL complex subunit 2 | 651 | 1.02E-51 | 68.85 | P:histone H4 acetylation; F:H4 histone acetyltransferase activity; C:histone acetyltransferase complex |
| Efet.01.210718.g471.t1 | mucin-2-like isoform X2 | 504 | 2.24E-08 | 51.17 | F:oligosaccharide binding |
| Efet.01.210718.g472.t1 | c-binding -like | 558 | 3.73E-16 | 58.05 | F:erythropoietin receptor binding; F:calcium ion binding; C:membrane; C:integral component of membrane; C:extracellular region; F:hormone activity |
| Efet.01.210718.g473.t1 | fibulin-1 isoform X1 | 567 | 2.16E-17 | 51 | F:protein binding; C:extracellular region part; P:negative regulation of cellular process |
| Efet.01.210731.g474.t1 | neuroligin- X-linked-like isoform X1 | 930 | 6.84E-17 | 49.88 | C:membrane; C:integral component of membrane |
| Efet.01.21075.g1556.t1 | WD and tetratricopeptide repeats 1-like | 396 | 9.39E-35 | 83.75 |  |
| Efet.01.210782.g476.t1 | galanin receptor type 1-like | 1074 | 2.85E-85 | 63.5 | F:G-protein coupled receptor activity; P:G-protein coupled receptor signaling pathway; C:integral component of membrane |
| Efet.01.210847.g480.t1 | neurofilament medium polypeptide-like | 258 | 3.52E-24 | 75.7 | F:structural molecule activity; C:intermediate filament |
| Efet.01.21091.g1558.t1 | tubulin epsilon chain isoform X2 | 585 | 5.08E-73 | 70.5 | F:GTP binding; F:GTPase activity; C:microtubule; P:microtubule-based process |
| Efet.01.210912.g482.t1 | zinc finger | 1503 | 1.83E-46 | 86.85 | F:histone acetyltransferase binding; F:RNA polymerase II regulatory region sequence-specific DNA binding; P:transcription from RNA polymerase II promoter; P:regulation of apoptotic process; P:regulation of transcription from RNA polymerase II promoter in response to hypoxia; P:positive regulation of transcription from RNA polymerase II promoter; C:nucleoplasm; P:negative regulation of transcription from RNA polymerase II promoter; P:T cell differentiation; P:negative regulation of canonical Wnt signaling pathway; F:transcriptional activator activity, RNA polymerase II core promoter proximal region sequence-specific binding; P:response to glucose; P:cellular response to gamma radiation; P:response to insulin; P:skeletal muscle cell differentiation; C:cytoplasm; F:metal ion binding; P:BMP signaling pathway; P:interleukin-1-mediated signaling pathway; P:regulation of protein sumoylation |
| Efet.01.210938.g483.t1 | potassium voltage-gated channel subfamily KQT member 4-like isoform X6 | 246 | 2.67E-29 | 85.85 | P:potassium ion transmembrane transport; C:voltage-gated potassium channel complex; F:delayed rectifier potassium channel activity |
| Efet.01.210973.g485.t1 | FH interacting FIP2-like | 1101 | 9.57E-29 | 56.75 | P:protein homooligomerization |
| Efet.01.210985.g486.t1 | ubiquitin domain-containing 2 | 435 | 1.92E-50 | 74.1 | F:ubiquitin binding |
| Efet.01.211089.g490.t1 | DENN domain-containing 5B-like isoform X4 | 1011 | 1.25E-56 | 54.6 |  |
| Efet.01.21118.g1560.t1 | ENL | 630 | 5.42E-39 | 66.2 | C:nucleus |
| Efet.01.211188.g491.t1 | UPF0183 C16orf70 homolog | 231 | 1.63E-17 | 86.45 |  |
| Efet.01.21119.g1561.t1 | acetylcholine receptor subunit alpha-like | 1260 | 0 | 59.2 | F:ion channel activity; P:ion transmembrane transport; C:synapse; C:membrane part; C:plasma membrane |
| Efet.01.21134.g1563.t1 | prickle 3 isoform X4 | 243 | 3.80E-20 | 72.85 | F:metal ion binding |
| Efet.01.211340.g494.t1 | serine threonine- phosphatase 6 regulatory ankyrin repeat subunit A-like isoform X4 | 1101 | 6.39E-15 | 49 |  |
| Efet.01.211440.g495.t1 | choline ethanolaminephosphotransferase 1 isoform X1 | 237 | 3.84E-14 | 76.95 | C:integral component of membrane; F:phosphotransferase activity, for other substituted phosphate groups; P:phospholipid biosynthetic process |
| Efet.01.211445.g496.t1 | dihydropyrimidinase-like isoform X1 | 258 | 2.68E-16 | 76.95 | F:hydrolase activity, acting on carbon-nitrogen (but not peptide) bonds; C:cytoplasm |
| Efet.01.211483.g498.t1 | neuronal acetylcholine receptor subunit alpha-10-like | 357 | 3.98E-48 | 81.35 | P:chemical synaptic transmission, postsynaptic; P:synaptic transmission, cholinergic; F:acetylcholine receptor activity; P:neuromuscular synaptic transmission; C:postsynaptic membrane; P:cation transmembrane transport; C:cell junction; F:extracellular ligand-gated ion channel activity; F:acetylcholine binding; C:acetylcholine-gated channel complex; P:response to nicotine |
| Efet.01.211491.g499.t1 | ras-responsive element-binding 1 | 1464 | 1.27E-15 | 81.7 | F:nucleic acid binding; F:metal ion binding |
| Efet.01.211512.g501.t1 | hypothetical protein HELRODRAFT_194138 | 840 | 4.87E-99 | 60.5 | F:actin binding; C:intracellular |
| Efet.01.211534.g503.t1 | hypothetical protein CAPTEDRAFT_207367 | 663 | 2.87E-07 | 70 |  |
| Efet.01.211540.g504.t1 | 39S ribosomal mitochondrial isoform X2 | 273 | 1.03E-35 | 76.55 | F:RNA binding; F:structural constituent of ribosome; P:cytoplasmic translation; P:mitochondrial translational initiation; P:mitochondrial translational elongation; F:transferase activity; C:mitochondrial large ribosomal subunit; C:mitochondrial inner membrane |
| Efet.01.211559.g505.t1 | 2-acylglycerol O-acyltransferase 1 | 300 | 6.49E-26 | 83.6 | F:2-acylglycerol O-acyltransferase activity; C:integral component of membrane; P:diacylglycerol biosynthetic process; C:endoplasmic reticulum; F:diacylglycerol O-acyltransferase activity |
| Efet.01.211583.g506.t1 | Toll-like receptor | 1470 | 1.89E-47 | 46.35 | P:signal transduction; C:membrane; C:integral component of membrane |
| Efet.01.211598.g507.t1 | neuropeptide SIFamide receptor-like | 498 | 1.41E-13 | 73.5 | P:neuropeptide signaling pathway; C:integral component of membrane; F:neuropeptide Y receptor activity |
| Efet.01.211660.g509.t1 | probable G- coupled receptor No9 | 1062 | 3.58E-68 | 57.6 | F:G-protein coupled receptor activity; P:G-protein coupled receptor signaling pathway; P:signal transduction; C:membrane; C:integral component of membrane; F:signal transducer activity |
| Efet.01.211726.g511.t1 | solute carrier family 25 member 44 | 744 | 5.83E-89 | 67.95 | C:membrane; P:transport |
| Efet.01.211785.g513.t1 | G- coupled receptor moody-like isoform X2 | 669 | 1.34E-21 | 53.5 | F:G-protein coupled receptor activity; P:G-protein coupled receptor signaling pathway; P:signal transduction; C:membrane; C:integral component of membrane; F:signal transducer activity |
| Efet.01.211818.g514.t1 | hypothetical protein HELRODRAFT_193020 | 402 | 9.85E-06 | 48 | C:membrane; C:integral component of membrane |
| Efet.01.211936.g515.t1 | unconventional myosin-VI-like | 249 | 5.67E-22 | 78.4 | C:myosin complex; F:ATP binding; F:actin binding; F:motor activity |
| Efet.01.21196.g1565.t1 | Serine-arginine 55 | 495 | 9.69E-20 | 91.8 | F:nucleotide binding; F:nucleic acid binding; C:integral component of membrane |
| Efet.01.21198.g1566.t1 | repulsive guidance molecule A | 480 | 9.73E-09 | 55.35 | F:BMP binding; P:iron ion homeostasis; F:coreceptor activity; P:positive regulation of transcription from RNA polymerase II promoter; F:receptor binding; C:plasma membrane; C:cell surface; P:negative regulation of BMP signaling pathway; P:negative regulation of transcription from RNA polymerase II promoter; P:cellular iron ion homeostasis; P:activin receptor signaling pathway; C:extracellular space; F:BMP receptor activity; P:cellular response to BMP stimulus; C:plasma membrane protein complex; P:BMP signaling pathway; C:HFE-transferrin receptor complex; C:membrane; F:transferrin receptor binding; C:integral component of membrane; F:glycoprotein binding; P:protein autoprocessing; C:BMP receptor complex |
| Efet.01.211982.g517.t1 | UPF0394 inner membrane -like | 537 | 6.78E-17 | 55.78 | C:membrane; C:integral component of membrane |
| Efet.01.211986.g518.t1 | forkhead box Q1-like | 1308 | 3.69E-51 | 80.5 | C:nucleus; F:sequence-specific DNA binding; F:RNA polymerase II transcription factor activity, sequence-specific DNA binding; P:regulation of transcription from RNA polymerase II promoter |
| Efet.01.21200.g1567.t1 | disulfide-isomerase A3-like | 567 | 1.97E-59 | 59.75 | F:protein disulfide isomerase activity; P:cell redox homeostasis; C:endoplasmic reticulum; F:isomerase activity |
| Efet.01.212011.g519.t1 | tripartite motif-containing 2-like isoform X3 | 909 | 4.12E-80 | 53.65 | F:zinc ion binding; F:metal ion binding; C:intracellular |
| Efet.01.212046.g520.t1 | hypothetical protein HELRODRAFT_109866 | 393 | 3.29E-18 | 53 | F:zinc ion binding; F:metal ion binding; C:intracellular |
| Efet.01.212159.g521.t1 | gastrula zinc finger -like | 1458 | 5.78E-69 | 50.65 | F:nucleic acid binding; F:metal ion binding |
| Efet.01.212159.g522.t1 | kelch 2 | 375 | 5.79E-08 | 54.45 | F:nucleic acid binding; F:metal ion binding |
| Efet.01.212161.g523.t1 | Zinc finger C3H1 type-like | 1722 | 6.78E-26 | 80.1 | F:metal ion binding |
| Efet.01.212172.g524.t1 | PREDICTED: cadherin-13 | 531 | 1.23E-07 | 41 |  |
| Efet.01.212181.g525.t1 | peptidyl-prolyl cis-trans isomerase FKBP62-like | 882 | 7.45E-88 | 52.05 | F:organic cyclic compound binding; F:heterocyclic compound binding; C:intracellular part; P:single-organism process; P:cellular process; C:membrane-bounded organelle |
| Efet.01.212188.g526.t1 | peptidyl-prolyl cis-trans isomerase FKBP4-like isoform X1 | 219 | 3.86E-14 | 69.38 | P:protein folding |
| Efet.01.212207.g528.t1 | E3 ubiquitin- ligase TRIP12 isoform X1 | 663 | 9.36E-86 | 65.3 | F:ubiquitin-protein transferase activity; F:zinc ion binding; F:ligase activity; P:protein ubiquitination |
| Efet.01.212235.g529.t1 | hypothetical protein HELRODRAFT_174875 | 2172 | 3.97E-06 | 90 |  |
| Efet.01.212268.g531.t1 | TP53-regulating kinase | 450 | 1.37E-57 | 72.25 | F:protein kinase activity; P:phosphorylation |
| Efet.01.212272.g532.t1 | hypothetical protein HELRODRAFT_170233 | 369 | 6.47E-32 | 67.15 | C:membrane |
| Efet.01.212307.g533.t1 | serine arginine repetitive matrix 1 | 930 | 8.27E-14 | 62.65 |  |
| Efet.01.212346.g534.t1 | hydroxylysine kinase-like | 369 | 2.45E-33 | 66.75 | C:membrane; C:integral component of membrane; F:protein kinase activity; F:transferase activity; P:protein phosphorylation |
| Efet.01.212390.g536.t1 | glutamyl aminopeptidase-like | 480 | 1.16E-17 | 55.85 | C:membrane; F:peptidase activity, acting on L-amino acid peptides |
| Efet.01.21242.g1569.t1 | FMRFamide receptor | 741 | 8.63E-39 | 53.95 | F:signal transducer activity |
| Efet.01.212473.g539.t1 | thrombospondin-type laminin G domain and EAR repeat-containing | 393 | 1.72E-28 | 67.1 | P:sensory perception of sound; F:G-protein coupled receptor activity; P:G-protein coupled receptor signaling pathway; P:signal transduction; C:membrane; C:integral component of membrane; F:signal transducer activity |
| Efet.01.212596.g542.t1 | arf-GAP domain and FG repeat-containing 1-like isoform X2 | 336 | 9.00E-16 | 63.05 | F:protein domain specific binding; F:GTPase activator activity; P:positive regulation of GTPase activity |
| Efet.01.212603.g543.t1 | glutamate decarboxylase 1-like | 303 | 5.24E-25 | 76.4 | F:pyridoxal phosphate binding; P:carboxylic acid metabolic process; F:carboxy-lyase activity |
| Efet.01.212638.g544.t1 | protocadherin Fat 1-like isoform X1 | 453 | 1.89E-25 | 62.12 | C:membrane |
| Efet.01.212656.g545.t1 | muscarinic acetylcholine receptor DM1 isoform X1 | 453 | 1.28E-48 | 72.8 | P:synaptic transmission, cholinergic; P:phospholipase C-activating G-protein coupled acetylcholine receptor signaling pathway; P:adenylate cyclase-inhibiting G-protein coupled acetylcholine receptor signaling pathway; C:integral component of membrane; F:G-protein coupled acetylcholine receptor activity; C:synapse; F:transferase activity |
| Efet.01.21275.g1571.t1 | angiotensin-converting enzyme isoform X3 | 405 | 1.60E-41 | 71.7 | P:positive regulation of molecular function; P:regulation of protein binding; C:membrane; P:metabolic process; P:regulation of gene expression; C:cell part; P:negative regulation of biological process; P:regulation of blood pressure; P:single-organism process; F:exopeptidase activity; P:positive regulation of peptidyl-tyrosine phosphorylation |
| Efet.01.212760.g546.t1 | 3-hydroxybutyrate dehydrogenase | 483 | 4.81E-71 | 72.85 | F:3-hydroxybutyrate dehydrogenase activity; P:oxidation-reduction process |
| Efet.01.212877.g547.t1 | calexcitin-2-like isoform X2 | 681 | 4.87E-22 | 65.55 | F:calcium ion binding |
| Efet.01.212886.g548.t1 | Exportin-5 | 300 | 3.30E-06 | 67 |  |
| Efet.01.212893.g549.t1 | Ras-related Rap-2c | 537 | 1.80E-84 | 83.5 | F:GTP binding; P:small GTPase mediated signal transduction; C:membrane; C:intracellular |
| Efet.01.212909.g551.t1 | dual serine threonine and tyrosine kinase-like | 378 | 9.55E-48 | 82.3 | F:ATP binding; F:protein serine/threonine kinase activity; P:protein phosphorylation |
| Efet.01.213034.g552.t1 | 5-hydroxytryptamine receptor | 3039 | 8.71E-34 | 72.2 | F:G-protein coupled receptor activity; P:signal transduction; C:integral component of membrane; C:plasma membrane; P:behavior |
| Efet.01.213051.g553.t1 | phosphate carrier mitochondrial-like | 291 | 1.01E-39 | 89.85 | P:transmembrane transport; C:integral component of membrane |
| Efet.01.213127.g554.t1 | proteasome subunit alpha type-7 | 495 | 3.86E-59 | 86.2 | C:nucleus; C:cytoplasm; C:proteasome core complex, alpha-subunit complex; F:threonine-type endopeptidase activity; P:ubiquitin-dependent protein catabolic process |
| Efet.01.21316.g1572.t1 | WD repeat-containing 47 isoform X2 | 345 | 1.17E-23 | 70.9 |  |
| Efet.01.21316.g1573.t1 | WD repeat-containing 47-like | 444 | 1.20E-38 | 72.05 |  |
| Efet.01.213227.g556.t1 | mitochondrial ATP synthase subunit b precursor | 420 | 6.18E-60 | 70 | C:mitochondrial proton-transporting ATP synthase complex, coupling factor F(o); F:hydrogen ion transmembrane transporter activity; P:ATP synthesis coupled proton transport |
| Efet.01.213261.g558.t1 | cytochrome P450 2U1-like | 678 | 2.99E-27 | 53.95 | F:oxidoreductase activity |
| Efet.01.213291.g559.t1 | zinc finger OZF-like | 1080 | 1.03E-27 | 48.9 | F:nucleic acid binding; F:metal ion binding |
| Efet.01.213291.g560.t1 | UPF0565 C2orf69 homolog | 591 | 7.85E-56 | 58.65 | C:extracellular region; F:molecular_function |
| Efet.01.213293.g561.t1 | zinc finger 236-like | 1107 | 1.14E-09 | 67.67 | F:nucleic acid binding; F:zinc ion binding; F:metal ion binding |
| Efet.01.213312.g562.t1 | receptor-type guanylate cyclase Gyc76C-like | 627 | 1.73E-68 | 59.5 | F:phosphorus-oxygen lyase activity; C:membrane; C:cell part; P:primary metabolic process; P:single-organism process; P:organic substance metabolic process; P:phosphate-containing compound metabolic process |
| Efet.01.213325.g563.t1 | activin receptor type-1-like | 846 | 7.73E-119 | 76.65 | P:chordate embryonic development; P:transforming growth factor beta receptor signaling pathway; P:mesoderm development; P:cardiac muscle cell fate commitment; P:positive regulation of osteoblast differentiation; P:positive regulation of transcription from RNA polymerase II promoter; P:pathway-restricted SMAD protein phosphorylation; F:transforming growth factor beta binding; P:activin receptor signaling pathway; F:ATP binding; F:activin binding; P:BMP signaling pathway; P:negative regulation of activin receptor signaling pathway; P:G1/S transition of mitotic cell cycle; F:peptide hormone binding; P:positive regulation of determination of dorsal identity; P:positive regulation of bone mineralization; F:protein homodimerization activity; P:mitral valve morphogenesis; P:anatomical structure formation involved in morphogenesis; P:embryonic heart tube morphogenesis; P:cell development; P:endocardial cushion cell fate commitment; P:atrial septum primum morphogenesis; P:gastrulation; F:SMAD binding; C:activin receptor complex; F:activin receptor activity, type I; P:negative regulation of extrinsic apoptotic signaling pathway; P:positive regulation of pathway-restricted SMAD protein phosphorylation; P:peptidyl-threonine phosphorylation; P:positive regulation of cell migration |
| Efet.01.213338.g564.t1 | collagen alpha-2(IX) chain isoform X7 | 435 | 3.03E-38 | 75.1 | C:collagen trimer; F:structural molecule activity; P:cell adhesion; C:extracellular matrix |
| Efet.01.213363.g567.t1 | FMRFamide receptor-like | 555 | 3.71E-24 | 70.36 | F:G-protein coupled peptide receptor activity; P:G-protein coupled receptor signaling pathway; C:integral component of membrane |
| Efet.01.213442.g568.t1 | ribosome biogenesis NSA2 homolog | 285 | 5.12E-41 | 93.55 | P:maturation of 5.8S rRNA; P:maturation of LSU-rRNA; C:nucleolus; C:preribosome, large subunit precursor |
| Efet.01.213491.g572.t1 | glycine dehydrogenase (aminomethyl-transferring) | 600 | 2.46E-125 | 70.9 | F:oxidoreductase activity; P:glycine catabolic process |
| Efet.01.2135.g188.t1 | Wnt-5b precursor | 330 | 6.00E-49 | 84.1 | P:negative regulation of canonical Wnt signaling pathway; C:extracellular space; F:frizzled binding; C:extracellular exosome; P:neuron differentiation; P:cell fate commitment; P:chondrocyte differentiation; P:Wnt signaling pathway; P:positive regulation of fat cell differentiation; C:proteinaceous extracellular matrix; C:cell surface; P:positive regulation of cell migration |
| Efet.01.213501.g573.t1 | peptidyl-prolyl cis-trans isomerase-like | 216 | 2.43E-14 | 88.6 | F:peptidyl-prolyl cis-trans isomerase activity; P:protein peptidyl-prolyl isomerization; P:protein folding |
| Efet.01.213526.g576.t1 | Niemann-Pick C1 | 708 | 1.89E-70 | 81.55 | P:viral entry into host cell; P:chordate embryonic development; P:protein glycosylation; C:Golgi apparatus; P:cholesterol homeostasis; P:establishment of protein localization to membrane; C:integral component of plasma membrane; P:adult walking behavior; C:endosome; C:extracellular exosome; P:response to hypoxia; P:autophagy; P:megakaryocyte development; P:membrane raft organization; F:cholesterol binding; C:endoplasmic reticulum; P:endocytosis; C:lysosomal membrane; C:nuclear envelope; P:cellular response to steroid hormone stimulus; C:perinuclear region of cytoplasm; F:lipid transporter activity; P:response to drug; C:membrane raft; P:negative regulation of macroautophagy; P:epiboly involved in gastrulation with mouth forming second; P:positive regulation of cholesterol homeostasis; P:cholesterol efflux; P:cellular response to low-density lipoprotein particle stimulus |
| Efet.01.213573.g578.t1 | histone-lysine N- H3 lysine-36 and H4 lysine-20 specific-like isoform X3 | 507 | 2.16E-37 | 61.6 | C:nucleus; F:zinc ion binding; P:histone lysine methylation; F:metal ion binding; F:histone-lysine N-methyltransferase activity; F:methyltransferase activity; F:transferase activity; P:methylation |
| Efet.01.213691.g581.t1 | sesquipedalian-1-like | 621 | 1.82E-57 | 66.25 |  |
| Efet.01.213709.g582.t1 | KN motif and ankyrin repeat domain-containing 1-like | 423 | 2.66E-33 | 69.5 |  |
| Efet.01.213720.g584.t1 | hypothetical protein HELRODRAFT_181224 | 543 | 7.20E-25 | 69 | C:membrane |
| Efet.01.213727.g585.t1 | Histone-lysine N-methyltransferase SETD2 | 2355 | 1.98E-44 | 52.15 | C:nucleus; P:histone lysine methylation; F:histone-lysine N-methyltransferase activity; P:regulation of transcription, DNA-templated; F:methyltransferase activity; C:chromosome; F:transferase activity; P:methylation |
| Efet.01.21376.g1575.t1 | monocarboxylate transporter 12-like | 1056 | 3.55E-43 | 55.5 | C:membrane |
| Efet.01.213854.g589.t1 | solute carrier family facilitated glucose transporter member 8 | 1449 | 8.29E-135 | 60.7 | C:integral component of membrane; P:hexose transmembrane transport; P:glucose transport; F:sugar transmembrane transporter activity |
| Efet.01.213946.g592.t1 | IWS1 homolog A-like | 426 | 1.48E-11 | 65.63 |  |
| Efet.01.213964.g593.t1 | band 7 AGAP004871-like | 231 | 3.50E-25 | 89.25 | C:integral component of membrane |
| Efet.01.213966.g595.t1 | interferon regulatory factor 6-like isoform X1 | 678 | 1.66E-06 | 62.33 | F:transcription factor activity, sequence-specific DNA binding; P:regulation of transcription, DNA-templated; F:regulatory region DNA binding |
| Efet.01.214036.g596.t1 | transmembrane 143-like | 699 | 4.97E-59 | 59.95 | C:membrane; C:integral component of membrane |
| Efet.01.214129.g597.t1 | hypothetical protein HELRODRAFT_160285 | 429 | 1.31E-07 | 53.25 | P:sodium ion transport; P:ion transport; F:sodium channel activity; C:membrane; C:integral component of membrane; P:sodium ion transmembrane transport; P:transport |
| Efet.01.214173.g598.t1 | intermediate filament b | 402 | 3.91E-10 | 79.64 | F:structural molecule activity; C:intermediate filament |
| Efet.01.214184.g599.t1 | muscarinic acetylcholine receptor M1-like | 2382 | 1.60E-99 | 70.5 | C:integral component of plasma membrane; F:G-protein coupled acetylcholine receptor activity; P:G-protein coupled acetylcholine receptor signaling pathway |
| Efet.01.214204.g600.t1 | serine arginine repetitive matrix 1-like | 435 | 1.92E-50 | 65 | F:insulin receptor binding |
| Efet.01.21422.g1576.t1 | Nuclear GTPase SLIP-GC | 579 | 5.59E-09 | 58 |  |
| Efet.01.214221.g601.t1 | kelch 13 | 1803 | 0 | 61.35 |  |
| Efet.01.214273.g603.t1 | tripartite motif-containing 45-like isoform X6 | 2082 | 2.47E-51 | 68.7 | F:zinc ion binding; F:metal ion binding; C:intracellular |
| Efet.01.214276.g604.t1 | autophagy-related 2 homolog B | 549 | 2.71E-46 | 58.4 | P:autophagy |
| Efet.01.214325.g605.t1 | FMRFamide receptor-like | 657 | 2.37E-34 | 59 | P:signal transduction; C:membrane; F:signal transducer activity |
| Efet.01.214388.g609.t1 | transient receptor potential cation channel subfamily M member 2-like isoform X3 | 630 | 1.93E-32 | 51.05 | P:transport |
| Efet.01.214397.g610.t1 | C-Jun-amino-terminal kinase-interacting 4 isoform X5 | 627 | 9.07E-58 | 68.05 |  |
| Efet.01.214444.g611.t1 | matrix metallo ase-16 | 579 | 5.65E-35 | 57.9 | F:hydrolase activity |
| Efet.01.214447.g612.t1 | tubulin alpha-1C chain | 1206 | 0 | 79.05 | F:GTP binding; C:cytoplasm; F:GTPase activity; F:structural constituent of cytoskeleton; C:microtubule; P:microtubule-based process |
| Efet.01.214501.g614.t1 | mitogen-activated kinase 7 | 540 | 1.01E-69 | 77.8 | P:negative regulation of response to cytokine stimulus; P:negative regulation of MAP kinase activity; C:PML body; P:negative regulation of oxidative stress-induced intrinsic apoptotic signaling pathway; P:regulation of angiogenesis; F:ATP binding; P:MAPK cascade; P:negative regulation of cAMP catabolic process; P:positive regulation of protein metabolic process; P:negative regulation of cyclic-nucleotide phosphodiesterase activity; C:cytosol; P:cellular response to laminar fluid shear stress; P:cellular response to hydrogen peroxide; P:negative regulation of endothelial cell apoptotic process; P:negative regulation of extrinsic apoptotic signaling pathway in absence of ligand; P:positive regulation of transcription from RNA polymerase II promoter in response to stress; P:negative regulation of inflammatory response; P:cell cycle; P:cellular response to transforming growth factor beta stimulus; P:cell differentiation; F:MAP kinase activity; P:negative regulation of heterotypic cell-cell adhesion; P:negative regulation of ERK5 cascade; F:mitogen-activated protein kinase binding; P:peptidyl-serine phosphorylation; P:cAMP-mediated signaling; P:negative regulation of NFAT protein import into nucleus |
| Efet.01.214524.g616.t1 | tripartite motif-containing 2-like isoform X5 | 1641 | 5.12E-12 | 43.4 | F:zinc ion binding; F:metal ion binding; P:regulation of apoptotic process; C:intracellular |
| Efet.01.21459.g1577.t1 | predicted protein | 420 | 1.76E-07 | 55 |  |
| Efet.01.214627.g618.t1 | RNA demethylase ALKBH5 | 762 | 2.51E-90 | 75.3 | P:mRNA export from nucleus; P:response to hypoxia; P:oxidative single-stranded RNA demethylation; C:nuclear speck; F:oxidoreductase activity, acting on paired donors, with incorporation or reduction of molecular oxygen, 2-oxoglutarate as one donor, and incorporation of one atom each of oxygen into both donors; P:spermatogenesis; F:oxidative RNA demethylase activity; P:mRNA processing; F:binding |
| Efet.01.214770.g620.t1 | ankyrin-3 isoform X1 | 480 | 3.69E-54 | 64 | P:neuromuscular junction development; P:sensory perception of sound; P:short-term memory; P:neuron cellular homeostasis; P:negative regulation of neuromuscular synaptic transmission; C:terminal bouton; P:axon extension; P:sensory perception of pain; C:neuromuscular junction; C:presynaptic periactive zone; P:microtubule cytoskeleton organization; P:positive regulation of synaptic growth at neuromuscular junction |
| Efet.01.21481.g1579.t1 | potassium voltage-gated channel eag-like | 267 | 6.47E-23 | 92.65 | F:phosphorelay sensor kinase activity; F:voltage-gated potassium channel activity; P:potassium ion transmembrane transport; P:phosphorelay signal transduction system; C:integral component of membrane; P:signal transduction by protein phosphorylation; C:intracellular |
| Efet.01.214833.g622.t1 | serine threonine- phosphatase 6 regulatory ankyrin repeat subunit B-like | 2982 | 0 | 56.9 |  |
| Efet.01.214849.g624.t1 | WD repeat-containing 70 | 1197 | 1.96E-173 | 75.5 | F:enzyme binding |
| Efet.01.214912.g625.t1 | zinc C2H2 type | 1464 | 1.03E-51 | 77.05 | C:nuclear speck; C:histone deacetylase complex; C:Golgi apparatus; P:negative regulation of programmed cell death; P:primary metabolic process; F:transcription factor activity, sequence-specific DNA binding; P:cellular macromolecule metabolic process; P:negative regulation of transcription, DNA-templated; P:positive regulation of transcription, DNA-templated; C:cytosol; P:regulation of cell cycle; C:aggresome; P:pronephric proximal tubule development; P:pronephric distal tubule development; F:protein homodimerization activity; P:multi-ciliated epithelial cell differentiation; P:embryonic neurocranium morphogenesis; P:negative regulation of JNK cascade; P:embryonic viscerocranium morphogenesis; F:DNA binding; P:proximal/distal pattern formation involved in pronephric nephron development; F:metal ion binding; F:histone-lysine N-methyltransferase activity; P:hematopoietic stem cell proliferation; P:negative regulation of transforming growth factor beta receptor signaling pathway |
| Efet.01.214982.g626.t1 | hypothetical protein CAPTEDRAFT_139224 | 492 | 1.54E-38 | 74.4 | P:transmembrane transport; C:integral component of membrane; F:transporter activity |
| Efet.01.21500.g1581.t1 | nucleoside diphosphate-linked moiety X motif mitochondrial | 804 | 4.24E-48 | 51.5 | F:hydrolase activity |
| Efet.01.215056.g627.t1 | echinoderm microtubule-associated -like 6 | 939 | 3.95E-121 | 75.9 | C:extracellular exosome; C:cytoplasm; F:catalytic activity; C:microtubule |
| Efet.01.215106.g628.t1 | RING finger 17-like | 729 | 3.45E-19 | 51 |  |
| Efet.01.215127.g629.t1 | bone morphogenetic 4 | 1440 | 1.36E-79 | 54.6 | P:inner ear development; P:anterior/posterior pattern specification; P:branching morphogenesis of an epithelial tube; P:regulation of morphogenesis of a branching structure; P:regulation of transmembrane receptor protein serine/threonine kinase signaling pathway; P:embryonic organ morphogenesis; P:heart morphogenesis; P:negative regulation of transcription, DNA-templated; P:BMP signaling pathway; P:mesenchymal cell differentiation; P:regulation of catalytic activity; P:mesoderm formation; P:regulation of biological quality; P:adenohypophysis development; P:regulation of apoptotic process; P:negative regulation of kidney development; P:positive regulation of ossification; P:positive regulation of molecular function; P:positive regulation of Wnt signaling pathway; P:positive regulation of neurogenesis; P:mesenchymal cell proliferation; P:cellular response to organic cyclic compound; P:positive regulation of cell migration; P:protein metabolic process; P:chordate embryonic development; P:regulation of cartilage development; P:telencephalon development; P:negative regulation of cell proliferation; P:hemopoiesis; P:positive regulation of cell proliferation; P:odontogenesis; C:extracellular region; P:positive regulation of transcription from RNA polymerase II promoter; P:glandular epithelial cell differentiation; P:cardiac muscle tissue development; P:appendage development; P:bone development; P:lung morphogenesis; P:regulation of muscle cell differentiation; P:nephron epithelium morphogenesis; P:cellular macromolecule metabolic process; P:blood vessel development; P:regulation of odontogenesis; P:negative regulation of cell differentiation; P:regulation of morphogenesis of an epithelium; P:bone mineralization; P:cardiocyte differentiation; P:positive regulation of MAPK cascade; P:cell development; F:receptor binding; P:skeletal system morphogenesis; P:response to stress; P:determination of left/right symmetry; P:ureteric bud development; P:muscle cell differentiation; P:cell fate commitment |
| Efet.01.215129.g630.t1 | platelet-derived growth factor subunit A isoform X1 | 645 | 2.84E-10 | 52.15 | F:growth factor activity; C:extracellular space; P:positive regulation of protein autophosphorylation; P:positive regulation of cell proliferation; C:membrane; C:integral component of membrane; F:growth factor receptor binding |
| Efet.01.215157.g631.t1 | family transcriptional regulator | 495 | 1.64E-103 | 83.25 | F:DNA binding; F:transcription factor activity, sequence-specific DNA binding; P:transcription, DNA-templated; P:regulation of transcription, DNA-templated |
| Efet.01.215157.g632.t1 | sensor histidine kinase | 1155 | 7.15E-146 | 88.2 | F:phosphorelay sensor kinase activity; P:phosphorelay signal transduction system; C:integral component of membrane; P:signal transduction by protein phosphorylation; C:intracellular |
| Efet.01.215157.g633.t1 | argininosuccinate lyase | 1467 | 0 | 94.05 | C:cytoplasm; P:arginine biosynthetic process via ornithine; F:argininosuccinate lyase activity |
| Efet.01.215159.g634.t1 | phosphatase 1 regulatory subunit 21 | 405 | 2.91E-16 | 72.4 | F:phosphatase binding; C:membrane |
| Efet.01.215166.g635.t1 | Katanin p80 WD40-containing subunit B1 | 258 | 1.27E-14 | 64.45 | C:microtubule organizing center; P:single-organism cellular process |
| Efet.01.215175.g636.t1 | mediator of RNA polymerase II transcription subunit 22-like | 459 | 1.12E-40 | 77.85 | C:mediator complex; F:RNA polymerase II transcription cofactor activity; P:regulation of transcription from RNA polymerase II promoter |
| Efet.01.2152.g189.t1 | hypothetical protein CAPTEDRAFT_225510 | 408 | 5.24E-08 | 50.2 | C:membrane; C:integral component of membrane; F:carbohydrate binding |
| Efet.01.215215.g637.t1 | multidrug resistance-associated 1-like isoform X1 | 300 | 3.36E-14 | 71.25 | F:nucleotide binding; F:ATPase activity, coupled to transmembrane movement of substances; P:transmembrane transport; C:membrane; P:single-organism transport |
| Efet.01.215251.g638.t1 | gap-Pol poly | 582 | 4.14E-40 | 52.65 | C:membrane; C:integral component of membrane |
| Efet.01.215264.g639.t1 | phosphatidylinositol 4-kinase beta-like | 453 | 1.34E-39 | 90.2 | P:phosphatidylinositol-mediated signaling; P:phosphatidylinositol phosphorylation; F:kinase activity; C:intracellular |
| Efet.01.2153.g190.t1 | hypothetical protein CAPTEDRAFT_91481 | 870 | 3.31E-08 | 94.5 | F:GTP binding; P:small GTPase mediated signal transduction; C:membrane; C:intracellular |
| Efet.01.215417.g640.t1 | ankyrin repeat | 909 | 4.23E-42 | 49.9 | C:membrane; C:integral component of membrane; F:methyltransferase activity; F:transferase activity; P:intracellular signal transduction; C:intracellular; P:methylation |
| Efet.01.215419.g641.t1 | FMRFamide receptor | 540 | 4.08E-15 | 51.14 | F:G-protein coupled receptor activity; P:G-protein coupled receptor signaling pathway; P:signal transduction; C:membrane; C:integral component of membrane; F:signal transducer activity |
| Efet.01.215456.g643.t1 | histone-lysine N-methyltransferase 2C-like | 258 | 2.29E-38 | 82.05 | F:histone methyltransferase activity (H3-K4 specific); P:multicellular organism growth; F:zinc ion binding; F:transferase activity, transferring acyl groups; P:transcription, DNA-templated; P:regulation of transcription, DNA-templated; C:MLL3/4 complex; P:single fertilization; P:eyelid development in camera-type eye; F:RNA binding; F:DNA binding; F:protein binding; P:positive regulation of fibroblast proliferation; P:histone H3-K4 methylation |
| Efet.01.215463.g644.t1 | FMRFamide receptor-like | 819 | 1.78E-63 | 60.4 | F:G-protein coupled receptor activity; C:membrane |
| Efet.01.215469.g645.t1 | hypothetical protein CAPTEDRAFT_144547 | 1803 | 1.15E-161 | 58.45 | C:membrane |
| Efet.01.2155.g191.t1 | laminin subunit beta-2 isoform X1 | 243 | 4.94E-16 | 74.9 | P:multicellular organism development |
| Efet.01.215501.g647.t1 | testis-specific serine threonine- kinase 3-like | 1182 | 6.63E-120 | 69.85 | F:ATP binding; C:integral component of membrane; F:protein serine/threonine kinase activity; P:protein phosphorylation |
| Efet.01.215576.g648.t1 | Serine threonine- kinase mig-15 | 690 | 1.04E-104 | 73.25 | F:signal transducer, downstream of receptor, with serine/threonine kinase activity; C:cytoplasm; P:protein phosphorylation |
| Efet.01.21559.g1584.t1 | zinc finger MYM-type 1-like | 603 | 1.29E-63 | 66.75 | F:nucleic acid binding |
| Efet.01.215595.g649.t1 | Round spermatid basic 1 | 2829 | 4.02E-63 | 73.1 | C:nucleus |
| Efet.01.215619.g650.t1 | peroxisomal enoyl- hydratase L-3-hydroxyacyl- | 405 | 2.50E-30 | 59.95 | F:catalytic activity; P:metabolic process |
| Efet.01.215662.g652.t1 | 1-acyl-sn-glycerol-3-phosphate acyltransferase epsilon | 303 | 1.03E-09 | 61 | F:transferase activity, transferring acyl groups; P:metabolic process; C:membrane; C:integral component of membrane; F:transferase activity |
| Efet.01.215751.g654.t1 | heat shock 70 kDa 13 | 261 | 4.77E-17 | 76.6 | F:ATP binding |
| Efet.01.215751.g655.t1 | Heat shock 70 kDa 13 | 567 | 7.12E-71 | 72 | F:nucleotide binding |
| Efet.01.215773.g656.t1 | cytoplasmic dynein 2 heavy chain 1 | 258 | 1.05E-30 | 75 | F:nucleotide binding; F:nucleoside-triphosphatase activity |
| Efet.01.215811.g657.t1 | neurogenic locus Notch | 639 | 3.82E-30 | 49.8 | P:regulation of developmental process; P:Notch signaling pathway; P:multicellular organism development; F:calcium ion binding; C:membrane; C:integral component of membrane; P:cell differentiation; P:regulation of transcription, DNA-templated; F:receptor activity |
| Efet.01.215816.g658.t1 | tyrosine- kinase SRK2-like isoform X2 | 393 | 4.69E-12 | 61.95 | P:peptidyl-tyrosine phosphorylation; F:protein tyrosine kinase activity; P:regulation of cellular process; F:binding; C:intracellular part; P:single-organism process; P:response to stimulus |
| Efet.01.21591.g1586.t1 | multidrug resistance 1-like | 345 | 1.20E-49 | 65.15 | F:ATP binding; F:ATPase activity, coupled to transmembrane movement of substances; P:transmembrane transport; C:integral component of membrane |
| Efet.01.215916.g659.t1 | cAMP-specific 3 ,5 -cyclic phosphodiesterase | 561 | 1.00E-59 | 88.25 | F:calmodulin-dependent cyclic-nucleotide phosphodiesterase activity; F:cGMP-inhibited cyclic-nucleotide phosphodiesterase activity; F:cGMP-stimulated cyclic-nucleotide phosphodiesterase activity; F:metal ion binding; P:signal transduction; F:photoreceptor cyclic-nucleotide phosphodiesterase activity; F:mannosyl-diinositol phosphorylceramide phospholipase activity; F:7,8-dihydro-D-neopterin 2',3'-cyclic phosphate phosphodiesterase activity; F:mannosyl-inositol phosphorylceramide phospholipase activity; F:3',5'-cyclic-AMP phosphodiesterase activity |
| Efet.01.215924.g660.t1 | hypothetical protein CAPTEDRAFT_225909 | 1167 | 1.95E-105 | 50.9 | C:membrane |
| Efet.01.216008.g661.t1 | neurexin-1 isoform X1 | 1707 | 1.88E-15 | 58.2 | F:metal ion binding; F:calcium ion binding; C:membrane; C:integral component of membrane; P:chemical synaptic transmission |
| Efet.01.216032.g662.t1 | neural-cadherin-like isoform X1 | 402 | 1.47E-25 | 50.1 | P:post-anal tail morphogenesis; P:neural tube development; P:cochlea development; P:heterophilic cell-cell adhesion via plasma membrane cell adhesion molecules; P:cell adhesion; P:neurogenesis; P:homophilic cell adhesion via plasma membrane adhesion molecules; C:plasma membrane; C:intracellular; P:ossification involved in bone maturation; P:heart morphogenesis; C:apical part of cell; P:kidney development; P:digestive tract development; P:branching involved in ureteric bud morphogenesis; F:calcium ion binding; C:membrane; C:integral component of membrane; P:hippo signaling; F:carbohydrate binding |
| Efet.01.216044.g663.t1 | hypothetical protein CAPTEDRAFT_167175 | 231 | 9.00E-24 | 86 |  |
| Efet.01.216065.g665.t1 | orexin receptor type 2-like | 1455 | 6.91E-54 | 65.75 | P:signal transduction; C:membrane; F:signal transducer activity |
| Efet.01.216081.g666.t1 | T-box transcription factor | 216 | 6.56E-12 | 57.35 | P:epithelial tube morphogenesis; P:aortic valve morphogenesis; F:RNA polymerase II regulatory region sequence-specific DNA binding; P:regulation of transcription, DNA-templated; P:anatomical structure formation involved in morphogenesis; P:embryonic heart tube development; P:negative regulation of cellular process; F:RNA polymerase II transcription coactivator activity; P:pulmonary vein morphogenesis; P:cellular process; P:atrial septum morphogenesis; P:outflow tract septum morphogenesis; P:mesenchyme morphogenesis; P:positive regulation of cellular process; P:embryonic morphogenesis; P:foramen ovale closure |
| Efet.01.216117.g667.t1 | hypothetical protein HELRODRAFT_183289 | 1896 | 2.25E-18 | 43 | C:membrane; C:integral component of membrane |
| Efet.01.216127.g668.t1 | water dikinase | 267 | 4.06E-23 | 70.9 | F:ATP binding |
| Efet.01.21617.g1587.t1 | chromobox homolog 2 | 246 | 6.78E-08 | 77.92 | C:nucleus |
| Efet.01.216175.g670.t1 | caspase-7 | 222 | 2.09E-11 | 83.3 | C:cytoplasm; P:proteolysis; P:apoptotic process; F:cysteine-type endopeptidase activity |
| Efet.01.216183.g671.t1 | transcription factor AP-1 | 474 | 9.84E-28 | 69.95 | P:response to cytokine; F:RNA polymerase II core promoter proximal region sequence-specific DNA binding; P:positive regulation of cell proliferation; P:response to radiation; P:response to cAMP; P:anatomical structure morphogenesis; P:animal organ development; P:transmembrane receptor protein serine/threonine kinase signaling pathway; P:positive regulation of transcription from RNA polymerase II promoter; P:modulation by host of viral transcription; P:response to mechanical stimulus; P:negative regulation of transcription from RNA polymerase II promoter; C:transcription factor complex; P:cellular response to stress; P:regulation of cell cycle; P:Ras protein signal transduction; P:positive regulation of cell differentiation; F:transcription factor binding; P:immune system process; P:regulation of molecular function; F:RNA polymerase II transcription factor activity, sequence-specific DNA binding; C:nuclear chromatin; F:transcription coactivator activity; P:regulation of protein phosphorylation; P:response to drug; P:negative regulation of apoptotic process; P:cell differentiation; P:circulatory system development; P:response to lipopolysaccharide; P:cellular response to hormone stimulus |
| Efet.01.2162.g192.t1 | transcription termination factor 2-like isoform X1 | 846 | 5.34E-75 | 68.95 | F:ATP binding |
| Efet.01.21624.g1588.t1 | protocadherin-7 isoform X4 | 639 | 1.08E-28 | 53.9 | P:animal organ development |
| Efet.01.21624.g1589.t1 | protocadherin-9 isoform X1 | 1461 | 6.53E-92 | 54.65 | F:calcium ion binding; C:membrane; C:integral component of membrane; P:cell adhesion; P:homophilic cell adhesion via plasma membrane adhesion molecules; C:plasma membrane |
| Efet.01.21625.g1590.t1 | cytochrome P450 2U1 | 249 | 1.17E-13 | 71.15 | F:oxidoreductase activity, acting on paired donors, with incorporation or reduction of molecular oxygen, reduced flavin or flavoprotein as one donor, and incorporation of one atom of oxygen; F:metal ion binding; C:endoplasmic reticulum; C:membrane part |
| Efet.01.216349.g678.t1 | pumilio homolog 2- | 219 | 2.25E-33 | 87.6 | F:RNA binding |
| Efet.01.216355.g679.t1 | F-box only 30-like | 1017 | 1.25E-35 | 72.25 | F:zinc ion binding; F:ubiquitin protein ligase activity; P:protein ubiquitination |
| Efet.01.216361.g681.t1 | allene oxide synthase-lipoxygenase -like | 603 | 4.87E-21 | 46.56 | F:catalytic activity |
| Efet.01.216373.g682.t1 | NADPH--cytochrome P450 reductase | 315 | 9.76E-32 | 66.2 | F:catalytic activity |
| Efet.01.216380.g683.t1 | FMRFamide receptor-like | 1236 | 1.14E-96 | 56.1 | F:G-protein coupled receptor activity; P:signal transduction; C:membrane |
| Efet.01.216626.g688.t1 | Ankyrin repeat | 846 | 1.51E-19 | 56.8 |  |
| Efet.01.216679.g690.t1 | neuroligin- X-linked-like isoform X1 | 546 | 8.68E-36 | 60.15 | C:membrane; C:integral component of membrane |
| Efet.01.216712.g691.t1 | eukaryotic elongation factor 2 kinase-like isoform X1 | 519 | 6.29E-16 | 61.1 | F:organic cyclic compound binding; F:heterocyclic compound binding; F:ion binding; P:cellular protein metabolic process; P:phosphorylation; F:protein serine/threonine kinase activity |
| Efet.01.216725.g692.t1 | hypothetical protein HELRODRAFT_75620 | 423 | 7.62E-07 | 47 |  |
| Efet.01.216812.g693.t1 | Epidermal growth factor | 258 | 1.17E-09 | 55 | P:peptidyl-tyrosine phosphorylation; F:ATP binding; F:transmembrane receptor protein tyrosine kinase activity; C:membrane; P:proteolysis; C:integral component of membrane; F:serine-type endopeptidase activity; F:serine-type peptidase activity; P:transmembrane receptor protein tyrosine kinase signaling pathway; F:peptidase activity; P:protein phosphorylation; F:hydrolase activity |
| Efet.01.216833.g694.t1 | probable G- coupled receptor 139 isoform X1 | 783 | 9.12E-52 | 48.25 | F:G-protein coupled receptor activity; P:G-protein coupled receptor signaling pathway; C:membrane; C:integral component of membrane |
| Efet.01.216860.g695.t1 | sacsin-like | 1266 | 7.76E-109 | 55.5 |  |
| Efet.01.21687.g1592.t1 | probable ATP-dependent RNA helicase | 243 | 1.78E-07 | 77.35 | P:sister chromatid cohesion; F:DNA-dependent ATPase activity; F:double-stranded DNA binding; F:hydrolase activity, acting on acid anhydrides, in phosphorus-containing anhydrides; C:spindle pole; F:ATP-dependent helicase activity; F:helicase activity; C:nucleolus; F:ATP-dependent DNA helicase activity; P:nucleobase-containing compound metabolic process; C:nucleoplasm; F:single-stranded DNA binding; F:nucleotide binding; F:nucleic acid binding; F:DNA binding; F:ATP binding; C:nucleus; C:extracellular exosome; C:nuclear chromatin; P:DNA duplex unwinding; C:midbody; F:hydrolase activity |
| Efet.01.216873.g696.t1 | potassium voltage-gated channel Shab | 567 | 1.79E-74 | 78.2 | F:voltage-gated potassium channel activity; P:potassium ion transmembrane transport; C:voltage-gated potassium channel complex; P:regulation of ion transmembrane transport; P:protein homooligomerization |
| Efet.01.216937.g699.t1 | suppressor of lurcher 1-like | 261 | 9.76E-22 | 65 | C:membrane |
| Efet.01.216967.g700.t1 | Cephalotocin receptor 1 | 609 | 3.80E-52 | 63.3 | P:G-protein coupled receptor signaling pathway; C:integral component of membrane; F:protein-hormone receptor activity |
| Efet.01.216971.g701.t1 | ubiquitin carboxyl-terminal hydrolase isozyme L3 | 225 | 9.71E-23 | 74.4 | F:thiol-dependent ubiquitin-specific protease activity; P:protein deubiquitination; P:Notch signaling pathway; C:cytoplasm; P:ubiquitin-dependent protein catabolic process |
| Efet.01.2170.g194.t1 | delta 2 | 396 | 2.67E-22 | 64.8 | C:integral component of membrane |
| Efet.01.217009.g702.t1 | cilia- and flagella-associated 47-like isoform X2 | 372 | 1.84E-45 | 73.75 |  |
| Efet.01.21701.g1593.t1 | tctex1 domain-containing 2-like | 549 | 2.09E-38 | 59.7 | P:biological_process; C:cellular_component; F:molecular_function |
| Efet.01.217012.g703.t1 | gastrula zinc finger -like | 846 | 4.35E-47 | 43.15 | F:binding |
| Efet.01.21702.g1594.t1 | monocarboxylate transporter 14-like | 1434 | 1.59E-80 | 46.25 | P:transmembrane transport; C:membrane; C:integral component of membrane |
| Efet.01.217080.g704.t1 | thioredoxin domain-containing 3 homolog isoform X2 | 408 | 3.30E-39 | 86.45 | C:cell; P:UTP biosynthetic process; P:CTP biosynthetic process; P:GTP biosynthetic process; P:nucleoside diphosphate phosphorylation; P:cell redox homeostasis; F:nucleoside diphosphate kinase activity |
| Efet.01.217082.g705.t1 | long-chain-fatty-acid-- ligase ACSBG2-like | 399 | 2.56E-52 | 75.2 | F:catalytic activity; P:metabolic process |
| Efet.01.217103.g706.t1 | ELAV 2 | 546 | 8.12E-20 | 67.1 | F:organic cyclic compound binding; F:heterocyclic compound binding |
| Efet.01.217124.g707.t1 | sodium channel type 2 subunit alpha-like | 327 | 5.96E-28 | 67.55 | P:regulation of postsynaptic membrane potential; P:regulation of ion transmembrane transport; C:voltage-gated sodium channel complex; P:sodium ion transmembrane transport; P:membrane depolarization during action potential; F:voltage-gated sodium channel activity; P:neuronal action potential |
| Efet.01.21733.g1595.t1 | neuralized PATS1 | 453 | 1.46E-18 | 55.45 | F:GTP binding; P:small GTPase mediated signal transduction; P:signal transduction; C:intracellular |
| Efet.01.217335.g716.t1 | fibrinogen 1 | 417 | 6.57E-17 | 55.5 |  |
| Efet.01.217376.g717.t1 | uncharacterized protein LOC111136903 isoform X3 | 219 | 9.03E-12 | 68.77 |  |
| Efet.01.217414.g718.t1 | FMRFamide receptor-like | 819 | 3.09E-59 | 55.15 | F:G-protein coupled receptor activity; F:G-protein coupled peptide receptor activity; P:G-protein coupled receptor signaling pathway; P:signal transduction; C:membrane; C:integral component of membrane; F:signal transducer activity |
| Efet.01.217422.g719.t1 | multidrug resistance-associated 1-like | 423 | 2.54E-32 | 58.4 | F:nucleotide binding; F:ATPase activity; F:ATP binding; F:ATPase activity, coupled to transmembrane movement of substances; P:transmembrane transport; C:membrane; C:integral component of membrane; P:transport; F:transporter activity |
| Efet.01.217531.g720.t1 | transport Sec24A-like isoform X2 | 498 | 9.23E-60 | 81.5 | F:zinc ion binding; P:ER to Golgi vesicle-mediated transport; P:intracellular protein transport; C:COPII vesicle coat |
| Efet.01.217538.g721.t1 | helicase ARIP4 | 276 | 2.21E-37 | 74.9 | F:ATP binding |
| Efet.01.217567.g722.t1 | PREDICTED: uncharacterized protein LOC105321710 isoform X1 | 1203 | 3.36E-99 | 62.05 |  |
| Efet.01.217618.g723.t1 | presequence mitochondrial | 834 | 1.40E-75 | 70.7 | F:catalytic activity |
| Efet.01.217633.g724.t1 | hypothetical protein CAPTEDRAFT_186249 | 411 | 5.81E-36 | 70.5 | C:integral component of membrane; F:carbohydrate binding |
| Efet.01.217634.g725.t1 | couch potato isoform X1 | 228 | 2.91E-12 | 69.55 | F:organic cyclic compound binding; F:heterocyclic compound binding |
| Efet.01.2179.g195.t1 | T-cell activation mitochondrial | 366 | 2.02E-33 | 62.75 |  |
| Efet.01.217932.g729.t1 | POU class transcription factor 1-like | 354 | 2.29E-22 | 66.43 | C:nucleus; F:DNA binding; F:transcription factor activity, sequence-specific DNA binding; P:multicellular organism development; P:regulation of transcription, DNA-templated; P:transcription from RNA polymerase II promoter |
| Efet.01.217935.g730.t1 | dolichyl-diphosphooligosaccharide-- glycosyltransferase subunit STT3B | 300 | 1.38E-20 | 81.75 | C:oligosaccharyltransferase complex; F:dolichyl-diphosphooligosaccharide-protein glycotransferase activity; F:ATP binding; F:heat shock protein binding; P:response to heat; F:metal ion binding; C:integral component of membrane; F:unfolded protein binding; P:protein N-linked glycosylation via asparagine; P:protein folding; P:post-translational protein modification |
| Efet.01.217968.g733.t1 | structural maintenance of chromosomes 4-like | 300 | 3.34E-23 | 69.05 | C:nucleus; F:ATP binding; P:chromosome organization; P:cell cycle process; C:chromosome |
| Efet.01.218019.g734.t1 | cadherin-related tumor suppressor | 1215 | 1.65E-56 | 44.9 | F:calcium ion binding; C:membrane; C:integral component of membrane; P:cell adhesion; P:homophilic cell adhesion via plasma membrane adhesion molecules; C:plasma membrane |
| Efet.01.218078.g738.t1 | 1-phosphatidylinositol 4,5-bisphosphate phosphodiesterase beta- | 426 | 1.31E-71 | 94.8 | C:nucleus; C:postsynaptic density; C:dendrite; F:phosphatidylinositol phospholipase C activity; C:smooth endoplasmic reticulum; F:calcium ion binding; P:lipid catabolic process; F:signal transducer activity; P:intracellular signal transduction |
| Efet.01.21816.g1598.t1 | and pleckstrin domain-containing | 591 | 1.53E-58 | 56.7 | P:regulation of Rho protein signal transduction; C:cytoskeleton; C:cytoplasm; F:Rho guanyl-nucleotide exchange factor activity; C:extrinsic component of membrane; F:cytoskeletal protein binding; P:positive regulation of GTPase activity |
| Efet.01.218220.g742.t1 | LIM homeobox Lhx1 | 459 | 2.25E-27 | 79.05 | C:nucleus; F:zinc ion binding; P:regulation of transcription, DNA-templated; F:sequence-specific DNA binding |
| Efet.01.218309.g745.t1 | beta-1-syntrophin-like isoform X1 | 489 | 2.62E-56 | 73.05 | P:synapse organization; C:dystrophin-associated glycoprotein complex; F:structural molecule activity |
| Efet.01.218335.g746.t1 | Bardet-Biedl syndrome 1 -like | 300 | 3.37E-24 | 66.15 | C:microtubule organizing center; C:ciliary part; F:receptor binding |
| Efet.01.218386.g748.t1 | Proteasome activator complex subunit 3 | 282 | 7.02E-47 | 90.25 | F:endopeptidase activator activity; P:regulation of proteasomal protein catabolic process; C:cytoplasm; P:regulation of G1/S transition of mitotic cell cycle; C:proteasome activator complex; P:positive regulation of endopeptidase activity |
| Efet.01.218392.g749.t1 | collagen alpha-1(XIV) chain-like | 474 | 2.17E-12 | 53.9 | F:calcium ion binding; C:membrane; C:integral component of membrane; C:proteinaceous extracellular matrix |
| Efet.01.218578.g751.t1 | probable ATP-dependent RNA helicase DHX58 | 1263 | 2.66E-34 | 57.05 | F:DNA binding; F:ATP binding; F:hydrolase activity, acting on acid anhydrides; P:regulation of apoptotic process; F:hydrolase activity |
| Efet.01.2186.g196.t1 | leucine-rich repeat lrrA-like | 2532 | 0 | 60.2 |  |
| Efet.01.218656.g753.t1 | hypothetical protein CAPTEDRAFT_206253 | 513 | 4.12E-09 | 46.88 | C:membrane; C:integral component of membrane; F:carbohydrate binding |
| Efet.01.218714.g756.t1 | glycerol-3-phosphate acyltransferase mitochondrial-like isoform X1 | 330 | 4.04E-06 | 60 |  |
| Efet.01.218715.g757.t1 | turtle-like isoform X1 | 573 | 1.10E-42 | 61.3 | C:membrane; C:integral component of membrane |
| Efet.01.218753.g759.t1 | kinase C and casein kinase substrate in neurons 1 isoform X3 | 438 | 5.88E-22 | 62.65 | C:endosome; P:regulation of endocytosis; P:phosphorylation; F:kinase activity |
| Efet.01.21880.g1600.t1 | Ankyrin repeat | 1068 | 5.38E-24 | 57.1 |  |
| Efet.01.21882.g1602.t1 | class E basic helix-loop-helix | 426 | 1.48E-29 | 93.7 | F:protein dimerization activity; P:neurogenesis; P:cell development; F:RNA polymerase II transcription factor activity, sequence-specific DNA binding; P:regulation of transcription from RNA polymerase II promoter |
| Efet.01.218844.g763.t1 | basement membrane-specific heparan sulfate proteoglycan core isoform X15 | 468 | 1.31E-21 | 57.4 |  |
| Efet.01.218940.g764.t1 | ankyrin-1-like isoform X2 | 1131 | 4.88E-36 | 56.5 | C:membrane; C:integral component of membrane; F:ADP binding |
| Efet.01.218983.g765.t1 | Stress-70 mitochondrial | 207 | 1.83E-36 | 93.3 | F:heat shock protein binding; C:myelin sheath; P:hemopoiesis; F:unfolded protein binding; C:nucleolus; P:protein folding; P:protein export from nucleus; P:response to toxic substance; C:mitochondrial nucleoid; F:RNA binding; F:ATP binding; C:extracellular exosome; F:ubiquitin protein ligase binding; C:focal adhesion; P:negative regulation of apoptotic process; P:cellular response to interleukin-1; F:fibroblast growth factor binding |
| Efet.01.21899.g1603.t1 | LDB2_CHICK ame: Full=LIM domain-binding 2 Short=LDB-2 ame: Full=Carboxyl-terminal LIM domain-binding 1 Short=CLIM-1 ame: Full=LIM domain-binding factor CLIM1 Short=cLdb2 | 270 | 3.26E-07 | 80.2 | P:regulation of kinase activity; C:cell leading edge; P:positive regulation of transcription from RNA polymerase II promoter; C:nucleus; C:transcription factor complex; F:transcription cofactor activity; P:hair follicle development; P:epithelial structure maintenance; P:positive regulation of cellular component biogenesis; P:somatic stem cell population maintenance; F:LIM domain binding; P:regulation of cell migration; F:enzyme binding |
| Efet.01.219011.g767.t1 | Transmembrane emp24 domain-containing 6 | 351 | 1.50E-07 | 45.62 | C:endoplasmic reticulum membrane; C:membrane; C:integral component of membrane; P:transport |
| Efet.01.219066.g769.t1 | probable domain-containing histone demethylation 2C isoform X3 | 483 | 5.29E-49 | 79.2 |  |
| Efet.01.2191.g198.t1 | clavaminate synthase At3g21360 | 1206 | 5.00E-84 | 52.6 | F:oxidoreductase activity; P:oxidation-reduction process |
| Efet.01.219166.g772.t1 | hypothetical protein HELRODRAFT_171423 | 1290 | 1.88E-09 | 57 | C:membrane; C:integral component of membrane |
| Efet.01.21918.g1604.t1 | cholecystokinin receptor-like | 1218 | 2.19E-58 | 47.9 | F:G-protein coupled receptor activity; P:G-protein coupled receptor signaling pathway; P:signal transduction; F:gastrin receptor activity; C:membrane; C:integral component of membrane; F:signal transducer activity |
| Efet.01.219186.g773.t1 | zinc finger 271-like | 1659 | 1.87E-114 | 53.8 | F:nucleic acid binding; F:metal ion binding; F:structural constituent of cuticle |
| Efet.01.21923.g1605.t1 | cytoplasmic dynein 1 light intermediate chain 2 | 471 | 4.33E-11 | 46 | F:microtubule motor activity; C:cytoplasmic dynein complex; P:microtubule-based movement |
| Efet.01.21925.g1606.t1 | serine threonine- kinase greatwall isoform X2 | 771 | 1.20E-34 | 82.95 | F:ATP binding; F:protein serine/threonine kinase activity; P:protein phosphorylation |
| Efet.01.219254.g777.t1 | polycystic kidney disease and receptor for egg jelly-related | 1803 | 5.01E-15 | 39.95 | P:detection of mechanical stimulus; F:calcium ion binding; F:calcium channel activity; C:membrane; C:integral component of membrane; P:calcium ion transmembrane transport |
| Efet.01.219282.g778.t1 | hypothetical protein YQE_05235, partial | 201 | 4.15E-06 | 83 |  |
| Efet.01.219288.g779.t1 | hypothetical protein HELRODRAFT_180213 | 531 | 3.04E-57 | 67.75 |  |
| Efet.01.21931.g1608.t1 | 1-phosphatidylinositol 4,5-bisphosphate phosphodiesterase gamma-1 | 399 | 8.56E-29 | 77.45 | F:phosphatidylinositol phospholipase C activity; P:phospholipid catabolic process; F:signal transducer activity; C:intracellular; P:intracellular signal transduction |
| Efet.01.219411.g782.t1 | orphan G- coupled receptor 38 | 612 | 1.29E-29 | 57.57 | P:signal transduction; F:signal transducer activity |
| Efet.01.219431.g783.t1 | Tight junction ZO- | 2160 | 4.31E-47 | 51.05 | P:regulation of apoptotic process |
| Efet.01.219431.g785.t1 | Receptor-interacting serine threonine- kinase 4 | 1056 | 3.82E-78 | 55.95 | F:nucleotide binding; F:ATP binding; F:protein kinase activity; P:phosphorylation; F:protein serine/threonine kinase activity; P:protein phosphorylation; F:kinase activity |
| Efet.01.219486.g786.t1 | probable Xaa-Pro aminopeptidase 3 | 927 | 9.78E-93 | 57 | C:extracellular exosome; F:metal ion binding; P:glomerular filtration; C:mitochondrion; P:proteolysis; P:protein processing; F:manganese ion binding; F:aminopeptidase activity; F:hydrolase activity |
| Efet.01.219507.g788.t1 | hypothetical protein CAPTEDRAFT_220857 | 456 | 1.18E-19 | 60 | C:membrane; C:integral component of membrane |
| Efet.01.219511.g789.t1 | hypothetical protein HELRODRAFT_191437 | 1203 | 3.38E-18 | 47.5 |  |
| Efet.01.219516.g790.t1 | 5-hydroxytryptamine receptor 2C-like | 1707 | 3.22E-44 | 72.4 | C:integral component of plasma membrane; P:G-protein coupled receptor signaling pathway; P:serotonin receptor signaling pathway; F:G-protein coupled serotonin receptor activity |
| Efet.01.219531.g791.t1 | sodium myo-inositol cotransporter 2 isoform X3 | 255 | 1.87E-08 | 65 |  |
| Efet.01.219531.g792.t1 | sodium myo-inositol cotransporter | 657 | 4.49E-60 | 74.95 | C:membrane; P:transport |
| Efet.01.219560.g793.t1 | outer membrane pmp20 precursor | 1500 | 1.39E-52 | 51 | C:membrane; C:integral component of membrane |
| Efet.01.219590.g795.t1 | tripartite motif-containing 2-like | 1428 | 0 | 68.2 | F:metal ion binding |
| Efet.01.219613.g796.t1 | poly-gamma-glutamate biosynthesis | 1128 | 5.81E-158 | 69.3 |  |
| Efet.01.219618.g797.t1 | low-density lipo receptor-related 6 | 531 | 9.96E-16 | 58.85 | P:chordate embryonic development; P:anterior/posterior pattern specification; P:regulation of transcription, DNA-templated; P:anatomical structure formation involved in morphogenesis; P:brain development; P:negative regulation of cellular process; C:membrane part; F:receptor activity; P:transport; C:plasma membrane; P:regulation of molecular function; P:animal organ morphogenesis; F:protein binding; P:positive regulation of cellular process; P:morphogenesis of an epithelium; P:embryonic morphogenesis; P:Wnt signaling pathway |
| Efet.01.219672.g798.t1 | cytoplasmic dynein 1 heavy chain 1 | 561 | 7.34E-98 | 68.5 | F:ATPase activity; F:ATP binding; F:microtubule motor activity; C:dynein complex; P:microtubule-based movement |
| Efet.01.21978.g1610.t1 | glycerate kinase-like isoform X2 | 996 | 9.50E-103 | 61.3 | C:cytoplasm; P:phosphorylation; P:protein phosphorylation; F:kinase activity; F:glycerate kinase activity |
| Efet.01.219832.g799.t1 | pumilio homolog 3-like | 303 | 5.64E-31 | 72 | F:RNA binding |
| Efet.01.219834.g800.t1 | zinc transporter ZIP1-like | 1230 | 3.32E-99 | 51.95 | P:transmembrane transport; C:membrane; C:integral component of membrane; P:kinetochore assembly; F:metal ion transmembrane transporter activity; P:metal ion transport; C:kinetochore |
| Efet.01.219841.g801.t1 | E3 ubiquitin- ligase TRIM33-like isoform X2 | 588 | 7.07E-25 | 71.2 | F:zinc ion binding |
| Efet.01.21987.g1611.t1 | kinesin KIF26B isoform X2 | 405 | 1.93E-41 | 81.25 | F:ATPase activity; P:protein localization; P:axon guidance; C:kinesin complex; P:regulation of cell growth by extracellular stimulus; P:microtubule-based movement; C:microtubule; F:microtubule binding; F:ATP binding; P:enteric nervous system development; F:microtubule motor activity; P:negative regulation of signal transduction; P:cytoskeleton-dependent intracellular transport; C:cytoplasm |
| Efet.01.21987.g1612.t1 | kinesin KIF26A | 426 | 4.89E-13 | 69.65 | F:binding |
| Efet.01.219902.g805.t1 | sex peptide receptor-like | 1194 | 4.84E-129 | 59.45 | F:G-protein coupled receptor activity; C:membrane |
| Efet.01.21993.g1615.t1 | beta-1,3-galactosyltransferase 5-like | 288 | 3.77E-11 | 55.25 | C:membrane; F:transferase activity |
| Efet.01.219977.g807.t1 | nuclear factor interleukin-3-regulated -like | 714 | 3.91E-09 | 68.25 | F:transcription factor activity, sequence-specific DNA binding |
| Efet.01.22003.g1616.t1 | CREB-binding -like | 291 | 1.69E-17 | 89.15 | F:zinc ion binding; C:nuclear body; F:core promoter proximal region sequence-specific DNA binding; F:chromatin binding; F:damaged DNA binding; F:MRF binding; C:histone acetyltransferase complex; P:negative regulation of transcription from RNA polymerase II promoter; F:transcriptional repressor activity, RNA polymerase II core promoter proximal region sequence-specific binding; F:histone acetyltransferase activity; F:p53 binding; C:nuclear chromatin; F:transcription coactivator activity; C:cytoplasm; F:RNA polymerase II transcription factor binding; P:positive regulation of transcription, DNA-templated; P:N-terminal peptidyl-lysine acetylation; P:cellular response to UV; F:transcriptional repressor activity, RNA polymerase II transcription factor binding; P:histone acetylation |
| Efet.01.220063.g809.t1 | hypothetical protein HELRODRAFT_171537 | 942 | 3.26E-15 | 51.5 |  |
| Efet.01.220076.g810.t1 | dynein heavy chain axonemal-like | 612 | 1.49E-44 | 67.65 | F:nucleotide binding |
| Efet.01.220076.g811.t1 | Dynein heavy chain axonemal | 222 | 2.63E-12 | 73.9 | F:ATPase activity; F:ATP binding; F:microtubule motor activity; C:dynein complex; P:microtubule-based movement |
| Efet.01.220119.g816.t1 | homeobox DBX1 | 375 | 1.56E-23 | 90.85 | C:nucleus; P:regulation of transcription, DNA-templated; F:sequence-specific DNA binding |
| Efet.01.220148.g818.t1 | ATP-dependent RNA helicase chloroplastic-like | 5169 | 2.95E-129 | 64.5 | F:organic cyclic compound binding; F:heterocyclic compound binding; F:ion binding; F:hydrolase activity |
| Efet.01.220282.g821.t1 | WD repeat-containing 52 | 534 | 2.64E-62 | 68.9 |  |
| Efet.01.220379.g823.t1 | neural cell adhesion molecule 2-like | 330 | 4.11E-16 | 49.5 | C:membrane; C:integral component of membrane; P:cell adhesion |
| Efet.01.2204.g199.t1 | unconventional myosin-VI- | 546 | 9.82E-53 | 81.2 | C:myosin complex; F:ATP binding; F:actin binding; F:motor activity |
| Efet.01.220412.g824.t1 | filamin-A-like isoform X1 | 474 | 2.67E-65 | 79.8 | F:actin filament binding; C:integral component of membrane |
| Efet.01.220462.g826.t1 | leucine-rich repeat-containing 24-like | 1875 | 1.28E-50 | 46.3 | C:membrane; C:integral component of membrane |
| Efet.01.220466.g829.t1 | hypothetical protein CAPTEDRAFT_221070 | 345 | 1.14E-08 | 54 |  |
| Efet.01.220466.g830.t1 | neural cell adhesion molecule 1 | 330 | 3.03E-07 | 60 |  |
| Efet.01.22048.g1617.t1 | peptidase M20 | 882 | 4.05E-152 | 90.1 | F:hydrolase activity, acting on carbon-nitrogen (but not peptide) bonds, in linear amidines; P:proteolysis; F:metallopeptidase activity |
| Efet.01.22048.g1618.t1 | FAD-dependent oxidoreductase | 1704 | 0 | 82.2 | F:oxidoreductase activity; P:oxidation-reduction process |
| Efet.01.22048.g1619.t1 | flagellin | 669 | 7.78E-44 | 68.21 | C:bacterial-type flagellum; P:bacterial-type flagellum organization |
| Efet.01.22048.g1620.t1 | flagellar cap | 1449 | 0 | 80.5 | P:bacterial-type flagellum organization; C:bacterial-type flagellum hook; P:cell adhesion; C:extracellular region |
| Efet.01.220561.g834.t1 | protocadherin-7-like isoform X3 | 612 | 2.15E-24 | 53.5 | F:calcium ion binding; C:membrane; C:integral component of membrane; P:cell adhesion; P:homophilic cell adhesion via plasma membrane adhesion molecules; C:plasma membrane |
| Efet.01.220571.g835.t1 | cytosolic carboxypeptidase 2 | 360 | 3.18E-38 | 81.65 | F:zinc ion binding; C:centriole; C:ciliary basal body; F:metallocarboxypeptidase activity; P:proteolysis; C:cytosol; P:protein side chain deglutamylation |
| Efet.01.220689.g839.t1 | hypothetical protein HELRODRAFT_170293 | 1077 | 0 | 52 |  |
| Efet.01.2207.g200.t1 | E3 ubiquitin- ligase TRIM56-like | 381 | 2.14E-31 | 57 | F:zinc ion binding; C:cytoplasm; F:metal ion binding; P:myeloid cell differentiation; F:ligase activity; P:primitive hemopoiesis; C:intracellular |
| Efet.01.220718.g841.t1 | PREDICTED: uncharacterized protein LOC106153441 | 705 | 1.07E-21 | 52.15 | C:membrane; C:integral component of membrane |
| Efet.01.22077.g1621.t1 | kinase C delta type-like isoform X1 | 255 | 3.42E-22 | 81.4 | F:ATP binding; F:zinc ion binding; P:protein phosphorylation; C:intracellular; P:intracellular signal transduction; F:protein kinase C activity |
| Efet.01.220780.g842.t1 | ankyrin repeat and SOCS box 12-like | 780 | 9.33E-43 | 49.6 | P:proteolysis; F:serine-type peptidase activity; F:serine-type endopeptidase activity; P:intracellular signal transduction; C:intracellular; F:serine-type exopeptidase activity |
| Efet.01.220781.g843.t1 | peroxidasin-like isoform X4 | 210 | 1.61E-14 | 72.17 | F:peroxidase activity; P:response to oxidative stress; P:cellular oxidant detoxification; P:oxidation-reduction process; F:heme binding |
| Efet.01.220853.g844.t1 | innexin unc-9-like isoform X2 | 879 | 3.49E-98 | 80 | C:gap junction; P:ion transport; P:transmembrane transport; C:integral component of membrane; P:cell communication by electrical coupling; C:synapse; F:gap junction channel activity; C:plasma membrane |
| Efet.01.220923.g846.t1 | Down syndrome cell adhesion molecule Dscam2 isoform X2 | 417 | 2.56E-47 | 55.3 | C:membrane; C:integral component of membrane |
| Efet.01.220926.g847.t1 | zinc finger 236-like | 522 | 1.89E-51 | 67.45 | F:metal ion binding |
| Efet.01.220965.g848.t1 | BTB POZ domain-containing 6 isoform X1 | 1059 | 4.26E-25 | 43.5 |  |
| Efet.01.220990.g850.t1 | ferredoxin | 258 | 1.16E-55 | 90.1 | F:metal ion binding; F:4 iron, 4 sulfur cluster binding |
| Efet.01.220990.g851.t1 | ABC transporter permease | 933 | 4.53E-151 | 90 | C:integral component of membrane; P:transport; C:plasma membrane |
| Efet.01.220990.g852.t1 | peptide ABC transporter permease | 714 | 1.63E-124 | 95.85 | C:integral component of membrane; P:transport; C:plasma membrane |
| Efet.01.221065.g853.t1 | trafficking particle complex subunit 11-like | 315 | 8.86E-12 | 66.45 | P:retina development in camera-type eye; P:liver development; C:membrane; C:integral component of membrane |
| Efet.01.221069.g854.t1 | retinoic acid receptor gamma isoform X3 | 273 | 1.34E-20 | 63.4 | C:intracellular organelle; F:DNA binding; F:transcription factor activity, sequence-specific DNA binding; P:signal transduction; F:signaling receptor activity; P:microtubule cytoskeleton organization |
| Efet.01.221169.g857.t1 | unc-93 homolog A-like | 1017 | 5.77E-54 | 59.65 | C:membrane |
| Efet.01.221179.g858.t1 | U2 snRNP-associated SURP motif-containing isoform X2 | 210 | 1.48E-14 | 79.9 | F:nucleotide binding; F:RNA binding; P:RNA processing |
| Efet.01.221190.g859.t1 | GPI ethanolamine phosphate transferase 3-like | 1125 | 4.17E-40 | 51.65 | F:catalytic activity; P:metabolic process; C:membrane; C:integral component of membrane; F:transferase activity |
| Efet.01.22120.g1622.t1 | G coupled receptor | 255 | 3.41E-11 | 77.5 | P:G-protein coupled receptor signaling pathway; C:integral component of membrane; F:vasopressin receptor activity |
| Efet.01.221223.g864.t1 | hypothetical protein CAPTEDRAFT_189062 | 504 | 1.59E-19 | 54.15 | F:oxidoreductase activity; P:metabolic process; C:membrane; C:integral component of membrane; P:oxidation-reduction process |
| Efet.01.221225.g865.t1 | numb-like isoform X1 | 546 | 5.61E-20 | 63.95 |  |
| Efet.01.221275.g868.t1 | epidermal growth factor receptor-like isoform X1 | 492 | 2.04E-21 | 57.25 | F:kinase activity; P:cellular process |
| Efet.01.221319.g869.t1 | diaphanous homolog 2-like isoform X2 | 288 | 9.71E-19 | 75.45 | F:actin binding; F:catalytic activity; P:metabolic process; P:actin filament organization; F:Rho GTPase binding; P:cell cycle |
| Efet.01.221350.g870.t1 | hypothetical protein HELRODRAFT_161832 | 2160 | 3.09E-06 | 76 |  |
| Efet.01.221406.g871.t1 | sex peptide receptor-like | 630 | 1.92E-54 | 66.3 | F:G-protein coupled receptor activity; C:membrane |
| Efet.01.221424.g872.t1 | translational activator GCN1 | 627 | 9.23E-96 | 88.35 | P:regulation of translation; F:protein kinase binding; P:regulation of protein kinase activity; F:ribosome binding; P:cellular response to stress; P:positive regulation of kinase activity; F:protein kinase regulator activity |
| Efet.01.221453.g874.t1 | hypothetical protein HELRODRAFT_181224 | 210 | 1.25E-22 | 76.67 | C:integral component of membrane |
| Efet.01.221473.g875.t1 | peroxisomal carnitine O-octanoyltransferase | 231 | 5.83E-18 | 68.7 | P:generation of precursor metabolites and energy; P:fatty acid beta-oxidation using acyl-CoA oxidase; P:medium-chain fatty acid metabolic process; P:coenzyme A metabolic process; C:peroxisomal matrix; F:receptor binding; F:carnitine O-octanoyltransferase activity; P:carnitine metabolic process |
| Efet.01.221522.g876.t1 | transport Sec31A isoform X1 | 1257 | 2.16E-61 | 54.65 | C:endomembrane system; C:cytoplasmic vesicle |
| Efet.01.221531.g877.t1 | CPLX_DROME ame: Full=Complexin Flags: Precursor | 243 | 2.98E-06 | 56 | P:regulation of neurotransmitter secretion; F:neurotransmitter transporter activity; P:synaptic growth at neuromuscular junction; C:presynaptic active zone; P:neuromuscular synaptic transmission; F:syntaxin binding; C:terminal bouton; C:membrane; C:cytosol; P:modulation of synaptic transmission; C:neuromuscular junction; P:synaptic vesicle exocytosis |
| Efet.01.221537.g878.t1 | tubulin-specific chaperone C | 858 | 6.75E-66 | 65.6 | P:cellular component organization; C:cell part; P:protein folding |
| Efet.01.221545.g879.t1 | mucin-4 isoform X2 | 1182 | 3.96E-22 | 41.8 | P:cell-matrix adhesion; F:calcium ion binding; C:membrane; F:extracellular matrix structural constituent; C:proteinaceous extracellular matrix |
| Efet.01.221565.g880.t1 | probable E3 ubiquitin- ligase HERC1 isoform X2 | 801 | 2.26E-15 | 43.86 | F:ubiquitin-protein transferase activity; F:ligase activity; P:protein ubiquitination |
| Efet.01.221591.g882.t1 | family | 678 | 3.59E-12 | 69.1 | F:transferase activity |
| Efet.01.22162.g1624.t1 | serine threonine- phosphatase 2A 55 kDa regulatory subunit B alpha isoform-like | 399 | 3.98E-79 | 92 | P:regulation of phosphoprotein phosphatase activity; C:protein phosphatase type 2A complex |
| Efet.01.221628.g884.t1 | 5-hydroxytryptamine receptor-like | 1437 | 5.05E-149 | 59 | P:signal transduction; C:membrane; F:signal transducer activity |
| Efet.01.221665.g887.t1 | tektin-3-like isoform X1 | 225 | 2.92E-07 | 73.57 |  |
| Efet.01.22167.g1625.t1 | hypothetical protein HELRODRAFT_175808 | 1203 | 2.93E-08 | 66 |  |
| Efet.01.221700.g888.t1 | beta-hexosaminidase subunit beta-like | 402 | 5.96E-20 | 53.9 | F:hydrolase activity |
| Efet.01.221778.g890.t1 | nuclear factor interleukin-3-regulated | 426 | 2.75E-09 | 65.45 | C:nucleus; F:transcriptional repressor activity, RNA polymerase II core promoter proximal region sequence-specific binding; F:sequence-specific DNA binding; P:circadian rhythm; P:regulation of transcription from RNA polymerase II promoter |
| Efet.01.221843.g891.t1 | transmembrane emp24 domain-containing 5-like | 414 | 8.41E-22 | 58 | C:membrane |
| Efet.01.221949.g893.t1 | WNT5A_AMBME ame: Full= Wnt-5a Flags: Precursor | 564 | 8.57E-78 | 76.8 | P:multicellular organism development; P:Wnt signaling pathway; F:receptor binding; C:proteinaceous extracellular matrix; P:positive regulation of cell migration |
| Efet.01.22196.g1626.t1 | FMRFamide-activated amiloride-sensitive sodium channel isoform X1 | 1509 | 6.99E-96 | 42.7 | P:sodium ion transport; P:ion transport; F:sodium channel activity; C:membrane; C:integral component of membrane; P:sodium ion transmembrane transport; P:transport |
| Efet.01.222118.g894.t1 | sex peptide receptor-like | 858 | 6.92E-78 | 60.85 | F:G-protein coupled receptor activity; C:membrane |
| Efet.01.222143.g895.t1 | V-type proton ATPase subunit G 1-like | 351 | 1.76E-26 | 74.55 | P:transport |
| Efet.01.222206.g898.t1 | hypothetical protein HELRODRAFT_189098 | 297 | 1.50E-20 | 59.75 | C:membrane; C:integral component of membrane; F:carbohydrate binding |
| Efet.01.222280.g901.t1 | cytoplasmic dynein 2 heavy chain 1 | 444 | 1.46E-38 | 78.95 | F:ATPase activity; F:ATP binding; F:microtubule motor activity; C:dynein complex; P:microtubule-based movement |
| Efet.01.222309.g902.t1 | rho GTPase-activating 190-like isoform X1 | 723 | 3.48E-75 | 72.6 | P:signal transduction |
| Efet.01.222382.g904.t1 | WD repeat-containing CG11141 | 228 | 2.82E-21 | 72.2 |  |
| Efet.01.222386.g905.t1 | nuclear hormone receptor family member daf-12-like | 291 | 9.79E-06 | 50 |  |
| Efet.01.222455.g906.t1 | probable N-acetylgalactosaminyltransferase 9 isoform X1 | 441 | 2.66E-14 | 64.67 | C:membrane; C:Golgi apparatus; F:transferase activity |
| Efet.01.222600.g910.t1 | gastrula zinc finger -like | 1287 | 2.61E-67 | 51.2 |  |
| Efet.01.222614.g911.t1 | synaptotagmin-17-like | 921 | 4.59E-95 | 73 | C:membrane; P:exocytosis |
| Efet.01.222636.g912.t1 | hypothetical protein CAPTEDRAFT_218762 | 273 | 2.03E-15 | 69 | C:membrane |
| Efet.01.222679.g913.t1 | Hikeshi isoform X3 | 270 | 1.14E-34 | 81.4 | C:nucleus; C:extracellular exosome; P:cellular response to heat; P:protein import into nucleus; P:Golgi organization; C:cytosol; C:cell junction; F:protein transporter activity; F:Hsp70 protein binding; P:lung development |
| Efet.01.2227.g201.t1 | plexin A3 | 1350 | 1.21E-63 | 53.05 | P:signal transduction; C:membrane; P:axon guidance |
| Efet.01.222759.g915.t1 | Regulator of G- signaling | 2067 | 1.14E-26 | 44.9 | C:cytoplasm; P:signal transduction; F:GTPase activator activity; P:regulation of catalytic activity; F:signal transducer activity, downstream of receptor; F:GTPase regulator activity; C:plasma membrane; P:positive regulation of GTPase activity |
| Efet.01.222770.g917.t1 | guanylate cyclase 32E-like | 705 | 5.77E-69 | 68.8 | F:ATP binding; P:cGMP biosynthetic process; C:integral component of membrane; F:guanylate cyclase activity; F:protein kinase activity; P:protein phosphorylation; C:intracellular; P:intracellular signal transduction |
| Efet.01.222803.g918.t1 | uncharacterized protein LOC107442137 | 1293 | 9.85E-06 | 55 |  |
| Efet.01.222808.g921.t1 | serine threonine- kinase PAK 3-like | 264 | 2.63E-27 | 73.8 | F:ATP binding; F:signal transducer, downstream of receptor, with serine/threonine kinase activity; C:cytoplasm; P:axonogenesis; P:protein phosphorylation; P:actin cytoskeleton organization; P:mitotic cell cycle |
| Efet.01.222847.g924.t1 | gonadotropin-releasing hormone receptor-like isoform X1 | 669 | 1.23E-55 | 68.1 | P:signal transduction; C:membrane; F:signal transducer activity |
| Efet.01.222892.g925.t1 | hypothetical protein CAPTEDRAFT_228921 | 360 | 1.08E-07 | 50 | C:membrane; C:integral component of membrane; F:carbohydrate binding |
| Efet.01.222898.g926.t1 | laminin subunit alpha | 528 | 1.45E-21 | 55 | P:regulation of embryonic development; P:cell adhesion; P:regulation of cell adhesion; P:regulation of cell migration; F:receptor binding |
| Efet.01.222917.g927.t1 | zinc finger MYND domain-containing 10-like | 387 | 5.43E-50 | 76.55 | P:cilium assembly; F:metal ion binding |
| Efet.01.222988.g928.t1 | polynucleotide 5 -hydroxyl-kinase NOL9 | 456 | 1.67E-06 | 53 |  |
| Efet.01.222995.g931.t1 | actin-binding anillin-like isoform X2 | 363 | 2.87E-21 | 60.35 |  |
| Efet.01.223054.g932.t1 | O-acetyl-ADP-ribose deacetylase 1 | 375 | 6.92E-55 | 76 | F:purine nucleoside binding; F:deacetylase activity; P:purine nucleoside metabolic process |
| Efet.01.223169.g935.t1 | hypothetical protein HELRODRAFT_194482 | 588 | 2.43E-104 | 62.8 | F:oxidoreductase activity; C:integral component of membrane; P:oxidation-reduction process |
| Efet.01.223219.g936.t1 | neuralized isoform X2 | 741 | 4.74E-40 | 70.05 | F:metal ion binding |
| Efet.01.223219.g937.t1 | neuralized-like isoform X1 | 840 | 1.02E-19 | 77.5 | F:zinc ion binding |
| Efet.01.223263.g938.t1 | Myosin heavy striated muscle | 762 | 1.87E-39 | 59.25 | F:binding |
| Efet.01.223291.g939.t1 | transmembrane 97 | 288 | 5.84E-23 | 69.4 | C:membrane; C:endoplasmic reticulum |
| Efet.01.223295.g940.t1 | malignant fibrous histiocytoma-amplified sequence 1 homolog isoform X1 | 2361 | 5.52E-142 | 52.95 | F:GTP binding; P:small GTPase mediated signal transduction; C:intracellular |
| Efet.01.223304.g942.t1 | solute carrier family 12 member 4-like isoform X2 | 402 | 3.26E-13 | 59 | F:potassium:chloride symporter activity; P:chloride transmembrane transport; C:integral component of membrane |
| Efet.01.223378.g943.t1 | hypothetical protein HELRODRAFT_161790 | 768 | 1.22E-22 | 51.45 | P:transport |
| Efet.01.223382.g944.t1 | usick-Kaufman Bardet-Biedl syndromes chaperonin | 813 | 1.36E-34 | 51.6 | F:ATP binding; P:cilium assembly; F:unfolded protein binding; P:protein folding |
| Efet.01.223416.g945.t1 | phosphatidylinositol 4-kinase alpha-like | 1185 | 1.39E-15 | 42.9 | P:phosphatidylinositol-mediated signaling; P:phosphatidylinositol phosphorylation; P:phosphorylation; F:transferase activity; F:kinase activity; C:intracellular |
| Efet.01.223452.g946.t1 | leucine-rich repeat-containing 15-like | 2514 | 2.49E-132 | 52.8 | C:membrane; C:integral component of membrane |
| Efet.01.223463.g947.t1 | T-complex 1 subunit zeta | 522 | 3.63E-90 | 79.45 | F:ATP binding; C:cytoplasm; F:unfolded protein binding; P:protein folding |
| Efet.01.223658.g950.t1 | AChain Structure Of The C Domain Of Synapsin Ia From Bovine Brain With Calcium Atp-Gamma-S Bound | 291 | 1.28E-29 | 83.45 | F:ATP binding; F:actin binding; F:catalytic activity; F:metal ion binding; C:synaptic vesicle; C:cell junction; C:Golgi apparatus; P:neurotransmitter secretion |
| Efet.01.223669.g951.t1 | 60 kDa SS-A Ro ribonucleo | 264 | 1.20E-09 | 64.4 | C:intracellular part; P:single-organism process |
| Efet.01.223676.g952.t1 | sodium potassium ATPase alpha | 513 | 1.43E-74 | 90.05 | P:potassium ion transport; F:ATP binding; P:sodium ion transport; F:sodium:potassium-exchanging ATPase activity; F:metal ion binding; P:ATP hydrolysis coupled transmembrane transport; C:integral component of membrane; P:establishment or maintenance of transmembrane electrochemical gradient |
| Efet.01.223681.g953.t1 | hypothetical protein CAPTEDRAFT_218417 | 2268 | 1.08E-157 | 45.2 | C:membrane; C:integral component of membrane |
| Efet.01.223694.g955.t1 | coiled-coil domain-containing 63-like | 585 | 2.49E-56 | 69.45 | C:outer dynein arm; P:outer dynein arm assembly |
| Efet.01.223744.g956.t1 | filamin-A-like isoform X3 | 258 | 5.77E-12 | 69.6 | F:actin filament binding |
| Efet.01.223757.g957.t1 | T family of potassium channels 7-like | 501 | 6.95E-28 | 53 | P:ion transport; P:potassium ion transmembrane transport; C:membrane; C:integral component of membrane; F:potassium channel activity; P:transport |
| Efet.01.223776.g959.t1 | ubiquitin thioesterase otulin-like | 432 | 3.08E-40 | 62.5 | C:membrane; C:integral component of membrane |
| Efet.01.22382.g1628.t1 | inactive tyrosine- kinase 7-like | 333 | 2.25E-33 | 66.3 | F:binding |
| Efet.01.223961.g963.t1 | probable serine threonine- kinase DDB_G0282963 isoform X2 | 2796 | 6.82E-19 | 55.4 | F:protein dimerization activity |
| Efet.01.224087.g966.t1 | E3 ubiquitin- ligase RNF19A | 936 | 1.44E-92 | 72.5 | F:ubiquitin-protein transferase activity; F:zinc ion binding; C:integral component of membrane; P:protein ubiquitination |
| Efet.01.224166.g970.t1 | Acyl- synthetase family member | 762 | 1.03E-39 | 53.25 | F:catalytic activity; P:metabolic process |
| Efet.01.224267.g972.t1 | outer dense fiber 3-like | 408 | 2.28E-64 | 77.8 |  |
| Efet.01.224295.g973.t1 | tektin-3-like isoform X1 | 231 | 6.45E-28 | 83.1 |  |
| Efet.01.224347.g974.t1 | activating signal cointegrator 1 complex subunit 3 | 1446 | 0 | 87.55 | F:nucleic acid binding; F:ATP binding; F:helicase activity |
| Efet.01.224394.g975.t1 | tyramine receptor 1 | 1074 | 2.98E-36 | 68.75 | C:integral component of plasma membrane; F:dopamine neurotransmitter receptor activity; P:dopamine receptor signaling pathway |
| Efet.01.22442.g1631.t1 | leucine-rich repeat and fibronectin type III domain-containing 1 | 1362 | 3.24E-124 | 54.7 | C:extracellular space; P:axonogenesis; C:membrane; C:integral component of membrane; C:proteinaceous extracellular matrix |
| Efet.01.224592.g981.t1 | beta,beta-carotene 15,15 -dioxygenase-like | 567 | 3.37E-31 | 57.75 | F:oxidoreductase activity, acting on single donors with incorporation of molecular oxygen, incorporation of two atoms of oxygen; P:oxidation-reduction process; C:cellular_component; F:monooxygenase activity |
| Efet.01.224599.g982.t1 | octopamine receptor beta-2R-like | 837 | 4.43E-35 | 79.6 | P:peptidyl-tyrosine phosphorylation; F:non-membrane spanning protein tyrosine kinase activity; F:adrenergic receptor activity; C:integral component of membrane; P:adrenergic receptor signaling pathway; P:serotonin receptor signaling pathway; F:G-protein coupled serotonin receptor activity; C:plasma membrane |
| Efet.01.224622.g983.t1 | octopamine receptor isoform X1 | 2676 | 2.26E-44 | 68.6 | P:signal transduction; F:signal transducer activity |
| Efet.01.224637.g984.t1 | hypothetical protein HELRODRAFT_183289 | 687 | 2.81E-10 | 48 | C:membrane; C:integral component of membrane |
| Efet.01.224683.g985.t1 | hypothetical protein CAPTEDRAFT_199444 | 315 | 5.86E-26 | 69 |  |
| Efet.01.224738.g989.t1 | hypothetical protein HELRODRAFT_174791 | 936 | 3.96E-55 | 61 | C:membrane; C:integral component of membrane |
| Efet.01.224739.g990.t1 | insulin-like growth factor 1 receptor | 345 | 1.51E-41 | 81.35 | F:ATP binding; P:cGMP biosynthetic process; P:peptidyl-tyrosine phosphorylation; F:protein tyrosine kinase activity; C:integral component of membrane; F:guanylate cyclase activity |
| Efet.01.224743.g992.t1 | mRNA export factor-like | 642 | 1.02E-108 | 80.4 | F:RNA binding; P:RNA export from nucleus; F:ubiquitin binding; P:protein import into nucleus; P:transcription-dependent tethering of RNA polymerase II gene DNA at nuclear periphery; C:nuclear pore |
| Efet.01.224777.g994.t1 | 1-phosphatidylinositol 4,5-bisphosphate phosphodiesterase beta-4 isoform X1 | 327 | 1.07E-29 | 96.1 | F:phosphatidylinositol phospholipase C activity; F:calcium ion binding; P:lipid catabolic process; F:signal transducer activity; C:intracellular; P:intracellular signal transduction |
| Efet.01.224857.g995.t1 | tetratricopeptide repeat 24 | 687 | 8.55E-19 | 50 | F:nucleotide binding; F:nucleic acid binding; C:nucleus; P:RNA splicing; F:RNA binding; C:cytoplasm; C:nuclear speck; P:RNA processing; P:mRNA processing; P:transport; P:mRNA transport |
| Efet.01.224874.g996.t1 | neuroligin- Y-linked-like | 507 | 1.15E-49 | 68.1 | C:membrane |
| Efet.01.224880.g997.t1 | Notch-regulated ankyrin repeat-containing | 426 | 5.65E-39 | 77.75 | P:Notch signaling pathway; P:branching involved in blood vessel morphogenesis; P:transcription, DNA-templated; P:regulation of cell-cell adhesion; P:regulation of canonical Wnt signaling pathway; P:negative regulation of multicellular organismal process; P:regulation of transcription, DNA-templated; P:negative regulation of developmental process; P:positive regulation of endothelial cell proliferation; P:blood vessel endothelial cell proliferation involved in sprouting angiogenesis; P:negative regulation of Notch signaling pathway; P:regulation of multicellular organismal development |
| Efet.01.22489.g1634.t1 | protocadherin-9-like | 1050 | 6.93E-66 | 59.7 | C:membrane; P:cell adhesion |
| Efet.01.22489.g1635.t1 | protocadherin beta-12 | 1614 | 4.98E-85 | 55.55 | F:calcium ion binding; C:membrane; C:integral component of membrane; P:cell adhesion; P:homophilic cell adhesion via plasma membrane adhesion molecules; C:plasma membrane |
| Efet.01.224902.g998.t1 | PCTP | 231 | 4.08E-13 | 68.1 | C:intercellular canaliculus; C:microvillus; P:positive regulation of peroxisome proliferator activated receptor signaling pathway; C:membrane; C:cytosol; P:bile acid secretion; F:lipid binding |
| Efet.01.224921.g999.t1 | phytochelatin synthase | 597 | 5.24E-135 | 89.5 | P:phytochelatin biosynthetic process; F:glutathione gamma-glutamylcysteinyltransferase activity; F:metal ion binding; P:response to metal ion |
| Efet.01.224929.g1000.t1 | inositol 1,4,5-trisphosphate receptor type 1- | 261 | 2.47E-21 | 86.35 | P:inositol phosphate-mediated signaling; C:integral component of membrane; F:inositol 1,4,5-trisphosphate-sensitive calcium-release channel activity; P:calcium ion transmembrane transport; C:endoplasmic reticulum |
| Efet.01.224947.g1001.t1 | zinc finger E-box-binding homeobox 2-like isoform X1 | 306 | 1.24E-29 | 84 | C:nucleus; F:metal ion binding; P:regulation of transcription, DNA-templated; F:sequence-specific DNA binding |
| Efet.01.224968.g1003.t1 | potassium voltage-gated channel Shal-like | 336 | 8.35E-11 | 73.25 | P:potassium ion transport; P:ion transmembrane transport; C:integral component of membrane; F:voltage-gated ion channel activity; F:potassium channel activity; P:cellular process |
| Efet.01.225027.g1004.t1 | atlastin-1 isoform X1 | 477 | 1.83E-31 | 73.25 | F:GTP binding; F:GTPase activity; C:integral component of membrane |
| Efet.01.22510.g1636.t1 | 29-kDa galactose-binding lectin | 447 | 1.22E-20 | 55.61 | F:actin filament binding; F:carbohydrate binding; F:protein binding, bridging |
| Efet.01.225135.g1008.t1 | sphingolipid delta(4)-desaturase DES1-like | 1290 | 1.56E-129 | 76.65 | F:sphingolipid delta-4 desaturase activity; C:integral component of membrane; P:oxidation-reduction process; P:sphingolipid biosynthetic process |
| Efet.01.225184.g1009.t1 | focal adhesion kinase 1-like isoform X1 | 237 | 1.79E-17 | 77.2 | P:signal complex assembly; P:peptidyl-tyrosine phosphorylation; F:protein tyrosine kinase activity; C:focal adhesion; P:signal transduction; F:signal transducer activity |
| Efet.01.225198.g1010.t1 | splicing factor 3A subunit 3 | 324 | 1.67E-45 | 83.1 | F:zinc ion binding; F:RNA binding; C:spliceosomal complex; P:hematopoietic stem cell differentiation; P:mRNA splicing, via spliceosome |
| Efet.01.22523.g1638.t1 | hypothetical protein CAPTEDRAFT_181469 | 606 | 3.97E-23 | 58.33 | P:ion transport; P:ion transmembrane transport; C:membrane; C:integral component of membrane; F:extracellular ligand-gated ion channel activity; P:transport |
| Efet.01.225406.g1015.t1 | HAUS augmin-like complex subunit 4 | 252 | 4.17E-20 | 69 | P:microtubule cytoskeleton organization |
| Efet.01.22543.g1639.t1 | probable G- coupled receptor 135 | 1446 | 8.93E-35 | 50.55 | F:G-protein coupled receptor activity; P:G-protein coupled receptor signaling pathway; P:signal transduction; C:membrane; C:integral component of membrane; F:signal transducer activity |
| Efet.01.225483.g1017.t1 | sodium potassium ATPase alpha | 306 | 1.20E-40 | 82.4 | C:integral component of plasma membrane; F:ATP binding; P:cellular potassium ion homeostasis; P:sodium ion export from cell; P:ATP hydrolysis coupled proton transport; F:sodium:potassium-exchanging ATPase activity; F:metal ion binding; C:intracellular membrane-bounded organelle; P:establishment or maintenance of transmembrane electrochemical gradient; P:cellular sodium ion homeostasis; P:potassium ion import |
| Efet.01.22549.g1640.t1 | hypothetical protein LOTGIDRAFT_175808 | 309 | 1.88E-08 | 57 |  |
| Efet.01.225494.g1018.t1 | hypothetical protein CAPTEDRAFT_225483 | 249 | 3.15E-12 | 54.45 | C:membrane; C:integral component of membrane; F:carbohydrate binding |
| Efet.01.225603.g1020.t1 | alanine aminotransferase 2-like isoform X1 | 396 | 1.44E-09 | 72.15 | F:pyridoxal phosphate binding; F:catalytic activity; P:biosynthetic process |
| Efet.01.225647.g1022.t1 | EF-hand domain-containing | 504 | 6.49E-13 | 49.71 | F:calcium ion binding |
| Efet.01.225708.g1023.t1 | lymphocyte-specific helicase-like | 669 | 4.41E-68 | 75.05 | C:nucleus; F:ATP binding; P:urogenital system development; P:DNA methylation; P:lymphocyte proliferation; P:pericentric heterochromatin assembly; F:chromatin binding; P:maintenance of DNA methylation; P:negative regulation of intrinsic apoptotic signaling pathway; C:pericentric heterochromatin; P:methylation-dependent chromatin silencing |
| Efet.01.225732.g1024.t1 | hypothetical protein | 786 | 1.24E-65 | 61.13 |  |
| Efet.01.225780.g1026.t1 | rho-associated kinase 1 isoform X1 | 360 | 1.01E-27 | 61.35 | F:protein serine/threonine kinase activity; F:binding; P:protein phosphorylation |
| Efet.01.225796.g1028.t1 | Holliday junction branch migration DNA helicase | 1077 | 0 | 86.15 | F:DNA binding; F:ATP binding; P:DNA repair; P:DNA duplex unwinding; P:DNA recombination; F:four-way junction helicase activity; P:SOS response |
| Efet.01.225823.g1029.t1 | pancreas duodenum homeobox 1-like isoform X1 | 696 | 2.89E-33 | 91.55 | C:nucleus; F:transcription factor activity, sequence-specific DNA binding; P:regulation of transcription, DNA-templated; F:sequence-specific DNA binding |
| Efet.01.225885.g1031.t1 | pro-interleukin-16 isoform X2 | 591 | 3.70E-08 | 69.67 |  |
| Efet.01.225893.g1032.t1 | neuronal acetylcholine receptor subunit alpha- | 594 | 2.49E-111 | 79.15 | P:chemical synaptic transmission, postsynaptic; P:synaptic transmission, cholinergic; F:acetylcholine receptor activity; C:postsynaptic membrane; F:calcium channel activity; P:calcium ion transmembrane transport; C:cell junction; F:extracellular ligand-gated ion channel activity; F:acetylcholine binding; C:acetylcholine-gated channel complex |
| Efet.01.22590.g1643.t1 | F-box only 22-like | 522 | 1.65E-12 | 45 |  |
| Efet.01.225929.g1033.t1 | coiled-coil domain-containing 105-like | 777 | 3.09E-47 | 55.4 |  |
| Efet.01.225954.g1034.t1 | protocadherin-9-like isoform X1 | 2655 | 3.55E-142 | 53.5 | F:calcium ion binding; C:membrane; C:integral component of membrane; P:cell adhesion; P:homophilic cell adhesion via plasma membrane adhesion molecules; C:plasma membrane |
| Efet.01.225973.g1035.t1 | cGMP-dependent kinase 1-like isoform X2 | 468 | 4.25E-37 | 72.1 | F:nucleotide binding; F:protein kinase activity |
| Efet.01.226048.g1039.t1 | FMRFamide receptor-like | 1086 | 2.21E-69 | 56.15 | F:signal transducer activity |
| Efet.01.226070.g1040.t1 | GTP-binding GEM | 585 | 2.12E-51 | 54.6 | F:nucleotide binding; F:GTP binding; P:signal transduction; P:small GTPase mediated signal transduction; C:membrane; C:intracellular |
| Efet.01.226079.g1043.t1 | homeobox zampogna-like | 849 | 4.10E-34 | 78.35 | C:nucleus; P:regulation of transcription, DNA-templated; F:sequence-specific DNA binding; F:hydrolase activity |
| Efet.01.226134.g1044.t1 | eukaryotic translation initiation factor 3 subunit D | 717 | 5.08E-102 | 85.25 | F:translation initiation factor activity; P:formation of translation preinitiation complex; C:eukaryotic 43S preinitiation complex; C:eukaryotic 48S preinitiation complex; P:regulation of translational initiation; C:eukaryotic translation initiation factor 3 complex |
| Efet.01.226191.g1047.t1 | Sulfite reductase flavo component | 852 | 1.27E-127 | 68.35 | F:thiamine pyrophosphate binding; F:FMN binding; F:iron ion binding; F:oxidoreductase activity, acting on the aldehyde or oxo group of donors; P:electron transport chain |
| Efet.01.22622.g1644.t1 | carbohydrate sulfotransferase 6-like | 1089 | 2.64E-20 | 48.7 | C:membrane; C:integral component of membrane; F:sulfotransferase activity |
| Efet.01.226287.g1049.t1 | lipoxygenase homology domain-containing 1 | 240 | 8.05E-11 | 72.15 | P:sensory perception of sound; C:stereocilium |
| Efet.01.226348.g1050.t1 | helicase with zinc finger domain 2 | 645 | 1.07E-46 | 63.5 | F:metal ion binding; C:membrane; F:ligand-dependent nuclear receptor transcription coactivator activity; P:regulation of nucleic acid-templated transcription; P:positive regulation of transcription from RNA polymerase II promoter; F:RNA binding |
| Efet.01.226399.g1052.t1 | Golgi resident GCP60-like isoform X2 | 393 | 7.81E-50 | 79.15 | F:fatty-acyl-CoA binding; C:integral component of membrane; P:transport |
| Efet.01.226426.g1053.t1 | hypothetical protein CAPTEDRAFT_222036 | 315 | 7.74E-21 | 60.57 | C:membrane; C:integral component of membrane |
| Efet.01.226428.g1054.t1 | short transient receptor potential channel 6 | 573 | 1.25E-96 | 78.25 | C:integral component of plasma membrane; F:store-operated calcium channel activity; P:manganese ion transport; P:regulation of cytosolic calcium ion concentration; P:calcium ion transmembrane transport; P:single fertilization |
| Efet.01.226444.g1055.t1 | hypothetical protein HELRODRAFT_62550 | 249 | 3.29E-07 | 96 |  |
| Efet.01.226463.g1057.t1 | disulfide-isomerase-like isoform X2 | 285 | 7.55E-18 | 71.5 | P:regulation of oxidative stress-induced intrinsic apoptotic signaling pathway; F:protein disulfide isomerase activity; P:metabolic process; C:endoplasmic reticulum; F:peptidyl-proline 4-dioxygenase activity; P:protein folding; P:single-organism process; P:response to endoplasmic reticulum stress |
| Efet.01.226540.g1059.t1 | allatostatin-A receptor-like | 564 | 1.64E-23 | 51.35 | F:G-protein coupled peptide receptor activity; P:G-protein coupled receptor signaling pathway |
| Efet.01.226550.g1060.t1 | hypothetical protein CAPTEDRAFT_222008 | 918 | 1.70E-13 | 64 | C:membrane |
| Efet.01.226555.g1061.t1 | S1 RNA-binding domain-containing 1-like | 534 | 2.05E-23 | 80.95 | F:translation elongation factor activity; P:nucleobase-containing compound metabolic process; P:translational elongation |
| Efet.01.226557.g1062.t1 | abnormal spindle-like microcephaly-associated homolog | 1971 | 2.74E-39 | 62.5 | P:spindle organization |
| Efet.01.226633.g1063.t1 | sacsin-like | 1608 | 4.35E-53 | 45.1 |  |
| Efet.01.22670.g1647.t1 | filamin-A-like isoform X1 | 879 | 5.84E-98 | 66 | F:actin binding |
| Efet.01.226728.g1067.t1 | Mo25 | 255 | 1.66E-38 | 95.05 |  |
| Efet.01.226824.g1070.t1 | protocadherin Fat 4 | 303 | 1.25E-14 | 59.8 | P:regulation of cellular process; C:membrane; C:cell part; P:cell adhesion; P:animal organ development; P:cellular process |
| Efet.01.226824.g1071.t1 | cadherin-related tumor suppressor | 1068 | 3.70E-48 | 54.1 | F:catalytic activity |
| Efet.01.226864.g1072.t1 | kelch 10 | 348 | 6.06E-29 | 64.85 | P:male genitalia morphogenesis; F:protein binding; P:male gonad development; C:cytoplasm; P:spermatid development; P:homeostasis of number of cells within a tissue; P:cell morphogenesis; P:fertilization |
| Efet.01.226885.g1073.t1 | Gamma-aminobutyric acid receptor subunit gamma- | 435 | 4.53E-22 | 70.2 | P:ion transmembrane transport; C:integral component of membrane; F:extracellular ligand-gated ion channel activity |
| Efet.01.226912.g1074.t1 | hypothetical protein CAPTEDRAFT_144547 | 2109 | 3.64E-116 | 54.45 | F:calcium ion binding; C:membrane; C:integral component of membrane; P:cell adhesion; P:homophilic cell adhesion via plasma membrane adhesion molecules; C:plasma membrane |
| Efet.01.226913.g1075.t1 | hydrocephalus-inducing -like | 768 | 3.89E-71 | 70.8 | P:cilium movement |
| Efet.01.226948.g1076.t1 | hypothetical protein CAPTEDRAFT_224679 | 255 | 1.97E-14 | 60.83 | F:zinc ion binding; F:metal ion binding |
| Efet.01.226954.g1077.t1 | hypothetical protein B5766_02655 | 2802 | 0 | 44.2 | C:membrane; C:integral component of membrane |
| Efet.01.226956.g1078.t1 | ATP-dependent 6-phosphofructokinase-like isoform X2 | 243 | 8.81E-19 | 57.1 | P:organophosphate metabolic process; P:carbohydrate derivative metabolic process; P:phosphorylation; F:binding; F:transferase activity |
| Efet.01.226961.g1079.t1 | neurabin-1 isoform X7 | 618 | 7.77E-83 | 91.35 |  |
| Efet.01.227020.g1082.t1 | serine protease | 1860 | 6.42E-15 | 43 |  |
| Efet.01.22707.g1652.t1 | PLANT CADMIUM RESISTANCE 3-like | 405 | 4.28E-35 | 66.15 | C:membrane; C:integral component of membrane |
| Efet.01.227089.g1086.t1 | ubiquitin-associated domain-containing 2-like | 294 | 7.51E-10 | 53.6 | C:membrane; C:integral component of membrane |
| Efet.01.2271.g203.t1 | glycerol-3-phosphate dehydrogenase 1 isoform X2 | 627 | 2.47E-83 | 71.75 | P:glycerol-3-phosphate metabolic process; C:cytoplasm; F:binding; F:glycerol-3-phosphate dehydrogenase activity; F:glycerol-3-phosphate dehydrogenase [NAD+] activity; P:intracellular accumulation of glycerol |
| Efet.01.227117.g1087.t1 | gastrula zinc finger -like | 861 | 1.91E-36 | 60.3 | F:nucleic acid binding; F:metal ion binding |
| Efet.01.227170.g1089.t1 | PREDICTED: uncharacterized protein LOC106870289 | 495 | 3.83E-21 | 57.5 |  |
| Efet.01.227313.g1092.t1 | nuclear factor 1 X-type-like isoform X1 | 474 | 8.96E-84 | 94.8 | P:negative regulation of transcription from RNA polymerase II promoter; C:nucleus; P:transcription, DNA-templated; P:DNA replication; P:positive regulation of transcription from RNA polymerase II promoter; F:RNA polymerase II transcription factor activity, sequence-specific DNA binding |
| Efet.01.227331.g1094.t1 | collagen alpha-1(I) chain-like isoform X1 | 246 | 4.41E-12 | 61.65 | C:collagen trimer; C:collagen type IX trimer |
| Efet.01.227482.g1096.t1 | endoribonuclease Dcr-1 | 1020 | 3.07E-100 | 61.7 | P:RNA metabolic process |
| Efet.01.227492.g1098.t1 | Potassium sodium hyperpolarization-activated cyclic nucleotide-gated channel 4 | 303 | 3.08E-22 | 91.6 | C:integral component of plasma membrane; F:voltage-gated potassium channel activity; P:regulation of membrane potential; P:potassium ion transmembrane transport |
| Efet.01.22752.g1653.t1 | fucose-1-phosphate guanylyltransferase | 1089 | 3.91E-72 | 55.6 | F:nucleotidyltransferase activity; F:transferase activity, transferring phosphorus-containing groups; F:transferase activity |
| Efet.01.227532.g1100.t1 | ribonuclease UK114 | 354 | 1.05E-37 | 78.85 | F:platinum binding; P:RNA phosphodiester bond hydrolysis, endonucleolytic; F:transition metal ion binding; F:protein homodimerization activity; P:response to salt; F:long-chain fatty acid binding; F:xenon atom binding; P:brain development; F:RNA binding; C:nucleus; C:peroxisome; C:extracellular exosome; P:regulation of translational termination; P:kidney development; C:mitochondrion; P:negative regulation of epithelial cell proliferation; C:cytosol; P:G1 to G0 transition; F:endoribonuclease activity, producing 3'-phosphomonoesters; P:response to lipid; P:negative regulation of translation; P:lung development |
| Efet.01.227538.g1101.t1 | cell death specification 2 | 366 | 2.52E-13 | 70.9 | F:transcription factor activity, sequence-specific DNA binding; P:regulation of transcription, DNA-templated; F:sequence-specific DNA binding |
| Efet.01.227552.g1103.t1 | antistasin-like isoform X1 | 801 | 2.58E-28 | 56.05 | P:negative regulation of catalytic activity; F:enzyme inhibitor activity |
| Efet.01.227590.g1105.t1 | adenosine deaminase CECR1-like | 1257 | 6.58E-127 | 61.45 | F:catalytic activity |
| Efet.01.227592.g1106.t1 | hypothetical protein HELRODRAFT_181360 | 219 | 3.40E-08 | 78.6 | F:nucleotide binding; F:RNA binding |
| Efet.01.22761.g1654.t1 | activator of 90 kDa heat shock ATPase homolog 1-like | 204 | 4.85E-23 | 75.35 | P:positive regulation of ATPase activity; F:chaperone binding; F:ATPase activator activity; P:response to stress |
| Efet.01.227631.g1108.t1 | lactadherin-like isoform X5 | 483 | 1.05E-85 | 86.85 |  |
| Efet.01.22765.g1655.t1 | RNA methyltransferase | 879 | 0 | 71.85 | F:transferase activity |
| Efet.01.227685.g1109.t1 | H ACA ribonucleo complex subunit 4-like | 549 | 8.16E-95 | 90.7 | P:cell proliferation; P:positive regulation of telomerase RNA localization to Cajal body; P:snRNA pseudouridine synthesis; P:positive regulation of establishment of protein localization to telomere; C:box H/ACA telomerase RNP complex; C:Cajal body; P:telomere maintenance via telomerase; F:pseudouridine synthase activity; P:mRNA pseudouridine synthesis; P:snoRNA guided rRNA pseudouridine synthesis; F:RNA binding; F:telomerase activity; C:box H/ACA snoRNP complex; P:box H/ACA snoRNA 3'-end processing; P:positive regulation of telomerase activity; F:protein binding; F:telomerase RNA binding; P:positive regulation of telomere maintenance via telomerase; C:viral nucleocapsid; P:telomerase RNA stabilization; F:box H/ACA snoRNA binding |
| Efet.01.227714.g1110.t1 | Ubiquitin carboxyl-terminal hydrolase 34 | 378 | 8.57E-45 | 76.2 | P:protein deubiquitination; C:integral component of membrane; F:thiol-dependent ubiquitinyl hydrolase activity; P:ubiquitin-dependent protein catabolic process |
| Efet.01.227748.g1112.t1 | T-box transcription factor | 363 | 1.57E-49 | 89.85 | P:cell proliferation; P:positive regulation of cardiac muscle cell proliferation; P:pulmonary valve formation; F:RNA polymerase II core promoter proximal region sequence-specific DNA binding; P:endocardial cushion formation; P:transcription from RNA polymerase II promoter; P:tricuspid valve development; F:RNA polymerase II transcription coactivator activity; P:positive regulation of transcription from RNA polymerase II promoter; F:RNA polymerase II activating transcription factor binding; P:dorsal/ventral pattern formation; P:negative regulation of transcription from RNA polymerase II promoter; P:outflow tract septum morphogenesis; F:transcriptional activator activity, RNA polymerase II core promoter proximal region sequence-specific binding; P:neuron migration; P:cardiac chamber formation; P:branching involved in blood vessel morphogenesis; P:muscle contraction; P:embryonic heart tube elongation; P:heart looping; P:endoderm formation; P:negative regulation of SMAD protein complex assembly; P:cardiac muscle tissue morphogenesis; P:aortic valve morphogenesis; P:blood circulation; P:visceral motor neuron differentiation; P:pulmonary vein morphogenesis; P:pericardium morphogenesis; P:atrial septum morphogenesis; C:nucleus; P:cardiac right ventricle morphogenesis; C:cytoplasm; P:foramen ovale closure; P:lateral mesoderm formation |
| Efet.01.22775.g1658.t1 | COP9 signalosome complex subunit 3 | 603 | 2.00E-88 | 84.3 | F:protein binding; C:cytoplasm; P:in utero embryonic development; C:COP9 signalosome; C:nucleoplasm; P:ubiquitin-dependent protein catabolic process |
| Efet.01.227770.g1115.t1 | WD repeat-containing 91 | 402 | 3.80E-42 | 71.7 |  |
| Efet.01.227789.g1117.t1 | hypothetical protein CAPTEDRAFT_197880 | 966 | 1.67E-06 | 50 |  |
| Efet.01.227800.g1118.t1 | UPF0577 KIAA1324-like | 564 | 4.32E-38 | 54.55 | C:membrane; C:integral component of membrane |
| Efet.01.227815.g1119.t1 | dynactin subunit 1-like isoform X11 | 318 | 2.84E-33 | 76.45 | C:dynactin complex; P:transport along microtubule |
| Efet.01.227858.g1121.t1 | rab GTPase-binding effector 1-like | 384 | 8.84E-11 | 64.5 | F:growth factor activity; F:metal ion binding; F:GTPase activator activity; P:endocytosis; P:positive regulation of GTPase activity |
| Efet.01.227861.g1122.t1 | myosin-VIIa isoform X2 | 678 | 1.56E-28 | 52.55 | F:nucleotide binding; C:myosin complex; F:ATP binding; C:cytoskeleton; F:actin binding; F:motor activity |
| Efet.01.22790.g1660.t1 | hypothetical protein HELRODRAFT_162227 | 285 | 4.57E-08 | 58 | F:ATP binding; P:regulation of apoptotic process; F:protein kinase activity; P:protein phosphorylation |
| Efet.01.227917.g1123.t1 | octopamine receptor Oamb-like | 1722 | 9.79E-24 | 55.05 | P:signal transduction; F:signal transducer activity |
| Efet.01.227928.g1124.t1 | inositol phosphorylceramide synthase catalytic subunit aur1-like | 894 | 1.65E-83 | 49.55 | F:inositol phosphoceramide synthase activity; C:inositol phosphoceramide synthase complex; C:membrane; C:integral component of membrane; P:inositolphosphoceramide metabolic process; C:Golgi apparatus; P:sphingolipid biosynthetic process |
| Efet.01.227961.g1125.t1 | puratrophin-1-like isoform X1 | 651 | 1.20E-41 | 77 | F:glycine-tRNA ligase activity; F:ATP binding; P:regulation of Rho protein signal transduction; P:glycyl-tRNA aminoacylation; C:cytoplasm; F:Rho guanyl-nucleotide exchange factor activity; P:positive regulation of GTPase activity |
| Efet.01.228010.g1126.t1 | ZEP1_MOUSE ame: Full=Zinc finger 40 ame: Full=Alpha A-crystallin-binding 1 Short=Alpha A-CRYBP1 ame: Full=Alpha A-crystallin-binding I ame: Full=Transcription factor alphaA-CRYBP1 | 405 | 2.54E-07 | 57 | F:RNA polymerase II distal enhancer sequence-specific DNA binding; P:negative regulation of transcription from RNA polymerase II promoter; C:nucleus; F:HMG box domain binding; C:mitochondrion; F:transcriptional repressor activity, RNA polymerase II distal enhancer sequence-specific binding; P:cellular process |
| Efet.01.22804.g1661.t1 | receptor-type tyrosine- phosphatase | 402 | 2.06E-18 | 58.3 |  |
| Efet.01.228094.g1128.t1 | regulator of nonsense transcripts 1 | 885 | 8.29E-113 | 93.65 | P:DNA repair; C:exon-exon junction complex; P:positive regulation of mRNA catabolic process; P:3'-UTR-mediated mRNA destabilization; C:P-body; C:supraspliceosomal complex; C:nucleoplasm; F:ATP-dependent RNA helicase activity; F:ATP binding; P:nuclear-transcribed mRNA catabolic process, nonsense-mediated decay; F:telomeric DNA binding; P:telomere maintenance via semi-conservative replication; C:cytosol; F:zinc ion binding; P:dosage compensation by inactivation of X chromosome; F:chromatin binding; C:chromatin; C:nuclear chromosome, telomeric region; F:RNA binding; P:mRNA export from nucleus; F:protein binding; P:regulation of translational termination; P:nuclear-transcribed mRNA catabolic process, endonucleolytic cleavage-dependent decay; P:cellular response to lipopolysaccharide; P:histone mRNA catabolic process; P:cellular response to interleukin-1 |
| Efet.01.228094.g1129.t1 | Regulator of nonsense transcripts 1 | 606 | 5.90E-105 | 92.2 | P:DNA repair; C:exon-exon junction complex; P:positive regulation of mRNA catabolic process; P:3'-UTR-mediated mRNA destabilization; C:P-body; C:supraspliceosomal complex; C:nucleoplasm; F:ATP-dependent RNA helicase activity; F:ATP binding; P:nuclear-transcribed mRNA catabolic process, nonsense-mediated decay; F:telomeric DNA binding; P:telomere maintenance via semi-conservative replication; C:cytosol; F:zinc ion binding; P:dosage compensation by inactivation of X chromosome; F:chromatin binding; C:chromatin; C:nuclear chromosome, telomeric region; F:RNA binding; P:mRNA export from nucleus; F:protein binding; P:regulation of translational termination; P:nuclear-transcribed mRNA catabolic process, endonucleolytic cleavage-dependent decay; P:cellular response to lipopolysaccharide; P:histone mRNA catabolic process; P:cellular response to interleukin-1 |
| Efet.01.228168.g1130.t1 | latent-transforming growth factor beta-binding 1 isoform X2 | 396 | 2.41E-17 | 56.7 | C:extracellular matrix; F:binding |
| Efet.01.228194.g1131.t1 | hypothetical protein HELRODRAFT_183479 | 939 | 6.64E-15 | 74.88 | F:nucleic acid binding; F:metal ion binding |
| Efet.01.228195.g1132.t1 | nuclear receptor-binding homolog isoform X1 | 336 | 7.76E-29 | 83.5 | F:ATP binding; F:protein kinase activity; P:protein phosphorylation |
| Efet.01.228204.g1133.t1 | Cephalotocin receptor 1 | 948 | 1.59E-63 | 65.7 | P:regulation of biological quality; F:G-protein coupled peptide receptor activity; P:response to organic substance; P:G-protein coupled receptor signaling pathway; P:response to endogenous stimulus; C:integral component of membrane; P:blood circulation; C:plasma membrane; F:protein-hormone receptor activity |
| Efet.01.228213.g1134.t1 | fukutin-related -like | 528 | 4.00E-16 | 61.7 | C:membrane; C:integral component of membrane |
| Efet.01.228215.g1135.t1 | Lipoxygenase homology domain-containing 1 | 411 | 2.40E-39 | 73.55 | P:detection of mechanical stimulus; F:calcium channel activity; C:membrane |
| Efet.01.228215.g1136.t1 | lipoxygenase homology domain-containing 1-like | 342 | 5.62E-32 | 65.7 |  |
| Efet.01.22823.g1666.t1 | T-box transcription factor | 306 | 4.30E-29 | 76.85 | P:positive regulation of cardiac muscle cell proliferation; P:pulmonary valve formation; F:RNA polymerase II regulatory region sequence-specific DNA binding; P:endocardial cushion formation; P:tricuspid valve development; F:RNA polymerase II transcription coactivator activity; F:RNA polymerase II activating transcription factor binding; P:dorsal/ventral pattern formation; P:negative regulation of transcription from RNA polymerase II promoter; P:outflow tract septum morphogenesis; F:transcription factor activity, sequence-specific DNA binding; P:system process; P:cardiac chamber formation; P:positive regulation of transcription, DNA-templated; P:heart looping; P:endoderm formation; P:cardiac muscle tissue morphogenesis; P:aortic valve morphogenesis; P:transcription, DNA-templated; P:visceral motor neuron differentiation; P:pulmonary vein morphogenesis; P:pericardium morphogenesis; P:atrial septum morphogenesis; C:nucleus; P:cardiac right ventricle morphogenesis; C:cytoplasm; C:membrane; P:foramen ovale closure; P:lateral mesoderm formation |
| Efet.01.228231.g1137.t1 | crumbs isoform X3 | 810 | 5.41E-18 | 48.1 | F:calcium ion binding; C:membrane; C:integral component of membrane |
| Efet.01.228264.g1140.t1 | transcriptional adapter 3-like | 720 | 7.15E-42 | 68.25 |  |
| Efet.01.228284.g1142.t1 | plasma membrane calcium-transporting ATPase 3- | 444 | 9.55E-45 | 73.25 | F:nucleotide binding; C:membrane; P:calcium ion transport; F:ion binding; C:cell part; C:membrane-bounded organelle; F:hydrolase activity |
| Efet.01.228296.g1143.t1 | lipoxygenase homology domain-containing 1-like isoform X2 | 312 | 4.04E-32 | 72.1 | P:sensory perception of sound; C:stereocilium |
| Efet.01.228315.g1144.t1 | ELAV 1 isoform X8 | 381 | 2.95E-51 | 82.8 | F:nucleotide binding; F:RNA binding |
| Efet.01.228341.g1145.t1 | catenin alpha-like | 222 | 1.42E-19 | 81.2 | F:actin filament binding; C:cell-cell adherens junction; P:epithelial cell-cell adhesion; F:cadherin binding; C:actin cytoskeleton; F:structural molecule activity; C:cytosol; P:actin filament organization; P:brain morphogenesis; F:beta-catenin binding |
| Efet.01.228410.g1147.t1 | hypothetical protein CAPTEDRAFT_224991 | 204 | 2.29E-18 | 66.45 | C:membrane |
| Efet.01.228460.g1148.t1 | Location of vulva defective 1 | 1230 | 3.98E-42 | 49.5 | P:detection of mechanical stimulus; F:calcium ion binding; F:calcium channel activity; C:membrane; C:integral component of membrane; P:calcium ion transmembrane transport |
| Efet.01.228505.g1150.t1 | hypothetical protein CAPTEDRAFT_209714 | 207 | 1.35E-21 | 68.5 | C:membrane |
| Efet.01.228573.g1151.t1 | hairy enhancer-of-split related with YRPW motif 1 | 330 | 4.28E-13 | 83.9 | C:nucleus; F:DNA binding; P:transcription, DNA-templated; P:regulation of transcription, DNA-templated; F:protein dimerization activity |
| Efet.01.22865.g1667.t1 | NCAM, partial | 612 | 7.02E-06 | 60 | P:positive regulation of cardiac muscle cell proliferation; C:myelin sheath; C:external side of plasma membrane; P:negative regulation of cell death; P:cell adhesion; F:heparin binding; C:neuronal cell body; P:thalamus development; P:learning or memory; P:axonal fasciculation; C:growth cone; P:positive regulation of calcium-mediated signaling; C:extracellular exosome; P:regulation of synaptic plasticity; P:multicellular organismal response to stress; P:regulation of exocyst assembly; P:homotypic cell-cell adhesion; C:axon; F:phosphatase binding; F:identical protein binding; P:neuron projection development; P:response to cocaine; P:cell surface receptor signaling pathway; P:neuron development; P:aging; C:plasma membrane; C:cell surface; C:cell-cell junction; F:protein binding; C:cytoplasm; P:response to drug; C:membrane; C:integral component of membrane; F:LRR domain binding; P:response to lead ion; P:regulation of sensory perception of pain; C:anchored component of membrane |
| Efet.01.228694.g1153.t1 | beta-1,3-galactosyl-O-glycosyl-glyco beta-1,6-N-acetylglucosaminyltransferase | 840 | 2.34E-12 | 56.05 | F:transferase activity, transferring glycosyl groups; F:acetylglucosaminyltransferase activity; C:membrane; F:transferase activity |
| Efet.01.228715.g1154.t1 | innexin unc-9-like isoform X6 | 309 | 3.50E-32 | 75.5 | C:gap junction; P:ion transport; C:integral component of membrane |
| Efet.01.22873.g1668.t1 | hypothetical protein HELRODRAFT_163906 | 444 | 1.55E-13 | 72.2 | F:nucleic acid binding |
| Efet.01.228778.g1155.t1 | mitochondrial malate dehydrogenase | 276 | 2.57E-40 | 85.05 | P:carbohydrate metabolic process; F:L-malate dehydrogenase activity; P:malate metabolic process; P:tricarboxylic acid cycle |
| Efet.01.228884.g1157.t1 | hypothetical protein LOTGIDRAFT_230536 | 513 | 7.39E-06 | 63 | C:membrane; C:integral component of membrane |
| Efet.01.228888.g1158.t1 | calmodulin 12 | 351 | 4.58E-25 | 69.6 | F:calcium ion binding |
| Efet.01.228899.g1159.t1 | 2-hydroxyacylsphingosine 1-beta-galactosyltransferase-like | 1047 | 4.14E-49 | 46.65 | F:transferase activity |
| Efet.01.228967.g1160.t1 | pleckstrin homology domain-containing family G member 7-like | 1398 | 1.38E-56 | 70.06 | P:regulation of Rho protein signal transduction; F:Rho guanyl-nucleotide exchange factor activity; P:positive regulation of GTPase activity |
| Efet.01.228988.g1161.t1 | tektin-2-like isoform X1 | 717 | 6.73E-69 | 70.55 |  |
| Efet.01.229002.g1162.t1 | netrin-1-like isoform X2 | 570 | 2.61E-60 | 74.1 | P:regulation of axon extension; C:integral component of membrane; P:olfactory bulb axon guidance; C:extracellular region; P:anterior commissure morphogenesis; P:dendrite development |
| Efet.01.229045.g1164.t1 | hypothetical protein CAPTEDRAFT_216159 | 1008 | 1.60E-87 | 52 | C:membrane; C:integral component of membrane |
| Efet.01.229079.g1165.t1 | Thrombospondin- | 288 | 6.20E-49 | 88.75 | F:calcium ion binding; P:cell adhesion; C:extracellular region |
| Efet.01.22919.g1670.t1 | FMRFamide receptor-like | 1044 | 5.60E-112 | 70.85 | F:G-protein coupled receptor activity; P:signal transduction; C:membrane |
| Efet.01.229219.g1168.t1 | Bardet-Biedl syndrome 7 homolog isoform X1 | 1731 | 2.88E-135 | 58.2 | C:organelle; C:cell projection |
| Efet.01.229228.g1169.t1 | Heparan-sulfate 6-O-sulfotransferase | 381 | 8.91E-28 | 62.85 | F:sulfotransferase activity |
| Efet.01.229254.g1172.t1 | DNA helicase MCM9-like | 987 | 6.11E-154 | 84.7 | P:female gamete generation; F:DNA binding; F:ATP binding; P:DNA replication; P:double-strand break repair via homologous recombination; C:MCM8-MCM9 complex |
| Efet.01.229254.g1173.t1 | DNA helicase MCM9 | 477 | 1.42E-47 | 76.3 | F:DNA binding; F:ATP binding; P:DNA replication |
| Efet.01.229264.g1174.t1 | heat shock 75 mitochondrial-like | 489 | 2.20E-60 | 75.45 | F:nucleic acid binding; F:ATP binding; F:unfolded protein binding; P:protein folding; P:response to stress |
| Efet.01.229279.g1175.t1 | Ubiquitin- ligase | 1392 | 5.94E-88 | 59.15 | F:ubiquitin protein ligase activity; F:protein binding; P:protein K48-linked ubiquitination; P:proteolysis; P:positive regulation of protein ubiquitination; P:regulation of protein ubiquitination involved in ubiquitin-dependent protein catabolic process; P:animal organ development; P:regulation of circadian rhythm; C:intracellular part; P:protein autoubiquitination |
| Efet.01.229456.g1177.t1 | exodeoxyribonuclease I | 918 | 5.82E-174 | 88.95 | F:nucleic acid binding; P:DNA repair; P:nucleic acid phosphodiester bond hydrolysis; F:exodeoxyribonuclease I activity |
| Efet.01.229517.g1178.t1 | serine--tRNA mitochondrial-like | 405 | 1.80E-07 | 90 | F:ATP binding; C:cytoplasm; F:serine-tRNA ligase activity; P:seryl-tRNA aminoacylation |
| Efet.01.22955.g1673.t1 | Suppressor of cytokine signaling 6 | 1254 | 3.21E-103 | 86.85 | C:cytoplasm; P:cytokine-mediated signaling pathway; F:protein kinase inhibitor activity; P:negative regulation of protein kinase activity; P:regulation of growth; P:intracellular signal transduction; P:negative regulation of JAK-STAT cascade |
| Efet.01.229555.g1179.t1 | neuroglian-like isoform X2 | 1116 | 1.58E-79 | 51.15 | C:membrane; C:integral component of membrane |
| Efet.01.229587.g1180.t1 | transport Sec24C-like isoform X1 | 261 | 2.67E-37 | 87.4 | F:zinc ion binding; P:ER to Golgi vesicle-mediated transport; P:intracellular protein transport; C:COPII vesicle coat |
| Efet.01.229658.g1182.t1 | one cut domain family member 2-like isoform X1 | 1167 | 1.05E-40 | 88.75 | F:RNA polymerase II core promoter proximal region sequence-specific DNA binding; P:transcription from RNA polymerase II promoter; P:epithelial cell development; P:anatomical structure morphogenesis; P:positive regulation of transcription from RNA polymerase II promoter; P:peripheral nervous system neuron development; C:nucleoplasm; F:transcriptional activator activity, RNA polymerase II core promoter proximal region sequence-specific binding; P:regulation of cell-matrix adhesion; P:liver development; P:cell fate commitment; C:actin cytoskeleton; P:endocrine pancreas development; P:negative regulation of transforming growth factor beta receptor signaling pathway; P:positive regulation of cell migration |
| Efet.01.229666.g1183.t1 | 7SK snRNA methylphosphate capping | 1449 | 7.74E-20 | 76 | F:methyltransferase activity; P:methylation |
| Efet.01.229679.g1185.t1 | myosin heavy striated muscle-like | 684 | 7.26E-26 | 73.5 | F:nucleotide binding; F:anion binding |
| Efet.01.229748.g1187.t1 | solute carrier family 12 member 2-like isoform X2 | 492 | 9.79E-11 | 57.67 | F:cation:chloride symporter activity; P:chloride transmembrane transport; C:integral component of membrane |
| Efet.01.22977.g1675.t1 | hypothetical protein HELRODRAFT_174886 | 615 | 1.87E-08 | 74.5 |  |
| Efet.01.229790.g1189.t1 | CUB and sushi domain-containing 1 | 285 | 2.16E-09 | 58.63 | P:startle response; P:glucose homeostasis; C:membrane; C:integral component of membrane |
| Efet.01.229812.g1191.t1 | inositol-trisphosphate 3-kinase A-like | 489 | 7.40E-40 | 57.35 | F:inositol-1,4,5-trisphosphate 3-kinase activity; P:phosphorylation; F:kinase activity |
| Efet.01.229837.g1192.t1 | max isoform X1 | 225 | 1.10E-08 | 74.2 | C:nucleus; F:transcription factor activity, sequence-specific DNA binding; C:integral component of membrane; P:regulation of transcription, DNA-templated; F:protein dimerization activity |
| Efet.01.229920.g1194.t1 | ecdysoneless homolog | 282 | 2.15E-14 | 84.05 | C:chloroplast; F:protein binding |
| Efet.01.23001.g1676.t1 | COUP transcription factor 2 isoform X3 | 762 | 1.77E-164 | 93.4 |  |
| Efet.01.230033.g1195.t1 | major vault | 390 | 2.53E-44 | 83.2 | P:eye development; P:neuron projection regeneration; P:brain development |
| Efet.01.230071.g1198.t1 | insulin-like growth factor-binding complex acid labile subunit | 1473 | 7.78E-78 | 46.7 | P:Roundabout signaling pathway; F:Roundabout binding; C:extracellular space; P:regulation of axonogenesis; C:membrane; C:integral component of membrane; P:otolith morphogenesis; F:heparin binding; C:proteinaceous extracellular matrix; P:axon extension involved in axon guidance |
| Efet.01.230071.g1199.t1 | leucine-rich repeat and transmembrane domain-containing 1 | 465 | 7.36E-22 | 59.8 | P:regulation of cellular process; C:membrane |
| Efet.01.230086.g1200.t1 | S-phase kinase-associated 2 isoform X1 | 942 | 8.65E-40 | 57.45 | P:phosphorylation; F:kinase activity |
| Efet.01.230096.g1201.t1 | echinoderm microtubule-associated -like CG42247 | 366 | 2.78E-19 | 75.55 | P:sensory perception of sound; P:melanotic encapsulation of foreign target; C:sensory dendrite; P:adult locomotory behavior; C:intracellular; P:intracellular signal transduction; F:microtubule binding |
| Efet.01.230121.g1202.t1 | chloride intracellular channel 2-like | 768 | 4.17E-131 | 66.9 | P:chloride transmembrane transport; F:chloride channel activity; P:negative regulation of ryanodine-sensitive calcium-release channel activity |
| Efet.01.230142.g1203.t1 | zinc finger 157-like isoform X1 | 1227 | 1.05E-11 | 45.2 | F:nucleic acid binding; C:nucleus; F:zinc ion binding; F:metal ion binding |
| Efet.01.230171.g1205.t1 | Tyrosinase domain containing | 402 | 1.88E-08 | 54.44 | F:metal ion binding; F:oxidoreductase activity; P:metabolic process; P:oxidation-reduction process |
| Efet.01.230226.g1206.t1 | cilia- and flagella-associated 58-like | 369 | 9.16E-07 | 67 |  |
| Efet.01.230369.g1208.t1 | neuronal calcium sensor 2-like | 234 | 3.46E-24 | 80.3 | F:calcium ion binding |
| Efet.01.230377.g1209.t1 | tyrosine- kinase SRK2-like isoform X2 | 615 | 1.17E-87 | 76 | F:ATP binding; P:peptidyl-tyrosine phosphorylation; F:non-membrane spanning protein tyrosine kinase activity; C:integral component of membrane |
| Efet.01.23041.g1679.t1 | PR domain zinc finger 14-like | 321 | 7.19E-30 | 76.45 | F:nucleic acid binding; F:metal ion binding |
| Efet.01.230423.g1210.t1 | tensin-3- | 393 | 1.62E-40 | 81.55 |  |
| Efet.01.23045.g1680.t1 | microtubule-actin cross-linking factor 1-like | 690 | 1.24E-15 | 48 | P:cell cycle arrest; C:cytoskeleton; F:calcium ion binding |
| Efet.01.230524.g1211.t1 | solute carrier organic anion transporter family member 4A1 | 261 | 1.44E-10 | 65.9 | P:ion transport; C:integral component of membrane; C:plasma membrane; F:transporter activity |
| Efet.01.230560.g1212.t1 | plasma membrane calcium-transporting ATPase 2 isoform X1 | 345 | 3.84E-25 | 81.55 | F:ATP binding; F:metal ion binding; C:integral component of membrane; P:calcium ion transmembrane transport; F:calcium-transporting ATPase activity |
| Efet.01.23060.g1681.t1 | nesprin-1-like isoform X1 | 642 | 1.31E-25 | 51.8 | C:nuclear envelope; F:actin filament binding; F:actin binding; P:cytoskeletal anchoring at nuclear membrane; C:LINC complex |
| Efet.01.230653.g1214.t1 | probable signal peptidase complex subunit 2 isoform X1 | 339 | 2.95E-18 | 73.5 | C:integral component of membrane; P:signal peptide processing; F:peptidase activity; C:signal peptidase complex |
| Efet.01.23072.g1682.t1 | growth arrest-specific 8-like | 282 | 1.32E-15 | 77.45 | P:cell motility; C:motile cilium |
| Efet.01.230751.g1217.t1 | hypothetical protein CAPTEDRAFT_130757 | 393 | 1.21E-11 | 68 |  |
| Efet.01.230803.g1219.t1 | desert hedgehog | 567 | 3.29E-27 | 54.6 | P:multicellular organism development; P:single-organism cellular process; F:binding |
| Efet.01.230816.g1220.t1 | alpha-catulin-like isoform X2 | 393 | 9.36E-41 | 60.8 | F:actin filament binding; F:cadherin binding; P:cell adhesion; P:Rho protein signal transduction; C:intracellular |
| Efet.01.230832.g1221.t1 | methenyltetrahydrofolate synthase domain-containing | 465 | 4.05E-31 | 63.85 | F:organic cyclic compound binding; F:heterocyclic compound binding |
| Efet.01.230836.g1222.t1 | Vesicular glutamate transporter | 333 | 1.22E-22 | 56.7 | P:ion transport; F:transmembrane transporter activity; C:neuron part; C:membrane; C:cytoplasmic vesicle; C:synapse |
| Efet.01.230978.g1225.t1 | F-actin-methionine sulfoxide oxidase MICAL3-like isoform X4 | 2610 | 1.10E-112 | 46.3 | F:zinc ion binding; F:metal ion binding |
| Efet.01.231.g29.t1 | hypothetical protein CAPTEDRAFT_193290 | 1611 | 9.48E-48 | 50 |  |
| Efet.01.231053.g1227.t1 | golgin subfamily A member 3-like | 2706 | 1.12E-70 | 60.9 | C:extrinsic component of Golgi membrane; P:spermatogenesis |
| Efet.01.231113.g1229.t1 | allatostatin-A receptor | 1293 | 7.28E-60 | 55 | P:signal transduction; F:signal transducer activity |
| Efet.01.231126.g1230.t1 | tyrosine- phosphatase non-receptor type 11-like isoform X2 | 447 | 1.77E-25 | 60.2 |  |
| Efet.01.23113.g1683.t1 | myosin heavy non-muscle-like isoform X1 | 222 | 4.44E-16 | 75.3 | F:binding |
| Efet.01.231136.g1231.t1 | leucine-rich repeats and immunoglobulin-like domains 3 | 1521 | 2.84E-45 | 46.6 | C:membrane; C:integral component of membrane |
| Efet.01.231146.g1232.t1 | vesicle transport through interaction with t-SNAREs homolog 1B-like | 249 | 2.70E-17 | 78.55 | P:cell proliferation; C:synaptic vesicle; C:Golgi apparatus; C:neuronal cell body; P:protein targeting to vacuole; C:endoplasmic reticulum membrane; C:cytosol; P:vesicle fusion with Golgi apparatus; F:chloride channel inhibitor activity; P:regulation of protein localization to plasma membrane; F:SNAP receptor activity; P:intra-Golgi vesicle-mediated transport; F:SNARE binding; P:retrograde transport, endosome to Golgi; C:lysosomal membrane; P:Golgi to vacuole transport; C:late endosome membrane; P:autophagosome maturation; P:ER to Golgi vesicle-mediated transport; C:perinuclear region of cytoplasm; C:ER to Golgi transport vesicle membrane; P:vesicle docking involved in exocytosis; C:integral component of membrane; C:SNARE complex; C:recycling endosome |
| Efet.01.231187.g1233.t1 | sperm flagellar 2 isoform X5 | 219 | 2.98E-17 | 84.95 | P:respiratory system development; C:sperm midpiece; P:spermatogenesis; P:multicellular organism aging; P:embryonic neurocranium morphogenesis; P:fertilization; C:Golgi apparatus; P:axoneme assembly; P:brain morphogenesis; C:manchette; P:immune system development |
| Efet.01.231203.g1234.t1 | adenylate cyclase type 5-like | 330 | 1.26E-28 | 78.5 | F:nucleotide binding; P:cAMP biosynthetic process; C:integral component of membrane; C:intracellular; C:plasma membrane; F:adenylate cyclase activity; P:intracellular signal transduction |
| Efet.01.231215.g1235.t1 | ras-related Rab-3 isoform X4 | 336 | 1.89E-40 | 94.6 | F:GTP binding; P:small GTPase mediated signal transduction; C:intracellular |
| Efet.01.231238.g1236.t1 | transitional endoplasmic reticulum ATPase- | 210 | 2.52E-14 | 94.15 | F:ATPase activity; P:positive regulation of ATP biosynthetic process; P:positive regulation of Lys63-specific deubiquitinase activity; P:flavin adenine dinucleotide catabolic process; C:cytosol; P:aggresome assembly; P:protein N-linked glycosylation via asparagine; P:activation of cysteine-type endopeptidase activity involved in apoptotic process; F:protein domain specific binding; F:protein phosphatase binding; P:protein homooligomerization; C:proteasome complex; F:ubiquitin-specific protease binding; P:ER to Golgi vesicle-mediated transport; C:VCP-NSFL1C complex; F:ubiquitin protein ligase binding; P:ATP metabolic process; P:viral genome replication; P:protein hexamerization; P:retrograde protein transport, ER to cytosol; P:positive regulation of proteasomal ubiquitin-dependent protein catabolic process; F:K48-linked polyubiquitin binding; C:Derlin-1 retrotranslocation complex; P:protein ubiquitination; P:chordate embryonic development; C:site of double-strand break; C:myelin sheath; P:positive regulation of oxidative phosphorylation; C:VCP-NPL4-UFD1 AAA ATPase complex; F:lipid binding; C:nucleoplasm; P:double-strand break repair; F:ATP binding; P:NADH metabolic process; P:positive regulation of mitochondrial membrane potential; C:extracellular exosome; P:locomotory behavior; P:endoplasmic reticulum unfolded protein response; P:regulation of protein ubiquitination involved in ubiquitin-dependent protein catabolic process; P:positive regulation of protein complex assembly; P:ER-associated misfolded protein catabolic process; P:error-free translesion synthesis; F:identical protein binding; F:BAT3 complex binding; F:MHC class I protein binding; F:ADP binding; P:regulation of aerobic respiration; F:RNA binding; C:lipid particle; C:perinuclear region of cytoplasm; C:integral component of membrane; F:deubiquitinase activator activity |
| Efet.01.231278.g1237.t1 | transportin-1-like isoform X2 | 873 | 2.70E-152 | 82.95 | C:nuclear membrane; C:cytoplasm; P:protein import into nucleus, translocation; P:NLS-bearing protein import into nucleus; F:protein transporter activity; C:nuclear periphery; F:Ran GTPase binding; P:protein import into nucleus, docking; F:nuclear localization sequence binding; P:ribosomal protein import into nucleus |
| Efet.01.231332.g1240.t1 | ER lumen -retaining receptor 2 isoform X1 | 312 | 4.96E-47 | 91.5 | C:endoplasmic reticulum membrane; C:cis-Golgi network; P:vesicle-mediated transport; P:protein transport; C:integral component of membrane; F:ER retention sequence binding; C:COPI-coated vesicle membrane; C:endoplasmic reticulum-Golgi intermediate compartment membrane; P:protein retention in ER lumen |
| Efet.01.231350.g1241.t1 | chitin synthase B | 615 | 2.11E-34 | 67.5 | C:membrane |
| Efet.01.231351.g1242.t1 | V-type proton ATPase 116 kDa subunit a | 246 | 3.29E-31 | 75.15 | C:vacuolar proton-transporting V-type ATPase, V0 domain; F:hydrogen ion transmembrane transporter activity; P:ATP hydrolysis coupled proton transport; C:integral component of membrane |
| Efet.01.231356.g1243.t1 | phytanoyl- hydroxylase-interacting -like | 1119 | 2.62E-28 | 54.1 |  |
| Efet.01.231384.g1245.t1 | Krueppel-like factor 11 | 1380 | 1.83E-09 | 64.43 | F:nucleic acid binding; F:metal ion binding |
| Efet.01.2314.g206.t1 | fibroblast growth factor receptor 2-like | 621 | 9.34E-60 | 73.8 | F:ATP binding; P:peptidyl-tyrosine phosphorylation; F:protein tyrosine kinase activity; C:integral component of membrane |
| Efet.01.231419.g1246.t1 | E3 ubiquitin- ligase UBR2 isoform X2 | 201 | 4.60E-13 | 77.2 | F:zinc ion binding; F:catalytic activity; P:protein catabolic process; P:cellular protein metabolic process; P:single-organism cellular process; P:negative regulation of cellular process; P:chromatin organization; C:intracellular organelle part; P:single organism reproductive process |
| Efet.01.231434.g1247.t1 | immunoglobulin superfamily member | 525 | 1.44E-28 | 59.95 | C:membrane; C:integral component of membrane |
| Efet.01.231464.g1249.t1 | fumarate hydratase class | 522 | 4.84E-108 | 91.7 | P:generation of precursor metabolites and energy; F:fumarate hydratase activity |
| Efet.01.231522.g1252.t1 | hypothetical protein CAPTEDRAFT_222844 | 3513 | 9.14E-15 | 45 | F:metal ion binding |
| Efet.01.231545.g1254.t1 | kinase D-interacting substrate of 220 kDa-like isoform X1 | 609 | 6.53E-14 | 66.75 |  |
| Efet.01.231594.g1257.t1 | unc-13 homolog D-like isoform X3 | 705 | 1.52E-36 | 54.65 |  |
| Efet.01.231613.g1259.t1 | high affinity cAMP-specific and IBMX-insensitive 3 ,5 -cyclic phosphodiesterase 8A | 219 | 1.06E-06 | 55.8 | P:signal transduction; F:metal ion binding; P:regulation of transcription, DNA-templated; F:phosphoric diester hydrolase activity; F:3',5'-cyclic-nucleotide phosphodiesterase activity; F:hydrolase activity |
| Efet.01.231624.g1260.t1 | Collagen alpha-5(VI) chain | 621 | 4.08E-36 | 57.95 | F:calcium ion binding; C:collagen trimer |
| Efet.01.231632.g1261.t1 | intraflagellar transport 122 homolog | 555 | 9.43E-50 | 76.95 | P:neural tube development; P:cilium assembly; P:smoothened signaling pathway; P:embryonic heart tube development; C:intracellular part; P:negative regulation of smoothened signaling pathway; P:protein localization to cilium; P:dorsal/ventral pattern formation; P:embryonic limb morphogenesis; C:motile cilium; C:ciliary part; C:membrane; C:intraciliary transport particle A; P:intraciliary retrograde transport |
| Efet.01.231687.g1263.t1 | growth differentiation factor 3 | 594 | 1.67E-24 | 51.75 | P:telencephalon development; P:hemopoiesis; P:positive regulation of cell proliferation; P:cell cycle process; P:negative regulation of cell cycle; P:negative regulation of steroid hormone biosynthetic process; P:ovulation cycle process; P:female gonad development; P:odontogenesis; P:epithelium development; P:protein phosphorylation; P:positive regulation of transcription from RNA polymerase II promoter; P:cardiac muscle tissue development; P:heart morphogenesis; P:negative regulation of transcription, DNA-templated; P:negative regulation of cell differentiation; P:BMP signaling pathway; P:regulation of follicle-stimulating hormone secretion; P:negative regulation of multicellular organismal process; P:formation of primary germ layer; P:muscle structure development; P:tissue morphogenesis; P:bone mineralization; P:cardiocyte differentiation; P:neuron differentiation; P:cell-cell signaling; C:cell part; P:positive regulation of cell differentiation; C:protein complex; P:positive regulation of MAPK cascade; P:regulation of myeloid cell differentiation; P:response to stress; P:positive regulation of ossification; C:extracellular space; P:positive regulation of catalytic activity; P:positive regulation of apoptotic process; P:epithelial to mesenchymal transition; P:regulation of ketone biosynthetic process; F:transmembrane receptor protein serine/threonine kinase binding; P:negative regulation of Wnt signaling pathway; P:regulation of organ morphogenesis; P:cartilage development |
| Efet.01.2317.g207.t1 | hypothetical protein HELRODRAFT_178970 | 393 | 5.05E-14 | 65 |  |
| Efet.01.231715.g1266.t1 | engrailed | 312 | 7.06E-48 | 87.25 | P:apoptotic process involved in morphogenesis; C:nucleus; P:midbrain development; P:midbrain-hindbrain boundary morphogenesis; P:regulation of transcription, DNA-templated; F:sequence-specific DNA binding; P:cell fate specification |
| Efet.01.231723.g1267.t1 | DNA replication licensing factor mcm7- | 297 | 9.58E-55 | 96.55 | F:ATPase activity; P:DNA replication initiation; P:regulation of DNA-dependent DNA replication initiation; F:chromatin binding; C:chromatin; C:MCM complex; P:DNA unwinding involved in DNA replication; P:cell cycle; C:nucleus; F:DNA binding; F:ATP binding; F:DNA helicase activity; F:protein binding; F:metal ion binding |
| Efet.01.231881.g1270.t1 | voltage-dependent calcium channel subunit alpha-2 delta-3-like | 666 | 9.73E-09 | 46.27 | C:membrane; C:integral component of membrane |
| Efet.01.231930.g1271.t1 | hypothetical protein CAPTEDRAFT_225483 | 240 | 2.35E-11 | 56.2 | C:membrane; C:integral component of membrane; F:carbohydrate binding |
| Efet.01.231993.g1273.t1 | hypothetical protein CAPTEDRAFT_198762 | 783 | 4.99E-19 | 66.4 | F:calcium-activated potassium channel activity; P:potassium ion transport; P:potassium ion transmembrane transport; C:membrane; C:integral component of membrane |
| Efet.01.2320.g208.t1 | ATP-binding cassette sub-family A member 2 | 537 | 5.94E-42 | 82.9 | P:negative regulation of beta-amyloid formation; P:cholesterol metabolic process; C:Golgi apparatus; P:positive regulation of phospholipid efflux; C:phagocytic cup; P:chloride transport; P:memory; F:ATP binding; C:endosome; P:positive regulation of cholesterol efflux; C:ruffle membrane; P:protein localization to nucleus; F:ATPase activity, coupled to transmembrane movement of substances; P:high-density lipoprotein particle assembly; P:apolipoprotein A-I-mediated signaling pathway; P:transmembrane transport; F:apolipoprotein A-I receptor activity; P:positive regulation of engulfment of apoptotic cell; P:regulation of intracellular cholesterol transport; P:positive regulation of beta-amyloid clearance; F:phospholipid-translocating ATPase activity; C:lysosomal membrane; P:negative regulation of amyloid precursor protein biosynthetic process; C:cell surface; P:regulation of transcription from RNA polymerase II promoter; F:ion channel activity; C:microtubule organizing center; P:positive regulation of ERK1 and ERK2 cascade; C:integral component of membrane; P:cholesterol efflux; P:peptide cross-linking; P:phospholipid efflux |
| Efet.01.23201.g1685.t1 | A disintegrin and metallo ase with thrombospondin motifs 16 | 216 | 1.42E-15 | 66.2 | P:macromolecule metabolic process; C:membrane; F:metallopeptidase activity |
| Efet.01.232020.g1274.t1 | dnaJ homolog subfamily C member 16-like | 459 | 1.04E-23 | 52.5 | C:cell; C:membrane; C:integral component of membrane; P:cell redox homeostasis |
| Efet.01.232025.g1275.t1 | Casein kinase I isoform gamma-3 | 423 | 1.06E-81 | 93.05 | P:regulation of cell shape; C:nucleus; F:ATP binding; C:cytoplasm; C:integral component of membrane; P:Wnt signaling pathway; F:protein serine/threonine kinase activity; P:endocytosis; P:peptidyl-serine phosphorylation |
| Efet.01.232124.g1276.t1 | somatostatin receptor type 3-like | 987 | 1.25E-10 | 49 | F:G-protein coupled receptor activity; P:G-protein coupled receptor signaling pathway; P:signal transduction; C:membrane; C:integral component of membrane; F:signal transducer activity |
| Efet.01.232163.g1278.t1 | Red | 945 | 3.92E-92 | 63.35 | C:nucleus |
| Efet.01.232238.g1280.t1 | tolloid 2 | 438 | 5.18E-18 | 50.4 | C:cytoplasm; C:membrane-bounded organelle |
| Efet.01.232250.g1282.t1 | anosmin-1 | 468 | 8.99E-33 | 60.85 | F:peptidase inhibitor activity; C:extracellular region; P:negative regulation of peptidase activity |
| Efet.01.23226.g1686.t1 | tyrosine phosphatase type IVA 1 | 408 | 4.94E-64 | 81.45 | P:protein dephosphorylation; F:protein tyrosine/serine/threonine phosphatase activity |
| Efet.01.232319.g1284.t1 | spectrin alpha non-erythrocytic 1-like isoform X1 | 975 | 1.20E-55 | 60.8 | F:actin binding; F:phospholipid binding |
| Efet.01.232364.g1285.t1 | serine arginine-rich splicing factor 7-like | 552 | 4.20E-56 | 74.65 | F:nucleotide binding; F:zinc ion binding; P:RNA splicing; C:extracellular exosome; F:protein binding; P:negative regulation of mRNA splicing, via spliceosome; P:transport; C:nucleoplasm; F:RNA binding |
| Efet.01.232471.g1288.t1 | frizzled1 2 7 | 1068 | 2.63E-103 | 64.95 | F:G-protein coupled receptor activity; F:Wnt-activated receptor activity; C:integral component of membrane; P:canonical Wnt signaling pathway; F:Wnt-protein binding; P:non-canonical Wnt signaling pathway |
| Efet.01.2325.g209.t1 | plexin-B | 681 | 4.92E-77 | 57.95 | P:biological regulation; P:single-organism process; P:cellular process; P:response to stimulus |
| Efet.01.232533.g1289.t1 | hypothetical protein CAPTEDRAFT_220096 | 1926 | 4.83E-08 | 73 | F:metal ion binding |
| Efet.01.2326.g210.t1 | monocarboxylate transporter 5 | 543 | 3.71E-27 | 74 | P:monocarboxylic acid transport; C:integral component of membrane; F:monocarboxylic acid transmembrane transporter activity |
| Efet.01.232700.g1294.t1 | potassium channel subfamily K member 12-like | 378 | 2.76E-38 | 65.15 | P:potassium ion transmembrane transport; C:integral component of membrane; F:potassium channel activity |
| Efet.01.232739.g1297.t1 | gamma-aminobutyric acid receptor alpha-like | 339 | 2.49E-17 | 78.1 | P:signal transduction; P:ion transmembrane transport; C:integral component of membrane; F:GABA-A receptor activity; C:cell junction; F:extracellular ligand-gated ion channel activity; C:synapse; C:plasma membrane |
| Efet.01.232754.g1298.t1 | influenza virus NS1A-binding homolog isoform X1 | 300 | 4.21E-16 | 67.2 | F:ubiquitin-protein transferase activity; C:Cul3-RING ubiquitin ligase complex; P:protein ubiquitination |
| Efet.01.232781.g1300.t1 | zinc finger 501-like | 1350 | 2.67E-19 | 64 | F:binding |
| Efet.01.232820.g1302.t1 | dopamine D2-like receptor | 387 | 3.51E-26 | 71.15 | F:dopamine neurotransmitter receptor activity, coupled via Gs; P:synaptic transmission, dopaminergic; P:G-protein coupled receptor signaling pathway; F:dopamine binding; C:integral component of membrane |
| Efet.01.232893.g1303.t1 | Ephrin type-A receptor | 546 | 2.01E-94 | 74.05 | F:nucleotide binding; F:protein tyrosine kinase activity; C:integral component of membrane; P:transmembrane receptor protein tyrosine kinase signaling pathway; P:protein phosphorylation |
| Efet.01.232930.g1304.t1 | follicular epithelium yolk subunit | 870 | 7.82E-87 | 66.1 |  |
| Efet.01.232948.g1305.t1 | hypothetical protein CAPTEDRAFT_223749 | 282 | 2.32E-18 | 57.5 | C:membrane; C:integral component of membrane |
| Efet.01.232962.g1306.t1 | Transcription initiation factor TFIID subunit | 246 | 7.70E-15 | 64.5 | C:membrane |
| Efet.01.232969.g1307.t1 | kelch 26 | 978 | 5.83E-98 | 69 | P:cell division; P:cell cycle process; C:cell part |
| Efet.01.232969.g1308.t1 | kelch 31 | 639 | 5.22E-85 | 62.15 | P:skeletal muscle tissue development; C:membrane; C:integral component of membrane; P:regulation of transcription, DNA-templated |
| Efet.01.232994.g1310.t1 | helicase with zinc finger domain 2-like isoform X1 | 285 | 6.05E-17 | 65.35 | F:metal ion binding; F:helicase activity |
| Efet.01.233069.g1312.t1 | Transmembrane 2 | 1071 | 2.93E-27 | 51.86 | F:DNA binding; C:nucleus; F:transcription factor activity, sequence-specific DNA binding; C:integral component of membrane; P:regulation of transcription, DNA-templated; F:sequence-specific DNA binding |
| Efet.01.233212.g1314.t1 | sugar ABC transporter substrate-binding | 408 | 1.34E-78 | 88.6 |  |
| Efet.01.23322.g1691.t1 | glutathione S-transferase family | 549 | 8.95E-27 | 73.8 | F:transferase activity |
| Efet.01.233319.g1315.t1 | 60S ribosomal L7 | 258 | 1.02E-23 | 72.95 | P:maturation of LSU-rRNA from tricistronic rRNA transcript (SSU-rRNA, 5.8S rRNA, LSU-rRNA); F:structural constituent of ribosome; P:cytoplasmic translation; C:cytosolic large ribosomal subunit; F:RNA binding |
| Efet.01.233410.g1316.t1 | BMA-KETN- isoform | 510 | 1.08E-30 | 49.25 |  |
| Efet.01.233474.g1318.t1 | girdin-like isoform X3 | 2568 | 3.19E-29 | 47.4 |  |
| Efet.01.233513.g1320.t1 | FMRFamide receptor-like | 1248 | 6.06E-51 | 50.7 | F:G-protein coupled receptor activity; P:G-protein coupled receptor signaling pathway; P:signal transduction; C:membrane; C:integral component of membrane; F:signal transducer activity |
| Efet.01.233538.g1321.t1 | hypothetical protein HELRODRAFT_181039 | 282 | 1.17E-30 | 63.6 | C:membrane; C:integral component of membrane; F:carbohydrate binding |
| Efet.01.233582.g1322.t1 | THAP domain-containing | 585 | 1.35E-60 | 70.55 | F:nucleic acid binding; F:transposase activity; P:transposition, DNA-mediated |
| Efet.01.23360.g1692.t1 | otopetrin-2-like isoform X1 | 1341 | 2.41E-79 | 47.95 | C:membrane; C:integral component of membrane |
| Efet.01.23372.g1693.t1 | sodium- and chloride-dependent glycine transporter 1-like isoform X1 | 234 | 3.14E-12 | 76.25 | P:neurotransmitter transport; F:neurotransmitter:sodium symporter activity; P:transmembrane transport; C:integral component of membrane |
| Efet.01.233779.g1327.t1 | tolloid 1 | 378 | 1.60E-27 | 59.25 | F:hydrolase activity |
| Efet.01.233781.g1328.t1 | cytochrome P450 2J2-like | 516 | 8.11E-22 | 62.85 | F:binding; F:monooxygenase activity |
| Efet.01.233789.g1329.t1 | serine threonine- phosphatase with EF-hands 2-like | 285 | 3.42E-18 | 76.25 | F:phosphoprotein phosphatase activity; P:detection of stimulus involved in sensory perception; F:iron ion binding; F:calcium ion binding; P:protein dephosphorylation; F:manganese ion binding |
| Efet.01.23383.g1694.t1 | myosin heavy striated muscle | 399 | 2.90E-47 | 82.6 | C:myosin complex; F:ATP binding; F:actin binding; F:motor activity |
| Efet.01.233855.g1330.t1 | Kyphoscoliosis peptidase | 390 | 1.16E-21 | 60.55 | P:regulation of apoptotic process |
| Efet.01.233871.g1331.t1 | hypothetical protein CAPTEDRAFT_167979 | 654 | 4.18E-13 | 75 | F:cysteine-type peptidase activity |
| Efet.01.233888.g1332.t1 | hypothetical protein HELRODRAFT_194228 | 846 | 5.69E-16 | 56 |  |
| Efet.01.233971.g1333.t1 | transcription factor mitochondrial | 495 | 2.31E-32 | 60.71 |  |
| Efet.01.233977.g1335.t1 | sarcoplasmic calcium-binding -like | 228 | 2.55E-12 | 65.09 | F:calcium ion binding |
| Efet.01.233990.g1336.t1 | neurexin-1 isoform X2 | 762 | 7.55E-64 | 61.05 |  |
| Efet.01.234082.g1339.t1 | 5 -AMP-activated kinase subunit gamma-2 isoform X6 | 483 | 2.74E-35 | 55.5 | P:phosphorylation; F:kinase activity |
| Efet.01.234090.g1340.t1 | G -activated inward rectifier potassium channel 3-like | 1473 | 6.02E-117 | 66.1 | P:ion transport |
| Efet.01.234109.g1343.t1 | centrosomal of 83 kDa-like | 750 | 5.17E-19 | 59.89 |  |
| Efet.01.234116.g1345.t1 | titin isoform X45 | 294 | 1.96E-10 | 57.05 |  |
| Efet.01.234126.g1346.t1 | alpha-1A adrenergic receptor-like | 294 | 9.75E-07 | 60 | F:G-protein coupled receptor activity; P:G-protein coupled receptor signaling pathway; C:integral component of membrane |
| Efet.01.234175.g1347.t1 | Ras-related Rab-33 | 405 | 7.00E-44 | 86.95 | F:GTP binding; F:sphingomyelin phosphodiesterase activity; P:small GTPase mediated signal transduction; P:regulation of autophagosome assembly; C:intracellular |
| Efet.01.234259.g1348.t1 | hypothetical protein HELRODRAFT_161987 | 915 | 5.74E-07 | 69 |  |
| Efet.01.234295.g1349.t1 | dual specificity phosphatase 22-A-like | 816 | 1.11E-24 | 61.55 | F:phosphatase activity; P:dephosphorylation |
| Efet.01.234304.g1350.t1 | ras-related Rab-27A | 468 | 5.45E-86 | 87.75 | P:multivesicular body organization; P:natural killer cell degranulation; C:Golgi apparatus; P:blood coagulation; C:photoreceptor outer segment; P:positive regulation of regulated secretory pathway; P:positive regulation of reactive oxygen species biosynthetic process; C:exocytic vesicle; F:GTP binding; C:dendrite; C:extracellular exosome; F:GTPase activity; F:GDP binding; P:antigen processing and presentation; C:multivesicular body membrane; P:melanosome transport; P:small GTPase mediated signal transduction; C:secretory granule; P:exosomal secretion; P:positive regulation of constitutive secretory pathway; P:cytotoxic T cell degranulation; C:lysosome; P:protein targeting; P:melanocyte differentiation; P:complement-dependent cytotoxicity; C:melanosome; P:positive regulation of gene expression; P:multivesicular body sorting pathway; P:positive regulation of phagocytosis; F:myosin V binding |
| Efet.01.234335.g1352.t1 | potassium voltage-gated channel Shaker-like | 1008 | 1.29E-118 | 79.45 | P:potassium ion transmembrane transport; C:voltage-gated potassium channel complex; F:delayed rectifier potassium channel activity; P:regulation of ion transmembrane transport; P:protein homooligomerization |
| Efet.01.234348.g1353.t1 | outer membrane | 2400 | 0 | 64.95 |  |
| Efet.01.234348.g1354.t1 | outer membrane | 1959 | 0 | 54.15 |  |
| Efet.01.23437.g1696.t1 | hypothetical protein HELRODRAFT_163113 | 426 | 9.68E-21 | 60.45 | C:membrane; C:integral component of membrane; F:carbohydrate binding |
| Efet.01.234379.g1355.t1 | probable elongator complex 3 | 453 | 4.52E-53 | 90.8 | F:iron-sulfur cluster binding; F:N-acetyltransferase activity |
| Efet.01.23438.g1697.t1 | hypothetical protein HELRODRAFT_160496 | 444 | 1.71E-15 | 62 | P:signal transduction |
| Efet.01.234414.g1359.t1 | discoidin domain-containing receptor 2-like isoform X1 | 504 | 1.10E-40 | 57.6 | F:ATP binding; P:peptidyl-tyrosine phosphorylation; C:integral component of plasma membrane; F:protein tyrosine kinase activity; C:membrane; C:integral component of membrane; F:protein tyrosine kinase collagen receptor activity; F:protein kinase activity; P:collagen-activated tyrosine kinase receptor signaling pathway; P:protein phosphorylation |
| Efet.01.234430.g1360.t1 | tiptop | 4437 | 4.44E-27 | 65.2 | P:regulation of gene expression |
| Efet.01.234439.g1361.t1 | UDP-glucuronosyltransferase 1-2 | 546 | 2.63E-24 | 49.95 | F:transferase activity |
| Efet.01.23444.g1698.t1 | E3 ubiquitin- ligase synoviolin B-like | 471 | 1.34E-31 | 88.85 | F:ubiquitin protein ligase activity involved in ERAD pathway; F:zinc ion binding; P:endoplasmic reticulum unfolded protein response; C:Hrd1p ubiquitin ligase complex; P:protein polyubiquitination; C:integral component of membrane; C:endoplasmic reticulum quality control compartment; F:unfolded protein binding; F:ubiquitin-specific protease binding; P:ER-associated ubiquitin-dependent protein catabolic process; C:Derlin-1 retrotranslocation complex; P:protein ubiquitination involved in ubiquitin-dependent protein catabolic process |
| Efet.01.234465.g1362.t1 | vacuolar sorting-associated 8 homolog | 1776 | 4.75E-113 | 52.55 | F:zinc ion binding; P:vesicle-mediated transport; F:metal ion binding; P:intracellular protein transport; C:intracellular |
| Efet.01.23454.g1699.t1 | regulator of G- signaling 22-like isoform X1 | 450 | 7.95E-28 | 69.05 | C:cytoplasm; F:GTPase activator activity; C:plasma membrane |
| Efet.01.234544.g1364.t1 | nesprin-1-like isoform X1 | 1536 | 9.63E-35 | 51.5 | C:nuclear envelope; F:actin filament binding; C:membrane; C:integral component of membrane; P:cytoskeletal anchoring at nuclear membrane; C:LINC complex |
| Efet.01.234586.g1366.t1 | g1 s-specific cyclin-d2 | 222 | 2.63E-15 | 70.35 | C:nucleus; P:cell cycle |
| Efet.01.234626.g1369.t1 | Chondroitin sulfate synthase 1 | 714 | 2.51E-49 | 61.55 | P:chondroitin sulfate biosynthetic process; F:UDP-glycosyltransferase activity; F:transferase activity, transferring hexosyl groups; C:membrane; P:positive regulation of smoothened signaling pathway; P:bone morphogenesis; P:chondrocyte development; P:sulfation; P:proximal/distal pattern formation |
| Efet.01.234637.g1370.t1 | axoneme-associated mst101(2)-like | 1515 | 2.32E-18 | 49.1 | P:RNA phosphodiester bond hydrolysis, endonucleolytic; F:nucleic acid binding; F:RNA-DNA hybrid ribonuclease activity; C:membrane; C:integral component of membrane |
| Efet.01.234683.g1371.t1 | phosphatase inhibitor 2-like | 507 | 2.92E-23 | 58.35 | F:protein phosphatase inhibitor activity; P:negative regulation of catalytic activity; P:regulation of phosphoprotein phosphatase activity; P:regulation of signal transduction |
| Efet.01.234778.g1373.t1 | huntingtin isoform X2 | 408 | 5.24E-22 | 69.35 | C:intracellular part |
| Efet.01.234778.g1374.t1 | Huntingtin | 669 | 1.06E-28 | 60.5 | C:intracellular part |
| Efet.01.23481.g1700.t1 | PREDICTED: uncharacterized protein LOC106153557 | 363 | 1.76E-07 | 78.5 |  |
| Efet.01.23482.g1701.t1 | hypothetical protein CAPTEDRAFT_185637 | 1293 | 5.21E-37 | 45 | C:membrane; C:integral component of membrane |
| Efet.01.234888.g1379.t1 | hypothetical protein CAPTEDRAFT_200575 | 1659 | 4.69E-134 | 52.45 | C:membrane; C:integral component of membrane |
| Efet.01.234900.g1380.t1 | pre-B-cell leukemia transcription factor 1-like isoform X3 | 381 | 3.16E-63 | 91.2 | C:nucleus; F:transcription factor activity, sequence-specific DNA binding; C:integral component of membrane; P:regulation of transcription, DNA-templated; F:sequence-specific DNA binding |
| Efet.01.234970.g1381.t1 | Glutamine-rich 2 | 621 | 1.88E-15 | 50.45 |  |
| Efet.01.234974.g1382.t1 | antimicrobial-like peptide PP-1 | 228 | 9.23E-20 | 76.91 | P:regulation of muscle contraction; P:killing of cells of other organism; P:defense response to fungus; C:troponin complex; P:defense response to bacterium |
| Efet.01.235037.g1383.t1 | cell adhesion molecule-related down-regulated by oncogenes | 420 | 8.67E-18 | 41.4 | P:positive regulation of cellular process; P:system development; P:cell differentiation |
| Efet.01.235102.g1385.t1 | gamma-aminobutyric acid receptor subunit beta-like | 372 | 1.79E-17 | 83.05 | P:signal transduction; P:ion transmembrane transport; C:integral component of membrane; F:GABA-A receptor activity; C:cell junction; F:extracellular ligand-gated ion channel activity; C:synapse; C:plasma membrane |
| Efet.01.235109.g1386.t1 | Transformation transcription domain-associated | 408 | 2.52E-45 | 79.25 | P:DNA repair; F:transmembrane transporter activity; C:membrane; P:histone acetylation; P:phosphorylation; F:kinase activity; C:histone acetyltransferase complex |
| Efet.01.235118.g1387.t1 | allatostatin-A receptor isoform X1 | 804 | 1.47E-61 | 56.45 | P:signal transduction; F:signal transducer activity |
| Efet.01.23518.g1702.t1 | Hau-cytoplasmic intermediate filament | 381 | 4.53E-29 | 79.45 | F:structural molecule activity; C:intermediate filament |
| Efet.01.235218.g1389.t1 | zinc finger CCHC domain-containing 8 homolog | 261 | 4.49E-07 | 53.67 |  |
| Efet.01.23527.g1703.t1 | nicotinamide nicotinic acid mononucleotide adenylyltransferase 1-like isoform X1 | 876 | 2.82E-21 | 73.75 | F:ATP binding; F:nicotinate-nucleotide adenylyltransferase activity; F:nicotinamide-nucleotide adenylyltransferase activity; P:NAD biosynthetic process |
| Efet.01.235339.g1391.t1 | cytoplasmic aconitate hydratase-like isoform X2 | 447 | 2.19E-64 | 74.75 | P:metabolic process; F:4 iron, 4 sulfur cluster binding; F:aconitate hydratase activity |
| Efet.01.235480.g1393.t1 | centrosomal of 131 kDa-like | 237 | 3.77E-12 | 76.6 | P:positive regulation of intracellular protein transport; C:nuclear membrane; C:intercellular bridge; F:protein homodimerization activity; P:regulation of centrosome duplication; C:centriolar satellite; P:positive regulation of cell proliferation; C:cilium; P:intraciliary transport involved in cilium assembly; F:protein complex binding |
| Efet.01.235621.g1395.t1 | abnormal spindle-like microcephaly-associated homolog | 3102 | 9.92E-33 | 77.85 | P:spindle organization |
| Efet.01.235632.g1396.t1 | type I cytoskeletal 9-like | 876 | 1.72E-11 | 44.75 | F:zinc ion binding; P:signal transduction; P:ion transmembrane transport; F:GABA-A receptor activity; F:extracellular ligand-gated ion channel activity; C:GABA-A receptor complex; C:synapse; P:transport; C:intracellular; C:plasma membrane; P:ion transport; F:metal ion binding; C:membrane; C:integral component of membrane; C:cell junction |
| Efet.01.235721.g1398.t1 | homeobox PKNOX2 isoform X1 | 519 | 4.47E-36 | 59.3 | F:DNA binding; C:nucleus; P:regulation of transcription, DNA-templated |
| Efet.01.23573.g1704.t1 | FMRFamide receptor-like | 414 | 5.72E-22 | 61.69 | F:G-protein coupled receptor activity; P:signal transduction; C:membrane |
| Efet.01.23573.g1705.t1 | FMRFamide receptor-like | 831 | 2.92E-38 | 56.05 | F:G-protein coupled receptor activity; F:G-protein coupled peptide receptor activity; P:G-protein coupled receptor signaling pathway; C:membrane; C:integral component of membrane |
| Efet.01.23574.g1706.t1 | arylacetamide deacetylase-like | 816 | 8.41E-56 | 54.4 | P:metabolic process; F:hydrolase activity |
| Efet.01.23576.g1707.t1 | arrestin domain-containing 2-like | 516 | 2.35E-16 | 62.15 | P:signal transduction |
| Efet.01.235778.g1400.t1 | tripartite motif-containing 3-like | 903 | 1.15E-16 | 45.25 | F:zinc ion binding; F:metal ion binding; C:intracellular |
| Efet.01.23579.g1708.t1 | Zinc finger 782 | 1242 | 2.36E-43 | 49.85 | F:nucleic acid binding; F:metal ion binding |
| Efet.01.235801.g1401.t1 | pre-B-cell leukemia transcription factor 4- | 279 | 3.91E-19 | 87.85 | C:nucleus; F:transcription factor activity, sequence-specific DNA binding; P:regulation of transcription, DNA-templated; F:sequence-specific DNA binding |
| Efet.01.235810.g1402.t1 | aminopeptidase N-like | 204 | 2.68E-11 | 73.75 | F:zinc ion binding; P:peptide catabolic process; P:proteolysis; C:cytosol; F:metalloaminopeptidase activity; F:peptide binding; C:cell wall-bounded periplasmic space; C:extracellular region; C:plasma membrane |
| Efet.01.23590.g1709.t1 | dopamine D2-like receptor | 312 | 6.76E-36 | 79.3 | C:integral component of plasma membrane; F:dopamine neurotransmitter receptor activity; P:dopamine receptor signaling pathway |
| Efet.01.23604.g1710.t1 | dynein heavy chain axonemal-like | 1140 | 3.93E-151 | 87.55 | F:ATPase activity; F:ATP binding; F:microtubule motor activity; C:dynein complex; P:microtubule-based movement |
| Efet.01.236040.g1404.t1 | hillarin isoform X1 | 951 | 1.33E-37 | 53.2 |  |
| Efet.01.23605.g1711.t1 | Kinesin costa | 210 | 7.48E-08 | 76.5 | F:nucleotide binding; F:ATPase activity; F:microtubule motor activity; C:kinesin complex; P:microtubule-based movement |
| Efet.01.236073.g1407.t1 | uncharacterized protein LOC111418253 | 1410 | 1.20E-114 | 58 | F:DNA binding; F:DNA-directed 5'-3' RNA polymerase activity; P:transcription, DNA-templated |
| Efet.01.236119.g1409.t1 | ankyrin repeat domain-containing 50-like | 882 | 8.17E-09 | 47 |  |
| Efet.01.23613.g1713.t1 | transient-receptor-potential | 261 | 1.89E-23 | 81 | F:calcium channel activity; C:integral component of membrane; P:calcium ion transmembrane transport |
| Efet.01.236147.g1410.t1 | hypothetical protein CAPTEDRAFT_197781 | 1734 | 5.96E-142 | 60.5 | C:membrane; C:integral component of membrane |
| Efet.01.236157.g1411.t1 | MIT domain-containing 1 | 333 | 1.38E-34 | 70.45 |  |
| Efet.01.236229.g1413.t1 | protocadherin-11 X-linked isoform X1 | 2763 | 5.49E-145 | 53.75 | F:calcium ion binding; C:membrane; C:integral component of membrane; P:cell adhesion; P:homophilic cell adhesion via plasma membrane adhesion molecules; C:plasma membrane |
| Efet.01.236273.g1414.t1 | 14-3-3 2 | 447 | 1.72E-37 | 96.7 | F:protein domain specific binding |
| Efet.01.23628.g1715.t1 | helicase domino-like isoform X6 | 264 | 1.18E-18 | 75.2 | F:ATP binding |
| Efet.01.236302.g1415.t1 | hypothetical protein CAPTEDRAFT_224013 | 582 | 1.28E-31 | 49 | F:thiolester hydrolase activity; P:fatty acid biosynthetic process |
| Efet.01.236322.g1416.t1 | discoidin domain-containing receptor 2-like isoform X1 | 432 | 7.54E-45 | 73.55 | F:protein tyrosine kinase activity; C:integral component of membrane; P:protein phosphorylation |
| Efet.01.236368.g1418.t1 | carcinine transporter-like | 297 | 1.33E-15 | 51.33 | P:transmembrane transport; F:transmembrane transporter activity; C:membrane; C:integral component of membrane; F:transporter activity |
| Efet.01.236380.g1419.t1 | testis-expressed sequence 2 isoform X1 | 2103 | 2.70E-51 | 46.85 | C:membrane; C:integral component of membrane |
| Efet.01.236483.g1421.t1 | sidekick-2 isoform X1 | 300 | 2.08E-17 | 59.45 | F:identical protein binding; P:synapse assembly; C:membrane; P:retina layer formation; C:synapse; P:homophilic cell adhesion via plasma membrane adhesion molecules |
| Efet.01.236525.g1423.t1 | zinc finger 37-like | 741 | 8.48E-38 | 48.9 | F:metal ion binding |
| Efet.01.236572.g1425.t1 | Nucleoporin GLE1 | 408 | 1.20E-33 | 63.3 | P:poly(A)+ mRNA export from nucleus; C:nuclear pore |
| Efet.01.236595.g1426.t1 | zinc finger 678-like | 675 | 1.24E-75 | 68.55 | F:binding |
| Efet.01.23662.g1717.t1 | T family of potassium channels 18-like | 1101 | 1.09E-18 | 62.4 | C:membrane |
| Efet.01.23675.g1718.t1 | Exosome complex exonuclease RRP44 | 459 | 4.96E-71 | 84.45 | P:nucleic acid phosphodiester bond hydrolysis; F:exonuclease activity |
| Efet.01.236756.g1428.t1 | KChain Model Of Alpha-Actinin Ch1 Bound To F-Actin | 288 | 3.53E-39 | 88.9 | P:transition between fast and slow fiber; P:response to denervation involved in regulation of muscle adaptation; P:negative regulation of oxidative phosphorylation; F:protein homodimerization activity; P:positive regulation of glucose catabolic process to lactate via pyruvate; P:negative regulation of glycolytic process; F:integrin binding; P:regulation of apoptotic process; P:positive regulation of skeletal muscle tissue growth; C:actin filament; P:regulation of aerobic respiration; F:ion channel binding; C:extracellular exosome; F:structural constituent of muscle; C:focal adhesion; F:actin binding; F:calcium ion binding; P:regulation of the force of skeletal muscle contraction; P:negative regulation of calcineurin-NFAT signaling cascade; C:cytosol; C:pseudopodium; P:focal adhesion assembly; P:skeletal muscle atrophy; P:muscle filament sliding |
| Efet.01.236783.g1429.t1 | T-box transcription factor TBX20-like | 273 | 6.16E-09 | 57.05 | F:DNA binding; C:nucleus; F:transcription factor activity, sequence-specific DNA binding; P:transcription, DNA-templated; P:regulation of transcription, DNA-templated |
| Efet.01.23681.g1719.t1 | TBC1 domain family member 31-like | 336 | 4.97E-31 | 82.05 | C:centrosome |
| Efet.01.236826.g1431.t1 | SUMO-specific isopeptidase USPL1 | 1575 | 6.90E-30 | 55.73 | P:cell proliferation; F:SUMO-specific isopeptidase activity; F:SUMO binding; P:protein desumoylation; C:Cajal body; P:Cajal body organization |
| Efet.01.236837.g1432.t1 | methylcytosine dioxygenase TET2 | 378 | 1.04E-13 | 80.95 | F:dioxygenase activity; F:DNA binding; F:zinc ion binding; P:oxidation-reduction process |
| Efet.01.236848.g1433.t1 | snRNA-activating complex subunit 1 | 381 | 4.33E-21 | 56 | C:intracellular membrane-bounded organelle |
| Efet.01.236887.g1435.t1 | cytochrome c-2 | 333 | 9.48E-63 | 86.65 | C:mitochondrial intermembrane space; F:metal ion binding; F:electron carrier activity; P:oxidation-reduction process; C:respiratory chain; F:heme binding |
| Efet.01.236977.g1439.t1 | metabotropic glutamate receptor 8-like | 516 | 1.42E-49 | 76.85 | F:G-protein coupled receptor activity; P:G-protein coupled receptor signaling pathway; C:integral component of membrane; C:plasma membrane |
| Efet.01.236986.g1440.t1 | FMRFamide receptor-like | 1209 | 3.38E-83 | 59.3 | F:G-protein coupled receptor activity; C:membrane |
| Efet.01.236996.g1441.t1 | nuclear receptor subfamily 2 group E member 1-like | 240 | 2.70E-12 | 74.05 | F:DNA binding; F:transcription factor activity, sequence-specific DNA binding; F:metal ion binding; P:signal transduction; F:signal transducer activity |
| Efet.01.237030.g1442.t1 | hypothetical protein HELRODRAFT_90889, partial | 279 | 9.96E-22 | 64.33 | F:cation:chloride symporter activity; P:chloride transmembrane transport; C:integral component of membrane |
| Efet.01.237034.g1443.t1 | G -activated inward rectifier potassium channel 3-like | 1659 | 1.69E-179 | 71.6 | P:potassium ion transmembrane transport; C:integral component of membrane; F:inward rectifier potassium channel activity; P:regulation of ion transmembrane transport |
| Efet.01.23708.g1721.t1 | fibrinogen alpha chain | 450 | 5.32E-12 | 53 |  |
| Efet.01.237111.g1447.t1 | dynein heavy chain axonemal-like | 1044 | 8.09E-92 | 58.95 | F:nucleotide binding; F:ATPase activity; F:ATP binding; F:microtubule motor activity; F:metal ion binding; C:dynein complex; P:microtubule-based movement |
| Efet.01.237184.g1450.t1 | protocadherin-16-like | 945 | 1.85E-35 | 51.8 | F:calcium ion binding; C:membrane; C:integral component of membrane; P:cell adhesion; P:homophilic cell adhesion via plasma membrane adhesion molecules; C:plasma membrane |
| Efet.01.237233.g1452.t1 | F-box At5g06550-like | 447 | 2.94E-24 | 63.85 | C:membrane; C:integral component of membrane |
| Efet.01.237253.g1453.t1 | transmembrane 184B-like | 498 | 1.11E-39 | 81.6 | C:integral component of membrane |
| Efet.01.237261.g1454.t1 | lysine-specific demethylase 6A- | 726 | 6.02E-117 | 85 | P:multicellular organism growth; F:chromatin DNA binding; F:identical protein binding; F:RNA polymerase II core promoter proximal region sequence-specific DNA binding; F:methyltransferase activity; C:MLL3/4 complex; F:dioxygenase activity; P:respiratory system process; P:heart morphogenesis; P:notochord morphogenesis; P:neural tube closure; F:metal ion binding; P:in utero embryonic development; P:positive regulation of gene expression; P:mesodermal cell differentiation; P:canonical Wnt signaling pathway; P:histone H3-K27 demethylation; P:oxidation-reduction process; P:cardiovascular system development; P:somite rostral/caudal axis specification; F:histone demethylase activity (H3-K27 specific); P:histone H3-K4 methylation |
| Efet.01.237262.g1455.t1 | Neuropeptide FF receptor 2 | 660 | 4.28E-26 | 57.3 | F:G-protein coupled receptor activity; P:signal transduction |
| Efet.01.23732.g1722.t1 | spectrin alpha chain-like isoform X3 | 1380 | 8.31E-173 | 75 | F:calcium ion binding |
| Efet.01.23732.g1723.t1 | spectrin alpha chain-like | 291 | 1.10E-39 | 86.2 | F:calcium ion binding |
| Efet.01.237412.g1458.t1 | hypothetical protein A3G93_12365 | 1038 | 1.59E-08 | 47 |  |
| Efet.01.237415.g1459.t1 | hypothetical protein | 279 | 3.38E-60 | 87.45 |  |
| Efet.01.237419.g1460.t1 | ceramide synthase 4 | 243 | 1.94E-11 | 77.8 | C:nucleus; F:DNA binding; C:integral component of membrane; F:sphingosine N-acyltransferase activity; P:ceramide biosynthetic process; C:endoplasmic reticulum |
| Efet.01.237570.g1464.t1 | MICOS complex subunit MIC19-like isoform X2 | 267 | 1.45E-11 | 59.1 | C:membrane; C:integral component of membrane |
| Efet.01.237592.g1466.t1 | FMRFamide receptor-like | 435 | 3.46E-18 | 67.57 | F:G-protein coupled receptor activity; C:membrane |
| Efet.01.237626.g1467.t1 | wings apart | 207 | 1.55E-08 | 72.5 |  |
| Efet.01.237637.g1468.t1 | N-fatty-acyl-amino acid synthase hydrolase -like | 423 | 4.05E-43 | 74.95 | C:membrane |
| Efet.01.237649.g1469.t1 | FAM166B isoform X1 | 219 | 3.47E-14 | 61.78 |  |
| Efet.01.237679.g1470.t1 | tubulin beta chain | 828 | 0 | 89 | F:GTP binding; C:cytoplasm; F:GTPase activity; F:structural constituent of cytoskeleton; C:microtubule; P:microtubule-based process |
| Efet.01.237725.g1471.t1 | actin cytoskeleton-regulatory complex PAN1-like | 405 | 6.16E-27 | 71.75 |  |
| Efet.01.237744.g1472.t1 | Mitochondrial thiamine pyrophosphate carrier | 312 | 2.25E-29 | 67.7 | C:nucleus; C:membrane; F:deoxynucleotide transmembrane transporter activity; P:positive regulation of defense response to virus by host; P:transport; P:xenophagy |
| Efet.01.237760.g1473.t1 | glutamate-gated chloride channel isoform X5 | 369 | 2.16E-11 | 59.3 | P:neuron projection morphogenesis; F:extracellular-glutamate-gated chloride channel activity; P:transport; C:plasma membrane |
| Efet.01.237874.g1474.t1 | hypothetical protein CAPTEDRAFT_228311 | 474 | 9.70E-26 | 57 | P:single organismal cell-cell adhesion; C:membrane; F:myosin binding; C:integral component of membrane |
| Efet.01.237893.g1475.t1 | zinc transporter ZIP10-like | 339 | 1.59E-24 | 65.85 | P:transmembrane transport; C:integral component of membrane; F:metal ion transmembrane transporter activity; P:metal ion transport |
| Efet.01.237901.g1476.t1 | beta-adrenergic receptor kinase 2-like | 288 | 2.47E-59 | 97.25 | F:ATP binding; F:G-protein coupled receptor kinase activity; P:signal transduction; P:protein phosphorylation |
| Efet.01.23797.g1726.t1 | hypothetical protein HELRODRAFT_189841 | 1005 | 1.81E-06 | 39 | C:membrane; C:integral component of membrane |
| Efet.01.238085.g1480.t1 | MAP kinase-activating death domain | 582 | 7.12E-89 | 81.3 | P:phosphorylation; F:kinase activity |
| Efet.01.238089.g1481.t1 | reelin-like | 627 | 2.43E-30 | 60.35 |  |
| Efet.01.238193.g1483.t1 | lactadherin-like isoform X5 | 294 | 1.30E-12 | 54.06 | F:oligosaccharide binding; C:membrane; C:integral component of membrane |
| Efet.01.238211.g1485.t1 | poly [ADP-ribose] polymerase 14-like isoform X2 | 348 | 3.09E-30 | 70.15 | F:transferase activity, transferring glycosyl groups |
| Efet.01.238231.g1486.t1 | zinc finger 665-like | 783 | 5.91E-47 | 60.35 | F:nucleic acid binding; P:biological_process; F:metal ion binding; C:blood microparticle; F:molecular_function |
| Efet.01.238309.g1487.t1 | histone-arginine methyltransferase CARMER isoform X2 | 597 | 2.97E-128 | 75.7 | F:methyltransferase activity; P:protein methylation |
| Efet.01.238348.g1489.t1 | hypothetical protein HELRODRAFT_168861 | 402 | 1.68E-14 | 57.67 | C:membrane; C:integral component of membrane |
| Efet.01.238394.g1490.t1 | BMP and activin membrane-bound inhibitor homolog | 363 | 2.48E-08 | 47.5 | C:membrane; C:integral component of membrane; P:positive regulation of canonical Wnt signaling pathway; P:negative regulation of transforming growth factor beta receptor signaling pathway |
| Efet.01.238405.g1491.t1 | tRNA wybutosine-synthesizing 5-like isoform X2 | 201 | 1.91E-11 | 68.2 | F:calcium ion binding |
| Efet.01.238417.g1492.t1 | neurexin-1 isoform X2 | 603 | 3.63E-46 | 64.4 | F:metal ion binding; C:integral component of membrane; P:chemical synaptic transmission |
| Efet.01.23843.g1728.t1 | somatostatin receptor type 5-like | 369 | 4.96E-29 | 70.95 | F:opioid receptor activity; P:opioid receptor signaling pathway; C:integral component of membrane |
| Efet.01.23844.g1729.t1 | hypothetical protein B5766_02725 | 477 | 9.70E-58 | 72.25 | C:membrane |
| Efet.01.238519.g1494.t1 | hypothetical protein HELRODRAFT_194061 | 285 | 4.64E-10 | 50 | F:GTP binding; P:small GTPase mediated signal transduction; C:intracellular |
| Efet.01.238548.g1495.t1 | E3 ubiquitin- ligase TRIM71-like | 879 | 2.93E-14 | 46.3 | F:zinc ion binding; P:signal transduction; P:ion transmembrane transport; F:GABA-A receptor activity; F:extracellular ligand-gated ion channel activity; C:GABA-A receptor complex; C:synapse; P:transport; C:intracellular; C:plasma membrane; F:ubiquitin-protein transferase activity; P:ion transport; F:metal ion binding; C:membrane; C:integral component of membrane; C:cell junction; P:protein ubiquitination |
| Efet.01.23857.g1730.t1 | mitochondrial import inner membrane translocase subunit TIM44-like | 291 | 8.84E-35 | 80.4 | P:protein import into mitochondrial matrix; F:chaperone binding; C:mitochondrial inner membrane |
| Efet.01.23859.g1732.t1 | sex peptide receptor-like | 279 | 1.75E-11 | 64.65 | F:rhodopsin kinase activity; F:G-protein coupled receptor activity; F:G-protein coupled peptide receptor activity; P:G-protein coupled receptor signaling pathway; C:membrane; C:integral component of membrane; F:transferase activity; P:protein phosphorylation |
| Efet.01.238657.g1497.t1 | FG-GAP repeat | 5430 | 0 | 57 | F:catalytic activity; F:carbohydrate binding |
| Efet.01.238657.g1498.t1 | FG-GAP repeat | 597 | 1.20E-33 | 76.71 | F:catalytic activity; F:carbohydrate binding |
| Efet.01.238666.g1500.t1 | asteroid homolog 1-like | 1884 | 2.53E-94 | 55.6 | P:nucleic acid phosphodiester bond hydrolysis; P:DNA repair; F:nuclease activity |
| Efet.01.238691.g1501.t1 | cyclin-dependent kinase inhibitor 1B | 417 | 1.64E-11 | 72.65 | P:negative regulation of cellular process; C:intracellular part; P:cellular process |
| Efet.01.238767.g1505.t1 | PREDICTED: uncharacterized protein LOC106151404 | 501 | 4.06E-35 | 56.88 |  |
| Efet.01.238964.g1507.t1 | hypothetical protein CAPTEDRAFT_217788 | 645 | 5.11E-37 | 54.6 |  |
| Efet.01.2390.g214.t1 | fat-like cadherin-related tumor suppressor homolog | 522 | 2.82E-22 | 59.05 | C:membrane |
| Efet.01.23903.g1735.t1 | hypothetical protein CAPTEDRAFT_185694 | 501 | 2.20E-36 | 62.5 |  |
| Efet.01.239057.g1508.t1 | TBC1 domain family member | 654 | 6.54E-72 | 66.95 |  |
| Efet.01.23906.g1736.t1 | CLIP-associating 1-B-like | 432 | 3.31E-40 | 73.25 | P:microtubule anchoring; P:Golgi organization; F:dystroglycan binding; P:establishment of spindle orientation; C:kinetochore; P:positive regulation of exocytosis; F:protein tyrosine kinase binding; P:cell division; P:regulation of epithelial to mesenchymal transition; C:trans-Golgi network; C:extracellular exosome; P:G2/M transition of mitotic cell cycle; C:focal adhesion; C:mitotic spindle; C:kinetochore microtubule; C:cytosol; C:ruffle membrane; P:exit from mitosis; P:positive regulation of epithelial cell migration; P:positive regulation of microtubule polymerization; P:cell migration; F:kinetochore binding; P:sister chromatid cohesion; P:regulation of gastrulation; P:negative regulation of wound healing, spreading of epidermal cells; C:centrosomal corona; P:establishment of mitotic spindle localization; P:microtubule nucleation; P:platelet-derived growth factor receptor-beta signaling pathway; P:establishment of protein localization to plasma membrane; P:negative regulation of microtubule depolymerization; P:establishment of epithelial cell polarity; C:cortical microtubule plus-end; F:actin filament binding; P:negative regulation of focal adhesion assembly; P:astral microtubule organization; P:positive regulation of extracellular matrix disassembly; P:regulation of axon extension; P:negative regulation of stress fiber assembly; P:microtubule bundle formation; C:axonal growth cone; P:vesicle targeting; F:microtubule plus-end binding; P:microtubule organizing center organization; C:basal cortex; P:positive regulation of basement membrane assembly involved in embryonic body morphogenesis; P:mitotic spindle assembly |
| Efet.01.239067.g1509.t1 | ATP-dependent RNA helicase mitochondrial | 720 | 2.38E-25 | 50.2 | P:RNA metabolic process |
| Efet.01.239185.g1511.t1 | GRB2-associated-binding 1-like isoform X2 | 360 | 2.02E-36 | 65.85 |  |
| Efet.01.239196.g1512.t1 | probable G- coupled receptor 139 | 1707 | 1.41E-44 | 63.15 | P:signal transduction; C:membrane; F:signal transducer activity |
| Efet.01.239197.g1513.t1 | probable G- coupled receptor 139 | 555 | 3.54E-41 | 62.6 | P:signal transduction; C:membrane; F:signal transducer activity |
| Efet.01.239235.g1514.t1 | carbohydrate sulfotransferase 4-like | 894 | 6.36E-56 | 43.95 | C:membrane; C:integral component of membrane; F:sulfotransferase activity |
| Efet.01.23924.g1737.t1 | uncharacterized protein LOC110452122 | 843 | 1.01E-49 | 56.9 |  |
| Efet.01.239270.g1517.t1 | zinc finger BED domain-containing 4-like | 657 | 1.18E-39 | 59 | F:nucleic acid binding; C:integral component of membrane; F:protein dimerization activity |
| Efet.01.23938.g1738.t1 | cytosolic Fe-S cluster assembly factor NARFL isoform X3 | 642 | 3.88E-86 | 68.05 | P:biological regulation; P:cellular process |
| Efet.01.239492.g1521.t1 | carbamoyl-phosphate synthase small subunit | 648 | 9.94E-96 | 78.3 | F:ATP binding; P:'de novo' pyrimidine nucleobase biosynthetic process; P:arginine biosynthetic process; P:glutamine metabolic process; F:carbamoyl-phosphate synthase (glutamine-hydrolyzing) activity; P:'de novo' UMP biosynthetic process |
| Efet.01.239605.g1522.t1 | Proto-oncogene tyrosine- kinase receptor Ret | 474 | 4.74E-59 | 80 | P:positive regulation of cellular protein metabolic process; C:intracellular organelle; P:peptidyl-tyrosine phosphorylation; P:tissue morphogenesis; P:signal transduction; P:neuron differentiation; P:tube development; P:developmental maturation; P:cell development; P:positive regulation of developmental process; P:epithelium development; P:regulation of signal transduction; C:plasma membrane; C:membrane-bounded organelle; F:ATP binding; F:protein tyrosine kinase activity; C:cytoplasm; P:kidney development; P:positive regulation of gene expression; P:positive regulation of multicellular organismal process; P:positive regulation of apoptotic process; C:integral component of membrane; P:regulation of neuron projection development; P:regulation of cell adhesion |
| Efet.01.239635.g1524.t1 | voltage-gated hydrogen channel 1-like | 501 | 3.08E-29 | 67.35 | P:transport |
| Efet.01.23966.g1740.t1 | small subunit processome component 20 homolog | 417 | 1.93E-23 | 58.6 | P:rRNA processing; C:nucleolus; F:RNA binding |
| Efet.01.239686.g1526.t1 | neuroligin- X-linked-like isoform X2 | 834 | 5.73E-108 | 64.3 | C:membrane |
| Efet.01.239691.g1527.t1 | insulin-like growth factor 2 mRNA-binding 3-A | 339 | 4.20E-12 | 68.1 | F:nucleic acid binding |
| Efet.01.23970.g1741.t1 | general transcription factor II-I repeat domain-containing 2-like | 1854 | 8.75E-143 | 55.45 | F:nucleic acid binding; F:DNA binding; C:cytoplasm; F:RNA polymerase II transcription factor activity, sequence-specific DNA binding; C:nucleoplasm; P:regulation of transcription from RNA polymerase II promoter |
| Efet.01.239760.g1528.t1 | sex peptide receptor-like | 1086 | 2.44E-64 | 56.15 | F:G-protein coupled peptide receptor activity; P:G-protein coupled receptor signaling pathway; C:integral component of membrane |
| Efet.01.239761.g1529.t1 | probable phosphatase 2C | 558 | 1.46E-98 | 87.6 | F:protein serine/threonine phosphatase activity; F:metal ion binding; P:protein dephosphorylation |
| Efet.01.2398.g216.t1 | DNA helicase INO80-like isoform X1 | 297 | 2.18E-08 | 61.5 | P:cellular process |
| Efet.01.239850.g1530.t1 | suppressor of cytokine signaling 5-like | 456 | 2.13E-80 | 90.05 | P:negative regulation of epidermal growth factor-activated receptor activity; P:epidermal growth factor receptor signaling pathway; P:positive regulation of proteasomal ubiquitin-dependent protein catabolic process; C:intracellular; P:JAK-STAT cascade |
| Efet.01.239860.g1532.t1 | B-cell receptor CD22 isoform X2 | 420 | 1.56E-09 | 57.2 | C:membrane; C:integral component of membrane |
| Efet.01.239919.g1534.t1 | tyramine receptor 1-like | 705 | 1.16E-36 | 69.15 | P:regulation of biological quality; P:G-protein coupled receptor signaling pathway; P:single-multicellular organism process; F:dopamine binding; P:response to alkaloid; P:regulation of G-protein coupled receptor protein signaling pathway; P:response to organic cyclic compound; P:response to oxygen-containing compound; P:behavior; P:response to ammonium ion; F:dopamine neurotransmitter receptor activity, coupled via Gs; P:synaptic transmission, dopaminergic; P:system process; P:negative regulation of signal transduction; C:integral component of membrane; P:regulation of primary metabolic process |
| Efet.01.24.g3.t1 | membrane-associated progesterone receptor component 1-like | 408 | 2.92E-28 | 75.4 | F:oxidoreductase activity; C:integral component of membrane; C:endomembrane system; F:heme binding |
| Efet.01.24001.g1743.t1 | hypothetical protein CAPTEDRAFT_201133 | 1146 | 3.63E-109 | 54.23 | C:membrane |
| Efet.01.240028.g1536.t1 | PREDICTED: uncharacterized protein LOC109476033 isoform X3 | 708 | 7.52E-19 | 47.25 | F:thiolester hydrolase activity; P:fatty acid biosynthetic process |
| Efet.01.24004.g1744.t1 | cilia- and flagella-associated 36 | 354 | 3.23E-33 | 65.6 |  |
| Efet.01.240113.g1539.t1 | homeobox Nkx- -like | 753 | 1.36E-27 | 73.05 | C:nucleus; P:regulation of transcription, DNA-templated; P:lymphocyte differentiation; F:sequence-specific DNA binding; P:anatomical structure morphogenesis |
| Efet.01.24015.g1745.t1 | peripheral-type benzodiazepine receptor-associated 1-like | 1002 | 2.65E-27 | 72.3 |  |
| Efet.01.240213.g1540.t1 | CASC3-like isoform X2 | 735 | 1.22E-09 | 74.75 | C:exon-exon junction complex; F:RNA binding; P:mRNA processing |
| Efet.01.240301.g1542.t1 | frizzled-4-like | 2022 | 2.84E-154 | 62.7 | F:transmembrane signaling receptor activity; C:membrane; P:Wnt signaling pathway |
| Efet.01.240304.g1543.t1 | Muscle M-line assembly unc- | 258 | 7.11E-10 | 61.8 | P:regulation of Rho protein signal transduction; F:Rho guanyl-nucleotide exchange factor activity; P:positive regulation of GTPase activity |
| Efet.01.2404.g218.t1 | oxidative stress-induced growth inhibitor 2-like | 1008 | 6.73E-99 | 56.9 | F:oxidoreductase activity; P:oxidation-reduction process |
| Efet.01.240487.g1553.t1 | sex peptide receptor-like | 1047 | 1.65E-98 | 69.35 | F:G-protein coupled receptor activity; C:membrane |
| Efet.01.24052.g1746.t1 | ATP-dependent Clp protease ATP-binding subunit | 453 | 7.60E-75 | 77.15 | F:ATP binding |
| Efet.01.24052.g1747.t1 | ATP-dependent endonuclease | 780 | 6.30E-100 | 81.55 | P:nucleic acid phosphodiester bond hydrolysis; F:endonuclease activity |
| Efet.01.24052.g1748.t1 | ATP-dependent endonuclease | 756 | 2.12E-110 | 74.75 | P:nucleic acid phosphodiester bond hydrolysis; F:endonuclease activity |
| Efet.01.24059.g1749.t1 | hypoxanthine-guanine phosphoribosyltransferase | 207 | 6.79E-15 | 89.6 | P:cytolysis; P:dopamine metabolic process; P:adenine salvage; P:hypoxanthine salvage; P:striatum development; P:central nervous system neuron development; P:GMP catabolic process; P:positive regulation of dopamine metabolic process; F:hypoxanthine phosphoribosyltransferase activity; P:cerebral cortex neuron differentiation; P:dendrite morphogenesis; F:nucleotide binding; F:magnesium ion binding; P:grooming behavior; C:extracellular exosome; P:locomotory behavior; P:protein homotetramerization; C:cytosol; F:guanine phosphoribosyltransferase activity; F:protein homodimerization activity; P:GMP salvage; P:IMP salvage; P:guanine salvage; P:response to amphetamine; P:lymphocyte proliferation |
| Efet.01.2406.g219.t1 | A disintegrin and metallo ase with thrombospondin motifs 6-like | 375 | 2.91E-30 | 54.3 | P:heart development; P:blood vessel development; F:metal ion binding; C:extracellular matrix; F:metallopeptidase activity |
| Efet.01.240723.g1557.t1 | kelch 10 | 498 | 1.53E-57 | 75.35 |  |
| Efet.01.240740.g1558.t1 | tripartite motif-containing 2 | 1566 | 9.78E-14 | 60.75 | F:zinc ion binding; F:metal ion binding; C:intracellular |
| Efet.01.240758.g1559.t1 | zinc finger CCHC domain-containing 8 homolog | 591 | 2.14E-06 | 60 |  |
| Efet.01.240853.g1562.t1 | cholesterol 7-alpha-monooxygenase-like | 1011 | 2.77E-100 | 55.25 | F:oxidoreductase activity; P:single-organism process |
| Efet.01.240865.g1563.t1 | gastrula zinc finger -like | 1779 | 2.48E-18 | 42.1 | F:nucleic acid binding; F:metal ion binding; P:regulation of transcription, DNA-templated; C:intracellular |
| Efet.01.240933.g1564.t1 | 3-deoxy-7-phosphoheptulonate synthase class II | 702 | 8.89E-169 | 94 | P:aromatic amino acid family biosynthetic process; F:3-deoxy-7-phosphoheptulonate synthase activity |
| Efet.01.241023.g1568.t1 | serine threonine- kinase PAK 1-like | 282 | 4.94E-28 | 80.5 | F:ATP binding; F:signal transducer, downstream of receptor, with serine/threonine kinase activity; C:cytoplasm; P:axonogenesis; P:actin filament organization; P:signal transduction by protein phosphorylation; P:mitotic cell cycle |
| Efet.01.241051.g1569.t1 | NEDD4-like E3 ubiquitin- ligase WWP1 | 669 | 3.58E-96 | 72.35 | P:viral entry into host cell; P:lipid storage; P:negative regulation of sequence-specific DNA binding transcription factor activity; P:regulation of potassium ion transmembrane transporter activity; P:protein ubiquitination involved in ubiquitin-dependent protein catabolic process; P:negative regulation of transcription from RNA polymerase II promoter; P:negative regulation of protein transport; C:nucleus; F:ubiquitin-protein transferase activity; P:proteasome-mediated ubiquitin-dependent protein catabolic process; C:extracellular exosome; P:regulation of membrane potential; C:ubiquitin ligase complex; C:cytoplasm; F:RNA polymerase II transcription factor binding; C:membrane; P:negative regulation of transporter activity; P:protein K63-linked ubiquitination; P:protein autoubiquitination |
| Efet.01.241097.g1570.t1 | mothers against decapentaplegic homolog 6 | 429 | 6.00E-33 | 58.55 | P:zygotic specification of dorsal/ventral axis; P:negative regulation of cell proliferation; F:RNA polymerase II core promoter proximal region sequence-specific DNA binding; P:animal organ development; P:fat cell differentiation; P:immune response; P:BMP signaling pathway; P:negative regulation of SMAD protein complex assembly; F:identical protein binding; P:negative regulation of pathway-restricted SMAD protein phosphorylation; C:protein complex; F:chromatin binding; P:negative regulation of BMP signaling pathway; C:nucleus; F:ubiquitin protein ligase binding; P:response to laminar fluid shear stress; F:transforming growth factor beta receptor, inhibitory cytoplasmic mediator activity; C:cytoplasm; P:negative regulation of apoptotic process; F:R-SMAD binding; F:type I activin receptor binding; P:circulatory system development; F:co-SMAD binding; F:I-SMAD binding; P:cell-substrate adhesion; F:type I transforming growth factor beta receptor binding; P:negative regulation of transforming growth factor beta receptor signaling pathway |
| Efet.01.24111.g1754.t1 | Innexin unc-9 | 273 | 1.07E-12 | 73 | C:gap junction; P:ion transport; C:integral component of membrane |
| Efet.01.241121.g1573.t1 | zinc finger C3H1 type-like 1 | 759 | 9.17E-39 | 78.5 | F:metal ion binding |
| Efet.01.241292.g1575.t1 | RAB6A-GEF complex partner 1-like | 405 | 3.69E-36 | 79.3 |  |
| Efet.01.241304.g1576.t1 | HEAT repeat-containing 4-like | 531 | 1.40E-21 | 50.2 |  |
| Efet.01.241387.g1580.t1 | Down syndrome cell adhesion molecule 1 homolog | 411 | 9.42E-08 | 54.5 |  |
| Efet.01.241486.g1582.t1 | arg8-vasotocin receptor-like | 462 | 1.57E-36 | 70.95 | C:integral component of plasma membrane; F:G-protein coupled receptor activity; P:signal transduction; P:response to peptide; F:peptide binding; P:cellular response to hormone stimulus |
| Efet.01.241488.g1583.t1 | serine threonine kinase | 471 | 1.15E-80 | 76 | C:membrane; F:protein kinase activity; P:phosphorylation |
| Efet.01.241537.g1584.t1 | junctophilin-1-like isoform X1 | 897 | 1.87E-124 | 78.05 | P:pseudouridine synthesis; F:RNA binding; F:pseudouridine synthase activity |
| Efet.01.241538.g1585.t1 | bone morphogenetic receptor type-2-like | 1659 | 1.19E-10 | 48.6 | F:protein kinase activity; P:transmembrane receptor protein serine/threonine kinase signaling pathway; F:protein serine/threonine kinase activity; P:protein phosphorylation; P:signal transduction by protein phosphorylation; F:nucleotide binding; F:ATP binding; F:signal transducer, downstream of receptor, with serine/threonine kinase activity; F:metal ion binding; C:membrane; C:integral component of membrane; P:phosphorylation; F:transferase activity; F:kinase activity |
| Efet.01.24163.g1756.t1 | ubiquitin carboxyl-terminal hydrolase 24-like | 627 | 1.28E-61 | 74.45 | P:protein deubiquitination; F:thiol-dependent ubiquitinyl hydrolase activity; P:ubiquitin-dependent protein catabolic process |
| Efet.01.2417.g220.t1 | potassium voltage-gated channel Shab-like | 597 | 1.11E-39 | 78.95 | F:voltage-gated potassium channel activity; P:potassium ion transmembrane transport; C:voltage-gated potassium channel complex; P:regulation of ion transmembrane transport; P:protein homooligomerization |
| Efet.01.241725.g1586.t1 | FMRFamide receptor-like | 1293 | 9.14E-42 | 49 | F:G-protein coupled receptor activity; F:G-protein coupled peptide receptor activity; P:G-protein coupled receptor signaling pathway; C:membrane; C:integral component of membrane |
| Efet.01.24177.g1757.t1 | gastrula zinc finger -like | 1011 | 5.02E-77 | 64.1 | F:nucleic acid binding; F:metal ion binding |
| Efet.01.241800.g1592.t1 | vacuolar sorting-associated 16 homolog | 333 | 2.65E-32 | 82 | P:regulation of vacuole fusion, non-autophagic; C:endosome; P:regulation of SNARE complex assembly; F:actin binding; P:vacuole organization; P:endosomal transport; P:intracellular protein transport; C:lysosomal membrane; C:HOPS complex |
| Efet.01.241839.g1593.t1 | Glutamyl aminopeptidase | 546 | 1.55E-12 | 51.05 | C:membrane; C:cell part; F:peptidase activity |
| Efet.01.241893.g1594.t1 | clavaminate synthase At3g21360 | 540 | 1.17E-43 | 59.9 | C:nucleus; F:oxidoreductase activity; P:oxidation-reduction process |
| Efet.01.24190.g1758.t1 | Poly [ADP-ribose] polymerase 14 | 507 | 3.24E-42 | 66.35 | F:transferase activity |
| Efet.01.241906.g1595.t1 | growth differentiation factor 3 | 1068 | 9.88E-18 | 55.4 | F:growth factor activity; P:regulation of epiboly involved in gastrulation with mouth forming second; C:membrane; C:integral component of membrane; P:regulation of nodal signaling pathway involved in determination of left/right asymmetry; P:heart looping; P:neuron development; C:extracellular region; P:growth |
| Efet.01.241930.g1596.t1 | uncharacterized protein C05D11.1-like | 204 | 2.29E-16 | 65.71 | F:catalytic activity; F:metal ion binding; P:proteolysis |
| Efet.01.241930.g1597.t1 | uncharacterized protein C05D11.1-like | 786 | 5.06E-47 | 57.35 | F:zinc ion binding; F:metal ion binding; F:catalytic activity; P:proteolysis; P:protein processing; F:metalloendopeptidase activity |
| Efet.01.241936.g1598.t1 | cyclic AMP-dependent transcription factor ATF-4 | 987 | 1.23E-17 | 74.9 | C:nucleus; C:cytoplasm; F:RNA polymerase II regulatory region sequence-specific DNA binding; P:brain development; P:negative regulation of cellular process; F:RNA polymerase II transcription factor activity, sequence-specific DNA binding; P:regulation of transcription from RNA polymerase II promoter |
| Efet.01.241940.g1599.t1 | Tetratricopeptide repeat 6 | 648 | 3.57E-17 | 49.26 | C:BBSome |
| Efet.01.242018.g1603.t1 | cytochrome P450 2J6-like | 390 | 1.07E-08 | 59 | F:oxidoreductase activity; F:binding |
| Efet.01.242029.g1604.t1 | ATP-dependent DNA helicase pif1 | 441 | 6.29E-27 | 61.1 | P:DNA repair; F:DNA helicase activity; P:DNA duplex unwinding; P:telomere maintenance |
| Efet.01.242037.g1605.t1 | Cytochrome c oxidase subunit mitochondrial | 213 | 2.73E-22 | 74 | F:cytochrome-c oxidase activity; C:integral component of membrane; P:hydrogen ion transmembrane transport |
| Efet.01.242062.g1608.t1 | atlastin- | 483 | 1.85E-26 | 55.35 | F:protein binding; C:endoplasmic reticulum tubular network |
| Efet.01.242070.g1610.t1 | secernin-2-like | 513 | 8.68E-38 | 57 | F:dipeptidase activity; P:proteolysis; P:exocytosis |
| Efet.01.242077.g1611.t1 | SUREJ_STRPU ame: Full=Sperm receptor for egg jelly Short=suREJ Flags: Precursor | 426 | 7.71E-06 | 51 | P:detection of mechanical stimulus; F:calcium ion binding; F:calcium channel activity; C:membrane; C:integral component of membrane; P:calcium ion transmembrane transport; F:carbohydrate binding; C:plasma membrane |
| Efet.01.242112.g1612.t1 | GRIP and coiled-coil domain-containing 1-like | 630 | 2.47E-27 | 56.35 | P:protein targeting to Golgi; C:Golgi apparatus; C:intracellular |
| Efet.01.242112.g1613.t1 | GRIP and coiled-coil domain-containing 1-like | 372 | 2.92E-18 | 64.15 | P:protein targeting to Golgi; C:Golgi apparatus; C:intracellular |
| Efet.01.242160.g1614.t1 | neuron navigator 2-like | 267 | 8.08E-37 | 81.1 | F:ATP binding |
| Efet.01.24223.g1759.t1 | TBC1 domain family member 10A-like | 333 | 2.28E-25 | 75.8 |  |
| Efet.01.242277.g1616.t1 | delta(3,5)-Delta(2,4)-dienoyl- mitochondrial | 489 | 3.09E-73 | 78.3 | C:extracellular exosome; C:mitochondrion; C:membrane; P:metabolic process; F:receptor binding; F:isomerase activity |
| Efet.01.242282.g1617.t1 | neuronal acetylcholine receptor subunit alpha-5-like isoform X1 | 222 | 1.02E-27 | 82.35 | C:postsynaptic membrane; P:cation transmembrane transport; C:integral component of membrane; C:cell junction; F:extracellular ligand-gated ion channel activity; P:isoprenoid biosynthetic process |
| Efet.01.242364.g1618.t1 | beta-1,3-galactosyl-O-glycosyl-glyco beta-1,6-N-acetylglucosaminyltransferase 3-like | 690 | 6.49E-83 | 70 | C:membrane |
| Efet.01.242458.g1621.t1 | SCY1 2 isoform X1 | 267 | 1.66E-19 | 82.9 | F:ATP binding; F:protein kinase activity; P:protein phosphorylation |
| Efet.01.24251.g1760.t1 | 39S ribosomal mitochondrial | 612 | 1.33E-43 | 64.85 | C:ribosome |
| Efet.01.24251.g1761.t1 | 3-hydroxyacyl- dehydrogenase type-2 | 585 | 2.52E-100 | 85.25 | F:3-hydroxyacyl-CoA dehydrogenase activity; C:mitochondrion; P:oxidation-reduction process; F:alcohol dehydrogenase (NAD) activity; F:RNA binding |
| Efet.01.242518.g1622.t1 | E3 ubiquitin- ligase RNF213-like | 504 | 1.04E-24 | 46.2 | F:nucleotide binding; F:ATPase activity; F:ubiquitin-protein transferase activity; F:ATP binding; F:zinc ion binding; P:protein ubiquitination |
| Efet.01.242568.g1625.t1 | alpha-actinin-1 isoform X3 | 204 | 9.33E-15 | 88.9 | F:actin filament binding; P:actin crosslink formation; F:calcium ion binding; P:actin filament bundle assembly |
| Efet.01.242597.g1626.t1 | tubulin beta-4B chain isoform X2 | 567 | 7.25E-113 | 99.95 | F:GTP binding; C:nucleus; C:myelin sheath; C:extracellular exosome; C:tubulin complex; C:cytoplasm; F:double-stranded RNA binding; F:GTPase activity; F:structural constituent of cytoskeleton; C:extracellular matrix; C:microtubule; P:microtubule-based process |
| Efet.01.24261.g1762.t1 | pyruvate kinase PKM-like | 1206 | 0 | 78.6 | F:magnesium ion binding; P:cellular response to insulin stimulus; F:pyruvate kinase activity; F:potassium ion binding; C:cytoplasm; P:glycolytic process; F:kinase activity |
| Efet.01.242647.g1628.t1 | discoidin domain-containing receptor 2-like isoform X1 | 534 | 1.43E-57 | 73.4 | F:ATP binding; P:peptidyl-tyrosine phosphorylation; F:protein tyrosine kinase activity; C:integral component of membrane |
| Efet.01.242710.g1629.t1 | hypothetical protein CAPTEDRAFT_202917 | 591 | 1.04E-13 | 49.2 | C:membrane; C:integral component of membrane |
| Efet.01.242783.g1633.t1 | resistance to inhibitors of cholinesterase 3-like | 837 | 1.12E-22 | 62 | C:membrane; C:integral component of membrane |
| Efet.01.242835.g1634.t1 | FMRFamide receptor | 795 | 3.36E-22 | 51 | F:G-protein coupled receptor activity; F:G-protein coupled peptide receptor activity; P:G-protein coupled receptor signaling pathway; C:membrane; C:integral component of membrane |
| Efet.01.242845.g1635.t1 | GPI mannosyltransferase 4 | 507 | 1.49E-20 | 64.1 | F:transferase activity, transferring glycosyl groups; C:membrane; C:integral component of membrane; F:transferase activity |
| Efet.01.24289.g1764.t1 | 26S proteasome non-ATPase regulatory subunit 11-like | 312 | 2.30E-32 | 84.2 | C:proteasome complex |
| Efet.01.242946.g1636.t1 | hypothetical protein CAPTEDRAFT_206253 | 459 | 2.55E-11 | 48.44 | C:membrane; C:integral component of membrane; F:carbohydrate binding |
| Efet.01.24295.g1766.t1 | ribosomal S6 kinase alpha-1 isoform X1 | 297 | 1.44E-27 | 78.2 | F:magnesium ion binding; F:ATP binding; F:protein serine/threonine kinase activity; P:protein phosphorylation; C:intracellular; P:intracellular signal transduction |
| Efet.01.242998.g1637.t1 | suppressor of lurcher 1-like isoform X1 | 237 | 1.04E-16 | 63.95 |  |
| Efet.01.24307.g1767.t1 | hypothetical protein HELRODRAFT_190718 | 975 | 1.02E-13 | 85 | P:signal transduction |
| Efet.01.243079.g1638.t1 | epidermal growth factor receptor isoform X1 | 636 | 1.61E-26 | 59.75 | P:cellular process |
| Efet.01.243125.g1641.t1 | peptidase C1 | 378 | 5.63E-33 | 74.45 | C:extracellular space; F:cysteine-type endopeptidase activity; P:cell adhesion; P:proteolysis involved in cellular protein catabolic process; C:lysosome |
| Efet.01.243146.g1642.t1 | tyrosine- kinase transmembrane receptor | 1596 | 1.76E-63 | 52.25 | F:nucleotide binding; F:ATP binding; C:membrane; C:integral component of membrane; P:transmembrane receptor protein tyrosine kinase signaling pathway; C:receptor complex; F:protein kinase activity; P:phosphorylation; F:Wnt-protein binding; P:protein phosphorylation; F:kinase activity |
| Efet.01.24322.g1768.t1 | hypothetical protein CAPTEDRAFT_218471 | 942 | 2.11E-25 | 49 | C:membrane; C:integral component of membrane |
| Efet.01.243235.g1646.t1 | Zinc finger | 231 | 2.11E-17 | 75.2 | F:binding |
| Efet.01.24328.g1769.t1 | calcium-translocating P-type PMCA-type | 246 | 3.89E-34 | 94.5 | F:ATP binding; F:metal ion binding; C:integral component of membrane; P:calcium ion transmembrane transport; F:calcium-transporting ATPase activity |
| Efet.01.243407.g1650.t1 | microspherule 1-like | 207 | 1.29E-12 | 82.6 | P:negative regulation of telomere maintenance via telomere lengthening; P:histone H4-K5 acetylation; F:histone acetyltransferase activity (H4-K16 specific); P:histone H4-K8 acetylation; F:histone acetyltransferase activity (H4-K8 specific); F:histone acetyltransferase activity (H4-K5 specific); P:histone H4-K16 acetylation; C:histone acetyltransferase complex; C:cytoplasm; C:actin cytoskeleton; C:cell junction; C:Ino80 complex; F:telomerase inhibitor activity; P:negative regulation of telomerase activity; C:MLL1 complex |
| Efet.01.243464.g1651.t1 | zinc finger 839-like | 702 | 9.41E-36 | 64.05 | F:metal ion binding |
| Efet.01.243473.g1652.t1 | hypothetical protein CAPTEDRAFT_222571 | 1749 | 1.67E-137 | 59.95 | C:membrane; C:integral component of membrane |
| Efet.01.243480.g1653.t1 | brain-specific homeobox POU domain 3 | 324 | 2.67E-34 | 82.7 | C:nucleus; F:transcription factor activity, sequence-specific DNA binding; P:transcription, DNA-templated; P:regulation of transcription, DNA-templated; F:sequence-specific DNA binding |
| Efet.01.243487.g1654.t1 | caspase 8 | 336 | 5.92E-15 | 60.75 | F:cysteine-type peptidase activity |
| Efet.01.243489.g1655.t1 | hypothetical protein CAPTEDRAFT_212909 | 705 | 2.63E-32 | 76 |  |
| Efet.01.24354.g1770.t1 | tumor p53-inducible nuclear 1 | 915 | 3.73E-18 | 60.15 | P:positive regulation of autophagy; P:positive regulation of transcription, DNA-templated; P:negative regulation of cell proliferation; P:regulation of autophagy; P:regulation of transcription, DNA-templated |
| Efet.01.243550.g1656.t1 | interferon-induced very large GTPase 1-like | 537 | 7.88E-14 | 52.29 | F:GTP binding |
| Efet.01.243623.g1659.t1 | chloroplast stem-loop binding of 41 kDa chloroplastic-like | 1179 | 2.24E-60 | 56.35 | F:coenzyme binding; F:catalytic activity; C:membrane; C:integral component of membrane |
| Efet.01.243669.g1660.t1 | filamin-A isoform X3 | 579 | 1.24E-41 | 62.35 |  |
| Efet.01.243707.g1661.t1 | monocarboxylate transporter 2-like | 492 | 3.30E-50 | 61.25 | P:transmembrane transport; C:membrane; C:integral component of membrane |
| Efet.01.243796.g1662.t1 | zinc finger OZF-like isoform X2 | 1872 | 3.41E-61 | 42.15 | F:nucleic acid binding; F:metal ion binding; F:methyltransferase activity; F:transferase activity; P:methylation |
| Efet.01.243797.g1663.t1 | hypothetical protein A3Q56_05864 | 633 | 8.28E-07 | 58 |  |
| Efet.01.243823.g1665.t1 | lysophosphatidic acid receptor 4-like | 243 | 9.62E-30 | 64.2 | P:signal transduction; C:membrane; F:signal transducer activity |
| Efet.01.243926.g1666.t1 | pre-mRNA-processing factor 17-like | 612 | 3.44E-25 | 85.95 | C:catalytic step 2 spliceosome; P:mRNA splicing, via spliceosome |
| Efet.01.243955.g1667.t1 | helicase carboxy-terminal domain | 2106 | 1.50E-176 | 54 | P:protein transport |
| Efet.01.243988.g1668.t1 | WD repeat and FYVE domain-containing 3 | 297 | 3.24E-08 | 78.3 | F:metal ion binding |
| Efet.01.244152.g1670.t1 | tyrosine- kinase Src42A-like isoform X1 | 450 | 6.09E-30 | 83.15 |  |
| Efet.01.244158.g1671.t1 | ecto-NOX disulfide-thiol exchanger 2-like | 315 | 2.01E-14 | 74.5 | F:nucleotide binding; F:nucleic acid binding |
| Efet.01.244165.g1672.t1 | Troponin T | 552 | 1.96E-38 | 59.3 | P:regulation of muscle contraction; C:troponin complex |
| Efet.01.244169.g1673.t1 | Na(+) H(+) exchange regulatory cofactor NHE-RF1 | 1044 | 5.51E-60 | 56.5 | F:phosphatase binding; P:sensory perception of sound; C:nucleus; P:negative regulation of phosphatidylinositol 3-kinase signaling; C:cytoplasm; C:stereocilium; C:apical plasma membrane |
| Efet.01.24419.g1772.t1 | alkaline ceramidase | 642 | 2.59E-47 | 56.9 | F:hydrolase activity, acting on carbon-nitrogen (but not peptide) bonds, in linear amides; C:membrane; C:integral component of membrane; P:ceramide metabolic process; F:hydrolase activity |
| Efet.01.244195.g1675.t1 | cation-independent mannose-6-phosphate receptor-like | 375 | 3.18E-24 | 57.15 | C:trans-Golgi network; C:cytoplasm; C:membrane; C:integral component of membrane; P:transport; F:transporter activity |
| Efet.01.244226.g1676.t1 | Casein kinase II subunit alpha | 285 | 3.19E-54 | 77.85 | P:mitotic spindle checkpoint; F:ATP binding; P:regulation of mitophagy; C:PcG protein complex; F:protein N-terminus binding; F:protein serine/threonine kinase activity; P:protein phosphorylation; P:positive regulation of protein targeting to mitochondrion |
| Efet.01.244246.g1678.t1 | F-box only 33 | 276 | 4.75E-13 | 56 |  |
| Efet.01.244290.g1679.t1 | GRB2-associated and regulator of MAPK -like | 1068 | 4.38E-12 | 58.45 |  |
| Efet.01.244598.g1680.t1 | Peptide-N(4)-(N-acetyl-beta-glucosaminyl)asparagine amidase | 1479 | 6.12E-164 | 64.25 | P:glycoprotein catabolic process; C:cytoplasm |
| Efet.01.244609.g1682.t1 | metabotropic glutamate receptor | 816 | 5.73E-54 | 52.05 | F:G-protein coupled receptor activity; P:G-protein coupled receptor signaling pathway; P:signal transduction; C:membrane; C:integral component of membrane; F:signal transducer activity; C:plasma membrane |
| Efet.01.244642.g1683.t1 | DNA polymerase eta-like | 474 | 9.63E-42 | 53.55 | P:DNA repair; F:nucleotidyltransferase activity; F:DNA-directed DNA polymerase activity; P:DNA biosynthetic process; F:transferase activity; F:damaged DNA binding |
| Efet.01.244652.g1684.t1 | putative prestalk protein | 246 | 3.57E-06 | 58 |  |
| Efet.01.244693.g1687.t1 | cell death specification 2-like | 501 | 5.26E-19 | 67.15 | F:transcription factor activity, sequence-specific DNA binding; P:transcription, DNA-templated; P:regulation of transcription, DNA-templated; F:sequence-specific DNA binding |
| Efet.01.244693.g1688.t1 | DNA-directed RNA polymerases and III subunit RPABC3 | 282 | 1.92E-48 | 86.2 | F:DNA-directed 5'-3' RNA polymerase activity; P:transcription, DNA-templated |
| Efet.01.244696.g1690.t1 | hypothetical protein HELRODRAFT_171537 | 966 | 8.22E-17 | 67 |  |
| Efet.01.24477.g1775.t1 | serine arginine repetitive matrix 1-like | 1467 | 4.22E-30 | 56.1 |  |
| Efet.01.244824.g1691.t1 | neuroglobin-like | 570 | 1.22E-31 | 64.2 | F:oxygen binding; P:oxygen transport; F:oxygen transporter activity; F:heme binding |
| Efet.01.244831.g1692.t1 | 3-hydroxy-3-methylglutaryl-coenzyme A reductase | 249 | 6.85E-18 | 92.6 | C:endoplasmic reticulum membrane; F:NADP binding; C:integral component of membrane; P:coenzyme A metabolic process; P:oxidation-reduction process; P:isoprenoid biosynthetic process; F:hydroxymethylglutaryl-CoA reductase (NADPH) activity |
| Efet.01.244879.g1694.t1 | trafficking particle complex subunit 13-like isoform X4 | 444 | 1.12E-26 | 77.7 |  |
| Efet.01.244973.g1696.t1 | troponin T | 237 | 2.18E-16 | 75.75 | P:regulation of muscle contraction; C:troponin complex |
| Efet.01.24505.g1777.t1 | titin isoform X2 | 1065 | 1.69E-54 | 45.5 |  |
| Efet.01.24505.g1778.t1 | titin isoform X2 | 7593 | 1.91E-59 | 60.25 |  |
| Efet.01.24509.g1779.t1 | dynein heavy chain axonemal-like isoform X1 | 1170 | 5.09E-156 | 84.3 | F:ATPase activity; F:ATP binding; F:microtubule motor activity; F:actin binding; C:dynein complex; P:phosphorylation; P:microtubule-based movement; F:kinase activity |
| Efet.01.245109.g1700.t1 | ras association domain-containing 1-like isoform X3 | 297 | 6.47E-14 | 66.15 | P:signal transduction |
| Efet.01.245206.g1702.t1 | filamin-A isoform X6 | 354 | 9.29E-40 | 67.15 | F:actin filament binding |
| Efet.01.245219.g1703.t1 | metallo-beta-lactamase domain-containing 2 | 654 | 1.21E-75 | 55.25 | C:extracellular exosome; F:metal ion binding; F:hydrolase activity |
| Efet.01.245278.g1704.t1 | netrin precursor | 366 | 8.42E-10 | 55.25 | C:extracellular region |
| Efet.01.24531.g1780.t1 | ELAV 1 isoform X3 | 699 | 1.69E-95 | 75.35 | F:nucleotide binding; F:RNA binding |
| Efet.01.245400.g1706.t1 | hypothetical protein CAPTEDRAFT_203582 | 780 | 7.45E-46 | 53.4 |  |
| Efet.01.245400.g1707.t1 | PREDICTED: uncharacterized protein LOC105849438 | 699 | 9.76E-41 | 60.4 |  |
| Efet.01.245408.g1708.t1 | 40S ribosomal S27-like | 249 | 2.33E-47 | 95.35 | F:structural constituent of ribosome; C:cytosolic small ribosomal subunit; F:metal ion binding; P:ribosomal small subunit assembly; P:translation |
| Efet.01.245427.g1709.t1 | synaptotagmin- | 594 | 2.19E-66 | 78.7 | C:presynaptic membrane; C:neuronal cell body; P:regulation of glucagon secretion; C:dendrite; F:phosphatidylinositol-4,5-bisphosphate binding; P:regulation of insulin secretion; C:early phagosome; F:phosphatidylserine binding; C:extracellular exosome; C:terminal bouton; F:calcium ion binding; C:cytosol; P:short-term synaptic potentiation; P:calcium ion regulated lysosome exocytosis; P:vesicle-mediated cholesterol transport; P:regulation of dopamine secretion; P:synaptic vesicle recycling; F:syntaxin binding; P:phagosome-lysosome fusion; P:positive regulation of calcium ion-dependent exocytosis; C:lysosomal membrane; F:calcium-dependent phospholipid binding; C:peroxisomal membrane; F:calmodulin binding; P:calcium ion-regulated exocytosis of neurotransmitter; P:plasma membrane repair; P:regulation of bone remodeling; C:integral component of membrane; C:cell junction; C:phagocytic vesicle membrane; C:synaptic vesicle membrane; C:dense core granule; F:clathrin binding; P:regulation of phagocytosis |
| Efet.01.245444.g1710.t1 | oocyte zinc finger 6-like | 1788 | 2.59E-21 | 44 | F:binding |
| Efet.01.245457.g1711.t1 | GDH 6PGL endoplasmic bifunctional | 402 | 7.74E-29 | 61.1 | P:small molecule metabolic process; P:carbohydrate metabolic process; F:catalytic activity |
| Efet.01.245458.g1712.t1 | solute carrier family 35 member E2-like | 240 | 3.12E-19 | 74.85 | C:membrane |
| Efet.01.245519.g1715.t1 | hypothetical protein HELRODRAFT_161987 | 1308 | 3.50E-12 | 72.63 | F:nucleic acid binding; F:metal ion binding |
| Efet.01.245570.g1717.t1 | Transmembrane 9 superfamily member | 240 | 1.64E-39 | 91.9 | C:integral component of membrane |
| Efet.01.2456.g222.t1 | nesprin-1-like isoform X1 | 1083 | 7.86E-29 | 53.1 |  |
| Efet.01.245601.g1718.t1 | protocadherin beta-10-like isoform X1 | 813 | 5.88E-13 | 51.65 | F:calcium ion binding; C:membrane; C:integral component of membrane; P:cell adhesion; P:homophilic cell adhesion via plasma membrane adhesion molecules; C:plasma membrane |
| Efet.01.245601.g1719.t1 | protocadherin alpha-10-like | 429 | 7.70E-18 | 57.8 | F:calcium ion binding; C:membrane; C:integral component of membrane; P:cell adhesion; P:homophilic cell adhesion via plasma membrane adhesion molecules; C:plasma membrane |
| Efet.01.245645.g1720.t1 | FMRFamide receptor-like | 438 | 8.52E-41 | 52.4 | F:signal transducer activity |
| Efet.01.245650.g1721.t1 | prolactin-releasing peptide receptor-like | 1152 | 3.56E-92 | 58.9 | F:G-protein coupled receptor activity; P:G-protein coupled receptor signaling pathway; C:membrane |
| Efet.01.24566.g1781.t1 | zinc finger 1 isoform X1 | 333 | 7.22E-22 | 69.45 | F:nucleic acid binding |
| Efet.01.24568.g1783.t1 | hypothetical protein HELRODRAFT_154876 | 498 | 1.11E-14 | 55.83 | F:transferase activity, transferring glycosyl groups |
| Efet.01.245862.g1725.t1 | FMRFamide receptor-like | 1287 | 9.36E-89 | 56.25 | F:G-protein coupled receptor activity; P:signal transduction; C:membrane |
| Efet.01.245886.g1726.t1 | atrial natriuretic peptide receptor 1-like | 237 | 6.68E-36 | 82.1 | F:ATP binding; P:cGMP biosynthetic process; C:integral component of membrane; F:guanylate cyclase activity; F:protein kinase activity; P:protein phosphorylation; C:intracellular; P:intracellular signal transduction |
| Efet.01.245917.g1728.t1 | cytoplasmic aconitate hydratase-like | 330 | 8.02E-10 | 56.3 | P:metabolic process; F:lyase activity; F:4 iron, 4 sulfur cluster binding; F:iron-sulfur cluster binding; F:aconitate hydratase activity |
| Efet.01.245982.g1731.t1 | Sorting nexin-12 | 207 | 1.29E-23 | 83.95 | C:endosome; P:vesicle organization; F:phosphatidylinositol binding; C:extrinsic component of membrane; P:endocytosis |
| Efet.01.24599.g1785.t1 | endoplasmin | 801 | 9.55E-158 | 85.85 | F:ATP binding; P:response to hypoxia; P:otolith tethering; F:unfolded protein binding; P:protein folding |
| Efet.01.246000.g1732.t1 | SCO-spondin | 450 | 2.90E-26 | 59.65 |  |
| Efet.01.246004.g1733.t1 | tektin-3-like isoform X1 | 942 | 3.03E-137 | 74.4 |  |
| Efet.01.246116.g1736.t1 | somatostatin receptor type 1-like | 675 | 1.45E-63 | 56 | F:G-protein coupled peptide receptor activity; P:G-protein coupled receptor signaling pathway; C:membrane |
| Efet.01.246142.g1737.t1 | vesicular glutamate transporter 2 isoform X3 | 891 | 5.45E-93 | 58.9 | C:membrane; C:cell part; P:chemical synaptic transmission; P:visual perception; P:transport |
| Efet.01.246146.g1739.t1 | lin-7 homolog B | 276 | 2.29E-45 | 97.15 | C:postsynaptic density; C:bicellular tight junction; C:presynapse; F:protein binding; P:protein localization to basolateral plasma membrane; P:maintenance of epithelial cell apical/basal polarity; C:postsynaptic membrane; P:protein transport; C:basolateral plasma membrane; C:neuron projection; P:exocytosis; P:neurotransmitter secretion |
| Efet.01.246206.g1744.t1 | hypothetical protein HELRODRAFT_192035 | 369 | 1.51E-17 | 67.6 | F:metal ion binding |
| Efet.01.246208.g1745.t1 | dynein heavy chain axonemal-like isoform X1 | 387 | 1.72E-14 | 64.15 | F:nucleotide binding |
| Efet.01.246214.g1747.t1 | multidrug resistance 1 | 444 | 4.08E-33 | 67.1 | F:nucleotide binding; F:ATPase activity; F:ATP binding; F:ATPase activity, coupled to transmembrane movement of substances; P:transmembrane transport; C:membrane; C:integral component of membrane; P:transport |
| Efet.01.246234.g1748.t1 | Coatomer subunit beta | 387 | 3.65E-63 | 89.9 | P:vesicle-mediated transport; F:structural molecule activity; C:Golgi membrane; P:intracellular protein transport; C:COPI vesicle coat |
| Efet.01.246246.g1749.t1 | CCAAT enhancer binding | 1221 | 2.49E-18 | 75.65 | F:transcription factor activity, sequence-specific DNA binding; P:transcription, DNA-templated; P:regulation of transcription, DNA-templated; F:sequence-specific DNA binding |
| Efet.01.246354.g1753.t1 | DCN1 1 | 639 | 3.68E-76 | 80.7 | F:ubiquitin-like protein binding; F:ubiquitin conjugating enzyme binding; P:positive regulation of ubiquitin-protein transferase activity; C:ubiquitin ligase complex; C:membrane; F:cullin family protein binding; P:protein neddylation |
| Efet.01.246377.g1754.t1 | multiple epidermal growth factor-like domains 8 | 489 | 1.88E-28 | 52.05 | F:calcium ion binding; C:membrane; C:integral component of membrane |
| Efet.01.246401.g1755.t1 | hypothetical protein CAPTEDRAFT_224152 | 498 | 5.58E-10 | 54 |  |
| Efet.01.246409.g1756.t1 | synaptotagmin 7a | 285 | 7.27E-24 | 82.8 | C:membrane; P:exocytosis |
| Efet.01.246440.g1757.t1 | neogenin isoform X2 | 459 | 1.11E-11 | 52.2 | C:membrane |
| Efet.01.246460.g1758.t1 | zinc finger 710 isoform X1 | 723 | 5.54E-29 | 48.05 | F:nucleic acid binding; C:nucleus; F:transcription cofactor activity; F:metal ion binding; P:regulation of transcription from RNA polymerase II promoter |
| Efet.01.246523.g1760.t1 | persulfide dioxygenase mitochondrial-like | 357 | 6.90E-08 | 76.53 |  |
| Efet.01.246546.g1762.t1 | KN motif and ankyrin repeat domain-containing 1-like isoform X1 | 1761 | 8.34E-34 | 45.2 |  |
| Efet.01.246588.g1763.t1 | 52 kDa repressor of the inhibitor of the kinase-like | 288 | 1.73E-09 | 66.05 | F:binding |
| Efet.01.246589.g1764.t1 | hypothetical protein CAPTEDRAFT_224745 | 480 | 1.95E-06 | 78 |  |
| Efet.01.24660.g1786.t1 | Bone morphogenetic 10 | 1011 | 1.85E-10 | 45.4 | P:positive regulation of cardiac muscle cell proliferation; P:regulation of cardiac muscle hypertrophy in response to stress; P:positive regulation of cartilage development; P:regulation of MAPK cascade; F:growth factor activity; P:ventricular cardiac muscle tissue morphogenesis; P:multicellular organism development; P:cell adhesion; P:pathway-restricted SMAD protein phosphorylation; C:extracellular region; F:hormone activity; P:heart trabecula formation; P:adult heart development; P:activin receptor signaling pathway; P:heart development; P:negative regulation of cell growth; P:negative regulation of cardiac muscle hypertrophy; P:positive regulation of transcription, DNA-templated; P:BMP signaling pathway; P:positive regulation of cardiac muscle hypertrophy; P:negative regulation of endothelial cell migration; P:positive regulation of cell proliferation involved in heart morphogenesis; F:telethonin binding; F:receptor serine/threonine kinase binding; P:atrial cardiac muscle tissue morphogenesis; P:positive regulation of sarcomere organization; F:transforming growth factor beta receptor binding; P:SMAD protein signal transduction; P:sarcomere organization; P:regulation of apoptotic process; F:cytokine activity; P:cardiac muscle cell proliferation; P:growth; C:cell surface; C:Z disc; C:extracellular space; C:cytoplasm; P:positive regulation of gene expression; P:ventricular cardiac muscle cell development; P:regulation of cardiac muscle contraction; P:positive regulation of pathway-restricted SMAD protein phosphorylation; P:negative regulation of cell migration |
| Efet.01.246645.g1767.t1 | kaptin | 288 | 2.04E-09 | 77.15 | F:actin binding; C:actin cytoskeleton; P:actin filament organization |
| Efet.01.246668.g1769.t1 | vacuolar sorting-associated VTA1 homolog isoform X2 | 582 | 1.44E-31 | 54.45 | C:extracellular exosome; C:cytoplasm; F:protein C-terminus binding; P:multivesicular body sorting pathway; C:cytosol; P:viral budding |
| Efet.01.246753.g1770.t1 | serine threonine kinase | 990 | 9.24E-84 | 67.65 | F:nucleotide binding; P:regulation of cell shape; C:nucleus; C:cytoplasm; P:Wnt signaling pathway; F:protein serine/threonine kinase activity; P:endocytosis; P:peptidyl-serine phosphorylation |
| Efet.01.246770.g1771.t1 | DCC-interacting 13-alpha-like | 510 | 3.66E-57 | 72.85 |  |
| Efet.01.246791.g1772.t1 | T family of potassium channels 18-like | 552 | 1.66E-44 | 61.94 | P:potassium ion transmembrane transport; C:integral component of membrane; F:potassium channel activity |
| Efet.01.246792.g1773.t1 | probable G- coupled receptor 158 | 369 | 3.40E-25 | 60.7 | C:membrane |
| Efet.01.246852.g1774.t1 | kelch 35 | 678 | 2.74E-14 | 46.7 |  |
| Efet.01.246867.g1775.t1 | transcription initiation factor TFIID subunit 4-like | 435 | 1.30E-11 | 84.15 | F:translation initiation factor activity; F:transcription factor activity, sequence-specific DNA binding; C:transcription factor TFIID complex; F:protein heterodimerization activity; P:regulation of transcription, DNA-templated; P:DNA-templated transcription, initiation; P:translational initiation |
| Efet.01.24698.g1789.t1 | DBF4 homolog B isoform X3 | 4986 | 6.31E-09 | 48 | F:protein kinase binding; F:zinc ion binding; P:positive regulation of nuclear cell cycle DNA replication; F:protein kinase activator activity; P:positive regulation of cell proliferation; C:nucleoplasm; F:nucleic acid binding; C:nucleus; C:cytoplasm; C:intracellular membrane-bounded organelle; P:activation of protein kinase activity; C:aggresome; P:positive regulation of G2/M transition of mitotic cell cycle |
| Efet.01.247036.g1776.t1 | rootletin-like isoform X3 | 450 | 2.38E-27 | 67 | C:ciliary rootlet; P:centrosome organization |
| Efet.01.247036.g1777.t1 | rootletin-like isoform X1 | 795 | 8.26E-13 | 53.15 |  |
| Efet.01.247061.g1778.t1 | zinc finger 674 isoform X1 | 1110 | 7.66E-38 | 47.65 | F:nucleic acid binding; F:metal ion binding; P:regulation of transcription, DNA-templated; C:intracellular |
| Efet.01.24712.g1790.t1 | hypothetical protein CAPTEDRAFT_193210 | 213 | 8.31E-07 | 53 | C:membrane; C:integral component of membrane |
| Efet.01.247142.g1782.t1 | translin isoform X2 | 204 | 3.64E-27 | 89.5 | C:nucleus; P:signal transduction; F:sequence-specific DNA binding |
| Efet.01.247164.g1784.t1 | zinc finger -like 1 | 345 | 6.46E-29 | 68.3 | F:metal ion binding; C:membrane |
| Efet.01.247181.g1785.t1 | tolloid 1 | 204 | 3.03E-17 | 73.6 | F:zinc ion binding; F:calcium ion binding; C:membrane; P:proteolysis; F:metalloendopeptidase activity |
| Efet.01.247293.g1788.t1 | lipoyltransferase mitochondrial | 516 | 2.79E-14 | 53.35 | C:mitochondrion; P:cellular protein modification process; F:transferase activity; P:protein lipoylation |
| Efet.01.24733.g1792.t1 | MAGUK p55 subfamily member 5 | 204 | 9.91E-33 | 90.8 | P:bicellular tight junction assembly; P:establishment of epithelial cell polarity |
| Efet.01.247396.g1791.t1 | histone-lysine N-methyltransferase NSD2 | 678 | 5.05E-12 | 60.75 | P:regulation of nucleobase-containing compound metabolic process; P:regulation of macromolecule metabolic process; P:histone lysine methylation; F:metal ion binding; F:methyltransferase activity; P:cardiac septum morphogenesis |
| Efet.01.247428.g1792.t1 | monocarboxylate transporter 12-like | 1413 | 3.96E-44 | 48.65 | P:transmembrane transport; C:membrane; P:monocarboxylic acid transport; C:integral component of membrane; F:monocarboxylic acid transmembrane transporter activity |
| Efet.01.247437.g1793.t1 | bicaudal D | 234 | 5.74E-14 | 79.1 | C:Golgi apparatus; P:transport |
| Efet.01.247437.g1794.t1 | bicaudal D-like isoform X1 | 2040 | 2.07E-157 | 60.3 | C:Golgi apparatus; P:transport |
| Efet.01.247441.g1795.t1 | Secernin-3 | 207 | 1.21E-17 | 79.1 | C:intracellular membrane-bounded organelle; F:dipeptidase activity; P:proteolysis; C:integral component of membrane; F:2 iron, 2 sulfur cluster binding; P:exocytosis |
| Efet.01.247483.g1797.t1 | dynein gamma flagellar outer arm-like | 882 | 1.93E-49 | 64.35 | F:nucleotide binding; F:ATP binding; F:microtubule motor activity; C:dynein complex; P:microtubule-based movement |
| Efet.01.247507.g1801.t1 | DDB1- and CUL4-associated factor 6-like | 279 | 3.64E-09 | 70.6 |  |
| Efet.01.247526.g1803.t1 | archaemetzincin-2-like | 327 | 1.45E-24 | 63.9 | F:zinc ion binding |
| Efet.01.247526.g1804.t1 | nucleolin-like isoform X1 | 576 | 3.71E-26 | 52.4 | F:organic cyclic compound binding; F:heterocyclic compound binding; C:organelle; C:intracellular part |
| Efet.01.247538.g1805.t1 | ankyrin repeat domain-containing 50-like | 495 | 2.38E-53 | 85.45 | F:2-alkenal reductase [NAD(P)] activity; P:oxidation-reduction process |
| Efet.01.247575.g1806.t1 | Potassium voltage-gated channel eag | 1041 | 4.94E-20 | 49.1 | P:transport |
| Efet.01.247606.g1807.t1 | galanin receptor type 2-like | 510 | 3.18E-11 | 55.33 | F:G-protein coupled receptor activity; P:G-protein coupled receptor signaling pathway; P:signal transduction; C:membrane; C:integral component of membrane; F:signal transducer activity |
| Efet.01.247624.g1809.t1 | cAMP-regulated phospho 21 isoform X6 | 333 | 9.93E-10 | 62.6 | F:nucleic acid binding; P:cellular response to heat |
| Efet.01.247644.g1812.t1 | histidine decarboxylase-like | 504 | 1.33E-22 | 70.32 | F:lyase activity; P:carboxylic acid metabolic process |
| Efet.01.24768.g1794.t1 | WD40 repeat domain-containing | 1122 | 5.81E-51 | 52.25 |  |
| Efet.01.247722.g1814.t1 | potassium voltage-gated channel Shaw-like isoform X1 | 441 | 1.76E-28 | 73.15 | F:voltage-gated potassium channel activity; P:potassium ion transmembrane transport; C:voltage-gated potassium channel complex; P:regulation of ion transmembrane transport |
| Efet.01.247787.g1816.t1 | hypothetical protein HELRODRAFT_193524 | 234 | 1.27E-23 | 71.55 | C:integral component of membrane; F:carbohydrate binding |
| Efet.01.247844.g1817.t1 | Chondroitin sulfate synthase | 708 | 3.79E-40 | 60.3 | C:Golgi cisterna membrane; F:acetylgalactosaminyltransferase activity |
| Efet.01.247864.g1818.t1 | mitogen-activated kinase kinase kinase 10-like isoform X1 | 207 | 2.75E-09 | 71.9 | F:ATP binding; F:protein kinase activity; P:protein phosphorylation |
| Efet.01.247875.g1819.t1 | galactose mutarotase | 969 | 0 | 69.5 | F:catalytic activity |
| Efet.01.248002.g1822.t1 | hypothetical protein HELRODRAFT_173596 | 333 | 7.82E-10 | 57.63 | C:membrane; C:integral component of membrane |
| Efet.01.248016.g1823.t1 | protocadherin beta-12 | 3201 | 0 | 55.25 | F:calcium ion binding; C:membrane; C:integral component of membrane; P:cell adhesion; P:homophilic cell adhesion via plasma membrane adhesion molecules; C:plasma membrane |
| Efet.01.248063.g1824.t1 | T family of potassium channels 18-like | 1611 | 4.22E-31 | 55.8 | P:ion transport; P:potassium ion transmembrane transport; C:membrane; C:integral component of membrane; F:potassium channel activity; P:transport |
| Efet.01.24823.g1795.t1 | hypothetical protein | 1080 | 2.34E-07 | 50 |  |
| Efet.01.24823.g1796.t1 | ubiquitin carboxyl-terminal hydrolase 16 isoform X1 | 579 | 2.90E-25 | 59.9 | P:cellular macromolecule metabolic process; P:proteolysis; F:cysteine-type peptidase activity; F:binding; P:regulation of biological process |
| Efet.01.248254.g1829.t1 | polycystic kidney disease 1-like 1 | 642 | 8.89E-24 | 48.4 | P:detection of mechanical stimulus; F:calcium ion binding; F:calcium channel activity; C:membrane; C:integral component of membrane; P:calcium ion transmembrane transport |
| Efet.01.248270.g1831.t1 | peptidyl-prolyl isomerase FKBP12 | 225 | 3.03E-22 | 79.15 | C:endoplasmic reticulum membrane; F:peptidyl-prolyl cis-trans isomerase activity; F:FK506 binding; P:chaperone-mediated protein folding; P:protein peptidyl-prolyl isomerization |
| Efet.01.248345.g1832.t1 | E3 ubiquitin- ligase LRSAM1-like | 255 | 1.39E-09 | 74.18 | F:zinc ion binding |
| Efet.01.248427.g1833.t1 | hypothetical protein HELRODRAFT_195072 | 630 | 8.86E-90 | 54.95 | C:membrane |
| Efet.01.248433.g1834.t1 | collagen alpha-1(XII) chain-like | 696 | 8.62E-11 | 48.8 | C:extracellular space; F:calcium ion binding; C:collagen trimer; C:proteinaceous extracellular matrix |
| Efet.01.2485.g224.t1 | brefeldin A-inhibited guanine nucleotide-exchange 1 isoform X1 | 291 | 3.32E-38 | 85.55 | F:ARF guanyl-nucleotide exchange factor activity; P:regulation of ARF protein signal transduction; P:positive regulation of GTPase activity |
| Efet.01.248502.g1836.t1 | CG6379- | 336 | 2.86E-18 | 70.25 | C:nucleus; P:7-methylguanosine mRNA capping; F:mRNA (nucleoside-2'-O-)-methyltransferase activity; P:cap1 mRNA methylation |
| Efet.01.248572.g1837.t1 | cAMP-dependent kinase type II regulatory subunit | 462 | 1.44E-53 | 78.35 | P:peptidyl-tyrosine phosphorylation; P:regulation of protein kinase activity; F:non-membrane spanning protein tyrosine kinase activity; F:cAMP binding; F:cAMP-dependent protein kinase regulator activity; C:cAMP-dependent protein kinase complex |
| Efet.01.248603.g1838.t1 | serine threonine- kinase WNK3- | 561 | 3.51E-25 | 56.8 | P:positive regulation of molecular function; F:protein serine/threonine kinase activity; P:protein phosphorylation; P:positive regulation of biological process; P:intracellular signal transduction |
| Efet.01.248652.g1842.t1 | tax1-binding 1 homolog isoform X2 | 903 | 1.06E-37 | 52.5 |  |
| Efet.01.248669.g1843.t1 | glutamate receptor NMDA 3A-like | 1059 | 3.18E-84 | 62.2 | C:postsynaptic membrane; P:ion transmembrane transport; C:integral component of membrane; C:cell junction; P:ionotropic glutamate receptor signaling pathway; F:ionotropic glutamate receptor activity; F:extracellular-glutamate-gated ion channel activity |
| Efet.01.248735.g1846.t1 | zinc finger 780A-like isoform X1 | 1815 | 1.18E-13 | 39.15 | F:nucleic acid binding; F:metal ion binding |
| Efet.01.248833.g1851.t1 | hypothetical protein CAPTEDRAFT_201920 | 231 | 6.72E-06 | 89 | P:inositol phosphate-mediated signaling; F:calcium channel activity; P:calcium ion transport; C:membrane; F:inositol 1,4,5-trisphosphate-sensitive calcium-release channel activity; P:calcium ion transmembrane transport; C:endoplasmic reticulum |
| Efet.01.248841.g1852.t1 | hypothetical protein CAPTEDRAFT_225483 | 735 | 5.40E-18 | 48.25 | C:membrane; C:integral component of membrane; F:carbohydrate binding |
| Efet.01.248855.g1853.t1 | TRAF3-interacting 1-like isoform X2 | 228 | 4.05E-18 | 78.15 | F:microtubule binding |
| Efet.01.248932.g1854.t1 | hypothetical protein CAPTEDRAFT_228772 | 807 | 4.56E-11 | 43.5 | C:membrane; C:integral component of membrane |
| Efet.01.248935.g1855.t1 | ribosomal S6 kinase delta-1-like | 1539 | 1.35E-33 | 64.75 | F:anion binding; P:phosphorylation; F:kinase activity |
| Efet.01.248937.g1856.t1 | FMRFamide receptor-like | 606 | 5.79E-38 | 51.85 | F:G-protein coupled receptor activity; F:G-protein coupled peptide receptor activity; P:G-protein coupled receptor signaling pathway; P:signal transduction; C:membrane; C:integral component of membrane; F:signal transducer activity |
| Efet.01.248938.g1857.t1 | neuronal calcium sensor 2-like | 213 | 4.64E-32 | 76.2 | F:calcium ion binding |
| Efet.01.248972.g1858.t1 | toll | 2880 | 0 | 45.7 | C:membrane |
| Efet.01.248993.g1859.t1 | sestrin-1 isoform X1 | 240 | 2.38E-29 | 83.2 | C:nucleus; P:regulation of response to reactive oxygen species |
| Efet.01.249014.g1860.t1 | polycystic kidney disease 1-like | 276 | 1.21E-10 | 64.1 | C:membrane |
| Efet.01.249103.g1862.t1 | uncharacterized protein LOC111129230 | 642 | 4.12E-25 | 50.2 |  |
| Efet.01.249133.g1863.t1 | hypothetical protein HELRODRAFT_162389 | 699 | 1.79E-36 | 68 |  |
| Efet.01.249155.g1865.t1 | substance-P receptor-like [Orbicella faveolata] | 630 | 1.47E-34 | 60.85 | F:G-protein coupled receptor activity; P:G-protein coupled receptor signaling pathway; C:membrane |
| Efet.01.249208.g1866.t1 | neuronal acetylcholine receptor subunit alpha-10-like | 366 | 2.06E-33 | 91.45 | C:postsynaptic membrane; P:cation transmembrane transport; C:integral component of membrane; C:cell junction; F:extracellular ligand-gated ion channel activity |
| Efet.01.249213.g1867.t1 | Dopamine D2-like | 957 | 7.22E-21 | 68.5 | C:integral component of plasma membrane; F:dopamine neurotransmitter receptor activity; P:dopamine receptor signaling pathway |
| Efet.01.24926.g1797.t1 | NAD-dependent deacetylase Sirt6-like | 414 | 6.22E-30 | 53.05 | F:NAD+ binding |
| Efet.01.249267.g1869.t1 | tubulin polyglutamylase TTLL13P-like | 411 | 1.90E-30 | 79 | P:cellular protein modification process |
| Efet.01.249309.g1871.t1 | 6-pyruvoyl tetrahydrobiopterin synthase | 408 | 2.21E-58 | 78.35 | P:queuosine biosynthetic process; F:metal ion binding; F:lyase activity |
| Efet.01.249313.g1872.t1 | hypothetical protein CAPTEDRAFT_226427 | 213 | 4.97E-15 | 67.5 | F:calcium ion binding; C:membrane; C:integral component of membrane |
| Efet.01.24933.g1798.t1 | dopey-1-like isoform X1 | 813 | 2.30E-41 | 54.2 |  |
| Efet.01.249521.g1874.t1 | thyrotropin-releasing hormone receptor-like | 726 | 3.11E-70 | 69.75 | F:G-protein coupled receptor activity; P:signal transduction; C:membrane |
| Efet.01.24958.g1802.t1 | DNA polymerase | 609 | 9.72E-59 | 70.9 |  |
| Efet.01.249598.g1875.t1 | coiled-coil domain-containing 51-like | 834 | 1.41E-35 | 60.5 | C:membrane; C:integral component of membrane |
| Efet.01.249691.g1876.t1 | sodium-dependent proline transporter-like | 303 | 4.03E-08 | 73.5 | P:neurotransmitter transport; F:neurotransmitter:sodium symporter activity; P:transmembrane transport; C:integral component of membrane |
| Efet.01.249755.g1877.t1 | calcium-binding and coiled-coil domain-containing 1-like | 333 | 9.99E-09 | 52 |  |
| Efet.01.249758.g1878.t1 | myosin heavy chain isoform A | 495 | 5.04E-27 | 75.7 | C:myosin complex; F:ATP binding; F:actin binding; P:metabolic process; F:amino acid binding; F:motor activity |
| Efet.01.249766.g1879.t1 | major facilitator superfamily domain-containing 6 | 1890 | 8.62E-178 | 57.05 | C:membrane; C:integral component of membrane |
| Efet.01.249769.g1880.t1 | O- case-like isoform X2 | 771 | 1.04E-95 | 79.65 |  |
| Efet.01.2498.g225.t1 | PREDICTED: uncharacterized protein LOC102806317 | 285 | 5.62E-13 | 62.73 | P:signal transduction |
| Efet.01.249870.g1883.t1 | tripartite motif-containing 2-like | 753 | 5.03E-15 | 46.5 | F:zinc ion binding; F:metal ion binding; C:intracellular |
| Efet.01.249907.g1884.t1 | YTH domain-containing family 2-like isoform X2 | 1383 | 9.48E-122 | 63.65 | F:RNA binding; C:membrane; C:integral component of membrane |
| Efet.01.249908.g1885.t1 | pyruvate dehydrogenase (acetyl-transferring) mitochondrial isoform X1 | 504 | 3.63E-19 | 76.65 | F:transferase activity, transferring phosphorus-containing groups |
| Efet.01.250016.g1.t1 | vacuolar sorting-associated | 249 | 1.20E-15 | 75.35 | P:vesicle-mediated transport; P:single-organism cellular process |
| Efet.01.25007.g1.t1 | substance-K receptor-like | 786 | 3.64E-73 | 69.3 | F:G-protein coupled receptor activity; P:G-protein coupled receptor signaling pathway; C:membrane |
| Efet.01.25013.g2.t1 | EF hand | 468 | 1.80E-43 | 66.85 | P:regulation of cellular process; C:cytoplasm; F:calcium ion binding |
| Efet.01.250161.g2.t1 | hypothetical protein HELRODRAFT_172521 | 315 | 1.03E-06 | 59 | C:membrane; C:integral component of membrane |
| Efet.01.250206.g3.t1 | nidogen and EGF-like domain-containing 1 isoform X1 | 1227 | 9.43E-45 | 69.4 | P:cell-matrix adhesion; C:extracellular exosome; F:calcium ion binding |
| Efet.01.250206.g4.t1 | hypothetical protein HELRODRAFT_165800 | 327 | 1.78E-10 | 68 |  |
| Efet.01.250254.g6.t1 | Retrovirus-related Pol poly from transposon 412 | 324 | 3.04E-37 | 64.55 | F:nucleic acid binding; F:aspartic-type endopeptidase activity; P:proteolysis; P:DNA integration |
| Efet.01.250343.g11.t1 | E3 ubiquitin- ligase Topors | 429 | 1.29E-17 | 75.65 | F:zinc ion binding |
| Efet.01.25039.g3.t1 | GTP cyclohydrolase 1 | 381 | 8.43E-32 | 72.25 | C:cytoplasm; F:GTP cyclohydrolase I activity; P:tetrahydrofolate biosynthetic process |
| Efet.01.250406.g12.t1 | zinc finger 84-like | 1728 | 2.24E-13 | 42.65 | F:nucleic acid binding; F:metal ion binding |
| Efet.01.250450.g13.t1 | exportin-5 | 279 | 2.15E-19 | 72.4 | P:intracellular protein transport; F:Ran GTPase binding; C:intracellular |
| Efet.01.250457.g14.t1 | rho GTPase-activating 17-like isoform X3 | 357 | 1.00E-22 | 72.55 | C:cytoplasm; P:signal transduction |
| Efet.01.25046.g4.t1 | polycystic kidney disease 2-like 1 isoform X1 | 348 | 2.07E-23 | 76.65 | F:calcium ion binding; C:integral component of membrane |
| Efet.01.25048.g5.t1 | muscle M-line assembly unc-89-like | 1203 | 7.35E-06 | 54 |  |
| Efet.01.250492.g15.t1 | hypothetical protein HELRODRAFT_166549 | 411 | 1.65E-35 | 62.33 | C:membrane; C:integral component of membrane |
| Efet.01.25057.g6.t1 | trithorax group osa-like isoform X7 | 1716 | 6.04E-107 | 69.45 | F:DNA binding; C:BAF-type complex; P:chromatin remodeling |
| Efet.01.25058.g7.t1 | KANK1_HUMAN ame: Full=KN motif and ankyrin repeat domain-containing 1 ame: Full=Ankyrin repeat domain-containing 15 ame: Full=Kidney ankyrin repeat-containing | 2331 | 1.32E-06 | 53 | P:negative regulation of Rho protein signal transduction; P:positive regulation of wound healing; P:transcription, DNA-templated; P:negative regulation of lamellipodium morphogenesis; P:negative regulation of substrate adhesion-dependent cell spreading; P:regulation of transcription, DNA-templated; F:beta-catenin binding; P:negative regulation of ruffle assembly; P:negative regulation of insulin receptor signaling pathway; C:cell projection; C:plasma membrane; C:nucleus; F:protein binding; P:negative regulation of actin filament polymerization; C:cytoplasm; P:negative regulation of neuron projection development; C:membrane; C:ruffle membrane; P:positive regulation of Wnt signaling pathway; P:positive regulation of catenin import into nucleus; P:negative regulation of cell migration; P:regulation of establishment of cell polarity |
| Efet.01.250649.g16.t1 | platelet-activating factor acetylhydrolase IB subunit gamma-like | 312 | 7.28E-38 | 70.45 | F:hydrolase activity |
| Efet.01.250659.g17.t1 | sulfate transporter-like | 1593 | 2.15E-106 | 58.8 | P:inorganic anion transport; F:inorganic anion transmembrane transporter activity; P:single-organism process |
| Efet.01.25068.g9.t1 | protocadherin beta-10-like isoform X1 | 2514 | 1.03E-140 | 52.75 | F:calcium ion binding; C:membrane; C:integral component of membrane; P:cell adhesion; P:homophilic cell adhesion via plasma membrane adhesion molecules; C:plasma membrane |
| Efet.01.250693.g19.t1 | cAMP-responsive element-binding -like | 234 | 6.19E-25 | 87.1 | F:transcription factor activity, sequence-specific DNA binding; P:regulation of transcription, DNA-templated; F:sequence-specific DNA binding |
| Efet.01.250728.g20.t1 | papilin-like isoform X1 | 825 | 2.48E-22 | 48.2 | F:chitin binding; F:serine-type endopeptidase inhibitor activity; P:chitin metabolic process; P:negative regulation of endopeptidase activity; C:extracellular region |
| Efet.01.250796.g23.t1 | protocadherin-11 X-linked | 2385 | 5.84E-141 | 53.8 | F:calcium ion binding; C:membrane; C:integral component of membrane; P:cell adhesion; P:homophilic cell adhesion via plasma membrane adhesion molecules; C:plasma membrane |
| Efet.01.250799.g24.t1 | synaptotagmin 46 | 339 | 2.35E-26 | 66.35 | F:protein binding; P:synaptic vesicle transport; P:synaptic vesicle cycle; C:membrane; P:regulation of neurotransmitter levels; P:regulation of secretion by cell; F:ion binding; C:cytoplasmic part; P:single-organism cellular process |
| Efet.01.25091.g10.t1 | beta-2 adrenergic receptor | 1107 | 1.84E-21 | 46 | P:positive regulation of cellular process; P:single-organism process; P:cellular process; P:response to stimulus; P:multicellular organismal process |
| Efet.01.251024.g29.t1 | Zinc finger 271 (Zinc finger 7) (HZF7) (Zinc finger ZNFphex133) (Epstein-Barr virus-induced zinc finger ) (ZNF-EB) (CT-ZFP48) (Zinc finger | 576 | 5.89E-42 | 73.5 | F:binding |
| Efet.01.251070.g32.t1 | ribosome biogenesis TSR3 homolog | 819 | 1.56E-90 | 74.5 | P:ribosome biogenesis |
| Efet.01.251124.g33.t1 | discoidin domain-containing receptor 2-like isoform X1 | 234 | 1.09E-09 | 75.9 | F:ATP binding; F:protein tyrosine kinase activity; F:oxidoreductase activity; C:integral component of membrane; C:endoplasmic reticulum; P:protein phosphorylation; P:single-organism process |
| Efet.01.251133.g34.t1 | AF484095_1munc18-1-interacting 1 | 984 | 4.11E-13 | 70.05 |  |
| Efet.01.251149.g36.t1 | mannosyl-oligosaccharide 1,2-alpha-mannosidase IB-like | 279 | 1.28E-33 | 83.9 | P:lung alveolus development; C:extracellular exosome; F:calcium ion binding; P:N-glycan processing; C:integral component of membrane; F:mannosyl-oligosaccharide 1,2-alpha-mannosidase activity; C:Golgi membrane; C:endoplasmic reticulum; P:respiratory gaseous exchange; P:Golgi apparatus mannose trimming |
| Efet.01.251162.g37.t1 | Retrovirus-related Pol poly from transposon | 1251 | 2.46E-12 | 58.25 | F:nucleic acid binding; F:zinc ion binding; F:aspartic-type endopeptidase activity; P:proteolysis; P:DNA integration |
| Efet.01.251185.g39.t1 | suppressor of cytokine signaling 2-like | 879 | 2.71E-36 | 57 | P:negative regulation of signal transduction; P:signal transduction |
| Efet.01.251212.g41.t1 | homeobox SIX4-like | 612 | 2.49E-102 | 90.4 | C:nucleus; P:regulation of transcription, DNA-templated; F:sequence-specific DNA binding |
| Efet.01.251242.g43.t1 | B-cell lymphoma leukemia 11A | 237 | 2.40E-18 | 81 | C:nucleus; F:transcription factor activity, sequence-specific DNA binding; F:metal ion binding; P:signal transduction; F:transcription regulatory region DNA binding; P:regulation of transcription, DNA-templated; P:transcription from RNA polymerase II promoter; F:sequence-specific DNA binding; P:neurogenesis |
| Efet.01.251256.g45.t1 | hepatocyte nuclear factor 6 | 408 | 5.25E-29 | 79.1 | C:nucleus; F:DNA binding; F:transcriptional activator activity, RNA polymerase II core promoter proximal region sequence-specific binding; P:system development; P:cell differentiation; P:transcription from RNA polymerase II promoter; P:positive regulation of transcription from RNA polymerase II promoter |
| Efet.01.25127.g13.t1 | leucine-rich repeat-containing 49-like isoform X1 | 705 | 3.51E-77 | 88.65 |  |
| Efet.01.251277.g46.t1 | ectonucleotide pyrophosphatase phosphodiesterase family member 5-like | 906 | 1.72E-89 | 58.1 | F:catalytic activity; C:membrane |
| Efet.01.251324.g49.t1 | sex peptide receptor-like | 807 | 1.63E-66 | 67.75 | F:G-protein coupled receptor activity; P:G-protein coupled receptor signaling pathway; C:membrane |
| Efet.01.251356.g50.t1 | 78 kDa glucose-regulated | 585 | 1.79E-81 | 92.5 | F:ATPase activity; P:IRE1-mediated unfolded protein response; P:PERK-mediated unfolded protein response; C:smooth endoplasmic reticulum; C:endoplasmic reticulum-Golgi intermediate compartment; C:endoplasmic reticulum lumen; P:cerebellar Purkinje cell layer development; P:nematode larval development; F:calcium ion binding; F:chaperone binding; P:positive regulation of protein ubiquitination; P:maintenance of protein localization in endoplasmic reticulum; F:glycoprotein binding; C:endoplasmic reticulum chaperone complex; C:COP9 signalosome; P:regulation of ATF6-mediated unfolded protein response; F:protein domain specific binding; P:regulation of IRE1-mediated unfolded protein response; P:cerebellum structural organization; P:regulation of PERK-mediated unfolded protein response; P:toxin transport; C:plasma membrane; P:cellular response to glucose starvation; F:ubiquitin protein ligase binding; C:melanosome; P:cellular response to manganese ion; C:integral component of endoplasmic reticulum membrane; C:midbody; P:positive regulation of cell migration; P:protein folding in endoplasmic reticulum; C:myelin sheath; P:locomotion; P:negative regulation of protein homodimerization activity; P:ER overload response; F:ATP binding; C:extracellular exosome; C:focal adhesion; P:regulation of protein folding in endoplasmic reticulum; P:cellular response to interleukin-4; P:cellular response to antibiotic; P:ATF6-mediated unfolded protein response; F:misfolded protein binding; P:germ cell development; P:body morphogenesis; F:unfolded protein binding; P:substantia nigra development; C:cell surface; P:positive regulation of transcription from RNA polymerase II promoter in response to endoplasmic reticulum stress; P:activation of signaling protein activity involved in unfolded protein response; P:gonad development; F:ribosome binding; P:negative regulation of apoptotic process; C:mitochondrion; P:ER-associated ubiquitin-dependent protein catabolic process; P:negative regulation of transforming growth factor beta receptor signaling pathway |
| Efet.01.251453.g52.t1 | transcription factor Sox-2-like | 732 | 3.31E-102 | 71.95 | C:nucleus; F:DNA binding; P:nervous system development; P:regulation of transcription, DNA-templated |
| Efet.01.251455.g53.t1 | AB hydrolase superfamily -like | 369 | 2.20E-10 | 57.1 | P:metabolic process; F:hydrolase activity |
| Efet.01.2515.g227.t1 | beta-glucuronidase isoform X2 | 414 | 3.26E-47 | 55.5 | F:hydrolase activity |
| Efet.01.251560.g56.t1 | tyrosine phosphatase | 864 | 1.04E-41 | 50.28 | P:protein dephosphorylation; F:phosphatase activity; P:dephosphorylation; F:protein tyrosine/serine/threonine phosphatase activity |
| Efet.01.251645.g59.t1 | argonaute-2-like isoform X3 | 420 | 6.00E-31 | 89.65 | F:nucleic acid binding |
| Efet.01.251664.g60.t1 | dynein heavy chain axonemal-like | 903 | 3.55E-64 | 60.15 | F:nucleotide binding; F:ATPase activity; F:ATP binding; F:microtubule motor activity; C:dynein complex; P:microtubule-based movement |
| Efet.01.251665.g61.t1 | kelch 2 | 459 | 1.33E-58 | 88.8 |  |
| Efet.01.251726.g62.t1 | hypothetical protein CAPTEDRAFT_228921 | 645 | 3.40E-16 | 52.3 | C:membrane; C:integral component of membrane; F:carbohydrate binding |
| Efet.01.251734.g63.t1 | hypothetical protein HELRODRAFT_184365 | 282 | 6.41E-16 | 61.85 | C:membrane; C:integral component of membrane; F:carbohydrate binding |
| Efet.01.251740.g65.t1 | neuroligin- X-linked-like | 870 | 6.31E-36 | 50.9 | C:membrane; C:integral component of membrane |
| Efet.01.251757.g67.t1 | xenotropic and polytropic retrovirus receptor 1 | 669 | 4.46E-66 | 68.4 | C:integral component of plasma membrane; F:G-protein coupled receptor activity; P:G-protein coupled receptor signaling pathway; P:cellular phosphate ion homeostasis |
| Efet.01.251816.g68.t1 | coiled-coil domain-containing 85C- | 681 | 7.87E-45 | 62.2 |  |
| Efet.01.251842.g70.t1 | hypothetical protein CRN43_01760 | 1107 | 2.41E-117 | 57.95 | F:metal ion binding |
| Efet.01.251998.g75.t1 | sentrin-specific protease 6 isoform X1 | 294 | 7.93E-21 | 62.05 | P:regulation of kinetochore assembly; P:protein desumoylation; P:proteolysis; F:SUMO-specific protease activity; F:cysteine-type peptidase activity |
| Efet.01.252050.g76.t1 | RCC1 domain-containing 1 | 1404 | 1.08E-59 | 54.4 | C:cytoplasm; C:plasma membrane |
| Efet.01.252160.g78.t1 | collagen alpha-6(VI) chain-like isoform X2 | 447 | 5.03E-07 | 58.75 |  |
| Efet.01.25223.g15.t1 | myosin-VIIa-like isoform X2 | 501 | 3.17E-68 | 78.85 | C:myosin complex; F:ATP binding; F:actin binding; C:integral component of membrane; F:motor activity |
| Efet.01.252247.g79.t1 | histidine ammonia-lyase | 684 | 6.30E-24 | 71.05 | P:histidine catabolic process; F:ammonia-lyase activity |
| Efet.01.252318.g80.t1 | fatty acyl- reductase 1-like | 390 | 2.70E-64 | 79.9 | C:integral component of membrane; P:oxidation-reduction process; F:fatty-acyl-CoA reductase (alcohol-forming) activity |
| Efet.01.252322.g81.t1 | somatostatin receptor type 2-like | 303 | 4.18E-20 | 73.5 | F:opioid receptor activity; P:opioid receptor signaling pathway; P:neuropeptide signaling pathway; C:integral component of membrane; F:neuropeptide Y receptor activity |
| Efet.01.252336.g82.t1 | GRAM domain-containing 4-like | 255 | 1.51E-10 | 69.45 | C:integral component of membrane |
| Efet.01.252382.g84.t1 | nascent polypeptide-associated complex subunit alpha-like | 417 | 4.49E-65 | 85.05 |  |
| Efet.01.252383.g85.t1 | Sey1p | 1119 | 4.01E-07 | 46 | F:GTP binding |
| Efet.01.252393.g86.t1 | MAGUK p55 subfamily member 5-like isoform X3 | 537 | 1.33E-68 | 82.4 | P:bicellular tight junction assembly; P:establishment of epithelial cell polarity |
| Efet.01.252395.g87.t1 | C-C chemokine receptor type 5-like isoform X2 | 1458 | 2.73E-28 | 48.5 | F:G-protein coupled receptor activity; P:G-protein coupled receptor signaling pathway; P:signal transduction; C:membrane; C:integral component of membrane; F:signal transducer activity |
| Efet.01.252426.g88.t1 | myosin heavy striated muscle-like isoform X25 | 936 | 4.32E-82 | 76.2 | C:myosin complex; F:ATP binding; F:actin binding; P:metabolic process; F:amino acid binding; F:motor activity |
| Efet.01.252450.g89.t1 | atypical chemokine receptor 3 | 738 | 2.19E-48 | 50.25 | F:G-protein coupled receptor activity; P:G-protein coupled receptor signaling pathway; C:membrane; C:cell part |
| Efet.01.252523.g92.t1 | microtubule-associated 6-like | 2970 | 2.46E-08 | 43.2 | C:membrane; C:integral component of membrane |
| Efet.01.25255.g16.t1 | primosomal N | 1461 | 0 | 90.1 | F:DNA binding; F:ATP binding; F:zinc ion binding; F:ATP-dependent DNA helicase activity; P:DNA unwinding involved in DNA replication; C:primosome complex; P:DNA replication, synthesis of RNA primer |
| Efet.01.25255.g17.t1 | inner membrane transmembrane | 1578 | 0 | 85.25 | C:integral component of membrane |
| Efet.01.25255.g18.t1 | shikimate kinase | 498 | 5.46E-97 | 85.55 | F:magnesium ion binding; F:shikimate kinase activity; F:ATP binding; P:aromatic amino acid family biosynthetic process; C:cytoplasm; P:chorismate biosynthetic process; P:phosphorylation |
| Efet.01.25255.g19.t1 | methionine synthase | 2712 | 0 | 90.35 | P:methionine biosynthetic process; P:pteridine-containing compound metabolic process; F:methionine synthase activity; F:zinc ion binding; F:cobalamin binding; C:intracellular; F:S-adenosylmethionine-homocysteine S-methyltransferase activity; P:methylation |
| Efet.01.25255.g20.t1 | transcriptional family | 645 | 5.45E-157 | 76.4 | F:DNA binding; P:transcription, DNA-templated; P:regulation of transcription, DNA-templated |
| Efet.01.25255.g21.t1 | 3-dehydroquinate synthase | 513 | 2.02E-91 | 83.15 | P:aromatic amino acid family biosynthetic process; F:3-dehydroquinate synthase activity; C:cytoplasm; P:chorismate biosynthetic process |
| Efet.01.25255.g22.t1 | 3-dehydroquinate synthase | 513 | 2.60E-95 | 78.4 | P:aromatic amino acid family biosynthetic process; F:3-dehydroquinate synthase activity; C:cytoplasm; P:chorismate biosynthetic process |
| Efet.01.25255.g23.t1 | hypothetical protein | 1143 | 0 | 55.78 |  |
| Efet.01.25255.g24.t1 | type IV pilus secretin | 2124 | 0 | 87.65 | P:pilus assembly; F:protein transporter activity; C:cell outer membrane; P:protein secretion |
| Efet.01.25255.g25.t1 | pilus assembly | 537 | 3.36E-125 | 89.1 | C:integral component of membrane |
| Efet.01.25255.g26.t1 | fimbrial | 492 | 3.61E-106 | 84.9 | C:integral component of membrane |
| Efet.01.25255.g27.t1 | pilus assembly | 1071 | 0 | 94.95 | P:cell cycle |
| Efet.01.25255.g28.t1 | penicillin-binding 1A | 2397 | 0 | 88.05 | C:integral component of membrane; F:penicillin binding; F:peptidoglycan glycosyltransferase activity |
| Efet.01.25255.g29.t1 | iron donor | 330 | 1.28E-76 | 92.25 | F:ferric iron binding; P:iron-sulfur cluster assembly |
| Efet.01.25255.g30.t1 | 2-hydroxy-acid oxidase | 1536 | 0 | 89.95 | F:oxidoreductase activity, acting on CH-OH group of donors; F:flavin adenine dinucleotide binding; P:oxidation-reduction process |
| Efet.01.25255.g31.t1 | quinolinate synthase | 951 | 0 | 93.1 | P:quinolinate biosynthetic process; F:metal ion binding; P:NAD biosynthetic process; F:4 iron, 4 sulfur cluster binding; F:transferase activity; F:quinolinate synthetase A activity |
| Efet.01.252654.g99.t1 | Dual specificity testis-specific kinase 2 | 618 | 5.66E-63 | 62 | F:ATP binding; F:metal ion binding; F:protein kinase activity; P:phosphorylation; P:protein phosphorylation; F:kinase activity; C:nucleoplasm |
| Efet.01.252661.g100.t1 | hypothetical protein CAPTEDRAFT_214693 | 828 | 9.35E-28 | 52.5 |  |
| Efet.01.25268.g34.t1 | octopamine receptor beta-3R | 273 | 1.19E-32 | 64.95 | C:integral component of plasma membrane; F:dopamine neurotransmitter receptor activity; F:octopamine receptor activity; P:dopamine receptor signaling pathway; P:positive regulation of adenylate cyclase activity involved in G-protein coupled receptor signaling pathway |
| Efet.01.25268.g35.t1 | 5-hydroxytryptamine receptor 2A | 426 | 5.99E-48 | 61.15 | C:integral component of plasma membrane; F:dopamine neurotransmitter receptor activity; P:dopamine receptor signaling pathway |
| Efet.01.25269.g36.t1 | GPI ethanolamine phosphate transferase 3-like | 765 | 1.33E-99 | 70 | C:endoplasmic reticulum membrane; P:GPI anchor biosynthetic process; F:mannose-ethanolamine phosphotransferase activity |
| Efet.01.25272.g37.t1 | glycogen-binding subunit 76A-like | 1074 | 1.37E-81 | 64.3 |  |
| Efet.01.252730.g101.t1 | isopentenyl-diphosphate delta-isomerase | 564 | 8.75E-18 | 67.05 | P:isoprenoid biosynthetic process; F:isomerase activity |
| Efet.01.252794.g102.t1 | roundabout homolog 2-like | 534 | 6.38E-16 | 56.15 |  |
| Efet.01.252799.g103.t1 | hypothetical protein HELRODRAFT_160048 | 390 | 4.47E-09 | 61 | P:neurotransmitter transport; F:neurotransmitter:sodium symporter activity; P:transmembrane transport; C:membrane; C:integral component of membrane; F:symporter activity; P:transport |
| Efet.01.252838.g104.t1 | hypothetical protein CAPTEDRAFT_225510 | 405 | 5.18E-09 | 47.25 | F:pyridoxal phosphate binding; P:tryptophan catabolic process; F:kynureninase activity; C:cytoplasm; F:catalytic activity; C:membrane; C:integral component of membrane; P:NAD biosynthetic process; F:carbohydrate binding |
| Efet.01.252868.g105.t1 | U4 U6 small nuclear ribonucleo Prp3 | 282 | 4.16E-28 | 51.85 | C:intracellular ribonucleoprotein complex; P:retina development in camera-type eye; C:U4/U6 x U5 tri-snRNP complex; C:viral nucleocapsid; P:mRNA processing; P:mRNA splicing, via spliceosome |
| Efet.01.252907.g106.t1 | heparan sulfate glucosamine 3-O-sulfotransferase | 576 | 2.71E-59 | 69.05 | F:sulfotransferase activity; F:transferase activity |
| Efet.01.252916.g107.t1 | SCO-spondin | 1404 | 1.68E-43 | 46.95 | C:extracellular space; F:catalytic activity; P:metabolic process; P:cell differentiation; P:nervous system development; P:cell adhesion; C:extracellular region |
| Efet.01.252949.g108.t1 | polypeptide N-acetylgalactosaminyltransferase 10 | 354 | 1.13E-45 | 79.7 | F:transferase activity, transferring glycosyl groups; C:integral component of membrane; P:protein glycosylation; C:Golgi membrane; F:carbohydrate binding |
| Efet.01.252956.g109.t1 | Homeobox prospero | 312 | 3.28E-52 | 85.9 | F:DNA binding; C:integral component of membrane |
| Efet.01.252963.g110.t1 | roundabout homolog 1-like isoform X1 | 468 | 2.50E-31 | 57.75 | C:membrane; C:integral component of membrane |
| Efet.01.252987.g113.t1 | E3 ubiquitin- ligase LRSAM1-like | 387 | 2.46E-10 | 54.45 |  |
| Efet.01.252992.g114.t1 | hypothetical protein OCBIM_22036063mg, partial | 279 | 7.98E-08 | 53 |  |
| Efet.01.253005.g115.t1 | titin-like isoform X12 | 2868 | 4.52E-08 | 50.93 | P:signal transduction |
| Efet.01.25304.g39.t1 | phosphatase 1B-like | 903 | 7.35E-152 | 81.7 | F:magnesium ion binding; F:protein serine/threonine phosphatase activity; C:cytoskeleton; P:protein dephosphorylation; F:manganese ion binding |
| Efet.01.253061.g117.t1 | mannan-binding lectin serine protease 2 | 243 | 2.11E-15 | 59.56 | C:membrane; C:integral component of membrane; F:carbohydrate binding |
| Efet.01.25307.g40.t1 | ankyrin-2-like isoform X8 | 885 | 8.81E-10 | 56.35 |  |
| Efet.01.253131.g120.t1 | hypothetical protein CAPTEDRAFT_200575 | 1824 | 0 | 51.25 | C:membrane |
| Efet.01.253138.g121.t1 | dmX 2 isoform X3 | 2274 | 3.78E-156 | 57.2 |  |
| Efet.01.253215.g122.t1 | RNA-dependent RNA polymerase 1 | 2559 | 1.52E-23 | 41.38 | F:RNA-directed 5'-3' RNA polymerase activity; P:transcription, RNA-templated; C:membrane; C:integral component of membrane |
| Efet.01.253256.g123.t1 | hypothetical protein HELRODRAFT_191344 | 1416 | 9.74E-06 | 68 |  |
| Efet.01.253258.g124.t1 | gamma-tubulin complex component 3 | 330 | 7.06E-27 | 77.85 | C:gamma-tubulin small complex; C:gamma-tubulin ring complex; C:equatorial microtubule organizing center; C:spindle pole; F:structural constituent of cytoskeleton; F:gamma-tubulin binding; P:meiotic nuclear division; C:microtubule; C:centrosome; F:microtubule minus-end binding; P:centrosome duplication; C:spindle pole body; P:cytoplasmic microtubule organization; P:interphase microtubule nucleation by interphase microtubule organizing center; P:mitotic spindle assembly |
| Efet.01.253272.g125.t1 | piggyBac transposable element-derived 4-like | 462 | 1.32E-33 | 56.85 |  |
| Efet.01.253272.g126.t1 | piggyBac transposable element-derived 4-like | 888 | 2.79E-55 | 65.4 |  |
| Efet.01.253281.g127.t1 | homeodomain-interacting kinase 2-like | 606 | 2.83E-110 | 84.15 | F:DNA binding; F:ATP binding; F:protein serine/threonine kinase activity; P:protein phosphorylation |
| Efet.01.253295.g128.t1 | phosphate carrier mitochondrial isoform X1 | 579 | 1.87E-79 | 86.35 | C:integral component of plasma membrane; P:generation of precursor metabolites and energy; C:myelin sheath; C:extracellular exosome; F:structural constituent of ribosome; F:phosphate ion carrier activity; F:symporter activity; C:nucleolus; F:protein complex binding; C:mitochondrial inner membrane; P:phosphate ion transmembrane transport; P:translation |
| Efet.01.253306.g129.t1 | voltage-dependent T-type calcium channel subunit alpha-1G | 309 | 9.35E-25 | 71.55 | P:calcium ion import; C:voltage-gated calcium channel complex; P:regulation of ion transmembrane transport; P:calcium ion transmembrane transport; F:low voltage-gated calcium channel activity |
| Efet.01.253314.g130.t1 | probable E3 ubiquitin- ligase HERC1 isoform X1 | 963 | 1.79E-13 | 62.3 | F:ubiquitin-protein transferase activity; P:cerebellar Purkinje cell differentiation; P:neuron projection development; P:negative regulation of autophagy; P:neuromuscular process controlling balance; F:ligase activity; P:protein ubiquitination |
| Efet.01.253320.g131.t1 | radixin isoform X3 | 306 | 1.50E-11 | 86.8 | P:regulation of cell shape; C:myelin sheath; C:cleavage furrow; P:regulation of actin filament bundle assembly; P:negative regulation of homotypic cell-cell adhesion; P:positive regulation of cellular protein catabolic process; P:regulation of organelle assembly; P:cellular response to thyroid hormone stimulus; C:extracellular exosome; C:focal adhesion; C:cell tip; C:microvillus; P:protein kinase A signaling; P:apical protein localization; P:negative regulation of cell size; C:ruffle; P:regulation of Rap protein signal transduction; C:stereocilium; C:cortical actin cytoskeleton; C:lamellipodium; F:protein domain specific binding; P:negative regulation of GTPase activity; F:protein homodimerization activity; P:positive regulation of G1/S transition of mitotic cell cycle; P:establishment of protein localization to plasma membrane; P:regulation of ruffle assembly; P:establishment of endothelial barrier; F:RNA binding; P:positive regulation of protein localization to early endosome; F:cadherin binding involved in cell-cell adhesion; C:T-tubule; P:barbed-end actin filament capping; C:extracellular space; F:actin binding; C:cell-cell adherens junction; P:positive regulation of gene expression; P:microvillus assembly; C:filopodium; P:positive regulation of early endosome to late endosome transport; C:midbody; C:extrinsic component of membrane; P:negative regulation of adherens junction organization; C:apical plasma membrane; F:protein kinase A binding; F:ATPase binding; P:positive regulation of cell migration |
| Efet.01.253424.g133.t1 | specificity 1 | 1932 | 8.31E-20 | 71.1 | F:nucleic acid binding; F:metal ion binding |
| Efet.01.25348.g41.t1 | kinesin KIF16B isoform X2 | 294 | 6.31E-19 | 68.3 | C:intracellular organelle; P:regulation of cellular process; F:phosphatidylinositol phosphate binding; C:cytoplasmic part; P:single-organism process; P:cellular process |
| Efet.01.2535.g228.t1 | hypothetical protein HELRODRAFT_190124 | 711 | 1.87E-18 | 62 | F:binding |
| Efet.01.2535.g229.t1 | hypothetical protein CAPTEDRAFT_220096 | 519 | 4.85E-15 | 75.8 | F:nucleic acid binding; F:metal ion binding |
| Efet.01.253638.g136.t1 | colorectal mutant cancer | 600 | 1.37E-36 | 80.05 |  |
| Efet.01.253655.g137.t1 | exportin-6 | 315 | 2.03E-36 | 76.8 | P:intracellular protein transport; F:Ran GTPase binding; C:intracellular |
| Efet.01.25367.g42.t1 | cytochrome P450 2C39 | 411 | 3.61E-08 | 61 | F:iron ion binding; F:oxidoreductase activity, acting on paired donors, with incorporation or reduction of molecular oxygen; C:membrane; C:integral component of membrane; P:oxidation-reduction process; F:heme binding |
| Efet.01.253715.g138.t1 | bestrophin-4-like isoform X1 | 492 | 8.77E-59 | 66.05 | C:integral component of membrane |
| Efet.01.253758.g139.t1 | insulin receptor-like | 636 | 5.01E-67 | 69.4 | F:protein kinase activity; F:binding; P:protein phosphorylation |
| Efet.01.253833.g141.t1 | RNA Polymerase | 537 | 1.82E-88 | 88.45 | F:DNA binding; F:DNA-directed 5'-3' RNA polymerase activity; P:transcription from RNA polymerase II promoter; C:DNA-directed RNA polymerase II, core complex |
| Efet.01.253844.g142.t1 | hypothetical protein CAPTEDRAFT_205084 | 345 | 2.69E-17 | 70 | C:cytoskeleton |
| Efet.01.253886.g143.t1 | probable G- coupled receptor 157 | 555 | 2.24E-28 | 58.85 | F:transmembrane signaling receptor activity; P:signal transduction; C:membrane |
| Efet.01.254056.g146.t1 | titin | 1080 | 1.11E-12 | 58.95 | P:skeletal muscle tissue development; F:cytoskeletal protein binding; P:skeletal myofibril assembly; P:cardiac muscle tissue development; C:sarcomere |
| Efet.01.254119.g149.t1 | 5-hydroxytryptamine receptor 2A | 345 | 5.05E-19 | 70.4 | P:signal transduction; C:membrane; F:signal transducer activity |
| Efet.01.254126.g150.t1 | kelch 38 | 1806 | 1.29E-43 | 43.35 | F:ubiquitin-protein transferase activity; C:Cul3-RING ubiquitin ligase complex; P:protein ubiquitination involved in ubiquitin-dependent protein catabolic process; P:protein ubiquitination |
| Efet.01.254136.g152.t1 | thyrotropin-releasing hormone receptor-like | 1041 | 9.87E-73 | 65.8 | F:G-protein coupled receptor activity; P:signal transduction; C:membrane |
| Efet.01.25416.g44.t1 | heparan sulfate glucosamine 3-O-sulfotransferase 6-like | 714 | 1.45E-11 | 88.7 | C:integral component of membrane; F:sulfotransferase activity |
| Efet.01.254268.g156.t1 | kelch 20 | 858 | 1.84E-108 | 69.95 | C:membrane |
| Efet.01.254268.g157.t1 | kelch 20 | 858 | 2.61E-120 | 67.1 | C:integral component of membrane |
| Efet.01.254293.g158.t1 | spermatid perinuclear RNA-binding isoform X1 | 315 | 3.84E-09 | 70.25 | F:RNA binding; P:spermatogenesis |
| Efet.01.254345.g159.t1 | class I SAM-dependent methyltransferase | 702 | 3.36E-42 | 45.2 | C:membrane; C:integral component of membrane |
| Efet.01.254372.g160.t1 | synaptotagmin-10 isoform X3 | 618 | 2.47E-41 | 67.4 | C:membrane |
| Efet.01.254374.g161.t1 | RER1 isoform X2 | 201 | 4.43E-17 | 86.05 | P:retrograde vesicle-mediated transport, Golgi to ER; C:integral component of membrane; P:sensory perception of pain; C:intracellular |
| Efet.01.2545.g230.t1 | hypothetical protein HELRODRAFT_191242 | 1281 | 7.39E-08 | 60 |  |
| Efet.01.2545.g232.t1 | hypothetical protein HELRODRAFT_191242 | 390 | 3.55E-18 | 59.5 |  |
| Efet.01.254528.g164.t1 | phosphatidylinositol-glycan-specific phospholipase D | 396 | 2.23E-10 | 66.25 | F:hydrolase activity, acting on ester bonds; P:positive regulation of cellular process; P:cellular response to organic substance; C:extracellular region; P:cellular response to oxygen-containing compound; C:intracellular part; P:single-organism process; C:membrane-bounded organelle |
| Efet.01.254586.g167.t1 | Charged multivesicular body 2b | 372 | 1.94E-40 | 69.55 | P:vacuolar transport; C:intracellular |
| Efet.01.25460.g46.t1 | lachesin-like isoform X1 | 264 | 2.03E-21 | 67.25 | P:peptidyl-tyrosine phosphorylation; F:transmembrane receptor protein tyrosine kinase activity; C:membrane; C:integral component of membrane; P:transmembrane receptor protein tyrosine kinase signaling pathway |
| Efet.01.254602.g168.t1 | transcription elongation factor SPT6 | 1125 | 5.59E-83 | 54.7 | F:nucleic acid binding; P:RNA metabolic process; P:regulation of developmental process; F:protein binding; P:cellular nitrogen compound biosynthetic process; P:negative regulation of macromolecule metabolic process; P:regulation of DNA-templated transcription, elongation; P:gene expression; P:regulation of transcription from RNA polymerase II promoter; P:negative regulation of cellular metabolic process; P:cellular macromolecule biosynthetic process |
| Efet.01.254619.g169.t1 | SET and MYND domain-containing 4-like | 216 | 6.27E-18 | 62.5 |  |
| Efet.01.254660.g170.t1 | AP-2 complex subunit alpha-2-like | 249 | 1.20E-36 | 90.95 | P:vesicle-mediated transport; C:clathrin adaptor complex; F:protein transporter activity; P:intracellular protein transport |
| Efet.01.254703.g171.t1 | zinc finger 28-like | 498 | 3.50E-10 | 67.4 | F:binding |
| Efet.01.254765.g174.t1 | ATP-dependent DNA helicase Q4-like | 336 | 4.05E-14 | 58.57 | F:nucleic acid binding; F:ATP binding; F:zinc ion binding; F:metal ion binding; F:helicase activity; F:hydrolase activity |
| Efet.01.25481.g47.t1 | calcium-activated potassium channel subunit alpha-1-like | 255 | 7.46E-07 | 70 |  |
| Efet.01.254833.g177.t1 | uncharacterized protein LOC109922783 | 846 | 6.60E-07 | 45 |  |
| Efet.01.254912.g178.t1 | phosphatidylinositol 4-kinase alpha-like isoform X1 | 381 | 2.02E-42 | 82.8 | P:phosphatidylinositol-mediated signaling; P:phosphatidylinositol phosphorylation; F:kinase activity; C:intracellular |
| Efet.01.254930.g179.t1 | Misshapen-like kinase 1 | 225 | 3.71E-18 | 94.55 | F:ATP binding; F:protein kinase activity; P:protein phosphorylation |
| Efet.01.254952.g180.t1 | sex peptide receptor-like | 477 | 2.68E-21 | 72.85 | F:G-protein coupled peptide receptor activity; P:G-protein coupled receptor signaling pathway; C:integral component of membrane |
| Efet.01.255064.g182.t1 | myosin heavy non-muscle | 600 | 1.23E-109 | 88.05 | C:myosin complex; F:ATP binding; F:motor activity |
| Efet.01.255178.g188.t1 | dehydrogenase reductase SDR family member 11 | 924 | 5.06E-66 | 56.85 | F:oxidoreductase activity; P:oxidation-reduction process; C:extracellular region; C:cellular_component |
| Efet.01.255191.g189.t1 | serine threonine- kinase Nek1 isoform X1 | 372 | 5.40E-17 | 57.5 | F:nucleotide binding; F:ATP binding; F:protein kinase activity; F:protein serine/threonine kinase activity; P:protein phosphorylation |
| Efet.01.255198.g190.t1 | Doublecortin domain-containing | 327 | 5.18E-19 | 61.9 | C:cell part |
| Efet.01.255222.g191.t1 | E3 ubiquitin- ligase HACE1 isoform X3 | 1317 | 3.87E-140 | 75.6 | P:Rac protein signal transduction; P:membrane fusion; P:protein K48-linked ubiquitination; P:transcription, DNA-templated; C:Golgi cisterna membrane; P:Golgi organization; P:regulation of transcription, DNA-templated; C:endoplasmic reticulum; P:cell cycle; P:protein ubiquitination involved in ubiquitin-dependent protein catabolic process; C:nucleus; F:ubiquitin-protein transferase activity; F:Rac GTPase binding; P:regulation of cell migration; F:Rab GTPase binding; F:ligase activity |
| Efet.01.255235.g192.t1 | gene 11-1 precursor | 3495 | 1.61E-17 | 47.63 |  |
| Efet.01.255258.g194.t1 | zinc finger Gfi-1-like | 762 | 3.04E-28 | 76.45 | P:regulation of macromolecule metabolic process; P:hemopoiesis; F:binding; P:regulation of primary metabolic process; P:regulation of cellular metabolic process |
| Efet.01.25528.g49.t1 | deoxyuridine 5 -triphosphate mitochondrial | 300 | 9.91E-52 | 87.4 | F:magnesium ion binding; C:extracellular exosome; F:dUTP diphosphatase activity; C:mitochondrion; P:dUMP biosynthetic process; C:nucleoplasm; F:RNA binding; P:dUTP catabolic process |
| Efet.01.25528.g50.t1 | procollagen-lysine,2-oxoglutarate 5-dioxygenase 3 | 255 | 1.10E-20 | 88.1 | F:L-ascorbic acid binding; F:iron ion binding; C:endoplasmic reticulum; P:oxidation-reduction process; F:procollagen-lysine 5-dioxygenase activity |
| Efet.01.255350.g195.t1 | anaphase-promoting complex subunit 7-like | 252 | 1.04E-10 | 66.7 |  |
| Efet.01.255458.g198.t1 | interferon-induced very large GTPase 1-like | 843 | 6.62E-08 | 44 | F:GTP binding |
| Efet.01.255502.g200.t1 | nachr subunit | 216 | 4.71E-26 | 82.25 | P:chemical synaptic transmission, postsynaptic; P:synaptic transmission, cholinergic; F:acetylcholine receptor activity; P:neuromuscular synaptic transmission; C:postsynaptic membrane; P:cation transmembrane transport; C:cell junction; F:extracellular ligand-gated ion channel activity; F:acetylcholine binding; C:acetylcholine-gated channel complex; P:response to nicotine |
| Efet.01.255509.g201.t1 | LRR and PYD domains-containing 5 | 2619 | 6.59E-50 | 49.9 |  |
| Efet.01.255548.g202.t1 | DENN domain-containing | 498 | 1.31E-15 | 57.75 |  |
| Efet.01.255638.g207.t1 | doublecortin domain-containing 5-like isoform X1 | 339 | 9.38E-22 | 67.65 | F:zinc ion binding; P:intracellular signal transduction; C:intracellular |
| Efet.01.255662.g208.t1 | transcription factor Sox-11-like | 612 | 1.34E-32 | 75.95 |  |
| Efet.01.2557.g234.t1 | solute carrier family facilitated glucose transporter member 2 | 561 | 2.22E-37 | 65.85 | P:liver development; C:membrane; F:D-glucose transmembrane transporter activity; P:positive regulation of peptide hormone secretion; P:sensory perception of pain; P:glucose import; P:brain development; P:adult chitin-containing cuticle pigmentation |
| Efet.01.255795.g211.t1 | telomere-associated RIF1 isoform X2 | 2235 | 7.71E-18 | 69.4 | P:negative regulation of transcription from RNA polymerase II promoter; C:chromosome, telomeric region; C:male pronucleus; P:stem cell population maintenance; F:protein binding; C:spindle; C:female pronucleus; P:double-strand break repair via nonhomologous end joining; P:positive regulation of double-strand break repair via nonhomologous end joining; C:plasma membrane; C:nucleoplasm |
| Efet.01.255862.g214.t1 | TKL kinase | 1041 | 1.19E-49 | 54.9 | P:peptidyl-tyrosine phosphorylation; F:ATP binding; F:G-protein coupled receptor activity; F:protein tyrosine kinase activity; P:G-protein coupled receptor signaling pathway; C:membrane; C:integral component of membrane; F:protein kinase activity; P:phosphorylation; P:protein phosphorylation; F:kinase activity |
| Efet.01.255995.g216.t1 | stoned-B-like | 2688 | 6.55E-120 | 51.5 | P:vesicle-mediated transport; C:clathrin adaptor complex; P:regulation of endocytosis; P:endocytosis; P:intracellular protein transport |
| Efet.01.256.g32.t1 | myosin light chain smooth muscle-like isoform X3 | 672 | 2.16E-35 | 54.6 | F:transferase activity |
| Efet.01.256034.g218.t1 | Phospholipase D2 | 3198 | 0 | 45.1 | C:membrane; C:integral component of membrane |
| Efet.01.256078.g221.t1 | Band 7 | 444 | 1.88E-36 | 63.4 | F:calcium ion binding; C:membrane; C:integral component of membrane |
| Efet.01.256116.g223.t1 | inhibitor of nuclear factor kappa-B kinase subunit beta | 228 | 1.33E-21 | 73 | P:regulation of establishment of endothelial barrier; P:I-kappaB phosphorylation; C:IkappaB kinase complex; F:IkappaB kinase activity; P:positive regulation of cell proliferation; P:anatomical structure morphogenesis; P:negative regulation of bicellular tight junction assembly; P:positive regulation of transcription from RNA polymerase II promoter; P:positive regulation of NF-kappaB transcription factor activity; C:cytoplasmic side of plasma membrane; P:cortical actin cytoskeleton organization; F:ATP binding; P:inflammatory response; F:protein heterodimerization activity; C:intracellular membrane-bounded organelle; P:positive regulation of neuron projection development; C:CD40 receptor complex; F:protein kinase binding; F:protein homodimerization activity; P:neuron projection development; P:establishment of protein localization to plasma membrane; P:positive regulation of neuron death; P:B cell homeostasis; P:regulation of phosphorylation; P:serine phosphorylation of STAT protein; F:scaffold protein binding; F:signal transducer, downstream of receptor, with serine/threonine kinase activity; P:tumor necrosis factor-mediated signaling pathway; P:negative regulation of myosin-light-chain-phosphatase activity; P:innate immune response; P:positive regulation of sodium ion transport; P:positive regulation of cation channel activity; P:interleukin-1-mediated signaling pathway |
| Efet.01.256146.g224.t1 | maestro heat-like repeat-containing family member 1 | 597 | 4.21E-41 | 59.05 | F:calcium ion binding; C:membrane; P:homophilic cell adhesion via plasma membrane adhesion molecules |
| Efet.01.256148.g225.t1 | interferon regulatory factor 1-like | 660 | 8.49E-32 | 63.3 | F:transcription factor activity, sequence-specific DNA binding; P:regulation of transcription, DNA-templated; F:regulatory region DNA binding |
| Efet.01.256176.g226.t1 | M-phase phospho 8-like isoform X9 | 810 | 6.24E-15 | 58.14 |  |
| Efet.01.256191.g227.t1 | hypothetical protein CAPTEDRAFT_227009 | 474 | 3.83E-16 | 51.5 | C:membrane; C:integral component of membrane |
| Efet.01.256284.g231.t1 | solute carrier organic anion transporter family member 5A1 | 342 | 1.96E-08 | 61.33 | C:membrane; C:integral component of membrane; P:transport; F:transporter activity |
| Efet.01.2563.g235.t1 | histone deacetylase 5 isoform X4 | 612 | 7.60E-44 | 85.5 | F:metal ion binding; P:transcription, DNA-templated; F:NAD-dependent histone deacetylase activity (H3-K14 specific); C:histone deacetylase complex; P:regulation of transcription, DNA-templated; P:histone H3 deacetylation |
| Efet.01.256385.g234.t1 | BAI1-associated 3-like isoform X1 | 384 | 1.53E-16 | 58.8 | C:membrane; C:integral component of membrane |
| Efet.01.25640.g53.t1 | transcription antitermination factor | 402 | 3.71E-70 | 66.67 |  |
| Efet.01.256409.g235.t1 | E3 ubiquitin- ligase SHPRH-like | 270 | 2.99E-07 | 68.17 |  |
| Efet.01.256409.g236.t1 | DEP domain-containing 5-like isoform X2 | 4845 | 7.16E-178 | 59.05 | P:intracellular signal transduction; C:intracellular |
| Efet.01.256420.g237.t1 | Ras-related Rab-9A | 600 | 1.63E-88 | 76.95 | F:GTP binding; P:small GTPase mediated signal transduction; C:intracellular |
| Efet.01.256427.g238.t1 | transcription factor 7-like 2 isoform X2 | 222 | 2.06E-16 | 85.4 | C:nucleus; P:Wnt signaling pathway; P:regulation of transcription from RNA polymerase II promoter |
| Efet.01.256455.g239.t1 | cholecystokinin receptor type A | 738 | 5.11E-27 | 48.4 | C:gap junction; F:G-protein coupled receptor activity; C:integral component of plasma membrane; F:G-protein coupled peptide receptor activity; P:neuropeptide signaling pathway; P:G-protein coupled receptor signaling pathway; P:signal transduction; C:membrane; C:integral component of membrane; P:phospholipase C-activating G-protein coupled receptor signaling pathway; F:signal transducer activity |
| Efet.01.256471.g240.t1 | amidohydrolase superfamily | 957 | 3.57E-75 | 54.7 | F:hydrolase activity, acting on carbon-nitrogen (but not peptide) bonds; F:hydrolase activity |
| Efet.01.256481.g241.t1 | leucine-rich repeat lrrA-like | 2454 | 0 | 61.3 |  |
| Efet.01.256488.g242.t1 | beta-adrenergic receptor kinase | 525 | 3.04E-67 | 72.5 | F:nucleotide binding; P:phosphorylation; F:protein serine/threonine kinase activity |
| Efet.01.256492.g243.t1 | Golgi to ER traffic 4 homolog | 528 | 2.03E-31 | 56.6 | C:BAT3 complex; P:protein insertion into ER membrane |
| Efet.01.25653.g55.t1 | Potassium voltage-gated channel subfamily A member 1 | 1302 | 4.25E-122 | 70 | P:positive regulation of membrane potential; P:cellular response to dopamine; P:positive regulation of circadian sleep/wake cycle, sleep; P:sensory perception of taste; P:regulation of synaptic activity; P:potassium ion transmembrane transport; P:detection of visible light; C:voltage-gated potassium channel complex; P:protein homooligomerization; P:larval locomotory behavior; P:learning or memory; F:voltage-gated potassium channel activity; P:proboscis extension reflex; P:axon extension; P:flight behavior; P:mating behavior, sex discrimination; P:courtship behavior; P:regulation of ion transmembrane transport; P:action potential; P:sleep; P:behavioral response to ether |
| Efet.01.256603.g246.t1 | cytoplasmic polyadenylation element-binding 1-like isoform X1 | 297 | 1.45E-28 | 88 | F:nucleotide binding; F:nucleic acid binding |
| Efet.01.256614.g247.t1 | T family of potassium channels 18-like | 483 | 4.36E-21 | 59.95 | P:transport |
| Efet.01.256670.g248.t1 | neurobeachin-like isoform X3 | 1623 | 1.63E-134 | 75.4 |  |
| Efet.01.256676.g249.t1 | mitogen-activated kinase 14B-like isoform X2 | 216 | 1.25E-11 | 84.55 | F:ATP binding; P:MAPK cascade; F:MAP kinase activity; C:intracellular |
| Efet.01.256703.g250.t1 | hypothetical protein HELRODRAFT_188788 | 933 | 1.71E-46 | 59 |  |
| Efet.01.25673.g57.t1 | afadin isoform X2 | 732 | 1.95E-13 | 67 | P:signal transduction |
| Efet.01.256778.g253.t1 | cysteine-rich 2-binding -like | 399 | 7.85E-06 | 79 |  |
| Efet.01.256781.g254.t1 | RL13_LUMRU ame: Full=60S ribosomal L13 | 207 | 1.88E-18 | 86.5 | F:structural constituent of ribosome; C:ribosome; P:translation |
| Efet.01.256804.g256.t1 | myotubularin-related 10-B | 525 | 1.69E-38 | 62.2 |  |
| Efet.01.25685.g58.t1 | transcription factor jun-B | 1077 | 2.76E-17 | 69.6 | P:multicellular organismal reproductive process; P:transcription, DNA-templated; F:RNA polymerase II core promoter proximal region sequence-specific DNA binding; P:female pregnancy; P:anatomical structure morphogenesis; P:positive regulation of cell differentiation; F:transcription factor binding; P:placenta development; P:positive regulation of transcription from RNA polymerase II promoter; P:regulation of cell proliferation; F:RNA polymerase II transcription factor activity, sequence-specific DNA binding; P:negative regulation of transcription from RNA polymerase II promoter; C:transcription factor complex; C:nuclear chromatin; F:transcription coactivator activity; P:blood vessel development; P:in utero embryonic development; P:regulation of cell death; P:cell differentiation; P:response to lipopolysaccharide; P:regulation of cell cycle |
| Efet.01.256918.g260.t1 | leucine zipper tumor suppressor 3-like | 1119 | 9.71E-07 | 49.25 | C:postsynaptic density |
| Efet.01.25692.g59.t1 | G -activated inward rectifier potassium channel 3-like | 948 | 1.44E-57 | 58.5 | P:transport |
| Efet.01.256928.g261.t1 | rho GTPase-activating 100F-like isoform X2 | 2142 | 2.43E-43 | 56.65 | P:signal transduction |
| Efet.01.256941.g262.t1 | zinc finger 704-like | 396 | 5.31E-15 | 86.45 | F:metal ion binding |
| Efet.01.257008.g263.t1 | serine threonine- kinase PLK1-like | 219 | 5.69E-31 | 83.55 | F:ATP binding; F:protein serine/threonine kinase activity; P:protein phosphorylation |
| Efet.01.257013.g264.t1 | dachsous | 654 | 2.96E-29 | 59.25 | F:oxidoreductase activity; F:calcium ion binding; C:membrane; C:integral component of membrane; P:cell adhesion; P:oxidation-reduction process; F:alcohol dehydrogenase (NAD) activity; P:homophilic cell adhesion via plasma membrane adhesion molecules; C:plasma membrane |
| Efet.01.257025.g265.t1 | hypothetical protein HELRODRAFT_182201 | 891 | 1.76E-47 | 49 |  |
| Efet.01.257031.g266.t1 | voltage-dependent calcium channel gamma-5 subunit-like | 591 | 6.37E-14 | 61.67 | C:integral component of membrane |
| Efet.01.257033.g267.t1 | mucin-12-like isoform X1 | 891 | 2.08E-68 | 76.7 | F:thyroid hormone receptor activity; C:nucleus; F:zinc ion binding; P:transcription, DNA-templated; F:steroid hormone receptor activity; P:regulation of transcription, DNA-templated; F:sequence-specific DNA binding; P:steroid hormone mediated signaling pathway; P:intracellular receptor signaling pathway |
| Efet.01.257112.g271.t1 | acetyl-coenzyme A synthetase 2- mitochondrial | 333 | 4.00E-32 | 75.75 | F:ATP binding; F:acetate-CoA ligase activity; F:AMP binding; C:integral component of membrane; P:acetyl-CoA biosynthetic process from acetate |
| Efet.01.25721.g60.t1 | potassium voltage-gated channel Shaker-like | 1359 | 4.40E-137 | 80.15 | P:potassium ion transmembrane transport; C:voltage-gated potassium channel complex; F:delayed rectifier potassium channel activity; P:regulation of ion transmembrane transport; P:protein homooligomerization |
| Efet.01.257254.g273.t1 | HHIP 1 isoform X1 | 321 | 2.61E-09 | 75.75 | F:catalytic activity; P:metabolic process |
| Efet.01.257263.g274.t1 | glycogen-binding subunit 76A-like | 2952 | 7.02E-58 | 54.85 |  |
| Efet.01.2573.g236.t1 | hypothetical protein CAPTEDRAFT_220856 | 492 | 6.95E-12 | 53.5 | F:structural molecule activity; C:intermediate filament |
| Efet.01.257315.g276.t1 | PDZ domain-containing GIPC3 | 345 | 1.37E-19 | 60.4 |  |
| Efet.01.257325.g277.t1 | cilia- and flagella-associated 58-like | 618 | 2.40E-41 | 71 | C:membrane; C:integral component of membrane |
| Efet.01.257337.g278.t1 | growth hormone secretagogue receptor type 1-like isoform X4 | 1098 | 3.23E-101 | 63.15 | C:membrane |
| Efet.01.257419.g280.t1 | Polycystic kidney disease 1-like 3 | 3990 | 2.26E-26 | 40.65 | P:detection of mechanical stimulus; F:calcium ion binding; F:calcium channel activity; C:membrane; C:integral component of membrane; P:calcium ion transmembrane transport |
| Efet.01.25755.g62.t1 | RING finger 225 | 1917 | 5.92E-08 | 54.29 | F:zinc ion binding; F:metal ion binding; C:membrane; C:integral component of membrane |
| Efet.01.257560.g281.t1 | Receptor-type tyrosine- phosphatase S | 525 | 9.31E-89 | 83.05 | P:establishment of endothelial intestinal barrier; P:negative regulation of cell proliferation; P:regulation of axon regeneration; F:heparin binding; C:neuronal cell body; P:homophilic cell adhesion via plasma membrane adhesion molecules; F:insulin receptor binding; P:negative regulation of epidermal growth factor receptor signaling pathway; P:positive regulation of neuron apoptotic process; P:spinal cord development; C:growth cone; P:negative regulation of cytokine-mediated signaling pathway; C:integral component of plasma membrane; P:negative regulation of neurotrophin TRK receptor signaling pathway; C:endosome; C:extracellular exosome; C:intracellular membrane-bounded organelle; P:cell migration; P:peptidyl-tyrosine dephosphorylation; P:positive regulation of dendrite morphogenesis; P:cerebellum development; C:excitatory synapse; F:chondroitin sulfate proteoglycan binding; P:negative regulation of receptor binding; P:chemical synaptic transmission; P:hippocampus development; F:transmembrane receptor protein tyrosine phosphatase activity; P:cerebral cortex development; F:phosphate ion binding; P:negative regulation of insulin receptor signaling pathway; P:corpus callosum development; P:regulation of synapse structure or activity; P:neuron projection regeneration; F:receptor tyrosine kinase binding; P:negative regulation of cell projection organization |
| Efet.01.257585.g282.t1 | kinesin KIF2A | 576 | 1.59E-90 | 85.65 | F:ATP binding; F:microtubule motor activity; P:microtubule-based movement; C:microtubule; F:microtubule binding |
| Efet.01.257601.g283.t1 | peregrin-like isoform X1 | 237 | 7.22E-08 | 81.95 | F:zinc ion binding |
| Efet.01.257628.g284.t1 | hypothetical protein CAPTEDRAFT_211921 | 237 | 1.11E-23 | 68.8 | F:pyridoxal phosphate binding; P:tryptophan catabolic process; C:cytoplasm; F:kynureninase activity; C:membrane; P:NAD biosynthetic process; F:carbohydrate binding |
| Efet.01.25768.g63.t1 | solute carrier family 26 member 6-like | 363 | 9.41E-14 | 64.65 | P:sulfate transport; P:transmembrane transport; C:membrane; F:sulfate transmembrane transporter activity |
| Efet.01.257694.g285.t1 | adenomatous polyposis coli -like isoform X1 | 2379 | 7.39E-86 | 74.05 | P:negative regulation of Wnt signaling pathway; F:beta-catenin binding |
| Efet.01.257764.g288.t1 | hypothetical protein CAPTEDRAFT_228619 | 504 | 4.26E-08 | 56 | C:membrane; C:integral component of membrane |
| Efet.01.257790.g289.t1 | protocadherin beta-16-like | 363 | 2.47E-16 | 60.7 | F:calcium ion binding; C:membrane; C:integral component of membrane; P:cell adhesion; P:homophilic cell adhesion via plasma membrane adhesion molecules; C:plasma membrane |
| Efet.01.257809.g290.t1 | G -coupled receptor kinase | 303 | 2.73E-48 | 92.7 | F:ATP binding; F:G-protein coupled receptor kinase activity; P:signal transduction; P:protein phosphorylation |
| Efet.01.257895.g292.t1 | pyruvate kinase PKM isoform X3 | 1425 | 5.10E-180 | 66.75 | P:pyruvate biosynthetic process; C:myelin sheath; P:glycolytic process; F:ADP binding; F:MHC class II protein complex binding; P:glucose metabolic process; P:ATP biosynthetic process; C:plasma membrane; F:RNA binding; P:skeletal muscle tissue regeneration; F:cadherin binding involved in cell-cell adhesion; P:cellular response to insulin stimulus; C:nucleus; F:pyruvate kinase activity; C:extracellular exosome; C:cell-cell adherens junction; F:metal ion binding; P:liver development; C:mitochondrion; C:cilium; C:extracellular matrix; P:animal organ regeneration; P:programmed cell death |
| Efet.01.258032.g296.t1 | kinesin KIF27 | 453 | 3.86E-10 | 60.7 | F:nucleotide binding; F:ATPase activity; F:ATP binding; F:microtubule motor activity; C:kinesin complex; P:microtubule-based movement; F:microtubule binding |
| Efet.01.258077.g297.t1 | hypothetical protein BSL78_05953 | 489 | 4.42E-19 | 81.55 |  |
| Efet.01.258130.g299.t1 | paramyosin-like isoform X2 | 207 | 9.19E-13 | 74.1 | C:myosin complex; C:cytoplasm; F:motor activity |
| Efet.01.258176.g301.t1 | Trafficking particle complex subunit 2 | 258 | 4.05E-48 | 87.85 | P:ER to Golgi vesicle-mediated transport; C:cytoplasm |
| Efet.01.258184.g302.t1 | flagellar motor | 741 | 2.02E-150 | 61.8 | C:membrane; C:integral component of membrane; C:cell outer membrane |
| Efet.01.258205.g303.t1 | family | 681 | 8.19E-88 | 86.4 | C:integral component of membrane; P:transport; F:transporter activity |
| Efet.01.258243.g304.t1 | aquaporin -like | 639 | 5.58E-36 | 67.85 | C:membrane |
| Efet.01.258373.g306.t1 | low-density lipo receptor-related 1-like | 840 | 1.13E-114 | 73.85 | F:calcium ion binding; C:integral component of membrane |
| Efet.01.258398.g307.t1 | dynein heavy chain axonemal-like isoform X1 | 249 | 9.93E-33 | 89.8 | F:ATPase activity; F:ATP binding; F:microtubule motor activity; F:actin binding; C:dynein complex; P:phosphorylation; P:microtubule-based movement; F:kinase activity |
| Efet.01.258409.g308.t1 | N,N -diacetylchitobiase-like isoform X2 | 252 | 3.17E-17 | 67 | P:carbohydrate metabolic process; C:membrane; C:integral component of membrane; F:carbohydrate binding; F:hydrolase activity, hydrolyzing O-glycosyl compounds; F:beta-N-acetylhexosaminidase activity; F:polysaccharide binding |
| Efet.01.258426.g310.t1 | Neuroligin- X-linked | 975 | 6.15E-76 | 50.05 | C:membrane; C:integral component of membrane |
| Efet.01.258463.g314.t1 | inactive tyrosine- kinase 7-like | 225 | 6.41E-24 | 72.55 | P:cellular macromolecule metabolic process; F:organic cyclic compound binding; F:heterocyclic compound binding; C:membrane; P:phosphorylation; P:primary metabolic process; F:kinase activity |
| Efet.01.258476.g315.t1 | urea carboxylase | 1875 | 0 | 74.2 | F:ATP binding; F:metal ion binding; F:lyase activity; F:biotin carboxylase activity; F:hydrolase activity |
| Efet.01.258504.g316.t1 | repulsive guidance molecule A-like | 480 | 8.24E-38 | 58.45 | C:membrane |
| Efet.01.258575.g319.t1 | X-box-binding 1 | 1545 | 9.19E-27 | 58.3 | P:positive regulation of cellular protein metabolic process; P:positive regulation of cell proliferation; F:transcription regulatory region DNA binding; F:sequence-specific DNA binding; P:negative regulation of intracellular signal transduction; P:regulation of anatomical structure morphogenesis; P:gland development; P:positive regulation of transcription from RNA polymerase II promoter; P:sterol homeostasis; P:positive regulation of response to stimulus; F:transcription factor activity, sequence-specific DNA binding; P:endoplasmic reticulum unfolded protein response; C:intracellular membrane-bounded organelle; P:positive regulation of multicellular organismal process; P:apoptotic process; P:cellular response to hexose stimulus; P:cellular response to peptide hormone stimulus; P:positive regulation of immunoglobulin production; P:regulation of protein modification process; P:positive regulation of protein secretion; P:intracellular signal transduction; P:cellular chemical homeostasis; P:epithelial cell maturation; P:transcription, DNA-templated; P:exocrine system development; P:negative regulation of response to endoplasmic reticulum stress; P:positive regulation of cell differentiation; C:cytoplasmic part; F:ubiquitin-like protein ligase binding; P:regulation of protein stability; P:cellular response to nutrient levels; P:negative regulation of apoptotic process; C:membrane; P:regulation of autophagy; P:glucose homeostasis; P:regulation of multicellular organismal development |
| Efet.01.258587.g320.t1 | hypothetical protein CAPTEDRAFT_213201 | 1254 | 1.91E-07 | 51.5 |  |
| Efet.01.258596.g321.t1 | dynein heavy chain axonemal-like | 939 | 1.01E-75 | 69.15 | F:binding |
| Efet.01.258659.g323.t1 | protocadherin-9 isoform X1 | 2934 | 0 | 52.55 | F:calcium ion binding; C:membrane; C:integral component of membrane; P:cell adhesion; P:homophilic cell adhesion via plasma membrane adhesion molecules; C:plasma membrane |
| Efet.01.258671.g324.t1 | hypothetical protein HELRODRAFT_183814 | 267 | 5.40E-06 | 53 | C:membrane; C:integral component of membrane |
| Efet.01.258721.g325.t1 | poly [ADP-ribose] polymerase 14-like | 522 | 9.25E-12 | 47.19 | F:nucleotide binding; F:nucleic acid binding; F:transferase activity, transferring glycosyl groups; F:transferase activity; F:NAD+ ADP-ribosyltransferase activity |
| Efet.01.258765.g328.t1 | Ankyrin repeat | 4038 | 8.22E-29 | 51.65 | C:nucleus; C:proteasome regulatory particle; P:proteasome regulatory particle assembly; P:proteolysis; C:cytosol |
| Efet.01.258767.g329.t1 | sodium potassium ATPase alpha | 690 | 4.13E-104 | 82.8 | P:potassium ion transport; F:ATP binding; P:sodium ion transport; F:sodium:potassium-exchanging ATPase activity; F:metal ion binding; P:ATP hydrolysis coupled transmembrane transport; C:integral component of membrane; P:establishment or maintenance of transmembrane electrochemical gradient |
| Efet.01.258775.g330.t1 | hypothetical protein CAPTEDRAFT_211035 | 537 | 8.66E-23 | 48.05 | C:membrane; C:integral component of membrane |
| Efet.01.258775.g331.t1 | beta-1,4-galactosyltransferase galt-1-like | 1038 | 7.29E-72 | 51.15 | C:membrane; C:integral component of membrane |
| Efet.01.258820.g332.t1 | nuclear pore complex Nup93-like | 312 | 1.09E-27 | 74.4 | P:protein import into nucleus; F:structural constituent of nuclear pore; P:nuclear pore complex assembly; P:poly(A)+ mRNA export from nucleus; C:nuclear pore |
| Efet.01.258920.g335.t1 | potassium voltage-gated channel Shaw-like isoform X1 | 579 | 7.60E-80 | 82.9 | F:voltage-gated potassium channel activity; P:potassium ion transmembrane transport; F:metal ion binding; C:voltage-gated potassium channel complex; P:regulation of ion transmembrane transport; P:protein homooligomerization |
| Efet.01.258939.g336.t1 | meiosis-specific coiled-coil domain-containing MEIOC-like isoform X1 | 978 | 8.60E-07 | 67.6 |  |
| Efet.01.258956.g337.t1 | potassium voltage-gated channel subfamily A member 1 isoform X1 | 552 | 3.34E-59 | 74.45 | P:potassium ion transmembrane transport; C:voltage-gated potassium channel complex; F:delayed rectifier potassium channel activity; P:regulation of ion transmembrane transport; P:protein homooligomerization |
| Efet.01.258956.g338.t1 | potassium voltage-gated channel Shaker isoform X1 | 909 | 2.85E-90 | 75.45 | F:voltage-gated potassium channel activity; P:potassium ion transmembrane transport; C:voltage-gated potassium channel complex; P:regulation of ion transmembrane transport; P:protein homooligomerization |
| Efet.01.258969.g339.t1 | TSSC4 | 1113 | 2.25E-12 | 49.5 | P:head kidney development |
| Efet.01.258970.g340.t1 | CREBRF homolog | 471 | 3.66E-50 | 69.35 | F:transcription factor activity, sequence-specific DNA binding; P:regulation of transcription, DNA-templated; F:sequence-specific DNA binding |
| Efet.01.258972.g342.t1 | phosphoenolpyruvate hydrolase family | 924 | 1.33E-177 | 81.45 | F:catalytic activity |
| Efet.01.258991.g343.t1 | mitogen-activated kinase kinase kinase 1 | 369 | 1.16E-21 | 73.7 | F:ATP binding; F:protein kinase binding; F:signal transducer, downstream of receptor, with serine/threonine kinase activity; F:zinc ion binding; P:cellular response to mechanical stimulus; C:cytoplasm; P:apoptotic process; C:integral component of membrane; P:signal transduction by protein phosphorylation |
| Efet.01.259040.g345.t1 | WD repeat-containing 19 isoform X1 | 456 | 6.36E-33 | 85.35 | C:photoreceptor connecting cilium; P:cilium assembly; P:embryonic camera-type eye development; P:cell morphogenesis; P:myotome development; P:embryonic cranial skeleton morphogenesis; P:ciliary receptor clustering involved in smoothened signaling pathway; C:plasma membrane; P:smoothened signaling pathway involved in dorsal/ventral neural tube patterning; C:nucleoplasm; P:embryonic limb morphogenesis; C:motile cilium; P:gonad development; P:ear morphogenesis; P:digestive system development; C:intraciliary transport particle A; P:neurological system process; P:intraciliary retrograde transport |
| Efet.01.259078.g348.t1 | RNA-binding MEX3B | 822 | 2.66E-29 | 85.45 | F:zinc ion binding; F:RNA binding |
| Efet.01.259084.g349.t1 | calcium calmodulin-dependent 3 ,5 -cyclic nucleotide phosphodiesterase 1A isoform X8 | 414 | 1.92E-08 | 67.25 |  |
| Efet.01.259108.g350.t1 | Kelch 1 | 249 | 1.48E-27 | 85.15 |  |
| Efet.01.259139.g351.t1 | 1-phosphatidylinositol 4,5-bisphosphate phosphodiesterase epsilon-1 | 1665 | 4.20E-20 | 55.5 | C:membrane; C:integral component of membrane; C:proteasome complex |
| Efet.01.259156.g352.t1 | acetylcholine receptor subunit alpha-like 1 | 666 | 1.09E-89 | 69.85 | C:postsynaptic membrane; P:cation transmembrane transport; C:integral component of membrane; C:cell junction; F:extracellular ligand-gated ion channel activity |
| Efet.01.259173.g353.t1 | XK-related 6 | 906 | 1.20E-24 | 59.85 | C:membrane |
| Efet.01.25925.g70.t1 | hypothetical protein HELRODRAFT_180969 | 555 | 3.74E-13 | 65 |  |
| Efet.01.25927.g71.t1 | sclerostin domain-containing 1-like | 372 | 1.60E-48 | 74.7 | C:extracellular space |
| Efet.01.259270.g354.t1 | SPRY domain-containing 3 | 732 | 4.06E-13 | 48.35 |  |
| Efet.01.259307.g356.t1 | beta-1,4-N-acetylgalactosaminyltransferase bre-4-like | 273 | 6.84E-18 | 72.75 | F:transferase activity; P:primary metabolic process; P:organic substance metabolic process |
| Efet.01.259315.g357.t1 | transcription factor 7-like 2 isoform X4 | 225 | 4.36E-18 | 68.9 | P:Wnt signaling pathway |
| Efet.01.259327.g358.t1 | interleukin 17 -like | 291 | 8.21E-06 | 56 |  |
| Efet.01.25937.g72.t1 | alpha-(1,6)-fucosyltransferase isoform X2 | 276 | 7.32E-31 | 71.75 | C:extracellular exosome; P:in utero embryonic development; P:N-glycan processing; C:integral component of membrane; P:N-glycan fucosylation; C:Golgi membrane; P:cell surface receptor signaling pathway; P:oligosaccharide biosynthetic process; P:protein N-linked glycosylation via asparagine; P:L-fucose catabolic process; P:GDP-L-fucose metabolic process; F:glycoprotein 6-alpha-L-fucosyltransferase activity |
| Efet.01.259386.g360.t1 | trichohyalin-like | 624 | 6.88E-06 | 58 |  |
| Efet.01.259400.g361.t1 | receptor-type guanylate cyclase gcy-5-like isoform X1 | 489 | 7.55E-43 | 55.35 | P:phosphorylation; P:primary metabolic process; F:kinase activity; P:organic substance metabolic process |
| Efet.01.25953.g73.t1 | leukocyte receptor cluster member 8 homolog | 225 | 1.29E-10 | 70.25 |  |
| Efet.01.259579.g366.t1 | dipeptidyl peptidase 9-like | 204 | 3.01E-10 | 79.7 | P:proteolysis; F:serine-type peptidase activity |
| Efet.01.259617.g368.t1 | metabotropic glutamate receptor 7-like isoform X1 | 2421 | 2.95E-121 | 50.65 | F:G-protein coupled receptor activity; C:presynapse; C:postsynapse; P:regulation of cellular process; F:glutamate receptor activity; P:cell communication; C:synaptic membrane; P:single organism signaling; C:neuron projection |
| Efet.01.259657.g369.t1 | ephrin type-B receptor 2- | 279 | 6.54E-29 | 73.3 | F:nucleotide binding; F:transmembrane receptor protein tyrosine kinase activity; C:integral component of membrane; P:transmembrane receptor protein tyrosine kinase signaling pathway; P:protein phosphorylation; F:hydrolase activity, acting on glycosyl bonds |
| Efet.01.259688.g371.t1 | transcription factor AP-2-epsilon-like isoform X2 | 450 | 7.01E-30 | 75.3 | C:nucleus; F:transcription factor activity, sequence-specific DNA binding; P:regulation of transcription, DNA-templated |
| Efet.01.2598.g238.t1 | Multidrug resistance-associated | 981 | 6.50E-107 | 56.9 | F:nucleotide binding; F:ATPase activity; F:ATP binding; F:ATPase activity, coupled to transmembrane movement of substances; P:transmembrane transport; C:membrane; C:integral component of membrane; P:transport; F:transporter activity |
| Efet.01.259865.g376.t1 | lysine-specific demethylase 4C-like | 759 | 6.34E-93 | 62.9 | F:metal ion binding; F:methyltransferase activity; P:methylation |
| Efet.01.260083.g380.t1 | polycystic kidney disease 1-like 2 | 1143 | 1.50E-47 | 50.8 |  |
| Efet.01.26010.g74.t1 | minor histocompatibility antigen H13 | 249 | 7.72E-18 | 77.7 | P:membrane protein proteolysis; C:Golgi-associated vesicle membrane; C:integral component of lumenal side of endoplasmic reticulum membrane; C:integral component of cytoplasmic side of endoplasmic reticulum membrane; P:signal peptide processing; F:aspartic endopeptidase activity, intramembrane cleaving; C:lysosomal membrane |
| Efet.01.260157.g382.t1 | gastrula zinc finger -like | 1266 | 1.21E-25 | 56.85 | F:metal ion binding |
| Efet.01.260176.g383.t1 | -tyrosine sulfotransferase 2 | 756 | 3.79E-88 | 72.45 | F:transferase activity; C:membrane-bounded organelle |
| Efet.01.260237.g384.t1 | hypothetical protein CAPTEDRAFT_226930 | 357 | 4.14E-12 | 76 |  |
| Efet.01.26034.g75.t1 | adenomatous polyposis coli down-regulated | 612 | 6.95E-24 | 52.7 | C:membrane |
| Efet.01.260430.g386.t1 | hypothetical protein CAPTEDRAFT_200340 | 516 | 2.27E-18 | 70.42 |  |
| Efet.01.260520.g388.t1 | nfx1-type zinc finger-containing 1 | 837 | 3.67E-75 | 65.95 | F:metal ion binding; F:NAD+ ADP-ribosyltransferase activity |
| Efet.01.260527.g389.t1 | hypothetical protein CAPTEDRAFT_129833, partial | 447 | 4.04E-11 | 72.67 |  |
| Efet.01.260531.g390.t1 | deoxynucleoside triphosphate triphosphohydrolase SAMHD1-like | 624 | 6.94E-10 | 55.75 | C:nucleus; P:regulation of innate immune response; P:defense response to virus; F:dGTPase activity; P:dGTP catabolic process |
| Efet.01.26059.g77.t1 | ATP-binding cassette sub-family G member 4-like isoform X1 | 429 | 1.90E-09 | 61 | F:ATPase activity; F:ATP binding; C:integral component of membrane |
| Efet.01.260593.g392.t1 | Gamma-aminobutyric acid receptor subunit gamma- | 261 | 7.39E-18 | 64.05 | P:transport |
| Efet.01.260600.g393.t1 | outer dense fiber 3 2 | 501 | 1.83E-51 | 52.5 | P:spermatid development; C:membrane; C:integral component of membrane |
| Efet.01.260611.g394.t1 | PREDICTED: uncharacterized protein LOC109470921 | 360 | 3.23E-13 | 63 |  |
| Efet.01.260625.g395.t1 | laminin subunit beta-2 | 204 | 1.17E-23 | 83.45 | P:astrocyte development; P:Schwann cell development; P:retina development in camera-type eye; P:axon guidance; F:integrin binding; C:synapse; P:axon extension involved in regeneration; P:visual perception; P:neuromuscular junction development; P:metanephric glomerular basement membrane development; C:extracellular exosome; C:laminin-3 complex; P:metanephric glomerular visceral epithelial cell development |
| Efet.01.260658.g398.t1 | myelin regulatory factor-like isoform X3 | 624 | 1.83E-71 | 69.65 | F:DNA binding; F:transcription factor activity, sequence-specific DNA binding; C:membrane; C:integral component of membrane; P:regulation of transcription, DNA-templated |
| Efet.01.260742.g400.t1 | spectrin alpha chain-like isoform X1 | 207 | 4.05E-32 | 83 | F:calcium ion binding |
| Efet.01.260747.g402.t1 | uncharacterized family 31 glucosidase KIAA1161-like | 1284 | 1.76E-100 | 58.6 | F:catalytic activity |
| Efet.01.260813.g406.t1 | serine protease P54 | 297 | 2.13E-14 | 58.5 | F:peptidase activity |
| Efet.01.26082.g80.t1 | hypothetical protein CAPTEDRAFT_229283 | 567 | 7.14E-26 | 72.5 | C:membrane |
| Efet.01.260829.g407.t1 | tRNA-splicing endonuclease subunit Sen54-like | 999 | 1.84E-07 | 40 |  |
| Efet.01.260885.g408.t1 | Potassium voltage-gated channel subfamily A member 2 | 342 | 2.33E-36 | 83.8 | P:potassium ion transmembrane transport; C:voltage-gated potassium channel complex; F:delayed rectifier potassium channel activity; P:regulation of ion transmembrane transport; P:protein homooligomerization |
| Efet.01.260885.g409.t1 | potassium voltage-gated channel subfamily A member 2 | 1047 | 2.58E-95 | 76 | P:potassium ion transmembrane transport; C:voltage-gated potassium channel complex; F:delayed rectifier potassium channel activity; P:regulation of ion transmembrane transport; P:protein homooligomerization |
| Efet.01.26096.g81.t1 | PREDICTED: uncharacterized protein LOC106161002 | 3753 | 1.40E-47 | 42.25 | P:detection of mechanical stimulus; F:calcium channel activity; F:calcium ion binding; C:membrane; C:integral component of membrane; P:calcium ion transmembrane transport |
| Efet.01.260967.g413.t1 | syntaxin-binding 1-like isoform X2 | 1140 | 0 | 78.2 | P:vesicle docking involved in exocytosis |
| Efet.01.261042.g414.t1 | transcription factor SOX-11-like | 228 | 4.03E-11 | 70.9 | C:nucleus |
| Efet.01.261097.g415.t1 | KN motif and ankyrin repeat domain-containing 1-like isoform X1 | 342 | 8.14E-31 | 72.25 | F:lysophospholipase activity; F:hydrolase activity |
| Efet.01.261110.g416.t1 | bactericidal permeability increasing | 870 | 2.20E-58 | 57.35 | C:extracellular space; F:lipid binding |
| Efet.01.261138.g418.t1 | Zinc finger ZIC | 900 | 5.87E-65 | 68.4 | P:central nervous system development; P:regulation of cellular process; F:binding; C:intracellular part; P:positive regulation of biological process |
| Efet.01.261191.g421.t1 | cytoplasmic FMR1-interacting 1 | 330 | 4.51E-37 | 76.85 | P:Rac protein signal transduction; P:Fc-gamma receptor signaling pathway involved in phagocytosis; C:neuron projection; F:actin filament binding; C:SCAR complex; P:vascular endothelial growth factor receptor signaling pathway; C:mRNA cap binding complex; C:perinuclear region of cytoplasm; C:extracellular exosome; C:focal adhesion; P:cognition; P:axon extension; P:ruffle organization; F:Rac GTPase binding; C:ruffle; C:cytosol; P:lamellipodium assembly; P:positive regulation of Arp2/3 complex-mediated actin nucleation; C:lamellipodium |
| Efet.01.26120.g84.t1 | RNA-binding 26 isoform X2 | 501 | 1.48E-09 | 62.89 | F:binding |
| Efet.01.261241.g422.t1 | hypothetical protein HELRODRAFT_171246 | 789 | 1.14E-10 | 50 | F:metal ion binding |
| Efet.01.261306.g424.t1 | membrane | 966 | 0 | 82.4 | C:integral component of membrane |
| Efet.01.261307.g426.t1 | Mu-like prophage host-nuclease inhibitor Gam | 462 | 4.90E-34 | 58.45 | P:DNA protection; F:double-stranded DNA binding |
| Efet.01.261307.g427.t1 | AAA domain | 711 | 4.83E-39 | 62.4 |  |
| Efet.01.26132.g85.t1 | hypothetical protein CAPTEDRAFT_219553 | 630 | 2.00E-39 | 53.7 | C:membrane; C:integral component of membrane; F:carbohydrate binding |
| Efet.01.261328.g429.t1 | myosin heavy chain isoform | 510 | 5.13E-37 | 67.75 | F:small molecule binding |
| Efet.01.261403.g431.t1 | 7-dehydrocholesterol reductase-like | 1248 | 1.09E-122 | 70 | C:membrane |
| Efet.01.26141.g86.t1 | serine- kinase ATM-like | 570 | 2.00E-51 | 85.85 | F:ATP binding; P:DNA damage checkpoint; P:DNA repair; P:replicative senescence; P:telomere maintenance; P:histone phosphorylation; F:protein serine/threonine kinase activity; P:response to ionizing radiation |
| Efet.01.261413.g432.t1 | sal 1 | 2307 | 8.34E-49 | 60.9 | F:nucleic acid binding; F:metal ion binding |
| Efet.01.26146.g87.t1 | tectonin beta-propeller repeat-containing 2 isoform X1 | 381 | 3.58E-10 | 53 |  |
| Efet.01.261483.g433.t1 | helicase with zinc finger domain 2-like | 1332 | 9.64E-57 | 46.75 | F:nucleotide binding; F:nucleic acid binding; F:5'-flap endonuclease activity; F:ATP binding; F:zinc ion binding; F:metal ion binding; F:single-stranded DNA-dependent ATPase activity; F:helicase activity; P:DNA replication, Okazaki fragment processing |
| Efet.01.261489.g434.t1 | dystrophin-like isoform X1 | 324 | 1.93E-15 | 59.4 | F:binding |
| Efet.01.261529.g436.t1 | Coiled-coil domain-containing 134 | 324 | 3.23E-11 | 50.65 | F:chitinase activity; F:hydrolase activity, acting on carbon-nitrogen (but not peptide) bonds; P:carbohydrate metabolic process; F:chitin binding; F:catalytic activity; P:chitin catabolic process; P:chitin metabolic process; C:extracellular region |
| Efet.01.26159.g89.t1 | tropomyosin isoform X1 | 354 | 1.86E-12 | 89.25 |  |
| Efet.01.261603.g437.t1 | class B secretin-like G- coupled receptor | 2319 | 7.53E-39 | 53.3 | F:G-protein coupled receptor activity; F:transmembrane signaling receptor activity; P:G-protein coupled receptor signaling pathway; C:membrane; C:integral component of membrane; P:cell surface receptor signaling pathway |
| Efet.01.26163.g90.t1 | polycystic kidney disease and receptor for egg jelly-related | 675 | 5.77E-06 | 49 | F:calcium ion binding; C:membrane; C:integral component of membrane |
| Efet.01.261757.g439.t1 | specificity 1 | 1038 | 2.33E-15 | 75.65 | F:nucleic acid binding; F:metal ion binding |
| Efet.01.26176.g92.t1 | unconventional myosin-X-like isoform X1 | 399 | 9.19E-40 | 59.6 | C:cytoskeleton |
| Efet.01.261863.g442.t1 | hypothetical protein CAPTEDRAFT_228560 | 234 | 3.16E-06 | 69 | F:ubiquitin-protein transferase activity; F:ligase activity; P:protein ubiquitination |
| Efet.01.261863.g443.t1 | probable E3 ubiquitin- ligase HERC1 isoform X2 | 939 | 6.01E-95 | 65.6 | F:ubiquitin-protein transferase activity; F:ligase activity; P:protein ubiquitination |
| Efet.01.261870.g444.t1 | multiple C2 and transmembrane domain-containing 1-like isoform X6 | 279 | 2.03E-22 | 69.9 | C:integral component of membrane |
| Efet.01.26194.g93.t1 | chondroitin sulfate N-acetylgalactosaminyltransferase 2-like | 282 | 1.34E-42 | 84.2 | C:Golgi cisterna membrane; C:integral component of membrane; F:acetylgalactosaminyltransferase activity |
| Efet.01.261972.g446.t1 | gastrula zinc finger -like | 3924 | 1.82E-128 | 63.55 | F:nucleic acid binding; F:transcription factor activity, sequence-specific DNA binding; F:metal ion binding; P:regulation of transcription, DNA-templated |
| Efet.01.262117.g449.t1 | hypothetical protein CAPTEDRAFT_227952 | 576 | 2.53E-19 | 49.3 | P:signal transduction |
| Efet.01.262124.g451.t1 | dual specificity phosphatase 14-like | 531 | 2.41E-73 | 74.2 | P:protein dephosphorylation; F:protein tyrosine/serine/threonine phosphatase activity |
| Efet.01.262143.g455.t1 | tripartite motif-containing 3-like | 1473 | 9.48E-160 | 48.45 | F:zinc ion binding; F:metal ion binding; C:intracellular |
| Efet.01.262148.g456.t1 | oligodendrocyte transcription factor 3-like | 987 | 3.98E-22 | 89.7 | F:transcription factor activity, sequence-specific DNA binding; P:neuron differentiation; P:regulation of transcription, DNA-templated; F:protein dimerization activity; P:myelination; P:oligodendrocyte cell fate specification; P:regulation of neuron differentiation |
| Efet.01.262195.g459.t1 | pilus assembly | 522 | 2.67E-68 | 85.2 | C:integral component of membrane |
| Efet.01.262205.g460.t1 | probable medium-chain specific acyl- mitochondrial | 228 | 2.14E-25 | 82.4 | F:acyl-CoA dehydrogenase activity; F:flavin adenine dinucleotide binding; P:oxidation-reduction process |
| Efet.01.262256.g461.t1 | Cyclic GMP-AMP synthase | 273 | 7.35E-14 | 53.75 |  |
| Efet.01.262287.g462.t1 | monocarboxylate transporter 13-like | 309 | 4.88E-31 | 55.45 | P:transmembrane transport; C:integral component of membrane |
| Efet.01.262309.g463.t1 | WD repeat-containing 86-like isoform X2 | 741 | 1.11E-78 | 65.65 |  |
| Efet.01.262332.g465.t1 | hypothetical protein HELRODRAFT_180640 | 333 | 2.81E-37 | 64.45 | C:membrane; C:integral component of membrane; F:carbohydrate binding |
| Efet.01.262367.g466.t1 | short transient receptor potential channel 3-like isoform X2 | 222 | 2.50E-14 | 72.35 | F:calcium channel activity; C:integral component of membrane; P:calcium ion transmembrane transport |
| Efet.01.262370.g467.t1 | lipoyltransferase mitochondrial | 240 | 2.17E-26 | 68.95 | P:cellular protein modification process |
| Efet.01.262431.g469.t1 | gamma-aminobutyric acid type B receptor subunit 2-like | 402 | 6.07E-19 | 55.67 | F:G-protein coupled receptor activity; F:G-protein coupled GABA receptor activity; P:G-protein coupled receptor signaling pathway; P:signal transduction; C:membrane; C:integral component of membrane; F:signal transducer activity |
| Efet.01.262435.g470.t1 | netrin receptor UNC5C-like isoform X3 | 255 | 7.25E-27 | 75.45 | P:signal transduction; C:integral component of membrane |
| Efet.01.262449.g471.t1 | zinc transporter ZIP10-like isoform X1 | 441 | 3.19E-33 | 65.85 | P:transmembrane transport; C:integral component of membrane; F:metal ion transmembrane transporter activity; P:metal ion transport |
| Efet.01.262461.g472.t1 | E3 ubiquitin- ligase TRIM56-like | 984 | 2.42E-21 | 44.95 | F:zinc ion binding; F:metal ion binding; C:intracellular |
| Efet.01.262545.g476.t1 | hypothetical protein CAPTEDRAFT_228268 | 1566 | 3.30E-12 | 68 | P:phosphorylation; F:kinase activity |
| Efet.01.26257.g96.t1 | grainyhead isoform X1 | 474 | 8.50E-29 | 78.6 |  |
| Efet.01.262645.g478.t1 | cyclic nucleotide-gated cation channel subunit A-like isoform X1 | 1089 | 2.39E-116 | 69.55 | C:integral component of plasma membrane; F:voltage-gated potassium channel activity; P:cation transport; P:regulation of membrane potential; P:ion transmembrane transport; F:intracellular cAMP activated cation channel activity; F:intracellular cGMP activated cation channel activity; F:cGMP binding |
| Efet.01.262709.g481.t1 | poly [ADP-ribose] polymerase 14-like | 399 | 1.46E-28 | 58.75 | F:transferase activity, transferring glycosyl groups; F:transferase activity; F:NAD+ ADP-ribosyltransferase activity |
| Efet.01.262746.g483.t1 | UNC93 | 1263 | 3.40E-80 | 61.2 | C:integral component of membrane |
| Efet.01.262752.g484.t1 | Visual system homeobox | 348 | 2.34E-10 | 67 | F:DNA binding |
| Efet.01.262790.g485.t1 | hypothetical protein HELRODRAFT_190695 | 1707 | 2.75E-10 | 51 |  |
| Efet.01.262822.g486.t1 | kinesin KIF2A | 339 | 4.32E-45 | 87.6 | F:ATP binding; F:microtubule motor activity; P:microtubule-based movement; C:microtubule; F:microtubule binding |
| Efet.01.262860.g492.t1 | ubiquinol-cytochrome c reductase cytochrome b subunit | 399 | 1.40E-67 | 87.25 | F:oxidoreductase activity; F:electron carrier activity; P:respiratory electron transport chain; C:integral component of membrane |
| Efet.01.262931.g494.t1 | dynein heavy chain axonemal | 360 | 1.07E-38 | 88.25 | F:ATPase activity; F:ATP binding; F:microtubule motor activity; C:integral component of membrane; C:dynein complex; P:microtubule-based movement |
| Efet.01.262936.g495.t1 | Potassium voltage-gated channel Shaker | 1320 | 2.77E-122 | 68.05 | P:potassium ion transport; P:transmembrane transport; C:integral component of membrane; F:voltage-gated ion channel activity; F:potassium channel activity; P:cellular process |
| Efet.01.26312.g99.t1 | NACHT domain- and WD repeat-containing 1-like | 1311 | 2.10E-22 | 44.2 |  |
| Efet.01.26319.g100.t1 | myosin heavy chain | 384 | 1.04E-16 | 69.45 | F:nucleotide binding; C:myosin complex; F:protein binding; F:anion binding |
| Efet.01.2633.g240.t1 | ELAV 3 isoform X1 | 726 | 1.22E-44 | 52.2 | F:nucleotide binding; F:nucleic acid binding; F:RNA binding |
| Efet.01.263316.g501.t1 | 28S ribosomal mitochondrial-like | 519 | 9.31E-82 | 81.55 | F:structural constituent of ribosome; P:positive regulation of macromitophagy in response to mitochondrial depolarization; P:positive regulation of defense response to virus by host; C:mitochondrial small ribosomal subunit; P:xenophagy; P:translation |
| Efet.01.263321.g502.t1 | Potassium voltage-gated channel Shal | 609 | 5.83E-126 | 90.6 | F:voltage-gated potassium channel activity; P:potassium ion transmembrane transport; C:voltage-gated potassium channel complex; P:regulation of ion transmembrane transport; P:protein homooligomerization |
| Efet.01.26335.g101.t1 | UPF0676 -like | 951 | 4.17E-65 | 59.85 | F:dioxygenase activity; F:iron ion binding; F:oxidoreductase activity; F:metal ion binding; P:oxidation-reduction process |
| Efet.01.263385.g504.t1 | limbic system-associated membrane -like | 498 | 2.68E-11 | 60.6 | C:membrane; C:integral component of membrane |
| Efet.01.263392.g505.t1 | protocadherin gamma-C5-like | 741 | 1.95E-25 | 53.75 | F:G-protein coupled receptor activity; F:transmembrane signaling receptor activity; P:G-protein coupled receptor signaling pathway; P:signal transduction; F:calcium ion binding; C:membrane; C:integral component of membrane; P:cell adhesion; P:cell surface receptor signaling pathway; P:homophilic cell adhesion via plasma membrane adhesion molecules; F:signal transducer activity; C:plasma membrane |
| Efet.01.263491.g507.t1 | disintegrin and metallo ase domain-containing 12-like isoform X2 | 450 | 2.76E-07 | 57.25 | C:membrane; P:proteolysis; C:integral component of membrane; F:metalloendopeptidase activity; F:metallopeptidase activity |
| Efet.01.26358.g103.t1 | glycerophosphocholine phosphodiesterase GPCPD1 isoform X1 | 1203 | 5.11E-82 | 57.2 | F:hydrolase activity |
| Efet.01.263580.g508.t1 | intermediate filament | 363 | 8.54E-18 | 62.35 | F:structural molecule activity; C:intermediate filament |
| Efet.01.263610.g509.t1 | plasma membrane calcium-transporting atpase, | 204 | 2.24E-12 | 77.15 | C:integral component of plasma membrane; F:ATP binding; F:metal ion binding; P:locomotion; C:intracellular membrane-bounded organelle; F:PDZ domain binding; P:regulation of cytosolic calcium ion concentration; P:calcium ion transmembrane transport; F:calcium-transporting ATPase activity; P:reproduction; P:positive regulation of multicellular organism growth |
| Efet.01.263663.g510.t1 | probable phospholipid-transporting ATPase IF isoform X7 | 363 | 4.24E-38 | 52.7 | F:binding |
| Efet.01.263703.g511.t1 | transmembrane 165-like | 444 | 8.57E-75 | 81.25 | C:integral component of membrane |
| Efet.01.26374.g104.t1 | paired box | 1038 | 1.54E-75 | 68.65 | C:nucleus; F:DNA binding; P:transcription, DNA-templated; P:multicellular organism development; P:regulation of transcription, DNA-templated |
| Efet.01.263746.g516.t1 | glyco -N-acetylgalactosamine 3-beta-galactosyltransferase 1 isoform X2 | 327 | 5.70E-37 | 72.75 | F:glycoprotein-N-acetylgalactosamine 3-beta-galactosyltransferase activity; P:O-glycan processing, core 1 |
| Efet.01.26381.g105.t1 | fibrillin-1-like isoform X1 | 609 | 6.07E-42 | 62.15 | F:oxidoreductase activity, acting on paired donors, with incorporation or reduction of molecular oxygen, reduced ascorbate as one donor, and incorporation of one atom of oxygen; F:copper ion binding; F:catalytic activity; F:calcium ion binding; P:oxidation-reduction process; F:extracellular matrix structural constituent; F:monooxygenase activity; C:proteinaceous extracellular matrix |
| Efet.01.263822.g519.t1 | PREDICTED: uncharacterized protein LOC105314754 | 927 | 8.43E-13 | 54.75 | P:DNA repair; F:ATP binding; F:DNA helicase activity; P:DNA duplex unwinding; P:telomere maintenance; F:helicase activity |
| Efet.01.263882.g522.t1 | leucine-rich repeat-containing 15-like | 903 | 4.22E-81 | 51.05 | C:membrane; C:integral component of membrane |
| Efet.01.263931.g523.t1 | Heterogeneous nuclear ribonucleo | 480 | 3.45E-60 | 64 | F:nucleotide binding; F:nucleic acid binding; C:intracellular ribonucleoprotein complex; C:viral nucleocapsid |
| Efet.01.263968.g525.t1 | sex peptide receptor-like | 420 | 1.73E-24 | 78.35 | F:G-protein coupled peptide receptor activity; P:G-protein coupled receptor signaling pathway; C:integral component of membrane |
| Efet.01.264096.g527.t1 | chondroitin sulfate proteoglycan 4-like | 435 | 4.77E-21 | 54.1 | C:membrane; C:integral component of membrane |
| Efet.01.264098.g528.t1 | neuroligin- X-linked-like isoform X1 | 954 | 7.07E-73 | 55.4 | C:membrane; C:integral component of membrane |
| Efet.01.264153.g530.t1 | nuclear hormone receptor FTZ-F1 beta-like | 708 | 2.37E-21 | 61.35 | C:nucleus; F:transcription factor activity, sequence-specific DNA binding; F:transition metal ion binding; P:metabolic process; P:regulation of transcription, DNA-templated; F:sequence-specific DNA binding; F:signal transducer activity; P:hormone-mediated signaling pathway |
| Efet.01.264161.g531.t1 | death domain-containing 1-like | 3207 | 3.55E-57 | 51.75 | P:signal transduction |
| Efet.01.264249.g534.t1 | phosphatidylinositol 5-phosphate 4-kinase type-2 alpha | 426 | 1.50E-36 | 62.05 | F:phosphatidylinositol phosphate kinase activity; P:phosphatidylinositol phosphorylation |
| Efet.01.264305.g535.t1 | coiled-coil domain-containing 74A-like isoform X1 | 321 | 3.14E-09 | 69.15 |  |
| Efet.01.264332.g536.t1 | peptidyl-prolyl cis-trans isomerase NIMA-interacting 4 | 246 | 1.40E-26 | 85.6 | F:isomerase activity |
| Efet.01.264435.g539.t1 | peritrophic membrane chitin binding | 522 | 2.36E-32 | 61.15 | F:catalytic activity; P:organic substance metabolic process |
| Efet.01.264440.g541.t1 | hypothetical protein CRN43_21720, partial | 276 | 1.21E-15 | 76 |  |
| Efet.01.264444.g542.t1 | hypothetical protein CAPTEDRAFT_195684 | 684 | 1.83E-50 | 59.6 | C:membrane; C:integral component of membrane |
| Efet.01.264453.g543.t1 | glycolipid 2-alpha-mannosyltransferase-like | 855 | 2.17E-79 | 59.5 | F:transferase activity |
| Efet.01.264468.g544.t1 | metabotropic glutamate receptor 7-like | 480 | 3.17E-15 | 57.1 |  |
| Efet.01.26450.g106.t1 | adenosylhomocysteinase | 1083 | 0 | 84.1 | P:S-adenosylmethionine cycle; C:cytosol; F:adenosylhomocysteinase activity; P:one-carbon metabolic process |
| Efet.01.264560.g547.t1 | AP-3 complex subunit delta-1-like isoform X2 | 279 | 5.04E-42 | 89.5 | P:vesicle-mediated transport; C:AP-3 adaptor complex; P:intracellular protein transport |
| Efet.01.264570.g548.t1 | FMRFamide receptor-like | 1311 | 9.73E-106 | 56.1 | F:G-protein coupled receptor activity; C:membrane |
| Efet.01.264585.g549.t1 | hypothetical protein | 2310 | 1.42E-06 | 45 |  |
| Efet.01.264626.g551.t1 | PKHH2_HUMAN ame: Full=Pleckstrin homology domain-containing family H member 2 | 222 | 1.35E-06 | 79 | F:identical protein binding; P:negative regulation of actin filament depolymerization; F:protein binding; C:cytoskeleton; F:actin binding; C:cytoplasm; C:membrane; C:cell projection; C:plasma membrane; C:cortical actin cytoskeleton; C:lamellipodium |
| Efet.01.264731.g555.t1 | class A basic helix-loop-helix 15-like isoform X1 | 849 | 6.74E-28 | 67.9 | F:protein dimerization activity |
| Efet.01.264832.g557.t1 | sonic hedgehog A | 273 | 9.88E-15 | 81.75 | C:extracellular space; P:intein-mediated protein splicing; P:multicellular organism development; C:integral component of membrane; P:cell-cell signaling; F:peptidase activity; C:plasma membrane |
| Efet.01.264935.g558.t1 | coiled-coil domain-containing 18 isoform X3 | 4287 | 1.87E-52 | 48.55 |  |
| Efet.01.265005.g560.t1 | condensin complex subunit 2-like | 417 | 3.01E-19 | 64.7 | P:organelle organization; P:cell cycle |
| Efet.01.26516.g108.t1 | turtle homolog B-like | 315 | 1.46E-16 | 56.25 | C:membrane; C:integral component of membrane |
| Efet.01.265233.g564.t1 | Short stature homeobox | 408 | 2.01E-27 | 87.4 | C:nucleus; P:regulation of transcription, DNA-templated; F:sequence-specific DNA binding |
| Efet.01.265246.g565.t1 | NF-kappa-B inhibitor epsilon | 285 | 7.69E-11 | 61.85 |  |
| Efet.01.265247.g566.t1 | Erythroid differentiation-related factor 1 | 531 | 2.07E-09 | 42.67 |  |
| Efet.01.265407.g567.t1 | synaptotagmin-14-like isoform X1 | 264 | 8.37E-28 | 81.4 | P:calcium ion-regulated exocytosis of neurotransmitter; F:syntaxin binding; P:vesicle fusion; F:calcium ion binding; C:integral component of membrane; P:phosphatidylinositol phosphorylation; F:clathrin binding; P:regulation of calcium ion-dependent exocytosis; F:1-phosphatidylinositol-4-phosphate 3-kinase activity; C:plasma membrane; F:calcium-dependent phospholipid binding |
| Efet.01.265556.g569.t1 | sodium channel para-like isoform X1 | 207 | 3.42E-16 | 65.5 | P:biological regulation; P:sodium ion transport; P:ion transmembrane transport; F:sodium channel activity; C:integral component of membrane; F:voltage-gated ion channel activity |
| Efet.01.265562.g570.t1 | pikachurin-like | 870 | 2.32E-28 | 62.9 | F:calcium ion binding; C:membrane; C:integral component of membrane |
| Efet.01.265565.g571.t1 | ankyrin repeat | 939 | 5.15E-40 | 57.7 | F:ubiquitin-protein transferase activity; P:protein ubiquitination |
| Efet.01.265569.g572.t1 | transmembrane 131-like isoform X2 | 360 | 1.75E-27 | 56.2 | C:membrane; C:integral component of membrane |
| Efet.01.265572.g573.t1 | synaptotagmin-16-like isoform X1 | 756 | 3.82E-09 | 58.05 | F:protein binding; P:vesicle-mediated transport; C:membrane; F:ion binding; P:single-organism cellular process |
| Efet.01.265645.g574.t1 | monocarboxylate transporter 5 | 492 | 9.38E-16 | 72 | P:transmembrane transport; C:integral component of membrane |
| Efet.01.265651.g575.t1 | low affinity cationic amino acid transporter | 282 | 4.22E-29 | 66.55 | C:membrane; C:integral component of membrane |
| Efet.01.265657.g576.t1 | tyrosine- kinase Src42A-like isoform X1 | 414 | 8.15E-35 | 76.95 | F:nucleotide binding; F:non-membrane spanning protein tyrosine kinase activity; P:innate immune response; P:cell differentiation; C:extrinsic component of cytoplasmic side of plasma membrane; P:transmembrane receptor protein tyrosine kinase signaling pathway; P:peptidyl-tyrosine autophosphorylation; F:receptor binding; P:regulation of cell proliferation |
| Efet.01.26568.g109.t1 | 5-hydroxytryptamine receptor 2B | 525 | 5.75E-35 | 61.1 | F:G-protein coupled receptor activity; P:G-protein coupled receptor signaling pathway; C:integral component of membrane |
| Efet.01.265733.g578.t1 | nesprin-1-like isoform X1 | 1500 | 1.32E-64 | 53.35 | C:nuclear envelope; F:actin filament binding; F:actin binding; P:cytoskeletal anchoring at nuclear membrane; C:LINC complex |
| Efet.01.265776.g579.t1 | hypothetical protein CAPTEDRAFT_227455 | 564 | 1.63E-07 | 43 |  |
| Efet.01.265891.g582.t1 | hypothetical protein HELRODRAFT_161987 | 1146 | 8.81E-15 | 77.85 | F:nucleic acid binding; F:metal ion binding |
| Efet.01.265923.g583.t1 | radial spoke head 3 homolog | 693 | 5.92E-34 | 85.35 |  |
| Efet.01.265946.g584.t1 | zinc finger 850-like | 1857 | 5.46E-53 | 38.5 | F:nucleic acid binding; F:metal ion binding |
| Efet.01.266003.g586.t1 | hypothetical protein CAPTEDRAFT_211986 | 258 | 4.86E-15 | 65 |  |
| Efet.01.266026.g587.t1 | cilia- and flagella-associated 157-like | 336 | 4.08E-24 | 76.4 |  |
| Efet.01.26606.g110.t1 | chondroitin sulfate synthase 2 | 282 | 4.85E-24 | 59.3 | C:Golgi cisterna membrane; F:acetylgalactosaminyltransferase activity |
| Efet.01.266089.g588.t1 | zinc finger 271-like isoform X2 | 243 | 4.77E-09 | 57.25 | P:nucleic acid metabolic process; P:covalent chromatin modification; F:protein binding; P:regulation of gene expression; F:sequence-specific DNA binding; C:intracellular part; P:regulation of primary metabolic process; P:regulation of cellular metabolic process; P:macromolecule methylation |
| Efet.01.266162.g589.t1 | hypothetical protein CAPTEDRAFT_206253 | 540 | 2.32E-14 | 52 | C:membrane; C:integral component of membrane; F:carbohydrate binding |
| Efet.01.266228.g590.t1 | glucosamine 6-phosphate N-acetyltransferase isoform X2 | 624 | 9.35E-51 | 59.9 | C:intracellular organelle; C:cytoplasmic part; F:N-acetyltransferase activity; C:membrane-bounded organelle |
| Efet.01.266248.g591.t1 | Gametogenetin-binding 2 | 384 | 3.35E-40 | 76.4 | C:nucleus; P:negative regulation of gene expression; F:protein binding; P:labyrinthine layer blood vessel development; C:cytoplasm; P:negative regulation of cell proliferation; P:cell differentiation |
| Efet.01.266274.g593.t1 | FAM46C-like | 1068 | 5.76E-101 | 67.65 |  |
| Efet.01.2663.g242.t1 | alpha-2C adrenergic receptor isoform X3 | 1044 | 5.10E-12 | 57.55 | F:G-protein coupled receptor activity; P:neuropeptide signaling pathway; P:G-protein coupled receptor signaling pathway; P:signal transduction; C:membrane; C:integral component of membrane; F:signal transducer activity; F:neuropeptide Y receptor activity |
| Efet.01.2663.g243.t1 | octopamine receptor 1 | 384 | 1.15E-13 | 64.15 | C:integral component of plasma membrane; P:neuropeptide signaling pathway; P:response to peptide; F:peptide binding; P:cellular response to hormone stimulus; F:neuropeptide Y receptor activity |
| Efet.01.266312.g594.t1 | F-box-like WD repeat-containing TBL1XR1 | 282 | 8.49E-55 | 94.65 |  |
| Efet.01.266364.g595.t1 | GPI mannosyltransferase 2 | 903 | 1.15E-42 | 53.3 | P:GPI anchor biosynthetic process; P:mannosylation; C:membrane; C:integral component of membrane; F:dolichyl-phosphate-mannose-glycolipid alpha-mannosyltransferase activity |
| Efet.01.26637.g115.t1 | E3 ubiquitin- ligase TRIM71-like isoform X2 | 1128 | 2.30E-19 | 49 | F:zinc ion binding; F:metal ion binding; C:intracellular |
| Efet.01.266383.g596.t1 | homeobox HMX3 | 681 | 2.32E-36 | 71.2 | C:nucleus; P:transcription, DNA-templated; P:nervous system development; P:cell differentiation; P:regulation of transcription, DNA-templated; F:sequence-specific DNA binding |
| Efet.01.266472.g600.t1 | unconventional myosin-XV-like isoform X1 | 726 | 5.65E-23 | 62.75 | F:nucleotide binding; C:myosin complex; F:ATP binding; C:cytoskeleton; F:actin binding; F:motor activity |
| Efet.01.266519.g602.t1 | NACHT and WD repeat domain-containing 1 | 786 | 1.18E-29 | 50.3 | C:membrane; C:integral component of membrane |
| Efet.01.266525.g603.t1 | nuclear receptor subfamily 5 group A member 2-like | 987 | 1.07E-50 | 44.95 | F:binding |
| Efet.01.266546.g604.t1 | synaptotagmin-6-like isoform X1 | 468 | 5.85E-21 | 57.7 | F:binding |
| Efet.01.266570.g605.t1 | dynein heavy chain domain-containing 1-like | 279 | 3.63E-16 | 65.7 | P:movement of cell or subcellular component; C:cell part; C:organelle |
| Efet.01.266587.g606.t1 | hydroxysteroid dehydrogenase 2 | 207 | 1.59E-19 | 79.15 | P:oxidation-reduction process; F:glucose 1-dehydrogenase [NAD(P)] activity |
| Efet.01.266611.g607.t1 | urea-proton symporter DUR3-like | 480 | 5.05E-18 | 56.1 | C:membrane; F:transporter activity |
| Efet.01.266670.g609.t1 | intraflagellar transport 81 homolog | 705 | 5.44E-65 | 71.55 | C:cilium |
| Efet.01.266681.g610.t1 | probable nuclear hormone receptor HR3 isoform X2 | 408 | 2.21E-22 | 66.1 | P:regulation of cellular process; F:binding; P:cellular process |
| Efet.01.2669.g244.t1 | hypothetical protein CAPTEDRAFT_223037 | 1539 | 1.25E-25 | 46 |  |
| Efet.01.266926.g615.t1 | dynein heavy chain axonemal-like isoform X3 | 498 | 5.15E-56 | 73.15 | F:microtubule motor activity; C:dynein complex; P:microtubule-based movement |
| Efet.01.266935.g616.t1 | potassium sodium hyperpolarization-activated cyclic nucleotide-gated channel 3-like isoform X1 | 396 | 3.05E-38 | 85.7 | F:voltage-gated potassium channel activity; P:potassium ion transmembrane transport; C:integral component of membrane |
| Efet.01.267025.g618.t1 | ER degradation-enhancing alpha-mannosidase 1 | 390 | 1.42E-58 | 83.25 | P:positive regulation of retrograde protein transport, ER to cytosol; P:IRE1-mediated unfolded protein response; P:trimming of terminal mannose on C branch; F:calcium ion binding; P:N-glycan processing; C:integral component of endoplasmic reticulum membrane; C:endoplasmic reticulum quality control compartment; F:mannosyl-oligosaccharide 1,2-alpha-mannosidase activity; F:glycoprotein binding; P:ER-associated ubiquitin-dependent protein catabolic process; P:mannose trimming involved in glycoprotein ERAD pathway; F:misfolded protein binding |
| Efet.01.267112.g623.t1 | carbon catabolite repressor 4 homolog 1-like | 1239 | 7.77E-17 | 60.6 |  |
| Efet.01.267118.g624.t1 | USP6 N-terminal isoform X1 | 1611 | 1.84E-15 | 66.25 |  |
| Efet.01.267193.g625.t1 | sodium hydrogen exchanger 3 isoform X1 | 330 | 7.91E-30 | 83.85 | F:sodium:proton antiporter activity; C:integral component of membrane; P:hydrogen ion transmembrane transport; P:sodium ion transmembrane transport; P:regulation of pH |
| Efet.01.26725.g119.t1 | hypothetical protein HELRODRAFT_130236, partial | 411 | 8.52E-08 | 62 | F:calcium ion binding; F:ribosome binding; C:membrane; C:integral component of membrane |
| Efet.01.267253.g626.t1 | gastrin cholecystokinin type B receptor-like | 726 | 1.64E-50 | 61 | P:signal transduction; F:signal transducer activity |
| Efet.01.267259.g627.t1 | fructose-bisphosphate aldolase-like | 477 | 5.82E-62 | 80.9 | P:glycolytic process; F:fructose-bisphosphate aldolase activity |
| Efet.01.267321.g628.t1 | kinesin unc-104 isoform X1 | 210 | 1.26E-28 | 91.4 | F:ATPase activity; F:ATP binding; F:microtubule motor activity; P:cytoskeleton-dependent intracellular transport; C:kinesin complex; P:microtubule-based movement; C:microtubule; F:microtubule binding |
| Efet.01.267323.g629.t1 | nuclear cap-binding subunit 1-like | 327 | 1.06E-30 | 56.9 | P:RNA metabolic process; C:nuclear cap binding complex; P:mRNA cis splicing, via spliceosome; F:RNA binding; F:RNA cap binding; P:mRNA transport |
| Efet.01.267355.g630.t1 | DUF4432 domain-containing | 369 | 9.09E-72 | 75.55 |  |
| Efet.01.26741.g120.t1 | Bromodomain and WD repeat-containing 3 | 345 | 3.05E-29 | 85.15 | P:regulation of cell shape; C:nucleus; P:cytoskeleton organization; P:regulation of transcription from RNA polymerase II promoter |
| Efet.01.26741.g122.t1 | bromodomain and WD repeat-containing 3-like | 375 | 1.16E-27 | 74.55 |  |
| Efet.01.267545.g633.t1 | uncharacterized protein LOC111036960 | 504 | 2.13E-32 | 56.75 | C:membrane; C:integral component of membrane |
| Efet.01.267554.g634.t1 | tRNA wybutosine-synthesizing 5-like | 567 | 2.27E-25 | 63.1 | F:calcium ion binding |
| Efet.01.267558.g635.t1 | tractin | 351 | 7.78E-09 | 75.33 | C:membrane |
| Efet.01.267586.g638.t1 | SEC23-interacting isoform X1 | 393 | 8.74E-07 | 65.2 | F:metal ion binding; F:RNA binding |
| Efet.01.267612.g639.t1 | KN motif and ankyrin repeat domain-containing | 2988 | 1.55E-11 | 75.25 |  |
| Efet.01.267626.g640.t1 | beta-N-acetylhexosaminidase | 1359 | 3.80E-64 | 45.85 | P:chitin catabolic process; P:metabolic process; C:periplasmic space; F:hydrolase activity, hydrolyzing O-glycosyl compounds; F:beta-N-acetylhexosaminidase activity; P:carbohydrate metabolic process; P:polysaccharide catabolic process; C:membrane; C:integral component of membrane; F:carbohydrate binding; F:polysaccharide binding; F:hydrolase activity; F:hydrolase activity, acting on glycosyl bonds |
| Efet.01.267648.g641.t1 | hemolysin-type calcium-binding region | 420 | 1.29E-58 | 72.7 | F:calcium ion binding; C:extracellular region; P:pathogenesis |
| Efet.01.267648.g642.t1 | Ig family | 867 | 1.35E-179 | 61.1 | F:calcium ion binding; C:extracellular region; P:pathogenesis |
| Efet.01.267648.g643.t1 | hemolysin-type calcium-binding region | 2430 | 0 | 61.35 | F:calcium ion binding; C:extracellular region; P:pathogenesis |
| Efet.01.267648.g644.t1 | type I secretion system permease ATPase | 2265 | 0 | 86.35 | F:ATP binding; F:ATPase activity, coupled to transmembrane movement of substances; P:proteolysis; C:integral component of membrane; P:protein secretion by the type I secretion system; F:protein transporter activity; F:peptidase activity; C:type I protein secretion system complex |
| Efet.01.267648.g645.t1 | family type I secretion periplasmic adaptor subunit | 1338 | 0 | 80.75 | C:integral component of membrane; F:protein transporter activity; P:protein secretion |
| Efet.01.267648.g646.t1 | family transcriptional regulator | 1068 | 0 | 80.4 | F:transcription factor activity, sequence-specific DNA binding; P:transcription, DNA-templated; P:regulation of transcription, DNA-templated; F:sequence-specific DNA binding |
| Efet.01.267648.g647.t1 | 4-hydroxybenzoate 3-monooxygenase | 1179 | 0 | 90.25 | F:4-hydroxybenzoate 3-monooxygenase activity; F:FAD binding; P:benzoate catabolic process; P:oxidation-reduction process |
| Efet.01.267648.g648.t1 | nuclear transport factor 2 family | 513 | 9.28E-87 | 77.1 |  |
| Efet.01.267648.g649.t1 | SMP-30 gluconolactonase LRE family | 900 | 0 | 82.15 | F:gluconolactonase activity |
| Efet.01.267648.g650.t1 | phosphogluconate dehydratase | 2340 | 0 | 92.6 | P:Entner-Doudoroff pathway through 6-phosphogluconate; F:phosphogluconate dehydratase activity |
| Efet.01.267648.g651.t1 | chaperonin | 1545 | 0 | 94.1 | F:ATP binding; C:cytoplasm; F:unfolded protein binding; P:protein refolding |
| Efet.01.267648.g652.t1 | co-chaperone | 339 | 4.35E-62 | 97.5 | F:ATP binding; C:cytoplasm; P:protein folding |
| Efet.01.2677.g245.t1 | glycine receptor subunit alpha-3-like isoform X1 | 408 | 6.46E-23 | 65.95 | P:ion transmembrane transport; C:integral component of membrane; C:cell junction; F:extracellular ligand-gated ion channel activity; C:synapse; C:plasma membrane |
| Efet.01.267712.g654.t1 | sodium potassium ATPase alpha | 525 | 2.04E-91 | 85.7 | P:potassium ion transport; F:ATP binding; P:sodium ion transport; F:sodium:potassium-exchanging ATPase activity; F:metal ion binding; P:ATP hydrolysis coupled transmembrane transport; C:integral component of membrane; P:establishment or maintenance of transmembrane electrochemical gradient |
| Efet.01.267720.g656.t1 | tyrosine- phosphatase non-receptor type 11-like | 285 | 1.67E-23 | 67.6 | F:protein tyrosine phosphatase activity; P:peptidyl-tyrosine dephosphorylation |
| Efet.01.267744.g657.t1 | adenylosuccinate synthetase isozyme 1 | 213 | 3.48E-21 | 84.65 | F:magnesium ion binding; F:GTP binding; P:'de novo' AMP biosynthetic process; P:IMP metabolic process; F:adenylosuccinate synthase activity; F:actin binding; C:cytoplasm; F:Rho GTPase binding; P:actin cytoskeleton organization |
| Efet.01.267756.g658.t1 | hypothetical protein CAPTEDRAFT_223740 | 579 | 5.55E-08 | 69 |  |
| Efet.01.267864.g659.t1 | mitochondrial ubiquitin ligase activator of NFKB 1 | 456 | 1.30E-25 | 56.55 | F:ubiquitin-protein transferase activity; F:zinc ion binding; F:metal ion binding; C:membrane; C:integral component of membrane; P:mitochondrion organization; F:ligase activity; P:protein ubiquitination |
| Efet.01.267975.g660.t1 | hypothetical protein CAPTEDRAFT_219378 | 327 | 6.05E-30 | 64 | C:membrane |
| Efet.01.268002.g662.t1 | hypothetical protein HELRODRAFT_188674 | 918 | 1.23E-52 | 46.9 | C:membrane; C:integral component of membrane |
| Efet.01.268016.g663.t1 | glycine receptor subunit alpha-3-like isoform X1 | 216 | 5.00E-16 | 65.85 | C:membrane; P:transport |
| Efet.01.268043.g665.t1 | sex peptide receptor-like | 1338 | 7.97E-130 | 68.6 | F:G-protein coupled receptor activity; P:G-protein coupled receptor signaling pathway; C:membrane |
| Efet.01.268108.g669.t1 | zinc finger 239-like | 1227 | 5.35E-29 | 45.5 | F:nucleic acid binding; F:transcription factor activity, sequence-specific DNA binding; F:metal ion binding; P:regulation of transcription, DNA-templated |
| Efet.01.268131.g670.t1 | specificity 1 | 1437 | 1.39E-19 | 80 | F:nucleic acid binding; F:metal ion binding |
| Efet.01.268174.g671.t1 | neuronal acetylcholine receptor subunit alpha-7 isoform X1 | 495 | 2.18E-40 | 58.1 | P:ion transport; C:membrane part; C:plasma membrane |
| Efet.01.268187.g672.t1 | hypothetical protein CAPTEDRAFT_209896 | 528 | 4.91E-12 | 50 | C:membrane; C:integral component of membrane |
| Efet.01.268196.g673.t1 | leucine-rich repeat serine threonine- kinase 1 | 843 | 1.89E-15 | 48 | P:positive regulation of cellular process; F:binding; C:intracellular |
| Efet.01.26825.g124.t1 | Atrial natriuretic peptide receptor | 633 | 6.88E-85 | 85.95 | C:guanylate cyclase complex, soluble; F:ATP binding; P:cGMP biosynthetic process; P:peptidyl-tyrosine phosphorylation; F:protein tyrosine kinase activity; C:integral component of membrane; F:guanylate cyclase activity; C:plasma membrane; F:adenylate cyclase activity; P:intracellular signal transduction |
| Efet.01.268287.g674.t1 | Cartilage oligomeric matrix | 381 | 3.62E-13 | 54.85 | F:calcium ion binding; C:membrane; C:integral component of membrane; P:cell adhesion; C:extracellular region |
| Efet.01.268321.g675.t1 | allatostatin-A receptor-like isoform X2 | 1032 | 2.87E-55 | 68.1 | P:signal transduction; C:membrane; F:signal transducer activity |
| Efet.01.268335.g676.t1 | hypothetical protein CAPTEDRAFT_217638 | 1530 | 2.62E-10 | 46 | P:lipid metabolic process; F:phosphoric diester hydrolase activity |
| Efet.01.268339.g677.t1 | stabilizer of axonemal microtubules 2-like isoform X4 | 1143 | 2.58E-48 | 50.8 | F:microtubule binding |
| Efet.01.268360.g678.t1 | Dual specificity | 1656 | 4.14E-51 | 61.3 | F:phosphoprotein phosphatase activity; P:protein dephosphorylation |
| Efet.01.268365.g679.t1 | targeting for Xklp2 isoform X1 | 516 | 1.23E-20 | 58.5 | C:microtubule cytoskeleton; C:intracellular organelle part |
| Efet.01.268394.g680.t1 | transmembrane and ubiquitin-like domain-containing 1 | 354 | 1.16E-28 | 57.75 | C:nucleus; C:cytoplasm; C:membrane; C:integral component of membrane |
| Efet.01.268411.g681.t1 | unconventional myosin-Ig | 837 | 1.36E-76 | 63.15 | P:localization; C:membrane; C:cell part; F:anion binding; P:single-organism process; P:cellular process |
| Efet.01.268506.g683.t1 | neuropeptides capa receptor-like | 972 | 1.47E-52 | 43 | F:G-protein coupled receptor activity; P:G-protein coupled receptor signaling pathway; P:signal transduction; C:membrane; C:integral component of membrane; F:signal transducer activity |
| Efet.01.268580.g685.t1 | sodium bicarbonate cotransporter 3 isoform X5 | 342 | 1.49E-21 | 72.8 | C:membrane; F:anion transmembrane transporter activity; P:anion transport |
| Efet.01.268628.g686.t1 | ankyrin repeat and EF-hand domain-containing 1 | 204 | 1.21E-07 | 80.9 | F:calcium ion binding |
| Efet.01.268657.g687.t1 | Fibroblast growth factor receptor substrate 2 | 1173 | 1.55E-31 | 66.05 | F:insulin receptor binding |
| Efet.01.268671.g688.t1 | DDRGK domain-containing 1 | 249 | 5.19E-28 | 84.2 | C:integral component of membrane |
| Efet.01.268685.g689.t1 | leucine-rich repeat-containing 9-like | 438 | 6.01E-17 | 66.8 | F:NAD+ ADP-ribosyltransferase activity |
| Efet.01.268685.g690.t1 | WD repeat-containing 36 | 390 | 3.06E-09 | 50.6 | C:nucleolus |
| Efet.01.268737.g692.t1 | inositol-tetrakisphosphate 1-kinase-like | 303 | 1.98E-11 | 73.75 | F:magnesium ion binding; P:inositol trisphosphate metabolic process; F:ATP binding; F:inositol tetrakisphosphate 1-kinase activity; F:inositol-1,3,4-trisphosphate 5-kinase activity; P:phosphorylation; F:inositol-1,3,4-trisphosphate 6-kinase activity; C:intracellular |
| Efet.01.26877.g127.t1 | regulator of G- signaling 12 | 441 | 1.17E-13 | 53.85 |  |
| Efet.01.268784.g693.t1 | von Willebrand factor D and EGF domain-containing -like | 870 | 6.86E-50 | 43.7 | C:membrane; C:integral component of membrane |
| Efet.01.268789.g694.t1 | DOMON domain-containing FRRS1L | 291 | 1.71E-14 | 58 | C:membrane; C:integral component of membrane |
| Efet.01.26879.g128.t1 | Sepiapterin reductase | 507 | 6.48E-43 | 59.4 | C:extracellular exosome; F:oxidoreductase activity, acting on the CH-OH group of donors, NAD or NADP as acceptor; C:cytoplasm; P:nitric oxide biosynthetic process; C:nucleoplasm |
| Efet.01.268792.g695.t1 | PREDICTED: uncharacterized protein LOC106176362 | 1476 | 1.34E-27 | 49.5 |  |
| Efet.01.268818.g696.t1 | Wnt-7b-like precursor | 282 | 4.66E-12 | 56.8 | P:regulation of cellular process; P:system development; P:cell-cell signaling; P:cell differentiation; P:cellular response to stimulus; C:extracellular region |
| Efet.01.268839.g697.t1 | Oxysterol-binding 1 | 426 | 1.12E-41 | 65.65 | P:lipid transport; P:transport |
| Efet.01.26888.g129.t1 | vigilin-like | 447 | 1.93E-24 | 57.45 | F:nucleic acid binding; F:RNA binding |
| Efet.01.268958.g701.t1 | turtle homolog B-like | 399 | 6.28E-32 | 62.7 | C:membrane; C:integral component of membrane |
| Efet.01.2690.g246.t1 | JNK-interacting 3 isoform X1 | 330 | 7.71E-34 | 79.55 | C:integral component of membrane; P:phosphorylation; F:kinase activity |
| Efet.01.269025.g704.t1 | multifunctional CCA addition repair | 681 | 3.55E-140 | 86.45 | F:magnesium ion binding; F:ATP binding; F:tRNA cytidylyltransferase activity; F:CTP:3'-cytidine-tRNA cytidylyltransferase activity; F:ATP:3'-cytidine-cytidine-tRNA adenylyltransferase activity; F:phosphatase activity; F:CTP:tRNA cytidylyltransferase activity; P:tRNA 3'-terminal CCA addition; P:RNA repair; F:tRNA binding; P:dephosphorylation; F:cyclic-nucleotide phosphodiesterase activity |
| Efet.01.269033.g705.t1 | FMRFamide receptor-like | 1086 | 4.61E-53 | 50.65 | F:G-protein coupled receptor activity; F:G-protein coupled peptide receptor activity; P:G-protein coupled receptor signaling pathway; C:membrane; C:integral component of membrane |
| Efet.01.269071.g706.t1 | insulin gene enhancer isl-1-like isoform X2 | 294 | 2.80E-08 | 66.36 | F:metal ion binding |
| Efet.01.269109.g709.t1 | spliceosome RNA helicase DDX39B | 402 | 1.24E-77 | 93.2 | P:mRNA export from nucleus; F:ATP binding; F:identical protein binding; C:cytoplasm; C:membrane; P:regulation of gene expression; P:RNA secondary structure unwinding; C:spliceosomal complex; P:cellular response to DNA damage stimulus; F:ATP-dependent RNA helicase activity; F:RNA binding; P:mRNA splicing, via spliceosome |
| Efet.01.269179.g711.t1 | BTB POZ domain-containing 6 isoform X1 | 375 | 1.56E-13 | 52.3 |  |
| Efet.01.269200.g713.t1 | major vault -like | 213 | 3.30E-25 | 87.55 | C:nucleus; C:intracellular ribonucleoprotein complex; C:cytoplasm |
| Efet.01.269272.g714.t1 | exportin-4 isoform X3 | 246 | 2.30E-10 | 71.4 |  |
| Efet.01.269316.g715.t1 | cGMP-dependent kinase 1-like isoform X7 | 324 | 4.09E-08 | 68 | C:membrane; C:integral component of membrane |
| Efet.01.269359.g716.t1 | hypothetical protein | 372 | 1.71E-63 | 99 |  |
| Efet.01.269359.g717.t1 | class II aldolase | 1173 | 3.25E-73 | 58.75 | F:oxidoreductase activity; F:sorbitol-6-phosphate 2-dehydrogenase activity; P:oxidation-reduction process |
| Efet.01.269389.g718.t1 | hypothetical protein CAPTEDRAFT_226182 | 210 | 1.34E-08 | 77 |  |
| Efet.01.269425.g720.t1 | bicyclomycin resistance | 1101 | 0 | 72.1 |  |
| Efet.01.269425.g721.t1 | sugar ABC transporter permease | 684 | 1.12E-123 | 80.45 | C:integral component of membrane; P:transport; C:plasma membrane |
| Efet.01.269425.g722.t1 | carbohydrate ABC transporter permease | 867 | 0 | 83.6 | F:substrate-specific transmembrane transporter activity; C:ATP-binding cassette (ABC) transporter complex, substrate-binding subunit-containing; P:transmembrane transport |
| Efet.01.269425.g723.t1 | ROK family transcriptional regulator | 909 | 1.49E-69 | 74.45 |  |
| Efet.01.269429.g724.t1 | laminin subunit alpha-2-like isoform X6 | 771 | 5.10E-13 | 46.2 | C:basement membrane; C:extracellular exosome; P:regulation of embryonic development; P:endodermal cell differentiation; P:cell adhesion; P:regulation of cell adhesion; P:regulation of cell migration; F:receptor binding; C:laminin-5 complex |
| Efet.01.269452.g725.t1 | Protocadherin Fat 4 | 855 | 7.76E-48 | 56.5 | F:calcium ion binding; C:membrane; C:integral component of membrane; P:cell adhesion; P:homophilic cell adhesion via plasma membrane adhesion molecules; C:plasma membrane |
| Efet.01.269453.g726.t1 | WD40-like Beta Propeller Repeat | 579 | 8.47E-117 | 57.45 |  |
| Efet.01.269597.g728.t1 | T family of potassium channels 18-like | 585 | 3.27E-18 | 52.19 | P:ion transport; P:potassium ion transmembrane transport; C:membrane; C:integral component of membrane; F:potassium channel activity; P:transport |
| Efet.01.269634.g729.t1 | transmembrane 168 isoform X1 | 1665 | 4.80E-100 | 61 | P:biological_process; C:membrane; C:integral component of membrane; C:transport vesicle; F:molecular_function |
| Efet.01.269642.g730.t1 | sialin-like | 228 | 1.12E-13 | 73.75 | P:transmembrane transport; C:integral component of membrane |
| Efet.01.269659.g731.t1 | BTB POZ domain-containing KCTD1 | 270 | 1.84E-13 | 94.9 | P:protein homooligomerization |
| Efet.01.269718.g733.t1 | galactose oxidase | 1122 | 0 | 52 |  |
| Efet.01.26975.g130.t1 | 26S proteasome non-ATPase regulatory subunit | 327 | 7.98E-55 | 86.2 | C:nucleus; P:proteasome-mediated ubiquitin-dependent protein catabolic process; C:proteasome regulatory particle, base subcomplex; F:polyubiquitin binding; C:cytosol; P:proteasome assembly |
| Efet.01.269807.g734.t1 | short transient receptor potential channel 3- | 396 | 1.32E-28 | 67.15 | P:transmembrane transport; F:calcium channel activity; C:integral component of membrane; P:metal ion transport |
| Efet.01.269841.g735.t1 | dynein heavy chain axonemal-like | 1296 | 0 | 84.8 | F:ATPase activity; F:ATP binding; F:microtubule motor activity; C:dynein complex; P:microtubule-based movement |
| Efet.01.269856.g736.t1 | turtle homolog A isoform X2 | 324 | 4.76E-33 | 61.35 | C:membrane; C:integral component of membrane |
| Efet.01.269859.g737.t1 | zinc finger 271 | 996 | 1.62E-89 | 64.55 | F:nucleic acid binding; F:transcription factor activity, sequence-specific DNA binding; F:metal ion binding; F:structural constituent of cuticle |
| Efet.01.26991.g131.t1 | beta-1,4-galactosyltransferase galt-1-like | 696 | 1.51E-54 | 56.45 | C:membrane; C:integral component of membrane |
| Efet.01.269911.g738.t1 | ankyrin repeat domain-containing 50-like | 1095 | 2.96E-121 | 63.35 |  |
| Efet.01.269912.g740.t1 | hypothetical protein CAPTEDRAFT_204194 | 369 | 2.16E-36 | 71.8 | C:membrane; P:transport |
| Efet.01.26992.g132.t1 | FMRFamide receptor-like | 1428 | 2.15E-28 | 54.8 | F:G-protein coupled receptor activity; F:G-protein coupled peptide receptor activity; P:G-protein coupled receptor signaling pathway; P:signal transduction; C:membrane; C:integral component of membrane; F:signal transducer activity |
| Efet.01.269936.g742.t1 | Wnt-5b-like | 420 | 1.86E-64 | 78.2 | P:multicellular organism development; C:membrane; P:Wnt signaling pathway; F:receptor binding; C:proteinaceous extracellular matrix |
| Efet.01.269997.g744.t1 | myosin heavy striated muscle-like isoform X2 | 468 | 2.48E-46 | 65.05 | F:binding |
| Efet.01.270153.g750.t1 | zinc finger 277 isoform X2 | 621 | 6.76E-25 | 47.8 | F:metal ion binding; F:RNA polymerase II core promoter sequence-specific DNA binding; P:cellular response to hydrogen peroxide; P:regulation of cellular senescence |
| Efet.01.27016.g133.t1 | transcription factor Sp5-like | 759 | 2.44E-52 | 77.9 | F:nucleic acid binding; P:post-anal tail morphogenesis; P:notochord development; F:metal ion binding; P:hindbrain development; P:anterior/posterior pattern specification; P:regulation of transcription, DNA-templated; P:Wnt signaling pathway; P:mesoderm formation |
| Efet.01.27021.g134.t1 | influenza virus NS1A-binding homolog A-like | 963 | 2.22E-45 | 50.85 |  |
| Efet.01.27021.g135.t1 | Influenza virus NS1A-binding | 225 | 3.74E-17 | 67.2 | C:actin cytoskeleton |
| Efet.01.270288.g752.t1 | inactive dipeptidyl peptidase 10-like | 216 | 1.63E-11 | 72.5 | P:proteolysis; C:membrane; C:integral component of membrane; F:serine-type peptidase activity; F:serine-type endopeptidase activity |
| Efet.01.270304.g753.t1 | glutathione gamma-glutamylcysteinyltransferase | 888 | 1.38E-43 | 53.45 | P:phytochelatin biosynthetic process; F:glutathione gamma-glutamylcysteinyltransferase activity; F:metal ion binding; P:response to metal ion; F:transferase activity |
| Efet.01.270461.g755.t1 | hypothetical protein CAPTEDRAFT_228921 | 360 | 5.69E-07 | 53.33 | C:membrane; C:integral component of membrane; F:carbohydrate binding |
| Efet.01.270578.g760.t1 | girdin isoform X1 | 534 | 9.82E-08 | 56.55 | P:regulation of actin cytoskeleton organization; F:actin binding; P:cell migration |
| Efet.01.270586.g761.t1 | Calpain B | 372 | 1.73E-40 | 72.15 | F:cysteine-type peptidase activity |
| Efet.01.270586.g762.t1 | calpain-A isoform X3 | 234 | 3.27E-13 | 69.75 | F:calcium ion binding; P:proteolysis; F:calcium-dependent cysteine-type endopeptidase activity; C:intracellular |
| Efet.01.270604.g763.t1 | hypothetical protein CAPTEDRAFT_224196 | 471 | 8.35E-19 | 58 | F:G-protein coupled receptor activity; F:transmembrane signaling receptor activity; P:G-protein coupled receptor signaling pathway; P:signal transduction; F:calcium ion binding; C:membrane; C:integral component of membrane; P:cell surface receptor signaling pathway; P:homophilic cell adhesion via plasma membrane adhesion molecules; F:signal transducer activity |
| Efet.01.270611.g764.t1 | eIF-2-alpha kinase activator GCN1 | 657 | 8.61E-34 | 54.4 | P:regulation of cellular process; P:cellular response to stimulus; C:intracellular |
| Efet.01.270661.g765.t1 | bifunctional 3 -phosphoadenosine 5 -phosphosulfate synthase 2 | 264 | 5.44E-18 | 89.05 | F:ATP binding; P:3'-phosphoadenosine 5'-phosphosulfate biosynthetic process; F:adenylylsulfate kinase activity; F:sulfate adenylyltransferase (ATP) activity; P:phosphorylation; P:sulfate assimilation |
| Efet.01.270738.g766.t1 | BMP family ABC transporter substrate-binding | 852 | 0 | 91.3 | C:plasma membrane |
| Efet.01.270738.g767.t1 | ABC transporter permease | 1080 | 4.76E-139 | 84.6 | C:integral component of membrane; P:transport; C:plasma membrane; F:transporter activity |
| Efet.01.270738.g768.t1 | ABC transporter permease | 888 | 4.58E-130 | 80.3 | C:integral component of membrane; P:transport; C:plasma membrane; F:transporter activity |
| Efet.01.270738.g769.t1 | cysteine hydrolase | 618 | 4.74E-145 | 92.1 | P:metabolic process; F:hydrolase activity |
| Efet.01.270738.g770.t1 | ABC transporter ATP-binding | 1359 | 0 | 79.85 | F:ATPase activity; F:ATP binding |
| Efet.01.270746.g771.t1 | transcription elongation factor SPT6-like isoform X4 | 297 | 8.43E-06 | 81 |  |
| Efet.01.270790.g772.t1 | cytochrome c oxidase subunit II | 1131 | 0 | 95.4 | F:cytochrome-c oxidase activity; F:copper ion binding; C:integral component of membrane; P:hydrogen ion transmembrane transport; P:electron transport chain; C:plasma membrane; C:respiratory chain; F:heme binding |
| Efet.01.270790.g773.t1 | cytochrome c oxidase subunit I | 1050 | 0 | 94.05 | F:cytochrome-c oxidase activity; P:oxidative phosphorylation; F:copper ion binding; F:iron ion binding; C:integral component of membrane; P:hydrogen ion transmembrane transport; P:electron transport chain; P:aerobic respiration; C:plasma membrane; C:respiratory chain; F:heme binding |
| Efet.01.270845.g776.t1 | DENN domain-containing 1A-like isoform X1 | 216 | 1.28E-26 | 77.4 |  |
| Efet.01.27086.g136.t1 | inactive rhomboid 1-like | 396 | 4.71E-27 | 54.65 | F:hydrolase activity |
| Efet.01.270897.g777.t1 | KRI1 homolog | 495 | 2.13E-08 | 72.43 | P:ribosome biogenesis; P:definitive hemopoiesis |
| Efet.01.270935.g779.t1 | PREDICTED: uncharacterized protein LOC106870838 | 630 | 8.54E-06 | 58 | F:metal ion binding |
| Efet.01.270979.g781.t1 | split ends-like isoform X1 | 9528 | 7.18E-29 | 72.1 | F:nucleotide binding; F:nucleic acid binding; C:nucleus; F:hydrolase activity, acting on glycosyl bonds; P:mRNA splicing, via spliceosome |
| Efet.01.271010.g782.t1 | F-box WD repeat-containing | 597 | 4.04E-116 | 93 | F:ubiquitin protein ligase activity; F:protein phosphorylated amino acid binding; P:regulation of proteasomal protein catabolic process; P:positive regulation of proteolysis; P:branching involved in mammary gland duct morphogenesis; P:positive regulation of circadian rhythm; P:negative regulation of transcription, DNA-templated; P:positive regulation of transcription, DNA-templated; P:regulation of canonical Wnt signaling pathway; C:cytosol; P:Wnt signaling pathway; P:mammary gland epithelial cell proliferation; C:SCF ubiquitin ligase complex; P:regulation of cell cycle; P:protein polyubiquitination; P:protein dephosphorylation; F:protein dimerization activity; F:beta-catenin binding; P:cell cycle; P:negative regulation of NF-kappaB import into nucleus; P:protein destabilization; C:nucleus; C:centrosome; P:rhythmic process; P:SCF-dependent proteasomal ubiquitin-dependent protein catabolic process; P:cellular response to organic cyclic compound |
| Efet.01.271014.g783.t1 | FMRFamide receptor | 1047 | 2.51E-76 | 56.35 | F:G-protein coupled receptor activity; P:signal transduction; C:membrane |
| Efet.01.271023.g784.t1 | 1-phosphatidylinositol 4,5-bisphosphate phosphodiesterase epsilon-1-like | 915 | 1.87E-22 | 53.05 | P:biological regulation; P:single-organism process |
| Efet.01.271034.g785.t1 | atonal homolog 1-like | 675 | 1.13E-13 | 81.5 | P:neuron differentiation; P:regulation of transcription, DNA-templated; F:protein dimerization activity; P:sensory organ development |
| Efet.01.271035.g786.t1 | atonal homolog 1 | 279 | 2.35E-15 | 82.55 | P:auditory receptor cell differentiation; P:regulation of transcription, DNA-templated; F:sequence-specific DNA binding; F:protein dimerization activity |
| Efet.01.271113.g787.t1 | tumor suppressor p53-binding 1-like | 420 | 3.32E-07 | 51.86 | P:DNA damage checkpoint; C:nucleus; F:histone binding; P:positive regulation of transcription from RNA polymerase II promoter |
| Efet.01.271151.g790.t1 | spire homolog 1-like isoform X2 | 564 | 2.32E-51 | 56.65 | C:cytoplasm |
| Efet.01.271166.g791.t1 | T family of potassium channels 7- | 1587 | 3.71E-18 | 78.7 | P:potassium ion transmembrane transport; C:integral component of membrane; F:potassium channel activity |
| Efet.01.271166.g792.t1 | T family of potassium channels 18-like | 510 | 1.88E-18 | 79.6 | P:potassium ion transmembrane transport; C:integral component of membrane; F:potassium channel activity |
| Efet.01.27122.g138.t1 | hypothetical protein CRN43_09195 | 279 | 2.03E-20 | 69.67 |  |
| Efet.01.271232.g793.t1 | FMRFamide receptor | 1209 | 1.16E-106 | 56.2 | F:signal transducer activity |
| Efet.01.271240.g794.t1 | neuron navigator 3 isoform X2 | 756 | 3.04E-63 | 71.1 | F:ATP binding |
| Efet.01.271245.g795.t1 | MAX gene-associated isoform X1 | 678 | 5.21E-06 | 54 |  |
| Efet.01.271328.g796.t1 | probable ATP-dependent RNA helicase YTHDC2 isoform X1 | 390 | 8.42E-42 | 78.15 | F:ATP binding; F:RNA binding; F:helicase activity |
| Efet.01.271355.g798.t1 | cleavage and polyadenylation specificity factor subunit 3 | 1098 | 0 | 84.8 | F:5'-3' exonuclease activity; P:mRNA export from nucleus; F:RNA binding; F:protein binding; C:mRNA cleavage and polyadenylation specificity factor complex; P:mRNA 3'-end processing by stem-loop binding and cleavage; P:termination of RNA polymerase II transcription; P:mRNA polyadenylation; F:endoribonuclease activity; P:mRNA splicing, via spliceosome; P:mRNA cleavage |
| Efet.01.271355.g799.t1 | Transmembrane 246 | 1191 | 6.07E-89 | 53.4 | C:membrane; C:integral component of membrane |
| Efet.01.271380.g800.t1 | Acetyl-coenzyme A transporter 1 | 576 | 5.86E-55 | 78.05 | P:negative regulation of BMP signaling pathway; P:central nervous system neuron axonogenesis; C:integral component of membrane; F:acetyl-CoA transporter activity; P:acetyl-CoA transport |
| Efet.01.27144.g139.t1 | hypothetical protein CAPTEDRAFT_216159 | 1233 | 6.84E-80 | 51.17 | C:membrane; C:integral component of membrane |
| Efet.01.271446.g803.t1 | family -linked outer membrane | 540 | 2.62E-101 | 69.35 | C:membrane |
| Efet.01.271454.g804.t1 | COUP transcription factor | 306 | 9.81E-36 | 98 | P:anterior/posterior pattern specification; P:blood vessel morphogenesis; F:sequence-specific DNA binding; P:radial pattern formation; P:steroid hormone mediated signaling pathway; P:maternal placenta development; P:negative regulation of transcription from RNA polymerase II promoter; P:neuron migration; P:forebrain development; F:RNA polymerase II transcription factor activity, ligand-activated sequence-specific DNA binding; P:positive regulation of transcription, DNA-templated; P:regulation of transcription involved in lymphatic endothelial cell fate commitment; P:trophoblast giant cell differentiation; F:steroid hormone receptor activity; P:negative regulation of endothelial cell migration; P:intracellular receptor signaling pathway; F:zinc ion binding; F:protein homodimerization activity; P:placenta blood vessel development; P:transcription, DNA-templated; P:limb development; P:fertilization; P:negative regulation of cyclin-dependent protein serine/threonine kinase activity; C:nucleus; F:retinoic acid binding; F:transcription corepressor activity; P:skeletal muscle tissue development; P:lipid metabolic process; P:response to estradiol; P:negative regulation of endothelial cell proliferation |
| Efet.01.271472.g805.t1 | hypothetical protein HELRODRAFT_191437 | 489 | 1.57E-08 | 45 |  |
| Efet.01.271475.g806.t1 | aquaporin -like | 807 | 2.30E-42 | 60.1 | C:membrane; P:transport; P:single-organism process; F:transporter activity |
| Efet.01.271584.g807.t1 | E3 ubiquitin- ligase NEURL1-like isoform X1 | 606 | 2.68E-31 | 54.85 | F:GTP binding; F:zinc ion binding; P:signal transduction; P:small GTPase mediated signal transduction; F:metal ion binding; C:intracellular |
| Efet.01.271589.g808.t1 | cholecystokinin receptor type A | 411 | 8.50E-29 | 71.45 | F:G-protein coupled receptor activity; P:signal transduction; C:membrane |
| Efet.01.271606.g810.t1 | pinin | 315 | 1.29E-20 | 73 | P:single organismal cell-cell adhesion; C:exon-exon junction complex; C:catalytic step 2 spliceosome; C:cytoplasm; C:nuclear speck; C:membrane; C:desmosome; P:RNA processing; F:RNA binding |
| Efet.01.271657.g811.t1 | interferon regulatory factor 2-binding -like | 804 | 6.00E-63 | 71.05 | F:metal ion binding; P:histone modification; C:Cdc73/Paf1 complex; P:regulation of transcription, DNA-templated |
| Efet.01.271704.g812.t1 | cytochrome P450 2U1 | 813 | 2.32E-46 | 54.35 | F:oxidoreductase activity; F:binding |
| Efet.01.271716.g813.t1 | nuclease HARBI1 | 624 | 4.90E-36 | 57.75 |  |
| Efet.01.271724.g814.t1 | Wnt-2b | 249 | 1.47E-26 | 82.75 | P:male gonad development; P:neuron differentiation; P:lung induction; P:gall bladder development; P:hepatic duct development; P:chondrocyte differentiation; P:positive regulation of canonical Wnt signaling pathway; P:cellular response to starvation; P:mesenchymal-epithelial cell signaling; P:forebrain regionalization; C:proteinaceous extracellular matrix; P:positive regulation of branching involved in ureteric bud morphogenesis; C:extracellular space; F:frizzled binding; P:angiogenesis; P:hepatocyte proliferation; P:cell fate commitment; P:swim bladder development; P:canonical Wnt signaling pathway; P:hematopoietic stem cell proliferation; P:regulation of Wnt signaling pathway involved in digestive tract morphogenesis; P:pancreas development |
| Efet.01.271737.g815.t1 | ammonia-dependent NAD(+) synthetase | 558 | 6.59E-98 | 78.15 | F:NAD+ synthase (glutamine-hydrolyzing) activity; F:ATP binding; P:NAD biosynthetic process; F:NAD+ synthase activity |
| Efet.01.271737.g816.t1 | 1-(5-phosphoribosyl)-5-((5-phosphoribosylamino)methylideneamino)imidazole-4-carboxamide isomerase | 657 | 7.15E-152 | 71.55 | F:isomerase activity |
| Efet.01.27178.g140.t1 | serine threonine- kinase TBK1 | 411 | 2.12E-13 | 54.25 | F:transferase activity |
| Efet.01.271797.g817.t1 | glutamate receptor 4 isoform X1 | 1212 | 2.09E-52 | 49.85 | P:ion transmembrane transport; C:synapse; F:receptor activity; F:ionotropic glutamate receptor activity; F:extracellular-glutamate-gated ion channel activity; P:transport; C:plasma membrane; F:ion channel activity; P:ion transport; C:postsynaptic membrane; C:membrane; C:integral component of membrane; C:cell junction; P:ionotropic glutamate receptor signaling pathway |
| Efet.01.271813.g818.t1 | N-acetylated-alpha-linked acidic dipeptidase | 447 | 9.22E-29 | 60.45 | F:carboxypeptidase activity; C:membrane; P:proteolysis; C:integral component of membrane |
| Efet.01.271830.g819.t1 | coronin-1B-like isoform X2 | 267 | 2.15E-13 | 71.84 |  |
| Efet.01.271851.g820.t1 | neuronal acetylcholine receptor subunit alpha-9-like | 582 | 2.15E-97 | 69.55 | C:postsynaptic membrane; P:cation transmembrane transport; C:integral component of membrane; C:cell junction; F:extracellular ligand-gated ion channel activity; P:single-organism process |
| Efet.01.271853.g821.t1 | FMRFamide receptor-like | 1104 | 1.68E-108 | 55.1 | F:G-protein coupled receptor activity; P:signal transduction; C:membrane |
| Efet.01.271866.g822.t1 | MFS transporter | 876 | 6.55E-141 | 76.45 | P:transmembrane transport; C:integral component of membrane; F:transporter activity |
| Efet.01.271876.g823.t1 | Solute carrier family 12 member 4 | 246 | 8.73E-13 | 77 | F:potassium:chloride symporter activity; P:chloride transmembrane transport; C:integral component of membrane |
| Efet.01.27196.g142.t1 | non-specific lipid-transfer | 531 | 3.96E-75 | 79.3 | F:transferase activity, transferring acyl groups other than amino-acyl groups; P:metabolic process |
| Efet.01.271974.g825.t1 | aromatic-L-amino-acid decarboxylase-like | 402 | 2.34E-18 | 70 | F:pyridoxal phosphate binding; P:cellular amino acid metabolic process; F:carboxy-lyase activity |
| Efet.01.271981.g826.t1 | RBM10_MOUSE ame: Full=RNA-binding 10 ame: Full=RNA-binding motif 10 | 270 | 3.50E-07 | 58 | P:negative regulation of transcription from RNA polymerase II promoter; P:positive regulation of smooth muscle cell apoptotic process; P:3'-UTR-mediated mRNA stabilization; F:organic cyclic compound binding; F:heterocyclic compound binding; P:negative regulation of cell proliferation; P:negative regulation of mRNA splicing, via spliceosome |
| Efet.01.271982.g827.t1 | ATP-binding cassette sub-family B member mitochondrial-like | 918 | 4.17E-112 | 75.25 | P:cellular iron ion homeostasis; F:ATP binding; F:ATPase activity, coupled to transmembrane movement of substances; P:transmembrane transport; C:integral component of membrane; P:single-organism transport; C:mitochondrial inner membrane |
| Efet.01.272052.g828.t1 | protocadherin-9-like isoform X1 | 426 | 1.02E-16 | 59.8 | C:membrane; P:cell adhesion |
| Efet.01.272100.g829.t1 | von Willebrand factor D and EGF domain-containing | 504 | 3.01E-29 | 59.5 | C:membrane |
| Efet.01.27213.g144.t1 | gamma-glutamyltranspeptidase 1 | 207 | 1.15E-17 | 67.4 | P:glutathione metabolic process; F:gamma-glutamyltransferase activity |
| Efet.01.272147.g830.t1 | glutamate decarboxylase | 318 | 2.51E-37 | 79.85 | F:pyridoxal phosphate binding; C:cytoplasm; C:integral component of membrane; C:synapse; P:carboxylic acid metabolic process; C:axon; F:carboxy-lyase activity |
| Efet.01.272199.g831.t1 | neuralized 4-like | 462 | 1.77E-49 | 48.55 | C:nucleus; C:cytoplasm; P:signal transduction |
| Efet.01.27222.g145.t1 | V-type proton ATPase catalytic subunit A | 618 | 4.69E-139 | 97.25 | F:ATP binding; P:ATP hydrolysis coupled proton transport; P:ATP metabolic process; F:proton-transporting ATPase activity, rotational mechanism; C:proton-transporting V-type ATPase, V1 domain |
| Efet.01.272262.g832.t1 | probable ATP-dependent RNA helicase DHX58 | 498 | 4.20E-34 | 61.3 | F:nucleic acid binding; F:DNA binding; F:ATP binding; F:hydrolase activity, acting on acid anhydrides; P:regulation of apoptotic process; F:helicase activity; F:hydrolase activity |
| Efet.01.272336.g833.t1 | dmX 2 isoform X1 | 591 | 2.69E-69 | 74.7 |  |
| Efet.01.272489.g836.t1 | dnaJ homolog subfamily C member 22-like | 321 | 1.55E-24 | 68.45 | C:integral component of membrane |
| Efet.01.272519.g837.t1 | mitogen-activated kinase kinase kinase 2-like | 501 | 2.63E-34 | 72.2 | F:ATP binding; F:protein kinase activity; P:protein phosphorylation |
| Efet.01.272611.g839.t1 | E3 ubiquitin- ligase TRIM56-like isoform X1 | 1110 | 2.83E-07 | 53 | F:zinc ion binding; F:metal ion binding; C:intracellular |
| Efet.01.272635.g840.t1 | filamin-A-like isoform X1 | 267 | 9.08E-36 | 75.4 | F:actin filament binding |
| Efet.01.272654.g841.t1 | eukaryotic translation initiation factor 4E-like isoform X1 | 414 | 8.74E-17 | 66.05 | F:translation initiation factor activity; C:cytoplasm; P:translational initiation |
| Efet.01.272665.g842.t1 | phytanoyl- hydroxylase-interacting -like isoform X2 | 582 | 5.12E-35 | 57.25 | C:membrane; C:integral component of membrane |
| Efet.01.272700.g844.t1 | proteasome subunit beta type-5-like | 420 | 5.86E-57 | 73.4 | P:proteolysis; F:endopeptidase activity; C:proteasome complex |
| Efet.01.272705.g845.t1 | zinc finger 710-like isoform X2 | 1191 | 1.08E-21 | 74.3 | F:nucleic acid binding; F:metal ion binding |
| Efet.01.272727.g846.t1 | solute carrier organic anion transporter family member 4C1 | 393 | 4.38E-10 | 63.6 | C:integral component of plasma membrane; C:extracellular exosome; P:sodium-independent organic anion transport; C:basolateral plasma membrane; P:single-organism process; F:sodium-independent organic anion transmembrane transporter activity |
| Efet.01.272755.g847.t1 | tigger transposable element-derived 4-like | 825 | 1.87E-56 | 65.3 | F:DNA binding |
| Efet.01.272756.g848.t1 | twitchin-like isoform X1 | 909 | 1.39E-118 | 56.85 | F:nucleotide binding; P:phosphorylation; F:kinase activity |
| Efet.01.272790.g849.t1 | gastrula zinc finger -like isoform X1 | 225 | 6.02E-06 | 53 |  |
| Efet.01.272795.g850.t1 | glutamyl aminopeptidase-like | 453 | 7.20E-16 | 54.4 | F:zinc ion binding; P:proteolysis; C:membrane; C:integral component of membrane; F:aminopeptidase activity; F:metallopeptidase activity |
| Efet.01.272874.g852.t1 | ankyrin repeat domain-containing 33B-like | 579 | 2.13E-33 | 64.5 |  |
| Efet.01.272987.g853.t1 | neuromedin-U receptor 2 | 516 | 1.41E-21 | 73.5 | F:G-protein coupled receptor activity; P:G-protein coupled receptor signaling pathway; C:membrane |
| Efet.01.2730.g248.t1 | BTB POZ domain-containing 6 | 993 | 6.50E-31 | 45.2 | P:regulation of neurogenesis |
| Efet.01.27300.g147.t1 | RNA polymerase II elongation factor ELL2 isoform X1 | 579 | 1.06E-07 | 65.6 | C:centrosome; C:transcription elongation factor complex; C:cytoplasm; P:snRNA transcription from RNA polymerase II promoter; C:nucleoplasm; P:transcription elongation from RNA polymerase II promoter |
| Efet.01.27301.g148.t1 | potassium voltage-gated channel Shal-like isoform X1 | 1161 | 0 | 83.3 | P:potassium ion transmembrane transport; C:voltage-gated potassium channel complex; F:delayed rectifier potassium channel activity; F:A-type (transient outward) potassium channel activity; P:regulation of ion transmembrane transport; P:protein homooligomerization |
| Efet.01.273055.g854.t1 | dynein heavy chain axonemal | 924 | 9.07E-122 | 82.5 | F:ATPase activity; F:ATP binding; F:microtubule motor activity; C:dynein complex; P:microtubule-based movement |
| Efet.01.273176.g859.t1 | dynein heavy chain axonemal | 258 | 3.30E-42 | 87.8 | F:ATPase activity; F:ATP binding; F:microtubule motor activity; C:dynein complex; P:microtubule-based movement |
| Efet.01.273189.g860.t1 | MOG interacting and ectopic P-granules 1-like | 2799 | 2.47E-23 | 48.4 | F:metal ion binding |
| Efet.01.273209.g861.t1 | probable serine threonine- kinase DDB_G0282963 | 231 | 1.03E-08 | 75.35 | F:ATP binding; F:metal ion binding; F:protein kinase activity; P:protein phosphorylation |
| Efet.01.273308.g863.t1 | tripartite motif-containing 2-like isoform X1 | 663 | 5.82E-13 | 63.25 | P:myeloid cell differentiation; P:primitive hemopoiesis |
| Efet.01.273345.g864.t1 | synaptotagmin-6-like isoform X1 | 501 | 1.70E-62 | 68.95 | C:exocytic vesicle; P:regulation of insulin secretion; P:calcium ion-regulated exocytosis of neurotransmitter; F:syntaxin binding; P:vesicle fusion; F:calcium ion binding; C:integral component of membrane; F:clathrin binding; P:regulation of calcium ion-dependent exocytosis; C:plasma membrane; F:calcium-dependent phospholipid binding |
| Efet.01.273368.g865.t1 | zinc metallo ase | 285 | 1.60E-08 | 61.6 |  |
| Efet.01.2734.g249.t1 | protocadherin Fat 4-like | 2334 | 2.55E-82 | 47.35 | F:catalytic activity |
| Efet.01.273513.g866.t1 | LCHN-like | 447 | 2.28E-73 | 74.8 | P:localization; C:cytoplasm |
| Efet.01.27357.g151.t1 | filamin-B isoform X3 | 210 | 1.34E-26 | 82.55 | F:identical protein binding; P:signal transduction; P:epithelial cell morphogenesis; C:plasma membrane; P:cytoskeletal anchoring at plasma membrane; F:RNA binding; P:keratinocyte development; F:actin filament binding; C:Z disc; C:extracellular exosome; C:brush border; C:focal adhesion; C:cell cortex; P:skeletal muscle tissue development; C:stress fiber; C:integral component of membrane; C:cytosol; P:actin cytoskeleton organization |
| Efet.01.273598.g868.t1 | unc-93 homolog A | 795 | 3.08E-49 | 64.65 | C:membrane |
| Efet.01.273643.g869.t1 | general transcription factor 3C polypeptide 3 | 2190 | 0 | 60.7 |  |
| Efet.01.273656.g870.t1 | GAS2 1 | 792 | 1.26E-75 | 64.3 | C:intracellular part |
| Efet.01.273770.g874.t1 | arrestin domain-containing 4-like | 1302 | 2.92E-61 | 48.85 | P:signal transduction |
| Efet.01.273772.g875.t1 | cGMP-inhibited 3 ,5 -cyclic phosphodiesterase A-like isoform X2 | 939 | 2.90E-18 | 46 | C:membrane; C:integral component of membrane |
| Efet.01.273788.g877.t1 | acyl- dehydrogenase family member mitochondrial-like | 393 | 2.65E-34 | 60.5 | F:coenzyme binding; F:oxidoreductase activity, acting on the CH-CH group of donors; P:oxidation-reduction process; F:anion binding |
| Efet.01.273837.g881.t1 | endothelin-converting enzyme-like 1 | 285 | 3.01E-10 | 62.05 | P:neuropeptide signaling pathway; P:proteolysis; C:membrane; C:integral component of membrane; F:metalloendopeptidase activity; F:metallopeptidase activity |
| Efet.01.273916.g882.t1 | otoferlin-like isoform X1 | 207 | 7.27E-08 | 67.57 | C:membrane; C:integral component of membrane |
| Efet.01.273997.g884.t1 | Transcription factor che-1 | 294 | 2.34E-21 | 66.05 | P:chemotaxis; P:response to salt; P:neuron differentiation; P:response to methionine; F:RNA polymerase II regulatory region sequence-specific DNA binding; P:response to lithium ion; P:response to alkaline pH; P:response to magnesium ion; P:positive regulation of transcription from RNA polymerase II promoter; P:chemosensory behavior; P:positive regulation of chemotaxis |
| Efet.01.273999.g885.t1 | C3 and PZP-like alpha-2-macroglobulin domain-containing 8 | 375 | 6.08E-13 | 54.2 | F:DNA binding; C:membrane; C:integral component of membrane; F:carbohydrate binding |
| Efet.01.2740.g250.t1 | AChain Structure Of Human Parp1 Catalytic Domain Bound To A Quinazoline- 4(1h,3h)-dione Inhibitor | 579 | 1.35E-93 | 72.8 | P:mitochondrial DNA repair; P:base-excision repair; P:protein sumoylation; P:negative regulation of ATP biosynthetic process; P:transforming growth factor beta receptor signaling pathway; P:signal transduction involved in regulation of gene expression; P:transcription from RNA polymerase II promoter; C:transcription factor complex; P:positive regulation of transcription regulatory region DNA binding; F:NAD binding; P:cellular response to superoxide; F:protein ADP-ribosylase activity; P:ATP generation from poly-ADP-D-ribose; P:positive regulation of cardiac muscle hypertrophy; P:macrophage differentiation; F:protein kinase binding; P:regulation of SMAD protein complex assembly; P:negative regulation of interleukin-17 secretion; F:histone deacetylase binding; P:nucleotide-excision repair, DNA incision, 3'-to lesion; P:nucleotide-excision repair, DNA incision, 5'-to lesion; P:nucleotide-excision repair, preincision complex stabilization; P:nucleotide-excision repair, preincision complex assembly; P:regulation of oxidative stress-induced neuron intrinsic apoptotic signaling pathway; P:regulation of growth rate; C:nuclear chromosome, telomeric region; C:nuclear envelope; F:metal ion binding; F:protein N-terminus binding; F:estrogen receptor binding; P:global genome nucleotide-excision repair; P:nucleotide-excision repair, DNA duplex unwinding; P:negative regulation of telomere maintenance via telomere lengthening; P:nucleotide-excision repair, DNA damage recognition; C:nucleolus; P:positive regulation of transcription from RNA polymerase II promoter; C:nucleoplasm; F:NAD+ ADP-ribosyltransferase activity; P:negative regulation of transcription from RNA polymerase II promoter; P:cellular response to insulin stimulus; P:DNA ligation involved in DNA repair; P:behavioral response to cocaine; P:positive regulation of mitochondrial depolarization; P:positive regulation of SMAD protein import into nucleus; F:identical protein binding; P:DNA damage response, detection of DNA damage; P:lagging strand elongation; P:positive regulation of myofibroblast differentiation; P:double-strand break repair via homologous recombination; P:telomere maintenance; P:cellular response to beta-amyloid; F:chromatin binding; F:transcription factor binding; P:positive regulation of neuron death; F:RNA binding; P:regulation of single strand break repair; F:DNA binding; P:regulation of DNA methylation; F:DNA ligase (ATP) activity; C:mitochondrion; C:membrane; F:R-SMAD binding; P:positive regulation of intracellular estrogen receptor signaling pathway; P:protein autoprocessing; P:protein poly-ADP-ribosylation |
| Efet.01.27432.g154.t1 | paired amphipathic helix Sin3a | 519 | 8.92E-27 | 55.6 | C:intracellular |
| Efet.01.274331.g887.t1 | eukaryotic translation initiation factor 5A | 222 | 5.79E-32 | 79.35 | F:translation initiation factor activity; P:positive regulation of translational termination; F:translation elongation factor activity; F:ribosome binding; P:translational frameshifting; P:translational initiation; P:positive regulation of translational elongation |
| Efet.01.274344.g888.t1 | 16 kDa calcium-binding | 255 | 1.62E-23 | 70.86 | F:calcium ion binding |
| Efet.01.27436.g155.t1 | fumarate mitochondrial | 1083 | 0 | 87.15 | C:extracellular exosome; P:homeostasis of number of cells within a tissue; C:mitochondrion; P:tricarboxylic acid cycle; F:fumarate hydratase activity; P:fumarate metabolic process; C:tricarboxylic acid cycle enzyme complex |
| Efet.01.274369.g890.t1 | DNA topoisomerase 2-alpha-like | 246 | 1.76E-13 | 84.15 | C:nucleoid; P:sister chromatid segregation; P:retinal ganglion cell axon guidance; C:DNA topoisomerase complex (ATP-hydrolyzing); P:chromosome condensation; F:DNA binding, bending; P:resolution of meiotic recombination intermediates; P:DNA topological change; P:mitotic DNA integrity checkpoint; P:positive regulation of transcription from RNA polymerase II promoter; C:synaptonemal complex; P:DNA unwinding involved in DNA replication; P:mitotic recombination; P:axon target recognition; F:magnesium ion binding; F:ATP binding; P:retina layer formation; F:DNA topoisomerase type II (ATP-hydrolyzing) activity |
| Efet.01.274389.g892.t1 | aquaporin -like | 603 | 2.53E-39 | 58.05 | C:membrane; C:integral component of membrane; P:transport; F:transporter activity |
| Efet.01.274434.g893.t1 | phosphorylase b kinase regulatory subunit beta-like | 429 | 2.35E-56 | 65.6 | F:catalytic activity; C:cell part |
| Efet.01.274540.g896.t1 | phosphatidylinositol 4-kinase alpha-like | 348 | 3.10E-67 | 85.4 | P:multi-organism membrane organization; P:viral RNA genome replication; P:phosphatidylinositol phosphorylation; P:viral replication complex formation and maintenance; C:plasma membrane; F:cadherin binding involved in cell-cell adhesion; F:ATP binding; C:extracellular exosome; C:focal adhesion; C:cytoplasm; C:cell-cell adherens junction; P:phosphatidylinositol-mediated signaling; C:viral replication complex; P:cell-cell adhesion; F:kinase activity |
| Efet.01.274544.g897.t1 | sacsin-like | 1104 | 1.16E-21 | 46.85 |  |
| Efet.01.274629.g901.t1 | cyclin-F-like isoform X3 | 363 | 2.19E-09 | 69.25 | C:nucleus; P:regulation of cell cycle |
| Efet.01.274687.g903.t1 | BTB POZ domain-containing 10-like | 228 | 1.16E-11 | 83.4 |  |
| Efet.01.274741.g907.t1 | neuroligin- X-linked-like isoform X1 | 891 | 5.77E-91 | 63 | C:membrane; C:integral component of membrane |
| Efet.01.274844.g912.t1 | mannan polymerase complex subunit mnn9-like | 1335 | 3.92E-93 | 59.05 | C:membrane; C:integral component of membrane |
| Efet.01.274856.g913.t1 | myosin heavy chain 95F isoform X3 | 435 | 3.82E-40 | 75.5 | C:myosin complex; F:ATP binding; F:actin binding; C:membrane; F:motor activity |
| Efet.01.274875.g914.t1 | probable G- coupled receptor 139 | 1182 | 2.05E-70 | 61.35 | F:G-protein coupled receptor activity; P:G-protein coupled receptor signaling pathway; C:integral component of membrane |
| Efet.01.274930.g916.t1 | solute carrier family 12 member 7 | 276 | 5.42E-32 | 81.2 |  |
| Efet.01.274959.g920.t1 | guanine nucleotide-binding subunit alpha-12-like | 1080 | 2.87E-179 | 78.3 | F:GTP binding; C:heterotrimeric G-protein complex; C:brush border membrane; F:GTPase activity; P:adenylate cyclase-modulating G-protein coupled receptor signaling pathway; F:G-protein beta/gamma-subunit complex binding; P:Rho protein signal transduction; F:signal transducer activity; F:D5 dopamine receptor binding |
| Efet.01.274962.g921.t1 | discoidin domain-containing receptor 2-like isoform X1 | 1284 | 2.17E-47 | 59.6 | F:ATP binding; P:peptidyl-tyrosine phosphorylation; C:integral component of plasma membrane; F:protein tyrosine kinase activity; C:membrane; C:integral component of membrane; F:protein tyrosine kinase collagen receptor activity; F:protein kinase activity; P:collagen-activated tyrosine kinase receptor signaling pathway; P:protein phosphorylation |
| Efet.01.274975.g922.t1 | tyrosine- kinase Tec-like isoform X1 | 2925 | 8.77E-99 | 56.2 | F:nucleotide binding; P:peptidyl-tyrosine phosphorylation; F:protein tyrosine kinase activity; P:regulation of cellular process; P:single-organism process; P:response to stimulus |
| Efet.01.274991.g923.t1 | Transmembrane 151B | 903 | 2.19E-18 | 50.7 | C:membrane; C:integral component of membrane |
| Efet.01.27500.g158.t1 | fibroblast growth factor receptor-like 1 | 258 | 1.07E-22 | 71.2 | C:membrane |
| Efet.01.275054.g924.t1 | G -coupled receptor | 345 | 4.70E-08 | 81.6 | P:G-protein coupled receptor signaling pathway; F:octopamine receptor activity; C:integral component of membrane |
| Efet.01.275103.g926.t1 | Brother of CDO | 267 | 2.91E-10 | 62.3 | C:integral component of plasma membrane; P:embryonic morphogenesis; C:membrane; C:integral component of membrane; P:smoothened signaling pathway; P:axon guidance; P:positive regulation of myoblast differentiation; P:positive regulation of macromitophagy in response to mitochondrial depolarization; P:regulation of neuron differentiation |
| Efet.01.275134.g927.t1 | outer membrane assembly factor | 1701 | 0 | 75.95 | C:outer membrane |
| Efet.01.275134.g928.t1 | VWA domain-containing | 1542 | 0 | 97.7 |  |
| Efet.01.275134.g929.t1 | AAA family ATPase | 1008 | 0 | 90.2 | F:ATP binding |
| Efet.01.275134.g930.t1 | cytochrome c4 | 552 | 1.12E-132 | 88.1 | F:iron ion binding; F:electron carrier activity; C:periplasmic space; F:heme binding |
| Efet.01.275134.g931.t1 | NADP-dependent oxidoreductase | 1020 | 0 | 94 | F:zinc ion binding; F:oxidoreductase activity; P:oxidation-reduction process |
| Efet.01.275134.g932.t1 | phenylacetic acid degradation | 372 | 1.37E-83 | 89.55 |  |
| Efet.01.275134.g933.t1 | gluconate 5-dehydrogenase | 843 | 1.13E-161 | 93.6 | F:gluconate 5-dehydrogenase activity; P:oxidation-reduction process |
| Efet.01.275134.g934.t1 | channel | 1341 | 0 | 88.45 | P:transport; C:outer membrane; F:transporter activity |
| Efet.01.275134.g935.t1 | sulfurtransferase | 336 | 1.47E-58 | 83.3 | F:transferase activity |
| Efet.01.275134.g936.t1 | protein-L-isoaspartate O-methyltransferase | 714 | 5.53E-155 | 87.9 | F:protein-L-isoaspartate (D-aspartate) O-methyltransferase activity; P:protein methylation |
| Efet.01.275134.g937.t1 | nodulation | 3180 | 0 | 90.45 | C:integral component of membrane; P:transport; F:transporter activity |
| Efet.01.275145.g938.t1 | hypothetical protein HELRODRAFT_178722 | 252 | 3.04E-11 | 78 | C:membrane; F:scavenger receptor activity; P:receptor-mediated endocytosis |
| Efet.01.275288.g942.t1 | FMRFamide receptor-like | 1245 | 3.46E-49 | 54 | F:G-protein coupled receptor activity; F:G-protein coupled peptide receptor activity; P:G-protein coupled receptor signaling pathway; P:signal transduction; C:membrane; C:integral component of membrane; F:signal transducer activity |
| Efet.01.275297.g944.t1 | hypothetical protein HELRODRAFT_78329 | 282 | 1.33E-06 | 71 | P:inorganic anion transport; P:ion transport; P:anion transmembrane transport; F:anion transmembrane transporter activity; C:membrane; C:integral component of membrane; F:inorganic anion exchanger activity; P:anion transport; P:transport; F:transporter activity |
| Efet.01.275326.g945.t1 | hypothetical protein PISMIDRAFT_56066, partial | 279 | 1.46E-06 | 59.42 |  |
| Efet.01.275471.g949.t1 | roundabout homolog 2- | 396 | 1.14E-16 | 55.5 | C:membrane; C:integral component of membrane |
| Efet.01.27554.g160.t1 | acid trehalase 1 | 270 | 9.26E-13 | 69.25 | C:cell wall; F:hydrolase activity |
| Efet.01.275645.g953.t1 | Retrovirus-related Pol poly from transposon | 522 | 9.26E-34 | 60.25 | F:nucleic acid binding; P:DNA integration |
| Efet.01.275664.g954.t1 | G -activated inward rectifier potassium channel 3-like | 1515 | 9.37E-159 | 75.75 | P:potassium ion transmembrane transport; C:integral component of membrane; F:inward rectifier potassium channel activity; P:regulation of ion transmembrane transport |
| Efet.01.275772.g955.t1 | SUMO-activating enzyme subunit 2 | 396 | 4.27E-12 | 68.6 | F:SUMO activating enzyme activity; P:protein sumoylation; C:cytosol; F:binding; C:SUMO activating enzyme complex |
| Efet.01.27584.g162.t1 | thyrotropin-releasing hormone receptor-like | 594 | 1.35E-18 | 55.95 | F:G-protein coupled receptor activity; P:G-protein coupled receptor signaling pathway; P:signal transduction; C:membrane; C:integral component of membrane; F:signal transducer activity |
| Efet.01.27587.g163.t1 | protocadherin-9-like isoform X1 | 2841 | 8.81E-162 | 53.85 | F:calcium ion binding; C:membrane; C:integral component of membrane; P:cell adhesion; P:homophilic cell adhesion via plasma membrane adhesion molecules; C:plasma membrane |
| Efet.01.275883.g957.t1 | ABC transporter permease | 2466 | 3.64E-157 | 87.2 | F:ATPase activity; F:ATP binding; C:integral component of membrane; P:peptide transport; C:plasma membrane |
| Efet.01.275883.g958.t1 | ABC transporter ATP-binding | 687 | 4.80E-161 | 77.4 | F:ATPase activity; F:ATP binding; P:peptide transport |
| Efet.01.275883.g959.t1 | ABC transporter substrate-binding | 1614 | 0 | 77.25 | P:transmembrane transport; C:ATP-binding cassette (ABC) transporter complex |
| Efet.01.275883.g960.t1 | quinone oxidoreductase | 2100 | 0 | 92.55 | F:zinc ion binding; F:oxidoreductase activity; P:oxidation-reduction process |
| Efet.01.275892.g961.t1 | DDB1- and CUL4-associated factor 5-like | 1083 | 9.70E-06 | 80 |  |
| Efet.01.275935.g964.t1 | protocadherin beta-18 | 1980 | 2.16E-133 | 57.05 | F:calcium ion binding; C:membrane; C:integral component of membrane; P:cell adhesion; P:homophilic cell adhesion via plasma membrane adhesion molecules; C:plasma membrane |
| Efet.01.275960.g965.t1 | acetylcholinesterase-like isoform X3 | 1215 | 3.52E-161 | 63.8 | C:membrane; F:hydrolase activity |
| Efet.01.275982.g966.t1 | otoferlin-like isoform X1 | 246 | 2.48E-17 | 81.15 | C:presynapse; F:calcium ion binding; C:integral component of membrane; C:cytosol; P:synaptic vesicle exocytosis |
| Efet.01.27600.g164.t1 | glutamine-rich 2-like isoform X4 | 969 | 1.85E-26 | 47 |  |
| Efet.01.276178.g969.t1 | baculoviral IAP repeat-containing 6-like isoform X2 | 945 | 2.04E-51 | 55.95 | P:regulation of cellular process; C:intracellular part; P:cellular process |
| Efet.01.276218.g970.t1 | vesicle-associated membrane -associated A | 213 | 4.20E-20 | 88.15 | C:microtubule cytoskeleton; P:COPII-coated vesicle budding; P:positive regulation of I-kappaB kinase/NF-kappaB signaling; C:Golgi membrane; C:endoplasmic reticulum membrane; C:bicellular tight junction; P:cell death; F:protein heterodimerization activity; P:sphingolipid biosynthetic process; P:membrane fusion; P:positive regulation by host of viral genome replication; P:signal transduction; P:neuron projection development; P:negative regulation by host of viral genome replication; P:endoplasmic reticulum organization; F:signal transducer activity; C:plasma membrane; F:microtubule binding; C:nuclear membrane; C:perinuclear region of cytoplasm; P:positive regulation by host of viral release from host cell; C:integral component of membrane; C:vesicle; C:endoplasmic reticulum exit site; P:protein localization to endoplasmic reticulum; F:FFAT motif binding; P:protein folding in endoplasmic reticulum |
| Efet.01.276228.g971.t1 | major facilitator superfamily domain-containing 12-like | 315 | 3.77E-33 | 78.45 | C:integral component of membrane |
| Efet.01.276265.g972.t1 | 60S ribosomal L10a | 372 | 3.28E-44 | 87.8 | F:RNA binding; F:structural constituent of ribosome; P:maturation of LSU-rRNA; C:cytosolic large ribosomal subunit; P:translation |
| Efet.01.276308.g974.t1 | hypothetical protein CAPTEDRAFT_223749 | 336 | 3.87E-22 | 64.88 | C:membrane; C:integral component of membrane |
| Efet.01.276381.g975.t1 | glutamate receptor kainate 2-like | 957 | 1.11E-25 | 53.7 | P:ion transmembrane transport; C:synapse; F:receptor activity; F:ionotropic glutamate receptor activity; F:extracellular-glutamate-gated ion channel activity; P:transport; C:plasma membrane; F:ion channel activity; P:ion transport; C:postsynaptic membrane; C:membrane; C:integral component of membrane; C:cell junction; P:ionotropic glutamate receptor signaling pathway |
| Efet.01.276421.g976.t1 | intraflagellar transport 172 homolog | 366 | 2.40E-37 | 61.95 | P:embryo development; P:regulation of cellular process; P:tube development; C:cilium; P:nervous system development; P:pattern specification process; P:animal organ development; P:epithelium development; P:cellular process |
| Efet.01.276451.g977.t1 | calcium-activated potassium channel slowpoke isoform X14 | 459 | 3.36E-24 | 62.05 | F:potassium channel activity; P:transport |
| Efet.01.276480.g979.t1 | insulinoma-associated 1a-like | 759 | 5.32E-26 | 77.05 | F:nucleic acid binding; F:metal ion binding |
| Efet.01.276504.g980.t1 | hypothetical protein HELRODRAFT_176332 | 570 | 9.76E-06 | 51 |  |
| Efet.01.276515.g981.t1 | cytosolic carboxypeptidase 5 isoform X1 | 2076 | 0 | 64 |  |
| Efet.01.27654.g166.t1 | transmembrane 26-like | 996 | 1.91E-19 | 82.55 | C:integral component of membrane |
| Efet.01.276624.g982.t1 | hypothetical protein CAPTEDRAFT_196175 | 960 | 1.92E-62 | 52.75 | C:membrane; C:integral component of membrane; F:sulfotransferase activity |
| Efet.01.276655.g983.t1 | sodium potassium ATPase alpha | 516 | 2.15E-65 | 80.65 | P:sodium ion export from cell; P:ATP hydrolysis coupled proton transport; F:sodium:potassium-exchanging ATPase activity; P:cellular sodium ion homeostasis; F:hydrogen:potassium-exchanging ATPase activity; F:magnesium ion binding; C:integral component of plasma membrane; F:ATP binding; P:cellular potassium ion homeostasis; C:extracellular space; C:intracellular membrane-bounded organelle; P:establishment or maintenance of transmembrane electrochemical gradient; P:potassium ion import |
| Efet.01.276696.g984.t1 | APOBEC1 complementation | 330 | 5.69E-24 | 78.9 | F:protein binding; P:mRNA metabolic process; C:cytoplasm; F:double-stranded RNA binding; F:single-stranded RNA binding; C:apolipoprotein B mRNA editing enzyme complex; P:protein stabilization; P:cytidine to uridine editing; C:nucleoplasm |
| Efet.01.27676.g167.t1 | serine threonine- kinase mos-like | 621 | 3.88E-23 | 63.1 | F:kinase activity |
| Efet.01.276854.g989.t1 | single-minded homolog 2-like | 267 | 2.60E-33 | 95.25 | P:negative regulation of transcription from RNA polymerase II promoter; F:DNA binding; F:protein heterodimerization activity; P:transcription, DNA-templated; P:embryonic pattern specification; P:nervous system development; P:cell differentiation; C:nucleoplasm; F:RNA polymerase II transcription factor activity, sequence-specific DNA binding; P:lung development |
| Efet.01.27686.g168.t1 | leukocyte elastase inhibitor-like | 480 | 9.84E-23 | 54.6 | C:extracellular space; F:serine-type endopeptidase inhibitor activity; C:extracellular exosome; P:negative regulation of endopeptidase activity; P:hematopoietic progenitor cell differentiation |
| Efet.01.27686.g169.t1 | serpin B8 isoform X3 | 936 | 3.10E-33 | 53.35 | F:peptidase inhibitor activity; C:extracellular space; F:serine-type endopeptidase inhibitor activity; C:extracellular exosome; C:cytoplasm; P:epithelial cell-cell adhesion; C:cytosol; P:negative regulation of endopeptidase activity; P:negative regulation of peptidase activity |
| Efet.01.276870.g991.t1 | zinc finger MIZ domain-containing 1 | 375 | 5.32E-14 | 69.3 | F:zinc ion binding |
| Efet.01.276883.g992.t1 | hypothetical protein HELRODRAFT_190034 | 2907 | 6.90E-21 | 68 |  |
| Efet.01.276917.g993.t1 | oxysterol-binding 1-like | 462 | 4.07E-58 | 69.35 | P:lipid transport; P:transport |
| Efet.01.276925.g994.t1 | helicase with zinc finger domain 2-like | 4530 | 2.33E-66 | 41.4 | F:nucleotide binding; F:nucleic acid binding; F:5'-flap endonuclease activity; F:ATP binding; F:zinc ion binding; F:metal ion binding; F:single-stranded DNA-dependent ATPase activity; F:ligand-dependent nuclear receptor transcription coactivator activity; P:regulation of nucleic acid-templated transcription; P:DNA replication, Okazaki fragment processing |
| Efet.01.276957.g997.t1 | disintegrin and metallo ase domain-containing 10 | 906 | 6.93E-65 | 56.6 | F:zinc ion binding; P:integrin-mediated signaling pathway; P:proteolysis; F:protein kinase activity; P:protein phosphorylation; F:metalloendopeptidase activity; F:metallopeptidase activity |
| Efet.01.277007.g998.t1 | ephrin type-B receptor 1-B-like isoform X7 | 240 | 1.43E-23 | 66.2 | F:nucleotide binding; F:protein tyrosine kinase activity; C:integral component of membrane; P:transmembrane receptor protein tyrosine kinase signaling pathway; P:protein phosphorylation |
| Efet.01.27707.g171.t1 | calpain-12 | 225 | 1.15E-08 | 64 | F:peptidase activity; P:transport |
| Efet.01.277079.g999.t1 | RHS repeat-associated core domain-containing | 822 | 1.92E-41 | 80.3 | P:self proteolysis; C:integral component of membrane |
| Efet.01.27710.g172.t1 | 5-hydroxytryptamine receptor 2A | 1341 | 3.68E-53 | 68.35 | C:integral component of plasma membrane; F:dopamine neurotransmitter receptor activity; P:dopamine receptor signaling pathway |
| Efet.01.277118.g1001.t1 | alpha-2-macroglobulin domain | 2361 | 0 | 73.3 | C:membrane |
| Efet.01.277136.g1002.t1 | hypothetical protein CAPTEDRAFT_221464 | 543 | 9.02E-06 | 43 | C:cell; P:cell redox homeostasis |
| Efet.01.277143.g1003.t1 | probable G- coupled receptor 139 isoform X1 | 252 | 1.18E-10 | 71.88 | F:G-protein coupled receptor activity; P:G-protein coupled receptor signaling pathway; C:integral component of membrane |
| Efet.01.27720.g173.t1 | neuronal acetylcholine receptor subunit alpha-10 | 993 | 1.81E-98 | 64.3 | C:postsynaptic membrane; P:cation transmembrane transport; C:integral component of membrane; C:cell junction; F:extracellular ligand-gated ion channel activity; P:chemical synaptic transmission; P:response to stimulus |
| Efet.01.277327.g1007.t1 | hypothetical protein CAPTEDRAFT_225483 | 288 | 1.05E-15 | 55.3 | C:membrane; C:integral component of membrane; F:carbohydrate binding |
| Efet.01.27735.g174.t1 | plexin-B isoform X1 | 1035 | 4.20E-53 | 58.95 | C:integral component of membrane; P:axon guidance; F:hydrolase activity; P:semaphorin-plexin signaling pathway |
| Efet.01.277426.g1011.t1 | acyl-coenzyme A oxidase peroxisomal | 1170 | 6.71E-132 | 82.65 | F:acyl-CoA dehydrogenase activity; C:peroxisome; F:acyl-CoA oxidase activity; P:fatty acid beta-oxidation using acyl-CoA dehydrogenase; F:fatty-acyl-CoA binding; F:flavin adenine dinucleotide binding; F:electron carrier activity; F:oxidoreductase activity, acting on the CH-CH group of donors, with a flavin as acceptor; P:lipid homeostasis |
| Efet.01.27744.g175.t1 | hypothetical protein BV898_09700 | 756 | 2.15E-26 | 62.3 | F:catalytic activity |
| Efet.01.277512.g1013.t1 | sex peptide receptor-like | 546 | 2.34E-29 | 71.1 | F:G-protein coupled receptor activity; C:membrane |
| Efet.01.277567.g1015.t1 | dynein heavy chain axonemal-like | 609 | 3.83E-54 | 82.1 | F:ATPase activity; F:ATP binding; F:microtubule motor activity; C:axonemal dynein complex; F:calcium ion binding; P:cilium movement; P:determination of liver left/right asymmetry |
| Efet.01.277577.g1017.t1 | 60S ribosomal L7a-like | 312 | 2.51E-27 | 87.6 | F:RNA binding; P:maturation of LSU-rRNA; C:cytosolic large ribosomal subunit; P:translation |
| Efet.01.27761.g178.t1 | non-structural maintenance of chromosomes element 3 homolog | 780 | 8.80E-66 | 63.3 | C:Smc5-Smc6 complex; P:positive regulation of protein ubiquitination |
| Efet.01.27763.g179.t1 | substance-K receptor-like isoform X1 | 840 | 5.22E-68 | 65.35 | P:signal transduction; F:signal transducer activity |
| Efet.01.277700.g1022.t1 | AChain Structural Basis For Hif-1alphaCBP RECOGNITION IN THE Cellular Hypoxic Response | 1605 | 4.08E-06 | 88.63 | P:response to estrogen; F:acetyltransferase activity; F:RNA polymerase II core promoter sequence-specific DNA binding; P:cellular response to nerve growth factor stimulus; F:RNA polymerase II core promoter proximal region sequence-specific DNA binding; P:transcription-coupled nucleotide-excision repair; P:intrinsic apoptotic signaling pathway in response to DNA damage by p53 class mediator; P:positive regulation of protein acetylation; P:positive regulation of sequence-specific DNA binding transcription factor activity; P:regulation of androgen receptor signaling pathway; P:positive regulation of cell growth; P:response to hypoxia; P:positive regulation of collagen biosynthetic process; P:positive regulation of transcription, DNA-templated; P:internal peptidyl-lysine acetylation; F:protein antigen binding; P:positive regulation of muscle atrophy; P:positive regulation of protein secretion; F:protein kinase binding; P:positive regulation of gene expression, epigenetic; P:protein-DNA complex assembly; P:positive regulation of axon extension; P:response to hydrogen peroxide; P:response to organic cyclic compound; F:core promoter proximal region DNA binding; C:nucleus; F:protein binding; F:androgen receptor binding; F:metal ion binding; P:positive regulation of cell death; F:peptide N-acetyltransferase activity; F:mitogen-activated protein kinase binding; P:cellular response to organic cyclic compound; P:response to tumor necrosis factor; P:positive regulation by host of viral transcription; F:transferase activity, transferring acyl groups; P:regulation of cellular response to heat; P:negative regulation of cell death; P:positive regulation of transcription from RNA polymerase II promoter; P:stimulatory C-type lectin receptor signaling pathway; P:heart development; F:transcription factor activity, sequence-specific DNA binding; P:protein kinase B signaling; F:antigen binding; P:positive regulation of cell size; P:liver development; P:response to cobalt ion; F:nuclear hormone receptor binding; F:glucocorticoid receptor binding; P:regulation of tubulin deacetylation; F:chromatin binding; F:damaged DNA binding; F:DNA binding; F:histone acetyltransferase activity; F:transcription cofactor activity; F:transcription coactivator activity; C:cytoplasm; P:cellular response to retinoic acid; P:skeletal muscle tissue development; P:positive regulation of protein phosphorylation; P:cellular response to mineralocorticoid stimulus; F:transferase activity; P:positive regulation of cellular metabolic process; P:cellular response to dexamethasone stimulus; P:negative regulation of cellular metabolic process; P:response to dexamethasone; P:positive regulation of protein binding; P:positive regulation of translation; F:protein C-terminus binding; P:transcription from RNA polymerase II promoter; P:regulation of transcription from RNA polymerase II promoter in response to hypoxia; F:transcriptional activator activity, RNA polymerase II transcription regulatory region sequence-specific binding; P:positive regulation of DNA binding; P:fat cell differentiation; P:memory; F:RNA polymerase II activating transcription factor binding; F:bHLH transcription factor binding; F:peroxisome proliferator activated receptor binding; C:transcription factor complex; F:p53 binding; P:response to glucose; P:response to retinoic acid; P:cellular response to glucose stimulus; P:digestive tract development; P:negative regulation of cysteine-type endopeptidase activity involved in apoptotic process; P:N-terminal peptidyl-lysine acetylation; P:apoptotic process; P:platelet formation; P:histone acetylation; P:regulation of cell cycle; F:protein complex binding; F:activating transcription factor binding; F:zinc ion binding; P:B cell differentiation; P:positive regulation of type I interferon production; P:regulation of signal transduction by p53 class mediator; P:circadian rhythm; C:chromatin; P:animal organ morphogenesis; F:NF-kappaB binding; F:lysine N-acetyltransferase activity, acting on acetyl phosphate as donor; P:Notch signaling pathway; F:RNA polymerase II transcription factor binding; P:somitogenesis; P:positive regulation of gene expression; P:regulation of autophagy; P:cellular response to cAMP; C:protein-DNA complex; P:cellular response to trichostatin A; P:positive regulation of histone acetylation; P:histone H4 acetylation; P:histone H3 acetylation; P:positive regulation of protein import into nucleus, translocation; P:histone H2B acetylation; P:positive regulation of glycoprotein biosynthetic process; P:nervous system development; P:positive regulation of proteolysis; P:beta-catenin-TCF complex assembly; F:core promoter binding; C:nucleoplasm; P:negative regulation of transcription from RNA polymerase II promoter; P:response to ethanol; P:DNA damage response, signal transduction by p53 class mediator resulting in cell cycle arrest; P:viral process; P:cellular response to UV; P:megakaryocyte development; P:response to fatty acid; P:cellular response to antibiotic; P:cellular response to hydrogen peroxide; P:lung development; F:chromatin DNA binding; P:positive regulation of sarcomere organization; P:regulation of angiotensin metabolic process; P:transcription, DNA-templated; P:cellular response to drug; P:internal protein amino acid acetylation; P:regulation of transcription, DNA-templated; P:positive regulation of transcription from RNA polymerase II promoter involved in unfolded protein response; P:protein acetylation; F:beta-catenin binding; F:transcription factor binding; P:cell cycle; C:histone acetyltransferase complex; P:negative regulation of miRNA metabolic process; F:pre-mRNA intronic binding; P:rhythmic process; F:SMAD binding; P:response to drug; P:response to calcium ion; P:protein stabilization |
| Efet.01.277850.g1026.t1 | prickle 3 isoform X4 | 570 | 6.06E-107 | 87 | F:zinc ion binding |
| Efet.01.277898.g1027.t1 | homeobox CDX-4 | 345 | 3.71E-22 | 82.9 | P:negative regulation of transcription from RNA polymerase II promoter; C:nucleus; P:anterior/posterior axis specification; F:transcriptional activator activity, RNA polymerase II core promoter proximal region sequence-specific binding; P:blood vessel development; F:RNA polymerase II core promoter proximal region sequence-specific DNA binding; P:cell differentiation; P:transcription from RNA polymerase II promoter; P:positive regulation of transcription from RNA polymerase II promoter; P:animal organ morphogenesis; P:labyrinthine layer development |
| Efet.01.27795.g181.t1 | tenascin-like isoform X4 | 1842 | 3.00E-34 | 47.1 | F:phosphoprotein phosphatase activity; F:protein tyrosine phosphatase activity; C:membrane; P:protein dephosphorylation; F:phosphatase activity; C:integral component of membrane; P:dephosphorylation; P:peptidyl-tyrosine dephosphorylation; F:hydrolase activity |
| Efet.01.277954.g1028.t1 | pilus assembly | 486 | 1.46E-70 | 63 |  |
| Efet.01.277975.g1029.t1 | tyrosine- kinase SRK2-like | 339 | 6.25E-29 | 75.95 | F:nucleotide binding; F:protein tyrosine kinase activity; P:protein phosphorylation |
| Efet.01.277980.g1030.t1 | cullin-4A | 300 | 3.08E-47 | 70.05 | F:ubiquitin protein ligase activity; P:nucleotide-excision repair, DNA duplex unwinding; P:nucleotide-excision repair, DNA damage recognition; P:hemopoiesis; P:transcription-coupled nucleotide-excision repair; P:positive regulation of cell proliferation; P:regulation of nucleotide-excision repair; C:nucleoplasm; C:extracellular exosome; P:histone H2A monoubiquitination; P:in utero embryonic development; P:somatic stem cell population maintenance; P:DNA damage response, detection of DNA damage; P:UV-damage excision repair; P:nucleotide-excision repair, DNA incision, 3'-to lesion; P:positive regulation of G1/S transition of mitotic cell cycle; P:nucleotide-excision repair, DNA incision, 5'-to lesion; P:nucleotide-excision repair, preincision complex stabilization; P:nucleotide-excision repair, preincision complex assembly; P:positive regulation of protein catabolic process; P:single-organism cellular process; F:damaged DNA binding; P:protein ubiquitination involved in ubiquitin-dependent protein catabolic process; P:proteasome-mediated ubiquitin-dependent protein catabolic process; F:ubiquitin protein ligase binding; C:cytoplasm; C:Cul4A-RING E3 ubiquitin ligase complex; C:Cul4B-RING E3 ubiquitin ligase complex; P:regulation of DNA damage checkpoint; P:global genome nucleotide-excision repair; P:negative regulation of granulocyte differentiation |
| Efet.01.277984.g1031.t1 | plastin-1 isoform X2 | 384 | 7.63E-40 | 66.4 | C:contractile ring; F:actin binding; F:calcium ion binding; C:cytosol; C:apical cortex; P:female meiosis chromosome segregation |
| Efet.01.278002.g1032.t1 | PH domain leucine-rich repeat-containing phosphatase 2-like isoform X3 | 1905 | 5.80E-55 | 62 | F:protein serine/threonine phosphatase activity; F:catalytic activity; P:protein dephosphorylation |
| Efet.01.278066.g1033.t1 | AChain Crystal Structure Of The Earthworm R-Type Lectin C-Half In Complex With Galnac | 216 | 1.94E-17 | 66.17 | F:carbohydrate binding |
| Efet.01.278081.g1034.t1 | cGMP-dependent kinase 1-like | 393 | 1.66E-30 | 63.88 | F:nucleotide binding; F:protein kinase activity |
| Efet.01.278104.g1035.t1 | ankyrin repeat | 2280 | 8.78E-13 | 47.2 |  |
| Efet.01.27813.g183.t1 | tyrosine phosphatase | 831 | 2.70E-84 | 56 | F:phosphatase activity; P:dephosphorylation |
| Efet.01.27816.g184.t1 | hypothetical protein CAPTEDRAFT_222159 | 576 | 1.69E-31 | 57.38 | C:membrane; C:integral component of membrane |
| Efet.01.278192.g1039.t1 | cleavage stimulation factor subunit 2-like | 414 | 1.31E-40 | 95.9 | F:nucleotide binding; F:RNA binding; C:mRNA cleavage and polyadenylation specificity factor complex; P:mRNA polyadenylation |
| Efet.01.27820.g185.t1 | cubilin- | 465 | 8.45E-29 | 62.25 | F:calcium ion binding; C:membrane; F:receptor activity; P:receptor-mediated endocytosis |
| Efet.01.278203.g1040.t1 | thymidine phosphorylase | 555 | 4.19E-39 | 63.1 | P:metabolic process; F:transferase activity, transferring pentosyl groups |
| Efet.01.278215.g1042.t1 | beta-(1,2)-xylosyltransferase-like | 981 | 9.77E-108 | 60.7 | F:transferase activity |
| Efet.01.278216.g1043.t1 | hypothetical protein HELRODRAFT_183479 | 1041 | 1.92E-15 | 71.22 | F:DNA binding |
| Efet.01.278227.g1044.t1 | dopamine D2-like receptor | 465 | 1.47E-27 | 77.9 | F:G-protein coupled receptor activity; P:G-protein coupled receptor signaling pathway; C:integral component of membrane |
| Efet.01.278263.g1045.t1 | C-Jun-amino-terminal kinase-interacting 4 isoform X7 | 756 | 3.84E-07 | 81.35 | P:vesicle-mediated transport; C:cytoplasm; F:kinesin binding; P:activation of JUN kinase activity; F:JUN kinase binding; F:MAP-kinase scaffold activity; F:receptor signaling complex scaffold activity |
| Efet.01.278273.g1046.t1 | sodium channel para-like isoform X1 | 288 | 1.74E-28 | 81.85 | P:regulation of postsynaptic membrane potential; F:calcium ion binding; P:regulation of ion transmembrane transport; C:voltage-gated sodium channel complex; P:sodium ion transmembrane transport; F:voltage-gated sodium channel activity |
| Efet.01.27830.g186.t1 | neuroglian-like isoform X1 | 204 | 5.19E-18 | 70.6 | C:integral component of membrane; F:transferase activity |
| Efet.01.278470.g1050.t1 | vacuolar sorting-associated 52 homolog | 507 | 4.35E-75 | 83.8 |  |
| Efet.01.27855.g188.t1 | hypothetical protein CAPTEDRAFT_223635 | 594 | 3.94E-43 | 57.33 | P:sensory perception of taste; F:taste receptor activity; C:membrane; C:integral component of membrane; P:detection of chemical stimulus involved in sensory perception of taste |
| Efet.01.27855.g189.t1 | gustatory receptor 68a-like | 552 | 1.68E-69 | 52.7 | P:sensory perception of taste; C:membrane |
| Efet.01.278685.g1053.t1 | kelch 9 | 1695 | 0 | 63.3 |  |
| Efet.01.278686.g1054.t1 | endothelin-converting enzyme 1 isoform X3 | 399 | 5.28E-45 | 61.35 | C:secretory granule; F:endopeptidase activity; P:regulation of system process; P:animal organ development; C:membrane part; F:binding; P:pharyngeal system development; P:regulation of blood pressure; P:regulation of signal transduction; C:plasma membrane; C:cell surface; P:hormone catabolic process; P:regulation of protein metabolic process; C:endosome; P:positive regulation of signaling; P:peptide catabolic process; C:intracellular membrane-bounded organelle; P:regulation of phosphate metabolic process; P:peptide hormone processing; P:positive regulation of cellular metabolic process |
| Efet.01.278716.g1055.t1 | mitochondrial aldehyde dehydrogenase | 468 | 2.41E-60 | 77.7 | F:aldehyde dehydrogenase (NAD) activity; P:carbohydrate metabolic process; C:extracellular exosome; C:mitochondrial matrix; F:aldehyde dehydrogenase [NAD(P)+] activity; F:electron carrier activity; P:ethanol oxidation |
| Efet.01.278771.g1057.t1 | orexin receptor type 2-like | 750 | 1.16E-33 | 51.6 | F:G-protein coupled receptor activity; P:G-protein coupled receptor signaling pathway; P:signal transduction; C:membrane; C:integral component of membrane; F:signal transducer activity |
| Efet.01.278788.g1062.t1 | Protocadherin-11 X-linked | 471 | 2.45E-37 | 59.05 | C:membrane |
| Efet.01.27883.g191.t1 | ATP-binding cassette sub-family A member 3 | 462 | 4.99E-48 | 72.05 | F:nucleotide binding; P:lipid transport; F:ATPase activity, coupled to transmembrane movement of substances; C:intracellular membrane-bounded organelle; C:membrane |
| Efet.01.278892.g1064.t1 | Cell death specification 2 | 1209 | 1.79E-19 | 80.6 | F:transcription factor activity, sequence-specific DNA binding; C:integral component of membrane; P:regulation of transcription, DNA-templated; F:sequence-specific DNA binding |
| Efet.01.278918.g1066.t1 | slit homolog 3 -like | 2349 | 1.68E-124 | 51.95 | C:membrane; C:integral component of membrane |
| Efet.01.278935.g1067.t1 | MFS transporter | 762 | 9.26E-127 | 81.9 | P:transmembrane transport; C:integral component of membrane; C:plasma membrane; F:transporter activity |
| Efet.01.278935.g1068.t1 | DNAase | 840 | 8.07E-132 | 89 | F:endodeoxyribonuclease activity, producing 5'-phosphomonoesters; P:DNA metabolic process |
| Efet.01.278935.g1069.t1 | sigma-54-dependent Fis family transcriptional regulator | 2121 | 0 | 73.1 | F:ATP binding; P:transcription, DNA-templated; P:phosphorelay signal transduction system; P:regulation of transcription, DNA-templated; F:sequence-specific DNA binding; F:transcription factor binding; C:intracellular |
| Efet.01.278935.g1070.t1 | cell filamentation Fic | 243 | 3.74E-44 | 90.3 |  |
| Efet.01.27904.g192.t1 | synaptotagmin 7d | 498 | 1.76E-43 | 80.45 | C:membrane; P:exocytosis |
| Efet.01.279123.g1072.t1 | probable cation-transporting ATPase 13A3 | 1083 | 2.42E-123 | 65.05 | F:binding |
| Efet.01.279191.g1074.t1 | axin | 720 | 1.55E-06 | 75 | C:beta-catenin destruction complex; P:signal transduction; P:Wnt signaling pathway; F:receptor signaling complex scaffold activity |
| Efet.01.279223.g1075.t1 | dynein heavy chain axonemal-like | 507 | 6.82E-18 | 68.95 | F:nucleotide binding |
| Efet.01.279225.g1076.t1 | forkhead box K2 | 768 | 1.28E-68 | 72.15 | C:nucleus; F:DNA binding; P:regulation of transcription, DNA-templated; F:RNA polymerase II transcription factor activity, sequence-specific DNA binding |
| Efet.01.279329.g1077.t1 | Voltage-dependent calcium channel type A subunit alpha- | 582 | 4.36E-79 | 79.6 | F:calcium ion binding; C:voltage-gated calcium channel complex; P:regulation of ion transmembrane transport; P:calcium ion transmembrane transport; F:voltage-gated calcium channel activity |
| Efet.01.279341.g1078.t1 | pikachurin-like | 258 | 1.55E-26 | 75.55 | F:calcium ion binding; C:membrane |
| Efet.01.279440.g1080.t1 | hypothetical protein HELRODRAFT_163113 | 240 | 3.87E-14 | 58.9 | C:membrane; C:integral component of membrane; F:carbohydrate binding |
| Efet.01.279443.g1081.t1 | hypothetical protein HELRODRAFT_83456 | 222 | 1.48E-08 | 64 | C:membrane |
| Efet.01.279473.g1082.t1 | long-chain fatty acid transport 4 | 1137 | 0 | 79.05 | P:metabolic process; C:integral component of membrane; P:single-organism process; F:fatty acid ligase activity |
| Efet.01.279510.g1083.t1 | neuroligin- Y-linked-like | 495 | 8.63E-73 | 56.6 | C:integral component of membrane |
| Efet.01.27958.g194.t1 | chondroitin sulfate proteoglycan 4-like | 1503 | 7.02E-53 | 60.85 | C:membrane |
| Efet.01.27960.g195.t1 | arf6 guanine nucleotide exchange factor | 246 | 1.02E-28 | 76.65 | F:ARF guanyl-nucleotide exchange factor activity; P:regulation of ARF protein signal transduction; F:phospholipid binding; P:positive regulation of GTPase activity |
| Efet.01.279647.g1086.t1 | Chorion peroxidase | 255 | 3.19E-25 | 76.9 | P:response to stimulus |
| Efet.01.279676.g1087.t1 | turtle homolog A- | 516 | 6.31E-28 | 62.4 | C:membrane; C:integral component of membrane |
| Efet.01.279682.g1088.t1 | ras-like GTP-binding Rho1 isoform X2 | 579 | 2.69E-128 | 95.25 | F:GTP binding; P:small GTPase mediated signal transduction; C:intracellular |
| Efet.01.279695.g1089.t1 | otoferlin isoform X10 | 315 | 2.02E-39 | 85.35 | C:presynapse; F:calcium ion binding; C:integral component of membrane; C:cytosol; P:synaptic vesicle exocytosis |
| Efet.01.279731.g1090.t1 | neuropilin-2 isoform X2 | 291 | 5.32E-11 | 57.65 | F:protein binding; P:neural crest cell migration; P:regulation of vascular endothelial growth factor receptor signaling pathway; P:protein homooligomerization; P:vasculogenesis |
| Efet.01.279753.g1093.t1 | metabotropic glutamate receptor | 1149 | 1.70E-148 | 76.05 | F:G-protein coupled receptor activity; P:G-protein coupled receptor signaling pathway; C:integral component of membrane; C:plasma membrane |
| Efet.01.279758.g1095.t1 | hypothetical protein CAPTEDRAFT_221645 | 1206 | 9.04E-06 | 56 | F:calcium ion binding; P:proteolysis; F:cysteine-type peptidase activity; F:peptidase activity; F:calcium-dependent cysteine-type endopeptidase activity; C:intracellular; F:hydrolase activity |
| Efet.01.279837.g1096.t1 | G- coupled receptor 54-like | 375 | 1.02E-07 | 74.5 | P:signal transduction; F:signal transducer activity |
| Efet.01.279910.g1098.t1 | CST complex subunit CTC1-like | 1491 | 5.99E-14 | 44.25 | F:single-stranded DNA binding |
| Efet.01.279930.g1099.t1 | ABC transporter ATP-binding | 846 | 1.10E-155 | 79.55 | F:ATPase activity; F:ATP binding; P:branched-chain amino acid transport; F:branched-chain amino acid transmembrane transporter activity |
| Efet.01.279930.g1100.t1 | fatty-acid-- ligase | 1872 | 0 | 91.25 | P:metabolic process; F:ligase activity |
| Efet.01.279930.g1101.t1 | ABC transporter substrate-binding | 336 | 6.44E-71 | 90.75 | P:amino acid transport |
| Efet.01.279945.g1102.t1 | suppressor of lurcher 1-like | 447 | 2.68E-38 | 63.35 |  |
| Efet.01.280042.g1103.t1 | beta-hexosaminidase subunit beta | 687 | 1.55E-26 | 48.06 | P:carbohydrate metabolic process; C:membrane; C:integral component of membrane; F:hydrolase activity, hydrolyzing O-glycosyl compounds; F:beta-N-acetylhexosaminidase activity |
| Efet.01.280092.g1104.t1 | probable E3 ubiquitin- ligase HERC1 | 1137 | 1.22E-151 | 88.95 | P:cerebellar Purkinje cell differentiation; P:neuron projection development; F:ARF guanyl-nucleotide exchange factor activity; C:Golgi apparatus; P:transport; P:positive regulation of GTPase activity; F:ubiquitin-protein transferase activity; P:negative regulation of autophagy; C:membrane; C:cytosol; F:ligase activity; P:neuromuscular process controlling balance; P:protein ubiquitination |
| Efet.01.280102.g1105.t1 | Sodium leak channel non-selective | 300 | 1.03E-36 | 83.15 | C:voltage-gated calcium channel complex; P:regulation of ion transmembrane transport; P:calcium ion transmembrane transport; F:voltage-gated calcium channel activity |
| Efet.01.280110.g1106.t1 | Eukaryotic translation initiation factor 4E | 234 | 1.87E-30 | 78.5 | F:translation initiation factor activity; C:cytoplasm; P:translational initiation |
| Efet.01.280112.g1107.t1 | F-box WD repeat-containing 7-like | 360 | 2.83E-36 | 76.9 | C:membrane |
| Efet.01.280114.g1108.t1 | Clavaminate synthase | 1500 | 4.51E-56 | 57.55 | C:membrane; C:integral component of membrane |
| Efet.01.280222.g1109.t1 | telomere length regulation TEL2 homolog | 1242 | 2.76E-53 | 48.05 |  |
| Efet.01.280222.g1110.t1 | telomere length regulation TEL2 homolog | 867 | 1.09E-44 | 50.95 |  |
| Efet.01.280239.g1112.t1 | Cyclic GMP-AMP synthase | 2904 | 1.59E-18 | 43.75 |  |
| Efet.01.280300.g1114.t1 | ATP-dependent RNA helicase | 222 | 7.36E-15 | 77.33 |  |
| Efet.01.28043.g197.t1 | hypothetical protein HELRODRAFT_192948 | 1215 | 2.81E-22 | 56 |  |
| Efet.01.28050.g198.t1 | SCY1 2 | 330 | 6.45E-38 | 69.95 | F:organic cyclic compound binding; F:heterocyclic compound binding; F:ion binding |
| Efet.01.280551.g1116.t1 | RING finger unkempt | 291 | 1.23E-37 | 88.7 | F:zinc ion binding |
| Efet.01.280566.g1118.t1 | PREDICTED: uncharacterized protein LOC106154093 isoform X3 | 378 | 2.63E-07 | 51.2 |  |
| Efet.01.280613.g1119.t1 | dual specificity phosphatase 26 | 879 | 8.18E-69 | 61.35 | P:regulation of macromolecule metabolic process; F:protein tyrosine phosphatase activity; C:intracellular part; P:peptidyl-tyrosine dephosphorylation; P:regulation of primary metabolic process; P:regulation of cellular metabolic process; C:membrane-bounded organelle; F:protein tyrosine/serine/threonine phosphatase activity |
| Efet.01.280661.g1121.t1 | hypothetical protein CAPTEDRAFT_187265 | 1482 | 5.50E-07 | 69 | F:nucleic acid binding; F:metal ion binding |
| Efet.01.28067.g199.t1 | hypothetical protein CAPTEDRAFT_225327 | 1245 | 1.79E-35 | 65.25 | C:membrane |
| Efet.01.280706.g1123.t1 | rho GTPase-activating 100F-like isoform X1 | 288 | 3.03E-07 | 60.5 | P:signal transduction |
| Efet.01.280752.g1124.t1 | vasopressin V1b receptor | 1065 | 2.77E-72 | 56 | P:regulation of biological quality; P:signal transduction; C:integral component of membrane; C:cell part; F:protein-hormone receptor activity; P:multicellular organismal process |
| Efet.01.280756.g1125.t1 | dnaJ homolog subfamily C member 9 | 687 | 2.66E-64 | 71.45 | C:nucleus; F:heat shock protein binding; C:extracellular space; C:cytoplasm; P:positive regulation of ATPase activity; C:plasma membrane |
| Efet.01.280876.g1126.t1 | tektin-3-like isoform X1 | 330 | 2.05E-13 | 78.16 |  |
| Efet.01.280891.g1127.t1 | Glypican-6 | 405 | 3.29E-42 | 71.8 | C:membrane |
| Efet.01.2809.g254.t1 | ETS domain-containing Elk-4-like isoform X2 | 1911 | 1.28E-08 | 62.15 | F:DNA binding; C:nucleus; F:transcription factor activity, sequence-specific DNA binding; P:regulation of transcription, DNA-templated; F:sequence-specific DNA binding |
| Efet.01.280918.g1128.t1 | Ankyrin repeat | 1077 | 3.54E-24 | 48.65 | C:membrane; C:integral component of membrane; P:intracellular signal transduction; C:intracellular |
| Efet.01.281035.g1131.t1 | mitogen-activated kinase kinase kinase 9 isoform X2 | 753 | 1.43E-06 | 50.33 | F:nucleotide binding; P:MAPK cascade; F:ATP binding; P:activation of MAPKK activity; P:protein autophosphorylation; F:protein kinase activity; F:MAP kinase kinase kinase activity; F:protein serine/threonine kinase activity; P:protein phosphorylation; C:intracellular |
| Efet.01.281065.g1133.t1 | SLIT-ROBO Rho GTPase-activating 1-like isoform X3 | 450 | 1.15E-57 | 67.7 | P:signal transduction |
| Efet.01.281074.g1134.t1 | hypothetical protein HELRODRAFT_162456 | 726 | 4.31E-20 | 64 | C:membrane; C:integral component of membrane |
| Efet.01.28109.g201.t1 | phosphatase 1 regulatory subunit 12A-like | 423 | 8.41E-47 | 67.25 |  |
| Efet.01.281370.g1138.t1 | potassium voltage-gated channel Shaw-like isoform X1 | 909 | 9.84E-122 | 83.05 | F:voltage-gated potassium channel activity; P:potassium ion transmembrane transport; F:metal ion binding; C:voltage-gated potassium channel complex; P:regulation of ion transmembrane transport; P:protein homooligomerization |
| Efet.01.281396.g1139.t1 | ankyrin repeat and SOCS box 12 | 555 | 8.92E-18 | 56.2 | P:intracellular signal transduction; C:intracellular; P:protein ubiquitination |
| Efet.01.28143.g205.t1 | Small conductance calcium-activated potassium channel | 507 | 1.37E-47 | 79 | F:calmodulin binding; P:potassium ion transmembrane transport; F:photoreceptor activity; C:integral component of membrane; F:small conductance calcium-activated potassium channel activity; C:neuronal cell body; P:regulation of membrane potential in photoreceptor cell; C:plasma membrane |
| Efet.01.281499.g1140.t1 | transient receptor potential cation channel subfamily V member 5-like | 1263 | 1.66E-156 | 50 | C:membrane; P:transport |
| Efet.01.281514.g1141.t1 | coiled-coil domain-containing 105-like | 387 | 3.67E-42 | 70.6 |  |
| Efet.01.281521.g1142.t1 | solute carrier organic anion transporter family member 4A1 isoform X2 | 600 | 7.12E-43 | 62.35 | C:integral component of plasma membrane; P:sodium-independent organic anion transport; F:thyroid hormone transmembrane transporter activity; F:sodium-independent organic anion transmembrane transporter activity; P:thyroid hormone transport |
| Efet.01.28159.g206.t1 | somatostatin receptor type 2-like | 861 | 2.12E-95 | 70 | F:G-protein coupled peptide receptor activity; P:G-protein coupled receptor signaling pathway; C:membrane |
| Efet.01.281594.g1143.t1 | ESF1 homolog | 513 | 2.69E-38 | 58.35 | F:nucleotide binding; C:nucleus; C:extracellular space; F:RNA binding |
| Efet.01.28160.g208.t1 | von Willebrand factor type EGF and pentraxin domain-containing 1 isoform X2 | 252 | 3.43E-12 | 59.05 | F:peptidase inhibitor activity; C:membrane; C:integral component of membrane; C:extracellular region; P:negative regulation of peptidase activity |
| Efet.01.281733.g1145.t1 | NACHT domain- and WD repeat-containing 1-like isoform X1 | 1077 | 5.94E-23 | 57.6 |  |
| Efet.01.281756.g1147.t1 | DNA topoisomerase 2-beta | 330 | 1.69E-52 | 86.85 | C:nucleoid; P:embryonic cleavage; P:sister chromatid segregation; F:drug binding; F:protein C-terminus binding; C:DNA topoisomerase complex (ATP-hydrolyzing); P:DNA ligation; F:DNA binding, bending; P:resolution of meiotic recombination intermediates; F:sequence-specific DNA binding; P:DNA topological change; P:mitotic DNA integrity checkpoint; C:nucleolus; P:regulation of circadian rhythm; P:positive regulation of transcription from RNA polymerase II promoter; P:hematopoietic progenitor cell differentiation; P:DNA unwinding involved in DNA replication; C:nucleoplasm; F:magnesium ion binding; F:ATP binding; P:neuron migration; P:forebrain development; F:ubiquitin binding; P:positive regulation of single stranded viral RNA replication via double stranded DNA intermediate; F:protein heterodimerization activity; P:axonogenesis; C:cytosol; P:apoptotic chromosome condensation; F:protein kinase C binding; F:protein homodimerization activity; F:histone deacetylase binding; C:nuclear chromosome; F:chromatin binding; P:cellular response to DNA damage stimulus; P:mitotic recombination; F:RNA binding; C:condensed chromosome; C:heterochromatin; P:rhythmic process; C:centriole; F:ribonucleoprotein complex binding; P:positive regulation of apoptotic process; F:DNA topoisomerase type II (ATP-hydrolyzing) activity |
| Efet.01.28183.g210.t1 | ras-like GTP-binding RHO | 336 | 1.57E-65 | 90.35 | F:GTP binding; P:wound healing, spreading of cells; P:small GTPase mediated signal transduction; P:skeletal muscle satellite cell migration; C:intracellular; C:plasma membrane |
| Efet.01.281832.g1148.t1 | 1-acyl-sn-glycerol-3-phosphate acyltransferase gamma isoform X1 | 465 | 4.49E-56 | 69.75 | F:transferase activity, transferring acyl groups; P:metabolic process; C:integral component of membrane |
| Efet.01.28192.g211.t1 | hypothetical protein HELRODRAFT_167757 | 945 | 4.32E-08 | 43 | C:membrane; C:integral component of membrane |
| Efet.01.281925.g1150.t1 | hypothetical protein CAPTEDRAFT_212892 | 237 | 1.08E-13 | 64.5 |  |
| Efet.01.281931.g1152.t1 | NACHT and WD repeat domain-containing 1 | 1170 | 2.36E-60 | 48.55 | F:nucleotide binding; F:ATP binding |
| Efet.01.281931.g1153.t1 | NACHT and WD repeat domain-containing 1 | 1425 | 2.16E-77 | 57.6 |  |
| Efet.01.281936.g1154.t1 | hypothetical protein CAPTEDRAFT_229337 | 576 | 2.51E-58 | 48.46 |  |
| Efet.01.282018.g1156.t1 | kinesin KIF17 | 654 | 3.37E-110 | 86.6 | F:ATP binding; P:cell projection organization; C:kinesin complex; P:neurogenesis; F:ATP-dependent microtubule motor activity, plus-end-directed; P:microtubule-based movement; C:microtubule; F:microtubule binding |
| Efet.01.282030.g1158.t1 | branched-chain amino acid ABC transporter substrate-binding | 453 | 4.51E-69 | 79.95 |  |
| Efet.01.282031.g1159.t1 | ABC transporter permease | 954 | 5.89E-177 | 93.7 | C:integral component of membrane; P:transport; C:plasma membrane; F:transporter activity |
| Efet.01.282031.g1160.t1 | ABC transporter permease | 2010 | 0 | 91.4 | C:integral component of membrane; P:transport; C:plasma membrane; F:transporter activity |
| Efet.01.282031.g1161.t1 | ABC transporter ATP-binding | 567 | 2.88E-125 | 89.15 | F:ATPase activity; F:ATP binding; F:transferase activity, transferring glycosyl groups |
| Efet.01.282031.g1162.t1 | 3-hydroxyacyl- dehydrogenase | 2055 | 0 | 87.8 | P:fatty acid beta-oxidation; F:3-hydroxyacyl-CoA dehydrogenase activity; F:enoyl-CoA hydratase activity |
| Efet.01.28205.g212.t1 | TGF-beta receptor type-1-like isoform X2 | 525 | 5.99E-41 | 82.2 | P:transforming growth factor beta receptor signaling pathway; P:cardiac muscle cell fate commitment; P:positive regulation of osteoblast differentiation; P:positive regulation of transcription from RNA polymerase II promoter; P:pathway-restricted SMAD protein phosphorylation; P:signal transduction by protein phosphorylation; F:transforming growth factor beta binding; P:activin receptor signaling pathway; F:ATP binding; P:smooth muscle cell differentiation; F:activin binding; C:apical part of cell; P:branching involved in blood vessel morphogenesis; P:in utero embryonic development; P:gastrulation with mouth forming second; P:BMP signaling pathway; P:negative regulation of activin receptor signaling pathway; P:G1/S transition of mitotic cell cycle; F:peptide hormone binding; P:mesoderm formation; P:positive regulation of determination of dorsal identity; P:positive regulation of bone mineralization; P:germ cell development; F:protein homodimerization activity; P:mitral valve morphogenesis; P:embryonic heart tube morphogenesis; P:pharyngeal system development; P:endocardial cushion cell fate commitment; F:transforming growth factor beta receptor activity, type I; P:atrial septum primum morphogenesis; P:determination of left/right symmetry; F:signal transducer, downstream of receptor, with serine/threonine kinase activity; F:SMAD binding; P:neural crest cell migration; F:metal ion binding; P:acute inflammatory response; C:activin receptor complex; P:cellular response to glucocorticoid stimulus; F:activin receptor activity, type I; P:negative regulation of extrinsic apoptotic signaling pathway; P:positive regulation of pathway-restricted SMAD protein phosphorylation; P:peptidyl-threonine phosphorylation; P:positive regulation of cell migration |
| Efet.01.282064.g1163.t1 | receptor-type guanylate cyclase daf-11 isoform X2 | 1455 | 4.14E-36 | 50.85 | F:nucleotide binding; P:cGMP biosynthetic process; F:phosphorus-oxygen lyase activity; C:membrane; C:integral component of membrane; F:lyase activity; F:guanylate cyclase activity; P:intracellular signal transduction; P:cyclic nucleotide biosynthetic process; C:intracellular |
| Efet.01.282080.g1164.t1 | Innexin unc-9 | 279 | 4.41E-19 | 65.55 | C:membrane; P:cell communication by electrical coupling; C:cell junction; C:synapse; F:gap junction channel activity; P:transport |
| Efet.01.28209.g213.t1 | hypothetical protein CAPTEDRAFT_201139 | 222 | 1.19E-07 | 82 |  |
| Efet.01.282098.g1165.t1 | muscarinic acetylcholine receptor M2 | 1734 | 2.84E-102 | 79.05 | C:integral component of plasma membrane; F:G-protein coupled acetylcholine receptor activity; P:G-protein coupled acetylcholine receptor signaling pathway; F:binding |
| Efet.01.282219.g1167.t1 | probable E3 ubiquitin- ligase HECTD4 | 1038 | 1.52E-93 | 68.1 | F:ubiquitin-protein transferase activity; F:ligase activity; P:protein ubiquitination |
| Efet.01.282230.g1168.t1 | fer3 | 441 | 7.77E-29 | 74.2 | F:protein dimerization activity |
| Efet.01.282308.g1169.t1 | oxidoreductase | 1101 | 0 | 75.4 | P:metabolic process |
| Efet.01.28231.g214.t1 | mediator of RNA polymerase II transcription subunit 7 | 561 | 3.65E-73 | 75.3 | C:mediator complex; F:RNA polymerase II transcription cofactor activity; P:regulation of transcription from RNA polymerase II promoter |
| Efet.01.28233.g215.t1 | hypoxia up-regulated 1-like isoform X1 | 861 | 4.75E-59 | 76.25 | F:ATP binding; C:membrane |
| Efet.01.28238.g216.t1 | ATP-binding cassette sub-family A member 3 | 330 | 5.97E-31 | 73.45 | F:nucleotide binding; P:lipid transport; F:ATPase activity, coupled to transmembrane movement of substances; C:intracellular membrane-bounded organelle; C:membrane |
| Efet.01.282394.g1172.t1 | zinc finger 239 isoform X3 | 1413 | 1.18E-67 | 56.2 | F:nucleic acid binding; P:biological_process; F:metal ion binding |
| Efet.01.282497.g1175.t1 | neuronal acetylcholine receptor subunit alpha-5-like isoform X1 | 507 | 6.39E-24 | 60.85 | P:ion transport; C:membrane |
| Efet.01.282511.g1177.t1 | potassium voltage-gated channel subfamily B member 1 | 327 | 2.22E-35 | 76.25 | F:voltage-gated potassium channel activity; P:potassium ion transmembrane transport; C:voltage-gated potassium channel complex; P:regulation of ion transmembrane transport; P:protein homooligomerization |
| Efet.01.28254.g217.t1 | ankyrin repeat | 1521 | 1.80E-36 | 45.05 | F:ADP binding; P:intracellular signal transduction; C:intracellular |
| Efet.01.282607.g1179.t1 | transmembrane 79-like | 435 | 9.00E-34 | 58.25 | C:membrane; C:integral component of membrane |
| Efet.01.282685.g1180.t1 | D(1C) dopamine receptor isoform X1 | 282 | 6.12E-08 | 69.1 | P:regulation of carbohydrate metabolic process; P:adenosine receptor signaling pathway; C:integral component of membrane; P:positive regulation of cAMP biosynthetic process; P:chemical synaptic transmission; P:sensory perception of pain; P:neurogenesis; F:G-protein coupled adenosine receptor activity; P:associative learning; P:negative regulation of calcium ion import |
| Efet.01.282713.g1181.t1 | hypothetical protein HELRODRAFT_165853 | 1251 | 3.24E-99 | 54.33 | C:membrane; C:integral component of membrane |
| Efet.01.282729.g1182.t1 | cGMP-inhibited 3 | 369 | 5.35E-52 | 83.85 | F:metal ion binding; P:signal transduction; F:3',5'-cyclic-nucleotide phosphodiesterase activity |
| Efet.01.282734.g1183.t1 | beta-lactamase 2-like | 810 | 6.78E-20 | 45.25 | C:membrane; C:integral component of membrane |
| Efet.01.282770.g1184.t1 | RYamide receptor-like | 261 | 2.03E-30 | 73.2 | F:G-protein coupled receptor activity; P:G-protein coupled receptor signaling pathway; C:membrane |
| Efet.01.282771.g1185.t1 | dual oxidase maturation factor 1-like | 630 | 3.43E-08 | 64.45 | C:membrane |
| Efet.01.282810.g1188.t1 | somatostatin receptor type 2-like | 441 | 7.86E-37 | 62.15 | F:neuropeptide receptor activity; P:G-protein coupled receptor signaling pathway; C:integral component of membrane |
| Efet.01.282912.g1189.t1 | neurobeachin-like isoform X2 | 225 | 9.30E-11 | 81.35 |  |
| Efet.01.282952.g1190.t1 | sulfhydryl oxidase 1-like | 687 | 9.66E-35 | 55.5 | F:organic cyclic compound binding; F:heterocyclic compound binding; C:cytoplasm; F:oxidoreductase activity; C:membrane; P:tRNA metabolic process; P:cellular amino acid metabolic process |
| Efet.01.28296.g220.t1 | von Willebrand factor A domain-containing 3B-like isoform X2 | 300 | 2.40E-24 | 79.1 |  |
| Efet.01.28296.g221.t1 | von Willebrand factor A domain-containing 3B-like | 471 | 5.89E-34 | 71.2 |  |
| Efet.01.282971.g1191.t1 | synaptotagmin 7a | 336 | 1.94E-47 | 81.7 | C:membrane; P:exocytosis |
| Efet.01.283023.g1193.t1 | alpha-2A adrenergic receptor | 1590 | 2.67E-96 | 78.4 | F:adrenergic receptor activity; C:integral component of membrane; P:adrenergic receptor signaling pathway; C:plasma membrane |
| Efet.01.283036.g1194.t1 | PREDICTED: uncharacterized protein LOC106181924 isoform X1 | 249 | 6.80E-15 | 65.4 |  |
| Efet.01.283038.g1195.t1 | E3 UFM1- ligase 1 | 273 | 5.10E-11 | 78.5 | P:negative regulation of NF-kappaB transcription factor activity; P:single-multicellular organism process; C:membrane; P:positive regulation of cell proliferation; P:cell differentiation; F:UFM1 transferase activity; P:regulation of cellular protein metabolic process; C:endoplasmic reticulum; P:response to stimulus; P:protein ufmylation |
| Efet.01.283047.g1197.t1 | hypothetical protein HELRODRAFT_159178 | 276 | 2.21E-25 | 72.5 | C:membrane; F:carbohydrate binding |
| Efet.01.283048.g1199.t1 | hypothetical protein HELRODRAFT_183479 | 1050 | 1.98E-16 | 77.33 | F:DNA binding; F:metal ion binding |
| Efet.01.283052.g1200.t1 | helicase with zinc finger domain 2-like | 3852 | 5.44E-56 | 49.3 | F:binding |
| Efet.01.283163.g1204.t1 | pygopus-like | 396 | 1.30E-46 | 89.15 | F:zinc ion binding |
| Efet.01.283182.g1205.t1 | serine arginine repetitive matrix 2 | 2904 | 6.53E-24 | 71.4 |  |
| Efet.01.283238.g1209.t1 | tubulin alpha-4 chain- | 225 | 5.98E-35 | 100 | F:GTP binding; C:cytoplasm; F:GTPase activity; F:structural constituent of cytoskeleton; C:microtubule; P:microtubule-based process |
| Efet.01.283238.g1210.t1 | tubulin alpha | 453 | 4.15E-95 | 100 | F:GTP binding; C:cytoplasm; F:GTPase activity; F:structural constituent of cytoskeleton; C:microtubule; P:microtubule-based process |
| Efet.01.283259.g1212.t1 | hypothetical protein CAPTEDRAFT_216159 | 618 | 6.21E-25 | 53.6 | C:membrane; C:integral component of membrane |
| Efet.01.283316.g1214.t1 | Kinesin heavy chain | 714 | 7.94E-107 | 86 | F:ATP binding; F:microtubule motor activity; C:cytoplasm; P:microtubule-based movement; C:microtubule; F:microtubule binding |
| Efet.01.283384.g1217.t1 | hypothetical protein CAPTEDRAFT_194734 | 279 | 3.05E-06 | 56.75 | C:membrane; C:integral component of membrane |
| Efet.01.283461.g1218.t1 | tripartite motif-containing 2-like isoform X3 | 2220 | 3.56E-132 | 53.3 | F:zinc ion binding; F:metal ion binding; C:intracellular |
| Efet.01.283523.g1219.t1 | Neuroligin- Y-linked | 435 | 4.27E-51 | 68.75 | C:membrane; C:integral component of membrane |
| Efet.01.28355.g223.t1 | E3 ubiquitin- ligase HUWE1-like | 588 | 3.77E-66 | 69.8 | C:nucleus; F:ubiquitin-protein transferase activity; C:cytoplasm; P:protein ubiquitination involved in ubiquitin-dependent protein catabolic process |
| Efet.01.283552.g1220.t1 | sodium-dependent glucose transporter 1C | 711 | 9.27E-25 | 44.3 | P:transmembrane transport; C:membrane; C:integral component of membrane |
| Efet.01.283563.g1221.t1 | hypothetical protein CAPTEDRAFT_201284, partial | 657 | 8.06E-16 | 44.71 | F:chitin binding; C:membrane; P:chitin metabolic process; C:integral component of membrane; C:extracellular region |
| Efet.01.283573.g1222.t1 | VPRBP isoform X1 | 462 | 1.13E-48 | 78.35 | C:nuclear part; P:single-organism cellular process; P:regulation of biological process; P:immune system development; P:protein ubiquitination |
| Efet.01.283583.g1223.t1 | Fatty acid hydroxylase domain-containing 2 | 375 | 4.16E-09 | 63.25 | F:iron ion binding; F:oxidoreductase activity; C:membrane; C:integral component of membrane; P:oxidation-reduction process; P:fatty acid biosynthetic process |
| Efet.01.283659.g1225.t1 | O-mannose kinase-like | 813 | 8.53E-79 | 65.4 | F:ATP binding; F:protein kinase activity; P:protein phosphorylation |
| Efet.01.283669.g1226.t1 | hypothetical protein HELRODRAFT_192374 | 561 | 4.86E-22 | 56 |  |
| Efet.01.283670.g1227.t1 | probable G- coupled receptor 139 isoform X1 | 1209 | 1.27E-132 | 60.45 | C:membrane |
| Efet.01.283687.g1229.t1 | Dual specificity tyrosine-phosphorylation-regulated kinase | 612 | 1.02E-33 | 80.65 | P:peptidyl-tyrosine phosphorylation; F:identical protein binding; P:negative regulation of DNA damage response, signal transduction by p53 class mediator; P:positive regulation of protein deacetylation; C:nuclear speck; P:nervous system development; P:protein autophosphorylation; F:protein serine/threonine kinase activity; P:circadian rhythm; F:protein self-association; F:ATP binding; F:protein serine/threonine/tyrosine kinase activity; C:intracellular ribonucleoprotein complex; F:non-membrane spanning protein tyrosine kinase activity; P:regulation of alternative mRNA splicing, via spliceosome; P:negative regulation of mRNA splicing, via spliceosome; F:tau protein binding; P:peptidyl-serine phosphorylation; P:peptidyl-threonine phosphorylation |
| Efet.01.283703.g1230.t1 | hypothetical protein CRN43_22640, partial | 417 | 1.31E-06 | 51.33 |  |
| Efet.01.2838.g255.t1 | follistatin-related 5-like | 459 | 8.35E-26 | 59.15 | F:calcium ion binding; C:membrane; P:protein glycosylation; F:galactosyltransferase activity |
| Efet.01.283861.g1234.t1 | dual serine threonine and tyrosine kinase-like | 498 | 2.98E-19 | 61.5 | F:nucleotide binding; F:protein kinase activity; P:phosphorylation |
| Efet.01.28387.g225.t1 | serine arginine-rich splicing factor 1A | 270 | 1.94E-28 | 79.1 | F:nucleotide binding; F:RNA binding |
| Efet.01.28395.g226.t1 | BMA-NMY- isoform c | 375 | 5.27E-49 | 79.8 | F:actin filament binding; C:myosin complex; F:ATP binding; F:pyridoxal phosphate binding; C:extracellular exosome; F:structural constituent of muscle; P:elastic fiber assembly; P:positive regulation of macromitophagy in response to mitochondrial depolarization; P:biosynthetic process; P:cardiac muscle fiber development; F:motor activity |
| Efet.01.283980.g1235.t1 | vesicle-fusing ATPase 1-like | 369 | 1.00E-56 | 86.75 | F:ATPase activity; F:ATP binding; P:Golgi vesicle docking; P:Golgi to plasma membrane protein transport; P:intra-Golgi vesicle-mediated transport; C:Golgi stack |
| Efet.01.2840.g256.t1 | phosphatase 1 regulatory subunit 3B-like isoform X2 | 1053 | 1.22E-64 | 63.15 | P:carbohydrate metabolic process; P:glycogen metabolic process |
| Efet.01.28400.g227.t1 | eukaryotic translation initiation factor 3 subunit L | 231 | 2.02E-13 | 81.9 | F:translation initiation factor activity; P:formation of translation preinitiation complex; C:eukaryotic 43S preinitiation complex; C:membrane; C:eukaryotic 48S preinitiation complex; P:regulation of translational initiation; C:fibrillar center; C:nucleoplasm; C:eukaryotic translation initiation factor 3 complex; F:RNA binding |
| Efet.01.28403.g228.t1 | acetolactate synthase | 207 | 2.01E-30 | 86.45 | F:magnesium ion binding; F:acetolactate synthase activity; F:thiamine pyrophosphate binding; F:benzoin aldolase activity |
| Efet.01.28403.g229.t1 | benzylsuccinyl- dehydrogenase | 1188 | 0 | 81.3 | F:acyl-CoA dehydrogenase activity; F:flavin adenine dinucleotide binding; P:oxidation-reduction process |
| Efet.01.28403.g230.t1 | family transcriptional regulator | 993 | 5.11E-132 | 80.1 | F:DNA binding; P:transcription, DNA-templated; P:regulation of transcription, DNA-templated |
| Efet.01.28403.g231.t1 | methionine repressor | 240 | 2.31E-49 | 90 |  |
| Efet.01.28403.g232.t1 | tripartite tricarboxylate transporter substrate binding | 1020 | 0 | 68.6 | C:outer membrane-bounded periplasmic space |
| Efet.01.28404.g233.t1 | SMG8 isoform X2 | 276 | 1.47E-12 | 73.1 | P:nuclear-transcribed mRNA catabolic process, nonsense-mediated decay |
| Efet.01.284049.g1237.t1 | potassium voltage-gated channel Shaw-like | 336 | 3.87E-08 | 65.45 | P:potassium ion transport; P:transmembrane transport; C:integral component of membrane; F:voltage-gated ion channel activity; F:potassium channel activity; P:cellular process |
| Efet.01.284072.g1238.t1 | Kinesin KIF11 | 291 | 3.00E-15 | 63.7 | C:microtubule cytoskeleton; F:microtubule motor activity; C:cytoskeletal part; P:single-organism cellular process; F:binding; P:microtubule-based process |
| Efet.01.284106.g1241.t1 | eukaryotic initiation factor 4A-III | 225 | 6.88E-44 | 99.3 | F:translation initiation factor activity; F:ATP binding; P:RNA splicing; C:catalytic step 2 spliceosome; P:regulation of gene expression; P:RNA secondary structure unwinding; P:translational initiation; F:ATP-dependent RNA helicase activity |
| Efet.01.284135.g1242.t1 | helicase SKI2W | 408 | 1.57E-19 | 56 | F:nucleic acid binding; F:ATP binding; C:nucleus; F:RNA binding; F:RNA helicase activity; C:Ski complex; F:helicase activity; C:nucleoplasm; F:hydrolase activity; P:RNA catabolic process |
| Efet.01.284175.g1243.t1 | copper oxidase | 714 | 2.34E-09 | 60 | F:copper ion binding; F:oxidoreductase activity; P:oxidation-reduction process |
| Efet.01.284186.g1244.t1 | Xylosyltransferase oxt | 492 | 3.50E-23 | 51.88 | P:kidney development; F:calcium ion binding; C:membrane; C:integral component of membrane |
| Efet.01.28426.g235.t1 | beta,beta-carotene 15,15 -dioxygenase-like | 1464 | 4.03E-116 | 56.2 | F:oxidoreductase activity, acting on single donors with incorporation of molecular oxygen, incorporation of two atoms of oxygen; C:membrane; C:integral component of membrane; P:oxidation-reduction process; F:monooxygenase activity |
| Efet.01.284287.g1245.t1 | serine threonine- kinase SIK3 isoform X1 | 381 | 6.30E-13 | 95.9 | C:nucleus; F:ATP binding; C:cytoplasm; F:protein serine/threonine kinase activity; P:protein phosphorylation; P:intracellular signal transduction |
| Efet.01.284289.g1246.t1 | cyclin-dependent kinase 17-like | 330 | 7.02E-30 | 53.1 | F:nucleotide binding; F:ATP binding; F:protein kinase activity; P:phosphorylation; F:protein serine/threonine kinase activity; P:protein phosphorylation; F:kinase activity |
| Efet.01.284319.g1247.t1 | hypothetical protein CAPTEDRAFT_207686 | 315 | 4.11E-12 | 62.4 | C:membrane |
| Efet.01.284368.g1248.t1 | Krueppel-like factor | 813 | 3.32E-47 | 90.2 | F:nucleic acid binding; F:metal ion binding |
| Efet.01.284585.g1251.t1 | DNA repair XRCC3 | 597 | 2.89E-15 | 75.9 | C:Rad51B-Rad51C-Rad51D-XRCC2 complex; F:DNA-dependent ATPase activity; P:meiotic DNA recombinase assembly; P:reciprocal meiotic recombination; F:double-stranded DNA binding; F:endodeoxyribonuclease activity; F:four-way junction DNA binding; P:strand invasion; P:mitotic recombination; F:single-stranded DNA binding; C:replication fork; F:recombinase activity; P:response to ionizing radiation |
| Efet.01.28462.g236.t1 | leucine-rich repeat and fibronectin type III domain-containing 1 | 1725 | 1.21E-167 | 55.1 | C:membrane; C:integral component of membrane |
| Efet.01.284662.g1253.t1 | Retinoic acid receptor RXR-alpha | 1191 | 9.10E-41 | 51 | F:binding |
| Efet.01.284677.g1254.t1 | membrane-associated guanylate WW and PDZ domain-containing 1-like isoform X1 | 267 | 5.36E-22 | 85.55 |  |
| Efet.01.28472.g238.t1 | protocadherin-9-like isoform X1 | 2676 | 1.39E-171 | 53.8 | F:calcium ion binding; C:membrane; C:integral component of membrane; P:cell adhesion; P:homophilic cell adhesion via plasma membrane adhesion molecules; C:plasma membrane |
| Efet.01.284784.g1255.t1 | spectrin beta chain isoform X1 | 387 | 5.02E-38 | 69.5 | F:binding |
| Efet.01.284823.g1258.t1 | T-cell leukemia homeobox 3 | 216 | 9.59E-20 | 84.8 | C:nucleus; P:regulation of transcription, DNA-templated; F:sequence-specific DNA binding |
| Efet.01.284847.g1259.t1 | aldo keto reductase | 912 | 7.14E-161 | 94.25 | F:oxidoreductase activity; P:oxidation-reduction process |
| Efet.01.284847.g1260.t1 | nucleotidyltransferase | 267 | 1.15E-39 | 96.55 | F:nucleotidyltransferase activity |
| Efet.01.284847.g1261.t1 | DUF86 domain-containing | 303 | 4.64E-65 | 96.1 | F:nucleotidyltransferase activity |
| Efet.01.284847.g1262.t1 | hypothetical protein | 396 | 8.69E-77 | 69.5 |  |
| Efet.01.284847.g1263.t1 | DNA invertase | 555 | 1.10E-96 | 99.85 | F:DNA binding; F:recombinase activity; P:DNA recombination |
| Efet.01.284847.g1264.t1 | type II toxin-antitoxin system family toxin | 822 | 1.66E-66 | 81.55 | F:transcription factor activity, sequence-specific DNA binding; P:regulation of cell growth; P:regulation of transcription, DNA-templated; F:toxin-antitoxin pair type II binding |
| Efet.01.284847.g1265.t1 | hypothetical protein | 327 | 3.12E-49 | 69 | C:membrane; C:integral component of membrane |
| Efet.01.284857.g1266.t1 | twist-related 2-like | 420 | 4.55E-49 | 75.4 | F:protein dimerization activity |
| Efet.01.284917.g1267.t1 | 14-3-3 zeta | 381 | 3.26E-49 | 86.95 | F:protein domain specific binding |
| Efet.01.28494.g239.t1 | cleavage stimulation factor subunit 3-like | 363 | 3.90E-28 | 71.9 | C:nucleus; P:mRNA processing |
| Efet.01.284982.g1268.t1 | O- case | 519 | 7.02E-11 | 44.25 |  |
| Efet.01.285003.g1269.t1 | Conserved oligomeric Golgi complex subunit 1 | 468 | 6.60E-21 | 56.5 | C:Golgi transport complex; P:intra-Golgi vesicle-mediated transport |
| Efet.01.285074.g1271.t1 | dimethylaniline monooxygenase [N-oxide-forming] 4 | 324 | 1.49E-21 | 69.4 | F:nucleotide binding; F:coenzyme binding; P:drug metabolic process; C:endoplasmic reticulum; P:oxidation-reduction process; C:organelle part; F:anion binding; C:membrane part; F:N,N-dimethylaniline monooxygenase activity |
| Efet.01.285095.g1272.t1 | FMRFamide receptor-like | 981 | 9.56E-74 | 53.7 | F:G-protein coupled receptor activity; F:G-protein coupled peptide receptor activity; P:G-protein coupled receptor signaling pathway; C:membrane; C:integral component of membrane |
| Efet.01.28516.g240.t1 | glutamate receptor NMDA 2B isoform X4 | 654 | 5.06E-09 | 50.2 | F:ion channel activity; C:membrane; F:receptor activity; P:transport |
| Efet.01.285205.g1276.t1 | ankyrin and armadillo repeat-containing -like | 576 | 6.93E-62 | 72.3 | C:nucleus; C:cytoplasm |
| Efet.01.28521.g241.t1 | NDNF | 1749 | 2.16E-81 | 50.15 | P:regulation of cellular process; F:glycosaminoglycan binding; P:angiogenesis; P:single-organism cellular process; C:extracellular region; P:positive regulation of biological process; P:response to stress |
| Efet.01.285214.g1277.t1 | AP-4 complex subunit epsilon-1-like isoform X2 | 252 | 3.20E-08 | 55.22 | C:membrane coat; P:vesicle-mediated transport; C:membrane; P:protein transport; P:intracellular protein transport; P:transport |
| Efet.01.285241.g1280.t1 | Sex muscle abnormal 5 | 264 | 5.57E-13 | 64.95 |  |
| Efet.01.285291.g1282.t1 | hypothetical protein CAPTEDRAFT_227150, partial | 1278 | 1.71E-81 | 53.8 | C:membrane; C:integral component of membrane |
| Efet.01.285291.g1283.t1 | hypothetical protein CAPTEDRAFT_227150, partial | 378 | 1.06E-16 | 66 | C:membrane; C:integral component of membrane |
| Efet.01.285291.g1284.t1 | hypothetical protein CAPTEDRAFT_227150, partial | 4053 | 0 | 45.75 | C:membrane; C:integral component of membrane |
| Efet.01.285433.g1286.t1 | neuroglian-like | 519 | 9.25E-32 | 53.85 | C:membrane; C:integral component of membrane |
| Efet.01.28547.g242.t1 | mitochondrial calcium uniporter regulator 1-like | 222 | 8.02E-35 | 69.3 | C:integral component of membrane; P:single-organism process; C:intracellular; P:mitochondrial calcium ion transport |
| Efet.01.285473.g1287.t1 | IQ and ubiquitin-like domain-containing | 999 | 4.80E-23 | 62.4 | C:motile cilium; P:cilium assembly; C:acrosomal vesicle; P:smoothened signaling pathway |
| Efet.01.285534.g1288.t1 | WASH complex subunit FAM21-like isoform X2 | 336 | 1.92E-06 | 60 |  |
| Efet.01.285707.g1291.t1 | sodium channel para isoform X6 | 303 | 1.73E-21 | 69.95 | P:regulation of postsynaptic membrane potential; F:calcium ion binding; P:regulation of ion transmembrane transport; C:voltage-gated sodium channel complex; P:sodium ion transmembrane transport; P:membrane depolarization during action potential; F:voltage-gated sodium channel activity; P:neuronal action potential |
| Efet.01.285725.g1292.t1 | shaker cognate | 543 | 1.97E-78 | 92 | F:voltage-gated potassium channel activity; P:potassium ion transmembrane transport; C:voltage-gated potassium channel complex; P:regulation of ion transmembrane transport; P:protein homooligomerization |
| Efet.01.285731.g1293.t1 | neuropeptide CCHamide-1 receptor-like | 849 | 2.11E-62 | 67.55 | F:G-protein coupled peptide receptor activity; P:G-protein coupled receptor signaling pathway; C:integral component of membrane |
| Efet.01.285802.g1294.t1 | Glyco 3-alpha-L-fucosyltransferase A | 264 | 1.78E-16 | 72.4 | P:glycosylation; F:transferase activity, transferring glycosyl groups; C:membrane; C:Golgi apparatus |
| Efet.01.285862.g1296.t1 | deleted in autism 1 | 1242 | 1.47E-119 | 57.2 | C:extracellular space; P:regulation of phosphatidylinositol 3-kinase signaling; C:Golgi membrane; P:cardiac muscle cell proliferation; C:COPI vesicle coat |
| Efet.01.285894.g1297.t1 | mitogen-activated kinase kinase kinase 11-like | 825 | 1.53E-106 | 77.3 | F:ATP binding; F:protein serine/threonine kinase activity; P:protein phosphorylation |
| Efet.01.2859.g257.t1 | PREDICTED: uncharacterized protein C45G9.7-like | 387 | 5.00E-42 | 57.7 | P:regulation of Wnt signaling pathway; P:Rho protein signal transduction; C:intracellular |
| Efet.01.28594.g244.t1 | TAPT1 homolog | 423 | 3.70E-11 | 63.2 | C:integral component of membrane |
| Efet.01.28595.g245.t1 | centrosomal of 192 kDa | 381 | 1.41E-22 | 61.45 |  |
| Efet.01.285950.g1299.t1 | Cyclin-dependent kinase 12 | 810 | 1.92E-27 | 53.3 | F:nucleotide binding; P:cell division; F:ATP binding; F:protein kinase activity; P:phosphorylation; P:protein phosphorylation; F:kinase activity |
| Efet.01.285973.g1301.t1 | tektin-3-like isoform X1 | 504 | 8.54E-51 | 60.55 |  |
| Efet.01.285982.g1302.t1 | transmembrane 249-like | 447 | 1.69E-14 | 50 | C:membrane; C:integral component of membrane |
| Efet.01.286014.g1303.t1 | chromatin assembly factor 1 subunit A-like | 1761 | 5.05E-40 | 49.25 | F:nucleic acid binding; F:DNA binding |
| Efet.01.28602.g247.t1 | Vacuolar sorting-associated 13A | 885 | 1.59E-30 | 52.15 | C:membrane; C:integral component of membrane |
| Efet.01.286071.g1304.t1 | glycine receptor subunit alpha-2-like | 204 | 1.30E-08 | 75.3 | P:synaptic transmission, glycinergic; P:neuropeptide signaling pathway; F:extracellular-glycine-gated chloride channel activity; P:chloride transmembrane transport; C:integral component of membrane; P:response to amino acid; C:cell junction; F:glycine binding; C:synapse; C:plasma membrane |
| Efet.01.286082.g1305.t1 | Transcription factor SOX- | 216 | 2.63E-15 | 81.9 | C:nucleus; F:DNA binding; F:transcription factor activity, sequence-specific DNA binding; P:regulation of transcription, DNA-templated; P:sex determination; P:neuron development |
| Efet.01.286185.g1308.t1 | AP-3 complex subunit delta-1-like | 276 | 4.37E-21 | 76.75 | P:vesicle-mediated transport; C:AP-3 adaptor complex; P:intracellular protein transport |
| Efet.01.286207.g1310.t1 | filamin-A isoform X1 | 651 | 1.35E-86 | 81.2 | F:actin filament binding |
| Efet.01.286235.g1312.t1 | hypothetical protein CAPTEDRAFT_215767 | 633 | 9.93E-60 | 63.67 |  |
| Efet.01.28632.g248.t1 | hypothetical protein CAPTEDRAFT_223635 | 1041 | 9.32E-27 | 42.5 | P:sensory perception of taste; F:taste receptor activity; C:membrane; C:integral component of membrane; P:detection of chemical stimulus involved in sensory perception of taste |
| Efet.01.286362.g1316.t1 | SKI SNO DAC family | 477 | 2.11E-45 | 79.95 | C:nucleus; C:transcription factor complex; F:transcription factor activity, RNA polymerase II core promoter sequence-specific binding involved in preinitiation complex assembly; P:multicellular organism development; P:regulation of transcription, DNA-templated |
| Efet.01.286375.g1317.t1 | leucine-rich repeat-containing 15-like | 1032 | 3.00E-58 | 52.65 | P:axonogenesis; C:membrane; C:integral component of membrane |
| Efet.01.286397.g1318.t1 | Beta-1,3-galactosyltransferase 1 | 1059 | 4.49E-83 | 53.5 | C:membrane; F:transferase activity |
| Efet.01.28650.g249.t1 | pancreas transcription factor 1 subunit alpha-like | 696 | 2.59E-33 | 82.8 | F:protein dimerization activity |
| Efet.01.286501.g1321.t1 | FMRFamide receptor-like | 1242 | 2.12E-118 | 56.55 | F:G-protein coupled receptor activity; C:membrane |
| Efet.01.286537.g1322.t1 | dynein heavy chain axonemal-like isoform X1 | 1392 | 0 | 87.3 | F:ATPase activity; F:ATP binding; F:microtubule motor activity; C:dynein complex; P:microtubule-based movement |
| Efet.01.286564.g1323.t1 | hypothetical protein CAPTEDRAFT_221435 | 552 | 4.18E-32 | 53.64 | C:membrane; C:integral component of membrane |
| Efet.01.28658.g250.t1 | transmembrane 9 superfamily member 4-like | 303 | 4.28E-16 | 92.9 | C:early endosome; P:regulation of intracellular pH; P:vacuolar proton-transporting V-type ATPase complex assembly; P:response to hypoxia; P:positive regulation of protein localization to cell surface; C:integral component of membrane; P:phagocytosis; P:positive regulation of protein exit from endoplasmic reticulum; C:Golgi apparatus; P:cell adhesion |
| Efet.01.286600.g1324.t1 | Alpha- sarcomeric | 429 | 2.14E-39 | 69.25 | F:actin binding; P:cellular component assembly; P:actin filament organization |
| Efet.01.286622.g1325.t1 | acid trehalase 1 | 636 | 1.57E-26 | 48.05 | F:G-protein coupled receptor activity; P:carbohydrate metabolic process; P:G-protein coupled receptor signaling pathway; C:integral component of membrane; F:hydrolase activity, hydrolyzing O-glycosyl compounds |
| Efet.01.28663.g251.t1 | ankyrin repeat domain-containing 12-like isoform X1 | 2463 | 8.60E-28 | 57.05 |  |
| Efet.01.286635.g1326.t1 | prolow-density lipo receptor-related 1-like | 648 | 2.97E-77 | 69.35 | C:membrane |
| Efet.01.286637.g1327.t1 | probable imidazolonepropionase | 675 | 3.05E-77 | 78.4 | F:hydrolase activity, acting on carbon-nitrogen (but not peptide) bonds, in cyclic amides; C:cytoplasm; P:histidine catabolic process to glutamate and formamide |
| Efet.01.28664.g252.t1 | allatostatin-A receptor | 1173 | 9.66E-63 | 56.7 | F:neuropeptide binding; P:signal transduction; C:membrane; F:hormone binding; F:signal transducer activity |
| Efet.01.286662.g1329.t1 | opioid-binding cell adhesion molecule homolog | 225 | 5.16E-17 | 70.5 | P:peptidyl-tyrosine phosphorylation; F:transmembrane receptor protein tyrosine kinase activity; P:transmembrane receptor protein tyrosine kinase signaling pathway |
| Efet.01.286667.g1330.t1 | vacuolar sorting-associated 13B-like | 702 | 6.07E-82 | 79.3 |  |
| Efet.01.286669.g1331.t1 | acetylcholinesterase | 285 | 8.22E-07 | 57.25 | F:methyl indole-3-acetate esterase activity; F:methyl salicylate esterase activity; F:Ser-tRNA(Thr) hydrolase activity; P:acetylcholine catabolic process; F:methyl jasmonate esterase activity; F:endosulfan lactone lactonase activity; F:Cys-tRNA(Pro) hydrolase activity; F:L-ascorbate 6-phosphate lactonase activity; F:Ala-tRNA(Pro) hydrolase activity; P:neurotransmitter catabolic process; C:synapse; F:acetylcholinesterase activity; C:extracellular region; F:cholinesterase activity; F:phospholipase activity; P:neuromuscular junction development; P:response to ethanol; P:response to glucose; F:retinyl-palmitate esterase activity; F:all-trans-retinyl-palmitate hydrolase, all-trans-retinol forming activity; F:Ser(Gly)-tRNA(Ala) hydrolase activity; C:neuromuscular junction; F:lipase activity; F:sulfolactone hydrolase activity; F:1-oxa-2-oxocycloheptane lactonase activity; F:butyrolactone hydrolase activity; F:3,4-dihydrocoumarin hydrolase activity; F:prenylcysteine methylesterase activity; P:neuron development; C:plasma membrane; P:myofibril assembly; C:extracellular space; P:response to amphetamine; C:membrane; P:regulation of synapse structure or activity; C:cell junction; F:mannosyl-oligosaccharide 1,3-alpha-mannosidase activity; F:carboxylic ester hydrolase activity; F:hydrolase activity; P:cellular response to methanol; F:mannosyl-oligosaccharide 1,6-alpha-mannosidase activity |
| Efet.01.286720.g1333.t1 | mediator of RNA polymerase II transcription subunit 13-like isoform X2 | 288 | 5.12E-28 | 75.5 | C:mediator complex; F:RNA polymerase II transcription cofactor activity; P:regulation of transcription from RNA polymerase II promoter |
| Efet.01.286820.g1336.t1 | serine threonine- kinase PAK 1-like isoform X3 | 729 | 2.83E-128 | 83.15 | F:ATP binding; P:actin filament organization; F:protein serine/threonine kinase activity; P:protein phosphorylation |
| Efet.01.28683.g253.t1 | protein-glutamate O-methyltransferase-like isoform X2 | 1266 | 4.58E-141 | 63.05 | F:tRNA (cytosine) methyltransferase activity; F:N-methyltransferase activity; F:C-methyltransferase activity; F:protein carboxyl O-methyltransferase activity; F:rRNA (cytosine) methyltransferase activity; P:methylation |
| Efet.01.286840.g1337.t1 | rap guanine nucleotide exchange factor 1-like isoform X3 | 630 | 1.33E-36 | 67.6 | P:biological regulation |
| Efet.01.286920.g1340.t1 | probable G- coupled receptor 139 isoform X1 | 1005 | 3.38E-50 | 53.75 | F:G-protein coupled receptor activity; P:G-protein coupled receptor signaling pathway; C:membrane; C:integral component of membrane |
| Efet.01.286960.g1341.t1 | cephalotocin receptor 1 | 804 | 5.73E-36 | 51 | F:G-protein coupled receptor activity; C:membrane |
| Efet.01.28708.g254.t1 | translocation SEC63 homolog | 375 | 1.67E-21 | 80.1 | P:SRP-dependent cotranslational protein targeting to membrane; P:nitrogen compound metabolic process; P:liver development; P:renal system development; C:integral component of membrane; P:multicellular organism aging; C:intracellular; P:posttranslational protein targeting to membrane; F:RNA binding |
| Efet.01.28713.g256.t1 | desumoylating isopeptidase 2-like | 339 | 3.61E-42 | 82.55 |  |
| Efet.01.287139.g1342.t1 | DNA-binding RFX2-like isoform X1 | 423 | 5.95E-65 | 88.25 | F:DNA binding; P:regulation of transcription, DNA-templated |
| Efet.01.287150.g1343.t1 | sterile alpha motif domain-containing 9-like | 1668 | 1.70E-108 | 52.3 |  |
| Efet.01.287175.g1344.t1 | BTB POZ domain-containing KCTD16 | 684 | 4.31E-117 | 78.3 | P:protein homooligomerization |
| Efet.01.287196.g1345.t1 | helicase-like transcription factor | 333 | 3.22E-39 | 82.75 | F:nucleic acid binding; F:ATP binding; F:zinc ion binding; P:cellular macromolecule metabolic process; F:helicase activity; P:primary metabolic process |
| Efet.01.28727.g259.t1 | gamma-glutamyltransferase isoform X5 | 408 | 3.71E-45 | 73.35 | P:glutathione metabolic process; F:gamma-glutamyltransferase activity |
| Efet.01.28728.g260.t1 | transcription factor AP-1 | 891 | 2.15E-40 | 51.75 | P:positive regulation of RNA biosynthetic process; P:response to organic substance; P:signal transduction; F:transcription regulatory region DNA binding; P:regulation of transcription, DNA-templated; P:regulation of cell proliferation; P:modulation by host of viral transcription; P:negative regulation of RNA biosynthetic process; C:nucleus; P:negative regulation of gene expression; F:protein binding; P:system development; P:positive regulation of gene expression; P:regulation of cellular component organization |
| Efet.01.287321.g1348.t1 | probable methyltransferase BMT2 homolog | 282 | 1.92E-14 | 66.6 | F:methyltransferase activity |
| Efet.01.287321.g1349.t1 | probable methyltransferase BTM2 homolog | 477 | 4.85E-10 | 68.42 | F:methyltransferase activity; P:methylation |
| Efet.01.287326.g1350.t1 | histone -like isoform X2 | 810 | 1.33E-16 | 83.3 | F:zinc ion binding; C:integral component of membrane |
| Efet.01.287350.g1351.t1 | mRNA-capping enzyme | 381 | 6.73E-34 | 66.3 | P:cellular macromolecule metabolic process; F:phosphatase activity; P:dephosphorylation; P:nucleobase-containing compound metabolic process |
| Efet.01.287358.g1352.t1 | hypothetical protein CAPTEDRAFT_195684 | 672 | 1.80E-49 | 59 | C:membrane; C:integral component of membrane |
| Efet.01.287395.g1355.t1 | tetratricopeptide repeat 17-like isoform X1 | 231 | 3.77E-23 | 81.7 |  |
| Efet.01.287459.g1356.t1 | mediator of RNA polymerase II transcription subunit 14-like | 468 | 1.53E-37 | 60 | C:mediator complex; F:RNA polymerase II transcription cofactor activity; P:regulation of transcription from RNA polymerase II promoter |
| Efet.01.287479.g1358.t1 | inactive serine threonine- kinase TEX14-like isoform X2 | 450 | 3.43E-09 | 47.5 | F:ATP binding; F:ARF guanyl-nucleotide exchange factor activity; P:regulation of ARF protein signal transduction; F:protein kinase activity; P:protein phosphorylation; P:positive regulation of GTPase activity |
| Efet.01.287526.g1361.t1 | D-aminoacylase | 1086 | 0 | 97.1 | F:dihydroorotase activity; F:N-acyl-D-amino-acid deacylase activity |
| Efet.01.287526.g1362.t1 | amino acid ABC transporter ATP-binding | 642 | 3.23E-133 | 88.75 | F:ATP binding; P:amino acid transmembrane transport; F:amino acid-transporting ATPase activity |
| Efet.01.287526.g1363.t1 | ABC transporter substrate-binding | 828 | 0 | 84.7 | C:membrane; P:ionotropic glutamate receptor signaling pathway; F:ionotropic glutamate receptor activity |
| Efet.01.287526.g1364.t1 | amino acid ABC transporter permease | 267 | 4.56E-53 | 90.1 | C:integral component of membrane; P:transport; C:plasma membrane; F:transporter activity |
| Efet.01.287526.g1365.t1 | amino acid ABC transporter permease | 606 | 1.51E-132 | 92.8 | C:integral component of membrane; P:transport; C:plasma membrane; F:transporter activity |
| Efet.01.287526.g1366.t1 | family transcriptional regulator | 597 | 1.58E-84 | 82 | F:DNA binding; F:transcription factor activity, sequence-specific DNA binding; P:transcription, DNA-templated; P:regulation of transcription, DNA-templated |
| Efet.01.287526.g1367.t1 | toxin | 384 | 9.64E-46 | 54.8 | F:ATP binding; P:phosphorylation; F:kinase activity |
| Efet.01.287526.g1368.t1 | glutamate--tRNA ligase | 1299 | 0 | 93.65 | F:ATP binding; C:cytoplasm; F:glutamate-tRNA ligase activity; F:tRNA binding; P:glutamyl-tRNA aminoacylation |
| Efet.01.287526.g1369.t1 | amidophosphoribosyltransferase | 1398 | 0 | 94.85 | F:magnesium ion binding; P:'de novo' IMP biosynthetic process; P:glutamine metabolic process; P:nucleoside metabolic process; F:amidophosphoribosyltransferase activity; P:purine nucleobase biosynthetic process |
| Efet.01.287526.g1370.t1 | colicin V synthesis | 444 | 4.01E-70 | 87.9 | C:integral component of membrane; P:toxin biosynthetic process |
| Efet.01.287526.g1371.t1 | sporulation | 681 | 2.45E-68 | 88.65 | P:cell division; F:peptidoglycan binding; C:integral component of membrane |
| Efet.01.287526.g1372.t1 | bifunctional folylpolyglutamate synthase dihydrofolate synthase | 1254 | 0 | 91.35 | F:tetrahydrofolylpolyglutamate synthase activity; F:ATP binding; P:tetrahydrofolylpolyglutamate biosynthetic process |
| Efet.01.287526.g1373.t1 | arsenate reductase | 408 | 2.71E-94 | 81.65 |  |
| Efet.01.287526.g1374.t1 | glycine zipper 2TM domain-containing | 645 | 1.03E-104 | 85.7 | C:outer membrane |
| Efet.01.28754.g261.t1 | ELM2 and SANT domain-containing 1-like | 1812 | 8.61E-06 | 72 |  |
| Efet.01.287664.g1376.t1 | CAP-Gly domain-containing linker 1-like isoform X1 | 435 | 1.77E-07 | 55 |  |
| Efet.01.287726.g1379.t1 | headcase isoform X1 | 921 | 1.86E-58 | 66.05 |  |
| Efet.01.28804.g263.t1 | gamma-aminobutyric acid type B receptor subunit 2-like | 378 | 7.56E-27 | 75.45 | F:G-protein coupled GABA receptor activity; P:G-protein coupled receptor signaling pathway; C:integral component of membrane |
| Efet.01.288047.g1383.t1 | piggyBac transposable element-derived 4-like | 468 | 1.75E-31 | 54.27 | C:membrane; C:integral component of membrane |
| Efet.01.28809.g264.t1 | nuclear receptor coactivator 5-like | 717 | 3.14E-10 | 59 | F:nucleotide binding; F:nucleic acid binding |
| Efet.01.288169.g1387.t1 | Tubulin alpha-3 | 294 | 3.01E-53 | 96.9 | F:GTP binding; C:cytoplasm; F:GTPase activity; F:structural constituent of cytoskeleton; C:microtubule; P:microtubule-based process |
| Efet.01.2882.g259.t1 | N-terminal Xaa-Pro-Lys N-methyltransferase 1 | 645 | 4.17E-61 | 64.2 | P:N-terminal protein amino acid methylation; F:methyltransferase activity; F:transferase activity; P:methylation |
| Efet.01.288255.g1390.t1 | stabilizer of axonemal microtubules 2-like isoform X4 | 435 | 2.36E-37 | 58.05 | F:microtubule binding |
| Efet.01.288262.g1391.t1 | MAGUK p55 subfamily member | 573 | 2.32E-10 | 51.33 | P:bicellular tight junction assembly; P:establishment of epithelial cell polarity |
| Efet.01.288284.g1392.t1 | phospholipase A-2-activating | 939 | 1.15E-90 | 69.4 | C:nucleus; P:inflammatory response; P:proteasome-mediated ubiquitin-dependent protein catabolic process; C:extracellular exosome; F:ubiquitin binding; C:cytoplasm; P:ubiquitin homeostasis; F:phospholipase A2 activator activity |
| Efet.01.288353.g1395.t1 | glycogen [starch] synthase | 477 | 9.29E-37 | 86.1 | P:glycogen biosynthetic process; F:glycogen (starch) synthase activity |
| Efet.01.28842.g265.t1 | FLNC_HUMAN ame: Full=Filamin-C Short=FLN-C Short=FLNc ame: Full=ABP-280 ame: Full=ABP-L ame: Full=Actin-binding ame: Full=Filamin-2 ame: Full=Gamma-filamin | 1365 | 1.93E-06 | 59.8 | P:wound healing, spreading of cells; C:sarcoplasm; P:cell projection organization; C:growth cone; P:actin crosslink formation; F:G-protein coupled receptor binding; P:actin filament-based process; C:brush border; C:sarcolemma; C:dendritic shaft; F:Rac GTPase binding; F:glycoprotein binding; P:positive regulation of integrin-mediated signaling pathway; F:Fc-gamma receptor I complex binding; P:mRNA transcription from RNA polymerase II promoter; F:protein kinase C binding; C:protein complex; P:receptor clustering; P:formation of radial glial scaffolds; P:positive regulation of substrate adhesion-dependent cell spreading; C:actin filament; F:ion channel binding; C:plasma membrane; F:actin filament binding; C:Z disc; C:nucleus; C:cell-cell junction; F:protein binding; C:cell-cell adherens junction; P:early endosome to late endosome transport; C:actin filament bundle; P:epithelial to mesenchymal transition; P:regulation of membrane repolarization during cardiac muscle cell action potential; P:semaphorin-plexin signaling pathway; P:adenylate cyclase-inhibiting dopamine receptor signaling pathway; P:positive regulation of I-kappaB kinase/NF-kappaB signaling; C:extracellular region; C:nucleolus; P:platelet degranulation; F:Ral GTPase binding; C:extracellular exosome; P:angiogenesis; F:GTPase binding; F:Rho GTPase binding; P:cell junction assembly; F:kinase binding; P:negative regulation of transcription from RNA polymerase I promoter; P:positive regulation of neuron migration; F:mu-type opioid receptor binding; C:cytoskeleton; C:cytoplasm; P:protein localization to cell surface; P:positive regulation of potassium ion transmembrane transport; P:negative regulation of protein catabolic process; C:neuronal cell body; C:apical dendrite; P:heart morphogenesis; F:ankyrin binding; P:negative regulation of neuron projection development; C:actin cytoskeleton; C:cytosol; F:protein complex binding; P:platelet aggregation; C:cortical cytoskeleton; P:cilium assembly; C:costamere; P:negative regulation of sequence-specific DNA binding transcription factor activity; F:signal transducer activity; P:cell-cell junction organization; P:establishment of protein localization; F:actin binding; F:small GTPase binding; P:regulation of cell migration; C:filamentous actin; P:protein localization to plasma membrane; P:mitotic spindle assembly; P:regulation of membrane repolarization during atrial cardiac muscle cell action potential; P:regulation of actin filament bundle assembly; P:positive regulation of transcription factor import into nucleus; C:trans-Golgi network; C:focal adhesion; P:cytoplasmic sequestering of protein; P:positive regulation of neural precursor cell proliferation; C:extracellular matrix; P:platelet activation; P:actin cytoskeleton reorganization; F:protein homodimerization activity; F:transcription factor binding; P:muscle fiber development; F:RNA binding; F:cadherin binding involved in cell-cell adhesion; C:perinuclear region of cytoplasm; P:blood vessel remodeling; F:SMAD binding; P:negative regulation of apoptotic process; C:cell cortex; C:membrane; F:cytoskeletal protein binding; C:Myb complex; P:protein stabilization; P:actin cytoskeleton organization; F:potassium channel regulator activity |
| Efet.01.28856.g266.t1 | hypothetical protein CAPTEDRAFT_212628 | 447 | 4.36E-07 | 55.67 | F:G-protein coupled receptor activity; P:G-protein coupled receptor signaling pathway; C:membrane; C:integral component of membrane |
| Efet.01.288638.g1400.t1 | SNF-related serine threonine- kinase-like | 579 | 9.20E-119 | 93.35 | C:nucleus; F:ATP binding; F:rhodopsin kinase activity; C:cytoplasm; F:protein serine/threonine kinase activity; P:protein phosphorylation; P:intracellular signal transduction |
| Efet.01.28865.g267.t1 | hypothetical protein HELRODRAFT_65821 | 231 | 3.28E-16 | 81.5 |  |
| Efet.01.288652.g1401.t1 | basigin-like isoform X1 | 513 | 1.84E-27 | 61 | P:peptidyl-tyrosine phosphorylation; F:transmembrane receptor protein tyrosine kinase activity; C:membrane; C:integral component of membrane; P:transmembrane receptor protein tyrosine kinase signaling pathway |
| Efet.01.288672.g1402.t1 | pellino isoform X2 | 906 | 2.67E-87 | 75.1 | P:Toll signaling pathway; F:ubiquitin-protein transferase activity; P:response to organic substance; P:regulation of macromolecule metabolic process; P:positive regulation of B cell activation; P:positive regulation of toll-like receptor signaling pathway; P:regulation of lymphocyte proliferation; P:protein polyubiquitination; P:negative regulation of T cell activation; P:regulation of primary metabolic process; P:regulation of cellular metabolic process |
| Efet.01.288723.g1404.t1 | glutamine--fructose-6-phosphate aminotransferase [isomerizing] 2- | 318 | 1.47E-44 | 86.7 | P:carbohydrate metabolic process; F:glutamine-fructose-6-phosphate transaminase (isomerizing) activity; P:carbohydrate derivative biosynthetic process; F:carbohydrate binding |
| Efet.01.288788.g1405.t1 | ATP-binding cassette sub-family A member 3-like | 423 | 4.29E-13 | 58 | C:cell part; P:transport |
| Efet.01.288795.g1407.t1 | PREDICTED: uncharacterized protein LOC106961309 isoform X3 | 762 | 2.27E-09 | 38 |  |
| Efet.01.288799.g1408.t1 | voltage-gated sodium channel alpha subunit | 297 | 2.69E-31 | 92.1 | P:regulation of postsynaptic membrane potential; P:regulation of ion transmembrane transport; C:voltage-gated sodium channel complex; P:sensory perception of pain; P:sodium ion transmembrane transport; F:voltage-gated sodium channel activity |
| Efet.01.288799.g1409.t1 | Sodium channel para | 534 | 2.14E-54 | 56.35 | F:ion channel activity; P:ion transport; P:transmembrane transport; C:membrane |
| Efet.01.288803.g1410.t1 | tyrosine- kinase transmembrane receptor Ror-like | 336 | 1.66E-21 | 64.55 | F:protein kinase activity; P:phosphorylation |
| Efet.01.288824.g1412.t1 | serine threonine- phosphatase 6 regulatory ankyrin repeat subunit A-like | 294 | 6.94E-37 | 83.05 | F:calcium channel activity; C:integral component of membrane; P:calcium ion transmembrane transport |
| Efet.01.288863.g1413.t1 | probable G- coupled receptor No18 | 1041 | 8.98E-36 | 66.95 | C:integral component of plasma membrane; F:dopamine neurotransmitter receptor activity; P:dopamine receptor signaling pathway |
| Efet.01.288887.g1414.t1 | octopamine receptor 1-like | 1209 | 3.71E-39 | 77.65 | C:integral component of plasma membrane; F:adrenergic receptor activity; P:adrenergic receptor signaling pathway; F:G-protein coupled acetylcholine receptor activity; P:G-protein coupled acetylcholine receptor signaling pathway |
| Efet.01.288887.g1415.t1 | octopamine receptor 1-like | 528 | 5.23E-24 | 74.55 | F:adrenergic receptor activity; C:integral component of membrane; P:adrenergic receptor signaling pathway; C:plasma membrane |
| Efet.01.288920.g1417.t1 | polycystic kidney disease 2-like 1 | 465 | 5.58E-52 | 71.8 | P:detection of mechanical stimulus; F:calcium channel activity; C:membrane |
| Efet.01.28894.g268.t1 | 2-deoxy-D-gluconate 3-dehydrogenase | 390 | 3.63E-69 | 77.5 | F:NAD binding; P:oxidation-reduction process; F:2-deoxy-D-gluconate 3-dehydrogenase activity |
| Efet.01.28898.g269.t1 | SPRY domain-containing SOCS box 4 isoform X3 | 600 | 6.35E-53 | 60 | F:protein binding |
| Efet.01.28903.g270.t1 | pumilio homolog 2 isoform X1 | 384 | 5.73E-15 | 61.7 |  |
| Efet.01.289040.g1419.t1 | ras-related Rab-3B | 237 | 2.22E-30 | 83.3 | P:small GTPase mediated signal transduction; C:synaptic vesicle; P:protein transport; C:cytoplasmic microtubule; C:zymogen granule; P:exocytosis; C:plasma membrane; P:regulation of vesicle size; P:positive regulation of regulated secretory pathway; F:GTP binding; C:perinuclear region of cytoplasm; C:extracellular exosome; P:positive regulation of dopamine uptake involved in synaptic transmission; P:bone resorption; F:GTPase activity; C:mitochondrion; F:GDP binding; C:cytosol; P:antigen processing and presentation; P:peptidyl-cysteine methylation; F:GTP-dependent protein binding; F:myosin V binding |
| Efet.01.289047.g1420.t1 | DNA methyltransferase 1-associated 1 | 219 | 1.27E-18 | 78.75 | P:histone H4 acetylation; F:DNA binding; P:DNA repair; P:chromatin remodeling; C:NuA4 histone acetyltransferase complex; P:histone H2A acetylation; P:negative regulation of transcription, DNA-templated; F:transcription corepressor activity; F:methyltransferase activity; P:methylation |
| Efet.01.28907.g271.t1 | neogenin isoform X3 | 465 | 5.13E-11 | 55.85 | P:iron ion homeostasis; C:integral component of plasma membrane; P:positive regulation of BMP signaling pathway; C:membrane; C:integral component of membrane; P:nervous system development; P:axon guidance; F:receptor activity |
| Efet.01.289089.g1421.t1 | dimethylaniline monooxygenase [N-oxide-forming] 5 | 618 | 2.47E-52 | 63.35 | C:cytoplasm; C:intracellular membrane-bounded organelle; C:membrane; P:metabolic process; F:monooxygenase activity |
| Efet.01.289089.g1422.t1 | dimethylaniline monooxygenase [N-oxide-forming] 5-like | 393 | 7.16E-28 | 65.45 | F:oxidoreductase activity; C:intracellular membrane-bounded organelle; C:membrane |
| Efet.01.289119.g1423.t1 | homeobox six1-like | 702 | 6.68E-115 | 87.15 | P:cell proliferation; C:nucleus; F:transcription factor activity, sequence-specific DNA binding; P:apoptotic process; P:regulation of transcription, DNA-templated; F:sequence-specific DNA binding; P:animal organ development |
| Efet.01.28919.g272.t1 | alpha-mannosidase 2-like | 2688 | 5.54E-146 | 68.15 | P:carbohydrate metabolic process; F:metal ion binding; C:membrane; F:mannosidase activity |
| Efet.01.289268.g1426.t1 | DC-STAMP domain-containing 2-like | 1383 | 2.41E-144 | 50 | C:membrane |
| Efet.01.289275.g1427.t1 | gelsolin 2 | 243 | 4.28E-22 | 89.9 | C:cytoskeleton; P:actin filament severing; F:actin binding; P:actin filament capping; C:cytoplasm; P:multicellular organism development; F:calcium ion binding; P:actin nucleation; P:actin filament polymerization; P:actin filament fragmentation |
| Efet.01.289367.g1429.t1 | ATP-binding cassette sub-family A member 2 isoform X2 | 1164 | 1.81E-122 | 64.9 |  |
| Efet.01.289390.g1430.t1 | dmX 2 isoform X1 | 489 | 9.29E-56 | 75.95 |  |
| Efet.01.289468.g1432.t1 | poly(ADP-ribose) glycohydrolase 1 isoform X1 | 780 | 1.31E-07 | 56.18 | P:carbohydrate metabolic process; F:poly(ADP-ribose) glycohydrolase activity; C:membrane; C:integral component of membrane; F:hydrolase activity |
| Efet.01.2895.g260.t1 | chloride channel 1 | 495 | 4.19E-11 | 68 | P:transport |
| Efet.01.28951.g274.t1 | serine threonine- kinase ULK2-like isoform X1 | 1011 | 9.17E-46 | 59.4 | F:kinase activity |
| Efet.01.289531.g1436.t1 | E3 ubiquitin- ligase | 381 | 1.14E-52 | 83.8 | F:zinc ion binding; C:integral component of membrane; F:ligase activity |
| Efet.01.289582.g1437.t1 | beta-1,3-galactosyltransferase 5-like | 1095 | 6.29E-31 | 53.9 | F:transferase activity, transferring glycosyl groups; C:membrane; C:integral component of membrane; P:protein glycosylation; F:galactosyltransferase activity; C:Golgi apparatus; C:Golgi membrane; F:transferase activity |
| Efet.01.289586.g1438.t1 | hypothetical protein CRN43_17955, partial | 360 | 2.62E-15 | 63 |  |
| Efet.01.28964.g275.t1 | follicular epithelium yolk subunit | 594 | 1.38E-24 | 61.1 |  |
| Efet.01.289710.g1442.t1 | probable serine threonine- kinase pats1 isoform X1 | 1665 | 3.06E-47 | 61.3 |  |
| Efet.01.28977.g276.t1 | sodium glucose cotransporter 4-like | 687 | 1.09E-12 | 57.3 |  |
| Efet.01.289879.g1443.t1 | CREB-binding isoform X1 | 903 | 2.44E-39 | 78.85 |  |
| Efet.01.289880.g1444.t1 | atrial natriuretic peptide receptor 1-like | 378 | 1.50E-42 | 73.85 | F:nucleotide binding; F:phosphorus-oxygen lyase activity; C:membrane; P:cyclic nucleotide biosynthetic process |
| Efet.01.2899.g261.t1 | dnaJ homolog subfamily B member 4 | 810 | 1.47E-79 | 73 | F:protein binding; C:cytoplasm; C:membrane-bounded organelle |
| Efet.01.289926.g1445.t1 | mannan-binding lectin serine protease 2-like | 618 | 4.91E-56 | 47.7 | C:membrane; C:integral component of membrane; F:carbohydrate binding |
| Efet.01.289987.g1447.t1 | probable G- coupled receptor 139 isoform X1 | 1254 | 5.90E-96 | 58.8 | C:membrane |
| Efet.01.290009.g1448.t1 | cilia- and flagella-associated 251-like | 237 | 2.46E-12 | 69.95 |  |
| Efet.01.29001.g278.t1 | hypothetical protein CAPTEDRAFT_222036 | 477 | 8.21E-11 | 62.8 |  |
| Efet.01.290010.g1449.t1 | kinesin unc-104 isoform X11 | 429 | 1.02E-09 | 55.85 |  |
| Efet.01.290064.g1450.t1 | hypothetical protein CAPTEDRAFT_225602 | 258 | 6.05E-07 | 68.33 | P:signal transduction |
| Efet.01.29013.g279.t1 | cytochrome P450 2J6-like | 576 | 3.06E-17 | 61.2 | F:monooxygenase activity |
| Efet.01.290133.g1453.t1 | beta-1,3-galactosyl-O-glycosyl-glyco beta-1,6-N-acetylglucosaminyltransferase-like | 231 | 1.77E-15 | 65.3 | F:acetylglucosaminyltransferase activity; C:membrane |
| Efet.01.290143.g1454.t1 | muskelin-like isoform X2 | 699 | 5.32E-87 | 65.65 |  |
| Efet.01.290205.g1458.t1 | SprT-like domain-containing protein Spartan, partial | 384 | 7.46E-62 | 85.25 | F:DNA binding; F:K63-linked polyubiquitin binding; C:nuclear speck; P:positive regulation of protein ubiquitination; P:response to UV; P:translesion synthesis |
| Efet.01.290247.g1459.t1 | Fibroblast growth factor receptor 2 | 855 | 1.01E-50 | 59.4 | P:phosphorylation; F:kinase activity |
| Efet.01.290317.g1462.t1 | conjugal transfer | 2418 | 6.20E-112 | 57.7 | F:DNA binding; C:membrane; C:integral component of membrane; P:unidirectional conjugation |
| Efet.01.290317.g1463.t1 | family conjugative transposon ATPase | 2520 | 3.74E-80 | 47.25 | F:ATP binding |
| Efet.01.290317.g1465.t1 | conjugative transposon | 654 | 2.89E-08 | 43.5 |  |
| Efet.01.290317.g1467.t1 | hypothetical protein | 1215 | 9.00E-11 | 46.5 |  |
| Efet.01.290323.g1468.t1 | Atrial natriuretic peptide receptor 2 | 1368 | 4.06E-40 | 49.45 | F:phosphorus-oxygen lyase activity; C:intracellular; P:cyclic nucleotide biosynthetic process; P:intracellular signal transduction |
| Efet.01.290452.g1472.t1 | Nucleolar MIF4G domain-containing 1 | 318 | 7.77E-06 | 57 | F:RNA binding |
| Efet.01.290544.g1473.t1 | discoidin domain-containing receptor 2-like isoform X1 | 705 | 3.69E-30 | 55.45 | F:ATP binding; P:peptidyl-tyrosine phosphorylation; F:protein tyrosine kinase activity; C:membrane; C:integral component of membrane; F:protein kinase activity; P:protein phosphorylation |
| Efet.01.290549.g1474.t1 | CDK5 and ABL1 enzyme substrate 1-like isoform X3 | 243 | 1.20E-27 | 74.3 | P:regulation of cell cycle |
| Efet.01.29056.g280.t1 | syntaxin-16 isoform X1 | 375 | 3.21E-18 | 70.45 | C:membrane part; P:transport |
| Efet.01.290601.g1475.t1 | pre-mRNA-processing factor 40 homolog A | 765 | 1.08E-32 | 72.65 |  |
| Efet.01.290620.g1476.t1 | hypothetical protein RFI_20426, partial | 2841 | 7.93E-06 | 60 |  |
| Efet.01.290621.g1477.t1 | catenin alpha-2-like isoform X2 | 528 | 5.89E-61 | 86.05 | F:actin filament binding; F:cadherin binding; P:cell adhesion |
| Efet.01.29077.g281.t1 | TOG array regulator of axonemal microtubules 1-like isoform X2 | 1521 | 2.35E-44 | 50.1 |  |
| Efet.01.29078.g282.t1 | repressor of RNA polymerase III transcription MAF1 homolog | 261 | 3.77E-16 | 79.95 | C:nucleus; P:transcription, DNA-templated; P:negative regulation of transcription from RNA polymerase III promoter |
| Efet.01.290804.g1479.t1 | soluble guanylate cyclase 88E-like | 570 | 8.13E-18 | 61.85 | F:nucleotide binding; F:phosphorus-oxygen lyase activity; P:cyclic nucleotide biosynthetic process |
| Efet.01.290813.g1481.t1 | Signal recognition particle subunit SRP68 | 816 | 6.02E-85 | 62.6 | F:RNA binding; C:intracellular part |
| Efet.01.290904.g1483.t1 | hypothetical protein CAPTEDRAFT_201913 | 759 | 5.54E-17 | 56 | F:G-protein coupled receptor activity; P:G-protein coupled receptor signaling pathway; C:membrane; C:integral component of membrane |
| Efet.01.290920.g1484.t1 | potassium sodium hyperpolarization-activated cyclic nucleotide-gated channel 2-like | 276 | 4.37E-11 | 69.2 | F:voltage-gated potassium channel activity; P:potassium ion transmembrane transport; C:integral component of membrane |
| Efet.01.290988.g1485.t1 | protocadherin beta-10-like isoform X1 | 2826 | 2.72E-172 | 56 | C:membrane |
| Efet.01.291000.g1486.t1 | lipoma HMGIC fusion partner-like 3 | 489 | 9.03E-24 | 61.55 | C:membrane; C:integral component of membrane |
| Efet.01.2912.g262.t1 | DNA polymerase alpha catalytic subunit-like | 297 | 5.50E-08 | 52.23 | P:DNA replication |
| Efet.01.291216.g1487.t1 | neurexin-1 isoform X3 | 816 | 3.17E-54 | 50.7 | C:presynaptic membrane; F:metal ion binding; F:calcium ion binding; C:integral component of membrane; P:chemical synaptic transmission |
| Efet.01.291261.g1488.t1 | T family of potassium channels 18-like | 846 | 4.11E-81 | 60 | P:transport |
| Efet.01.291324.g1489.t1 | uncharacterized zinc finger 814 | 858 | 7.62E-30 | 47.7 | F:nucleic acid binding; F:metal ion binding; P:regulation of transcription, DNA-templated; C:intracellular |
| Efet.01.291348.g1490.t1 | calcium calmodulin-dependent 3 ,5 -cyclic nucleotide phosphodiesterase 1A isoform X8 | 777 | 1.42E-07 | 73.85 | F:metal ion binding; P:signal transduction; P:sensory perception of smell; C:cilium; F:phosphoric diester hydrolase activity; F:3',5'-cyclic-nucleotide phosphodiesterase activity; F:hydrolase activity |
| Efet.01.291362.g1491.t1 | protocadherin-9-like isoform X1 | 2781 | 6.02E-106 | 50.45 | F:calcium ion binding; C:membrane; C:integral component of membrane; P:cell adhesion; P:homophilic cell adhesion via plasma membrane adhesion molecules; C:plasma membrane |
| Efet.01.291445.g1493.t1 | MAGUK p55 subfamily member 5-like isoform X3 | 933 | 4.47E-27 | 45.25 | C:membrane; C:integral component of membrane; P:bicellular tight junction assembly; P:establishment of epithelial cell polarity |
| Efet.01.29146.g286.t1 | NACHT and WD repeat domain-containing 2-like | 513 | 8.11E-21 | 49.56 |  |
| Efet.01.29146.g287.t1 | NACHT and WD repeat domain-containing 2-like | 1878 | 1.11E-135 | 51.94 | F:acetylglucosaminyltransferase activity; C:membrane; P:glycosaminoglycan biosynthetic process; F:transferase activity; F:protein xylosyltransferase activity |
| Efet.01.291484.g1495.t1 | hypothetical protein CAPTEDRAFT_195684 | 693 | 5.92E-60 | 59.57 | C:membrane; C:integral component of membrane |
| Efet.01.291539.g1496.t1 | glycogen-binding subunit 76A-like | 693 | 1.43E-12 | 51.9 |  |
| Efet.01.291553.g1497.t1 | hypothetical protein HELRODRAFT_165757 | 231 | 1.36E-07 | 100 | F:ATP binding |
| Efet.01.291609.g1499.t1 | tyrosine- kinase CSK-like | 291 | 2.18E-14 | 76 | F:ATP binding; P:peptidyl-tyrosine phosphorylation; F:non-membrane spanning protein tyrosine kinase activity |
| Efet.01.291648.g1500.t1 | OTOL1_ONCKE ame: Full=Otolin-1 Flags: Precursor | 705 | 7.77E-06 | 52 | C:cell; P:otolith mineralization; C:collagen trimer; C:extracellular region; F:molecular_function |
| Efet.01.291697.g1501.t1 | furry-like isoform X1 | 498 | 6.18E-34 | 74.3 | C:membrane |
| Efet.01.291724.g1502.t1 | hypothetical protein CAPTEDRAFT_219019 | 345 | 6.08E-20 | 63.1 | C:membrane |
| Efet.01.291727.g1503.t1 | hypothetical protein CAPTEDRAFT_227219 | 354 | 1.39E-12 | 72 |  |
| Efet.01.291804.g1506.t1 | FTS and Hook-interacting | 378 | 2.84E-19 | 65.45 |  |
| Efet.01.29184.g288.t1 | endoplasmic reticulum aminopeptidase 2 isoform X1 | 624 | 7.70E-10 | 49.75 | F:zinc ion binding; P:proteolysis; C:membrane; C:integral component of membrane; F:metallopeptidase activity |
| Efet.01.291881.g1507.t1 | hypothetical protein CAPTEDRAFT_224991 | 219 | 2.36E-24 | 65 | C:integral component of membrane; F:carbohydrate binding |
| Efet.01.291977.g1508.t1 | hypothetical protein CAPTEDRAFT_219563 | 297 | 2.07E-08 | 60 | C:membrane; C:integral component of membrane |
| Efet.01.292013.g1509.t1 | alpha-galactosidase A isoform X2 | 399 | 5.76E-14 | 52.55 | F:catalytic activity |
| Efet.01.292064.g1511.t1 | hypothetical protein CAPTEDRAFT_225483 | 405 | 1.41E-21 | 58.05 | C:membrane; C:integral component of membrane; F:carbohydrate binding |
| Efet.01.29207.g290.t1 | tyrosine- kinase JAK2 | 822 | 2.40E-30 | 43.05 | P:response to organic substance; P:cell surface receptor signaling pathway; F:binding; F:transferase activity; P:protein phosphorylation |
| Efet.01.29214.g291.t1 | A-kinase anchor mitochondrial-like | 402 | 5.63E-20 | 69.1 | C:nucleus; P:phosphorylation; C:nucleolus; F:kinase activity |
| Efet.01.292188.g1514.t1 | UPF0016 domain-containing | 522 | 5.98E-105 | 94.3 | C:integral component of membrane |
| Efet.01.292188.g1515.t1 | homoserine O-acetyltransferase | 1128 | 0 | 96.75 | F:homoserine O-acetyltransferase activity; P:methionine biosynthetic process; C:cytoplasm |
| Efet.01.292188.g1516.t1 | methionine biosynthesis | 498 | 3.42E-119 | 96.85 |  |
| Efet.01.292188.g1517.t1 | cyclase | 750 | 9.09E-143 | 77.95 | F:arylformamidase activity; P:tryptophan catabolic process to kynurenine |
| Efet.01.292223.g1518.t1 | receptor tyrosine phosphatase | 543 | 1.63E-49 | 60.85 | F:phosphatase activity; P:dephosphorylation |
| Efet.01.292246.g1519.t1 | alkali metal ion proton exchanger 3 | 435 | 8.67E-06 | 47 | P:transmembrane transport; F:solute:proton antiporter activity; F:antiporter activity; F:sodium:proton antiporter activity; P:hydrogen ion transmembrane transport; P:transport; P:regulation of pH; P:sodium ion transport; P:ion transport; P:cation transport; C:membrane; C:integral component of membrane; P:sodium ion transmembrane transport |
| Efet.01.292258.g1520.t1 | crooked neck 1 | 357 | 1.58E-39 | 78.45 | C:prespliceosome; C:catalytic step 2 spliceosome; C:post-mRNA release spliceosomal complex; C:precatalytic spliceosome; C:catalytic step 1 spliceosome; P:positive regulation of macromitophagy in response to mitochondrial depolarization; C:Prp19 complex; P:positive regulation of defense response to virus by host; P:xenophagy; P:spliceosomal complex assembly; F:RNA binding |
| Efet.01.292262.g1521.t1 | hypothetical protein CAPTEDRAFT_216764 | 972 | 2.21E-30 | 62.33 |  |
| Efet.01.292300.g1522.t1 | PREDICTED: uncharacterized protein LOC106172092 | 2595 | 2.15E-20 | 54.33 |  |
| Efet.01.29236.g292.t1 | serine arginine repetitive matrix 1 | 990 | 4.41E-12 | 57.67 |  |
| Efet.01.292388.g1524.t1 | actin-related 2 3 complex subunit 1A | 447 | 1.33E-79 | 78.8 | P:Arp2/3 complex-mediated actin nucleation; C:Arp2/3 protein complex |
| Efet.01.292550.g1527.t1 | lambda-crystallin homolog isoform X3 | 483 | 7.71E-43 | 65.1 | C:nucleus; C:cytosol; F:L-gulonate 3-dehydrogenase activity; P:single-organism metabolic process; F:NAD+ binding |
| Efet.01.292570.g1528.t1 | one cut domain family member 2-like isoform X1 | 264 | 1.15E-35 | 84.1 | C:nucleus; F:DNA binding; P:transcription, DNA-templated; P:regulation of transcription, DNA-templated |
| Efet.01.292607.g1530.t1 | nucleobindin-2-like isoform X1 | 495 | 1.66E-31 | 59.35 | F:calcium ion binding; C:membrane; C:integral component of membrane |
| Efet.01.292670.g1532.t1 | myogenic factor 5 | 1134 | 3.19E-14 | 72.8 | C:nucleus; P:skeletal muscle cell differentiation; P:transcription, DNA-templated; F:RNA polymerase II core promoter proximal region sequence-specific DNA binding; F:transcription factor activity, RNA polymerase II distal enhancer sequence-specific binding; P:positive regulation of myoblast fusion; F:transcriptional activator activity, RNA polymerase II transcription regulatory region sequence-specific binding; P:positive regulation of myoblast differentiation; P:positive regulation of skeletal muscle fiber development; P:regulation of transcription from RNA polymerase II promoter; P:animal organ morphogenesis |
| Efet.01.292681.g1533.t1 | Dynein heavy chain axonemal | 327 | 1.85E-43 | 80 | F:ATP binding; F:microtubule motor activity; F:calcium ion binding; C:dynein complex; P:microtubule-based movement |
| Efet.01.29274.g295.t1 | MLX-interacting -like | 768 | 4.25E-38 | 53.75 | P:regulation of transcription, DNA-templated |
| Efet.01.292766.g1535.t1 | Cysteine and histidine-rich 1 homolog | 219 | 4.04E-06 | 63.17 | F:ubiquitin-protein transferase activity; F:zinc ion binding; F:metal ion binding; P:protein ubiquitination |
| Efet.01.292819.g1536.t1 | PREDICTED: uncharacterized protein LOC100891675 | 540 | 9.28E-13 | 52.4 |  |
| Efet.01.292914.g1538.t1 | Dual specificity phosphatase 7 | 453 | 7.77E-14 | 56.25 | F:phosphoprotein phosphatase activity |
| Efet.01.292958.g1540.t1 | probable serine carboxypeptidase CPVL | 504 | 2.44E-59 | 75.9 | F:carboxypeptidase activity |
| Efet.01.29296.g298.t1 | universal stress in QAH OAS sulfhydrylase 3 region | 369 | 1.29E-14 | 64.13 | P:response to stress |
| Efet.01.293073.g1541.t1 | vacuolar sorting-associated 13D-like | 201 | 1.47E-19 | 73 | P:protein retention in Golgi apparatus; C:extrinsic component of membrane; P:protein targeting to vacuole; C:intracellular |
| Efet.01.29309.g300.t1 | peptidylglycine alpha-amidating monooxygenase | 264 | 1.48E-19 | 64.55 | F:peptidase inhibitor activity; P:negative regulation of peptidase activity |
| Efet.01.293104.g1542.t1 | proton-gated ion channel subunit pbo-5-like isoform X1 | 246 | 7.06E-24 | 71.35 | P:ion transmembrane transport; C:integral component of membrane; F:extracellular ligand-gated ion channel activity |
| Efet.01.293106.g1543.t1 | synaptotagmin-4-like isoform X2 | 870 | 7.71E-52 | 66.15 | P:biological regulation; F:protein binding; P:vesicle-mediated transport; C:membrane; F:ion binding; C:cell part; P:single-organism cellular process |
| Efet.01.293225.g1546.t1 | helix-loop-helix 13- | 594 | 5.87E-38 | 79.25 | F:protein dimerization activity |
| Efet.01.29330.g301.t1 | potassium voltage-gated channel Shaw-like isoform X1 | 297 | 2.87E-15 | 65.9 | F:ion channel activity; P:ion transport; C:membrane |
| Efet.01.293373.g1547.t1 | eukaryotic translation initiation factor 3 subunit C-like | 657 | 1.99E-66 | 74.5 | F:RNA binding; C:translation preinitiation complex; P:translational initiation |
| Efet.01.293421.g1549.t1 | hypothetical protein CAPTEDRAFT_217918 | 675 | 2.13E-14 | 50.8 | C:membrane; C:integral component of membrane; F:carbohydrate binding |
| Efet.01.293437.g1550.t1 | filamin-A-like isoform X6 | 336 | 2.55E-30 | 63.85 | F:actin binding |
| Efet.01.293477.g1552.t1 | peptide chain release factor 1 | 315 | 1.30E-50 | 94.6 | P:translational termination; C:cytoplasm; F:translation release factor activity, codon specific |
| Efet.01.293477.g1553.t1 | transcription termination factor Rho | 1284 | 5.35E-88 | 95.5 | F:ATP binding; F:RNA binding; F:RNA-dependent ATPase activity; P:regulation of transcription, DNA-templated; F:helicase activity; P:DNA-templated transcription, termination |
| Efet.01.293547.g1554.t1 | neurexin-1a-like isoform X1 | 615 | 6.03E-53 | 58.95 | C:membrane; P:single-organism cellular process |
| Efet.01.293581.g1555.t1 | tubby-related 4-like | 558 | 6.02E-25 | 53.2 | P:intracellular signal transduction; C:intracellular |
| Efet.01.293590.g1556.t1 | metabotropic glutamate receptor 4 isoform X2 | 2202 | 0 | 53.55 | F:transmembrane signaling receptor activity; P:signal transduction; C:membrane part; C:plasma membrane |
| Efet.01.293646.g1559.t1 | MAATS1 isoform X1 | 600 | 8.53E-46 | 68.95 |  |
| Efet.01.29370.g302.t1 | hypothetical protein HELRODRAFT_188674 | 1101 | 2.14E-82 | 49.3 | C:membrane; C:integral component of membrane |
| Efet.01.293717.g1562.t1 | hypothetical protein HELRODRAFT_166009 | 444 | 1.75E-07 | 75 |  |
| Efet.01.293755.g1564.t1 | hypothetical protein HELRODRAFT_167673 | 207 | 7.33E-06 | 85 |  |
| Efet.01.293791.g1567.t1 | amyloid beta A4 isoform X1 | 270 | 2.78E-20 | 66.75 | P:macromolecule metabolic process; F:peptidase inhibitor activity; F:transition metal ion binding; F:carbohydrate derivative binding; C:extracellular matrix; P:negative regulation of peptidase activity; F:metallopeptidase activity |
| Efet.01.293816.g1570.t1 | family transporter | 855 | 6.11E-167 | 72.55 | F:catalytic activity; C:membrane |
| Efet.01.293873.g1572.t1 | ankyrin repeat | 1182 | 2.33E-11 | 42.6 |  |
| Efet.01.293879.g1573.t1 | Fatty-acid amide hydrolase 2 | 423 | 1.60E-27 | 63.25 | F:DNA binding; C:nucleus; P:transcription, DNA-templated; C:membrane; C:integral component of membrane; P:regulation of transcription, DNA-templated; F:protein dimerization activity; F:carbon-nitrogen ligase activity, with glutamine as amido-N-donor; F:hydrolase activity |
| Efet.01.293893.g1574.t1 | cytoplasmic dynein 2 heavy chain 1-like | 921 | 2.16E-104 | 69.95 | F:nucleotide binding; F:ATPase activity; F:ATP binding; F:microtubule motor activity; C:dynein complex; P:microtubule-based movement |
| Efet.01.294086.g1576.t1 | ras-related Rap-2c-like | 282 | 2.27E-44 | 88.7 | F:GTP binding; P:small GTPase mediated signal transduction; C:membrane; C:intracellular |
| Efet.01.294156.g1577.t1 | probable G- coupled receptor 139 isoform X1 | 1086 | 8.48E-95 | 59.9 | F:G-protein coupled receptor activity; P:G-protein coupled receptor signaling pathway; C:membrane; C:integral component of membrane |
| Efet.01.29417.g303.t1 | cAMP-responsive element-binding -like | 645 | 2.17E-23 | 76.15 | F:transcription factor activity, sequence-specific DNA binding; P:regulation of transcription, DNA-templated; F:sequence-specific DNA binding |
| Efet.01.294182.g1578.t1 | peroxisomal coenzyme A diphosphatase NUDT7 | 897 | 4.35E-48 | 57.75 | F:magnesium ion binding; P:nucleoside diphosphate metabolic process; C:membrane; F:hydrolase activity, acting on acid anhydrides, in phosphorus-containing anhydrides; C:integral component of membrane; F:manganese ion binding; F:hydrolase activity |
| Efet.01.29423.g304.t1 | estrogen sulfotransferase-like isoform X3 | 330 | 1.59E-21 | 65.45 | F:transferase activity |
| Efet.01.29434.g305.t1 | monocarboxylate transporter 12-like | 597 | 5.54E-39 | 57.2 | P:transmembrane transport; C:membrane; P:monocarboxylic acid transport; C:integral component of membrane; F:monocarboxylic acid transmembrane transporter activity |
| Efet.01.294358.g1581.t1 | hypothetical protein HELRODRAFT_178410 | 420 | 1.64E-12 | 82 |  |
| Efet.01.294470.g1582.t1 | E3 ubiquitin- ligase RNF103 | 2223 | 0 | 55.2 | F:ubiquitin-protein transferase activity; F:zinc ion binding; F:metal ion binding; C:membrane; C:integral component of membrane; C:endoplasmic reticulum; P:ER-associated ubiquitin-dependent protein catabolic process; F:ligase activity; P:protein ubiquitination |
| Efet.01.294500.g1583.t1 | vacuolar sorting-associated 41 homolog | 252 | 1.11E-11 | 76.9 | F:zinc ion binding; P:vesicle-mediated transport; P:regulation of SNARE complex assembly; C:CORVET complex; F:GTPase binding; C:late endosome; P:vacuole fusion, non-autophagic; P:protein targeting to vacuole; C:HOPS complex |
| Efet.01.294516.g1584.t1 | alpha-ketoglutarate-dependent dioxygenase alkB homolog mitochondrial-like | 495 | 2.51E-13 | 73.6 | P:single-organism process; P:cellular process |
| Efet.01.294644.g1585.t1 | serine arginine repetitive matrix 1 | 1146 | 8.51E-16 | 60 |  |
| Efet.01.29465.g307.t1 | protogenin B-like | 261 | 2.62E-11 | 57.4 | F:transferase activity |
| Efet.01.294664.g1586.t1 | ras-related Rab-3A | 312 | 1.07E-42 | 96.1 | F:GTP binding; P:small GTPase mediated signal transduction; C:intracellular |
| Efet.01.294674.g1588.t1 | chromodomain-helicase-DNA-binding 7 isoform X1 | 1122 | 5.19E-23 | 67.8 | F:hydrolase activity, acting on acid anhydrides |
| Efet.01.294804.g1591.t1 | chloride channel B-like isoform X1 | 570 | 2.28E-69 | 76.8 | P:regulation of anion transmembrane transport; P:ion transmembrane transport; C:integral component of membrane; P:chloride transport; F:voltage-gated chloride channel activity |
| Efet.01.294922.g1593.t1 | dopamine receptor 1-like | 576 | 1.27E-63 | 77.3 | C:integral component of plasma membrane; F:dopamine neurotransmitter receptor activity, coupled via Gs; P:synaptic transmission, dopaminergic; F:dopamine binding; P:dopamine receptor signaling pathway; P:serotonin receptor signaling pathway; F:G-protein coupled serotonin receptor activity; F:transferase activity |
| Efet.01.294922.g1594.t1 | dopamine receptor 1 | 540 | 1.11E-48 | 64 | F:G-protein coupled receptor activity; P:G-protein coupled receptor signaling pathway; C:integral component of membrane |
| Efet.01.294932.g1595.t1 | cysteinyl leukotriene receptor 2-like | 1227 | 1.39E-45 | 49.94 | F:G-protein coupled receptor activity; F:G-protein coupled peptide receptor activity; P:G-protein coupled receptor signaling pathway; P:signal transduction; C:membrane; C:integral component of membrane; F:signal transducer activity |
| Efet.01.29499.g309.t1 | cytoplasmic FMR1-interacting -like | 393 | 6.67E-40 | 75.95 | P:Rac protein signal transduction; P:negative regulation of synaptic vesicle recycling; C:neuronal cell body; P:cellular response to insulin stimulus; F:RNA 7-methylguanosine cap binding; C:mRNA cap binding complex; C:extracellular exosome; C:focal adhesion; P:cognition; C:terminal bouton; C:dendritic spine; F:Rac GTPase binding; F:protein complex binding; P:response to electrical stimulus; C:lamellipodium; C:excitatory synapse; P:positive regulation of axon extension; P:positive regulation of ruffle assembly; P:positive regulation of dendrite development; F:profilin binding; C:SCAR complex; C:dendritic growth cone; C:perinuclear region of cytoplasm; C:axonal growth cone; P:axon extension; C:filopodium tip; P:lamellipodium assembly; P:positive regulation of Arp2/3 complex-mediated actin nucleation; C:central region of growth cone; C:peripheral region of growth cone |
| Efet.01.295038.g1597.t1 | Down syndrome cell adhesion molecule 1 isoform X2 | 486 | 5.08E-21 | 49.1 | C:membrane; C:integral component of membrane |
| Efet.01.295038.g1598.t1 | roundabout homolog 1- | 237 | 1.21E-08 | 55 | C:membrane; C:integral component of membrane |
| Efet.01.295180.g1600.t1 | ataxin-3 | 468 | 2.65E-15 | 58.3 | F:thiol-dependent ubiquitin-specific protease activity; P:protein deubiquitination; F:omega peptidase activity; P:proteolysis |
| Efet.01.295261.g1601.t1 | ataxin-7 isoform X2 | 1968 | 7.77E-07 | 73.25 | C:nucleus; P:regulation of cellular metabolic process |
| Efet.01.29538.g310.t1 | reticulon-4 receptor-like 2 | 930 | 2.96E-09 | 50.7 | C:cytoplasm; P:cytokine-mediated signaling pathway; C:membrane; C:integral component of membrane; F:protein kinase inhibitor activity; P:negative regulation of protein kinase activity; P:negative regulation of JAK-STAT cascade |
| Efet.01.2954.g263.t1 | nuclear hormone receptor HR96-like isoform X3 | 1245 | 1.45E-153 | 75.05 | F:DNA binding; F:transcription factor activity, sequence-specific DNA binding; F:metal ion binding; P:signal transduction; F:signaling receptor activity |
| Efet.01.295601.g1604.t1 | vacuolar -sorting-associated 25 | 300 | 1.69E-22 | 72.6 | P:protein targeting to vacuole involved in ubiquitin-dependent protein catabolic process via the multivesicular body sorting pathway; F:protein homodimerization activity; C:ESCRT II complex; F:structural molecule activity |
| Efet.01.295634.g1605.t1 | transcription factor HIVEP3 | 573 | 2.47E-22 | 67.5 |  |
| Efet.01.295656.g1606.t1 | E3 ubiquitin- ligase TRIM56-like | 1518 | 1.87E-11 | 41.6 | F:zinc ion binding; F:metal ion binding; C:intracellular |
| Efet.01.29580.g312.t1 | Threonine synthase-like 1 | 558 | 1.88E-49 | 62.4 | C:cytoplasm; C:intracellular membrane-bounded organelle; P:cellular amino acid metabolic process |
| Efet.01.295853.g1610.t1 | Outer membrane transport (OMPP1 ) | 453 | 6.94E-100 | 53.9 |  |
| Efet.01.295933.g1612.t1 | histamine H2 receptor-like | 1233 | 7.73E-147 | 61.65 | C:membrane |
| Efet.01.295983.g1613.t1 | dopey-1-like isoform X1 | 546 | 4.70E-16 | 60.7 |  |
| Efet.01.29599.g313.t1 | 2-oxoglutarate mitochondrial-like isoform X1 | 828 | 2.74E-143 | 80.5 | F:thiamine pyrophosphate binding; F:oxoglutarate dehydrogenase (succinyl-transferring) activity; P:tricarboxylic acid cycle |
| Efet.01.29605.g315.t1 | WD repeat-containing 20 isoform X2 | 267 | 1.51E-25 | 70.6 |  |
| Efet.01.296052.g1614.t1 | Zinc finger and BTB domain-containing 24 | 1704 | 2.85E-76 | 64.3 | F:binding |
| Efet.01.296107.g1615.t1 | titin-like isoform X1 | 1440 | 2.95E-29 | 45.65 | P:regulation of Rho protein signal transduction; F:Rho guanyl-nucleotide exchange factor activity; P:positive regulation of GTPase activity |
| Efet.01.29615.g316.t1 | Troponin T | 477 | 2.76E-35 | 70.85 | P:defense response to other organism; P:regulation of muscle contraction; P:killing of cells of other organism; C:troponin complex |
| Efet.01.296151.g1616.t1 | RIB43A-like with coiled-coils 2 | 261 | 6.07E-18 | 78.3 |  |
| Efet.01.296178.g1617.t1 | hypothetical protein HELRODRAFT_194410 | 267 | 3.62E-08 | 68 | C:membrane; C:integral component of membrane |
| Efet.01.296194.g1618.t1 | RNA helicase Mov10l1-like | 570 | 2.77E-10 | 62.5 |  |
| Efet.01.296216.g1621.t1 | FMRFamide receptor-like | 1161 | 2.47E-67 | 56.85 | F:signal transducer activity |
| Efet.01.296245.g1622.t1 | SH3 and PX domain-containing 2A-like isoform X7 | 543 | 2.69E-38 | 55.05 | F:phosphatidylinositol binding |
| Efet.01.296254.g1623.t1 | 33 kDa inner dynein arm light axonemal | 381 | 2.46E-64 | 92.4 |  |
| Efet.01.2963.g265.t1 | myelin regulatory factor-like isoform X6 | 636 | 2.21E-41 | 69.45 | F:DNA binding; F:transcription factor activity, sequence-specific DNA binding; C:membrane; C:integral component of membrane; P:regulation of transcription, DNA-templated |
| Efet.01.296312.g1625.t1 | ephrin-B2-like isoform X2 | 306 | 2.24E-18 | 59.63 | C:membrane |
| Efet.01.296318.g1626.t1 | partitioning defective 3 homolog | 837 | 3.23E-21 | 55.55 | C:bicellular tight junction; C:cell-cell junction; C:cell junction |
| Efet.01.296404.g1629.t1 | bactericidal permeability-increasing -like | 1548 | 2.79E-25 | 42.75 | C:extracellular space; F:lipid binding |
| Efet.01.296407.g1630.t1 | Polypeptide N-acetylgalactosaminyltransferase | 735 | 1.06E-41 | 71.2 | F:transferase activity |
| Efet.01.296467.g1631.t1 | allatostatin-A receptor-like | 924 | 7.95E-52 | 60.6 | F:G-protein coupled receptor activity; P:G-protein coupled receptor signaling pathway; C:membrane |
| Efet.01.296469.g1632.t1 | protocadherin-like wing polarity stan | 3513 | 0 | 65.3 | F:transmembrane signaling receptor activity; P:signal transduction; C:membrane; P:cell adhesion |
| Efet.01.296517.g1634.t1 | c-Jun N-terminal kinase | 252 | 7.96E-17 | 91 | F:ATP binding; P:MAPK cascade; F:MAP kinase activity; C:intracellular |
| Efet.01.296519.g1635.t1 | helicase | 705 | 1.34E-114 | 86.85 | F:helicase activity |
| Efet.01.296519.g1636.t1 | sulfate ABC transporter substrate-binding | 3540 | 0 | 92.5 |  |
| Efet.01.296560.g1638.t1 | exostosin-like 2 | 480 | 2.85E-07 | 55.33 |  |
| Efet.01.29663.g317.t1 | lethal(2) giant larvae homolog 1-like isoform X2 | 855 | 4.73E-65 | 64.75 |  |
| Efet.01.296729.g1643.t1 | metal-dependent hydrolase [Candidatus Planktophila versatilis] | 291 | 1.57E-54 | 61.3 | F:hydrolase activity |
| Efet.01.296729.g1644.t1 | class 1b ribonucleoside-diphosphate reductase subunit beta | 801 | 0 | 97 | F:ribonucleoside-diphosphate reductase activity, thioredoxin disulfide as acceptor; F:metal ion binding; P:DNA replication; P:deoxyribonucleoside diphosphate metabolic process; P:deoxyribonucleotide biosynthetic process; P:oxidation-reduction process; C:ribonucleoside-diphosphate reductase complex |
| Efet.01.296729.g1645.t1 | class 1b ribonucleoside-diphosphate reductase subunit alpha | 2175 | 0 | 91.45 | F:ATP binding; F:ribonucleoside-diphosphate reductase activity, thioredoxin disulfide as acceptor; P:DNA replication; P:oxidation-reduction process |
| Efet.01.296729.g1646.t1 | glutaredoxin | 447 | 1.06E-48 | 93.6 | C:cell; F:protein disulfide oxidoreductase activity; F:electron carrier activity; P:cell redox homeostasis; P:oxidation-reduction process |
| Efet.01.296738.g1648.t1 | TSC22 domain family 1-like | 273 | 6.40E-14 | 79.7 | F:transcription factor activity, sequence-specific DNA binding; P:regulation of transcription, DNA-templated |
| Efet.01.296775.g1650.t1 | 3 beta-hydroxysteroid dehydrogenase Delta 5-- | 366 | 7.77E-19 | 58.31 |  |
| Efet.01.296779.g1651.t1 | UPF0748 - | 501 | 1.46E-49 | 65.85 | C:membrane; C:integral component of membrane; C:outer membrane-bounded periplasmic space |
| Efet.01.296862.g1653.t1 | hypothetical protein CAPTEDRAFT_196175 | 870 | 1.44E-44 | 56.15 | C:membrane; C:integral component of membrane; F:sulfotransferase activity |
| Efet.01.296873.g1654.t1 | dihydroorotase | 852 | 4.01E-117 | 70.35 | F:hydrolase activity, acting on carbon-nitrogen (but not peptide) bonds; F:metal ion binding; P:pyrimidine nucleotide biosynthetic process |
| Efet.01.29692.g318.t1 | complex I intermediate-associated mitochondrial-like | 390 | 6.45E-31 | 61.25 |  |
| Efet.01.29692.g319.t1 | serine arginine-rich splicing factor 7-like isoform X2 | 660 | 3.61E-22 | 67.7 | P:mRNA export from nucleus; P:insulin receptor signaling pathway; C:nuclear speck; F:phospholipase binding; P:regulation of mRNA splicing, via spliceosome; P:mRNA 3'-end processing; P:termination of RNA polymerase II transcription; F:RNA binding; P:mRNA splicing, via spliceosome |
| Efet.01.296993.g1657.t1 | long-chain-fatty-acid-- ligase ACSBG2-like | 558 | 4.76E-65 | 74.6 | F:catalytic activity |
| Efet.01.297112.g1659.t1 | tachykinin-like peptides receptor 99D | 366 | 3.76E-16 | 66.5 | F:neuropeptide receptor activity; P:G-protein coupled receptor signaling pathway; C:membrane |
| Efet.01.297153.g1661.t1 | acyl- synthetase family member 4-like | 621 | 2.45E-09 | 43.5 |  |
| Efet.01.297209.g1662.t1 | guanylyl cyclase-activating 2-like | 240 | 1.65E-07 | 61.9 | F:nucleotide binding; F:ATP binding; F:metal ion binding; F:calcium ion binding; F:protein kinase activity; F:protein serine/threonine kinase activity; P:protein phosphorylation |
| Efet.01.297271.g1665.t1 | PREDICTED: uncharacterized protein KIAA0825-like | 636 | 1.89E-11 | 62.4 |  |
| Efet.01.297281.g1666.t1 | GPI mannosyltransferase 3-like | 375 | 1.71E-22 | 59.2 | C:membrane |
| Efet.01.297332.g1667.t1 | prolactin-releasing peptide receptor-like | 1173 | 2.09E-118 | 71 | F:G-protein coupled receptor activity; P:G-protein coupled receptor signaling pathway; C:membrane |
| Efet.01.297339.g1668.t1 | serum paraoxonase arylesterase 2-like | 270 | 4.37E-19 | 62.9 | F:hydrolase activity, acting on ester bonds |
| Efet.01.29735.g324.t1 | hypothetical protein HELRODRAFT_166711 | 807 | 4.34E-105 | 59.25 | C:integral component of membrane |
| Efet.01.297493.g1672.t1 | achaete-scute homolog 4-like | 615 | 7.16E-15 | 65.05 | F:DNA binding; F:protein dimerization activity |
| Efet.01.29751.g327.t1 | hypothetical protein CAPTEDRAFT_19210 | 315 | 3.23E-08 | 63 |  |
| Efet.01.297556.g1673.t1 | cubilin-like isoform X1 | 747 | 9.35E-06 | 53 |  |
| Efet.01.297618.g1674.t1 | leucine-rich repeat-containing 74B | 396 | 2.96E-17 | 61.3 | F:calcium ion binding |
| Efet.01.297647.g1675.t1 | IQ and AAA domain-containing 1-like | 294 | 4.73E-10 | 83.5 | F:ATP binding |
| Efet.01.297726.g1677.t1 | sacsin-like | 10029 | 0 | 56.05 |  |
| Efet.01.297736.g1681.t1 | chromodomain Y | 231 | 4.11E-10 | 70.63 | C:nucleus; F:catalytic activity; P:metabolic process; C:membrane; C:integral component of membrane |
| Efet.01.297755.g1682.t1 | ras GEF | 1599 | 2.97E-12 | 72.1 | C:integral component of membrane |
| Efet.01.29780.g329.t1 | acetylcholinesterase-like isoform X3 | 1503 | 0 | 68.9 | C:membrane; F:cholinesterase activity |
| Efet.01.29783.g330.t1 | polypyrimidine tract-binding 1-like | 246 | 4.24E-16 | 68.45 | F:nucleotide binding; C:nucleus; F:RNA binding; P:regulation of cell differentiation; P:mRNA processing |
| Efet.01.297848.g1683.t1 | receptor-type tyrosine- phosphatase epsilon-like | 498 | 4.55E-10 | 46.83 |  |
| Efet.01.297866.g1684.t1 | steroid hormone receptor ERR2-like isoform X1 | 276 | 4.08E-28 | 68.2 | C:nucleus; F:transcription factor activity, sequence-specific DNA binding; F:zinc ion binding; P:transcription, DNA-templated; F:steroid hormone receptor activity; P:regulation of transcription, DNA-templated; F:sequence-specific DNA binding; P:steroid hormone mediated signaling pathway |
| Efet.01.297994.g1685.t1 | tricarboxylate transport mitochondrial | 264 | 4.68E-37 | 88.95 | P:transmembrane transport; C:integral component of membrane |
| Efet.01.298057.g1686.t1 | zinc finger 366-like | 285 | 5.39E-13 | 55.35 | F:nucleic acid binding; F:metal ion binding |
| Efet.01.298057.g1687.t1 | hypothetical protein HELRODRAFT_183479 | 1104 | 3.79E-18 | 77 | F:DNA binding; F:metal ion binding |
| Efet.01.29812.g332.t1 | hypothetical protein CAPTEDRAFT_220098 | 912 | 7.77E-08 | 70.14 | F:nucleic acid binding; F:metal ion binding |
| Efet.01.298297.g1691.t1 | rab GTPase-activating 1- | 486 | 2.71E-08 | 77.67 |  |
| Efet.01.298328.g1692.t1 | serine threonine- phosphatase 2A 55 kDa regulatory subunit B beta isoform-like isoform X2 | 900 | 1.47E-79 | 58.05 | F:protein serine/threonine phosphatase activity; P:protein dephosphorylation; P:regulation of phosphoprotein phosphatase activity; C:protein phosphatase type 2A complex |
| Efet.01.298345.g1693.t1 | mitochondrial ribosome-associated GTPase 2-like | 465 | 1.36E-58 | 75.55 | F:ion binding |
| Efet.01.298396.g1694.t1 | syntaxin isoform X2 | 387 | 8.52E-55 | 93.55 | P:membrane fusion; P:vesicle-mediated transport; C:integral component of membrane; F:SNAP receptor activity; F:SNARE binding; P:regulation of exocytosis; P:intracellular protein transport; C:intracellular |
| Efet.01.298402.g1695.t1 | unc-13 homolog B- | 222 | 1.16E-32 | 93.25 | F:metal ion binding; F:diacylglycerol binding; P:chemical synaptic transmission; C:intracellular; P:intracellular signal transduction |
| Efet.01.298454.g1697.t1 | von Willebrand factor D and EGF domain-containing -like | 390 | 4.30E-20 | 56.05 | F:calcium ion binding; C:membrane; C:integral component of membrane |
| Efet.01.298471.g1698.t1 | voltage-gated hydrogen channel 1-like | 423 | 2.74E-20 | 68.5 | F:ion channel activity; P:ion transport; P:transmembrane transport; C:membrane |
| Efet.01.298538.g1699.t1 | Elongation factor 1-alpha 2 | 726 | 5.50E-153 | 85.95 | F:GTP binding; F:protein kinase binding; C:myelin sheath; F:translation elongation factor activity; F:GTPase activity; P:positive regulation of apoptotic process; P:translational elongation; C:eukaryotic translation elongation factor 1 complex; P:positive regulation of lipid kinase activity |
| Efet.01.298575.g1700.t1 | anoctamin-5-like isoform X1 | 603 | 1.28E-10 | 60.8 | C:membrane; C:integral component of membrane; F:protein dimerization activity |
| Efet.01.298688.g1703.t1 | Mitochondrial dynamic MID51 | 870 | 2.37E-15 | 57.8 | C:membrane; C:integral component of membrane |
| Efet.01.298689.g1704.t1 | zinc C2H2 | 480 | 1.93E-16 | 68.9 | F:binding |
| Efet.01.298696.g1705.t1 | hypothetical protein CRN43_03380, partial | 462 | 1.58E-09 | 86 |  |
| Efet.01.298707.g1706.t1 | dynein heavy chain axonemal-like | 741 | 6.41E-125 | 75.9 | F:ATPase activity; F:ATP binding; F:microtubule motor activity; C:dynein complex; P:microtubule-based movement |
| Efet.01.298724.g1707.t1 | retinol dehydrogenase 13-like isoform X3 | 246 | 6.95E-15 | 65.17 | C:integral component of membrane |
| Efet.01.298804.g1710.t1 | phosphoglycerate kinase | 258 | 4.10E-17 | 73.35 | F:ATP binding; F:phosphoglycerate kinase activity; P:glycolytic process |
| Efet.01.298810.g1711.t1 | protocadherin Fat 1 isoform X1 | 222 | 1.35E-16 | 79.9 | P:single organismal cell-cell adhesion; P:establishment or maintenance of cell polarity; P:actin filament organization; P:homophilic cell adhesion via plasma membrane adhesion molecules; C:plasma membrane; C:nucleus; C:cell-cell junction; C:perinuclear region of cytoplasm; C:extracellular exosome; C:focal adhesion; F:calcium ion binding; C:integral component of membrane; C:filopodium; C:lamellipodium |
| Efet.01.298908.g1713.t1 | deltex isoform X2 | 858 | 1.56E-11 | 60.5 | F:zinc ion binding; F:metal ion binding |
| Efet.01.298969.g1715.t1 | 25S rRNA (cytosine-C(5))-methyltransferase nop2-like | 240 | 8.18E-24 | 81.85 | F:RNA binding; P:rRNA processing; F:S-adenosylmethionine-dependent methyltransferase activity; P:methylation |
| Efet.01.299005.g1716.t1 | somatostatin receptor type 2-like | 1029 | 6.78E-74 | 50.9 | F:G-protein coupled receptor activity; P:G-protein coupled receptor signaling pathway |
| Efet.01.299048.g1717.t1 | TNF receptor-associated factor 4 | 237 | 1.18E-13 | 58.8 | F:ubiquitin-protein transferase activity; F:zinc ion binding; F:metal ion binding; P:protein ubiquitination |
| Efet.01.299058.g1718.t1 | sex comb on midleg 2 isoform X2 | 312 | 6.34E-38 | 83.45 | C:nucleus; P:regulation of transcription, DNA-templated |
| Efet.01.29911.g334.t1 | BTB POZ domain-containing 17-like isoform X8 | 258 | 2.02E-15 | 71.75 |  |
| Efet.01.299116.g1721.t1 | uncharacterized protein Dsimw501_GD15724 | 1056 | 4.90E-07 | 41.67 | F:nucleic acid binding; F:metal ion binding |
| Efet.01.299194.g1723.t1 | sarcoplasmic calcium binding isoform 2 | 396 | 3.07E-16 | 45.86 | F:calcium ion binding |
| Efet.01.29921.g335.t1 | HEAT repeat-containing 6 | 729 | 2.09E-31 | 51.9 |  |
| Efet.01.299223.g1724.t1 | FAM69C-like | 504 | 1.38E-37 | 56 | C:membrane; C:integral component of membrane |
| Efet.01.299311.g1727.t1 | hypothetical protein HELRODRAFT_162763 | 297 | 3.46E-19 | 63.05 | C:membrane |
| Efet.01.299359.g1728.t1 | mucin-2-like isoform X1 | 1632 | 1.94E-41 | 45.2 | P:cell-matrix adhesion; F:calcium ion binding; C:membrane; C:integral component of membrane; F:extracellular matrix structural constituent; C:proteinaceous extracellular matrix |
| Efet.01.299403.g1729.t1 | septin-11-like isoform X2 | 228 | 8.83E-13 | 82.35 | F:GTP binding; F:calcium ion binding |
| Efet.01.29951.g336.t1 | collagen alpha-3(VI) chain-like isoform X1 | 1932 | 5.64E-117 | 49.05 | F:calcium ion binding |
| Efet.01.299547.g1731.t1 | bactericidal permeability increasing | 633 | 4.42E-28 | 51.7 | C:extracellular space; F:lipid binding |
| Efet.01.299564.g1732.t1 | short transient receptor potential channel 6 | 414 | 1.19E-81 | 90.7 | C:integral component of plasma membrane; F:store-operated calcium channel activity; P:manganese ion transport; P:regulation of cytosolic calcium ion concentration; P:calcium ion transmembrane transport; P:single fertilization |
| Efet.01.299597.g1733.t1 | gamma-tubulin complex component 5 isoform X2 | 342 | 3.52E-33 | 71.3 | C:microtubule organizing center |
| Efet.01.299613.g1735.t1 | DNA-binding RFX2- | 255 | 2.06E-39 | 85.4 | C:nucleus; F:DNA binding; P:regulation of transcription, DNA-templated |
| Efet.01.299695.g1736.t1 | hypothetical protein CAPTEDRAFT_222656 | 342 | 2.01E-20 | 57.05 | F:pyridoxal phosphate binding; P:tryptophan catabolic process; C:cytoplasm; F:kynureninase activity; F:catalytic activity; C:membrane; C:integral component of membrane; P:NAD biosynthetic process; F:carbohydrate binding |
| Efet.01.299728.g1737.t1 | GPC3 5 | 399 | 3.13E-10 | 64 |  |
| Efet.01.299777.g1739.t1 | CMY2 MIR ACT EC family class c beta-lactamase | 324 | 1.10E-11 | 61.55 | P:response to antibiotic; P:antibiotic catabolic process; C:outer membrane-bounded periplasmic space; F:beta-lactamase activity; F:hydrolase activity |
| Efet.01.299802.g1740.t1 | kelch 24 | 1728 | 8.37E-44 | 44.75 |  |
| Efet.01.299821.g1742.t1 | Rho GTPase-activating 100F | 297 | 9.73E-33 | 81.1 | P:signal transduction |
| Efet.01.299885.g1743.t1 | hypothetical protein CAPTEDRAFT_168270 | 939 | 1.22E-64 | 48.9 |  |
| Efet.01.299910.g1744.t1 | dynein beta flagellar outer arm-like isoform X5 | 219 | 2.64E-07 | 72.1 | F:microtubule motor activity; C:motile cilium; P:microtubule-based movement |
| Efet.01.29999.g339.t1 | sonic hedgehog | 573 | 5.06E-31 | 54.5 | P:chordate embryonic development; P:neuron differentiation; P:embryonic morphogenesis; P:tube development; P:pattern specification process; P:embryonic organ development; P:brain development; P:epithelium development; P:regulation of biological process; P:animal organ morphogenesis |
| Efet.01.300.g34.t1 | fer3 | 645 | 1.04E-14 | 74.7 | C:nucleus; F:DNA binding; P:negative regulation of transcription, DNA-templated; P:nervous system development; P:cell differentiation; F:protein dimerization activity |
| Efet.01.300048.g1.t1 | Zinc finger 395 | 549 | 4.23E-45 | 60.25 | F:metal ion binding |
| Efet.01.300101.g3.t1 | zinc finger SWIM domain-containing 1-like | 984 | 7.47E-42 | 47.6 |  |
| Efet.01.300117.g5.t1 | EF hand | 258 | 3.83E-27 | 71.1 | C:myosin complex; F:calcium ion binding |
| Efet.01.300138.g6.t1 | proton-coupled folate transporter | 447 | 1.33E-31 | 57.95 | P:ion transport; P:nitrogen compound transport; C:membrane; C:cell part; P:single-organism transport; P:organic substance transport |
| Efet.01.30014.g340.t1 | CAD | 300 | 1.53E-47 | 90.25 | F:aspartate carbamoyltransferase activity; F:zinc ion binding; F:dihydroorotase activity; P:glutamine metabolic process; P:protein autophosphorylation; F:protein kinase activity; F:carbamoyl-phosphate synthase (glutamine-hydrolyzing) activity; P:'de novo' UMP biosynthetic process; F:UTP binding; F:ATP binding; P:'de novo' pyrimidine nucleobase biosynthetic process; C:mitochondrion; F:aspartate binding; C:cytosol; C:nuclear matrix; P:peptidyl-threonine phosphorylation |
| Efet.01.300204.g8.t1 | type-1 angiotensin II receptor-like isoform X1 | 546 | 9.58E-32 | 62.2 | F:G-protein coupled receptor activity; P:G-protein coupled receptor signaling pathway; P:signal transduction; C:membrane; C:integral component of membrane; F:signal transducer activity |
| Efet.01.300366.g11.t1 | histamine H1 receptor | 1461 | 2.14E-46 | 46.25 | P:system process; P:signal transduction; C:membrane; F:signal transducer activity |
| Efet.01.30037.g342.t1 | DNA-binding Ikaros | 831 | 4.98E-22 | 75.4 | F:nucleic acid binding; F:metal ion binding |
| Efet.01.300383.g13.t1 | serine threonine- kinase MARK1-like | 273 | 1.37E-51 | 92.4 | C:microtubule cytoskeleton; P:cytoskeleton organization; F:protein serine/threonine kinase activity; P:protein phosphorylation; C:plasma membrane; F:magnesium ion binding; F:tau-protein kinase activity; F:ATP binding; F:phosphatidylinositol-4,5-bisphosphate binding; F:phosphatidylserine binding; P:neuron migration; C:cytoplasm; P:Wnt signaling pathway; F:phosphatidic acid binding; P:intracellular signal transduction |
| Efet.01.300388.g14.t1 | Peptidyl-prolyl cis-trans isomerase | 327 | 9.50E-50 | 88.6 | F:nucleotide binding; F:nucleic acid binding; F:peptidyl-prolyl cis-trans isomerase activity; P:protein peptidyl-prolyl isomerization; P:protein folding |
| Efet.01.300396.g15.t1 | hypothetical protein CRN43_08700, partial | 321 | 4.72E-72 | 100 |  |
| Efet.01.300440.g17.t1 | nicotinate phosphoribosyltransferase | 378 | 9.09E-86 | 98.2 | F:transferase activity, transferring glycosyl groups; P:nucleoside metabolic process |
| Efet.01.300440.g18.t1 | adenylosuccinate synthase | 1377 | 0 | 95.75 | F:magnesium ion binding; F:GTP binding; P:'de novo' AMP biosynthetic process; F:adenylosuccinate synthase activity; C:cytoplasm |
| Efet.01.300440.g19.t1 | ATP phosphoribosyltransferase regulatory subunit | 1149 | 0 | 92.85 | F:aminoacyl-tRNA ligase activity; F:transferase activity, transferring glycosyl groups; C:cytoplasm; P:histidine biosynthetic process |
| Efet.01.300440.g20.t1 | xylulokinase | 591 | 6.29E-93 | 78.1 | P:xylulose metabolic process; F:xylulokinase activity; P:carbohydrate phosphorylation |
| Efet.01.300516.g21.t1 | leucine-rich repeat-containing 24-like | 1833 | 6.80E-53 | 46.95 | C:membrane; C:integral component of membrane |
| Efet.01.300577.g22.t1 | ubiquitin-conjugating enzyme E2 J1 | 282 | 5.93E-23 | 95.25 | P:negative regulation of retrograde protein transport, ER to cytosol; F:ubiquitin protein ligase binding; F:ubiquitin conjugating enzyme activity; P:spermatid development; C:integral component of membrane; P:protein N-linked glycosylation via asparagine; P:ER-associated ubiquitin-dependent protein catabolic process; P:regulation of tumor necrosis factor biosynthetic process; P:protein ubiquitination |
| Efet.01.300726.g23.t1 | dynein heavy chain axonemal-like | 261 | 2.99E-34 | 79.45 | F:ATPase activity; F:ATP binding; F:microtubule motor activity; C:dynein complex; P:microtubule-based movement |
| Efet.01.300789.g25.t1 | zinc finger Xfin-like | 2136 | 5.00E-15 | 73.67 | F:nucleic acid binding; F:metal ion binding |
| Efet.01.300799.g26.t1 | DUF1016 domain-containing | 1065 | 0 | 91.65 |  |
| Efet.01.300799.g27.t1 | Reverse transcriptase | 2607 | 0 | 48.05 | F:hydrolase activity, acting on carbon-nitrogen (but not peptide) bonds; P:nitrogen compound metabolic process |
| Efet.01.300799.g28.t1 | ATP phosphoribosyltransferase regulatory subunit | 3948 | 0 | 79.25 | F:nucleic acid binding; F:transferase activity, transferring glycosyl groups; F:methyltransferase activity; P:methylation |
| Efet.01.300799.g29.t1 | helicase | 2859 | 0 | 95.9 | F:ATP binding; F:helicase activity |
| Efet.01.300799.g30.t1 | very short patch repair endonuclease | 363 | 2.45E-84 | 86.3 | P:nucleic acid phosphodiester bond hydrolysis; F:endonuclease activity; P:mismatch repair |
| Efet.01.300799.g31.t1 | DNA (cytosine-5-)-methyltransferase | 1224 | 0 | 93.25 | F:DNA (cytosine-5-)-methyltransferase activity; P:C-5 methylation of cytosine; P:DNA restriction-modification system |
| Efet.01.300799.g32.t1 | universal stress | 444 | 9.03E-78 | 94.35 | C:cytoplasm; P:response to stress |
| Efet.01.300799.g33.t1 | copper-translocating P-type ATPase | 2457 | 0 | 83.65 | F:nucleotide binding; F:cation-transporting ATPase activity; F:metal ion binding; P:cation transmembrane transport; C:integral component of membrane; P:metal ion transport; C:plasma membrane |
| Efet.01.300799.g34.t1 | cytochrome-c cbb3-type subunit I | 1461 | 0 | 95.55 | F:cytochrome-c oxidase activity; F:iron ion binding; C:integral component of membrane; P:hydrogen ion transmembrane transport; C:plasma membrane respiratory chain complex IV; P:aerobic respiration; F:heme binding |
| Efet.01.300799.g35.t1 | cytochrome-c cbb3-type subunit II | 564 | 2.49E-124 | 94.1 | F:cytochrome-c oxidase activity; F:iron ion binding; C:integral component of membrane; P:hydrogen ion transmembrane transport; P:aerobic respiration; F:heme binding |
| Efet.01.300799.g36.t1 | cytochrome-c cbb3-type subunit III | 915 | 0 | 90.75 | P:oxidative phosphorylation; P:proton transport; F:iron ion binding; F:oxidoreductase activity; F:electron carrier activity; C:integral component of membrane; P:oxidation-reduction process; C:plasma membrane; C:respiratory chain; F:heme binding |
| Efet.01.300799.g37.t1 | cytochrome c oxidase accessory | 1500 | 0 | 85.7 | C:integral component of membrane |
| Efet.01.300802.g38.t1 | hypothetical protein CAPTEDRAFT_215417 | 294 | 7.40E-14 | 67 | P:ion transport; C:membrane |
| Efet.01.300814.g39.t1 | hypothetical protein CAPTEDRAFT_223749 | 264 | 2.79E-22 | 63.29 | C:membrane; C:integral component of membrane |
| Efet.01.300927.g41.t1 | structural maintenance of chromosomes 5-like | 243 | 1.51E-17 | 69.4 | P:DNA repair; C:intracellular ribonucleoprotein complex; P:DNA recombination; C:intracellular non-membrane-bounded organelle |
| Efet.01.3010.g266.t1 | sodium channel type 5 subunit alpha-like | 234 | 1.20E-16 | 87.45 | P:regulation of postsynaptic membrane potential; F:calcium ion binding; P:regulation of ion transmembrane transport; C:voltage-gated sodium channel complex; P:sensory perception of pain; P:sodium ion transmembrane transport; F:voltage-gated sodium channel activity |
| Efet.01.30100.g344.t1 | SPRY domain-containing 3-like | 429 | 8.85E-38 | 70.1 |  |
| Efet.01.301100.g43.t1 | immunoglobulin superfamily DCC subclass member 4 isoform X1 | 492 | 1.73E-16 | 58.1 |  |
| Efet.01.301245.g45.t1 | peroxisomal biogenesis factor 19 | 282 | 5.83E-24 | 76.1 | C:peroxisome; P:metabolic process; F:hydrolase activity |
| Efet.01.301322.g46.t1 | DUF2723 domain-containing | 651 | 2.53E-136 | 62.7 | C:membrane; C:integral component of membrane; F:transferase activity |
| Efet.01.301334.g47.t1 | hypothetical protein CAPTEDRAFT_179200 | 282 | 1.02E-06 | 59 |  |
| Efet.01.301345.g48.t1 | annexin A4 | 393 | 4.08E-54 | 79.7 | F:calcium ion binding; F:calcium-dependent phospholipid binding |
| Efet.01.301388.g49.t1 | hypothetical protein CAPTEDRAFT_228921 | 627 | 2.85E-16 | 51.65 | C:membrane; C:integral component of membrane; F:carbohydrate binding |
| Efet.01.301428.g50.t1 | tRNA-dihydrouridine(16 17) synthase [NAD(P)(+)]-like | 261 | 1.76E-30 | 66.55 | F:tRNA dihydrouridine synthase activity; P:tRNA dihydrouridine synthesis; F:flavin adenine dinucleotide binding; P:oxidation-reduction process |
| Efet.01.301530.g52.t1 | disulfide isomerase | 285 | 3.90E-14 | 70.2 | C:cell; F:isomerase activity |
| Efet.01.301609.g56.t1 | inter-alpha-trypsin inhibitor heavy chain H3-like isoform X1 | 204 | 2.04E-11 | 61.25 | F:serine-type endopeptidase inhibitor activity; P:negative regulation of endopeptidase activity; P:hyaluronan metabolic process |
| Efet.01.301613.g57.t1 | phosphoglucomutase-1 isoform X2 | 345 | 1.67E-33 | 81.45 | F:magnesium ion binding; F:phosphoglucomutase activity; P:glycogen biosynthetic process; P:galactose catabolic process; C:cytosol; P:glucose metabolic process |
| Efet.01.301718.g60.t1 | E3 ubiquitin- ligase RNF19A | 555 | 2.75E-38 | 61 | F:protein binding; F:transferase activity; C:intracellular part; P:protein ubiquitination |
| Efet.01.301737.g61.t1 | hypothetical protein IIQ_02519, partial | 285 | 2.02E-06 | 66.5 |  |
| Efet.01.30190.g349.t1 | hypothetical protein CAPTEDRAFT_176129 | 1524 | 1.20E-09 | 84 |  |
| Efet.01.30192.g350.t1 | borderless isoform X2 | 450 | 5.76E-16 | 57.6 | C:membrane; C:integral component of membrane |
| Efet.01.301942.g66.t1 | calmodulin isoform X1 | 501 | 2.92E-90 | 94.95 | F:calcium ion binding |
| Efet.01.302008.g67.t1 | tyramine receptor 1-like | 2205 | 1.14E-100 | 68.5 | F:G-protein coupled receptor activity; P:G-protein coupled receptor signaling pathway; C:integral component of membrane |
| Efet.01.30203.g351.t1 | Nipped-B- | 375 | 7.25E-20 | 59.5 | F:protein binding; P:embryonic morphogenesis; P:mitotic sister chromatid cohesion; P:regulation of gene expression; P:regulation of multicellular organismal process; P:positive regulation of biological process; P:animal organ morphogenesis |
| Efet.01.302034.g68.t1 | proteasome subunit beta type 7 precursor | 351 | 2.98E-16 | 61.5 | C:intracellular part; F:hydrolase activity |
| Efet.01.302086.g69.t1 | sarcoplasmic calcium-binding -like | 228 | 2.96E-16 | 61.25 | F:calcium ion binding |
| Efet.01.302089.g70.t1 | WD repeat-containing 97-like isoform X2 | 639 | 4.70E-16 | 55.3 |  |
| Efet.01.302150.g71.t1 | FAM46C-like isoform X2 | 1281 | 2.42E-103 | 65.15 |  |
| Efet.01.302161.g72.t1 | T-box transcription factor TBX20 | 408 | 1.03E-09 | 58 | F:DNA binding; P:cardiac chamber morphogenesis; P:morphogenesis of an epithelium; P:heart valve development; P:regulation of transcription, DNA-templated; P:anatomical structure formation involved in morphogenesis; P:embryonic heart tube development; P:cellular process; P:cardiac septum development |
| Efet.01.302220.g74.t1 | homeobox HMX3 | 951 | 1.28E-57 | 80.05 | C:nucleus; P:posterior lateral line development; P:inner ear morphogenesis; P:regulation of transcription, DNA-templated; F:sequence-specific DNA binding |
| Efet.01.30223.g352.t1 | sodium-dependent multivitamin transporter | 267 | 3.72E-14 | 57.2 | C:membrane; P:transport |
| Efet.01.302232.g75.t1 | E3 ubiquitin- ligase Zswim2-like | 336 | 7.77E-22 | 79.65 | F:zinc ion binding |
| Efet.01.302257.g76.t1 | inositol-3-phosphate synthase 1-A-like | 678 | 3.21E-118 | 86.6 | F:inositol-3-phosphate synthase activity; P:inositol biosynthetic process; P:phospholipid biosynthetic process |
| Efet.01.302279.g77.t1 | hypothetical protein CAPTEDRAFT_222036 | 306 | 5.02E-18 | 60.19 | C:membrane; C:integral component of membrane |
| Efet.01.30229.g353.t1 | 60S ribosomal L3 | 480 | 3.68E-13 | 71.65 | C:intracellular ribonucleoprotein complex |
| Efet.01.30244.g354.t1 | beta-adrenergic receptor kinase 2-like | 216 | 1.71E-17 | 97.4 | P:viral entry into host cell; P:negative regulation of striated muscle contraction; P:cardiac muscle contraction; P:tachykinin receptor signaling pathway; P:follicle-stimulating hormone signaling pathway; F:ATP binding; P:response to oxidative stress; P:heart development; C:caveola; P:negative regulation of the force of heart contraction by chemical signal; F:beta-adrenergic receptor kinase activity; C:dendritic shaft; C:dendritic spine; F:Edg-2 lysophosphatidic acid receptor binding; C:axon; P:response to organic cyclic compound; P:positive regulation of catecholamine secretion; P:desensitization of G-protein coupled receptor protein signaling pathway; C:cytoplasm; F:G-protein coupled receptor kinase activity; P:viral genome replication; P:peptidyl-serine phosphorylation; C:basolateral plasma membrane; P:receptor internalization; C:apical plasma membrane; P:peptidyl-threonine phosphorylation |
| Efet.01.302504.g81.t1 | neuropeptide Y receptor-like | 870 | 8.79E-100 | 69.95 | P:neuropeptide signaling pathway; C:integral component of membrane; P:tachykinin receptor signaling pathway; C:plasma membrane; F:tachykinin receptor activity; F:neuropeptide Y receptor activity |
| Efet.01.302542.g83.t1 | Toll-like receptor | 1416 | 3.85E-88 | 48.65 | P:Toll signaling pathway; P:Roundabout signaling pathway; P:signal transduction; P:zygotic specification of dorsal/ventral axis; P:response to fungus; F:heparin binding; P:positive regulation of antibacterial peptide biosynthetic process; C:proteinaceous extracellular matrix; P:axon extension involved in axon guidance; F:Roundabout binding; C:extracellular space; F:transmembrane signaling receptor activity; P:regulation of axonogenesis; C:membrane; C:integral component of membrane; P:defense response to Gram-positive bacterium |
| Efet.01.302544.g84.t1 | Mitochondrial dicarboxylate carrier | 537 | 3.45E-69 | 81.75 | P:transmembrane transport; C:integral component of membrane |
| Efet.01.30262.g355.t1 | adenosine deaminase CECR1-like | 1098 | 6.92E-118 | 60.75 | C:extracellular space; F:deaminase activity |
| Efet.01.302674.g85.t1 | hypothetical protein COB15_04330 | 690 | 1.85E-06 | 46 |  |
| Efet.01.30272.g356.t1 | Bone morphogenetic receptor type- | 591 | 4.24E-107 | 85.4 | F:ATP binding; F:signal transducer, downstream of receptor, with serine/threonine kinase activity; F:metal ion binding; C:integral component of membrane; P:transmembrane receptor protein serine/threonine kinase signaling pathway; P:signal transduction by protein phosphorylation; F:transmembrane receptor protein serine/threonine kinase activity |
| Efet.01.302759.g87.t1 | hypothetical protein HELRODRAFT_190322 | 501 | 5.48E-10 | 69 | F:binding |
| Efet.01.302773.g88.t1 | casein kinase II subunit alpha isoform X2 | 444 | 5.44E-76 | 92.3 | F:ATP binding; F:RNA polymerase II carboxy-terminal domain kinase activity; P:protein phosphorylation |
| Efet.01.302791.g89.t1 | kinase C and casein kinase substrate in neurons 1 isoform X2 | 450 | 3.81E-43 | 67.4 | P:phosphorylation; F:kinase activity |
| Efet.01.302866.g90.t1 | dual 3 ,5 -cyclic-AMP and -GMP phosphodiesterase 11-like isoform X1 | 915 | 1.54E-64 | 66.75 | F:hydrolase activity |
| Efet.01.30289.g358.t1 | NHL repeat containing | 1395 | 2.39E-06 | 44.67 |  |
| Efet.01.303006.g93.t1 | prostacyclin synthase-like | 429 | 7.84E-62 | 72.35 | C:endoplasmic reticulum membrane; F:iron ion binding; F:oxidoreductase activity, acting on paired donors, with incorporation or reduction of molecular oxygen; C:integral component of membrane; P:oxidation-reduction process; F:monooxygenase activity; F:heme binding |
| Efet.01.303057.g94.t1 | histone acetyltransferase p300 | 435 | 4.31E-63 | 80.25 | P:positive regulation by host of viral transcription; P:histone H4 acetylation; P:positive regulation of protein binding; P:histone H2B acetylation; P:response to estrogen; F:protein C-terminus binding; F:RNA polymerase II core promoter sequence-specific DNA binding; P:transcription from RNA polymerase II promoter; P:intrinsic apoptotic signaling pathway in response to DNA damage by p53 class mediator; F:transcriptional activator activity, RNA polymerase II transcription regulatory region sequence-specific binding; P:fat cell differentiation; P:positive regulation of sequence-specific DNA binding transcription factor activity; P:regulation of androgen receptor signaling pathway; F:RNA polymerase II activating transcription factor binding; P:negative regulation of transcription from RNA polymerase II promoter; C:transcription factor complex; P:heart development; F:p53 binding; P:response to hypoxia; P:N-terminal peptidyl-lysine acetylation; P:platelet formation; P:cellular response to UV; P:megakaryocyte development; P:lung development; F:chromatin DNA binding; F:zinc ion binding; P:B cell differentiation; P:regulation of tubulin deacetylation; P:positive regulation of transcription from RNA polymerase II promoter involved in unfolded protein response; F:beta-catenin binding; P:circadian rhythm; F:damaged DNA binding; C:histone acetyltransferase complex; P:animal organ morphogenesis; F:histone acetyltransferase activity; F:lysine N-acetyltransferase activity, acting on acetyl phosphate as donor; F:pre-mRNA intronic binding; F:transcription coactivator activity; F:androgen receptor binding; C:cytoplasm; P:somitogenesis; P:skeletal muscle tissue development; P:protein stabilization |
| Efet.01.303093.g95.t1 | leucine zipper tumor suppressor 2 homolog isoform X1 | 1356 | 1.02E-12 | 58.79 |  |
| Efet.01.303139.g97.t1 | tRNA-dihydrouridine(20a 20b) synthase [NAD(P)+]-like isoform X2 | 345 | 7.83E-30 | 68.1 | F:tRNA dihydrouridine synthase activity; P:metabolic process; C:cytosol |
| Efet.01.303203.g98.t1 | katanin p60 ATPase-containing subunit A1 | 501 | 3.75E-91 | 88.95 | C:spindle pole; P:mitotic nuclear division; F:microtubule-severing ATPase activity; C:microtubule; F:microtubule binding; P:cell division; C:nucleus; F:ATP binding; C:centrosome; P:microtubule severing; C:cytoplasm; F:protein heterodimerization activity; C:midbody |
| Efet.01.303226.g99.t1 | RNA-directed DNA polymerase from mobile element jockey-like | 477 | 1.69E-10 | 60.5 |  |
| Efet.01.303259.g102.t1 | heat shock 70 kDa 4L-like | 285 | 1.00E-07 | 69.6 | F:nucleotide binding; F:ATP binding |
| Efet.01.303296.g103.t1 | E3 ubiquitin- ligase HECTD1-like | 423 | 1.80E-77 | 95.35 | P:negative regulation of protein localization to plasma membrane; F:ubiquitin protein ligase activity; P:heart valve development; P:ventricular septum development; P:protein ubiquitination involved in ubiquitin-dependent protein catabolic process; C:nucleus; F:protein binding; P:neural tube closure; C:cytoplasm; F:metal ion binding; P:aorta development; P:natural killer cell differentiation; P:trophoblast giant cell differentiation; P:spongiotrophoblast differentiation; P:protein K63-linked ubiquitination; F:ligase activity; P:protein autoubiquitination |
| Efet.01.303314.g104.t1 | dynactin subunit 1-like isoform X11 | 363 | 4.80E-29 | 69.7 |  |
| Efet.01.303320.g105.t1 | electroneutral sodium bicarbonate exchanger 1-like isoform X1 | 240 | 1.69E-13 | 72.95 | P:inorganic anion transport; P:anion transmembrane transport; C:integral component of membrane; F:inorganic anion exchanger activity |
| Efet.01.303476.g109.t1 | DNA polymerase epsilon subunit 4 | 228 | 1.06E-15 | 70.55 | F:protein heterodimerization activity |
| Efet.01.30349.g360.t1 | probable leucine--tRNA mitochondrial | 633 | 1.43E-14 | 48.4 | F:catalytic activity |
| Efet.01.303499.g111.t1 | tyrosine- phosphatase Lar-like isoform X1 | 327 | 1.78E-17 | 62.95 |  |
| Efet.01.303502.g112.t1 | dystrophin-like isoform X1 | 342 | 3.10E-24 | 74.95 |  |
| Efet.01.303542.g115.t1 | glycine receptor subunit alpha-3-like isoform X1 | 351 | 1.40E-25 | 65 | P:ion transmembrane transport; C:integral component of membrane; C:cell junction; F:extracellular ligand-gated ion channel activity; C:synapse; C:plasma membrane |
| Efet.01.303580.g116.t1 | transcription factor AP-1 | 1014 | 3.07E-36 | 51.65 | P:positive regulation of RNA biosynthetic process; P:response to organic substance; F:transcription regulatory region DNA binding; P:nervous system development; P:cell surface receptor signaling pathway; P:animal organ development; P:single-organism cellular process; P:regulation of cell proliferation; P:negative regulation of RNA biosynthetic process; P:regulation of transcription from RNA polymerase II promoter; C:nucleus; P:negative regulation of gene expression; F:transcription factor activity, sequence-specific DNA binding; F:protein binding; P:positive regulation of gene expression; P:response to metal ion; C:macromolecular complex |
| Efet.01.303580.g117.t1 | ubiquitin-related modifier 1 | 297 | 1.95E-42 | 82.95 | C:extracellular exosome; C:cytosol; F:sulfurtransferase activity; P:tRNA wobble uridine modification; P:protein urmylation; P:tRNA thio-modification |
| Efet.01.303598.g118.t1 | cell number regulator 10-like | 342 | 2.26E-21 | 66.9 | C:membrane |
| Efet.01.303664.g120.t1 | LIM homeobox | 345 | 7.88E-14 | 92.6 | F:zinc ion binding; P:inner ear development; P:motor neuron axon guidance; P:medial motor column neuron differentiation; P:transcription, DNA-templated; P:spinal cord association neuron differentiation; F:sequence-specific DNA binding; P:placenta development; P:positive regulation of transcription from RNA polymerase II promoter; P:spinal cord motor neuron cell fate specification; P:animal organ morphogenesis; P:ventral spinal cord interneuron specification; P:pituitary gland development; C:nucleus; C:transcription factor complex; F:transcription factor activity, RNA polymerase II transcription factor binding; P:negative regulation of apoptotic process |
| Efet.01.303680.g121.t1 | TALPID3 -like isoform X2 | 333 | 4.08E-09 | 82 |  |
| Efet.01.303700.g123.t1 | hypothetical protein CAPTEDRAFT_202967 | 3447 | 2.23E-34 | 57 |  |
| Efet.01.303724.g124.t1 | Tyrosine- phosphatase non-receptor type | 273 | 5.06E-14 | 78.05 | F:SH3/SH2 adaptor activity; P:inner ear development; P:regulation of phosphatidylinositol 3-kinase signaling; P:epidermal growth factor receptor signaling pathway; P:phosphatidylinositol-3-phosphate biosynthetic process; P:triglyceride metabolic process; P:fibroblast growth factor receptor signaling pathway; P:phosphatidylinositol phosphorylation; F:insulin receptor binding; F:phosphatidylinositol-4,5-bisphosphate 3-kinase activity; P:negative regulation of growth hormone secretion; P:face morphogenesis; P:hormone-mediated signaling pathway; P:microvillus organization; P:activation of MAPK activity; P:axonogenesis; F:phospholipase binding; P:platelet formation; C:cytosol; F:protein domain specific binding; P:multicellular organismal reproductive process; P:genitalia development; P:platelet-derived growth factor receptor signaling pathway; C:protein complex; P:regulation of protein export from nucleus; P:heart jogging; P:positive regulation of mitotic cell cycle; C:nucleus; F:D1 dopamine receptor binding; P:DNA damage checkpoint; P:neurotrophin TRK receptor signaling pathway; P:phosphatidylinositol-mediated signaling; F:insulin receptor substrate binding; P:glucose homeostasis; P:ephrin receptor signaling pathway; F:receptor tyrosine kinase binding; P:multicellular organism growth; P:organ growth; P:regulation of multicellular organism growth; P:positive regulation of glucose import in response to insulin stimulus; P:Bergmann glial cell differentiation; P:negative regulation of cell death; P:negative regulation of insulin secretion; P:convergent extension involved in gastrulation; P:convergent extension involved in axis elongation; P:cerebellar cortex formation; P:integrin-mediated signaling pathway; P:negative regulation of cortisol secretion; P:megakaryocyte development; F:peptide hormone receptor binding; P:abortive mitotic cell cycle; P:atrioventricular canal development; P:platelet activation; P:peptidyl-tyrosine dephosphorylation; P:T cell costimulation; P:intestinal epithelial cell migration; P:regulation of type I interferon-mediated signaling pathway; P:regulation of protein complex assembly; P:positive regulation of hormone secretion; P:hormone metabolic process; P:embryonic viscerocranium morphogenesis; P:negative regulation of cell adhesion mediated by integrin; F:non-membrane spanning protein tyrosine phosphatase activity; P:homeostasis of number of cells within a tissue; C:mitochondrion; P:positive regulation of ERK1 and ERK2 cascade; P:leukocyte migration; F:1-phosphatidylinositol-3-kinase activity |
| Efet.01.303737.g125.t1 | KH domain- RNA- signal transduction-associated 3 | 648 | 1.35E-30 | 52.05 | F:RNA binding; F:protein binding; C:membrane; P:regulation of gene expression; P:regulation of cellular macromolecule biosynthetic process; P:regulation of nitrogen compound metabolic process; C:intracellular part; P:single-organism process; P:positive regulation of biological process; P:regulation of primary metabolic process; P:cellular process |
| Efet.01.303743.g126.t1 | tetratricopeptide repeat 6 | 561 | 2.10E-45 | 63.15 | C:BBSome |
| Efet.01.303772.g127.t1 | Golgi resident GCP60-like isoform X1 | 852 | 3.41E-78 | 78.1 | F:fatty-acyl-CoA binding; C:integral component of membrane; P:transport |
| Efet.01.30378.g362.t1 | Chitin synthase 1 | 390 | 1.72E-23 | 61.55 | F:transferase activity, transferring hexosyl groups; C:membrane; C:integral component of membrane |
| Efet.01.303797.g129.t1 | stum homolog | 261 | 7.97E-15 | 54.33 | C:membrane; C:integral component of membrane |
| Efet.01.30380.g363.t1 | mab-21-like | 996 | 0 | 83.9 |  |
| Efet.01.303844.g131.t1 | potassium voltage-gated channel Shab-like | 876 | 5.34E-85 | 85.4 | F:voltage-gated potassium channel activity; P:potassium ion transmembrane transport; C:voltage-gated potassium channel complex; P:regulation of ion transmembrane transport; P:protein homooligomerization |
| Efet.01.303864.g134.t1 | EGF-like domain-containing | 399 | 3.42E-14 | 50.58 | F:calcium ion binding; C:membrane; C:integral component of membrane |
| Efet.01.303916.g136.t1 | fibrillin-2-like isoform X2 | 666 | 1.69E-77 | 64.95 | F:calcium ion binding; F:extracellular matrix structural constituent; C:proteinaceous extracellular matrix |
| Efet.01.303950.g137.t1 | huntingtin isoform X3 | 906 | 1.17E-10 | 62.05 | C:nucleus; F:protein binding; P:regulation of cellular process; P:establishment of localization; C:endomembrane system; C:cytoplasmic part; C:intracellular organelle part; P:single-organism process; P:cellular process |
| Efet.01.303971.g138.t1 | SRSF kinase 3-like isoform X1 | 231 | 2.25E-32 | 95.3 | P:positive regulation of cell proliferation; C:nucleolus; F:protein serine/threonine kinase activity; P:protein phosphorylation; P:spliceosomal complex assembly; C:nucleoplasm; F:RNA binding; F:magnesium ion binding; F:ATP binding; C:cytoplasm; P:positive regulation of viral genome replication; P:negative regulation of viral genome replication; P:intracellular signal transduction |
| Efet.01.304010.g139.t1 | rap guanine nucleotide exchange factor 4-like | 468 | 1.16E-18 | 61.6 | P:signal transduction |
| Efet.01.304054.g141.t1 | Hau-cytoplasmic intermediate filament | 318 | 6.91E-12 | 70.18 | F:structural molecule activity; C:intermediate filament |
| Efet.01.304139.g143.t1 | headcase isoform X1 | 1335 | 6.89E-94 | 57.05 |  |
| Efet.01.304160.g144.t1 | electron transfer flavo -ubiquinone mitochondrial | 321 | 1.12E-36 | 89.95 | P:response to oxidative stress; C:integral component of mitochondrial inner membrane; P:fatty acid beta-oxidation using acyl-CoA dehydrogenase; F:electron carrier activity; F:electron-transferring-flavoprotein dehydrogenase activity; F:ubiquinone binding; F:4 iron, 4 sulfur cluster binding; P:electron transport chain |
| Efet.01.304172.g145.t1 | arylamine N- pineal gland isozyme NAT-3-like | 633 | 6.41E-24 | 48.7 | F:acetyltransferase activity; P:metabolic process |
| Efet.01.304253.g147.t1 | leucine-rich repeat-containing 63-like isoform X2 | 702 | 2.73E-21 | 58.1 |  |
| Efet.01.304280.g148.t1 | Voltage-dependent T-type calcium channel subunit alpha-1H | 264 | 2.85E-15 | 80.05 | P:calcium ion import; C:voltage-gated calcium channel complex; P:regulation of ion transmembrane transport; P:calcium ion transmembrane transport; F:low voltage-gated calcium channel activity |
| Efet.01.304293.g149.t1 | DNA-directed RNA polymerases and III subunit RPABC1 | 222 | 2.87E-42 | 97.8 | F:DNA binding; F:DNA-directed 5'-3' RNA polymerase activity; P:transcription from RNA polymerase II promoter; C:DNA-directed RNA polymerase II, core complex |
| Efet.01.304378.g151.t1 | tripartite motif-containing 2-like | 855 | 1.32E-16 | 49 | F:zinc ion binding; P:signal transduction; F:metal ion binding; P:regulation of apoptotic process; C:intracellular |
| Efet.01.304410.g153.t1 | FMRFamide receptor-like | 1107 | 2.02E-58 | 51.3 | F:G-protein coupled receptor activity; F:G-protein coupled peptide receptor activity; P:G-protein coupled receptor signaling pathway; P:signal transduction; C:membrane; C:integral component of membrane; F:signal transducer activity |
| Efet.01.30444.g366.t1 | IQ domain-containing D-like | 903 | 5.47E-13 | 61 |  |
| Efet.01.30445.g367.t1 | heat shock 70 kDa 12A-like isoform X2 | 435 | 1.35E-51 | 65.9 |  |
| Efet.01.304486.g157.t1 | zinc finger 511-like | 429 | 3.29E-30 | 57.05 | F:metal ion binding |
| Efet.01.304629.g158.t1 | hypothetical protein HELRODRAFT_191982 | 639 | 3.36E-19 | 55 | F:transferase activity, transferring glycosyl groups; C:membrane; C:integral component of membrane; P:Wnt signaling pathway; P:negative regulation of Wnt signaling pathway; F:beta-catenin binding |
| Efet.01.30465.g370.t1 | fibroblast growth factor receptor-like 1 | 1149 | 2.09E-26 | 66.8 | P:ventricular system development |
| Efet.01.30468.g371.t1 | cGMP-dependent isozyme 2 forms cD5 T2 | 279 | 5.12E-09 | 78.8 | F:ATP binding; F:cGMP-dependent protein kinase activity; P:protein phosphorylation; F:cGMP binding |
| Efet.01.304838.g159.t1 | unconventional myosin-VIIa | 231 | 8.67E-15 | 68.8 | C:photoreceptor connecting cilium; P:auditory receptor cell stereocilium organization; C:myosin complex; F:protein domain specific binding; F:purine ribonucleoside binding; C:synapse; F:anion binding; P:intracellular protein transport; P:visual perception; C:photoreceptor outer segment; F:motor activity; P:sensory perception of sound; F:adenyl ribonucleotide binding; F:actin binding; C:microvillus; C:melanosome; P:phagolysosome assembly; P:post-embryonic animal organ morphogenesis; P:pigment granule transport; C:stereocilium; C:photoreceptor inner segment; F:protein complex binding; C:apical plasma membrane |
| Efet.01.304956.g163.t1 | GTP-binding Di-Ras1 | 249 | 3.45E-28 | 81.65 | F:GTP binding; P:small GTPase mediated signal transduction; C:membrane; C:intracellular |
| Efet.01.30497.g372.t1 | probable beta-D-xylosidase 2 | 645 | 1.68E-53 | 67.2 | F:hydrolase activity |
| Efet.01.305117.g164.t1 | ankyrin-3-like isoform X15 | 1041 | 1.97E-18 | 63.9 |  |
| Efet.01.305137.g165.t1 | turtle homolog A-like | 306 | 1.13E-22 | 62.05 | C:membrane; C:integral component of membrane |
| Efet.01.305147.g167.t1 | beta-1,3-galactosyltransferase 1-like | 1476 | 2.53E-136 | 64.9 | F:transferase activity, transferring glycosyl groups; C:membrane; C:Golgi apparatus |
| Efet.01.305150.g168.t1 | cyclin-dependent kinase | 480 | 2.63E-73 | 92.65 | P:phosphorylation of RNA polymerase II C-terminal domain; P:regulation of MAP kinase activity; F:RNA polymerase II carboxy-terminal domain kinase activity; P:regulation of mitotic nuclear division; C:nuclear speck; C:nuclear cyclin-dependent protein kinase holoenzyme complex; P:hemopoiesis; P:positive regulation of cell proliferation; P:protein autophosphorylation; C:Golgi apparatus; F:cyclin-dependent protein serine/threonine kinase activity; C:nucleolus; F:RNA binding; P:cell division; F:ATP binding; C:extracellular space; C:cyclin K-CDK12 complex; C:cyclin K-CDK13 complex; P:alternative mRNA splicing, via spliceosome; P:regulation of RNA splicing; P:viral process; F:cyclin binding |
| Efet.01.305235.g169.t1 | dTDP-4-dehydrorhamnose reductase | 972 | 0 | 86.7 | P:oxidation-reduction process; F:dTDP-4-dehydrorhamnose reductase activity |
| Efet.01.305235.g170.t1 | glucose-1-phosphate thymidylyltransferase | 1254 | 0 | 88.95 | F:glucose-1-phosphate thymidylyltransferase activity; F:metal ion binding; P:extracellular polysaccharide biosynthetic process |
| Efet.01.305235.g171.t1 | erythromycin biosynthesis sensory transduction eryC1 | 459 | 4.04E-84 | 73.6 | F:transaminase activity |
| Efet.01.305235.g172.t1 | 1 family aminotransferase | 237 | 8.05E-46 | 66.7 | F:transaminase activity |
| Efet.01.305235.g173.t1 | Glycosyl group 2 family | 504 | 6.58E-70 | 73.5 | C:integral component of membrane; F:transferase activity |
| Efet.01.305235.g174.t1 | glycosyltransferase | 327 | 2.55E-45 | 75.6 | C:integral component of membrane; F:transferase activity |
| Efet.01.305235.g175.t1 | isomerase | 519 | 9.60E-108 | 77.1 | F:transferase activity; F:isomerase activity |
| Efet.01.305248.g176.t1 | -tyrosine sulfotransferase 1-like | 744 | 8.33E-87 | 70.65 | F:transferase activity |
| Efet.01.305267.g178.t1 | FMRFamide receptor-like | 1170 | 3.80E-134 | 65.25 | F:G-protein coupled receptor activity; C:membrane |
| Efet.01.305275.g179.t1 | G- coupled receptor moody-like | 1401 | 3.75E-89 | 48.6 | F:G-protein coupled receptor activity; P:signal transduction; C:integral component of membrane |
| Efet.01.305383.g180.t1 | carboxyvinyl-carboxyphosphonate phosphorylmutase | 492 | 2.62E-100 | 87.15 | F:lyase activity |
| Efet.01.305383.g181.t1 | tripartite tricarboxylate transporter family receptor | 963 | 0 | 90.6 | C:outer membrane-bounded periplasmic space |
| Efet.01.305383.g182.t1 | gfo Idh family oxidoreductase | 1092 | 0 | 78.85 | F:oxidoreductase activity; P:oxidation-reduction process |
| Efet.01.305383.g183.t1 | sugar phosphate isomerase epimerase | 912 | 0 | 85.55 | F:isomerase activity |
| Efet.01.30539.g374.t1 | phosphatase Slingshot isoform X3 | 3570 | 1.50E-46 | 76.85 | F:DNA binding; F:protein tyrosine phosphatase activity; P:peptidyl-tyrosine dephosphorylation; F:protein tyrosine/serine/threonine phosphatase activity |
| Efet.01.305495.g185.t1 | hypothetical protein CAPTEDRAFT_187428 | 339 | 7.32E-25 | 61.5 |  |
| Efet.01.305590.g186.t1 | ABC transporter permease | 309 | 6.05E-47 | 65.4 | C:membrane |
| Efet.01.305590.g187.t1 | hypothetical protein B5766_09380 | 588 | 8.93E-62 | 84 | C:membrane |
| Efet.01.305590.g188.t1 | sugar ABC transporter substrate-binding | 1104 | 0 | 57.05 | C:membrane; C:integral component of membrane |
| Efet.01.305590.g189.t1 | short-chain dehydrogenase | 1242 | 0 | 65.35 | F:3-oxoacyl-[acyl-carrier-protein] reductase (NADPH) activity; F:oxidoreductase activity; P:oxidation-reduction process |
| Efet.01.305634.g191.t1 | ADAM family mig-17 | 312 | 1.20E-17 | 54 | F:chitin binding; P:proteolysis; P:chitin metabolic process; C:extracellular region; F:metalloendopeptidase activity; F:metallopeptidase activity |
| Efet.01.305661.g193.t1 | gliding motility-associated C-terminal domain-containing | 534 | 7.17E-126 | 57.65 |  |
| Efet.01.305662.g194.t1 | GRB2-associated and regulator of MAPK -like | 429 | 1.39E-17 | 61.3 |  |
| Efet.01.305666.g196.t1 | 5-hydroxytryptamine receptor 1A-like | 474 | 2.43E-44 | 82.9 | C:integral component of plasma membrane; P:neuropeptide signaling pathway; F:adrenergic receptor activity; P:adrenergic receptor signaling pathway; F:G-protein coupled acetylcholine receptor activity; P:G-protein coupled acetylcholine receptor signaling pathway; F:neuropeptide Y receptor activity |
| Efet.01.305731.g198.t1 | TNF receptor-associated factor | 723 | 2.50E-90 | 82.75 | P:Toll signaling pathway; C:cytoplasmic side of plasma membrane; C:endosome; P:regulation of interferon-beta production; P:apoptotic process; C:cytosol; C:CD40 receptor complex; F:thioesterase binding; F:ligase activity; F:protein kinase binding; P:negative regulation of type I interferon production; F:zinc ion binding; F:protein phosphatase binding; P:negative regulation of NF-kappaB transcription factor activity; P:regulation of proteolysis; F:tumor necrosis factor receptor binding; P:regulation of apoptotic process; F:signal transducer activity; F:ubiquitin-protein transferase activity; P:tumor necrosis factor-mediated signaling pathway; F:ubiquitin protein ligase binding; P:innate immune response; C:mitochondrion; P:TRIF-dependent toll-like receptor signaling pathway; P:protein ubiquitination; P:regulation of defense response to virus |
| Efet.01.305761.g200.t1 | zinc finger 596-like | 903 | 4.18E-59 | 57.25 | F:nucleic acid binding; F:metal ion binding |
| Efet.01.305978.g202.t1 | protein-tyrosine sulfotransferase 1-like isoform X2 | 237 | 3.39E-15 | 72.45 | C:membrane; F:transferase activity |
| Efet.01.306049.g203.t1 | hypothetical protein HELRODRAFT_190744 | 270 | 1.50E-27 | 80 |  |
| Efet.01.30611.g380.t1 | AChain Snapshots Of The Rna Processing Factor Scaf8 Bound To Different Phosphorylated Forms Of The Carboxy-terminal Domain Of Rna-polymerase Ii | 822 | 9.77E-37 | 82.35 | F:nucleotide binding; P:RNA splicing; F:protein C-terminus binding; F:RNA polymerase core enzyme binding; C:spliceosomal complex; C:nuclear matrix; P:mRNA processing; C:nucleoplasm; F:RNA binding |
| Efet.01.306163.g204.t1 | photoreceptor outer segment membrane glyco 2-like | 321 | 2.57E-29 | 66.25 | C:integral component of membrane |
| Efet.01.306217.g206.t1 | arylesterase | 582 | 2.77E-121 | 91.95 | F:arylesterase activity |
| Efet.01.306219.g207.t1 | FMRFamide receptor | 1320 | 5.53E-77 | 55.55 | F:G-protein coupled receptor activity; F:G-protein coupled peptide receptor activity; P:G-protein coupled receptor signaling pathway; C:membrane; C:integral component of membrane |
| Efet.01.306368.g208.t1 | WD repeat-containing 5B | 399 | 3.43E-09 | 62 | C:membrane; C:integral component of membrane |
| Efet.01.30637.g381.t1 | dopamine receptor 1-like isoform X1 | 2019 | 1.36E-56 | 60.5 | P:signal transduction; F:signal transducer activity |
| Efet.01.306434.g210.t1 | NFX1-type zinc finger-containing 1-like | 987 | 8.78E-52 | 65.8 | C:nucleus; F:transcription factor activity, sequence-specific DNA binding; F:zinc ion binding; P:regulation of transcription, DNA-templated |
| Efet.01.306437.g211.t1 | neogenin isoform X1 | 429 | 1.00E-15 | 50.55 | P:iron ion homeostasis; P:positive regulation of BMP signaling pathway; C:integral component of plasma membrane; C:membrane; C:integral component of membrane; P:nervous system development; P:axon guidance; F:receptor activity |
| Efet.01.30644.g382.t1 | mitochondrial genome maintenance exonuclease 1 | 276 | 2.37E-09 | 78.6 | P:mitochondrial DNA repair; F:single-stranded DNA exodeoxyribonuclease activity; F:endonuclease activity; C:mitochondrion; P:mitochondrial DNA replication |
| Efet.01.30651.g383.t1 | hypothetical protein CAPTEDRAFT_220151 | 531 | 5.74E-24 | 50 |  |
| Efet.01.306527.g213.t1 | cholecystokinin receptor type A-like | 402 | 5.60E-28 | 84.65 | P:G-protein coupled receptor signaling pathway; F:gastrin receptor activity; C:integral component of membrane |
| Efet.01.30684.g388.t1 | 25-hydroxycholesterol 7-alpha-hydroxylase-like | 1392 | 0 | 60.6 | P:regulation of cellular process; F:oxidoreductase activity; F:metal ion binding; P:lipid metabolic process; C:endoplasmic reticulum; P:single-organism cellular process; C:membrane part; P:response to stimulus |
| Efet.01.306927.g215.t1 | 28S ribosomal mitochondrial | 243 | 6.12E-15 | 72.59 | F:structural constituent of ribosome; C:mitochondrion; C:ribosome; P:translation |
| Efet.01.306959.g217.t1 | tolloid 1 | 258 | 1.82E-10 | 58.83 | C:membrane; C:integral component of membrane |
| Efet.01.30699.g390.t1 | Coatomer subunit beta | 288 | 5.14E-26 | 79.7 | P:vesicle-mediated transport; F:structural molecule activity; C:Golgi membrane; P:intracellular protein transport; C:COPI vesicle coat |
| Efet.01.307.g35.t1 | otoferlin-like isoform X3 | 531 | 3.37E-51 | 70.3 | C:membrane; C:cell part; C:synapse |
| Efet.01.307000.g218.t1 | nachr subunit | 594 | 3.09E-77 | 78 | P:synaptic transmission, cholinergic; F:acetylcholine receptor activity; P:neuromuscular synaptic transmission; C:postsynaptic membrane; P:signal transduction; P:cation transmembrane transport; C:cell junction; F:extracellular ligand-gated ion channel activity; F:acetylcholine binding; C:acetylcholine-gated channel complex; P:response to nicotine |
| Efet.01.307049.g220.t1 | cadherin EGF LAG seven-pass G-type receptor 2-like isoform X3 | 285 | 7.50E-28 | 78.65 | F:G-protein coupled receptor activity; P:G-protein coupled receptor signaling pathway; F:calcium ion binding; C:integral component of membrane; P:cell surface receptor signaling pathway; P:homophilic cell adhesion via plasma membrane adhesion molecules; C:plasma membrane |
| Efet.01.307104.g222.t1 | Epidermal growth factor receptor | 435 | 9.82E-21 | 59.9 | P:epidermal growth factor receptor signaling pathway; P:negative regulation of protein catabolic process; P:protein autophosphorylation; P:positive regulation of production of miRNAs involved in gene silencing by miRNA; P:animal organ development; P:single-organism metabolic process; P:positive regulation of cell growth; C:multivesicular body, internal vesicle lumen; F:protein heterodimerization activity; P:regulation of nitric-oxide synthase activity; P:positive regulation of epithelial cell proliferation; P:intracellular signal transduction; P:positive regulation of DNA repair; F:protein phosphatase binding; C:receptor complex; P:single-organism cellular process; F:epidermal growth factor-activated receptor activity; C:early endosome membrane; F:actin filament binding; C:nucleus; C:endocytic vesicle; F:ubiquitin protein ligase binding; P:localization; P:cellular component organization; C:basolateral plasma membrane; P:positive regulation of protein kinase B signaling; P:positive regulation of cell migration; P:cell proliferation; P:positive regulation of MAP kinase activity; P:positive regulation of nitric oxide biosynthetic process; F:heterocyclic compound binding; P:positive regulation of DNA replication; P:positive regulation of transcription from RNA polymerase II promoter; P:positive regulation of cyclin-dependent protein serine/threonine kinase activity involved in G1/S transition of mitotic cell cycle; P:regulation of peptidyl-tyrosine phosphorylation; P:response to UV-A; P:cellular response to estradiol stimulus; C:focal adhesion; C:plasma membrane protein complex; P:positive regulation of phospholipase activity; P:positive regulation of catenin import into nucleus; P:single organismal cell-cell adhesion; F:identical protein binding; F:chromatin binding; C:cell surface; F:organic cyclic compound binding; P:negative regulation of apoptotic process; C:membrane raft; P:positive regulation of ERK1 and ERK2 cascade; F:nitric-oxide synthase regulator activity |
| Efet.01.307113.g223.t1 | hypothetical protein HELRODRAFT_166632 | 723 | 1.20E-07 | 77.67 | F:metal ion binding |
| Efet.01.30727.g392.t1 | innexin unc-9 isoform X6 | 726 | 8.60E-60 | 58.9 | C:gap junction; P:ion transport; C:integral component of membrane; C:plasma membrane |
| Efet.01.30727.g393.t1 | innexin unc-9-like isoform X2 | 648 | 1.12E-79 | 63.1 | C:membrane; C:cell junction; P:transport |
| Efet.01.307398.g225.t1 | solute carrier family 28 member 3-like | 273 | 1.82E-11 | 59.33 | F:nucleoside transmembrane transporter activity; C:membrane; C:integral component of membrane; P:nucleoside transmembrane transport |
| Efet.01.30746.g394.t1 | junctophilin-1-like isoform X1 | 648 | 5.68E-54 | 66 | P:pseudouridine synthesis; F:RNA binding; P:RNA modification; F:pseudouridine synthase activity |
| Efet.01.307480.g226.t1 | tachykinin-like peptides receptor 99D | 405 | 5.63E-09 | 68.3 | C:integral component of membrane; P:tachykinin receptor signaling pathway; C:plasma membrane; F:tachykinin receptor activity |
| Efet.01.30749.g396.t1 | tubulin beta-4B chain | 954 | 7.14E-169 | 92 | F:GTP binding; C:nucleus; C:myelin sheath; C:extracellular exosome; C:tubulin complex; C:cytoplasm; F:double-stranded RNA binding; F:GTPase activity; F:structural constituent of cytoskeleton; C:extracellular matrix; C:microtubule; P:microtubule-based process |
| Efet.01.30764.g398.t1 | filamin-C-like isoform X1 | 288 | 6.61E-44 | 79.8 | F:actin filament binding |
| Efet.01.307677.g230.t1 | zinc finger | 285 | 1.81E-52 | 93.2 | P:positive regulation of histone acetylation; P:negative regulation of intrinsic apoptotic signaling pathway in response to DNA damage; P:palate development; P:cell migration involved in endocardial cushion formation; P:positive regulation of fat cell differentiation; F:sequence-specific DNA binding; P:epithelial to mesenchymal transition involved in endocardial cushion formation; P:epithelium development; P:regulation of osteoblast differentiation; P:sensory perception of sound; P:negative regulation of keratinocyte proliferation; P:white fat cell differentiation; F:transcriptional repressor activity, RNA polymerase II core promoter proximal region sequence-specific binding; P:negative regulation of cell adhesion involved in substrate-bound cell migration; P:negative regulation of anoikis; P:negative regulation of vitamin D receptor signaling pathway; P:pigmentation; F:transcription factor activity, RNA polymerase II distal enhancer sequence-specific binding; P:negative regulation of vitamin D biosynthetic process; P:osteoblast differentiation; P:cellular response to ionizing radiation; P:regulation of chemokine production; P:canonical Wnt signaling pathway; P:regulation of bicellular tight junction assembly; P:negative regulation of catenin import into nucleus; P:regulation of branching involved in salivary gland morphogenesis; P:negative regulation of extrinsic apoptotic signaling pathway in absence of ligand; P:negative regulation of DNA damage response, signal transduction by p53 class mediator; P:neural crest cell development; P:cartilage morphogenesis; P:negative regulation of cell-cell adhesion by negative regulation of transcription from RNA polymerase II promoter; F:chromatin binding; P:negative regulation of canonical Wnt signaling pathway; C:nuclear chromatin; P:Notch signaling pathway; C:cytoplasm; F:metal ion binding; P:negative regulation of stem cell proliferation; P:cellular response to epidermal growth factor stimulus; P:desmosome disassembly; P:negative regulation of chondrocyte differentiation; P:positive regulation of cell migration |
| Efet.01.307720.g231.t1 | Serine protease inhibitor Kazal-type 9 | 363 | 4.89E-10 | 55.78 | F:chitinase activity; F:hydrolase activity, acting on carbon-nitrogen (but not peptide) bonds; P:carbohydrate metabolic process; F:chitin binding; F:catalytic activity; P:chitin catabolic process; C:extracellular region |
| Efet.01.307770.g232.t1 | Calmodulin | 1374 | 1.74E-12 | 53.9 | F:transferase activity, transferring acyl groups; F:1-acylglycerophosphocholine O-acyltransferase activity; F:calcium ion binding; F:transferase activity |
| Efet.01.307809.g233.t1 | thymidylate synthase | 759 | 2.35E-151 | 88.65 | F:thymidylate synthase activity; C:nucleus; C:mitochondrial matrix; P:retina development in camera-type eye; P:response to organophosphorus; P:tetrahydrofolate interconversion; P:dTMP biosynthetic process; C:mitochondrial inner membrane; P:methylation |
| Efet.01.307993.g236.t1 | betaine-aldehyde dehydrogenase | 663 | 1.11E-92 | 87.9 | F:betaine-aldehyde dehydrogenase activity; F:aldehyde dehydrogenase (quinone) activity; P:oxidation-reduction process |
| Efet.01.30802.g400.t1 | TRPM8_RAT ame: Full=Transient receptor potential cation channel subfamily M member 8 ame: Full=Cold menthol receptor 1 | 294 | 3.74E-06 | 62 | C:external side of plasma membrane; C:endoplasmic reticulum membrane; P:response to cold; P:ion transport; P:protein homotetramerization; P:cation transport; P:calcium ion transport; P:calcium ion transmembrane transport; P:thermoception; P:protein homotrimerization; P:sensory perception of temperature stimulus; P:response to stimulus; P:transmembrane transport; F:protein homodimerization activity; P:response to temperature stimulus; P:ion transmembrane transport; F:calcium channel activity; C:endoplasmic reticulum; P:transport; C:plasma membrane; P:cellular calcium ion homeostasis; F:ion channel activity; F:protein binding; C:membrane raft; C:membrane; C:integral component of membrane; P:detection of temperature stimulus |
| Efet.01.308044.g238.t1 | histamine H1 receptor-like | 408 | 4.86E-34 | 81.4 | C:integral component of plasma membrane; F:G-protein coupled acetylcholine receptor activity; P:G-protein coupled acetylcholine receptor signaling pathway |
| Efet.01.308049.g240.t1 | zinc finger and BTB domain-containing 49-like | 1035 | 1.84E-62 | 81.65 | F:nucleic acid binding; F:metal ion binding |
| Efet.01.308074.g241.t1 | Acyl- dehydrogenase | 1773 | 1.37E-180 | 60.05 | F:acyl-CoA dehydrogenase activity; F:oxidoreductase activity, acting on the CH-CH group of donors; F:flavin adenine dinucleotide binding; F:oxidoreductase activity; P:metabolic process; P:oxidation-reduction process |
| Efet.01.308074.g242.t1 | family transcriptional regulator | 309 | 1.25E-24 | 90.25 | F:DNA binding |
| Efet.01.308074.g243.t1 | recombinase family | 1053 | 0 | 83.3 | F:DNA binding; F:recombinase activity; P:DNA recombination |
| Efet.01.308089.g245.t1 | transmembrane and coiled-coil domains 2 isoform X1 | 771 | 1.99E-25 | 70.3 | C:membrane |
| Efet.01.3081.g268.t1 | trunk-like isoform X3 | 858 | 6.13E-28 | 53.6 |  |
| Efet.01.30813.g401.t1 | nuclear factor interleukin-3-regulated -like | 540 | 1.96E-24 | 76.4 | P:negative regulation of transcription from RNA polymerase II promoter; C:nucleus; F:transcriptional repressor activity, RNA polymerase II core promoter proximal region sequence-specific binding; P:circadian regulation of gene expression; F:sequence-specific DNA binding |
| Efet.01.308226.g247.t1 | FAM114A2 | 861 | 1.08E-33 | 56.45 |  |
| Efet.01.308244.g248.t1 | heat shock 70 kDa 12A-like | 2385 | 5.31E-47 | 89 | F:ATP binding; C:extracellular exosome |
| Efet.01.308265.g250.t1 | serine threonine- kinase PAK | 495 | 4.15E-76 | 86.65 | F:ATP binding; F:protein serine/threonine kinase activity; P:protein phosphorylation |
| Efet.01.308301.g252.t1 | transglycosylase | 2181 | 0 | 66.4 | F:carboxypeptidase activity; C:membrane; P:proteolysis; C:integral component of membrane; F:penicillin binding |
| Efet.01.308311.g253.t1 | fasciclin-2 isoform X4 | 1395 | 9.52E-67 | 46.75 | C:membrane; C:integral component of membrane; P:cell adhesion |
| Efet.01.30832.g402.t1 | GDP-mannose 4,6 dehydratase-like | 399 | 5.72E-81 | 92.65 | P:GDP-mannose metabolic process; F:GDP-mannose 4,6-dehydratase activity |
| Efet.01.308350.g256.t1 | testis-specific serine threonine- kinase 2 | 384 | 6.75E-20 | 64.55 | C:intracellular organelle; F:ATP binding; C:cytoplasm; F:protein serine/threonine kinase activity; P:protein phosphorylation; P:single-organism process; C:membrane-bounded organelle |
| Efet.01.308434.g260.t1 | peptidyl-prolyl cis-trans isomerase FKBP4 | 1248 | 2.58E-161 | 65.85 | F:protein binding; F:organic cyclic compound binding; F:heterocyclic compound binding; C:cytoplasm; C:intracellular membrane-bounded organelle; P:protein folding; C:intracellular organelle part; P:single-organism process; F:isomerase activity |
| Efet.01.308459.g263.t1 | zinc finger MIZ domain-containing 1 isoform X1 | 438 | 1.96E-60 | 73.15 | F:zinc ion binding |
| Efet.01.308534.g264.t1 | SCY1 2 | 345 | 5.23E-27 | 64 | F:ATP binding; F:protein kinase activity; P:protein phosphorylation |
| Efet.01.308573.g267.t1 | Patatin-like phospholipase domain-containing 7 | 375 | 7.11E-26 | 64.9 | C:membrane; P:lipid metabolic process |
| Efet.01.308577.g268.t1 | ABC transporter substrate-binding | 1005 | 0 | 53.85 | P:carbohydrate transport; P:transport; F:transporter activity |
| Efet.01.30859.g404.t1 | thyroid receptor-interacting 11-like isoform X7 | 2238 | 1.09E-120 | 57.95 | P:protein targeting to Golgi; C:intracellular |
| Efet.01.308667.g271.t1 | Deoxynucleotidyltransferase terminal-interacting 2 | 210 | 2.55E-20 | 80 | C:nucleolus; F:transferase activity; F:RNA binding |
| Efet.01.308685.g272.t1 | voltage-dependent calcium channel subunit alpha-2 delta-3 isoform X1 | 348 | 5.05E-21 | 55.85 | C:membrane |
| Efet.01.30873.g407.t1 | helicase with zinc finger domain 2-like isoform X1 | 1164 | 1.29E-63 | 53.35 | F:metal ion binding; F:helicase activity |
| Efet.01.308745.g273.t1 | Adenylate kinase isoenzyme 5 | 441 | 1.37E-18 | 66.15 | F:transferase activity |
| Efet.01.308804.g275.t1 | AP-3 complex subunit mu-1 | 333 | 6.03E-58 | 96.25 | P:vesicle-mediated transport; C:clathrin adaptor complex; P:intracellular protein transport |
| Efet.01.308844.g276.t1 | hypothetical protein CAPTEDRAFT_216885 | 675 | 1.62E-06 | 49 | F:G-protein coupled peptide receptor activity; P:G-protein coupled receptor signaling pathway; C:membrane; C:integral component of membrane |
| Efet.01.308898.g277.t1 | dual specificity tyrosine-phosphorylation-regulated kinase 1A | 309 | 3.53E-06 | 94.25 | F:protein serine/threonine/tyrosine kinase activity; P:protein autophosphorylation; P:protein phosphorylation |
| Efet.01.308920.g278.t1 | ankyrin repeat | 1323 | 4.74E-61 | 58.75 |  |
| Efet.01.308953.g280.t1 | homeobox SIX6 | 642 | 6.87E-94 | 89.35 | C:nucleus; F:DNA binding; P:eye development; F:protein binding; P:transcription from RNA polymerase II promoter; F:transcriptional activator activity, RNA polymerase II distal enhancer sequence-specific binding; P:brain development; P:visual perception; P:positive regulation of transcription from RNA polymerase II promoter; P:animal organ morphogenesis |
| Efet.01.308959.g281.t1 | bactericidal permeability-increasing | 420 | 2.61E-07 | 51.33 |  |
| Efet.01.308968.g282.t1 | hypothetical protein CAPTEDRAFT_226427 | 303 | 2.43E-24 | 62.33 | F:nucleic acid binding; F:protein dimerization activity |
| Efet.01.308972.g283.t1 | bcl-2 1 | 582 | 1.32E-54 | 57.65 | P:regulation of cellular process; C:membrane |
| Efet.01.30909.g408.t1 | dual specificity phosphatase 8 isoform X1 | 786 | 1.51E-09 | 71.75 | P:inactivation of MAPK activity; C:nucleus; C:cytoplasm; F:protein tyrosine phosphatase activity; P:peptidyl-tyrosine dephosphorylation; F:MAP kinase tyrosine/serine/threonine phosphatase activity |
| Efet.01.30910.g409.t1 | sodium channel para | 438 | 1.88E-17 | 56.65 | F:ion channel activity; P:ion transport; P:transmembrane transport; C:membrane |
| Efet.01.309150.g285.t1 | AFTIN_HUMAN ame: Full=Aftiphilin | 273 | 9.02E-07 | 71 | P:intracellular transport; C:AP-1 adaptor complex; C:cytosol; F:clathrin binding; C:nucleoplasm |
| Efet.01.309240.g288.t1 | N-alpha-acetyltransferase 40 isoform X2 | 333 | 3.19E-41 | 75.95 | P:lipid metabolic process; F:N-acetyltransferase activity |
| Efet.01.309334.g290.t1 | cAMP-dependent kinase regulatory subunit | 525 | 8.40E-32 | 63.8 | P:phosphorylation; F:kinase activity |
| Efet.01.309348.g291.t1 | aspartate cytoplasmic | 564 | 2.23E-78 | 76.9 | F:pyridoxal phosphate binding; F:L-phenylalanine:2-oxoglutarate aminotransferase activity; P:cellular amino acid metabolic process; P:biosynthetic process; F:L-aspartate:2-oxoglutarate aminotransferase activity |
| Efet.01.30935.g411.t1 | pleckstrin homology domain-containing family G member 3-like isoform X1 | 525 | 1.18E-13 | 68.95 | P:biological regulation; C:membrane |
| Efet.01.309352.g292.t1 | hemoglobin subunit mu-like | 603 | 2.94E-40 | 56.9 | F:binding |
| Efet.01.309444.g297.t1 | polycomb Asx-like | 888 | 1.92E-13 | 84 | F:DNA binding |
| Efet.01.309464.g298.t1 | hypothetical protein HELRODRAFT_194052 | 1125 | 1.02E-43 | 53.5 | C:membrane; C:integral component of membrane |
| Efet.01.309477.g299.t1 | evolutionarily conserved signaling intermediate in Toll mitochondrial-like isoform X2 | 699 | 1.48E-89 | 66.3 |  |
| Efet.01.30948.g412.t1 | Sterol-4-alpha-carboxylate 3- decarboxylating | 282 | 1.80E-31 | 76.15 | F:oxidoreductase activity, acting on the CH-OH group of donors, NAD or NADP as acceptor; C:membrane; P:single-organism metabolic process |
| Efet.01.309519.g301.t1 | ceramide kinase-like | 348 | 5.33E-31 | 60.4 | P:phosphorylation; F:kinase activity |
| Efet.01.309552.g302.t1 | trimethylguanosine synthase-like | 951 | 1.93E-80 | 86.5 | P:RNA methylation; P:7-methylguanosine RNA capping; F:methyltransferase activity |
| Efet.01.309640.g304.t1 | intraflagellar transport 80 homolog | 318 | 2.63E-40 | 81.15 | C:centrosome; C:motile cilium; C:intraciliary transport particle B |
| Efet.01.309643.g305.t1 | Major egg antigen | 726 | 1.91E-63 | 51.9 |  |
| Efet.01.309708.g306.t1 | plasma membrane calcium-transporting ATPase 2 isoform X1 | 1461 | 0 | 82.1 | F:ATP binding; F:metal ion binding; C:integral component of membrane; P:calcium ion transmembrane transport; F:calcium-transporting ATPase activity |
| Efet.01.309708.g307.t1 | plasma membrane calcium-transporting ATPase 1-like | 459 | 1.42E-38 | 65 | F:nucleotide binding; P:ion transport; C:membrane; F:ion binding; F:hydrolase activity |
| Efet.01.30976.g413.t1 | bromodomain adjacent to zinc finger domain 2B-like | 846 | 1.29E-16 | 57.85 | F:DNA binding; C:nucleus; F:zinc ion binding; F:metal ion binding |
| Efet.01.309809.g310.t1 | ubiquitin conjugation factor E4 A | 291 | 3.46E-30 | 79.85 | C:ubiquitin ligase complex; F:ubiquitin-ubiquitin ligase activity; P:ubiquitin-dependent protein catabolic process; P:protein ubiquitination |
| Efet.01.30994.g414.t1 | erythroid differentiation-related factor 1 | 204 | 2.89E-20 | 83.45 |  |
| Efet.01.309957.g315.t1 | hypothetical protein CAPTEDRAFT_209450 | 660 | 1.67E-18 | 62 |  |
| Efet.01.310032.g316.t1 | BTG2-like | 504 | 9.85E-35 | 63.4 |  |
| Efet.01.310041.g317.t1 | PREDICTED: formin-J-like | 717 | 1.20E-09 | 71 |  |
| Efet.01.310052.g318.t1 | tyrosinase 1 | 213 | 2.32E-12 | 63.65 | F:monophenol monooxygenase activity; F:oxidoreductase activity; F:metal ion binding; P:metabolic process; P:oxidation-reduction process; C:extracellular region |
| Efet.01.310054.g320.t1 | Cell surface glyco 1 | 645 | 2.14E-16 | 48.63 |  |
| Efet.01.3101.g270.t1 | hypoxia-inducible factor 1-alpha-like | 396 | 1.30E-31 | 61.7 | P:negative regulation of growth; P:retina vasculature development in camera-type eye; P:negative regulation of mesenchymal cell apoptotic process; P:embryonic hemopoiesis; C:nuclear speck; P:visual learning; P:lactate metabolic process; P:positive regulation of erythrocyte differentiation; P:cellular iron ion homeostasis; C:transcription factor complex; P:negative regulation of bone mineralization; C:motile cilium; F:transcriptional activator activity, RNA polymerase II core promoter proximal region sequence-specific binding; F:transcription factor activity, RNA polymerase II transcription factor binding; P:cardiac ventricle morphogenesis; P:negative regulation of thymocyte apoptotic process; P:positive regulation of vascular endothelial growth factor receptor signaling pathway; P:regulation of catalytic activity; P:heart looping; P:oxygen homeostasis; P:negative regulation of TOR signaling; F:E-box binding; P:collagen metabolic process; P:positive regulation of macroautophagy; F:histone deacetylase binding; P:positive regulation of neuroblast proliferation; F:protein dimerization activity; P:neural fold elevation formation; P:hemoglobin biosynthetic process; P:cerebral cortex development; P:connective tissue replacement involved in inflammatory response wound healing; P:neural crest cell migration; P:hypoxia-inducible factor-1alpha signaling pathway; P:glucose homeostasis; P:epithelial to mesenchymal transition; P:cartilage development; P:muscle cell cellular homeostasis; P:epithelial cell differentiation involved in mammary gland alveolus development; P:outflow tract morphogenesis; F:sequence-specific DNA binding; P:lactation; P:positive regulation of transcription from RNA polymerase II promoter; P:embryonic placenta development; P:negative regulation of neuron apoptotic process; P:elastin metabolic process; P:angiogenesis; P:B-1 B cell homeostasis; P:positive regulation of epithelial cell migration; P:positive regulation of insulin secretion involved in cellular response to glucose stimulus; P:negative regulation of reactive oxygen species metabolic process; P:response to muscle activity; P:intestinal epithelial cell maturation; P:regulation of transcription from RNA polymerase II promoter in response to oxidative stress; P:transcription, DNA-templated; P:regulation of glycolytic process; P:digestive tract morphogenesis; P:positive regulation of mitophagy; P:regulation of aerobic respiration; C:cytoplasm; P:dopaminergic neuron differentiation; P:iris morphogenesis; P:regulation of cytokine production |
| Efet.01.310107.g321.t1 | inositol 1,4,5-trisphosphate receptor type 3- | 405 | 3.39E-52 | 72.2 | P:inositol phosphate-mediated signaling; C:membrane; F:inositol 1,4,5-trisphosphate-sensitive calcium-release channel activity; P:calcium ion transmembrane transport; C:endoplasmic reticulum |
| Efet.01.31016.g415.t1 | hypothetical protein CAPTEDRAFT_209963, partial | 480 | 1.47E-06 | 71 |  |
| Efet.01.310168.g323.t1 | syntaxin-binding 1-like isoform X2 | 267 | 1.38E-37 | 86.2 | P:vesicle docking involved in exocytosis |
| Efet.01.310294.g326.t1 | tyramine beta-hydroxylase-like | 579 | 4.07E-12 | 57.4 | F:oxidoreductase activity, acting on paired donors, with incorporation or reduction of molecular oxygen, reduced ascorbate as one donor, and incorporation of one atom of oxygen; F:copper ion binding; F:catalytic activity; C:membrane; C:integral component of membrane; P:oxidation-reduction process; F:monooxygenase activity |
| Efet.01.310446.g329.t1 | DNA-directed RNA polymerase I subunit RPA1 | 477 | 6.39E-47 | 70.1 | F:nucleotidyltransferase activity; F:binding |
| Efet.01.310452.g330.t1 | neuronal acetylcholine receptor subunit alpha-5-like isoform X1 | 426 | 1.65E-73 | 82.25 | C:postsynaptic membrane; P:cation transmembrane transport; C:integral component of membrane; C:cell junction; F:extracellular ligand-gated ion channel activity |
| Efet.01.310488.g331.t1 | Wnt-9b | 402 | 2.19E-23 | 58.15 | P:single-organism cellular process; C:extracellular region; P:single-organism developmental process |
| Efet.01.31049.g418.t1 | fibrinolytic enzyme | 378 | 4.07E-70 | 82.65 | P:proteolysis; F:serine-type endopeptidase activity; P:blood coagulation; P:phosphorylation; P:fibrinolysis; F:kinase activity |
| Efet.01.310530.g332.t1 | activating signal cointegrator 1 | 705 | 1.19E-58 | 73 | C:nucleus; F:zinc ion binding; P:regulation of transcription, DNA-templated |
| Efet.01.310554.g334.t1 | Forkhead box O | 1716 | 6.87E-09 | 77.25 | C:nucleus; F:transcription factor activity, sequence-specific DNA binding; P:transcription, DNA-templated; P:regulation of transcription, DNA-templated; F:sequence-specific DNA binding |
| Efet.01.310608.g335.t1 | WD repeat-containing 64-like | 258 | 2.61E-08 | 63.29 |  |
| Efet.01.310610.g336.t1 | dynein heavy chain axonemal-like | 273 | 1.01E-24 | 82.1 | F:ATPase activity; F:ATP binding; F:microtubule motor activity; C:axonemal dynein complex; P:cilium movement |
| Efet.01.310656.g339.t1 | Bardet-Biedl syndrome 12 | 633 | 1.98E-11 | 42.05 | F:ATP binding; P:intraciliary transport; P:photoreceptor cell maintenance; P:chaperone-mediated protein complex assembly; C:cilium; P:negative regulation of fat cell differentiation; P:eating behavior |
| Efet.01.310657.g340.t1 | potassium voltage-gated channel subfamily A member 1 | 999 | 2.87E-119 | 69.7 | P:regulation of biological quality; P:response to external stimulus; P:potassium ion transmembrane transport; C:voltage-gated potassium channel complex; C:cell projection part; F:delayed rectifier potassium channel activity; P:nervous system development; P:regulation of ion transmembrane transport; P:protein homooligomerization; P:neurological system process; C:axon |
| Efet.01.31068.g421.t1 | transducin beta 3 | 261 | 1.63E-18 | 57.25 | C:nucleus |
| Efet.01.310684.g341.t1 | mitochondrial import inner membrane translocase subunit Tim8 A | 306 | 1.00E-31 | 75.25 | F:metal ion binding; C:mitochondrion; P:protein transport |
| Efet.01.31073.g422.t1 | suppressor of lurcher 1-like | 279 | 1.21E-25 | 68.3 | C:integral component of membrane |
| Efet.01.310784.g343.t1 | Calcium-dependent kinase C | 216 | 3.69E-17 | 91.6 | P:central nervous system neuron axonogenesis; P:negative regulation of glial cell apoptotic process; P:regulation of platelet aggregation; C:neuronal cell body; P:positive regulation of angiogenesis; C:photoreceptor outer segment; P:learning or memory; P:positive regulation of exocytosis; P:inactivation of MAPK activity; C:dendrite; P:desmosome assembly; C:apical part of cell; P:positive regulation of macrophage differentiation; C:cytosol; P:positive regulation of cardiac muscle hypertrophy; P:positive regulation of synapse assembly; P:negative regulation of translation; C:axon; F:zinc ion binding; P:peptidyl-serine autophosphorylation; P:positive regulation of inflammatory response; C:protein complex; P:response to reactive oxygen species; P:negative regulation of insulin receptor signaling pathway; C:plasma membrane; P:positive regulation of mitotic cell cycle; C:nucleus; P:cellular response to carbohydrate stimulus; P:response to estradiol; P:response to interleukin-1; P:positive regulation of lipopolysaccharide-mediated signaling pathway; P:negative regulation of cell proliferation; P:chondrocyte differentiation; P:cell adhesion; P:intrinsic apoptotic signaling pathway; P:positive regulation of cell adhesion; P:regulation of receptor-mediated endocytosis; P:negative regulation of heart contraction; P:positive regulation of smooth muscle cell proliferation; F:ATP binding; P:response to ethanol; P:regulation of peptidyl-tyrosine phosphorylation; C:extracellular exosome; P:angiogenesis; P:regulation of muscle contraction; P:induction of positive chemotaxis; P:negative regulation of glucose import; C:endoplasmic reticulum; F:calcium-dependent protein kinase C activity; P:positive regulation of blood vessel endothelial cell migration; P:cellular calcium ion homeostasis; P:regulation of the force of heart contraction; P:positive regulation of dense core granule biogenesis; C:perinuclear region of cytoplasm; P:histone H3-T6 phosphorylation; C:mitochondrion; C:membrane raft; P:positive regulation of ERK1 and ERK2 cascade; P:neutrophil chemotaxis; P:positive regulation of endothelial cell proliferation; F:enzyme binding; F:histone kinase activity (H3-T6 specific) |
| Efet.01.310814.g344.t1 | hypothetical protein HELRODRAFT_191437 | 1155 | 7.76E-11 | 44 |  |
| Efet.01.310815.g345.t1 | Cytoplasmic aconitate hydratase | 201 | 6.42E-21 | 97.25 | F:iron-responsive element binding; P:response to iron(II) ion; P:post-embryonic development; P:citrate metabolic process; C:endoplasmic reticulum; C:Golgi apparatus; F:aconitate hydratase activity; P:regulation of translation; P:cellular iron ion homeostasis; P:intestinal absorption; C:extracellular exosome; C:mitochondrion; C:cytosol; F:4 iron, 4 sulfur cluster binding |
| Efet.01.31083.g423.t1 | laminin subunit beta-1-like | 459 | 1.18E-17 | 54 | C:basement membrane; P:system development; P:basement membrane assembly |
| Efet.01.310939.g346.t1 | glutamate receptor | 354 | 4.93E-57 | 86.45 | C:postsynaptic membrane; P:ion transmembrane transport; C:integral component of membrane; C:cell junction; P:ionotropic glutamate receptor signaling pathway; F:ionotropic glutamate receptor activity; F:extracellular-glutamate-gated ion channel activity |
| Efet.01.310941.g347.t1 | Plasma membrane calcium-transporting ATPase | 456 | 5.48E-24 | 80.3 | F:ATP binding; F:metal ion binding; C:integral component of membrane; P:calcium ion transmembrane transport; F:calcium-transporting ATPase activity |
| Efet.01.310948.g348.t1 | sortilin-related receptor-like isoform X1 | 390 | 1.64E-25 | 62.7 | C:membrane; C:integral component of membrane; C:Golgi apparatus; P:transport |
| Efet.01.310959.g349.t1 | growth differentiation factor 11 | 291 | 1.33E-34 | 68 | P:regulation of response to stimulus; P:negative regulation of myoblast proliferation; P:cellular response to stimulus; P:negative regulation of satellite cell differentiation; F:receptor binding; P:negative regulation of skeletal muscle satellite cell proliferation |
| Efet.01.311069.g351.t1 | protocadherin-11 X-linked-like | 435 | 1.39E-06 | 87 |  |
| Efet.01.311103.g352.t1 | hypothetical protein CAPTEDRAFT_195855 | 225 | 4.72E-07 | 64 |  |
| Efet.01.311103.g353.t1 | internalin-A-like isoform X4 | 948 | 4.48E-35 | 50.75 | C:membrane; C:integral component of membrane |
| Efet.01.311134.g354.t1 | hypothetical protein HELRODRAFT_167810 | 7980 | 1.49E-17 | 42 |  |
| Efet.01.311240.g357.t1 | hypothetical protein CAPTEDRAFT_184805 | 297 | 1.13E-12 | 52 | C:membrane; C:integral component of membrane |
| Efet.01.311269.g358.t1 | DNA mismatch repair Msh6 | 384 | 5.80E-36 | 76.5 | P:somatic recombination of immunoglobulin gene segments; P:reciprocal meiotic recombination; C:nuclear chromosome; P:response to UV; P:intrinsic apoptotic signaling pathway; P:meiotic mismatch repair; F:four-way junction DNA binding; F:damaged DNA binding; P:negative regulation of DNA recombination; F:ATP binding; F:protein binding; P:maintenance of DNA repeat elements; C:cytoplasm; C:MutSalpha complex; F:guanine/thymine mispair binding; F:single base insertion or deletion binding |
| Efet.01.311335.g360.t1 | hypothetical protein HELRODRAFT_159538 | 360 | 2.03E-07 | 54 |  |
| Efet.01.311362.g363.t1 | hypothetical protein CAPTEDRAFT_149978 | 201 | 1.23E-07 | 70 | C:membrane; C:integral component of membrane |
| Efet.01.3114.g271.t1 | fibroblast growth factor receptor 2-like | 1362 | 2.22E-170 | 76.55 | F:ATP binding; P:peptidyl-tyrosine phosphorylation; F:protein tyrosine kinase activity; C:integral component of membrane |
| Efet.01.311412.g364.t1 | RNA-binding 45 | 618 | 3.07E-22 | 54.3 | F:nucleotide binding; F:nucleic acid binding |
| Efet.01.311414.g365.t1 | phytanoyl- hydroxylase-interacting -like | 651 | 4.68E-42 | 59.15 |  |
| Efet.01.311511.g367.t1 | mitochondrial import inner membrane translocase subunit Tim29 | 621 | 5.30E-51 | 61.1 |  |
| Efet.01.311516.g368.t1 | peptidyl-prolyl cis-trans isomerase B-like | 267 | 1.21E-25 | 69.95 | P:cellular process; F:isomerase activity |
| Efet.01.311559.g369.t1 | neuroendocrine convertase 2 | 471 | 4.48E-74 | 73 | P:proteolysis; F:serine-type endopeptidase activity |
| Efet.01.311566.g370.t1 | FMRFamide receptor-like | 489 | 3.84E-34 | 60.55 | F:G-protein coupled receptor activity; C:membrane |
| Efet.01.311749.g374.t1 | SZT2 isoform B | 423 | 1.59E-32 | 70.85 | C:peroxisome |
| Efet.01.31175.g427.t1 | unconventional myosin-XV-like isoform X1 | 420 | 9.85E-50 | 76.15 | C:myosin complex; F:ATP binding; F:motor activity |
| Efet.01.31176.g428.t1 | IS481 family transposase | 606 | 9.03E-76 | 67.7 | F:nucleic acid binding; F:DNA binding; P:DNA integration |
| Efet.01.311777.g375.t1 | E3 ubiquitin- ligase HECTD1-like | 1626 | 7.93E-101 | 61.15 | F:ubiquitin-protein transferase activity; F:metal ion binding; F:ligase activity; P:protein ubiquitination |
| Efet.01.31180.g429.t1 | ribonuclease E G | 555 | 1.65E-118 | 97.65 | F:magnesium ion binding; P:RNA phosphodiester bond hydrolysis, endonucleolytic; F:zinc ion binding; F:RNA binding; C:cytoplasm; P:tRNA processing; F:ribonuclease E activity; P:rRNA processing; P:mRNA catabolic process; C:cytoplasmic side of plasma membrane; F:endoribonuclease activity |
| Efet.01.31180.g430.t1 | ribosomal large subunit pseudouridine synthase C | 1533 | 1.43E-165 | 81.95 | P:pseudouridine synthesis; F:RNA binding; F:pseudouridine synthase activity |
| Efet.01.311866.g376.t1 | potassium voltage-gated channel Shaw-like | 681 | 2.49E-62 | 74.3 | F:voltage-gated potassium channel activity; P:potassium ion transmembrane transport; C:voltage-gated potassium channel complex; P:regulation of ion transmembrane transport; P:protein homooligomerization |
| Efet.01.311931.g377.t1 | hypothetical protein HELRODRAFT_176894 | 606 | 5.36E-10 | 48 | F:calcium ion binding; C:membrane; C:integral component of membrane; P:cell adhesion; P:homophilic cell adhesion via plasma membrane adhesion molecules; C:plasma membrane |
| Efet.01.311939.g378.t1 | T family of potassium channels 18-like | 486 | 2.21E-39 | 58.75 | P:ion transport; C:membrane |
| Efet.01.31198.g431.t1 | zinc finger and BTB domain-containing 24 | 1494 | 1.96E-75 | 53.15 | F:binding |
| Efet.01.312.g36.t1 | voltage-dependent T-type calcium channel subunit alpha-1I-like | 402 | 1.71E-49 | 91.95 | P:calcium ion import; C:voltage-gated calcium channel complex; P:regulation of ion transmembrane transport; P:calcium ion transmembrane transport; F:low voltage-gated calcium channel activity |
| Efet.01.312008.g380.t1 | cholesterol 7-alpha-monooxygenase | 828 | 1.78E-87 | 54.4 | F:oxidoreductase activity; C:intracellular membrane-bounded organelle; C:membrane; C:cytoplasmic part; F:binding; P:single-organism metabolic process |
| Efet.01.312042.g381.t1 | phosphatidate photoreceptor-specific-like isoform X1 | 276 | 3.47E-29 | 86.65 | C:endoplasmic reticulum membrane; F:phosphatidate cytidylyltransferase activity; C:integral component of membrane; P:CDP-diacylglycerol biosynthetic process; P:phosphatidylglycerol biosynthetic process |
| Efet.01.312054.g382.t1 | multivalent antigen sjGAPDH-97 | 243 | 2.03E-15 | 66.45 | C:myosin complex |
| Efet.01.312134.g383.t1 | probable cationic amino acid transporter | 1881 | 6.09E-41 | 46.15 | C:membrane; C:integral component of membrane; F:amino acid transmembrane transporter activity; P:amino acid transmembrane transport |
| Efet.01.312141.g384.t1 | tektin-3-like isoform X1 | 330 | 2.15E-18 | 64.35 |  |
| Efet.01.312197.g385.t1 | solute carrier family 15 member 1 | 393 | 6.82E-11 | 65.3 | F:neutral amino acid transmembrane transporter activity |
| Efet.01.312282.g386.t1 | hypothetical protein CAPTEDRAFT_209714 | 624 | 2.74E-39 | 52.15 | C:membrane; C:integral component of membrane; F:carbohydrate binding |
| Efet.01.312306.g387.t1 | Tyrosine- kinase RYK | 408 | 1.99E-11 | 60.25 | F:Wnt-activated receptor activity; P:negative regulation of axon extension involved in axon guidance; P:midbrain dopaminergic neuron differentiation; P:skeletal system morphogenesis; C:plasma membrane; P:corpus callosum development; C:nucleus; F:protein tyrosine kinase activity; F:frizzled binding; P:planar cell polarity pathway involved in axon guidance; C:cytoplasm; P:chemorepulsion of dopaminergic neuron axon; F:Wnt-protein binding; P:commissural neuron axon guidance |
| Efet.01.312379.g389.t1 | ryanodine receptor | 246 | 9.87E-18 | 91.3 | F:ryanodine-sensitive calcium-release channel activity; F:calcium ion binding; C:integral component of membrane; P:release of sequestered calcium ion into cytosol; C:intracellular |
| Efet.01.312463.g391.t1 | zinc finger 271 | 969 | 8.04E-61 | 55.5 | F:nucleic acid binding; F:metal ion binding |
| Efet.01.312464.g392.t1 | Transcription initiation factor IIB | 228 | 6.51E-20 | 88.45 | F:translation initiation factor activity; F:zinc ion binding; P:regulation of transcription, DNA-templated; P:DNA-templated transcription, initiation; F:TBP-class protein binding; P:translational initiation |
| Efet.01.312465.g393.t1 | elongation of very long chain fatty acids 4-like | 324 | 1.17E-15 | 76.95 | C:integral component of membrane; F:transferase activity; P:fatty acid biosynthetic process |
| Efet.01.31249.g435.t1 | partitioning defective 3 homolog | 954 | 1.14E-25 | 52.9 |  |
| Efet.01.312576.g394.t1 | 14-3-3 epsilon | 660 | 2.23E-113 | 83.9 | F:protein domain specific binding |
| Efet.01.312578.g395.t1 | potassium channel | 1230 | 1.74E-93 | 60.1 | F:ion channel activity; P:transport |
| Efet.01.312634.g398.t1 | glutathione S-transferase theta-1-like | 318 | 4.65E-31 | 64.9 | F:transferase activity |
| Efet.01.312671.g399.t1 | homeobox SIX1-like | 915 | 2.84E-54 | 69.55 | F:DNA binding |
| Efet.01.31268.g437.t1 | mitogen-activated kinase kinase kinase 9-like | 540 | 2.88E-21 | 52.45 | F:ATP binding; F:protein kinase activity; P:phosphorylation; F:protein serine/threonine kinase activity; P:protein phosphorylation; F:kinase activity |
| Efet.01.312693.g400.t1 | FAD-linked sulfhydryl oxidase ALR | 462 | 2.74E-19 | 76.25 | C:cytoplasm; P:animal organ development; P:oxidation-reduction process; F:binding; F:thiol oxidase activity |
| Efet.01.312728.g402.t1 | kelch 21 | 1776 | 0 | 55.55 |  |
| Efet.01.312745.g405.t1 | hypothetical protein CAPTEDRAFT_210277 | 855 | 1.25E-58 | 71 |  |
| Efet.01.31275.g439.t1 | DNA repair RAD52 homolog | 981 | 3.92E-29 | 87.15 | C:nucleus; P:double-strand break repair via single-strand annealing; P:DNA recombinase assembly |
| Efet.01.312832.g406.t1 | hypothetical protein HELRODRAFT_179757 | 408 | 4.90E-11 | 53.5 |  |
| Efet.01.312839.g407.t1 | beta-1,4-galactosyltransferase galt-1-like | 1245 | 1.33E-77 | 52.95 | C:membrane; C:integral component of membrane |
| Efet.01.312852.g408.t1 | insulin-like growth factor 1 receptor | 510 | 8.25E-31 | 63.7 | F:protein kinase activity; F:binding; P:protein phosphorylation |
| Efet.01.312948.g411.t1 | Dual specificity phosphatase 23 | 534 | 2.45E-50 | 64.8 | F:phosphoprotein phosphatase activity; P:protein dephosphorylation; C:intracellular part; C:membrane-bounded organelle |
| Efet.01.312955.g412.t1 | leucine-rich repeat and immunoglobulin-like domain-containing nogo receptor-interacting 3 | 2523 | 1.57E-81 | 44.85 | F:phosphoprotein phosphatase activity; P:axonogenesis; P:protein dephosphorylation; C:membrane; C:integral component of membrane; F:hydrolase activity |
| Efet.01.312980.g413.t1 | solute carrier family 25 member 45-like | 438 | 1.98E-25 | 57.55 | P:transmembrane transport; F:structural constituent of ribosome; C:membrane; C:integral component of membrane; P:phosphorylation; F:kinase activity; P:transport; P:translation |
| Efet.01.313009.g414.t1 | polyadenylate-binding 2 | 276 | 3.15E-48 | 95.5 | C:nuclear inclusion body; P:positive regulation of polynucleotide adenylyltransferase activity; F:poly(A) binding; F:protein self-association; P:mRNA polyadenylation; F:RNA binding; F:nucleotide binding; F:RNA polymerase binding; P:MAPK cascade; C:intracellular ribonucleoprotein complex; P:cellular response to lipopolysaccharide; C:cytoplasm; P:poly(A)+ mRNA export from nucleus |
| Efet.01.313025.g415.t1 | neurogenic locus notch homolog 1 | 324 | 6.04E-24 | 69.25 | F:catalytic activity; P:single-organism process; P:cellular process |
| Efet.01.313055.g417.t1 | thyrotropin-releasing hormone receptor | 324 | 5.16E-51 | 68.9 | F:G-protein coupled receptor activity; P:G-protein coupled receptor signaling pathway; C:integral component of membrane |
| Efet.01.31309.g441.t1 | glutaredoxin 2 isoform X2 | 336 | 9.30E-40 | 72.75 | C:cell; F:protein disulfide oxidoreductase activity; F:electron carrier activity; P:circulatory system development; P:cell redox homeostasis; P:animal organ development; P:oxidation-reduction process |
| Efet.01.313196.g420.t1 | G -activated inward rectifier potassium channel 3-like | 1491 | 1.68E-133 | 67.2 | P:potassium ion transport; C:membrane; F:voltage-gated ion channel activity |
| Efet.01.313207.g422.t1 | chitotriosidase-1 isoform X3 | 270 | 1.05E-07 | 50.8 | F:chitinase activity; P:carbohydrate metabolic process; C:extracellular space; F:chitin binding; P:chitin catabolic process; P:chitin metabolic process; C:extracellular region; F:hydrolase activity, hydrolyzing O-glycosyl compounds; F:endochitinase activity |
| Efet.01.313227.g423.t1 | NACHT and WD repeat domain-containing 2-like | 2856 | 0 | 64.8 |  |
| Efet.01.313260.g424.t1 | alpha-N-acetylglucosaminidase | 489 | 1.58E-28 | 52.5 | F:nucleotide binding; F:ATP binding; F:protein kinase activity; P:phosphorylation; F:transferase activity; F:protein serine/threonine kinase activity; P:protein phosphorylation; F:kinase activity |
| Efet.01.313497.g426.t1 | splicing factor U2AF 35 kDa subunit-like | 237 | 1.89E-30 | 66.05 | C:nucleus; F:organic cyclic compound binding; F:heterocyclic compound binding |
| Efet.01.313518.g428.t1 | Zinc transporter ZIP1 | 1101 | 5.07E-79 | 63.45 | C:membrane; P:transport |
| Efet.01.313562.g429.t1 | hypothetical protein CAPTEDRAFT_183585 | 348 | 8.53E-19 | 66.75 | C:membrane |
| Efet.01.313685.g432.t1 | hypothetical protein XENTR_v90026278mg | 678 | 5.95E-08 | 48.33 |  |
| Efet.01.3137.g273.t1 | multiple C2 and transmembrane domain-containing 1-like isoform X1 | 837 | 2.00E-37 | 80.2 | C:integral component of membrane |
| Efet.01.313716.g433.t1 | myelin transcription factor 1 isoform X2 | 1092 | 7.07E-33 | 61.65 |  |
| Efet.01.313787.g435.t1 | serine threonine- kinase TAO1-like | 696 | 1.25E-32 | 66.15 | P:MAPK cascade; P:mitotic G2 DNA damage checkpoint; P:positive regulation of JUN kinase activity; F:protein binding; C:cell part; P:protein autophosphorylation; F:protein serine/threonine kinase activity; P:negative regulation of JNK cascade; F:protein kinase inhibitor activity |
| Efet.01.31382.g444.t1 | ATP-dependent RNA helicase cgh-1-like | 444 | 1.37E-47 | 86.7 | F:nucleic acid binding; F:ATP binding; F:helicase activity |
| Efet.01.313861.g437.t1 | Zinc finger 541 | 402 | 7.17E-15 | 79.7 | F:nucleic acid binding; F:metal ion binding |
| Efet.01.313864.g438.t1 | titin-like isoform X1 | 1737 | 3.08E-77 | 50.7 | F:nucleotide binding; P:regulation of Rho protein signal transduction; F:ATP binding; C:membrane; C:integral component of membrane; F:protein kinase activity; F:Rho guanyl-nucleotide exchange factor activity; P:phosphorylation; F:transferase activity; P:protein phosphorylation; F:kinase activity; P:positive regulation of GTPase activity |
| Efet.01.313865.g439.t1 | adenosine kinase 2 | 1134 | 8.30E-55 | 53.05 | P:AMP biosynthetic process; F:adenosine kinase activity; P:purine ribonucleoside salvage; F:phosphotransferase activity, alcohol group as acceptor |
| Efet.01.313896.g440.t1 | hypothetical protein CAPTEDRAFT_222036 | 261 | 6.60E-18 | 64.4 | C:membrane; C:integral component of membrane |
| Efet.01.313946.g441.t1 | transmembrane 184B-like | 606 | 1.92E-33 | 78.05 | C:integral component of membrane; P:transport; F:transporter activity |
| Efet.01.31396.g445.t1 | matrix metallo ase-2-like | 312 | 1.04E-15 | 53.85 | F:hydrolase activity |
| Efet.01.31401.g446.t1 | bromodomain adjacent to zinc finger domain 1A isoform X3 | 234 | 7.91E-06 | 65 |  |
| Efet.01.314076.g442.t1 | potassium sodium hyperpolarization-activated cyclic nucleotide-gated channel 2-like | 345 | 3.34E-13 | 86.05 | C:integral component of plasma membrane; F:voltage-gated potassium channel activity; P:regulation of membrane potential; P:potassium ion transmembrane transport |
| Efet.01.314102.g443.t1 | E3 ubiquitin- ligase | 402 | 1.45E-49 | 62.1 | F:zinc ion binding; F:metal ion binding; F:ligase activity |
| Efet.01.314190.g445.t1 | hypothetical protein CAPTEDRAFT_228921 | 702 | 1.09E-42 | 52.25 | C:membrane; C:integral component of membrane; F:carbohydrate binding |
| Efet.01.314202.g446.t1 | splicing factor 45-like | 477 | 1.59E-14 | 56.69 | F:organic cyclic compound binding; F:heterocyclic compound binding; F:catalytic activity |
| Efet.01.314262.g447.t1 | ddi1 2 | 504 | 2.88E-13 | 51.08 | F:nucleic acid binding; F:zinc ion binding; F:aspartic-type endopeptidase activity; F:ubiquitin binding; P:proteolysis |
| Efet.01.31435.g447.t1 | capicua homolog isoform X1 | 342 | 1.91E-30 | 74 |  |
| Efet.01.314370.g450.t1 | hypothetical protein CAPTEDRAFT_222844 | 1629 | 1.45E-19 | 44 | F:metal ion binding |
| Efet.01.314371.g451.t1 | hypothetical protein CAPTEDRAFT_222844 | 1629 | 1.45E-19 | 44 | F:metal ion binding |
| Efet.01.3144.g274.t1 | hypothetical protein HELRODRAFT_162600 | 465 | 2.02E-06 | 65 |  |
| Efet.01.314457.g454.t1 | pilus assembly | 669 | 2.99E-97 | 50.86 | C:integral component of membrane; P:type IV pilus biogenesis |
| Efet.01.314463.g455.t1 | epidermal growth factor receptor-like isoform X1 | 372 | 1.60E-28 | 62.1 | F:protein kinase activity; P:phosphorylation |
| Efet.01.314490.g456.t1 | TonB-dependent receptor | 1953 | 2.86E-06 | 68 | C:membrane; C:cell outer membrane; F:receptor activity; P:transport |
| Efet.01.314492.g457.t1 | hypothetical protein HELRODRAFT_179757 | 408 | 7.19E-06 | 46 |  |
| Efet.01.314519.g458.t1 | ras-related Rab-3 isoform X4 | 381 | 7.11E-40 | 90.7 | F:GTP binding; C:extracellular exosome; P:small GTPase mediated signal transduction; C:mitochondrion; P:peptidyl-cysteine methylation; C:zymogen granule; F:myosin V binding; P:positive regulation of regulated secretory pathway |
| Efet.01.314596.g459.t1 | collagen alpha-1(III) chain-like | 363 | 7.06E-13 | 59.56 | F:calcium ion binding |
| Efet.01.31463.g449.t1 | aquaporin -like | 825 | 4.50E-105 | 66.3 | C:integral component of membrane; P:transport; F:transporter activity |
| Efet.01.314642.g461.t1 | MOB kinase activator-like 3 | 345 | 3.75E-40 | 85.9 | P:phosphorylation; F:kinase activity |
| Efet.01.314672.g462.t1 | protocadherin-16 | 1206 | 1.04E-47 | 56.2 | P:animal organ development |
| Efet.01.314685.g463.t1 | neuroglian isoform X1 | 276 | 1.95E-10 | 58.1 | P:regulation of female receptivity; P:motor neuron axon guidance; P:epidermal growth factor receptor signaling pathway; P:male courtship behavior; P:neuron cell-cell adhesion; P:axon ensheathment; P:dendrite morphogenesis; P:nerve maturation; F:calcium ion binding; P:melanotic encapsulation of foreign target; P:cell adhesion involved in heart morphogenesis; P:female courtship behavior; P:imaginal disc morphogenesis; P:mushroom body development; P:regulation of tube size, open tracheal system; P:synapse organization; C:cytoneme; P:establishment of glial blood-brain barrier; C:membrane part; C:plasma membrane; P:photoreceptor cell axon guidance; P:axon extension; P:septate junction assembly; P:central complex development; C:filopodium; C:pleated septate junction |
| Efet.01.314741.g465.t1 | RING finger 44 isoform X1 | 207 | 3.49E-14 | 74.65 | F:metal ion binding |
| Efet.01.314762.g466.t1 | PREDICTED: uncharacterized protein LOC102806254 | 405 | 2.51E-07 | 59 |  |
| Efet.01.314815.g468.t1 | hypothetical protein CAPTEDRAFT_222265 | 753 | 4.87E-24 | 62.67 | F:calcium-activated potassium channel activity; P:potassium ion transport; P:potassium ion transmembrane transport; C:membrane; C:integral component of membrane |
| Efet.01.314858.g469.t1 | 3-hydroxybutyryl- dehydrogenase | 924 | 0 | 88.5 | F:carnitine 3-dehydrogenase activity; C:cytoplasm; F:3-hydroxyacyl-CoA dehydrogenase activity; F:NAD binding; P:oxidation-reduction process; P:fatty acid metabolic process; P:carnitine catabolic process |
| Efet.01.314858.g470.t1 | 4-hydroxybenzoyl- thioesterase [Burkholderia stagnalis] | 426 | 1.02E-92 | 83.45 |  |
| Efet.01.314901.g471.t1 | cysteine and histidine-rich 1-like | 342 | 8.17E-42 | 67.55 | F:metal ion binding |
| Efet.01.314923.g472.t1 | glyceraldehyde-3-phosphate | 333 | 1.99E-43 | 90 | P:oxidation-reduction process; F:glyceraldehyde-3-phosphate dehydrogenase (NAD+) (phosphorylating) activity |
| Efet.01.314930.g473.t1 | discoidin domain-containing receptor 2-like isoform X1 | 630 | 9.14E-65 | 73.35 | F:protein tyrosine kinase activity; C:integral component of membrane; P:protein phosphorylation |
| Efet.01.314946.g474.t1 | hypothetical protein CAPTEDRAFT_202552 | 1218 | 2.93E-29 | 62 | C:membrane; C:integral component of membrane |
| Efet.01.31497.g450.t1 | Gawky-like isoform X1 | 783 | 1.22E-15 | 75.75 | F:nucleotide binding; F:nucleic acid binding |
| Efet.01.31498.g451.t1 | TNFAIP3-interacting 2 | 423 | 2.94E-06 | 55 |  |
| Efet.01.315097.g475.t1 | NF-kappa-B inhibitor 1 | 264 | 3.77E-19 | 59.8 |  |
| Efet.01.315146.g476.t1 | F-box LRR-repeat 8-like | 1155 | 3.17E-115 | 60.55 |  |
| Efet.01.315253.g478.t1 | protocadherin-18 isoform X2 | 1551 | 1.25E-65 | 51.65 | F:calcium ion binding; C:membrane; C:integral component of membrane; P:nervous system development; P:cell adhesion; P:homophilic cell adhesion via plasma membrane adhesion molecules; C:plasma membrane |
| Efet.01.315256.g479.t1 | Thyrotropin-releasing hormone receptor | 879 | 1.24E-25 | 51.8 | F:G-protein coupled receptor activity; P:G-protein coupled receptor signaling pathway; C:membrane; C:integral component of membrane |
| Efet.01.31526.g453.t1 | C3 and PZP-like alpha-2-macroglobulin domain-containing 8 isoform X2 | 345 | 6.12E-09 | 53.5 | C:extracellular space; C:extracellular region |
| Efet.01.315325.g481.t1 | hypothetical protein HELRODRAFT_181224 | 624 | 1.77E-28 | 75.67 | C:membrane |
| Efet.01.315337.g482.t1 | GRAM domain-containing 1B-like isoform X1 | 321 | 1.68E-18 | 64.9 | C:membrane; C:integral component of membrane |
| Efet.01.315351.g483.t1 | teneurin-m isoform X1 | 207 | 1.60E-11 | 58.1 | C:membrane; C:integral component of membrane |
| Efet.01.315370.g484.t1 | ATP-binding cassette sub-family D member 3 | 435 | 9.04E-58 | 78.6 | C:peroxisome; F:ATP binding; F:ATPase activity, coupled to transmembrane movement of substances; F:protein homodimerization activity; P:transmembrane transport; C:integral component of membrane; P:peroxisomal long-chain fatty acid import; F:long-chain fatty acid transporter activity |
| Efet.01.315385.g485.t1 | 1-phosphatidylinositol 4,5-bisphosphate phosphodiesterase epsilon-1-like | 300 | 2.31E-15 | 61.6 | P:biological regulation; P:single-organism process |
| Efet.01.315403.g486.t1 | pumilio homolog 2 isoform X5 | 300 | 1.85E-11 | 56.95 |  |
| Efet.01.315456.g487.t1 | ATP-binding cassette sub-family A member 1 | 264 | 5.26E-21 | 82.25 | F:ATPase activity; C:integral component of plasma membrane; F:ATP binding; P:phagocytosis, engulfment; P:reverse cholesterol transport; F:apolipoprotein A-I binding; P:cholesterol efflux; F:transporter activity |
| Efet.01.315476.g488.t1 | chitin synthase | 723 | 7.79E-67 | 59 | F:transferase activity, transferring hexosyl groups; C:integral component of membrane; F:binding |
| Efet.01.31548.g456.t1 | FAM193A-like isoform X1 | 1941 | 1.58E-07 | 73.73 |  |
| Efet.01.315484.g489.t1 | alpha- sarcomeric-like isoform X1 | 471 | 1.11E-75 | 84.75 | F:actin filament binding; P:actin crosslink formation; F:calcium ion binding; P:actin filament bundle assembly |
| Efet.01.315491.g490.t1 | ADP-ribosylation factor-binding GGA1-like | 456 | 2.52E-55 | 81.35 | C:trans-Golgi network; F:ADP-ribosylation factor binding; P:vesicle-mediated transport; C:clathrin adaptor complex; P:intracellular protein transport |
| Efet.01.315501.g491.t1 | oxidation resistance 1-like isoform X2 | 315 | 3.11E-18 | 80.9 |  |
| Efet.01.315502.g492.t1 | dual specificity tyrosine-phosphorylation-regulated kinase 2 isoform X2 | 1089 | 9.79E-166 | 82.1 | P:peptidyl-tyrosine phosphorylation; P:smoothened signaling pathway; P:intrinsic apoptotic signaling pathway in response to DNA damage by p53 class mediator; F:protein serine/threonine kinase activity; P:positive regulation of glycogen biosynthetic process; C:nucleoplasm; F:magnesium ion binding; F:ATP binding; F:protein tyrosine kinase activity; F:protein serine/threonine/tyrosine kinase activity; C:ubiquitin ligase complex; C:intracellular ribonucleoprotein complex; F:ubiquitin binding; C:cytoplasm; F:manganese ion binding; P:negative regulation of NFAT protein import into nucleus |
| Efet.01.315508.g493.t1 | 14-3-3 epsilon | 375 | 7.99E-68 | 92.15 | F:protein domain specific binding |
| Efet.01.315538.g496.t1 | DNA methylase | 1011 | 1.05E-16 | 59.1 | F:nucleotide binding; F:DNA binding; F:ATP binding; P:DNA methylation; F:N-methyltransferase activity; F:helicase activity |
| Efet.01.315538.g497.t1 | DNA methylase | 987 | 6.35E-39 | 56.8 | F:nucleotide binding; F:ATP binding |
| Efet.01.315538.g498.t1 | Snf2 family | 1614 | 3.92E-44 | 48.45 | F:nucleotide binding; F:nucleic acid binding; F:DNA binding; F:ATP binding; F:helicase activity; F:methyltransferase activity; P:methylation; F:hydrolase activity |
| Efet.01.315581.g500.t1 | FMRFamide receptor-like | 1119 | 1.07E-89 | 53.4 | F:signal transducer activity |
| Efet.01.315696.g503.t1 | homeotic female sterile-like isoform X1 | 522 | 8.76E-12 | 54 |  |
| Efet.01.315709.g504.t1 | neural-cadherin-like | 348 | 2.50E-33 | 66.4 | F:calcium ion binding; C:integral component of membrane; P:homophilic cell adhesion via plasma membrane adhesion molecules; C:plasma membrane |
| Efet.01.315758.g505.t1 | hypothetical protein CAPTEDRAFT_208702 | 576 | 8.59E-32 | 50.95 | C:membrane; C:integral component of membrane |
| Efet.01.315786.g506.t1 | S phase cyclin A-associated in the endoplasmic reticulum-like isoform X2 | 417 | 9.20E-31 | 70 | F:binding |
| Efet.01.31585.g458.t1 | heparan-sulfate 6-O-sulfotransferase 3-like | 366 | 3.10E-32 | 71.9 | C:integral component of plasma membrane; P:heparan sulfate proteoglycan biosynthetic process, enzymatic modification; P:lung alveolus development; F:heparan sulfate 6-O-sulfotransferase activity; P:labyrinthine layer blood vessel development; P:angiogenesis; P:glycosaminoglycan biosynthetic process; C:Golgi membrane; P:neuron development |
| Efet.01.315871.g507.t1 | hypothetical protein HELRODRAFT_105536 | 612 | 3.61E-07 | 83 |  |
| Efet.01.315883.g508.t1 | dnaJ homolog subfamily A member 2 | 291 | 3.82E-35 | 80.55 | F:ATP binding; F:heat shock protein binding; P:response to heat; C:extracellular exosome; F:metal ion binding; F:chaperone binding; C:cytosol; F:unfolded protein binding; P:protein refolding |
| Efet.01.31589.g459.t1 | guanylyl cyclase GC-E-like | 384 | 2.60E-09 | 63.33 |  |
| Efet.01.31592.g460.t1 | hypothetical protein HELRODRAFT_154876 | 522 | 3.81E-12 | 55.33 | F:transferase activity, transferring glycosyl groups; F:transferase activity, transferring hexosyl groups; P:metabolic process; C:membrane; F:glucuronosyltransferase activity; C:integral component of membrane; F:transferase activity |
| Efet.01.315963.g511.t1 | metal tolerance 9-like | 714 | 1.71E-47 | 71.7 | P:cation transmembrane transport; C:integral component of membrane; F:cation transmembrane transporter activity |
| Efet.01.315983.g512.t1 | sacsin-like | 2586 | 1.36E-52 | 45.8 |  |
| Efet.01.316009.g513.t1 | phospholipid-transporting ATPase ID-like isoform X1 | 279 | 2.66E-19 | 64.7 | F:ion binding |
| Efet.01.316166.g517.t1 | diamine acetyltransferase 2 | 258 | 2.69E-12 | 70.55 | P:polyamine acetylation; F:N-acetyltransferase activity; P:ammonium ion metabolic process |
| Efet.01.31638.g465.t1 | Zinc metallo ase dpy-31 | 555 | 1.00E-21 | 55.35 | P:cuticle development involved in collagen and cuticulin-based cuticle molting cycle; F:metal ion binding; F:peptidase activity |
| Efet.01.316410.g520.t1 | dihydropyrimidinase isoform X1 | 264 | 1.03E-24 | 84.6 | C:extracellular exosome; C:cytoplasm; P:uracil catabolic process; F:phosphoprotein binding; F:dihydropyrimidinase activity |
| Efet.01.316496.g521.t1 | hypothetical protein HELRODRAFT_167086 | 624 | 2.67E-08 | 51 |  |
| Efet.01.316542.g522.t1 | structural maintenance of chromosomes 4-like | 366 | 1.20E-09 | 66.43 | F:ATP binding; C:nucleus; P:chromosome organization; C:chromosome |
| Efet.01.316608.g523.t1 | uncharacterized protein C1orf158 homolog | 441 | 6.27E-30 | 57.7 |  |
| Efet.01.316608.g524.t1 | orexin receptor type 2-like | 726 | 7.76E-46 | 48.6 | P:cellular process |
| Efet.01.31663.g467.t1 | ribosome biogenesis bop1-A-like | 507 | 1.23E-45 | 64.6 | P:ribosomal large subunit biogenesis; C:preribosome; P:rRNA processing; C:nucleolus |
| Efet.01.31664.g468.t1 | flap endonuclease GEN homolog 1 | 387 | 5.07E-55 | 86.7 | F:DNA binding; P:nucleic acid phosphodiester bond hydrolysis; C:centrosome; C:extracellular exosome; P:resolution of mitotic recombination intermediates; P:regulation of centrosome duplication; P:positive regulation of mitotic cell cycle spindle assembly checkpoint; P:double-strand break repair via homologous recombination; F:crossover junction endodeoxyribonuclease activity |
| Efet.01.316652.g526.t1 | dynein heavy chain axonemal-like | 306 | 5.08E-27 | 71.7 | F:nucleotide binding; F:ion binding |
| Efet.01.316681.g527.t1 | heparan-sulfate 6-O-sulfotransferase 1 | 549 | 1.07E-32 | 65.55 | C:integral component of membrane; F:sulfotransferase activity; F:transferase activity |
| Efet.01.316780.g530.t1 | Laminin G domain-containing | 1104 | 0 | 67.6 | F:catalytic activity |
| Efet.01.316780.g531.t1 | Laminin G domain-containing | 1299 | 0 | 61.2 | F:catalytic activity |
| Efet.01.316780.g532.t1 | sugar ABC transporter permease | 384 | 3.65E-60 | 76.1 | C:integral component of membrane; P:transport; C:plasma membrane |
| Efet.01.31679.g469.t1 | G -activated inward rectifier potassium channel 3-like | 1641 | 1.53E-171 | 72.8 | C:integral component of plasma membrane; F:inward rectifier potassium channel activity; P:regulation of ion transmembrane transport; P:potassium ion import |
| Efet.01.316795.g533.t1 | phosphatase mitochondrial-like | 642 | 5.98E-44 | 68.85 | F:phosphoprotein phosphatase activity; F:cation binding |
| Efet.01.316830.g535.t1 | sidekick isoform X1 | 1737 | 5.15E-93 | 59 | F:rhodopsin kinase activity; C:membrane; C:integral component of membrane; P:regulation of apoptotic process; F:transferase activity; P:protein phosphorylation |
| Efet.01.316852.g537.t1 | UPF0469 KIAA0907 homolog isoform X2 | 336 | 1.98E-08 | 86.2 | C:nucleus; F:RNA binding |
| Efet.01.316952.g541.t1 | calmodulin | 222 | 1.17E-21 | 59.75 | F:calcium ion binding |
| Efet.01.31699.g470.t1 | cytochrome P450 10-like | 207 | 5.75E-16 | 63.72 | F:oxidoreductase activity; F:metal ion binding |
| Efet.01.317115.g545.t1 | dehydrogenase reductase SDR family member 4-like | 309 | 5.03E-25 | 72.6 | F:oxidoreductase activity; P:oxidation-reduction process |
| Efet.01.31715.g472.t1 | metaxin-1 isoform X2 | 426 | 1.12E-16 | 61.8 | C:membrane |
| Efet.01.317268.g547.t1 | polycystic kidney disease and receptor for egg jelly-related | 603 | 5.33E-09 | 46.78 | P:detection of mechanical stimulus; F:calcium ion binding; F:calcium channel activity; C:membrane; C:integral component of membrane; P:calcium ion transmembrane transport |
| Efet.01.31730.g473.t1 | lethal(2) giant larvae homolog 1-like | 237 | 1.41E-09 | 69.1 |  |
| Efet.01.317314.g549.t1 | Cat eye syndrome critical region 6 | 915 | 9.52E-13 | 54.15 | C:membrane; C:integral component of membrane |
| Efet.01.317494.g551.t1 | Activated RNA polymerase II transcriptional coactivator p15 | 408 | 1.31E-21 | 50.75 | F:DNA binding; F:transcription coactivator activity; P:regulation of transcription, DNA-templated |
| Efet.01.317513.g553.t1 | cytochrome P450 2U1-like | 951 | 4.90E-15 | 53.1 | F:monooxygenase activity |
| Efet.01.317552.g554.t1 | tyrosine--tRNA cytoplasmic | 387 | 2.84E-65 | 89.85 | P:signal transduction; F:interleukin-8 receptor binding; F:tRNA binding; P:cellular response to DNA damage stimulus; F:signal transducer activity; F:RNA binding; F:valine-tRNA ligase activity; C:nucleus; F:tyrosine-tRNA ligase activity; F:ATP binding; C:extracellular space; P:tyrosyl-tRNA aminoacylation; P:valyl-tRNA aminoacylation; P:apoptotic process; C:cytosol |
| Efet.01.317671.g556.t1 | hypothetical protein CAPTEDRAFT_223820 | 375 | 4.03E-26 | 63 |  |
| Efet.01.317673.g557.t1 | centromere kinetochore zw10 homolog | 687 | 2.34E-71 | 67.55 | P:mitotic cell cycle checkpoint; P:vesicle-mediated transport; P:regulation of mitotic cell cycle phase transition; P:regulation of mitotic nuclear division; C:spindle; P:mitotic sister chromatid segregation; P:protein localization; C:endoplasmic reticulum; C:kinetochore; P:establishment of localization in cell; P:establishment of organelle localization |
| Efet.01.317765.g561.t1 | ZBED8-like | 237 | 1.98E-11 | 70.7 |  |
| Efet.01.317832.g562.t1 | laminin subunit alpha | 285 | 1.44E-13 | 62.75 | P:regulation of biological process |
| Efet.01.317845.g563.t1 | tetratricopeptide repeat 28 | 1542 | 2.51E-103 | 59.25 | C:centrosome; C:midbody; P:regulation of mitotic cell cycle |
| Efet.01.317854.g564.t1 | hypothetical protein CAPTEDRAFT_226618 | 270 | 2.62E-11 | 64 | F:DNA binding; C:nucleus; F:transcription factor activity, sequence-specific DNA binding; P:transcription, DNA-templated; P:regulation of transcription, DNA-templated |
| Efet.01.317872.g566.t1 | hypothetical protein CAPTEDRAFT_129802 | 252 | 9.95E-10 | 57 |  |
| Efet.01.317876.g567.t1 | puromycin-sensitive aminopeptidase-like isoform X2 | 825 | 3.50E-136 | 74.95 | F:zinc ion binding; P:proteolysis; F:metallopeptidase activity |
| Efet.01.31806.g476.t1 | Hepatocyte nuclear factor 6 | 1017 | 9.35E-70 | 76.4 | C:nucleus; F:DNA binding; F:transcription factor activity, sequence-specific DNA binding; P:positive regulation of cellular process; P:transcription, DNA-templated; P:cell differentiation; P:hematopoietic or lymphoid organ development; P:tissue development; P:regulation of transcription from RNA polymerase II promoter; P:pancreas development |
| Efet.01.318150.g569.t1 | hypothetical protein HELRODRAFT_192523 | 2025 | 2.87E-168 | 49.11 | C:membrane; C:integral component of membrane |
| Efet.01.318200.g571.t1 | forkhead box C1-A-like | 396 | 1.48E-13 | 84.05 | P:negative regulation of angiogenesis; P:ventricular cardiac muscle tissue morphogenesis; F:RNA polymerase II regulatory region sequence-specific DNA binding; F:DNA binding, bending; P:odontogenesis of dentin-containing tooth; P:negative regulation of apoptotic process involved in outflow tract morphogenesis; P:positive regulation of hematopoietic stem cell differentiation; P:positive regulation of transcription from RNA polymerase II promoter; C:nucleoplasm; P:paraxial mesoderm formation; P:skeletal system development; P:ossification; P:in utero embryonic development; P:lacrimal gland development; F:transcription factor activity, RNA polymerase II distal enhancer sequence-specific binding; P:lymph vessel development; P:camera-type eye development; P:glycosaminoglycan metabolic process; P:negative regulation of lymphangiogenesis; P:transcription, DNA-templated; P:neural crest cell development; P:vascular endothelial growth factor signaling pathway; P:negative regulation of mitotic cell cycle; P:artery morphogenesis; P:regulation of organ growth; F:transcription factor binding; P:brain development; P:germ cell migration; P:embryonic heart tube development; P:cardiac muscle cell proliferation; C:nuclear heterochromatin; P:ovarian follicle development; P:vascular endothelial growth factor receptor signaling pathway; P:blood vessel remodeling; P:Notch signaling pathway; C:cytoplasm; P:ureteric bud development; P:somitogenesis; P:regulation of blood vessel size; P:maintenance of lens transparency; P:collagen fibril organization |
| Efet.01.318267.g574.t1 | coiled-coil domain-containing 93-like | 243 | 1.34E-33 | 92.15 | P:Golgi to plasma membrane transport; C:intracellular |
| Efet.01.31827.g477.t1 | hypothetical protein CAPTEDRAFT_219098 | 927 | 1.06E-08 | 74 |  |
| Efet.01.31833.g478.t1 | RAD50-interacting 1 | 255 | 1.12E-12 | 83.25 | P:regulation of ER to Golgi vesicle-mediated transport; C:Dsl1/NZR complex; P:Golgi vesicle transport; P:regulation of signal transduction involved in mitotic G2 DNA damage checkpoint |
| Efet.01.318392.g575.t1 | opioid growth factor receptor | 429 | 3.33E-20 | 63.3 | C:membrane; F:receptor activity |
| Efet.01.318395.g576.t1 | cadherin-4-like isoform X2 | 282 | 9.96E-15 | 72.05 | C:membrane; P:cell adhesion |
| Efet.01.318420.g577.t1 | tripartite motif-containing 2-like isoform X3 | 633 | 3.44E-17 | 48.05 | F:zinc ion binding; F:metal ion binding; C:intracellular |
| Efet.01.318550.g579.t1 | Wilms tumor 1-interacting homolog | 1200 | 1.66E-16 | 46.75 | F:zinc ion binding |
| Efet.01.318554.g580.t1 | kelch 20 | 1008 | 7.77E-117 | 59.8 | C:membrane; C:integral component of membrane |
| Efet.01.318693.g583.t1 | anaphase-promoting complex subunit 1-like | 558 | 1.44E-33 | 62.5 | C:anaphase-promoting complex |
| Efet.01.318728.g584.t1 | ras family member 10B | 252 | 1.36E-12 | 71.3 | F:nucleotide binding; P:signal transduction |
| Efet.01.318733.g585.t1 | CST complex subunit TEN1 | 276 | 5.41E-16 | 64.4 | F:DNA binding; C:CST complex; P:regulation of DNA metabolic process |
| Efet.01.318739.g586.t1 | probable global transcription activator SNF2L2 isoform X1 | 1080 | 1.04E-73 | 77.8 | F:ATPase activity; C:SWI/SNF complex; F:ATP binding; F:histone binding; P:ATP-dependent chromatin remodeling; P:regulation of transcription, DNA-templated |
| Efet.01.318747.g587.t1 | ABC transporter substrate-binding | 783 | 5.61E-177 | 91.2 | C:outer membrane-bounded periplasmic space |
| Efet.01.318747.g588.t1 | succinylglutamate desuccinylase | 855 | 3.20E-150 | 88.4 | F:hydrolase activity, acting on ester bonds; F:metal ion binding; P:metabolic process |
| Efet.01.318747.g589.t1 | acetyl- carboxyl transferase | 207 | 7.27E-43 | 84.95 | F:transferase activity |
| Efet.01.318747.g590.t1 | segregation condensation A | 705 | 1.44E-142 | 92.6 | P:cell division; C:cytoplasm; P:chromosome segregation; P:cell cycle |
| Efet.01.318747.g591.t1 | L-aspartate oxidase | 1587 | 0 | 94.55 | F:L-aspartate:fumarate oxidoreductase activity; C:cytoplasm; P:NAD biosynthetic process; P:oxidation-reduction process; F:L-aspartate oxidase activity |
| Efet.01.318747.g592.t1 | 7-carboxy-7-deazaguanine synthase | 639 | 1.23E-125 | 91.6 | F:magnesium ion binding; P:queuosine biosynthetic process; F:S-adenosyl-L-methionine binding; F:4 iron, 4 sulfur cluster binding; F:carbon-nitrogen lyase activity |
| Efet.01.318747.g593.t1 | adenosine deaminase | 1053 | 0 | 93.6 | F:adenine deaminase activity; F:zinc ion binding; P:adenine catabolic process; P:hypoxanthine salvage; P:nucleotide metabolic process |
| Efet.01.318747.g594.t1 | L-fuconate dehydratase | 1281 | 0 | 91.8 | F:L-fuconate dehydratase activity; P:cellular amino acid catabolic process |
| Efet.01.318747.g595.t1 | FAA hydrolase family | 828 | 0 | 89.1 | P:metabolic process; F:5-oxopent-3-ene-1,2,5-tricarboxylate decarboxylase activity |
| Efet.01.318747.g596.t1 | family transcriptional regulator | 942 | 0 | 92.85 | P:carbohydrate transport |
| Efet.01.318747.g597.t1 | ABC transporter permease | 954 | 4.95E-136 | 93 | C:integral component of membrane; P:carbohydrate transport; C:plasma membrane; F:transporter activity |
| Efet.01.318747.g598.t1 | sugar ABC transporter ATP-binding | 1416 | 0 | 88.6 | F:ATP binding; P:monosaccharide transport; F:monosaccharide-transporting ATPase activity; P:carbohydrate transmembrane transport; C:plasma membrane |
| Efet.01.318747.g599.t1 | NAD(P)-dependent oxidoreductase | 753 | 2.16E-159 | 85.15 |  |
| Efet.01.318747.g600.t1 | family transcriptional regulator | 762 | 2.97E-156 | 85.85 | F:DNA binding; P:transcription, DNA-templated; P:regulation of transcription, DNA-templated |
| Efet.01.318747.g601.t1 | alkyl hydroperoxide reductase subunit F | 1608 | 0 | 89.2 | C:cell; F:protein disulfide oxidoreductase activity; F:flavin adenine dinucleotide binding; F:NAD binding; F:electron carrier activity; F:alkyl hydroperoxide reductase activity; P:cell redox homeostasis; P:oxidation-reduction process; P:response to reactive oxygen species |
| Efet.01.318747.g602.t1 | peroxiredoxin | 576 | 1.96E-139 | 96.35 | F:peroxidase activity; P:response to oxidative stress; P:cellular oxidant detoxification; P:oxidation-reduction process; F:peroxiredoxin activity |
| Efet.01.318747.g603.t1 | phosphonate utilization associated transcriptional regulator | 1047 | 6.59E-168 | 75.25 | F:DNA binding; F:transcription factor activity, sequence-specific DNA binding; P:transcription, DNA-templated; P:regulation of transcription, DNA-templated |
| Efet.01.318747.g604.t1 | phosphonoacetate hydrolase | 1038 | 0 | 85.85 | F:phosphonoacetate hydrolase activity; P:metabolic process |
| Efet.01.31878.g479.t1 | Myophilin | 291 | 4.64E-31 | 66.5 | F:oxidoreductase activity; F:binding |
| Efet.01.318823.g606.t1 | neural-cadherin-like isoform X2 | 447 | 1.64E-60 | 68.05 | F:calcium ion binding; C:integral component of membrane; P:homophilic cell adhesion via plasma membrane adhesion molecules; C:plasma membrane |
| Efet.01.318882.g607.t1 | Zinc finger SWIM domain-containing 8 | 945 | 3.02E-22 | 48.33 |  |
| Efet.01.31892.g481.t1 | gonadotropin-releasing hormone receptor-like isoform X2 | 711 | 2.67E-64 | 72.95 | F:G-protein coupled receptor activity; P:G-protein coupled receptor signaling pathway; C:integral component of membrane |
| Efet.01.318970.g609.t1 | tribbles homolog 2-like | 339 | 4.93E-36 | 71.75 | C:nucleus; P:regulation of MAP kinase activity; P:heart looping; P:positive regulation of proteasomal ubiquitin-dependent protein catabolic process; F:mitogen-activated protein kinase kinase binding |
| Efet.01.31900.g482.t1 | microtubule-associated futsch-like | 1659 | 1.62E-11 | 68 |  |
| Efet.01.319144.g612.t1 | hypothetical protein HELRODRAFT_93529 | 330 | 3.67E-14 | 60.33 | F:ATP binding; F:catalytic activity; F:metal ion binding; C:synaptic vesicle; P:neurotransmitter secretion |
| Efet.01.319179.g615.t1 | chaperonin | 1485 | 0 | 96.95 | F:ATP binding; C:cytoplasm; F:unfolded protein binding; P:protein refolding |
| Efet.01.319214.g616.t1 | acetylglucosamine transferase | 1434 | 0 | 80.9 | F:transferase activity |
| Efet.01.319214.g617.t1 | sigma-54-dependent Fis family transcriptional regulator | 2013 | 0 | 87.2 | F:ATP binding; P:transcription, DNA-templated; P:phosphorelay signal transduction system; P:regulation of transcription, DNA-templated; F:sequence-specific DNA binding; F:transcription factor binding; C:intracellular |
| Efet.01.319248.g618.t1 | membrane magnesium transporter 1 | 324 | 1.23E-46 | 76.05 | C:integral component of membrane |
| Efet.01.319321.g620.t1 | Rho guanine nucleotide exchange factor 17 | 717 | 1.80E-63 | 64.95 | P:regulation of Rho protein signal transduction; F:Rho guanyl-nucleotide exchange factor activity; P:positive regulation of GTPase activity |
| Efet.01.319387.g623.t1 | tubulin polymerization-promoting family member 2-like | 723 | 7.81E-38 | 59.85 | F:tubulin binding; P:microtubule bundle formation; P:microtubule polymerization |
| Efet.01.319467.g624.t1 | hypothetical protein HELRODRAFT_183251 | 537 | 8.46E-17 | 62.5 | C:membrane; C:integral component of membrane |
| Efet.01.319486.g625.t1 | far upstream element-binding 3 isoform X5 | 315 | 5.32E-22 | 56.8 | F:nucleic acid binding |
| Efet.01.319606.g627.t1 | nucleolar 6-like isoform X2 | 666 | 3.85E-24 | 54.95 | C:nuclear part; C:macromolecular complex |
| Efet.01.319627.g628.t1 | solute carrier family 12 member 6 isoform X3 | 240 | 8.93E-36 | 86.5 | F:potassium:chloride symporter activity; P:chloride transmembrane transport; C:integral component of membrane |
| Efet.01.31973.g487.t1 | tyrosinase tyr-3 | 1323 | 9.90E-59 | 50.45 | F:metal ion binding; F:oxidoreductase activity; P:metabolic process; P:apoptotic process; P:oxidation-reduction process; F:monooxygenase activity |
| Efet.01.319767.g634.t1 | Transient receptor potential-gamma | 351 | 3.23E-45 | 77.95 | F:calcium channel activity; C:integral component of membrane; P:calcium ion transmembrane transport |
| Efet.01.319861.g636.t1 | prolactin-releasing peptide receptor-like | 345 | 2.28E-43 | 80.75 | P:neuropeptide signaling pathway; C:integral component of membrane; F:transferase activity; F:neuropeptide Y receptor activity |
| Efet.01.319872.g637.t1 | radial spoke head 9 homolog | 822 | 9.57E-74 | 62.35 | P:single-organism cellular process; P:microtubule-based process |
| Efet.01.319873.g638.t1 | WD repeat-containing 81-like isoform X1 | 297 | 2.55E-16 | 64.1 |  |
| Efet.01.319952.g639.t1 | FMRFamide receptor-like | 1164 | 2.47E-81 | 58.9 | F:G-protein coupled receptor activity; F:G-protein coupled peptide receptor activity; P:G-protein coupled receptor signaling pathway; C:membrane; C:integral component of membrane |
| Efet.01.32007.g488.t1 | dynein heavy chain axonemal-like | 540 | 4.24E-21 | 64.2 | F:nucleotide binding; F:nucleoside-triphosphatase activity |
| Efet.01.32009.g489.t1 | pleckstrin homology domain-containing family J member 1-like | 1569 | 1.82E-38 | 61.1 |  |
| Efet.01.320189.g641.t1 | guanine nucleotide-binding G(I) G(S) G(T) subunit beta-2 | 201 | 1.29E-29 | 84.8 | P:cell proliferation; C:myelin sheath; P:retina development in camera-type eye; C:dendrite; P:phototransduction, visible light; C:extracellular exosome; C:focal adhesion; F:spectrin binding; P:cardiac muscle cell apoptotic process; F:GTPase activity; F:GTPase binding; P:cellular response to hypoxia; F:protein complex binding; F:alkylglycerophosphoethanolamine phosphodiesterase activity; P:protein heterotrimerization; P:sensory perception of taste; P:phospholipase C-activating G-protein coupled receptor signaling pathway; F:signal transducer activity; C:lysosomal membrane; C:photoreceptor outer segment membrane; P:positive regulation of cytosolic calcium ion concentration; C:extracellular space; C:heterotrimeric G-protein complex; C:perinuclear region of cytoplasm; F:type 1 angiotensin receptor binding; C:cell body; C:photoreceptor inner segment |
| Efet.01.320250.g643.t1 | glutamate receptor 8 | 660 | 1.37E-96 | 70.4 | C:postsynaptic membrane; P:ion transmembrane transport; C:integral component of membrane; C:cell junction; P:ionotropic glutamate receptor signaling pathway; F:ionotropic glutamate receptor activity; F:extracellular-glutamate-gated ion channel activity |
| Efet.01.320499.g646.t1 | Potassium voltage-gated channel subfamily A member 2 | 954 | 2.41E-145 | 75.75 | P:potassium ion transmembrane transport; C:voltage-gated potassium channel complex; F:delayed rectifier potassium channel activity; P:regulation of ion transmembrane transport; P:protein homooligomerization |
| Efet.01.320499.g647.t1 | potassium voltage-gated channel Shaker-like | 573 | 3.77E-09 | 76.79 | F:voltage-gated potassium channel activity; P:potassium ion transmembrane transport; C:voltage-gated potassium channel complex; P:regulation of ion transmembrane transport; P:protein homooligomerization |
| Efet.01.320524.g648.t1 | E3 ubiquitin- ligase TRIP12 isoform X1 | 237 | 1.48E-26 | 87.05 | F:ubiquitin-protein transferase activity; F:zinc ion binding; F:ligase activity; P:protein ubiquitination |
| Efet.01.320634.g650.t1 | delphilin-like isoform X2 | 228 | 7.29E-18 | 71.5 |  |
| Efet.01.320638.g651.t1 | collagen alpha-1(XIV) chain- | 627 | 2.24E-56 | 66.35 |  |
| Efet.01.320812.g657.t1 | potassium voltage-gated channel Shaw-like isoform X1 | 753 | 2.33E-86 | 83.05 | F:voltage-gated potassium channel activity; P:potassium ion transmembrane transport; F:metal ion binding; C:voltage-gated potassium channel complex; P:regulation of ion transmembrane transport; P:protein homooligomerization |
| Efet.01.320887.g659.t1 | adhesion G -coupled receptor A1 isoform X1 | 870 | 2.53E-28 | 51.3 | P:regulation of cellular process; P:single-organism process |
| Efet.01.32090.g491.t1 | insulin-like growth factor-binding complex acid labile subunit | 1815 | 1.83E-64 | 45.2 | P:sensory perception of sound; C:extracellular exosome; C:membrane; P:innervation; C:integral component of membrane; P:otolith morphogenesis; C:insulin-like growth factor ternary complex; C:nucleoplasm |
| Efet.01.320901.g660.t1 | 26S proteasome non-ATPase regulatory subunit 4-like | 339 | 1.07E-18 | 68.95 | C:proteasome regulatory particle, base subcomplex; C:proteasome complex; P:ubiquitin-dependent protein catabolic process |
| Efet.01.320949.g662.t1 | repulsive guidance molecule A-like | 438 | 9.95E-33 | 57.5 | C:membrane; C:integral component of membrane |
| Efet.01.32095.g492.t1 | phosphatidylinositol 4,5-bisphosphate 3-kinase catalytic subunit alpha isoform-like | 477 | 1.20E-39 | 68.9 | C:phosphatidylinositol 3-kinase complex; P:regulation of collateral sprouting in absence of injury; P:phosphatidylinositol metabolic process; P:phosphorylation; F:phosphatidylinositol-4,5-bisphosphate 3-kinase activity; F:1-phosphatidylinositol-4-phosphate 3-kinase activity; C:plasma membrane; F:1-phosphatidylinositol-3-kinase activity |
| Efet.01.32095.g493.t1 | phosphatidylinositol 4,5-bisphosphate 3-kinase catalytic subunit alpha isoform-like | 363 | 6.43E-58 | 82.2 | P:phosphatidylinositol-mediated signaling; P:phosphatidylinositol phosphorylation; F:kinase activity; C:intracellular |
| Efet.01.321.g37.t1 | FMRFamide receptor-like | 1158 | 3.48E-65 | 55.6 | F:G-protein coupled receptor activity; F:G-protein coupled peptide receptor activity; P:G-protein coupled receptor signaling pathway; C:membrane; C:integral component of membrane |
| Efet.01.32105.g494.t1 | hypothetical protein HELRODRAFT_81331 | 303 | 2.26E-12 | 73.33 |  |
| Efet.01.321180.g665.t1 | kinase DC2 | 342 | 3.07E-20 | 77.55 | F:ATP binding; F:protein serine/threonine kinase activity; P:protein phosphorylation |
| Efet.01.321189.g666.t1 | neuropeptide FF receptor 1-like | 525 | 1.41E-33 | 51.55 | F:G-protein coupled receptor activity; P:signal transduction; C:membrane |
| Efet.01.321268.g668.t1 | neuropeptide Y receptor-like | 417 | 1.45E-26 | 71.9 | P:neuropeptide signaling pathway; C:integral component of membrane; P:tachykinin receptor signaling pathway; C:plasma membrane; F:tachykinin receptor activity; F:neuropeptide Y receptor activity |
| Efet.01.32134.g496.t1 | solute carrier family 12 member 2-like isoform X1 | 762 | 7.83E-45 | 70.25 | F:cation:chloride symporter activity; P:chloride transmembrane transport; C:integral component of membrane |
| Efet.01.321531.g672.t1 | sialin-like | 606 | 2.83E-62 | 67.6 | P:transmembrane transport; C:membrane; C:integral component of membrane |
| Efet.01.32170.g498.t1 | hypothetical protein CAPTEDRAFT_139004, partial | 735 | 3.99E-82 | 66.8 | F:actin binding |
| Efet.01.321709.g675.t1 | hypothetical protein CAPTEDRAFT_228037 | 372 | 9.44E-12 | 53 |  |
| Efet.01.321720.g676.t1 | Guanine nucleotide-binding G(q) subunit | 315 | 4.12E-62 | 99.6 | F:GTP binding; F:G-protein coupled receptor binding; P:G-protein coupled receptor signaling pathway; F:GTPase activity; F:G-protein beta/gamma-subunit complex binding; F:signal transducer activity; C:lysosomal membrane |
| Efet.01.321727.g677.t1 | ribonuclease 3-like isoform X2 | 429 | 6.27E-44 | 82.75 | P:RNA phosphodiester bond hydrolysis, endonucleolytic; F:RNA binding; F:ribonuclease III activity; P:rRNA catabolic process; P:RNA processing |
| Efet.01.32174.g499.t1 | SWI SNF-related matrix-associated actin-dependent regulator of chromatin subfamily D member 1 isoform X2 | 777 | 6.61E-150 | 87.15 | C:SWI/SNF complex; F:protein complex scaffold; F:transcription coactivator activity; P:cellular response to fatty acid; C:npBAF complex; P:nervous system development; C:nBAF complex; F:chromatin binding; P:chromatin-mediated maintenance of transcription; F:receptor binding; P:nucleosome disassembly; P:regulation of transcription from RNA polymerase II promoter |
| Efet.01.321798.g679.t1 | Protocadherin Fat 4 | 951 | 6.52E-50 | 58.4 | C:membrane |
| Efet.01.321826.g680.t1 | 4-hydroxy-tetrahydrodipicolinate synthase | 750 | 1.23E-153 | 95.05 | C:cytoplasm; P:lysine biosynthetic process via diaminopimelate; F:4-hydroxy-tetrahydrodipicolinate synthase; P:diaminopimelate biosynthetic process |
| Efet.01.321860.g681.t1 | ATP-dependent RNA helicase chloroplastic-like isoform X2 | 681 | 1.48E-25 | 45.85 | F:nucleotide binding; F:nucleic acid binding; F:ubiquitin-protein transferase activity; F:ATP binding; F:zinc ion binding; F:metal ion binding; F:ATP-dependent helicase activity; F:helicase activity; F:hydrolase activity; P:protein ubiquitination |
| Efet.01.32188.g500.t1 | AGAP004427-PA | 429 | 1.47E-07 | 74 | P:cellular protein modification process |
| Efet.01.321963.g683.t1 | family transcriptional regulator | 249 | 1.10E-49 | 79.05 | F:DNA binding; F:transcription factor activity, sequence-specific DNA binding; P:transcription, DNA-templated; P:regulation of transcription, DNA-templated |
| Efet.01.321975.g684.t1 | clavaminate synthase At3g21360 | 540 | 1.38E-39 | 59 | C:nucleus; F:oxidoreductase activity; P:oxidation-reduction process |
| Efet.01.32199.g501.t1 | dystonin-like isoform X1 | 621 | 2.15E-82 | 75.45 | F:ATPase activity; P:cell cycle arrest; F:actin binding; F:calcium ion binding; P:Wnt signaling pathway; F:microtubule binding |
| Efet.01.322080.g686.t1 | somatostatin receptor type 2 | 984 | 1.77E-45 | 55.6 | F:G-protein coupled receptor activity; P:G-protein coupled receptor signaling pathway; C:membrane; C:cell part |
| Efet.01.322086.g687.t1 | extracellular tyrosine- kinase PKDCC-like | 315 | 8.68E-33 | 61.85 | C:membrane; F:protein kinase activity; P:protein phosphorylation |
| Efet.01.322098.g688.t1 | sodium channel para-like isoform X1 | 405 | 4.40E-16 | 56.45 | F:ion channel activity; P:ion transport |
| Efet.01.322100.g689.t1 | rootletin-like isoform X2 | 1200 | 3.00E-76 | 67.3 | C:ciliary rootlet; P:centrosome organization |
| Efet.01.322106.g690.t1 | eukaryotic translation initiation factor 3 subunit J-like isoform X5 | 246 | 4.90E-09 | 76.09 |  |
| Efet.01.322116.g691.t1 | FMRFamide receptor-like | 441 | 2.31E-33 | 73.95 | F:G-protein coupled receptor activity; P:G-protein coupled receptor signaling pathway; C:integral component of membrane |
| Efet.01.32214.g502.t1 | acyl- thioesterase 1 | 207 | 1.05E-20 | 69.75 | F:lysophospholipase activity; C:extracellular exosome; F:palmitoyl-(protein) hydrolase activity; C:mitochondrion; P:protein depalmitoylation; C:cytosol; P:negative regulation of Golgi to plasma membrane protein transport |
| Efet.01.322182.g692.t1 | protocadherin gamma-B1-like | 1386 | 1.17E-52 | 53.15 | P:multicellular organism development; P:cell-cell signaling; P:cell adhesion; F:peptidase activity; P:homophilic cell adhesion via plasma membrane adhesion molecules; C:extracellular region; C:plasma membrane; C:extracellular space; F:calcium ion binding; C:membrane; P:proteolysis; C:integral component of membrane; F:hydrolase activity |
| Efet.01.322182.g693.t1 | protocadherin alpha-5- | 804 | 2.06E-41 | 58.9 | F:calcium ion binding; C:membrane; C:integral component of membrane; P:cell adhesion; P:homophilic cell adhesion via plasma membrane adhesion molecules; C:plasma membrane |
| Efet.01.32219.g503.t1 | actin-related 3 | 513 | 4.06E-102 | 82.25 | F:ATP binding; P:Arp2/3 complex-mediated actin nucleation; C:Arp2/3 protein complex |
| Efet.01.322329.g697.t1 | FMRFamide receptor-like | 1035 | 2.33E-34 | 53.85 | F:G-protein coupled receptor activity; F:G-protein coupled peptide receptor activity; P:G-protein coupled receptor signaling pathway; P:signal transduction; C:membrane; C:integral component of membrane; F:signal transducer activity |
| Efet.01.322348.g698.t1 | cat eye syndrome critical region 5 homolog | 408 | 6.66E-42 | 65.4 |  |
| Efet.01.32238.g505.t1 | DNA-directed RNA polymerase III subunit RPC5 | 1095 | 4.45E-77 | 61.7 | F:RNA polymerase III activity; C:nucleus; F:DNA-directed 5'-3' RNA polymerase activity; P:transcription from RNA polymerase III promoter; P:transcription, DNA-templated; C:DNA-directed RNA polymerase III complex |
| Efet.01.322383.g699.t1 | sucrase- intestinal-like | 549 | 1.35E-08 | 51.33 | P:carbohydrate metabolic process; F:catalytic activity; P:metabolic process; C:membrane; C:integral component of membrane; F:carbohydrate binding; F:hydrolase activity, hydrolyzing O-glycosyl compounds; F:hydrolase activity; F:hydrolase activity, acting on glycosyl bonds |
| Efet.01.32239.g506.t1 | mitochondrial heat shock 70 kDa | 396 | 3.19E-71 | 95.1 | F:ATP binding; F:unfolded protein binding; P:protein folding |
| Efet.01.322415.g700.t1 | hypothetical protein HELRODRAFT_164068 | 1596 | 4.75E-08 | 87 |  |
| Efet.01.322441.g701.t1 | phospholipase membrane-associated-like | 291 | 6.32E-23 | 55 | F:hydrolase activity, acting on ester bonds |
| Efet.01.322458.g702.t1 | furry homolog isoform X1 | 264 | 9.50E-17 | 73.15 | C:membrane |
| Efet.01.322538.g703.t1 | protocadherin beta-15 isoform X1 | 1824 | 5.92E-118 | 49.3 | F:calcium ion binding; C:membrane; C:integral component of membrane; P:cell adhesion; P:homophilic cell adhesion via plasma membrane adhesion molecules; C:plasma membrane |
| Efet.01.32254.g507.t1 | cilia- and flagella-associated 45-like | 372 | 1.53E-08 | 76.5 |  |
| Efet.01.322612.g704.t1 | hypothetical protein HELRODRAFT_183781 | 321 | 1.40E-09 | 54.5 | P:regulation of Rho protein signal transduction; F:Rho guanyl-nucleotide exchange factor activity; P:positive regulation of GTPase activity |
| Efet.01.322679.g705.t1 | cobalamin (vitamin b12) biosynthesis cbix | 7236 | 9.15E-08 | 45.75 |  |
| Efet.01.322707.g706.t1 | solute carrier family 12 member 4-like isoform X1 | 321 | 9.46E-54 | 84.55 | F:potassium:chloride symporter activity; P:chloride transmembrane transport; C:integral component of membrane |
| Efet.01.32281.g508.t1 | A disintegrin and metallo ase with thrombospondin motifs 6 | 231 | 9.78E-16 | 75.05 | P:heart development; P:blood vessel development; C:extracellular matrix; F:metallopeptidase activity |
| Efet.01.322867.g708.t1 | PREDICTED: uncharacterized protein LOC102463496 | 744 | 4.02E-125 | 68.2 | F:nucleic acid binding; F:protein dimerization activity |
| Efet.01.32294.g509.t1 | glycosyltransferase LARGE1 | 948 | 1.17E-147 | 78.25 | P:muscle cell cellular homeostasis; F:acetylglucosaminyltransferase activity; C:integral component of Golgi membrane; F:glucuronosyltransferase activity; F:UDP-xylosyltransferase activity; F:manganese ion binding; P:N-acetylglucosamine metabolic process; P:skeletal muscle organ development; P:glycosphingolipid biosynthetic process; P:protein O-linked mannosylation; P:skeletal muscle tissue regeneration |
| Efet.01.322944.g710.t1 | casein kinase I isoform alpha | 834 | 8.35E-152 | 88.2 | P:regulation of cell shape; C:nuclear speck; P:Golgi organization; P:intermediate filament cytoskeleton organization; C:condensed chromosome kinetochore; F:peptide binding; F:magnesium ion binding; P:cell division; F:ATP binding; C:keratin filament; C:intracellular ribonucleoprotein complex; C:mRNA cleavage and polyadenylation specificity factor complex; C:cytosol; P:Wnt signaling pathway; F:glycoprotein binding; P:cell morphogenesis; P:mitotic nuclear division; P:regulation of GTP binding; F:protein serine/threonine kinase activity; C:beta-catenin destruction complex; P:negative regulation of canonical Wnt signaling pathway; C:centrosome; C:membrane; P:phagocytosis; F:phosphoprotein binding; P:peptidyl-serine phosphorylation; P:positive regulation of proteasomal ubiquitin-dependent protein catabolic process |
| Efet.01.322947.g711.t1 | prickle 2 | 465 | 1.28E-56 | 72.85 | F:metal ion binding |
| Efet.01.322978.g712.t1 | cytoplasmic dynein 1 heavy chain 1-like isoform X2 | 531 | 2.26E-75 | 87.25 | F:ATPase activity; F:ATP binding; F:microtubule motor activity; C:dynein complex; P:microtubule-based movement |
| Efet.01.323044.g713.t1 | DNA excision repair ERCC-6-like | 738 | 9.69E-45 | 78.75 | F:ATP binding; P:cellular response to DNA damage stimulus |
| Efet.01.32308.g510.t1 | probable calcium-binding CML23 | 294 | 2.25E-30 | 59.65 | F:calcium ion binding |
| Efet.01.323111.g715.t1 | forkhead box M1 isoform X1 | 975 | 1.15E-11 | 72.75 | C:nucleus; F:sequence-specific DNA binding; F:RNA polymerase II transcription factor activity, sequence-specific DNA binding; P:regulation of transcription from RNA polymerase II promoter |
| Efet.01.323165.g718.t1 | Diphosphoinositol polyphosphate phosphohydrolase 1 | 219 | 3.24E-09 | 78.6 | C:nucleus; P:inositol phosphate metabolic process; C:cytoplasm; P:lipid homeostasis; P:glucose homeostasis; P:response to starvation; F:diphosphoinositol-polyphosphate diphosphatase activity; P:regulation of insulin receptor signaling pathway; P:nucleotide metabolic process |
| Efet.01.32323.g513.t1 | ankyrin repeat | 816 | 4.16E-50 | 51.7 | F:catalytic activity; C:membrane; C:integral component of membrane; P:nucleoside metabolic process; P:intracellular signal transduction; C:intracellular |
| Efet.01.323234.g720.t1 | cilia- and flagella-associated 47-like isoform X3 | 414 | 1.27E-21 | 70.35 |  |
| Efet.01.323242.g721.t1 | DNA-binding SATB1 | 312 | 4.50E-34 | 82.85 | C:nucleus; F:sequence-specific DNA binding; P:regulation of transcription from RNA polymerase II promoter |
| Efet.01.323272.g722.t1 | elongation factor mitochondrial-like | 1365 | 3.80E-176 | 74 | F:nucleotide binding; C:cytoskeleton; F:translation elongation factor activity; F:GTPase activity; C:mitochondrion; P:mitochondrial translational elongation; C:intracellular organelle part |
| Efet.01.32340.g514.t1 | type II secretion system | 447 | 1.68E-97 | 77.6 | C:integral component of membrane; F:protein transporter activity; P:protein secretion by the type II secretion system; C:type II protein secretion system complex |
| Efet.01.32340.g515.t1 | general secretion pathway | 342 | 4.99E-73 | 70.45 | C:integral component of membrane |
| Efet.01.32340.g516.t1 | general secretion pathway | 378 | 5.66E-82 | 61.05 | C:integral component of membrane |
| Efet.01.32340.g517.t1 | general secretion pathway | 627 | 2.17E-77 | 78.6 | C:integral component of membrane; P:protein secretion |
| Efet.01.32340.g518.t1 | general secretion pathway | 1482 | 3.89E-72 | 63.35 | P:extracellular transport; C:integral component of membrane; C:extracellular region |
| Efet.01.323400.g725.t1 | calpain-5-like isoform X1 | 204 | 5.92E-25 | 82.95 | P:regulation of cytoskeleton organization; P:proteolysis; F:calcium-dependent cysteine-type endopeptidase activity; C:intracellular |
| Efet.01.323450.g726.t1 | death-associated kinase 1-like isoform X2 | 1137 | 7.73E-61 | 52.2 | P:signal transduction |
| Efet.01.323555.g728.t1 | type III glutamate--ammonia ligase | 309 | 3.87E-26 | 66.7 | P:nitrogen compound metabolic process; F:glutamate-ammonia ligase activity |
| Efet.01.32366.g520.t1 | bone morphogenetic receptor type-1B isoform X2 | 1548 | 0 | 69.4 | P:positive regulation of cartilage development; P:endochondral bone morphogenesis; P:positive regulation of cell proliferation; P:chondrocyte development; P:transmembrane receptor protein serine/threonine kinase signaling pathway; P:negative regulation of chondrocyte proliferation; P:signal transduction by protein phosphorylation; F:transmembrane receptor protein serine/threonine kinase activity; F:ATP binding; F:signal transducer, downstream of receptor, with serine/threonine kinase activity; F:metal ion binding; P:positive regulation of transcription, DNA-templated; P:negative regulation of apoptotic process; C:integral component of membrane; P:proteoglycan biosynthetic process |
| Efet.01.32368.g521.t1 | isocitrate dehydrogenase [NADP] cytoplasmic | 336 | 6.25E-54 | 89.8 | F:protein homodimerization activity; P:regulation of phospholipid catabolic process; P:hemopoiesis; F:isocitrate dehydrogenase (NADP+) activity; P:isocitrate metabolic process; P:tricarboxylic acid cycle; P:regulation of phospholipid biosynthetic process; F:receptor binding; P:2-oxoglutarate metabolic process; F:magnesium ion binding; C:peroxisome; P:response to oxidative stress; C:extracellular exosome; F:NAD binding; P:glutathione metabolic process; C:mitochondrion; C:cytosol |
| Efet.01.32369.g522.t1 | phosphatase 1 regulatory subunit 3B | 1059 | 9.28E-36 | 53.3 | P:carbohydrate metabolic process; P:glycogen metabolic process |
| Efet.01.323702.g730.t1 | synaptotagmin-14-like isoform X4 | 717 | 1.63E-37 | 56.45 | C:membrane; C:integral component of membrane; P:exocytosis |
| Efet.01.32371.g523.t1 | bactericidal permeability-increasing -like | 774 | 3.99E-56 | 49.95 | C:extracellular space; P:defense response to Gram-negative bacterium; P:innate immune response; F:lipid binding; P:acute-phase response; F:lipopolysaccharide binding |
| Efet.01.323721.g731.t1 | WD repeat-containing 81-like isoform X2 | 366 | 5.84E-25 | 70.9 |  |
| Efet.01.323739.g732.t1 | SH2B adapter 1-like | 444 | 2.54E-34 | 57.05 | F:signaling adaptor activity; P:signal transduction; P:positive regulation of signal transduction; F:signal transducer activity; P:intracellular signal transduction; C:intracellular |
| Efet.01.323740.g733.t1 | hypothetical protein CAPTEDRAFT_219019 | 486 | 2.15E-17 | 48.41 | C:membrane; C:integral component of membrane; F:carbohydrate binding |
| Efet.01.323764.g734.t1 | cartilage matrix -like | 336 | 5.09E-23 | 63.95 | C:collagen trimer |
| Efet.01.323787.g737.t1 | hypothetical protein CAPTEDRAFT_218728 | 285 | 7.04E-20 | 65.8 | C:membrane; C:integral component of membrane; F:carbohydrate binding |
| Efet.01.323828.g738.t1 | Collagen alpha-4(VI) chain | 1026 | 8.26E-43 | 51.75 | F:calcium ion binding; C:collagen trimer; C:membrane; C:integral component of membrane; C:proteinaceous extracellular matrix |
| Efet.01.323858.g739.t1 | Potassium voltage-gated channel Shaker | 852 | 3.13E-51 | 92.25 | F:voltage-gated potassium channel activity; P:potassium ion transmembrane transport; C:voltage-gated potassium channel complex; P:regulation of ion transmembrane transport; P:protein homooligomerization |
| Efet.01.3239.g276.t1 | neuralized 2 | 585 | 4.12E-20 | 47.2 | P:intracellular signal transduction; C:intracellular |
| Efet.01.323909.g741.t1 | lysine--tRNA ligase | 783 | 7.52E-106 | 73 | F:nucleotide binding; P:tRNA aminoacylation for protein translation; F:aminoacyl-tRNA ligase activity; F:metal ion binding |
| Efet.01.323909.g742.t1 | lysine--tRNA ligase | 825 | 1.64E-121 | 87.35 | F:magnesium ion binding; F:nucleic acid binding; F:ATP binding; C:cytoplasm; F:lysine-tRNA ligase activity; P:lysyl-tRNA aminoacylation |
| Efet.01.323949.g743.t1 | kinesin light chain-like isoform X7 | 258 | 3.91E-37 | 96.95 | F:microtubule motor activity; C:kinesin complex |
| Efet.01.324006.g744.t1 | NACHT domain- and WD repeat-containing 1-like isoform X1 | 846 | 5.59E-74 | 61.1 |  |
| Efet.01.324176.g746.t1 | Neurogenic locus Notch | 564 | 2.62E-23 | 58.35 | P:blood vessel morphogenesis; P:cardiac ventricle development; P:regulation of anatomical structure morphogenesis; P:endothelium development; P:cardiac chamber morphogenesis; P:positive regulation of multicellular organismal process; P:negative regulation of response to stimulus; P:tube morphogenesis; P:muscle structure development; P:cellular response to chemical stimulus; P:response to organic substance; P:regulation of gliogenesis; P:coronary vasculature development; P:anatomical structure formation involved in morphogenesis; P:positive regulation of cell differentiation; P:cell development; P:positive regulation of signal transduction; C:plasma membrane; P:regulation of cell proliferation; P:regulation of transcription from RNA polymerase II promoter; P:epithelial cell differentiation; F:DNA binding; F:protein binding; P:urogenital system development; P:Notch signaling pathway; P:morphogenesis of an epithelium; P:embryonic morphogenesis; P:striated muscle tissue development; P:negative regulation of neurogenesis; P:regulation of cell migration |
| Efet.01.324191.g747.t1 | DNA polymerase epsilon catalytic subunit A | 396 | 1.27E-66 | 86.35 | F:nucleotide binding; F:DNA binding; F:3'-5' exonuclease activity; P:DNA repair; P:nucleic acid phosphodiester bond hydrolysis; F:DNA-directed DNA polymerase activity; F:zinc ion binding; P:DNA replication; P:DNA biosynthetic process; C:epsilon DNA polymerase complex |
| Efet.01.32422.g524.t1 | glutathione S-transferase | 636 | 6.76E-131 | 82.6 | F:transferase activity |
| Efet.01.32422.g525.t1 | dioxygenase | 414 | 4.65E-88 | 93.5 | F:dioxygenase activity; P:oxidation-reduction process |
| Efet.01.32422.g526.t1 | death-on-curing | 432 | 1.41E-18 | 88.15 | P:phosphorylation; F:kinase activity |
| Efet.01.32422.g527.t1 | pyridoxal phosphate-dependent aminotransferase | 537 | 1.70E-107 | 95.9 | F:pyridoxal phosphate binding; F:transaminase activity; P:biosynthetic process |
| Efet.01.324245.g750.t1 | ATPase-dependent protease | 540 | 4.79E-06 | 69 |  |
| Efet.01.32427.g528.t1 | altronate hydrolase | 1215 | 9.51E-171 | 73.9 | F:lyase activity |
| Efet.01.32438.g529.t1 | spermatogenesis-associated 2 | 789 | 1.46E-13 | 54.15 | C:intracellular part |
| Efet.01.324406.g753.t1 | xyloside xylosyltransferase 1-like | 459 | 1.48E-13 | 64.67 | F:transferase activity, transferring glycosyl groups; F:transferase activity |
| Efet.01.324488.g754.t1 | toll-like receptor 4 | 2010 | 4.02E-79 | 45.55 | P:signal transduction; C:membrane; C:integral component of membrane |
| Efet.01.324510.g755.t1 | Dopamine D2-like receptor | 372 | 4.01E-14 | 91.55 | C:integral component of plasma membrane; F:dopamine neurotransmitter receptor activity; P:adenylate cyclase-inhibiting dopamine receptor signaling pathway |
| Efet.01.324563.g756.t1 | Casein kinase I isoform gamma- | 273 | 4.46E-31 | 84.55 | P:regulation of cell shape; C:nucleus; F:ATP binding; C:cytoplasm; P:Wnt signaling pathway; F:protein serine/threonine kinase activity; P:endocytosis; P:peptidyl-serine phosphorylation |
| Efet.01.324653.g758.t1 | rho-associated kinase 2 isoform X2 | 576 | 1.81E-36 | 68.5 | F:binding; F:kinase activity; P:cellular process |
| Efet.01.324693.g759.t1 | hypothetical protein CAPTEDRAFT_222036 | 405 | 1.72E-32 | 60.82 | C:membrane; C:integral component of membrane |
| Efet.01.324717.g760.t1 | mechanosensory 2-like | 531 | 6.82E-32 | 90.4 | C:integral component of membrane |
| Efet.01.324724.g761.t1 | hypothetical protein CAPTEDRAFT_149791 | 222 | 5.85E-09 | 60 | P:intracellular protein transport; F:Ran GTPase binding; C:intracellular |
| Efet.01.324800.g762.t1 | probable E3 ubiquitin- ligase HECTD4 | 510 | 1.32E-84 | 83.05 | C:nucleus; F:ubiquitin-protein transferase activity; C:cytoplasm; P:glucose homeostasis; C:integral component of membrane; P:glucose metabolic process; F:ligase activity; P:protein ubiquitination involved in ubiquitin-dependent protein catabolic process |
| Efet.01.324805.g763.t1 | kelch 24 | 1044 | 1.88E-09 | 45.15 |  |
| Efet.01.324821.g764.t1 | zinc finger SWIM domain-containing 8-like | 921 | 3.71E-52 | 82.5 | F:zinc ion binding |
| Efet.01.324841.g765.t1 | somatostatin receptor type 2 | 810 | 7.91E-69 | 57.35 | P:adenylate cyclase-inhibiting G-protein coupled receptor signaling pathway; P:neuropeptide signaling pathway; C:integral component of membrane; C:plasma membrane; F:somatostatin receptor activity |
| Efet.01.32487.g531.t1 | HIV Tat-specific factor 1 | 444 | 4.33E-12 | 65.4 | F:nucleic acid binding |
| Efet.01.324944.g766.t1 | Tuftelin-interacting 11 | 804 | 3.72E-58 | 55.9 | C:nuclear part |
| Efet.01.324963.g767.t1 | sodium potassium adenosine | 234 | 1.09E-22 | 76.6 | P:potassium ion transport; F:ATP binding; P:sodium ion transport; F:sodium:potassium-exchanging ATPase activity; F:metal ion binding; P:ATP hydrolysis coupled transmembrane transport; C:integral component of membrane; P:establishment or maintenance of transmembrane electrochemical gradient |
| Efet.01.325050.g769.t1 | hypothetical protein HELRODRAFT_181005 | 207 | 2.49E-17 | 80 |  |
| Efet.01.325095.g770.t1 | titin isoform X2 | 744 | 1.71E-81 | 75.55 |  |
| Efet.01.325105.g771.t1 | doublesex- and mab-3-related transcription factor 2 isoform X2 | 495 | 7.51E-33 | 98.3 | F:protein homodimerization activity; P:transcription, DNA-templated; F:RNA polymerase II regulatory region sequence-specific DNA binding; F:core promoter proximal region sequence-specific DNA binding; P:positive regulation of transcription from RNA polymerase II promoter; P:embryonic skeletal system development; P:left/right pattern formation; C:nucleus; F:transcription factor activity, sequence-specific DNA binding; P:sex differentiation; F:metal ion binding; P:regulation of somitogenesis; P:positive regulation of myotome development |
| Efet.01.32512.g532.t1 | F-box only 47-like | 399 | 8.24E-41 | 65.3 |  |
| Efet.01.325157.g773.t1 | ubiquitin ATG12 | 270 | 1.41E-24 | 71.6 | P:autophagosome assembly; P:nucleophagy; C:pre-autophagosomal structure membrane; C:Atg12-Atg5-Atg16 complex; P:C-terminal protein lipidation; F:Atg8 ligase activity; P:mitophagy |
| Efet.01.325190.g774.t1 | DNA-directed RNA polymerase III subunit RPC5 | 630 | 1.89E-20 | 50.15 | F:RNA polymerase III activity; C:nucleus; F:DNA-directed 5'-3' RNA polymerase activity; P:transcription from RNA polymerase III promoter; P:transcription, DNA-templated; C:DNA-directed RNA polymerase III complex |
| Efet.01.325254.g775.t1 | hypothetical protein HELRODRAFT_164499 | 570 | 9.54E-12 | 53.71 | P:transport |
| Efet.01.325268.g776.t1 | AFG3 2 | 357 | 4.18E-48 | 87.1 | F:zinc ion binding; P:regulation of multicellular organism growth; P:righting reflex; P:mitochondrial calcium uptake; P:cristae formation; P:protein complex assembly; P:mitochondrial fusion; P:myelination; F:ATP-dependent peptidase activity; F:metalloendopeptidase activity; F:microtubule-severing ATPase activity; P:nerve development; P:muscle fiber development; C:m-AAA complex; P:neuromuscular junction development; P:protein import into mitochondrial intermembrane space; F:ATP binding; P:axonogenesis; P:mitochondrial calcium ion homeostasis; P:mitochondrial protein processing |
| Efet.01.325363.g777.t1 | CCAAT enhancer-binding beta-like | 456 | 8.46E-20 | 68.35 | F:transcription factor activity, sequence-specific DNA binding; P:transcription, DNA-templated; P:regulation of transcription, DNA-templated; F:sequence-specific DNA binding |
| Efet.01.325378.g778.t1 | transcription initiation factor TFIID subunit 4-like | 510 | 8.59E-49 | 67.7 | F:translation initiation factor activity; F:transcription factor activity, sequence-specific DNA binding; C:transcription factor TFIID complex; F:protein heterodimerization activity; P:regulation of transcription, DNA-templated; P:DNA-templated transcription, initiation; P:translational initiation |
| Efet.01.325390.g779.t1 | tRNA-dihydrouridine(20) synthase [NAD(P)+]-like isoform X1 | 528 | 6.92E-53 | 67.65 | F:tRNA dihydrouridine synthase activity; P:metabolic process; C:cytosol |
| Efet.01.325440.g781.t1 | probable glycosyltransferase STELLO1 | 999 | 9.01E-106 | 53.95 | C:membrane; C:integral component of membrane |
| Efet.01.325491.g782.t1 | U1 small nuclear ribonucleo A-like | 282 | 5.83E-42 | 95.65 | F:nucleotide binding; F:protein binding; C:catalytic step 2 spliceosome; F:snRNP binding; C:viral nucleocapsid; F:snRNA stem-loop binding; C:U2 snRNP; C:nucleolus; C:nucleoplasm; P:mRNA splicing, via spliceosome |
| Efet.01.325515.g784.t1 | plexin-A4-like | 729 | 6.39E-30 | 55.35 | P:regulation of cellular process; P:cellular component organization; P:generation of neurons; P:response to stimulus |
| Efet.01.325542.g785.t1 | gastrula zinc finger -like | 1023 | 4.87E-26 | 44.2 | F:metal ion binding |
| Efet.01.325602.g787.t1 | gastrula zinc finger -like | 819 | 1.79E-75 | 58.95 | F:nucleic acid binding; F:metal ion binding |
| Efet.01.325606.g788.t1 | Zn peptidase [Mycobacterium abscessus abscessus] | 603 | 4.20E-74 | 70.85 | F:sequence-specific DNA binding |
| Efet.01.325607.g789.t1 | FAD-binding oxidoreductase | 735 | 5.88E-174 | 78.25 | F:oxidoreductase activity, acting on CH-OH group of donors; F:flavin adenine dinucleotide binding; P:oxidation-reduction process |
| Efet.01.325607.g790.t1 | family transcriptional regulator | 603 | 9.49E-141 | 77.85 | F:DNA binding; P:transcription, DNA-templated; P:regulation of transcription, DNA-templated |
| Efet.01.325607.g791.t1 | family transcriptional regulator | 813 | 0 | 68.55 | F:DNA binding; F:transcription factor activity, sequence-specific DNA binding; P:transcription, DNA-templated; P:regulation of transcription, DNA-templated |
| Efet.01.325607.g792.t1 | glutamate-5-semialdehyde dehydrogenase | 1290 | 0 | 92.15 | F:NADP binding; C:cytoplasm; F:glutamate-5-semialdehyde dehydrogenase activity; P:oxidation-reduction process; P:L-proline biosynthetic process |
| Efet.01.325607.g793.t1 | DNA polymerase III subunit delta | 1056 | 0 | 93.75 | C:DNA polymerase III complex; F:DNA binding; F:DNA-directed DNA polymerase activity; P:DNA replication; P:DNA biosynthetic process |
| Efet.01.325668.g794.t1 | MAM and LDL-receptor class A domain-containing 1-like isoform X2 | 216 | 1.20E-09 | 57.95 | C:membrane; C:integral component of membrane; F:scavenger receptor activity; P:receptor-mediated endocytosis |
| Efet.01.3258.g277.t1 | uncharacterized protein C12orf29 homolog | 999 | 1.67E-100 | 60.75 | P:biological_process; C:cellular_component; F:molecular_function |
| Efet.01.325919.g797.t1 | zinc finger 239-like | 660 | 6.66E-21 | 57.25 | F:nucleic acid binding; F:metal ion binding |
| Efet.01.325938.g798.t1 | glycerol-3-phosphate dehydrogenase oxidase | 690 | 6.23E-134 | 75.25 | P:glycerol-3-phosphate metabolic process; C:glycerol-3-phosphate dehydrogenase complex; P:oxidation-reduction process; F:glycerol-3-phosphate dehydrogenase activity |
| Efet.01.32594.g533.t1 | sugar ABC transporter substrate-binding | 288 | 4.60E-57 | 60.64 |  |
| Efet.01.325968.g799.t1 | protocadherin-11 X-linked isoform X2 | 2715 | 3.34E-151 | 55 | F:calcium ion binding; C:membrane; C:integral component of membrane; P:cell adhesion; P:homophilic cell adhesion via plasma membrane adhesion molecules; C:plasma membrane |
| Efet.01.326084.g804.t1 | GRIP and coiled-coil domain-containing 1 | 459 | 7.70E-23 | 54.4 | C:intracellular |
| Efet.01.326123.g805.t1 | myosin light chain smooth muscle-like | 384 | 5.37E-36 | 80.4 | F:ATP binding; F:protein serine/threonine kinase activity; P:protein phosphorylation |
| Efet.01.326134.g806.t1 | baculoviral IAP repeat-containing 6-like isoform X1 | 1572 | 1.94E-45 | 54.2 | F:ubiquitin-protein transferase activity; P:apoptotic process; P:regulation of cytokinesis; P:protein ubiquitination |
| Efet.01.326202.g808.t1 | follistatin-related 5-like | 660 | 8.20E-47 | 52.8 | F:calcium ion binding; C:membrane; P:protein glycosylation; F:galactosyltransferase activity |
| Efet.01.326237.g809.t1 | atrial natriuretic peptide receptor 1-like | 705 | 2.33E-12 | 50.3 | F:catalytic activity; P:primary metabolic process; P:single-organism process; P:organic substance metabolic process; P:phosphate-containing compound metabolic process |
| Efet.01.32625.g534.t1 | E3 SUMO- ligase | 261 | 1.03E-39 | 88.3 | P:negative regulation of transcription from RNA polymerase II promoter; F:zinc ion binding; F:ubiquitin protein ligase binding; C:PML body; P:protein sumoylation; P:positive regulation of protein sumoylation; F:transcription factor binding; P:fat cell differentiation; F:SUMO ligase activity; P:positive regulation of proteasomal ubiquitin-dependent protein catabolic process; F:ligase activity; P:regulation of cell proliferation |
| Efet.01.326361.g810.t1 | hypothetical protein CAPTEDRAFT_205572 | 834 | 9.05E-62 | 69.5 | C:membrane |
| Efet.01.326363.g811.t1 | kelch 10 | 291 | 1.26E-40 | 83.1 |  |
| Efet.01.326409.g813.t1 | TNF receptor-associated factor 6-like isoform X2 | 426 | 3.28E-46 | 71.35 | P:cellular macromolecule metabolic process; P:regulation of cellular process; F:metal ion binding; P:cellular response to stimulus; P:primary metabolic process |
| Efet.01.326467.g816.t1 | serine threonine- phosphatase | 492 | 1.43E-85 | 84.15 | F:protein serine/threonine phosphatase activity; P:protein dephosphorylation |
| Efet.01.3265.g278.t1 | WD repeat and FYVE domain-containing 3 | 354 | 2.03E-48 | 84.8 | F:metal ion binding |
| Efet.01.326544.g819.t1 | vesicle-trafficking SEC22b-like isoform X2 | 228 | 4.03E-12 | 77.2 | P:ER to Golgi vesicle-mediated transport; C:bounding membrane of organelle; C:integral component of membrane; F:SNAP receptor activity; C:SNARE complex; C:endoplasmic reticulum; C:Golgi apparatus; F:SNARE binding; C:intracellular organelle part; P:exocytosis; P:vesicle fusion with Golgi apparatus |
| Efet.01.326574.g820.t1 | farnesyltransferase geranylgeranyltransferase type-1 subunit alpha | 624 | 2.76E-97 | 76.15 | F:protein prenyltransferase activity; P:protein prenylation |
| Efet.01.32672.g536.t1 | Nucleolar RNA helicase 2 | 384 | 1.34E-22 | 61.75 | F:organic cyclic compound binding; F:heterocyclic compound binding; F:hydrolase activity |
| Efet.01.326730.g824.t1 | retinal degeneration B | 546 | 1.23E-21 | 74.5 | F:metal ion binding; P:transport; C:intracellular |
| Efet.01.32675.g537.t1 | kelch 10 | 210 | 3.13E-28 | 83 |  |
| Efet.01.326824.g826.t1 | spire homolog 1-like isoform X1 | 294 | 3.60E-20 | 69.67 | P:vesicle-mediated transport; F:actin binding; P:actin nucleation |
| Efet.01.326891.g828.t1 | hypothetical protein CAPTEDRAFT_194097 | 1071 | 1.84E-80 | 57.17 | C:membrane; F:cysteine-type peptidase activity; P:single-organism process; P:cellular process |
| Efet.01.326961.g829.t1 | domain-containing [Micromonospora yangpuensis] | 1149 | 4.36E-24 | 63.3 |  |
| Efet.01.327.g38.t1 | vacuolar sorting-associated 13B | 282 | 2.50E-19 | 69 | P:biological_process; P:protein transport; C:cellular_component; P:transport; F:molecular_function |
| Efet.01.327.g39.t1 | vacuolar sorting-associated 13B-like | 1284 | 8.85E-52 | 49.7 |  |
| Efet.01.327016.g832.t1 | low-density lipo receptor-related 1B-like | 444 | 1.77E-26 | 54.6 | F:calcium ion binding; C:membrane; C:integral component of membrane |
| Efet.01.327027.g833.t1 | mesoderm induction early response 1-like | 492 | 6.19E-07 | 72 |  |
| Efet.01.327033.g834.t1 | ATP-dependent RNA helicase DDX3X-like isoform X2 | 234 | 2.04E-28 | 93 | F:nucleic acid binding; C:cytoplasmic ribonucleoprotein granule; F:ATP binding; P:regulation of gene expression; P:RNA secondary structure unwinding; P:translational initiation; F:ATP-dependent RNA helicase activity |
| Efet.01.327140.g836.t1 | protocadherin-11 X-linked-like | 906 | 4.58E-76 | 59.85 | C:membrane |
| Efet.01.327351.g838.t1 | hypothetical protein CAPTEDRAFT_225615 | 933 | 6.53E-21 | 46.8 |  |
| Efet.01.327375.g839.t1 | phosphoethanolamine N-methyltransferase 3-like isoform X2 | 306 | 1.06E-25 | 68.35 | P:metabolic process; F:methyltransferase activity; F:transferase activity; P:methylation |
| Efet.01.3274.g279.t1 | U4 U6 small nuclear ribonucleo Prp31 | 444 | 4.27E-65 | 85.35 | C:nuclear speck; C:U4atac snRNP; C:Cajal body; C:U2-type spliceosomal complex; C:U4 snRNP; F:RNA binding; P:spliceosomal tri-snRNP complex assembly; P:snoRNA localization; F:protein binding; C:precatalytic spliceosome; C:U4/U6 x U5 tri-snRNP complex; F:snRNP binding; P:ribonucleoprotein complex localization; C:viral nucleocapsid; F:U4atac snRNA binding; C:MLL1 complex; F:U4 snRNA binding |
| Efet.01.327429.g842.t1 | acetolactate synthase | 369 | 5.46E-44 | 94 | F:magnesium ion binding; F:thiamine pyrophosphate binding; F:catalytic activity; C:integral component of membrane; P:embryonic cranial skeleton morphogenesis |
| Efet.01.32743.g538.t1 | dynamin-1 | 417 | 5.39E-55 | 86.55 | F:GTP binding; P:peroxisome fission; F:GTPase activity; P:mitochondrial fission |
| Efet.01.327540.g845.t1 | RAB6A-GEF complex partner 1-like | 516 | 1.03E-23 | 56.4 |  |
| Efet.01.327588.g848.t1 | actin depolymerizing | 324 | 1.39E-35 | 59.71 | F:actin binding; C:intracellular |
| Efet.01.327682.g850.t1 | hypothetical protein CAPTEDRAFT_220779 | 489 | 3.56E-37 | 59.05 | F:calcium ion binding |
| Efet.01.327761.g853.t1 | histone acetyltransferase KAT6B isoform X1 | 636 | 8.68E-11 | 50.8 |  |
| Efet.01.327848.g854.t1 | vacuolar sorting-associated 13B-like | 330 | 2.27E-09 | 65.38 |  |
| Efet.01.327891.g858.t1 | probable cation-transporting ATPase 13A3 | 207 | 2.09E-33 | 89.4 |  |
| Efet.01.32790.g540.t1 | serine threonine- kinase 10-like isoform X1 | 1218 | 2.25E-09 | 63.6 | F:nucleotide binding; F:ATP binding; F:protein kinase activity; P:phosphorylation; F:protein serine/threonine kinase activity; P:protein phosphorylation; F:kinase activity |
| Efet.01.327929.g859.t1 | Cytosolic purine 5 - | 450 | 2.23E-17 | 71.35 | F:5'-nucleotidase activity; F:metal ion binding; P:metabolic process; P:cellular process |
| Efet.01.327991.g862.t1 | integrator complex subunit 7 | 486 | 1.27E-32 | 61.1 |  |
| Efet.01.328055.g863.t1 | Tropomyosin- isoforms 9A A B | 282 | 2.10E-18 | 77.45 |  |
| Efet.01.32808.g541.t1 | Contactin-associated 1 | 630 | 1.37E-78 | 67.1 | C:integral component of membrane |
| Efet.01.328117.g865.t1 | disintegrin and metallo ase domain-containing 12-like isoform X2 | 279 | 2.46E-24 | 67.9 | C:membrane; F:metallopeptidase activity |
| Efet.01.328121.g866.t1 | ubiquitin-associated domain-containing 1-like | 360 | 1.92E-19 | 67 |  |
| Efet.01.32822.g543.t1 | clusterin-associated 1 isoform X2 | 501 | 4.32E-11 | 83.6 | P:floor plate formation; P:smoothened signaling pathway; P:axoneme assembly; P:left/right pattern formation; C:axoneme; C:nucleoplasm; C:centrosome; C:ciliary tip; P:neural tube closure; P:intraciliary transport; C:ciliary base; P:heart looping; C:intraciliary transport particle B |
| Efet.01.328321.g870.t1 | suppression of tumorigenicity 5 isoform X1 | 216 | 4.97E-11 | 59.25 | C:membrane; C:integral component of membrane |
| Efet.01.328402.g872.t1 | hypothetical protein HELRODRAFT_160748 | 567 | 2.11E-16 | 51.8 | F:calcium ion binding; C:membrane; C:integral component of membrane |
| Efet.01.32847.g544.t1 | hypothetical protein HELRODRAFT_169090 | 1116 | 3.90E-48 | 48.67 | F:chitin binding; C:membrane; P:chitin metabolic process; C:integral component of membrane; C:extracellular region |
| Efet.01.328523.g875.t1 | plasma membrane calcium-transporting ATPase 2 isoform X1 | 327 | 1.12E-21 | 80.15 | F:ATP binding; F:metal ion binding; C:integral component of membrane; P:calcium ion transmembrane transport; F:calcium-transporting ATPase activity |
| Efet.01.328568.g877.t1 | Aminopeptidase Y ( Leu preference) | 285 | 8.24E-61 | 72.8 | F:hydrolase activity |
| Efet.01.328571.g878.t1 | spectrin beta chain-like | 444 | 8.04E-54 | 78.3 | F:actin binding; C:integral component of membrane; C:Golgi apparatus; F:structural constituent of cytoskeleton; F:phospholipid binding; C:spectrin; P:transport |
| Efet.01.32862.g546.t1 | triple functional domain -like isoform X1 [Orbicella faveolata] | 249 | 1.58E-23 | 65.15 | F:ATP binding; P:regulation of Rho protein signal transduction; C:integral component of membrane; F:protein kinase activity; F:Rho guanyl-nucleotide exchange factor activity; P:protein phosphorylation; P:positive regulation of GTPase activity |
| Efet.01.328623.g879.t1 | NAD(P)H-hydrate epimerase-like isoform X1 | 243 | 1.17E-21 | 80.25 | F:nucleotide binding; F:metal ion binding; C:mitochondrion; F:NADHX epimerase activity; F:NADPHX epimerase activity |
| Efet.01.328642.g880.t1 | ER degradation-enhancing alpha-mannosidase 3 isoform X1 | 579 | 1.43E-70 | 61.55 | P:protein metabolic process; P:response to organic substance; P:cellular macromolecule metabolic process; P:single-organism cellular process; P:single-organism metabolic process; P:response to endoplasmic reticulum stress; F:hydrolase activity |
| Efet.01.328646.g881.t1 | lethal(2) giant larvae homolog 1-like | 567 | 4.05E-63 | 74.95 |  |
| Efet.01.3287.g280.t1 | filamin-interacting FAM101B | 723 | 2.88E-19 | 54 |  |
| Efet.01.328721.g883.t1 | serine threonine- kinase TAO1 | 621 | 9.72E-33 | 58.45 | F:nucleotide binding; F:ATP binding; F:signal transducer, downstream of receptor, with serine/threonine kinase activity; C:cytoplasm; F:protein kinase activity; P:protein phosphorylation; P:signal transduction by protein phosphorylation |
| Efet.01.328763.g884.t1 | neuronal acetylcholine receptor subunit alpha-3-like | 609 | 2.76E-100 | 75.2 | C:postsynaptic membrane; P:cation transmembrane transport; C:integral component of membrane; C:cell junction; F:extracellular ligand-gated ion channel activity; P:chemical synaptic transmission; P:response to stimulus |
| Efet.01.328790.g886.t1 | potassium sodium hyperpolarization-activated cyclic nucleotide-gated channel 2-like | 276 | 2.44E-12 | 79.2 | C:integral component of plasma membrane; F:voltage-gated potassium channel activity; P:regulation of membrane potential; P:potassium ion transmembrane transport |
| Efet.01.328857.g891.t1 | sex peptide receptor-like | 537 | 2.04E-19 | 59.4 | F:G-protein coupled receptor activity; F:G-protein coupled peptide receptor activity; P:neuropeptide signaling pathway; P:G-protein coupled receptor signaling pathway; C:membrane; C:integral component of membrane |
| Efet.01.32896.g549.t1 | potassium channel subfamily K member 18-like isoform X3 | 1242 | 1.39E-41 | 75.4 | C:integral component of plasma membrane; P:potassium ion transmembrane transport; P:stabilization of membrane potential; F:potassium ion leak channel activity |
| Efet.01.328976.g892.t1 | voltage-dependent L-type calcium channel subunit beta-2 | 204 | 6.06E-19 | 93.05 | C:voltage-gated calcium channel complex; P:calcium ion transmembrane transport; F:voltage-gated calcium channel activity |
| Efet.01.32906.g550.t1 | zinc finger CCHC domain-containing 2-like | 537 | 1.33E-35 | 62.35 | F:binding |
| Efet.01.329083.g893.t1 | hypothetical protein CAPTEDRAFT_211035 | 828 | 1.02E-52 | 50.3 | C:membrane; C:integral component of membrane |
| Efet.01.329087.g894.t1 | vascular endothelial growth factor receptor kdr-like | 489 | 4.11E-08 | 46.65 | C:membrane; C:integral component of membrane |
| Efet.01.32914.g551.t1 | pseudouridylate synthase 7 homolog | 612 | 4.82E-39 | 58.2 | F:nucleic acid binding; P:pseudouridine synthesis; F:RNA binding; P:RNA modification; C:membrane; C:integral component of membrane; F:pseudouridine synthase activity |
| Efet.01.329232.g895.t1 | hypothetical protein CAPTEDRAFT_193210 | 381 | 5.49E-14 | 58 | C:membrane; C:integral component of membrane |
| Efet.01.329241.g896.t1 | sialin-like | 438 | 1.24E-23 | 48.3 | C:membrane; P:transport |
| Efet.01.329347.g899.t1 | polyubiquitin-like | 972 | 1.45E-09 | 56.25 | F:structural constituent of ribosome; C:cytoplasm; C:ribosome; P:translation |
| Efet.01.329399.g900.t1 | zinc finger 239-like | 1221 | 6.25E-55 | 51.45 | F:nucleic acid binding; F:metal ion binding |
| Efet.01.329563.g902.t1 | chromodomain-helicase-DNA-binding 8-like isoform X1 | 303 | 5.03E-41 | 75.3 | C:nucleus; F:ATP binding; F:hydrolase activity, acting on acid anhydrides |
| Efet.01.329622.g905.t1 | U-box domain-containing 35-like | 1749 | 3.41E-79 | 47.45 | P:MAPK cascade; F:ATP binding; P:activation of MAPKK activity; F:protein kinase activity; F:MAP kinase kinase kinase activity; P:protein phosphorylation; C:intracellular |
| Efet.01.329633.g906.t1 | CWF19 1 isoform X2 | 357 | 7.67E-15 | 70.95 | F:catalytic activity |
| Efet.01.329669.g907.t1 | neurogenic locus notch | 651 | 3.40E-52 | 55.65 | C:membrane; C:integral component of membrane |
| Efet.01.329708.g908.t1 | neurexin-3 isoform X5 | 699 | 4.18E-29 | 57.2 |  |
| Efet.01.329834.g911.t1 | amyloid -binding 2-like | 243 | 1.71E-12 | 67.65 |  |
| Efet.01.32984.g555.t1 | Vesicular inhibitory amino acid transporter | 354 | 7.81E-10 | 50 | C:membrane; C:integral component of membrane |
| Efet.01.329982.g913.t1 | innexin unc-9-like isoform X2 | 258 | 1.70E-22 | 69 | C:membrane; C:cell junction; P:transport |
| Efet.01.329982.g914.t1 | innexin unc-9 isoform X6 | 957 | 6.57E-126 | 66.25 | C:membrane; C:cell junction; P:transport |
| Efet.01.330238.g918.t1 | vacuolar sorting-associated 13D isoform X1 | 1914 | 3.73E-56 | 49.45 | F:hydrolase activity, acting on ester bonds |
| Efet.01.330239.g919.t1 | vacuolar sorting-associated 13D-like | 1086 | 4.96E-14 | 49.83 | F:hydrolase activity, acting on ester bonds |
| Efet.01.330259.g920.t1 | fem-1 homolog C-like | 1980 | 2.61E-152 | 56.1 |  |
| Efet.01.330277.g921.t1 | palmitoyltransferase akr1 | 279 | 1.60E-23 | 59.7 | F:transferase activity, transferring acyl groups; F:zinc ion binding; F:protein-cysteine S-palmitoyltransferase activity; F:metal ion binding; P:transcription, DNA-templated; C:membrane; P:apoptotic process; C:integral component of membrane; F:transcription factor binding; F:transferase activity |
| Efet.01.330278.g922.t1 | zinc finger 665-like | 1020 | 1.47E-61 | 67.6 | F:binding |
| Efet.01.330287.g923.t1 | Transmembrane 246 | 1209 | 1.15E-112 | 54.4 | C:membrane; C:integral component of membrane |
| Efet.01.330374.g924.t1 | Sentrin-specific protease 2 | 1563 | 1.10E-83 | 52.1 | F:zinc ion binding; F:metal ion binding |
| Efet.01.330512.g927.t1 | tubulin gamma-1 chain | 450 | 1.13E-28 | 89.45 | F:GTP binding; F:GTPase activity; P:microtubule nucleation; C:gamma-tubulin complex; P:cytoplasmic microtubule organization; C:microtubule |
| Efet.01.330515.g928.t1 | neuralized 4 | 465 | 1.50E-56 | 75.05 |  |
| Efet.01.330517.g929.t1 | hypothetical protein LOTGIDRAFT_123956 | 222 | 1.42E-07 | 61 | F:catalytic activity |
| Efet.01.33053.g557.t1 | neuronal acetylcholine receptor subunit alpha-10-like | 357 | 2.16E-33 | 70.85 | P:ion transmembrane transport; C:integral component of membrane; F:extracellular ligand-gated ion channel activity |
| Efet.01.330552.g931.t1 | ankyrin repeat domain-containing 11-like | 666 | 1.00E-12 | 49.6 |  |
| Efet.01.330561.g934.t1 | beta-1,4-N-acetylgalactosaminyltransferase bre-4-like | 237 | 6.74E-08 | 72.8 | P:carbohydrate metabolic process; F:transferase activity, transferring glycosyl groups |
| Efet.01.330589.g935.t1 | hypothetical protein CAPTEDRAFT_175577 | 432 | 9.25E-09 | 67 | C:cytoskeleton |
| Efet.01.330621.g936.t1 | NADPH--cytochrome P450 reductase-like | 762 | 1.02E-138 | 80.85 | C:endoplasmic reticulum membrane; F:FMN binding; F:NADPH-hemoprotein reductase activity; C:integral component of membrane; P:oxidation-reduction process |
| Efet.01.330623.g937.t1 | elongator complex 1 | 378 | 1.77E-48 | 80.35 | F:G-protein coupled receptor activity; P:G-protein coupled receptor signaling pathway; C:integral component of membrane; C:Elongator holoenzyme complex; P:regulation of transcription, DNA-templated; P:tRNA wobble uridine modification |
| Efet.01.33073.g558.t1 | mothers against decapentaplegic homolog 5 | 513 | 1.15E-59 | 67.75 | C:nucleus; F:DNA binding; P:cellular macromolecule metabolic process; P:cellular response to growth factor stimulus; P:cellular response to endogenous stimulus; P:regulation of transcription, DNA-templated; P:animal organ development; C:protein complex; P:transmembrane receptor protein serine/threonine kinase signaling pathway; P:primary metabolic process |
| Efet.01.330778.g938.t1 | 1-phosphatidylinositol 4,5-bisphosphate phosphodiesterase classes I and II | 447 | 1.36E-38 | 68.4 | P:single-organism process; F:hydrolase activity |
| Efet.01.330894.g939.t1 | WD repeat-containing 78 | 297 | 4.01E-24 | 84.6 |  |
| Efet.01.3309.g282.t1 | nucleosome-remodeling factor subunit NURF301-like isoform X1 | 675 | 9.70E-13 | 61.63 | F:zinc ion binding; F:metal ion binding |
| Efet.01.33094.g559.t1 | sphingosine kinase 2 isoform X2 | 507 | 5.18E-14 | 47.15 | P:phosphorylation; F:kinase activity |
| Efet.01.330998.g940.t1 | kelch 8 isoform X1 | 771 | 1.37E-73 | 72.6 | P:protein ubiquitination |
| Efet.01.330998.g941.t1 | kelch 8 isoform X1 | 483 | 1.66E-30 | 63.45 | C:Cul3-RING ubiquitin ligase complex; P:protein ubiquitination |
| Efet.01.331001.g942.t1 | speriolin isoform X2 | 360 | 5.54E-11 | 53.55 | P:biological_process; C:centrosome; F:protein binding; C:cellular_component; F:molecular_function |
| Efet.01.331133.g944.t1 | probable G- coupled receptor No18 | 906 | 6.57E-109 | 63.1 | F:G-protein coupled receptor activity; P:G-protein coupled receptor signaling pathway; C:integral component of membrane |
| Efet.01.331228.g945.t1 | Potassium channel subfamily K member 9 | 351 | 3.85E-38 | 74.55 | C:integral component of plasma membrane; P:potassium ion transmembrane transport; P:stabilization of membrane potential; F:potassium ion leak channel activity |
| Efet.01.331282.g946.t1 | coiled-coil domain-containing 96-like | 618 | 5.03E-59 | 73.95 |  |
| Efet.01.331361.g947.t1 | C-ets-2 isoform X2 | 423 | 1.92E-63 | 88.1 | P:transcription, DNA-templated; F:RNA polymerase II core promoter proximal region sequence-specific DNA binding; P:mesoderm development; P:positive regulation of transcription from RNA polymerase II promoter; C:plasma membrane; C:nucleoplasm; P:negative regulation of transcription from RNA polymerase II promoter; F:transcriptional repressor activity, RNA polymerase II core promoter proximal region sequence-specific binding; P:skeletal system development; C:cytoplasm; P:ectodermal cell fate commitment; F:glucocorticoid receptor binding; P:primitive streak formation |
| Efet.01.331421.g949.t1 | exocyst complex component 2-like isoform X1 | 243 | 1.86E-12 | 68.05 | P:Golgi to plasma membrane transport; C:exocyst |
| Efet.01.33146.g561.t1 | heat shock cognate 71 kDa | 1947 | 0 | 95.85 | C:myelin sheath; F:C3HC4-type RING finger domain binding; C:late endosome; C:Prp19 complex; P:late endosomal microautophagy; F:G-protein coupled receptor binding; F:ATP binding; F:phosphatidylserine binding; P:response to heat; C:extracellular exosome; P:negative regulation of supramolecular fiber organization; C:focal adhesion; C:intracellular ribonucleoprotein complex; P:blood vessel development; P:negative regulation of transcription, DNA-templated; P:positive regulation of mRNA splicing, via spliceosome; C:blood microparticle; C:cytosol; P:regulation of cell cycle; F:heat shock protein binding; P:chaperone mediated protein folding requiring cofactor; F:unfolded protein binding; P:response to cadmium ion; F:MHC class II protein complex binding; P:protein refolding; F:ATPase activity, coupled; F:RNA binding; C:nucleus; F:ubiquitin protein ligase binding; P:regulation of protein stability; C:ubiquitin ligase complex; C:membrane; P:ATP metabolic process; P:clathrin coat disassembly |
| Efet.01.331480.g951.t1 | centromere-associated E | 393 | 2.69E-06 | 89.5 |  |
| Efet.01.331580.g952.t1 | hypothetical protein CRN43_14440 | 324 | 2.76E-17 | 57 |  |
| Efet.01.331747.g957.t1 | hypothetical protein CAPTEDRAFT_223974 | 498 | 5.64E-07 | 63 |  |
| Efet.01.331755.g958.t1 | thyrotropin-releasing hormone receptor-like | 363 | 5.81E-29 | 79.05 | F:thyrotropin-releasing hormone receptor activity; P:G-protein coupled receptor signaling pathway; C:integral component of membrane |
| Efet.01.332.g40.t1 | ankyrin repeat | 2538 | 5.43E-13 | 43.85 | F:catalytic activity; P:nucleoside metabolic process |
| Efet.01.332031.g961.t1 | suppressor of cytokine signaling 2-like | 549 | 3.48E-27 | 57.15 | P:negative regulation of cellular process |
| Efet.01.332053.g963.t1 | kelch 24 | 750 | 1.15E-13 | 46.4 |  |
| Efet.01.332072.g964.t1 | Zinc finger MYM-type 3 | 759 | 1.36E-48 | 73.85 | F:zinc ion binding |
| Efet.01.332080.g965.t1 | septin-7-like isoform X2 | 393 | 6.00E-69 | 93.3 | F:GTP binding |
| Efet.01.33232.g562.t1 | Voltage-dependent anion-selective channel | 282 | 9.58E-24 | 74.3 | F:voltage-gated anion channel activity; P:regulation of anion transmembrane transport; P:transmembrane transport; C:integral component of membrane; C:mitochondrial outer membrane; P:anion transport |
| Efet.01.332327.g968.t1 | C-type lectin mannose-binding isoform isoform X3 | 387 | 6.65E-36 | 47.17 | C:membrane; P:proteolysis; C:integral component of membrane; F:serine-type peptidase activity; F:carbohydrate binding |
| Efet.01.332444.g969.t1 | glycine receptor subunit alpha-2-like isoform X3 | 321 | 8.85E-06 | 56 |  |
| Efet.01.332529.g973.t1 | pyrokinin-1 receptor-like isoform X2 | 1089 | 1.06E-86 | 56.1 | F:G-protein coupled receptor activity; F:G-protein coupled peptide receptor activity; P:G-protein coupled receptor signaling pathway; C:membrane; C:integral component of membrane |
| Efet.01.332549.g975.t1 | SZT2-like isoform X1 | 1029 | 1.30E-14 | 49.67 | C:peroxisome |
| Efet.01.33257.g563.t1 | beta-1,3-galactosyltransferase 1-like | 702 | 3.43E-55 | 58.9 | F:transferase activity, transferring glycosyl groups; C:membrane; C:integral component of membrane; P:protein glycosylation; F:galactosyltransferase activity; C:Golgi apparatus; C:Golgi membrane; F:transferase activity |
| Efet.01.332591.g976.t1 | centrosomal of 135 kDa-like isoform X1 | 576 | 2.22E-38 | 66.75 | P:centriole replication; P:centriole-centriole cohesion |
| Efet.01.33261.g564.t1 | Neuralized 1A | 618 | 6.48E-16 | 58.89 | F:zinc ion binding; F:metal ion binding |
| Efet.01.33261.g565.t1 | Neuralized 1A | 648 | 1.94E-42 | 55 | F:zinc ion binding; F:metal ion binding |
| Efet.01.332623.g977.t1 | hypothetical protein HELRODRAFT_165611 | 585 | 8.52E-48 | 56.8 | P:axonogenesis; C:membrane; C:integral component of membrane; C:plasma membrane; C:anchored component of plasma membrane |
| Efet.01.332661.g979.t1 | hypothetical protein CAPTEDRAFT_221622 | 342 | 1.75E-22 | 55.67 | C:membrane; C:integral component of membrane |
| Efet.01.332717.g981.t1 | carboxypeptidase B-like | 336 | 8.09E-18 | 57.2 | F:binding |
| Efet.01.332748.g983.t1 | solute carrier family 25 member 45 isoform X3 | 297 | 3.28E-09 | 55.45 | P:transmembrane transport; F:structural constituent of ribosome; C:mitochondrion; C:membrane; C:integral component of membrane; P:transport; P:translation |
| Efet.01.332778.g984.t1 | PREDICTED: uncharacterized protein LOC106961309 isoform X1 | 762 | 1.03E-08 | 39.14 |  |
| Efet.01.332814.g985.t1 | kinesin-II 95 kDa subunit-like | 270 | 1.51E-19 | 95.6 | F:ATP binding; F:microtubule motor activity; P:microtubule-based movement; C:microtubule; F:microtubule binding |
| Efet.01.332901.g987.t1 | 5-hydroxytryptamine receptor-like | 1365 | 5.05E-147 | 59.15 | P:signal transduction; C:membrane; F:signal transducer activity |
| Efet.01.33291.g569.t1 | tyrosine- phosphatase 13- | 936 | 2.19E-167 | 88.8 | F:protein tyrosine phosphatase activity; P:neuron differentiation; F:cell adhesion molecule binding; P:presynaptic membrane assembly; C:integral component of membrane; P:heterophilic cell-cell adhesion via plasma membrane cell adhesion molecules; F:receptor binding; P:peptidyl-tyrosine dephosphorylation; P:negative regulation of JAK-STAT cascade; P:positive regulation of dendrite morphogenesis; P:regulation of immune response |
| Efet.01.332914.g988.t1 | polycystic kidney disease 1-like | 345 | 5.30E-12 | 55.5 |  |
| Efet.01.332935.g989.t1 | PREDICTED: uncharacterized protein LOC106158513 | 447 | 4.23E-14 | 57 |  |
| Efet.01.33299.g570.t1 | dopamine D2-like receptor | 978 | 6.84E-26 | 71.55 | C:integral component of plasma membrane; F:dopamine neurotransmitter receptor activity; P:dopamine receptor signaling pathway |
| Efet.01.33301.g571.t1 | vang 2 | 1689 | 5.81E-172 | 65.75 | C:membrane |
| Efet.01.33302.g572.t1 | peptide-methionine (S)-S-oxide reductase | 312 | 4.60E-43 | 83.1 | P:protein repair; P:response to oxidative stress; P:cellular protein modification process; F:peptide-methionine (S)-S-oxide reductase activity; P:oxidation-reduction process |
| Efet.01.33314.g573.t1 | annexin B11-like isoform X2 | 552 | 5.20E-52 | 64.05 | F:calcium ion binding; F:calcium-dependent phospholipid binding |
| Efet.01.333148.g993.t1 | probable G- coupled receptor 19 | 1020 | 3.98E-62 | 62.35 | F:G-protein coupled receptor activity; P:G-protein coupled receptor signaling pathway; P:signal transduction; C:membrane; C:integral component of membrane; F:signal transducer activity |
| Efet.01.333335.g1000.t1 | limbic system-associated membrane -like isoform X4 | 465 | 2.41E-31 | 74.1 | C:membrane; P:cellular process |
| Efet.01.333397.g1001.t1 | class B secretin-like G- coupled receptor | 1518 | 2.73E-32 | 40.3 | F:G-protein coupled receptor activity; F:transmembrane signaling receptor activity; P:immune response; P:G-protein coupled receptor signaling pathway; C:membrane; C:integral component of membrane; F:scavenger receptor activity; P:cell surface receptor signaling pathway; P:receptor-mediated endocytosis; F:polysaccharide binding |
| Efet.01.333397.g1002.t1 | G- coupled receptor Mth2 isoform X4 | 1005 | 2.45E-29 | 48.05 | F:G-protein coupled receptor activity; F:transmembrane signaling receptor activity; P:immune response; P:G-protein coupled receptor signaling pathway; C:membrane; C:integral component of membrane; F:scavenger receptor activity; P:cell surface receptor signaling pathway; P:receptor-mediated endocytosis; F:polysaccharide binding |
| Efet.01.333555.g1004.t1 | ADAM 17-like protease | 297 | 5.55E-14 | 58.9 | F:zinc ion binding; P:proteolysis; C:membrane; C:integral component of membrane; F:peptidase activity; F:metalloendopeptidase activity; F:metallopeptidase activity |
| Efet.01.333597.g1005.t1 | THAP domain-containing 1-like | 900 | 1.69E-08 | 59.9 | F:nucleic acid binding; F:DNA binding; F:DNA topoisomerase type I activity; C:chromosome; P:DNA topological change |
| Efet.01.333614.g1006.t1 | serine threonine- kinase LATS1-like | 234 | 1.80E-41 | 92.5 | F:ATP binding; F:structural constituent of ribosome; P:G1/S transition of mitotic cell cycle; P:positive regulation of apoptotic process; P:mitotic nuclear division; P:hippo signaling; P:regulation of organ growth; C:ribosome; F:protein serine/threonine kinase activity; P:peptidyl-serine phosphorylation; P:convergent extension involved in gastrulation; P:translation |
| Efet.01.333751.g1007.t1 | netrin-1-like isoform X4 | 990 | 4.48E-112 | 67.1 | C:extracellular region |
| Efet.01.333784.g1008.t1 | dynein heavy chain axonemal-like isoform X1 | 645 | 6.27E-62 | 73.4 | F:microtubule motor activity; P:microtubule-based movement |
| Efet.01.333909.g1012.t1 | peptidylprolyl isomerase domain and WD repeat-containing 1 | 249 | 9.91E-33 | 82.05 | F:peptidyl-prolyl cis-trans isomerase activity; C:catalytic step 2 spliceosome; P:protein peptidyl-prolyl isomerization; P:protein folding |
| Efet.01.333967.g1014.t1 | hemolin-like | 309 | 2.19E-06 | 57 |  |
| Efet.01.333984.g1015.t1 | plexin-A2-like isoform X1 | 1080 | 1.51E-39 | 49 | P:regulation of cellular process; P:single-organism process; P:cellular process; P:response to stimulus |
| Efet.01.334013.g1016.t1 | inactive rhomboid 1-like | 441 | 7.01E-07 | 65 |  |
| Efet.01.334050.g1017.t1 | hydrocephalus-inducing -like | 507 | 6.40E-29 | 57.7 | P:cilium movement |
| Efet.01.334119.g1019.t1 | ankyrin repeat | 1158 | 1.07E-24 | 43 | F:catalytic activity; P:nucleoside metabolic process |
| Efet.01.334180.g1022.t1 | actin beta gamma 1 | 552 | 1.78E-99 | 85 | F:ATP binding |
| Efet.01.334215.g1023.t1 | hypothetical protein CRN43_19005, partial | 303 | 2.95E-12 | 91 |  |
| Efet.01.334278.g1024.t1 | junction-mediating and -regulatory | 705 | 8.90E-79 | 61.9 | F:actin binding; F:transcription coactivator activity; P:Arp2/3 complex-mediated actin nucleation; P:'de novo' actin filament nucleation; P:regulation of transcription from RNA polymerase II promoter |
| Efet.01.334417.g1026.t1 | NADH dehydrogenase [ubiquinone] iron-sulfur mitochondrial | 261 | 1.32E-23 | 96.4 | C:membrane; F:NADH dehydrogenase (ubiquinone) activity; P:oxidation-reduction process; F:4 iron, 4 sulfur cluster binding |
| Efet.01.33442.g578.t1 | solute carrier family 22 member 8 | 249 | 1.09E-06 | 65.5 | P:transmembrane transport; F:transmembrane transporter activity; C:membrane; C:integral component of membrane |
| Efet.01.334432.g1027.t1 | coiled-coil domain-containing 149-like | 258 | 6.54E-19 | 68.2 |  |
| Efet.01.334438.g1028.t1 | chromosome-associated kinesin KIF4A-like | 342 | 1.77E-30 | 85.45 | C:kinesin complex; C:chromosome; F:ATP-dependent microtubule motor activity, plus-end-directed; P:microtubule-based movement; C:nucleoplasm; C:microtubule; F:microtubule binding; F:DNA binding; F:ATP binding; P:mitotic spindle midzone assembly; C:cytoplasm; P:mitotic cytokinesis; P:lipid metabolic process; C:integral component of membrane; C:midbody; F:phosphoric diester hydrolase activity |
| Efet.01.334473.g1029.t1 | ankyrin repeat and KH domain-containing mask-like | 1371 | 1.36E-118 | 60.3 | P:intracellular signal transduction; C:intracellular |
| Efet.01.334496.g1030.t1 | delta 2 | 666 | 1.04E-49 | 46.44 | F:calcium ion binding; C:membrane; C:integral component of membrane |
| Efet.01.334564.g1034.t1 | Deleted in malignant brain tumors 1 | 231 | 1.34E-22 | 70.5 | C:membrane; F:scavenger receptor activity; P:receptor-mediated endocytosis |
| Efet.01.334588.g1035.t1 | adenosine monophosphate- transferase FICD homolog | 1011 | 1.07E-172 | 75.65 | C:membrane |
| Efet.01.334603.g1036.t1 | acetylcholine receptor subunit alpha-like isoform X2 | 552 | 3.41E-110 | 84.9 | C:postsynaptic membrane; P:cation transmembrane transport; C:integral component of membrane; C:cell junction; F:extracellular ligand-gated ion channel activity |
| Efet.01.334617.g1037.t1 | PREDICTED: uncharacterized protein LOC107358098 | 546 | 7.55E-34 | 71.6 | F:metal ion binding |
| Efet.01.334636.g1038.t1 | tyrosine- kinase PR2 isoform X1 | 222 | 1.72E-16 | 71.4 | F:protein tyrosine kinase activity; P:salivary gland cell autophagic cell death; P:protein phosphorylation |
| Efet.01.33471.g579.t1 | anoctamin-5 isoform X3 | 207 | 9.29E-27 | 75.55 | C:integral component of membrane; C:cell part; F:protein dimerization activity |
| Efet.01.33473.g580.t1 | 6-phosphofructokinase type | 345 | 1.25E-55 | 87.85 | F:ATP binding; C:cytoplasm; F:metal ion binding; P:fructose 6-phosphate metabolic process; P:glycolytic process through fructose-6-phosphate; F:6-phosphofructokinase activity |
| Efet.01.33477.g581.t1 | iron zinc purple acid phosphatase | 222 | 5.07E-14 | 66.3 | F:hydrolase activity |
| Efet.01.334791.g1041.t1 | atrial natriuretic peptide receptor 1-like isoform X2 | 906 | 4.81E-50 | 61.6 | F:lyase activity; P:primary metabolic process; P:single-organism process; P:organic substance metabolic process; P:phosphate-containing compound metabolic process |
| Efet.01.334792.g1042.t1 | EGF-like domain-containing | 639 | 1.57E-98 | 55.12 | C:integral component of membrane |
| Efet.01.334837.g1043.t1 | hypothetical protein CAPTEDRAFT_222036 | 366 | 1.01E-32 | 63.06 | C:membrane; C:integral component of membrane |
| Efet.01.334852.g1044.t1 | yippee-like 5 | 372 | 5.13E-72 | 91 | F:metal ion binding; P:oxidation-reduction process; F:peptide-methionine (R)-S-oxide reductase activity |
| Efet.01.334916.g1046.t1 | cyclin-dependent kinase 5 activator 2 | 432 | 5.68E-15 | 73.15 | P:positive regulation of protein serine/threonine kinase activity; C:cyclin-dependent protein kinase 5 holoenzyme complex; F:cyclin-dependent protein kinase 5 activator activity |
| Efet.01.33492.g582.t1 | Arrestin domain-containing 3 | 492 | 2.29E-34 | 66 |  |
| Efet.01.334920.g1047.t1 | peregrin | 522 | 2.91E-72 | 74.15 | F:metal ion binding |
| Efet.01.334973.g1049.t1 | hypothetical protein CAPTEDRAFT_222036 | 657 | 1.90E-43 | 55.12 | C:membrane; C:integral component of membrane |
| Efet.01.33505.g583.t1 | meiosis-specific coiled-coil domain-containing MEIOC-like | 444 | 5.62E-33 | 71.2 | C:membrane; C:integral component of membrane |
| Efet.01.33507.g584.t1 | gamma-aminobutyric acid receptor alpha-like | 270 | 4.56E-33 | 75.6 | P:signal transduction; P:ion transmembrane transport; C:integral component of membrane; F:GABA-A receptor activity; C:cell junction; F:extracellular ligand-gated ion channel activity; C:synapse; C:plasma membrane |
| Efet.01.335070.g1051.t1 | E3 ubiquitin- ligase TRIM71-like | 1332 | 1.68E-14 | 52.75 | F:ubiquitin-protein transferase activity; F:ubiquitin protein ligase activity; F:zinc ion binding; C:cytoplasm; F:metal ion binding; C:intracellular; P:protein ubiquitination |
| Efet.01.335091.g1053.t1 | citron Rho-interacting kinase-like | 429 | 1.54E-20 | 59.15 |  |
| Efet.01.33512.g586.t1 | neurexin-1-like isoform X1 | 669 | 4.64E-35 | 52.75 |  |
| Efet.01.335240.g1054.t1 | hypothetical protein CAPTEDRAFT_219353 | 672 | 1.16E-39 | 61.5 |  |
| Efet.01.335353.g1057.t1 | rho GTPase-activating 100F-like isoform X1 | 600 | 1.82E-40 | 61.9 | P:signal transduction |
| Efet.01.335373.g1058.t1 | polybromo-1 isoform X1 | 651 | 7.93E-48 | 61.75 | F:nucleic acid binding; F:chromatin binding |
| Efet.01.335408.g1059.t1 | FMRFamide receptor-like | 1182 | 1.35E-101 | 57.55 | F:G-protein coupled receptor activity; P:signal transduction; C:membrane |
| Efet.01.335490.g1062.t1 | cephalotocin receptor 2-like | 792 | 1.50E-48 | 57.85 | F:G-protein coupled receptor activity; P:G-protein coupled receptor signaling pathway; P:signal transduction; C:membrane; C:integral component of membrane; F:signal transducer activity |
| Efet.01.335506.g1063.t1 | bark beetle-like isoform X1 | 336 | 1.69E-33 | 59 | C:membrane; F:scavenger receptor activity; P:receptor-mediated endocytosis |
| Efet.01.335530.g1064.t1 | Ankyrin repeat and SOCS box 8 | 249 | 9.69E-20 | 64.5 | C:cytoplasm; F:2-alkenal reductase [NAD(P)] activity; F:oxidoreductase activity; P:oxidation-reduction process; P:intracellular signal transduction; C:intracellular; P:protein ubiquitination |
| Efet.01.3356.g287.t1 | spectrin beta chain | 210 | 6.45E-18 | 94.85 | P:vesicle transport along actin filament; P:protein localization to bicellular tight junction; P:protein transport; F:RNA polymerase II regulatory region sequence-specific DNA binding; P:negative regulation of cellular component movement; P:positive regulation of NIK/NF-kappaB signaling; P:regulation of nucleic acid-templated transcription; C:neuron projection; F:retinoic acid receptor binding; P:actin crosslink formation; P:retinoic acid receptor signaling pathway; C:extracellular exosome; C:focal adhesion; C:intracellular ribonucleoprotein complex; F:calcium ion binding; C:stress fiber; P:peroxisome proliferator activated receptor signaling pathway; C:cortical cytoskeleton; F:chromatin DNA binding; F:protein homodimerization activity; P:negative regulation of substrate adhesion-dependent cell spreading; P:regulation of apoptotic process; C:protein complex; F:ion channel binding; F:nucleoside binding; F:RNA binding; P:positive regulation of pinocytosis; F:actin filament binding; C:nucleus; C:Z disc; C:extracellular space; C:cell-cell junction; C:perinuclear region of cytoplasm; F:protein N-terminus binding; C:integral component of membrane; F:ligand-dependent nuclear receptor transcription coactivator activity; P:bicellular tight junction assembly; C:pseudopodium; P:actin filament bundle assembly; P:positive regulation of cell migration |
| Efet.01.335699.g1066.t1 | hypothetical protein HELRODRAFT_184091 | 252 | 4.78E-16 | 56.17 | C:membrane; C:integral component of membrane |
| Efet.01.33576.g590.t1 | N-alpha-acetyltransferase 30 | 819 | 3.10E-09 | 68.9 | C:NatC complex; P:N-terminal protein amino acid acetylation; F:peptide alpha-N-acetyltransferase activity |
| Efet.01.335772.g1068.t1 | orexin receptor type 2-like | 513 | 7.61E-32 | 73.4 | C:integral component of plasma membrane; F:G-protein coupled peptide receptor activity; P:G-protein coupled receptor signaling pathway; P:response to peptide; F:peptide binding; P:cellular response to hormone stimulus; F:transferase activity |
| Efet.01.335919.g1070.t1 | family transcriptional regulator | 345 | 6.13E-50 | 83.55 | F:DNA binding; F:transcription factor activity, sequence-specific DNA binding; P:transcription, DNA-templated; P:regulation of transcription, DNA-templated |
| Efet.01.33597.g591.t1 | hypothetical protein CAPTEDRAFT_221576 | 333 | 2.04E-09 | 72 |  |
| Efet.01.336099.g1074.t1 | NEDD4-like E3 ubiquitin- ligase WWP2 | 252 | 8.24E-37 | 90.4 | F:ubiquitin-protein transferase activity; F:ligase activity; P:protein ubiquitination |
| Efet.01.3361.g288.t1 | CAFF_RIFPA ame: Full=Fibril-forming collagen alpha chain | 822 | 3.68E-15 | 58 | C:extracellular region |
| Efet.01.336132.g1075.t1 | sacsin-like | 855 | 4.77E-15 | 45.25 |  |
| Efet.01.336354.g1078.t1 | hypothetical protein CRN43_10635, partial | 324 | 2.17E-06 | 63 |  |
| Efet.01.336401.g1079.t1 | nuclear hormone receptor HR96-like isoform X8 | 762 | 6.39E-95 | 78.85 | F:thyroid hormone receptor activity; C:nucleus; F:zinc ion binding; P:transcription, DNA-templated; F:steroid hormone receptor activity; P:regulation of transcription, DNA-templated; F:sequence-specific DNA binding; P:steroid hormone mediated signaling pathway; P:intracellular receptor signaling pathway |
| Efet.01.336475.g1080.t1 | calponin-2 isoform X3 | 933 | 2.45E-174 | 80.15 | F:protein binding |
| Efet.01.33662.g593.t1 | T-complex 11 1 | 339 | 1.45E-25 | 77.2 | C:integral component of membrane |
| Efet.01.336665.g1083.t1 | vacuolar sorting-associated 35-like | 354 | 2.00E-56 | 92.85 | C:retromer complex; P:protein transport; C:cytosol; F:protein transporter activity; P:retrograde transport, endosome to Golgi |
| Efet.01.336817.g1086.t1 | active breakpoint cluster region-related | 222 | 2.22E-11 | 71.1 | P:system process; P:signal transduction; C:membrane; F:guanyl-nucleotide exchange factor activity; P:negative regulation of response to stimulus; C:cell part; P:animal organ development; P:regulation of vesicle-mediated transport; P:negative regulation of immune system process; P:negative regulation of cell migration |
| Efet.01.336823.g1087.t1 | pyruvate kinase PKM-like isoform X3 | 1731 | 0 | 76.15 | F:magnesium ion binding; F:pyruvate kinase activity; F:potassium ion binding; P:glycolytic process; F:kinase activity |
| Efet.01.336837.g1088.t1 | EH domain-containing 1 | 1008 | 0 | 89.85 | F:GTP binding; F:ATPase activity; P:lipid tube assembly involved in organelle fission; F:protein domain specific binding; F:calcium ion binding; P:embryo development ending in birth or egg hatching; P:positive regulation of necrotic cell death; C:recycling endosome; P:meiotic nuclear division; P:endocytic recycling |
| Efet.01.33687.g595.t1 | cyclic nucleotide-gated cation channel alpha-3-like | 975 | 4.89E-124 | 73.25 | P:potassium ion transport; C:integral component of plasma membrane; F:voltage-gated potassium channel activity; P:regulation of membrane potential; P:ion transmembrane transport |
| Efet.01.336941.g1090.t1 | G -activated inward rectifier potassium channel 3 isoform X5 | 1854 | 1.83E-95 | 58.3 | P:transport |
| Efet.01.336965.g1091.t1 | dimethylaniline monooxygenase [N-oxide-forming] 5-like | 1179 | 4.95E-140 | 62.25 | F:oxidoreductase activity; C:membrane; C:membrane-bounded organelle |
| Efet.01.33698.g596.t1 | OTU domain-containing 7B-like | 1668 | 6.96E-14 | 55 | F:DNA binding; P:protein K11-linked deubiquitination; F:zinc ion binding; F:peptidase activity |
| Efet.01.33715.g598.t1 | Short transient receptor potential channel 3 | 570 | 4.93E-97 | 88.5 | F:calcium channel activity; C:integral component of membrane; P:calcium ion transmembrane transport |
| Efet.01.33715.g599.t1 | short transient receptor potential channel 3- | 492 | 1.38E-18 | 73.55 | F:calcium channel activity; C:integral component of membrane; P:calcium ion transmembrane transport |
| Efet.01.33719.g600.t1 | RING finger 11 | 207 | 1.39E-28 | 86.3 | F:zinc ion binding |
| Efet.01.3372.g290.t1 | proto-oncogene tyrosine- kinase receptor Ret | 405 | 2.85E-31 | 68 | P:phosphorylation; F:kinase activity |
| Efet.01.33724.g602.t1 | sodium hydrogen exchanger 7 isoform X3 | 267 | 9.86E-11 | 94.89 | F:sodium:proton antiporter activity; C:integral component of membrane; P:hydrogen ion transmembrane transport; P:sodium ion transmembrane transport; P:regulation of pH |
| Efet.01.337246.g1097.t1 | histidine utilization | 297 | 1.73E-46 | 74.15 |  |
| Efet.01.33727.g603.t1 | nuclear mitotic apparatus 1-like | 1083 | 1.26E-12 | 49.39 |  |
| Efet.01.337302.g1098.t1 | structural maintenance of chromosomes 1A-like | 411 | 4.36E-48 | 79.2 | C:nucleus; F:ATP binding; P:DNA repair; P:mitotic sister chromatid cohesion; C:chromosome; F:chromatin binding |
| Efet.01.337317.g1099.t1 | netrin-1-like isoform X1 | 1218 | 8.60E-114 | 65.8 | C:membrane; C:integral component of membrane; C:extracellular region |
| Efet.01.337386.g1100.t1 | E3 ubiquitin- ligase SH3RF1-like | 342 | 5.48E-07 | 47 |  |
| Efet.01.337448.g1101.t1 | cytoplasmic dynein 1 heavy chain 1-like | 276 | 8.65E-42 | 71.7 | F:nucleotide binding; F:nucleoside-triphosphatase activity |
| Efet.01.337504.g1102.t1 | WD repeat and FYVE domain-containing 3-like isoform X1 | 306 | 1.81E-33 | 67.85 | F:metal ion binding |
| Efet.01.337578.g1106.t1 | zinc transporter ZIP4 | 330 | 1.43E-32 | 72.6 | C:integral component of plasma membrane; F:zinc ion transmembrane transporter activity; P:cellular zinc ion homeostasis; P:zinc II ion transmembrane import |
| Efet.01.337646.g1107.t1 | signal-induced proliferation-associated 1 2 | 1440 | 4.44E-38 | 49.1 | P:regulation of small GTPase mediated signal transduction; P:regulation of GTPase activity; F:GTPase activator activity; P:actin cytoskeleton reorganization; P:positive regulation of GTPase activity; P:regulation of dendrite morphogenesis |
| Efet.01.33769.g605.t1 | transcription initiation SPT3 homolog | 423 | 2.11E-33 | 58.25 | F:protein heterodimerization activity; P:transcription from RNA polymerase II promoter |
| Efet.01.33771.g606.t1 | EAA2_MOUSE ame: Full=Excitatory amino acid transporter 2 ame: Full=GLT-1 ame: Full=Sodium-dependent glutamate aspartate transporter 2 ame: Full=Solute carrier family 1 member 2 | 366 | 5.08E-08 | 91.71 | C:presynaptic membrane; P:multicellular organism growth; P:L-glutamate transmembrane transport; P:glutamate secretion; P:telencephalon development; P:L-glutamate import; P:response to wounding; P:response to amino acid; P:chemical synaptic transmission; P:positive regulation of glucose import; F:high-affinity glutamate transmembrane transporter activity; P:visual behavior; C:cell surface; C:integral component of plasma membrane; P:cellular response to extracellular stimulus; F:protein binding; C:dendritic shaft; P:response to drug; C:dendritic spine; P:multicellular organism aging; C:axolemma; P:D-aspartate import; P:adult behavior; F:glutamate:sodium symporter activity |
| Efet.01.337798.g1110.t1 | hypothetical protein CAPTEDRAFT_204392 | 4047 | 3.16E-11 | 49 |  |
| Efet.01.3378.g291.t1 | mediator of RNA polymerase II transcription subunit 23-like | 627 | 1.42E-79 | 70.45 | C:transcription factor complex; F:protein binding; F:transcription coactivator activity; P:transcription initiation from RNA polymerase II promoter; C:nucleoplasm; P:regulation of transcription from RNA polymerase II promoter |
| Efet.01.33810.g608.t1 | elongation of very long chain fatty acids 6-like | 879 | 2.90E-114 | 72 | C:membrane; P:fatty acid metabolic process |
| Efet.01.338133.g1114.t1 | GDP-fucose transporter 1-like | 375 | 1.17E-49 | 75.25 | C:integral component of membrane |
| Efet.01.338161.g1115.t1 | orexin receptor type 2-like | 1338 | 4.11E-68 | 54.05 | F:G-protein coupled receptor activity; P:G-protein coupled receptor signaling pathway; P:signal transduction; C:membrane; C:integral component of membrane; F:signal transducer activity |
| Efet.01.338163.g1116.t1 | dynein heavy chain axonemal-like | 222 | 7.75E-20 | 81.95 | F:ATPase activity; F:ATP binding; F:microtubule motor activity; C:dynein complex; P:microtubule-based movement |
| Efet.01.338189.g1117.t1 | fukutin-related -like | 939 | 5.46E-31 | 57.05 | C:membrane; C:integral component of membrane; F:transferase activity |
| Efet.01.338200.g1118.t1 | heat shock 90 | 1029 | 1.26E-38 | 75.1 | F:ATPase activity; P:positive regulation of nitric oxide biosynthetic process; P:regulation of cellular response to heat; P:response to antibiotic; C:lysosomal lumen; P:Fc-gamma receptor signaling pathway involved in phagocytosis; P:response to unfolded protein; C:nucleoplasm; F:ATP binding; P:response to cold; F:protein tyrosine kinase activity; P:response to heat; C:extracellular exosome; P:G2/M transition of mitotic cell cycle; F:GTPase binding; C:cytosol; C:cell projection membrane; P:regulation of protein ubiquitination; P:regulation of nitric-oxide synthase activity; P:ERBB2 signaling pathway; P:chaperone-mediated autophagy; F:protein homodimerization activity; F:histone deacetylase binding; P:single-multicellular organism process; P:regulation of protein complex assembly; F:unfolded protein binding; F:MHC class II protein complex binding; P:protein refolding; P:receptor-mediated endocytosis; P:protein unfolding; F:RNA binding; P:vascular endothelial growth factor receptor signaling pathway; F:TPR domain binding; P:protein import into mitochondrial outer membrane; C:endocytic vesicle lumen; C:neuron part; P:chaperone-mediated protein complex assembly; F:pyrimidine ribonucleoside binding; F:nitric-oxide synthase regulator activity; F:pyrimidine ribonucleotide binding; P:protein stabilization |
| Efet.01.338257.g1119.t1 | NACHT domain- and WD repeat-containing 1-like isoform X1 | 2199 | 2.21E-35 | 49.2 | F:nucleotide binding; F:ATP binding |
| Efet.01.338282.g1120.t1 | Smaug homolog 2 isoform X3 | 225 | 1.43E-18 | 68.55 | P:posttranscriptional regulation of gene expression; C:intracellular organelle; P:RNA metabolic process; F:protein binding; C:cytoplasm; F:mRNA binding; P:cerebellar neuron development; P:regulation of cellular macromolecule biosynthetic process; P:regulation of nitrogen compound metabolic process; P:regulation of primary metabolic process |
| Efet.01.338309.g1121.t1 | plexin-A1 isoform X2 | 297 | 1.42E-36 | 81.05 | F:structural constituent of ribosome; C:integral component of membrane; F:semaphorin receptor activity; C:ribosome; P:translation; P:semaphorin-plexin signaling pathway |
| Efet.01.33837.g611.t1 | docking 5 isoform X2 | 303 | 3.48E-21 | 69.25 | P:regulation of neurotrophin TRK receptor signaling pathway; P:single-organism process |
| Efet.01.338426.g1122.t1 | glutamate receptor 2- | 561 | 1.21E-32 | 52.65 | F:ion channel activity; C:membrane; F:receptor activity; P:transport |
| Efet.01.338451.g1123.t1 | tyrosine- kinase src42a | 219 | 3.76E-29 | 84.1 | F:ATP binding; P:peptidyl-tyrosine phosphorylation; F:non-membrane spanning protein tyrosine kinase activity |
| Efet.01.338477.g1124.t1 | Dynein heavy chain axonemal | 384 | 2.64E-25 | 53.75 | F:nucleotide binding; F:ATPase activity; F:ATP binding; F:microtubule motor activity; C:dynein complex; P:microtubule-based movement |
| Efet.01.33854.g612.t1 | EH domain-containing 3 | 669 | 4.37E-70 | 65.85 | F:GTP binding; F:calcium ion binding; P:endocytic recycling; C:intracellular |
| Efet.01.33865.g613.t1 | stAR-related lipid transfer mitochondrial-like isoform X2 | 675 | 1.29E-07 | 59.6 | F:lipid binding |
| Efet.01.338654.g1126.t1 | Rho-related GTP-binding | 534 | 8.15E-56 | 66.45 | P:regulation of cell shape; P:Rac protein signal transduction; F:GTPase activity; P:G1/S transition of mitotic cell cycle; C:cell part; F:binding; P:actin cytoskeleton organization |
| Efet.01.338659.g1127.t1 | Nuclear hormone receptor FTZ-F1 beta | 474 | 2.78E-35 | 67.7 | F:DNA binding; P:regulation of cellular process; F:metal ion binding; P:primary metabolic process; P:organic substance metabolic process; P:cellular process; F:hydrolase activity, acting on glycosyl bonds |
| Efet.01.338669.g1128.t1 | mediator complex subunit | 1236 | 2.22E-49 | 58.45 | P:regulation of transcription, DNA-templated |
| Efet.01.338710.g1129.t1 | outer membrane assembly factor | 1188 | 4.14E-150 | 73.05 | C:outer membrane |
| Efet.01.338710.g1130.t1 | outer membrane assembly factor | 570 | 5.16E-80 | 70.1 | C:outer membrane |
| Efet.01.338710.g1131.t1 | outer membrane chaperone Skp | 294 | 2.21E-61 | 83.55 | F:unfolded protein binding |
| Efet.01.338758.g1133.t1 | spondin-2 isoform X1 | 555 | 1.44E-31 | 47.95 | P:response to other organism; P:defense response; P:immune system process |
| Efet.01.338778.g1134.t1 | hypothetical protein B5766_06525 | 513 | 9.99E-20 | 100 |  |
| Efet.01.338850.g1136.t1 | orphan G- coupled receptor 38 | 408 | 8.62E-09 | 67 | F:G-protein coupled receptor activity; P:G-protein coupled receptor signaling pathway; C:membrane |
| Efet.01.338861.g1137.t1 | bone morphogenetic 2-like | 1095 | 1.77E-72 | 54.2 | P:chordate embryonic development; P:regulation of MAPK cascade; P:negative regulation of cell proliferation; P:hemopoiesis; P:nervous system development; P:branching morphogenesis of an epithelial tube; P:kidney epithelium development; P:regulation of morphogenesis of a branching structure; P:regulation of transmembrane receptor protein serine/threonine kinase signaling pathway; P:appendage development; P:lung morphogenesis; P:embryonic organ morphogenesis; P:heart development; P:blood vessel development; P:negative regulation of cell differentiation; P:mesonephros development; P:BMP signaling pathway; P:positive regulation of multicellular organismal process; P:mesoderm formation; P:regulation of morphogenesis of an epithelium; P:regulation of apoptotic process; P:positive regulation of cell differentiation; P:cell development; P:negative regulation of kidney development; P:positive regulation of signal transduction; F:receptor binding; P:regionalization; P:skeletal system morphogenesis; P:determination of left/right symmetry; P:positive regulation of protein phosphorylation; P:negative regulation of macromolecule metabolic process; P:regulation of organ morphogenesis; P:negative regulation of cellular metabolic process |
| Efet.01.33888.g615.t1 | hypothetical protein CAPTEDRAFT_222265 | 714 | 2.82E-18 | 69 | P:potassium ion transport; C:membrane |
| Efet.01.338972.g1138.t1 | double C2-like domain-containing beta isoform X1 | 318 | 1.38E-48 | 87.3 | C:presynapse; P:calcium ion-regulated exocytosis of neurotransmitter; P:positive regulation of vesicle fusion; F:calcium ion binding; C:membrane; P:positive regulation of insulin secretion; P:intracellular protein transport; F:Rab GTPase binding; P:positive regulation of calcium ion-dependent exocytosis; C:intracellular; F:calcium-dependent phospholipid binding |
| Efet.01.339069.g1139.t1 | segmentation Runt-like | 369 | 1.38E-41 | 79.45 | C:nucleus; F:DNA binding; F:ATP binding; F:transcription factor activity, sequence-specific DNA binding; P:regulation of transcription, DNA-templated |
| Efet.01.339111.g1140.t1 | tyrosine- phosphatase non-receptor type 4-like isoform X4 | 687 | 2.73E-104 | 82.95 | C:cytoskeleton; F:protein tyrosine phosphatase activity; P:peptidyl-tyrosine dephosphorylation |
| Efet.01.33912.g616.t1 | 2-phosphoxylose phosphatase 1 isoform X1 | 1281 | 6.22E-101 | 49.25 | P:dephosphorylation; F:acid phosphatase activity |
| Efet.01.339128.g1141.t1 | BMP family ABC transporter substrate-binding | 531 | 1.44E-97 | 81 | C:plasma membrane |
| Efet.01.339172.g1142.t1 | exostosin-like 3 | 2025 | 0 | 56.3 | P:heparan sulfate proteoglycan biosynthetic process; F:acetylglucosaminyltransferase activity; C:membrane; C:integral component of membrane; P:glycosaminoglycan biosynthetic process; C:endoplasmic reticulum |
| Efet.01.339274.g1147.t1 | sorting nexin-13-like isoform X3 | 339 | 4.04E-30 | 67.15 | F:binding |
| Efet.01.339291.g1148.t1 | pre-mRNA-processing-splicing factor 8 | 276 | 1.32E-34 | 94.8 | F:second spliceosomal transesterification activity; F:U6 snRNA binding; F:U5 snRNA binding; F:pre-mRNA intronic binding; C:catalytic step 2 spliceosome; F:U1 snRNA binding; C:U5 snRNP; F:U2 snRNA binding; P:spliceosomal tri-snRNP complex assembly |
| Efet.01.339291.g1149.t1 | pre-mRNA-processing-splicing factor 8 | 249 | 3.12E-27 | 99.9 | C:U5 snRNP; F:RNA binding; P:spliceosomal tri-snRNP complex assembly; F:second spliceosomal transesterification activity; F:U6 snRNA binding; F:U5 snRNA binding; F:pre-mRNA intronic binding; C:catalytic step 2 spliceosome; P:cellular response to lipopolysaccharide; F:K63-linked polyubiquitin binding; F:U1 snRNA binding; C:membrane; P:cellular response to tumor necrosis factor; F:U2 snRNA binding |
| Efet.01.339351.g1151.t1 | neuronal acetylcholine receptor subunit alpha-7 isoform X1 | 420 | 1.85E-51 | 61.6 | C:postsynaptic membrane; P:cation transmembrane transport; C:integral component of membrane; C:cell junction; F:extracellular ligand-gated ion channel activity |
| Efet.01.339368.g1152.t1 | hypothetical protein CAPTEDRAFT_222036 | 453 | 3.33E-18 | 56.46 | C:membrane; C:integral component of membrane |
| Efet.01.339372.g1153.t1 | hypothetical protein CAPTEDRAFT_220792 | 408 | 8.72E-14 | 53 |  |
| Efet.01.339383.g1156.t1 | O-linked-mannose beta-1,4-N-acetylglucosaminyltransferase 2 | 1839 | 7.24E-170 | 62.05 | F:transferase activity, transferring glycosyl groups; P:protein O-linked glycosylation; P:nervous system development; P:cell differentiation; C:endoplasmic reticulum |
| Efet.01.339464.g1159.t1 | hypothetical protein HELRODRAFT_154876 | 423 | 1.02E-13 | 57.75 | F:transferase activity, transferring glycosyl groups |
| Efet.01.339505.g1160.t1 | vacuolar sorting-associated 8 homolog | 852 | 7.39E-70 | 67.8 | C:early endosome; F:protein binding; P:endosomal vesicle fusion |
| Efet.01.339583.g1161.t1 | cadherin | 615 | 4.71E-79 | 70 | F:ATP binding |
| Efet.01.339664.g1163.t1 | methylthioribose-1-phosphate isomerase | 405 | 8.93E-60 | 79.75 | F:translation initiation factor activity; F:identical protein binding; C:cytoplasm; P:L-methionine salvage from S-adenosylmethionine; P:L-methionine salvage from methylthioadenosine; F:S-methyl-5-thioribose-1-phosphate isomerase activity; C:nucleolus; P:translational initiation |
| Efet.01.33967.g619.t1 | hypothetical protein CAPTEDRAFT_226654 | 390 | 4.56E-10 | 84 | F:calcium ion binding; C:integral component of membrane; P:homophilic cell adhesion via plasma membrane adhesion molecules; C:plasma membrane |
| Efet.01.33967.g620.t1 | neural-cadherin-like isoform X1 | 261 | 5.87E-34 | 62.1 | F:calcium ion binding; C:integral component of membrane; P:homophilic cell adhesion via plasma membrane adhesion molecules; C:plasma membrane |
| Efet.01.339742.g1164.t1 | mitochondrial intermembrane space import and assembly 40 | 264 | 9.24E-23 | 70.1 |  |
| Efet.01.339751.g1165.t1 | dmX 2 isoform X2 | 1779 | 1.49E-151 | 57.6 |  |
| Efet.01.33977.g621.t1 | heterochromatin 1-binding 3-like | 570 | 6.62E-14 | 65.18 | C:chromosome; F:binding; P:chromatin organization |
| Efet.01.339772.g1168.t1 | neuroglian isoform X2 | 261 | 1.30E-24 | 58.8 | P:cytokine-mediated signaling pathway; C:membrane; F:cytokine receptor activity |
| Efet.01.339851.g1169.t1 | hypothetical protein HELRODRAFT_175390 | 480 | 3.51E-07 | 64 |  |
| Efet.01.339857.g1170.t1 | retinoic acid receptor RXR-like isoform X7 | 402 | 9.98E-68 | 88.25 | C:nucleus; F:transcription factor activity, sequence-specific DNA binding; F:zinc ion binding; P:transcription, DNA-templated; F:steroid hormone receptor activity; F:retinal binding; P:regulation of transcription, DNA-templated; F:sequence-specific DNA binding; P:steroid hormone mediated signaling pathway |
| Efet.01.339877.g1171.t1 | frizzled-5-like | 1866 | 0 | 72.45 | C:intracellular organelle; F:Wnt-activated receptor activity; P:forebrain neuron differentiation; P:embryonic axis specification; P:spinal cord radial glial cell differentiation; C:membrane-bounded organelle; P:positive regulation of response to stimulus; F:G-protein coupled receptor activity; P:vasculature development; P:canonical Wnt signaling pathway; F:Wnt-protein binding; P:spinal cord oligodendrocyte cell differentiation; C:cytoplasmic part; P:regulation of signal transduction; P:regulation of cell proliferation; P:eye field cell fate commitment involved in camera-type eye formation; P:positive regulation of macromolecule metabolic process; C:integral component of membrane; C:endomembrane system; P:regulation of mitochondrion organization; P:positive regulation of cellular metabolic process; P:positive regulation of cytokine production; P:regulation of primary metabolic process; P:non-canonical Wnt signaling pathway; F:enzyme binding |
| Efet.01.339932.g1173.t1 | hypothetical protein LOTGIDRAFT_229075 | 279 | 1.63E-09 | 68.5 |  |
| Efet.01.340109.g1174.t1 | lysocardiolipin acyltransferase 1-like | 252 | 1.51E-16 | 68.25 | F:transferase activity, transferring acyl groups; P:metabolic process; C:integral component of membrane |
| Efet.01.340129.g1175.t1 | sphingosine-1-phosphate lyase-like | 318 | 1.41E-34 | 70.55 | F:lyase activity |
| Efet.01.340194.g1176.t1 | histone-lysine N-methyltransferase NSD2-like | 2007 | 1.33E-20 | 54.95 | C:nucleus; F:zinc ion binding; P:histone lysine methylation; F:metal ion binding; F:histone-lysine N-methyltransferase activity; F:methyltransferase activity; F:transferase activity; P:methylation |
| Efet.01.340441.g1179.t1 | zinc finger 782 isoform X2 | 801 | 5.12E-59 | 55 | F:nucleic acid binding; F:metal ion binding |
| Efet.01.340450.g1180.t1 | hypothetical protein HELRODRAFT_188599 | 246 | 5.64E-08 | 85 | F:calcium ion binding; C:integral component of membrane; P:homophilic cell adhesion via plasma membrane adhesion molecules; C:plasma membrane |
| Efet.01.34054.g624.t1 | adenosine deaminase CECR1-A-like | 381 | 5.54E-16 | 59.65 | P:blood vessel endothelial cell differentiation; P:nucleobase-containing compound biosynthetic process; P:purine ribonucleoside metabolic process; P:purine-containing compound biosynthetic process; C:extracellular region; P:single-organism biosynthetic process; F:receptor binding; P:neutrophil differentiation; F:hydrolase activity; F:deaminase activity |
| Efet.01.34061.g626.t1 | hypothetical protein CAPTEDRAFT_214196 | 927 | 1.81E-26 | 56.2 | C:membrane; C:integral component of membrane |
| Efet.01.34062.g627.t1 | 1-phosphatidylinositol 4,5-bisphosphate phosphodiesterase beta-4-like isoform X1 | 576 | 1.52E-45 | 55.75 | P:single-organism process |
| Efet.01.34064.g628.t1 | XIAP-associated factor 1 | 480 | 5.70E-25 | 67 | P:apoptotic process |
| Efet.01.340783.g1183.t1 | restin homolog isoform X15 | 1911 | 3.30E-12 | 55.9 |  |
| Efet.01.340790.g1185.t1 | hypothetical protein CAPTEDRAFT_225890 | 423 | 3.21E-19 | 63 |  |
| Efet.01.340797.g1186.t1 | acid-sensing ion channel 1-like | 543 | 1.44E-10 | 54.91 |  |
| Efet.01.340867.g1187.t1 | gliding motility-associated C-terminal domain-containing | 876 | 9.08E-07 | 44 |  |
| Efet.01.34107.g630.t1 | DNA double-strand break repair rad50 ATPase, | 315 | 1.37E-08 | 74 | C:centrosome; C:spindle pole; P:positive regulation of cytokinesis; C:microtubule |
| Efet.01.341071.g1189.t1 | protocadherin beta-10-like isoform X1 | 792 | 8.48E-30 | 53.7 | F:calcium ion binding; C:membrane; C:integral component of membrane; P:cell adhesion; P:homophilic cell adhesion via plasma membrane adhesion molecules; C:plasma membrane |
| Efet.01.34118.g631.t1 | Adhesive plaque matrix | 1239 | 4.70E-14 | 41 |  |
| Efet.01.341256.g1192.t1 | cyclin B | 921 | 4.94E-18 | 70.3 | C:nucleus; C:intracellular non-membrane-bounded organelle; P:positive regulation of cell cycle; P:mitotic nuclear division; C:intracellular organelle part; P:regulation of cell cycle process |
| Efet.01.341339.g1193.t1 | FMRFamide receptor-like | 600 | 4.07E-17 | 52.33 | F:G-protein coupled receptor activity; P:G-protein coupled receptor signaling pathway; C:membrane; C:integral component of membrane |
| Efet.01.341377.g1194.t1 | neurexin-2-like isoform X1 | 315 | 3.67E-20 | 65.35 | F:metal ion binding; C:membrane; C:integral component of membrane; P:chemical synaptic transmission |
| Efet.01.341388.g1196.t1 | class I SAM-dependent methyltransferase | 1338 | 1.76E-105 | 50.3 | C:membrane; C:integral component of membrane |
| Efet.01.341508.g1197.t1 | charged multivesicular body 4c-like | 201 | 4.03E-23 | 92.25 | P:vacuolar transport; C:intracellular |
| Efet.01.341567.g1198.t1 | ATP-binding cassette sub-family G member 4 | 534 | 8.29E-34 | 65.3 | F:nucleotide binding; F:ATPase activity; C:membrane |
| Efet.01.341587.g1199.t1 | sodium bicarbonate transporter 11 isoform X1 | 258 | 8.10E-24 | 71.85 | C:integral component of plasma membrane; P:regulation of intracellular pH; P:sodium ion transmembrane transport; F:inorganic anion exchanger activity; P:borate transmembrane transport |
| Efet.01.341607.g1200.t1 | polycystic kidney disease 1-like 2 | 636 | 5.39E-44 | 58.4 | F:calcium ion binding; C:membrane; C:integral component of membrane |
| Efet.01.34168.g632.t1 | phosphatase 2A regulatory subunit | 531 | 1.49E-91 | 89.6 | P:positive regulation of extrinsic apoptotic signaling pathway in absence of ligand; P:protein complex assembly; P:female meiotic division; C:protein phosphatase type 2A complex; C:chromosome, centromeric region; P:regulation of meiotic cell cycle process involved in oocyte maturation; F:protein serine/threonine phosphatase activity; P:peptidyl-serine dephosphorylation; C:extracellular exosome; F:antigen binding; F:[phosphorylase] phosphatase activity; F:protein heterodimerization activity; P:meiotic spindle elongation; C:cytosol; P:regulation of phosphoprotein phosphatase activity; P:mitotic sister chromatid separation; P:meiotic sister chromatid cohesion, centromeric |
| Efet.01.341693.g1201.t1 | calumenin-B-like isoform X1 | 297 | 5.62E-14 | 59.6 | F:calcium ion binding; C:membrane; C:integral component of membrane |
| Efet.01.341779.g1202.t1 | ankyrin repeat and EF-hand domain-containing 1-like | 303 | 1.12E-26 | 63.9 | F:calcium ion binding |
| Efet.01.341791.g1203.t1 | hypothetical protein HELRODRAFT_182774 | 225 | 1.13E-08 | 64.75 | C:membrane; C:integral component of membrane |
| Efet.01.34187.g634.t1 | low choriolytic enzyme-like | 228 | 2.91E-16 | 69.6 | F:metal ion binding; F:metallopeptidase activity |
| Efet.01.341904.g1205.t1 | glutaminase liver mitochondrial isoform X7 | 1218 | 0 | 75.5 | F:glutaminase activity; P:glutamate biosynthetic process; C:mitochondrial matrix; P:glutamine catabolic process |
| Efet.01.341935.g1207.t1 | extracellular sulfatase Sulf-2 isoform X1 | 339 | 3.14E-50 | 84.15 | P:metabolic process; F:N-acetylglucosamine-6-sulfatase activity |
| Efet.01.34194.g635.t1 | WD repeat-containing 66 | 582 | 3.06E-82 | 78.25 |  |
| Efet.01.3420.g295.t1 | cilia- and flagella-associated 57-like | 1338 | 7.65E-108 | 65.8 |  |
| Efet.01.34200.g636.t1 | DENN domain-containing 1A-like isoform X3 | 2772 | 2.02E-35 | 48.8 |  |
| Efet.01.342019.g1208.t1 | rab GTPase-binding effector 1-like | 303 | 1.42E-11 | 83.65 | F:growth factor activity; F:metal ion binding; F:GTPase activator activity; P:endocytosis; P:positive regulation of GTPase activity |
| Efet.01.342045.g1209.t1 | hypothetical protein HELRODRAFT_175680 | 1032 | 5.27E-27 | 62.25 | P:regulation of apoptotic process |
| Efet.01.3421.g296.t1 | beta-1,3-galactosyltransferase 1-like | 1563 | 8.24E-84 | 61.2 | C:membrane; F:transferase activity |
| Efet.01.342115.g1210.t1 | POU domain CF1A | 888 | 6.54E-44 | 69.8 | F:DNA binding; C:nucleus; F:transcription factor activity, sequence-specific DNA binding; P:transcription, DNA-templated; P:regulation of transcription, DNA-templated; F:sequence-specific DNA binding; P:brain development; P:regulation of transcription from RNA polymerase II promoter |
| Efet.01.34215.g637.t1 | hypothetical protein CAPTEDRAFT_227405 | 1068 | 9.84E-73 | 55.85 | C:membrane; C:integral component of membrane |
| Efet.01.34225.g639.t1 | homeodomain-interacting kinase 2-like isoform X1 | 1125 | 0 | 82.2 | F:DNA binding; F:ATP binding; F:protein serine/threonine kinase activity; P:protein phosphorylation |
| Efet.01.3425.g297.t1 | serine threonine- kinase pim-1 | 330 | 6.87E-42 | 81.3 | F:ATP binding; P:negative regulation of apoptotic process; F:protein serine/threonine kinase activity; P:protein phosphorylation |
| Efet.01.34250.g640.t1 | neurexin-1-like isoform X1 | 504 | 2.04E-37 | 58 | F:metal ion binding; F:calcium ion binding; C:membrane; C:integral component of membrane; P:chemical synaptic transmission |
| Efet.01.342663.g1213.t1 | tyrosine- phosphatase Lar-like isoform X1 | 516 | 6.48E-25 | 50.95 | F:hydrolase activity |
| Efet.01.34275.g641.t1 | Insulin-like peptide receptor | 759 | 4.80E-08 | 55.78 | P:peptidyl-tyrosine phosphorylation; P:cGMP biosynthetic process; P:G-protein coupled receptor signaling pathway; F:lyase activity; F:guanylate cyclase activity; F:protein kinase activity; P:protein phosphorylation; F:nucleotide binding; F:ATP binding; F:protein tyrosine kinase activity; F:G-protein coupled GABA receptor activity; C:membrane; C:integral component of membrane; P:phosphorylation; F:kinase activity |
| Efet.01.342816.g1218.t1 | tetratricopeptide repeat mitochondrial-like | 936 | 4.90E-55 | 58.05 |  |
| Efet.01.342857.g1219.t1 | succinyl- ligase [ADP-forming] subunit mitochondrial | 339 | 2.02E-29 | 76.4 | F:ATP binding; C:myelin sheath; C:extracellular exosome; F:metal ion binding; C:mitochondrion; P:metabolic process; F:ligase activity |
| Efet.01.342858.g1220.t1 | FAM65A-like isoform X1 | 1206 | 8.69E-42 | 54.6 |  |
| Efet.01.342881.g1221.t1 | GPI mannosyltransferase 1-like | 726 | 2.88E-43 | 66.15 | C:membrane; F:transferase activity |
| Efet.01.342908.g1222.t1 | hypothetical protein CAPTEDRAFT_207925 | 315 | 2.76E-13 | 65.5 | C:membrane; C:integral component of membrane |
| Efet.01.342951.g1224.t1 | induced myeloid leukemia cell differentiation Mcl-1 homolog | 339 | 2.48E-19 | 55.4 | F:protein homodimerization activity; F:protein heterodimerization activity; C:membrane; C:integral component of membrane; P:regulation of apoptotic process; P:intrinsic apoptotic signaling pathway in response to DNA damage; P:extrinsic apoptotic signaling pathway in absence of ligand; C:mitochondrial outer membrane |
| Efet.01.343203.g1225.t1 | neogenin isoform X2 | 660 | 4.91E-43 | 66.15 | P:regulation of dendrite development; C:dendrite; C:perivitelline space; P:motor neuron axon guidance; F:netrin receptor activity; P:salivary gland boundary specification; P:axon midline choice point recognition; C:neuronal cell body; P:dendrite guidance; C:plasma membrane; C:axon; P:glial cell migration |
| Efet.01.343215.g1226.t1 | vacuolar sorting-associated 13A-like | 231 | 1.34E-19 | 64.3 | C:membrane; C:integral component of membrane |
| Efet.01.343304.g1229.t1 | glutamate receptor kainate 2 isoform X2 | 633 | 5.46E-35 | 51.7 | C:membrane |
| Efet.01.343316.g1230.t1 | probable E3 ubiquitin- ligase makorin-2 | 303 | 1.03E-11 | 56.15 | F:binding |
| Efet.01.343433.g1234.t1 | clustered mitochondria homolog | 630 | 4.66E-42 | 63.95 | F:RNA binding; C:cytoplasm; P:intracellular distribution of mitochondria; C:membrane; C:integral component of membrane; P:mitochondrion organization |
| Efet.01.343447.g1235.t1 | calcium-activated potassium channel subunit beta-3 | 726 | 3.13E-56 | 49.35 | C:integral component of membrane; P:single-organism process; P:cellular process |
| Efet.01.343449.g1236.t1 | secreted frizzled-related 5-like | 597 | 3.20E-12 | 59.05 | P:closure of optic fissure; P:positive regulation of BMP signaling pathway; P:forebrain development; P:liver morphogenesis; P:endodermal digestive tract morphogenesis; P:Wnt signaling pathway; P:regulation of Wnt signaling pathway involved in digestive tract morphogenesis; P:positive regulation of Wnt signaling pathway; P:embryonic retina morphogenesis in camera-type eye |
| Efet.01.3436.g298.t1 | dual specificity tyrosine-phosphorylation-regulated kinase 1B isoform X2 | 339 | 5.57E-35 | 74.2 | F:nucleotide binding; P:ncRNA processing; F:transcription coactivator activity; P:positive regulation of transcription, DNA-templated; P:myoblast fusion; F:protein kinase activity; P:protein phosphorylation; P:adipose tissue development |
| Efet.01.343607.g1240.t1 | matrix metallo ase-14 isoform X5 | 222 | 3.08E-12 | 70.15 | F:metal ion binding; F:peptidase activity |
| Efet.01.34363.g643.t1 | beta-catenin 1 | 360 | 2.84E-52 | 81.65 |  |
| Efet.01.343704.g1241.t1 | guanine nucleotide-binding subunit alpha-11 isoform X1 | 222 | 1.21E-28 | 97.95 | F:GTPase activator activity; P:post-embryonic development; P:G-protein coupled acetylcholine receptor signaling pathway; P:negative regulation of protein kinase activity; P:positive regulation of GTPase activity; C:photoreceptor outer segment; P:glutamate receptor signaling pathway; P:maternal behavior; P:embryonic digit morphogenesis; F:GTP binding; C:dendrite; P:heart development; P:developmental pigmentation; P:phototransduction, visible light; C:extracellular exosome; P:skeletal system development; P:entrainment of circadian clock; F:GTPase activity; P:action potential; F:alkylglycerophosphoethanolamine phosphodiesterase activity; P:platelet activation; P:regulation of catenin import into nucleus; P:protein heterotrimerization; P:activation of phospholipase C activity; P:adenylate cyclase-activating G-protein coupled receptor signaling pathway; P:phospholipase C-activating dopamine receptor signaling pathway; F:signal transducer activity; C:lysosomal membrane; C:plasma membrane; P:regulation of melanocyte differentiation; P:forebrain neuron development; C:nuclear membrane; F:metal ion binding; F:type 2A serotonin receptor binding; C:cell body; F:G-protein beta/gamma-subunit complex binding; P:neuron remodeling; P:protein stabilization |
| Efet.01.343710.g1242.t1 | lanC 2 | 246 | 1.16E-13 | 68.25 |  |
| Efet.01.343801.g1245.t1 | clavaminate synthase At3g21360 | 921 | 1.37E-59 | 58.6 | C:nucleus; F:oxidoreductase activity; P:oxidation-reduction process |
| Efet.01.343822.g1246.t1 | Cyclic nucleotide-gated channel cone photoreceptor subunit | 1356 | 1.07E-135 | 66.55 | C:integral component of plasma membrane; F:voltage-gated potassium channel activity; P:cation transport; P:regulation of membrane potential; P:ion transmembrane transport; F:intracellular cAMP activated cation channel activity; F:intracellular cGMP activated cation channel activity; F:cGMP binding |
| Efet.01.34383.g644.t1 | C-myc promoter-binding -like | 807 | 1.72E-16 | 75.45 |  |
| Efet.01.343830.g1247.t1 | glutamate receptor 3- | 315 | 1.52E-25 | 82.65 | C:postsynaptic membrane; P:ion transmembrane transport; C:integral component of membrane; C:cell junction; P:ionotropic glutamate receptor signaling pathway; F:ionotropic glutamate receptor activity; F:extracellular-glutamate-gated ion channel activity |
| Efet.01.343851.g1248.t1 | intermediate filament | 393 | 5.77E-20 | 60.1 | F:structural molecule activity; C:intermediate filament |
| Efet.01.343852.g1249.t1 | Acetylcholine receptor subunit beta-like 1 | 717 | 1.77E-67 | 64.35 | F:ion channel activity; P:ion transmembrane transport; C:synapse; C:membrane part; C:plasma membrane |
| Efet.01.343951.g1250.t1 | Cationic amino acid transporter 4 | 1638 | 3.83E-114 | 61.3 | C:membrane; C:integral component of membrane; F:amino acid transmembrane transporter activity; P:amino acid transmembrane transport |
| Efet.01.344.g41.t1 | titin isoform X2 | 1071 | 1.25E-70 | 63.95 |  |
| Efet.01.344.g42.t1 | titin-like isoform X3 | 765 | 1.29E-19 | 45.35 |  |
| Efet.01.34405.g647.t1 | GTP-binding Di-Ras2-like | 687 | 4.13E-57 | 55.95 | F:nucleotide binding; F:GTP binding; P:signal transduction; P:small GTPase mediated signal transduction; C:membrane; C:intracellular |
| Efet.01.34431.g649.t1 | Dual specificity tyrosine-phosphorylation-regulated kinase 2 | 1629 | 0 | 82.9 | C:nucleus; F:ATP binding; F:metal ion binding; P:signal transduction; C:macromolecular complex; F:protein serine/threonine kinase activity; P:protein phosphorylation; P:cellular response to DNA damage stimulus |
| Efet.01.344418.g1255.t1 | monocarboxylate transporter 12-like | 351 | 3.38E-09 | 50 | P:transmembrane transport; F:hydrolase activity, acting on acid anhydrides, in phosphorus-containing anhydrides; F:ATP-dependent helicase activity; F:helicase activity; F:ATP-dependent DNA helicase activity; P:nucleobase-containing compound metabolic process; F:nucleotide binding; F:nucleic acid binding; F:DNA binding; C:nucleus; F:ATP binding; P:DNA duplex unwinding; C:membrane; C:integral component of membrane; F:hydrolase activity |
| Efet.01.344506.g1257.t1 | beta,beta-carotene 15,15 -dioxygenase isoform X1 | 273 | 1.78E-32 | 78.35 | F:beta-carotene 15,15'-monooxygenase activity; P:retinal metabolic process; P:oxidation-reduction process; F:monooxygenase activity; P:beta-carotene metabolic process |
| Efet.01.34460.g650.t1 | wee1 kinase 1-A | 249 | 9.43E-25 | 76.45 | F:magnesium ion binding; C:nucleus; F:ATP binding; P:peptidyl-tyrosine phosphorylation; F:non-membrane spanning protein tyrosine kinase activity; P:mitotic nuclear division; F:protein serine/threonine kinase activity |
| Efet.01.344693.g1260.t1 | ATP-binding cassette sub-family A member 3-like | 441 | 2.31E-14 | 63.8 | P:lipid transport; F:ATPase activity, coupled to transmembrane movement of substances; C:intracellular membrane-bounded organelle |
| Efet.01.344713.g1262.t1 | WD repeat-containing alr2800 | 2121 | 8.54E-126 | 46.33 |  |
| Efet.01.344761.g1263.t1 | FMRFamide receptor-like | 1344 | 3.23E-50 | 51.05 | F:G-protein coupled receptor activity; F:G-protein coupled peptide receptor activity; P:G-protein coupled receptor signaling pathway; C:membrane; C:integral component of membrane |
| Efet.01.344802.g1264.t1 | serine threonine- kinase DCLK2 isoform X1 | 255 | 9.97E-47 | 92.3 | F:ATP binding; F:protein serine/threonine kinase activity; P:protein phosphorylation; C:intracellular; P:intracellular signal transduction |
| Efet.01.34487.g651.t1 | nucleoside diphosphate kinase 7-like | 513 | 1.55E-50 | 70.95 | F:nucleotide binding; P:pyrimidine ribonucleoside triphosphate biosynthetic process; P:pyrimidine ribonucleotide biosynthetic process; P:pyrimidine ribonucleoside biosynthetic process; F:nucleoside diphosphate kinase activity; P:phosphorylation; C:intracellular |
| Efet.01.34490.g652.t1 | kinase DC2 | 306 | 3.15E-20 | 91.55 | F:ATP binding; F:protein serine/threonine kinase activity; P:protein phosphorylation |
| Efet.01.344990.g1266.t1 | cytochrome P450 4c3 | 204 | 1.05E-09 | 78.65 | F:iron ion binding; F:oxidoreductase activity, acting on paired donors, with incorporation or reduction of molecular oxygen; C:integral component of membrane; P:oxidation-reduction process; F:monooxygenase activity; F:heme binding |
| Efet.01.345004.g1267.t1 | wiskott-Aldrich syndrome family member 2-like isoform X2 | 1710 | 1.71E-12 | 66.33 | C:cytoskeleton; F:actin binding; P:actin cytoskeleton organization |
| Efet.01.345072.g1270.t1 | huntingtin-like isoform X2 | 603 | 4.90E-45 | 75.3 | C:nucleus; C:cytoplasm; C:membrane; F:transcription factor binding |
| Efet.01.345122.g1272.t1 | cadherin-23 isoform X2 | 648 | 9.18E-26 | 51.05 | P:cell projection organization; P:inner ear receptor cell development; C:organelle; C:cell projection; P:sensory perception of mechanical stimulus; P:hair cell differentiation |
| Efet.01.345177.g1273.t1 | segment polarity dishevelled homolog DVL-3 isoform X4 | 1131 | 6.61E-28 | 54.9 | P:signal transduction |
| Efet.01.345196.g1274.t1 | kinase brain isozyme | 297 | 6.03E-32 | 79.95 | F:ATP binding; F:zinc ion binding; P:protein phosphorylation; C:intracellular; P:intracellular signal transduction; F:protein kinase C activity |
| Efet.01.345219.g1275.t1 | piggyBac transposable element-derived 3-like | 1284 | 2.15E-90 | 64 |  |
| Efet.01.345236.g1276.t1 | Carbohydrate sulfotransferase | 567 | 2.93E-47 | 65.3 | P:skeletal system development; P:regulation of cellular process; P:embryonic morphogenesis; F:sulfotransferase activity; P:animal organ development; P:single-organism cellular process; P:primary metabolic process; P:single-organism metabolic process; P:organic substance metabolic process |
| Efet.01.345255.g1277.t1 | calcium-independent phospholipase A2-gamma isoform X1 | 249 | 2.02E-32 | 79.05 | P:lipid metabolic process; C:integral component of membrane |
| Efet.01.345303.g1278.t1 | hypothetical protein CAPTEDRAFT_169938 | 240 | 1.58E-07 | 77 | C:integral component of membrane |
| Efet.01.34534.g655.t1 | leukocyte elastase inhibitor-like isoform X1 | 978 | 1.53E-76 | 63.4 | C:extracellular space; F:serine-type endopeptidase inhibitor activity; P:negative regulation of endopeptidase activity |
| Efet.01.34535.g656.t1 | allatostatin-A receptor-like | 783 | 3.41E-59 | 64.25 | F:G-protein coupled receptor activity; P:transmembrane transport; P:G-protein coupled receptor signaling pathway; P:signal transduction; C:membrane; C:integral component of membrane; F:metal ion transmembrane transporter activity; P:metal ion transport; F:signal transducer activity |
| Efet.01.345403.g1280.t1 | potassium voltage-gated channel Shaw-like | 381 | 1.06E-50 | 81.8 | F:voltage-gated potassium channel activity; P:potassium ion transmembrane transport; C:voltage-gated potassium channel complex; P:protein homooligomerization |
| Efet.01.345467.g1283.t1 | hypothetical protein CAPTEDRAFT_92948 | 567 | 3.42E-11 | 65 | P:ion transport; C:membrane |
| Efet.01.345501.g1284.t1 | omega-3 fatty acid endoplasmic reticulum-like | 837 | 7.01E-127 | 71.35 | P:lipid metabolic process; C:integral component of membrane |
| Efet.01.345591.g1287.t1 | hypothetical protein CAPTEDRAFT_224281 | 621 | 3.06E-29 | 52.36 | C:membrane; C:integral component of membrane |
| Efet.01.34560.g657.t1 | ral guanine nucleotide dissociation stimulator-like 1 | 471 | 8.75E-31 | 69.3 | P:signal transduction |
| Efet.01.345643.g1289.t1 | FAM210A | 429 | 6.30E-09 | 68.5 | C:membrane |
| Efet.01.345646.g1290.t1 | hypothetical protein LOTGIDRAFT_116422 | 528 | 1.57E-47 | 68.45 | C:membrane |
| Efet.01.345667.g1291.t1 | solute carrier family 12 member 9 | 252 | 6.24E-17 | 69.45 | C:membrane; P:transport |
| Efet.01.345685.g1292.t1 | gamma-aminobutyric acid type B receptor subunit 2-like | 363 | 3.94E-30 | 59.15 | F:signal transducer activity |
| Efet.01.345709.g1293.t1 | lactase-phlorizin hydrolase | 435 | 1.56E-17 | 74.05 | P:response to iron(II) ion; F:beta-glucosidase activity; P:response to nutrient; F:lactase activity; P:glycosyl compound metabolic process; C:integral component of plasma membrane; P:response to ethanol; P:response to hypoxia; C:brush border; P:response to drug; F:transferase activity; P:response to lead ion; P:response to nickel cation |
| Efet.01.345709.g1294.t1 | lactase-phlorizin hydrolase-like | 213 | 5.01E-12 | 66.8 | F:beta-glucosidase activity; P:glycosyl compound metabolic process |
| Efet.01.34578.g658.t1 | Glycine receptor subunit alpha-3 | 255 | 1.23E-10 | 57.18 | C:membrane; P:transport |
| Efet.01.345963.g1297.t1 | 60 kDa neurofilament -like | 492 | 1.16E-22 | 66.95 | F:structural molecule activity; C:intermediate filament |
| Efet.01.345971.g1298.t1 | headcase isoform X1 | 1410 | 3.99E-124 | 59.25 |  |
| Efet.01.345973.g1299.t1 | V-type proton ATPase subunit D-like | 303 | 5.70E-35 | 90.4 | F:ATPase activity, coupled to transmembrane movement of substances; P:transmembrane transport |
| Efet.01.346087.g1301.t1 | tropomyosin-2-like isoform X6 | 291 | 2.49E-25 | 73.05 |  |
| Efet.01.346145.g1302.t1 | Golgi-associated plant pathogenesis-related 1 | 210 | 1.58E-06 | 61.33 | C:extracellular region |
| Efet.01.346168.g1303.t1 | hypothetical protein CRN43_17300 | 375 | 5.00E-06 | 66 |  |
| Efet.01.34619.g660.t1 | specificity 1 | 1440 | 1.64E-13 | 78.2 | F:nucleic acid binding; F:metal ion binding |
| Efet.01.346234.g1304.t1 | uncharacterized protein LOC110466304 | 273 | 4.09E-15 | 63.86 | C:membrane; C:integral component of membrane |
| Efet.01.346237.g1305.t1 | Bcl-2-related ovarian killer | 522 | 1.45E-36 | 60.35 | C:intracellular membrane-bounded organelle; C:membrane; P:apoptotic process; P:positive regulation of apoptotic process; C:cytoplasmic part |
| Efet.01.34624.g661.t1 | DDB1- and CUL4-associated factor 12-like | 342 | 6.64E-11 | 75.1 | C:Cul4-RING E3 ubiquitin ligase complex |
| Efet.01.346242.g1306.t1 | Cyclic GMP-AMP synthase | 1077 | 3.90E-24 | 53.35 |  |
| Efet.01.346290.g1307.t1 | calcyphosin isoform X2 | 282 | 3.59E-18 | 70.5 | F:calcium ion binding |
| Efet.01.346491.g1309.t1 | GTP-binding Rhes | 435 | 2.13E-31 | 64.45 | F:nucleotide binding; P:signal transduction |
| Efet.01.346511.g1310.t1 | cytochrome P450 2G1-like | 366 | 2.33E-12 | 67.5 | F:arachidonic acid epoxygenase activity; P:epoxygenase P450 pathway; F:steroid hydroxylase activity; F:binding |
| Efet.01.34663.g664.t1 | jagunal | 423 | 1.41E-55 | 62 | C:endoplasmic reticulum membrane; C:integral component of membrane; P:endoplasmic reticulum organization |
| Efet.01.3468.g299.t1 | chondroitin sulfate synthase 2 | 423 | 5.16E-25 | 75.95 | C:membrane |
| Efet.01.3468.g300.t1 | chondroitin sulfate synthase 2-like | 621 | 8.14E-72 | 63.35 | C:membrane |
| Efet.01.346812.g1316.t1 | Conserved oligomeric Golgi complex subunit 3 | 231 | 1.94E-13 | 69.35 | C:cis-Golgi network; F:metal ion binding; C:membrane; P:intracellular protein transport |
| Efet.01.346871.g1317.t1 | O- case-like | 825 | 1.45E-49 | 50.05 | P:glycoprotein metabolic process; F:beta-N-acetylglucosaminidase activity |
| Efet.01.346921.g1319.t1 | sodium potassium-transporting atpase subunit beta-2 | 219 | 5.69E-32 | 73.05 | P:potassium ion transport; P:sodium ion transport; C:sodium:potassium-exchanging ATPase complex |
| Efet.01.347007.g1320.t1 | suppressor of cytokine signaling 2-like | 1287 | 3.53E-35 | 57.5 | P:negative regulation of cellular process |
| Efet.01.34703.g665.t1 | dynein gamma flagellar outer arm-like | 840 | 3.30E-27 | 51.35 | F:nucleotide binding; F:ATP binding; F:microtubule motor activity; C:dynein complex; P:microtubule-based movement |
| Efet.01.34704.g666.t1 | hypothetical protein CAPTEDRAFT_120325, partial | 549 | 1.51E-15 | 47 | C:membrane; C:integral component of membrane |
| Efet.01.347050.g1321.t1 | cation channel sperm-associated 2 | 537 | 1.38E-13 | 53.35 | F:ion channel activity; C:membrane; P:transport |
| Efet.01.347085.g1323.t1 | ATP-binding cassette sub-family G member 4-like isoform X1 | 288 | 7.20E-35 | 88.6 | F:ATP binding; F:sterol-transporting ATPase activity; F:zinc ion binding; C:integral component of membrane; P:cholesterol transport; C:plasma membrane; F:cholesterol transporter activity |
| Efet.01.347232.g1324.t1 | ral guanine nucleotide dissociation stimulator-like 1 | 537 | 9.40E-28 | 59.15 | P:biological regulation |
| Efet.01.347236.g1325.t1 | hypothetical protein HELRODRAFT_81331 | 366 | 2.71E-11 | 87 |  |
| Efet.01.347237.g1327.t1 | hypothetical protein | 1182 | 2.23E-08 | 46 |  |
| Efet.01.347255.g1329.t1 | tubulin beta chain isoform X1 | 927 | 1.70E-153 | 78 | F:GTP binding; F:GTPase activity; F:structural constituent of cytoskeleton; C:microtubule; P:microtubule-based process |
| Efet.01.347287.g1330.t1 | neurexin-3 isoform X1 | 888 | 1.68E-54 | 54.8 | C:presynaptic membrane; F:metal ion binding; F:calcium ion binding; C:membrane; C:integral component of membrane; P:chemical synaptic transmission |
| Efet.01.34730.g667.t1 | WD repeat-containing 36 | 240 | 2.36E-17 | 76.2 | C:small-subunit processome; P:regulation of biological quality; P:single-multicellular organism process; P:rRNA processing |
| Efet.01.347335.g1333.t1 | Polypeptide N-acetylgalactosaminyltransferase 5 | 648 | 3.45E-31 | 75.85 | F:transferase activity, transferring glycosyl groups; C:integral component of membrane; P:protein glycosylation; C:Golgi membrane; F:carbohydrate binding |
| Efet.01.34738.g668.t1 | mediator of RNA polymerase II transcription subunit 16 | 1998 | 8.36E-170 | 58.25 | C:nucleus; F:transcription coactivator activity; P:positive regulation of transcription, DNA-templated; P:transcription, DNA-templated; C:mediator complex; P:regulation of transcription, DNA-templated |
| Efet.01.347406.g1335.t1 | zinc finger homeobox 4-like isoform X1 | 4599 | 3.96E-169 | 58.2 | F:nucleic acid binding |
| Efet.01.347409.g1336.t1 | furry isoform X3 | 303 | 6.14E-12 | 69.85 | P:cellular component organization; P:anatomical structure development; C:cell part; P:cellular developmental process |
| Efet.01.347437.g1337.t1 | serrate RNA effector molecule homolog isoform X1 | 426 | 3.25E-34 | 78.35 | F:nucleotide binding; F:nucleic acid binding; C:membrane |
| Efet.01.347451.g1339.t1 | transcription factor LBX1 | 570 | 4.51E-26 | 78.25 | C:nucleus; C:transcription factor complex; F:transcription factor activity, sequence-specific DNA binding; P:neuron fate determination; P:negative regulation of cell proliferation; P:spinal cord motor neuron differentiation; P:negative regulation of neuron differentiation; P:heart looping; F:sequence-specific DNA binding; P:regulation of transcription from RNA polymerase II promoter involved in spinal cord association neuron specification; P:regulation of Wnt signaling pathway; P:convergent extension |
| Efet.01.347475.g1341.t1 | beta-galactosidase-1 2 | 2052 | 4.67E-174 | 59.85 | F:hydrolase activity, acting on glycosyl bonds |
| Efet.01.347501.g1342.t1 | Cytochrome P450 4V2 | 351 | 9.59E-32 | 67.75 | F:iron ion binding; F:oxidoreductase activity, acting on paired donors, with incorporation or reduction of molecular oxygen; P:oxidation-reduction process; F:monooxygenase activity; F:heme binding |
| Efet.01.347523.g1343.t1 | potassium voltage-gated channel subfamily H member 1-like | 480 | 1.30E-49 | 77.4 | F:phosphorelay sensor kinase activity; F:voltage-gated potassium channel activity; P:potassium ion transmembrane transport; P:phosphorelay signal transduction system; C:integral component of membrane; P:signal transduction by protein phosphorylation; C:intracellular |
| Efet.01.347534.g1344.t1 | speckle-type POZ -like | 273 | 1.12E-44 | 81.15 |  |
| Efet.01.347557.g1345.t1 | INO80 complex subunit B | 243 | 1.10E-13 | 67.3 | P:chromatin remodeling; P:regulation of transcription, DNA-templated; C:Ino80 complex |
| Efet.01.347811.g1349.t1 | neuroligin- X-linked-like isoform X1 | 558 | 2.85E-62 | 68.3 | C:membrane |
| Efet.01.347820.g1350.t1 | KIAA0556-like isoform X5 | 558 | 1.99E-29 | 65.05 |  |
| Efet.01.34783.g673.t1 | acetylcholinesterase-like isoform X3 | 1587 | 0 | 65.25 | F:cholinesterase activity |
| Efet.01.347874.g1353.t1 | gastrula zinc finger -like | 849 | 6.14E-69 | 60.45 |  |
| Efet.01.347929.g1355.t1 | hypothetical protein CAPTEDRAFT_208831 | 648 | 1.12E-17 | 48.56 | C:membrane; C:integral component of membrane |
| Efet.01.347981.g1357.t1 | cysteine and histidine-rich 1-like | 543 | 1.33E-65 | 70.95 | F:metal ion binding |
| Efet.01.348.g43.t1 | matrix metallo ase-14 | 516 | 2.63E-10 | 55.38 | F:hydrolase activity |
| Efet.01.348014.g1358.t1 | hypothetical protein CAPTEDRAFT_21078 | 381 | 1.05E-14 | 58.6 | C:membrane; C:integral component of membrane; P:transport; F:transporter activity |
| Efet.01.348061.g1359.t1 | SMP-30 gluconolactonase LRE family | 201 | 7.99E-29 | 70.05 |  |
| Efet.01.348098.g1360.t1 | hypothetical protein CAPTEDRAFT_216867 | 414 | 1.15E-08 | 54 | C:membrane; C:integral component of membrane; F:carbohydrate binding |
| Efet.01.34812.g674.t1 | leucine-rich repeat neuronal 1-like | 1362 | 1.09E-14 | 45.5 | C:membrane; C:integral component of membrane |
| Efet.01.348247.g1362.t1 | Low-density lipo receptor-related 4 | 411 | 2.45E-22 | 57.45 | C:membrane; C:integral component of membrane |
| Efet.01.348247.g1363.t1 | low-density lipo receptor-related 4-like | 363 | 1.68E-14 | 55.2 | C:membrane; C:integral component of membrane |
| Efet.01.348252.g1365.t1 | tyrosine- kinase SYK-like | 585 | 3.35E-35 | 56.3 | F:binding; F:kinase activity |
| Efet.01.348325.g1367.t1 | G- coupled receptor GRL101-like isoform X1 | 633 | 3.13E-76 | 77.35 | P:G-protein coupled receptor signaling pathway; C:integral component of membrane; F:protein-hormone receptor activity |
| Efet.01.348381.g1369.t1 | hypothetical protein HELRODRAFT_162325 | 432 | 5.11E-48 | 64 | C:membrane |
| Efet.01.3484.g302.t1 | cilia- and flagella-associated 52 | 720 | 6.80E-77 | 69.05 |  |
| Efet.01.348626.g1373.t1 | hypothetical protein CAPTEDRAFT_222036 | 399 | 2.58E-18 | 51.25 | C:membrane; C:integral component of membrane |
| Efet.01.348645.g1374.t1 | tetraspanin-1 | 276 | 5.40E-10 | 62.4 | C:membrane; C:integral component of membrane |
| Efet.01.34868.g676.t1 | programmed cell death 6-like isoform X2 | 237 | 6.67E-39 | 88.6 | F:calcium ion binding |
| Efet.01.34869.g677.t1 | peroxisomal acyl-coenzyme A oxidase 1 | 513 | 2.45E-47 | 58.15 | F:oxidoreductase activity, acting on the CH-CH group of donors; C:cytoplasm; C:intracellular membrane-bounded organelle; P:oxidation-reduction process; F:binding; P:fatty acid metabolic process |
| Efet.01.348756.g1376.t1 | degenerin unc-105 | 297 | 5.66E-27 | 65.65 | P:sodium ion transport; C:membrane |
| Efet.01.348929.g1379.t1 | UPF0676 -like | 786 | 8.81E-28 | 50.4 | F:dioxygenase activity; F:iron ion binding; F:oxidoreductase activity; F:metal ion binding; P:oxidation-reduction process |
| Efet.01.349054.g1381.t1 | inositol 1,4,5-trisphosphate receptor type 1-like | 336 | 4.44E-43 | 72.05 | P:inositol phosphate-mediated signaling; C:integral component of membrane; F:inositol 1,4,5-trisphosphate-sensitive calcium-release channel activity; P:calcium ion transmembrane transport; C:endoplasmic reticulum |
| Efet.01.349160.g1383.t1 | MULE and FLYWCH domain containing | 1410 | 1.26E-145 | 61.85 |  |
| Efet.01.349167.g1384.t1 | transmembrane and TPR repeat-containing 4-like | 417 | 1.82E-22 | 72.1 | C:membrane |
| Efet.01.349194.g1385.t1 | BTB POZ domain-containing 6-like | 1137 | 8.81E-21 | 47 |  |
| Efet.01.349227.g1387.t1 | DNA damage-binding 2 | 234 | 5.85E-12 | 65.55 | P:DNA repair; C:macromolecular complex; C:intracellular part; P:protein ubiquitination |
| Efet.01.349300.g1389.t1 | Transposase IS4 family {ECO:0000313 | 1077 | 2.69E-118 | 69.85 | F:nucleic acid binding |
| Efet.01.349344.g1391.t1 | spectrin beta chain-like isoform X1 | 534 | 2.16E-91 | 88.85 | F:actin binding; F:structural constituent of cytoskeleton; F:phospholipid binding; C:spectrin |
| Efet.01.349370.g1392.t1 | hypothetical protein CAPTEDRAFT_225483 | 291 | 6.40E-12 | 58.55 | C:membrane; C:integral component of membrane; F:carbohydrate binding |
| Efet.01.349537.g1393.t1 | polyphosphate kinase 1 | 903 | 0 | 83.6 | F:ATP binding; C:polyphosphate kinase complex; P:phosphorylation; F:polyphosphate kinase activity; P:polyphosphate biosynthetic process |
| Efet.01.349578.g1394.t1 | spondin-1-like isoform X2 | 246 | 1.58E-07 | 67.83 |  |
| Efet.01.349609.g1395.t1 | testis-expressed sequence 264 | 276 | 1.96E-16 | 67.65 | C:extracellular exosome; C:membrane; C:integral component of membrane |
| Efet.01.349656.g1396.t1 | Retrovirus-related Pol poly from transposon | 420 | 1.29E-23 | 66.75 | F:nucleic acid binding; C:membrane; C:integral component of membrane; P:DNA integration |
| Efet.01.349746.g1398.t1 | acetylcholine receptor subunit alpha-like | 1164 | 3.74E-109 | 67.1 | P:ion transport; C:membrane |
| Efet.01.349801.g1399.t1 | sarcoplasmic calcium-binding -like | 228 | 4.41E-18 | 66.05 | F:calcium ion binding |
| Efet.01.349854.g1401.t1 | nuclear hormone receptor HR96-like isoform X8 | 747 | 6.84E-51 | 77.75 | F:thyroid hormone receptor activity; C:nucleus; F:DNA binding; F:metal ion binding; P:transcription, DNA-templated; F:steroid hormone receptor activity; P:regulation of transcription, DNA-templated; P:steroid hormone mediated signaling pathway; P:intracellular receptor signaling pathway |
| Efet.01.34989.g678.t1 | pituitary-specific positive transcription factor 1-like | 1029 | 6.41E-28 | 58.25 | C:nucleus; F:transcription factor activity, sequence-specific DNA binding; P:transcription, DNA-templated; P:positive regulation of transcription, DNA-templated; F:sequence-specific DNA binding; F:transcription factor binding; P:somatotropin secreting cell differentiation |
| Efet.01.349899.g1403.t1 | nuclear factor 1 X-type isoform X7 | 939 | 9.63E-35 | 78.1 | C:nucleus; F:transcription factor activity, sequence-specific DNA binding; P:transcription, DNA-templated; P:DNA replication; P:regulation of transcription, DNA-templated |
| Efet.01.35000.g679.t1 | hypothetical protein HELRODRAFT_182047 | 837 | 5.23E-22 | 65.5 | C:integral component of membrane |
| Efet.01.350027.g1.t1 | NYN domain-containing | 561 | 1.51E-13 | 52.11 | F:nucleic acid binding; F:zinc ion binding; F:metal ion binding |
| Efet.01.350055.g2.t1 | sax-3 isoform X2 | 462 | 2.68E-13 | 54.05 | C:membrane; C:integral component of membrane |
| Efet.01.35008.g680.t1 | hypothetical protein HELRODRAFT_194550 | 1671 | 1.42E-80 | 72 | F:zinc ion binding; C:Smc5-Smc6 complex; P:double-strand break repair via homologous recombination; F:SUMO transferase activity |
| Efet.01.35015.g681.t1 | leucine-rich repeat serine threonine- kinase 1-like | 498 | 5.09E-29 | 61.35 | F:GTP binding; F:ATP binding; P:small GTPase mediated signal transduction; F:protein kinase activity; P:protein phosphorylation; C:intracellular |
| Efet.01.350181.g6.t1 | asunder homolog isoform X2 | 2070 | 0 | 68.95 |  |
| Efet.01.350225.g7.t1 | cortactin-binding 2 | 813 | 6.55E-07 | 58.25 |  |
| Efet.01.35023.g682.t1 | armadillo repeat-containing 5-like | 2766 | 3.15E-12 | 50.08 |  |
| Efet.01.350325.g9.t1 | pyruvate dehydrogenase [acetyl-transferring]-phosphatase mitochondrial | 1518 | 0 | 63.15 | F:phosphoprotein phosphatase activity; F:cation binding |
| Efet.01.350391.g10.t1 | acid trehalase 1 | 729 | 6.59E-75 | 53.95 | P:carbohydrate metabolic process; F:G-protein coupled receptor activity; P:G-protein coupled receptor signaling pathway; F:catalytic activity; C:cell wall; C:membrane; C:integral component of membrane; F:hydrolase activity |
| Efet.01.35041.g683.t1 | hypothetical protein HELRODRAFT_178970 | 1629 | 1.18E-30 | 72 |  |
| Efet.01.350436.g12.t1 | 1-phosphatidylinositol 4,5-bisphosphate phosphodiesterase epsilon-1-like | 495 | 4.03E-29 | 47.6 | P:single-organism process |
| Efet.01.350464.g13.t1 | hypothetical protein CAPTEDRAFT_221826 | 420 | 6.08E-09 | 53 | C:membrane; C:integral component of membrane; F:carbohydrate binding |
| Efet.01.350495.g15.t1 | hypothetical protein | 972 | 2.22E-06 | 41 |  |
| Efet.01.350502.g16.t1 | hypothetical protein HELRODRAFT_192853 | 822 | 4.09E-10 | 58.5 | C:membrane; C:integral component of membrane |
| Efet.01.350605.g17.t1 | telomerase component 1 | 453 | 1.34E-12 | 60.8 |  |
| Efet.01.350607.g18.t1 | ribosomal S6 kinase alpha-5 isoform X3 | 357 | 1.45E-38 | 62.15 | F:nucleotide binding; F:protein kinase activity; P:phosphorylation |
| Efet.01.350654.g20.t1 | Retrovirus-related Pol poly from transposon | 270 | 1.05E-10 | 65.25 |  |
| Efet.01.350740.g21.t1 | universal stress | 429 | 3.62E-45 | 63 | C:cytoplasm; P:response to stress |
| Efet.01.350771.g22.t1 | pyruvate mitochondrial isoform X1 | 426 | 4.28E-64 | 88.65 | F:DNA binding; F:ATP binding; P:gluconeogenesis; F:pyruvate carboxylase activity; F:metal ion binding; F:biotin binding; F:biotin carboxylase activity; P:pyruvate metabolic process |
| Efet.01.350816.g23.t1 | F-box LRR-repeat 16 | 978 | 9.22E-79 | 58.95 |  |
| Efet.01.350874.g25.t1 | band 5 | 414 | 3.74E-32 | 83.05 | C:cytoskeleton; C:cytoplasm; C:extrinsic component of membrane; F:cytoskeletal protein binding |
| Efet.01.350892.g26.t1 | coiled-coil domain-containing 130 | 375 | 5.45E-65 | 87.4 | C:membrane; F:galactosylgalactosylxylosylprotein 3-beta-glucuronosyltransferase activity |
| Efet.01.350900.g27.t1 | casein kinase I isoform X2 | 984 | 0 | 92.4 | P:regulation of cell shape; C:nucleus; F:ATP binding; C:cytoplasm; P:phagocytosis; P:Wnt signaling pathway; F:protein serine/threonine kinase activity; P:peptidyl-serine phosphorylation |
| Efet.01.350955.g28.t1 | MULTISPECIES: hypothetical protein | 816 | 1.93E-50 | 52.2 | C:membrane; C:integral component of membrane |
| Efet.01.350955.g29.t1 | DEAD DEAH box helicase | 1245 | 1.21E-163 | 97 | F:ATP binding; F:helicase activity |
| Efet.01.350955.g30.t1 | DEAD DEAH box helicase | 495 | 1.97E-62 | 92.45 | F:ATP binding; F:helicase activity |
| Efet.01.350955.g31.t1 | O-methyl transferase family | 306 | 1.88E-57 | 80.45 |  |
| Efet.01.350988.g32.t1 | EH domain-containing 1 | 771 | 5.05E-90 | 66.7 | F:GTP binding; F:calcium ion binding; P:endocytic recycling; C:intracellular |
| Efet.01.350991.g33.t1 | Zinc finger | 1482 | 4.65E-76 | 57.7 | F:nucleic acid binding; F:metal ion binding |
| Efet.01.351006.g34.t1 | 5 (3 )- mitochondrial-like | 237 | 2.74E-06 | 77 |  |
| Efet.01.351135.g37.t1 | zinc finger 878-like isoform X3 | 927 | 1.58E-30 | 76.45 | F:binding |
| Efet.01.351182.g38.t1 | zinc finger 239 isoform X3 | 1488 | 1.94E-74 | 52.55 | F:nucleic acid binding; F:DNA binding; P:biological_process; F:metal ion binding |
| Efet.01.351277.g40.t1 | sodium channel type 4 subunit alpha B-like isoform X1 | 342 | 2.84E-17 | 55.65 | P:biological regulation; F:ion channel activity; P:ion transport; C:integral component of membrane; C:intrinsic component of plasma membrane; P:single-organism process; C:axon; P:cellular process |
| Efet.01.35143.g687.t1 | nucleolar 6 | 645 | 9.60E-40 | 58.25 | C:nucleus; C:mitochondrion; C:nucleolus; C:condensed nuclear chromosome; F:RNA binding |
| Efet.01.351434.g42.t1 | interferon regulatory factor 2 | 954 | 3.46E-12 | 61.1 | P:cell proliferation; P:transcription, DNA-templated; F:RNA polymerase II regulatory region sequence-specific DNA binding; P:regulation of transcription, DNA-templated; P:transcription from RNA polymerase II promoter; F:regulatory region DNA binding; F:transcriptional activator activity, RNA polymerase II transcription regulatory region sequence-specific binding; P:positive regulation of transcription from RNA polymerase II promoter; C:nucleoplasm; F:DNA binding; C:nucleus; F:transcription factor activity, sequence-specific DNA binding; C:focal adhesion; C:cytoplasm |
| Efet.01.351450.g43.t1 | histone-lysine N-methyltransferase SETD1A | 594 | 3.23E-14 | 60.05 |  |
| Efet.01.351507.g44.t1 | 2-hydroxyacylsphingosine 1-beta-galactosyltransferase-like | 1017 | 5.46E-44 | 49.3 | F:transferase activity |
| Efet.01.351600.g46.t1 | unconventional myosin-Ie- | 351 | 2.87E-51 | 87.75 | C:myosin complex; F:ATP binding; F:actin binding; F:motor activity |
| Efet.01.351623.g48.t1 | kinase C delta type-like isoform X1 | 621 | 8.49E-47 | 61.75 | F:kinase activity |
| Efet.01.351649.g49.t1 | glypican-6-like isoform X1 | 567 | 3.06E-57 | 70 | C:membrane |
| Efet.01.351721.g50.t1 | constitutive coactivator of PPAR-gamma 1 homolog isoform X1 | 732 | 6.31E-46 | 57.25 | C:nucleus |
| Efet.01.35173.g688.t1 | heterogeneous nuclear ribonucleo H-like | 351 | 6.50E-23 | 67.85 | F:organic cyclic compound binding; F:heterocyclic compound binding |
| Efet.01.351798.g54.t1 | bacitracin ABC transporter | 528 | 4.21E-41 | 72.65 | C:membrane |
| Efet.01.351847.g55.t1 | coiled-coil domain-containing 78-like isoform X1 | 402 | 1.83E-24 | 67.95 | F:binding |
| Efet.01.351983.g58.t1 | translin-associated X-like | 342 | 2.19E-37 | 75.15 | F:sequence-specific DNA binding |
| Efet.01.35201.g689.t1 | mRNA decay activator ZFP36L2-A-like | 870 | 2.91E-34 | 70.1 | F:metal ion binding |
| Efet.01.352075.g61.t1 | probable E3 ubiquitin- ligase HERC1 | 342 | 1.31E-10 | 62.75 | F:ubiquitin-protein transferase activity; F:ligase activity; P:protein ubiquitination |
| Efet.01.352234.g65.t1 | bile acid receptor isoform X5 | 324 | 4.17E-09 | 66 | P:regulation of cellular process; F:binding; P:cellular process |
| Efet.01.352247.g66.t1 | proton-coupled folate transporter-like | 891 | 4.05E-07 | 53 |  |
| Efet.01.35225.g691.t1 | methyltransferase 22 | 264 | 1.07E-16 | 60.35 |  |
| Efet.01.35238.g692.t1 | tyrosine sulfotransferase | 204 | 5.92E-07 | 67.8 | F:transferase activity |
| Efet.01.352391.g68.t1 | nuclear factor erythroid 2-related factor 1-like isoform X1 | 915 | 1.12E-67 | 65.85 | P:positive regulation of transcription, DNA-templated; F:sequence-specific DNA binding; C:intracellular part; P:regulation of transcription from RNA polymerase II promoter |
| Efet.01.35241.g693.t1 | SGT1 homolog | 237 | 4.80E-30 | 91 | C:nucleus; P:positive regulation by host of symbiont catalytic activity; P:regulation of protein stability; C:cytoplasm; C:protein complex |
| Efet.01.352477.g69.t1 | hypothetical protein CAPTEDRAFT_218417 | 1380 | 2.49E-67 | 43.94 | C:membrane; C:integral component of membrane |
| Efet.01.352616.g72.t1 | MAP kinase-activating death domain | 660 | 1.68E-15 | 84.17 | P:phosphorylation; F:kinase activity |
| Efet.01.352630.g73.t1 | Kyphoscoliosis peptidase | 1569 | 3.33E-136 | 49.65 |  |
| Efet.01.352643.g74.t1 | Serine threonine- phosphatase BSU1 | 282 | 1.00E-41 | 83.7 | F:phosphoprotein phosphatase activity; F:calcium ion binding; P:protein dephosphorylation |
| Efet.01.35276.g694.t1 | suppressor of lurcher 1-like | 246 | 1.73E-18 | 71.35 |  |
| Efet.01.35281.g695.t1 | follistatin-related 5-like | 636 | 1.87E-21 | 47.6 | F:calcium ion binding |
| Efet.01.352825.g76.t1 | nesprin-1 isoform G | 798 | 2.59E-47 | 52.35 | C:nuclear envelope; F:actin filament binding; C:membrane; C:integral component of membrane; P:cytoskeletal anchoring at nuclear membrane; C:LINC complex |
| Efet.01.352843.g77.t1 | probable E3 ubiquitin- ligase HECTD4 | 699 | 1.02E-60 | 66.55 | F:ubiquitin-protein transferase activity; F:ligase activity; P:protein ubiquitination |
| Efet.01.352855.g78.t1 | acetylcholine receptor subunit beta-type unc-29 | 1320 | 3.45E-72 | 53.8 | P:cell communication; C:membrane; P:single organism signaling; P:transport |
| Efet.01.352960.g80.t1 | suppressor of lurcher 1-like | 696 | 2.95E-64 | 59.2 | C:membrane; C:integral component of membrane |
| Efet.01.353008.g81.t1 | acetylcholine receptor subunit beta-like 1 | 243 | 1.52E-20 | 85 | P:chemical synaptic transmission, postsynaptic; P:synaptic transmission, cholinergic; F:acetylcholine receptor activity; P:neuromuscular synaptic transmission; C:postsynaptic membrane; P:cation transmembrane transport; C:cell junction; F:extracellular ligand-gated ion channel activity; F:acetylcholine binding; C:acetylcholine-gated channel complex; P:response to nicotine |
| Efet.01.353129.g82.t1 | chondroitin sulfate proteoglycan 4-like | 1902 | 5.74E-83 | 47.25 | C:integral component of plasma membrane; C:membrane; C:integral component of membrane; P:cell migration |
| Efet.01.353129.g83.t1 | chondroitin sulfate proteoglycan 4-like | 915 | 1.54E-47 | 52.4 | C:membrane; C:integral component of membrane |
| Efet.01.353158.g85.t1 | zinc finger homeobox 4-like isoform X1 | 603 | 1.84E-39 | 57.65 | F:binding |
| Efet.01.353188.g86.t1 | tripartite motif-containing 2-like | 1950 | 6.84E-21 | 49.7 | F:zinc ion binding; P:signal transduction; P:ion transmembrane transport; F:GABA-A receptor activity; F:extracellular ligand-gated ion channel activity; C:GABA-A receptor complex; C:synapse; P:transport; C:intracellular; C:plasma membrane; P:ion transport; F:metal ion binding; C:membrane; C:integral component of membrane; C:cell junction |
| Efet.01.35319.g696.t1 | titin-like isoform X5 | 6387 | 2.90E-22 | 57 |  |
| Efet.01.353192.g87.t1 | mitogen-activated kinase kinase kinase 11-like isoform X1 | 387 | 2.74E-29 | 76.8 | F:ATP binding; F:protein serine/threonine kinase activity; P:protein phosphorylation |
| Efet.01.353199.g88.t1 | inactive tyrosine- kinase 7-like | 288 | 3.11E-16 | 60.1 | P:peptidyl-tyrosine phosphorylation; F:protein tyrosine kinase activity; P:regulation of cellular process; P:single-organism cellular process; C:membrane part; F:binding; P:response to stimulus |
| Efet.01.353207.g89.t1 | cyclic nucleotide-gated olfactory channel | 285 | 3.76E-32 | 76.55 | F:ion channel activity; P:ion transmembrane transport; C:integral component of membrane |
| Efet.01.353298.g93.t1 | ABC transporter permease | 780 | 3.79E-157 | 87.55 | C:integral component of membrane; P:transport; C:plasma membrane |
| Efet.01.353298.g94.t1 | ABC transporter permease | 852 | 1.99E-161 | 93.25 | C:integral component of membrane; P:transport; C:plasma membrane |
| Efet.01.353298.g95.t1 | family transcriptional regulator | 672 | 2.74E-123 | 90.45 | F:DNA binding; F:transcription factor activity, sequence-specific DNA binding; P:transcription, DNA-templated; P:regulation of transcription, DNA-templated |
| Efet.01.353298.g96.t1 | Asp Glu racemase | 753 | 3.92E-110 | 86.65 | F:racemase activity, acting on amino acids and derivatives; P:nitrogen compound metabolic process |
| Efet.01.353298.g97.t1 | death-on-curing | 321 | 3.90E-66 | 83.15 | P:phosphorylation; F:kinase activity |
| Efet.01.353298.g98.t1 | endonuclease III | 450 | 4.10E-103 | 89.1 | F:DNA binding; P:nucleic acid phosphodiester bond hydrolysis; P:base-excision repair; F:metal ion binding; F:DNA-(apurinic or apyrimidinic site) lyase activity; F:DNA N-glycosylase activity; F:4 iron, 4 sulfur cluster binding |
| Efet.01.353333.g99.t1 | AP-3 complex subunit beta-2 | 240 | 9.18E-23 | 69.85 | C:membrane coat; P:protein transport |
| Efet.01.353382.g100.t1 | ubiquitin carboxyl-terminal hydrolase 31 | 966 | 7.42E-65 | 78.8 | P:protein deubiquitination; F:thiol-dependent ubiquitinyl hydrolase activity; P:ubiquitin-dependent protein catabolic process |
| Efet.01.35339.g697.t1 | ATP-dependent helicase brm isoform X1 | 213 | 1.59E-25 | 91.7 | F:ATPase activity; C:SWI/SNF complex; F:ATP binding; F:histone binding; P:ATP-dependent chromatin remodeling; P:regulation of transcription, DNA-templated |
| Efet.01.353580.g102.t1 | MLP1 homolog isoform X1 | 480 | 1.21E-11 | 63.55 | C:extracellular space; F:catalytic activity; P:regulation of autophagy |
| Efet.01.353580.g103.t1 | Coiled-coil domain-containing 165 | 489 | 7.27E-11 | 58.7 |  |
| Efet.01.353580.g104.t1 | golgin subfamily A member 4-like isoform X2 | 606 | 6.16E-18 | 51.75 | C:extracellular space; P:regulation of autophagy |
| Efet.01.353591.g105.t1 | kinesin KIF20A | 843 | 7.44E-14 | 61 | F:nucleotide binding; F:ATP binding; F:microtubule motor activity; P:microtubule-based movement; F:microtubule binding; C:microtubule |
| Efet.01.353605.g106.t1 | regulator of G- signaling 22-like isoform X1 | 801 | 2.22E-31 | 71.95 | P:biological regulation; C:cytoplasm; F:GTPase activator activity; C:plasma membrane |
| Efet.01.353655.g107.t1 | lysine-specific demethylase 2A-like isoform X2 | 510 | 4.89E-19 | 88.4 | F:DNA binding; F:zinc ion binding; F:iron ion binding; F:methyltransferase activity; P:methylation |
| Efet.01.353701.g108.t1 | zinc finger basonuclin-1-like isoform X2 | 2817 | 6.64E-110 | 68.05 | F:nucleic acid binding; F:metal ion binding |
| Efet.01.353784.g109.t1 | voltage sensitive sodium channel | 309 | 9.11E-32 | 67.45 | F:ion channel activity; P:ion transport; P:transmembrane transport; C:membrane |
| Efet.01.353798.g110.t1 | capicua homolog isoform X1 | 819 | 7.86E-51 | 47.6 | C:transcription factor TFIIA complex; P:transcription initiation from RNA polymerase II promoter |
| Efet.01.353816.g111.t1 | hypothetical protein HELRODRAFT_159817 | 600 | 1.98E-08 | 72 | C:nucleus; F:protein heterodimerization activity; P:DNA-templated transcription, initiation |
| Efet.01.353892.g112.t1 | Ceramide glucosyltransferase | 243 | 1.26E-10 | 62.85 | F:transferase activity, transferring glycosyl groups; P:glucosylceramide biosynthetic process; F:ceramide glucosyltransferase activity; C:membrane; C:integral component of membrane; F:transferase activity |
| Efet.01.3539.g303.t1 | hypothetical protein CAPTEDRAFT_209614 | 408 | 9.83E-22 | 62 |  |
| Efet.01.353900.g113.t1 | von Willebrand factor D and EGF domain-containing | 882 | 2.57E-11 | 49.4 | P:carbohydrate metabolic process; F:calcium ion binding; C:membrane; C:integral component of membrane; F:carbohydrate binding; F:hydrolase activity, hydrolyzing O-glycosyl compounds |
| Efet.01.354022.g117.t1 | hypothetical protein CAPTEDRAFT_226428 | 570 | 3.04E-20 | 52.5 | F:calcium ion binding; C:membrane; C:integral component of membrane |
| Efet.01.354027.g118.t1 | fatty acyl- hydrolase medium chain- | 279 | 1.93E-20 | 52.45 | P:metabolic process; C:membrane; C:integral component of membrane; F:hydrolase activity |
| Efet.01.354041.g121.t1 | Long-chain fatty acid transport | 447 | 4.30E-42 | 68.95 | F:catalytic activity |
| Efet.01.354314.g123.t1 | dimethylaniline monooxygenase [N-oxide-forming] 5-like | 573 | 8.57E-33 | 85.6 | C:endoplasmic reticulum membrane; F:NADP binding; F:flavin adenine dinucleotide binding; C:integral component of membrane; P:drug metabolic process; P:oxidation-reduction process; C:organelle membrane; F:N,N-dimethylaniline monooxygenase activity |
| Efet.01.354363.g124.t1 | allatostatin-A receptor-like isoform X1 | 1254 | 1.10E-68 | 59.7 | F:G-protein coupled receptor activity; P:G-protein coupled receptor signaling pathway; P:signal transduction; C:membrane; C:integral component of membrane; F:signal transducer activity |
| Efet.01.354377.g125.t1 | stAR-related lipid transfer 13 isoform X1 | 225 | 1.85E-29 | 80.1 | P:endothelial tube lumen extension; P:signal transduction; F:lipid binding; P:endothelial cell migration; P:negative regulation of cell migration involved in sprouting angiogenesis |
| Efet.01.354441.g126.t1 | malignant fibrous histiocytoma-amplified sequence 1-like isoform X2 | 1155 | 6.49E-64 | 48.6 | F:GTP binding; C:cytoplasm; P:small GTPase mediated signal transduction; P:apoptotic process; F:cysteine-type endopeptidase activity involved in apoptotic process |
| Efet.01.354456.g127.t1 | extracellular signal-regulated kinase 2-like isoform X2 | 282 | 3.03E-38 | 94 | F:ATP binding; P:MAPK cascade; F:MAP kinase activity; C:intracellular |
| Efet.01.354504.g128.t1 | Low-density lipo receptor-related 4 | 477 | 9.29E-46 | 54.55 | C:membrane |
| Efet.01.354513.g129.t1 | neurogenic differentiation factor 4-like [Orbicella faveolata] | 432 | 6.92E-16 | 71.75 | F:protein dimerization activity |
| Efet.01.354562.g131.t1 | probable G- coupled receptor | 1158 | 3.42E-63 | 55.95 | F:G-protein coupled receptor activity; F:G-protein coupled peptide receptor activity; P:G-protein coupled receptor signaling pathway; C:membrane; C:integral component of membrane |
| Efet.01.354564.g132.t1 | prickle 1 | 1806 | 3.26E-38 | 82.5 | F:zinc ion binding; P:neuron projection development; P:determination of digestive tract left/right asymmetry; P:cell migration in hindbrain; P:convergent extension involved in gastrulation; C:cell; P:establishment of mitotic spindle orientation; P:neuron migration; P:cell migration involved in gastrulation; P:neural retina development; P:Kupffer's vesicle development; P:heart looping; P:Wnt signaling pathway; P:intrahepatic bile duct development; P:determination of liver left/right asymmetry |
| Efet.01.354576.g133.t1 | Atrial natriuretic peptide receptor 1 | 393 | 3.64E-15 | 54.55 | F:catalytic activity; P:cellular process |
| Efet.01.354578.g134.t1 | fumarylacetoacetate hydrolase | 393 | 6.88E-72 | 81.05 | P:metabolic process; F:hydrolase activity |
| Efet.01.354578.g135.t1 | family transcriptional regulator | 1158 | 0 | 73.3 | F:DNA binding; F:transcription factor activity, sequence-specific DNA binding; P:transcription, DNA-templated; P:regulation of transcription, DNA-templated |
| Efet.01.354737.g136.t1 | microtubule-associated serine threonine- kinase 3 isoform X2 | 513 | 2.23E-39 | 61.57 | C:membrane; C:integral component of membrane; P:phosphorylation; F:kinase activity |
| Efet.01.354842.g138.t1 | tudor domain-containing 1 | 414 | 5.07E-12 | 53.45 | F:metal ion binding |
| Efet.01.354879.g139.t1 | otoferlin-like isoform X2 | 342 | 7.83E-30 | 84.1 | C:integral component of membrane |
| Efet.01.354962.g140.t1 | FMRFamide receptor-like | 1245 | 6.12E-74 | 56.45 | F:G-protein coupled receptor activity; P:signal transduction; C:membrane |
| Efet.01.355061.g144.t1 | tyrosine phosphatase type IVA 1 | 417 | 8.28E-28 | 75.95 | P:protein dephosphorylation; F:protein tyrosine/serine/threonine phosphatase activity |
| Efet.01.355116.g145.t1 | sperm flagellar 2-like isoform X1 | 456 | 8.73E-40 | 62.7 | C:motile cilium |
| Efet.01.355154.g146.t1 | tyrosine- kinase FRK | 459 | 2.52E-65 | 76 | F:ATP binding; P:peptidyl-tyrosine phosphorylation; F:non-membrane spanning protein tyrosine kinase activity |
| Efet.01.355250.g147.t1 | adhesin | 741 | 1.39E-97 | 67.4 |  |
| Efet.01.35526.g700.t1 | epidermal growth factor receptor-like isoform X1 | 435 | 9.67E-21 | 61.05 | F:protein kinase activity; P:phosphorylation |
| Efet.01.355263.g148.t1 | phosphatase 1 regulatory subunit 16A-like | 294 | 2.02E-24 | 76.45 | F:hydrolase activity |
| Efet.01.35528.g702.t1 | glyco 3-alpha-L-fucosyltransferase A isoform X1 | 732 | 4.13E-68 | 61.85 | F:transferase activity |
| Efet.01.35531.g703.t1 | gastrula zinc finger -like | 1137 | 3.60E-25 | 44.1 | F:nucleic acid binding; C:nucleus; F:metal ion binding; P:regulation of transcription, DNA-templated |
| Efet.01.35531.g704.t1 | zinc finger | 1062 | 8.07E-23 | 54.3 | F:nucleic acid binding; F:metal ion binding |
| Efet.01.355453.g149.t1 | Poly [ADP-ribose] polymerase 4 | 396 | 4.41E-40 | 67.45 | P:cellular macromolecule metabolic process; C:membrane; F:transferase activity; P:primary metabolic process |
| Efet.01.355459.g150.t1 | pre-mRNA-splicing factor ATP-dependent RNA helicase DHX16 | 738 | 1.30E-19 | 67 | F:organic cyclic compound binding; F:heterocyclic compound binding; P:cellular process; F:hydrolase activity |
| Efet.01.355500.g152.t1 | Inositol phosphoceramide mannosyltransferase 3 | 726 | 4.85E-13 | 55.35 | C:membrane; C:integral component of membrane |
| Efet.01.355537.g154.t1 | hypothetical protein PV09_03562 [Verruconis gallopava] | 1734 | 5.33E-06 | 72 | F:zinc ion binding; P:regulation of chromatin silencing at silent mating-type cassette; P:regulation of transcription, DNA-templated; P:regulation of chromatin silencing at centromere; C:mitotic spindle pole body; P:chromatin silencing at centromere outer repeat region; P:regulation of chromatin assembly or disassembly; F:RNA polymerase II transcription factor activity, sequence-specific DNA binding; C:nuclear heterochromatin; P:regulation of transcription from RNA polymerase II promoter; C:nucleus; C:chromosome, centromeric outer repeat region; P:chromatin remodeling; F:metal ion binding; P:regulation of extent of heterochromatin assembly; P:negative regulation of extent of heterochromatin assembly |
| Efet.01.355548.g155.t1 | UDP-glucuronosyltransferase 2C1-like | 1467 | 4.80E-88 | 54.2 | F:transferase activity |
| Efet.01.355570.g156.t1 | Brain-derived neurotrophic factor | 438 | 9.24E-43 | 60.25 | C:integral component of membrane; F:receptor binding |
| Efet.01.355588.g158.t1 | Conserved oligomeric Golgi complex subunit 5 | 510 | 7.73E-35 | 74.65 | C:Golgi transport complex; P:intra-Golgi vesicle-mediated transport |
| Efet.01.355625.g160.t1 | probable cytochrome P450 49a1 isoform X1 | 342 | 5.54E-41 | 53 | F:oxidoreductase activity; F:metal ion binding |
| Efet.01.355709.g161.t1 | centromere kinetochore zw10 homolog | 204 | 2.85E-22 | 70.95 | C:nucleus; P:mitotic nuclear division; C:chromosome, centromeric region |
| Efet.01.355738.g162.t1 | dynein heavy chain axonemal-like | 387 | 9.09E-76 | 93.85 | F:ATPase activity; F:ATP binding; F:microtubule motor activity; C:dynein complex; P:microtubule-based movement |
| Efet.01.355783.g163.t1 | leucine-rich repeat neuronal 3-like | 1935 | 2.35E-78 | 48.4 | F:phosphoprotein phosphatase activity; P:axonogenesis; C:membrane; P:protein dephosphorylation; C:integral component of membrane; F:hydrolase activity |
| Efet.01.355840.g164.t1 | nuclear migration nudC isoform X2 | 261 | 1.14E-32 | 91 |  |
| Efet.01.355886.g165.t1 | phosphatase 1 regulatory subunit 15A | 249 | 6.70E-11 | 61.5 |  |
| Efet.01.356039.g166.t1 | telomerase reverse transcriptase | 3486 | 4.38E-68 | 50.9 | C:transferase complex, transferring phosphorus-containing groups; P:regulation of biological quality; P:positive regulation of metabolic process; P:negative regulation of cellular process; P:organelle organization; C:chromosome, telomeric region; P:negative regulation of gene expression; F:RNA-directed DNA polymerase activity; P:RNA metabolic process; F:RNA binding; F:protein binding; C:intracellular ribonucleoprotein complex; P:positive regulation of cellular process; C:cytoplasm; P:negative regulation of response to stimulus; C:nuclear lumen; P:DNA biosynthetic process |
| Efet.01.35606.g705.t1 | anoctamin-1-like isoform X10 | 834 | 3.09E-41 | 69.4 | C:integral component of membrane; F:protein dimerization activity |
| Efet.01.356087.g167.t1 | hypothetical protein HELRODRAFT_183986 | 2637 | 9.49E-06 | 53 |  |
| Efet.01.356095.g168.t1 | solute carrier organic anion transporter family member 5A1-like isoform X1 | 408 | 4.10E-15 | 49.15 | C:membrane |
| Efet.01.356144.g170.t1 | 39S ribosomal mitochondrial | 600 | 3.25E-74 | 61 | C:intracellular organelle; C:cytoplasmic part |
| Efet.01.356215.g172.t1 | hypothetical protein CAPTEDRAFT_197722 | 297 | 2.92E-13 | 62 | C:membrane; C:integral component of membrane |
| Efet.01.35625.g707.t1 | cell number regulator 10-like | 327 | 1.02E-37 | 68.65 | C:membrane |
| Efet.01.356276.g174.t1 | adiponectin receptor 1 | 360 | 1.96E-17 | 71.75 | F:hormone binding; P:adiponectin-activated signaling pathway; C:plasma membrane |
| Efet.01.356286.g175.t1 | ACHA3_CHICK ame: Full=Neuronal acetylcholine receptor subunit alpha-3 Flags: Precursor | 294 | 2.43E-07 | 62 | P:ion transport; F:protein binding; P:neuromuscular synaptic transmission; F:transmembrane receptor activity; C:integral component of membrane; P:response to nicotine; C:plasma membrane part |
| Efet.01.356310.g176.t1 | protocadherin gamma-C5- | 465 | 6.90E-06 | 52 |  |
| Efet.01.356320.g177.t1 | gastrula zinc finger -like isoform X2 | 741 | 1.34E-58 | 61.3 | F:nucleic acid binding; F:metal ion binding |
| Efet.01.356392.g179.t1 | neuroglian-like isoform X3 | 309 | 6.83E-11 | 59 | C:membrane; C:integral component of membrane |
| Efet.01.356415.g180.t1 | sodium channel para-like isoform X1 | 693 | 5.74E-48 | 70.35 | P:biological regulation; P:sodium ion transport; P:ion transmembrane transport; F:sodium channel activity; C:integral component of membrane; F:voltage-gated ion channel activity |
| Efet.01.356458.g182.t1 | cGMP-specific 3 ,5 -cyclic phosphodiesterase isoform X1 | 891 | 1.68E-45 | 60 | F:metal ion binding; P:signal transduction; F:phosphoric diester hydrolase activity; F:3',5'-cyclic-nucleotide phosphodiesterase activity; F:hydrolase activity |
| Efet.01.356495.g183.t1 | dynein heavy chain axonemal-like isoform X2 | 573 | 1.35E-60 | 67.1 | F:nucleotide binding; F:ATPase activity; F:ATP binding; F:microtubule motor activity; C:axonemal dynein complex; C:dynein complex; P:cilium movement; P:microtubule-based movement |
| Efet.01.356551.g184.t1 | lipoyltransferase mitochondrial | 450 | 4.79E-50 | 72 | F:transferase activity, transferring acyl groups other than amino-acyl groups; C:cytoplasm; P:protein lipoylation |
| Efet.01.35659.g710.t1 | dynein assembly factor with WDR repeat domains 1-like | 447 | 9.82E-54 | 72.6 | P:heart development; P:determination of left/right symmetry; P:response to stimulus |
| Efet.01.356609.g185.t1 | phosphorylase b kinase gamma catalytic skeletal muscle heart isoform | 465 | 1.15E-20 | 62.95 | F:kinase activity |
| Efet.01.356647.g186.t1 | 39S ribosomal mitochondrial | 417 | 1.26E-52 | 76.35 | F:structural constituent of ribosome |
| Efet.01.35665.g711.t1 | protocadherin beta-16-like | 1119 | 1.94E-40 | 50.6 | F:calcium ion binding; C:membrane; C:integral component of membrane; P:cell adhesion; P:homophilic cell adhesion via plasma membrane adhesion molecules; C:plasma membrane |
| Efet.01.356683.g187.t1 | mitochondrial intermediate peptidase-like | 1095 | 8.93E-142 | 71.3 | F:metallopeptidase activity |
| Efet.01.356741.g189.t1 | acid-sensing ion channel 1C-like isoform X2 | 441 | 3.53E-06 | 50 |  |
| Efet.01.356750.g191.t1 | F-box only 9-like | 495 | 1.89E-23 | 69.65 | C:membrane; C:integral component of membrane |
| Efet.01.356784.g192.t1 | hypothetical protein CAPTEDRAFT_227502 | 657 | 3.43E-06 | 65 | P:cell proliferation; F:SUMO-specific isopeptidase activity; F:SUMO binding; P:protein desumoylation; C:Cajal body; P:Cajal body organization |
| Efet.01.356924.g194.t1 | calmodulin-A-like isoform X1 | 354 | 4.07E-22 | 65.45 | F:calcium ion binding |
| Efet.01.356975.g196.t1 | hypothetical protein HELRODRAFT_191274 | 249 | 8.57E-19 | 62.95 | C:membrane; C:integral component of membrane; F:carbohydrate binding |
| Efet.01.357018.g197.t1 | DNA-binding inhibitor ID-2 | 435 | 3.96E-18 | 71.45 | P:prostate gland epithelium morphogenesis; P:endodermal digestive tract morphogenesis; P:cellular senescence; P:negative regulation of neuron differentiation; P:negative regulation of astrocyte differentiation; P:embryonic digestive tract morphogenesis; P:fat cell differentiation; P:regulation of circadian rhythm; P:positive regulation of osteoblast differentiation; P:positive regulation of transcription from RNA polymerase II promoter; P:prostate gland stromal morphogenesis; P:negative regulation of oligodendrocyte differentiation; F:bHLH transcription factor binding; P:cerebral cortex neuron differentiation; P:negative regulation of transcription from RNA polymerase II promoter; P:positive regulation of smooth muscle cell proliferation; P:central nervous system myelination; P:neuroblast proliferation; P:neuron fate commitment; P:G1/S transition of mitotic cell cycle; P:negative regulation of neural precursor cell proliferation; P:positive regulation of cell cycle arrest; P:transcription, DNA-templated; P:seminal vesicle morphogenesis; P:negative regulation of fat cell differentiation; F:protein dimerization activity; P:hippocampus development; C:protein complex; P:circadian rhythm; P:positive regulation of blood pressure; C:nucleus; C:cytoplasm; F:RNA polymerase II transcription factor binding; P:cellular protein localization |
| Efet.01.357075.g199.t1 | E3 ubiquitin- ligase TRIM56-like | 1131 | 7.61E-29 | 47 | F:zinc ion binding; F:metal ion binding; C:intracellular |
| Efet.01.357077.g200.t1 | Plasma alpha-L-fucosidase | 381 | 9.03E-47 | 76.85 | F:alpha-L-fucosidase activity; P:fucose metabolic process |
| Efet.01.357087.g201.t1 | visinin-like | 249 | 2.74E-16 | 60.85 | P:regulation of cellular process; C:intracellular part |
| Efet.01.35715.g713.t1 | arrestin domain-containing 3-like | 732 | 1.48E-41 | 52.3 | P:signal transduction |
| Efet.01.35722.g714.t1 | hypothetical protein CAPTEDRAFT_194437 | 324 | 1.16E-10 | 76.33 |  |
| Efet.01.35723.g715.t1 | niemann-Pick C1 -like | 294 | 2.66E-18 | 62.55 | P:lipid transport; F:lipid transporter activity; F:calcium ion binding; C:membrane; C:integral component of membrane |
| Efet.01.35723.g716.t1 | Niemann-Pick C1 -like | 828 | 1.12E-64 | 54.1 | P:single-organism process |
| Efet.01.357282.g204.t1 | hypothetical protein CAPTEDRAFT_219540 | 1299 | 7.76E-140 | 62.1 |  |
| Efet.01.357317.g207.t1 | TAR DNA-binding 43-like | 735 | 1.38E-58 | 83.7 | P:negative regulation by host of viral transcription; F:identical protein binding; P:RNA splicing; P:3'-UTR-mediated mRNA stabilization; C:nuclear speck; F:double-stranded DNA binding; P:transcription from RNA polymerase II promoter; C:interchromatin granule; F:transcriptional activator activity, RNA polymerase II distal enhancer sequence-specific binding; P:mRNA processing; P:positive regulation of transcription from RNA polymerase II promoter; F:mRNA 3'-UTR binding; F:nucleotide binding; C:perichromatin fibrils; C:cytoplasm; P:negative regulation of protein phosphorylation; C:integral component of membrane; P:nuclear fragmentation involved in apoptotic nuclear change; P:nuclear inner membrane organization; P:positive regulation of insulin secretion; P:regulation of cell cycle; P:response to endoplasmic reticulum stress |
| Efet.01.357335.g208.t1 | Na(+) H(+) exchange regulatory cofactor NHE-RF1-like | 516 | 2.31E-53 | 79.3 |  |
| Efet.01.357349.g209.t1 | serine threonine- kinase NIM1-like | 279 | 7.84E-23 | 83.8 | F:magnesium ion binding; C:nucleus; F:ATP binding; C:cytoplasm; C:integral component of membrane; F:protein serine/threonine kinase activity; P:protein phosphorylation; P:intracellular signal transduction |
| Efet.01.357630.g211.t1 | low-density lipo receptor-related 12 | 837 | 1.28E-20 | 52.71 | C:membrane; C:integral component of membrane |
| Efet.01.357662.g213.t1 | 1-phosphatidylinositol-4,5-bisphosphate phosphodiesterase classes I and | 291 | 2.36E-07 | 75.67 | P:single-organism process |
| Efet.01.357665.g214.t1 | transcription factor E2F7 | 522 | 4.46E-11 | 61.45 | P:system development; P:regulation of transcription, DNA-templated; P:single-organism cellular process; P:negative regulation of cellular process; F:binding |
| Efet.01.357773.g217.t1 | RING finger 150-like | 555 | 1.46E-35 | 59.5 | F:zinc ion binding; F:metal ion binding; C:membrane; C:integral component of membrane |
| Efet.01.35780.g719.t1 | ATP-binding cassette sub-family C member 9-like | 771 | 1.88E-28 | 52.2 | F:transmembrane transporter activity; F:catalytic activity; P:transport |
| Efet.01.357824.g219.t1 | microtubule-actin cross-linking factor 1 | 660 | 1.40E-16 | 54.2 | C:cytoskeleton |
| Efet.01.357831.g220.t1 | hypothetical protein A0U90_03040 | 231 | 6.05E-06 | 54 |  |
| Efet.01.357932.g223.t1 | FMRFamide receptor-like | 1269 | 1.23E-51 | 50.45 | F:G-protein coupled receptor activity; F:G-protein coupled peptide receptor activity; P:G-protein coupled receptor signaling pathway; P:signal transduction; C:membrane; C:integral component of membrane; F:signal transducer activity |
| Efet.01.35804.g720.t1 | FACT complex subunit spt16 isoform X1 | 258 | 2.76E-23 | 82 |  |
| Efet.01.358041.g224.t1 | bone morphogenetic 10 | 831 | 8.83E-26 | 68.6 | P:positive regulation of cardiac muscle cell proliferation; P:positive regulation of cartilage development; P:ventricular cardiac muscle tissue morphogenesis; P:regulation of transcription, DNA-templated; C:extracellular region; P:pathway-restricted SMAD protein phosphorylation; F:receptor binding; P:cardiac muscle cell proliferation; P:heart trabecula formation; P:adult heart development; C:cytoplasm; P:negative regulation of cardiac muscle hypertrophy; P:positive regulation of gene expression; P:ventricular cardiac muscle cell development; P:positive regulation of cell proliferation involved in heart morphogenesis; P:positive regulation of pathway-restricted SMAD protein phosphorylation; P:negative regulation of cell migration; P:regulation of cellular component organization; P:atrial cardiac muscle tissue morphogenesis |
| Efet.01.35808.g721.t1 | succinate dehydrogenase [ubiquinone] flavo mitochondrial-like | 303 | 2.90E-57 | 92.2 | P:anaerobic respiration; C:mitochondrial respiratory chain complex II, succinate dehydrogenase complex (ubiquinone); F:succinate dehydrogenase (ubiquinone) activity; F:flavin adenine dinucleotide binding; F:electron carrier activity; P:tricarboxylic acid cycle; P:mitochondrial electron transport, succinate to ubiquinone; P:succinate metabolic process; P:transport |
| Efet.01.358091.g225.t1 | sodium:proton antiporter | 825 | 3.71E-108 | 85.6 | F:ATPase activity; F:ATP binding; F:metal ion binding; F:iron-sulfur cluster binding |
| Efet.01.358120.g227.t1 | nesprin-1-like isoform X5 | 354 | 1.68E-17 | 63.6 |  |
| Efet.01.358124.g229.t1 | 1,4-alpha-glucan-branching enzyme | 411 | 1.03E-73 | 88.3 | P:G-protein coupled receptor signaling pathway; F:aminopeptidase activity; F:cation binding; P:cell surface receptor signaling pathway; C:ribosome; F:hydrolase activity, hydrolyzing O-glycosyl compounds; P:translation; F:G-protein coupled receptor activity; P:glycogen biosynthetic process; F:1,4-alpha-glucan branching enzyme activity; F:structural constituent of ribosome; P:proteolysis; C:integral component of membrane |
| Efet.01.35822.g722.t1 | SSUH2 homolog | 381 | 9.96E-22 | 70.95 | F:heat shock protein binding; F:unfolded protein binding |
| Efet.01.3584.g307.t1 | hypothetical protein CAPTEDRAFT_199139 | 312 | 6.96E-21 | 71.45 | C:membrane |
| Efet.01.358491.g235.t1 | polycystic kidney disease 2-like 1 isoform X1 | 294 | 3.90E-18 | 73.5 | C:membrane |
| Efet.01.358609.g238.t1 | PDZ DHR GLGF domain | 1572 | 3.10E-34 | 71.15 | C:membrane; P:Wnt signaling pathway |
| Efet.01.358613.g239.t1 | bactericidal permeability-increasing -like | 1605 | 3.72E-25 | 45.15 | C:extracellular space; F:lipid binding |
| Efet.01.35862.g723.t1 | slowpoke potassium channel family member SLO-1 | 261 | 1.07E-35 | 83.65 | F:voltage-gated potassium channel activity; P:potassium ion transmembrane transport; C:voltage-gated potassium channel complex; F:large conductance calcium-activated potassium channel activity |
| Efet.01.358680.g242.t1 | U2 snRNP-associated SURP motif-containing -like | 435 | 5.15E-12 | 69.25 | F:nucleotide binding; F:RNA binding; P:RNA processing |
| Efet.01.358688.g243.t1 | kinase C epsilon type-like | 240 | 8.56E-26 | 67.5 | F:nucleotide binding; F:ion binding; P:phosphorylation; F:protein serine/threonine kinase activity; P:primary metabolic process; P:single-organism process; P:organic substance metabolic process |
| Efet.01.358710.g244.t1 | hypothetical protein CAPTEDRAFT_228532 | 330 | 1.50E-12 | 61 | F:ATP binding; F:protein kinase activity; P:protein phosphorylation |
| Efet.01.358765.g246.t1 | dnaJ homolog subfamily C member 7-like isoform X2 | 498 | 5.39E-61 | 85.25 |  |
| Efet.01.358861.g247.t1 | hypothetical protein CAPTEDRAFT_219915 | 840 | 1.05E-26 | 46.4 | F:thiolester hydrolase activity; P:fatty acid biosynthetic process |
| Efet.01.35889.g725.t1 | hypothetical protein CAPTEDRAFT_226205 | 675 | 2.07E-10 | 63 | F:nucleotide binding; F:ATP binding; F:protein kinase activity; P:protein phosphorylation |
| Efet.01.358935.g248.t1 | Cyclic GMP-AMP synthase | 912 | 9.74E-17 | 53 |  |
| Efet.01.358943.g249.t1 | neuroblastoma-amplified sequence | 522 | 1.66E-59 | 78.85 | C:endoplasmic reticulum |
| Efet.01.358961.g250.t1 | hypothetical protein CAPTEDRAFT_229371 | 1713 | 1.56E-09 | 63 | P:negative regulation of cell migration; P:negative regulation of G2/M transition of mitotic cell cycle |
| Efet.01.359090.g251.t1 | allatostatin-A receptor-like | 795 | 3.06E-58 | 63.75 | F:G-protein coupled receptor activity; P:G-protein coupled receptor signaling pathway; P:signal transduction; C:membrane; C:integral component of membrane; F:signal transducer activity |
| Efet.01.35920.g726.t1 | hypothetical protein HELRODRAFT_166585 | 243 | 5.01E-26 | 68 | C:membrane |
| Efet.01.359266.g254.t1 | histone deacetylase complex subunit SAP30L | 333 | 1.44E-25 | 67.6 | F:DNA binding; C:nucleus; F:metal ion binding; P:transcription, DNA-templated; P:regulation of transcription, DNA-templated |
| Efet.01.359293.g255.t1 | spectrin beta chain-like isoform X1 | 4248 | 0 | 63.4 | F:actin binding; F:structural constituent of cytoskeleton; F:phospholipid binding; C:spectrin |
| Efet.01.35930.g727.t1 | condensin complex subunit 3-like | 552 | 2.91E-29 | 64.55 | C:intracellular part |
| Efet.01.3594.g308.t1 | RNA-binding 40 | 1524 | 1.30E-53 | 63.35 | C:nuclear part; F:RNA binding |
| Efet.01.359441.g257.t1 | probable cation-transporting ATPase 13A3 isoform X2 | 300 | 1.62E-36 | 86.4 | F:ATPase activity; F:ATP binding; P:cation transport; F:metal ion binding; C:integral component of membrane |
| Efet.01.35947.g729.t1 | cAMP-dependent beta-catalytic | 342 | 3.80E-39 | 91.9 | F:ATP binding; F:protein serine/threonine kinase activity; P:protein phosphorylation |
| Efet.01.359526.g258.t1 | POU class transcription factor | 612 | 5.56E-32 | 77.3 | P:multicellular organism development |
| Efet.01.35953.g730.t1 | 26S proteasome non-ATPase regulatory subunit 1-like | 600 | 1.50E-113 | 89.1 | P:regulation of catalytic activity; P:regulation of protein catabolic process; C:proteasome complex; F:enzyme regulator activity |
| Efet.01.359578.g259.t1 | E3 ubiquitin- ligase Bre1-like isoform X1 | 639 | 1.29E-85 | 78.7 | F:zinc ion binding; F:ligase activity |
| Efet.01.359592.g260.t1 | neurexin-1-like isoform X1 | 324 | 1.68E-08 | 57.83 | F:metal ion binding; C:integral component of membrane; P:chemical synaptic transmission |
| Efet.01.359643.g261.t1 | PREDICTED: uncharacterized protein LOC100378520 | 498 | 9.49E-10 | 57.33 | C:membrane; C:integral component of membrane |
| Efet.01.359651.g262.t1 | Kyphoscoliosis peptidase | 492 | 6.43E-63 | 56.65 | F:zinc ion binding |
| Efet.01.359684.g263.t1 | zinc finger 236-like isoform X2 | 639 | 6.66E-20 | 60.25 | F:nucleic acid binding; F:metal ion binding |
| Efet.01.359719.g264.t1 | sarcoplasmic calcium binding isoform 2 | 267 | 2.63E-21 | 81 | F:calcium ion binding |
| Efet.01.359719.g265.t1 | sarcoplasmic calcium-binding -like | 339 | 1.33E-26 | 66.1 | F:calcium ion binding |
| Efet.01.359740.g267.t1 | E3 ubiquitin- ligase TRIM9-like isoform X1 | 927 | 2.05E-51 | 52.45 | F:zinc ion binding; F:metal ion binding; F:ligase activity; C:intracellular |
| Efet.01.359746.g268.t1 | polypeptide N-acetylgalactosaminyltransferase 5-like isoform X1 | 351 | 1.72E-30 | 69 | C:membrane |
| Efet.01.35976.g733.t1 | BLOC-1-related complex subunit 5-like | 663 | 1.87E-68 | 67.95 |  |
| Efet.01.359903.g271.t1 | alpha beta hydrolase domain-containing 17B-like | 684 | 1.16E-63 | 88.15 | F:hydrolase activity |
| Efet.01.359947.g272.t1 | crumbs-like isoform X2 | 642 | 1.16E-11 | 54.56 | F:calcium ion binding; C:membrane; C:integral component of membrane |
| Efet.01.35997.g735.t1 | cytoplasmic dynein 2 heavy chain 1-like | 438 | 6.86E-44 | 74.75 | F:nucleoside-triphosphatase activity |
| Efet.01.35997.g736.t1 | cytoplasmic dynein 2 heavy chain 1-like | 1173 | 6.67E-92 | 86.05 | F:ATPase activity; F:ATP binding; F:microtubule motor activity; C:dynein complex; P:microtubule-based movement |
| Efet.01.360231.g275.t1 | fasciclin-2 isoform X1 | 219 | 2.91E-07 | 59.6 | C:membrane; C:integral component of membrane; P:cell adhesion |
| Efet.01.360271.g276.t1 | hypothetical protein HELRODRAFT_184091 | 249 | 1.14E-17 | 63.4 | C:membrane; C:integral component of membrane |
| Efet.01.360327.g278.t1 | hypothetical protein CAPTEDRAFT_72731, partial | 1038 | 8.95E-45 | 56.7 | C:nucleus; C:cytoplasm; P:signal transduction |
| Efet.01.360349.g279.t1 | 5 -nucleotidase-like | 402 | 3.09E-34 | 72.05 | C:membrane; F:binding; F:hydrolase activity |
| Efet.01.360377.g280.t1 | hypothetical protein HELRODRAFT_180703 | 360 | 4.50E-07 | 57 |  |
| Efet.01.360459.g281.t1 | probable muscarinic acetylcholine receptor gar-2 | 1758 | 8.70E-59 | 61.85 | C:integral component of plasma membrane; F:G-protein coupled acetylcholine receptor activity; P:G-protein coupled acetylcholine receptor signaling pathway |
| Efet.01.360473.g283.t1 | carbohydrate sulfotransferase 3-like | 279 | 8.15E-28 | 71.25 | C:integral component of membrane; F:sulfotransferase activity |
| Efet.01.360588.g284.t1 | bcl-2 1 | 501 | 1.68E-31 | 55.65 | C:membrane; C:integral component of membrane; P:regulation of apoptotic process |
| Efet.01.360655.g288.t1 | Transcription factor SOX- | 993 | 1.34E-37 | 59.9 | F:DNA binding; P:positive regulation of macromolecule metabolic process; P:nervous system development; P:regulation of transcription, DNA-templated; P:animal organ development; P:single-organism cellular process; P:positive regulation of cellular metabolic process; C:intracellular part |
| Efet.01.360703.g289.t1 | EF-hand domain-containing 1 | 246 | 3.85E-18 | 84.3 | F:calcium ion binding |
| Efet.01.360769.g292.t1 | beta-catenin 1 | 456 | 2.48E-48 | 85.55 |  |
| Efet.01.360802.g294.t1 | hypothetical protein CAPTEDRAFT_228121 | 486 | 8.35E-29 | 80 | F:G-protein coupled receptor activity; P:G-protein coupled receptor signaling pathway; C:integral component of membrane |
| Efet.01.361028.g297.t1 | zinc finger homeobox 4-like isoform X1 | 762 | 4.54E-58 | 66.7 | F:nucleic acid binding |
| Efet.01.361028.g298.t1 | zinc finger homeobox 3 isoform X3 | 2211 | 7.49E-70 | 69.45 | F:nucleic acid binding |
| Efet.01.361038.g299.t1 | UDP-galactose translocator-like isoform X3 | 678 | 8.04E-59 | 63.25 | F:UDP-galactose transmembrane transporter activity; P:UDP-galactose transport; P:CMP-N-acetylneuraminate transport; C:membrane; F:UDP-glucuronic acid transmembrane transporter activity; F:UDP-xylose transmembrane transporter activity; F:UDP-N-acetylgalactosamine transmembrane transporter activity; P:galactose transport; P:UDP-N-acetylgalactosamine transport; C:Golgi medial cisterna; F:CMP-N-acetylneuraminate transmembrane transporter activity |
| Efet.01.361240.g300.t1 | 116 kDa U5 small nuclear ribonucleo component-like | 240 | 8.84E-41 | 93.65 | F:GTP binding; F:GTPase activity |
| Efet.01.361298.g301.t1 | sodium-dependent glucose transporter 1A-like | 1137 | 2.26E-36 | 48.39 | P:transmembrane transport; C:membrane; C:integral component of membrane; P:carbohydrate transport |
| Efet.01.361313.g302.t1 | OTU domain-containing 7B-like | 249 | 1.90E-13 | 76.5 | F:thiol-dependent ubiquitin-specific protease activity; C:nucleus; P:protein K11-linked deubiquitination; P:protein deubiquitination involved in ubiquitin-dependent protein catabolic process; P:immune response; C:cytoplasm; F:K63-linked polyubiquitin binding; P:protein K63-linked deubiquitination; P:protein K48-linked deubiquitination |
| Efet.01.361526.g308.t1 | tenascin-like isoform X3 | 297 | 6.20E-10 | 62.95 | P:signal transduction; C:membrane; F:signal transducer activity |
| Efet.01.361627.g310.t1 | FMRFamide receptor | 1098 | 1.87E-83 | 54.1 | F:signal transducer activity |
| Efet.01.361676.g311.t1 | 39S ribosomal mitochondrial | 237 | 3.68E-13 | 71.15 | F:structural constituent of ribosome; C:mitochondrial large ribosomal subunit |
| Efet.01.361705.g312.t1 | phosphatase 1E | 1098 | 3.46E-11 | 62.55 | P:dephosphorylation; F:hydrolase activity |
| Efet.01.361764.g313.t1 | phospholipase A-2-activating | 333 | 8.89E-12 | 57.2 | P:inflammatory response; C:extracellular exosome; P:positive regulation of catalytic activity; P:proteolysis; F:metalloendopeptidase activity; F:phospholipase A2 activator activity; F:metallopeptidase activity |
| Efet.01.361767.g315.t1 | Heparan sulfate glucosamine 3-O-sulfotransferase | 606 | 4.54E-91 | 72.45 | C:membrane; F:transferase activity |
| Efet.01.361770.g316.t1 | A disintegrin and metallo ase with thrombospondin motifs 7-like | 255 | 6.22E-12 | 63.07 | F:zinc ion binding; P:integrin-mediated signaling pathway; P:proteolysis; C:extracellular matrix; F:metalloendopeptidase activity; C:proteinaceous extracellular matrix; F:metallopeptidase activity |
| Efet.01.361799.g317.t1 | staphylococcal nuclease domain-containing 1-like | 264 | 3.06E-40 | 82.75 | F:GTP binding; F:transcription cofactor activity; P:posttranscriptional gene silencing by RNA; P:small GTPase mediated signal transduction; C:membrane; C:RISC complex; P:regulation of nucleic acid-templated transcription |
| Efet.01.361805.g318.t1 | transmembrane 17B-like | 291 | 3.89E-13 | 69.79 | C:membrane |
| Efet.01.361871.g320.t1 | Transmembrane GTPase Marf | 360 | 7.82E-57 | 85.4 | F:GTP binding; F:GTPase activity; P:mitochondrial fusion; P:negative regulation of smooth muscle cell proliferation; C:integral component of membrane; C:mitochondrial outer membrane |
| Efet.01.361885.g321.t1 | hypothetical protein [Paraburkholderia caryophylli] | 537 | 2.75E-07 | 42.33 |  |
| Efet.01.361892.g322.t1 | ankyrin-1-like | 1926 | 9.11E-56 | 46.95 |  |
| Efet.01.362119.g325.t1 | Two pore potassium channel sup-9 | 315 | 1.17E-06 | 78.9 | P:ion transport; P:potassium ion transmembrane transport; C:membrane; C:integral component of membrane; F:potassium channel activity; P:transport |
| Efet.01.36215.g744.t1 | dynein heavy chain axonemal-like | 609 | 2.69E-67 | 64.35 | F:nucleotide binding; F:ATPase activity; F:ATP binding; F:microtubule motor activity; C:axonemal dynein complex; C:dynein complex; P:cilium movement; P:microtubule-based movement |
| Efet.01.362172.g326.t1 | serine threonine- kinase tousled-like 2 isoform X2 | 483 | 3.30E-69 | 64.55 | F:nucleotide binding; F:protein kinase activity; P:phosphorylation |
| Efet.01.362212.g328.t1 | thyrotropin-releasing hormone receptor-like | 297 | 4.66E-31 | 78.25 | F:thyrotropin-releasing hormone receptor activity; P:G-protein coupled receptor signaling pathway; C:integral component of membrane |
| Efet.01.36229.g745.t1 | pleckstrin homology domain-containing family G member 5-like isoform X1 | 312 | 2.61E-22 | 53.15 | P:regulation of Rho protein signal transduction; C:membrane; C:integral component of membrane; F:Rho guanyl-nucleotide exchange factor activity; P:positive regulation of GTPase activity |
| Efet.01.362386.g330.t1 | transmembrane channel 7 | 342 | 1.63E-07 | 64 | C:membrane; C:integral component of membrane |
| Efet.01.36239.g746.t1 | laminin subunit gamma-1 | 339 | 2.53E-35 | 76.2 | C:basement membrane; P:system development; P:basement membrane assembly |
| Efet.01.362443.g332.t1 | succinate dehydrogenase assembly factor mitochondrial | 249 | 3.96E-29 | 69.8 | P:biological_process; C:cellular_component; F:molecular_function |
| Efet.01.36246.g747.t1 | hypothetical protein CAPTEDRAFT_225483 | 507 | 4.60E-20 | 57.2 | C:membrane; C:integral component of membrane; F:carbohydrate binding |
| Efet.01.362539.g333.t1 | ankyrin repeat and KH domain-containing 1-like | 831 | 7.63E-10 | 50 |  |
| Efet.01.362556.g334.t1 | FMRFamide receptor | 777 | 1.62E-19 | 59.25 | C:membrane; P:protein dephosphorylation |
| Efet.01.362570.g335.t1 | glutamic acid-rich -like | 417 | 7.17E-09 | 60.2 |  |
| Efet.01.362600.g336.t1 | DNA RNA-binding KIN17 | 819 | 1.84E-69 | 63.05 | C:nucleus; P:DNA replication; F:double-stranded DNA binding; P:cellular response to DNA damage stimulus |
| Efet.01.362708.g339.t1 | partitioning defective 3 homolog isoform X10 | 939 | 6.32E-07 | 63.25 |  |
| Efet.01.362713.g340.t1 | hypothetical protein CAPTEDRAFT_201931 | 1503 | 1.36E-49 | 49.67 | C:membrane; C:integral component of membrane |
| Efet.01.362718.g341.t1 | hypothetical protein CAPTEDRAFT_224946 | 471 | 1.03E-21 | 60.09 |  |
| Efet.01.362724.g342.t1 | PREDICTED: utrophin-like, partial | 309 | 4.50E-07 | 61 |  |
| Efet.01.362816.g345.t1 | serine threonine- kinase 31 isoform X3 | 507 | 5.31E-22 | 55.1 | F:nucleotide binding; F:nucleic acid binding; F:ATP binding; F:protein kinase activity; P:protein phosphorylation |
| Efet.01.362821.g346.t1 | hypothetical protein CAPTEDRAFT_164574 | 234 | 6.29E-06 | 55 | C:nucleus; P:RNA processing; P:mRNA processing |
| Efet.01.36288.g750.t1 | sterol regulatory element-binding 2 | 1203 | 1.28E-12 | 70.9 | P:regulation of transport; C:cytoplasm; C:intracellular membrane-bounded organelle; F:transcription regulatory region DNA binding; F:protein dimerization activity; P:negative regulation of biological process; P:positive regulation of biological process; P:regulation of transcription from RNA polymerase II promoter |
| Efet.01.362941.g349.t1 | beta-1,4-galactosyltransferase galt-1-like | 456 | 8.94E-23 | 64.6 | C:membrane |
| Efet.01.362941.g350.t1 | beta-1,4-galactosyltransferase galt-1-like | 303 | 1.12E-09 | 59.25 | C:membrane; C:integral component of membrane |
| Efet.01.3630.g309.t1 | multiple epidermal growth factor-like domains 6 | 273 | 8.41E-20 | 68.25 | F:calcium ion binding |
| Efet.01.363018.g352.t1 | penicillin-binding 2 | 669 | 3.33E-72 | 72.5 | F:penicillin binding |
| Efet.01.363018.g353.t1 | hypothetical protein B5766_10585 | 387 | 2.04E-63 | 70.5 | C:membrane |
| Efet.01.363215.g354.t1 | GNAT family N-acetyltransferase | 270 | 1.08E-46 | 95.9 | F:N-acetyltransferase activity |
| Efet.01.36322.g755.t1 | paramyosin-like isoform X1 | 519 | 4.87E-48 | 81.05 | C:myosin filament; C:myofibril; F:motor activity |
| Efet.01.36323.g756.t1 | insulin-like growth factor-binding complex acid labile subunit | 4065 | 4.12E-32 | 45.15 | P:peptidyl-tyrosine phosphorylation; F:ATP binding; F:protein tyrosine kinase activity; C:membrane; C:integral component of membrane; F:protein kinase activity; P:phosphorylation; P:protein phosphorylation; F:kinase activity |
| Efet.01.363390.g356.t1 | calcium-independent kinase C isoform X1 | 330 | 3.75E-13 | 82.2 | F:ATP binding; F:metal ion binding; C:membrane; P:protein phosphorylation; C:intracellular; P:intracellular signal transduction; F:protein kinase C activity |
| Efet.01.363465.g358.t1 | dystonin isoform X1 | 630 | 2.37E-78 | 76.3 |  |
| Efet.01.363465.g359.t1 | dystonin isoform X1 | 330 | 2.62E-27 | 80.6 | F:ATPase activity; P:cell cycle arrest; F:actin binding; F:calcium ion binding; P:Wnt signaling pathway; F:microtubule binding |
| Efet.01.363551.g361.t1 | discoidin domain-containing receptor 2-like isoform X1 | 1476 | 5.48E-94 | 54.55 | F:ATP binding; P:peptidyl-tyrosine phosphorylation; F:protein tyrosine kinase activity; C:membrane; C:integral component of membrane; F:protein kinase activity; P:protein phosphorylation |
| Efet.01.363563.g362.t1 | platelet glyco Ib alpha chain-like isoform X2 | 1011 | 7.18E-87 | 47.1 | P:biological regulation |
| Efet.01.363635.g364.t1 | GRB2-associated-binding 1-like | 342 | 1.86E-18 | 57 |  |
| Efet.01.363684.g365.t1 | coiled-coil domain-containing | 495 | 3.59E-52 | 83.5 |  |
| Efet.01.363721.g366.t1 | hypothetical protein [Flavobacterium gilvum] | 483 | 2.34E-10 | 56 |  |
| Efet.01.363757.g367.t1 | endolytic transglycosylase | 1236 | 2.56E-115 | 80.75 | F:nucleic acid binding |
| Efet.01.363772.g368.t1 | glycogen debranching | 930 | 3.00E-169 | 57.65 | F:catalytic activity; P:glycogen catabolic process; F:amylo-alpha-1,6-glucosidase activity; F:4-alpha-glucanotransferase activity |
| Efet.01.363782.g369.t1 | testis-specific serine threonine- kinase 1 | 441 | 3.50E-54 | 65.4 | C:nucleus; F:ATP binding; C:cytoplasm; F:protein serine/threonine kinase activity; P:protein phosphorylation; P:intracellular signal transduction |
| Efet.01.36380.g758.t1 | mediator of RNA polymerase II transcription subunit 18 | 282 | 1.76E-34 | 79.75 | C:mediator complex; F:RNA polymerase II transcription cofactor activity; P:regulation of transcription from RNA polymerase II promoter |
| Efet.01.363882.g372.t1 | trunk | 342 | 1.20E-26 | 57.85 |  |
| Efet.01.363886.g373.t1 | heavy metal translocating P-type ATPase | 351 | 2.27E-54 | 75.75 | F:nucleotide binding; C:membrane; F:ion binding; P:metal ion transport; F:hydrolase activity |
| Efet.01.363890.g374.t1 | guanine nucleotide-binding G(k) subunit | 213 | 3.44E-33 | 92.25 | C:photoreceptor connecting cilium; P:adenylate cyclase-inhibiting G-protein coupled receptor signaling pathway; P:cell proliferation; P:regulation of cAMP-mediated signaling; P:sensory perception of umami taste; P:retina development in camera-type eye; P:positive regulation of cell proliferation; P:G-protein coupled acetylcholine receptor signaling pathway; P:gamma-aminobutyric acid signaling pathway; C:nucleoplasm; F:magnesium ion binding; F:GTP binding; P:cell division; C:dendrite; P:activation of MAPKK activity; C:extracellular exosome; F:GTPase activity; F:GDP binding; C:cytosol; P:cellular response to electrical stimulus; P:regulation of calcium ion transport; P:intracellular signal transduction; P:detection of light stimulus involved in visual perception; P:negative regulation of synaptic transmission; P:cellular response to forskolin; P:regulation of heart contraction; P:eye photoreceptor cell development; F:signal transducer activity; C:lysosomal membrane; P:cell cycle; C:centrosome; P:adenosine receptor signaling pathway; C:heterotrimeric G-protein complex; C:cell body; C:membrane raft; C:integral component of membrane; C:midbody; F:G-protein beta/gamma-subunit complex binding; F:G-protein coupled serotonin receptor binding |
| Efet.01.363928.g375.t1 | hypothetical protein HELRODRAFT_169693 | 273 | 1.77E-19 | 70 |  |
| Efet.01.363997.g379.t1 | steroid 17-alpha-hydroxylase 17,20 lyase-like | 1584 | 1.62E-150 | 55.85 | F:iron ion binding; F:oxidoreductase activity; F:oxidoreductase activity, acting on paired donors, with incorporation or reduction of molecular oxygen; F:metal ion binding; C:membrane; C:integral component of membrane; P:oxidation-reduction process; F:monooxygenase activity; F:heme binding |
| Efet.01.364017.g380.t1 | nuclear receptor subfamily 0 group B member 1 | 240 | 2.67E-06 | 60 | F:DNA binding; C:nucleus; P:transcription, DNA-templated; F:steroid hormone receptor activity; P:regulation of transcription, DNA-templated; P:steroid hormone mediated signaling pathway |
| Efet.01.36410.g759.t1 | Cohesin subunit SA- | 243 | 7.32E-34 | 91.15 | P:cell division; P:chromosome segregation; F:transcriptional activator activity, RNA polymerase II transcription regulatory region sequence-specific binding; P:mitotic nuclear division; F:chromatin binding; C:chromatin; C:nucleoplasm |
| Efet.01.364108.g383.t1 | kelch 17 | 408 | 1.19E-07 | 53.5 | F:ubiquitin-protein transferase activity; C:Cul3-RING ubiquitin ligase complex; P:protein ubiquitination |
| Efet.01.364268.g385.t1 | FMRFamide receptor-like | 918 | 3.47E-60 | 46 | F:G-protein coupled receptor activity; F:G-protein coupled peptide receptor activity; P:G-protein coupled receptor signaling pathway; P:signal transduction; C:membrane; C:integral component of membrane; F:signal transducer activity |
| Efet.01.364338.g388.t1 | UPF0462 C4orf33 homolog | 294 | 2.20E-26 | 69.45 |  |
| Efet.01.364375.g389.t1 | Metalloprotease | 315 | 1.90E-42 | 86.85 | P:proteolysis; F:metallopeptidase activity |
| Efet.01.364419.g391.t1 | hypothetical protein HELRODRAFT_81234, partial | 429 | 1.79E-19 | 51.05 |  |
| Efet.01.364437.g392.t1 | A disintegrin and metallo ase with thrombospondin motifs 7-like isoform X1 | 444 | 9.73E-41 | 74.3 | F:zinc ion binding; P:proteolysis; F:metalloendopeptidase activity; C:proteinaceous extracellular matrix |
| Efet.01.36447.g761.t1 | RGWamide neuropeptide precursor | 525 | 2.20E-10 | 54.5 | P:neuropeptide signaling pathway; F:neuropeptide hormone activity; C:extracellular region; P:hormone-mediated signaling pathway |
| Efet.01.364528.g393.t1 | Trimeric intracellular cation channel type B | 246 | 1.48E-09 | 73.45 | P:monovalent inorganic cation transport; F:cation channel activity; P:cation transmembrane transport; C:integral component of membrane |
| Efet.01.364584.g394.t1 | importin subunit alpha-4 | 300 | 1.45E-39 | 91.3 | P:NLS-bearing protein import into nucleus; C:cytosol; F:protein transporter activity; F:nuclear localization sequence binding; C:nuclear pore; C:nucleoplasm |
| Efet.01.36464.g763.t1 | ATP-binding cassette sub-family A member 3-like | 2106 | 4.39E-139 | 60 | F:nucleotide binding; F:ATPase activity; F:ATP binding; C:membrane; C:integral component of membrane; P:transport; F:transporter activity |
| Efet.01.364737.g397.t1 | protocadherin-9 isoform X3 | 2640 | 1.93E-115 | 51.05 | C:growth cone; C:cell-cell contact zone; F:calcium ion binding; C:membrane; C:integral component of membrane; P:cell adhesion; P:homophilic cell adhesion via plasma membrane adhesion molecules; C:plasma membrane |
| Efet.01.364785.g398.t1 | dynein heavy chain axonemal-like | 411 | 1.08E-56 | 75.5 | F:nucleotide binding; F:microtubule motor activity; C:cell part; C:organelle; P:microtubule-based movement |
| Efet.01.364812.g399.t1 | unc-93 homolog A-like | 1860 | 1.39E-51 | 62.15 | C:integral component of membrane |
| Efet.01.364850.g400.t1 | ATP-binding cassette sub-family A member 3 | 822 | 9.47E-43 | 55.2 | F:nucleotide binding; F:ATPase activity; F:ATP binding; C:membrane; C:integral component of membrane; P:transport; F:transporter activity |
| Efet.01.36487.g764.t1 | synaptotagmin 7d | 315 | 3.87E-27 | 76.15 | C:presynaptic membrane; C:neuronal cell body; P:regulation of glucagon secretion; C:dendrite; F:phosphatidylinositol-4,5-bisphosphate binding; P:regulation of insulin secretion; C:early phagosome; F:phosphatidylserine binding; C:extracellular exosome; C:terminal bouton; F:calcium ion binding; C:cytosol; P:short-term synaptic potentiation; P:calcium ion regulated lysosome exocytosis; P:vesicle-mediated cholesterol transport; P:regulation of dopamine secretion; P:synaptic vesicle recycling; F:syntaxin binding; P:phagosome-lysosome fusion; P:positive regulation of calcium ion-dependent exocytosis; C:lysosomal membrane; F:calcium-dependent phospholipid binding; C:peroxisomal membrane; F:calmodulin binding; P:calcium ion-regulated exocytosis of neurotransmitter; P:plasma membrane repair; P:regulation of bone remodeling; C:integral component of membrane; C:cell junction; C:phagocytic vesicle membrane; C:synaptic vesicle membrane; C:dense core granule; F:clathrin binding; P:regulation of phagocytosis |
| Efet.01.3649.g311.t1 | STE STE11 SSK kinase | 456 | 6.94E-13 | 54.75 | F:nucleotide binding; F:ATP binding; P:activation of MAPKK activity; C:cytoplasm; F:protein kinase activity; F:MAP kinase kinase kinase activity; P:phosphorylation; P:protein phosphorylation; F:kinase activity; P:stress-activated MAPK cascade |
| Efet.01.364911.g401.t1 | AP-2 complex subunit beta-like | 267 | 1.07E-36 | 96.1 | C:clathrin-coated pit; C:clathrin adaptor complex; F:protein transporter activity; P:intracellular protein transport; P:endocytosis; C:plasma membrane |
| Efet.01.364933.g402.t1 | voltage-gated sodium channel | 387 | 7.88E-28 | 70.7 | P:regulation of postsynaptic membrane potential; P:regulation of ion transmembrane transport; C:voltage-gated sodium channel complex; P:sodium ion transmembrane transport; F:voltage-gated sodium channel activity |
| Efet.01.365169.g407.t1 | tyrosine- phosphatase non-receptor type 21-like | 2529 | 5.08E-113 | 69.4 | F:transcription cofactor activity; C:cytoplasm; F:protein tyrosine phosphatase activity; P:negative regulation of cell proliferation; P:protein dephosphorylation; P:lymphangiogenesis; F:receptor tyrosine kinase binding; P:regulation of protein export from nucleus; C:nucleoplasm |
| Efet.01.365207.g408.t1 | protein JBTS17-like | 1326 | 1.85E-20 | 44.21 |  |
| Efet.01.365252.g409.t1 | PTHB1-like isoform X2 | 696 | 5.07E-24 | 67.4 | C:cilium |
| Efet.01.365259.g411.t1 | hypothetical protein B5766_03860 | 375 | 1.12E-10 | 98.5 |  |
| Efet.01.365267.g413.t1 | transmembrane 131-like isoform X1 | 309 | 1.13E-24 | 75.4 | C:membrane; C:integral component of membrane |
| Efet.01.365305.g414.t1 | Wnt-4 isoform X2 | 453 | 1.17E-91 | 83.45 | C:extracellular space; F:frizzled binding; P:neuron differentiation; P:cell fate commitment; P:Wnt signaling pathway; C:proteinaceous extracellular matrix |
| Efet.01.365325.g415.t1 | DUF3108 domain-containing | 888 | 1.56E-144 | 89 |  |
| Efet.01.365325.g416.t1 | DUF3567 domain-containing | 303 | 1.39E-67 | 87.05 |  |
| Efet.01.365325.g417.t1 | peroxiredoxin | 624 | 2.13E-115 | 97.65 | F:antioxidant activity; F:oxidoreductase activity; P:cellular oxidant detoxification; P:oxidation-reduction process |
| Efet.01.365418.g419.t1 | hypothetical protein HELRODRAFT_191437 | 594 | 2.68E-13 | 47 |  |
| Efet.01.365501.g421.t1 | MBL fold metallo-hydrolase | 969 | 0 | 94.7 | F:hydrolase activity |
| Efet.01.365501.g422.t1 | DNA mismatch repair | 732 | 1.31E-161 | 93 | F:ATP binding; P:mismatch repair; F:mismatched DNA binding; F:damaged DNA binding |
| Efet.01.365522.g423.t1 | dual specificity tyrosine-phosphorylation-regulated kinase 2 isoform X1 | 489 | 9.25E-91 | 89.35 | F:ATP binding; F:protein serine/threonine kinase activity; P:protein phosphorylation |
| Efet.01.365616.g425.t1 | Kyphoscoliosis peptidase | 537 | 1.58E-07 | 58.75 |  |
| Efet.01.365633.g426.t1 | poly [ADP-ribose] polymerase 14-like | 1611 | 3.98E-19 | 41.95 |  |
| Efet.01.365656.g428.t1 | multidrug resistance-associated 1 isoform X1 | 414 | 2.47E-59 | 82.35 | F:ATP binding; F:ATPase activity, coupled to transmembrane movement of substances; P:transmembrane transport; C:integral component of membrane |
| Efet.01.365657.g429.t1 | lambda-crystallin homolog | 234 | 5.35E-12 | 66.2 | F:oxidoreductase activity; P:single-organism metabolic process |
| Efet.01.365988.g434.t1 | transmembrane and coiled-coil domains 2-like isoform X5 | 267 | 2.06E-28 | 84.75 | C:integral component of membrane |
| Efet.01.366129.g435.t1 | fibroblast growth factor receptor 2-like isoform X1 | 462 | 2.31E-51 | 76.65 | F:nucleotide binding; F:protein tyrosine kinase activity; C:membrane; P:protein phosphorylation |
| Efet.01.366245.g437.t1 | EH domain-binding 1-like isoform X1 | 1887 | 7.48E-40 | 74.5 |  |
| Efet.01.366296.g439.t1 | TAR DNA-binding 43 | 264 | 5.71E-30 | 89.5 | F:nucleotide binding; F:DNA binding; C:intracellular ribonucleoprotein complex; C:viral nucleocapsid |
| Efet.01.366307.g440.t1 | phosphatidylinositol 3,4,5-trisphosphate 5-phosphatase 1-like | 492 | 1.06E-11 | 57.5 |  |
| Efet.01.36631.g769.t1 | Mo25 | 285 | 1.73E-51 | 94.75 |  |
| Efet.01.366320.g441.t1 | lamin Dm0 | 822 | 5.03E-44 | 57.05 | F:structural molecule activity; C:intermediate filament |
| Efet.01.366337.g442.t1 | serine threonine- kinase DCLK2 isoform X1 | 504 | 7.88E-39 | 72.2 | F:ATP binding; F:protein serine/threonine kinase activity; P:protein phosphorylation; C:intracellular; P:intracellular signal transduction |
| Efet.01.366469.g446.t1 | hypothetical protein HELRODRAFT_166464 | 333 | 9.13E-25 | 70.1 | C:integral component of membrane; F:carbohydrate binding |
| Efet.01.366483.g447.t1 | zinc finger SNAI2-like | 354 | 3.33E-58 | 89.5 | F:nucleic acid binding; F:metal ion binding |
| Efet.01.366620.g449.t1 | probable G- coupled receptor 52 | 879 | 2.47E-69 | 55.85 | F:G-protein coupled receptor activity; P:G-protein coupled receptor signaling pathway; C:membrane; C:integral component of membrane |
| Efet.01.36672.g770.t1 | hypothetical protein CAPTEDRAFT_210308 | 1443 | 2.59E-18 | 45.5 |  |
| Efet.01.36673.g771.t1 | Monocarboxylate transporter 12 | 312 | 7.09E-43 | 78.7 | P:transmembrane transport; C:integral component of membrane |
| Efet.01.366845.g454.t1 | glycogen debranching enzyme isoform X2 | 288 | 2.57E-35 | 79.2 | P:glycogen biosynthetic process; P:glycogen catabolic process; F:amylo-alpha-1,6-glucosidase activity; F:4-alpha-glucanotransferase activity |
| Efet.01.366862.g455.t1 | dmX 2 | 543 | 1.69E-24 | 60.9 |  |
| Efet.01.366886.g456.t1 | peroxidasin homolog | 1137 | 5.92E-139 | 70.85 | F:peroxidase activity; P:response to oxidative stress; P:cellular oxidant detoxification; P:oxidation-reduction process; F:heme binding |
| Efet.01.366963.g459.t1 | tripartite motif-containing 2-like | 843 | 8.78E-10 | 45.9 | F:zinc ion binding; P:signal transduction; P:ion transmembrane transport; F:GABA-A receptor activity; F:extracellular ligand-gated ion channel activity; C:GABA-A receptor complex; C:synapse; P:transport; C:intracellular; C:plasma membrane; P:ion transport; F:metal ion binding; C:membrane; C:integral component of membrane; C:cell junction |
| Efet.01.36697.g772.t1 | hypothetical protein CAPTEDRAFT_191266 | 588 | 1.47E-47 | 57 | C:membrane |
| Efet.01.367055.g460.t1 | PREDICTED: uncharacterized protein LOC107078209 | 243 | 3.04E-12 | 76.5 |  |
| Efet.01.367109.g461.t1 | dynein heavy chain axonemal-like | 456 | 5.69E-73 | 88.7 | F:ATPase activity; F:ATP binding; C:motile cilium; F:microtubule motor activity; C:dynein complex; P:microtubule-based movement |
| Efet.01.36722.g774.t1 | coiled-coil domain-containing 73-like | 432 | 3.28E-11 | 64.7 |  |
| Efet.01.367291.g466.t1 | 1-phosphatidylinositol 4,5-bisphosphate phosphodiesterase epsilon-1 | 375 | 8.45E-51 | 76.15 | F:phosphoric diester hydrolase activity; P:intracellular signal transduction |
| Efet.01.367334.g467.t1 | spectrin alpha non-erythrocytic 1 isoform X3 | 243 | 2.01E-12 | 80.65 | F:actin binding; F:phospholipid binding |
| Efet.01.367455.g468.t1 | potassium voltage-gated channel subfamily H member 2 isoform X5 | 924 | 4.45E-152 | 88.85 | F:phosphorelay sensor kinase activity; F:voltage-gated potassium channel activity; P:potassium ion transmembrane transport; P:phosphorelay signal transduction system; C:integral component of membrane; P:signal transduction by protein phosphorylation; C:intracellular |
| Efet.01.367470.g470.t1 | centrosomal of 95 kDa-like | 321 | 2.85E-29 | 81.8 | C:centrosome; F:zinc ion binding; C:spindle pole |
| Efet.01.367505.g472.t1 | acetyl- | 618 | 3.42E-95 | 87.25 | P:malonyl-CoA biosynthetic process; P:acetyl-CoA metabolic process; P:lipid homeostasis; F:biotin carboxylase activity; P:multicellular organismal protein metabolic process; C:nucleolus; P:fatty acid biosynthetic process; P:carnitine shuttle; P:cellular response to prostaglandin E stimulus; P:tissue homeostasis; F:ATP binding; C:extracellular exosome; F:protein binding; P:biotin metabolic process; P:protein homotetramerization; F:acetyl-CoA carboxylase activity; F:metal ion binding; C:mitochondrion; C:actin cytoskeleton; P:long-chain fatty-acyl-CoA biosynthetic process; C:cytosol; P:positive regulation of cellular metabolic process |
| Efet.01.367527.g473.t1 | Neuroendocrine convertase 2 | 342 | 8.07E-51 | 69.7 | P:proteolysis; F:serine-type endopeptidase activity |
| Efet.01.367531.g474.t1 | TNFAIP3-interacting 2-like | 360 | 3.18E-09 | 59.33 |  |
| Efet.01.367559.g475.t1 | bactericidal permeability-increasing -like | 324 | 1.02E-07 | 53 | C:extracellular space; F:lipid binding |
| Efet.01.367589.g476.t1 | testicular haploid expressed gene -like | 630 | 1.63E-14 | 51.2 |  |
| Efet.01.367658.g477.t1 | serine threonine- kinase TAO1-like isoform X1 | 366 | 1.30E-24 | 77.9 | F:ATP binding; F:protein serine/threonine kinase activity; P:protein phosphorylation |
| Efet.01.367906.g480.t1 | specificity 1 | 1497 | 4.50E-14 | 74.15 | F:nucleic acid binding; F:metal ion binding |
| Efet.01.368018.g483.t1 | hypothetical protein HELRODRAFT_174918 | 1278 | 4.15E-89 | 55.4 | C:extracellular space |
| Efet.01.36804.g778.t1 | slit homolog 3 | 2247 | 1.53E-15 | 48.7 | P:single-organism process; P:cellular process |
| Efet.01.368091.g486.t1 | glutamyl aminopeptidase | 324 | 7.87E-06 | 55 |  |
| Efet.01.36816.g779.t1 | ubinuclein-1 isoform X2 | 522 | 3.31E-44 | 67.9 | C:nucleus; C:bicellular tight junction; P:negative regulation of phosphatase activity; P:DNA replication-independent nucleosome assembly |
| Efet.01.368173.g489.t1 | FAST kinase domain-containing 5-like isoform X1 | 822 | 9.29E-18 | 48.78 |  |
| Efet.01.368213.g490.t1 | hypothetical protein HELRODRAFT_190011 | 270 | 1.96E-08 | 82 |  |
| Efet.01.36823.g780.t1 | sacsin-like | 2355 | 4.18E-23 | 44.5 |  |
| Efet.01.368253.g491.t1 | alpha- sarcomeric | 270 | 7.64E-24 | 86.6 |  |
| Efet.01.36827.g781.t1 | isoform B | 561 | 2.84E-16 | 56.3 | F:zinc ion binding; F:metal ion binding |
| Efet.01.368319.g492.t1 | Death domain-containing 1 | 2322 | 6.31E-56 | 47.35 | P:signal transduction |
| Efet.01.368337.g494.t1 | toll isoform X1 | 3240 | 7.87E-149 | 42.15 | P:signal transduction; C:membrane; C:integral component of membrane |
| Efet.01.368485.g497.t1 | Dual specificity phosphatase | 405 | 2.38E-33 | 61.9 | F:phosphoprotein phosphatase activity; P:dephosphorylation |
| Efet.01.368523.g498.t1 | glutamate receptor 4-like | 390 | 4.61E-70 | 83.85 | C:postsynaptic membrane; P:ion transmembrane transport; C:integral component of membrane; C:cell junction; P:ionotropic glutamate receptor signaling pathway; F:ionotropic glutamate receptor activity; F:extracellular-glutamate-gated ion channel activity |
| Efet.01.368540.g499.t1 | nephrin-like isoform X3 | 324 | 2.09E-18 | 64.1 | C:membrane; C:integral component of membrane |
| Efet.01.368595.g500.t1 | transmembrane 6 superfamily member 1-like | 255 | 5.00E-07 | 60.2 | C:membrane; C:integral component of membrane; C:lysosomal membrane |
| Efet.01.368609.g501.t1 | NTF2-related export | 282 | 1.47E-14 | 71.7 | P:transport; C:intracellular |
| Efet.01.368652.g502.t1 | glutathione gamma-glutamylcysteinyltransferase 1-like | 666 | 7.03E-142 | 76.75 | P:phytochelatin biosynthetic process; F:glutathione gamma-glutamylcysteinyltransferase activity; F:metal ion binding; P:response to metal ion |
| Efet.01.368667.g503.t1 | NACHT and WD repeat domain-containing 2-like | 3639 | 1.03E-145 | 60.1 | F:GDP-dissociation inhibitor activity; C:Rab-protein geranylgeranyltransferase complex; P:small GTPase mediated signal transduction; F:oxidoreductase activity; P:regulation of catalytic activity; P:oxidation-reduction process; P:protein geranylgeranylation; P:intracellular protein transport |
| Efet.01.368667.g504.t1 | hypothetical protein CAPTEDRAFT_223662 | 1401 | 8.07E-71 | 44.25 |  |
| Efet.01.368702.g506.t1 | dyslexia-associated KIAA0319 homolog | 972 | 1.21E-81 | 64.1 | C:membrane |
| Efet.01.368764.g508.t1 | D(1B) dopamine receptor | 1257 | 1.12E-45 | 43.05 | F:dopamine neurotransmitter receptor activity; C:integral component of plasma membrane; F:G-protein coupled receptor activity; P:G-protein coupled receptor signaling pathway; P:signal transduction; C:membrane; C:integral component of membrane; P:dopamine receptor signaling pathway; P:positive regulation of adenylate cyclase activity involved in G-protein coupled receptor signaling pathway; P:vasodilation; F:signal transducer activity |
| Efet.01.368848.g510.t1 | single-strand selective monofunctional uracil DNA glycosylase isoform X2 | 693 | 2.08E-22 | 71.85 | F:protein binding; F:oxidized pyrimidine nucleobase lesion DNA N-glycosylase activity; F:single-strand selective uracil DNA N-glycosylase activity; C:nucleolus; C:nucleoplasm; P:depyrimidination |
| Efet.01.368853.g511.t1 | dnaJ homolog subfamily C member 13-like isoform X1 | 657 | 6.93E-55 | 78.05 | F:DNA binding; C:nucleus |
| Efet.01.369016.g514.t1 | cartilage matrix | 642 | 5.64E-07 | 46 |  |
| Efet.01.369069.g516.t1 | serine threonine- kinase H1 homolog | 1308 | 0 | 74.55 | F:nucleotide binding; F:protein kinase activity; P:phosphorylation |
| Efet.01.36909.g787.t1 | nuclear receptor subfamily 0 group B member 1-like | 387 | 1.09E-06 | 55.67 | F:DNA binding; C:nucleus; P:transcription, DNA-templated; F:steroid hormone receptor activity; P:regulation of transcription, DNA-templated; P:steroid hormone mediated signaling pathway |
| Efet.01.369119.g519.t1 | DChain Conformational Landscape Of The P28-bound Human Proteasome Regulatory Particle | 783 | 5.19E-107 | 78.05 | F:ATP binding; C:proteasome regulatory particle, base subcomplex; C:membrane; C:cytosolic proteasome complex; C:nuclear proteasome complex; F:peptidase activity; F:TBP-class protein binding; P:ER-associated ubiquitin-dependent protein catabolic process; P:positive regulation of proteasomal protein catabolic process; F:proteasome-activating ATPase activity; C:nucleoplasm; P:positive regulation of RNA polymerase II transcriptional preinitiation complex assembly |
| Efet.01.369134.g520.t1 | teneurin-m-like isoform X8 | 222 | 1.84E-06 | 78.45 |  |
| Efet.01.369241.g521.t1 | zinc finger 271-like | 1323 | 3.32E-49 | 51.7 | F:nucleic acid binding; F:metal ion binding; P:regulation of transcription, DNA-templated; C:intracellular |
| Efet.01.369276.g523.t1 | coronin-6 isoform X1 | 228 | 3.53E-42 | 85.45 | F:actin filament binding; P:adult somatic muscle development; C:mitotic spindle; C:actin cytoskeleton; P:defense response to fungus; P:actin cytoskeleton organization |
| Efet.01.369320.g524.t1 | hypothetical protein CAPTEDRAFT_168270 | 1311 | 6.45E-106 | 51.3 |  |
| Efet.01.369358.g525.t1 | hypothetical protein HELRODRAFT_183182 | 426 | 5.35E-38 | 54.33 |  |
| Efet.01.36937.g788.t1 | 40S ribosomal S15Aa | 303 | 1.99E-56 | 92.55 | C:extracellular exosome; F:structural constituent of ribosome; C:cytosolic small ribosomal subunit; C:mitochondrion; C:membrane; P:positive regulation of cell proliferation; P:positive regulation of cell cycle; C:extracellular matrix; P:response to virus; F:RNA binding; P:translation |
| Efet.01.369443.g526.t1 | hypothetical protein HELRODRAFT_160582 | 453 | 1.61E-19 | 56 |  |
| Efet.01.369469.g528.t1 | fibroblast growth factor receptor 3-like | 336 | 2.59E-31 | 86.1 | F:ATP binding; P:peptidyl-tyrosine phosphorylation; F:protein tyrosine kinase activity; C:integral component of membrane |
| Efet.01.369489.g529.t1 | discoidin domain-containing receptor 2-like isoform X1 | 558 | 3.31E-58 | 70.5 | F:protein tyrosine kinase activity; C:integral component of membrane; P:protein phosphorylation |
| Efet.01.369509.g531.t1 | kazrin-like isoform X4 | 639 | 4.61E-25 | 61.7 |  |
| Efet.01.369522.g532.t1 | glutamate receptor kainate 3-like | 276 | 5.72E-14 | 67.5 | F:ion channel activity; C:membrane; F:receptor activity; P:transport |
| Efet.01.369565.g533.t1 | nitric oxide-associated 1 | 1179 | 1.41E-52 | 66.65 | F:GTP binding; F:GTPase activity; C:mitochondrion; P:ribosome biogenesis |
| Efet.01.369578.g534.t1 | acid-sensing ion channel 3-like | 249 | 1.64E-09 | 53.71 | P:sodium ion transport; P:ion transport; F:sodium channel activity; C:membrane; C:integral component of membrane; P:sodium ion transmembrane transport; P:transport |
| Efet.01.369652.g535.t1 | transmembrane and coiled-coil domain-containing 1 | 300 | 1.72E-59 | 90.2 | C:integral component of membrane |
| Efet.01.369675.g536.t1 | gamma-aminobutyric acid type B receptor subunit 1-like | 432 | 6.64E-17 | 68.2 | F:G-protein coupled GABA receptor activity; P:G-protein coupled receptor signaling pathway; C:integral component of membrane |
| Efet.01.369719.g537.t1 | forkhead box K1-like | 396 | 2.16E-48 | 78.95 | C:nucleus; F:sequence-specific DNA binding; F:RNA polymerase II transcription factor activity, sequence-specific DNA binding; P:regulation of transcription from RNA polymerase II promoter |
| Efet.01.369812.g539.t1 | Hemicentin-1 | 570 | 1.37E-35 | 59.4 | F:calcium ion binding; C:membrane; P:cell adhesion |
| Efet.01.369822.g540.t1 | carbamoylphosphate | 567 | 2.75E-86 | 86.85 | F:ATP binding; F:catalytic activity; F:metal ion binding; P:nitrogen compound metabolic process |
| Efet.01.369900.g542.t1 | type I restriction endonuclease subunit R | 2535 | 0 | 93 | F:DNA binding; F:ATP binding; P:nucleic acid phosphodiester bond hydrolysis; F:Type I site-specific deoxyribonuclease activity; F:helicase activity; P:DNA restriction-modification system |
| Efet.01.369900.g543.t1 | MFS transporter | 1431 | 0 | 76.95 | P:transmembrane transport; C:integral component of membrane |
| Efet.01.369900.g544.t1 | ribbon-helix-helix family | 297 | 1.52E-64 | 87.65 | P:regulation of transcription, DNA-templated |
| Efet.01.369900.g545.t1 | type II toxin-antitoxin system family toxin | 567 | 8.68E-37 | 88.45 | F:sequence-specific DNA binding |
| Efet.01.369901.g546.t1 | ribbon-helix-helix family | 507 | 1.02E-41 | 87.9 | P:regulation of transcription, DNA-templated |
| Efet.01.369901.g547.t1 | MFS transporter | 1431 | 0 | 76.95 | P:transmembrane transport; C:integral component of membrane |
| Efet.01.369901.g548.t1 | type I restriction endonuclease subunit R | 2535 | 0 | 93 | F:DNA binding; F:ATP binding; P:nucleic acid phosphodiester bond hydrolysis; F:Type I site-specific deoxyribonuclease activity; F:helicase activity; P:DNA restriction-modification system |
| Efet.01.369903.g549.t1 | uridine-cytidine kinase-like 1 | 840 | 5.50E-108 | 84.7 | F:ATP binding; F:uridine kinase activity; P:CTP salvage; P:phosphorylation; P:UMP salvage |
| Efet.01.369905.g550.t1 | neuroligin- Y-linked | 738 | 1.19E-53 | 70.8 | C:membrane |
| Efet.01.36995.g789.t1 | transportin-1-like isoform X1 | 459 | 2.69E-80 | 93.6 | P:intracellular protein transport; F:Ran GTPase binding; C:intracellular |
| Efet.01.36997.g790.t1 | Dynein heavy chain axonemal | 201 | 4.29E-12 | 78.1 | F:nucleotide binding; F:microtubule motor activity; C:dynein complex; P:microtubule-based movement |
| Efet.01.369989.g552.t1 | serine threonine- phosphatase 4 regulatory subunit 4-like isoform X7 | 225 | 8.10E-11 | 70.8 |  |
| Efet.01.37000.g791.t1 | Dynein heavy chain | 381 | 1.42E-60 | 85.55 | F:ATPase activity; F:ATP binding; F:microtubule motor activity; C:dynein complex; P:microtubule-based movement |
| Efet.01.370035.g554.t1 | enoyl- hydratase | 780 | 4.40E-160 | 90.8 | P:metabolic process; F:enoyl-CoA hydratase activity; F:isomerase activity |
| Efet.01.370035.g555.t1 | peptidase M61 | 1779 | 0 | 83.3 |  |
| Efet.01.370035.g556.t1 | disulfide isomerase | 654 | 7.76E-116 | 90.15 | C:cell; P:cell redox homeostasis; F:isomerase activity |
| Efet.01.370035.g557.t1 | ubiquinone biosynthesis | 1854 | 0 | 82.4 | F:FAD binding; F:oxidoreductase activity, acting on paired donors, with incorporation or reduction of molecular oxygen, NAD(P)H as one donor, and incorporation of one atom of oxygen; P:oxidation-reduction process; P:ubiquinone biosynthetic process |
| Efet.01.370047.g558.t1 | myosin heavy non-muscle-like | 216 | 8.12E-23 | 74.3 | F:nucleotide binding |
| Efet.01.370064.g559.t1 | dynein heavy chain axonemal-like | 939 | 8.22E-165 | 82.8 | F:ATPase activity; F:ATP binding; F:microtubule motor activity; C:dynein complex; P:microtubule-based movement |
| Efet.01.370163.g560.t1 | ADP-ribosylation factor 4C | 576 | 3.20E-78 | 78.9 | F:GTP binding; P:small GTPase mediated signal transduction; C:intracellular |
| Efet.01.37018.g793.t1 | helicase domino-like | 651 | 2.95E-13 | 60.55 |  |
| Efet.01.370192.g561.t1 | ATP-binding cassette sub-family G member 2 | 603 | 1.04E-61 | 66.05 | F:nucleotide binding; F:ATPase activity; F:ATP binding; C:membrane; C:integral component of membrane |
| Efet.01.370247.g564.t1 | Tubulin beta-2C chain | 531 | 2.02E-109 | 99.9 | F:GTP binding; C:cytoplasm; F:GTPase activity; F:structural constituent of cytoskeleton; C:microtubule; P:microtubule-based process |
| Efet.01.37027.g794.t1 | glycogen liver form-like | 303 | 7.91E-44 | 79.95 | F:glycogen phosphorylase activity; F:adenyl ribonucleotide binding; F:protein binding; P:glycogen metabolic process; F:purine ribonucleoside binding; P:necroptotic process; C:cell part; F:anion binding |
| Efet.01.370312.g566.t1 | G T mismatch-specific thymine DNA glycosylase | 213 | 2.20E-14 | 76.45 | F:protein kinase C binding; P:embryo development; F:SUMO binding; P:negative regulation of chromatin binding; F:protein homodimerization activity; C:PML body; P:base-excision repair, AP site formation; F:pyrimidine-specific mismatch base pair DNA N-glycosylase activity; P:DNA demethylation; P:mismatch repair; F:RNA polymerase II transcription cofactor activity; P:negative regulation of protein binding; C:plasma membrane; P:regulation of gene expression, epigenetic; P:negative regulation of transcription from RNA polymerase II promoter; F:uracil DNA N-glycosylase activity; F:mismatched DNA binding |
| Efet.01.37032.g795.t1 | ATP-binding cassette sub-family B member mitochondrial | 504 | 1.85E-26 | 59.15 | F:nucleotide binding; F:ATPase activity; F:ATP binding; F:ATPase activity, coupled to transmembrane movement of substances; P:transmembrane transport; C:membrane; C:integral component of membrane; P:transport |
| Efet.01.37033.g796.t1 | hypothetical protein HELRODRAFT_194109 | 540 | 3.75E-24 | 82 | C:integral component of membrane |
| Efet.01.370469.g569.t1 | NT-3 growth factor receptor-like | 663 | 5.03E-33 | 62.15 | F:nucleotide binding; F:protein tyrosine kinase activity; C:integral component of membrane; P:protein phosphorylation |
| Efet.01.37056.g797.t1 | acid-sensing ion channel 1-like isoform X1 | 1014 | 5.89E-35 | 53.85 | P:transport |
| Efet.01.370593.g571.t1 | homeobox H55 | 276 | 5.17E-32 | 87 | C:nucleus; F:transcription factor activity, sequence-specific DNA binding; P:transcription, DNA-templated; P:multicellular organism development; P:regulation of transcription, DNA-templated; F:sequence-specific DNA binding |
| Efet.01.370612.g572.t1 | rho guanine nucleotide exchange factor 11-like isoform X2 | 285 | 3.76E-19 | 64.1 | P:regulation of Rho protein signal transduction; F:Rho guanyl-nucleotide exchange factor activity; P:positive regulation of GTPase activity |
| Efet.01.370634.g573.t1 | hypothetical protein CAPTEDRAFT_216629 | 3234 | 4.27E-143 | 43.15 | C:membrane; C:integral component of membrane |
| Efet.01.370637.g574.t1 | 3-methylitaconate isomerase | 1182 | 0 | 66.7 | F:methylitaconate delta-isomerase activity |
| Efet.01.370637.g575.t1 | nonspecific lipid-transfer family | 987 | 0 | 79.75 | F:transferase activity, transferring acyl groups other than amino-acyl groups; P:metabolic process |
| Efet.01.370637.g576.t1 | enoyl- hydratase | 714 | 8.68E-154 | 79.65 | P:metabolic process; F:lyase activity; F:isomerase activity |
| Efet.01.370637.g577.t1 | family | 1011 | 0 | 68.85 | F:lyase activity |
| Efet.01.370637.g578.t1 | transferase | 1146 | 0 | 66.65 | F:catalytic activity |
| Efet.01.370637.g579.t1 | CoA-transferase family III family protein 7 | 606 | 4.09E-141 | 69.6 | F:transferase activity |
| Efet.01.370691.g580.t1 | leukocyte receptor cluster member 9-like | 1128 | 3.90E-69 | 50.6 |  |
| Efet.01.370733.g581.t1 | homeobox cut-like isoform X4 | 2925 | 2.24E-96 | 60 | F:DNA binding; C:nucleus; P:transcription, DNA-templated; P:regulation of transcription, DNA-templated; F:sequence-specific DNA binding |
| Efet.01.370798.g584.t1 | leucine-rich repeat serine threonine- kinase 1 isoform X4 | 561 | 1.51E-15 | 53.55 | F:GTP binding; F:ATP binding; P:small GTPase mediated signal transduction; F:protein kinase activity; P:protein phosphorylation; C:intracellular |
| Efet.01.370808.g585.t1 | hypothetical protein HELRODRAFT_183289 | 1536 | 1.54E-11 | 38.5 | C:membrane; C:integral component of membrane |
| Efet.01.370925.g587.t1 | TPR repeat-containing DDB_G0287407 | 741 | 7.75E-36 | 68.95 |  |
| Efet.01.370959.g588.t1 | ceramide synthase 6-like | 204 | 5.53E-14 | 70.1 | C:membrane; F:sphingosine N-acyltransferase activity; P:ceramide biosynthetic process; C:endoplasmic reticulum |
| Efet.01.370996.g589.t1 | Vesicular glutamate transporter 1 | 228 | 4.22E-09 | 61.6 | P:transmembrane transport; C:membrane; C:integral component of membrane |
| Efet.01.371006.g590.t1 | follicular epithelium yolk subunit | 663 | 2.65E-28 | 53.35 |  |
| Efet.01.371070.g592.t1 | LRR and PYD domains-containing 14-like isoform X4 | 2067 | 1.75E-77 | 55.55 |  |
| Efet.01.371075.g593.t1 | RNA-binding 15B | 1689 | 3.83E-102 | 60.3 | F:nucleotide binding; F:nucleic acid binding; C:nucleus; P:intracellular signal transduction; C:intracellular; P:mRNA splicing, via spliceosome |
| Efet.01.3711.g312.t1 | intron-binding aquarius | 249 | 2.61E-30 | 85.8 | C:spliceosomal complex; P:mRNA splicing, via spliceosome |
| Efet.01.371110.g595.t1 | phosphofurin acidic cluster sorting 2 | 357 | 2.91E-06 | 78 |  |
| Efet.01.371157.g596.t1 | hypothetical protein CAPTEDRAFT_198404 | 1554 | 1.02E-83 | 51.65 |  |
| Efet.01.371278.g597.t1 | alpha-2C adrenergic receptor-like | 1629 | 3.58E-35 | 70.25 | F:G-protein coupled receptor activity; P:G-protein coupled receptor signaling pathway; C:membrane |
| Efet.01.371294.g598.t1 | glycine receptor subunit alpha-3-like isoform X1 | 390 | 2.61E-29 | 68.55 | P:ion transmembrane transport; C:integral component of membrane; C:cell junction; F:extracellular ligand-gated ion channel activity; C:synapse; C:plasma membrane |
| Efet.01.371309.g599.t1 | potassium voltage-gated channel subfamily H member 7-like isoform X3 | 372 | 3.96E-23 | 67.4 | P:ion transport; P:transmembrane transport; P:cellular process |
| Efet.01.37132.g800.t1 | thyrotropin-releasing hormone receptor-like | 354 | 1.61E-24 | 70.95 | F:thyrotropin-releasing hormone receptor activity; P:G-protein coupled receptor signaling pathway; C:integral component of membrane |
| Efet.01.37135.g802.t1 | dexamethasone-induced Ras-related 1 isoform X2 | 441 | 2.85E-22 | 74.2 | F:GTP binding; F:protein binding; P:G-protein coupled receptor signaling pathway; P:small GTPase mediated signal transduction; C:intracellular membrane-bounded organelle; F:GTPase activity; C:membrane; C:cytoplasmic part |
| Efet.01.371355.g602.t1 | low-density lipo receptor-related 2-like | 441 | 8.12E-26 | 62.9 | F:calcium ion binding; P:proteolysis; C:membrane; C:integral component of membrane; F:serine-type endopeptidase activity |
| Efet.01.371376.g603.t1 | growth arrest-specific 1-like | 939 | 1.49E-35 | 46.5 | C:membrane; C:integral component of membrane |
| Efet.01.371413.g604.t1 | tripartite motif-containing 3-like | 1182 | 1.17E-10 | 56.7 | P:carbohydrate metabolic process; F:zinc ion binding; F:oxidoreductase activity, acting on the CH-OH group of donors, quinone or similar compound as acceptor; F:quinone binding; F:metal ion binding; P:oxidation-reduction process; C:intracellular |
| Efet.01.371414.g605.t1 | tripartite motif-containing 3-like | 1182 | 1.17E-10 | 56.7 | P:carbohydrate metabolic process; F:zinc ion binding; F:oxidoreductase activity, acting on the CH-OH group of donors, quinone or similar compound as acceptor; F:quinone binding; F:metal ion binding; P:oxidation-reduction process; C:intracellular |
| Efet.01.371415.g606.t1 | Planctomycete cytochrome C | 786 | 1.80E-167 | 83.1 | F:electron carrier activity; F:heme binding |
| Efet.01.371415.g607.t1 | DUF1501 domain-containing | 270 | 3.28E-35 | 91.85 | F:catalytic activity; P:metabolic process |
| Efet.01.371428.g609.t1 | glutamate receptor | 261 | 5.47E-22 | 71.9 | P:ion transport; F:transmembrane signaling receptor activity; F:extracellular ligand-gated ion channel activity; C:synapse; C:membrane part; C:plasma membrane |
| Efet.01.371471.g610.t1 | NHL repeat containing | 582 | 8.54E-10 | 55.67 | C:membrane; C:integral component of membrane |
| Efet.01.371545.g611.t1 | hypothetical protein CAPTEDRAFT_26965, partial | 417 | 4.36E-33 | 57.95 |  |
| Efet.01.371551.g612.t1 | glutathione S-transferase A-like | 267 | 5.31E-30 | 75.65 | F:transferase activity |
| Efet.01.3717.g313.t1 | DNA-binding SATB2 | 375 | 7.33E-11 | 64.42 | F:DNA binding; C:nucleus |
| Efet.01.37180.g805.t1 | LRR and PYD domains-containing 12-like isoform X2 | 795 | 7.21E-14 | 47.83 |  |
| Efet.01.371801.g613.t1 | Metabotropic glutamate receptor | 1002 | 1.03E-90 | 78.3 | C:presynaptic membrane; C:integral component of plasma membrane; F:G-protein coupled receptor activity; P:negative regulation of adenylate cyclase activity; C:postsynapse; F:glutamate receptor activity; P:adenylate cyclase-inhibiting G-protein coupled glutamate receptor signaling pathway; P:chemical synaptic transmission; C:neuron projection; P:regulation of synaptic transmission, glutamatergic |
| Efet.01.371875.g615.t1 | aminopeptidase N-like | 465 | 5.26E-14 | 51.9 | F:zinc ion binding; C:membrane; P:proteolysis; C:integral component of membrane; F:aminopeptidase activity; F:metallopeptidase activity; F:hydrolase activity |
| Efet.01.371952.g617.t1 | myosin light chain smooth muscle-like | 477 | 1.18E-41 | 69.7 | F:nucleotide binding; P:phosphorylation; F:protein serine/threonine kinase activity |
| Efet.01.371981.g618.t1 | double-stranded RNA-specific editase 1 isoform X2 | 1077 | 1.92E-132 | 71.95 | P:RNA metabolic process; F:RNA binding; C:nuclear lumen; F:adenosine deaminase activity; P:negative regulation of cellular process |
| Efet.01.372209.g623.t1 | hypothetical protein HELRODRAFT_179042 | 309 | 7.64E-08 | 60 |  |
| Efet.01.372232.g624.t1 | neuronal acetylcholine receptor subunit alpha-10-like | 363 | 4.03E-54 | 81.95 | C:postsynaptic membrane; P:cation transmembrane transport; C:integral component of membrane; C:cell junction; F:extracellular ligand-gated ion channel activity |
| Efet.01.372242.g625.t1 | 7-dehydrocholesterol reductase-like | 1143 | 4.64E-168 | 73.7 | C:membrane |
| Efet.01.372247.g626.t1 | muscle calcium channel subunit alpha-1-like | 495 | 4.05E-33 | 65.1 | P:ion transmembrane transport; F:cation channel activity; C:integral component of membrane; F:voltage-gated ion channel activity; F:metal ion transmembrane transporter activity; P:metal ion transport |
| Efet.01.37225.g806.t1 | mediator of RNA polymerase II transcription subunit 14 isoform X1 | 849 | 5.19E-48 | 58 | C:core mediator complex; P:intracellular steroid hormone receptor signaling pathway; C:mediator complex; F:RNA polymerase II transcription cofactor activity; P:regulation of transcription from RNA polymerase II promoter |
| Efet.01.37227.g807.t1 | serine threonine- kinase WNK3 | 282 | 6.45E-13 | 71.4 | P:regulation of biological quality; P:positive regulation of cation transmembrane transport; P:positive regulation of cellular process; P:positive regulation of sodium ion transport; C:cell junction; F:protein kinase activity; P:protein phosphorylation; P:positive regulation of ion transmembrane transporter activity; P:negative regulation of biological process; P:positive regulation of rubidium ion transport |
| Efet.01.3723.g314.t1 | PREDICTED: uncharacterized protein LOC105317617 | 1458 | 2.42E-51 | 57 | F:nucleic acid binding; P:nucleic acid phosphodiester bond hydrolysis; F:3'-5' exonuclease activity; P:nucleobase-containing compound metabolic process |
| Efet.01.372426.g628.t1 | somatostatin receptor type 2-like | 1119 | 1.31E-105 | 62.4 | F:G-protein coupled peptide receptor activity; P:G-protein coupled receptor signaling pathway; C:membrane |
| Efet.01.37251.g810.t1 | gastrula zinc finger -like | 1401 | 5.35E-73 | 56.8 | F:nucleic acid binding; F:metal ion binding |
| Efet.01.372525.g629.t1 | peripheral plasma membrane CASK isoform X6 | 300 | 1.96E-17 | 69.2 | F:ATP binding; F:protein kinase activity; P:protein phosphorylation |
| Efet.01.372580.g631.t1 | mynd domain | 1026 | 2.79E-08 | 47.31 | F:metal ion binding |
| Efet.01.372596.g633.t1 | somatomedin-B and thrombospondin type-1 domain-containing -like | 345 | 2.69E-10 | 52.75 | P:immune response; F:scavenger receptor activity; P:receptor-mediated endocytosis; F:polysaccharide binding |
| Efet.01.372604.g634.t1 | tyrosinase tyr-3 | 762 | 2.29E-33 | 50.35 | F:oxidoreductase activity; F:metal ion binding; P:metabolic process; P:oxidation-reduction process |
| Efet.01.372691.g636.t1 | dnaJ homolog subfamily C member 30-like | 741 | 3.76E-30 | 57.25 | C:membrane; C:integral component of membrane |
| Efet.01.372742.g637.t1 | sodium-dependent phosphate transport 2B | 1431 | 6.67E-122 | 70.4 | F:inorganic phosphate transmembrane transporter activity; F:sodium:phosphate symporter activity; F:sodium-dependent phosphate transmembrane transporter activity; C:brush border; P:phosphate ion transport; C:cytoplasm; F:ion binding; C:vesicle; P:inorganic ion transmembrane transport; C:apical plasma membrane; P:cellular phosphate ion homeostasis |
| Efet.01.372751.g639.t1 | myTH4 domain | 687 | 4.61E-66 | 68.35 | F:nucleotide binding; C:cytoskeleton |
| Efet.01.372774.g641.t1 | cAMP-specific 3 ,5 -cyclic phosphodiesterase | 600 | 2.33E-69 | 81.35 | C:postsynaptic density; F:drug binding; C:synaptic vesicle; C:gamma-tubulin complex; C:dendritic spine; P:positive regulation of interferon-gamma production; P:negative regulation of peptidyl-serine phosphorylation; C:cytosol; P:regulation of cardiac muscle cell contraction; C:excitatory synapse; F:ion channel binding; F:3',5'-cyclic-AMP phosphodiesterase activity; P:regulation of ryanodine-sensitive calcium-release channel activity; C:Z disc; F:ubiquitin protein ligase binding; F:metal ion binding; P:regulation of cAMP metabolic process; P:cellular response to cAMP; C:apical plasma membrane; P:positive regulation of interleukin-5 production; P:multicellular organism growth; P:regulation of release of sequestered calcium ion into cytosol by sarcoplasmic reticulum; C:voltage-gated calcium channel complex; P:negative regulation of insulin secretion; P:regulation of high voltage-gated calcium channel activity; P:regulation of cell communication by electrical coupling involved in cardiac conduction; P:negative regulation of heart contraction; P:smooth muscle contraction; P:cellular response to epinephrine stimulus; C:cilium; P:T cell receptor signaling pathway; F:beta-2 adrenergic receptor binding; P:positive regulation of interleukin-2 production; P:adrenergic receptor signaling pathway involved in positive regulation of heart rate; P:cellular response to drug; F:gamma-tubulin binding; P:cAMP catabolic process; P:neutrophil homeostasis; P:aging; P:establishment of endothelial barrier; P:negative regulation of relaxation of cardiac muscle; F:scaffold protein binding; C:extracellular space; C:centrosome; P:cellular response to lipopolysaccharide; P:neutrophil chemotaxis; F:cAMP binding; P:cAMP-mediated signaling; F:ATPase binding |
| Efet.01.372827.g642.t1 | hypothetical protein CAPTEDRAFT_197375 | 324 | 3.45E-16 | 52.33 | C:membrane; C:integral component of membrane |
| Efet.01.372855.g643.t1 | helicase ARIP4-like | 330 | 3.41E-57 | 88.95 | F:translation initiation factor activity; F:ATP binding; F:helicase activity; P:translational initiation; C:eukaryotic translation initiation factor 3 complex |
| Efet.01.372911.g644.t1 | vesicular inhibitory amino acid transporter-like | 1290 | 0 | 70.3 | C:membrane; F:amino acid transmembrane transporter activity; P:amino acid transmembrane transport |
| Efet.01.37294.g811.t1 | rotatin isoform X1 | 969 | 1.09E-47 | 53.65 | C:centrosome; C:ciliary basal body; P:cilium organization; C:membrane; C:integral component of membrane; P:determination of left/right symmetry |
| Efet.01.372944.g645.t1 | hypothetical protein CAPTEDRAFT_218862 | 312 | 2.96E-38 | 65.67 |  |
| Efet.01.37300.g812.t1 | ribosome-binding factor mitochondrial | 969 | 7.22E-22 | 59.85 | P:rRNA processing |
| Efet.01.37305.g814.t1 | enolase 3-2 | 381 | 8.25E-72 | 94.8 | P:gluconeogenesis; C:myelin sheath; P:transcription, DNA-templated; P:response to virus; P:canonical glycolysis; C:phosphopyruvate hydratase complex; F:RNA binding; F:magnesium ion binding; F:phosphopyruvate hydratase activity; P:negative regulation of transcription from RNA polymerase II promoter; C:nucleus; F:DNA binding; C:extracellular space; F:transcription factor activity, sequence-specific DNA binding; C:extracellular exosome; P:positive regulation of binding; F:transcription corepressor activity; F:protein heterodimerization activity; P:in utero embryonic development; P:negative regulation of cell growth; F:GTPase binding; C:M band; C:extrinsic component of plasma membrane |
| Efet.01.373055.g646.t1 | ATP-binding cassette sub-family A member 3-like | 420 | 3.65E-39 | 72.75 | F:nucleotide binding; P:lipid transport; F:ATPase activity, coupled to transmembrane movement of substances; C:intracellular membrane-bounded organelle; C:membrane |
| Efet.01.37320.g815.t1 | cAMP-dependent kinase type II regulatory subunit-like | 927 | 1.34E-114 | 66.35 | F:protein binding; P:behavioral response to cocaine; C:cytoplasm; P:locomotor rhythm; P:axon guidance; P:phosphorylation; P:behavioral response to ethanol; F:transferase activity; C:plasma membrane |
| Efet.01.37326.g816.t1 | substance-P receptor-like isoform X1 | 432 | 5.47E-27 | 59.05 | F:G-protein coupled receptor activity; P:neuropeptide signaling pathway; P:G-protein coupled receptor signaling pathway; P:signal transduction; C:membrane; C:integral component of membrane; F:signal transducer activity; F:neuropeptide Y receptor activity |
| Efet.01.373276.g647.t1 | Inhibitor of apoptosis | 549 | 1.44E-09 | 41.5 | P:regulation of programmed cell death |
| Efet.01.373378.g650.t1 | -tyrosine sulfotransferase 1-like | 450 | 5.67E-49 | 74.4 | C:membrane; C:Golgi apparatus; F:protein-tyrosine sulfotransferase activity; P:peptidyl-tyrosine sulfation |
| Efet.01.373448.g652.t1 | GLUG motif-containing | 663 | 1.55E-71 | 67.4 |  |
| Efet.01.37345.g818.t1 | uncharacterized protein LOC110460117 isoform X2 | 636 | 3.17E-30 | 50.15 |  |
| Efet.01.37345.g819.t1 | Neurexin-2-alpha | 1359 | 2.63E-109 | 53.45 |  |
| Efet.01.373468.g653.t1 | oxygen-independent coproporphyrinogen III oxidase | 1296 | 0 | 92.9 | C:cytoplasm; F:coproporphyrinogen oxidase activity; F:metal ion binding; F:coproporphyrinogen dehydrogenase activity; P:oxidation-reduction process; F:4 iron, 4 sulfur cluster binding; P:protoporphyrinogen IX biosynthetic process |
| Efet.01.373488.g654.t1 | alsin-like isoform X1 | 852 | 2.57E-23 | 48.25 | C:neuron part; F:guanyl-nucleotide exchange factor activity; P:regulation of catalytic activity; P:cellular component organization; P:single-organism cellular process; C:organelle; C:cell projection; C:intracellular part; P:regulation of biological process |
| Efet.01.373495.g655.t1 | lysophospholipid acyltransferase 7-like | 216 | 4.29E-08 | 58 |  |
| Efet.01.37351.g820.t1 | Tubulin epsilon chain | 909 | 1.96E-56 | 59.2 | C:membrane; C:integral component of membrane |
| Efet.01.373603.g657.t1 | AN1-type zinc finger 3-like isoform X1 | 237 | 1.78E-06 | 80.67 | F:DNA binding; F:zinc ion binding; F:metal ion binding |
| Efet.01.373610.g658.t1 | UDP-N-acetylglucosamine--N-acetylmuramyl-(pentapeptide) pyrophosphoryl-undecaprenol N-acetylglucosamine transferase | 381 | 1.17E-26 | 79.8 | P:regulation of cell shape; P:cell division; P:carbohydrate metabolic process; F:UDP-N-acetyl-D-glucosamine:N-acetylmuramoyl-L-alanyl-D-glutamyl-meso-2,6-diaminopimelyl-D-alanyl-D-alanine-diphosphoundecaprenol 4-beta-N-acetylglucosaminlytransferase activity; P:peptidoglycan biosynthetic process; P:cell wall organization; C:plasma membrane; F:undecaprenyldiphospho-muramoylpentapeptide beta-N-acetylglucosaminyltransferase activity; P:cell cycle; P:lipid glycosylation |
| Efet.01.373679.g659.t1 | Chromosome 14 open reading frame 119 | 333 | 8.31E-29 | 66.9 | C:mitochondrion |
| Efet.01.373741.g660.t1 | tyrosine- phosphatase Lar-like isoform X1 | 378 | 3.63E-25 | 65.85 | F:phosphoprotein phosphatase activity; P:protein dephosphorylation |
| Efet.01.373754.g661.t1 | filamin-A-like isoform X1 | 1089 | 3.49E-20 | 45.1 | F:actin filament binding; F:actin binding |
| Efet.01.373783.g662.t1 | mitochondrial inner membrane OXA1L-like isoform X2 | 345 | 2.65E-39 | 54.5 | C:membrane; C:integral component of membrane; P:protein insertion into membrane |
| Efet.01.373825.g663.t1 | gamma-aminobutyric acid receptor subunit beta-like | 285 | 2.42E-12 | 68.2 | C:membrane; P:transport |
| Efet.01.373950.g664.t1 | tribbles homolog 2-like | 483 | 9.56E-49 | 74.5 | C:nucleus; P:regulation of MAP kinase activity; P:negative regulation of cellular process; P:positive regulation of proteasomal ubiquitin-dependent protein catabolic process; F:mitogen-activated protein kinase kinase binding |
| Efet.01.37398.g822.t1 | tyramine receptor 1 | 1338 | 4.87E-132 | 62.35 | P:signal transduction; C:membrane; F:signal transducer activity |
| Efet.01.374274.g668.t1 | kintoun isoform X1 | 1083 | 1.36E-24 | 59.65 |  |
| Efet.01.37455.g825.t1 | plectin-like isoform X4 | 969 | 6.01E-76 | 61.05 | C:cytoskeleton |
| Efet.01.374558.g673.t1 | thyrotropin-releasing hormone receptor | 1137 | 1.70E-70 | 51 | F:G-protein coupled receptor activity; P:G-protein coupled receptor signaling pathway; P:signal transduction; C:membrane; C:integral component of membrane; F:signal transducer activity |
| Efet.01.3748.g318.t1 | hypothetical protein HELRODRAFT_63031 | 255 | 7.26E-09 | 100 | C:nucleus; P:transcription, DNA-templated; P:regulation of transcription, DNA-templated; F:protein dimerization activity |
| Efet.01.374803.g678.t1 | teneurin-m-like isoform X1 | 1764 | 9.32E-147 | 57.25 | P:self proteolysis |
| Efet.01.374844.g679.t1 | RING finger 17 | 1029 | 3.16E-12 | 64.75 |  |
| Efet.01.374880.g680.t1 | prolactin-releasing peptide receptor-like | 909 | 1.38E-64 | 62.95 | P:signal transduction; F:signal transducer activity |
| Efet.01.374889.g681.t1 | insulin-like growth factor-binding complex acid labile subunit | 1161 | 2.52E-35 | 48.9 | P:negative regulation of establishment of protein localization to plasma membrane; C:extracellular exosome; F:collagen binding; C:membrane; F:fibronectin binding; C:integral component of membrane; P:receptor-mediated virion attachment to host cell; F:laminin binding; P:positive regulation of cell migration |
| Efet.01.374937.g682.t1 | neurogenic differentiation factor 1-like | 1008 | 2.70E-25 | 78.35 | P:regulation of transcription, DNA-templated; F:protein dimerization activity; P:neuron development |
| Efet.01.37497.g826.t1 | hypothetical protein HELRODRAFT_190994 | 366 | 1.70E-20 | 67.2 | P:metabolic process |
| Efet.01.3750.g319.t1 | PH domain leucine-rich repeat-containing phosphatase 2-like isoform X3 | 318 | 1.36E-39 | 78.2 | F:catalytic activity |
| Efet.01.375164.g684.t1 | serine palmitoyltransferase 2-like | 435 | 1.01E-38 | 88.05 | C:endoplasmic reticulum membrane; F:pyridoxal phosphate binding; F:serine C-palmitoyltransferase activity; C:integral component of membrane; P:biosynthetic process; P:sphingolipid metabolic process |
| Efet.01.375186.g685.t1 | Wnt-4 | 447 | 6.02E-67 | 75.45 | P:multicellular organism development; C:membrane; P:Wnt signaling pathway; F:receptor binding; C:proteinaceous extracellular matrix |
| Efet.01.3752.g320.t1 | lactase-phlorizin hydrolase-like | 756 | 5.39E-54 | 58.15 | P:metabolic process; F:hydrolase activity, hydrolyzing O-glycosyl compounds |
| Efet.01.375277.g687.t1 | somatostatin receptor type 2-like | 1107 | 5.04E-76 | 49.85 | F:G-protein coupled receptor activity; P:G-protein coupled receptor signaling pathway |
| Efet.01.375403.g689.t1 | hypothetical protein CAPTEDRAFT_226428 | 1107 | 1.73E-45 | 55.5 | F:calcium ion binding; C:membrane; C:integral component of membrane |
| Efet.01.375426.g690.t1 | Voltage-dependent calcium channel gamma-5 subunit | 201 | 1.50E-11 | 68.91 | P:calcium ion transport; C:integral component of membrane |
| Efet.01.375573.g692.t1 | zinc finger 90 isoform X3 | 1206 | 1.39E-44 | 47.7 | F:binding |
| Efet.01.375689.g693.t1 | polypeptide N-acetylgalactosaminyltransferase 13-like isoform X1 | 414 | 3.79E-47 | 86.95 | F:transferase activity, transferring glycosyl groups; C:integral component of membrane; P:protein glycosylation; C:Golgi membrane; F:carbohydrate binding |
| Efet.01.375701.g694.t1 | ectopic P granules 5 homolog | 201 | 1.76E-11 | 69.13 | P:autophagy |
| Efet.01.375715.g695.t1 | ABC transporter permease | 600 | 5.10E-105 | 76 | C:membrane |
| Efet.01.375767.g696.t1 | hypothetical protein HELRODRAFT_188786 | 1263 | 6.51E-23 | 66.67 | C:membrane |
| Efet.01.375887.g700.t1 | ATP-dependent helicase | 696 | 4.39E-164 | 81.2 | F:ATP binding; F:helicase activity; F:protein serine/threonine kinase activity; P:protein phosphorylation |
| Efet.01.375927.g702.t1 | cholecystokinin receptor-like | 423 | 2.13E-24 | 76.85 | P:G-protein coupled receptor signaling pathway; F:gastrin receptor activity; C:integral component of membrane; F:transferase activity |
| Efet.01.37594.g831.t1 | E3 ubiquitin- ligase HECW1 isoform X1 | 708 | 2.04E-61 | 62.25 | F:ubiquitin-protein transferase activity; C:intracellular part; P:protein ubiquitination |
| Efet.01.375976.g703.t1 | hypothetical protein CAPTEDRAFT_222467 | 258 | 4.99E-13 | 70 | F:pyridoxal phosphate binding; F:catalytic activity |
| Efet.01.376044.g705.t1 | uncharacterized protein LOC106461344 | 471 | 1.82E-06 | 65 |  |
| Efet.01.376094.g706.t1 | GRB2-associated and regulator of MAPK -like | 687 | 3.74E-12 | 60.85 |  |
| Efet.01.376215.g707.t1 | neuroligin- Y-linked isoform X1 | 333 | 4.27E-22 | 67.6 | C:integral component of plasma membrane; P:postsynaptic membrane assembly; F:neurexin family protein binding; P:neuron cell-cell adhesion; P:modulation of synaptic transmission; C:synapse; F:receptor activity; F:carboxylic ester hydrolase activity; C:cell surface |
| Efet.01.376288.g709.t1 | TPA_inf: p63gamma | 285 | 1.26E-15 | 66.2 | F:DNA binding; C:nucleus; F:transcription factor activity, sequence-specific DNA binding; P:protein tetramerization; P:apoptotic process; F:transcription regulatory region DNA binding; P:regulation of transcription, DNA-templated |
| Efet.01.3763.g321.t1 | metabotropic glutamate receptor 2-like | 882 | 9.36E-44 | 56.2 | F:G-protein coupled receptor activity; P:G-protein coupled receptor signaling pathway; P:signal transduction; C:membrane; C:integral component of membrane; F:signal transducer activity |
| Efet.01.376308.g710.t1 | neurogenic locus notch homolog 1-like | 453 | 3.07E-20 | 64.35 | P:single-organism process |
| Efet.01.376380.g711.t1 | mab-21-like | 951 | 1.78E-140 | 79.9 |  |
| Efet.01.37641.g832.t1 | hypothetical protein HELRODRAFT_182778 | 399 | 8.12E-08 | 47 |  |
| Efet.01.376469.g712.t1 | probable S-acyltransferase 23 | 312 | 5.48E-18 | 67.1 | F:transferase activity |
| Efet.01.376471.g713.t1 | hypothetical protein HELRODRAFT_170343 | 1563 | 5.01E-25 | 70.25 | F:binding |
| Efet.01.376498.g715.t1 | E3 ubiquitin- ligase DTX3L | 807 | 4.06E-33 | 48.3 | F:zinc ion binding; F:metal ion binding |
| Efet.01.376552.g716.t1 | shTK domain | 405 | 6.70E-13 | 64.45 | P:metabolic process |
| Efet.01.376597.g718.t1 | carbohydrate sulfotransferase 1-like | 1092 | 3.50E-28 | 50.15 | C:membrane; C:integral component of membrane; F:sulfotransferase activity |
| Efet.01.376603.g719.t1 | eukaryotic translation initiation factor 3 subunit C-like isoform X1 | 420 | 3.15E-64 | 85.35 | F:translation initiation factor activity; P:formation of translation preinitiation complex; P:positive regulation of translation; C:eukaryotic translation initiation factor 3 complex, eIF3m; C:eukaryotic 43S preinitiation complex; F:ribosome binding; C:cytosol; C:eukaryotic 48S preinitiation complex; F:translation initiation factor binding; P:regulation of translational initiation; F:RNA binding; P:positive regulation of mRNA binding |
| Efet.01.376621.g720.t1 | hypothetical protein CAPTEDRAFT_228538 | 579 | 1.96E-07 | 50 |  |
| Efet.01.37663.g833.t1 | Krueppel-like factor 5 | 270 | 3.24E-20 | 85.3 | C:nucleus; F:DNA binding; F:transcription factor activity, sequence-specific DNA binding; F:zinc ion binding; F:transcription coactivator activity; P:transcription, DNA-templated; P:positive regulation of transcription, DNA-templated; P:axon guidance; P:regulation of transcription from RNA polymerase II promoter; P:dendrite morphogenesis |
| Efet.01.376672.g721.t1 | Neuropeptide FF receptor 2 | 228 | 1.38E-20 | 66.8 | C:integral component of plasma membrane; F:neuropeptide binding; P:neuropeptide signaling pathway; P:G-protein coupled receptor signaling pathway, coupled to cyclic nucleotide second messenger; C:neuron projection; F:neuropeptide Y receptor activity |
| Efet.01.376824.g722.t1 | kinesin light chain | 507 | 2.33E-51 | 78.8 | F:microtubule motor activity; C:cytoplasm; C:kinesin complex; C:microtubule |
| Efet.01.376846.g723.t1 | S-adenosylmethionine synthase isoform type-2-like | 588 | 1.15E-91 | 90.95 | C:nucleus; F:ATP binding; F:metal ion binding; C:cytosol; P:S-adenosylmethionine biosynthetic process; F:methionine adenosyltransferase activity; P:one-carbon metabolic process; P:methionine metabolic process |
| Efet.01.37685.g834.t1 | novel [Xenopus tropicalis] | 1104 | 5.18E-84 | 52.15 |  |
| Efet.01.37687.g835.t1 | alpha-1,3-mannosyl-glyco 4-beta-N-acetylglucosaminyltransferase C-like | 1191 | 6.33E-147 | 62.1 | F:transferase activity, transferring hexosyl groups; P:primary metabolic process; P:organic substance metabolic process |
| Efet.01.376922.g726.t1 | Nucleoporin NSP1 ame: Full=Nuclear pore NSP1 ame: Full=Nucleoskeletal ame: Full=p110 | 813 | 1.17E-13 | 46.43 | C:nuclear pore nuclear basket; P:intracellular transport; F:neuropeptide hormone activity; F:nucleocytoplasmic transporter activity; P:transcription-dependent tethering of RNA polymerase II gene DNA at nuclear periphery; C:intracellular; C:nuclear pore central transport channel; C:nuclear pore; P:ribosomal large subunit export from nucleus; P:protein import into nucleus; P:NLS-bearing protein import into nucleus; F:structural constituent of nuclear pore; P:poly(A)+ mRNA export from nucleus |
| Efet.01.377032.g727.t1 | hypothetical protein CAPTEDRAFT_193384 | 246 | 6.81E-11 | 71.5 | P:macromolecule metabolic process; F:hydrolase activity |
| Efet.01.37704.g837.t1 | leucine-rich repeat and immunoglobulin-like domain-containing nogo receptor-interacting 3 | 579 | 3.44E-13 | 54.05 | P:axonogenesis; C:membrane; C:integral component of membrane |
| Efet.01.37705.g838.t1 | toll-like receptor Tollo | 798 | 2.23E-16 | 54.05 | P:Toll signaling pathway; F:transmembrane signaling receptor activity; P:signal transduction; P:zygotic specification of dorsal/ventral axis; C:membrane; C:integral component of membrane; P:response to fungus; P:positive regulation of antibacterial peptide biosynthetic process; P:defense response to Gram-positive bacterium |
| Efet.01.377106.g728.t1 | hypothetical protein HELRODRAFT_162084 | 3219 | 6.63E-06 | 64 |  |
| Efet.01.377118.g729.t1 | fibrinogen and fibronectin | 471 | 1.54E-06 | 71.89 |  |
| Efet.01.37717.g839.t1 | nidogen-1-like isoform X2 | 1002 | 1.11E-58 | 51.2 | F:peroxidase activity; F:iodide peroxidase activity; P:cell-matrix adhesion; F:oxidoreductase activity; F:calcium ion binding; P:cellular oxidant detoxification; P:oxidation-reduction process |
| Efet.01.377262.g732.t1 | headcase isoform X2 | 1344 | 9.72E-67 | 66.55 |  |
| Efet.01.377271.g735.t1 | TPA: SCO-spondin | 777 | 4.17E-19 | 45.8 | F:catalytic activity; P:metabolic process; P:cell differentiation; P:nervous system development |
| Efet.01.377309.g736.t1 | serine threonine- kinase TBK1 | 366 | 5.07E-28 | 57.05 | P:positive regulation of cellular process; F:binding |
| Efet.01.377322.g737.t1 | peripheral plasma membrane CASK-like | 246 | 7.16E-36 | 90.95 | C:presynaptic membrane; P:calcium ion import; P:GDP metabolic process; P:cell adhesion; P:GMP metabolic process; F:guanylate kinase activity; C:nucleolus; P:protein phosphorylation; P:positive regulation of transcription from RNA polymerase II promoter; C:nuclear lamina; P:negative regulation of keratinocyte proliferation; F:ATP binding; C:basement membrane; C:focal adhesion; F:neurexin family protein binding; C:actin cytoskeleton; C:cytosol; C:ciliary membrane; P:negative regulation of wound healing; P:negative regulation of cellular response to growth factor stimulus; F:protein serine/threonine kinase activity; P:neurotransmitter secretion; P:positive regulation of calcium ion import; F:calmodulin binding; C:cell-cell junction; P:negative regulation of cell-matrix adhesion; C:integral component of membrane; C:nuclear matrix; C:vesicle; C:basolateral plasma membrane |
| Efet.01.377327.g738.t1 | caspase recruitment domain-containing 9 | 609 | 6.58E-40 | 59.1 | P:regulation of interleukin-6 biosynthetic process; P:response to peptidoglycan; P:response to muramyl dipeptide; P:positive regulation of NF-kappaB transcription factor activity; P:regulation of tumor necrosis factor biosynthetic process; P:regulation of interleukin-2 biosynthetic process; P:response to exogenous dsRNA; P:defense response to virus; P:positive regulation of tumor necrosis factor production; P:defense response to Gram-positive bacterium; P:RNA phosphodiester bond hydrolysis, endonucleolytic; F:protein domain specific binding; F:protein homodimerization activity; P:response to fungus; P:regulation of apoptotic process; P:I-kappaB kinase/NF-kappaB signaling; P:immune system process; F:nucleic acid binding; F:RNA-DNA hybrid ribonuclease activity; F:protein binding; P:positive regulation of innate immune response; C:cytoplasm; P:positive regulation of JNK cascade; P:innate immune response; P:response to drug; F:CARD domain binding; P:positive regulation of interleukin-6 production; P:positive regulation of stress-activated MAPK cascade |
| Efet.01.377408.g740.t1 | diguanylate cyclase | 411 | 1.87E-78 | 91.7 | F:methyltransferase activity; P:methylation |
| Efet.01.3775.g322.t1 | innexin unc-9-like | 609 | 7.36E-62 | 54.75 | C:gap junction; P:ion transport; P:transmembrane transport; C:membrane; C:integral component of membrane; P:cell communication by electrical coupling; C:cell junction; F:gap junction channel activity; C:synapse; P:transport; C:plasma membrane |
| Efet.01.377513.g741.t1 | Polypeptide N-acetylgalactosaminyltransferase 5 | 258 | 1.27E-12 | 68.35 | F:transferase activity, transferring glycosyl groups; C:membrane |
| Efet.01.377589.g743.t1 | sex peptide receptor-like | 1125 | 3.10E-114 | 66.15 | F:G-protein coupled receptor activity; F:G-protein coupled peptide receptor activity; P:G-protein coupled receptor signaling pathway; C:membrane; C:integral component of membrane |
| Efet.01.377762.g744.t1 | phosphatidylinositol 4-kinase type 2-alpha | 258 | 1.51E-08 | 68.9 | P:phosphorylation; F:kinase activity |
| Efet.01.377804.g745.t1 | calcium-transporting ATPase type 2C member 1-like isoform X1 | 246 | 2.09E-40 | 91.25 | C:integral component of plasma membrane; F:ATP binding; F:manganese-transporting ATPase activity; F:metal ion binding; P:manganese ion transmembrane transport; P:calcium ion transmembrane transport; C:Golgi membrane; F:calcium-transporting ATPase activity; P:cellular calcium ion homeostasis |
| Efet.01.377814.g746.t1 | calcium-dependent secretion activator 1- | 624 | 1.42E-18 | 85.4 | C:presynapse; P:dense core granule exocytosis; C:cytosol; P:synaptic vesicle exocytosis |
| Efet.01.377886.g747.t1 | myosin heavy striated muscle isoform X1 | 741 | 9.13E-49 | 73.35 | F:nucleotide binding; F:actin binding; F:anion binding |
| Efet.01.377895.g748.t1 | E3 ubiquitin- ligase HERC2-like isoform X1 | 666 | 8.46E-86 | 66.5 | F:ubiquitin-protein transferase activity; F:zinc ion binding; F:metal ion binding; F:ligase activity; P:protein ubiquitination |
| Efet.01.3779.g323.t1 | 5-hydroxytryptamine receptor-like | 1467 | 1.17E-138 | 60.5 | P:signal transduction; C:membrane; F:signal transducer activity |
| Efet.01.37794.g846.t1 | hypothetical protein CAPTEDRAFT_221325 | 399 | 1.65E-09 | 53 |  |
| Efet.01.37807.g850.t1 | S phase cyclin A-associated in the endoplasmic reticulum-like isoform X2 | 660 | 1.73E-34 | 65.05 | F:nucleic acid binding; F:zinc ion binding |
| Efet.01.378076.g749.t1 | E3 ubiquitin- ligase DZIP3 | 4146 | 0 | 44.5 |  |
| Efet.01.378160.g753.t1 | microtubule-actin cross-linking factor isoforms 1 2 3 5-like | 1026 | 3.85E-51 | 54.8 | P:cell cycle arrest; C:cytoskeleton; F:calcium ion binding |
| Efet.01.378313.g756.t1 | Solute carrier family 13 member 2 | 423 | 2.43E-18 | 84.1 | P:sodium ion transport; P:transmembrane transport; C:integral component of membrane; F:transporter activity |
| Efet.01.37834.g853.t1 | unc-45 homolog B | 807 | 5.23E-59 | 68.5 |  |
| Efet.01.37836.g854.t1 | unc-13 homolog B-like isoform X9 | 363 | 4.15E-14 | 62 |  |
| Efet.01.378411.g757.t1 | NT-3 growth factor receptor-like | 429 | 5.61E-37 | 59.7 | F:nucleotide binding; F:protein tyrosine kinase activity; F:signaling receptor activity; C:integral component of membrane; P:cell surface receptor signaling pathway; P:protein phosphorylation; P:defense response |
| Efet.01.37851.g855.t1 | substance-K receptor-like | 891 | 6.38E-78 | 72.4 | P:neuropeptide signaling pathway; C:integral component of membrane; F:neuropeptide Y receptor activity |
| Efet.01.378586.g760.t1 | N-sulphoglucosamine sulphohydrolase | 396 | 1.98E-50 | 71.25 | F:hydrolase activity |
| Efet.01.378716.g761.t1 | aconitate hydratase | 489 | 2.49E-65 | 87.6 | P:tricarboxylic acid cycle; F:4 iron, 4 sulfur cluster binding; F:aconitate hydratase activity |
| Efet.01.378716.g762.t1 | aconitate hydratase | 315 | 2.70E-50 | 91.9 | P:tricarboxylic acid cycle; F:4 iron, 4 sulfur cluster binding; F:aconitate hydratase activity |
| Efet.01.378733.g763.t1 | random slug 5-like | 651 | 9.04E-62 | 64.85 |  |
| Efet.01.378747.g764.t1 | UDP-N-acetylmuramoyl-tripeptide--D-alanyl-D-alanine ligase | 225 | 3.41E-13 | 83.75 | P:regulation of cell shape; P:cell division; F:ATP binding; C:cytoplasm; P:peptidoglycan biosynthetic process; P:cell wall organization; F:UDP-N-acetylmuramoyl-tripeptide-D-alanyl-D-alanine ligase activity; P:cell cycle |
| Efet.01.378747.g765.t1 | UDP-N-acetylmuramoyl-L-alanyl-D-glutamate--2,6-diaminopimelate ligase | 789 | 1.69E-144 | 79.3 | F:magnesium ion binding; F:tetrahydrofolylpolyglutamate synthase activity; P:regulation of cell shape; P:cell division; F:ATP binding; C:cytoplasm; P:peptidoglycan biosynthetic process; P:cell wall organization; P:tetrahydrofolylpolyglutamate biosynthetic process; P:cell cycle |
| Efet.01.378778.g767.t1 | family transcriptional regulator | 1023 | 1.83E-150 | 89.1 | F:DNA binding; P:transcription, DNA-templated; P:regulation of transcription, DNA-templated |
| Efet.01.378778.g768.t1 | thioesterase | 420 | 1.19E-99 | 90.8 |  |
| Efet.01.378778.g769.t1 | short-chain dehydrogenase | 801 | 3.77E-164 | 96.3 | F:oxidoreductase activity; P:oxidation-reduction process |
| Efet.01.378778.g770.t1 | electron transfer flavo -ubiquinone oxidoreductase | 1701 | 0 | 93.35 | F:electron-transferring-flavoprotein dehydrogenase activity; P:oxidation-reduction process; F:iron-sulfur cluster binding |
| Efet.01.378802.g772.t1 | hypothetical protein CAPTEDRAFT_204751 | 441 | 4.48E-07 | 61 | F:G-protein coupled receptor activity; P:G-protein coupled receptor signaling pathway; C:membrane; C:integral component of membrane |
| Efet.01.37888.g857.t1 | ubiquitin- ligase E3C-like | 456 | 1.16E-73 | 86.3 | C:nucleus; F:ubiquitin-protein transferase activity; C:cytoplasm; F:ligase activity; P:protein ubiquitination involved in ubiquitin-dependent protein catabolic process |
| Efet.01.37890.g858.t1 | Calsyntenin-1 | 252 | 4.96E-18 | 61.2 | F:calcium ion binding; C:membrane; C:integral component of membrane; P:homophilic cell adhesion via plasma membrane adhesion molecules |
| Efet.01.378907.g775.t1 | cytoskeleton-associated 5 isoform X2 | 219 | 1.54E-16 | 82.7 | P:spindle organization; P:sister chromatid cohesion; C:microtubule plus-end; C:spindle pole; P:mitotic nuclear division; C:gamma-tubulin complex; P:cell division; C:centrosome; F:protein binding; P:G2/M transition of mitotic cell cycle; P:centrosome organization; C:membrane; C:cytosol; P:RNA transport; P:establishment or maintenance of microtubule cytoskeleton polarity |
| Efet.01.378919.g776.t1 | DNA helicase | 1185 | 0 | 89.1 | F:nucleic acid binding; F:ATP binding; P:DNA repair; F:ATP-dependent 3'-5' DNA helicase activity; P:DNA replication; P:DNA duplex unwinding; P:DNA recombination; P:SOS response; C:intracellular |
| Efet.01.378958.g777.t1 | GS homeobox 1 | 276 | 3.69E-18 | 77.15 | C:nucleus; P:regulation of transcription, DNA-templated; F:sequence-specific DNA binding |
| Efet.01.378975.g778.t1 | WD40-like Beta Propeller Repeat | 705 | 8.73E-58 | 64.95 | C:membrane |
| Efet.01.37904.g859.t1 | von Willebrand factor D and EGF domain-containing -like | 714 | 3.42E-07 | 51.33 |  |
| Efet.01.379140.g779.t1 | Angiotensin-converting enzyme | 417 | 4.65E-07 | 64.4 | F:carboxypeptidase activity; F:metal ion binding; F:peptidyl-dipeptidase activity; C:membrane; P:proteolysis; F:peptidase activity; F:metallopeptidase activity; F:hydrolase activity |
| Efet.01.37915.g860.t1 | helicase SKI2W | 291 | 1.90E-34 | 88.9 | F:ATP binding; F:RNA binding; F:RNA helicase activity; P:RNA catabolic process |
| Efet.01.379162.g780.t1 | gremlin-2-like isoform X2 | 420 | 3.19E-34 | 63.9 | P:biological regulation |
| Efet.01.3792.g324.t1 | casein kinase I | 972 | 0 | 91.4 | P:regulation of cell shape; C:nucleus; F:ATP binding; C:cytoplasm; P:phagocytosis; P:Wnt signaling pathway; F:protein serine/threonine kinase activity; P:peptidyl-serine phosphorylation |
| Efet.01.379243.g781.t1 | phosphatase 1B-like | 885 | 3.80E-158 | 81.85 | F:magnesium ion binding; F:protein serine/threonine phosphatase activity; P:protein dephosphorylation; F:manganese ion binding |
| Efet.01.379348.g783.t1 | otopetrin-2-like isoform X3 | 741 | 3.14E-32 | 62.15 | C:membrane; C:integral component of membrane |
| Efet.01.379381.g784.t1 | protocadherin beta-15 isoform X1 | 540 | 8.01E-28 | 59.7 | C:membrane |
| Efet.01.379455.g785.t1 | S-adenosyl-L-methionine-dependent methyltransferase | 711 | 7.11E-42 | 52.05 | P:biological_process; C:cytoplasm; F:methyltransferase activity; F:transferase activity; C:cellular_component; F:molecular_function; P:methylation |
| Efet.01.37955.g861.t1 | axin-1-like | 990 | 6.18E-64 | 57.8 | C:beta-catenin destruction complex; P:pseudouridine synthesis; F:RNA binding; P:signal transduction; P:RNA modification; F:pseudouridine synthase activity; F:receptor signaling complex scaffold activity |
| Efet.01.379572.g786.t1 | RNA-binding Nova-1 isoform X2 | 1026 | 5.36E-42 | 49.7 | F:nucleic acid binding; F:RNA binding; F:hydrolase activity |
| Efet.01.379695.g787.t1 | Protocadherin-9 | 3024 | 2.06E-180 | 56.4 | F:calcium ion binding; C:membrane; C:integral component of membrane; P:cell adhesion; P:homophilic cell adhesion via plasma membrane adhesion molecules; C:plasma membrane |
| Efet.01.379736.g788.t1 | Voltage-dependent T-type calcium channel subunit alpha- | 330 | 1.06E-34 | 90.55 | P:calcium ion import; C:voltage-gated calcium channel complex; P:regulation of ion transmembrane transport; P:calcium ion transmembrane transport; F:low voltage-gated calcium channel activity |
| Efet.01.379755.g790.t1 | double-strand-break repair rad21 homolog | 705 | 1.11E-16 | 68.5 | C:nuclear chromosome |
| Efet.01.379815.g792.t1 | MOG interacting and ectopic P-granules 1-like | 2061 | 2.34E-47 | 46.1 | F:metal ion binding |
| Efet.01.379821.g793.t1 | mothers against decapentaplegic homolog 6-like | 690 | 3.18E-11 | 41.3 | F:DNA binding; C:transcription factor complex; C:nucleus; F:transcription factor activity, sequence-specific DNA binding; C:cytoplasm; P:transcription, DNA-templated; P:transforming growth factor beta receptor signaling pathway; P:regulation of transcription, DNA-templated; C:intracellular |
| Efet.01.379966.g796.t1 | family -linked outer membrane | 744 | 1.71E-119 | 60.4 | C:membrane; F:receptor activity; P:transport |
| Efet.01.380002.g797.t1 | homeobox-containing 1-like | 528 | 3.69E-17 | 82.7 | C:nucleus; F:DNA binding; P:positive regulation of transcription, DNA-templated |
| Efet.01.38006.g862.t1 | splicing suppressor of white-apricot homolog isoform X1 | 420 | 1.26E-18 | 76.5 | P:mRNA 5'-splice site recognition; F:RNA binding; F:protein binding; P:negative regulation of mRNA splicing, via spliceosome |
| Efet.01.38007.g864.t1 | hypothetical protein HELRODRAFT_185969 | 246 | 1.85E-11 | 66 | C:membrane; P:transport |
| Efet.01.380171.g798.t1 | somatostatin receptor type 2-like | 636 | 5.87E-74 | 64.9 | F:G-protein coupled peptide receptor activity; P:G-protein coupled receptor signaling pathway; C:membrane |
| Efet.01.380192.g799.t1 | FMRFamide receptor-like | 1233 | 8.19E-105 | 57.95 | F:G-protein coupled receptor activity; P:signal transduction; C:membrane |
| Efet.01.38025.g865.t1 | Hepatic leukemia factor | 318 | 3.65E-25 | 79.55 | F:transcription factor activity, sequence-specific DNA binding; P:regulation of transcription, DNA-templated; F:sequence-specific DNA binding |
| Efet.01.380348.g800.t1 | hypothetical protein CAPTEDRAFT_221374 | 1716 | 9.21E-33 | 52.95 |  |
| Efet.01.380390.g801.t1 | FMRFamide receptor-like | 1353 | 4.14E-57 | 53.85 | F:signal transducer activity |
| Efet.01.380433.g802.t1 | zinc finger 271-like | 771 | 5.46E-58 | 54.75 | F:nucleic acid binding; F:metal ion binding; C:membrane; C:integral component of membrane; P:oxidation-reduction process; F:fatty-acyl-CoA reductase (alcohol-forming) activity |
| Efet.01.380627.g806.t1 | monocarboxylate transporter 12-B isoform X1 [Zeugodacus cucurbitae] | 579 | 5.11E-61 | 66.45 | P:transmembrane transport; C:integral component of membrane |
| Efet.01.380739.g808.t1 | tyrosine- phosphatase corkscrew isoform X1 | 216 | 1.68E-14 | 69.65 | F:phosphatase activity; P:dephosphorylation |
| Efet.01.38077.g866.t1 | hypothetical protein HELRODRAFT_179880 | 345 | 7.96E-24 | 64 |  |
| Efet.01.38090.g867.t1 | tyrosine- phosphatase Lar isoform X1 | 351 | 3.37E-43 | 88.3 | P:establishment of endothelial intestinal barrier; C:extracellular exosome; F:protein binding; F:protein tyrosine phosphatase activity; P:cerebellum development; C:integral component of membrane; P:cell adhesion; P:hippocampus development; P:cerebral cortex development; P:peptidyl-tyrosine dephosphorylation; P:spinal cord development; P:corpus callosum development |
| Efet.01.380911.g810.t1 | protocadherin-9 isoform X1 | 2826 | 3.28E-176 | 55.55 | F:calcium ion binding; C:membrane; C:integral component of membrane; P:cell adhesion; P:homophilic cell adhesion via plasma membrane adhesion molecules; C:plasma membrane |
| Efet.01.381077.g814.t1 | integrator complex subunit 1 isoform X2 | 459 | 2.25E-16 | 59.35 | P:snRNA processing; P:U2 snRNA 3'-end processing; C:membrane; C:integrator complex |
| Efet.01.38111.g868.t1 | la-related CG11505 isoform X1 | 1368 | 1.85E-40 | 81.65 | F:nucleotide binding |
| Efet.01.381141.g816.t1 | cytochrome P450 1A1 | 1563 | 1.96E-102 | 55.9 | C:cell; F:oxidoreductase activity |
| Efet.01.381231.g818.t1 | gonadotropin-releasing hormone II receptor-like | 1047 | 4.38E-53 | 53.35 | C:membrane; P:cellular response to stimulus; F:signal transducer activity |
| Efet.01.38135.g871.t1 | 5-hydroxytryptamine receptor 1 | 1044 | 2.57E-15 | 42.6 | F:G-protein coupled receptor activity; P:cell communication; P:single organism signaling |
| Efet.01.38140.g872.t1 | hypothetical protein CAPTEDRAFT_220792 | 3681 | 1.09E-09 | 64.5 |  |
| Efet.01.38145.g873.t1 | tolloid 1 | 210 | 2.21E-09 | 69.8 | C:membrane |
| Efet.01.381507.g821.t1 | ARM repeat-containing | 1845 | 1.76E-27 | 41.15 | P:protein import into nucleus |
| Efet.01.381535.g823.t1 | ATP-binding cassette sub-family G member 1-like isoform X1 | 603 | 3.28E-104 | 84.2 | F:ATPase activity; F:ATP binding; F:zinc ion binding; C:integral component of membrane |
| Efet.01.381633.g826.t1 | glycosaminoglycan xylosylkinase | 324 | 7.43E-33 | 57.45 | C:membrane |
| Efet.01.381665.g827.t1 | Hsp20 alpha crystallin family | 429 | 5.76E-94 | 74.9 |  |
| Efet.01.38168.g875.t1 | spectrin alpha non-erythrocytic 1-like | 438 | 3.71E-80 | 90.15 | F:calcium ion binding |
| Efet.01.381741.g830.t1 | tRNA (cytosine(34)-C(5))-methyltransferase | 888 | 7.67E-20 | 66.5 | C:nucleus; F:tRNA (cytosine-5-)-methyltransferase activity; C:cytoplasm; F:tRNA binding; P:tRNA methylation |
| Efet.01.381745.g831.t1 | parafibromin | 285 | 1.46E-17 | 50.55 | P:histone modification; C:Cdc73/Paf1 complex; P:transcription elongation from RNA polymerase II promoter |
| Efet.01.381746.g832.t1 | voltage-dependent calcium channel gamma-5 subunit-like | 375 | 9.12E-17 | 60.25 | C:membrane; P:calcium ion transport; C:integral component of membrane |
| Efet.01.381769.g834.t1 | unconventional myosin-XVIIIa isoform X1 | 1122 | 3.82E-24 | 67.6 | C:myosin complex; F:ATP binding; F:motor activity |
| Efet.01.381785.g835.t1 | protocadherin beta-12 | 2859 | 2.90E-133 | 54 | F:calcium ion binding; C:membrane; C:integral component of membrane; P:cell adhesion; P:homophilic cell adhesion via plasma membrane adhesion molecules; C:plasma membrane |
| Efet.01.3818.g325.t1 | probable G- coupled receptor 139 | 1302 | 4.28E-68 | 56.55 | P:signal transduction; C:membrane; F:signal transducer activity |
| Efet.01.381835.g836.t1 | neurexin-1a-like isoform X1 | 870 | 5.63E-43 | 51.7 | F:metal ion binding; F:calcium ion binding; C:membrane; C:integral component of membrane; P:chemical synaptic transmission |
| Efet.01.381855.g837.t1 | TPA_inf: Pax-beta paired domain | 327 | 2.85E-21 | 75.6 | F:DNA binding; C:nucleus; P:transcription, DNA-templated; P:multicellular organism development; P:regulation of transcription, DNA-templated |
| Efet.01.381944.g839.t1 | GTP-binding Rhes | 420 | 3.91E-31 | 65.9 | F:nucleotide binding; P:signal transduction |
| Efet.01.382066.g844.t1 | POU class transcription factor 2 | 273 | 3.87E-21 | 93.5 | C:nucleus; F:DNA binding; F:transcription factor activity, sequence-specific DNA binding; P:multicellular organism development; P:regulation of transcription, DNA-templated; P:transcription from RNA polymerase II promoter |
| Efet.01.382128.g845.t1 | nucleolar transcription factor 1-A-like isoform X1 | 378 | 2.40E-08 | 74.5 | F:RNA polymerase I upstream control element sequence-specific DNA binding; F:RNA polymerase I CORE element sequence-specific DNA binding; F:protein binding; P:transcription initiation from RNA polymerase I promoter; P:transcription elongation from RNA polymerase I promoter; P:termination of RNA polymerase I transcription; C:nucleolus; P:regulation of glucose mediated signaling pathway; C:nucleoplasm; F:RNA binding; P:positive regulation of transcription from RNA polymerase I promoter |
| Efet.01.382178.g846.t1 | hypothetical protein CAPTEDRAFT_216764 | 636 | 2.42E-10 | 60 |  |
| Efet.01.382181.g847.t1 | E3 ubiquitin- ligase | 918 | 1.83E-30 | 51.4 | F:SUMO binding; F:zinc ion binding; P:spermatogenesis; P:cellular response to DNA damage stimulus; C:mitochondrial inner membrane; F:ubiquitin-protein transferase activity; C:nucleus; F:ubiquitin protein ligase binding; C:cytoplasm; F:metal ion binding; C:membrane; F:ligase activity; P:protein ubiquitination |
| Efet.01.382181.g848.t1 | E3 ubiquitin- ligase HERC2-like | 399 | 1.46E-14 | 92.75 | C:nucleus; F:ubiquitin-protein transferase activity; F:zinc ion binding; C:cytoplasm; F:ligase activity; P:protein ubiquitination involved in ubiquitin-dependent protein catabolic process |
| Efet.01.38223.g876.t1 | spectrin beta chain isoform X1 | 1407 | 4.41E-178 | 65.75 | F:binding |
| Efet.01.38228.g877.t1 | supervillin-like isoform X5 | 909 | 1.74E-64 | 53.95 | F:actin filament binding; F:actin binding; P:cytoskeleton organization |
| Efet.01.382296.g849.t1 | Acidic leucine-rich nuclear phospho 32 family member A | 774 | 1.33E-32 | 84.4 |  |
| Efet.01.38230.g878.t1 | DNA damage-induced apoptosis suppressor | 285 | 1.04E-15 | 58.55 |  |
| Efet.01.38231.g879.t1 | zinc finger 423-like | 1719 | 7.46E-81 | 48.95 | F:metal ion binding |
| Efet.01.38232.g880.t1 | suppressor APC domain-containing 2 | 489 | 9.96E-20 | 63.45 |  |
| Efet.01.382360.g851.t1 | pecanex 1 | 231 | 7.49E-33 | 86.05 | C:integral component of membrane |
| Efet.01.382434.g852.t1 | tctex1 domain-containing 1-like | 381 | 8.65E-06 | 57 |  |
| Efet.01.382542.g853.t1 | paraplegin | 375 | 3.85E-24 | 74.2 | F:ATP binding; C:membrane; P:proteolysis; F:metalloendopeptidase activity |
| Efet.01.38271.g882.t1 | CD109 antigen-like | 663 | 7.27E-56 | 56.4 | C:extracellular exosome; F:endopeptidase inhibitor activity; C:blood microparticle; P:negative regulation of cellular process; P:multicellular organismal process |
| Efet.01.382746.g854.t1 | tripartite motif-containing 2-like | 423 | 3.55E-06 | 56 |  |
| Efet.01.382751.g855.t1 | hypothetical protein HELRODRAFT_181039 | 285 | 7.29E-33 | 66.2 | F:binding |
| Efet.01.382764.g856.t1 | suppressor of lurcher 1-like | 327 | 1.17E-23 | 74.8 |  |
| Efet.01.382787.g857.t1 | tudor domain-containing 1-like isoform X4 | 1623 | 7.33E-18 | 45.9 | C:intracellular ribonucleoprotein complex; C:cytoplasm; P:single-organism cellular process; F:binding; P:single organism reproductive process; P:multicellular organismal process |
| Efet.01.382798.g858.t1 | heat shock HSP 90-alpha-like | 465 | 2.43E-19 | 51.25 |  |
| Efet.01.382798.g859.t1 | transmembrane 9-like isoform X1 | 240 | 6.03E-11 | 71.75 | C:integral component of membrane |
| Efet.01.382856.g861.t1 | ubiquitin carboxyl-terminal hydrolase 47 isoform X1 | 351 | 9.29E-23 | 64.85 | F:thiol-dependent ubiquitin-specific protease activity; P:positive regulation of cell growth; P:monoubiquitinated protein deubiquitination; P:base-excision repair; P:negative regulation of transcription, DNA-templated; C:cytoplasm; P:response to drug; P:negative regulation of cysteine-type endopeptidase activity involved in apoptotic process; C:SCF ubiquitin ligase complex; F:WD40-repeat domain binding; P:negative regulation of G2/M transition of mitotic cell cycle |
| Efet.01.382875.g863.t1 | Runt-related transcription factor 1 | 363 | 1.57E-29 | 77.15 | C:nucleus; F:DNA binding; F:ATP binding; F:transcription factor activity, sequence-specific DNA binding; P:regulation of transcription, DNA-templated |
| Efet.01.382878.g864.t1 | neural-cadherin-like isoform X2 | 1119 | 8.65E-57 | 45.9 | F:calcium ion binding; C:membrane; C:integral component of membrane; P:cell adhesion; P:homophilic cell adhesion via plasma membrane adhesion molecules; C:plasma membrane |
| Efet.01.383034.g865.t1 | zinc finger MYND domain-containing 11-like isoform X1 | 327 | 4.08E-24 | 67.95 | P:negative regulation of transcription from RNA polymerase II promoter; C:nucleus; F:transcription cofactor activity; F:histone binding; F:metal ion binding; F:RNA polymerase II regulatory region sequence-specific DNA binding; F:chromatin binding |
| Efet.01.383036.g866.t1 | High mobility group DSP1 | 351 | 8.98E-43 | 82.65 | C:nucleus; F:DNA binding; P:regulation of cell differentiation; F:2-alkenal reductase [NAD(P)] activity; P:spermatogenesis; P:regulation of transcription, DNA-templated; P:oxidation-reduction process |
| Efet.01.38304.g883.t1 | Phosphoinositide 3-kinase regulatory subunit 4 | 2940 | 0 | 55.15 | C:macromolecular complex; C:organelle; F:kinase activity; P:cellular process |
| Efet.01.38304.g884.t1 | phosphoinositide 3-kinase regulatory subunit 4-like | 813 | 1.66E-36 | 63.2 | C:endosome; P:cellular macromolecule metabolic process; P:regulation of cellular process; P:autophagy; C:phosphatidylinositol 3-kinase complex, class III; F:protein kinase activity; P:vacuolar transport; P:phosphorylation; C:axoneme; P:cellular response to glucose starvation |
| Efet.01.383041.g867.t1 | cilia- and flagella-associated 53-like | 702 | 4.56E-40 | 69 | F:DNA binding; P:DNA metabolic process |
| Efet.01.383063.g868.t1 | phosphatase 1F | 240 | 3.99E-12 | 71.55 | P:positive regulation of growth; P:peptidyl-threonine dephosphorylation; F:calmodulin-dependent protein phosphatase activity; P:cellular response to drug; P:negative regulation of protein kinase activity by regulation of protein phosphorylation; F:cation binding; P:intrinsic apoptotic signaling pathway; C:protein complex; P:positive regulation of cysteine-type endopeptidase activity involved in apoptotic process; F:protein binding; P:positive regulation of focal adhesion assembly; P:negative regulation of transcription, DNA-templated; P:positive regulation of stress fiber assembly; P:positive regulation of gene expression; P:negative regulation of peptidyl-serine phosphorylation; C:cytosol; P:positive regulation of epithelial cell migration; P:positive regulation of chemotaxis |
| Efet.01.38316.g886.t1 | methyltransferase 14 homolog | 411 | 1.02E-50 | 93.3 | F:methyltransferase activity; P:nucleobase-containing compound metabolic process; P:methylation |
| Efet.01.38317.g887.t1 | electroneutral sodium bicarbonate exchanger 1-like | 306 | 4.27E-32 | 70.5 | P:inorganic anion transport; P:anion transmembrane transport; C:integral component of membrane; F:inorganic anion exchanger activity |
| Efet.01.383263.g872.t1 | dynein heavy chain axonemal-like | 735 | 7.50E-106 | 84.15 | F:ATPase activity; F:ATP binding; F:microtubule motor activity; C:dynein complex; P:microtubule-based movement |
| Efet.01.383414.g874.t1 | serine threonine- phosphatase 4 regulatory subunit 1-like isoform X2 | 519 | 2.68E-13 | 64.05 |  |
| Efet.01.383428.g875.t1 | calcium calmodulin-dependent kinase kinase 1 isoform X1 | 450 | 1.23E-29 | 71.15 | F:ATP binding; F:protein serine/threonine kinase activity; P:protein phosphorylation |
| Efet.01.383545.g876.t1 | rho GTPase-activating 39- | 477 | 4.15E-64 | 77.9 | C:cytoskeleton; P:signal transduction; P:regulation of small GTPase mediated signal transduction; F:GTPase activator activity; C:cytosol |
| Efet.01.383583.g878.t1 | hypothetical protein HELRODRAFT_109524 | 336 | 3.18E-14 | 76.5 | F:ARF guanyl-nucleotide exchange factor activity; P:regulation of ARF protein signal transduction; P:positive regulation of GTPase activity |
| Efet.01.383613.g879.t1 | proteoglycan 4-like isoform X2 | 471 | 3.14E-28 | 60.35 |  |
| Efet.01.383626.g880.t1 | 1-phosphatidylinositol 4,5-bisphosphate phosphodiesterase classes I and II-like | 357 | 4.98E-15 | 59.75 | P:single-organism process; F:hydrolase activity |
| Efet.01.383634.g881.t1 | hypothetical protein HELRODRAFT_89291, partial | 627 | 4.00E-10 | 62.33 | F:ion channel activity; P:ion transport; P:transmembrane transport; C:membrane |
| Efet.01.383813.g884.t1 | AChain Crystal Structures Of Glur2 Ligand-binding-domain In Complex With Glutamate And Positive Allosteric Modulators | 777 | 1.14E-62 | 58.9 | C:AMPA glutamate receptor complex; F:identical protein binding; F:kainate selective glutamate receptor activity; P:positive regulation of synaptic transmission; F:excitatory extracellular ligand-gated ion channel activity; C:neuronal cell body; C:protein complex; C:cytoplasmic part; F:AMPA glutamate receptor activity; C:intracellular organelle part; C:membrane-bounded organelle; P:establishment of protein localization; P:ion transport; C:dendritic shaft; P:protein tetramerization; C:dendritic spine; F:PDZ domain binding; P:response to lithium ion; P:ionotropic glutamate receptor signaling pathway; P:receptor internalization; P:regulation of synaptic transmission, glutamatergic; P:regulation of receptor recycling |
| Efet.01.383830.g885.t1 | transcription factor hamlet-like | 2088 | 2.59E-58 | 83.45 | F:nucleic acid binding; F:metal ion binding; P:regulation of transcription, DNA-templated |
| Efet.01.38392.g890.t1 | Ser Thr | 321 | 3.09E-26 | 67.35 | F:protein kinase activity |
| Efet.01.383942.g886.t1 | zinc finger MYM-type 1-like | 1011 | 9.72E-92 | 68.7 | F:nucleic acid binding |
| Efet.01.384014.g887.t1 | bicaudal D-like isoform X1 | 2088 | 3.22E-138 | 58.8 | C:Golgi apparatus; P:transport |
| Efet.01.384035.g888.t1 | Krueppel homolog 1-like isoform X1 | 1803 | 1.65E-37 | 49.15 | F:nucleic acid binding; F:metal ion binding |
| Efet.01.384045.g889.t1 | heat shock 70 kDa 4 | 339 | 2.17E-14 | 80.5 | F:ATP binding |
| Efet.01.38405.g891.t1 | dystroglycan | 2574 | 3.68E-61 | 51.8 | C:membrane |
| Efet.01.384283.g892.t1 | microtubule-associated tumor suppressor candidate 2-like isoform X3 | 447 | 9.18E-07 | 81 |  |
| Efet.01.384324.g894.t1 | atrial natriuretic peptide receptor A | 294 | 7.56E-44 | 79.75 | F:ATP binding; P:cGMP biosynthetic process; P:peptidyl-tyrosine phosphorylation; F:protein tyrosine kinase activity; C:integral component of membrane; F:guanylate cyclase activity; C:intracellular; P:intracellular signal transduction |
| Efet.01.38436.g893.t1 | hypothetical protein CAPTEDRAFT_219225 | 522 | 7.66E-07 | 55 | F:DNA binding; C:transcription factor TFIIIB complex; P:regulation of transcription from RNA polymerase III promoter; F:TFIIIB-type transcription factor activity |
| Efet.01.38438.g895.t1 | octopamine receptor 2-like | 1893 | 4.51E-93 | 67.05 | F:G-protein coupled receptor activity; P:G-protein coupled receptor signaling pathway; C:integral component of membrane |
| Efet.01.38447.g896.t1 | membrane metallo-endopeptidase-like 1 | 465 | 6.37E-66 | 90.95 | P:proteolysis; C:integral component of membrane; P:olfactory learning; F:metalloendopeptidase activity |
| Efet.01.38448.g897.t1 | tyrosine- kinase SRK2-like isoform X1 | 297 | 2.96E-37 | 79.55 | F:ATP binding; P:peptidyl-tyrosine phosphorylation; F:non-membrane spanning protein tyrosine kinase activity; P:innate immune response |
| Efet.01.38448.g898.t1 | tyrosine- kinase Yes isoform X2 | 348 | 1.79E-44 | 63.65 | C:intracellular organelle; P:transmembrane receptor protein tyrosine kinase signaling pathway; P:peptidyl-tyrosine autophosphorylation; F:receptor binding; P:regulation of cell proliferation; C:membrane-bounded organelle; F:nucleotide binding; F:non-membrane spanning protein tyrosine kinase activity; P:positive regulation of macromolecule metabolic process; C:cytoplasm; P:innate immune response; P:cellular response to growth factor stimulus; P:cellular response to endogenous stimulus; P:cell differentiation; C:extrinsic component of cytoplasmic side of plasma membrane; P:positive regulation of cellular metabolic process; P:regulation of primary metabolic process |
| Efet.01.384495.g896.t1 | potassium sodium hyperpolarization-activated cyclic nucleotide-gated channel 3-like isoform X1 | 1074 | 1.50E-13 | 55.55 | P:transport |
| Efet.01.384505.g897.t1 | PREDICTED: uncharacterized protein LOC107333961 | 741 | 1.64E-10 | 53.5 |  |
| Efet.01.38455.g899.t1 | hypothetical protein HELRODRAFT_191885 | 1320 | 2.71E-07 | 48 |  |
| Efet.01.384600.g898.t1 | BTB POZ domain-containing KCTD16 | 852 | 1.56E-106 | 67.2 | P:protein homooligomerization |
| Efet.01.384610.g900.t1 | hypothetical protein CAPTEDRAFT_199465, partial | 1404 | 1.16E-09 | 41 |  |
| Efet.01.384679.g902.t1 | homeobox domain | 378 | 6.64E-07 | 96.8 | C:nucleus; F:zinc ion binding; P:regulation of transcription, DNA-templated; F:sequence-specific DNA binding |
| Efet.01.384680.g903.t1 | synaptotagmin-6-like isoform X1 | 537 | 6.29E-27 | 62.05 | P:biological regulation; C:membrane; C:cell part; F:binding |
| Efet.01.384685.g904.t1 | phosphatase 2C-like domain-containing 1 | 1923 | 9.11E-74 | 50.6 | F:protein serine/threonine phosphatase activity; F:catalytic activity; P:protein dephosphorylation |
| Efet.01.384777.g909.t1 | NACHT and WD repeat domain-containing 2-like | 333 | 1.21E-08 | 55.3 |  |
| Efet.01.3848.g327.t1 | trithorax group osa-like isoform X2 | 2097 | 9.96E-48 | 65.75 | F:DNA binding; C:BAF-type complex; P:chromatin remodeling |
| Efet.01.38482.g904.t1 | regulator of nonsense transcripts 2-like | 1659 | 8.59E-83 | 61.9 | F:RNA binding |
| Efet.01.38489.g905.t1 | aminopeptidase N isoform X1 | 279 | 9.29E-37 | 84.5 | F:zinc ion binding; P:proteolysis; C:integral component of membrane; F:metallopeptidase activity |
| Efet.01.384950.g910.t1 | kyphoscoliosis peptidase | 1368 | 1.73E-57 | 57.35 |  |
| Efet.01.38505.g907.t1 | acetolactate synthase | 306 | 1.52E-20 | 79.95 | F:magnesium ion binding; F:thiamine pyrophosphate binding; C:integral component of membrane; P:embryonic cranial skeleton morphogenesis; F:transferase activity |
| Efet.01.385050.g912.t1 | capicua homolog isoform X1 | 2337 | 3.32E-68 | 48.65 |  |
| Efet.01.385103.g916.t1 | ufm1-specific protease 2-like | 552 | 9.21E-48 | 81.3 | P:proteolysis; F:peptidase activity |
| Efet.01.385389.g922.t1 | hypothetical protein HELRODRAFT_190294 | 576 | 5.46E-06 | 59 | P:transmembrane transport; C:membrane; C:integral component of membrane |
| Efet.01.385677.g926.t1 | cilia- and flagella-associated 44-like isoform X2 | 459 | 6.40E-53 | 84.6 |  |
| Efet.01.38594.g908.t1 | N(G),N(G)-dimethylarginine dimethylaminohydrolase 1 | 264 | 5.03E-32 | 70.35 | F:hydrolase activity |
| Efet.01.385949.g930.t1 | potassium voltage-gated channel subfamily H member 7-like isoform X3 | 492 | 4.79E-45 | 81.85 | F:voltage-gated potassium channel activity; F:ATP binding; P:potassium ion transmembrane transport; C:integral component of membrane; F:adenylylsulfate kinase activity; F:sulfate adenylyltransferase (ATP) activity; P:phosphorylation; P:sulfate assimilation |
| Efet.01.386139.g933.t1 | complement C1q tumor necrosis factor-related 2-like | 582 | 1.49E-79 | 64.7 |  |
| Efet.01.386354.g935.t1 | transformation transcription domain-associated -like | 330 | 7.25E-48 | 70.4 | C:nucleus; P:histone acetylation; P:phosphorylation; F:kinase activity; C:histone acetyltransferase complex |
| Efet.01.386404.g936.t1 | neuronal acetylcholine receptor subunit alpha-7-like isoform X1 | 213 | 4.04E-07 | 83.4 | C:postsynaptic membrane; P:cation transmembrane transport; C:integral component of membrane; C:cell junction; F:extracellular ligand-gated ion channel activity |
| Efet.01.386431.g937.t1 | restriction endonuclease subunit R | 480 | 1.38E-64 | 91.2 | F:DNA binding; F:ATP binding; P:nucleic acid phosphodiester bond hydrolysis; P:DNA metabolic process; F:Type III site-specific deoxyribonuclease activity |
| Efet.01.386562.g938.t1 | PREDICTED: uncharacterized protein LOC106180140 | 369 | 3.97E-15 | 56.8 |  |
| Efet.01.386635.g940.t1 | LIM domain kinase 1 isoform X2 | 246 | 3.01E-15 | 96.55 | F:ATP binding; F:zinc ion binding; F:protein kinase activity; P:protein phosphorylation |
| Efet.01.386675.g941.t1 | radixin isoform X1 | 201 | 1.73E-27 | 92.55 | P:negative regulation of homotypic cell-cell adhesion; P:positive regulation of cellular protein catabolic process; F:cell adhesion molecule binding; C:secretory granule membrane; C:cell tip; F:double-stranded RNA binding; P:protein kinase A signaling; P:apical protein localization; P:regulation of Rap protein signal transduction; C:cortical actin cytoskeleton; C:lamellipodium; F:protein kinase binding; F:protein domain specific binding; P:negative regulation of GTPase activity; P:regulation of ruffle assembly; P:positive regulation of protein localization to early endosome; C:nucleus; F:actin binding; P:actin filament capping; P:positive regulation of gene expression; P:positive regulation of early endosome to late endosome transport; C:pseudopodium; C:basolateral plasma membrane; P:positive regulation of podosome assembly; C:apical plasma membrane; P:regulation of lymphocyte migration; F:protein kinase A binding; P:positive regulation of cell migration; P:regulation of cell shape; C:myelin sheath; C:cleavage furrow; P:regulation of actin filament bundle assembly; P:leukocyte cell-cell adhesion; C:cytoplasmic side of plasma membrane; P:cellular response to testosterone stimulus; P:cellular response to thyroid hormone stimulus; C:extracellular exosome; C:focal adhesion; C:blood microparticle; P:establishment of epithelial cell apical/basal polarity; P:negative regulation of cell size; C:ruffle; C:stereocilium; F:protein homodimerization activity; P:positive regulation of G1/S transition of mitotic cell cycle; P:establishment of protein localization to plasma membrane; P:gland morphogenesis; P:membrane to membrane docking; F:receptor binding; P:establishment of endothelial barrier; F:RNA binding; C:filopodium membrane; C:T-tubule; C:microvillus membrane; C:perinuclear region of cytoplasm; C:uropod; P:microvillus assembly; P:leukocyte migration; C:extrinsic component of membrane; P:negative regulation of adherens junction organization; F:ATPase binding |
| Efet.01.38674.g910.t1 | rho GTPase-activating 24 isoform X1 | 1440 | 1.82E-59 | 59.5 | C:focal adhesion; P:activation of GTPase activity; F:GTPase activator activity; P:negative regulation of Rac protein signal transduction; P:wound healing, spreading of epidermal cells; P:negative regulation of ruffle assembly |
| Efet.01.38674.g911.t1 | rho GTPase-activating 24-like | 477 | 9.50E-25 | 70.35 | P:signal transduction; F:GTPase activator activity; P:positive regulation of GTPase activity |
| Efet.01.386744.g942.t1 | G -activated inward rectifier potassium channel 3-like | 1275 | 1.65E-97 | 78.15 | P:potassium ion transmembrane transport; C:integral component of membrane; F:inward rectifier potassium channel activity; P:regulation of ion transmembrane transport |
| Efet.01.386779.g943.t1 | transcriptional repressor YY1 | 321 | 1.84E-64 | 92.25 | P:spermatogenesis; P:anterior/posterior pattern specification; P:cellular response to DNA damage stimulus; P:RNA localization; P:negative regulation of transcription from RNA polymerase II promoter; C:transcription factor complex; F:transcriptional repressor activity, RNA polymerase II core promoter proximal region sequence-specific binding; F:DNA binding; P:camera-type eye morphogenesis; F:metal ion binding; P:chromosome organization; P:positive regulation of gene expression; C:PcG protein complex; P:response to UV-C |
| Efet.01.38685.g912.t1 | glycogen debranching enzyme-like isoform X2 | 2763 | 0 | 61.15 | P:carbohydrate metabolic process; F:catalytic activity |
| Efet.01.38692.g913.t1 | AChain Structural Determinants For Improved Thermal Stability Of Designed Ankyrin Repeat s With A Redesigned C- Capping | 561 | 2.68E-26 | 50.65 | F:nucleic acid binding; F:RNA binding |
| Efet.01.38692.g914.t1 | ankyrin repeat | 1167 | 6.76E-32 | 47.25 |  |
| Efet.01.38692.g915.t1 | serine threonine- phosphatase 6 regulatory ankyrin repeat subunit A- | 1209 | 1.30E-51 | 49.8 |  |
| Efet.01.387.g45.t1 | hypothetical protein CAPTEDRAFT_198395 | 603 | 1.39E-06 | 42 |  |
| Efet.01.387012.g945.t1 | nodal modulator 1-like | 645 | 2.00E-66 | 63.6 | C:membrane; C:integral component of membrane; F:carbohydrate binding |
| Efet.01.38704.g916.t1 | probable G- coupled receptor 139 | 1176 | 4.41E-55 | 53.2 | F:signal transducer activity |
| Efet.01.387053.g946.t1 | Zinc finger domain containing | 984 | 5.21E-56 | 57.7 | F:metal ion binding |
| Efet.01.3871.g329.t1 | GTP-binding REM 1-like | 402 | 3.64E-12 | 73.8 | F:GTP binding; P:small GTPase mediated signal transduction; C:membrane; C:intracellular |
| Efet.01.387159.g947.t1 | E3 ubiquitin- ligase listerin | 1179 | 4.99E-97 | 58.35 | F:zinc ion binding; F:metal ion binding; F:ligase activity |
| Efet.01.387168.g949.t1 | transmembrane 184B- | 234 | 9.27E-06 | 60 |  |
| Efet.01.387330.g953.t1 | transport and Golgi organization 6 homolog | 687 | 5.09E-16 | 51.55 | P:biological_process; C:membrane; C:integral component of membrane; C:cellular_component; F:molecular_function |
| Efet.01.387336.g954.t1 | gamma-aminobutyric acid receptor subunit beta-like | 390 | 7.91E-21 | 55.1 | C:membrane; F:GABA receptor activity; F:extracellular ligand-gated ion channel activity |
| Efet.01.38734.g917.t1 | p53 tumor suppressor | 249 | 1.13E-07 | 53.14 | F:DNA binding; C:nucleus; F:transcription factor activity, sequence-specific DNA binding; P:protein tetramerization; P:apoptotic process; F:transcription regulatory region DNA binding; P:regulation of transcription, DNA-templated |
| Efet.01.38735.g918.t1 | guanine nucleotide-binding G(i) subunit alpha-2 | 276 | 2.15E-52 | 80.8 | C:photoreceptor connecting cilium; P:adenylate cyclase-inhibiting G-protein coupled receptor signaling pathway; P:cell proliferation; P:sensory perception of umami taste; P:retina development in camera-type eye; P:positive regulation of cell proliferation; P:G-protein coupled acetylcholine receptor signaling pathway; P:gamma-aminobutyric acid signaling pathway; C:nucleoplasm; F:GTP binding; P:cell division; C:dendrite; F:G-protein coupled receptor binding; P:activation of MAPKK activity; C:extracellular exosome; F:GTPase activity; C:cytosol; P:cellular response to electrical stimulus; P:regulation of calcium ion transport; P:intracellular signal transduction; P:detection of light stimulus involved in visual perception; P:negative regulation of synaptic transmission; P:regulation of heart contraction; P:eye photoreceptor cell development; F:signal transducer activity; P:cell cycle; C:centrosome; P:adenosine receptor signaling pathway; C:heterotrimeric G-protein complex; F:metal ion binding; C:cell body; C:membrane raft; C:midbody; F:G-protein beta/gamma-subunit complex binding |
| Efet.01.38747.g919.t1 | leucine-rich repeat serine threonine- kinase 1 | 489 | 3.37E-14 | 70.4 | P:cellular macromolecule metabolic process; F:purine ribonucleoside triphosphate binding; F:purine ribonucleoside binding; P:cellular response to stimulus; P:phosphorylation; P:primary metabolic process; F:kinase activity; F:purine ribonucleotide binding |
| Efet.01.387480.g956.t1 | thiamine-binding | 489 | 2.13E-52 | 90 |  |
| Efet.01.387480.g957.t1 | DUF2029 domain-containing | 465 | 9.83E-62 | 76.9 | F:transferase activity, transferring hexosyl groups; C:integral component of membrane |
| Efet.01.387540.g959.t1 | regulator of G- signaling 8-like isoform X3 | 1221 | 2.31E-59 | 62.15 | C:cytoplasm; F:GTPase activator activity; P:regulation of G-protein coupled receptor protein signaling pathway; C:plasma membrane |
| Efet.01.387735.g963.t1 | Serine threonine- phosphatase 2A regulatory subunit B subunit alpha | 1008 | 3.35E-07 | 56 | F:calcium ion binding |
| Efet.01.387738.g964.t1 | ZZ-type zinc finger-containing 3-like | 267 | 2.17E-14 | 68.45 | F:metal ion binding |
| Efet.01.387933.g966.t1 | mitochondrial fission process 1-like | 255 | 6.53E-31 | 78.55 |  |
| Efet.01.387979.g969.t1 | inactive tyrosine- kinase 7 | 360 | 5.32E-18 | 58.9 | P:morphogenesis of an epithelium; P:animal organ development; P:single-organism cellular process; F:kinase activity; P:response to stimulus |
| Efet.01.388037.g971.t1 | cAMP-dependent kinase type II regulatory subunit-like | 474 | 6.86E-46 | 55.95 | F:nucleotide binding; P:regulation of protein kinase activity; P:regulation of protein phosphorylation; C:membrane; C:integral component of membrane; F:cAMP binding; P:phosphorylation; F:cAMP-dependent protein kinase regulator activity; F:kinase activity; C:cAMP-dependent protein kinase complex |
| Efet.01.388135.g972.t1 | family nutrient-binding outer membrane lipo | 504 | 7.62E-85 | 60.55 |  |
| Efet.01.388165.g973.t1 | E3 ubiquitin- ligase MYCBP2-like | 246 | 1.25E-17 | 83.3 | F:zinc ion binding; F:metal ion binding |
| Efet.01.38820.g921.t1 | sarcalumenin isoform X2 | 441 | 1.76E-36 | 62.7 | P:response to muscle activity involved in regulation of muscle adaptation; P:store-operated calcium entry |
| Efet.01.38824.g922.t1 | BTB POZ domain-containing 17-like isoform X8 | 1242 | 6.28E-161 | 75.6 |  |
| Efet.01.388345.g975.t1 | Calmodulin-binding transcription activator 1 | 204 | 8.74E-40 | 98 | C:nucleus; F:DNA binding |
| Efet.01.388403.g976.t1 | bark beetle-like isoform X1 | 315 | 3.66E-30 | 57.46 | C:membrane |
| Efet.01.38849.g923.t1 | C kinase 98E | 318 | 5.78E-12 | 94.9 | F:ATP binding; F:metal ion binding; P:peptidyl-serine phosphorylation; C:intracellular; P:intracellular signal transduction; F:protein kinase C activity |
| Efet.01.388532.g979.t1 | arrestin domain-containing 4-like | 621 | 1.98E-44 | 57.2 |  |
| Efet.01.38862.g924.t1 | Proton-associated sugar transporter A | 222 | 3.30E-11 | 78.35 | P:transmembrane transport; C:integral component of membrane |
| Efet.01.38869.g926.t1 | thioredoxin domain-containing 15-like | 666 | 1.33E-22 | 66.45 | C:cell; C:membrane; C:integral component of membrane; P:cell redox homeostasis |
| Efet.01.388695.g983.t1 | substance-P receptor-like | 1134 | 5.56E-29 | 46.4 | C:gap junction; F:G-protein coupled receptor activity; P:G-protein coupled receptor signaling pathway; P:signal transduction; C:membrane; C:integral component of membrane; F:signal transducer activity |
| Efet.01.388698.g984.t1 | cyclic nucleotide-gated cation channel alpha-3-like | 384 | 2.69E-42 | 87.6 | C:integral component of plasma membrane; F:voltage-gated potassium channel activity; P:regulation of membrane potential; P:potassium ion transmembrane transport; F:cGMP-dependent protein kinase activity; P:protein phosphorylation |
| Efet.01.38870.g927.t1 | unc-45 homolog B-like | 408 | 1.47E-50 | 69.4 |  |
| Efet.01.388710.g985.t1 | unc-93 homolog A-like | 1047 | 2.56E-65 | 61.85 | C:membrane |
| Efet.01.38882.g928.t1 | Zinc finger CW-type PWWP domain 2 | 342 | 1.96E-18 | 58.7 | F:DNA binding; F:zinc ion binding |
| Efet.01.388854.g986.t1 | spermatogenesis-associated 4-like | 771 | 1.54E-52 | 62.6 |  |
| Efet.01.38901.g930.t1 | lebercilin | 381 | 3.62E-19 | 65.1 |  |
| Efet.01.389203.g989.t1 | hypothetical protein HELRODRAFT_166723 | 1752 | 2.47E-13 | 44 |  |
| Efet.01.389213.g990.t1 | troponin C-like | 519 | 4.29E-39 | 54 | F:calcium ion binding |
| Efet.01.389221.g991.t1 | 1-phosphatidylinositol 4,5-bisphosphate phosphodiesterase beta-4-like | 435 | 1.41E-44 | 75.35 | C:nucleus; C:postsynaptic density; C:dendrite; F:phosphatidylinositol phospholipase C activity; C:smooth endoplasmic reticulum; F:calcium ion binding; P:lipid catabolic process; P:negative regulation of potassium ion transport; F:mitogen-activated protein kinase binding; F:signal transducer activity; P:intracellular signal transduction |
| Efet.01.389262.g992.t1 | prickle 3 isoform X2 | 225 | 1.41E-24 | 71.7 | P:anterior/posterior axis specification; F:metal ion binding; P:establishment of planar polarity; C:plasma membrane |
| Efet.01.389401.g993.t1 | phospholipid-transporting ATPase ID-like isoform X1 | 594 | 3.36E-14 | 57.4 | F:binding |
| Efet.01.389414.g994.t1 | zinc finger BED domain-containing 4-like | 1431 | 2.23E-76 | 53.2 | F:nucleic acid binding; F:DNA binding; F:protein dimerization activity |
| Efet.01.389533.g996.t1 | DNA annealing helicase and endonuclease ZRANB3-like isoform X2 | 570 | 5.22E-57 | 80.7 | F:annealing helicase activity; P:DNA repair; P:nucleic acid phosphodiester bond hydrolysis; F:zinc ion binding; C:nuclear replication fork; P:DNA strand renaturation; P:response to UV; P:DNA rewinding; F:endodeoxyribonuclease activity; P:negative regulation of DNA recombination; F:ATP binding; P:replication fork protection; F:K63-linked polyubiquitin binding; P:replication fork processing |
| Efet.01.389560.g997.t1 | TATA element modulatory factor | 255 | 1.57E-21 | 81.45 | P:regulation of proteasomal protein catabolic process; P:luteinizing hormone secretion; P:spermatid nucleus differentiation; P:protein neddylation; C:endoplasmic reticulum; C:Golgi apparatus; F:small protein activating enzyme activity; P:defense response to bacterium; F:ATP binding; P:acrosome assembly; P:negative regulation of gene expression; P:flagellated sperm motility; P:positive regulation of testosterone secretion; P:negative regulation of apoptotic process; F:acid-amino acid ligase activity; P:positive regulation of cytokine production; P:Leydig cell differentiation; P:cellular response to organic cyclic compound |
| Efet.01.389586.g999.t1 | AP-1 complex subunit beta-1-like isoform X1 | 528 | 3.44E-84 | 77.55 | C:endolysosome membrane; F:protein transporter activity; F:signal sequence binding; P:intracellular protein transport; P:microtubule-based movement; P:negative regulation of epidermal growth factor receptor signaling pathway; P:cardiac septum development; P:clathrin-dependent endocytosis; P:blood vessel development; P:clathrin coat assembly; P:regulation of defense response to virus by virus; P:ephrin receptor signaling pathway; C:cytosol; F:clathrin adaptor activity; P:Wnt signaling pathway, planar cell polarity pathway; C:AP-2 adaptor complex; P:antigen processing and presentation of exogenous peptide antigen via MHC class II |
| Efet.01.389886.g1003.t1 | potassium channel subfamily K member 6-like | 465 | 3.39E-06 | 65 |  |
| Efet.01.389950.g1005.t1 | histamine H2 receptor-like | 1680 | 4.65E-121 | 56.65 | P:signal transduction; F:signal transducer activity |
| Efet.01.389997.g1006.t1 | retinoic acid receptor RXR-like isoform X7 | 567 | 1.55E-10 | 58.27 | F:binding |
| Efet.01.39004.g936.t1 | zinc finger 782-like isoform X1 | 684 | 1.18E-17 | 52.4 | F:nucleic acid binding; F:metal ion binding; P:regulation of transcription, DNA-templated; C:intracellular |
| Efet.01.390208.g1010.t1 | porin | 924 | 0 | 62.15 |  |
| Efet.01.390288.g1011.t1 | protocadherin gamma-B1 | 1581 | 1.74E-22 | 57.65 | F:calcium ion binding; C:membrane; C:integral component of membrane; P:cell adhesion; P:homophilic cell adhesion via plasma membrane adhesion molecules; C:plasma membrane |
| Efet.01.390398.g1013.t1 | atrial natriuretic peptide receptor 3-like | 822 | 7.72E-54 | 57.3 | C:membrane; C:integral component of membrane |
| Efet.01.390429.g1014.t1 | isoaspartyl peptidase L-asparaginase-like | 438 | 9.65E-26 | 63.15 | F:hydrolase activity |
| Efet.01.39045.g939.t1 | multiple C2 and transmembrane domain-containing 1-like isoform X5 | 2361 | 0 | 72.1 | C:membrane |
| Efet.01.390489.g1015.t1 | zinc finger 91 | 681 | 4.61E-54 | 52.75 | F:nucleic acid binding; C:nucleus; F:metal ion binding; P:regulation of transcription, DNA-templated |
| Efet.01.39055.g940.t1 | hypothetical protein HELRODRAFT_161507 | 456 | 7.64E-23 | 76.33 |  |
| Efet.01.39069.g941.t1 | neutral alpha-glucosidase AB-like | 411 | 7.45E-25 | 65.6 | F:hydrolase activity |
| Efet.01.390709.g1017.t1 | Retrovirus-related Pol poly from transposon | 543 | 4.76E-12 | 49.55 | C:mitochondrion |
| Efet.01.390738.g1019.t1 | FMRFamide receptor-like | 747 | 1.13E-51 | 72.55 | F:G-protein coupled receptor activity; P:G-protein coupled receptor signaling pathway; C:integral component of membrane |
| Efet.01.390740.g1020.t1 | 26S protease regulatory subunit 4 | 441 | 7.80E-90 | 97.05 | F:ATP binding; C:proteasome regulatory particle, base subcomplex; C:cytosolic proteasome complex; C:nuclear proteasome complex; F:TBP-class protein binding; P:ER-associated ubiquitin-dependent protein catabolic process; P:positive regulation of proteasomal protein catabolic process; F:proteasome-activating ATPase activity; P:positive regulation of RNA polymerase II transcriptional preinitiation complex assembly |
| Efet.01.390799.g1023.t1 | sprouty- EVH1 domain-containing 2-like isoform X2 | 816 | 4.06E-36 | 54.45 | P:multicellular organism development; C:membrane; P:regulation of signal transduction |
| Efet.01.3908.g331.t1 | Baculoviral IAP repeat-containing 6 | 483 | 1.15E-25 | 55.8 | P:regulation of cellular process; C:intracellular part; P:cellular process |
| Efet.01.390862.g1024.t1 | octopamine receptor Oamb isoform X1 | 912 | 5.82E-41 | 60.95 | F:G-protein coupled receptor activity; F:adrenergic receptor activity; P:G-protein coupled receptor signaling pathway; P:signal transduction; C:membrane; C:integral component of membrane; P:adrenergic receptor signaling pathway; F:signal transducer activity; C:plasma membrane |
| Efet.01.390882.g1026.t1 | RHS repeat-associated core domain-containing | 1335 | 0 | 61.85 | P:self proteolysis; P:intein-mediated protein splicing; F:calcium ion binding; P:cell adhesion |
| Efet.01.391058.g1029.t1 | hypothetical protein CAPTEDRAFT_221435 | 924 | 9.08E-63 | 54.15 | C:membrane; C:integral component of membrane |
| Efet.01.39110.g943.t1 | receptor-type guanylate cyclase Gyc76C-like | 495 | 1.45E-25 | 61.05 | F:lyase activity; P:primary metabolic process; P:single-organism process; P:organic substance metabolic process; P:phosphate-containing compound metabolic process |
| Efet.01.391130.g1031.t1 | hypothetical protein HELRODRAFT_136819, partial | 288 | 1.04E-12 | 63 |  |
| Efet.01.391218.g1032.t1 | cytochrome P450 4F6-like | 450 | 1.83E-33 | 72.9 | F:iron ion binding; F:oxidoreductase activity, acting on paired donors, with incorporation or reduction of molecular oxygen; C:membrane; P:oxidation-reduction process; F:monooxygenase activity; F:heme binding |
| Efet.01.39133.g945.t1 | acid ceramidase-like | 552 | 7.98E-88 | 81.25 | F:ceramidase activity; P:lipid metabolic process; P:collateral sprouting; C:lysosome; F:NAD+ ADP-ribosyltransferase activity |
| Efet.01.391376.g1035.t1 | tektin-4-like | 528 | 9.54E-73 | 77.3 |  |
| Efet.01.391377.g1036.t1 | tripartite motif-containing 45-like isoform X1 | 246 | 1.18E-15 | 77.05 | F:metal ion binding |
| Efet.01.39151.g946.t1 | nuclear hormone receptor HR96 isoform X11 | 1029 | 8.09E-109 | 60.1 | P:regulation of cellular process; F:binding; P:cellular process |
| Efet.01.391562.g1038.t1 | potassium voltage-gated channel subfamily H member 8 isoform X2 | 684 | 8.59E-69 | 73.65 | F:phosphorelay sensor kinase activity; F:voltage-gated potassium channel activity; P:potassium ion transmembrane transport; P:phosphorelay signal transduction system; C:integral component of membrane; P:signal transduction by protein phosphorylation; C:intracellular |
| Efet.01.391587.g1039.t1 | STAGA complex 65 subunit gamma | 390 | 4.28E-24 | 68 |  |
| Efet.01.391594.g1040.t1 | family transporter | 357 | 3.21E-34 | 80.3 | F:catalytic activity; C:membrane |
| Efet.01.391668.g1041.t1 | hypothetical protein CAPTEDRAFT_223958 | 288 | 8.48E-18 | 69.67 | C:membrane |
| Efet.01.391709.g1042.t1 | Repulsive guidance molecule A | 207 | 4.72E-16 | 73.25 |  |
| Efet.01.391769.g1044.t1 | double-stranded RNA-binding Staufen homolog 2 isoform X1 | 531 | 1.83E-30 | 81.9 |  |
| Efet.01.391891.g1046.t1 | cAMP-dependent kinase regulatory subunit | 306 | 7.21E-32 | 72.55 |  |
| Efet.01.391903.g1047.t1 | cytochrome P450 26A1-like | 351 | 3.95E-17 | 59.45 | F:oxidoreductase activity; F:binding |
| Efet.01.392011.g1050.t1 | matrilin-2-like | 273 | 2.17E-06 | 53 |  |
| Efet.01.39202.g948.t1 | aminopeptidase N-like | 438 | 2.60E-09 | 63.15 | C:membrane; C:cell part; F:binding; P:organic substance metabolic process; F:metallopeptidase activity |
| Efet.01.392068.g1055.t1 | hypothetical protein CAPTEDRAFT_191938 | 351 | 1.56E-17 | 67.71 | C:membrane |
| Efet.01.392229.g1056.t1 | fizzy-related homolog | 294 | 2.28E-12 | 71.21 | F:ubiquitin-protein transferase activator activity; P:regulation of catalytic activity; F:anaphase-promoting complex binding |
| Efet.01.392261.g1057.t1 | leucine rich repeat | 390 | 8.34E-24 | 61.5 |  |
| Efet.01.392315.g1058.t1 | sex peptide receptor-like | 1011 | 1.20E-106 | 66.05 | F:G-protein coupled receptor activity; C:membrane |
| Efet.01.392321.g1059.t1 | dimmed-like | 630 | 2.37E-08 | 58.05 | F:protein dimerization activity |
| Efet.01.392378.g1060.t1 | ERC 2-like | 417 | 9.62E-15 | 76.05 | C:presynaptic active zone |
| Efet.01.392422.g1061.t1 | GTPase | 747 | 1.04E-153 | 86 | F:magnesium ion binding; F:GTP binding; C:cytoplasm; F:GTPase activity |
| Efet.01.392427.g1062.t1 | CoA-binding protein | 651 | 1.12E-60 | 51.95 | F:ATP binding; F:cofactor binding; F:ligase activity |
| Efet.01.392427.g1063.t1 | reactive intermediate imine deaminase | 222 | 2.94E-42 | 90.95 | F:hydrolase activity |
| Efet.01.392427.g1064.t1 | peptide ABC transporter | 846 | 2.20E-121 | 90.65 | P:transmembrane transport; C:ATP-binding cassette (ABC) transporter complex |
| Efet.01.392444.g1065.t1 | hypothetical protein CAPTEDRAFT_215352 | 522 | 1.84E-06 | 73 | C:membrane; C:integral component of membrane |
| Efet.01.39256.g949.t1 | hypothetical protein CAPTEDRAFT_154023 | 351 | 4.26E-07 | 40 | P:RNA phosphodiester bond hydrolysis, endonucleolytic; F:RNA binding; F:ribonuclease T2 activity |
| Efet.01.39258.g950.t1 | G- coupled receptor 98 | 1404 | 1.49E-38 | 52.65 | F:G-protein coupled receptor activity; F:transmembrane signaling receptor activity; P:G-protein coupled receptor signaling pathway; P:cell communication; C:membrane; C:integral component of membrane; P:cell surface receptor signaling pathway |
| Efet.01.392596.g1067.t1 | WW domain-binding 11-like | 792 | 1.15E-26 | 66.7 | P:RNA processing |
| Efet.01.392631.g1068.t1 | mab-21-like 2 | 996 | 2.80E-12 | 53.73 | C:membrane; C:integral component of membrane |
| Efet.01.39264.g951.t1 | hypothetical protein HELRODRAFT_192948 | 1251 | 7.15E-38 | 52 |  |
| Efet.01.392656.g1069.t1 | innexin 2 | 405 | 5.51E-23 | 51.3 | C:gap junction; P:ion transport; P:transmembrane transport; C:membrane; C:integral component of membrane; P:cell communication by electrical coupling; C:cell junction; F:gap junction channel activity; C:synapse; P:transport; C:plasma membrane |
| Efet.01.392705.g1071.t1 | phosphatidylinositol 5-phosphate 4-kinase type-2 alpha-like | 261 | 2.66E-24 | 93.25 | F:phosphatidylinositol phosphate kinase activity; P:phosphatidylinositol phosphorylation |
| Efet.01.392803.g1072.t1 | dopamine D2-like receptor | 894 | 3.75E-28 | 48.5 | F:dopamine neurotransmitter receptor activity; C:integral component of plasma membrane; F:G-protein coupled receptor activity; P:peptidyl-tyrosine phosphorylation; F:non-membrane spanning protein tyrosine kinase activity; P:G-protein coupled receptor signaling pathway; P:signal transduction; C:membrane; C:integral component of membrane; P:dopamine receptor signaling pathway; F:transferase activity; F:signal transducer activity |
| Efet.01.392905.g1073.t1 | ribosomal S24 | 432 | 3.87E-44 | 92.6 | F:nucleotide binding; P:maturation of SSU-rRNA from tricistronic rRNA transcript (SSU-rRNA, 5.8S rRNA, LSU-rRNA); F:structural constituent of ribosome; C:cytosolic small ribosomal subunit; P:translation |
| Efet.01.39292.g952.t1 | hypothetical protein HELRODRAFT_190034 | 4353 | 7.74E-12 | 59 |  |
| Efet.01.392970.g1074.t1 | hypothetical protein CAPTEDRAFT_228927 | 222 | 5.72E-08 | 62 | C:membrane; C:integral component of membrane; P:regulation of kainate selective glutamate receptor activity |
| Efet.01.393047.g1075.t1 | rabenosyn-5 | 375 | 9.06E-21 | 66.05 | P:vacuole inheritance; C:cytoplasmic side of endosome membrane; P:endosomal vesicle fusion; P:endocytosis; F:Rab GTPase binding; P:Golgi to endosome transport; P:Golgi to vacuole transport |
| Efet.01.393087.g1077.t1 | SPRY domain-containing SOCS box 1-like | 720 | 9.92E-117 | 78 | C:intracellular; P:intracellular signal transduction |
| Efet.01.39321.g953.t1 | SGT1 homolog isoform X1 | 288 | 9.80E-21 | 72.9 | P:positive regulation by host of symbiont catalytic activity; F:protein binding; P:regulation of protein stability; C:ubiquitin ligase complex; C:cytosol; P:mitotic nuclear division; C:kinetochore |
| Efet.01.39329.g954.t1 | nucleolin-like isoform X1 | 816 | 1.37E-37 | 52.55 | F:nucleotide binding; F:nucleic acid binding; F:DNA binding; C:nucleus; F:RNA binding; C:nucleolus |
| Efet.01.393322.g1080.t1 | outer membrane assembly factor | 570 | 6.84E-110 | 93.8 | P:Gram-negative-bacterium-type cell outer membrane assembly; C:integral component of membrane; C:cell outer membrane; P:protein insertion into membrane |
| Efet.01.393322.g1081.t1 | helicase | 2235 | 0 | 77.05 | F:DNA binding; F:ATP binding; P:DNA duplex unwinding; F:ATP-dependent DNA helicase activity; P:nucleobase-containing compound metabolic process |
| Efet.01.393322.g1082.t1 | serine threonine phosphatase | 831 | 0 | 86.95 | F:protein serine/threonine phosphatase activity; P:protein dephosphorylation |
| Efet.01.393322.g1083.t1 | hypothetical protein | 270 | 5.76E-25 | 73.5 |  |
| Efet.01.393322.g1084.t1 | Asp Glu hydantoin racemase | 696 | 1.82E-167 | 72.1 |  |
| Efet.01.393322.g1085.t1 | FAD-binding oxidoreductase | 1389 | 0 | 77.45 | F:oxidoreductase activity, acting on CH-OH group of donors; F:flavin adenine dinucleotide binding; P:oxidation-reduction process |
| Efet.01.393322.g1086.t1 | 2-dehydro-3-deoxyglucarate aldolase 4-hydroxy-2-oxoheptanedioate aldolase | 468 | 8.30E-103 | 67.75 | F:catalytic activity; F:metal ion binding |
| Efet.01.393459.g1088.t1 | Ankyrin repeat and SOCS box 2 | 1395 | 4.99E-95 | 49.05 | C:membrane; C:integral component of membrane |
| Efet.01.393459.g1089.t1 | Ankyrin repeat and SOCS box 2 | 615 | 5.68E-56 | 61.55 | C:integral component of membrane |
| Efet.01.39355.g955.t1 | RIB43A-like with coiled-coils 2 | 207 | 8.99E-12 | 80.88 |  |
| Efet.01.39361.g956.t1 | nidogen and EGF-like domain-containing 1 | 375 | 3.58E-38 | 69.55 | F:calcium ion binding |
| Efet.01.393694.g1091.t1 | BTB POZ domain-containing 6-like | 666 | 8.64E-15 | 44.8 |  |
| Efet.01.393748.g1093.t1 | mesencephalic astrocyte-derived neurotrophic factor | 294 | 1.24E-47 | 79.35 | C:nucleus; C:integral component of membrane; F:RNA binding |
| Efet.01.393823.g1094.t1 | CREB-binding -like isoform X1 | 219 | 6.70E-29 | 82.4 | C:nucleus; F:histone acetyltransferase activity; F:zinc ion binding; F:transcription cofactor activity; P:regulation of transcription, DNA-templated; P:histone acetylation |
| Efet.01.393846.g1096.t1 | orphan G- coupled receptor 51 | 1002 | 1.09E-40 | 51.75 | F:G-protein coupled receptor activity; F:G-protein coupled peptide receptor activity; P:G-protein coupled receptor signaling pathway; C:membrane; C:integral component of membrane |
| Efet.01.393927.g1097.t1 | prolyl-tRNA synthetase associated domain-containing 1-like | 408 | 4.54E-56 | 75.05 | F:catalytic activity |
| Efet.01.393956.g1098.t1 | hypothetical protein CAPTEDRAFT_226428 | 630 | 3.52E-48 | 50.33 | F:calcium ion binding; C:membrane; C:integral component of membrane |
| Efet.01.393956.g1099.t1 | hypothetical protein CAPTEDRAFT_226428 | 288 | 4.00E-21 | 72.5 | C:integral component of membrane |
| Efet.01.39405.g958.t1 | calcineurin subunit B type 2 | 417 | 9.02E-24 | 89.35 | F:calcium ion binding |
| Efet.01.394157.g1100.t1 | hypothetical protein CAPTEDRAFT_220050 | 288 | 6.26E-08 | 52 | F:acetylglucosaminyltransferase activity; P:protein glycosylation |
| Efet.01.394194.g1101.t1 | DEP domain-containing | 255 | 1.98E-15 | 60.7 | P:signal transduction; P:intracellular signal transduction; C:intracellular |
| Efet.01.39424.g959.t1 | coiled-coil domain-containing 180-like isoform X3 | 360 | 7.01E-19 | 81 |  |
| Efet.01.39440.g960.t1 | Coiled-coil domain-containing 158 | 1242 | 2.78E-121 | 56.75 | C:membrane; C:integral component of membrane |
| Efet.01.39440.g961.t1 | hypothetical protein CAPTEDRAFT_185637 | 828 | 5.71E-112 | 47.85 | C:integral component of membrane |
| Efet.01.394446.g1104.t1 | FTS and Hook-interacting -like isoform X1 | 681 | 2.69E-22 | 50.55 |  |
| Efet.01.394449.g1105.t1 | CUGBP Elav-like family member 2 isoform X3 | 330 | 7.19E-16 | 76.7 | F:nucleotide binding; F:RNA binding |
| Efet.01.394560.g1107.t1 | delta-1-pyrroline-5-carboxylate synthase | 369 | 2.42E-53 | 79 | F:glutamate 5-kinase activity; C:cytoplasm; F:glutamate-5-semialdehyde dehydrogenase activity; P:proline biosynthetic process; P:oxidation-reduction process; P:phosphorylation |
| Efet.01.394691.g1109.t1 | zinc finger 598 | 2289 | 9.22E-25 | 50.3 | P:biological_process; F:zinc ion binding; F:metal ion binding; F:RNA binding |
| Efet.01.394810.g1110.t1 | O-acetylhomoserine aminocarboxypropyltransferase | 1392 | 0 | 88.3 | F:pyridoxal phosphate binding; P:'de novo' L-methionine biosynthetic process; F:O-acetylhomoserine aminocarboxypropyltransferase activity |
| Efet.01.394810.g1111.t1 | alpha beta hydrolase | 564 | 3.21E-85 | 90.1 | F:hydrolase activity |
| Efet.01.394810.g1112.t1 | tRNA-processing RNAse BN | 1287 | 0 | 91.1 | C:integral component of plasma membrane |
| Efet.01.394817.g1113.t1 | lon protease mitochondrial-like | 681 | 4.87E-11 | 85.2 | F:ATP binding; F:mitochondrial light strand promoter anti-sense binding; P:misfolded or incompletely synthesized protein catabolic process; C:mitochondrial matrix; P:chaperone-mediated protein complex assembly; F:serine-type endopeptidase activity; F:ATP-dependent peptidase activity; P:mitochondrion organization; F:single-stranded DNA binding |
| Efet.01.394969.g1115.t1 | nesprin-1 isoform X8 | 1179 | 5.23E-34 | 46.4 | C:nuclear envelope; F:actin filament binding; F:actin binding; P:cytoskeletal anchoring at nuclear membrane; C:LINC complex |
| Efet.01.394969.g1116.t1 | nesprin-1 isoform X1 | 741 | 6.46E-13 | 54.05 | C:nuclear envelope; F:actin filament binding; F:actin binding; P:cytoskeletal anchoring at nuclear membrane; C:LINC complex |
| Efet.01.39498.g964.t1 | Glycine receptor subunit alpha- | 321 | 3.50E-32 | 79.15 | P:synaptic transmission, glycinergic; C:somatodendritic compartment; F:extracellular-glycine-gated chloride channel activity; C:glycine-gated chloride channel complex; P:chloride transmembrane transport; P:response to amino acid; P:protein homooligomerization; C:cell projection; F:glycine-gated chloride ion channel activity; P:chemical synaptic transmission, postsynaptic; P:neuropeptide signaling pathway; C:postsynaptic membrane; C:cell junction; F:glycine binding |
| Efet.01.394995.g1117.t1 | ras-related Rab-10-like | 204 | 1.39E-29 | 90.45 | F:GTP binding; P:small GTPase mediated signal transduction; C:intracellular |
| Efet.01.395045.g1118.t1 | insulin-like growth factor 2 mRNA-binding 3-A | 342 | 1.90E-19 | 70 | F:nucleotide binding; F:RNA binding |
| Efet.01.395065.g1119.t1 | brain-specific angiogenesis inhibitor 3 | 216 | 5.11E-07 | 57.9 | P:regulation of cellular process; P:single-organism process; P:cellular process |
| Efet.01.39513.g965.t1 | hypothetical protein CAPTEDRAFT_227484 | 261 | 1.08E-11 | 67.5 | F:thyroid hormone receptor activity; C:nucleus; F:DNA binding; C:cytoskeleton; P:microtubule bundle formation; P:transcription, DNA-templated; F:steroid hormone receptor activity; P:cytokinesis; P:regulation of transcription, DNA-templated; P:steroid hormone mediated signaling pathway; F:microtubule binding; P:intracellular receptor signaling pathway |
| Efet.01.395219.g1122.t1 | hypothetical protein CAPTEDRAFT_213611 | 573 | 1.14E-14 | 56 | F:thyroxine 5'-deiodinase activity; F:oxidoreductase activity; P:oxidation-reduction process; P:hormone biosynthetic process |
| Efet.01.395258.g1123.t1 | mab-21-like 1-like | 1545 | 3.22E-12 | 51.8 | C:membrane; C:integral component of membrane |
| Efet.01.395303.g1124.t1 | DCN1 3 | 861 | 1.65E-95 | 70.3 |  |
| Efet.01.395351.g1125.t1 | sortilin isoform X1 | 351 | 7.31E-07 | 63 | C:membrane; C:integral component of membrane; P:cellular response to xenobiotic stimulus; C:Golgi apparatus; P:transport |
| Efet.01.395395.g1126.t1 | Sodium-dependent glucose transporter | 696 | 2.50E-09 | 60.29 | P:transmembrane transport; C:membrane; C:integral component of membrane; P:carbohydrate transport |
| Efet.01.395446.g1127.t1 | intraflagellar transport 56-like | 228 | 1.34E-06 | 79.3 | C:ciliary tip; C:centrosome; P:intraciliary transport; C:ciliary basal body; P:spermatid development; P:protein transport; C:cilium; P:smoothened signaling pathway; C:intraciliary transport particle B; P:transport; C:cell projection |
| Efet.01.395506.g1129.t1 | eukaryotic translation initiation factor 4 gamma 1 isoform X4 | 1188 | 1.10E-68 | 55.1 | F:translation initiation factor activity; F:RNA binding; P:translational initiation |
| Efet.01.395589.g1130.t1 | hypothetical protein CGI_10028239 | 408 | 6.33E-07 | 58.5 | F:nucleic acid binding; F:zinc ion binding |
| Efet.01.395589.g1131.t1 | Retrovirus-related Pol poly from transposon | 438 | 9.06E-15 | 68.55 |  |
| Efet.01.395721.g1133.t1 | family transcriptional regulator | 639 | 1.20E-52 | 60.45 | F:DNA binding; F:transcription factor activity, sequence-specific DNA binding; P:transcription, DNA-templated; P:regulation of transcription, DNA-templated |
| Efet.01.395805.g1135.t1 | acetylcholinesterase-like isoform X3 | 1650 | 0 | 69.7 | C:integral component of membrane; F:cholinesterase activity |
| Efet.01.395810.g1136.t1 | type-1 angiotensin II receptor-associated | 306 | 1.24E-14 | 59.75 | C:membrane; C:intracellular part |
| Efet.01.39587.g969.t1 | thimet oligopeptidase | 264 | 1.51E-07 | 73.15 | F:metallopeptidase activity |
| Efet.01.395894.g1138.t1 | basement membrane-specific heparan sulfate proteoglycan core -like | 459 | 1.50E-26 | 54.4 | F:calcium ion binding |
| Efet.01.396105.g1140.t1 | dynein light chain Tctex-type 1 | 375 | 6.44E-59 | 86.7 |  |
| Efet.01.396152.g1143.t1 | alpha-2B adrenergic receptor-like | 651 | 1.45E-31 | 52.05 | F:G-protein coupled receptor activity; P:G-protein coupled receptor signaling pathway; P:signal transduction; C:membrane; C:integral component of membrane; F:signal transducer activity |
| Efet.01.39616.g970.t1 | MAGUK p55 subfamily member 5 | 351 | 1.26E-48 | 81.25 | P:GDP metabolic process; P:bicellular tight junction assembly; P:GMP metabolic process; P:establishment of epithelial cell polarity; F:guanylate kinase activity; P:phosphorylation |
| Efet.01.396187.g1144.t1 | atrial natriuretic peptide receptor 1-like | 354 | 5.30E-25 | 59.05 | F:catalytic activity; C:membrane; P:cellular process |
| Efet.01.396232.g1145.t1 | cartilage matrix -like | 705 | 1.82E-21 | 52.6 | F:calcium ion binding; C:collagen trimer; C:membrane; C:integral component of membrane; C:proteinaceous extracellular matrix |
| Efet.01.396285.g1150.t1 | RILP 1 isoform X2 | 369 | 2.43E-30 | 74.9 |  |
| Efet.01.396287.g1151.t1 | tachykinin-like peptides receptor 99D | 270 | 1.82E-41 | 85.6 | F:rhodopsin kinase activity; P:neuropeptide signaling pathway; C:integral component of membrane; P:protein phosphorylation; P:tachykinin receptor signaling pathway; C:plasma membrane; F:tachykinin receptor activity; F:neuropeptide Y receptor activity |
| Efet.01.396319.g1152.t1 | gastrula zinc finger -like | 3084 | 1.17E-37 | 47.3 | F:nucleic acid binding; F:metal ion binding |
| Efet.01.39634.g971.t1 | glyceraldehyde-3-phosphate dehydrogenase | 561 | 4.05E-102 | 88.85 | F:NADP binding; C:cytoplasm; F:NAD binding; P:glycolytic process; P:oxidation-reduction process; P:glucose metabolic process; F:glyceraldehyde-3-phosphate dehydrogenase (NAD+) (phosphorylating) activity |
| Efet.01.396400.g1156.t1 | cell death abnormality 1-like | 360 | 4.18E-07 | 69 |  |
| Efet.01.396462.g1157.t1 | otopetrin-1-like isoform X1 | 1182 | 2.36E-49 | 50.95 | C:membrane |
| Efet.01.396570.g1158.t1 | gastrula zinc finger -like | 1056 | 5.58E-32 | 47.25 | F:nucleic acid binding; F:metal ion binding |
| Efet.01.396584.g1160.t1 | ankyrin repeat domain-containing 40-like | 423 | 1.02E-32 | 68 |  |
| Efet.01.396629.g1161.t1 | substance-K receptor | 312 | 1.83E-11 | 54.6 | P:cellular process |
| Efet.01.396685.g1162.t1 | transporter arsB | 507 | 1.06E-34 | 56 | P:transport |
| Efet.01.39673.g973.t1 | ATP-binding cassette sub-family A member 3-like | 369 | 7.29E-22 | 68.75 | F:nucleotide binding; P:lipid transport; F:ATPase activity, coupled to transmembrane movement of substances; C:intracellular membrane-bounded organelle; C:membrane |
| Efet.01.39673.g974.t1 | ATP-binding cassette sub-family A member 3-like | 957 | 1.39E-54 | 55.55 | F:nucleotide binding; F:ATPase activity; P:lipid transport; F:ATP binding; F:ATPase activity, coupled to transmembrane movement of substances; P:transmembrane transport; C:intracellular membrane-bounded organelle; C:membrane; C:integral component of membrane; P:transport; F:transporter activity |
| Efet.01.396818.g1164.t1 | myosin heavy chain type | 363 | 2.55E-54 | 85.35 | C:myosin complex; F:ATP binding; F:actin binding; P:metabolic process; F:amino acid binding; F:motor activity |
| Efet.01.396858.g1165.t1 | BTB POZ domain-containing KCTD7 | 1671 | 9.65E-53 | 59.75 | P:protein homooligomerization |
| Efet.01.39690.g975.t1 | O-acetyl-ADP-ribose deacetylase MACROD1 isoform X1 | 2277 | 4.94E-09 | 59.85 |  |
| Efet.01.396930.g1168.t1 | Neuropeptides capa receptor | 483 | 6.26E-48 | 73 | P:neuropeptide signaling pathway; C:integral component of membrane; F:neuromedin U receptor activity |
| Efet.01.39695.g976.t1 | tachykinin-like peptides receptor 99D isoform X3 | 534 | 2.78E-28 | 71.3 | P:neuropeptide signaling pathway; C:integral component of membrane; F:neuropeptide Y receptor activity |
| Efet.01.396998.g1170.t1 | HEAT repeat-containing 5B-like isoform X4 | 693 | 5.82E-90 | 83.5 |  |
| Efet.01.397003.g1171.t1 | Retrovirus-related Pol poly from transposon | 1437 | 2.12E-25 | 65 | F:binding |
| Efet.01.397003.g1172.t1 | Retrovirus-related Pol poly from transposon | 438 | 3.30E-52 | 73.8 | P:macromolecule metabolic process; F:metal ion binding; P:primary metabolic process |
| Efet.01.397003.g1173.t1 | Retrovirus-related Pol poly from transposon | 222 | 6.06E-10 | 87.05 | F:nucleic acid binding; F:zinc ion binding; F:aspartic-type endopeptidase activity; P:proteolysis; P:DNA integration |
| Efet.01.397003.g1174.t1 | Retrovirus-related Pol poly from transposon | 1167 | 2.40E-32 | 54.9 | F:nucleic acid binding; F:zinc ion binding; F:aspartic-type endopeptidase activity; P:proteolysis; P:DNA integration |
| Efet.01.397083.g1175.t1 | baculoviral IAP repeat-containing 6-like | 297 | 1.72E-30 | 72.2 | F:ubiquitin-protein transferase activity; C:microtubule cytoskeleton; C:cytoskeletal part; P:negative regulation of apoptotic process; P:cellular protein modification process; C:endomembrane system; C:cytoplasmic part; P:regulation of signal transduction; P:embryonic placenta development; P:regulation of cell proliferation; C:membrane-bounded organelle |
| Efet.01.39720.g978.t1 | epidermal growth | 1368 | 2.31E-17 | 61.25 | C:extracellular region part |
| Efet.01.397305.g1178.t1 | neuropeptide SIFamide receptor-like | 720 | 3.04E-39 | 61.8 | F:G-protein coupled receptor activity; P:signal transduction |
| Efet.01.397340.g1179.t1 | poly [ADP-ribose] polymerase 12-like | 876 | 1.69E-46 | 52.6 | F:nucleic acid binding; F:metal ion binding; F:NAD+ ADP-ribosyltransferase activity |
| Efet.01.397488.g1180.t1 | kinase C delta type-like isoform X1 | 201 | 1.21E-07 | 75.4 | F:nucleotide binding; F:ATP binding; F:metal ion binding; F:protein kinase activity; P:phosphorylation; F:transferase activity; F:protein serine/threonine kinase activity; P:protein phosphorylation; F:kinase activity; P:intracellular signal transduction; C:intracellular; F:protein kinase C activity |
| Efet.01.397511.g1181.t1 | PREDICTED: uncharacterized protein LOC106961309 isoform X9 | 387 | 6.44E-07 | 38 |  |
| Efet.01.397522.g1182.t1 | G- coupled receptor 98 | 1293 | 5.14E-46 | 44.6 | P:G-protein coupled receptor signaling pathway; P:cell communication; P:cell surface receptor signaling pathway; C:receptor complex; C:cell surface; P:maintenance of animal organ identity; P:sensory perception of sound; F:G-protein coupled receptor activity; F:transmembrane signaling receptor activity; C:extracellular exosome; C:cytoplasm; P:photoreceptor cell maintenance; F:calcium ion binding; C:membrane; C:integral component of membrane; P:sensory perception of light stimulus; P:neurological system process |
| Efet.01.397573.g1183.t1 | WD G-beta repeat | 234 | 2.69E-48 | 97.35 | P:cell proliferation; C:myelin sheath; P:sensory perception of taste; P:retina development in camera-type eye; P:phospholipase C-activating G-protein coupled receptor signaling pathway; F:signal transducer activity; C:lysosomal membrane; C:photoreceptor outer segment membrane; P:positive regulation of cytosolic calcium ion concentration; C:dendrite; P:phototransduction, visible light; C:heterotrimeric G-protein complex; C:extracellular exosome; F:type 1 angiotensin receptor binding; F:spectrin binding; P:cardiac muscle cell apoptotic process; F:GTPase activity; F:GTPase binding; C:cell body; P:cellular response to hypoxia; C:photoreceptor inner segment; F:protein complex binding; F:alkylglycerophosphoethanolamine phosphodiesterase activity; P:protein heterotrimerization |
| Efet.01.397634.g1184.t1 | hypothetical protein HELRODRAFT_191657 | 330 | 3.83E-07 | 54 | C:membrane; C:integral component of membrane |
| Efet.01.3977.g334.t1 | transcription factor Sp8 | 1290 | 6.33E-60 | 75.6 | F:nucleic acid binding; F:transcription factor activity, sequence-specific DNA binding; F:metal ion binding |
| Efet.01.397822.g1186.t1 | tctex1 domain-containing 4 | 213 | 1.90E-17 | 63.8 | C:nucleus; C:microtubule organizing center; C:acrosomal vesicle; C:axoneme |
| Efet.01.397879.g1187.t1 | hypothetical protein CRN43_00585, partial | 282 | 1.43E-07 | 57 |  |
| Efet.01.397926.g1189.t1 | UDP-glucose 4-epimerase | 351 | 1.24E-45 | 70.85 | F:catalytic activity |
| Efet.01.397960.g1192.t1 | polycystic kidney disease 1-like 2 | 1395 | 1.02E-09 | 45 |  |
| Efet.01.39802.g980.t1 | calcyphosin isoform X1 [Xenopus tropicalis] | 216 | 2.69E-09 | 67.88 | F:calcium ion binding |
| Efet.01.398129.g1195.t1 | cyclin-dependent kinase 12-like | 435 | 6.37E-26 | 64.55 | F:kinase activity; P:cellular process |
| Efet.01.398213.g1196.t1 | ubiquinol-cytochrome-c reductase complex assembly factor 2 | 360 | 9.02E-35 | 70.2 | C:mitochondrial part; C:intracellular organelle lumen; P:regulation of primary metabolic process; P:regulation of cellular metabolic process |
| Efet.01.398300.g1197.t1 | malate dehydrogenase 1B | 1065 | 4.56E-85 | 60.7 | F:oxidoreductase activity; P:single-organism cellular process; P:single-organism metabolic process; P:organic substance metabolic process |
| Efet.01.398328.g1198.t1 | rab3 GTPase-activating non-catalytic subunit | 753 | 3.21E-27 | 49.95 | P:regulation of GTPase activity |
| Efet.01.398399.g1201.t1 | growth inhibitor | 411 | 8.48E-06 | 86 | F:DNA binding |
| Efet.01.398399.g1202.t1 | superoxide dismutase | 558 | 8.48E-101 | 89.2 | F:metal ion binding; P:removal of superoxide radicals; P:oxidation-reduction process; F:superoxide dismutase activity |
| Efet.01.39842.g982.t1 | hypothetical protein HELRODRAFT_162203 | 606 | 4.53E-08 | 67 | F:G-protein coupled receptor activity; F:G-protein coupled peptide receptor activity; P:G-protein coupled receptor signaling pathway; C:membrane; C:integral component of membrane |
| Efet.01.398470.g1204.t1 | phosphatidate mitochondrial | 981 | 2.86E-121 | 70 | F:phosphatidate cytidylyltransferase activity; C:extrinsic component of mitochondrial inner membrane; P:cardiolipin biosynthetic process |
| Efet.01.398517.g1206.t1 | DDB1- and CUL4-associated factor 1-like isoform X1 | 234 | 3.91E-14 | 77.45 | P:protein ubiquitination |
| Efet.01.398616.g1207.t1 | hypothetical protein CAPTEDRAFT_192595 | 393 | 1.25E-08 | 58 |  |
| Efet.01.398617.g1208.t1 | PREDICTED: uncharacterized protein LOC108671210 | 468 | 3.99E-28 | 60 |  |
| Efet.01.398671.g1209.t1 | Glycylpeptide N-tetradecanoyltransferase 2 | 285 | 1.04E-34 | 68.4 | C:cytoplasm; F:glycylpeptide N-tetradecanoyltransferase activity; P:N-terminal peptidyl-glycine N-myristoylation |
| Efet.01.398743.g1210.t1 | serine arginine repetitive matrix 1 | 858 | 8.04E-17 | 64.15 |  |
| Efet.01.398766.g1211.t1 | centrosomal of 295 kDa isoform X1 | 453 | 4.45E-11 | 57.55 |  |
| Efet.01.398824.g1212.t1 | bactericidal permeability increasing | 1161 | 1.91E-28 | 44.75 | C:extracellular space; F:lipid binding |
| Efet.01.398837.g1213.t1 | zinc knuckle domain | 366 | 6.37E-21 | 55.55 | P:organelle fission; F:binding |
| Efet.01.398923.g1214.t1 | latrophilin Cirl isoform X1 | 396 | 2.11E-40 | 49.2 | C:integral component of membrane; F:carbohydrate binding |
| Efet.01.398970.g1216.t1 | hypothetical protein CAPTEDRAFT_229158 | 1737 | 3.14E-19 | 78 |  |
| Efet.01.398980.g1217.t1 | hypothetical protein HELRODRAFT_166386 | 300 | 3.13E-08 | 82 | P:calcium ion transport; C:integral component of membrane |
| Efet.01.399093.g1219.t1 | protocadherin beta-10-like isoform X1 | 2757 | 1.29E-173 | 55.75 | C:membrane |
| Efet.01.399157.g1220.t1 | thyrotropin-releasing hormone receptor-like | 354 | 2.41E-34 | 75.85 | F:thyrotropin-releasing hormone receptor activity; P:G-protein coupled receptor signaling pathway; C:integral component of membrane |
| Efet.01.399173.g1221.t1 | dynein heavy chain axonemal-like | 261 | 5.39E-38 | 84.85 | F:ATPase activity; F:ATP binding; F:microtubule motor activity; C:dynein complex; P:microtubule-based movement |
| Efet.01.3992.g336.t1 | allatostatin-A receptor-like | 1269 | 9.05E-97 | 61.55 | P:signal transduction; F:signal transducer activity |
| Efet.01.39921.g984.t1 | bifunctional glutamate proline--tRNA ligase- | 768 | 3.81E-124 | 74.1 | P:protein complex assembly; P:glutamyl-tRNA aminoacylation; P:prolyl-tRNA aminoacylation; C:GAIT complex; F:ATP binding; C:intracellular ribonucleoprotein complex; F:proline-tRNA ligase activity; F:GTPase binding; C:membrane; F:glutamate-tRNA ligase activity; F:RNA stem-loop binding; C:cytosol; C:aminoacyl-tRNA synthetase multienzyme complex; P:cellular response to interferon-gamma; P:negative regulation of translation |
| Efet.01.399210.g1223.t1 | PREDICTED: uncharacterized protein LOC106873077 | 966 | 1.21E-11 | 66.85 | F:zinc ion binding |
| Efet.01.399257.g1224.t1 | clavaminate synthase At3g21360 | 1005 | 7.71E-87 | 57.8 | C:nucleus; F:oxidoreductase activity; P:oxidation-reduction process |
| Efet.01.399322.g1225.t1 | probable ATP-dependent RNA helicase DHX35 | 2109 | 0 | 79.75 | F:ATP binding; C:catalytic step 2 spliceosome; C:cytoplasm; P:RNA processing; F:ATP-dependent RNA helicase activity; F:RNA binding |
| Efet.01.399326.g1226.t1 | U5 small nuclear ribonucleo 200 kDa helicase- | 822 | 2.17E-152 | 88.35 | F:identical protein binding; P:negative regulation of cell proliferation; C:U5 snRNP; C:nucleoplasm; F:ATP-dependent RNA helicase activity; F:RNA binding; P:mRNA splicing, via spliceosome; F:ATP binding; C:catalytic step 2 spliceosome; C:integral component of membrane; P:osteoblast differentiation; C:viral nucleocapsid; P:negative regulation of TOR signaling |
| Efet.01.399406.g1227.t1 | substance-K receptor-like | 1152 | 2.03E-10 | 45.65 | P:cellular response to stimulus |
| Efet.01.399460.g1228.t1 | Kyphoscoliosis peptidase | 1551 | 1.54E-11 | 41.17 |  |
| Efet.01.399471.g1229.t1 | radial spoke head 4 homolog A-like | 237 | 1.10E-21 | 83.1 |  |
| Efet.01.399482.g1230.t1 | small heat shock p36-like isoform X1 | 1164 | 6.79E-85 | 53.25 | F:nucleic acid binding |
| Efet.01.399571.g1234.t1 | hypothetical protein CAPTEDRAFT_222036 | 498 | 2.40E-16 | 54.7 | C:membrane; C:integral component of membrane |
| Efet.01.399580.g1235.t1 | Forkhead box B1 | 423 | 3.42E-49 | 96.45 | C:nucleus; F:transcription factor activity, sequence-specific DNA binding; P:transcription, DNA-templated; P:regulation of transcription, DNA-templated; F:sequence-specific DNA binding |
| Efet.01.399586.g1236.t1 | ryncolin-2-like [Orbicella faveolata] | 567 | 9.80E-85 | 69.65 |  |
| Efet.01.39963.g985.t1 | collagen alpha-3(VI) chain-like isoform X8 | 705 | 7.97E-30 | 54.85 | F:calcium ion binding; C:membrane; C:integral component of membrane; C:proteinaceous extracellular matrix |
| Efet.01.399656.g1238.t1 | hypothetical protein HELRODRAFT_168860 | 321 | 4.60E-18 | 60 | C:membrane; C:integral component of membrane |
| Efet.01.399733.g1239.t1 | innexin unc-9-like isoform X6 | 498 | 6.81E-96 | 76.2 | C:gap junction; P:ion transport; P:transmembrane transport; C:integral component of membrane; P:cell communication by electrical coupling; C:synapse; F:gap junction channel activity; C:plasma membrane |
| Efet.01.399789.g1240.t1 | E3 ubiquitin- ligase TRIM71-like | 1182 | 2.65E-29 | 47.7 | F:ubiquitin-protein transferase activity; F:zinc ion binding; F:metal ion binding; C:intracellular; P:protein ubiquitination |
| Efet.01.399855.g1242.t1 | UTP--glucose-1-phosphate uridylyltransferase | 267 | 2.37E-24 | 95.9 | P:UDP-glucose metabolic process; P:biosynthetic process; F:UTP:glucose-1-phosphate uridylyltransferase activity |
| Efet.01.39989.g986.t1 | hypothetical protein HELRODRAFT_175028 | 270 | 4.34E-24 | 63.75 | C:membrane; F:binding |
| Efet.01.39991.g987.t1 | Pleckstrin homology domain-containing family G member 7 | 267 | 5.27E-16 | 78.85 | P:regulation of Rho protein signal transduction; F:Rho guanyl-nucleotide exchange factor activity; P:positive regulation of GTPase activity |
| Efet.01.39998.g988.t1 | gamma-glutamylcyclotransferase CG2811 isoform X1 | 315 | 1.84E-11 | 49.2 | F:transferase activity |
| Efet.01.400070.g1.t1 | hypothetical protein CAPTEDRAFT_195152 | 765 | 4.21E-09 | 64 | C:membrane; C:integral component of membrane |
| Efet.01.400102.g2.t1 | epidermal growth factor receptor isoform X1 | 597 | 4.27E-34 | 61.5 | F:kinase activity; P:cellular process |
| Efet.01.400110.g3.t1 | hypothetical protein HELRODRAFT_191437 | 855 | 3.11E-13 | 46 |  |
| Efet.01.40012.g989.t1 | hypothetical protein HELRODRAFT_109714 | 1416 | 7.33E-08 | 86.5 |  |
| Efet.01.40014.g991.t1 | tubulin polymerization-promoting family member 2-like | 288 | 2.38E-31 | 84.4 |  |
| Efet.01.40026.g992.t1 | insulin-like growth factor-binding complex acid labile subunit | 1263 | 5.07E-51 | 47.85 | P:neurotrophin signaling pathway; C:integral component of plasma membrane; P:signal transduction; F:neurotrophin receptor activity; C:membrane; C:integral component of membrane; P:axon guidance |
| Efet.01.400310.g7.t1 | fer3 | 1032 | 7.57E-19 | 84 | F:protein dimerization activity |
| Efet.01.40033.g993.t1 | ATP-dependent Clp protease ATP-binding subunit clpX- mitochondrial | 468 | 1.31E-66 | 80.6 | F:ATP binding; C:mitochondrial matrix; P:protein catabolic process; P:proteolysis; F:ATP-dependent peptidase activity |
| Efet.01.400774.g11.t1 | sodium potassium-transporting ATPase subunit alpha isoform X1 | 324 | 8.90E-39 | 81.9 | P:potassium ion transport; F:ATP binding; P:sodium ion transport; F:sodium:potassium-exchanging ATPase activity; F:metal ion binding; P:ATP hydrolysis coupled transmembrane transport; C:integral component of membrane; P:establishment or maintenance of transmembrane electrochemical gradient |
| Efet.01.400806.g14.t1 | beta-1,4-galactosyltransferase galt-1-like | 516 | 6.04E-10 | 49 | C:membrane; C:integral component of membrane |
| Efet.01.400875.g17.t1 | ABC transporter ATP-binding | 603 | 4.75E-69 | 73.5 | F:nucleotide binding |
| Efet.01.400895.g18.t1 | hypothetical protein CRN43_14440 | 360 | 6.52E-28 | 67.5 |  |
| Efet.01.401055.g20.t1 | neurogenic locus notch homolog 1-like isoform X2 | 2181 | 6.87E-174 | 48.2 | F:G-protein coupled receptor activity; F:transmembrane signaling receptor activity; P:G-protein coupled receptor signaling pathway; P:signal transduction; F:calcium ion binding; C:membrane; C:integral component of membrane; P:cell surface receptor signaling pathway; P:homophilic cell adhesion via plasma membrane adhesion molecules; F:signal transducer activity |
| Efet.01.401112.g21.t1 | reverse transcriptase-like | 351 | 8.95E-08 | 56 |  |
| Efet.01.401130.g23.t1 | probable serine threonine- kinase DDB_G0267686 isoform X1 | 654 | 3.69E-07 | 50 | P:signal transduction |
| Efet.01.401177.g25.t1 | Calcyphosin | 420 | 2.83E-57 | 80.05 | F:calcium ion binding |
| Efet.01.401223.g27.t1 | p53-induced death domain-containing 1-like isoform X2 | 210 | 5.66E-09 | 60 | P:signal transduction |
| Efet.01.401292.g28.t1 | twitchin-like isoform X1 | 462 | 2.16E-62 | 62.45 | F:ATP binding; F:protein kinase activity; P:protein phosphorylation |
| Efet.01.401337.g29.t1 | acetolactate synthase large subunit | 465 | 6.03E-47 | 94.1 | F:thiamine pyrophosphate binding; F:catalytic activity |
| Efet.01.401337.g30.t1 | acetolactate synthase large subunit | 486 | 1.12E-58 | 80.15 | F:thiamine pyrophosphate binding; F:catalytic activity |
| Efet.01.401337.g31.t1 | ribose-5-phosphate isomerase | 603 | 2.79E-139 | 93.7 | P:pentose-phosphate shunt, non-oxidative branch; F:ribose-5-phosphate isomerase activity |
| Efet.01.401473.g33.t1 | cytochrome P450 4F22 | 324 | 8.64E-28 | 73.5 | F:oxidoreductase activity; F:metal ion binding; C:membrane |
| Efet.01.401518.g34.t1 | spectrin beta chain-like isoform X1 | 567 | 1.58E-76 | 75.6 | F:actin binding; F:structural constituent of cytoskeleton; F:phospholipid binding; C:spectrin |
| Efet.01.401565.g36.t1 | hypothetical protein CAPTEDRAFT_216764 | 1764 | 7.03E-82 | 48 |  |
| Efet.01.401571.g37.t1 | ERGIC-53 | 252 | 6.23E-14 | 64 | C:intracellular organelle; C:cytoplasmic part; C:membrane-bounded organelle |
| Efet.01.401578.g38.t1 | hypothetical protein | 495 | 8.26E-23 | 49.25 |  |
| Efet.01.401587.g39.t1 | SRSF kinase 3-like isoform X2 | 1239 | 3.73E-85 | 82 | F:ATP binding; F:protein serine/threonine kinase activity; P:protein phosphorylation |
| Efet.01.40160.g995.t1 | deoxycytidylate deaminase isoform X2 | 396 | 3.12E-63 | 86.6 | F:zinc ion binding; F:hydrolase activity |
| Efet.01.401607.g40.t1 | hypothetical protein CRN43_14440 | 270 | 6.33E-17 | 66.5 |  |
| Efet.01.401646.g41.t1 | FMRFamide receptor-like | 1434 | 4.02E-42 | 53.85 | F:G-protein coupled receptor activity; F:G-protein coupled peptide receptor activity; P:G-protein coupled receptor signaling pathway; P:signal transduction; C:membrane; C:integral component of membrane; F:signal transducer activity |
| Efet.01.401694.g43.t1 | probable phospholipid-transporting ATPase IF isoform X5 | 384 | 2.35E-15 | 67.15 | F:nucleotide binding; P:membrane organization; F:metal ion binding; C:membrane; C:Golgi apparatus part; P:phospholipid transport; F:hydrolase activity |
| Efet.01.40170.g996.t1 | somatostatin receptor type 2-like | 501 | 5.17E-38 | 57.9 | F:neuropeptide receptor activity; P:G-protein coupled receptor signaling pathway; C:integral component of membrane; C:cell part |
| Efet.01.401700.g44.t1 | ankyrin repeat and EF-hand domain-containing 1 | 432 | 1.91E-28 | 67 | F:calcium ion binding |
| Efet.01.401729.g45.t1 | uncharacterized protein LOC110454948 | 384 | 6.65E-06 | 59 |  |
| Efet.01.401737.g46.t1 | tyramine receptor 1 | 1275 | 1.76E-135 | 62.55 | P:signal transduction; F:signal transducer activity |
| Efet.01.401777.g48.t1 | neuropeptide CCHamide-1 receptor-like | 570 | 4.88E-42 | 82.55 | P:neuropeptide signaling pathway; C:integral component of membrane; F:neuropeptide Y receptor activity |
| Efet.01.40181.g998.t1 | transmembrane 19 | 612 | 6.14E-74 | 76.85 | C:integral component of membrane |
| Efet.01.401863.g49.t1 | bcl-2 homologous antagonist killer-like | 252 | 1.04E-15 | 65 | P:positive regulation of apoptotic process |
| Efet.01.401892.g51.t1 | hypothetical protein HELRODRAFT_176332 | 522 | 5.32E-10 | 49 |  |
| Efet.01.401970.g52.t1 | tubulin beta chain | 828 | 7.80E-105 | 92.55 | F:GTP binding; C:cytoplasm; F:GTPase activity; F:structural constituent of cytoskeleton; C:microtubule; P:microtubule-based process |
| Efet.01.40205.g999.t1 | ELAV 1 isoform X6 | 273 | 1.14E-15 | 73.2 | F:nucleotide binding; F:RNA binding |
| Efet.01.402068.g53.t1 | ankyrin-2-like isoform X24 | 822 | 5.17E-44 | 62 |  |
| Efet.01.40207.g1000.t1 | hypothetical protein HELRODRAFT_191872 | 231 | 1.20E-09 | 63 |  |
| Efet.01.40211.g1001.t1 | 6-phosphofructo-2-kinase fructose-2,6-bisphosphatase-like isoform X1 | 297 | 3.90E-36 | 79.85 | F:ATP binding; C:integral component of membrane; P:fructose 2,6-bisphosphate metabolic process; P:fructose metabolic process; P:carbohydrate phosphorylation; F:6-phosphofructo-2-kinase activity |
| Efet.01.402143.g54.t1 | Potassium voltage-gated channel Shaker | 747 | 9.55E-71 | 75.9 | P:potassium ion transport; P:transmembrane transport; C:integral component of membrane; F:voltage-gated ion channel activity; F:potassium channel activity; P:cellular process |
| Efet.01.402163.g56.t1 | PR domain zinc finger 13 | 1050 | 2.42E-57 | 72.45 | F:nucleic acid binding; F:metal ion binding |
| Efet.01.4022.g337.t1 | 60 kDa neurofilament -like | 828 | 2.35E-39 | 53.8 | F:structural molecule activity; C:intermediate filament |
| Efet.01.40236.g1002.t1 | dispatched homolog 1-like | 312 | 3.79E-14 | 60 | P:retinal ganglion cell axon guidance; P:inner ear development; P:skeletal muscle tissue development; P:anterior/posterior pattern specification; P:adenohypophysis development; P:smoothened signaling pathway; P:blood circulation; P:muscle cell fate specification; P:embryonic cranial skeleton morphogenesis |
| Efet.01.40236.g1003.t1 | dispatched homolog 1 isoform X1 | 642 | 7.71E-52 | 77.15 | P:retinal ganglion cell axon guidance; P:inner ear development; F:peptide transporter activity; P:embryonic pattern specification; P:anterior/posterior pattern specification; P:adenohypophysis development; P:smoothened signaling pathway; P:blood circulation; P:muscle cell fate specification; P:embryonic cranial skeleton morphogenesis; P:diaphragm development; P:patched ligand maturation; P:determination of left/right symmetry; P:dorsal/ventral pattern formation; P:skeletal muscle tissue development; C:integral component of membrane; P:peptide transport |
| Efet.01.402538.g66.t1 | chondroitin sulfate proteoglycan 4-like | 1911 | 3.87E-66 | 48.85 | C:membrane; C:integral component of membrane |
| Efet.01.402544.g68.t1 | adenylate kinase 7 | 789 | 5.78E-79 | 67.55 | F:ATP binding; C:membrane; C:integral component of membrane; P:phosphorylation; F:nucleobase-containing compound kinase activity; F:kinase activity; P:nucleobase-containing compound metabolic process |
| Efet.01.402647.g69.t1 | BTB POZ domain-containing 10-like | 834 | 4.51E-82 | 70.2 |  |
| Efet.01.402762.g71.t1 | NEDD4-like E3 ubiquitin- ligase WWP1 isoform X1 | 228 | 7.19E-14 | 83.65 | C:nucleus; F:ubiquitin protein ligase activity; P:proteasome-mediated ubiquitin-dependent protein catabolic process; C:extracellular exosome; P:negative regulation of transcription, DNA-templated; C:cytoplasm; F:ligase activity; P:protein ubiquitination involved in ubiquitin-dependent protein catabolic process |
| Efet.01.402768.g72.t1 | follistatin-related 5-like | 684 | 8.18E-27 | 59.5 | P:single-organism cellular process |
| Efet.01.402871.g74.t1 | iron dicitrate transport regulator | 288 | 1.39E-11 | 66.2 | C:membrane; C:integral component of membrane |
| Efet.01.402876.g75.t1 | potassium voltage-gated channel Shal isoform X2 | 306 | 6.10E-27 | 82.2 |  |
| Efet.01.402896.g77.t1 | rRNA methyltransferase mitochondrial | 423 | 1.37E-14 | 54.05 | P:metabolic process |
| Efet.01.402937.g78.t1 | Pyruvate kinase isozymes M1 M2 | 738 | 1.24E-132 | 80.45 | F:magnesium ion binding; F:pyruvate kinase activity; F:potassium ion binding; P:glycolytic process; F:kinase activity |
| Efet.01.402937.g79.t1 | pyruvate kinase PKM isoform X3 | 987 | 2.05E-128 | 77.2 | F:magnesium ion binding; C:extracellular vesicle; F:pyruvate kinase activity; F:potassium ion binding; C:cytoplasm; C:intracellular membrane-bounded organelle; P:glycolytic process; F:kinase activity |
| Efet.01.40298.g1007.t1 | arf-GAP with dual PH domain-containing 2 isoform X5 | 426 | 1.37E-40 | 58.1 | F:GTPase activator activity; P:positive regulation of GTPase activity |
| Efet.01.4030.g340.t1 | YTH domain family 2 | 1359 | 2.42E-106 | 71.05 | F:RNA binding |
| Efet.01.403004.g80.t1 | otopetrin-3-like isoform X3 | 684 | 4.28E-32 | 55.3 | C:membrane; C:integral component of membrane |
| Efet.01.403034.g81.t1 | follicular epithelium yolk subunit | 420 | 8.06E-26 | 59.25 |  |
| Efet.01.403070.g82.t1 | tryptophan--tRNA cytoplasmic | 1227 | 0 | 85.85 | C:nucleus; F:ATP binding; C:extracellular exosome; C:cytoplasm; P:regulation of angiogenesis; P:tryptophanyl-tRNA aminoacylation; F:tryptophan-tRNA ligase activity |
| Efet.01.403089.g83.t1 | E3 ubiquitin- ligase RNF126 isoform X1 | 306 | 6.36E-09 | 62.9 | C:nucleus; F:zinc ion binding; F:ubiquitin protein ligase activity; P:proteasome-mediated ubiquitin-dependent protein catabolic process; P:protein polyubiquitination; P:protein ubiquitination involved in ubiquitin-dependent protein catabolic process |
| Efet.01.403117.g85.t1 | predicted protein | 2886 | 6.75E-06 | 47 |  |
| Efet.01.40313.g1009.t1 | turtle homolog B | 309 | 1.92E-22 | 57.3 | C:membrane; C:integral component of membrane |
| Efet.01.403178.g86.t1 | kinesin unc-104 isoform X1 | 285 | 2.09E-35 | 86.55 | F:ATP binding; F:microtubule motor activity; P:microtubule-based movement; F:microtubule binding |
| Efet.01.4032.g341.t1 | neuroglobin | 234 | 2.93E-32 | 61.3 | F:oxygen binding; P:oxygen transport; F:oxygen transporter activity; F:heme binding |
| Efet.01.40328.g1010.t1 | glucosamine-6-phosphate isomerase isoform X1 | 402 | 1.01E-60 | 89.8 | P:carbohydrate metabolic process; C:cytoplasm; F:glucosamine-6-phosphate deaminase activity; P:N-acetylglucosamine metabolic process; F:hydrolase activity |
| Efet.01.403289.g87.t1 | adenine nucleotide | 435 | 1.25E-84 | 88.95 | P:transmembrane transport; F:structural constituent of ribosome; C:integral component of membrane; C:mitochondrial inner membrane; F:transporter activity; P:translation |
| Efet.01.403298.g88.t1 | alpha-L-fucosidase | 861 | 5.70E-180 | 81.4 | F:alpha-L-fucosidase activity; P:fucose metabolic process |
| Efet.01.40338.g1011.t1 | prickle 2 isoform X4 | 411 | 1.20E-58 | 82.45 | F:zinc ion binding |
| Efet.01.403416.g89.t1 | 5-hydroxytryptamine receptor 2B | 465 | 1.88E-12 | 81.4 | P:G-protein coupled receptor signaling pathway; C:integral component of membrane; P:serotonin receptor signaling pathway; F:G-protein coupled serotonin receptor activity |
| Efet.01.403421.g90.t1 | TRPM8 channel-associated factor homolog | 591 | 3.95E-21 | 48.21 |  |
| Efet.01.403421.g91.t1 | TRPM8 channel-associated factor homolog | 426 | 1.56E-13 | 60.5 | C:membrane; C:integral component of membrane |
| Efet.01.40348.g1013.t1 | arfaptin-2 isoform X1 | 474 | 1.13E-73 | 82.6 | F:protein domain specific binding |
| Efet.01.40359.g1014.t1 | double-stranded RNA-specific editase 1 isoform X2 | 1224 | 1.30E-08 | 43.9 | F:RNA binding; P:RNA processing; F:adenosine deaminase activity |
| Efet.01.403601.g93.t1 | radixin isoform X3 | 444 | 7.51E-67 | 68.05 | P:regulation of cellular localization; C:myelin sheath; P:regulation of cell size; C:plasma membrane part; P:cellular response to thyroid hormone stimulus; C:apical part of cell; C:microvillus; P:apical protein localization; C:ruffle; C:stereocilium; C:cortical actin cytoskeleton; C:lamellipodium; F:protein domain specific binding; F:protein homodimerization activity; P:positive regulation of G1/S transition of mitotic cell cycle; C:extracellular region part; P:establishment of protein localization; C:adherens junction; F:actin binding; P:positive regulation of macromolecule metabolic process; P:actin filament capping; P:regulation of GTPase activity; P:microvillus assembly; C:filopodium; C:extrinsic component of membrane |
| Efet.01.403654.g95.t1 | homeobox orthopedia-like | 627 | 1.07E-34 | 53 | F:DNA binding; C:nucleus; P:multicellular organism development; P:regulation of transcription, DNA-templated; F:sequence-specific DNA binding |
| Efet.01.403655.g96.t1 | speract receptor-like | 672 | 1.62E-15 | 47.4 | F:G-protein coupled receptor activity; F:G-protein coupled GABA receptor activity; P:G-protein coupled receptor signaling pathway; C:membrane; C:integral component of membrane |
| Efet.01.403658.g97.t1 | briggsae CBR-LNTL-1 | 954 | 2.26E-70 | 62.45 |  |
| Efet.01.403666.g98.t1 | zinc finger 423-like isoform X3 | 351 | 8.72E-07 | 56.4 | F:nucleic acid binding; F:metal ion binding |
| Efet.01.403912.g100.t1 | Guanine nucleotide-binding subunit beta-5 | 345 | 1.34E-49 | 90.25 | F:histone acetyltransferase activity; P:signal transduction; P:histone acetylation |
| Efet.01.403932.g101.t1 | nesprin-1-like isoform X1 | 483 | 1.02E-21 | 62.8 | C:nuclear envelope; F:actin filament binding; F:actin binding; P:cytoskeletal anchoring at nuclear membrane; C:LINC complex |
| Efet.01.403933.g102.t1 | centrosomal of 131 kDa-like | 246 | 1.23E-19 | 80 | C:cilium; P:intraciliary transport involved in cilium assembly |
| Efet.01.4040.g342.t1 | neurogenic locus delta | 1053 | 5.75E-35 | 63.55 | P:Notch signaling pathway; P:multicellular organism development; P:cell communication; F:calcium ion binding; C:membrane; C:integral component of membrane |
| Efet.01.40401.g1015.t1 | synaptotagmin 7 | 291 | 4.99E-40 | 79.75 | C:integral component of membrane; P:exocytosis |
| Efet.01.404091.g104.t1 | RNA-binding 41-like | 600 | 2.94E-07 | 74 |  |
| Efet.01.404132.g105.t1 | DNA repair RAD51 homolog 1 isoform X2 | 285 | 1.87E-56 | 94.05 | P:response to X-ray; C:site of double-strand break; C:PML body; P:reciprocal meiotic recombination; F:protein C-terminus binding; F:double-stranded DNA binding; P:strand displacement; C:nucleolus; F:endodeoxyribonuclease activity; P:DNA synthesis involved in DNA repair; P:DNA recombinase assembly; P:response to toxic substance; P:chromosome organization involved in meiotic cell cycle; P:DNA unwinding involved in DNA replication; F:single-stranded DNA binding; F:ATP binding; F:DNA polymerase binding; P:mitotic recombination-dependent replication fork processing; P:telomere maintenance via telomere lengthening; P:positive regulation of DNA ligation; P:cellular response to cisplatin; F:identical protein binding; P:protein homooligomerization; C:lateral element; P:telomere maintenance via recombination; P:response to etoposide; F:chromatin binding; C:chromatin; F:four-way junction DNA binding; P:strand invasion; C:nuclear chromosome, telomeric region; P:cellular response to gamma radiation; C:perinuclear region of cytoplasm; C:microtubule organizing center; C:mitochondrial matrix; F:recombinase activity; P:regulation of protein phosphorylation; P:response to drug; P:replication-born double-strand break repair via sister chromatid exchange; F:single-stranded DNA-dependent ATPase activity; P:cellular response to hydroxyurea; P:regulation of double-strand break repair via homologous recombination; P:cellular response to camptothecin |
| Efet.01.404361.g108.t1 | octopamine receptor beta-2R-like | 402 | 1.80E-27 | 75.2 | F:adrenergic receptor activity; C:integral component of membrane; P:adrenergic receptor signaling pathway; C:plasma membrane |
| Efet.01.404361.g109.t1 | octopamine receptor beta-2R-like isoform X1 | 882 | 6.54E-64 | 49.05 | F:G-protein coupled receptor activity; F:adrenergic receptor activity; P:G-protein coupled receptor signaling pathway; P:signal transduction; C:membrane; C:integral component of membrane; P:adrenergic receptor signaling pathway; F:signal transducer activity; C:plasma membrane |
| Efet.01.404400.g110.t1 | trachealess isoform X1 | 1317 | 1.13E-17 | 47.15 | P:regulation of transcription, DNA-templated |
| Efet.01.40449.g1019.t1 | solute carrier family 12 member 1-like | 390 | 1.56E-19 | 62.7 | F:cation:chloride symporter activity; P:chloride transmembrane transport; C:integral component of membrane |
| Efet.01.404637.g114.t1 | H6S1B_DANRE ame: Full=Heparan-sulfate 6-O-sulfotransferase 1-B Short=HS 6-OST-1B | 330 | 3.88E-06 | 54 | F:trans-3,4-dihydrodiolphenanthrene sulfotransferase activity; F:4-phenanthrol sulfotransferase activity; P:heparan sulfate proteoglycan biosynthetic process, enzymatic modification; F:2-phenanthrol sulfotransferase activity; F:9-phenanthrol sulfotransferase activity; F:hydroxyjasmonate sulfotransferase activity; F:heparan sulfate 2-O-sulfotransferase activity; F:sulfotransferase activity; F:galactose 3-O-sulfotransferase activity; F:heparan sulfate 6-O-sulfotransferase activity; F:N-acetylgalactosamine 4-O-sulfotransferase activity; F:phenanthrol sulfotransferase activity; C:membrane; C:integral component of membrane; F:N-acetylglucosamine 6-O-sulfotransferase activity; F:1-hydroxypyrene sulfotransferase activity; F:HNK-1 sulfotransferase activity; F:transferase activity; F:trans-9R,10R-dihydrodiolphenanthrene sulfotransferase activity; F:cholesterol sulfotransferase activity; F:proteoglycan sulfotransferase activity; F:3-phenanthrol sulfotransferase activity; F:1-phenanthrol sulfotransferase activity |
| Efet.01.404684.g117.t1 | hypothetical protein CAPTEDRAFT_214964 | 516 | 3.88E-42 | 66.4 |  |
| Efet.01.4048.g344.t1 | Tob1-like | 1086 | 2.45E-59 | 71.35 |  |
| Efet.01.404876.g118.t1 | hypothetical protein CAPTEDRAFT_121881, partial | 363 | 2.27E-12 | 59 | F:ubiquitin-protein transferase activity; C:membrane; C:integral component of membrane; F:ligase activity; P:protein ubiquitination |
| Efet.01.404906.g119.t1 | acyl- desaturase-like isoform X2 | 402 | 1.43E-67 | 78.4 | F:oxidoreductase activity, acting on paired donors, with oxidation of a pair of donors resulting in the reduction of molecular oxygen to two molecules of water; C:integral component of membrane; P:oxidation-reduction process; P:fatty acid biosynthetic process |
| Efet.01.40499.g1021.t1 | FH1 FH2 domain-containing 3-like | 1101 | 1.88E-51 | 71.8 |  |
| Efet.01.40504.g1022.t1 | nuclear hormone receptor HR96-like isoform X2 | 957 | 5.42E-107 | 63.2 | C:nucleus; F:transcription factor activity, sequence-specific DNA binding; F:zinc ion binding; P:transcription, DNA-templated; F:steroid hormone receptor activity; P:regulation of transcription, DNA-templated; F:sequence-specific DNA binding; P:steroid hormone mediated signaling pathway |
| Efet.01.405067.g120.t1 | glycine cleavage system T | 207 | 9.45E-18 | 84.5 |  |
| Efet.01.405072.g121.t1 | condensin complex subunit 2-like | 525 | 4.48E-27 | 59.8 | P:single-organism cellular process |
| Efet.01.405079.g122.t1 | speract receptor-like | 678 | 1.73E-20 | 48.4 | P:signal transduction; F:phosphorus-oxygen lyase activity; C:membrane; P:primary metabolic process; P:organic substance metabolic process; P:phosphate-containing compound metabolic process |
| Efet.01.40511.g1023.t1 | SET-like | 747 | 8.08E-119 | 85.65 | C:nucleus; P:nucleosome assembly |
| Efet.01.405153.g124.t1 | structural maintenance of chromosomes 4-like | 558 | 2.34E-87 | 86.7 | C:nucleus; F:ATP binding; P:chromosome organization; C:chromosome |
| Efet.01.405236.g126.t1 | T family of potassium channels 18-like | 609 | 1.77E-37 | 77.75 | P:potassium ion transmembrane transport; C:integral component of membrane; F:potassium channel activity |
| Efet.01.40525.g1024.t1 | urea-proton symporter DUR3-like | 1296 | 4.49E-82 | 50.75 | C:membrane; P:transport; F:transporter activity |
| Efet.01.405260.g127.t1 | potassium channel | 300 | 3.76E-42 | 83.45 | F:voltage-gated potassium channel activity; P:potassium ion transmembrane transport; C:voltage-gated potassium channel complex; P:regulation of ion transmembrane transport; P:protein homooligomerization |
| Efet.01.405260.g128.t1 | potassium voltage-gated channel subfamily A member 1-like | 807 | 2.70E-102 | 72.6 | P:regulation of dopamine secretion; P:potassium ion transmembrane transport; C:voltage-gated potassium channel complex; F:delayed rectifier potassium channel activity; P:protein homooligomerization; C:plasma membrane region; C:endoplasmic reticulum; C:neuronal cell body membrane; C:juxtaparanode region of axon; C:perikaryon; C:axon terminus; C:dendrite; F:protein binding; P:regulation of ion transmembrane transport; P:sensory perception of pain; C:lamellipodium; P:neuronal action potential |
| Efet.01.40530.g1025.t1 | Endothelin-converting enzyme 2 | 492 | 7.02E-50 | 63.5 | F:catalytic activity; P:metabolic process |
| Efet.01.40532.g1026.t1 | zinc finger CCCH domain-containing 13-like isoform X1 | 1221 | 6.31E-20 | 47.3 | F:metal ion binding |
| Efet.01.405406.g130.t1 | chloroplast stem-loop binding of 41 kDa chloroplastic-like | 528 | 2.79E-52 | 57.35 | F:coenzyme binding; F:catalytic activity; C:membrane; C:integral component of membrane |
| Efet.01.405644.g137.t1 | ubiquitin carboxyl-terminal hydrolase 20 isoform X1 | 2769 | 0 | 59.6 | P:proteolysis; P:cellular protein metabolic process; F:cysteine-type peptidase activity |
| Efet.01.405697.g139.t1 | hypothetical protein CAPTEDRAFT_225694 | 303 | 4.61E-06 | 54 |  |
| Efet.01.40572.g1027.t1 | condensin complex subunit 3-like | 639 | 6.76E-52 | 55.7 | C:intracellular non-membrane-bounded organelle |
| Efet.01.405737.g142.t1 | sodium-independent sulfate anion transporter-like | 297 | 1.32E-22 | 67.45 | F:secondary active sulfate transmembrane transporter activity; C:integral component of membrane; P:sulfate transmembrane transport |
| Efet.01.405816.g144.t1 | hypothetical protein HELRODRAFT_176714 | 543 | 2.71E-06 | 58 | F:ATP binding; C:membrane; C:integral component of membrane; F:protein kinase activity; P:protein phosphorylation |
| Efet.01.40583.g1029.t1 | serine threonine- kinase SIK1 | 234 | 9.33E-12 | 61.55 | F:protein kinase binding; F:heterocyclic compound binding; F:ion binding; P:cellular amino acid biosynthetic process; F:protein serine/threonine kinase activity; P:negative regulation of biological process; C:intracellular part; P:regulation of sodium ion transport; P:cellular macromolecule metabolic process; F:organic cyclic compound binding; P:phosphorylation; P:regulation of cellular biosynthetic process; P:regulation of primary metabolic process; P:response to stimulus |
| Efet.01.405852.g146.t1 | circadian locomoter output cycles kaput-like isoform X4 | 1359 | 1.88E-26 | 52.56 | C:nucleus; P:transcription, DNA-templated; P:regulation of transcription, DNA-templated; F:protein dimerization activity |
| Efet.01.40587.g1030.t1 | VWFA and cache domain-containing 1-like | 540 | 3.53E-31 | 62.65 | C:membrane; C:integral component of membrane |
| Efet.01.405886.g147.t1 | Gamma-aminobutyric acid receptor subunit gamma- | 537 | 8.15E-78 | 70.65 | P:ion transmembrane transport; C:integral component of membrane; F:extracellular ligand-gated ion channel activity |
| Efet.01.40595.g1031.t1 | heparan sulfate glucosamine 3-O-sulfotransferase 5 | 678 | 7.48E-98 | 71.55 | P:heparan sulfate proteoglycan biosynthetic process, enzymatic modification; F:protein binding; P:regulation of viral entry into host cell; P:negative regulation of coagulation; C:integral component of membrane; F:[heparan sulfate]-glucosamine 3-sulfotransferase 1 activity; P:glycosaminoglycan biosynthetic process; C:Golgi membrane; F:3'-phosphoadenosine 5'-phosphosulfate binding; P:protein sulfation |
| Efet.01.40624.g1033.t1 | alpha-amylase 1 | 552 | 5.25E-36 | 55.9 | F:hydrolase activity |
| Efet.01.40627.g1034.t1 | rho GTPase-activating 39-like isoform X3 | 1560 | 5.17E-49 | 59.65 | C:cytoskeleton; P:signal transduction |
| Efet.01.406286.g153.t1 | glutamine synthetase-like | 321 | 2.04E-24 | 83.25 | F:ATP binding; P:glutamine biosynthetic process; F:glutamate-ammonia ligase activity |
| Efet.01.406317.g154.t1 | ATP-dependent RNA helicase DDX42 | 504 | 9.73E-43 | 80.9 | F:nucleic acid binding; F:ATP binding; F:helicase activity |
| Efet.01.406349.g155.t1 | hypothetical protein CAPTEDRAFT_152802 | 495 | 1.54E-11 | 56 | C:membrane; C:integral component of membrane |
| Efet.01.406366.g156.t1 | transcriptional repressor CTCF-like | 1500 | 0 | 80.3 | F:nucleic acid binding; F:metal ion binding |
| Efet.01.406381.g157.t1 | cytoplasmic aconitate hydratase-like isoform X2 | 453 | 8.83E-86 | 68.15 | F:lyase activity; F:iron-sulfur cluster binding |
| Efet.01.40641.g1035.t1 | hypothetical protein CAPTEDRAFT_186654 | 612 | 7.99E-23 | 76.5 | F:G-protein coupled receptor activity; P:G-protein coupled receptor signaling pathway; C:integral component of membrane |
| Efet.01.40641.g1036.t1 | hypothetical protein CAPTEDRAFT_216107 | 678 | 3.29E-39 | 56.33 | F:G-protein coupled receptor activity; P:G-protein coupled receptor signaling pathway; C:membrane; C:integral component of membrane |
| Efet.01.406411.g158.t1 | cell surface | 330 | 1.05E-11 | 57.3 | F:zinc ion binding; F:metal ion binding |
| Efet.01.406419.g159.t1 | Myosin heavy striated muscle | 555 | 2.70E-40 | 68 | C:intracellular non-membrane-bounded organelle; F:binding |
| Efet.01.40656.g1037.t1 | tripartite motif-containing 45-like isoform X6 | 1020 | 7.08E-147 | 82.9 | F:zinc ion binding; C:intracellular |
| Efet.01.406564.g160.t1 | rhizopine-binding [Burkholderia stagnalis] | 1335 | 1.06E-127 | 62 | P:carbohydrate transport |
| Efet.01.406570.g161.t1 | Location of vulva defective 1 | 222 | 8.55E-15 | 75.15 | P:detection of mechanical stimulus; F:calcium channel activity; C:membrane |
| Efet.01.406577.g162.t1 | neurexin-1 isoform X1 | 615 | 2.03E-29 | 54.75 |  |
| Efet.01.40659.g1039.t1 | hypothetical protein CAPTEDRAFT_182794 | 954 | 4.35E-95 | 63.5 | F:calcium ion binding |
| Efet.01.40659.g1040.t1 | PREDICTED: uncharacterized protein LOC106153608 | 576 | 6.60E-71 | 67.2 | F:calcium ion binding |
| Efet.01.406600.g163.t1 | neurogenic locus notch | 906 | 6.85E-140 | 70.85 | C:membrane; C:integral component of membrane |
| Efet.01.40661.g1041.t1 | RNA-binding Nova-1 | 978 | 7.17E-39 | 52.85 | F:nucleic acid binding; F:RNA binding; F:hydrolase activity |
| Efet.01.406615.g164.t1 | C3 and PZP-like alpha-2-macroglobulin domain-containing 8 | 789 | 1.27E-48 | 55.95 | F:catalytic activity; P:nucleoside metabolic process |
| Efet.01.406639.g165.t1 | orexin receptor type 2-like | 639 | 9.91E-28 | 59.8 | P:signal transduction; C:membrane; F:signal transducer activity |
| Efet.01.406957.g167.t1 | spectrin alpha non-erythrocytic 1-like isoform X1 | 255 | 1.97E-22 | 81.25 | F:actin binding; F:phospholipid binding |
| Efet.01.407039.g168.t1 | cyclin-dependent kinase 2-associated 1-like | 423 | 2.07E-33 | 81.85 | P:phosphorylation; F:kinase activity |
| Efet.01.40704.g1046.t1 | laminin subunit gamma-1-like | 1593 | 2.82E-87 | 49.3 | C:basement membrane; P:system development; P:basement membrane assembly |
| Efet.01.40709.g1047.t1 | ATP-binding cassette sub-family A member 2-like | 1713 | 1.59E-160 | 61.05 | P:transport |
| Efet.01.407315.g172.t1 | integrator complex subunit 11-like | 321 | 4.36E-21 | 66.65 |  |
| Efet.01.407332.g173.t1 | gremlin-2-like isoform X2 | 570 | 1.15E-40 | 71.9 | C:membrane |
| Efet.01.407536.g174.t1 | hypothetical protein HELRODRAFT_171077 | 507 | 2.71E-06 | 74 | P:transmembrane transport; C:membrane; C:integral component of membrane |
| Efet.01.407585.g175.t1 | hypothetical protein CAPTEDRAFT_185095 | 309 | 1.41E-20 | 68 |  |
| Efet.01.407607.g176.t1 | MKL myocardin 2 isoform X3 | 1980 | 2.90E-09 | 78.4 |  |
| Efet.01.407630.g177.t1 | zinc finger 271-like | 819 | 2.54E-75 | 61.75 | F:nucleic acid binding; F:metal ion binding |
| Efet.01.40766.g1050.t1 | bifunctional (p)ppGpp synthetase guanosine-3 ,5 -bis(diphosphate) 3 -pyrophosphohydrolase | 1098 | 0 | 94.45 | P:guanosine tetraphosphate metabolic process; P:phosphorylation; F:amino acid binding; F:kinase activity |
| Efet.01.407661.g179.t1 | ankyrin and armadillo repeat-containing -like | 522 | 4.00E-38 | 65.35 | C:intracellular part |
| Efet.01.407751.g181.t1 | hypothetical protein B5766_02170 | 276 | 1.11E-49 | 90.4 |  |
| Efet.01.407760.g182.t1 | paired box Pax-7- | 486 | 4.93E-37 | 75.65 | C:nucleus; P:chordate embryonic development; P:skeletal system development; P:positive regulation of macromolecule metabolic process; P:transcription, DNA-templated; P:neuron differentiation; P:skeletal muscle tissue development; P:cell fate commitment; P:regulation of transcription, DNA-templated; F:sequence-specific DNA binding; P:positive regulation of cellular metabolic process |
| Efet.01.407768.g183.t1 | synaptic vesicle membrane VAT-1 homolog- | 606 | 6.09E-67 | 77.8 | F:zinc ion binding; F:oxidoreductase activity; P:oxidation-reduction process |
| Efet.01.407773.g185.t1 | atrial natriuretic peptide receptor 1-like | 345 | 6.17E-13 | 69.3 |  |
| Efet.01.407777.g186.t1 | coiled-coil domain-containing 63 isoform X1 | 411 | 9.76E-15 | 66.82 | C:outer dynein arm; P:outer dynein arm assembly |
| Efet.01.407777.g187.t1 | coiled-coil domain-containing 63-like | 237 | 4.35E-17 | 84.15 | C:outer dynein arm; P:outer dynein arm assembly |
| Efet.01.40778.g1051.t1 | Kyphoscoliosis peptidase | 1458 | 1.71E-177 | 58.85 | F:zinc ion binding; F:metal ion binding |
| Efet.01.407831.g188.t1 | testis-specific serine threonine- kinase 5-like | 489 | 3.23E-54 | 61.65 | P:phosphorylation; F:kinase activity |
| Efet.01.407854.g189.t1 | DUF3416 domain-containing | 1449 | 0 | 82.55 | F:transferase activity, transferring hexosyl groups; P:alpha-glucan biosynthetic process; F:hydrolase activity, hydrolyzing O-glycosyl compounds |
| Efet.01.407854.g190.t1 | META domain-containing | 453 | 9.63E-65 | 58.35 |  |
| Efet.01.407944.g191.t1 | tyrosine- kinase Abl-like isoform X2 | 1170 | 5.71E-19 | 53.25 | F:protein kinase activity; P:phosphorylation |
| Efet.01.407946.g192.t1 | uncharacterized protein LOC108179280 | 870 | 5.68E-67 | 60.4 |  |
| Efet.01.407960.g194.t1 | insulin-like receptor isoform X1 | 225 | 1.48E-40 | 90.45 | F:ATP binding; P:peptidyl-tyrosine phosphorylation; F:transmembrane receptor protein tyrosine kinase activity; C:integral component of membrane; P:transmembrane receptor protein tyrosine kinase signaling pathway |
| Efet.01.407967.g195.t1 | WD repeat-containing WRAP73 | 984 | 3.32E-131 | 75.65 | C:microtubule organizing center |
| Efet.01.407970.g196.t1 | histone-lysine N-methyltransferase 2E isoform X1 | 810 | 2.50E-22 | 70.65 | P:positive regulation of cellular process; F:metal ion binding; P:cellular response to stimulus; F:methyltransferase activity; P:single-organism cellular process; P:primary metabolic process; P:immune system process; C:nucleoplasm; P:macromolecule methylation |
| Efet.01.40799.g1053.t1 | splicing factor U2AF 50 kDa subunit-like | 507 | 2.49E-56 | 75.1 | F:nucleic acid binding |
| Efet.01.408.g47.t1 | Cys-loop ligand-gated ionic channel | 630 | 1.59E-55 | 70.4 | P:ion transport; C:membrane |
| Efet.01.408156.g198.t1 | REST corepressor 3-like | 351 | 6.92E-07 | 75 |  |
| Efet.01.408157.g199.t1 | BTB POZ domain-containing 9-like | 234 | 2.31E-22 | 87.75 |  |
| Efet.01.408352.g200.t1 | E3 ubiquitin- ligase TRIM56-like | 945 | 3.80E-20 | 44.85 | F:zinc ion binding; F:metal ion binding; C:intracellular |
| Efet.01.408472.g202.t1 | coiled-coil domain-containing 146-like | 384 | 1.35E-38 | 72.65 |  |
| Efet.01.408488.g203.t1 | zinc finger 239-like | 990 | 3.04E-82 | 63.7 | F:nucleic acid binding; F:metal ion binding; F:methyltransferase activity; F:transferase activity; P:methylation |
| Efet.01.408561.g204.t1 | hypothetical protein CAPTEDRAFT_218172 | 1125 | 3.11E-89 | 54.3 | C:membrane; C:integral component of membrane |
| Efet.01.408562.g205.t1 | zinc finger 366-like | 354 | 2.94E-36 | 63.1 | P:regulation of cellular process; F:binding |
| Efet.01.408648.g206.t1 | ubiquitin thioesterase trabid | 357 | 4.55E-21 | 86.25 | F:thiol-dependent ubiquitin-specific protease activity; C:nucleus; F:zinc ion binding; P:protein deubiquitination involved in ubiquitin-dependent protein catabolic process; P:protein K33-linked deubiquitination; P:cytoskeleton organization; C:cytoplasm; F:K63-linked polyubiquitin binding; P:positive regulation of Wnt signaling pathway; P:cell migration; P:protein K29-linked deubiquitination |
| Efet.01.408769.g209.t1 | serine threonine- kinase PAK 1-like isoform X3 | 477 | 1.16E-66 | 81.85 | F:ATP binding; F:signal transducer, downstream of receptor, with serine/threonine kinase activity; C:cytoplasm; P:axonogenesis; P:actin filament organization; P:signal transduction by protein phosphorylation; P:mitotic cell cycle |
| Efet.01.40889.g1056.t1 | eukaryotic translation initiation factor 4E-binding 1 | 282 | 2.87E-14 | 64.55 | P:regulation of cellular process; C:cytoplasm; F:binding; P:single-organism process; P:cellular process |
| Efet.01.408894.g210.t1 | sodium hydrogen | 213 | 4.40E-20 | 72.25 | F:sodium:proton antiporter activity; C:integral component of membrane; P:hydrogen ion transmembrane transport; P:sodium ion transmembrane transport; P:regulation of pH |
| Efet.01.408985.g211.t1 | arrestin domain-containing 3-like | 222 | 9.54E-17 | 69.05 | P:signal transduction |
| Efet.01.40904.g1057.t1 | trimeric intracellular cation channel type B | 330 | 8.20E-21 | 65.4 | P:monovalent inorganic cation transport; F:cation channel activity; P:cation transmembrane transport; C:membrane; C:integral component of membrane |
| Efet.01.409390.g215.t1 | hypothetical protein CAPTEDRAFT_168270 | 888 | 2.52E-124 | 58.95 |  |
| Efet.01.409392.g216.t1 | cytoplasmic polyadenylation element-binding 1 isoform X1 | 360 | 1.87E-38 | 86.45 | C:postsynaptic density; P:positive regulation of translation; P:negative regulation of cell proliferation; C:P-body; C:neuronal cell body; C:nucleoplasm; C:growth cone; F:nucleotide binding; P:cellular response to insulin stimulus; C:dendrite; P:cellular response to amino acid stimulus; C:messenger ribonucleoprotein complex; C:postsynaptic membrane; P:positive regulation of neuron projection development; P:cellular response to hypoxia; P:mRNA transport; F:translation repressor activity, nucleic acid binding; F:translation factor activity, RNA binding; P:mRNA processing; P:translation; C:centrosome; C:perinuclear region of cytoplasm; F:mRNA 3'-UTR AU-rich region binding; P:positive regulation of nitric-oxide synthase biosynthetic process; P:cellular response to lipopolysaccharide; F:metal ion binding; F:ribosome binding; C:cell junction; P:negative regulation of cytoplasmic translation; P:positive regulation of cell migration |
| Efet.01.409452.g217.t1 | WD repeat and FYVE domain-containing 3-like isoform X1 | 300 | 5.57E-21 | 56.35 | F:metal ion binding; C:endomembrane system; C:extrinsic component of membrane; F:phospholipid binding |
| Efet.01.4098.g345.t1 | follistatin-A-like isoform X1 | 711 | 4.05E-51 | 50.95 | P:biological_process; C:cellular_component |
| Efet.01.409839.g220.t1 | forkhead box l2 | 1578 | 1.61E-55 | 74.1 | C:nucleus; F:transcription factor activity, sequence-specific DNA binding; P:regulation of transcription, DNA-templated; P:transcription from RNA polymerase II promoter; F:sequence-specific DNA binding |
| Efet.01.409866.g221.t1 | hypothetical protein CAPTEDRAFT_208069 | 207 | 9.68E-18 | 72 |  |
| Efet.01.410014.g222.t1 | metal cation ZIP family | 348 | 1.05E-12 | 68.9 | F:zinc ion transmembrane transporter activity; P:zinc II ion transmembrane transport; C:plasma membrane |
| Efet.01.410157.g223.t1 | cysteine dioxygenase type 1 | 234 | 2.54E-10 | 69.65 | F:dioxygenase activity; F:metal ion binding |
| Efet.01.41018.g1059.t1 | glutamate receptor NMDA 2B-like isoform X1 | 834 | 1.21E-14 | 44.55 | P:ion transmembrane transport; C:synapse; F:receptor activity; F:ionotropic glutamate receptor activity; F:extracellular-glutamate-gated ion channel activity; P:transport; C:plasma membrane; F:ion channel activity; P:ion transport; C:postsynaptic membrane; C:membrane; C:integral component of membrane; C:cell junction; P:ionotropic glutamate receptor signaling pathway |
| Efet.01.41021.g1060.t1 | hypothetical protein CRN43_14440 | 219 | 4.11E-07 | 52 |  |
| Efet.01.410272.g225.t1 | uncharacterized protein LOC111117935 | 357 | 1.50E-09 | 62.83 | P:proteolysis; F:metallopeptidase activity |
| Efet.01.410278.g226.t1 | protocadherin-9 isoform X1 | 2232 | 1.94E-87 | 47.5 | F:calcium ion binding; C:membrane; C:integral component of membrane; P:cell adhesion; P:homophilic cell adhesion via plasma membrane adhesion molecules; C:plasma membrane |
| Efet.01.410401.g229.t1 | voltage-dependent calcium channel subunit alpha-2 delta-2-like | 648 | 3.40E-109 | 71.75 | C:membrane |
| Efet.01.410425.g231.t1 | family transcriptional regulator | 732 | 6.41E-172 | 55.95 | F:DNA binding; F:transcription factor activity, sequence-specific DNA binding; P:transcription, DNA-templated; P:regulation of transcription, DNA-templated |
| Efet.01.410425.g232.t1 | hypothetical protein B5766_08020 | 225 | 8.05E-43 | 100 |  |
| Efet.01.41049.g1062.t1 | hypothetical protein CAPTEDRAFT_222036 | 651 | 4.73E-40 | 53.12 | C:membrane; C:integral component of membrane |
| Efet.01.410524.g234.t1 | 5 exonuclease Apollo-like | 1560 | 2.95E-69 | 56.85 | P:DNA metabolic process; P:cellular response to DNA damage stimulus |
| Efet.01.410526.g236.t1 | cholecystokinin receptor type A-like | 945 | 4.32E-37 | 50.55 | F:G-protein coupled receptor activity; C:integral component of plasma membrane; P:G-protein coupled receptor signaling pathway; P:signal transduction; C:membrane; C:integral component of membrane; P:cell surface receptor signaling pathway; F:signal transducer activity |
| Efet.01.410633.g238.t1 | IQ domain-containing H-like isoform X2 | 249 | 4.81E-18 | 86.75 |  |
| Efet.01.410669.g239.t1 | procollagen C-endopeptidase enhancer 1 | 354 | 5.75E-33 | 52.65 | F:peptidase activator activity; P:G-protein coupled receptor signaling pathway; C:collagen trimer; F:heparin binding; C:extracellular region; F:G-protein coupled receptor activity; C:extracellular space; C:extracellular exosome; F:collagen binding; C:membrane; P:proteolysis; C:integral component of membrane; P:positive regulation of peptidase activity; C:extracellular matrix |
| Efet.01.410699.g240.t1 | cadherin-related tumor suppressor- | 408 | 9.80E-11 | 56.83 | F:calcium ion binding; C:membrane; C:integral component of membrane; P:homophilic cell adhesion via plasma membrane adhesion molecules |
| Efet.01.410808.g241.t1 | polyadenylate-binding 4-like | 261 | 9.61E-32 | 96 | P:positive regulation of translation; F:protein C-terminus binding; F:poly(A) binding; F:poly(U) RNA binding; F:mRNA 3'-UTR binding; P:mRNA splicing, via spliceosome; F:nucleotide binding; C:dendrite; P:heart development; C:messenger ribonucleoprotein complex; C:extracellular exosome; C:focal adhesion; C:catalytic step 2 spliceosome; P:nuclear-transcribed mRNA catabolic process, nonsense-mediated decay; C:cytosol; P:positive regulation of viral genome replication; C:cytoplasmic stress granule; F:translation activator activity; P:translational initiation; P:positive regulation of nuclear-transcribed mRNA poly(A) tail shortening; P:nuclear-transcribed mRNA poly(A) tail shortening; P:mRNA polyadenylation; C:membrane; P:mRNA stabilization; P:gene silencing by RNA; P:negative regulation of nuclear-transcribed mRNA catabolic process, nonsense-mediated decay |
| Efet.01.410846.g242.t1 | pyroglutamylated RFamide peptide receptor-like isoform X2 | 474 | 3.44E-29 | 62.8 | P:signal transduction; F:signal transducer activity |
| Efet.01.410874.g244.t1 | ABC transporter ATP-binding | 384 | 1.21E-80 | 87.6 | F:ATP binding; F:ATPase activity, coupled to transmembrane movement of substances; P:transmembrane transport; C:integral component of membrane |
| Efet.01.410874.g245.t1 | MFS transporter | 546 | 6.12E-79 | 76.7 | C:membrane |
| Efet.01.411005.g248.t1 | caspase-10 isoform X1 | 345 | 3.99E-15 | 58 | F:cysteine-type endopeptidase activity |
| Efet.01.411017.g249.t1 | ankyrin-3 isoform X1 | 1557 | 5.07E-178 | 50.95 | P:intracellular signal transduction; C:intracellular |
| Efet.01.411027.g250.t1 | hypothetical protein CAPTEDRAFT_199139 | 267 | 4.41E-18 | 62.25 | C:membrane; C:integral component of membrane; F:carbohydrate binding |
| Efet.01.411119.g252.t1 | hypothetical protein CAPTEDRAFT_220765 | 378 | 5.26E-15 | 73 | C:membrane |
| Efet.01.411192.g254.t1 | glypican-5 isoform X2 | 678 | 4.61E-51 | 60.1 | F:heparan sulfate proteoglycan binding; C:membrane; C:plasma membrane; C:proteinaceous extracellular matrix; C:anchored component of membrane |
| Efet.01.411267.g255.t1 | Valine--tRNA ligase | 735 | 9.70E-81 | 65.25 | F:nucleotide binding; P:tRNA aminoacylation for protein translation; F:aminoacyl-tRNA ligase activity; C:cytoplasmic part |
| Efet.01.411322.g257.t1 | WD repeat-containing 86-like isoform X2 | 870 | 2.18E-95 | 72.3 |  |
| Efet.01.411371.g258.t1 | ecdysone-induced 74EF-like | 1128 | 1.37E-33 | 48.08 | C:membrane; C:integral component of membrane |
| Efet.01.411379.g259.t1 | hypothetical protein CAPTEDRAFT_151708 | 264 | 3.68E-10 | 91 | C:integral component of membrane |
| Efet.01.41153.g1063.t1 | hypothetical protein HELRODRAFT_173396 | 786 | 1.20E-11 | 59.33 | F:G-protein coupled receptor activity; P:G-protein coupled receptor signaling pathway; C:membrane; C:integral component of membrane |
| Efet.01.411637.g260.t1 | type I restriction endonuclease subunit R | 2007 | 0 | 90.7 | F:DNA binding; F:ATP binding; P:nucleic acid phosphodiester bond hydrolysis; F:Type I site-specific deoxyribonuclease activity; F:helicase activity; P:DNA restriction-modification system |
| Efet.01.411659.g262.t1 | moonraker-like isoform X2 | 288 | 5.51E-11 | 67.75 | P:centriole replication |
| Efet.01.411717.g263.t1 | hypothetical protein CAPTEDRAFT_191992 | 1809 | 5.52E-38 | 44 | C:membrane; C:integral component of membrane |
| Efet.01.411886.g264.t1 | Recombinase | 615 | 2.49E-48 | 61.55 | F:DNA binding; F:recombinase activity; P:DNA recombination |
| Efet.01.411977.g266.t1 | 1-phosphatidylinositol 4,5-bisphosphate phosphodiesterase epsilon-1-like isoform X2 | 537 | 2.58E-11 | 51.33 | F:phosphatidylinositol phospholipase C activity; P:small GTPase mediated signal transduction; P:signal transduction; F:guanyl-nucleotide exchange factor activity; P:lipid metabolic process; F:phosphoric diester hydrolase activity; F:signal transducer activity; P:intracellular signal transduction; P:positive regulation of GTPase activity; C:intracellular |
| Efet.01.411977.g267.t1 | 1-phosphatidylinositol 4,5-bisphosphate phosphodiesterase epsilon-1-like | 555 | 6.72E-40 | 64.95 | P:signal transduction |
| Efet.01.412103.g270.t1 | porphobilinogen synthase | 705 | 3.81E-95 | 66.95 | F:catalytic activity |
| Efet.01.412104.g271.t1 | hypothetical protein CAPTEDRAFT_178598 | 204 | 1.15E-07 | 66 | P:peptidyl-tyrosine phosphorylation; F:ATP binding; F:protein tyrosine kinase activity; F:protein kinase activity; P:protein phosphorylation |
| Efet.01.41213.g1064.t1 | AP-1 complex subunit gamma-1 | 738 | 3.62E-84 | 74.45 | C:microtubule cytoskeleton; F:protein C-terminus binding; F:protein transporter activity; P:positive regulation of natural killer cell degranulation; P:microtubule cytoskeleton organization; P:intracellular protein transport; P:Golgi to lysosome transport; C:trans-Golgi network; C:perinuclear region of cytoplasm; F:collagen binding; F:kinesin binding; P:endosome to melanosome transport; C:cytosol; C:clathrin adaptor complex; C:recycling endosome; C:clathrin-coated vesicle; F:Rab GTPase binding; F:GTP-dependent protein binding |
| Efet.01.412135.g272.t1 | mitogen-activated kinase kinase kinase 4 isoform X2 | 1377 | 1.20E-26 | 50.35 | F:signal transducer, downstream of receptor, with serine/threonine kinase activity; P:response to abiotic stimulus; P:positive regulation of protein phosphorylation; P:cellular component organization; P:cellular response to stress; P:single-organism cellular process; F:binding; P:protein phosphorylation; P:intracellular signal transduction; P:cellular response to chemical stimulus |
| Efet.01.412154.g273.t1 | Eukaryotic initiation factor 4A-III | 279 | 3.14E-50 | 71.25 | F:translation initiation factor activity; F:ATP binding; P:RNA splicing; C:catalytic step 2 spliceosome; P:regulation of gene expression; P:RNA secondary structure unwinding; P:translational initiation; F:ATP-dependent RNA helicase activity |
| Efet.01.41229.g1065.t1 | FMRFamide receptor-like | 1167 | 4.42E-92 | 56.15 | F:G-protein coupled receptor activity; P:signal transduction; C:membrane |
| Efet.01.41235.g1066.t1 | 3-isopropylmalate dehydratase large subunit | 894 | 4.44E-131 | 71.35 | F:lyase activity; F:iron-sulfur cluster binding; P:branched-chain amino acid biosynthetic process |
| Efet.01.41241.g1067.t1 | cGMP-gated cation channel alpha-1 | 357 | 4.53E-15 | 69.95 | F:ion channel activity; P:ion transmembrane transport; C:membrane part; C:photoreceptor outer segment |
| Efet.01.412424.g275.t1 | S9 family peptidase | 1548 | 0 | 67.65 | P:proteolysis; F:serine-type endopeptidase activity; F:serine-type peptidase activity |
| Efet.01.412429.g276.t1 | hypothetical protein | 2112 | 1.09E-10 | 39.86 |  |
| Efet.01.41244.g1068.t1 | dynein heavy chain axonemal-like isoform X1 | 972 | 3.57E-72 | 73.35 | F:nucleotide binding; F:nucleoside-triphosphatase activity; C:intracellular; P:cellular process |
| Efet.01.41246.g1069.t1 | gamma-glutamyltranspeptidase 1 isoform X1 | 507 | 1.00E-42 | 70.1 | P:sulfur compound biosynthetic process; P:glutathione metabolic process; C:membrane; P:organonitrogen compound biosynthetic process; P:single-organism process; P:regulation of biological process |
| Efet.01.41250.g1070.t1 | Krueppel-like factor 2 | 1365 | 1.97E-48 | 89.7 | F:nucleic acid binding; F:metal ion binding |
| Efet.01.412509.g277.t1 | family transporter | 909 | 6.39E-160 | 93.35 | C:integral component of membrane |
| Efet.01.412509.g278.t1 | aromatic ring-hydroxylating dioxygenase subunit alpha | 1053 | 0 | 92.4 | P:aromatic compound catabolic process; F:iron ion binding; F:oxidoreductase activity, acting on paired donors, with incorporation or reduction of molecular oxygen, NAD(P)H as one donor, and incorporation of two atoms of oxygen into one donor; P:oxidation-reduction process; F:2 iron, 2 sulfur cluster binding |
| Efet.01.412509.g279.t1 | exodeoxyribonuclease VII small subunit | 234 | 2.87E-34 | 93.3 | P:DNA catabolic process; P:nucleic acid phosphodiester bond hydrolysis; C:cytoplasm; C:exodeoxyribonuclease VII complex; F:exodeoxyribonuclease VII activity |
| Efet.01.412509.g280.t1 | polyprenyl synthetase family | 642 | 2.96E-100 | 74.45 | P:isoprenoid biosynthetic process; F:transferase activity |
| Efet.01.412509.g281.t1 | 1-deoxy-D-xylulose-5-phosphate synthase | 1896 | 0 | 94.1 | F:magnesium ion binding; F:thiamine pyrophosphate binding; F:1-deoxy-D-xylulose-5-phosphate synthase activity; P:terpenoid biosynthetic process; P:thiamine biosynthetic process; P:1-deoxy-D-xylulose 5-phosphate biosynthetic process |
| Efet.01.412551.g282.t1 | histamine H1 receptor-like | 438 | 2.42E-37 | 79.2 | C:integral component of plasma membrane; F:G-protein coupled acetylcholine receptor activity; P:G-protein coupled acetylcholine receptor signaling pathway |
| Efet.01.412555.g285.t1 | sodium-driven chloride bicarbonate exchanger | 312 | 6.32E-21 | 71.75 | P:inorganic anion transport; P:anion transmembrane transport; C:integral component of membrane; F:inorganic anion exchanger activity |
| Efet.01.41257.g1072.t1 | liprin-alpha-1-like isoform X2 | 591 | 8.29E-56 | 81 |  |
| Efet.01.412692.g288.t1 | neuropeptide S receptor-like | 585 | 2.15E-44 | 68.95 | F:G-protein coupled receptor activity; P:response to organic substance; P:G-protein coupled receptor signaling pathway; P:response to endogenous stimulus; C:integral component of membrane; C:plasma membrane |
| Efet.01.412773.g289.t1 | probable G- coupled receptor CG31760 | 366 | 6.44E-36 | 76.5 | C:membrane |
| Efet.01.41283.g1074.t1 | breakpoint cluster region -like | 240 | 7.76E-23 | 59 | P:biological regulation |
| Efet.01.412879.g292.t1 | DTW domain-containing 1 | 339 | 4.66E-08 | 66.75 |  |
| Efet.01.412888.g293.t1 | FMRFamide receptor-like | 1032 | 2.15E-72 | 52.65 | F:G-protein coupled receptor activity; F:G-protein coupled peptide receptor activity; P:G-protein coupled receptor signaling pathway; P:signal transduction; C:membrane; C:integral component of membrane; F:signal transducer activity |
| Efet.01.412895.g294.t1 | mRNA-decapping enzyme 1A-like | 423 | 4.59E-25 | 58.9 | C:intracellular organelle; C:cytoplasm; P:positive regulation of catalytic activity; P:deadenylation-dependent decapping of nuclear-transcribed mRNA; F:enzyme activator activity |
| Efet.01.413048.g295.t1 | zinc finger basonuclin-2 isoform X2 | 1491 | 3.05E-31 | 90.1 | F:nucleic acid binding; F:metal ion binding |
| Efet.01.41308.g1077.t1 | probable G- coupled receptor No18 | 900 | 4.56E-09 | 52.25 | F:G-protein coupled receptor activity; P:G-protein coupled receptor signaling pathway; P:signal transduction; C:membrane; C:integral component of membrane; F:signal transducer activity |
| Efet.01.413150.g296.t1 | esterase OVCA2-like | 282 | 1.47E-27 | 77.45 | C:membrane |
| Efet.01.413202.g299.t1 | FMRFamide receptor-like | 1266 | 1.02E-101 | 56.7 | F:G-protein coupled receptor activity; P:signal transduction; C:membrane |
| Efet.01.413355.g303.t1 | hypothetical protein CAPTEDRAFT_188760 | 489 | 1.34E-15 | 63 | C:membrane; C:integral component of membrane |
| Efet.01.413420.g305.t1 | CREB ATF bZIP transcription factor-like | 981 | 1.84E-17 | 50.25 | C:nucleus; F:transcription factor activity, sequence-specific DNA binding; P:regulation of transcription, DNA-templated; F:sequence-specific DNA binding; P:regulation of sequence-specific DNA binding transcription factor activity |
| Efet.01.413431.g306.t1 | PP2C-like domain-containing | 432 | 6.46E-42 | 82.4 | F:catalytic activity |
| Efet.01.413593.g307.t1 | CLIP-associating | 267 | 3.03E-29 | 82.9 |  |
| Efet.01.413703.g308.t1 | hypothetical protein CAPTEDRAFT_226428 | 291 | 2.77E-24 | 65.75 | C:membrane |
| Efet.01.413810.g309.t1 | short-chain dehydrogenase | 759 | 2.65E-142 | 72.4 | F:oxidoreductase activity; P:oxidation-reduction process |
| Efet.01.413810.g310.t1 | acyl- dehydrogenase | 1113 | 0 | 88.3 | F:acyl-CoA dehydrogenase activity; F:flavin adenine dinucleotide binding; P:oxidation-reduction process |
| Efet.01.413810.g311.t1 | 3-hydroxybutyryl- dehydrogenase | 834 | 0 | 84.15 | F:3-hydroxyacyl-CoA dehydrogenase activity; F:3-hydroxybutyryl-CoA dehydrogenase activity; P:oxidation-reduction process; F:NAD+ binding; P:fatty acid metabolic process |
| Efet.01.413810.g312.t1 | Enoyl- hydratase | 1317 | 1.56E-148 | 82.5 | P:metabolic process; F:enoyl-CoA hydratase activity |
| Efet.01.413810.g313.t1 | short-chain dehydrogenase | 780 | 6.52E-155 | 79.05 | F:3-oxoacyl-[acyl-carrier-protein] reductase (NADPH) activity; P:oxidation-reduction process |
| Efet.01.413810.g314.t1 | putattive exported | 978 | 0 | 70.2 | C:outer membrane-bounded periplasmic space |
| Efet.01.413810.g315.t1 | tripartite tricarboxylate transporter substrate binding | 951 | 0 | 63.2 | C:outer membrane-bounded periplasmic space |
| Efet.01.413810.g316.t1 | acyl dehydratase | 444 | 1.10E-106 | 86.75 |  |
| Efet.01.413810.g317.t1 | thiolase | 1110 | 0 | 86.25 | F:transferase activity, transferring acyl groups other than amino-acyl groups; P:metabolic process |
| Efet.01.413810.g318.t1 | acyl- dehydrogenase | 915 | 0 | 75.75 | F:acyl-CoA dehydrogenase activity; F:flavin adenine dinucleotide binding; P:oxidation-reduction process |
| Efet.01.413810.g319.t1 | nucleic-acid-binding containing a Zn-ribbon | 336 | 2.24E-76 | 72.4 |  |
| Efet.01.413810.g320.t1 | enoyl- hydratase | 699 | 5.58E-121 | 74.15 | P:metabolic process; F:isomerase activity |
| Efet.01.413810.g321.t1 | transferase | 2538 | 0 | 77.95 | F:formyl-CoA transferase activity |
| Efet.01.413810.g322.t1 | pilus assembly | 270 | 3.65E-30 | 75.85 | C:integral component of membrane |
| Efet.01.413810.g323.t1 | Flp pilus-assembly G-like | 1287 | 0 | 67.75 | C:integral component of membrane |
| Efet.01.413886.g324.t1 | LIM domain transcription factor -like | 228 | 8.68E-21 | 80.95 | F:zinc ion binding; F:transferase activity |
| Efet.01.41390.g1080.t1 | acetylcholine receptor subunit alpha-type acr-16-like | 513 | 6.69E-23 | 58.2 | C:membrane; P:transport |
| Efet.01.414.g50.t1 | High mobility group DSP1 | 492 | 5.45E-83 | 79.85 | C:nucleus; F:DNA binding; P:regulation of cell differentiation; P:spermatogenesis; C:integral component of membrane; P:regulation of transcription, DNA-templated |
| Efet.01.414004.g327.t1 | potassium channel subfamily T member 2 | 423 | 1.65E-09 | 67.67 | F:calcium-activated potassium channel activity; P:potassium ion transport; P:potassium ion transmembrane transport; C:membrane; C:integral component of membrane |
| Efet.01.414066.g328.t1 | orphan G- coupled receptor 56 | 888 | 1.01E-92 | 56.5 | P:signal transduction; C:membrane |
| Efet.01.414074.g329.t1 | hypothetical protein CAPTEDRAFT_147499 | 264 | 2.31E-09 | 66 | P:cytokine-mediated signaling pathway; C:membrane; F:cytokine receptor activity |
| Efet.01.41424.g1081.t1 | hypothetical protein HELRODRAFT_162456 | 1086 | 3.89E-22 | 44 | C:membrane; C:integral component of membrane |
| Efet.01.414252.g332.t1 | hypothetical protein HELRODRAFT_190746 | 1245 | 4.58E-13 | 52 |  |
| Efet.01.414256.g333.t1 | hypothetical protein CAPTEDRAFT_220857 | 264 | 1.46E-16 | 60.15 | C:membrane; C:integral component of membrane |
| Efet.01.414305.g334.t1 | hypothetical protein CAPTEDRAFT_144043 | 204 | 6.11E-07 | 70 | C:membrane; P:transport |
| Efet.01.41442.g1082.t1 | potassium voltage-gated channel subfamily A member 1 isoform X1 | 1650 | 0 | 81.65 | F:voltage-gated potassium channel activity; P:potassium ion transmembrane transport; C:voltage-gated potassium channel complex; P:regulation of ion transmembrane transport; P:protein homooligomerization |
| Efet.01.414475.g338.t1 | ankyrin repeat | 5916 | 4.05E-39 | 50.8 |  |
| Efet.01.41463.g1085.t1 | dynein beta flagellar outer arm-like | 498 | 2.37E-42 | 66.35 |  |
| Efet.01.414772.g341.t1 | tubulin delta chain isoform X2 | 480 | 2.73E-42 | 67.9 | F:nucleotide binding; C:centriole; P:cellular process; C:nucleoplasm; P:multicellular organismal process; P:single-organism developmental process |
| Efet.01.414910.g343.t1 | clathrin light chain B isoform X1 | 213 | 1.31E-09 | 77.35 | P:vesicle-mediated transport; C:clathrin coat of trans-Golgi network vesicle; C:clathrin coat of coated pit; F:structural molecule activity; P:intracellular protein transport |
| Efet.01.415015.g345.t1 | WD repeat-containing on Y chromosome-like isoform X3 | 252 | 3.49E-23 | 70.6 | F:calcium ion binding |
| Efet.01.415059.g346.t1 | wiskott-Aldrich syndrome family member 2-like | 1482 | 1.57E-25 | 67.4 | C:cytoskeleton; F:actin binding; P:actin cytoskeleton organization |
| Efet.01.415242.g350.t1 | E3 ubiquitin- ligase TRIM71-like | 1350 | 1.11E-25 | 44.95 |  |
| Efet.01.415266.g351.t1 | hypothetical protein HELRODRAFT_160282 | 309 | 8.54E-16 | 65 | C:membrane |
| Efet.01.415307.g354.t1 | sphingosine kinase 1-like | 789 | 1.43E-39 | 57.55 | P:heart morphogenesis; P:cell migration to the midline involved in heart development; P:phosphorylation; F:kinase activity; P:embryonic viscerocranium morphogenesis |
| Efet.01.415327.g355.t1 | golgin subfamily A member 4-like | 7428 | 1.18E-10 | 51.67 |  |
| Efet.01.41539.g1089.t1 | tetraspanin-11 | 270 | 4.79E-19 | 63.3 | C:integral component of membrane |
| Efet.01.415394.g357.t1 | acidic leucine-rich nuclear phospho 32 family member A isoform X1 | 285 | 4.75E-07 | 80.4 |  |
| Efet.01.4154.g347.t1 | hypothetical protein CAPTEDRAFT_229053 | 747 | 1.06E-51 | 58.7 |  |
| Efet.01.415585.g359.t1 | hypothetical protein HELRODRAFT_167866 | 279 | 3.91E-10 | 85 | C:integral component of membrane |
| Efet.01.415633.g360.t1 | vacuolar sorting-associated 13A-like | 465 | 3.09E-11 | 53.5 |  |
| Efet.01.415664.g361.t1 | phospholipase A-2-activating | 336 | 1.12E-11 | 56.05 |  |
| Efet.01.415691.g362.t1 | inactive tyrosine- kinase 7-like isoform X2 | 243 | 4.98E-07 | 63 |  |
| Efet.01.415691.g363.t1 | inactive tyrosine- kinase 7-like isoform X1 | 342 | 1.27E-06 | 63 |  |
| Efet.01.415711.g365.t1 | hypothetical protein B5766_06380 | 339 | 3.71E-41 | 74 |  |
| Efet.01.415756.g367.t1 | vesicular acetylcholine transporter | 1704 | 0 | 73.55 | P:drug transport; P:transmembrane transport; C:integral component of membrane |
| Efet.01.415758.g368.t1 | gamma-aminobutyric acid receptor alpha-like | 243 | 1.96E-29 | 80.5 | P:signal transduction; P:chloride transmembrane transport; C:integral component of membrane; F:GABA-A receptor activity; C:cell junction; F:extracellular ligand-gated ion channel activity; C:synapse; F:chloride channel activity; C:plasma membrane |
| Efet.01.415812.g369.t1 | DUF4290 domain-containing | 384 | 1.10E-64 | 76.6 |  |
| Efet.01.415847.g370.t1 | short transient receptor potential channel 6 | 288 | 6.00E-41 | 69.4 | P:transmembrane transport; F:calcium channel activity; C:integral component of membrane; P:metal ion transport; P:single-organism process |
| Efet.01.415860.g371.t1 | patronin isoform X1 | 2811 | 2.35E-24 | 71.6 | F:calmodulin binding; F:spectrin binding; P:neuron projection development; F:microtubule binding |
| Efet.01.41590.g1092.t1 | DUF1016 domain-containing | 555 | 5.57E-131 | 85.25 |  |
| Efet.01.41602.g1094.t1 | E3 ubiquitin- ligase Su(dx)-like isoform X1 | 453 | 1.75E-96 | 95.5 | P:viral entry into host cell; F:ubiquitin protein ligase activity; P:signal transduction; P:ion transmembrane transport; C:plasma membrane; P:protein ubiquitination involved in ubiquitin-dependent protein catabolic process; P:T cell differentiation; C:nucleus; P:proteasome-mediated ubiquitin-dependent protein catabolic process; C:extracellular exosome; F:protein binding; P:central nervous system development; C:ubiquitin ligase complex; P:negative regulation of transcription, DNA-templated; C:integral component of membrane; C:cytosol; F:ligase activity; P:lung development |
| Efet.01.41609.g1095.t1 | pumilio homolog 2-like isoform X1 | 879 | 5.18E-129 | 74.6 | F:binding |
| Efet.01.41609.g1096.t1 | pumilio homolog 2-like isoform X1 | 366 | 5.73E-14 | 75.71 | F:RNA binding |
| Efet.01.416099.g378.t1 | ras-related Rab-11A-like | 303 | 1.06E-39 | 97.2 | F:GTP binding; P:small GTPase mediated signal transduction; C:intracellular |
| Efet.01.416103.g379.t1 | tropomodulin-2 | 552 | 2.41E-09 | 77.8 | P:neuron-neuron synaptic transmission; P:actin filament organization; P:erythrocyte development; F:tropomyosin binding; P:learning or memory; P:positive regulation of G-protein coupled receptor protein signaling pathway; C:growth cone; P:myofibril assembly; C:striated muscle thin filament; F:actin binding; P:pointed-end actin filament capping; P:muscle contraction; C:integral component of membrane |
| Efet.01.416118.g380.t1 | galanin receptor type 2-like isoform X3 | 1089 | 3.10E-104 | 60.1 | P:G-protein coupled receptor signaling pathway; C:membrane; P:positive regulation of calcineurin-NFAT signaling cascade; F:signal transducer activity |
| Efet.01.416122.g381.t1 | IS66 family transposase | 1644 | 0 | 82.05 |  |
| Efet.01.416122.g382.t1 | hypothetical protein | 450 | 1.91E-70 | 74.07 |  |
| Efet.01.416122.g383.t1 | N-acetyltransferase | 663 | 1.49E-126 | 63.45 | C:membrane |
| Efet.01.416122.g384.t1 | tmRNA | 570 | 2.30E-114 | 76.8 |  |
| Efet.01.416122.g385.t1 | hypothetical protein | 378 | 8.05E-69 | 82.85 |  |
| Efet.01.416122.g386.t1 | hypothetical protein | 351 | 1.57E-61 | 68.95 |  |
| Efet.01.41614.g1097.t1 | polycystic kidney disease 2-like 1 | 309 | 4.39E-23 | 71.5 | F:calcium ion binding; C:integral component of membrane |
| Efet.01.416194.g387.t1 | BTB POZ domain-containing 17 | 1407 | 0 | 61.65 |  |
| Efet.01.416197.g388.t1 | hypothetical protein CAPTEDRAFT_192864 | 1386 | 4.29E-16 | 49.5 |  |
| Efet.01.416263.g390.t1 | ras-related Rab-39B-like | 645 | 1.51E-122 | 82.1 | F:GTP binding; P:small GTPase mediated signal transduction; C:intracellular |
| Efet.01.416481.g393.t1 | hypothetical protein HELRODRAFT_190314 | 876 | 4.14E-69 | 67.75 |  |
| Efet.01.416495.g394.t1 | alpha-ketoglutarate-dependent dioxygenase alkB homolog 4 isoform X2 | 327 | 7.33E-30 | 77.85 | C:contractile ring; F:actin binding; P:protein demethylation; P:actomyosin structure organization; F:oxidoreductase activity, acting on paired donors, with incorporation or reduction of molecular oxygen, 2-oxoglutarate as one donor, and incorporation of one atom each of oxygen into both donors; P:oxidation-reduction process; C:midbody; C:intracellular part; P:cleavage furrow ingression |
| Efet.01.416518.g395.t1 | tubby-related 4 isoform X4 | 1584 | 2.87E-20 | 53.3 | P:intracellular signal transduction; C:intracellular |
| Efet.01.416574.g396.t1 | potassium voltage-gated channel Shaker-like | 1038 | 2.33E-114 | 74.65 | P:potassium ion transmembrane transport; C:voltage-gated potassium channel complex; F:delayed rectifier potassium channel activity; P:regulation of ion transmembrane transport; P:protein homooligomerization |
| Efet.01.416653.g397.t1 | zinc finger CCCH domain-containing 15 | 318 | 6.33E-31 | 85.65 | C:nucleus; C:cytoplasm; F:metal ion binding; P:cytokine-mediated signaling pathway |
| Efet.01.416746.g399.t1 | basement membrane-specific heparan sulfate proteoglycan core -like | 558 | 7.89E-25 | 53.5 | F:calcium ion binding |
| Efet.01.416771.g400.t1 | supervillin-like isoform X5 | 354 | 1.87E-28 | 69.3 | F:actin binding |
| Efet.01.416801.g401.t1 | transcription initiation factor IIE subunit beta | 444 | 1.84E-46 | 75.25 | F:DNA binding; F:translation initiation factor activity; P:regulation of transcription, DNA-templated; C:transcription factor TFIIE complex; P:translational initiation; P:transcription initiation from RNA polymerase II promoter |
| Efet.01.416867.g402.t1 | NFX1-type zinc finger-containing 1-like | 2538 | 9.74E-67 | 42.45 | C:nucleus; F:transcription factor activity, sequence-specific DNA binding; F:zinc ion binding; P:regulation of transcription, DNA-templated |
| Efet.01.416880.g403.t1 | 28S ribosomal mitochondrial-like | 207 | 6.96E-21 | 66.75 | F:structural constituent of ribosome; C:ribosome; F:rRNA binding; P:translation |
| Efet.01.4170.g349.t1 | leucine-rich repeat and calponin homology domain-containing 2-like | 303 | 2.20E-19 | 66.85 |  |
| Efet.01.417019.g404.t1 | titin-like isoform X1 | 471 | 5.12E-18 | 57.75 | P:regulation of kinase activity; P:peptidyl-tyrosine phosphorylation; P:protein autophosphorylation; P:cell surface receptor signaling pathway; C:cytoplasmic part; P:immune system process; C:membrane-bounded organelle; P:positive regulation of response to stimulus; F:protein tyrosine kinase activity; C:cytoskeleton; P:regulation of localization; P:positive regulation of cellular process; C:membrane; P:regulation of primary metabolic process; F:immunoglobulin receptor binding; P:regulation of immune response |
| Efet.01.41703.g1101.t1 | calcium-activated potassium channel subunit alpha-1-like isoform X1 | 465 | 6.27E-33 | 62.3 | P:ion transport; F:gated channel activity; C:integral component of membrane; F:potassium channel activity |
| Efet.01.417042.g406.t1 | epoxide hydrolase 1 | 249 | 3.65E-18 | 72.3 | C:membrane; F:hydrolase activity |
| Efet.01.417053.g407.t1 | family transcriptional regulator | 927 | 0 | 89.85 | F:DNA binding; P:carbohydrate metabolic process; F:transcription factor activity, sequence-specific DNA binding; P:regulation of transcription, DNA-templated; F:carbohydrate binding |
| Efet.01.417053.g408.t1 | GTP pyrophosphokinase | 1935 | 0 | 86.2 | F:GTP diphosphokinase activity; P:guanosine tetraphosphate metabolic process; P:phosphorylation; F:amino acid binding; F:kinase activity |
| Efet.01.417096.g409.t1 | arrestin domain-containing 3-like | 291 | 1.62E-20 | 70.8 | F:protein phosphatase 2B binding |
| Efet.01.417196.g412.t1 | hypothetical protein CAPTEDRAFT_188407 | 408 | 8.31E-14 | 50 | C:membrane; C:integral component of membrane |
| Efet.01.417565.g416.t1 | hypothetical protein CAPTEDRAFT_216629 | 2559 | 2.15E-129 | 44 | C:membrane; C:integral component of membrane |
| Efet.01.417659.g418.t1 | NAD(P) mitochondrial-like | 657 | 9.45E-83 | 76.9 | F:NADP binding; P:proton transport; F:NAD binding; C:integral component of membrane; P:NADPH regeneration; F:NAD(P)+ transhydrogenase (AB-specific) activity; C:mitochondrial inner membrane |
| Efet.01.417662.g419.t1 | Cytosolic carboxypeptidase 2 | 813 | 3.68E-32 | 47.2 |  |
| Efet.01.41772.g1103.t1 | activin receptor type-2A | 1014 | 1.16E-78 | 51.6 | F:kinase activity |
| Efet.01.41772.g1104.t1 | calcyphosin isoform X1 | 225 | 1.24E-18 | 71.35 | F:calcium ion binding |
| Efet.01.41773.g1105.t1 | low-density lipo receptor-related 1-like | 888 | 3.84E-67 | 53.65 | F:calcium ion binding; C:membrane; C:integral component of membrane |
| Efet.01.417863.g423.t1 | hypothetical protein CAPTEDRAFT_223814 | 477 | 7.93E-27 | 63.5 |  |
| Efet.01.417928.g424.t1 | hypothetical protein CAPTEDRAFT_222540 | 702 | 5.10E-09 | 57 | C:membrane; C:integral component of membrane; F:scavenger receptor activity; P:receptor-mediated endocytosis |
| Efet.01.41794.g1106.t1 | vacuolar sorting-associated 13A-like | 426 | 7.90E-13 | 61.8 | C:membrane; C:integral component of membrane |
| Efet.01.418043.g426.t1 | Transposon Ty3-I Gag-Pol poly | 681 | 8.91E-18 | 46.7 |  |
| Efet.01.418103.g427.t1 | PREDICTED: uncharacterized protein LOC106172853 | 837 | 7.66E-18 | 49.7 | C:membrane; C:integral component of membrane |
| Efet.01.41847.g1107.t1 | Collagen alpha-5(VI) chain | 576 | 1.39E-46 | 61.85 | F:calcium ion binding; C:collagen trimer |
| Efet.01.41850.g1108.t1 | GPI ethanolamine phosphate transferase 2-like | 390 | 4.61E-26 | 64.25 | C:integral component of membrane |
| Efet.01.418529.g432.t1 | sodium potassium-transporting ATPase subunit beta-like | 957 | 4.32E-148 | 59.55 | P:monovalent inorganic cation transport; C:integral component of membrane; P:metal ion transport |
| Efet.01.41853.g1109.t1 | alkyl aryl-sulfatase BDS1-like | 849 | 5.80E-52 | 68.75 | C:membrane; C:integral component of membrane; F:protein dimerization activity; F:hydrolase activity |
| Efet.01.418553.g433.t1 | nesprin-1-like isoform X1 | 312 | 2.43E-29 | 78.5 |  |
| Efet.01.418601.g435.t1 | zinc finger C3H1 type-like 1 isoform X2 | 1110 | 4.83E-34 | 76.05 | F:metal ion binding |
| Efet.01.418977.g439.t1 | ras GTPase-activating -binding 2-like isoform X2 | 240 | 5.04E-22 | 84.15 | F:nucleotide binding; F:nucleic acid binding; P:transport; C:intracellular |
| Efet.01.419081.g442.t1 | neogenin isoform X4 | 378 | 6.31E-33 | 76.9 | C:integral component of plasma membrane; P:positive regulation of BMP signaling pathway; P:iron ion homeostasis; P:axon guidance; F:receptor activity |
| Efet.01.419168.g443.t1 | nuclear hormone receptor HR96-like isoform X8 | 282 | 1.04E-19 | 83.5 | C:nucleus; F:transcription factor activity, sequence-specific DNA binding; F:zinc ion binding; P:transcription, DNA-templated; P:regulation of transcription, DNA-templated; F:sequence-specific DNA binding; P:steroid hormone mediated signaling pathway |
| Efet.01.419196.g445.t1 | hypothetical protein HELRODRAFT_133020, partial | 1521 | 1.23E-23 | 50.14 | F:metal ion binding |
| Efet.01.419320.g447.t1 | CREB-binding -like | 315 | 4.29E-31 | 93.05 | F:histone acetyltransferase activity; F:zinc ion binding; F:transcription coactivator activity; P:regulation of transcription, DNA-templated; P:histone acetylation; C:histone acetyltransferase complex |
| Efet.01.419403.g450.t1 | F5 8 type C domain-containing | 831 | 4.11E-102 | 63.6 | F:catalytic activity |
| Efet.01.419436.g451.t1 | hypothetical protein RF11_10812 | 597 | 2.65E-07 | 62 |  |
| Efet.01.419529.g452.t1 | leucine-rich repeat-containing 70 | 1068 | 2.39E-69 | 47.6 | C:membrane; C:integral component of membrane |
| Efet.01.419552.g453.t1 | amino acid ABC transporter permease | 792 | 2.88E-128 | 72.8 | C:membrane |
| Efet.01.419552.g454.t1 | ABC transporter substrate-binding | 780 | 0 | 95.65 |  |
| Efet.01.419552.g455.t1 | dihydrodipicolinate synthase family | 897 | 0 | 97.6 | C:cytoplasm; P:lysine biosynthetic process via diaminopimelate; F:4-hydroxy-tetrahydrodipicolinate synthase; P:diaminopimelate biosynthetic process |
| Efet.01.419625.g457.t1 | ABC transporter permease | 969 | 0 | 93.1 | C:integral component of membrane; P:transport; C:plasma membrane; F:transporter activity |
| Efet.01.419625.g458.t1 | sugar ABC transporter ATP-binding | 618 | 3.34E-131 | 93.5 | F:ATPase activity; F:ATP binding |
| Efet.01.419625.g459.t1 | or transport | 453 | 2.87E-89 | 89.15 | F:monosaccharide binding; P:monosaccharide metabolic process; F:isomerase activity |
| Efet.01.41969.g1111.t1 | DIS3-like exonuclease 2 isoform X1 | 2529 | 2.04E-175 | 64.35 | C:intracellular organelle; P:nucleic acid phosphodiester bond hydrolysis; F:exonuclease activity; P:ncRNA catabolic process; P:nuclear-transcribed mRNA catabolic process, exonucleolytic; C:macromolecular complex; C:cytoplasmic part; F:binding; P:single-organism process |
| Efet.01.419729.g461.t1 | basement membrane-specific heparan sulfate proteoglycan core -like | 219 | 3.74E-13 | 63.45 | F:calcium ion binding |
| Efet.01.419761.g464.t1 | titin isoform X2 | 1062 | 2.76E-40 | 61.9 |  |
| Efet.01.41981.g1112.t1 | pyruvate mitochondrial-like isoform X1 | 822 | 5.94E-128 | 83.05 | F:DNA binding; F:ATP binding; P:gluconeogenesis; F:pyruvate carboxylase activity; F:metal ion binding; F:biotin binding; F:biotin carboxylase activity; P:pyruvate metabolic process |
| Efet.01.419835.g466.t1 | N,N -diacetylchitobiase | 477 | 1.93E-39 | 64.6 | P:carbohydrate metabolic process; C:membrane; C:integral component of membrane; F:hydrolase activity, hydrolyzing O-glycosyl compounds; F:carbohydrate binding; F:beta-N-acetylhexosaminidase activity; F:polysaccharide binding |
| Efet.01.419835.g467.t1 | N,N -diacetylchitobiase | 417 | 2.37E-30 | 64.7 | P:carbohydrate metabolic process; F:carbohydrate binding; F:hydrolase activity, hydrolyzing O-glycosyl compounds; F:beta-N-acetylhexosaminidase activity; F:polysaccharide binding |
| Efet.01.419939.g471.t1 | limbic system-associated membrane -like isoform X4 | 309 | 1.16E-29 | 71.7 | C:membrane; P:cellular process |
| Efet.01.419944.g472.t1 | rolling stone-like | 843 | 8.66E-36 | 51 | C:membrane; C:integral component of membrane |
| Efet.01.419997.g474.t1 | prickle isoform X1 | 1680 | 2.46E-30 | 73 | F:metal ion binding |
| Efet.01.420057.g475.t1 | enkurin domain-containing 1 | 429 | 3.48E-16 | 71.55 | C:microtubule cytoskeleton |
| Efet.01.420075.g476.t1 | cytochrome P450 | 456 | 2.90E-26 | 57.15 | F:oxidoreductase activity; F:binding |
| Efet.01.420107.g477.t1 | E3 ubiquitin- ligase MYCBP2-like isoform X2 | 288 | 7.07E-24 | 56.85 | F:zinc ion binding; F:metal ion binding; F:ligase activity |
| Efet.01.42027.g1114.t1 | CD109 antigen-like | 252 | 8.64E-11 | 59.75 | C:extracellular space; F:endopeptidase inhibitor activity; P:negative regulation of endopeptidase activity; C:extracellular region |
| Efet.01.420374.g483.t1 | E3 ubiquitin- ligase TRIP12 isoform X1 | 357 | 5.95E-08 | 65 | F:catalytic activity |
| Efet.01.420410.g484.t1 | ubiquitin carboxyl-terminal hydrolase 25 isoform X2 | 444 | 8.03E-42 | 72.1 | P:proteolysis; P:cellular protein metabolic process |
| Efet.01.420435.g485.t1 | kyphoscoliosis peptidase | 1986 | 0 | 63.2 |  |
| Efet.01.42046.g1115.t1 | serine arginine repetitive matrix 1-like | 372 | 1.19E-07 | 70.4 |  |
| Efet.01.420546.g488.t1 | cholecystokinin receptor type A-like | 381 | 1.62E-36 | 74.6 | F:G-protein coupled receptor activity; P:G-protein coupled receptor signaling pathway; C:integral component of membrane; F:transferase activity |
| Efet.01.420673.g490.t1 | Voltage-dependent L-type calcium channel subunit alpha- | 360 | 5.67E-54 | 91.25 | C:voltage-gated calcium channel complex; P:regulation of ion transmembrane transport; P:calcium ion transmembrane transport; F:voltage-gated calcium channel activity |
| Efet.01.420676.g491.t1 | sodium channel para-like isoform X1 | 219 | 6.90E-17 | 77.45 | P:regulation of postsynaptic membrane potential; F:calcium ion binding; P:regulation of ion transmembrane transport; C:voltage-gated sodium channel complex; P:sodium ion transmembrane transport; P:membrane depolarization during action potential; F:voltage-gated sodium channel activity; P:neuronal action potential |
| Efet.01.420722.g494.t1 | hypothetical protein CAPTEDRAFT_111747 | 270 | 1.95E-09 | 75 | P:ion transmembrane transport; C:integral component of membrane; F:extracellular ligand-gated ion channel activity |
| Efet.01.420782.g496.t1 | serine threonine- kinase roco4 | 3723 | 0 | 50.75 |  |
| Efet.01.420818.g497.t1 | zinc finger OZF-like | 333 | 2.44E-09 | 48.4 | F:nucleic acid binding; F:metal ion binding |
| Efet.01.4209.g350.t1 | kinetochore-associated 1 | 927 | 2.57E-38 | 59.15 | C:intracellular non-membrane-bounded organelle |
| Efet.01.42090.g1116.t1 | capicua homolog isoform X1 | 1092 | 1.05E-24 | 53.85 | C:transcription factor TFIIA complex; P:transcription initiation from RNA polymerase II promoter |
| Efet.01.421050.g502.t1 | zinc finger Xfin-like | 2673 | 2.71E-10 | 77.6 | F:nucleic acid binding; F:metal ion binding; C:integral component of membrane |
| Efet.01.421057.g503.t1 | casein kinase I isoform alpha | 558 | 1.66E-77 | 91.6 | P:regulation of cell shape; C:nuclear speck; P:Golgi organization; P:intermediate filament cytoskeleton organization; C:condensed chromosome kinetochore; F:peptide binding; F:magnesium ion binding; P:cell division; F:ATP binding; C:keratin filament; C:intracellular ribonucleoprotein complex; C:mRNA cleavage and polyadenylation specificity factor complex; C:cytosol; P:Wnt signaling pathway; F:glycoprotein binding; P:cell morphogenesis; P:mitotic nuclear division; P:regulation of GTP binding; F:protein serine/threonine kinase activity; C:beta-catenin destruction complex; P:negative regulation of canonical Wnt signaling pathway; C:centrosome; C:membrane; P:phagocytosis; F:phosphoprotein binding; P:peptidyl-serine phosphorylation; P:positive regulation of proteasomal ubiquitin-dependent protein catabolic process |
| Efet.01.42106.g1117.t1 | hypothetical protein HELRODRAFT_195025 | 303 | 3.12E-18 | 63.4 | C:membrane; C:integral component of membrane; F:carbohydrate binding |
| Efet.01.421099.g505.t1 | transcriptional regulator ERG homolog | 405 | 5.33E-12 | 77.7 | C:nucleus; F:transcription factor activity, sequence-specific DNA binding; P:regulation of transcription, DNA-templated; F:sequence-specific DNA binding |
| Efet.01.421142.g506.t1 | hypothetical protein CAPTEDRAFT_219306 | 1635 | 2.20E-18 | 55.57 | C:membrane; C:integral component of membrane |
| Efet.01.421207.g509.t1 | Calcium-independent kinase C | 360 | 4.20E-48 | 76.8 | F:ATP binding; F:metal ion binding; P:protein phosphorylation; C:intracellular; P:intracellular signal transduction; F:protein kinase C activity |
| Efet.01.42123.g1118.t1 | hypothetical protein CAPTEDRAFT_226428 | 636 | 2.32E-30 | 53.5 | F:calcium ion binding; C:membrane; C:integral component of membrane |
| Efet.01.421288.g510.t1 | hypothetical protein HELRODRAFT_183479 | 855 | 2.46E-14 | 72.26 | F:nucleic acid binding; F:metal ion binding |
| Efet.01.42130.g1119.t1 | PREDICTED: uncharacterized protein LOC109061345 | 228 | 9.54E-06 | 65 |  |
| Efet.01.421324.g512.t1 | glycogen phosphorylase | 414 | 2.05E-69 | 83.2 | F:glycogen phosphorylase activity; P:carbohydrate metabolic process; F:pyridoxal phosphate binding |
| Efet.01.421339.g513.t1 | bifunctional 3 -phosphoadenosine 5 -phosphosulfate synthase 2 | 234 | 2.15E-38 | 88.55 | F:ATP binding; P:3'-phosphoadenosine 5'-phosphosulfate biosynthetic process; F:adenylylsulfate kinase activity; F:sulfate adenylyltransferase (ATP) activity; P:blood coagulation; P:phosphorylation; P:sulfate assimilation; P:bone development |
| Efet.01.42139.g1121.t1 | Peripheral plasma membrane | 384 | 1.44E-53 | 90.35 | C:presynaptic membrane; P:calcium ion import; F:protein C-terminus binding; P:GDP metabolic process; P:cell adhesion; P:GMP metabolic process; F:guanylate kinase activity; C:nucleolus; P:protein phosphorylation; P:intracellular protein transport; P:positive regulation of transcription from RNA polymerase II promoter; C:nuclear lamina; P:negative regulation of keratinocyte proliferation; C:dendrite; F:ATP binding; C:basement membrane; C:focal adhesion; F:neurexin family protein binding; C:actin cytoskeleton; F:PDZ domain binding; C:cytosol; C:ciliary membrane; F:protein complex binding; P:negative regulation of wound healing; P:negative regulation of cellular response to growth factor stimulus; C:protein complex; F:protein serine/threonine kinase activity; P:neurotransmitter secretion; P:positive regulation of calcium ion import; F:calmodulin binding; C:cell-cell junction; P:negative regulation of cell-matrix adhesion; C:nuclear matrix; C:vesicle; C:basolateral plasma membrane |
| Efet.01.421392.g514.t1 | Glutamate--cysteine ligase regulatory subunit | 486 | 5.86E-53 | 72.25 | C:glutamate-cysteine ligase complex; P:glutathione biosynthetic process; P:cysteine metabolic process; F:glutamate-cysteine ligase activity; P:negative regulation of neuron apoptotic process; P:response to oxidative stress; P:glutamate metabolic process; P:apoptotic mitochondrial changes; F:protein heterodimerization activity; P:regulation of blood vessel size; P:response to drug; P:positive regulation of glutamate-cysteine ligase activity; P:response to nitrosative stress; P:negative regulation of extrinsic apoptotic signaling pathway; F:enzyme regulator activity; P:regulation of mitochondrial depolarization; F:glutamate-cysteine ligase catalytic subunit binding |
| Efet.01.42154.g1122.t1 | follistatin-related 5-like | 570 | 3.94E-18 | 55.05 | F:calcium ion binding; C:membrane; P:protein glycosylation; F:galactosyltransferase activity |
| Efet.01.421560.g515.t1 | ankyrin repeat | 2562 | 2.82E-08 | 41.33 | C:membrane; C:integral component of membrane |
| Efet.01.421566.g516.t1 | splicing regulatory glutamine lysine-rich 1-like | 1749 | 3.62E-111 | 65.55 | F:nucleotide binding; F:nucleic acid binding |
| Efet.01.421611.g517.t1 | transcriptional activator Pur-beta isoform X3 | 246 | 6.32E-23 | 82.5 |  |
| Efet.01.42165.g1124.t1 | neurabin-1 isoform X1 | 1323 | 2.47E-16 | 69.7 |  |
| Efet.01.421681.g518.t1 | atrial natriuretic peptide receptor 1-like | 387 | 3.79E-13 | 52.25 | P:single-organism process; P:cellular process |
| Efet.01.421789.g519.t1 | arabinose ABC transporter permease | 453 | 1.54E-66 | 93.25 | C:integral component of membrane |
| Efet.01.421900.g520.t1 | tctex1 domain-containing 1-B | 363 | 5.83E-22 | 62.55 |  |
| Efet.01.42192.g1125.t1 | G T mismatch-specific thymine DNA glycosylase | 393 | 2.14E-14 | 78.6 | F:DNA binding; P:DNA repair; F:DNA N-glycosylase activity |
| Efet.01.422049.g523.t1 | MAM and LDL-receptor class A domain-containing 2-like | 339 | 1.26E-12 | 52.3 | F:catalytic activity; P:nucleoside metabolic process; F:methyltransferase activity; F:transferase activity; P:methylation |
| Efet.01.42217.g1127.t1 | phosphatase and actin regulator 1- | 360 | 3.98E-15 | 64.5 |  |
| Efet.01.422248.g526.t1 | NADH dehydrogenase [ubiquinone] 1 alpha subcomplex subunit mitochondrial | 456 | 1.07E-54 | 79.55 | F:NADH dehydrogenase activity; F:coenzyme binding; P:ubiquinone-6 biosynthetic process |
| Efet.01.42235.g1128.t1 | udp-n-acetylglucosamine--peptide n-acetylglucosaminyltransferase | 1779 | 1.46E-123 | 64 | C:integral component of membrane |
| Efet.01.422489.g529.t1 | probable tubulin polyglutamylase TTLL2 | 291 | 9.87E-09 | 53.2 | P:cellular protein modification process |
| Efet.01.422630.g531.t1 | phosphofurin acidic cluster sorting 2-like isoform X13 | 441 | 3.38E-29 | 69.35 |  |
| Efet.01.422683.g533.t1 | insulin-like growth factor 1 receptor | 249 | 1.39E-28 | 78.55 | F:ATP binding; P:cGMP biosynthetic process; P:peptidyl-tyrosine phosphorylation; F:protein tyrosine kinase activity; F:G-protein coupled GABA receptor activity; P:G-protein coupled receptor signaling pathway; C:integral component of membrane; F:guanylate cyclase activity |
| Efet.01.422696.g534.t1 | monocarboxylate transporter 12-like | 264 | 9.50E-14 | 65.7 | C:membrane; P:transport |
| Efet.01.4227.g352.t1 | c-binding -like | 411 | 8.63E-23 | 62.95 |  |
| Efet.01.42283.g1129.t1 | muscarinic acetylcholine receptor M5-like | 1209 | 4.30E-99 | 75.6 | P:gastric acid secretion; C:integral component of plasma membrane; P:synaptic transmission, cholinergic; P:phospholipase C-activating G-protein coupled acetylcholine receptor signaling pathway; P:adenylate cyclase-inhibiting G-protein coupled acetylcholine receptor signaling pathway; F:G-protein coupled acetylcholine receptor activity; C:synapse; F:transferase activity |
| Efet.01.42283.g1130.t1 | muscarinic acetylcholine receptor M3 | 840 | 3.06E-17 | 69.4 | P:gastric acid secretion; C:integral component of plasma membrane; P:regulation of biological quality; F:G-protein coupled acetylcholine receptor activity; P:G-protein coupled acetylcholine receptor signaling pathway; C:synapse; P:regulation of smooth muscle contraction |
| Efet.01.422855.g539.t1 | aquaporin-8-like isoform X3 | 426 | 5.61E-08 | 57.6 | C:membrane; C:integral component of membrane; P:transport; F:transporter activity |
| Efet.01.422899.g540.t1 | cytochrome C | 408 | 3.70E-77 | 77.05 | F:oxidoreductase activity; P:metabolic process; F:binding |
| Efet.01.42290.g1131.t1 | interferon-induced very large GTPase 1-like | 819 | 3.73E-40 | 46.1 | F:GTP binding |
| Efet.01.42291.g1132.t1 | interferon-induced very large GTPase 1-like | 1056 | 5.35E-23 | 51.05 | F:GTP binding |
| Efet.01.422931.g541.t1 | importin-4-like isoform X2 | 735 | 9.25E-11 | 57.13 | P:intracellular protein transport; F:Ran GTPase binding; C:intracellular |
| Efet.01.422950.g542.t1 | hypothetical protein CAPTEDRAFT_187682 | 708 | 2.46E-55 | 73.75 |  |
| Efet.01.423054.g543.t1 | DNA topoisomerase 2-beta isoform X2 | 366 | 1.88E-24 | 79.9 | F:magnesium ion binding; C:nucleus; F:ATP binding; F:DNA binding, bending; F:DNA topoisomerase type II (ATP-hydrolyzing) activity; P:DNA topological change |
| Efet.01.423076.g544.t1 | adenosine kinase | 480 | 5.21E-21 | 67.3 | F:deaminase activity |
| Efet.01.423130.g547.t1 | BTB POZ domain-containing 6-like | 294 | 2.79E-11 | 53.1 |  |
| Efet.01.423212.g548.t1 | nicotinamide phosphoribosyltransferase-like | 471 | 1.84E-72 | 84.4 | F:nicotinate-nucleotide diphosphorylase (carboxylating) activity; P:NAD biosynthetic process |
| Efet.01.423283.g550.t1 | TSP1_XENLA ame: Full=Thrombospondin-1 Flags: Precursor | 930 | 5.21E-06 | 52 | P:negative regulation of angiogenesis; C:platelet alpha granule; C:sarcoplasmic reticulum; P:cell adhesion; F:heparin binding; C:endoplasmic reticulum; C:extracellular region; P:response to unfolded protein; C:proteinaceous extracellular matrix; F:extracellular matrix binding; C:extracellular space; C:basement membrane; P:inflammatory response; F:protein binding; F:calcium ion binding; C:extracellular matrix; P:positive regulation of synapse assembly |
| Efet.01.423326.g551.t1 | hypothetical protein LOTGIDRAFT_118013 | 714 | 1.23E-06 | 71.5 |  |
| Efet.01.423368.g554.t1 | hypothetical protein HELRODRAFT_177006 | 438 | 1.87E-11 | 59 |  |
| Efet.01.423413.g555.t1 | PR domain zinc finger 1-like isoform X4 | 825 | 5.56E-50 | 68.1 | F:binding; C:intracellular part |
| Efet.01.423456.g556.t1 | hypothetical protein CRN43_19745, partial | 459 | 1.66E-18 | 56.33 |  |
| Efet.01.423483.g557.t1 | hypothetical protein HELRODRAFT_183479 | 681 | 2.98E-09 | 79.75 | F:nucleic acid binding; F:metal ion binding |
| Efet.01.423486.g558.t1 | 1Chain Fitted Coordinates For Lumbricus Terrestris Hemoglobin Cryo-em Complex (emd-2627) | 324 | 8.71E-09 | 91 |  |
| Efet.01.423585.g559.t1 | plasma membrane calcium-transporting ATPase 2 isoform X1 | 483 | 6.33E-62 | 80.85 | F:ATP binding; F:metal ion binding; C:integral component of membrane; P:calcium ion transmembrane transport; F:calcium-transporting ATPase activity |
| Efet.01.4236.g353.t1 | dynein heavy chain axonemal-like isoform X1 | 609 | 1.52E-79 | 84.7 | F:ATPase activity; F:ATP binding; F:microtubule motor activity; C:axonemal dynein complex; P:cilium or flagellum-dependent cell motility; P:microtubule-based movement |
| Efet.01.4236.g354.t1 | dynein heavy chain axonemal | 327 | 4.61E-50 | 87.8 | F:ATPase activity; F:ATP binding; F:microtubule motor activity; C:axonemal dynein complex; P:cilium or flagellum-dependent cell motility; P:microtubule-based movement |
| Efet.01.4236.g355.t1 | dynein heavy chain axonemal-like isoform X1 | 693 | 6.28E-120 | 86.25 | F:ATPase activity; F:ATP binding; F:microtubule motor activity; C:axonemal dynein complex; P:cilium or flagellum-dependent cell motility; P:microtubule-based movement |
| Efet.01.423677.g560.t1 | gastrula zinc finger -like | 669 | 4.51E-18 | 46.2 | P:multicellular organism development; P:regulation of transcription, DNA-templated; F:binding; P:positive regulation of biological process |
| Efet.01.423728.g562.t1 | FMRFamide receptor-like | 555 | 1.34E-28 | 54.75 | F:G-protein coupled receptor activity; F:G-protein coupled peptide receptor activity; P:G-protein coupled receptor signaling pathway; P:signal transduction; C:membrane; C:integral component of membrane; F:signal transducer activity |
| Efet.01.423747.g563.t1 | UPF0489 C5orf22 homolog | 1125 | 3.58E-60 | 47.45 | C:membrane; C:integral component of membrane |
| Efet.01.423747.g564.t1 | beta-1,3-galactosyltransferase 6-like | 999 | 3.46E-112 | 66.35 | C:membrane; C:Golgi apparatus part; F:galactosyltransferase activity; P:macromolecule biosynthetic process; P:carbohydrate derivative biosynthetic process |
| Efet.01.423962.g568.t1 | gonadotropin-releasing hormone receptor-like isoform X1 | 540 | 2.13E-47 | 68.9 | P:signal transduction; C:membrane; F:signal transducer activity |
| Efet.01.423964.g569.t1 | tyrosine- kinase transmembrane receptor Ror | 324 | 5.83E-37 | 78.9 | F:ATP binding; P:peptidyl-tyrosine phosphorylation; F:protein tyrosine kinase activity; C:integral component of membrane |
| Efet.01.424113.g571.t1 | alpha-1 2-mannosidase | 513 | 1.71E-72 | 74.25 | P:carbohydrate metabolic process; F:catalytic activity; F:carbohydrate binding |
| Efet.01.424158.g572.t1 | zinc finger 658 isoform X1 | 2373 | 2.17E-55 | 50.5 | F:nucleic acid binding; F:metal ion binding |
| Efet.01.424172.g573.t1 | ABC transporter ATP-binding | 216 | 4.64E-40 | 73.25 | F:ATPase activity; F:ATP binding |
| Efet.01.424172.g574.t1 | family transcriptional regulator | 855 | 1.09E-164 | 61.5 | F:DNA binding; P:transcription, DNA-templated; P:regulation of transcription, DNA-templated |
| Efet.01.424172.g575.t1 | DNA-binding | 402 | 1.34E-77 | 68 | F:DNA binding |
| Efet.01.424172.g576.t1 | tripartite tricarboxylate transporter substrate binding | 1002 | 0 | 87.45 | C:outer membrane-bounded periplasmic space |
| Efet.01.424198.g578.t1 | GTP-binding GEM | 309 | 1.08E-08 | 49.31 | F:GTP binding; F:nucleotide binding; P:signal transduction; P:small GTPase mediated signal transduction; C:membrane; C:intracellular |
| Efet.01.424495.g583.t1 | E3 ubiquitin- ligase RBBP6-like | 231 | 1.43E-20 | 84.75 | F:nucleic acid binding; C:nucleus; F:ubiquitin-protein transferase activity; F:zinc ion binding; P:protein ubiquitination |
| Efet.01.424579.g584.t1 | PREDICTED: uncharacterized protein LOC108242774 | 426 | 4.98E-06 | 51 |  |
| Efet.01.424604.g585.t1 | ankyrin repeat | 1014 | 1.36E-51 | 52.5 | P:intracellular signal transduction; C:intracellular |
| Efet.01.424659.g586.t1 | hypothetical protein CAPTEDRAFT_199065 | 444 | 7.74E-08 | 69.5 |  |
| Efet.01.42467.g1139.t1 | RING FYVE PHD zinc finger | 747 | 1.62E-08 | 51.55 | F:zinc ion binding; F:metal ion binding; C:membrane; C:integral component of membrane |
| Efet.01.42470.g1140.t1 | fat-like cadherin-related tumor suppressor homolog | 2637 | 1.09E-153 | 50.8 | F:calcium ion binding; C:membrane; C:integral component of membrane; P:cell adhesion; P:homophilic cell adhesion via plasma membrane adhesion molecules; C:plasma membrane |
| Efet.01.424716.g587.t1 | AF-9 isoform X1 | 1584 | 2.02E-09 | 73.15 | P:regulation of transcription, DNA-templated |
| Efet.01.42473.g1141.t1 | transient receptor potential cation channel subfamily M member 2-like isoform X3 | 564 | 2.88E-33 | 65.65 | P:ion transport; P:transmembrane transport |
| Efet.01.424735.g588.t1 | zinc finger 271 | 828 | 7.04E-51 | 65 | F:nucleic acid binding; F:metal ion binding |
| Efet.01.424750.g589.t1 | RNA-directed DNA polymerase from transposon BS | 636 | 1.83E-17 | 51.65 |  |
| Efet.01.424836.g590.t1 | hypothetical protein HELRODRAFT_185352 | 312 | 7.72E-14 | 65.8 | C:membrane; C:integral component of membrane; P:transport; F:transporter activity |
| Efet.01.424910.g592.t1 | mab-21-like | 987 | 5.69E-92 | 72.4 |  |
| Efet.01.42494.g1142.t1 | hypothetical protein HELRODRAFT_169408 | 588 | 7.15E-10 | 70 | F:G-protein coupled receptor activity; P:G-protein coupled receptor signaling pathway; P:signal transduction; C:membrane; C:integral component of membrane; F:signal transducer activity |
| Efet.01.424959.g593.t1 | E3 ubiquitin- ligase RNF213 | 2739 | 2.84E-97 | 50 | F:nucleotide binding; F:ATPase activity; F:ATP binding |
| Efet.01.425067.g594.t1 | stress-activated kinase JNK-like isoform X1 | 378 | 3.69E-47 | 91.55 | F:ATP binding; P:MAPK cascade; F:MAP kinase activity; C:intracellular |
| Efet.01.425111.g596.t1 | ROK family | 630 | 1.17E-118 | 66.95 |  |
| Efet.01.425126.g597.t1 | ubiquitin thioesterase zranb1-B-like | 1119 | 5.50E-78 | 51.05 | P:cellular process |
| Efet.01.425207.g600.t1 | calmodulin B | 246 | 1.22E-28 | 81 | F:phosphatidylinositol 3-kinase binding; F:protein domain specific binding; F:2-alkenal reductase [NAD(P)] activity; C:voltage-gated potassium channel complex; F:calcium-dependent protein binding; F:adenylate cyclase binding; F:ion channel binding; C:growth cone; C:nucleus; F:type 3 metabotropic glutamate receptor binding; P:G2/M transition of mitotic cell cycle; C:cytoplasm; F:calcium ion binding; F:protein N-terminus binding; P:oxidation-reduction process; F:nitric-oxide synthase regulator activity; F:nitric-oxide synthase binding |
| Efet.01.425302.g601.t1 | dynein beta ciliary-like | 594 | 5.58E-110 | 88.05 | F:ATPase activity; F:ATP binding; C:motile cilium; F:microtubule motor activity; C:cytoplasm; P:cell projection organization; C:integral component of membrane; C:dynein complex; P:microtubule-based movement; C:microtubule |
| Efet.01.425304.g602.t1 | ng-dimethylarginine dimethylaminohydrolase | 345 | 1.09E-52 | 68.8 | F:hydrolase activity |
| Efet.01.425351.g603.t1 | peroxiredoxin-1 isoform X1 | 222 | 1.98E-32 | 92.35 | F:peroxidase activity; P:cellular oxidant detoxification; P:oxidation-reduction process; F:peroxiredoxin activity |
| Efet.01.425430.g604.t1 | calcium-activated potassium channel slowpoke-like isoform X4 | 492 | 9.89E-61 | 80.95 | F:calcium-activated potassium channel activity; F:voltage-gated potassium channel activity; P:potassium ion transmembrane transport; C:voltage-gated potassium channel complex |
| Efet.01.42547.g1143.t1 | homeobox posterior hox 2 | 351 | 1.46E-23 | 82.65 | C:nucleus; P:regulation of transcription, DNA-templated; F:sequence-specific DNA binding |
| Efet.01.425484.g605.t1 | cilia- and flagella-associated 97 isoform X2 | 1149 | 3.86E-11 | 56.15 |  |
| Efet.01.425514.g606.t1 | potassium sodium hyperpolarization-activated cyclic nucleotide-gated channel 3-like isoform X1 | 441 | 4.19E-40 | 92 | F:voltage-gated potassium channel activity; P:potassium ion transmembrane transport; C:integral component of membrane |
| Efet.01.425516.g607.t1 | translation initiation factor eIF-2B subunit delta-like | 228 | 2.72E-17 | 68.8 | P:negative regulation of translational initiation; P:L-methionine salvage from methylthioadenosine; F:guanyl-nucleotide exchange factor activity; F:S-methyl-5-thioribose-1-phosphate isomerase activity; C:eukaryotic translation initiation factor 2B complex |
| Efet.01.42557.g1144.t1 | voltage-dependent T-type calcium channel subunit alpha-1H-like | 624 | 8.72E-18 | 94 | P:calcium ion import; C:voltage-gated calcium channel complex; P:regulation of ion transmembrane transport; P:calcium ion transmembrane transport; F:low voltage-gated calcium channel activity |
| Efet.01.42558.g1145.t1 | sodium channel type 4 subunit alpha B-like isoform X4 | 252 | 1.84E-40 | 67.45 | P:regulation of postsynaptic membrane potential; F:calcium ion binding; P:regulation of ion transmembrane transport; C:voltage-gated sodium channel complex; P:sodium ion transmembrane transport; F:voltage-gated sodium channel activity |
| Efet.01.425604.g609.t1 | transcription factor Sox-2-like | 762 | 2.06E-36 | 51.2 | F:DNA binding; C:nucleus; F:transcription factor activity, sequence-specific DNA binding; P:nervous system development; P:regulation of transcription, DNA-templated; P:neuron development; P:sex determination |
| Efet.01.425662.g611.t1 | cholecystokinin receptor type A-like | 636 | 5.72E-46 | 70.7 | P:signal transduction; C:membrane; F:signal transducer activity |
| Efet.01.425716.g613.t1 | bifunctional heparan sulfate N-deacetylase N-sulfotransferase | 1194 | 1.78E-106 | 69.4 | F:sulfotransferase activity |
| Efet.01.425734.g614.t1 | 28S ribosomal mitochondrial-like | 324 | 5.10E-13 | 58.33 |  |
| Efet.01.42584.g1146.t1 | Kyphoscoliosis peptidase | 1290 | 9.38E-118 | 56.45 | F:zinc ion binding; F:metal ion binding |
| Efet.01.42591.g1147.t1 | PR domain zinc finger 2 | 2118 | 1.69E-18 | 47.55 | F:nucleic acid binding; F:metal ion binding |
| Efet.01.426142.g622.t1 | IQ and ubiquitin-like domain-containing | 423 | 9.79E-50 | 73.65 |  |
| Efet.01.426153.g623.t1 | rolling stone-like | 423 | 1.61E-06 | 55 | C:membrane; C:integral component of membrane |
| Efet.01.426161.g624.t1 | Kinase DC2 | 315 | 1.40E-39 | 86.8 | F:ATP binding; F:cAMP-dependent protein kinase activity; P:protein phosphorylation |
| Efet.01.426224.g626.t1 | hemicentin-1-like isoform X2 | 450 | 3.36E-24 | 85.65 | P:cell division; C:basement membrane; C:extracellular exosome; C:cell cortex; F:calcium ion binding; C:cell junction; P:cell cycle |
| Efet.01.426232.g627.t1 | methyltransferase 16 | 720 | 2.54E-93 | 69.65 | F:tRNA (adenine) methyltransferase activity; F:O-methyltransferase activity; F:tRNA (cytosine) methyltransferase activity; F:protein methyltransferase activity; F:arginine N-methyltransferase activity; F:C-methyltransferase activity; F:rRNA (adenine-N6-)-methyltransferase activity; F:tRNA (guanine) methyltransferase activity; F:rRNA (cytosine) methyltransferase activity; P:methylation |
| Efet.01.426237.g628.t1 | tyrosine- phosphatase non-receptor type 9 | 489 | 1.47E-14 | 76.25 | F:phosphoprotein phosphatase activity; P:protein dephosphorylation |
| Efet.01.426391.g633.t1 | polycystin-2 isoform X2 | 390 | 3.29E-39 | 84.95 | P:cell cycle arrest; P:neural tube development; P:JAK-STAT cascade; P:dorsal/ventral pattern formation; P:spinal cord development; P:detection of nodal flow; F:calcium-induced calcium release activity; P:cellular response to fluid shear stress; F:calcium ion binding; P:lymphangiogenesis; C:integral component of cytoplasmic side of endoplasmic reticulum membrane; P:heart looping; P:release of sequestered calcium ion into cytosol; P:regulation of collagen biosynthetic process; C:lamellipodium; P:renal artery morphogenesis; P:regulation of postsynaptic membrane potential; P:placenta blood vessel development; P:potassium ion transmembrane transport; F:HLH domain binding; C:polycystin complex; F:voltage-gated calcium channel activity; F:ion channel binding; F:voltage-gated sodium channel activity; P:heart jogging; P:negative regulation of ryanodine-sensitive calcium-release channel activity; C:cell-cell junction; P:regulation of cAMP metabolic process; P:aorta development; P:mesonephric duct development; P:proximal convoluted tubule development; F:phosphoprotein binding; P:metanephric cortex development; C:filamentous actin; C:basal cortex; P:positive regulation of inositol 1,4,5-trisphosphate-sensitive calcium-release channel activity; P:metanephric cortical collecting duct development; P:metanephric part of ureteric bud development; P:metanephric ascending thin limb development; P:positive regulation of nitric oxide biosynthetic process; P:cytoplasmic sequestering of transcription factor; P:positive regulation of transcription from RNA polymerase II promoter; P:embryonic placenta development; P:positive regulation of cyclin-dependent protein serine/threonine kinase activity involved in G1/S transition of mitotic cell cycle; C:integral component of plasma membrane; C:basal plasma membrane; P:metanephric S-shaped body morphogenesis; C:extracellular exosome; P:cellular response to osmotic stress; C:mitotic spindle; C:integral component of lumenal side of endoplasmic reticulum membrane; P:negative regulation of G1/S transition of mitotic cell cycle; P:positive regulation of cell cycle arrest; P:determination of liver left/right asymmetry; P:metanephric smooth muscle tissue development; F:protein homodimerization activity; C:ciliary basal body; F:actinin binding; F:receptor binding; F:potassium channel activity; P:regulation of cell proliferation; P:detection of mechanical stimulus; P:metanephric mesenchyme development; P:cellular response to hydrostatic pressure; P:pronephros morphogenesis; P:metanephric distal tubule development; P:branching involved in ureteric bud morphogenesis; P:sodium ion transmembrane transport; P:regulation of cardiac muscle contraction by calcium ion signaling; F:ATPase binding |
| Efet.01.426413.g634.t1 | zinc finger ush | 564 | 3.57E-10 | 48.3 | F:nucleic acid binding; F:metal ion binding |
| Efet.01.426420.g637.t1 | sulfhydryl oxidase 1-like | 378 | 2.27E-28 | 58.35 | F:organic cyclic compound binding; F:heterocyclic compound binding; F:oxidoreductase activity; C:membrane; P:tRNA metabolic process; P:cellular amino acid metabolic process; C:cytoplasmic part |
| Efet.01.426499.g639.t1 | lebercilin | 534 | 9.51E-17 | 63.7 |  |
| Efet.01.426513.g640.t1 | hypothetical protein CAPTEDRAFT_210308 | 969 | 1.52E-70 | 51.33 |  |
| Efet.01.42652.g1150.t1 | tripartite motif-containing 45-like isoform X1 | 2499 | 9.97E-38 | 60.6 |  |
| Efet.01.42663.g1151.t1 | B-cell lymphoma leukemia 11A-like | 1905 | 2.11E-46 | 86.9 | C:nucleus; F:transcription factor activity, sequence-specific DNA binding; F:metal ion binding; P:signal transduction; F:transcription regulatory region DNA binding; P:regulation of transcription, DNA-templated; P:transcription from RNA polymerase II promoter; F:sequence-specific DNA binding; P:neurogenesis |
| Efet.01.426760.g643.t1 | short chain dehydrogenase | 360 | 9.22E-37 | 83.55 |  |
| Efet.01.426803.g644.t1 | mitochondrial carrier Rim2 | 366 | 1.52E-48 | 77.6 | P:transcription from mitochondrial promoter; P:transmembrane transport; P:locomotion; P:mitochondrion distribution; C:mitochondrion; C:integral component of membrane; P:cellular respiration; P:deoxynucleotide transport |
| Efet.01.426910.g645.t1 | glycosyltransferase family 2 | 951 | 0 | 67.25 | F:transferase activity |
| Efet.01.426977.g646.t1 | long-chain-fatty-acid-- ligase 3-like | 1032 | 4.97E-147 | 70.35 | F:catalytic activity; C:membrane |
| Efet.01.42717.g1152.t1 | mannosyl-oligosaccharide glucosidase-like | 1863 | 0 | 71.5 | C:endoplasmic reticulum membrane; P:carbohydrate metabolic process; P:protein N-linked glycosylation; F:mannosyl-oligosaccharide glucosidase activity |
| Efet.01.427237.g649.t1 | 1-phosphatidylinositol 4,5-bisphosphate phosphodiesterase beta-4 isoform X1 | 375 | 1.88E-17 | 72.55 | P:signal transduction; P:lipid metabolic process; F:phosphoric diester hydrolase activity |
| Efet.01.427246.g651.t1 | Blastula protease 10 | 906 | 7.50E-111 | 49.85 | F:zinc ion binding; F:metal ion binding; P:proteolysis; F:peptidase activity; F:metalloendopeptidase activity; F:metallopeptidase activity; F:hydrolase activity |
| Efet.01.427307.g652.t1 | hypothetical protein | 582 | 8.85E-118 | 71.44 |  |
| Efet.01.427307.g653.t1 | outer membrane | 807 | 1.92E-144 | 66.2 |  |
| Efet.01.427428.g655.t1 | cyclic nucleotide-gated cation channel alpha-3 | 474 | 1.40E-38 | 66.7 | F:nucleotide binding; P:biological regulation; F:protein binding; P:cation transport; P:ion transmembrane transport; C:integral component of membrane; P:response to chemical; C:neuronal cell body; F:intracellular cyclic nucleotide activated cation channel activity; P:retinal cone cell development; C:photoreceptor outer segment; C:plasma membrane part |
| Efet.01.42747.g1153.t1 | transcription factor Maf | 918 | 6.11E-17 | 68 | F:protein homodimerization activity; P:inner ear development; P:regulation of chondrocyte differentiation; F:double-stranded DNA binding; F:transcriptional activator activity, RNA polymerase II transcription regulatory region sequence-specific binding; F:sequence-specific DNA binding; P:cell development; P:positive regulation of transcription from RNA polymerase II promoter; P:negative regulation of transcription from RNA polymerase II promoter; C:nucleus; C:cytoplasm; F:protein heterodimerization activity; P:cytokine production; P:lens fiber cell differentiation |
| Efet.01.42748.g1154.t1 | mab-21-like 2 | 900 | 2.70E-15 | 50.5 | C:membrane; C:integral component of membrane |
| Efet.01.427521.g656.t1 | hypothetical protein CAPTEDRAFT_219768 | 336 | 6.15E-10 | 60 | C:membrane; C:integral component of membrane |
| Efet.01.42754.g1155.t1 | elongator complex 5 | 900 | 7.21E-27 | 47.35 | C:Elongator holoenzyme complex |
| Efet.01.427593.g657.t1 | histidinol-phosphate transaminase | 228 | 2.21E-33 | 68.8 | F:pyridoxal phosphate binding; F:transaminase activity; P:biosynthetic process |
| Efet.01.427593.g658.t1 | cyclase | 954 | 1.14E-174 | 74.6 | F:arylformamidase activity; P:tryptophan catabolic process to kynurenine |
| Efet.01.427593.g659.t1 | tripartite tricarboxylate transporter substrate binding | 825 | 0 | 75.1 | C:outer membrane-bounded periplasmic space |
| Efet.01.427593.g660.t1 | NAD(P)-dependent oxidoreductase | 831 | 0 | 83.65 | F:oxidoreductase activity; P:oxidation-reduction process |
| Efet.01.427593.g661.t1 | acyl- synthetase | 1422 | 0 | 74.1 | P:metabolic process; F:o-succinylbenzoate-CoA ligase activity |
| Efet.01.427593.g662.t1 | tripartite tricarboxylate transporter substrate binding | 813 | 0 | 72.2 | C:outer membrane-bounded periplasmic space |
| Efet.01.427593.g663.t1 | family transcriptional regulator | 984 | 0 | 69.8 | F:DNA binding; F:transcription factor activity, sequence-specific DNA binding; P:transcription, DNA-templated; P:regulation of transcription, DNA-templated |
| Efet.01.427593.g664.t1 | 4-oxalocrotonate tautomerase | 321 | 3.43E-26 | 94.45 | P:cellular aromatic compound metabolic process; F:isomerase activity |
| Efet.01.427593.g665.t1 | 4-hydroxy-2-oxovalerate aldolase | 981 | 0 | 91.35 | P:aromatic compound catabolic process; F:manganese ion binding; F:4-hydroxy-2-oxovalerate aldolase activity |
| Efet.01.427593.g666.t1 | acetaldehyde dehydrogenase (acetylating) | 930 | 0 | 96.4 | P:aromatic compound catabolic process; F:NAD binding; F:acetaldehyde dehydrogenase (acetylating) activity; P:oxidation-reduction process |
| Efet.01.427593.g667.t1 | tripartite tricarboxylate transporter substrate binding | 441 | 2.64E-61 | 76.75 | C:outer membrane-bounded periplasmic space |
| Efet.01.427593.g668.t1 | 2-oxo-3-hexenedioate decarboxylase | 687 | 4.34E-141 | 83.8 | P:metabolic process; F:4-oxalocrotonate decarboxylase activity |
| Efet.01.427593.g669.t1 | 2-oxopent-4-enoate hydratase | 654 | 4.54E-153 | 95.85 | P:metabolic process; F:4-oxalocrotonate decarboxylase activity; F:2-oxopent-4-enoate hydratase activity |
| Efet.01.427593.g670.t1 | 2-hydroxy-6-oxo-2,4-heptadienoate hydrolase | 648 | 1.53E-150 | 90.45 | C:integral component of membrane; F:2-hydroxymuconate-semialdehyde hydrolase activity |
| Efet.01.427593.g671.t1 | 2-hydroxymuconic semialdehyde dehydrogenase | 1080 | 0 | 94.8 | F:betaine-aldehyde dehydrogenase activity; P:oxidation-reduction process |
| Efet.01.427595.g672.t1 | TAR DNA-binding 43 isoform X2 | 282 | 2.79E-24 | 81.7 | F:nucleotide binding; F:nucleic acid binding |
| Efet.01.42763.g1157.t1 | filamin-A-like isoform X2 | 1980 | 1.12E-78 | 42.5 | F:actin binding |
| Efet.01.427672.g673.t1 | intraflagellar transport 81 homolog | 345 | 4.01E-28 | 61.2 | P:pronephros development; F:tubulin binding; P:intraciliary transport; C:cilium; C:intraciliary transport particle B; P:regulation of TOR signaling |
| Efet.01.4277.g357.t1 | receptor-type tyrosine- phosphatase delta- | 570 | 2.07E-56 | 62.5 | C:membrane; F:phosphatase activity; P:brain development; P:single-organism cellular process; P:dephosphorylation |
| Efet.01.427722.g675.t1 | nucleolar complex 2 homolog | 390 | 2.70E-13 | 51.85 | C:nuclear part; F:protein binding; P:negative regulation of cellular process; P:cellular process |
| Efet.01.427790.g677.t1 | plectin-like isoform X4 | 939 | 1.74E-52 | 58.6 | C:cytoskeleton |
| Efet.01.427828.g678.t1 | tektin-4-like | 678 | 7.87E-58 | 72.25 |  |
| Efet.01.42798.g1158.t1 | importin-7-like isoform X2 | 486 | 6.77E-36 | 79.55 | C:nuclear envelope; F:SMAD binding; F:histone binding; P:signal transduction; P:protein import into nucleus; P:innate immune response; C:membrane; C:cytosol; F:protein transporter activity; F:Ran GTPase binding; C:nucleoplasm |
| Efet.01.428050.g682.t1 | Cephalotocin receptor 1 | 1149 | 1.72E-80 | 56.65 | P:signal transduction; C:membrane; F:protein-hormone receptor activity |
| Efet.01.42817.g1160.t1 | cortactin-binding 2-like isoform X2 | 318 | 1.95E-21 | 66.9 |  |
| Efet.01.428175.g684.t1 | hypothetical protein BSL78_17803 | 726 | 5.71E-47 | 55.85 |  |
| Efet.01.42823.g1161.t1 | acetylserotonin O-methyltransferase | 261 | 8.53E-21 | 59.7 | F:methyltransferase activity |
| Efet.01.42824.g1162.t1 | gamma-aminobutyric acid type B receptor subunit 2 | 1110 | 7.74E-10 | 64.65 | P:regulation of glucose metabolic process; F:G-protein coupled GABA receptor activity; P:G-protein coupled receptor signaling pathway; C:membrane; C:integral component of membrane; P:sleep |
| Efet.01.428255.g686.t1 | neuromedin-U receptor 2-like | 630 | 2.79E-25 | 70.75 | P:neuropeptide signaling pathway; C:integral component of membrane; F:neuromedin U receptor activity |
| Efet.01.42827.g1163.t1 | microtubule-actin cross-linking factor isoforms 1 2 3 5-like | 1017 | 2.38E-28 | 52.5 | C:cytoskeleton |
| Efet.01.428287.g687.t1 | sugar ABC transporter permease | 540 | 4.06E-99 | 81.15 | C:integral component of membrane; P:transport; C:plasma membrane |
| Efet.01.428304.g688.t1 | cold shock domain-containing E1-like | 345 | 4.90E-31 | 72.95 | F:DNA binding; P:regulation of transcription, DNA-templated |
| Efet.01.428314.g689.t1 | zinc finger C2HC domain-containing 1B | 504 | 2.28E-30 | 68.2 |  |
| Efet.01.428329.g690.t1 | monocarboxylate transporter 12-B isoform X1 [Zeugodacus cucurbitae] | 1917 | 2.18E-88 | 50.55 | P:transmembrane transport; C:membrane; C:integral component of membrane |
| Efet.01.428348.g691.t1 | diguanylate cyclase | 633 | 6.22E-111 | 83.15 | F:dipeptidase activity; P:proteolysis |
| Efet.01.42836.g1164.t1 | cyclic GMP-AMP synthase-like | 1020 | 2.39E-16 | 46.4 |  |
| Efet.01.42847.g1165.t1 | solute carrier family 12 member 4-like isoform X1 | 231 | 4.15E-26 | 72.85 | F:cation:chloride symporter activity; P:chloride transmembrane transport; C:integral component of membrane |
| Efet.01.428470.g692.t1 | cephalotocin receptor 2-like | 831 | 2.35E-57 | 59.15 | C:membrane; P:cellular response to stimulus |
| Efet.01.428573.g694.t1 | hypothetical protein HELRODRAFT_175590 | 234 | 3.36E-06 | 54 |  |
| Efet.01.428610.g696.t1 | neurexin-1a-like isoform X1 | 525 | 2.05E-36 | 59.5 | C:membrane |
| Efet.01.428617.g697.t1 | laminin subunit alpha-3 isoform X4 | 246 | 3.10E-06 | 51 | P:regulation of embryonic development; P:cell adhesion; P:regulation of cell adhesion; P:regulation of cell migration; F:receptor binding |
| Efet.01.42862.g1167.t1 | alkyl aryl-sulfatase BDS1-like | 1041 | 2.84E-83 | 54.5 | C:membrane; C:integral component of membrane; F:protein dimerization activity |
| Efet.01.42862.g1169.t1 | MBL fold metallo-hydrolase | 429 | 1.49E-23 | 70.8 | F:protein dimerization activity |
| Efet.01.428633.g698.t1 | titin-like isoform X2 | 576 | 5.35E-52 | 66.3 |  |
| Efet.01.428663.g700.t1 | NPC2 homolog | 270 | 1.52E-19 | 70.65 |  |
| Efet.01.428700.g701.t1 | UDP-N-acetylhexosamine pyrophosphorylase-like isoform X1 | 288 | 1.55E-18 | 62.9 | P:metabolic process; F:uridylyltransferase activity |
| Efet.01.428708.g702.t1 | follistatin-related 5-like isoform X2 | 294 | 9.36E-15 | 55.1 | F:calcium ion binding |
| Efet.01.428715.g703.t1 | glutamate receptor 3- | 486 | 5.11E-25 | 60.05 | C:postsynapse; F:transmembrane signaling receptor activity; C:integral component of membrane; C:plasma membrane region; F:extracellular ligand-gated ion channel activity; C:neuron projection; P:transport; P:single-organism process; P:cellular process |
| Efet.01.428810.g704.t1 | TBC1 domain family member 16-like | 711 | 8.86E-90 | 78.05 |  |
| Efet.01.428848.g705.t1 | hormonally up-regulated neu tumor-associated kinase | 504 | 9.15E-60 | 85.35 | C:nucleus; F:ATP binding; C:cytoplasm; F:protein serine/threonine kinase activity; P:protein phosphorylation; P:intracellular signal transduction |
| Efet.01.428860.g706.t1 | myotubularin-related 1 | 258 | 4.06E-43 | 73.55 | F:phosphatase activity; P:dephosphorylation |
| Efet.01.428867.g707.t1 | cholinesterase isoform X2 | 822 | 1.62E-56 | 51.8 | C:extracellular region; F:hydrolase activity |
| Efet.01.428900.g708.t1 | Ankyrin repeat and fibronectin type-III domain-containing 1 | 882 | 1.11E-78 | 62.5 | P:signal transduction |
| Efet.01.428927.g710.t1 | G T mismatch-specific thymine DNA glycosylase | 756 | 3.35E-19 | 71.45 | C:nucleus; F:DNA binding; F:uracil DNA N-glycosylase activity; P:base-excision repair, AP site formation; F:pyrimidine-specific mismatch base pair DNA N-glycosylase activity |
| Efet.01.42893.g1170.t1 | beta-1,3-galactosyl-O-glycosyl-glyco beta-1,6-N-acetylglucosaminyltransferase-like | 843 | 2.22E-71 | 69.95 | C:membrane |
| Efet.01.42898.g1171.t1 | cGMP-dependent kinase 1-like | 417 | 4.28E-69 | 92.8 | F:ATP binding; F:cGMP-dependent protein kinase activity; P:protein phosphorylation; F:cGMP binding |
| Efet.01.429104.g712.t1 | Neuropeptide FF receptor 2 | 861 | 8.29E-60 | 64.9 | P:signal transduction; F:signal transducer activity |
| Efet.01.429114.g713.t1 | argininosuccinate lyase | 528 | 1.07E-118 | 94.35 | C:cytoplasm; P:arginine biosynthetic process via ornithine; F:argininosuccinate lyase activity |
| Efet.01.429122.g715.t1 | 30S ribosomal S20 | 402 | 3.83E-55 | 97.9 | F:structural constituent of ribosome; C:ribosome; F:rRNA binding; P:translation |
| Efet.01.429177.g716.t1 | chloride intracellular channel 2-like | 729 | 3.36E-109 | 67 | P:chloride transmembrane transport; F:chloride channel activity; P:negative regulation of ryanodine-sensitive calcium-release channel activity |
| Efet.01.429193.g717.t1 | RNA polymerase subunit sigma | 570 | 4.42E-76 | 69.8 | P:transcription, DNA-templated |
| Efet.01.429232.g719.t1 | Zinc transporter ZIP1 | 1047 | 4.05E-50 | 54.2 | C:membrane; P:transport |
| Efet.01.429296.g720.t1 | calcitonin gene-related peptide type 1 receptor-like | 630 | 5.48E-20 | 46.65 | F:G-protein coupled receptor activity; F:transmembrane signaling receptor activity; P:G-protein coupled receptor signaling pathway; P:signal transduction; C:membrane; C:integral component of membrane; P:cell surface receptor signaling pathway; F:signal transducer activity |
| Efet.01.429319.g721.t1 | hypothetical protein CAPTEDRAFT_228448 | 459 | 5.20E-18 | 69.5 | F:RNA binding; P:RNA processing; F:adenosine deaminase activity |
| Efet.01.429340.g722.t1 | nuclear hormone receptor HR96-like isoform X8 | 1185 | 2.56E-118 | 70.4 | F:DNA binding; F:transcription factor activity, sequence-specific DNA binding; F:metal ion binding; P:signal transduction; F:signaling receptor activity |
| Efet.01.429353.g723.t1 | neuronal acetylcholine receptor subunit alpha-7-like | 201 | 7.59E-31 | 87.45 | C:postsynaptic membrane; P:cation transmembrane transport; C:integral component of membrane; C:cell junction; F:extracellular ligand-gated ion channel activity |
| Efet.01.429413.g724.t1 | unc-93 homolog A-like | 1710 | 2.65E-88 | 52.4 | P:transmembrane transport; C:membrane; C:integral component of membrane |
| Efet.01.429497.g727.t1 | sulfate adenylyltransferase small subunit | 2496 | 0 | 90.75 | P:sulfate reduction; F:sulfate adenylyltransferase (ATP) activity |
| Efet.01.429511.g728.t1 | multiple epidermal growth factor-like domains 8 | 1296 | 5.49E-117 | 59.8 | F:calcium ion binding; C:membrane; C:integral component of membrane |
| Efet.01.429708.g729.t1 | filamin-A-like isoform X1 | 207 | 3.07E-22 | 79.9 | F:actin filament binding |
| Efet.01.429926.g733.t1 | N(G),N(G)-dimethylarginine dimethylaminohydrolase 1-like | 345 | 5.12E-53 | 70.05 | F:dimethylargininase activity; F:hydrolase activity |
| Efet.01.430067.g735.t1 | NEDD8-conjugating enzyme UBE2F-like isoform X2 | 234 | 2.64E-18 | 81.25 | C:nucleus; F:ATP binding; F:ubiquitin protein ligase activity; F:ubiquitin protein ligase binding; C:cytoplasm; P:protein K63-linked ubiquitination; P:postreplication repair |
| Efet.01.430081.g736.t1 | sclerostin domain-containing 1-like | 432 | 2.91E-52 | 71.4 | C:extracellular space; P:multicellular organism development |
| Efet.01.430145.g737.t1 | hypothetical protein CAPTEDRAFT_203099 | 1419 | 1.00E-09 | 63 | C:membrane; C:integral component of membrane |
| Efet.01.430165.g738.t1 | protocadherin gamma-B5-like | 687 | 1.38E-47 | 58.7 | C:membrane |
| Efet.01.430343.g742.t1 | disheveled-associated activator of morphogenesis 1 | 789 | 5.80E-60 | 74 | F:actin binding; C:cytoskeletal part; C:membrane; C:actin cytoskeleton; F:Rho GTPase binding; P:actin cytoskeleton organization |
| Efet.01.430435.g744.t1 | guanylate-binding 1-like | 1056 | 5.67E-32 | 50.75 | F:protein binding; P:negative regulation of cellular process |
| Efet.01.43045.g1174.t1 | translin-associated factor X-interacting 1-like | 504 | 7.00E-14 | 59.86 | F:calcium ion binding |
| Efet.01.430547.g746.t1 | type I-E CRISPR-associated Cse1 | 315 | 3.23E-59 | 98 |  |
| Efet.01.430559.g747.t1 | succinyl- ligase [ADP-forming] subunit mitochondrial | 768 | 2.64E-101 | 74.2 | F:nucleotide binding; F:catalytic activity; F:ion binding |
| Efet.01.430569.g748.t1 | muscarinic acetylcholine receptor M1-like | 1914 | 5.13E-86 | 73.55 | C:integral component of plasma membrane; F:G-protein coupled acetylcholine receptor activity; P:G-protein coupled acetylcholine receptor signaling pathway |
| Efet.01.430627.g749.t1 | DNA-directed RNA polymerase III subunit RPC1-like | 426 | 1.98E-41 | 75.95 | F:RNA polymerase III activity; F:DNA binding; P:transcription from RNA polymerase III promoter; C:DNA-directed RNA polymerase III complex |
| Efet.01.43074.g1175.t1 | guanine nucleotide-binding G(o) subunit alpha | 342 | 1.84E-69 | 94.6 | P:cell adhesion involved in heart morphogenesis; P:sensory perception of sweet taste; P:asymmetric cell division; P:behavioral response to starvation; P:negative regulation of synaptic growth at neuromuscular junction; P:adenylate cyclase-modulating G-protein coupled receptor signaling pathway; P:establishment of glial blood-brain barrier; F:signal transducer activity; F:GTP binding; P:cortical actin cytoskeleton organization; P:ventral cord development; P:negative regulation of dendrite morphogenesis; C:heterotrimeric G-protein complex; F:metal ion binding; F:GTPase activity; P:septate junction assembly; P:establishment of imaginal disc-derived wing hair orientation; P:Wnt signaling pathway; F:G-protein beta/gamma-subunit complex binding; P:calcium-mediated signaling; P:detection of temperature stimulus involved in sensory perception of pain |
| Efet.01.430820.g752.t1 | aldolase | 411 | 1.94E-61 | 74.95 |  |
| Efet.01.430820.g753.t1 | hydroxypyruvate isomerase | 801 | 9.86E-173 | 83.45 | F:hydroxypyruvate isomerase activity |
| Efet.01.430820.g754.t1 | ABC transporter substrate-binding | 981 | 0 | 84.3 | C:outer membrane-bounded periplasmic space |
| Efet.01.430820.g755.t1 | amidase | 534 | 8.21E-119 | 87 | F:amidase activity; F:transferase activity; F:carbon-nitrogen ligase activity, with glutamine as amido-N-donor |
| Efet.01.430820.g756.t1 | amidase | 429 | 1.63E-57 | 81.05 | F:amidase activity; F:transferase activity; F:carbon-nitrogen ligase activity, with glutamine as amido-N-donor |
| Efet.01.430846.g757.t1 | IS110 family transposase | 1041 | 2.87E-136 | 72.45 | F:DNA binding; F:transposase activity; P:transposition, DNA-mediated |
| Efet.01.430853.g758.t1 | pyridine nucleotide-disulfide oxidoreductase | 912 | 1.98E-165 | 89 | P:oxidation-reduction process; F:ferredoxin-NADP+ reductase activity |
| Efet.01.430892.g759.t1 | E3 ubiquitin- ligase TRIP12-like isoform X1 | 801 | 1.74E-55 | 63.8 | F:ubiquitin-protein transferase activity; F:zinc ion binding; F:ligase activity; P:protein ubiquitination |
| Efet.01.430896.g760.t1 | LPP20 lipo | 624 | 6.91E-94 | 66.7 |  |
| Efet.01.4311.g358.t1 | hypothetical protein CAPTEDRAFT_225085 | 1275 | 1.19E-17 | 58.5 | C:membrane; C:integral component of membrane |
| Efet.01.431156.g764.t1 | voltage-gated sodium channel | 210 | 6.01E-18 | 69.85 | P:biological regulation; P:sodium ion transport; P:ion transmembrane transport; F:sodium channel activity; C:integral component of membrane; F:voltage-gated ion channel activity |
| Efet.01.43121.g1176.t1 | E3 ubiquitin- ligase TRIM71-like | 516 | 7.87E-12 | 52.19 | F:zinc ion binding; F:metal ion binding; C:intracellular |
| Efet.01.431242.g766.t1 | hypothetical protein CAPTEDRAFT_136251, partial | 231 | 7.03E-08 | 66 | P:intracellular signal transduction; C:intracellular |
| Efet.01.431304.g767.t1 | DNA-binding SATB2 | 813 | 1.24E-16 | 68.91 | F:DNA binding |
| Efet.01.43142.g1178.t1 | frizzled- | 861 | 1.64E-73 | 68.9 | F:transmembrane signaling receptor activity; C:membrane; P:cell surface receptor signaling pathway |
| Efet.01.43158.g1179.t1 | hypothetical protein HELRODRAFT_188786 | 372 | 3.46E-10 | 76 | C:membrane |
| Efet.01.431626.g772.t1 | alpha-N-acetylglucosaminidase-like isoform X2 | 252 | 1.42E-11 | 66.45 | F:kinase activity |
| Efet.01.431777.g774.t1 | sodium calcium exchanger 3-like | 1818 | 0 | 75.15 | P:cell communication; P:calcium ion transport; C:integral component of membrane; P:sodium ion transmembrane transport; F:calcium:sodium antiporter activity |
| Efet.01.431861.g775.t1 | forkhead box D3 | 1113 | 8.78E-50 | 88.85 | C:nucleus; P:transcription, DNA-templated; P:regulation of transcription, DNA-templated; F:sequence-specific DNA binding; F:RNA polymerase II transcription factor activity, sequence-specific DNA binding |
| Efet.01.432.g51.t1 | MOG interacting and ectopic P-granules 1-like | 3081 | 2.65E-40 | 44.5 | F:metal ion binding |
| Efet.01.432187.g777.t1 | cytoplasmic tyrosine- kinase BMX isoform X1 | 870 | 4.68E-59 | 62.2 | F:protein kinase activity; P:phosphorylation |
| Efet.01.432193.g778.t1 | turtle isoform X1 | 288 | 8.78E-08 | 49.93 |  |
| Efet.01.432204.g779.t1 | arginine N-methyltransferase 9 | 474 | 4.22E-36 | 62.55 | P:biological_process; F:methyltransferase activity; F:transferase activity; P:protein methylation; C:cellular_component; F:molecular_function; P:methylation |
| Efet.01.432304.g781.t1 | transcriptional coactivator YAP1- | 471 | 1.06E-09 | 54.33 |  |
| Efet.01.432410.g782.t1 | sex peptide receptor-like | 489 | 6.48E-50 | 70.85 | F:G-protein coupled peptide receptor activity; P:G-protein coupled receptor signaling pathway; C:integral component of membrane |
| Efet.01.432422.g784.t1 | Protocadherin Fat 4 | 525 | 6.24E-40 | 56.7 | F:calcium ion binding; C:membrane; C:integral component of membrane; P:cell adhesion; P:homophilic cell adhesion via plasma membrane adhesion molecules; C:plasma membrane |
| Efet.01.432532.g786.t1 | coenzyme Q-binding COQ10 homolog mitochondrial-like | 207 | 4.02E-17 | 72 |  |
| Efet.01.432533.g787.t1 | rho GTPase-activating 100F-like isoform X2 | 966 | 5.37E-52 | 65.7 | C:cytoplasm; P:regulation of GTPase activity |
| Efet.01.432687.g788.t1 | hypothetical protein CAPTEDRAFT_222036 | 222 | 1.17E-15 | 63.4 | C:membrane; C:integral component of membrane |
| Efet.01.432741.g789.t1 | innexin unc-9 isoform X6 | 498 | 1.63E-102 | 76.3 | C:gap junction; P:ion transport; P:transmembrane transport; C:integral component of membrane; P:cell communication by electrical coupling; C:synapse; F:gap junction channel activity; C:plasma membrane |
| Efet.01.43285.g1186.t1 | hypothetical protein CAPTEDRAFT_211886 | 756 | 6.40E-36 | 49.6 | C:membrane; C:integral component of membrane; F:carbohydrate binding |
| Efet.01.432886.g793.t1 | transmembrane 94 | 885 | 1.90E-13 | 87.6 | F:nucleotide binding; C:integral component of membrane |
| Efet.01.43291.g1187.t1 | vacuolar sorting-associated 13D | 1317 | 9.39E-51 | 50.9 |  |
| Efet.01.432910.g795.t1 | focadhesin isoform X1 | 213 | 7.18E-12 | 65.9 |  |
| Efet.01.432918.g796.t1 | hypothetical protein HELRODRAFT_182760 | 1260 | 2.19E-39 | 66 |  |
| Efet.01.432939.g798.t1 | histamine H3 receptor-like | 1230 | 9.79E-59 | 53.4 | F:G-protein coupled receptor activity; C:membrane; P:G-protein coupled acetylcholine receptor signaling pathway |
| Efet.01.432961.g799.t1 | sodium-driven chloride bicarbonate exchanger | 579 | 1.42E-37 | 51.15 | P:ion transport |
| Efet.01.433090.g802.t1 | mannosyl-oligosaccharide 1,2-alpha-mannosidase IA-like | 639 | 1.53E-19 | 57.75 | C:membrane; F:hydrolase activity |
| Efet.01.433154.g803.t1 | plasma membrane calcium-transporting ATPase 1- | 876 | 9.26E-119 | 89.2 | F:ATP binding; F:metal ion binding; C:integral component of membrane; P:calcium ion transmembrane transport; F:calcium-transporting ATPase activity |
| Efet.01.433171.g804.t1 | cation-independent mannose-6-phosphate receptor | 747 | 5.95E-20 | 59.1 | C:cytoplasm; C:membrane; C:integral component of membrane; P:transport; F:transporter activity |
| Efet.01.433197.g805.t1 | beta-1,4-N-acetylgalactosaminyltransferase bre-4-like isoform X1 | 705 | 7.29E-32 | 83 | P:carbohydrate metabolic process; F:transferase activity, transferring glycosyl groups |
| Efet.01.433228.g807.t1 | DNA repair RAD50-like | 768 | 2.21E-31 | 56.05 | F:ATPase activity; P:DNA repair; C:nucleus; P:telomere maintenance; C:Mre11 complex |
| Efet.01.43323.g1188.t1 | coiled-coil domain-containing 157-like | 549 | 6.78E-25 | 75.1 |  |
| Efet.01.43341.g1190.t1 | nidogen and EGF-like domain-containing 1 | 567 | 1.13E-25 | 62.2 | P:single-organism process; C:proteinaceous extracellular matrix |
| Efet.01.433467.g810.t1 | neuropeptide SIFamide receptor-like | 822 | 2.54E-53 | 64.75 | F:neuropeptide receptor activity; P:G-protein coupled receptor signaling pathway; C:membrane |
| Efet.01.43354.g1192.t1 | WD repeat-containing 66-like | 210 | 9.41E-10 | 67.55 |  |
| Efet.01.43363.g1193.t1 | serine threonine- kinase 10-like isoform X3 | 666 | 8.45E-37 | 72.65 | F:ATP binding; F:protein serine/threonine kinase activity; P:protein phosphorylation |
| Efet.01.433640.g814.t1 | hypothetical protein CAPTEDRAFT_210438 | 285 | 1.65E-35 | 61.4 |  |
| Efet.01.433650.g815.t1 | WD repeat-containing 90 | 324 | 2.39E-37 | 57.95 |  |
| Efet.01.43376.g1194.t1 | glutaryl- mitochondrial-like | 747 | 1.37E-124 | 86.9 | F:acyl-CoA dehydrogenase activity; F:flavin adenine dinucleotide binding; P:oxidation-reduction process |
| Efet.01.433836.g818.t1 | histone-lysine N-methyltransferase SUV39H2 | 690 | 2.35E-68 | 66.05 | P:negative regulation of transcription from RNA polymerase II promoter; P:chromatin remodeling; F:protein binding; F:metal ion binding; F:histone methyltransferase activity (H3-K9 specific); P:chromatin assembly or disassembly; P:cellular response to hypoxia; C:chromatin; P:histone H3-K9 methylation; C:nucleoplasm |
| Efet.01.43390.g1195.t1 | tctex1 domain-containing 1-like | 330 | 1.31E-19 | 58.85 | C:axoneme |
| Efet.01.433957.g819.t1 | ubiquitin carboxyl-terminal hydrolase 1-like | 915 | 4.03E-06 | 70 |  |
| Efet.01.433992.g820.t1 | electroneutral sodium bicarbonate exchanger 1-like | 753 | 5.86E-57 | 60.55 | P:anion transport; F:transporter activity |
| Efet.01.434.g52.t1 | serine threonine- kinase SBK1-like | 1095 | 1.20E-138 | 71.95 | F:ATP binding; F:protein serine/threonine kinase activity; P:protein phosphorylation |
| Efet.01.434072.g822.t1 | cilia- and flagella-associated 20 | 249 | 1.93E-37 | 100 | P:protein polyglutamylation; C:extracellular exosome; P:cilium assembly; C:centriole; C:ciliary basal body; P:regulation of cilium beat frequency involved in ciliary motility; P:positive regulation of feeding behavior; C:nucleoplasm; C:microtubule; F:RNA binding; P:positive regulation of cell motility |
| Efet.01.434077.g823.t1 | E3 ubiquitin- ligase UBR5-like | 753 | 4.15E-122 | 84.6 | F:ubiquitin-protein transferase activity; F:zinc ion binding; F:RNA binding; F:ubiquitin binding; F:ligase activity; P:protein ubiquitination |
| Efet.01.43410.g1197.t1 | Tyrosine- kinase | 1473 | 2.44E-37 | 85.55 | F:ATP binding; P:peptidyl-tyrosine phosphorylation; F:non-membrane spanning protein tyrosine kinase activity |
| Efet.01.434114.g824.t1 | calmodulin 4 | 372 | 6.82E-15 | 59.8 | F:calcium ion binding |
| Efet.01.434336.g825.t1 | Tudor domain-containing 1 | 2535 | 3.54E-77 | 44.8 |  |
| Efet.01.434359.g826.t1 | branched-chain amino acid ABC transporter substrate-binding | 1239 | 9.73E-136 | 53.25 | P:amino acid transport |
| Efet.01.434359.g827.t1 | 3-phosphoglycerate dehydrogenase | 1125 | 0 | 96.95 | F:oxidoreductase activity, acting on the CH-OH group of donors, NAD or NADP as acceptor; F:NAD binding; P:oxidation-reduction process |
| Efet.01.434360.g828.t1 | CREB-regulated transcription coactivator 1-like isoform X2 | 270 | 2.09E-16 | 73.85 | C:intracellular part |
| Efet.01.43442.g1198.t1 | dynein heavy chain axonemal | 474 | 2.44E-68 | 86.55 | F:ATPase activity; F:ATP binding; F:microtubule motor activity; C:dynein complex; P:microtubule-based movement |
| Efet.01.434454.g829.t1 | zinc finger MYM-type 4-like | 1935 | 1.95E-94 | 56.6 | F:zinc ion binding |
| Efet.01.434538.g831.t1 | 60 kDa neurofilament -like | 387 | 2.88E-40 | 86.1 | F:structural molecule activity; C:intermediate filament |
| Efet.01.43469.g1200.t1 | transcription factor hamlet-like | 660 | 5.04E-54 | 82.2 | F:nucleic acid binding; F:metal ion binding; P:regulation of transcription, DNA-templated |
| Efet.01.434709.g833.t1 | G- coupled receptor 161 | 1065 | 3.61E-12 | 41.8 | P:cellular process; P:response to stimulus |
| Efet.01.434863.g836.t1 | myosin heavy chain | 744 | 2.33E-32 | 68.8 | F:small molecule binding |
| Efet.01.434985.g837.t1 | 50S ribosomal L4 | 465 | 1.65E-92 | 85.05 | F:structural constituent of ribosome; C:ribosome; F:rRNA binding; P:translation |
| Efet.01.434985.g838.t1 | 50S ribosomal L3 | 606 | 6.67E-132 | 94.6 | F:structural constituent of ribosome; C:ribosome; F:rRNA binding; P:translation |
| Efet.01.434985.g839.t1 | 30S ribosomal S10 | 309 | 3.55E-67 | 99.45 | F:structural constituent of ribosome; C:ribosome; F:tRNA binding; P:translation |
| Efet.01.43509.g1201.t1 | nuclear speckle splicing regulatory 1 isoform X1 | 1140 | 3.37E-23 | 64.1 | P:nucleocytoplasmic transport; F:protein binding; C:intracellular ribonucleoprotein complex; P:regulation of alternative mRNA splicing, via spliceosome; C:nuclear speck; F:mRNA binding; P:developmental process |
| Efet.01.43510.g1202.t1 | RING finger nhl-1-like | 990 | 8.75E-09 | 43 |  |
| Efet.01.435123.g841.t1 | FAM207A isoform X2 | 513 | 1.04E-13 | 56.82 |  |
| Efet.01.435187.g842.t1 | ADP-ribosylation factor-like | 432 | 5.78E-62 | 78.6 | F:GTP binding; P:small GTPase mediated signal transduction; C:intracellular |
| Efet.01.435236.g843.t1 | polycystic kidney disease 2-like 1 | 402 | 5.90E-25 | 59.05 | P:detection of mechanical stimulus; F:calcium ion binding; F:calcium channel activity; C:membrane; C:integral component of membrane; P:calcium ion transmembrane transport |
| Efet.01.435286.g844.t1 | rab GDP dissociation inhibitor alpha | 351 | 2.43E-28 | 83.5 | F:Rab GDP-dissociation inhibitor activity; F:oxidoreductase activity; P:small GTPase mediated signal transduction; P:protein transport; P:regulation of catalytic activity; P:oxidation-reduction process; C:intracellular |
| Efet.01.43558.g1203.t1 | PDZ domain-containing GIPC3 | 321 | 1.41E-40 | 85.25 |  |
| Efet.01.43560.g1204.t1 | mannosyl-oligosaccharide 1,2-alpha-mannosidase MNS3-like | 276 | 6.46E-31 | 75.1 | F:calcium ion binding; C:membrane; P:metabolic process; F:mannosyl-oligosaccharide 1,2-alpha-mannosidase activity; C:Golgi apparatus |
| Efet.01.435664.g846.t1 | sushi domain-containing 4-like | 399 | 1.70E-12 | 50.45 | C:membrane; C:integral component of membrane |
| Efet.01.435784.g848.t1 | hillarin isoform X1 | 369 | 1.44E-44 | 65 |  |
| Efet.01.435849.g849.t1 | hypothetical protein HELRODRAFT_109965 | 351 | 1.39E-08 | 83 | P:regulation of postsynaptic membrane potential; P:regulation of ion transmembrane transport; C:voltage-gated sodium channel complex; P:sodium ion transmembrane transport; F:voltage-gated sodium channel activity |
| Efet.01.435887.g850.t1 | gamma-aminobutyric acid type B receptor subunit 2 isoform X1 | 396 | 1.21E-31 | 69.1 | C:membrane; F:signal transducer activity |
| Efet.01.435949.g852.t1 | zinc finger | 975 | 8.31E-26 | 49.6 | F:nucleic acid binding; F:metal ion binding |
| Efet.01.43596.g1205.t1 | mitogen-activated kinase kinase kinase 9-like | 396 | 4.53E-19 | 66.15 | F:protein kinase activity; P:protein phosphorylation |
| Efet.01.436057.g853.t1 | Baculoviral IAP repeat-containing 6 | 1764 | 1.41E-22 | 47.65 | F:ubiquitin-protein transferase activity; P:apoptotic process; P:regulation of cytokinesis; P:protein ubiquitination |
| Efet.01.436067.g854.t1 | PREDICTED: uncharacterized protein LOC106153608 | 540 | 1.33E-36 | 60.8 | F:calcium ion binding |
| Efet.01.43607.g1206.t1 | plexin | 507 | 1.38E-71 | 90.9 | P:motor neuron axon guidance; F:GTPase activator activity; F:heparin binding; P:Ras protein signal transduction; F:axon guidance receptor activity; C:intracellular; P:positive regulation of GTPase activity; C:integral component of plasma membrane; P:sensory neuron axon guidance; P:semaphorin-plexin signaling pathway involved in regulation of photoreceptor cell axon guidance; F:semaphorin receptor activity; P:sensory perception of pain; P:axon midline choice point recognition; F:14-3-3 protein binding |
| Efet.01.436083.g855.t1 | DUF2662 domain-containing | 513 | 1.95E-118 | 76.85 |  |
| Efet.01.43615.g1207.t1 | dopamine receptor 2-like | 1542 | 1.41E-148 | 63.15 | F:G-protein coupled receptor activity; P:G-protein coupled receptor signaling pathway; C:membrane |
| Efet.01.43616.g1208.t1 | leukocyte elastase inhibitor | 285 | 1.87E-10 | 54.2 | F:peptidase inhibitor activity; C:extracellular space; F:serine-type endopeptidase inhibitor activity; C:cytoplasm; P:negative regulation of endopeptidase activity; P:negative regulation of peptidase activity |
| Efet.01.436160.g856.t1 | SZT2 isoform X2 | 1605 | 2.01E-19 | 55.13 | C:peroxisome |
| Efet.01.43618.g1209.t1 | heat shock 70 kDa 12A-like isoform X2 | 1440 | 4.75E-54 | 73.2 | C:membrane |
| Efet.01.436194.g857.t1 | phosphoglycerate mutase | 705 | 4.76E-139 | 90 | P:gluconeogenesis; P:glycolytic process; F:2,3-bisphosphoglycerate-dependent phosphoglycerate mutase activity |
| Efet.01.436197.g858.t1 | hypothetical protein B5766_04330 | 1212 | 7.79E-154 | 67.95 |  |
| Efet.01.436238.g859.t1 | hypothetical protein CRN43_22895 | 330 | 5.29E-19 | 63 |  |
| Efet.01.436285.g860.t1 | ETS-related transcription factor Elf-3-like | 534 | 4.91E-08 | 60.6 | F:DNA binding; C:nucleus; F:transcription factor activity, sequence-specific DNA binding; P:regulation of transcription, DNA-templated; F:sequence-specific DNA binding |
| Efet.01.436318.g861.t1 | ectonucleoside triphosphate diphosphohydrolase 8-like isoform X2 | 204 | 1.59E-11 | 62.15 | C:membrane; C:integral component of membrane; F:hydrolase activity |
| Efet.01.436356.g862.t1 | forkhead box L1 | 831 | 6.28E-42 | 90.7 | C:nucleus; P:heart development; F:transcription factor activity, sequence-specific DNA binding; P:Peyer's patch morphogenesis; P:transcription, DNA-templated; P:visceral mesoderm-endoderm interaction involved in midgut development; P:regulation of transcription, DNA-templated; F:sequence-specific DNA binding; P:regulation of Wnt signaling pathway; P:proteoglycan biosynthetic process |
| Efet.01.43637.g1210.t1 | regulator of cytokinesis 1 isoform X1 | 678 | 1.69E-31 | 57.7 |  |
| Efet.01.436441.g864.t1 | turtle homolog A | 276 | 6.87E-27 | 66.4 | C:inhibitory synapse; C:dendrite; P:regulation of synapse organization; F:protein binding involved in cell-cell adhesion; C:membrane; C:integral component of membrane; P:homophilic cell adhesion via plasma membrane adhesion molecules; C:axon; P:dendrite development |
| Efet.01.436447.g866.t1 | Microtubule-actin cross-linking factor 1 | 1410 | 9.54E-45 | 48.35 | P:cell cycle arrest; C:cytoskeleton; F:actin binding; F:calcium ion binding; C:membrane; C:integral component of membrane |
| Efet.01.436505.g867.t1 | transcriptional repressor | 468 | 1.17E-75 | 83.25 | F:DNA binding; F:transcription factor activity, sequence-specific DNA binding; F:metal ion binding; P:regulation of transcription, DNA-templated |
| Efet.01.436505.g868.t1 | very short patch repair endonuclease | 456 | 3.88E-104 | 82.5 | P:nucleic acid phosphodiester bond hydrolysis; F:endonuclease activity; P:mismatch repair |
| Efet.01.436562.g869.t1 | AP-3 complex subunit beta-2-like isoform X1 | 1200 | 5.84E-97 | 89.05 | P:vesicle-mediated transport; C:clathrin adaptor complex; C:AP-3 adaptor complex; P:intracellular protein transport |
| Efet.01.436613.g870.t1 | cytochrome C | 438 | 1.67E-87 | 74.4 | F:electron carrier activity; C:membrane; C:integral component of membrane; F:heme binding |
| Efet.01.436613.g871.t1 | 6-bladed beta-propeller | 957 | 0 | 88.1 |  |
| Efet.01.436778.g873.t1 | Gamma-aminobutyric acid receptor subunit gamma- | 342 | 4.58E-41 | 69 | P:ion transmembrane transport; C:integral component of membrane; F:extracellular ligand-gated ion channel activity |
| Efet.01.436802.g876.t1 | vigilin-like | 465 | 3.68E-66 | 82.9 | F:cadherin binding involved in cell-cell adhesion; C:nucleus; P:lipid transport; C:cytoplasm; C:cell-cell adherens junction; C:high-density lipoprotein particle; P:cholesterol metabolic process; P:cell-cell adhesion; F:RNA binding |
| Efet.01.436833.g877.t1 | DNA annealing helicase and endonuclease ZRANB3 | 1422 | 1.10E-11 | 68.65 | F:ion binding |
| Efet.01.43699.g1212.t1 | ubiquitin carboxyl-terminal hydrolase CYLD-like isoform X1 | 1059 | 2.00E-50 | 58.05 | P:cellular process; F:hydrolase activity |
| Efet.01.43699.g1213.t1 | ubiquitin carboxyl-terminal hydrolase CYLD isoform X1 | 1299 | 1.35E-45 | 46.05 | P:biological regulation; P:response to stress; F:hydrolase activity |
| Efet.01.436993.g878.t1 | origin recognition complex subunit 1 | 486 | 1.36E-62 | 81.45 | P:cell division; F:ATP binding; P:DNA replication initiation; P:chordate embryonic development; P:regulation of multicellular organism growth; P:response to yeast; F:chromatin binding |
| Efet.01.437065.g879.t1 | thyrotropin-releasing hormone receptor | 1185 | 1.22E-36 | 48.45 | F:G-protein coupled receptor activity; P:G-protein coupled receptor signaling pathway; P:signal transduction; C:membrane; C:integral component of membrane; F:signal transducer activity |
| Efet.01.437078.g880.t1 | pyruvate dehydrogenase phosphatase regulatory mitochondrial-like | 357 | 9.85E-37 | 70.05 | C:cytoplasm; F:oxidoreductase activity; C:mitochondrion; P:oxidation-reduction process |
| Efet.01.437089.g881.t1 | hypothetical protein CAPTEDRAFT_224303 | 267 | 1.15E-14 | 59.5 | C:membrane; C:integral component of membrane |
| Efet.01.437367.g885.t1 | hemocyte -glutamine gamma-glutamyltransferase-like | 417 | 7.48E-09 | 60.13 | F:protein-glutamine gamma-glutamyltransferase activity; F:metal ion binding; P:peptide cross-linking |
| Efet.01.437384.g886.t1 | DNA repair complementing XP-C cells | 414 | 1.90E-22 | 54.65 | C:nuclear part; F:DNA binding; P:DNA repair |
| Efet.01.43739.g1216.t1 | filamin - | 342 | 2.60E-44 | 87.6 | F:actin filament binding |
| Efet.01.437536.g888.t1 | COX assembly mitochondrial homolog | 375 | 3.20E-32 | 73.15 | C:mitochondrion |
| Efet.01.437564.g890.t1 | polypeptide N-acetylgalactosaminyltransferase 1-like | 789 | 1.94E-95 | 55 | C:membrane; C:Golgi apparatus; F:transferase activity |
| Efet.01.437682.g891.t1 | dystrophin-like isoform X1 | 252 | 7.36E-11 | 64.22 | F:binding |
| Efet.01.437753.g893.t1 | family transcriptional regulator | 771 | 0 | 76.8 | F:DNA binding; F:transcription factor activity, sequence-specific DNA binding; P:transcription, DNA-templated; P:regulation of transcription, DNA-templated |
| Efet.01.437777.g895.t1 | ral GTPase-activating subunit beta isoform X1 | 501 | 9.17E-36 | 70 | P:regulation of small GTPase mediated signal transduction; F:GTPase activator activity; P:positive regulation of GTPase activity |
| Efet.01.43785.g1217.t1 | zinc finger MYND domain-containing 10-like | 480 | 1.07E-38 | 69.2 | F:metal ion binding |
| Efet.01.437861.g897.t1 | family transcriptional regulator | 1107 | 0 | 75.1 | F:DNA binding; F:transcription factor activity, sequence-specific DNA binding; P:transcription, DNA-templated; P:regulation of transcription, DNA-templated |
| Efet.01.437929.g898.t1 | zinc phosphodiesterase ELAC 2-like isoform X2 | 348 | 1.59E-07 | 73 | P:tRNA processing |
| Efet.01.437978.g900.t1 | SNW domain-containing 1-like | 354 | 5.40E-47 | 76.55 | C:spliceosomal complex; P:mRNA splicing, via spliceosome |
| Efet.01.438019.g901.t1 | carbohydrate sulfotransferase 11 | 663 | 2.98E-67 | 63.3 | P:skeletal system development; P:embryonic morphogenesis; F:sulfotransferase activity; P:animal organ development; P:primary metabolic process; P:single-organism metabolic process; P:organic substance metabolic process |
| Efet.01.438125.g903.t1 | DET1 homolog | 1680 | 0 | 81.6 |  |
| Efet.01.438239.g906.t1 | nicolin-1-like isoform X3 | 660 | 4.14E-62 | 58.35 |  |
| Efet.01.438273.g907.t1 | phosphatidylinositol 5-phosphate 4-kinase type-2 beta-like | 303 | 1.28E-36 | 80.4 | F:nucleotide binding; F:protein binding; F:1-phosphatidylinositol-4-phosphate 5-kinase activity; F:1-phosphatidylinositol-5-phosphate 4-kinase activity; C:membrane; P:phosphatidylinositol phosphorylation; P:positive regulation of autophagosome assembly; C:autophagosome; C:nucleoplasm |
| Efet.01.438451.g909.t1 | pentatricopeptide repeat-containing mitochondrial | 348 | 3.23E-13 | 67.65 | C:mitochondrial matrix; C:membrane; P:tRNA 3'-end processing; C:integral component of membrane; F:tRNA binding |
| Efet.01.438619.g913.t1 | probable methylmalonate-semialdehyde dehydrogenase [acylating] mitochondrial | 918 | 1.29E-138 | 81.1 | F:methylmalonate-semialdehyde dehydrogenase (acylating) activity; P:oxidation-reduction process |
| Efet.01.438635.g914.t1 | septin-5-like isoform X4 | 201 | 6.05E-14 | 81.95 | F:GTP binding; P:cell division; P:cell cycle |
| Efet.01.438647.g915.t1 | adenylate cyclase type 10-like | 4200 | 1.89E-140 | 46.1 | F:phosphorus-oxygen lyase activity; P:intracellular signal transduction; C:intracellular; P:cyclic nucleotide biosynthetic process |
| Efet.01.43870.g1219.t1 | tyrosine- kinase BTK | 2847 | 6.37E-169 | 52.7 | F:nucleotide binding; P:peptidyl-tyrosine phosphorylation; F:protein tyrosine kinase activity; P:signal transduction; F:anion binding; C:intracellular part; P:immune system process |
| Efet.01.438803.g920.t1 | protocadherin-11 X-linked-like isoform X2 | 2622 | 1.06E-159 | 54.25 | F:calcium ion binding; C:membrane; C:integral component of membrane; P:cell adhesion; P:homophilic cell adhesion via plasma membrane adhesion molecules; C:plasma membrane |
| Efet.01.438810.g921.t1 | NAD-dependent deacetylase sirtuin-6 | 282 | 5.90E-29 | 83.3 | F:NAD-dependent histone deacetylase activity (H3-K9 specific); P:base-excision repair; P:protein ADP-ribosylation; P:negative regulation of cell proliferation; P:negative regulation of glucose import; P:histone H3-K9 modification; P:negative regulation of glycolytic process; F:chromatin binding; C:nucleoplasm; P:protein destabilization; C:nuclear telomeric heterochromatin; P:negative regulation of transcription, DNA-templated; F:transcription corepressor activity; F:NAD(P)+-protein-arginine ADP-ribosyltransferase activity; P:positive regulation of fibroblast proliferation; P:positive regulation of stem cell proliferation; P:glucose homeostasis; P:regulation of double-strand break repair via homologous recombination; P:histone H3-K9 deacetylation; F:NAD+ binding |
| Efet.01.43891.g1220.t1 | alpha-aminoadipic semialdehyde dehydrogenase | 312 | 6.30E-28 | 71.2 | P:metabolic process; F:oxidoreductase activity, acting on the aldehyde or oxo group of donors, NAD or NADP as acceptor |
| Efet.01.43904.g1221.t1 | radixin isoform X4 | 306 | 2.73E-46 | 90.65 | P:establishment or maintenance of apical/basal cell polarity; P:negative regulation of homotypic cell-cell adhesion; P:positive regulation of cellular protein catabolic process; F:cell adhesion molecule binding; P:regulation of organelle assembly; P:cortical microtubule organization; P:sphingosine-1-phosphate signaling pathway; P:cellular protein complex localization; C:brush border; C:microvillus; P:protein kinase A signaling; P:apical protein localization; P:regulation of Rap protein signal transduction; C:cytosol; P:positive regulation of protein secretion; C:cortical actin cytoskeleton; C:lamellipodium; F:protein domain specific binding; P:negative regulation of GTPase activity; P:regulation of ruffle assembly; C:actin filament; C:cytoplasmic side of apical plasma membrane; P:positive regulation of protein localization to early endosome; F:actin filament binding; P:intestinal D-glucose absorption; P:actin filament capping; P:protein localization to cell cortex; P:phosphatidylinositol-mediated signaling; P:positive regulation of gene expression; P:cellular response to cAMP; C:filopodium; P:positive regulation of early endosome to late endosome transport; P:receptor internalization; P:actin filament bundle assembly; P:positive regulation of cell migration; P:regulation of cell shape; C:TCR signalosome; C:myelin sheath; C:cleavage furrow; P:regulation of actin filament bundle assembly; P:terminal web assembly; C:nucleolus; C:plasma membrane raft; P:leukocyte cell-cell adhesion; P:positive regulation of multicellular organism growth; C:immunological synapse; P:negative regulation of transcription from RNA polymerase II promoter; P:cellular response to thyroid hormone stimulus; C:endosome; C:extracellular exosome; C:focal adhesion; P:establishment of centrosome localization; P:negative regulation of cell size; C:ruffle; C:stereocilium; P:actin cytoskeleton reorganization; P:negative regulation of interleukin-2 secretion; F:protein homodimerization activity; P:positive regulation of G1/S transition of mitotic cell cycle; C:ciliary basal body; P:positive regulation of establishment of protein localization to plasma membrane; P:establishment of protein localization to plasma membrane; P:negative regulation of p38MAPK cascade; P:membrane to membrane docking; P:filopodium assembly; P:establishment of endothelial barrier; F:S100 protein binding; F:microtubule binding; F:RNA binding; P:astral microtubule organization; C:extracellular space; C:perinuclear region of cytoplasm; P:negative regulation of T cell receptor signaling pathway; C:uropod; P:negative regulation of ERK1 and ERK2 cascade; F:protein kinase A regulatory subunit binding; P:microvillus assembly; F:protein kinase A catalytic subunit binding; P:regulation of microvillus length; C:extrinsic component of membrane; P:negative regulation of adherens junction organization; F:ATPase binding |
| Efet.01.439123.g924.t1 | electroneutral sodium bicarbonate exchanger 1-like isoform X1 | 477 | 1.73E-38 | 69.35 | P:inorganic anion transport; P:anion transmembrane transport; C:integral component of membrane; F:inorganic anion exchanger activity |
| Efet.01.439171.g925.t1 | C3 and PZP-like alpha-2-macroglobulin domain-containing 8 | 564 | 9.48E-26 | 52.05 | F:catalytic activity; C:membrane; C:integral component of membrane; P:nucleoside metabolic process |
| Efet.01.439176.g926.t1 | peregrin-like isoform X1 | 501 | 1.82E-07 | 49.7 | F:zinc ion binding; F:metal ion binding |
| Efet.01.439279.g927.t1 | tubulin beta-4B chain | 735 | 5.94E-132 | 92 | F:GTP binding; C:nucleus; C:myelin sheath; C:extracellular exosome; C:tubulin complex; C:cytoplasm; F:double-stranded RNA binding; F:GTPase activity; F:structural constituent of cytoskeleton; C:extracellular matrix; C:microtubule; P:microtubule-based process |
| Efet.01.43932.g1223.t1 | UV-stimulated scaffold A-like | 882 | 3.92E-64 | 60 | P:transcription-coupled nucleotide-excision repair; P:response to UV; C:chromosome; F:RNA polymerase II core binding; P:protein ubiquitination |
| Efet.01.43932.g1224.t1 | UV-stimulated scaffold A | 1593 | 5.77E-59 | 69.1 | P:transcription-coupled nucleotide-excision repair; P:response to UV; C:chromosome; F:RNA polymerase II core binding; P:protein ubiquitination |
| Efet.01.439340.g928.t1 | DUF2188 domain-containing | 231 | 6.48E-48 | 92.35 |  |
| Efet.01.439340.g929.t1 | dienelactone hydrolase | 684 | 4.15E-59 | 54.85 | F:hydrolase activity |
| Efet.01.439340.g930.t1 | tRNA glutamyl-Q(34) synthetase | 750 | 2.61E-176 | 80.2 | F:ATP binding; F:aminoacyl-tRNA ligase activity; F:zinc ion binding; C:cytoplasm; P:tRNA aminoacylation; P:tRNA modification; P:translation |
| Efet.01.439340.g931.t1 | PLP-dependent aminotransferase family | 1098 | 0 | 74.3 | F:DNA binding; F:transcription factor activity, sequence-specific DNA binding; F:pyridoxal phosphate binding; F:catalytic activity; P:transcription, DNA-templated; P:regulation of transcription, DNA-templated |
| Efet.01.439345.g932.t1 | hypothetical protein CAPTEDRAFT_224314 | 552 | 5.97E-06 | 57 | F:zinc ion binding; F:metal ion binding |
| Efet.01.439351.g933.t1 | beta-1,3-galactosyltransferase 1-like | 1572 | 1.11E-42 | 50.5 | F:transferase activity, transferring glycosyl groups; C:membrane; C:integral component of membrane; P:protein glycosylation; F:galactosyltransferase activity; C:Golgi apparatus; C:Golgi membrane; F:transferase activity |
| Efet.01.439429.g934.t1 | dynein beta ciliary-like | 417 | 1.25E-16 | 77.85 | F:ATPase activity; F:ATP binding; F:microtubule motor activity; C:cilium; C:dynein complex; P:microtubule-based movement |
| Efet.01.439429.g935.t1 | dynein beta ciliary-like | 420 | 2.60E-28 | 73.2 | F:nucleotide binding; C:microtubule cytoskeleton; C:cytoskeletal part; C:cilium; P:single-organism cellular process; F:motor activity |
| Efet.01.439498.g936.t1 | structural maintenance of chromosomes 1A-like | 492 | 6.68E-43 | 74.15 | C:nucleus; F:ATP binding; P:DNA repair; P:mitotic sister chromatid cohesion; C:chromosome; F:chromatin binding |
| Efet.01.439588.g938.t1 | superkiller viralicidic activity 2-like 2 | 414 | 9.64E-65 | 81.85 | F:ATP binding; P:RNA splicing; P:maturation of 5.8S rRNA; C:catalytic step 2 spliceosome; P:mRNA processing; C:exosome (RNase complex); F:ATP-dependent RNA helicase activity; F:RNA binding; P:RNA catabolic process |
| Efet.01.439592.g939.t1 | histone deacetylase 11-like | 222 | 1.99E-23 | 82.75 | P:protein deacetylation; P:cellular response to DNA damage stimulus; F:protein deacetylase activity |
| Efet.01.439642.g940.t1 | bifunctional phosphoribosylaminoimidazolecarboxamide formyltransferase IMP cyclohydrolase | 471 | 2.32E-35 | 80.4 | F:IMP cyclohydrolase activity; P:'de novo' IMP biosynthetic process; F:phosphoribosylaminoimidazolecarboxamide formyltransferase activity |
| Efet.01.43974.g1225.t1 | Hau-cytoplasmic intermediate filament | 327 | 9.01E-14 | 89.4 | F:structural molecule activity; C:intermediate filament |
| Efet.01.43976.g1226.t1 | zinc finger FYVE domain-containing 16-like isoform X1 | 858 | 7.49E-24 | 87.6 | F:metal ion binding |
| Efet.01.439764.g941.t1 | NAD-dependent DNA ligase | 804 | 0 | 92.55 | F:dioxygenase activity; F:DNA binding; P:DNA repair; F:DNA ligase (NAD+) activity; F:metal ion binding; P:DNA replication; P:DNA ligation; P:oxidation-reduction process |
| Efet.01.439764.g942.t1 | DNA polymerase | 297 | 3.65E-39 | 89.1 |  |
| Efet.01.439787.g943.t1 | mitochondrial-processing peptidase subunit beta | 258 | 4.35E-37 | 85.1 | F:zinc ion binding; C:mitochondrial respiratory chain complex III; P:protein processing; P:mitochondrial electron transport, ubiquinol to cytochrome c; P:aerobic respiration; F:metalloendopeptidase activity |
| Efet.01.439848.g944.t1 | NHL repeat containing | 867 | 3.21E-13 | 46 | F:zinc ion binding; F:metal ion binding; P:proteolysis; P:regulation of apoptotic process; F:peptidase activity; C:intracellular |
| Efet.01.439866.g945.t1 | coiled-coil domain-containing 77-like | 405 | 3.17E-34 | 71.9 |  |
| Efet.01.439915.g946.t1 | ankyrin-3-like isoform X4 | 705 | 1.08E-55 | 65.25 |  |
| Efet.01.439950.g948.t1 | Calpain- isoform A | 432 | 7.22E-65 | 78.85 | P:determination of adult lifespan; P:myoblast fusion; P:larval locomotory behavior; C:neuronal cell body; F:calcium-dependent cysteine-type endopeptidase activity; P:cuticle development; C:cytoplasm; F:calcium ion binding; C:actin cytoskeleton; P:phagocytosis; P:protein autoprocessing; P:neuron remodeling; P:BMP signaling pathway involved in spinal cord dorsal/ventral patterning; P:defense response to fungus |
| Efet.01.43997.g1227.t1 | trans-1,2-dihydrobenzene-1,2-diol dehydrogenase-like isoform X1 | 381 | 2.04E-25 | 77.9 | F:oxidoreductase activity; P:oxidation-reduction process |
| Efet.01.439984.g949.t1 | excinuclease ABC subunit | 282 | 8.01E-55 | 84.05 | F:DNA binding; P:nucleic acid phosphodiester bond hydrolysis; F:excinuclease ABC activity; C:excinuclease repair complex; C:cytoplasm; P:nucleotide-excision repair; P:SOS response |
| Efet.01.440039.g951.t1 | pre translocase subunit | 510 | 6.79E-95 | 75.9 | F:nucleotide binding; F:metal ion binding; P:DNA duplex unwinding; P:DNA-dependent DNA replication; F:helicase activity |
| Efet.01.440292.g955.t1 | Serine threonine- kinase ATR | 222 | 6.27E-25 | 91 | P:DNA repair; P:positive regulation of DNA damage response, signal transduction by p53 class mediator; C:PML body; P:replicative senescence; P:positive regulation of telomerase catalytic core complex assembly; P:protein autophosphorylation; C:Golgi apparatus; P:negative regulation of DNA replication; F:protein serine/threonine kinase activity; P:embryo implantation; F:MutSalpha complex binding; P:establishment of RNA localization to telomere; C:XY body; F:DNA binding; F:ATP binding; P:DNA damage checkpoint; F:MutLalpha complex binding; P:cellular response to gamma radiation; P:regulation of protein binding; P:chromosome organization; P:protein localization to chromosome, telomeric region; P:positive regulation of telomere maintenance via telomerase; P:cellular response to UV; P:peptidyl-serine phosphorylation |
| Efet.01.440373.g959.t1 | ubiquitin carboxyl-terminal hydrolase 7 isoform X2 | 336 | 2.80E-25 | 78.1 | P:protein deubiquitination; C:integral component of membrane; F:thiol-dependent ubiquitinyl hydrolase activity; P:ubiquitin-dependent protein catabolic process |
| Efet.01.440432.g961.t1 | tyrosine- phosphatase Lar-like | 390 | 1.44E-17 | 54.05 | F:phosphoprotein phosphatase activity; F:protein tyrosine phosphatase activity; P:protein dephosphorylation; C:membrane; F:phosphatase activity; C:integral component of membrane; P:dephosphorylation; P:peptidyl-tyrosine dephosphorylation; F:hydrolase activity |
| Efet.01.44048.g1230.t1 | coiled-coil domain-containing 40-like isoform X1 | 501 | 7.33E-37 | 70.85 |  |
| Efet.01.440538.g964.t1 | sulfate ABC transporter permease subunit | 414 | 2.60E-55 | 78.6 | C:integral component of membrane; P:sulfate transmembrane transport; F:ATPase-coupled sulfate transmembrane transporter activity |
| Efet.01.440538.g965.t1 | sulfate ABC transporter ATP-binding | 1005 | 7.85E-129 | 89.6 | F:ATP binding; P:sulfate transmembrane transport; F:ATPase-coupled sulfate transmembrane transporter activity; C:ATP-binding cassette (ABC) transporter complex |
| Efet.01.440538.g966.t1 | disulfide bond formation B | 534 | 2.98E-71 | 88.45 | F:protein disulfide oxidoreductase activity; F:electron carrier activity; C:integral component of membrane; P:oxidation-reduction process; C:plasma membrane |
| Efet.01.440538.g967.t1 | amino-acid N-acetyltransferase | 1347 | 0 | 95.25 | P:arginine biosynthetic process; C:cytoplasm; F:acetyl-CoA:L-glutamate N-acetyltransferase activity |
| Efet.01.440538.g968.t1 | family | 840 | 1.34E-173 | 70.4 |  |
| Efet.01.440538.g969.t1 | family transcriptional regulator | 1056 | 0 | 66.7 | F:transcription factor activity, sequence-specific DNA binding; P:transcription, DNA-templated; P:regulation of transcription, DNA-templated; F:sequence-specific DNA binding |
| Efet.01.440538.g970.t1 | ATP-grasp domain-containing | 1014 | 0 | 57.05 | F:ATP binding; F:metal ion binding |
| Efet.01.440538.g971.t1 | MFS transporter | 927 | 6.08E-177 | 70.8 | C:integral component of membrane |
| Efet.01.440538.g972.t1 | ATP-dependent RNA helicase | 1776 | 0 | 90.65 | F:nucleic acid binding; F:ATP binding; F:ATP-dependent RNA helicase activity |
| Efet.01.440538.g973.t1 | ATP-dependent RNA helicase | 2079 | 0 | 85.6 | F:nucleic acid binding; F:ATP binding; F:ATP-dependent RNA helicase activity |
| Efet.01.440538.g974.t1 | long-chain fatty acid-- ligase | 1695 | 0 | 89.6 | P:metabolic process; F:ligase activity |
| Efet.01.440538.g975.t1 | carnitine dehydratase | 1617 | 0 | 86.75 | F:formyl-CoA transferase activity |
| Efet.01.440538.g976.t1 | acyl- dehydrogenase | 453 | 3.64E-99 | 95.05 | F:acyl-CoA dehydrogenase activity; F:flavin adenine dinucleotide binding; F:butyryl-CoA dehydrogenase activity; P:oxidation-reduction process |
| Efet.01.440538.g977.t1 | acyl- dehydrogenase | 567 | 1.91E-86 | 94.85 | F:acyl-CoA dehydrogenase activity; F:flavin adenine dinucleotide binding; F:butyryl-CoA dehydrogenase activity; P:oxidation-reduction process |
| Efet.01.440538.g978.t1 | acyl :acetate 3-ketoacid transferase | 300 | 4.82E-32 | 93.4 | F:acetate CoA-transferase activity; P:ketone body catabolic process |
| Efet.01.440538.g979.t1 | acyl :acetate 3-ketoacid transferase | 1248 | 0 | 92.45 | F:acetate CoA-transferase activity; P:ketone body catabolic process |
| Efet.01.440538.g980.t1 | alanine-phosphoribitol ligase | 1662 | 0 | 90.7 | F:oxidoreductase activity, acting on CH-OH group of donors; F:flavin adenine dinucleotide binding; P:oxidation-reduction process |
| Efet.01.440538.g981.t1 | sugar ABC transporter substrate-binding | 984 | 0 | 90.85 | C:membrane |
| Efet.01.440538.g982.t1 | ABC transporter permease | 948 | 0 | 93.35 | C:integral component of membrane; P:monosaccharide transport; F:monosaccharide-transporting ATPase activity; C:plasma membrane |
| Efet.01.440538.g983.t1 | sugar ABC transporter ATP-binding | 459 | 2.66E-86 | 88.7 | F:ATPase activity; F:ATP binding; P:carbohydrate transport |
| Efet.01.44055.g1231.t1 | histone-lysine N-methyltransferase NSD2 isoform X1 | 279 | 1.45E-42 | 86.2 | C:nucleus; F:zinc ion binding; F:histone-lysine N-methyltransferase activity; P:embryonic skeletal system development; P:histone H3-K36 methylation; P:cartilage development |
| Efet.01.440683.g987.t1 | sugar ABC transporter ATP-binding | 537 | 1.70E-101 | 79.05 | F:ATPase activity; F:ATP binding |
| Efet.01.440807.g989.t1 | zinc finger MYM-type 1-like | 420 | 1.87E-19 | 70.25 | F:nucleic acid binding; F:protein dimerization activity |
| Efet.01.440815.g990.t1 | inositol-tetrakisphosphate 1-kinase-like | 216 | 5.79E-12 | 65.4 | F:ion binding; F:phosphotransferase activity, alcohol group as acceptor; F:kinase activity |
| Efet.01.44085.g1232.t1 | midasin | 285 | 1.99E-19 | 58.25 | C:intermediate filament cytoskeleton; C:cytoplasm; C:membrane; P:rRNA processing; C:nucleolus; P:ribosomal large subunit assembly; C:preribosome, large subunit precursor |
| Efet.01.44090.g1234.t1 | uncharacterized protein LOC111001920 | 1251 | 1.73E-60 | 65.5 |  |
| Efet.01.440903.g992.t1 | calcium-dependent secretion activator 1- | 579 | 1.04E-101 | 86.9 | C:presynapse; P:dense core granule exocytosis; C:cytosol; P:synaptic vesicle exocytosis |
| Efet.01.440935.g1000.t1 | Cro Cl family transcriptional regulator | 1446 | 0 | 79.6 | P:regulation of transcription, DNA-templated; F:sequence-specific DNA binding |
| Efet.01.440935.g1001.t1 | 2-methylcitrate synthase | 1320 | 0 | 93.2 | C:cytoplasm; P:tricarboxylic acid cycle; F:transferase activity, transferring acyl groups, acyl groups converted into alkyl on transfer |
| Efet.01.440935.g1002.t1 | UDP-N-acetylglucosamine 1-carboxyvinyltransferase | 2028 | 0 | 93.2 | P:regulation of cell shape; P:cell division; F:UDP-N-acetylglucosamine 1-carboxyvinyltransferase activity; C:cytoplasm; P:peptidoglycan biosynthetic process; P:UDP-N-acetylgalactosamine biosynthetic process; P:cell wall organization; P:cell cycle |
| Efet.01.440935.g1003.t1 | ABC transporter ATP-binding | 1623 | 0 | 94.3 | F:ATPase activity; F:ATP binding |
| Efet.01.440935.g1004.t1 | 3-hydroxyacyl- dehydrogenase | 2088 | 0 | 81.05 | P:fatty acid beta-oxidation; F:3-hydroxyacyl-CoA dehydrogenase activity; F:enoyl-CoA hydratase activity |
| Efet.01.440935.g993.t1 | trifunctional transcriptional regulator proline dehydrogenase L-glutamate gamma-semialdehyde dehydrogenase | 1419 | 0 | 87.65 | F:DNA binding; F:1-pyrroline-5-carboxylate dehydrogenase activity; F:transcription factor activity, sequence-specific DNA binding; F:proline dehydrogenase activity; P:transcription, DNA-templated; P:regulation of transcription, DNA-templated; P:proline biosynthetic process; P:oxidation-reduction process; F:oxidoreductase activity, acting on the aldehyde or oxo group of donors, NAD or NADP as acceptor; P:proline catabolic process to glutamate |
| Efet.01.440935.g994.t1 | family transcriptional regulator | 474 | 2.47E-101 | 92.05 | F:transcription factor activity, sequence-specific DNA binding; P:transcription, DNA-templated; P:regulation of transcription, DNA-templated; F:sequence-specific DNA binding; C:intracellular |
| Efet.01.440935.g995.t1 | MFS transporter | 1200 | 0 | 80.25 | P:transmembrane transport; C:integral component of membrane |
| Efet.01.440935.g996.t1 | 4-oxalocrotonate tautomerase | 258 | 4.12E-50 | 66.05 | F:catalytic activity; P:metabolic process |
| Efet.01.440935.g997.t1 | exodeoxyribonuclease III | 777 | 0 | 93.3 | F:DNA binding; P:DNA repair; P:nucleic acid phosphodiester bond hydrolysis; F:endonuclease activity; C:intracellular |
| Efet.01.440935.g998.t1 | 5-methyltetrahydropteroyltriglutamate--homocysteine S-methyltransferase | 2097 | 0 | 84.35 | P:methionine biosynthetic process; F:zinc ion binding; F:5-methyltetrahydropteroyltriglutamate-homocysteine S-methyltransferase activity; P:methylation |
| Efet.01.440935.g999.t1 | family transcriptional regulator | 915 | 0 | 87.6 | F:DNA binding; F:transcription factor activity, sequence-specific DNA binding; P:transcription, DNA-templated; P:regulation of transcription, DNA-templated |
| Efet.01.44102.g1235.t1 | uncharacterized aminotransferase -like | 612 | 4.75E-60 | 57.6 | F:catalytic activity |
| Efet.01.441093.g1006.t1 | unc-93 homolog A-like | 1911 | 5.33E-84 | 53.6 | P:transmembrane transport; C:membrane; C:integral component of membrane |
| Efet.01.44111.g1237.t1 | centrosomal of 290 kDa-like | 357 | 9.74E-10 | 68 | C:nucleus; C:centrosome; P:cilium assembly |
| Efet.01.441139.g1007.t1 | Homeobox orthopedia | 522 | 8.83E-28 | 84.2 | C:nucleus; C:membrane; P:regulation of transcription, DNA-templated; F:sequence-specific DNA binding |
| Efet.01.441173.g1009.t1 | family nutrient uptake outer membrane | 615 | 3.07E-66 | 66.65 |  |
| Efet.01.441227.g1011.t1 | dual 3 ,5 -cyclic-AMP and -GMP phosphodiesterase 11A-like | 1272 | 3.06E-52 | 61.2 | F:hydrolase activity |
| Efet.01.441232.g1012.t1 | hypothetical protein CAPTEDRAFT_207931 | 801 | 3.12E-15 | 71.75 | C:integral component of membrane |
| Efet.01.441255.g1013.t1 | nucleoredoxin-like | 261 | 1.30E-17 | 66.8 | C:cell; P:cell redox homeostasis |
| Efet.01.441327.g1016.t1 | 3-beta-hydroxysteroid-Delta(8),Delta(7)-isomerase | 342 | 5.87E-40 | 73.8 | F:cholestenol delta-isomerase activity; P:hemopoiesis; C:integral component of membrane; C:endoplasmic reticulum; F:C-8 sterol isomerase activity; P:sterol biosynthetic process |
| Efet.01.441429.g1018.t1 | cartilage matrix -like | 630 | 3.85E-20 | 56.5 | F:calcium ion binding; C:collagen trimer |
| Efet.01.44149.g1238.t1 | Collagen alpha-1(XII) chain | 495 | 7.24E-08 | 64.08 | F:calcium ion binding; C:membrane; C:integral component of membrane |
| Efet.01.441490.g1019.t1 | Wnt- | 426 | 1.19E-21 | 78.1 | P:multicellular organism development; C:integral component of membrane; P:Wnt signaling pathway; F:receptor binding; C:proteinaceous extracellular matrix |
| Efet.01.441504.g1020.t1 | 5 -AMP-activated kinase subunit gamma-2 isoform X2 | 357 | 6.28E-22 | 55 | P:regulation of protein phosphorylation; F:binding |
| Efet.01.441530.g1021.t1 | ATP-dependent RNA helicase DDX42 | 357 | 1.17E-15 | 70.65 | F:nucleic acid binding; F:ATP binding; F:helicase activity |
| Efet.01.441611.g1023.t1 | SPRY domain-containing SOCS box 1-like | 789 | 1.56E-136 | 85.3 | C:intracellular; P:intracellular signal transduction |
| Efet.01.441739.g1025.t1 | hypothetical protein LOTGIDRAFT_103177, partial | 279 | 1.52E-06 | 75 |  |
| Efet.01.441748.g1026.t1 | monocarboxylate transporter 13-like isoform X1 | 849 | 8.83E-45 | 52.2 | C:integral component of plasma membrane; P:transmembrane transport; C:membrane; C:integral component of membrane; P:plasma membrane lactate transport; F:lactate transmembrane transporter activity |
| Efet.01.441784.g1027.t1 | hypothetical protein HELRODRAFT_92217 | 555 | 3.88E-29 | 59.4 |  |
| Efet.01.441807.g1028.t1 | FMRFamide receptor | 1539 | 9.69E-44 | 51.9 | F:G-protein coupled receptor activity; P:G-protein coupled receptor signaling pathway; P:signal transduction; C:membrane; C:integral component of membrane; F:signal transducer activity |
| Efet.01.441817.g1029.t1 | Poly [ADP-ribose] polymerase 12 | 861 | 3.09E-36 | 47.45 | F:metal ion binding; F:NAD+ ADP-ribosyltransferase activity |
| Efet.01.441947.g1031.t1 | serine threonine- kinase PAK 3-like | 273 | 2.07E-35 | 80.35 | F:protein kinase binding; F:identical protein binding; P:negative regulation of cysteine-type endopeptidase activity involved in execution phase of apoptosis; P:protein autophosphorylation; P:signal transduction by protein phosphorylation; P:mitotic cell cycle; P:dendritic spine development; C:nucleoplasm; F:cadherin binding involved in cell-cell adhesion; F:ATP binding; F:signal transducer, downstream of receptor, with serine/threonine kinase activity; C:cytoplasm; C:cell-cell adherens junction; P:axonogenesis; F:protein tyrosine kinase activator activity; P:positive regulation of extrinsic apoptotic signaling pathway; P:peptidyl-serine phosphorylation; P:actin cytoskeleton organization; P:cellular response to organic cyclic compound; P:positive regulation of peptidyl-tyrosine phosphorylation |
| Efet.01.442011.g1033.t1 | serine threonine- kinase LATS2-like | 1062 | 2.65E-16 | 69.35 | F:nucleotide binding; C:nucleus; P:G1/S transition of mitotic cell cycle; P:positive regulation of apoptotic process; C:spindle pole; P:hippo signaling; P:regulation of organ growth; F:protein serine/threonine kinase activity; P:peptidyl-serine phosphorylation; P:convergent extension involved in gastrulation |
| Efet.01.44203.g1239.t1 | solute carrier family 15 member 4-like | 420 | 2.59E-20 | 58.7 | P:oligopeptide transport; C:membrane; C:integral component of membrane; P:transport; F:transporter activity |
| Efet.01.442087.g1036.t1 | solute carrier family 22 member 15-like | 699 | 1.43E-74 | 58.65 | F:transmembrane transporter activity; C:integral component of membrane |
| Efet.01.442105.g1037.t1 | inorganic phosphate cotransporter | 231 | 1.37E-15 | 65.4 | C:membrane |
| Efet.01.442147.g1038.t1 | gamma-aminobutyric acid receptor subunit beta-like | 234 | 9.49E-22 | 75.35 | P:signal transduction; P:ion transmembrane transport; C:integral component of membrane; F:GABA-A receptor activity; C:cell junction; C:GABA-A receptor complex; F:extracellular ligand-gated ion channel activity; C:synapse; P:chloride transport; C:plasma membrane |
| Efet.01.442188.g1039.t1 | hypothetical protein | 417 | 2.38E-06 | 53 |  |
| Efet.01.442188.g1040.t1 | hypothetical protein LR48_Vigan10s001700 | 303 | 5.47E-08 | 50 |  |
| Efet.01.442193.g1041.t1 | E3 ubiquitin- ligase HECTD1-like isoform X2 | 984 | 7.29E-126 | 69.6 | F:catalytic activity |
| Efet.01.442201.g1042.t1 | macoilin-like isoform X2 | 906 | 6.62E-29 | 61.6 | C:membrane |
| Efet.01.44229.g1240.t1 | tripartite motif-containing 66-like isoform X1 | 447 | 1.56E-15 | 68.05 | F:metal ion binding |
| Efet.01.442571.g1049.t1 | hypothetical protein CAPTEDRAFT_228177 | 999 | 1.82E-86 | 55.65 | C:membrane; C:integral component of membrane; P:cellular developmental process |
| Efet.01.442625.g1052.t1 | mitochondrial ribonuclease P 3 | 1260 | 2.14E-15 | 49.85 | C:mitochondrion; C:mitochondrial nucleoid |
| Efet.01.442655.g1053.t1 | family transcriptional regulator | 744 | 1.13E-164 | 84.2 | F:transcription factor activity, sequence-specific DNA binding; P:transcription, DNA-templated; P:regulation of transcription, DNA-templated; F:sequence-specific DNA binding |
| Efet.01.442655.g1054.t1 | tripartite tricarboxylate transporter substrate binding | 996 | 0 | 69.15 | C:outer membrane-bounded periplasmic space |
| Efet.01.442655.g1055.t1 | 3-ketoacyl-ACP reductase [Burkholderia stagnalis] | 771 | 1.60E-162 | 75.45 |  |
| Efet.01.442655.g1056.t1 | aldo keto reductase | 1158 | 0 | 85.5 | F:pyridoxal 4-dehydrogenase activity; P:oxidation-reduction process |
| Efet.01.442696.g1057.t1 | PREDICTED: uncharacterized protein LOC106163611 | 1008 | 2.91E-80 | 61.35 |  |
| Efet.01.44276.g1242.t1 | solute carrier family 23 member 1-like | 213 | 2.00E-06 | 78 |  |
| Efet.01.442887.g1062.t1 | histone-lysine N-methyltransferase ASH1L-like isoform X10 | 540 | 2.71E-68 | 73.35 | F:metal ion binding; F:methyltransferase activity; P:methylation |
| Efet.01.442976.g1065.t1 | BTB and MATH domain-containing 38-like | 663 | 2.67E-76 | 56.85 |  |
| Efet.01.44302.g1245.t1 | zinc finger 883-like | 1632 | 6.16E-31 | 45.95 | F:nucleic acid binding; F:metal ion binding |
| Efet.01.443028.g1066.t1 | Ecdysone-induced 74EF isoform A | 1197 | 4.77E-33 | 82.8 | C:nucleus; F:transcription factor activity, sequence-specific DNA binding; P:regulation of transcription, DNA-templated; F:sequence-specific DNA binding |
| Efet.01.443045.g1067.t1 | alpha-1A adrenergic receptor-like | 792 | 3.86E-95 | 64.45 | F:G-protein coupled receptor activity; P:G-protein coupled receptor signaling pathway; C:integral component of membrane |
| Efet.01.443110.g1068.t1 | PREDICTED: uncharacterized protein LOC106151284, partial | 633 | 1.54E-16 | 73.05 | C:integral component of membrane |
| Efet.01.443238.g1069.t1 | zinc finger FYVE domain-containing 1-like | 1248 | 3.44E-21 | 54.15 | F:zinc ion binding; F:metal ion binding; C:intracellular |
| Efet.01.443364.g1070.t1 | protocadherin beta-16-like | 1128 | 2.32E-51 | 52.15 | F:calcium ion binding; C:membrane; C:integral component of membrane; P:cell adhesion; P:homophilic cell adhesion via plasma membrane adhesion molecules; C:plasma membrane |
| Efet.01.443439.g1071.t1 | tumor p63-regulated gene 1 | 537 | 1.02E-56 | 63.45 | F:identical protein binding; C:extracellular exosome |
| Efet.01.443453.g1072.t1 | zygotic DNA replication licensing factor mcm6-B-like | 213 | 6.98E-18 | 92.55 | C:nucleus; F:DNA binding; F:ATP binding; P:DNA replication initiation; F:DNA helicase activity; P:DNA duplex unwinding; C:MCM complex |
| Efet.01.44352.g1247.t1 | hypothetical protein HELRODRAFT_160513 | 1524 | 9.08E-13 | 59 |  |
| Efet.01.443535.g1073.t1 | F-box leucine rich repeat | 687 | 1.49E-27 | 51.45 |  |
| Efet.01.443551.g1074.t1 | pre-mRNA-splicing factor ATP-dependent RNA helicase PRP1 | 510 | 1.23E-99 | 93.45 | C:U12-type spliceosomal complex; F:ATP binding; F:protein binding; C:cytoplasm; F:double-stranded RNA binding; P:response to alkaloid; C:nucleolus; P:response to toxic substance; C:U2-type post-mRNA release spliceosomal complex; F:ATP-dependent RNA helicase activity; F:RNA binding; P:mRNA splicing, via spliceosome |
| Efet.01.443588.g1075.t1 | angiotensin-converting enzyme-like | 294 | 1.41E-37 | 79.85 | F:NAD+ kinase activity; F:carboxypeptidase activity; F:metal ion binding; F:peptidyl-dipeptidase activity; P:proteolysis; C:integral component of membrane; P:NAD metabolic process; P:phosphorylation; P:NADP biosynthetic process; F:metallopeptidase activity |
| Efet.01.44359.g1248.t1 | probable 39S ribosomal mitochondrial isoform X2 | 261 | 5.07E-21 | 68.25 | C:intracellular organelle; C:cytoplasmic part |
| Efet.01.443609.g1078.t1 | hypothetical protein CAPTEDRAFT_203895 | 258 | 1.20E-13 | 60.65 | C:membrane; C:integral component of membrane; F:carbohydrate binding |
| Efet.01.44371.g1249.t1 | probable splicing factor 3B subunit 5 | 258 | 2.61E-55 | 95.75 | C:precatalytic spliceosome; P:spermatogenesis; C:U2 snRNP; P:mRNA splicing, via spliceosome |
| Efet.01.443879.g1081.t1 | vesicle transport USE1-like | 342 | 2.25E-15 | 59.15 | F:nucleotide binding; C:myosin complex; F:ATP binding; P:signal transduction; F:metal ion binding; F:GTPase activator activity; C:membrane; C:integral component of membrane; C:endoplasmic reticulum; P:intracellular signal transduction; P:positive regulation of GTPase activity; F:motor activity |
| Efet.01.44391.g1250.t1 | insulin-like growth factor 2 mRNA-binding 1 isoform X3 | 216 | 2.31E-10 | 68.05 | F:nucleotide binding; P:posttranscriptional regulation of gene expression; C:cytoplasm; F:mRNA binding; C:macromolecular complex |
| Efet.01.443911.g1082.t1 | UDP-N-acetylhexosamine pyrophosphorylase-like | 474 | 1.61E-49 | 73.4 | F:identical protein binding; F:UDP-N-acetylglucosamine diphosphorylase activity; C:cytosol; F:carbohydrate binding; C:plasma membrane; P:UDP-N-acetylglucosamine biosynthetic process; C:nucleoplasm |
| Efet.01.443977.g1085.t1 | FMRFamide receptor-like | 1293 | 2.04E-71 | 52.05 | F:signal transducer activity |
| Efet.01.443983.g1086.t1 | TANC2-like isoform X4 | 1443 | 3.08E-148 | 76.35 | F:zinc ion binding |
| Efet.01.444098.g1087.t1 | FMRFamide receptor-like | 594 | 7.73E-38 | 59.86 | F:G-protein coupled receptor activity; C:membrane |
| Efet.01.444142.g1088.t1 | polypeptide N-acetylgalactosaminyltransferase 10 | 369 | 1.01E-42 | 76.2 | F:transferase activity, transferring glycosyl groups; C:integral component of membrane; P:protein glycosylation; C:Golgi membrane; F:carbohydrate binding |
| Efet.01.444232.g1089.t1 | homeobox Nkx- | 933 | 3.87E-29 | 78.7 | P:oligodendrocyte development; P:ventral spinal cord interneuron fate determination; P:negative regulation of neuron differentiation; P:positive regulation of neuron differentiation; F:sequence-specific DNA binding; P:positive regulation of transcription from RNA polymerase II promoter; P:positive regulation of sequence-specific DNA binding transcription factor activity; P:spinal cord oligodendrocyte cell fate specification; P:positive regulation of oligodendrocyte differentiation; F:transcription factor activity, sequence-specific DNA binding; P:pancreatic A cell fate commitment; P:type B pancreatic cell fate commitment; P:neuron fate specification; P:pancreatic PP cell fate commitment; P:digestive tract development; P:floor plate formation; P:astrocyte differentiation; P:spinal cord motor neuron differentiation; P:smoothened signaling pathway; F:chromatin binding; F:transcription factor binding; F:core promoter proximal region DNA binding; P:type B pancreatic cell development; C:nucleus; F:transcription coactivator activity |
| Efet.01.444254.g1090.t1 | homeobox engrailed-1-B-like | 480 | 2.53E-18 | 76.45 | C:growth cone; P:positive regulation of phosphorylation; C:nucleus; P:positive regulation of translation; C:filopodium; P:regulation of transcription, DNA-templated; P:segment polarity determination; P:axon guidance; F:sequence-specific DNA binding; P:negative regulation of neuron death; P:brain development; C:lamellipodium |
| Efet.01.444264.g1091.t1 | hypothetical protein HELRODRAFT_171280 | 750 | 4.73E-10 | 68 | C:membrane; C:integral component of membrane |
| Efet.01.44441.g1252.t1 | hypothetical protein CAPTEDRAFT_218471 | 903 | 2.51E-10 | 65 | C:membrane; C:integral component of membrane |
| Efet.01.444576.g1093.t1 | proteasome subunit alpha type-5 | 207 | 1.23E-29 | 92.7 | C:nucleus; C:cytoplasm; C:proteasome core complex, alpha-subunit complex; F:threonine-type endopeptidase activity; P:ubiquitin-dependent protein catabolic process |
| Efet.01.444657.g1094.t1 | FMRFamide receptor-like | 1320 | 3.01E-75 | 53.6 | F:G-protein coupled receptor activity; F:G-protein coupled peptide receptor activity; P:G-protein coupled receptor signaling pathway; P:signal transduction; C:membrane; C:integral component of membrane; F:signal transducer activity |
| Efet.01.444678.g1095.t1 | bifunctional glutamine-synthetase adenylyltransferase deadenyltransferase | 606 | 1.28E-78 | 91 | F:ATP binding; F:[glutamate-ammonia-ligase] adenylyltransferase activity |
| Efet.01.44468.g1254.t1 | dachsous 2 (Drosophila) isoform CRA_b | 1476 | 6.23E-33 | 46.65 | P:biological_process; P:protein dephosphorylation; P:cell adhesion; P:homophilic cell adhesion via plasma membrane adhesion molecules; C:cellular_component; F:molecular_function; C:plasma membrane; P:polysaccharide catabolic process; F:protein tyrosine phosphatase activity; F:calcium ion binding; C:membrane; C:integral component of membrane; F:phosphatase activity; P:dephosphorylation; F:carbohydrate binding; P:peptidyl-tyrosine dephosphorylation; F:hydrolase activity |
| Efet.01.44468.g1255.t1 | protocadherin alpha-3- | 1056 | 2.67E-47 | 55.05 | F:calcium ion binding; C:membrane; C:integral component of membrane; P:cell adhesion; P:homophilic cell adhesion via plasma membrane adhesion molecules; C:plasma membrane |
| Efet.01.444733.g1098.t1 | lysophospholipid acyltransferase 7-like | 261 | 3.95E-11 | 67.19 | C:membrane; F:transferase activity |
| Efet.01.444780.g1100.t1 | adenosine 3 -phospho 5 -phosphosulfate transporter 1-like | 906 | 2.20E-112 | 72.05 | P:transmembrane transport; C:intracellular membrane-bounded organelle; C:integral component of membrane; C:endomembrane system; C:cytoplasmic part; C:intracellular organelle part |
| Efet.01.44481.g1257.t1 | Neuroligin- X-linked | 309 | 2.57E-34 | 83.35 | C:integral component of plasma membrane; P:postsynaptic membrane assembly; F:neurexin family protein binding; P:metabolic process; P:neuron cell-cell adhesion; P:modulation of synaptic transmission; C:synapse; F:receptor activity; F:carboxylic ester hydrolase activity; C:cell surface |
| Efet.01.444843.g1102.t1 | vacuolar sorting-associated 53 homolog | 699 | 1.65E-71 | 63.55 | C:cytoplasmic part |
| Efet.01.444919.g1104.t1 | inhibitor of Bruton tyrosine kinase isoform X2 | 870 | 4.96E-70 | 69.4 | P:phosphorylation; F:kinase activity |
| Efet.01.444948.g1105.t1 | family | 720 | 9.90E-139 | 55.5 | P:transport; F:transporter activity |
| Efet.01.444984.g1107.t1 | von Willebrand factor type EGF and pentraxin domain-containing 1-like | 624 | 3.19E-16 | 46.75 | F:calcium ion binding; C:membrane; C:integral component of membrane; F:hydrolase activity |
| Efet.01.44507.g1259.t1 | proline-rich transmembrane 4-like | 2106 | 5.07E-13 | 43.75 | C:membrane; C:integral component of membrane |
| Efet.01.445086.g1109.t1 | Paired amphipathic helix (PAH2) superfamily | 1110 | 2.26E-12 | 57.7 | C:nucleus; P:regulation of transcription, DNA-templated |
| Efet.01.445097.g1110.t1 | 2-hydroxy-3-oxopropionate reductase | 885 | 0 | 88.45 | F:phosphogluconate dehydrogenase (decarboxylating) activity; F:NAD binding; P:oxidation-reduction process; F:2-hydroxy-3-oxopropionate reductase activity |
| Efet.01.445097.g1111.t1 | aspartate aminotransferase family | 1281 | 0 | 91.1 | F:pyridoxal phosphate binding; F:transaminase activity |
| Efet.01.445097.g1112.t1 | glutamine ABC transporter substrate-binding | 828 | 2.20E-120 | 88.65 | C:membrane; P:ionotropic glutamate receptor signaling pathway; F:ionotropic glutamate receptor activity; P:transport |
| Efet.01.445097.g1113.t1 | glutamine ABC transporter permease | 531 | 1.47E-93 | 91.05 | C:integral component of membrane; P:transport; C:plasma membrane; F:transporter activity |
| Efet.01.445097.g1114.t1 | glutamine ABC transporter ATP-binding | 450 | 2.72E-100 | 92.3 | F:ATP binding; P:amino acid transmembrane transport; F:amino acid-transporting ATPase activity |
| Efet.01.445097.g1115.t1 | peptidase C45 | 960 | 0 | 73.2 |  |
| Efet.01.445097.g1116.t1 | iron-sulfur cluster carrier | 1092 | 0 | 95.4 | F:ATPase activity; F:ATP binding; F:metal ion binding; F:iron-sulfur cluster binding |
| Efet.01.445097.g1117.t1 | N-acetylmuramoyl-L-alanine amidase | 510 | 7.41E-97 | 91.85 | P:peptidoglycan catabolic process; F:N-acetylmuramoyl-L-alanine amidase activity |
| Efet.01.445119.g1118.t1 | APC family permease | 948 | 9.42E-176 | 86.7 | C:integral component of membrane; F:amino acid transmembrane transporter activity; P:amino acid transmembrane transport |
| Efet.01.445197.g1119.t1 | Lysine-specific demethylase | 516 | 1.98E-27 | 66.75 | P:negative regulation of transcription, DNA-templated; F:oxidoreductase activity; P:histone H3-K4 demethylation; F:histone demethylase activity (H3-K4 specific); F:binding; C:nucleoplasm |
| Efet.01.445217.g1120.t1 | methylated-DNA-- -cysteine methyltransferase-like | 513 | 6.94E-31 | 60.95 | P:DNA repair; F:methyltransferase activity |
| Efet.01.445227.g1121.t1 | potassium voltage-gated channel subfamily A member 1 isoform X1 | 1005 | 1.88E-130 | 88.25 | F:voltage-gated potassium channel activity; P:potassium ion transmembrane transport; C:voltage-gated potassium channel complex; P:regulation of ion transmembrane transport; P:protein homooligomerization |
| Efet.01.445279.g1123.t1 | poly [ADP-ribose] polymerase 12-like | 630 | 1.29E-37 | 71 | F:binding; F:NAD+ ADP-ribosyltransferase activity |
| Efet.01.44535.g1262.t1 | hypothetical protein HELRODRAFT_160497 | 366 | 4.77E-29 | 80 | C:integral component of membrane |
| Efet.01.445355.g1125.t1 | Kyphoscoliosis peptidase | 966 | 1.15E-72 | 59.7 |  |
| Efet.01.44536.g1263.t1 | hypothetical protein CAPTEDRAFT_221016 | 315 | 1.96E-39 | 74.55 | C:integral component of membrane |
| Efet.01.445400.g1127.t1 | renin receptor-like | 213 | 4.65E-22 | 77.2 | P:biological regulation; C:integral component of membrane; F:receptor activity |
| Efet.01.445406.g1128.t1 | arylsulfatase B-like | 432 | 4.07E-12 | 61.7 | F:catalytic activity; F:sulfuric ester hydrolase activity; P:metabolic process |
| Efet.01.44544.g1264.t1 | PCNA-interacting partner | 846 | 8.62E-56 | 52.5 | C:intracellular part |
| Efet.01.445459.g1129.t1 | Transmembrane 145 | 555 | 8.98E-53 | 62.75 | P:G-protein coupled receptor signaling pathway; C:membrane; C:integral component of membrane; P:response to pheromone |
| Efet.01.44551.g1265.t1 | unconventional myosin-Ib isoform X6 | 462 | 4.28E-42 | 71.5 | F:nucleotide binding |
| Efet.01.445529.g1131.t1 | ankyrin repeat domain-containing 50-like | 1404 | 1.53E-41 | 59.25 | P:phosphorylation; F:kinase activity |
| Efet.01.44565.g1267.t1 | transcription factor E2F5 | 462 | 1.11E-09 | 54.8 |  |
| Efet.01.445722.g1135.t1 | coilin-like isoform X1 | 315 | 5.34E-08 | 58 |  |
| Efet.01.445984.g1137.t1 | gastrula zinc finger -like | 1602 | 1.71E-60 | 52.35 | F:nucleic acid binding; P:biological_process; F:metal ion binding; C:cellular_component; F:molecular_function |
| Efet.01.446.g53.t1 | titin-like isoform X48 | 2211 | 4.68E-127 | 53.05 |  |
| Efet.01.446018.g1138.t1 | neuropeptide precursor | 540 | 1.18E-13 | 55 | P:neuropeptide signaling pathway |
| Efet.01.446074.g1139.t1 | filamin-C-like isoform X1 | 444 | 2.16E-37 | 81.55 | F:actin filament binding |
| Efet.01.446089.g1140.t1 | sorbitol dehydrogenase-like | 330 | 1.38E-23 | 66 | F:zinc ion binding; F:metal ion binding; F:oxidoreductase activity; P:oxidation-reduction process |
| Efet.01.446102.g1141.t1 | sodium channel type 4 subunit alpha B-like isoform X1 | 300 | 1.30E-29 | 69 | P:regulation of postsynaptic membrane potential; F:calcium ion binding; P:regulation of ion transmembrane transport; C:voltage-gated sodium channel complex; P:sodium ion transmembrane transport; P:membrane depolarization during action potential; F:voltage-gated sodium channel activity; P:neuronal action potential |
| Efet.01.446106.g1142.t1 | CUB domain-containing 2 | 234 | 1.98E-10 | 59.15 | F:calcium ion binding; C:membrane; F:receptor activity; P:receptor-mediated endocytosis |
| Efet.01.446132.g1143.t1 | tolloid 1 | 684 | 1.69E-08 | 51 | F:calcium ion binding; C:membrane; C:integral component of membrane |
| Efet.01.44619.g1269.t1 | R3H domain-containing 4 | 780 | 2.43E-44 | 52 | F:nucleic acid binding; C:membrane; C:integral component of membrane |
| Efet.01.446197.g1146.t1 | uncharacterized acetyltransferase - | 393 | 1.39E-09 | 49 |  |
| Efet.01.44625.g1270.t1 | hypothetical protein HELRODRAFT_181350 | 219 | 1.75E-09 | 69 | C:membrane; C:integral component of membrane |
| Efet.01.446255.g1147.t1 | hypothetical protein CAPTEDRAFT_223880 | 234 | 1.08E-09 | 75.8 |  |
| Efet.01.44631.g1272.t1 | hypothetical protein HELRODRAFT_184293 | 237 | 8.78E-06 | 67 | F:G-protein coupled receptor activity; P:G-protein coupled receptor signaling pathway; C:membrane; C:integral component of membrane |
| Efet.01.446335.g1148.t1 | carnitine O-palmitoyltransferase liver isoform-like isoform X1 | 249 | 2.48E-09 | 81.3 |  |
| Efet.01.44643.g1273.t1 | PR domain zinc finger 13 | 531 | 4.92E-32 | 95.05 | P:negative regulation of transcription from RNA polymerase II promoter; C:nucleus; F:DNA binding; F:metal ion binding; P:transcription, DNA-templated; F:histone methyltransferase activity; P:histone methylation; P:neurogenesis; F:chromatin binding |
| Efet.01.446475.g1150.t1 | alpha-2A adrenergic receptor-like | 774 | 2.84E-50 | 76.3 | F:adrenergic receptor activity; C:integral component of membrane; P:adrenergic receptor signaling pathway; C:plasma membrane |
| Efet.01.446475.g1151.t1 | alpha-2C adrenergic receptor-like | 1110 | 4.58E-27 | 78.65 | C:integral component of plasma membrane; F:adrenergic receptor activity; P:adrenergic receptor signaling pathway; F:G-protein coupled acetylcholine receptor activity; P:G-protein coupled acetylcholine receptor signaling pathway; F:transferase activity |
| Efet.01.446498.g1153.t1 | sacsin-like | 480 | 4.55E-25 | 65.55 |  |
| Efet.01.44655.g1274.t1 | Alpha-(1,3)-fucosyltransferase C | 684 | 4.82E-09 | 47.85 | F:transferase activity, transferring glycosyl groups; F:3-galactosyl-N-acetylglucosaminide 4-alpha-L-fucosyltransferase activity; F:fucosyltransferase activity; C:Golgi cisterna membrane; P:fucosylation; C:membrane; C:integral component of membrane; P:protein glycosylation; C:Golgi apparatus; F:transferase activity |
| Efet.01.44655.g1275.t1 | Alpha--fucosyltransferase C | 576 | 9.74E-38 | 58.05 | F:transferase activity, transferring glycosyl groups; F:fucosyltransferase activity; C:Golgi cisterna membrane; P:fucosylation; C:membrane; C:integral component of membrane; P:protein glycosylation; C:Golgi apparatus; F:transferase activity |
| Efet.01.446673.g1155.t1 | Roundabout | 318 | 1.78E-37 | 70.85 |  |
| Efet.01.44674.g1276.t1 | bicaudal D-like | 1965 | 0 | 65.1 | C:Golgi apparatus; P:transport |
| Efet.01.44676.g1278.t1 | hypothetical protein CAPTEDRAFT_32685, partial | 2436 | 7.52E-121 | 52.8 | F:calcium ion binding; C:membrane; C:integral component of membrane; P:cell adhesion; P:homophilic cell adhesion via plasma membrane adhesion molecules; C:plasma membrane |
| Efet.01.446910.g1156.t1 | coiled-coil domain-containing 83-like | 279 | 1.09E-12 | 76.5 |  |
| Efet.01.44697.g1279.t1 | tumor p63-regulated gene 1 | 492 | 5.34E-45 | 62.2 |  |
| Efet.01.44699.g1281.t1 | testis-expressed sequence 9 | 747 | 4.22E-84 | 76 |  |
| Efet.01.447295.g1159.t1 | VWA domain-containing | 1632 | 4.62E-08 | 58 |  |
| Efet.01.44746.g1283.t1 | 17-beta-hydroxysteroid dehydrogenase type 6 | 486 | 1.46E-26 | 54.9 | F:oxidoreductase activity; C:membrane; C:integral component of membrane; P:oxidation-reduction process |
| Efet.01.447463.g1161.t1 | IS21 family transposase | 1071 | 0 | 83.2 | F:nucleic acid binding; P:DNA integration |
| Efet.01.44749.g1284.t1 | inositol 1,4,5-trisphosphate receptor type 2- | 516 | 2.27E-103 | 79.65 | P:inositol phosphate-mediated signaling; C:integral component of membrane; F:inositol 1,4,5-trisphosphate-sensitive calcium-release channel activity; P:calcium ion transmembrane transport; C:endoplasmic reticulum |
| Efet.01.447531.g1162.t1 | uncharacterized protein LOC111123490 | 288 | 3.49E-26 | 60.55 | C:membrane; C:integral component of membrane |
| Efet.01.44757.g1286.t1 | multidrug resistance-associated 5 isoform X2 | 975 | 1.74E-63 | 68.4 | P:transport |
| Efet.01.44764.g1287.t1 | hypothetical protein CAPTEDRAFT_215348 | 717 | 3.23E-16 | 62 |  |
| Efet.01.447682.g1165.t1 | uncharacterized protein LOC110983230 | 696 | 1.15E-22 | 46.5 | C:membrane; C:integral component of membrane |
| Efet.01.44771.g1288.t1 | hypothetical protein CAPTEDRAFT_189004 | 978 | 9.87E-54 | 50.7 | C:membrane; C:integral component of membrane |
| Efet.01.447754.g1167.t1 | multivalent antigen sj23-97 | 375 | 1.01E-33 | 68.05 | C:myosin complex; F:motor activity |
| Efet.01.447765.g1168.t1 | microtubule-actin cross-linking factor isoforms 1 2 3 5-like | 297 | 3.20E-13 | 63.22 | C:cytoskeleton |
| Efet.01.447789.g1170.t1 | sex peptide receptor-like | 1251 | 1.63E-125 | 63.75 | F:G-protein coupled receptor activity; C:membrane |
| Efet.01.44786.g1289.t1 | Forkhead box F1 | 987 | 3.10E-52 | 84.8 | C:nucleus; P:transcription, DNA-templated; F:transcription factor activity, RNA polymerase II distal enhancer sequence-specific binding; F:sequence-specific DNA binding; P:regulation of transcription from RNA polymerase II promoter |
| Efet.01.447897.g1172.t1 | Zinc finger 271 (Zinc finger 7) (HZF7) (Zinc finger ZNFphex133) (Epstein-Barr virus-induced zinc finger ) (ZNF-EB) (CT-ZFP48) (Zinc finger | 624 | 1.86E-51 | 60.8 | F:nucleic acid binding; F:metal ion binding |
| Efet.01.447904.g1173.t1 | sestrin homolog isoform X2 | 369 | 4.02E-27 | 68.95 | C:nucleus; P:regulation of response to reactive oxygen species |
| Efet.01.448018.g1174.t1 | hypothetical protein CRN43_01760 | 660 | 2.91E-74 | 59.1 | F:metal ion binding |
| Efet.01.448052.g1175.t1 | major surface trophozoite antigen 11-like | 831 | 3.50E-15 | 41.13 |  |
| Efet.01.448112.g1177.t1 | 3-oxoacyl-[acyl-carrier- ] mitochondrial | 954 | 3.32E-123 | 77.6 | F:transferase activity, transferring acyl groups other than amino-acyl groups; C:integral component of membrane; P:fatty acid biosynthetic process |
| Efet.01.448174.g1178.t1 | fat-like cadherin-related tumor suppressor | 1296 | 2.96E-98 | 53.7 | F:calcium ion binding; C:membrane; C:integral component of membrane; P:cell adhesion; P:homophilic cell adhesion via plasma membrane adhesion molecules; C:plasma membrane |
| Efet.01.44841.g1293.t1 | mannosyl-oligosaccharide 1,2-alpha-mannosidase IA-like | 615 | 5.09E-67 | 83.3 | P:N-glycan processing; F:mannosyl-oligosaccharide 1,2-alpha-mannosidase activity; C:Golgi membrane; C:endoplasmic reticulum; F:starch alpha-glucosidase activity; F:dextrin alpha-glucosidase activity; F:lytic transglycosylase activity; F:amylase activity; F:hexosaminidase activity; F:calcium ion binding; F:fucosidase activity; F:trehalase activity; C:integral component of membrane; F:galactosidase activity; F:glucan endo-1,4-beta-glucosidase activity; F:beta-glucanase activity; F:beta-6-sulfate-N-acetylglucosaminidase activity |
| Efet.01.44845.g1294.t1 | afadin-like isoform X4 | 465 | 8.91E-33 | 74.4 | P:signal transduction |
| Efet.01.448462.g1181.t1 | hypothetical protein HELRODRAFT_179880 | 294 | 1.40E-16 | 72 |  |
| Efet.01.448496.g1183.t1 | suppressor of cytokine signaling 5-like | 1485 | 7.22E-99 | 87.85 | F:zinc ion binding; P:epidermal growth factor receptor signaling pathway; P:positive regulation of proteasomal ubiquitin-dependent protein catabolic process; C:intracellular; P:JAK-STAT cascade |
| Efet.01.448498.g1184.t1 | hypothetical protein CAPTEDRAFT_211304 | 780 | 5.40E-50 | 52.78 | C:membrane; C:integral component of membrane |
| Efet.01.448506.g1185.t1 | hypothetical protein HELRODRAFT_160944 | 807 | 6.25E-92 | 55.33 |  |
| Efet.01.448506.g1186.t1 | serine threonine- kinase roco4 | 2805 | 0 | 47.65 | F:GTP binding; P:small GTPase mediated signal transduction; C:intracellular |
| Efet.01.4486.g361.t1 | calreticulin | 594 | 9.54E-60 | 80.1 | P:cell cycle arrest; P:negative regulation of intracellular steroid hormone receptor signaling pathway; P:positive regulation of cell proliferation; P:cellular senescence; C:external side of plasma membrane; P:negative regulation of neuron differentiation; P:positive regulation of cell cycle; F:integrin binding; P:positive regulation of DNA replication; C:polysome; P:negative regulation of transcription from RNA polymerase II promoter; P:cortical actin cytoskeleton organization; C:endoplasmic reticulum lumen; C:extracellular exosome; C:focal adhesion; F:calcium ion binding; P:negative regulation of retinoic acid receptor signaling pathway; P:positive regulation of dendritic cell chemotaxis; C:cytosol; F:mRNA binding; F:glycoprotein binding; F:carbohydrate binding; P:protein localization to nucleus; C:MHC class I peptide loading complex; P:regulation of meiotic nuclear division; F:unfolded protein binding; P:positive regulation of substrate adhesion-dependent cell spreading; P:protein folding; P:protein export from nucleus; C:nucleus; C:extracellular space; C:perinuclear region of cytoplasm; F:ubiquitin protein ligase binding; P:peptide antigen assembly with MHC class I protein complex; F:androgen receptor binding; P:positive regulation of gene expression; P:positive regulation of phagocytosis; P:protein stabilization |
| Efet.01.4486.g362.t1 | leukocyte receptor cluster member 1 | 372 | 4.14E-12 | 66.6 |  |
| Efet.01.448761.g1189.t1 | GTP-binding 128up | 321 | 1.49E-28 | 84.35 | C:polysome; F:GTP binding; C:nucleus; P:cytoplasmic translation; C:cytosol |
| Efet.01.44891.g1295.t1 | dynein heavy chain axonemal-like | 648 | 5.62E-98 | 79.2 | F:ATPase activity; F:ATP binding; F:microtubule motor activity; C:dynein complex; P:microtubule-based movement |
| Efet.01.448981.g1192.t1 | zinc finger 271-like | 588 | 1.97E-20 | 53.2 | F:nucleic acid binding; C:nucleus; F:transcription factor activity, sequence-specific DNA binding; F:zinc ion binding; F:metal ion binding; P:regulation of transcription, DNA-templated |
| Efet.01.44913.g1296.t1 | transient receptor potential cation channel subfamily M member 3-like | 303 | 1.27E-24 | 77.1 | F:ion channel activity; P:ion transmembrane transport; P:protein tetramerization; C:integral component of membrane |
| Efet.01.449155.g1195.t1 | sodium-dependent glucose transporter 1-like | 858 | 1.10E-59 | 59.45 | P:transmembrane transport; C:membrane |
| Efet.01.449160.g1196.t1 | heat shock factor 2-like | 528 | 9.97E-16 | 73.65 | F:DNA binding; C:membrane |
| Efet.01.449179.g1197.t1 | TBC1 domain family member 19-like isoform X1 | 1626 | 0 | 78.7 | C:integral component of membrane |
| Efet.01.449198.g1199.t1 | histone-lysine N-methyltransferase 2C isoform X1 | 483 | 1.06E-15 | 58.3 |  |
| Efet.01.449263.g1200.t1 | inwardly rectifying k+ channel | 252 | 5.04E-23 | 72.45 | P:potassium ion transmembrane transport; C:integral component of membrane; F:inward rectifier potassium channel activity; P:regulation of ion transmembrane transport |
| Efet.01.449263.g1201.t1 | G -activated inward rectifier potassium channel 3-like | 867 | 9.85E-78 | 75.8 | C:integral component of plasma membrane; F:inward rectifier potassium channel activity; P:regulation of ion transmembrane transport; P:potassium ion import |
| Efet.01.449436.g1203.t1 | cyclin-dependent kinase 12 | 591 | 6.40E-38 | 69.45 | F:nucleotide binding; F:protein kinase activity; P:phosphorylation |
| Efet.01.449489.g1205.t1 | FAA hydrolase family | 615 | 1.67E-148 | 84.8 | P:metabolic process; F:hydrolase activity; F:isomerase activity |
| Efet.01.449531.g1207.t1 | hypothetical protein BRAFLDRAFT_93720 | 1542 | 1.37E-06 | 48 |  |
| Efet.01.44962.g1299.t1 | Proteasome subunit beta type-3 | 408 | 1.70E-75 | 91 | C:extracellular exosome; C:proteasome core complex; C:mitochondrion; C:actin cytoskeleton; C:Golgi apparatus; F:threonine-type endopeptidase activity; P:proteolysis involved in cellular protein catabolic process; C:nucleoplasm |
| Efet.01.449666.g1209.t1 | 1-acyl-sn-glycerol-3-phosphate acyltransferase | 237 | 6.99E-50 | 85.3 | F:transferase activity, transferring acyl groups; P:metabolic process; C:integral component of membrane |
| Efet.01.449761.g1210.t1 | probable ATP-dependent RNA helicase DHX58 | 990 | 2.36E-34 | 57.7 | F:nucleic acid binding; F:DNA binding; F:ATP binding; F:hydrolase activity, acting on acid anhydrides; P:regulation of apoptotic process; F:helicase activity; F:hydrolase activity |
| Efet.01.449767.g1211.t1 | reelin-like | 279 | 6.43E-34 | 74.25 | P:peptidyl-tyrosine phosphorylation; P:spinal cord patterning; P:axon guidance; P:learning or memory; P:response to pain; C:dendrite; P:neuron migration; F:protein serine/threonine/tyrosine kinase activity; P:postsynaptic membrane organization; P:regulation of synaptic plasticity; P:regulation of neurotransmitter receptor activity; P:localization within membrane; P:positive regulation of synaptic transmission; P:cerebral cortex tangential migration; F:binding; P:regulation of glutamate receptor signaling pathway; C:extracellular space; P:positive regulation of small GTPase mediated signal transduction; C:cytoplasm; P:positive regulation of protein kinase activity; P:positive regulation of cellular component organization; P:protein localization to membrane; P:positive regulation of neurogenesis; P:glial cell differentiation; P:regulation of cation channel activity |
| Efet.01.449769.g1212.t1 | enhancer of sevenless 2B | 216 | 1.03E-13 | 78.63 |  |
| Efet.01.449787.g1213.t1 | hypothetical protein HELRODRAFT_159334 | 438 | 1.43E-06 | 69 | F:carbohydrate binding |
| Efet.01.449832.g1215.t1 | carbonic anhydrase-related 10 isoform X2 | 261 | 7.14E-20 | 86.55 |  |
| Efet.01.449884.g1217.t1 | interferon-induced very large GTPase 1-like | 7893 | 0 | 51.5 | F:GTP binding |
| Efet.01.44995.g1301.t1 | NACHT and WD repeat domain-containing 2 | 804 | 3.43E-32 | 55 |  |
| Efet.01.44995.g1302.t1 | NACHT and WD repeat domain-containing 2-like | 1446 | 3.09E-30 | 49.5 |  |
| Efet.01.449958.g1219.t1 | flagellar hook-length control | 1533 | 6.36E-09 | 39.6 |  |
| Efet.01.449969.g1221.t1 | 4-hydroxy-3-methylbut-2-enyl diphosphate reductase | 744 | 4.78E-131 | 95.1 | P:dimethylallyl diphosphate biosynthetic process; F:metal ion binding; P:isopentenyl diphosphate biosynthetic process, methylerythritol 4-phosphate pathway; F:3 iron, 4 sulfur cluster binding; P:oxidation-reduction process; F:4-hydroxy-3-methylbut-2-en-1-yl diphosphate reductase activity; P:terpenoid biosynthetic process |
| Efet.01.450124.g1.t1 | cAMP-dependent kinase regulatory subunit | 1266 | 1.07E-23 | 54.9 | P:phosphorylation; F:kinase activity |
| Efet.01.450196.g5.t1 | methionine adenosyltransferase | 321 | 7.23E-70 | 93.1 | F:magnesium ion binding; F:ATP binding; C:cytoplasm; P:S-adenosylmethionine biosynthetic process; F:methionine adenosyltransferase activity; P:one-carbon metabolic process |
| Efet.01.450321.g9.t1 | Cytochrome P450 | 936 | 2.04E-29 | 44 | F:monooxygenase activity |
| Efet.01.450396.g10.t1 | ral guanine nucleotide dissociation stimulator-like 1 | 567 | 1.19E-56 | 64.25 | P:biological regulation |
| Efet.01.450404.g11.t1 | zinc finger RNA-binding isoform X1 | 1341 | 5.75E-30 | 75.1 | F:nucleic acid binding; F:zinc ion binding |
| Efet.01.450424.g12.t1 | hypothetical protein B5766_02205 | 489 | 5.80E-72 | 100 |  |
| Efet.01.450446.g13.t1 | transferase | 741 | 7.99E-141 | 69.6 | F:catalytic activity; F:transferase activity |
| Efet.01.450481.g14.t1 | Cytoplasmic FMR1-interacting | 246 | 3.25E-38 | 69.7 |  |
| Efet.01.450490.g15.t1 | cilia- and flagella-associated 65-like isoform X1 | 267 | 2.51E-22 | 70.85 |  |
| Efet.01.450516.g16.t1 | deformed epidermal autoregulatory factor 1 | 687 | 7.81E-20 | 61.05 | F:DNA binding; C:nucleus; F:transcription coactivator activity; F:RNA polymerase II core promoter proximal region sequence-specific DNA binding; P:regulation of nucleic acid-templated transcription; P:regulation of transcription from RNA polymerase II promoter |
| Efet.01.450529.g18.t1 | probable G- coupled receptor 139 | 1185 | 8.81E-60 | 61.9 | F:G-protein coupled receptor activity; P:G-protein coupled receptor signaling pathway; P:signal transduction; C:membrane; C:integral component of membrane; F:signal transducer activity |
| Efet.01.450560.g19.t1 | potassium voltage-gated channel Shaw-like isoform X1 | 591 | 6.12E-56 | 64.1 | F:ion channel activity; P:ion transport; C:membrane |
| Efet.01.450597.g22.t1 | RNA-binding Nova-1 isoform X2 | 777 | 2.61E-35 | 45.95 | F:nucleic acid binding; F:RNA binding; F:hydrolase activity |
| Efet.01.450618.g23.t1 | hypothetical protein CAPTEDRAFT_226427 | 1962 | 1.80E-18 | 50.5 | F:calcium ion binding; C:membrane; C:integral component of membrane |
| Efet.01.45076.g1308.t1 | thyrotropin-releasing hormone receptor-like | 582 | 1.20E-58 | 72.35 | F:thyrotropin-releasing hormone receptor activity; P:G-protein coupled receptor signaling pathway; C:integral component of membrane; F:transferase activity |
| Efet.01.450822.g26.t1 | espinas-like isoform X1 | 354 | 2.38E-29 | 75.95 | F:zinc ion binding |
| Efet.01.45092.g1309.t1 | UBX domain-containing 11-like isoform X1 | 228 | 3.50E-21 | 84.25 |  |
| Efet.01.451142.g30.t1 | PREDICTED: uncharacterized protein C8orf74 homolog | 594 | 4.69E-20 | 48 |  |
| Efet.01.451187.g31.t1 | cartilage matrix -like isoform X2 | 387 | 3.05E-16 | 56.25 | F:calcium ion binding; C:membrane; F:scavenger receptor activity; P:receptor-mediated endocytosis |
| Efet.01.451476.g34.t1 | cAMP-dependent kinase regulatory subunit | 756 | 1.78E-12 | 59.14 | P:phosphorylation; F:kinase activity |
| Efet.01.451498.g35.t1 | RING finger 150-like | 426 | 7.73E-20 | 54.35 | F:zinc ion binding; F:metal ion binding; C:membrane; C:integral component of membrane |
| Efet.01.451554.g36.t1 | DNA-binding Ikaros-like | 780 | 3.96E-39 | 73.8 | F:nucleic acid binding; F:metal ion binding |
| Efet.01.451589.g38.t1 | DNA-directed RNA polymerase III subunit RPC9 | 360 | 6.65E-23 | 68.25 | F:RNA polymerase III activity; F:organic cyclic compound binding; F:heterocyclic compound binding; P:transcription initiation from RNA polymerase III promoter; C:DNA-directed RNA polymerase III complex |
| Efet.01.451660.g39.t1 | phosphatidylcholine translocator ABCB4 isoform X6 | 594 | 4.21E-69 | 72.05 | P:positive regulation of phospholipid transport; F:ATPase activity, coupled to transmembrane movement of substances; P:transmembrane transport; P:antigen processing and presentation of exogenous protein antigen via MHC class Ib, TAP-dependent; P:positive regulation of antigen processing and presentation of peptide antigen via MHC class I; P:response to organic cyclic compound; F:phosphatidylcholine transporter activity; P:antigen processing and presentation of endogenous peptide antigen via MHC class I via ER pathway, TAP-dependent; P:response to oxygen-containing compound; C:plasma membrane; F:ATP binding; P:antigen processing and presentation of endogenous peptide antigen via MHC class Ib via ER pathway, TAP-dependent; P:lipid transport; P:regulation of membrane lipid distribution; C:integral component of membrane; P:organic anion transport |
| Efet.01.451680.g40.t1 | Golgin subfamily A member 1 | 480 | 8.28E-10 | 46.25 |  |
| Efet.01.451716.g41.t1 | myostatin | 369 | 1.86E-07 | 53.8 | P:regulation of cellular process; F:binding; P:negative regulation of biological process; P:cellular process; P:response to stimulus |
| Efet.01.451717.g42.t1 | zinc finger CCCH domain-containing 7A | 663 | 2.03E-25 | 49.25 | F:metal ion binding; F:helicase activity |
| Efet.01.451759.g43.t1 | centaurin gamma homolog | 483 | 6.64E-49 | 90.9 | F:GTP binding; P:small GTPase mediated signal transduction; F:GTPase activator activity; C:intracellular; P:positive regulation of GTPase activity |
| Efet.01.451768.g44.t1 | protocadherin Fat 4-like | 276 | 3.01E-37 | 66.25 | F:calcium ion binding; C:integral component of membrane; P:homophilic cell adhesion via plasma membrane adhesion molecules; C:plasma membrane |
| Efet.01.451909.g46.t1 | adenylate cyclase type 1 | 759 | 3.63E-40 | 60.25 | P:signal transduction; C:membrane; F:lyase activity |
| Efet.01.451953.g47.t1 | mitochondrial ubiquitin ligase activator of nfkb 1-A | 345 | 1.10E-19 | 53.55 | P:positive regulation of cellular process; C:intracellular membrane-bounded organelle; C:membrane; F:acid-amino acid ligase activity; C:cytoplasmic part; P:negative regulation of cellular process; F:binding; P:organelle organization; P:regulation of signal transduction; P:regulation of cellular component organization |
| Efet.01.451967.g49.t1 | bactericidal permeability-increasing | 1359 | 3.50E-27 | 42.45 | P:negative regulation of biological process |
| Efet.01.451977.g50.t1 | PREDICTED: tenascin-X-like | 219 | 1.44E-09 | 54.4 | F:phosphoprotein phosphatase activity; F:protein tyrosine phosphatase activity; P:protein dephosphorylation; C:membrane; F:phosphatase activity; C:integral component of membrane; P:dephosphorylation; P:peptidyl-tyrosine dephosphorylation; F:hydrolase activity |
| Efet.01.452003.g51.t1 | monocarboxylate transporter 12 | 1032 | 8.13E-51 | 42.7 | P:transmembrane transport; C:membrane; C:integral component of membrane |
| Efet.01.452012.g52.t1 | Cephalotocin receptor 1 | 1167 | 2.11E-73 | 56.15 | P:signal transduction; C:membrane; F:protein-hormone receptor activity |
| Efet.01.452089.g53.t1 | hypothetical protein CAPTEDRAFT_206977 | 765 | 7.89E-20 | 44.27 | F:chitin binding; C:membrane; P:chitin metabolic process; C:integral component of membrane; C:extracellular region |
| Efet.01.452136.g54.t1 | RNA-binding 38 isoform X3 | 309 | 9.43E-55 | 93.9 | P:cell cycle arrest; P:RNA splicing; P:3'-UTR-mediated mRNA stabilization; P:negative regulation of cell proliferation; P:mRNA processing; F:mRNA 3'-UTR binding; F:nucleotide binding; P:DNA damage response, signal transduction by p53 class mediator resulting in transcription of p21 class mediator; C:nucleus; P:DNA damage response, signal transduction by p53 class mediator resulting in cell cycle arrest; P:regulation of RNA splicing; P:cell differentiation; C:cytosol; P:regulation of myotube differentiation |
| Efet.01.452252.g56.t1 | Low-density lipo receptor-related 4 | 948 | 1.81E-79 | 51.9 | P:regulation of canonical Wnt signaling pathway; P:BMP signaling pathway; F:calcium ion binding; C:membrane; C:integral component of membrane; P:animal organ development |
| Efet.01.45234.g1311.t1 | WD repeat-containing 37-like | 357 | 6.23E-56 | 87.05 | P:maturation of 5.8S rRNA; P:maturation of LSU-rRNA; C:PeBoW complex; C:preribosome, large subunit precursor |
| Efet.01.45234.g1312.t1 | hypothetical protein CAPTEDRAFT_226930 | 1143 | 3.94E-13 | 70 |  |
| Efet.01.452416.g58.t1 | isoform A | 621 | 1.52E-36 | 49.5 | C:membrane; C:integral component of membrane |
| Efet.01.452593.g59.t1 | Inositol phosphoceramide mannosyltransferase 3 | 942 | 1.75E-123 | 56.05 | C:membrane |
| Efet.01.45278.g1315.t1 | PREDICTED: uncharacterized protein LOC106811571 | 1053 | 2.16E-13 | 52.5 | P:pseudouridine synthesis; F:RNA binding; P:RNA modification; F:pseudouridine synthase activity |
| Efet.01.452803.g60.t1 | hypothetical protein HELRODRAFT_166386 | 204 | 1.04E-11 | 92 | P:calcium ion transport; C:integral component of membrane |
| Efet.01.452829.g63.t1 | C-1-tetrahydrofolate cytoplasmic-like | 324 | 1.41E-35 | 81.15 | F:ATP binding; F:formate-tetrahydrofolate ligase activity; C:cytosol; P:10-formyltetrahydrofolate biosynthetic process; P:oxidation-reduction process; P:purine nucleobase biosynthetic process; P:one-carbon metabolic process; F:methenyltetrahydrofolate cyclohydrolase activity; F:methylenetetrahydrofolate dehydrogenase (NADP+) activity |
| Efet.01.45284.g1316.t1 | glutamate receptor 2 | 276 | 1.35E-27 | 72.3 | C:postsynaptic membrane; P:ion transmembrane transport; C:integral component of membrane; C:cell junction; P:ionotropic glutamate receptor signaling pathway; F:ionotropic glutamate receptor activity; F:extracellular-glutamate-gated ion channel activity |
| Efet.01.452884.g65.t1 | CREB-regulated transcription coactivator 1-like isoform X2 | 204 | 2.31E-15 | 74.15 | C:intracellular part |
| Efet.01.452987.g67.t1 | stress-70 mitochondrial-like | 204 | 2.90E-36 | 97.55 | F:heat shock protein binding; C:myelin sheath; P:hemopoiesis; F:unfolded protein binding; C:nucleolus; P:protein folding; P:protein export from nucleus; P:response to toxic substance; C:mitochondrial nucleoid; F:RNA binding; F:ATP binding; C:extracellular exosome; F:ubiquitin protein ligase binding; C:focal adhesion; P:negative regulation of apoptotic process; P:cellular response to interleukin-1; F:fibroblast growth factor binding |
| Efet.01.453054.g68.t1 | UPF0468 | 327 | 5.15E-67 | 98.1 | P:protein polyglutamylation; C:extracellular exosome; P:cilium assembly; C:centriole; C:ciliary basal body; P:regulation of cilium beat frequency involved in ciliary motility; P:positive regulation of feeding behavior; C:nucleoplasm; C:microtubule; F:RNA binding; P:positive regulation of cell motility |
| Efet.01.453249.g70.t1 | bile salt-activated lipase | 273 | 4.77E-11 | 53.6 | C:extracellular space; P:acetylcholine catabolic process; P:metabolic process; C:membrane; F:neurexin family protein binding; C:integral component of membrane; C:neuromuscular junction; F:acetylcholinesterase activity; F:hydrolase activity |
| Efet.01.45325.g1318.t1 | PREDICTED: uncharacterized protein LOC100892708 | 258 | 4.10E-07 | 61.33 |  |
| Efet.01.453264.g71.t1 | calsyntenin-1 | 480 | 8.34E-48 | 67.95 | F:calcium ion binding; C:membrane; C:integral component of membrane; P:homophilic cell adhesion via plasma membrane adhesion molecules |
| Efet.01.453348.g72.t1 | glutamate receptor kainate 2 | 255 | 4.79E-11 | 76.9 | C:postsynaptic membrane; P:ion transmembrane transport; C:integral component of membrane; C:cell junction; P:ionotropic glutamate receptor signaling pathway; F:ionotropic glutamate receptor activity; F:extracellular-glutamate-gated ion channel activity |
| Efet.01.453351.g73.t1 | mucin-2-like isoform X1 | 480 | 5.86E-15 | 50.1 | P:cell differentiation; P:nervous system development |
| Efet.01.453392.g74.t1 | junctophilin-1-like isoform X1 | 462 | 5.30E-48 | 81.15 | P:pseudouridine synthesis; F:RNA binding; F:pseudouridine synthase activity |
| Efet.01.453439.g76.t1 | innexin unc-9-like isoform X1 | 741 | 2.20E-47 | 64.7 | C:membrane; P:cell communication by electrical coupling; C:synapse; F:gap junction channel activity; P:transport |
| Efet.01.453507.g77.t1 | chromodomain-helicase-DNA-binding 1-like | 684 | 2.46E-45 | 57.4 | F:core promoter sequence-specific DNA binding; F:DNA binding; C:nucleus; F:ATP binding; P:regulation of transcription, DNA-templated |
| Efet.01.453586.g84.t1 | MULTISPECIES: hypothetical protein | 645 | 2.80E-07 | 46 |  |
| Efet.01.453586.g86.t1 | family conjugative transposon ATPase | 2424 | 1.49E-76 | 46.5 |  |
| Efet.01.453586.g87.t1 | conjugal transfer | 2100 | 1.50E-111 | 59 | C:membrane; C:integral component of membrane |
| Efet.01.453629.g90.t1 | GTP-binding ypt1 | 315 | 3.75E-16 | 58.3 | C:intracellular membrane-bounded organelle; C:cytoplasmic part |
| Efet.01.453828.g91.t1 | hypothetical protein CAPTEDRAFT_202797 | 996 | 1.51E-44 | 58 |  |
| Efet.01.45398.g1319.t1 | dual specificity phosphatase 16-like isoform X1 | 927 | 9.37E-09 | 47.4 |  |
| Efet.01.454059.g95.t1 | hypothetical protein CAPTEDRAFT_186249 | 213 | 9.86E-06 | 57 | C:membrane; C:integral component of membrane; F:carbohydrate binding |
| Efet.01.454251.g96.t1 | O-phosphoseryl-tRNA(Sec) selenium transferase | 360 | 1.74E-17 | 65.6 | P:selenocysteinyl-tRNA(Sec) biosynthetic process; F:RNA binding; C:cytoplasm; F:catalytic activity; F:transferase activity; F:tRNA binding; F:transferase activity, transferring selenium-containing groups; P:translation |
| Efet.01.45427.g1322.t1 | regulator of G- signaling 20 isoform X3 | 276 | 2.02E-45 | 88.65 | C:postsynaptic density; F:protein kinase binding; F:GTPase activator activity; P:Wnt signaling pathway involved in somitogenesis; P:regulation of transcription, DNA-templated; P:dorsal/ventral axis specification; C:cytoplasmic microtubule; F:beta-catenin binding; P:regulation of G-protein coupled receptor protein signaling pathway; C:plasma membrane; P:positive regulation of GTPase activity; C:beta-catenin destruction complex; P:negative regulation of canonical Wnt signaling pathway; C:nucleus; C:trans-Golgi network; P:cell death; C:cell cortex; F:armadillo repeat domain binding; P:cell differentiation; P:regulation of catenin import into nucleus; P:cellular response to organic cyclic compound |
| Efet.01.454313.g97.t1 | 1-acyl-sn-glycerol-3-phosphate acyltransferase | 264 | 1.90E-26 | 70.3 | F:transferase activity, transferring acyl groups |
| Efet.01.454345.g99.t1 | forkhead Box | 837 | 6.37E-27 | 82.7 | C:nucleus; F:sequence-specific DNA binding; F:RNA polymerase II transcription factor activity, sequence-specific DNA binding; P:regulation of transcription from RNA polymerase II promoter |
| Efet.01.45444.g1324.t1 | Fibrinogen 1 | 933 | 4.28E-61 | 66.75 |  |
| Efet.01.45445.g1325.t1 | solute carrier family 35 member B1 | 411 | 5.00E-23 | 82.6 | P:transmembrane transport; C:integral component of membrane |
| Efet.01.454463.g101.t1 | neuralized 2 | 927 | 6.83E-96 | 63.25 | P:intracellular signal transduction; C:intracellular |
| Efet.01.454467.g102.t1 | prepilin peptidase | 450 | 2.54E-70 | 92.85 | F:aspartic-type endopeptidase activity; P:proteolysis; C:integral component of membrane; F:methyltransferase activity; C:plasma membrane; P:methylation |
| Efet.01.454467.g103.t1 | dephospho- kinase | 555 | 1.36E-104 | 82.85 | F:ATP binding; C:cytoplasm; F:dephospho-CoA kinase activity; P:coenzyme A biosynthetic process; P:phosphorylation |
| Efet.01.454467.g104.t1 | cell division | 615 | 1.63E-134 | 90.5 | P:FtsZ-dependent cytokinesis; C:cytoplasm |
| Efet.01.454467.g105.t1 | NUDIX domain-containing | 372 | 1.90E-83 | 91.85 | F:hydrolase activity |
| Efet.01.454467.g106.t1 | AAA family ATPase | 873 | 0 | 93.1 |  |
| Efet.01.454467.g107.t1 | bifunctional ornithine acetyltransferase N-acetylglutamate synthase | 936 | 0 | 89.2 | F:glutamate N-acetyltransferase activity; P:arginine biosynthetic process; C:cytoplasm; F:acetyl-CoA:L-glutamate N-acetyltransferase activity |
| Efet.01.454593.g109.t1 | vacuolar sorting-associated 13A-like | 249 | 2.88E-08 | 86.8 | C:membrane; C:integral component of membrane |
| Efet.01.454599.g110.t1 | retrovirus-related Pol poly from type-1 retrotransposable element R2 | 312 | 5.25E-24 | 63.36 | C:membrane; F:binding |
| Efet.01.454754.g113.t1 | G- coupled receptor GRL101-like isoform X1 | 654 | 3.53E-79 | 74.5 | P:G-protein coupled receptor signaling pathway; C:integral component of membrane; F:protein-hormone receptor activity |
| Efet.01.454754.g114.t1 | G- coupled receptor GRL101-like | 330 | 1.36E-33 | 72.9 | P:G-protein coupled receptor signaling pathway; C:integral component of membrane; F:protein-hormone receptor activity |
| Efet.01.454759.g115.t1 | kelch 5 isoform X3 | 264 | 4.60E-38 | 84.95 | F:ubiquitin-protein transferase activity; C:cytoskeleton; F:actin binding; C:cytoplasm; C:Cul3-RING ubiquitin ligase complex; P:protein ubiquitination |
| Efet.01.454838.g118.t1 | ubiquitin carboxyl-terminal hydrolase 5 isoform X2 | 267 | 1.23E-39 | 86.3 | F:thiol-dependent ubiquitin-specific protease activity; F:zinc ion binding; F:ubiquitin binding; P:positive regulation of proteasomal ubiquitin-dependent protein catabolic process; P:ubiquitin-dependent protein catabolic process; P:protein K48-linked deubiquitination |
| Efet.01.4549.g364.t1 | Innexin unc-9 | 948 | 1.29E-117 | 69.4 | C:membrane; C:cell junction; P:transport |
| Efet.01.45501.g1327.t1 | EvxA, partial | 597 | 4.73E-29 | 53 |  |
| Efet.01.455065.g122.t1 | sex peptide receptor-like | 951 | 7.82E-100 | 66.6 | F:G-protein coupled receptor activity; F:G-protein coupled peptide receptor activity; P:G-protein coupled receptor signaling pathway; C:membrane; C:integral component of membrane |
| Efet.01.455077.g124.t1 | probable helicase with zinc finger domain | 834 | 3.07E-91 | 67.5 | F:ion binding; F:hydrolase activity |
| Efet.01.455088.g125.t1 | tyrosine- phosphatase non-receptor type 2-like | 270 | 9.19E-06 | 68 |  |
| Efet.01.455137.g126.t1 | alpha-ketoglutarate-dependent dioxygenase alkB homolog 6-like | 525 | 1.23E-41 | 63.35 | F:oxidoreductase activity; P:oxidation-reduction process |
| Efet.01.455318.g128.t1 | mediator of RNA polymerase II transcription subunit 26-like | 1161 | 3.94E-36 | 61 | F:DNA binding; C:nucleus; P:transcription, DNA-templated |
| Efet.01.455354.g129.t1 | valine--tRNA ligase-like | 363 | 3.94E-36 | 70.7 | F:valine-tRNA ligase activity; F:ATP binding; P:valyl-tRNA aminoacylation; C:cytoplasm; P:regulation of translational fidelity; C:membrane; F:aminoacyl-tRNA editing activity |
| Efet.01.455377.g132.t1 | Internalin-J precursor | 1200 | 1.65E-49 | 60.45 |  |
| Efet.01.455382.g133.t1 | GTPase Era | 939 | 0 | 94.8 | F:GTP binding; C:cytoplasm; F:GTPase activity; P:ribosomal small subunit biogenesis; F:small ribosomal subunit rRNA binding; C:plasma membrane |
| Efet.01.455382.g134.t1 | carnitine dehydratase | 1152 | 0 | 89.55 | F:catalytic activity |
| Efet.01.455382.g135.t1 | hydroxymethylglutaryl- lyase | 885 | 0 | 79.75 | F:hydroxymethylglutaryl-CoA lyase activity |
| Efet.01.455382.g136.t1 | tripartite tricarboxylate transporter substrate binding | 843 | 4.96E-145 | 93.1 | C:outer membrane-bounded periplasmic space |
| Efet.01.455393.g139.t1 | diacylglycerol kinase delta-like isoform X2 | 339 | 1.08E-10 | 73.58 | F:nucleotide binding; P:signal transduction; F:kinase activity |
| Efet.01.455421.g140.t1 | bicaudal D-related homolog | 270 | 1.67E-07 | 71.4 |  |
| Efet.01.455425.g141.t1 | LIM homeobox Lhx3-like | 330 | 1.67E-45 | 63.1 | F:binding |
| Efet.01.455491.g142.t1 | RNA exonuclease 1 homolog | 342 | 3.31E-34 | 87.7 | F:nucleic acid binding; P:nucleic acid phosphodiester bond hydrolysis; F:exonuclease activity |
| Efet.01.455498.g143.t1 | calmodulin | 366 | 2.18E-46 | 68.35 | F:calcium ion binding; C:intracellular part |
| Efet.01.455545.g144.t1 | sugar phosphate isomerase epimerase | 276 | 1.10E-35 | 96 | P:nucleic acid phosphodiester bond hydrolysis; F:endonuclease activity |
| Efet.01.455567.g146.t1 | adenylate kinase 7-like | 258 | 3.79E-28 | 84.3 | F:ATP binding; C:integral component of membrane; P:phosphorylation; F:nucleobase-containing compound kinase activity; P:nucleobase-containing compound metabolic process |
| Efet.01.45584.g1330.t1 | septin-1 | 723 | 1.11E-84 | 79.05 | F:GTP binding; P:cell division; P:cell cycle |
| Efet.01.455995.g151.t1 | type I cytoskeletal 9-like | 465 | 4.84E-47 | 59.55 | F:metal ion binding |
| Efet.01.45600.g1331.t1 | short transient receptor potential channel 6 | 456 | 1.29E-66 | 85.25 | C:integral component of plasma membrane; F:store-operated calcium channel activity; P:manganese ion transport; P:regulation of cytosolic calcium ion concentration; P:calcium ion transmembrane transport; P:single fertilization |
| Efet.01.456088.g152.t1 | dual specificity kinase TTK | 264 | 1.72E-17 | 65.25 | P:cell cycle process; F:protein kinase activity; P:phosphorylation; P:regulation of cell cycle |
| Efet.01.456275.g157.t1 | adenylate kinase 9-like | 393 | 1.34E-48 | 62.3 | F:ATP binding; P:phosphorylation; F:nucleobase-containing compound kinase activity; P:nucleobase-containing compound metabolic process; F:kinase activity |
| Efet.01.456290.g158.t1 | cytochrome P450 1A5-like | 1434 | 1.28E-126 | 56.1 | F:oxidoreductase activity, acting on paired donors, with incorporation or reduction of molecular oxygen; C:intracellular membrane-bounded organelle; F:binding; F:monooxygenase activity; P:single-organism metabolic process |
| Efet.01.45649.g1333.t1 | fork head domain-containing crocodile-like | 696 | 1.20E-60 | 81.6 | C:nucleus; F:transcription factor activity, sequence-specific DNA binding; P:transcription, DNA-templated; P:regulation of transcription, DNA-templated; F:sequence-specific DNA binding |
| Efet.01.456769.g165.t1 | octopamine receptor 2-like | 1098 | 1.02E-91 | 68.8 | F:G-protein coupled receptor activity; P:G-protein coupled receptor signaling pathway; C:integral component of membrane |
| Efet.01.456769.g166.t1 | alpha-2B adrenergic receptor-like | 270 | 2.97E-40 | 71.1 | F:G-protein coupled receptor activity; P:G-protein coupled receptor signaling pathway; C:integral component of membrane; F:transferase activity |
| Efet.01.456779.g167.t1 | acid-sensing ion channel 5-like | 1344 | 3.35E-29 | 57.44 | P:ion transport |
| Efet.01.456841.g168.t1 | laminin subunit beta-1-like | 420 | 1.20E-27 | 67.3 | F:catalytic activity |
| Efet.01.45686.g1337.t1 | tumor p53-inducible nuclear 2 | 1347 | 4.74E-10 | 74.35 | P:autophagosome assembly; C:nucleus; P:response to abiotic stimulus; P:cellular response to organic substance; P:positive regulation of transcription, DNA-templated; P:regulation of autophagy; P:positive regulation of apoptotic process; P:single-organism cellular process; P:negative regulation of cellular process; P:cellular response to oxygen-containing compound; C:autophagosome; P:response to stress |
| Efet.01.456881.g169.t1 | hypothetical protein HELRODRAFT_160941 | 657 | 5.27E-06 | 47 | F:aspartic-type endopeptidase activity; P:proteolysis |
| Efet.01.456900.g170.t1 | ABC transporter ATP-binding | 237 | 1.33E-37 | 83.65 | F:ATPase activity; F:ATP binding |
| Efet.01.456900.g171.t1 | ABC transporter ATP-binding | 576 | 9.47E-134 | 94 | F:ATPase activity; F:ATP binding; P:branched-chain amino acid transport; F:branched-chain amino acid transmembrane transporter activity |
| Efet.01.456900.g172.t1 | 30S ribosomal S21 | 213 | 4.96E-26 | 99.7 | F:structural constituent of ribosome; C:ribosome; P:translation |
| Efet.01.456900.g173.t1 | glutamyl-tRNA amidotransferase | 312 | 2.51E-38 | 74.15 | F:carbon-nitrogen ligase activity, with glutamine as amido-N-donor |
| Efet.01.45695.g1338.t1 | intraflagellar transport 172 homolog | 1308 | 3.47E-104 | 48 | P:regulation of cellular process; C:cilium; P:animal organ development; P:cellular process |
| Efet.01.456993.g176.t1 | hypothetical protein HELRODRAFT_175128 | 417 | 8.69E-10 | 51 |  |
| Efet.01.457023.g178.t1 | homeobox Nkx- | 678 | 3.97E-32 | 78.7 | P:floor plate formation; C:nucleus; F:transcription factor activity, sequence-specific DNA binding; P:transcription, DNA-templated; F:double-stranded DNA binding; P:regulation of transcription, DNA-templated; F:sequence-specific DNA binding; P:ventral spinal cord interneuron differentiation; P:respiratory tube development |
| Efet.01.457030.g179.t1 | universal minicircle sequence binding (UMSBP) | 1242 | 1.02E-24 | 47.85 | F:binding |
| Efet.01.457042.g180.t1 | unc-79 homolog | 687 | 2.07E-50 | 57.55 |  |
| Efet.01.457053.g181.t1 | trimethyllysine mitochondrial | 801 | 2.95E-80 | 62.3 | F:oxidoreductase activity |
| Efet.01.457074.g182.t1 | RING finger nhl-1 | 531 | 5.04E-33 | 53.15 | F:zinc ion binding; F:metal ion binding; C:intracellular |
| Efet.01.45714.g1339.t1 | matrix-remodeling-associated 7-like | 426 | 2.77E-22 | 73.4 | C:integral component of membrane |
| Efet.01.457146.g185.t1 | Dimethylaniline monooxygenase [N-oxide-forming] 2 | 231 | 8.56E-23 | 80 | C:endoplasmic reticulum membrane; F:NADP binding; F:flavin adenine dinucleotide binding; C:integral component of membrane; P:drug metabolic process; P:oxidation-reduction process; C:organelle membrane; F:N,N-dimethylaniline monooxygenase activity |
| Efet.01.45722.g1341.t1 | hypothetical protein HELRODRAFT_190749 | 270 | 7.92E-16 | 67.33 | P:response to stress |
| Efet.01.457296.g192.t1 | helicase with zinc finger domain 2-like isoform X1 | 573 | 1.89E-15 | 46.17 | F:metal ion binding; F:helicase activity |
| Efet.01.45731.g1342.t1 | calcium-independent kinase C | 417 | 5.44E-50 | 77.25 | F:ATP binding; F:metal ion binding; P:peptidyl-serine phosphorylation; C:intracellular; P:intracellular signal transduction; F:protein kinase C activity |
| Efet.01.457356.g195.t1 | T family of potassium channels 18-like | 432 | 5.29E-28 | 57.78 | P:transport |
| Efet.01.457380.g196.t1 | cell division | 1083 | 0 | 70.55 | F:ATP binding |
| Efet.01.457411.g199.t1 | tektin-3-like isoform X1 | 294 | 1.02E-40 | 79.95 |  |
| Efet.01.457479.g200.t1 | LIM domain containing | 504 | 4.66E-23 | 62.25 | F:zinc ion binding; F:metal ion binding |
| Efet.01.45754.g1343.t1 | methylcrotonoyl- carboxylase beta mitochondrial | 543 | 1.50E-107 | 93.6 | C:mitochondrion; F:methylcrotonoyl-CoA carboxylase activity |
| Efet.01.457601.g202.t1 | growth hormone secretagogue receptor type 1-like isoform X4 | 366 | 3.93E-29 | 65.35 | C:membrane |
| Efet.01.457601.g203.t1 | probable G- coupled receptor 139 isoform X2 | 747 | 1.04E-68 | 64.55 | C:membrane |
| Efet.01.45767.g1344.t1 | CAP-Gly domain-containing linker 1-like isoform X1 | 822 | 3.37E-53 | 58.6 |  |
| Efet.01.45769.g1345.t1 | tRNA wybutosine-synthesizing 2 homolog | 1065 | 1.64E-72 | 76.15 | P:tRNA processing; F:transferase activity |
| Efet.01.457697.g207.t1 | poly [ADP-ribose] polymerase 14-like isoform X1 | 408 | 8.40E-53 | 70.4 | F:transferase activity |
| Efet.01.457776.g213.t1 | translation initiation factor eIF-2B subunit delta | 417 | 1.79E-21 | 73.45 | P:negative regulation of translational initiation; P:L-methionine salvage from methylthioadenosine; F:guanyl-nucleotide exchange factor activity; F:S-methyl-5-thioribose-1-phosphate isomerase activity; C:eukaryotic translation initiation factor 2B complex |
| Efet.01.457823.g214.t1 | hypothetical protein CAPTEDRAFT_40304, partial | 810 | 2.99E-07 | 60 |  |
| Efet.01.457868.g215.t1 | 5-hydroxytryptamine receptor-like | 1260 | 2.09E-118 | 62.35 | F:G-protein coupled receptor activity; P:signal transduction; C:integral component of membrane; C:plasma membrane |
| Efet.01.457883.g216.t1 | transmembrane 151B-like | 1671 | 2.87E-83 | 74.1 | C:membrane |
| Efet.01.457968.g218.t1 | nucleo TPR-like | 309 | 1.68E-09 | 82 |  |
| Efet.01.457996.g219.t1 | hypothetical protein CAPTEDRAFT_189267 | 267 | 9.60E-10 | 59.67 |  |
| Efet.01.458077.g220.t1 | zinc finger fyve domain-containing 9 | 213 | 6.80E-14 | 63.4 | F:metal ion binding |
| Efet.01.458128.g221.t1 | muscleblind isoform X8 | 237 | 1.37E-25 | 81.7 | F:metal ion binding |
| Efet.01.458138.g222.t1 | uncharacterized protein LOC110447848 | 741 | 1.84E-16 | 50.5 |  |
| Efet.01.458150.g223.t1 | serine arginine repetitive matrix 1-like | 1542 | 9.04E-56 | 64.7 |  |
| Efet.01.4582.g366.t1 | mitoferrin-1 isoform X3 | 492 | 3.77E-58 | 77.05 | C:membrane |
| Efet.01.45821.g1346.t1 | titin-like isoform X10 | 1359 | 2.63E-51 | 61.95 | P:signal transduction |
| Efet.01.45822.g1347.t1 | ATP-dependent rna helicase dbp2 [Rhodotorula toruloides NP11] | 210 | 2.70E-21 | 84.1 | F:nucleic acid binding; P:nuclear polyadenylation-dependent mRNA catabolic process; F:ATP binding; P:nuclear-transcribed mRNA catabolic process, nonsense-mediated decay; C:mitochondrion; C:integral component of membrane; P:RNA secondary structure unwinding; C:cytosol; P:rRNA processing; C:nucleolus; P:messenger ribonucleoprotein complex assembly; F:ATP-dependent RNA helicase activity |
| Efet.01.45824.g1348.t1 | 2-acylglycerol O-acyltransferase 2 | 615 | 8.53E-76 | 70.8 | F:transferase activity, transferring acyl groups other than amino-acyl groups; C:membrane; P:lipid metabolic process |
| Efet.01.458246.g227.t1 | alpha-L-rhamnosidase | 645 | 8.28E-134 | 59.65 | F:hydrolase activity, acting on glycosyl bonds |
| Efet.01.45826.g1349.t1 | probable G- coupled receptor No9 isoform X1 | 330 | 1.90E-08 | 70.15 | P:G-protein coupled receptor signaling pathway; C:membrane; F:G-protein coupled amine receptor activity |
| Efet.01.458277.g228.t1 | tRNA-splicing ligase homolog | 1053 | 0 | 93.85 | F:DNA binding; F:ATP binding; P:DNA repair; F:DNA-dependent ATPase activity; F:metal ion binding; P:tRNA splicing, via endonucleolytic cleavage and ligation; C:tRNA-splicing ligase complex; F:RNA ligase (ATP) activity |
| Efet.01.458309.g229.t1 | beta-1,4-galactosyltransferase galt-1-like | 1014 | 3.38E-93 | 49.65 | C:membrane; C:integral component of membrane |
| Efet.01.458360.g230.t1 | troponin slow skeletal and cardiac muscles-like | 225 | 2.93E-17 | 59.25 | C:contractile fiber; F:metal ion binding; P:single-multicellular organism process; C:intracellular membrane-bounded organelle; P:regulation of muscle contraction; C:actin cytoskeleton; F:cytoskeletal protein binding; P:striated muscle contraction; C:intracellular organelle part |
| Efet.01.458423.g233.t1 | homeobox Nkx- -like | 1074 | 1.95E-36 | 89.5 | P:oligodendrocyte development; P:ventral spinal cord interneuron fate determination; P:response to progesterone; P:negative regulation of neuron differentiation; P:positive regulation of neuron differentiation; F:sequence-specific DNA binding; P:positive regulation of transcription from RNA polymerase II promoter; P:positive regulation of sequence-specific DNA binding transcription factor activity; P:spinal cord oligodendrocyte cell fate specification; C:nucleoplasm; P:optic nerve development; P:positive regulation of oligodendrocyte differentiation; F:transcription factor activity, sequence-specific DNA binding; P:pancreatic A cell fate commitment; P:response to glucose; P:type B pancreatic cell fate commitment; P:neuron fate specification; P:pancreatic PP cell fate commitment; P:digestive tract development; P:astrocyte differentiation; P:transcription, DNA-templated; P:spinal cord motor neuron differentiation; P:smoothened signaling pathway; F:chromatin binding; F:transcription factor binding; P:brain development; F:core promoter proximal region DNA binding; P:type B pancreatic cell development; F:transcription coactivator activity |
| Efet.01.458432.g234.t1 | Glyco 3-alpha-L-fucosyltransferase A | 288 | 2.09E-31 | 73.55 | F:fucosyltransferase activity; C:Golgi cisterna membrane; P:fucosylation; C:integral component of membrane; P:protein glycosylation |
| Efet.01.458437.g235.t1 | GTP-binding GEM | 255 | 6.27E-12 | 59.05 | F:GTP binding; P:small GTPase mediated signal transduction; C:membrane; C:intracellular |
| Efet.01.458460.g236.t1 | turtle-like isoform X2 | 264 | 2.97E-24 | 60.85 | C:membrane; C:integral component of membrane |
| Efet.01.458503.g238.t1 | hypothetical protein CAPTEDRAFT_176233 | 294 | 3.70E-16 | 64.13 | C:membrane; C:integral component of membrane |
| Efet.01.458510.g239.t1 | RNA-binding 42 | 1029 | 1.40E-50 | 85.3 | F:nucleotide binding; F:nucleic acid binding; P:lens development in camera-type eye |
| Efet.01.458517.g240.t1 | sal 1 | 2562 | 2.63E-68 | 53.95 | F:nucleic acid binding; F:metal ion binding |
| Efet.01.458546.g241.t1 | C-type lectin | 678 | 9.29E-18 | 56.75 | F:carbohydrate binding |
| Efet.01.458583.g242.t1 | ABC transporter ATP-binding | 624 | 4.11E-124 | 74 | F:ATPase activity; F:ATP binding |
| Efet.01.458583.g243.t1 | ABC transporter ATP-binding | 735 | 6.79E-139 | 74.95 | F:ATPase activity; F:ATP binding; P:peptide transport |
| Efet.01.458583.g244.t1 | ABC transporter permease | 588 | 2.17E-100 | 83.65 | C:integral component of membrane; P:transport; C:plasma membrane |
| Efet.01.458583.g245.t1 | ABC transporter permease | 864 | 1.48E-116 | 72.75 | C:integral component of membrane; P:transport; C:plasma membrane |
| Efet.01.458583.g246.t1 | peptide ABC transporter substrate-binding | 1557 | 0 | 76.8 | P:transmembrane transport; C:ATP-binding cassette (ABC) transporter complex |
| Efet.01.458709.g249.t1 | Dynein heavy chain axonemal | 354 | 1.13E-56 | 71.25 | F:nucleotide binding; F:ATPase activity; F:ATP binding; F:microtubule motor activity; C:dynein complex; P:microtubule-based movement |
| Efet.01.458788.g250.t1 | Methylenetetrahydrofolate reductase | 315 | 1.25E-12 | 69.4 | P:tetrahydrofolate interconversion; P:oxidation-reduction process; F:methylenetetrahydrofolate reductase (NAD(P)H) activity; P:methionine metabolic process |
| Efet.01.458795.g251.t1 | ras 3 | 381 | 1.34E-11 | 70.65 | F:nucleotide binding; P:signal transduction |
| Efet.01.45882.g1350.t1 | F-box LRR-repeat 2-like isoform X8 | 414 | 8.29E-31 | 84.7 |  |
| Efet.01.459009.g255.t1 | hypothetical protein CAPTEDRAFT_89034 | 543 | 1.85E-40 | 58.1 | F:iron ion binding; F:oxidoreductase activity; F:oxidoreductase activity, acting on paired donors, with incorporation or reduction of molecular oxygen; F:metal ion binding; C:membrane; C:integral component of membrane; P:oxidation-reduction process; F:monooxygenase activity; F:heme binding |
| Efet.01.459053.g257.t1 | calmodulin 4 | 300 | 2.35E-35 | 71.05 | F:calcium ion binding |
| Efet.01.459059.g258.t1 | cell filamentation Fic | 1059 | 3.52E-156 | 89.85 |  |
| Efet.01.45924.g1351.t1 | glycogen [starch] synthase-like | 210 | 4.01E-22 | 88.6 | P:glycogen biosynthetic process; F:glycogen (starch) synthase activity |
| Efet.01.459294.g262.t1 | beta-amyloid | 474 | 2.03E-37 | 65.65 | F:transition metal ion binding; C:membrane; C:integral component of membrane; F:heparin binding |
| Efet.01.4595.g367.t1 | hypothetical protein HELRODRAFT_188674 | 1071 | 6.72E-60 | 49.5 | C:membrane; C:integral component of membrane |
| Efet.01.459504.g265.t1 | MLX-interacting isoform X1 | 327 | 1.00E-12 | 53.35 | P:regulation of transcription, DNA-templated |
| Efet.01.459527.g266.t1 | uncharacterized protein LOC110450097 | 372 | 2.84E-07 | 59 |  |
| Efet.01.459729.g271.t1 | sodium glucose cotransporter 4-like | 618 | 4.25E-39 | 52.65 | C:integral component of plasma membrane; P:transmembrane transport; C:membrane; C:integral component of membrane; F:symporter activity; P:transport; F:transporter activity |
| Efet.01.459793.g272.t1 | helicase SKI2W-like | 564 | 8.14E-83 | 83.9 | F:ATP binding; F:RNA binding; C:Ski complex; P:nuclear-transcribed mRNA catabolic process, 3'-5' exonucleolytic nonsense-mediated decay; C:nucleoplasm; F:ATP-dependent RNA helicase activity |
| Efet.01.45981.g1353.t1 | hypothetical protein CAPTEDRAFT_191290 | 234 | 4.03E-06 | 51 |  |
| Efet.01.459913.g274.t1 | epoxide hydrolase 1-like | 285 | 9.67E-18 | 68.8 | F:catalytic activity |
| Efet.01.45994.g1354.t1 | Rho GTPase-activating 20 | 939 | 4.23E-31 | 74.55 | P:signal transduction |
| Efet.01.459990.g275.t1 | BTG1 | 489 | 2.44E-46 | 64.45 | C:nucleus; C:cytoplasm; P:negative regulation of cell proliferation; P:positive regulation of fibroblast apoptotic process; P:positive regulation of myoblast differentiation; P:protein methylation; P:positive regulation of endothelial cell differentiation; P:positive regulation of angiogenesis; F:enzyme binding |
| Efet.01.46000.g1355.t1 | dystrophin-like isoform X11 | 393 | 1.21E-26 | 69.65 | F:metal ion binding |
| Efet.01.460004.g277.t1 | copper-transporting ATPase 1 isoform X2 | 399 | 7.89E-08 | 76.35 | F:cation-transporting ATPase activity; C:trans-Golgi network transport vesicle; C:late endosome; F:nucleotide binding; P:copper ion transport; C:trans-Golgi network; C:perinuclear region of cytoplasm; P:cellular copper ion homeostasis; F:copper ion binding; P:positive regulation of oxidoreductase activity; P:cation transmembrane transport; C:integral component of membrane; C:basolateral plasma membrane; F:copper-dependent protein binding |
| Efet.01.460015.g279.t1 | isocitrate dehydrogenase [NAD] subunit mitochondrial | 705 | 1.05E-64 | 71.95 | F:magnesium ion binding; F:isocitrate dehydrogenase (NAD+) activity; F:NAD binding; C:mitochondrion; P:tricarboxylic acid cycle |
| Efet.01.460071.g281.t1 | muscarinic acetylcholine receptor M1-like | 1017 | 8.81E-80 | 64.75 | C:integral component of plasma membrane; F:G-protein coupled acetylcholine receptor activity; P:G-protein coupled acetylcholine receptor signaling pathway |
| Efet.01.460097.g282.t1 | inorganic phosphate cotransporter | 372 | 4.09E-13 | 64.4 | P:transmembrane transport; F:transmembrane transporter activity; C:integral component of membrane; P:anion transport |
| Efet.01.460117.g283.t1 | hypothetical protein HELRODRAFT_168105 | 1542 | 9.63E-14 | 61 |  |
| Efet.01.460118.g284.t1 | protocadherin beta-12 | 1491 | 7.72E-96 | 58.05 | C:membrane |
| Efet.01.460118.g285.t1 | protocadherin beta-10-like isoform X1 | 1188 | 7.03E-34 | 50.05 | F:calcium ion binding; C:membrane; C:integral component of membrane; P:cell adhesion; P:homophilic cell adhesion via plasma membrane adhesion molecules; C:plasma membrane |
| Efet.01.46014.g1357.t1 | Phospholysine phosphohistidine inorganic pyrophosphate phosphatase | 330 | 9.14E-26 | 59.5 | P:metabolic process; C:intracellular part; F:hydrolase activity |
| Efet.01.46015.g1358.t1 | Pancreatic alpha-amylase | 249 | 7.53E-17 | 79 | F:alpha-amylase activity; P:carbohydrate metabolic process; F:cation binding |
| Efet.01.460181.g288.t1 | collagen alpha-1(IV) chain | 267 | 1.69E-44 | 84.95 | C:collagen trimer; F:extracellular matrix structural constituent; C:proteinaceous extracellular matrix |
| Efet.01.460200.g289.t1 | hypothetical protein B5766_08930 | 534 | 7.92E-62 | 75 |  |
| Efet.01.460320.g296.t1 | polypeptide N-acetylgalactosaminyltransferase 13-like | 201 | 3.50E-35 | 93.2 | F:transferase activity, transferring glycosyl groups; C:integral component of membrane; P:protein glycosylation; C:Golgi membrane; F:carbohydrate binding |
| Efet.01.460344.g297.t1 | cyclin-dependent kinase inhibitor 1-like | 822 | 1.29E-07 | 62 |  |
| Efet.01.460381.g298.t1 | Hau-cytoplasmic intermediate filament | 225 | 1.90E-13 | 68.5 | F:structural molecule activity; C:intermediate filament |
| Efet.01.460391.g299.t1 | hypothetical protein HELRODRAFT_188786 | 1245 | 1.45E-17 | 71.5 | C:integral component of membrane |
| Efet.01.4605.g369.t1 | pyruvate kinase | 204 | 9.77E-15 | 68.1 | F:magnesium ion binding; F:pyruvate kinase activity; F:potassium ion binding; P:glycolytic process; F:kinase activity |
| Efet.01.460527.g301.t1 | hypothetical protein CAPTEDRAFT_223168 | 615 | 5.78E-24 | 58.1 | C:membrane; C:integral component of membrane |
| Efet.01.460565.g302.t1 | myosin heavy chain isoform | 522 | 7.83E-46 | 79.75 | C:myosin complex; F:motor activity |
| Efet.01.460671.g303.t1 | FMRFamide receptor-like | 1173 | 3.40E-113 | 54.65 | F:G-protein coupled receptor activity; P:signal transduction; C:membrane |
| Efet.01.460724.g304.t1 | Ankyrin repeat domain-containing 33B | 279 | 1.94E-16 | 73.3 |  |
| Efet.01.460797.g306.t1 | TRL4 interactor with leucine rich repeat area | 729 | 1.86E-11 | 46.75 | F:phosphoprotein phosphatase activity; F:G-protein coupled receptor activity; F:transmembrane signaling receptor activity; P:G-protein coupled receptor signaling pathway; P:signal transduction; C:membrane; P:protein dephosphorylation; C:integral component of membrane; P:cell surface receptor signaling pathway; F:signal transducer activity; F:hydrolase activity |
| Efet.01.46083.g1361.t1 | small conductance calcium-activated potassium channel -like | 528 | 1.18E-20 | 54.4 | F:calmodulin binding; P:potassium ion transmembrane transport; C:integral component of membrane; F:small conductance calcium-activated potassium channel activity |
| Efet.01.460897.g308.t1 | peptidase Imelysin | 597 | 1.33E-116 | 54.5 |  |
| Efet.01.461029.g313.t1 | ubiquitin thioesterase OTU1-like | 735 | 2.62E-76 | 71.2 | F:metal ion binding |
| Efet.01.461078.g314.t1 | solute carrier family 10 member 6 | 378 | 1.37E-10 | 55.45 | P:sodium-dependent organic anion transport; F:sodium-dependent organic anion transmembrane transporter activity; C:membrane; C:integral component of membrane; C:plasma membrane |
| Efet.01.461125.g316.t1 | acetylcholine receptor subunit beta-like 1 | 666 | 9.93E-09 | 55 | C:membrane; P:transport |
| Efet.01.46116.g1363.t1 | E3 ubiquitin- ligase SIAH1-like | 279 | 1.34E-24 | 93.4 | C:nucleus; F:ubiquitin-protein transferase activity; F:zinc ion binding; P:multicellular organism development; F:ligase activity; P:ubiquitin-dependent protein catabolic process; P:protein ubiquitination |
| Efet.01.461181.g318.t1 | sprouty- EVH1 domain-containing 1- | 1092 | 6.79E-18 | 57.85 | P:inactivation of MAPK activity; C:intracellular organelle; F:protein kinase binding; F:protein serine/threonine kinase inhibitor activity; P:positive regulation of DNA damage response, signal transduction by p53 class mediator; P:regulation of protein deacetylation; C:cytoplasm; P:negative regulation of peptidyl-threonine phosphorylation; F:stem cell factor receptor binding; C:membrane-bounded organelle |
| Efet.01.461230.g319.t1 | sorting nexin-21-like | 639 | 1.96E-70 | 62.9 | F:phosphatidylinositol binding |
| Efet.01.461264.g320.t1 | Mi- | 870 | 9.85E-99 | 89.55 | F:DNA binding; F:ATP binding; P:spindle organization; F:zinc ion binding; P:centrosome organization; C:NuRD complex; F:ATP-dependent helicase activity; P:regulation of transcription, DNA-templated; P:chromatin organization |
| Efet.01.46128.g1364.t1 | N-acetyltransferase ESCO2 | 291 | 1.15E-10 | 66.3 | P:single-organism process |
| Efet.01.461385.g321.t1 | coelomic cytolytic factor 1 | 237 | 1.06E-46 | 78.9 | P:carbohydrate metabolic process; F:hydrolase activity, hydrolyzing O-glycosyl compounds |
| Efet.01.461616.g325.t1 | rho GTPase-activating 100F-like isoform X1 | 1083 | 2.20E-58 | 57.9 | P:signal transduction |
| Efet.01.461630.g327.t1 | coiled-coil domain-containing 186-like isoform X1 | 471 | 2.25E-16 | 63.13 |  |
| Efet.01.461650.g328.t1 | voltage-gated sodium | 279 | 1.83E-44 | 82.25 | P:regulation of postsynaptic membrane potential; P:regulation of ion transmembrane transport; C:voltage-gated sodium channel complex; P:sodium ion transmembrane transport; F:voltage-gated sodium channel activity |
| Efet.01.461767.g329.t1 | hypothetical protein CAPTEDRAFT_226428 | 873 | 6.54E-58 | 44.38 | F:calcium ion binding; C:membrane; C:integral component of membrane |
| Efet.01.461905.g331.t1 | potassium sodium hyperpolarization-activated cyclic nucleotide-gated channel 4 | 213 | 3.14E-24 | 85.2 | F:voltage-gated potassium channel activity; P:potassium ion transmembrane transport; C:integral component of membrane |
| Efet.01.461951.g332.t1 | hypothetical protein CAPTEDRAFT_211986 | 216 | 1.38E-16 | 68.13 |  |
| Efet.01.46204.g1366.t1 | germ cell-less -like 1 | 483 | 3.59E-20 | 71.05 | C:nuclear part; F:protein binding; F:ion binding; P:cellular process; P:multicellular organismal process; P:single-organism developmental process |
| Efet.01.46208.g1367.t1 | hypothetical protein CAPTEDRAFT_225957 | 405 | 7.70E-08 | 49.78 | C:membrane; C:integral component of membrane; F:carbohydrate binding |
| Efet.01.462085.g336.t1 | hypothetical protein CAPTEDRAFT_220081 | 873 | 2.45E-07 | 60.67 | F:GTP binding; F:nucleotide binding; P:signal transduction; P:small GTPase mediated signal transduction; C:membrane; C:intracellular |
| Efet.01.462103.g337.t1 | monocarboxylate transporter 12-like isoform X1 | 576 | 2.19E-46 | 60.37 | C:membrane |
| Efet.01.462288.g338.t1 | atrial natriuretic peptide receptor 1-like | 378 | 2.65E-41 | 74.95 | F:ATP binding; P:cGMP biosynthetic process; C:integral component of membrane; F:guanylate cyclase activity; F:protein kinase activity; P:protein phosphorylation; C:intracellular; P:intracellular signal transduction |
| Efet.01.462344.g341.t1 | ADP-ribosyl cyclase cyclic ADP-ribose hydrolase 1 | 312 | 1.64E-11 | 59.15 | F:NAD+ nucleosidase activity |
| Efet.01.462427.g343.t1 | bark beetle | 438 | 2.10E-25 | 66.2 | C:membrane; F:scavenger receptor activity; P:receptor-mediated endocytosis |
| Efet.01.462434.g344.t1 | Transmembrane 26 | 663 | 3.73E-26 | 77.5 | C:integral component of membrane |
| Efet.01.46247.g1368.t1 | WD repeat-containing 49 | 252 | 5.55E-13 | 78.8 |  |
| Efet.01.462517.g346.t1 | Ribosome biogenesis | 249 | 4.95E-06 | 64 | C:nucleus; P:ribosome biogenesis; P:rRNA processing; C:nucleolus; C:nucleoplasm |
| Efet.01.462758.g349.t1 | suppressor of hairless | 420 | 2.34E-54 | 95.95 | C:nucleus; F:RNA polymerase II core promoter proximal region sequence-specific DNA binding; F:transcription factor activity, RNA polymerase II core promoter proximal region sequence-specific binding; P:regulation of transcription from RNA polymerase II promoter |
| Efet.01.462834.g350.t1 | replication factor C subunit 4 | 429 | 1.98E-41 | 71.75 | F:DNA clamp loader activity; P:retina development in camera-type eye; P:DNA-dependent DNA replication; C:DNA replication factor C complex |
| Efet.01.462860.g351.t1 | beta-1,3-galactosyltransferase brn | 1101 | 3.51E-150 | 60.1 | C:integral component of membrane; P:protein glycosylation; F:galactosyltransferase activity; C:Golgi membrane |
| Efet.01.462929.g353.t1 | myosin heavy chain type | 204 | 4.86E-33 | 88.65 | C:myosin complex; F:ATP binding; F:actin binding; F:motor activity |
| Efet.01.462960.g354.t1 | IQ and ubiquitin-like domain-containing | 306 | 4.30E-24 | 68.45 |  |
| Efet.01.46298.g1370.t1 | glutathione S-transferase omega-like 2-like | 972 | 3.52E-169 | 78.8 |  |
| Efet.01.463131.g356.t1 | dynein heavy chain axonemal-like | 231 | 2.32E-40 | 86.15 | F:ATPase activity; F:ATP binding; F:microtubule motor activity; C:dynein complex; P:microtubule-based movement |
| Efet.01.46318.g1371.t1 | hypothetical protein HELRODRAFT_162600 | 630 | 3.49E-27 | 67 |  |
| Efet.01.46334.g1372.t1 | hypothetical protein CAPTEDRAFT_192274 | 1770 | 2.53E-25 | 58 |  |
| Efet.01.463340.g359.t1 | T-cell acute lymphocytic leukemia | 570 | 4.52E-36 | 79.6 | C:nucleus; F:RNA polymerase II regulatory region sequence-specific DNA binding; F:protein dimerization activity; F:RNA polymerase II transcription factor activity, sequence-specific DNA binding; P:regulation of transcription from RNA polymerase II promoter |
| Efet.01.463370.g360.t1 | phosphate starvation-inducible | 843 | 3.74E-180 | 90.95 | F:ATP binding |
| Efet.01.463370.g361.t1 | Holliday junction branch migration | 576 | 1.45E-98 | 91.7 | F:DNA binding; F:ATP binding; P:DNA repair; P:DNA duplex unwinding; P:DNA recombination; C:Holliday junction helicase complex; F:four-way junction helicase activity; P:SOS response |
| Efet.01.463370.g362.t1 | peptidase S1 | 1137 | 0 | 73.8 | P:proteolysis; F:serine-type endopeptidase activity |
| Efet.01.463370.g363.t1 | Holliday junction branch migration DNA helicase | 1635 | 0 | 94.9 | F:DNA binding; F:ATP binding; P:DNA repair; P:DNA duplex unwinding; P:DNA recombination; F:four-way junction helicase activity; P:SOS response |
| Efet.01.463370.g364.t1 | DUF2818 domain-containing | 306 | 1.98E-52 | 89.35 | C:integral component of membrane |
| Efet.01.463371.g365.t1 | hypothetical protein HELRODRAFT_171065 | 387 | 1.24E-20 | 61.67 | C:membrane; C:integral component of membrane |
| Efet.01.463449.g366.t1 | SWI SNF-related matrix-associated actin-dependent regulator of chromatin subfamily A member | 390 | 6.21E-81 | 94.8 | F:ATPase activity; P:embryo development; P:covalent chromatin modification; F:nucleosome binding; C:nuclear replication fork; F:helicase activity; P:DNA-templated transcription, initiation; C:nucleolus; C:chromatin silencing complex; P:nucleosome assembly; P:double-strand break repair; C:condensed chromosome; F:DNA binding; F:ATP binding; F:histone binding; P:chromatin silencing at rDNA; P:positive regulation of transcription, DNA-templated; P:ATP-dependent chromatin remodeling; P:nucleosome positioning; C:RSF complex; C:NURF complex |
| Efet.01.46367.g1374.t1 | tubulin polyglutamylase ttll6-like isoform X1 | 249 | 1.32E-11 | 81 | P:cellular protein modification process |
| Efet.01.463735.g368.t1 | unconventional myosin-Id-like | 375 | 4.64E-39 | 71.3 | F:nucleotide binding |
| Efet.01.463792.g370.t1 | Cyclic GMP-AMP synthase | 2103 | 3.75E-24 | 47.85 |  |
| Efet.01.4639.g371.t1 | ankyrin repeat domain-containing 6-like | 1275 | 4.35E-142 | 63.15 | P:intracellular signal transduction; C:intracellular |
| Efet.01.463936.g372.t1 | sodium potassium ATPase alpha | 558 | 3.12E-78 | 81.95 | P:potassium ion transport; F:ATP binding; P:sodium ion transport; F:sodium:potassium-exchanging ATPase activity; F:metal ion binding; P:ATP hydrolysis coupled transmembrane transport; C:integral component of membrane; P:establishment or maintenance of transmembrane electrochemical gradient |
| Efet.01.463965.g374.t1 | metallophosphoesterase domain-containing 1 | 312 | 1.10E-26 | 69.65 | P:biological_process; C:cellular_component; F:molecular_function; F:hydrolase activity |
| Efet.01.46406.g1377.t1 | histone chaperone asf1 | 657 | 7.47E-101 | 81.05 | C:nucleus; P:chromatin assembly or disassembly |
| Efet.01.464105.g375.t1 | filamin-A isoform X6 | 360 | 1.43E-41 | 66.65 | F:actin filament binding |
| Efet.01.464146.g376.t1 | vacuolar fusion MON1 homolog A-like | 387 | 1.18E-28 | 68.55 | P:vesicle-mediated transport; C:endomembrane system |
| Efet.01.46427.g1380.t1 | ras and EF-hand domain-containing homolog | 678 | 2.48E-25 | 53.8 | F:GTP binding; P:small GTPase mediated signal transduction; F:calcium ion binding; C:intracellular |
| Efet.01.46431.g1381.t1 | MORC family CW-type zinc finger 2-like | 471 | 1.96E-38 | 60.55 | F:zinc ion binding |
| Efet.01.464383.g379.t1 | DUF898 domain-containing | 969 | 4.04E-103 | 62.9 | C:integral component of membrane |
| Efet.01.464530.g380.t1 | NAD(P)-dependent oxidoreductase | 360 | 1.73E-65 | 85.3 | F:phosphogluconate dehydrogenase (decarboxylating) activity; F:NAD binding; P:oxidation-reduction process |
| Efet.01.464530.g381.t1 | tripartite tricarboxylate transporter substrate binding | 867 | 0 | 78.2 | C:outer membrane-bounded periplasmic space |
| Efet.01.464530.g382.t1 | FAD-binding oxidoreductase | 1026 | 0 | 84.85 | F:L-pipecolate oxidase activity; P:oxidation-reduction process |
| Efet.01.464530.g383.t1 | L-cystine import ATP-binding | 1272 | 1.19E-154 | 90.95 | F:ATP binding; C:integral component of membrane; C:plasma membrane; P:amino acid transmembrane transport; F:amino acid-transporting ATPase activity |
| Efet.01.464530.g384.t1 | ABC transporter substrate-binding | 762 | 1.90E-174 | 86.05 | C:membrane; P:ionotropic glutamate receptor signaling pathway; F:ionotropic glutamate receptor activity |
| Efet.01.464530.g385.t1 | XRE family transcriptional regulator | 432 | 1.24E-88 | 80.6 | F:sequence-specific DNA binding |
| Efet.01.464584.g387.t1 | alpha-adducin isoform X1 | 213 | 2.79E-16 | 69.95 |  |
| Efet.01.464587.g388.t1 | neurochondrin-like | 1053 | 9.82E-06 | 67 |  |
| Efet.01.464633.g389.t1 | hypothetical protein HELRODRAFT_165596 | 276 | 1.82E-10 | 82.25 | C:integral component of membrane |
| Efet.01.464678.g391.t1 | prolyl endopeptidase FAP-like | 210 | 1.95E-17 | 66.05 | C:membrane |
| Efet.01.464713.g392.t1 | dynein regulatory complex subunit 7-like | 609 | 1.96E-19 | 68 |  |
| Efet.01.464751.g394.t1 | ras association domain-containing 1-like isoform X1 | 669 | 1.32E-53 | 61.45 | P:signal transduction; P:intracellular signal transduction; C:intracellular |
| Efet.01.464893.g397.t1 | serine threonine- kinase MRCK alpha-like isoform X1 | 711 | 6.74E-35 | 62.05 |  |
| Efet.01.464979.g400.t1 | ruvB-like helicase 1 | 246 | 2.78E-37 | 97.6 | C:nucleus; F:ATP binding; P:DNA repair; F:ATP-dependent 5'-3' DNA helicase activity; P:transcription, DNA-templated; P:DNA duplex unwinding; P:regulation of transcription, DNA-templated |
| Efet.01.464992.g401.t1 | Cs neurogenin like | 816 | 1.38E-29 | 76.6 | P:neuron differentiation; F:transcription factor activity, transcription factor binding; P:positive regulation of neuron differentiation; F:protein dimerization activity; F:chromatin binding; C:perikaryon; P:positive regulation of transcription from RNA polymerase II promoter; P:positive regulation of sequence-specific DNA binding transcription factor activity; C:intracellular; F:transcription factor activity, sequence-specific DNA binding; P:positive regulation of exit from mitosis; P:inner ear morphogenesis; P:cell fate commitment; F:E-box binding |
| Efet.01.465072.g403.t1 | potassium voltage-gated channel subfamily B member 2-like isoform X5 | 753 | 1.42E-23 | 80.55 | F:voltage-gated potassium channel activity; P:potassium ion transmembrane transport; C:voltage-gated potassium channel complex; P:regulation of ion transmembrane transport; P:protein homooligomerization |
| Efet.01.465183.g404.t1 | menin-like isoform X1 | 1257 | 5.01E-58 | 67.65 | C:nucleus; F:DNA binding; P:negative regulation of cellular process; C:intracellular organelle part |
| Efet.01.46526.g1382.t1 | WD repeat and SOCS box-containing 1 | 675 | 1.05E-74 | 68.05 | P:intracellular signal transduction; C:intracellular |
| Efet.01.465352.g405.t1 | dnaJ homolog subfamily C member 13-like isoform X1 | 291 | 7.18E-15 | 87.6 | F:GDP-dissociation inhibitor activity; P:endosome organization; P:small GTPase mediated signal transduction; C:lysosomal membrane; P:regulation of early endosome to recycling endosome transport; C:early endosome membrane; C:extracellular exosome; F:oxidoreductase activity; P:regulation of catalytic activity; P:osteoblast differentiation; P:oxidation-reduction process; C:WASH complex; P:regulation of early endosome to late endosome transport |
| Efet.01.465358.g406.t1 | organic cation transporter -like | 291 | 2.49E-26 | 66.15 | F:transmembrane transporter activity; C:integral component of membrane; P:transport |
| Efet.01.46536.g1383.t1 | ELAV 1 isoform X2 | 1026 | 1.24E-142 | 60.4 | F:nucleic acid binding |
| Efet.01.465638.g410.t1 | gamma-aminobutyric acid receptor subunit beta-like isoform X1 | 405 | 2.18E-17 | 83 | P:signal transduction; P:ion transmembrane transport; C:integral component of membrane; F:GABA-A receptor activity; C:cell junction; F:extracellular ligand-gated ion channel activity; C:synapse; C:plasma membrane |
| Efet.01.46569.g1385.t1 | gastrula zinc finger -like | 648 | 7.02E-48 | 69.35 | F:binding |
| Efet.01.465725.g413.t1 | myosin heavy chain isoform | 375 | 4.13E-20 | 80.85 | C:myosin complex; F:ATP binding; F:actin binding; P:metabolic process; F:amino acid binding; F:motor activity |
| Efet.01.465731.g414.t1 | actin-7-like isoform X1 | 207 | 2.60E-10 | 65.6 |  |
| Efet.01.465744.g415.t1 | 3-isopropylmalate dehydrogenase | 1071 | 0 | 88.75 | F:magnesium ion binding; F:NAD binding; P:oxidation-reduction process; F:3-isopropylmalate dehydrogenase activity |
| Efet.01.465744.g416.t1 | tripartite tricarboxylate transporter substrate binding | 1209 | 0 | 67.9 | C:outer membrane-bounded periplasmic space |
| Efet.01.465744.g417.t1 | ribonuclease R | 2271 | 0 | 90.5 | F:exoribonuclease II activity; P:RNA phosphodiester bond hydrolysis, exonucleolytic; F:RNA binding; C:cytoplasm |
| Efet.01.465744.g418.t1 | bifunctional ADP-dependent NAD(P)H-hydrate dehydratase NAD(P)H-hydrate epimerase | 696 | 5.12E-74 | 86.85 | F:ATP binding; F:metal ion binding; P:phosphorylation; F:kinase activity; F:isomerase activity; F:ADP-dependent NAD(P)H-hydrate dehydratase activity |
| Efet.01.465744.g419.t1 | bifunctional ADP-dependent NAD(P)H-hydrate dehydratase NAD(P)H-hydrate epimerase | 810 | 1.94E-118 | 77.95 | F:ATP binding; F:metal ion binding; P:phosphorylation; F:kinase activity; F:isomerase activity; F:ADP-dependent NAD(P)H-hydrate dehydratase activity |
| Efet.01.465744.g420.t1 | amidase | 594 | 1.54E-107 | 85.85 | F:amidase activity; F:carbon-nitrogen ligase activity, with glutamine as amido-N-donor |
| Efet.01.46575.g1386.t1 | serine threonine- kinase MRCK alpha-like isoform X4 | 486 | 2.58E-66 | 83.7 | F:ATP binding; F:metal ion binding; F:protein serine/threonine kinase activity; P:protein phosphorylation; C:intracellular; P:actin cytoskeleton reorganization; P:intracellular signal transduction |
| Efet.01.465750.g421.t1 | hypothetical protein HELRODRAFT_159335 | 321 | 4.41E-09 | 59.5 | C:membrane; C:integral component of membrane; F:carbohydrate binding |
| Efet.01.465758.g422.t1 | hypothetical protein HELRODRAFT_188889 | 498 | 5.35E-37 | 89.5 |  |
| Efet.01.4658.g372.t1 | PTPRD_MOUSE ame: Full=Receptor-type tyrosine- phosphatase delta Short= -tyrosine phosphatase delta Short=R-PTP-delta Flags: Precursor | 468 | 3.72E-06 | 90 | P:neuron differentiation; P:protein dephosphorylation; F:cell adhesion molecule binding; P:heterophilic cell-cell adhesion via plasma membrane cell adhesion molecules; F:receptor binding; P:negative regulation of JAK-STAT cascade; F:phosphoprotein phosphatase activity; F:protein binding; F:protein tyrosine phosphatase activity; P:presynaptic membrane assembly; C:membrane; F:phosphatase activity; C:integral component of membrane; P:dephosphorylation; P:positive regulation of dendrite morphogenesis; P:regulation of immune response; F:hydrolase activity |
| Efet.01.465852.g423.t1 | endothelin-converting enzyme-like 1 | 516 | 2.10E-47 | 62.65 | C:integral component of plasma membrane; P:neuropeptide signaling pathway; F:metallopeptidase activity |
| Efet.01.46588.g1387.t1 | PREDICTED: uncharacterized protein LOC105437692 | 1755 | 1.48E-178 | 63.7 |  |
| Efet.01.46589.g1388.t1 | gastrula zinc finger -like isoform X1 | 1455 | 2.83E-112 | 58.7 | F:nucleic acid binding; F:transcription factor activity, sequence-specific DNA binding; F:metal ion binding |
| Efet.01.465938.g424.t1 | 39S ribosomal mitochondrial | 420 | 1.38E-52 | 71.3 | F:structural constituent of ribosome; F:rRNA binding; C:ribosome; P:translation |
| Efet.01.466010.g425.t1 | Potassium voltage-gated channel Shab | 702 | 4.62E-96 | 71.5 | P:potassium ion transport; P:transmembrane transport; F:delayed rectifier potassium channel activity; C:integral component of membrane; P:cellular process |
| Efet.01.466010.g427.t1 | hypothetical protein CRN43_21100 | 354 | 6.97E-06 | 54 |  |
| Efet.01.46602.g1389.t1 | PREDICTED: uncharacterized protein LOC106153553 | 774 | 5.37E-80 | 59.69 | F:nucleic acid binding; F:ATP binding; F:ATP-dependent helicase activity; F:helicase activity; F:hydrolase activity |
| Efet.01.466062.g429.t1 | Zinc finger MYM-type 4 | 429 | 1.34E-38 | 79.35 | F:DNA binding; F:zinc ion binding; P:cytoskeleton organization; P:regulation of cell morphogenesis; C:cytoplasm; P:multicellular organism development; C:nucleoplasm; F:RNA polymerase II transcription factor activity, sequence-specific DNA binding |
| Efet.01.466081.g431.t1 | hypothetical protein CAPTEDRAFT_188563 | 1125 | 1.78E-22 | 48 | F:G-protein coupled receptor activity; P:G-protein coupled receptor signaling pathway; P:signal transduction; C:membrane; C:integral component of membrane; F:signal transducer activity |
| Efet.01.466101.g432.t1 | somatostatin receptor type 2-like | 879 | 3.29E-80 | 63.55 | F:G-protein coupled peptide receptor activity; P:G-protein coupled receptor signaling pathway; C:membrane |
| Efet.01.466185.g433.t1 | myomodulin receptor 2 | 777 | 5.26E-55 | 52.45 | F:G-protein coupled receptor activity; P:signal transduction; C:membrane |
| Efet.01.466200.g434.t1 | FK506-binding 59-like isoform X1 | 369 | 1.14E-22 | 73.95 | C:endoplasmic reticulum membrane; F:peptidyl-prolyl cis-trans isomerase activity; F:FK506 binding; P:chaperone-mediated protein folding |
| Efet.01.466221.g435.t1 | mediator of RNA polymerase II transcription subunit 13-like | 324 | 8.94E-10 | 64.1 | C:mediator complex; F:RNA polymerase II transcription cofactor activity; P:regulation of transcription from RNA polymerase II promoter |
| Efet.01.466400.g436.t1 | hypothetical protein B5766_00670 | 288 | 4.88E-62 | 64.13 |  |
| Efet.01.466400.g437.t1 | family toxin-antitoxin system | 795 | 8.68E-59 | 69.35 |  |
| Efet.01.466400.g438.t1 | haloacid dehalogenase | 288 | 3.85E-29 | 79.05 | F:hydrolase activity |
| Efet.01.466476.g439.t1 | potassium voltage-gated channel subfamily A member 3-like | 1311 | 0 | 73.15 | F:voltage-gated potassium channel activity; P:potassium ion transmembrane transport; C:voltage-gated potassium channel complex; P:regulation of ion transmembrane transport; P:protein homooligomerization |
| Efet.01.466552.g440.t1 | PREDICTED: uncharacterized protein LOC105321710 isoform X1 | 1437 | 2.68E-42 | 59.45 |  |
| Efet.01.46657.g1394.t1 | CD109 antigen-like isoform X2 | 465 | 4.13E-38 | 58.55 | C:extracellular space; F:endopeptidase inhibitor activity; F:RNA binding; P:negative regulation of endopeptidase activity; C:extracellular region |
| Efet.01.466628.g442.t1 | lanosterol 14-alpha demethylase | 354 | 1.33E-54 | 83.5 | F:iron ion binding; P:response to yeast; C:integral component of membrane; P:cholesterol biosynthetic process via 24,25-dihydrolanosterol; C:endoplasmic reticulum; F:methyltransferase activity; P:oxidation-reduction process; F:sterol 14-demethylase activity; P:demethylation; C:plasma membrane; F:heme binding; P:methylation |
| Efet.01.466792.g446.t1 | 28S ribosomal mitochondrial-like | 873 | 4.49E-33 | 52.4 | C:ribosome |
| Efet.01.466842.g447.t1 | D-threo-3-hydroxyaspartate dehydratase-like | 351 | 2.12E-22 | 56.45 |  |
| Efet.01.466859.g448.t1 | PREDICTED: uncharacterized protein LOC106156013 | 750 | 3.35E-36 | 53.4 | C:membrane; C:integral component of membrane |
| Efet.01.466943.g452.t1 | titin-like isoform X12 | 207 | 1.85E-11 | 73.85 |  |
| Efet.01.467082.g453.t1 | galactoside 2-alpha-L-fucosyltransferase 2-like isoform X2 | 537 | 2.15E-14 | 52.11 | P:carbohydrate metabolic process; F:transferase activity, transferring glycosyl groups; P:fucosylation; C:membrane; F:transferase activity; F:galactoside 2-alpha-L-fucosyltransferase activity |
| Efet.01.467105.g454.t1 | copper homeostasis cutC homolog isoform X1 | 741 | 1.31E-96 | 78.85 | F:copper ion binding; C:cytoplasm; P:protein tetramerization; C:nucleolus; P:copper ion homeostasis |
| Efet.01.46711.g1396.t1 | maltase- intestinal-like | 342 | 5.62E-25 | 66.6 | P:metabolic process; F:hydrolase activity, acting on glycosyl bonds |
| Efet.01.467137.g455.t1 | hypothetical protein CAPTEDRAFT_91481 | 585 | 3.21E-08 | 88.67 | F:GTP binding; P:small GTPase mediated signal transduction; C:membrane; C:intracellular |
| Efet.01.467151.g456.t1 | TELO2-interacting 1 homolog | 426 | 8.73E-14 | 56.5 | C:TORC2 complex; C:cytoplasm; C:TORC1 complex; P:regulation of TOR signaling |
| Efet.01.467188.g460.t1 | calcium-independent kinase C isoform X1 | 363 | 3.81E-40 | 71.6 | F:nucleotide binding; F:ion binding; P:phosphorylation; F:protein serine/threonine kinase activity |
| Efet.01.46719.g1397.t1 | hypothetical protein CAPTEDRAFT_97992 | 453 | 7.38E-46 | 65.15 | C:membrane |
| Efet.01.467253.g461.t1 | transcription factor AP-1-like | 903 | 3.22E-19 | 49.85 | F:DNA binding |
| Efet.01.467295.g462.t1 | FAD-binding monooxygenase | 1302 | 0 | 77.4 | F:FAD binding; P:oxidation-reduction process; F:monooxygenase activity |
| Efet.01.467295.g463.t1 | VOC family | 384 | 3.28E-61 | 75.7 | F:hydrolase activity |
| Efet.01.467324.g465.t1 | tripartite motif-containing 2-like | 2040 | 0 | 72.7 | F:zinc ion binding; C:intracellular |
| Efet.01.467423.g467.t1 | short transient receptor potential channel 3-like | 387 | 2.86E-20 | 66.15 | F:ion channel activity; P:ion transport; P:transmembrane transport; C:membrane |
| Efet.01.467469.g468.t1 | relaxase | 369 | 5.61E-40 | 60.15 |  |
| Efet.01.467490.g469.t1 | cysteine desulfurase | 891 | 1.51E-78 | 76.6 | F:catalytic activity |
| Efet.01.4675.g374.t1 | Root phototropism 2 | 480 | 8.53E-29 | 52.33 |  |
| Efet.01.467568.g471.t1 | hypothetical protein CAPTEDRAFT_228921 | 387 | 2.56E-13 | 50.55 | C:membrane; C:integral component of membrane; F:carbohydrate binding |
| Efet.01.467593.g472.t1 | hypothetical protein HELRODRAFT_194269 | 258 | 2.46E-07 | 56.5 |  |
| Efet.01.46774.g1399.t1 | atrial natriuretic peptide receptor 1-like | 213 | 8.56E-18 | 76.54 | F:ATP binding; P:cGMP biosynthetic process; C:integral component of membrane; F:guanylate cyclase activity; F:protein kinase activity; P:protein phosphorylation; C:intracellular; P:intracellular signal transduction |
| Efet.01.46775.g1400.t1 | hypothetical protein HELRODRAFT_168336 | 393 | 3.03E-08 | 81 | P:proteolysis; C:integral component of membrane |
| Efet.01.467801.g475.t1 | 5-hydroxytryptamine receptor 2B | 465 | 1.88E-12 | 81.4 | P:G-protein coupled receptor signaling pathway; C:integral component of membrane; P:serotonin receptor signaling pathway; F:G-protein coupled serotonin receptor activity |
| Efet.01.467887.g476.t1 | RNA-binding single-stranded-interacting | 219 | 4.52E-20 | 84.75 | F:nucleotide binding; F:RNA binding |
| Efet.01.46790.g1401.t1 | cell cycle control 50A-like | 426 | 8.05E-24 | 68.05 | C:membrane |
| Efet.01.467938.g477.t1 | hypothetical protein HELRODRAFT_169095 | 255 | 5.43E-06 | 54 |  |
| Efet.01.467973.g478.t1 | protocadherin Fat 1-like | 462 | 4.45E-32 | 64.55 | F:calcium ion binding |
| Efet.01.46800.g1402.t1 | bark beetle isoform X1 | 366 | 1.67E-33 | 58.9 | C:membrane |
| Efet.01.468161.g480.t1 | PH domain leucine-rich repeat-containing phosphatase 2-like isoform X3 | 528 | 1.06E-34 | 73.7 | F:catalytic activity |
| Efet.01.468232.g482.t1 | DNA-binding Ikaros-like | 4785 | 6.32E-12 | 57 | F:metal ion binding |
| Efet.01.468278.g485.t1 | MULTISPECIES: family secretion | 1656 | 0 | 57.6 | C:membrane; C:integral component of membrane |
| Efet.01.46837.g1404.t1 | frizzled-1-like | 1509 | 0 | 72.75 | F:transmembrane signaling receptor activity; F:protein binding; C:membrane; P:cell differentiation; P:regulation of transcription, DNA-templated; P:canonical Wnt signaling pathway; P:negative regulation of cellular process; P:positive regulation of biological process; P:ventricular septum morphogenesis; P:tissue development |
| Efet.01.468387.g486.t1 | polycystic kidney disease 2-like 1 isoform X1 | 387 | 2.65E-52 | 77.65 | F:calcium ion binding; C:integral component of membrane |
| Efet.01.468407.g488.t1 | Jade-1 | 2529 | 1.37E-42 | 67.55 | P:negative regulation of canonical Wnt signaling pathway; P:histone H3 acetylation; C:ciliary basal body; F:metal ion binding; P:histone H4-K5 acetylation; C:cytosol; P:negative regulation of G1/S transition of mitotic cell cycle; P:histone H4-K12 acetylation; P:histone H4-K8 acetylation; F:RNA polymerase II transcription coactivator activity; C:plasma membrane; C:histone acetyltransferase complex |
| Efet.01.468431.g489.t1 | SMC-Scp complex subunit | 489 | 2.93E-105 | 88.2 | P:cell division; C:cytoplasm; P:chromosome separation |
| Efet.01.46849.g1405.t1 | serine threonine- kinase OSR1-like | 306 | 5.34E-32 | 71.65 | F:nucleotide binding; P:wing disc development; P:protein localization to cell cortex; C:cytoplasm; P:signal transduction; P:establishment of blood-nerve barrier; P:chitin-based embryonic cuticle biosynthetic process; P:sensory perception of pain; P:protein autophosphorylation; P:axon ensheathment; F:protein serine/threonine kinase activity |
| Efet.01.468501.g490.t1 | calsyntenin-2-like | 462 | 7.64E-20 | 52.9 | F:calcium ion binding; C:membrane; C:integral component of membrane; P:homophilic cell adhesion via plasma membrane adhesion molecules |
| Efet.01.468525.g491.t1 | E3 ubiquitin- ligase TRIM71-like | 828 | 3.66E-18 | 49.13 | P:signal transduction; P:regulation of apoptotic process |
| Efet.01.468581.g493.t1 | calcium-transporting ATPase sarcoplasmic endoplasmic reticulum type-like isoform X4 | 339 | 1.67E-56 | 91.1 | F:ATP binding; F:metal ion binding; C:integral component of membrane; P:calcium ion transmembrane transport; F:calcium-transporting ATPase activity; C:sarcoplasmic reticulum membrane |
| Efet.01.468601.g494.t1 | hypothetical protein HELRODRAFT_185513 | 204 | 3.90E-24 | 83 |  |
| Efet.01.468616.g495.t1 | serine threonine- kinase DCLK1-like isoform X3 | 843 | 1.55E-57 | 65.45 | F:protein binding; F:protein serine/threonine kinase activity; P:protein phosphorylation; C:intracellular part; P:single-organism process |
| Efet.01.468627.g496.t1 | pilus assembly | 3114 | 0 | 62.05 |  |
| Efet.01.468633.g497.t1 | acyltransferase | 939 | 1.83E-129 | 75.7 | F:transferase activity, transferring acyl groups; C:membrane; P:metabolic process |
| Efet.01.468658.g498.t1 | CSMD2_HUMAN ame: Full=CUB and sushi domain-containing 2 ame: Full=CUB and sushi multiple domains 2 | 390 | 7.74E-06 | 57 | C:membrane; C:integral component of membrane; C:plasma membrane |
| Efet.01.468693.g500.t1 | bifunctional (p)ppGpp synthetase guanosine-3 ,5 -bis(diphosphate) 3 -pyrophosphohydrolase | 501 | 8.64E-92 | 92.2 | F:GTP diphosphokinase activity; P:guanosine tetraphosphate metabolic process; P:phosphorylation; F:amino acid binding; F:kinase activity |
| Efet.01.468726.g502.t1 | hypothetical protein B5766_07350 | 255 | 6.72E-56 | 71 |  |
| Efet.01.468732.g503.t1 | elongation factor Tu | 540 | 5.77E-63 | 80.05 | F:GTP binding; F:translation elongation factor activity; C:cytoplasm; F:GTPase activity; P:translational elongation |
| Efet.01.468866.g504.t1 | cat eye syndrome critical region 6 homolog | 522 | 1.17E-08 | 51.33 | C:membrane; C:integral component of membrane |
| Efet.01.468984.g506.t1 | Nuclear receptor corepressor | 267 | 1.23E-18 | 78 | C:nucleus; F:DNA binding |
| Efet.01.46903.g1406.t1 | small GTP-binding domain | 297 | 6.54E-29 | 68.65 | P:signal transduction; P:cell morphogenesis; P:cell growth mode switching, budding to filamentous; P:filamentous growth of a population of unicellular organisms in response to biotic stimulus; P:cell budding; P:reproductive process; P:cell separation after cytokinesis; P:positive regulation of filamentous growth of a population of unicellular organisms in response to biotic stimulus; C:intracellular organelle part; P:pathogenesis; F:GTP binding; P:hyphal growth; C:cellular bud tip; C:cell septum; C:cellular bud neck; P:regulation of cell growth; C:membrane; P:establishment of cell polarity; C:hyphal tip polarisome; C:spitzenkorper |
| Efet.01.469050.g508.t1 | suppressor of lurcher 1-like | 240 | 2.82E-09 | 67.09 | C:membrane |
| Efet.01.469089.g509.t1 | hypothetical protein CAPTEDRAFT_222024 | 489 | 8.61E-33 | 61 | C:membrane; C:integral component of membrane; F:phosphotransferase activity, for other substituted phosphate groups; P:phospholipid biosynthetic process |
| Efet.01.469150.g511.t1 | tartrate-resistant acid phosphatase type 5-like | 402 | 2.60E-40 | 70.3 | F:hydrolase activity |
| Efet.01.469171.g512.t1 | piggyBac transposable element-derived 3-like | 399 | 4.99E-35 | 56.2 |  |
| Efet.01.469285.g515.t1 | heparan sulfate glucosamine 3-O-sulfotransferase | 525 | 9.42E-88 | 79.15 | C:integral component of membrane; F:sulfotransferase activity |
| Efet.01.469334.g516.t1 | Wnt-5b-like | 426 | 9.51E-77 | 71.6 | C:extracellular space; F:frizzled binding; P:neuron differentiation; P:cell fate commitment; P:Wnt signaling pathway; C:proteinaceous extracellular matrix |
| Efet.01.469369.g517.t1 | substrate import-associated zinc metallohydrolase lipo | 1560 | 0 | 70.4 |  |
| Efet.01.46938.g1410.t1 | tyramine receptor 1-like | 1401 | 6.38E-62 | 48.5 | P:signal transduction; F:signal transducer activity |
| Efet.01.46945.g1411.t1 | 39S ribosomal mitochondrial | 408 | 3.34E-27 | 63.85 | F:structural constituent of ribosome; C:mitochondrial ribosome; P:translation |
| Efet.01.469460.g518.t1 | probable phosphorylase b kinase regulatory subunit alpha isoform X2 | 1008 | 1.27E-96 | 73.65 | F:calmodulin binding; P:glycogen metabolic process; F:transferase activity |
| Efet.01.46947.g1412.t1 | Inactive phospholipase C 2 | 327 | 3.78E-19 | 61 | P:signal transduction; P:lipid metabolic process; F:phosphoric diester hydrolase activity |
| Efet.01.46947.g1413.t1 | endothelin-converting enzyme 1-like | 852 | 6.57E-48 | 48.85 | P:proteolysis; C:membrane; C:integral component of membrane; F:metalloendopeptidase activity; F:metallopeptidase activity |
| Efet.01.46950.g1414.t1 | cGMP-specific 3 ,5 -cyclic phosphodiesterase isoform X1 | 651 | 8.33E-37 | 63.35 | P:signal transduction; F:metal ion binding; F:phosphoric diester hydrolase activity; F:3',5'-cyclic-nucleotide phosphodiesterase activity; F:hydrolase activity |
| Efet.01.469585.g521.t1 | dynein heavy chain axonemal-like | 513 | 6.94E-60 | 82.9 | F:ATPase activity; F:ATP binding; F:microtubule motor activity; C:dynein complex; P:microtubule-based movement |
| Efet.01.469589.g522.t1 | chloride transport 6-like isoform X2 | 564 | 3.70E-20 | 72.3 | P:regulation of anion transmembrane transport; P:ion transmembrane transport; C:integral component of membrane; P:chloride transport; F:voltage-gated chloride channel activity |
| Efet.01.469592.g523.t1 | NC domain-containing | 495 | 3.55E-16 | 57.75 | P:RNA phosphodiester bond hydrolysis, endonucleolytic; F:nucleic acid binding; F:RNA-DNA hybrid ribonuclease activity; C:membrane; C:integral component of membrane |
| Efet.01.469651.g524.t1 | FMRFamide receptor-like | 1194 | 7.76E-77 | 55.3 | F:G-protein coupled receptor activity; F:G-protein coupled peptide receptor activity; P:G-protein coupled receptor signaling pathway; C:membrane; C:integral component of membrane |
| Efet.01.469672.g525.t1 | trafficking particle complex subunit 12-like | 1281 | 3.70E-18 | 62.55 | C:intracellular organelle; P:establishment of localization; F:endopeptidase activity; P:regulation of biological process |
| Efet.01.46971.g1415.t1 | misato homolog 1 | 411 | 1.62E-13 | 54.15 | F:GTPase activity |
| Efet.01.47013.g1417.t1 | raf homolog serine threonine- kinase phl | 897 | 9.02E-47 | 57.5 | P:signal transduction; F:ion binding; P:phosphorylation; F:kinase activity |
| Efet.01.470219.g535.t1 | protocadherin-19 isoform X1 | 1539 | 4.49E-37 | 43.95 | F:calcium ion binding; C:membrane; C:integral component of membrane; P:cell adhesion; P:homophilic cell adhesion via plasma membrane adhesion molecules; P:brain development; C:plasma membrane |
| Efet.01.470289.g538.t1 | PREDICTED: uncharacterized protein LOC109088671 isoform X1 | 426 | 9.27E-28 | 59.3 |  |
| Efet.01.470455.g540.t1 | glycine receptor subunit alpha-3-like isoform X1 | 498 | 8.53E-28 | 83.95 | F:transmitter-gated ion channel activity; P:chloride transmembrane transport; C:external side of plasma membrane; P:protein heterooligomerization; P:regulation of respiratory gaseous exchange by neurological system process; C:perikaryon; C:chloride channel complex; C:integral component of plasma membrane; C:dendrite; P:adult walking behavior; P:cellular response to amino acid stimulus; P:cellular response to zinc ion; P:startle response; C:postsynaptic membrane; P:muscle contraction; F:glycine binding; P:action potential; C:inhibitory synapse; P:synaptic transmission, glycinergic; F:zinc ion binding; P:righting reflex; F:extracellular-glycine-gated chloride channel activity; P:acrosome reaction; P:protein homooligomerization; C:endoplasmic reticulum; P:visual perception; P:cellular response to ethanol; P:neuromuscular process controlling posture; P:neuropeptide signaling pathway; P:negative regulation of transmission of nerve impulse; F:protein binding; F:taurine binding; P:inhibitory postsynaptic potential; P:positive regulation of acrosome reaction; C:cell junction |
| Efet.01.470533.g542.t1 | 72 kDa inositol polyphosphate 5-phosphatase | 201 | 2.32E-16 | 71.2 | C:membrane; F:hydrolase activity; P:phosphatidylinositol dephosphorylation |
| Efet.01.470540.g543.t1 | dynein heavy chain axonemal-like isoform X1 | 432 | 1.22E-74 | 88.6 | F:ATPase activity; F:ATP binding; F:microtubule motor activity; C:dynein complex; P:microtubule-based movement |
| Efet.01.470636.g546.t1 | acetylcholine receptor subunit alpha-like isoform X1 | 1059 | 3.95E-128 | 70.9 | C:postsynaptic membrane; P:cation transmembrane transport; C:integral component of membrane; C:cell junction; F:extracellular ligand-gated ion channel activity |
| Efet.01.470660.g547.t1 | ADP-ribose mitochondrial | 243 | 1.78E-12 | 62.3 | F:hydrolase activity |
| Efet.01.4707.g375.t1 | hypothetical protein CAPTEDRAFT_191217 | 831 | 1.66E-64 | 62 | P:transport |
| Efet.01.47076.g1419.t1 | probable palmitoyltransferase ZDHHC4 isoform X1 | 270 | 3.30E-19 | 57.85 | F:transferase activity |
| Efet.01.470851.g549.t1 | inactive tyrosine- kinase 7 | 405 | 1.84E-36 | 79.4 | P:lung-associated mesenchyme development; P:peptidyl-tyrosine phosphorylation; P:planar cell polarity pathway involved in neural tube closure; P:cochlea morphogenesis; P:ventricular septum development; P:coronary vasculature development; P:cell adhesion; P:convergent extension; P:axis elongation; C:integral component of plasma membrane; F:ATP binding; F:coreceptor activity involved in Wnt signaling pathway, planar cell polarity pathway; P:wound healing; C:cell-cell junction; C:focal adhesion; F:transmembrane receptor protein tyrosine kinase activity; P:cellular response to retinoic acid; P:establishment of planar polarity; P:establishment of epithelial cell apical/basal polarity; P:canonical Wnt signaling pathway; P:positive regulation of neuron projection development; P:cell migration; P:actin cytoskeleton reorganization |
| Efet.01.470864.g551.t1 | dynein heavy chain axonemal | 447 | 1.41E-58 | 84.1 | F:ATP binding; F:microtubule motor activity; F:calcium ion binding; C:dynein complex; P:microtubule-based movement |
| Efet.01.470890.g552.t1 | plexin-B-like isoform X1 | 1251 | 1.76E-39 | 48.85 | P:regulation of cellular process; P:single-organism process; P:cellular process; P:response to stimulus |
| Efet.01.4709.g376.t1 | Inositol 1,4,5-trisphosphate receptor type | 471 | 1.41E-46 | 89.35 | C:myelin sheath; P:sensory perception of umami taste; C:sarcoplasmic reticulum; P:sensory perception of sweet taste; P:protein heterooligomerization; C:neuronal cell body; C:nucleolus; P:memory; P:calcium ion transport into cytosol; C:nucleoplasm; C:endoplasmic reticulum membrane; C:integral component of plasma membrane; C:dendrite; C:secretory granule membrane; C:brush border; C:apical part of cell; F:calcium ion binding; P:sensory perception of bitter taste; P:calcium ion transmembrane transport; P:long-term synaptic potentiation; P:G-protein coupled receptor signaling pathway; F:inositol 1,4,5-trisphosphate-sensitive calcium-release channel activity; F:phosphatidylinositol binding; P:protein homooligomerization; F:inositol hexakisphosphate binding; C:nuclear outer membrane; C:receptor complex; F:inositol 1,3,4,5 tetrakisphosphate binding; C:perinuclear region of cytoplasm; F:protein binding; P:inositol phosphate-mediated signaling; P:response to calcium ion; P:cellular response to cAMP; F:inositol 1,4,5 trisphosphate binding |
| Efet.01.470916.g553.t1 | TANC2 isoform X3 | 342 | 1.02E-17 | 69 | F:zinc ion binding |
| Efet.01.471008.g554.t1 | lanC 3 isoform X3 | 273 | 7.90E-22 | 73.7 | C:membrane |
| Efet.01.47104.g1420.t1 | probable multidrug resistance-associated lethal(2)03659 | 312 | 2.59E-22 | 75.7 | F:nucleotide binding; F:ATPase activity; C:membrane; P:transport |
| Efet.01.471073.g555.t1 | sarcoplasmic calcium-binding -like | 351 | 1.39E-25 | 60.45 | F:calcium ion binding |
| Efet.01.471128.g556.t1 | amino acid amidase | 837 | 1.14E-169 | 69.3 | F:hydrolase activity |
| Efet.01.47121.g1422.t1 | leucine-rich repeat-containing 4B | 2013 | 2.96E-21 | 41.3 | C:cytoplasm; P:axonogenesis; P:cytokine-mediated signaling pathway; C:membrane; C:integral component of membrane; C:cerebellar mossy fiber; P:positive regulation of synapse assembly; F:protein kinase inhibitor activity; F:receptor binding; P:negative regulation of protein kinase activity; P:negative regulation of JAK-STAT cascade |
| Efet.01.471269.g557.t1 | hypothetical protein CAPTEDRAFT_224996 | 693 | 1.59E-08 | 45 |  |
| Efet.01.471289.g558.t1 | calcium-binding 5 | 246 | 6.24E-17 | 65.35 | F:calcium ion binding |
| Efet.01.47130.g1423.t1 | NFX1-type zinc finger-containing 1-like | 1422 | 4.20E-101 | 57.6 | C:nucleus; F:ATP binding; F:transcription factor activity, sequence-specific DNA binding; F:zinc ion binding; F:metal ion binding; P:regulation of transcription, DNA-templated |
| Efet.01.471393.g560.t1 | branched-chain amino acid ABC transporter permease | 675 | 4.79E-91 | 95.25 | C:integral component of membrane; P:transport; C:plasma membrane; F:transporter activity |
| Efet.01.471393.g561.t1 | branched-chain amino acid ABC transporter permease | 867 | 1.38E-145 | 85.25 | C:integral component of membrane; P:antibiotic transport; C:plasma membrane; F:transporter activity |
| Efet.01.471393.g562.t1 | DEAD DEAH box helicase | 1203 | 0 | 73.4 | F:nucleotide binding; F:hydrolase activity |
| Efet.01.471393.g563.t1 | oligoribonuclease | 579 | 1.45E-142 | 89.3 | F:nucleic acid binding; P:RNA phosphodiester bond hydrolysis, exonucleolytic; C:cytoplasm; F:exoribonuclease activity, producing 5'-phosphomonoesters |
| Efet.01.471393.g564.t1 | peptidase M48 | 1473 | 5.72E-168 | 88.6 | P:CAAX-box protein processing; C:integral component of membrane; F:metalloendopeptidase activity |
| Efet.01.471393.g565.t1 | ribosome small subunit-dependent GTPase A | 945 | 0 | 91.05 | F:GTP binding; F:metal ion binding; F:GTPase activity |
| Efet.01.471393.g566.t1 | cobalamin biosynthesis | 564 | 1.61E-128 | 93.95 | F:threonine-phosphate decarboxylase activity; F:cobalamin-transporting ATPase activity; P:vitamin transmembrane transport; C:integral component of membrane; P:cobalamin biosynthetic process; P:cobalamin transport; C:plasma membrane |
| Efet.01.471393.g567.t1 | coenzyme A pyrophosphatase | 732 | 4.69E-175 | 88.65 | F:hydrolase activity |
| Efet.01.471393.g568.t1 | family transcriptional regulator | 825 | 3.56E-175 | 72.4 | F:DNA binding; P:transcription, DNA-templated; P:regulation of transcription, DNA-templated |
| Efet.01.471393.g569.t1 | receptor family ligand-binding | 1095 | 0 | 70.5 | P:amino acid transport |
| Efet.01.471393.g570.t1 | branched-chain amino acid ABC transporter permease | 819 | 2.52E-143 | 67.2 | C:membrane |
| Efet.01.471393.g571.t1 | branched-chain amino acid ABC transporter permease | 798 | 1.26E-132 | 68.95 | C:membrane |
| Efet.01.471393.g572.t1 | ABC transporter ATP-binding | 471 | 2.50E-78 | 73.55 | F:ATPase activity; F:ATP binding |
| Efet.01.471393.g573.t1 | ABC transporter ATP-binding | 261 | 8.55E-52 | 76.45 | F:ATPase activity; F:ATP binding |
| Efet.01.471393.g574.t1 | ABC transporter ATP-binding | 267 | 1.73E-26 | 82.75 | F:ATPase activity; F:ATP binding |
| Efet.01.471393.g575.t1 | NAD(P)-dependent oxidoreductase | 978 | 3.76E-154 | 66.85 | F:oxidoreductase activity; P:oxidation-reduction process |
| Efet.01.471393.g576.t1 | amidase | 1710 | 0 | 75.55 | F:amidase activity; F:carbon-nitrogen ligase activity, with glutamine as amido-N-donor |
| Efet.01.471393.g577.t1 | acetyltransferase (GNAT) domain | 465 | 4.50E-110 | 73.15 | F:N-acetyltransferase activity |
| Efet.01.471518.g579.t1 | rho-related racA-like | 300 | 4.95E-30 | 81.85 | F:GTP binding; P:small GTPase mediated signal transduction; C:intracellular |
| Efet.01.471543.g580.t1 | regulator of cytokinesis 1-like isoform X2 | 264 | 6.86E-17 | 66.75 | P:microtubule cytoskeleton organization |
| Efet.01.471635.g581.t1 | Glutamate receptor 1 | 279 | 4.25E-15 | 61.7 | F:ion channel activity; C:membrane; F:receptor activity; P:transport |
| Efet.01.472084.g589.t1 | agrin-like | 303 | 9.85E-20 | 67.15 | P:G-protein coupled acetylcholine receptor signaling pathway; P:receptor clustering; F:laminin binding; C:plasma membrane |
| Efet.01.47213.g1427.t1 | rho GTPase-activating 21-like isoform X3 | 1752 | 1.04E-09 | 77.85 | P:biological regulation |
| Efet.01.472219.g591.t1 | neuralized 4 | 264 | 8.97E-23 | 75.2 |  |
| Efet.01.472222.g592.t1 | solute carrier family 12 member 4 isoform X3 | 330 | 9.57E-57 | 79.65 | F:potassium:chloride symporter activity; C:integral component of plasma membrane; F:protein kinase binding; P:potassium ion transmembrane transport; P:chloride transmembrane transport; F:potassium ion symporter activity; P:chemical synaptic transmission; P:cellular hypotonic salinity response; C:lysosomal membrane; P:cell volume homeostasis |
| Efet.01.472236.g594.t1 | hypothetical protein HELRODRAFT_73348 | 318 | 3.24E-07 | 65 | C:membrane; P:transport |
| Efet.01.472242.g595.t1 | family transcriptional regulator | 372 | 3.93E-53 | 59.5 | F:DNA binding; F:transcription factor activity, sequence-specific DNA binding; P:transcription, DNA-templated; P:regulation of transcription, DNA-templated |
| Efet.01.472242.g596.t1 | aminotransferase class V-fold PLP-dependent enzyme | 1113 | 0 | 67.6 | F:catalytic activity |
| Efet.01.472242.g597.t1 | sugar ABC transporter substrate-binding | 975 | 0 | 74.45 | P:transport; F:transporter activity |
| Efet.01.472242.g598.t1 | sugar ABC transporter permease | 783 | 1.48E-166 | 87.75 | C:integral component of membrane; P:transport; C:plasma membrane |
| Efet.01.472242.g599.t1 | carbohydrate ABC transporter permease | 366 | 3.47E-80 | 95.65 | C:integral component of membrane; P:transport; C:plasma membrane |
| Efet.01.472242.g600.t1 | 3-oxoacyl-ACP reductase | 771 | 1.10E-169 | 65.25 | F:oxidoreductase activity; F:gluconate 5-dehydrogenase activity; P:oxidation-reduction process |
| Efet.01.47225.g1428.t1 | Microtubule-associated futsch | 5265 | 4.45E-84 | 54.7 | P:microtubule cytoskeleton organization; F:microtubule binding; C:microtubule |
| Efet.01.47225.g1429.t1 | microtubule-associated futsch | 651 | 7.10E-16 | 69.45 | F:protein binding |
| Efet.01.472269.g601.t1 | anthrax edema factor in complex with calmodulin and pyrophosphate | 213 | 1.74E-22 | 89.7 | F:phosphatidylinositol 3-kinase binding; F:ligand-gated ion channel activity; P:positive regulation of DNA binding; P:regulation of rhodopsin mediated signaling pathway; P:positive regulation of GTPase activity; C:growth cone; P:positive regulation of protein autophosphorylation; C:calcium channel complex; P:positive regulation of nitric-oxide synthase activity; F:protein phosphatase activator activity; F:calcium ion binding; F:phospholipase binding; C:cytosol; F:titin binding; F:thioesterase binding; F:N-terminal myristoylation domain binding; F:nitric-oxide synthase binding; F:protein kinase binding; F:protein domain specific binding; P:G-protein coupled receptor signaling pathway; C:voltage-gated potassium channel complex; P:ion transmembrane transport; F:Ras guanyl-nucleotide exchange factor activity; P:Wnt signaling pathway, calcium modulating pathway; P:positive regulation of ryanodine-sensitive calcium-release channel activity; F:ion channel binding; P:negative regulation of ryanodine-sensitive calcium-release channel activity; C:spindle microtubule; P:detection of calcium ion; P:response to corticosterone; P:positive regulation of phosphoprotein phosphatase activity; C:sarcomere; P:inositol phosphate metabolic process; F:inositol-1,4,5-trisphosphate 3-kinase activity; P:Fc-epsilon receptor signaling pathway; P:regulation of high voltage-gated calcium channel activity; P:regulation of cell communication by electrical coupling involved in cardiac conduction; C:nucleoplasm; P:platelet degranulation; P:MAPK cascade; P:positive regulation of cyclic-nucleotide phosphodiesterase activity; C:extracellular exosome; P:G2/M transition of mitotic cell cycle; P:muscle contraction; P:regulation of cytokinesis; P:activation of adenylate cyclase activity; F:protein serine/threonine kinase activator activity; C:spindle pole; F:calcium-dependent protein binding; F:adenylate cyclase binding; P:glycogen catabolic process; P:substantia nigra development; P:regulation of heart rate; C:centrosome; F:type 3 metabotropic glutamate receptor binding; P:response to amphetamine; P:negative regulation of peptidyl-threonine phosphorylation; P:positive regulation of peptidyl-threonine phosphorylation; F:nitric-oxide synthase regulator activity; P:regulation of cardiac muscle contraction by regulation of the release of sequestered calcium ion; P:positive regulation of protein serine/threonine kinase activity |
| Efet.01.472289.g602.t1 | protein-tyrosine sulfotransferase isoform X1 | 264 | 1.36E-11 | 70.05 | F:protein-tyrosine sulfotransferase activity; P:peptidyl-tyrosine sulfation |
| Efet.01.472391.g605.t1 | steroid hormone receptor ERR1 | 483 | 3.11E-14 | 66.55 | F:zinc ion binding; P:transcription, DNA-templated; P:multicellular organism development; F:RNA polymerase II core promoter proximal region sequence-specific DNA binding; F:steroid binding; P:positive regulation of transcription from RNA polymerase II promoter; P:steroid hormone mediated signaling pathway; P:negative regulation of transcription from RNA polymerase II promoter; C:nucleus; F:transcriptional repressor activity, RNA polymerase II core promoter proximal region sequence-specific binding; F:transcriptional activator activity, RNA polymerase II core promoter proximal region sequence-specific binding; P:regulation of cell differentiation; C:integral component of membrane; F:steroid hormone receptor activity; P:regulation of ossification |
| Efet.01.472399.g606.t1 | Planctomycete cytochrome C | 225 | 4.25E-43 | 79.95 | F:electron carrier activity; F:heme binding |
| Efet.01.47240.g1431.t1 | axoneme-associated mst101(2)-like | 642 | 3.59E-12 | 51.38 | P:RNA phosphodiester bond hydrolysis, endonucleolytic; F:nucleic acid binding; F:RNA-DNA hybrid ribonuclease activity; C:membrane; C:integral component of membrane |
| Efet.01.472460.g608.t1 | glycogen brain form | 465 | 3.22E-74 | 70.65 | F:glycogen phosphorylase activity; F:pyridoxal phosphate binding; C:cytoplasm; P:glycogen catabolic process |
| Efet.01.472493.g610.t1 | DNA excision repair ERCC-6-like | 333 | 1.98E-36 | 80.45 | F:ATP binding; C:integral component of membrane |
| Efet.01.472549.g611.t1 | peptidyl-prolyl cis-trans isomerase-like | 369 | 1.43E-30 | 67.55 | P:cellular process; F:isomerase activity |
| Efet.01.472620.g613.t1 | nitroreductase | 1260 | 2.86E-119 | 74.05 | F:oxidoreductase activity; P:oxidation-reduction process |
| Efet.01.472620.g614.t1 | VIT family | 717 | 1.18E-152 | 71.45 | C:membrane |
| Efet.01.472620.g615.t1 | TIGR03364 family FAD-dependent oxidoreductase | 297 | 4.17E-53 | 71.7 | F:oxidoreductase activity; P:oxidation-reduction process |
| Efet.01.472687.g617.t1 | growth hormone secretagogue receptor type 1-like | 1215 | 3.98E-119 | 54.2 | F:signal transducer activity |
| Efet.01.472695.g619.t1 | ATP-dependent Clp protease ATP-binding subunit clpX- mitochondrial isoform X1 | 255 | 3.31E-17 | 75.65 | F:ATP binding; C:mitochondrial matrix; P:protein catabolic process; P:proteolysis; P:erythrocyte development; F:ATP-dependent peptidase activity |
| Efet.01.472791.g620.t1 | titin-like isoform X1 | 1407 | 1.11E-87 | 54.05 |  |
| Efet.01.472935.g622.t1 | EF-hand domain-containing 1-like | 411 | 3.07E-43 | 69 | C:motile cilium; F:calcium ion binding |
| Efet.01.473007.g625.t1 | follicular epithelium yolk subunit | 207 | 7.03E-14 | 67.05 |  |
| Efet.01.47302.g1433.t1 | neuroglian isoform X1 | 360 | 1.30E-14 | 54.15 | C:membrane; C:integral component of membrane; P:regulation of apoptotic process |
| Efet.01.473430.g628.t1 | E3 ubiquitin- ligase RNF31-like | 1254 | 4.82E-40 | 47.75 | F:ubiquitin-protein transferase activity; F:zinc ion binding; F:metal ion binding; C:LUBAC complex; C:intracellular; P:protein ubiquitination |
| Efet.01.47347.g1435.t1 | hypothetical protein CAPTEDRAFT_218417 | 2025 | 6.86E-82 | 44.05 |  |
| Efet.01.47349.g1436.t1 | beta-1,3-galactosyltransferase 1-like | 765 | 1.05E-24 | 61.3 | P:protein metabolic process; F:transferase activity, transferring glycosyl groups; C:membrane; C:Golgi apparatus; P:single-organism cellular process; C:intracellular organelle part |
| Efet.01.47349.g1437.t1 | beta-1,3-galactosyltransferase 1-like | 330 | 1.59E-07 | 57.88 | F:transferase activity, transferring glycosyl groups; C:membrane; C:integral component of membrane; P:protein glycosylation; F:galactosyltransferase activity; C:Golgi apparatus; C:Golgi membrane; F:transferase activity |
| Efet.01.47357.g1438.t1 | probable muscarinic acetylcholine receptor gar-2 | 612 | 3.08E-42 | 76.35 | C:integral component of plasma membrane; F:G-protein coupled acetylcholine receptor activity; P:G-protein coupled acetylcholine receptor signaling pathway |
| Efet.01.47357.g1439.t1 | muscarinic acetylcholine receptor M1-like | 1533 | 2.43E-38 | 75.55 | F:G-protein coupled receptor activity; P:G-protein coupled receptor signaling pathway; C:integral component of membrane |
| Efet.01.473583.g630.t1 | survival motor neuron | 240 | 1.46E-07 | 77.2 | C:nucleus; F:RNA binding; C:cytoplasm; P:mRNA processing |
| Efet.01.473681.g633.t1 | potassium channel kcnq | 279 | 1.12E-29 | 80.95 | F:voltage-gated potassium channel activity; P:potassium ion transmembrane transport; C:voltage-gated potassium channel complex |
| Efet.01.47379.g1440.t1 | atypical kinase C | 381 | 4.76E-71 | 88.15 | F:ATP binding; F:metal ion binding; P:protein phosphorylation; C:intracellular; P:intracellular signal transduction; F:protein kinase C activity |
| Efet.01.47403.g1441.t1 | excitatory amino acid transporter 1-like | 234 | 5.74E-07 | 73.15 | C:membrane; F:symporter activity; P:transport |
| Efet.01.474209.g638.t1 | UDP-glucuronosyltransferase 2A1-like | 1668 | 1.33E-116 | 53.95 | F:transferase activity |
| Efet.01.47426.g1442.t1 | phosphatase and actin regulator 2-like isoform X4 | 222 | 3.67E-21 | 88.65 | C:integral component of membrane |
| Efet.01.474317.g639.t1 | FMRFamide receptor-like | 1074 | 1.29E-51 | 53.55 | F:G-protein coupled receptor activity; F:G-protein coupled peptide receptor activity; P:G-protein coupled receptor signaling pathway; C:membrane; C:integral component of membrane |
| Efet.01.474362.g640.t1 | zinc finger 596-like isoform X1 | 768 | 2.61E-42 | 53.75 | F:nucleic acid binding; F:metal ion binding |
| Efet.01.474390.g641.t1 | Bardet-Biedl syndrome 1 -like isoform X3 | 228 | 1.59E-31 | 83.1 | P:neural precursor cell proliferation; P:Golgi to plasma membrane protein transport; P:cellular lipid metabolic process; P:brain morphogenesis; P:striatum development; P:fat cell differentiation; F:patched binding; P:protein localization to cilium; C:axoneme; F:RNA polymerase II repressing transcription factor binding; C:motile cilium; P:neuron migration; P:photoreceptor cell maintenance; P:sensory perception of smell; P:dendrite development; C:BBSome; C:ciliary basal body; P:photoreceptor cell morphogenesis; P:regulation of cilium beat frequency involved in ciliary motility; P:fertilization; P:non-motile cilium assembly; P:hippocampus development; P:microtubule cytoskeleton organization; P:cerebral cortex development; P:olfactory behavior; P:hormone metabolic process; P:ventricular system development; C:centrosome; F:smoothened binding; C:integral component of membrane; F:phosphoprotein binding; P:adult behavior; P:intraciliary retrograde transport; P:cartilage development |
| Efet.01.47442.g1443.t1 | retinoic acid receptor RXR-alpha-B-like | 417 | 1.38E-33 | 81.45 | F:thyroid hormone receptor activity; C:nucleus; F:zinc ion binding; P:transcription, DNA-templated; F:steroid hormone receptor activity; P:regulation of transcription, DNA-templated; F:sequence-specific DNA binding; P:steroid hormone mediated signaling pathway; P:intracellular receptor signaling pathway |
| Efet.01.474601.g644.t1 | hypothetical protein HELRODRAFT_104231 | 810 | 4.42E-08 | 69 |  |
| Efet.01.47478.g1446.t1 | hypothetical protein CAPTEDRAFT_195367 | 327 | 7.80E-07 | 52 | C:membrane; C:integral component of membrane |
| Efet.01.474909.g646.t1 | metabotropic glutamate receptor 3-like | 2016 | 2.16E-120 | 49.65 | F:G-protein coupled receptor activity; P:G-protein coupled receptor signaling pathway; P:signal transduction; C:membrane; C:integral component of membrane; F:signal transducer activity; C:plasma membrane |
| Efet.01.474932.g647.t1 | sulfate transporter | 2247 | 0 | 92.1 | F:secondary active sulfate transmembrane transporter activity; C:integral component of membrane; P:sulfate transmembrane transport |
| Efet.01.474932.g648.t1 | hypothetical protein Veis_3286 | 378 | 1.22E-50 | 98 | C:integral component of membrane |
| Efet.01.474936.g649.t1 | SCL-interrupting locus | 252 | 8.25E-22 | 74.8 | C:centrosome; C:cytoplasm; P:cell cycle process; P:microtubule cytoskeleton organization |
| Efet.01.475008.g652.t1 | hypothetical protein HELRODRAFT_171217 | 228 | 4.21E-13 | 65 |  |
| Efet.01.475036.g653.t1 | innexin unc-9-like isoform X2 | 879 | 1.04E-73 | 69.95 | C:membrane; C:cell junction; P:transport |
| Efet.01.475053.g654.t1 | phosphofurin acidic cluster sorting 2-like isoform X1 | 204 | 4.31E-11 | 89.05 | P:protein targeting to plasma membrane; C:intracellular |
| Efet.01.47515.g1447.t1 | dual specificity mitogen-activated kinase kinase 6 | 609 | 9.67E-69 | 79.55 | P:positive regulation of prostaglandin secretion; C:intracellular organelle; F:ATP binding; F:protein kinase binding; P:MAPK cascade; P:activation of MAPK activity; F:signal transducer, downstream of receptor, with serine/threonine kinase activity; P:positive regulation of nitric-oxide synthase biosynthetic process; C:cytoplasm; P:positive regulation of apoptotic process; F:MAP kinase kinase activity; P:cardiac muscle contraction |
| Efet.01.475266.g656.t1 | tribbles homolog 2 | 600 | 3.80E-37 | 64.5 | P:regulation of cellular protein metabolic process; F:binding |
| Efet.01.475314.g659.t1 | growth differentiation factor 8 | 861 | 7.81E-52 | 50.9 | F:growth factor activity; C:extracellular region; P:growth |
| Efet.01.475381.g660.t1 | probable G- coupled receptor 139 | 819 | 4.93E-31 | 54.15 | F:G-protein coupled receptor activity; P:G-protein coupled receptor signaling pathway; P:signal transduction; C:membrane; C:integral component of membrane; F:signal transducer activity |
| Efet.01.475381.g661.t1 | probable G- coupled receptor 139 | 417 | 3.48E-13 | 58.45 | F:G-protein coupled receptor activity; P:G-protein coupled receptor signaling pathway; P:signal transduction; C:membrane; C:integral component of membrane; F:signal transducer activity |
| Efet.01.475523.g665.t1 | glycosyl transferase | 417 | 7.36E-49 | 80.8 | C:integral component of membrane; F:transferase activity |
| Efet.01.47558.g1448.t1 | ELAV 4 isoform X4 | 399 | 1.93E-51 | 84.65 | F:nucleotide binding; F:RNA binding |
| Efet.01.475649.g667.t1 | Soluble calcium-activated nucleotidase 1 | 873 | 5.88E-85 | 65 | F:nucleoside-diphosphatase activity; P:cellular process |
| Efet.01.4757.g380.t1 | 14-3-3 epsilon | 717 | 4.57E-125 | 80.85 | F:protein domain specific binding |
| Efet.01.475707.g669.t1 | matrix metallo ase-19 | 210 | 2.22E-27 | 77.3 | F:zinc ion binding; F:calcium ion binding; C:membrane; P:proteolysis; C:extracellular matrix; F:metalloendopeptidase activity |
| Efet.01.475796.g670.t1 | Plexin-B | 339 | 1.39E-41 | 77.6 | C:integral component of membrane; F:semaphorin receptor activity; P:semaphorin-plexin signaling pathway |
| Efet.01.475948.g671.t1 | Collagen alpha-5(VI) chain | 468 | 2.17E-32 | 60.2 | F:calcium ion binding; C:collagen trimer |
| Efet.01.47595.g1449.t1 | retinoic acid receptor RXR-alpha-B-like | 480 | 4.26E-57 | 71.35 | F:DNA binding; F:RNA polymerase II transcription factor activity, ligand-activated sequence-specific DNA binding; F:metal ion binding; F:signaling receptor activity; P:intracellular receptor signaling pathway |
| Efet.01.476004.g672.t1 | striatin-3 isoform X1 | 216 | 1.20E-17 | 70.6 |  |
| Efet.01.476046.g673.t1 | GTP-binding Di-Ras2 | 390 | 3.10E-64 | 86.5 | F:GTP binding; P:positive regulation of MAP kinase activity; P:small GTPase mediated signal transduction; C:intracellular; C:plasma membrane |
| Efet.01.476292.g674.t1 | 4-hydroxy-tetrahydrodipicolinate reductase | 261 | 1.73E-47 | 77.3 | F:NADP binding; F:oxidoreductase activity, acting on CH or CH2 groups, NAD or NADP as acceptor; C:cytoplasm; F:NAD binding; P:lysine biosynthetic process via diaminopimelate; F:4-hydroxy-tetrahydrodipicolinate reductase; P:oxidation-reduction process; P:diaminopimelate biosynthetic process |
| Efet.01.47630.g1450.t1 | dynein heavy chain axonemal-like isoform X1 | 531 | 1.51E-79 | 87.25 | F:ATPase activity; F:ATP binding; F:microtubule motor activity; C:axonemal dynein complex; P:cilium or flagellum-dependent cell motility; P:microtubule-based movement |
| Efet.01.476494.g676.t1 | MFS transporter | 1254 | 0 | 91.3 | P:transmembrane transport; C:integral component of membrane |
| Efet.01.476494.g677.t1 | sulfatase | 1488 | 0 | 58.05 | F:sulfuric ester hydrolase activity; P:metabolic process |
| Efet.01.476525.g679.t1 | AN1-type zinc finger 6 isoform X1 | 234 | 4.13E-09 | 75.35 | F:DNA binding; F:zinc ion binding |
| Efet.01.476543.g680.t1 | tripartite motif-containing 45 isoform X1 | 960 | 3.28E-07 | 45.42 | F:zinc ion binding; C:intracellular |
| Efet.01.47657.g1451.t1 | rna-binding mex3b | 459 | 5.04E-07 | 85.8 | F:nucleic acid binding; F:AU-rich element binding; C:nucleus; F:RNA binding; C:perinuclear region of cytoplasm; F:RNA binding |
| Efet.01.476603.g681.t1 | rho GTPase-activating 190-like isoform X1 | 246 | 9.21E-23 | 71.2 | C:cytoplasm; P:signal transduction; P:regulation of GTPase activity |
| Efet.01.476793.g685.t1 | Retrovirus-related Pol poly from transposon 412 | 243 | 1.12E-07 | 60.67 |  |
| Efet.01.476829.g686.t1 | myosin light chain smooth muscle-like | 534 | 3.91E-68 | 86.35 | F:ATP binding; F:protein serine/threonine kinase activity; P:protein phosphorylation |
| Efet.01.476973.g687.t1 | calmodulin 1 | 558 | 1.94E-29 | 62.7 | F:calcium ion binding |
| Efet.01.476997.g688.t1 | oocyte zinc finger 6-like | 1059 | 1.51E-59 | 50.2 | F:nucleic acid binding; F:metal ion binding |
| Efet.01.476997.g689.t1 | zinc finger 208-like | 5253 | 0 | 44.95 |  |
| Efet.01.477074.g690.t1 | sprouty homolog 3 | 1335 | 7.95E-27 | 61.95 | P:axon development; P:multicellular organism development; C:membrane; P:regulation of signal transduction |
| Efet.01.47711.g1452.t1 | N,N-dimethylformamidase | 267 | 1.12E-51 | 81.5 |  |
| Efet.01.47711.g1453.t1 | Transposase DDE domain group 1 | 279 | 1.91E-14 | 88.25 |  |
| Efet.01.47719.g1455.t1 | trichohyalin-like isoform X7 | 366 | 6.70E-11 | 64.6 | F:zinc ion binding; F:metal ion binding |
| Efet.01.477269.g692.t1 | EF-hand domain-containing family member C2 | 726 | 2.05E-23 | 45.35 | P:biological_process; F:calcium ion binding; C:cellular_component |
| Efet.01.477273.g693.t1 | tyrosine- kinase PR2-like isoform X1 | 666 | 8.42E-15 | 54.35 | F:nucleotide binding; F:protein tyrosine kinase activity; P:protein phosphorylation |
| Efet.01.477289.g694.t1 | ATP synthase subunit mitochondrial | 399 | 5.39E-31 | 69.9 | F:nucleotide binding; P:hydrogen ion transmembrane transport; C:proton-transporting two-sector ATPase complex, catalytic domain; P:ATP biosynthetic process; F:hydrolase activity, acting on acid anhydrides, catalyzing transmembrane movement of substances |
| Efet.01.477310.g695.t1 | LIX1 | 285 | 4.10E-47 | 88.1 |  |
| Efet.01.477390.g698.t1 | sugar ABC transporter permease | 597 | 1.60E-115 | 93.1 | C:integral component of membrane; P:transport; C:plasma membrane |
| Efet.01.4774.g381.t1 | guanylyl cyclase GC-E-like | 291 | 2.98E-25 | 72.4 | F:nucleotide binding; F:phosphorus-oxygen lyase activity; C:membrane; P:cyclic nucleotide biosynthetic process |
| Efet.01.477434.g699.t1 | eukaryotic translation initiation factor isoform CRA_c | 399 | 3.75E-87 | 97.45 | P:negative regulation of RNA-directed 5'-3' RNA polymerase activity; P:translational initiation; P:regulation of translational initiation; F:ATP-dependent RNA helicase activity; F:RNA binding; F:translation initiation factor activity; F:ATP binding; C:perinuclear region of cytoplasm; C:extracellular exosome; F:protein binding; F:double-stranded RNA binding; C:membrane; P:RNA secondary structure unwinding; C:extracellular matrix |
| Efet.01.477481.g700.t1 | homeobox cut-like 1 isoform X2 | 1425 | 2.41E-72 | 63.9 | C:nucleus; F:DNA binding |
| Efet.01.477481.g701.t1 | homeobox cut-like 1 isoform X2 | 1092 | 9.12E-76 | 59.8 | F:DNA binding; C:nucleus; P:transcription, DNA-templated; P:regulation of transcription, DNA-templated; F:sequence-specific DNA binding |
| Efet.01.477540.g706.t1 | FMRFamide receptor-like | 963 | 9.92E-85 | 53.9 | F:signal transducer activity |
| Efet.01.477544.g707.t1 | B-cell lymphoma 3 | 1707 | 4.51E-42 | 61.35 | P:positive regulation of translation; P:transcription, DNA-templated; P:anatomical structure development; P:intrinsic apoptotic signaling pathway in response to DNA damage by p53 class mediator; F:transcription factor binding; P:I-kappaB kinase/NF-kappaB signaling; P:response to virus; P:regulation of NF-kappaB import into nucleus; C:nucleoplasm; P:single-organism developmental process; P:negative regulation of interleukin-8 biosynthetic process; C:intercellular bridge; P:regulation of DNA binding; P:immune response; P:negative regulation of transcription, DNA-templated; C:cytoplasm; P:positive regulation of transcription, DNA-templated; P:negative regulation of apoptotic process; P:protein import into nucleus, translocation; P:DNA damage response, signal transduction by p53 class mediator; C:Bcl3/NF-kappaB2 complex; P:response to UV-C; F:protein binding, bridging; C:Bcl3-Bcl10 complex |
| Efet.01.477548.g709.t1 | zinc finger 236-like isoform X3 | 288 | 8.50E-24 | 80.65 | F:nucleic acid binding; F:metal ion binding |
| Efet.01.477558.g710.t1 | dynein heavy chain axonemal-like | 222 | 6.89E-35 | 93.3 | F:ATPase activity; F:ATP binding; F:microtubule motor activity; C:integral component of membrane; C:dynein complex; P:microtubule-based movement |
| Efet.01.477628.g711.t1 | collagen alpha-6(VI) chain-like | 360 | 1.02E-18 | 56.5 | F:calcium ion binding |
| Efet.01.477677.g712.t1 | glutamate--cysteine ligase | 1395 | 0 | 86.95 | F:ATP binding; P:glutathione biosynthetic process; F:glutamate-cysteine ligase activity |
| Efet.01.477677.g713.t1 | conserved hypothetical protein | 306 | 1.52E-21 | 89.1 |  |
| Efet.01.47771.g1456.t1 | magnesium transporter NIPA2-like isoform X3 | 585 | 1.12E-49 | 76.15 | C:integral component of membrane; F:magnesium ion transmembrane transporter activity; P:magnesium ion transmembrane transport |
| Efet.01.477781.g715.t1 | forkhead box A | 1533 | 1.33E-67 | 70.4 | F:DNA binding; F:protein binding |
| Efet.01.477826.g716.t1 | flocculation FLO11-like isoform X2 | 723 | 2.08E-27 | 50.59 | C:centrosome; P:mitotic spindle organization; C:ciliary base; P:cytoplasmic microtubule organization |
| Efet.01.477877.g717.t1 | clavaminate synthase At3g21360 | 378 | 2.88E-27 | 64.75 | C:nucleus |
| Efet.01.477895.g718.t1 | dynein heavy chain axonemal-like | 747 | 2.03E-126 | 90.1 | F:ATPase activity; F:ATP binding; F:microtubule motor activity; F:actin binding; C:dynein complex; P:phosphorylation; P:microtubule-based movement; F:kinase activity |
| Efet.01.478012.g719.t1 | hypothetical protein CAPTEDRAFT_115450 | 246 | 1.35E-12 | 60.5 | P:G-protein coupled acetylcholine receptor signaling pathway; P:receptor clustering; F:laminin binding; C:plasma membrane |
| Efet.01.478046.g720.t1 | uncharacterized protein LOC110452651 | 603 | 6.52E-23 | 56.9 | F:catalytic activity; P:metabolic process; C:membrane; C:integral component of membrane |
| Efet.01.478130.g721.t1 | Lipoxygenase homology domain-containing | 291 | 2.21E-11 | 70.4 | P:sensory perception of sound; C:stereocilium |
| Efet.01.47816.g1458.t1 | homeobox SIX6 | 393 | 2.11E-80 | 95.65 | C:nucleus; F:DNA binding; P:eye development; P:transcription from RNA polymerase II promoter; F:transcriptional activator activity, RNA polymerase II distal enhancer sequence-specific binding; P:brain development; P:visual perception; P:positive regulation of transcription from RNA polymerase II promoter; P:animal organ morphogenesis |
| Efet.01.478212.g723.t1 | Neuronal acetylcholine receptor subunit alpha- | 399 | 3.74E-46 | 77.85 | P:chemical synaptic transmission, postsynaptic; P:synaptic transmission, cholinergic; F:acetylcholine receptor activity; P:neuromuscular synaptic transmission; C:postsynaptic membrane; P:cation transmembrane transport; C:cell junction; F:extracellular ligand-gated ion channel activity; F:acetylcholine binding; C:acetylcholine-gated channel complex; P:response to nicotine |
| Efet.01.478316.g724.t1 | Actin-binding anillin | 282 | 2.03E-42 | 78.95 | C:actomyosin contractile ring; F:actin binding; P:glomerular visceral epithelial cell migration; P:mitotic cytokinesis; P:regulation of exit from mitosis; P:septin ring assembly; C:nucleoplasm |
| Efet.01.478397.g725.t1 | 28S ribosomal mitochondrial | 909 | 4.31E-122 | 67.7 | C:ribosome; C:intracellular organelle part |
| Efet.01.478434.g726.t1 | beta-1,3-galactosyltransferase 1-like | 1488 | 1.16E-52 | 55.15 | F:transferase activity, transferring glycosyl groups; C:membrane; C:integral component of membrane; P:protein glycosylation; F:galactosyltransferase activity; C:Golgi apparatus; C:Golgi membrane; F:transferase activity |
| Efet.01.478569.g728.t1 | neuralized isoform X2 | 525 | 1.68E-29 | 62.35 | F:zinc ion binding; F:metal ion binding |
| Efet.01.47857.g1459.t1 | leucine-rich repeat-containing 49-like isoform X2 | 309 | 1.65E-23 | 76.3 |  |
| Efet.01.478646.g729.t1 | short transient receptor potential channel 6 | 396 | 4.36E-18 | 66.54 | F:calcium channel activity; C:integral component of membrane; P:calcium ion transmembrane transport; P:single-organism process |
| Efet.01.47875.g1460.t1 | MOB kinase activator-like 2 isoform X2 | 432 | 2.47E-14 | 67.75 |  |
| Efet.01.478766.g731.t1 | Transcriptional repressor scratch 1 | 387 | 1.34E-46 | 72.05 | F:nucleic acid binding; F:metal ion binding |
| Efet.01.479006.g732.t1 | carnosine N-methyltransferase | 447 | 8.81E-51 | 82.65 |  |
| Efet.01.479080.g734.t1 | hypothetical protein CAPTEDRAFT_198655 | 954 | 3.62E-15 | 73 |  |
| Efet.01.479239.g739.t1 | DBF4 homolog B isoform X1 | 510 | 1.79E-06 | 59 |  |
| Efet.01.479283.g741.t1 | tripartite motif-containing 71 (TRIM71) | 1077 | 9.31E-09 | 47 | F:zinc ion binding; F:metal ion binding; C:membrane; C:integral component of membrane; C:intracellular |
| Efet.01.479285.g742.t1 | cat eye syndrome critical region 6-like | 507 | 4.39E-11 | 63.29 | C:membrane; C:integral component of membrane |
| Efet.01.479285.g743.t1 | cat eye syndrome critical region 6-like | 594 | 1.65E-13 | 49.27 | C:membrane; C:integral component of membrane |
| Efet.01.479290.g744.t1 | abscission ut checkpoint regulator-like isoform X2 | 420 | 7.47E-08 | 48.33 |  |
| Efet.01.479325.g745.t1 | T9SS C-terminal target domain-containing | 1095 | 5.24E-44 | 53.56 |  |
| Efet.01.479337.g747.t1 | export cytoplasm | 318 | 6.23E-62 | 94.45 | F:ATP binding; P:protein targeting; C:cytoplasm; P:intracellular protein transmembrane transport; P:protein import; C:plasma membrane |
| Efet.01.479364.g748.t1 | sodium-coupled neutral amino acid transporter 7 | 408 | 1.44E-36 | 66.15 | F:L-amino acid transmembrane transporter activity; P:cation transport; P:L-alpha-amino acid transmembrane transport; F:neutral amino acid transmembrane transporter activity; C:membrane; F:cation transmembrane transporter activity; P:neutral amino acid transport |
| Efet.01.479453.g749.t1 | zinc finger 236-like isoform X2 | 2100 | 5.39E-13 | 60.45 | F:nucleic acid binding; F:metal ion binding |
| Efet.01.479751.g753.t1 | hypothetical protein HELRODRAFT_194765 | 318 | 5.00E-56 | 74.25 |  |
| Efet.01.47978.g1461.t1 | hypothetical protein LOTGIDRAFT_235628 | 423 | 1.63E-07 | 50 |  |
| Efet.01.47983.g1462.t1 | bifunctional arginine demethylase and lysyl-hydroxylase PSR | 204 | 9.48E-11 | 65.6 | F:methyltransferase activity; P:methylation |
| Efet.01.479869.g754.t1 | UMP kinase | 585 | 5.65E-115 | 82.05 | F:ATP binding; P:nucleotide phosphorylation; C:cytoplasm; P:'de novo' CTP biosynthetic process; F:UMP kinase activity |
| Efet.01.47993.g1463.t1 | coiled-coil domain-containing 171-like | 726 | 5.52E-28 | 52.5 |  |
| Efet.01.479953.g756.t1 | Modification methylase | 555 | 4.28E-91 | 75.2 | F:DNA (cytosine-5-)-methyltransferase activity; P:C-5 methylation of cytosine; P:DNA restriction-modification system |
| Efet.01.48003.g1464.t1 | PH and SEC7 domain-containing 4-like isoform X2 | 1122 | 5.26E-06 | 96 | F:ARF guanyl-nucleotide exchange factor activity; P:regulation of ARF protein signal transduction; F:phospholipid binding; P:positive regulation of GTPase activity |
| Efet.01.48009.g1466.t1 | titin isoform X2 | 432 | 2.46E-19 | 55.9 |  |
| Efet.01.480190.g759.t1 | transformation transcription domain-associated | 357 | 3.33E-53 | 80.1 | P:histone acetylation; P:phosphorylation; F:kinase activity; C:histone acetyltransferase complex |
| Efet.01.480232.g760.t1 | hypothetical protein HELRODRAFT_119038, partial | 438 | 9.13E-16 | 77.67 | P:regulation of postsynaptic membrane potential; P:regulation of ion transmembrane transport; C:voltage-gated sodium channel complex; P:sodium ion transmembrane transport; F:voltage-gated sodium channel activity |
| Efet.01.480239.g761.t1 | hypothetical protein HELRODRAFT_178970 | 300 | 1.70E-10 | 86 |  |
| Efet.01.480258.g762.t1 | 60S ribosomal L26-like | 285 | 1.95E-27 | 88.15 | F:structural constituent of ribosome; P:cytoplasmic translation; P:ribosomal large subunit biogenesis; C:cytosolic large ribosomal subunit |
| Efet.01.480432.g763.t1 | Neuropeptide FF receptor 2 | 570 | 1.14E-49 | 57.95 | F:G-protein coupled receptor activity; P:G-protein coupled receptor signaling pathway; C:membrane |
| Efet.01.48054.g1469.t1 | ruvB-like helicase 1 | 270 | 7.99E-48 | 95.6 | C:nucleus; F:ATP binding; P:DNA repair; F:ATP-dependent 5'-3' DNA helicase activity; P:transcription, DNA-templated; P:DNA duplex unwinding; P:regulation of transcription, DNA-templated |
| Efet.01.480590.g768.t1 | sugar ABC transporter permease | 309 | 3.58E-66 | 91 | C:integral component of membrane; P:transport; C:plasma membrane |
| Efet.01.48071.g1470.t1 | receptor-type tyrosine- phosphatase kappa-like isoform X2 | 816 | 8.33E-07 | 43.13 | F:phosphoprotein phosphatase activity; F:protein tyrosine phosphatase activity; P:protein dephosphorylation; C:membrane; F:phosphatase activity; C:integral component of membrane; P:dephosphorylation; P:peptidyl-tyrosine dephosphorylation; F:hydrolase activity |
| Efet.01.480730.g771.t1 | Collagen alpha-1(IV) | 405 | 6.92E-41 | 84.7 | P:renal tubule morphogenesis; P:brain development; C:collagen type IV trimer; F:platelet-derived growth factor binding; P:retinal blood vessel morphogenesis; P:neuromuscular junction development; P:epithelial cell differentiation; C:endoplasmic reticulum lumen; P:cellular response to amino acid stimulus; P:branching involved in blood vessel morphogenesis; P:basement membrane organization; P:collagen catabolic process; F:extracellular matrix constituent conferring elasticity |
| Efet.01.48092.g1472.t1 | mitochondrial ATP synthase subunit 9 precursor | 429 | 5.79E-30 | 81.45 | F:hydrogen ion transmembrane transporter activity; P:ATP hydrolysis coupled proton transport; C:proton-transporting ATP synthase complex, coupling factor F(o); C:integral component of membrane; P:ATP synthesis coupled proton transport |
| Efet.01.48095.g1473.t1 | nucleosome-remodeling factor subunit BPTF-like | 678 | 1.24E-71 | 73.4 | F:metal ion binding |
| Efet.01.481185.g774.t1 | PA14 domain-containing | 756 | 4.43E-06 | 47 |  |
| Efet.01.481211.g775.t1 | Golgi SNAP receptor complex member 2-like | 510 | 7.57E-66 | 71.3 | C:membrane |
| Efet.01.48123.g1475.t1 | hypothetical protein CAPTEDRAFT_193210 | 216 | 1.38E-11 | 64 | C:membrane; C:integral component of membrane |
| Efet.01.481316.g777.t1 | TSC22 domain family 1-like | 1326 | 3.10E-25 | 53.85 |  |
| Efet.01.481364.g778.t1 | cAMP-specific 3 ,5 -cyclic phosphodiesterase | 507 | 1.43E-52 | 82.55 | P:multicellular organism growth; P:regulation of release of sequestered calcium ion into cytosol by sarcoplasmic reticulum; F:drug binding; C:voltage-gated calcium channel complex; P:regulation of cell communication by electrical coupling involved in cardiac conduction; P:negative regulation of heart contraction; P:smooth muscle contraction; P:cellular response to epinephrine stimulus; P:positive regulation of interferon-gamma production; P:negative regulation of peptidyl-serine phosphorylation; C:cytosol; P:T cell receptor signaling pathway; F:beta-2 adrenergic receptor binding; P:positive regulation of interleukin-2 production; P:adrenergic receptor signaling pathway involved in positive regulation of heart rate; P:regulation of cardiac muscle cell contraction; P:cAMP catabolic process; P:aging; F:ion channel binding; P:establishment of endothelial barrier; P:negative regulation of relaxation of cardiac muscle; F:3',5'-cyclic-AMP phosphodiesterase activity; P:regulation of ryanodine-sensitive calcium-release channel activity; F:scaffold protein binding; C:centrosome; F:ubiquitin protein ligase binding; P:cellular response to lipopolysaccharide; F:metal ion binding; P:regulation of cAMP metabolic process; P:cellular response to cAMP; P:neutrophil chemotaxis; F:cAMP binding; C:apical plasma membrane; P:cAMP-mediated signaling; F:ATPase binding; P:positive regulation of interleukin-5 production |
| Efet.01.481492.g780.t1 | glutamate receptor 3 isoform X4 | 210 | 9.39E-14 | 95.1 | C:AMPA glutamate receptor complex; C:postsynaptic membrane; P:ion transmembrane transport; C:cell junction; F:AMPA glutamate receptor activity; P:ionotropic glutamate receptor signaling pathway; F:extracellular-glutamate-gated ion channel activity; C:nucleoplasm |
| Efet.01.481500.g781.t1 | PA14 domain-containing | 1290 | 2.21E-07 | 51.33 |  |
| Efet.01.481519.g782.t1 | bcl-2 2 | 420 | 8.77E-29 | 52.1 | P:regulation of cellular process; C:membrane |
| Efet.01.481699.g783.t1 | Tubulin epsilon chain | 690 | 2.09E-45 | 54.3 | C:membrane; C:integral component of membrane |
| Efet.01.481701.g784.t1 | interferon-related developmental regulator 1-like | 354 | 2.72E-15 | 61.15 | C:nucleus |
| Efet.01.48175.g1477.t1 | HEAT repeat-containing 5B-like isoform X4 | 687 | 3.71E-27 | 74.45 |  |
| Efet.01.48178.g1478.t1 | hypothetical protein CAPTEDRAFT_227575 | 600 | 6.97E-07 | 58 |  |
| Efet.01.48186.g1479.t1 | secreted frizzled-related 5-like | 423 | 2.39E-55 | 83.25 | F:G-protein coupled receptor activity; F:Wnt-activated receptor activity; P:G-protein coupled receptor signaling pathway; P:multicellular organism development; C:integral component of membrane; P:canonical Wnt signaling pathway; C:extracellular region; F:Wnt-protein binding; P:non-canonical Wnt signaling pathway |
| Efet.01.48196.g1481.t1 | 4-coumarate-- ligase 1- | 207 | 2.62E-10 | 63.8 | F:catalytic activity; P:metabolic process |
| Efet.01.481974.g789.t1 | short transient receptor potential channel | 435 | 1.46E-15 | 63.9 | C:integral component of plasma membrane; F:store-operated calcium channel activity; P:transmembrane transport; P:manganese ion transport; P:regulation of cytosolic calcium ion concentration; P:single fertilization |
| Efet.01.481998.g790.t1 | Testis-expressed sequence 10 | 789 | 4.52E-15 | 47.1 | C:MLL1 complex |
| Efet.01.482042.g791.t1 | RNA 3 -terminal phosphate cyclase | 270 | 4.25E-13 | 67.08 | F:catalytic activity; P:ribosome biogenesis; P:RNA processing; C:nucleolus |
| Efet.01.482212.g794.t1 | phospholipid-transporting ATPase ID-like isoform X1 | 438 | 2.53E-37 | 67.7 | F:ion binding |
| Efet.01.482393.g798.t1 | cDNA sequence isoform CRA_b | 492 | 1.35E-19 | 50.45 | P:regulation of cellular process; P:animal organ development |
| Efet.01.482491.g799.t1 | fatty acid-binding heart | 318 | 3.18E-15 | 62.9 | F:lipid binding |
| Efet.01.48252.g1483.t1 | malonyl- -acyl carrier mitochondrial-like | 651 | 9.10E-41 | 79.15 | F:transferase activity; P:tRNA modification |
| Efet.01.48268.g1484.t1 | angiomotin isoform X1 | 243 | 1.03E-19 | 78.1 | P:negative regulation of angiogenesis; C:external side of plasma membrane; P:vasculogenesis; P:positive regulation of embryonic development; P:chemotaxis; C:bicellular tight junction; P:in utero embryonic development; P:gastrulation with mouth forming second; C:stress fiber; C:ruffle; C:COP9 signalosome; C:lamellipodium; P:negative regulation of GTPase activity; F:angiostatin binding; C:actin filament; F:receptor activity; P:negative regulation of vascular permeability; P:blood vessel endothelial cell migration; P:establishment of cell polarity involved in ameboidal cell migration; P:positive regulation of blood vessel endothelial cell migration; C:endocytic vesicle; P:cell migration involved in gastrulation; P:regulation of small GTPase mediated signal transduction; C:integral component of membrane; P:hippo signaling; P:actin cytoskeleton organization; P:cellular protein localization |
| Efet.01.482699.g801.t1 | solute carrier family 35 member E1 homolog | 321 | 6.86E-37 | 78.2 | C:integral component of membrane; P:carbohydrate transmembrane transport |
| Efet.01.482711.g802.t1 | Dopamine D2-like receptor | 273 | 3.11E-18 | 80.9 | C:integral component of plasma membrane; F:dopamine neurotransmitter receptor activity, coupled via Gs; P:synaptic transmission, dopaminergic; F:dopamine binding; P:dopamine receptor signaling pathway |
| Efet.01.482748.g803.t1 | transmembrane protease serine 9 | 339 | 6.28E-13 | 60.6 | F:peptidase activity |
| Efet.01.482760.g804.t1 | COMM domain-containing 2-like | 240 | 5.64E-27 | 74 |  |
| Efet.01.482762.g805.t1 | MICAL 1 isoform X1 | 2115 | 3.09E-06 | 62.33 | F:zinc ion binding; F:metal ion binding; F:Rab GTPase binding; P:endocytic recycling; C:intracellular |
| Efet.01.48277.g1485.t1 | cytochrome P450 4V2 | 246 | 1.12E-07 | 75 | F:oxidoreductase activity; F:metal ion binding; C:membrane |
| Efet.01.482853.g807.t1 | glycosyl hydrolase | 948 | 3.68E-106 | 60.3 | F:hydrolase activity |
| Efet.01.482874.g808.t1 | armadillo repeat-containing 4 | 249 | 2.57E-14 | 66.45 | C:nucleus; C:cytoplasm; P:multicellular organism development; C:organelle part; P:transport |
| Efet.01.483017.g809.t1 | uncharacterized protein LOC110986198 | 1365 | 1.83E-32 | 45.15 | F:zinc ion binding |
| Efet.01.483038.g810.t1 | serine-rich adhesin for platelets-like | 4665 | 7.53E-13 | 65.5 |  |
| Efet.01.483133.g811.t1 | Four and a half LIM domains 2 | 222 | 3.03E-26 | 82.95 | F:zinc ion binding |
| Efet.01.483140.g812.t1 | upstream stimulatory factor 2-like isoform X3 | 303 | 6.08E-10 | 61.3 | F:protein dimerization activity |
| Efet.01.483186.g813.t1 | hypothetical protein CAPTEDRAFT_117978, partial | 243 | 1.88E-09 | 76 | F:nucleotide binding; F:ATPase activity; C:membrane; P:transport |
| Efet.01.48331.g1486.t1 | tektin A1 | 576 | 9.87E-68 | 76 |  |
| Efet.01.483335.g814.t1 | hypothetical protein CAPTEDRAFT_206809 | 513 | 1.02E-06 | 79 |  |
| Efet.01.483370.g816.t1 | sperm flagellar 2-like isoform X1 | 267 | 6.56E-19 | 68.5 | C:motile cilium |
| Efet.01.483378.g817.t1 | palmitoyltransferase ZDHHC22-like | 954 | 6.71E-61 | 51.4 | F:transferase activity, transferring acyl groups; F:protein-cysteine S-palmitoyltransferase activity; F:zinc ion binding; F:metal ion binding; C:membrane; C:integral component of membrane; F:transferase activity |
| Efet.01.483397.g818.t1 | Neuroligin- X- | 525 | 5.84E-50 | 68.05 | C:membrane; C:integral component of membrane |
| Efet.01.483441.g820.t1 | leucine-rich repeat-containing G- coupled receptor 4-like | 2571 | 3.85E-38 | 45.35 | P:peptidyl-tyrosine phosphorylation; F:ATP binding; F:protein tyrosine kinase activity; C:membrane; C:integral component of membrane; F:protein kinase activity; P:phosphorylation; P:protein phosphorylation; F:kinase activity |
| Efet.01.483470.g822.t1 | dopamine D2-like receptor | 408 | 2.92E-19 | 79.45 | C:integral component of plasma membrane; F:dopamine neurotransmitter receptor activity; P:anesthesia-resistant memory; P:dopamine receptor signaling pathway; F:G-protein coupled amine receptor activity |
| Efet.01.483529.g823.t1 | leucine-rich repeat serine threonine- kinase 1 | 249 | 3.73E-22 | 64.95 | F:purine ribonucleoside triphosphate binding; F:purine ribonucleoside binding; F:purine ribonucleotide binding; P:cellular process |
| Efet.01.483556.g824.t1 | hypothetical protein HELRODRAFT_160914 | 363 | 1.06E-34 | 60 | P:regulation of Rho protein signal transduction; F:Rho guanyl-nucleotide exchange factor activity; P:positive regulation of GTPase activity |
| Efet.01.483559.g825.t1 | acetyl- carboxylase biotin carboxylase subunit | 285 | 1.58E-59 | 91.35 | F:ATP binding; F:acetyl-CoA carboxylase activity; F:metal ion binding; F:biotin carboxylase activity |
| Efet.01.4836.g383.t1 | Chloride intracellular channel 2 | 342 | 2.13E-41 | 56.69 |  |
| Efet.01.483637.g827.t1 | Bromodomain and WD repeat-containing 3 | 459 | 6.80E-49 | 78.4 | P:regulation of cell shape; P:cytoskeleton organization |
| Efet.01.483648.g828.t1 | probable phospholipid-transporting ATPase IIB isoform X3 | 216 | 2.43E-24 | 83.55 | F:magnesium ion binding; F:ATP binding; C:trans-Golgi network; P:phospholipid translocation; C:integral component of membrane; F:phospholipid-translocating ATPase activity |
| Efet.01.483652.g829.t1 | homeobox six1-like | 684 | 1.98E-80 | 76.55 | F:DNA binding; P:single-organism process |
| Efet.01.483749.g833.t1 | PREDICTED: protein pecanex-like | 459 | 8.86E-06 | 45 |  |
| Efet.01.483853.g834.t1 | V-type proton ATPase catalytic subunit | 489 | 2.39E-76 | 98.9 | F:ATP binding; P:potassium ion transmembrane transport; P:ATP hydrolysis coupled proton transport; P:ATP metabolic process; C:integral component of membrane; F:proton-transporting ATPase activity, rotational mechanism; C:proton-transporting V-type ATPase, V1 domain; F:potassium channel activity |
| Efet.01.483904.g835.t1 | reverse transcriptase | 2487 | 1.37E-17 | 54.3 | F:nucleic acid binding; F:RNA-directed DNA polymerase activity; F:zinc ion binding; P:RNA-dependent DNA biosynthetic process |
| Efet.01.484071.g837.t1 | histamine H3 receptor-like | 603 | 2.26E-40 | 51.25 | F:G-protein coupled receptor activity; P:G-protein coupled receptor signaling pathway |
| Efet.01.484082.g838.t1 | 28S ribosomal mitochondrial | 480 | 2.79E-56 | 82.75 | F:structural constituent of ribosome; C:mitochondrion; C:small ribosomal subunit; F:RNA binding; P:translation |
| Efet.01.484317.g840.t1 | sodium-dependent noradrenaline transporter | 420 | 3.96E-19 | 77.05 | C:integral component of plasma membrane; P:serotonin uptake; C:presynapse; F:neurotransmitter:sodium symporter activity; F:serotonin transmembrane transporter activity; P:transmembrane transport; P:chemical synaptic transmission; C:neuron projection |
| Efet.01.48433.g1490.t1 | rho GTPase-activating 7 isoform X2 | 522 | 6.44E-19 | 57.7 | P:signal transduction; F:lipid binding |
| Efet.01.484428.g843.t1 | dolichyl-diphosphooligosaccharide-- glycosyltransferase subunit 1-like | 258 | 1.08E-28 | 76.75 | C:oligosaccharyltransferase complex; F:dolichyl-diphosphooligosaccharide-protein glycotransferase activity; C:integral component of membrane; P:protein N-linked glycosylation via asparagine |
| Efet.01.484479.g844.t1 | cyclin-dependent serine threonine- kinase DDB_G0272797 | 438 | 1.34E-06 | 61.5 |  |
| Efet.01.484534.g845.t1 | sex peptide receptor-like | 1209 | 8.03E-119 | 67.85 | F:G-protein coupled receptor activity; P:G-protein coupled receptor signaling pathway; C:membrane |
| Efet.01.484544.g846.t1 | sex peptide receptor-like | 1119 | 2.18E-87 | 63.1 | F:G-protein coupled receptor activity; F:G-protein coupled peptide receptor activity; P:G-protein coupled receptor signaling pathway; C:membrane; C:integral component of membrane |
| Efet.01.48455.g1492.t1 | microtubule-associated serine threonine- kinase 3 isoform X2 | 300 | 8.40E-26 | 92.35 | F:magnesium ion binding; F:ATP binding; F:protein serine/threonine kinase activity; P:protein phosphorylation |
| Efet.01.484637.g849.t1 | serine threonine- phosphatase 4 regulatory subunit 1 isoform X5 | 564 | 1.72E-24 | 51.25 | P:signal transduction; P:regulation of catalytic activity; C:protein phosphatase 4 complex |
| Efet.01.484663.g850.t1 | dynein heavy chain axonemal-like | 291 | 6.23E-13 | 59.89 | F:nucleotide binding; F:ATPase activity; F:ATP binding; F:microtubule motor activity; C:dynein complex; C:sperm principal piece; P:microtubule-based movement |
| Efet.01.484797.g852.t1 | biotin-- ligase-like | 1053 | 3.26E-37 | 43.15 | P:cellular protein modification process; F:biotin-[acetyl-CoA-carboxylase] ligase activity; F:ligase activity |
| Efet.01.484831.g853.t1 | potassium voltage-gated channel Shaw-like | 315 | 8.58E-33 | 66.85 | F:voltage-gated potassium channel activity; P:potassium ion transmembrane transport; C:voltage-gated potassium channel complex; P:regulation of ion transmembrane transport; P:protein homooligomerization |
| Efet.01.484960.g855.t1 | hypothetical protein HELRODRAFT_195030 | 459 | 5.84E-07 | 84 |  |
| Efet.01.485014.g857.t1 | ankyrin repeat and BTB POZ domain-containing 1-like | 207 | 8.88E-18 | 78.5 |  |
| Efet.01.48507.g1493.t1 | elongation of very long chain fatty acids 4-like | 558 | 7.24E-49 | 62.2 | C:integral component of membrane; F:transferase activity; P:fatty acid biosynthetic process |
| Efet.01.485094.g858.t1 | death-associated kinase 1-like | 717 | 4.01E-28 | 49.35 | P:signal transduction; P:phosphorylation; F:kinase activity |
| Efet.01.485141.g859.t1 | Ras-specific guanine nucleotide-releasing factor 2 | 240 | 1.57E-38 | 89.9 | P:regulation of Rho protein signal transduction; P:small GTPase mediated signal transduction; C:integral component of membrane; F:Rho guanyl-nucleotide exchange factor activity; C:intracellular; P:positive regulation of GTPase activity |
| Efet.01.485210.g861.t1 | FMRFamide receptor-like | 987 | 8.04E-49 | 49.95 | F:G-protein coupled receptor activity; F:G-protein coupled peptide receptor activity; P:G-protein coupled receptor signaling pathway; P:signal transduction; C:membrane; C:integral component of membrane; F:signal transducer activity |
| Efet.01.485253.g862.t1 | F-actin-uncapping LRRC16A isoform X1 | 321 | 1.39E-23 | 67.3 | P:positive regulation of cellular component organization |
| Efet.01.485300.g863.t1 | lysoplasmalogenase TMEM86A | 540 | 4.06E-74 | 72.65 | C:membrane |
| Efet.01.485381.g867.t1 | DNA repair and recombination RAD54B | 294 | 1.25E-16 | 61.9 | P:response to stimulus |
| Efet.01.485390.g869.t1 | fasciculation and elongation zeta-2-like | 408 | 1.56E-27 | 61.35 |  |
| Efet.01.485391.g870.t1 | Actin-like | 618 | 3.52E-42 | 56.05 | C:npBAF complex; P:ATP-dependent chromatin remodeling |
| Efet.01.485525.g871.t1 | hypothetical protein CAPTEDRAFT_208825 | 288 | 1.15E-22 | 69 |  |
| Efet.01.485585.g872.t1 | USP6 N-terminal | 423 | 7.92E-15 | 65.4 |  |
| Efet.01.485619.g874.t1 | calmodulin-alpha-like isoform X4 | 507 | 4.64E-24 | 71.75 | P:positive regulation of phosphate metabolic process; P:positive regulation of molecular function; P:regulation of biological quality; C:microtubule cytoskeleton; C:cytoskeletal part; F:calcium ion binding; F:myosin binding; P:regulation of primary metabolic process; C:membrane-bounded organelle; F:enzyme binding |
| Efet.01.485734.g876.t1 | beta-1,3-galactosyl-O-glycosyl-glyco beta-1,6-N-acetylglucosaminyltransferase 3-like | 744 | 3.71E-57 | 56.55 | F:transferase activity |
| Efet.01.48578.g1495.t1 | acid sphingomyelinase-like phosphodiesterase 3b | 465 | 3.98E-28 | 55.85 | C:extracellular space; F:sphingomyelin phosphodiesterase activity; C:membrane; C:integral component of membrane; P:sphingomyelin catabolic process; F:hydrolase activity |
| Efet.01.485993.g877.t1 | hepatocyte growth factor receptor-like | 303 | 1.77E-40 | 86.05 | F:ATP binding; P:peptidyl-tyrosine phosphorylation; F:protein tyrosine kinase activity; P:integrin-mediated signaling pathway; C:integral component of membrane |
| Efet.01.48605.g1496.t1 | protocadherin gamma-B1 | 711 | 2.63E-19 | 53.5 | F:calcium ion binding; C:membrane; C:integral component of membrane; P:cell adhesion; P:homophilic cell adhesion via plasma membrane adhesion molecules; C:plasma membrane |
| Efet.01.486108.g879.t1 | oxysterol-binding 1 isoform X4 | 573 | 8.43E-63 | 81.5 | P:lipid transport; F:Goodpasture-antigen-binding protein kinase activity; P:protein phosphorylation |
| Efet.01.486124.g880.t1 | RNA-directed DNA polymerase from mobile element jockey-like | 399 | 1.84E-17 | 61.1 |  |
| Efet.01.486245.g882.t1 | tribbles homolog 2-like | 411 | 1.46E-50 | 75.45 | P:positive regulation of myeloid leukocyte differentiation; C:nucleus; P:regulation of MAP kinase activity; F:protein kinase activity; P:protein phosphorylation; P:positive regulation of proteasomal ubiquitin-dependent protein catabolic process; F:enzyme regulator activity; P:negative regulation of protein kinase activity; P:single-organism process; P:response to stimulus; F:mitogen-activated protein kinase kinase binding; P:regulation of granulocyte differentiation |
| Efet.01.486296.g884.t1 | Cat eye syndrome critical region 5 | 411 | 1.16E-24 | 51.2 | C:mitochondrion |
| Efet.01.486310.g885.t1 | valine--tRNA ligase | 1173 | 6.43E-131 | 65.25 | F:ligase activity; P:translation |
| Efet.01.486375.g886.t1 | allene oxide synthase-lipoxygenase -like | 375 | 1.79E-37 | 68.71 | F:oxidoreductase activity, acting on single donors with incorporation of molecular oxygen, incorporation of two atoms of oxygen; F:metal ion binding; F:catalase activity; P:cellular oxidant detoxification; P:oxidation-reduction process; F:heme binding |
| Efet.01.48638.g1498.t1 | Chitin synthase 6 | 918 | 5.65E-44 | 46.33 | F:transferase activity, transferring hexosyl groups; C:membrane; C:integral component of membrane |
| Efet.01.486596.g887.t1 | serine hydrolase | 624 | 4.01E-96 | 65.9 | C:extracellular region |
| Efet.01.486596.g888.t1 | Fis family transcriptional regulator | 339 | 8.41E-57 | 64.25 | F:ATP binding; P:transcription, DNA-templated; P:regulation of transcription, DNA-templated; F:sequence-specific DNA binding; F:transcription factor binding |
| Efet.01.486631.g889.t1 | Low-density lipo receptor-related 2 | 249 | 3.54E-15 | 60.75 | C:intracellular organelle; C:endomembrane system; P:circulatory system development; P:animal organ development; C:cytoplasmic part; C:membrane part; C:membrane-bounded organelle |
| Efet.01.486631.g890.t1 | Low-density lipo receptor-related 2 | 726 | 2.73E-61 | 65.3 | F:calcium ion binding; C:membrane; C:integral component of membrane |
| Efet.01.48664.g1499.t1 | FMRFamide receptor-like | 1356 | 6.35E-126 | 58.65 | F:G-protein coupled receptor activity; C:membrane |
| Efet.01.48666.g1500.t1 | NADH dehydrogenase [ubiquinone] 1 alpha subcomplex subunit 13 | 219 | 9.05E-23 | 65.6 | C:integral component of membrane |
| Efet.01.486768.g891.t1 | mitochondrial ATP synthase subunit b precursor | 378 | 4.45E-15 | 72.9 | C:mitochondrion |
| Efet.01.486864.g892.t1 | FMRFamide receptor-like | 1131 | 8.95E-78 | 60.75 | F:G-protein coupled receptor activity; F:G-protein coupled peptide receptor activity; P:G-protein coupled receptor signaling pathway; C:membrane; C:integral component of membrane |
| Efet.01.486960.g895.t1 | endothelin-converting enzyme 1-like isoform X2 | 912 | 5.38E-12 | 55.6 | F:nucleotide binding; F:nucleic acid binding; C:membrane; P:proteolysis; C:integral component of membrane; P:peptide hormone processing; F:metalloendopeptidase activity; F:metallopeptidase activity |
| Efet.01.486977.g896.t1 | sestrin-1-like isoform X1 | 264 | 1.14E-29 | 75.05 | C:nucleus; P:regulation of response to reactive oxygen species |
| Efet.01.486994.g897.t1 | sex peptide receptor-like | 1230 | 1.20E-112 | 65.65 | F:nucleic acid binding; F:G-protein coupled receptor activity; F:ATP binding; F:G-protein coupled peptide receptor activity; P:neuropeptide signaling pathway; P:G-protein coupled receptor signaling pathway; C:membrane; C:integral component of membrane; F:helicase activity; F:hydrolase activity |
| Efet.01.487066.g898.t1 | dorsal-ventral patterning tolloid isoform X1 | 633 | 3.55E-26 | 52.75 | F:peptidase activity |
| Efet.01.48712.g1501.t1 | cilia- and flagella-associated 52 | 978 | 1.92E-128 | 59.7 | F:protein binding |
| Efet.01.487337.g900.t1 | TKL kinase | 981 | 7.47E-64 | 58.15 | F:kinase activity |
| Efet.01.48741.g1502.t1 | sesquipedalian-1-like | 357 | 2.75E-38 | 84.95 | F:nucleic acid binding; F:protein dimerization activity |
| Efet.01.487410.g902.t1 | polysaccharide deacetylase family | 453 | 3.70E-49 | 75.55 | F:hydrolase activity, acting on carbon-nitrogen (but not peptide) bonds; P:carbohydrate metabolic process; F:cellulase activity |
| Efet.01.487417.g903.t1 | isocitrate dehydrogenase [NADP] mitochondrial-like | 534 | 3.10E-111 | 91.5 | F:magnesium ion binding; F:NAD binding; F:isocitrate dehydrogenase (NADP+) activity; P:isocitrate metabolic process; P:tricarboxylic acid cycle |
| Efet.01.48750.g1504.t1 | bicaudal C homolog 1 isoform X4 | 561 | 1.26E-31 | 74.95 | F:RNA binding; P:multicellular organism development |
| Efet.01.487512.g905.t1 | 30S ribosomal S7 | 243 | 1.04E-34 | 94.85 | F:structural constituent of ribosome; C:small ribosomal subunit; F:tRNA binding; F:rRNA binding; P:translation |
| Efet.01.48752.g1505.t1 | mitochondrial ribosome-associated GTPase 2 isoform X4 | 525 | 1.55E-48 | 65.95 | C:integral component of membrane; C:plasma membrane |
| Efet.01.48752.g1506.t1 | E3 ubiquitin- ligase MARCH6 | 1533 | 3.65E-72 | 68.3 | C:membrane; F:enzyme binding |
| Efet.01.487536.g906.t1 | hypothetical protein HELRODRAFT_185969 | 204 | 1.14E-07 | 73 | P:ion transport; C:membrane |
| Efet.01.487632.g908.t1 | sodium-potassium adenosine | 489 | 5.49E-82 | 89.8 | P:potassium ion transport; F:ATP binding; P:sodium ion transport; F:sodium:potassium-exchanging ATPase activity; F:metal ion binding; P:ATP hydrolysis coupled transmembrane transport; C:integral component of membrane; P:establishment or maintenance of transmembrane electrochemical gradient |
| Efet.01.487711.g909.t1 | phosphatase 1G | 240 | 3.23E-11 | 66.3 | F:protein serine/threonine phosphatase activity; F:metal ion binding; P:protein dephosphorylation |
| Efet.01.487733.g910.t1 | treslin isoform X1 | 735 | 2.19E-24 | 49.25 | C:nucleus; P:DNA replication; P:mitotic DNA replication checkpoint; P:response to ionizing radiation |
| Efet.01.487738.g911.t1 | hypothetical protein HELRODRAFT_171537 | 1776 | 9.21E-23 | 44.5 |  |
| Efet.01.487749.g912.t1 | oxysterol-binding -related 2-like | 372 | 3.93E-26 | 68.05 | C:intracellular organelle; P:lipid transport; C:membrane-bounded organelle |
| Efet.01.487768.g913.t1 | peptide chain release factor N(5)-glutamine methyltransferase | 816 | 5.46E-114 | 61 | F:transferase activity |
| Efet.01.488005.g917.t1 | calcium-dependent secretion activator 1- | 375 | 1.10E-67 | 92.3 | C:presynapse; P:dense core granule exocytosis; C:cytosol; P:synaptic vesicle exocytosis |
| Efet.01.488025.g919.t1 | Golgi-associated plant pathogenesis-related 1 | 252 | 1.68E-13 | 60.2 | C:extracellular region |
| Efet.01.488232.g923.t1 | Inhibitor of apoptosis | 543 | 1.74E-09 | 40.7 | F:zinc ion binding; F:metal ion binding; P:regulation of apoptotic process |
| Efet.01.48825.g1511.t1 | cation channel sperm-associated 2-like isoform X1 | 723 | 9.98E-58 | 62 | P:cation transport; F:gated channel activity; P:ion transmembrane transport; C:membrane; F:cation channel activity |
| Efet.01.48839.g1512.t1 | hypothetical protein CAPTEDRAFT_220760 | 528 | 1.88E-09 | 55.5 |  |
| Efet.01.48842.g1513.t1 | L-rhamnonate dehydratase | 351 | 9.42E-31 | 74.55 | F:catalytic activity; P:metabolic process |
| Efet.01.48843.g1514.t1 | CMT1A duplicated region transcript 1 -like | 1203 | 7.47E-85 | 54.75 |  |
| Efet.01.488482.g925.t1 | ADP-ribosylation factor 6 | 465 | 2.85E-85 | 92.15 | P:protein localization to cell surface; C:myelin sheath; P:regulation of Rac protein signal transduction; P:protein localization to endosome; P:protein transport; C:Golgi apparatus; F:GTP binding; P:cortical actin cytoskeleton organization; P:regulation of filopodium assembly; C:extracellular exosome; P:vesicle-mediated transport; C:focal adhesion; C:ruffle; F:thioesterase binding; P:small GTPase mediated signal transduction; P:positive regulation of establishment of protein localization to plasma membrane; P:regulation of dendritic spine development; P:establishment of epithelial cell polarity; P:hepatocyte apoptotic process; C:filopodium membrane; C:endocytic vesicle; C:cell cortex; P:ruffle organization; F:protein N-terminus binding; P:positive regulation of actin filament polymerization; P:epiboly involved in gastrulation with mouth forming second; C:recycling endosome membrane; P:intrahepatic bile duct development; F:hydrolase activity; P:myeloid cell apoptotic process |
| Efet.01.488518.g926.t1 | M-phase inducer phosphatase | 243 | 3.43E-15 | 70.6 | P:protein dephosphorylation |
| Efet.01.488568.g928.t1 | carbohydrate kinase | 1038 | 0 | 76.95 | P:phosphorylation; F:phosphotransferase activity, alcohol group as acceptor; F:kinase activity |
| Efet.01.488568.g929.t1 | PIN domain-containing | 384 | 3.66E-54 | 72.2 | P:nucleic acid phosphodiester bond hydrolysis; F:metal ion binding; F:nuclease activity |
| Efet.01.488568.g930.t1 | amidohydrolase family | 1248 | 0 | 81.05 | F:hydrolase activity, acting on carbon-nitrogen (but not peptide) bonds |
| Efet.01.488568.g931.t1 | MFS transporter | 1371 | 0 | 79.65 | P:transmembrane transport; F:transmembrane transporter activity; C:integral component of membrane |
| Efet.01.488594.g932.t1 | forkhead box | 1176 | 3.15E-51 | 78.85 | C:nucleus; P:negative regulation of gene expression; F:transcription factor activity, sequence-specific DNA binding; P:autonomic nervous system development; P:transcription, DNA-templated; P:neural crest cell development; P:regulation of transcription, DNA-templated; F:sequence-specific DNA binding; P:pigment cell differentiation; P:cartilage development |
| Efet.01.488732.g934.t1 | hypothetical protein CRN43_04285 | 735 | 4.58E-168 | 47.65 |  |
| Efet.01.488732.g935.t1 | aspartate kinase | 843 | 0 | 75.95 | F:aspartate kinase activity; P:lysine biosynthetic process via diaminopimelate; P:phosphorylation |
| Efet.01.48881.g1516.t1 | probable C-mannosyltransferase DPY19L3 isoform X3 | 300 | 4.29E-11 | 82.3 | P:protein C-linked glycosylation via 2'-alpha-mannosyl-L-tryptophan; C:nuclear inner membrane; F:mannosyltransferase activity; C:integral component of membrane |
| Efet.01.488926.g941.t1 | Golgi phospho 3-like | 408 | 8.72E-79 | 91.65 | F:phosphatidylinositol-4-phosphate binding; C:intracellular; P:Golgi vesicle transport |
| Efet.01.488935.g942.t1 | nuclear hormone receptor HR96 isoform X11 | 435 | 1.34E-51 | 87.85 | F:thyroid hormone receptor activity; C:nucleus; F:zinc ion binding; P:transcription, DNA-templated; F:steroid hormone receptor activity; P:regulation of transcription, DNA-templated; F:sequence-specific DNA binding; P:steroid hormone mediated signaling pathway; P:intracellular receptor signaling pathway |
| Efet.01.488987.g944.t1 | PREDICTED: uncharacterized protein LOC106181814 | 804 | 1.11E-25 | 55.15 |  |
| Efet.01.489035.g945.t1 | inositol phosphorylceramide synthase catalytic subunit aur1-like | 660 | 8.98E-39 | 45.85 | C:membrane; C:integral component of membrane; C:Golgi apparatus |
| Efet.01.48911.g1517.t1 | ileal sodium bile acid cotransporter-like | 726 | 2.21E-14 | 53.65 | C:integral component of plasma membrane; C:membrane; C:integral component of membrane; F:organic acid transmembrane transporter activity; P:organic acid transmembrane transport |
| Efet.01.48913.g1518.t1 | nesprin-1-like isoform X3 | 3774 | 1.58E-11 | 51 |  |
| Efet.01.489168.g946.t1 | neuroglian isoform X1 | 426 | 2.88E-22 | 56.55 | F:nucleic acid binding; P:cytokine-mediated signaling pathway; C:membrane; C:integral component of membrane; F:protein dimerization activity; F:cytokine receptor activity |
| Efet.01.489283.g947.t1 | serine threonine- kinase NLK-like isoform X2 | 555 | 2.37E-75 | 76.15 | F:nucleotide binding; F:protein serine/threonine kinase activity; P:protein phosphorylation |
| Efet.01.48933.g1519.t1 | tripartite motif-containing 3-like | 3255 | 7.37E-104 | 47.75 | F:zinc ion binding; F:metal ion binding; C:intracellular |
| Efet.01.489389.g948.t1 | zinc finger OZF-like | 423 | 2.54E-22 | 56.55 | F:nucleic acid binding; F:DNA binding; F:metal ion binding |
| Efet.01.489453.g949.t1 | PRKC apoptosis WT1 regulator -like isoform X2 | 354 | 2.81E-17 | 67.25 | P:apoptotic process |
| Efet.01.48950.g1520.t1 | patched domain-containing 2-like | 1425 | 1.65E-65 | 57.1 | C:membrane; C:integral component of membrane; P:smoothened signaling pathway |
| Efet.01.489579.g952.t1 | alpha-tocopherol transfer -like isoform X3 | 387 | 9.98E-17 | 52.9 | P:transport; F:transporter activity; C:intracellular |
| Efet.01.48966.g1521.t1 | branched-chain-amino-acid aminotransferase 2 | 363 | 1.37E-61 | 57.75 | F:catalytic activity; P:metabolic process |
| Efet.01.489687.g954.t1 | transmembrane and coiled-coil domain-containing 3 | 852 | 6.56E-13 | 59.75 | P:transport |
| Efet.01.489704.g955.t1 | voltage-gated hydrogen channel 1-like | 489 | 9.90E-37 | 65.05 | P:transport |
| Efet.01.489798.g956.t1 | ABC transporter substrate-binding | 636 | 3.10E-96 | 78.75 | P:amino acid transport |
| Efet.01.489798.g957.t1 | ABC transporter substrate-binding | 795 | 5.96E-72 | 57.75 | P:amino acid transport |
| Efet.01.489806.g958.t1 | ATP-binding cassette sub-family C member 8-like isoform X3 | 231 | 7.64E-27 | 77.7 | P:potassium ion transport; F:ATP binding; F:sulfonylurea receptor activity; F:ATPase activity, coupled to transmembrane movement of substances; P:transmembrane transport; P:signal transduction; C:integral component of membrane |
| Efet.01.489945.g959.t1 | sFRP1 2 5 | 642 | 2.08E-11 | 60 | P:multicellular organism development; P:Wnt signaling pathway; C:extracellular region |
| Efet.01.49002.g1523.t1 | TRPM8 channel-associated factor homolog | 2085 | 1.01E-134 | 49.95 | C:membrane; C:integral component of membrane |
| Efet.01.490025.g961.t1 | DNA adenine methylase | 714 | 1.17E-176 | 85 | F:nucleic acid binding; P:DNA methylation on adenine; F:site-specific DNA-methyltransferase (adenine-specific) activity |
| Efet.01.49027.g1525.t1 | segment polarity dishevelled homolog DVL-3-like isoform X1 | 1125 | 8.96E-103 | 65.6 | P:Wnt signaling pathway; P:intracellular signal transduction; C:intracellular |
| Efet.01.49033.g1526.t1 | teneurin-m-like isoform X1 | 678 | 1.34E-81 | 67.55 | F:catalytic activity; C:membrane; C:integral component of membrane |
| Efet.01.490393.g964.t1 | multidrug resistance-associated 4-like isoform X1 | 360 | 8.66E-37 | 82.15 |  |
| Efet.01.490542.g965.t1 | multiple C2 and transmembrane domain-containing 1-like isoform X2 | 396 | 1.12E-09 | 68 | C:membrane |
| Efet.01.490568.g966.t1 | T family of potassium channels 18-like | 627 | 7.45E-29 | 57.2 | P:ion transport; P:potassium ion transmembrane transport; C:membrane; C:integral component of membrane; F:potassium channel activity; P:transport |
| Efet.01.490573.g967.t1 | chaoptin isoform X1 | 2052 | 4.06E-79 | 43.85 | P:Roundabout signaling pathway; F:Roundabout binding; C:extracellular space; P:regulation of axonogenesis; C:membrane; C:integral component of membrane; F:heparin binding; C:proteinaceous extracellular matrix; P:axon extension involved in axon guidance |
| Efet.01.49067.g1528.t1 | unconventional myosin-Va-like | 747 | 1.99E-18 | 59.85 | C:myosin complex; F:ATP binding; F:motor activity |
| Efet.01.49068.g1529.t1 | cAMP-specific 3 ,5 -cyclic phosphodiesterase 4D isoform X4 | 693 | 5.24E-87 | 64.35 | F:metal ion binding; P:signal transduction; F:3',5'-cyclic-nucleotide phosphodiesterase activity |
| Efet.01.4907.g385.t1 | cAMP-dependent kinase regulatory subunit | 333 | 3.71E-41 | 85.45 | P:negative regulation of cAMP-dependent protein kinase activity; P:sarcomere organization; P:negative regulation of activated T cell proliferation; F:cAMP-dependent protein kinase regulator activity; P:protein phosphorylation; C:plasma membrane raft; P:cardiac muscle cell proliferation; C:cAMP-dependent protein kinase complex; C:immunological synapse; F:cAMP-dependent protein kinase inhibitor activity; P:animal organ morphogenesis; F:ubiquitin protein ligase binding; C:cytoplasm; F:protein kinase A catalytic subunit binding; C:neuromuscular junction; F:cAMP binding; P:mesoderm formation; C:nucleotide-activated protein kinase complex |
| Efet.01.49085.g1530.t1 | prolactin-releasing peptide receptor-like | 531 | 3.59E-47 | 65.7 | P:neuropeptide signaling pathway; C:integral component of membrane; F:neuropeptide Y receptor activity |
| Efet.01.490850.g970.t1 | hypothetical protein HELRODRAFT_171217 | 432 | 4.93E-34 | 67 |  |
| Efet.01.490872.g971.t1 | UDP-N-acetylmuramoyl-L-alanine--D-glutamate ligase | 279 | 6.31E-45 | 73.33 |  |
| Efet.01.490890.g972.t1 | Cyclin-I | 591 | 9.20E-14 | 52.3 | P:regulation of cell cycle; C:cellular_component; F:molecular_function |
| Efet.01.490897.g973.t1 | FGFR3_HUMAN ame: Full=Fibroblast growth factor receptor 3 Short=FGFR-3 ame: CD_antigen=CD333 Flags: Precursor | 276 | 3.39E-06 | 52 | P:peptidyl-tyrosine phosphorylation; P:regulation of phosphatidylinositol 3-kinase signaling; P:phosphatidylinositol-3-phosphate biosynthetic process; P:phosphatidylinositol phosphorylation; P:protein autophosphorylation; P:fibroblast growth factor receptor signaling pathway; C:Golgi apparatus; F:phosphatidylinositol-4,5-bisphosphate 3-kinase activity; P:positive regulation of GTPase activity; P:JAK-STAT cascade; P:skeletal system development; P:apoptotic process; P:negative regulation of developmental growth; P:positive regulation of phosphatidylinositol 3-kinase activity; F:Ras guanyl-nucleotide exchange factor activity; P:cell-cell signaling; F:protein kinase activity; C:plasma membrane; C:nucleus; F:protein binding; P:endochondral bone growth; P:phosphatidylinositol-mediated signaling; P:endochondral ossification; P:chondrocyte proliferation; F:fibroblast growth factor binding; C:transport vesicle; P:positive regulation of tyrosine phosphorylation of Stat3 protein; P:fibroblast growth factor receptor apoptotic signaling pathway; P:positive regulation of cell proliferation; P:bone morphogenesis; P:chondrocyte differentiation; C:extracellular region; P:protein phosphorylation; P:positive regulation of tyrosine phosphorylation of Stat1 protein; F:nucleotide binding; F:fibroblast growth factor-activated receptor activity; F:ATP binding; C:integral component of plasma membrane; P:MAPK cascade; F:protein tyrosine kinase activity; C:focal adhesion; F:transmembrane receptor protein tyrosine kinase activity; P:positive regulation of phospholipase activity; C:cytoplasmic vesicle; P:phosphorylation; P:bone mineralization; C:endoplasmic reticulum; P:positive regulation of MAPK cascade; C:cell surface; C:cytoplasm; C:membrane; P:positive regulation of ERK1 and ERK2 cascade; C:integral component of membrane; F:transferase activity; P:bone maturation; F:kinase activity; F:1-phosphatidylinositol-3-kinase activity |
| Efet.01.49096.g1531.t1 | octopamine receptor beta-2R-like | 504 | 5.68E-28 | 59.25 | F:G-protein coupled receptor activity; P:signal transduction |
| Efet.01.491040.g976.t1 | 26S proteasome non-ATPase regulatory subunit 1-like | 597 | 4.94E-57 | 89.15 | P:regulation of catalytic activity; C:integral component of membrane; P:regulation of protein catabolic process; C:proteasome complex; F:enzyme regulator activity |
| Efet.01.491090.g977.t1 | FAM91A1 | 225 | 1.88E-18 | 73.1 | C:cellular_component |
| Efet.01.491091.g978.t1 | Muscarinic acetylcholine receptor DM1 | 1920 | 1.77E-78 | 75.8 | P:gastric acid secretion; C:integral component of plasma membrane; P:synaptic transmission, cholinergic; P:phospholipase C-activating G-protein coupled acetylcholine receptor signaling pathway; P:adenylate cyclase-inhibiting G-protein coupled acetylcholine receptor signaling pathway; F:G-protein coupled acetylcholine receptor activity; C:synapse |
| Efet.01.491357.g979.t1 | D-hexose-6-phosphate mutarotase | 465 | 2.55E-83 | 63.05 | P:carbohydrate metabolic process; F:catalytic activity; F:carbohydrate binding; F:isomerase activity |
| Efet.01.491361.g980.t1 | oxysterol-binding 1-like | 360 | 4.79E-43 | 75.5 | P:lipid transport |
| Efet.01.491388.g981.t1 | dynein heavy chain axonemal | 291 | 6.41E-40 | 86.75 | F:ATPase activity; F:ATP binding; F:microtubule motor activity; C:dynein complex; P:microtubule-based movement |
| Efet.01.491472.g982.t1 | hypothetical protein CAPTEDRAFT_228177 | 1011 | 4.28E-72 | 51.2 | C:membrane; C:integral component of membrane; P:cellular developmental process |
| Efet.01.491516.g983.t1 | G -activated inward rectifier potassium channel 3-like | 729 | 4.47E-65 | 84.2 | P:potassium ion transmembrane transport; C:integral component of membrane; F:inward rectifier potassium channel activity; P:regulation of ion transmembrane transport |
| Efet.01.49153.g1534.t1 | very low-density lipo receptor-like isoform X3 | 789 | 3.90E-46 | 54.7 | F:calcium ion binding; C:membrane; C:integral component of membrane |
| Efet.01.491537.g985.t1 | hypothetical protein CAPTEDRAFT_225404 | 1401 | 1.72E-10 | 44.29 | C:membrane; C:integral component of membrane |
| Efet.01.491574.g986.t1 | ATP-binding cassette sub-family A member 1-like | 366 | 3.53E-16 | 54.5 | F:ATPase activity; P:transmembrane transport; F:transmembrane transporter activity; C:intracellular part; C:plasma membrane part; F:nucleotide binding; P:lipid transport; F:protein binding; C:integral component of membrane; P:cellular component organization; P:regulation of biological process; P:response to stimulus; P:multicellular organismal process |
| Efet.01.491627.g987.t1 | glycosylphosphatidylinositol anchor attachment 1 | 246 | 2.29E-06 | 70 | C:membrane; C:integral component of membrane; P:attachment of GPI anchor to protein; C:GPI-anchor transamidase complex |
| Efet.01.491629.g988.t1 | fibroblast growth factor receptor | 396 | 7.72E-23 | 60.9 | F:protein tyrosine kinase activity; P:phosphorylation |
| Efet.01.49163.g1536.t1 | hypothetical protein HELRODRAFT_191437 | 771 | 1.88E-20 | 47 |  |
| Efet.01.49166.g1537.t1 | phosphatase 1 regulatory subunit 37 | 537 | 1.22E-20 | 88.8 | F:phosphatase binding; P:negative regulation of phosphatase activity |
| Efet.01.491721.g989.t1 | homeobox | 429 | 3.51E-26 | 74.05 | F:DNA binding |
| Efet.01.491732.g990.t1 | leucine-rich repeat 1 | 471 | 5.62E-42 | 55.55 | F:isomerase activity |
| Efet.01.49176.g1538.t1 | cyclin-dependent kinase 5 activator 1 | 756 | 8.81E-64 | 78.5 | F:nucleic acid binding; F:metal ion binding; P:positive regulation of protein serine/threonine kinase activity; C:cyclin-dependent protein kinase 5 holoenzyme complex; F:cyclin-dependent protein kinase 5 activator activity |
| Efet.01.49179.g1539.t1 | multiple epidermal growth factor-like domains 8 | 924 | 1.13E-59 | 49.4 | P:pattern specification process |
| Efet.01.49188.g1540.t1 | cilia- and flagella-associated 47-like isoform X2 | 207 | 7.84E-22 | 80.65 |  |
| Efet.01.491896.g994.t1 | split ends-like | 372 | 7.59E-13 | 66.95 | F:organic cyclic compound binding; F:heterocyclic compound binding |
| Efet.01.492009.g996.t1 | class I SAM-dependent rRNA methyltransferase | 1101 | 1.57E-174 | 70.45 | P:metabolic process; F:transferase activity |
| Efet.01.49202.g1541.t1 | trafficking particle complex subunit 8 isoform X4 | 471 | 7.26E-14 | 62.1 |  |
| Efet.01.49211.g1543.t1 | lysosomal-trafficking regulator-like isoform X4 | 2052 | 2.41E-07 | 46.33 | P:lysosome organization |
| Efet.01.492121.g997.t1 | response regulator | 636 | 5.20E-99 | 89.5 | P:phosphorelay signal transduction system; C:intracellular |
| Efet.01.492121.g998.t1 | DNA-binding response regulator | 633 | 5.16E-150 | 94.8 | F:DNA binding; P:transcription, DNA-templated; P:phosphorelay signal transduction system; P:regulation of transcription, DNA-templated; C:intracellular |
| Efet.01.492121.g999.t1 | histidine kinase | 1089 | 0 | 82.3 | F:phosphorelay sensor kinase activity; F:ATP binding; P:phosphorelay signal transduction system; C:integral component of membrane; F:protein dimerization activity; P:signal transduction by protein phosphorylation; C:intracellular |
| Efet.01.492122.g1000.t1 | GTP-binding Rhes | 378 | 6.43E-42 | 89.5 | F:GTP binding; P:small GTPase mediated signal transduction; C:membrane; C:intracellular |
| Efet.01.49215.g1544.t1 | pre-mRNA-splicing factor ATP-dependent RNA helicase DHX16 | 213 | 7.89E-28 | 97.5 | F:ATP binding; C:cytoplasm; P:endodermal cell differentiation; C:spliceosomal complex; F:ATP-dependent RNA helicase activity; F:RNA binding; P:mRNA splicing, via spliceosome |
| Efet.01.492220.g1002.t1 | fumarylacetoacetase | 228 | 1.90E-11 | 81.05 | P:aromatic amino acid family metabolic process; C:integral component of membrane; F:fumarylacetoacetase activity |
| Efet.01.492264.g1003.t1 | homeobox goosecoid-like | 249 | 1.15E-20 | 88.2 | C:nucleus; P:regulation of transcription, DNA-templated; F:sequence-specific DNA binding |
| Efet.01.492353.g1005.t1 | follicular epithelium yolk subunit | 447 | 3.15E-21 | 66 |  |
| Efet.01.492369.g1006.t1 | ral guanine nucleotide dissociation stimulator-like 1 | 351 | 2.14E-35 | 65 | P:small GTPase mediated signal transduction; P:signal transduction; F:guanyl-nucleotide exchange factor activity; F:Ral guanyl-nucleotide exchange factor activity; C:intracellular; P:positive regulation of GTPase activity |
| Efet.01.49245.g1546.t1 | homeobox cut-like | 339 | 3.81E-31 | 76.95 | F:DNA binding; C:nucleus |
| Efet.01.492485.g1007.t1 | 4-hydroxybutyrate coenzyme A transferase-like | 288 | 4.52E-19 | 86.6 | P:acetyl-CoA metabolic process; F:transferase activity |
| Efet.01.492504.g1008.t1 | family RNA methyltransferase | 309 | 6.38E-56 | 73.1 | P:RNA metabolic process; F:methyltransferase activity; P:methylation |
| Efet.01.492615.g1009.t1 | E3 ubiquitin- ligase TRIM9-like | 993 | 1.28E-81 | 64.2 | F:zinc ion binding; F:metal ion binding; F:ligase activity; C:intracellular |
| Efet.01.49266.g1547.t1 | homeobox onecut | 684 | 4.43E-49 | 78.3 | C:nucleus; F:DNA binding |
| Efet.01.4927.g386.t1 | polypeptide N-acetylgalactosaminyltransferase 5-like isoform X2 | 1707 | 2.03E-124 | 48.2 | F:transferase activity, transferring glycosyl groups; C:membrane; C:integral component of membrane; P:protein glycosylation; C:Golgi apparatus; C:Golgi membrane; F:transferase activity; F:carbohydrate binding |
| Efet.01.492718.g1010.t1 | voltage-dependent calcium channel subunit alpha-2 delta-2-like | 291 | 1.49E-39 | 83.5 |  |
| Efet.01.492745.g1011.t1 | high affinity cGMP-specific 3 ,5 -cyclic phosphodiesterase 9A-like | 1578 | 0 | 70 | F:phosphoric diester hydrolase activity |
| Efet.01.492854.g1014.t1 | cephalosporin hydroxylase | 468 | 6.54E-97 | 89.7 | P:lipid biosynthetic process; F:methyltransferase activity; P:methylation |
| Efet.01.492908.g1017.t1 | matrix metallopeptidase-21-like | 306 | 2.18E-15 | 66.05 | F:metal ion binding; F:metallopeptidase activity |
| Efet.01.492942.g1018.t1 | tyrosine- kinase Src42A-like | 213 | 5.05E-36 | 88.25 | F:ATP binding; P:peptidyl-tyrosine phosphorylation; F:non-membrane spanning protein tyrosine kinase activity |
| Efet.01.492983.g1019.t1 | insulin-like growth factor-binding complex acid labile subunit | 777 | 5.54E-30 | 52.55 | C:extracellular region part; P:single-organism process |
| Efet.01.493.g55.t1 | paladin-like isoform X2 | 222 | 5.36E-20 | 86.1 | C:nucleus; F:DNA binding; C:cytoplasm; F:protein tyrosine phosphatase activity; F:calcium ion binding; C:integral component of membrane; C:nucleosome; P:dephosphorylation; P:nucleosome assembly |
| Efet.01.493038.g1020.t1 | beta-1,4-N-acetylgalactosaminyltransferase bre-4-like isoform X1 | 237 | 8.76E-32 | 83.1 | P:carbohydrate metabolic process; F:transferase activity, transferring glycosyl groups; C:integral component of membrane |
| Efet.01.493058.g1021.t1 | family transcriptional regulator | 537 | 1.57E-119 | 84.35 | F:DNA binding; F:transcription factor activity, sequence-specific DNA binding; P:transcription, DNA-templated; P:regulation of transcription, DNA-templated |
| Efet.01.493110.g1023.t1 | neuronal acetylcholine receptor subunit alpha-10-like | 216 | 1.15E-07 | 70.57 | F:ion channel activity; P:ion transport; C:postsynaptic membrane; P:ion transmembrane transport; C:membrane; P:cation transmembrane transport; C:integral component of membrane; C:cell junction; F:extracellular ligand-gated ion channel activity; C:synapse; P:transport; C:plasma membrane |
| Efet.01.493135.g1025.t1 | rap guanine nucleotide exchange factor 2-like isoform X13 | 255 | 1.02E-13 | 55.45 | P:small GTPase mediated signal transduction; P:signal transduction; F:guanyl-nucleotide exchange factor activity; C:intracellular; P:positive regulation of GTPase activity |
| Efet.01.493204.g1028.t1 | Potassium sodium hyperpolarization-activated cyclic nucleotide-gated channel 2 | 972 | 7.79E-18 | 83.3 | F:voltage-gated potassium channel activity; P:potassium ion transmembrane transport; C:integral component of membrane |
| Efet.01.49324.g1550.t1 | Chitobiase precursor | 789 | 8.51E-37 | 47.55 | P:carbohydrate metabolic process; P:metabolic process; F:hydrolase activity, hydrolyzing O-glycosyl compounds; F:carbohydrate binding; F:beta-N-acetylhexosaminidase activity; F:polysaccharide binding; F:hydrolase activity, acting on glycosyl bonds; F:hydrolase activity |
| Efet.01.49324.g1551.t1 | N,N -diacetylchitobiase | 1041 | 1.69E-114 | 62.75 | F:binding |
| Efet.01.49329.g1552.t1 | hypothetical protein CAPTEDRAFT_193290 | 1308 | 8.55E-33 | 67.33 |  |
| Efet.01.493319.g1030.t1 | mothers against decapentaplegic homolog 6-like | 621 | 8.12E-61 | 68.5 | P:regulation of cellular process; C:intracellular part; P:cellular process |
| Efet.01.493463.g1033.t1 | Asparagine-rich zinc finger AZF1 | 273 | 6.49E-11 | 61.85 | F:nucleic acid binding; P:regulation of cellular process |
| Efet.01.493503.g1035.t1 | structural maintenance of chromosomes flexible hinge domain-containing 1 | 384 | 7.48E-15 | 57.8 | C:Barr body; C:chromosome, telomeric region; F:ATP binding; P:chromosome organization; P:dosage compensation by inactivation of X chromosome; C:chromosome; P:inactivation of X chromosome by DNA methylation |
| Efet.01.493620.g1036.t1 | adenylate cyclase type 5-like | 249 | 5.48E-11 | 77.15 | F:nucleotide binding; P:adenylate cyclase-inhibiting G-protein coupled receptor signaling pathway; P:cAMP biosynthetic process; C:integral component of membrane; P:adenylate cyclase-activating G-protein coupled receptor signaling pathway; C:intracellular; C:plasma membrane; F:adenylate cyclase activity; P:cAMP-mediated signaling |
| Efet.01.493623.g1037.t1 | energy transducer | 420 | 5.49E-53 | 62.12 | F:siderophore transmembrane transporter activity; P:siderophore transmembrane transport; P:siderophore transport; C:membrane; P:protein transport; C:integral component of membrane; F:energy transducer activity; C:outer membrane-bounded periplasmic space; P:transport; C:plasma membrane |
| Efet.01.493660.g1039.t1 | stAR-related lipid transfer mitochondrial | 378 | 7.03E-20 | 79.6 | F:lipid binding |
| Efet.01.493760.g1042.t1 | zinc finger 500 isoform X1 | 1104 | 8.46E-16 | 70.15 | F:nucleic acid binding; F:transcription factor activity, sequence-specific DNA binding; C:cytoplasm; F:metal ion binding; P:transcription, DNA-templated; P:regulation of transcription, DNA-templated; P:animal organ development; P:determination of left/right symmetry; C:nucleoplasm |
| Efet.01.49383.g1553.t1 | roundabout homolog 1-like isoform X1 | 795 | 6.35E-61 | 49.6 | P:Roundabout signaling pathway; P:kidney development; C:membrane; C:integral component of membrane; P:axon guidance; C:axolemma |
| Efet.01.49391.g1555.t1 | hypothetical protein CAPTEDRAFT_223958 | 324 | 3.36E-10 | 69 | C:membrane; C:integral component of membrane |
| Efet.01.494038.g1045.t1 | 2-oxoglutarate dehydrogenase E1 component | 1098 | 0 | 71.8 | F:oxidoreductase activity, acting on the aldehyde or oxo group of donors, disulfide as acceptor; P:oxidation-reduction process |
| Efet.01.494042.g1046.t1 | NAD(P)H-quinone oxidoreductase | 669 | 7.27E-98 | 92.5 | F:zinc ion binding; F:oxidoreductase activity; P:oxidation-reduction process |
| Efet.01.494042.g1047.t1 | triose-phosphate isomerase | 762 | 4.42E-134 | 81.1 | P:gluconeogenesis; C:cytoplasm; P:pentose-phosphate shunt; P:glycolytic process; F:triose-phosphate isomerase activity |
| Efet.01.494042.g1048.t1 | NADH-quinone oxidoreductase subunit A | 411 | 4.93E-79 | 96.55 | F:quinone binding; C:integral component of membrane; F:NADH dehydrogenase (ubiquinone) activity; P:oxidation-reduction process; P:transport; C:plasma membrane |
| Efet.01.494042.g1049.t1 | NADH dehydrogenase | 480 | 1.33E-101 | 98.15 | F:quinone binding; F:iron ion binding; F:NADH dehydrogenase (ubiquinone) activity; P:oxidation-reduction process; F:4 iron, 4 sulfur cluster binding; P:transport; C:plasma membrane |
| Efet.01.494042.g1050.t1 | NADH-quinone oxidoreductase subunit C | 474 | 2.41E-100 | 91.1 | F:quinone binding; F:NADH dehydrogenase (ubiquinone) activity; P:oxidation-reduction process; P:transport; C:plasma membrane |
| Efet.01.494042.g1051.t1 | NADH dehydrogenase | 1254 | 0 | 96.3 | F:NADH dehydrogenase (quinone) activity; F:quinone binding; F:NAD binding; P:oxidation-reduction process; P:transport; C:plasma membrane |
| Efet.01.494042.g1052.t1 | NADH oxidoreductase (quinone) subunit F | 1743 | 0 | 94.25 | F:FMN binding; F:metal ion binding; F:NAD binding; C:integral component of membrane; F:NADH dehydrogenase (ubiquinone) activity; P:oxidation-reduction process; F:4 iron, 4 sulfur cluster binding |
| Efet.01.494042.g1053.t1 | NADH-quinone oxidoreductase subunit G | 678 | 3.28E-163 | 97 | F:metal ion binding; F:electron carrier activity; C:membrane; F:NADH dehydrogenase (ubiquinone) activity; F:4 iron, 4 sulfur cluster binding; P:ATP synthesis coupled electron transport |
| Efet.01.49434.g1557.t1 | kinesin heavy chain isoform X1 | 276 | 1.23E-13 | 78.8 | F:ATP binding; P:cytoskeleton-dependent intracellular transport; C:cytoplasm; P:protein localization; P:axon guidance; C:kinesin complex; F:ATP-dependent microtubule motor activity, plus-end-directed; P:microtubule-based movement; C:microtubule; F:microtubule binding |
| Efet.01.494344.g1055.t1 | Multiple sugar-binding precursor | 1029 | 1.48E-168 | 81.45 |  |
| Efet.01.494378.g1056.t1 | calcyphosin- | 327 | 2.35E-15 | 62.05 | F:calcium ion binding |
| Efet.01.494422.g1057.t1 | myosin-VIIa isoform X1 | 561 | 4.49E-109 | 91.7 | C:myosin complex; F:ATP binding; F:actin binding; F:motor activity |
| Efet.01.494448.g1058.t1 | thyrotropin-releasing hormone receptor-like | 1011 | 2.26E-63 | 73.7 | F:thyrotropin-releasing hormone receptor activity; P:G-protein coupled receptor signaling pathway; C:integral component of membrane; F:transferase activity |
| Efet.01.49451.g1558.t1 | tight junction ZO-1 isoform X8 | 4587 | 1.16E-41 | 51.2 | C:membrane; C:integral component of membrane |
| Efet.01.49457.g1559.t1 | E3 ubiquitin- ligase MARCH4-like | 789 | 2.64E-25 | 53.2 | F:zinc ion binding; F:metal ion binding; C:membrane; C:integral component of membrane; F:ligase activity |
| Efet.01.494659.g1062.t1 | RAD50-interacting 1 isoform X1 | 270 | 2.25E-17 | 69.95 | C:intracellular organelle; C:cytoplasmic part |
| Efet.01.494676.g1063.t1 | hypothetical protein B5766_08220 | 246 | 2.70E-24 | 100 |  |
| Efet.01.494704.g1064.t1 | sodium hydrogen exchanger 3-like isoform X3 | 1077 | 1.26E-08 | 53.75 |  |
| Efet.01.494785.g1066.t1 | cell division control 42 homolog | 264 | 1.07E-12 | 56.25 | F:GTP binding; P:small GTPase mediated signal transduction; C:intracellular |
| Efet.01.494798.g1067.t1 | leucine-rich repeat and immunoglobulin-like domain-containing nogo receptor-interacting 2 isoform X1 | 1128 | 2.20E-60 | 55 | C:membrane; C:integral component of membrane |
| Efet.01.49492.g1561.t1 | piezo-type mechanosensitive ion channel component 2-like | 261 | 3.38E-08 | 59.33 | F:mechanically-gated ion channel activity; P:ion transmembrane transport; C:membrane; C:integral component of membrane |
| Efet.01.49492.g1562.t1 | piezo-type mechanosensitive ion channel component 1-like | 501 | 8.18E-29 | 62.85 | F:mechanically-gated ion channel activity; P:ion transmembrane transport; C:membrane; C:integral component of membrane |
| Efet.01.494920.g1069.t1 | BAG domain-containing Samui isoform X2 | 1137 | 2.18E-17 | 67.75 | P:biological regulation; F:protein binding; C:cell part |
| Efet.01.495083.g1071.t1 | cyclic nucleotide-gated cation channel alpha-3 | 1551 | 0 | 71.2 | C:integral component of plasma membrane; F:voltage-gated potassium channel activity; P:cation transport; P:regulation of membrane potential; P:ion transmembrane transport; F:intracellular cAMP activated cation channel activity; F:intracellular cGMP activated cation channel activity; F:cGMP binding |
| Efet.01.495120.g1072.t1 | Lymphoid-specific helicase | 216 | 5.25E-14 | 75.7 | F:ATP binding; P:multicellular organism development; P:negative regulation of apoptotic process; F:helicase activity; P:chromatin silencing; C:chromosome, centromeric region; P:DNA metabolic process; P:macromolecule modification |
| Efet.01.495296.g1074.t1 | DNA primase large subunit-like | 408 | 2.44E-26 | 68.75 | F:DNA-directed 5'-3' RNA polymerase activity; P:DNA replication; F:iron-sulfur cluster binding |
| Efet.01.495380.g1076.t1 | helicase carboxy-terminal domain | 1758 | 3.89E-88 | 56.3 | P:protein targeting; F:ATP binding; C:membrane; C:integral component of membrane; F:helicase activity; P:intracellular protein transport; P:protein import; C:intracellular |
| Efet.01.495458.g1077.t1 | telomerase component 1-like | 918 | 1.85E-06 | 62 |  |
| Efet.01.495480.g1078.t1 | hypothetical protein B5766_00055 | 522 | 4.49E-106 | 92 |  |
| Efet.01.49571.g1565.t1 | interferon-induced GTP-binding Mx1-like | 624 | 2.31E-12 | 56.8 | F:nucleotide binding; F:GTP binding; F:GTPase activity |
| Efet.01.495764.g1081.t1 | rab5 GDP GTP exchange factor | 222 | 4.72E-22 | 74.85 | F:DNA binding; F:zinc ion binding |
| Efet.01.495764.g1082.t1 | rab5 GDP GTP exchange factor-like isoform X1 | 321 | 5.53E-20 | 66.17 | F:binding |
| Efet.01.49581.g1566.t1 | DNA polymerase subunit gamma-1-like | 627 | 4.08E-38 | 59.7 | C:mitochondrion; F:binding; P:DNA metabolic process; P:cellular macromolecule biosynthetic process |
| Efet.01.495820.g1084.t1 | copine-8-like isoform X1 | 384 | 3.82E-51 | 85.45 |  |
| Efet.01.496047.g1086.t1 | nuclear pore complex Nup98-Nup96-like isoform X1 | 279 | 3.32E-07 | 80.71 | P:transport; C:nuclear pore |
| Efet.01.496128.g1088.t1 | outer membrane | 441 | 5.67E-96 | 63.75 |  |
| Efet.01.496178.g1089.t1 | fukutin-related -like | 579 | 6.18E-17 | 58.05 | C:membrane; C:integral component of membrane |
| Efet.01.496235.g1090.t1 | high affinity cAMP-specific and IBMX-insensitive 3 ,5 -cyclic phosphodiesterase 8B isoform X1 | 315 | 4.75E-16 | 59.55 | F:metal ion binding; P:signal transduction; F:phosphoric diester hydrolase activity; F:3',5'-cyclic-nucleotide phosphodiesterase activity; F:hydrolase activity |
| Efet.01.49627.g1568.t1 | B-cell CLL lymphoma 9 -like isoform X1 | 4110 | 1.80E-10 | 63 |  |
| Efet.01.496299.g1091.t1 | FMRFamide receptor-like | 1359 | 2.88E-61 | 53.2 | F:G-protein coupled receptor activity; F:G-protein coupled peptide receptor activity; P:G-protein coupled receptor signaling pathway; C:membrane; C:integral component of membrane |
| Efet.01.49641.g1572.t1 | hypothetical protein HELRODRAFT_167810 | 6753 | 3.39E-14 | 51 |  |
| Efet.01.496426.g1092.t1 | homeobox Nkx- -like | 507 | 5.46E-61 | 72.85 | C:nucleus; P:regulation of transcription, DNA-templated; F:sequence-specific DNA binding |
| Efet.01.496456.g1093.t1 | dual specificity phosphatase 14-like | 696 | 3.82E-70 | 66.15 | P:protein dephosphorylation; C:striated muscle myosin thick filament; F:protein tyrosine/serine/threonine phosphatase activity |
| Efet.01.496468.g1094.t1 | dynein heavy chain axonemal | 342 | 4.77E-11 | 80.4 | F:ATPase activity; F:ATP binding; F:microtubule motor activity; P:microtubule-based movement |
| Efet.01.49647.g1573.t1 | transmembrane 115 | 885 | 3.40E-107 | 70.55 | C:membrane |
| Efet.01.496484.g1095.t1 | PREDICTED: uncharacterized protein LOC106817274 | 1038 | 9.67E-06 | 37 |  |
| Efet.01.496508.g1096.t1 | poly [ADP-ribose] polymerase 14-like | 897 | 8.41E-11 | 49.75 |  |
| Efet.01.496510.g1097.t1 | hypothetical protein CAPTEDRAFT_219915 | 747 | 1.47E-28 | 47.7 | F:thiolester hydrolase activity; P:fatty acid biosynthetic process |
| Efet.01.496516.g1098.t1 | golgin subfamily A member 4-like isoform X4 | 315 | 8.55E-11 | 67.9 | P:protein targeting to Golgi; C:intracellular |
| Efet.01.496522.g1099.t1 | fibrinogen 1 | 387 | 2.37E-08 | 60.1 | C:membrane; C:integral component of membrane |
| Efet.01.496527.g1100.t1 | 1-phosphatidylinositol 4,5-bisphosphate phosphodiesterase classes I and II-like | 234 | 3.03E-23 | 89.5 | F:phosphatidylinositol phospholipase C activity; F:calcium ion binding; P:lipid catabolic process; F:signal transducer activity; C:intracellular; P:intracellular signal transduction |
| Efet.01.496564.g1102.t1 | mitochondrial ubiquitin ligase activator of nfkb 1-A-like | 555 | 5.21E-33 | 52.85 | F:ubiquitin-protein transferase activity; F:zinc ion binding; F:metal ion binding; C:membrane; C:integral component of membrane; P:mitochondrion organization; P:protein ubiquitination |
| Efet.01.49666.g1574.t1 | plectin-like isoform X4 | 327 | 9.39E-26 | 64.4 | C:cytoskeleton; C:membrane; C:integral component of membrane |
| Efet.01.49668.g1575.t1 | strawberry notch homolog 1 | 1467 | 1.15E-169 | 69.3 | P:biological_process; P:regulation of transcription, DNA-templated; C:cellular_component; F:molecular_function |
| Efet.01.496743.g1105.t1 | collagen alpha-3(VI) chain-like isoform X6 | 1596 | 1.61E-81 | 55.5 | F:calcium ion binding |
| Efet.01.496820.g1106.t1 | glutamate receptor delta-1 isoform X1 | 210 | 1.66E-14 | 72.1 | P:ion transport; F:transmembrane signaling receptor activity; F:extracellular ligand-gated ion channel activity; C:synapse; C:membrane part; C:plasma membrane |
| Efet.01.496822.g1108.t1 | gastrula zinc finger -like | 1422 | 1.14E-60 | 53.5 | F:nucleic acid binding; F:metal ion binding |
| Efet.01.496825.g1109.t1 | nucleoside hydrolase | 849 | 1.21E-85 | 78.1 | F:hydrolase activity, hydrolyzing N-glycosyl compounds; P:metabolic process |
| Efet.01.49683.g1576.t1 | discoidin domain-containing receptor 2-like isoform X1 | 1257 | 2.35E-43 | 70.5 | C:membrane; F:protein kinase activity; P:protein phosphorylation |
| Efet.01.496900.g1111.t1 | filamin-A-like isoform X1 | 564 | 3.42E-75 | 67.8 | F:actin filament binding |
| Efet.01.496985.g1113.t1 | helicase MOV-10 | 1734 | 0 | 67.55 | F:organic cyclic compound binding; F:heterocyclic compound binding |
| Efet.01.497043.g1114.t1 | G -regulated inducer of neurite outgrowth 3 | 576 | 3.73E-12 | 72 |  |
| Efet.01.497157.g1116.t1 | twitchin isoform X7 | 351 | 9.42E-45 | 75.35 | F:ATP binding; F:protein kinase activity; P:protein phosphorylation |
| Efet.01.497208.g1117.t1 | retinol dehydrogenase 12-like | 216 | 3.22E-24 | 79.1 | C:membrane |
| Efet.01.497237.g1118.t1 | serine threonine- kinase DCLK2 isoform X1 | 438 | 1.08E-25 | 68.15 | P:phosphorylation; F:kinase activity |
| Efet.01.497364.g1120.t1 | Outer dense fiber 3 | 477 | 2.68E-42 | 64.6 |  |
| Efet.01.497414.g1121.t1 | Tetratricopeptide repeat 28 | 318 | 6.67E-12 | 59.5 |  |
| Efet.01.497455.g1122.t1 | serine threonine- kinase PAK | 363 | 1.14E-46 | 81.1 | P:positive regulation of JUN kinase activity; P:negative regulation of cell proliferation involved in contact inhibition; P:Fc-epsilon receptor signaling pathway; P:protein autophosphorylation; C:Golgi apparatus; P:Fc-gamma receptor signaling pathway involved in phagocytosis; P:branching morphogenesis of an epithelial tube; P:exocytosis; P:mitotic cell cycle; P:stimulatory C-type lectin receptor signaling pathway; P:cellular response to insulin stimulus; C:dendrite; F:ATP binding; P:MAPK cascade; P:wound healing; P:response to hypoxia; C:focal adhesion; P:axonogenesis; P:apoptotic process; C:cytosol; C:ruffle membrane; C:axon; C:invadopodium; P:actin cytoskeleton reorganization; P:T cell receptor signaling pathway; P:positive regulation of peptidyl-serine phosphorylation; F:protein kinase binding; P:T cell costimulation; C:Z disc; C:nuclear membrane; F:signal transducer, downstream of receptor, with serine/threonine kinase activity; F:collagen binding; P:positive regulation of stress fiber assembly; P:ephrin receptor signaling pathway; P:positive regulation of intracellular estrogen receptor signaling pathway; C:intercalated disc; C:filamentous actin; P:positive regulation of cell migration |
| Efet.01.497469.g1123.t1 | hydroxypyruvate isomerase | 807 | 2.96E-101 | 68.9 | F:hydroxypyruvate isomerase activity; F:isomerase activity |
| Efet.01.497534.g1125.t1 | gliding motility-associated C-terminal domain-containing | 1107 | 7.92E-133 | 47.95 |  |
| Efet.01.497535.g1126.t1 | frizzled-5 | 1878 | 0 | 65.1 | P:apoptotic process involved in morphogenesis; F:Wnt-activated receptor activity; P:positive regulation of JUN kinase activity; P:regulation of chorionic trophoblast cell proliferation; P:regulation of mitophagy; P:negative regulation of cell proliferation; P:Wnt signaling pathway involved in dorsal/ventral axis specification; C:Golgi apparatus; P:positive regulation of transcription from RNA polymerase II promoter; C:bicellular tight junction; C:clathrin-coated endocytic vesicle membrane; P:angiogenesis; P:regulation of canonical Wnt signaling pathway; P:positive regulation of interferon-gamma production; P:post-embryonic camera-type eye development; P:Wnt signaling pathway, planar cell polarity pathway; P:regulation of bicellular tight junction assembly; F:Wnt-protein binding; P:cellular response to molecule of bacterial origin; F:protein kinase binding; P:anterior/posterior axis specification, embryo; P:T cell differentiation in thymus; P:branching involved in labyrinthine layer morphogenesis; P:Wnt signaling pathway, calcium modulating pathway; P:cell maturation; P:syncytiotrophoblast cell differentiation involved in labyrinthine layer development; P:beta-catenin destruction complex disassembly; C:plasma membrane; F:beta-amyloid binding; P:positive regulation of protein targeting to mitochondrion; C:cell surface; C:early endosome membrane; P:synapse assembly; C:perinuclear region of cytoplasm; F:ubiquitin protein ligase binding; P:labyrinthine layer blood vessel development; P:chorionic trophoblast cell differentiation; P:positive regulation of T cell cytokine production; P:embryonic camera-type eye morphogenesis; P:Spemann organizer formation |
| Efet.01.497567.g1127.t1 | unc-13 homolog B-like | 417 | 2.79E-80 | 91.4 | F:metal ion binding; F:diacylglycerol binding; P:chemical synaptic transmission; C:intracellular; P:intracellular signal transduction |
| Efet.01.49765.g1577.t1 | voltage-dependent T-type calcium channel subunit alpha-1H-like | 495 | 2.15E-82 | 89.45 | P:calcium ion import; C:voltage-gated calcium channel complex; P:regulation of ion transmembrane transport; P:calcium ion transmembrane transport; F:low voltage-gated calcium channel activity |
| Efet.01.497671.g1129.t1 | hypothetical protein CAPTEDRAFT_193210 | 312 | 3.77E-11 | 73 | C:membrane |
| Efet.01.4977.g387.t1 | PREDICTED: uncharacterized protein LOC101235528 | 414 | 2.77E-12 | 65.5 |  |
| Efet.01.497711.g1130.t1 | PIN domain-containing | 408 | 1.51E-79 | 74.2 |  |
| Efet.01.497711.g1131.t1 | family transcriptional regulator | 912 | 0 | 91.95 | F:DNA binding; F:transcription factor activity, sequence-specific DNA binding; P:transcription, DNA-templated; P:regulation of transcription, DNA-templated |
| Efet.01.497711.g1132.t1 | MFS transporter | 987 | 0 | 93 | C:outer membrane-bounded periplasmic space |
| Efet.01.497719.g1133.t1 | histidine--tRNA ligase | 285 | 3.38E-35 | 58.5 | F:histidine-tRNA ligase activity; F:nucleotide binding; P:tRNA aminoacylation for protein translation; F:ATP binding; F:aminoacyl-tRNA ligase activity; P:histidyl-tRNA aminoacylation; C:cytoplasm; F:ligase activity; P:translation |
| Efet.01.497753.g1134.t1 | leukocyte receptor cluster member 8 homolog | 1557 | 3.05E-06 | 73.25 |  |
| Efet.01.497755.g1135.t1 | citrate lyase subunit beta mitochondrial | 558 | 2.43E-69 | 70.6 | F:catalytic activity; F:metal ion binding |
| Efet.01.49776.g1578.t1 | hypothetical protein CAPTEDRAFT_206253 | 384 | 8.03E-21 | 56.83 | C:membrane; C:integral component of membrane; F:carbohydrate binding |
| Efet.01.497777.g1136.t1 | golgin subfamily A member 4-like isoform X3 | 582 | 3.21E-36 | 76.25 | C:extracellular space; P:regulation of autophagy |
| Efet.01.497787.g1137.t1 | neuronal acetylcholine receptor subunit alpha-10-like isoform X3 | 309 | 6.79E-14 | 79.8 | C:postsynaptic membrane; P:cation transmembrane transport; C:integral component of membrane; C:cell junction; F:extracellular ligand-gated ion channel activity |
| Efet.01.497840.g1138.t1 | hypothetical protein HELRODRAFT_160822 | 1107 | 1.10E-09 | 62.5 |  |
| Efet.01.497887.g1139.t1 | mitofusin-2-like isoform X1 | 381 | 7.52E-28 | 68.95 | F:GTP binding; F:GTPase activity; P:mitochondrial fusion; P:negative regulation of smooth muscle cell proliferation; C:integral component of membrane; C:mitochondrial outer membrane |
| Efet.01.497993.g1141.t1 | malectin-B-like isoform X1 | 201 | 7.01E-12 | 83.15 | C:integral component of membrane |
| Efet.01.49800.g1581.t1 | four and a half LIM domains 2 isoform X4 | 864 | 2.99E-51 | 61.4 | F:zinc ion binding; F:metal ion binding |
| Efet.01.498155.g1143.t1 | RUN domain-containing 1-like isoform X1 | 255 | 9.54E-21 | 85.95 |  |
| Efet.01.498215.g1144.t1 | transcriptional enhancer factor TEF-5-like | 216 | 3.20E-23 | 78.15 | F:DNA binding; F:transcription factor activity, sequence-specific DNA binding; P:regulation of transcription, DNA-templated; P:hippo signaling; C:intracellular |
| Efet.01.4984.g388.t1 | colorectal mutant cancer -like isoform X1 | 354 | 1.90E-36 | 83.75 |  |
| Efet.01.498609.g1146.t1 | hypothetical protein B5766_07610 | 522 | 3.84E-72 | 63.7 | C:membrane; C:integral component of membrane |
| Efet.01.498672.g1150.t1 | amiloride-sensitive sodium channel subunit alpha-like isoform X1 | 678 | 2.30E-10 | 75.65 | F:sodium channel activity; C:integral component of membrane; P:sodium ion transmembrane transport |
| Efet.01.498867.g1153.t1 | ETS pointed isoform X1 | 570 | 1.06E-06 | 76.22 | F:DNA binding; C:nucleus; F:transcription factor activity, sequence-specific DNA binding; P:cell differentiation; P:regulation of transcription, DNA-templated; F:sequence-specific DNA binding; F:RNA polymerase II transcription factor activity, sequence-specific DNA binding; P:regulation of transcription from RNA polymerase II promoter |
| Efet.01.498885.g1154.t1 | small G signaling modulator 1 isoform X1 | 285 | 3.54E-25 | 84.35 |  |
| Efet.01.49894.g1582.t1 | plasma membrane calcium-transporting ATPase 2-like | 480 | 2.87E-25 | 78.55 | F:ATP binding; F:metal ion binding; C:integral component of membrane; P:calcium ion transmembrane transport; C:cell part; F:calcium-transporting ATPase activity |
| Efet.01.49916.g1583.t1 | substance-P receptor-like isoform X1 | 576 | 2.25E-25 | 77.05 | P:neuropeptide signaling pathway; C:integral component of membrane; F:neuropeptide Y receptor activity |
| Efet.01.499170.g1157.t1 | forkhead box B1 | 480 | 3.93E-46 | 74.85 | F:DNA binding; P:chordate embryonic development; P:midbrain development; P:regulation of cellular process; P:forebrain cell migration; P:anatomical structure morphogenesis; P:mammillary body development; P:epithelium development |
| Efet.01.499285.g1158.t1 | Voltage-dependent T-type calcium channel subunit alpha- | 291 | 8.16E-20 | 94.4 | P:calcium ion import; C:voltage-gated calcium channel complex; P:regulation of ion transmembrane transport; P:calcium ion transmembrane transport; F:low voltage-gated calcium channel activity |
| Efet.01.499369.g1159.t1 | EH domain-binding 1-like isoform X1 | 510 | 1.72E-22 | 61.75 |  |
| Efet.01.499463.g1163.t1 | membrane | 636 | 1.65E-81 | 76.3 | C:integral component of membrane |
| Efet.01.499463.g1164.t1 | glutamine synthetase | 1419 | 0 | 83.25 | P:nitrogen compound metabolic process; F:glutamate-ammonia ligase activity |
| Efet.01.499594.g1166.t1 | solute carrier organic anion transporter family member 4C1 | 792 | 8.92E-12 | 45.83 | P:ion transport; C:membrane; C:integral component of membrane; P:transport; F:transporter activity; C:plasma membrane |
| Efet.01.499720.g1168.t1 | Probable 3-oxoacyl-(Acyl-carrier- ) synthase II | 309 | 1.24E-45 | 73.25 | P:metabolic process; F:transferase activity |
| Efet.01.499848.g1169.t1 | serine threonine kinase-like domain-containing STKLD1 isoform X1 | 276 | 1.86E-09 | 72 |  |
| Efet.01.499920.g1170.t1 | repulsive guidance molecule A-like | 735 | 2.11E-29 | 51.65 | C:membrane |
| Efet.01.499945.g1171.t1 | Ubiquitin carboxyl-terminal hydrolase CYLD | 1059 | 4.66E-18 | 44.85 | P:protein deubiquitination; P:signal transduction; P:apoptotic process; C:cytosol; C:intracellular; F:thiol-dependent ubiquitinyl hydrolase activity; F:hydrolase activity |
| Efet.01.499985.g1173.t1 | hypothetical protein B5766_11315 | 705 | 3.08E-71 | 66 |  |
| Efet.01.50002.g1.t1 | SH3 domain-binding 5 isoform X4 | 1179 | 3.01E-22 | 61.05 | P:regulation of cellular process; C:intracellular |
| Efet.01.500023.g1.t1 | threonine dehydratase | 570 | 1.56E-103 | 75.8 | F:lyase activity; P:branched-chain amino acid biosynthetic process |
| Efet.01.500045.g2.t1 | Glutamate decarboxylase 2 | 243 | 5.45E-24 | 85.45 | C:presynaptic membrane; C:inhibitory synapse; P:social behavior; F:pyridoxal phosphate binding; C:clathrin-sculpted gamma-aminobutyric acid transport vesicle membrane; P:locomotory exploration behavior; F:glutamate decarboxylase activity; C:Golgi membrane; P:glutamate decarboxylation to succinate; P:neurotransmitter biosynthetic process; P:gamma-aminobutyric acid biosynthetic process; C:axon terminus; P:neurotransmitter secretion; C:presynaptic active zone; F:protein heterodimerization activity; C:cell cortex; P:protein-pyridoxal-5-phosphate linkage; P:response to drug; C:mitochondrion; F:protein N-terminus binding; C:cell junction; F:glutamate binding; C:axon |
| Efet.01.50005.g2.t1 | xaa-Pro dipeptidase-like | 420 | 1.13E-37 | 67.25 | F:metal ion binding; F:hydrolase activity |
| Efet.01.5001.g389.t1 | dynein heavy chain axonemal | 1134 | 9.78E-107 | 67.75 | F:nucleotide binding; F:ATPase activity; F:ATP binding; F:microtubule motor activity; C:membrane; C:integral component of membrane; C:dynein complex; P:microtubule-based movement |
| Efet.01.500190.g5.t1 | ankyrin repeat | 1152 | 2.60E-66 | 56.2 |  |
| Efet.01.50029.g3.t1 | M-phase inducer phosphatase-like | 468 | 2.34E-36 | 66.9 | P:regulation of mitotic cell cycle, embryonic; P:protein dephosphorylation; P:regulation of G2/M transition of mitotic cell cycle; P:positive regulation of mitotic cell cycle |
| Efet.01.500297.g6.t1 | XRP2 | 450 | 1.80E-19 | 45.2 | C:intracellular organelle; F:protein binding; C:membrane; C:cytoplasmic part; C:cell projection; P:transport; P:cellular process; C:membrane-bounded organelle |
| Efet.01.50040.g4.t1 | allatostatin-A receptor-like | 882 | 1.29E-50 | 62.35 | F:G-protein coupled receptor activity; P:G-protein coupled receptor signaling pathway; C:integral component of membrane |
| Efet.01.500434.g7.t1 | probable G- coupled receptor 139 | 1344 | 6.01E-98 | 59.3 | F:G-protein coupled receptor activity; P:G-protein coupled receptor signaling pathway; P:signal transduction; C:membrane; C:integral component of membrane; F:signal transducer activity |
| Efet.01.500525.g11.t1 | arginine vasopressin receptor | 417 | 6.21E-13 | 54.2 | P:signal transduction; C:membrane; F:protein-hormone receptor activity |
| Efet.01.500598.g12.t1 | nascent polypeptide-associated complex subunit alpha isoform X2 | 318 | 2.89E-19 | 73.85 | P:heart trabecula morphogenesis; P:cardiac ventricle development; P:positive regulation of skeletal muscle tissue growth; P:positive regulation of transcription from RNA polymerase II promoter involved in heart development; P:transport; P:negative regulation of transcription from RNA polymerase II promoter involved in heart development; P:translation; P:skeletal muscle tissue regeneration; C:nucleus; C:nascent polypeptide-associated complex; C:extracellular exosome; F:protein binding; F:transcription coactivator activity; P:regulation of skeletal muscle fiber development; P:positive regulation of cell proliferation involved in heart morphogenesis; P:negative regulation of striated muscle cell apoptotic process |
| Efet.01.500602.g13.t1 | DNA-directed RNA polymerase subunit beta | 1653 | 0 | 85.7 | F:DNA binding; F:DNA-directed 5'-3' RNA polymerase activity; P:transcription, DNA-templated |
| Efet.01.500646.g14.t1 | hypothetical protein CAPTEDRAFT_220023 | 510 | 4.58E-10 | 60 |  |
| Efet.01.500652.g15.t1 | DNA translocase | 408 | 1.67E-71 | 93.95 | P:cell division; F:DNA binding; F:ATP binding; C:integral component of membrane; P:chromosome segregation; C:plasma membrane; P:cell cycle |
| Efet.01.500689.g16.t1 | ecto-NOX disulfide-thiol exchanger 2-like | 681 | 1.14E-52 | 77.5 | F:nucleotide binding; F:nucleic acid binding |
| Efet.01.500791.g18.t1 | prohormone 4 | 390 | 7.16E-19 | 64.2 |  |
| Efet.01.500837.g20.t1 | antitoxin | 303 | 1.52E-45 | 86.95 | F:DNA binding; P:regulation of transcription, DNA-templated |
| Efet.01.500837.g21.t1 | mobilization A domain | 264 | 3.85E-43 | 68.8 |  |
| Efet.01.50091.g6.t1 | Serine arginine-rich splicing factor | 1131 | 1.40E-11 | 82.9 | F:nucleotide binding; F:nucleic acid binding; C:integral component of membrane |
| Efet.01.500982.g23.t1 | myxovirus resistance | 270 | 1.78E-36 | 93.15 | C:postsynaptic density; P:sperm ejaculation; P:positive regulation of filopodium assembly; C:Golgi apparatus; P:dynamin family protein polymerization involved in mitochondrial fission; C:microtubule; F:GTP binding; P:clathrin-dependent endocytosis; C:extracellular exosome; F:GTPase activity; C:cytosol; C:cytoplasmic vesicle; F:type 1 metabotropic glutamate receptor binding; F:nitric-oxide synthase binding; C:axon; P:negative regulation of dendritic spine morphogenesis; P:membrane fusion; P:filopodium assembly; C:plasma membrane; C:dendritic spine head; F:microtubule binding; P:mitochondrial fission; F:type 5 metabotropic glutamate receptor binding; P:synapse assembly; C:mitochondrial membrane; C:synaptic cleft; C:perinuclear region of cytoplasm; C:photoreceptor inner segment; P:receptor internalization |
| Efet.01.501168.g26.t1 | transcription termination factor mitochondrial-like | 501 | 2.55E-38 | 65.5 | P:transcription from mitochondrial promoter; P:positive regulation of mitochondrial translation; C:mitochondrion; P:mitochondrial translation; P:mitochondrial DNA replication; P:regulation of cell cycle; F:small ribosomal subunit rRNA binding; P:regulation of transcription from mitochondrial promoter; P:mitochondrial ribosome assembly |
| Efet.01.50118.g10.t1 | hypothetical protein CAPTEDRAFT_190719 | 495 | 5.10E-15 | 60 |  |
| Efet.01.501260.g28.t1 | lysine-specific demethylase 3B-like isoform X5 | 288 | 1.33E-25 | 66.1 | F:methyltransferase activity; P:methylation |
| Efet.01.501312.g30.t1 | Short transient receptor potential channel 3 | 633 | 2.42E-87 | 84.75 | C:integral component of plasma membrane; F:store-operated calcium channel activity; P:manganese ion transport; P:regulation of cytosolic calcium ion concentration; P:calcium ion transmembrane transport; P:single fertilization |
| Efet.01.501418.g33.t1 | monocarboxylate transporter 12-like | 1005 | 9.48E-32 | 45.15 | P:transmembrane transport; C:membrane; C:integral component of membrane |
| Efet.01.501502.g35.t1 | sex peptide receptor-like | 1107 | 9.98E-137 | 66 | F:G-protein coupled peptide receptor activity; P:G-protein coupled receptor signaling pathway; C:integral component of membrane |
| Efet.01.501666.g37.t1 | hypothetical protein HELRODRAFT_171705 | 1128 | 1.25E-17 | 51 | F:G-protein coupled receptor activity; F:G-protein coupled peptide receptor activity; P:G-protein coupled receptor signaling pathway; C:membrane; C:integral component of membrane |
| Efet.01.501763.g39.t1 | homeobox prospero-like isoform X1 | 1926 | 1.06E-19 | 48 | F:DNA binding |
| Efet.01.5018.g390.t1 | neuron navigator 2-like | 201 | 1.11E-26 | 89.9 |  |
| Efet.01.501813.g41.t1 | hypothetical protein HELRODRAFT_185802 | 972 | 2.00E-76 | 65.45 | F:actin binding; C:intracellular part; P:actin cytoskeleton organization |
| Efet.01.501884.g42.t1 | phospholipid-transporting ATPase ID-like isoform X1 | 300 | 1.42E-15 | 84.4 | F:magnesium ion binding; F:ATP binding; P:phospholipid translocation; C:integral component of membrane; F:phospholipid-translocating ATPase activity; F:chromatin binding |
| Efet.01.501923.g43.t1 | hypothetical protein CAPTEDRAFT_188765 | 999 | 2.19E-12 | 50.25 | C:Golgi membrane; P:COPII vesicle coating |
| Efet.01.501982.g44.t1 | vigilin-like | 201 | 4.51E-11 | 77.45 | F:RNA binding |
| Efet.01.502010.g45.t1 | hypothetical protein LOTGIDRAFT_169247 | 921 | 6.65E-06 | 77 |  |
| Efet.01.502019.g46.t1 | hypothetical protein BSL78_19101 | 1959 | 1.26E-116 | 62.2 |  |
| Efet.01.502020.g47.t1 | glycosaminoglycan xylosylkinase | 399 | 8.16E-19 | 72.7 | P:cellular metabolic process |
| Efet.01.502060.g49.t1 | Translation initiation factor IF-2 | 201 | 1.38E-17 | 76.25 | C:membrane |
| Efet.01.502067.g50.t1 | ABC transporter ATP-binding | 987 | 1.56E-178 | 68 | F:ATPase activity; F:ATP binding; P:transmembrane transport; C:ATP-binding cassette (ABC) transporter complex; F:hydrolase activity, acting on acid anhydrides, catalyzing transmembrane movement of substances |
| Efet.01.50213.g17.t1 | fibroblast growth factor receptor 2-like isoform X3 | 594 | 3.37E-49 | 58.25 | F:protein kinase activity; P:cellular process |
| Efet.01.502147.g51.t1 | neural-cadherin 2 | 486 | 1.76E-38 | 56.9 | C:membrane; P:cell adhesion |
| Efet.01.50215.g18.t1 | neurogenic locus Notch -like | 369 | 2.56E-28 | 66.3 | C:membrane; P:regulation of biological process; P:cellular process; P:single-organism developmental process |
| Efet.01.502300.g52.t1 | TLC domain-containing 2-like | 495 | 2.05E-40 | 66.95 | C:membrane |
| Efet.01.502348.g53.t1 | armadillo repeat-containing 4 | 273 | 3.09E-14 | 84.9 |  |
| Efet.01.502375.g54.t1 | hemolysin III | 621 | 1.84E-131 | 84.45 | F:DNA binding; P:cytolysis; C:integral component of membrane |
| Efet.01.502403.g55.t1 | cyclin-dependent kinase 12-like | 471 | 1.07E-16 | 64.31 |  |
| Efet.01.502405.g56.t1 | dynamin-binding isoform X2 | 339 | 1.59E-17 | 59.9 | P:regulation of cellular process; F:guanyl-nucleotide exchange factor activity |
| Efet.01.50244.g20.t1 | Ras-like GTP-binding | 540 | 4.25E-72 | 70.15 | F:GTP binding; P:small GTPase mediated signal transduction; C:intracellular |
| Efet.01.502484.g58.t1 | Nuclear receptor subfamily 2 group E member 1 | 360 | 1.07E-15 | 76.2 | C:nucleus; F:zinc ion binding; F:RNA polymerase II transcription factor activity, ligand-activated sequence-specific DNA binding; P:transcription, DNA-templated; F:steroid hormone receptor activity; P:regulation of transcription, DNA-templated; F:sequence-specific DNA binding; P:steroid hormone mediated signaling pathway; P:intracellular receptor signaling pathway |
| Efet.01.502532.g60.t1 | teneurin-3-like isoform X8 | 222 | 2.06E-24 | 70.15 |  |
| Efet.01.50257.g22.t1 | cullin-3-like isoform X1 | 666 | 2.70E-86 | 84.75 | F:ubiquitin protein ligase activity; F:ubiquitin protein ligase binding; C:cullin-RING ubiquitin ligase complex; P:protein ubiquitination involved in ubiquitin-dependent protein catabolic process |
| Efet.01.502615.g62.t1 | voltage-sensitive sodium | 297 | 1.52E-43 | 83 | F:voltage-gated potassium channel activity; P:regulation of postsynaptic membrane potential; P:potassium ion transmembrane transport; C:voltage-gated potassium channel complex; F:calcium ion binding; P:regulation of ion transmembrane transport; C:voltage-gated sodium channel complex; P:sodium ion transmembrane transport; F:voltage-gated sodium channel activity |
| Efet.01.502676.g64.t1 | hypothetical protein CAPTEDRAFT_158069 | 480 | 5.12E-11 | 56 | F:DNA binding; C:nucleus; P:multicellular organism development; P:regulation of transcription, DNA-templated; F:sequence-specific DNA binding |
| Efet.01.50274.g23.t1 | coiled-coil domain-containing 170-like | 219 | 6.24E-21 | 78.33 |  |
| Efet.01.502741.g67.t1 | zinc finger 202 | 357 | 5.74E-10 | 50.45 | F:nucleic acid binding; F:metal ion binding |
| Efet.01.502787.g69.t1 | ras-related Rap-1b-like | 732 | 4.78E-58 | 69.9 | F:nucleotide binding; P:signal transduction |
| Efet.01.502789.g70.t1 | type IX secretion system membrane family | 519 | 2.56E-40 | 54.95 |  |
| Efet.01.50279.g24.t1 | metabotropic glutamate receptor 7-like | 1302 | 1.91E-48 | 52.45 | F:G-protein coupled receptor activity; P:G-protein coupled receptor signaling pathway; C:membrane; C:integral component of membrane |
| Efet.01.502802.g71.t1 | DNA primase | 408 | 5.48E-37 | 68.75 |  |
| Efet.01.502821.g72.t1 | [Naegleria gruberi] | 249 | 5.04E-15 | 70 | F:calcium ion binding |
| Efet.01.502829.g73.t1 | aconitase | 279 | 2.97E-23 | 82.35 | C:plasmodesma; F:copper ion binding; F:mRNA 5'-UTR binding; C:mitochondrion; C:apoplast; P:citrate metabolic process; C:cytosol; P:isocitrate metabolic process; F:4 iron, 4 sulfur cluster binding; C:vacuole; C:plasma membrane; F:aconitate hydratase activity |
| Efet.01.502836.g74.t1 | D-aspartate oxidase-like | 429 | 6.83E-30 | 65.95 | F:oxidoreductase activity; P:single-organism metabolic process |
| Efet.01.502860.g75.t1 | Retrovirus-related Pol poly from transposon | 708 | 2.10E-26 | 62.25 | F:nucleic acid binding; F:zinc ion binding; F:aspartic-type endopeptidase activity; F:metal ion binding; P:proteolysis |
| Efet.01.503151.g76.t1 | NADP-dependent malic enzyme | 732 | 3.41E-129 | 82 | F:oxaloacetate decarboxylase activity; F:transferase activity, transferring acyl groups; P:malate metabolic process; F:NAD binding; C:cytosol; F:malate dehydrogenase (decarboxylating) (NAD+) activity; F:manganese ion binding; P:oxidation-reduction process; F:malate dehydrogenase (decarboxylating) (NADP+) activity |
| Efet.01.503172.g77.t1 | tripartite motif-containing 2-like | 1143 | 9.94E-32 | 47.2 | F:zinc ion binding; F:metal ion binding; C:intracellular |
| Efet.01.50323.g28.t1 | hypothetical protein CAPTEDRAFT_223749 | 342 | 1.16E-21 | 65.53 | C:membrane; C:integral component of membrane |
| Efet.01.503313.g79.t1 | Homeobox prospero | 264 | 6.36E-14 | 85.05 | F:DNA binding |
| Efet.01.503314.g80.t1 | nicotinic acetylcholine receptor subunit | 357 | 6.45E-43 | 59.05 | C:postsynaptic membrane; P:cell communication; P:cation transmembrane transport; C:integral component of membrane; C:cell junction; F:extracellular ligand-gated ion channel activity; P:single organism signaling |
| Efet.01.50347.g29.t1 | insulin gene enhancer ISL-1 | 210 | 2.87E-28 | 89.3 | C:nucleus; F:zinc ion binding; P:regulation of transcription, DNA-templated; F:sequence-specific DNA binding |
| Efet.01.503482.g81.t1 | transcriptional regulator ATRX-like | 744 | 6.16E-67 | 74.35 | C:membrane |
| Efet.01.503541.g83.t1 | endopeptidase La | 2451 | 0 | 95.75 | F:ATP binding; P:misfolded or incompletely synthesized protein catabolic process; C:cytoplasm; F:serine-type endopeptidase activity; P:cellular response to stress; F:sequence-specific DNA binding; F:ATP-dependent peptidase activity |
| Efet.01.503541.g84.t1 | integrase | 1455 | 0 | 78.7 | F:DNA binding; P:DNA recombination; P:DNA integration |
| Efet.01.503541.g85.t1 | IS66 family transposase | 1521 | 0 | 91.95 |  |
| Efet.01.503541.g86.t1 | transposase | 288 | 4.76E-59 | 95.05 |  |
| Efet.01.503541.g87.t1 | DUF3732 domain-containing | 1311 | 0 | 77.5 |  |
| Efet.01.50358.g30.t1 | mu-type opioid receptor-like | 954 | 1.01E-91 | 53.6 | F:signal transducer activity |
| Efet.01.5036.g391.t1 | hepatocyte nuclear factor 3-beta-like | 1203 | 1.05E-50 | 69.05 | F:DNA binding; F:transcription factor activity, sequence-specific DNA binding; F:protein binding; P:system development; P:regulation of transcription, DNA-templated |
| Efet.01.503608.g89.t1 | zinc finger and SCAN domain-containing 21-like | 1191 | 7.01E-17 | 80.18 | F:nucleic acid binding; F:metal ion binding |
| Efet.01.503634.g90.t1 | cGMP-specific 3 ,5 -cyclic phosphodiesterase | 213 | 1.10E-10 | 69.65 | F:metal ion binding; P:signal transduction; C:membrane; F:3',5'-cyclic-nucleotide phosphodiesterase activity |
| Efet.01.503635.g91.t1 | rho GTPase-activating 39 isoform X3 | 1512 | 1.06E-12 | 46.4 | C:cytoskeleton; P:signal transduction; C:membrane; C:integral component of membrane |
| Efet.01.503659.g92.t1 | unconventional myosin-Va-like isoform X2 | 279 | 4.88E-27 | 76.2 | F:nucleotide binding; F:actin binding; C:membrane |
| Efet.01.503764.g95.t1 | serine-rich adhesin for platelets-like isoform X1 | 1665 | 3.75E-16 | 55.4 | C:nucleus; F:transcription coactivator activity; P:regulation of nucleic acid-templated transcription |
| Efet.01.503874.g96.t1 | twinkle mitochondrial-like | 564 | 2.80E-73 | 65.95 | F:ATP binding; F:DNA helicase activity; P:DNA replication; P:DNA duplex unwinding; F:5'-3' DNA helicase activity; F:single-stranded DNA binding |
| Efet.01.503875.g97.t1 | histone -like | 312 | 1.11E-61 | 98.2 | F:RNA polymerase II distal enhancer sequence-specific DNA binding; P:positive regulation of cell growth; C:extracellular exosome; F:histone binding; F:protein heterodimerization activity; F:RNA polymerase II core promoter sequence-specific DNA binding; F:nucleosomal DNA binding; C:nuclear nucleosome; C:protein complex; P:DNA replication-independent nucleosome assembly |
| Efet.01.503980.g101.t1 | cat eye syndrome critical region 5 isoform X1 | 318 | 2.40E-16 | 52.27 | C:mitochondrion |
| Efet.01.504035.g102.t1 | hypothetical protein CAPTEDRAFT_224386 | 261 | 6.69E-20 | 92 |  |
| Efet.01.504138.g103.t1 | hypothetical protein CAPTEDRAFT_223749 | 456 | 1.26E-17 | 55.8 | C:membrane; C:integral component of membrane |
| Efet.01.504191.g104.t1 | AMP deaminase 2 isoform X2 | 273 | 1.04E-42 | 89.95 | F:AMP deaminase activity; P:IMP salvage |
| Efet.01.504193.g105.t1 | tyrosine- phosphatase non-receptor type 1-like | 222 | 4.77E-14 | 83.1 | P:IRE1-mediated unfolded protein response; P:negative regulation of MAP kinase activity; P:insulin receptor signaling pathway; P:positive regulation of protein tyrosine kinase activity; F:ephrin receptor binding; P:activation of JUN kinase activity; P:negative regulation of endoplasmic reticulum stress-induced intrinsic apoptotic signaling pathway; C:cytoplasmic side of endoplasmic reticulum membrane; C:early endosome; C:sorting endosome; P:negative regulation of vascular endothelial growth factor receptor signaling pathway; P:regulation of intracellular protein transport; P:regulation of endocytosis; P:actin cytoskeleton reorganization; F:zinc ion binding; P:platelet-derived growth factor receptor-beta signaling pathway; P:negative regulation of PERK-mediated unfolded protein response; P:regulation of hepatocyte growth factor receptor signaling pathway; C:plasma membrane; F:RNA binding; P:peptidyl-tyrosine dephosphorylation involved in inactivation of protein kinase activity; P:positive regulation of receptor catabolic process; F:protein tyrosine phosphatase activity; P:negative regulation of ERK1 and ERK2 cascade; C:integral component of membrane; F:receptor tyrosine kinase binding |
| Efet.01.50432.g33.t1 | PREDICTED: uncharacterized protein LOC107603859 isoform X1 | 222 | 4.85E-07 | 63 |  |
| Efet.01.504368.g107.t1 | dynein heavy chain axonemal-like isoform X1 | 309 | 1.11E-33 | 88.55 | F:microtubule motor activity; C:dynein complex; P:microtubule-based movement |
| Efet.01.504417.g109.t1 | exonuclease 3 -5 domain-containing 1 | 213 | 1.84E-08 | 65.4 | F:nucleic acid binding; P:nucleic acid phosphodiester bond hydrolysis; F:3'-5' exonuclease activity; F:exonuclease activity; P:nucleobase-containing compound metabolic process |
| Efet.01.504423.g110.t1 | proto-oncogene tyrosine- kinase ROS isoform X2 | 246 | 1.14E-14 | 61.9 | F:kinase activity; P:cellular process |
| Efet.01.50443.g34.t1 | hypothetical protein CAPTEDRAFT_208831 | 579 | 8.61E-10 | 54 | C:membrane; C:integral component of membrane |
| Efet.01.504459.g111.t1 | bacillithiol system redox-active | 345 | 1.08E-77 | 69.35 |  |
| Efet.01.504526.g112.t1 | DNA alkylation response | 1443 | 0 | 87.35 | F:acyl-CoA dehydrogenase activity; F:flavin adenine dinucleotide binding; P:oxidation-reduction process |
| Efet.01.504526.g113.t1 | amino acid permease | 1197 | 0 | 77.6 | C:integral component of membrane; F:amino acid transmembrane transporter activity; P:amino acid transmembrane transport |
| Efet.01.504526.g114.t1 | GNAT family N-acetyltransferase | 867 | 0 | 84.5 | F:N-acetyltransferase activity; F:hydrolase activity |
| Efet.01.504526.g115.t1 | alpha beta hydrolase | 1431 | 0 | 89 | P:metabolic process; F:hydrolase activity |
| Efet.01.504526.g116.t1 | pilus assembly | 918 | 0 | 69.8 | C:integral component of membrane |
| Efet.01.504526.g117.t1 | pilus assembly | 3381 | 0 | 66.55 | C:integral component of membrane |
| Efet.01.504526.g118.t1 | type IV pilus assembly | 678 | 3.57E-142 | 71.95 | C:integral component of membrane |
| Efet.01.50455.g35.t1 | trachealess-like isoform X2 | 1347 | 1.48E-09 | 46.19 |  |
| Efet.01.504560.g119.t1 | zinc finger ZIC 4-like | 690 | 9.62E-46 | 66.7 | F:binding |
| Efet.01.504602.g120.t1 | Heparan sulfate glucosamine 3-O-sulfotransferase 3A1 | 645 | 1.42E-85 | 71 | C:integral component of plasma membrane; P:heparan sulfate proteoglycan biosynthetic process, enzymatic modification; F:[heparan sulfate]-glucosamine 3-sulfotransferase 1 activity; P:glycosaminoglycan biosynthetic process; C:Golgi membrane |
| Efet.01.504674.g122.t1 | metabotropic glutamate receptor 8-like | 948 | 1.06E-141 | 71.45 | F:G-protein coupled receptor activity; P:G-protein coupled receptor signaling pathway; C:integral component of membrane; C:plasma membrane |
| Efet.01.504811.g125.t1 | hypothetical protein OCBIM_22024763mg | 855 | 1.08E-39 | 49.25 |  |
| Efet.01.504906.g126.t1 | unconventional myosin-Va-like | 408 | 1.53E-17 | 75.35 | F:nucleotide binding; F:actin binding |
| Efet.01.50491.g37.t1 | hypothetical protein HELRODRAFT_160513 | 2922 | 2.02E-23 | 63 |  |
| Efet.01.504963.g127.t1 | vacuolar sorting-associated 13D-like | 291 | 3.49E-16 | 67.95 | F:hydrolase activity, acting on ester bonds; C:membrane; C:integral component of membrane |
| Efet.01.505101.g130.t1 | cGMP-dependent isozyme 2 forms cD4 T1 T3A T3B-like isoform X1 | 369 | 2.44E-10 | 65.95 | F:ATP binding; F:cGMP-dependent protein kinase activity; P:protein phosphorylation; F:cGMP binding |
| Efet.01.505168.g131.t1 | hypothetical protein HELRODRAFT_191140 | 1017 | 1.24E-37 | 88.75 |  |
| Efet.01.50518.g38.t1 | fibronectin type III domain-containing | 738 | 1.76E-61 | 52.3 |  |
| Efet.01.505184.g132.t1 | neogenin isoform X2 | 453 | 2.19E-24 | 56.5 | P:iron ion homeostasis; C:integral component of plasma membrane; P:positive regulation of BMP signaling pathway; C:membrane; C:integral component of membrane; P:nervous system development; P:axon guidance; F:receptor activity |
| Efet.01.505280.g134.t1 | abhydrolase domain-containing 2 | 309 | 9.98E-22 | 62.75 | C:membrane; C:integral component of membrane; F:hydrolase activity |
| Efet.01.50534.g40.t1 | citron Rho-interacting kinase-like | 1200 | 2.26E-60 | 56.5 | F:nucleotide binding; F:ATP binding; F:metal ion binding; F:protein kinase activity; P:phosphorylation; F:transferase activity; F:protein serine/threonine kinase activity; P:protein phosphorylation; F:kinase activity; P:intracellular signal transduction; C:intracellular |
| Efet.01.505346.g135.t1 | melanopsin isoform X2 | 2589 | 3.21E-56 | 49.15 | P:signal transduction; C:membrane; F:signal transducer activity |
| Efet.01.505417.g136.t1 | discoidin domain-containing receptor 2-like isoform X1 | 609 | 1.95E-60 | 70.85 | C:membrane; F:protein kinase activity; P:protein phosphorylation |
| Efet.01.505442.g137.t1 | glycosyl transferase | 1119 | 4.36E-109 | 57.2 | F:transferase activity, transferring hexosyl groups; P:metabolic process; F:lyase activity |
| Efet.01.505473.g138.t1 | helicase SRCAP- | 207 | 8.63E-41 | 95.45 |  |
| Efet.01.505477.g139.t1 | cGMP-specific 3 ,5 -cyclic phosphodiesterase isoform X1 | 885 | 1.23E-100 | 71.9 | F:phosphoric diester hydrolase activity |
| Efet.01.50559.g42.t1 | microtubule-actin cross-linking factor isoforms 1 2 3 5-like | 669 | 6.75E-34 | 72.8 | P:cell cycle arrest; F:calcium ion binding; F:cytoskeletal protein binding |
| Efet.01.50566.g44.t1 | Oxidative stress-responsive serine-rich 1 | 780 | 1.50E-08 | 64.55 |  |
| Efet.01.505702.g143.t1 | trypsin-3-like isoform X1 | 717 | 3.36E-64 | 64.15 | F:serine-type peptidase activity |
| Efet.01.505751.g144.t1 | breakpoint cluster region -like | 288 | 1.28E-19 | 67.05 | P:signal transduction; C:membrane; F:guanyl-nucleotide exchange factor activity; F:GTPase activator activity; P:positive regulation of GTPase activity |
| Efet.01.505809.g145.t1 | hypothetical protein HELRODRAFT_174990 | 312 | 6.63E-17 | 64 |  |
| Efet.01.50593.g45.t1 | hypothetical protein CAPTEDRAFT_192540 | 297 | 4.70E-33 | 67 |  |
| Efet.01.5060.g392.t1 | glutamate receptor 1-like isoform X2 | 282 | 1.30E-33 | 83.15 | C:postsynaptic membrane; P:ion transmembrane transport; C:integral component of membrane; C:cell junction; P:ionotropic glutamate receptor signaling pathway; F:ionotropic glutamate receptor activity; F:extracellular-glutamate-gated ion channel activity |
| Efet.01.506033.g151.t1 | mitogen-activated kinase 8-like | 261 | 2.47E-30 | 94.5 | P:positive regulation of cyclase activity; P:positive regulation of glial cell apoptotic process; P:type B pancreatic cell apoptotic process; P:negative regulation of protein binding; P:JUN phosphorylation; P:inflammatory response; P:ossification; P:positive regulation of transcription, DNA-templated; P:cellular response to nitric oxide; C:cytosol; P:regulation of protein ubiquitination; P:activation of cysteine-type endopeptidase activity involved in apoptotic process; F:mitogen-activated protein kinase kinase kinase binding; P:positive regulation of microtubule polymerization; P:positive regulation of chemokine production; C:axon; F:histone deacetylase regulator activity; F:histone deacetylase binding; P:positive regulation of prostaglandin biosynthetic process; P:regulation of protein localization; P:response to osmotic stress; P:positive regulation of neuroblast proliferation; C:nucleus; P:positive regulation of nitric-oxide synthase biosynthetic process; C:dendrite cytoplasm; F:JUN kinase activity; P:release of cytochrome c from mitochondria; P:positive regulation of cardiac muscle cell apoptotic process; C:vesicle; P:positive regulation of macrophage derived foam cell differentiation; P:peptidyl-serine phosphorylation; P:positive regulation of podosome assembly; P:peptidyl-threonine phosphorylation; P:positive regulation of nitric oxide biosynthetic process; P:positive regulation of DNA replication; P:regulation of neuron differentiation; C:perikaryon; P:regulation of circadian rhythm; P:neuronal stem cell population maintenance; P:positive regulation of neuron apoptotic process; C:podosome; P:dendrite morphogenesis; P:positive regulation of prostaglandin secretion; F:ATP binding; P:neuron migration; P:response to heat; P:determination of dorsal identity; P:programmed necrotic cell death; P:cellular response to UV; P:cellular response to hydrogen peroxide; P:positive regulation of determination of dorsal identity; P:positive regulation of neuron migration; P:positive regulation of cell morphogenesis involved in differentiation; P:positive regulation of deacetylase activity; P:response to cadmium ion; F:transcription factor binding; P:regulation of centrosome cycle; F:cysteine-type endopeptidase activator activity involved in apoptotic process; P:regulation of JNK cascade; P:rhythmic process; P:cellular response to lipopolysaccharide; P:response to drug; P:negative regulation of apoptotic process; C:mitochondrion; P:positive regulation of protein phosphorylation; F:kinesin binding; P:positive regulation of apoptotic signaling pathway |
| Efet.01.506052.g152.t1 | thyrotropin-releasing hormone receptor-like | 1215 | 6.73E-65 | 54.35 | F:G-protein coupled receptor activity; F:G-protein coupled peptide receptor activity; P:G-protein coupled receptor signaling pathway; P:signal transduction; C:membrane; C:integral component of membrane; F:signal transducer activity |
| Efet.01.506059.g154.t1 | NAD(P)H-hydrate epimerase-like | 255 | 8.26E-26 | 76.85 | C:mitochondrion; F:binding; F:NADHX epimerase activity; F:NADPHX epimerase activity |
| Efet.01.506188.g155.t1 | FAD-dependent oxidoreductase | 1341 | 0 | 71.8 | F:oxidoreductase activity; P:oxidation-reduction process |
| Efet.01.506188.g156.t1 | NAD(P)-dependent oxidoreductase | 765 | 3.03E-151 | 63.2 | F:3-oxoacyl-[acyl-carrier-protein] reductase (NADPH) activity; F:oxidoreductase activity; P:oxidation-reduction process |
| Efet.01.506188.g157.t1 | ROK family | 933 | 6.52E-164 | 57.4 | P:glucose 6-phosphate metabolic process; F:glucokinase activity; P:phosphorylation; F:transferase activity; F:kinase activity |
| Efet.01.506188.g158.t1 | family transcriptional regulator | 495 | 1.26E-110 | 64.15 | F:DNA binding; F:transcription factor activity, sequence-specific DNA binding; P:transcription, DNA-templated; P:regulation of transcription, DNA-templated |
| Efet.01.506207.g159.t1 | potassium channel subfamily K member 2-like isoform X1 | 321 | 6.13E-16 | 57.75 | F:nucleotide binding; F:ATP binding; F:microtubule motor activity; P:ion transport; P:potassium ion transmembrane transport; C:membrane; C:integral component of membrane; P:microtubule-based movement; F:potassium channel activity; P:transport; F:microtubule binding |
| Efet.01.506318.g161.t1 | transmembrane 164-like | 495 | 1.10E-28 | 66 | C:membrane |
| Efet.01.506324.g162.t1 | glutamine-rich 2-like isoform X4 | 216 | 2.90E-14 | 63.22 |  |
| Efet.01.506340.g163.t1 | ankyrin repeat and SAM domain-containing 6-like | 855 | 4.95E-87 | 70.9 |  |
| Efet.01.506406.g165.t1 | tripartite motif-containing 2-like isoform X3 | 1083 | 1.53E-29 | 51.35 | F:zinc ion binding; P:transmembrane transport; F:metal ion binding; C:membrane; C:integral component of membrane; P:transport; C:intracellular |
| Efet.01.50643.g46.t1 | serine threonine- kinase STK11 isoform X2 | 483 | 1.21E-52 | 79.4 | P:cell cycle arrest; C:TCR signalosome; P:TCR signalosome assembly; F:protein kinase activator activity; P:protein autophosphorylation; P:regulation of dendrite morphogenesis; F:magnesium ion binding; P:cellular response to UV-B; F:ATP binding; P:dendrite extension; F:p53 binding; C:extracellular exosome; P:positive regulation of axonogenesis; P:negative regulation of cell growth; P:vasculature development; P:axonogenesis; C:cytosol; P:activation of protein kinase activity; P:canonical Wnt signaling pathway; P:positive regulation of peptidyl-tyrosine phosphorylation; P:T cell receptor signaling pathway; P:response to ionizing radiation; P:Golgi localization; P:negative regulation of epithelial cell proliferation involved in prostate gland development; P:positive regulation of protein localization to nucleus; P:spermatid development; P:anoikis; P:positive thymic T cell selection; F:protein serine/threonine kinase activity; P:cellular response to DNA damage stimulus; P:tissue homeostasis; C:nucleus; P:regulation of protein kinase B signaling; P:negative regulation of lipid biosynthetic process; P:intrinsic apoptotic signaling pathway by p53 class mediator; C:mitochondrion; C:membrane; P:glucose homeostasis; F:LRR domain binding; P:establishment of cell polarity; P:regulation of Wnt signaling pathway; P:positive regulation of protein serine/threonine kinase activity; P:positive regulation of transforming growth factor beta receptor signaling pathway |
| Efet.01.506539.g168.t1 | 29-kDa galactose-binding lectin | 471 | 2.41E-51 | 60.95 | F:actin filament binding; F:carbohydrate binding; F:protein binding, bridging |
| Efet.01.50666.g47.t1 | calmodulin isoform X2 | 222 | 1.97E-15 | 81.75 | F:phosphatidylinositol 3-kinase binding; P:regulation of release of sequestered calcium ion into cytosol by sarcoplasmic reticulum; C:growth cone; P:positive regulation of cyclic-nucleotide phosphodiesterase activity; C:calcium channel complex; C:extracellular exosome; P:G2/M transition of mitotic cell cycle; P:positive regulation of cyclic nucleotide metabolic process; F:protein phosphatase activator activity; F:calcium ion binding; F:phospholipase binding; P:regulation of cytokinesis; F:titin binding; F:thioesterase binding; F:N-terminal myristoylation domain binding; F:nitric-oxide synthase binding; F:protein kinase binding; F:protein domain specific binding; C:voltage-gated potassium channel complex; C:spindle pole; F:calcium-dependent protein binding; P:positive regulation of ryanodine-sensitive calcium-release channel activity; F:adenylate cyclase binding; F:ion channel binding; P:substantia nigra development; C:nucleus; C:spindle microtubule; P:regulation of heart rate; P:detection of calcium ion; C:centrosome; F:type 3 metabotropic glutamate receptor binding; P:positive regulation of phosphoprotein phosphatase activity; F:nitric-oxide synthase regulator activity; P:regulation of cardiac muscle contraction; C:sarcomere |
| Efet.01.506751.g171.t1 | Inactive tyrosine- kinase 7 | 498 | 9.86E-59 | 70.75 | P:lung-associated mesenchyme development; P:planar cell polarity pathway involved in neural tube closure; P:cochlea morphogenesis; P:ventricular septum development; P:coronary vasculature development; P:protein phosphorylation; P:convergent extension; C:plasma membrane; P:axis elongation; F:ATP binding; P:wound healing; C:cell-cell junction; C:focal adhesion; F:transmembrane receptor protein tyrosine kinase activity; P:cellular response to retinoic acid; P:establishment of planar polarity; P:establishment of epithelial cell apical/basal polarity; C:integral component of membrane; P:canonical Wnt signaling pathway; P:positive regulation of neuron projection development; P:cell migration; P:actin cytoskeleton reorganization |
| Efet.01.50680.g48.t1 | dual specificity phosphatase 14-like | 522 | 6.94E-74 | 75.8 | P:inactivation of MAPK activity; F:protein tyrosine phosphatase activity; P:peptidyl-tyrosine dephosphorylation; F:MAP kinase tyrosine/serine/threonine phosphatase activity |
| Efet.01.506828.g172.t1 | nitrate reductase | 1014 | 0 | 85.45 | F:electron carrier activity; F:molybdenum ion binding; P:oxidation-reduction process; F:4 iron, 4 sulfur cluster binding; F:nitrate reductase (NADH) activity |
| Efet.01.506828.g173.t1 | toxin-antitoxin system antitoxin TIGR02293 family | 543 | 1.01E-57 | 75.35 |  |
| Efet.01.506828.g174.t1 | RES domain-containing | 426 | 2.09E-61 | 76.7 |  |
| Efet.01.506840.g175.t1 | conserved repeat domain-containing | 513 | 1.09E-83 | 59.7 | C:membrane; C:integral component of membrane |
| Efet.01.506840.g176.t1 | hydantoinase oxoprolinase family | 630 | 3.38E-100 | 82.05 | F:5-oxoprolinase (ATP-hydrolyzing) activity |
| Efet.01.506845.g177.t1 | ATP-binding cassette sub-family A member 3 | 507 | 6.83E-53 | 77.8 | F:ATP binding; P:lipid transport; F:ATPase activity, coupled to transmembrane movement of substances; C:intracellular membrane-bounded organelle; C:integral component of membrane |
| Efet.01.506957.g178.t1 | low-density lipo receptor-related 6-like | 324 | 4.11E-40 | 79.45 | F:Wnt-activated receptor activity; P:negative regulation of smooth muscle cell apoptotic process; C:Golgi apparatus; C:neuronal cell body; C:synapse; P:positive regulation of sequence-specific DNA binding transcription factor activity; P:thalamus development; P:face morphogenesis; C:caveola; F:frizzled binding; F:coreceptor activity involved in canonical Wnt signaling pathway; C:cytosol; F:Wnt-protein binding; F:apolipoprotein binding; P:embryonic pattern specification; F:low-density lipoprotein receptor activity; P:positive regulation of canonical Wnt signaling pathway; P:chemical synaptic transmission; C:receptor complex; P:cerebral cortex development; P:toxin transport; P:response to peptide hormone; P:pericardium morphogenesis; P:cerebellum morphogenesis; C:early endosome membrane; C:Wnt-Frizzled-LRP5/6 complex; P:neural crest formation; P:protein localization to plasma membrane; C:Wnt signalosome; P:receptor-mediated endocytosis involved in cholesterol transport; P:external genitalia morphogenesis; P:palate development; P:Wnt signaling pathway involved in dorsal/ventral axis specification; P:positive regulation of cell cycle; P:odontogenesis of dentin-containing tooth; P:canonical Wnt signaling pathway involved in neural crest cell differentiation; P:Wnt signaling pathway involved in midbrain dopaminergic neuron differentiation; C:extracellular region; P:positive regulation of transcription from RNA polymerase II promoter; P:convergent extension; P:neural tube closure; P:canonical Wnt signaling pathway involved in regulation of cell proliferation; P:cellular response to cholesterol; P:positive regulation of Wnt signaling pathway involved in dorsal/ventral axis specification; P:single organismal cell-cell adhesion; F:protein homodimerization activity; P:axis elongation involved in somitogenesis; P:Wnt signaling pathway involved in somitogenesis; P:midbrain-hindbrain boundary development; C:endoplasmic reticulum; P:beta-catenin destruction complex disassembly; P:embryonic retina morphogenesis in camera-type eye; C:cell surface; P:positive regulation of cytosolic calcium ion concentration; P:trachea cartilage morphogenesis; P:negative regulation of canonical Wnt signaling pathway; F:kinase inhibitor activity; C:integral component of membrane; P:negative regulation of protein serine/threonine kinase activity; F:toxin transporter activity; P:primitive streak formation |
| Efet.01.506989.g180.t1 | voltage-dependent calcium channel type A subunit alpha-1-like | 1059 | 5.91E-17 | 60.85 | F:ion channel activity; P:ion transport |
| Efet.01.506990.g181.t1 | usherin | 300 | 2.66E-17 | 68.55 | F:protein binding; P:system development; C:cell projection part; C:stereocilium bundle; C:plasma membrane region; C:organelle part; C:intracellular part |
| Efet.01.506996.g182.t1 | 6-bladed beta-propeller | 843 | 6.52E-23 | 48.6 | F:zinc ion binding; P:signal transduction; F:metal ion binding; P:regulation of apoptotic process; P:phosphorylation; F:transferase activity; F:kinase activity; C:intracellular |
| Efet.01.507007.g183.t1 | hypothetical protein CAPTEDRAFT_201721 | 279 | 3.01E-15 | 62 |  |
| Efet.01.50714.g49.t1 | hypothetical protein CAPTEDRAFT_191853 | 666 | 4.83E-13 | 61.22 |  |
| Efet.01.507147.g184.t1 | glycogen muscle form-like isoform X1 | 360 | 4.46E-56 | 86.45 | F:glycogen phosphorylase activity; P:carbohydrate metabolic process; F:pyridoxal phosphate binding |
| Efet.01.50738.g50.t1 | 40S ribosomal S7 | 360 | 1.03E-70 | 93.55 | C:small-subunit processome; C:cytosolic small ribosomal subunit; F:poly(U) RNA binding; P:rRNA processing; C:nucleolus; P:translational initiation; C:nucleoplasm; F:RNA binding; P:viral transcription; C:extracellular exosome; F:protein binding; P:SRP-dependent cotranslational protein targeting to membrane; C:microtubule organizing center; C:focal adhesion; F:structural constituent of ribosome; P:nuclear-transcribed mRNA catabolic process, nonsense-mediated decay; P:neural tube closure; C:membrane; P:ribosomal small subunit biogenesis; P:cell differentiation; C:extracellular matrix; C:90S preribosome |
| Efet.01.507395.g191.t1 | tyrosine- phosphatase non-receptor type 4-like isoform X1 | 336 | 5.86E-40 | 82.9 | F:protein tyrosine phosphatase activity; P:peptidyl-tyrosine dephosphorylation |
| Efet.01.507448.g192.t1 | octopamine receptor | 1188 | 3.79E-143 | 65.15 | F:G-protein coupled receptor activity; P:G-protein coupled receptor signaling pathway; C:membrane |
| Efet.01.50745.g51.t1 | transmembrane 145 | 1458 | 3.96E-39 | 53.8 | C:membrane; C:integral component of membrane |
| Efet.01.507497.g193.t1 | condensin complex subunit 1-like isoform X1 | 285 | 2.13E-34 | 81.1 | P:cell division; P:meiotic chromosome condensation; P:mitotic chromosome condensation; F:histone binding; C:condensed chromosome, centromeric region; C:nuclear condensin complex; F:chromatin binding; P:chromosome separation |
| Efet.01.507545.g196.t1 | iron dicitrate transport regulator | 474 | 1.30E-105 | 80 |  |
| Efet.01.507549.g197.t1 | netrin receptor UNC5D | 267 | 3.37E-17 | 61.22 | P:signal transduction; C:membrane; C:integral component of membrane |
| Efet.01.507557.g198.t1 | hypothetical protein CAPTEDRAFT_225510 | 342 | 5.19E-20 | 55.7 | C:membrane; C:integral component of membrane; F:carbohydrate binding |
| Efet.01.507686.g200.t1 | PREDICTED: protein FAM81A-like | 276 | 8.32E-11 | 68.5 |  |
| Efet.01.5077.g393.t1 | CD63 antigen | 534 | 4.76E-09 | 47.65 | C:membrane; C:integral component of membrane |
| Efet.01.507761.g201.t1 | alpha-1B adrenergic receptor | 1020 | 2.13E-88 | 63.75 | P:signal transduction; C:membrane; F:signal transducer activity |
| Efet.01.507789.g202.t1 | Uncharacterized F-box LRR-repeat | 1029 | 1.19E-50 | 49.1 | P:single-organism process |
| Efet.01.507841.g203.t1 | limbic system-associated membrane -like isoform X1 | 507 | 2.21E-31 | 76.05 |  |
| Efet.01.50798.g54.t1 | hypothetical protein CAPTEDRAFT_209645 | 1083 | 1.30E-15 | 82 | C:integral component of membrane |
| Efet.01.50798.g55.t1 | hypothetical protein CAPTEDRAFT_209645 | 1224 | 2.44E-127 | 51 | C:membrane; C:integral component of membrane |
| Efet.01.507997.g205.t1 | low-density lipo receptor-related 6-like | 279 | 3.75E-34 | 78.9 | P:regulation of insulin secretion involved in cellular response to glucose stimulus; F:Wnt-activated receptor activity; P:Wnt signaling pathway involved in dorsal/ventral axis specification; C:receptor complex; P:osteoblast development; P:cholesterol homeostasis; C:plasma membrane; P:bone marrow development; P:retinal blood vessel morphogenesis; P:adipose tissue development; P:liver development; P:regulation of canonical Wnt signaling pathway; C:integral component of membrane; P:canonical Wnt signaling pathway; F:Wnt-protein binding |
| Efet.01.508013.g206.t1 | DC-STAMP domain-containing 2-like | 1023 | 7.54E-81 | 48.8 | F:zinc ion binding; C:membrane; C:integral component of membrane |
| Efet.01.508013.g207.t1 | hypothetical protein CAPTEDRAFT_146494 | 591 | 1.58E-17 | 58.67 | F:zinc ion binding; C:membrane; C:integral component of membrane |
| Efet.01.508022.g208.t1 | Membrane carboxypeptidase (penicillin-binding ) | 2568 | 0 | 71.85 | C:membrane; C:integral component of membrane; F:penicillin binding |
| Efet.01.508022.g209.t1 | metallophosphatase | 366 | 4.45E-59 | 83.2 | F:hydrolase activity |
| Efet.01.508022.g210.t1 | UDP-glucose GDP-mannose dehydrogenase family | 324 | 1.74E-65 | 84.55 | F:NAD binding; P:polysaccharide biosynthetic process; F:UDP-glucose 6-dehydrogenase activity; P:oxidation-reduction process |
| Efet.01.508047.g211.t1 | methenyltetrahydrofolate synthase domain-containing isoform X1 | 384 | 1.00E-16 | 63.25 | F:organic cyclic compound binding; F:heterocyclic compound binding |
| Efet.01.508062.g212.t1 | hypothetical protein HELRODRAFT_190729 | 657 | 9.97E-09 | 59.5 |  |
| Efet.01.508265.g216.t1 | neural-cadherin-like isoform X1 | 930 | 4.69E-136 | 57.2 | F:calcium ion binding; C:integral component of membrane; P:homophilic cell adhesion via plasma membrane adhesion molecules; C:plasma membrane |
| Efet.01.508377.g217.t1 | probable G- coupled receptor 139 | 1149 | 1.06E-43 | 48.15 | P:signal transduction; C:membrane; F:signal transducer activity |
| Efet.01.50848.g58.t1 | Ribosomal L35A | 210 | 4.58E-27 | 86 | F:structural constituent of ribosome; P:cytoplasmic translation; P:ribosomal large subunit biogenesis; C:cytosolic large ribosomal subunit |
| Efet.01.508523.g219.t1 | acetylcholinesterase isoform X1 | 1752 | 0 | 64 | F:cholinesterase activity |
| Efet.01.508540.g220.t1 | fibrinolytic enzyme | 252 | 6.59E-24 | 69.8 | P:metabolic process; F:serine-type peptidase activity |
| Efet.01.508554.g221.t1 | protocadherin-11 X-linked-like | 2691 | 5.32E-125 | 54.15 | F:calcium ion binding; C:membrane; C:integral component of membrane; P:cell adhesion; P:homophilic cell adhesion via plasma membrane adhesion molecules; C:plasma membrane |
| Efet.01.508578.g222.t1 | ribosome small subunit-dependent GTPase A | 285 | 9.61E-62 | 78.05 | F:GTP binding; F:metal ion binding; F:GTPase activity |
| Efet.01.508580.g223.t1 | transforming acidic coiled-coil-containing 1-like isoform X1 | 225 | 4.29E-14 | 82.5 |  |
| Efet.01.508606.g224.t1 | sugar ABC transporter ATP-binding | 327 | 6.34E-53 | 63.9 | F:hydrolase activity |
| Efet.01.508634.g225.t1 | Ras-related Rab-33 | 546 | 4.81E-20 | 82.9 | F:GTP binding; P:small GTPase mediated signal transduction; P:regulation of autophagosome assembly; C:intracellular |
| Efet.01.50864.g59.t1 | neurabin-1-like isoform X7 | 462 | 3.64E-25 | 89 |  |
| Efet.01.508646.g226.t1 | MFS transporter | 1032 | 4.88E-125 | 82.35 | P:transmembrane transport; F:transmembrane transporter activity; C:integral component of membrane |
| Efet.01.508646.g227.t1 | family transcriptional regulator | 726 | 1.81E-163 | 84.35 | F:DNA binding; F:transcription factor activity, sequence-specific DNA binding; P:transcription, DNA-templated; P:regulation of transcription, DNA-templated |
| Efet.01.508646.g228.t1 | tripartite tricarboxylate transporter family receptor | 783 | 6.31E-124 | 70.95 | F:lyase activity; C:outer membrane-bounded periplasmic space |
| Efet.01.508689.g229.t1 | cytoplasmic dynein 2 heavy chain 1-like | 1005 | 3.68E-157 | 83.35 | F:ATPase activity; F:ATP binding; F:microtubule motor activity; C:dynein complex; P:microtubule-based movement |
| Efet.01.508698.g230.t1 | transcriptional regulator | 336 | 2.52E-61 | 84.55 | F:transcription factor activity, sequence-specific DNA binding; P:transcription, DNA-templated; P:regulation of transcription, DNA-templated; F:sequence-specific DNA binding; C:intracellular |
| Efet.01.508993.g234.t1 | neuroligin- Y-linked-like | 513 | 3.49E-45 | 64.85 | C:membrane; C:integral component of membrane |
| Efet.01.509026.g235.t1 | methyl- -binding domain 2-like | 540 | 5.79E-65 | 74.9 | P:negative regulation of transcription from RNA polymerase II promoter; C:nucleus; F:methyl-CpG binding; P:methylation-dependent chromatin silencing |
| Efet.01.50906.g62.t1 | probable D-tyrosyl-tRNA(Tyr) deacylase 2 | 567 | 9.56E-65 | 71.05 | F:D-tyrosyl-tRNA(Tyr) deacylase activity; C:cytoplasm; F:aminoacyl-tRNA editing activity; P:tRNA metabolic process |
| Efet.01.50910.g63.t1 | sodium potassium-transporting ATPase subunit alpha-2-like | 471 | 4.50E-80 | 78.15 | F:nucleotide binding; P:hyperosmotic salinity response; P:transmembrane transport; P:monovalent inorganic cation transport; F:sodium:potassium-exchanging ATPase activity; C:membrane; F:ion binding; P:response to growth hormone; P:metal ion transport |
| Efet.01.509178.g236.t1 | leucine-rich repeat-containing 15-like | 1338 | 8.61E-35 | 46.8 |  |
| Efet.01.509347.g238.t1 | CCR4-NOT transcription complex subunit 10-like | 255 | 1.47E-28 | 73.85 |  |
| Efet.01.509455.g240.t1 | dynamin-2-like isoform X1 | 207 | 2.78E-29 | 84.25 | F:GTP binding; P:clathrin-dependent endocytosis; F:GTPase activity; C:microtubule |
| Efet.01.509552.g241.t1 | discoidin domain-containing receptor 2-like isoform X1 | 291 | 1.69E-30 | 72.3 | F:ATP binding; P:peptidyl-tyrosine phosphorylation; P:negative regulation of dendrite morphogenesis; F:protein tyrosine kinase activity; C:integral component of membrane; P:detection of temperature stimulus involved in sensory perception of pain |
| Efet.01.50957.g65.t1 | epsin-2-like isoform X5 | 795 | 2.31E-87 | 86.75 | C:clathrin coat of endocytic vesicle; P:endocytosis |
| Efet.01.509585.g242.t1 | polycystic kidney disease 1-like 2 | 549 | 2.22E-30 | 71.15 | C:integral component of membrane |
| Efet.01.5096.g394.t1 | gonadotropin-releasing hormone II receptor-like | 1320 | 9.75E-132 | 65.3 | F:G-protein coupled peptide receptor activity; P:response to organic substance; P:G-protein coupled receptor signaling pathway; P:response to endogenous stimulus; C:integral component of membrane; C:plasma membrane |
| Efet.01.509666.g243.t1 | SWI SNF complex subunit SMARCC1-like | 348 | 1.24E-34 | 69.9 | C:BAF-type complex; P:chromatin remodeling |
| Efet.01.50974.g66.t1 | TCDD-inducible poly [ADP-ribose] polymerase-like | 1347 | 3.16E-14 | 68.15 | F:transferase activity, transferring glycosyl groups; F:methyltransferase activity |
| Efet.01.50978.g67.t1 | probable G- coupled receptor 139 isoform X1 | 972 | 1.47E-54 | 60.2 | F:G-protein coupled receptor activity; P:G-protein coupled receptor signaling pathway; C:membrane; C:integral component of membrane |
| Efet.01.50989.g68.t1 | kinase C-binding NELL2-like | 378 | 2.48E-06 | 54 |  |
| Efet.01.510009.g245.t1 | zinc finger 239-like | 1623 | 2.41E-25 | 62.05 | F:nucleic acid binding; F:metal ion binding |
| Efet.01.510023.g246.t1 | kinase C isoform X1 | 288 | 5.33E-23 | 86.9 | F:ATP binding; F:metal ion binding; P:protein phosphorylation; C:intracellular; P:intracellular signal transduction; F:protein kinase C activity |
| Efet.01.5102.g395.t1 | echinoderm microtubule-associated -like 2 isoform X1 | 354 | 6.25E-11 | 68.14 | P:intracellular signal transduction; C:intracellular |
| Efet.01.51022.g70.t1 | helicase with zinc finger domain 2-like | 648 | 3.99E-45 | 61.55 | F:binding |
| Efet.01.51025.g72.t1 | omega-3 fatty acid endoplasmic reticulum-like | 1173 | 1.38E-169 | 69.3 | C:membrane |
| Efet.01.510335.g250.t1 | cyclic GMP-AMP synthase-like | 720 | 1.38E-39 | 54.25 |  |
| Efet.01.510339.g251.t1 | family -linked outer membrane | 660 | 2.78E-83 | 69.25 | C:membrane; C:cell outer membrane; F:receptor activity; P:transport |
| Efet.01.510339.g252.t1 | Fe2+-dicitrate membrane component | 312 | 1.80E-66 | 55.7 | C:membrane; C:integral component of membrane |
| Efet.01.510371.g253.t1 | hypothetical protein CAPTEDRAFT_124119 | 456 | 2.41E-07 | 77 |  |
| Efet.01.510379.g254.t1 | acetylornithine deacetylase | 867 | 1.52E-93 | 76.75 | P:metabolic process; F:hydrolase activity |
| Efet.01.510405.g256.t1 | myosin heavy skeletal muscle-like | 324 | 3.95E-13 | 63.2 | C:myosin complex; F:motor activity |
| Efet.01.510405.g257.t1 | paramyosin | 549 | 4.17E-43 | 81.35 | C:myosin filament; C:myofibril; F:motor activity |
| Efet.01.51052.g74.t1 | calsyntenin-1-like isoform X2 | 204 | 1.50E-10 | 65.9 | C:membrane |
| Efet.01.51052.g75.t1 | calsyntenin-1 isoform X2 | 474 | 4.49E-10 | 62.45 | C:membrane |
| Efet.01.510654.g259.t1 | microtubule-actin cross-linking factor 1-like isoform X8 | 993 | 9.44E-39 | 46.75 | C:cytoskeleton |
| Efet.01.511186.g264.t1 | site-specific DNA-methyltransferase | 573 | 1.86E-110 | 91.65 | F:DNA binding; F:N-methyltransferase activity; P:DNA methylation |
| Efet.01.511237.g265.t1 | peptidase S8 and S53 subtilisin kexin sedolisin | 456 | 1.17E-59 | 67.88 | P:proteolysis; F:serine-type endopeptidase activity |
| Efet.01.51129.g77.t1 | collagen alpha-1(XII) chain-like | 1248 | 4.00E-89 | 51.35 | F:calcium ion binding; C:collagen trimer |
| Efet.01.511297.g266.t1 | sarcoplasmic calcium-binding | 315 | 5.95E-12 | 63 | F:calcium ion binding |
| Efet.01.511346.g268.t1 | family metallo-endopeptidase | 444 | 9.01E-70 | 53.7 |  |
| Efet.01.511346.g269.t1 | dihydroxy-acid dehydratase | 813 | 2.20E-103 | 83.5 | F:dihydroxy-acid dehydratase activity; P:metabolic process |
| Efet.01.511404.g270.t1 | zinc finger 711-like | 822 | 2.50E-07 | 48.25 | F:nucleic acid binding; F:metal ion binding |
| Efet.01.51146.g78.t1 | hypothetical protein LOTGIDRAFT_237861 | 369 | 2.83E-12 | 54 |  |
| Efet.01.51149.g79.t1 | plexin-A2 isoform X1 | 714 | 2.11E-13 | 60.95 | P:signal transduction; C:membrane; C:integral component of membrane; F:semaphorin receptor activity; P:semaphorin-plexin signaling pathway |
| Efet.01.5115.g396.t1 | vesicular glutamate transporter 2 | 336 | 5.21E-12 | 52.45 | P:transmembrane transport; F:transmembrane transporter activity; C:membrane; C:integral component of membrane; C:synaptic vesicle membrane; P:anion transport |
| Efet.01.511550.g271.t1 | hypothetical protein CAPTEDRAFT_211677 | 471 | 2.69E-17 | 53 |  |
| Efet.01.511666.g274.t1 | NACHT and WD repeat domain-containing 2-like | 1467 | 1.12E-142 | 60.15 |  |
| Efet.01.51173.g80.t1 | cyclic nucleotide-gated channel rod photoreceptor subunit alpha-like | 1587 | 8.51E-123 | 66.05 | P:potassium ion transport; F:ion channel activity; P:ion transmembrane transport; C:integral component of membrane |
| Efet.01.511760.g275.t1 | hypothetical protein CAPTEDRAFT_219441 | 1791 | 3.12E-09 | 71 | F:DNA binding; C:nucleus |
| Efet.01.51183.g81.t1 | aquaporin -like | 279 | 7.96E-41 | 65 | C:integral component of membrane; P:transport; F:transporter activity |
| Efet.01.511854.g278.t1 | lysine-specific demethylase 4C-like | 1242 | 3.33E-07 | 76.22 | C:nucleus; F:zinc ion binding; C:integral component of membrane; F:methyltransferase activity; P:methylation |
| Efet.01.511922.g279.t1 | ATP-dependent Clp protease proteolytic mitochondrial | 636 | 2.72E-130 | 90.95 | P:proteolysis; F:serine-type endopeptidase activity |
| Efet.01.511938.g280.t1 | SRSF kinase 3 isoform X1 | 696 | 3.10E-14 | 76.65 | F:nucleotide binding; F:ion binding; F:protein kinase activity; P:regulation of viral genome replication; P:phosphorylation; C:intracellular part; P:single-organism process |
| Efet.01.511940.g281.t1 | Transposase Tn5 dimerisation domain-containing | 744 | 1.07E-111 | 85.9 | F:nucleic acid binding |
| Efet.01.512081.g283.t1 | Cat eye syndrome critical region | 390 | 1.29E-06 | 48 | C:mitochondrion |
| Efet.01.512325.g285.t1 | prostatic acid phosphatase isoform X2 | 345 | 7.67E-39 | 74.85 | F:identical protein binding; P:positive regulation of adenosine receptor signaling pathway; F:5'-nucleotidase activity; P:thiamine metabolic process; C:lysosomal membrane; C:plasma membrane; C:nucleus; C:extracellular space; C:extracellular exosome; F:lysophosphatidic acid phosphatase activity; P:adenosine metabolic process; C:vesicle membrane; F:thiamine phosphate phosphatase activity; C:endomembrane system; C:cytoplasmic vesicle; P:dephosphorylation; P:regulation of sensory perception of pain |
| Efet.01.512340.g287.t1 | SCY1 2 isoform X1 | 453 | 2.51E-41 | 77.25 | F:ATP binding; C:integral component of membrane; F:protein kinase activity; P:protein phosphorylation |
| Efet.01.512343.g288.t1 | 4 -phosphopantetheinyl transferase | 774 | 2.06E-152 | 57.9 | F:magnesium ion binding; F:holo-[acyl-carrier-protein] synthase activity |
| Efet.01.512343.g289.t1 | thioesterase | 516 | 4.73E-95 | 65.65 | F:hydrolase activity, acting on ester bonds; C:integral component of membrane; P:biosynthetic process |
| Efet.01.512410.g290.t1 | ribonuclease activity regulator | 582 | 1.31E-137 | 92.25 | F:oxaloacetate decarboxylase activity; F:4-hydroxy-4-methyl-2-oxoglutarate aldolase activity; F:methyltransferase activity; F:4-hydroxy-2-oxoglutarate aldolase activity; P:methylation |
| Efet.01.512410.g291.t1 | tripartite tricarboxylate transporter substrate binding | 534 | 9.34E-65 | 90.55 | C:outer membrane-bounded periplasmic space |
| Efet.01.512410.g292.t1 | lactoylglutathione lyase | 627 | 2.93E-106 | 93.95 | F:lyase activity |
| Efet.01.512410.g293.t1 | cystathionine beta-lyase | 1281 | 0 | 86.6 | F:pyridoxal phosphate binding; F:cystathionine beta-lyase activity |
| Efet.01.512556.g298.t1 | hypothetical protein HELRODRAFT_170638 | 564 | 1.87E-08 | 68 |  |
| Efet.01.512594.g299.t1 | GTP-binding Rhes-like | 726 | 8.67E-90 | 68.05 | F:nucleotide binding; P:signal transduction |
| Efet.01.51275.g82.t1 | regulator of nonsense transcripts 1-like | 762 | 7.31E-146 | 90.4 | P:DNA repair; C:exon-exon junction complex; F:zinc ion binding; P:DNA replication; C:supraspliceosomal complex; F:chromatin binding; C:chromatin; C:nucleoplasm; F:ATP-dependent RNA helicase activity; F:RNA binding; F:DNA binding; F:ATP binding; P:regulation of translational termination; P:nuclear-transcribed mRNA catabolic process, nonsense-mediated decay; C:cytoplasm; P:histone mRNA catabolic process |
| Efet.01.512803.g301.t1 | Transcription factor jun- | 1062 | 8.61E-15 | 72.4 | F:DNA binding; P:regulation of transcription, DNA-templated |
| Efet.01.512876.g303.t1 | alpha- sarcomeric isoform X1 | 246 | 1.39E-20 | 77.75 | F:actin binding; F:calcium ion binding |
| Efet.01.512899.g304.t1 | centrosomal of 104 kDa-like isoform X2 | 285 | 7.60E-21 | 70.7 |  |
| Efet.01.513005.g305.t1 | probable G- coupled receptor 139 | 1164 | 7.42E-56 | 51.95 | F:signal transducer activity |
| Efet.01.513011.g306.t1 | splicing factor 3B subunit 2-like | 1344 | 0 | 86.5 | C:U12-type spliceosomal complex; C:catalytic step 2 spliceosome; C:precatalytic spliceosome; C:U2-type spliceosomal complex; C:U2 snRNP; P:mRNA splicing, via spliceosome |
| Efet.01.513018.g308.t1 | hypothetical protein HELRODRAFT_167756 | 705 | 4.11E-09 | 88 | C:integral component of membrane |
| Efet.01.513032.g309.t1 | 2 ,5 -phosphodiesterase | 240 | 7.48E-33 | 76.75 |  |
| Efet.01.513147.g311.t1 | hypothetical protein CAPTEDRAFT_200340 | 252 | 4.70E-14 | 64 |  |
| Efet.01.513153.g312.t1 | probable G- coupled receptor 158 | 369 | 3.31E-37 | 67.9 | F:G-protein coupled receptor activity; P:G-protein coupled receptor signaling pathway; C:integral component of membrane |
| Efet.01.513169.g313.t1 | C3 and PZP-like alpha-2-macroglobulin domain-containing 8 | 504 | 3.37E-21 | 53.75 | F:catalytic activity; C:membrane; C:integral component of membrane; P:nucleoside metabolic process |
| Efet.01.513258.g314.t1 | double-strand break repair MRE11-like | 1068 | 1.07E-167 | 84 | F:3'-5' exonuclease activity; P:nucleic acid phosphodiester bond hydrolysis; P:meiotic cell cycle; F:endonuclease activity; F:manganese ion binding; C:Mre11 complex; P:intra-S DNA damage checkpoint; P:double-strand break repair |
| Efet.01.513298.g316.t1 | phenylacetic acid degradation | 480 | 7.98E-99 | 80.45 | F:thiolester hydrolase activity |
| Efet.01.513298.g317.t1 | phenylacetate-- ligase | 1320 | 0 | 88.95 | F:nucleotide binding; F:phenylacetate-CoA ligase activity; P:phenylacetate catabolic process |
| Efet.01.513298.g318.t1 | 1,2-phenylacetyl- epoxidase subunit A | 792 | 3.77E-180 | 91.55 | F:phenylacetyl-CoA 1,2-epoxidase activity; P:oxidation-reduction process; P:phenylacetate catabolic process |
| Efet.01.51332.g83.t1 | hypothetical protein HELRODRAFT_180676 | 2907 | 0 | 51.75 | C:membrane; C:integral component of membrane |
| Efet.01.513358.g320.t1 | hypothetical protein HELRODRAFT_171759 | 753 | 6.54E-10 | 79.86 | F:nucleic acid binding; F:metal ion binding |
| Efet.01.51343.g85.t1 | ATP-citrate synthase isoform X1 | 417 | 3.36E-56 | 86.3 | F:ATP binding; F:ATP citrate synthase activity; C:cytoplasm; F:metal ion binding; F:cofactor binding; P:acetyl-CoA biosynthetic process; P:lipid metabolic process; P:citrate metabolic process |
| Efet.01.513435.g323.t1 | tyrosine- kinase SRK3 | 222 | 9.55E-12 | 67 | F:nucleotide binding; F:protein tyrosine kinase activity; P:protein phosphorylation |
| Efet.01.51355.g86.t1 | transmembrane and coiled-coil domains 2-like isoform X5 | 432 | 8.61E-20 | 91.45 | C:integral component of membrane |
| Efet.01.51377.g89.t1 | TATA-binding -associated factor 172 | 381 | 1.37E-22 | 72.75 | F:ATP binding |
| Efet.01.513780.g327.t1 | potassium channel subfamily K member 16 | 402 | 1.09E-07 | 62.15 | P:potassium ion transport; F:protein binding; F:potassium channel activity; C:plasma membrane |
| Efet.01.5138.g397.t1 | 60S ribosomal L19 | 300 | 9.06E-34 | 88.55 | F:structural constituent of ribosome; C:cytosolic large ribosomal subunit; P:translation |
| Efet.01.513874.g329.t1 | hypothetical protein HELRODRAFT_160275 | 813 | 9.26E-26 | 49 |  |
| Efet.01.51388.g91.t1 | Nuclear receptor subfamily 0 group B member 1 | 1488 | 7.97E-29 | 56.83 | F:DNA binding; C:nucleus; P:transcription, DNA-templated; P:regulation of transcription, DNA-templated; F:steroid hormone receptor activity; P:steroid hormone mediated signaling pathway; P:water homeostasis |
| Efet.01.513953.g331.t1 | probable phospholipid-transporting ATPase IF isoform X5 | 387 | 1.43E-13 | 52.45 |  |
| Efet.01.513966.g332.t1 | SAGA-associated factor 29 homolog | 219 | 7.41E-26 | 85.15 | P:histone H3 acetylation; C:SAGA-type complex; F:methylated histone binding; C:Ada2/Gcn5/Ada3 transcription activator complex |
| Efet.01.5142.g398.t1 | Collagen alpha-4(VI) chain | 699 | 1.65E-06 | 46 | C:collagen trimer |
| Efet.01.514247.g333.t1 | histidine kinase | 861 | 1.26E-160 | 87.6 | F:phosphorelay sensor kinase activity; F:ATP binding; P:phosphorelay signal transduction system; C:integral component of membrane; P:signal transduction by protein phosphorylation; C:intracellular; P:peptidyl-histidine phosphorylation |
| Efet.01.514247.g334.t1 | 3-hydroxybutyryl- dehydrogenase | 1458 | 0 | 83.25 | F:3-hydroxyacyl-CoA dehydrogenase activity; P:oxidation-reduction process; F:NAD+ binding; P:fatty acid metabolic process |
| Efet.01.514247.g335.t1 | family transcriptional regulator | 990 | 0 | 79.75 | F:transcription factor activity, sequence-specific DNA binding; P:transcription, DNA-templated; P:regulation of transcription, DNA-templated; F:sequence-specific DNA binding |
| Efet.01.514247.g336.t1 | glycosyl transferase | 2178 | 0 | 89.4 | F:transferase activity |
| Efet.01.514247.g337.t1 | glycosyl transferase | 813 | 5.33E-174 | 91.3 | C:integral component of membrane; F:transferase activity |
| Efet.01.514247.g338.t1 | branched-chain amino acid aminotransferase | 894 | 0 | 96.2 | F:L-leucine transaminase activity; F:L-valine transaminase activity; P:branched-chain amino acid metabolic process; F:L-isoleucine transaminase activity |
| Efet.01.514247.g339.t1 | glycerate kinase | 327 | 1.46E-65 | 87.05 | C:integral component of membrane; P:phosphorylation; F:kinase activity |
| Efet.01.514247.g340.t1 | Fe3+ spermidine putrescine ABC transporter ATP-binding | 477 | 1.14E-86 | 94.2 | F:ATPase activity; F:ATP binding; P:transmembrane transport; C:ATP-binding cassette (ABC) transporter complex; F:hydrolase activity, acting on acid anhydrides, catalyzing transmembrane movement of substances |
| Efet.01.514247.g341.t1 | ABC transporter ATP-binding | 564 | 9.09E-88 | 79.75 | F:ATPase activity; F:ATP binding; P:transmembrane transport; C:ATP-binding cassette (ABC) transporter complex; F:hydrolase activity, acting on acid anhydrides, catalyzing transmembrane movement of substances |
| Efet.01.514247.g342.t1 | ABC transporter substrate-binding | 879 | 1.41E-147 | 98.15 |  |
| Efet.01.514275.g343.t1 | histone-lysine N-methyltransferase ASH1L-like isoform X10 | 216 | 3.96E-36 | 89 | C:nucleus; F:DNA binding; F:zinc ion binding; P:histone lysine methylation; F:histone-lysine N-methyltransferase activity; F:chromatin binding |
| Efet.01.514315.g345.t1 | DNA internalization-related competence Rec2 | 786 | 1.28E-69 | 89.05 | C:integral component of membrane; P:establishment of competence for transformation; F:hydrolase activity |
| Efet.01.51437.g92.t1 | dual specificity phosphatase 10-like | 1041 | 3.94E-23 | 63.1 | P:protein dephosphorylation; F:protein tyrosine/serine/threonine phosphatase activity |
| Efet.01.514423.g348.t1 | probable G- coupled receptor No9 | 399 | 5.01E-27 | 83.3 | F:adrenergic receptor activity; F:octopamine receptor activity; C:integral component of membrane; P:adrenergic receptor signaling pathway; C:plasma membrane |
| Efet.01.514432.g349.t1 | hypothetical protein CAPTEDRAFT_228921 | 414 | 8.03E-29 | 64.15 | C:membrane; F:binding |
| Efet.01.514471.g350.t1 | dmX 2 | 1554 | 1.03E-83 | 49.4 |  |
| Efet.01.51455.g93.t1 | tektin-1-like | 591 | 4.06E-65 | 73.8 |  |
| Efet.01.514559.g351.t1 | indolepyruvate ferredoxin oxidoreductase | 3582 | 0 | 88.3 | F:thiamine pyrophosphate binding; F:indolepyruvate ferredoxin oxidoreductase activity; P:oxidation-reduction process |
| Efet.01.514559.g352.t1 | tripartite tricarboxylate transporter substrate binding | 822 | 0 | 65.2 | C:outer membrane-bounded periplasmic space |
| Efet.01.514559.g353.t1 | tripartite tricarboxylate transporter substrate binding | 429 | 3.89E-91 | 81.15 | C:outer membrane-bounded periplasmic space |
| Efet.01.514565.g354.t1 | neuronal acetylcholine receptor subunit alpha-9-like | 525 | 2.19E-86 | 73.55 | C:postsynaptic membrane; P:cation transmembrane transport; C:integral component of membrane; C:cell junction; F:extracellular ligand-gated ion channel activity |
| Efet.01.514566.g355.t1 | beta-1-syntrophin-like | 624 | 5.80E-55 | 53 | F:nucleotide binding; F:ATPase activity; F:ATP binding; P:synapse organization; P:positive regulation of viral budding via host ESCRT complex; P:DNA replication; C:dystrophin-associated glycoprotein complex; P:endosomal transport; F:structural molecule activity; C:nuclear origin of replication recognition complex; C:intracellular |
| Efet.01.51463.g94.t1 | proteasomal ubiquitin receptor ADRM1- | 417 | 1.19E-31 | 76.85 | F:endopeptidase activator activity; C:integral component of plasma membrane; F:proteasome binding; C:cytoplasm; C:proteasome complex; F:protease binding; P:proteasome assembly; C:nucleoplasm; P:transcription elongation from RNA polymerase II promoter |
| Efet.01.514711.g356.t1 | Neuropilin and tolloid 1 | 453 | 2.33E-18 | 63.65 | P:regulation of biological quality; P:modulation of synaptic transmission; C:synapse |
| Efet.01.514760.g357.t1 | transmembrane 127 | 519 | 2.51E-11 | 56.8 | P:negative regulation of cell proliferation; C:membrane; C:integral component of membrane; P:negative regulation of TOR signaling |
| Efet.01.51493.g97.t1 | kelch domain-containing 4 | 492 | 1.43E-20 | 65.9 |  |
| Efet.01.514979.g359.t1 | hypothetical protein CAPTEDRAFT_206253 | 552 | 3.36E-34 | 51.35 | C:membrane; C:integral component of membrane; F:carbohydrate binding |
| Efet.01.514983.g360.t1 | methylsterol monooxygenase 1 | 324 | 4.23E-40 | 76.75 | F:iron ion binding; F:oxidoreductase activity; C:integral component of membrane; P:oxidation-reduction process; P:fatty acid biosynthetic process |
| Efet.01.515.g56.t1 | ras-related Rab-39B | 789 | 3.00E-46 | 57 | F:protein binding; C:late endosome; C:Golgi apparatus; C:neuronal cell body; C:synapse |
| Efet.01.51500.g98.t1 | potassium voltage-gated channel subfamily H member 4 | 960 | 8.84E-81 | 73.55 | F:phosphorelay sensor kinase activity; F:voltage-gated potassium channel activity; P:potassium ion transmembrane transport; P:phosphorelay signal transduction system; C:integral component of membrane; P:signal transduction by protein phosphorylation; C:intracellular |
| Efet.01.515021.g361.t1 | tryptophan synthase subunit beta | 321 | 1.61E-71 | 85.35 | F:tryptophan synthase activity; P:tryptophan biosynthetic process |
| Efet.01.515048.g362.t1 | TonB-dependent receptor | 903 | 1.27E-164 | 52.9 | F:phosphatidylinositol phospholipase C activity; P:signal transduction; C:membrane; P:lipid metabolic process; F:receptor activity; P:transport; P:intracellular signal transduction; C:intracellular |
| Efet.01.515095.g364.t1 | UDP- c:betaGal beta-1,3-N-acetylglucosaminyltransferase 1 | 1077 | 5.64E-136 | 72.15 | F:transferase activity, transferring glycosyl groups |
| Efet.01.515187.g366.t1 | glycine receptor subunit alpha-3-like isoform X1 | 309 | 3.03E-42 | 73.2 | P:ion transport; C:membrane |
| Efet.01.51521.g99.t1 | filamin-A-like | 372 | 1.35E-47 | 86.5 | F:actin filament binding |
| Efet.01.515244.g368.t1 | hypothetical protein CAPTEDRAFT_224202 | 342 | 5.24E-10 | 61.33 | F:calcium ion binding; C:membrane; P:homophilic cell adhesion via plasma membrane adhesion molecules |
| Efet.01.515250.g369.t1 | innexin unc-9 isoform X6 | 498 | 8.46E-62 | 67.25 | C:membrane; P:cell communication by electrical coupling; C:cell junction; C:synapse; F:gap junction channel activity; P:transport |
| Efet.01.515254.g370.t1 | mannosyl-oligosaccharide glucosidase-like | 519 | 6.16E-55 | 68.75 | C:endoplasmic reticulum membrane; P:protein N-linked glycosylation; F:mannosyl-oligosaccharide glucosidase activity |
| Efet.01.51527.g100.t1 | lysosomal aspartic protease | 453 | 1.04E-50 | 52.6 | F:aspartic-type endopeptidase activity; P:proteolysis; F:peptidase activity; C:lysosome; F:hydrolase activity |
| Efet.01.515327.g371.t1 | sprouty- EVH1 domain-containing 2-like isoform X2 | 723 | 6.79E-48 | 53.8 | P:multicellular organism development; C:membrane; P:regulation of signal transduction |
| Efet.01.515427.g375.t1 | homeobox Hmx | 891 | 2.12E-08 | 50.15 | F:DNA binding; C:nucleus; P:regulation of transcription, DNA-templated; F:sequence-specific DNA binding |
| Efet.01.515479.g376.t1 | hypothetical protein HELRODRAFT_162325 | 363 | 4.55E-19 | 60.33 | F:calcium-activated potassium channel activity; P:potassium ion transport; P:potassium ion transmembrane transport; C:membrane; C:integral component of membrane |
| Efet.01.515497.g377.t1 | hypothetical protein HELRODRAFT_180676 | 3189 | 0 | 47.75 | C:membrane; C:integral component of membrane |
| Efet.01.515599.g378.t1 | PDZ domain-containing 2-like isoform X1 | 735 | 1.40E-09 | 66.7 |  |
| Efet.01.515650.g379.t1 | bacterioferritin | 477 | 9.20E-112 | 94.35 | P:cellular iron ion homeostasis; C:cell; F:ferric iron binding; P:iron ion transport |
| Efet.01.515650.g380.t1 | cytoplasmic | 681 | 9.94E-167 | 59.25 |  |
| Efet.01.515650.g381.t1 | molecular chaperone | 1146 | 0 | 94.6 | F:ATP binding; F:heat shock protein binding; F:zinc ion binding; P:response to heat; C:cytoplasm; P:DNA replication; F:unfolded protein binding; P:protein folding |
| Efet.01.515650.g382.t1 | molecular chaperone | 1962 | 0 | 94.8 | F:ATP binding; F:unfolded protein binding; P:protein folding |
| Efet.01.515650.g383.t1 | nucleotide exchange factor | 546 | 9.33E-80 | 94.7 | F:protein homodimerization activity; C:cytoplasm; F:chaperone binding; P:regulation of catalytic activity; P:protein folding; F:adenyl-nucleotide exchange factor activity |
| Efet.01.515650.g384.t1 | 30S ribosomal S6 | 366 | 3.57E-63 | 86.15 | F:structural constituent of ribosome; C:ribosome; F:rRNA binding; P:translation |
| Efet.01.515650.g385.t1 | 30S ribosomal S18 | 681 | 3.81E-52 | 98.6 | F:structural constituent of ribosome; C:ribosome; F:rRNA binding; P:translation |
| Efet.01.515650.g386.t1 | replicative DNA helicase | 1431 | 0 | 93.45 | F:DNA binding; F:ATP binding; F:DNA helicase activity; P:DNA duplex unwinding; C:primosome complex; P:DNA replication, synthesis of RNA primer |
| Efet.01.515650.g387.t1 | hypothetical protein | 360 | 6.02E-62 | 73.15 | C:integral component of membrane |
| Efet.01.515650.g388.t1 | phosphate starvation-inducible | 1302 | 0 | 94.1 | F:ATP binding |
| Efet.01.515674.g389.t1 | hypothetical protein CAPTEDRAFT_196175 | 1269 | 1.05E-66 | 50.75 | C:membrane; C:integral component of membrane; F:sulfotransferase activity |
| Efet.01.515782.g391.t1 | ADAMTS 1 | 216 | 3.30E-22 | 71.3 |  |
| Efet.01.515787.g392.t1 | hypothetical protein CAPTEDRAFT_211886 | 603 | 2.00E-38 | 49.3 | C:membrane; C:integral component of membrane; F:carbohydrate binding |
| Efet.01.515933.g393.t1 | IS110 family transposase | 1065 | 2.96E-138 | 76.65 | F:DNA binding; F:transposase activity; P:transposition, DNA-mediated |
| Efet.01.515933.g394.t1 | MFS transporter | 1278 | 0 | 71.7 | C:membrane; F:transporter activity |
| Efet.01.515933.g395.t1 | NAD-dependent succinate-semialdehyde dehydrogenase | 915 | 0 | 71.1 | F:oxidoreductase activity |
| Efet.01.515945.g396.t1 | serine threonine- kinase BRSK2-like | 453 | 2.24E-10 | 95 | F:nucleotide binding; F:ATP binding; F:protein kinase activity; P:phosphorylation; F:protein serine/threonine kinase activity; P:protein phosphorylation; F:kinase activity |
| Efet.01.515957.g397.t1 | cullin-1 | 432 | 2.71E-38 | 58.25 | F:ubiquitin protein ligase activity; F:ubiquitin protein ligase binding; C:cullin-RING ubiquitin ligase complex; C:SCF ubiquitin ligase complex; P:protein ubiquitination involved in ubiquitin-dependent protein catabolic process; P:ubiquitin-dependent protein catabolic process |
| Efet.01.515990.g398.t1 | Cytoplasmic NCK1 | 444 | 3.14E-25 | 77.05 |  |
| Efet.01.516019.g399.t1 | hypothetical protein CAPTEDRAFT_221350 | 1989 | 4.31E-53 | 56.4 |  |
| Efet.01.516124.g401.t1 | WD repeat-containing 17 | 522 | 1.58E-33 | 63.8 | P:biological_process; C:membrane; C:integral component of membrane; C:cellular_component; F:molecular_function |
| Efet.01.516197.g403.t1 | heparan sulfate glucosamine 3-O-sulfotransferase 5 | 1047 | 1.03E-94 | 75.25 | P:protein metabolic process; P:cellular macromolecule metabolic process; P:sulfur compound metabolic process; C:membrane; F:sulfotransferase activity; P:regulation of biological process |
| Efet.01.516205.g404.t1 | BTG1 | 483 | 5.83E-41 | 65.9 | P:positive regulation of cellular process; P:positive regulation of developmental process |
| Efet.01.516274.g405.t1 | hypothetical protein CAPTEDRAFT_226428 | 372 | 2.02E-22 | 59.45 | C:membrane |
| Efet.01.516309.g406.t1 | PREDICTED: uncharacterized protein LOC105845334 | 1443 | 1.33E-99 | 55.55 | F:DNA binding; F:DNA-directed 5'-3' RNA polymerase activity; P:transcription, DNA-templated |
| Efet.01.51631.g104.t1 | potassium voltage-gated channel Shab-like | 648 | 6.19E-79 | 78.6 | F:voltage-gated potassium channel activity; P:potassium ion transmembrane transport; C:voltage-gated potassium channel complex; P:regulation of ion transmembrane transport; P:protein homooligomerization |
| Efet.01.516348.g407.t1 | U11 U12 small nuclear ribonucleo 35 kDa | 777 | 1.68E-71 | 76.25 | F:nucleotide binding; F:nucleic acid binding; C:intracellular ribonucleoprotein complex; C:viral nucleocapsid |
| Efet.01.516480.g411.t1 | discoidin domain-containing receptor 2-like isoform X1 | 501 | 1.98E-59 | 79.6 | C:integral component of plasma membrane; F:ATP binding; P:peptidyl-tyrosine phosphorylation; F:protein tyrosine kinase collagen receptor activity; P:collagen-activated tyrosine kinase receptor signaling pathway |
| Efet.01.516565.g413.t1 | acetylcholinesterase-like isoform X3 | 1530 | 0 | 68.25 | C:membrane; F:cholinesterase activity |
| Efet.01.516580.g414.t1 | obscurin isoform X3 | 717 | 7.67E-08 | 41.75 |  |
| Efet.01.516583.g415.t1 | DNA primase | 327 | 3.16E-44 | 79.6 | F:magnesium ion binding; F:DNA primase activity; F:DNA binding; F:zinc ion binding; C:primosome complex; P:DNA replication, synthesis of RNA primer |
| Efet.01.5166.g400.t1 | Biotin-- | 459 | 7.17E-49 | 68.9 | P:cell proliferation; F:biotin-[propionyl-CoA-carboxylase (ATP-hydrolyzing)] ligase activity; F:protein homodimerization activity; F:biotin binding; C:chromatin; C:nuclear lamina; P:biotin metabolic process; P:histone biotinylation; C:mitochondrion; P:response to biotin; C:cytosol; C:nuclear matrix; F:enzyme binding |
| Efet.01.51664.g106.t1 | sorting nexin-6-like | 351 | 9.40E-28 | 76.35 | C:endosome; P:vesicle organization; F:phosphatidylinositol binding; C:extrinsic component of membrane; P:endocytosis |
| Efet.01.5167.g401.t1 | endothelin-converting enzyme 1-like isoform X2 | 675 | 4.72E-34 | 52.85 | P:proteolysis; C:membrane; C:integral component of membrane; P:peptide hormone processing; F:metalloendopeptidase activity; F:metallopeptidase activity |
| Efet.01.516705.g419.t1 | molecular chaperone | 564 | 7.92E-75 | 75.05 | F:ATP binding; F:unfolded protein binding; P:protein folding |
| Efet.01.51691.g107.t1 | nuclear envelope integral membrane 1-like | 333 | 9.31E-24 | 68.7 | C:membrane |
| Efet.01.516989.g421.t1 | gastrula zinc finger -like | 1548 | 1.37E-59 | 50.6 | F:nucleic acid binding; P:biological_process; F:metal ion binding |
| Efet.01.51702.g109.t1 | glycine receptor subunit alpha-2 | 372 | 1.36E-22 | 49.3 | P:ion transport; C:membrane |
| Efet.01.517044.g422.t1 | carboxypeptidase N subunit 2 | 1326 | 5.07E-52 | 49.1 | F:DNA binding; C:nucleus; C:extracellular exosome; P:transcription, DNA-templated; C:blood microparticle; P:regulation of transcription, DNA-templated; F:protein dimerization activity |
| Efet.01.517051.g423.t1 | von Willebrand factor type EGF and pentraxin domain-containing | 363 | 8.17E-28 | 70.4 | F:peptidase inhibitor activity; C:extracellular region; P:negative regulation of peptidase activity |
| Efet.01.517078.g424.t1 | EF-hand calcium-binding domain-containing 6-like | 336 | 3.76E-08 | 58.33 | F:calcium ion binding |
| Efet.01.517085.g425.t1 | hypothetical protein HELRODRAFT_191437 | 756 | 2.30E-18 | 49 | P:signal transduction; P:regulation of apoptotic process |
| Efet.01.517156.g426.t1 | cysteine--tRNA ligase | 495 | 9.80E-65 | 72.25 | F:nucleotide binding; P:tRNA aminoacylation for protein translation; F:aminoacyl-tRNA ligase activity; F:metal ion binding |
| Efet.01.517235.g427.t1 | hypothetical protein CAPTEDRAFT_216189 | 2415 | 8.30E-14 | 43 |  |
| Efet.01.51739.g110.t1 | sex peptide receptor-like | 1179 | 9.26E-113 | 65.65 | F:G-protein coupled receptor activity; C:membrane |
| Efet.01.517413.g428.t1 | eyes absent homolog 1-like isoform X14 | 351 | 9.22E-39 | 79.7 | F:protein tyrosine phosphatase activity; F:metal ion binding; P:multicellular organism development; P:regulation of transcription, DNA-templated; P:peptidyl-tyrosine dephosphorylation |
| Efet.01.51744.g112.t1 | histone acetyltransferase p300-like | 825 | 8.14E-74 | 58.4 | P:histone H4-K12 acetylation; P:histone H4-K8 acetylation; P:R7 cell differentiation; P:positive regulation of transcription from RNA polymerase II promoter; F:histone acetyltransferase activity (H3-K27 specific); P:glial cell migration; C:polytene chromosome; P:circadian regulation of gene expression; F:histone methyltransferase binding; P:locomotor rhythm; P:ovarian follicle cell development; P:thermosensory behavior; P:histone H3-K4 methylation; F:histone methyltransferase activity (H3-K4 specific); P:regulation of mitotic nuclear division; C:histone methyltransferase complex; P:smoothened signaling pathway; C:protein complex; F:transcription factor binding; F:histone acetyltransferase activity (H3-K18 specific); P:R3/R4 cell fate commitment; P:histone H3-K18 acetylation; P:histone H3-K27 acetylation; P:neurotransmitter secretion; C:histone acetyltransferase complex; P:DNA replication checkpoint; P:synapse assembly; P:regulation of glucose metabolic process; F:transcription coactivator activity; P:sensory perception of pain |
| Efet.01.51746.g113.t1 | hypothetical protein HELRODRAFT_189103 | 225 | 3.93E-19 | 75 |  |
| Efet.01.517467.g429.t1 | centrosomal of 63 kDa-like isoform X6 | 540 | 4.43E-20 | 68.6 |  |
| Efet.01.517616.g431.t1 | Planctomycete cytochrome C | 360 | 6.31E-56 | 91.9 | F:electron carrier activity; F:heme binding |
| Efet.01.517660.g433.t1 | family -linked outer membrane | 1557 | 0 | 74.75 | C:membrane; F:receptor activity; P:transport |
| Efet.01.517765.g436.t1 | zinc finger 1 | 1488 | 2.06E-26 | 56.7 | F:nucleic acid binding; F:metal ion binding |
| Efet.01.517829.g437.t1 | transcription factor Sox-11-like isoform X2 | 978 | 1.57E-32 | 57.9 |  |
| Efet.01.5179.g403.t1 | multidrug resistance 1 | 546 | 1.76E-37 | 64.8 | F:catalytic activity |
| Efet.01.517926.g439.t1 | Complement factor H | 831 | 1.45E-14 | 55.67 | C:membrane; C:integral component of membrane |
| Efet.01.518002.g440.t1 | Collagen alpha-4(VI) chain | 477 | 5.08E-13 | 55.3 | F:chitin binding; F:calcium ion binding; C:collagen trimer; P:chitin metabolic process; C:extracellular region |
| Efet.01.518008.g441.t1 | bestrophin homolog 22-like | 276 | 3.39E-11 | 66.35 | C:integral component of membrane |
| Efet.01.51805.g115.t1 | SET and MYND domain-containing 4-like | 1560 | 2.55E-113 | 53.45 |  |
| Efet.01.51805.g116.t1 | guanine nucleotide exchange factor MSS4 | 657 | 3.79E-45 | 71.9 | P:biological regulation |
| Efet.01.518124.g443.t1 | rho guanine nucleotide exchange factor 16 | 213 | 2.41E-08 | 61.55 | P:regulation of Rho protein signal transduction; F:Rho guanyl-nucleotide exchange factor activity; P:positive regulation of GTPase activity |
| Efet.01.518403.g445.t1 | IST1 homolog isoform X1 | 414 | 8.05E-06 | 57 |  |
| Efet.01.518467.g446.t1 | calcium calmodulin-dependent 3 ,5 -cyclic nucleotide phosphodiesterase 1A-like isoform X6 | 294 | 7.09E-17 | 81.15 | F:metal ion binding; P:signal transduction; C:integral component of membrane; F:3',5'-cyclic-nucleotide phosphodiesterase activity |
| Efet.01.518526.g447.t1 | T family of potassium channels 7-like | 918 | 1.84E-56 | 57.5 | P:transport |
| Efet.01.518706.g449.t1 | zinc finger 622-like | 276 | 1.75E-13 | 62.35 | F:metal ion binding |
| Efet.01.51872.g121.t1 | dynein beta flagellar outer arm-like isoform X15 | 1167 | 7.56E-44 | 53.2 |  |
| Efet.01.518735.g450.t1 | E3 ubiquitin- ligase TRIM56-like | 1203 | 6.14E-17 | 43.85 | F:zinc ion binding; F:RNA binding; F:metal ion binding; C:intracellular |
| Efet.01.518738.g451.t1 | thiol ase | 267 | 6.97E-11 | 69.1 | P:proteolysis; F:cysteine-type peptidase activity |
| Efet.01.51876.g122.t1 | rho GTPase-activating 100F-like isoform X1 | 573 | 2.75E-51 | 70.45 | P:signal transduction |
| Efet.01.518799.g452.t1 | Adenylyl cyclase class-3 4 guanylyl cyclase domain containing | 402 | 3.31E-14 | 72.45 | F:nucleotide binding; F:phosphorus-oxygen lyase activity; C:membrane; P:phosphorylation; F:kinase activity; P:cyclic nucleotide biosynthetic process |
| Efet.01.51880.g123.t1 | WD repeat-containing 5-like | 270 | 1.48E-14 | 66.08 | F:ATPase activity; F:cation-transporting ATPase activity; P:cellular calcium ion homeostasis; F:nucleotide binding; F:ATP binding; C:integral component of plasma membrane; P:cation transport; F:metal ion binding; C:intracellular membrane-bounded organelle; C:membrane; P:cation transmembrane transport; C:integral component of membrane; F:hydrolase activity |
| Efet.01.518849.g454.t1 | Krueppel-like factor 15 | 1164 | 9.64E-44 | 82.2 | F:nucleic acid binding; F:metal ion binding |
| Efet.01.518908.g455.t1 | casein kinase I isoform gamma-3-like isoform X10 | 486 | 2.68E-98 | 97.2 | F:ATP binding; F:protein serine/threonine kinase activity; P:protein phosphorylation |
| Efet.01.51895.g124.t1 | hypothetical protein CAPTEDRAFT_223749 | 249 | 1.08E-20 | 61.41 | C:membrane; C:integral component of membrane |
| Efet.01.518974.g456.t1 | vacuolar sorting-associated 8 homolog | 2082 | 0 | 80.95 | F:zinc ion binding; P:vesicle-mediated transport; P:intracellular protein transport; C:intracellular |
| Efet.01.518981.g457.t1 | phosducin | 348 | 1.80E-31 | 71.9 | C:cell; P:cell redox homeostasis |
| Efet.01.51907.g126.t1 | cytochrome P450 1A1-like | 1521 | 9.95E-106 | 53.55 | F:monooxygenase activity |
| Efet.01.519211.g459.t1 | DNA ligase 3-like | 330 | 1.69E-37 | 80.05 | P:DNA ligation involved in DNA repair; C:nucleus; F:DNA binding; F:ATP binding; F:zinc ion binding; F:DNA ligase (ATP) activity; P:DNA replication; P:DNA recombination; P:DNA biosynthetic process; P:mitochondrion organization; P:double-strand break repair |
| Efet.01.51926.g127.t1 | histone-lysine N-methyltransferase 2E-like isoform X1 | 804 | 8.67E-48 | 72.45 | F:zinc ion binding; F:transferase activity |
| Efet.01.519399.g463.t1 | transcription factor 19 | 336 | 1.21E-23 | 64.45 |  |
| Efet.01.519408.g464.t1 | inosine-5 -monophosphate dehydrogenase 1b isoform X1 | 435 | 4.80E-26 | 79.25 | F:nucleotide binding; C:cytoplasm; F:metal ion binding; F:IMP dehydrogenase activity; P:oxidation-reduction process; P:GMP biosynthetic process |
| Efet.01.519525.g466.t1 | glucose-6-phosphate 1- | 492 | 1.96E-72 | 83.75 | F:NADP binding; P:pentose-phosphate shunt; P:oxidation-reduction process; P:glucose metabolic process; F:glucose-6-phosphate dehydrogenase activity |
| Efet.01.519544.g467.t1 | helicase ARIP4-like | 321 | 2.32E-12 | 70 | F:ATP binding |
| Efet.01.519583.g468.t1 | Uncharacterized membrane | 675 | 1.88E-127 | 76.9 | C:integral component of membrane |
| Efet.01.519583.g469.t1 | DUF4233 domain-containing | 480 | 7.96E-107 | 70.85 | C:membrane |
| Efet.01.519606.g470.t1 | FMRFamide receptor-like | 1146 | 6.65E-80 | 53.9 | F:signal transducer activity |
| Efet.01.519674.g471.t1 | beta-galactosidase | 1644 | 8.49E-138 | 57.9 | P:metabolic process; F:hydrolase activity, hydrolyzing O-glycosyl compounds |
| Efet.01.51975.g132.t1 | reelin-like | 831 | 1.61E-58 | 53.95 | C:membrane; C:integral component of membrane |
| Efet.01.519797.g473.t1 | DUF4062 domain-containing | 402 | 5.59E-90 | 66.4 | P:signal transduction |
| Efet.01.520.g57.t1 | regulating synaptic membrane exocytosis 2-like | 327 | 1.83E-12 | 78.2 | P:insulin secretion; P:positive regulation of dendrite extension; P:spontaneous neurotransmitter secretion; P:positive regulation of inhibitory postsynaptic potential; F:ion channel binding; P:calcium ion-regulated exocytosis of neurotransmitter; C:extracellular exosome; C:presynaptic active zone; P:positive regulation of gene expression; C:membrane; P:positive regulation of excitatory postsynaptic potential; P:regulation of exocytosis; P:cAMP-mediated signaling |
| Efet.01.52006.g133.t1 | E3 ubiquitin- ligase UBR5 | 354 | 6.63E-41 | 77.65 | C:nucleus; F:ubiquitin-protein transferase activity; F:zinc ion binding; F:RNA binding; F:ubiquitin binding; C:cytoplasm; F:ligase activity; P:protein ubiquitination involved in ubiquitin-dependent protein catabolic process |
| Efet.01.520061.g477.t1 | Zinc finger homeobox 4 | 3477 | 4.77E-149 | 61.75 | F:nucleic acid binding |
| Efet.01.52008.g134.t1 | hypothetical protein HELRODRAFT_154876 | 531 | 5.58E-12 | 60 | F:transferase activity |
| Efet.01.520113.g478.t1 | transcriptional regulator | 465 | 5.15E-49 | 98.4 | P:transcription, DNA-templated; P:regulation of catalytic activity; P:regulation of transcription, DNA-templated; P:regulation of nitrogen utilization; F:enzyme regulator activity |
| Efet.01.520113.g479.t1 | hypothetical protein | 711 | 5.15E-128 | 84.65 |  |
| Efet.01.520113.g480.t1 | ATP-dependent protease | 1464 | 0 | 93.3 | F:DNA binding; F:ATP binding; P:DNA replication; P:proteolysis; F:peptidase activity |
| Efet.01.520113.g481.t1 | family transcriptional regulator | 960 | 0 | 68.85 | F:DNA binding; F:transcription factor activity, sequence-specific DNA binding; P:transcription, DNA-templated; P:regulation of transcription, DNA-templated |
| Efet.01.520113.g482.t1 | family | 591 | 1.37E-114 | 69.15 | F:lyase activity |
| Efet.01.52024.g135.t1 | hypothetical protein HELRODRAFT_189125 | 534 | 2.88E-24 | 57 | C:membrane; C:integral component of membrane |
| Efet.01.52027.g136.t1 | axonemal dynein light intermediate polypeptide 1 | 273 | 2.31E-40 | 87.8 | C:cytoplasm; C:filopodium; F:dynein heavy chain binding; C:axoneme |
| Efet.01.520291.g483.t1 | adenylate kinase mitochondrial-like | 222 | 4.03E-29 | 90.55 | F:adenylate kinase activity; F:ATP binding; P:nucleotide phosphorylation; C:mitochondrial intermembrane space; P:ADP biosynthetic process; P:AMP metabolic process; P:ATP metabolic process; C:integral component of membrane; C:cytosol |
| Efet.01.520421.g485.t1 | hypothetical protein CAPTEDRAFT_223749 | 216 | 4.14E-13 | 66.5 | C:membrane; C:integral component of membrane |
| Efet.01.520470.g486.t1 | probable ubiquitin carboxyl-terminal hydrolase FAF-X isoform X1 | 1395 | 5.88E-135 | 75.35 | P:protein deubiquitination; C:cytoplasm; P:cell migration; F:thiol-dependent ubiquitinyl hydrolase activity; P:ubiquitin-dependent protein catabolic process |
| Efet.01.520514.g488.t1 | sperm-associated antigen 17-like | 702 | 2.15E-20 | 54.3 |  |
| Efet.01.52053.g137.t1 | syntaxin-binding 5-like | 423 | 2.61E-32 | 60.85 | P:vesicle-mediated transport; C:integral component of membrane |
| Efet.01.52059.g138.t1 | TSC22 domain family 1-like | 1059 | 6.02E-08 | 53.33 |  |
| Efet.01.520621.g491.t1 | type II toxin-antitoxin system family toxin | 645 | 1.38E-72 | 59.85 |  |
| Efet.01.52068.g140.t1 | formin isoform A | 273 | 1.70E-13 | 72.4 | F:actin binding; P:cellular component organization; P:branch fusion, open tracheal system |
| Efet.01.520921.g494.t1 | retinol dehydrogenase 7-like | 420 | 1.21E-18 | 60.7 | F:oxidoreductase activity; C:membrane; C:integral component of membrane; P:oxidation-reduction process |
| Efet.01.520989.g495.t1 | pituitary homeobox x isoform X3 | 453 | 3.44E-30 | 64.05 | F:DNA binding; C:nucleus; F:transcription factor activity, sequence-specific DNA binding; P:multicellular organism development; P:regulation of transcription, DNA-templated; F:sequence-specific DNA binding |
| Efet.01.521053.g496.t1 | E3 ubiquitin- ligase TRIM56-like | 1011 | 1.14E-29 | 46.95 | F:zinc ion binding; F:RNA binding; F:metal ion binding; C:intracellular |
| Efet.01.521124.g497.t1 | hypothetical protein CAPTEDRAFT_179956 | 429 | 2.93E-42 | 64.6 | C:membrane; C:integral component of membrane |
| Efet.01.521131.g498.t1 | potassium voltage-gated channel subfamily A member | 972 | 6.54E-121 | 70.5 | F:voltage-gated potassium channel activity; P:potassium ion transmembrane transport; C:voltage-gated potassium channel complex; P:regulation of ion transmembrane transport; P:protein homooligomerization |
| Efet.01.521254.g500.t1 | eIF-2-alpha kinase GCN2-like | 252 | 3.51E-12 | 64.35 | F:nucleotide binding; F:ATP binding; F:protein kinase activity; P:protein phosphorylation |
| Efet.01.521270.g501.t1 | gastrulation brain homeobox | 402 | 3.58E-41 | 71.55 | F:DNA binding; P:regulation of biological process |
| Efet.01.521297.g504.t1 | leucine-rich repeat neuronal 1 | 1053 | 5.25E-30 | 49.85 | P:axonogenesis; C:membrane; C:integral component of membrane; P:positive regulation of synapse assembly |
| Efet.01.521309.g505.t1 | predicted protein | 1278 | 5.96E-07 | 66 |  |
| Efet.01.521411.g506.t1 | MBL fold metallo-hydrolase | 372 | 4.14E-72 | 69.2 | F:hydrolase activity |
| Efet.01.52147.g142.t1 | mediator of RNA polymerase II transcription subunit 25 isoform X4 | 636 | 1.15E-18 | 59.9 | P:palate development |
| Efet.01.52148.g143.t1 | autophagy-related 13 homolog isoform X2 | 1368 | 9.98E-75 | 65.3 | P:autophagy; C:ATG1/ULK1 kinase complex |
| Efet.01.521545.g508.t1 | penicillin-binding 4-like isoform X1 | 729 | 5.73E-07 | 42 |  |
| Efet.01.521651.g509.t1 | ETS-related transcription factor Elf-3-like | 231 | 7.19E-07 | 74.91 | F:DNA binding; P:signal transduction; C:membrane; F:signal transducer activity |
| Efet.01.521674.g510.t1 | low-density lipo receptor class A domain-containing 2-like isoform X2 | 279 | 1.62E-24 | 67.58 | C:membrane; C:integral component of membrane |
| Efet.01.521753.g511.t1 | ankyrin repeat and EF-hand domain-containing 1-like | 1071 | 1.20E-80 | 52.9 | F:calcium ion binding |
| Efet.01.521816.g512.t1 | family transcriptional regulator | 213 | 2.64E-17 | 87.75 | F:DNA binding; F:transcription factor activity, sequence-specific DNA binding; P:transcription, DNA-templated; P:regulation of transcription, DNA-templated |
| Efet.01.521843.g513.t1 | hypothetical protein HELRODRAFT_170293 | 573 | 3.31E-80 | 54.2 |  |
| Efet.01.52190.g147.t1 | lysophosphatidic acid receptor 4-like | 1008 | 6.43E-78 | 59.7 | F:G-protein coupled receptor activity; P:G-protein coupled receptor signaling pathway; P:protein dephosphorylation; C:integral component of membrane |
| Efet.01.52193.g148.t1 | glutathione-specific gamma-glutamylcyclotransferase 2 | 555 | 2.06E-18 | 65.55 | F:nucleotide binding; F:nucleic acid binding |
| Efet.01.52197.g149.t1 | double-stranded RNA-specific adenosine deaminase-like isoform X2 | 510 | 3.62E-28 | 53.9 | F:RNA binding; F:double-stranded RNA adenosine deaminase activity; P:RNA processing; F:adenosine deaminase activity |
| Efet.01.521972.g516.t1 | hypothetical protein CAPTEDRAFT_195379 | 741 | 1.81E-10 | 66 | C:membrane; C:integral component of membrane |
| Efet.01.521979.g517.t1 | sex peptide receptor-like | 1206 | 2.74E-122 | 67.2 | F:G-protein coupled receptor activity; C:membrane |
| Efet.01.522033.g519.t1 | alkaline phosphatase family | 315 | 4.34E-35 | 77.65 | F:catalytic activity; P:metabolic process |
| Efet.01.522129.g520.t1 | serine threonine- kinase PLK1-like | 255 | 7.34E-16 | 62.75 | F:kinase activity |
| Efet.01.522438.g523.t1 | hypothetical protein CAPTEDRAFT_217918 | 462 | 2.41E-08 | 67 | C:membrane |
| Efet.01.522487.g524.t1 | heat shock beta-1-like isoform X2 | 1035 | 5.30E-33 | 54.75 | C:nucleus; C:cytoplasm; F:metal ion binding; F:structural constituent of eye lens |
| Efet.01.52249.g150.t1 | gonadotropin-releasing hormone II receptor-like | 1251 | 3.17E-141 | 62.6 | F:G-protein coupled peptide receptor activity; P:response to organic substance; P:G-protein coupled receptor signaling pathway; P:response to endogenous stimulus; C:integral component of membrane; C:plasma membrane |
| Efet.01.52250.g151.t1 | glutamate receptor kainate 2 isoform X1 | 240 | 2.07E-12 | 61.95 | P:ion transport; F:transmembrane signaling receptor activity; F:extracellular ligand-gated ion channel activity; C:synapse; C:membrane part; C:plasma membrane |
| Efet.01.522587.g527.t1 | plexin-A1 isoform X1 | 567 | 5.64E-10 | 40.15 | P:signal transduction; C:membrane; C:integral component of membrane; F:semaphorin receptor activity; P:semaphorin-plexin signaling pathway |
| Efet.01.522670.g529.t1 | protocadherin-18-like isoform X2 | 1356 | 3.18E-56 | 54.95 | F:calcium ion binding; C:membrane; C:integral component of membrane; P:nervous system development; P:cell adhesion; P:homophilic cell adhesion via plasma membrane adhesion molecules; C:plasma membrane |
| Efet.01.522687.g530.t1 | tyrosine- phosphatase Lar-like isoform X1 | 465 | 5.71E-55 | 70.85 | F:phosphoprotein phosphatase activity; P:protein dephosphorylation |
| Efet.01.52273.g152.t1 | 1-phosphatidylinositol 4,5-bisphosphate phosphodiesterase beta-1 | 417 | 3.75E-17 | 62.3 | P:regulation of cellular process; P:single-organism process; C:intracellular; F:hydrolase activity |
| Efet.01.52277.g153.t1 | filamin-A-like isoform X1 | 1362 | 5.33E-157 | 67.15 | F:actin binding |
| Efet.01.52278.g154.t1 | probable cytochrome P450 CYP44 | 267 | 1.19E-22 | 66.85 | F:oxidoreductase activity; F:binding |
| Efet.01.522832.g532.t1 | MAP microtubule affinity-regulating kinase 3-like | 276 | 2.82E-46 | 86.3 | C:microtubule cytoskeleton; P:microtubule cytoskeleton organization; F:protein serine/threonine kinase activity; P:protein phosphorylation; C:plasma membrane; F:magnesium ion binding; F:tau-protein kinase activity; F:ATP binding; F:phosphatidylinositol-4,5-bisphosphate binding; F:phosphatidylserine binding; P:neuron migration; C:cytoplasm; P:Wnt signaling pathway; P:establishment of cell polarity; F:phosphatidic acid binding; P:intracellular signal transduction |
| Efet.01.52284.g155.t1 | 2-oxoglutarate mitochondrial isoform X5 | 435 | 4.27E-55 | 74.5 | F:thiamine pyrophosphate binding; C:oxoglutarate dehydrogenase complex; F:oxoglutarate dehydrogenase (succinyl-transferring) activity; C:mitochondrion; C:cytosol; P:tricarboxylic acid cycle |
| Efet.01.522848.g533.t1 | molybdate-anion transporter-like | 720 | 4.85E-56 | 64.15 | C:membrane |
| Efet.01.522868.g534.t1 | F-box only 11 | 360 | 1.90E-19 | 83.15 | F:zinc ion binding; C:ubiquitin ligase complex; P:protein ubiquitination |
| Efet.01.522934.g535.t1 | Programmed cell death 6-interacting | 630 | 9.20E-29 | 57.6 |  |
| Efet.01.5230.g405.t1 | hypothetical protein HELRODRAFT_183289 | 1149 | 4.15E-09 | 50 | C:membrane; C:integral component of membrane |
| Efet.01.5230.g406.t1 | hypothetical protein HELRODRAFT_183289 | 1236 | 7.96E-20 | 47 | C:membrane; C:integral component of membrane |
| Efet.01.523046.g537.t1 | RING finger and SPRY domain-containing 1 | 540 | 4.41E-55 | 74.35 | F:metal ion binding |
| Efet.01.52310.g156.t1 | Glutaminyl-peptide cyclotransferase | 525 | 2.68E-58 | 67.65 | F:catalytic activity |
| Efet.01.523186.g538.t1 | cadherin-23 isoform X3 | 300 | 7.23E-12 | 45.95 | P:inner ear receptor cell differentiation; C:stereocilium bundle; C:organelle part; F:binding; C:cell projection; P:sensory perception; P:animal organ morphogenesis |
| Efet.01.52330.g157.t1 | tektin-3-like isoform X1 | 903 | 1.23E-52 | 60.9 |  |
| Efet.01.523305.g539.t1 | microtubule-associated serine threonine- kinase 3-like isoform X3 | 1746 | 5.93E-23 | 54.55 | F:nucleotide binding; F:magnesium ion binding; F:ATP binding; F:protein kinase activity; P:phosphorylation; F:transferase activity; F:protein serine/threonine kinase activity; P:protein phosphorylation; F:kinase activity |
| Efet.01.52341.g158.t1 | rap guanine nucleotide exchange factor 6-like isoform X1 | 285 | 1.09E-24 | 74.15 | P:signal transduction |
| Efet.01.523430.g540.t1 | Krueppel-like factor 15 | 1392 | 6.26E-46 | 87.55 | F:nucleic acid binding; F:metal ion binding |
| Efet.01.52345.g159.t1 | fatty acid-binding heart | 315 | 1.43E-16 | 66.8 | P:multicellular organism development; F:lipid binding; P:regulation of biological process; P:cellular process |
| Efet.01.5235.g408.t1 | ankyrin repeat | 1269 | 2.31E-21 | 53.65 | C:membrane; C:integral component of membrane |
| Efet.01.523509.g541.t1 | Major egg antigen (p40) | 909 | 1.75E-63 | 56.4 | F:nucleic acid binding |
| Efet.01.523524.g543.t1 | T9SS C-terminal target domain-containing | 822 | 2.30E-51 | 54.6 | P:peptidoglycan catabolic process; F:N-acetylmuramoyl-L-alanine amidase activity; F:hydrolase activity |
| Efet.01.523548.g544.t1 | degenerin unc-105 | 516 | 1.64E-41 | 62.15 | P:sodium ion transport; C:membrane |
| Efet.01.523793.g548.t1 | paired box Pax- | 948 | 8.88E-59 | 85.2 | C:nucleus; F:DNA binding; F:transcription factor activity, sequence-specific DNA binding; P:transcription, DNA-templated; P:regulation of transcription, DNA-templated; P:animal organ morphogenesis |
| Efet.01.523797.g549.t1 | hypothetical protein CAPTEDRAFT_218417 | 2286 | 4.84E-108 | 39.33 |  |
| Efet.01.52384.g163.t1 | keratin-associated 9-1-like | 1167 | 2.85E-06 | 45 |  |
| Efet.01.52387.g164.t1 | protocadherin Fat 1 isoform X1 | 1359 | 2.31E-29 | 51.55 | C:cell part |
| Efet.01.523923.g552.t1 | branched-chain amino acid ABC transporter permease | 918 | 3.24E-150 | 79.95 | C:integral component of membrane; P:transport; C:plasma membrane; F:transporter activity |
| Efet.01.523923.g553.t1 | amino acid ABC transporter substrate-binding | 1089 | 0 | 78.95 | P:amino acid transport |
| Efet.01.523923.g554.t1 | hydroxymethylglutaryl- lyase | 594 | 2.21E-111 | 85.2 | F:hydroxymethylglutaryl-CoA lyase activity; P:carboxylic acid metabolic process; F:transferase activity, transferring acyl groups, acyl groups converted into alkyl on transfer |
| Efet.01.523923.g555.t1 | transferase | 1221 | 0 | 89.3 | F:transferase activity |
| Efet.01.523927.g556.t1 | SusC/RagA family TonB-linked outer membrane protein | 1020 | 2.13E-137 | 74.45 | C:membrane; F:receptor activity; P:transport |
| Efet.01.523999.g557.t1 | enoyl- hydratase | 729 | 8.17E-176 | 87.05 | P:metabolic process; F:enoyl-CoA hydratase activity |
| Efet.01.523999.g558.t1 | osmotically inducible | 333 | 1.42E-64 | 81.4 |  |
| Efet.01.523999.g559.t1 | AMP-dependent synthetase | 1194 | 0 | 87.85 | F:phenylacetate-CoA ligase activity; P:metabolic process |
| Efet.01.524000.g560.t1 | intersectin-1 isoform X1 | 297 | 1.07E-23 | 65.25 | P:biological regulation; F:calcium ion binding |
| Efet.01.52406.g165.t1 | glutathione S-transferase C-terminal domain-containing -like | 990 | 1.58E-93 | 67 | F:transferase activity |
| Efet.01.524115.g561.t1 | hypothetical protein HELRODRAFT_194873 | 513 | 2.42E-18 | 52.67 | C:cytoskeleton; C:membrane; C:integral component of membrane |
| Efet.01.524143.g562.t1 | coiled-coil domain-containing 170 | 492 | 1.48E-15 | 58.15 |  |
| Efet.01.52418.g166.t1 | kelch 18 | 342 | 5.10E-57 | 85.75 |  |
| Efet.01.524241.g563.t1 | Dorsal-ventral patterning tolloid | 282 | 3.90E-06 | 64.33 | C:membrane; C:integral component of membrane |
| Efet.01.524250.g564.t1 | chemotaxis | 978 | 0 | 96.15 | F:phosphorelay sensor kinase activity; F:ATP binding; P:chemotaxis; C:cytoplasm; P:phosphorelay signal transduction system; P:signal transduction by protein phosphorylation; P:peptidyl-histidine phosphorylation |
| Efet.01.524250.g565.t1 | chemotaxis | 489 | 4.20E-98 | 91.35 | P:chemotaxis; P:signal transduction; F:signal transducer activity |
| Efet.01.524250.g566.t1 | chemotaxis | 891 | 0 | 87.75 | F:protein-glutamate O-methyltransferase activity; P:protein methylation |
| Efet.01.524250.g567.t1 | chemotaxis | 750 | 5.21E-136 | 83.35 | P:chemotaxis; F:protein-glutamine glutaminase activity |
| Efet.01.524274.g568.t1 | family transporter | 1353 | 0 | 75.9 | F:oxidoreductase activity, acting on CH-OH group of donors; F:flavin adenine dinucleotide binding; C:integral component of membrane; P:oxidation-reduction process |
| Efet.01.52428.g167.t1 | DNA methylase | 2268 | 8.42E-47 | 50.45 | F:nucleotide binding; F:nucleic acid binding; F:DNA binding; F:ATP binding; F:methyltransferase activity; F:helicase activity; F:transferase activity; P:methylation; F:hydrolase activity |
| Efet.01.52428.g168.t1 | DNA (cytosine-5-)-methyltransferase | 1554 | 5.13E-69 | 55 | F:DNA (cytosine-5-)-methyltransferase activity; P:C-5 methylation of cytosine; F:methyltransferase activity; F:transferase activity; P:DNA restriction-modification system; P:methylation |
| Efet.01.524324.g570.t1 | malignant fibrous histiocytoma-amplified sequence 1 homolog | 798 | 3.24E-82 | 72.75 |  |
| Efet.01.524381.g571.t1 | hypothetical protein LOTGIDRAFT_138086, partial | 414 | 1.75E-06 | 93 | F:metal ion binding; P:intracellular protein transport; F:Rab GTPase binding; C:intracellular |
| Efet.01.524439.g573.t1 | metastasis suppressor 1-like isoform X3 | 234 | 9.13E-17 | 71.9 | P:cellular component organization; P:single-organism cellular process |
| Efet.01.52447.g169.t1 | T family of potassium channels 18-like | 282 | 7.14E-29 | 55.2 | C:integral component of plasma membrane; P:ion transport; P:potassium ion transmembrane transport; C:membrane; C:integral component of membrane; F:potassium channel activity; P:stabilization of membrane potential; P:transport; F:potassium ion leak channel activity |
| Efet.01.52462.g171.t1 | ankyrin repeat and EF-hand domain-containing 1-like | 897 | 2.14E-55 | 50.3 | F:calcium ion binding |
| Efet.01.524679.g576.t1 | ras association domain-containing 1-like | 216 | 4.95E-06 | 83 |  |
| Efet.01.524684.g577.t1 | type I glyceraldehyde-3-phosphate dehydrogenase | 315 | 3.08E-66 | 92 | F:NADP binding; F:NAD binding; P:oxidation-reduction process; P:glucose metabolic process; F:oxidoreductase activity, acting on the aldehyde or oxo group of donors, NAD or NADP as acceptor |
| Efet.01.524733.g578.t1 | probable N-acetylgalactosaminyltransferase 9 isoform X1 | 366 | 8.63E-17 | 66.2 | C:membrane; C:Golgi apparatus; F:transferase activity |
| Efet.01.524796.g581.t1 | aminoglycoside phosphotransferase (APT) family | 906 | 1.93E-55 | 65.35 |  |
| Efet.01.524813.g582.t1 | glutamate racemase | 207 | 2.81E-38 | 80.65 | P:regulation of cell shape; F:glutamate racemase activity; P:peptidoglycan biosynthetic process; P:cell wall organization |
| Efet.01.524844.g583.t1 | Poly [ADP-ribose] polymerase 15 | 558 | 5.54E-11 | 67.6 | F:transferase activity |
| Efet.01.52485.g172.t1 | UTP--glucose-1-phosphate uridylyltransferase isoform X3 | 336 | 1.58E-45 | 86.4 | C:nucleus; F:identical protein binding; C:extracellular exosome; C:cytoplasm; F:metal ion binding; P:UDP-glucose metabolic process; F:UTP:glucose-1-phosphate uridylyltransferase activity |
| Efet.01.524891.g584.t1 | hillarin isoform X1 | 1353 | 9.71E-176 | 65.75 |  |
| Efet.01.52506.g174.t1 | Regulating synaptic membrane exocytosis 2 | 645 | 1.76E-51 | 82.95 | F:metal ion binding; P:intracellular protein transport; F:Rab GTPase binding; C:intracellular |
| Efet.01.525091.g586.t1 | cytochrome P450 4F4-like | 294 | 1.13E-26 | 72.6 | F:iron ion binding; F:oxidoreductase activity, acting on paired donors, with incorporation or reduction of molecular oxygen; C:integral component of membrane; P:oxidation-reduction process; F:monooxygenase activity; F:heme binding |
| Efet.01.525220.g588.t1 | coup transcription | 573 | 1.38E-44 | 81.75 | C:nucleus; F:zinc ion binding; P:response to retinoic acid; F:RNA polymerase II transcription factor activity, ligand-activated sequence-specific DNA binding; P:transcription, DNA-templated; F:steroid hormone receptor activity; P:regulation of transcription, DNA-templated; F:sequence-specific DNA binding; P:steroid hormone mediated signaling pathway; P:intracellular receptor signaling pathway |
| Efet.01.525268.g589.t1 | uncharacterized protein LOC111197578 | 759 | 3.79E-18 | 49.67 |  |
| Efet.01.52531.g176.t1 | E3 ubiquitin- ligase Topors-like | 1956 | 7.24E-43 | 48.95 | F:zinc ion binding; F:metal ion binding |
| Efet.01.525310.g590.t1 | dachsous-like | 468 | 1.04E-52 | 68.7 | F:calcium ion binding; C:integral component of membrane; P:homophilic cell adhesion via plasma membrane adhesion molecules; C:plasma membrane |
| Efet.01.525310.g591.t1 | dachsous-like | 225 | 2.57E-14 | 62.2 | C:membrane |
| Efet.01.525325.g592.t1 | otoferlin isoform X2 | 369 | 1.07E-29 | 62.85 | C:membrane |
| Efet.01.525420.g594.t1 | Retrovirus-related Pol poly | 345 | 2.26E-11 | 56.55 | F:nucleic acid binding; P:DNA integration |
| Efet.01.52547.g177.t1 | two pore potassium channel sup-9-like | 891 | 2.05E-77 | 69.15 | P:potassium ion transmembrane transport; C:integral component of membrane; F:potassium channel activity |
| Efet.01.525567.g598.t1 | glutathione gamma-glutamylcysteinyltransferase | 543 | 3.34E-24 | 52.1 | P:phytochelatin biosynthetic process; F:glutathione gamma-glutamylcysteinyltransferase activity; F:metal ion binding; C:membrane; C:integral component of membrane; P:response to metal ion |
| Efet.01.525574.g599.t1 | hypothetical protein LOTGIDRAFT_123206, partial | 264 | 5.31E-06 | 62 |  |
| Efet.01.525686.g600.t1 | beta-2 adrenergic receptor-like | 1356 | 3.24E-123 | 66.75 | P:G-protein coupled receptor signaling pathway; C:integral component of membrane; P:serotonin receptor signaling pathway; F:G-protein coupled serotonin receptor activity |
| Efet.01.52571.g179.t1 | hypothetical protein CAPTEDRAFT_99765 | 309 | 8.49E-22 | 63.67 | C:membrane; P:transport |
| Efet.01.525779.g602.t1 | V-type proton ATPase 116 kDa subunit a isoform 1-like isoform X1 | 258 | 1.69E-23 | 70.05 | F:hydrogen ion transmembrane transporter activity; P:ATP hydrolysis coupled proton transport; C:integral component of membrane; C:proton-transporting V-type ATPase, V0 domain |
| Efet.01.526064.g603.t1 | glucarate dehydratase | 834 | 5.65E-153 | 76.2 | F:lyase activity |
| Efet.01.526064.g604.t1 | conserved hypothetical alanine-rich | 633 | 3.55E-123 | 45.75 |  |
| Efet.01.526064.g605.t1 | 3-methyl-2-oxobutanoate hydroxymethyltransferase | 414 | 8.30E-64 | 91.4 | P:pantothenate biosynthetic process; F:3-methyl-2-oxobutanoate hydroxymethyltransferase activity; C:cytoplasm; F:metal ion binding; F:methyltransferase activity; P:methylation |
| Efet.01.526119.g606.t1 | neurexin 3 isoform X11 | 348 | 1.83E-11 | 51.95 | P:synapse assembly; F:protein binding; C:membrane; C:protein complex; F:calcium channel regulator activity; P:neurotransmitter secretion |
| Efet.01.52612.g182.t1 | DC-STAMP domain-containing 2-like | 768 | 4.69E-33 | 51.5 | C:membrane |
| Efet.01.526202.g608.t1 | 3-isopropylmalate dehydratase | 1776 | 0 | 73.95 | F:hydro-lyase activity |
| Efet.01.526202.g609.t1 | family transcriptional regulator | 609 | 7.86E-132 | 66.25 | F:DNA binding; F:transcription factor activity, sequence-specific DNA binding; P:transcription, DNA-templated; P:regulation of transcription, DNA-templated |
| Efet.01.526202.g610.t1 | 3-isopropylmalate dehydratase small subunit | 582 | 2.38E-129 | 77.9 | P:leucine biosynthetic process; C:3-isopropylmalate dehydratase complex; F:3-isopropylmalate dehydratase activity; F:isomerase activity |
| Efet.01.526223.g611.t1 | alpha beta hydrolase | 462 | 1.84E-103 | 76.45 | C:membrane |
| Efet.01.526232.g612.t1 | sialin-like | 396 | 5.56E-24 | 69.2 | F:phosphate ion transmembrane transporter activity; P:excitatory postsynaptic potential; P:transmembrane transport; P:L-glutamate import; C:excitatory synapse; C:cerebellar mossy fiber; F:symporter activity; F:extracellular-glutamate-gated chloride channel activity; P:sequestering of neurotransmitter; P:long-term memory; P:sodium ion transport; C:presynaptic active zone; P:synaptic vesicle lumen acidification; C:synaptic vesicle membrane; P:synaptic transmission, glutamatergic |
| Efet.01.526276.g613.t1 | hypothetical protein HELRODRAFT_171005 | 2325 | 1.46E-06 | 85 | F:phosphorus-oxygen lyase activity; P:intracellular signal transduction; C:intracellular; P:cyclic nucleotide biosynthetic process |
| Efet.01.526314.g614.t1 | WD repeat-containing mio | 2700 | 0 | 61.5 | C:intracellular membrane-bounded organelle |
| Efet.01.526328.g615.t1 | Tyrosine- kinase | 288 | 1.20E-27 | 71.5 | F:nucleotide binding; F:protein tyrosine kinase activity; C:membrane; P:protein phosphorylation |
| Efet.01.52633.g183.t1 | prolactin-releasing peptide receptor-like | 720 | 4.46E-56 | 66.25 | F:G-protein coupled receptor activity; P:G-protein coupled receptor signaling pathway; C:integral component of membrane |
| Efet.01.52636.g184.t1 | lamin Dm0-like | 453 | 1.98E-25 | 74.8 | C:nuclear envelope; F:oxidoreductase activity; C:membrane; C:intermediate filament |
| Efet.01.526468.g616.t1 | D-amino acid dehydrogenase | 924 | 1.05E-162 | 78.8 | F:D-amino-acid dehydrogenase activity; P:oxidation-reduction process |
| Efet.01.526559.g617.t1 | hypothetical protein CAPTEDRAFT_208030 | 1236 | 4.39E-152 | 60 | F:RNA binding; P:immune system process; P:response to stimulus |
| Efet.01.526563.g618.t1 | calcium calmodulin-dependent 3 ,5 -cyclic nucleotide phosphodiesterase 1A-like isoform X3 | 273 | 2.01E-22 | 86.05 | F:metal ion binding; P:signal transduction; C:integral component of membrane; F:3',5'-cyclic-nucleotide phosphodiesterase activity |
| Efet.01.526612.g619.t1 | NT-3 growth factor | 597 | 2.55E-27 | 52.15 | F:transferase activity |
| Efet.01.52677.g186.t1 | beta-1,4-galactosyltransferase galt-1-like | 360 | 1.77E-17 | 61.4 | C:membrane; C:integral component of membrane |
| Efet.01.526798.g621.t1 | bactericidal permeability-increasing -like | 627 | 2.17E-24 | 48.55 | C:extracellular space; P:defense response to Gram-negative bacterium; P:innate immune response; F:lipid binding; P:acute-phase response; F:lipopolysaccharide binding |
| Efet.01.5268.g410.t1 | ribosomal S6 kinase alpha-5-like | 396 | 5.25E-48 | 82.45 | P:positive regulation of histone acetylation; P:histone H3-S28 phosphorylation; F:histone kinase activity (H3-S10 specific); P:positive regulation of transcription from RNA polymerase II promoter; P:histone H3-S10 phosphorylation; C:nucleoplasm; F:magnesium ion binding; F:ATP binding; F:mitogen-activated protein kinase p38 binding; C:cytoplasm; P:interleukin-1-mediated signaling pathway; P:positive regulation of histone phosphorylation; P:intracellular signal transduction |
| Efet.01.5268.g411.t1 | ribosomal S6 kinase alpha-5-like | 1212 | 2.59E-36 | 65.2 | F:nucleotide binding; F:ion binding; P:phosphorylation; F:protein serine/threonine kinase activity |
| Efet.01.52685.g188.t1 | Glutamine-dependent NAD(+) synthetase | 540 | 5.54E-84 | 82.05 | F:hydrolase activity, acting on carbon-nitrogen (but not peptide) bonds; F:NAD+ synthase (glutamine-hydrolyzing) activity; F:ATP binding; P:NAD biosynthetic process |
| Efet.01.526873.g622.t1 | IQ motif and SEC7 domain-containing 1 | 279 | 6.23E-18 | 66.95 | P:biological regulation |
| Efet.01.52691.g189.t1 | low-density lipo receptor | 306 | 7.77E-18 | 60.05 | P:receptor-mediated endocytosis involved in cholesterol transport; F:calcium ion binding; C:membrane; C:integral component of membrane; C:intracellular; F:hydrolase activity |
| Efet.01.526998.g623.t1 | DUF3291 domain-containing | 876 | 0 | 70.55 |  |
| Efet.01.527063.g627.t1 | hypothetical protein CAPTEDRAFT_93209, partial | 261 | 8.22E-08 | 66 |  |
| Efet.01.527451.g628.t1 | peptidase M48 | 1518 | 0 | 65.25 | F:peptidase activity |
| Efet.01.527451.g629.t1 | hypothetical protein CRN43_08900, partial | 1548 | 6.90E-169 | 52.3 | C:membrane; C:integral component of membrane |
| Efet.01.5275.g412.t1 | N-alpha-acetyltransferase 20 | 351 | 1.99E-62 | 87.2 | F:N-acetyltransferase activity |
| Efet.01.52751.g192.t1 | rhodopsin | 876 | 5.58E-97 | 68.3 | P:signal transduction; C:membrane; F:signaling receptor activity |
| Efet.01.527516.g630.t1 | hemicentin-1-like isoform X2 | 513 | 2.76E-18 | 62.1 | F:calcium ion binding; C:membrane; C:integral component of membrane |
| Efet.01.527555.g631.t1 | serine arginine repetitive matrix 1 | 771 | 4.67E-11 | 57.36 |  |
| Efet.01.52756.g193.t1 | AChain Structural Determinants For Improved Thermal Stability Of Designed Ankyrin Repeat s With A Redesigned C- Capping | 1107 | 1.18E-11 | 67.6 |  |
| Efet.01.52760.g194.t1 | sodium bicarbonate transporter 11 isoform X3 | 843 | 1.13E-75 | 69.8 | C:integral component of plasma membrane; P:regulation of intracellular pH; P:sodium ion transmembrane transport; F:inorganic anion exchanger activity; P:borate transmembrane transport |
| Efet.01.527608.g632.t1 | protocadherin beta-15- | 591 | 3.10E-19 | 52.1 | F:calcium ion binding; C:membrane; C:integral component of membrane; P:cell adhesion; P:homophilic cell adhesion via plasma membrane adhesion molecules; C:plasma membrane |
| Efet.01.527677.g633.t1 | prolactin-releasing peptide receptor-like | 828 | 8.38E-67 | 65.75 | F:G-protein coupled receptor activity; P:G-protein coupled receptor signaling pathway; C:integral component of membrane |
| Efet.01.527724.g634.t1 | hypothetical protein CAPTEDRAFT_218786 | 1254 | 1.45E-06 | 57 | P:intracellular signal transduction; C:intracellular |
| Efet.01.527774.g635.t1 | hypothetical protein CAPTEDRAFT_225483 | 249 | 4.35E-28 | 66.65 | C:integral component of membrane; F:carbohydrate binding |
| Efet.01.527797.g636.t1 | dynein heavy chain axonemal-like isoform X1 | 474 | 1.04E-58 | 78.6 | F:ATPase activity; F:ATP binding; F:microtubule motor activity; F:actin binding; C:dynein complex; P:phosphorylation; P:microtubule-based movement; F:kinase activity |
| Efet.01.527797.g637.t1 | dynein heavy chain axonemal-like isoform X1 | 444 | 2.20E-37 | 75.5 | F:ATPase activity; F:ATP binding; F:microtubule motor activity; F:actin binding; C:dynein complex; P:phosphorylation; P:microtubule-based movement; F:kinase activity |
| Efet.01.527809.g638.t1 | cGMP-dependent kinase 1-like | 291 | 1.35E-07 | 65 | F:kinase activity |
| Efet.01.52786.g196.t1 | partitioning defective 6 homolog beta-like | 912 | 4.05E-104 | 77.3 |  |
| Efet.01.528100.g640.t1 | transcription factor Sox-11-like isoform X2 | 1236 | 5.51E-14 | 96.1 |  |
| Efet.01.528129.g641.t1 | hypothetical protein CAPTEDRAFT_207686 | 906 | 3.03E-42 | 48.05 | F:carbohydrate binding |
| Efet.01.528131.g642.t1 | cholecystokinin receptor type A-like | 315 | 2.39E-07 | 72.33 | F:G-protein coupled receptor activity; P:G-protein coupled receptor signaling pathway; C:integral component of membrane |
| Efet.01.528348.g645.t1 | cytoglobin-2-like isoform X1 | 543 | 1.62E-29 | 53.1 | F:binding |
| Efet.01.528379.g646.t1 | transient receptor potential-gamma | 399 | 2.14E-51 | 77.6 | F:calcium channel activity; C:integral component of membrane; P:calcium ion transmembrane transport |
| Efet.01.528516.g647.t1 | cAMP-dependent kinase 9 | 255 | 4.45E-32 | 82.1 | F:ATP binding; F:protein serine/threonine kinase activity; P:protein phosphorylation |
| Efet.01.528560.g649.t1 | hypothetical protein CAPTEDRAFT_152410 | 201 | 6.37E-12 | 69 | F:oxidoreductase activity; F:binding |
| Efet.01.528680.g651.t1 | uncharacterized protein LOC110252048 | 408 | 3.74E-16 | 50.47 |  |
| Efet.01.528741.g654.t1 | signal recognition particle-docking | 1098 | 1.32E-145 | 92.8 | F:GTP binding; P:SRP-dependent cotranslational protein targeting to membrane; C:cytoplasm; F:GTPase activity; C:intrinsic component of plasma membrane |
| Efet.01.528741.g655.t1 | DUF1631 domain-containing | 1530 | 0 | 68.2 |  |
| Efet.01.528741.g656.t1 | energy-dependent translational throttle | 1662 | 0 | 97.35 | F:ATPase activity; F:ATP binding |
| Efet.01.528741.g657.t1 | RNA helicase | 576 | 1.92E-65 | 95.95 | F:nucleic acid binding; F:ATP binding; C:cytoplasm; P:ribosome assembly; F:ATP-dependent RNA helicase activity |
| Efet.01.528754.g658.t1 | tyramine receptor 1 | 1497 | 6.19E-90 | 52.8 | F:G-protein coupled receptor activity; P:G-protein coupled receptor signaling pathway; C:integral component of membrane |
| Efet.01.528798.g659.t1 | mitochondrial import inner membrane translocase subunit Tim8 | 249 | 1.07E-35 | 80.05 | F:metal ion binding; C:mitochondrion; C:membrane; P:protein transport; P:cation transmembrane transport; F:metal ion transmembrane transporter activity; P:metal ion transport |
| Efet.01.528809.g660.t1 | potassium voltage-gated channel eag | 243 | 1.41E-40 | 93.1 | F:phosphorelay sensor kinase activity; F:voltage-gated potassium channel activity; P:potassium ion transmembrane transport; P:phosphorelay signal transduction system; C:integral component of membrane; P:signal transduction by protein phosphorylation; C:intracellular |
| Efet.01.528809.g661.t1 | potassium voltage-gated channel subfamily H member 5- | 474 | 8.28E-28 | 82.3 | F:phosphorelay sensor kinase activity; C:integral component of plasma membrane; F:voltage-gated potassium channel activity; F:calmodulin binding; P:regulation of membrane potential; P:potassium ion transmembrane transport; P:phosphorelay signal transduction system; P:regulation of ion transmembrane transport; P:signal transduction by protein phosphorylation; C:intracellular; P:regulation of G2/M transition of mitotic cell cycle; C:cell surface |
| Efet.01.528832.g662.t1 | proton-associated sugar transporter A-like | 1725 | 6.40E-79 | 61.95 | P:transmembrane transport; C:membrane; C:integral component of membrane |
| Efet.01.52885.g199.t1 | BTB POZ and MATH domain-containing 1-like | 396 | 5.69E-13 | 55.45 |  |
| Efet.01.52888.g200.t1 | forkhead box D3 | 762 | 1.72E-45 | 84.95 | P:negative regulation of transcription from RNA polymerase II promoter; C:nucleus; P:in utero embryonic development; P:transcription, DNA-templated; F:sequence-specific DNA binding; P:positive regulation of transcription from RNA polymerase II promoter; F:RNA polymerase II transcription factor activity, sequence-specific DNA binding |
| Efet.01.528953.g663.t1 | polypeptide N-acetylgalactosaminyltransferase 1-like | 1602 | 5.30E-114 | 48.45 | P:protein O-linked glycosylation; C:membrane; C:cytoplasmic part; F:binding; F:transferase activity |
| Efet.01.528996.g666.t1 | P-loop containing nucleoside triphosphate hydrolase | 1302 | 6.65E-162 | 51.05 | C:membrane |
| Efet.01.529081.g667.t1 | transmembrane 151B-like | 408 | 3.23E-23 | 55.95 | C:membrane; C:integral component of membrane |
| Efet.01.529093.g668.t1 | Gamma-aminobutyric acid receptor subunit beta-like | 252 | 2.33E-39 | 89.15 | P:signal transduction; P:ion transmembrane transport; C:integral component of membrane; F:GABA-A receptor activity; C:cell junction; F:extracellular ligand-gated ion channel activity; C:synapse; C:plasma membrane |
| Efet.01.529096.g669.t1 | Serine threonine- kinase | 1632 | 1.29E-65 | 80.6 | F:ATP binding; P:mitotic nuclear division; P:hippo signaling; P:protein phosphorylation; C:intracellular; F:protein kinase C activity |
| Efet.01.529116.g670.t1 | uncharacterized protein LOC111120670 | 1071 | 8.46E-113 | 62.8 | P:intracellular signal transduction; C:intracellular |
| Efet.01.529125.g671.t1 | WYL domain-containing | 573 | 4.94E-54 | 72.85 | F:DNA binding |
| Efet.01.529136.g672.t1 | Xaa-Pro aminopeptidase | 792 | 2.48E-170 | 79.55 | P:proteolysis; F:aminopeptidase activity; F:manganese ion binding |
| Efet.01.529153.g673.t1 | CBS domain-containing | 204 | 6.70E-09 | 67.4 | F:catalytic activity |
| Efet.01.529195.g674.t1 | ADAMTS 1 isoform X1 | 909 | 6.16E-18 | 46.05 | F:zinc ion binding; P:proteolysis; F:peptidase activity; C:proteinaceous extracellular matrix; F:metallopeptidase activity |
| Efet.01.529220.g675.t1 | NAD(P) FAD-dependent oxidoreductase | 819 | 1.09E-152 | 67.95 | F:oxidoreductase activity; P:oxidation-reduction process |
| Efet.01.529220.g676.t1 | cyclase | 498 | 5.88E-115 | 89.15 | F:arylformamidase activity; P:tryptophan catabolic process to kynurenine |
| Efet.01.529220.g677.t1 | Indolepyruvate ferredoxin oxidoreductase | 870 | 0 | 88.95 | F:thiamine pyrophosphate binding; F:indolepyruvate ferredoxin oxidoreductase activity; P:oxidation-reduction process |
| Efet.01.52926.g203.t1 | hypothetical protein CAPTEDRAFT_185637 | 3084 | 0 | 50.85 | C:membrane; C:integral component of membrane |
| Efet.01.529298.g679.t1 | 2-aminoethanethiol dioxygenase-like | 765 | 4.05E-46 | 53.1 | F:dioxygenase activity; F:oxidoreductase activity, acting on single donors with incorporation of molecular oxygen, incorporation of two atoms of oxygen; C:mitochondrion; P:oxidation-reduction process |
| Efet.01.529311.g680.t1 | E3 ubiquitin- ligase BRE1B | 240 | 5.80E-23 | 89.1 | F:zinc ion binding; F:ligase activity |
| Efet.01.52933.g204.t1 | E3 ubiquitin- ligase PDZRN3-like | 951 | 1.91E-20 | 57.65 |  |
| Efet.01.529331.g681.t1 | hypothetical protein CAPTEDRAFT_181469 | 567 | 5.06E-22 | 45.75 | F:ion channel activity; P:ion transport; C:postsynaptic membrane; P:ion transmembrane transport; C:membrane; P:cation transmembrane transport; C:integral component of membrane; F:extracellular ligand-gated ion channel activity; C:cell junction; C:synapse; P:transport; C:plasma membrane |
| Efet.01.529344.g682.t1 | Retrovirus-related Pol poly from transposon | 366 | 1.53E-28 | 63.45 |  |
| Efet.01.529348.g683.t1 | copine-3 isoform X1 | 471 | 1.39E-79 | 79.05 | F:calcium-dependent protein binding; F:protein serine/threonine kinase activity; P:protein phosphorylation; C:plasma membrane; F:calcium-dependent phospholipid binding; F:RNA binding; C:nucleus; C:extracellular exosome; C:focal adhesion; P:cellular response to calcium ion; P:cellular response to growth factor stimulus; C:cytosol; F:receptor tyrosine kinase binding; P:ERBB2 signaling pathway; P:positive regulation of cell migration |
| Efet.01.529496.g684.t1 | G- coupled receptor 84 | 282 | 2.56E-22 | 65.8 | C:integral component of plasma membrane; F:G-protein coupled peptide receptor activity; P:neuropeptide signaling pathway; P:phospholipase C-activating G-protein coupled receptor signaling pathway |
| Efet.01.529529.g687.t1 | succinate dehydrogenase iron-sulfur subunit | 495 | 2.15E-113 | 91.5 | F:succinate dehydrogenase (ubiquinone) activity; F:metal ion binding; F:electron carrier activity; F:3 iron, 4 sulfur cluster binding; P:tricarboxylic acid cycle; F:4 iron, 4 sulfur cluster binding; F:2 iron, 2 sulfur cluster binding |
| Efet.01.529529.g688.t1 | succinate dehydrogenase flavo subunit | 1545 | 0 | 72.7 | P:generation of precursor metabolites and energy; F:oxidoreductase activity, acting on the CH-CH group of donors; P:oxidation-reduction process; P:single-organism cellular process |
| Efet.01.529529.g689.t1 | succinate cytochrome b556 subunit | 465 | 1.28E-68 | 91.75 | C:succinate dehydrogenase complex; C:integral component of membrane; P:tricarboxylic acid cycle; F:succinate dehydrogenase activity |
| Efet.01.52954.g205.t1 | ubiquitin carboxyl-terminal hydrolase 31-like | 1542 | 1.96E-71 | 52.4 | P:protein deubiquitination; F:thiol-dependent ubiquitinyl hydrolase activity; P:ubiquitin-dependent protein catabolic process; F:hydrolase activity |
| Efet.01.529558.g690.t1 | ATP-dependent DNA helicase | 1587 | 0 | 71.35 | F:ATP binding |
| Efet.01.529578.g692.t1 | hypothetical protein CAPTEDRAFT_108085, partial | 1395 | 1.00E-11 | 45.67 |  |
| Efet.01.52967.g206.t1 | cytochrome P450 family | 318 | 5.33E-38 | 73.2 | F:oxidoreductase activity; F:metal ion binding; C:membrane |
| Efet.01.529764.g693.t1 | SZT2-like isoform X1 | 924 | 8.23E-53 | 57.5 | C:peroxisome; F:galactosylceramide sulfotransferase activity; C:integral component of membrane; P:glycolipid biosynthetic process; C:Golgi apparatus |
| Efet.01.52981.g207.t1 | WD repeat-containing 7 | 843 | 3.11E-38 | 57.65 |  |
| Efet.01.52984.g208.t1 | PREDICTED: uncharacterized protein KIAA0825-like | 486 | 5.03E-15 | 55.3 |  |
| Efet.01.530060.g3.t1 | somatostatin receptor type 2-like | 273 | 7.53E-10 | 63.2 | F:G-protein coupled peptide receptor activity; P:G-protein coupled receptor signaling pathway; C:integral component of membrane |
| Efet.01.530098.g4.t1 | voltage-dependent T-type calcium channel subunit alpha-1H-like | 471 | 1.47E-12 | 50.67 | F:ion channel activity; P:ion transport |
| Efet.01.530141.g5.t1 | adenylate cyclase type 6 | 855 | 8.17E-32 | 58.55 | P:single-organism process; P:cellular process |
| Efet.01.530151.g6.t1 | A-kinase anchor mitochondrial-like | 258 | 2.75E-21 | 66.15 | F:binding |
| Efet.01.53017.g209.t1 | microtubule-associated futsch | 1644 | 2.88E-52 | 78.25 | P:microtubule cytoskeleton organization; C:microtubule; F:microtubule binding |
| Efet.01.530307.g8.t1 | EGF-like domain containing | 219 | 2.54E-23 | 63.85 | C:membrane; P:cell adhesion |
| Efet.01.530327.g9.t1 | hypothetical protein CAPTEDRAFT_196447 | 360 | 1.01E-06 | 48 |  |
| Efet.01.53041.g211.t1 | titin-like isoform X1 | 1254 | 9.07E-23 | 43.5 |  |
| Efet.01.530517.g13.t1 | trichohyalin-like isoform X1 | 471 | 5.87E-49 | 70.15 |  |
| Efet.01.530573.g14.t1 | 3-oxoacyl-ACP reductase | 900 | 5.24E-121 | 88.35 | F:oxidoreductase activity; P:oxidation-reduction process |
| Efet.01.530573.g15.t1 | benzoyl- 2,3-epoxidase subunit | 546 | 4.92E-102 | 95.8 | F:metal ion binding; P:oxidation-reduction process; F:4 iron, 4 sulfur cluster binding; F:ferredoxin-NADP+ reductase activity |
| Efet.01.530573.g16.t1 | benzoyl- oxygenase subunit B | 1506 | 0 | 92 | F:oxidoreductase activity; P:oxidation-reduction process |
| Efet.01.530573.g17.t1 | benzoyl- -dihydrodiol lyase | 1722 | 0 | 90.85 | P:metabolic process; F:enoyl-CoA hydratase activity |
| Efet.01.530573.g18.t1 | transcriptional regulator | 981 | 0 | 91.4 | F:magnesium ion binding; F:shikimate kinase activity; F:ATP binding; P:aromatic amino acid family biosynthetic process; C:cytoplasm; P:chorismate biosynthetic process; F:sequence-specific DNA binding; P:phosphorylation |
| Efet.01.530573.g19.t1 | benzoate- ligase family | 1569 | 0 | 89.9 | F:ATP binding; F:benzoate-CoA ligase activity; P:metabolic process |
| Efet.01.530573.g20.t1 | twin-arginine translocation pathway signal | 636 | 6.33E-116 | 85.6 | C:outer membrane-bounded periplasmic space |
| Efet.01.530573.g21.t1 | twin-arginine translocation pathway signal | 372 | 3.91E-67 | 89.15 | C:outer membrane-bounded periplasmic space |
| Efet.01.530574.g22.t1 | 3-oxoadipyl- thiolase | 354 | 1.71E-72 | 96.95 | F:acetyl-CoA C-acyltransferase activity; P:3,4-dihydroxybenzoate catabolic process |
| Efet.01.530574.g23.t1 | threonine dehydratase | 1710 | 0 | 92.65 | P:isoleucine biosynthetic process; F:pyridoxal phosphate binding; F:L-threonine ammonia-lyase activity |
| Efet.01.530574.g24.t1 | excinuclease ABC subunit A | 4155 | 0 | 86.6 | F:ATPase activity; F:DNA binding; F:ATP binding; P:nucleic acid phosphodiester bond hydrolysis; F:excinuclease ABC activity; C:excinuclease repair complex; C:cytoplasm; F:metal ion binding; P:nucleotide-excision repair |
| Efet.01.530627.g25.t1 | family transcriptional regulator | 492 | 1.08E-56 | 75.9 | F:DNA binding; F:transcription factor activity, sequence-specific DNA binding; P:transcription, DNA-templated; P:regulation of transcription, DNA-templated |
| Efet.01.530744.g27.t1 | galactose-1-phosphate uridylyltransferase | 267 | 1.37E-32 | 93.2 | F:zinc ion binding; P:galactose metabolic process; F:UDP-glucose:hexose-1-phosphate uridylyltransferase activity |
| Efet.01.530863.g29.t1 | hypothetical protein B5766_09670 | 912 | 0 | 53.95 |  |
| Efet.01.530881.g30.t1 | tripartite tricarboxylate transporter substrate binding | 942 | 0 | 83.7 | C:outer membrane-bounded periplasmic space |
| Efet.01.530916.g31.t1 | adenosylhomocysteinase | 459 | 9.15E-85 | 93.05 | C:cytoplasm; F:adenosylhomocysteinase activity; P:one-carbon metabolic process |
| Efet.01.530991.g32.t1 | sex peptide receptor-like | 1125 | 3.01E-139 | 65.2 | F:G-protein coupled peptide receptor activity; P:G-protein coupled receptor signaling pathway; C:integral component of membrane |
| Efet.01.531003.g33.t1 | visual pigment-like receptor peropsin | 2871 | 5.04E-29 | 49.55 | P:signal transduction; C:membrane; F:signaling receptor activity |
| Efet.01.53101.g212.t1 | 5-hydroxytryptamine receptor 1A-alpha-like | 840 | 1.68E-37 | 52.55 | P:signal transduction; C:membrane; F:signal transducer activity |
| Efet.01.531010.g34.t1 | interference hedgehog-like isoform X1 | 786 | 5.61E-22 | 55.9 | C:membrane; C:integral component of membrane |
| Efet.01.531034.g35.t1 | LIM homeobox transcription factor 1-alpha-like | 222 | 4.55E-06 | 59 |  |
| Efet.01.531039.g36.t1 | Potassium channel subfamily K member | 936 | 1.52E-98 | 65.15 | P:transport |
| Efet.01.531107.g37.t1 | hypothetical protein CAPTEDRAFT_204666, partial | 342 | 3.33E-09 | 85.67 | C:integral component of membrane |
| Efet.01.531113.g38.t1 | misato homolog 1-like | 258 | 1.68E-07 | 62.5 |  |
| Efet.01.531129.g39.t1 | Tetratricopeptide repeat 28 | 4314 | 5.47E-61 | 48.85 |  |
| Efet.01.531211.g41.t1 | somatostatin receptor type 2-like | 630 | 5.81E-54 | 66.95 | F:G-protein coupled receptor activity; P:G-protein coupled receptor signaling pathway |
| Efet.01.531365.g43.t1 | 28S ribosomal mitochondrial | 939 | 1.98E-30 | 52.33 | F:structural constituent of ribosome; C:mitochondrion; C:ribosome; P:translation |
| Efet.01.531365.g44.t1 | hypothetical protein CAPTEDRAFT_192093, partial | 684 | 1.70E-09 | 81 | F:metal ion binding |
| Efet.01.531405.g45.t1 | ABC transporter ATP-binding | 384 | 5.37E-59 | 69.9 | F:nucleotide binding; F:ATPase activity; F:ATP binding |
| Efet.01.531412.g46.t1 | recombinase family | 438 | 3.00E-48 | 88 | F:DNA binding; F:recombinase activity; P:DNA recombination |
| Efet.01.531427.g47.t1 | soluble calcium-activated nucleotidase 1 isoform X2 | 348 | 8.54E-43 | 79.45 | F:calcium ion binding; C:integral component of membrane; F:pyrophosphatase activity |
| Efet.01.53144.g213.t1 | turtle isoform X2 | 483 | 7.73E-31 | 61.75 | C:membrane; C:integral component of membrane |
| Efet.01.531474.g48.t1 | ADP-ribosylation factor 4C | 534 | 1.31E-76 | 77.75 | F:GTP binding; P:small GTPase mediated signal transduction; C:intracellular |
| Efet.01.531542.g49.t1 | cytochrome c oxidase subunit mitochondrial-like | 267 | 5.68E-46 | 88.1 | F:cytochrome-c oxidase activity; P:hydrogen ion transmembrane transport; C:mitochondrial inner membrane |
| Efet.01.531592.g51.t1 | tektin-3-like isoform X1 | 354 | 8.29E-22 | 72 |  |
| Efet.01.53167.g214.t1 | Retrovirus-related Pol poly from transposon | 408 | 1.64E-35 | 62.9 | F:nucleic acid binding; F:zinc ion binding; F:aspartic-type endopeptidase activity; F:metal ion binding; P:proteolysis; P:DNA integration |
| Efet.01.531896.g54.t1 | helicase with zinc finger domain 2-like isoform X3 | 507 | 7.58E-39 | 61.5 | F:binding |
| Efet.01.5319.g417.t1 | hypothetical protein CAPTEDRAFT_226868 | 765 | 3.46E-09 | 64 | P:signal transduction; F:signal transducer activity |
| Efet.01.531999.g55.t1 | tyrosine--tRNA ligase | 549 | 2.54E-97 | 84.6 | F:tyrosine-tRNA ligase activity; F:ATP binding; P:tyrosyl-tRNA aminoacylation; F:RNA binding; C:cytoplasm |
| Efet.01.53205.g215.t1 | GRB2-associated and regulator of MAPK 2-like isoform X3 | 639 | 1.69E-13 | 59.65 |  |
| Efet.01.532050.g59.t1 | ABC transporter ATP-binding | 330 | 1.22E-57 | 82.6 | F:ATPase activity; F:ATP binding |
| Efet.01.532098.g60.t1 | filamin-A-like isoform X1 | 534 | 1.60E-72 | 76.15 | F:actin filament binding |
| Efet.01.532116.g61.t1 | nuclear receptor-binding factor 2-like | 432 | 8.91E-16 | 51 | P:autophagy |
| Efet.01.532148.g63.t1 | 3 -5 exonuclease | 348 | 1.55E-23 | 97.5 | F:nucleic acid binding; F:hydrolase activity |
| Efet.01.532148.g64.t1 | DNA polymerase III subunit epsilon | 438 | 5.02E-92 | 75.8 | F:nucleic acid binding; P:nucleic acid phosphodiester bond hydrolysis; F:exonuclease activity |
| Efet.01.532148.g65.t1 | DUF1902 domain-containing | 477 | 4.68E-35 | 75.1 |  |
| Efet.01.532148.g66.t1 | hypothetical protein | 249 | 1.23E-33 | 87.33 |  |
| Efet.01.532166.g67.t1 | somatostatin receptor type 5-like | 1428 | 7.45E-99 | 60.55 | F:G-protein coupled receptor activity; P:G-protein coupled receptor signaling pathway |
| Efet.01.532173.g69.t1 | ATP-binding cassette sub-family C member 9 | 501 | 4.72E-17 | 64.95 | C:ATP-sensitive potassium channel complex; F:sulfonylurea receptor activity; F:identical protein binding; P:potassium ion import; F:potassium channel regulator activity |
| Efet.01.532190.g70.t1 | tumor p73-like | 348 | 2.62E-21 | 66 | C:nucleus; F:transcription factor activity, sequence-specific DNA binding; P:protein tetramerization; P:apoptotic process; F:transcription regulatory region DNA binding; P:regulation of transcription, DNA-templated |
| Efet.01.53220.g216.t1 | hypothetical protein CAPTEDRAFT_229175 | 2124 | 3.56E-25 | 47 |  |
| Efet.01.53225.g217.t1 | plectin-like isoform X4 | 783 | 1.15E-73 | 63.05 | C:cytoskeleton |
| Efet.01.532340.g71.t1 | syntaxin-18 isoform X2 | 450 | 7.89E-12 | 72.7 | C:endoplasmic reticulum membrane; F:protein binding; P:retrograde vesicle-mediated transport, Golgi to ER; P:endoplasmic reticulum membrane organization; P:protein transport; C:SNARE complex; P:positive regulation of organelle assembly; P:positive regulation of ER to Golgi vesicle-mediated transport; C:Golgi apparatus; P:regulation of Golgi organization |
| Efet.01.532488.g73.t1 | glycosylasparaginase | 810 | 4.06E-153 | 80.9 | F:N4-(beta-N-acetylglucosaminyl)-L-asparaginase activity |
| Efet.01.532488.g74.t1 | family transcriptional regulator | 690 | 2.62E-151 | 63.45 | F:DNA binding; F:transcription factor activity, sequence-specific DNA binding; P:transcription, DNA-templated; P:regulation of transcription, DNA-templated |
| Efet.01.53253.g219.t1 | Sonic hedgehog | 204 | 2.09E-30 | 90.95 | C:extracellular space; P:intein-mediated protein splicing; P:multicellular organism development; C:integral component of membrane; P:cell-cell signaling; F:peptidase activity; C:plasma membrane |
| Efet.01.532534.g75.t1 | Neuropeptide FF receptor 2 | 675 | 3.11E-60 | 65.5 | F:neuropeptide receptor activity; P:G-protein coupled receptor signaling pathway; C:membrane |
| Efet.01.532551.g76.t1 | glycine N-methyltransferase | 210 | 8.93E-35 | 89.75 | F:glycine N-methyltransferase activity; P:methylation |
| Efet.01.532577.g78.t1 | sarcoplasmic calcium-binding -like | 330 | 1.03E-08 | 67.5 | F:calcium ion binding |
| Efet.01.532591.g79.t1 | hypothetical protein B5766_07985 | 225 | 1.32E-45 | 100 |  |
| Efet.01.532597.g80.t1 | mpv17 2 | 621 | 1.32E-47 | 59.45 | C:membrane; C:integral component of membrane |
| Efet.01.5326.g418.t1 | protocadherin beta-10-like isoform X1 | 2325 | 1.18E-174 | 56.1 | F:calcium ion binding; C:membrane; C:integral component of membrane; P:cell adhesion; P:homophilic cell adhesion via plasma membrane adhesion molecules; C:plasma membrane |
| Efet.01.532658.g83.t1 | innexin 1 | 1395 | 1.19E-153 | 61.35 | C:membrane; P:cell communication by electrical coupling; C:synapse; F:gap junction channel activity; P:transport |
| Efet.01.532667.g84.t1 | sterile alpha and TIR motif-containing 1-like isoform X10 | 1494 | 1.00E-117 | 70 | P:signal transduction |
| Efet.01.532811.g86.t1 | cyclin-dependent kinase 5 activator 1 | 1131 | 1.58E-72 | 79.25 | P:positive regulation of protein serine/threonine kinase activity; C:cyclin-dependent protein kinase 5 holoenzyme complex; F:cyclin-dependent protein kinase 5 activator activity |
| Efet.01.532816.g87.t1 | DNA repair XRCC2 | 1119 | 1.27E-56 | 55.9 | F:DNA binding; P:DNA recombination; P:response to stimulus |
| Efet.01.532831.g88.t1 | gastrula zinc finger -like | 1674 | 2.22E-99 | 57.1 | F:nucleic acid binding; F:transcription factor activity, sequence-specific DNA binding; F:metal ion binding |
| Efet.01.532868.g90.t1 | atlastin isoform X4 | 399 | 3.24E-27 | 63.75 |  |
| Efet.01.53289.g220.t1 | tether containing UBX domain for GLUT4 | 201 | 4.40E-11 | 66.9 | P:signal transduction; F:signal transducer activity, downstream of receptor |
| Efet.01.532920.g91.t1 | DUF1804 domain-containing | 378 | 6.67E-29 | 64.4 | F:ATP binding; P:viral capsid assembly |
| Efet.01.532983.g93.t1 | neural-cadherin 2 | 687 | 5.67E-68 | 64.75 | F:calcium ion binding; C:integral component of membrane; P:homophilic cell adhesion via plasma membrane adhesion molecules; C:plasma membrane |
| Efet.01.532989.g94.t1 | 2-hydroxyacylsphingosine 1-beta-galactosyltransferase-like | 1416 | 3.34E-88 | 53.85 | F:transferase activity |
| Efet.01.532993.g95.t1 | matrix metallo ase-2-like | 342 | 7.41E-18 | 70.75 | F:metal ion binding; F:metallopeptidase activity |
| Efet.01.532996.g100.t1 | pilus assembly | 2529 | 0 | 61.2 |  |
| Efet.01.532996.g101.t1 | pilus assembly | 654 | 1.33E-132 | 70 |  |
| Efet.01.532996.g102.t1 | pilus assembly | 1050 | 0 | 64.4 |  |
| Efet.01.532996.g96.t1 | enamine deaminase | 321 | 1.33E-71 | 91.85 |  |
| Efet.01.532996.g97.t1 | pilus assembly | 612 | 1.87E-124 | 66.8 | F:protein transporter activity; P:protein secretion by the type II secretion system; C:type II protein secretion system complex |
| Efet.01.532996.g98.t1 | pilus assembly | 795 | 1.78E-101 | 61.8 | C:integral component of membrane; P:type IV pilus biogenesis |
| Efet.01.532996.g99.t1 | pilus assembly | 765 | 5.55E-177 | 67.45 | C:integral component of membrane |
| Efet.01.533093.g104.t1 | cadherin domain | 879 | 7.45E-14 | 54.35 | F:calcium ion binding; C:membrane; C:integral component of membrane; P:cell adhesion; P:homophilic cell adhesion via plasma membrane adhesion molecules; C:plasma membrane |
| Efet.01.53310.g223.t1 | sodium channel type 4 subunit alpha B-like isoform X1 | 1101 | 2.46E-12 | 69.55 | P:biological regulation; P:sodium ion transport; P:ion transmembrane transport; F:sodium channel activity; C:integral component of membrane; F:voltage-gated ion channel activity |
| Efet.01.533149.g105.t1 | acyl- dehydrogenase | 1437 | 0 | 78.5 | F:acyl-CoA dehydrogenase activity; F:flavin adenine dinucleotide binding; P:oxidation-reduction process |
| Efet.01.533149.g106.t1 | 3-oxoadipate -transferase | 246 | 6.54E-33 | 73.95 | P:metabolic process; F:acetate CoA-transferase activity; F:3-oxoadipate CoA-transferase activity |
| Efet.01.533149.g107.t1 | 3-alpha,7-alpha,12-alpha-trihydroxy-5-beta-cholest-24-enoyl- hydratase | 552 | 2.51E-82 | 75.15 |  |
| Efet.01.533149.g108.t1 | 3-hydroxyacyl- dehydrogenase | 843 | 0 | 87.95 | F:oxidoreductase activity; P:oxidation-reduction process |
| Efet.01.533149.g109.t1 | thiolase | 651 | 6.10E-119 | 76.55 | F:transferase activity, transferring acyl groups other than amino-acyl groups; P:metabolic process |
| Efet.01.533149.g110.t1 | tripartite tricarboxylate transporter substrate binding | 915 | 0 | 81.75 | C:outer membrane-bounded periplasmic space |
| Efet.01.533149.g111.t1 | chloride channel | 270 | 2.71E-33 | 93.25 | P:regulation of anion transmembrane transport; P:ion transmembrane transport; C:integral component of membrane; P:chloride transport; C:plasma membrane; F:voltage-gated chloride channel activity |
| Efet.01.533149.g112.t1 | transposase | 309 | 2.25E-10 | 87.9 | P:transport |
| Efet.01.533149.g113.t1 | acetyl- carboxylase carboxyltransferase subunit | 1650 | 0 | 82.2 | F:propionyl-CoA carboxylase activity; F:methylcrotonoyl-CoA carboxylase activity; F:transferase activity |
| Efet.01.533149.g114.t1 | tripartite tricarboxylate transporter substrate binding | 612 | 1.31E-111 | 68.5 | C:outer membrane-bounded periplasmic space |
| Efet.01.533203.g115.t1 | eukaryotic translation initiation factor 2D isoform X2 | 483 | 1.33E-35 | 63.15 | F:translation initiation factor activity; F:RNA binding; P:translational initiation |
| Efet.01.533219.g116.t1 | AChain Quisqualate Bound To The D655a Mutant Of The Ligand Binding Domain Of Glua3 | 495 | 1.33E-37 | 58.05 | C:AMPA glutamate receptor complex; C:postsynaptic density; F:identical protein binding; F:kainate selective glutamate receptor activity; C:neuronal cell body; C:protein complex; F:AMPA glutamate receptor activity; P:establishment of protein localization; P:ion transport; C:dendritic shaft; P:protein tetramerization; C:dendritic spine; F:PDZ domain binding; P:response to lithium ion; C:kainate selective glutamate receptor complex; P:positive regulation of synaptic transmission, glutamatergic; P:receptor internalization; P:regulation of receptor recycling |
| Efet.01.533220.g117.t1 | aldehyde dehydrogenase family 8 member A1 isoform X1 | 228 | 1.75E-06 | 74.44 | F:oxidoreductase activity; P:metabolic process; P:oxidation-reduction process; F:oxidoreductase activity, acting on the aldehyde or oxo group of donors, NAD or NADP as acceptor |
| Efet.01.53324.g224.t1 | FMRFamide receptor-like | 729 | 2.97E-44 | 56.7 | F:G-protein coupled receptor activity; P:G-protein coupled receptor signaling pathway; P:cell communication; F:calcium ion binding; C:membrane; C:integral component of membrane |
| Efet.01.533247.g118.t1 | dynein gamma flagellar outer arm-like | 222 | 1.80E-18 | 69.85 | F:nucleotide binding |
| Efet.01.533278.g119.t1 | dynein heavy chain axonemal | 435 | 2.25E-30 | 71.1 | F:nucleotide binding; P:movement of cell or subcellular component; C:dynein complex; F:nucleoside-triphosphatase activity |
| Efet.01.533365.g122.t1 | Tyrosine- kinase transmembrane receptor Ror2 | 243 | 2.18E-07 | 50.8 | P:sodium ion transport; P:peptidyl-tyrosine phosphorylation; F:ATP binding; F:protein tyrosine kinase activity; P:ion transport; F:sodium channel activity; C:membrane; C:integral component of membrane; P:sodium ion transmembrane transport; F:protein kinase activity; P:protein phosphorylation; P:transport |
| Efet.01.533383.g123.t1 | family transporter | 705 | 1.42E-112 | 74.55 | C:integral component of membrane |
| Efet.01.533383.g124.t1 | serine threonine- kinase | 825 | 0 | 67.8 | P:phosphorylation; F:kinase activity |
| Efet.01.533383.g125.t1 | transcriptional regulator | 471 | 1.08E-112 | 95.15 | F:DNA binding; F:zinc ion binding; C:cytoplasm; P:transcription, DNA-templated; P:positive regulation of transcription, DNA-templated; P:bacterial-type flagellum organization; P:regulation of bacterial-type flagellum assembly |
| Efet.01.533383.g126.t1 | flagellar transcriptional regulator | 351 | 4.59E-77 | 86.65 | F:DNA binding; P:bacterial-type flagellum assembly; C:cytoplasm; P:transcription, DNA-templated; P:positive regulation of transcription, DNA-templated; P:regulation of bacterial-type flagellum assembly |
| Efet.01.533383.g127.t1 | D-aminoacylase | 1236 | 0 | 90.1 | F:N-acyl-D-amino-acid deacylase activity |
| Efet.01.533383.g128.t1 | peptide ABC transporter substrate-binding | 987 | 1.43E-163 | 93.35 | F:ATPase activity; F:ATP binding; P:peptide transport |
| Efet.01.533383.g129.t1 | ABC transporter ATP-binding | 999 | 0 | 88.15 | F:ATPase activity; F:ATP binding; P:peptide transport |
| Efet.01.533495.g130.t1 | DUF4190 domain-containing | 558 | 6.49E-102 | 65.93 | C:membrane; C:integral component of membrane |
| Efet.01.533528.g131.t1 | neuroligin- Y-linked-like | 546 | 1.94E-42 | 66.65 | C:membrane; C:integral component of membrane |
| Efet.01.53353.g226.t1 | corticotropin-releasing factor receptor 2-like | 462 | 6.92E-26 | 46.85 | F:G-protein coupled receptor activity; F:transmembrane signaling receptor activity; P:G-protein coupled receptor signaling pathway; P:signal transduction; C:membrane; C:integral component of membrane; P:cell surface receptor signaling pathway; F:signal transducer activity |
| Efet.01.533609.g132.t1 | energy-dependent translational throttle | 417 | 1.70E-28 | 84.7 | F:ATPase activity; F:ATP binding |
| Efet.01.533610.g133.t1 | pre translocase subunit | 240 | 1.25E-20 | 86.85 | F:DNA binding; F:ATP binding; F:zinc ion binding; F:ATP-dependent DNA helicase activity; P:DNA unwinding involved in DNA replication; C:primosome complex; P:DNA replication, synthesis of RNA primer |
| Efet.01.533616.g135.t1 | hypothetical protein HELRODRAFT_133020, partial | 768 | 9.11E-18 | 71 | F:metal ion binding |
| Efet.01.533790.g136.t1 | probable palmitoyltransferase ZDHHC24 | 633 | 1.92E-81 | 64.65 | F:transferase activity, transferring acyl groups; F:metal ion binding; C:membrane |
| Efet.01.533832.g138.t1 | ankyrin repeat | 438 | 1.82E-35 | 58.9 | F:zinc ion binding; F:metal ion binding |
| Efet.01.533836.g139.t1 | D-alanyl-D-alanine carboxypeptidase | 399 | 1.57E-62 | 78.45 | F:serine-type D-Ala-D-Ala carboxypeptidase activity; C:membrane; P:proteolysis |
| Efet.01.533904.g140.t1 | isochorismatase domain-containing mitochondrial-like | 213 | 4.58E-23 | 82.75 | F:catalytic activity; P:metabolic process |
| Efet.01.533919.g141.t1 | WASH complex subunit FAM21C-like | 543 | 6.83E-08 | 73.65 | C:WASH complex |
| Efet.01.534125.g144.t1 | 17-beta-hydroxysteroid dehydrogenase 13-like isoform X1 | 360 | 8.75E-19 | 67.05 | C:membrane |
| Efet.01.534227.g145.t1 | solute carrier family 25 member 44-like | 723 | 1.17E-62 | 63.75 | P:transmembrane transport; C:membrane; C:integral component of membrane; P:transport |
| Efet.01.534400.g147.t1 | SPATA45 homolog | 462 | 2.29E-07 | 60.5 |  |
| Efet.01.53441.g229.t1 | zinc finger 40 isoform X3 | 924 | 1.78E-49 | 69.35 | F:RNA polymerase II distal enhancer sequence-specific DNA binding; P:negative regulation of transcription from RNA polymerase II promoter; C:nucleus; F:zinc ion binding; F:HMG box domain binding; P:signal transduction; P:multicellular organism development; C:mitochondrion; P:transcription from RNA polymerase II promoter; F:transcriptional repressor activity, RNA polymerase II distal enhancer sequence-specific binding |
| Efet.01.534710.g149.t1 | hypothetical protein CAPTEDRAFT_187752 | 450 | 2.10E-10 | 62 | F:zinc ion binding; F:metal ion binding; C:intracellular |
| Efet.01.534728.g150.t1 | octopamine receptor 2-like | 1794 | 3.81E-101 | 65.35 | F:G-protein coupled receptor activity; P:G-protein coupled receptor signaling pathway; C:integral component of membrane |
| Efet.01.534784.g152.t1 | G T mismatch-specific thymine DNA glycosylase | 825 | 2.52E-19 | 80.65 | C:nucleus; F:DNA binding; F:uracil DNA N-glycosylase activity; P:base-excision repair, AP site formation; F:pyrimidine-specific mismatch base pair DNA N-glycosylase activity |
| Efet.01.53479.g231.t1 | NADH dehydrogenase [ubiquinone] iron-sulfur mitochondrial | 369 | 5.67E-46 | 68.9 | C:mitochondrion; C:membrane; P:oxidation-reduction process; P:cellular metabolic process |
| Efet.01.534802.g153.t1 | 2-hydroxyacylsphingosine 1-beta-galactosyltransferase isoform X2 | 966 | 3.87E-42 | 54.1 | F:transferase activity |
| Efet.01.534802.g154.t1 | UDP-glucuronosyltransferase 2C1-like isoform X1 | 429 | 3.78E-27 | 66.25 | F:transferase activity, transferring hexosyl groups; C:membrane |
| Efet.01.534821.g155.t1 | hypothetical protein CAPTEDRAFT_187072 | 1032 | 1.16E-19 | 46.38 | F:G-protein coupled receptor activity; F:G-protein coupled peptide receptor activity; P:G-protein coupled receptor signaling pathway; C:membrane; C:integral component of membrane |
| Efet.01.534866.g156.t1 | ornithine decarboxylase antizyme | 366 | 3.62E-21 | 53.55 | F:nucleic acid binding; F:ornithine decarboxylase inhibitor activity; P:negative regulation of catalytic activity; F:metal ion binding; C:membrane; C:integral component of membrane |
| Efet.01.534932.g158.t1 | Four and a half LIM domains 2 | 270 | 1.97E-16 | 66.15 | F:zinc ion binding |
| Efet.01.534940.g159.t1 | axin-1-like isoform X2 | 843 | 9.92E-06 | 50 |  |
| Efet.01.534987.g160.t1 | cell division | 255 | 1.91E-34 | 89.15 | P:FtsZ-dependent cytokinesis; C:cytoplasm; P:cell septum assembly |
| Efet.01.534987.g161.t1 | family pyridoxal phosphate-dependent enzyme | 345 | 2.51E-58 | 78.45 |  |
| Efet.01.535023.g163.t1 | piccolo isoform X4 | 1359 | 2.23E-50 | 55.7 | P:synapse assembly; C:presynaptic active zone; C:integral component of membrane |
| Efet.01.535133.g167.t1 | tensin-4-like isoform X6 | 555 | 1.60E-30 | 58.2 |  |
| Efet.01.53522.g233.t1 | DNA cross-link repair 1A | 2454 | 4.82E-141 | 71.35 | P:protection from non-homologous end joining at telomere; P:interstrand cross-link repair; F:5'-3' exodeoxyribonuclease activity; P:double-strand break repair via nonhomologous end joining; F:damaged DNA binding; C:nuclear chromosome, telomeric region |
| Efet.01.53523.g234.t1 | hypothetical protein CAPTEDRAFT_219019 | 630 | 2.23E-08 | 51.25 | C:membrane; C:integral component of membrane; F:carbohydrate binding |
| Efet.01.5353.g419.t1 | innexin unc-9-like isoform X2 | 633 | 1.56E-71 | 70.15 | C:gap junction; P:ion transport; C:integral component of membrane; P:cell communication by electrical coupling; C:synapse; F:gap junction channel activity |
| Efet.01.53530.g235.t1 | integrator complex subunit 4-like | 207 | 5.46E-28 | 89.4 | P:snRNA processing; C:integral component of membrane; P:ionotropic glutamate receptor signaling pathway; F:ionotropic glutamate receptor activity; C:integrator complex |
| Efet.01.535313.g170.t1 | ras-related Rab-3 isoform X2 | 291 | 2.10E-26 | 83.05 | F:GTP binding; P:small GTPase mediated signal transduction; C:intracellular |
| Efet.01.535341.g172.t1 | gamma-butyrobetaine dioxygenase-like isoform X1 | 1404 | 1.20E-142 | 64.1 | F:oxidoreductase activity; P:oxidation-reduction process |
| Efet.01.53546.g236.t1 | piggyBac transposable element-derived 4-like | 474 | 1.52E-26 | 52.6 |  |
| Efet.01.535511.g175.t1 | pap2 superfamily | 954 | 7.79E-62 | 47.95 | F:inositol phosphoceramide synthase activity; C:inositol phosphoceramide synthase complex; C:membrane; C:integral component of membrane; P:inositolphosphoceramide metabolic process; C:Golgi apparatus; P:sphingolipid biosynthetic process |
| Efet.01.535536.g176.t1 | glutamic acid-rich -like | 525 | 1.27E-16 | 60.67 | F:metal ion binding |
| Efet.01.535585.g177.t1 | turtle homolog A isoform X2 | 327 | 1.13E-30 | 61.85 | C:inhibitory synapse; C:dendrite; P:regulation of synapse organization; F:protein binding involved in cell-cell adhesion; C:membrane; C:integral component of membrane; P:homophilic cell adhesion via plasma membrane adhesion molecules; C:axon; P:dendrite development |
| Efet.01.535660.g178.t1 | Cyclic GMP-AMP synthase | 1032 | 1.19E-16 | 48.55 |  |
| Efet.01.535726.g180.t1 | 40S ribosomal S5 | 387 | 1.21E-85 | 99.9 | C:cytosolic small ribosomal subunit; P:regulation of translational fidelity; P:ribosomal small subunit assembly; P:rRNA processing; P:translational initiation; C:nucleoplasm; P:viral transcription; C:extracellular exosome; F:protein binding; P:SRP-dependent cotranslational protein targeting to membrane; C:focal adhesion; F:structural constituent of ribosome; P:nuclear-transcribed mRNA catabolic process, nonsense-mediated decay; C:membrane; F:mRNA binding; C:extracellular matrix; F:rRNA binding |
| Efet.01.535757.g181.t1 | dynein heavy chain axonemal-like isoform X2 | 351 | 5.40E-24 | 71.7 | F:nucleotide binding; F:ion binding; F:nucleoside-triphosphatase activity |
| Efet.01.535801.g182.t1 | atrial natriuretic peptide receptor 1-like isoform X1 | 228 | 4.29E-15 | 73.15 | F:ATP binding; P:cGMP biosynthetic process; C:integral component of membrane; F:guanylate cyclase activity; F:protein kinase activity; P:protein phosphorylation; C:intracellular; P:intracellular signal transduction |
| Efet.01.535817.g183.t1 | BTB POZ domain-containing KCTD9 | 1188 | 8.50E-166 | 72.25 | P:cellular process |
| Efet.01.53595.g237.t1 | rho GTPase-activating 39 isoform X2 | 609 | 1.61E-99 | 76.35 | C:cytoskeleton; P:signal transduction; C:integral component of membrane |
| Efet.01.53613.g239.t1 | acetyl- carboxylase-like | 339 | 6.11E-47 | 85.6 | F:ATP binding; F:acetyl-CoA carboxylase activity; F:metal ion binding; F:biotin carboxylase activity; P:fatty acid biosynthetic process |
| Efet.01.536169.g187.t1 | matrix metallopeptidase-21-like | 531 | 9.71E-64 | 68 | P:determination of heart left/right asymmetry; F:metal ion binding; P:coronary vasculature development; C:extracellular region; P:hematopoietic progenitor cell differentiation; F:metallopeptidase activity |
| Efet.01.536200.g188.t1 | RAD50-interacting 1-like | 474 | 8.84E-52 | 71.85 | C:endoplasmic reticulum; P:Golgi vesicle transport |
| Efet.01.536258.g189.t1 | peroxisomal sarcosine oxidase | 387 | 7.01E-08 | 61.5 | C:peroxisome; F:L-pipecolate oxidase activity; F:oxidoreductase activity; C:membrane; C:integral component of membrane; F:sarcosine oxidase activity; P:tetrahydrofolate metabolic process; P:oxidation-reduction process; F:receptor binding; P:L-lysine catabolic process to acetyl-CoA via L-pipecolate |
| Efet.01.536272.g190.t1 | spectrin beta chain-like | 249 | 6.05E-20 | 83.95 | F:actin binding; F:structural constituent of cytoskeleton; F:phospholipid binding; C:spectrin |
| Efet.01.536272.g191.t1 | spectrin beta chain-like isoform X1 | 444 | 1.50E-52 | 81.45 | F:actin binding; F:structural constituent of cytoskeleton; F:phospholipid binding; C:spectrin |
| Efet.01.536310.g192.t1 | hypothetical protein CAPTEDRAFT_91192 | 420 | 3.51E-28 | 57.6 | P:transmembrane transport; C:membrane; C:integral component of membrane; P:transport; F:transporter activity |
| Efet.01.53633.g240.t1 | serine threonine- kinase pim-1-like | 651 | 9.66E-81 | 81.1 | F:ATP binding; P:camera-type eye morphogenesis; C:cytoplasm; P:negative regulation of apoptotic process; P:protein autophosphorylation; F:calmodulin-dependent protein kinase activity; P:optokinetic behavior |
| Efet.01.536338.g193.t1 | hypothetical protein HELRODRAFT_161031 | 309 | 1.87E-08 | 74 |  |
| Efet.01.536338.g194.t1 | transmembrane 145 | 846 | 3.17E-37 | 55.8 |  |
| Efet.01.536388.g195.t1 | cilia- and flagella-associated 47 | 213 | 3.06E-14 | 72.3 | C:motile cilium |
| Efet.01.536439.g197.t1 | atrial natriuretic peptide receptor 1- | 303 | 5.28E-20 | 80.85 | C:guanylate cyclase complex, soluble; F:ATP binding; P:cGMP biosynthetic process; C:integral component of membrane; P:receptor guanylyl cyclase signaling pathway; F:guanylate cyclase activity; F:protein kinase activity; P:protein phosphorylation; C:plasma membrane; F:adenylate cyclase activity; P:intracellular signal transduction; F:natriuretic peptide receptor activity |
| Efet.01.536494.g199.t1 | mitochondrial ubiquitin ligase activator of nfkb 1-A-like | 225 | 2.38E-06 | 68 |  |
| Efet.01.536532.g200.t1 | 5-hydroxytryptamine receptor 2C-like | 1848 | 4.27E-37 | 69.15 | F:G-protein coupled receptor activity; P:signal transduction; C:integral component of membrane |
| Efet.01.53657.g241.t1 | liprin-alpha-1-like isoform X1 | 876 | 2.01E-90 | 74.4 | P:synapse organization; C:synapse |
| Efet.01.536717.g201.t1 | 2,3,4,5-tetrahydropyridine-2,6-dicarboxylate N-succinyltransferase | 564 | 3.05E-49 | 91.85 | F:magnesium ion binding; C:cytoplasm; P:lysine biosynthetic process via diaminopimelate; F:2,3,4,5-tetrahydropyridine-2,6-dicarboxylate N-succinyltransferase activity; P:diaminopimelate biosynthetic process |
| Efet.01.536738.g202.t1 | cilia- and flagella-associated 46-like | 384 | 8.47E-14 | 60.1 |  |
| Efet.01.536832.g203.t1 | disks large homolog 5-like | 363 | 6.79E-25 | 66.5 | P:anatomical structure development; P:cellular process; P:single-organism developmental process |
| Efet.01.53685.g244.t1 | tripartite motif-containing 2-like | 807 | 7.39E-09 | 46.2 |  |
| Efet.01.53691.g247.t1 | zinc finger with KRAB and SCAN domains 5 isoform X1 | 2157 | 6.41E-16 | 38.9 | F:nucleic acid binding; C:nucleus; F:transcription factor activity, sequence-specific DNA binding; F:metal ion binding; P:transcription, DNA-templated; P:regulation of transcription, DNA-templated; C:intracellular |
| Efet.01.537099.g205.t1 | phospho-N-acetylmuramoyl-pentapeptide-transferase | 504 | 7.18E-85 | 90.2 | P:regulation of cell shape; P:cell division; F:UDP-N-acetylmuramoyl-L-alanyl-D-glutamyl-meso-2,6-diaminopimelyl-D-alanyl-D-alanine:undecaprenyl-phosphate transferase activity; P:peptidoglycan biosynthetic process; C:integral component of membrane; P:cell wall organization; C:plasma membrane; P:cell cycle; F:phospho-N-acetylmuramoyl-pentapeptide-transferase activity |
| Efet.01.537193.g207.t1 | glutamate--tRNA ligase | 840 | 1.73E-169 | 88.75 | F:ATP binding; C:cytoplasm; F:glutamate-tRNA ligase activity; F:tRNA binding; P:glutamyl-tRNA aminoacylation |
| Efet.01.537256.g209.t1 | neurocan core | 288 | 7.12E-18 | 55.3 | F:carbohydrate binding |
| Efet.01.537305.g210.t1 | PREDICTED: uncharacterized protein LOC106867206 | 726 | 8.12E-47 | 52.35 |  |
| Efet.01.537432.g211.t1 | probable G- coupled receptor No9 | 897 | 1.64E-45 | 64.25 | P:G-protein coupled receptor signaling pathway; F:octopamine receptor activity; C:integral component of membrane |
| Efet.01.537432.g212.t1 | octopamine receptor Oamb | 786 | 4.42E-39 | 67.95 | F:adrenergic receptor activity; C:integral component of membrane; P:adrenergic receptor signaling pathway; C:plasma membrane |
| Efet.01.537473.g214.t1 | E3 ubiquitin- ligase TRIM71-like | 1914 | 4.47E-30 | 43.2 | F:zinc ion binding; F:metal ion binding; C:intracellular |
| Efet.01.537492.g215.t1 | T family of potassium channels 18-like | 1143 | 3.94E-39 | 63.8 | P:ion transport; C:membrane |
| Efet.01.537525.g216.t1 | gonadotropin-releasing hormone receptor | 315 | 1.21E-13 | 59 | F:G-protein coupled receptor activity; P:G-protein coupled receptor signaling pathway; P:signal transduction; C:membrane; C:integral component of membrane; F:signal transducer activity |
| Efet.01.537568.g217.t1 | neuronal acetylcholine receptor subunit alpha-5-like isoform X2 | 351 | 1.19E-24 | 83.5 | C:postsynaptic membrane; P:cation transmembrane transport; C:integral component of membrane; C:cell junction; F:extracellular ligand-gated ion channel activity |
| Efet.01.537660.g220.t1 | ribosomal S6 kinase alpha-2 | 225 | 1.36E-16 | 87.35 | F:magnesium ion binding; C:nucleus; F:ATP binding; C:cytoplasm; F:protein serine/threonine kinase activity; P:protein phosphorylation; P:intracellular signal transduction |
| Efet.01.537674.g221.t1 | discoidin domain-containing receptor 2-like | 306 | 3.21E-28 | 70.2 | F:transmembrane receptor protein tyrosine kinase activity; C:integral component of membrane; P:protein phosphorylation |
| Efet.01.537684.g222.t1 | branched-chain amino acid ABC transporter permease | 246 | 1.89E-47 | 97.2 | C:integral component of membrane; P:transport; C:plasma membrane; F:transporter activity |
| Efet.01.537684.g223.t1 | metal-dependent hydrolase | 693 | 2.57E-42 | 64.15 | C:membrane |
| Efet.01.537695.g224.t1 | hypothetical protein CAPTEDRAFT_206253 | 519 | 5.41E-35 | 49.42 | C:membrane; C:integral component of membrane; F:carbohydrate binding |
| Efet.01.537781.g226.t1 | LIM domain transcription factor | 312 | 1.52E-14 | 74.25 | F:zinc ion binding |
| Efet.01.537791.g227.t1 | rho GTPase-activating gacF isoform X1 | 582 | 9.09E-29 | 58.15 |  |
| Efet.01.537894.g229.t1 | virilizer homolog | 399 | 4.14E-30 | 63.17 |  |
| Efet.01.537894.g230.t1 | virilizer homolog | 252 | 2.37E-18 | 63.6 |  |
| Efet.01.53795.g252.t1 | sidekick-2-like isoform X3 | 297 | 2.11E-09 | 59.2 |  |
| Efet.01.537957.g231.t1 | carboxypeptidase N subunit 2-like | 1335 | 3.15E-36 | 52.35 | C:membrane; C:integral component of membrane |
| Efet.01.53797.g253.t1 | Mitogen-activated kinase kinase kinase 9 | 831 | 9.40E-116 | 80.1 | F:ATP binding; F:protein serine/threonine kinase activity; P:protein phosphorylation |
| Efet.01.537981.g232.t1 | somatostatin receptor type 2-like | 1044 | 1.09E-58 | 56.25 | F:G-protein coupled receptor activity; P:G-protein coupled receptor signaling pathway |
| Efet.01.538065.g234.t1 | hypothetical protein CAPTEDRAFT_212380 | 537 | 6.75E-10 | 55 | F:chitin binding; P:chitin metabolic process; C:extracellular region |
| Efet.01.538077.g235.t1 | tektin-3-like isoform X1 | 321 | 1.60E-06 | 71.7 |  |
| Efet.01.538092.g236.t1 | Testis-expressed sequence 2 | 1164 | 1.20E-68 | 53.25 | C:membrane; C:integral component of membrane |
| Efet.01.538213.g237.t1 | hyperpolarizaion-activated cyclic nucleotide-gated cation channel | 387 | 1.99E-35 | 84.05 | F:voltage-gated potassium channel activity; P:potassium ion transmembrane transport; C:integral component of membrane |
| Efet.01.538252.g238.t1 | monocarboxylate transporter 7-like | 798 | 3.92E-35 | 69.7 | C:membrane; P:transport |
| Efet.01.538409.g239.t1 | mitogen-activated kinase 4 | 408 | 7.25E-15 | 58.7 | F:kinase activity |
| Efet.01.538415.g240.t1 | E3 ubiquitin- ligase TRIM71-like | 735 | 1.53E-11 | 49.6 | F:ubiquitin-protein transferase activity; F:zinc ion binding; F:metal ion binding; C:Cul3-RING ubiquitin ligase complex; P:phosphorylation; F:transferase activity; F:kinase activity; C:intracellular; P:protein ubiquitination |
| Efet.01.538473.g242.t1 | hypothetical protein CRN43_10635, partial | 354 | 3.29E-10 | 63.5 |  |
| Efet.01.538552.g244.t1 | ribulose-phosphate 3-epimerase | 522 | 1.53E-99 | 75.95 | P:carbohydrate metabolic process; F:metal ion binding; P:pentose-phosphate shunt; F:ribulose-phosphate 3-epimerase activity |
| Efet.01.53863.g254.t1 | Periodic tryptophan | 561 | 3.38E-62 | 71.9 |  |
| Efet.01.53864.g255.t1 | basic leucine zipper and W2 domain-containing 1-like isoform X1 | 318 | 2.66E-33 | 73.75 | C:membrane; C:integral component of membrane |
| Efet.01.538680.g245.t1 | ras association domain-containing 8 isoform X1 | 1011 | 4.99E-33 | 85.55 | P:signal transduction |
| Efet.01.538715.g246.t1 | carbohydrate FGGY family | 615 | 1.15E-134 | 68.65 | P:metabolic process; F:transferase activity, transferring phosphorus-containing groups |
| Efet.01.538773.g247.t1 | leucine--tRNA ligase | 567 | 3.40E-71 | 87.7 | F:ATP binding; P:leucyl-tRNA aminoacylation; F:leucine-tRNA ligase activity; C:cytoplasm; P:regulation of translational fidelity; F:aminoacyl-tRNA editing activity |
| Efet.01.538906.g250.t1 | complement receptor type 2-like | 711 | 2.35E-20 | 44.4 |  |
| Efet.01.538974.g251.t1 | PAX3- and PAX7-binding | 612 | 7.98E-59 | 70.5 | C:nucleus; P:positive regulation of histone methylation; F:RNA polymerase II core promoter proximal region sequence-specific DNA binding; C:cytosol; P:positive regulation of myoblast proliferation; F:transcription factor binding; P:positive regulation of transcription from RNA polymerase II promoter; P:regulation of skeletal muscle satellite cell proliferation |
| Efet.01.53900.g258.t1 | aquaporin -like isoform X1 | 471 | 1.04E-23 | 48.8 | C:membrane; C:integral component of membrane; P:transport; F:transporter activity |
| Efet.01.539033.g253.t1 | phosphoribosylformylglycinamidine synthase | 1110 | 5.90E-134 | 66 | P:small molecule metabolic process; P:organonitrogen compound metabolic process; P:single-organism cellular process; F:binding; P:primary metabolic process; P:cellular metabolic process |
| Efet.01.539084.g254.t1 | atrial natriuretic peptide receptor 1-like | 279 | 1.60E-20 | 83.05 | F:ATP binding; P:cGMP biosynthetic process; C:integral component of membrane; F:guanylate cyclase activity; F:protein kinase activity; P:protein phosphorylation; C:intracellular; P:intracellular signal transduction |
| Efet.01.539170.g256.t1 | lipoxygenase homology domain-containing 1-like | 282 | 2.41E-13 | 69.1 |  |
| Efet.01.539243.g258.t1 | hypothetical protein Veis_3255 | 723 | 1.14E-130 | 77.35 |  |
| Efet.01.53955.g260.t1 | hypothetical protein CAPTEDRAFT_215767 | 288 | 3.65E-26 | 78.5 |  |
| Efet.01.539567.g261.t1 | forkhead box N3-like isoform X1 | 597 | 6.09E-50 | 67.25 | F:DNA binding; C:nucleus; F:transcription factor activity, sequence-specific DNA binding; P:transcription, DNA-templated; P:regulation of transcription, DNA-templated; F:sequence-specific DNA binding |
| Efet.01.53959.g261.t1 | hypothetical protein HELRODRAFT_170181 | 1641 | 5.17E-16 | 49.5 |  |
| Efet.01.539676.g262.t1 | AChain Crystal structure of the human filamin A Ig-like domains 20-21 in complex with migfilin peptide | 402 | 8.59E-35 | 76.7 | P:protein localization to cell surface; P:wound healing, spreading of cells; P:adenylate cyclase-inhibiting dopamine receptor signaling pathway; P:positive regulation of I-kappaB kinase/NF-kappaB signaling; P:negative regulation of protein catabolic process; P:positive regulation of transcription factor import into nucleus; C:nucleolus; P:platelet degranulation; C:dendrite; F:G-protein coupled receptor binding; P:actin crosslink formation; C:extracellular exosome; F:Ral GTPase binding; C:focal adhesion; P:cytoplasmic sequestering of protein; F:Rac GTPase binding; C:cytosol; F:glycoprotein binding; P:cell junction assembly; F:Fc-gamma receptor I complex binding; P:platelet aggregation; P:positive regulation of integrin-mediated signaling pathway; F:kinase binding; P:actin cytoskeleton reorganization; P:negative regulation of transcription from RNA polymerase I promoter; F:protein homodimerization activity; P:negative regulation of sequence-specific DNA binding transcription factor activity; F:transcription factor binding; P:receptor clustering; P:positive regulation of substrate adhesion-dependent cell spreading; F:signal transducer activity; C:plasma membrane; F:RNA binding; F:actin filament binding; P:establishment of protein localization; C:cell-cell junction; C:cell cortex; P:negative regulation of apoptotic process; C:Myb complex; C:filamentous actin; P:regulation of cell migration; P:protein stabilization; P:semaphorin-plexin signaling pathway; P:mitotic spindle assembly |
| Efet.01.539695.g263.t1 | gluconate 5-dehydrogenase | 582 | 1.74E-97 | 69.55 | F:oxidoreductase activity; F:gluconate 5-dehydrogenase activity; P:oxidation-reduction process |
| Efet.01.53973.g262.t1 | probable 28S rRNA (cytosine-C(5))-methyltransferase | 783 | 8.78E-26 | 62.35 | C:nucleus; F:methyltransferase activity; F:transferase activity; C:nucleolus; F:RNA binding; P:methylation |
| Efet.01.539730.g264.t1 | amidohydrolase family | 333 | 4.47E-73 | 84.75 | F:hydrolase activity, acting on carbon-nitrogen (but not peptide) bonds; F:proline dipeptidase activity; P:proteolysis |
| Efet.01.539731.g265.t1 | hypothetical protein CAPTEDRAFT_197857 | 900 | 4.36E-16 | 53 | F:metal ion binding; P:regulation of gene expression |
| Efet.01.539731.g266.t1 | tiptop isoform X2 | 1296 | 5.14E-39 | 58.25 | P:regulation of gene expression |
| Efet.01.539738.g267.t1 | EF-hand domain-containing family member C2-like | 243 | 8.57E-20 | 76.05 | F:calcium ion binding |
| Efet.01.539751.g268.t1 | neuronal acetylcholine receptor subunit alpha-7-like | 414 | 9.61E-08 | 55 | F:ion channel activity; P:ion transport; C:postsynaptic membrane; P:ion transmembrane transport; C:membrane; P:cation transmembrane transport; C:integral component of membrane; F:extracellular ligand-gated ion channel activity; C:cell junction; C:synapse; P:transport; C:plasma membrane |
| Efet.01.53984.g263.t1 | atlastin-2 isoform X2 | 237 | 5.07E-09 | 63.45 | F:nucleotide binding; C:membrane |
| Efet.01.539861.g270.t1 | tyrosine- phosphatase Lar-like isoform X1 | 285 | 7.92E-08 | 64.33 | F:phosphoprotein phosphatase activity; F:protein tyrosine phosphatase activity; C:membrane; P:protein dephosphorylation; F:phosphatase activity; C:integral component of membrane; P:dephosphorylation; P:peptidyl-tyrosine dephosphorylation; F:hydrolase activity |
| Efet.01.54004.g265.t1 | glycogen phosphorylase | 465 | 1.55E-49 | 67.05 | F:transferase activity, transferring glycosyl groups |
| Efet.01.540063.g271.t1 | 30S ribosomal S1 | 294 | 3.17E-62 | 82.85 | F:RNA binding; F:structural constituent of ribosome; C:ribosome; P:translation |
| Efet.01.54008.g268.t1 | collagen | 219 | 2.06E-08 | 60.45 |  |
| Efet.01.540229.g275.t1 | glutamyl aminopeptidase-like | 207 | 1.10E-22 | 66.25 | C:membrane; F:peptidase activity, acting on L-amino acid peptides |
| Efet.01.540295.g276.t1 | polyadenylate-binding 2-B isoform X1 | 261 | 9.93E-07 | 71.25 | F:organic cyclic compound binding; F:heterocyclic compound binding |
| Efet.01.540391.g278.t1 | sprouty- EVH1 domain-containing 2 isoform X1 | 1308 | 7.82E-14 | 52.3 | P:regulation of cellular process |
| Efet.01.540423.g280.t1 | collagen alpha-1(II) chain- | 264 | 8.14E-06 | 92 |  |
| Efet.01.54051.g269.t1 | dynein heavy chain axonemal isoform X2 | 1077 | 1.96E-152 | 77.25 | C:microtubule cytoskeleton; C:cytoskeletal part; F:nucleoside-triphosphatase activity |
| Efet.01.540547.g281.t1 | ankyrin domain | 282 | 1.99E-10 | 57.82 | F:structural constituent of ribosome; C:membrane; C:integral component of membrane; C:ribosome; C:intracellular; P:translation |
| Efet.01.540563.g282.t1 | Alcohol dehydrogenase class-3 | 396 | 1.48E-68 | 86.3 | F:zinc ion binding; C:cytoplasm; F:S-(hydroxymethyl)glutathione dehydrogenase activity; P:ethanol oxidation; F:alcohol dehydrogenase activity, zinc-dependent |
| Efet.01.540613.g284.t1 | unc-80 homolog | 474 | 8.47E-61 | 72.55 |  |
| Efet.01.540625.g285.t1 | uncharacterized F-box LRR-repeat -like isoform X2 | 375 | 1.11E-63 | 81.9 |  |
| Efet.01.540946.g289.t1 | coiled-coil domain-containing 173-like | 516 | 5.64E-13 | 61.29 |  |
| Efet.01.540949.g290.t1 | 60 kDa neurofilament -like | 630 | 4.42E-41 | 66.05 | F:structural molecule activity; C:intermediate filament |
| Efet.01.541011.g291.t1 | epidermal growth factor receptor-like isoform X2 | 213 | 2.20E-12 | 80.25 | F:ATP binding; P:peptidyl-tyrosine phosphorylation; F:transmembrane receptor protein tyrosine kinase activity; C:integral component of membrane; P:transmembrane receptor protein tyrosine kinase signaling pathway |
| Efet.01.541109.g293.t1 | negative elongation factor B-like | 501 | 1.85E-65 | 79.2 | C:nucleus; F:translation elongation factor activity; P:negative regulation of transcription, DNA-templated; P:translational elongation |
| Efet.01.541120.g294.t1 | neuronal acetylcholine receptor subunit alpha-10-like | 570 | 3.53E-46 | 79.5 | C:postsynaptic membrane; P:cation transmembrane transport; C:integral component of membrane; C:cell junction; F:extracellular ligand-gated ion channel activity |
| Efet.01.541286.g295.t1 | centrosomal of 135 kDa-like isoform X1 | 519 | 1.94E-72 | 83.05 | C:centriole; F:protein C-terminus binding; P:centriole replication; P:centriole-centriole cohesion |
| Efet.01.541357.g296.t1 | hypothetical protein RF11_14163 | 411 | 4.77E-16 | 58.65 | C:membrane; C:integral component of membrane |
| Efet.01.541473.g297.t1 | gamma-tubulin complex component 6-like isoform X2 | 411 | 3.55E-14 | 57.82 | C:microtubule organizing center; P:microtubule nucleation; C:spindle pole; F:gamma-tubulin binding; P:microtubule cytoskeleton organization |
| Efet.01.54148.g271.t1 | coiled-coil domain-containing 146 isoform X2 | 399 | 8.16E-10 | 61.9 | C:motile cilium |
| Efet.01.541560.g298.t1 | discoidin domain-containing receptor 2-like isoform X1 | 231 | 4.38E-28 | 75.1 | C:integral component of plasma membrane; F:ATP binding; P:peptidyl-tyrosine phosphorylation; F:protein tyrosine kinase collagen receptor activity; P:collagen-activated tyrosine kinase receptor signaling pathway |
| Efet.01.54157.g274.t1 | calcium-activated potassium channel slowpoke isoform X40 | 312 | 1.88E-29 | 63.8 | P:ion transport; C:integral component of membrane; F:potassium channel activity |
| Efet.01.541570.g299.t1 | Calbindin-32 | 267 | 2.03E-19 | 80.35 | F:nucleic acid binding; F:calcium ion binding |
| Efet.01.54158.g275.t1 | calcium-activated potassium channel slowpoke isoform X40 | 312 | 1.88E-29 | 63.8 | P:ion transport; C:integral component of membrane; F:potassium channel activity |
| Efet.01.541621.g301.t1 | collagen alpha-1(XIX) chain isoform X4 | 261 | 5.18E-06 | 64 |  |
| Efet.01.541637.g302.t1 | agrin-like | 474 | 9.00E-39 | 59.45 | F:calcium ion binding |
| Efet.01.541664.g304.t1 | ISXO2-like domain-containing | 321 | 1.82E-17 | 61 |  |
| Efet.01.541676.g305.t1 | Dual specificity phosphatase 4 | 516 | 5.63E-22 | 66.65 | P:inactivation of MAPK activity; F:protein tyrosine phosphatase activity; P:peptidyl-tyrosine dephosphorylation; F:MAP kinase tyrosine/serine/threonine phosphatase activity |
| Efet.01.541684.g306.t1 | hemoglobin subunit mu-like | 687 | 3.63E-54 | 62.15 | F:binding; P:transport |
| Efet.01.54171.g277.t1 | dopamine D2-like receptor | 981 | 3.01E-19 | 76.6 | C:integral component of plasma membrane; F:dopamine neurotransmitter receptor activity, coupled via Gs; P:synaptic transmission, dopaminergic; F:dopamine binding; P:dopamine receptor signaling pathway |
| Efet.01.54188.g278.t1 | Plastin-2 | 252 | 2.31E-22 | 64.3 | F:actin binding; F:calcium ion binding; C:membrane |
| Efet.01.54210.g279.t1 | maternal embryonic leucine zipper kinase-like | 654 | 4.62E-11 | 71.95 | F:nucleotide binding; C:nucleus; P:peptidyl-tyrosine phosphorylation; F:non-membrane spanning protein tyrosine kinase activity; C:cytoplasm; F:protein serine/threonine kinase activity; P:intracellular signal transduction |
| Efet.01.542240.g309.t1 | Ras-like GTP-binding | 429 | 8.53E-49 | 68.3 | P:wound healing, spreading of cells; P:mitotic cell cycle process; P:small GTPase mediated signal transduction; P:regulation of intracellular signal transduction; P:cellular component assembly; P:nervous system development; P:skeletal muscle satellite cell migration; P:cell surface receptor signaling pathway; P:animal organ development; C:cytoplasmic part; P:microtubule cytoskeleton organization; P:negative regulation of cellular process; F:binding; P:regulation of actin cytoskeleton organization; P:ameboidal-type cell migration; P:positive regulation of cellular process; P:positive regulation of catalytic activity; C:cell junction; C:vesicle; P:actin cytoskeleton organization; C:cell periphery; P:regulation of multicellular organismal development |
| Efet.01.542241.g310.t1 | calcium-dependent cysteine | 255 | 7.03E-16 | 66.9 | C:cytoplasm; P:proteolysis; F:calcium-dependent cysteine-type endopeptidase activity |
| Efet.01.542389.g311.t1 | signal transducer and activator of transcription | 216 | 7.24E-18 | 68.9 | C:nucleus; F:DNA binding; F:transcription factor activity, sequence-specific DNA binding; C:cytoplasm; P:transcription, DNA-templated; P:signal transduction; P:regulation of transcription, DNA-templated; F:signal transducer activity |
| Efet.01.54241.g281.t1 | Kyphoscoliosis peptidase | 744 | 5.35E-29 | 48.5 |  |
| Efet.01.542499.g313.t1 | UPF0462 C4orf33 homolog | 222 | 8.23E-13 | 79.3 |  |
| Efet.01.542525.g314.t1 | beta-1,4-galactosyltransferase galt-1-like | 492 | 4.11E-11 | 48.7 | C:membrane; C:integral component of membrane |
| Efet.01.542536.g315.t1 | DNA polymerase III subunit delta | 1185 | 6.69E-179 | 81.5 | F:DNA binding; P:DNA replication |
| Efet.01.542672.g317.t1 | PC-esterase domain-containing 1A-like | 753 | 2.53E-30 | 59.5 | F:hydrolase activity, acting on ester bonds |
| Efet.01.542699.g319.t1 | heavy metal translocating P-type ATPase | 867 | 3.36E-158 | 66 | P:cation transport; C:membrane; F:hydrolase activity |
| Efet.01.542750.g320.t1 | ubiA prenyltransferase domain-containing 1 homolog | 999 | 1.20E-126 | 76.1 | C:membrane; F:transferase activity |
| Efet.01.542780.g321.t1 | serine threonine- kinase 10-like | 234 | 2.93E-12 | 69.7 | F:nucleotide binding; F:protein serine/threonine kinase activity |
| Efet.01.54285.g282.t1 | Alpha beta hydrolase domain-containing 17C | 675 | 1.30E-114 | 80.2 | F:hydrolase activity |
| Efet.01.542867.g322.t1 | DH31-like receptor 2 | 1134 | 5.67E-74 | 55.6 | P:signal transduction; F:signal transducer activity |
| Efet.01.542925.g323.t1 | hypothetical protein CAPTEDRAFT_227906 | 465 | 4.80E-32 | 48 |  |
| Efet.01.542986.g324.t1 | F-box only 22 | 432 | 9.58E-26 | 63.45 | P:regulation of cellular process |
| Efet.01.54319.g285.t1 | gliomedin-like isoform X2 | 846 | 8.66E-105 | 60.55 | P:regulation of cellular process |
| Efet.01.543267.g326.t1 | multiple C2 and transmembrane domain-containing 1 isoform X6 | 204 | 2.61E-10 | 83.22 | C:integral component of membrane |
| Efet.01.54330.g286.t1 | hypothetical protein CAPTEDRAFT_211886 | 312 | 4.25E-07 | 53 | C:membrane; C:integral component of membrane; F:carbohydrate binding |
| Efet.01.543309.g327.t1 | spondin-1-like isoform X1 | 297 | 5.44E-10 | 63 | F:serine-type endopeptidase inhibitor activity; P:immune response; C:membrane; C:integral component of membrane; F:scavenger receptor activity; P:negative regulation of endopeptidase activity; P:receptor-mediated endocytosis; F:polysaccharide binding |
| Efet.01.543313.g328.t1 | plectin-like isoform X4 | 405 | 4.37E-32 | 58.95 | C:cytoskeleton; C:membrane; C:integral component of membrane |
| Efet.01.543362.g329.t1 | A disintegrin and metallo ase with thrombospondin motifs 7-like | 504 | 9.99E-20 | 52.75 | F:zinc ion binding; P:integrin-mediated signaling pathway; P:proteolysis; F:peptidase activity; C:extracellular matrix; F:metalloendopeptidase activity; C:proteinaceous extracellular matrix; F:metallopeptidase activity |
| Efet.01.543473.g331.t1 | fibrinogen 1 | 420 | 4.88E-22 | 57.3 |  |
| Efet.01.543615.g333.t1 | voltage-gated sodium channel alpha subunit isoform | 288 | 1.10E-35 | 79.3 | P:regulation of postsynaptic membrane potential; F:calcium ion binding; P:regulation of ion transmembrane transport; C:voltage-gated sodium channel complex; P:sodium ion transmembrane transport; F:voltage-gated sodium channel activity |
| Efet.01.543629.g334.t1 | ATP ADP translocase | 810 | 2.76E-74 | 64 | C:membrane; C:integral component of membrane |
| Efet.01.543655.g336.t1 | guanylate cyclase 2G-like | 213 | 5.48E-17 | 74.65 | C:guanylate cyclase complex, soluble; F:nucleotide binding; P:cGMP biosynthetic process; P:signal transduction; F:guanylate cyclase activity; C:plasma membrane; F:adenylate cyclase activity |
| Efet.01.543821.g337.t1 | hypothetical protein CAPTEDRAFT_227170 | 282 | 3.18E-25 | 65 | C:membrane; F:binding |
| Efet.01.543955.g340.t1 | neural-cadherin 2 | 609 | 7.53E-95 | 62.1 | P:cell communication; F:calcium ion binding; C:integral component of membrane; P:homophilic cell adhesion via plasma membrane adhesion molecules; P:single organism signaling; C:plasma membrane; P:response to stimulus |
| Efet.01.544010.g341.t1 | potassium voltage-gated channel subfamily H member 8-like | 261 | 7.57E-29 | 82.9 | F:phosphorelay sensor kinase activity; F:voltage-gated potassium channel activity; P:potassium ion transmembrane transport; P:phosphorelay signal transduction system; C:integral component of membrane; P:signal transduction by protein phosphorylation; C:intracellular |
| Efet.01.544055.g342.t1 | voltage-dependent T-type calcium channel subunit alpha-1G | 456 | 8.80E-17 | 70.6 | P:calcium ion import; C:voltage-gated calcium channel complex; P:regulation of ion transmembrane transport; P:calcium ion transmembrane transport; F:low voltage-gated calcium channel activity |
| Efet.01.54406.g289.t1 | ras-specific guanine nucleotide-releasing factor 2-like | 345 | 7.61E-26 | 71.2 | P:regulation of cellular process; F:guanyl-nucleotide exchange factor activity |
| Efet.01.544123.g343.t1 | potassium sodium hyperpolarization-activated cyclic nucleotide-gated channel 3-like isoform X2 | 1239 | 1.31E-13 | 60.75 | P:transport |
| Efet.01.5443.g422.t1 | ankyrin repeat domain-containing 50-like | 2073 | 0 | 72.8 | P:retrograde transport, endosome to plasma membrane; P:intracellular protein transport |
| Efet.01.544334.g345.t1 | Serine threonine- kinase pim-3 | 345 | 4.50E-33 | 80.25 | F:ATP binding; C:cytoplasm; P:negative regulation of apoptotic process; P:protein autophosphorylation; F:protein serine/threonine kinase activity; P:regulation of mitotic cell cycle |
| Efet.01.544383.g346.t1 | tyrosine- kinase RYK-like isoform X2 | 285 | 3.28E-06 | 64 | F:ATP binding; C:membrane; C:integral component of membrane; F:protein kinase activity; P:phosphorylation; P:protein phosphorylation; F:kinase activity |
| Efet.01.544428.g347.t1 | prohormone | 267 | 8.15E-33 | 91.35 | P:proteolysis; F:serine-type endopeptidase activity |
| Efet.01.544463.g348.t1 | zinc finger 665-like | 1539 | 1.99E-119 | 59.5 | F:nucleic acid binding; F:metal ion binding |
| Efet.01.544502.g350.t1 | leucine-rich repeat-containing 24-like | 2103 | 1.06E-55 | 47.55 | C:membrane; C:integral component of membrane |
| Efet.01.544550.g351.t1 | potassium voltage-gated channel eag isoform X12 | 390 | 1.15E-44 | 90.5 | F:phosphorelay sensor kinase activity; F:voltage-gated potassium channel activity; P:potassium ion transmembrane transport; P:phosphorelay signal transduction system; C:integral component of membrane; P:signal transduction by protein phosphorylation; C:intracellular |
| Efet.01.544590.g352.t1 | titin isoform X1 | 1203 | 7.59E-08 | 54 | F:nucleotide binding; P:regulation of Rho protein signal transduction; F:ATP binding; C:membrane; C:integral component of membrane; F:protein kinase activity; F:Rho guanyl-nucleotide exchange factor activity; P:phosphorylation; F:transferase activity; P:protein phosphorylation; F:kinase activity; P:positive regulation of GTPase activity |
| Efet.01.5446.g423.t1 | histamine H2 receptor-like | 1251 | 2.81E-111 | 54.6 | F:G-protein coupled receptor activity; P:signal transduction; C:membrane |
| Efet.01.54462.g290.t1 | hypothetical protein CAPTEDRAFT_211886 | 681 | 2.16E-18 | 44.45 | C:membrane; C:integral component of membrane; F:carbohydrate binding |
| Efet.01.544715.g353.t1 | ferredoxin--NADP reductase | 660 | 5.86E-156 | 98.35 | P:oxidation-reduction process; F:ferredoxin-NADP+ reductase activity |
| Efet.01.544715.g354.t1 | enamidase | 1749 | 0 | 98.9 | F:enamidase activity |
| Efet.01.544715.g355.t1 | family | 414 | 1.37E-69 | 89.25 | P:polysaccharide biosynthetic process; C:integral component of membrane; P:transport |
| Efet.01.544763.g357.t1 | leucine-rich repeat-containing 9-like | 363 | 8.66E-30 | 77.6 |  |
| Efet.01.54488.g293.t1 | carbohydrate sulfotransferase 1-like | 978 | 7.80E-66 | 47.7 | C:membrane; C:integral component of membrane; F:sulfotransferase activity |
| Efet.01.54489.g294.t1 | hypothetical protein HELRODRAFT_191437 | 891 | 8.65E-19 | 46 |  |
| Efet.01.54493.g296.t1 | chloride channel B-like | 372 | 2.30E-09 | 64.67 | P:ion transport |
| Efet.01.544969.g360.t1 | short-chain dehydrogenase | 786 | 1.53E-173 | 89.3 | F:oxidoreductase activity; P:oxidation-reduction process |
| Efet.01.544969.g361.t1 | LPS export ABC transporter periplasmic | 513 | 9.00E-120 | 85.25 | C:integral component of plasma membrane; F:lipopolysaccharide transmembrane transporter activity; P:lipopolysaccharide transport |
| Efet.01.544969.g362.t1 | arabinose-5-phosphate isomerase | 1404 | 0 | 93.3 | P:carbohydrate metabolic process; F:metal ion binding; F:arabinose-5-phosphate isomerase activity; F:carbohydrate binding |
| Efet.01.545219.g366.t1 | craniofacial development 2-like | 522 | 1.27E-39 | 63.3 | P:nucleic acid phosphodiester bond hydrolysis; F:RNA-directed DNA polymerase activity; F:endonuclease activity; P:RNA-dependent DNA biosynthetic process |
| Efet.01.545223.g367.t1 | 6-phosphogluconate dehydrogenase | 951 | 0 | 80.4 | F:phosphogluconate dehydrogenase (decarboxylating) activity; P:pentose-phosphate shunt; P:oxidation-reduction process |
| Efet.01.545291.g368.t1 | family cyclic di-GMP-binding | 960 | 1.68E-107 | 97.6 | P:regulation of cell shape; P:cell division; C:cytoplasm; F:flavin adenine dinucleotide binding; P:peptidoglycan biosynthetic process; F:UDP-N-acetylmuramate dehydrogenase activity; P:oxidation-reduction process; P:cell wall organization; P:cell cycle |
| Efet.01.545291.g369.t1 | peptidase A2 | 570 | 3.38E-106 | 77.6 |  |
| Efet.01.545291.g370.t1 | phosphodiesterase | 387 | 6.37E-86 | 93.15 | F:hydrolase activity |
| Efet.01.545291.g371.t1 | glycerol-3-phosphate acyltransferase | 690 | 3.79E-87 | 85.65 | C:integral component of membrane; F:acyl-phosphate glycerol-3-phosphate acyltransferase activity; P:phospholipid biosynthetic process; C:plasma membrane |
| Efet.01.545302.g372.t1 | UTP--glucose-1-phosphate uridylyltransferase | 891 | 0 | 94 | P:UDP-glucose metabolic process; P:biosynthetic process; F:UTP:glucose-1-phosphate uridylyltransferase activity |
| Efet.01.545302.g373.t1 | valine--tRNA ligase | 2919 | 0 | 86.75 | F:valine-tRNA ligase activity; F:ATP binding; P:valyl-tRNA aminoacylation; C:cytoplasm; P:regulation of translational fidelity; F:aminoacyl-tRNA editing activity |
| Efet.01.545302.g374.t1 | VOC family | 759 | 1.37E-137 | 92.25 | F:dioxygenase activity; P:aromatic compound catabolic process; F:ferrous iron binding; P:oxidation-reduction process |
| Efet.01.545302.g375.t1 | permease of the drug metabolite transporter (DMT) superfamily | 759 | 2.35E-102 | 80.05 | C:integral component of membrane |
| Efet.01.545302.g376.t1 | MFS transporter [Caballeronia catudaia] | 1107 | 1.05E-85 | 58.75 |  |
| Efet.01.545341.g377.t1 | FMRFamide-activated amiloride-sensitive sodium channel-like | 1761 | 2.04E-81 | 48.35 | P:sodium ion transport; P:ion transport; F:sodium channel activity; C:membrane; C:integral component of membrane; P:sodium ion transmembrane transport; P:transport |
| Efet.01.545519.g379.t1 | meckelin-like | 213 | 1.15E-10 | 74.38 | P:negative regulation of centrosome duplication; C:integral component of membrane; C:MKS complex |
| Efet.01.54552.g299.t1 | glutaminase liver mitochondrial-like | 1455 | 0 | 74.85 | F:glutaminase activity; P:glutamate biosynthetic process; C:mitochondrial matrix; P:glutamine catabolic process |
| Efet.01.545522.g380.t1 | acid-sensing ion channel 1-like | 618 | 1.11E-14 | 57.25 | P:sodium ion transport; C:membrane |
| Efet.01.545525.g381.t1 | Protocadherin Fat 4 | 789 | 1.20E-58 | 56.6 | C:membrane |
| Efet.01.54559.g301.t1 | hypothetical protein CAPTEDRAFT_186249 | 501 | 1.67E-46 | 47.6 | C:membrane; C:integral component of membrane; F:carbohydrate binding |
| Efet.01.545625.g382.t1 | one cut domain family member 2 | 1092 | 3.93E-50 | 90.85 | P:B cell differentiation; P:endoderm development; P:transcription from RNA polymerase II promoter; P:spleen development; F:transcriptional activator activity, RNA polymerase II transcription regulatory region sequence-specific binding; P:epithelial cell development; P:anatomical structure morphogenesis; P:glucose metabolic process; P:positive regulation of transcription from RNA polymerase II promoter; C:nucleus; F:DNA binding; P:regulation of cell-matrix adhesion; P:Notch signaling pathway; P:liver development; P:cell fate commitment; P:endocrine pancreas development; P:negative regulation of transforming growth factor beta receptor signaling pathway; P:positive regulation of cell migration |
| Efet.01.545636.g384.t1 | Zinc finger ZIC 4 | 459 | 4.58E-33 | 92.1 | C:nucleus; F:metal ion binding; F:RNA polymerase II regulatory region sequence-specific DNA binding |
| Efet.01.545657.g385.t1 | transcriptional coactivator YAP1 | 453 | 6.14E-19 | 51.65 | C:membrane; C:integral component of membrane |
| Efet.01.54566.g302.t1 | utrophin isoform X5 | 330 | 2.37E-23 | 61.2 | F:metal ion binding; F:cytoskeletal protein binding; C:plasma membrane |
| Efet.01.54581.g303.t1 | somatostatin receptor type 5 | 483 | 6.17E-08 | 66.38 | F:G-protein coupled receptor activity; P:G-protein coupled receptor signaling pathway |
| Efet.01.545867.g387.t1 | twitchin-like isoform X1 | 399 | 4.42E-17 | 61.45 | F:nucleotide binding; F:ATP binding; F:protein kinase activity; P:phosphorylation; F:transferase activity; P:protein phosphorylation; F:kinase activity |
| Efet.01.545876.g388.t1 | von Willebrand factor A domain-containing 3A-like | 201 | 7.24E-24 | 79.15 |  |
| Efet.01.545951.g389.t1 | noggin-2-like | 579 | 1.19E-60 | 57.1 | P:negative regulation of BMP signaling pathway; P:negative regulation of cell differentiation |
| Efet.01.54597.g304.t1 | MORC family CW-type zinc finger 2-like | 426 | 3.02E-60 | 79.45 | F:zinc ion binding |
| Efet.01.546036.g390.t1 | DUF4292 domain-containing | 357 | 4.34E-64 | 66.88 |  |
| Efet.01.546084.g391.t1 | sodium potassium ATPase alpha | 201 | 1.87E-31 | 97.9 | F:ATP binding; F:metal ion binding; C:integral component of membrane; F:hydrolase activity |
| Efet.01.546195.g394.t1 | ATP-binding | 408 | 4.99E-91 | 83.1 | F:protein serine/threonine kinase activity; P:protein phosphorylation |
| Efet.01.546195.g395.t1 | liver stage | 1461 | 0 | 66.4 | C:membrane |
| Efet.01.546196.g396.t1 | FLYWCH-type zinc finger-containing 1 | 810 | 7.90E-47 | 62.55 |  |
| Efet.01.546202.g397.t1 | DNA gyrase subunit A | 291 | 7.15E-44 | 93.65 | F:DNA binding; F:ATP binding; C:cytoplasm; P:DNA-dependent DNA replication; C:chromosome; F:DNA topoisomerase type II (ATP-hydrolyzing) activity; P:DNA topological change |
| Efet.01.546558.g399.t1 | ATP-binding cassette sub-family A member 3 | 243 | 4.29E-10 | 70.05 | F:ATPase activity; F:ATP binding; C:integral component of membrane; P:single-organism transport; F:transporter activity |
| Efet.01.546654.g401.t1 | RNA polymerase-associated RTF1 homolog | 315 | 2.04E-14 | 86.1 | P:negative regulation of transcription from RNA polymerase II promoter; P:positive regulation of transcription elongation from RNA polymerase II promoter; P:histone H3-K4 trimethylation; P:stem cell population maintenance; P:endodermal cell fate commitment; P:positive regulation of histone H3-K4 methylation; C:Cdc73/Paf1 complex; C:nucleolus; F:single-stranded DNA binding; F:RNA binding; P:transcription elongation from RNA polymerase II promoter |
| Efet.01.546668.g402.t1 | elongation factor Tu | 942 | 1.61E-121 | 89.95 | F:GTP binding; F:translation elongation factor activity; C:cytoplasm; F:GTPase activity; P:translational elongation |
| Efet.01.546674.g403.t1 | xylulokinase | 753 | 2.15E-106 | 91.1 | P:xylulose metabolic process; F:xylulokinase activity; P:carbohydrate phosphorylation |
| Efet.01.546674.g404.t1 | family transcriptional regulator | 1608 | 0 | 92.25 | F:transcription factor activity, sequence-specific DNA binding; P:transcription, DNA-templated; P:regulation of transcription, DNA-templated; F:sequence-specific DNA binding |
| Efet.01.546787.g407.t1 | non-canonical purine NTP HAM1 family | 600 | 1.29E-139 | 90.85 | F:nucleotide binding; P:purine nucleotide metabolic process; F:metal ion binding; P:nucleoside triphosphate catabolic process; F:nucleoside-triphosphatase activity; F:nucleoside-triphosphate diphosphatase activity |
| Efet.01.546787.g408.t1 | serine threonine phosphatase | 840 | 0 | 86.05 | F:protein serine/threonine phosphatase activity; P:protein dephosphorylation |
| Efet.01.546787.g409.t1 | serine threonine kinase | 1023 | 0 | 94.9 | F:ATP binding; F:protein serine/threonine kinase activity; P:protein phosphorylation |
| Efet.01.546787.g410.t1 | family | 756 | 9.14E-129 | 71.1 |  |
| Efet.01.546787.g411.t1 | site-specific integrase | 1368 | 0 | 64.7 | F:DNA binding; P:DNA recombination; P:DNA integration |
| Efet.01.546787.g412.t1 | type II toxin-antitoxin system family toxin | 1239 | 0 | 88.1 | P:phosphorylation; F:kinase activity |
| Efet.01.546787.g413.t1 | RNA 2 -phosphotransferase | 2460 | 0 | 65.55 | F:transferase activity |
| Efet.01.546787.g414.t1 | integrase | 531 | 5.10E-74 | 75.55 | F:DNA binding; C:membrane; P:DNA recombination; P:DNA integration |
| Efet.01.546898.g417.t1 | ribosome-recycling mitochondrial | 888 | 4.95E-62 | 68.7 | P:translation |
| Efet.01.546943.g418.t1 | type II secretion system | 1593 | 0 | 89.9 | F:ATP binding; F:protein transporter activity; P:protein secretion by the type II secretion system; C:type II protein secretion system complex |
| Efet.01.547070.g420.t1 | spliceosome RNA helicase DDX39B | 528 | 2.83E-74 | 92.75 | P:positive regulation of translation; C:nuclear speck; P:termination of RNA polymerase II transcription; F:ATP-dependent protein binding; F:ATP-dependent RNA helicase activity; F:ATP binding; P:positive regulation of vascular smooth muscle cell proliferation; P:viral mRNA export from host cell nucleus; P:liver development; F:protein complex binding; P:positive regulation of DNA biosynthetic process; P:positive regulation of DNA-templated transcription, elongation; F:U4 snRNA binding; F:identical protein binding; C:spliceosomal complex; C:transcription export complex; P:cellular response to DNA damage stimulus; C:U6 snRNP; P:spliceosomal complex assembly; C:U4 snRNP; F:RNA binding; F:U6 snRNA binding; C:cytoplasm; C:membrane; P:RNA secondary structure unwinding; C:nuclear matrix; P:mRNA 3'-end processing; P:negative regulation of DNA damage checkpoint; P:positive regulation of cell growth involved in cardiac muscle cell development |
| Efet.01.547096.g421.t1 | eukaryotic elongation factor 2 kinase | 240 | 1.48E-28 | 75.4 | P:protein autophosphorylation; F:translation factor activity, RNA binding; P:cellular response to oxygen-containing compound; P:translational elongation; P:response to peptide hormone; P:response to stress; F:elongation factor-2 kinase activity; F:ATP binding; P:positive regulation of nervous system development; F:calmodulin binding; F:calcium ion binding; P:positive regulation of cellular component organization; C:cytosol; P:cellular response to organonitrogen compound |
| Efet.01.547136.g422.t1 | TBC1 domain family member 2B | 486 | 1.24E-28 | 79.4 | P:regulation of vesicle fusion; P:activation of GTPase activity; F:GTPase activator activity; C:integral component of membrane; C:endomembrane system; P:intracellular protein transport; F:Rab GTPase binding; C:intracellular |
| Efet.01.547139.g423.t1 | sonic hedgehog | 972 | 1.33E-95 | 63.25 | C:extracellular space; P:intein-mediated protein splicing; P:multicellular organism development; F:metal ion binding; P:proteolysis; C:membrane; P:cell-cell signaling; F:peptidase activity; C:extracellular region; C:plasma membrane; F:hydrolase activity |
| Efet.01.54730.g315.t1 | armadillo repeat-containing 4-like | 645 | 1.22E-44 | 69.6 | C:intracellular organelle; P:multicellular organism development; C:organelle part |
| Efet.01.547320.g424.t1 | Zinc finger 622 | 465 | 3.13E-27 | 63.25 | P:positive regulation of cellular process; F:binding |
| Efet.01.547414.g425.t1 | neurexin-1a-like isoform X1 | 432 | 5.69E-37 | 65.3 | P:chemical synaptic transmission; C:membrane part |
| Efet.01.547445.g428.t1 | hypothetical protein HELRODRAFT_176473 | 720 | 2.96E-29 | 58.65 | C:membrane; C:integral component of membrane |
| Efet.01.547460.g429.t1 | 60S ribosomal L18 | 270 | 1.07E-08 | 87.83 | F:RNA binding; F:structural constituent of ribosome; C:cytosolic large ribosomal subunit; P:translation |
| Efet.01.547603.g434.t1 | protocadherin gamma-C5- | 1299 | 4.69E-117 | 60.85 | C:membrane |
| Efet.01.54767.g319.t1 | striatin-interacting 1 homolog isoform X2 | 249 | 5.80E-38 | 86.25 |  |
| Efet.01.54768.g320.t1 | NACHT and WD repeat domain-containing 2-like | 306 | 1.06E-13 | 59.86 |  |
| Efet.01.547803.g438.t1 | venom -2 | 225 | 9.57E-13 | 56.86 | C:membrane; C:integral component of membrane |
| Efet.01.547849.g439.t1 | inactive tyrosine- kinase 7 isoform X3 | 486 | 8.20E-49 | 66.7 | P:lung-associated mesenchyme development; P:planar cell polarity pathway involved in neural tube closure; P:cochlea morphogenesis; P:ventricular septum development; P:coronary vasculature development; P:protein phosphorylation; P:convergent extension; C:plasma membrane; P:axis elongation; F:ATP binding; P:wound healing; C:cell-cell junction; F:transmembrane receptor protein tyrosine kinase activity; P:establishment of planar polarity; P:establishment of epithelial cell apical/basal polarity |
| Efet.01.54803.g324.t1 | Polypeptide N-acetylgalactosaminyltransferase 5 | 504 | 2.28E-52 | 51.85 | F:transferase activity, transferring glycosyl groups; C:membrane; C:integral component of membrane; P:protein glycosylation; C:Golgi apparatus; C:Golgi membrane; F:transferase activity; F:carbohydrate binding |
| Efet.01.54808.g325.t1 | tripartite motif-containing 45-like isoform X6 | 2259 | 1.30E-90 | 63.75 | F:zinc ion binding; F:metal ion binding; C:intracellular |
| Efet.01.548151.g441.t1 | retrovirus-related Pol poly from transposon | 258 | 1.48E-45 | 74 | F:nucleic acid binding; F:RNA-directed DNA polymerase activity; F:transcription factor activity, sequence-specific DNA binding; F:zinc ion binding; P:DNA integration; P:regulation of transcription, DNA-templated; P:RNA-dependent DNA biosynthetic process |
| Efet.01.548169.g442.t1 | tumor necrosis factor alpha-induced 8 isoform X1 | 498 | 6.41E-60 | 74.85 | C:cytoplasm |
| Efet.01.548172.g443.t1 | mucin-17-like isoform X1 | 744 | 2.34E-35 | 64.85 | P:developmental process |
| Efet.01.548173.g444.t1 | chondroitin sulfate synthase 3 | 837 | 7.01E-83 | 71.4 | P:chondroitin sulfate biosynthetic process; F:N-acetylgalactosaminyl-proteoglycan 3-beta-glucuronosyltransferase activity; P:retina development in camera-type eye; P:sensory organ morphogenesis; F:glucuronosyl-N-acetylgalactosaminyl-proteoglycan 4-beta-N-acetylgalactosaminyltransferase activity; C:Golgi membrane |
| Efet.01.548217.g446.t1 | ectonucleoside triphosphate diphosphohydrolase 1-like | 366 | 1.61E-35 | 63.9 | C:membrane; C:integral component of membrane; F:hydrolase activity |
| Efet.01.548251.g447.t1 | histone | 552 | 2.29E-110 | 98 | F:histone deacetylase activity; P:histone deacetylation |
| Efet.01.548286.g448.t1 | myosin-M heavy chain-like | 1125 | 2.59E-35 | 65.4 | P:regulation of Rho protein signal transduction; F:Rho guanyl-nucleotide exchange factor activity; P:positive regulation of GTPase activity |
| Efet.01.548297.g449.t1 | HEAT repeat-containing 4-like | 315 | 1.19E-21 | 58.7 |  |
| Efet.01.548329.g450.t1 | histidine phosphatase family | 579 | 1.82E-70 | 71.85 |  |
| Efet.01.548516.g452.t1 | GPI-anchor transamidase | 399 | 1.31E-55 | 84.3 | F:GPI-anchor transamidase activity; P:proteolysis; F:peptidase activity; P:attachment of GPI anchor to protein; C:GPI-anchor transamidase complex |
| Efet.01.548599.g454.t1 | type II IV secretion system | 1380 | 0 | 80.7 | F:ATP binding; P:transport |
| Efet.01.548599.g455.t1 | type II secretion system F family | 1221 | 9.90E-157 | 76.6 | C:integral component of membrane |
| Efet.01.548599.g456.t1 | Flagellar hook-length control | 3741 | 0 | 63.6 | F:calcium ion binding; C:membrane |
| Efet.01.548599.g457.t1 | glyoxalase | 774 | 2.22E-164 | 53.25 | F:dioxygenase activity; P:oxidation-reduction process |
| Efet.01.548599.g458.t1 | excinuclease ABC subunit A | 3096 | 0 | 92.05 | F:ATPase activity; F:DNA binding; F:ATP binding; P:nucleic acid phosphodiester bond hydrolysis; F:zinc ion binding; F:excinuclease ABC activity; C:excinuclease repair complex; C:cytoplasm; P:nucleotide-excision repair; P:SOS response |
| Efet.01.548599.g459.t1 | family transcriptional regulator | 816 | 3.05E-173 | 83.2 | F:DNA binding; F:transcription factor activity, sequence-specific DNA binding; P:transcription, DNA-templated; C:integral component of membrane; P:regulation of transcription, DNA-templated |
| Efet.01.548599.g460.t1 | choline dehydrogenase | 1452 | 0 | 80 | F:flavin adenine dinucleotide binding; P:oxidation-reduction process; F:choline dehydrogenase activity |
| Efet.01.548599.g461.t1 | ABC transporter ATP-binding | 2115 | 0 | 71.7 | F:ATPase activity; F:ATP binding; C:integral component of membrane; P:transport; C:plasma membrane; F:transporter activity |
| Efet.01.548630.g462.t1 | neurogenic locus notch homolog 1-like isoform X1 | 846 | 2.17E-37 | 55.65 | C:extracellular region |
| Efet.01.5487.g426.t1 | T-box transcription factor | 504 | 3.47E-78 | 91.4 | P:positive regulation of cardiac muscle cell proliferation; P:pharynx development; F:RNA polymerase II core promoter proximal region sequence-specific DNA binding; P:palate development; P:developmental growth involved in morphogenesis; P:cellular senescence; P:cardiac muscle tissue development; P:regulation of transcription from RNA polymerase II promoter involved in myocardial precursor cell differentiation; P:negative regulation of heart looping; P:embryonic digit morphogenesis; P:negative regulation of transcription from RNA polymerase II promoter; P:outflow tract septum morphogenesis; P:endocardial cushion morphogenesis; C:transcription factor complex; F:transcriptional repressor activity, RNA polymerase II core promoter proximal region sequence-specific binding; P:atrioventricular canal development; P:mammary placode formation; P:transcription, DNA-templated; P:negative regulation of cardiac chamber formation; P:muscle cell fate determination; P:embryonic heart tube development; P:pharyngeal system development; C:nucleus; P:aorta morphogenesis; F:protein binding; P:Notch signaling pathway; P:embryonic camera-type eye morphogenesis |
| Efet.01.548703.g463.t1 | spectrin alpha non-erythrocytic 1-like isoform X1 | 363 | 5.71E-47 | 81.4 | F:actin binding; F:phospholipid binding |
| Efet.01.548779.g464.t1 | uncharacterized transporter slc- -like isoform X1 | 240 | 4.46E-16 | 71.9 | C:membrane |
| Efet.01.548789.g465.t1 | actin-binding LIM 2-like isoform X2 | 252 | 4.64E-19 | 73.9 | P:Roundabout signaling pathway; F:Roundabout binding; C:extracellular space; F:metal ion binding; P:regulation of axonogenesis; C:membrane; F:heparin binding; P:transport; C:proteinaceous extracellular matrix; P:axon extension involved in axon guidance |
| Efet.01.54879.g328.t1 | glutamate receptor NMDA 2B-like | 495 | 4.96E-66 | 74.15 | C:postsynaptic membrane; P:ion transmembrane transport; C:integral component of membrane; C:cell junction; P:ionotropic glutamate receptor signaling pathway; F:ionotropic glutamate receptor activity; F:extracellular-glutamate-gated ion channel activity |
| Efet.01.548842.g467.t1 | codanin-1-like | 210 | 2.32E-06 | 71.5 |  |
| Efet.01.549066.g468.t1 | hypothetical protein HELRODRAFT_159178 | 315 | 2.46E-41 | 71 | C:membrane; F:carbohydrate binding |
| Efet.01.549089.g470.t1 | hypothetical protein CAPTEDRAFT_225957 | 420 | 2.20E-11 | 46.67 | C:membrane; C:integral component of membrane; F:carbohydrate binding |
| Efet.01.5491.g428.t1 | carotenoid cleavage dioxygenase 8 homolog chloroplastic-like | 213 | 1.12E-08 | 50.85 | F:oxidoreductase activity, acting on single donors with incorporation of molecular oxygen, incorporation of two atoms of oxygen; P:oxidation-reduction process |
| Efet.01.549120.g471.t1 | IQ motif and SEC7 domain-containing 1 isoform X2 | 1359 | 1.35E-25 | 80.9 | F:ARF guanyl-nucleotide exchange factor activity; P:regulation of ARF protein signal transduction; P:positive regulation of GTPase activity |
| Efet.01.549137.g472.t1 | iron dicitrate transport regulator | 234 | 3.76E-49 | 67.7 | C:membrane; C:integral component of membrane |
| Efet.01.549162.g473.t1 | sorting nexin | 459 | 1.08E-55 | 74.75 | F:protein homodimerization activity; F:dynactin binding; F:phosphatidylinositol binding; P:intracellular protein transport; P:endocytosis; P:retrograde transport, endosome to Golgi; C:early endosome membrane; P:vesicle organization; P:negative regulation of transcription, DNA-templated; C:tubular endosome; C:intracellular membrane-bounded organelle; C:retromer complex; C:extrinsic component of membrane; F:type I transforming growth factor beta receptor binding; P:negative regulation of transforming growth factor beta receptor signaling pathway |
| Efet.01.549171.g474.t1 | Apoptosis regulator Bcl-2 | 1128 | 1.97E-13 | 48.15 | F:protein homodimerization activity; F:protein heterodimerization activity; C:membrane; C:integral component of membrane; P:regulation of apoptotic process; P:intrinsic apoptotic signaling pathway in response to DNA damage; P:extrinsic apoptotic signaling pathway in absence of ligand; P:negative regulation of intrinsic apoptotic signaling pathway; C:mitochondrial outer membrane |
| Efet.01.549184.g475.t1 | zinc transporter ZIP1-like | 1221 | 2.69E-29 | 57.65 | P:transmembrane transport; C:membrane; F:metal ion transmembrane transporter activity; P:metal ion transport |
| Efet.01.549223.g477.t1 | transcriptional regulator | 783 | 1.50E-126 | 71 |  |
| Efet.01.549255.g478.t1 | FAST kinase domain-containing 1-like | 1653 | 2.25E-29 | 48 | F:protein kinase activity; P:protein phosphorylation |
| Efet.01.549303.g479.t1 | potassium sodium hyperpolarization-activated cyclic nucleotide-gated channel 4 | 459 | 1.39E-68 | 75.8 | C:integral component of plasma membrane; F:voltage-gated potassium channel activity; P:regulation of membrane potential; P:potassium ion transmembrane transport |
| Efet.01.54939.g330.t1 | rab5 GDP GTP exchange factor-like | 921 | 2.15E-95 | 79.1 | F:DNA binding; F:zinc ion binding; C:integral component of membrane |
| Efet.01.54943.g331.t1 | Potassium voltage-gated channel Shal | 357 | 1.95E-15 | 81.5 | F:voltage-gated potassium channel activity; P:potassium ion transmembrane transport; C:voltage-gated potassium channel complex; P:regulation of ion transmembrane transport; P:protein homooligomerization |
| Efet.01.549455.g480.t1 | alpha-amlyase | 210 | 2.39E-10 | 72.6 | P:metabolic process; F:cation binding; F:hydrolase activity, acting on glycosyl bonds |
| Efet.01.549512.g481.t1 | neural-cadherin-like isoform X2 | 483 | 1.91E-35 | 52.05 | F:calcium ion binding; C:membrane; C:integral component of membrane; P:cell adhesion; P:homophilic cell adhesion via plasma membrane adhesion molecules; C:plasma membrane |
| Efet.01.549594.g483.t1 | plexin A3 | 978 | 2.29E-48 | 53.55 | P:signal transduction; C:membrane; F:semaphorin receptor activity; C:integral component of membrane; P:semaphorin-plexin signaling pathway |
| Efet.01.549679.g484.t1 | 4-hydroxybutyrate coenzyme A transferase-like | 1542 | 9.63E-116 | 73.85 | P:acetyl-CoA metabolic process; C:mitochondrion; C:cytosol; F:CoA-transferase activity; F:hydrolase activity |
| Efet.01.549689.g485.t1 | zinc finger 862-like | 1554 | 7.12E-79 | 52.5 | F:nucleic acid binding; F:transposase activity; P:transposition, DNA-mediated |
| Efet.01.54970.g333.t1 | potassium voltage-gated channel Shab-like | 894 | 1.11E-96 | 86.65 | F:voltage-gated potassium channel activity; P:potassium ion transmembrane transport; C:voltage-gated potassium channel complex; P:regulation of ion transmembrane transport; P:protein homooligomerization |
| Efet.01.549718.g486.t1 | adenylate kinase isoenzyme 1 isoform X2 | 696 | 5.04E-100 | 82.05 | P:cell cycle arrest; F:uridylate kinase activity; P:ADP biosynthetic process; P:nucleoside triphosphate biosynthetic process; P:nucleoside diphosphate phosphorylation; F:nucleoside diphosphate kinase activity; P:pyrimidine nucleotide biosynthetic process; C:plasma membrane; C:outer dense fiber; F:adenylate kinase activity; F:cytidylate kinase activity; F:ATP binding; C:extracellular exosome; P:'de novo' pyrimidine nucleobase biosynthetic process; C:cytoplasm; P:AMP metabolic process; P:ATP metabolic process |
| Efet.01.549740.g487.t1 | hypothetical protein HELRODRAFT_176332 | 726 | 8.93E-11 | 44 |  |
| Efet.01.549763.g488.t1 | irregular chiasm C-roughest -like isoform X1 | 357 | 3.37E-27 | 58.1 | C:membrane; C:integral component of membrane |
| Efet.01.54977.g335.t1 | RING finger 145-like | 2247 | 8.42E-168 | 58.5 | P:protein ubiquitination |
| Efet.01.549827.g490.t1 | FMRFamide receptor-like | 1119 | 1.18E-51 | 53.45 | F:G-protein coupled receptor activity; F:G-protein coupled peptide receptor activity; P:G-protein coupled receptor signaling pathway; C:membrane; C:integral component of membrane |
| Efet.01.54985.g336.t1 | hypothetical protein CAPTEDRAFT_205745 | 1956 | 2.39E-16 | 73 | F:chitin binding; P:chitin metabolic process; C:extracellular region |
| Efet.01.549909.g491.t1 | splicing factor U2af 38 kDa | 273 | 3.72E-22 | 94.8 | F:nucleotide binding; C:U2AF; F:RNA binding; F:metal ion binding; P:mRNA splicing, via spliceosome |
| Efet.01.54995.g337.t1 | SH3 and multiple ankyrin repeat domains 3-like isoform X3 | 681 | 1.57E-42 | 72.95 |  |
| Efet.01.550043.g494.t1 | hypothetical protein CAPTEDRAFT_180887 | 504 | 4.33E-44 | 71.95 | C:membrane |
| Efet.01.550084.g495.t1 | ABC transporter ATP-binding | 1317 | 1.33E-150 | 74.55 | F:ATPase activity; F:ATP binding |
| Efet.01.550084.g496.t1 | ABC transporter permease | 633 | 4.52E-68 | 77.8 | C:integral component of membrane; P:transport; C:plasma membrane |
| Efet.01.550127.g498.t1 | FAD NAD(P)-binding oxidoreductase | 1188 | 0 | 79.8 | F:oxidoreductase activity; P:oxidation-reduction process |
| Efet.01.550127.g499.t1 | ABC transporter permease | 738 | 1.89E-147 | 95.15 | C:integral component of membrane; P:transport; C:plasma membrane |
| Efet.01.550127.g500.t1 | ABC transporter permease | 747 | 7.07E-140 | 85.05 | C:integral component of membrane; P:transport; C:plasma membrane |
| Efet.01.550127.g501.t1 | Fe3+ spermidine putrescine ABC transporter ATP-binding | 1098 | 0 | 90.5 | F:ATPase activity; F:ATP binding; P:transmembrane transport; C:ATP-binding cassette (ABC) transporter complex; F:hydrolase activity, acting on acid anhydrides, catalyzing transmembrane movement of substances |
| Efet.01.550127.g502.t1 | aldehyde dehydrogenase | 612 | 4.70E-70 | 78.45 | F:oxidoreductase activity; F:electron carrier activity; P:oxidation-reduction process; F:heme binding |
| Efet.01.550134.g503.t1 | sex peptide receptor-like | 1212 | 3.98E-117 | 68.7 | F:G-protein coupled receptor activity; C:membrane |
| Efet.01.550166.g504.t1 | DNA-binding | 288 | 3.81E-60 | 81.95 |  |
| Efet.01.550200.g506.t1 | methyltransferase C9orf114 homolog isoform X3 | 333 | 6.29E-41 | 82.45 | P:cell division; F:methyltransferase activity; C:kinetochore; P:cell cycle; F:RNA binding; P:methylation |
| Efet.01.550202.g507.t1 | peptide nickel transport system substrate-binding | 1296 | 0 | 70.7 | P:transmembrane transport; C:ATP-binding cassette (ABC) transporter complex |
| Efet.01.550271.g508.t1 | PEP-CTERM sorting domain-containing | 1134 | 0 | 83.35 |  |
| Efet.01.550271.g509.t1 | tripartite-type tricarboxylate transporter receptor subunit | 987 | 0 | 89.35 | C:outer membrane-bounded periplasmic space |
| Efet.01.550271.g510.t1 | MFS transporter | 846 | 0 | 92.95 | C:outer membrane-bounded periplasmic space |
| Efet.01.550271.g511.t1 | chromosome segregation SMC | 1056 | 0 | 72.65 | F:ATP binding |
| Efet.01.550271.g512.t1 | cyclopropane-fatty-acyl-phospholipid synthase | 993 | 0 | 89.2 | F:cyclopropane-fatty-acyl-phospholipid synthase activity; P:lipid biosynthetic process; P:methylation |
| Efet.01.550298.g513.t1 | exocyst complex component 4-like | 900 | 8.66E-57 | 61.35 | P:transport |
| Efet.01.550400.g516.t1 | xylosyltransferase 2 | 867 | 6.07E-60 | 56.75 | P:macromolecule biosynthetic process; P:carbohydrate derivative biosynthetic process |
| Efet.01.550416.g517.t1 | neuromedin-U receptor 2-like | 627 | 1.30E-17 | 65.95 | F:G-protein coupled receptor activity; P:G-protein coupled receptor signaling pathway; C:membrane |
| Efet.01.550440.g518.t1 | hypothetical protein CAPTEDRAFT_223558 | 990 | 1.89E-14 | 67 |  |
| Efet.01.55045.g338.t1 | importin-13-like | 654 | 7.53E-19 | 54.2 | C:nuclear membrane; P:protein import into nucleus; F:protein transporter activity; P:intracellular protein transport; F:Ran GTPase binding; C:intracellular; F:nuclear localization sequence binding |
| Efet.01.550518.g519.t1 | hypothetical protein CRN43_01210 | 552 | 1.29E-120 | 56.33 |  |
| Efet.01.55071.g339.t1 | hypothetical protein CAPTEDRAFT_168270 | 1704 | 4.81E-95 | 51.7 |  |
| Efet.01.55071.g340.t1 | transmembrane 53-like isoform X2 | 1026 | 4.78E-31 | 49.35 |  |
| Efet.01.550788.g521.t1 | histone-lysine N- H3 lysine-36 and H4 lysine-20 specific | 1026 | 3.21E-18 | 62.45 | F:transferase activity |
| Efet.01.550874.g522.t1 | hypoxia up-regulated 1 | 522 | 3.06E-79 | 67.85 | F:nucleotide binding |
| Efet.01.550984.g524.t1 | neuronal acetylcholine receptor subunit beta-2-like | 588 | 3.71E-26 | 43.88 | P:ion transport; P:ion transmembrane transport; C:membrane; C:integral component of membrane; F:extracellular ligand-gated ion channel activity; P:transport |
| Efet.01.550998.g525.t1 | mannose-1-phosphate guanyltransferase beta | 297 | 1.22E-46 | 88.4 | F:GTP binding; P:GDP-mannose biosynthetic process; P:protein glycosylation; F:mannose-1-phosphate guanylyltransferase activity; P:muscle fiber development |
| Efet.01.55101.g343.t1 | E3 ubiquitin- ligase MYCBP2- | 1551 | 1.47E-179 | 71.5 | C:microtubule cytoskeleton; F:protein homodimerization activity; P:cellular macromolecule metabolic process; F:metal ion binding; P:regulation of protein localization; P:regulation of cytoskeleton organization; C:membrane; P:central nervous system projection neuron axonogenesis; P:negative regulation of protein catabolic process; P:primary metabolic process; C:axon; P:branchiomotor neuron axon guidance |
| Efet.01.551139.g526.t1 | magnesium transporter | 621 | 6.59E-73 | 72.65 | P:magnesium ion transport; C:membrane; P:cation transmembrane transport; F:cation transmembrane transporter activity |
| Efet.01.551171.g527.t1 | voltage-dependent calcium channel gamma-5 subunit-like | 873 | 1.03E-21 | 68.88 | P:calcium ion transport; C:integral component of membrane |
| Efet.01.551303.g529.t1 | methylmalonyl- carboxyltransferase | 573 | 1.27E-129 | 98.2 | F:acetyl-CoA carboxylase activity; F:transferase activity; P:fatty acid biosynthetic process; C:acetyl-CoA carboxylase complex |
| Efet.01.551303.g530.t1 | class I SAM-dependent RNA methyltransferase | 255 | 7.37E-14 | 88.25 | F:RNA methyltransferase activity; P:RNA methylation; P:RNA processing; F:methyltransferase activity; F:transferase activity; P:methylation |
| Efet.01.551358.g531.t1 | vang 1 | 306 | 6.75E-08 | 54.83 | P:multicellular organism development; C:membrane; C:integral component of membrane |
| Efet.01.551491.g534.t1 | band 3 anion transport -like isoform X1 | 276 | 6.12E-26 | 79.45 | P:inorganic anion transport; C:integral component of plasma membrane; P:regulation of intracellular pH; P:anion transmembrane transport; F:inorganic anion exchanger activity; P:bicarbonate transport |
| Efet.01.551505.g535.t1 | pantetheine-phosphate adenylyltransferase | 471 | 7.65E-112 | 84.1 | F:ATP binding; C:cytoplasm; P:coenzyme A biosynthetic process; F:pantetheine-phosphate adenylyltransferase activity |
| Efet.01.551547.g538.t1 | tight junction ZO-2 isoform X2 | 5421 | 9.89E-58 | 51.95 |  |
| Efet.01.55161.g345.t1 | sex peptide receptor-like | 627 | 1.60E-55 | 71.3 | F:G-protein coupled receptor activity; C:membrane |
| Efet.01.551655.g542.t1 | ribonucleo RB97D isoform X1 | 429 | 6.14E-18 | 73.25 | F:nucleotide binding; F:nucleic acid binding; C:intracellular ribonucleoprotein complex; C:viral nucleocapsid |
| Efet.01.551753.g544.t1 | Ig-like domain-containing | 1719 | 1.75E-86 | 47.6 | F:carbohydrate binding |
| Efet.01.551753.g545.t1 | tRNA uridine-5-carboxymethylaminomethyl(34) synthesis enzyme | 1443 | 0 | 84.1 | C:cytoplasm; F:oxidoreductase activity; F:flavin adenine dinucleotide binding; P:oxidation-reduction process; P:tRNA wobble uridine modification |
| Efet.01.551788.g546.t1 | hypothetical protein CAPTEDRAFT_224550 | 396 | 8.01E-13 | 61 | P:signal transduction; C:membrane; C:integral component of membrane |
| Efet.01.5518.g429.t1 | NADH dehydrogenase [ubiquinone] iron-sulfur mitochondrial | 753 | 5.57E-80 | 66.45 | C:mitochondrial respiratory chain complex I; F:NADH dehydrogenase activity; P:metabolic process; P:negative regulation of cellular process |
| Efet.01.551856.g547.t1 | hypothetical protein CAPTEDRAFT_158449 | 462 | 1.21E-06 | 80 | P:cation transport; P:cation transmembrane transport; C:membrane; C:integral component of membrane; F:cation transmembrane transporter activity |
| Efet.01.551887.g548.t1 | cyclic GMP-AMP synthase-like | 930 | 1.51E-18 | 47.6 | C:membrane; C:integral component of membrane |
| Efet.01.551910.g549.t1 | tripartite motif-containing 2-like | 2019 | 2.58E-42 | 48.05 | F:zinc ion binding; F:metal ion binding; C:intracellular |
| Efet.01.552029.g552.t1 | carboxypeptidase D-like | 516 | 2.55E-39 | 68.5 | F:carboxypeptidase activity; C:intracellular membrane-bounded organelle; C:membrane; P:proteolysis |
| Efet.01.55204.g346.t1 | coiled-coil domain-containing 57-like | 588 | 2.10E-25 | 72.05 |  |
| Efet.01.552138.g553.t1 | BTB POZ domain-containing KCTD6 | 234 | 1.66E-18 | 69.9 | P:protein homooligomerization |
| Efet.01.552145.g554.t1 | cAMP-dependent kinase catalytic subunit | 441 | 3.87E-51 | 65 | C:nucleus; F:ATP binding; P:regulation of cellular process; C:cytoplasm; P:angiogenesis; P:endothelial cell proliferation; P:cell differentiation; F:cAMP-dependent protein kinase activity; P:cell adhesion; P:animal organ development; P:protein phosphorylation |
| Efet.01.552187.g556.t1 | catenin beta-like | 1011 | 2.52E-174 | 88.55 | F:alpha-catenin binding; C:nucleus; C:cytoskeleton; F:protein phosphatase binding; C:cytoplasm; P:signal transduction; F:cadherin binding; C:catenin complex; P:cell adhesion; P:adherens junction assembly; F:signal transducer activity |
| Efet.01.552216.g557.t1 | ecdysone receptor | 330 | 1.48E-43 | 72.8 | C:nucleus; F:zinc ion binding; P:transcription, DNA-templated; P:ecdysone receptor-mediated signaling pathway; P:regulation of transcription, DNA-templated; F:sequence-specific DNA binding; F:steroid binding; F:ecdysteroid hormone receptor activity |
| Efet.01.552276.g558.t1 | guanylate cyclase soluble subunit beta-1 | 852 | 1.39E-44 | 55.6 | F:lyase activity; P:single-organism process; P:cellular process |
| Efet.01.552303.g559.t1 | Cytochrome P450 10 | 231 | 5.49E-15 | 72.8 | F:iron ion binding; F:oxidoreductase activity, acting on paired donors, with incorporation or reduction of molecular oxygen; P:oxidation-reduction process; F:monooxygenase activity; F:heme binding |
| Efet.01.552317.g560.t1 | Sodium-driven chloride bicarbonate exchanger | 330 | 1.93E-32 | 69.55 | P:inorganic anion transport; P:anion transmembrane transport; C:integral component of membrane; F:inorganic anion exchanger activity |
| Efet.01.552381.g562.t1 | KN motif and ankyrin repeat domain-containing 1 isoform X3 | 390 | 1.74E-06 | 69.67 | C:membrane; C:integral component of membrane |
| Efet.01.55244.g347.t1 | transmembrane 136 isoform X2 | 594 | 1.87E-30 | 59.2 | C:membrane; C:integral component of membrane |
| Efet.01.552532.g566.t1 | serine protease | 348 | 9.69E-10 | 71.2 | P:proteolysis; F:serine-type endopeptidase activity |
| Efet.01.552607.g568.t1 | cytochrome C oxidase subunit II | 681 | 1.20E-156 | 75.7 | F:cytochrome-c oxidase activity; F:copper ion binding; C:integral component of membrane; P:hydrogen ion transmembrane transport; P:electron transport chain; C:plasma membrane; C:respiratory chain |
| Efet.01.552701.g570.t1 | omega-amidase NIT2 | 324 | 3.02E-28 | 74 | F:hydrolase activity, acting on carbon-nitrogen (but not peptide) bonds |
| Efet.01.552742.g571.t1 | allatostatin-A receptor-like | 534 | 1.97E-25 | 71.35 | P:signal transduction; C:membrane; F:signal transducer activity; P:transport |
| Efet.01.552889.g573.t1 | acyl-coenzyme A synthetase mitochondrial-like isoform X2 | 234 | 4.02E-23 | 74.65 | P:acyl-CoA metabolic process; F:acyl-CoA ligase activity; C:mitochondrial matrix; F:binding; P:fatty acid biosynthetic process; F:fatty-acyl-CoA synthase activity; F:fatty acid ligase activity |
| Efet.01.55309.g350.t1 | EGF-like repeat and discoidin I-like domain-containing 3 | 375 | 7.42E-08 | 61.25 | C:membrane; C:integral component of membrane |
| Efet.01.553112.g576.t1 | rhabdomeric opsin 3 | 705 | 8.79E-63 | 65.55 | P:signal transduction; C:membrane; F:signaling receptor activity |
| Efet.01.553238.g577.t1 | forkhead box J3 | 321 | 5.12E-15 | 66.4 | C:nucleus; P:transcription, DNA-templated; P:positive regulation of transcription, DNA-templated; F:sequence-specific DNA binding; F:RNA polymerase II transcription factor activity, sequence-specific DNA binding; P:regulation of transcription from RNA polymerase II promoter |
| Efet.01.553257.g578.t1 | transketolase | 444 | 1.76E-85 | 91.3 | F:catalytic activity; P:metabolic process |
| Efet.01.553264.g579.t1 | serine threonine- kinase RIO2 | 435 | 1.50E-59 | 79.1 | F:ATP binding; F:protein serine/threonine kinase activity; P:protein phosphorylation |
| Efet.01.553292.g580.t1 | DNA alkylation response | 1731 | 0 | 82.9 | F:acyl-CoA dehydrogenase activity; F:flavin adenine dinucleotide binding; P:oxidation-reduction process |
| Efet.01.553292.g581.t1 | tripartite tricarboxylate transporter substrate binding | 852 | 0 | 87.5 | C:outer membrane-bounded periplasmic space |
| Efet.01.553292.g582.t1 | MBL fold metallo-hydrolase | 1800 | 0 | 83.95 | F:hydrolase activity |
| Efet.01.553364.g584.t1 | Dipeptidyl peptidase 1 | 225 | 2.73E-10 | 58 | P:proteolysis; F:cysteine-type peptidase activity; F:peptidase activity; F:hydrolase activity |
| Efet.01.553365.g585.t1 | PREDICTED: uncharacterized protein LOC106170360 | 207 | 2.91E-10 | 66 | C:nucleus; F:methyltransferase activity; F:transferase activity; P:methylation |
| Efet.01.553365.g587.t1 | Histone-lysine N-methyltransferase 2A | 1200 | 9.89E-10 | 50.4 |  |
| Efet.01.553377.g588.t1 | la-related 6-like | 1473 | 1.13E-101 | 74.65 | F:nucleotide binding; F:nucleic acid binding; P:cellular macromolecule metabolic process; P:primary metabolic process; C:intracellular part |
| Efet.01.553408.g589.t1 | beta-N-acetylhexosaminidase | 837 | 0 | 87.4 | P:regulation of cell shape; P:cell division; P:carbohydrate metabolic process; C:cytoplasm; P:peptidoglycan turnover; P:peptidoglycan biosynthetic process; P:cell wall organization; F:beta-N-acetylhexosaminidase activity; P:cell cycle |
| Efet.01.553408.g590.t1 | beta-ketoacyl-ACP reductase | 705 | 3.00E-172 | 98 | F:3-oxoacyl-[acyl-carrier-protein] reductase (NADPH) activity; C:cytoplasm; P:poly-hydroxybutyrate biosynthetic process; P:oxidation-reduction process; F:acetoacetyl-CoA reductase activity |
| Efet.01.553408.g591.t1 | efflux RND transporter periplasmic adaptor subunit | 627 | 1.71E-72 | 92.5 | P:transmembrane transport; C:membrane |
| Efet.01.553421.g592.t1 | 3-isopropylmalate dehydratase large subunit | 831 | 2.61E-99 | 91.9 | F:metal ion binding; P:leucine biosynthetic process; F:4 iron, 4 sulfur cluster binding; F:3-isopropylmalate dehydratase activity |
| Efet.01.553463.g594.t1 | RNA-directed DNA polymerase from transposon BS | 387 | 2.10E-12 | 71.6 | F:RNA-directed DNA polymerase activity; F:RNA binding; P:RNA-dependent DNA biosynthetic process |
| Efet.01.553483.g595.t1 | histone-lysine N-methyltransferase 2D-like [Zeugodacus cucurbitae] | 699 | 4.34E-13 | 65.8 | F:transferase activity |
| Efet.01.553529.g596.t1 | roundabout homolog 4 | 321 | 1.05E-16 | 62.4 | P:system development |
| Efet.01.553639.g597.t1 | ABC transporter permease | 1695 | 5.10E-120 | 72.8 | C:integral component of membrane; P:transport; C:plasma membrane |
| Efet.01.553639.g598.t1 | dihydroxy-acid dehydratase | 3360 | 0 | 90 | P:transmembrane transport; F:dihydroxy-acid dehydratase activity; P:metabolic process; F:xylonate dehydratase activity; C:ATP-binding cassette (ABC) transporter complex |
| Efet.01.553639.g599.t1 | NAD(P)-dependent oxidoreductase | 756 | 7.98E-155 | 79.3 | F:D-xylose 1-dehydrogenase (NAD) activity; P:oxidation-reduction process |
| Efet.01.553639.g600.t1 | beta-glucosidase | 2409 | 0 | 80.3 | P:carbohydrate metabolic process; F:hydrolase activity, hydrolyzing O-glycosyl compounds |
| Efet.01.553639.g601.t1 | glycoside hydrolase | 1482 | 0 | 75.8 | P:carbohydrate metabolic process; F:hydrolase activity, hydrolyzing O-glycosyl compounds |
| Efet.01.553639.g602.t1 | ABC transporter substrate-binding | 2529 | 0 | 83.55 | P:transport; F:transporter activity |
| Efet.01.553639.g603.t1 | sn-glycerol-3-phosphate ABC transporter ATP-binding | 402 | 6.44E-51 | 90.9 | F:ATP binding; P:glycerol-3-phosphate transport; P:transmembrane transport; F:glycerol-3-phosphate-transporting ATPase activity; P:glycerophosphodiester transport; P:carbohydrate transport; C:ATP-binding cassette (ABC) transporter complex |
| Efet.01.55365.g355.t1 | NACHT and WD repeat domain-containing 2-like | 489 | 2.67E-43 | 57.75 |  |
| Efet.01.55373.g356.t1 | probable G- coupled receptor 139 | 783 | 7.44E-39 | 60.6 | P:signal transduction; C:membrane; F:signal transducer activity |
| Efet.01.55373.g357.t1 | probable G- coupled receptor 139 | 390 | 7.81E-22 | 63.55 | P:signal transduction; C:membrane; F:signal transducer activity |
| Efet.01.55385.g358.t1 | Retrovirus-related Pol poly from transposon | 501 | 6.29E-08 | 45.83 | F:nucleic acid binding; F:zinc ion binding; F:metal ion binding |
| Efet.01.553917.g605.t1 | leucine-rich repeat-containing 52-like | 834 | 4.12E-08 | 54.7 | C:membrane; C:integral component of membrane |
| Efet.01.554123.g606.t1 | hypothetical protein CAPTEDRAFT_226427 | 714 | 4.11E-44 | 48.53 | F:calcium ion binding; C:membrane; C:integral component of membrane |
| Efet.01.554142.g607.t1 | signal recognition particle | 525 | 8.22E-91 | 91.05 | F:GTP binding; P:SRP-dependent cotranslational protein targeting to membrane; C:signal recognition particle; F:GTPase activity; F:7S RNA binding |
| Efet.01.554143.g608.t1 | nucleosome-remodeling factor subunit NURF301-like isoform X1 | 1221 | 4.44E-76 | 65.1 | F:zinc ion binding; F:metal ion binding |
| Efet.01.554267.g609.t1 | microcystin degradation | 1185 | 0 | 85.3 |  |
| Efet.01.554294.g612.t1 | neuronal acetylcholine receptor subunit alpha-5-like isoform X1 | 468 | 2.91E-28 | 67.2 | F:ion channel activity; P:ion transmembrane transport; C:synapse; C:membrane part; C:plasma membrane |
| Efet.01.554376.g613.t1 | singed | 675 | 2.07E-99 | 69.1 | F:actin binding; C:intracellular part |
| Efet.01.554457.g614.t1 | mitogen-activated kinase kinase kinase 10-like isoform X2 | 363 | 2.94E-29 | 64.7 | F:protein kinase activity; P:phosphorylation |
| Efet.01.554460.g615.t1 | myosin-VIIa | 285 | 7.93E-08 | 56.65 | F:binding; C:intracellular part; P:animal organ morphogenesis |
| Efet.01.554463.g616.t1 | Pre-mRNA cleavage complex 2 Pcf11 | 228 | 1.76E-13 | 71.65 | C:cytoplasm; C:mRNA cleavage factor complex; F:mRNA binding; P:termination of RNA polymerase II transcription; F:RNA polymerase II core binding; P:mRNA polyadenylation; P:mRNA cleavage |
| Efet.01.554557.g617.t1 | PREDICTED: uncharacterized protein LOC109061345 | 243 | 2.75E-06 | 61 |  |
| Efet.01.554586.g618.t1 | acyl- transferase | 456 | 2.11E-100 | 68.45 | F:transferase activity |
| Efet.01.554586.g619.t1 | crotonase | 702 | 8.27E-149 | 92.5 | F:1,4-dihydroxy-2-naphthoyl-CoA synthase activity; P:metabolic process; F:isomerase activity |
| Efet.01.554608.g621.t1 | DNA methyltransferase 1-associated 1-like isoform X2 | 213 | 1.59E-08 | 79.67 | P:histone H4 acetylation; P:DNA repair; P:chromatin remodeling; C:NuA4 histone acetyltransferase complex; P:histone H2A acetylation; P:negative regulation of transcription, DNA-templated |
| Efet.01.55463.g359.t1 | hypothetical protein LOTGIDRAFT_112762 | 2166 | 6.68E-06 | 60 | C:membrane; C:integral component of membrane |
| Efet.01.554660.g622.t1 | 6-phosphofructokinase | 477 | 1.87E-107 | 84.55 | F:ATP binding; C:cytoplasm; F:metal ion binding; P:fructose 6-phosphate metabolic process; P:glycolytic process through fructose-6-phosphate; P:carbohydrate phosphorylation; F:6-phosphofructokinase activity |
| Efet.01.554745.g623.t1 | dynein heavy chain axonemal | 264 | 2.10E-36 | 82.15 | F:ATPase activity; F:ATP binding; F:microtubule motor activity; C:dynein complex; P:microtubule-based movement |
| Efet.01.554757.g626.t1 | PIN domain-containing | 285 | 3.82E-20 | 88.15 | F:magnesium ion binding; P:RNA phosphodiester bond hydrolysis; F:ribonuclease activity |
| Efet.01.554764.g627.t1 | hypothetical protein B5766_02500 | 450 | 7.77E-92 | 54.9 |  |
| Efet.01.554796.g628.t1 | potassium voltage-gated channel Shab isoform X3 | 219 | 4.54E-10 | 77.3 | P:potassium ion transport; P:transmembrane transport; C:integral component of membrane; F:voltage-gated ion channel activity; F:potassium channel activity; P:cellular process |
| Efet.01.554957.g630.t1 | uncharacterized protein LOC107447601, partial | 666 | 1.08E-30 | 54.25 | F:zinc ion binding; F:aspartic-type endopeptidase activity; F:metal ion binding; P:proteolysis |
| Efet.01.554999.g631.t1 | hypothetical protein CAPTEDRAFT_196347 | 708 | 4.10E-09 | 79 |  |
| Efet.01.555069.g632.t1 | T family of potassium channels 7-like | 405 | 1.21E-30 | 77.95 | C:integral component of plasma membrane; P:potassium ion transmembrane transport; P:stabilization of membrane potential; F:potassium ion leak channel activity |
| Efet.01.555072.g633.t1 | voltage-dependent calcium channel gamma-5 subunit-like | 222 | 3.84E-16 | 65.4 | P:ion transport; C:membrane |
| Efet.01.555104.g634.t1 | nuclear envelope integral membrane 1-like | 327 | 1.06E-15 | 61.05 | C:nuclear envelope; P:hermaphrodite genitalia development; P:nematode larval development; P:embryo development ending in birth or egg hatching; C:membrane; C:integral component of membrane; P:reproduction |
| Efet.01.555240.g637.t1 | F0F1 ATP synthase subunit alpha | 1179 | 0 | 90.8 | C:proton-transporting ATP synthase complex, catalytic core F(1); F:ATP binding; P:ATP hydrolysis coupled proton transport; F:proton-transporting ATPase activity, rotational mechanism; C:plasma membrane; F:proton-transporting ATP synthase activity, rotational mechanism; P:plasma membrane ATP synthesis coupled proton transport |
| Efet.01.55527.g361.t1 | sestrin homolog isoform X3 | 291 | 3.23E-17 | 57.55 |  |
| Efet.01.55530.g362.t1 | Protocadherin-11 X-linked | 768 | 7.75E-42 | 55.75 | F:calcium ion binding; C:membrane; C:integral component of membrane; P:cell adhesion; P:homophilic cell adhesion via plasma membrane adhesion molecules; C:plasma membrane |
| Efet.01.555350.g639.t1 | A disintegrin and metallo ase with thrombospondin motifs 7 isoform X1 | 771 | 6.49E-15 | 52.35 | F:zinc ion binding; P:integrin-mediated signaling pathway; P:proteolysis; C:membrane; C:integral component of membrane; C:extracellular matrix; F:carbohydrate binding; F:metalloendopeptidase activity; C:proteinaceous extracellular matrix; F:metallopeptidase activity |
| Efet.01.555357.g640.t1 | tRNA dihydrouridine synthase | 948 | 1.31E-130 | 87.15 | F:tRNA dihydrouridine synthase activity; P:tRNA dihydrouridine synthesis; F:flavin adenine dinucleotide binding; P:oxidation-reduction process |
| Efet.01.555360.g641.t1 | hypothetical protein CAPTEDRAFT_211208 | 279 | 7.35E-08 | 63 | P:signal transduction; C:membrane; C:integral component of membrane |
| Efet.01.555392.g642.t1 | stoned-B-like | 717 | 2.05E-112 | 70.9 | P:vesicle-mediated transport |
| Efet.01.555546.g645.t1 | rho GDP-dissociation inhibitor 2 isoform X1 | 444 | 2.96E-55 | 56.7 | C:cytoplasm; F:Rho GDP-dissociation inhibitor activity; P:regulation of catalytic activity |
| Efet.01.555586.g647.t1 | transcription factor SOX-15 | 684 | 4.32E-15 | 69.7 |  |
| Efet.01.555692.g648.t1 | T family of potassium channels 7-like | 915 | 3.10E-38 | 61.75 | C:membrane; P:transport |
| Efet.01.555710.g649.t1 | transmembrane 145 | 1473 | 8.48E-39 | 58.9 | P:G-protein coupled receptor signaling pathway; C:membrane; C:integral component of membrane; P:response to pheromone |
| Efet.01.555721.g650.t1 | transmembrane 161B | 912 | 8.02E-100 | 65.5 | C:membrane |
| Efet.01.555974.g655.t1 | Voltage-gated hydrogen channel 1 | 381 | 1.68E-39 | 67.65 | C:integral component of membrane; P:transport |
| Efet.01.556035.g656.t1 | hypothetical protein HELRODRAFT_180238 | 516 | 2.56E-06 | 63.5 | P:sodium ion transport; P:ion transport; F:sodium channel activity; C:membrane; C:integral component of membrane; P:sodium ion transmembrane transport; P:transport |
| Efet.01.556088.g657.t1 | myosin heavy chain | 372 | 1.59E-15 | 66.7 | F:small molecule binding |
| Efet.01.556107.g658.t1 | 2-phosphoxylose phosphatase 1-like | 846 | 3.33E-55 | 51.55 | P:dephosphorylation; F:acid phosphatase activity |
| Efet.01.556123.g659.t1 | kelch 35 | 690 | 3.29E-19 | 50 | F:ubiquitin-protein transferase activity; C:Cul3-RING ubiquitin ligase complex; P:protein ubiquitination |
| Efet.01.556127.g660.t1 | hypothetical protein CRN43_16135 | 777 | 2.73E-176 | 47.3 |  |
| Efet.01.556127.g661.t1 | valine--tRNA ligase | 708 | 1.09E-123 | 81.4 | F:valine-tRNA ligase activity; F:ATP binding; P:valyl-tRNA aminoacylation; C:cytoplasm; P:regulation of translational fidelity; F:aminoacyl-tRNA editing activity |
| Efet.01.556159.g662.t1 | hypothetical protein CAPTEDRAFT_214871 | 291 | 2.46E-07 | 58 | C:membrane; C:integral component of membrane |
| Efet.01.55619.g363.t1 | hypothetical protein CAPTEDRAFT_211886 | 435 | 1.90E-12 | 53 | C:membrane; C:integral component of membrane; F:carbohydrate binding |
| Efet.01.55624.g364.t1 | hypothetical protein CAPTEDRAFT_226427 | 441 | 1.55E-20 | 62.3 | F:binding |
| Efet.01.55629.g365.t1 | sodium-dependent multivitamin transporter isoform X1 | 501 | 1.22E-27 | 72.75 | P:transmembrane transport; C:integral component of membrane; F:transporter activity |
| Efet.01.556294.g664.t1 | transferase | 651 | 6.90E-134 | 80.8 | P:carbohydrate metabolic process; F:transferase activity |
| Efet.01.556322.g665.t1 | hypothetical protein B5766_00685 | 318 | 8.02E-35 | 100 |  |
| Efet.01.556322.g666.t1 | ABC transporter permease | 1083 | 0 | 59 |  |
| Efet.01.556379.g669.t1 | glutamine ABC transporter substrate-binding | 612 | 8.37E-96 | 86.65 | C:membrane; P:ionotropic glutamate receptor signaling pathway; F:ionotropic glutamate receptor activity; P:transport |
| Efet.01.556379.g670.t1 | hybrid sensor histidine kinase response regulator | 2202 | 0 | 85.45 | F:phosphorelay sensor kinase activity; P:chemotaxis; C:cytoplasm; P:phosphorelay signal transduction system; P:signal transduction by protein phosphorylation; P:peptidyl-histidine phosphorylation |
| Efet.01.556410.g671.t1 | general secretion pathway | 600 | 2.94E-48 | 62.5 | P:extracellular transport; C:extracellular region |
| Efet.01.556487.g672.t1 | transmembrane mitochondrial-like | 516 | 9.39E-56 | 70.85 | C:integral component of membrane |
| Efet.01.556705.g673.t1 | 5-hydroxytryptamine receptor 2C-like | 1374 | 8.98E-29 | 59.1 | P:G-protein coupled receptor signaling pathway; C:integral component of membrane; P:serotonin receptor signaling pathway; F:G-protein coupled serotonin receptor activity |
| Efet.01.556746.g674.t1 | plasma membrane calcium-transporting ATPase 2 isoform X1 | 201 | 3.70E-20 | 87 | F:ATP binding; F:metal ion binding; C:integral component of membrane; P:calcium ion transmembrane transport; F:calcium-transporting ATPase activity |
| Efet.01.556872.g676.t1 | integrase | 504 | 1.83E-74 | 81.15 | P:DNA metabolic process |
| Efet.01.55694.g370.t1 | transcription factor Sp9-like | 942 | 3.90E-61 | 75.45 | F:nucleic acid binding; F:metal ion binding |
| Efet.01.556962.g678.t1 | dynein heavy chain axonemal-like | 792 | 4.70E-128 | 90.6 | F:microtubule motor activity; F:calcium ion binding; C:dynein complex; P:microtubule-based movement |
| Efet.01.55698.g371.t1 | type I inositol 3,4-bisphosphate 4-phosphatase-like isoform X2 | 348 | 4.95E-08 | 51.13 | P:protein deubiquitination; F:hydrolase activity; F:thiol-dependent ubiquitinyl hydrolase activity; P:ubiquitin-dependent protein catabolic process |
| Efet.01.55702.g372.t1 | cytochrome P450 2J6-like isoform X2 | 894 | 5.53E-50 | 52.9 | F:oxidoreductase activity |
| Efet.01.557101.g679.t1 | dynein heavy chain axonemal-like isoform X1 | 630 | 8.60E-86 | 69.8 | F:ATPase activity; F:microtubule motor activity; C:axonemal dynein complex; C:dynein complex; P:cilium movement; P:microtubule-based movement |
| Efet.01.557109.g681.t1 | phosphatase PAP2 family | 330 | 2.43E-34 | 73 | C:membrane |
| Efet.01.557109.g682.t1 | elongation factor P | 564 | 4.70E-131 | 95.95 | F:translation elongation factor activity; C:cytoplasm; P:translational elongation |
| Efet.01.557161.g684.t1 | tyrosinase 1 | 594 | 3.92E-16 | 46.85 | F:oxidoreductase activity; F:metal ion binding; P:metabolic process; P:oxidation-reduction process |
| Efet.01.557284.g686.t1 | KN motif and ankyrin repeat domain-containing 1 | 342 | 1.13E-15 | 70.65 |  |
| Efet.01.557312.g687.t1 | hypothetical protein HELRODRAFT_190584 | 351 | 8.21E-20 | 62 | F:nucleotide binding; F:nucleic acid binding; P:lipid metabolic process; C:membrane; C:integral component of membrane; F:transferase activity; P:fatty acid biosynthetic process; P:fatty acid metabolic process |
| Efet.01.557378.g688.t1 | hypothetical protein CAPTEDRAFT_189452 | 609 | 1.32E-09 | 47 | P:intracellular signal transduction; C:intracellular |
| Efet.01.557426.g689.t1 | PREDICTED: uncharacterized protein LOC106163300 | 339 | 4.50E-21 | 69.9 | C:cytoplasm; C:membrane; F:S-adenosylmethionine-dependent methyltransferase activity; P:methylation |
| Efet.01.557490.g691.t1 | methylcrotonoyl- carboxylase | 1200 | 0 | 69.1 | F:ATP binding; F:metal ion binding; F:biotin carboxylase activity |
| Efet.01.557503.g692.t1 | bestrophin homolog 22-like | 237 | 1.73E-21 | 80.3 | C:chloride channel complex; C:integral component of plasma membrane; P:chloride transmembrane transport; P:sensory perception of smell; C:cilium; F:chloride channel activity; P:membrane depolarization |
| Efet.01.557638.g693.t1 | beta carbonic anhydrase 1-like | 249 | 3.64E-40 | 80.4 | F:zinc ion binding; F:carbonate dehydratase activity |
| Efet.01.557665.g694.t1 | FAD-binding oxidoreductase | 1185 | 0 | 89.45 | F:D-amino-acid dehydrogenase activity; F:sarcosine oxidase activity; P:oxidation-reduction process |
| Efet.01.557665.g695.t1 | N-formylglutamate deformylase | 813 | 0 | 77.3 | P:peptidoglycan catabolic process; F:N-acetylmuramoyl-L-alanine amidase activity |
| Efet.01.557665.g696.t1 | electron transfer flavo beta subunit | 666 | 4.86E-122 | 97 | F:electron carrier activity |
| Efet.01.557665.g697.t1 | electron transfer flavo alpha subunit apo | 933 | 8.92E-167 | 83.15 | F:flavin adenine dinucleotide binding; F:electron carrier activity |
| Efet.01.557665.g698.t1 | FAD-binding oxidoreductase | 396 | 7.13E-89 | 75.4 | F:D-lactate dehydrogenase (cytochrome) activity; F:flavin adenine dinucleotide binding; P:oxidation-reduction process |
| Efet.01.557665.g699.t1 | FAD-binding oxidoreductase | 960 | 4.24E-152 | 75.4 | F:D-lactate dehydrogenase (cytochrome) activity; F:flavin adenine dinucleotide binding; P:oxidation-reduction process |
| Efet.01.55780.g375.t1 | atrial natriuretic peptide receptor 1-like isoform X1 | 741 | 3.40E-44 | 60.65 | F:lyase activity; P:primary metabolic process; P:single-organism process; P:organic substance metabolic process; P:phosphate-containing compound metabolic process |
| Efet.01.557838.g701.t1 | pilus assembly | 2652 | 0 | 81.4 | C:integral component of membrane |
| Efet.01.55784.g376.t1 | bark beetle-like | 429 | 2.51E-33 | 57.05 | C:membrane |
| Efet.01.557911.g703.t1 | gfo Idh family oxidoreductase | 420 | 6.53E-57 | 80 | F:oxidoreductase activity; P:oxidation-reduction process |
| Efet.01.557920.g704.t1 | meningioma expressed antigen 5 (hyaluronidase) isoform CRA_c | 324 | 5.53E-07 | 58.5 | P:protein deglycosylation; C:nucleus; C:membrane; C:cytosol; F:beta-N-acetylglucosaminidase activity; P:N-acetylglucosamine metabolic process; P:histone acetylation |
| Efet.01.558052.g705.t1 | folliculin-interacting 1 isoform X2 | 1380 | 2.55E-10 | 85.5 |  |
| Efet.01.558121.g706.t1 | lysozyme | 345 | 1.86E-22 | 65.5 | F:lysozyme activity |
| Efet.01.55841.g377.t1 | pilus assembly | 1302 | 4.19E-128 | 62.4 | C:membrane; C:integral component of membrane |
| Efet.01.55841.g378.t1 | IS3 family transposase | 321 | 9.75E-29 | 68.1 | F:nucleic acid binding; P:DNA integration |
| Efet.01.558489.g708.t1 | Testis-expressed sequence 33 | 537 | 1.74E-08 | 61.33 |  |
| Efet.01.558516.g709.t1 | Importin-9 | 267 | 4.18E-16 | 81.2 | F:zinc ion binding; P:intracellular protein transport; F:Ran GTPase binding; C:intracellular |
| Efet.01.558562.g710.t1 | serine protease | 891 | 4.44E-146 | 75.75 | C:membrane; F:peptidase activity |
| Efet.01.558574.g711.t1 | tripartite motif-containing 2-like | 1968 | 3.83E-12 | 46.17 | F:zinc ion binding; F:metal ion binding; C:intracellular |
| Efet.01.558699.g712.t1 | adenosine kinase | 1170 | 0 | 61.8 | F:transferase activity |
| Efet.01.558703.g713.t1 | ATP-binding cassette sub-family F member 2 | 600 | 3.53E-92 | 85.45 | F:ATPase activity; C:nucleus; F:DNA binding; F:ATP binding; F:protein heterodimerization activity; C:nucleosome |
| Efet.01.558740.g714.t1 | ADP-ribosylation factor 4C | 528 | 3.71E-88 | 82.3 | F:GTP binding; P:small GTPase mediated signal transduction; C:intracellular |
| Efet.01.558742.g715.t1 | DUF898 domain-containing | 1383 | 0 | 62.75 | C:integral component of membrane |
| Efet.01.558772.g716.t1 | hypothetical protein CAPTEDRAFT_196622 | 1131 | 1.87E-12 | 59 |  |
| Efet.01.558794.g717.t1 | histamine H3 receptor-like | 1314 | 9.17E-56 | 50.35 | P:signal transduction; F:signal transducer activity |
| Efet.01.558796.g718.t1 | BTB POZ domain-containing 1-like | 1560 | 1.55E-155 | 84.1 | C:P-body |
| Efet.01.559102.g723.t1 | galanin receptor type 2 | 375 | 1.40E-29 | 65.2 | F:G-protein coupled receptor activity; P:G-protein coupled receptor signaling pathway; C:integral component of membrane |
| Efet.01.559162.g724.t1 | monocarboxylate transporter 12-like | 759 | 4.34E-45 | 49.1 | P:transmembrane transport; C:membrane; C:integral component of membrane |
| Efet.01.559164.g725.t1 | TRC8 homolog | 1410 | 1.90E-139 | 67.3 | F:zinc ion binding; F:metal ion binding; C:membrane; C:integral component of membrane |
| Efet.01.559199.g727.t1 | serine hydrolase | 213 | 4.54E-10 | 72.8 |  |
| Efet.01.559264.g729.t1 | Zinc finger | 498 | 1.58E-52 | 72.15 | F:nucleic acid binding; F:metal ion binding |
| Efet.01.559328.g731.t1 | efflux RND transporter periplasmic adaptor subunit | 333 | 3.78E-53 | 64.5 |  |
| Efet.01.559401.g732.t1 | phosphopyruvate hydratase | 1026 | 0 | 70.8 | F:magnesium ion binding; F:phosphopyruvate hydratase activity; P:glycolytic process; C:extracellular region; C:phosphopyruvate hydratase complex; C:cell surface |
| Efet.01.559401.g733.t1 | family toxin | 387 | 5.59E-71 | 77.65 |  |
| Efet.01.559401.g734.t1 | addiction module antitoxin | 282 | 1.14E-48 | 82.1 | P:pathogenesis; F:hydrolase activity |
| Efet.01.559401.g735.t1 | toxin | 363 | 1.69E-78 | 73.05 | F:DNA binding |
| Efet.01.559401.g736.t1 | type II toxin-antitoxin system family | 264 | 4.70E-53 | 88.3 | F:toxic substance binding; P:single-species biofilm formation; F:bacterial-type RNA polymerase core promoter proximal region sequence-specific DNA binding |
| Efet.01.559401.g737.t1 | type II toxin-antitoxin system family toxin | 216 | 4.22E-42 | 85.1 | F:hydrolase activity |
| Efet.01.559401.g738.t1 | hypothetical protein | 222 | 2.15E-34 | 90.4 |  |
| Efet.01.559401.g739.t1 | outer membrane | 3393 | 0 | 56.55 |  |
| Efet.01.559401.g740.t1 | outer membrane | 606 | 1.08E-108 | 56.25 |  |
| Efet.01.559562.g742.t1 | molecular chaperone | 693 | 7.35E-111 | 77.45 | F:unfolded protein binding; P:protein folding |
| Efet.01.5596.g433.t1 | nuclear hormone receptor FTZ-F1 | 222 | 7.12E-10 | 81.6 | C:nucleus; F:transcription factor activity, sequence-specific DNA binding; F:zinc ion binding; P:transcription, DNA-templated; F:steroid hormone receptor activity; P:regulation of transcription, DNA-templated; F:sequence-specific DNA binding; P:steroid hormone mediated signaling pathway |
| Efet.01.559606.g743.t1 | dynein heavy chain axonemal-like | 222 | 1.63E-23 | 81.85 | F:ATPase activity; F:ATP binding; F:microtubule motor activity; C:dynein complex; P:microtubule-based movement |
| Efet.01.559694.g744.t1 | prolow-density lipo receptor-related 1 isoform X1 | 573 | 1.36E-07 | 52.13 | C:membrane; C:integral component of membrane |
| Efet.01.559723.g746.t1 | tryptophan--tRNA mitochondrial | 405 | 4.41E-42 | 72.05 | F:nucleotide binding; P:tRNA aminoacylation for protein translation; F:aminoacyl-tRNA ligase activity; C:cytoplasm |
| Efet.01.559739.g747.t1 | hypothetical protein CAPTEDRAFT_199934 | 537 | 1.52E-08 | 80 |  |
| Efet.01.559811.g748.t1 | plexin-A2 isoform X1 | 1110 | 5.62E-23 | 47.35 | P:signal transduction; C:membrane; C:integral component of membrane; F:semaphorin receptor activity; P:semaphorin-plexin signaling pathway |
| Efet.01.559874.g749.t1 | dynein heavy chain axonemal-like isoform X1 | 621 | 8.56E-88 | 83.95 | F:ATP binding; F:microtubule motor activity; C:dynein complex; P:microtubule-based movement |
| Efet.01.560128.g1.t1 | CTP synthase | 354 | 3.04E-64 | 92.3 | F:ATP binding; P:'de novo' CTP biosynthetic process; P:glutamine metabolic process; F:CTP synthase activity |
| Efet.01.560128.g2.t1 | CTP synthase | 312 | 4.12E-31 | 90.1 | F:ATP binding; P:'de novo' CTP biosynthetic process; P:glutamine metabolic process; F:CTP synthase activity |
| Efet.01.560210.g3.t1 | 30S ribosomal S20 | 564 | 2.97E-40 | 88.85 | F:structural constituent of ribosome; C:ribosome; F:rRNA binding; P:translation |
| Efet.01.56024.g383.t1 | plasma membrane calcium-transporting ATPase 4 | 210 | 1.02E-08 | 61.55 | P:ion transport; F:binding |
| Efet.01.560299.g7.t1 | early growth response | 1008 | 5.03E-40 | 87.4 | C:nucleus; F:DNA binding; F:metal ion binding; P:transcription, DNA-templated; P:regulation of transcription, DNA-templated |
| Efet.01.560312.g9.t1 | nucleolar 58 | 435 | 4.59E-61 | 81.85 | C:box C/D snoRNP complex; C:small-subunit processome; P:rRNA modification; F:snoRNA binding |
| Efet.01.560378.g10.t1 | vacuolar segregation PEP7-like isoform X1 | 354 | 3.69E-10 | 56.4 |  |
| Efet.01.560453.g13.t1 | aquaporin-3-like [Orbicella faveolata] | 414 | 3.69E-46 | 69.4 | C:integral component of plasma membrane; F:glycerol channel activity; P:cellular water homeostasis; P:ion transmembrane transport; F:water channel activity |
| Efet.01.560581.g14.t1 | hypothetical protein CAPTEDRAFT_226928 | 321 | 5.23E-13 | 61 |  |
| Efet.01.560587.g15.t1 | G1 S-specific cyclin-D2-like | 279 | 8.46E-19 | 79.45 | C:nucleus; P:cell cycle |
| Efet.01.560773.g16.t1 | E3 ubiquitin- ligase RNF31-like | 645 | 7.33E-06 | 53 |  |
| Efet.01.560830.g17.t1 | ABC transporter ATP-binding | 501 | 5.93E-102 | 93.25 | F:ATPase activity; F:ATP binding; P:peptide transport |
| Efet.01.560830.g18.t1 | ABC transporter ATP-binding | 330 | 9.38E-69 | 98.95 | F:ATPase activity; F:ATP binding; P:peptide transport |
| Efet.01.560830.g19.t1 | ABC transporter permease | 834 | 1.78E-152 | 98.15 | C:integral component of membrane; P:transport; C:plasma membrane |
| Efet.01.56104.g386.t1 | telomeric repeat-binding factor 2-like | 2262 | 2.13E-16 | 47.33 | F:DNA binding; F:protein homodimerization activity; F:telomeric DNA binding |
| Efet.01.561085.g21.t1 | TIGR00730 family Rossman fold | 273 | 5.96E-58 | 93.75 | F:hydrolase activity, hydrolyzing N-glycosyl compounds; P:cytokinin biosynthetic process |
| Efet.01.561085.g22.t1 | pyridoxine 5 -phosphate synthase | 1425 | 0 | 80.8 | P:pyridoxine biosynthetic process; C:cytoplasm; F:pyridoxine 5'-phosphate synthase activity; P:DNA metabolic process; P:cellular response to DNA damage stimulus |
| Efet.01.561135.g23.t1 | UDP-glucose:glyco glucosyltransferase 1-like isoform X1 | 411 | 1.47E-30 | 55.3 | F:transferase activity, transferring glycosyl groups; P:UDP-glucosylation; P:protein glycosylation; F:UDP-glucose:glycoprotein glucosyltransferase activity; F:transferase activity |
| Efet.01.56125.g387.t1 | hypothetical protein CAPTEDRAFT_200338 | 990 | 6.62E-26 | 50 |  |
| Efet.01.561295.g24.t1 | leucine-rich repeat and immunoglobulin-like domain-containing nogo receptor-interacting 3 | 1518 | 6.03E-106 | 46 | C:membrane; C:integral component of membrane |
| Efet.01.561311.g25.t1 | kelch 15 | 1620 | 2.44E-140 | 54.85 | F:ubiquitin-protein transferase activity; C:Cul3-RING ubiquitin ligase complex; P:protein ubiquitination |
| Efet.01.561343.g26.t1 | follistatin-related 5-like | 219 | 4.37E-11 | 59.25 |  |
| Efet.01.56147.g388.t1 | Cytosolic purine 5 -nucleotidase | 579 | 3.56E-46 | 68.2 | F:5'-nucleotidase activity; F:metal ion binding; P:dephosphorylation |
| Efet.01.561521.g27.t1 | golgin subfamily B member 1-like | 1707 | 7.84E-08 | 64 |  |
| Efet.01.56158.g389.t1 | glycine receptor subunit alpha-3-like isoform X1 | 309 | 1.67E-26 | 70.05 | P:ion transmembrane transport; C:integral component of membrane; C:cell junction; F:extracellular ligand-gated ion channel activity; C:synapse; C:plasma membrane |
| Efet.01.561604.g28.t1 | dynein beta ciliary-like | 396 | 1.34E-67 | 86.85 | F:ATPase activity; F:ATP binding; F:microtubule motor activity; C:dynein complex; P:microtubule-based movement |
| Efet.01.56162.g390.t1 | THEMIS isoform X2 | 903 | 2.93E-10 | 45.27 | C:nucleus; C:cell-cell junction; P:negative T cell selection; C:cytoplasm; C:COP9 signalosome; P:positive T cell selection; P:T cell receptor signaling pathway |
| Efet.01.561711.g30.t1 | probable ergosterol biosynthetic 28 | 516 | 4.35E-54 | 73.15 | C:membrane |
| Efet.01.561737.g31.t1 | 5 -AMP-activated kinase subunit gamma-2 isoform X2 | 291 | 2.64E-10 | 59.55 | P:phosphorylation; F:kinase activity |
| Efet.01.561853.g32.t1 | NADP-dependent oxidoreductase | 1002 | 1.22E-165 | 73.95 | F:oxidoreductase activity |
| Efet.01.561853.g33.t1 | family transcriptional regulator | 252 | 2.46E-38 | 73.1 | F:DNA binding |
| Efet.01.561907.g36.t1 | type IX secretion system membrane family | 894 | 5.96E-122 | 65.6 |  |
| Efet.01.561911.g37.t1 | One cut domain family member | 234 | 5.71E-19 | 76.5 | C:nucleus; F:DNA binding; F:transcriptional activator activity, RNA polymerase II core promoter proximal region sequence-specific binding; P:system development; P:cell differentiation; P:transcription from RNA polymerase II promoter; P:positive regulation of transcription from RNA polymerase II promoter |
| Efet.01.56193.g393.t1 | PREDICTED: uncharacterized protein LOC106061064 | 993 | 4.29E-07 | 47 | P:binding of sperm to zona pellucida; C:membrane; C:integral component of membrane; C:extracellular region |
| Efet.01.562032.g38.t1 | insulin-like growth factor-binding complex acid labile subunit | 2124 | 2.90E-102 | 48.25 | C:membrane; C:integral component of membrane |
| Efet.01.562142.g40.t1 | zinc finger ZXDC-like | 366 | 6.77E-41 | 70.65 | F:binding |
| Efet.01.562188.g41.t1 | glutamate mitochondrial | 588 | 1.15E-34 | 88.3 | F:GTP binding; F:ATP binding; P:tricarboxylic acid metabolic process; C:mitochondrial matrix; P:glutamine metabolic process; F:glutamate dehydrogenase [NAD(P)+] activity; P:oxidation-reduction process; P:positive regulation of insulin secretion; C:mitochondrial inner membrane |
| Efet.01.562218.g42.t1 | octopamine receptor beta-2R-like isoform X1 | 780 | 1.20E-39 | 69.2 | F:adrenergic receptor activity; C:integral component of membrane; P:adrenergic receptor signaling pathway; C:plasma membrane |
| Efet.01.562244.g43.t1 | FAD-binding oxidoreductase | 471 | 1.00E-61 | 91.15 | F:oxidoreductase activity, acting on CH-OH group of donors; F:flavin adenine dinucleotide binding; P:oxidation-reduction process |
| Efet.01.562244.g44.t1 | Asp Glu racemase | 660 | 6.26E-140 | 78.3 | F:racemase activity, acting on amino acids and derivatives; P:nitrogen compound metabolic process |
| Efet.01.56236.g394.t1 | importin-4-like isoform X1 | 321 | 2.37E-12 | 60.35 | P:intracellular protein transport; F:Ran GTPase binding; C:intracellular |
| Efet.01.562385.g46.t1 | Fic family | 339 | 4.19E-49 | 77.45 |  |
| Efet.01.562494.g49.t1 | 16S rRNA (guanine(966)-N(2))-methyltransferase | 924 | 2.46E-115 | 74.35 | F:transferase activity; P:methylation |
| Efet.01.562513.g51.t1 | carbohydrate ABC transporter permease | 261 | 1.76E-52 | 66.05 | C:membrane |
| Efet.01.562540.g52.t1 | N-acetylated-alpha-linked acidic dipeptidase 2-like | 1440 | 1.25E-101 | 73.25 | C:membrane |
| Efet.01.562555.g53.t1 | multiple PDZ domain | 789 | 2.41E-43 | 78.75 |  |
| Efet.01.562557.g54.t1 | tektin-3-like isoform X1 | 507 | 3.55E-83 | 82.4 |  |
| Efet.01.562590.g55.t1 | nanos homolog 3 | 600 | 4.65E-24 | 64.35 | F:zinc ion binding; F:RNA binding |
| Efet.01.5626.g434.t1 | cilia- and flagella-associated 44-like isoform X2 | 513 | 1.96E-09 | 53.22 |  |
| Efet.01.562647.g56.t1 | leucine-rich repeat-containing 14-like | 315 | 5.71E-32 | 64.22 |  |
| Efet.01.562677.g58.t1 | E3 ubiquitin- ligase TRIM71-like | 1548 | 7.03E-07 | 47 | F:zinc ion binding; F:metal ion binding; C:intracellular |
| Efet.01.562681.g59.t1 | voltage-dependent T-type calcium channel subunit alpha-1G-like isoform X11 | 231 | 2.95E-35 | 86.8 | P:calcium ion import; C:voltage-gated calcium channel complex; P:regulation of ion transmembrane transport; P:calcium ion transmembrane transport; F:low voltage-gated calcium channel activity |
| Efet.01.562821.g61.t1 | hypothetical protein CAPTEDRAFT_200978 | 741 | 3.16E-43 | 55.4 | C:membrane; C:integral component of membrane |
| Efet.01.562840.g62.t1 | hypothetical protein B5766_01185 | 522 | 4.84E-63 | 67.5 | C:membrane; C:integral component of membrane |
| Efet.01.562840.g63.t1 | cell filamentation Fic | 582 | 3.17E-96 | 80.4 |  |
| Efet.01.562840.g64.t1 | NUDIX domain-containing | 447 | 7.73E-85 | 81.95 | F:hydrolase activity |
| Efet.01.562840.g65.t1 | thiosulfate reductase cytochrome subunit beta (membrane anchoring ) | 924 | 0 | 74.15 | F:electron carrier activity; P:respiratory electron transport chain; C:integral component of membrane |
| Efet.01.562842.g66.t1 | Cubilin | 270 | 1.70E-18 | 75.25 | F:calcium ion binding; C:membrane; F:receptor activity; P:receptor-mediated endocytosis |
| Efet.01.562928.g67.t1 | FAM173B-like | 621 | 4.12E-76 | 69.3 | C:membrane |
| Efet.01.562940.g68.t1 | T family of potassium channels 18-like | 351 | 1.05E-11 | 67.6 | P:ion transport; P:potassium ion transmembrane transport; C:membrane; C:integral component of membrane; F:potassium channel activity; P:transport |
| Efet.01.562940.g69.t1 | T family of potassium channels 7-like | 537 | 2.44E-34 | 76.55 | C:integral component of plasma membrane; P:potassium ion transmembrane transport; P:stabilization of membrane potential; F:potassium ion leak channel activity |
| Efet.01.562974.g70.t1 | Glycine receptor subunit alpha- | 327 | 6.15E-49 | 84.3 | P:chemical synaptic transmission, postsynaptic; C:postsynapse; F:extracellular-glycine-gated chloride channel activity; C:glycine-gated chloride channel complex; P:chloride transmembrane transport; F:neurotransmitter receptor activity; C:cell junction; F:glycine binding |
| Efet.01.56299.g397.t1 | Copper-transporting ATPase 1 | 429 | 9.54E-59 | 64.25 | P:cation transport; F:binding |
| Efet.01.563018.g72.t1 | guanine nucleotide-binding G(12) alpha subunit | 528 | 5.08E-59 | 71.65 | C:heterotrimeric G-protein complex; C:brush border membrane; F:GTPase activity; F:guanyl nucleotide binding; P:adenylate cyclase-modulating G-protein coupled receptor signaling pathway; F:G-protein beta/gamma-subunit complex binding; P:Rho protein signal transduction; F:signal transducer activity; F:D5 dopamine receptor binding |
| Efet.01.563063.g73.t1 | hypothetical protein CAPTEDRAFT_225510 | 258 | 2.57E-25 | 69.9 | P:cellular aromatic compound metabolic process; P:small molecule metabolic process; P:heterocycle metabolic process; P:organic cyclic compound metabolic process; F:catalytic activity; C:integral component of membrane; P:organonitrogen compound metabolic process; P:cellular nitrogen compound metabolic process; P:single-organism cellular process; F:carbohydrate binding; P:primary metabolic process |
| Efet.01.56313.g398.t1 | FH1 FH2 domain-containing 1 isoform X6 | 327 | 2.10E-06 | 48 |  |
| Efet.01.563201.g74.t1 | amino acid ABC transporter permease | 666 | 2.26E-143 | 82.85 | C:integral component of membrane; P:transport; C:plasma membrane; F:transporter activity |
| Efet.01.563201.g75.t1 | acetolactate synthase | 1689 | 0 | 83.2 | F:magnesium ion binding; F:acetolactate synthase activity; F:thiamine pyrophosphate binding |
| Efet.01.563201.g76.t1 | thiamine pyrophosphate-binding | 342 | 3.89E-61 | 87 | F:magnesium ion binding; F:acetolactate synthase activity; F:thiamine pyrophosphate binding |
| Efet.01.563201.g77.t1 | thiamine pyrophosphate-binding | 1140 | 0 | 91.2 | F:magnesium ion binding; F:acetolactate synthase activity; F:thiamine pyrophosphate binding |
| Efet.01.563201.g78.t1 | amino acid ABC transporter substrate-binding | 915 | 2.03E-180 | 90.3 |  |
| Efet.01.563201.g79.t1 | aldehyde dehydrogenase family | 1536 | 0 | 88.5 | P:oxidation-reduction process; F:oxidoreductase activity, acting on the aldehyde or oxo group of donors, NAD or NADP as acceptor |
| Efet.01.563201.g80.t1 | hypothetical protein ABS43_15020 | 720 | 3.81E-122 | 88.15 | P:biological_process; C:cellular_component; F:molecular_function |
| Efet.01.563201.g81.t1 | recombinase family | 381 | 1.49E-26 | 89.65 | F:DNA binding; F:recombinase activity; P:DNA recombination |
| Efet.01.563201.g82.t1 | recombinase family | 240 | 7.37E-17 | 64.2 | F:DNA binding; F:recombinase activity; P:DNA recombination |
| Efet.01.563201.g83.t1 | hypothetical protein | 216 | 4.86E-35 | 87 |  |
| Efet.01.563201.g84.t1 | isoprenylcysteine carboxylmethyltransferase family | 696 | 4.50E-94 | 60.1 | C:integral component of membrane |
| Efet.01.563201.g85.t1 | short-chain dehydrogenase | 1392 | 5.21E-109 | 69.35 | F:oxidoreductase activity; P:oxidation-reduction process |
| Efet.01.563201.g86.t1 | hypothetical protein | 552 | 1.04E-50 | 57.8 | F:L-ascorbic acid binding; F:iron ion binding; F:oxidoreductase activity; F:oxidoreductase activity, acting on paired donors, with incorporation or reduction of molecular oxygen, 2-oxoglutarate as one donor, and incorporation of one atom each of oxygen into both donors; P:oxidation-reduction process |
| Efet.01.563201.g87.t1 | mangotoxin biosynthesis-involved | 792 | 3.30E-94 | 69.25 |  |
| Efet.01.563201.g88.t1 | ISAs1 family transposase | 420 | 1.77E-61 | 73.75 | F:DNA binding; F:transposase activity; P:transposition, DNA-mediated |
| Efet.01.563201.g89.t1 | ISAs1 family transposase | 1281 | 3.65E-120 | 78.9 | F:DNA binding; F:transposase activity; P:transposition, DNA-mediated |
| Efet.01.563201.g90.t1 | 4-hydroxy-4-methyl-2-oxoglutarate aldolase | 369 | 9.90E-80 | 90.85 | F:methyltransferase activity; P:methylation |
| Efet.01.563201.g91.t1 | carbon-nitrogen hydrolase family | 831 | 0 | 89.45 | F:transferase activity, transferring acyl groups; P:nitrogen compound metabolic process; F:nitrilase activity |
| Efet.01.563201.g92.t1 | membrane | 1233 | 0 | 86.75 | F:aspartic-type endopeptidase activity; F:electron carrier activity; P:proteolysis; C:integral component of membrane; F:heme binding |
| Efet.01.563229.g93.t1 | glucose-repressible alcohol dehydrogenase transcriptional effector-like | 510 | 2.65E-24 | 57.6 | F:hydrolase activity |
| Efet.01.563261.g95.t1 | split ends-like isoform X1 | 273 | 8.00E-10 | 66.5 | F:organic cyclic compound binding; F:heterocyclic compound binding |
| Efet.01.563262.g96.t1 | hypothetical protein HELRODRAFT_193047 | 261 | 4.02E-29 | 74 | F:calcium ion binding |
| Efet.01.563274.g97.t1 | PHD finger | 498 | 9.42E-15 | 69.85 | F:zinc ion binding; F:metal ion binding |
| Efet.01.5633.g436.t1 | E3 ubiquitin- ligase TRIM71- | 1044 | 2.01E-19 | 49 |  |
| Efet.01.563308.g99.t1 | and PH domain-containing 1-like isoform X1 | 840 | 1.29E-55 | 75.9 | P:regulation of Rho protein signal transduction; F:Rho guanyl-nucleotide exchange factor activity; P:positive regulation of GTPase activity |
| Efet.01.563393.g101.t1 | alpha beta hydrolase | 888 | 0 | 73.8 | F:hydrolase activity |
| Efet.01.563393.g102.t1 | family transcriptional regulator | 537 | 2.92E-129 | 82.2 | F:DNA binding; F:transcription factor activity, sequence-specific DNA binding; P:transcription, DNA-templated; P:regulation of transcription, DNA-templated |
| Efet.01.563393.g103.t1 | uracil-DNA glycosylase | 765 | 1.35E-150 | 82.7 | F:uracil DNA N-glycosylase activity; P:base-excision repair; C:cytoplasm |
| Efet.01.563393.g104.t1 | amino acid permease | 1569 | 0 | 84.65 | C:integral component of membrane; F:amino acid transmembrane transporter activity; P:amino acid transmembrane transport |
| Efet.01.5634.g438.t1 | small conductance calcium-activated potassium channel -like | 825 | 1.07E-24 | 56 | F:calcium-activated potassium channel activity; P:potassium ion transport; P:carbohydrate metabolic process; F:calmodulin binding; P:potassium ion transmembrane transport; C:membrane; C:integral component of membrane; F:small conductance calcium-activated potassium channel activity; F:hydrolase activity, hydrolyzing O-glycosyl compounds |
| Efet.01.563613.g108.t1 | hypothetical protein CAPTEDRAFT_222036 | 252 | 1.42E-20 | 65.25 | C:membrane; C:integral component of membrane |
| Efet.01.563632.g109.t1 | mannosyltransferase | 585 | 4.69E-98 | 61.35 | C:membrane; C:integral component of membrane |
| Efet.01.563632.g110.t1 | MFS transporter | 567 | 2.12E-67 | 62.9 | C:membrane |
| Efet.01.56365.g399.t1 | kinase D-interacting substrate of 220 kDa-like | 288 | 5.52E-09 | 81.11 | C:integral component of membrane |
| Efet.01.563706.g112.t1 | plexin-A1 | 327 | 7.52E-12 | 56 | P:neuron projection guidance; C:nucleus; C:extracellular exosome; P:regulation of locomotion; C:cytoplasm; P:signal transduction; P:regulation of cellular component movement; F:receptor activity; C:plasma membrane |
| Efet.01.563715.g114.t1 | signal recognition particle-docking | 279 | 4.60E-48 | 89.7 | F:GTP binding; P:cell division; P:SRP-dependent cotranslational protein targeting to membrane; C:cytoplasm; F:GTPase activity; C:intrinsic component of plasma membrane |
| Efet.01.563785.g116.t1 | VWA domain-containing | 1539 | 1.48E-180 | 71.3 | C:integral component of membrane |
| Efet.01.563785.g117.t1 | VWA domain-containing | 810 | 1.86E-177 | 72.05 |  |
| Efet.01.563785.g118.t1 | 3-oxoacid -transferase | 624 | 2.83E-144 | 83.2 | P:metabolic process; F:3-oxoadipate CoA-transferase activity |
| Efet.01.563792.g119.t1 | sugar phosphate isomerase epimerase | 210 | 2.69E-42 | 70.9 | F:isomerase activity |
| Efet.01.563792.g120.t1 | pyridoxamine 5 -phosphate oxidase family | 477 | 8.12E-94 | 70.95 | F:oxidoreductase activity; P:single-organism metabolic process |
| Efet.01.563814.g121.t1 | ornithine decarboxylase-like isoform X1 | 279 | 1.50E-35 | 80.3 | F:ornithine decarboxylase activator activity; C:cytoplasm; P:putrescine biosynthetic process from ornithine; F:ornithine decarboxylase activity |
| Efet.01.563840.g122.t1 | FMRFamide receptor-like | 1122 | 6.30E-67 | 56.25 | F:G-protein coupled receptor activity; P:G-protein coupled receptor signaling pathway; P:cell communication; F:calcium ion binding; C:membrane; C:integral component of membrane |
| Efet.01.563913.g124.t1 | specificity 1 | 1365 | 2.19E-09 | 73.9 | F:binding |
| Efet.01.563917.g125.t1 | ABC transporter | 1308 | 1.55E-157 | 73.05 | C:integral component of membrane; C:plasma membrane |
| Efet.01.563923.g126.t1 | hypothetical protein HELRODRAFT_159178 | 246 | 1.60E-23 | 73.05 | C:integral component of membrane; F:carbohydrate binding |
| Efet.01.563953.g127.t1 | RING zinc finger-containing | 510 | 9.01E-25 | 60.95 | F:zinc ion binding; F:metal ion binding |
| Efet.01.56401.g400.t1 | coadhesin-like isoform X2 | 765 | 1.20E-10 | 68.35 | P:negative regulation of angiogenesis; F:calcium ion binding; P:cell adhesion; F:heparin binding; C:extracellular matrix; C:extracellular region |
| Efet.01.564043.g128.t1 | tyrosine- phosphatase Lar-like isoform X1 | 252 | 5.12E-34 | 84.25 | F:protein tyrosine phosphatase activity; C:integral component of membrane; P:peptidyl-tyrosine dephosphorylation |
| Efet.01.564055.g129.t1 | recombinase family | 405 | 1.65E-64 | 76 | F:DNA binding; F:recombinase activity; P:DNA recombination |
| Efet.01.564183.g130.t1 | hypothetical protein CAPTEDRAFT_220857 | 405 | 9.95E-15 | 53.2 |  |
| Efet.01.564228.g131.t1 | major facilitator superfamily domain-containing 3-like | 276 | 1.61E-15 | 65.95 | C:integral component of membrane; C:cell part; P:transport; F:transporter activity |
| Efet.01.564232.g132.t1 | innexin 4 | 627 | 1.98E-66 | 65.5 | C:membrane; C:cell junction; P:transport |
| Efet.01.56425.g401.t1 | Ras-related Rab-33 isoform X3 | 417 | 1.35E-41 | 75.35 | F:GTP binding; P:small GTPase mediated signal transduction; C:intracellular |
| Efet.01.56431.g402.t1 | CAP-Gly domain-containing linker 1-like isoform X1 | 207 | 2.89E-08 | 72.05 | C:microtubule |
| Efet.01.5644.g439.t1 | arpin | 681 | 4.34E-75 | 64.1 |  |
| Efet.01.564454.g133.t1 | vacuolar sorting-associated 13D-like | 399 | 1.63E-19 | 54.15 | F:hydrolase activity, acting on ester bonds |
| Efet.01.564507.g134.t1 | transcription intermediary factor 1-alpha-like isoform X1 | 1200 | 6.59E-40 | 63.5 | F:zinc ion binding; F:catalytic activity; C:intracellular |
| Efet.01.564563.g135.t1 | flagellar motor switch | 459 | 8.33E-35 | 93.1 | P:chemotaxis; P:bacterial-type flagellum-dependent cell motility; C:plasma membrane; C:bacterial-type flagellum basal body; F:motor activity |
| Efet.01.564657.g138.t1 | cell surface | 1032 | 0 | 62.8 |  |
| Efet.01.564691.g139.t1 | AAA family ATPase | 321 | 1.64E-72 | 70.95 | F:DNA binding; P:DNA replication; F:helicase activity |
| Efet.01.564720.g140.t1 | Aldolase | 993 | 1.39E-78 | 62.3 | P:glycolytic process; F:fructose-bisphosphate aldolase activity |
| Efet.01.564763.g145.t1 | solute carrier family 22 member 2-like | 429 | 2.91E-36 | 60 | C:membrane; F:transporter activity |
| Efet.01.564796.g146.t1 | hydroxypyruvate isomerase | 561 | 4.93E-92 | 86.2 | F:hydroxypyruvate isomerase activity |
| Efet.01.564797.g147.t1 | ATP-dependent serine peptidase containing a PDZ domain | 267 | 3.14E-47 | 69.55 | P:protein metabolic process; C:membrane; F:peptidase activity, acting on L-amino acid peptides |
| Efet.01.564839.g148.t1 | Ubiquitin carboxyl-terminal hydrolase 4 | 1014 | 6.69E-14 | 47.33 | P:protein deubiquitination; F:zinc ion binding; F:thiol-dependent ubiquitinyl hydrolase activity; P:ubiquitin-dependent protein catabolic process; F:hydrolase activity |
| Efet.01.56486.g403.t1 | dimethylaniline monooxygenase [N-oxide-forming] 5 | 687 | 3.38E-61 | 64 | C:membrane; P:metabolic process; C:endoplasmic reticulum; F:monooxygenase activity |
| Efet.01.564890.g149.t1 | KIBRA-like isoform X4 | 666 | 4.75E-13 | 45.29 |  |
| Efet.01.564901.g150.t1 | Sperm-associated antigen 17 | 399 | 4.37E-29 | 61.2 | F:metal ion binding |
| Efet.01.564916.g151.t1 | enhancer of polycomb homolog 1 | 312 | 1.18E-33 | 63.2 | C:Piccolo NuA4 histone acetyltransferase complex; P:regulation of transcription from RNA polymerase II promoter |
| Efet.01.564919.g153.t1 | glycine receptor subunit alpha-3-like isoform X1 | 615 | 1.58E-74 | 65.3 | P:ion transmembrane transport; P:neuromuscular process; C:external side of plasma membrane; P:response to amino acid; F:extracellular ligand-gated ion channel activity; P:regulation of system process; C:endoplasmic reticulum; F:cation binding; C:neuronal cell body; C:synapse; F:anion binding; P:cellular response to oxygen-containing compound; C:neuron projection; P:chloride transport; P:chemical synaptic transmission, postsynaptic; F:transmembrane signaling receptor activity; P:response to external stimulus; P:regulation of membrane potential; P:cellular response to organic substance; C:integral component of membrane; C:cell junction; P:response to alcohol |
| Efet.01.5650.g440.t1 | shaker-related potassium channel tsha2-like | 1107 | 8.42E-173 | 64.55 | P:positive regulation of membrane potential; P:cellular response to dopamine; P:positive regulation of circadian sleep/wake cycle, sleep; P:transmembrane transport; P:regulation of synaptic activity; P:detection of visible light; C:voltage-gated potassium channel complex; P:larval locomotory behavior; P:potassium ion transport; F:voltage-gated potassium channel activity; P:proboscis extension reflex; P:axon extension; P:flight behavior; P:mating behavior, sex discrimination; P:courtship behavior; P:action potential; P:sleep; P:neurological system process |
| Efet.01.565008.g155.t1 | hypothetical protein CAPTEDRAFT_166277 | 600 | 2.69E-09 | 63 | P:ion transport; C:membrane; C:integral component of membrane |
| Efet.01.565040.g156.t1 | tripartite motif-containing 2-like | 855 | 4.59E-11 | 46.75 | F:zinc ion binding; P:signal transduction; P:ion transmembrane transport; F:GABA-A receptor activity; F:extracellular ligand-gated ion channel activity; C:GABA-A receptor complex; C:synapse; P:transport; C:intracellular; C:plasma membrane; P:ion transport; F:metal ion binding; C:membrane; C:integral component of membrane; C:cell junction |
| Efet.01.565049.g157.t1 | rRNA pseudouridine synthase | 840 | 1.99E-163 | 80.15 | P:pseudouridine synthesis; F:RNA binding; F:pseudouridine synthase activity |
| Efet.01.565049.g158.t1 | molecular chaperone | 1935 | 0 | 87 | F:ATP binding; C:cytoplasm; F:unfolded protein binding; P:protein folding; P:response to stress |
| Efet.01.565049.g159.t1 | serine threonine kinase | 933 | 0 | 89.8 | F:magnesium ion binding; F:ATP binding; C:cytoplasm; F:protein serine/threonine kinase activity; P:protein phosphorylation; P:response to stress |
| Efet.01.565049.g160.t1 | type II toxin-antitoxin system family toxin | 567 | 3.21E-45 | 80.05 | P:nucleic acid phosphodiester bond hydrolysis; F:endonuclease activity |
| Efet.01.565049.g161.t1 | F0F1 ATP synthase subunit epsilon | 420 | 1.21E-73 | 96.85 | C:proton-transporting ATP synthase complex, catalytic core F(1); F:ATP binding; F:proton-transporting ATPase activity, rotational mechanism; C:plasma membrane; F:proton-transporting ATP synthase activity, rotational mechanism; P:plasma membrane ATP synthesis coupled proton transport |
| Efet.01.565049.g162.t1 | F0F1 ATP synthase subunit beta | 1416 | 0 | 98.05 | C:proton-transporting ATP synthase complex, catalytic core F(1); F:ATP binding; P:ATP hydrolysis coupled proton transport; C:plasma membrane; F:proton-transporting ATP synthase activity, rotational mechanism; P:plasma membrane ATP synthesis coupled proton transport |
| Efet.01.565049.g163.t1 | F0F1 ATP synthase subunit gamma | 879 | 0 | 97.15 | C:proton-transporting ATP synthase complex, catalytic core F(1); F:ATP binding; F:proton-transporting ATPase activity, rotational mechanism; C:plasma membrane; F:proton-transporting ATP synthase activity, rotational mechanism; P:plasma membrane ATP synthesis coupled proton transport |
| Efet.01.565049.g164.t1 | F0F1 ATP synthase subunit alpha | 270 | 5.37E-45 | 89.35 | C:proton-transporting ATP synthase complex, catalytic core F(1); F:ATP binding; P:ATP hydrolysis coupled proton transport; F:proton-transporting ATPase activity, rotational mechanism; C:plasma membrane; F:proton-transporting ATP synthase activity, rotational mechanism; P:plasma membrane ATP synthesis coupled proton transport |
| Efet.01.565049.g165.t1 | F0F1 ATP synthase subunit alpha | 1215 | 0 | 99.05 | C:proton-transporting ATP synthase complex, catalytic core F(1); F:ATP binding; P:ATP hydrolysis coupled proton transport; F:proton-transporting ATPase activity, rotational mechanism; C:plasma membrane; F:proton-transporting ATP synthase activity, rotational mechanism; P:plasma membrane ATP synthesis coupled proton transport |
| Efet.01.565049.g166.t1 | F0F1 ATP synthase subunit delta | 321 | 8.59E-67 | 89.9 | C:proton-transporting ATP synthase complex, catalytic core F(1); C:plasma membrane; F:proton-transporting ATP synthase activity, rotational mechanism; P:plasma membrane ATP synthesis coupled proton transport |
| Efet.01.565049.g167.t1 | F0F1 ATP synthase subunit B | 408 | 1.97E-62 | 94.2 | C:proton-transporting ATP synthase complex, coupling factor F(o); C:integral component of membrane; C:plasma membrane; F:proton-transporting ATP synthase activity, rotational mechanism; P:plasma membrane ATP synthesis coupled proton transport |
| Efet.01.565049.g168.t1 | F0F1 ATP synthase subunit C | 270 | 4.03E-33 | 95.25 | F:hydrogen ion transmembrane transporter activity; P:ATP hydrolysis coupled proton transport; C:proton-transporting ATP synthase complex, coupling factor F(o); C:integral component of membrane; F:lipid binding; C:plasma membrane; P:ATP synthesis coupled proton transport; F:hydrolase activity |
| Efet.01.565049.g169.t1 | F0F1 ATP synthase subunit A | 1302 | 9.15E-171 | 92.95 | C:proton-transporting ATP synthase complex, coupling factor F(o); C:integral component of membrane; C:plasma membrane; F:proton-transporting ATP synthase activity, rotational mechanism; P:plasma membrane ATP synthesis coupled proton transport |
| Efet.01.565049.g170.t1 | DUF493 domain-containing | 324 | 1.99E-72 | 87.4 |  |
| Efet.01.565049.g171.t1 | lipoyl(octanoyl) transferase | 639 | 1.55E-139 | 86.5 | F:octanoyltransferase activity; C:cytoplasm; F:lipoyl(octanoyl) transferase activity; P:cellular protein modification process; F:ligase activity; P:lipoate biosynthetic process |
| Efet.01.565056.g172.t1 | kinase | 693 | 9.30E-110 | 59.55 | P:phosphorylation; F:transferase activity; F:phosphotransferase activity, alcohol group as acceptor; F:kinase activity |
| Efet.01.565066.g173.t1 | Retrovirus-related Pol poly | 456 | 2.62E-34 | 59.5 | F:binding |
| Efet.01.56510.g405.t1 | kinesin Klp98A isoform X1 | 369 | 8.70E-44 | 79.2 | F:ATPase activity; F:ATP binding; F:microtubule motor activity; P:vesicle-mediated transport; P:cytoskeleton-dependent intracellular transport; F:phosphatidylinositol binding; C:kinesin complex; P:microtubule-based movement; F:microtubule binding |
| Efet.01.56518.g406.t1 | T-box transcription factor | 354 | 6.11E-64 | 69.6 | P:transcription, DNA-templated; P:cell communication; P:regulation of transcription, DNA-templated; P:negative regulation of developmental process; P:negative regulation of cellular process; P:single organism signaling; P:tissue development; P:cellular developmental process; C:nucleus; F:DNA binding; F:transcription factor activity, sequence-specific DNA binding; P:heart morphogenesis; P:positive regulation of cellular process; P:embryonic morphogenesis |
| Efet.01.565205.g176.t1 | exocyst complex component 7-like | 222 | 8.21E-22 | 71.95 | P:exocytosis; C:exocyst |
| Efet.01.565209.g177.t1 | cilia- and flagella-associated 61-like | 225 | 7.45E-10 | 68.45 | C:membrane |
| Efet.01.565223.g178.t1 | small VCP p97-interacting -like | 231 | 7.26E-07 | 67 |  |
| Efet.01.565253.g179.t1 | DNA annealing helicase and endonuclease ZRANB3-like isoform X2 | 273 | 3.84E-09 | 73.67 | F:nucleic acid binding; F:ATP binding; P:nucleic acid phosphodiester bond hydrolysis; F:zinc ion binding; F:endonuclease activity |
| Efet.01.565302.g180.t1 | GDH 6PGL endoplasmic bifunctional | 378 | 5.68E-37 | 69.8 | F:NADP binding; P:oxidation-reduction process; P:glucose metabolic process; F:glucose-6-phosphate dehydrogenase activity |
| Efet.01.565356.g181.t1 | solute carrier family facilitated glucose transporter member 5 | 795 | 1.36E-73 | 61.95 | C:integral component of plasma membrane; F:fructose binding; P:cellular response to fructose stimulus; C:sarcolemma; P:fructose import across plasma membrane; F:fructose transmembrane transporter activity |
| Efet.01.565356.g182.t1 | solute carrier family facilitated glucose transporter member 1-like | 801 | 4.50E-50 | 54.5 | F:substrate-specific transmembrane transporter activity; P:transmembrane transport; F:transmembrane transporter activity; C:membrane; C:integral component of membrane; P:carbohydrate transport; P:transport; F:transporter activity |
| Efet.01.565394.g185.t1 | kelch 2 | 249 | 5.43E-32 | 86.15 | F:ubiquitin-protein transferase activity; P:ion homeostasis; C:Cul3-RING ubiquitin ligase complex; P:protein ubiquitination |
| Efet.01.565440.g186.t1 | delta-1-pyrroline-5-carboxylate synthase | 654 | 6.72E-40 | 65.3 | F:glutamate 5-kinase activity; C:cytoplasm; F:glutamate-5-semialdehyde dehydrogenase activity; P:proline biosynthetic process; P:oxidation-reduction process; P:phosphorylation |
| Efet.01.565468.g188.t1 | hypothetical protein CAPTEDRAFT_221456 | 309 | 1.57E-44 | 67.6 | C:membrane; C:integral component of membrane |
| Efet.01.56548.g408.t1 | germinal-center associated nuclear -like | 624 | 2.54E-19 | 47.35 | F:nucleotide binding; F:nucleic acid binding |
| Efet.01.5657.g441.t1 | dynamin-2 isoform X7 | 270 | 7.89E-12 | 83.6 | F:GTP binding; P:clathrin-dependent endocytosis; F:GTPase activity |
| Efet.01.565724.g189.t1 | amino acid ABC transporter permease | 477 | 2.67E-95 | 75.15 | C:integral component of membrane; P:transport; C:plasma membrane; F:transporter activity |
| Efet.01.565724.g190.t1 | amino acid permease ATP-binding | 840 | 1.44E-158 | 78.75 | F:ATP binding; P:amino acid transmembrane transport; F:amino acid-transporting ATPase activity |
| Efet.01.565724.g191.t1 | cystathionine beta-lyase | 1059 | 0 | 74.35 | F:pyridoxal phosphate binding; C:cytoplasm; P:cellular amino acid metabolic process; F:cystathionine beta-lyase activity |
| Efet.01.565819.g192.t1 | ELAV 2 | 1170 | 1.41E-64 | 70.05 | F:nucleotide binding; F:RNA binding; P:regulation of neurogenesis |
| Efet.01.565865.g194.t1 | isoleucine--tRNA ligase | 1002 | 0 | 84.6 | P:isoleucyl-tRNA aminoacylation; F:ATP binding; F:zinc ion binding; F:isoleucine-tRNA ligase activity; C:cytoplasm; P:regulation of translational fidelity; F:aminoacyl-tRNA editing activity |
| Efet.01.565869.g195.t1 | short-chain dehydrogenase | 381 | 2.99E-82 | 88.4 | F:3-oxoacyl-[acyl-carrier-protein] reductase (NADPH) activity; P:oxidation-reduction process |
| Efet.01.565977.g196.t1 | Integrase catalytic region | 570 | 8.83E-85 | 82.3 | F:nucleic acid binding; P:DNA integration |
| Efet.01.565977.g197.t1 | hypothetical protein AC240_08855 | 315 | 8.67E-36 | 78.4 |  |
| Efet.01.56602.g410.t1 | geranylgeranyl transferase type-2 subunit alpha-like | 453 | 2.64E-71 | 76.65 | F:zinc ion binding; C:Rab-protein geranylgeranyltransferase complex; P:protein geranylgeranylation; F:Rab geranylgeranyltransferase activity |
| Efet.01.566024.g200.t1 | hypothetical protein HELRODRAFT_171246 | 1344 | 1.78E-38 | 54 | F:metal ion binding |
| Efet.01.56603.g411.t1 | HEAT repeat-containing 6 | 546 | 7.44E-22 | 48.2 | F:RNA binding |
| Efet.01.56613.g412.t1 | p53-induced death domain-containing 1-like isoform X1 | 798 | 1.31E-18 | 46.25 | P:signal transduction |
| Efet.01.566177.g203.t1 | DNA-binding SATB2 | 1386 | 3.49E-27 | 76.25 | C:nucleus; F:DNA binding |
| Efet.01.566197.g205.t1 | calmodulin 3 | 267 | 1.08E-06 | 63 | C:extracellular exosome; F:calcium ion binding |
| Efet.01.566222.g206.t1 | ABC transporter permease | 1104 | 0 | 82.3 | C:integral component of membrane; P:monosaccharide transport; F:monosaccharide-transporting ATPase activity; C:plasma membrane |
| Efet.01.566222.g207.t1 | family transcriptional regulator | 969 | 0 | 85.8 | P:carbohydrate transport |
| Efet.01.566222.g208.t1 | D-ribose transporter ATP-binding | 1551 | 0 | 92.55 | F:ATP binding; P:monosaccharide transport; F:monosaccharide-transporting ATPase activity; P:carbohydrate transmembrane transport; C:plasma membrane |
| Efet.01.566222.g209.t1 | ABC transporter substrate-binding | 984 | 0 | 88.05 | C:outer membrane-bounded periplasmic space |
| Efet.01.566222.g210.t1 | rhizopine-binding | 342 | 1.53E-51 | 92.25 | P:carbohydrate transport |
| Efet.01.566223.g211.t1 | cytochrome P450 | 288 | 1.45E-23 | 65.65 | F:oxygen binding; F:arachidonic acid epoxygenase activity; P:renal water homeostasis; P:leukotriene metabolic process; P:pressure natriuresis; P:sodium ion homeostasis; F:leukotriene-B4 20-monooxygenase activity; F:heme binding; C:endoplasmic reticulum membrane; P:omega-hydroxylase P450 pathway; C:extracellular exosome; P:epoxygenase P450 pathway; F:iron ion binding; P:oxidation-reduction process; F:alkane 1-monooxygenase activity; P:positive regulation of icosanoid secretion; C:apical plasma membrane |
| Efet.01.566366.g214.t1 | glutathione synthase | 678 | 1.79E-120 | 79.45 | F:ATP binding; F:metal ion binding |
| Efet.01.56646.g413.t1 | autophagy-related 13 homolog isoform X2 | 876 | 1.59E-25 | 48.35 | P:autophagy; C:ATG1/ULK1 kinase complex |
| Efet.01.566481.g216.t1 | Potassium voltage-gated channel Shal | 993 | 3.44E-177 | 88.25 | F:voltage-gated potassium channel activity; P:potassium ion transmembrane transport; C:voltage-gated potassium channel complex; P:regulation of ion transmembrane transport; P:protein homooligomerization |
| Efet.01.56656.g414.t1 | gastrula zinc finger -like | 1158 | 1.52E-81 | 55.25 | F:nucleic acid binding; F:metal ion binding |
| Efet.01.56668.g415.t1 | muscle M-line assembly unc-89 isoform X3 | 1059 | 1.48E-71 | 47.35 | F:nucleotide binding; P:regulation of Rho protein signal transduction; F:ATP binding; C:membrane; C:integral component of membrane; F:protein kinase activity; F:Rho guanyl-nucleotide exchange factor activity; P:phosphorylation; F:transferase activity; P:protein phosphorylation; F:kinase activity; P:positive regulation of GTPase activity |
| Efet.01.566699.g219.t1 | peptidase M1 | 429 | 1.83E-99 | 64.65 | F:zinc ion binding; P:proteolysis; F:metallopeptidase activity |
| Efet.01.566725.g221.t1 | hydroxymethylglutaryl- lyase | 912 | 0 | 92.95 | F:hydroxymethylglutaryl-CoA lyase activity; F:transferase activity |
| Efet.01.566725.g222.t1 | 2-hydroxychromene-2-carboxylate isomerase | 1746 | 4.79E-116 | 88.3 | F:protein disulfide oxidoreductase activity; P:oxidation-reduction process |
| Efet.01.566725.g223.t1 | MFS transporter | 387 | 4.18E-47 | 99.05 | P:transmembrane transport; F:transmembrane transporter activity; C:integral component of membrane |
| Efet.01.566728.g224.t1 | DNA helicase | 417 | 1.23E-48 | 71.15 | F:nucleic acid binding; F:helicase activity |
| Efet.01.566758.g226.t1 | pyridoxine pyridoxamine 5 -phosphate oxidase chloroplastic | 231 | 1.18E-09 | 67.95 | F:oxidoreductase activity; P:single-organism metabolic process |
| Efet.01.566787.g227.t1 | chitin synthase | 666 | 2.84E-23 | 45.75 | F:nucleotide binding; C:myosin complex; F:ATP binding; F:transferase activity, transferring hexosyl groups; F:actin binding; C:membrane; C:integral component of membrane; F:motor activity |
| Efet.01.566853.g230.t1 | hypothetical protein CAPTEDRAFT_218417 | 2388 | 8.32E-166 | 44.8 | C:membrane; C:integral component of membrane |
| Efet.01.566908.g231.t1 | hypothetical protein CAPTEDRAFT_113772, partial | 324 | 5.47E-06 | 75 |  |
| Efet.01.56698.g416.t1 | collagen alpha-1(XV) chain-like | 735 | 9.72E-27 | 58 | C:collagen trimer; F:structural molecule activity; P:cell adhesion; C:extracellular matrix |
| Efet.01.567084.g233.t1 | myosin binding isoform P | 384 | 2.05E-38 | 70.1 | P:dorsal closure; P:germline ring canal formation; P:oogenesis; P:axon extension; P:regulation of locomotor rhythm; C:apical junction complex; P:imaginal disc-derived wing hair organization; P:regulation of compound eye photoreceptor development; C:apical cortex |
| Efet.01.567101.g234.t1 | transcriptional regulator | 510 | 6.89E-64 | 68.55 | F:DNA binding; F:transcription factor activity, sequence-specific DNA binding; P:transcription, DNA-templated; P:regulation of transcription, DNA-templated; C:intracellular |
| Efet.01.567153.g235.t1 | gfo Idh family oxidoreductase | 342 | 6.15E-74 | 75.25 | F:oxidoreductase activity; C:integral component of membrane; P:oxidation-reduction process |
| Efet.01.567190.g237.t1 | nicotinamide phosphoribosyltransferase-like | 273 | 8.19E-36 | 82.35 | F:nicotinate-nucleotide diphosphorylase (carboxylating) activity; P:NAD biosynthetic process |
| Efet.01.567234.g238.t1 | Ephrin type-A receptor 4-A | 366 | 5.10E-07 | 52.4 | F:transferase activity; P:cellular process |
| Efet.01.56738.g418.t1 | hypothetical protein HELRODRAFT_194331 | 279 | 2.46E-10 | 67.5 | C:membrane |
| Efet.01.567384.g241.t1 | hypothetical protein B5766_09665 | 234 | 7.13E-20 | 100 |  |
| Efet.01.567384.g242.t1 | ribokinase | 234 | 4.44E-19 | 76.95 |  |
| Efet.01.56739.g419.t1 | cullin-2-like isoform X1 | 234 | 2.78E-24 | 82.95 | F:ubiquitin protein ligase activity; F:ubiquitin protein ligase binding; C:Cul2-RING ubiquitin ligase complex; P:protein ubiquitination involved in ubiquitin-dependent protein catabolic process |
| Efet.01.567399.g243.t1 | lipopolysaccharide ABC transporter substrate-binding | 246 | 2.75E-09 | 69.7 | P:transport; P:single-organism process |
| Efet.01.567501.g245.t1 | molecular chaperone | 1587 | 0 | 75.4 | F:nucleotide binding |
| Efet.01.567546.g246.t1 | gonadotropin-releasing hormone II receptor-like | 294 | 1.17E-25 | 74.75 | P:G-protein coupled receptor signaling pathway; C:integral component of membrane; F:vasopressin receptor activity; C:plasma membrane |
| Efet.01.567719.g251.t1 | endoribonuclease Dicer-like | 1527 | 1.62E-151 | 60.6 | P:regulation of muscle cell apoptotic process; P:peripheral nervous system myelin formation; P:post-embryonic development; P:regulation of oligodendrocyte differentiation; P:branching morphogenesis of an epithelial tube; P:meiotic spindle organization; P:nerve development; P:defense response to virus; P:regulation of cell cycle; P:hair follicle cell proliferation; P:production of siRNA involved in RNA interference; P:embryonic hindlimb morphogenesis; P:positive regulation of miRNA metabolic process; F:miRNA binding; P:stem cell population maintenance; P:reproductive structure development; P:RNA phosphodiester bond hydrolysis; P:zygote asymmetric cell division; P:regulation of Notch signaling pathway; P:inner ear receptor cell development; P:cerebral cortex development; P:chromatin silencing at centromere outer repeat region; F:protein binding; P:negative regulation of Schwann cell proliferation; P:mRNA stabilization; P:cardiac muscle cell development; P:hair follicle morphogenesis; P:regulation of inflammatory response; P:cartilage development; P:multicellular organism growth; C:micro-ribonucleoprotein complex; P:positive regulation of Schwann cell differentiation; P:neuron projection morphogenesis; P:regulation of regulatory T cell differentiation; P:spleen development; P:regulation of viral genome replication; P:regulation of neuron differentiation; P:positive regulation of transcription from RNA polymerase II promoter; C:neuron projection; P:negative regulation of transcription from RNA polymerase II promoter; P:spindle assembly; P:angiogenesis; P:regulation of stem cell differentiation; P:myoblast differentiation involved in skeletal muscle regeneration; P:lung development; P:olfactory bulb interneuron differentiation; P:pre-miRNA processing; P:ribonucleoprotein complex assembly; P:spinal cord motor neuron differentiation; P:ganglion development; P:cardiac neural crest cell development involved in outflow tract morphogenesis; P:intestinal epithelial cell development; P:regulation of enamel mineralization; F:ribonuclease III activity; C:cytoplasm; P:regulation of protein phosphorylation; P:positive regulation of myelination |
| Efet.01.567762.g253.t1 | basal body-orientation factor 1-like | 402 | 1.79E-19 | 75.3 | C:ciliary basal body |
| Efet.01.567964.g255.t1 | probable ATP-dependent RNA helicase DDX52 | 591 | 5.25E-61 | 72.35 | F:nucleic acid binding; C:nucleus; P:RNA secondary structure unwinding; P:rRNA processing; F:ATP-dependent RNA helicase activity |
| Efet.01.567995.g256.t1 | G -activated inward rectifier potassium channel 3-like | 1203 | 9.00E-103 | 75.7 | P:potassium ion transmembrane transport; C:integral component of membrane; F:inward rectifier potassium channel activity; P:regulation of ion transmembrane transport |
| Efet.01.567999.g257.t1 | DUF2236 domain-containing | 348 | 8.15E-19 | 77.43 |  |
| Efet.01.568048.g259.t1 | Cytochrome P450 2J6 | 516 | 7.90E-12 | 55.55 | F:oxidoreductase activity; F:binding |
| Efet.01.568065.g260.t1 | splicing factor 3B subunit 1-like isoform X2 | 507 | 1.46E-90 | 93.55 | C:U12-type spliceosomal complex; P:melanocyte differentiation; C:catalytic step 2 spliceosome; P:neural crest cell development; F:mRNA binding; C:U2-type prespliceosome; P:regulation of apoptotic process; C:U2 snRNP; P:spliceosomal complex assembly |
| Efet.01.568083.g261.t1 | aminomethyl-transferring glycine dehydrogenase | 588 | 4.29E-100 | 77.95 | F:glycine dehydrogenase (decarboxylating) activity; P:oxidation-reduction process; F:transferase activity; P:glycine catabolic process |
| Efet.01.568083.g262.t1 | class II fructose-bisphosphate aldolase | 639 | 1.10E-129 | 87.4 | F:zinc ion binding; P:glycolytic process; F:fructose-bisphosphate aldolase activity |
| Efet.01.56810.g423.t1 | high affinity cGMP-specific 3 ,5 -cyclic phosphodiesterase 9A-like | 384 | 2.29E-22 | 87.9 | F:metal ion binding; P:signal transduction; F:3',5'-cyclic-nucleotide phosphodiesterase activity |
| Efet.01.568134.g263.t1 | glycine dehydrogenase (aminomethyl-transferring) | 915 | 2.13E-179 | 87.6 | P:glycine decarboxylation via glycine cleavage system; F:glycine dehydrogenase (decarboxylating) activity; P:oxidation-reduction process |
| Efet.01.568134.g264.t1 | CPBP family intramembrane metalloprotease | 540 | 1.79E-84 | 72.15 | P:proteolysis; C:integral component of membrane; F:peptidase activity |
| Efet.01.568134.g265.t1 | RNA polymerase sigma factor | 246 | 1.60E-18 | 66 | F:DNA binding; F:sigma factor activity; F:transcription factor activity, sequence-specific DNA binding; P:regulation of transcription, DNA-templated; P:DNA-templated transcription, initiation |
| Efet.01.568145.g267.t1 | hypothetical protein CRN43_08080, partial | 387 | 8.66E-43 | 78.65 |  |
| Efet.01.568152.g268.t1 | serine threonine- phosphatase 6 regulatory ankyrin repeat subunit B-like | 1524 | 3.58E-78 | 53.15 |  |
| Efet.01.568360.g272.t1 | two pore potassium channel sup-9-like | 534 | 6.81E-68 | 74.85 | P:ion transport; C:membrane |
| Efet.01.56837.g425.t1 | hypothetical protein CAPTEDRAFT_159249 | 582 | 2.37E-06 | 57 | P:transmembrane transport; F:calcium channel activity; P:transport; C:intracellular; P:cellular calcium ion homeostasis; F:ion channel activity; C:cell; F:ryanodine-sensitive calcium-release channel activity; P:ion transport; F:calcium ion binding; C:membrane; P:calcium ion transport; C:integral component of membrane; P:calcium ion transmembrane transport; P:release of sequestered calcium ion into cytosol |
| Efet.01.568488.g274.t1 | histidinol-phosphate transaminase | 780 | 9.41E-94 | 75.95 | F:pyridoxal phosphate binding; P:histidine biosynthetic process; F:histidinol-phosphate transaminase activity |
| Efet.01.568488.g275.t1 | pyruvate dehydrogenase (acetyl-transferring) E1 component subunit alpha | 987 | 0 | 85.6 | F:oxidoreductase activity, acting on the aldehyde or oxo group of donors, disulfide as acceptor; P:oxidation-reduction process |
| Efet.01.568488.g276.t1 | alpha-ketoacid dehydrogenase subunit beta | 1077 | 0 | 88.9 | F:catalytic activity; P:metabolic process |
| Efet.01.568497.g277.t1 | TonB-dependent receptor | 1560 | 2.31E-153 | 64.5 | C:membrane; C:cell outer membrane; F:receptor activity; P:transport |
| Efet.01.568497.g278.t1 | ribosome biogenesis GTPase Der | 1308 | 0 | 83.75 | F:GTP binding; P:ribosome biogenesis |
| Efet.01.568502.g279.t1 | solute carrier family riboflavin member 3 | 1440 | 7.36E-90 | 54.3 | C:integral component of plasma membrane; P:riboflavin transport; C:membrane; C:integral component of membrane; F:riboflavin transporter activity |
| Efet.01.568601.g281.t1 | sodium iodide cotransporter-like isoform X1 | 420 | 2.15E-09 | 55.25 | P:transport |
| Efet.01.568690.g282.t1 | family tellurite resistance | 447 | 9.75E-81 | 56.45 |  |
| Efet.01.56873.g426.t1 | cytochrome b5 | 228 | 1.73E-07 | 83.17 | F:metal ion binding; C:integral component of membrane; F:heme binding |
| Efet.01.568886.g285.t1 | ATPase family AAA domain-containing 1-B-like | 450 | 5.00E-33 | 60.05 | C:intracellular membrane-bounded organelle; C:membrane; P:single-organism process; F:hydrolase activity |
| Efet.01.56889.g427.t1 | hypothetical protein CAPTEDRAFT_201143 | 288 | 9.57E-15 | 61.67 | P:regulation of apoptotic process |
| Efet.01.56912.g428.t1 | octopamine receptor beta-2R-like | 1413 | 1.84E-152 | 66.75 | P:G-protein coupled receptor signaling pathway; C:membrane; F:G-protein coupled amine receptor activity |
| Efet.01.569311.g290.t1 | kinesin KIF9 | 528 | 1.83E-32 | 79.95 | F:ATPase activity; P:extracellular matrix disassembly; P:organelle disassembly; C:kinesin complex; F:protein dimerization activity; P:microtubule-based movement; C:podosome; C:microtubule; F:microtubule binding; F:ATP binding; F:microtubule motor activity; C:cytoplasm; C:vesicle; P:regulation of podosome assembly |
| Efet.01.569313.g291.t1 | cation transporter | 300 | 1.12E-39 | 88.65 | P:cation transmembrane transport; C:integral component of membrane; F:cation transmembrane transporter activity |
| Efet.01.569313.g292.t1 | family transcriptional regulator | 426 | 1.75E-90 | 70.7 | F:DNA binding; F:transcription factor activity, sequence-specific DNA binding; P:transcription, DNA-templated; P:regulation of transcription, DNA-templated |
| Efet.01.569313.g293.t1 | ABC transporter ATP-binding | 1974 | 0 | 77.1 | F:ATPase activity; F:ATP binding |
| Efet.01.569313.g294.t1 | ABC transporter permease | 441 | 8.10E-72 | 87.45 | C:integral component of membrane; P:transport; C:plasma membrane |
| Efet.01.569313.g295.t1 | thiamine pyrophosphate-binding | 1869 | 0 | 60.7 | F:magnesium ion binding; F:thiamine pyrophosphate binding; F:catalytic activity |
| Efet.01.569313.g296.t1 | short-chain dehydrogenase | 354 | 1.22E-76 | 73.15 | F:oxidoreductase activity; P:oxidation-reduction process |
| Efet.01.569313.g297.t1 | tripartite tricarboxylate transporter substrate binding | 1005 | 0 | 71.75 | C:outer membrane-bounded periplasmic space |
| Efet.01.569313.g298.t1 | NAD(P)-dependent oxidoreductase | 588 | 1.59E-140 | 71.6 | F:oxidoreductase activity; P:oxidation-reduction process |
| Efet.01.56943.g431.t1 | hypothetical protein CAPTEDRAFT_219804 | 2373 | 2.16E-15 | 61.5 |  |
| Efet.01.569449.g300.t1 | anoctamin-10-like isoform X3 | 681 | 9.44E-67 | 66.9 | C:membrane |
| Efet.01.569509.g301.t1 | potassium voltage-gated channel subfamily H member 1- | 315 | 3.22E-21 | 88.85 | F:phosphorelay sensor kinase activity; F:voltage-gated potassium channel activity; P:potassium ion transmembrane transport; P:phosphorelay signal transduction system; C:integral component of membrane; P:signal transduction by protein phosphorylation; C:intracellular |
| Efet.01.569625.g306.t1 | importin-4-like isoform X2 | 840 | 1.93E-56 | 59.45 | P:protein import into nucleus; C:intracellular |
| Efet.01.569634.g307.t1 | cytoplasmic FMR1-interacting | 699 | 5.40E-56 | 53 |  |
| Efet.01.569638.g309.t1 | alpha-taxilin-like isoform X3 | 393 | 6.84E-38 | 75.45 | F:syntaxin binding |
| Efet.01.569658.g310.t1 | pyrimidine-nucleoside phosphorylase | 279 | 3.46E-27 | 83.5 | F:phosphorylase activity; P:pyrimidine nucleobase metabolic process; F:thymidine phosphorylase activity; F:pyrimidine-nucleoside phosphorylase activity; P:pyrimidine nucleoside metabolic process |
| Efet.01.569707.g311.t1 | MFS transporter | 252 | 2.98E-32 | 76.85 | P:transmembrane transport; C:integral component of membrane |
| Efet.01.569748.g313.t1 | transcription termination antitermination | 237 | 2.48E-30 | 84.95 | P:DNA-templated transcription, elongation; P:regulation of DNA-templated transcription, elongation; P:transcription antitermination; P:DNA-templated transcription, termination |
| Efet.01.56975.g432.t1 | E3 ubiquitin- ligase HECTD1 | 657 | 5.99E-78 | 73.95 | F:catalytic activity |
| Efet.01.569805.g314.t1 | glutamate receptor 1 | 552 | 1.28E-32 | 52.4 | C:postsynaptic membrane; P:ion transmembrane transport; C:integral component of membrane; C:cell junction; P:ionotropic glutamate receptor signaling pathway; F:ionotropic glutamate receptor activity; F:extracellular-glutamate-gated ion channel activity |
| Efet.01.569832.g315.t1 | hypothetical protein CAPTEDRAFT_187300 | 348 | 3.05E-23 | 63.15 | F:pyridoxal phosphate binding; P:tryptophan catabolic process; C:cytoplasm; F:kynureninase activity; F:catalytic activity; C:membrane; C:integral component of membrane; P:NAD biosynthetic process; F:carbohydrate binding |
| Efet.01.569865.g317.t1 | shTK domain | 552 | 1.52E-19 | 44.5 | F:oxidoreductase activity; F:metal ion binding; P:metabolic process; P:oxidation-reduction process |
| Efet.01.569897.g318.t1 | hypothetical protein CAPTEDRAFT_194388 | 336 | 1.38E-20 | 62.25 | C:membrane; C:integral component of membrane |
| Efet.01.5699.g445.t1 | carbohydrate sulfotransferase 3-like | 957 | 4.31E-69 | 47.55 | C:membrane; C:integral component of membrane; F:sulfotransferase activity |
| Efet.01.569918.g321.t1 | long-chain fatty acid-- ligase | 1695 | 0 | 91.5 | P:long-chain fatty acid metabolic process; F:long-chain fatty acid-CoA ligase activity |
| Efet.01.569918.g322.t1 | ABC transporter ATP-binding | 558 | 7.23E-133 | 94.65 | F:ATPase activity; F:ATP binding |
| Efet.01.569918.g323.t1 | branched-chain amino acid ABC transporter permease | 867 | 4.79E-162 | 95.55 | C:integral component of membrane; P:transport; C:plasma membrane; F:transporter activity |
| Efet.01.569935.g324.t1 | transcriptional regulator | 354 | 1.54E-68 | 71.85 | F:ATP binding; F:helicase activity |
| Efet.01.569967.g326.t1 | 16S rRNA (cytidine(1402)-2 -O)-methyltransferase | 303 | 6.50E-29 | 77.1 | C:cytoplasm; F:rRNA (cytosine-2'-O-)-methyltransferase activity; P:enzyme-directed rRNA 2'-O-methylation |
| Efet.01.569985.g327.t1 | DNA-directed RNA polymerase I subunit RPA2-like | 279 | 1.13E-10 | 63.75 | C:nucleus; F:DNA-directed 5'-3' RNA polymerase activity |
| Efet.01.57.g4.t1 | hypothetical protein CAPTEDRAFT_219486 | 216 | 1.16E-12 | 55.89 | F:zinc ion binding; F:carboxypeptidase activity; F:metallocarboxypeptidase activity; P:proteolysis |
| Efet.01.57000.g434.t1 | acidic mammalian chitinase-like | 246 | 8.82E-17 | 70.7 | P:chitin metabolic process; F:hydrolase activity, hydrolyzing O-glycosyl compounds |
| Efet.01.57005.g435.t1 | tctex1 domain-containing 1 | 459 | 1.93E-21 | 59.5 |  |
| Efet.01.57008.g436.t1 | hypothetical protein CAPTEDRAFT_141249 | 261 | 6.97E-06 | 70 | F:oxidoreductase activity, acting on paired donors, with incorporation or reduction of molecular oxygen, reduced ascorbate as one donor, and incorporation of one atom of oxygen; F:copper ion binding; F:catalytic activity; P:oxidation-reduction process; F:monooxygenase activity |
| Efet.01.570091.g328.t1 | somatostatin receptor type 4 | 738 | 2.20E-52 | 68.9 | C:integral component of plasma membrane; F:neuropeptide binding; P:neuropeptide signaling pathway; P:G-protein coupled receptor signaling pathway, coupled to cyclic nucleotide second messenger; P:negative regulation of cell proliferation; P:cellular response to glucocorticoid stimulus; P:chemical synaptic transmission; C:neuron projection; F:somatostatin receptor activity |
| Efet.01.570125.g330.t1 | SDA1 homolog isoform X3 | 267 | 3.52E-07 | 44.8 | P:ribosomal large subunit export from nucleus; C:nucleus; C:cell; P:ribosomal large subunit biogenesis; P:protein transport; P:ribosome biogenesis; C:nucleolus; P:actin cytoskeleton organization; P:transport; F:molecular_function |
| Efet.01.570134.g331.t1 | mitochondrial isoform X2 | 426 | 1.59E-51 | 77.7 | C:cell; F:protein disulfide oxidoreductase activity; P:glycerol ether metabolic process; P:cell redox homeostasis; P:oxidation-reduction process |
| Efet.01.570164.g334.t1 | ABC transporter substrate-binding | 582 | 2.17E-115 | 78.35 |  |
| Efet.01.57017.g437.t1 | homeobox slou- | 858 | 1.60E-42 | 64.8 | F:DNA binding; C:nucleus; P:regulation of transcription, DNA-templated; F:sequence-specific DNA binding |
| Efet.01.570409.g335.t1 | cyclic-nucleotide-gated cation | 255 | 2.79E-49 | 95.05 | C:integral component of plasma membrane; F:voltage-gated potassium channel activity; P:regulation of membrane potential; P:potassium ion transmembrane transport; F:cGMP-dependent protein kinase activity; P:protein phosphorylation |
| Efet.01.570622.g337.t1 | golgin subfamily A member 2-like isoform X2 | 528 | 3.26E-18 | 48.43 | C:Golgi apparatus |
| Efet.01.570656.g339.t1 | hypothetical protein CRN43_14440 | 291 | 1.74E-08 | 61 |  |
| Efet.01.570663.g340.t1 | transcriptional regulator ATRX-like | 423 | 8.59E-67 | 82.55 | F:DNA binding; F:ATP binding; C:integral component of membrane; C:nucleosome; P:nucleosome assembly |
| Efet.01.570699.g341.t1 | cyclic nucleotide-gated cation channel beta-1-like | 417 | 7.67E-09 | 75.29 | F:ion channel activity; P:ion transport; P:transmembrane transport; P:ion transmembrane transport; C:membrane; C:integral component of membrane; P:transport |
| Efet.01.570764.g343.t1 | homeobox goosecoid-like | 231 | 9.33E-16 | 87.7 | C:nucleus; P:regulation of transcription, DNA-templated; F:sequence-specific DNA binding |
| Efet.01.57085.g440.t1 | gastrula zinc finger -like | 1854 | 1.24E-19 | 42 | F:binding |
| Efet.01.57085.g441.t1 | zinc finger 711-like | 1131 | 3.07E-09 | 42.65 | F:metal ion binding |
| Efet.01.570898.g346.t1 | RNA-directed DNA polymerase from mobile element jockey-like | 468 | 1.03E-30 | 53.75 |  |
| Efet.01.570912.g347.t1 | ABC transporter G family member 9 isoform X1 | 336 | 1.42E-09 | 55.5 | F:nucleotide binding; F:ATPase activity; F:ATP binding; F:ATPase activity, coupled to transmembrane movement of substances; F:zinc ion binding; P:transmembrane transport; C:membrane; C:integral component of membrane; C:plasma membrane |
| Efet.01.570920.g348.t1 | leucine-rich repeat-containing 70 | 1305 | 1.30E-54 | 51.1 | P:axonogenesis; C:membrane; C:integral component of membrane |
| Efet.01.57096.g442.t1 | ubiquitin-40S ribosomal S27a | 369 | 6.11E-48 | 75.95 | P:transcription-coupled nucleotide-excision repair; P:interstrand cross-link repair; P:transforming growth factor beta receptor signaling pathway; P:fibroblast growth factor receptor signaling pathway; P:JNK cascade; P:regulation of transcription from RNA polymerase II promoter in response to hypoxia; P:rRNA processing; P:negative regulation of epidermal growth factor receptor signaling pathway; P:positive regulation of NF-kappaB transcription factor activity; P:viral transcription; P:activation of MAPK activity; P:MyD88-dependent toll-like receptor signaling pathway; P:regulation of necrotic cell death; P:intracellular transport of virus; P:nuclear-transcribed mRNA catabolic process, nonsense-mediated decay; C:endocytic vesicle membrane; P:negative regulation of type I interferon production; P:nucleotide-excision repair, DNA incision, 5'-to lesion; P:ion transmembrane transport; P:nucleotide-excision repair, preincision complex assembly; P:positive regulation of canonical Wnt signaling pathway; P:regulation of signal transduction by p53 class mediator; P:NIK/NF-kappaB signaling; P:nucleotide-excision repair, DNA gap filling; P:translational initiation; C:plasma membrane; P:protein ubiquitination involved in ubiquitin-dependent protein catabolic process; P:tumor necrosis factor-mediated signaling pathway; F:protein binding; P:Notch signaling pathway; F:structural constituent of ribosome; P:anaphase-promoting complex-dependent catabolic process; P:global genome nucleotide-excision repair; P:TRIF-dependent toll-like receptor signaling pathway; P:nucleotide-excision repair, DNA duplex unwinding; C:cytosolic small ribosomal subunit; P:nucleotide-excision repair, DNA damage recognition; P:Fc-epsilon receptor signaling pathway; P:positive regulation of I-kappaB kinase/NF-kappaB signaling; P:positive regulation of epidermal growth factor receptor signaling pathway; P:error-prone translesion synthesis; P:regulation of mRNA stability; C:nucleolus; P:positive regulation of transcription from RNA polymerase II promoter; C:nucleoplasm; P:stimulatory C-type lectin receptor signaling pathway; P:negative regulation of transcription from RNA polymerase II promoter; C:extracellular exosome; P:G2/M transition of mitotic cell cycle; P:SRP-dependent cotranslational protein targeting to membrane; P:DNA damage response, signal transduction by p53 class mediator resulting in cell cycle arrest; P:endosomal transport; P:nucleotide-binding oligomerization domain containing signaling pathway; P:virion assembly; P:Wnt signaling pathway, planar cell polarity pathway; P:ERBB2 signaling pathway; P:macroautophagy; P:T cell receptor signaling pathway; P:error-free translesion synthesis; P:DNA damage response, detection of DNA damage; P:protein polyubiquitination; P:I-kappaB kinase/NF-kappaB signaling; F:RNA binding; P:negative regulation of canonical Wnt signaling pathway; C:extracellular space; P:glycogen biosynthetic process; P:negative regulation of apoptotic process; P:innate immune response; P:regulation of tumor necrosis factor-mediated signaling pathway; P:positive regulation of apoptotic process; C:endosome membrane; P:positive regulation of ubiquitin-protein ligase activity involved in regulation of mitotic cell cycle transition; P:negative regulation of transforming growth factor beta receptor signaling pathway; P:negative regulation of ubiquitin-protein ligase activity involved in mitotic cell cycle |
| Efet.01.570960.g349.t1 | hypothetical protein HELRODRAFT_193047 | 201 | 4.87E-30 | 81.5 | F:calcium ion binding |
| Efet.01.570967.g350.t1 | transcription termination factor Rho | 279 | 4.16E-58 | 95.2 | F:ATP binding; F:RNA binding; F:RNA-dependent ATPase activity; P:regulation of transcription, DNA-templated; F:helicase activity; P:DNA-templated transcription, termination |
| Efet.01.570987.g351.t1 | hypothetical protein HELRODRAFT_176332 | 684 | 1.66E-14 | 60 |  |
| Efet.01.5710.g446.t1 | cell division cycle 7-related kinase-like | 723 | 1.71E-20 | 57.25 | F:ATP binding; F:protein serine/threonine kinase activity; P:protein phosphorylation |
| Efet.01.57104.g443.t1 | Galactoside 2-alpha-L-fucosyltransferase 1 | 639 | 8.82E-20 | 50.2 | P:carbohydrate metabolic process; F:transferase activity, transferring glycosyl groups; P:fucosylation; C:membrane; C:integral component of membrane; F:transferase activity; F:galactoside 2-alpha-L-fucosyltransferase activity |
| Efet.01.571076.g354.t1 | peptidyl-prolyl cis-trans isomerase D | 1056 | 0 | 59.6 | F:peptidyl-prolyl cis-trans isomerase activity; C:membrane; C:integral component of membrane; P:protein peptidyl-prolyl isomerization; F:isomerase activity |
| Efet.01.571102.g355.t1 | ABC transporter ATP-binding | 885 | 1.20E-59 | 60.65 | F:nucleotide binding; F:ATPase activity; F:ATP binding |
| Efet.01.571127.g356.t1 | insulin-like growth factor-binding -related 1 | 327 | 1.22E-07 | 53.65 | F:actin binding; F:calcium ion binding; P:actin cytoskeleton organization; P:cell migration |
| Efet.01.571139.g357.t1 | pre-mRNA-splicing factor 38B-like | 882 | 1.40E-30 | 82.4 | C:precatalytic spliceosome |
| Efet.01.571141.g358.t1 | organic solvent tolerance | 1245 | 2.98E-141 | 67.45 |  |
| Efet.01.571142.g359.t1 | glycosyltransferase family 2 | 462 | 4.25E-49 | 56.67 | C:membrane; C:integral component of membrane |
| Efet.01.57120.g444.t1 | NAD(P) mitochondrial-like | 423 | 2.48E-77 | 87.15 | P:reactive oxygen species metabolic process; F:NADP binding; C:mitochondrial respiratory chain; F:NAD binding; F:NAD(P)+ transhydrogenase (B-specific) activity; C:integral component of membrane; P:hydrogen ion transmembrane transport; P:NADPH regeneration; P:tricarboxylic acid cycle; P:cell redox homeostasis; F:NAD(P)+ transhydrogenase (AB-specific) activity |
| Efet.01.571236.g360.t1 | E3 ubiquitin- ligase RNF14 | 456 | 2.18E-12 | 73.65 | F:catalytic activity; F:metal ion binding |
| Efet.01.571246.g361.t1 | hypothetical protein CAPTEDRAFT_202379 | 1338 | 7.69E-52 | 57 | F:G-protein coupled receptor activity; F:G-protein coupled peptide receptor activity; P:G-protein coupled receptor signaling pathway; C:membrane; C:integral component of membrane |
| Efet.01.571338.g363.t1 | F-box LRR-repeat 17 | 558 | 3.23E-16 | 58.15 |  |
| Efet.01.571369.g364.t1 | dehydrogenase | 600 | 1.12E-53 | 71.95 | F:catalytic activity; P:metabolic process |
| Efet.01.571376.g365.t1 | fructose-bisphosphate aldolase | 339 | 1.89E-43 | 78 | F:aldehyde-lyase activity; P:carbohydrate metabolic process; F:zinc ion binding |
| Efet.01.57151.g445.t1 | Kyphoscoliosis peptidase | 447 | 5.90E-21 | 60.6 |  |
| Efet.01.571546.g368.t1 | unc-50 homolog | 204 | 1.41E-13 | 81.1 | C:integral component of Golgi membrane; P:transport |
| Efet.01.571572.g369.t1 | mitogen-activated kinase kinase kinase | 243 | 4.25E-25 | 78.75 | F:ATP binding; F:protein kinase activity; P:protein phosphorylation |
| Efet.01.571578.g370.t1 | T family of potassium channels 18-like | 615 | 3.90E-18 | 77.75 | P:potassium ion transmembrane transport; C:integral component of membrane; F:potassium channel activity |
| Efet.01.571578.g371.t1 | T family of potassium channels 18-like | 813 | 8.67E-62 | 78.85 | C:integral component of plasma membrane; P:potassium ion transmembrane transport; P:stabilization of membrane potential; F:potassium ion leak channel activity |
| Efet.01.57160.g446.t1 | sacsin-like | 8268 | 0 | 52.9 |  |
| Efet.01.57160.g447.t1 | sacsin-like | 1590 | 0 | 69.3 |  |
| Efet.01.57160.g448.t1 | sacsin-like | 2133 | 2.95E-159 | 50.35 |  |
| Efet.01.571676.g373.t1 | prospero homeobox 1-like | 2778 | 8.02E-09 | 45.67 | F:DNA binding |
| Efet.01.571687.g374.t1 | hypothetical protein CAPTEDRAFT_227143 | 237 | 6.69E-09 | 57 | C:membrane; C:integral component of membrane; F:carbohydrate binding |
| Efet.01.5717.g447.t1 | C163B_HUMAN ame: Full=Scavenger receptor cysteine-rich type 1 M160 ame: Full=CD163 antigen-like 1 ame: CD_antigen=CD163b Flags: Precursor | 264 | 1.89E-07 | 76 | C:membrane; P:positive regulation of macromitophagy in response to mitochondrial depolarization; P:positive regulation of defense response to virus by host; P:xenophagy |
| Efet.01.571795.g377.t1 | glycine alpha 2 subunit,- | 384 | 4.09E-11 | 71.5 | P:ion transmembrane transport; C:integral component of membrane; C:cell junction; F:extracellular ligand-gated ion channel activity; C:synapse; C:plasma membrane |
| Efet.01.571837.g378.t1 | hypothetical protein CAPTEDRAFT_211416 | 738 | 1.16E-09 | 60 |  |
| Efet.01.571840.g379.t1 | twin-arginine translocase subunit | 765 | 5.28E-66 | 83.05 | C:integral component of plasma membrane; C:TAT protein transport complex; F:protein transmembrane transporter activity; P:protein transport by the Tat complex |
| Efet.01.571841.g380.t1 | methylated-DNA--[ ]-cysteine S-methyltransferase | 495 | 6.43E-118 | 75.1 | P:metabolic process; F:methyltransferase activity |
| Efet.01.571927.g381.t1 | GRB2-associated and regulator of MAPK -like | 678 | 2.18E-13 | 55.94 | F:calcium ion binding |
| Efet.01.571939.g382.t1 | diphthine methyl ester synthase | 213 | 3.57E-34 | 88.15 | P:peptidyl-diphthamide biosynthetic process from peptidyl-histidine; F:diphthine synthase activity; P:methylation |
| Efet.01.57206.g450.t1 | nudC domain-containing 3-like | 633 | 1.50E-76 | 77.7 |  |
| Efet.01.572068.g383.t1 | neuropeptide Y receptor type 2 | 348 | 3.46E-14 | 65.8 | P:G-protein coupled receptor signaling pathway; C:integral component of membrane; F:neuropeptide Y receptor activity |
| Efet.01.572116.g384.t1 | tripartite motif-containing 3-like | 504 | 7.95E-10 | 49.2 | F:zinc ion binding; F:metal ion binding; C:intracellular |
| Efet.01.572225.g385.t1 | somatostatin receptor type 2-like | 738 | 3.94E-80 | 66.25 | F:G-protein coupled peptide receptor activity; P:G-protein coupled receptor signaling pathway; C:membrane |
| Efet.01.572246.g386.t1 | EH_Signature domain-containing | 1029 | 3.20E-107 | 59.25 |  |
| Efet.01.572295.g388.t1 | Na(+) H(+) exchange regulatory cofactor NHE-RF1 | 405 | 1.92E-28 | 63.85 |  |
| Efet.01.57234.g451.t1 | Golgi apparatus 1 | 258 | 2.10E-13 | 67.85 | P:regulation of cellular process; C:Golgi membrane; F:fibroblast growth factor binding |
| Efet.01.572354.g390.t1 | potassium voltage-gated channel subfamily H member 7-like isoform X3 | 327 | 4.08E-31 | 76.1 | F:voltage-gated potassium channel activity; P:potassium ion transmembrane transport; C:integral component of membrane |
| Efet.01.572470.g393.t1 | GTP-binding GEM | 384 | 1.89E-26 | 59.95 | F:GTP binding; P:small GTPase mediated signal transduction; C:membrane; C:intracellular |
| Efet.01.572476.g394.t1 | ribonuclease J | 432 | 1.00E-69 | 95.95 | P:RNA phosphodiester bond hydrolysis, endonucleolytic; F:5'-3' exoribonuclease activity; P:RNA phosphodiester bond hydrolysis, exonucleolytic; F:zinc ion binding; F:RNA binding; C:cytoplasm; P:rRNA processing; F:endoribonuclease activity |
| Efet.01.572488.g395.t1 | peptide ABC transporter | 276 | 1.18E-38 | 96.3 | P:transmembrane transport; C:ATP-binding cassette (ABC) transporter complex |
| Efet.01.572648.g396.t1 | aquaporin -like | 549 | 5.65E-42 | 61.05 | C:membrane; C:integral component of membrane; P:transport; F:transporter activity |
| Efet.01.572761.g400.t1 | neuropeptide Y receptor-like | 615 | 2.24E-29 | 75.75 | P:neuropeptide signaling pathway; C:integral component of membrane; F:transferase activity; P:tachykinin receptor signaling pathway; C:plasma membrane; F:tachykinin receptor activity; F:neuropeptide Y receptor activity |
| Efet.01.572798.g401.t1 | mitochondrial chaperone BCS1-like | 627 | 5.12E-110 | 84 | F:ATP binding; P:mitochondrial respiratory chain complex IV assembly; P:mitochondrial respiratory chain complex III assembly; C:mitochondrion; C:integral component of membrane; P:mitochondrial respiratory chain complex I assembly |
| Efet.01.572840.g402.t1 | family | 543 | 1.62E-56 | 65 |  |
| Efet.01.57291.g452.t1 | Beta-hexosaminidase | 516 | 1.21E-37 | 70.3 | P:carbohydrate metabolic process; F:hydrolase activity, hydrolyzing O-glycosyl compounds; F:carbohydrate binding; F:beta-N-acetylhexosaminidase activity; F:polysaccharide binding |
| Efet.01.57291.g453.t1 | beta-N-acetylglucosaminidase [Photobacterium aquae] | 792 | 7.53E-66 | 66 | P:carbohydrate metabolic process; F:hydrolase activity, hydrolyzing O-glycosyl compounds; F:carbohydrate binding; F:beta-N-acetylhexosaminidase activity; F:polysaccharide binding |
| Efet.01.572982.g404.t1 | hypothetical protein | 828 | 8.83E-07 | 69 |  |
| Efet.01.573104.g405.t1 | tetraspanin-13-like isoform X2 | 270 | 1.89E-14 | 80.05 | C:integral component of plasma membrane; P:cell surface receptor signaling pathway |
| Efet.01.573127.g406.t1 | potassium voltage-gated channel subfamily H member 4 | 363 | 6.25E-36 | 77.95 | F:phosphorelay sensor kinase activity; F:voltage-gated potassium channel activity; P:potassium ion transmembrane transport; P:phosphorelay signal transduction system; C:integral component of membrane; P:signal transduction by protein phosphorylation; C:intracellular |
| Efet.01.573129.g407.t1 | hypothetical protein HELRODRAFT_159178 | 237 | 1.93E-17 | 63.65 | C:membrane; C:integral component of membrane; F:carbohydrate binding |
| Efet.01.57315.g455.t1 | Zinc metallo ase nas-13 | 591 | 5.29E-18 | 47.65 | F:zinc ion binding; P:transmembrane transport; F:metal ion binding; F:calcium ion binding; P:proteolysis; C:membrane; C:integral component of membrane; F:peptidase activity; F:metalloendopeptidase activity; F:metallopeptidase activity; F:hydrolase activity |
| Efet.01.573159.g408.t1 | ABC-2 type transport system permease | 213 | 1.97E-31 | 69.4 | C:membrane; C:integral component of membrane |
| Efet.01.573202.g409.t1 | DNA-directed RNA polymerase subunit beta | 2715 | 0 | 83.95 | F:DNA binding; F:DNA-directed 5'-3' RNA polymerase activity; P:transcription, DNA-templated |
| Efet.01.57321.g456.t1 | echinoderm microtubule-associated -like 2 isoform X1 | 468 | 6.67E-29 | 64.55 | C:intracellular; P:intracellular signal transduction |
| Efet.01.57321.g457.t1 | echinoderm microtubule-associated -like CG42247 isoform X1 | 1017 | 4.30E-26 | 76.45 | C:intracellular; P:intracellular signal transduction |
| Efet.01.573338.g410.t1 | -like C-terminal region | 429 | 2.38E-40 | 73.35 | C:membrane |
| Efet.01.573348.g411.t1 | hypothetical protein CSA86_00010 | 255 | 8.14E-06 | 76 |  |
| Efet.01.573348.g412.t1 | calcium binding hemolysin (Modular ) (fragment) | 1662 | 0 | 84.9 | F:calcium ion binding |
| Efet.01.573373.g413.t1 | myosin heavy non-muscle-like | 276 | 2.85E-26 | 87.4 | F:actin filament binding; C:myosin complex; F:ATP binding; F:motor activity |
| Efet.01.57346.g458.t1 | proton-coupled folate transporter-like | 1515 | 5.16E-101 | 55.15 | P:transmembrane transport; C:membrane; C:integral component of membrane; F:transporter activity |
| Efet.01.573469.g414.t1 | E3 ubiquitin- ligase HERC2-like isoform X1 | 1236 | 1.56E-88 | 61.4 | F:ubiquitin-protein transferase activity; F:zinc ion binding; F:metal ion binding; F:ligase activity; P:protein ubiquitination |
| Efet.01.573477.g415.t1 | Mitogen-activated kinase kinase kinase 9 | 816 | 2.33E-119 | 81.1 | F:ATP binding; F:protein serine/threonine kinase activity; P:protein phosphorylation |
| Efet.01.573518.g416.t1 | DNA-directed RNA polymerase subunit beta | 2196 | 0 | 95.65 | F:DNA binding; F:DNA-directed 5'-3' RNA polymerase activity; F:ribonucleoside binding; P:transcription, DNA-templated |
| Efet.01.573518.g417.t1 | 50S ribosomal L7 L12 | 375 | 6.35E-46 | 92.9 | F:structural constituent of ribosome; C:ribosome; P:translation |
| Efet.01.573518.g418.t1 | 50S ribosomal L10 | 414 | 2.86E-73 | 90.4 | F:structural constituent of ribosome; P:ribosome biogenesis; C:ribosome; F:large ribosomal subunit rRNA binding; P:translation |
| Efet.01.573518.g419.t1 | 50S ribosomal L1 | 666 | 4.12E-141 | 95.6 | P:regulation of translation; F:structural constituent of ribosome; C:large ribosomal subunit; F:tRNA binding; F:rRNA binding; P:translation |
| Efet.01.573518.g420.t1 | 50S ribosomal L11 | 432 | 2.60E-96 | 98.75 | F:structural constituent of ribosome; C:ribosome; F:large ribosomal subunit rRNA binding; P:translation |
| Efet.01.573518.g421.t1 | transcription termination antitermination | 786 | 6.14E-144 | 90.6 | P:DNA-templated transcription, elongation; P:regulation of DNA-templated transcription, elongation; P:transcription antitermination; P:DNA-templated transcription, termination |
| Efet.01.57354.g460.t1 | enhancer of filamentation 1 | 2220 | 9.03E-19 | 53.2 |  |
| Efet.01.573548.g422.t1 | kinase C and casein kinase substrate in neurons 1-like | 216 | 9.62E-16 | 68.5 | P:plasma membrane tubulation; C:endosome; P:neuron projection morphogenesis; P:actin filament organization; P:neuron development; P:regulation of endocytosis |
| Efet.01.573678.g425.t1 | Retinal guanylyl cyclase | 207 | 5.81E-30 | 86.8 | C:guanylate cyclase complex, soluble; F:ATP binding; P:cGMP biosynthetic process; C:integral component of membrane; F:guanylate cyclase activity; F:protein kinase activity; P:protein phosphorylation; C:plasma membrane; F:adenylate cyclase activity; P:intracellular signal transduction |
| Efet.01.573694.g426.t1 | Low-density lipo receptor-related 1B | 567 | 2.85E-09 | 46.25 | F:calcium ion binding; C:membrane; C:integral component of membrane |
| Efet.01.573711.g427.t1 | two pore potassium channel sup-9-like | 642 | 1.48E-73 | 83.25 | P:potassium ion transmembrane transport; C:integral component of membrane; F:potassium channel activity |
| Efet.01.573720.g428.t1 | hypothetical protein HELRODRAFT_176714 | 750 | 1.28E-36 | 49.55 | F:ATP binding; F:oxidoreductase activity; F:calcium ion binding; C:membrane; C:integral component of membrane; P:oxidation-reduction process; F:protein kinase activity; P:protein phosphorylation; F:histamine N-methyltransferase activity; P:methylation |
| Efet.01.573753.g429.t1 | centrosome-associated 350 isoform X1 | 576 | 6.31E-15 | 56.7 | C:centrosome; P:microtubule anchoring; F:microtubule binding |
| Efet.01.573757.g430.t1 | serine threonine- kinase tousled-like 2 isoform X4 | 312 | 1.10E-31 | 82.85 | F:ATP binding; F:protein serine/threonine kinase activity; P:protein phosphorylation |
| Efet.01.57379.g461.t1 | Ras-related Rab-33 isoform X2 | 432 | 1.56E-45 | 78.7 | F:GTP binding; P:small GTPase mediated signal transduction; P:regulation of autophagosome assembly; C:intracellular |
| Efet.01.573857.g431.t1 | U2 snRNP-associated SURP motif-containing | 372 | 5.20E-48 | 90 | F:nucleotide binding; F:RNA binding; C:integral component of membrane; P:RNA processing |
| Efet.01.573904.g432.t1 | glutamate synthase large subunit | 243 | 8.21E-26 | 91.6 | P:glutamate biosynthetic process; F:glutamate synthase (ferredoxin) activity; P:oxidation-reduction process; F:glutamate synthase (NADPH) activity |
| Efet.01.574013.g433.t1 | probable G- coupled receptor 139 | 1209 | 4.46E-45 | 44.45 | F:G-protein coupled receptor activity; F:G-protein coupled peptide receptor activity; P:G-protein coupled receptor signaling pathway; P:signal transduction; C:membrane; C:integral component of membrane; F:signal transducer activity |
| Efet.01.574025.g435.t1 | CKAP5_XENLA ame: Full=Cytoskeleton-associated 5-A ame: Full=Microtubule-associated 215 kDa ame: Full=XMAP215 | 429 | 3.97E-06 | 51 |  |
| Efet.01.574039.g436.t1 | hypothetical protein CAPTEDRAFT_210438 | 438 | 2.07E-47 | 62 |  |
| Efet.01.574081.g437.t1 | hypothetical protein CAPTEDRAFT_218901 | 294 | 4.03E-06 | 49 | F:hormone activity |
| Efet.01.574138.g440.t1 | von Willebrand factor type EGF and pentraxin domain-containing 1-like | 300 | 9.37E-17 | 72.2 | F:peptidase inhibitor activity; C:extracellular region; P:negative regulation of peptidase activity |
| Efet.01.574156.g441.t1 | enolase-phosphatase E1 | 792 | 1.13E-61 | 59.3 | P:cellular metabolic process |
| Efet.01.574275.g443.t1 | cytochrome P450 10-like | 390 | 1.57E-30 | 63.25 | F:oxidoreductase activity; F:binding |
| Efet.01.574299.g444.t1 | orphan G- coupled receptor 7 | 681 | 1.73E-45 | 53.55 | F:G-protein coupled receptor activity; F:G-protein coupled peptide receptor activity; P:G-protein coupled receptor signaling pathway; P:signal transduction; C:membrane; C:integral component of membrane; F:signal transducer activity |
| Efet.01.574299.g445.t1 | orphan G- coupled receptor 7 | 366 | 1.31E-06 | 59 | F:G-protein coupled receptor activity; F:G-protein coupled peptide receptor activity; P:G-protein coupled receptor signaling pathway; C:membrane; C:integral component of membrane |
| Efet.01.574313.g446.t1 | FMRFamide receptor-like | 1569 | 4.30E-123 | 56.2 | F:G-protein coupled receptor activity; P:G-protein coupled receptor signaling pathway; C:integral component of membrane |
| Efet.01.574366.g449.t1 | TonB-dependent receptor | 438 | 1.06E-69 | 59.6 | C:membrane; F:receptor activity; P:transport |
| Efet.01.57437.g463.t1 | methyltransferase 22 | 759 | 1.63E-33 | 57.15 |  |
| Efet.01.574379.g451.t1 | sugar ABC transporter permease | 900 | 1.74E-173 | 78.05 | C:integral component of membrane; P:transport; C:plasma membrane |
| Efet.01.57438.g464.t1 | neurogenic differentiation factor 1-like | 1200 | 9.12E-46 | 80.2 | P:amacrine cell differentiation; P:inner ear development; C:RNA polymerase II transcription factor complex; P:insulin secretion; F:RNA polymerase II core promoter proximal region sequence-specific DNA binding; P:anterior/posterior pattern specification; P:signal transduction involved in regulation of gene expression; P:transcription from RNA polymerase II promoter; P:positive regulation of neuron differentiation; F:RNA polymerase II transcription coactivator activity; P:positive regulation of transcription from RNA polymerase II promoter; P:positive regulation of sequence-specific DNA binding transcription factor activity; C:nucleoplasm; F:RNA polymerase II activating transcription factor binding; P:embryonic organ morphogenesis; P:nucleocytoplasmic transport; P:regulation of insulin secretion; F:transcriptional activator activity, RNA polymerase II core promoter proximal region sequence-specific binding; P:pancreatic A cell fate commitment; P:regulation of cell cycle arrest; P:positive regulation of transcription regulatory region DNA binding; F:protein heterodimerization activity; P:pancreatic PP cell fate commitment; P:cellular response to glucose stimulus; P:negative regulation of type B pancreatic cell apoptotic process; F:E-box binding; P:nitric oxide mediated signal transduction; P:cerebellum development; F:chromatin binding; P:neuron development; P:regulation of intestinal epithelial structure maintenance; P:dentate gyrus development; P:negative regulation of JAK-STAT cascade; C:cytoplasm; P:response to drug; P:positive regulation of apoptotic process |
| Efet.01.574381.g452.t1 | outer membrane assembly factor | 717 | 5.95E-128 | 88.15 | P:Gram-negative-bacterium-type cell outer membrane assembly; C:cell outer membrane; P:protein insertion into membrane |
| Efet.01.574381.g453.t1 | ribosome biogenesis GTPase Der | 1377 | 0 | 90.4 | F:GTP binding; P:ribosome biogenesis |
| Efet.01.574381.g454.t1 | GTPase | 1164 | 0 | 91.9 | F:magnesium ion binding; F:GTP binding; C:cytoplasm; F:GTPase activity |
| Efet.01.574381.g455.t1 | protease activity modulator | 1434 | 0 | 84.45 | C:integral component of membrane |
| Efet.01.574381.g456.t1 | protease modulator | 681 | 1.38E-122 | 96.55 | P:proteolysis; C:integral component of membrane; F:peptidase activity; P:regulation of peptidase activity |
| Efet.01.57445.g465.t1 | ubiquitin carboxyl-terminal hydrolase 1 isoform X1 | 351 | 2.62E-16 | 57.25 | P:protein deubiquitination; F:thiol-dependent ubiquitinyl hydrolase activity; P:ubiquitin-dependent protein catabolic process |
| Efet.01.574548.g459.t1 | hypothetical protein | 486 | 1.25E-09 | 55 |  |
| Efet.01.574697.g462.t1 | ankyrin repeat domain-containing 17-like | 1101 | 8.64E-07 | 41 |  |
| Efet.01.574697.g463.t1 | ankyrin repeat domain-containing 17-like | 2763 | 0 | 85.35 | F:RNA binding |
| Efet.01.57472.g466.t1 | Disintegrin and metallo ase domain-containing 10 | 828 | 2.82E-08 | 45.5 | F:zinc ion binding; P:integrin-mediated signaling pathway; P:Notch signaling pathway; P:membrane protein ectodomain proteolysis; P:proteolysis; C:membrane; C:integral component of membrane; F:metalloendopeptidase activity; C:intracellular; F:metallopeptidase activity |
| Efet.01.57472.g467.t1 | zinc finger 271-like | 1209 | 2.81E-131 | 62.1 | F:nucleic acid binding; F:metal ion binding |
| Efet.01.574733.g464.t1 | haloacid dehalogenase-like hydrolase domain-containing 3 | 534 | 2.39E-57 | 59.7 | P:metabolic process; C:mitochondrion; F:hydrolase activity |
| Efet.01.57474.g468.t1 | hypothetical protein CAPTEDRAFT_103345, partial | 225 | 1.14E-06 | 75 | F:nucleotide binding; F:ATP binding; F:microtubule motor activity; P:microtubule-based movement; F:microtubule binding; C:microtubule |
| Efet.01.574744.g465.t1 | FAD-dependent oxidoreductase | 1005 | 5.47E-179 | 74.7 | F:oxidoreductase activity; C:integral component of membrane; P:oxidation-reduction process |
| Efet.01.574744.g466.t1 | dihydrodipicolinate synthase family | 816 | 6.53E-168 | 85.2 | P:metabolic process; F:lyase activity |
| Efet.01.574744.g467.t1 | endoribonuclease L-PSP family | 405 | 9.42E-93 | 78.45 | F:hydrolase activity |
| Efet.01.574744.g468.t1 | glyoxalase bleomycin resistance dioxygenase family | 432 | 1.11E-82 | 63.3 | F:dioxygenase activity; P:oxidation-reduction process |
| Efet.01.574744.g469.t1 | sodium:solute symporter family | 1497 | 0 | 83.35 | P:transmembrane transport; C:integral component of membrane; F:transporter activity |
| Efet.01.574744.g470.t1 | XRE family transcriptional regulator | 369 | 9.72E-84 | 72 | F:sequence-specific DNA binding |
| Efet.01.574744.g471.t1 | hydrolase Nlp P60 | 513 | 1.07E-91 | 90.05 | F:hydrolase activity |
| Efet.01.574761.g472.t1 | Tudor domain-containing 1 | 1482 | 1.32E-36 | 47.05 | C:intracellular part |
| Efet.01.574820.g473.t1 | M13 family peptidase | 993 | 5.34E-102 | 67.95 | F:hydrolase activity |
| Efet.01.574879.g474.t1 | hypothetical protein HELRODRAFT_164372 | 456 | 3.73E-08 | 52 |  |
| Efet.01.574964.g475.t1 | type I restriction endonuclease subunit R | 237 | 2.37E-19 | 87.9 | F:DNA binding; F:ATP binding; P:nucleic acid phosphodiester bond hydrolysis; F:Type I site-specific deoxyribonuclease activity; F:helicase activity; P:DNA restriction-modification system |
| Efet.01.574972.g476.t1 | hypothetical protein CAPTEDRAFT_157479 | 216 | 1.14E-06 | 67 | P:cell adhesion |
| Efet.01.575010.g477.t1 | hypothetical protein HELRODRAFT_184858 | 282 | 1.37E-12 | 86.67 | C:cytoskeleton; F:actin binding |
| Efet.01.575043.g478.t1 | GRIP and coiled-coil domain-containing 2-like | 231 | 1.58E-25 | 82.5 | P:protein targeting to Golgi; C:intracellular |
| Efet.01.575046.g479.t1 | dolichyl-diphosphooligosaccharide-- glycosyltransferase subunit STT3B-like | 207 | 4.68E-10 | 87.2 | C:oligosaccharyltransferase complex; F:dolichyl-diphosphooligosaccharide-protein glycotransferase activity; P:glycoprotein catabolic process; C:integral component of membrane; P:protein N-linked glycosylation via asparagine; P:co-translational protein modification; P:ER-associated ubiquitin-dependent protein catabolic process; P:response to unfolded protein; P:post-translational protein modification |
| Efet.01.575077.g481.t1 | hypothetical protein CAPTEDRAFT_195684 | 267 | 1.58E-14 | 58.67 |  |
| Efet.01.575084.g482.t1 | phosphatidylinositol 4,5-bisphosphate 3-kinase catalytic subunit delta isoform | 255 | 6.76E-30 | 74.95 | P:phosphatidylinositol-mediated signaling; P:phosphatidylinositol phosphorylation; F:kinase activity; C:intracellular |
| Efet.01.575168.g483.t1 | alginate lyase | 930 | 4.58E-145 | 70.85 | F:lyase activity; C:periplasmic space |
| Efet.01.575298.g484.t1 | cell division | 705 | 1.76E-167 | 72.95 | P:cell division; C:cytoplasm; P:cell cycle |
| Efet.01.575355.g485.t1 | hypothetical protein HELRODRAFT_92753, partial | 1149 | 3.27E-14 | 46.5 |  |
| Efet.01.575390.g486.t1 | glucosamine-6-phosphate deaminase | 906 | 2.21E-157 | 85.5 | P:carbohydrate metabolic process; F:glucosamine-6-phosphate deaminase activity; P:N-acetylglucosamine metabolic process |
| Efet.01.575390.g487.t1 | formyltetrahydrofolate deformylase | 1731 | 0 | 87.65 | P:'de novo' IMP biosynthetic process; F:amino acid binding; F:hydroxymethyl-, formyl- and related transferase activity; P:one-carbon metabolic process; F:formyltetrahydrofolate deformylase activity |
| Efet.01.5754.g450.t1 | serine threonine- phosphatase 4 regulatory subunit 3 isoform X1 | 444 | 2.80E-56 | 80.9 | C:nucleus; C:cytoskeleton; F:protein binding; C:cytoplasm; P:protein dephosphorylation; C:protein phosphatase 4 complex; P:positive regulation of gluconeogenesis |
| Efet.01.575406.g488.t1 | methylmalonic aciduria and homocystinuria type D mitochondrial-like | 351 | 4.17E-45 | 68.65 | P:cobalamin metabolic process |
| Efet.01.575408.g489.t1 | tctex1 domain-containing 2-like | 648 | 8.57E-36 | 62.65 |  |
| Efet.01.575430.g490.t1 | A30BL_HUMAN ame: Full= ankyrin repeat domain-containing 30B-like | 807 | 3.00E-07 | 49 |  |
| Efet.01.575483.g492.t1 | BTB POZ domain-containing 3-like | 303 | 3.08E-12 | 53.67 |  |
| Efet.01.575505.g493.t1 | Ankyrin repeat and SOCS box 2 | 468 | 4.01E-25 | 56 | C:membrane; C:integral component of membrane |
| Efet.01.575546.g494.t1 | enoyl- hydratase | 327 | 3.33E-48 | 73.65 | P:metabolic process; F:enoyl-CoA hydratase activity |
| Efet.01.575546.g495.t1 | ABC transporter substrate-binding | 1566 | 0 | 60.15 | P:transmembrane transport; C:ATP-binding cassette (ABC) transporter complex |
| Efet.01.575546.g496.t1 | ABC transporter permease | 447 | 1.99E-67 | 77.15 | C:integral component of membrane; P:transport; C:plasma membrane |
| Efet.01.575546.g497.t1 | ABC transporter permease | 765 | 1.58E-146 | 69.4 | C:integral component of membrane; P:transport; C:plasma membrane |
| Efet.01.575546.g498.t1 | ABC transporter ATP-binding | 795 | 1.07E-150 | 76.7 | F:ATPase activity; F:ATP binding; P:peptide transport |
| Efet.01.575546.g499.t1 | peptide ABC transporter ATP-binding | 807 | 0 | 72 | F:ATPase activity; F:ATP binding; P:peptide transport |
| Efet.01.575546.g500.t1 | IS630 family transposase | 408 | 6.46E-94 | 90.5 | F:DNA binding |
| Efet.01.575546.g501.t1 | IS630 family transposase | 210 | 2.22E-25 | 86.85 | F:DNA binding |
| Efet.01.57569.g474.t1 | poly [ADP-ribose] polymerase 14-like | 399 | 3.06E-15 | 56 | F:nucleotide binding; F:nucleic acid binding; F:transferase activity, transferring glycosyl groups; F:transferase activity; F:NAD+ ADP-ribosyltransferase activity |
| Efet.01.575699.g504.t1 | brain tumor -like | 2358 | 2.34E-172 | 55.7 | F:zinc ion binding; F:metal ion binding; C:intracellular |
| Efet.01.575700.g505.t1 | ABC transporter permease | 912 | 0 | 91.4 | C:integral component of membrane; P:transport; C:plasma membrane |
| Efet.01.575763.g507.t1 | short stature homeobox | 438 | 2.25E-25 | 91.35 | C:nucleus; P:regulation of transcription, DNA-templated; F:sequence-specific DNA binding |
| Efet.01.575783.g508.t1 | rho GTPase-activating 12 isoform X9 | 705 | 9.42E-06 | 63 | P:signal transduction |
| Efet.01.575796.g509.t1 | set1 Ash2 histone methyltransferase complex subunit ASH2-like | 255 | 2.62E-29 | 75.9 |  |
| Efet.01.575817.g510.t1 | aryldialkylphosphatase | 1026 | 0 | 77 | F:zinc ion binding; P:catabolic process |
| Efet.01.57582.g477.t1 | zinc finger SWIM domain-containing 8-like | 501 | 8.07E-53 | 69.8 | F:zinc ion binding |
| Efet.01.575832.g511.t1 | ELAV 1 isoform X1 | 351 | 1.05E-48 | 87 | F:nucleotide binding; P:regulation of mRNA processing; C:cytoplasm; P:male courtship behavior; F:mRNA binding; P:sensory perception of pain; F:poly(U) RNA binding |
| Efet.01.575886.g512.t1 | allatostatin-A receptor-like | 1218 | 3.15E-63 | 63.75 | F:G-protein coupled receptor activity; P:G-protein coupled receptor signaling pathway; P:signal transduction; C:membrane; C:integral component of membrane; F:signal transducer activity |
| Efet.01.575925.g514.t1 | adenylate cyclase type 9- | 816 | 1.72E-132 | 78.35 | F:nucleotide binding; F:phosphorus-oxygen lyase activity; C:integral component of membrane; C:intracellular; P:cyclic nucleotide biosynthetic process; P:intracellular signal transduction |
| Efet.01.5761.g452.t1 | FMRFamide receptor-like | 627 | 2.53E-54 | 58.8 | F:G-protein coupled receptor activity; P:signal transduction; C:membrane |
| Efet.01.576160.g517.t1 | Potassium voltage-gated channel Shal | 480 | 1.01E-72 | 88.95 | F:voltage-gated potassium channel activity; P:potassium ion transmembrane transport; C:voltage-gated potassium channel complex; P:regulation of ion transmembrane transport; P:protein homooligomerization |
| Efet.01.576188.g518.t1 | tkl kinase | 408 | 1.44E-12 | 61.55 | F:ATP binding; F:protein kinase activity; P:phosphorylation; P:protein phosphorylation; F:kinase activity; P:transport; F:transporter activity |
| Efet.01.576298.g522.t1 | hypothetical protein CAPTEDRAFT_222036 | 594 | 7.52E-29 | 48.67 | C:membrane; C:integral component of membrane |
| Efet.01.576304.g523.t1 | malignant fibrous histiocytoma-amplified sequence 1 homolog | 798 | 8.65E-88 | 67.45 |  |
| Efet.01.576403.g526.t1 | E3 ubiquitin- ligase UNKL isoform X2 | 282 | 7.21E-37 | 75.5 | F:metal ion binding |
| Efet.01.576405.g527.t1 | doublesex- and mab-3-related transcription factor A2-like | 492 | 1.03E-32 | 86.2 | C:nucleus; F:transcription factor activity, sequence-specific DNA binding; P:regulation of transcription, DNA-templated; F:sequence-specific DNA binding |
| Efet.01.576455.g528.t1 | ABC transporter ATP-binding | 945 | 0 | 73.65 | F:ATP binding; F:ATPase activity, coupled to transmembrane movement of substances; P:transmembrane transport; C:integral component of membrane |
| Efet.01.576471.g530.t1 | aminopeptidase N-like | 735 | 1.58E-06 | 46 |  |
| Efet.01.576541.g531.t1 | semaphorin-5B-like | 201 | 5.11E-11 | 93.8 | C:extracellular space; P:negative chemotaxis; P:neural crest cell migration; C:integral component of membrane; P:negative regulation of axon extension involved in axon guidance; F:chemorepellent activity; F:semaphorin receptor binding; P:positive regulation of cell migration; P:semaphorin-plexin signaling pathway |
| Efet.01.576557.g532.t1 | dihydroxy-acid dehydratase | 219 | 1.41E-40 | 90.95 | F:dihydroxy-acid dehydratase activity; F:L-arabinonate dehydratase activity; P:metabolic process |
| Efet.01.576585.g533.t1 | leucine-rich repeat serine threonine- kinase 1 isoform X1 | 612 | 7.40E-26 | 51.2 | P:metabolic process; P:single-organism process; P:cellular process |
| Efet.01.576621.g534.t1 | translation initiation factor IF-2 | 300 | 1.18E-42 | 74.25 | F:nucleotide binding; C:intracellular; P:translation |
| Efet.01.576621.g535.t1 | ribosome-binding factor A | 225 | 2.38E-44 | 85.95 | C:cytoplasm; P:rRNA processing |
| Efet.01.576662.g536.t1 | limbic system-associated membrane -like isoform X4 | 285 | 4.08E-32 | 72.2 | C:membrane; P:cellular process |
| Efet.01.576698.g537.t1 | growth hormone secretagogue receptor type 1 | 1101 | 2.11E-27 | 45.75 | F:G-protein coupled receptor activity; P:G-protein coupled receptor signaling pathway; P:signal transduction; C:membrane; C:integral component of membrane; F:signal transducer activity |
| Efet.01.57679.g479.t1 | growth hormone secretagogue receptor type 1-like | 1044 | 2.12E-49 | 51.45 | F:G-protein coupled receptor activity; P:signal transduction; C:membrane |
| Efet.01.576819.g539.t1 | hypothetical protein | 327 | 1.61E-58 | 63.1 |  |
| Efet.01.576819.g540.t1 | BMC domain-containing | 285 | 4.64E-28 | 88.9 |  |
| Efet.01.576819.g541.t1 | microcompartment | 627 | 3.36E-145 | 85.45 |  |
| Efet.01.576819.g542.t1 | 3-oxoacyl-ACP reductase | 597 | 1.70E-139 | 87.2 |  |
| Efet.01.576819.g543.t1 | APC family permease | 549 | 3.46E-126 | 80.7 | C:integral component of membrane; F:amino acid transmembrane transporter activity; P:amino acid transmembrane transport |
| Efet.01.576819.g544.t1 | serine kinase | 765 | 2.77E-95 | 66.9 | F:kinase activity |
| Efet.01.576819.g545.t1 | transcriptional regulator | 957 | 4.06E-152 | 67.1 | F:DNA binding; F:transcription factor activity, sequence-specific DNA binding; P:transcription, DNA-templated; P:regulation of transcription, DNA-templated; C:intracellular |
| Efet.01.57686.g480.t1 | sodium bicarbonate transporter 11 isoform X1 | 312 | 8.60E-17 | 96.3 | P:inorganic anion transport; C:integral component of membrane; F:inorganic anion exchanger activity |
| Efet.01.576893.g548.t1 | hypothetical protein CAPTEDRAFT_201600, partial | 921 | 1.06E-14 | 46 | F:transferase activity, transferring hexosyl groups; C:membrane; C:integral component of membrane |
| Efet.01.576906.g549.t1 | helicase | 369 | 1.68E-13 | 58.65 |  |
| Efet.01.576941.g551.t1 | Programmed cell death 5 | 300 | 1.49E-19 | 75.55 | F:DNA binding |
| Efet.01.576944.g553.t1 | neuropeptide Y receptor type 5-like | 813 | 2.61E-56 | 62.7 | F:G-protein coupled receptor activity; P:G-protein coupled receptor signaling pathway; C:membrane |
| Efet.01.577003.g555.t1 | beta-glucosidase | 411 | 1.27E-75 | 67.65 | F:hydrolase activity, hydrolyzing O-glycosyl compounds |
| Efet.01.577022.g556.t1 | alkyl aryl-sulfatase BDS1-like | 1248 | 8.03E-99 | 57.8 | C:membrane; C:integral component of membrane; F:protein dimerization activity |
| Efet.01.57705.g481.t1 | vascular endothelial growth factor receptor 1 | 369 | 2.89E-10 | 55.2 | P:positive regulation of cellular process; F:transmembrane receptor protein tyrosine kinase activity; C:membrane; P:cellular response to stimulus; P:phosphorylation; P:single-organism process |
| Efet.01.577062.g558.t1 | 3-(3-hydroxyphenyl)propionate hydroxylase | 1752 | 0 | 72.8 | F:FAD binding; P:oxidation-reduction process; F:monooxygenase activity |
| Efet.01.577062.g559.t1 | family transcriptional regulator | 765 | 5.69E-173 | 68.85 | F:DNA binding; P:transcription, DNA-templated; P:regulation of transcription, DNA-templated |
| Efet.01.577062.g560.t1 | glyoxalase | 276 | 5.34E-59 | 76.7 | F:dioxygenase activity; P:oxidation-reduction process |
| Efet.01.577151.g561.t1 | glutamate-gated chloride channel-like isoform X1 | 240 | 1.85E-06 | 52 |  |
| Efet.01.577184.g563.t1 | hypothetical protein HELRODRAFT_163113 | 252 | 2.74E-11 | 54.95 | C:membrane; C:integral component of membrane; F:carbohydrate binding |
| Efet.01.577234.g565.t1 | E3 ubiquitin- ligase TRIM71-like | 417 | 5.52E-15 | 52.2 | F:zinc ion binding; P:signal transduction; P:ion transmembrane transport; F:GABA-A receptor activity; F:extracellular ligand-gated ion channel activity; C:GABA-A receptor complex; C:synapse; P:transport; C:intracellular; C:plasma membrane; P:ion transport; F:metal ion binding; C:membrane; C:integral component of membrane; C:cell junction |
| Efet.01.577276.g566.t1 | vacuolar sorting-associated 13B isoform X1 | 807 | 5.72E-41 | 54.2 |  |
| Efet.01.577276.g567.t1 | vacuolar sorting-associated 13B-like | 396 | 4.54E-19 | 59.55 |  |
| Efet.01.577373.g569.t1 | phospholipase | 366 | 1.41E-78 | 88.95 | C:membrane; P:lipid metabolic process; F:phospholipase activity |
| Efet.01.577373.g570.t1 | tRNA guanosine(34) transglycosylase Tgt | 1182 | 0 | 92.55 | P:queuosine biosynthetic process; F:metal ion binding; P:tRNA modification; F:queuine tRNA-ribosyltransferase activity |
| Efet.01.577373.g571.t1 | hypothetical protein | 420 | 9.12E-98 | 65.8 |  |
| Efet.01.577373.g572.t1 | hypothetical protein | 1281 | 0 | 60.25 |  |
| Efet.01.577373.g573.t1 | ribonuclease HI | 333 | 1.99E-72 | 98.25 | F:magnesium ion binding; F:nucleic acid binding; P:RNA phosphodiester bond hydrolysis, endonucleolytic; F:RNA-DNA hybrid ribonuclease activity; C:cytoplasm; P:RNA catabolic process |
| Efet.01.577373.g574.t1 | Methyltransferase type 11 | 756 | 4.06E-137 | 76.05 | F:methyltransferase activity; P:methylation |
| Efet.01.577373.g575.t1 | hydroxyacylglutathione hydrolase | 777 | 2.11E-177 | 86.8 | F:hydroxyacylglutathione hydrolase activity; F:zinc ion binding; P:methylglyoxal catabolic process to D-lactate via S-lactoyl-glutathione; P:antibiotic catabolic process; F:beta-lactamase activity |
| Efet.01.577373.g576.t1 | lytic transglycosylase | 1224 | 0 | 92.75 | P:peptidoglycan metabolic process; C:membrane; F:lytic transglycosylase activity |
| Efet.01.577373.g577.t1 | ABC transporter substrate-binding | 1068 | 0 | 84.7 |  |
| Efet.01.577373.g578.t1 | glutathione metabolism | 405 | 1.18E-58 | 82.1 | C:integral component of membrane |
| Efet.01.577373.g579.t1 | ribulose-phosphate 3-epimerase | 567 | 2.60E-89 | 90.5 | P:carbohydrate metabolic process; F:metal ion binding; P:pentose-phosphate shunt; F:ribulose-phosphate 3-epimerase activity |
| Efet.01.577373.g580.t1 | Co2+ Mg2+ efflux | 537 | 8.23E-82 | 74.85 |  |
| Efet.01.577373.g581.t1 | sodium:proton exchanger | 1263 | 0 | 92.6 | F:solute:proton antiporter activity; C:integral component of membrane; P:hydrogen ion transmembrane transport |
| Efet.01.577373.g582.t1 | glutamate--cysteine ligase | 1017 | 0 | 96.5 | F:ATP binding; P:cellular modified amino acid biosynthetic process; F:glutamate-cysteine ligase activity |
| Efet.01.577373.g583.t1 | thiamine-phosphate kinase | 597 | 1.38E-80 | 82.65 | F:magnesium ion binding; F:ATP binding; F:thiamine-phosphate kinase activity; P:phosphorylation; P:thiamine diphosphate biosynthetic process; P:thiamine biosynthetic process |
| Efet.01.577373.g584.t1 | damage-inducible | 423 | 8.40E-95 | 89.75 |  |
| Efet.01.577373.g585.t1 | sigma 28 | 2175 | 0 | 66.65 | P:transcription, DNA-templated |
| Efet.01.577373.g586.t1 | ATP-binding | 1449 | 0 | 87.5 |  |
| Efet.01.577373.g587.t1 | endonuclease | 2826 | 0 | 83.85 | P:nucleic acid phosphodiester bond hydrolysis; F:endonuclease activity |
| Efet.01.577373.g588.t1 | PD-(D E)XK motif | 492 | 7.33E-27 | 83.5 |  |
| Efet.01.577373.g589.t1 | abortive phage infection | 1725 | 0 | 92.05 |  |
| Efet.01.577373.g590.t1 | very short patch repair endonuclease | 441 | 1.30E-77 | 80.2 | P:nucleic acid phosphodiester bond hydrolysis; F:endonuclease activity; P:mismatch repair |
| Efet.01.577373.g591.t1 | DNA cytosine methyltransferase | 1671 | 0 | 81.7 | F:DNA (cytosine-5-)-methyltransferase activity; P:C-5 methylation of cytosine |
| Efet.01.577373.g592.t1 | RNA methyltransferase | 1101 | 0 | 89.75 | F:RNA binding; F:methyltransferase activity; P:methylation |
| Efet.01.577373.g593.t1 | RNA polymerase sigma-54 factor | 1464 | 0 | 91.3 | F:DNA binding; F:sigma factor activity; F:transcription factor activity, sequence-specific DNA binding; F:DNA-directed 5'-3' RNA polymerase activity; P:regulation of transcription, DNA-templated; P:DNA-templated transcription, initiation |
| Efet.01.577373.g594.t1 | LPS export ABC transporter ATP-binding | 600 | 7.09E-110 | 95.5 | F:ATP binding; P:sulfate transmembrane transport; F:ATPase-coupled sulfate transmembrane transporter activity; C:ATP-binding cassette (ABC) transporter complex |
| Efet.01.577430.g596.t1 | ABC transporter permease | 741 | 9.30E-66 | 81.3 | C:integral component of membrane; P:transport; C:plasma membrane; F:transporter activity |
| Efet.01.577451.g597.t1 | diguanylate cyclase | 342 | 8.75E-56 | 86.55 | C:integral component of membrane; P:transport; C:plasma membrane |
| Efet.01.577451.g598.t1 | N-acetylmuramic acid 6-phosphate etherase | 627 | 4.40E-77 | 73.05 | F:lyase activity; P:amino sugar catabolic process |
| Efet.01.577451.g599.t1 | anhydro-N-acetylmuramic acid kinase | 708 | 6.10E-80 | 74.15 | F:nucleotide binding; P:carbohydrate derivative metabolic process; F:transferase activity, transferring phosphorus-containing groups; P:cellular metabolic process |
| Efet.01.577467.g600.t1 | mannonate dehydratase | 342 | 5.22E-76 | 78.95 | P:glucuronate catabolic process; F:mannonate dehydratase activity |
| Efet.01.57753.g482.t1 | hypothetical protein CAPTEDRAFT_159664 | 477 | 4.76E-07 | 64 | F:structural constituent of ribosome; C:ribosome; C:intracellular; P:translation |
| Efet.01.577572.g602.t1 | innexin unc-9-like isoform X2 | 774 | 1.18E-118 | 68.15 | C:gap junction; P:ion transport; C:integral component of membrane; C:plasma membrane |
| Efet.01.577572.g603.t1 | innexin unc-9-like isoform X2 | 486 | 8.79E-72 | 65.6 | C:membrane; C:cell junction; P:transport |
| Efet.01.577578.g604.t1 | family transcriptional regulator | 1017 | 1.71E-123 | 77.8 | F:hydrolase activity, acting on carbon-nitrogen (but not peptide) bonds; F:DNA binding; P:carbohydrate metabolic process; F:transcription factor activity, sequence-specific DNA binding; P:transcription, DNA-templated; P:regulation of transcription, DNA-templated |
| Efet.01.577578.g605.t1 | chitin deacetylase | 237 | 3.28E-23 | 82.45 | F:hydrolase activity, acting on carbon-nitrogen (but not peptide) bonds; P:carbohydrate metabolic process |
| Efet.01.577664.g607.t1 | ribonuclease N | 411 | 2.02E-92 | 82.1 | P:RNA phosphodiester bond hydrolysis, endonucleolytic; F:RNA binding; F:endoribonuclease activity |
| Efet.01.577664.g608.t1 | barnase inhibitor | 510 | 8.07E-121 | 93.65 |  |
| Efet.01.577664.g609.t1 | 16S rRNA (adenine(1518)-N(6) adenine(1519)-N(6))-dimethyltransferase | 720 | 8.11E-145 | 85.1 | F:RNA binding; F:16S rRNA (adenine(1518)-N(6)/adenine(1519)-N(6))-dimethyltransferase activity; C:cytoplasm; P:rRNA methylation; F:rRNA (adenine-N6,N6-)-dimethyltransferase activity |
| Efet.01.577664.g610.t1 | molecular chaperone | 606 | 8.80E-138 | 90.25 | F:peptidyl-prolyl cis-trans isomerase activity; P:Gram-negative-bacterium-type cell outer membrane assembly; P:maintenance of stationary phase; P:chaperone mediated protein folding requiring cofactor; C:outer membrane-bounded periplasmic space; F:peptide binding; F:unfolded protein binding; P:protein peptidyl-prolyl isomerization; P:protein stabilization |
| Efet.01.577665.g611.t1 | phosphoglycerate kinase | 216 | 5.75E-26 | 82.7 | F:ATP binding; C:cytoplasm; F:phosphoglycerate kinase activity; P:glycolytic process |
| Efet.01.577753.g612.t1 | outer membrane | 546 | 1.07E-117 | 67.55 |  |
| Efet.01.577753.g613.t1 | cytosine deaminase | 1185 | 0 | 85.15 | F:cytosine deaminase activity; F:N-isopropylammelide isopropylaminohydrolase activity |
| Efet.01.57776.g484.t1 | neurexin-1 isoform X1 | 315 | 1.62E-13 | 76.4 | F:metal ion binding; C:integral component of membrane; P:chemical synaptic transmission |
| Efet.01.577820.g614.t1 | leucine-rich repeat-containing 58-like | 564 | 1.96E-60 | 67.35 | C:membrane; C:integral component of membrane |
| Efet.01.577832.g615.t1 | oxidoreductase | 768 | 1.53E-180 | 82.15 | F:oxidoreductase activity; P:oxidation-reduction process |
| Efet.01.577872.g616.t1 | 50S ribosomal L5 | 249 | 2.20E-41 | 76.9 | F:structural constituent of ribosome; C:ribosome; F:tRNA binding; F:rRNA binding; P:translation |
| Efet.01.577873.g617.t1 | ABC transporter | 867 | 0 | 80.2 |  |
| Efet.01.577929.g618.t1 | tRNA-specific adenosine deaminase 2 | 414 | 2.40E-54 | 74.75 | F:metal ion binding; F:tRNA-specific adenosine-34 deaminase activity; C:tRNA-specific adenosine-34 deaminase complex; P:tRNA modification |
| Efet.01.577933.g619.t1 | histone-lysine N-methyltransferase NSD2-like | 270 | 8.10E-14 | 81.25 | C:nucleus; F:zinc ion binding; P:histone lysine methylation; F:histone-lysine N-methyltransferase activity |
| Efet.01.577981.g620.t1 | synaptotagmin 4 | 369 | 3.82E-46 | 73.55 | C:membrane |
| Efet.01.57809.g486.t1 | hypothetical protein CAPTEDRAFT_198404 | 2520 | 2.33E-45 | 51.2 |  |
| Efet.01.578120.g621.t1 | bone morphogenetic 2-like | 447 | 7.08E-18 | 91.95 | F:growth factor activity; C:extracellular region; P:growth |
| Efet.01.578127.g622.t1 | titin-like isoform X2 | 276 | 9.31E-28 | 74.7 | F:nucleotide binding; P:phosphorylation; F:kinase activity |
| Efet.01.578167.g623.t1 | 6-hydroxynicotinate reductase | 402 | 2.46E-65 | 91.55 | F:6-hydroxynicotinate reductase activity; P:oxidation-reduction process |
| Efet.01.578167.g624.t1 | 6-hydroxynicotinate reductase | 2109 | 1.76E-139 | 89.65 | F:6-hydroxynicotinate reductase activity; P:oxidation-reduction process |
| Efet.01.578167.g625.t1 | peptide synthetase | 531 | 2.16E-106 | 91.2 |  |
| Efet.01.578167.g626.t1 | ABC transporter substrate-binding | 531 | 1.63E-83 | 96.4 | P:amino acid transport |
| Efet.01.578167.g627.t1 | ABC transporter substrate-binding | 582 | 3.68E-130 | 95.5 | P:amino acid transport |
| Efet.01.578168.g628.t1 | amino acid synthesis family | 531 | 8.58E-109 | 92.15 |  |
| Efet.01.578168.g629.t1 | Amino acid synthesis | 573 | 1.05E-132 | 98.1 |  |
| Efet.01.578168.g630.t1 | UPF0280 family | 930 | 7.63E-163 | 87.25 |  |
| Efet.01.57817.g487.t1 | multiple C2 and transmembrane domain-containing 1-like isoform X1 | 330 | 2.15E-27 | 74.4 | C:membrane |
| Efet.01.578197.g631.t1 | muscle calcium channel subunit alpha-1-like | 204 | 1.20E-29 | 81.4 | F:high voltage-gated calcium channel activity; C:voltage-gated calcium channel complex; P:regulation of ion transmembrane transport; P:calcium ion transmembrane transport; P:membrane depolarization during action potential |
| Efet.01.578243.g632.t1 | phosphoribosylaminoimidazolesuccinocarboxamide synthase | 480 | 6.85E-67 | 79.55 | F:ATP binding; F:phosphoribosylaminoimidazolesuccinocarboxamide synthase activity; P:'de novo' IMP biosynthetic process |
| Efet.01.57826.g489.t1 | Translation initiation factor eIF-2B subunit | 960 | 1.55E-62 | 50.9 | F:translation initiation factor activity; F:nucleotidyltransferase activity; P:biosynthetic process; P:translational initiation |
| Efet.01.578374.g634.t1 | hypothetical protein B5766_02130 | 357 | 3.91E-66 | 71.5 |  |
| Efet.01.578409.g635.t1 | doublecortin domain-containing 2-like | 273 | 3.44E-24 | 61.25 | P:regulation of cellular process |
| Efet.01.578459.g637.t1 | excinuclease ABC subunit | 510 | 1.73E-105 | 87.1 | F:ATPase activity; F:DNA binding; F:ATP binding; P:nucleic acid phosphodiester bond hydrolysis; F:zinc ion binding; F:excinuclease ABC activity; C:excinuclease repair complex; C:cytoplasm; P:nucleotide-excision repair; P:SOS response |
| Efet.01.578471.g638.t1 | tyramine receptor 1-like | 1338 | 1.63E-68 | 53.85 | F:G-protein coupled receptor activity; P:G-protein coupled receptor signaling pathway; C:membrane |
| Efet.01.578494.g639.t1 | potassium voltage-gated channel Shaw-like isoform X1 | 528 | 1.18E-72 | 83.05 | F:voltage-gated potassium channel activity; P:potassium ion transmembrane transport; C:voltage-gated potassium channel complex; P:regulation of ion transmembrane transport; P:protein homooligomerization |
| Efet.01.57853.g491.t1 | nicastrin-like isoform X2 | 684 | 2.74E-57 | 61.9 | C:membrane; C:integral component of membrane; P:protein processing |
| Efet.01.578540.g640.t1 | multidrug ABC transporter ATP-binding | 927 | 0 | 78.85 | F:ATPase activity; F:ATP binding |
| Efet.01.57859.g492.t1 | peroxisome proliferator-activated receptor gamma-like isoform X6 | 399 | 6.60E-31 | 68.2 | C:nucleus; F:transcription factor activity, sequence-specific DNA binding; F:zinc ion binding; P:transcription, DNA-templated; F:steroid hormone receptor activity; P:regulation of transcription, DNA-templated; F:sequence-specific DNA binding; P:steroid hormone mediated signaling pathway |
| Efet.01.578612.g642.t1 | ABC transporter | 1032 | 0 | 79 | F:ATPase activity; F:ATP binding |
| Efet.01.578612.g643.t1 | 50S ribosomal L3 N(5)-glutamine methyltransferase | 921 | 0 | 87.4 | F:nucleic acid binding; F:protein methyltransferase activity; C:ribosome; P:protein methylation; P:DNA methylation on adenine; F:site-specific DNA-methyltransferase (adenine-specific) activity |
| Efet.01.578612.g644.t1 | succinyl-diaminopimelate desuccinylase | 1083 | 0 | 89.35 | F:zinc ion binding; P:lysine biosynthetic process via diaminopimelate; F:succinyl-diaminopimelate desuccinylase activity; F:cobalt ion binding; P:diaminopimelate biosynthetic process |
| Efet.01.578612.g645.t1 | type IV pili twitching motility | 1167 | 0 | 91.3 | F:ATP binding; P:transport |
| Efet.01.578612.g646.t1 | 2,3,4,5-tetrahydropyridine-2,6-dicarboxylate N-succinyltransferase | 399 | 4.07E-49 | 98.7 | C:cytoplasm; P:lysine biosynthetic process via diaminopimelate; F:2,3,4,5-tetrahydropyridine-2,6-dicarboxylate N-succinyltransferase activity; P:diaminopimelate biosynthetic process |
| Efet.01.578612.g647.t1 | succinyldiaminopimelate transaminase | 1230 | 0 | 87.35 | F:pyridoxal phosphate binding; P:lysine biosynthetic process via diaminopimelate; F:succinyldiaminopimelate transaminase activity; F:N2-acetyl-L-ornithine:2-oxoglutarate 5-aminotransferase activity |
| Efet.01.578625.g648.t1 | High-affnity carbon uptake Hat | 336 | 2.00E-74 | 57.95 | C:membrane; C:integral component of membrane |
| Efet.01.578651.g649.t1 | four-carbon acid sugar kinase family | 1272 | 0 | 89.75 |  |
| Efet.01.57887.g493.t1 | cadherin-like and PC-esterase domain-containing 1 isoform X1 | 435 | 2.93E-35 | 64.2 |  |
| Efet.01.578902.g652.t1 | beta-1,3-galactosyl-O-glycosyl-glyco beta-1,6-N-acetylglucosaminyltransferase-like | 528 | 1.83E-85 | 74.75 | F:acetylglucosaminyltransferase activity; C:membrane |
| Efet.01.579120.g655.t1 | heat shock 70 kDa | 453 | 3.34E-42 | 67 |  |
| Efet.01.579135.g656.t1 | neuronal acetylcholine receptor subunit alpha-7-like | 321 | 1.06E-40 | 75.95 | C:postsynaptic membrane; P:cation transmembrane transport; C:integral component of membrane; C:cell junction; F:extracellular ligand-gated ion channel activity |
| Efet.01.579185.g657.t1 | XK-related 4-like | 747 | 2.68E-38 | 60.05 | C:membrane |
| Efet.01.579347.g660.t1 | protocadherin gamma-B5-like | 267 | 2.05E-14 | 64.9 | C:membrane |
| Efet.01.579357.g661.t1 | deleted in autism 1 homolog | 1389 | 2.44E-62 | 50.35 | C:extracellular space; P:regulation of phosphatidylinositol 3-kinase signaling; C:membrane; C:integral component of membrane; P:cardiac muscle cell proliferation; F:molecular_function; C:COPI vesicle coat |
| Efet.01.57948.g495.t1 | hypothetical protein CAPTEDRAFT_226348 | 300 | 3.27E-15 | 86 |  |
| Efet.01.579669.g663.t1 | low-density lipo receptor-related 2-like | 783 | 4.20E-70 | 67.1 | F:calcium ion binding; C:membrane; C:integral component of membrane |
| Efet.01.579669.g664.t1 | low-density lipo receptor-related 2-like | 438 | 4.34E-45 | 76.1 | C:membrane; F:transferase activity |
| Efet.01.57969.g496.t1 | voltage-dependent calcium channel gamma-5 subunit-like | 621 | 5.40E-22 | 59.56 | P:calcium ion transport; C:membrane; C:integral component of membrane |
| Efet.01.579690.g666.t1 | Heparan sulfate glucosamine 3-O-sulfotransferase 2 | 453 | 8.73E-24 | 83.45 | C:heterotrimeric G-protein complex; P:G-protein coupled receptor signaling pathway; C:integral component of membrane; F:sulfotransferase activity; F:signal transducer activity |
| Efet.01.57980.g497.t1 | glutamate synthase [NADPH] isoform X1 | 453 | 5.25E-12 | 44.25 | P:glutamate biosynthetic process; F:oxidoreductase activity, acting on the CH-NH2 group of donors; F:catalytic activity; P:nitrogen compound metabolic process; F:oxidoreductase activity; P:metabolic process; P:oxidation-reduction process; F:glutamate synthase activity |
| Efet.01.57989.g498.t1 | kinesin light chain 2 isoform X2 | 897 | 7.24E-57 | 62.85 |  |
| Efet.01.57997.g499.t1 | CLIP-associating 2-like isoform X3 | 318 | 4.87E-23 | 76.9 | F:kinetochore binding; C:kinetochore microtubule; F:microtubule plus-end binding; C:cytoplasmic microtubule |
| Efet.01.579992.g671.t1 | ATP-dependent Clp protease ATP-binding subunit | 660 | 2.20E-108 | 94.25 | F:ATP binding; P:proteolysis; F:peptidase activity |
| Efet.01.580112.g674.t1 | CAP-Gly domain-containing linker 1 isoform X12 | 2406 | 4.27E-21 | 58.95 |  |
| Efet.01.580136.g676.t1 | family nutrient uptake outer membrane | 498 | 4.24E-50 | 62.9 |  |
| Efet.01.580212.g678.t1 | glycosyl transferase | 783 | 3.61E-33 | 65.75 | F:transferase activity |
| Efet.01.580237.g679.t1 | pyruvate:ferredoxin (flavodoxin) oxidoreductase | 3948 | 0 | 68.45 | F:thiamine pyrophosphate binding; F:FMN binding; F:iron ion binding; F:oxidoreductase activity, acting on the aldehyde or oxo group of donors; C:mitochondrion; P:cellular respiration; P:electron transport chain; P:pyruvate metabolic process |
| Efet.01.580335.g682.t1 | sodium glucose cotransporter 4 | 531 | 7.43E-14 | 53.05 | C:integral component of plasma membrane; C:extracellular exosome; P:transmembrane transport; C:membrane; C:integral component of membrane; F:symporter activity; P:transport; F:transporter activity |
| Efet.01.580463.g683.t1 | dihydroxyacetone kinase | 1443 | 0 | 60.3 | P:glycerol metabolic process; F:glycerone kinase activity; P:phosphorylation; F:transferase activity; F:kinase activity |
| Efet.01.580499.g685.t1 | cell division | 669 | 7.48E-127 | 82.5 | F:GTP binding; P:FtsZ-dependent cytokinesis; P:protein polymerization; C:cell division site; C:cytoplasm; F:GTPase activity |
| Efet.01.580499.g686.t1 | cell division | 765 | 1.38E-137 | 83.8 | P:regulation of cell shape; P:FtsZ-dependent cytokinesis; C:cell division site; C:cytoplasmic side of plasma membrane |
| Efet.01.58055.g502.t1 | solute carrier family 13 member 2-like | 417 | 5.69E-10 | 80.6 | P:sodium ion transport; P:transmembrane transport; C:integral component of membrane; F:transporter activity |
| Efet.01.580583.g687.t1 | LPS export ABC transporter ATP-binding | 606 | 4.79E-111 | 87.85 | F:ATPase activity; F:ATP binding; P:transmembrane transport; C:ATP-binding cassette (ABC) transporter complex |
| Efet.01.580633.g689.t1 | S-adenosyl-l-methionine hydroxide adenosyltransferase | 309 | 4.04E-17 | 55.35 | F:transferase activity |
| Efet.01.580633.g690.t1 | aspartate aminotransferase family | 1011 | 0 | 72.35 | F:transferase activity |
| Efet.01.580648.g691.t1 | ribosomal L15 | 327 | 6.30E-60 | 84.95 | C:nucleus; C:extracellular exosome; F:structural constituent of ribosome; P:cytoplasmic translation; C:membrane; C:cytosolic large ribosomal subunit; F:RNA binding |
| Efet.01.58075.g503.t1 | forkhead box P1 isoform X3 | 255 | 9.41E-34 | 86.7 | C:nucleus; F:transcription factor activity, sequence-specific DNA binding; P:transcription, DNA-templated; P:regulation of transcription, DNA-templated; F:sequence-specific DNA binding |
| Efet.01.580788.g692.t1 | eIF-2-alpha kinase GCN2 isoform X1 | 636 | 1.60E-53 | 68.75 | F:nucleotide binding; P:cellular response to stress; F:protein kinase activity; P:regulation of translational initiation; P:protein phosphorylation |
| Efet.01.580827.g693.t1 | labial homeodomain 3 | 627 | 1.58E-10 | 91 | C:nucleus; P:regulation of transcription, DNA-templated; F:sequence-specific DNA binding |
| Efet.01.58092.g504.t1 | target of rapamycin complex subunit lst8-like | 357 | 6.90E-36 | 75.9 |  |
| Efet.01.580939.g697.t1 | dihydrolipoyl dehydrogenase | 870 | 4.78E-157 | 86.9 | C:cell; F:flavin adenine dinucleotide binding; P:glycolytic process; P:cell redox homeostasis; P:oxidation-reduction process; F:dihydrolipoyl dehydrogenase activity |
| Efet.01.580959.g698.t1 | cilia- and flagella-associated 44-like isoform X2 | 453 | 1.38E-45 | 76.55 |  |
| Efet.01.580979.g699.t1 | WD repeat-containing 86-like isoform X2 | 945 | 2.23E-103 | 58.95 |  |
| Efet.01.581030.g700.t1 | microtubule-actin cross-linking factor isoforms 1 2 3 5-like | 318 | 5.36E-17 | 83.6 | P:cell cycle arrest; C:cytoskeleton; F:calcium ion binding |
| Efet.01.581047.g703.t1 | CBS domain-containing | 699 | 6.93E-44 | 56.25 |  |
| Efet.01.581087.g704.t1 | MFS transporter | 429 | 1.37E-90 | 84.35 | P:transmembrane transport; C:integral component of membrane; C:plasma membrane |
| Efet.01.581185.g705.t1 | helix-loop-helix 1 | 732 | 5.45E-18 | 85.55 | C:nucleus; F:RNA polymerase II regulatory region sequence-specific DNA binding; P:transcription from RNA polymerase II promoter; F:transcriptional activator activity, RNA polymerase II transcription regulatory region sequence-specific binding; F:protein dimerization activity; P:positive regulation of transcription from RNA polymerase II promoter |
| Efet.01.581218.g707.t1 | prolyl 4-hydroxylase subunit alpha-1-like | 354 | 2.98E-29 | 70.6 | F:procollagen-proline 4-dioxygenase activity; F:L-ascorbic acid binding; F:oxidoreductase activity, acting on single donors with incorporation of molecular oxygen, incorporation of two atoms of oxygen; F:iron ion binding; C:integral component of membrane; C:endoplasmic reticulum; P:peptidyl-proline hydroxylation; P:oxidation-reduction process |
| Efet.01.581255.g708.t1 | hypothetical protein HELRODRAFT_190695 | 1239 | 7.84E-46 | 58 |  |
| Efet.01.581396.g709.t1 | phosphoribosylglycinamide formyltransferase | 582 | 5.07E-124 | 69.25 | P:biosynthetic process; F:transferase activity |
| Efet.01.581396.g710.t1 | hypothetical protein CRN43_03330 | 582 | 1.11E-141 | 61.6 |  |
| Efet.01.5814.g456.t1 | INO80 complex subunit B | 240 | 7.76E-07 | 86.5 |  |
| Efet.01.581400.g711.t1 | high affinity cAMP-specific and IBMX-insensitive 3 ,5 -cyclic phosphodiesterase 8A isoform X2 | 675 | 5.22E-92 | 68.9 | F:metal ion binding; P:signal transduction; F:3',5'-cyclic-nucleotide phosphodiesterase activity |
| Efet.01.581436.g713.t1 | substance-P receptor-like | 438 | 4.94E-42 | 65.4 | F:G-protein coupled receptor activity; P:G-protein coupled receptor signaling pathway; C:membrane |
| Efet.01.581510.g715.t1 | cAMP phosphodiesterase | 810 | 1.74E-109 | 70.1 | F:nucleotide binding; F:metal ion binding; F:3',5'-cyclic-AMP phosphodiesterase activity |
| Efet.01.581510.g716.t1 | DUF2721 domain-containing | 387 | 1.50E-70 | 83.1 | C:integral component of membrane |
| Efet.01.581510.g717.t1 | phosphoserine phosphatase | 795 | 0 | 92.35 | F:phosphoserine phosphatase activity; P:L-serine biosynthetic process; P:dephosphorylation |
| Efet.01.581510.g718.t1 | amidohydrolase | 1215 | 0 | 94.35 | F:hippurate hydrolase activity; P:metabolic process |
| Efet.01.581510.g719.t1 | ABC transporter permease | 1152 | 0 | 90.45 |  |
| Efet.01.581510.g720.t1 | molybdopterin oxidoreductase | 2067 | 0 | 89.6 | F:oxidoreductase activity; F:electron carrier activity; F:molybdenum ion binding; P:oxidation-reduction process; F:iron-sulfur cluster binding |
| Efet.01.581551.g721.t1 | glutamate receptor kainate 2-like | 360 | 6.59E-11 | 82.6 | C:postsynaptic membrane; P:ion transmembrane transport; C:integral component of membrane; C:cell junction; P:ionotropic glutamate receptor signaling pathway; F:ionotropic glutamate receptor activity; F:extracellular-glutamate-gated ion channel activity |
| Efet.01.581588.g723.t1 | phosphoenolpyruvate carboxylase | 1278 | 0 | 89.45 | F:magnesium ion binding; P:oxaloacetate metabolic process; P:tricarboxylic acid cycle; P:carbon fixation; F:phosphoenolpyruvate carboxylase activity |
| Efet.01.581598.g724.t1 | neuroligin- X-linked-like isoform X1 | 840 | 4.33E-83 | 58.35 | C:membrane; C:integral component of membrane |
| Efet.01.581638.g725.t1 | tripartite motif-containing 3-like | 867 | 6.64E-09 | 52.55 | F:zinc ion binding; F:metal ion binding; C:intracellular |
| Efet.01.581651.g726.t1 | tRNA (N6-threonylcarbamoyladenosine(37)-N6)-methyltransferase | 243 | 9.17E-11 | 77.7 | F:methyltransferase activity; P:methylation |
| Efet.01.581651.g727.t1 | DMSO reductase | 1056 | 2.09E-143 | 85.5 | C:integral component of membrane; P:anaerobic electron transport chain |
| Efet.01.581684.g728.t1 | ABC transporter ATP-binding | 2346 | 0 | 81.75 | F:ATPase activity; F:ATP binding |
| Efet.01.581723.g729.t1 | hybrid sensor histidine kinase response regulator | 597 | 5.33E-80 | 67.85 | F:organic cyclic compound binding; F:heterocyclic compound binding; P:signal transduction; F:transferase activity, transferring phosphorus-containing groups; P:cellular metabolic process |
| Efet.01.581723.g730.t1 | DNA-binding response regulator | 852 | 0 | 73.15 | F:DNA binding; P:regulation of cellular process; P:cellular process |
| Efet.01.58175.g506.t1 | hypothetical protein HELRODRAFT_167348 | 651 | 2.20E-31 | 48.13 | P:L-arabinose metabolic process; F:calcium ion binding; C:membrane; C:integral component of membrane; F:scavenger receptor activity; F:alpha-L-arabinofuranosidase activity; P:cell adhesion; P:homophilic cell adhesion via plasma membrane adhesion molecules; P:receptor-mediated endocytosis; C:plasma membrane |
| Efet.01.581759.g731.t1 | SAM-dependent methyltransferase | 978 | 0 | 89.35 |  |
| Efet.01.581759.g732.t1 | short chain dehydrogenase | 648 | 4.14E-125 | 87.65 |  |
| Efet.01.581774.g733.t1 | alpha beta hydrolase | 1182 | 0 | 79.1 | F:hydrolase activity |
| Efet.01.581774.g734.t1 | 3-isopropylmalate dehydratase large subunit | 2409 | 0 | 75.35 | F:metal ion binding; P:leucine biosynthetic process; F:4 iron, 4 sulfur cluster binding; F:3-isopropylmalate dehydratase activity |
| Efet.01.581774.g735.t1 | 2-dehydropantoate 2-reductase | 891 | 0 | 80.9 | P:pantothenate biosynthetic process; F:NADP binding; C:cytoplasm; P:oxidation-reduction process; F:2-dehydropantoate 2-reductase activity |
| Efet.01.581774.g736.t1 | 3-isopropylmalate dehydratase small subunit | 603 | 5.82E-144 | 77.55 | P:leucine biosynthetic process; C:3-isopropylmalate dehydratase complex; F:3-isopropylmalate dehydratase activity |
| Efet.01.581794.g737.t1 | hypothetical protein CAPTEDRAFT_172041 | 324 | 1.53E-08 | 68 | C:basement membrane; P:system development; P:basement membrane assembly |
| Efet.01.581844.g740.t1 | hypothetical protein CAPTEDRAFT_215030 | 1026 | 9.62E-23 | 50 | C:membrane; C:integral component of membrane |
| Efet.01.581851.g741.t1 | acid-sensing ion channel 1-like | 360 | 3.17E-14 | 56.6 | P:ion transport |
| Efet.01.581892.g742.t1 | hypothetical protein CRN43_14530 | 366 | 3.62E-55 | 76.05 |  |
| Efet.01.581933.g743.t1 | zinc finger Noc | 1806 | 5.20E-21 | 56.3 | F:nucleic acid binding; F:metal ion binding |
| Efet.01.582019.g745.t1 | allatostatin-A receptor-like | 1251 | 4.10E-62 | 55.8 | P:signal transduction; F:signal transducer activity |
| Efet.01.582094.g747.t1 | Serine threonine- phosphatase 6 regulatory ankyrin repeat subunit | 597 | 5.03E-87 | 86.1 |  |
| Efet.01.58211.g510.t1 | Homeobox Nkx- | 717 | 4.70E-16 | 52.2 | P:central nervous system development; P:regulation of cell differentiation; P:cell fate commitment; P:regulation of gene expression; P:generation of neurons; F:binding; P:endocrine pancreas development; P:positive regulation of biological process |
| Efet.01.58214.g511.t1 | sodium channel type 4 subunit alpha B-like isoform X2 | 861 | 1.16E-99 | 77.45 | P:regulation of postsynaptic membrane potential; F:calcium ion binding; P:regulation of ion transmembrane transport; C:voltage-gated sodium channel complex; P:sodium ion transmembrane transport; F:voltage-gated sodium channel activity |
| Efet.01.582142.g749.t1 | POU class transcription factor 2 | 582 | 3.76E-75 | 88.95 | P:retinal ganglion cell axon guidance; P:inner ear development; P:retina development in camera-type eye; C:nuclear speck; P:spermatogenesis; F:RNA polymerase II core promoter proximal region sequence-specific DNA binding; P:regulation of signal transduction by p53 class mediator; P:transcription from RNA polymerase II promoter; P:positive regulation of cell differentiation; F:chromatin binding; P:positive regulation of transcription from RNA polymerase II promoter; P:negative regulation of transcription from RNA polymerase II promoter; P:sensory perception of sound; F:transcriptional repressor activity, RNA polymerase II core promoter proximal region sequence-specific binding; P:MAPK cascade; F:transcriptional activator activity, RNA polymerase II core promoter proximal region sequence-specific binding; P:axon extension; P:intracellular estrogen receptor signaling pathway; P:neuromuscular process controlling balance |
| Efet.01.582173.g750.t1 | ecdysone-induced isoform B- | 654 | 1.11E-08 | 75 |  |
| Efet.01.582174.g751.t1 | cyclic nucleotide-gated cation channel alpha-3-like | 738 | 6.35E-94 | 83.5 | P:potassium ion transmembrane transport; F:protein C-terminus binding; P:signal transduction; C:transmembrane transporter complex; F:cGMP-dependent protein kinase activity; P:response to cAMP; F:intracellular cAMP activated cation channel activity; C:perikaryon; P:protein phosphorylation; P:visual perception; F:intracellular cGMP activated cation channel activity; C:photoreceptor outer segment membrane; C:integral component of plasma membrane; C:dendrite; F:voltage-gated potassium channel activity; P:regulation of membrane potential; C:cytoplasm; P:response to corticosteroid; P:response to magnesium ion; F:cGMP binding |
| Efet.01.582239.g753.t1 | septum formation inhibitor Maf | 579 | 5.93E-99 | 81 | C:cytoplasm |
| Efet.01.582375.g755.t1 | mechanosensitive ion channel | 609 | 1.61E-99 | 76.3 | C:membrane |
| Efet.01.582383.g756.t1 | hypothetical protein HELRODRAFT_159474 | 468 | 2.00E-38 | 64 | F:translation elongation factor activity; P:translational elongation; C:intracellular |
| Efet.01.582387.g757.t1 | hypothetical protein | 261 | 1.96E-19 | 60.15 |  |
| Efet.01.582422.g760.t1 | forkhead box J3 isoform X1 | 447 | 3.16E-14 | 55.7 | F:DNA binding; C:nucleus; F:transcription factor activity, sequence-specific DNA binding; P:transcription, DNA-templated; P:regulation of transcription, DNA-templated; F:sequence-specific DNA binding; F:RNA polymerase II transcription factor activity, sequence-specific DNA binding |
| Efet.01.582589.g765.t1 | carbohydrate ABC transporter permease | 828 | 8.07E-165 | 67.4 | C:membrane |
| Efet.01.582665.g767.t1 | glutamyl-tRNA reductase | 300 | 1.45E-61 | 65.5 | P:tetrapyrrole biosynthetic process |
| Efet.01.582696.g768.t1 | RNA polymerase II-associated 3 | 462 | 1.17E-25 | 68.1 |  |
| Efet.01.582699.g769.t1 | EF-hand domain-containing family member C2-like | 1029 | 3.04E-167 | 77.7 | F:calcium ion binding |
| Efet.01.5827.g458.t1 | galactoside 2-alpha-L-fucosyltransferase 2-like | 1014 | 1.59E-101 | 57.2 | F:transferase activity |
| Efet.01.582753.g770.t1 | excinuclease ABC subunit B | 1152 | 0 | 72.2 | F:nucleotide binding; P:DNA repair; F:nuclease activity |
| Efet.01.582998.g773.t1 | hypothetical protein CAPTEDRAFT_146855 | 249 | 6.20E-07 | 70 | F:lyase activity; P:primary metabolic process; P:single-organism process; P:organic substance metabolic process; P:phosphate-containing compound metabolic process |
| Efet.01.58303.g513.t1 | neurexin-1a-like isoform X1 | 477 | 7.34E-44 | 68 | C:membrane part |
| Efet.01.583254.g776.t1 | isoform C | 441 | 2.70E-25 | 55.17 | C:membrane; C:integral component of membrane |
| Efet.01.5833.g459.t1 | von Willebrand factor A domain-containing 2-like | 951 | 4.24E-60 | 57.5 | F:chitin binding; P:chitin metabolic process; C:extracellular region |
| Efet.01.583339.g777.t1 | exosome component 10 | 459 | 1.08E-08 | 78.75 | P:nucleic acid phosphodiester bond hydrolysis; C:extracellular region; C:nucleolus; C:nuclear lamina; P:mRNA polyadenylation; F:nucleotide binding; F:nucleic acid binding; C:nuclear exosome (RNase complex); F:3'-5' exonuclease activity; P:regulation of gene expression; P:defense response to virus; P:nuclear RNA surveillance; F:carboxylic ester hydrolase activity |
| Efet.01.583457.g780.t1 | polybromo-1-like isoform X17 | 324 | 1.36E-10 | 61.25 | F:zinc ion binding; F:chromatin binding; C:intracellular |
| Efet.01.58354.g514.t1 | cytochrome P450 2U1-like | 783 | 7.12E-21 | 46.4 | F:iron ion binding; F:metal ion binding; F:oxidoreductase activity, acting on paired donors, with incorporation or reduction of molecular oxygen; F:oxidoreductase activity; F:steroid hydroxylase activity; C:membrane; C:integral component of membrane; P:oxidation-reduction process; F:monooxygenase activity; F:heme binding |
| Efet.01.583543.g782.t1 | carboxypeptidase B-like | 345 | 3.96E-06 | 51 | F:zinc ion binding; F:carboxypeptidase activity; F:metallocarboxypeptidase activity; P:proteolysis |
| Efet.01.583549.g783.t1 | prolactin-releasing peptide receptor-like | 408 | 3.77E-56 | 79.5 | P:neuropeptide signaling pathway; C:integral component of membrane; F:neuropeptide Y receptor activity |
| Efet.01.58361.g515.t1 | hypothetical protein HELRODRAFT_188711 | 3099 | 4.72E-25 | 48.5 |  |
| Efet.01.583614.g784.t1 | 2-oxoglutarate dehydrogenase E1 component | 1158 | 0 | 89 | F:thiamine pyrophosphate binding; F:oxoglutarate dehydrogenase (succinyl-transferring) activity; P:tricarboxylic acid cycle |
| Efet.01.583627.g785.t1 | aspartate carbamoyltransferase catalytic subunit | 261 | 3.14E-51 | 96.75 | F:aspartate carbamoyltransferase activity; P:'de novo' pyrimidine nucleobase biosynthetic process; P:cellular amino acid metabolic process; F:amino acid binding; P:'de novo' UMP biosynthetic process |
| Efet.01.583642.g787.t1 | Retrovirus-related Pol poly from transposon | 822 | 3.26E-47 | 60.2 | F:nucleic acid binding |
| Efet.01.583658.g788.t1 | transmembrane 132C-like | 855 | 1.46E-48 | 45 | C:membrane; C:integral component of membrane |
| Efet.01.583669.g789.t1 | phospholipase | 576 | 7.32E-134 | 64.3 | F:hydrolase activity |
| Efet.01.583689.g790.t1 | cyclin-dependent kinase inhibitor 1B | 351 | 1.88E-08 | 65.3 | P:positive regulation of cellular process; P:negative regulation of cell proliferation; P:negative regulation of macromolecule metabolic process; P:regulation of cell death; P:cell cycle process; P:negative regulation of cell cycle; P:regulation of cellular protein metabolic process; P:negative regulation of kinase activity; C:intracellular part; P:cellular response to chemical stimulus |
| Efet.01.58382.g516.t1 | BMA-SLO- isoform e | 210 | 1.79E-10 | 76.95 | F:calcium-activated potassium channel activity; P:potassium ion transport; P:transmembrane transport; C:integral component of membrane |
| Efet.01.583822.g792.t1 | RHS repeat-associated core domain-containing | 780 | 2.17E-30 | 46.17 | P:self proteolysis |
| Efet.01.583853.g793.t1 | rho GTPase-activating 24 isoform X1 | 1026 | 8.30E-55 | 63.15 | C:focal adhesion; P:activation of GTPase activity; F:GTPase activator activity; P:negative regulation of Rac protein signal transduction; P:wound healing, spreading of epidermal cells; P:negative regulation of ruffle assembly |
| Efet.01.583872.g794.t1 | 30S ribosomal S2 | 492 | 3.62E-81 | 80.05 | F:structural constituent of ribosome; C:small ribosomal subunit; P:translation |
| Efet.01.58397.g517.t1 | V-type proton ATPase catalytic subunit A | 282 | 2.18E-46 | 87.4 | F:ATP binding; P:ATP hydrolysis coupled proton transport; P:ATP metabolic process; F:proton-transporting ATPase activity, rotational mechanism; C:proton-transporting V-type ATPase, V1 domain |
| Efet.01.58408.g519.t1 | bone morphogenetic 10 | 387 | 3.16E-32 | 85.5 | F:growth factor activity; C:integral component of membrane; C:extracellular region; P:artery development; P:growth |
| Efet.01.584181.g796.t1 | sodium channel | 384 | 2.21E-46 | 80 | P:regulation of postsynaptic membrane potential; P:mRNA metabolic process; F:calcium ion binding; P:regulation of ion transmembrane transport; C:voltage-gated sodium channel complex; F:methyltransferase activity; P:sodium ion transmembrane transport; F:voltage-gated sodium channel activity; P:methylation |
| Efet.01.584237.g797.t1 | ras family member 10B | 318 | 1.15E-32 | 66.6 | F:nucleotide binding; P:signal transduction |
| Efet.01.584281.g799.t1 | sigma-54-dependent Fis family transcriptional regulator | 468 | 3.51E-91 | 85.35 | F:DNA binding; F:ATP binding; P:transcription, DNA-templated; P:phosphorelay signal transduction system; P:regulation of transcription, DNA-templated; F:transcription factor binding; C:intracellular |
| Efet.01.584286.g800.t1 | excinuclease ABC subunit | 222 | 1.13E-22 | 90.35 | F:DNA binding; F:ATP binding; P:nucleic acid phosphodiester bond hydrolysis; F:excinuclease ABC activity; C:cytoplasm; P:nucleotide-excision repair; F:helicase activity; P:SOS response |
| Efet.01.584287.g801.t1 | FAD-binding oxidoreductase | 327 | 3.85E-60 | 82.55 | F:oxidoreductase activity, acting on CH-OH group of donors; F:flavin adenine dinucleotide binding; P:oxidation-reduction process |
| Efet.01.584413.g804.t1 | cyclin-dependent kinase-like 2 isoform X2 | 321 | 5.48E-11 | 74.3 | F:nucleotide binding; P:phosphorylation; F:protein serine/threonine kinase activity |
| Efet.01.584473.g805.t1 | piggyBac transposable element-derived 4-like | 354 | 2.51E-11 | 65.41 | C:membrane |
| Efet.01.5845.g460.t1 | DNA (cytosine-5)-methyltransferase 1 | 822 | 2.96E-155 | 87.9 | F:zinc ion binding; P:C-5 methylation of cytosine; F:chromatin binding; P:lens development in camera-type eye; C:nucleus; F:DNA binding; F:DNA (cytosine-5-)-methyltransferase activity; P:regulation of DNA methylation; P:digestive tract development; P:retina layer formation; P:exocrine pancreas development; P:intrahepatic bile duct development; P:histone H3-K9 methylation; P:cartilage development |
| Efet.01.5845.g461.t1 | DNA (cytosine-5)-methyltransferase 1-like | 1740 | 9.24E-161 | 65.3 | F:binding; F:transferase activity |
| Efet.01.5845.g462.t1 | DNA (cytosine-5)-methyltransferase 1-like | 1059 | 5.14E-98 | 72.5 | C:nucleus; F:DNA binding; F:DNA (cytosine-5-)-methyltransferase activity; F:zinc ion binding; P:C-5 methylation of cytosine; F:chromatin binding |
| Efet.01.584515.g806.t1 | peptide deformylase | 273 | 1.10E-31 | 91.95 | F:iron ion binding; F:peptide deformylase activity; P:translation |
| Efet.01.584598.g808.t1 | arginyl-tRNA synthetase | 360 | 5.44E-16 | 67.4 | F:nucleotide binding; P:tRNA aminoacylation for protein translation; F:aminoacyl-tRNA ligase activity; C:cytoplasm |
| Efet.01.584650.g810.t1 | cysteine--tRNA cytoplasmic isoform X2 | 261 | 1.41E-37 | 90.75 | F:ATP binding; P:lipid transport; F:cysteine-tRNA ligase activity; P:cysteinyl-tRNA aminoacylation |
| Efet.01.584674.g811.t1 | GTP-binding GEM-like | 324 | 9.56E-07 | 57.6 | F:GTP binding; F:nucleotide binding; P:small GTPase mediated signal transduction; P:signal transduction; C:membrane; C:intracellular |
| Efet.01.5847.g463.t1 | PREDICTED: uncharacterized protein LOC106961309 isoform X4 | 645 | 1.83E-11 | 42.56 |  |
| Efet.01.584704.g813.t1 | ACP S-malonyltransferase | 459 | 8.14E-73 | 76.4 | F:catalytic activity |
| Efet.01.58473.g521.t1 | gonadotropin-releasing hormone II receptor-like | 861 | 5.74E-30 | 52.65 | F:G-protein coupled receptor activity; P:neuropeptide signaling pathway; P:G-protein coupled receptor signaling pathway; P:signal transduction; C:membrane; C:integral component of membrane; F:signal transducer activity; F:neuropeptide Y receptor activity |
| Efet.01.58477.g522.t1 | leucine-rich repeat-containing 24-like | 2661 | 1.24E-51 | 49.05 | C:membrane; C:integral component of membrane |
| Efet.01.5848.g464.t1 | filamin-C-like isoform X1 | 489 | 1.14E-51 | 85.6 | F:actin filament binding |
| Efet.01.58480.g523.t1 | USP6 N-terminal | 270 | 2.08E-25 | 77 | P:regulation of vesicle fusion; P:activation of GTPase activity; F:GTPase activator activity; C:integral component of membrane; C:endomembrane system; P:intracellular protein transport; F:Rab GTPase binding; C:intracellular |
| Efet.01.584882.g815.t1 | multidrug resistance-associated 1-like | 279 | 2.01E-30 | 81.3 | F:ATP binding; F:ATPase activity, coupled to transmembrane movement of substances; P:transmembrane transport; C:integral component of membrane |
| Efet.01.584901.g816.t1 | LMBR1 domain-containing 2-B-like | 261 | 1.04E-06 | 57.08 | C:membrane; C:integral component of membrane |
| Efet.01.585016.g820.t1 | RHS repeat-associated core domain-containing | 1668 | 0 | 64.3 | P:self proteolysis |
| Efet.01.585033.g821.t1 | 4-carboxymuconolactone decarboxylase | 378 | 5.99E-88 | 88.55 | F:4-carboxymuconolactone decarboxylase activity; P:cellular oxidant detoxification; P:oxidation-reduction process; F:peroxiredoxin activity |
| Efet.01.585033.g822.t1 | beta-D-hydroxybutyrate dehydrogenase | 357 | 3.63E-75 | 50.5 | F:oxidoreductase activity; F:3-hydroxybutyrate dehydrogenase activity; P:oxidation-reduction process |
| Efet.01.585033.g823.t1 | tripartite tricarboxylate transporter substrate binding | 969 | 0 | 78 | C:outer membrane-bounded periplasmic space |
| Efet.01.585033.g824.t1 | NAD(P)-dependent oxidoreductase | 906 | 0 | 79.85 | F:phosphogluconate dehydrogenase (decarboxylating) activity; F:NAD binding; P:oxidation-reduction process; F:3-hydroxyisobutyrate dehydrogenase activity |
| Efet.01.585033.g825.t1 | NIPSNAP family | 327 | 5.39E-75 | 89.35 |  |
| Efet.01.585033.g826.t1 | cupin domain-containing | 984 | 2.06E-92 | 80.75 | P:regulation of transcription, DNA-templated; F:isomerase activity |
| Efet.01.585033.g827.t1 | family transcriptional regulator | 879 | 1.18E-125 | 89.15 | F:DNA binding; F:transcription factor activity, sequence-specific DNA binding; P:transcription, DNA-templated; P:regulation of transcription, DNA-templated |
| Efet.01.585033.g828.t1 | phosphonate ABC permease | 801 | 2.49E-150 | 87.55 | C:integral component of plasma membrane; P:organic phosphonate transport; F:organic phosphonate transmembrane transporter activity |
| Efet.01.585033.g829.t1 | phosphonate ABC transporter substrate-binding | 903 | 0 | 91.25 | P:transmembrane transport; P:organic phosphonate transport; F:organic phosphonate transmembrane transporter activity; C:ATP-binding cassette (ABC) transporter complex |
| Efet.01.585033.g830.t1 | phosphonate ABC transporter ATP-binding | 891 | 1.33E-176 | 90.85 | F:ATP binding; P:organic phosphonate transport; P:organophosphate ester transport; F:ATPase-coupled organic phosphonate transmembrane transporter activity; C:ATP-binding cassette (ABC) transporter complex |
| Efet.01.585143.g834.t1 | dexamethasone-induced Ras-related 1 isoform X2 | 429 | 8.11E-43 | 88.5 | F:GTP binding; P:small GTPase mediated signal transduction; C:membrane; C:intracellular |
| Efet.01.585200.g835.t1 | bestrophin-4 | 288 | 4.51E-27 | 71.15 | C:integral component of membrane |
| Efet.01.58522.g524.t1 | probable rRNA-processing EBP2 | 396 | 2.06E-11 | 75.05 | F:protein binding; P:ribosomal large subunit biogenesis; C:nuclear periphery; P:rRNA processing; C:nucleolus; C:preribosome, large subunit precursor; F:RNA binding |
| Efet.01.585438.g838.t1 | baculoviral IAP repeat-containing 6-like isoform X2 | 756 | 6.85E-46 | 71.1 | P:cellular process |
| Efet.01.585463.g839.t1 | family transcriptional regulator | 921 | 0 | 79.2 | F:DNA binding; F:transcription factor activity, sequence-specific DNA binding; P:transcription, DNA-templated; P:regulation of transcription, DNA-templated |
| Efet.01.585463.g840.t1 | ABC transporter ATP-binding | 303 | 7.01E-62 | 95.05 | F:ATPase activity; F:ATP binding; P:branched-chain amino acid transport; F:branched-chain amino acid transmembrane transporter activity |
| Efet.01.585463.g841.t1 | ABC transporter ATP-binding | 633 | 1.50E-143 | 78.65 | F:ATPase activity; F:ATP binding |
| Efet.01.585463.g842.t1 | high-affinity branched-chain amino acid ABC transporter permease | 1071 | 0 | 94.85 | F:ATP binding; C:integral component of membrane; P:transport; C:plasma membrane; F:transporter activity |
| Efet.01.585463.g843.t1 | high-affinity branched-chain amino acid ABC transporter permease | 927 | 0 | 92 | C:integral component of membrane; P:transport; C:plasma membrane; F:transporter activity |
| Efet.01.585463.g844.t1 | branched-chain amino acid ABC transporter substrate-binding | 1119 | 0 | 93.15 | P:amino acid transport |
| Efet.01.585463.g845.t1 | hypothetical protein | 390 | 2.56E-26 | 93 |  |
| Efet.01.585463.g846.t1 | microcystin LR degradation | 1476 | 0 | 80.9 | F:metal ion binding; P:proteolysis; F:metallopeptidase activity |
| Efet.01.585463.g847.t1 | peptide ABC transporter permease | 807 | 1.08E-166 | 83.85 | P:transmembrane transport; C:ATP-binding cassette (ABC) transporter complex |
| Efet.01.585542.g851.t1 | Phosphatidylinositide phosphatase SAC2 | 267 | 6.02E-18 | 68.2 | F:phosphoric ester hydrolase activity |
| Efet.01.5856.g465.t1 | Hau-cytoplasmic intermediate filament | 315 | 1.62E-29 | 80.8 | F:structural molecule activity; C:intermediate filament |
| Efet.01.585656.g852.t1 | DNA-protecting | 1335 | 0 | 73.1 | P:cellular process |
| Efet.01.58573.g525.t1 | sphingosine kinase 2-like | 294 | 5.07E-22 | 67.85 | F:transferase activity |
| Efet.01.585749.g854.t1 | turtle isoform X1 | 279 | 2.27E-32 | 68.35 | C:membrane |
| Efet.01.58581.g526.t1 | homeobox CHOX-CAD-like isoform X2 | 426 | 3.47E-10 | 83.15 | C:nucleus; P:anterior/posterior axis specification; F:transcription factor activity, sequence-specific DNA binding; F:RNA polymerase II regulatory region sequence-specific DNA binding; P:cell differentiation; P:regulation of transcription from RNA polymerase II promoter; P:animal organ morphogenesis |
| Efet.01.58582.g527.t1 | PREDICTED: uncharacterized protein KIAA0556 | 492 | 1.51E-19 | 57.95 |  |
| Efet.01.585823.g855.t1 | hypothetical protein HELRODRAFT_162325 | 393 | 1.37E-26 | 66 | F:calcium-activated potassium channel activity; P:potassium ion transport; P:potassium ion transmembrane transport; C:membrane; C:integral component of membrane |
| Efet.01.58585.g528.t1 | galanin receptor type 2-like | 912 | 1.22E-08 | 53 | C:gap junction; F:G-protein coupled receptor activity; P:G-protein coupled receptor signaling pathway; P:signal transduction; C:membrane; C:integral component of membrane; F:signal transducer activity |
| Efet.01.585884.g856.t1 | hypothetical protein HELRODRAFT_161680 | 375 | 2.46E-12 | 52 | F:calcium ion binding |
| Efet.01.58590.g529.t1 | octopamine receptor 2-like | 1710 | 3.86E-84 | 66.35 | F:G-protein coupled receptor activity; P:G-protein coupled receptor signaling pathway; C:integral component of membrane |
| Efet.01.585951.g858.t1 | T family of potassium channels 18 | 531 | 2.32E-25 | 81.25 | P:potassium ion transmembrane transport; C:integral component of membrane; F:potassium channel activity |
| Efet.01.585955.g860.t1 | fez family zinc finger 2-like | 2319 | 1.03E-106 | 87.7 | F:nucleic acid binding; F:metal ion binding |
| Efet.01.58600.g531.t1 | hypothetical protein CAPTEDRAFT_226348 | 291 | 1.50E-29 | 64.88 |  |
| Efet.01.586035.g862.t1 | unconventional myosin-XVIIIa-like [Orbicella faveolata] | 360 | 1.78E-08 | 75.6 |  |
| Efet.01.586079.g863.t1 | rab GTPase-activating 1-like isoform X1 | 534 | 4.28E-38 | 71.3 | F:GTPase activator activity; P:cell cycle; P:positive regulation of GTPase activity |
| Efet.01.586087.g864.t1 | phospholipid-transporting ATPase ID-like | 231 | 2.53E-31 | 87.3 | F:magnesium ion binding; F:ATP binding; P:phospholipid translocation; P:Golgi organization; C:integral component of membrane; C:Golgi apparatus; F:phospholipid-translocating ATPase activity; C:plasma membrane |
| Efet.01.586089.g865.t1 | Anopheles gambiae PEST AGAP012902- | 513 | 8.17E-24 | 59.2 | C:membrane; C:integral component of membrane |
| Efet.01.586124.g866.t1 | dioxygenase | 441 | 3.02E-34 | 62.2 | F:dioxygenase activity; P:cellular aromatic compound metabolic process; F:oxidoreductase activity, acting on single donors with incorporation of molecular oxygen; F:zinc ion binding; F:oxidoreductase activity; F:metal ion binding; F:ferrous iron binding; P:oxidation-reduction process |
| Efet.01.58621.g535.t1 | Fanconi anemia core complex-associated 100 | 2106 | 2.80E-12 | 39.55 | P:DNA repair; C:Fanconi anaemia nuclear complex |
| Efet.01.586210.g868.t1 | SCBP2-like protein, partial | 348 | 1.20E-19 | 53.8 | F:calcium ion binding |
| Efet.01.586228.g869.t1 | metalloprotease | 1815 | 0 | 88.85 | P:proteolysis; F:metallopeptidase activity |
| Efet.01.586228.g870.t1 | 3-deoxy-7-phosphoheptulonate synthase | 1242 | 0 | 95.5 | P:aromatic amino acid family biosynthetic process; F:3-deoxy-7-phosphoheptulonate synthase activity; P:chorismate biosynthetic process |
| Efet.01.586228.g871.t1 | family transcriptional regulator | 675 | 2.48E-145 | 84.2 | F:DNA binding; F:transcription factor activity, sequence-specific DNA binding; P:transcription, DNA-templated; P:regulation of transcription, DNA-templated |
| Efet.01.586228.g872.t1 | short-chain dehydrogenase | 774 | 3.52E-133 | 79.05 | F:oxidoreductase activity |
| Efet.01.586228.g873.t1 | TRAP transporter large permease | 399 | 3.95E-34 | 78.5 | C:integral component of membrane |
| Efet.01.586228.g874.t1 | TRAP transporter large permease | 213 | 3.71E-15 | 93.4 | C:integral component of membrane |
| Efet.01.586232.g875.t1 | AAA family ATPase | 573 | 9.22E-60 | 78.95 |  |
| Efet.01.586233.g876.t1 | SIS domain-containing | 507 | 2.19E-102 | 86.6 | P:carbohydrate metabolic process; F:carbohydrate binding; F:isomerase activity |
| Efet.01.586304.g878.t1 | carboxypeptidase regulatory-like domain-containing | 429 | 3.92E-71 | 74.9 |  |
| Efet.01.586314.g879.t1 | FAA hydrolase family | 864 | 9.48E-20 | 94 | P:metabolic process; F:lyase activity |
| Efet.01.586323.g880.t1 | phospholipid-transporting ATPase ID-like isoform X2 | 267 | 3.43E-10 | 59.67 | F:binding |
| Efet.01.586480.g883.t1 | neuropeptide SIFamide receptor-like | 603 | 8.30E-49 | 59.2 | F:G-protein coupled receptor activity; P:G-protein coupled receptor signaling pathway; C:membrane |
| Efet.01.586576.g885.t1 | isoform A | 909 | 4.90E-70 | 50.65 | P:regulation of cellular process; P:cellular component organization; P:generation of neurons; P:response to stimulus |
| Efet.01.586718.g886.t1 | ankyrin repeat and kinase domain-containing 1 | 1077 | 1.12E-10 | 45 | F:ATP binding; F:protein kinase activity; P:protein phosphorylation |
| Efet.01.586731.g887.t1 | Wnt- | 231 | 9.12E-14 | 62.55 | P:regulation of cellular process; C:extracellular region; P:single-organism process |
| Efet.01.58675.g539.t1 | toll-like receptor 13 | 612 | 7.60E-24 | 50.85 | P:signal transduction; C:membrane; C:integral component of membrane |
| Efet.01.58685.g540.t1 | neuronal cell adhesion molecule isoform X22 | 1479 | 3.02E-67 | 46.7 | C:membrane; C:integral component of membrane |
| Efet.01.586884.g890.t1 | AChain Crystal Structure Of The Usp7 hausp Catalytic Domain | 771 | 1.06E-106 | 72.55 | P:regulation of telomere capping; C:nuclear body; F:protein homodimerization activity; P:monoubiquitinated protein deubiquitination; F:protein C-terminus binding; P:negative regulation of NF-kappaB transcription factor activity; P:transcription-coupled nucleotide-excision repair; F:cysteine-type endopeptidase activity; F:transcription factor binding; F:thiol-dependent ubiquitin-specific protease activity; C:XY body; F:p53 binding; F:ubiquitin protein ligase binding; P:positive regulation of apoptotic process; C:cytosol; P:maintenance of DNA methylation; P:protein stabilization |
| Efet.01.5869.g466.t1 | cytosolic beta-glucosidase | 219 | 1.39E-18 | 71.2 | P:carbohydrate metabolic process; C:integral component of membrane; F:hydrolase activity, hydrolyzing O-glycosyl compounds |
| Efet.01.586990.g891.t1 | ubiquitin domain-containing | 303 | 4.68E-14 | 71.95 | F:RNA binding |
| Efet.01.587005.g892.t1 | cell division | 381 | 1.82E-52 | 73.75 | P:cell division; C:cytoplasm; P:cell cycle |
| Efet.01.587022.g893.t1 | AT-rich interactive domain-containing 2 | 282 | 9.68E-21 | 66.25 | F:DNA binding; C:membrane; C:integral component of membrane |
| Efet.01.587031.g894.t1 | ABC transporter ATP-binding | 1497 | 1.80E-90 | 62.35 | F:nucleotide binding; F:ATPase activity; F:ATP binding; F:lyase activity; F:hydrolase activity |
| Efet.01.587031.g895.t1 | bifunctional UDP-N-acetylglucosamine diphosphorylase glucosamine-1-phosphate N-acetyltransferase | 570 | 6.69E-100 | 81.75 | F:magnesium ion binding; P:regulation of cell shape; F:UDP-N-acetylglucosamine diphosphorylase activity; C:cytoplasm; P:cell morphogenesis; P:peptidoglycan biosynthetic process; F:glucosamine-1-phosphate N-acetyltransferase activity; P:lipopolysaccharide biosynthetic process; P:lipid A biosynthetic process; P:cell wall organization; P:UDP-N-acetylglucosamine biosynthetic process |
| Efet.01.587031.g896.t1 | ribose-phosphate pyrophosphokinase | 918 | 3.42E-179 | 94.85 | F:magnesium ion binding; F:ATP binding; P:ribonucleoside monophosphate biosynthetic process; C:cytoplasm; P:nucleotide biosynthetic process; F:ribose phosphate diphosphokinase activity; P:phosphorylation; F:kinase activity; P:5-phosphoribose 1-diphosphate biosynthetic process |
| Efet.01.587058.g898.t1 | C2 domain-containing 5-like isoform X1 | 333 | 1.85E-09 | 57.5 |  |
| Efet.01.587100.g901.t1 | rho GTPase-activating 20 isoform X2 | 1050 | 3.38E-06 | 55 |  |
| Efet.01.5872.g467.t1 | titin-like isoform X10 | 459 | 1.26E-52 | 85.8 |  |
| Efet.01.587295.g903.t1 | disheveled-associated activator of morphogenesis 1 isoform X2 | 303 | 4.15E-22 | 66.9 | P:positive regulation of sarcomere organization; C:sarcoplasm; P:mushroom body development; P:regulation of tube architecture, open tracheal system; P:positive regulation of filopodium assembly; P:axon guidance; C:striated muscle thin filament; C:Z disc; F:actin binding; C:axonal growth cone; P:axon extension; P:regulation of axonogenesis; P:positive regulation of actin filament polymerization; P:muscle thin filament assembly; P:actin filament bundle assembly |
| Efet.01.587298.g904.t1 | hypothetical protein CAPTEDRAFT_227412 | 315 | 7.94E-30 | 66.44 |  |
| Efet.01.587351.g905.t1 | hypothetical protein HELRODRAFT_192560 | 246 | 2.34E-09 | 69 | F:actin filament binding |
| Efet.01.587612.g907.t1 | DNA polymerase delta subunit 2 | 1182 | 2.00E-146 | 71.95 | P:DNA metabolic process; P:cellular macromolecule biosynthetic process |
| Efet.01.58768.g545.t1 | IS110 family transposase | 627 | 3.23E-137 | 87.85 | F:DNA binding; F:transposase activity; P:transposition, DNA-mediated |
| Efet.01.587803.g914.t1 | zinc finger 708-like | 336 | 3.60E-25 | 54.6 | F:nucleic acid binding; F:transcription factor activity, sequence-specific DNA binding; F:metal ion binding; P:regulation of transcription, DNA-templated; C:intracellular |
| Efet.01.587840.g915.t1 | coiled-coil domain-containing 42 homolog | 201 | 1.22E-12 | 80.85 |  |
| Efet.01.587858.g916.t1 | ribosome biogenesis GTPase Der | 1278 | 0 | 94.95 | F:GTP binding; P:ribosome biogenesis |
| Efet.01.587920.g919.t1 | sarcosine oxidase subunit alpha family | 1833 | 0 | 73.55 | F:sarcosine oxidase activity; P:tetrahydrofolate metabolic process; P:oxidation-reduction process; F:aminomethyltransferase activity; P:methylation |
| Efet.01.587924.g920.t1 | TRAP transporter large permease | 414 | 7.63E-74 | 79.45 | C:integral component of membrane |
| Efet.01.587924.g921.t1 | C4-dicarboxylate ABC transporter substrate-binding | 903 | 0 | 75.55 | C:outer membrane-bounded periplasmic space; P:transport |
| Efet.01.587924.g922.t1 | short-chain dehydrogenase | 687 | 1.40E-157 | 85.2 | F:diacetyl reductase ((S)-acetoin forming) activity; F:3-oxoacyl-[acyl-carrier-protein] reductase (NADPH) activity; P:oxidation-reduction process |
| Efet.01.587924.g923.t1 | fumarate reductase | 1524 | 0 | 75.05 | F:oxidoreductase activity; P:oxidation-reduction process |
| Efet.01.587924.g924.t1 | DNA-binding response regulator | 918 | 1.36E-140 | 94.05 | F:DNA binding; P:transcription, DNA-templated; P:phosphorelay signal transduction system; P:regulation of transcription, DNA-templated; C:intracellular |
| Efet.01.587924.g925.t1 | response regulator receiver sensor signal transduction histidine kinase | 1053 | 0 | 87.75 | F:phosphorelay sensor kinase activity; P:phosphorelay signal transduction system; C:integral component of membrane; F:protein dimerization activity; P:signal transduction by protein phosphorylation; C:intracellular |
| Efet.01.587924.g926.t1 | hypothetical protein | 447 | 6.12E-103 | 90.8 |  |
| Efet.01.587924.g927.t1 | Fis family transcriptional regulator | 363 | 5.58E-82 | 95.9 | F:DNA binding; P:phosphorelay signal transduction system; C:intracellular |
| Efet.01.58798.g547.t1 | SWI SNF-related | 519 | 1.85E-14 | 68.45 |  |
| Efet.01.588154.g932.t1 | ABC transporter ATP-binding | 699 | 4.50E-152 | 89.45 | F:ATPase activity; F:ATP binding; P:transmembrane transport; C:ATP-binding cassette (ABC) transporter complex; F:hydrolase activity, acting on acid anhydrides, catalyzing transmembrane movement of substances |
| Efet.01.588154.g933.t1 | ABC transporter permease | 618 | 2.52E-138 | 82.95 | C:integral component of membrane; P:transport; C:plasma membrane |
| Efet.01.588154.g934.t1 | ABC transporter permease | 777 | 1.02E-169 | 81.6 | C:integral component of membrane; P:transport; C:plasma membrane |
| Efet.01.588154.g935.t1 | ABC transporter substrate-binding | 981 | 0 | 79.6 |  |
| Efet.01.588154.g936.t1 | 4-hydroxy-tetrahydrodipicolinate synthase | 897 | 0 | 74.6 | C:cytoplasm; P:lysine biosynthetic process via diaminopimelate; F:4-hydroxy-tetrahydrodipicolinate synthase; P:diaminopimelate biosynthetic process |
| Efet.01.588154.g937.t1 | 1-pyrroline-5-carboxylate dehydrogenase | 1317 | 0 | 82.05 | F:1-pyrroline-5-carboxylate dehydrogenase activity; P:glutamate biosynthetic process; P:proline biosynthetic process; P:oxidation-reduction process; F:oxidoreductase activity, acting on the aldehyde or oxo group of donors, NAD or NADP as acceptor |
| Efet.01.58817.g548.t1 | myotubularin-related 6-like | 321 | 5.97E-40 | 77.1 | F:protein tyrosine phosphatase activity; F:metal ion binding; P:peptidyl-tyrosine dephosphorylation |
| Efet.01.588245.g940.t1 | sugar ABC transporter permease | 327 | 2.70E-57 | 68.25 | C:integral component of membrane; P:transport; C:plasma membrane |
| Efet.01.588274.g941.t1 | piezo-type mechanosensitive ion channel component 2-like | 486 | 3.68E-40 | 64 | C:membrane |
| Efet.01.588296.g942.t1 | hypothetical protein HELRODRAFT_174549 | 243 | 7.89E-06 | 52 |  |
| Efet.01.58830.g549.t1 | 60S acidic ribosomal P2 | 237 | 1.29E-17 | 85.8 | F:structural constituent of ribosome; P:cytoplasmic translation; C:cytosolic large ribosomal subunit; F:large ribosomal subunit rRNA binding; P:translational elongation; C:preribosome, large subunit precursor |
| Efet.01.58830.g550.t1 | peptide-methionine (S)-S-oxide reductase | 489 | 9.41E-71 | 79 | P:protein repair; P:response to oxidative stress; P:cellular protein modification process; F:peptide-methionine (S)-S-oxide reductase activity; P:oxidation-reduction process |
| Efet.01.588350.g944.t1 | zinc finger 598 | 651 | 4.87E-59 | 67.45 | F:zinc ion binding |
| Efet.01.588418.g945.t1 | DNA-directed RNA polymerase subunit beta | 1053 | 0 | 88.7 | F:DNA binding; F:DNA-directed 5'-3' RNA polymerase activity; F:ribonucleoside binding; P:transcription, DNA-templated |
| Efet.01.588431.g946.t1 | collagen alpha-1(XII) chain-like | 510 | 6.19E-25 | 57.5 | F:calcium ion binding |
| Efet.01.588479.g947.t1 | FAM135A-like isoform X7 | 897 | 6.28E-06 | 59 |  |
| Efet.01.58850.g552.t1 | calumenin isoform X1 | 657 | 1.18E-56 | 70.65 | F:oxidoreductase activity; F:2-alkenal reductase [NAD(P)] activity; F:calcium ion binding; P:oxidation-reduction process |
| Efet.01.588565.g948.t1 | acetylcholine receptor subunit alpha-type acr-16-like isoform X4 | 351 | 1.46E-42 | 76.15 | C:postsynaptic membrane; P:cation transmembrane transport; C:integral component of membrane; C:cell junction; F:extracellular ligand-gated ion channel activity |
| Efet.01.588642.g949.t1 | progesterone-induced-blocking factor 1 | 360 | 2.24E-18 | 67.2 | C:centrosome; F:protein binding; P:cellular localization; P:organelle assembly; P:regulation of tyrosine phosphorylation of STAT protein; P:positive regulation of protein phosphorylation; P:single-organism organelle organization; P:mitotic nuclear division; P:regulation of cytokine production; P:negative regulation of cellular metabolic process; P:positive regulation of JAK-STAT cascade |
| Efet.01.588686.g950.t1 | FMRFamide receptor-like | 987 | 4.81E-57 | 51.8 | F:signal transducer activity |
| Efet.01.58869.g555.t1 | kinesin KIF14 isoform X1 | 771 | 5.65E-112 | 79 | F:ATP binding; F:microtubule motor activity; P:microtubule-based movement; F:microtubule binding |
| Efet.01.588731.g951.t1 | toxin-antitoxin toxin component | 303 | 6.69E-55 | 87.25 | P:quorum sensing; P:negative regulation of translation; F:hydrolase activity |
| Efet.01.588731.g952.t1 | antitoxin | 351 | 6.67E-71 | 91.2 | F:sequence-specific DNA binding |
| Efet.01.588731.g953.t1 | family addiction module antitoxin | 261 | 1.43E-47 | 90.65 | F:toxic substance binding; F:DNA-directed 5'-3' RNA polymerase activity; P:single-species biofilm formation; P:RNA biosynthetic process; F:bacterial-type RNA polymerase core promoter proximal region sequence-specific DNA binding |
| Efet.01.588782.g954.t1 | SIS domain-containing | 552 | 1.57E-126 | 71.6 | P:carbohydrate metabolic process; F:carbohydrate binding; F:isomerase activity |
| Efet.01.588783.g955.t1 | beta-1,3-galactosyltransferase 1-like | 984 | 1.17E-86 | 56.25 | F:transferase activity, transferring glycosyl groups; C:membrane; C:integral component of membrane; P:protein glycosylation; F:galactosyltransferase activity; C:Golgi membrane; C:Golgi apparatus; F:transferase activity |
| Efet.01.588929.g956.t1 | hypothetical protein | 234 | 1.42E-12 | 57.67 |  |
| Efet.01.588946.g958.t1 | sodium calcium exchanger 1- | 393 | 1.56E-43 | 69.15 | P:transmembrane transport; C:membrane; P:metal ion transport |
| Efet.01.588961.g959.t1 | pleiotropic regulator 1-like | 243 | 4.10E-28 | 90 | C:catalytic step 2 spliceosome; C:precatalytic spliceosome; C:DNA replication factor A complex; C:Prp19 complex; C:Cul4-RING E3 ubiquitin ligase complex; P:mRNA splicing, via spliceosome |
| Efet.01.58900.g557.t1 | IGHMBP2 family helicase | 339 | 1.05E-22 | 76.1 | F:organic cyclic compound binding; F:heterocyclic compound binding; F:hydrolase activity |
| Efet.01.58901.g558.t1 | RIMS2_MOUSE ame: Full=Regulating synaptic membrane exocytosis 2 ame: Full=Rab-3-interacting molecule 2 Short=RIM 2 ame: Full=Rab-3-interacting 2 | 837 | 7.18E-06 | 60 | C:presynaptic membrane; P:insulin secretion; P:positive regulation of dendrite extension; C:synapse; P:positive regulation of inhibitory postsynaptic potential; P:intracellular protein transport; C:neuron projection; P:exocytosis; P:neurotransmitter transport; C:extracellular exosome; C:presynaptic active zone; F:protein heterodimerization activity; F:protein domain specific binding; P:spontaneous neurotransmitter secretion; C:protein complex; P:transport; F:ion channel binding; P:neurotransmitter secretion; C:intracellular; P:calcium ion-regulated exocytosis of neurotransmitter; P:regulation of membrane potential; F:protein binding; F:metal ion binding; P:positive regulation of gene expression; P:cell differentiation; P:regulation of synaptic vesicle exocytosis; C:cell junction; P:calcium ion regulated exocytosis; P:positive regulation of excitatory postsynaptic potential; P:regulation of exocytosis; F:Rab GTPase binding; P:cAMP-mediated signaling |
| Efet.01.589035.g962.t1 | FAD-binding oxidoreductase | 411 | 2.38E-47 | 72.2 | F:oxidoreductase activity, acting on CH-OH group of donors; F:flavin adenine dinucleotide binding; P:oxidation-reduction process; F:iron-sulfur cluster binding |
| Efet.01.589108.g963.t1 | Transposon Ty3-G Gag-Pol poly | 2877 | 0 | 59.25 | F:binding |
| Efet.01.589149.g964.t1 | Rab GDP dissociation inhibitor alpha | 666 | 8.54E-99 | 79.2 | F:Rab GDP-dissociation inhibitor activity; F:oxidoreductase activity; P:small GTPase mediated signal transduction; P:protein transport; P:regulation of catalytic activity; P:oxidation-reduction process; F:transferase activity; C:intracellular |
| Efet.01.589173.g965.t1 | ABC transporter ATP-binding | 744 | 1.53E-92 | 77.85 | F:ATPase activity; F:ATP binding |
| Efet.01.589191.g966.t1 | Leucine rich repeat | 1821 | 4.40E-56 | 53 |  |
| Efet.01.589229.g967.t1 | hypothetical protein HELRODRAFT_168308 | 567 | 1.17E-26 | 58 |  |
| Efet.01.58927.g559.t1 | glucoside xylosyltransferase 1-like isoform X2 | 477 | 7.89E-59 | 70.5 | F:transferase activity, transferring glycosyl groups |
| Efet.01.589358.g970.t1 | neutral cholesterol ester hydrolase 1-like | 477 | 3.48E-45 | 59.9 | P:metabolic process; C:membrane; C:integral component of membrane; F:carboxylic ester hydrolase activity; F:hydrolase activity |
| Efet.01.58937.g560.t1 | TBRG4-like isoform X2 | 1221 | 7.09E-37 | 51.44 | C:mitochondrion; P:cellular respiration; F:protein kinase activity; P:protein phosphorylation |
| Efet.01.589431.g971.t1 | solute carrier family 41 member 1 | 222 | 1.35E-07 | 78 | P:transmembrane transport; P:cation transport; C:membrane; P:cation transmembrane transport; C:integral component of membrane; F:cation transmembrane transporter activity |
| Efet.01.589440.g972.t1 | cell wall AWA1-like | 750 | 1.18E-27 | 46.15 |  |
| Efet.01.589441.g973.t1 | rho GTPase-activating 44- | 432 | 2.71E-08 | 70.63 | C:cytoplasm; P:signal transduction |
| Efet.01.589587.g976.t1 | Patatin-like phospholipase domain-containing 7 | 459 | 1.87E-71 | 71.25 | C:membrane; P:metabolic process |
| Efet.01.58960.g561.t1 | neuroblastoma-amplified sequence | 285 | 4.47E-15 | 65.8 |  |
| Efet.01.589615.g978.t1 | plexin-a4 isoform x3 | 972 | 2.10E-54 | 57.4 | F:semaphorin receptor activity; P:semaphorin-plexin signaling pathway |
| Efet.01.589679.g980.t1 | disintegrin and metallo ase domain-containing 10 isoform X1 | 351 | 5.31E-16 | 81.75 | F:RNA-directed DNA polymerase activity; F:zinc ion binding; P:integrin-mediated signaling pathway; F:RNA binding; P:Notch signaling pathway; P:membrane protein ectodomain proteolysis; C:integral component of membrane; P:RNA-dependent DNA biosynthetic process; F:metalloendopeptidase activity |
| Efet.01.589719.g981.t1 | family transcriptional regulator | 600 | 1.02E-93 | 52.2 | F:DNA binding; F:transcription factor activity, sequence-specific DNA binding; P:transcription, DNA-templated; P:regulation of transcription, DNA-templated |
| Efet.01.58974.g562.t1 | extracellular hemoglobin linker L4 subunit precursor | 273 | 1.10E-14 | 83 |  |
| Efet.01.589747.g982.t1 | hypothetical protein CRN43_20725, partial | 276 | 4.09E-58 | 68.33 |  |
| Efet.01.589748.g983.t1 | ribosome small subunit-dependent GTPase A | 1149 | 3.57E-100 | 79.95 | F:GTP binding; F:metal ion binding; F:GTPase activity |
| Efet.01.58977.g563.t1 | nuclear hormone receptor HR96-like isoform X2 | 1302 | 1.48E-121 | 61.55 | C:nucleus; F:transcription factor activity, sequence-specific DNA binding; F:zinc ion binding; P:transcription, DNA-templated; F:steroid hormone receptor activity; P:regulation of transcription, DNA-templated; F:sequence-specific DNA binding; P:steroid hormone mediated signaling pathway |
| Efet.01.589877.g987.t1 | kelch diablo | 1221 | 2.89E-76 | 55.75 |  |
| Efet.01.589904.g988.t1 | centrosomal of 19 kDa | 291 | 1.58E-13 | 80.15 | C:centriole; C:ciliary basal body; C:spindle pole |
| Efet.01.589955.g990.t1 | forkhead box N3-like isoform X1 | 642 | 1.15E-80 | 69.3 | F:transcription factor activity, sequence-specific DNA binding; P:regulation of transcription, DNA-templated; F:sequence-specific DNA binding |
| Efet.01.590049.g991.t1 | kinase C-binding NELL1-like isoform X1 | 690 | 8.18E-09 | 51.95 | F:calcium ion binding; C:membrane; C:integral component of membrane; F:extracellular matrix structural constituent; C:proteinaceous extracellular matrix |
| Efet.01.59006.g564.t1 | retinol dehydrogenase 7-like | 504 | 8.03E-27 | 69.15 | C:membrane; P:metabolic process; F:metallopeptidase activity |
| Efet.01.590083.g992.t1 | plectin-like isoform X4 | 783 | 6.78E-86 | 64.5 | C:cytoskeleton |
| Efet.01.5901.g471.t1 | PHD finger 3 | 732 | 9.56E-13 | 46.25 | F:zinc ion binding; P:transcription, DNA-templated; F:metal ion binding |
| Efet.01.590143.g993.t1 | DNA-binding response regulator | 567 | 8.18E-115 | 84.15 | F:DNA binding; P:transcription, DNA-templated; P:phosphorelay signal transduction system; P:regulation of transcription, DNA-templated; C:intracellular |
| Efet.01.590143.g994.t1 | sensor histidine kinase | 1095 | 0 | 72.7 | F:nucleotide binding; P:signal transduction; C:membrane; P:phosphorylation; F:signal transducer activity; F:kinase activity |
| Efet.01.590156.g995.t1 | phosphoribosylformylglycinamidine cyclo-ligase | 1032 | 0 | 83.35 | F:ligase activity |
| Efet.01.590200.g1000.t1 | PIN domain-containing | 354 | 2.09E-66 | 70.45 | P:nucleic acid phosphodiester bond hydrolysis; F:metal ion binding; F:nuclease activity |
| Efet.01.590200.g1001.t1 | family DNA-binding domain-containing | 261 | 1.13E-54 | 79.7 | F:DNA binding |
| Efet.01.590200.g1002.t1 | 1-acyl-sn-glycerol-3-phosphate acyltransferase | 759 | 4.66E-154 | 79.15 | F:transferase activity, transferring acyl groups; P:metabolic process |
| Efet.01.590200.g1003.t1 | dihydroorotase | 978 | 0 | 90 | F:metal ion binding; F:dihydroorotase activity; P:pyrimidine nucleotide biosynthetic process |
| Efet.01.590200.g996.t1 | two-component sensor histidine kinase | 447 | 1.10E-89 | 77.15 | F:phosphorelay sensor kinase activity; F:ATP binding; P:phosphorelay signal transduction system; P:signal transduction by protein phosphorylation; C:intracellular |
| Efet.01.590200.g997.t1 | family integral membrane | 807 | 9.58E-119 | 95.15 | C:integral component of membrane |
| Efet.01.590200.g998.t1 | iron ABC transporter permease | 1611 | 0 | 80.2 | C:integral component of membrane; P:transport; C:plasma membrane |
| Efet.01.590200.g999.t1 | ABC transporter ATP-binding | 1089 | 0 | 85.3 | F:ATPase activity; F:ATP binding; P:transmembrane transport; C:ATP-binding cassette (ABC) transporter complex; F:hydrolase activity, acting on acid anhydrides, catalyzing transmembrane movement of substances |
| Efet.01.590334.g1006.t1 | transcription-repair coupling factor | 411 | 7.52E-67 | 92.75 | F:ATP binding; C:cytoplasm; P:transcription-coupled nucleotide-excision repair, DNA damage recognition; P:regulation of transcription, DNA-templated; F:helicase activity; F:damaged DNA binding |
| Efet.01.590339.g1007.t1 | lactadherin-like isoform X5 | 564 | 7.20E-83 | 66.2 |  |
| Efet.01.59035.g566.t1 | probable ATP-dependent RNA helicase DDX4 | 339 | 1.66E-42 | 78 | F:nucleic acid binding; F:ATP binding; C:membrane; F:helicase activity |
| Efet.01.590436.g1009.t1 | vacuolar sorting-associated 13B | 1182 | 1.77E-20 | 51.35 |  |
| Efet.01.590479.g1013.t1 | methyltransferase | 276 | 1.19E-56 | 84.3 | F:O-methyltransferase activity; P:methylation |
| Efet.01.59060.g568.t1 | dual specificity tyrosine-phosphorylation-regulated kinase 1B | 378 | 9.17E-18 | 75.75 | F:nucleotide binding; P:anatomical structure development; F:protein kinase activity; P:cellular process; P:single-organism developmental process |
| Efet.01.59070.g569.t1 | IQ and ubiquitin-like domain-containing | 1110 | 3.28E-146 | 69.8 |  |
| Efet.01.590768.g1016.t1 | thrombospondin-type laminin G domain and EAR repeat-containing | 378 | 3.20E-15 | 54.8 |  |
| Efet.01.59077.g570.t1 | peroxisomal membrane 2-like | 342 | 1.86E-21 | 49.2 | C:membrane |
| Efet.01.590828.g1018.t1 | oxidoreductase | 825 | 2.25E-121 | 68.7 | F:binding |
| Efet.01.590974.g1022.t1 | SWI SNF-related matrix-associated actin-dependent regulator of chromatin subfamily B member 1 isoform X1 | 411 | 6.49E-71 | 88.45 | P:chromatin remodeling; C:nuclear chromosome |
| Efet.01.59101.g572.t1 | 5-hydroxytryptamine receptor 1A-like | 1134 | 3.25E-41 | 57.75 | F:G-protein coupled receptor activity; P:G-protein coupled receptor signaling pathway; C:membrane |
| Efet.01.591014.g1024.t1 | ROK family transcriptional regulator | 636 | 8.13E-118 | 67.5 |  |
| Efet.01.591056.g1025.t1 | potassium channel subfamily K member 6 | 249 | 7.32E-12 | 63.8 | P:potassium ion transport; C:integral component of membrane; F:potassium channel activity |
| Efet.01.591126.g1028.t1 | homeobox prospero isoform X1 | 213 | 1.10E-28 | 99.55 | P:axonogenesis involved in innervation; P:male courtship behavior; P:protein localization; P:axon guidance; P:regulation of retinal cone cell fate specification; P:compound eye cone cell fate commitment; C:microtubule associated complex; P:negative regulation of transcription from RNA polymerase II promoter; F:transcriptional repressor activity, RNA polymerase II core promoter proximal region sequence-specific binding; P:negative regulation of axonogenesis; P:negative regulation of neuroblast proliferation; P:dendrite guidance; P:cell fate specification; P:asymmetric neuroblast division resulting in ganglion mother cell formation; P:sensory perception of taste; P:transcription, DNA-templated; P:R7 cell fate commitment; P:regulation of R7 cell differentiation; P:neuroblast fate determination; P:peripheral nervous system development; C:apical cortex; P:brain development; C:plasma membrane; P:ganglion mother cell fate determination; C:nucleus; F:DNA binding; P:ventral cord development; P:synapse assembly; P:positive regulation of gene expression; C:integral component of membrane; P:phagocytosis; P:G1 to G0 transition; C:basal cortex; P:glial cell differentiation; P:regulation of protein localization to nucleus |
| Efet.01.591235.g1030.t1 | peptide ABC transporter ATP-binding | 957 | 1.08E-134 | 94.25 | F:ATPase activity; F:ATP binding; P:peptide transport |
| Efet.01.59126.g574.t1 | homeobox Hox-C6 isoform X2 | 645 | 2.29E-30 | 75.45 | C:nucleus; F:transcription factor activity, sequence-specific DNA binding; P:multicellular organism development; P:regulation of transcription, DNA-templated; F:sequence-specific DNA binding |
| Efet.01.591301.g1031.t1 | NUDIX hydrolase | 552 | 1.93E-133 | 93.3 | F:hydrolase activity |
| Efet.01.591301.g1032.t1 | 5-carboxymethyl-2-hydroxymuconate isomerase | 696 | 7.48E-160 | 88 | P:metabolic process; F:hydrolase activity; F:isomerase activity |
| Efet.01.591301.g1033.t1 | maleylacetoacetate isomerase | 765 | 4.06E-127 | 80.7 | P:aromatic amino acid family metabolic process; C:cytoplasm; F:maleylacetoacetate isomerase activity |
| Efet.01.591301.g1034.t1 | laccase | 633 | 8.24E-107 | 75.65 |  |
| Efet.01.591301.g1035.t1 | class I poly(R)-hydroxyalkanoic acid synthase | 1821 | 0 | 86.4 | F:transferase activity, transferring acyl groups; C:cytoplasm; P:poly-hydroxybutyrate biosynthetic process |
| Efet.01.591317.g1036.t1 | transcription factor Ovo-like 1 | 1281 | 1.17E-16 | 73.05 | C:nucleus; F:metal ion binding; P:regulation of transcription, DNA-templated; F:sequence-specific DNA binding |
| Efet.01.59142.g575.t1 | calmodulin | 312 | 3.04E-22 | 65.7 | F:calcium ion binding |
| Efet.01.59144.g576.t1 | UDP-glucose 6-dehydrogenase-like | 279 | 9.87E-34 | 76.6 | C:nucleus; P:carbohydrate metabolic process; F:NAD binding; P:UDP-glucuronate biosynthetic process; F:UDP-glucose 6-dehydrogenase activity; P:glycosaminoglycan biosynthetic process; C:cytosol; P:oxidation-reduction process |
| Efet.01.591473.g1037.t1 | zinc finger 347 isoform X2 | 801 | 4.42E-27 | 49.15 | F:nucleic acid binding; F:metal ion binding; P:regulation of transcription, DNA-templated; C:intracellular |
| Efet.01.591585.g1038.t1 | transmembrane 74B | 534 | 1.74E-13 | 58.4 | P:autophagy; C:membrane; C:integral component of membrane |
| Efet.01.591615.g1039.t1 | cAMP-dependent kinase type II regulatory subunit isoform X2 | 744 | 1.36E-106 | 72.7 | F:nucleotide binding; P:regulation of protein phosphorylation |
| Efet.01.591691.g1040.t1 | rho GTPase-activating 190 isoform X1 | 432 | 3.01E-63 | 83.55 | F:GTP binding; P:small GTPase mediated signal transduction; C:intracellular |
| Efet.01.591718.g1044.t1 | transketolase | 480 | 2.75E-77 | 87.2 | F:oxidoreductase activity, acting on the aldehyde or oxo group of donors, disulfide as acceptor; P:oxidation-reduction process |
| Efet.01.591789.g1045.t1 | monocarboxylate transporter 12-like | 882 | 3.32E-15 | 67.7 | C:membrane; P:transport |
| Efet.01.591817.g1046.t1 | type III restriction enzyme | 909 | 4.46E-174 | 69.65 | F:DNA binding; F:ATP binding; P:DNA metabolic process; F:Type III site-specific deoxyribonuclease activity; F:hydrolase activity |
| Efet.01.591854.g1047.t1 | signal peptidase I | 285 | 7.79E-30 | 87.9 | P:proteolysis; C:integral component of membrane; F:serine-type peptidase activity |
| Efet.01.591855.g1048.t1 | titin-like isoform X1 | 1245 | 4.24E-102 | 55.8 | F:nucleotide binding; F:ATP binding; F:protein kinase activity; P:phosphorylation; F:transferase activity; P:protein phosphorylation; F:kinase activity |
| Efet.01.591858.g1049.t1 | nicotinate-nucleotide diphosphorylase (carboxylating) | 810 | 2.74E-126 | 84.85 | F:nicotinate-nucleotide diphosphorylase (carboxylating) activity; P:NAD biosynthetic process |
| Efet.01.591858.g1050.t1 | 3-methyl-2-oxobutanoate hydroxymethyltransferase | 1572 | 7.75E-174 | 91.75 | P:pantothenate biosynthetic process; F:3-methyl-2-oxobutanoate hydroxymethyltransferase activity; C:cytoplasm; F:metal ion binding; F:methyltransferase activity; P:methylation |
| Efet.01.591877.g1051.t1 | hypothetical protein B5766_05145 | 231 | 7.12E-19 | 76.2 | C:membrane; C:integral component of membrane |
| Efet.01.5919.g472.t1 | 60 kDa neurofilament -like | 444 | 1.74E-12 | 93.4 | F:structural molecule activity; C:intermediate filament |
| Efet.01.591923.g1052.t1 | ABC transporter ATP-binding | 303 | 2.41E-47 | 92.95 | F:ATPase activity; F:ATP binding |
| Efet.01.591949.g1053.t1 | mitogen-activated kinase kinase kinase 10-like | 708 | 4.72E-99 | 68.2 | F:ATP binding; F:protein serine/threonine kinase activity; P:protein phosphorylation |
| Efet.01.592015.g1054.t1 | FERM domain-containing 8 isoform X1 | 324 | 9.69E-21 | 62.3 | F:nucleic acid binding; C:cytoskeleton |
| Efet.01.592054.g1056.t1 | NAD(P)-dependent alcohol dehydrogenase | 429 | 5.10E-94 | 76.65 | F:oxidoreductase activity; F:metal ion binding |
| Efet.01.592057.g1057.t1 | cell surface | 861 | 2.52E-143 | 59 |  |
| Efet.01.592057.g1058.t1 | cell surface | 819 | 0 | 71.8 |  |
| Efet.01.592074.g1059.t1 | ADP-ribosylglycohydrolase family | 1083 | 0 | 71.6 |  |
| Efet.01.592077.g1060.t1 | dihydrodipicolinate synthase family | 1104 | 4.18E-143 | 77.05 | F:catalytic activity |
| Efet.01.592090.g1061.t1 | formate dehydrogenase | 1092 | 0 | 88.75 | F:oxidoreductase activity; F:molybdenum ion binding; P:oxidation-reduction process; F:iron-sulfur cluster binding |
| Efet.01.592090.g1062.t1 | DNA-binding transcriptional family | 972 | 0 | 85.95 | F:DNA binding; F:transcription factor activity, sequence-specific DNA binding; P:transcription, DNA-templated; P:regulation of transcription, DNA-templated |
| Efet.01.592091.g1063.t1 | L-ribulose-5-phosphate 4-epimerase | 432 | 4.10E-76 | 65.25 |  |
| Efet.01.592151.g1064.t1 | ubiquitin carboxyl-terminal hydrolase 31 | 924 | 2.27E-68 | 76.6 | P:protein deubiquitination; F:thiol-dependent ubiquitinyl hydrolase activity; P:ubiquitin-dependent protein catabolic process |
| Efet.01.592185.g1065.t1 | Ubiquitin carboxyl-terminal hydrolase 40 | 282 | 4.10E-10 | 60.16 | P:protein deubiquitination; C:nucleus; C:cytoplasm; P:ubiquitin-dependent protein catabolic process; F:thiol-dependent ubiquitinyl hydrolase activity; F:hydrolase activity |
| Efet.01.592294.g1068.t1 | inositol monophosphatase | 921 | 1.49E-161 | 90.7 | F:inositol monophosphate 1-phosphatase activity; P:phosphatidylinositol phosphorylation; F:inositol monophosphate 3-phosphatase activity; P:inositol phosphate dephosphorylation; F:inositol monophosphate 4-phosphatase activity |
| Efet.01.592294.g1069.t1 | transcriptional regulator | 450 | 2.02E-102 | 89.3 | F:DNA binding; F:ATP binding; F:zinc ion binding; P:negative regulation of transcription, DNA-templated; P:transcription, DNA-templated |
| Efet.01.592294.g1070.t1 | serine hydroxymethyltransferase | 1287 | 0 | 90.55 | F:pyridoxal phosphate binding; C:cytoplasm; P:glycine biosynthetic process from serine; F:glycine hydroxymethyltransferase activity; P:tetrahydrofolate interconversion; F:methyltransferase activity; P:methylation |
| Efet.01.592294.g1071.t1 | methylcrotonoyl- carboxylase | 1608 | 0 | 95.1 | F:propionyl-CoA carboxylase activity |
| Efet.01.592296.g1072.t1 | calsyntenin-1 | 318 | 1.85E-21 | 71.25 | C:membrane |
| Efet.01.59243.g579.t1 | uncharacterized protein LOC110232929 | 366 | 3.19E-14 | 57.2 |  |
| Efet.01.592441.g1074.t1 | hypothetical protein CAPTEDRAFT_200392 | 612 | 6.28E-09 | 58.6 | C:membrane; C:integral component of membrane |
| Efet.01.592563.g1076.t1 | ADP-forming succinate-- ligase subunit beta | 2232 | 0 | 92.75 | F:magnesium ion binding; F:ATP binding; F:manganese ion binding; P:tricarboxylic acid cycle; F:succinate-CoA ligase (ADP-forming) activity |
| Efet.01.592582.g1077.t1 | DUF2783 domain-containing | 240 | 3.50E-18 | 91.8 |  |
| Efet.01.592582.g1078.t1 | hypothetical protein | 618 | 5.64E-135 | 74.3 |  |
| Efet.01.592582.g1079.t1 | peptide ABC transporter | 3213 | 0 | 81.8 | P:transmembrane transport; C:ATP-binding cassette (ABC) transporter complex |
| Efet.01.592582.g1080.t1 | ABC transporter permease | 804 | 5.54E-127 | 68.6 | C:membrane |
| Efet.01.592582.g1081.t1 | ABC transporter substrate-binding | 1194 | 0 | 71.55 | P:carbohydrate transport |
| Efet.01.592650.g1082.t1 | hypothetical protein HELRODRAFT_170685 | 390 | 3.83E-07 | 61 | F:metal ion binding; F:calcium ion binding |
| Efet.01.592653.g1083.t1 | ABC transporter ATP-binding | 1620 | 0 | 85.75 | F:ATPase activity; F:ATP binding |
| Efet.01.592653.g1084.t1 | xanthine dehydrogenase small subunit | 1110 | 0 | 85.1 | F:xanthine dehydrogenase activity; F:oxidoreductase activity, acting on CH-OH group of donors; F:xanthine oxidase activity; F:metal ion binding; F:flavin adenine dinucleotide binding; F:electron carrier activity; P:oxidation-reduction process; F:2 iron, 2 sulfur cluster binding |
| Efet.01.592685.g1085.t1 | FMRFamide receptor-like | 1185 | 1.30E-65 | 51.75 | F:G-protein coupled receptor activity; F:G-protein coupled peptide receptor activity; P:G-protein coupled receptor signaling pathway; P:signal transduction; C:membrane; C:integral component of membrane; F:signal transducer activity |
| Efet.01.592788.g1090.t1 | RRP5 homolog | 282 | 2.38E-22 | 63.75 | F:nucleic acid binding; C:nucleus; C:cytosol; P:RNA processing; F:transcription factor binding; P:mRNA processing; F:RNA binding |
| Efet.01.5928.g473.t1 | ragulator complex LAMTOR5 homolog | 297 | 1.33E-18 | 61.3 | C:cytoplasm; P:negative regulation of cysteine-type endopeptidase activity involved in apoptotic process; P:negative regulation of apoptotic process; P:viral genome replication; C:Ragulator complex |
| Efet.01.59281.g580.t1 | collagen alpha-1(XI) chain-like | 498 | 5.26E-14 | 51 | P:tissue homeostasis; C:collagen trimer; P:chondrocyte differentiation; F:extracellular matrix structural constituent; P:skeletal system morphogenesis; C:proteinaceous extracellular matrix |
| Efet.01.592891.g1093.t1 | haloacid dehalogenase subfamily variant 3 with third motif having DD or ED | 711 | 3.21E-71 | 74.85 | P:metabolic process; F:hydrolase activity |
| Efet.01.592962.g1094.t1 | EF-hand domain-containing 1-like | 270 | 2.33E-39 | 83.45 | F:calcium ion binding |
| Efet.01.59298.g583.t1 | hypothetical protein HELRODRAFT_81674 | 552 | 4.77E-45 | 62.15 |  |
| Efet.01.593134.g1096.t1 | short-chain dehydrogenase | 399 | 1.30E-73 | 80.7 |  |
| Efet.01.59329.g584.t1 | nipped-B isoform X1 | 1131 | 3.03E-105 | 76.85 | P:mitotic sister chromatid cohesion; P:regulation of gene expression; F:chromatin binding |
| Efet.01.5934.g474.t1 | agrin-like isoform X1 | 570 | 4.19E-37 | 66.1 | F:calcium ion binding |
| Efet.01.593431.g1098.t1 | mitochondrial ATP synthase subunit delta precursor | 342 | 5.49E-33 | 79.2 | C:mitochondrial proton-transporting ATP synthase complex, catalytic core F(1); F:proton-transporting ATPase activity, rotational mechanism; P:ATP synthesis coupled proton transport; F:proton-transporting ATP synthase activity, rotational mechanism |
| Efet.01.59347.g585.t1 | interleukin 17 | 354 | 4.18E-09 | 58 | P:inflammatory response; F:cytokine activity; C:extracellular region |
| Efet.01.593514.g1099.t1 | carbohydrate kinase | 489 | 2.36E-99 | 75.4 | P:metabolic process; F:transferase activity, transferring phosphorus-containing groups |
| Efet.01.593574.g1100.t1 | type I methionyl aminopeptidase | 951 | 1.28E-128 | 81.05 | P:protein initiator methionine removal; F:metal ion binding; P:proteolysis; F:metalloaminopeptidase activity |
| Efet.01.593574.g1101.t1 | 30S ribosomal S11 | 402 | 2.14E-68 | 92.9 | F:structural constituent of ribosome; C:ribosome; F:rRNA binding; P:translation |
| Efet.01.593574.g1102.t1 | 30S ribosomal S4 | 549 | 1.22E-130 | 86.05 | F:structural constituent of ribosome; C:small ribosomal subunit; F:rRNA binding; P:translation |
| Efet.01.593574.g1103.t1 | DNA-directed RNA polymerase subunit alpha | 348 | 4.66E-42 | 78.7 | F:DNA binding; F:DNA-directed 5'-3' RNA polymerase activity; P:transcription, DNA-templated; F:protein dimerization activity |
| Efet.01.59365.g586.t1 | tachykinin-like peptides receptor 99D | 522 | 1.14E-33 | 74.7 | C:integral component of membrane; P:tachykinin receptor signaling pathway; C:plasma membrane; F:tachykinin receptor activity |
| Efet.01.593811.g1105.t1 | orotate phosphoribosyltransferase | 585 | 7.90E-88 | 92.6 | F:magnesium ion binding; P:'de novo' UMP biosynthetic process; F:orotate phosphoribosyltransferase activity |
| Efet.01.59384.g587.t1 | DUF1446 domain-containing | 1356 | 0 | 87 |  |
| Efet.01.59384.g588.t1 | tripartite tricarboxylate transporter substrate binding | 741 | 1.56E-155 | 88.55 | C:outer membrane-bounded periplasmic space |
| Efet.01.59384.g589.t1 | family transcriptional regulator | 843 | 0 | 84.25 | F:DNA binding; F:transcription factor activity, sequence-specific DNA binding; P:transcription, DNA-templated; P:regulation of transcription, DNA-templated |
| Efet.01.59384.g590.t1 | bifunctional rhamnulose-1-phosphate aldolase short-chain dehydrogenase | 1995 | 0 | 81.6 |  |
| Efet.01.59384.g591.t1 | L-rhamnose catabolism isomerase | 1287 | 0 | 88.35 | F:isomerase activity |
| Efet.01.59384.g592.t1 | rhamnose ABC transporter substrate-binding | 666 | 6.80E-64 | 62.35 | P:rhamnose transport; C:ATP-binding cassette (ABC) transporter complex |
| Efet.01.59384.g593.t1 | rhamnose ABC transporter substrate-binding | 246 | 7.38E-50 | 88.35 | P:rhamnose transport; C:ATP-binding cassette (ABC) transporter complex |
| Efet.01.59384.g594.t1 | sugar ABC transporter ATP-binding | 1350 | 0 | 84.25 | F:ATP binding; P:monosaccharide transport; F:monosaccharide-transporting ATPase activity |
| Efet.01.59384.g595.t1 | ABC transporter permease | 489 | 1.28E-60 | 83.8 | C:integral component of membrane; P:monosaccharide transport; F:monosaccharide-transporting ATPase activity; C:plasma membrane |
| Efet.01.59385.g596.t1 | vacuolar sorting-associated 37B | 372 | 5.07E-09 | 54 |  |
| Efet.01.594044.g1108.t1 | FMRFamide receptor-like | 648 | 3.59E-30 | 55.45 | F:signal transducer activity |
| Efet.01.594083.g1109.t1 | SYS1 homolog | 273 | 1.16E-35 | 73.35 | C:integral component of membrane; C:Golgi membrane |
| Efet.01.594126.g1110.t1 | fumarylacetoacetate hydrolase | 615 | 7.05E-100 | 94.9 | P:metabolic process; F:hydrolase activity |
| Efet.01.594190.g1111.t1 | Calcineurin subunit B type | 273 | 4.14E-42 | 97.15 | F:calcium ion binding |
| Efet.01.594275.g1112.t1 | urocanate hydratase | 726 | 4.06E-156 | 95.1 | C:cytoplasm; F:urocanate hydratase activity; P:histidine catabolic process to glutamate and formate; P:histidine catabolic process to glutamate and formamide |
| Efet.01.594275.g1113.t1 | histidine ammonia-lyase | 1023 | 0 | 88 | C:cytoplasm; F:histidine ammonia-lyase activity; P:histidine catabolic process to glutamate and formate; P:histidine catabolic process to glutamate and formamide |
| Efet.01.594275.g1114.t1 | histidine utilization repressor | 717 | 1.64E-172 | 94.65 | F:DNA binding; F:transcription factor activity, sequence-specific DNA binding; P:histidine metabolic process; P:negative regulation of transcription, DNA-templated; P:transcription, DNA-templated |
| Efet.01.594275.g1115.t1 | sulfonate ABC transporter ATP-binding | 540 | 2.35E-127 | 94.4 | F:ATPase activity; F:ATP binding |
| Efet.01.594275.g1116.t1 | ABC transporter permease | 639 | 1.06E-107 | 89.1 | C:integral component of membrane; P:transport; C:plasma membrane |
| Efet.01.594275.g1117.t1 | ABC transporter permease | 423 | 7.03E-95 | 91.7 |  |
| Efet.01.594302.g1118.t1 | dynein beta ciliary-like | 489 | 1.25E-63 | 87.1 | F:ATPase activity; F:ATP binding; C:motile cilium; F:microtubule motor activity; C:cytoplasm; P:cell projection organization; C:dynein complex; P:microtubule-based movement; C:microtubule |
| Efet.01.594316.g1120.t1 | tRNA (adenosine(37)-N6)-threonylcarbamoyltransferase complex transferase subunit | 705 | 2.53E-120 | 81.75 | C:cytoplasm; F:iron ion binding; P:proteolysis; P:tRNA threonylcarbamoyladenosine modification; F:metalloendopeptidase activity; F:N(6)-L-threonylcarbamoyladenine synthase |
| Efet.01.594332.g1121.t1 | F-BAR domain only 2 isoform X3 | 471 | 1.79E-29 | 81.4 |  |
| Efet.01.594427.g1122.t1 | plectin-like isoform X4 | 903 | 1.26E-74 | 63.05 | C:cytoskeleton |
| Efet.01.594479.g1124.t1 | hypothetical protein HELRODRAFT_161529 | 300 | 3.94E-32 | 63.7 | C:membrane |
| Efet.01.594479.g1125.t1 | hypothetical protein CAPTEDRAFT_225510 | 417 | 3.02E-07 | 52 | C:membrane; C:integral component of membrane; F:carbohydrate binding |
| Efet.01.594585.g1126.t1 | 40S ribosomal S3a-like | 456 | 1.48E-73 | 89.4 | F:structural constituent of ribosome; C:cytosolic small ribosomal subunit; P:translation |
| Efet.01.594594.g1127.t1 | rRNA methyltransferase | 291 | 8.14E-61 | 85.65 | F:RNA methyltransferase activity; P:RNA methylation; F:RNA binding; P:RNA processing |
| Efet.01.594594.g1128.t1 | rRNA methyltransferase | 456 | 1.91E-90 | 80.75 | F:RNA methyltransferase activity; P:RNA methylation; F:RNA binding; C:cytoplasm; P:tRNA processing |
| Efet.01.59460.g598.t1 | Smoothelin 1 | 399 | 2.82E-28 | 77.55 |  |
| Efet.01.594804.g1129.t1 | IS256 family transposase | 603 | 5.37E-61 | 75.4 | F:DNA binding; F:transposase activity; P:transposition, DNA-mediated |
| Efet.01.594835.g1131.t1 | 2-oxoglutarate E2 dihydrolipoamide succinyltransferase | 549 | 3.08E-47 | 92.55 | F:transferase activity, transferring acyl groups; P:metabolic process |
| Efet.01.59485.g600.t1 | ras-specific guanine nucleotide-releasing factor 1 isoform X3 | 243 | 3.36E-24 | 75.25 | P:small GTPase mediated signal transduction; F:guanyl-nucleotide exchange factor activity; P:regulation of Ral protein signal transduction; C:intracellular; P:positive regulation of GTPase activity |
| Efet.01.594862.g1132.t1 | Exocyst complex component 2 | 1698 | 3.15E-25 | 64.35 | P:Golgi to plasma membrane transport; C:exocyst |
| Efet.01.594922.g1134.t1 | transcriptional regulator | 624 | 2.19E-150 | 72.3 | F:DNA binding; F:transcription factor activity, sequence-specific DNA binding; P:transcription, DNA-templated; P:regulation of transcription, DNA-templated; C:intracellular |
| Efet.01.595011.g1138.t1 | N-alpha-acetyltransferase auxiliary subunit | 246 | 2.25E-21 | 87.3 | F:transferase activity |
| Efet.01.595218.g1141.t1 | heme ABC transporter ATP-binding | 558 | 7.07E-65 | 68.4 | F:nucleotide binding; F:ATPase activity; C:membrane; P:transport; F:transporter activity |
| Efet.01.595235.g1143.t1 | neuroligin- X-linked-like | 303 | 3.03E-08 | 56.67 | P:metabolic process; C:membrane; C:integral component of membrane; F:hydrolase activity |
| Efet.01.595240.g1144.t1 | amiloride-sensitive sodium channel subunit gamma-like isoform X2 | 609 | 8.57E-07 | 56.75 | P:transport |
| Efet.01.59526.g601.t1 | bicaudal C homolog 1 | 417 | 9.72E-13 | 78.6 | F:RNA binding |
| Efet.01.595267.g1145.t1 | hypothetical protein CAPTEDRAFT_215274 | 1089 | 4.18E-32 | 54.55 | P:signal transduction |
| Efet.01.595396.g1149.t1 | folate-binding | 306 | 9.31E-38 | 59.45 | F:methyltransferase activity; F:transferase activity; P:methylation |
| Efet.01.595396.g1150.t1 | folate-binding | 774 | 8.82E-105 | 68.85 | F:methyltransferase activity; F:transferase activity; P:methylation |
| Efet.01.595421.g1151.t1 | sugar ABC transporter permease | 813 | 3.51E-141 | 79.05 | C:integral component of membrane; P:transport; C:plasma membrane |
| Efet.01.595421.g1152.t1 | sugar ABC transporter substrate-binding | 771 | 8.67E-124 | 58.7 | P:transport; F:transporter activity |
| Efet.01.595431.g1154.t1 | sugar ABC transporter permease | 750 | 1.08E-115 | 71.3 | C:membrane |
| Efet.01.595454.g1155.t1 | Spermatogenesis-associated 13 | 327 | 5.62E-15 | 72.05 | P:regulation of cellular process; F:guanyl-nucleotide exchange factor activity |
| Efet.01.595474.g1156.t1 | leukocyte elastase inhibitor isoform X2 | 564 | 3.57E-44 | 61.6 | F:serine-type endopeptidase inhibitor activity; C:extracellular region part; P:regulation of protein catabolic process |
| Efet.01.595491.g1158.t1 | dipeptide ABC transporter ATP-binding | 957 | 0 | 81.25 | F:ATPase activity; F:ATP binding; P:peptide transport |
| Efet.01.595491.g1159.t1 | AMP-dependent synthetase | 2832 | 0 | 80.75 | F:acetyl-CoA C-acyltransferase activity; F:oxidoreductase activity; F:lyase activity; P:oxidation-reduction process; F:ligase activity; P:fatty acid metabolic process; F:isomerase activity |
| Efet.01.59551.g603.t1 | TPR repeat-containing DDB_G0287407-like | 2973 | 1.89E-128 | 52.4 | F:ATP binding |
| Efet.01.59556.g604.t1 | Autophagy-related 9A | 636 | 5.80E-06 | 56 |  |
| Efet.01.595595.g1162.t1 | C4-dicarboxylate ABC transporter substrate-binding | 897 | 0 | 94.65 | C:outer membrane-bounded periplasmic space; P:transport |
| Efet.01.595618.g1163.t1 | sarcoplasmic calcium-binding -like | 318 | 2.70E-11 | 55.55 | F:nucleotide binding; F:ATP binding; C:nucleus; C:cytoplasm; F:calcium ion binding; F:protein kinase activity; F:protein serine/threonine kinase activity; P:protein phosphorylation; C:plasma membrane |
| Efet.01.595633.g1164.t1 | EVE domain-containing | 339 | 3.43E-77 | 74.5 |  |
| Efet.01.595633.g1165.t1 | guanosine-3 ,5 -bis(diphosphate) 3 -pyrophosphohydrolase | 2529 | 0 | 90.9 | F:GTP diphosphokinase activity; P:guanosine tetraphosphate metabolic process; F:amino acid binding; F:hydrolase activity |
| Efet.01.595633.g1166.t1 | guanylate kinase | 714 | 2.52E-154 | 95.45 | F:ATP binding; C:cytoplasm; P:GDP metabolic process; P:GMP metabolic process; F:guanylate kinase activity; P:phosphorylation |
| Efet.01.595633.g1167.t1 | DNA helicase | 1035 | 4.94E-102 | 86.6 | F:DNA binding; F:ATP binding; P:DNA duplex unwinding; F:ATP-dependent DNA helicase activity |
| Efet.01.595640.g1168.t1 | hypothetical protein B5766_12140 | 201 | 1.53E-12 | 96 |  |
| Efet.01.595643.g1169.t1 | transient-receptor-potential | 207 | 1.11E-12 | 70 | F:calcium channel activity; C:integral component of membrane; P:calcium ion transmembrane transport |
| Efet.01.595647.g1170.t1 | sugar kinase | 687 | 2.31E-108 | 79.85 | P:phosphorylation; F:kinase activity |
| Efet.01.59570.g605.t1 | atrial natriuretic peptide receptor 1-like | 258 | 5.97E-18 | 67.4 | F:nucleotide binding; F:phosphorus-oxygen lyase activity; C:membrane; P:cyclic nucleotide biosynthetic process |
| Efet.01.595746.g1174.t1 | peflin-like | 306 | 2.05E-20 | 77.5 | C:cytoplasm; F:calcium ion binding; P:proteolysis; F:calcium-dependent cysteine-type endopeptidase activity |
| Efet.01.595749.g1175.t1 | tRNA (guanosine(37)-N1)-methyltransferase | 432 | 3.70E-84 | 55.65 | P:metabolic process; F:transferase activity |
| Efet.01.59586.g606.t1 | 40S ribosomal SA | 369 | 2.52E-24 | 59.25 | C:ribosome |
| Efet.01.595883.g1177.t1 | coiled-coil domain-containing 40-like | 390 | 1.41E-20 | 65.25 |  |
| Efet.01.59590.g607.t1 | tumor necrosis factor ligand superfamily member 10-like | 579 | 5.44E-06 | 47 |  |
| Efet.01.596027.g1180.t1 | chaperonin | 939 | 3.55E-174 | 90.9 | F:ATP binding; C:cytoplasm; F:unfolded protein binding; P:protein refolding |
| Efet.01.596048.g1181.t1 | 6-phosphofructokinase | 693 | 5.36E-168 | 83.45 | F:diphosphate-fructose-6-phosphate 1-phosphotransferase activity; P:fructose 6-phosphate metabolic process; P:glycolytic process through fructose-6-phosphate; P:carbohydrate phosphorylation; F:6-phosphofructokinase activity |
| Efet.01.596156.g1183.t1 | AI-2E family transporter | 840 | 5.66E-115 | 72.1 | C:membrane |
| Efet.01.596181.g1185.t1 | NAD-dependent DNA ligase | 759 | 2.57E-115 | 73.7 | F:binding; P:DNA metabolic process; P:cellular response to DNA damage stimulus; F:ligase activity |
| Efet.01.596340.g1186.t1 | hypothetical protein CAPTEDRAFT_198655 | 669 | 3.93E-10 | 57 |  |
| Efet.01.596342.g1187.t1 | vesicle-trafficking SEC22a | 348 | 3.55E-34 | 69.1 | C:membrane |
| Efet.01.596412.g1189.t1 | ribonucleoside-diphosphate reductase subunit alpha | 588 | 2.22E-101 | 84.3 | F:ATP binding; F:ribonucleoside-diphosphate reductase activity, thioredoxin disulfide as acceptor; P:DNA replication; P:oxidation-reduction process |
| Efet.01.59646.g611.t1 | fat-like cadherin-related tumor suppressor homolog | 579 | 5.17E-06 | 51 |  |
| Efet.01.596473.g1190.t1 | hypothetical protein HELRODRAFT_161431 | 1620 | 3.27E-20 | 50.5 |  |
| Efet.01.596554.g1191.t1 | unc-80 homolog | 231 | 1.35E-22 | 77.3 |  |
| Efet.01.596555.g1192.t1 | peroxiredoxin 6 | 327 | 1.16E-58 | 87.05 | F:peroxidase activity; F:phospholipase A2 activity; P:response to oxidative stress; C:extracellular space; C:extracellular exosome; F:ubiquitin protein ligase binding; C:cytoplasm; C:membrane; P:phospholipid catabolic process; P:cellular oxidant detoxification; P:oxidation-reduction process; F:peroxiredoxin activity |
| Efet.01.596611.g1193.t1 | heavy chain smooth muscle | 369 | 2.73E-54 | 82.8 | P:cardiac muscle fiber development; F:motor activity; F:actin filament binding; F:ATP binding; F:calmodulin binding; C:extracellular exosome; F:structural constituent of muscle; P:elastic fiber assembly; C:muscle myosin complex; C:melanosome; P:smooth muscle contraction; C:myosin filament; C:cytosol; P:skeletal muscle myosin thick filament assembly; P:positive regulation of macromitophagy in response to mitochondrial depolarization |
| Efet.01.596935.g1198.t1 | leucine--tRNA ligase | 267 | 4.70E-29 | 89.95 | F:ATP binding; P:leucyl-tRNA aminoacylation; F:leucine-tRNA ligase activity; C:cytoplasm; P:regulation of translational fidelity; F:aminoacyl-tRNA editing activity |
| Efet.01.596941.g1199.t1 | ATP-binding cassette sub-family A member 3-like | 225 | 2.29E-12 | 61.8 | P:transport |
| Efet.01.59707.g612.t1 | inactive gamma-glutamyltranspeptidase 2 isoform X1 | 333 | 3.42E-43 | 75.7 | P:zymogen activation; C:perinuclear region of cytoplasm; C:extracellular exosome; P:leukotriene biosynthetic process; C:anchored component of external side of plasma membrane; F:glutathione hydrolase activity; P:xenobiotic metabolic process; P:glutathione biosynthetic process; C:endoplasmic reticulum; F:gamma-glutamyltransferase activity |
| Efet.01.59710.g613.t1 | G- coupled receptor 158 | 549 | 6.02E-71 | 73.5 | C:integral component of membrane |
| Efet.01.59716.g614.t1 | B-type nuclear lamin | 432 | 1.92E-11 | 74.35 | F:structural molecule activity; C:intermediate filament |
| Efet.01.597178.g1200.t1 | lipo signal peptidase | 480 | 1.46E-108 | 76.55 | F:aspartic-type endopeptidase activity; P:proteolysis; C:integral component of membrane; C:plasma membrane |
| Efet.01.597232.g1201.t1 | achaete-scute homolog 1b-like | 975 | 1.96E-11 | 72.25 | F:DNA binding; P:regulation of cellular process; P:nervous system development; F:protein dimerization activity; P:animal organ development |
| Efet.01.597264.g1202.t1 | pyruvate carboxylase | 381 | 5.61E-29 | 98.5 | F:ATP binding; P:gluconeogenesis; F:pyruvate carboxylase activity; F:metal ion binding; F:biotin binding; F:biotin carboxylase activity; P:pyruvate metabolic process |
| Efet.01.597291.g1203.t1 | multiple exostoses | 921 | 5.51E-113 | 69.45 | F:heparan sulfate N-acetylglucosaminyltransferase activity; P:heparan sulfate proteoglycan biosynthetic process, polysaccharide chain biosynthetic process; F:protein homodimerization activity; P:signal transduction; F:glucuronosyl-N-acetylglucosaminyl-proteoglycan 4-alpha-N-acetylglucosaminyltransferase activity; F:N-acetylglucosaminyl-proteoglycan 4-beta-glucuronosyltransferase activity; P:glycosaminoglycan biosynthetic process; P:nervous system development; C:Golgi membrane; P:skeletal system development; F:protein heterodimerization activity; P:ossification; C:integral component of endoplasmic reticulum membrane |
| Efet.01.597386.g1205.t1 | peptide chain release factor 1 | 645 | 7.25E-147 | 84.05 | P:translational termination; C:cytoplasm; F:translation release factor activity, codon specific |
| Efet.01.5975.g475.t1 | otoferlin-like isoform X2 | 375 | 7.45E-42 | 76.4 | C:cell part; C:synapse |
| Efet.01.597508.g1207.t1 | nitrate ABC transporter ATP-binding | 372 | 3.06E-80 | 97.3 | F:ATPase activity; F:ATP binding; F:nitrate transmembrane transporter activity; C:membrane; P:nitrate transport |
| Efet.01.597508.g1208.t1 | nitrite reductase large subunit | 2493 | 0 | 87.45 | F:NADP binding; F:metal ion binding; F:flavin adenine dinucleotide binding; P:oxidation-reduction process; F:4 iron, 4 sulfur cluster binding; F:nitrite reductase [NAD(P)H] activity; F:heme binding; P:nitrate assimilation |
| Efet.01.597509.g1209.t1 | von Willebrand factor type EGF and pentraxin domain-containing 1-like | 564 | 1.01E-33 | 50.6 | F:calcium ion binding; C:membrane; C:integral component of membrane; F:chromatin binding |
| Efet.01.597541.g1210.t1 | epidermal growth | 402 | 3.56E-32 | 65.7 | C:proteinaceous extracellular matrix |
| Efet.01.597547.g1211.t1 | molybdenum ABC transporter permease subunit | 621 | 1.06E-89 | 93.35 | F:molybdate ion transmembrane transporter activity; C:integral component of membrane; C:plasma membrane; P:molybdate ion transport |
| Efet.01.597547.g1212.t1 | molybdate ABC transporter substrate-binding | 783 | 1.57E-159 | 87.15 | F:ATPase-coupled molybdate transmembrane transporter activity; C:outer membrane-bounded periplasmic space; P:molybdate ion transport |
| Efet.01.597547.g1213.t1 | fatty acid hydroxylase | 819 | 0 | 88.1 | F:iron ion binding; F:oxidoreductase activity; C:integral component of membrane; P:oxidation-reduction process; P:fatty acid biosynthetic process |
| Efet.01.597630.g1215.t1 | 30S ribosomal S12 methylthiotransferase | 1053 | 0 | 94.2 | P:peptidyl-L-beta-methylthioaspartic acid biosynthetic process from peptidyl-aspartic acid; F:iron ion binding; P:RNA modification; F:4 iron, 4 sulfur cluster binding; C:ribosome; F:transferase activity |
| Efet.01.597630.g1216.t1 | polyhydroxyalkanoate synthesis repressor | 408 | 9.48E-82 | 85.45 |  |
| Efet.01.597687.g1217.t1 | E3 ubiquitin- ligase MSL2 | 1242 | 4.30E-15 | 68.6 | F:zinc ion binding; F:ubiquitin protein ligase activity; P:protein ubiquitination |
| Efet.01.597700.g1218.t1 | ATP-dependent endonuclease | 615 | 1.72E-74 | 71.75 | P:nucleic acid metabolic process; F:organic cyclic compound binding; F:heterocyclic compound binding |
| Efet.01.597702.g1219.t1 | strictosidine synthase | 330 | 7.90E-52 | 81.5 | F:strictosidine synthase activity; P:biosynthetic process |
| Efet.01.597702.g1220.t1 | sugar ABC transporter ATP-binding | 1503 | 0 | 87.9 | F:ATP binding; P:monosaccharide transport; F:monosaccharide-transporting ATPase activity |
| Efet.01.597702.g1221.t1 | alcohol dehydrogenase | 810 | 7.20E-158 | 86 | F:zinc ion binding; C:integral component of membrane; P:oxidation-reduction process; F:alcohol dehydrogenase (NAD) activity |
| Efet.01.597702.g1222.t1 | membrane | 1536 | 0 | 76.65 | P:transmembrane transport; C:integral component of membrane |
| Efet.01.597702.g1223.t1 | aminotransferase class V-fold PLP-dependent enzyme | 1182 | 0 | 73.75 | F:transaminase activity |
| Efet.01.597702.g1224.t1 | alpha-aspartyl dipeptidase Peptidase E | 735 | 1.13E-174 | 70.75 | F:dipeptidase activity; P:proteolysis; F:serine-type peptidase activity |
| Efet.01.597702.g1225.t1 | DUF1177 domain-containing | 882 | 0 | 77.4 |  |
| Efet.01.597702.g1226.t1 | DNA-binding response regulator | 1911 | 0 | 61.6 | F:phosphorelay sensor kinase activity; F:DNA binding; P:transcription, DNA-templated; P:phosphorelay signal transduction system; C:integral component of membrane; P:regulation of transcription, DNA-templated; F:protein dimerization activity; P:signal transduction by protein phosphorylation; C:intracellular |
| Efet.01.597702.g1227.t1 | gamma-aminobutyraldehyde dehydrogenase | 1071 | 0 | 70.9 | F:betaine-aldehyde dehydrogenase activity; P:oxidation-reduction process |
| Efet.01.597702.g1228.t1 | ABC transporter permease | 747 | 8.39E-145 | 83.15 | C:integral component of membrane; P:transport; C:plasma membrane |
| Efet.01.597702.g1229.t1 | ABC transporter permease | 1017 | 0 | 73.1 | C:integral component of membrane; P:transport; C:plasma membrane |
| Efet.01.597702.g1230.t1 | ABC transporter ATP-binding | 681 | 7.02E-133 | 87.95 | F:ATP binding; P:polyamine transmembrane transport; F:polyamine-transporting ATPase activity; C:ATP-binding cassette (ABC) transporter complex |
| Efet.01.597702.g1231.t1 | spermidine putrescine ABC transporter substrate-binding | 1125 | 0 | 80.85 | F:polyamine binding; C:periplasmic space; P:polyamine transport |
| Efet.01.597702.g1232.t1 | family transcriptional regulator | 867 | 0 | 72.7 | F:DNA binding; F:transcription factor activity, sequence-specific DNA binding; P:transcription, DNA-templated; P:regulation of transcription, DNA-templated |
| Efet.01.597702.g1233.t1 | glycolate oxidase iron-sulfur subunit | 1368 | 0 | 86.85 | F:metal ion binding; P:oxidation-reduction process; F:4 iron, 4 sulfur cluster binding |
| Efet.01.597702.g1234.t1 | glycolate oxidase subunit | 285 | 1.27E-31 | 70.7 | F:oxidoreductase activity, acting on CH-OH group of donors; F:flavin adenine dinucleotide binding; P:oxidation-reduction process |
| Efet.01.597763.g1235.t1 | SWI SNF-related matrix-associated actin-dependent regulator of chromatin subfamily A member 5- | 468 | 2.03E-67 | 85.85 | C:nucleus; F:DNA binding; F:ATP binding; F:nucleosome binding; P:ATP-dependent chromatin remodeling; F:hydrolase activity, acting on acid anhydrides, in phosphorus-containing anhydrides |
| Efet.01.597770.g1236.t1 | 5-methyltetrahydropteroyltriglutamate--homocysteine S-methyltransferase | 528 | 1.68E-109 | 75.5 | P:methionine biosynthetic process; F:zinc ion binding; F:5-methyltetrahydropteroyltriglutamate-homocysteine S-methyltransferase activity; P:methylation |
| Efet.01.597775.g1237.t1 | potassium voltage-gated channel subfamily H member 7-like isoform X3 | 369 | 5.39E-33 | 86.85 | F:voltage-gated potassium channel activity; P:potassium ion transmembrane transport; C:integral component of membrane |
| Efet.01.597811.g1238.t1 | lysosomal-trafficking regulator | 438 | 6.59E-24 | 57.9 | P:lysosome organization; C:microtubule cytoskeleton; P:pigmentation; P:endosome to lysosome transport via multivesicular body sorting pathway; P:natural killer cell mediated cytotoxicity |
| Efet.01.597855.g1239.t1 | histamine H2 receptor-like | 1059 | 8.55E-74 | 62 | C:membrane; P:cellular process |
| Efet.01.59787.g618.t1 | BTB POZ domain-containing 3 isoform X2 | 1467 | 8.73E-77 | 56.85 | C:cytoplasm; P:nervous system development; P:cell differentiation |
| Efet.01.597981.g1241.t1 | hypothetical protein CAPTEDRAFT_216159 | 966 | 1.44E-53 | 50 | C:membrane; C:integral component of membrane |
| Efet.01.598022.g1242.t1 | polycystic kidney disease 2 | 414 | 1.26E-46 | 67.9 | C:membrane |
| Efet.01.598101.g1245.t1 | sperm-associated antigen 6 | 381 | 2.34E-57 | 85.35 |  |
| Efet.01.59816.g620.t1 | 5-hydroxytryptamine receptor 4-like | 1104 | 4.33E-07 | 44.95 | F:G-protein coupled receptor activity; P:G-protein coupled receptor signaling pathway; P:signal transduction; C:membrane; C:integral component of membrane; P:serotonin receptor signaling pathway; F:G-protein coupled serotonin receptor activity; F:signal transducer activity; F:trace-amine receptor activity |
| Efet.01.598182.g1246.t1 | dihydroxyacetone kinase subunit L | 729 | 2.94E-104 | 76.1 | F:kinase activity; P:cellular metabolic process |
| Efet.01.598213.g1247.t1 | hypothetical protein HELRODRAFT_176694 | 714 | 5.77E-48 | 60.6 | C:membrane |
| Efet.01.598431.g1250.t1 | spire homolog 2 | 405 | 2.69E-08 | 71.4 | P:vesicle-mediated transport; F:actin binding; P:actin nucleation; P:cell cycle process; C:cell part |
| Efet.01.598493.g1252.t1 | neuronal acetylcholine receptor subunit alpha-3-like | 231 | 4.34E-17 | 73.75 | P:ion transmembrane transport; C:integral component of membrane; F:extracellular ligand-gated ion channel activity; C:synapse; C:plasma membrane |
| Efet.01.598541.g1253.t1 | Dynein light chain cytoplasmic | 270 | 2.00E-60 | 100 | C:nucleus; C:P granule; C:dynein complex; P:microtubule-based process |
| Efet.01.598556.g1254.t1 | hepatocyte growth factor receptor-like | 489 | 9.29E-09 | 52.67 | P:signal transduction; C:membrane; F:semaphorin receptor activity; C:integral component of membrane; P:semaphorin-plexin signaling pathway |
| Efet.01.598556.g1255.t1 | plexin-A2-like | 840 | 1.19E-35 | 52.95 | P:signal transduction; P:cellular component organization; P:generation of neurons |
| Efet.01.598581.g1256.t1 | plexin-B | 891 | 9.16E-57 | 48.7 | P:signal transduction; P:regulation of GTPase activity; C:membrane; F:semaphorin receptor activity; C:integral component of membrane; P:axon guidance; P:semaphorin-plexin signaling pathway |
| Efet.01.598590.g1257.t1 | hypothetical protein CRN43_09195 | 312 | 7.54E-12 | 67.5 |  |
| Efet.01.598638.g1258.t1 | coiled-coil domain-containing 96 | 687 | 7.13E-32 | 61.55 |  |
| Efet.01.598670.g1260.t1 | ABC transporter ATP-binding | 228 | 1.43E-41 | 80.45 | F:ATPase activity; F:ATP binding; P:peptide transport |
| Efet.01.598713.g1261.t1 | transcriptional regulator | 1239 | 2.87E-165 | 71.7 | F:ATP binding |
| Efet.01.598816.g1262.t1 | voltage-dependent T-type calcium channel subunit alpha-1H-like | 2004 | 4.12E-26 | 75.75 | P:calcium ion import; C:voltage-gated calcium channel complex; P:regulation of ion transmembrane transport; P:calcium ion transmembrane transport; F:low voltage-gated calcium channel activity |
| Efet.01.598835.g1263.t1 | SET domain-containing | 507 | 9.81E-65 | 70.65 |  |
| Efet.01.598861.g1265.t1 | hypothetical protein CAPTEDRAFT_211886 | 660 | 4.03E-25 | 53.45 | F:carbohydrate binding |
| Efet.01.598881.g1266.t1 | family | 912 | 0 | 94.8 | F:ATP binding |
| Efet.01.598881.g1267.t1 | class II fumarate hydratase | 717 | 1.39E-97 | 75 | C:cytoplasm; F:lyase activity; P:carboxylic acid metabolic process |
| Efet.01.598882.g1268.t1 | [acyl-carrier- ] S-malonyltransferase | 951 | 2.29E-169 | 90 | F:[acyl-carrier-protein] S-malonyltransferase activity; P:metabolic process |
| Efet.01.598882.g1269.t1 | 3-oxoacyl-ACP reductase | 594 | 2.89E-102 | 94.15 | F:3-oxoacyl-[acyl-carrier-protein] reductase (NADPH) activity; F:NAD binding; P:oxidation-reduction process; P:fatty acid biosynthetic process |
| Efet.01.598882.g1270.t1 | beta-ketoacyl-[acyl-carrier- ] synthase II | 1338 | 0 | 95.3 | F:beta-ketoacyl-acyl-carrier-protein synthase II activity; P:fatty acid biosynthetic process |
| Efet.01.598882.g1271.t1 | RNA polymerase sigma factor | 627 | 1.07E-138 | 94.45 | F:DNA binding; F:sigma factor activity; F:transcription factor activity, sequence-specific DNA binding; P:regulation of transcription, DNA-templated; P:DNA-templated transcription, initiation |
| Efet.01.598882.g1272.t1 | anti-anti-sigma factor | 603 | 4.35E-65 | 63 | F:sigma factor antagonist activity; P:regulation of nucleic acid-templated transcription |
| Efet.01.598885.g1273.t1 | GIY-YIG catalytic domain-containing | 1290 | 1.26E-134 | 70.55 |  |
| Efet.01.598981.g1274.t1 | ABC transporter permease | 864 | 4.07E-160 | 79.75 | C:integral component of membrane; P:transport; C:plasma membrane |
| Efet.01.598981.g1275.t1 | choline dehydrogenase | 630 | 1.75E-145 | 79.6 | F:oxidoreductase activity, acting on CH-OH group of donors; F:flavin adenine dinucleotide binding; P:oxidation-reduction process |
| Efet.01.599013.g1276.t1 | scribble homolog | 207 | 7.52E-15 | 67.3 |  |
| Efet.01.599013.g1277.t1 | scribble homolog | 255 | 7.57E-16 | 74.1 | P:regulation of cellular process |
| Efet.01.599043.g1279.t1 | hypothetical protein CAPTEDRAFT_210308 | 639 | 2.20E-26 | 57 |  |
| Efet.01.599117.g1282.t1 | MFS transporter | 552 | 9.72E-57 | 72.2 | P:transmembrane transport; C:integral component of membrane; F:transporter activity |
| Efet.01.599142.g1283.t1 | 7SK snRNA methylphosphate capping enzyme-like | 978 | 2.44E-40 | 56.65 | F:methyltransferase activity; P:methylation |
| Efet.01.599149.g1284.t1 | Glioma pathogenesis-related | 240 | 5.20E-10 | 56.95 | P:biological_process; C:extracellular region; C:cellular_component; F:molecular_function |
| Efet.01.599153.g1285.t1 | type-1 angiotensin II receptor-like isoform X1 | 303 | 2.32E-18 | 62.2 | F:G-protein coupled receptor activity; P:G-protein coupled receptor signaling pathway; C:integral component of membrane |
| Efet.01.599318.g1286.t1 | catenin alpha-2-like isoform X3 | 273 | 2.01E-35 | 89.15 | F:actin filament binding; F:cadherin binding; P:cell adhesion |
| Efet.01.599343.g1287.t1 | membrane | 483 | 5.57E-101 | 63 | C:membrane; C:integral component of membrane |
| Efet.01.59935.g621.t1 | spliceosome RNA helicase DDX39B | 519 | 9.46E-51 | 94.5 | P:positive regulation of translation; C:nuclear speck; P:termination of RNA polymerase II transcription; F:ATP-dependent protein binding; F:ATP-dependent RNA helicase activity; F:ATP binding; P:positive regulation of vascular smooth muscle cell proliferation; P:viral mRNA export from host cell nucleus; P:liver development; F:protein complex binding; P:positive regulation of DNA biosynthetic process; P:positive regulation of DNA-templated transcription, elongation; F:U4 snRNA binding; F:identical protein binding; C:spliceosomal complex; C:transcription export complex; P:cellular response to DNA damage stimulus; C:U6 snRNP; P:spliceosomal complex assembly; C:U4 snRNP; F:RNA binding; F:U6 snRNA binding; C:cytoplasm; P:RNA secondary structure unwinding; C:nuclear matrix; P:mRNA 3'-end processing; P:negative regulation of DNA damage checkpoint; P:positive regulation of cell growth involved in cardiac muscle cell development |
| Efet.01.599360.g1288.t1 | beta-1,3-galactosyltransferase 1-like | 1047 | 1.22E-62 | 64.95 | F:transferase activity, transferring glycosyl groups; C:membrane; C:Golgi apparatus |
| Efet.01.599369.g1289.t1 | ras-specific guanine nucleotide-releasing factor 2-like | 279 | 2.73E-33 | 77.55 | P:regulation of cellular process; C:membrane; F:guanyl-nucleotide exchange factor activity |
| Efet.01.599423.g1291.t1 | heparan sulfate glucosamine 3-O-sulfotransferase 3B1-like | 726 | 4.50E-23 | 80.55 | C:membrane; F:sulfotransferase activity |
| Efet.01.599453.g1292.t1 | Transposon Ty3-G Gag-Pol poly | 498 | 1.00E-12 | 54.5 | F:nucleic acid binding; F:zinc ion binding; P:DNA integration |
| Efet.01.599543.g1293.t1 | phosphatidate cytidylyltransferase | 936 | 5.38E-165 | 55.7 | F:nucleotidyltransferase activity; F:phosphatidate cytidylyltransferase activity; C:membrane; C:integral component of membrane; F:transferase activity, transferring phosphorus-containing groups; P:CDP-diacylglycerol biosynthetic process; F:transferase activity |
| Efet.01.599544.g1294.t1 | hypothetical protein LOTGIDRAFT_159467 | 306 | 5.39E-06 | 65 | C:membrane; C:integral component of membrane |
| Efet.01.599551.g1295.t1 | Structural maintenance of chromosomes flexible hinge domain-containing | 300 | 5.42E-29 | 67.7 | F:ATP binding; P:chromosome organization; C:chromosome |
| Efet.01.599553.g1296.t1 | 2 ,3 -cyclic-nucleotide 3 -phosphodiesterase | 753 | 3.74E-11 | 55.8 | C:membrane; P:cyclic nucleotide catabolic process; F:2',3'-cyclic-nucleotide 3'-phosphodiesterase activity |
| Efet.01.599555.g1297.t1 | protogenin A-like | 363 | 3.96E-24 | 57.5 | P:multicellular organism development; C:membrane; C:integral component of membrane |
| Efet.01.59965.g624.t1 | LIM domain transcription factor -like | 327 | 3.28E-11 | 56.85 | F:zinc ion binding; F:metal ion binding |
| Efet.01.599706.g1300.t1 | calmodulin-binding transcription activator 2-like isoform X1 | 459 | 1.20E-21 | 59.7 |  |
| Efet.01.599744.g1301.t1 | sacsin-like | 1020 | 2.86E-25 | 46.8 |  |
| Efet.01.599744.g1302.t1 | PREDICTED: sacsin-like | 1275 | 1.54E-09 | 43.5 |  |
| Efet.01.599761.g1303.t1 | 1-pyrroline-5-carboxylate dehydrogenase | 501 | 3.14E-88 | 85.55 | F:1-pyrroline-5-carboxylate dehydrogenase activity; F:transcription factor activity, sequence-specific DNA binding; F:proline dehydrogenase activity; C:integral component of membrane; P:regulation of transcription, DNA-templated; P:oxidation-reduction process; F:oxidoreductase activity, acting on the aldehyde or oxo group of donors, NAD or NADP as acceptor; P:proline catabolic process to glutamate |
| Efet.01.599761.g1304.t1 | 1-pyrroline-5-carboxylate dehydrogenase | 687 | 9.84E-48 | 59.75 | F:oxidoreductase activity |
| Efet.01.599818.g1305.t1 | TANC2 isoform X4 | 447 | 2.50E-49 | 72.9 | F:zinc ion binding |
| Efet.01.599842.g1307.t1 | sex peptide receptor-like isoform X5 | 219 | 1.41E-10 | 79.75 | F:G-protein coupled peptide receptor activity; P:G-protein coupled receptor signaling pathway; C:integral component of membrane |
| Efet.01.599924.g1308.t1 | DNA polymerase I | 210 | 2.41E-28 | 91.6 | F:DNA binding; P:nucleic acid phosphodiester bond hydrolysis; F:DNA-directed DNA polymerase activity; F:nuclease activity; P:DNA-dependent DNA replication; P:DNA biosynthetic process |
| Efet.01.599952.g1309.t1 | hypothetical protein N864_15835 | 294 | 1.03E-14 | 83 |  |
| Efet.01.599964.g1312.t1 | zinc finger 397 isoform X3 | 1056 | 1.12E-07 | 47.46 | F:nucleic acid binding; F:metal ion binding |
| Efet.01.600045.g1.t1 | Replication factor C subunit 4 | 327 | 9.99E-47 | 85.65 | F:DNA binding; F:ATP binding; P:DNA replication |
| Efet.01.60019.g626.t1 | N(G),N(G)-dimethylarginine dimethylaminohydrolase 1-like | 333 | 1.86E-51 | 69.05 | F:hydrolase activity |
| Efet.01.600263.g2.t1 | nucleolar 4-like | 465 | 4.73E-17 | 70.05 | P:cellular process |
| Efet.01.600278.g3.t1 | DNA helicase | 273 | 3.47E-18 | 79.9 | F:nucleotide binding; P:cellular response to stress; F:ATP-dependent DNA helicase activity; P:DNA metabolic process |
| Efet.01.600309.g4.t1 | bifunctional demethylmenaquinone methyltransferase 2-methoxy-6-polyprenyl-1,4-benzoquinol methylase | 369 | 7.93E-77 | 71.8 | P:metabolic process; F:transferase activity |
| Efet.01.600309.g5.t1 | polyprenyl synthetase family | 873 | 1.83E-152 | 85.3 | P:isoprenoid biosynthetic process; F:transferase activity |
| Efet.01.600309.g6.t1 | geranylgeranyl pyrophosphate synthase | 225 | 3.28E-31 | 74.92 | P:isoprenoid biosynthetic process; F:transferase activity |
| Efet.01.600309.g7.t1 | prepilin peptidase | 516 | 7.25E-62 | 64 |  |
| Efet.01.600309.g8.t1 | family transcriptional regulator | 1149 | 0 | 60.3 | F:DNA binding; F:transcription factor activity, sequence-specific DNA binding; P:transcription, DNA-templated; P:regulation of transcription, DNA-templated |
| Efet.01.60034.g627.t1 | cytoplasmic dynein 2 heavy chain 1-like | 693 | 7.54E-109 | 85.3 | F:ATPase activity; F:ATP binding; F:microtubule motor activity; C:dynein complex; P:microtubule-based movement |
| Efet.01.600376.g9.t1 | Leukocyte elastase inhibitor | 561 | 1.85E-16 | 57.05 | C:extracellular space; F:serine-type endopeptidase inhibitor activity; P:proteolysis; C:membrane; C:integral component of membrane; P:negative regulation of endopeptidase activity; F:peptidase activity |
| Efet.01.600388.g10.t1 | serine threonine- kinase WNK1- | 513 | 1.50E-63 | 79.15 | P:posterior lateral line neuromast development; F:protein serine/threonine kinase inhibitor activity; P:regulation of ion homeostasis; F:protein kinase activator activity; P:DNA integration; P:protein autophosphorylation; P:signal transduction by trans-phosphorylation; F:magnesium ion binding; F:ATP binding; P:ion transport; P:angiogenesis; C:cytosol; P:intracellular signal transduction; F:chloride channel inhibitor activity; F:phosphatase binding; F:protein kinase binding; P:negative regulation of pancreatic juice secretion; P:positive regulation of systemic arterial blood pressure; P:neuron development; F:protein serine/threonine kinase activity; P:transposition, DNA-mediated; F:DNA binding; P:negative regulation of phosphatase activity; F:potassium channel inhibitor activity; C:membrane; P:negative regulation of sodium ion transport; P:positive regulation of ion transmembrane transporter activity; P:negative regulation of protein serine/threonine kinase activity; P:peptidyl-threonine phosphorylation |
| Efet.01.600452.g12.t1 | RNA polymerase sigma-70 factor | 603 | 8.76E-125 | 65.2 | F:DNA binding; F:sigma factor activity; F:transcription factor activity, sequence-specific DNA binding; P:transcription, DNA-templated; P:regulation of transcription, DNA-templated; P:DNA-templated transcription, initiation |
| Efet.01.600467.g13.t1 | ryanodine receptor 44F-like | 534 | 8.10E-79 | 74.5 | F:ryanodine-sensitive calcium-release channel activity; F:calcium ion binding; C:integral component of membrane; P:release of sequestered calcium ion into cytosol; C:intracellular |
| Efet.01.600501.g14.t1 | Lipoxygenase homology domain-containing 1 | 459 | 1.97E-28 | 66.7 | P:system development; C:ciliary part; C:neuron part; P:single-organism cellular process; C:intracellular |
| Efet.01.600504.g15.t1 | dynein heavy chain axonemal-like | 336 | 1.85E-36 | 86.75 | F:ATPase activity; F:ATP binding; F:microtubule motor activity; C:dynein complex; P:microtubule-based movement |
| Efet.01.60054.g628.t1 | hypothetical protein HELRODRAFT_177878 | 771 | 6.15E-41 | 49.45 | C:membrane; C:integral component of membrane; F:carbohydrate binding |
| Efet.01.600648.g17.t1 | roundabout homolog 1-like isoform X1 | 270 | 1.53E-09 | 72.95 | C:membrane |
| Efet.01.600650.g18.t1 | glycine--tRNA ligase | 933 | 0 | 92.25 | F:glycine-tRNA ligase activity; F:ATP binding; P:glycyl-tRNA aminoacylation; C:cytoplasm |
| Efet.01.600650.g19.t1 | Enterochelin esterase | 960 | 0 | 70.3 | C:membrane |
| Efet.01.600657.g20.t1 | transferase | 1443 | 0 | 80.5 | F:formyl-CoA transferase activity |
| Efet.01.600657.g21.t1 | enoyl- hydratase | 549 | 2.58E-98 | 63.75 | P:metabolic process; F:isomerase activity |
| Efet.01.600657.g22.t1 | acyl- dehydrogenase | 354 | 5.94E-37 | 75.4 | F:isovaleryl-CoA dehydrogenase activity; F:flavin adenine dinucleotide binding; P:oxidation-reduction process |
| Efet.01.600657.g23.t1 | acyl- dehydrogenase | 933 | 0 | 82.65 | F:isovaleryl-CoA dehydrogenase activity; F:flavin adenine dinucleotide binding; P:oxidation-reduction process |
| Efet.01.600657.g24.t1 | 2,4-dienoyl- reductase | 879 | 1.10E-172 | 78.1 | F:2,4-dienoyl-CoA reductase (NADPH) activity; F:trans-2-enoyl-CoA reductase (NADPH) activity; P:oxidation-reduction process |
| Efet.01.600657.g25.t1 | family transcriptional regulator | 762 | 3.15E-139 | 68.35 | F:DNA binding; P:transcription, DNA-templated; P:regulation of transcription, DNA-templated |
| Efet.01.600657.g26.t1 | acyl- dehydrogenase | 1674 | 0 | 91.35 | F:acyl-CoA dehydrogenase activity; F:flavin adenine dinucleotide binding; P:oxidation-reduction process |
| Efet.01.600693.g27.t1 | family -linked outer membrane | 786 | 6.88E-151 | 74.2 | C:membrane; F:receptor activity; P:transport |
| Efet.01.600704.g28.t1 | non-canonical purine NTP HAM1 family | 579 | 4.72E-91 | 77.35 | F:nucleotide binding; P:purine nucleotide metabolic process; F:metal ion binding; P:nucleoside triphosphate catabolic process; F:nucleoside-triphosphatase activity; F:nucleoside-triphosphate diphosphatase activity |
| Efet.01.600704.g29.t1 | cation transporter | 459 | 2.86E-30 | 80.8 | P:cation transmembrane transport; C:integral component of membrane; F:cation transmembrane transporter activity; C:plasma membrane |
| Efet.01.600711.g30.t1 | monovalent cation H+ antiporter subunit D | 1050 | 0 | 91.65 | C:integral component of membrane; F:lyase activity |
| Efet.01.600719.g31.t1 | B-box type zinc finger ncl-1-like | 492 | 1.67E-55 | 74.85 | F:metal ion binding |
| Efet.01.600738.g32.t1 | Transmembrane 5 | 336 | 3.05E-27 | 63.55 | C:integral component of membrane |
| Efet.01.600779.g34.t1 | cysteine desulfurase | 372 | 3.44E-26 | 87.65 | F:catalytic activity |
| Efet.01.600797.g35.t1 | Uncharacterized membrane DUF308 family | 330 | 8.32E-53 | 56.32 | C:membrane; C:integral component of membrane |
| Efet.01.60080.g629.t1 | mitochondrial import receptor subunit TOM70 | 240 | 3.72E-20 | 86 | C:extracellular exosome; C:mitochondrion; C:integral component of membrane |
| Efet.01.600908.g36.t1 | Wnt-6 isoform X2 | 258 | 3.01E-13 | 63.15 | P:regulation of cellular process; C:extracellular region part; P:cell-cell signaling; P:cell differentiation; P:animal organ development; F:receptor binding |
| Efet.01.600917.g37.t1 | acetate-- ligase | 741 | 8.64E-144 | 70.9 | F:adenyl ribonucleotide binding; F:purine ribonucleoside binding; P:metabolic process; F:cation binding; F:anion binding; F:ligase activity |
| Efet.01.600917.g38.t1 | cation transporter | 1083 | 1.67E-164 | 78.55 | P:cation transmembrane transport; C:integral component of membrane; F:cation transmembrane transporter activity; C:plasma membrane |
| Efet.01.600917.g39.t1 | glycosyl transferase family 1 | 1026 | 0 | 72.9 | F:transferase activity |
| Efet.01.600925.g40.t1 | GPI mannosyltransferase 3 | 303 | 2.76E-11 | 70.3 | C:endoplasmic reticulum membrane; P:GPI anchor biosynthetic process; F:mannosyltransferase activity |
| Efet.01.600946.g41.t1 | asteroid homolog 1-like | 828 | 1.99E-37 | 54.6 | P:nucleic acid phosphodiester bond hydrolysis; P:DNA repair; F:nuclease activity |
| Efet.01.600975.g42.t1 | low-density lipo receptor-related 4 | 594 | 3.07E-08 | 46.33 | C:membrane; C:integral component of membrane |
| Efet.01.600986.g43.t1 | tyrosine- kinase JAK2 | 720 | 6.26E-13 | 42.75 | P:peptidyl-tyrosine phosphorylation; F:protein kinase activity; P:protein phosphorylation; F:nucleotide binding; F:ATP binding; F:protein tyrosine kinase activity; C:cytoskeleton; F:non-membrane spanning protein tyrosine kinase activity; F:histone binding; C:membrane; P:phosphorylation; F:transferase activity; F:kinase activity; P:intracellular signal transduction |
| Efet.01.601035.g44.t1 | pyroglutamylated RFamide peptide receptor-like | 603 | 5.13E-25 | 61.5 | F:G-protein coupled receptor activity; P:signal transduction; C:membrane |
| Efet.01.601035.g45.t1 | hypothetical protein CAPTEDRAFT_89696 | 576 | 3.57E-10 | 54 | F:G-protein coupled receptor activity; P:G-protein coupled receptor signaling pathway; C:membrane; C:integral component of membrane |
| Efet.01.601050.g46.t1 | RNA polymerase-associated RTF1 homolog | 264 | 4.57E-20 | 79.25 | F:DNA binding; P:histone modification; C:Cdc73/Paf1 complex; P:transcription elongation from RNA polymerase II promoter |
| Efet.01.601081.g47.t1 | potassium voltage-gated channel subfamily H member 8 | 288 | 1.60E-30 | 84.1 | F:phosphorelay sensor kinase activity; F:voltage-gated potassium channel activity; P:potassium ion transmembrane transport; P:phosphorelay signal transduction system; C:integral component of membrane; P:signal transduction by protein phosphorylation; C:intracellular |
| Efet.01.601084.g48.t1 | E3 ubiquitin- ligase HECTD1-like | 414 | 3.01E-73 | 85.05 | P:negative regulation of protein localization to plasma membrane; F:ubiquitin protein ligase activity; P:heart valve development; P:ventricular septum development; P:protein ubiquitination involved in ubiquitin-dependent protein catabolic process; C:nucleus; F:protein binding; P:neural tube closure; C:cytoplasm; F:metal ion binding; P:aorta development; P:natural killer cell differentiation; P:trophoblast giant cell differentiation; P:spongiotrophoblast differentiation; P:protein K63-linked ubiquitination; F:ligase activity; P:protein autoubiquitination |
| Efet.01.60114.g630.t1 | zinc finger 678-like | 1077 | 9.07E-88 | 64.45 | F:nucleic acid binding; F:metal ion binding |
| Efet.01.601156.g50.t1 | hypothetical protein B5766_09955 | 468 | 3.05E-29 | 67.5 |  |
| Efet.01.601188.g53.t1 | DNA excision repair ERCC-8-like | 624 | 4.34E-56 | 62.75 |  |
| Efet.01.601191.g54.t1 | PREDICTED: uncharacterized protein LOC105340518 isoform X2 | 2154 | 4.05E-09 | 59.67 |  |
| Efet.01.601193.g55.t1 | methionine--tRNA ligase | 1809 | 0 | 91.9 | F:ATP binding; F:methionine-tRNA ligase activity; C:cytoplasm; F:metal ion binding; P:methionyl-tRNA aminoacylation; F:tRNA binding |
| Efet.01.601193.g56.t1 | patatin | 1065 | 0 | 93.95 | P:lipid metabolic process |
| Efet.01.601193.g57.t1 | RNA polymerase factor sigma-32 | 1782 | 0 | 96.45 | P:transcription initiation from bacterial-type RNA polymerase promoter; F:DNA binding; F:sigma factor activity; F:transcription factor activity, sequence-specific DNA binding; F:zinc ion binding; P:response to heat; C:cytoplasm; P:regulation of transcription, DNA-templated; P:sporulation resulting in formation of a cellular spore |
| Efet.01.601193.g58.t1 | family | 354 | 2.61E-78 | 79.3 | F:hydrolase activity |
| Efet.01.601193.g59.t1 | D-amino acid dehydrogenase | 1002 | 0 | 76.85 | F:D-amino-acid dehydrogenase activity; P:oxidation-reduction process |
| Efet.01.601193.g60.t1 | ABC transporter substrate-binding | 1653 | 0 | 80.35 | P:amino acid transport |
| Efet.01.601193.g61.t1 | branched-chain amino acid ABC transporter permease | 894 | 2.25E-141 | 83.4 | C:integral component of membrane; P:transport; C:plasma membrane; F:transporter activity |
| Efet.01.601193.g62.t1 | ABC transporter ATP-binding | 1428 | 3.35E-144 | 74 | F:ATPase activity; F:ATP binding; C:integral component of membrane; P:single-organism transport; C:plasma membrane; F:transporter activity; P:organic substance transport |
| Efet.01.601193.g63.t1 | ABC transporter ATP-binding | 498 | 2.58E-110 | 86.1 | F:ATP binding; P:polyamine transmembrane transport; F:polyamine-transporting ATPase activity |
| Efet.01.601239.g65.t1 | site-specific tyrosine recombinase | 291 | 1.96E-52 | 80.6 | P:cell division; F:DNA binding; C:cytoplasm; P:DNA integration; P:chromosome segregation; F:tyrosine-based site-specific recombinase activity; P:cell cycle; P:transposition, DNA-mediated |
| Efet.01.60127.g631.t1 | hairy enhancer-of-split related with YRPW motif 1-like | 615 | 1.38E-30 | 60.65 | C:nucleus; F:DNA binding; P:transcription, DNA-templated; P:regulation of transcription, DNA-templated; F:protein dimerization activity |
| Efet.01.601319.g66.t1 | protocadherin beta-18 | 2319 | 0 | 57.7 | C:membrane |
| Efet.01.6014.g477.t1 | Transcription elongation factor SPT6 | 1314 | 9.21E-163 | 80 | P:chromatin maintenance; P:RNA splicing; C:transcription elongation factor complex; P:chromatin silencing; P:positive regulation of transcription elongation from RNA polymerase II promoter; P:regulation of muscle cell differentiation; P:nucleosome organization; F:transcription factor activity, sequence-specific DNA binding; P:chromatin remodeling; F:translation elongation factor activity; P:viral process; P:otic vesicle development; P:regulation of isotype switching; P:regulation of mRNA export from nucleus; P:mRNA transport; P:mRNA transcription from RNA polymerase II promoter; P:negative regulation of histone H3-K27 methylation; F:nucleosome binding; P:mRNA processing; P:embryonic heart tube development; P:translational elongation; F:RNA binding; F:transcription factor activity, core RNA polymerase II binding; F:DNA binding; P:regulation of mRNA processing; F:histone binding; P:somitogenesis; C:transcriptionally active chromatin |
| Efet.01.601407.g69.t1 | hypothetical protein CAPTEDRAFT_226428 | 1602 | 3.34E-57 | 48 | F:calcium ion binding; C:membrane; C:integral component of membrane |
| Efet.01.601480.g71.t1 | 1,4-dihydroxy-2-naphthoate polyprenyltransferase | 900 | 5.51E-144 | 74.5 | F:prenyltransferase activity; C:integral component of membrane; C:plasma membrane |
| Efet.01.601635.g73.t1 | gamma-aminobutyric acid receptor alpha- | 537 | 1.61E-07 | 55.8 |  |
| Efet.01.601832.g75.t1 | dynein beta ciliary-like | 594 | 1.03E-105 | 85.1 | F:ATPase activity; F:ATP binding; C:motile cilium; F:microtubule motor activity; C:cytoplasm; P:cell projection organization; C:integral component of membrane; C:dynein complex; P:microtubule-based movement; C:microtubule |
| Efet.01.601834.g76.t1 | glutamate receptor-interacting 1 isoform X2 | 924 | 8.26E-32 | 74.3 | C:dendrite; C:membrane raft; P:protein localization; C:recycling endosome; F:receptor binding; C:plasma membrane; F:receptor signaling complex scaffold activity; P:dendrite development |
| Efet.01.601873.g77.t1 | type II toxin-antitoxin system family toxin | 561 | 1.35E-66 | 76.45 |  |
| Efet.01.601873.g78.t1 | XRE family transcriptional regulator | 348 | 5.52E-24 | 74.6 | F:sequence-specific DNA binding |
| Efet.01.601873.g79.t1 | toxin | 1347 | 0 | 90.25 | F:transferase activity |
| Efet.01.601873.g81.t1 | DUF1845 domain-containing | 921 | 6.24E-126 | 87.95 |  |
| Efet.01.601873.g82.t1 | hypothetical protein | 495 | 1.06E-59 | 69.45 |  |
| Efet.01.601896.g83.t1 | calcium calmodulin-dependent 3 ,5 -cyclic nucleotide phosphodiesterase 1A-like isoform X1 | 363 | 8.71E-15 | 74.9 | F:metal ion binding; P:signal transduction; F:3',5'-cyclic-nucleotide phosphodiesterase activity |
| Efet.01.601977.g85.t1 | Mastermind 1 | 351 | 6.91E-10 | 64 | F:transcription coactivator activity; P:positive regulation of transcription of Notch receptor target |
| Efet.01.602021.g86.t1 | oxidoreductase | 249 | 1.54E-24 | 90.2 | F:FAD binding; F:pentaerythritol trinitrate reductase activity; F:FMN binding; C:integral component of membrane; F:NADPH dehydrogenase activity; F:trichloro-p-hydroquinone reductive dehalogenase activity; P:oxidation-reduction process |
| Efet.01.602021.g87.t1 | oxidoreductase | 393 | 3.60E-86 | 86.3 | F:FAD binding; F:pentaerythritol trinitrate reductase activity; F:FMN binding; C:integral component of membrane; F:NADPH dehydrogenase activity; F:anthraniloyl-CoA monooxygenase activity; F:trichloro-p-hydroquinone reductive dehalogenase activity; P:oxidation-reduction process |
| Efet.01.602039.g89.t1 | Inositol phosphoceramide mannosyltransferase 3 | 558 | 5.41E-08 | 58.36 | C:membrane; C:integral component of membrane |
| Efet.01.602122.g91.t1 | iron dicitrate transport regulator | 423 | 2.15E-42 | 61.65 | C:membrane; C:integral component of membrane |
| Efet.01.60216.g634.t1 | Zinc finger SWIM domain-containing 8 | 381 | 1.56E-07 | 58.43 |  |
| Efet.01.602169.g92.t1 | DNA-binding Ikaros-like isoform X4 | 1599 | 3.83E-22 | 77.65 | P:regulation of B cell proliferation; C:nucleus; F:identical protein binding; C:cytoplasm; F:metal ion binding; F:protein heterodimerization activity; F:RNA polymerase II regulatory region sequence-specific DNA binding; P:regulation of B cell differentiation; F:transcriptional activator activity, RNA polymerase II transcription regulatory region sequence-specific binding; P:positive regulation of transcription from RNA polymerase II promoter |
| Efet.01.602313.g94.t1 | sugar ABC transporter ATP-binding | 897 | 2.29E-119 | 79.65 | F:ATPase activity; F:ATP binding |
| Efet.01.602342.g96.t1 | Neuroglobin | 345 | 1.49E-07 | 74 | F:binding; P:transport |
| Efet.01.602454.g98.t1 | voltage-dependent calcium channel gamma-5 subunit-like | 423 | 6.48E-19 | 67.4 | C:membrane |
| Efet.01.602456.g100.t1 | leucine-rich repeat and coiled-coil domain-containing 1 | 1617 | 1.75E-56 | 59.65 | C:intracellular part |
| Efet.01.602456.g99.t1 | leucine-rich repeat and coiled-coil domain-containing 1 | 1500 | 2.47E-80 | 61.35 | P:biological_process; F:molecular_function |
| Efet.01.60251.g636.t1 | translation initiation factor IF- mitochondrial isoform X2 | 501 | 4.67E-62 | 72.4 | F:nucleotide binding |
| Efet.01.602573.g102.t1 | transposase | 462 | 3.00E-100 | 85.25 | F:DNA binding |
| Efet.01.602573.g103.t1 | family transcriptional regulator | 840 | 2.90E-118 | 90.65 | F:DNA binding; F:transcription factor activity, sequence-specific DNA binding; P:transcription, DNA-templated; P:regulation of transcription, DNA-templated |
| Efet.01.602579.g104.t1 | EH domain-binding 1-like isoform X1 | 330 | 7.38E-19 | 69.2 |  |
| Efet.01.602628.g105.t1 | twitchin isoform X2 | 600 | 8.08E-59 | 59.7 | F:nucleotide binding; F:ATP binding; F:protein kinase activity; P:phosphorylation; F:transferase activity; P:protein phosphorylation; F:kinase activity |
| Efet.01.602630.g106.t1 | domain-containing | 720 | 3.63E-131 | 74.75 | F:catalytic activity; P:phosphorelay signal transduction system; P:metabolic process; C:intracellular |
| Efet.01.602643.g107.t1 | Kv channel-interacting 4-like isoform X2 | 219 | 2.75E-26 | 80.7 | F:calcium ion binding |
| Efet.01.602647.g108.t1 | type I DNA topoisomerase | 657 | 5.99E-107 | 77.65 | F:magnesium ion binding; F:DNA binding; F:DNA topoisomerase type I activity; P:DNA topological change |
| Efet.01.602649.g109.t1 | restriction endonuclease subunit S | 450 | 3.03E-62 | 88.55 | F:DNA binding; P:nucleic acid phosphodiester bond hydrolysis; F:N-methyltransferase activity; P:DNA methylation; F:endonuclease activity |
| Efet.01.602653.g110.t1 | DNA topoisomerase I | 456 | 9.43E-53 | 89.55 | F:magnesium ion binding; F:DNA binding; F:DNA topoisomerase type I activity; P:DNA topological change |
| Efet.01.602679.g111.t1 | glycogen-binding subunit 76A-like | 1188 | 3.89E-93 | 64.3 |  |
| Efet.01.602728.g112.t1 | COP9 signalosome complex subunit 1 | 231 | 3.67E-21 | 85.95 | C:COP9 signalosome; P:COP9 signalosome assembly |
| Efet.01.602794.g114.t1 | FMRFamide receptor-like | 1191 | 1.16E-108 | 54.9 | F:signal transducer activity |
| Efet.01.602830.g115.t1 | ATP-binding cassette sub-family A member 3-like | 834 | 4.87E-35 | 51.9 | F:nucleotide binding; F:ATPase activity; F:ATP binding; C:extracellular space; C:membrane; C:integral component of membrane; C:alveolar lamellar body; P:transport; C:plasma membrane; F:transporter activity |
| Efet.01.602849.g116.t1 | STE20-like serine threonine- kinase isoform X3 | 276 | 5.72E-41 | 83.4 | F:ATP binding; F:signal transducer, downstream of receptor, with serine/threonine kinase activity; C:cytoplasm; P:signal transduction by protein phosphorylation |
| Efet.01.60291.g640.t1 | neurogenic locus notch homolog 2-like | 768 | 2.58E-10 | 49.75 | F:chitin binding; F:calcium ion binding; C:collagen trimer; C:membrane; P:chitin metabolic process; C:integral component of membrane; C:extracellular region |
| Efet.01.60297.g641.t1 | zinc C2H2 | 273 | 1.30E-31 | 83.8 | P:negative regulation of transcription from RNA polymerase II promoter; C:nucleus; F:metal ion binding; P:transcription, DNA-templated; P:axon guidance; F:sequence-specific DNA binding; P:positive regulation of axon extension |
| Efet.01.602975.g118.t1 | hypothetical protein B5766_11380 | 729 | 2.48E-113 | 49.3 |  |
| Efet.01.603005.g119.t1 | AAA family ATPase | 1851 | 0 | 84.7 | F:ATP binding |
| Efet.01.603005.g120.t1 | rhodanese-like domain-containing | 435 | 1.71E-20 | 58.7 |  |
| Efet.01.603047.g121.t1 | fibrillin-2 isoform X1 | 366 | 1.45E-38 | 65.05 | P:sequestering of TGFbeta in extracellular matrix; P:embryonic limb morphogenesis; F:protein binding; F:calcium ion binding; C:microfibril; P:positive regulation of osteoblast differentiation; P:bone trabecula formation; P:positive regulation of bone mineralization |
| Efet.01.603047.g122.t1 | fibrillin-2-like | 405 | 9.55E-41 | 73.9 | P:regulation of cellular response to growth factor stimulus; P:anatomical structure morphogenesis; C:extracellular matrix; F:extracellular matrix structural constituent |
| Efet.01.603056.g123.t1 | ABC transporter ATP-binding permease | 1671 | 0 | 89.95 | F:ATP binding; F:ATPase activity, coupled to transmembrane movement of substances; P:transmembrane transport; C:integral component of membrane |
| Efet.01.603066.g124.t1 | hypothetical protein HELRODRAFT_68144, partial | 216 | 8.05E-06 | 71 |  |
| Efet.01.603079.g125.t1 | DNA primase | 657 | 3.64E-83 | 81.55 | F:magnesium ion binding; F:DNA primase activity; F:DNA binding; F:zinc ion binding; C:primosome complex; P:DNA replication, synthesis of RNA primer |
| Efet.01.603122.g126.t1 | thioredoxin 4A | 429 | 1.24E-99 | 96.35 | C:spliceosomal complex; P:mRNA splicing, via spliceosome |
| Efet.01.603152.g127.t1 | F-box WD repeat-containing | 318 | 8.09E-51 | 95.65 |  |
| Efet.01.60321.g642.t1 | chondroitin sulfate proteoglycan 4-like | 2907 | 1.74E-122 | 52 | C:integral component of plasma membrane; C:membrane; C:integral component of membrane; P:cell migration |
| Efet.01.603263.g128.t1 | coiled-coil domain-containing 148-like | 411 | 6.63E-17 | 61 | C:membrane; C:integral component of membrane |
| Efet.01.603287.g129.t1 | lactadherin-like isoform X5 | 381 | 4.41E-60 | 71.25 |  |
| Efet.01.60330.g643.t1 | STE20-like serine threonine- kinase | 558 | 1.07E-18 | 63.65 | F:nucleotide binding; F:ATP binding; F:protein kinase activity; F:protein serine/threonine kinase activity; P:protein phosphorylation |
| Efet.01.603337.g130.t1 | zinc finger 271-like isoform X2 | 3072 | 1.56E-57 | 52.8 | F:nucleic acid binding; F:metal ion binding |
| Efet.01.603394.g132.t1 | ATP-dependent exonuclease C subunit | 666 | 3.29E-93 | 74.7 |  |
| Efet.01.603471.g135.t1 | coronin-7 isoform X1 | 384 | 7.29E-39 | 53.75 |  |
| Efet.01.603579.g138.t1 | helicase with zinc finger domain 2-like | 3168 | 7.47E-111 | 50.55 | F:binding |
| Efet.01.603622.g139.t1 | polybromo-1-like isoform X2 | 417 | 8.46E-19 | 79.85 | C:BAF-type complex; P:chromatin remodeling; F:protein binding; P:negative regulation of cell proliferation; C:nuclear chromosome; P:mitotic nuclear division; F:chromatin binding; C:nucleoplasm |
| Efet.01.6037.g478.t1 | sodium potassium-transporting ATPase subunit alpha- | 306 | 2.36E-48 | 85.7 | P:sodium ion export from cell; P:ATP hydrolysis coupled proton transport; P:cardiocyte differentiation; F:sodium:potassium-exchanging ATPase activity; F:alpha-D-galactofuranose transporter activity; P:embryonic body morphogenesis; P:cellular sodium ion homeostasis; P:midbrain-hindbrain boundary development; P:brain morphogenesis; P:embryonic heart tube morphogenesis; P:determination of left/right symmetry; P:cell migration involved in heart formation; P:ventricular system development; C:integral component of plasma membrane; F:ATP binding; P:cellular potassium ion homeostasis; F:metal ion binding; P:otolith development; C:intracellular membrane-bounded organelle; P:otic vesicle formation; P:extracellular matrix organization; P:potassium ion import; P:bract development |
| Efet.01.603747.g140.t1 | interferon regulatory factor 4-like isoform X1 | 393 | 2.26E-15 | 67.55 | P:regulation of cellular process |
| Efet.01.603758.g141.t1 | dopamine D2-like receptor | 1116 | 1.08E-25 | 79.05 | C:integral component of plasma membrane; F:dopamine neurotransmitter receptor activity; P:dopamine receptor signaling pathway; F:transferase activity |
| Efet.01.603784.g142.t1 | serine threonine- phosphatase 2A 56 kDa regulatory subunit gamma isoform-like isoform X2 | 348 | 3.26E-44 | 83.1 | P:signal transduction; P:regulation of phosphoprotein phosphatase activity; C:protein phosphatase type 2A complex |
| Efet.01.603794.g143.t1 | Translation initiation factor eIF-2B subunit epsilon | 1464 | 2.07E-65 | 48.05 | F:translation initiation factor activity; F:nucleotidyltransferase activity; P:biosynthetic process; P:translational initiation |
| Efet.01.603871.g144.t1 | peptidase S8 | 1593 | 0 | 69.25 | F:peptidase activity |
| Efet.01.603871.g145.t1 | hypothetical protein CRN43_05950, partial | 999 | 1.73E-152 | 84 |  |
| Efet.01.603871.g146.t1 | ATPase | 978 | 1.69E-127 | 71.85 |  |
| Efet.01.603887.g147.t1 | RNA polymerase sigma-70 factor | 765 | 1.23E-152 | 60.4 | F:DNA binding; F:sigma factor activity; F:transcription factor activity, sequence-specific DNA binding; P:transcription, DNA-templated; P:regulation of transcription, DNA-templated; P:DNA-templated transcription, initiation |
| Efet.01.603890.g148.t1 | tincar-like isoform X1 | 1839 | 5.98E-35 | 49 | C:membrane; C:integral component of membrane |
| Efet.01.603923.g149.t1 | ferrochelatase | 888 | 1.87E-180 | 70.5 | P:porphyrin-containing compound biosynthetic process; F:lyase activity |
| Efet.01.604040.g150.t1 | alpha-2C adrenergic receptor-like | 1110 | 1.59E-25 | 74.15 | F:adrenergic receptor activity; C:integral component of membrane; P:adrenergic receptor signaling pathway; C:plasma membrane |
| Efet.01.604040.g151.t1 | alpha-2C adrenergic receptor-like | 471 | 4.79E-35 | 75.3 | F:adrenergic receptor activity; C:integral component of membrane; P:adrenergic receptor signaling pathway; C:plasma membrane |
| Efet.01.60412.g644.t1 | lysosomal-associated transmembrane 4A-like isoform X2 | 207 | 1.95E-07 | 70.4 | C:membrane; C:integral component of membrane |
| Efet.01.604212.g154.t1 | zinc protease | 1395 | 4.58E-145 | 92.45 | F:metal ion binding; P:proteolysis; F:peptidase activity |
| Efet.01.604212.g155.t1 | pantetheine-phosphate adenylyltransferase | 504 | 5.50E-118 | 95.1 | F:ATP binding; C:cytoplasm; P:coenzyme A biosynthetic process; F:pantetheine-phosphate adenylyltransferase activity |
| Efet.01.604212.g156.t1 | diaminopimelate decarboxylase | 1029 | 0 | 80.3 | F:diaminopimelate decarboxylase activity; F:pyridoxal phosphate binding; P:lysine biosynthetic process via diaminopimelate |
| Efet.01.604212.g157.t1 | nitrate ABC transporter substrate-binding | 909 | 0 | 89.2 |  |
| Efet.01.604222.g159.t1 | MinD-like ATPase involved in chromosome partitioning or flagellar assembly | 813 | 5.68E-123 | 54.71 |  |
| Efet.01.604243.g160.t1 | family transcriptional regulator | 1047 | 0 | 73.9 | F:DNA binding; F:transcription factor activity, sequence-specific DNA binding; P:transcription, DNA-templated; P:regulation of transcription, DNA-templated |
| Efet.01.604243.g161.t1 | ABC transporter permease | 672 | 6.80E-115 | 82.35 | C:integral component of membrane; P:transport; C:plasma membrane |
| Efet.01.604243.g162.t1 | ABC transporter permease | 558 | 9.22E-97 | 88.05 | C:integral component of membrane; P:transport; C:plasma membrane |
| Efet.01.604243.g163.t1 | ABC transporter ATP-binding | 798 | 0 | 82.35 | F:ATPase activity; F:ATP binding |
| Efet.01.604269.g164.t1 | histidinol dehydrogenase | 927 | 0 | 90.35 | F:zinc ion binding; F:NAD binding; P:oxidation-reduction process; P:histidine biosynthetic process; F:histidinol dehydrogenase activity |
| Efet.01.604269.g165.t1 | NAD(P)-dependent oxidoreductase | 423 | 3.17E-94 | 87.65 | F:oxidoreductase activity; P:oxidation-reduction process |
| Efet.01.60429.g645.t1 | Cation transporter family | 594 | 5.38E-86 | 75.1 | C:postsynaptic membrane; P:cation transmembrane transport; C:integral component of membrane; C:cell junction; F:extracellular ligand-gated ion channel activity |
| Efet.01.60444.g646.t1 | sex peptide receptor-like | 1044 | 5.07E-62 | 61.25 | F:G-protein coupled receptor activity; C:membrane |
| Efet.01.604603.g169.t1 | family -linked outer membrane | 1974 | 3.47E-150 | 60.4 | C:membrane; F:receptor activity; P:transport; C:outer membrane |
| Efet.01.604633.g170.t1 | serine threonine- phosphatase 2A 56 kDa regulatory subunit alpha isoform | 336 | 2.92E-55 | 89.35 | P:negative regulation of establishment of protein localization to plasma membrane; P:signal transduction; C:protein phosphatase type 2A complex; C:chromosome, centromeric region; C:nucleus; C:Z disc; C:membrane; P:positive regulation of protein dephosphorylation; C:M band; P:regulation of phosphoprotein phosphatase activity; F:kinase binding; F:protein phosphatase regulator activity; P:negative regulation of lipid kinase activity |
| Efet.01.604636.g171.t1 | EGF-like repeat and discoidin I-like domain-containing 3 | 720 | 5.94E-16 | 45.3 | F:oligosaccharide binding; F:calcium ion binding; C:membrane; P:homophilic cell adhesion via plasma membrane adhesion molecules |
| Efet.01.604656.g173.t1 | Ankyrin repeat | 1098 | 1.79E-19 | 47.7 |  |
| Efet.01.604680.g174.t1 | hyperpolarizaion-activated cyclic nucleotide-gated cation channel | 396 | 3.16E-26 | 68.65 | P:potassium ion transport; F:ion channel activity; P:ion transmembrane transport; C:integral component of membrane |
| Efet.01.604684.g175.t1 | FMRFamide receptor-like | 561 | 2.92E-26 | 49.5 | F:G-protein coupled receptor activity; P:signal transduction; C:membrane |
| Efet.01.604766.g177.t1 | GTP-binding | 441 | 4.28E-70 | 93.1 | F:GTP binding; F:GTPase activity |
| Efet.01.604801.g179.t1 | Dual specificity phosphatase | 429 | 3.97E-33 | 67.8 | F:phosphatase activity; P:dephosphorylation |
| Efet.01.604816.g180.t1 | hypothetical protein CAPTEDRAFT_222159 | 507 | 2.55E-32 | 55.25 | C:membrane |
| Efet.01.604828.g181.t1 | metabotropic glutamate receptor 7-like isoform X2 | 2142 | 1.71E-99 | 48.2 | F:G-protein coupled receptor activity; P:G-protein coupled receptor signaling pathway; P:signal transduction; C:membrane; C:integral component of membrane; F:signal transducer activity; C:plasma membrane |
| Efet.01.604849.g182.t1 | family | 408 | 6.71E-30 | 45.2 | P:transport; F:transporter activity |
| Efet.01.604870.g183.t1 | shikimate dehydrogenase | 798 | 1.50E-97 | 71.15 | F:oxidoreductase activity |
| Efet.01.604910.g184.t1 | NADH-quinone oxidoreductase subunit N | 828 | 2.91E-51 | 60.25 | F:oxidoreductase activity; C:membrane |
| Efet.01.60492.g649.t1 | cAMP-dependent kinase catalytic subunit (PKA) | 1707 | 5.19E-07 | 53.5 | F:nucleotide binding; F:ATP binding; F:protein kinase activity; P:phosphorylation; F:protein serine/threonine kinase activity; P:protein phosphorylation; F:kinase activity |
| Efet.01.604964.g185.t1 | cobalamin biosynthesis | 516 | 2.81E-53 | 62.4 | P:cobalamin biosynthetic process; F:sirohydrochlorin cobaltochelatase activity |
| Efet.01.604979.g186.t1 | O-antigen teichoic acid export membrane | 912 | 6.06E-65 | 59.67 | C:membrane |
| Efet.01.605198.g191.t1 | hypothetical protein CAPTEDRAFT_202552 | 1335 | 2.28E-13 | 79.75 | C:integral component of membrane |
| Efet.01.60520.g650.t1 | SH3 and multiple ankyrin repeat domains 3 isoform X4 | 231 | 1.50E-06 | 85 |  |
| Efet.01.60523.g651.t1 | Zinc carboxypeptidase A 1 | 252 | 3.20E-15 | 69.85 | F:carboxypeptidase activity |
| Efet.01.60525.g652.t1 | atrial natriuretic peptide receptor 3-like | 744 | 1.79E-10 | 48 |  |
| Efet.01.605259.g192.t1 | conserved oligomeric Golgi complex subunit 5-like | 474 | 2.92E-52 | 80.6 | C:Golgi transport complex; P:intra-Golgi vesicle-mediated transport |
| Efet.01.605275.g196.t1 | galactoside 2-alpha-L-fucosyltransferase 2-like | 1068 | 8.45E-48 | 48.55 | P:carbohydrate metabolic process; F:transferase activity, transferring glycosyl groups; P:fucosylation; C:membrane; C:integral component of membrane; F:transferase activity; F:galactoside 2-alpha-L-fucosyltransferase activity |
| Efet.01.605295.g198.t1 | Xaa-Pro aminopeptidase | 459 | 1.42E-82 | 80.65 | P:proteolysis; F:aminopeptidase activity; F:manganese ion binding |
| Efet.01.605308.g199.t1 | OTU domain-containing 7B-like isoform X1 | 450 | 3.10E-39 | 63.2 | F:DNA binding; C:nucleus; P:protein K11-linked deubiquitination; F:zinc ion binding; F:peptidase activity; P:mRNA transport |
| Efet.01.605357.g201.t1 | lipo -releasing system transmembrane subunit | 435 | 9.49E-75 | 95.25 | F:lipoprotein transporter activity; P:cell division; C:integral component of membrane; C:plasma membrane; P:lipoprotein transport |
| Efet.01.605357.g202.t1 | lipo -releasing system ATP-binding | 483 | 1.71E-76 | 73.9 | F:nucleotide binding; P:transport; C:plasma membrane; F:hydrolase activity |
| Efet.01.605357.g203.t1 | acetoacetate-- ligase | 597 | 1.81E-114 | 92.65 | F:acetoacetate-CoA ligase activity; P:lipid metabolic process |
| Efet.01.605364.g204.t1 | MBL fold hydrolase | 333 | 4.38E-35 | 78.45 | F:hydrolase activity |
| Efet.01.605364.g205.t1 | catabolic enzyme | 252 | 8.47E-49 | 61.75 | F:lyase activity |
| Efet.01.605365.g206.t1 | OHCU decarboxylase | 603 | 1.62E-132 | 86.4 | P:allantoin biosynthetic process; F:hydrolase activity, acting on carbon-nitrogen (but not peptide) bonds, in linear amidines; F:N-carbamoyl-L-amino-acid hydrolase activity |
| Efet.01.605365.g207.t1 | allantoinase | 972 | 0 | 91.2 | F:hydrolase activity, acting on carbon-nitrogen (but not peptide) bonds; P:carbohydrate metabolic process |
| Efet.01.605365.g208.t1 | hydroxyisourate hydrolase | 351 | 1.19E-66 | 90.65 | F:hydroxyisourate hydrolase activity; P:purine nucleobase metabolic process |
| Efet.01.605365.g209.t1 | MFS transporter | 1281 | 0 | 83.4 | P:transmembrane transport; C:integral component of membrane |
| Efet.01.605365.g210.t1 | imidazole glycerol phosphate synthase subunit | 774 | 2.27E-157 | 89.6 | C:cytoplasm; F:lyase activity; F:imidazoleglycerol-phosphate synthase activity; P:histidine biosynthetic process |
| Efet.01.605365.g211.t1 | 1-(5-phosphoribosyl)-5-[(5-phosphoribosylamino)methylideneamino]imidazole-4-carboxamide isomerase | 738 | 4.12E-174 | 97.15 | C:cytoplasm; F:1-(5-phosphoribosyl)-5-[(5-phosphoribosylamino)methylideneamino]imidazole-4-carboxamide isomerase activity; P:histidine biosynthetic process |
| Efet.01.605365.g212.t1 | imidazole glycerol phosphate synthase subunit | 669 | 2.43E-162 | 92.15 | C:cytoplasm; P:glutamine metabolic process; F:imidazoleglycerol-phosphate synthase activity; P:histidine biosynthetic process |
| Efet.01.605365.g213.t1 | imidazoleglycerol-phosphate dehydratase | 1275 | 3.95E-131 | 89.45 | F:imidazoleglycerol-phosphate dehydratase activity; F:pyridoxal phosphate binding; F:L-phenylalanine:2-oxoglutarate aminotransferase activity; C:cytoplasm; P:histidine biosynthetic process; F:histidinol-phosphate transaminase activity |
| Efet.01.605365.g214.t1 | nucleotidyl transferase ii ii toxin family | 762 | 3.32E-121 | 63.95 | F:transferase activity |
| Efet.01.605365.g215.t1 | S-adenosylhomocysteine hydrolase | 426 | 1.21E-62 | 69.65 |  |
| Efet.01.605365.g216.t1 | family transcriptional regulator | 543 | 2.68E-30 | 53.4 | F:DNA binding; F:transcription factor activity, sequence-specific DNA binding; P:transcription, DNA-templated; P:regulation of transcription, DNA-templated |
| Efet.01.605426.g218.t1 | Leucine-rich repeat and fibronectin type III domain-containing 1 | 1383 | 2.43E-144 | 54.9 | C:membrane |
| Efet.01.605428.g219.t1 | coiled-coil domain-containing lobo homolog | 507 | 4.61E-11 | 51.4 |  |
| Efet.01.605435.g220.t1 | purine-nucleoside phosphorylase | 474 | 4.30E-56 | 89.4 | P:nucleoside metabolic process; F:purine-nucleoside phosphorylase activity |
| Efet.01.605483.g221.t1 | GGDEF domain-containing | 1542 | 0 | 62.85 | C:integral component of membrane |
| Efet.01.605483.g222.t1 | phosphonate metabolism 1,5-bisphosphokinase (PRPP-forming) | 567 | 5.42E-108 | 74.65 | F:ATPase activity; F:ATP binding; F:ribose 1,5-bisphosphate phosphokinase activity; P:organic phosphonate metabolic process; P:carbohydrate phosphorylation; P:5-phosphoribose 1-diphosphate biosynthetic process |
| Efet.01.605483.g223.t1 | DUF1045 domain-containing | 504 | 5.09E-79 | 88.1 |  |
| Efet.01.605483.g224.t1 | carbon-phosphorus lyase complex subunit | 2133 | 0 | 83.35 | F:lyase activity; P:organic phosphonate metabolic process |
| Efet.01.605483.g225.t1 | carbon-phosphorus lyase | 840 | 6.80E-175 | 97 | F:metal ion binding; P:alkylphosphonate transport; F:alpha-D-ribose 1-methylphosphonate 5-phosphate C-P-lyase activity; F:4 iron, 4 sulfur cluster binding; P:organic phosphonate catabolic process |
| Efet.01.605483.g226.t1 | alpha-D-ribose 1-methylphosphonate 5-triphosphate diphosphatase | 1665 | 1.85E-131 | 86.25 | F:hydrolase activity, acting on carbon-nitrogen (but not peptide) bonds; F:ATPase activity; F:ATP binding; F:lyase activity; P:organic phosphonate catabolic process |
| Efet.01.605501.g228.t1 | DNA topoisomerase (ATP-hydrolyzing) subunit B | 489 | 6.54E-78 | 90.5 | F:magnesium ion binding; F:DNA binding; F:ATP binding; C:cytoplasm; P:DNA-dependent DNA replication; C:chromosome; F:DNA topoisomerase type II (ATP-hydrolyzing) activity; P:DNA topological change |
| Efet.01.605603.g231.t1 | tyrosine- kinase BTK isoform X1 | 3231 | 3.93E-164 | 58 | P:signal transduction; C:membrane; F:protein kinase activity; F:binding; P:protein phosphorylation; C:intracellular part |
| Efet.01.605603.g232.t1 | L-asparaginase-like | 285 | 2.34E-15 | 57.7 | P:cellular amino acid metabolic process; F:asparaginase activity |
| Efet.01.605637.g233.t1 | E3 ubiquitin- ligase RNF213 | 321 | 1.08E-10 | 62.15 | F:ATPase activity; F:ubiquitin-protein transferase activity; F:zinc ion binding; P:protein ubiquitination |
| Efet.01.605638.g234.t1 | zinc finger MIZ domain-containing 2 | 447 | 8.93E-70 | 91.15 | F:zinc ion binding; C:mitochondrion; C:nuclear replication fork; F:ligand-dependent nuclear receptor transcription coactivator activity; P:positive regulation of transcription from RNA polymerase II promoter; C:nucleoplasm |
| Efet.01.605673.g235.t1 | Fe-S cluster assembly ATPase | 507 | 1.09E-119 | 93.3 | F:ATPase activity; F:ATP binding; P:transport |
| Efet.01.605755.g236.t1 | glycoside hydrolase family 3 | 603 | 2.04E-113 | 58.45 | P:metabolic process; F:hydrolase activity, hydrolyzing O-glycosyl compounds |
| Efet.01.605763.g237.t1 | cartilage matrix | 333 | 1.61E-20 | 63.6 | C:collagen trimer; F:calcium ion binding |
| Efet.01.60578.g653.t1 | unconventional myosin-XVIIIa-like | 1176 | 8.90E-44 | 60.1 | C:myosin complex; F:ATP binding; F:motor activity |
| Efet.01.605795.g238.t1 | xylose isomerase | 342 | 1.27E-46 | 43.5 | F:isomerase activity |
| Efet.01.605795.g239.t1 | family transcriptional regulator | 924 | 1.78E-127 | 51.3 |  |
| Efet.01.605802.g240.t1 | lactam utilization | 780 | 3.51E-174 | 86.25 | P:carbohydrate metabolic process; F:catalytic activity |
| Efet.01.605802.g241.t1 | acetyl- carboxylase biotin carboxyl carrier subunit | 246 | 1.92E-49 | 77.75 | F:acetyl-CoA carboxylase activity; P:fatty acid biosynthetic process; C:acetyl-CoA carboxylase complex |
| Efet.01.605843.g244.t1 | sodium potassium-transporting ATPase subunit alpha-1-like isoform X1 [Orbicella faveolata] | 285 | 3.62E-49 | 89.65 | P:potassium ion transport; F:ATP binding; P:sodium ion transport; F:sodium:potassium-exchanging ATPase activity; F:metal ion binding; P:ATP hydrolysis coupled transmembrane transport; C:integral component of membrane; P:establishment or maintenance of transmembrane electrochemical gradient |
| Efet.01.605872.g245.t1 | aspartate aminotransferase family | 753 | 1.16E-137 | 94.75 | F:pyridoxal phosphate binding; F:transaminase activity |
| Efet.01.605884.g246.t1 | S49 family peptidase | 312 | 3.51E-48 | 96.25 | P:proteolysis; C:integral component of membrane; F:peptidase activity |
| Efet.01.605884.g247.t1 | peptidase S49 | 306 | 5.40E-59 | 90.9 | P:proteolysis; C:integral component of membrane; F:peptidase activity |
| Efet.01.605884.g249.t1 | FHA domain-containing | 558 | 2.46E-95 | 71.3 |  |
| Efet.01.605974.g251.t1 | DNA ligase 4 | 297 | 8.00E-13 | 68.2 | F:nucleotide binding; P:DNA ligation involved in DNA repair; F:DNA ligase (ATP) activity; C:DNA-dependent protein kinase-DNA ligase 4 complex; C:DNA ligase IV complex; P:lagging strand elongation; C:cytoplasm; P:nucleotide-excision repair, DNA gap filling; P:double-strand break repair via nonhomologous end joining |
| Efet.01.606065.g252.t1 | zinc finger 26-like isoform X3 | 1611 | 8.08E-47 | 51.45 | F:nucleic acid binding; F:metal ion binding |
| Efet.01.60607.g655.t1 | Sodium-independent sulfate anion transporter | 282 | 2.67E-08 | 58.67 | P:transport |
| Efet.01.606117.g253.t1 | serine threonine- kinase PLK1 | 300 | 2.86E-37 | 84.35 | P:mitotic spindle assembly checkpoint; C:nucleolus; C:synaptonemal complex; P:negative regulation of transcription from RNA polymerase II promoter; F:ATP binding; P:G2/M transition of mitotic cell cycle; P:positive regulation of ubiquitin-protein transferase activity; P:regulation of protein binding; P:mitotic cytokinesis; P:G2 DNA damage checkpoint; F:anaphase-promoting complex binding; F:protein kinase binding; P:regulation of mitotic spindle assembly; C:spindle pole; C:condensed nuclear chromosome outer kinetochore; F:protein serine/threonine kinase activity; C:chromatin; P:negative regulation of cyclin-dependent protein serine/threonine kinase activity; F:microtubule binding; P:protein destabilization; P:establishment of protein localization; C:spindle microtubule; C:centrosome; C:spindle midzone; P:microtubule bundle formation; P:synaptonemal complex disassembly; P:centrosome organization; C:cytoplasm; P:protein localization to chromatin; P:negative regulation of apoptotic process; P:homologous chromosome segregation; P:mitotic sister chromatid segregation; P:positive regulation of peptidyl-threonine phosphorylation; C:midbody; P:female meiosis chromosome segregation; P:peptidyl-serine phosphorylation; P:positive regulation of proteasomal ubiquitin-dependent protein catabolic process; P:protein ubiquitination |
| Efet.01.606285.g260.t1 | aldehyde dehydrogenase | 1437 | 0 | 84.25 | P:oxidation-reduction process; F:lactaldehyde dehydrogenase activity |
| Efet.01.606285.g261.t1 | Rieske (2Fe-2S) | 588 | 2.82E-121 | 68.65 | F:oxidoreductase activity; F:metal ion binding; C:membrane; P:oxidation-reduction process; F:2 iron, 2 sulfur cluster binding |
| Efet.01.606285.g262.t1 | PQQ-dependent methanol ethanol family | 1680 | 0 | 83.95 | F:oxidoreductase activity, acting on CH-OH group of donors; F:calcium ion binding; C:membrane; P:oxidation-reduction process |
| Efet.01.606285.g263.t1 | sigma-54-dependent Fis family transcriptional regulator | 384 | 5.55E-62 | 68.25 | F:ATP binding; P:transcription, DNA-templated; P:phosphorelay signal transduction system; P:regulation of transcription, DNA-templated; F:sequence-specific DNA binding; F:transcription factor binding; C:intracellular |
| Efet.01.606285.g264.t1 | sigma-54-dependent Fis family transcriptional regulator | 1404 | 0 | 66.15 | F:ATP binding; P:transcription, DNA-templated; P:phosphorelay signal transduction system; P:regulation of transcription, DNA-templated; F:sequence-specific DNA binding; F:transcription factor binding; C:intracellular |
| Efet.01.606285.g265.t1 | alpha beta hydrolase | 969 | 0 | 77.2 | F:hydrolase activity |
| Efet.01.606285.g266.t1 | alpha beta hydrolase | 444 | 2.58E-97 | 72.6 | F:hydrolase activity |
| Efet.01.606285.g267.t1 | family transcriptional regulator | 1239 | 0 | 64.85 | F:DNA binding; P:transcription, DNA-templated; P:regulation of transcription, DNA-templated |
| Efet.01.606285.g268.t1 | porin | 1044 | 0 | 59.45 | P:transmembrane transport; F:porin activity; C:membrane; P:transport |
| Efet.01.606285.g269.t1 | pimeloyl- dehydrogenase small subunit | 1689 | 0 | 78.6 | F:acyl-CoA dehydrogenase activity; F:flavin adenine dinucleotide binding; P:oxidation-reduction process |
| Efet.01.606285.g270.t1 | pimeloyl- dehydrogenase large subunit | 540 | 3.33E-92 | 78 | F:acyl-CoA dehydrogenase activity; F:flavin adenine dinucleotide binding; P:oxidation-reduction process |
| Efet.01.606285.g271.t1 | 3-hydroxyacyl- dehydrogenase | 1647 | 0 | 84.25 | P:fatty acid beta-oxidation; F:3-hydroxyacyl-CoA dehydrogenase activity; F:3-hydroxybutyryl-CoA epimerase activity; F:dodecenoyl-CoA delta-isomerase activity; F:enoyl-CoA hydratase activity |
| Efet.01.60629.g657.t1 | neuralized 4 | 405 | 6.09E-28 | 87.2 |  |
| Efet.01.606340.g272.t1 | prolactin-releasing peptide receptor-like | 546 | 1.12E-31 | 63.75 | P:neuropeptide signaling pathway; C:integral component of membrane; F:neuropeptide Y receptor activity |
| Efet.01.606395.g274.t1 | delta 1 | 1053 | 1.19E-42 | 56.75 | P:regulation of cellular process; P:cell communication; P:multicellular organism development; C:membrane |
| Efet.01.606399.g275.t1 | homeobox cut-like isoform X4 | 492 | 2.10E-50 | 78.45 | C:nucleus; P:transcription, DNA-templated; P:regulation of transcription, DNA-templated; F:sequence-specific DNA binding |
| Efet.01.6064.g479.t1 | Calcium-responsive transcription factor | 1689 | 1.17E-116 | 50.75 |  |
| Efet.01.606404.g276.t1 | acyl- dehydrogenase | 612 | 8.27E-126 | 66.35 | F:oxidoreductase activity, acting on the CH-CH group of donors; P:single-organism metabolic process |
| Efet.01.606404.g277.t1 | acyl- dehydrogenase | 1176 | 0 | 83.35 | F:acyl-CoA dehydrogenase activity; C:peroxisome; F:acyl-CoA oxidase activity; P:fatty acid beta-oxidation; F:flavin adenine dinucleotide binding |
| Efet.01.606486.g279.t1 | glucokinase | 1014 | 0 | 79.85 | F:ATP binding; P:glucose 6-phosphate metabolic process; P:glycolytic process; F:glucokinase activity |
| Efet.01.606486.g280.t1 | 2-alkenal reductase | 1185 | 0 | 87.6 | F:2-alkenal reductase [NAD(P)] activity; P:proteolysis; F:serine-type endopeptidase activity; P:oxidation-reduction process |
| Efet.01.606486.g281.t1 | Nif3-like dinuclear metal center hexameric | 759 | 1.51E-154 | 90.2 |  |
| Efet.01.606493.g282.t1 | PD-(D E)XK nuclease family | 744 | 6.39E-142 | 67.4 | F:helicase activity |
| Efet.01.606524.g283.t1 | prickle 3 isoform X2 | 1287 | 5.98E-38 | 72.45 | F:metal ion binding |
| Efet.01.606599.g285.t1 | chromatin-remodeling complex ATPase chain Iswi | 213 | 4.53E-25 | 97.35 | F:ATPase activity; F:DNA binding; F:ATP binding; F:nucleosome binding; P:ATP-dependent chromatin remodeling; F:helicase activity; C:NURF complex |
| Efet.01.606668.g286.t1 | GTP-binding Rhes | 654 | 1.02E-42 | 59.05 | F:nucleotide binding; F:GTP binding; P:small GTPase mediated signal transduction; P:signal transduction; C:membrane; C:intracellular |
| Efet.01.606690.g287.t1 | ATP-dependent Clp protease adaptor | 282 | 2.21E-37 | 84.75 | P:protein catabolic process; P:proteolysis; F:peptidase activity |
| Efet.01.606853.g288.t1 | PREDICTED: uncharacterized protein LOC101849946 | 597 | 8.22E-06 | 48 |  |
| Efet.01.606919.g290.t1 | rho GTPase-activating 39-like isoform X9 | 378 | 1.71E-41 | 77.45 | C:membrane |
| Efet.01.606945.g291.t1 | RNA polymerase sigma-70 factor | 402 | 6.38E-38 | 60 | F:sigma factor activity; F:DNA binding; F:transcription factor activity, sequence-specific DNA binding; P:transcription, DNA-templated; P:regulation of transcription, DNA-templated; P:DNA-templated transcription, initiation |
| Efet.01.606970.g292.t1 | rapamycin-insensitive companion of mTOR isoform X3 | 219 | 9.14E-12 | 65.25 | P:regulation of response to stimulus; P:positive regulation of cellular process; P:regulation of protein phosphorylation; P:single-organism process; P:cellular process |
| Efet.01.60699.g661.t1 | diacylglycerol kinase zeta-like isoform X6 | 288 | 1.95E-10 | 86.25 | F:ATP binding; F:diacylglycerol kinase activity; P:phosphorylation; P:protein kinase C-activating G-protein coupled receptor signaling pathway |
| Efet.01.60700.g662.t1 | PDZ domain-containing 7 | 519 | 5.51E-22 | 57.75 | P:establishment of protein localization; P:auditory receptor cell stereocilium organization; C:nucleus; C:USH2 complex; C:extracellular space; F:protein homodimerization activity; F:protein heterodimerization activity; C:cilium; C:stereocilia ankle link; P:detection of mechanical stimulus involved in sensory perception of sound; P:auditory receptor cell development |
| Efet.01.607027.g294.t1 | Cleft lip and palate transmembrane 1 | 390 | 4.53E-11 | 61.95 | C:membrane; P:apoptotic process; C:integral component of membrane |
| Efet.01.60706.g663.t1 | monocarboxylate transporter 9-like isoform X3 | 870 | 1.55E-44 | 48.1 | C:integral component of plasma membrane; P:transmembrane transport; C:membrane; C:integral component of membrane; P:plasma membrane lactate transport; F:lactate transmembrane transporter activity |
| Efet.01.607077.g295.t1 | embryonic polarity dorsal-like isoform X1 | 381 | 5.43E-38 | 79.05 | C:nucleus; F:DNA binding; F:transcription factor activity, sequence-specific DNA binding; C:cytoplasm; P:regulation of transcription, DNA-templated |
| Efet.01.60717.g665.t1 | phosphatidylcholine-sterol acyltransferase | 240 | 8.80E-30 | 79.25 | F:O-acyltransferase activity; P:lipid metabolic process |
| Efet.01.607171.g296.t1 | cilia- and flagella-associated 45-like | 501 | 1.26E-36 | 75.2 |  |
| Efet.01.607352.g298.t1 | hypothetical protein CAPTEDRAFT_222268 | 912 | 1.50E-42 | 60 |  |
| Efet.01.607401.g300.t1 | NACHT domain- and WD repeat-containing 1-like isoform X1 | 3333 | 2.26E-96 | 55.75 |  |
| Efet.01.607412.g302.t1 | FAD-binding oxidoreductase | 2682 | 0 | 84.2 | F:oxidoreductase activity; P:oxidation-reduction process |
| Efet.01.607412.g303.t1 | FAD-binding oxidoreductase | 1815 | 0 | 76.65 | F:oxidoreductase activity; C:integral component of membrane; P:oxidation-reduction process; P:transport; C:plasma membrane |
| Efet.01.607412.g304.t1 | ABC transporter ATP-binding | 1851 | 0 | 82.15 | F:ATP binding; F:substrate-specific transmembrane transporter activity; F:ATPase activity, coupled to transmembrane movement of substances; P:ion transport; P:transmembrane transport; P:polyamine transport; C:ATP-binding cassette (ABC) transporter complex |
| Efet.01.607412.g305.t1 | branched-chain amino acid ABC transporter substrate-binding | 1038 | 0 | 82.9 |  |
| Efet.01.607412.g306.t1 | transcriptional regulator | 735 | 9.92E-162 | 69.15 | F:DNA binding; F:transcription factor activity, sequence-specific DNA binding; P:transcription, DNA-templated; P:regulation of transcription, DNA-templated |
| Efet.01.607412.g307.t1 | pyruvate kinase | 441 | 1.84E-93 | 95.25 | F:magnesium ion binding; F:pyruvate kinase activity; F:potassium ion binding; P:glycolytic process; F:kinase activity |
| Efet.01.607412.g308.t1 | pyruvate kinase | 969 | 9.13E-173 | 95.4 | F:magnesium ion binding; F:pyruvate kinase activity; F:potassium ion binding; P:glycolytic process; F:kinase activity |
| Efet.01.607412.g309.t1 | Xaa-Pro aminopeptidase | 1401 | 0 | 91.15 | P:proteolysis; F:aminopeptidase activity; F:manganese ion binding |
| Efet.01.607412.g310.t1 | nucleotidyltransferase family | 735 | 2.32E-134 | 83.45 | F:transferase activity |
| Efet.01.607412.g311.t1 | transketolase | 1983 | 0 | 87.15 | F:transketolase activity; F:metal ion binding; P:metabolic process |
| Efet.01.607412.g312.t1 | type I glyceraldehyde-3-phosphate dehydrogenase | 1002 | 0 | 94.7 | F:NADP binding; F:NAD binding; P:oxidation-reduction process; P:glucose metabolic process; F:oxidoreductase activity, acting on the aldehyde or oxo group of donors, NAD or NADP as acceptor |
| Efet.01.607412.g313.t1 | antibiotic biosynthesis monooxygenase | 363 | 7.24E-83 | 70.2 | P:oxidation-reduction process; F:monooxygenase activity |
| Efet.01.60746.g667.t1 | chromatin modification-related EAF7- | 759 | 1.80E-29 | 53.4 |  |
| Efet.01.607469.g314.t1 | slit homolog 2 -like isoform X2 | 258 | 2.91E-20 | 75.65 | F:calcium ion binding; C:integral component of membrane; C:extracellular region |
| Efet.01.60754.g669.t1 | disintegrin and metallo ase domain-containing 12-like | 267 | 2.24E-07 | 60.2 | P:positive regulation of T cell differentiation in thymus; C:phagolysosome; P:regulation of cell-cell adhesion; F:cell adhesion molecule binding; P:positive regulation of cell adhesion; F:metalloendopeptidase activity; P:positive regulation of NF-kappaB transcription factor activity; P:negative regulation of neuron apoptotic process; C:podosome; P:positive regulation of eosinophil migration; P:positive regulation of acute inflammatory response; C:integral component of plasma membrane; P:inflammatory response; P:positive regulation of fibronectin-dependent thymocyte migration; P:positive regulation of membrane protein ectodomain proteolysis; P:activation of MAPK activity involved in innate immune response; P:lymphocyte chemotaxis; P:angiogenesis; F:calcium ion binding; C:specific granule; C:alpha9-beta1 integrin-ADAM8 complex; C:tertiary granule; P:positive regulation of thymocyte apoptotic process; P:cellular response to hypoxia; C:dense core granule membrane; P:positive regulation of neutrophil extravasation; P:single organismal cell-cell adhesion; F:zinc ion binding; P:cell morphogenesis; P:positive regulation of inflammatory response; P:positive regulation of cellular extravasation; C:plasma membrane; F:metallopeptidase activity; C:cell surface; C:alpha9-beta1 integrin-vascular cell adhesion molecule-1 complex; P:positive regulation of T cell migration; P:leukocyte migration involved in inflammatory response; C:cytoplasm; P:positive regulation of bone resorption; C:membrane; P:positive regulation of tumor necrosis factor (ligand) superfamily member 11 production; P:proteolysis; C:integral component of membrane; P:positive regulation of protein kinase B signaling |
| Efet.01.607560.g316.t1 | tumor necrosis factor alpha-induced 3 | 1224 | 5.86E-27 | 55.85 | P:protein deubiquitination; P:regulation of protein metabolic process; P:response to organic substance; F:protein binding; P:negative regulation of signal transduction; P:regulation of innate immune response; P:regulation of response to external stimulus; P:negative regulation of cytokine production; P:negative regulation of immune system process; P:response to oxygen-containing compound; P:regulation of cellular metabolic process |
| Efet.01.607589.g318.t1 | hypothetical protein CRN43_16865 | 339 | 1.42E-76 | 75.85 |  |
| Efet.01.607618.g320.t1 | neuropeptide receptor 15-like | 447 | 2.75E-31 | 76.9 | F:thyrotropin-releasing hormone receptor activity; P:G-protein coupled receptor signaling pathway; C:integral component of membrane |
| Efet.01.607698.g321.t1 | peptide ABC transporter substrate-binding | 498 | 6.67E-109 | 76.25 | F:nucleotide binding; P:transport |
| Efet.01.607707.g322.t1 | neuronal calcium sensor | 282 | 1.02E-12 | 87 | F:calcium ion binding |
| Efet.01.607739.g323.t1 | prolactin-releasing peptide receptor-like | 1236 | 5.16E-138 | 72.25 | P:neuropeptide signaling pathway; C:integral component of membrane; F:neuropeptide Y receptor activity |
| Efet.01.607756.g324.t1 | Lrp family transcriptional regulator | 384 | 4.64E-34 | 91.2 | F:transcription factor activity, sequence-specific DNA binding; P:transcription, DNA-templated; P:regulation of transcription, DNA-templated; F:sequence-specific DNA binding; C:intracellular |
| Efet.01.607780.g325.t1 | cGMP-inhibited 3 ,5 -cyclic phosphodiesterase A-like | 798 | 1.47E-39 | 55.1 | C:membrane; C:integral component of membrane |
| Efet.01.607855.g327.t1 | glycosyl hydrolase | 675 | 7.85E-100 | 79.1 | F:hydrolase activity |
| Efet.01.607939.g328.t1 | hypothetical protein B5766_10250 | 885 | 5.78E-172 | 64.25 |  |
| Efet.01.60800.g672.t1 | -tyrosine sulfotransferase 2 | 840 | 1.96E-108 | 72.45 | C:Golgi apparatus; F:protein-tyrosine sulfotransferase activity; P:peptidyl-tyrosine sulfation |
| Efet.01.608010.g330.t1 | Charged multivesicular body 2a | 279 | 1.85E-26 | 99 | C:nuclear envelope; C:late endosome membrane; P:protein transport; P:vacuolar transport; P:exit from mitosis; P:nuclear envelope reassembly |
| Efet.01.608016.g331.t1 | Brefeldin A-inhibited guanine nucleotide-exchange | 291 | 1.74E-06 | 56 | F:ARF guanyl-nucleotide exchange factor activity; P:regulation of ARF protein signal transduction; P:positive regulation of GTPase activity |
| Efet.01.608021.g332.t1 | vitamin K epoxide reductase complex subunit 1 1 | 219 | 1.86E-10 | 70.2 | C:integral component of membrane |
| Efet.01.608039.g333.t1 | beta-1,3-galactosyl-O-glycosyl-glyco beta-1,6-N-acetylglucosaminyltransferase 3-like | 207 | 8.71E-18 | 69.1 | F:acetylglucosaminyltransferase activity; C:membrane |
| Efet.01.608042.g335.t1 | G- coupled receptor Mth-like | 1149 | 4.63E-62 | 55.35 | F:G-protein coupled receptor activity; F:transmembrane signaling receptor activity; P:immune response; P:G-protein coupled receptor signaling pathway; C:membrane; C:integral component of membrane; F:scavenger receptor activity; P:cell surface receptor signaling pathway; P:receptor-mediated endocytosis; F:polysaccharide binding |
| Efet.01.608047.g336.t1 | zinc chelation | 801 | 1.32E-179 | 60 |  |
| Efet.01.608047.g337.t1 | transcriptional regulator | 306 | 8.54E-66 | 96.75 | F:DNA binding |
| Efet.01.608047.g338.t1 | addiction module toxin | 303 | 2.23E-63 | 96.2 | P:nucleic acid phosphodiester bond hydrolysis; F:endonuclease activity |
| Efet.01.608071.g339.t1 | Oxysterol-binding -related 8 | 333 | 6.03E-15 | 64.82 | C:membrane |
| Efet.01.608072.g340.t1 | homeobox Meis1-like | 357 | 2.13E-19 | 58 | F:DNA binding; C:nucleus; P:regulation of transcription, DNA-templated |
| Efet.01.608091.g341.t1 | WD repeat-containing on Y chromosome-like isoform X3 | 444 | 5.52E-62 | 80 | F:calcium ion binding |
| Efet.01.608093.g342.t1 | octopamine receptor | 1884 | 1.12E-87 | 72.9 | P:G-protein coupled receptor signaling pathway; F:octopamine receptor activity; C:integral component of membrane |
| Efet.01.6081.g481.t1 | KIAA0100-like isoform X1 | 396 | 1.37E-23 | 54.25 |  |
| Efet.01.60829.g676.t1 | guanine nucleotide-binding G(q) subunit alpha | 210 | 4.26E-35 | 89.4 | F:GTP binding; F:G-protein coupled receptor binding; P:G-protein coupled receptor signaling pathway; F:GTPase activity; F:G-protein beta/gamma-subunit complex binding; F:signal transducer activity |
| Efet.01.608299.g343.t1 | reelin-like | 486 | 3.17E-34 | 63.95 |  |
| Efet.01.608349.g346.t1 | NAD(P)-dependent oxidoreductase | 930 | 2.40E-173 | 88.05 |  |
| Efet.01.608389.g348.t1 | DUF4845 domain-containing | 357 | 1.15E-64 | 77.4 | C:integral component of membrane |
| Efet.01.608389.g349.t1 | signal peptidase I | 747 | 0 | 93.55 | P:proteolysis; C:integral component of membrane; F:serine-type peptidase activity |
| Efet.01.608389.g350.t1 | elongation factor 4 | 1809 | 0 | 98.05 | F:GTP binding; P:positive regulation of translation; F:translation elongation factor activity; F:GTPase activity; F:ribosome binding; P:translational elongation; C:plasma membrane |
| Efet.01.608389.g351.t1 | serine peptidase | 1515 | 0 | 82 | P:proteolysis; F:serine-type endopeptidase activity |
| Efet.01.608389.g352.t1 | transcriptional regulator | 816 | 0 | 90.55 |  |
| Efet.01.608424.g353.t1 | ATP-dependent DNA helicase | 399 | 2.62E-34 | 72.9 | F:nucleotide binding; F:helicase activity |
| Efet.01.608446.g354.t1 | mucin-17-like | 1683 | 1.69E-100 | 58.95 | P:regulation of endocytosis; P:endocytosis |
| Efet.01.608520.g356.t1 | FAR1-RELATED SEQUENCE 3- | 615 | 6.17E-18 | 46.63 | F:zinc ion binding |
| Efet.01.608639.g358.t1 | cholecystokinin receptor type A-like | 546 | 2.16E-24 | 56.15 | F:G-protein coupled receptor activity; P:G-protein coupled receptor signaling pathway; P:signal transduction; C:membrane; C:integral component of membrane; F:signal transducer activity |
| Efet.01.60866.g678.t1 | SUMO-activating enzyme subunit 1 isoform X2 | 567 | 3.22E-54 | 66.05 | F:protein binding |
| Efet.01.608718.g361.t1 | xylose isomerase | 1167 | 0 | 83.2 | F:magnesium ion binding; C:cytoplasm; F:xylose isomerase activity; P:pentose-phosphate shunt; P:D-xylose metabolic process |
| Efet.01.608730.g362.t1 | Multidrug efflux pump subunit | 462 | 6.09E-42 | 84.15 | C:integral component of membrane; P:transport; F:transporter activity |
| Efet.01.608766.g363.t1 | glutamate receptor 1 isoform X2 | 285 | 3.92E-11 | 66 | C:membrane; P:transport |
| Efet.01.608804.g365.t1 | cadherin-related neuronal receptor constant | 1221 | 5.68E-70 | 56.45 | C:membrane |
| Efet.01.608807.g366.t1 | somatostatin receptor type 2-like | 282 | 5.00E-22 | 67.1 | F:G-protein coupled peptide receptor activity; P:G-protein coupled receptor signaling pathway; C:membrane |
| Efet.01.60885.g679.t1 | Laminin subunit alpha- | 528 | 2.29E-29 | 57.55 | P:regulation of embryonic development; P:cell adhesion; P:regulation of cell adhesion; P:regulation of cell migration; F:receptor binding |
| Efet.01.608879.g367.t1 | 2-succinyl-5-enolpyruvyl-6-hydroxy-3-cyclohexene-1-carboxylate synthase | 564 | 3.25E-82 | 77.4 | F:magnesium ion binding; F:thiamine pyrophosphate binding; F:2-succinyl-5-enolpyruvyl-6-hydroxy-3-cyclohexene-1-carboxylic-acid synthase activity; F:manganese ion binding; P:menaquinone biosynthetic process |
| Efet.01.608879.g368.t1 | 2-succinyl-5-enolpyruvyl-6-hydroxy-3-cyclohexene-1-carboxylic-acid synthase | 507 | 1.41E-102 | 71.05 | F:metal ion binding; F:transferase activity |
| Efet.01.608883.g369.t1 | neuronal acetylcholine receptor subunit alpha-10-like | 219 | 1.90E-38 | 96.1 | C:postsynaptic membrane; P:cation transmembrane transport; C:integral component of membrane; C:cell junction; F:extracellular ligand-gated ion channel activity |
| Efet.01.608889.g370.t1 | golgin subfamily A member 4-like isoform X3 | 1332 | 1.69E-43 | 64.25 | C:membrane; C:integral component of membrane |
| Efet.01.608919.g371.t1 | acyl- synthetase | 378 | 1.02E-56 | 84.1 | P:metabolic process; F:o-succinylbenzoate-CoA ligase activity |
| Efet.01.608984.g372.t1 | alpha-2B adrenergic receptor | 1959 | 9.81E-104 | 78.65 | F:adrenergic receptor activity; C:integral component of membrane; P:adrenergic receptor signaling pathway; C:plasma membrane |
| Efet.01.609062.g374.t1 | hypothetical protein B5766_10835 | 204 | 1.11E-34 | 96 |  |
| Efet.01.609092.g377.t1 | 3-oxoacyl-ACP reductase | 786 | 4.35E-139 | 79.8 |  |
| Efet.01.609092.g378.t1 | MFS transporter | 993 | 1.22E-124 | 87.7 | P:transmembrane transport; C:integral component of membrane |
| Efet.01.609092.g379.t1 | phosphoribosylaminoimidazolesuccinocarboxamide synthase | 606 | 1.14E-106 | 71.8 | F:ATP binding; F:phosphoribosylaminoimidazolesuccinocarboxamide synthase activity; P:'de novo' IMP biosynthetic process |
| Efet.01.609097.g380.t1 | phosphatase PP2A 55 kDa regulatory subunit | 492 | 2.28E-84 | 90.85 | P:regulation of phosphoprotein phosphatase activity; C:protein phosphatase type 2A complex |
| Efet.01.609219.g381.t1 | CREB-binding isoform X3 | 654 | 1.28E-102 | 84.15 | F:histone acetyltransferase activity; F:zinc ion binding; F:transcription coactivator activity; P:regulation of transcription, DNA-templated; P:histone acetylation; C:histone acetyltransferase complex |
| Efet.01.609276.g382.t1 | crossover junction endodeoxyribonuclease | 549 | 4.44E-127 | 93.35 | F:magnesium ion binding; F:nucleic acid binding; P:DNA repair; P:nucleic acid phosphodiester bond hydrolysis; P:DNA recombination; F:crossover junction endodeoxyribonuclease activity |
| Efet.01.609276.g383.t1 | Crp Fnr family transcriptional regulator | 711 | 4.87E-170 | 65.05 | F:DNA binding; P:transcription, DNA-templated; P:regulation of transcription, DNA-templated |
| Efet.01.609276.g384.t1 | aminotransferase class V-fold PLP-dependent enzyme | 1146 | 0 | 72.1 | F:transaminase activity |
| Efet.01.609276.g385.t1 | twin-arginine translocation pathway signal | 1002 | 0 | 69.7 | C:outer membrane-bounded periplasmic space |
| Efet.01.609276.g386.t1 | tripartite tricarboxylate transporter substrate binding | 903 | 0 | 77.75 | C:outer membrane-bounded periplasmic space |
| Efet.01.609276.g387.t1 | dehydratase | 396 | 6.76E-90 | 83.9 | C:fatty acid synthase complex; P:oxidation-reduction process; P:fatty acid biosynthetic process; F:fatty acid synthase activity |
| Efet.01.609276.g388.t1 | family dehydratase | 453 | 8.81E-95 | 73.3 |  |
| Efet.01.609276.g389.t1 | acyl- dehydrogenase | 1209 | 0 | 88.75 | F:acyl-CoA dehydrogenase activity; F:flavin adenine dinucleotide binding; P:oxidation-reduction process |
| Efet.01.609276.g390.t1 | acyl- synthetase | 900 | 0 | 83.35 | P:long-chain fatty acid metabolic process; F:long-chain fatty acid-CoA ligase activity |
| Efet.01.609276.g391.t1 | acyl- synthetase | 507 | 5.91E-90 | 76.6 | P:metabolic process; F:ligase activity |
| Efet.01.609276.g392.t1 | thiolase | 1152 | 0 | 79.4 | F:transferase activity, transferring acyl groups other than amino-acyl groups; P:metabolic process |
| Efet.01.609276.g393.t1 | short-chain dehydrogenase | 804 | 3.29E-139 | 63.25 | F:oxidoreductase activity; P:oxidation-reduction process |
| Efet.01.609276.g394.t1 | family transcriptional regulator | 1548 | 0 | 67.3 | F:DNA binding; P:transcription, DNA-templated; P:regulation of transcription, DNA-templated |
| Efet.01.609276.g395.t1 | family transcriptional regulator | 1041 | 3.87E-151 | 81.95 | F:DNA binding; F:identical protein binding; P:transcription, DNA-templated; P:regulation of transcription, DNA-templated |
| Efet.01.609276.g396.t1 | tripartite tricarboxylate transporter substrate binding | 1047 | 0 | 77.45 | F:lyase activity; C:outer membrane-bounded periplasmic space |
| Efet.01.609276.g397.t1 | family transcriptional regulator | 765 | 5.28E-175 | 73.45 | F:DNA binding; P:transcription, DNA-templated; P:regulation of transcription, DNA-templated |
| Efet.01.609276.g398.t1 | hemerythrin | 387 | 4.43E-62 | 69.2 | F:metal ion binding |
| Efet.01.609353.g399.t1 | phospholipase D1-like | 561 | 1.32E-83 | 75.1 | P:cell motility; F:phosphatidylinositol binding; C:Golgi apparatus; P:inositol lipid-mediated signaling; F:phospholipase D activity; P:regulation of cell migration; P:regulation of phagocytosis; P:phosphatidic acid biosynthetic process |
| Efet.01.609438.g402.t1 | dachsous-like | 1113 | 1.80E-57 | 50.2 | F:oxidoreductase activity; F:calcium ion binding; C:membrane; C:integral component of membrane; P:cell adhesion; P:oxidation-reduction process; F:alcohol dehydrogenase (NAD) activity; P:homophilic cell adhesion via plasma membrane adhesion molecules; C:plasma membrane |
| Efet.01.60946.g682.t1 | Acetylcholine receptor subunit alpha-type acr-7 | 387 | 3.16E-13 | 51.65 | C:membrane |
| Efet.01.60946.g683.t1 | zinc finger 518A isoform X1 | 1248 | 4.45E-10 | 48.3 | F:nucleic acid binding; F:DNA binding; C:nucleus; F:transcription factor activity, sequence-specific DNA binding; P:transcription, DNA-templated; F:metal ion binding; P:regulation of transcription, DNA-templated; F:RNA polymerase II transcription factor activity, sequence-specific DNA binding; P:regulation of transcription from RNA polymerase II promoter |
| Efet.01.609482.g403.t1 | ankyrin and armadillo repeat-containing -like | 1533 | 1.36E-121 | 58.2 | C:nucleus; C:cytoplasm |
| Efet.01.609513.g404.t1 | Tau-tubulin kinase 1 | 2076 | 5.52E-22 | 68.35 | F:ATP binding; F:protein kinase activity; P:protein phosphorylation |
| Efet.01.609514.g405.t1 | ABC transporter ATP-binding | 870 | 0 | 90.15 | F:ATPase activity; F:ATP binding |
| Efet.01.609514.g406.t1 | ABC transporter ATP-binding | 288 | 9.99E-44 | 89.1 | F:ATPase activity; F:ATP binding |
| Efet.01.609516.g407.t1 | potassium voltage-gated channel Shab-like | 813 | 1.33E-67 | 73.25 | F:voltage-gated potassium channel activity; P:potassium ion transmembrane transport; C:voltage-gated potassium channel complex; P:protein homooligomerization |
| Efet.01.609535.g408.t1 | hypothetical protein CAPTEDRAFT_226427 | 297 | 1.10E-27 | 63.25 | F:nucleic acid binding; F:protein dimerization activity |
| Efet.01.60963.g684.t1 | acetylcholinesterase-like isoform X2 | 297 | 7.12E-15 | 63.2 | C:membrane; C:integral component of membrane; F:cholinesterase activity; F:hydrolase activity |
| Efet.01.609640.g412.t1 | hypothetical protein HELRODRAFT_191437 | 411 | 1.72E-09 | 49 |  |
| Efet.01.60966.g685.t1 | teneurin-m isoform X1 | 894 | 6.26E-65 | 54.4 | P:self proteolysis; C:membrane; C:integral component of membrane |
| Efet.01.609804.g415.t1 | serum response factor | 747 | 1.22E-68 | 88.35 | C:nucleus; F:DNA binding; P:transcription, DNA-templated; P:regulation of transcription, DNA-templated; F:protein dimerization activity |
| Efet.01.609819.g416.t1 | phospholipid-transporting ATPase ID isoform X7 | 756 | 2.12E-20 | 61.15 | F:nucleotide binding; F:metal ion binding; C:membrane; P:cellular component organization; P:phospholipid transport; F:hydrolase activity |
| Efet.01.609830.g417.t1 | sugar phosphate isomerase epimerase | 462 | 1.02E-91 | 86.85 | P:nucleic acid phosphodiester bond hydrolysis; F:endonuclease activity; F:isomerase activity |
| Efet.01.609830.g418.t1 | tartrate dehydrogenase | 1644 | 0 | 82.75 | F:magnesium ion binding; F:NAD binding; F:tartrate dehydrogenase activity; P:oxidation-reduction process; F:3-isopropylmalate dehydrogenase activity |
| Efet.01.609830.g419.t1 | aldolase | 807 | 6.16E-121 | 61.3 | F:6-deoxy-6-sulfofructose-1-phosphate aldolase activity; F:catalytic activity; F:lyase activity; P:6-sulfoquinovose(1-) catabolic process |
| Efet.01.609830.g420.t1 | sulfofructose kinase | 909 | 1.78E-160 | 56.8 | F:transferase activity |
| Efet.01.609830.g421.t1 | carbohydrate ABC transporter permease | 897 | 2.78E-96 | 86.4 | C:integral component of membrane; P:transport; C:plasma membrane |
| Efet.01.609830.g422.t1 | sugar ABC transporter substrate-binding | 744 | 1.50E-138 | 60.3 | P:carbohydrate transport; P:transport; F:transporter activity |
| Efet.01.609920.g423.t1 | ephrin type-B receptor 1-B-like isoform X4 | 441 | 1.81E-68 | 84.85 | F:ATP binding; P:peptidyl-tyrosine phosphorylation; F:protein tyrosine kinase activity; P:ephrin receptor signaling pathway; C:integral component of membrane |
| Efet.01.609961.g424.t1 | threonine--tRNA ligase | 993 | 0 | 75.65 | F:nucleotide binding; P:tRNA aminoacylation for protein translation; F:aminoacyl-tRNA ligase activity; F:ion binding |
| Efet.01.609991.g425.t1 | hypothetical protein B5766_12400 | 432 | 2.60E-62 | 100 |  |
| Efet.01.609996.g426.t1 | tripartite motif-containing 66 | 816 | 2.60E-11 | 46.65 | F:zinc ion binding; F:metal ion binding; C:aggresome; C:intracellular; C:nucleoplasm |
| Efet.01.61.g5.t1 | Solute carrier family facilitated glucose transporter member 8 | 1035 | 1.83E-100 | 62.25 | F:substrate-specific transmembrane transporter activity; P:transmembrane transport; C:membrane; P:hexose transport |
| Efet.01.610044.g428.t1 | Synaptopodin 2 | 1023 | 1.35E-49 | 47.8 |  |
| Efet.01.610105.g430.t1 | CDP-diacylglycerol--glycerol-3-phosphate 3-phosphatidyltransferase | 483 | 2.03E-89 | 66.25 | F:transferase activity |
| Efet.01.610173.g432.t1 | ankyrin repeat and EF-hand domain-containing 1-like | 492 | 2.76E-16 | 62.45 | F:calcium ion binding |
| Efet.01.610211.g433.t1 | receptor-type tyrosine- phosphatase T-like | 402 | 5.12E-42 | 69.15 | F:protein tyrosine phosphatase activity; C:integral component of membrane; P:peptidyl-tyrosine dephosphorylation |
| Efet.01.610308.g435.t1 | glycerol acyltransferase | 1884 | 0 | 83.6 | F:transferase activity, transferring acyl groups; P:transmembrane transport; P:metabolic process; C:integral component of membrane |
| Efet.01.610319.g436.t1 | double-stranded RNA-specific editase 1 | 348 | 1.23E-15 | 62.65 | P:regulation of cellular process; C:intracellular part |
| Efet.01.610340.g437.t1 | methylmalonate-semialdehyde dehydrogenase ( acylating) | 1029 | 0 | 85.9 | F:methylmalonate-semialdehyde dehydrogenase (acylating) activity; P:oxidation-reduction process |
| Efet.01.610340.g438.t1 | family transcriptional regulator | 486 | 2.03E-64 | 65.1 | F:DNA binding; F:transcription factor activity, sequence-specific DNA binding; P:transcription, DNA-templated; P:regulation of transcription, DNA-templated |
| Efet.01.610445.g439.t1 | dixin-like isoform X2 | 519 | 1.84E-17 | 64.7 | P:forebrain development; C:cytoplasm; P:Wnt signaling pathway |
| Efet.01.61049.g689.t1 | Kinase suppressor of Ras 2 | 378 | 6.29E-29 | 66.4 | P:phosphorylation; F:kinase activity |
| Efet.01.610504.g441.t1 | hypothetical protein CAPTEDRAFT_194088, partial | 729 | 4.30E-15 | 71 |  |
| Efet.01.610558.g443.t1 | tripartite motif-containing 3 isoform X1 | 801 | 3.97E-49 | 49.75 | F:zinc ion binding |
| Efet.01.610577.g444.t1 | S9 family peptidase | 429 | 1.89E-96 | 85.05 | P:proteolysis; F:serine-type endopeptidase activity; F:serine-type exopeptidase activity |
| Efet.01.610580.g445.t1 | HEAT repeat-containing 5B | 321 | 5.05E-45 | 73.45 | C:extracellular exosome; F:protein binding; C:membrane |
| Efet.01.610689.g448.t1 | predicted protein | 1035 | 6.14E-55 | 53.9 | P:nucleic acid phosphodiester bond hydrolysis; F:metal ion binding; F:endonuclease activity; C:membrane; C:integral component of membrane; P:mismatch repair |
| Efet.01.610725.g449.t1 | PREDICTED: protein inturned-like, partial | 645 | 2.01E-09 | 48 |  |
| Efet.01.610741.g450.t1 | hypothetical protein B5766_02085 | 528 | 5.97E-74 | 91.14 |  |
| Efet.01.610801.g451.t1 | Methyltransferase type 12 | 876 | 6.79E-173 | 79.1 | F:methyltransferase activity; P:methylation |
| Efet.01.610801.g452.t1 | acyl- dehydrogenase | 1116 | 0 | 92.4 | F:oxidoreductase activity, acting on the CH-CH group of donors; F:flavin adenine dinucleotide binding; P:oxidation-reduction process |
| Efet.01.610801.g453.t1 | pimeloyl- dehydrogenase large subunit | 1122 | 0 | 92 | F:acyl-CoA dehydrogenase activity; F:flavin adenine dinucleotide binding; P:oxidation-reduction process |
| Efet.01.610863.g454.t1 | dihydrodipicolinate synthetase | 813 | 1.93E-148 | 69.6 | F:catalytic activity; P:metabolic process; F:lyase activity |
| Efet.01.610888.g455.t1 | pre translocase subunit | 486 | 1.30E-105 | 79.55 | F:ATP binding; P:protein targeting; C:cytoplasm; P:intracellular protein transmembrane transport; F:helicase activity; P:protein import; C:plasma membrane; C:thylakoid |
| Efet.01.610904.g457.t1 | ribonuclease R | 759 | 2.05E-87 | 75.75 | F:nucleic acid binding; P:RNA phosphodiester bond hydrolysis; F:exonuclease activity; F:ribonuclease activity |
| Efet.01.610904.g458.t1 | ribonuclease R | 600 | 6.98E-81 | 77.75 | F:exoribonuclease II activity; P:RNA phosphodiester bond hydrolysis, exonucleolytic; F:RNA binding; C:cytoplasm |
| Efet.01.610922.g459.t1 | hypothetical protein CRN43_22895 | 384 | 1.23E-12 | 64.5 |  |
| Efet.01.610928.g460.t1 | 50S ribosomal L5 | 525 | 2.03E-72 | 90.8 | F:structural constituent of ribosome; C:ribosome; F:tRNA binding; F:rRNA binding; P:translation |
| Efet.01.610928.g461.t1 | 50S ribosomal L14 | 369 | 8.75E-80 | 98.8 | F:structural constituent of ribosome; C:large ribosomal subunit; F:rRNA binding; P:translation |
| Efet.01.610928.g462.t1 | 30S ribosomal S17 | 324 | 7.71E-59 | 84.5 | F:structural constituent of ribosome; C:ribosome; F:rRNA binding; P:translation |
| Efet.01.61095.g691.t1 | Lipoamide acyltransferase component of branched-chain alpha-keto acid dehydrogenase mitochondrial | 234 | 2.73E-27 | 85.6 | F:transferase activity, transferring acyl groups; P:metabolic process |
| Efet.01.611056.g464.t1 | FAM204A-like isoform X2 | 693 | 5.94E-24 | 59.65 | P:biological_process; C:cellular_component; F:molecular_function |
| Efet.01.611100.g465.t1 | neurogenic locus notch homolog 1 isoform X2 | 984 | 3.24E-72 | 51.85 | F:G-protein coupled receptor activity; F:transmembrane signaling receptor activity; P:G-protein coupled receptor signaling pathway; P:signal transduction; F:calcium ion binding; C:membrane; C:integral component of membrane; P:cell surface receptor signaling pathway; P:homophilic cell adhesion via plasma membrane adhesion molecules; F:signal transducer activity |
| Efet.01.611210.g467.t1 | peroxiredoxin | 444 | 2.23E-47 | 80.3 | F:peroxidase activity; P:response to oxidative stress; P:cellular oxidant detoxification; P:oxidation-reduction process; F:peroxiredoxin activity |
| Efet.01.611215.g468.t1 | ribonuclease Y | 753 | 3.63E-139 | 88.3 | P:RNA phosphodiester bond hydrolysis, endonucleolytic; F:RNA binding; C:integral component of membrane; P:mRNA catabolic process; C:plasma membrane; F:endoribonuclease activity |
| Efet.01.611231.g469.t1 | I-type lectin 3 | 243 | 2.77E-17 | 67.43 | C:membrane |
| Efet.01.611250.g470.t1 | Potassium voltage-gated channel Shab | 1077 | 1.65E-78 | 78.55 | P:potassium ion transmembrane transport; C:voltage-gated potassium channel complex; F:delayed rectifier potassium channel activity; P:regulation of ion transmembrane transport; P:protein homooligomerization |
| Efet.01.611343.g471.t1 | acetylornithine deacetylase | 1224 | 0 | 84.1 | F:zinc ion binding; P:arginine biosynthetic process; C:cytoplasm; F:acetylornithine deacetylase activity; F:cobalt ion binding |
| Efet.01.611343.g472.t1 | saccharopine dehydrogenase | 1158 | 0 | 89.4 | F:oxidoreductase activity; P:oxidation-reduction process |
| Efet.01.611343.g473.t1 | family transcriptional regulator | 453 | 1.67E-101 | 95.4 | F:transcription factor activity, sequence-specific DNA binding; P:transcription, DNA-templated; P:regulation of transcription, DNA-templated; F:sequence-specific DNA binding; C:intracellular |
| Efet.01.611343.g474.t1 | tRNA dihydrouridine(20 20a) synthase | 975 | 0 | 88.8 | F:FMN binding; F:tRNA dihydrouridine synthase activity; P:tRNA dihydrouridine synthesis; F:flavin adenine dinucleotide binding; P:oxidation-reduction process; F:tRNA binding |
| Efet.01.611343.g475.t1 | polyhydroxyalkanoate depolymerase | 981 | 0 | 94.35 |  |
| Efet.01.611353.g476.t1 | plexin-A2-like isoform X1 | 540 | 3.39E-57 | 74.35 | P:signal transduction; C:membrane |
| Efet.01.6114.g483.t1 | NACHT and WD repeat domain-containing 2-like | 4053 | 0 | 54.1 |  |
| Efet.01.61151.g693.t1 | cGMP-dependent kinase alpha isozyme | 339 | 2.83E-16 | 65.75 | P:cGMP-mediated signaling; P:relaxation of vascular smooth muscle; F:cGMP-dependent protein kinase activity; C:Golgi apparatus; P:regulation of blood circulation; P:negative regulation of cellular process; P:protein phosphorylation; P:regulation of ion transport; P:positive regulation of biological process; C:plasma membrane; P:regulation of molecular function; P:neuron migration; F:protein binding; P:forebrain development; F:cGMP binding; P:regulation of cellular biosynthetic process; P:regulation of primary metabolic process; P:dendrite development |
| Efet.01.611514.g478.t1 | CX3C chemokine receptor 1-like | 1263 | 3.71E-19 | 46.1 | F:G-protein coupled receptor activity; P:G-protein coupled receptor signaling pathway; P:signal transduction; C:membrane; C:integral component of membrane; F:signal transducer activity |
| Efet.01.611552.g480.t1 | Piezo-type mechanosensitive ion channel component 2 | 360 | 4.80E-15 | 68.05 | F:mechanically-gated ion channel activity; P:ion transmembrane transport; C:integral component of membrane |
| Efet.01.611577.g481.t1 | orai-2-like isoform X2 | 489 | 9.08E-33 | 72.9 | F:store-operated calcium channel activity; P:store-operated calcium entry; C:integral component of membrane |
| Efet.01.61158.g694.t1 | coiled-coil domain-containing 61 isoform X1 | 624 | 1.59E-14 | 70.6 |  |
| Efet.01.611588.g482.t1 | hypothetical protein HELRODRAFT_183479 | 1044 | 7.75E-18 | 72.35 | F:nucleic acid binding; F:metal ion binding |
| Efet.01.611631.g483.t1 | N-acetyltransferase | 570 | 7.90E-125 | 81.4 | F:N-acetyltransferase activity |
| Efet.01.611631.g484.t1 | UTP--glucose-1-phosphate uridylyltransferase | 825 | 0 | 86.75 | P:UDP-glucose metabolic process; P:biosynthetic process; F:UTP:glucose-1-phosphate uridylyltransferase activity |
| Efet.01.611702.g485.t1 | heat-inducible transcriptional repressor | 1020 | 0 | 81.1 | F:DNA binding; P:negative regulation of transcription, DNA-templated; P:transcription, DNA-templated |
| Efet.01.611795.g488.t1 | NAD(P) FAD-dependent oxidoreductase | 2184 | 0 | 75.95 | F:oxidoreductase activity; P:oxidation-reduction process |
| Efet.01.611795.g489.t1 | family transcriptional regulator | 984 | 0 | 62.4 | F:transcription factor activity, sequence-specific DNA binding; P:transcription, DNA-templated; P:regulation of transcription, DNA-templated; F:sequence-specific DNA binding |
| Efet.01.611795.g490.t1 | alpha beta hydrolase | 666 | 1.10E-156 | 77.95 | F:transferase activity; F:hydrolase activity |
| Efet.01.611795.g491.t1 | NAD(P) FAD-dependent oxidoreductase | 291 | 1.89E-15 | 87.5 | F:oxidoreductase activity; P:oxidation-reduction process |
| Efet.01.611807.g492.t1 | dihydroxy-acid dehydratase | 654 | 1.72E-96 | 72.4 | F:lyase activity |
| Efet.01.611807.g493.t1 | 3-oxoacyl-ACP reductase | 600 | 7.37E-53 | 66.8 | F:oxidoreductase activity; P:oxidation-reduction process |
| Efet.01.611807.g494.t1 | sn-glycerol-3-phosphate ABC transporter ATP-binding | 1131 | 3.87E-124 | 72.5 | F:nucleotide binding; C:membrane |
| Efet.01.611807.g495.t1 | carbohydrate ABC transporter permease | 828 | 8.26E-122 | 84.25 | C:integral component of membrane; P:transport; C:plasma membrane |
| Efet.01.611807.g496.t1 | ABC transporter permease | 954 | 5.23E-102 | 76.65 | C:membrane |
| Efet.01.611807.g497.t1 | ABC transporter substrate-binding | 1143 | 2.42E-154 | 68.05 | P:carbohydrate transport |
| Efet.01.611807.g498.t1 | tripartite tricarboxylate transporter substrate binding | 684 | 2.27E-130 | 85.6 | C:outer membrane-bounded periplasmic space |
| Efet.01.611807.g499.t1 | dihydroxy-acid dehydratase | 705 | 1.49E-144 | 98.55 | F:dihydroxy-acid dehydratase activity; P:metabolic process |
| Efet.01.611807.g500.t1 | dihydroxy-acid dehydratase | 804 | 1.60E-167 | 90.85 | F:dihydroxy-acid dehydratase activity; P:metabolic process |
| Efet.01.611807.g501.t1 | family transcriptional regulator | 819 | 2.21E-136 | 83.7 | F:DNA binding; F:transcription factor activity, sequence-specific DNA binding; P:transcription, DNA-templated; P:regulation of transcription, DNA-templated |
| Efet.01.611807.g502.t1 | dihydrodipicolinate synthase family | 918 | 0 | 77.6 | P:metabolic process; F:lyase activity |
| Efet.01.611807.g503.t1 | family transcriptional regulator | 690 | 6.18E-159 | 76.85 | F:DNA binding; F:transcription factor activity, sequence-specific DNA binding; P:transcription, DNA-templated; P:regulation of transcription, DNA-templated |
| Efet.01.611807.g504.t1 | ABC transporter substrate-binding | 1299 | 0 | 87.5 |  |
| Efet.01.611807.g505.t1 | branched-chain amino acid ABC transporter permease | 891 | 1.07E-124 | 95.65 | C:integral component of membrane; P:transport; C:plasma membrane; F:transporter activity |
| Efet.01.611807.g506.t1 | ABC transporter | 2397 | 0 | 88.15 | F:ATPase activity; F:ATP binding; C:integral component of membrane; P:carbohydrate transport; C:plasma membrane; F:transporter activity |
| Efet.01.611807.g507.t1 | 3-hydroxyacyl- dehydrogenase | 1632 | 0 | 83.3 | F:3-hydroxyacyl-CoA dehydrogenase activity; F:3-hydroxybutyrate dehydrogenase activity; F:3-hydroxybutyryl-CoA dehydrogenase activity; P:oxidation-reduction process; F:NAD+ binding; P:fatty acid metabolic process |
| Efet.01.611807.g508.t1 | 3-keto-5-aminohexanoate cleavage | 870 | 0 | 87.45 | P:L-lysine catabolic process to acetate; F:transferase activity |
| Efet.01.611895.g509.t1 | diaphanous-related formin 2 | 234 | 8.07E-10 | 83.2 | F:actin binding; F:catalytic activity; P:metabolic process; P:actin filament organization; F:Rho GTPase binding; P:cell cycle |
| Efet.01.612007.g511.t1 | acetyl- carboxylase carboxyltransferase subunit | 873 | 3.31E-153 | 90.9 | F:propionyl-CoA carboxylase activity; F:transferase activity |
| Efet.01.61208.g698.t1 | zinc finger 1 isoform X1 | 210 | 2.22E-20 | 87.15 | C:nucleus; F:metal ion binding; P:regulation of transcription, DNA-templated; F:sequence-specific DNA binding |
| Efet.01.612091.g512.t1 | gamma-glutamyltransferase | 450 | 1.03E-67 | 87.15 | P:glutathione metabolic process; F:gamma-glutamyltransferase activity |
| Efet.01.612091.g513.t1 | tripartite tricarboxylate transporter substrate binding | 933 | 0 | 74.95 | C:outer membrane-bounded periplasmic space |
| Efet.01.612091.g514.t1 | family 10 | 1206 | 0 | 58.05 | F:metal ion binding; P:proteolysis; F:metallopeptidase activity |
| Efet.01.612091.g515.t1 | amino acid ABC transporter | 789 | 5.04E-154 | 67 | P:transport; F:transporter activity |
| Efet.01.612091.g516.t1 | amino acid ABC transporter permease | 711 | 1.00E-141 | 83.6 | C:integral component of membrane; P:transport; C:plasma membrane; F:transporter activity |
| Efet.01.612091.g517.t1 | ABC transporter | 639 | 6.13E-129 | 80.7 | C:integral component of membrane; P:transport; C:plasma membrane; F:transporter activity |
| Efet.01.612091.g518.t1 | oligopeptidase A | 456 | 5.68E-92 | 91.1 | F:metal ion binding; P:proteolysis; F:metalloendopeptidase activity |
| Efet.01.612091.g519.t1 | oligopeptidase A | 1230 | 0 | 89.4 | F:metal ion binding; P:proteolysis; F:metalloendopeptidase activity |
| Efet.01.612091.g520.t1 | bifunctional methylenetetrahydrofolate dehydrogenase methenyltetrahydrofolate cyclohydrolase | 762 | 9.09E-156 | 76.8 | P:methionine biosynthetic process; P:purine nucleotide biosynthetic process; P:folic acid-containing compound biosynthetic process; P:tetrahydrofolate interconversion; P:oxidation-reduction process; P:histidine biosynthetic process; F:methenyltetrahydrofolate cyclohydrolase activity; F:methylenetetrahydrofolate dehydrogenase (NADP+) activity |
| Efet.01.612091.g521.t1 | DNA-binding response regulator | 627 | 3.40E-151 | 97.35 | F:DNA binding; P:transcription, DNA-templated; P:phosphorelay signal transduction system; P:regulation of transcription, DNA-templated; C:intracellular |
| Efet.01.612104.g522.t1 | class II aldolase | 852 | 6.76E-172 | 85.1 |  |
| Efet.01.612104.g523.t1 | 5-guanidino-2-oxopentanoate decarboxylase | 498 | 3.30E-88 | 76.95 | F:magnesium ion binding; F:thiamine pyrophosphate binding; F:catalytic activity |
| Efet.01.612104.g524.t1 | 5-guanidino-2-oxopentanoate decarboxylase | 1140 | 0 | 79.1 | F:magnesium ion binding; F:thiamine pyrophosphate binding; F:catalytic activity |
| Efet.01.612113.g525.t1 | nucleoside hydrolase | 396 | 7.57E-90 | 60.95 | F:hydrolase activity, hydrolyzing N-glycosyl compounds; F:hydrolase activity |
| Efet.01.612122.g526.t1 | porin | 480 | 9.53E-93 | 71.7 | C:integral component of membrane |
| Efet.01.612122.g527.t1 | tripartite tricarboxylate transporter substrate binding | 594 | 1.53E-82 | 66.25 | C:outer membrane-bounded periplasmic space |
| Efet.01.612122.g528.t1 | transferase | 957 | 0 | 65.95 | F:transferase activity |
| Efet.01.612151.g529.t1 | peptidase M16 | 840 | 2.34E-90 | 69.3 | F:metal ion binding; F:catalytic activity |
| Efet.01.612154.g530.t1 | carbohydrate sulfotransferase 5-like | 834 | 3.62E-49 | 45.2 | C:membrane; C:integral component of membrane; F:sulfotransferase activity |
| Efet.01.612196.g531.t1 | phosphoenolpyruvate carboxykinase (GTP) | 492 | 3.39E-100 | 91.65 | F:GTP binding; P:gluconeogenesis; F:phosphoenolpyruvate carboxykinase (GTP) activity; C:cytoplasm; F:manganese ion binding; P:phosphorylation; F:kinase activity |
| Efet.01.612230.g532.t1 | acyl- carboxylase subunit beta | 270 | 7.56E-30 | 99.9 | F:acetyl-CoA carboxylase activity; F:transferase activity; P:fatty acid biosynthetic process; C:acetyl-CoA carboxylase complex |
| Efet.01.612275.g534.t1 | gliding motility lipo | 486 | 1.90E-77 | 91.85 | P:phosphorylation; F:kinase activity |
| Efet.01.612277.g535.t1 | DUF349 domain-containing | 636 | 3.59E-100 | 68.75 |  |
| Efet.01.612295.g536.t1 | ATP-dependent DNA helicase | 423 | 4.35E-68 | 70 | F:ATP binding; F:helicase activity |
| Efet.01.612310.g538.t1 | DBH-like monooxygenase 1 | 243 | 4.34E-07 | 60.67 | F:oxidoreductase activity, acting on paired donors, with incorporation or reduction of molecular oxygen, reduced ascorbate as one donor, and incorporation of one atom of oxygen; F:copper ion binding; F:catalytic activity; P:oxidation-reduction process; F:monooxygenase activity |
| Efet.01.612348.g539.t1 | neuron navigator 2-like isoform X3 | 258 | 1.82E-38 | 84.95 | F:ATP binding |
| Efet.01.61236.g699.t1 | ATP-dependent RNA helicase chloroplastic-like | 249 | 1.03E-19 | 59.65 | F:ion binding; F:hydrolase activity |
| Efet.01.612380.g540.t1 | neurofascin-like isoform X1 | 414 | 1.64E-12 | 53.65 |  |
| Efet.01.612448.g542.t1 | DNA mismatch repair | 774 | 1.51E-100 | 71.25 | P:DNA repair; F:organic cyclic compound binding; F:heterocyclic compound binding |
| Efet.01.612449.g543.t1 | FMRFamide receptor-like | 954 | 9.28E-77 | 52.75 | F:G-protein coupled receptor activity; F:G-protein coupled peptide receptor activity; P:G-protein coupled receptor signaling pathway; P:signal transduction; C:membrane; C:integral component of membrane; F:signal transducer activity |
| Efet.01.61248.g700.t1 | ATP-dependent RNA helicase TDRD9 | 327 | 2.87E-43 | 79.65 | C:nucleus; F:ATP binding; F:metal ion binding; P:male meiosis; P:spermatogenesis; P:DNA methylation involved in gamete generation; P:fertilization; P:RNA processing; P:piRNA metabolic process; C:piP-body; F:ATP-dependent RNA helicase activity; F:RNA binding |
| Efet.01.612518.g544.t1 | hypothetical protein B5766_01300 | 537 | 4.91E-109 | 69.29 |  |
| Efet.01.612518.g545.t1 | cytochrome C biogenesis | 261 | 6.64E-51 | 76.95 | C:integral component of membrane |
| Efet.01.612518.g546.t1 | cytochrome C biogenesis | 1173 | 0 | 80.3 | C:integral component of membrane |
| Efet.01.612518.g547.t1 | cytochrome C biogenesis | 738 | 7.15E-125 | 78.9 | C:integral component of membrane; P:oxidation-reduction process; P:cytochrome complex assembly |
| Efet.01.612521.g548.t1 | pre translocase subunit | 405 | 1.44E-61 | 89.85 | P:protein targeting; P:intracellular protein transmembrane transport; C:integral component of membrane; P:protein transport by the Sec complex; C:intracellular; C:plasma membrane |
| Efet.01.612521.g549.t1 | 50S ribosomal L15 | 264 | 9.98E-55 | 95.75 | F:structural constituent of ribosome; C:large ribosomal subunit; F:rRNA binding; P:translation |
| Efet.01.612521.g550.t1 | 30S ribosomal S5 | 438 | 2.90E-53 | 95.7 | F:structural constituent of ribosome; C:small ribosomal subunit; F:rRNA binding; P:translation |
| Efet.01.612521.g551.t1 | 50S ribosomal L6 | 537 | 2.49E-121 | 92.95 | F:structural constituent of ribosome; C:ribosome; F:rRNA binding; P:translation |
| Efet.01.612525.g552.t1 | EF-hand calcium-binding domain-containing 5-like isoform X1 | 585 | 2.31E-40 | 69.65 | F:calcium ion binding |
| Efet.01.612527.g553.t1 | vigilin-like | 348 | 5.16E-27 | 85.8 | F:RNA binding; C:cytoplasm |
| Efet.01.612565.g554.t1 | glucosamine-6-phosphate deaminase | 516 | 8.50E-123 | 80.9 |  |
| Efet.01.612643.g556.t1 | tripartite motif-containing 3-like | 1176 | 3.06E-09 | 46.3 | F:transferase activity, transferring glycosyl groups; F:zinc ion binding; P:signal transduction; F:sialyltransferase activity; P:embryonic body morphogenesis; P:protein glycosylation; P:regulation of apoptotic process; C:Golgi apparatus; C:intracellular; F:metal ion binding; C:membrane; C:integral component of membrane; P:sialylation; F:transferase activity |
| Efet.01.612664.g557.t1 | Chain length determinant [Chryseolinea serpens] | 360 | 2.60E-76 | 73.33 | C:membrane; C:integral component of membrane; P:lipopolysaccharide biosynthetic process |
| Efet.01.612672.g558.t1 | Bacteriocin- or -Associated | 324 | 1.91E-35 | 62.4 | C:membrane; C:integral component of membrane |
| Efet.01.612693.g559.t1 | gfo Idh family oxidoreductase | 321 | 8.31E-60 | 86.75 | F:inositol 2-dehydrogenase activity; C:integral component of membrane; P:oxidation-reduction process |
| Efet.01.612721.g561.t1 | Protocadherin Fat 4 | 729 | 6.56E-34 | 50.05 | F:calcium ion binding; C:membrane; C:integral component of membrane; P:cell adhesion; P:homophilic cell adhesion via plasma membrane adhesion molecules; C:plasma membrane |
| Efet.01.612737.g562.t1 | lysine-specific demethylase 3B-like isoform X5 | 288 | 4.13E-22 | 70.95 |  |
| Efet.01.612787.g564.t1 | diaminopimelate epimerase | 402 | 1.61E-74 | 77.25 | F:diaminopimelate epimerase activity; C:cytoplasm; P:lysine biosynthetic process via diaminopimelate |
| Efet.01.612787.g565.t1 | DUF484 domain-containing | 558 | 2.40E-93 | 84.5 |  |
| Efet.01.612803.g566.t1 | DUF2017 domain-containing | 387 | 1.13E-76 | 52.65 |  |
| Efet.01.61281.g703.t1 | egl nine homolog 1 isoform X1 | 1641 | 7.66E-86 | 67.25 | F:ion binding |
| Efet.01.612830.g567.t1 | hypothetical protein CRN43_17050 | 678 | 2.91E-165 | 50.6 | C:membrane; C:integral component of membrane |
| Efet.01.612844.g568.t1 | Kyphoscoliosis peptidase | 1179 | 1.57E-122 | 55.3 | F:zinc ion binding; F:metal ion binding |
| Efet.01.612881.g569.t1 | apolipo N-acyltransferase | 861 | 0 | 76.05 | F:transferase activity, transferring acyl groups; C:membrane; P:metabolic process; F:hydrolase activity |
| Efet.01.612891.g570.t1 | phosphoglycerate dehydrogenase | 573 | 2.04E-80 | 84.25 | F:phosphoserine phosphatase activity; F:NAD binding; F:phosphoglycerate dehydrogenase activity; P:L-serine biosynthetic process; P:oxidation-reduction process; F:amino acid binding; P:dephosphorylation |
| Efet.01.612929.g571.t1 | cAMP-dependent kinase regulatory subunit | 282 | 7.13E-29 | 71.5 | P:phosphorylation; F:kinase activity |
| Efet.01.612952.g572.t1 | DUF2156 domain-containing | 1359 | 0 | 69.45 | C:membrane |
| Efet.01.612959.g573.t1 | hypothetical protein CRN43_08015 | 273 | 2.68E-57 | 100 |  |
| Efet.01.612968.g574.t1 | ankyrin repeat | 1398 | 5.56E-44 | 51.2 | F:nucleotide binding; F:ATP binding; F:catalytic activity; P:nucleoside metabolic process; F:protein kinase activity; P:phosphorylation; P:protein phosphorylation; F:kinase activity |
| Efet.01.612999.g575.t1 | serine--tRNA ligase | 414 | 7.88E-85 | 84.2 | P:selenocysteinyl-tRNA(Sec) biosynthetic process; F:ATP binding; C:cytoplasm; P:selenocysteine biosynthetic process; F:serine-tRNA ligase activity; P:seryl-tRNA aminoacylation |
| Efet.01.613026.g577.t1 | NADPH-dependent FMN reductase | 765 | 1.63E-119 | 93.1 | F:FMN binding; F:oxidoreductase activity; F:electron carrier activity; P:oxidation-reduction process |
| Efet.01.613026.g578.t1 | Xaa-Pro aminopeptidase | 1386 | 0 | 63.7 | F:hydrolase activity |
| Efet.01.613026.g579.t1 | multidrug ABC transporter permease | 801 | 7.09E-141 | 86.6 | C:integral component of membrane; P:transport; C:plasma membrane |
| Efet.01.613026.g580.t1 | branched-chain amino acid ABC transporter substrate-binding | 2421 | 0 | 80.65 | C:integral component of membrane; P:transport; C:plasma membrane |
| Efet.01.613038.g581.t1 | 30S ribosomal S17 | 201 | 1.92E-33 | 91.8 | F:structural constituent of ribosome; C:ribosome; F:rRNA binding; P:translation |
| Efet.01.613088.g584.t1 | ABC transporter substrate-binding | 405 | 5.09E-38 | 87.45 |  |
| Efet.01.613088.g585.t1 | TonB-dependent receptor | 1608 | 0 | 74.35 | C:cell outer membrane; F:receptor activity; P:transport |
| Efet.01.613112.g586.t1 | neuropeptide receptor 15-like | 447 | 1.64E-42 | 71.15 | F:G-protein coupled receptor activity; P:G-protein coupled receptor signaling pathway; C:integral component of membrane |
| Efet.01.613186.g587.t1 | thioredoxin family | 291 | 5.93E-63 | 70.9 | F:catalytic activity |
| Efet.01.613186.g588.t1 | DUF1573 domain-containing | 396 | 9.78E-93 | 61.5 |  |
| Efet.01.613322.g590.t1 | metal-dependent transcriptional regulator | 834 | 2.53E-161 | 83 | F:DNA binding; F:transcription factor activity, sequence-specific DNA binding; F:transition metal ion binding; P:regulation of transcription, DNA-templated; F:protein dimerization activity |
| Efet.01.613371.g591.t1 | ataxin-1 | 912 | 1.48E-09 | 63.75 | C:nuclear inclusion body; P:excitatory postsynaptic potential; P:positive regulation of glial cell proliferation; P:adult locomotory behavior; P:visual learning; C:protein complex; F:chromatin binding; P:positive regulation of transcription from RNA polymerase II promoter; P:negative regulation of insulin-like growth factor receptor signaling pathway; C:nucleoplasm; P:negative regulation of phosphorylation; P:negative regulation of transcription from RNA polymerase II promoter; P:lung alveolus development; F:protein binding; F:single-stranded RNA binding |
| Efet.01.6134.g485.t1 | Actin-binding anillin | 1059 | 5.40E-89 | 61.8 |  |
| Efet.01.613416.g592.t1 | hypothetical protein | 609 | 3.63E-138 | 87.45 |  |
| Efet.01.613416.g593.t1 | phosphoglycerate kinase | 693 | 1.36E-156 | 90.9 | P:phosphorylation; F:kinase activity; F:isomerase activity |
| Efet.01.613416.g594.t1 | Rieske (2Fe-2S) | 1032 | 0 | 90.95 | F:dioxygenase activity; F:metal ion binding; P:oxidation-reduction process; F:2 iron, 2 sulfur cluster binding |
| Efet.01.613416.g595.t1 | UDP-N-acetylmuramate:L-alanyl-gamma-D-glutamyl-meso-diaminopimelate ligase | 1398 | 0 | 86.6 | P:regulation of cell shape; P:cell division; F:ATP binding; P:peptidoglycan turnover; P:peptidoglycan biosynthetic process; F:acid-amino acid ligase activity; P:cell wall organization; P:cell cycle |
| Efet.01.613416.g596.t1 | hypothetical protein | 597 | 4.81E-140 | 88.15 |  |
| Efet.01.613416.g597.t1 | family disulfide reductase | 279 | 3.33E-56 | 81.6 | C:cell; F:antioxidant activity; F:ubiquinol-cytochrome-c reductase activity; C:integral component of membrane; P:cellular oxidant detoxification; P:hydrogen ion transmembrane transport; P:cell redox homeostasis; P:oxidation-reduction process |
| Efet.01.613416.g598.t1 | acetyl- carboxylase biotin carboxyl carrier | 333 | 7.67E-53 | 98.3 | F:transcription factor activity, sequence-specific DNA binding; F:acetyl-CoA carboxylase activity; P:DNA replication; P:regulation of transcription, DNA-templated; P:fatty acid biosynthetic process; C:acetyl-CoA carboxylase complex |
| Efet.01.613416.g599.t1 | acetyl- carboxylase biotin carboxylase subunit | 1263 | 0 | 96.1 | F:ATP binding; F:acetyl-CoA carboxylase activity; F:metal ion binding; F:biotin carboxylase activity |
| Efet.01.613416.g600.t1 | ribosomal L11 methyltransferase | 444 | 3.90E-72 | 86.2 | F:protein methyltransferase activity; C:ribosome; P:protein methylation |
| Efet.01.613416.g601.t1 | DUF3426 domain-containing | 762 | 5.21E-80 | 86.9 | C:integral component of membrane |
| Efet.01.613416.g602.t1 | carbohydrate kinase family | 897 | 0 | 90.9 | P:phosphorylation; F:phosphotransferase activity, alcohol group as acceptor; F:kinase activity |
| Efet.01.613416.g603.t1 | hypothetical protein Veis_1494 | 567 | 3.55E-06 | 100 | C:membrane; C:integral component of membrane |
| Efet.01.613416.g604.t1 | ribonucleotide-diphosphate reductase subunit beta | 1200 | 0 | 90.4 | F:ribonucleoside-diphosphate reductase activity, thioredoxin disulfide as acceptor; F:metal ion binding; P:DNA replication; P:deoxyribonucleoside diphosphate metabolic process; C:integral component of membrane; P:oxidation-reduction process |
| Efet.01.613416.g605.t1 | ribonucleoside-diphosphate reductase subunit alpha | 1035 | 0 | 90.6 | F:ATP binding; F:ribonucleoside-diphosphate reductase activity, thioredoxin disulfide as acceptor; P:DNA replication; P:oxidation-reduction process |
| Efet.01.613416.g606.t1 | ribonucleoside-diphosphate reductase subunit alpha | 1680 | 0 | 90.75 | F:ATP binding; F:ribonucleoside-diphosphate reductase activity, thioredoxin disulfide as acceptor; P:DNA replication; P:oxidation-reduction process |
| Efet.01.613416.g607.t1 | sigma-54-dependent Fis family transcriptional regulator | 3174 | 0 | 87.1 | F:phosphorelay sensor kinase activity; F:ATP binding; P:transcription, DNA-templated; P:phosphorelay signal transduction system; C:integral component of membrane; P:regulation of transcription, DNA-templated; F:sequence-specific DNA binding; F:transcription factor binding; P:signal transduction by protein phosphorylation; C:intracellular |
| Efet.01.613416.g608.t1 | cytochrome c assembly | 696 | 1.39E-101 | 93.85 | C:integral component of membrane; P:cytochrome complex assembly; F:heme binding |
| Efet.01.613416.g609.t1 | signal recognition particle | 1365 | 0 | 96.85 | F:GTP binding; P:SRP-dependent cotranslational protein targeting to membrane; C:signal recognition particle; F:GTPase activity; F:7S RNA binding |
| Efet.01.613416.g610.t1 | pilus assembly | 1731 | 0 | 62.35 | C:membrane; C:integral component of membrane |
| Efet.01.613419.g611.t1 | transcriptional regulator | 435 | 2.30E-59 | 63.95 |  |
| Efet.01.613439.g613.t1 | phosphomevalonate kinase-like | 252 | 5.09E-25 | 69.75 | C:peroxisome; P:isopentenyl diphosphate biosynthetic process, mevalonate pathway; P:cholesterol biosynthetic process; F:phosphomevalonate kinase activity |
| Efet.01.613487.g614.t1 | Fis family transcriptional regulator | 1071 | 0 | 73.5 | F:ATP binding; P:transcription, DNA-templated; P:regulation of transcription, DNA-templated; F:sequence-specific DNA binding; F:transcription factor binding |
| Efet.01.613504.g616.t1 | glutamate synthase large subunit | 489 | 1.07E-79 | 77.3 | F:oxidoreductase activity, acting on the CH-NH2 group of donors; P:nitrogen compound metabolic process; P:single-organism metabolic process |
| Efet.01.613649.g617.t1 | sorting nexin | 297 | 3.85E-26 | 80.6 | F:phosphatidylinositol binding; P:intracellular protein transport; C:intracellular |
| Efet.01.613715.g619.t1 | hypothetical protein CAPTEDRAFT_172510 | 1578 | 1.67E-11 | 68.75 | F:nucleotide binding; F:protein kinase activity; P:protein phosphorylation |
| Efet.01.613800.g620.t1 | insulinoma-associated 1a-like | 1563 | 4.34E-53 | 72.25 | F:binding |
| Efet.01.613800.g621.t1 | hypothetical protein CAPTEDRAFT_219688 | 381 | 3.49E-08 | 57 | F:nucleic acid binding; F:metal ion binding |
| Efet.01.613817.g622.t1 | retinoblastoma 1 | 372 | 1.53E-22 | 61.7 | P:regulation of cellular process; C:intracellular part; C:membrane-bounded organelle |
| Efet.01.613856.g624.t1 | vacuolar sorting-associated 13A-like | 249 | 3.14E-23 | 70.1 | C:membrane; C:integral component of membrane |
| Efet.01.613884.g625.t1 | cytoplasmic dynein 1 heavy chain 1- | 849 | 3.71E-165 | 91.05 | F:ATPase activity; F:ATP binding; F:microtubule motor activity; C:dynein complex; P:microtubule-based movement |
| Efet.01.613959.g627.t1 | kinase C-like | 264 | 2.59E-19 | 97.9 | F:ATP binding; F:zinc ion binding; P:protein phosphorylation; C:intracellular; P:intracellular signal transduction; F:protein kinase C activity |
| Efet.01.61400.g707.t1 | endothelin-converting enzyme 1-like isoform X1 | 240 | 8.18E-10 | 64.4 | P:proteolysis; C:membrane; C:integral component of membrane; F:metalloendopeptidase activity; F:metallopeptidase activity |
| Efet.01.614177.g630.t1 | FMN-binding glutamate synthase family | 342 | 4.44E-48 | 74.85 | P:glutamate biosynthetic process; P:oxidation-reduction process; F:glutamate synthase activity |
| Efet.01.614177.g631.t1 | amino acid ABC transporter substrate-binding | 732 | 1.72E-109 | 69.95 | P:amino acid transport |
| Efet.01.614177.g632.t1 | ABC transporter ATP-binding | 909 | 3.02E-142 | 78.95 | F:ATPase activity; F:ATP binding |
| Efet.01.614177.g633.t1 | ABC transporter ATP-binding | 669 | 1.41E-154 | 91.95 | F:ATPase activity; F:ATP binding |
| Efet.01.614177.g634.t1 | branched-chain amino acid ABC transporter permease | 732 | 5.22E-141 | 88.25 | C:integral component of membrane; P:transport; C:plasma membrane; F:transporter activity |
| Efet.01.614177.g635.t1 | branched-chain amino acid ABC transporter permease | 1512 | 0 | 86.15 | C:integral component of membrane; P:transport; C:plasma membrane; F:transporter activity |
| Efet.01.614177.g636.t1 | N,N-dimethylformamidase | 1602 | 0 | 76.15 |  |
| Efet.01.61418.g708.t1 | ELAV 1 isoform X6 | 1083 | 1.42E-38 | 91.1 | F:nucleotide binding; F:RNA binding |
| Efet.01.614215.g637.t1 | hypothetical protein B5766_00800 | 810 | 8.28E-162 | 66.35 |  |
| Efet.01.614272.g638.t1 | RHS repeat | 3048 | 0 | 51.75 | P:self proteolysis; P:intein-mediated protein splicing; C:membrane; C:integral component of membrane |
| Efet.01.614272.g639.t1 | fibronectin type III domain-containing | 2394 | 0 | 73.2 |  |
| Efet.01.61428.g710.t1 | Wnt-6 isoform X2 | 234 | 5.06E-15 | 57.2 | P:multicellular organism development |
| Efet.01.614287.g640.t1 | retinol dehydrogenase 2 | 342 | 1.96E-22 | 65.55 | F:catalytic activity |
| Efet.01.614331.g641.t1 | ATP-dependent zinc metalloprotease | 765 | 7.61E-128 | 94.9 | F:ATPase activity; P:cell division; F:ATP binding; F:zinc ion binding; P:protein catabolic process; P:proteolysis; C:integral component of membrane; F:metalloendopeptidase activity; C:plasma membrane |
| Efet.01.614335.g642.t1 | hypothetical protein | 423 | 7.75E-06 | 63 |  |
| Efet.01.614356.g643.t1 | helicase with zinc finger domain 2- | 249 | 1.55E-06 | 57 |  |
| Efet.01.614411.g644.t1 | glucose-regulated 94 | 363 | 9.49E-57 | 93.45 | F:ATP binding; F:unfolded protein binding; P:protein folding; P:response to stress |
| Efet.01.614449.g645.t1 | TPR repeat-containing DDB_G0287407-like isoform X1 | 276 | 2.24E-09 | 51.19 |  |
| Efet.01.614463.g646.t1 | acyl- dehydrogenase | 1137 | 0 | 82.85 | F:acyl-CoA dehydrogenase activity; F:flavin adenine dinucleotide binding; F:butyryl-CoA dehydrogenase activity; P:oxidation-reduction process |
| Efet.01.614463.g647.t1 | ABC transporter permease | 972 | 8.72E-166 | 86.1 | C:integral component of membrane; P:transport; C:plasma membrane; F:transporter activity |
| Efet.01.614463.g648.t1 | ABC transporter substrate-binding | 1053 | 0 | 89.1 | F:monosaccharide transmembrane transporter activity; C:integral component of membrane; P:monosaccharide transport; C:plasma membrane |
| Efet.01.614463.g649.t1 | ABC transporter permease | 213 | 3.04E-30 | 84.95 | F:strictosidine synthase activity; C:integral component of membrane; P:biosynthetic process; P:transport; C:plasma membrane; F:transporter activity |
| Efet.01.614469.g650.t1 | Gastrula zinc finger | 522 | 9.72E-29 | 56.9 | F:nucleic acid binding; P:DNA integration |
| Efet.01.614500.g651.t1 | EF-hand domain-containing D2 | 330 | 6.24E-13 | 52.75 | F:calcium ion binding; C:membrane; C:integral component of membrane |
| Efet.01.614564.g653.t1 | elongation factor G | 1305 | 0 | 90.55 | F:GTP binding; F:translation elongation factor activity; C:cytoplasm; F:GTPase activity; P:translational elongation |
| Efet.01.614594.g654.t1 | peptidase M23 | 342 | 2.24E-78 | 54.75 |  |
| Efet.01.614627.g655.t1 | glutamine--fructose-6-phosphate transaminase (isomerizing) | 1311 | 0 | 85.5 | P:carbohydrate metabolic process; C:cytoplasm; F:glutamine-fructose-6-phosphate transaminase (isomerizing) activity; P:glutamine metabolic process; P:carbohydrate derivative biosynthetic process; F:carbohydrate binding |
| Efet.01.614640.g656.t1 | alpha-hydroxy-acid oxidizing | 483 | 1.22E-62 | 93.9 | F:FMN binding; F:oxidoreductase activity; P:oxidation-reduction process |
| Efet.01.614640.g657.t1 | alpha-hydroxy-acid oxidizing | 297 | 5.78E-59 | 86.9 | F:FMN binding; F:oxidoreductase activity; P:oxidation-reduction process |
| Efet.01.614640.g658.t1 | dolichol-phosphate mannosyltransferase | 285 | 7.26E-60 | 79.3 | F:transferase activity, transferring glycosyl groups |
| Efet.01.614674.g659.t1 | heat shock 70 kDa 12A | 372 | 3.68E-49 | 77.5 | F:nucleotide binding; C:extracellular exosome; P:blood vessel morphogenesis |
| Efet.01.614702.g660.t1 | O-methyltransferase | 642 | 8.63E-105 | 80.75 | F:caffeoyl-CoA O-methyltransferase activity; P:methylation |
| Efet.01.614715.g661.t1 | coiled-coil domain-containing 63-like | 417 | 2.17E-14 | 59.6 | C:outer dynein arm; P:outer dynein arm assembly |
| Efet.01.614719.g662.t1 | family transcriptional regulator | 651 | 4.66E-106 | 58.85 | F:DNA binding; F:transcription factor activity, sequence-specific DNA binding; P:transcription, DNA-templated; P:regulation of transcription, DNA-templated |
| Efet.01.614719.g663.t1 | glycine dehydrogenase subunit 2 | 2355 | 0 | 79.1 | F:glycine dehydrogenase (decarboxylating) activity; P:oxidation-reduction process; P:glycine catabolic process |
| Efet.01.614719.g664.t1 | aminomethyl-transferring glycine dehydrogenase | 504 | 7.25E-111 | 79 | F:glycine dehydrogenase (decarboxylating) activity; P:oxidation-reduction process; P:glycine catabolic process |
| Efet.01.614731.g665.t1 | family transcriptional regulator | 879 | 4.36E-176 | 84.55 | F:DNA binding; F:transcription factor activity, sequence-specific DNA binding; P:transcription, DNA-templated; P:regulation of transcription, DNA-templated |
| Efet.01.614731.g666.t1 | ABC transporter permease | 585 | 6.65E-91 | 88.95 | C:integral component of membrane; P:transport; C:plasma membrane |
| Efet.01.614731.g667.t1 | peptide nickel transport system permease | 768 | 1.96E-143 | 88.1 | C:integral component of membrane; P:transport; C:plasma membrane |
| Efet.01.614779.g670.t1 | inner nuclear membrane Man1 | 1617 | 1.11E-40 | 50.45 | F:nucleotide binding; P:negative regulation of BMP signaling pathway; C:nuclear inner membrane; C:integral component of nuclear inner membrane; P:negative regulation of activin receptor signaling pathway; C:membrane; C:integral component of membrane; P:negative regulation of transforming growth factor beta receptor signaling pathway |
| Efet.01.614783.g672.t1 | FMRFamide receptor-like | 1185 | 9.14E-115 | 55.35 | F:G-protein coupled receptor activity; P:signal transduction; C:membrane |
| Efet.01.614802.g673.t1 | PREDICTED: uncharacterized protein LOC109109510 | 426 | 4.15E-24 | 65.85 |  |
| Efet.01.614863.g674.t1 | Transmembrane protease serine 3 | 258 | 1.78E-11 | 58.25 | C:membrane; P:proteolysis; P:regulation of transcription, DNA-templated; F:peptidase activity; P:negative regulation of biological process |
| Efet.01.614947.g675.t1 | family transporter | 975 | 0 | 58.75 | C:integral component of membrane |
| Efet.01.614947.g676.t1 | family | 741 | 1.94E-91 | 92.3 |  |
| Efet.01.614947.g677.t1 | DNA internalization-related competence Rec2 | 759 | 5.61E-150 | 81.95 | C:integral component of membrane; P:establishment of competence for transformation |
| Efet.01.614988.g679.t1 | hypothetical protein HELRODRAFT_159178 | 264 | 1.02E-27 | 74.75 | C:integral component of membrane; F:carbohydrate binding |
| Efet.01.615088.g680.t1 | cysteine desulfuration | 441 | 2.35E-72 | 80.2 |  |
| Efet.01.615102.g681.t1 | hydrolase RBBP9 | 570 | 4.88E-69 | 72.15 | C:nucleus |
| Efet.01.615132.g682.t1 | gliding motility-associated C-terminal domain-containing | 1536 | 3.85E-135 | 54.9 |  |
| Efet.01.615141.g683.t1 | acid-sensing ion channel 1-like | 384 | 2.99E-13 | 56.79 | P:ion transport |
| Efet.01.61527.g714.t1 | beta-hexosaminidase subunit beta-like | 531 | 7.15E-26 | 60.65 | F:hydrolase activity, acting on glycosyl bonds |
| Efet.01.615273.g686.t1 | outer membrane | 756 | 3.87E-122 | 56.55 |  |
| Efet.01.61532.g715.t1 | intraflagellar transport 88 homolog isoform X2 | 453 | 7.86E-45 | 81.95 | C:cilium |
| Efet.01.615474.g691.t1 | peregrin isoform X1 | 885 | 6.02E-133 | 76.25 | P:histone H3 acetylation; F:metal ion binding; P:positive regulation of transcription, DNA-templated; C:membrane; C:MOZ/MORF histone acetyltransferase complex |
| Efet.01.615480.g692.t1 | low-density lipo receptor-related 2 | 366 | 1.20E-13 | 50.45 | F:calcium ion binding; C:membrane; C:integral component of membrane |
| Efet.01.615518.g693.t1 | adenosine deaminase | 471 | 3.75E-40 | 62.45 | F:metal ion binding; F:deaminase activity; F:hydrolase activity |
| Efet.01.615518.g694.t1 | hypothetical protein B5766_04620 | 1071 | 8.20E-126 | 57.45 | C:membrane; C:integral component of membrane |
| Efet.01.61556.g717.t1 | tyrosine- kinase RYK-like isoform X2 | 420 | 1.50E-30 | 63.6 | F:ATP binding; C:membrane; C:integral component of membrane; F:protein kinase activity; P:protein phosphorylation |
| Efet.01.615563.g696.t1 | Transmembrane 126A | 483 | 1.22E-15 | 52.25 | C:membrane; C:integral component of membrane |
| Efet.01.615722.g697.t1 | pre translocase subunit | 1212 | 0 | 91.45 | F:ATP binding; P:protein targeting; C:cytoplasm; P:intracellular protein transmembrane transport; C:thylakoid membrane; F:helicase activity; P:protein import; C:plasma membrane |
| Efet.01.615736.g698.t1 | non-ribosomal peptide synthetase | 1398 | 0 | 50.65 | F:hydrolase activity, acting on ester bonds; F:catalytic activity; P:metabolic process; P:biosynthetic process; F:phosphopantetheine binding |
| Efet.01.615753.g699.t1 | NYN domain-containing | 894 | 4.58E-22 | 52.85 | F:nucleic acid binding; F:ATP binding; F:zinc ion binding; F:metal ion binding; F:protein kinase activity; P:protein phosphorylation |
| Efet.01.615793.g700.t1 | outer membrane | 285 | 6.40E-48 | 74.95 | P:transport; F:transporter activity |
| Efet.01.6158.g487.t1 | semaphorin-5A-like isoform X1 | 270 | 2.05E-20 | 70.1 | P:regulation of locomotion; P:system development; P:locomotion; C:membrane; P:cell differentiation; P:regulation of cellular component movement; P:response to stimulus |
| Efet.01.615802.g701.t1 | hypothetical protein | 237 | 3.06E-11 | 62.9 |  |
| Efet.01.615846.g703.t1 | MFS transporter | 1089 | 4.84E-79 | 58.95 | C:membrane coat; P:transmembrane transport; P:vesicle-mediated transport; C:membrane; C:integral component of membrane; P:intracellular protein transport |
| Efet.01.615846.g704.t1 | 7-cyano-7-deazaguanine synthase | 249 | 1.35E-42 | 81.4 | F:ATP binding; F:metal ion binding; F:ligase activity |
| Efet.01.615846.g705.t1 | N-acetyl-gamma-glutamyl-phosphate reductase | 1191 | 6.48E-179 | 92.5 | F:N-acetyl-gamma-glutamyl-phosphate reductase activity; P:arginine biosynthetic process; C:cytoplasm; F:NAD binding; F:protein dimerization activity; P:oxidation-reduction process |
| Efet.01.615846.g706.t1 | repressor | 453 | 4.49E-54 | 67.55 | P:cellular response to stress |
| Efet.01.615846.g707.t1 | Acyl- synthetase (AMP-forming) AMP-acid ligase II | 1470 | 0 | 64.55 | F:catalytic activity |
| Efet.01.615846.g708.t1 | conserved hypothetical transmembrane | 1032 | 4.79E-114 | 64.3 | C:membrane |
| Efet.01.615846.g709.t1 | Acetylornithine succinyldiaminopimelate putrescine aminotransferase | 2781 | 0 | 74.25 | F:transferase activity |
| Efet.01.615910.g711.t1 | metal tolerance 11-like | 294 | 6.59E-46 | 86.85 | P:cation transmembrane transport; C:integral component of membrane; F:cation transmembrane transporter activity |
| Efet.01.616135.g713.t1 | LPP20 lipo | 207 | 2.78E-38 | 83.15 |  |
| Efet.01.61614.g718.t1 | Kelch 8 | 396 | 6.53E-17 | 58.25 | F:nucleic acid binding; F:ubiquitin-protein transferase activity; F:metal ion binding; C:Cul3-RING ubiquitin ligase complex; P:protein ubiquitination |
| Efet.01.61615.g719.t1 | tumor necrosis factor alpha-induced 3-like | 1206 | 2.52E-09 | 47.1 | P:protein deubiquitination |
| Efet.01.61619.g720.t1 | baculoviral IAP repeat-containing 6 isoform X2 | 210 | 2.25E-08 | 62.3 | F:ubiquitin-protein transferase activity; P:apoptotic process; P:regulation of cytokinesis; P:protein ubiquitination |
| Efet.01.616211.g714.t1 | hypothetical protein UZ12_BCD005002796 | 1212 | 1.33E-92 | 55.85 |  |
| Efet.01.616213.g715.t1 | hypothetical protein HELRODRAFT_158794 | 651 | 2.49E-09 | 64 | C:membrane; C:integral component of membrane |
| Efet.01.616228.g716.t1 | monovalent cation H+ antiporter subunit A | 1371 | 2.79E-168 | 87.95 | F:NADH dehydrogenase (quinone) activity; C:integral component of membrane; P:oxidation-reduction process |
| Efet.01.616228.g717.t1 | Helix-hairpin-helix motif | 354 | 1.26E-43 | 87.4 |  |
| Efet.01.616228.g718.t1 | lipopolysaccharide assembly | 570 | 1.10E-95 | 91.8 | P:regulation of lipid biosynthetic process; F:iron ion binding; C:integral component of membrane; P:lipopolysaccharide metabolic process; C:intrinsic component of the cytoplasmic side of the plasma membrane |
| Efet.01.61623.g721.t1 | neural-cadherin-like isoform X1 | 642 | 2.85E-83 | 60.7 | F:calcium ion binding; C:integral component of membrane; P:homophilic cell adhesion via plasma membrane adhesion molecules; C:plasma membrane |
| Efet.01.616238.g719.t1 | Arrestin domain-containing 2 | 2181 | 5.77E-07 | 50.83 |  |
| Efet.01.616322.g721.t1 | piezo-type mechanosensitive ion channel component 2-like isoform X2 | 216 | 5.85E-16 | 86.9 | F:mechanically-gated ion channel activity; P:ion transmembrane transport; C:integral component of membrane |
| Efet.01.616332.g722.t1 | ATP-dependent 6-phosphofructokinase | 732 | 1.83E-115 | 81.3 | F:ATP binding; F:diphosphate-fructose-6-phosphate 1-phosphotransferase activity; C:cytoplasm; F:metal ion binding; P:fructose 6-phosphate metabolic process; P:glycolytic process through fructose-6-phosphate; P:carbohydrate phosphorylation; F:6-phosphofructokinase activity |
| Efet.01.61634.g722.t1 | synapsin | 303 | 4.98E-30 | 82.1 | F:ATP binding; F:catalytic activity; F:metal ion binding; C:synaptic vesicle; P:neurotransmitter secretion |
| Efet.01.61636.g723.t1 | nuclear hormone receptor HR96-like isoform X3 | 402 | 2.56E-45 | 86 | C:nucleus; F:DNA binding; P:transcription, DNA-templated; F:steroid hormone receptor activity; P:regulation of transcription, DNA-templated; P:steroid hormone mediated signaling pathway |
| Efet.01.616429.g723.t1 | transaldolase | 276 | 3.57E-27 | 82.65 | P:carbohydrate metabolic process; F:sedoheptulose-7-phosphate:D-glyceraldehyde-3-phosphate glyceronetransferase activity; C:cytoplasm; P:pentose-phosphate shunt |
| Efet.01.616453.g724.t1 | sugar ABC transporter substrate-binding | 288 | 1.10E-51 | 78.1 |  |
| Efet.01.616477.g726.t1 | RNA polymerase sigma-54 factor | 594 | 1.46E-48 | 74.1 | F:DNA binding; F:sigma factor activity; F:transcription factor activity, sequence-specific DNA binding; F:DNA-directed 5'-3' RNA polymerase activity; P:regulation of transcription, DNA-templated; P:DNA-templated transcription, initiation |
| Efet.01.616518.g727.t1 | general transcription factor IIH subunit 1 | 654 | 4.16E-37 | 55.85 | P:transcription, DNA-templated; C:core TFIIH complex; P:nucleotide-excision repair |
| Efet.01.616557.g728.t1 | leucine zipper tumor suppressor 2 homolog | 309 | 8.51E-07 | 67 |  |
| Efet.01.616615.g730.t1 | ABC transporter permease | 885 | 1.13E-160 | 91.55 | C:integral component of membrane; P:transport; C:plasma membrane |
| Efet.01.616615.g731.t1 | polyamine ABC transporter permease | 483 | 4.17E-81 | 94.15 | C:integral component of membrane; P:transport; C:plasma membrane |
| Efet.01.616615.g732.t1 | NAD-dependent succinate-semialdehyde dehydrogenase | 2982 | 0 | 92.65 | F:succinate-semialdehyde dehydrogenase [NAD(P)+] activity; P:gamma-aminobutyric acid catabolic process; P:oxidation-reduction process |
| Efet.01.616615.g733.t1 | acyl- dehydrogenase | 1161 | 0 | 90.65 | F:isovaleryl-CoA dehydrogenase activity; F:flavin adenine dinucleotide binding; P:oxidation-reduction process |
| Efet.01.616615.g734.t1 | acyl- dehydrogenase | 837 | 1.21E-142 | 75.65 | F:oxidoreductase activity, acting on the CH-CH group of donors; F:flavin adenine dinucleotide binding; P:oxidation-reduction process |
| Efet.01.616622.g735.t1 | DNA repair | 366 | 3.51E-50 | 72.8 | P:DNA metabolic process; P:cellular response to DNA damage stimulus |
| Efet.01.616625.g736.t1 | hypothetical protein CAPTEDRAFT_191415 | 558 | 4.00E-14 | 66 | C:membrane; C:integral component of membrane |
| Efet.01.61674.g725.t1 | eukaryotic translation initiation factor 3 subunit L-like | 450 | 4.68E-65 | 84.15 | F:translation initiation factor activity; P:formation of translation preinitiation complex; C:eukaryotic 43S preinitiation complex; C:eukaryotic 48S preinitiation complex; P:regulation of translational initiation; C:eukaryotic translation initiation factor 3 complex |
| Efet.01.61675.g726.t1 | tyrosine- kinase PR2 isoform X3 | 627 | 1.10E-08 | 79.65 | F:ATP binding; P:peptidyl-tyrosine phosphorylation; F:transmembrane receptor protein tyrosine kinase activity; C:membrane; P:transmembrane receptor protein tyrosine kinase signaling pathway |
| Efet.01.616805.g739.t1 | hypothetical protein HELRODRAFT_193047 | 204 | 3.45E-33 | 74.5 | F:calcium ion binding |
| Efet.01.616830.g741.t1 | dachsous-like | 2259 | 1.16E-107 | 48.9 | F:calcium ion binding; C:membrane; C:integral component of membrane; P:cell adhesion; P:homophilic cell adhesion via plasma membrane adhesion molecules; C:plasma membrane |
| Efet.01.616864.g743.t1 | transcriptional regulator | 447 | 3.38E-63 | 89.1 | F:DNA binding; C:cytoplasm; P:transcription, DNA-templated; P:regulation of transcription, DNA-templated |
| Efet.01.616898.g744.t1 | CTP synthase | 840 | 3.47E-158 | 72.3 | F:nucleotide binding; P:pyrimidine nucleotide biosynthetic process; F:ligase activity |
| Efet.01.616899.g745.t1 | 5 -3 exonuclease | 615 | 1.64E-133 | 83.45 | F:DNA binding; P:nucleic acid phosphodiester bond hydrolysis; F:exonuclease activity |
| Efet.01.61693.g727.t1 | centrosomal of 135 kDa-like isoform X1 | 738 | 4.59E-54 | 64.7 | P:centriole replication; P:centriole-centriole cohesion |
| Efet.01.616936.g747.t1 | 30S ribosomal S16 | 336 | 9.05E-27 | 84.5 | F:structural constituent of ribosome; C:ribosome; P:translation |
| Efet.01.617012.g748.t1 | oxysterol-binding -related 2 | 288 | 1.00E-25 | 68.45 | P:lipid transport; P:transport |
| Efet.01.617036.g749.t1 | leucine--tRNA cytoplasmic | 399 | 1.06E-36 | 79.05 | F:ATP binding; P:leucyl-tRNA aminoacylation; F:leucine-tRNA ligase activity; P:regulation of translational fidelity; F:aminoacyl-tRNA editing activity; C:cytosol |
| Efet.01.617117.g750.t1 | glutamine amidotransferase | 423 | 4.55E-95 | 72.25 | F:catalytic activity |
| Efet.01.617179.g752.t1 | ryanodine receptor-like | 363 | 4.62E-28 | 76.9 | F:ryanodine-sensitive calcium-release channel activity; F:calcium ion binding; C:integral component of membrane; P:release of sequestered calcium ion into cytosol; C:intracellular |
| Efet.01.617194.g753.t1 | insulin-like growth factor-binding complex acid labile subunit | 969 | 1.35E-79 | 48.65 | F:protein binding |
| Efet.01.617238.g755.t1 | NADH:ubiquinone reductase (Na(+)-transporting) subunit F | 612 | 6.28E-106 | 60.35 | F:oxidoreductase activity; C:membrane; F:iron-sulfur cluster binding; P:single-organism metabolic process |
| Efet.01.617238.g756.t1 | hypothetical protein | 924 | 0 | 61.4 |  |
| Efet.01.617238.g757.t1 | ABC transporter permease | 483 | 1.26E-105 | 93.2 | C:integral component of membrane; P:transport; C:plasma membrane |
| Efet.01.617238.g758.t1 | nitrate ABC transporter ATP-binding | 837 | 0 | 89.5 | F:ATPase activity; F:ATP binding |
| Efet.01.617238.g759.t1 | taurine ABC transporter permease | 975 | 0 | 91.15 | P:thiamine biosynthetic process |
| Efet.01.617238.g760.t1 | ribokinase | 534 | 5.38E-57 | 80.8 | F:ribokinase activity; P:D-ribose metabolic process; P:carbohydrate phosphorylation |
| Efet.01.617251.g761.t1 | type II secretion system | 360 | 7.54E-67 | 94.05 | C:integral component of membrane; F:protein transporter activity; P:protein secretion by the type II secretion system; C:type II protein secretion system complex |
| Efet.01.617251.g762.t1 | type II secretion system | 546 | 8.59E-61 | 96.1 | C:integral component of membrane; F:protein transporter activity; P:protein secretion by the type II secretion system; C:type II protein secretion system complex |
| Efet.01.617251.g763.t1 | general secretion pathway C | 348 | 3.66E-43 | 89 | C:integral component of membrane |
| Efet.01.617251.g764.t1 | amidohydrolase | 372 | 7.54E-28 | 75.45 | F:hydrolase activity; P:nitrate assimilation |
| Efet.01.617251.g765.t1 | family transcriptional regulator | 654 | 4.15E-71 | 76.8 | F:DNA binding; F:transcription factor activity, sequence-specific DNA binding; P:regulation of transcription, DNA-templated |
| Efet.01.617254.g766.t1 | FAD-binding oxidoreductase | 894 | 2.26E-167 | 66.05 | F:oxidoreductase activity |
| Efet.01.617285.g767.t1 | suppressor of cytokine signaling 2-like | 384 | 4.54E-21 | 58.75 | P:negative regulation of cellular process |
| Efet.01.617300.g768.t1 | F-box LRR-repeat 18-like | 930 | 1.02E-21 | 51.33 |  |
| Efet.01.617305.g769.t1 | family transcriptional regulator | 687 | 1.92E-115 | 59.6 |  |
| Efet.01.617314.g770.t1 | T-box -like isoform X1 | 303 | 7.24E-29 | 91.85 | C:nucleus; F:DNA binding; F:transcription factor activity, sequence-specific DNA binding; P:transcription, DNA-templated; P:regulation of transcription, DNA-templated |
| Efet.01.617320.g771.t1 | 3 -5 exonuclease | 612 | 4.65E-61 | 71.15 | F:nucleic acid binding; P:nucleic acid phosphodiester bond hydrolysis; F:exonuclease activity |
| Efet.01.617322.g772.t1 | collagen alpha-1(XXVIII) chain-like | 465 | 2.97E-12 | 51 | C:collagen trimer |
| Efet.01.617364.g773.t1 | dynein heavy chain axonemal | 474 | 1.54E-56 | 85.05 | F:ATPase activity; F:ATP binding; F:microtubule motor activity; C:dynein complex; P:microtubule-based movement |
| Efet.01.617369.g774.t1 | anhydro-N-acetylmuramic acid kinase | 597 | 1.55E-26 | 68.7 | P:carbohydrate derivative metabolic process; F:transferase activity, transferring phosphorus-containing groups |
| Efet.01.617383.g775.t1 | nuclease mitochondrial-like | 1074 | 1.88E-124 | 69.7 | C:membrane; F:binding; F:hydrolase activity |
| Efet.01.617415.g776.t1 | hypothetical protein HELRODRAFT_163906 | 471 | 1.14E-13 | 56.67 | F:nucleic acid binding |
| Efet.01.617415.g777.t1 | hypothetical protein CAPTEDRAFT_210373 | 420 | 9.10E-23 | 70.75 | F:nucleic acid binding |
| Efet.01.617461.g778.t1 | AAA family ATPase | 615 | 2.56E-70 | 84.2 | F:ATP binding |
| Efet.01.617477.g779.t1 | ABC transporter permease | 372 | 9.16E-44 | 67.75 | C:membrane |
| Efet.01.617477.g780.t1 | sugar ABC transporter ATP-binding | 690 | 1.72E-160 | 68.3 | F:hydrolase activity |
| Efet.01.617490.g781.t1 | extracellular serine threonine CG31145 isoform X2 | 204 | 1.15E-23 | 69.7 | C:membrane; C:integral component of membrane |
| Efet.01.617535.g783.t1 | amino acid permease | 291 | 2.06E-34 | 73 | C:membrane |
| Efet.01.617535.g784.t1 | amino acid permease | 858 | 4.09E-180 | 72.05 | C:membrane; C:integral component of membrane; F:amino acid transmembrane transporter activity; P:amino acid transmembrane transport |
| Efet.01.617546.g785.t1 | phospholipase c epsilon | 375 | 3.45E-10 | 53.5 | P:biological regulation; P:single-organism process |
| Efet.01.617606.g786.t1 | aldehyde dehydrogenase | 2277 | 0 | 84.15 | F:oxidoreductase activity; F:electron carrier activity; P:oxidation-reduction process; F:heme binding |
| Efet.01.617630.g787.t1 | acyl- dehydrogenase | 1614 | 0 | 84.25 | F:acyl-CoA dehydrogenase activity; F:flavin adenine dinucleotide binding; P:oxidation-reduction process |
| Efet.01.617638.g788.t1 | stAR-related lipid transfer 5-like | 225 | 1.37E-17 | 59.7 | F:lipid binding |
| Efet.01.617871.g791.t1 | ubiquitin conjugation factor E4 A | 516 | 7.78E-32 | 56.5 | F:ubiquitin-protein transferase activity; C:ubiquitin ligase complex; F:ubiquitin-ubiquitin ligase activity; P:ubiquitin-dependent protein catabolic process; P:protein ubiquitination |
| Efet.01.617979.g792.t1 | transcription factor Sox-19b-like | 882 | 2.11E-25 | 46.3 | P:multicellular organism development |
| Efet.01.618025.g793.t1 | FAD NAD(P)-binding oxidoreductase | 996 | 0 | 76.35 | F:oxidoreductase activity; P:oxidation-reduction process |
| Efet.01.618025.g794.t1 | ABC transporter permease | 816 | 1.65E-119 | 70.85 | C:integral component of membrane; P:transport; C:plasma membrane; F:transporter activity |
| Efet.01.618025.g795.t1 | ABC transporter permease | 2514 | 0 | 64.6 | F:ATPase activity; F:ATP binding; C:integral component of membrane; P:transport; C:plasma membrane; F:transporter activity |
| Efet.01.618025.g796.t1 | family transcriptional regulator | 1119 | 0 | 78.45 |  |
| Efet.01.618025.g797.t1 | inner-membrane translocator | 321 | 5.74E-74 | 66 |  |
| Efet.01.618025.g798.t1 | large subunit of N,N-dimethylformamidase | 2262 | 0 | 65.55 |  |
| Efet.01.618025.g799.t1 | tRNA (guanosine(46)-N7)-methyltransferase | 834 | 2.22E-157 | 82.8 | P:RNA (guanine-N7)-methylation; F:tRNA (guanine-N7-)-methyltransferase activity; P:tRNA modification |
| Efet.01.618025.g800.t1 | diguanylate cyclase | 1050 | 0 | 89.55 |  |
| Efet.01.618025.g801.t1 | alanine--tRNA ligase | 2619 | 0 | 92.6 | F:ATP binding; F:zinc ion binding; C:cytoplasm; F:alanine-tRNA ligase activity; P:alanyl-tRNA aminoacylation; F:tRNA binding |
| Efet.01.618030.g802.t1 | L-arabinose isomerase | 705 | 1.28E-145 | 84.25 | C:cytoplasm; F:manganese ion binding; F:L-arabinose isomerase activity; P:L-arabinose catabolic process to xylulose 5-phosphate |
| Efet.01.61804.g730.t1 | patatin | 915 | 0 | 83.6 | P:lipid metabolic process |
| Efet.01.61804.g731.t1 | phosphoribosyl-ATP diphosphatase | 405 | 5.01E-80 | 86.7 | F:ATP binding; F:phosphoribosyl-ATP diphosphatase activity; C:cytoplasm; P:histidine biosynthetic process |
| Efet.01.61804.g732.t1 | phosphoribosyl-AMP cyclohydrolase | 324 | 1.76E-61 | 93.95 | F:magnesium ion binding; F:zinc ion binding; F:phosphoribosyl-AMP cyclohydrolase activity; C:cytoplasm; P:histidine biosynthetic process |
| Efet.01.61804.g733.t1 | formyltetrahydrofolate deformylase | 717 | 3.51E-142 | 86.6 | P:'de novo' IMP biosynthetic process; F:amino acid binding; F:hydroxymethyl-, formyl- and related transferase activity; P:one-carbon metabolic process; F:formyltetrahydrofolate deformylase activity |
| Efet.01.61804.g734.t1 | heme-binding uptake (Tiki superfamily) | 462 | 1.26E-87 | 78.7 | C:integral component of membrane |
| Efet.01.61804.g735.t1 | DUF4440 domain-containing | 516 | 2.74E-81 | 87.1 |  |
| Efet.01.618071.g803.t1 | WD repeat-containing 24 | 564 | 7.31E-35 | 64.4 |  |
| Efet.01.618083.g804.t1 | glutamine amidotransferase | 1191 | 3.61E-125 | 85.35 | P:glutamine metabolic process; F:transferase activity |
| Efet.01.618112.g805.t1 | hypothetical protein HELRODRAFT_170818 | 489 | 1.29E-43 | 59.35 | C:membrane; C:integral component of membrane |
| Efet.01.618138.g807.t1 | transcriptional regulator | 342 | 3.56E-43 | 81 | F:DNA binding; F:transcription factor activity, sequence-specific DNA binding; P:transcription, DNA-templated; P:regulation of transcription, DNA-templated; C:intracellular |
| Efet.01.618138.g808.t1 | galactonate dehydratase | 438 | 1.16E-84 | 82.45 | P:cellular amino acid catabolic process; F:galactonate dehydratase activity |
| Efet.01.618209.g811.t1 | peptidase M23 | 324 | 2.89E-45 | 66.45 |  |
| Efet.01.618270.g812.t1 | surfeit locus 4-like | 321 | 5.16E-36 | 81.8 | F:GTP binding; P:small GTPase mediated signal transduction; C:integral component of membrane; C:intracellular |
| Efet.01.618287.g813.t1 | sugar ABC transporter | 411 | 2.99E-73 | 80.3 | C:integral component of membrane; P:transport; C:plasma membrane |
| Efet.01.618287.g814.t1 | sugar ABC transporter | 2448 | 0 | 85.85 | C:integral component of membrane; P:carbohydrate transport; C:plasma membrane; F:transporter activity |
| Efet.01.618287.g815.t1 | ABC transporter ATP-binding | 597 | 6.60E-127 | 85.15 | F:ATP binding; P:glycerol-3-phosphate transport; P:transmembrane transport; F:glycerol-3-phosphate-transporting ATPase activity; C:ATP-binding cassette (ABC) transporter complex |
| Efet.01.618313.g818.t1 | bifunctional glutamate N-acetyltransferase amino-acid acetyltransferase | 588 | 1.06E-105 | 84.35 | F:glutamate N-acetyltransferase activity; P:arginine biosynthetic process; C:cytoplasm; F:acetyl-CoA:L-glutamate N-acetyltransferase activity |
| Efet.01.618343.g819.t1 | chromosomal replication initiator | 477 | 5.48E-54 | 95.45 | F:DNA replication origin binding; F:ATP binding; P:DNA replication initiation; C:cytoplasm; P:regulation of DNA replication |
| Efet.01.618343.g820.t1 | DNA polymerase III subunit beta | 2217 | 0 | 88.5 | C:DNA polymerase III complex; F:DNA binding; F:3'-5' exonuclease activity; P:nucleic acid phosphodiester bond hydrolysis; F:DNA-directed DNA polymerase activity; C:cytoplasm; P:DNA replication; P:DNA biosynthetic process |
| Efet.01.618376.g822.t1 | chromosome segregation SMC | 864 | 1.56E-139 | 68.75 | F:organic cyclic compound binding; F:heterocyclic compound binding; P:chromosome organization |
| Efet.01.61838.g736.t1 | laminin subunit alpha-1 | 1143 | 5.43E-85 | 47.95 | C:membrane; C:integral component of membrane |
| Efet.01.618382.g823.t1 | dienelactone hydrolase | 321 | 5.81E-60 | 71.55 | F:hydrolase activity |
| Efet.01.618401.g824.t1 | 3-keto-5-aminohexanoate cleavage | 870 | 0 | 87.45 | P:L-lysine catabolic process to acetate; F:transferase activity |
| Efet.01.618401.g825.t1 | 3-hydroxyacyl- dehydrogenase | 1632 | 0 | 83.3 | F:3-hydroxyacyl-CoA dehydrogenase activity; F:3-hydroxybutyrate dehydrogenase activity; F:3-hydroxybutyryl-CoA dehydrogenase activity; P:oxidation-reduction process; F:NAD+ binding; P:fatty acid metabolic process |
| Efet.01.618401.g826.t1 | ABC transporter | 2397 | 0 | 89.8 | F:ATPase activity; F:ATP binding; C:integral component of membrane; P:carbohydrate transport; C:plasma membrane; F:transporter activity |
| Efet.01.618401.g827.t1 | branched-chain amino acid ABC transporter permease | 891 | 1.07E-124 | 95.65 | C:integral component of membrane; P:transport; C:plasma membrane; F:transporter activity |
| Efet.01.618401.g828.t1 | ABC transporter substrate-binding | 1299 | 0 | 87.5 |  |
| Efet.01.618401.g829.t1 | family transcriptional regulator | 690 | 6.18E-159 | 76.85 | F:DNA binding; F:transcription factor activity, sequence-specific DNA binding; P:transcription, DNA-templated; P:regulation of transcription, DNA-templated |
| Efet.01.618414.g830.t1 | nitrous oxide reductase family maturation | 915 | 3.51E-129 | 73.45 |  |
| Efet.01.618419.g831.t1 | sugar phosphate isomerase epimerase | 807 | 0 | 77.7 | F:catalytic activity |
| Efet.01.61843.g737.t1 | hypothetical protein HELRODRAFT_176722 | 5949 | 3.04E-68 | 67 | F:metal ion binding |
| Efet.01.618477.g833.t1 | racemase | 861 | 1.34E-147 | 76.5 | F:metal ion binding; F:catalytic activity; P:metabolic process |
| Efet.01.618477.g834.t1 | hypothetical protein B5766_01535 | 561 | 3.15E-132 | 54.2 |  |
| Efet.01.618479.g835.t1 | 2-dehydro-3-deoxyglucarate aldolase | 486 | 3.84E-59 | 66.6 | F:metal ion binding; F:catalytic activity; F:lyase activity |
| Efet.01.618480.g837.t1 | alpha-1A adrenergic receptor-like | 1152 | 7.72E-95 | 74.7 | F:adrenergic receptor activity; P:regulation of muscle contraction; C:integral component of membrane; P:adrenergic receptor signaling pathway; P:regulation of blood circulation; C:plasma membrane |
| Efet.01.618559.g838.t1 | copper-transporting ATPase 1-like isoform X2 | 1227 | 1.02E-54 | 49.55 | F:nucleotide binding; P:cation transport; F:copper ion binding; F:cation-transporting ATPase activity; F:metal ion binding; C:membrane; P:cation transmembrane transport; C:integral component of membrane; P:metal ion transport; F:hydrolase activity |
| Efet.01.618629.g839.t1 | kunitz-type protease inhibitor 3 | 831 | 7.03E-07 | 54.65 | F:peptidase inhibitor activity; F:serine-type endopeptidase inhibitor activity; F:zinc ion binding; P:proteolysis; P:negative regulation of endopeptidase activity; F:peptidase activity; C:extracellular matrix; C:extracellular region; F:metalloendopeptidase activity; P:negative regulation of peptidase activity; C:proteinaceous extracellular matrix; F:metallopeptidase activity |
| Efet.01.61863.g739.t1 | FMRFamide receptor-like | 657 | 1.71E-59 | 55.9 | F:signal transducer activity |
| Efet.01.618695.g841.t1 | carbamoyl-phosphate synthase large subunit | 642 | 1.49E-105 | 91.45 | F:magnesium ion binding; F:ATP binding; P:arginine biosynthetic process; F:manganese ion binding; F:carbamoyl-phosphate synthase (glutamine-hydrolyzing) activity; P:'de novo' UMP biosynthetic process |
| Efet.01.618725.g842.t1 | serine threonine kinase | 1797 | 1.73E-26 | 54.5 | F:metal ion binding; C:membrane; C:integral component of membrane; C:proteasome complex; P:phosphorylation; F:protein serine/threonine kinase activity; P:protein phosphorylation; F:kinase activity |
| Efet.01.618726.g843.t1 | gastrula zinc finger -like | 2463 | 1.62E-46 | 51.2 | F:nucleic acid binding; F:metal ion binding |
| Efet.01.618741.g844.t1 | polycystic kidney disease and receptor for egg jelly-related | 510 | 4.84E-13 | 48.7 | P:detection of mechanical stimulus; F:calcium channel activity; F:calcium ion binding; C:membrane; C:integral component of membrane; P:calcium ion transmembrane transport; P:regulation of apoptotic process |
| Efet.01.618756.g845.t1 | deleted in autism 1 homolog isoform X1 | 552 | 1.41E-12 | 52.6 | C:membrane; C:integral component of membrane; P:regulation of signal transduction |
| Efet.01.618818.g846.t1 | carbohydrate ABC transporter permease | 870 | 2.19E-155 | 75.25 |  |
| Efet.01.618821.g847.t1 | 5-methyltetrahydropteroyltriglutamate--homocysteine S-methyltransferase | 270 | 1.64E-35 | 91 | P:methionine biosynthetic process; F:zinc ion binding; F:5-methyltetrahydropteroyltriglutamate-homocysteine S-methyltransferase activity; P:methylation |
| Efet.01.618826.g848.t1 | bromodomain adjacent to zinc finger domain 2B isoform X1 | 345 | 3.17E-40 | 79.9 | C:polytene chromosome; F:DNA binding; F:histone acetyltransferase activity; F:zinc ion binding; C:ISWI-type complex; P:ATP-dependent chromatin remodeling; P:nervous system development; P:histone acetylation; F:transcription factor binding; P:positive regulation of transcription from RNA polymerase II promoter |
| Efet.01.618835.g849.t1 | hypothetical protein CRN43_13620, partial | 429 | 6.47E-72 | 59.4 | P:self proteolysis |
| Efet.01.618848.g850.t1 | WD repeat-containing 63 | 270 | 3.67E-31 | 81.55 |  |
| Efet.01.618860.g851.t1 | titin-like isoform X5 | 510 | 6.72E-35 | 58.35 |  |
| Efet.01.618905.g853.t1 | lon protease homolog peroxisomal | 606 | 3.11E-55 | 63.15 | F:peptidase activity, acting on L-amino acid peptides; P:proteolysis; P:cellular process |
| Efet.01.618943.g854.t1 | proton-coupled folate transporter-like | 1239 | 1.71E-127 | 47.95 | P:transmembrane transport; C:membrane; C:integral component of membrane |
| Efet.01.61898.g740.t1 | zinc finger homeobox 4-like isoform X1 | 771 | 4.13E-26 | 55.45 | F:binding |
| Efet.01.619063.g855.t1 | family transcriptional regulator | 1287 | 0 | 62.05 |  |
| Efet.01.61926.g744.t1 | Nucleoside diphosphate kinase | 825 | 2.53E-51 | 61 | P:ribonucleotide biosynthetic process; P:ribonucleoside triphosphate biosynthetic process; P:ribonucleoside biosynthetic process |
| Efet.01.619296.g856.t1 | nucleoside hydrolase | 552 | 1.89E-70 | 68.35 | F:hydrolase activity |
| Efet.01.619389.g858.t1 | DUF2917 domain-containing | 504 | 7.37E-77 | 69.2 |  |
| Efet.01.619389.g859.t1 | family transcriptional regulator | 942 | 3.71E-177 | 90.25 | F:DNA binding; F:transcription factor activity, sequence-specific DNA binding; P:transcription, DNA-templated; P:regulation of transcription, DNA-templated |
| Efet.01.619389.g860.t1 | tyrosine--tRNA ligase | 1233 | 0 | 93 | F:tyrosine-tRNA ligase activity; F:ATP binding; P:tyrosyl-tRNA aminoacylation; F:RNA binding; C:cytoplasm |
| Efet.01.619389.g861.t1 | peptidase M23 | 1389 | 0 | 92.9 | C:integral component of membrane |
| Efet.01.619389.g862.t1 | anhydro-N-acetylmuramic acid kinase | 1110 | 0 | 84.9 | F:ATP binding; P:carbohydrate metabolic process; P:peptidoglycan turnover; P:amino sugar metabolic process; P:phosphorylation; F:phosphotransferase activity, alcohol group as acceptor; F:kinase activity; P:transport; F:transporter activity; P:1,6-anhydro-N-acetyl-beta-muramic acid catabolic process |
| Efet.01.619389.g863.t1 | cell shape determination | 399 | 2.03E-89 | 89.6 |  |
| Efet.01.619389.g864.t1 | hypothetical protein | 723 | 1.55E-129 | 79.7 | C:integral component of membrane |
| Efet.01.619389.g865.t1 | 50S ribosomal L13 | 567 | 9.40E-109 | 96.4 | F:structural constituent of ribosome; C:ribosome; P:translation |
| Efet.01.619389.g866.t1 | peptidase M48 | 669 | 4.63E-141 | 91.1 | P:proteolysis; F:metalloendopeptidase activity |
| Efet.01.619442.g867.t1 | hypothetical protein CAPTEDRAFT_224679 | 957 | 5.94E-90 | 50.38 | C:membrane; F:transferase activity; P:cellular metabolic process |
| Efet.01.619447.g868.t1 | serine threonine- kinase tousled-like 2 isoform X1 | 306 | 2.00E-41 | 93.85 | F:ATP binding; F:protein serine/threonine kinase activity; P:protein phosphorylation |
| Efet.01.619478.g870.t1 | dynein heavy chain axonemal | 213 | 1.65E-27 | 86.5 | F:ATPase activity; C:inner dynein arm; F:ATP binding; F:microtubule motor activity; C:cytoplasm; F:calcium ion binding; P:cilium or flagellum-dependent cell motility; P:cilium movement; P:inner dynein arm assembly; P:determination of liver left/right asymmetry; C:microtubule |
| Efet.01.619569.g871.t1 | titin-like isoform X5 | 321 | 1.16E-34 | 76.8 | P:mitotic chromosome condensation; F:muscle alpha-actinin binding; F:protease binding; P:protein phosphorylation; P:cardiac muscle fiber development; P:cardiac muscle contraction; F:protein self-association; C:condensed nuclear chromosome; F:structural molecule activity conferring elasticity; F:nucleotide binding; P:platelet degranulation; C:striated muscle thin filament; F:protein tyrosine kinase activity; C:extracellular exosome; F:calcium ion binding; C:cytosol; P:skeletal muscle myosin thick filament assembly; P:cardiac myofibril assembly; C:M band; P:detection of muscle stretch; F:telethonin binding; P:cardiac muscle tissue morphogenesis; P:muscle filament sliding; F:protein kinase binding; F:identical protein binding; P:regulation of protein kinase activity; P:skeletal muscle thin filament assembly; P:sarcomere organization; F:protein serine/threonine kinase activity; P:cardiac muscle hypertrophy; P:sarcomerogenesis; F:actin filament binding; C:Z disc; F:calmodulin binding; F:structural constituent of muscle; C:muscle myosin complex; P:response to calcium ion |
| Efet.01.619581.g872.t1 | O-acetylhomoserine aminocarboxypropyltransferase cysteine synthase | 615 | 6.86E-103 | 92.9 | F:pyridoxal phosphate binding; P:'de novo' L-methionine biosynthetic process; F:O-acetylhomoserine aminocarboxypropyltransferase activity |
| Efet.01.619642.g873.t1 | uncharacterized protein LOC107455767 | 750 | 2.66E-20 | 58.85 | P:signal transduction; C:membrane; C:integral component of membrane |
| Efet.01.619725.g874.t1 | glass-like | 1212 | 3.29E-58 | 97.3 | F:DNA binding; F:metal ion binding; P:DNA integration; P:transposition, DNA-mediated |
| Efet.01.61974.g746.t1 | uncharacterized protein LOC111044999 | 318 | 1.16E-10 | 82.9 |  |
| Efet.01.620007.g877.t1 | hypothetical protein CAPTEDRAFT_109895 | 750 | 1.11E-76 | 61.55 | C:membrane; C:integral component of membrane; C:outer membrane-bounded periplasmic space |
| Efet.01.620063.g878.t1 | DUF1343 domain-containing | 705 | 3.13E-150 | 69.85 |  |
| Efet.01.62007.g750.t1 | dysferlin-like isoform X3 | 555 | 1.49E-40 | 67.5 | C:membrane |
| Efet.01.620076.g879.t1 | secretion system | 1032 | 1.18E-174 | 80.45 | C:integral component of membrane |
| Efet.01.620076.g880.t1 | pilus assembly | 660 | 1.89E-118 | 86.95 | C:integral component of membrane |
| Efet.01.620076.g881.t1 | secretion system E | 1338 | 0 | 90.95 | F:ATP binding; P:transport |
| Efet.01.620132.g882.t1 | bile acid:sodium symporter family | 1005 | 0 | 84.8 | C:integral component of membrane |
| Efet.01.620132.g883.t1 | dimethylmenaquinone methyltransferase | 861 | 2.82E-130 | 88.55 | F:methyltransferase activity; P:methylation |
| Efet.01.620144.g884.t1 | nucleoid-associated family | 201 | 1.86E-20 | 78.65 | F:DNA binding; C:bacterial nucleoid; C:cytoplasm |
| Efet.01.620235.g885.t1 | ADP-ribosylglycohydrolase | 270 | 7.45E-40 | 83.35 | F:hydrolase activity |
| Efet.01.620235.g886.t1 | family transcriptional regulator | 663 | 3.39E-121 | 63.8 | F:DNA binding; F:transcription factor activity, sequence-specific DNA binding; P:transcription, DNA-templated; P:regulation of transcription, DNA-templated |
| Efet.01.620235.g887.t1 | PIN domain-containing | 651 | 2.24E-53 | 78.5 | F:DNA binding |
| Efet.01.620235.g888.t1 | nucleotidyl transferase ii ii toxin family | 873 | 1.70E-176 | 74.35 |  |
| Efet.01.620247.g889.t1 | netrin-3 [Xenopus tropicalis] | 300 | 7.57E-23 | 65.85 | C:extracellular region |
| Efet.01.620294.g890.t1 | allatostatin-A receptor-like | 717 | 2.09E-49 | 59.55 | F:G-protein coupled receptor activity; P:G-protein coupled receptor signaling pathway; P:signal transduction; C:membrane; C:integral component of membrane; F:signal transducer activity |
| Efet.01.62034.g752.t1 | chromodomain-helicase-DNA-binding 4-like | 537 | 7.85E-61 | 87.6 | F:DNA binding; F:ATP binding; P:spindle organization; F:zinc ion binding; P:centrosome organization; C:NuRD complex; F:ATP-dependent helicase activity; P:regulation of transcription, DNA-templated; P:chromatin organization |
| Efet.01.620396.g892.t1 | tripartite motif-containing 2-like | 1299 | 3.01E-99 | 46.65 |  |
| Efet.01.620402.g893.t1 | interferon-induced very large GTPase 1 | 1437 | 2.67E-44 | 48.25 | F:GTP binding |
| Efet.01.620404.g894.t1 | ATP-dependent DNA helicase PIF1-like | 615 | 6.80E-38 | 62.85 | F:organic cyclic compound binding; F:heterocyclic compound binding; F:helicase activity; P:DNA metabolic process; P:organelle organization |
| Efet.01.620408.g895.t1 | DNA polymerase I | 369 | 2.10E-58 | 88.1 | F:DNA binding; P:nucleic acid phosphodiester bond hydrolysis; F:DNA-directed DNA polymerase activity; F:nuclease activity; P:DNA-dependent DNA replication; P:DNA biosynthetic process |
| Efet.01.620435.g896.t1 | pilus assembly | 1695 | 0 | 68.15 |  |
| Efet.01.620496.g897.t1 | bacteriocin biosynthesis cyclodehydratase domain-containing | 339 | 7.20E-73 | 68.55 |  |
| Efet.01.620549.g898.t1 | phospholipase D1 isoform X1 | 1074 | 3.31E-08 | 91.88 | F:phosphatidylinositol binding; P:inositol lipid-mediated signaling; F:phospholipase D activity; P:regulation of cell migration; C:intracellular; P:regulation of phagocytosis; P:phosphatidic acid biosynthetic process |
| Efet.01.620559.g899.t1 | endonuclease exonuclease phosphatase family | 351 | 2.12E-74 | 60.6 | C:membrane; C:integral component of membrane |
| Efet.01.620559.g900.t1 | sugar ABC transporter substrate-binding | 1317 | 0 | 61.2 | P:transport; F:transporter activity |
| Efet.01.62056.g753.t1 | proline dehydrogenase mitochondrial isoform X1 | 345 | 5.26E-22 | 86.2 | F:proline dehydrogenase activity; F:calcium ion binding; P:oxidation-reduction process; P:proline catabolic process |
| Efet.01.620660.g902.t1 | hypothetical protein HELRODRAFT_168921 | 435 | 7.78E-32 | 58.4 | C:membrane; C:integral component of membrane |
| Efet.01.620686.g903.t1 | POU class transcription factor 2 | 1149 | 3.20E-80 | 76.75 | P:inner ear receptor cell differentiation; P:transcription, DNA-templated; P:retina development in camera-type eye; F:RNA polymerase II core promoter proximal region sequence-specific DNA binding; P:regulation of signal transduction by p53 class mediator; P:axon guidance; P:positive regulation of cell differentiation; P:positive regulation of transcription from RNA polymerase II promoter; C:nucleoplasm; P:negative regulation of transcription from RNA polymerase II promoter; P:sensory perception of sound; F:transcriptional repressor activity, RNA polymerase II core promoter proximal region sequence-specific binding; P:MAPK cascade; F:transcriptional activator activity, RNA polymerase II core promoter proximal region sequence-specific binding; P:intracellular estrogen receptor signaling pathway |
| Efet.01.620724.g904.t1 | glucoamylase family | 588 | 4.36E-93 | 83.5 |  |
| Efet.01.620747.g905.t1 | citrate (Si)-synthase | 1332 | 0 | 79.2 | F:citrate (Si)-synthase activity; C:cytoplasm; P:tricarboxylic acid cycle |
| Efet.01.620750.g906.t1 | 2-oxoglutarate dehydrogenase E1 component | 1608 | 0 | 96.45 | F:thiamine pyrophosphate binding; F:oxoglutarate dehydrogenase (succinyl-transferring) activity; P:tricarboxylic acid cycle |
| Efet.01.62079.g755.t1 | Spermatogenesis-associated 13 | 357 | 1.62E-16 | 62.3 | P:regulation of Rho protein signal transduction; F:guanyl-nucleotide exchange factor activity; F:Rho guanyl-nucleotide exchange factor activity; P:intracellular signal transduction; P:positive regulation of GTPase activity; C:intracellular |
| Efet.01.620809.g908.t1 | serine protease | 471 | 1.72E-65 | 55.1 | C:membrane; P:proteolysis; C:integral component of membrane; F:serine-type endopeptidase activity; P:toxin biosynthetic process |
| Efet.01.620822.g909.t1 | calreticulin | 798 | 1.39E-126 | 92.45 | F:calcium ion binding; C:endoplasmic reticulum; F:unfolded protein binding; P:protein folding |
| Efet.01.620860.g911.t1 | TonB-dependent siderophore receptor | 1554 | 0 | 88.85 | F:iron ion binding; P:siderophore transport; C:cell outer membrane; F:receptor activity |
| Efet.01.620881.g913.t1 | family nutrient uptake outer membrane | 354 | 7.79E-77 | 73.65 |  |
| Efet.01.62090.g756.t1 | PREDICTED: uncharacterized protein LOC101858864 | 303 | 2.74E-33 | 91.05 | F:metal ion binding |
| Efet.01.621075.g915.t1 | Transformation transcription domain-associated | 375 | 1.65E-16 | 88.6 | P:DNA repair; F:substrate-specific transmembrane transporter activity; P:transmembrane transport; C:integral component of membrane; P:histone acetylation; P:phosphorylation; F:kinase activity; C:histone acetyltransferase complex |
| Efet.01.621126.g916.t1 | hypothetical protein HELRODRAFT_181938 | 270 | 3.17E-06 | 71 |  |
| Efet.01.621224.g917.t1 | DNA-directed RNA polymerase subunit beta | 2052 | 0 | 85.5 | F:DNA binding; F:DNA-directed 5'-3' RNA polymerase activity; F:ribonucleoside binding; P:transcription, DNA-templated |
| Efet.01.621228.g918.t1 | long-chain fatty acid transport 4-like | 300 | 1.42E-41 | 82.4 | F:catalytic activity; P:metabolic process; C:integral component of membrane |
| Efet.01.621233.g919.t1 | family heavy metal efflux RND transporter | 357 | 1.79E-70 | 90.2 | P:cation transmembrane transport; C:integral component of membrane; F:cation transmembrane transporter activity |
| Efet.01.621259.g920.t1 | LTA synthase family | 252 | 4.38E-19 | 63.5 | F:catalytic activity; F:metal ion binding; F:sulfuric ester hydrolase activity; P:metabolic process; C:membrane; C:integral component of membrane |
| Efet.01.621298.g922.t1 | ribosome-binding factor A | 426 | 1.44E-37 | 74.95 | C:cytoplasm; P:rRNA processing |
| Efet.01.62130.g757.t1 | UPF0183 C16orf70 homolog | 267 | 1.05E-08 | 67.6 |  |
| Efet.01.621328.g923.t1 | class II fructose-bisphosphate aldolase | 777 | 2.45E-157 | 91.25 | F:zinc ion binding; P:glycolytic process; F:fructose-bisphosphate aldolase activity |
| Efet.01.621328.g924.t1 | hypothetical protein B5766_09000 | 288 | 1.01E-60 | 60.65 | C:membrane; C:integral component of membrane |
| Efet.01.62134.g758.t1 | hypothetical protein OCBIM_22028527mg, partial | 288 | 1.02E-14 | 61.7 |  |
| Efet.01.621343.g926.t1 | ABC transporter ATP-binding | 384 | 1.08E-66 | 74.85 | F:nucleotide binding |
| Efet.01.621349.g927.t1 | tripartite tricarboxylate transporter substrate binding | 900 | 5.48E-150 | 68.85 | C:outer membrane-bounded periplasmic space |
| Efet.01.621349.g928.t1 | sulfoacetaldehyde dehydrogenase | 1254 | 0 | 92.4 | F:succinate-semialdehyde dehydrogenase [NAD(P)+] activity; F:acetaldehyde dehydrogenase (acetylating) activity; P:oxidation-reduction process |
| Efet.01.621349.g929.t1 | long-chain fatty acid-- ligase | 1353 | 0 | 86.3 | P:long-chain fatty acid metabolic process; F:long-chain fatty acid-CoA ligase activity |
| Efet.01.621349.g930.t1 | choline dehydrogenase | 1698 | 0 | 92.7 | F:flavin adenine dinucleotide binding; P:oxidation-reduction process; F:choline dehydrogenase activity |
| Efet.01.621374.g933.t1 | ephrin type-B receptor 3- | 354 | 2.46E-30 | 72.05 | F:nucleotide binding; F:protein tyrosine kinase activity; C:integral component of membrane; P:transmembrane receptor protein tyrosine kinase signaling pathway; P:protein phosphorylation |
| Efet.01.621374.g934.t1 | ephrin type-B receptor 1-B-like isoform X4 | 345 | 3.98E-19 | 57.85 | F:transferase activity; P:cellular process |
| Efet.01.62138.g759.t1 | coatomer subunit delta | 372 | 2.29E-19 | 80.3 | P:cerebellar Purkinje cell layer maturation; F:protein binding; P:retrograde vesicle-mediated transport, Golgi to ER; P:pigmentation; P:protein transport; C:Golgi membrane; C:endoplasmic reticulum; P:intra-Golgi vesicle-mediated transport; P:adult locomotory behavior; C:COPI vesicle coat; F:RNA binding |
| Efet.01.62138.g760.t1 | coatomer subunit delta-like | 258 | 1.25E-37 | 94.3 | P:cerebellar Purkinje cell layer maturation; P:retrograde vesicle-mediated transport, Golgi to ER; P:pigmentation; P:protein transport; C:endoplasmic reticulum; P:adult locomotory behavior; C:COPI vesicle coat; F:RNA binding |
| Efet.01.621466.g935.t1 | F-box LRR-repeat 21 | 1404 | 1.97E-58 | 43.75 | C:nucleus; P:rhythmic process; C:cytoplasm; P:entrainment of circadian clock by photoperiod; C:cytosol; C:SCF ubiquitin ligase complex; P:protein ubiquitination |
| Efet.01.621517.g938.t1 | Transcription factor SUM-1 | 867 | 3.20E-18 | 79.7 | P:muscle organ development; C:nucleus; F:DNA binding; P:regulation of transcription, DNA-templated; F:protein dimerization activity |
| Efet.01.621603.g941.t1 | ABC transporter ATP-binding | 807 | 3.63E-154 | 81.3 | F:ATP binding; F:ATPase activity, coupled to transmembrane movement of substances; P:transmembrane transport; C:integral component of membrane |
| Efet.01.621630.g942.t1 | spermidine putrescine ABC transporter substrate-binding | 303 | 2.76E-64 | 82.15 | F:polyamine binding; C:periplasmic space; P:polyamine transport |
| Efet.01.621650.g944.t1 | sodium calcium exchanger 1-like isoform X1 | 726 | 5.72E-102 | 74.15 | P:cell communication; P:calcium ion transport; C:integral component of membrane; P:sodium ion transmembrane transport; F:calcium:sodium antiporter activity |
| Efet.01.621685.g947.t1 | acetyl- propionyl- carboxylase subunit alpha | 207 | 4.87E-34 | 87.8 | F:ATP binding; F:metal ion binding; F:biotin carboxylase activity |
| Efet.01.621690.g948.t1 | N-acetylglucosamine kinase-like BadF-type ATPase | 921 | 0 | 53.75 |  |
| Efet.01.621705.g950.t1 | bifunctional nicotinamidase pyrazinamidase | 798 | 3.89E-130 | 99.85 | F:nicotinamidase activity; P:metabolic process |
| Efet.01.621705.g951.t1 | ABC transporter ATP-binding | 621 | 5.51E-149 | 98.85 | F:ATPase activity; F:ATP binding |
| Efet.01.621705.g952.t1 | ABC transporter ATP-binding | 792 | 0 | 97.4 | F:ATPase activity; F:ATP binding |
| Efet.01.621744.g953.t1 | ankyrin repeat | 261 | 6.18E-18 | 86.05 |  |
| Efet.01.621789.g955.t1 | hypothetical protein OCBIM_22010820mg | 525 | 1.15E-10 | 57.5 |  |
| Efet.01.621879.g958.t1 | specificity 1 | 918 | 6.06E-11 | 75.8 | F:nucleic acid binding; F:metal ion binding |
| Efet.01.621913.g960.t1 | oxidoreductase | 867 | 3.04E-100 | 54.65 | F:oxidoreductase activity; P:metabolic process; P:oxidation-reduction process |
| Efet.01.621926.g961.t1 | sex peptide receptor-like | 609 | 3.95E-40 | 70.65 | F:G-protein coupled peptide receptor activity; P:G-protein coupled receptor signaling pathway; C:integral component of membrane |
| Efet.01.621926.g962.t1 | sex peptide receptor-like | 639 | 5.27E-52 | 63.4 | F:G-protein coupled receptor activity; C:membrane |
| Efet.01.621967.g963.t1 | hypothetical protein BRAFLDRAFT_76566 | 510 | 5.25E-06 | 51 | C:membrane; C:integral component of membrane |
| Efet.01.621993.g964.t1 | cilia- and flagella-associated 54- | 615 | 3.11E-29 | 53.75 |  |
| Efet.01.62204.g764.t1 | multiple PDZ domain -like isoform X3 | 549 | 1.11E-25 | 61.65 |  |
| Efet.01.622071.g965.t1 | tripartite motif-containing 2-like | 726 | 5.71E-70 | 71.35 | F:metal ion binding |
| Efet.01.622077.g966.t1 | acetylcholinesterase isoform X2 | 312 | 2.31E-40 | 76.5 | C:extracellular space; P:ion transport; C:membrane; F:carboxylic ester hydrolase activity |
| Efet.01.62210.g765.t1 | death domain-containing CRADD | 267 | 6.22E-16 | 66.55 | P:regulation of cellular process |
| Efet.01.62214.g766.t1 | helicase with zinc finger domain 2 | 708 | 2.77E-07 | 51.5 | F:nucleotide binding; F:nucleic acid binding; F:5'-flap endonuclease activity; F:ATP binding; F:zinc ion binding; F:metal ion binding; F:single-stranded DNA-dependent ATPase activity; P:DNA replication, Okazaki fragment processing |
| Efet.01.622143.g967.t1 | glycoside hydrolase family 92 | 288 | 4.38E-37 | 90.3 | P:carbohydrate metabolic process; F:catalytic activity; F:carbohydrate binding |
| Efet.01.622143.g968.t1 | glycoside hydrolase family 92 | 519 | 1.01E-122 | 83.55 | P:carbohydrate metabolic process; F:carbohydrate binding; F:hydrolase activity |
| Efet.01.622144.g969.t1 | ABC transporter permease | 777 | 9.75E-68 | 66.62 | C:membrane |
| Efet.01.622158.g971.t1 | uncharacterized protein LOC110452122 | 1530 | 7.55E-89 | 54.6 |  |
| Efet.01.622173.g972.t1 | ABC transporter permease | 873 | 4.14E-148 | 86.7 | C:integral component of membrane; P:transport; C:plasma membrane |
| Efet.01.622184.g973.t1 | ABC transporter ATP-binding | 438 | 1.28E-25 | 56.75 | F:organic cyclic compound binding; F:heterocyclic compound binding |
| Efet.01.622187.g974.t1 | phosphate ABC transporter ATP-binding | 378 | 1.29E-50 | 94.2 | F:inorganic phosphate transmembrane transporter activity; F:ATP binding; F:ATPase-coupled phosphate ion transmembrane transporter activity; C:ATP-binding cassette (ABC) transporter complex; P:phosphate ion transmembrane transport |
| Efet.01.622209.g975.t1 | hypothetical protein CAPTEDRAFT_202568 | 243 | 4.07E-07 | 51 | P:sodium ion transport; P:ion transport; F:sodium channel activity; C:membrane; C:integral component of membrane; P:sodium ion transmembrane transport; P:transport |
| Efet.01.622218.g976.t1 | zinc finger 333 | 897 | 4.00E-24 | 70.14 | F:nucleic acid binding; F:metal ion binding |
| Efet.01.622230.g977.t1 | major facilitator superfamily domain-containing 6 isoform X1 | 471 | 8.79E-18 | 51 | C:membrane; C:integral component of membrane |
| Efet.01.622256.g978.t1 | arabinose ABC transporter permease | 390 | 6.51E-86 | 95.35 | C:integral component of membrane |
| Efet.01.622256.g979.t1 | uroporphyrinogen decarboxylase | 540 | 5.46E-107 | 95.65 | F:uroporphyrinogen decarboxylase activity; C:cytoplasm; P:protoporphyrinogen IX biosynthetic process |
| Efet.01.622471.g981.t1 | hypothetical protein HELRODRAFT_194269 | 279 | 8.63E-06 | 55 |  |
| Efet.01.622543.g982.t1 | protocadherin beta-10-like isoform X1 | 2853 | 0 | 56.35 | F:calcium ion binding; C:membrane; C:integral component of membrane; P:cell adhesion; P:homophilic cell adhesion via plasma membrane adhesion molecules; C:plasma membrane |
| Efet.01.622554.g984.t1 | phosphatase | 363 | 3.84E-49 | 84.65 | F:protein serine/threonine phosphatase activity; P:protein dephosphorylation; C:integral component of membrane |
| Efet.01.62256.g768.t1 | aldehyde dehydrogenase family 3 member B1 isoform X1 | 369 | 8.26E-28 | 86.05 | F:aldehyde dehydrogenase (NAD) activity; P:aldehyde catabolic process; F:3-chloroallyl aldehyde dehydrogenase activity; C:extracellular exosome; F:aldehyde dehydrogenase [NAD(P)+] activity; C:integral component of membrane; C:cytosol; P:cellular response to oxidative stress; P:oxidation-reduction process; C:plasma membrane |
| Efet.01.622583.g985.t1 | Site-specific recombinase | 657 | 1.01E-111 | 58.9 | F:DNA binding; P:DNA integration; P:DNA recombination |
| Efet.01.622583.g986.t1 | family transcriptional regulator | 378 | 2.01E-56 | 78.1 | F:DNA binding; P:DNA recombination; P:DNA integration |
| Efet.01.622583.g987.t1 | integrase | 1449 | 0 | 80.45 | F:DNA binding; C:cytoplasm; P:DNA recombination; P:DNA integration |
| Efet.01.622583.g988.t1 | hypothetical protein HMPREF1004_02335 | 411 | 9.46E-60 | 93.85 | P:biological_process; C:cellular_component; F:molecular_function |
| Efet.01.622583.g989.t1 | hypothetical protein ABS84_10000 | 645 | 2.90E-137 | 93.35 | P:biological_process; C:cellular_component; F:molecular_function |
| Efet.01.622583.g990.t1 | PIN domain-containing | 339 | 1.04E-67 | 84.65 | F:magnesium ion binding; P:RNA phosphodiester bond hydrolysis; F:serine O-acetyltransferase activity; F:ribonuclease activity |
| Efet.01.622588.g991.t1 | hypothetical protein | 1056 | 0 | 94 |  |
| Efet.01.622588.g992.t1 | N-acetyltransferase | 474 | 1.17E-112 | 78.3 | F:N-acetyltransferase activity |
| Efet.01.622588.g993.t1 | Transposase | 840 | 9.93E-113 | 69.8 | F:nucleic acid binding; F:catalytic activity |
| Efet.01.62261.g769.t1 | caspase 8 | 405 | 3.12E-38 | 58.95 | P:regulation of cellular process; P:proteolysis; P:apoptotic process; F:cysteine-type endopeptidase activity; P:cellular response to stimulus; C:intracellular part |
| Efet.01.622611.g995.t1 | signal peptide | 1083 | 0 | 71.25 |  |
| Efet.01.622611.g996.t1 | branched-chain amino acid ABC transporter permease | 624 | 6.24E-147 | 64.25 | C:integral component of membrane |
| Efet.01.622640.g997.t1 | diacylglycerol kinase delta-like isoform X2 | 321 | 1.02E-54 | 92.65 | F:ATP binding; F:diacylglycerol kinase activity; P:phosphorylation; P:protein kinase C-activating G-protein coupled receptor signaling pathway; C:intracellular; P:intracellular signal transduction |
| Efet.01.622654.g1000.t1 | outer membrane | 375 | 2.26E-21 | 77.65 |  |
| Efet.01.622654.g998.t1 | outer membrane | 969 | 1.03E-171 | 57.45 |  |
| Efet.01.622654.g999.t1 | hypothetical protein | 753 | 8.75E-151 | 70.88 |  |
| Efet.01.62271.g770.t1 | KAT8 regulatory NSL complex subunit 3 isoform X1 | 342 | 6.54E-25 | 65.5 |  |
| Efet.01.622836.g1003.t1 | transmembrane 132E-like | 852 | 2.71E-49 | 60 | C:membrane; C:integral component of membrane |
| Efet.01.622836.g1004.t1 | transmembrane 132C | 543 | 1.72E-15 | 49.14 | C:membrane; C:integral component of membrane |
| Efet.01.622882.g1005.t1 | alpha-glucosidase | 267 | 9.28E-11 | 83.65 | P:carbohydrate metabolic process; F:catalytic activity |
| Efet.01.622883.g1006.t1 | chromatin-remodeling complex ATPase chain Iswi | 219 | 4.33E-41 | 97.2 | F:ATPase activity; F:DNA binding; F:ATP binding; F:nucleosome binding; P:ATP-dependent chromatin remodeling; C:NURF complex |
| Efet.01.622929.g1008.t1 | dynein heavy chain axonemal-like isoform X1 | 1056 | 5.72E-06 | 63 |  |
| Efet.01.622937.g1009.t1 | agrin-like | 567 | 1.08E-46 | 60.35 | P:G-protein coupled acetylcholine receptor signaling pathway; P:receptor clustering; F:laminin binding; C:plasma membrane |
| Efet.01.62294.g771.t1 | bactericidal permeability increasing | 1341 | 0 | 54 | F:lipid binding |
| Efet.01.623094.g1010.t1 | G- coupled receptor | 1053 | 1.03E-98 | 65.45 | F:G-protein coupled receptor activity; P:G-protein coupled receptor signaling pathway; C:membrane |
| Efet.01.62310.g772.t1 | sacsin-like isoform X1 | 357 | 1.81E-11 | 58.6 |  |
| Efet.01.623105.g1012.t1 | iron dicitrate transport regulator | 993 | 1.24E-122 | 60.3 | C:membrane; C:integral component of membrane |
| Efet.01.623153.g1013.t1 | ribonuclease HI | 462 | 6.19E-90 | 89.75 | F:magnesium ion binding; F:nucleic acid binding; P:RNA phosphodiester bond hydrolysis, endonucleolytic; F:RNA-DNA hybrid ribonuclease activity; C:cytoplasm; P:RNA catabolic process |
| Efet.01.623153.g1014.t1 | aminoacyl-tRNA hydrolase | 588 | 3.34E-111 | 83.1 | C:cytoplasm; F:aminoacyl-tRNA hydrolase activity; P:translation |
| Efet.01.62320.g773.t1 | kelch 17 | 990 | 5.07E-127 | 70.75 | F:ubiquitin-protein transferase activity; C:Cul3-RING ubiquitin ligase complex |
| Efet.01.623231.g1015.t1 | Dystonin | 1104 | 2.53E-08 | 50 |  |
| Efet.01.623247.g1016.t1 | UDP-galactose translocator-like isoform X3 | 597 | 1.80E-74 | 79.5 | P:proton transport; C:integral component of membrane; P:carbohydrate transport; F:sugar:proton symporter activity; C:Golgi membrane |
| Efet.01.623265.g1017.t1 | dedicator of cytokinesis 7-like isoform X2 | 291 | 1.28E-16 | 75.8 | P:small GTPase mediated signal transduction; F:guanyl-nucleotide exchange factor activity; C:intracellular; P:positive regulation of GTPase activity |
| Efet.01.623336.g1020.t1 | hypothetical protein CAPTEDRAFT_226428 | 651 | 2.78E-13 | 62 | F:calcium ion binding; C:membrane; C:integral component of membrane |
| Efet.01.623419.g1021.t1 | FAD-dependent oxidoreductase domain-containing 1-like | 294 | 3.02E-21 | 58.9 | F:oxidoreductase activity; P:oxidation-reduction process |
| Efet.01.623527.g1027.t1 | bifunctional [glutamate--ammonia ligase]-adenylyl-L-tyrosine phosphorylase [glutamate--ammonia-ligase] adenylyltransferase | 2496 | 0 | 90.25 | F:magnesium ion binding; F:ATP binding; F:[glutamate-ammonia-ligase] adenylyltransferase activity; F:ligase activity |
| Efet.01.623527.g1028.t1 | efflux RND transporter periplasmic adaptor subunit | 1221 | 0 | 84.2 | P:transmembrane transport; C:membrane |
| Efet.01.62369.g777.t1 | ATP-binding cassette sub-family B member mitochondrial-like | 432 | 7.38E-22 | 74.75 | F:ATP binding; F:ATPase activity, coupled to transmembrane movement of substances; P:transmembrane transport; C:integral component of membrane; C:mitochondrial inner membrane |
| Efet.01.62370.g778.t1 | Growth arrest-specific 8 | 270 | 5.78E-09 | 82.5 | C:motile cilium; P:flagellated sperm motility; P:positive regulation of smoothened signaling pathway; P:muscle cell development; C:Golgi apparatus; F:Rab GTPase binding; P:cellular protein localization; C:microtubule |
| Efet.01.623745.g1030.t1 | sugar kinase | 324 | 2.27E-48 | 64.55 | P:metabolic process; F:phosphotransferase activity, alcohol group as acceptor; F:kinase activity |
| Efet.01.623795.g1031.t1 | endopeptidase La | 1683 | 0 | 80.95 | F:ATP binding; P:misfolded or incompletely synthesized protein catabolic process; C:cytoplasm; F:serine-type endopeptidase activity; P:cellular response to stress; F:sequence-specific DNA binding; F:ATP-dependent peptidase activity |
| Efet.01.623825.g1033.t1 | beta-galactosidase | 1011 | 2.28E-159 | 82.3 | P:carbohydrate metabolic process; F:beta-galactosidase activity |
| Efet.01.623840.g1034.t1 | Oxysterol-binding 1 | 249 | 1.48E-15 | 82.4 |  |
| Efet.01.623866.g1035.t1 | phosphorylase b kinase regulatory subunit beta-like isoform X1 | 438 | 2.59E-25 | 79.55 | F:phosphorylase kinase activity; C:phosphorylase kinase complex; F:calmodulin binding; P:glycogen metabolic process |
| Efet.01.623868.g1036.t1 | hypothetical protein CAPTEDRAFT_211416 | 483 | 6.64E-09 | 67 |  |
| Efet.01.623940.g1038.t1 | 14-3-3 epsilon | 453 | 5.02E-76 | 88.7 | P:regulation of membrane repolarization; P:neuron migration; P:negative regulation of peptidyl-serine dephosphorylation; C:extracellular exosome; C:focal adhesion; F:protein heterodimerization activity; F:phosphoserine binding; F:protein domain specific binding; F:histone deacetylase binding; P:hippocampus development; P:cerebral cortex development; P:regulation of potassium ion transmembrane transporter activity; F:MHC class II protein complex binding; F:ion channel binding; P:substantia nigra development; F:RNA binding; F:cadherin binding involved in cell-cell adhesion; P:protein targeting; F:ubiquitin protein ligase binding; C:cell-cell adherens junction; C:melanosome; C:mitochondrion; C:membrane; P:negative regulation of protein serine/threonine kinase activity; P:cell-cell adhesion; F:potassium channel regulator activity |
| Efet.01.623950.g1039.t1 | bifunctional D-altronate D-mannonate dehydratase | 606 | 5.34E-125 | 96.35 | P:cellular amino acid catabolic process; F:catalytic activity; F:metal ion binding |
| Efet.01.623961.g1040.t1 | LTR-retrotransposon skipper | 273 | 4.15E-07 | 61.83 | F:nucleic acid binding; F:ATP binding; F:zinc ion binding; P:DNA integration; F:protein kinase activity; P:protein phosphorylation |
| Efet.01.623992.g1041.t1 | family transporter | 699 | 3.01E-130 | 85.1 | C:integral component of membrane |
| Efet.01.623992.g1042.t1 | adenosine deaminase | 2385 | 0 | 77.1 | F:transcription factor activity, sequence-specific DNA binding; F:zinc ion binding; F:DNA-3-methyladenine glycosylase activity; F:DNA-7-methylguanine glycosylase activity; P:base-excision repair; P:transcription, DNA-templated; P:regulation of transcription, DNA-templated; F:sequence-specific DNA binding; F:methylated-DNA-[protein]-cysteine S-methyltransferase activity; F:DNA-7-methyladenine glycosylase activity; F:DNA-3-methylguanine glycosylase activity; P:methylation |
| Efet.01.623992.g1043.t1 | phosphomannomutase phosphoglucomutase | 1392 | 0 | 86.2 | F:magnesium ion binding; P:carbohydrate metabolic process; F:phosphoglucomutase activity; F:phosphomannomutase activity |
| Efet.01.623992.g1044.t1 | 3-deoxy-D-manno-octulosonic acid transferase | 1206 | 0 | 86.45 | F:transferase activity |
| Efet.01.623992.g1045.t1 | aconitase subunit 1 | 1158 | 0 | 79.95 | F:aconitate hydratase activity |
| Efet.01.623992.g1046.t1 | DUF126 domain-containing | 300 | 9.91E-56 | 73.95 | F:transferase activity, transferring phosphorus-containing groups; P:phosphorylation; F:aconitate hydratase activity |
| Efet.01.623992.g1047.t1 | Phasin (PHA-granule associated ) | 549 | 7.69E-97 | 95.15 |  |
| Efet.01.623992.g1048.t1 | Fe(3+) ABC transporter substrate-binding | 1062 | 0 | 92.3 | F:metal ion binding; P:transport; F:transporter activity |
| Efet.01.623992.g1049.t1 | 3-hydroxyacyl- dehydrogenase | 738 | 3.05E-136 | 95.25 | F:oxidoreductase activity; P:oxidation-reduction process |
| Efet.01.623992.g1050.t1 | long-chain fatty acid-- ligase | 1737 | 0 | 93.2 | P:metabolic process; F:ligase activity |
| Efet.01.623992.g1051.t1 | magnesium transporter | 987 | 0 | 90.45 | P:cobalt ion transport; F:cobalt ion transmembrane transporter activity; C:integral component of membrane; F:magnesium ion transmembrane transporter activity; P:magnesium ion transmembrane transport; C:plasma membrane |
| Efet.01.623992.g1052.t1 | ACP synthase | 396 | 4.19E-85 | 63.75 | F:magnesium ion binding; C:cytoplasm; P:fatty acid biosynthetic process; F:holo-[acyl-carrier-protein] synthase activity |
| Efet.01.623996.g1053.t1 | outer membrane | 1062 | 1.29E-163 | 53.4 | F:calcium ion binding; C:extracellular region; P:pathogenesis |
| Efet.01.624000.g1054.t1 | mediator of RNA polymerase II transcription subunit 17 | 576 | 1.25E-43 | 63.1 | C:mediator complex; F:RNA polymerase II transcription cofactor activity; P:regulation of transcription from RNA polymerase II promoter |
| Efet.01.624010.g1055.t1 | protein-disulfide reductase | 1137 | 1.38E-95 | 76.05 | C:integral component of membrane; P:cell redox homeostasis; P:oxidation-reduction process; P:cytochrome complex assembly; C:plasma membrane |
| Efet.01.624063.g1057.t1 | hypothetical protein CRN43_02475 | 399 | 1.54E-69 | 91 |  |
| Efet.01.624068.g1058.t1 | 3-phosphoshikimate 1-carboxyvinyltransferase | 525 | 5.30E-39 | 72.1 | P:cellular amino acid biosynthetic process; F:transferase activity, transferring alkyl or aryl (other than methyl) groups |
| Efet.01.624068.g1059.t1 | DNA-binding transcriptional family | 411 | 1.34E-24 | 62.4 | F:DNA binding; F:transcription factor activity, sequence-specific DNA binding; P:transcription, DNA-templated; P:regulation of transcription, DNA-templated |
| Efet.01.624118.g1061.t1 | major facilitator superfamily domain-containing 6-A-like | 705 | 3.55E-37 | 54.35 | P:ion transport; P:proton transport; P:ATP hydrolysis coupled proton transport; F:hydrogen ion transmembrane transporter activity; C:membrane; C:integral component of membrane; C:proton-transporting V-type ATPase, V0 domain; P:transport |
| Efet.01.624119.g1062.t1 | spectrin alpha chain isoform X1 | 393 | 1.81E-60 | 71.75 | F:calcium ion binding |
| Efet.01.62413.g779.t1 | NACHT and WD repeat domain-containing 2-like | 5037 | 0 | 58.4 |  |
| Efet.01.624178.g1063.t1 | microtubule-actin cross-linking factor 1-like isoform X7 | 606 | 5.70E-41 | 70 | F:binding |
| Efet.01.624203.g1064.t1 | IS5 IS1182 family transposase | 777 | 5.49E-83 | 66.5 | F:DNA binding; F:transposase activity; P:transposition, DNA-mediated |
| Efet.01.624241.g1065.t1 | prune homolog 2-like isoform X1 | 450 | 2.40E-13 | 65.75 |  |
| Efet.01.6243.g491.t1 | Guanine nucleotide-binding subunit alpha-12 | 717 | 7.60E-60 | 80.4 | P:regulation of cell shape; P:adenylate cyclase-modulating G-protein coupled receptor signaling pathway; P:Rho protein signal transduction; F:signal transducer activity; P:embryonic digit morphogenesis; F:GTP binding; F:G-protein coupled receptor binding; C:heterotrimeric G-protein complex; C:focal adhesion; P:in utero embryonic development; F:GTPase activity; P:cell differentiation; F:G-protein beta/gamma-subunit complex binding; P:regulation of proteasomal ubiquitin-dependent protein catabolic process; P:regulation of fibroblast migration; P:regulation of TOR signaling |
| Efet.01.624318.g1066.t1 | plasma membrane calcium-transporting ATPase 2-like | 297 | 1.97E-25 | 90.9 | F:ATP binding; F:metal ion binding; C:integral component of membrane; P:calcium ion transmembrane transport; F:calcium-transporting ATPase activity |
| Efet.01.624326.g1067.t1 | ROK family | 669 | 2.70E-99 | 64.95 |  |
| Efet.01.624331.g1068.t1 | sugar ABC transporter substrate-binding | 1233 | 0 | 77.25 | P:transport; F:transporter activity |
| Efet.01.624347.g1069.t1 | SAM-dependent DNA methyltransferase | 1563 | 0 | 89.7 | F:DNA binding; F:N-methyltransferase activity; P:DNA methylation on adenine; F:site-specific DNA-methyltransferase (adenine-specific) activity |
| Efet.01.624347.g1070.t1 | 3-oxoacyl-ACP reductase | 762 | 0 | 95.65 | F:3-oxoacyl-[acyl-carrier-protein] reductase (NADPH) activity; P:oxidation-reduction process |
| Efet.01.624347.g1071.t1 | aldehyde dehydrogenase | 1434 | 0 | 94.5 | F:aldehyde dehydrogenase (NAD) activity; P:oxidation-reduction process |
| Efet.01.624397.g1072.t1 | collagen alpha-1(XIV) chain- | 738 | 2.12E-45 | 55.4 | F:calcium ion binding; C:collagen trimer |
| Efet.01.624524.g1073.t1 | PREDICTED: uncharacterized protein LOC106055922 | 516 | 4.40E-09 | 61 |  |
| Efet.01.624573.g1075.t1 | methyltransferase 24 | 261 | 1.55E-32 | 58.1 | C:membrane; C:integral component of membrane |
| Efet.01.624613.g1076.t1 | hypothetical protein | 861 | 7.70E-114 | 71.05 | F:catalytic activity |
| Efet.01.62471.g780.t1 | RNA-binding MEX3B-like | 1518 | 4.44E-50 | 77.7 | F:zinc ion binding; F:RNA binding |
| Efet.01.624818.g1078.t1 | ABC transporter ATP-binding | 258 | 1.11E-50 | 87.5 | F:ATPase activity; F:ATP binding |
| Efet.01.624818.g1079.t1 | branched-chain amino acid ABC transporter substrate-binding | 1062 | 0 | 82 | P:amino acid transport |
| Efet.01.624818.g1080.t1 | family transcriptional regulator | 912 | 9.63E-161 | 68.3 | F:DNA binding; F:transcription factor activity, sequence-specific DNA binding; P:transcription, DNA-templated; P:regulation of transcription, DNA-templated |
| Efet.01.624912.g1082.t1 | inositol monophosphatase | 477 | 4.50E-81 | 63.15 | F:catalytic activity |
| Efet.01.624933.g1084.t1 | ATP-binding | 468 | 3.07E-41 | 80.4 | F:ATP binding |
| Efet.01.624933.g1085.t1 | tRNA-(MS[2]IO[6]A)-hydroxylase ( )-like | 723 | 3.35E-93 | 49.35 |  |
| Efet.01.624933.g1086.t1 | ATP-dependent helicase | 876 | 1.16E-137 | 75.4 | F:nucleotide binding; F:hydrolase activity |
| Efet.01.624968.g1087.t1 | hypothetical protein LOTGIDRAFT_235976 | 276 | 5.08E-06 | 60 |  |
| Efet.01.624973.g1089.t1 | hypothetical protein CAPTEDRAFT_195412, partial | 405 | 1.04E-10 | 74 | P:cilium movement |
| Efet.01.625010.g1090.t1 | transcriptional regulator | 903 | 0 | 90.75 | F:DNA binding; F:transcription factor activity, sequence-specific DNA binding; P:transcription, DNA-templated; P:regulation of transcription, DNA-templated |
| Efet.01.625010.g1091.t1 | LPS export ABC transporter permease | 2136 | 0 | 90.6 | P:transmembrane transport; C:ATP-binding cassette (ABC) transporter complex |
| Efet.01.625096.g1095.t1 | periodic tryptophan 1 homolog | 1272 | 1.07E-98 | 62.35 | C:Golgi apparatus; C:nucleolus |
| Efet.01.6251.g492.t1 | thyrotropin-releasing hormone receptor-like | 1107 | 9.92E-25 | 49.9 | P:G-protein coupled receptor signaling pathway; C:membrane; F:signal transducer activity |
| Efet.01.625114.g1096.t1 | gastrin-releasing peptide receptor-like | 1074 | 8.56E-87 | 60.35 | F:G-protein coupled receptor activity; P:signal transduction |
| Efet.01.625120.g1098.t1 | prolactin-releasing peptide receptor-like | 1215 | 1.77E-84 | 60.65 | P:signal transduction; F:signal transducer activity |
| Efet.01.625145.g1099.t1 | outer membrane | 270 | 1.98E-45 | 57.15 |  |
| Efet.01.625145.g1100.t1 | N-acetyltransferase | 537 | 2.90E-48 | 90.15 | P:nucleic acid phosphodiester bond hydrolysis; F:endonuclease activity; P:phosphorylation; F:N-acetyltransferase activity; F:kinase activity |
| Efet.01.625145.g1101.t1 | DUF1778 domain-containing | 282 | 1.53E-44 | 94.85 | P:regulation of transcription, DNA-templated; F:methyltransferase activity; P:methylation |
| Efet.01.625145.g1102.t1 | addiction module toxin | 387 | 5.12E-82 | 94.65 |  |
| Efet.01.625145.g1103.t1 | transposase | 363 | 2.02E-61 | 89 | F:DNA binding; F:transposase activity; P:transposition, DNA-mediated |
| Efet.01.625146.g1104.t1 | Potassium voltage-gated channel subfamily A member 2 | 1632 | 0 | 79.2 | P:potassium ion transmembrane transport; C:voltage-gated potassium channel complex; F:delayed rectifier potassium channel activity; P:regulation of ion transmembrane transport; P:protein homooligomerization |
| Efet.01.625202.g1106.t1 | mitochondrial ATP synthase subunit beta precursor | 309 | 2.17E-65 | 97.75 | C:proton-transporting ATP synthase complex, catalytic core F(1); F:ATP binding; P:ATP hydrolysis coupled proton transport; P:ATP synthesis coupled proton transport; F:proton-transporting ATP synthase activity, rotational mechanism |
| Efet.01.625219.g1107.t1 | aminopeptidase P family | 1110 | 0 | 82.7 | F:hydrolase activity |
| Efet.01.625229.g1108.t1 | ATP-dependent DNA helicase | 393 | 7.76E-26 | 58.3 | F:ATP binding |
| Efet.01.625229.g1109.t1 | transcriptional regulator | 246 | 4.78E-25 | 71.29 | F:ATP binding |
| Efet.01.625229.g1110.t1 | DUF4062 domain-containing | 555 | 1.08E-127 | 68.7 |  |
| Efet.01.625264.g1111.t1 | superoxide dismutase | 678 | 4.09E-134 | 76.45 | P:superoxide metabolic process; F:oxidoreductase activity |
| Efet.01.625368.g1112.t1 | beta-1,4-galactosyltransferase 4 | 609 | 2.56E-14 | 56.85 | P:regulation of acrosome reaction; P:negative regulation of cell proliferation; P:protein glycosylation; C:Golgi apparatus part; P:epithelial cell development; P:cell adhesion; P:branching morphogenesis of an epithelial tube; P:regulation of cellular component movement; P:response to toxic substance; P:positive regulation of Notch signaling pathway; P:formation of infection structure on or near host; P:lactose biosynthetic process; P:angiogenesis involved in wound healing; P:positive regulation of apoptotic process involved in mammary gland involution; P:penetration of zona pellucida; P:positive regulation of epithelial cell proliferation involved in wound healing; P:galactose metabolic process; P:development of secondary sexual characteristics; C:membrane part; C:plasma membrane; C:cell surface; F:beta-N-acetylglucosaminylglycopeptide beta-1,4-galactosyltransferase activity; C:extracellular space; P:binding of sperm to zona pellucida; P:mammary gland development; P:acute inflammatory response; P:extracellular matrix organization; P:leukocyte migration; F:cytoskeletal protein binding; F:beta-1,4-N-acetylgalactosaminyltransferase activity |
| Efet.01.625433.g1114.t1 | carbon monoxide dehydrogenase | 654 | 7.41E-108 | 94.25 | F:oxidoreductase activity; F:metal ion binding; F:electron carrier activity; P:oxidation-reduction process; F:iron-sulfur cluster binding |
| Efet.01.625433.g1115.t1 | carbon monoxide dehydrogenase | 2409 | 0 | 92.15 | F:xanthine dehydrogenase activity; P:oxidation-reduction process |
| Efet.01.625433.g1116.t1 | carbon monoxide dehydrogenase | 687 | 3.91E-146 | 93.45 | F:oxidoreductase activity, acting on CH-OH group of donors; F:carbon-monoxide dehydrogenase (acceptor) activity; F:flavin adenine dinucleotide binding; P:oxidation-reduction process |
| Efet.01.625433.g1117.t1 | tripartite tricarboxylate transporter substrate binding | 1044 | 0 | 85.15 | C:outer membrane-bounded periplasmic space |
| Efet.01.625458.g1118.t1 | fumarate hydratase | 201 | 1.10E-30 | 95.85 | P:generation of precursor metabolites and energy; F:fumarate hydratase activity |
| Efet.01.625494.g1119.t1 | ribosome maturation factor | 579 | 6.20E-127 | 86.95 | F:ribosome binding; P:ribosomal small subunit biogenesis; P:rRNA processing; C:ribosome |
| Efet.01.625494.g1120.t1 | 30S ribosomal S16 | 252 | 1.07E-53 | 96 | F:structural constituent of ribosome; C:ribosome; P:translation |
| Efet.01.625531.g1123.t1 | APOBEC1 complementation factor-like isoform X2 | 696 | 5.09E-122 | 88.95 | F:nucleotide binding; F:single-stranded RNA binding; C:apolipoprotein B mRNA editing enzyme complex; P:mRNA modification |
| Efet.01.625602.g1125.t1 | family transporter | 378 | 4.63E-64 | 77 | C:integral component of membrane |
| Efet.01.625602.g1126.t1 | benzoate transporter | 816 | 1.16E-135 | 86.1 | F:benzoate transporter activity; C:integral component of membrane; P:benzoate transport |
| Efet.01.625620.g1127.t1 | hypothetical protein CRN43_10290, partial | 252 | 7.31E-06 | 55 |  |
| Efet.01.625693.g1129.t1 | RNA-dependent RNA polymerase 1 | 1143 | 5.50E-77 | 65.2 | F:RNA-directed 5'-3' RNA polymerase activity; P:transcription, RNA-templated |
| Efet.01.625757.g1132.t1 | DNA recombination | 732 | 4.52E-92 | 77.15 | C:integral component of membrane |
| Efet.01.625818.g1135.t1 | homeotic spalt-major-like isoform X2 | 1116 | 1.74E-54 | 70.1 | F:nucleic acid binding; F:metal ion binding |
| Efet.01.626070.g1138.t1 | slit homolog 2 -like isoform X3 | 267 | 1.51E-24 | 68.5 | P:regulation of cellular process; C:extracellular region part; F:ion binding; P:generation of neurons; P:response to stimulus |
| Efet.01.626131.g1141.t1 | hypothetical protein CAPTEDRAFT_189093 | 357 | 7.18E-06 | 54 | P:sodium ion transport; P:ion transport; F:sodium channel activity; C:membrane; C:integral component of membrane; P:sodium ion transmembrane transport; P:transport |
| Efet.01.626144.g1142.t1 | chymotrypsin-like elastase family member 1 | 720 | 4.26E-25 | 49.5 | C:cytoplasm; P:proteolysis; F:serine-type peptidase activity; F:serine-type endopeptidase activity; F:peptidase activity; F:hydrolase activity |
| Efet.01.626208.g1144.t1 | hypothetical protein | 366 | 1.65E-81 | 88.15 |  |
| Efet.01.626208.g1145.t1 | aminotransferase | 1230 | 0 | 95.25 | F:pyridoxal phosphate binding; F:L-alanine:2-oxoglutarate aminotransferase activity; P:biosynthetic process |
| Efet.01.626208.g1146.t1 | homoserine dehydrogenase | 1023 | 0 | 86.7 | P:methionine biosynthetic process; P:isoleucine biosynthetic process; F:NADP binding; P:threonine biosynthetic process; P:oxidation-reduction process; F:amino acid binding; F:homoserine dehydrogenase activity |
| Efet.01.626208.g1147.t1 | threonine synthase | 2022 | 0 | 87.45 | F:threonine synthase activity |
| Efet.01.626208.g1148.t1 | molybdopterin molybdenumtransferase | 585 | 2.11E-118 | 79.75 | P:molybdopterin cofactor biosynthetic process |
| Efet.01.626245.g1149.t1 | 4-phosphopantetheinyl transferase | 744 | 2.04E-156 | 52.05 | F:magnesium ion binding; F:holo-[acyl-carrier-protein] synthase activity |
| Efet.01.626245.g1150.t1 | bifunctional demethylmenaquinone methyltransferase 2-methoxy-6-polyprenyl-1,4-benzoquinol methylase | 741 | 0 | 93.2 | F:2-octaprenyl-6-methoxy-1,4-benzoquinone methylase activity; P:menaquinone biosynthetic process; P:aerobic respiration; P:ubiquinone biosynthetic process; F:2-polyprenyl-6-methoxy-1,4-benzoquinone methyltransferase activity; P:methylation |
| Efet.01.626245.g1151.t1 | Tim44 domain-containing | 654 | 3.33E-128 | 80.75 | C:integral component of membrane |
| Efet.01.626245.g1152.t1 | ubiquinone biosynthesis | 498 | 6.23E-73 | 90.25 |  |
| Efet.01.626245.g1153.t1 | ubiquinone biosynthesis regulatory kinase | 1317 | 0 | 85.9 | F:ATP binding; F:oxidoreductase activity; C:integral component of membrane; F:protein kinase activity; P:oxidation-reduction process; P:protein phosphorylation; P:ubiquinone biosynthetic process; C:plasma membrane; P:regulation of ubiquinone biosynthetic process |
| Efet.01.626245.g1154.t1 | DUF502 domain-containing | 912 | 8.81E-130 | 96.15 | C:integral component of membrane |
| Efet.01.626245.g1155.t1 | aspartate--tRNA ligase | 1764 | 0 | 94.4 | F:nucleic acid binding; F:ATP binding; P:tRNA aminoacylation for protein translation; F:aspartate-tRNA(Asn) ligase activity; C:cytoplasm; F:aspartate-tRNA ligase activity |
| Efet.01.626245.g1156.t1 | endonuclease | 738 | 4.90E-176 | 93.5 | P:nucleic acid phosphodiester bond hydrolysis; F:exonuclease activity; F:endonuclease activity |
| Efet.01.626245.g1157.t1 | cardiolipin synthase | 1431 | 0 | 88.3 | F:cardiolipin synthase activity; C:plasma membrane; P:cardiolipin biosynthetic process |
| Efet.01.626245.g1158.t1 | GTP cyclohydrolase | 702 | 7.82E-172 | 95.15 | C:cytoplasm; F:GTP cyclohydrolase I activity; P:tetrahydrofolate biosynthetic process |
| Efet.01.626245.g1159.t1 | blue light sensor | 243 | 1.89E-46 | 83.75 | F:FAD binding; F:blue light photoreceptor activity; P:blue light signaling pathway |
| Efet.01.626245.g1160.t1 | acetyl- acetyltransferase | 1023 | 0 | 90.05 | F:acetyl-CoA C-acetyltransferase activity; P:metabolic process |
| Efet.01.626280.g1161.t1 | gastrula zinc finger -like | 792 | 3.07E-49 | 61.35 | F:nucleic acid binding; F:metal ion binding |
| Efet.01.626322.g1162.t1 | Helix-hairpin-helix motif-containing | 552 | 3.53E-54 | 68.65 | C:membrane; C:integral component of membrane |
| Efet.01.62636.g783.t1 | voltage-dependent T-type calcium channel subunit alpha-1G-like | 525 | 5.80E-28 | 92.85 | P:calcium ion import; C:voltage-gated calcium channel complex; P:regulation of ion transmembrane transport; P:calcium ion transmembrane transport; F:low voltage-gated calcium channel activity |
| Efet.01.626369.g1163.t1 | cholesterol 7-desaturase-like | 501 | 3.84E-28 | 61.3 | F:iron-sulfur cluster binding |
| Efet.01.626474.g1166.t1 | cystathionine beta-lyase | 294 | 4.82E-26 | 91.5 | F:iron ion binding; F:electron carrier activity; C:integral component of membrane; F:lyase activity; F:heme binding |
| Efet.01.626536.g1167.t1 | AChain Structural Determinants For Improved Thermal Stability Of Designed Ankyrin Repeat s With A Redesigned C- Capping | 306 | 5.94E-21 | 61.5 |  |
| Efet.01.626647.g1168.t1 | microtubule-associated serine threonine- kinase 3-like | 480 | 1.36E-46 | 77.7 | F:magnesium ion binding; F:ATP binding; F:protein serine/threonine kinase activity; P:protein phosphorylation |
| Efet.01.626661.g1169.t1 | SAM-dependent methyltransferase | 384 | 2.07E-88 | 70.7 | P:metabolic process; F:methyltransferase activity; F:transferase activity; P:methylation |
| Efet.01.626673.g1170.t1 | hypothetical protein CAPTEDRAFT_219169 | 945 | 5.98E-14 | 39.56 | F:chitin binding; C:membrane; P:chitin metabolic process; C:integral component of membrane; C:extracellular region |
| Efet.01.626729.g1172.t1 | ATP-dependent Clp protease ATP-binding subunit | 1398 | 0 | 93.75 | F:ATP binding; P:protein metabolic process |
| Efet.01.626762.g1173.t1 | hypothetical protein CRN43_09585 | 489 | 1.83E-07 | 50 |  |
| Efet.01.626803.g1174.t1 | RHS repeat-associated core domain-containing | 666 | 1.08E-35 | 57 | P:self proteolysis; F:metallopeptidase activity |
| Efet.01.62684.g788.t1 | charged multivesicular body 1b-like | 354 | 1.73E-55 | 92.15 | P:protein transport; P:vacuolar transport; C:intracellular |
| Efet.01.62690.g789.t1 | probable ATP-dependent RNA helicase DDX58 | 1500 | 4.13E-95 | 54.9 | F:nucleic acid binding; P:regulation of cellular process; P:positive regulation of cytokine production; P:response to stimulus |
| Efet.01.626977.g1176.t1 | Organic solvent tolerance | 891 | 6.08E-166 | 63.85 |  |
| Efet.01.626983.g1177.t1 | tricarballylate dehydrogenase | 1263 | 0 | 91.4 | F:oxidoreductase activity; P:oxidation-reduction process |
| Efet.01.626983.g1178.t1 | family transcriptional regulator | 888 | 1.87E-140 | 84.2 | F:DNA binding; F:transcription factor activity, sequence-specific DNA binding; P:transcription, DNA-templated; P:regulation of transcription, DNA-templated |
| Efet.01.626983.g1179.t1 | family transcriptional regulator | 930 | 0 | 71.8 | F:DNA binding; P:carbohydrate metabolic process; F:transcription factor activity, sequence-specific DNA binding; P:regulation of transcription, DNA-templated; F:carbohydrate binding |
| Efet.01.626983.g1180.t1 | FAD-binding oxidoreductase | 1293 | 0 | 77.75 | F:oxidoreductase activity, acting on CH-OH group of donors; F:flavin adenine dinucleotide binding; P:oxidation-reduction process |
| Efet.01.626983.g1181.t1 | glucose-6-phosphate isomerase | 606 | 2.07E-125 | 74.2 | F:glucose-6-phosphate isomerase activity; P:gluconeogenesis; C:cytoplasm; P:glycolytic process |
| Efet.01.626983.g1182.t1 | shikimate dehydrogenase | 891 | 0 | 80.05 |  |
| Efet.01.626983.g1183.t1 | ABC transporter substrate-binding | 906 | 0 | 84.45 |  |
| Efet.01.626983.g1184.t1 | sugar ABC transporter ATP-binding | 1356 | 0 | 73.55 | F:ATP binding; F:monosaccharide transmembrane transporter activity; F:carbohydrate-transporting ATPase activity; P:monosaccharide transport; C:plasma membrane |
| Efet.01.626983.g1185.t1 | ABC transporter permease | 867 | 8.61E-176 | 80.7 | C:integral component of membrane; P:transport; C:plasma membrane; F:transporter activity |
| Efet.01.626983.g1186.t1 | pentose kinase | 1443 | 0 | 73.7 | P:carbohydrate metabolic process; P:phosphorylation; F:phosphotransferase activity, alcohol group as acceptor; F:kinase activity |
| Efet.01.626983.g1187.t1 | HAD family phosphatase | 522 | 1.33E-120 | 63 | P:metabolic process; F:hydrolase activity |
| Efet.01.626983.g1188.t1 | 3-oxoacyl-ACP reductase | 651 | 2.35E-158 | 85.75 | F:oxidoreductase activity; P:oxidation-reduction process |
| Efet.01.626983.g1189.t1 | MFS transporter | 612 | 5.93E-82 | 86.4 | P:transmembrane transport; F:transmembrane transporter activity; C:integral component of membrane |
| Efet.01.626983.g1190.t1 | MFS transporter | 816 | 4.56E-93 | 93.35 | P:transmembrane transport; F:transmembrane transporter activity; C:integral component of membrane |
| Efet.01.626983.g1191.t1 | Ribulose-5-phosphate 4-epimerase Fuculose-1-phosphate aldolase | 807 | 5.42E-164 | 85.35 |  |
| Efet.01.626991.g1192.t1 | TonB-dependent receptor | 1161 | 1.83E-120 | 60.2 | C:membrane; C:cell outer membrane; F:receptor activity; P:transport |
| Efet.01.627060.g1195.t1 | cell division | 1059 | 0 | 93.95 | P:cell division; F:penicillin binding; F:peptidoglycan glycosyltransferase activity |
| Efet.01.627068.g1196.t1 | short-chain dehydrogenase | 594 | 7.15E-83 | 65.2 | F:oxidoreductase activity |
| Efet.01.6271.g494.t1 | plexin-A2 isoform X1 | 972 | 4.26E-49 | 55.75 | P:signal transduction; C:membrane; P:axon guidance |
| Efet.01.627107.g1199.t1 | hypothetical protein HELRODRAFT_184146 | 231 | 3.31E-10 | 55.67 | F:nucleic acid binding; F:metal ion binding |
| Efet.01.627110.g1200.t1 | piggyBac transposable element-derived 4-like | 753 | 5.64E-40 | 65.25 | C:membrane; C:integral component of membrane |
| Efet.01.62716.g791.t1 | sodium potassium-transporting ATPase subunit alpha-like isoform X2 | 1314 | 0 | 79.8 | P:potassium ion transport; F:ATP binding; P:sodium ion transport; F:sodium:potassium-exchanging ATPase activity; F:metal ion binding; P:ATP hydrolysis coupled transmembrane transport; C:integral component of membrane; P:establishment or maintenance of transmembrane electrochemical gradient |
| Efet.01.627199.g1201.t1 | B-cell lymphoma leukemia 11A isoform X3 | 2349 | 1.17E-40 | 74.75 | C:nucleus; F:transcription factor activity, sequence-specific DNA binding; F:metal ion binding; P:signal transduction; F:transcription regulatory region DNA binding; P:regulation of transcription, DNA-templated; P:transcription from RNA polymerase II promoter; F:sequence-specific DNA binding; P:neurogenesis |
| Efet.01.627326.g1205.t1 | furry homolog isoform X8 | 204 | 5.07E-12 | 68.85 | C:membrane; C:integral component of membrane |
| Efet.01.627410.g1207.t1 | 2-C-methyl-D-erythritol 2,4-cyclodiphosphate synthase | 273 | 1.06E-36 | 87.85 | F:metal ion binding; P:isopentenyl diphosphate biosynthetic process, methylerythritol 4-phosphate pathway; F:2-C-methyl-D-erythritol 2,4-cyclodiphosphate synthase activity; P:terpenoid biosynthetic process |
| Efet.01.627431.g1208.t1 | sulfatase-modifying factor | 369 | 2.30E-33 | 79.95 |  |
| Efet.01.627489.g1209.t1 | ferredoxin | 741 | 9.69E-108 | 87.8 | F:sarcosine oxidase activity; F:methyltransferase activity; P:tetrahydrofolate metabolic process; P:oxidation-reduction process; P:methylation |
| Efet.01.627540.g1211.t1 | IMP dehydrogenase | 609 | 1.36E-123 | 91.55 | F:nucleotide binding; F:metal ion binding; F:IMP dehydrogenase activity; P:oxidation-reduction process; P:GMP biosynthetic process |
| Efet.01.627540.g1212.t1 | molecular chaperone | 324 | 6.25E-58 | 97.95 | F:ATP binding; C:cytoplasm; P:protein folding |
| Efet.01.627560.g1213.t1 | gastrula zinc finger -like | 699 | 1.48E-49 | 67.05 | F:nucleic acid binding; F:metal ion binding |
| Efet.01.627621.g1216.t1 | hypothetical protein CAPTEDRAFT_224550 | 300 | 1.88E-13 | 69.5 | C:membrane |
| Efet.01.627635.g1217.t1 | family | 462 | 2.00E-94 | 67.6 | C:membrane; C:integral component of membrane; C:cell outer membrane |
| Efet.01.627769.g1219.t1 | SMC-Scp complex subunit | 561 | 3.34E-104 | 62.65 | P:single-organism cellular process |
| Efet.01.627817.g1220.t1 | aminopeptidase N | 975 | 1.04E-75 | 62.85 | F:peptidase activity, acting on L-amino acid peptides |
| Efet.01.627823.g1221.t1 | AP-1 complex subunit mu-1 | 303 | 3.91E-59 | 92.35 | P:secretory granule organization; C:synaptic vesicle; F:protein transporter activity; P:intracellular protein transport; P:negative regulation of Notch signaling pathway; P:compound eye development; P:neurotransmitter secretion; P:synaptic vesicle coating; C:AP-1 adaptor complex; C:transcription factor AP-1 complex; P:sensory perception of pain; F:clathrin binding; P:negative regulation of endocytic recycling |
| Efet.01.627824.g1222.t1 | family transcriptional regulator | 774 | 0 | 57.75 | F:DNA binding; F:transcription factor activity, sequence-specific DNA binding; P:transcription, DNA-templated; P:regulation of transcription, DNA-templated |
| Efet.01.627824.g1223.t1 | ATP-binding | 540 | 3.94E-125 | 91.5 | F:ATP binding |
| Efet.01.62795.g792.t1 | phenylalanine--tRNA mitochondrial-like | 462 | 3.82E-41 | 69 | F:nucleotide binding; P:tRNA aminoacylation for protein translation; F:aminoacyl-tRNA ligase activity; C:cytoplasm; F:ion binding |
| Efet.01.628006.g1225.t1 | Wnt-7b-like isoform X4 | 468 | 1.77E-63 | 72.6 | C:extracellular region; P:single-organism process |
| Efet.01.628009.g1226.t1 | XRE family transcriptional regulator | 207 | 2.19E-39 | 75.85 | F:DNA binding |
| Efet.01.628009.g1227.t1 | squalene synthase | 825 | 0 | 88.25 | P:carotenoid biosynthetic process; F:geranylgeranyl-diphosphate geranylgeranyltransferase activity; F:farnesyl-diphosphate farnesyltransferase activity; F:squalene synthase activity |
| Efet.01.628031.g1228.t1 | bifunctional pyr operon transcriptional regulator uracil phosphoribosyltransferase | 372 | 2.29E-34 | 73.75 | F:transferase activity, transferring glycosyl groups; P:nucleobase-containing compound metabolic process |
| Efet.01.62805.g793.t1 | low-density lipo receptor-related 2-like | 558 | 3.72E-49 | 66.1 | C:membrane |
| Efet.01.628051.g1229.t1 | spectrin beta chain-like isoform X1 | 450 | 1.95E-59 | 83.2 | F:actin binding; F:structural constituent of cytoskeleton; F:phospholipid binding; C:spectrin |
| Efet.01.628078.g1230.t1 | T-box transcription factor TBX20 | 660 | 6.54E-72 | 84.4 | P:cell proliferation; P:positive regulation of cardiac muscle cell proliferation; P:pulmonary valve formation; F:RNA polymerase II core promoter proximal region sequence-specific DNA binding; P:endocardial cushion formation; P:transcription from RNA polymerase II promoter; P:tricuspid valve development; F:RNA polymerase II transcription coactivator activity; P:positive regulation of transcription from RNA polymerase II promoter; F:RNA polymerase II activating transcription factor binding; P:dorsal/ventral pattern formation; P:negative regulation of transcription from RNA polymerase II promoter; P:outflow tract septum morphogenesis; P:regulation of epithelial to mesenchymal transition; F:transcriptional activator activity, RNA polymerase II core promoter proximal region sequence-specific binding; P:neuron migration; P:cardiac chamber formation; P:branching involved in blood vessel morphogenesis; P:muscle contraction; P:embryonic heart tube elongation; P:heart looping; P:endoderm formation; P:negative regulation of SMAD protein complex assembly; P:cardiac muscle tissue morphogenesis; P:aortic valve morphogenesis; P:blood circulation; P:visceral motor neuron differentiation; P:pulmonary vein morphogenesis; P:pericardium morphogenesis; P:atrial septum morphogenesis; C:nucleus; P:cardiac right ventricle morphogenesis; C:cytoplasm; P:foramen ovale closure; P:lateral mesoderm formation |
| Efet.01.628138.g1232.t1 | long-chain fatty acid-- ligase | 597 | 7.75E-92 | 88.5 | P:long-chain fatty acid metabolic process; F:long-chain fatty acid-CoA ligase activity |
| Efet.01.628190.g1233.t1 | homeobox posterior hox 2 | 252 | 4.30E-29 | 86.8 | C:nucleus; P:regulation of transcription, DNA-templated; F:sequence-specific DNA binding |
| Efet.01.628200.g1235.t1 | SCO-spondin | 489 | 5.99E-28 | 57.75 | P:cell differentiation; P:nervous system development |
| Efet.01.628226.g1236.t1 | thyrotropin-releasing hormone receptor | 1167 | 5.98E-25 | 53.9 | F:G-protein coupled receptor activity; P:neuropeptide signaling pathway; P:G-protein coupled receptor signaling pathway; P:signal transduction; C:membrane; C:integral component of membrane; F:signal transducer activity |
| Efet.01.628251.g1237.t1 | gfo Idh family oxidoreductase | 591 | 3.67E-120 | 70.8 | F:oxidoreductase activity; P:oxidation-reduction process |
| Efet.01.628256.g1238.t1 | proline--tRNA ligase | 474 | 2.23E-100 | 85.85 | F:ATP binding; C:cytoplasm; P:regulation of translational fidelity; F:proline-tRNA ligase activity; F:aminoacyl-tRNA editing activity; P:prolyl-tRNA aminoacylation |
| Efet.01.62833.g795.t1 | diacylglycerol kinase beta-like isoform X1 | 372 | 2.18E-20 | 62.9 | F:binding; F:transferase activity; P:cellular process |
| Efet.01.628375.g1239.t1 | ABC transporter ATP-binding | 654 | 3.02E-139 | 68.85 | F:nucleotide binding; F:ATPase activity; F:ATP binding |
| Efet.01.628433.g1240.t1 | methylmalonyl- carboxyltransferase | 1584 | 0 | 88.75 | F:methylmalonyl-CoA carboxytransferase activity; F:propionyl-CoA carboxylase activity |
| Efet.01.628433.g1241.t1 | allophanate hydrolase | 1734 | 0 | 79.65 | F:carbon-nitrogen ligase activity, with glutamine as amido-N-donor; F:hydrolase activity |
| Efet.01.628433.g1242.t1 | ABC transporter substrate-binding | 1008 | 0 | 85.75 |  |
| Efet.01.628433.g1243.t1 | ABC transporter permease | 753 | 3.25E-141 | 92.7 | C:integral component of membrane; P:transport; C:plasma membrane |
| Efet.01.628433.g1244.t1 | nitrate ABC transporter ATP-binding | 612 | 4.88E-132 | 92.1 | F:ATPase activity; F:ATP binding |
| Efet.01.628451.g1245.t1 | family permease | 501 | 9.05E-104 | 64.75 | C:membrane; C:integral component of membrane |
| Efet.01.628471.g1246.t1 | Serine threonine- kinase | 387 | 1.66E-56 | 97.95 | C:cleavage furrow; P:spleen development; P:negative regulation of protein kinase activity; P:renal system process; F:ATP binding; C:endosome; P:regulation of germinal center formation; P:negative regulation of B cell proliferation; P:regulation of immunoglobulin production; F:Rac GTPase binding; F:GTP-Rho binding; P:epithelial cell migration; F:protein kinase C binding; F:histone deacetylase binding; P:signal transduction; C:protein complex; F:chromatin binding; P:B cell homeostasis; P:hyperosmotic response; P:B cell apoptotic process; P:regulation of transcription from RNA polymerase II promoter; C:nucleus; F:androgen receptor binding; F:histone binding; F:ligand-dependent nuclear receptor transcription coactivator activity; P:regulation of cell motility; C:midbody; P:histone H3-T11 phosphorylation; F:histone kinase activity (H3-T11 specific) |
| Efet.01.628482.g1247.t1 | branched-chain amino acid ABC transporter permease | 915 | 7.46E-118 | 96.55 | C:integral component of membrane; P:transport; C:plasma membrane; F:transporter activity |
| Efet.01.628482.g1248.t1 | branched-chain amino acid ABC transporter permease | 885 | 5.06E-126 | 92.95 | C:integral component of membrane; P:transport; C:plasma membrane; F:transporter activity |
| Efet.01.628482.g1249.t1 | ABC transporter permease | 1185 | 0 | 96.35 |  |
| Efet.01.628518.g1251.t1 | XPRLamide neuropeptide precursor | 618 | 3.52E-97 | 99.5 | P:neuropeptide signaling pathway |
| Efet.01.628537.g1253.t1 | type II secretion system | 2217 | 0 | 74.4 | F:protein transporter activity; P:protein secretion by the type II secretion system; C:cell outer membrane; C:type II protein secretion system complex |
| Efet.01.628537.g1254.t1 | cell surface | 2025 | 0 | 56.15 |  |
| Efet.01.628537.g1255.t1 | peptidylprolyl isomerase | 840 | 1.03E-155 | 70.9 | F:peptidyl-prolyl cis-trans isomerase activity; C:integral component of membrane; P:protein peptidyl-prolyl isomerization |
| Efet.01.628537.g1256.t1 | ABC transporter ATP-binding | 1542 | 0 | 78.8 | F:ATP binding; P:carbohydrate derivative transport; F:teichoic-acid-transporting ATPase activity |
| Efet.01.628537.g1257.t1 | type II toxin-antitoxin system family toxin | 1212 | 0 | 64.15 | P:phosphorylation; F:kinase activity |
| Efet.01.62855.g796.t1 | argonaute-2-like isoform X4 | 219 | 1.09E-21 | 83.7 | F:nucleic acid binding; C:integral component of membrane |
| Efet.01.628685.g1259.t1 | ABC transporter permease | 897 | 0 | 97.35 | C:integral component of membrane; P:carbohydrate transport; C:plasma membrane |
| Efet.01.628685.g1260.t1 | sugar ABC transporter permease | 735 | 1.09E-175 | 97.4 | C:integral component of membrane; P:carbohydrate transport; C:plasma membrane |
| Efet.01.628685.g1261.t1 | small integral membrane | 297 | 8.59E-61 | 89.4 | C:integral component of membrane |
| Efet.01.628685.g1262.t1 | ABC transporter substrate-binding | 1749 | 0 | 93.55 | P:carbohydrate transport; F:transporter activity |
| Efet.01.628687.g1263.t1 | urea carboxylase | 357 | 3.02E-41 | 88.65 | F:ATP binding; F:urea carboxylase activity; F:metal ion binding; F:lyase activity; F:biotin carboxylase activity |
| Efet.01.628698.g1264.t1 | transcription elongation regulator 1-like | 684 | 4.47E-64 | 88.95 |  |
| Efet.01.62878.g798.t1 | metabotropic glutamate receptor | 963 | 8.90E-50 | 52.1 | F:G-protein coupled receptor activity; P:G-protein coupled receptor signaling pathway; P:signal transduction; C:membrane; C:integral component of membrane; F:signal transducer activity; C:plasma membrane |
| Efet.01.629029.g1272.t1 | meiosis-specific coiled-coil domain-containing MEIOC-like | 348 | 1.07E-28 | 69.65 | C:membrane; C:integral component of membrane |
| Efet.01.62903.g799.t1 | calsyntenin-1-like isoform X1 | 366 | 6.97E-08 | 51.33 | F:calcium ion binding; C:membrane; C:integral component of membrane; P:homophilic cell adhesion via plasma membrane adhesion molecules |
| Efet.01.629049.g1273.t1 | urea ABC transporter ATP-binding | 693 | 4.21E-131 | 92.6 | F:ATPase activity; F:ATP binding |
| Efet.01.629153.g1274.t1 | family ATPase | 735 | 1.00E-83 | 65.1 | F:ATPase activity; F:ATP binding |
| Efet.01.629153.g1275.t1 | lactate dehydrogenase | 501 | 2.75E-113 | 93.65 | F:L-lactate dehydrogenase activity; P:oxidation-reduction process |
| Efet.01.629153.g1276.t1 | C4-dicarboxylate ABC transporter permease | 1446 | 0 | 92.8 | C:integral component of membrane |
| Efet.01.629299.g1278.t1 | hypothetical protein CAPTEDRAFT_221435 | 651 | 2.00E-25 | 52.95 | C:membrane; C:integral component of membrane |
| Efet.01.629306.g1279.t1 | metal transporter CNNM4-like | 543 | 2.81E-13 | 70.2 |  |
| Efet.01.629309.g1280.t1 | collagen alpha-1(I) chain-like isoform X1 | 414 | 1.37E-23 | 57.8 | C:collagen trimer; C:collagen type IX trimer |
| Efet.01.629312.g1281.t1 | Methylsterol monooxygenase 1 | 219 | 4.26E-30 | 84.75 | F:iron ion binding; C:integral component of membrane; P:oxidation-reduction process; P:fatty acid biosynthetic process; F:monooxygenase activity |
| Efet.01.629329.g1282.t1 | ATP-dependent DNA helicase | 864 | 1.24E-135 | 79.6 | F:nucleic acid binding; F:ATP binding; P:DNA repair; P:DNA duplex unwinding; P:DNA recombination; F:ATP-dependent DNA helicase activity |
| Efet.01.629329.g1283.t1 | tRNA preQ1(34) S-adenosylmethionine ribosyltransferase-isomerase | 1101 | 0 | 87.5 | P:queuosine biosynthetic process; C:cytoplasm; C:integral component of membrane; F:transferase activity; F:isomerase activity |
| Efet.01.629329.g1284.t1 | C4-dicarboxylate ABC transporter substrate-binding | 975 | 0 | 91.7 | C:outer membrane-bounded periplasmic space; P:transport |
| Efet.01.629329.g1285.t1 | TRAP transporter small permease | 411 | 5.57E-65 | 89.65 | C:integral component of membrane |
| Efet.01.629329.g1286.t1 | C4-dicarboxylate ABC transporter permease | 927 | 4.76E-150 | 87.3 | C:integral component of membrane |
| Efet.01.629351.g1288.t1 | Wnt-11b-2-like | 447 | 1.10E-37 | 69.8 | C:membrane; C:extracellular region; P:single-organism process |
| Efet.01.629376.g1290.t1 | MFS transporter | 1308 | 0 | 78.15 | P:transmembrane transport; C:integral component of membrane |
| Efet.01.629376.g1291.t1 | amino acid ABC transporter substrate-binding | 1104 | 0 | 94.5 | P:amino acid transport |
| Efet.01.629376.g1292.t1 | ABC transporter | 2850 | 0 | 79.5 | F:ATPase activity; F:ATP binding; C:integral component of membrane; P:transport; C:plasma membrane; F:transporter activity |
| Efet.01.629376.g1293.t1 | ABC transporter ATP-binding | 594 | 1.43E-137 | 92.8 | F:ATPase activity; F:ATP binding |
| Efet.01.629376.g1294.t1 | alpha beta hydrolase | 645 | 3.48E-105 | 81.45 |  |
| Efet.01.629376.g1295.t1 | hypothetical protein | 948 | 0 | 86.15 |  |
| Efet.01.629376.g1296.t1 | penicillin-binding activator | 618 | 3.57E-131 | 97.85 |  |
| Efet.01.629376.g1297.t1 | DUF1425 domain-containing | 354 | 7.54E-80 | 91.8 |  |
| Efet.01.629510.g1298.t1 | type I methionyl aminopeptidase | 222 | 9.93E-43 | 87.05 | P:protein initiator methionine removal; F:metal ion binding; P:proteolysis; F:metalloaminopeptidase activity |
| Efet.01.629521.g1299.t1 | transcriptional regulator | 591 | 2.50E-140 | 78.8 |  |
| Efet.01.629577.g1301.t1 | tail-specific protease | 864 | 2.75E-98 | 72.65 | F:peptidase activity |
| Efet.01.629634.g1302.t1 | tripartite motif-containing 2-like isoform X1 | 540 | 1.85E-33 | 55.7 | F:ubiquitin-protein transferase activity; F:protein binding; C:intracellular |
| Efet.01.629639.g1303.t1 | family transcriptional regulator | 816 | 1.01E-118 | 75.45 | F:DNA binding |
| Efet.01.629639.g1304.t1 | pyruvate dehydrogenase (acetyl-transferring) homodimeric type | 264 | 1.27E-42 | 88.35 | F:pyruvate dehydrogenase (acetyl-transferring) activity; P:oxidation-reduction process |
| Efet.01.629703.g1306.t1 | 4-hydroxy-3-methylbut-2-en-1-yl diphosphate synthase | 513 | 1.45E-64 | 94.05 | F:iron ion binding; P:isopentenyl diphosphate biosynthetic process, methylerythritol 4-phosphate pathway; P:oxidation-reduction process; F:4 iron, 4 sulfur cluster binding; F:4-hydroxy-3-methylbut-2-en-1-yl diphosphate synthase activity; P:terpenoid biosynthetic process |
| Efet.01.629703.g1307.t1 | 4-hydroxy-3-methylbut-2-en-1-yl diphosphate synthase | 858 | 3.64E-114 | 90.4 | F:iron ion binding; P:isopentenyl diphosphate biosynthetic process, methylerythritol 4-phosphate pathway; P:oxidation-reduction process; F:4 iron, 4 sulfur cluster binding; F:4-hydroxy-3-methylbut-2-en-1-yl diphosphate synthase activity; P:terpenoid biosynthetic process |
| Efet.01.629714.g1308.t1 | potassium voltage-gated channel subfamily KQT member 1-like isoform X1 | 729 | 1.32E-13 | 56 | P:ion transport; C:membrane; F:potassium channel activity |
| Efet.01.629720.g1309.t1 | ABC transporter permease | 381 | 6.93E-80 | 93.55 | C:integral component of membrane; P:transport; C:plasma membrane |
| Efet.01.629740.g1310.t1 | carbohydrate ABC transporter permease | 594 | 1.19E-74 | 88.75 | C:integral component of membrane; P:transport; C:plasma membrane |
| Efet.01.629741.g1311.t1 | alcohol dehydrogenase | 255 | 3.92E-28 | 78.63 | F:zinc ion binding; F:oxidoreductase activity; P:oxidation-reduction process |
| Efet.01.629741.g1312.t1 | family transcriptional regulator | 675 | 5.90E-160 | 55.95 | F:DNA binding; F:transcription factor activity, sequence-specific DNA binding; P:transcription, DNA-templated; P:regulation of transcription, DNA-templated; F:alanine racemase activity; F:isomerase activity |
| Efet.01.629756.g1313.t1 | dynein heavy chain axonemal | 282 | 2.87E-32 | 92.35 | F:ATPase activity; F:ATP binding; F:microtubule motor activity; C:dynein complex; C:sperm principal piece; P:microtubule-based movement |
| Efet.01.629799.g1314.t1 | Coiled-coil domain-containing 65 | 336 | 1.94E-12 | 74.7 |  |
| Efet.01.629929.g1316.t1 | follicular epithelium yolk subunit | 444 | 4.94E-27 | 62.8 |  |
| Efet.01.630034.g1.t1 | disks large homolog 2-like isoform X1 | 207 | 3.13E-12 | 74.5 | P:purine ribonucleotide metabolic process; P:guanosine-containing compound metabolic process; F:transferase activity |
| Efet.01.630065.g2.t1 | RNA pseudouridylate synthase domain-containing 2-like | 270 | 2.91E-15 | 76.8 | F:RNA binding; P:tRNA pseudouridine synthesis; C:mitochondrion; F:pseudouridine synthase activity; F:deaminase activity |
| Efet.01.630075.g4.t1 | PIF1 helicase [Mycobacterium abscessus abscessus] | 813 | 4.40E-154 | 71.65 | F:helicase activity |
| Efet.01.630075.g5.t1 | PIF1 helicase [Mycobacterium abscessus abscessus] | 636 | 6.23E-109 | 79.55 | F:helicase activity; P:nucleobase-containing compound metabolic process |
| Efet.01.630132.g6.t1 | neuronal acetylcholine receptor subunit alpha-5-like isoform X1 | 336 | 3.87E-44 | 79.8 | C:postsynaptic membrane; P:cation transmembrane transport; C:integral component of membrane; C:cell junction; F:extracellular ligand-gated ion channel activity |
| Efet.01.630200.g10.t1 | ester lyase | 849 | 0 | 89.7 | F:citryl-CoA lyase activity; F:metal ion binding |
| Efet.01.630200.g11.t1 | tripartite tricarboxylate transporter substrate binding | 927 | 0 | 76.7 | C:outer membrane-bounded periplasmic space |
| Efet.01.630200.g12.t1 | ABC transporter substrate-binding | 1053 | 0 | 94 | C:outer membrane-bounded periplasmic space |
| Efet.01.630200.g13.t1 | DUF2189 domain-containing | 969 | 8.22E-164 | 92.8 | C:integral component of membrane |
| Efet.01.630200.g7.t1 | acyl- dehydrogenase | 396 | 7.96E-84 | 92.6 | F:acyl-CoA dehydrogenase activity; F:flavin adenine dinucleotide binding; F:butyryl-CoA dehydrogenase activity; P:oxidation-reduction process |
| Efet.01.630200.g8.t1 | family dehydratase | 393 | 6.06E-92 | 86.8 |  |
| Efet.01.630200.g9.t1 | Formyl- transferase | 801 | 2.36E-136 | 82.4 | F:formyl-CoA transferase activity |
| Efet.01.63028.g802.t1 | kinesin unc-104 isoform X1 | 243 | 9.78E-14 | 66.65 | F:binding |
| Efet.01.630355.g16.t1 | serine threonine- kinase roco4 | 3561 | 0 | 46.55 | F:GTP binding; P:small GTPase mediated signal transduction; C:intracellular |
| Efet.01.63036.g803.t1 | potassium voltage-gated channel Shal-like | 1161 | 0 | 83.9 | F:voltage-gated potassium channel activity; P:potassium ion transmembrane transport; C:voltage-gated potassium channel complex; P:regulation of ion transmembrane transport; P:protein homooligomerization |
| Efet.01.630370.g17.t1 | hypothetical protein HELRODRAFT_188009 | 321 | 7.36E-18 | 58 | C:membrane; C:integral component of membrane |
| Efet.01.630379.g18.t1 | OTU domain-containing 7B-like isoform X1 | 1767 | 1.30E-15 | 47.8 | F:DNA binding; P:protein K11-linked deubiquitination; F:zinc ion binding; F:peptidase activity |
| Efet.01.630390.g19.t1 | translation initiation factor IF-2 | 1356 | 0 | 96.5 | F:GTP binding; F:translation initiation factor activity; C:cytoplasm; F:GTPase activity; P:translational initiation |
| Efet.01.630390.g20.t1 | tRNA pseudouridine(55) synthase | 996 | 0 | 81.9 | F:RNA binding; P:tRNA pseudouridine synthesis; F:pseudouridine synthase activity |
| Efet.01.630390.g21.t1 | translational GTPase | 1620 | 0 | 82.9 | F:GTP binding; F:GTPase activity |
| Efet.01.630390.g22.t1 | family transporter | 945 | 1.54E-162 | 90.7 | C:integral component of membrane |
| Efet.01.630390.g23.t1 | 3-hydroxyisobutyryl- hydrolase | 993 | 0 | 79.45 | F:3-hydroxyisobutyryl-CoA hydrolase activity; F:isomerase activity |
| Efet.01.630390.g24.t1 | BFD (2Fe-2S)-binding domain-containing | 393 | 7.96E-31 | 92 | F:oxidoreductase activity; P:oxidation-reduction process |
| Efet.01.630441.g25.t1 | Protocadherin- | 768 | 2.15E-28 | 50.55 | F:oxidoreductase activity; F:calcium ion binding; C:membrane; C:integral component of membrane; P:cell adhesion; P:oxidation-reduction process; F:alcohol dehydrogenase (NAD) activity; P:homophilic cell adhesion via plasma membrane adhesion molecules; C:plasma membrane |
| Efet.01.630521.g28.t1 | tartrate dehydrogenase | 1089 | 0 | 94.7 | F:magnesium ion binding; F:D-malate dehydrogenase (decarboxylating) activity; F:NAD binding; F:tartrate dehydrogenase activity; P:oxidation-reduction process; F:tartrate decarboxylase activity |
| Efet.01.630521.g29.t1 | family transcriptional regulator | 924 | 0 | 88.5 | F:DNA binding; F:transcription factor activity, sequence-specific DNA binding; P:transcription, DNA-templated; P:regulation of transcription, DNA-templated; F:carbonate dehydratase activity |
| Efet.01.630521.g30.t1 | family transcriptional regulator | 561 | 8.73E-121 | 74.2 | F:DNA binding; F:transcription factor activity, sequence-specific DNA binding; P:transcription, DNA-templated; P:regulation of transcription, DNA-templated |
| Efet.01.630521.g31.t1 | ABC transporter ATP-binding | 942 | 0 | 74.05 | F:ATPase activity; F:ATP binding; P:transmembrane transport; P:polyamine transport; C:ATP-binding cassette (ABC) transporter complex; F:hydrolase activity, acting on acid anhydrides, catalyzing transmembrane movement of substances |
| Efet.01.630521.g32.t1 | ABC transporter substrate-binding | 1023 | 0 | 82.5 |  |
| Efet.01.630521.g33.t1 | spermidine putrescine transport system permease | 669 | 8.74E-110 | 82 | C:integral component of membrane; P:transport; C:plasma membrane |
| Efet.01.630521.g34.t1 | ABC transporter permease | 699 | 2.19E-138 | 83.85 | C:integral component of membrane; P:transport; C:plasma membrane |
| Efet.01.630546.g35.t1 | hydroxypyruvate isomerase | 771 | 0 | 89.5 | F:hydroxypyruvate isomerase activity |
| Efet.01.630546.g36.t1 | inositol 2-dehydrogenase | 270 | 1.46E-44 | 83.1 | F:inositol 2-dehydrogenase activity; P:oxidation-reduction process |
| Efet.01.630546.g37.t1 | ABC transporter | 1341 | 1.68E-158 | 93.7 | C:integral component of membrane; P:monosaccharide transport; F:monosaccharide-transporting ATPase activity; C:plasma membrane |
| Efet.01.630546.g38.t1 | rhizopine-binding | 801 | 0 | 81.4 | F:3-dehydroquinate dehydratase activity; P:carbohydrate transport |
| Efet.01.630546.g39.t1 | oxidoreductase [Burkholderia stagnalis] | 1173 | 0 | 91.05 | F:inositol 2-dehydrogenase activity; P:oxidation-reduction process |
| Efet.01.630546.g40.t1 | family transporter | 858 | 1.95E-140 | 64.9 | C:integral component of membrane |
| Efet.01.630546.g41.t1 | DUF2000 domain-containing | 438 | 6.45E-101 | 65 |  |
| Efet.01.630546.g42.t1 | NADH-dependent dehydrogenase | 1158 | 0 | 82.65 | F:oxidoreductase activity; P:oxidation-reduction process |
| Efet.01.630546.g43.t1 | sugar phosphate isomerase epimerase | 948 | 0 | 81.1 | F:isomerase activity |
| Efet.01.630546.g44.t1 | PIG-L family deacetylase | 555 | 3.81E-119 | 54.45 |  |
| Efet.01.630546.g45.t1 | sugar ABC transporter ATP-binding | 555 | 7.84E-107 | 59.6 | F:ATPase activity; F:ATP binding |
| Efet.01.630546.g46.t1 | ABC transporter permease | 957 | 0 | 70 | C:integral component of membrane; P:transport; C:plasma membrane; F:transporter activity |
| Efet.01.630557.g47.t1 | chitin synthase | 1674 | 1.62E-136 | 58.2 | C:myosin complex; F:ATP binding; F:transferase activity, transferring hexosyl groups; C:membrane; C:integral component of membrane; F:motor activity |
| Efet.01.630569.g48.t1 | sugar ABC transporter substrate-binding | 243 | 8.28E-47 | 69.67 | P:transport; F:transporter activity |
| Efet.01.630610.g50.t1 | integrator complex subunit 6-like | 549 | 1.55E-13 | 54.1 | P:snRNA processing; C:actin cytoskeleton; C:nucleoplasm; C:integrator complex |
| Efet.01.630616.g51.t1 | Alstrom syndrome 1 | 453 | 2.74E-14 | 65.95 | P:regulation of cellular process; P:cell development; P:multicellular organismal process |
| Efet.01.630636.g52.t1 | 4-aminobutyrate--2-oxoglutarate transaminase | 378 | 2.81E-19 | 91.65 | F:pyridoxal phosphate binding; F:4-aminobutyrate transaminase activity; P:gamma-aminobutyric acid metabolic process |
| Efet.01.630637.g53.t1 | ribosome silencing factor | 399 | 3.54E-74 | 76.45 | C:cytoplasm; P:mature ribosome assembly; P:negative regulation of translation; P:negative regulation of ribosome biogenesis |
| Efet.01.630701.g54.t1 | tripartite motif-containing 3-like | 642 | 1.19E-28 | 53.15 | F:zinc ion binding; F:metal ion binding; C:intracellular |
| Efet.01.630711.g55.t1 | non-ribosomal peptide synthetase | 429 | 8.68E-32 | 51.65 | F:D-alanine-poly(phosphoribitol) ligase activity; F:hydrolase activity, acting on ester bonds; F:catalytic activity; P:metabolic process; P:biosynthetic process; F:phosphopantetheine binding; F:ligase activity |
| Efet.01.630711.g56.t1 | GNAT family N-acetyltransferase | 231 | 4.01E-13 | 66.3 | F:transferase activity |
| Efet.01.630725.g57.t1 | aspartate aminotransferase family | 606 | 3.32E-122 | 89.25 | F:pyridoxal phosphate binding; F:beta-alanine-pyruvate transaminase activity |
| Efet.01.630738.g59.t1 | hypothetical protein HELRODRAFT_169090 | 1233 | 1.57E-55 | 47.5 | F:chitin binding; P:chitin metabolic process; C:membrane; C:integral component of membrane; C:extracellular region |
| Efet.01.630767.g61.t1 | ABC transporter substrate-binding | 459 | 3.93E-81 | 68.15 | P:transport; F:transporter activity |
| Efet.01.630768.g62.t1 | tripartite tricarboxylate transporter substrate binding | 1272 | 0 | 86.95 | C:outer membrane-bounded periplasmic space |
| Efet.01.630768.g63.t1 | family | 1407 | 0 | 81.65 | F:lyase activity |
| Efet.01.630768.g64.t1 | ester lyase | 366 | 1.59E-57 | 75.45 | F:catalytic activity; F:metal ion binding |
| Efet.01.630768.g65.t1 | tripartite tricarboxylate transporter substrate binding | 1032 | 0 | 89.2 | C:outer membrane-bounded periplasmic space |
| Efet.01.630768.g66.t1 | cupin domain-containing | 441 | 1.48E-90 | 76 |  |
| Efet.01.630768.g67.t1 | tripartite tricarboxylate transporter substrate binding | 786 | 1.14E-158 | 64 | C:outer membrane-bounded periplasmic space |
| Efet.01.630774.g68.t1 | ABC transporter ATP-binding | 1044 | 0 | 87.45 | F:ATPase activity; F:ATP binding; P:peptide transport |
| Efet.01.6308.g495.t1 | piggyBac transposable element-derived 4-like | 855 | 2.77E-36 | 61.3 | C:membrane; C:integral component of membrane |
| Efet.01.630814.g70.t1 | bcl-2 homologous antagonist killer-like | 261 | 1.57E-18 | 65.5 | P:positive regulation of apoptotic process |
| Efet.01.630858.g72.t1 | malonyl -ACP transacylase | 345 | 3.55E-40 | 59.1 | P:metabolic process; F:transferase activity |
| Efet.01.630858.g73.t1 | malonate decarboxylase holo-[acyl-carrier- ] synthase | 387 | 9.12E-66 | 59.1 | F:nucleotidyltransferase activity |
| Efet.01.630858.g74.t1 | biotin-independent malonate decarboxylase subunit gamma | 714 | 4.76E-94 | 77.25 | F:ligase activity |
| Efet.01.630860.g75.t1 | MFS transporter | 1227 | 0 | 90.3 | P:transmembrane transport; C:integral component of membrane; F:transporter activity |
| Efet.01.630860.g76.t1 | alcohol dehydrogenase | 1098 | 0 | 84.65 | F:lactaldehyde reductase activity; F:metal ion binding; P:oxidation-reduction process; F:alcohol dehydrogenase (NAD) activity |
| Efet.01.630860.g77.t1 | family transcriptional regulator | 885 | 0 | 82.85 | F:DNA binding; F:transcription factor activity, sequence-specific DNA binding; P:transcription, DNA-templated; P:regulation of transcription, DNA-templated |
| Efet.01.630860.g78.t1 | aspartate aminotransferase family | 1341 | 0 | 88.85 | F:pyridoxal phosphate binding; F:4-aminobutyrate transaminase activity; F:N2-acetyl-L-ornithine:2-oxoglutarate 5-aminotransferase activity |
| Efet.01.630860.g79.t1 | NAD-dependent succinate-semialdehyde dehydrogenase | 1479 | 0 | 90.95 | F:succinate-semialdehyde dehydrogenase [NAD(P)+] activity; P:oxidation-reduction process; F:lactaldehyde dehydrogenase activity |
| Efet.01.630860.g80.t1 | Lrp family transcriptional regulator | 237 | 9.05E-52 | 93.15 | F:transcription factor activity, sequence-specific DNA binding; P:transcription, DNA-templated; P:regulation of transcription, DNA-templated; F:sequence-specific DNA binding; C:intracellular |
| Efet.01.630860.g81.t1 | N-alpha-acetyl diaminobutyric acid deacetylase | 924 | 0 | 85.9 | F:hydrolase activity, acting on ester bonds; F:metal ion binding; P:metabolic process |
| Efet.01.630860.g82.t1 | ectoine hydrolase | 1116 | 0 | 94.3 | P:proteolysis; F:aminopeptidase activity |
| Efet.01.630860.g83.t1 | ornithine cyclodeaminase | 867 | 0 | 86.15 | F:ornithine cyclodeaminase activity |
| Efet.01.630860.g84.t1 | hydroxyectoine utilization dehydratase | 867 | 4.27E-178 | 83.45 | F:pyridoxal phosphate binding; P:cellular amino acid metabolic process; F:L-threonine ammonia-lyase activity |
| Efet.01.630860.g85.t1 | PLP-dependent aminotransferase family | 1284 | 0 | 92.2 | F:DNA binding; F:transcription factor activity, sequence-specific DNA binding; F:pyridoxal phosphate binding; P:transcription, DNA-templated; F:transaminase activity; P:regulation of transcription, DNA-templated |
| Efet.01.630860.g86.t1 | ectoine hydroxyectoine ABC transporter substrate-binding | 924 | 1.80E-156 | 86.6 | F:ectoine binding; F:ectoine transmembrane transporter activity; P:ectoine transport; C:plasma membrane |
| Efet.01.630860.g87.t1 | polar amino acid ABC transporter inner membrane subunit | 669 | 4.23E-129 | 90.1 | C:integral component of membrane; P:transport; C:plasma membrane; F:transporter activity |
| Efet.01.630860.g88.t1 | ectoine hydroxyectoine ABC transporter permease subunit | 540 | 1.19E-125 | 94.15 | C:integral component of membrane; P:transport; C:plasma membrane; F:transporter activity |
| Efet.01.630860.g89.t1 | ectoine hydroxyectoine ABC transporter ATP-binding | 825 | 0 | 91.8 | F:ATP binding; P:ferric iron transport; F:ferric-transporting ATPase activity; P:amino acid transmembrane transport; F:amino acid-transporting ATPase activity |
| Efet.01.630860.g90.t1 | magnesium cobalt efflux | 981 | 2.06E-179 | 83.1 | F:oxidoreductase activity, acting on CH-OH group of donors; F:flavin adenine dinucleotide binding; P:oxidation-reduction process |
| Efet.01.630860.g91.t1 | apolipo N-acyltransferase | 1578 | 0 | 79.25 | F:hydrolase activity, acting on carbon-nitrogen (but not peptide) bonds; P:nitrogen compound metabolic process; C:integral component of membrane; F:N-acyltransferase activity; P:lipoprotein biosynthetic process; C:plasma membrane |
| Efet.01.630860.g92.t1 | 3-hydroxyacyl-[acyl-carrier- ] dehydratase | 531 | 2.23E-115 | 97.15 | F:3-hydroxyoctanoyl-[acyl-carrier-protein] dehydratase activity; C:cytoplasm; F:3-hydroxydecanoyl-[acyl-carrier-protein] dehydratase activity; P:fatty acid biosynthetic process; F:isomerase activity |
| Efet.01.630860.g93.t1 | beta-ketoacyl-[acyl-carrier- ] synthase I | 1203 | 0 | 95.6 | F:3-oxoacyl-[acyl-carrier-protein] synthase activity; P:metabolic process |
| Efet.01.630860.g94.t1 | glycine--tRNA ligase subunit alpha | 948 | 0 | 94.95 | F:glycine-tRNA ligase activity; F:ATP binding; P:glycyl-tRNA aminoacylation; C:cytoplasm |
| Efet.01.630860.g95.t1 | glycine--tRNA ligase subunit beta | 1929 | 0 | 86.7 | F:glycine-tRNA ligase activity; F:ATP binding; P:glycyl-tRNA aminoacylation; F:arginine-tRNA ligase activity; C:cytoplasm; P:arginyl-tRNA aminoacylation |
| Efet.01.630860.g96.t1 | histidinol phosphate phosphatase | 621 | 8.09E-101 | 91.7 | C:cytoplasm; F:phosphatase activity; P:dephosphorylation |
| Efet.01.630860.g97.t1 | acyl-phosphate glycerol 3-phosphate acyltransferase | 777 | 2.20E-137 | 85.75 | F:transferase activity, transferring acyl groups; P:metabolic process; C:integral component of membrane |
| Efet.01.630903.g99.t1 | cartilage matrix -like | 498 | 1.30E-25 | 57.6 | C:collagen trimer; F:calcium ion binding |
| Efet.01.63094.g804.t1 | 2-hydroxyacylsphingosine 1-beta-galactosyltransferase-like | 1011 | 8.58E-75 | 59.9 | F:transferase activity, transferring glycosyl groups |
| Efet.01.630972.g101.t1 | OTU domain-containing | 1569 | 1.07E-50 | 48.9 | F:peptidase activity |
| Efet.01.630975.g102.t1 | transcriptional family | 768 | 1.12E-132 | 72.7 | C:membrane |
| Efet.01.630993.g103.t1 | phosphate ABC transporter permease subunit | 222 | 2.01E-39 | 93.65 | F:inorganic phosphate transmembrane transporter activity; P:transmembrane transport; P:phosphate ion transport; C:integral component of membrane; C:plasma membrane |
| Efet.01.631106.g105.t1 | DUF4442 domain-containing | 399 | 1.63E-41 | 67.95 | C:membrane; C:integral component of membrane |
| Efet.01.631188.g106.t1 | 5-oxoprolinase-like isoform X1 | 972 | 1.35E-140 | 85.05 | F:hydrolase activity |
| Efet.01.631230.g107.t1 | acid-sensing ion channel 1-like | 876 | 3.12E-08 | 57.15 | P:sodium ion transport; P:ion transport; F:sodium channel activity; C:membrane; C:integral component of membrane; P:sodium ion transmembrane transport; P:transport |
| Efet.01.631289.g110.t1 | ATP-dependent DNA helicase | 1509 | 0 | 84.05 | F:DNA binding; F:ATP binding; P:DNA duplex unwinding; F:ATP-dependent DNA helicase activity; P:nucleobase-containing compound metabolic process |
| Efet.01.631292.g111.t1 | UDP-glucose 4-epimerase | 1062 | 0 | 84.25 | F:coenzyme binding; F:UDP-glucose 4-epimerase activity; P:galactose metabolic process |
| Efet.01.631326.g112.t1 | hypothetical protein HELRODRAFT_165884 | 264 | 3.57E-07 | 86 |  |
| Efet.01.63135.g808.t1 | hypothetical protein CAPTEDRAFT_226428 | 1089 | 7.47E-98 | 50.2 | F:calcium ion binding; C:membrane; C:integral component of membrane |
| Efet.01.631384.g114.t1 | uncharacterized protein LOC110464501 | 432 | 4.97E-57 | 71.75 |  |
| Efet.01.631414.g115.t1 | zinc finger 431-like | 3087 | 1.51E-33 | 39.35 | F:nucleic acid binding; C:nucleus; F:metal ion binding; P:regulation of transcription, DNA-templated |
| Efet.01.631415.g116.t1 | DUF2079 domain-containing | 318 | 1.28E-46 | 67.5 | C:membrane; C:integral component of membrane |
| Efet.01.631477.g117.t1 | carbohydrate ABC transporter permease | 687 | 1.43E-84 | 77.55 | C:integral component of membrane; P:transport; C:plasma membrane |
| Efet.01.631524.g118.t1 | trichohyalin-like isoform X1 | 246 | 2.85E-13 | 72.17 | P:regulation of microtubule polymerization or depolymerization |
| Efet.01.631564.g119.t1 | peptidyl-prolyl cis-trans isomerase-like | 261 | 2.22E-18 | 69.58 | P:cellular process; F:isomerase activity |
| Efet.01.631579.g120.t1 | aminotransferase | 1047 | 1.25E-174 | 81.7 | F:pyridoxal phosphate binding; F:L-phenylalanine:2-oxoglutarate aminotransferase activity; P:biosynthetic process; F:L-aspartate:2-oxoglutarate aminotransferase activity |
| Efet.01.631580.g121.t1 | hypothetical protein HELRODRAFT_172421 | 756 | 1.18E-102 | 69.75 | F:catalytic activity; P:nucleoside metabolic process |
| Efet.01.631607.g122.t1 | Peptidoglycan-binding | 396 | 5.61E-38 | 88.7 |  |
| Efet.01.631607.g123.t1 | Peptidoglycan-binding | 666 | 3.21E-134 | 82.65 |  |
| Efet.01.631607.g124.t1 | DNA protecting | 1188 | 0 | 81.25 | P:DNA mediated transformation |
| Efet.01.631607.g125.t1 | DUF1631 domain-containing | 2289 | 0 | 77.1 |  |
| Efet.01.631607.g126.t1 | translocase subunit | 930 | 0 | 92.6 | P:protein targeting; F:P-P-bond-hydrolysis-driven protein transmembrane transporter activity; P:intracellular protein transmembrane transport; C:integral component of membrane; P:protein transport by the Sec complex; C:intracellular; C:plasma membrane |
| Efet.01.631607.g127.t1 | translocase subunit | 1911 | 0 | 91.4 | P:protein targeting; F:P-P-bond-hydrolysis-driven protein transmembrane transporter activity; P:intracellular protein transmembrane transport; C:integral component of membrane; P:protein transport by the Sec complex; C:intracellular; C:plasma membrane |
| Efet.01.631607.g128.t1 | pre translocase subunit | 276 | 9.96E-57 | 86.85 | C:integral component of membrane |
| Efet.01.631644.g129.t1 | OCTopamine Receptor (GPCR) | 930 | 2.89E-14 | 48.85 | P:G-protein coupled receptor signaling pathway; C:membrane; P:regulation of response to stress; F:G-protein coupled amine receptor activity |
| Efet.01.631678.g130.t1 | 3 family IMP dehydrogenase-related | 726 | 1.84E-128 | 83.75 | F:IMP dehydrogenase activity; P:oxidation-reduction process |
| Efet.01.631737.g131.t1 | short-chain dehydrogenase | 438 | 2.58E-79 | 91.6 |  |
| Efet.01.631741.g132.t1 | enoyl- hydratase | 789 | 0 | 92.1 | P:metabolic process; F:enoyl-CoA hydratase activity; F:isomerase activity |
| Efet.01.631741.g133.t1 | transposase | 546 | 1.69E-64 | 72.95 | F:DNA binding |
| Efet.01.631741.g134.t1 | Mobile element | 495 | 1.21E-86 | 82.85 |  |
| Efet.01.631766.g135.t1 | D-xylose ABC transporter ATP-binding | 609 | 1.50E-39 | 90.35 | F:ATPase activity; F:ATP binding |
| Efet.01.631813.g136.t1 | aspartate aminotransferase | 915 | 1.11E-150 | 68.25 | F:transaminase activity |
| Efet.01.631813.g137.t1 | alpha beta hydrolase | 882 | 8.40E-176 | 90.25 | F:oxidoreductase activity; F:arylesterase activity; P:oxidation-reduction process |
| Efet.01.631813.g138.t1 | Zn-dependent hydrolase | 1209 | 0 | 84.35 | F:hydrolase activity, acting on carbon-nitrogen (but not peptide) bonds, in linear amidines; P:metabolic process; F:N-carbamoyl-L-amino-acid hydrolase activity |
| Efet.01.631865.g140.t1 | alanine--tRNA ligase | 936 | 1.84E-134 | 71.15 | F:nucleic acid binding; F:ligase activity, forming aminoacyl-tRNA and related compounds; P:cellular macromolecule metabolic process; F:ion binding; P:organonitrogen compound metabolic process; P:cellular nitrogen compound metabolic process; P:primary metabolic process |
| Efet.01.631865.g141.t1 | alanine--tRNA ligase | 1014 | 7.46E-140 | 70.15 | F:nucleotide binding; F:aminoacyl-tRNA ligase activity; F:RNA binding; F:metal ion binding; P:tRNA aminoacylation; P:translation |
| Efet.01.631940.g142.t1 | sugar ABC transporter permease | 333 | 5.36E-33 | 80.5 | C:integral component of membrane; P:monosaccharide transport; F:monosaccharide-transporting ATPase activity; C:plasma membrane |
| Efet.01.631942.g143.t1 | dynein heavy chain domain-containing 1-like | 285 | 6.90E-24 | 73.8 | F:nucleotide binding |
| Efet.01.631968.g144.t1 | ankyrin repeat RF_0381 | 318 | 7.38E-07 | 61 |  |
| Efet.01.63199.g810.t1 | hypothetical protein HELRODRAFT_190044 | 378 | 3.12E-10 | 82 | F:ryanodine-sensitive calcium-release channel activity; F:calcium ion binding; C:integral component of membrane; P:release of sequestered calcium ion into cytosol; C:intracellular |
| Efet.01.632046.g146.t1 | glycoside hydrolase | 483 | 1.27E-40 | 83.45 | P:carbohydrate metabolic process; F:hydrolase activity, hydrolyzing O-glycosyl compounds |
| Efet.01.632079.g147.t1 | ceramide glucosyltransferase | 255 | 3.86E-13 | 71.6 | P:glucosylceramide biosynthetic process; F:ceramide glucosyltransferase activity; C:integral component of membrane |
| Efet.01.632102.g148.t1 | cytochrome P450 2G1 | 222 | 6.82E-08 | 68.1 | F:oxidoreductase activity; F:binding |
| Efet.01.632142.g149.t1 | serine threonine- kinase Tao isoform X1 | 270 | 2.86E-13 | 64.65 | P:phosphorylation; F:protein serine/threonine kinase activity |
| Efet.01.632184.g152.t1 | Structural maintenance of chromosomes 1A | 213 | 1.32E-22 | 74.7 | P:glycosylation; F:transferase activity, transferring glycosyl groups; P:cellular macromolecule metabolic process; P:chromosome organization; C:membrane; C:Golgi apparatus; P:single-organism cellular process; F:binding; P:primary metabolic process |
| Efet.01.632298.g153.t1 | very low-density lipo receptor isoform X1 | 462 | 1.90E-09 | 45.8 | F:calcium ion binding; C:membrane; C:integral component of membrane |
| Efet.01.632328.g154.t1 | hypothetical protein CAPTEDRAFT_205041 | 402 | 9.64E-11 | 48 | F:zinc ion binding; F:chitin binding; P:proteolysis; P:chitin metabolic process; C:extracellular region; F:metalloendopeptidase activity |
| Efet.01.632329.g155.t1 | early growth response 1-like isoform X1 | 3147 | 1.99E-48 | 74.55 | F:nucleic acid binding; F:metal ion binding |
| Efet.01.63236.g812.t1 | Inositol 1,4,5-trisphosphate receptor type 1 | 288 | 4.39E-15 | 88.45 | P:inositol phosphate-mediated signaling; C:integral component of membrane; F:inositol 1,4,5-trisphosphate-sensitive calcium-release channel activity; P:calcium ion transmembrane transport; C:endoplasmic reticulum |
| Efet.01.63237.g813.t1 | IQ domain-containing E-like isoform X1 | 345 | 1.57E-19 | 69.05 |  |
| Efet.01.632423.g157.t1 | hybrid sensor histidine kinase response regulator | 1584 | 0 | 87.25 | F:phosphorelay sensor kinase activity; F:ATP binding; P:phosphorelay signal transduction system; P:regulation of transcription, DNA-templated; P:signal transduction by protein phosphorylation; C:intracellular; P:peptidyl-histidine phosphorylation |
| Efet.01.632468.g160.t1 | protoporphyrinogen oxidase | 288 | 3.65E-34 | 81.5 | P:porphyrin-containing compound biosynthetic process; F:oxygen-dependent protoporphyrinogen oxidase activity; P:oxidation-reduction process |
| Efet.01.632504.g161.t1 | NChain Single-particle Cryo-em Using Alignment By Classification (abc): The Structure Of Lumbricus Terrestris Hemoglobin | 255 | 1.24E-23 | 81.25 |  |
| Efet.01.63252.g814.t1 | patatin-like phospholipase domain-containing 7 isoform X1 | 3342 | 0 | 63.9 | P:metabolic process; P:developmental process; P:single-organism process |
| Efet.01.632627.g163.t1 | cationic amino acid transporter 4 | 1536 | 1.20E-120 | 74.3 | C:integral component of plasma membrane; F:L-amino acid transmembrane transporter activity; F:antiporter activity; P:L-amino acid transport; P:amino acid transmembrane transport |
| Efet.01.6327.g498.t1 | ectonucleoside triphosphate diphosphohydrolase 4 isoform X1 | 267 | 2.68E-19 | 65.75 | C:intracellular membrane-bounded organelle; C:bounding membrane of organelle; C:cytoplasmic part; C:intracellular organelle part; C:integral component of organelle membrane; F:hydrolase activity |
| Efet.01.632722.g165.t1 | HEX_VIBVL ame: Full=Beta-hexosaminidase ame: Full=Beta-N-acetylhexosaminidase ame: Full=Chitobiase ame: Full=N-acetyl-beta-glucosaminidase | 231 | 2.34E-20 | 69.75 | P:carbohydrate metabolic process; F:hydrolase activity, hydrolyzing O-glycosyl compounds; F:carbohydrate binding |
| Efet.01.632756.g166.t1 | 4-hydroxythreonine-4-phosphate dehydrogenase [Burkholderia stagnalis] | 441 | 4.35E-100 | 79.55 | F:magnesium ion binding; P:pyridoxal phosphate biosynthetic process; F:zinc ion binding; P:pyridoxine biosynthetic process; C:cytoplasm; F:NAD binding; F:4-hydroxythreonine-4-phosphate dehydrogenase activity; P:oxidation-reduction process; F:cobalt ion binding |
| Efet.01.632756.g167.t1 | sugar ABC transporter substrate-binding | 867 | 0 | 78.3 | P:carbohydrate transport |
| Efet.01.632756.g168.t1 | ABC transporter permease | 999 | 0 | 92.25 | C:integral component of membrane; P:transport; C:plasma membrane; F:transporter activity |
| Efet.01.632756.g169.t1 | sugar ABC transporter ATP-binding | 1254 | 0 | 83.6 | F:ATP binding; P:monosaccharide transport; F:monosaccharide-transporting ATPase activity |
| Efet.01.632756.g170.t1 | 40-residue YVTN family beta-propeller repeat | 963 | 0 | 82.2 |  |
| Efet.01.632756.g171.t1 | family transcriptional regulator | 843 | 6.27E-174 | 69.2 | F:DNA binding; F:transcription factor activity, sequence-specific DNA binding; F:catalytic activity; P:transcription, DNA-templated; P:regulation of transcription, DNA-templated |
| Efet.01.632798.g172.t1 | trifunctional transcriptional regulator proline dehydrogenase L-glutamate gamma-semialdehyde dehydrogenase | 1155 | 0 | 89.3 | F:DNA binding; F:1-pyrroline-5-carboxylate dehydrogenase activity; F:transcription factor activity, sequence-specific DNA binding; F:proline dehydrogenase activity; P:transcription, DNA-templated; P:regulation of transcription, DNA-templated; P:proline biosynthetic process; P:oxidation-reduction process; F:oxidoreductase activity, acting on the aldehyde or oxo group of donors, NAD or NADP as acceptor; P:proline catabolic process to glutamate |
| Efet.01.632850.g174.t1 | bifunctional (p)ppGpp synthetase guanosine-3 ,5 -bis(diphosphate) 3 -pyrophosphohydrolase | 279 | 1.01E-52 | 92.1 | P:guanosine tetraphosphate metabolic process; P:phosphorylation; F:amino acid binding; F:kinase activity |
| Efet.01.632855.g175.t1 | leucyl aminopeptidase | 651 | 2.88E-91 | 83.35 | C:cytoplasm; P:proteolysis; F:metalloexopeptidase activity; F:aminopeptidase activity; F:manganese ion binding |
| Efet.01.63288.g816.t1 | FMRFamide receptor-like | 612 | 1.66E-53 | 68.9 | F:G-protein coupled peptide receptor activity; P:G-protein coupled receptor signaling pathway; C:integral component of membrane |
| Efet.01.632909.g176.t1 | neuroglian isoform X1 | 684 | 2.07E-16 | 46.25 | C:membrane; C:integral component of membrane |
| Efet.01.632957.g177.t1 | SCBP2-like protein, partial | 423 | 6.27E-15 | 52 | F:calcium ion binding |
| Efet.01.63299.g817.t1 | inhibitor of growth 5 | 480 | 2.04E-26 | 80.15 | F:zinc ion binding; P:negative regulation of cell proliferation; C:MOZ/MORF histone acetyltransferase complex; P:histone acetylation |
| Efet.01.632992.g178.t1 | 2Fe-2S ferredoxin | 360 | 2.09E-51 | 92.25 |  |
| Efet.01.632992.g179.t1 | alpha beta hydrolase | 645 | 5.41E-137 | 84 | C:integral component of membrane; F:hydrolase activity |
| Efet.01.633035.g180.t1 | neuron navigator 2 | 699 | 3.85E-18 | 53.4 | P:multicellular organismal process |
| Efet.01.633150.g181.t1 | galectin | 267 | 1.38E-07 | 62.9 | F:carbohydrate binding |
| Efet.01.633156.g182.t1 | family transcriptional regulator | 456 | 2.93E-51 | 66.85 | C:cytoplasm; P:regulation of DNA repair |
| Efet.01.633190.g184.t1 | NAD-dependent succinate-semialdehyde dehydrogenase | 768 | 5.99E-144 | 85.65 | P:oxidation-reduction process; F:oxidoreductase activity, acting on the aldehyde or oxo group of donors, NAD or NADP as acceptor |
| Efet.01.633237.g185.t1 | solute carrier family riboflavin member 3 | 1092 | 9.34E-27 | 50.05 | P:sensory perception of sound; C:integral component of plasma membrane; P:riboflavin transport; P:cellular response to heat; C:membrane; C:integral component of membrane; P:transport; F:riboflavin transporter activity; C:plasma membrane |
| Efet.01.633242.g186.t1 | tetratricopeptide repeat 22 | 2196 | 1.07E-10 | 46 | F:nucleotide binding; P:tRNA aminoacylation for protein translation; F:ATP binding; F:aminoacyl-tRNA ligase activity |
| Efet.01.633266.g187.t1 | NCK-interacting with SH3 domain-like | 471 | 4.64E-51 | 67.45 |  |
| Efet.01.633356.g190.t1 | translation initiation factor IF-2 | 327 | 1.71E-46 | 99.45 | F:GTP binding; F:translation initiation factor activity; C:cytoplasm; F:GTPase activity; P:translational initiation |
| Efet.01.633406.g191.t1 | WD repeat and FYVE domain-containing 3 | 258 | 1.43E-29 | 80.85 | F:metal ion binding |
| Efet.01.63345.g819.t1 | serine threonine- phosphatase 6 regulatory ankyrin repeat subunit C-like | 309 | 2.48E-24 | 63.2 | P:cellular process |
| Efet.01.633502.g192.t1 | dTDP-glucose 4,6-dehydratase | 531 | 1.20E-65 | 92.95 | F:oxidoreductase activity; F:dTDP-glucose 4,6-dehydratase activity; P:nucleotide-sugar metabolic process; P:oxidation-reduction process; C:viral capsid |
| Efet.01.633502.g193.t1 | twin-arginine translocase subunit | 465 | 2.00E-58 | 94.4 | C:integral component of plasma membrane; C:TAT protein transport complex; F:protein transmembrane transporter activity; P:protein transport by the Tat complex |
| Efet.01.633502.g194.t1 | Sec-independent translocase | 471 | 5.64E-86 | 80.15 | C:integral component of plasma membrane; C:TAT protein transport complex; F:protein transmembrane transporter activity; P:protein secretion; P:protein transport by the Tat complex |
| Efet.01.633502.g195.t1 | pre translocase subunit | 225 | 1.35E-36 | 83.3 | C:integral component of plasma membrane; C:TAT protein transport complex; F:protein transmembrane transporter activity; P:protein secretion; P:protein transport by the Tat complex |
| Efet.01.633502.g196.t1 | histidine triad nucleotide-binding | 357 | 1.43E-80 | 96.1 | F:hydrolase activity |
| Efet.01.63352.g820.t1 | sperm-tail PG-rich repeat-containing 2-like | 657 | 7.00E-52 | 59.35 |  |
| Efet.01.633528.g197.t1 | Quinolinate synthase | 858 | 1.08E-134 | 73.55 | P:quinolinate biosynthetic process; C:cytoplasm; F:metal ion binding; P:NAD biosynthetic process; F:4 iron, 4 sulfur cluster binding; F:quinolinate synthetase A activity; F:transferase activity, transferring alkyl or aryl (other than methyl) groups |
| Efet.01.633530.g198.t1 | hypothetical protein CAPTEDRAFT_226427 | 318 | 1.04E-12 | 53.17 | F:calcium ion binding; C:membrane; C:integral component of membrane |
| Efet.01.633552.g199.t1 | NAD-dependent DNA ligase | 1143 | 0 | 83.15 | F:dioxygenase activity; F:DNA binding; P:DNA repair; F:DNA ligase (NAD+) activity; F:metal ion binding; P:DNA replication; P:DNA ligation; P:oxidation-reduction process |
| Efet.01.633584.g200.t1 | serine threonine kinase | 438 | 4.91E-73 | 87.1 | F:ATP binding; C:integral component of membrane; F:protein serine/threonine kinase activity; P:protein phosphorylation |
| Efet.01.633623.g201.t1 | anoctamin-4-like | 276 | 4.60E-16 | 86.65 | C:integral component of membrane; F:protein dimerization activity |
| Efet.01.633726.g203.t1 | L-arabinose isomerase | 786 | 7.86E-156 | 85.45 | C:cytoplasm; F:manganese ion binding; F:L-arabinose isomerase activity; P:L-arabinose catabolic process to xylulose 5-phosphate |
| Efet.01.633778.g205.t1 | FAM214A-like isoform X1 | 261 | 2.97E-22 | 76.7 |  |
| Efet.01.63380.g822.t1 | RNA-binding fusilli | 294 | 5.10E-17 | 65.55 | F:nucleotide binding; F:nucleic acid binding |
| Efet.01.633829.g207.t1 | urea ABC transporter | 2286 | 0 | 77.95 |  |
| Efet.01.633829.g208.t1 | urea ABC transporter | 339 | 5.33E-74 | 76.75 |  |
| Efet.01.633829.g209.t1 | branched-chain amino acid ABC transporter permease | 393 | 1.46E-65 | 83.55 | C:integral component of membrane; P:transport; C:plasma membrane; F:transporter activity |
| Efet.01.633829.g210.t1 | ABC transporter ATP-binding | 408 | 1.08E-64 | 77.85 | F:ATPase activity; F:ATP binding |
| Efet.01.633829.g211.t1 | ABC transporter ATP-binding | 297 | 4.87E-62 | 79.4 | F:ATPase activity; F:ATP binding |
| Efet.01.633829.g212.t1 | amidase | 2313 | 0 | 70.45 | F:ATPase activity; F:ATP binding; F:carbon-nitrogen ligase activity, with glutamine as amido-N-donor |
| Efet.01.633829.g213.t1 | AMP-binding | 1311 | 0 | 76.35 | P:metabolic process; F:ligase activity |
| Efet.01.633831.g214.t1 | ribulokinase | 705 | 5.24E-109 | 82.45 | F:ATP binding; F:ribulokinase activity; P:carbohydrate phosphorylation; P:L-arabinose catabolic process to xylulose 5-phosphate |
| Efet.01.633887.g215.t1 | Mg chelatase | 444 | 7.23E-51 | 77.4 |  |
| Efet.01.633887.g216.t1 | ATP-binding | 312 | 2.54E-33 | 78.7 | F:DNA binding; F:ATP binding; P:DNA replication |
| Efet.01.633913.g217.t1 | dolichyl-diphosphooligosaccharide-- glycosyltransferase subunit 1-like | 699 | 2.66E-101 | 83.4 | C:oligosaccharyltransferase complex; F:dolichyl-diphosphooligosaccharide-protein glycotransferase activity; C:integral component of membrane; P:protein N-linked glycosylation via asparagine |
| Efet.01.633916.g218.t1 | hypothetical protein | 945 | 0 | 80.45 | P:carbohydrate metabolic process; F:catalytic activity; F:carbohydrate binding |
| Efet.01.633916.g219.t1 | enoyl- hydratase isomerase family | 705 | 2.25E-164 | 91.7 | P:metabolic process; F:enoyl-CoA hydratase activity; F:isomerase activity |
| Efet.01.633916.g220.t1 | transferase | 1077 | 0 | 93.5 | F:alpha-methylacyl-CoA racemase activity; F:formyl-CoA transferase activity |
| Efet.01.633916.g221.t1 | NAD(P)-dependent oxidoreductase | 1416 | 0 | 93.6 | F:oxidoreductase activity; P:oxidation-reduction process |
| Efet.01.633916.g222.t1 | sugar ABC transporter ATP-binding | 1467 | 0 | 92.3 | F:ATP binding; P:monosaccharide transport; F:monosaccharide-transporting ATPase activity; P:carbohydrate transmembrane transport; C:plasma membrane |
| Efet.01.633916.g223.t1 | ABC transporter permease | 945 | 0 | 92.05 | C:integral component of membrane; P:transport; C:plasma membrane; F:transporter activity |
| Efet.01.633916.g224.t1 | sugar ABC transporter substrate-binding | 1086 | 0 | 90.9 | P:carbohydrate transport |
| Efet.01.633916.g225.t1 | ATP-dependent chaperone | 2694 | 0 | 96.95 | F:ATP binding; P:response to heat; C:cytoplasm; P:protein processing; F:peptidase activity |
| Efet.01.633916.g226.t1 | coniferyl-aldehyde dehydrogenase | 204 | 7.07E-13 | 100 | P:cellular aldehyde metabolic process; F:aldehyde dehydrogenase [NAD(P)+] activity; P:oxidation-reduction process |
| Efet.01.633916.g227.t1 | coniferyl aldehyde dehydrogenase | 882 | 1.28E-153 | 83.7 | P:cellular aldehyde metabolic process; F:aldehyde dehydrogenase [NAD(P)+] activity; P:oxidation-reduction process |
| Efet.01.633916.g228.t1 | GNAT family N-acetyltransferase | 561 | 6.86E-134 | 95.8 | F:N-acetyltransferase activity |
| Efet.01.633916.g229.t1 | membrane | 597 | 3.55E-114 | 86.5 |  |
| Efet.01.633916.g230.t1 | periplasmic lipo involved in iron transport | 291 | 1.40E-64 | 81.6 |  |
| Efet.01.633916.g231.t1 | 4Fe-4S binding domain-containing | 969 | 0 | 83.3 | C:integral component of membrane |
| Efet.01.633941.g232.t1 | dynein heavy chain axonemal-like | 297 | 2.46E-29 | 82 | F:ATPase activity; C:inner dynein arm; F:ATP binding; F:microtubule motor activity; C:cytoplasm; F:calcium ion binding; P:cilium or flagellum-dependent cell motility; F:protein kinase activity; P:cilium movement; P:protein phosphorylation; P:inner dynein arm assembly; C:microtubule |
| Efet.01.633957.g233.t1 | 3-isopropylmalate dehydrogenase | 579 | 4.03E-86 | 83.9 | F:magnesium ion binding; C:cytoplasm; F:NAD binding; P:leucine biosynthetic process; P:oxidation-reduction process; F:3-isopropylmalate dehydrogenase activity |
| Efet.01.63396.g823.t1 | prolactin-releasing peptide receptor-like | 846 | 1.50E-46 | 56.5 | F:G-protein coupled receptor activity; P:G-protein coupled receptor signaling pathway; C:membrane |
| Efet.01.634043.g235.t1 | Neuropeptide FF receptor 2 | 738 | 5.79E-58 | 62.85 | F:neuropeptide receptor activity; P:G-protein coupled receptor signaling pathway; C:membrane |
| Efet.01.634060.g236.t1 | D-alanyl-D-alanine carboxypeptidase | 405 | 3.24E-80 | 68.4 | P:proteolysis; C:membrane; C:integral component of membrane; F:peptidase activity |
| Efet.01.634085.g237.t1 | dihydroxy-acid dehydratase | 537 | 3.33E-97 | 82.7 | F:dihydroxy-acid dehydratase activity; P:metabolic process |
| Efet.01.634160.g238.t1 | tripartite motif-containing 2-like isoform X1 | 954 | 6.44E-14 | 49.1 | F:metal ion binding |
| Efet.01.634160.g239.t1 | kelch 35 | 819 | 6.15E-11 | 41 | P:germinal center formation; F:ubiquitin-protein transferase activity; C:Cul3-RING ubiquitin ligase complex; P:protein ubiquitination; P:B cell receptor signaling pathway |
| Efet.01.634208.g240.t1 | neuroglian-like | 465 | 1.16E-22 | 49.1 | C:membrane; C:integral component of membrane |
| Efet.01.634241.g241.t1 | DNA polymerase III subunit chi | 321 | 5.95E-69 | 82.3 | F:DNA binding; F:DNA-directed DNA polymerase activity; P:DNA replication; P:DNA biosynthetic process |
| Efet.01.634241.g242.t1 | leucyl aminopeptidase | 576 | 1.77E-110 | 90.8 | C:cytoplasm; P:proteolysis; F:metalloexopeptidase activity; F:aminopeptidase activity; F:manganese ion binding |
| Efet.01.63428.g826.t1 | SCY1 2 | 495 | 1.34E-35 | 69.4 | C:membrane; P:phosphorylation; F:kinase activity |
| Efet.01.634400.g244.t1 | AAA family ATPase | 219 | 2.82E-25 | 83.75 |  |
| Efet.01.634415.g245.t1 | RNA helicase | 603 | 1.23E-123 | 96.3 | F:nucleic acid binding; F:ATP binding; C:cytoplasm; P:ribosome assembly; F:ATP-dependent RNA helicase activity |
| Efet.01.634438.g246.t1 | glutamine--fructose-6-phosphate transaminase (isomerizing) | 1080 | 0 | 84.95 | P:carbohydrate metabolic process; C:cytoplasm; F:glutamine-fructose-6-phosphate transaminase (isomerizing) activity; P:glutamine metabolic process; P:carbohydrate derivative biosynthetic process; F:carbohydrate binding |
| Efet.01.634438.g247.t1 | holo-ACP synthase | 369 | 1.10E-62 | 73.7 | F:metal ion binding; F:transferase activity; P:fatty acid metabolic process |
| Efet.01.634561.g250.t1 | probable G- coupled receptor No9 | 882 | 2.80E-40 | 48.65 | F:G-protein coupled receptor activity; F:adrenergic receptor activity; P:G-protein coupled receptor signaling pathway; P:signal transduction; C:membrane; C:integral component of membrane; P:adrenergic receptor signaling pathway; F:signal transducer activity; C:plasma membrane |
| Efet.01.634612.g251.t1 | M23 family peptidase | 231 | 2.50E-46 | 79.55 |  |
| Efet.01.634682.g254.t1 | SMG8 isoform X2 | 273 | 2.36E-12 | 56.1 | P:nuclear-transcribed mRNA catabolic process, nonsense-mediated decay |
| Efet.01.634691.g255.t1 | DUF4870 domain-containing | 264 | 4.53E-55 | 85 |  |
| Efet.01.6347.g501.t1 | Glucose dehydrogenase [acceptor] | 363 | 1.47E-40 | 64.85 | F:oxidoreductase activity |
| Efet.01.634726.g256.t1 | lin-9 homolog isoform X2 | 255 | 5.24E-20 | 75.65 | P:transcription, DNA-templated; C:transcriptional repressor complex; P:cell cycle |
| Efet.01.634752.g257.t1 | family transcriptional regulator | 897 | 2.77E-161 | 87.4 | F:DNA binding; F:transcription factor activity, sequence-specific DNA binding; P:transcription, DNA-templated; P:regulation of transcription, DNA-templated; C:intracellular |
| Efet.01.634752.g258.t1 | ABC transporter | 696 | 5.20E-108 | 88.4 | F:ATPase activity; F:ATP binding; P:transmembrane transport; P:carbohydrate transport; C:ATP-binding cassette (ABC) transporter complex; F:hydrolase activity, acting on acid anhydrides, catalyzing transmembrane movement of substances |
| Efet.01.634752.g259.t1 | ABC transporter ATP-binding | 387 | 1.56E-84 | 97.25 | F:ATP binding; P:glycerol-3-phosphate transport; P:transmembrane transport; F:glycerol-3-phosphate-transporting ATPase activity; P:carbohydrate transport; C:ATP-binding cassette (ABC) transporter complex |
| Efet.01.634762.g260.t1 | potassium voltage-gated channel subfamily H member 2 isoform X2 | 549 | 3.84E-78 | 86.8 | F:phosphorelay sensor kinase activity; C:integral component of plasma membrane; F:voltage-gated potassium channel activity; P:regulation of membrane potential; P:potassium ion transmembrane transport; P:phosphorelay signal transduction system; P:signal transduction by protein phosphorylation; C:intracellular |
| Efet.01.634809.g261.t1 | DUF2804 domain-containing | 1062 | 0 | 65.95 | F:hydrolase activity |
| Efet.01.634840.g262.t1 | serine-rich adhesin for platelets-like isoform X1 | 2124 | 9.87E-18 | 82.95 | C:nucleus; F:transcription coactivator activity; P:regulation of nucleic acid-templated transcription |
| Efet.01.6349.g502.t1 | Retrovirus-related Pol poly from transposon TNT 1-94 | 501 | 1.87E-20 | 66.6 | C:membrane |
| Efet.01.634939.g264.t1 | sugar ABC transporter substrate-binding | 900 | 0 | 97.05 | P:carbohydrate transport; F:transporter activity |
| Efet.01.634939.g265.t1 | sugar ABC transporter permease | 1515 | 2.99E-171 | 92.45 | C:integral component of membrane; P:carbohydrate transport; C:plasma membrane |
| Efet.01.634939.g266.t1 | ABC transporter ATP-binding | 861 | 1.02E-156 | 73.35 | F:ATPase activity; F:ATP binding; P:transmembrane transport; C:ATP-binding cassette (ABC) transporter complex; F:hydrolase activity, acting on acid anhydrides, catalyzing transmembrane movement of substances |
| Efet.01.634939.g267.t1 | D-arabinitol 4-dehydrogenase | 507 | 3.89E-103 | 85.15 | F:coenzyme binding; F:oxidoreductase activity; P:oxidation-reduction process |
| Efet.01.634939.g268.t1 | mannitol dehydrogenase family | 612 | 1.48E-137 | 88.35 | F:coenzyme binding; F:oxidoreductase activity; P:oxidation-reduction process |
| Efet.01.634953.g269.t1 | abortive phage resistance | 726 | 8.78E-95 | 75.75 |  |
| Efet.01.635019.g270.t1 | gliding motility | 681 | 4.82E-117 | 74.75 | C:integral component of membrane |
| Efet.01.63503.g830.t1 | hypothetical protein HELRODRAFT_83456 | 210 | 1.37E-10 | 67 | C:membrane; C:integral component of membrane |
| Efet.01.635054.g271.t1 | glutamate receptor 3-like | 501 | 4.22E-83 | 79.9 | C:postsynaptic membrane; P:ion transmembrane transport; C:integral component of membrane; C:cell junction; P:ionotropic glutamate receptor signaling pathway; F:ionotropic glutamate receptor activity; F:extracellular-glutamate-gated ion channel activity |
| Efet.01.635092.g273.t1 | phospholipid-transporting ATPase | 207 | 3.19E-26 | 91.1 | F:magnesium ion binding; F:ATP binding; P:phospholipid translocation; P:Golgi organization; C:integral component of membrane; C:Golgi apparatus; F:phospholipid-translocating ATPase activity; C:plasma membrane |
| Efet.01.63510.g831.t1 | extracellular hemoglobin linker L4 subunit precursor | 321 | 7.06E-14 | 73 |  |
| Efet.01.63515.g832.t1 | glutamate dehydrogenase mitochondrial-like | 255 | 4.01E-38 | 87.4 | F:transferase activity, transferring hexosyl groups; F:oxidoreductase activity, acting on the CH-NH2 group of donors, NAD or NADP as acceptor; P:cellular amino acid metabolic process; P:oxidation-reduction process |
| Efet.01.63522.g833.t1 | phosphatase 1 regulatory subunit 3C | 1584 | 1.23E-09 | 44.15 | P:carbohydrate metabolic process; P:glycogen metabolic process; P:regulation of catalytic activity; F:protein phosphatase regulator activity |
| Efet.01.63528.g834.t1 | junctophilin-1-like isoform X1 | 1275 | 0 | 80.15 |  |
| Efet.01.635430.g277.t1 | baculoviral IAP repeat-containing 7-like | 825 | 3.17E-19 | 57.6 |  |
| Efet.01.635436.g278.t1 | PREDICTED: uncharacterized protein LOC106175668 | 702 | 1.38E-08 | 49 |  |
| Efet.01.63556.g836.t1 | noggin-2-like isoform X2 | 921 | 2.71E-42 | 51.85 | C:membrane; C:integral component of membrane |
| Efet.01.635568.g280.t1 | porin | 837 | 6.25E-121 | 74.85 | P:transport |
| Efet.01.635568.g281.t1 | ABC transporter substrate-binding | 834 | 0 | 91.15 | C:outer membrane-bounded periplasmic space |
| Efet.01.635582.g282.t1 | 14-3-3 2 | 318 | 7.12E-52 | 92.4 | F:protein domain specific binding |
| Efet.01.635605.g283.t1 | membrane-associated [Mycetocola miduiensis] | 315 | 7.82E-53 | 73.25 | C:membrane |
| Efet.01.635643.g284.t1 | bifunctional 3,4-dihydroxy-2-butanone-4-phosphate synthase GTP cyclohydrolase II | 822 | 0 | 73.95 | F:nucleotide binding; F:ion binding; F:hydrolase activity |
| Efet.01.635649.g285.t1 | family | 729 | 2.86E-52 | 65.75 | C:membrane |
| Efet.01.635688.g288.t1 | Smaug homolog 1- | 660 | 4.68E-12 | 77.9 | C:dendrite; F:protein binding; P:positive regulation of translation; C:cytoplasm; F:translation repressor activity; C:cell junction; P:regulation of transcription, DNA-templated; C:synapse; P:negative regulation of translation; F:RNA binding |
| Efet.01.6357.g503.t1 | tyrosine- phosphatase Lar-like isoform X1 | 465 | 6.94E-54 | 72.5 | F:phosphoprotein phosphatase activity; P:protein dephosphorylation |
| Efet.01.635754.g290.t1 | hypothetical protein B5766_08855 | 927 | 0 | 62.67 | C:membrane; C:integral component of membrane |
| Efet.01.635754.g291.t1 | ABC transporter ATP-binding | 684 | 4.12E-137 | 65.55 | F:hydrolase activity |
| Efet.01.635836.g292.t1 | sugar ABC transporter substrate-binding | 525 | 9.63E-42 | 77.5 | P:transport; F:transporter activity |
| Efet.01.635868.g293.t1 | ATP-binding cassette sub-family A member 3-like | 303 | 3.00E-13 | 68.6 | P:lipid transport; F:ATPase activity, coupled to transmembrane movement of substances; C:intracellular membrane-bounded organelle |
| Efet.01.635880.g294.t1 | Glutamate receptor kainate | 204 | 2.75E-27 | 77.9 | P:ion transport; C:integral component of membrane; P:ionotropic glutamate receptor signaling pathway; F:ionotropic glutamate receptor activity |
| Efet.01.635927.g295.t1 | transposase | 228 | 1.45E-21 | 80.75 | F:nucleic acid binding; P:DNA integration |
| Efet.01.635927.g296.t1 | alpha beta hydrolase | 885 | 1.19E-141 | 63.7 | P:metabolic process; F:hydrolase activity |
| Efet.01.635927.g297.t1 | IS5 IS1182 family transposase | 333 | 1.43E-52 | 82.25 | F:DNA binding; F:transposase activity; P:transposition, DNA-mediated |
| Efet.01.635927.g298.t1 | transposase | 495 | 1.01E-92 | 87.3 | F:DNA binding; F:transposase activity; P:transposition, DNA-mediated |
| Efet.01.635927.g299.t1 | family transcriptional regulator | 792 | 5.16E-146 | 81.7 | F:DNA binding; F:transcription factor activity, sequence-specific DNA binding; P:transcription, DNA-templated; P:regulation of transcription, DNA-templated |
| Efet.01.635927.g300.t1 | glyoxalase bleomycin resistance dioxygenase | 435 | 4.39E-72 | 66.85 | F:dioxygenase activity; P:oxidation-reduction process |
| Efet.01.635927.g301.t1 | ABC transporter | 1038 | 0 | 72.75 | F:nucleotide binding; F:ATPase activity; P:transport; F:hydrolase activity, acting on acid anhydrides, catalyzing transmembrane movement of substances |
| Efet.01.635927.g302.t1 | ABC transporter substrate-binding | 1335 | 0 | 72.45 | P:transport |
| Efet.01.635927.g303.t1 | sugar ABC transporter permease | 1701 | 9.20E-153 | 78.25 | C:membrane; P:transport |
| Efet.01.635927.g304.t1 | alpha beta hydrolase | 1242 | 3.94E-129 | 56.15 | F:hydrolase activity |
| Efet.01.635927.g305.t1 | 3-hydroxyisobutyrate dehydrogenase | 858 | 1.11E-154 | 89.25 | F:phosphogluconate dehydrogenase (decarboxylating) activity; F:NAD binding; P:oxidation-reduction process; F:3-hydroxyisobutyrate dehydrogenase activity |
| Efet.01.635927.g306.t1 | four-carbon acid sugar kinase family | 1548 | 3.24E-141 | 84.1 | P:phosphorylation; F:kinase activity |
| Efet.01.635927.g307.t1 | hydroxypyruvate isomerase | 810 | 2.98E-148 | 85.95 | F:hydroxypyruvate isomerase activity |
| Efet.01.635927.g308.t1 | IS6 family transposase | 738 | 2.71E-137 | 87.95 | F:nucleic acid binding |
| Efet.01.635927.g309.t1 | hypothetical protein L810_1857 | 303 | 8.56E-06 | 49 |  |
| Efet.01.635927.g310.t1 | transposase | 825 | 6.30E-132 | 78.2 |  |
| Efet.01.635927.g311.t1 | transposase | 1560 | 0 | 66.75 | F:nucleic acid binding; P:DNA integration |
| Efet.01.635929.g312.t1 | coiled-coil domain-containing 42 homolog | 240 | 5.17E-31 | 88.2 | C:integral component of membrane |
| Efet.01.635962.g313.t1 | hypothetical protein HELRODRAFT_190300 | 201 | 5.01E-12 | 81 | F:GTP binding; F:ATP binding; P:small GTPase mediated signal transduction; F:protein kinase activity; P:protein phosphorylation; C:intracellular |
| Efet.01.636111.g317.t1 | Na(+) H(+) exchange regulatory cofactor NHE-RF1 | 477 | 6.68E-48 | 76.85 |  |
| Efet.01.636184.g319.t1 | septation ring formation regulator | 279 | 2.54E-30 | 88.8 | P:FtsZ-dependent cytokinesis; C:integral component of plasma membrane; C:cell division site |
| Efet.01.636187.g320.t1 | homeodomain transcription factor | 237 | 1.24E-26 | 82.85 | C:integral component of membrane |
| Efet.01.636270.g322.t1 | hypothetical protein CAPTEDRAFT_219019 | 330 | 9.16E-40 | 64.95 | C:integral component of membrane; F:carbohydrate binding |
| Efet.01.636323.g323.t1 | transcriptional regulator | 564 | 1.43E-119 | 88.4 | F:DNA binding; P:regulation of transcription, DNA-templated |
| Efet.01.636339.g324.t1 | recombinase | 708 | 4.54E-91 | 96.05 | F:ATP binding; P:DNA repair; F:DNA-dependent ATPase activity; C:cytoplasm; P:DNA recombination; P:SOS response; F:damaged DNA binding; F:single-stranded DNA binding |
| Efet.01.636358.g326.t1 | family transporter | 933 | 0 | 68.3 | C:integral component of membrane |
| Efet.01.636372.g327.t1 | RIP metalloprotease | 333 | 3.43E-52 | 93.2 | F:metal ion binding; P:proteolysis; C:integral component of membrane; F:metalloendopeptidase activity; C:plasma membrane |
| Efet.01.636372.g328.t1 | outer membrane assembly factor | 2655 | 0 | 93.95 | P:Gram-negative-bacterium-type cell outer membrane assembly; C:integral component of membrane; C:cell outer membrane; P:protein insertion into membrane |
| Efet.01.636372.g329.t1 | UDP-3-O-(3-hydroxymyristoyl)glucosamine N-acyltransferase | 540 | 7.60E-97 | 92.8 | P:lipid A biosynthetic process; F:N-acyltransferase activity |
| Efet.01.636372.g330.t1 | 3-hydroxyacyl-[acyl-carrier- ] dehydratase | 567 | 2.82E-98 | 96.6 | F:3-hydroxyoctanoyl-[acyl-carrier-protein] dehydratase activity; C:cytoplasm; P:lipid A biosynthetic process; P:fatty acid biosynthetic process |
| Efet.01.636379.g331.t1 | molecular chaperone | 828 | 4.45E-170 | 89.55 | F:ATP binding; F:unfolded protein binding; P:protein folding |
| Efet.01.636379.g332.t1 | molecular chaperone | 888 | 3.13E-168 | 97.7 | F:ATP binding; F:unfolded protein binding; P:protein folding |
| Efet.01.636391.g333.t1 | RNA-binding 38 | 222 | 6.65E-27 | 91.45 | F:nucleotide binding; F:nucleic acid binding |
| Efet.01.636406.g334.t1 | Serine phosphatase regulator of sigma subunit | 276 | 8.42E-58 | 69.9 | F:catalytic activity |
| Efet.01.63643.g838.t1 | E3 ubiquitin- ligase TM129 | 231 | 6.77E-33 | 81.35 | C:integral component of membrane |
| Efet.01.636439.g335.t1 | hypothetical protein CRN43_16260 | 480 | 1.45E-87 | 77.5 |  |
| Efet.01.636592.g337.t1 | EF-hand domain-containing family member C2-like | 390 | 5.46E-41 | 72.95 | F:calcium ion binding |
| Efet.01.636598.g338.t1 | thiosulfate transporter subunit | 1749 | 0 | 93.85 | P:sulfate transmembrane transport; F:ATPase-coupled sulfate transmembrane transporter activity |
| Efet.01.636598.g339.t1 | DUF4236 domain-containing | 288 | 1.66E-50 | 77.15 | C:integral component of membrane |
| Efet.01.636598.g340.t1 | peroxidase | 642 | 1.98E-152 | 94.6 | F:peroxidase activity; P:cellular oxidant detoxification; P:oxidation-reduction process; F:peroxiredoxin activity |
| Efet.01.636598.g341.t1 | tetratricopeptide repeat-containing | 1092 | 0 | 90.7 |  |
| Efet.01.636624.g342.t1 | hypothetical protein | 579 | 6.91E-59 | 71.35 |  |
| Efet.01.636627.g343.t1 | cytochrome C oxidase Cbb3 | 534 | 6.53E-52 | 85.5 | F:cytochrome-c oxidase activity; F:iron ion binding; C:integral component of membrane; P:hydrogen ion transmembrane transport; C:plasma membrane respiratory chain complex IV; P:aerobic respiration; F:heme binding |
| Efet.01.636679.g345.t1 | PREDICTED: uncharacterized protein LOC106151497 isoform X1 | 2655 | 1.58E-17 | 46.83 |  |
| Efet.01.636719.g346.t1 | hypothetical protein | 300 | 1.60E-30 | 71.18 |  |
| Efet.01.636758.g347.t1 | multidrug resistance-associated 1-like | 237 | 6.88E-22 | 80 | F:ATP binding; F:ATPase activity, coupled to transmembrane movement of substances; P:transmembrane transport; C:integral component of membrane |
| Efet.01.63676.g840.t1 | hypothetical protein CAPTEDRAFT_223239 | 675 | 7.57E-09 | 47 |  |
| Efet.01.636780.g348.t1 | ubiquitin-conjugating enzyme E2 G1 | 264 | 2.81E-34 | 89.85 | F:ATP binding; C:integral component of membrane |
| Efet.01.636814.g349.t1 | ribonuclease II | 1968 | 0 | 79.7 | C:myosin complex; F:ATP binding; F:motor activity |
| Efet.01.636814.g350.t1 | energy transducer | 744 | 8.46E-152 | 90.15 | P:transport |
| Efet.01.636814.g351.t1 | monofunctional biosynthetic peptidoglycan transglycosylase | 756 | 2.86E-141 | 83.85 | P:regulation of cell shape; C:peptidoglycan-based cell wall; P:peptidoglycan biosynthetic process; C:integral component of membrane; P:cell wall organization; F:transferase activity, transferring pentosyl groups; C:plasma membrane |
| Efet.01.636814.g353.t1 | hypothetical protein Veis_2199 | 1062 | 1.56E-21 | 89 |  |
| Efet.01.636814.g354.t1 | Fibronectin type III domain | 3702 | 0 | 60.4 |  |
| Efet.01.636825.g355.t1 | calmodulin 5 | 300 | 1.83E-26 | 60.65 | F:calcium ion binding |
| Efet.01.636947.g356.t1 | sphingomyelin phosphodiesterase-like | 237 | 2.27E-06 | 59 | F:sphingomyelin phosphodiesterase activity; P:metabolic process; P:lipid metabolic process; P:sphingomyelin catabolic process; F:hydrolase activity; F:hydrolase activity, acting on glycosyl bonds |
| Efet.01.636957.g357.t1 | bile acid beta-glucosidase | 534 | 1.03E-117 | 71.75 | F:catalytic activity |
| Efet.01.636970.g358.t1 | Lysine-specific demethylase | 465 | 4.72E-71 | 85.75 | C:nucleus; F:zinc ion binding; C:integral component of membrane; F:methyltransferase activity; P:methylation |
| Efet.01.637025.g361.t1 | 50S ribosomal L21 | 441 | 4.26E-55 | 91.85 | F:structural constituent of ribosome; C:ribosome; F:rRNA binding; P:translation |
| Efet.01.63707.g841.t1 | crossover junction endonuclease EME1 | 252 | 3.74E-18 | 72.4 | P:nucleic acid metabolic process; C:intracellular part |
| Efet.01.637081.g362.t1 | hypothetical protein CAPTEDRAFT_188461 | 531 | 3.46E-12 | 64 | C:membrane; C:integral component of membrane |
| Efet.01.637108.g363.t1 | ABC transporter substrate-binding | 870 | 0 | 70.85 | P:transport; F:transporter activity |
| Efet.01.637131.g364.t1 | O-succinylbenzoate synthase | 876 | 0 | 74.3 | P:small molecule metabolic process; F:metal ion binding; F:lyase activity; P:single-organism cellular process; P:cellular metabolic process; P:organic substance metabolic process |
| Efet.01.637131.g365.t1 | c-type cytochrome biogenesis | 627 | 1.68E-95 | 68.9 | C:membrane; C:integral component of membrane; P:cytochrome complex assembly; F:heme binding |
| Efet.01.637136.g366.t1 | A-kinase anchor 17A-like | 810 | 8.94E-137 | 80.7 | F:nucleotide binding |
| Efet.01.63716.g842.t1 | Fibronectin type-III domain-containing 3a | 285 | 2.41E-12 | 63.4 | C:membrane; C:integral component of membrane |
| Efet.01.637227.g367.t1 | myelin regulatory factor | 234 | 1.83E-27 | 82.2 | C:endoplasmic reticulum membrane; C:nucleus; F:DNA binding; F:transcription factor activity, sequence-specific DNA binding; P:transcription, DNA-templated; P:central nervous system myelin maintenance; P:positive regulation of transcription, DNA-templated; P:proteolysis; C:integral component of membrane; F:peptidase activity; P:positive regulation of myelination |
| Efet.01.637250.g368.t1 | ATP-dependent zinc metalloprotease | 747 | 5.41E-121 | 81.1 | F:ATPase activity; P:cell division; F:ATP binding; F:zinc ion binding; P:protein catabolic process; P:proteolysis; C:integral component of membrane; C:thylakoid membrane; F:metalloendopeptidase activity; C:plasma membrane |
| Efet.01.637303.g370.t1 | translocase subunit | 576 | 3.30E-72 | 92.6 | P:protein targeting; F:P-P-bond-hydrolysis-driven protein transmembrane transporter activity; P:intracellular protein transmembrane transport; C:integral component of membrane; P:protein transport by the Sec complex; C:intracellular; C:plasma membrane |
| Efet.01.637329.g371.t1 | 3Chain Fitted Coordinates For Lumbricus Terrestris Hemoglobin Cryo-em Complex (emd-2627) | 405 | 4.56E-52 | 66.3 | C:hemoglobin complex; F:oxygen binding; F:iron ion binding; P:oxygen transport; C:extracellular region; F:oxygen transporter activity; F:heme binding |
| Efet.01.637344.g372.t1 | probable cation-transporting ATPase 13A3 isoform X2 | 270 | 2.75E-18 | 77.9 | F:ATPase activity; F:ATP binding; P:cation transport; F:metal ion binding; C:integral component of membrane |
| Efet.01.637415.g374.t1 | Collagen alpha-1(XII) chain | 405 | 1.17E-15 | 51.29 | F:chitin binding; F:calcium ion binding; P:chitin metabolic process; C:extracellular region |
| Efet.01.637419.g375.t1 | spectrin beta chain-like isoform X1 | 450 | 9.78E-40 | 76.7 | F:actin binding; F:structural constituent of cytoskeleton; F:phospholipid binding; C:spectrin |
| Efet.01.637446.g377.t1 | elongation factor 4 | 288 | 4.20E-50 | 88.4 | F:GTP binding; P:positive regulation of translation; F:translation elongation factor activity; F:GTPase activity; F:ribosome binding; P:translational elongation; C:plasma membrane |
| Efet.01.637471.g378.t1 | lipoyl mitochondrial | 249 | 1.94E-17 | 83.75 | F:calcium ion binding; C:mitochondrion; F:lipoate synthase activity; F:4 iron, 4 sulfur cluster binding; P:lipoate biosynthetic process; P:protein lipoylation |
| Efet.01.637557.g379.t1 | UDP-glucose 6-dehydrogenase | 483 | 2.07E-79 | 87.3 | C:nucleus; F:NAD binding; P:polysaccharide biosynthetic process; P:UDP-glucuronate biosynthetic process; F:UDP-glucose 6-dehydrogenase activity; P:glycosaminoglycan biosynthetic process; C:cytosol; P:oxidation-reduction process |
| Efet.01.63758.g845.t1 | calcium-dependent kinase C-like isoform X4 | 297 | 3.51E-49 | 84.3 | P:positive regulation of I-kappaB kinase/NF-kappaB signaling; P:B cell activation; P:positive regulation of NF-kappaB transcription factor activity; P:positive regulation of angiogenesis; F:ATP binding; C:extracellular exosome; P:response to hypoxia; P:positive regulation of vascular endothelial growth factor receptor signaling pathway; P:calcium ion transport; P:intracellular signal transduction; P:B cell receptor signaling pathway; F:protein kinase C binding; F:zinc ion binding; F:chromatin binding; F:calcium channel regulator activity; C:plasma membrane; F:protein kinase C activity; P:regulation of transcription from RNA polymerase II promoter; P:cellular calcium ion homeostasis; P:positive regulation of B cell receptor signaling pathway; C:nucleus; P:negative regulation of glucose transport; F:androgen receptor binding; P:histone H3-T6 phosphorylation; C:cytoplasm; F:histone binding; P:cellular response to carbohydrate stimulus; F:ligand-dependent nuclear receptor transcription coactivator activity; P:peptidyl-serine phosphorylation; F:histone kinase activity (H3-T6 specific) |
| Efet.01.6376.g505.t1 | BAG family molecular chaperone regulator 3-like | 243 | 1.17E-19 | 70.8 | F:chaperone binding |
| Efet.01.637639.g381.t1 | excinuclease ABC subunit | 453 | 1.68E-75 | 92.55 | F:ATPase activity; F:DNA binding; F:ATP binding; P:nucleic acid phosphodiester bond hydrolysis; F:excinuclease ABC activity; C:excinuclease repair complex; C:cytoplasm; F:metal ion binding; P:nucleotide-excision repair |
| Efet.01.637705.g382.t1 | TPM_BRABE ame: Full=Tropomyosin | 240 | 4.07E-07 | 67 |  |
| Efet.01.637720.g383.t1 | 5-hydroxytryptamine receptor 2C-like | 417 | 3.60E-19 | 67.7 | P:cGMP biosynthetic process; F:drug binding; P:regulation of appetite; P:dopamine receptor signaling pathway; P:feeding behavior; P:regulation of corticotropin-releasing hormone secretion; P:positive regulation of fat cell differentiation; P:behavioral fear response; F:serotonin binding; C:integral component of plasma membrane; F:Gq/11-coupled serotonin receptor activity; F:dopamine neurotransmitter receptor activity; P:phospholipase C-activating serotonin receptor signaling pathway; P:locomotory behavior; P:response to drug; P:positive regulation of cytosolic calcium ion concentration involved in phospholipase C-activating G-protein coupled signaling pathway; P:positive regulation of ERK1 and ERK2 cascade; F:1-(4-iodo-2,5-dimethoxyphenyl)propan-2-amine binding; C:cytosol; P:positive regulation of phosphatidylinositol biosynthetic process; P:release of sequestered calcium ion into cytosol; P:regulation of neurological system process |
| Efet.01.63779.g846.t1 | leucine zipper tumor suppressor 2 homolog | 672 | 1.94E-16 | 62.25 |  |
| Efet.01.637797.g385.t1 | Endo-beta-1,4-mannanase | 243 | 8.56E-13 | 61.85 | P:metabolic process; F:hydrolase activity, hydrolyzing O-glycosyl compounds |
| Efet.01.63782.g847.t1 | hypothetical protein HELRODRAFT_183282 | 207 | 2.29E-15 | 64.85 | C:membrane |
| Efet.01.637843.g386.t1 | cell division | 228 | 2.34E-43 | 92.7 | F:ATP binding |
| Efet.01.637843.g387.t1 | cyanase | 828 | 4.80E-100 | 94 | F:DNA binding; F:cyanate hydratase activity; P:cyanate metabolic process |
| Efet.01.637843.g388.t1 | nitrate ABC transporter substrate-binding | 792 | 1.21E-165 | 84.7 |  |
| Efet.01.637884.g389.t1 | Four and a half LIM domains 2 | 207 | 1.58E-24 | 73.6 | F:zinc ion binding |
| Efet.01.637912.g390.t1 | uncharacterized protein LOC107453613 | 243 | 2.72E-22 | 70.6 |  |
| Efet.01.637995.g392.t1 | N-fatty-acyl-amino acid synthase hydrolase -like | 273 | 1.79E-08 | 63 | F:carboxypeptidase activity; P:metabolic process; P:proteolysis; F:hydrolase activity |
| Efet.01.637997.g393.t1 | alanine--tRNA ligase | 411 | 2.85E-72 | 92.05 | F:ATP binding; F:zinc ion binding; C:cytoplasm; F:alanine-tRNA ligase activity; P:alanyl-tRNA aminoacylation; F:tRNA binding |
| Efet.01.638.g62.t1 | hypothetical protein CAPTEDRAFT_191217 | 549 | 1.38E-33 | 57 | P:transport |
| Efet.01.638023.g394.t1 | MFS transporter | 336 | 1.56E-33 | 91.8 | P:transmembrane transport; C:integral component of membrane; F:transporter activity |
| Efet.01.638023.g395.t1 | hydro-lyase | 819 | 3.28E-167 | 84.9 |  |
| Efet.01.638023.g396.t1 | family transcriptional regulator | 981 | 0 | 60.45 | F:DNA binding; F:transcription factor activity, sequence-specific DNA binding; P:transcription, DNA-templated; P:regulation of transcription, DNA-templated |
| Efet.01.638023.g397.t1 | fructose-1,6-bisphosphate aldolase | 1386 | 0 | 94.1 | F:zinc ion binding; P:glycolytic process; F:fructose-bisphosphate aldolase activity |
| Efet.01.638023.g398.t1 | NAD(P)(+) transhydrogenase (Re Si-specific) subunit alpha | 1131 | 0 | 91.35 | P:oxidation-reduction process; F:NAD(P)+ transhydrogenase (AB-specific) activity |
| Efet.01.638023.g399.t1 | NAD(P) transhydrogenase subunit alpha | 327 | 5.43E-52 | 93.15 | C:integral component of membrane |
| Efet.01.638026.g400.t1 | aspartate aminotransferase | 276 | 2.59E-54 | 83.65 | F:pyridoxal phosphate binding; F:transaminase activity; P:biosynthetic process |
| Efet.01.638026.g401.t1 | recombination factor | 936 | 0 | 96.75 | F:DNA binding; F:ATP binding; P:DNA replication |
| Efet.01.638026.g402.t1 | outer membrane lipo carrier | 591 | 4.89E-117 | 85.35 | F:lipoprotein transporter activity; C:periplasmic space; P:lipoprotein transport |
| Efet.01.638026.g403.t1 | cell division | 2388 | 0 | 89 | P:cell division; F:DNA binding; F:ATP binding; C:integral component of membrane; P:chromosome segregation; C:plasma membrane; P:cell cycle |
| Efet.01.638026.g404.t1 | thioredoxin-disulfide reductase | 981 | 0 | 95.35 | C:cytoplasm; P:removal of superoxide radicals; F:thioredoxin-disulfide reductase activity; P:oxidation-reduction process |
| Efet.01.638026.g405.t1 | 50S ribosomal L28 | 489 | 6.41E-40 | 99 | F:structural constituent of ribosome; C:ribosome; P:translation |
| Efet.01.638026.g406.t1 | type II secretion system | 1389 | 0 | 96 | F:ATP binding; P:transport |
| Efet.01.638026.g407.t1 | CDP-diacylglycerol--serine O-phosphatidyltransferase | 837 | 0 | 94.8 | C:integral component of membrane; P:phospholipid biosynthetic process; F:CDP-diacylglycerol-serine O-phosphatidyltransferase activity |
| Efet.01.638026.g408.t1 | DEAD DEAH box helicase | 3180 | 0 | 91 | F:helicase activity |
| Efet.01.638026.g409.t1 | DUF1016 domain-containing | 1065 | 0 | 93 |  |
| Efet.01.638026.g410.t1 | lactate dehydrogenase | 2772 | 0 | 83.8 | F:DNA binding; F:N-methyltransferase activity; P:DNA methylation |
| Efet.01.638026.g411.t1 | ketol-acid reductoisomerase | 1017 | 0 | 97.8 | P:isoleucine biosynthetic process; P:valine biosynthetic process; P:oxidation-reduction process; F:ketol-acid reductoisomerase activity; F:isomerase activity |
| Efet.01.638026.g412.t1 | acetolactate synthase small subunit | 492 | 7.43E-112 | 98.65 | F:acetolactate synthase activity; F:amino acid binding; P:branched-chain amino acid biosynthetic process |
| Efet.01.638026.g413.t1 | acetolactate large biosynthetic type | 1875 | 0 | 95.15 | F:magnesium ion binding; F:acetolactate synthase activity; F:thiamine pyrophosphate binding; P:isoleucine biosynthetic process; F:flavin adenine dinucleotide binding; P:valine biosynthetic process |
| Efet.01.638026.g414.t1 | RNA polymerase sigma factor | 453 | 3.59E-107 | 93.2 | F:DNA binding; F:sigma factor activity; F:transcription factor activity, sequence-specific DNA binding; F:DNA-directed 5'-3' RNA polymerase activity; P:regulation of transcription, DNA-templated; P:DNA-templated transcription, initiation |
| Efet.01.638040.g415.t1 | interferon-induced very large GTPase 1-like isoform X1 | 435 | 2.10E-09 | 51.1 |  |
| Efet.01.638044.g416.t1 | ferredoxin | 468 | 3.66E-29 | 74.05 | F:oxidoreductase activity; P:single-organism metabolic process |
| Efet.01.638070.g417.t1 | yippee-like 5 | 366 | 6.22E-65 | 89.25 | F:metal ion binding; P:oxidation-reduction process; F:peptide-methionine (R)-S-oxide reductase activity |
| Efet.01.638084.g418.t1 | calbindin-32-like isoform X2 | 381 | 1.05E-31 | 72.45 | F:calcium ion binding |
| Efet.01.638173.g419.t1 | peregrin-like isoform X12 | 210 | 2.45E-15 | 81.85 | F:zinc ion binding |
| Efet.01.638213.g421.t1 | neuroligin- X-linked-like isoform X1 | 603 | 1.76E-14 | 46.75 | P:metabolic process; C:membrane; C:integral component of membrane; F:hydrolase activity |
| Efet.01.638287.g424.t1 | hypothetical protein HELRODRAFT_116840 | 243 | 2.50E-06 | 70 | C:membrane; C:integral component of membrane; C:Golgi membrane |
| Efet.01.638356.g425.t1 | hypothetical protein CAPTEDRAFT_199134 | 1308 | 2.76E-86 | 50.5 | P:transport |
| Efet.01.638404.g426.t1 | Por secretion system C-terminal sorting domain-containing | 453 | 2.72E-65 | 56.75 | P:proteolysis; F:metallopeptidase activity |
| Efet.01.638425.g427.t1 | flagellar export | 303 | 3.34E-29 | 99 | C:bacterial-type flagellum; P:bacterial-type flagellum-dependent cell motility |
| Efet.01.638425.g428.t1 | flagellar export ATPase | 978 | 0 | 87.4 | F:ATPase activity; F:ATP binding; P:proton transport; P:bacterial-type flagellum assembly; C:cytoplasm; P:ATP metabolic process; P:biosynthetic process; P:protein secretion by the type III secretion system; P:bacterial-type flagellum-dependent cell motility; C:type III protein secretion system complex |
| Efet.01.638425.g429.t1 | flagellar assembly | 477 | 5.49E-72 | 88.55 |  |
| Efet.01.638425.g430.t1 | flagellar motor switch | 996 | 0 | 94.65 | P:chemotaxis; C:bacterial-type flagellum; P:bacterial-type flagellum-dependent cell motility; F:motor activity |
| Efet.01.638425.g431.t1 | flagellar M-ring | 1593 | 0 | 85.85 | C:bacterial-type flagellum basal body, MS ring; C:integral component of membrane; P:bacterial-type flagellum-dependent cell motility; F:motor activity |
| Efet.01.638425.g432.t1 | flagellar hook-basal body complex | 324 | 2.22E-50 | 88.95 | F:structural molecule activity; P:bacterial-type flagellum-dependent cell motility; C:bacterial-type flagellum basal body; F:motor activity |
| Efet.01.638425.g433.t1 | flagellar | 246 | 4.59E-50 | 80.3 |  |
| Efet.01.638425.g434.t1 | flagellar export chaperone | 441 | 4.45E-88 | 85.25 | C:bacterial-type flagellum; P:bacterial-type flagellum assembly; C:cytosol |
| Efet.01.638425.g435.t1 | taurine ABC transporter substrate-binding | 1062 | 0 | 82.75 |  |
| Efet.01.638425.g436.t1 | ABC transporter permease | 438 | 6.35E-97 | 96.6 | C:integral component of membrane; P:transport; C:plasma membrane |
| Efet.01.638425.g437.t1 | ATP-binding | 705 | 4.07E-171 | 89.15 | F:ATPase activity; F:ATP binding |
| Efet.01.638425.g438.t1 | FAD-binding oxidoreductase | 1251 | 0 | 83.35 | F:oxidoreductase activity; P:oxidation-reduction process |
| Efet.01.638425.g439.t1 | hypothetical protein | 246 | 2.91E-08 | 85 |  |
| Efet.01.638426.g440.t1 | family | 210 | 4.46E-26 | 77.1 | F:ATP binding |
| Efet.01.63846.g851.t1 | PREDICTED: uncharacterized protein LOC106160369 | 1302 | 9.73E-153 | 63.7 | F:catalytic activity; P:metabolic process; C:membrane; C:integral component of membrane |
| Efet.01.638466.g441.t1 | solute carrier family facilitated glucose transporter member 8 | 1119 | 3.26E-114 | 63.55 | C:integral component of membrane; P:hexose transmembrane transport; P:glucose transport; F:sugar transmembrane transporter activity |
| Efet.01.638470.g442.t1 | sodium channel para-like isoform X3 | 303 | 2.56E-21 | 59.5 | P:regulation of postsynaptic membrane potential; P:regulation of ion transmembrane transport; C:voltage-gated sodium channel complex; P:sodium ion transmembrane transport; F:voltage-gated sodium channel activity |
| Efet.01.63857.g854.t1 | sodium-dependent phosphate transport chloroplastic-like | 333 | 3.52E-32 | 82.4 | P:transmembrane transport; C:integral component of membrane |
| Efet.01.638674.g445.t1 | ABC transporter permease | 930 | 1.01E-164 | 96.8 | C:integral component of membrane; P:transport; C:plasma membrane; F:transporter activity |
| Efet.01.638674.g446.t1 | ABC transporter ATP-binding | 858 | 9.53E-158 | 96.9 | F:ATP binding; C:integral component of membrane; P:transport; C:plasma membrane; F:transporter activity |
| Efet.01.638674.g447.t1 | ABC transporter ATP-binding | 216 | 6.83E-26 | 89.05 | F:ATPase activity; F:ATP binding |
| Efet.01.638678.g448.t1 | methyl-accepting chemotaxis | 1386 | 1.95E-169 | 82.65 | P:chemotaxis; P:signal transduction; C:integral component of membrane; F:signal transducer activity |
| Efet.01.638678.g449.t1 | DNA topoisomerase IV subunit A | 2358 | 0 | 93.85 | F:DNA binding; F:ATP binding; P:chromosome segregation; C:chromosome; F:DNA topoisomerase type II (ATP-hydrolyzing) activity; P:DNA topological change; C:extrinsic component of plasma membrane |
| Efet.01.638678.g450.t1 | magnesium-translocating P-type ATPase | 2568 | 0 | 78.6 | F:ATP binding; F:magnesium-importing ATPase activity; F:metal ion binding; P:phospholipid translocation; C:integral component of membrane; F:phospholipid-translocating ATPase activity; P:magnesium ion transmembrane transport |
| Efet.01.638678.g451.t1 | tripartite tricarboxylate transporter | 1494 | 0 | 95.1 | C:integral component of membrane |
| Efet.01.638678.g452.t1 | tripartite tricarboxylate transporter | 366 | 2.17E-55 | 91.5 | C:integral component of membrane |
| Efet.01.638678.g453.t1 | C4-dicarboxylate ABC transporter substrate-binding | 924 | 0 | 93.4 | C:outer membrane-bounded periplasmic space |
| Efet.01.638679.g454.t1 | DNA methyltransferase | 885 | 0 | 84.3 | F:nucleic acid binding; F:methyltransferase activity; P:nucleobase-containing compound metabolic process; P:methylation |
| Efet.01.638679.g455.t1 | restriction endonuclease | 480 | 5.55E-86 | 79.65 | F:magnesium ion binding; F:DNA binding; P:nucleic acid phosphodiester bond hydrolysis; F:Type II site-specific deoxyribonuclease activity; P:DNA restriction-modification system |
| Efet.01.638679.g456.t1 | type VII secretion | 297 | 5.62E-62 | 78.85 |  |
| Efet.01.638679.g457.t1 | DNA segregation ATPase S-DNA-T family | 1086 | 5.49E-136 | 58.8 | F:nucleotide binding; P:cell division; F:DNA binding; F:ATP binding; C:membrane; C:integral component of membrane |
| Efet.01.638685.g459.t1 | Homeobox ARX | 813 | 3.30E-12 | 98.35 | P:transcription, DNA-templated; P:cell proliferation in forebrain; P:globus pallidus development; P:positive regulation of organ growth; P:axon guidance; P:embryonic olfactory bulb interneuron precursor migration; P:cerebral cortex GABAergic interneuron migration; F:transcriptional repressor activity, RNA polymerase II distal enhancer sequence-specific binding; F:chromatin binding; P:cerebral cortex tangential migration; P:regulation of cell proliferation; F:RNA polymerase II distal enhancer sequence-specific DNA binding; P:negative regulation of transcription from RNA polymerase II promoter; C:nucleus; P:lipid digestion; P:positive regulation of gene expression; P:epithelial cell fate commitment |
| Efet.01.638688.g460.t1 | fibroblast growth factor receptor 2-like isoform X1 | 273 | 1.30E-20 | 74.9 | F:ATP binding; P:peptidyl-tyrosine phosphorylation; P:regulation of cellular process; F:transmembrane receptor protein tyrosine kinase activity; C:integral component of membrane |
| Efet.01.63869.g855.t1 | proton myo-inositol cotransporter-like | 603 | 2.90E-40 | 72.25 | C:integral component of plasma membrane; F:sugar:proton symporter activity; P:hexose transmembrane transport; P:glucose import; F:glucose transmembrane transporter activity |
| Efet.01.638713.g461.t1 | phosphoribosylformylglycinamidine cyclo-ligase | 249 | 4.52E-38 | 96.2 | F:ATP binding; C:cytoplasm; P:'de novo' IMP biosynthetic process; F:phosphoribosylformylglycinamidine cyclo-ligase activity |
| Efet.01.638713.g462.t1 | amidophosphoribosyltransferase | 1011 | 0 | 89.95 | F:magnesium ion binding; P:'de novo' IMP biosynthetic process; P:glutamine metabolic process; P:nucleoside metabolic process; F:amidophosphoribosyltransferase activity; P:purine nucleobase biosynthetic process |
| Efet.01.638730.g463.t1 | otopetrin-2-like isoform X2 | 756 | 4.19E-47 | 64.5 | C:membrane; C:integral component of membrane |
| Efet.01.638776.g465.t1 | bifunctional aminodeoxychorismate synthase component I aminotransferase | 1683 | 0 | 80.15 | F:catalytic activity; P:folic acid-containing compound biosynthetic process |
| Efet.01.638780.g466.t1 | FMRFamide receptor-like | 1173 | 3.84E-75 | 54 | F:G-protein coupled receptor activity; F:G-protein coupled peptide receptor activity; P:G-protein coupled receptor signaling pathway; P:signal transduction; C:membrane; C:integral component of membrane; F:signal transducer activity |
| Efet.01.638828.g467.t1 | Conserved containing a Zn-ribbon-like possibly RNA-binding | 348 | 2.41E-60 | 57.75 |  |
| Efet.01.638871.g469.t1 | divalent metal cation transporter | 444 | 2.18E-51 | 78.75 | C:integral component of membrane; P:transport; C:plasma membrane; F:transporter activity |
| Efet.01.638872.g470.t1 | inorganic triphosphatase | 381 | 3.37E-45 | 69.95 | P:cAMP biosynthetic process; F:lyase activity; F:adenylate cyclase activity |
| Efet.01.638872.g471.t1 | GDSL family lipase | 1278 | 6.28E-75 | 74.95 |  |
| Efet.01.638872.g472.t1 | 4-hydroxybenzoate octaprenyltransferase | 717 | 4.86E-132 | 89.35 | C:integral component of membrane; F:4-hydroxybenzoate octaprenyltransferase activity; P:ubiquinone biosynthetic process; C:plasma membrane |
| Efet.01.638872.g473.t1 | family transcriptional regulator | 906 | 2.59E-112 | 67.9 |  |
| Efet.01.638881.g474.t1 | PKD domain-containing | 363 | 2.03E-68 | 59 | F:catalytic activity |
| Efet.01.638894.g475.t1 | dihydroxy-acid dehydratase | 273 | 5.64E-41 | 92 | F:dihydroxy-acid dehydratase activity; P:metabolic process |
| Efet.01.638894.g476.t1 | dihydroxy-acid dehydratase | 720 | 2.60E-151 | 92.7 | F:dihydroxy-acid dehydratase activity; P:metabolic process |
| Efet.01.638894.g477.t1 | tripartite tricarboxylate transporter substrate binding | 999 | 0 | 82.8 | C:outer membrane-bounded periplasmic space |
| Efet.01.638894.g478.t1 | glutaredoxin 3 | 261 | 2.43E-55 | 91.7 | C:cell; F:protein disulfide oxidoreductase activity; F:electron carrier activity; P:cell redox homeostasis; P:oxidation-reduction process |
| Efet.01.638894.g479.t1 | protein-export protein SecB | 459 | 1.23E-95 | 93.6 | C:cytoplasm; P:protein tetramerization; P:protein transport; F:unfolded protein binding; P:protein folding |
| Efet.01.63891.g856.t1 | ankyrin repeat and MYND domain-containing 2 | 1347 | 4.08E-129 | 68.5 | F:metal ion binding; C:cilium; C:cell projection |
| Efet.01.638933.g480.t1 | Retrovirus-related Pol poly from transposon opus | 459 | 1.11E-31 | 60.15 |  |
| Efet.01.63900.g857.t1 | zinc finger | 792 | 1.28E-28 | 61.6 | F:nucleic acid binding; F:metal ion binding |
| Efet.01.63903.g858.t1 | Transcription factor ETV6 | 240 | 2.68E-19 | 67.3 | C:nucleus; F:DNA binding; P:cell differentiation; F:RNA polymerase II transcription factor activity, sequence-specific DNA binding; P:regulation of transcription from RNA polymerase II promoter |
| Efet.01.639058.g482.t1 | MFS transporter | 795 | 9.43E-168 | 73.45 | C:membrane; P:transport |
| Efet.01.639145.g484.t1 | hypothetical protein HELRODRAFT_192107 | 285 | 1.80E-09 | 86 |  |
| Efet.01.639162.g485.t1 | family heavy metal efflux RND transporter | 1521 | 0 | 78.95 | P:cation transmembrane transport; C:integral component of membrane; F:cation transmembrane transporter activity |
| Efet.01.639165.g486.t1 | splicing factor 3B subunit 1-like isoform X2 | 351 | 1.28E-65 | 91.55 |  |
| Efet.01.639184.g488.t1 | afadin-like isoform X4 | 207 | 2.29E-13 | 78.15 | P:signal transduction |
| Efet.01.639207.g489.t1 | FAD-dependent oxidoreductase | 294 | 5.10E-62 | 94.6 | F:oxidoreductase activity; P:oxidation-reduction process |
| Efet.01.639207.g490.t1 | peptide ABC transporter substrate-binding | 1593 | 0 | 93.3 | P:transmembrane transport; C:ATP-binding cassette (ABC) transporter complex |
| Efet.01.639213.g491.t1 | nuclear migration nudC | 333 | 7.16E-14 | 70.9 |  |
| Efet.01.639260.g494.t1 | conserved hypothetical protein | 1221 | 0 | 92.75 |  |
| Efet.01.639260.g495.t1 | ferric iron uptake transcriptional regulator | 726 | 1.09E-97 | 95.4 | F:DNA binding; F:transcription factor activity, sequence-specific DNA binding; F:metal ion binding; P:regulation of transcription, DNA-templated |
| Efet.01.639260.g496.t1 | HPr kinase phosphorylase | 1239 | 0 | 96.4 | F:phosphorelay sensor kinase activity; F:magnesium ion binding; P:regulation of carbohydrate metabolic process; F:ATP binding; F:protein serine/threonine/tyrosine kinase activity; P:phosphorelay signal transduction system; F:protein serine/threonine kinase activity; P:signal transduction by protein phosphorylation; C:intracellular |
| Efet.01.639260.g497.t1 | ribosomal subunit interface | 336 | 1.45E-62 | 98.2 | C:ribosome; P:primary metabolic process |
| Efet.01.639260.g498.t1 | ATP-dependent DNA helicase Rep | 2181 | 0 | 89.15 | F:ATP binding; F:3'-5' exonuclease activity; P:nucleic acid phosphodiester bond hydrolysis; F:ATP-dependent DNA helicase activity; P:DNA unwinding involved in DNA replication; F:single-stranded DNA binding |
| Efet.01.639297.g499.t1 | pseudouridine-5 -phosphate glycosidase | 309 | 5.96E-43 | 79 | P:pseudouridine synthesis; F:metal ion binding; P:nucleobase catabolic process; F:pseudouridylate synthase activity; F:hydrolase activity, acting on glycosyl bonds |
| Efet.01.639343.g500.t1 | sugar ABC transporter ATP-binding | 1542 | 0 | 58.7 | P:transport; F:hydrolase activity |
| Efet.01.639356.g501.t1 | lytic transglycosylase | 933 | 1.20E-99 | 80.25 | C:integral component of membrane |
| Efet.01.639356.g502.t1 | hypothetical protein Veis_3630 | 312 | 1.54E-54 | 99 |  |
| Efet.01.639356.g503.t1 | DUF349 domain-containing | 2604 | 0 | 75.45 |  |
| Efet.01.639369.g504.t1 | nodulation | 2436 | 0 | 87.25 | C:integral component of membrane; P:transport; F:transporter activity |
| Efet.01.639369.g505.t1 | undecaprenyl-diphosphate phosphatase | 792 | 0 | 92.55 | P:regulation of cell shape; P:peptidoglycan biosynthetic process; C:integral component of membrane; P:response to antibiotic; F:undecaprenyl-diphosphatase activity; P:cell wall organization; P:dephosphorylation; C:plasma membrane |
| Efet.01.639369.g506.t1 | plasmid stabilization | 330 | 5.47E-64 | 87.1 | C:integral component of membrane |
| Efet.01.639369.g507.t1 | family transcriptional regulator | 267 | 5.60E-56 | 95.4 | P:regulation of transcription, DNA-templated |
| Efet.01.639382.g508.t1 | centrosomal of 95 kDa | 204 | 3.92E-10 | 75.6 | C:centrosome; C:spindle pole |
| Efet.01.639396.g509.t1 | transient receptor potential cation channel subfamily V member 6- | 408 | 1.16E-21 | 63.3 | C:perinuclear region of cytoplasm; C:non-motile cilium; F:calcium channel activity; P:ion transmembrane transport; C:membrane; P:sensory perception of smell; P:olfactory learning; C:neuronal cell body; P:hyperosmotic response; P:response to mechanical stimulus; P:negative adaptation of signaling pathway |
| Efet.01.639465.g512.t1 | tripartite motif-containing 3-like | 1914 | 9.22E-75 | 46.8 | F:zinc ion binding; F:metal ion binding; P:phosphorylation; F:transferase activity; F:kinase activity; C:intracellular |
| Efet.01.639525.g514.t1 | ABC transporter | 675 | 1.32E-132 | 79.2 | F:ATP binding; F:heme-transporting ATPase activity; C:ATP-binding cassette (ABC) transporter complex; P:heme transport |
| Efet.01.639525.g515.t1 | iron ABC transporter permease | 597 | 1.41E-80 | 85.35 | C:integral component of membrane; P:transport; C:plasma membrane; F:transporter activity |
| Efet.01.639527.g516.t1 | casein kinase I isoform delta-like isoform X1 | 789 | 8.67E-109 | 95.4 | F:ATP binding; F:protein serine/threonine kinase activity; P:protein phosphorylation |
| Efet.01.639531.g517.t1 | hypothetical protein HELRODRAFT_187893, partial | 786 | 1.66E-12 | 61 |  |
| Efet.01.639535.g518.t1 | DNA repair | 1203 | 2.49E-112 | 77.4 | P:nucleic acid phosphodiester bond hydrolysis; F:exonuclease activity |
| Efet.01.639535.g520.t1 | hypothetical protein | 285 | 5.84E-39 | 67.25 |  |
| Efet.01.639535.g521.t1 | GTPase | 693 | 2.11E-84 | 59.25 |  |
| Efet.01.639535.g522.t1 | hypothetical protein | 261 | 1.04E-14 | 60 |  |
| Efet.01.639535.g524.t1 | DNA repair exonuclease | 873 | 1.42E-110 | 82.15 | P:nucleic acid phosphodiester bond hydrolysis; F:exonuclease activity |
| Efet.01.639572.g525.t1 | nucleolar gtp-binding 1 | 702 | 3.57E-41 | 52.33 | F:GTP binding; P:ribosomal subunit export from nucleus; C:mitotic spindle; P:ribosomal large subunit biogenesis; P:rRNA processing; P:assembly of large subunit precursor of preribosome; C:preribosome, large subunit precursor |
| Efet.01.639609.g526.t1 | glycerol-3-phosphate dehydrogenase oxidase | 357 | 4.97E-34 | 69.25 | P:metabolic process; F:sn-glycerol-3-phosphate:ubiquinone oxidoreductase activity |
| Efet.01.639641.g528.t1 | IQ domain-containing D | 438 | 5.52E-08 | 66.56 |  |
| Efet.01.63965.g861.t1 | FAM43A-like | 852 | 9.39E-89 | 71.6 | C:membrane |
| Efet.01.639657.g529.t1 | family transcriptional regulator | 594 | 2.08E-138 | 50.5 | F:DNA binding; F:transcription factor activity, sequence-specific DNA binding; P:transcription, DNA-templated; P:regulation of transcription, DNA-templated |
| Efet.01.639657.g530.t1 | zinc-binding alcohol dehydrogenase | 309 | 4.46E-40 | 66.25 | F:metal ion binding |
| Efet.01.639657.g531.t1 | D-ribose ABC transporter substrate-binding | 858 | 8.28E-145 | 54.9 |  |
| Efet.01.639669.g532.t1 | octopamine receptor-like | 264 | 2.45E-21 | 65.85 | F:G-protein coupled receptor activity; P:G-protein coupled receptor signaling pathway; C:integral component of membrane |
| Efet.01.639706.g534.t1 | transcription elongation factor | 285 | 6.72E-45 | 85.4 | F:RNA polymerase binding; F:DNA binding; F:translation elongation factor activity; P:transcription, DNA-templated; P:regulation of DNA-templated transcription, elongation; P:translational elongation |
| Efet.01.63972.g862.t1 | atherin-like isoform X1 | 855 | 2.59E-22 | 70.15 | F:metal ion binding |
| Efet.01.639728.g536.t1 | receptor-type tyrosine- phosphatase eta-like isoform X1 | 2709 | 0 | 50.35 | F:hydrolase activity |
| Efet.01.639737.g537.t1 | teneurin-1 isoform X1 | 891 | 1.56E-54 | 52.55 | P:signal transduction; C:membrane; C:integral component of membrane |
| Efet.01.639761.g538.t1 | 6-hydroxynicotinate reductase | 1347 | 0 | 97 |  |
| Efet.01.639788.g539.t1 | extracellular tyrosine- kinase PKDCC-like | 1398 | 8.92E-71 | 56.6 | F:oxidoreductase activity; F:metal ion binding; C:membrane; P:metabolic process; P:cellular process |
| Efet.01.639914.g542.t1 | RNA helicase | 1038 | 0 | 90.8 | F:nucleic acid binding; F:ATP binding; F:helicase activity |
| Efet.01.639931.g544.t1 | C4-dicarboxylate ABC transporter substrate-binding | 702 | 1.57E-113 | 88.15 | C:integral component of membrane |
| Efet.01.639963.g546.t1 | AMP-dependent synthetase | 2928 | 0 | 76.75 | F:oxo-acid-lyase activity; F:nucleotidyltransferase activity; F:isochorismate synthase activity; P:biosynthetic process; F:ligase activity |
| Efet.01.639963.g547.t1 | class I SAM-dependent methyltransferase | 621 | 1.45E-146 | 75.4 | F:methyltransferase activity; P:biosynthetic process; P:methylation |
| Efet.01.639970.g548.t1 | Nipped-B- | 246 | 5.08E-15 | 83.85 | P:mitotic sister chromatid cohesion; P:regulation of gene expression; F:chromatin binding |
| Efet.01.640020.g549.t1 | cytoplasmic 1 | 480 | 5.54E-76 | 83.05 | F:ATP binding; C:cytoskeleton; C:focal adhesion; C:dense body; C:plasma membrane |
| Efet.01.640021.g550.t1 | 3-hydroxyacyl- dehydrogenase | 447 | 1.63E-52 | 77.45 | F:oxidoreductase activity; P:oxidation-reduction process; P:fatty acid metabolic process |
| Efet.01.640024.g551.t1 | ABC transporter ATP-binding | 513 | 6.92E-95 | 69.2 | F:organic cyclic compound binding; F:heterocyclic compound binding |
| Efet.01.640027.g552.t1 | hypothetical protein CAPTEDRAFT_38953, partial | 249 | 1.75E-13 | 71.5 | P:regulation of apoptotic process |
| Efet.01.640044.g553.t1 | guanylate-binding 1-like | 375 | 4.33E-22 | 70.6 | F:nucleotide binding |
| Efet.01.640048.g554.t1 | peptidylprolyl isomerase | 669 | 8.18E-65 | 90.55 | F:peptidyl-prolyl cis-trans isomerase activity; P:protein peptidyl-prolyl isomerization; P:protein folding |
| Efet.01.640048.g555.t1 | cupin domain-containing | 1179 | 0 | 80.95 |  |
| Efet.01.640054.g556.t1 | two-component response regulator | 525 | 1.39E-123 | 90.4 | F:DNA binding; P:transcription, DNA-templated; P:phosphorelay signal transduction system; P:regulation of transcription, DNA-templated; C:intracellular |
| Efet.01.640058.g557.t1 | titin-like isoform X1 | 624 | 5.32E-46 | 60.45 |  |
| Efet.01.640112.g558.t1 | DNA-directed RNA polymerase subunit beta | 384 | 3.21E-69 | 98.6 | F:DNA binding; F:DNA-directed 5'-3' RNA polymerase activity; F:ribonucleoside binding; P:transcription, DNA-templated |
| Efet.01.640141.g559.t1 | filamentation induced by cAMP fic | 573 | 1.71E-117 | 65.7 |  |
| Efet.01.640198.g561.t1 | turtle homolog B | 357 | 1.42E-41 | 65.3 | C:membrane; C:integral component of membrane |
| Efet.01.640207.g562.t1 | transcription factor SOX-11-like | 1032 | 1.30E-26 | 64.75 | F:2-alkenal reductase [NAD(P)] activity; P:oxidation-reduction process |
| Efet.01.64021.g864.t1 | peptidyl-prolyl cis-trans isomerase NIMA-interacting 4 | 225 | 4.55E-19 | 72.15 | C:extracellular exosome; F:protein binding; C:mitochondrial matrix; C:spindle; F:double-stranded DNA binding; C:preribosome; F:bent DNA binding; P:rRNA processing; C:nucleolus; F:isomerase activity; F:RNA binding |
| Efet.01.640215.g563.t1 | tagatose 1,6-diphosphate aldolase | 906 | 0 | 58.4 | F:tagatose-bisphosphate aldolase activity |
| Efet.01.640242.g565.t1 | sugar ABC transporter permease | 774 | 2.20E-129 | 84.9 | C:integral component of membrane; P:transport; C:plasma membrane; F:transporter activity |
| Efet.01.640251.g566.t1 | Fe-S cluster assembly | 408 | 1.11E-79 | 99.95 | P:iron-sulfur cluster assembly |
| Efet.01.640254.g567.t1 | ABC transporter permease | 405 | 1.17E-74 | 64.25 | C:membrane |
| Efet.01.640254.g568.t1 | peptide chain release factor N(5)-glutamine methyltransferase | 1158 | 0 | 69.2 | F:methyltransferase activity; P:methylation |
| Efet.01.640370.g569.t1 | family Raf kinase inhibitor | 552 | 1.41E-100 | 77 | P:phosphorylation; F:kinase activity |
| Efet.01.640454.g572.t1 | NUDIX hydrolase | 549 | 9.22E-92 | 76.25 | F:hydrolase activity |
| Efet.01.640475.g574.t1 | nitrogen regulation NR(I) | 1383 | 0 | 85.6 | F:ATP binding; P:transcription, DNA-templated; P:phosphorelay signal transduction system; P:regulation of transcription, DNA-templated; F:sequence-specific DNA binding; P:regulation of nitrogen utilization; F:transcription factor binding; C:intracellular; F:phosphorelay response regulator activity |
| Efet.01.640475.g575.t1 | PAS domain-containing sensor histidine kinase | 1092 | 0 | 95.15 | F:phosphorelay sensor kinase activity; F:ATP binding; P:phosphorelay signal transduction system; P:signal transduction by protein phosphorylation; C:intracellular |
| Efet.01.640475.g576.t1 | signal peptide | 225 | 1.09E-42 | 92.6 |  |
| Efet.01.64051.g865.t1 | neural-cadherin-like isoform X1 | 567 | 2.78E-50 | 57.2 | C:membrane |
| Efet.01.640585.g577.t1 | autophagy-related 2 homolog B-like | 312 | 1.86E-08 | 67.39 | P:autophagy |
| Efet.01.640602.g578.t1 | hypothetical protein ECANGB1_216 | 264 | 7.69E-08 | 60 |  |
| Efet.01.640607.g579.t1 | sorting nexin-13-like isoform X1 | 201 | 2.65E-23 | 85.45 | C:integral component of membrane; F:phosphatidylinositol binding |
| Efet.01.640619.g581.t1 | proline racemase | 639 | 3.99E-126 | 80.25 |  |
| Efet.01.640642.g582.t1 | multidrug ABC transporter ATP-binding | 498 | 6.64E-89 | 75.4 | F:nucleotide binding; F:ATPase activity; C:membrane; P:transport |
| Efet.01.640707.g584.t1 | Deleted in malignant brain tumors 1 | 1218 | 6.19E-39 | 49.05 | C:membrane; F:scavenger receptor activity; P:receptor-mediated endocytosis |
| Efet.01.640715.g585.t1 | prickle 3 isoform X2 | 237 | 2.95E-14 | 75.4 | F:metal ion binding |
| Efet.01.640737.g586.t1 | Sugar (pentulose or hexulose) kinase | 456 | 4.60E-53 | 67.45 | P:carbohydrate metabolic process; P:phosphorylation; F:phosphotransferase activity, alcohol group as acceptor; F:kinase activity |
| Efet.01.640737.g587.t1 | L-rhamnose mutarotase | 294 | 3.97E-65 | 79.35 | F:racemase and epimerase activity, acting on carbohydrates and derivatives; C:cytoplasm; P:rhamnose metabolic process |
| Efet.01.640737.g588.t1 | ABC transporter permease | 963 | 1.37E-139 | 81.7 | C:integral component of membrane; P:monosaccharide transport; F:monosaccharide-transporting ATPase activity; C:plasma membrane |
| Efet.01.640787.g589.t1 | DUF488 domain-containing | 237 | 1.61E-27 | 64.95 |  |
| Efet.01.640793.g591.t1 | 78 kDa glucose-regulated | 705 | 1.68E-123 | 93.6 | F:ATPase activity; P:IRE1-mediated unfolded protein response; P:PERK-mediated unfolded protein response; C:smooth endoplasmic reticulum; C:endoplasmic reticulum-Golgi intermediate compartment; C:endoplasmic reticulum lumen; P:cerebellar Purkinje cell layer development; F:calcium ion binding; F:chaperone binding; P:positive regulation of protein ubiquitination; P:maintenance of protein localization in endoplasmic reticulum; F:glycoprotein binding; C:endoplasmic reticulum chaperone complex; C:COP9 signalosome; P:regulation of ATF6-mediated unfolded protein response; F:protein domain specific binding; P:regulation of IRE1-mediated unfolded protein response; P:cerebellum structural organization; P:regulation of PERK-mediated unfolded protein response; P:toxin transport; C:plasma membrane; P:cellular response to glucose starvation; F:ubiquitin protein ligase binding; C:melanosome; P:cellular response to manganese ion; C:integral component of endoplasmic reticulum membrane; C:midbody; P:positive regulation of cell migration; P:protein folding in endoplasmic reticulum; C:myelin sheath; P:negative regulation of protein homodimerization activity; P:ER overload response; F:ATP binding; C:extracellular exosome; C:focal adhesion; P:regulation of protein folding in endoplasmic reticulum; P:cellular response to interleukin-4; P:cellular response to antibiotic; P:ATF6-mediated unfolded protein response; F:misfolded protein binding; F:unfolded protein binding; P:substantia nigra development; C:cell surface; P:positive regulation of transcription from RNA polymerase II promoter in response to endoplasmic reticulum stress; P:activation of signaling protein activity involved in unfolded protein response; F:ribosome binding; P:negative regulation of apoptotic process; C:mitochondrion; P:ER-associated ubiquitin-dependent protein catabolic process; P:negative regulation of transforming growth factor beta receptor signaling pathway |
| Efet.01.6408.g508.t1 | aquaporin-9-like | 210 | 1.03E-16 | 69.25 | C:membrane |
| Efet.01.640805.g592.t1 | unconventional myosin-XVIIIa isoform X1 | 210 | 4.99E-13 | 75.55 | F:nucleotide binding |
| Efet.01.640829.g594.t1 | carbohydrate kinase | 987 | 0 | 77.2 | P:metabolic process; F:transferase activity, transferring phosphorus-containing groups |
| Efet.01.640829.g595.t1 | ribose ABC transporter substrate-binding | 252 | 4.50E-48 | 83.7 |  |
| Efet.01.640833.g596.t1 | LIM domain-binding 2 isoform X2 | 363 | 4.17E-12 | 60.45 | P:biological regulation |
| Efet.01.640851.g598.t1 | potassium voltage-gated channel subfamily A member 1-like isoform X1 | 1233 | 5.83E-151 | 82.95 | F:voltage-gated potassium channel activity; P:potassium ion transmembrane transport; C:voltage-gated potassium channel complex; P:regulation of ion transmembrane transport; P:protein homooligomerization |
| Efet.01.640857.g599.t1 | carbohydrate ABC transporter permease | 207 | 3.85E-23 | 75.5 | C:integral component of membrane; P:transport; C:plasma membrane |
| Efet.01.640857.g600.t1 | sugar ABC transporter permease | 414 | 1.47E-63 | 59.3 | C:membrane; C:integral component of membrane; P:transport; C:plasma membrane |
| Efet.01.640917.g601.t1 | NADPH:quinone reductase [Burkholderia stagnalis] | 558 | 6.81E-104 | 88.1 | P:L-lysine catabolic process to acetate; F:transferase activity |
| Efet.01.640917.g602.t1 | glycine betaine ABC transporter substrate-binding | 951 | 0 | 91.4 | P:choline transport; F:choline binding; C:periplasmic space; F:transporter activity |
| Efet.01.640944.g603.t1 | hypothetical protein HELRODRAFT_190469 | 228 | 3.69E-23 | 84 | P:proteolysis; F:serine-type peptidase activity |
| Efet.01.640954.g604.t1 | acid-sensing ion channel 1-like | 612 | 2.87E-08 | 52.92 | P:sodium ion transport; P:ion transport; F:sodium channel activity; C:membrane; C:integral component of membrane; P:sodium ion transmembrane transport; P:transport |
| Efet.01.64099.g868.t1 | cartilage matrix -like isoform X2 | 240 | 3.31E-09 | 59.91 | F:calcium ion binding; C:membrane; C:integral component of membrane |
| Efet.01.641023.g606.t1 | thyrotropin-releasing hormone receptor-like | 1866 | 1.90E-28 | 45.29 | F:G-protein coupled receptor activity; P:neuropeptide signaling pathway; P:G-protein coupled receptor signaling pathway; P:signal transduction; C:membrane; C:integral component of membrane; F:signal transducer activity |
| Efet.01.641084.g607.t1 | TKL kinase | 1755 | 1.82E-43 | 47.8 | P:peptidyl-tyrosine phosphorylation; F:ATP binding; F:protein tyrosine kinase activity; C:membrane; C:integral component of membrane; F:protein kinase activity; P:phosphorylation; P:protein phosphorylation; F:kinase activity |
| Efet.01.641113.g608.t1 | CTR1_RAT ame: Full=High affinity cationic amino acid transporter 1 Short=CAT-1 Short=CAT1 ame: Full=Ecotropic retroviral leukemia receptor ame: Full=Ecotropic retrovirus receptor Short= ame: Full=Solute carrier family 7 member 1 ame: Full=System Y+ basic amino acid transporter | 246 | 1.27E-06 | 82.4 | P:L-ornithine transmembrane transport; F:L-lysine transmembrane transporter activity; P:L-alpha-amino acid transmembrane transport; F:antiporter activity; F:basic amino acid transmembrane transporter activity; P:L-lysine transmembrane transport; F:amino acid transmembrane transporter activity; F:arginine transmembrane transporter activity; P:transport; P:amino acid transport; P:arginine transmembrane transport; P:amino acid transmembrane transport; C:plasma membrane; C:integral component of plasma membrane; F:protein binding; F:L-ornithine transmembrane transporter activity; P:arginine transport; P:lysine transport; C:membrane; P:basic amino acid transmembrane transport; C:integral component of membrane; P:ornithine transport; P:regulation of TOR signaling |
| Efet.01.641141.g610.t1 | hypothetical protein HELRODRAFT_166723 | 840 | 1.74E-19 | 57 |  |
| Efet.01.641186.g611.t1 | Alpha-1A adrenergic receptor | 1959 | 3.69E-28 | 43.5 | F:G-protein coupled receptor activity; P:G-protein coupled receptor signaling pathway; C:membrane; C:integral component of membrane |
| Efet.01.641209.g612.t1 | hypothetical protein CRN43_07125, partial | 324 | 9.12E-25 | 71.2 |  |
| Efet.01.641218.g613.t1 | malonyl- decarboxylase | 435 | 1.19E-77 | 81.15 | P:fatty acid biosynthetic process; F:malonyl-CoA decarboxylase activity |
| Efet.01.641218.g614.t1 | ABC transporter substrate-binding | 984 | 0 | 92.95 | C:outer membrane-bounded periplasmic space |
| Efet.01.641218.g615.t1 | malonyl- synthase | 2622 | 0 | 88.8 | P:metabolic process; F:ligase activity |
| Efet.01.641218.g616.t1 | transcription termination antitermination | 1485 | 0 | 97.15 | F:nucleotide binding; F:transcription factor activity, sequence-specific DNA binding; F:translation elongation factor activity; C:cytoplasm; P:transcription antitermination; P:DNA-templated transcription, termination; P:translational elongation |
| Efet.01.641242.g617.t1 | 30S ribosomal S9 | 438 | 6.67E-49 | 82.25 | F:structural constituent of ribosome; C:ribosome; P:translation |
| Efet.01.641291.g619.t1 | trigger factor | 1281 | 0 | 87.2 | P:cell division; F:peptidyl-prolyl cis-trans isomerase activity; C:cytoplasm; P:protein transport; P:protein peptidyl-prolyl isomerization; P:protein folding; P:cell cycle |
| Efet.01.641291.g620.t1 | ATP-dependent Clp protease ATP-binding subunit | 1269 | 0 | 91.75 | F:ATP binding; F:zinc ion binding; P:proteolysis; P:protein transport; F:serine-type endopeptidase activity; F:protein dimerization activity; F:unfolded protein binding; P:protein folding |
| Efet.01.641352.g624.t1 | receptor-type guanylate cyclase Gyc76C-like | 207 | 5.70E-09 | 77.43 | F:ATP binding; P:cGMP biosynthetic process; C:integral component of membrane; F:guanylate cyclase activity; F:protein kinase activity; P:protein phosphorylation; C:intracellular; P:intracellular signal transduction |
| Efet.01.641366.g625.t1 | beta-1,3-galactosyl-O-glycosyl-glyco beta-1,6-N-acetylglucosaminyltransferase 3-like | 420 | 8.83E-43 | 58.1 | F:acetylglucosaminyltransferase activity; C:membrane |
| Efet.01.64144.g872.t1 | cytochrome c oxidase subunit mitochondrial-like | 291 | 2.36E-50 | 79.55 | F:cytochrome-c oxidase activity; P:hydrogen ion transmembrane transport; P:oxidation-reduction process; C:mitochondrial envelope |
| Efet.01.641462.g627.t1 | acyclic terpene utilization family | 891 | 0 | 84.45 | F:transferase activity |
| Efet.01.641462.g628.t1 | ABC transporter substrate-binding | 975 | 0 | 86.25 | F:lyase activity; C:outer membrane-bounded periplasmic space |
| Efet.01.641462.g629.t1 | sigma-54-dependent Fis family transcriptional regulator | 978 | 0 | 83.75 | F:ATP binding; P:transcription, DNA-templated; P:phosphorelay signal transduction system; P:regulation of transcription, DNA-templated; F:sequence-specific DNA binding; F:transcription factor binding; C:intracellular |
| Efet.01.641462.g630.t1 | 3-phosphoglycerate dehydrogenase | 543 | 1.78E-120 | 86.95 | F:oxidoreductase activity, acting on the CH-OH group of donors, NAD or NADP as acceptor; F:NAD binding; P:oxidation-reduction process |
| Efet.01.641519.g631.t1 | family -linked outer membrane | 489 | 2.39E-55 | 68.4 | C:membrane; F:receptor activity; P:transport |
| Efet.01.641589.g632.t1 | myosin regulatory light chain 12B-like | 210 | 2.35E-14 | 76.55 | F:calcium ion binding |
| Efet.01.641591.g633.t1 | hypothetical protein B5766_11660 | 246 | 1.19E-31 | 100 |  |
| Efet.01.641615.g634.t1 | acyl- dehydrogenase | 1032 | 5.39E-179 | 76 | F:oxidoreductase activity, acting on the CH-CH group of donors; P:metabolic process |
| Efet.01.641682.g637.t1 | muscle calcium channel subunit alpha-1-like | 225 | 3.45E-08 | 86.1 | C:postsynaptic density; F:calmodulin binding; C:voltage-gated calcium channel complex; P:regulation of ion transmembrane transport; P:calcium ion transmembrane transport; C:cytosol; F:alpha-actinin binding; F:voltage-gated calcium channel activity; P:positive regulation of cytosolic calcium ion concentration |
| Efet.01.64170.g874.t1 | Helix-loop-helix 1 | 417 | 1.50E-06 | 85.1 | C:membrane; C:integral component of membrane; F:protein dimerization activity |
| Efet.01.64177.g875.t1 | cytochrome c | 396 | 8.26E-13 | 82.05 | F:structural constituent of ribosome; F:metal ion binding; F:electron carrier activity; C:mitochondrion; P:oxidation-reduction process; C:ribosome; C:respiratory chain; F:heme binding; P:translation |
| Efet.01.641815.g642.t1 | galactose mutarotase | 588 | 3.23E-52 | 80 | P:hexose metabolic process; F:aldose 1-epimerase activity; F:carbohydrate binding |
| Efet.01.641828.g645.t1 | tektin-3-like isoform X1 | 594 | 1.99E-57 | 74.85 |  |
| Efet.01.641849.g646.t1 | malate synthase G | 795 | 1.12E-167 | 73.7 | F:transferase activity, transferring acyl groups; P:carboxylic acid metabolic process; P:primary metabolic process |
| Efet.01.641953.g647.t1 | 30S ribosomal S5 | 507 | 4.39E-69 | 96.45 | F:structural constituent of ribosome; C:small ribosomal subunit; F:rRNA binding; P:translation |
| Efet.01.641969.g649.t1 | family transcriptional regulator | 609 | 1.02E-89 | 57.85 |  |
| Efet.01.642062.g651.t1 | DNA topoisomerase IV subunit B | 1017 | 5.53E-158 | 84.95 | F:DNA binding; F:ATP binding; F:DNA topoisomerase type II (ATP-hydrolyzing) activity; P:DNA topological change |
| Efet.01.64207.g876.t1 | anoctamin-8 isoform X1 | 462 | 6.71E-37 | 73.7 | C:integral component of membrane; C:cell part; P:chloride transport |
| Efet.01.642071.g652.t1 | transferase | 1149 | 0 | 85.5 | F:catalytic activity |
| Efet.01.642072.g653.t1 | imidazole glycerol phosphate synthase subunit | 600 | 4.51E-90 | 68.95 | F:catalytic activity; P:cellular amino acid biosynthetic process |
| Efet.01.642087.g654.t1 | ABC transporter ATP-binding | 993 | 0 | 63.8 | F:ATPase activity; F:ATP binding; P:peptide transport |
| Efet.01.642087.g655.t1 | peptide ABC transporter ATP-binding | 480 | 9.24E-107 | 77.55 | F:ATPase activity; F:ATP binding; P:peptide transport |
| Efet.01.642094.g656.t1 | phosphate acetyltransferase | 303 | 1.02E-50 | 91.2 | F:phosphate acetyltransferase activity; P:metabolic process; F:phosphate butyryltransferase activity |
| Efet.01.642094.g657.t1 | sulfoacetaldehyde acetyltransferase | 1818 | 0 | 95.25 | F:magnesium ion binding; F:thiamine pyrophosphate binding; F:sulfoacetaldehyde acetyltransferase activity; P:taurine catabolic process; C:cytoplasm |
| Efet.01.642094.g658.t1 | family transcriptional regulator | 915 | 0 | 92.1 | F:DNA binding; P:transcription, DNA-templated; P:regulation of transcription, DNA-templated |
| Efet.01.642117.g659.t1 | family | 333 | 1.96E-67 | 65.1 | P:polysaccharide biosynthetic process; C:integral component of membrane; P:transport |
| Efet.01.64212.g877.t1 | cilia- and flagella-associated 45-like | 363 | 1.05E-13 | 76.59 |  |
| Efet.01.642123.g660.t1 | ABC transporter substrate-binding | 477 | 1.34E-90 | 90.75 | F:arogenate dehydratase activity |
| Efet.01.642123.g661.t1 | FAD-binding oxidoreductase | 1317 | 0 | 73.3 | F:D-amino-acid dehydrogenase activity; P:oxidation-reduction process |
| Efet.01.642123.g662.t1 | family transcriptional regulator | 828 | 9.86E-168 | 81.65 | F:transcription factor activity, sequence-specific DNA binding; P:transcription, DNA-templated; P:regulation of transcription, DNA-templated; F:sequence-specific DNA binding |
| Efet.01.642123.g663.t1 | DNA repair | 1656 | 0 | 92.8 | F:ATP binding; P:DNA repair; F:DNA-dependent ATPase activity; F:metal ion binding; P:proteolysis; F:serine-type endopeptidase activity; F:ATP-dependent peptidase activity; F:damaged DNA binding |
| Efet.01.642123.g664.t1 | tagatose 1,6-diphosphate aldolase | 1029 | 0 | 75.9 | F:tagatose-bisphosphate aldolase activity |
| Efet.01.642123.g665.t1 | sugar kinase | 750 | 8.74E-111 | 73.7 | F:ribokinase activity; P:D-ribose metabolic process; P:carbohydrate phosphorylation |
| Efet.01.642123.g666.t1 | sn-glycerol-3-phosphate ABC transporter ATP-binding | 1038 | 0 | 73.75 | F:ATP binding; P:glycerol-3-phosphate transport; P:transmembrane transport; F:glycerol-3-phosphate-transporting ATPase activity; C:ATP-binding cassette (ABC) transporter complex |
| Efet.01.642123.g667.t1 | alcohol dehydrogenase | 972 | 1.70E-168 | 85.9 | F:zinc ion binding; P:oxidation-reduction process; F:L-threonine 3-dehydrogenase activity |
| Efet.01.642149.g668.t1 | sulfate adenylyltransferase subunit | 378 | 1.19E-79 | 92 | F:ATP binding; P:sulfate reduction; F:sulfate adenylyltransferase (ATP) activity |
| Efet.01.642149.g669.t1 | sulfate adenylyltransferase | 201 | 4.70E-29 | 97.85 | F:GTP binding; F:ATP binding; P:hydrogen sulfide biosynthetic process; F:GTPase activity; F:sulfate adenylyltransferase (ATP) activity |
| Efet.01.642191.g670.t1 | Small conductance calcium-activated potassium channel | 240 | 1.06E-22 | 89.75 | F:calmodulin binding; P:potassium ion transmembrane transport; C:integral component of membrane; F:small conductance calcium-activated potassium channel activity; C:neuronal cell body; C:plasma membrane |
| Efet.01.642283.g671.t1 | 60S ribosomal L13 | 294 | 4.06E-30 | 83.15 | F:RNA binding; F:structural constituent of ribosome; C:cytosolic large ribosomal subunit; P:regulation of cell cycle; P:translation |
| Efet.01.642313.g673.t1 | PR domain zinc finger | 411 | 1.44E-24 | 60.6 | P:negative regulation of transcription from RNA polymerase II promoter; C:nucleus; F:transcriptional repressor activity, RNA polymerase II core promoter proximal region sequence-specific binding; P:developmental process involved in reproduction; P:heart development; F:protein binding; C:cytoplasm; F:RNA polymerase II core promoter proximal region sequence-specific DNA binding; P:cell differentiation; P:single organism reproductive process |
| Efet.01.642328.g674.t1 | potassium voltage-gated channel subfamily H member 1- | 240 | 5.84E-33 | 92.5 | F:voltage-gated potassium channel activity; P:potassium ion transmembrane transport; C:integral component of membrane |
| Efet.01.642357.g676.t1 | glycosyl transferase family 2 | 3126 | 0 | 73.4 | F:transferase activity |
| Efet.01.642390.g678.t1 | ribonucleoside-diphosphate adenosylcobalamin-dependent | 2328 | 0 | 88.3 | F:ATP binding; F:ribonucleoside-diphosphate reductase activity, thioredoxin disulfide as acceptor; F:cobalamin binding; P:DNA replication; P:oxidation-reduction process |
| Efet.01.642390.g679.t1 | flavin reductase | 444 | 2.12E-81 | 85.65 | F:FMN binding; F:oxidoreductase activity; P:oxidation-reduction process |
| Efet.01.642392.g680.t1 | cell surface | 2004 | 0 | 63.65 |  |
| Efet.01.642395.g681.t1 | ABC transporter | 822 | 4.10E-169 | 87.65 | F:ATPase activity; F:ATP binding |
| Efet.01.642395.g682.t1 | cob(I)yrinic acid a,c-diamide adenosyltransferase | 795 | 4.96E-135 | 94.35 | F:ATP binding; F:cob(I)yrinic acid a,c-diamide adenosyltransferase activity; P:cobalamin biosynthetic process |
| Efet.01.642415.g684.t1 | elongation factor P | 666 | 1.18E-119 | 71.8 | F:translation elongation factor activity; C:cytoplasm; P:translational elongation |
| Efet.01.64243.g878.t1 | structural maintenance of chromosomes 3-like | 1026 | 6.57E-142 | 71.65 | C:intracellular organelle; P:chromosome organization; C:membrane; F:binding |
| Efet.01.642485.g685.t1 | family transcriptional regulator | 1185 | 0 | 64.55 | F:DNA binding; F:transcription factor activity, sequence-specific DNA binding; P:transcription, DNA-templated; P:regulation of transcription, DNA-templated |
| Efet.01.642485.g686.t1 | porin | 1581 | 0 | 73.45 | F:porin activity; P:transmembrane transport; C:membrane |
| Efet.01.642485.g687.t1 | NADPH-dependent oxidoreductase | 270 | 3.47E-44 | 71.15 | F:oxidoreductase activity; P:oxidation-reduction process |
| Efet.01.642485.g688.t1 | NADPH-dependent FMN reductase | 222 | 4.77E-33 | 73.05 | F:oxidoreductase activity; P:oxidation-reduction process |
| Efet.01.642485.g689.t1 | ABC transporter substrate-binding | 1089 | 0 | 66.55 | P:amino acid transport |
| Efet.01.642485.g690.t1 | branched-chain amino acid ABC transporter permease | 576 | 1.40E-57 | 78.09 | C:integral component of membrane; P:transport; C:plasma membrane; F:transporter activity |
| Efet.01.642485.g691.t1 | branched-chain amino acid ABC transporter permease | 873 | 3.83E-126 | 70 | C:integral component of membrane; P:transport; C:plasma membrane; F:transporter activity |
| Efet.01.642524.g692.t1 | biotin transporter | 501 | 6.73E-51 | 67.5 | F:electron carrier activity; F:molybdenum ion binding; P:oxidation-reduction process; F:trimethylamine-N-oxide reductase (cytochrome c) activity |
| Efet.01.642524.g693.t1 | pre translocase | 2010 | 0 | 86.6 |  |
| Efet.01.642550.g694.t1 | CRISPR type aferr-associated csf3 | 624 | 8.66E-37 | 45.7 |  |
| Efet.01.642550.g695.t1 | type IV CRISPR-associated Csf2 | 1050 | 2.46E-72 | 49.4 |  |
| Efet.01.642550.g696.t1 | hypothetical protein | 762 | 2.58E-50 | 57 |  |
| Efet.01.642550.g697.t1 | ATP-dependent DNA helicase | 2292 | 6.52E-131 | 45.1 | F:nucleic acid binding; F:ATP binding; F:hydrolase activity, acting on acid anhydrides, in phosphorus-containing anhydrides; F:ATP-dependent helicase activity; F:helicase activity; P:nucleobase-containing compound metabolic process |
| Efet.01.642550.g698.t1 | Glucose-6-phosphate isomerase | 2835 | 0 | 85.75 | F:glucose-6-phosphate isomerase activity; P:gluconeogenesis; C:cytoplasm; P:glycolytic process |
| Efet.01.642550.g699.t1 | glucose-6-phosphate dehydrogenase | 1455 | 0 | 95.65 | F:NADP binding; P:pentose-phosphate shunt; P:oxidation-reduction process; P:glucose metabolic process; F:glucose-6-phosphate dehydrogenase activity |
| Efet.01.642550.g700.t1 | DNA mismatch repair | 2514 | 0 | 82.05 | F:ATP binding; P:mismatch repair; F:mismatched DNA binding |
| Efet.01.642550.g701.t1 | MFS transporter | 1461 | 0 | 91.75 | P:transmembrane transport; C:integral component of membrane; F:transporter activity |
| Efet.01.642550.g702.t1 | tRNA (adenosine(37)-N6)-dimethylallyltransferase | 669 | 7.00E-104 | 77.15 | F:ATP binding; P:tRNA processing; F:tRNA dimethylallyltransferase activity |
| Efet.01.642550.g703.t1 | UDP-2,3-diacylglucosamine diphosphatase | 801 | 2.47E-155 | 81.75 | C:cytoplasm; F:pyrophosphatase activity; P:lipid A biosynthetic process |
| Efet.01.642550.g704.t1 | cyclophilin | 507 | 2.30E-119 | 93.9 | F:peptidyl-prolyl cis-trans isomerase activity; P:protein peptidyl-prolyl isomerization; P:protein folding |
| Efet.01.642550.g705.t1 | Peptidylprolyl isomerase | 549 | 7.66E-115 | 91.9 | F:peptidyl-prolyl cis-trans isomerase activity; P:protein peptidyl-prolyl isomerization; P:protein folding |
| Efet.01.642550.g706.t1 | DUF2157 domain-containing | 1092 | 0 | 74.45 | C:integral component of membrane |
| Efet.01.642630.g707.t1 | fumarylacetoacetate hydrolase | 288 | 6.64E-25 | 80.45 | P:metabolic process; F:hydrolase activity |
| Efet.01.642630.g708.t1 | enoyl- hydratase isomerase family | 435 | 2.08E-76 | 84.35 | F:catalytic activity; P:metabolic process |
| Efet.01.642649.g709.t1 | cytochrome oxidase subunit III | 636 | 3.15E-68 | 68.6 | C:membrane |
| Efet.01.642711.g710.t1 | sex peptide receptor-like | 417 | 4.04E-44 | 70.65 | F:G-protein coupled peptide receptor activity; P:G-protein coupled receptor signaling pathway; C:integral component of membrane |
| Efet.01.642713.g711.t1 | bifunctional phosphoribosyl-AMP cyclohydrolase phosphoribosyl-ATP diphosphatase | 516 | 1.12E-123 | 79.8 | F:ATP binding; F:phosphoribosyl-ATP diphosphatase activity; F:phosphoribosyl-AMP cyclohydrolase activity; C:cytoplasm; P:histidine biosynthetic process |
| Efet.01.642722.g712.t1 | ABC transporter ATP-binding | 672 | 2.95E-118 | 88.15 | F:ATPase activity; F:ATP binding; P:transmembrane transport; C:ATP-binding cassette (ABC) transporter complex; F:hydrolase activity, acting on acid anhydrides, catalyzing transmembrane movement of substances |
| Efet.01.642722.g713.t1 | sugar ABC transporter | 822 | 1.01E-158 | 86.25 | C:integral component of membrane; P:transport; C:plasma membrane |
| Efet.01.642722.g714.t1 | sugar ABC transporter permease | 795 | 9.62E-154 | 83.8 | C:integral component of membrane; P:transport; C:plasma membrane |
| Efet.01.642722.g715.t1 | sugar ABC transporter | 1278 | 0 | 77 | P:transport; F:transporter activity |
| Efet.01.642722.g716.t1 | oxidoreductase | 771 | 2.48E-137 | 73.3 | F:oxidoreductase activity; P:oxidation-reduction process |
| Efet.01.642722.g717.t1 | family transcriptional regulator | 444 | 1.22E-83 | 75.9 | F:DNA binding; F:transcription factor activity, sequence-specific DNA binding; P:transcription, DNA-templated; P:regulation of transcription, DNA-templated |
| Efet.01.642783.g719.t1 | uncharacterized protein LOC111418265 | 333 | 6.39E-08 | 56.5 |  |
| Efet.01.642823.g720.t1 | DUF490 domain-containing | 2763 | 0 | 65.9 | C:integral component of membrane |
| Efet.01.642832.g721.t1 | magnesium and cobalt transport | 594 | 5.05E-122 | 78.85 | P:cobalt ion transport; F:cobalt ion transmembrane transporter activity; C:integral component of membrane; F:magnesium ion transmembrane transporter activity; P:magnesium ion transmembrane transport; C:plasma membrane |
| Efet.01.642832.g722.t1 | rRNA methyltransferase | 732 | 3.12E-148 | 83.9 | F:RNA methyltransferase activity; P:RNA methylation; F:RNA binding; P:RNA processing |
| Efet.01.642837.g723.t1 | elongation factor P maturation arginine rhamnosyltransferase | 330 | 1.08E-13 | 71.5 |  |
| Efet.01.642837.g724.t1 | elongation factor P maturation arginine rhamnosyltransferase | 285 | 3.15E-42 | 87.95 |  |
| Efet.01.642837.g725.t1 | lactoylglutathione lyase | 462 | 1.84E-109 | 88 | F:metal ion binding; F:lactoylglutathione lyase activity |
| Efet.01.642837.g726.t1 | aryl-sulfate sulfotransferase | 375 | 1.36E-86 | 80.5 | F:transferase activity |
| Efet.01.642837.g727.t1 | GMC family oxidoreductase | 2088 | 0 | 90.85 | F:oxidoreductase activity, acting on CH-OH group of donors; F:flavin adenine dinucleotide binding; P:oxidation-reduction process |
| Efet.01.642837.g728.t1 | dihydrodipicolinate synthase family | 819 | 0 | 89.6 | P:metabolic process; F:2-dehydro-3-deoxy-L-arabinonate dehydratase activity |
| Efet.01.642837.g729.t1 | cytochrome C signal peptide | 999 | 0 | 77.8 | F:electron carrier activity; F:heme binding |
| Efet.01.642837.g730.t1 | L-rhamnonate dehydratase | 1197 | 0 | 93.45 | P:cellular amino acid catabolic process; F:metal ion binding; F:L-rhamnonate dehydratase activity |
| Efet.01.642859.g732.t1 | hypothetical protein A3F76_05550 | 675 | 8.88E-56 | 53.5 |  |
| Efet.01.642859.g733.t1 | Uncharacterised | 783 | 7.64E-31 | 71.85 | F:transferase activity |
| Efet.01.642859.g734.t1 | hypothetical protein [Diaphorobacter polyhydroxybutyrativorans] | 225 | 1.12E-20 | 73 |  |
| Efet.01.642859.g735.t1 | hypothetical protein [Diaphorobacter polyhydroxybutyrativorans] | 342 | 1.89E-29 | 74.75 |  |
| Efet.01.642948.g737.t1 | DUF4432 domain-containing | 531 | 6.59E-108 | 57.35 | P:carbohydrate metabolic process; F:catalytic activity; F:carbohydrate binding |
| Efet.01.642964.g738.t1 | FAD synthase-like | 990 | 3.65E-146 | 69.4 | F:catalytic activity; P:metabolic process |
| Efet.01.643.g63.t1 | HEAT repeat-containing 1 | 948 | 8.88E-15 | 44.4 | C:intracellular part |
| Efet.01.643008.g740.t1 | hypothetical protein HELRODRAFT_163906 | 375 | 7.88E-15 | 71 |  |
| Efet.01.643008.g741.t1 | major egg antigen-like isoform X2 | 417 | 8.66E-24 | 68.43 | F:nucleic acid binding |
| Efet.01.643011.g742.t1 | beta keto-acyl synthase | 714 | 1.15E-99 | 72.1 | F:catalytic activity |
| Efet.01.643012.g743.t1 | ATP-dependent chaperone | 894 | 1.97E-122 | 84.85 | F:ATP binding; P:response to heat; C:cytoplasm; P:protein processing; F:peptidase activity |
| Efet.01.643018.g745.t1 | two-component sensor histidine kinase | 804 | 8.85E-85 | 84.9 | F:phosphorelay sensor kinase activity; F:ATP binding; P:phosphorelay signal transduction system; C:integral component of membrane; P:signal transduction by protein phosphorylation; C:intracellular |
| Efet.01.643024.g746.t1 | two-component system response regulator | 366 | 1.66E-83 | 98.05 | F:DNA binding; P:phosphorelay signal transduction system; C:intracellular |
| Efet.01.643024.g747.t1 | chemotaxis | 2304 | 0 | 87.05 | P:chemotaxis; P:signal transduction; C:integral component of membrane; F:signal transducer activity |
| Efet.01.643024.g748.t1 | hybrid sensor histidine kinase response regulator | 1311 | 0 | 83.25 | F:phosphorelay sensor kinase activity; P:chemotaxis; C:cytoplasm; P:phosphorelay signal transduction system; P:signal transduction by protein phosphorylation; P:peptidyl-histidine phosphorylation |
| Efet.01.643024.g749.t1 | hybrid sensor histidine kinase response regulator | 1476 | 1.31E-75 | 89.9 | F:phosphorelay sensor kinase activity; P:chemotaxis; C:cytoplasm; P:phosphorelay signal transduction system; P:signal transduction by protein phosphorylation; P:peptidyl-histidine phosphorylation |
| Efet.01.643051.g750.t1 | AMP deaminase 2-like isoform X3 | 378 | 1.59E-09 | 46.57 | F:AMP deaminase activity; P:IMP salvage; P:purine ribonucleoside monophosphate biosynthetic process; F:deaminase activity |
| Efet.01.643060.g751.t1 | glycosyl transferase | 747 | 8.54E-91 | 56.8 | C:membrane; C:integral component of membrane; F:transferase activity |
| Efet.01.643060.g752.t1 | glycosyl transferase family 2 | 1023 | 0 | 66.25 | F:transferase activity, transferring glycosyl groups; F:transferase activity |
| Efet.01.643060.g753.t1 | dTDP-4-dehydrorhamnose reductase | 837 | 7.86E-170 | 65.95 |  |
| Efet.01.643064.g754.t1 | DNA topoisomerase (ATP-hydrolyzing) subunit B | 1623 | 0 | 89.4 | F:magnesium ion binding; F:DNA binding; F:ATP binding; C:cytoplasm; P:DNA-dependent DNA replication; C:chromosome; F:DNA topoisomerase type II (ATP-hydrolyzing) activity; P:DNA topological change |
| Efet.01.643067.g755.t1 | DNA topoisomerase I | 282 | 2.00E-24 | 77 | F:magnesium ion binding; F:DNA binding; F:DNA topoisomerase type I activity; P:DNA topological change |
| Efet.01.643097.g756.t1 | M23 family peptidase | 870 | 6.17E-155 | 74.4 |  |
| Efet.01.643097.g757.t1 | cobyric acid synthase | 1011 | 1.27E-137 | 92 | F:cobalamin-transporting ATPase activity; P:vitamin transmembrane transport; P:glutamine metabolic process; P:cobalamin biosynthetic process; P:cobalamin transport |
| Efet.01.643099.g758.t1 | 3-mercaptopyruvate sulfurtransferase | 693 | 1.58E-154 | 74.4 | F:3-mercaptopyruvate sulfurtransferase activity |
| Efet.01.643099.g759.t1 | penicillin acylase family | 2172 | 0 | 79.7 | P:antibiotic biosynthetic process; F:penicillin amidase activity |
| Efet.01.643099.g760.t1 | hypothetical protein Veis_3276 | 225 | 3.79E-07 | 57 |  |
| Efet.01.643099.g761.t1 | transcriptional regulator | 1077 | 0 | 92.1 | P:phosphorylation; F:kinase activity |
| Efet.01.643099.g762.t1 | ABC transporter ATP-binding | 1353 | 0 | 91.7 | F:ATPase activity; F:ATP binding; P:carbohydrate transport |
| Efet.01.643099.g763.t1 | ABC transporter permease | 888 | 5.34E-151 | 87.2 | C:integral component of membrane; P:transport; C:plasma membrane; F:transporter activity |
| Efet.01.64311.g880.t1 | CLEC16A-like isoform X8 | 486 | 2.12E-14 | 61.55 |  |
| Efet.01.643111.g764.t1 | phosphoheptose isomerase | 594 | 4.28E-79 | 80.5 | P:carbohydrate metabolic process; F:zinc ion binding; F:D-sedoheptulose 7-phosphate isomerase activity; C:cytoplasm; P:D-glycero-D-manno-heptose 7-phosphate biosynthetic process; F:carbohydrate binding |
| Efet.01.643150.g765.t1 | ATP-dependent DNA helicase | 984 | 2.61E-143 | 88.9 | F:DNA binding; F:ATP binding; P:DNA duplex unwinding; F:ATP-dependent DNA helicase activity |
| Efet.01.643183.g766.t1 | protocadherin beta-18 isoform X3 | 1194 | 2.17E-52 | 50.8 | F:calcium ion binding; C:membrane; C:integral component of membrane; P:cell adhesion; P:homophilic cell adhesion via plasma membrane adhesion molecules; C:plasma membrane |
| Efet.01.643213.g767.t1 | Tumor p63-regulated gene 1 | 306 | 2.99E-20 | 53.35 | C:cytoplasm; C:membrane; C:integral component of membrane |
| Efet.01.643237.g768.t1 | GSK3-beta interaction -like | 231 | 1.45E-15 | 68.35 |  |
| Efet.01.643291.g769.t1 | pyruvate kinase | 558 | 6.07E-53 | 94.85 | F:magnesium ion binding; F:pyruvate kinase activity; F:potassium ion binding; P:glycolytic process; F:kinase activity |
| Efet.01.643324.g771.t1 | EH domain-containing 3-like | 465 | 1.18E-73 | 83.45 | F:GTP binding; F:calcium ion binding; C:intracellular; P:endocytic recycling |
| Efet.01.643389.g773.t1 | filamin-A-like isoform X6 | 249 | 8.54E-18 | 65.5 | F:actin filament binding |
| Efet.01.643393.g774.t1 | sugar ABC transporter permease | 213 | 1.28E-37 | 87.55 | C:integral component of membrane; P:transport; C:plasma membrane |
| Efet.01.643393.g775.t1 | sugar ABC transporter substrate-binding | 468 | 2.93E-103 | 81.05 | P:transport; F:transporter activity |
| Efet.01.643394.g776.t1 | TrpB-like pyridoxal phosphate-dependent enzyme | 1269 | 0 | 88.05 | F:tryptophan synthase activity; F:pyridoxal phosphate binding; P:tryptophan biosynthetic process |
| Efet.01.643394.g777.t1 | phosphate regulon sensor histidine kinase | 1740 | 0 | 92.2 | F:phosphorelay sensor kinase activity; F:ATP binding; P:phosphorelay signal transduction system; C:integral component of membrane; P:regulation of transcription, DNA-templated; P:signal transduction by protein phosphorylation; C:intracellular; P:peptidyl-histidine phosphorylation |
| Efet.01.643394.g778.t1 | phosphate transport system regulatory | 639 | 1.31E-147 | 95.05 | P:negative regulation of phosphate metabolic process; P:phosphate ion transport; C:cytoplasm; P:cellular phosphate ion homeostasis |
| Efet.01.643394.g779.t1 | phosphate ABC transporter ATP-binding | 786 | 4.89E-173 | 95.5 | F:inorganic phosphate transmembrane transporter activity; F:ATP binding; F:ATPase-coupled phosphate ion transmembrane transporter activity; C:ATP-binding cassette (ABC) transporter complex; P:phosphate ion transmembrane transport |
| Efet.01.643394.g780.t1 | phosphate ABC permease | 294 | 6.24E-59 | 97.05 | C:integral component of plasma membrane; F:inorganic phosphate transmembrane transporter activity; P:phosphate ion transmembrane transport |
| Efet.01.643394.g781.t1 | phosphate ABC permease | 354 | 3.22E-49 | 98.15 | C:integral component of plasma membrane; F:inorganic phosphate transmembrane transporter activity; P:phosphate ion transmembrane transport |
| Efet.01.643394.g782.t1 | phosphate ABC transporter permease subunit | 1017 | 0 | 95.45 | F:inorganic phosphate transmembrane transporter activity; P:transmembrane transport; P:phosphate ion transport; C:integral component of membrane; C:plasma membrane |
| Efet.01.643394.g783.t1 | phosphate ABC transporter substrate-binding | 1038 | 0 | 94.5 | F:phosphate ion binding; C:ATP-binding cassette (ABC) transporter complex; P:phosphate ion transmembrane transport |
| Efet.01.643394.g784.t1 | exopolyphosphatase | 1482 | 0 | 91.6 |  |
| Efet.01.643394.g785.t1 | polyphosphate kinase 1 | 1023 | 0 | 94.6 | F:ATP binding; C:polyphosphate kinase complex; P:phosphorylation; F:polyphosphate kinase activity; P:polyphosphate biosynthetic process |
| Efet.01.643464.g786.t1 | DNA-binding response regulator | 879 | 8.17E-152 | 85.6 | F:DNA binding; P:transcription, DNA-templated; P:phosphorelay signal transduction system; P:regulation of transcription, DNA-templated; C:intracellular |
| Efet.01.643464.g787.t1 | family transcriptional regulator | 786 | 0 | 69.6 | F:DNA binding; P:transcription, DNA-templated; P:regulation of transcription, DNA-templated |
| Efet.01.643464.g788.t1 | maltose ABC transporter permease | 777 | 9.44E-175 | 87.2 | C:integral component of membrane; P:transport; C:plasma membrane |
| Efet.01.643464.g789.t1 | sugar ABC transporter substrate-binding | 2040 | 0 | 78.75 | P:carbohydrate transport; F:transporter activity |
| Efet.01.643464.g790.t1 | Oxidoreductase | 735 | 2.11E-177 | 71.65 | F:oxidoreductase activity; P:oxidation-reduction process |
| Efet.01.643464.g791.t1 | amidohydrolase | 876 | 0 | 65.75 | F:hydrolase activity |
| Efet.01.643464.g792.t1 | amine oxidase | 366 | 1.99E-85 | 87.1 | F:aldehyde dehydrogenase (NAD) activity; C:integral component of membrane; P:oxidation-reduction process |
| Efet.01.643464.g793.t1 | transferase | 1074 | 0 | 77.55 | F:transferase activity |
| Efet.01.643464.g794.t1 | ABC transporter substrate-binding | 651 | 2.56E-122 | 70.8 | P:transport; F:transporter activity |
| Efet.01.643464.g795.t1 | mandelate racemase | 1152 | 0 | 79.65 | P:cellular amino acid catabolic process; F:catalytic activity; F:metal ion binding |
| Efet.01.643464.g796.t1 | L-rhamnose mutarotase | 291 | 1.24E-66 | 83.05 | F:racemase and epimerase activity, acting on carbohydrates and derivatives; C:cytoplasm |
| Efet.01.643464.g797.t1 | XRE family transcriptional regulator | 675 | 4.27E-135 | 88.2 | P:phosphorelay signal transduction system; F:sequence-specific DNA binding; C:intracellular |
| Efet.01.643464.g798.t1 | PAS domain-containing sensor histidine kinase | 2202 | 0 | 92.25 | F:phosphorelay sensor kinase activity; F:ATP binding; P:phosphorelay signal transduction system; C:integral component of membrane; P:signal transduction by protein phosphorylation; C:intracellular |
| Efet.01.643464.g799.t1 | DUF4390 domain-containing | 429 | 1.01E-97 | 83.4 |  |
| Efet.01.643474.g800.t1 | dopamine D2-like receptor | 477 | 6.01E-25 | 85 | F:G-protein coupled receptor activity; P:G-protein coupled receptor signaling pathway; C:integral component of membrane |
| Efet.01.643497.g801.t1 | GGDEF domain-containing | 1266 | 0 | 87.9 | F:phosphorelay sensor kinase activity; P:phosphorelay signal transduction system; P:signal transduction by protein phosphorylation; C:intracellular |
| Efet.01.643497.g802.t1 | choline ABC transporter substrate-binding | 951 | 0 | 84.95 | P:choline transport; F:choline binding; C:periplasmic space; F:transporter activity |
| Efet.01.643497.g803.t1 | formaldehyde glutathione-independent | 1044 | 0 | 93.75 | F:zinc ion binding; F:oxidoreductase activity; P:oxidation-reduction process |
| Efet.01.643497.g804.t1 | choline dehydrogenase | 1614 | 0 | 75.95 | P:glycine betaine biosynthetic process from choline; F:flavin adenine dinucleotide binding; P:oxidation-reduction process; F:choline dehydrogenase activity |
| Efet.01.643497.g805.t1 | L-serine ammonia-lyase | 1320 | 0 | 80.85 | F:L-serine ammonia-lyase activity; P:gluconeogenesis; F:4 iron, 4 sulfur cluster binding |
| Efet.01.643497.g806.t1 | prolipo diacylglyceryl transferase | 834 | 4.06E-158 | 89.5 | C:integral component of membrane; F:phosphatidylglycerol-prolipoprotein diacylglyceryl transferase activity; P:lipoprotein biosynthetic process; C:plasma membrane; P:protein lipoylation |
| Efet.01.643497.g807.t1 | dihydroxy-acid dehydratase | 1707 | 0 | 96 | P:isoleucine biosynthetic process; F:dihydroxy-acid dehydratase activity; F:metal ion binding; P:valine biosynthetic process; F:4 iron, 4 sulfur cluster binding |
| Efet.01.643497.g808.t1 | small Trp-rich | 249 | 5.19E-40 | 81.15 | C:integral component of membrane |
| Efet.01.643528.g810.t1 | cytoplasmic NCK2-like | 1008 | 2.36E-96 | 63.5 |  |
| Efet.01.643536.g811.t1 | cell division ATP-binding | 513 | 1.29E-61 | 75.1 | F:ATPase activity; P:cell division; F:ATP binding |
| Efet.01.643576.g812.t1 | cilia- and flagella-associated 44-like isoform X2 | 312 | 1.64E-18 | 61.95 |  |
| Efet.01.643605.g813.t1 | HMG box | 1407 | 1.75E-32 | 63.8 | P:developmental process |
| Efet.01.643637.g816.t1 | MMPL family transporter | 222 | 3.20E-31 | 86.75 | C:integral component of membrane |
| Efet.01.643637.g817.t1 | MMPL family transporter | 972 | 0 | 66.5 | C:membrane; C:integral component of membrane |
| Efet.01.643637.g818.t1 | 23S rRNA (guanosine(2251)-2 -O)-methyltransferase | 663 | 2.94E-136 | 90.55 | F:RNA methyltransferase activity; P:RNA methylation; F:RNA binding; P:RNA processing |
| Efet.01.643650.g819.t1 | DUF262 domain-containing | 996 | 0 | 73.2 |  |
| Efet.01.643669.g820.t1 | 2-hydroxyacid dehydrogenase | 354 | 2.26E-62 | 65 | F:oxidoreductase activity |
| Efet.01.643674.g821.t1 | Na+ H+ antiporter subunit E | 537 | 1.73E-37 | 84.9 | P:cation transmembrane transport; C:integral component of membrane; F:cation transmembrane transporter activity |
| Efet.01.643674.g822.t1 | K+ H+ antiporter subunit F | 237 | 1.82E-26 | 94.75 | P:ion transmembrane transport; F:ion transmembrane transporter activity; C:integral component of membrane |
| Efet.01.643674.g823.t1 | SsrA-binding protein | 474 | 1.58E-98 | 93.8 | F:RNA binding; C:cytoplasm; P:trans-translation |
| Efet.01.643674.g824.t1 | ubiquinone-binding | 789 | 4.64E-73 | 87.45 |  |
| Efet.01.643674.g825.t1 | DUF4124 domain-containing | 456 | 2.12E-42 | 73.45 |  |
| Efet.01.643704.g826.t1 | phosphoribosylformylglycinamidine synthase subunit | 1182 | 0 | 79.3 | F:magnesium ion binding; F:ATP binding; C:cytoplasm; P:'de novo' IMP biosynthetic process; F:phosphoribosylformylglycinamidine synthase activity |
| Efet.01.643731.g827.t1 | 16S rRNA (uracil(1498)-N(3))-methyltransferase | 228 | 9.78E-41 | 80.9 | C:cytoplasm; F:methyltransferase activity; P:rRNA processing; P:methylation |
| Efet.01.643740.g828.t1 | hypothetical protein CRN43_04345, partial | 1173 | 0 | 58.1 |  |
| Efet.01.643749.g829.t1 | hypothetical protein CRN43_14440 | 345 | 2.03E-09 | 61.5 |  |
| Efet.01.643824.g831.t1 | Hypothetical protein CBG16001 | 315 | 1.70E-16 | 66.05 |  |
| Efet.01.64385.g883.t1 | PREDICTED: caveolin-3-like | 279 | 4.76E-06 | 64 |  |
| Efet.01.643860.g833.t1 | acid-sensing ion channel 4-like | 618 | 5.29E-25 | 67 | P:sodium ion transport; C:membrane |
| Efet.01.643870.g834.t1 | SET and MYND domain-containing 4-like | 306 | 5.11E-17 | 60.25 |  |
| Efet.01.643898.g835.t1 | 3-oxoadipate -transferase | 477 | 1.25E-103 | 95.5 | P:metabolic process; F:3-oxoadipate CoA-transferase activity |
| Efet.01.643898.g836.t1 | 3-oxoadipyl- thiolase | 1092 | 0 | 96.15 | F:acetyl-CoA C-acetyltransferase activity; P:3,4-dihydroxybenzoate catabolic process; F:3-oxoadipyl-CoA thiolase activity |
| Efet.01.643898.g837.t1 | hypothetical protein | 582 | 1.98E-49 | 64.4 |  |
| Efet.01.643898.g838.t1 | hypothetical protein | 828 | 1.61E-133 | 80.55 |  |
| Efet.01.643898.g839.t1 | hypothetical protein | 417 | 2.04E-96 | 61.08 | C:membrane; C:integral component of membrane |
| Efet.01.643898.g840.t1 | family transcriptional regulator | 1005 | 5.94E-180 | 69.45 | F:DNA binding; F:transcription factor activity, sequence-specific DNA binding; P:transcription, DNA-templated; P:regulation of transcription, DNA-templated |
| Efet.01.643898.g841.t1 | phytanoyl- dioxygenase | 1884 | 0 | 74.65 | F:inositol 2-dehydrogenase activity; F:dioxygenase activity; P:oxidation-reduction process |
| Efet.01.643898.g842.t1 | tripartite tricarboxylate transporter substrate binding | 816 | 3.48E-172 | 72.05 | C:outer membrane-bounded periplasmic space |
| Efet.01.643899.g843.t1 | ATP-dependent RNA helicase | 1152 | 0 | 97.35 | F:nucleic acid binding; F:ATP binding; F:helicase activity |
| Efet.01.643899.g844.t1 | ornithine cyclodeaminase | 315 | 1.14E-35 | 85.1 | F:ornithine cyclodeaminase activity |
| Efet.01.643922.g846.t1 | branched-chain amino acid ABC transporter permease | 762 | 1.32E-64 | 91.8 | C:integral component of membrane; P:transport; C:plasma membrane; F:transporter activity |
| Efet.01.643922.g847.t1 | branched-chain amino acid ABC transporter permease | 555 | 3.57E-56 | 79.4 | C:integral component of membrane; P:transport; C:plasma membrane; F:transporter activity |
| Efet.01.643929.g848.t1 | hypothetical protein [Diaphorobacter polyhydroxybutyrativorans] | 525 | 6.98E-42 | 72.4 | C:integral component of membrane |
| Efet.01.643929.g849.t1 | hypothetical protein BA022_08525 | 393 | 7.54E-30 | 80.8 |  |
| Efet.01.64393.g884.t1 | hemicentin-1-like isoform X1 | 270 | 8.62E-10 | 68.6 | F:calcium ion binding |
| Efet.01.643930.g850.t1 | sterol regulatory element-binding 1 | 597 | 4.71E-30 | 59.35 | C:membrane; C:integral component of membrane; F:protein dimerization activity |
| Efet.01.643992.g852.t1 | cytochrome C | 846 | 3.19E-53 | 56.05 | F:electron carrier activity; C:membrane; C:integral component of membrane; F:heme binding |
| Efet.01.644010.g854.t1 | cystathionine gamma-synthase | 1308 | 0 | 92.65 | F:cystathionine gamma-synthase activity; F:pyridoxal phosphate binding; C:integral component of membrane |
| Efet.01.644010.g855.t1 | isochorismatase | 609 | 3.07E-118 | 88.8 | P:metabolic process; F:hydrolase activity |
| Efet.01.644010.g856.t1 | propionate-- ligase | 1866 | 0 | 88.1 | P:propionate catabolic process, 2-methylcitrate cycle; C:integral component of membrane; F:propionate-CoA ligase activity |
| Efet.01.644010.g857.t1 | hypothetical protein | 483 | 1.84E-113 | 63.3 |  |
| Efet.01.644010.g858.t1 | DUF1444 domain-containing | 960 | 0 | 66.65 |  |
| Efet.01.644010.g859.t1 | hypothetical protein BGO63_19145 | 585 | 7.25E-69 | 63.45 |  |
| Efet.01.644096.g863.t1 | dTDP-glucose 4,6-dehydratase | 840 | 0 | 81.05 | F:dTDP-glucose 4,6-dehydratase activity; P:nucleotide-sugar metabolic process; C:viral capsid |
| Efet.01.644096.g864.t1 | adenylosuccinate lyase | 894 | 2.09E-134 | 82.1 | F:N6-(1,2-dicarboxyethyl)AMP AMP-lyase (fumarate-forming) activity; P:'de novo' AMP biosynthetic process; P:'de novo' IMP biosynthetic process; F:(S)-2-(5-amino-1-(5-phospho-D-ribosyl)imidazole-4-carboxamido)succinate AMP-lyase (fumarate-forming) activity |
| Efet.01.644100.g865.t1 | oxidative stress-induced growth inhibitor 1 | 1044 | 1.36E-74 | 56.3 | P:negative regulation of cell growth; F:oxidoreductase activity; P:positive regulation of apoptotic process; P:oxidation-reduction process |
| Efet.01.644107.g866.t1 | hypothetical protein HELRODRAFT_180640 | 210 | 6.28E-23 | 67.15 | F:carbohydrate binding |
| Efet.01.644110.g867.t1 | carbohydrate ABC transporter permease | 519 | 6.34E-87 | 76.75 | C:integral component of membrane; P:transport; C:plasma membrane |
| Efet.01.644162.g869.t1 | hypothetical protein HELRODRAFT_166765 | 360 | 2.12E-32 | 67.3 | P:cellular aromatic compound metabolic process; P:small molecule metabolic process; P:heterocycle metabolic process; P:organic cyclic compound metabolic process; F:catalytic activity; C:membrane; P:organonitrogen compound metabolic process; P:cellular nitrogen compound metabolic process; P:single-organism cellular process; F:binding; P:primary metabolic process |
| Efet.01.644201.g871.t1 | glyco 3-alpha-L-fucosyltransferase A-like | 912 | 2.20E-20 | 50.8 | F:transferase activity, transferring glycosyl groups; F:glycoprotein 3-alpha-L-fucosyltransferase activity; F:fucosyltransferase activity; C:Golgi cisterna membrane; P:fucosylation; C:membrane; C:integral component of membrane; P:protein glycosylation; C:Golgi apparatus; F:transferase activity |
| Efet.01.64429.g885.t1 | Pre-mRNA-processing-splicing factor 8 | 834 | 0 | 94.25 | C:U5 snRNP; F:RNA binding; P:spliceosomal tri-snRNP complex assembly; F:second spliceosomal transesterification activity; F:U6 snRNA binding; F:U5 snRNA binding; F:pre-mRNA intronic binding; C:catalytic step 2 spliceosome; P:cellular response to lipopolysaccharide; F:K63-linked polyubiquitin binding; F:U1 snRNA binding; C:membrane; P:cellular response to tumor necrosis factor; F:U2 snRNA binding |
| Efet.01.644334.g873.t1 | neuronal acetylcholine receptor subunit alpha-10-like isoform X3 | 282 | 6.32E-12 | 72.35 | C:postsynaptic membrane; P:cation transmembrane transport; C:integral component of membrane; C:cell junction; F:extracellular ligand-gated ion channel activity |
| Efet.01.64436.g886.t1 | sulfurtransferase | 1434 | 0 | 82.6 | F:thiosulfate sulfurtransferase activity |
| Efet.01.64436.g887.t1 | metal-dependent enzyme of the double-stranded beta helix superfamily | 342 | 3.61E-76 | 93.6 |  |
| Efet.01.64436.g888.t1 | transcriptional regulator | 861 | 3.86E-173 | 94.35 | F:DNA binding; F:transcription factor activity, sequence-specific DNA binding; P:transcription, DNA-templated; P:regulation of transcription, DNA-templated |
| Efet.01.644388.g875.t1 | ABC-type glycerol-3-phosphate transport substrate-binding | 294 | 9.03E-62 | 72 |  |
| Efet.01.644427.g876.t1 | ribonucleoside-diphosphate reductase subunit alpha | 654 | 2.19E-119 | 86.1 | F:ATP binding; F:ribonucleoside-diphosphate reductase activity, thioredoxin disulfide as acceptor; P:DNA replication; P:oxidation-reduction process |
| Efet.01.644464.g878.t1 | DNA polymerase III subunit alpha | 2490 | 0 | 81.55 | F:DNA binding; F:3'-5' exonuclease activity; P:nucleic acid phosphodiester bond hydrolysis; F:DNA-directed DNA polymerase activity; C:cytoplasm; P:DNA replication; P:DNA biosynthetic process |
| Efet.01.644492.g879.t1 | ADP-dependent phosphofructokinase glucokinase | 687 | 3.13E-148 | 62.45 | P:carbohydrate metabolic process; F:phosphotransferase activity, alcohol group as acceptor |
| Efet.01.644507.g881.t1 | DEAD DEAH box helicase | 2034 | 0 | 83.85 | F:DNA binding; F:ATP binding; F:helicase activity |
| Efet.01.644507.g882.t1 | family transcriptional regulator | 1176 | 3.17E-160 | 61.2 | F:DNA binding; F:transcription factor activity, sequence-specific DNA binding; P:transcription, DNA-templated; P:regulation of transcription, DNA-templated |
| Efet.01.644507.g883.t1 | thiosulfate sulfurtransferase | 1713 | 0 | 65.95 |  |
| Efet.01.644507.g884.t1 | cysteine dioxygenase | 576 | 4.52E-135 | 66.55 | F:dioxygenase activity; P:oxidation-reduction process |
| Efet.01.644581.g887.t1 | TonB-dependent receptor | 771 | 2.99E-165 | 53.9 | C:membrane; C:cell outer membrane; F:receptor activity; P:transport |
| Efet.01.644595.g888.t1 | epidermal growth factor receptor-like | 582 | 5.22E-21 | 61.2 | F:protein kinase activity; P:phosphorylation |
| Efet.01.644657.g889.t1 | hypothetical protein LOTGIDRAFT_155849 | 582 | 9.97E-23 | 48.67 | F:DNA binding; F:DNA-directed 5'-3' RNA polymerase activity; P:transcription, DNA-templated |
| Efet.01.644691.g890.t1 | ABC transporter permease | 666 | 1.49E-75 | 81.6 | C:integral component of membrane; P:transport |
| Efet.01.644693.g891.t1 | transcription factor Sox-12-like | 1362 | 5.42E-39 | 67.7 |  |
| Efet.01.644732.g892.t1 | hemoglobin subunit mu-like | 207 | 7.19E-13 | 64.05 | F:binding |
| Efet.01.644746.g893.t1 | DUF883 domain-containing | 393 | 4.51E-33 | 94.6 | F:aldehyde dehydrogenase (quinone) activity; C:integral component of membrane; P:oxidation-reduction process; F:oxidoreductase activity, acting on the aldehyde or oxo group of donors, NAD or NADP as acceptor |
| Efet.01.644746.g894.t1 | phage holin family | 306 | 9.69E-48 | 81.25 | C:integral component of membrane |
| Efet.01.644746.g895.t1 | dihydroorotate dehydrogenase (quinone) | 1083 | 0 | 89.35 | P:'de novo' pyrimidine nucleobase biosynthetic process; C:cytoplasm; F:dihydroorotate dehydrogenase activity; P:oxidation-reduction process; P:'de novo' UMP biosynthetic process; C:plasma membrane |
| Efet.01.644747.g896.t1 | RND transporter | 1872 | 0 | 82.8 | C:integral component of membrane; P:transport; F:transporter activity |
| Efet.01.644765.g897.t1 | solute carrier organic anion transporter family member 4A1-like | 354 | 1.25E-25 | 75.8 | C:integral component of plasma membrane; P:sodium-independent organic anion transport; F:sodium-independent organic anion transmembrane transporter activity |
| Efet.01.644812.g900.t1 | family DNA-binding domain-containing | 336 | 1.60E-71 | 90.1 | F:DNA binding |
| Efet.01.644812.g901.t1 | Zn-dependent hydrolase | 441 | 3.54E-93 | 93.3 | F:hydrolase activity, acting on carbon-nitrogen (but not peptide) bonds, in linear amidines; P:metabolic process; F:N-carbamoyl-L-amino-acid hydrolase activity |
| Efet.01.644827.g902.t1 | ankyrin repeat and EF-hand domain-containing 1 | 297 | 3.01E-14 | 77.5 | F:calcium ion binding |
| Efet.01.644844.g903.t1 | autophagy-related 2 homolog B isoform X1 | 492 | 1.56E-23 | 55.05 | P:autophagy |
| Efet.01.644901.g904.t1 | dynein heavy chain axonemal-like | 444 | 1.91E-26 | 77.1 | F:ATPase activity; F:ATP binding; F:microtubule motor activity; C:dynein complex; P:microtubule-based movement |
| Efet.01.644910.g905.t1 | hypothetical protein HELRODRAFT_166765 | 204 | 2.78E-16 | 66.55 | P:cellular aromatic compound metabolic process; P:small molecule metabolic process; P:heterocycle metabolic process; P:organic cyclic compound metabolic process; F:catalytic activity; C:membrane; P:organonitrogen compound metabolic process; P:cellular nitrogen compound metabolic process; P:single-organism cellular process; F:binding; P:primary metabolic process |
| Efet.01.64498.g890.t1 | Prefoldin subunit 3 | 219 | 7.48E-16 | 71.5 | C:polysome; F:tubulin binding; P:tubulin complex assembly; C:cytoplasm; C:prefoldin complex; P:protein folding; P:microtubule-based process |
| Efet.01.644987.g909.t1 | ABC transporter permease | 894 | 1.75E-115 | 78.4 | C:integral component of membrane; P:transport; C:plasma membrane; F:transporter activity |
| Efet.01.644987.g910.t1 | ABC transporter ATP-binding | 1374 | 0 | 78.3 | F:ATPase activity; F:ATP binding |
| Efet.01.644987.g911.t1 | ABC transporter permease | 816 | 1.49E-98 | 82.25 | C:integral component of membrane; P:transport; C:plasma membrane; F:transporter activity |
| Efet.01.644987.g912.t1 | ABC transporter substrate-binding | 873 | 1.82E-154 | 71.75 |  |
| Efet.01.644987.g913.t1 | dehydrogenase | 618 | 9.35E-77 | 71.65 | F:zinc ion binding; F:oxidoreductase activity; P:oxidation-reduction process |
| Efet.01.644987.g914.t1 | glyoxalase bleomycin resistance dioxygenase family | 540 | 9.68E-77 | 74.5 | F:lyase activity |
| Efet.01.644987.g915.t1 | aldo keto reductase | 1056 | 0 | 87.2 | F:pyridoxal 4-dehydrogenase activity; P:oxidation-reduction process |
| Efet.01.644987.g916.t1 | dehydrogenase | 1899 | 1.59E-155 | 77.15 | F:oxidoreductase activity; P:oxidation-reduction process |
| Efet.01.644987.g917.t1 | alanine-phosphoribitol ligase | 1485 | 0 | 81.05 | F:oxidoreductase activity, acting on CH-OH group of donors; F:flavin adenine dinucleotide binding; P:oxidation-reduction process |
| Efet.01.644987.g918.t1 | sorbosone dehydrogenase | 540 | 6.83E-89 | 69.65 | F:oxidoreductase activity; P:metabolic process; P:oxidation-reduction process; F:oxidoreductase activity, acting on the aldehyde or oxo group of donors, NAD or NADP as acceptor |
| Efet.01.644990.g919.t1 | ABC transporter ATP-binding | 966 | 0 | 61.95 | F:nucleotide binding; F:ATPase activity; F:ATP binding |
| Efet.01.645006.g920.t1 | tubulin polymerization-promoting family member 2 | 525 | 8.87E-56 | 55.75 | F:tubulin binding; P:microtubule bundle formation; C:cytoplasm; F:calcium ion binding; P:microtubule polymerization |
| Efet.01.645029.g921.t1 | serine threonine- kinase mos-like | 651 | 4.45E-45 | 73.65 | F:ATP binding; F:protein serine/threonine kinase activity; P:protein phosphorylation |
| Efet.01.645040.g922.t1 | ribosomal- -alanine N-acetyltransferase | 519 | 6.42E-106 | 88.9 | P:N-terminal protein amino acid acetylation; C:ribosome; F:N-acetyltransferase activity |
| Efet.01.645063.g924.t1 | Serine threonine- kinase WNK3 | 216 | 4.47E-23 | 83 | F:ATP binding; F:protein serine/threonine kinase activity; P:protein phosphorylation |
| Efet.01.645075.g925.t1 | tyrosine- kinase Src42A-like | 405 | 8.85E-68 | 90.35 | F:ATP binding; P:peptidyl-tyrosine phosphorylation; F:non-membrane spanning protein tyrosine kinase activity |
| Efet.01.645105.g926.t1 | type II secretion system | 759 | 1.67E-47 | 74.05 | C:integral component of membrane; F:protein transporter activity; P:protein secretion by the type II secretion system; C:type II protein secretion system complex |
| Efet.01.645105.g927.t1 | type II secretion system | 438 | 2.28E-71 | 76.35 | C:integral component of membrane; F:protein transporter activity; P:protein secretion by the type II secretion system; C:type II protein secretion system complex |
| Efet.01.645105.g928.t1 | general secretion pathway | 2022 | 0 | 75.95 | P:polysaccharide catabolic process; F:carbohydrate binding; P:protein secretion; C:outer membrane |
| Efet.01.645105.g929.t1 | secretion system X translation initiation factor | 492 | 4.21E-14 | 67.8 | F:translation initiation factor activity; P:translational initiation |
| Efet.01.645105.g930.t1 | secretion system X transmembrane 2 | 222 | 1.86E-16 | 69.4 | C:membrane |
| Efet.01.645105.g931.t1 | hypothetical protein | 1206 | 1.22E-57 | 55.35 | C:membrane; C:integral component of membrane |
| Efet.01.645117.g932.t1 | transcription factor Sox-14-like | 555 | 2.12E-06 | 89.25 | P:regulation of transcription, DNA-templated |
| Efet.01.645133.g933.t1 | flagellar biosynthesis | 297 | 1.34E-51 | 79.3 | P:bacterial-type flagellum organization; C:integral component of membrane |
| Efet.01.645133.g934.t1 | flagellar biosynthetic | 702 | 2.14E-142 | 96.3 | P:bacterial-type flagellum organization; C:integral component of membrane; P:protein secretion; C:plasma membrane; C:bacterial-type flagellum basal body |
| Efet.01.645133.g935.t1 | flagellar biosynthetic | 258 | 1.14E-42 | 92.1 | C:motile cilium; P:bacterial-type flagellum assembly; C:integral component of membrane; P:protein secretion |
| Efet.01.645138.g936.t1 | family transcriptional regulator | 915 | 9.34E-173 | 83.6 | F:DNA binding; F:transcription factor activity, sequence-specific DNA binding; P:transcription, DNA-templated; P:regulation of transcription, DNA-templated |
| Efet.01.645138.g937.t1 | tripartite tricarboxylate transporter substrate binding | 351 | 1.28E-46 | 86.7 | C:outer membrane-bounded periplasmic space |
| Efet.01.645138.g938.t1 | tripartite tricarboxylate transporter substrate binding | 354 | 1.38E-59 | 86.75 | C:outer membrane-bounded periplasmic space |
| Efet.01.645138.g939.t1 | ABC transporter ATP-binding | 324 | 2.08E-25 | 97.1 | F:ATPase activity; F:ATP binding |
| Efet.01.645138.g940.t1 | ABC transporter ATP-binding | 525 | 2.30E-107 | 92.35 | F:ATPase activity; F:ATP binding |
| Efet.01.645138.g941.t1 | branched-chain amino acid ABC transporter permease | 843 | 6.64E-98 | 90.6 | C:integral component of membrane; P:transport; C:plasma membrane; F:transporter activity |
| Efet.01.645138.g942.t1 | ABC transporter substrate-binding | 1869 | 0 | 94.2 | P:amino acid transport |
| Efet.01.645138.g943.t1 | family transporter | 906 | 1.11E-167 | 82.95 | C:integral component of membrane |
| Efet.01.645138.g944.t1 | phosphoesterase | 615 | 6.97E-145 | 89.4 | F:nucleic acid binding |
| Efet.01.645147.g945.t1 | sarcoplasmic calcium-binding | 225 | 1.40E-22 | 65.05 | F:calcium ion binding |
| Efet.01.645152.g946.t1 | receptor-type guanylate cyclase Gyc76C-like | 459 | 1.58E-17 | 58.6 | F:lyase activity; P:primary metabolic process; P:single-organism process; P:organic substance metabolic process; P:phosphate-containing compound metabolic process |
| Efet.01.645197.g948.t1 | elongation factor 4 | 855 | 5.95E-80 | 95.95 | F:GTP binding; P:positive regulation of translation; F:translation elongation factor activity; F:GTPase activity; F:ribosome binding; P:translational elongation; C:plasma membrane |
| Efet.01.645199.g949.t1 | DUF58 domain-containing | 1077 | 3.61E-151 | 60.75 |  |
| Efet.01.645199.g950.t1 | family ATPase | 954 | 0 | 73.6 | F:ATPase activity; F:ATP binding |
| Efet.01.645199.g951.t1 | methanol dehydrogenase | 276 | 3.16E-47 | 86.6 | F:methanol ferricytochrome-c oxidoreductase activity; P:methanol oxidation; F:alcohol dehydrogenase (NAD) activity; F:ethanol cytochrome-c oxidoreductase activity; F:2-chloroethanol cytochrome-c oxidoreductase activity |
| Efet.01.645199.g952.t1 | cytochrome c(L) periplasmic | 411 | 3.76E-97 | 77 | F:iron ion binding; F:oxidoreductase activity; F:electron carrier activity; C:periplasmic space; F:heme binding |
| Efet.01.645199.g953.t1 | methanol oxidation system | 678 | 2.71E-159 | 73.5 | C:periplasmic space; P:methanol catabolic process |
| Efet.01.645199.g954.t1 | PQQ-dependent methanol ethanol family | 1743 | 0 | 86.5 | F:methanol ferricytochrome-c oxidoreductase activity; F:calcium ion binding; C:membrane; C:outer membrane-bounded periplasmic space; P:methanol oxidation; F:alcohol dehydrogenase (acceptor) activity; F:ethanol cytochrome-c oxidoreductase activity; F:2-chloroethanol cytochrome-c oxidoreductase activity |
| Efet.01.645199.g955.t1 | family transcriptional regulator | 1047 | 0 | 61.7 | F:transcription factor activity, sequence-specific DNA binding; P:transcription, DNA-templated; P:regulation of transcription, DNA-templated; F:sequence-specific DNA binding |
| Efet.01.645199.g956.t1 | hydantoinase oxoprolinase family | 1989 | 0 | 65.75 | F:hydrolase activity |
| Efet.01.645199.g957.t1 | hydantoinase oxoprolinase family | 1158 | 0 | 81.1 | F:5-oxoprolinase (ATP-hydrolyzing) activity; F:ligase activity |
| Efet.01.645236.g959.t1 | outer dense fiber 2-like isoform X3 | 207 | 2.76E-06 | 79 |  |
| Efet.01.645263.g960.t1 | triose-phosphate isomerase | 726 | 5.58E-128 | 75.75 | P:gluconeogenesis; C:cytoplasm; P:pentose-phosphate shunt; P:glycolytic process; F:triose-phosphate isomerase activity |
| Efet.01.645268.g961.t1 | arabinose ABC transporter substrate-binding | 918 | 0 | 80.3 | P:L-arabinose transport; C:periplasmic space; F:hydrolase activity |
| Efet.01.645268.g962.t1 | L-arabinose ABC transporter ATP-binding | 1563 | 0 | 82.3 | F:ATP binding; P:monosaccharide transport; F:monosaccharide-transporting ATPase activity |
| Efet.01.645268.g963.t1 | L-arabinose ABC transporter permease | 948 | 0 | 91.1 | C:integral component of membrane; P:monosaccharide transport; F:monosaccharide-transporting ATPase activity; C:plasma membrane |
| Efet.01.645268.g964.t1 | short-chain dehydrogenase | 720 | 8.71E-148 | 88.75 | F:oxidoreductase activity; P:oxidation-reduction process |
| Efet.01.645268.g965.t1 | 2-dehydro-3-deoxy-6-phosphogalactonate aldolase | 372 | 7.12E-57 | 79.85 | P:metabolic process; F:2-dehydro-3-deoxy-6-phosphogalactonate aldolase activity |
| Efet.01.645268.g966.t1 | galactonate dehydratase | 1086 | 0 | 85.6 | F:magnesium ion binding; P:cellular amino acid catabolic process; F:galactonate dehydratase activity; P:D-galactonate catabolic process |
| Efet.01.645268.g967.t1 | glutathione S-transferase | 693 | 2.02E-170 | 93.35 | F:glutathione transferase activity |
| Efet.01.645268.g968.t1 | integrase | 309 | 2.59E-46 | 89.95 | F:nucleic acid binding; P:DNA integration |
| Efet.01.645268.g969.t1 | tripartite tricarboxylate transporter substrate binding | 531 | 1.11E-73 | 86.65 | C:outer membrane-bounded periplasmic space |
| Efet.01.645275.g970.t1 | hypothetical protein | 597 | 4.97E-126 | 67.45 |  |
| Efet.01.645275.g971.t1 | Flp pilus assembly | 921 | 0 | 74.6 | C:integral component of membrane |
| Efet.01.645275.g972.t1 | type II and III secretion system | 1266 | 0 | 86.55 | F:protein transporter activity; P:protein secretion |
| Efet.01.645279.g973.t1 | nucleolar 4-like | 420 | 7.43E-26 | 69.2 |  |
| Efet.01.645281.g974.t1 | polyribonucleotide nucleotidyltransferase | 795 | 3.33E-134 | 83.25 | F:magnesium ion binding; P:RNA phosphodiester bond hydrolysis, exonucleolytic; F:3'-5'-exoribonuclease activity; F:RNA binding; C:cytoplasm; P:RNA processing; P:mRNA catabolic process; F:polyribonucleotide nucleotidyltransferase activity |
| Efet.01.645338.g975.t1 | urease subunit alpha | 900 | 3.62E-125 | 84.35 | P:urea catabolic process; C:cytoplasm; F:nickel cation binding; F:urease activity |
| Efet.01.645362.g977.t1 | solute carrier family 15 member 4-like | 765 | 8.73E-39 | 56.9 | P:oligopeptide transport; C:membrane; C:integral component of membrane; P:transport; F:transporter activity |
| Efet.01.645367.g978.t1 | cyclic nucleotide-gated cation channel alpha-3 | 219 | 6.24E-17 | 69.45 | P:cation transmembrane transport; C:integral component of membrane; F:intracellular cyclic nucleotide activated cation channel activity |
| Efet.01.645400.g980.t1 | hypothetical protein CAPTEDRAFT_224725 | 390 | 2.19E-25 | 58.89 | C:integral component of membrane |
| Efet.01.645412.g981.t1 | RNA polymerase sigma-70 ECF subfamily | 312 | 1.70E-55 | 69.55 | F:sigma factor activity; F:DNA binding; F:transcription factor activity, sequence-specific DNA binding; P:transcription, DNA-templated; P:regulation of transcription, DNA-templated; P:DNA-templated transcription, initiation |
| Efet.01.645413.g982.t1 | PRKCA-binding -like isoform X2 | 204 | 1.28E-15 | 81.1 | F:protein domain specific binding |
| Efet.01.645424.g983.t1 | dihydropyrimidinase | 1464 | 0 | 86.15 | C:cytoplasm; F:dihydropyrimidinase activity |
| Efet.01.645424.g984.t1 | polar amino acid ABC transporter substrate-binding | 723 | 3.97E-166 | 87 |  |
| Efet.01.645424.g985.t1 | amino acid ABC transporter ATP-binding | 612 | 1.03E-114 | 84.2 | F:ATP binding; P:amino acid transmembrane transport; F:amino acid-transporting ATPase activity |
| Efet.01.645424.g986.t1 | amino acid ABC transporter permease | 468 | 5.52E-86 | 89.2 | C:integral component of membrane; P:transport; C:plasma membrane; F:transporter activity |
| Efet.01.645424.g987.t1 | amino acid ABC transporter permease | 612 | 1.72E-102 | 87.8 | C:integral component of membrane; P:transport; C:plasma membrane; F:transporter activity |
| Efet.01.645478.g988.t1 | glutamate receptor 4-like | 321 | 2.50E-56 | 84.25 | C:postsynaptic membrane; P:ion transmembrane transport; C:integral component of membrane; C:cell junction; P:ionotropic glutamate receptor signaling pathway; F:ionotropic glutamate receptor activity; F:extracellular-glutamate-gated ion channel activity |
| Efet.01.645500.g989.t1 | family | 828 | 0 | 91.25 | F:ATPase activity; F:ATP binding |
| Efet.01.645500.g990.t1 | amino acid ABC transporter substrate-binding | 3336 | 0 | 96.35 | P:amino acid transport |
| Efet.01.645500.g991.t1 | DUF4209 domain-containing | 1674 | 0 | 79.75 |  |
| Efet.01.645507.g992.t1 | peroxidasin-like isoform X4 | 213 | 1.72E-08 | 63 |  |
| Efet.01.645508.g993.t1 | phosphatidate phosphatase LPIN1-like isoform X1 | 234 | 5.63E-20 | 87.55 |  |
| Efet.01.645589.g994.t1 | 3-oxoacyl-ACP reductase | 774 | 3.71E-160 | 88.35 |  |
| Efet.01.645629.g995.t1 | DUF2079 domain-containing | 1125 | 0 | 52.2 | C:membrane; C:integral component of membrane |
| Efet.01.645717.g999.t1 | BTB POZ domain-containing 6 isoform X1 | 996 | 2.34E-26 | 44.7 |  |
| Efet.01.645758.g1002.t1 | hypothetical protein CAPTEDRAFT_220517 | 756 | 1.51E-14 | 53 |  |
| Efet.01.645778.g1003.t1 | voltage-dependent calcium channel gamma-5 subunit-like | 294 | 4.84E-17 | 75.91 | P:calcium ion transport; C:integral component of membrane |
| Efet.01.645850.g1006.t1 | DUF1844 domain-containing | 849 | 2.80E-46 | 86.5 |  |
| Efet.01.64587.g891.t1 | sodium channel type 4 subunit alpha B-like isoform X1 | 342 | 5.90E-17 | 74.05 | P:regulation of postsynaptic membrane potential; F:calcium ion binding; P:regulation of ion transmembrane transport; C:voltage-gated sodium channel complex; P:sodium ion transmembrane transport; P:membrane depolarization during action potential; F:voltage-gated sodium channel activity; P:neuronal action potential |
| Efet.01.645930.g1007.t1 | DUF2029 domain-containing | 222 | 1.32E-32 | 83.5 | F:transferase activity, transferring hexosyl groups; C:membrane; C:integral component of membrane |
| Efet.01.645930.g1008.t1 | ABC transporter permease | 642 | 5.85E-103 | 64.2 | C:integral component of membrane; C:plasma membrane |
| Efet.01.645947.g1010.t1 | NAD+ synthase | 1014 | 2.13E-153 | 84.7 | F:hydrolase activity, acting on carbon-nitrogen (but not peptide) bonds; F:NAD+ synthase (glutamine-hydrolyzing) activity; F:ATP binding; P:NAD biosynthetic process |
| Efet.01.645947.g1011.t1 | N-acetyltransferase | 1254 | 0 | 80.3 |  |
| Efet.01.645947.g1012.t1 | transferase | 657 | 3.20E-116 | 94.6 | F:formyl-CoA transferase activity |
| Efet.01.645947.g1013.t1 | acyl- dehydrogenase | 1368 | 0 | 88.7 | F:acyl-CoA dehydrogenase activity; F:flavin adenine dinucleotide binding; P:oxidation-reduction process |
| Efet.01.645947.g1014.t1 | chemotaxis | 699 | 8.87E-149 | 87.9 |  |
| Efet.01.645947.g1015.t1 | DNA-binding response regulator | 261 | 9.86E-53 | 94.2 | F:DNA binding; P:transcription, DNA-templated; P:phosphorelay signal transduction system; P:regulation of transcription, DNA-templated; C:intracellular |
| Efet.01.645971.g1016.t1 | cyclohexanecarboxylate- ligase | 1590 | 0 | 83.95 | P:metabolic process; F:o-succinylbenzoate-CoA ligase activity |
| Efet.01.645971.g1017.t1 | cyclohexanecarboxyl- dehydrogenase | 1020 | 0 | 92.45 | F:acyl-CoA dehydrogenase activity; F:flavin adenine dinucleotide binding; P:oxidation-reduction process |
| Efet.01.645971.g1018.t1 | 2-ketocyclohexanecarboxyl- hydrolase | 783 | 0 | 95.9 | F:1,4-dihydroxy-2-naphthoyl-CoA synthase activity; P:menaquinone biosynthetic process; F:hydrolase activity |
| Efet.01.645971.g1019.t1 | 2-hydroxycyclohexanecarboxyl- dehydrogenase | 657 | 6.55E-129 | 90.05 | F:3-oxoacyl-[acyl-carrier-protein] reductase (NADPH) activity; P:oxidation-reduction process |
| Efet.01.645993.g1020.t1 | hypothetical protein CAPTEDRAFT_104243, partial | 645 | 7.87E-08 | 78 | F:ion channel activity; P:ion transmembrane transport; C:synapse; C:membrane part; C:plasma membrane |
| Efet.01.645994.g1021.t1 | family nutrient-binding outer membrane lipo | 945 | 1.28E-160 | 77.25 |  |
| Efet.01.646005.g1022.t1 | dedicator of cytokinesis 1-like | 207 | 4.08E-21 | 73.95 | P:cytoskeleton organization; P:small GTPase mediated signal transduction; F:guanyl-nucleotide exchange factor activity; F:GTPase activator activity; P:cell migration; C:intracellular; P:positive regulation of GTPase activity |
| Efet.01.646006.g1023.t1 | FAD-binding oxidoreductase | 453 | 2.54E-70 | 86.5 | F:oxidoreductase activity, acting on CH-OH group of donors; F:flavin adenine dinucleotide binding; F:alkylglycerone-phosphate synthase activity; P:lipid biosynthetic process; P:oxidation-reduction process |
| Efet.01.646009.g1024.t1 | enoyl- hydratase | 819 | 2.52E-156 | 81.85 | P:metabolic process; F:enoyl-CoA hydratase activity |
| Efet.01.646009.g1025.t1 | family transcriptional regulator | 420 | 6.16E-95 | 90.15 | F:DNA binding; F:transcription factor activity, sequence-specific DNA binding; P:transcription, DNA-templated; P:regulation of transcription, DNA-templated |
| Efet.01.646025.g1027.t1 | transcriptional contains HTH domain | 681 | 3.24E-46 | 51 |  |
| Efet.01.646033.g1028.t1 | conditioned medium factor receptor 1-like | 231 | 2.16E-25 | 73.4 | F:FAD binding; F:oxidoreductase activity; C:integral component of membrane; P:oxidation-reduction process |
| Efet.01.646046.g1029.t1 | PX domain | 240 | 2.88E-27 | 87.1 | F:phosphatidylinositol binding |
| Efet.01.646057.g1030.t1 | unsaturated rhamnogalacturonyl hydrolase | 1068 | 0 | 61.25 | F:catalytic activity |
| Efet.01.646057.g1031.t1 | hypothetical protein B5766_00800 | 561 | 2.89E-125 | 52.9 |  |
| Efet.01.646092.g1032.t1 | sodium-translocating pyrophosphatase | 2082 | 0 | 94.05 | F:magnesium ion binding; C:integral component of plasma membrane; F:inorganic diphosphatase activity; P:proton transport; P:transmembrane transport; F:hydrogen-translocating pyrophosphatase activity |
| Efet.01.646099.g1033.t1 | probable methyltransferase 15 homolog | 597 | 2.13E-61 | 68.35 | F:transferase activity |
| Efet.01.646105.g1034.t1 | plasma membrane calcium-transporting ATPase 2 isoform X1 | 288 | 3.06E-29 | 82.85 |  |
| Efet.01.646112.g1035.t1 | carboxypeptidase D-like | 270 | 9.39E-12 | 68.2 | F:carboxypeptidase activity; F:zinc ion binding; F:metallocarboxypeptidase activity; P:proteolysis |
| Efet.01.646129.g1036.t1 | ATP-binding | 246 | 8.89E-26 | 90.6 | F:ATP binding |
| Efet.01.646144.g1037.t1 | calcium-activated potassium channel | 318 | 1.68E-34 | 85.35 | F:voltage-gated potassium channel activity; P:potassium ion transmembrane transport; C:voltage-gated potassium channel complex; F:large conductance calcium-activated potassium channel activity |
| Efet.01.646145.g1038.t1 | serine arginine-rich splicing factor 7-like isoform X2 | 273 | 3.44E-30 | 78.3 | F:nucleotide binding; F:nucleic acid binding; F:zinc ion binding |
| Efet.01.646149.g1039.t1 | nitrite reductase | 1569 | 0 | 92.45 | F:oxidoreductase activity; F:metal ion binding; P:oxidation-reduction process; F:4 iron, 4 sulfur cluster binding; F:heme binding |
| Efet.01.646149.g1040.t1 | amino acid transporter | 576 | 3.93E-63 | 71.1 | C:integral component of membrane; P:amino acid transport; C:plasma membrane |
| Efet.01.646149.g1041.t1 | alanine racemase | 1167 | 0 | 87.6 | F:pyridoxal phosphate binding; F:alanine racemase activity; P:D-alanine biosynthetic process |
| Efet.01.646149.g1042.t1 | lysophospholipid transporter | 1095 | 0 | 91.6 | P:transmembrane transport; C:integral component of membrane |
| Efet.01.646149.g1043.t1 | outer membrane | 426 | 1.40E-30 | 80.95 |  |
| Efet.01.646149.g1044.t1 | plasmid stabilization | 300 | 3.52E-66 | 88.4 |  |
| Efet.01.646163.g1045.t1 | 60S ribosomal L8 | 264 | 2.54E-46 | 92 | F:RNA binding; F:structural constituent of ribosome; C:large ribosomal subunit; P:translation |
| Efet.01.646178.g1046.t1 | ABC transporter substrate-binding | 219 | 1.02E-40 | 79.85 | P:carbohydrate transport |
| Efet.01.646179.g1047.t1 | ABC transporter permease | 1347 | 2.07E-91 | 86.3 | C:integral component of membrane; P:transport; C:plasma membrane; F:transporter activity |
| Efet.01.646179.g1048.t1 | DUF1993 domain-containing | 510 | 2.11E-118 | 80.5 |  |
| Efet.01.646179.g1049.t1 | gluconolactonase | 933 | 0 | 90.4 | F:gluconolactonase activity |
| Efet.01.646179.g1050.t1 | malate lactate ureidoglycolate dehydrogenase | 912 | 0 | 89.25 | F:oxidoreductase activity; P:oxidation-reduction process |
| Efet.01.646179.g1051.t1 | UDP-3-O-[3-hydroxymyristoyl] N-acetylglucosamine deacetylase | 924 | 0 | 96.1 | F:UDP-3-O-[3-hydroxymyristoyl] N-acetylglucosamine deacetylase activity; F:transferase activity, transferring acyl groups; F:UDP-3-O-acyl-N-acetylglucosamine deacetylase activity; F:metal ion binding; P:lipid A biosynthetic process |
| Efet.01.646179.g1052.t1 | family | 267 | 3.45E-56 | 89.25 | F:transferase activity |
| Efet.01.646256.g1057.t1 | dynein heavy chain axonemal-like | 297 | 6.06E-38 | 87.4 | F:ATPase activity; F:ATP binding; F:microtubule motor activity; C:axonemal dynein complex; P:cilium movement |
| Efet.01.646269.g1059.t1 | hypothetical protein LOTGIDRAFT_166880 | 408 | 3.08E-08 | 52 | F:ligand-dependent nuclear receptor transcription coactivator activity; P:regulation of nucleic acid-templated transcription |
| Efet.01.646274.g1060.t1 | formimidoylglutamate deiminase | 2163 | 0 | 79.65 | F:formimidoylglutamate deiminase activity |
| Efet.01.646274.g1061.t1 | N-formylglutamate deformylase | 864 | 4.84E-177 | 88.4 | P:peptidoglycan catabolic process; F:N-acetylmuramoyl-L-alanine amidase activity |
| Efet.01.646274.g1062.t1 | serine--tRNA ligase | 1317 | 0 | 91.4 | P:selenocysteinyl-tRNA(Sec) biosynthetic process; F:ATP binding; C:cytoplasm; P:selenocysteine biosynthetic process; F:serine-tRNA ligase activity; P:seryl-tRNA aminoacylation |
| Efet.01.646274.g1063.t1 | hypothetical protein | 288 | 8.51E-63 | 77 |  |
| Efet.01.646274.g1064.t1 | family transcriptional regulator | 879 | 0 | 65.95 | F:DNA binding; F:transcription factor activity, sequence-specific DNA binding; P:transcription, DNA-templated; P:regulation of transcription, DNA-templated |
| Efet.01.646274.g1065.t1 | PIN domain-containing | 312 | 5.00E-65 | 86.95 | F:magnesium ion binding; P:RNA phosphodiester bond hydrolysis; F:ribonuclease activity |
| Efet.01.646274.g1066.t1 | plasmid stabilization | 255 | 1.77E-41 | 92.1 | F:DNA binding; P:regulation of transcription, DNA-templated |
| Efet.01.646274.g1067.t1 | 3-oxoacyl-ACP reductase | 606 | 1.47E-128 | 89.2 | F:diacetyl reductase ((S)-acetoin forming) activity; P:oxidation-reduction process |
| Efet.01.646280.g1068.t1 | L-serine ammonia-lyase | 846 | 5.45E-150 | 73.2 | F:lyase activity |
| Efet.01.646280.g1069.t1 | sarcosine oxidase subunit gamma | 657 | 3.14E-143 | 59.05 |  |
| Efet.01.646283.g1070.t1 | PREDICTED: uncharacterized protein LOC106060435 | 528 | 1.60E-07 | 49 |  |
| Efet.01.646300.g1071.t1 | cilia- and flagella-associated 157-like | 279 | 8.65E-30 | 84.8 | C:integral component of membrane |
| Efet.01.646312.g1072.t1 | DUF445 domain-containing | 495 | 7.44E-79 | 85.95 | C:integral component of membrane |
| Efet.01.646331.g1073.t1 | TELO2-interacting 1 homolog | 276 | 1.08E-12 | 67.4 |  |
| Efet.01.646337.g1075.t1 | protocadherin-17 isoform X3 | 1491 | 3.79E-75 | 55.55 | F:calcium ion binding; C:membrane; C:integral component of membrane; P:cell adhesion; P:homophilic cell adhesion via plasma membrane adhesion molecules; C:plasma membrane |
| Efet.01.646364.g1076.t1 | glutamine amidotransferase | 717 | 1.08E-165 | 77.7 | F:catalytic activity; P:cobalamin biosynthetic process |
| Efet.01.64637.g892.t1 | transcription factor Sox-1a-like | 792 | 1.57E-31 | 79.4 | P:nervous system development; P:regulation of transcription, DNA-templated |
| Efet.01.64641.g893.t1 | CREB3 regulatory factor | 789 | 1.04E-07 | 67.1 | F:transcription factor activity, sequence-specific DNA binding; P:regulation of transcription, DNA-templated; F:sequence-specific DNA binding |
| Efet.01.646417.g1077.t1 | 60S ribosomal L12-like | 357 | 7.06E-48 | 95.55 | F:structural constituent of ribosome; C:cytosolic large ribosomal subunit; F:rRNA binding; P:ribosomal large subunit assembly; P:translation |
| Efet.01.646419.g1078.t1 | T family of potassium channels 7-like | 327 | 1.04E-12 | 69.35 | P:ion transport; C:membrane |
| Efet.01.646421.g1079.t1 | gfo Idh family oxidoreductase | 654 | 1.13E-156 | 47.3 | F:oxidoreductase activity; P:metabolic process; P:oxidation-reduction process |
| Efet.01.646431.g1080.t1 | alpha-L-fucosidase | 1209 | 0 | 75.8 | P:carbohydrate metabolic process |
| Efet.01.646436.g1081.t1 | RND transporter | 1332 | 0 | 79.65 | C:integral component of membrane; F:lipid binding; P:transport; C:plasma membrane; F:transporter activity |
| Efet.01.646436.g1082.t1 | efflux RND transporter periplasmic adaptor subunit | 1269 | 0 | 82.6 | P:transmembrane transport; C:integral component of membrane |
| Efet.01.646437.g1083.t1 | cold-shock | 204 | 1.89E-41 | 93.05 | F:DNA binding; C:cytoplasm; P:transcription, DNA-templated; P:regulation of transcription, DNA-templated |
| Efet.01.646487.g1085.t1 | PAS domain-containing sensor histidine kinase | 1542 | 0 | 86.15 | F:phosphorelay sensor kinase activity; F:ATP binding; P:phosphorelay signal transduction system; C:integral component of membrane; P:regulation of transcription, DNA-templated; P:signal transduction by protein phosphorylation; C:intracellular |
| Efet.01.646487.g1086.t1 | DNA-binding response regulator | 498 | 3.47E-97 | 94 | F:DNA binding; P:transcription, DNA-templated; P:phosphorelay signal transduction system; P:regulation of transcription, DNA-templated; P:phosphorylation; F:kinase activity; C:intracellular |
| Efet.01.646487.g1087.t1 | transglycosylase | 981 | 0 | 87.5 | P:peptidoglycan turnover; F:hydrolase activity, hydrolyzing O-glycosyl compounds; C:outer membrane |
| Efet.01.646487.g1089.t1 | tripartite tricarboxylate transporter substrate binding | 681 | 5.64E-129 | 73.05 | C:outer membrane-bounded periplasmic space |
| Efet.01.646487.g1090.t1 | diaminopropionate ammonia-lyase | 1194 | 0 | 86.7 | F:diaminopropionate ammonia-lyase activity; F:pyridoxal phosphate binding |
| Efet.01.646487.g1091.t1 | family translocator | 633 | 2.12E-132 | 62.3 | C:integral component of membrane; P:amino acid transport; C:plasma membrane |
| Efet.01.646487.g1092.t1 | translation initiation factor IF-1 | 228 | 7.98E-48 | 98.9 | F:translation initiation factor activity; C:cytoplasm; F:ribosome binding; F:rRNA binding; P:translational initiation |
| Efet.01.646487.g1093.t1 | ABC transporter permease | 1419 | 0 | 93.9 |  |
| Efet.01.646487.g1094.t1 | branched-chain amino acid ABC transporter permease | 864 | 7.12E-149 | 91.9 | C:integral component of membrane; P:transport; C:plasma membrane; F:transporter activity |
| Efet.01.646487.g1095.t1 | branched-chain amino acid ABC transporter permease | 897 | 3.90E-160 | 90.95 | C:integral component of membrane; P:transport; C:plasma membrane; F:transporter activity |
| Efet.01.646487.g1096.t1 | ABC transporter ATP-binding | 612 | 7.95E-146 | 89.6 | F:ATPase activity; F:ATP binding |
| Efet.01.646487.g1097.t1 | ABC transporter ATP-binding | 708 | 1.73E-169 | 92.2 | F:ATPase activity; F:ATP binding |
| Efet.01.646487.g1098.t1 | NAD(P)-dependent oxidoreductase | 828 | 1.92E-170 | 87.25 | F:3-oxoacyl-[acyl-carrier-protein] reductase (NADPH) activity; P:oxidation-reduction process |
| Efet.01.646487.g1099.t1 | ABC transporter substrate-binding | 1014 | 0 | 84.85 | C:integral component of membrane; C:outer membrane-bounded periplasmic space; P:transport |
| Efet.01.646487.g1100.t1 | membrane | 1629 | 0 | 75.3 | C:integral component of membrane |
| Efet.01.646487.g1101.t1 | 16S rRNA (uracil(1498)-N(3))-methyltransferase | 822 | 1.06E-159 | 77.8 | C:cytoplasm; F:methyltransferase activity; P:rRNA processing; P:methylation |
| Efet.01.646487.g1102.t1 | aminoglycoside phosphotransferase | 462 | 3.67E-104 | 94.9 | F:transferase activity |
| Efet.01.646556.g1103.t1 | pyruvate mitochondrial isoform X1 | 270 | 3.85E-51 | 88.45 | F:DNA binding; F:ATP binding; P:gluconeogenesis; P:habituation; F:pyruvate carboxylase activity; F:metal ion binding; F:biotin binding; F:biotin carboxylase activity; P:pyruvate metabolic process |
| Efet.01.646577.g1104.t1 | histidine phosphatase family | 438 | 8.22E-73 | 66.35 |  |
| Efet.01.646577.g1105.t1 | phosphate phosphite phosphonate ABC transporter substrate-binding | 921 | 1.86E-179 | 70.6 | P:transmembrane transport; C:ATP-binding cassette (ABC) transporter complex |
| Efet.01.646642.g1106.t1 | Ferrochelatase | 1278 | 0 | 89.6 | F:ferrochelatase activity; C:cytoplasm; F:metal ion binding; P:heme biosynthetic process |
| Efet.01.646642.g1107.t1 | transcriptional regulator | 1866 | 0 | 89.95 | F:ATP binding |
| Efet.01.646642.g1108.t1 | recombinase family | 252 | 1.59E-16 | 69.45 | F:DNA binding; F:recombinase activity; P:DNA recombination |
| Efet.01.646642.g1109.t1 | DUF1016 domain-containing | 990 | 9.41E-138 | 86.85 |  |
| Efet.01.646642.g1110.t1 | restriction endonuclease | 2484 | 0 | 84.2 |  |
| Efet.01.646642.g1111.t1 | tripartite tricarboxylate transporter substrate binding | 828 | 3.17E-172 | 65.4 | C:outer membrane-bounded periplasmic space |
| Efet.01.646642.g1112.t1 | family transporter | 849 | 1.96E-121 | 80.35 | C:integral component of membrane |
| Efet.01.646642.g1113.t1 | autotransporter domain-containing | 1344 | 2.38E-52 | 56.7 |  |
| Efet.01.646642.g1114.t1 | AAA family ATPase | 1311 | 0 | 92.65 | F:ATP binding; P:protein catabolic process; P:proteolysis; F:serine-type endopeptidase activity; F:ATP-dependent peptidase activity |
| Efet.01.646642.g1115.t1 | monothiol Grx4 family | 318 | 4.97E-72 | 98.3 | C:cell; F:protein disulfide oxidoreductase activity; F:metal ion binding; F:electron carrier activity; P:cell redox homeostasis; P:oxidation-reduction process; F:2 iron, 2 sulfur cluster binding |
| Efet.01.646649.g1116.t1 | carbohydrate kinase | 465 | 1.37E-83 | 74.6 | F:transferase activity, transferring phosphorus-containing groups |
| Efet.01.646649.g1117.t1 | aspartate aminotransferase | 522 | 3.66E-94 | 80.1 | F:pyridoxal phosphate binding; F:transaminase activity; P:biosynthetic process |
| Efet.01.646649.g1118.t1 | pyridoxal phosphate-dependent aminotransferase | 555 | 4.56E-107 | 86.3 | F:pyridoxal phosphate binding; F:L-phenylalanine:2-oxoglutarate aminotransferase activity; P:biosynthetic process; F:L-aspartate:2-oxoglutarate aminotransferase activity |
| Efet.01.64671.g894.t1 | Paired mesoderm homeobox 2 | 477 | 1.87E-08 | 56 | F:DNA binding; C:nucleus; P:multicellular organism development; P:regulation of transcription, DNA-templated; F:sequence-specific DNA binding |
| Efet.01.646715.g1120.t1 | TIGR00645 family | 1170 | 1.23E-113 | 81.1 | C:integral component of membrane; C:plasma membrane |
| Efet.01.646722.g1121.t1 | UPF0182 family | 972 | 1.83E-159 | 88.95 | C:integral component of membrane; C:plasma membrane |
| Efet.01.646730.g1123.t1 | PREDICTED: uncharacterized protein LOC105845334 | 1476 | 3.29E-98 | 57.15 | F:DNA binding; F:DNA-directed 5'-3' RNA polymerase activity; P:transcription, DNA-templated |
| Efet.01.646781.g1125.t1 | xylulokinase | 459 | 3.80E-89 | 68.5 | F:transferase activity |
| Efet.01.646781.g1126.t1 | glycerol-1-phosphate dehydrogenase [NAD(P)+] | 471 | 1.33E-99 | 59.75 |  |
| Efet.01.646833.g1128.t1 | dual specificity phosphatase 19 | 402 | 7.35E-53 | 65.05 | P:protein dephosphorylation; F:protein tyrosine/serine/threonine phosphatase activity |
| Efet.01.646847.g1130.t1 | udp-n-acetylglucosamine--peptide n-acetylglucosaminyltransferase | 738 | 1.54E-72 | 56.75 | F:transferase activity, transferring glycosyl groups; C:membrane; C:integral component of membrane; F:transferase activity |
| Efet.01.646875.g1131.t1 | dual specificity tyrosine-phosphorylation-regulated kinase 1A-like | 231 | 3.77E-15 | 86.1 | F:ATP binding; F:protein serine/threonine kinase activity; P:protein phosphorylation |
| Efet.01.646950.g1134.t1 | PREDICTED: uncharacterized protein LOC106156013 | 450 | 1.21E-21 | 51.45 | C:membrane; C:integral component of membrane |
| Efet.01.646963.g1135.t1 | strictosidine synthase | 1107 | 0 | 65.15 | F:strictosidine synthase activity; P:biosynthetic process |
| Efet.01.646963.g1136.t1 | sugar ABC transporter ATP-binding | 1452 | 0 | 90.3 | F:ATPase activity; F:ATP binding |
| Efet.01.646963.g1137.t1 | methylmalonate-semialdehyde dehydrogenase ( acylating) | 1524 | 0 | 93.65 | F:methylmalonate-semialdehyde dehydrogenase (acylating) activity; P:oxidation-reduction process |
| Efet.01.646963.g1138.t1 | gamma-glutamyltransferase | 1779 | 0 | 79.75 | P:glutathione metabolic process; F:gamma-glutamyltransferase activity; F:hydrolase activity |
| Efet.01.646963.g1139.t1 | tripartite tricarboxylate transporter substrate binding | 966 | 0 | 83.65 | C:outer membrane-bounded periplasmic space |
| Efet.01.646963.g1140.t1 | type II secretion system | 1959 | 0 | 74.3 | F:peptidoglycan binding; F:protein transporter activity; P:protein secretion by the type II secretion system; C:cell outer membrane; C:type II protein secretion system complex |
| Efet.01.646963.g1141.t1 | general secretion pathway | 315 | 3.66E-29 | 68.55 | C:membrane |
| Efet.01.646973.g1142.t1 | spermidine putrescine ABC transporter substrate-binding | 1035 | 0 | 61.3 |  |
| Efet.01.646973.g1143.t1 | spermidine putrescine transport system permease | 438 | 7.98E-56 | 77 | C:membrane |
| Efet.01.647031.g1146.t1 | microtubule-actin cross-linking factor 1 isoform X4 | 2355 | 2.65E-39 | 41.2 | C:cytoskeleton |
| Efet.01.64704.g896.t1 | short transient receptor potential channel 6 | 1053 | 2.82E-103 | 72.4 | C:integral component of plasma membrane; F:store-operated calcium channel activity; P:manganese ion transport; P:regulation of cytosolic calcium ion concentration; P:calcium ion transmembrane transport; P:single fertilization |
| Efet.01.647040.g1148.t1 | coiled-coil domain-containing 14 isoform X2 | 618 | 2.36E-06 | 72 |  |
| Efet.01.647059.g1149.t1 | Creatininase | 678 | 6.68E-154 | 82.1 | F:creatininase activity |
| Efet.01.647059.g1150.t1 | ABC transporter permease | 975 | 0 | 79 | C:integral component of membrane; P:transport; C:plasma membrane |
| Efet.01.647059.g1151.t1 | ABC transporter permease | 852 | 4.83E-133 | 80.5 | C:integral component of membrane; P:transport; C:plasma membrane |
| Efet.01.647059.g1152.t1 | hypothetical protein | 1098 | 0 | 75.35 | C:integral component of membrane |
| Efet.01.647059.g1153.t1 | peptidase C39 | 768 | 6.10E-130 | 77 | F:ATP binding; P:proteolysis; C:integral component of membrane; F:peptidase activity |
| Efet.01.647059.g1154.t1 | ABC transporter ATP-binding | 885 | 0 | 78.6 | F:ATPase activity; F:ATP binding; P:cation transport; P:peptide transport |
| Efet.01.647059.g1155.t1 | ABC transporter ATP-binding | 957 | 9.43E-127 | 86.95 | F:ATPase activity; F:ATP binding; P:peptide transport |
| Efet.01.647059.g1156.t1 | peptide ABC transporter substrate-binding | 2325 | 0 | 70.5 | P:transmembrane transport; C:ATP-binding cassette (ABC) transporter complex |
| Efet.01.647059.g1157.t1 | Zn protease | 969 | 1.12E-26 | 85.3 | P:proteolysis; F:peptidase activity; P:phosphorylation; F:kinase activity |
| Efet.01.64706.g897.t1 | insulin-like growth factor 2 mRNA-binding 1 isoform X3 | 687 | 7.29E-54 | 63.35 | F:nucleotide binding; F:RNA binding |
| Efet.01.647075.g1158.t1 | gamma-tubulin complex component 6 | 240 | 7.26E-09 | 62.4 | C:microtubule organizing center; P:nuclear division; P:microtubule nucleation; P:cell cycle process; F:microtubule binding |
| Efet.01.64708.g898.t1 | DNA damage-binding | 810 | 2.53E-107 | 80.6 | P:UV-damage excision repair; P:regulation of mitotic cell cycle phase transition; P:positive regulation of viral release from host cell; P:nucleotide-excision repair; F:damaged DNA binding; P:positive regulation by virus of viral protein levels in host cell; C:nuclear chromosome, telomeric region; P:protein ubiquitination involved in ubiquitin-dependent protein catabolic process; C:nucleoplasm; C:extracellular space; P:proteasome-mediated ubiquitin-dependent protein catabolic process; C:extracellular exosome; F:protein binding; P:histone H2A monoubiquitination; C:cytoplasm; P:negative regulation of apoptotic process; C:Cul4A-RING E3 ubiquitin ligase complex; C:Cul4B-RING E3 ubiquitin ligase complex; P:Wnt signaling pathway; P:positive regulation of viral genome replication; P:interaction with symbiont |
| Efet.01.647154.g1159.t1 | penicillin acylase family | 318 | 2.61E-65 | 71.95 | P:antibiotic biosynthetic process; F:penicillin amidase activity |
| Efet.01.647165.g1160.t1 | heat shock 70 kDa cognate 3 | 459 | 1.95E-57 | 92.45 | F:ATPase activity; P:IRE1-mediated unfolded protein response; P:PERK-mediated unfolded protein response; C:smooth endoplasmic reticulum; C:endoplasmic reticulum-Golgi intermediate compartment; C:endoplasmic reticulum lumen; P:cerebellar Purkinje cell layer development; F:calcium ion binding; F:chaperone binding; P:positive regulation of protein ubiquitination; P:maintenance of protein localization in endoplasmic reticulum; F:glycoprotein binding; C:endoplasmic reticulum chaperone complex; C:COP9 signalosome; P:regulation of ATF6-mediated unfolded protein response; F:protein domain specific binding; P:regulation of IRE1-mediated unfolded protein response; P:cerebellum structural organization; P:regulation of PERK-mediated unfolded protein response; P:toxin transport; C:plasma membrane; P:cellular response to glucose starvation; F:ubiquitin protein ligase binding; C:melanosome; P:cellular response to manganese ion; C:integral component of endoplasmic reticulum membrane; C:midbody; P:positive regulation of cell migration; P:protein folding in endoplasmic reticulum; C:myelin sheath; P:negative regulation of protein homodimerization activity; P:ER overload response; F:ATP binding; C:extracellular exosome; C:focal adhesion; P:regulation of protein folding in endoplasmic reticulum; P:cellular response to interleukin-4; P:cellular response to antibiotic; P:ATF6-mediated unfolded protein response; F:misfolded protein binding; F:unfolded protein binding; P:substantia nigra development; C:cell surface; P:positive regulation of transcription from RNA polymerase II promoter in response to endoplasmic reticulum stress; P:activation of signaling protein activity involved in unfolded protein response; F:ribosome binding; P:negative regulation of apoptotic process; C:mitochondrion; P:ER-associated ubiquitin-dependent protein catabolic process; P:negative regulation of transforming growth factor beta receptor signaling pathway |
| Efet.01.647241.g1162.t1 | ABC transporter permease | 255 | 1.26E-27 | 75.75 | C:integral component of membrane; P:transport; C:plasma membrane |
| Efet.01.647241.g1163.t1 | ABC transporter permease | 495 | 2.51E-92 | 85.05 | C:integral component of membrane; P:transport; C:plasma membrane |
| Efet.01.647278.g1165.t1 | FAD-dependent oxidoreductase | 609 | 1.40E-133 | 73.85 | F:oxidoreductase activity; P:oxidation-reduction process |
| Efet.01.647321.g1167.t1 | hypothetical protein HELRODRAFT_162358 | 300 | 1.37E-30 | 81 |  |
| Efet.01.647324.g1169.t1 | maltose phosphorylase | 534 | 8.19E-74 | 80.6 | P:carbohydrate metabolic process; F:carbohydrate binding; F:hydrolase activity |
| Efet.01.647387.g1174.t1 | carbohydrate sulfotransferase 3-like | 204 | 4.58E-21 | 69.3 | C:membrane |
| Efet.01.647395.g1175.t1 | replicative DNA helicase | 546 | 3.45E-126 | 93.05 | F:DNA binding; F:ATP binding; F:DNA helicase activity; P:DNA duplex unwinding; C:primosome complex; P:DNA replication, synthesis of RNA primer |
| Efet.01.647425.g1176.t1 | Dihydroxy-acid dehydratase | 624 | 1.32E-101 | 82.65 | P:isoleucine biosynthetic process; F:dihydroxy-acid dehydratase activity; F:metal ion binding; P:valine biosynthetic process; F:4 iron, 4 sulfur cluster binding |
| Efet.01.647444.g1177.t1 | glutamine synthetase | 1377 | 0 | 87.25 | P:glutamine biosynthetic process; F:glutamate-ammonia ligase activity |
| Efet.01.647444.g1178.t1 | carboxymuconolactone decarboxylase family | 552 | 2.54E-111 | 68.05 | P:cellular oxidant detoxification; P:oxidation-reduction process; F:peroxiredoxin activity |
| Efet.01.647444.g1179.t1 | ABC transporter substrate-binding | 1416 | 0 | 61.4 | P:transmembrane transport; C:ATP-binding cassette (ABC) transporter complex |
| Efet.01.647470.g1182.t1 | hydroxyacid dehydrogenase | 483 | 4.24E-70 | 92.4 | F:oxidoreductase activity, acting on CH-OH group of donors; F:flavin adenine dinucleotide binding; P:oxidation-reduction process |
| Efet.01.64750.g899.t1 | Kyphoscoliosis peptidase | 555 | 1.06E-28 | 55.8 |  |
| Efet.01.647513.g1183.t1 | hypothetical protein CAPTEDRAFT_226428 | 441 | 1.74E-15 | 67 | C:membrane; C:integral component of membrane |
| Efet.01.647551.g1185.t1 | ribulokinase | 1317 | 0 | 85.15 | F:ATP binding; F:ribulokinase activity; P:carbohydrate phosphorylation; P:L-arabinose catabolic process to xylulose 5-phosphate |
| Efet.01.647551.g1186.t1 | type I pantothenate kinase | 768 | 2.08E-157 | 83.65 | F:ATP binding; C:cytoplasm; P:coenzyme A biosynthetic process; F:pantothenate kinase activity; P:phosphorylation |
| Efet.01.647569.g1187.t1 | expressed conserved | 1725 | 2.92E-128 | 53.95 |  |
| Efet.01.647596.g1188.t1 | pre-mRNA-processing factor 6-like | 393 | 1.88E-66 | 92.9 | C:catalytic step 2 spliceosome; C:U4/U6 x U5 tri-snRNP complex; P:spliceosomal tri-snRNP complex assembly |
| Efet.01.647603.g1190.t1 | Zinc finger 37 | 1560 | 3.14E-50 | 51.65 | F:nucleic acid binding; C:nucleus; F:zinc ion binding; F:metal ion binding; C:membrane; C:integral component of membrane |
| Efet.01.647660.g1194.t1 | hypothetical protein CRN43_19050, partial | 213 | 9.47E-06 | 65 |  |
| Efet.01.647709.g1195.t1 | potassium voltage-gated channel subfamily H member 8-like | 1395 | 2.65E-07 | 40 |  |
| Efet.01.647720.g1196.t1 | dehydrogenase | 513 | 1.94E-68 | 90.15 | F:zinc ion binding; F:S-(hydroxymethyl)glutathione dehydrogenase activity; P:oxidation-reduction process |
| Efet.01.647734.g1197.t1 | helicase with zinc finger domain 2 | 1092 | 1.03E-08 | 48.55 | F:nucleotide binding; F:ATP binding; F:zinc ion binding; F:metal ion binding; F:ligand-dependent nuclear receptor transcription coactivator activity; F:helicase activity; P:regulation of nucleic acid-templated transcription; C:intracellular |
| Efet.01.647742.g1198.t1 | 25S rRNA (cytosine-C(5))-methyltransferase nop2-like | 321 | 3.88E-33 | 73.4 | P:metabolic process; F:methyltransferase activity |
| Efet.01.647774.g1199.t1 | dipeptidase | 420 | 2.69E-83 | 79.15 | P:metabolic process; F:hydrolase activity |
| Efet.01.647774.g1200.t1 | iron-sulfur cluster insertion | 369 | 1.01E-56 | 94.8 | F:structural molecule activity; F:iron-sulfur cluster binding; P:iron-sulfur cluster assembly |
| Efet.01.647786.g1201.t1 | ADP-ribosylglycohydrolase family | 486 | 6.91E-96 | 75.3 |  |
| Efet.01.647897.g1203.t1 | mandelamide amidase | 816 | 3.87E-113 | 89.85 | F:mandelamide amidase activity; F:carbon-nitrogen ligase activity, with glutamine as amido-N-donor |
| Efet.01.647897.g1204.t1 | ABC transporter substrate-binding | 963 | 0 | 84.9 | P:thiamine biosynthetic process |
| Efet.01.647897.g1205.t1 | family transport system permease | 1410 | 1.43E-140 | 92.55 | F:ATPase activity; F:ATP binding; C:integral component of membrane; P:transport; C:plasma membrane |
| Efet.01.647979.g1207.t1 | ER lumen retaining receptor | 273 | 1.81E-38 | 86.9 | C:endoplasmic reticulum membrane; P:protein transport; C:integral component of membrane; F:ER retention sequence binding; P:protein retention in ER lumen |
| Efet.01.647987.g1208.t1 | amidase | 936 | 6.85E-153 | 73.95 | F:carbon-nitrogen ligase activity, with glutamine as amido-N-donor; F:hydrolase activity |
| Efet.01.647987.g1209.t1 | ABC transporter substrate-binding | 1509 | 0 | 88.65 | P:transmembrane transport; C:ATP-binding cassette (ABC) transporter complex |
| Efet.01.647987.g1210.t1 | peptide ABC transporter ATP-binding | 849 | 1.88E-143 | 85.45 | F:ATPase activity; F:ATP binding; P:peptide transport |
| Efet.01.647987.g1211.t1 | ABC transporter ATP-binding | 411 | 1.53E-89 | 86.25 | F:ATPase activity; F:ATP binding; P:peptide transport |
| Efet.01.647991.g1212.t1 | E3 ubiquitin- ligase RNF34-like | 252 | 3.64E-07 | 65.17 | F:zinc ion binding; F:metal ion binding; F:ligase activity |
| Efet.01.648009.g1213.t1 | glutamate receptor 3-like | 378 | 1.32E-45 | 82.75 | C:AMPA glutamate receptor complex; C:postsynaptic density; C:extracellular vesicle; P:ion transmembrane transport; C:neuronal cell body; F:AMPA glutamate receptor activity; F:extracellular-glutamate-gated ion channel activity; C:nucleoplasm; C:dendrite; F:protein binding; C:postsynaptic membrane; P:regulation of synapse structure or activity; C:cell junction; C:kainate selective glutamate receptor complex; P:positive regulation of synaptic transmission, glutamatergic; P:ionotropic glutamate receptor signaling pathway |
| Efet.01.648013.g1214.t1 | transposase | 276 | 3.40E-44 | 96 | F:DNA binding; F:transposase activity; P:transposition, DNA-mediated |
| Efet.01.648013.g1215.t1 | 2-aminoethylphosphonate ABC transporter substrate-binding | 1029 | 0 | 80.35 | F:metal ion binding |
| Efet.01.648013.g1216.t1 | 2-aminoethylphosphonate ABC transporter ATP-binding | 1089 | 0 | 78 | F:ATPase activity; F:ATP binding; P:transmembrane transport; F:hydrolase activity, acting on acid anhydrides, catalyzing transmembrane movement of substances |
| Efet.01.648013.g1217.t1 | 2-aminoethylphosphonate ABC transporter permease subunit | 1581 | 0 | 82.9 | C:integral component of membrane; P:transport; C:plasma membrane |
| Efet.01.648013.g1218.t1 | 2-aminoethylphosphonate--pyruvate transaminase | 1044 | 0 | 84.95 | F:2-aminoethylphosphonate-pyruvate transaminase activity; P:organic phosphonate catabolic process |
| Efet.01.648013.g1219.t1 | arginase | 1035 | 0 | 89.55 | P:arginine metabolic process; F:metal ion binding; F:arginase activity |
| Efet.01.648041.g1221.t1 | PREDICTED: uncharacterized protein C2orf42 | 702 | 4.92E-94 | 70.65 | F:zinc ion binding |
| Efet.01.648156.g1224.t1 | family transcriptional regulator | 630 | 1.23E-148 | 72.1 | F:DNA binding; F:transcription factor activity, sequence-specific DNA binding; P:transcription, DNA-templated; P:regulation of transcription, DNA-templated |
| Efet.01.648189.g1226.t1 | RNA polymerase sigma-70 factor | 324 | 1.07E-19 | 65.7 | F:sigma factor activity; F:DNA binding; F:transcription factor activity, sequence-specific DNA binding; P:transcription, DNA-templated; P:regulation of transcription, DNA-templated; P:DNA-templated transcription, initiation |
| Efet.01.648194.g1227.t1 | family cell cycle | 663 | 4.84E-135 | 89.55 | P:cell division; C:integral component of membrane |
| Efet.01.648290.g1229.t1 | cell division | 660 | 3.66E-129 | 68 | C:membrane |
| Efet.01.648296.g1230.t1 | WD repeat-containing 54 | 306 | 1.62E-17 | 65.9 |  |
| Efet.01.648372.g1232.t1 | asparagine synthase (glutamine-hydrolyzing) | 1107 | 0 | 64.25 | P:asparagine biosynthetic process; F:asparagine synthase (glutamine-hydrolyzing) activity |
| Efet.01.648372.g1233.t1 | cytidyltransferase | 1059 | 0 | 82.55 | P:biosynthetic process; F:transferase activity; F:hydrolase activity |
| Efet.01.648372.g1234.t1 | methionine adenosyltransferase | 1146 | 0 | 91.6 | F:magnesium ion binding; F:ATP binding; C:cytoplasm; P:S-adenosylmethionine biosynthetic process; F:methionine adenosyltransferase activity; P:one-carbon metabolic process |
| Efet.01.648372.g1235.t1 | lipid A biosynthesis acyltransferase | 672 | 2.07E-123 | 91.85 | F:transferase activity, transferring acyl groups; C:integral component of membrane |
| Efet.01.648372.g1236.t1 | lipid A biosynthesis acyltransferase | 522 | 1.62E-120 | 91.1 | F:transferase activity, transferring acyl groups; C:integral component of membrane |
| Efet.01.648373.g1237.t1 | site-specific tyrosine recombinase | 633 | 2.75E-95 | 81.8 | P:cell division; F:DNA binding; C:cytoplasm; P:DNA integration; P:chromosome segregation; F:tyrosine-based site-specific recombinase activity; P:cell cycle; P:transposition, DNA-mediated |
| Efet.01.648399.g1238.t1 | histone-lysine N-methyltransferase ASH1L-like isoform X10 | 243 | 1.95E-17 | 66.05 | F:binding |
| Efet.01.648426.g1239.t1 | 60S ribosomal | 315 | 9.50E-28 | 85.8 | F:structural constituent of ribosome; P:cytoplasmic translation; C:cytosolic large ribosomal subunit; P:ribosomal large subunit assembly |
| Efet.01.648435.g1240.t1 | hypothetical protein CAPTEDRAFT_65598, partial | 654 | 1.62E-07 | 47 | C:membrane; C:integral component of membrane |
| Efet.01.648489.g1242.t1 | cell surface | 2934 | 8.38E-134 | 41.65 | C:membrane; C:integral component of membrane |
| Efet.01.648525.g1245.t1 | gamma-aminobutyric acid receptor subunit beta-like isoform X1 | 258 | 3.36E-17 | 73.15 | P:signal transduction; P:ion transmembrane transport; C:integral component of membrane; F:GABA-A receptor activity; C:cell junction; F:extracellular ligand-gated ion channel activity; C:synapse; C:plasma membrane |
| Efet.01.648535.g1246.t1 | baculoviral IAP repeat-containing 6-like | 297 | 1.72E-30 | 72.2 | F:ubiquitin-protein transferase activity; C:microtubule cytoskeleton; C:cytoskeletal part; P:negative regulation of apoptotic process; P:cellular protein modification process; C:endomembrane system; C:cytoplasmic part; P:regulation of signal transduction; P:embryonic placenta development; P:regulation of cell proliferation; C:membrane-bounded organelle |
| Efet.01.648552.g1247.t1 | hypothetical protein HELRODRAFT_161987 | 717 | 4.38E-11 | 60.67 | F:nucleic acid binding; F:metal ion binding |
| Efet.01.648641.g1250.t1 | FAST kinase domain-containing 3 | 1065 | 2.03E-84 | 50.95 | F:metal ion binding; P:proteolysis; F:protein kinase activity; F:aminopeptidase activity; F:manganese ion binding; P:protein phosphorylation; F:hydrolase activity |
| Efet.01.648654.g1251.t1 | ATP-dependent Clp protease proteolytic subunit | 576 | 5.46E-101 | 87.55 | C:cytoplasm; P:proteolysis; F:serine-type endopeptidase activity |
| Efet.01.648654.g1252.t1 | trigger factor | 270 | 8.60E-40 | 69.55 | P:cellular process |
| Efet.01.648669.g1254.t1 | lisH domain and HEAT repeat-containing KIAA1468 homolog isoform X1 | 3576 | 0 | 63.35 | P:biological_process; C:cellular_component; F:molecular_function |
| Efet.01.648671.g1255.t1 | gfo Idh family oxidoreductase | 786 | 6.71E-71 | 73.65 | F:oxidoreductase activity; P:oxidation-reduction process |
| Efet.01.648671.g1256.t1 | ABC transporter permease | 594 | 5.19E-115 | 80.3 | C:integral component of membrane; P:transport; C:plasma membrane; F:transporter activity |
| Efet.01.648705.g1258.t1 | phosphoenolpyruvate carboxykinase (GTP) | 303 | 1.27E-61 | 90.4 | F:GTP binding; P:gluconeogenesis; F:phosphoenolpyruvate carboxykinase (GTP) activity; C:cytoplasm; F:manganese ion binding; P:phosphorylation; F:kinase activity |
| Efet.01.648705.g1259.t1 | XRE family transcriptional regulator | 267 | 3.65E-46 | 83.4 | F:sequence-specific DNA binding |
| Efet.01.648718.g1261.t1 | cold shock domain-containing E1-like | 525 | 5.73E-33 | 50.6 | C:CRD-mediated mRNA stability complex; F:nucleic acid binding; F:DNA binding; C:cytoplasm; P:regulation of transcription, DNA-templated; C:Golgi apparatus; P:nuclear-transcribed mRNA catabolic process, no-go decay; C:mitochondrial inner membrane; C:plasma membrane; F:RNA binding |
| Efet.01.648719.g1262.t1 | peptidyl-prolyl cis-trans isomerase CYP63-like isoform X1 | 357 | 6.11E-23 | 61.25 | C:membrane; C:integral component of membrane |
| Efet.01.64877.g902.t1 | short transient receptor potential channel 3-like | 531 | 7.31E-09 | 77.13 | F:ion channel activity; P:ion transport; P:transmembrane transport; F:calcium channel activity; C:membrane; C:integral component of membrane; P:calcium ion transmembrane transport; P:transport |
| Efet.01.648797.g1265.t1 | 50S ribosomal L21 | 312 | 1.18E-66 | 96.3 | F:structural constituent of ribosome; C:ribosome; F:rRNA binding; P:translation |
| Efet.01.648797.g1266.t1 | GTPase | 1092 | 0 | 89.7 | F:magnesium ion binding; F:GTP binding; C:cytoplasm; F:GTPase activity; P:ribosome biogenesis |
| Efet.01.648797.g1267.t1 | glutamate 5-kinase | 1143 | 0 | 94.6 | F:ATP binding; F:RNA binding; F:glutamate 5-kinase activity; C:cytoplasm; P:phosphorylation; P:L-proline biosynthetic process |
| Efet.01.648797.g1268.t1 | peptidase | 831 | 0 | 95.85 | P:proteolysis; C:proteasome complex; F:peptidase activity |
| Efet.01.648797.g1269.t1 | lipopolysaccharide assembly | 216 | 4.57E-43 | 100 | P:regulation of lipid biosynthetic process; F:iron ion binding; C:integral component of membrane; P:lipopolysaccharide metabolic process; C:intrinsic component of the cytoplasmic side of the plasma membrane |
| Efet.01.648808.g1271.t1 | paired box Pax-6- | 447 | 2.62E-57 | 86.65 | P:regulation of transcription from RNA polymerase II promoter involved in somatic motor neuron fate commitment; P:regulation of transcription from RNA polymerase II promoter involved in spinal cord motor neuron fate specification; P:retina development in camera-type eye; F:RNA polymerase II core promoter sequence-specific DNA binding; P:regulation of transcription from RNA polymerase II promoter involved in ventral spinal cord interneuron specification; F:RNA polymerase II core promoter proximal region sequence-specific DNA binding; P:forebrain anterior/posterior pattern specification; P:signal transduction involved in regulation of gene expression; P:transcription from RNA polymerase II promoter; P:axon guidance; P:forebrain dorsal/ventral pattern formation; P:dorsal/ventral axis specification; F:transcriptional repressor activity, RNA polymerase II transcription regulatory region sequence-specific binding; P:lens development in camera-type eye; P:cerebral cortex regionalization; F:transcriptional activator activity, RNA polymerase II core promoter proximal region sequence-specific binding; P:lacrimal gland development; P:cell fate determination; F:protein kinase binding; P:forebrain-midbrain boundary formation; F:histone acetyltransferase binding; P:commitment of neuronal cell to specific neuron type in forebrain; P:eye photoreceptor cell development; P:positive regulation of neuroblast proliferation; P:smoothened signaling pathway; P:pituitary gland development; P:keratinocyte differentiation; C:nuclear chromatin; F:ubiquitin protein ligase binding; P:positive regulation of epithelial cell differentiation; P:glucose homeostasis; P:negative regulation of epithelial cell proliferation; P:protein localization to organelle; P:regulation of cell migration; P:protein ubiquitination; F:HMG box domain binding; P:response to wounding; P:negative regulation of neuron differentiation; P:oligodendrocyte cell fate specification; P:positive regulation of transcription from RNA polymerase II promoter; C:nucleoplasm; P:salivary gland morphogenesis; P:negative regulation of transcription from RNA polymerase II promoter; P:neuron migration; P:blood vessel development; P:type B pancreatic cell differentiation; P:negative regulation of neural precursor cell proliferation; P:astrocyte differentiation; P:regulation of asymmetric cell division; F:chromatin binding; F:transcription factor binding; P:visual perception; P:cornea development in camera-type eye; P:pancreatic A cell development; P:regulation of timing of cell differentiation; F:ubiquitin-protein transferase activity; P:establishment of mitotic spindle orientation; C:cytoplasm; P:negative regulation of protein phosphorylation; P:embryonic camera-type eye morphogenesis; F:R-SMAD binding; F:co-SMAD binding; P:iris morphogenesis |
| Efet.01.648865.g1274.t1 | hypothetical protein HELRODRAFT_172085 | 621 | 1.01E-45 | 63.67 | P:signal transduction |
| Efet.01.648921.g1276.t1 | potassium voltage-gated channel Shaw-like | 1050 | 1.61E-84 | 70.75 | P:potassium ion transport; F:voltage-gated potassium channel activity; P:transmembrane transport; C:integral component of membrane; P:cellular process |
| Efet.01.648953.g1277.t1 | ABC transporter permease | 1674 | 0 | 83.8 | F:strictosidine synthase activity; C:integral component of membrane; P:monosaccharide transport; F:monosaccharide-transporting ATPase activity; P:biosynthetic process; C:plasma membrane |
| Efet.01.648999.g1279.t1 | diacylglycerol kinase | 252 | 3.34E-27 | 93.5 | C:integral component of membrane; P:phospholipid biosynthetic process; F:diacylglycerol kinase activity; P:phosphorylation |
| Efet.01.648999.g1280.t1 | RDD family | 456 | 2.35E-90 | 89.2 | C:integral component of membrane |
| Efet.01.649023.g1281.t1 | universal stress | 795 | 7.20E-96 | 67.2 | P:response to stress |
| Efet.01.64906.g904.t1 | GATA-type zinc finger 1 isoform X2 | 939 | 2.40E-26 | 56.1 | F:binding; P:single-organism process; P:cellular process |
| Efet.01.649106.g1283.t1 | oxidoreductase | 246 | 7.36E-49 | 82.3 | F:oxidoreductase activity; P:oxidation-reduction process |
| Efet.01.649106.g1284.t1 | oxidoreductase | 387 | 5.10E-45 | 79.85 | F:oxidoreductase activity; P:oxidation-reduction process |
| Efet.01.649156.g1286.t1 | zinc finger E-box-binding homeobox zag-1 isoform X1 | 1095 | 2.50E-17 | 74.7 | F:nucleic acid binding |
| Efet.01.649195.g1287.t1 | Major egg antigen | 849 | 8.72E-53 | 52.4 | F:nucleic acid binding |
| Efet.01.649206.g1288.t1 | DNA-3-methyladenine glycosylase I | 573 | 2.22E-116 | 77.3 | F:DNA-3-methyladenine glycosylase activity; P:base-excision repair |
| Efet.01.649206.g1289.t1 | arginine--tRNA ligase | 1032 | 0 | 72.3 | F:nucleotide binding; P:tRNA aminoacylation for protein translation; F:aminoacyl-tRNA ligase activity |
| Efet.01.649251.g1292.t1 | J domain-containing DDB_G0295729-like isoform X1 | 390 | 4.75E-11 | 75.18 |  |
| Efet.01.649268.g1293.t1 | lysosomal-trafficking regulator-like | 459 | 1.50E-07 | 51 |  |
| Efet.01.649271.g1294.t1 | hypothetical protein CAPTEDRAFT_214871 | 348 | 2.14E-13 | 54 | C:membrane; C:integral component of membrane |
| Efet.01.649284.g1296.t1 | BTB POZ domain-containing 3 isoform X2 | 978 | 8.19E-34 | 44.8 | P:regulation of neurogenesis |
| Efet.01.649310.g1298.t1 | helicase with zinc finger domain 2-like | 231 | 1.40E-06 | 56.33 |  |
| Efet.01.649352.g1299.t1 | probable phospholipid-transporting ATPase IA isoform X3 | 312 | 2.50E-42 | 87.65 | F:magnesium ion binding; F:ATP binding; P:phospholipid translocation; C:integral component of membrane; F:phospholipid-translocating ATPase activity |
| Efet.01.64936.g905.t1 | speedy C isoform X1 | 357 | 9.01E-09 | 64.65 | F:protein kinase binding; P:positive regulation of cyclin-dependent protein serine/threonine kinase activity; P:multicellular organism development; P:mRNA processing; P:cell cycle; P:mRNA polyadenylation; P:meiotic G2/MI transition; P:activation of MAPK activity; C:nucleus; P:meiotic cell cycle; P:oocyte maturation; F:cyclin binding; P:positive regulation of meiotic cell cycle |
| Efet.01.649364.g1300.t1 | magnesium transporter | 597 | 2.53E-102 | 92.85 | P:transmembrane transport; C:integral component of membrane; F:metal ion transmembrane transporter activity; P:metal ion transport |
| Efet.01.649364.g1301.t1 | cyclic pyranopterin monophosphate synthase | 489 | 5.66E-111 | 90.25 | P:Mo-molybdopterin cofactor biosynthetic process; F:hydrolase activity |
| Efet.01.649364.g1302.t1 | pilus assembly | 537 | 9.90E-79 | 84.7 | C:integral component of membrane |
| Efet.01.649393.g1305.t1 | glycine receptor subunit alpha-3-like isoform X1 | 216 | 1.20E-25 | 68.6 | P:ion transmembrane transport; C:integral component of membrane; C:cell junction; F:extracellular ligand-gated ion channel activity; C:synapse; C:plasma membrane |
| Efet.01.649414.g1306.t1 | sequestosome 1 | 336 | 1.73E-17 | 57.35 | P:regulation of cellular process; C:autophagosome; P:cellular process; F:enzyme binding |
| Efet.01.649418.g1307.t1 | sex peptide receptor-like | 1467 | 2.82E-92 | 52.8 | F:G-protein coupled peptide receptor activity; P:G-protein coupled receptor signaling pathway; C:integral component of membrane |
| Efet.01.649448.g1308.t1 | tyrosine- phosphatase Lar-like isoform X1 | 258 | 1.19E-12 | 68.35 | F:protein tyrosine phosphatase activity; P:peptidyl-tyrosine dephosphorylation |
| Efet.01.649449.g1309.t1 | neurexin-3-like isoform X5 | 723 | 2.09E-48 | 57.2 | C:membrane; C:integral component of membrane |
| Efet.01.649450.g1310.t1 | rho GTPase-activating 39-like | 393 | 8.28E-57 | 78.2 | C:cytoskeleton; P:signal transduction; P:regulation of small GTPase mediated signal transduction; F:GTPase activator activity; C:cytosol |
| Efet.01.649466.g1311.t1 | 1 family IMP dehydrogenase-related | 1410 | 0 | 78.9 | P:purine nucleotide biosynthetic process; F:IMP dehydrogenase activity; P:oxidation-reduction process |
| Efet.01.649470.g1312.t1 | fibrinogen 1 | 366 | 8.98E-17 | 57 |  |
| Efet.01.649471.g1313.t1 | PD-(D E)XK nuclease family | 2760 | 0 | 73.65 | F:hydrolase activity |
| Efet.01.649471.g1314.t1 | thioredoxin | 336 | 2.73E-75 | 88.7 | C:cell; F:protein disulfide oxidoreductase activity; P:glycerol ether metabolic process; P:cell redox homeostasis; P:oxidation-reduction process |
| Efet.01.649481.g1317.t1 | bifunctional 5,10-methylene-tetrahydrofolate dehydrogenase 5,10-methylene-tetrahydrofolate cyclohydrolase | 546 | 2.58E-98 | 78.45 | P:methionine biosynthetic process; P:purine nucleotide biosynthetic process; P:folic acid-containing compound biosynthetic process; P:tetrahydrofolate interconversion; P:oxidation-reduction process; P:histidine biosynthetic process; F:methenyltetrahydrofolate cyclohydrolase activity; F:methylenetetrahydrofolate dehydrogenase (NADP+) activity |
| Efet.01.649488.g1318.t1 | histamine H2 receptor-like | 1320 | 4.29E-79 | 57.5 | F:G-protein coupled receptor activity; P:G-protein coupled receptor signaling pathway; C:membrane; C:integral component of membrane |
| Efet.01.649489.g1319.t1 | zinc finger homeobox 4-like isoform X1 | 4503 | 5.26E-133 | 50.1 | F:binding |
| Efet.01.649489.g1320.t1 | ZSA5B_HUMAN ame: Full=Zinc finger and SCAN domain-containing 5B | 606 | 5.18E-06 | 54 | F:nucleic acid binding; F:DNA binding; C:nucleus; F:transcription factor activity, sequence-specific DNA binding; P:transcription, DNA-templated; F:metal ion binding; P:regulation of transcription, DNA-templated; F:RNA polymerase II transcription factor activity, sequence-specific DNA binding; P:regulation of transcription from RNA polymerase II promoter |
| Efet.01.649492.g1321.t1 | calcitonin gene-related peptide type 1 receptor-like isoform X1 | 273 | 1.68E-09 | 71.69 | P:G-protein coupled receptor signaling pathway; F:calcitonin receptor activity; C:integral component of membrane; P:cell surface receptor signaling pathway |
| Efet.01.649494.g1322.t1 | peptide-methionine (R)-S-oxide reductase | 414 | 9.69E-62 | 93.1 | P:protein repair; P:response to oxidative stress; F:zinc ion binding; P:oxidation-reduction process; F:peptide-methionine (R)-S-oxide reductase activity |
| Efet.01.649494.g1323.t1 | cell envelope-related transcriptional attenuator | 549 | 6.93E-85 | 59.75 | F:aspartic-type endopeptidase activity; C:membrane; P:proteolysis; C:integral component of membrane |
| Efet.01.649499.g1324.t1 | PREDICTED: uncharacterized protein LOC106153529 | 393 | 8.58E-14 | 53.95 |  |
| Efet.01.649506.g1325.t1 | zinc finger 423-like | 1674 | 1.37E-115 | 58.9 | F:nucleic acid binding; F:metal ion binding |
| Efet.01.649511.g1326.t1 | peptidase M14 | 1056 | 0 | 87.35 | F:zinc ion binding; F:metallocarboxypeptidase activity; P:proteolysis |
| Efet.01.649511.g1327.t1 | spermidine putrescine ABC transporter substrate-binding | 1053 | 0 | 77.2 | F:polyamine binding; C:periplasmic space; P:polyamine transport |
| Efet.01.649511.g1328.t1 | ABC transporter permease | 231 | 7.44E-44 | 91.8 | C:integral component of membrane; P:transport; C:plasma membrane |
| Efet.01.649562.g1329.t1 | muscarinic acetylcholine receptor M1-like | 285 | 1.86E-39 | 78.15 | C:integral component of plasma membrane; F:G-protein coupled acetylcholine receptor activity; P:G-protein coupled acetylcholine receptor signaling pathway |
| Efet.01.649580.g1330.t1 | TRAP transporter large permease | 1221 | 1.13E-145 | 65.5 | C:membrane; C:integral component of membrane |
| Efet.01.649586.g1331.t1 | roundabout homolog 1-like isoform X1 | 228 | 2.84E-26 | 74.85 | C:integral component of membrane |
| Efet.01.649588.g1332.t1 | flagellar hook-associated | 1236 | 0 | 87.35 | P:bacterial-type flagellum assembly; C:bacterial-type flagellum hook; F:structural molecule activity; P:bacterial-type flagellum-dependent cell motility |
| Efet.01.649588.g1333.t1 | flagellar rod assembly muramidase | 363 | 1.48E-24 | 73.55 | P:bacterial-type flagellum assembly; F:amidase activity; F:hydrolase activity, acting on glycosyl bonds |
| Efet.01.649588.g1334.t1 | flagellar biosynthesis | 1158 | 0 | 91.85 | F:structural molecule activity; C:outer membrane-bounded periplasmic space; P:bacterial-type flagellum-dependent cell motility; C:bacterial-type flagellum basal body, distal rod, P ring |
| Efet.01.649588.g1335.t1 | flagellar biosynthesis | 999 | 1.20E-103 | 85.2 | C:cell outer membrane; P:bacterial-type flagellum-dependent cell motility; C:bacterial-type flagellum basal body, distal rod, L ring; F:motor activity |
| Efet.01.649588.g1336.t1 | flagellar basal-body rod | 783 | 7.99E-176 | 94.8 | P:bacterial-type flagellum-dependent cell motility; C:bacterial-type flagellum basal body, distal rod |
| Efet.01.649588.g1337.t1 | flagellar basal-body rod | 717 | 1.68E-166 | 90.4 | C:bacterial-type flagellum basal body, rod; P:bacterial-type flagellum-dependent cell motility |
| Efet.01.649588.g1338.t1 | flagellar hook | 477 | 2.75E-85 | 74.2 | P:bacterial-type flagellum-dependent cell motility; C:bacterial-type flagellum basal body |
| Efet.01.649588.g1339.t1 | flagellar biosynthesis | 564 | 2.93E-76 | 92.45 | P:bacterial-type flagellum-dependent cell motility; C:bacterial-type flagellum basal body |
| Efet.01.649588.g1340.t1 | flagellar biosynthesis | 711 | 8.20E-124 | 87.75 | P:bacterial-type flagellum organization |
| Efet.01.649588.g1341.t1 | flagellar basal body rod | 405 | 7.03E-91 | 93.55 | C:bacterial-type flagellum basal body, rod; P:bacterial-type flagellum-dependent cell motility |
| Efet.01.649588.g1342.t1 | flagellar basal body rod | 426 | 1.48E-94 | 76.75 | C:bacterial-type flagellum basal body, rod; P:bacterial-type flagellum-dependent cell motility |
| Efet.01.649588.g1343.t1 | flagellar basal body P-ring formation | 975 | 3.84E-112 | 78.55 | P:bacterial-type flagellum organization; C:periplasmic space |
| Efet.01.649588.g1344.t1 | arginine--tRNA ligase | 2862 | 0 | 86.4 | F:ATP binding; F:arginine-tRNA ligase activity; C:cytoplasm; P:arginyl-tRNA aminoacylation |
| Efet.01.649593.g1345.t1 | interferon-induced very large GTPase 1-like | 2370 | 2.89E-19 | 43.85 | F:GTP binding |
| Efet.01.649669.g1347.t1 | low-density lipo receptor-related 5- | 390 | 4.96E-49 | 84.1 |  |
| Efet.01.64968.g908.t1 | malate cytoplasmic | 315 | 2.33E-36 | 85.95 | P:carbohydrate metabolic process; F:L-malate dehydrogenase activity; P:malate metabolic process; P:tricarboxylic acid cycle |
| Efet.01.64969.g909.t1 | DC-STAMP domain-containing 2-like | 1935 | 3.46E-42 | 52.75 | F:zinc ion binding; C:membrane; C:integral component of membrane |
| Efet.01.649709.g1349.t1 | peptide chain release factor 3 | 372 | 1.35E-59 | 90.55 | F:GTP binding; P:translational termination; P:regulation of translational termination; C:cytoplasm; F:GTPase activity; F:translation release factor activity, codon specific |
| Efet.01.64976.g910.t1 | cyclin-Y | 489 | 1.07E-46 | 90 | P:cell division; C:nucleus; F:protein kinase binding; P:positive regulation of cyclin-dependent protein serine/threonine kinase activity; C:extracellular exosome; F:cyclin-dependent protein serine/threonine kinase regulator activity; P:G2/M transition of mitotic cell cycle; C:cytoplasmic cyclin-dependent protein kinase holoenzyme complex; P:regulation of canonical Wnt signaling pathway; P:Wnt signaling pathway; C:plasma membrane |
| Efet.01.649786.g1350.t1 | ribonucleoside-diphosphate reductase | 573 | 1.25E-99 | 93.15 | F:oxidoreductase activity; P:deoxyribonucleoside diphosphate metabolic process; C:integral component of membrane; P:oxidation-reduction process |
| Efet.01.649821.g1351.t1 | PTS cellobiose transporter subunit IIB | 378 | 3.10E-84 | 71.35 | C:cytoplasm; P:phosphoenolpyruvate-dependent sugar phosphotransferase system; F:protein-N(PI)-phosphohistidine-sugar phosphotransferase activity; P:carbohydrate transmembrane transport |
| Efet.01.649821.g1352.t1 | PTS sorbitol transporter subunit IIB | 903 | 1.86E-154 | 82 | C:integral component of membrane; P:phosphoenolpyruvate-dependent sugar phosphotransferase system; F:protein-N(PI)-phosphohistidine-sugar phosphotransferase activity; P:carbohydrate transmembrane transport; F:D-glucosamine PTS permease activity |
| Efet.01.649821.g1353.t1 | PTS galactitol transporter subunit IIB | 204 | 5.82E-39 | 94.6 | P:phosphoenolpyruvate-dependent sugar phosphotransferase system; F:protein-N(PI)-phosphohistidine-sugar phosphotransferase activity; P:carbohydrate transmembrane transport |
| Efet.01.649821.g1354.t1 | PTS sugar transporter subunit IIA | 447 | 6.18E-90 | 78.65 | P:transport; F:transporter activity |
| Efet.01.649821.g1355.t1 | PTS sugar transporter subunit IIC | 1530 | 0 | 92.05 | F:galactitol transmembrane transporter activity; P:galactitol transport; C:integral component of membrane; P:phosphoenolpyruvate-dependent sugar phosphotransferase system |
| Efet.01.649821.g1356.t1 | phosphoenolpyruvate-- phosphotransferase | 2028 | 0 | 75.85 | C:cytoplasm; F:metal ion binding; P:phosphoenolpyruvate-dependent sugar phosphotransferase system; P:phosphorylation; F:phosphoenolpyruvate-protein phosphotransferase activity; F:kinase activity |
| Efet.01.649821.g1357.t1 | PTS sorbitol transporter subunit IIC | 555 | 1.84E-127 | 83.35 | C:integral component of membrane; P:phosphoenolpyruvate-dependent sugar phosphotransferase system; F:phosphotransferase activity, alcohol group as acceptor |
| Efet.01.649821.g1358.t1 | hypothetical protein | 639 | 1.74E-149 | 49.65 |  |
| Efet.01.649916.g1359.t1 | ABC transporter permease | 387 | 7.41E-68 | 77.35 | C:integral component of membrane; P:transport; C:plasma membrane; F:transporter activity |
| Efet.01.649916.g1360.t1 | amino acid ABC transporter ATP-binding | 1197 | 6.11E-140 | 73.7 | F:ATP binding; C:integral component of membrane; C:plasma membrane; P:amino acid transmembrane transport; F:amino acid-transporting ATPase activity |
| Efet.01.649916.g1361.t1 | ABC transporter substrate-binding | 798 | 7.57E-174 | 68.8 | P:transport; F:transporter activity |
| Efet.01.649916.g1362.t1 | family transcriptional regulator | 720 | 1.83E-137 | 63.8 | F:DNA binding; P:carbohydrate metabolic process; F:transcription factor activity, sequence-specific DNA binding; P:regulation of transcription, DNA-templated; F:carbohydrate binding |
| Efet.01.649916.g1363.t1 | NYN domain-containing | 834 | 3.44E-174 | 64.8 |  |
| Efet.01.649940.g1364.t1 | cysteine--tRNA ligase | 399 | 8.10E-78 | 65.8 | F:ion binding; F:ligase activity; P:translation |
| Efet.01.649998.g1366.t1 | ketopantoate reductase family | 1032 | 0 | 73.55 | P:pantothenate biosynthetic process; F:NADP binding; C:cytoplasm; P:oxidation-reduction process; F:2-dehydropantoate 2-reductase activity |
| Efet.01.649998.g1367.t1 | mannitol dehydrogenase family | 2586 | 0 | 69.7 | F:coenzyme binding; F:oxidoreductase activity; P:oxidation-reduction process; P:phosphorylation; F:phosphotransferase activity, alcohol group as acceptor; F:kinase activity |
| Efet.01.65.g6.t1 | glycogen phosphorylase | 510 | 2.96E-75 | 78.75 | F:glycogen phosphorylase activity; F:pyridoxal phosphate binding; C:cytoplasm; P:glycogen catabolic process |
| Efet.01.650005.g1.t1 | 1-phosphatidylinositol 4,5-bisphosphate phosphodiesterase zeta-1 | 360 | 1.03E-07 | 78.3 | F:phosphatidylinositol phospholipase C activity; P:calcium ion transport; P:lipid catabolic process; C:cytosol; P:egg activation; F:signal transducer activity; P:intracellular signal transduction |
| Efet.01.650045.g2.t1 | hypothetical protein HELRODRAFT_194269 | 240 | 9.14E-13 | 65 |  |
| Efet.01.650077.g3.t1 | hypothetical protein HELRODRAFT_62642 | 393 | 4.63E-14 | 73.33 |  |
| Efet.01.650091.g5.t1 | synthetase | 258 | 8.25E-43 | 87.25 | P:metabolic process; F:CoA-transferase activity |
| Efet.01.650091.g6.t1 | synthetase | 462 | 2.20E-103 | 82.65 | P:metabolic process; F:glutaconate CoA-transferase activity |
| Efet.01.650091.g7.t1 | cysteine dioxygenase | 594 | 2.29E-137 | 80.35 | F:cysteine dioxygenase activity; F:iron ion binding; P:oxidation-reduction process |
| Efet.01.650091.g8.t1 | family transcriptional regulator | 879 | 0 | 73.5 | F:DNA binding; F:transcription factor activity, sequence-specific DNA binding; P:transcription, DNA-templated; P:regulation of transcription, DNA-templated |
| Efet.01.650091.g9.t1 | acyl- dehydrogenase | 1194 | 0 | 80.15 | F:acyl-CoA dehydrogenase activity; F:flavin adenine dinucleotide binding; P:oxidation-reduction process |
| Efet.01.650175.g11.t1 | tripartite motif-containing 45-like isoform X1 | 486 | 1.49E-67 | 91.65 | F:zinc ion binding; C:intracellular |
| Efet.01.650206.g12.t1 | YY1-associated factor 2-like | 357 | 9.58E-19 | 94.55 | F:zinc ion binding; P:forebrain development; P:negative regulation of apoptotic process |
| Efet.01.650262.g16.t1 | 3-ketoacyl-ACP reductase [Rhodococcus hoagii] | 462 | 7.63E-90 | 64.15 | F:oxidoreductase activity; P:oxidation-reduction process |
| Efet.01.650262.g17.t1 | gfo Idh family oxidoreductase | 1044 | 0 | 64.5 | F:oxidoreductase activity; P:oxidation-reduction process |
| Efet.01.650275.g18.t1 | class II fumarate hydratase | 432 | 3.26E-90 | 86.75 | P:tricarboxylic acid cycle; F:fumarate hydratase activity; P:fumarate metabolic process; C:tricarboxylic acid cycle enzyme complex |
| Efet.01.650275.g19.t1 | aminopeptidase | 627 | 6.59E-132 | 93.8 | F:metal ion binding; P:proteolysis; F:aminopeptidase activity |
| Efet.01.650297.g20.t1 | allophanate hydrolase | 810 | 2.46E-133 | 86.5 | F:hydrolase activity |
| Efet.01.650311.g21.t1 | glycosyl transferase family 2 | 345 | 6.43E-48 | 62.1 | F:transferase activity |
| Efet.01.650311.g22.t1 | dolichyl-phosphate-mannose-- mannosyltransferase | 1629 | 0 | 54.6 | C:integral component of membrane |
| Efet.01.650311.g23.t1 | class I SAM-dependent methyltransferase | 1272 | 1.26E-137 | 82.4 | F:methyltransferase activity; P:methylation |
| Efet.01.650311.g24.t1 | L-rhamnonate dehydratase | 1269 | 0 | 93.5 | F:magnesium ion binding; P:cellular amino acid catabolic process; F:L-rhamnonate dehydratase activity |
| Efet.01.650311.g25.t1 | MFS transporter | 1305 | 0 | 85.05 | P:transmembrane transport; C:integral component of membrane |
| Efet.01.650311.g26.t1 | NAD(P)-dependent oxidoreductase | 777 | 0 | 84.95 | F:3-oxoacyl-[acyl-carrier-protein] reductase (NADPH) activity; P:oxidation-reduction process |
| Efet.01.650311.g27.t1 | family transcriptional regulator | 459 | 4.12E-103 | 85.9 | F:DNA binding; F:transcription factor activity, sequence-specific DNA binding; P:transcription, DNA-templated; P:regulation of transcription, DNA-templated |
| Efet.01.650311.g28.t1 | Rrf2 family transcriptional regulator | 621 | 4.25E-104 | 69.2 |  |
| Efet.01.650311.g29.t1 | acyl- synthetase | 1836 | 0 | 77.5 | P:metabolic process; F:ligase activity |
| Efet.01.650311.g30.t1 | ABC transporter ATP-binding | 1257 | 0 | 78.1 | F:ATP binding |
| Efet.01.650311.g31.t1 | ABC transporter ATP-binding | 759 | 5.01E-113 | 79.75 | F:ATPase activity; F:ATP binding |
| Efet.01.650311.g32.t1 | ABC transporter ATP-binding | 807 | 4.93E-117 | 77.05 | F:ATPase activity; F:ATP binding |
| Efet.01.650311.g33.t1 | branched-chain amino acid ABC transporter permease | 873 | 2.50E-93 | 80.15 | C:integral component of membrane; P:transport; C:plasma membrane; F:transporter activity |
| Efet.01.650311.g34.t1 | branched-chain amino acid ABC transporter permease | 1023 | 1.11E-113 | 76.05 | C:integral component of membrane; P:transport; C:plasma membrane; F:transporter activity |
| Efet.01.650311.g35.t1 | Enoyl- hydratase | 549 | 2.78E-58 | 69.35 | P:metabolic process; F:enoyl-CoA hydratase activity |
| Efet.01.650311.g36.t1 | Enoyl- hydratase | 801 | 1.27E-127 | 77.1 | P:metabolic process; F:enoyl-CoA hydratase activity |
| Efet.01.650329.g37.t1 | Spermatogenesis-associated 13 | 282 | 1.53E-35 | 82.25 | P:regulation of Rho protein signal transduction; F:Rho guanyl-nucleotide exchange factor activity; C:intracellular; P:intracellular signal transduction; P:positive regulation of GTPase activity |
| Efet.01.650336.g38.t1 | peptidase S14 | 330 | 1.80E-36 | 65.45 | F:ATP binding |
| Efet.01.650336.g39.t1 | Hin recombinase | 435 | 2.31E-59 | 78.29 | F:DNA binding |
| Efet.01.650338.g40.t1 | Domain of uncharacterised function (DUF955) [Mycobacterium abscessus abscessus] | 633 | 3.58E-142 | 77.6 | F:DNA binding |
| Efet.01.650338.g41.t1 | L-arabinose isomerase | 546 | 2.85E-107 | 90.45 | C:cytoplasm; F:manganese ion binding; F:L-arabinose isomerase activity; P:L-arabinose catabolic process to xylulose 5-phosphate |
| Efet.01.650357.g43.t1 | Transcriptional component of viral defense system | 468 | 2.34E-81 | 67.7 |  |
| Efet.01.650357.g44.t1 | nucleotidyl transferase ii ii toxin family | 717 | 3.63E-151 | 77.8 |  |
| Efet.01.650357.g45.t1 | ATP-dependent helicase | 333 | 1.50E-56 | 83.5 | F:ATP binding; C:integral component of membrane; F:helicase activity |
| Efet.01.650357.g46.t1 | family thioesterase | 201 | 3.49E-23 | 100 |  |
| Efet.01.65039.g911.t1 | neuropeptide FF receptor 2-like | 462 | 7.88E-31 | 65.2 | F:G-protein coupled receptor activity; P:G-protein coupled receptor signaling pathway; C:integral component of membrane |
| Efet.01.650455.g50.t1 | sugar ABC transporter substrate-binding | 483 | 3.93E-75 | 74.35 |  |
| Efet.01.650480.g52.t1 | pre translocase subunit | 2754 | 0 | 89.1 | F:ATP binding; P:protein targeting; C:cytoplasm; F:metal ion binding; P:intracellular protein transmembrane transport; P:protein import; C:plasma membrane |
| Efet.01.650480.g53.t1 | peptidase M23 | 1101 | 0 | 92.55 | C:integral component of membrane |
| Efet.01.650480.g54.t1 | protease specificity-enhancing factor | 531 | 1.52E-82 | 91.8 |  |
| Efet.01.650480.g55.t1 | glutathione S-transferase | 612 | 1.18E-147 | 99.45 | F:maleylacetoacetate isomerase activity; F:transferase activity |
| Efet.01.650480.g56.t1 | cytochrome c | 579 | 6.32E-142 | 94.65 | F:electron carrier activity; C:integral component of membrane; F:heme binding |
| Efet.01.650480.g57.t1 | cytochrome b | 1308 | 0 | 94.5 | F:ubiquinol-cytochrome-c reductase activity; F:electron carrier activity; C:respiratory chain complex III; P:hydrogen ion transmembrane transport; P:mitochondrial electron transport, ubiquinol to cytochrome c |
| Efet.01.650480.g58.t1 | ubiquinol-cytochrome c reductase iron-sulfur subunit | 600 | 1.96E-130 | 93.1 | F:ubiquinol-cytochrome-c reductase activity; F:metal ion binding; C:integral component of membrane; P:hydrogen ion transmembrane transport; P:oxidation-reduction process; F:2 iron, 2 sulfur cluster binding |
| Efet.01.650480.g59.t1 | 4-hydroxythreonine-4-phosphate dehydrogenase | 1038 | 0 | 83.45 | F:magnesium ion binding; P:pyridoxal phosphate biosynthetic process; F:zinc ion binding; P:pyridoxine biosynthetic process; C:cytoplasm; F:NAD binding; F:4-hydroxythreonine-4-phosphate dehydrogenase activity; P:oxidation-reduction process; F:cobalt ion binding |
| Efet.01.650480.g60.t1 | TM2 domain-containing | 360 | 2.34E-66 | 89.35 | C:integral component of membrane |
| Efet.01.650480.g61.t1 | N-acetyltransferase | 546 | 1.65E-114 | 92.75 | F:N-acetyltransferase activity |
| Efet.01.650481.g62.t1 | heavy metal translocating P-type ATPase | 726 | 2.04E-87 | 75.7 | F:cation-transporting ATPase activity; C:membrane; P:cation transmembrane transport; P:metal ion transport; F:binding |
| Efet.01.65051.g912.t1 | tubby homolog | 642 | 2.88E-51 | 81.45 | C:cilium; F:phosphatidylinositol binding; P:protein localization to cilium |
| Efet.01.650542.g64.t1 | oxidoreductase | 363 | 5.53E-52 | 74 | F:metal ion binding; F:electron carrier activity; F:phthalate 4,5-dioxygenase activity; P:oxidation-reduction process; F:2 iron, 2 sulfur cluster binding |
| Efet.01.650542.g65.t1 | oxidoreductase | 342 | 8.26E-74 | 62.35 | F:metal ion binding; F:electron carrier activity; F:phthalate 4,5-dioxygenase activity; P:oxidation-reduction process; F:2 iron, 2 sulfur cluster binding |
| Efet.01.650580.g66.t1 | beta-N-acetylhexosaminidase | 738 | 4.31E-122 | 70.45 | P:metabolic process; F:hydrolase activity, hydrolyzing O-glycosyl compounds |
| Efet.01.650606.g67.t1 | spectrin alpha non-erythrocytic 1-like | 546 | 6.52E-58 | 74.85 | F:binding |
| Efet.01.65064.g914.t1 | vacuolar sorting-associated 51 homolog | 342 | 3.81E-28 | 74.7 | P:regulation of Rho protein signal transduction; F:Rho guanyl-nucleotide exchange factor activity; P:positive regulation of GTPase activity |
| Efet.01.650644.g71.t1 | ethylmalonyl- decarboxylase-like | 387 | 6.53E-22 | 60.2 | F:catalytic activity; P:metabolic process |
| Efet.01.650654.g73.t1 | UDP-N-acetylmuramate--L-alanine ligase | 879 | 7.86E-173 | 81.8 | P:regulation of cell shape; P:cell division; F:ATP binding; C:cytoplasm; P:peptidoglycan biosynthetic process; F:UDP-N-acetylmuramate-L-alanine ligase activity; P:cell wall organization; P:cell cycle |
| Efet.01.650655.g74.t1 | calmodulin isoform X1 | 240 | 7.91E-45 | 96.7 | F:calcium ion binding |
| Efet.01.650664.g75.t1 | allatostatin-A receptor-like | 351 | 2.85E-11 | 74.5 | F:opioid receptor activity; P:opioid receptor signaling pathway; C:integral component of membrane |
| Efet.01.65072.g915.t1 | Centromere N | 366 | 2.52E-09 | 63 |  |
| Efet.01.650742.g77.t1 | histone-lysine N-methyltransferase SETD1- | 1563 | 1.64E-06 | 58 | F:nucleotide binding; F:nucleic acid binding |
| Efet.01.650749.g78.t1 | aldehyde dehydrogenase | 306 | 1.59E-60 | 77.2 | F:aldehyde dehydrogenase (NAD) activity; P:oxidation-reduction process |
| Efet.01.650749.g79.t1 | nucleotidyl transferase ii ii toxin family | 702 | 1.25E-137 | 72.25 |  |
| Efet.01.650749.g80.t1 | hypothetical protein | 612 | 8.61E-130 | 92.1 |  |
| Efet.01.650749.g81.t1 | OST-HTH LOTUS domain | 540 | 8.15E-59 | 75.6 |  |
| Efet.01.650749.g82.t1 | peptidylprolyl isomerase | 1650 | 0 | 85.85 | F:peptidyl-prolyl cis-trans isomerase activity; C:integral component of membrane; P:protein peptidyl-prolyl isomerization |
| Efet.01.650749.g83.t1 | DNA-binding HU | 318 | 2.69E-68 | 97.7 | F:DNA binding; P:chromosome condensation |
| Efet.01.650749.g84.t1 | tartrate dehydrogenase | 1095 | 0 | 96.75 | F:magnesium ion binding; F:NAD binding; F:tartrate dehydrogenase activity; P:oxidation-reduction process |
| Efet.01.650749.g85.t1 | CDP-diacylglycerol--glycerol-3-phosphate 3-phosphatidyltransferase | 567 | 3.60E-132 | 94.45 | C:integral component of membrane; P:phospholipid biosynthetic process; F:CDP-diacylglycerol-glycerol-3-phosphate 3-phosphatidyltransferase activity |
| Efet.01.650749.g86.t1 | Excinuclease ABC subunit C | 1782 | 0 | 82.8 | F:DNA binding; P:nucleic acid phosphodiester bond hydrolysis; F:excinuclease ABC activity; C:excinuclease repair complex; C:cytoplasm; P:nucleotide-excision repair; P:SOS response |
| Efet.01.650749.g87.t1 | ABC transporter substrate-binding | 1041 | 0 | 89.5 | C:outer membrane-bounded periplasmic space |
| Efet.01.650788.g89.t1 | la-related 6-like | 1386 | 8.53E-68 | 70.05 | F:nucleotide binding; F:nucleic acid binding; P:cellular macromolecule metabolic process; P:primary metabolic process; C:intracellular part |
| Efet.01.650818.g90.t1 | 1-deoxy-D-xylulose-5-phosphate synthase | 687 | 2.67E-122 | 88.5 | F:magnesium ion binding; F:thiamine pyrophosphate binding; F:1-deoxy-D-xylulose-5-phosphate synthase activity; P:terpenoid biosynthetic process; P:thiamine biosynthetic process; P:1-deoxy-D-xylulose 5-phosphate biosynthetic process |
| Efet.01.650832.g91.t1 | BTB and MATH domain-containing 38-like | 279 | 3.60E-36 | 66.1 |  |
| Efet.01.650854.g92.t1 | PREDICTED: uncharacterized protein LOC105217475 | 228 | 7.70E-08 | 62.17 |  |
| Efet.01.650868.g93.t1 | amidohydrolase | 393 | 5.13E-57 | 74.95 | P:metabolic process; F:hydrolase activity |
| Efet.01.650872.g94.t1 | myomodulin receptor 2 | 1125 | 2.41E-87 | 54.3 | F:G-protein coupled receptor activity; F:G-protein coupled peptide receptor activity; P:G-protein coupled receptor signaling pathway; P:signal transduction; C:membrane; C:integral component of membrane; F:signal transducer activity |
| Efet.01.650931.g98.t1 | flagellar hook-associated | 288 | 2.11E-55 | 93.45 | P:bacterial-type flagellum assembly; C:bacterial-type flagellum hook; F:structural molecule activity; P:bacterial-type flagellum-dependent cell motility |
| Efet.01.650931.g99.t1 | flagellar hook-associated 3 | 1269 | 0 | 84.3 | C:bacterial-type flagellum filament; C:bacterial-type flagellum hook; F:structural molecule activity; P:bacterial-type flagellum-dependent cell motility |
| Efet.01.650981.g103.t1 | Inorganic phosphate cotransporter | 321 | 9.42E-19 | 70.2 | C:membrane |
| Efet.01.65099.g917.t1 | FMRFamide receptor-like | 1008 | 3.14E-41 | 58.2 | F:G-protein coupled receptor activity; P:signal transduction; C:membrane |
| Efet.01.651000.g104.t1 | ATP synthase F0 subunit B | 324 | 6.60E-64 | 66.1 | P:proton transport; C:membrane; P:ATP biosynthetic process; C:intracellular part |
| Efet.01.65104.g918.t1 | 39S ribosomal mitochondrial | 510 | 1.98E-33 | 55.1 | C:myosin complex; F:RNA binding; C:ribosome; F:motor activity |
| Efet.01.651068.g106.t1 | retrovirus-related Pol poly from transposon 412 | 2514 | 0 | 65.95 | F:binding |
| Efet.01.651068.g107.t1 | hypothetical protein BSL78_10941 | 1632 | 1.35E-23 | 44.05 |  |
| Efet.01.651088.g109.t1 | AAA family ATPase | 795 | 2.44E-176 | 95.3 | F:ATPase activity; F:ATP binding |
| Efet.01.651088.g110.t1 | histone deacetylase superfamily | 285 | 1.17E-36 | 82.8 |  |
| Efet.01.651136.g112.t1 | 23S rRNA (pseudouridine(1915)-N(3))-methyltransferase | 468 | 1.38E-80 | 90.85 | C:cytoplasm; F:rRNA (pseudouridine-N3-)-methyltransferase activity; P:rRNA methylation |
| Efet.01.651136.g113.t1 | ribosome silencing factor | 702 | 4.94E-97 | 95.6 | C:cytoplasm; P:mature ribosome assembly; P:negative regulation of translation; P:negative regulation of ribosome biogenesis |
| Efet.01.651136.g114.t1 | fumarylacetoacetate hydrolase | 1035 | 0 | 82.6 | P:metabolic process; F:hydrolase activity |
| Efet.01.651136.g115.t1 | DUF1156 domain-containing | 1212 | 2.69E-178 | 74.9 | P:DNA methylation on adenine; F:site-specific DNA-methyltransferase (adenine-specific) activity |
| Efet.01.651136.g116.t1 | -class molecular chaperone with C-terminal Zn finger domain | 1053 | 0 | 78.85 |  |
| Efet.01.651136.g117.t1 | NYN domain-containing | 573 | 1.49E-92 | 79.5 | P:oxidation-reduction process; F:monooxygenase activity |
| Efet.01.651136.g118.t1 | RNA helicase | 375 | 1.16E-78 | 88.65 | F:DNA binding; F:ATP binding; F:helicase activity |
| Efet.01.651136.g119.t1 | RNA helicase | 2961 | 0 | 86.3 | F:DNA binding; F:ATP binding; F:helicase activity |
| Efet.01.651136.g120.t1 | NADH:ubiquinone oxidoreductase subunit N | 1470 | 0 | 92.55 | F:quinone binding; C:integral component of membrane; F:NADH dehydrogenase (ubiquinone) activity; P:ATP synthesis coupled electron transport; P:transport; C:plasma membrane |
| Efet.01.651136.g121.t1 | NADH-quinone oxidoreductase subunit M | 1416 | 0 | 94.2 | P:mitochondrial electron transport, NADH to ubiquinone; C:integral component of membrane; F:NADH dehydrogenase (ubiquinone) activity |
| Efet.01.651136.g122.t1 | NADH-quinone oxidoreductase subunit L | 2022 | 0 | 90.65 | C:integral component of membrane; F:NADH dehydrogenase (ubiquinone) activity; P:ATP synthesis coupled electron transport |
| Efet.01.651136.g123.t1 | NADH-quinone oxidoreductase subunit K | 249 | 2.69E-44 | 87.4 | F:NADH dehydrogenase (quinone) activity; F:quinone binding; C:integral component of membrane; P:ATP synthesis coupled electron transport; P:transport; C:plasma membrane |
| Efet.01.651136.g124.t1 | NADH:ubiquinone oxidoreductase subunit J | 672 | 1.37E-128 | 91.65 | C:integral component of membrane; F:NADH dehydrogenase (ubiquinone) activity; P:oxidation-reduction process |
| Efet.01.651136.g125.t1 | NADH-quinone oxidoreductase subunit I | 234 | 6.59E-50 | 98.8 | F:NADH dehydrogenase (quinone) activity; F:quinone binding; F:iron ion binding; P:oxidation-reduction process; F:4 iron, 4 sulfur cluster binding; C:plasma membrane |
| Efet.01.651141.g126.t1 | isocitrate lyase | 1311 | 0 | 97.85 | F:isocitrate lyase activity; P:carboxylic acid metabolic process |
| Efet.01.651141.g127.t1 | threonine--tRNA ligase | 1953 | 0 | 91.7 | F:ATP binding; C:cytoplasm; F:metal ion binding; F:threonine-tRNA ligase activity; P:threonyl-tRNA aminoacylation |
| Efet.01.651141.g128.t1 | translation initiation factor IF-3 | 540 | 4.88E-96 | 98.8 | F:translation initiation factor activity; C:cytoplasm; P:translational initiation |
| Efet.01.651141.g129.t1 | 50S ribosomal L20 | 360 | 1.07E-77 | 99.25 | F:structural constituent of ribosome; C:ribosome; F:rRNA binding; P:ribosomal large subunit assembly; P:translation |
| Efet.01.651141.g130.t1 | phenylalanine--tRNA ligase subunit alpha | 1053 | 0 | 95.35 | F:magnesium ion binding; F:ATP binding; C:cytoplasm; F:phenylalanine-tRNA ligase activity; P:phenylalanyl-tRNA aminoacylation; F:tRNA binding |
| Efet.01.651141.g131.t1 | phenylalanine--tRNA ligase subunit beta | 1539 | 0 | 89.3 | F:magnesium ion binding; F:ATP binding; C:cytoplasm; P:tRNA processing; F:phenylalanine-tRNA ligase activity; P:phenylalanyl-tRNA aminoacylation; F:tRNA binding |
| Efet.01.651148.g132.t1 | type II DNA modification enzyme | 4200 | 0 | 68.9 | F:DNA binding; F:methyltransferase activity; P:DNA modification; P:methylation |
| Efet.01.651148.g133.t1 | phosphoglycerate dehydrogenase | 1230 | 0 | 93.25 | F:NAD binding; F:phosphoglycerate dehydrogenase activity; P:L-serine biosynthetic process; P:oxidation-reduction process; F:amino acid binding |
| Efet.01.651148.g134.t1 | glycerophosphodiester phosphodiesterase | 834 | 0 | 84.7 | P:lipid metabolic process; F:glycerophosphodiester phosphodiesterase activity |
| Efet.01.651148.g135.t1 | porin | 999 | 0 | 82.9 |  |
| Efet.01.651148.g136.t1 | ABC transporter substrate-binding | 1581 | 0 | 93.05 | P:transmembrane transport; C:ATP-binding cassette (ABC) transporter complex |
| Efet.01.651148.g137.t1 | ABC transporter permease | 954 | 0 | 94.85 | C:integral component of membrane; P:transport; C:plasma membrane |
| Efet.01.651148.g138.t1 | amidohydrolase | 972 | 0 | 95.3 | F:hippurate hydrolase activity; P:metabolic process |
| Efet.01.651148.g139.t1 | ABC transporter substrate-binding | 1581 | 0 | 91.9 | P:transmembrane transport; C:ATP-binding cassette (ABC) transporter complex |
| Efet.01.651148.g140.t1 | ABC transporter permease | 849 | 8.39E-172 | 94.55 | C:integral component of membrane; P:transport; C:plasma membrane |
| Efet.01.651148.g141.t1 | ABC transporter ATP-binding | 1989 | 0 | 93.3 | F:ATPase activity; F:ATP binding; P:peptide transport |
| Efet.01.651148.g142.t1 | polyphosphate kinase 1 | 483 | 3.23E-86 | 88.2 | F:ATP binding; C:polyphosphate kinase complex; P:phosphorylation; F:polyphosphate kinase activity; P:polyphosphate biosynthetic process |
| Efet.01.651181.g144.t1 | bromodomain-containing DDB_G0280777-like isoform X1 | 468 | 4.31E-30 | 57.95 | F:protein binding; C:cytoplasm; P:transcription, DNA-templated; C:nuclear speck; P:regulation of transcription, DNA-templated; F:RNA binding |
| Efet.01.651187.g145.t1 | GRB2-associated-binding 1-like isoform X4 | 249 | 2.93E-13 | 67.4 |  |
| Efet.01.651203.g146.t1 | MULTISPECIES: hypothetical protein | 1425 | 0 | 80.65 |  |
| Efet.01.651204.g147.t1 | cell filamentation Fic | 393 | 4.53E-54 | 87.8 | F:DNA binding |
| Efet.01.651204.g148.t1 | restriction endonuclease subunit S | 1149 | 5.17E-143 | 68.45 | F:DNA binding; P:nucleic acid phosphodiester bond hydrolysis; F:endonuclease activity; P:DNA modification |
| Efet.01.651205.g149.t1 | hypothetical protein BGO47_06270 | 225 | 5.54E-16 | 77.4 | F:N-acetyltransferase activity |
| Efet.01.651229.g150.t1 | TIGR00730 family Rossman fold | 306 | 5.42E-65 | 86.2 | F:hydrolase activity, hydrolyzing N-glycosyl compounds; P:cytokinin biosynthetic process |
| Efet.01.651229.g151.t1 | transcriptional regulator | 372 | 2.27E-33 | 96.3 | P:transcription, DNA-templated; P:regulation of catalytic activity; P:regulation of transcription, DNA-templated; P:regulation of nitrogen utilization; F:enzyme regulator activity |
| Efet.01.651229.g152.t1 | NAD+ synthase | 201 | 7.41E-37 | 97.3 | F:hydrolase activity, acting on carbon-nitrogen (but not peptide) bonds; F:NAD+ synthase (glutamine-hydrolyzing) activity; F:ATP binding; P:NAD biosynthetic process |
| Efet.01.65125.g920.t1 | hypothetical protein CAPTEDRAFT_228538 | 645 | 2.79E-07 | 46 |  |
| Efet.01.651263.g153.t1 | Membrane-associated protease regulator of activity | 1170 | 2.88E-170 | 59.4 | F:hydrolase activity |
| Efet.01.651263.g154.t1 | 1-deoxy-D-xylulose-5-phosphate reductoisomerase | 573 | 1.72E-111 | 84.05 | F:1-deoxy-D-xylulose-5-phosphate reductoisomerase activity; F:metal ion binding; F:NADPH binding; P:isopentenyl diphosphate biosynthetic process, methylerythritol 4-phosphate pathway; P:oxidation-reduction process; P:terpenoid biosynthetic process; F:isomerase activity |
| Efet.01.651267.g155.t1 | sarcoplasmic calcium-binding -like | 249 | 8.45E-12 | 66.8 | F:calcium ion binding |
| Efet.01.651287.g156.t1 | discoidin domain-containing receptor 2-like isoform X1 | 339 | 1.92E-38 | 77.9 | C:integral component of plasma membrane; F:ATP binding; P:peptidyl-tyrosine phosphorylation; F:protein tyrosine kinase collagen receptor activity; P:collagen-activated tyrosine kinase receptor signaling pathway |
| Efet.01.651338.g158.t1 | hypothetical protein CAPTEDRAFT_223749 | 279 | 7.22E-25 | 64 | C:membrane; C:integral component of membrane |
| Efet.01.651372.g161.t1 | nucleotidyl transferase ii ii toxin family | 405 | 1.51E-41 | 63.5 |  |
| Efet.01.651372.g162.t1 | hypothetical protein | 972 | 4.27E-125 | 64.25 |  |
| Efet.01.651412.g164.t1 | water and ammonia transporting aquaporin | 360 | 2.61E-19 | 88 | C:integral component of membrane; P:transport; F:transporter activity |
| Efet.01.651429.g165.t1 | 4-hydroxy-2-oxoheptanedioate aldolase | 609 | 1.22E-86 | 64.55 | F:catalytic activity; F:metal ion binding; C:membrane; C:integral component of membrane; F:lyase activity |
| Efet.01.651429.g166.t1 | tripartite tricarboxylate transporter substrate binding | 966 | 5.39E-136 | 68.3 | F:catalytic activity; C:outer membrane-bounded periplasmic space |
| Efet.01.651429.g167.t1 | SMP-30 gluconolactonase LRE family | 807 | 0 | 72.6 | F:hydrolase activity |
| Efet.01.651429.g168.t1 | phosphate ABC transporter substrate-binding | 801 | 3.73E-111 | 67.55 |  |
| Efet.01.651429.g169.t1 | Two-component system response regulator | 513 | 3.48E-54 | 93.5 | F:DNA binding; P:transcription, DNA-templated; P:phosphorelay signal transduction system; P:regulation of transcription, DNA-templated; C:intracellular |
| Efet.01.651438.g170.t1 | family transcriptional regulator | 408 | 7.84E-77 | 54.06 | F:DNA binding; F:transcription factor activity, sequence-specific DNA binding; P:transcription, DNA-templated; P:regulation of transcription, DNA-templated |
| Efet.01.651441.g171.t1 | MBL fold metallo-hydrolase | 336 | 3.08E-72 | 90 | F:hydrolase activity |
| Efet.01.651441.g172.t1 | c-type cytochrome biogenesis | 1206 | 0 | 91.2 | C:integral component of membrane; P:cytochrome complex assembly; F:heme binding |
| Efet.01.651441.g173.t1 | cytochrome c biogenesis | 2124 | 0 | 86.55 | C:integral component of membrane |
| Efet.01.651441.g174.t1 | cytochrome c4 | 639 | 1.66E-134 | 90.75 | F:iron ion binding; F:electron carrier activity; C:periplasmic space; F:heme binding |
| Efet.01.651441.g175.t1 | family ribosome biogenesis GTP-binding | 744 | 7.96E-164 | 93.8 | F:magnesium ion binding; F:GTP binding; P:cell division; F:GTPase activity; P:cell cycle |
| Efet.01.651501.g179.t1 | Arabinoxylan arabinofuranohydrolase precursor | 933 | 0 | 74.05 |  |
| Efet.01.651515.g180.t1 | Penicillin amidase | 1713 | 0 | 79.45 | P:antibiotic biosynthetic process; F:penicillin amidase activity |
| Efet.01.651515.g181.t1 | acyl- dehydrogenase | 1275 | 0 | 96.55 | F:acyl-CoA dehydrogenase activity; F:flavin adenine dinucleotide binding; P:oxidation-reduction process |
| Efet.01.651515.g182.t1 | phosphotransferase family | 981 | 0 | 94.2 | F:transferase activity |
| Efet.01.651515.g183.t1 | Crp Fnr family transcriptional regulator | 720 | 6.41E-162 | 86.85 | F:DNA binding; P:transcription, DNA-templated; P:regulation of transcription, DNA-templated |
| Efet.01.651515.g184.t1 | thiamine phosphate synthase | 900 | 1.86E-161 | 84.65 | F:thiamine-phosphate diphosphorylase activity; P:thiamine diphosphate biosynthetic process; P:thiamine biosynthetic process |
| Efet.01.651518.g185.t1 | thiol reductant ABC exporter subunit | 1296 | 0 | 61.15 | F:nucleotide binding; F:ATPase activity; C:membrane; P:transport |
| Efet.01.65153.g922.t1 | epidermal growth factor receptor-like isoform X1 | 906 | 6.49E-118 | 69.9 | F:protein kinase activity; P:phosphorylation |
| Efet.01.651547.g187.t1 | sodium-coupled monocarboxylate transporter 1-like | 393 | 3.15E-33 | 74.25 | C:integral component of plasma membrane; P:biotin transport; P:pantothenate transmembrane transport; F:sodium-dependent multivitamin transmembrane transporter activity |
| Efet.01.651566.g188.t1 | ABC transporter permease | 696 | 8.93E-129 | 59 | C:membrane; C:integral component of membrane; P:transport; F:transporter activity; C:plasma membrane |
| Efet.01.651567.g189.t1 | riboflavin synthase | 702 | 3.81E-158 | 84.8 | F:riboflavin synthase activity; F:oxidoreductase activity; P:oxidation-reduction process |
| Efet.01.651568.g190.t1 | DUF58 domain-containing | 348 | 5.80E-74 | 72.45 |  |
| Efet.01.651575.g191.t1 | D-ala-D-ala transporter subunit | 1011 | 1.97E-162 | 83.6 | C:integral component of membrane; P:transport; C:plasma membrane |
| Efet.01.651575.g192.t1 | peptide ABC transporter permease | 927 | 0 | 83.55 | C:integral component of membrane; P:transport; C:plasma membrane |
| Efet.01.651575.g193.t1 | peptide ABC transporter substrate-binding | 1548 | 0 | 76.25 | P:transmembrane transport; C:ATP-binding cassette (ABC) transporter complex |
| Efet.01.651575.g194.t1 | family transcriptional regulator | 810 | 0 | 66.25 | F:DNA binding; P:carbohydrate metabolic process; F:transcription factor activity, sequence-specific DNA binding; P:regulation of transcription, DNA-templated; F:carbohydrate binding |
| Efet.01.651575.g195.t1 | glutamate-1-semialdehyde 2,1-aminomutase | 705 | 2.05E-138 | 80.1 | F:pyridoxal phosphate binding; F:transaminase activity |
| Efet.01.651575.g196.t1 | IS110 family transposase | 300 | 9.31E-65 | 84.1 | F:DNA binding; F:transposase activity; P:transposition, DNA-mediated |
| Efet.01.651575.g197.t1 | IS110 family transposase | 468 | 1.11E-62 | 62.75 | F:DNA binding; F:transposase activity; P:transposition, DNA-mediated |
| Efet.01.651575.g198.t1 | family transcriptional regulator | 735 | 5.27E-158 | 76.75 | F:DNA binding; F:transcription factor activity, sequence-specific DNA binding; P:transcription, DNA-templated; P:regulation of transcription, DNA-templated |
| Efet.01.651575.g199.t1 | N,N-dimethylformamidase | 1413 | 0 | 64.6 |  |
| Efet.01.651586.g200.t1 | histone acetyltransferase p300-like | 408 | 5.49E-11 | 62.2 | F:transferase activity |
| Efet.01.651609.g202.t1 | ADP,ATP carrier mitochondrial-like | 564 | 1.87E-107 | 88.25 | P:transmembrane transport; F:structural constituent of ribosome; C:integral component of membrane; C:mitochondrial inner membrane; F:transporter activity; P:translation |
| Efet.01.651631.g203.t1 | alanine dehydrogenase | 570 | 5.79E-103 | 88.25 | F:alanine dehydrogenase activity; P:oxidation-reduction process; P:L-alanine catabolic process |
| Efet.01.651659.g207.t1 | potassium sodium hyperpolarization-activated cyclic nucleotide-gated channel 2-like | 291 | 2.92E-46 | 91.5 | F:voltage-gated potassium channel activity; P:potassium ion transmembrane transport; C:integral component of membrane |
| Efet.01.651713.g208.t1 | hypothetical protein CAPTEDRAFT_221558 | 294 | 5.32E-06 | 65 | F:nucleoside transmembrane transporter activity; P:transmembrane transport; C:membrane; C:integral component of membrane; P:nucleoside transmembrane transport; F:monoamine transmembrane transporter activity; P:transport; P:monoamine transport |
| Efet.01.651762.g211.t1 | ABC transporter substrate-binding | 765 | 3.20E-167 | 93.2 | C:integral component of membrane; P:transport; C:plasma membrane |
| Efet.01.651762.g212.t1 | sugar ABC transporter substrate-binding | 1122 | 0 | 83.15 | P:transport; F:transporter activity |
| Efet.01.65177.g924.t1 | von Willebrand factor type EGF and pentraxin domain-containing 1-like | 252 | 4.50E-19 | 70.25 | F:serine-type peptidase activity |
| Efet.01.651784.g213.t1 | peptidoglycan-binding | 696 | 6.17E-144 | 59.2 | C:membrane; C:integral component of membrane |
| Efet.01.651805.g214.t1 | Transcriptional i Type IV TA system | 627 | 4.78E-35 | 71.2 |  |
| Efet.01.651810.g215.t1 | biopolymer transporter | 348 | 1.08E-74 | 84.3 | P:protein transport; C:integral component of membrane; C:plasma membrane; F:transporter activity |
| Efet.01.651810.g216.t1 | Cell division and transport-associated | 708 | 1.02E-133 | 83.85 | P:cell division; P:protein transport; C:integral component of membrane; F:protein transporter activity |
| Efet.01.651810.g217.t1 | transcription antitermination factor | 669 | 4.60E-69 | 86.25 | F:RNA binding; P:regulation of transcription, DNA-templated; P:DNA-templated transcription, termination |
| Efet.01.651810.g218.t1 | 3,4-dihydroxy-2-butanone 4-phosphate synthase | 1122 | 0 | 91.15 | F:metal ion binding; P:riboflavin biosynthetic process; F:3,4-dihydroxy-2-butanone-4-phosphate synthase activity |
| Efet.01.651810.g219.t1 | NADPH-dependent 7-cyano-7-deazaguanine reductase | 810 | 6.12E-162 | 76 | F:preQ1 synthase activity; P:queuosine biosynthetic process; C:cytoplasm; P:oxidation-reduction process; F:oxidoreductase activity, acting on other nitrogenous compounds as donors, with NAD or NADP as acceptor |
| Efet.01.651810.g220.t1 | peroxide stress | 783 | 1.43E-173 | 88.45 |  |
| Efet.01.651812.g221.t1 | histone deacetylase complex subunit SAP18 | 291 | 6.39E-30 | 79.3 |  |
| Efet.01.651815.g222.t1 | acriflavin resistance | 1140 | 5.33E-159 | 78.7 | C:integral component of membrane; P:transport; F:transporter activity |
| Efet.01.651816.g223.t1 | MFS transporter | 576 | 1.18E-50 | 90.5 | P:transmembrane transport; C:integral component of membrane |
| Efet.01.651816.g224.t1 | phosphocarrier HPr | 270 | 8.54E-45 | 95.35 | C:cytoplasm; P:phosphoenolpyruvate-dependent sugar phosphotransferase system; F:protein serine/threonine kinase activity; P:protein phosphorylation |
| Efet.01.651816.g225.t1 | phosphoenolpyruvate-- phosphotransferase | 1779 | 0 | 93.65 | C:cytoplasm; F:metal ion binding; P:phosphoenolpyruvate-dependent sugar phosphotransferase system; P:phosphorylation; F:phosphoenolpyruvate-protein phosphotransferase activity; F:kinase activity |
| Efet.01.651816.g226.t1 | DNA-binding response regulator | 615 | 9.48E-29 | 61.8 | F:DNA binding; P:transcription, DNA-templated; P:phosphorelay signal transduction system; P:regulation of transcription, DNA-templated; C:intracellular |
| Efet.01.651816.g227.t1 | lipo signal peptide | 768 | 5.18E-166 | 57.55 | F:1-alkyl-2-acetylglycerophosphocholine esterase activity; P:lipid catabolic process |
| Efet.01.651816.g228.t1 | serine O-acetyltransferase | 585 | 9.12E-67 | 76.35 | F:transferase activity, transferring acyl groups |
| Efet.01.651825.g229.t1 | farnesyltransferase geranylgeranyltransferase type-1 subunit alpha | 390 | 3.10E-45 | 87.75 | F:protein prenyltransferase activity; P:protein prenylation |
| Efet.01.651871.g231.t1 | protocadherin-like wing polarity stan isoform X2 | 357 | 5.76E-31 | 72.35 | F:G-protein coupled receptor activity; P:G-protein coupled receptor signaling pathway; F:calcium ion binding; C:integral component of membrane; P:cell surface receptor signaling pathway; P:homophilic cell adhesion via plasma membrane adhesion molecules; C:plasma membrane |
| Efet.01.651897.g233.t1 | potassium voltage-gated channel subfamily A member 1 | 603 | 1.10E-46 | 81.5 | C:presynaptic membrane; P:regulation of dopamine secretion; P:potassium ion transmembrane transport; C:voltage-gated potassium channel complex; F:delayed rectifier potassium channel activity; P:neuromuscular process; P:protein homooligomerization; P:optic nerve structural organization; C:neuronal cell body membrane; C:juxtaparanode region of axon; P:brain development; C:perikaryon; C:axon terminus; C:endoplasmic reticulum membrane; C:dendrite; C:endosome; F:protein binding; P:regulation of circadian sleep/wake cycle, non-REM sleep; P:regulation of ion transmembrane transport; C:cell junction; P:sensory perception of pain; C:lamellipodium membrane; P:neuronal action potential; P:detection of mechanical stimulus involved in sensory perception |
| Efet.01.651921.g234.t1 | protocadherin-8 isoform X2 | 339 | 2.93E-17 | 63.65 | C:membrane |
| Efet.01.651935.g237.t1 | innexin 1 | 1308 | 8.39E-130 | 62.3 | C:membrane; P:cell communication by electrical coupling; C:synapse; F:gap junction channel activity; P:transport |
| Efet.01.651939.g238.t1 | family | 228 | 2.63E-36 | 92.95 |  |
| Efet.01.651939.g239.t1 | tripartite tricarboxylate transporter substrate binding | 912 | 0 | 86.95 | C:outer membrane-bounded periplasmic space |
| Efet.01.651939.g240.t1 | flavin reductase family | 537 | 7.60E-106 | 62.65 | F:FMN binding; F:oxidoreductase activity; P:oxidation-reduction process |
| Efet.01.651940.g241.t1 | teicoplanin resistance | 1512 | 6.21E-127 | 80.4 | C:integral component of membrane |
| Efet.01.651940.g242.t1 | metal ABC transporter substrate-binding | 1518 | 0 | 93.95 |  |
| Efet.01.651940.g243.t1 | ABC transporter ATP-binding | 573 | 2.55E-133 | 93.35 | F:ATPase activity; F:ATP binding |
| Efet.01.651940.g244.t1 | ABC transporter permease | 426 | 9.04E-76 | 84.65 | C:integral component of membrane; P:transport; C:plasma membrane |
| Efet.01.651940.g245.t1 | ABC transporter permease | 363 | 1.38E-55 | 92.4 | C:integral component of membrane; P:transport; C:plasma membrane |
| Efet.01.651940.g246.t1 | cyclase | 807 | 0 | 96.05 | F:arylformamidase activity; P:tryptophan catabolic process to kynurenine |
| Efet.01.651940.g247.t1 | delta-aminolevulinic acid dehydratase | 1077 | 0 | 92.65 | F:porphobilinogen synthase activity; P:porphyrin-containing compound biosynthetic process; F:metal ion binding |
| Efet.01.651940.g248.t1 | magnesium transporter | 348 | 1.10E-41 | 91.6 | P:transmembrane transport; C:integral component of membrane; F:metal ion transmembrane transporter activity; P:metal ion transport |
| Efet.01.651945.g249.t1 | hypothetical protein CAPTEDRAFT_214964 | 462 | 1.95E-24 | 53.45 |  |
| Efet.01.651978.g250.t1 | DNA-binding response regulator | 606 | 4.76E-88 | 67.45 | P:regulation of cellular process; P:cellular process |
| Efet.01.651984.g251.t1 | BTB POZ domain-containing 17-like | 612 | 2.03E-31 | 51.9 | C:membrane; C:integral component of membrane |
| Efet.01.652001.g253.t1 | interferon-induced very large GTPase 1 | 468 | 1.63E-19 | 59.73 | F:GTP binding |
| Efet.01.652023.g255.t1 | hypothetical protein HELRODRAFT_181005 | 228 | 4.54E-25 | 79.6 |  |
| Efet.01.652033.g256.t1 | hypothetical protein CGI_10028476 | 315 | 2.33E-18 | 69.25 |  |
| Efet.01.652103.g258.t1 | cytochrome c oxidase subunit I | 243 | 6.83E-29 | 96.5 | F:cytochrome-c oxidase activity; P:oxidative phosphorylation; F:copper ion binding; F:iron ion binding; C:integral component of membrane; P:hydrogen ion transmembrane transport; P:electron transport chain; P:aerobic respiration; C:plasma membrane; C:respiratory chain; F:heme binding |
| Efet.01.652108.g259.t1 | sugar ABC transporter substrate-binding | 837 | 9.95E-172 | 81.9 |  |
| Efet.01.652108.g260.t1 | ABC transporter permease | 987 | 4.10E-137 | 84.4 | C:integral component of membrane; P:monosaccharide transport; F:monosaccharide-transporting ATPase activity; C:plasma membrane |
| Efet.01.652108.g261.t1 | Cobyrinic acid a,c-diamide synthase | 615 | 4.36E-139 | 89.9 | F:ATP binding |
| Efet.01.652152.g262.t1 | MFS transporter | 654 | 5.43E-55 | 74.5 | C:membrane |
| Efet.01.652175.g263.t1 | Leucine-rich repeat-containing 47 | 387 | 1.27E-14 | 52.25 | F:RNA binding; F:phenylalanine-tRNA ligase activity |
| Efet.01.65218.g925.t1 | leucine-rich repeat-containing 70 | 1494 | 4.62E-121 | 48.6 |  |
| Efet.01.652207.g264.t1 | Protein-disulfide isomerase | 444 | 9.08E-65 | 70.2 | C:membrane; C:integral component of membrane |
| Efet.01.652215.g265.t1 | phosphoethanolamine transferase | 1731 | 0 | 85.05 | F:sulfuric ester hydrolase activity; P:metabolic process; C:integral component of membrane |
| Efet.01.652215.g266.t1 | hypothetical protein | 597 | 1.54E-111 | 69.44 |  |
| Efet.01.652216.g267.t1 | ATP-dependent Clp protease ATP-binding subunit | 846 | 0 | 86.15 | F:ATP binding; P:proteolysis; F:peptidase activity |
| Efet.01.652239.g268.t1 | gonadotropin-releasing hormone II receptor-like | 390 | 6.20E-23 | 70.35 | F:G-protein coupled peptide receptor activity; P:G-protein coupled receptor signaling pathway; C:membrane |
| Efet.01.652240.g269.t1 | bifunctional UDP-N-acetylmuramoyl-tripeptide:D-alanyl-D-alanine ligase alanine racemase | 288 | 1.09E-56 | 66.6 | F:catalytic activity |
| Efet.01.652283.g271.t1 | glutamyl-tRNA reductase | 1326 | 0 | 87.05 | F:NADP binding; F:glutamyl-tRNA reductase activity; P:oxidation-reduction process; P:protoporphyrinogen IX biosynthetic process |
| Efet.01.652283.g272.t1 | peptide chain release factor 1 | 996 | 0 | 92.25 | P:translational termination; C:cytoplasm; F:translation release factor activity, codon specific |
| Efet.01.652283.g273.t1 | SIR2 family | 1230 | 0 | 63.35 |  |
| Efet.01.652283.g274.t1 | DUF853 domain-containing | 1230 | 1.74E-167 | 74.3 | F:helicase activity |
| Efet.01.652307.g275.t1 | hypothetical protein CAPTEDRAFT_210637 | 816 | 1.74E-18 | 50 |  |
| Efet.01.652309.g277.t1 | branched-chain amino acid ABC transporter permease | 222 | 2.18E-27 | 96.9 | C:integral component of membrane; P:transport; C:plasma membrane; F:transporter activity |
| Efet.01.652309.g278.t1 | ABC transporter permease | 1002 | 0 | 96.3 |  |
| Efet.01.652309.g279.t1 | ABC transporter ATP-binding | 804 | 0 | 95.8 | F:ATPase activity; F:ATP binding |
| Efet.01.652314.g280.t1 | FAD-dependent oxidoreductase | 885 | 0 | 79 | F:oxidoreductase activity; P:oxidation-reduction process |
| Efet.01.652346.g281.t1 | apoptosis-inducing factor mitochondrial isoform X3 | 393 | 1.31E-52 | 80.95 | C:cell; F:flavin adenine dinucleotide binding; C:integral component of membrane; F:protein dimerization activity; P:cell redox homeostasis; P:oxidation-reduction process; F:ferredoxin-NAD+ reductase activity |
| Efet.01.652354.g282.t1 | macrolide ABC transporter ATP-binding | 723 | 1.96E-67 | 63.3 | C:membrane |
| Efet.01.652384.g284.t1 | peptidase S15 | 2019 | 0 | 74.2 | P:proteolysis; F:dipeptidyl-peptidase activity |
| Efet.01.652384.g285.t1 | N-ethylammeline chlorohydrolase | 2607 | 0 | 80.15 | F:N-isopropylammelide isopropylaminohydrolase activity |
| Efet.01.652384.g286.t1 | amidohydrolase | 1452 | 0 | 85.6 | F:hydrolase activity, acting on carbon-nitrogen (but not peptide) bonds |
| Efet.01.652384.g287.t1 | ABC transporter ATP-binding | 939 | 0 | 75.35 | F:ATPase activity; F:ATP binding; P:peptide transport |
| Efet.01.652384.g288.t1 | ABC transporter ATP-binding | 813 | 0 | 80.55 | F:ATPase activity; F:ATP binding; P:peptide transport |
| Efet.01.652384.g289.t1 | ABC transporter permease | 924 | 0 | 83.05 | C:integral component of membrane; P:transport; C:plasma membrane |
| Efet.01.652384.g290.t1 | ABC transporter permease | 966 | 0 | 85.15 | C:integral component of membrane; P:transport; C:plasma membrane |
| Efet.01.652384.g291.t1 | ABC transporter substrate-binding | 1164 | 9.94E-142 | 78.2 | P:transmembrane transport; C:ATP-binding cassette (ABC) transporter complex |
| Efet.01.652384.g292.t1 | ABC transporter substrate-binding | 645 | 1.65E-119 | 66.15 | P:transmembrane transport; C:ATP-binding cassette (ABC) transporter complex |
| Efet.01.652384.g293.t1 | ABC transporter substrate-binding | 1446 | 0 | 76.25 | P:transmembrane transport; C:ATP-binding cassette (ABC) transporter complex |
| Efet.01.65243.g927.t1 | CChain Conserved Mechanisms Of Microtubule-stimulated Adp Atp And Force Generation In Transport Kinesins | 804 | 2.51E-160 | 72.8 | F:ATPase activity; P:anterograde axonal transport; P:retrograde vesicle-mediated transport, Golgi to ER; P:cranial nerve development; C:Golgi apparatus; C:kinesin complex; P:posterior lateral line system development; C:microtubule; F:microtubule binding; F:RNA binding; F:ATP binding; F:microtubule motor activity; P:cellular component organization; P:myeloid cell differentiation |
| Efet.01.652439.g295.t1 | myosin heavy striated muscle-like isoform X6 | 810 | 6.19E-93 | 84 | C:myosin complex; F:ATP binding; F:actin binding; P:metabolic process; F:amino acid binding; F:motor activity |
| Efet.01.652440.g296.t1 | phosphoglycerate kinase | 528 | 1.49E-83 | 83.9 | F:ATP binding; C:cytoplasm; F:phosphoglycerate kinase activity; P:glycolytic process |
| Efet.01.652440.g297.t1 | branched-chain amino acid transporter | 336 | 7.45E-64 | 87.65 | C:integral component of membrane |
| Efet.01.652441.g298.t1 | arrestin domain-containing 3-like | 288 | 1.75E-37 | 80.35 | P:signal transduction |
| Efet.01.652442.g299.t1 | ferredoxin--NADP reductase | 660 | 1.37E-158 | 92.3 | F:metal ion binding; P:oxidation-reduction process; F:ferredoxin-NADP+ reductase activity |
| Efet.01.652442.g300.t1 | family transcriptional regulator | 693 | 2.29E-143 | 93.5 | F:DNA binding; P:transcription, DNA-templated; P:regulation of transcription, DNA-templated |
| Efet.01.652442.g301.t1 | enamidase | 1149 | 0 | 92.05 | F:enamidase activity |
| Efet.01.652442.g302.t1 | family transporter | 201 | 1.40E-32 | 77.3 | C:integral component of membrane |
| Efet.01.652442.g303.t1 | family transporter | 387 | 7.17E-57 | 81.6 | C:integral component of membrane |
| Efet.01.652446.g304.t1 | fem-1 homolog B | 1938 | 0 | 68.6 | P:regulation of cellular process; P:epithelium development; C:intracellular part; P:cellular process; P:single-organism developmental process |
| Efet.01.652453.g305.t1 | Carnitine O-palmitoyltransferase mitochondrial | 420 | 1.34E-61 | 79.45 | F:transferase activity, transferring acyl groups; C:mitochondrion; C:nucleolus; C:nucleoplasm |
| Efet.01.652468.g307.t1 | furin | 543 | 1.47E-10 | 55.35 | P:regulation of protein metabolic process; P:proteolysis; F:peptidase activity; P:single-organism process; P:cellular process |
| Efet.01.652475.g308.t1 | F-box WD repeat-containing 7 | 453 | 4.77E-80 | 91.05 | P:positive regulation of protein ubiquitination involved in ubiquitin-dependent protein catabolic process; P:regulation of mitophagy; P:negative regulation of RNA polymerase II regulatory region sequence-specific DNA binding; F:phosphothreonine binding; C:Golgi apparatus; P:vasculogenesis; P:positive regulation of epidermal growth factor-activated receptor activity; C:nucleolus; P:negative regulation of Notch signaling pathway; P:positive regulation of proteasomal protein catabolic process; C:nucleoplasm; P:regulation of cell cycle G1/S phase transition; P:negative regulation of SREBP signaling pathway; F:transcription factor activity, sequence-specific DNA binding; P:positive regulation of ubiquitin-protein transferase activity; F:ubiquitin-protein transferase activator activity; P:viral process; P:cellular response to UV; C:cytosol; C:SCF ubiquitin ligase complex; P:lung development; P:sister chromatid cohesion; F:identical protein binding; P:regulation of cell migration involved in sprouting angiogenesis; P:positive regulation of oxidative stress-induced neuron intrinsic apoptotic signaling pathway; P:protein polyubiquitination; P:lipid homeostasis; C:endoplasmic reticulum; C:protein complex; P:cellular response to DNA damage stimulus; P:positive regulation of protein targeting to mitochondrion; C:Parkin-FBXW7-Cul1 ubiquitin ligase complex; P:protein destabilization; F:ubiquitin-protein transferase activity; C:perinuclear region of cytoplasm; F:ubiquitin protein ligase binding; P:Notch signaling pathway; P:negative regulation of triglyceride biosynthetic process; C:mitochondrion; P:positive regulation of ERK1 and ERK2 cascade; C:integral component of membrane; P:SCF-dependent proteasomal ubiquitin-dependent protein catabolic process; F:cyclin binding; P:negative regulation of DNA endoreduplication; P:protein stabilization; F:protein binding, bridging; P:negative regulation of hepatocyte proliferation; P:regulation of lipid storage |
| Efet.01.652520.g310.t1 | hypothetical protein CRN43_05890 | 621 | 1.42E-148 | 84 |  |
| Efet.01.652534.g312.t1 | cornichon homolog 4 | 228 | 1.95E-26 | 83.2 | P:ER to Golgi vesicle-mediated transport; C:integral component of membrane; C:endoplasmic reticulum; C:endoplasmic reticulum-Golgi intermediate compartment; F:CCR5 chemokine receptor binding |
| Efet.01.652554.g313.t1 | tyrosine- phosphatase Lar | 318 | 3.06E-13 | 65.55 | F:phosphatase activity; P:dephosphorylation |
| Efet.01.652565.g314.t1 | microtubule-actin cross-linking factor 1-like isoform X7 | 1317 | 2.16E-72 | 54.1 | P:cell cycle arrest; C:cytoskeleton; F:actin binding; F:calcium ion binding |
| Efet.01.652587.g315.t1 | pseudouridine synthase | 696 | 1.93E-153 | 90.2 | P:pseudouridine synthesis; F:RNA binding; F:pseudouridine synthase activity |
| Efet.01.652587.g316.t1 | SMC-Scp complex subunit | 651 | 2.84E-125 | 93.4 | P:cell division; C:cytoplasm; P:chromosome separation |
| Efet.01.652587.g317.t1 | rRNA pseudouridine synthase | 567 | 6.85E-117 | 98.05 | P:pseudouridine synthesis; F:RNA binding; F:pseudouridine synthase activity |
| Efet.01.652588.g318.t1 | DNA repair | 789 | 4.90E-33 | 77.85 | P:nucleic acid phosphodiester bond hydrolysis; F:exonuclease activity |
| Efet.01.652588.g319.t1 | hypothetical protein A3F76_05525 | 309 | 1.24E-32 | 65.6 |  |
| Efet.01.652588.g320.t1 | Plasmid stabilization system antitoxin | 261 | 2.83E-41 | 86.15 |  |
| Efet.01.652588.g321.t1 | hypothetical protein [Diaphorobacter polyhydroxybutyrativorans] | 336 | 1.64E-48 | 62.95 |  |
| Efet.01.652588.g322.t1 | IS110 family transposase | 573 | 1.38E-97 | 82.9 | F:DNA binding; F:transposase activity; P:transposition, DNA-mediated |
| Efet.01.652632.g323.t1 | hypothetical protein CAPTEDRAFT_222844 | 762 | 7.08E-15 | 43 | F:metal ion binding |
| Efet.01.652644.g326.t1 | enabled isoform X5 | 360 | 7.29E-25 | 60.15 |  |
| Efet.01.652656.g328.t1 | transmembrane and TPR repeat-containing 2 isoform X2 | 1026 | 3.90E-53 | 53.85 | C:endoplasmic reticulum membrane; P:calcium ion homeostasis; C:membrane; C:integral component of membrane; C:endoplasmic reticulum |
| Efet.01.652659.g329.t1 | nickel resistance | 450 | 5.85E-56 | 85.55 | F:DNA binding; F:metal ion binding; P:regulation of transcription, DNA-templated |
| Efet.01.652659.g330.t1 | nickel cobalt efflux | 606 | 7.54E-126 | 67.1 | F:nickel cation transmembrane transporter activity; F:metal ion binding; C:integral component of membrane; P:nickel cation transmembrane transport; C:plasma membrane |
| Efet.01.652659.g331.t1 | ribosome biogenesis GTPase | 642 | 6.13E-119 | 94.15 | F:GTP binding; C:cytoplasm |
| Efet.01.652659.g332.t1 | glutamine--fructose-6-phosphate transaminase (isomerizing) | 711 | 4.38E-106 | 79.3 | P:carbohydrate metabolic process; C:cytoplasm; F:glutamine-fructose-6-phosphate transaminase (isomerizing) activity; P:glutamine metabolic process; P:carbohydrate derivative biosynthetic process; F:carbohydrate binding |
| Efet.01.652674.g333.t1 | otoferlin isoform X9 | 453 | 1.08E-36 | 66.6 | C:membrane |
| Efet.01.652693.g334.t1 | DUF2029 domain-containing | 750 | 4.18E-138 | 63.2 | F:transferase activity, transferring hexosyl groups; C:membrane; C:integral component of membrane |
| Efet.01.652703.g335.t1 | phosphate dikinase | 1326 | 0 | 79.9 | F:ATP binding; F:metal ion binding; F:pyruvate, phosphate dikinase activity; P:phosphorylation; F:kinase activity; P:pyruvate metabolic process |
| Efet.01.652729.g336.t1 | alpha beta hydrolase | 357 | 1.20E-74 | 71.8 | F:catalytic activity |
| Efet.01.652748.g339.t1 | glutaminase | 1062 | 0 | 75.5 | F:catalytic activity |
| Efet.01.65277.g928.t1 | zinc finger ZIC 4 isoform X2 | 756 | 2.71E-38 | 85.2 | C:nucleus; F:metal ion binding; F:RNA polymerase II regulatory region sequence-specific DNA binding |
| Efet.01.652812.g342.t1 | turtle homolog A- | 390 | 1.41E-32 | 63.85 | C:membrane; C:integral component of membrane |
| Efet.01.652822.g343.t1 | transmembrane 62-like | 381 | 2.42E-15 | 55.57 | C:membrane |
| Efet.01.652843.g345.t1 | D-alanyl-D-alanine dipeptidase | 303 | 3.15E-64 | 75.3 | F:zinc ion binding; F:dipeptidase activity; C:cell wall; P:proteolysis; P:cell wall organization; F:metallopeptidase activity |
| Efet.01.652843.g346.t1 | tripartite tricarboxylate transporter substrate binding | 252 | 1.79E-36 | 90.85 | F:lyase activity; C:outer membrane-bounded periplasmic space |
| Efet.01.652843.g347.t1 | 4-hydroxy-tetrahydrodipicolinate synthase | 1809 | 0 | 90.7 | P:metabolic process; F:lyase activity |
| Efet.01.652858.g348.t1 | A G-specific adenine glycosylase | 1029 | 0 | 84.45 | F:DNA binding; P:base-excision repair; F:DNA N-glycosylase activity; F:4 iron, 4 sulfur cluster binding |
| Efet.01.652858.g349.t1 | RNase adaptor | 837 | 0 | 93.55 | F:GTP binding; F:ATP binding |
| Efet.01.652858.g350.t1 | DNA repair | 2508 | 0 | 88.5 | F:ATP binding; P:DNA repair; P:DNA recombination |
| Efet.01.652858.g351.t1 | heat-inducible transcriptional repressor | 870 | 0 | 96.1 | F:DNA binding; P:negative regulation of transcription, DNA-templated; P:transcription, DNA-templated |
| Efet.01.652858.g352.t1 | 3-isopropylmalate dehydratase large subunit | 1299 | 0 | 88.55 | F:metal ion binding; P:leucine biosynthetic process; F:4 iron, 4 sulfur cluster binding; F:3-isopropylmalate dehydratase activity; F:isomerase activity |
| Efet.01.652858.g353.t1 | 3-isopropylmalate dehydratase small subunit | 651 | 8.57E-156 | 91.8 | P:leucine biosynthetic process; C:3-isopropylmalate dehydratase complex; F:3-isopropylmalate dehydratase activity |
| Efet.01.652858.g354.t1 | 3-isopropylmalate dehydrogenase | 1074 | 0 | 95.9 | F:magnesium ion binding; C:cytoplasm; F:NAD binding; P:leucine biosynthetic process; P:oxidation-reduction process; F:3-isopropylmalate dehydrogenase activity |
| Efet.01.652858.g355.t1 | dihydrodipicolinate synthase family | 1158 | 0 | 96.25 | P:metabolic process; F:4-hydroxy-tetrahydrodipicolinate synthase |
| Efet.01.652860.g356.t1 | beta keto-acyl synthase | 1359 | 0 | 53 |  |
| Efet.01.652863.g357.t1 | conjugative coupling factor PFGI-1 class | 1629 | 0 | 93.05 | C:integral component of membrane |
| Efet.01.652863.g358.t1 | Iron-containing redox enzyme | 288 | 2.06E-12 | 80.73 |  |
| Efet.01.652863.g359.t1 | family transporter | 894 | 1.74E-116 | 67.55 | C:membrane |
| Efet.01.652863.g360.t1 | NAD(P)-dependent oxidoreductase | 879 | 2.00E-122 | 64.35 | F:oxidoreductase activity, acting on the CH-OH group of donors, NAD or NADP as acceptor |
| Efet.01.652863.g361.t1 | family transcriptional regulator | 891 | 4.24E-161 | 80.1 | F:DNA binding; F:transcription factor activity, sequence-specific DNA binding; P:transcription, DNA-templated; C:integral component of membrane; P:regulation of transcription, DNA-templated |
| Efet.01.652863.g362.t1 | lipopolysaccharide transport periplasmic | 501 | 1.79E-59 | 89.05 | P:Gram-negative-bacterium-type cell outer membrane assembly; C:periplasmic space; P:lipopolysaccharide transport; F:lipopolysaccharide binding |
| Efet.01.652898.g364.t1 | family | 348 | 4.51E-38 | 84.6 | P:carbohydrate metabolic process; F:catalytic activity |
| Efet.01.652898.g365.t1 | carboxylase | 831 | 9.69E-170 | 68.5 | F:urea carboxylase activity; F:lyase activity |
| Efet.01.652898.g366.t1 | allophanate hydrolase subunit 1 | 750 | 7.56E-158 | 68.8 | F:hydrolase activity |
| Efet.01.652898.g367.t1 | diguanylate phosphodiesterase | 2808 | 0 | 89.25 | F:phosphorelay sensor kinase activity; P:phosphorelay signal transduction system; C:integral component of membrane; P:signal transduction by protein phosphorylation; C:intracellular |
| Efet.01.652898.g368.t1 | ornithine-acyl-ACP acyltransferase | 858 | 0 | 89 | F:transferase activity, transferring acyl groups |
| Efet.01.652898.g369.t1 | creatininase | 849 | 1.32E-171 | 84.85 |  |
| Efet.01.652898.g370.t1 | Ala-tRNA(Pro) hydrolase | 759 | 2.94E-151 | 89.8 | F:nucleic acid binding; F:ATP binding; F:alanine-tRNA ligase activity; P:alanyl-tRNA aminoacylation; F:hydrolase activity |
| Efet.01.652898.g371.t1 | histidine phosphatase family | 675 | 1.46E-160 | 85.9 | P:phosphorylation; F:kinase activity |
| Efet.01.652898.g372.t1 | 2-aminoadipate aminotransferase | 678 | 2.32E-115 | 77.55 | F:pyridoxal phosphate binding; F:transaminase activity; P:biosynthetic process |
| Efet.01.652898.g373.t1 | lysine transporter | 540 | 4.97E-101 | 87.7 | C:integral component of membrane; P:amino acid transport; C:plasma membrane |
| Efet.01.652898.g374.t1 | PLP-dependent aminotransferase family | 555 | 3.68E-56 | 71.14 | F:DNA binding; F:transcription factor activity, sequence-specific DNA binding; F:pyridoxal phosphate binding; F:catalytic activity; P:transcription, DNA-templated; F:transaminase activity; P:regulation of transcription, DNA-templated; P:biosynthetic process; F:transferase activity |
| Efet.01.652898.g375.t1 | sensor histidine kinase | 1392 | 0 | 90.2 | F:phosphorelay sensor kinase activity; F:ATP binding; P:phosphorelay signal transduction system; C:integral component of membrane; P:signal transduction by protein phosphorylation; C:intracellular |
| Efet.01.652898.g376.t1 | DNA-binding response regulator | 681 | 1.89E-143 | 96.35 | F:DNA binding; P:transcription, DNA-templated; P:phosphorelay signal transduction system; P:regulation of transcription, DNA-templated; C:intracellular |
| Efet.01.652898.g377.t1 | family transcriptional regulator | 423 | 3.78E-93 | 92.1 | F:DNA binding; F:transcription factor activity, sequence-specific DNA binding; P:transcription, DNA-templated; P:regulation of transcription, DNA-templated |
| Efet.01.652898.g378.t1 | recombinase | 807 | 3.60E-157 | 98.1 | F:ATP binding; P:DNA repair; F:DNA-dependent ATPase activity; C:cytoplasm; P:DNA recombination; P:SOS response; F:damaged DNA binding; F:single-stranded DNA binding |
| Efet.01.652903.g379.t1 | hypothetical protein CAPTEDRAFT_225085 | 1020 | 1.11E-34 | 52.5 | C:membrane |
| Efet.01.652914.g380.t1 | dynein heavy chain axonemal-like isoform X1 | 327 | 2.70E-37 | 74.6 | F:nucleotide binding; P:movement of cell or subcellular component; C:dynein complex; F:nucleoside-triphosphatase activity |
| Efet.01.652915.g381.t1 | neurocalcin homolog | 378 | 1.11E-73 | 88.75 | C:extracellular exosome; F:protein binding; F:calcium ion binding; C:membrane |
| Efet.01.652956.g383.t1 | VOC family | 207 | 3.27E-40 | 70.4 | F:methyltransferase activity; P:methylation |
| Efet.01.652956.g384.t1 | DNA helicase | 279 | 2.23E-50 | 85.75 | F:nucleic acid binding; F:ATP binding; P:DNA repair; F:ATP-dependent 3'-5' DNA helicase activity; P:DNA replication; P:DNA duplex unwinding; P:DNA recombination; P:SOS response; C:intracellular |
| Efet.01.652972.g385.t1 | transcriptional regulator | 708 | 1.67E-108 | 71.95 | F:RNA binding; F:double-stranded RNA adenosine deaminase activity |
| Efet.01.652977.g386.t1 | PAS domain-containing sensor histidine kinase | 1752 | 0 | 94.05 | F:phosphorelay sensor kinase activity; F:ATP binding; P:phosphorelay signal transduction system; C:integral component of membrane; P:regulation of transcription, DNA-templated; P:signal transduction by protein phosphorylation; C:intracellular |
| Efet.01.652977.g387.t1 | pyruvate dehydrogenase (acetyl-transferring) homodimeric type | 2604 | 0 | 86.95 | F:pyruvate dehydrogenase (acetyl-transferring) activity; P:oxidation-reduction process |
| Efet.01.652980.g388.t1 | snRNA-activating complex subunit 1-like isoform X1 | 264 | 9.46E-10 | 67 |  |
| Efet.01.652985.g389.t1 | iron transporter | 510 | 5.80E-114 | 67.05 | C:membrane; C:integral component of membrane |
| Efet.01.653018.g391.t1 | U1 small nuclear ribonucleo 70 kDa | 540 | 4.80E-15 | 91.5 | F:nucleotide binding; F:nucleic acid binding; C:intracellular ribonucleoprotein complex; C:viral nucleocapsid |
| Efet.01.653028.g392.t1 | RNA pseudouridine synthase | 1182 | 2.07E-132 | 75.3 | P:RNA modification |
| Efet.01.653063.g393.t1 | C-terminal domain | 1146 | 0 | 79.35 | F:transferase activity, transferring acyl groups other than amino-acyl groups; P:metabolic process |
| Efet.01.653063.g394.t1 | nucleic-acid-binding containing a Zn-ribbon | 372 | 1.81E-82 | 79.9 |  |
| Efet.01.653063.g395.t1 | enoyl- hydratase isomerase family | 810 | 3.33E-150 | 74.45 | P:metabolic process; F:enoyl-CoA hydratase activity |
| Efet.01.653063.g396.t1 | tripartite tricarboxylate transporter substrate binding | 1071 | 5.51E-138 | 82.5 | F:lyase activity; C:outer membrane-bounded periplasmic space |
| Efet.01.653068.g397.t1 | zinc finger 271-like | 1497 | 5.03E-125 | 59.15 | F:nucleic acid binding; F:transcription factor activity, sequence-specific DNA binding; F:metal ion binding; F:calcium ion binding; C:membrane; C:integral component of membrane; P:regulation of transcription, DNA-templated; C:cellular_component; F:structural constituent of cuticle |
| Efet.01.653099.g398.t1 | serine threonine- phosphatase 6 regulatory ankyrin repeat subunit A-like | 1113 | 1.50E-56 | 52 | P:intracellular signal transduction; C:intracellular |
| Efet.01.653128.g400.t1 | hypothetical protein HELRODRAFT_160275 | 567 | 9.94E-17 | 64 |  |
| Efet.01.653146.g402.t1 | NADH dehydrogenase [ubiquinone] 1 alpha subcomplex subunit 6 | 255 | 9.11E-15 | 83.7 | F:NADH dehydrogenase activity; C:integral component of membrane; P:oxidation-reduction process |
| Efet.01.653157.g403.t1 | Glycogen debranching enzyme | 219 | 9.39E-14 | 68.5 | P:carbohydrate metabolic process; F:catalytic activity |
| Efet.01.653171.g404.t1 | heme-copper oxidase subunit III | 531 | 6.61E-122 | 89.35 | F:cytochrome-c oxidase activity; C:integral component of membrane; P:hydrogen ion transmembrane transport; C:plasma membrane; P:aerobic electron transport chain |
| Efet.01.65318.g931.t1 | AP-2 complex subunit alpha | 225 | 8.88E-11 | 72.25 | P:synaptic vesicle endocytosis; C:clathrin-coated pit; C:clathrin adaptor complex; F:protein transporter activity; P:intracellular protein transport; C:plasma membrane |
| Efet.01.653184.g406.t1 | methionine--tRNA ligase | 840 | 9.45E-175 | 84.95 | F:ATP binding; F:methionine-tRNA ligase activity; C:cytoplasm; P:methionyl-tRNA aminoacylation |
| Efet.01.653210.g407.t1 | laminin subunit gamma-1-like | 408 | 1.02E-28 | 49.5 | P:astrocyte development; C:basement membrane; P:cell morphogenesis involved in differentiation; P:retina development in camera-type eye; P:visual perception |
| Efet.01.653215.g408.t1 | type II citrate synthase | 714 | 4.82E-163 | 94.2 | F:citrate (Si)-synthase activity; C:cytoplasm; P:tricarboxylic acid cycle |
| Efet.01.653238.g410.t1 | Sytalpha, partial | 315 | 4.58E-08 | 65.33 | P:regulation of insulin secretion; C:membrane; C:integral component of membrane; P:regulation of calcium ion-dependent exocytosis |
| Efet.01.653239.g411.t1 | plasma kallikrein | 231 | 1.65E-26 | 82.65 | F:oxidoreductase activity, acting on paired donors, with incorporation or reduction of molecular oxygen; P:positive regulation of fibrinolysis; P:extracellular matrix disassembly; F:serine-type endopeptidase activity; P:blood coagulation, intrinsic pathway; P:fibrinolysis; C:plasma membrane; F:heme binding; P:Factor XII activation; C:extracellular space; P:liver regeneration; C:extracellular exosome; F:protein binding; F:iron ion binding; P:plasminogen activation; P:oxidation-reduction process |
| Efet.01.653283.g412.t1 | aquaporin-10-like isoform X3 | 279 | 1.31E-30 | 76.5 | C:membrane |
| Efet.01.653311.g414.t1 | cell filamentation Fic | 366 | 1.18E-44 | 76.3 |  |
| Efet.01.653312.g415.t1 | peptidase S41 | 1212 | 0 | 87.15 | P:proteolysis; F:serine-type peptidase activity |
| Efet.01.653312.g416.t1 | phosphoglyceromutase | 738 | 5.74E-162 | 87.55 | P:gluconeogenesis; P:glycolytic process; F:2,3-bisphosphoglycerate-dependent phosphoglycerate mutase activity |
| Efet.01.653325.g417.t1 | DNA polymerase III subunit alpha | 2718 | 0 | 91.75 | F:DNA binding; F:3'-5' exonuclease activity; P:nucleic acid phosphodiester bond hydrolysis; F:DNA-directed DNA polymerase activity; C:cytoplasm; P:DNA replication; P:DNA biosynthetic process |
| Efet.01.653325.g418.t1 | ATP-dependent Clp protease adapter | 360 | 4.06E-69 | 95 | P:protein catabolic process; P:proteolysis; F:peptidase activity |
| Efet.01.653325.g419.t1 | ATP-dependent Clp protease ATP-binding subunit | 2223 | 0 | 93.2 | F:ATP binding; P:proteolysis; F:ATP-dependent peptidase activity |
| Efet.01.653346.g420.t1 | branched-chain amino acid ABC transporter substrate-binding | 1143 | 5.31E-127 | 90.65 | P:amino acid transport |
| Efet.01.653350.g421.t1 | carbohydrate ABC transporter permease | 549 | 6.30E-80 | 65.45 | C:membrane |
| Efet.01.653365.g422.t1 | galanin receptor type 1-like | 519 | 1.51E-40 | 59.8 | P:signal transduction; F:signal transducer activity |
| Efet.01.653365.g423.t1 | allatostatin-A receptor-like | 606 | 1.77E-41 | 65.65 | F:G-protein coupled peptide receptor activity; P:G-protein coupled receptor signaling pathway; C:integral component of membrane |
| Efet.01.6534.g511.t1 | RNA-binding squid-like isoform X2 | 576 | 2.55E-28 | 78.25 | F:nucleotide binding; F:nucleic acid binding; C:intracellular ribonucleoprotein complex; C:viral nucleocapsid |
| Efet.01.653401.g424.t1 | beta keto-acyl synthase | 1125 | 0 | 52.05 | F:catalytic activity; P:metabolic process; F:transferase activity |
| Efet.01.65341.g933.t1 | tachykinin-like peptides receptor 99D isoform X1 | 459 | 1.05E-30 | 70.45 | P:neuropeptide signaling pathway; C:integral component of membrane; F:neuropeptide Y receptor activity |
| Efet.01.653425.g427.t1 | Thyrotropin-releasing hormone receptor | 723 | 9.43E-33 | 55.85 | P:signal transduction; C:membrane; F:signal transducer activity |
| Efet.01.653445.g428.t1 | [ -PII] uridylyltransferase | 1992 | 0 | 91.9 | P:nitrogen compound metabolic process; F:[protein-PII] uridylyltransferase activity; P:regulation of nitrogen utilization; F:phosphoric diester hydrolase activity; F:amino acid binding |
| Efet.01.653445.g429.t1 | peptide deformylase | 561 | 1.99E-116 | 88.45 | F:iron ion binding; F:peptide deformylase activity; P:translation |
| Efet.01.653460.g430.t1 | dihydrolipoyl dehydrogenase | 375 | 1.11E-83 | 83.35 | C:cell; F:flavin adenine dinucleotide binding; P:glycolytic process; P:cell redox homeostasis; P:oxidation-reduction process; F:dihydrolipoyl dehydrogenase activity |
| Efet.01.653460.g431.t1 | dihydrolipoyl dehydrogenase | 735 | 1.02E-85 | 74.35 | F:oxidoreductase activity, acting on a sulfur group of donors, NAD(P) as acceptor; P:single-organism cellular process; P:single-organism metabolic process |
| Efet.01.653479.g432.t1 | glyoxylate hydroxypyruvate reductase A | 1011 | 0 | 76.45 | F:NAD binding; P:oxidation-reduction process |
| Efet.01.653479.g433.t1 | DNA polymerase subunit gamma and tau | 1821 | 0 | 75.75 | C:DNA polymerase III complex; F:DNA binding; F:ATP binding; F:DNA-directed DNA polymerase activity; P:DNA replication; P:DNA biosynthetic process |
| Efet.01.653479.g434.t1 | nucleoid-associated family | 303 | 1.48E-55 | 97.1 | F:DNA binding; C:bacterial nucleoid; C:cytoplasm |
| Efet.01.653479.g435.t1 | recombination | 510 | 1.90E-118 | 92.75 | F:DNA binding; P:DNA repair; F:metal ion binding; P:DNA recombination |
| Efet.01.653479.g436.t1 | FAD-dependent oxidoreductase | 1812 | 0 | 88.95 | F:oxidoreductase activity; P:oxidation-reduction process |
| Efet.01.653516.g439.t1 | DNA helicase | 1569 | 0 | 67.85 | F:nucleic acid binding; F:helicase activity |
| Efet.01.65352.g938.t1 | COMM domain-containing 2-like | 375 | 1.61E-47 | 73.85 |  |
| Efet.01.65352.g939.t1 | plectin-like isoform X4 | 1050 | 1.97E-50 | 63.9 | C:cytoskeleton |
| Efet.01.653526.g440.t1 | DUF3299 domain-containing | 555 | 3.25E-111 | 76.2 | F:transferase activity, transferring acyl groups, acyl groups converted into alkyl on transfer |
| Efet.01.653526.g441.t1 | peptide ABC transporter permease | 1188 | 0 | 79.4 | C:integral component of membrane; C:plasma membrane |
| Efet.01.653526.g442.t1 | methionine ABC transporter ATP-binding | 498 | 2.26E-98 | 81.15 | F:ATPase activity; F:ATP binding |
| Efet.01.653526.g443.t1 | metal ABC transporter substrate-binding | 597 | 4.98E-119 | 70.7 |  |
| Efet.01.653526.g444.t1 | family transcriptional regulator | 636 | 2.51E-103 | 92.25 | F:DNA binding; F:transcription factor activity, sequence-specific DNA binding; P:transcription, DNA-templated; P:regulation of transcription, DNA-templated |
| Efet.01.653526.g445.t1 | methylmalonyl- mutase | 414 | 4.07E-49 | 95.7 | F:metal ion binding; F:cobalamin binding; P:metabolic process; F:methylmalonyl-CoA mutase activity |
| Efet.01.653541.g447.t1 | pyrroloquinoline quinone biosynthesis | 600 | 4.39E-144 | 91 | P:pyrroloquinoline quinone biosynthetic process; P:oxidation-reduction process; F:pyrroloquinoline-quinone synthase activity |
| Efet.01.653541.g448.t1 | pyrroloquinoline quinone biosynthesis | 915 | 0 | 82.4 | P:pyrroloquinoline quinone biosynthetic process; P:transport |
| Efet.01.653541.g449.t1 | pyrroloquinoline quinone precursor peptide | 381 | 9.97E-49 | 81.05 | P:pyrroloquinoline quinone biosynthetic process |
| Efet.01.653541.g450.t1 | family ATPase | 1005 | 0 | 85.5 | F:ATPase activity; F:ATP binding |
| Efet.01.653548.g452.t1 | vesicle-fusing ATPase 1-like | 300 | 2.53E-57 | 96.4 | F:ATP binding |
| Efet.01.653575.g453.t1 | ATP-dependent helicase | 747 | 5.52E-84 | 59.55 | F:nucleotide binding; F:helicase activity |
| Efet.01.653575.g454.t1 | AAA family ATPase | 1161 | 1.70E-180 | 65.35 | P:nucleic acid phosphodiester bond hydrolysis; F:ATP binding; F:endonuclease activity |
| Efet.01.653584.g455.t1 | nucleoside diphosphate kinase homolog 5-like | 345 | 1.18E-37 | 78.55 | P:UTP biosynthetic process; P:CTP biosynthetic process; P:GTP biosynthetic process; P:negative regulation of oxidative stress-induced intrinsic apoptotic signaling pathway; P:spermatid differentiation; P:nucleoside diphosphate phosphorylation; F:nucleoside diphosphate kinase activity; C:intracellular |
| Efet.01.653594.g457.t1 | family nutrient-binding outer membrane lipo | 510 | 3.84E-104 | 68.4 |  |
| Efet.01.653599.g458.t1 | bifunctional aspartate kinase homoserine dehydrogenase I | 846 | 6.48E-135 | 80.1 | F:NADP binding; F:aspartate kinase activity; P:aspartate family amino acid biosynthetic process; P:oxidation-reduction process; P:phosphorylation; F:amino acid binding; F:homoserine dehydrogenase activity |
| Efet.01.653613.g459.t1 | amino acid ABC transporter substrate-binding | 264 | 2.24E-21 | 94.3 |  |
| Efet.01.653613.g460.t1 | amino acid ABC transporter substrate-binding | 639 | 1.53E-153 | 96 |  |
| Efet.01.653615.g461.t1 | L-aspartate oxidase | 681 | 4.42E-128 | 68.55 | F:oxidoreductase activity, acting on the CH-NH2 group of donors; P:pyridine nucleotide biosynthetic process |
| Efet.01.653626.g462.t1 | phospholipid methyltransferase | 294 | 1.03E-62 | 75.8 | P:rRNA methylation; F:rRNA (adenine-N6,N6-)-dimethyltransferase activity |
| Efet.01.653626.g463.t1 | U32 family peptidase | 1362 | 0 | 87.35 | P:proteolysis; F:peptidase activity |
| Efet.01.653626.g464.t1 | 5-methyltetrahydrofolate--homocysteine methyltransferase | 987 | 0 | 94.85 | P:methionine biosynthetic process; F:methionine synthase activity; P:methylation |
| Efet.01.653626.g465.t1 | family transcriptional regulator | 1023 | 0 | 85.8 | F:DNA binding; F:transcription factor activity, sequence-specific DNA binding; P:transcription, DNA-templated; C:integral component of membrane; P:regulation of transcription, DNA-templated |
| Efet.01.653635.g466.t1 | glutamine--fructose-6-phosphate transaminase (isomerizing) | 285 | 8.01E-34 | 89.2 | P:carbohydrate metabolic process; C:cytoplasm; F:glutamine-fructose-6-phosphate transaminase (isomerizing) activity; P:glutamine metabolic process; P:carbohydrate derivative biosynthetic process; F:carbohydrate binding |
| Efet.01.653635.g467.t1 | bifunctional methylenetetrahydrofolate dehydrogenase methenyltetrahydrofolate cyclohydrolase | 549 | 1.79E-106 | 88.85 | P:methionine biosynthetic process; P:purine nucleotide biosynthetic process; P:folic acid-containing compound biosynthetic process; P:tetrahydrofolate interconversion; P:oxidation-reduction process; P:histidine biosynthetic process; F:methenyltetrahydrofolate cyclohydrolase activity; F:methylenetetrahydrofolate dehydrogenase (NADP+) activity |
| Efet.01.653647.g468.t1 | adenosylhomocysteinase | 513 | 2.16E-121 | 88.9 | C:cytoplasm; F:adenosylhomocysteinase activity; P:one-carbon metabolic process |
| Efet.01.653655.g469.t1 | hypothetical protein HELRODRAFT_175049 | 216 | 2.99E-07 | 65 | F:calcium ion binding |
| Efet.01.653719.g470.t1 | branched-chain amino acid ABC transporter substrate-binding | 594 | 8.27E-115 | 93.8 |  |
| Efet.01.653719.g471.t1 | molybdopterin converting factor subunit 1 | 252 | 4.83E-52 | 91.15 | F:nucleotide binding; P:Mo-molybdopterin cofactor biosynthetic process |
| Efet.01.653735.g473.t1 | serine dehydratase | 900 | 5.64E-165 | 93.35 | F:L-threonine ammonia-lyase activity |
| Efet.01.653787.g475.t1 | PREDICTED: mucin-5AC | 213 | 1.30E-08 | 62.25 | C:extracellular space; C:extracellular exosome; C:cytoplasm; P:phosphatidylinositol-mediated signaling; P:maintenance of lens transparency; C:extracellular region |
| Efet.01.653800.g476.t1 | ABC transporter permease | 324 | 4.26E-54 | 97.7 | C:integral component of membrane; P:transport; C:plasma membrane; F:transporter activity |
| Efet.01.653800.g477.t1 | ABC transporter permease | 876 | 1.55E-163 | 87.45 | C:integral component of membrane; P:transport; C:plasma membrane; F:transporter activity |
| Efet.01.653800.g478.t1 | enoyl- hydratase | 666 | 5.20E-107 | 89.25 | P:metabolic process; F:enoyl-CoA hydratase activity; F:isomerase activity |
| Efet.01.653844.g480.t1 | phosphoglycerate dehydrogenase | 1320 | 0 | 77.15 | F:oxidoreductase activity; F:NAD binding; P:oxidation-reduction process |
| Efet.01.653844.g481.t1 | 2-keto-4-pentenoate hydratase | 942 | 3.34E-179 | 70.2 | F:catalytic activity; P:metabolic process |
| Efet.01.653844.g482.t1 | gamma-glutamyltransferase | 1671 | 0 | 86.3 | P:glutathione metabolic process; F:gamma-glutamyltransferase activity |
| Efet.01.653844.g483.t1 | hypothetical protein Veis_3443 | 249 | 3.93E-20 | 70.2 |  |
| Efet.01.653852.g484.t1 | uncharacterized protein LOC110451963 | 510 | 2.74E-08 | 60 |  |
| Efet.01.653859.g486.t1 | DUF3800 domain-containing | 669 | 5.50E-105 | 60.9 |  |
| Efet.01.653876.g487.t1 | SCAI isoform X2 | 228 | 1.48E-25 | 88.5 | F:transcription corepressor activity; P:transcription, DNA-templated; P:negative regulation of nucleic acid-templated transcription |
| Efet.01.653904.g488.t1 | quino dehydrogenase-associated ABC transporter substrate-binding | 876 | 0 | 77.1 |  |
| Efet.01.653904.g489.t1 | methanol dehydrogenase | 405 | 7.38E-77 | 82.3 | F:electron carrier activity; F:heme binding |
| Efet.01.653904.g490.t1 | PQQ-dependent methanol ethanol family | 1827 | 0 | 86.15 | F:oxidoreductase activity, acting on CH-OH group of donors; F:calcium ion binding; C:membrane; C:outer membrane-bounded periplasmic space; P:oxidation-reduction process |
| Efet.01.653904.g491.t1 | tripartite tricarboxylate transporter substrate binding | 1428 | 0 | 75.7 | C:outer membrane-bounded periplasmic space |
| Efet.01.653904.g492.t1 | DUF521 domain-containing | 1089 | 0 | 84.55 | F:aconitate hydratase activity |
| Efet.01.653904.g493.t1 | DUF126 domain-containing | 219 | 1.70E-44 | 89.7 | F:transferase activity, transferring phosphorus-containing groups; P:phosphorylation; F:aconitate hydratase activity |
| Efet.01.653915.g494.t1 | RNA-binding | 333 | 2.84E-70 | 82.35 | P:pseudouridine synthesis; F:RNA binding; F:pseudouridine synthase activity |
| Efet.01.653926.g495.t1 | E3 ubiquitin- ligase ZNRF3-like | 888 | 1.35E-17 | 71.4 | F:zinc ion binding; C:integral component of membrane |
| Efet.01.653933.g496.t1 | adenylate kinase 9-like isoform X9 | 270 | 2.29E-23 | 82.6 | F:ATP binding; P:phosphorylation; F:nucleobase-containing compound kinase activity; P:nucleobase-containing compound metabolic process |
| Efet.01.653968.g497.t1 | family transcriptional regulator | 297 | 1.02E-57 | 85 | F:DNA binding; F:transcription factor activity, sequence-specific DNA binding; P:transcription, DNA-templated; P:regulation of transcription, DNA-templated |
| Efet.01.653968.g498.t1 | aspartate aminotransferase | 1038 | 0 | 85.9 | F:pyridoxal phosphate binding; F:L-phenylalanine:2-oxoglutarate aminotransferase activity; P:biosynthetic process; F:L-aspartate:2-oxoglutarate aminotransferase activity |
| Efet.01.653968.g499.t1 | tripartite tricarboxylate transporter substrate binding | 930 | 0 | 89.15 | C:outer membrane-bounded periplasmic space |
| Efet.01.653968.g500.t1 | tripartite tricarboxylate transporter substrate binding | 846 | 0 | 80.9 | C:outer membrane-bounded periplasmic space |
| Efet.01.653968.g501.t1 | hydroxyacid dehydrogenase | 954 | 2.29E-151 | 87.4 | F:oxidoreductase activity, acting on the CH-OH group of donors, NAD or NADP as acceptor; F:NAD binding; P:oxidation-reduction process |
| Efet.01.653968.g502.t1 | ABC transporter substrate-binding | 483 | 1.18E-20 | 82.38 | C:outer membrane-bounded periplasmic space |
| Efet.01.653968.g503.t1 | family transcriptional regulator | 1002 | 0 | 81.85 | F:DNA binding; F:transcription factor activity, sequence-specific DNA binding; P:transcription, DNA-templated; P:regulation of transcription, DNA-templated |
| Efet.01.653968.g504.t1 | 5-oxoprolinase | 780 | 2.85E-105 | 80.9 | F:5-oxoprolinase (ATP-hydrolyzing) activity |
| Efet.01.653970.g505.t1 | sodium-dependent multivitamin transporter-like | 306 | 3.90E-19 | 65.4 | P:transmembrane transport; P:monocarboxylic acid transport; C:integral component of membrane; P:cofactor transport; P:vitamin transport; F:transporter activity; P:amide transport |
| Efet.01.65398.g942.t1 | hypothetical protein CAPTEDRAFT_217462 | 408 | 4.74E-18 | 66.95 | C:membrane |
| Efet.01.653992.g506.t1 | homoserine kinase | 696 | 7.01E-69 | 88.95 | F:homoserine kinase activity; F:ATP binding; P:threonine biosynthetic process; P:phosphorylation |
| Efet.01.653992.g507.t1 | DNA polymerase I | 2835 | 0 | 84.65 | F:DNA binding; F:3'-5' exonuclease activity; P:nucleic acid phosphodiester bond hydrolysis; F:DNA-directed DNA polymerase activity; P:DNA-dependent DNA replication; P:DNA biosynthetic process |
| Efet.01.653992.g508.t1 | dicarboxylate amino acid:cation symporter | 1314 | 0 | 93.95 | P:dicarboxylic acid transport; P:transmembrane transport; C:integral component of membrane; F:symporter activity; C:plasma membrane |
| Efet.01.653992.g509.t1 | Thiolase | 711 | 6.46E-169 | 91.35 | F:propanoyl-CoA C-acyltransferase activity; P:metabolic process |
| Efet.01.653992.g510.t1 | long-chain fatty acid-- ligase | 1557 | 0 | 85.2 | P:metabolic process; F:ligase activity |
| Efet.01.653992.g511.t1 | hypothetical protein | 414 | 1.29E-95 | 75.67 |  |
| Efet.01.653992.g512.t1 | hypothetical protein | 660 | 9.11E-119 | 77.5 |  |
| Efet.01.653992.g513.t1 | acetone carboxylase subunit alpha | 2043 | 0 | 65.15 | F:catalytic activity |
| Efet.01.653992.g514.t1 | hydantoinase oxoprolinase family | 822 | 0 | 67.55 | F:5-oxoprolinase (ATP-hydrolyzing) activity |
| Efet.01.653998.g515.t1 | uroporphyrinogen III synthase | 645 | 1.98E-126 | 83.3 | P:tetrapyrrole biosynthetic process; F:uroporphyrinogen-III synthase activity |
| Efet.01.6540.g513.t1 | E3 ubiquitin- ligase HUWE1-like isoform X1 | 1701 | 1.30E-119 | 61.5 | F:ubiquitin-protein transferase activity; C:membrane; C:integral component of membrane; F:ligase activity; P:protein ubiquitination |
| Efet.01.654000.g516.t1 | Krueppel-like factor 15 | 1356 | 7.43E-52 | 85.5 | F:nucleic acid binding; F:metal ion binding |
| Efet.01.654015.g518.t1 | hypothetical protein HELRODRAFT_174718 | 273 | 3.56E-23 | 64.15 |  |
| Efet.01.654019.g519.t1 | T family of potassium channels 18-like | 429 | 1.89E-31 | 61.3 | P:transport |
| Efet.01.654027.g520.t1 | hypothetical protein | 228 | 6.56E-41 | 72.73 |  |
| Efet.01.654027.g521.t1 | hypothetical protein | 1266 | 0 | 65.4 |  |
| Efet.01.654034.g522.t1 | acetyl- carboxylase biotin carboxyl carrier subunit | 213 | 4.80E-36 | 69.55 |  |
| Efet.01.654034.g523.t1 | biotin carboxylase | 1164 | 0 | 75.25 | F:ATP binding; F:metal ion binding; F:biotin carboxylase activity |
| Efet.01.654034.g524.t1 | methylmalonyl- carboxyltransferase | 1491 | 0 | 85.55 | F:methylmalonyl-CoA carboxytransferase activity; F:propionyl-CoA carboxylase activity |
| Efet.01.654034.g525.t1 | tripartite tricarboxylate transporter substrate binding | 672 | 4.27E-117 | 66.25 | C:outer membrane-bounded periplasmic space |
| Efet.01.654034.g526.t1 | family transcriptional regulator | 726 | 1.32E-124 | 79.05 | F:DNA binding; P:transcription, DNA-templated; P:regulation of transcription, DNA-templated |
| Efet.01.654034.g527.t1 | aldehyde dehydrogenase | 1434 | 0 | 84.25 | F:betaine-aldehyde dehydrogenase activity; P:oxidation-reduction process |
| Efet.01.654034.g528.t1 | tripartite tricarboxylate transporter substrate binding | 1857 | 0 | 78.95 | C:outer membrane-bounded periplasmic space |
| Efet.01.654034.g529.t1 | tricarballylate dehydrogenase | 1080 | 0 | 91.35 | F:oxidoreductase activity; P:oxidation-reduction process |
| Efet.01.654034.g530.t1 | tricarballylate utilization | 651 | 2.07E-99 | 83.15 | C:integral component of membrane; F:iron-sulfur cluster binding |
| Efet.01.654050.g532.t1 | roundabout homolog 2- | 378 | 1.10E-20 | 57.65 | P:single-organism process |
| Efet.01.654066.g533.t1 | family transcriptional regulator | 570 | 4.11E-14 | 88.7 |  |
| Efet.01.654067.g534.t1 | phosphoribosylformylglycinamidine synthase | 2103 | 0 | 81.15 | F:ATP binding; C:cytoplasm; F:metal ion binding; P:'de novo' IMP biosynthetic process; P:glutamine metabolic process; F:phosphoribosylformylglycinamidine synthase activity |
| Efet.01.654079.g535.t1 | Kinesin KIF27 | 351 | 2.56E-07 | 72 | F:nucleotide binding |
| Efet.01.654120.g536.t1 | DNA-directed RNA polymerase subunit beta | 966 | 0 | 81.6 | F:DNA binding; F:DNA-directed 5'-3' RNA polymerase activity; F:ribonucleoside binding; P:transcription, DNA-templated |
| Efet.01.654123.g537.t1 | cellulase | 651 | 4.62E-72 | 76.8 | P:metabolic process; F:hydrolase activity, hydrolyzing O-glycosyl compounds |
| Efet.01.654159.g538.t1 | Neuromedin-U receptor 1 | 294 | 1.58E-21 | 74.4 | P:neuropeptide signaling pathway; C:integral component of membrane; F:neuromedin U receptor activity |
| Efet.01.654163.g540.t1 | histidine kinase | 597 | 3.23E-66 | 71.1 | P:phosphorylation; F:kinase activity |
| Efet.01.654179.g541.t1 | nicotinic acetylcholine receptor subunit | 711 | 2.49E-09 | 55.2 | C:membrane; P:transport |
| Efet.01.654191.g542.t1 | cytoplasmic [Paeniglutamicibacter gangotriensis] | 630 | 5.46E-52 | 61.65 |  |
| Efet.01.654191.g543.t1 | bacteriocin biosynthesis cyclodehydratase domain-containing | 546 | 8.14E-11 | 44.73 |  |
| Efet.01.654200.g545.t1 | multifunctional oxoglutarate decarboxylase oxoglutarate dehydrogenase thiamine pyrophosphate-binding subunit dihydrolipoyllysine-residue succinyltransferase subunit | 1014 | 0 | 88.05 | F:thiamine pyrophosphate binding; F:transferase activity, transferring acyl groups; F:2-oxoglutarate decarboxylase activity; F:oxoglutarate dehydrogenase (succinyl-transferring) activity; P:tricarboxylic acid cycle |
| Efet.01.654200.g546.t1 | multifunctional oxoglutarate decarboxylase oxoglutarate dehydrogenase thiamine pyrophosphate-binding subunit dihydrolipoyllysine-residue succinyltransferase subunit | 759 | 9.50E-160 | 91.3 | F:thiamine pyrophosphate binding; F:transferase activity, transferring acyl groups; F:2-oxoglutarate decarboxylase activity; F:oxoglutarate dehydrogenase (succinyl-transferring) activity; P:tricarboxylic acid cycle |
| Efet.01.654241.g548.t1 | ribonuclease D | 720 | 7.85E-80 | 81.15 | F:nucleotide binding; F:nucleic acid binding; F:3'-5' exonuclease activity; P:nucleic acid phosphodiester bond hydrolysis; C:intracellular |
| Efet.01.654241.g549.t1 | acetyl- C-acyltransferase | 516 | 5.83E-60 | 86.75 | F:transferase activity, transferring acyl groups other than amino-acyl groups; P:metabolic process |
| Efet.01.654251.g551.t1 | transcriptional regulator ATRX-like | 213 | 1.97E-16 | 80.3 | F:ATP binding; C:membrane |
| Efet.01.654253.g552.t1 | hypothetical protein BSL78_26032 | 372 | 3.01E-37 | 70.35 | F:metal ion binding |
| Efet.01.654253.g553.t1 | retrovirus-related Pol poly from transposon | 477 | 1.07E-63 | 62 |  |
| Efet.01.654257.g554.t1 | sarcosine oxidase subunit beta family | 1023 | 0 | 85.8 | F:sarcosine oxidase activity; P:tetrahydrofolate metabolic process; P:oxidation-reduction process |
| Efet.01.654257.g555.t1 | Fe3+ spermidine putrescine ABC transporter ATP-binding | 1080 | 1.02E-153 | 93.75 | F:ATP binding; P:polyamine transmembrane transport; F:polyamine-transporting ATPase activity; C:ATP-binding cassette (ABC) transporter complex |
| Efet.01.654264.g557.t1 | family transcriptional regulator | 1014 | 2.48E-136 | 70.85 | F:DNA binding; F:transcription factor activity, sequence-specific DNA binding; P:transcription, DNA-templated; P:regulation of transcription, DNA-templated |
| Efet.01.654268.g558.t1 | glycosyl hydrolase | 654 | 4.83E-142 | 68.3 | F:hydrolase activity |
| Efet.01.654303.g559.t1 | hydroxymethylbilane synthase | 942 | 3.51E-131 | 89.05 | F:hydroxymethylbilane synthase activity; P:protoporphyrinogen IX biosynthetic process |
| Efet.01.654309.g560.t1 | alpha-(1,3)-fucosyltransferase 11 | 1062 | 4.95E-86 | 72.35 | P:glycosylation; F:fucosyltransferase activity; C:Golgi membrane |
| Efet.01.654310.g561.t1 | tRNA lysidine(34) synthetase | 933 | 3.15E-150 | 85.1 | F:ATP binding; F:ligase activity, forming carbon-nitrogen bonds; C:cytoplasm; P:tRNA modification |
| Efet.01.654310.g562.t1 | acetyl- carboxylase carboxyltransferase subunit alpha | 1650 | 0 | 97.65 | F:ATP binding; P:malonyl-CoA biosynthetic process; F:acetyl-CoA carboxylase activity; F:transferase activity; P:fatty acid biosynthetic process; C:acetyl-CoA carboxylase complex |
| Efet.01.654310.g563.t1 | cysteine--tRNA ligase | 1383 | 0 | 92.1 | F:ATP binding; F:zinc ion binding; C:cytoplasm; F:cysteine-tRNA ligase activity; P:cysteinyl-tRNA aminoacylation |
| Efet.01.654310.g564.t1 | family transporter | 1281 | 0 | 93.75 | F:oxidoreductase activity, acting on CH-OH group of donors; F:flavin adenine dinucleotide binding; C:integral component of membrane; P:oxidation-reduction process |
| Efet.01.654310.g565.t1 | small ligand-binding sensory domain FIST | 1185 | 0 | 86.05 |  |
| Efet.01.654310.g566.t1 | hypothetical protein | 756 | 2.90E-134 | 71.75 |  |
| Efet.01.654310.g567.t1 | copper chaperone PCu(A)C | 483 | 8.59E-105 | 77.5 |  |
| Efet.01.654316.g568.t1 | succinyl- --3-ketoacid- transferase | 330 | 5.88E-74 | 95.25 | F:3-oxoacid CoA-transferase activity; P:metabolic process |
| Efet.01.654316.g569.t1 | transferase subunit B | 621 | 1.76E-145 | 96.4 | P:metabolic process; F:acetate CoA-transferase activity |
| Efet.01.654316.g570.t1 | alpha beta hydrolase | 846 | 1.18E-172 | 73.45 |  |
| Efet.01.654316.g571.t1 | ATPase (AAA+ superfamily) | 2625 | 0 | 69.4 | F:DNA binding; C:integral component of membrane |
| Efet.01.654316.g572.t1 | transcription termination factor Rho | 2193 | 0 | 98.9 | F:ATP binding; F:RNA binding; F:RNA-dependent ATPase activity; P:regulation of transcription, DNA-templated; F:helicase activity; P:DNA-templated transcription, termination |
| Efet.01.654333.g573.t1 | Diaminobutyrate--2-oxoglutarate aminotransferase | 840 | 3.55E-114 | 67.5 | F:transaminase activity |
| Efet.01.654333.g574.t1 | anthranilate synthase component I | 795 | 1.01E-84 | 85.95 | F:oxo-acid-lyase activity; P:biosynthetic process |
| Efet.01.654333.g575.t1 | PLP-dependent aminotransferase family | 1407 | 0 | 79.25 | F:DNA binding; F:transcription factor activity, sequence-specific DNA binding; F:pyridoxal phosphate binding; P:transcription, DNA-templated; F:transaminase activity; P:regulation of transcription, DNA-templated |
| Efet.01.654333.g576.t1 | 2-methylcitrate dehydratase | 1857 | 0 | 77.9 | F:lyase activity |
| Efet.01.654333.g577.t1 | acyl- dehydrogenase | 495 | 2.67E-107 | 91.95 | F:acyl-CoA dehydrogenase activity; F:flavin adenine dinucleotide binding; F:butyryl-CoA dehydrogenase activity; P:oxidation-reduction process |
| Efet.01.65437.g945.t1 | E3 ubiquitin- ligase TRIM71-like | 912 | 3.27E-20 | 46.71 |  |
| Efet.01.654371.g581.t1 | hypothetical protein HELRODRAFT_194765 | 354 | 5.33E-44 | 66.8 | C:membrane; C:integral component of membrane |
| Efet.01.65439.g946.t1 | testis-expressed sequence 15 isoform X1 | 1161 | 1.49E-87 | 53.25 | P:biological regulation; P:meiotic nuclear division |
| Efet.01.654391.g582.t1 | DNA-directed RNA polymerase subunit beta | 3885 | 0 | 96.1 | F:DNA binding; F:DNA-directed 5'-3' RNA polymerase activity; P:transcription, DNA-templated |
| Efet.01.654391.g583.t1 | RNA polymerase subunit | 660 | 2.95E-148 | 96.35 | F:DNA binding; F:DNA-directed 5'-3' RNA polymerase activity; F:ribonucleoside binding; P:transcription, DNA-templated |
| Efet.01.654391.g584.t1 | DNA-directed RNA polymerase subunit beta | 987 | 1.03E-173 | 96.2 | F:DNA binding; F:DNA-directed 5'-3' RNA polymerase activity; F:ribonucleoside binding; P:transcription, DNA-templated |
| Efet.01.654418.g585.t1 | yaeq family | 444 | 2.98E-89 | 93.4 |  |
| Efet.01.654418.g586.t1 | ATP-dependent RNA helicase | 1350 | 0 | 91.2 | F:nucleic acid binding; F:ATP binding; F:helicase activity |
| Efet.01.654418.g587.t1 | AMP nucleosidase | 1542 | 0 | 95 | P:AMP salvage; F:AMP nucleosidase activity |
| Efet.01.654418.g588.t1 | alpha beta hydrolase | 1020 | 0 | 90.05 | F:hydrolase activity |
| Efet.01.654418.g589.t1 | esterase | 945 | 0 | 81.5 |  |
| Efet.01.654418.g590.t1 | peptide chain release factor 2 | 1146 | 0 | 92.55 | P:translational termination; C:cytoplasm; F:translation release factor activity, codon specific |
| Efet.01.654418.g591.t1 | aminopeptidase N | 2772 | 0 | 85.45 | F:zinc ion binding; P:proteolysis; F:aminopeptidase activity; F:metallopeptidase activity |
| Efet.01.654418.g592.t1 | fructose 1,6-bisphosphatase | 1008 | 0 | 93.7 | F:magnesium ion binding; P:gluconeogenesis; C:cytoplasm; F:fructose 1,6-bisphosphate 1-phosphatase activity; P:dephosphorylation |
| Efet.01.654418.g593.t1 | carbamoyl-phosphate synthase small subunit | 1218 | 0 | 90.55 | F:ATP binding; P:'de novo' pyrimidine nucleobase biosynthetic process; P:arginine biosynthetic process; P:glutamine metabolic process; F:carbamoyl-phosphate synthase (glutamine-hydrolyzing) activity; P:'de novo' UMP biosynthetic process |
| Efet.01.654418.g594.t1 | carbamoyl-phosphate synthase large subunit | 3105 | 0 | 96.6 | F:magnesium ion binding; F:ATP binding; P:arginine biosynthetic process; F:manganese ion binding; F:carbamoyl-phosphate synthase (glutamine-hydrolyzing) activity; P:'de novo' UMP biosynthetic process |
| Efet.01.654418.g595.t1 | transcription elongation factor | 477 | 3.03E-107 | 94.85 | F:RNA polymerase binding; F:DNA binding; F:translation elongation factor activity; P:transcription, DNA-templated; P:regulation of DNA-templated transcription, elongation; P:translational elongation |
| Efet.01.654418.g596.t1 | DUF4149 domain-containing | 435 | 1.98E-82 | 86.95 | C:integral component of membrane |
| Efet.01.654418.g597.t1 | nodulation factor ABC transporter ATP-binding | 966 | 0 | 88 | F:ATPase activity; F:ATP binding; P:transport; C:ATP-binding cassette (ABC) transporter complex; F:transporter activity; P:nodulation |
| Efet.01.654418.g598.t1 | nodulation | 843 | 4.18E-175 | 88.3 | P:oligosaccharide transport; C:integral component of membrane; C:plasma membrane; F:transporter activity; P:nodulation |
| Efet.01.654418.g599.t1 | tripartite tricarboxylate transporter substrate binding | 1389 | 0 | 84.25 | C:outer membrane-bounded periplasmic space |
| Efet.01.654418.g600.t1 | galactarate dehydratase | 1572 | 0 | 92.65 | P:galactarate catabolic process; F:galactarate dehydratase activity |
| Efet.01.654418.g601.t1 | galactose mutarotase | 246 | 1.32E-40 | 87.75 | P:hexose metabolic process; F:aldose 1-epimerase activity; F:carbohydrate binding |
| Efet.01.654418.g602.t1 | NYN domain-containing | 645 | 1.35E-117 | 70.15 | F:helicase activity |
| Efet.01.654418.g603.t1 | tripartite tricarboxylate transporter substrate binding | 936 | 0 | 84.05 | C:outer membrane-bounded periplasmic space |
| Efet.01.654418.g604.t1 | acetyl- acetyltransferase | 1239 | 0 | 84.5 | F:formyl-CoA transferase activity |
| Efet.01.654418.g605.t1 | acyl- dehydrogenase | 1104 | 1.16E-169 | 71.4 | F:acyl-CoA dehydrogenase activity; F:flavin adenine dinucleotide binding; P:oxidation-reduction process |
| Efet.01.654418.g606.t1 | acyl- dehydrogenase | 1101 | 1.73E-118 | 68.4 | F:acyl-CoA dehydrogenase activity; F:flavin adenine dinucleotide binding; P:oxidation-reduction process |
| Efet.01.654418.g607.t1 | enoyl- hydratase | 810 | 6.08E-161 | 76.6 | P:metabolic process; F:isomerase activity |
| Efet.01.65442.g947.t1 | LPS biosynthesis | 1830 | 0 | 89.25 | P:response to organic substance; P:Gram-negative-bacterium-type cell outer membrane assembly; C:integral component of membrane; C:cell outer membrane; P:lipopolysaccharide transport |
| Efet.01.654437.g608.t1 | hydrogenase | 549 | 3.68E-89 | 93.95 | C:integral component of membrane |
| Efet.01.654468.g610.t1 | Cadherin-87A | 306 | 5.62E-16 | 57.05 | F:calcium ion binding; C:membrane; C:integral component of membrane; P:cell adhesion; P:homophilic cell adhesion via plasma membrane adhesion molecules; C:plasma membrane |
| Efet.01.654469.g611.t1 | dihydrolipoyl dehydrogenase | 699 | 9.64E-92 | 77.85 | C:cell; F:flavin adenine dinucleotide binding; P:glycolytic process; P:cell redox homeostasis; P:oxidation-reduction process; F:dihydrolipoyl dehydrogenase activity |
| Efet.01.654469.g612.t1 | dihydrolipoyllysine-residue succinyltransferase | 1464 | 0 | 76.1 | F:transferase activity, transferring acyl groups; P:carboxylic acid metabolic process; P:primary metabolic process |
| Efet.01.654486.g616.t1 | DNA gyrase subunit A | 1194 | 0 | 94.9 | F:DNA binding; F:ATP binding; C:cytoplasm; P:DNA-dependent DNA replication; C:chromosome; F:DNA topoisomerase type II (ATP-hydrolyzing) activity; P:DNA topological change |
| Efet.01.654492.g617.t1 | importin-4-like | 324 | 1.70E-25 | 65.4 | P:intracellular protein transport; F:Ran GTPase binding; C:intracellular |
| Efet.01.654516.g620.t1 | eukaryotic translation initiation factor 3 subunit H-like | 333 | 1.81E-31 | 76.15 | F:translation initiation factor activity; P:formation of translation preinitiation complex; C:eukaryotic 43S preinitiation complex; C:eukaryotic 48S preinitiation complex; C:eukaryotic translation initiation factor 3 complex |
| Efet.01.654521.g621.t1 | beta-ketoacyl-ACP synthase | 1365 | 0 | 63.1 | P:metabolic process; F:transferase activity |
| Efet.01.654521.g622.t1 | 3-oxoacyl-(acyl-carrier- ) synthase | 1146 | 0 | 57.9 | P:metabolic process; F:transferase activity |
| Efet.01.654521.g623.t1 | hypothetical protein | 963 | 0 | 54.95 | F:catalytic activity; P:metabolic process |
| Efet.01.654538.g625.t1 | copper-translocating P-type ATPase | 396 | 5.33E-80 | 91.4 | F:nucleotide binding; F:cation-transporting ATPase activity; F:metal ion binding; P:cation transmembrane transport; C:integral component of membrane; P:metal ion transport; C:plasma membrane |
| Efet.01.654539.g626.t1 | hypothetical protein CAPTEDRAFT_187911 | 264 | 1.77E-12 | 68 | C:membrane; C:integral component of membrane |
| Efet.01.654545.g627.t1 | tyrosine- phosphatase non-receptor type 9 isoform X2 | 213 | 3.09E-23 | 75.2 | F:protein tyrosine phosphatase activity; P:peptidyl-tyrosine dephosphorylation |
| Efet.01.654558.g628.t1 | DNA helicase | 828 | 7.33E-74 | 79.65 | F:DNA binding; F:ATP binding; P:DNA duplex unwinding; F:ATP-dependent DNA helicase activity |
| Efet.01.654569.g630.t1 | thioesterase | 384 | 6.69E-86 | 88.35 |  |
| Efet.01.654569.g631.t1 | transglutaminase | 669 | 3.95E-136 | 81.35 |  |
| Efet.01.654569.g632.t1 | Fic family | 1002 | 0 | 84.5 | P:cell division |
| Efet.01.654591.g633.t1 | 4-oxalomesaconate tautomerase | 756 | 8.88E-145 | 84.85 |  |
| Efet.01.654591.g634.t1 | family transcriptional regulator | 1002 | 2.42E-167 | 96.45 | F:DNA binding; F:transcription factor activity, sequence-specific DNA binding; P:transcription, DNA-templated; P:regulation of transcription, DNA-templated |
| Efet.01.654591.g635.t1 | tRNA 2-thiouridine(34) synthase | 1002 | 0 | 87.95 | F:ATP binding; C:cytoplasm; F:methyltransferase activity; F:sulfurtransferase activity; F:tRNA binding; P:tRNA modification; P:methylation |
| Efet.01.654591.g636.t1 | DNA helicase II | 1815 | 0 | 83.55 | F:nucleotide binding; F:helicase activity |
| Efet.01.654591.g637.t1 | single-stranded DNA-binding | 522 | 3.67E-80 | 84.2 | P:DNA repair; P:DNA replication; P:DNA recombination; F:single-stranded DNA binding |
| Efet.01.654591.g638.t1 | MFS transporter | 1191 | 0 | 88.6 | P:transmembrane transport; C:integral component of membrane; F:transporter activity |
| Efet.01.654597.g639.t1 | phosphoribosylformylglycinamidine synthase | 1326 | 0 | 82.9 | F:ATP binding; C:cytoplasm; F:metal ion binding; P:'de novo' IMP biosynthetic process; P:glutamine metabolic process; F:phosphoribosylformylglycinamidine synthase activity |
| Efet.01.654597.g640.t1 | type I methionyl aminopeptidase | 462 | 1.18E-92 | 94.35 | P:protein initiator methionine removal; F:metal ion binding; P:proteolysis; F:metalloaminopeptidase activity |
| Efet.01.654620.g642.t1 | malate:quinone oxidoreductase | 1032 | 0 | 85.25 | F:malate dehydrogenase (menaquinone) activity; F:malate dehydrogenase (quinone) activity; P:tricarboxylic acid cycle |
| Efet.01.654640.g643.t1 | hypothetical protein CAPTEDRAFT_220792 | 669 | 3.48E-38 | 60.33 |  |
| Efet.01.654656.g645.t1 | toluene tolerance family | 765 | 1.17E-105 | 85.5 |  |
| Efet.01.654656.g646.t1 | ABC transporter | 627 | 1.84E-133 | 86.3 | C:membrane |
| Efet.01.654656.g647.t1 | outer membrane lipid asymmetry maintenance | 471 | 8.31E-82 | 88.85 | C:integral component of membrane; P:phospholipid transport |
| Efet.01.654656.g648.t1 | ABC transporter permease | 720 | 8.21E-118 | 95.95 | F:ATP binding; P:transport; C:ATP-binding cassette (ABC) transporter complex |
| Efet.01.654656.g649.t1 | ABC transporter ATP-binding | 834 | 1.02E-174 | 92.4 | F:ATPase activity; F:ATP binding |
| Efet.01.654656.g650.t1 | glutamate synthase subunit beta | 1455 | 0 | 88.4 | P:glutamate biosynthetic process; F:glutamate synthase (NADH) activity; P:oxidation-reduction process; F:glutamate synthase (NADPH) activity; F:iron-sulfur cluster binding |
| Efet.01.654656.g651.t1 | glutamate synthase subunit alpha | 375 | 5.81E-71 | 92.1 | P:glutamate biosynthetic process; F:glutamate synthase (NADH) activity; P:oxidation-reduction process |
| Efet.01.654656.g652.t1 | glutamate synthase subunit alpha | 4341 | 0 | 95.05 | P:glutamate biosynthetic process; F:glutamate synthase (NADH) activity; P:oxidation-reduction process |
| Efet.01.654656.g653.t1 | transposase | 696 | 1.50E-143 | 88.8 | F:DNA binding; F:transposase activity; P:transposition, DNA-mediated |
| Efet.01.654656.g654.t1 | RNA pyrophosphohydrolase | 666 | 1.49E-161 | 90 | F:metal ion binding; F:hydrolase activity, acting on acid anhydrides, in phosphorus-containing anhydrides |
| Efet.01.654656.g655.t1 | proline--tRNA ligase | 2346 | 0 | 88.6 | F:ATP binding; C:cytoplasm; P:regulation of translational fidelity; F:proline-tRNA ligase activity; F:aminoacyl-tRNA editing activity; P:prolyl-tRNA aminoacylation |
| Efet.01.654656.g656.t1 | methyltransferase | 591 | 6.66E-140 | 87.6 | F:methyltransferase activity; P:methylation |
| Efet.01.654656.g657.t1 | family transcriptional regulator | 768 | 2.67E-161 | 90.4 | F:DNA binding; F:transcription factor activity, sequence-specific DNA binding; P:transcription, DNA-templated; P:regulation of transcription, DNA-templated |
| Efet.01.654656.g658.t1 | 3-hydroxyacyl- dehydrogenase | 840 | 0 | 91.35 | F:oxidoreductase activity; P:oxidation-reduction process |
| Efet.01.654656.g659.t1 | family transcriptional regulator | 1002 | 0 | 74.95 | F:DNA binding; F:transcription factor activity, sequence-specific DNA binding; P:transcription, DNA-templated; P:regulation of transcription, DNA-templated |
| Efet.01.654656.g660.t1 | MFS transporter | 1128 | 0 | 87.5 | P:transmembrane transport; C:integral component of membrane |
| Efet.01.654656.g661.t1 | formate dehydrogenase | 1557 | 0 | 88.65 | F:FMN binding; F:NADH dehydrogenase (ubiquinone) activity; P:oxidation-reduction process; F:4 iron, 4 sulfur cluster binding |
| Efet.01.654656.g662.t1 | formate dehydrogenase subunit alpha | 2763 | 0 | 89.85 | F:electron carrier activity; F:molybdenum ion binding; F:formate dehydrogenase (NAD+) activity; P:oxidation-reduction process; F:4 iron, 4 sulfur cluster binding; P:formate metabolic process |
| Efet.01.654656.g663.t1 | formate dehydrogenase family accessory | 834 | 0 | 75.4 | C:cytoplasm; F:sulfurtransferase activity; P:Mo-molybdopterin cofactor biosynthetic process; P:transport; F:sulfur carrier activity |
| Efet.01.654656.g664.t1 | helicase | 1092 | 3.39E-130 | 72.85 | F:organic cyclic compound binding; F:heterocyclic compound binding |
| Efet.01.654656.g665.t1 | DEAD DEAH box helicase | 4308 | 0 | 66.7 | F:nucleotide binding; F:nucleic acid binding; F:ATP binding; F:helicase activity; F:hydrolase activity |
| Efet.01.654656.g666.t1 | DUF4338 domain-containing | 2439 | 0 | 73.9 |  |
| Efet.01.654656.g667.t1 | hypothetical protein BURPSPAST_Y0111 | 2316 | 0 | 65.36 |  |
| Efet.01.654656.g668.t1 | DNA polymerase III subunit epsilon | 714 | 1.08E-168 | 88.7 | F:DNA binding; F:DNA-directed DNA polymerase activity; P:DNA replication; P:DNA biosynthetic process |
| Efet.01.654656.g669.t1 | Fe-S assembly chaperone | 1869 | 0 | 91.3 | F:ATPase activity; F:ATP binding; F:unfolded protein binding; P:protein folding; P:iron-sulfur cluster assembly |
| Efet.01.654656.g670.t1 | Fe-S assembly co-chaperone | 537 | 2.77E-101 | 85.3 | F:chaperone binding; P:protein folding; P:protein oligomerization |
| Efet.01.654656.g671.t1 | iron-sulfur cluster assembly | 324 | 1.73E-61 | 98.9 | F:iron ion binding; F:structural molecule activity; F:iron-sulfur cluster binding; P:iron-sulfur cluster assembly |
| Efet.01.654656.g672.t1 | subfamily cysteine desulfurase | 1617 | 0 | 84.8 | F:cysteine desulfurase activity; F:pyridoxal phosphate binding; P:[2Fe-2S] cluster assembly; C:cytoplasm; F:metal ion binding; F:2 iron, 2 sulfur cluster binding |
| Efet.01.654656.g673.t1 | excinuclease ABC subunit B | 2151 | 0 | 93.5 | F:DNA binding; F:ATP binding; P:nucleic acid phosphodiester bond hydrolysis; F:excinuclease ABC activity; C:cytoplasm; P:nucleotide-excision repair; F:helicase activity; P:SOS response |
| Efet.01.654656.g674.t1 | aromatic amino acid aminotransferase | 1086 | 0 | 83.55 | F:pyridoxal phosphate binding; F:aromatic-amino-acid:2-oxoglutarate aminotransferase activity; F:L-phenylalanine:2-oxoglutarate aminotransferase activity; P:cellular amino acid metabolic process; P:biosynthetic process |
| Efet.01.654656.g675.t1 | family transcriptional regulator | 864 | 0 | 84 | F:DNA binding; P:transcription, DNA-templated; P:regulation of transcription, DNA-templated |
| Efet.01.654656.g676.t1 | 4-hydroxy-tetrahydrodipicolinate synthase | 906 | 0 | 87.25 | C:cytoplasm; P:lysine biosynthetic process via diaminopimelate; F:4-hydroxy-tetrahydrodipicolinate synthase; P:diaminopimelate biosynthetic process |
| Efet.01.654656.g677.t1 | ABC transporter permease | 1545 | 0 | 84.25 | C:integral component of membrane; P:transport; C:plasma membrane |
| Efet.01.654656.g678.t1 | spermidine putrescine ABC transporter ATP-binding | 1113 | 0 | 81.5 | F:ATP binding; P:polyamine transmembrane transport; F:polyamine-transporting ATPase activity; C:ATP-binding cassette (ABC) transporter complex |
| Efet.01.654656.g679.t1 | ABC transporter substrate-binding | 831 | 0 | 88.7 |  |
| Efet.01.654656.g680.t1 | FAD-binding oxidoreductase | 1482 | 0 | 81.2 | F:oxidoreductase activity, acting on CH-OH group of donors; F:flavin adenine dinucleotide binding; P:oxidation-reduction process |
| Efet.01.654669.g681.t1 | thyrotropin-releasing hormone receptor-like | 1209 | 2.74E-80 | 70.15 | F:G-protein coupled receptor activity; P:signal transduction; C:membrane; F:transferase activity |
| Efet.01.654678.g682.t1 | hypothetical protein CAPTEDRAFT_96860, partial | 1332 | 7.51E-103 | 55.65 | F:calcium ion binding; C:membrane; C:integral component of membrane; P:cell adhesion; P:homophilic cell adhesion via plasma membrane adhesion molecules; C:plasma membrane |
| Efet.01.654699.g684.t1 | FMRFamide-activated amiloride-sensitive sodium channel-like isoform X2 | 1704 | 0 | 49.25 | P:sodium ion transport; C:membrane |
| Efet.01.654738.g688.t1 | Tetratricopeptide repeat-containing | 1503 | 0 | 60.1 | P:RNA processing |
| Efet.01.654744.g689.t1 | TIGR02099 family | 1218 | 0 | 74.95 | C:membrane |
| Efet.01.654756.g690.t1 | family transcriptional regulator | 720 | 6.83E-145 | 73.35 | F:DNA binding; F:transcription factor activity, sequence-specific DNA binding; P:transcription, DNA-templated; P:regulation of transcription, DNA-templated; C:intracellular |
| Efet.01.654756.g691.t1 | ABC transporter ATP-binding | 1173 | 7.61E-112 | 66.25 | F:ATPase activity; F:ATP binding |
| Efet.01.654756.g692.t1 | ABC transporter ATP-binding | 435 | 7.81E-98 | 81.25 | F:ATPase activity; F:ATP binding |
| Efet.01.654769.g693.t1 | ATP-dependent acyl- ligase | 570 | 5.90E-118 | 93.8 | P:metabolic process; F:ligase activity |
| Efet.01.654769.g694.t1 | thiolase family | 585 | 3.77E-106 | 96.8 | F:transferase activity, transferring acyl groups other than amino-acyl groups; P:metabolic process |
| Efet.01.654769.g695.t1 | thiolase family | 540 | 1.03E-108 | 94.95 | F:transferase activity, transferring acyl groups other than amino-acyl groups; P:metabolic process |
| Efet.01.654774.g696.t1 | phosphatase Slingshot homolog 2-like isoform X2 | 201 | 2.70E-16 | 74.05 | F:DNA binding; F:protein tyrosine phosphatase activity; P:peptidyl-tyrosine dephosphorylation; F:protein tyrosine/serine/threonine phosphatase activity |
| Efet.01.654783.g697.t1 | hypothetical protein CAPTEDRAFT_219019 | 408 | 1.83E-22 | 51.9 | C:membrane; C:integral component of membrane; F:carbohydrate binding |
| Efet.01.654787.g698.t1 | Peptidase family M23 | 666 | 4.91E-133 | 58.64 |  |
| Efet.01.654851.g700.t1 | FAD-dependent cmnm(5)s(2)U34 oxidoreductase | 1068 | 1.11E-118 | 77.35 | F:oxidoreductase activity, acting on the CH-NH group of donors; C:cytoplasm; F:flavin adenine dinucleotide binding; P:tRNA wobble base modification; P:oxidation-reduction process; F:tRNA (5-methylaminomethyl-2-thiouridylate)-methyltransferase activity; P:tRNA methylation |
| Efet.01.654851.g701.t1 | Carboxymethylenebutenolidase | 576 | 6.56E-62 | 84.2 | F:carboxymethylenebutenolidase activity |
| Efet.01.654851.g702.t1 | amino acid ABC transporter substrate-binding | 720 | 5.23E-160 | 93.95 |  |
| Efet.01.654851.g703.t1 | glutamate ABC transporter permease | 786 | 1.27E-160 | 92.25 | P:amino acid transport; C:ATP-binding cassette (ABC) transporter complex; F:transporter activity |
| Efet.01.654851.g704.t1 | amino acid ABC transporter permease | 534 | 9.11E-109 | 87.1 | P:amino acid transport; C:ATP-binding cassette (ABC) transporter complex; F:transporter activity |
| Efet.01.654851.g705.t1 | amino acid ABC transporter ATP-binding | 765 | 4.26E-176 | 92.25 | F:ATP binding; C:ATP-binding cassette (ABC) transporter complex; P:amino acid transmembrane transport; F:amino acid-transporting ATPase activity |
| Efet.01.654851.g706.t1 | dihydroorotase | 1044 | 0 | 90.45 | F:zinc ion binding; F:dihydroorotase activity; P:'de novo' UMP biosynthetic process; P:pyrimidine nucleobase biosynthetic process |
| Efet.01.654851.g707.t1 | zinc metalloprotease | 879 | 1.12E-148 | 84.4 | F:zinc ion binding; P:proteolysis; C:integral component of membrane; F:metalloendopeptidase activity; C:plasma membrane |
| Efet.01.654851.g708.t1 | anthranilate synthase component I | 1440 | 0 | 96.7 | P:tryptophan biosynthetic process; F:anthranilate synthase activity |
| Efet.01.654851.g709.t1 | type 1 glutamine amidotransferase | 573 | 1.60E-136 | 93.65 | P:glutamine metabolic process; F:transferase activity; F:anthranilate synthase activity |
| Efet.01.654851.g710.t1 | anthranilate phosphoribosyltransferase | 1029 | 0 | 89.4 | F:magnesium ion binding; P:tryptophan biosynthetic process; F:anthranilate phosphoribosyltransferase activity |
| Efet.01.654868.g711.t1 | Site-specific recombinase | 1155 | 1.17E-140 | 72.65 | F:DNA binding; P:DNA recombination; P:DNA integration |
| Efet.01.654895.g712.t1 | tRNA 2-selenouridine(34) synthase | 1101 | 0 | 63.1 | F:transferase activity; P:cellular metabolic process |
| Efet.01.65491.g950.t1 | doublecortin domain-containing 2C | 393 | 6.28E-26 | 61.9 | P:regulation of cellular process |
| Efet.01.654936.g716.t1 | bifunctional aconitate hydratase 2 2-methylisocitrate dehydratase | 930 | 0 | 86.75 | F:metal ion binding; C:cytosol; P:tricarboxylic acid cycle; F:2-methylisocitrate dehydratase activity; F:4 iron, 4 sulfur cluster binding; F:aconitate hydratase activity |
| Efet.01.654940.g717.t1 | neutral and basic amino acid transport rBAT-like | 459 | 9.56E-28 | 64.5 | P:carbohydrate metabolic process; F:catalytic activity; C:integral component of membrane |
| Efet.01.654942.g718.t1 | hypothetical protein HELRODRAFT_166464 | 324 | 1.58E-40 | 66.6 | C:integral component of membrane; F:carbohydrate binding |
| Efet.01.654946.g719.t1 | PLP-dependent aminotransferase family | 555 | 5.26E-105 | 80.15 | F:pyridoxal phosphate binding; F:L-phenylalanine:2-oxoglutarate aminotransferase activity; P:biosynthetic process; F:L-aspartate:2-oxoglutarate aminotransferase activity |
| Efet.01.654946.g720.t1 | chemotaxis | 975 | 0 | 94.85 | P:chemotaxis; P:phosphorelay signal transduction system; F:signal transducer activity; C:intracellular |
| Efet.01.654946.g721.t1 | NAD(P)-dependent oxidoreductase | 288 | 3.01E-46 | 87.3 | F:coenzyme binding; F:catalytic activity |
| Efet.01.654956.g722.t1 | fibrinogen A | 387 | 2.44E-19 | 63.8 |  |
| Efet.01.654965.g723.t1 | low-specificity L-threonine aldolase | 798 | 1.39E-178 | 91.1 | P:cellular amino acid metabolic process; F:threonine aldolase activity |
| Efet.01.654965.g724.t1 | family pyridoxal phosphate enzyme | 447 | 1.46E-58 | 85.75 |  |
| Efet.01.654965.g725.t1 | twitching motility | 966 | 0 | 96.35 | F:ATP binding; P:transport |
| Efet.01.654965.g726.t1 | Crp Fnr family transcriptional regulator | 663 | 2.47E-137 | 87.05 |  |
| Efet.01.654965.g727.t1 | type IV pili twitching motility | 1137 | 0 | 94.35 | F:ATP binding; P:transport |
| Efet.01.654965.g728.t1 | oxidoreductase | 843 | 1.08E-157 | 93.9 | F:phosphogluconate dehydrogenase (decarboxylating) activity; F:NAD binding; P:oxidation-reduction process |
| Efet.01.654965.g729.t1 | NYN domain-containing | 630 | 2.29E-150 | 79.95 |  |
| Efet.01.654969.g730.t1 | aspartate-semialdehyde dehydrogenase | 1101 | 0 | 93.55 | P:isoleucine biosynthetic process; F:N-acetyl-gamma-glutamyl-phosphate reductase activity; F:NADP binding; P:'de novo' L-methionine biosynthetic process; C:cytoplasm; F:NAD binding; P:lysine biosynthetic process via diaminopimelate; P:threonine biosynthetic process; F:aspartate-semialdehyde dehydrogenase activity; F:protein dimerization activity; P:oxidation-reduction process; P:diaminopimelate biosynthetic process |
| Efet.01.654969.g731.t1 | diguanylate cyclase | 2157 | 0 | 82.7 | C:integral component of membrane |
| Efet.01.654987.g732.t1 | acetyl propionyl methylcrotonyl- carboxylase subunit alpha | 1827 | 0 | 79.85 | F:ATP binding; F:metal ion binding; F:biotin carboxylase activity; F:methylcrotonoyl-CoA carboxylase activity |
| Efet.01.654993.g734.t1 | hybrid-cluster NAD(P)-dependent oxidoreductase | 1194 | 0 | 69.1 | F:oxidoreductase activity; F:metal ion binding; F:electron carrier activity; P:oxidation-reduction process; F:2 iron, 2 sulfur cluster binding |
| Efet.01.654993.g735.t1 | aromatic ring-hydroxylating dioxygenase subunit alpha | 1266 | 0 | 83.65 | P:aromatic compound catabolic process; F:iron ion binding; F:oxidoreductase activity, acting on paired donors, with incorporation or reduction of molecular oxygen, NAD(P)H as one donor, and incorporation of two atoms of oxygen into one donor; P:oxidation-reduction process; F:2 iron, 2 sulfur cluster binding |
| Efet.01.655025.g737.t1 | N-acetylgalactosamine kinase-like | 387 | 1.12E-44 | 74.5 | F:nucleotide binding; P:carbohydrate metabolic process; P:phosphorylation; F:phosphotransferase activity, alcohol group as acceptor; F:kinase activity |
| Efet.01.655042.g739.t1 | serine threonine kinase | 537 | 1.39E-87 | 84.55 | F:protein serine/threonine kinase activity; P:protein phosphorylation |
| Efet.01.655044.g740.t1 | collagen alpha-1(XII) chain-like | 318 | 1.21E-07 | 55.67 | C:collagen trimer |
| Efet.01.655057.g743.t1 | calcium-dependent kinase 1 | 201 | 5.67E-26 | 64.95 | F:calcium ion binding |
| Efet.01.655059.g744.t1 | C-type lectin | 558 | 1.61E-23 | 60 | F:carbohydrate binding |
| Efet.01.655076.g746.t1 | electron transfer flavo subunit alpha family | 672 | 3.51E-104 | 74.45 | F:flavin adenine dinucleotide binding; F:electron carrier activity |
| Efet.01.655095.g748.t1 | hypothetical protein CAPTEDRAFT_211327, partial | 702 | 4.17E-29 | 58.6 |  |
| Efet.01.655096.g749.t1 | hypothetical protein CAPTEDRAFT_208208 | 537 | 7.99E-15 | 45.25 | C:membrane; C:integral component of membrane; F:carbohydrate binding |
| Efet.01.655098.g750.t1 | ABC transporter permease | 459 | 1.75E-84 | 77.35 | C:integral component of membrane; P:transport; C:plasma membrane |
| Efet.01.655098.g751.t1 | ABC transporter ATP-binding | 1098 | 2.19E-123 | 67.2 | F:ATP binding; P:transmembrane transport; F:polyamine-transporting ATPase activity; P:polyamine transport; C:ATP-binding cassette (ABC) transporter complex |
| Efet.01.655098.g752.t1 | NAD synthetase | 813 | 2.00E-170 | 96.4 | F:NADP binding; C:integral component of membrane; P:oxidation-reduction process; F:NAD(P)+ transhydrogenase (AB-specific) activity; C:plasma membrane |
| Efet.01.655099.g753.t1 | agrin-like isoform X3 | 243 | 4.05E-28 | 73.7 | C:integral component of membrane; F:arylesterase activity |
| Efet.01.655110.g755.t1 | glutamate-1-semialdehyde 2,1-aminomutase | 546 | 1.45E-109 | 73.5 | F:transferase activity |
| Efet.01.655112.g756.t1 | mandelate racemase muconate lactonizing enzyme family | 1011 | 0 | 65.15 | F:galactonate dehydratase activity; F:metal ion binding; F:catalytic activity; P:metabolic process; F:lyase activity |
| Efet.01.655128.g757.t1 | cell division | 588 | 1.40E-115 | 91.65 | P:cell division; F:penicillin binding; F:peptidoglycan glycosyltransferase activity |
| Efet.01.655128.g758.t1 | 16S rRNA (cytosine(1402)-N(4))-methyltransferase | 801 | 1.38E-159 | 90.85 | C:cytoplasm; P:rRNA base methylation; F:rRNA (cytosine-N4-)-methyltransferase activity |
| Efet.01.655128.g759.t1 | cell division cell wall cluster transcriptional repressor | 438 | 1.95E-89 | 95.7 | C:nucleoid; P:cell division; F:DNA binding; F:transcription factor activity, sequence-specific DNA binding; C:cytoplasm; P:transcription, DNA-templated; P:regulation of transcription, DNA-templated |
| Efet.01.655128.g760.t1 | heat shock protein HslVU, ATPase subunit HslU | 1326 | 0 | 94.45 | F:ATPase activity; F:ATP binding; C:HslUV protease complex; F:peptidase activity, acting on L-amino acid peptides; P:proteolysis; P:protein unfolding |
| Efet.01.655128.g761.t1 | ATP-dependent protease subunit | 621 | 3.24E-97 | 86.35 | C:HslUV protease complex; F:metal ion binding; C:proteasome core complex; F:threonine-type endopeptidase activity; P:proteolysis involved in cellular protein catabolic process |
| Efet.01.655128.g762.t1 | STAS domain-containing | 1509 | 0 | 77 |  |
| Efet.01.655128.g763.t1 | RNA polymerase-binding | 951 | 3.45E-150 | 96.85 | F:zinc ion binding; C:cytoplasm; P:regulation of gene expression |
| Efet.01.655128.g764.t1 | cobalamin biosynthesis | 1164 | 0 | 90.45 |  |
| Efet.01.655129.g765.t1 | vacuolar sorting-associated 8 homolog | 816 | 2.74E-82 | 69.75 | F:metal ion binding; P:transport |
| Efet.01.655193.g770.t1 | ABC transporter ATP-binding | 450 | 1.29E-90 | 84.05 | F:ATPase activity; F:ATP binding; P:transmembrane transport; P:carbohydrate transport; C:ATP-binding cassette (ABC) transporter complex; F:hydrolase activity, acting on acid anhydrides, catalyzing transmembrane movement of substances |
| Efet.01.655193.g771.t1 | ABC transporter ATP-binding | 1095 | 0 | 74.4 | F:ATPase activity; F:ATP binding; P:transport |
| Efet.01.655193.g772.t1 | carbohydrate ABC transporter permease | 690 | 1.57E-113 | 80.5 | C:integral component of membrane; P:transport; C:plasma membrane |
| Efet.01.655224.g773.t1 | cytochrome c oxidase subunit I | 363 | 4.26E-78 | 92.45 | F:cytochrome-c oxidase activity; P:oxidative phosphorylation; F:copper ion binding; F:iron ion binding; C:integral component of membrane; P:hydrogen ion transmembrane transport; P:electron transport chain; P:aerobic respiration; C:plasma membrane; C:respiratory chain; F:heme binding |
| Efet.01.655224.g774.t1 | cytochrome c oxidase assembly | 603 | 4.87E-114 | 79.9 | F:copper ion binding; C:integral component of membrane |
| Efet.01.655224.g775.t1 | cytochrome c oxidase subunit 3 | 882 | 0 | 93.45 | F:cytochrome-c oxidase activity; C:integral component of membrane; P:hydrogen ion transmembrane transport; C:plasma membrane; P:aerobic electron transport chain |
| Efet.01.655224.g776.t1 | DUF2909 domain-containing | 237 | 1.07E-47 | 88.95 | C:integral component of membrane |
| Efet.01.655234.g777.t1 | hypothetical protein CAPTEDRAFT_205572 | 471 | 7.60E-11 | 71 | C:membrane; C:integral component of membrane; P:cell adhesion; P:tissue regeneration |
| Efet.01.655245.g779.t1 | leucine-rich repeat SHOC-2- | 960 | 2.77E-07 | 66.33 |  |
| Efet.01.655265.g780.t1 | hypothetical protein CAPTEDRAFT_228538 | 531 | 9.43E-14 | 49.5 | P:regulation of cell growth; C:membrane; C:integral component of membrane; C:extracellular region; F:insulin-like growth factor binding |
| Efet.01.655269.g781.t1 | hypothetical protein CAPTEDRAFT_212909 | 1374 | 3.29E-27 | 78.5 |  |
| Efet.01.655272.g783.t1 | probable glycosyltransferase STELLO1 | 1887 | 0 | 56.5 | C:membrane; C:integral component of membrane |
| Efet.01.655274.g784.t1 | calmodulin 3 | 450 | 5.34E-60 | 65.35 | F:calcium ion binding |
| Efet.01.655275.g785.t1 | kelch 24 | 1647 | 1.08E-70 | 46.5 | F:ubiquitin-protein transferase activity; C:Cul3-RING ubiquitin ligase complex; P:regulation of kainate selective glutamate receptor activity; P:protein ubiquitination |
| Efet.01.655281.g786.t1 | Fis family transcriptional regulator | 234 | 4.17E-50 | 92.6 | P:regulation of transcription, DNA-templated; F:sequence-specific DNA binding |
| Efet.01.655281.g787.t1 | bifunctional phosphoribosylaminoimidazolecarboxamide formyltransferase IMP cyclohydrolase | 1605 | 0 | 89.75 | F:IMP cyclohydrolase activity; P:'de novo' IMP biosynthetic process; F:phosphoribosylaminoimidazolecarboxamide formyltransferase activity |
| Efet.01.655281.g788.t1 | glutamate-1-semialdehyde-2,1-aminomutase | 936 | 2.01E-164 | 74.6 | F:pyridoxal phosphate binding; C:cytoplasm; F:transaminase activity; F:glutamate-1-semialdehyde 2,1-aminomutase activity; P:protoporphyrinogen IX biosynthetic process |
| Efet.01.655281.g789.t1 | hydroxymethylpyrimidine phosphomethylpyrimidine kinase | 951 | 1.71E-153 | 93.4 | F:ATP binding; P:phosphorylation; F:kinase activity |
| Efet.01.655285.g790.t1 | sugar ABC transporter ATP-binding | 648 | 4.62E-144 | 94.95 | F:ATPase activity; F:ATP binding; P:transmembrane transport; C:ATP-binding cassette (ABC) transporter complex; F:hydrolase activity, acting on acid anhydrides, catalyzing transmembrane movement of substances |
| Efet.01.655285.g791.t1 | DUF4032 domain-containing | 891 | 0 | 90.4 | F:ATP binding; C:membrane; P:phosphorylation; F:phosphotransferase activity, alcohol group as acceptor; F:kinase activity |
| Efet.01.655299.g793.t1 | fatty-acid-- ligase | 1122 | 9.00E-152 | 58.85 | F:catalytic activity; P:metabolic process; C:membrane; C:integral component of membrane; F:ligase activity |
| Efet.01.655301.g794.t1 | 26S proteasome non-ATPase regulatory subunit 12 | 276 | 8.11E-46 | 90.55 | P:proteasome-mediated ubiquitin-dependent protein catabolic process; C:cytoplasm; C:proteasome regulatory particle, lid subcomplex; C:nuclear proteasome complex |
| Efet.01.655312.g795.t1 | hypothetical protein HELRODRAFT_191437 | 1101 | 5.23E-09 | 44 |  |
| Efet.01.655317.g796.t1 | solute carrier family 15 member 2 isoform X2 | 258 | 1.08E-06 | 57 |  |
| Efet.01.655322.g797.t1 | discoidin domain-containing receptor 2-like isoform X1 | 558 | 6.72E-65 | 71.9 | F:ATP binding; P:peptidyl-tyrosine phosphorylation; F:protein tyrosine kinase activity; C:integral component of membrane |
| Efet.01.655329.g798.t1 | hypothetical protein HELRODRAFT_160537 | 228 | 6.65E-26 | 70.05 | C:integral component of membrane; F:carbohydrate binding |
| Efet.01.655346.g799.t1 | hypothetical protein OCBIM_22028527mg, partial | 282 | 6.30E-11 | 57.95 |  |
| Efet.01.655360.g800.t1 | Acetylcholine receptor subunit beta-like 2 | 309 | 4.66E-21 | 63.3 | P:ion transport; C:membrane |
| Efet.01.655361.g801.t1 | unc-79 homolog | 390 | 3.69E-39 | 65.9 | C:membrane; C:integral component of membrane |
| Efet.01.655365.g802.t1 | Dynein heavy chain axonemal | 393 | 7.63E-60 | 89.65 | F:ATPase activity; F:ATP binding; F:microtubule motor activity; C:dynein complex; P:microtubule-based movement |
| Efet.01.655365.g803.t1 | dynein heavy chain axonemal-like | 372 | 1.94E-61 | 86.2 | F:ATPase activity; F:ATP binding; F:microtubule motor activity; C:dynein complex; P:microtubule-based movement |
| Efet.01.655372.g805.t1 | doublesex- and mab-3-related transcription factor 3 | 249 | 8.42E-13 | 82.3 | C:nucleus; F:transcription factor activity, sequence-specific DNA binding; P:regulation of transcription, DNA-templated; F:sequence-specific DNA binding |
| Efet.01.655392.g806.t1 | MFS transporter | 1473 | 0 | 77.7 | C:integral component of membrane |
| Efet.01.655399.g808.t1 | bifunctional aconitate hydratase 2 2-methylisocitrate dehydratase | 915 | 0 | 91.4 | F:metal ion binding; C:cytosol; P:tricarboxylic acid cycle; F:2-methylisocitrate dehydratase activity; F:4 iron, 4 sulfur cluster binding; F:aconitate hydratase activity |
| Efet.01.655399.g809.t1 | hypothetical protein | 357 | 1.56E-65 | 88.25 |  |
| Efet.01.655399.g810.t1 | malate dehydrogenase | 987 | 0 | 93.65 | P:carbohydrate metabolic process; F:L-malate dehydrogenase activity; P:malate metabolic process; P:tricarboxylic acid cycle |
| Efet.01.655399.g811.t1 | family transcriptional regulator | 774 | 4.38E-168 | 83.8 | F:DNA binding; F:transcription factor activity, sequence-specific DNA binding; P:transcription, DNA-templated; P:regulation of transcription, DNA-templated |
| Efet.01.655399.g812.t1 | succinate cytochrome b556 subunit | 474 | 1.62E-79 | 73.3 | C:succinate dehydrogenase complex; F:succinate dehydrogenase (ubiquinone) activity; C:integral component of membrane; P:tricarboxylic acid cycle |
| Efet.01.655399.g813.t1 | succinate hydrophobic membrane anchor | 369 | 5.35E-79 | 90.3 | F:succinate dehydrogenase (ubiquinone) activity; F:metal ion binding; C:integral component of membrane; P:tricarboxylic acid cycle; F:heme binding |
| Efet.01.655399.g814.t1 | succinate dehydrogenase flavo subunit | 1806 | 0 | 95.1 | F:succinate dehydrogenase (ubiquinone) activity; F:flavin adenine dinucleotide binding; P:tricarboxylic acid cycle; P:electron transport chain; C:plasma membrane |
| Efet.01.655399.g815.t1 | succinate dehydrogenase iron-sulfur subunit | 705 | 7.61E-164 | 95.7 | F:succinate dehydrogenase (ubiquinone) activity; F:metal ion binding; F:electron carrier activity; F:3 iron, 4 sulfur cluster binding; P:tricarboxylic acid cycle; F:4 iron, 4 sulfur cluster binding; F:2 iron, 2 sulfur cluster binding |
| Efet.01.655405.g816.t1 | Thrombospondin type-1 domain-containing | 1095 | 6.37E-26 | 42.69 | C:membrane; C:integral component of membrane |
| Efet.01.655421.g817.t1 | serine threonine- kinase roco4 | 2952 | 2.52E-145 | 42.08 |  |
| Efet.01.655428.g818.t1 | ribonuclease P component | 324 | 6.30E-38 | 72.55 | F:RNA binding; P:RNA phosphodiester bond hydrolysis; P:tRNA processing; F:endonuclease activity |
| Efet.01.655428.g819.t1 | membrane insertase | 273 | 3.36E-37 | 83.6 | C:integral component of membrane; P:protein insertion into membrane |
| Efet.01.655428.g820.t1 | 16S rRNA (guanine(527)-N(7))-methyltransferase | 564 | 1.11E-102 | 76.35 | F:rRNA methyltransferase activity; P:rRNA methylation |
| Efet.01.655446.g821.t1 | hypothetical protein CAPTEDRAFT_195684 | 528 | 6.23E-38 | 59 |  |
| Efet.01.655451.g822.t1 | ABC transporter | 435 | 2.74E-81 | 78.45 | F:ATPase activity; F:ATP binding |
| Efet.01.655451.g823.t1 | ABC transporter permease | 603 | 4.42E-107 | 67.65 | C:integral component of membrane |
| Efet.01.655451.g824.t1 | ABC transporter permease | 750 | 5.02E-142 | 70.55 | C:integral component of membrane; P:transport |
| Efet.01.655474.g825.t1 | prolow-density lipo receptor-related 1 | 420 | 1.39E-12 | 49.9 | F:calcium ion binding; C:membrane; C:integral component of membrane |
| Efet.01.65548.g954.t1 | eukaryotic translation initiation factor 4E-like isoform X1 | 246 | 3.20E-18 | 67.3 | F:translation initiation factor activity; C:cytoplasm; P:translational initiation |
| Efet.01.65551.g955.t1 | jmjC domain-containing histone demethylation 1-like | 807 | 6.06E-17 | 67.25 | F:binding |
| Efet.01.655549.g826.t1 | Ficolin B | 225 | 1.90E-25 | 73.8 | P:cell surface pattern recognition receptor signaling pathway; P:protein localization to cell surface; F:G-protein coupled receptor binding; F:signaling pattern recognition receptor activity; P:G-protein coupled receptor signaling pathway; P:negative regulation of viral entry into host cell; P:innate immune response; F:sialic acid binding; C:extrinsic component of external side of plasma membrane; P:positive regulation of interleukin-8 secretion; P:recognition of apoptotic cell |
| Efet.01.655573.g829.t1 | repressor | 678 | 7.04E-137 | 88.6 | F:DNA binding; P:DNA repair; P:negative regulation of transcription, DNA-templated; P:transcription, DNA-templated; P:DNA replication; P:proteolysis; F:serine-type endopeptidase activity; P:SOS response |
| Efet.01.655573.g830.t1 | serine threonine dehydratase | 1143 | 6.78E-161 | 85 | F:pyridoxal phosphate binding; P:cellular amino acid metabolic process; F:L-threonine ammonia-lyase activity |
| Efet.01.655573.g831.t1 | urea ABC transporter substrate-binding | 1281 | 0 | 95.15 | P:amino acid transport |
| Efet.01.655573.g832.t1 | urea ABC transporter permease subunit | 903 | 0 | 90 | C:integral component of membrane; P:transport; C:plasma membrane; F:transporter activity |
| Efet.01.655573.g833.t1 | urea ABC transporter ATP-binding | 1578 | 4.33E-164 | 90.15 | F:ATPase activity; F:ATP binding |
| Efet.01.655573.g834.t1 | urea ABC transporter ATP-binding subunit | 459 | 1.69E-92 | 73.05 | F:nucleotide binding |
| Efet.01.655573.g835.t1 | acylamide amidohydrolase | 414 | 1.32E-94 | 98.7 | F:transferase activity, transferring acyl groups; P:nitrogen compound metabolic process; F:amidase activity |
| Efet.01.655584.g836.t1 | IQ motif and SEC7 domain-containing 2-like isoform X10 | 1362 | 3.71E-14 | 67.9 |  |
| Efet.01.655585.g837.t1 | somatomedin-B and thrombospondin type-1 domain-containing -like | 339 | 8.97E-08 | 57.05 | P:immune response; F:scavenger receptor activity; P:receptor-mediated endocytosis; F:polysaccharide binding |
| Efet.01.655622.g840.t1 | branched-chain amino acid ABC transporter permease | 696 | 8.59E-95 | 90.45 | C:integral component of membrane |
| Efet.01.655622.g841.t1 | methionyl-tRNA formyltransferase | 726 | 1.12E-166 | 80.1 | F:methionyl-tRNA formyltransferase activity; P:conversion of methionyl-tRNA to N-formyl-methionyl-tRNA; P:translational initiation |
| Efet.01.655622.g842.t1 | peptide deformylase | 513 | 9.10E-117 | 90.4 | F:iron ion binding; F:peptide deformylase activity; P:translation |
| Efet.01.655648.g843.t1 | small conductance calcium-activated potassium channel SK splice variant | 525 | 5.40E-66 | 82.45 | F:calmodulin binding; P:potassium ion transmembrane transport; C:integral component of membrane; F:small conductance calcium-activated potassium channel activity; C:neuronal cell body; C:plasma membrane |
| Efet.01.655650.g844.t1 | sugar ABC transporter permease | 594 | 1.94E-81 | 81.75 | C:integral component of membrane; P:transport; C:plasma membrane |
| Efet.01.655650.g845.t1 | ABC transporter substrate-binding | 636 | 2.75E-136 | 80.75 | P:transport; F:transporter activity |
| Efet.01.655669.g848.t1 | cell filamentation Fic | 519 | 8.26E-115 | 68.85 |  |
| Efet.01.655669.g849.t1 | aldose 1-epimerase | 759 | 4.56E-172 | 63.45 | P:carbohydrate metabolic process; F:carbohydrate binding; F:isomerase activity |
| Efet.01.655687.g850.t1 | energy-dependent translational throttle | 1329 | 0 | 78.45 | F:ATPase activity; F:ATP binding |
| Efet.01.655750.g855.t1 | aspartate aminotransferase family | 393 | 3.23E-87 | 94.55 | F:pyridoxal phosphate binding; F:beta-alanine-pyruvate transaminase activity |
| Efet.01.655750.g856.t1 | methylmalonate-semialdehyde dehydrogenase ( acylating) | 1557 | 0 | 90.55 | F:methylmalonate-semialdehyde dehydrogenase (acylating) activity; P:oxidation-reduction process |
| Efet.01.655750.g857.t1 | class II aldolase | 789 | 0 | 91.5 | F:lyase activity |
| Efet.01.655750.g858.t1 | tripartite tricarboxylate transporter substrate binding | 996 | 3.63E-180 | 89.65 | C:outer membrane-bounded periplasmic space |
| Efet.01.655755.g859.t1 | amino acid deaminase | 1092 | 0 | 85.2 | F:lyase activity |
| Efet.01.655756.g860.t1 | flagellar motor | 366 | 3.07E-80 | 90.65 | P:protein transport; C:integral component of membrane; F:protein transporter activity |
| Efet.01.655756.g861.t1 | biopolymer transporter | 348 | 3.97E-66 | 93 | P:protein transport; C:integral component of membrane; C:plasma membrane; F:transporter activity |
| Efet.01.655756.g862.t1 | tetraacyldisaccharide 4 -kinase | 1122 | 0 | 71.2 | F:ATP binding; C:membrane; P:lipid A biosynthetic process; P:phosphorylation; F:tetraacyldisaccharide 4'-kinase activity |
| Efet.01.655756.g863.t1 | 3-deoxy-manno-octulosonate cytidylyltransferase | 750 | 1.39E-154 | 82.5 | F:3-deoxy-manno-octulosonate cytidylyltransferase activity; P:keto-3-deoxy-D-manno-octulosonic acid biosynthetic process; C:cytoplasm; P:CMP-keto-3-deoxy-D-manno-octulosonic acid biosynthetic process |
| Efet.01.655756.g864.t1 | adenylate kinase | 558 | 9.16E-132 | 94.3 | P:AMP salvage; F:adenylate kinase activity; F:ATP binding; P:nucleotide phosphorylation; C:cytoplasm |
| Efet.01.655756.g865.t1 | L-asparaginase | 1101 | 0 | 80.5 | P:cellular amino acid metabolic process; F:asparaginase activity |
| Efet.01.655756.g866.t1 | DUF2807 domain-containing | 300 | 4.84E-62 | 67.21 |  |
| Efet.01.655759.g868.t1 | rRNA adenine dimethylase | 243 | 1.10E-36 | 100 |  |
| Efet.01.655759.g869.t1 | class II aldolase family | 474 | 7.61E-110 | 83.75 | F:L-fuculose-phosphate aldolase activity; F:methyltransferase activity; P:methylation |
| Efet.01.655767.g870.t1 | energy-dependent translational throttle | 786 | 1.20E-162 | 92.75 | F:ATPase activity; F:ATP binding |
| Efet.01.655767.g871.t1 | energy-dependent translational throttle | 501 | 1.13E-116 | 91.7 | F:ATPase activity; F:ATP binding |
| Efet.01.655777.g873.t1 | Enamidase | 705 | 1.20E-156 | 66.1 | P:cofactor catabolic process; F:enamidase activity |
| Efet.01.655791.g874.t1 | haloacid dehalogenase | 810 | 1.43E-135 | 80.05 |  |
| Efet.01.655791.g875.t1 | endonuclease | 285 | 4.79E-59 | 95.55 | F:DNA binding; P:nucleic acid phosphodiester bond hydrolysis; C:cytoplasm; P:DNA metabolic process; F:single-stranded DNA endodeoxyribonuclease activity |
| Efet.01.655798.g876.t1 | serine threonine- phosphatase 2A 55 kDa regulatory subunit B delta isoform isoform X2 | 405 | 5.30E-49 | 76.9 | P:regulation of phosphoprotein phosphatase activity; C:protein phosphatase type 2A complex |
| Efet.01.655798.g877.t1 | tubulin delta chain-like | 1290 | 4.52E-130 | 63.85 | F:nucleotide binding; F:GTP binding; F:GTPase activity; F:structural constituent of cytoskeleton; C:microtubule; P:microtubule-based process |
| Efet.01.655821.g879.t1 | deacetylase | 528 | 3.15E-122 | 92.35 |  |
| Efet.01.655821.g880.t1 | electron transfer flavo subunit beta family | 642 | 2.12E-120 | 96.25 | F:electron carrier activity |
| Efet.01.655821.g881.t1 | electron transfer flavo subunit alpha | 948 | 2.76E-175 | 93.8 | F:flavin adenine dinucleotide binding; F:electron carrier activity |
| Efet.01.655821.g882.t1 | acyl- dehydrogenase | 1548 | 0 | 86.95 | F:acyl-CoA dehydrogenase activity; F:flavin adenine dinucleotide binding; P:oxidation-reduction process |
| Efet.01.655821.g883.t1 | nitronate monooxygenase | 951 | 0 | 95.05 | F:dioxygenase activity; F:nitronate monooxygenase activity; P:oxidation-reduction process |
| Efet.01.655821.g884.t1 | 2-hydroxy-3-oxopropionate reductase | 846 | 6.64E-174 | 80 | F:phosphogluconate dehydrogenase (decarboxylating) activity; F:NAD binding; P:oxidation-reduction process; F:3-hydroxyisobutyrate dehydrogenase activity |
| Efet.01.655821.g885.t1 | aldehyde dehydrogenase | 1458 | 0 | 81.65 | F:betaine-aldehyde dehydrogenase activity; P:oxidation-reduction process |
| Efet.01.655821.g886.t1 | tricarballylate utilization | 237 | 1.35E-43 | 86.4 | C:integral component of membrane; F:iron-sulfur cluster binding |
| Efet.01.655836.g887.t1 | peptidoglycan-associated lipo | 513 | 2.23E-91 | 65.55 | C:membrane |
| Efet.01.655851.g888.t1 | PREDICTED: uncharacterized protein KIAA1109-like isoform X2 | 840 | 3.92E-43 | 53.8 | C:membrane; C:integral component of membrane |
| Efet.01.655878.g891.t1 | DNA-binding SATB2 | 1041 | 1.75E-48 | 80.55 | C:nucleus; F:DNA binding |
| Efet.01.655882.g892.t1 | Lymphoid-specific helicase | 921 | 1.16E-11 | 74.6 | F:nucleotide binding; F:protein binding; P:lymphocyte proliferation; P:multicellular organism development; P:pericentric heterochromatin assembly; P:maintenance of DNA methylation; C:pericentric heterochromatin; P:cell cycle; F:hydrolase activity; P:methylation-dependent chromatin silencing |
| Efet.01.655920.g893.t1 | acyl- reductase | 597 | 1.08E-88 | 70.65 | P:metabolic process |
| Efet.01.655926.g895.t1 | microtubule-associated futsch | 681 | 3.43E-34 | 70.6 | P:microtubule cytoskeleton organization; F:microtubule binding; C:microtubule |
| Efet.01.655933.g897.t1 | gfo Idh family oxidoreductase | 402 | 9.12E-60 | 75.8 | F:phthalate 4,5-cis-dihydrodiol dehydrogenase activity; P:oxidation-reduction process |
| Efet.01.655933.g898.t1 | oxidoreductase | 939 | 0 | 79.8 | F:metal ion binding; F:electron carrier activity; F:phthalate 4,5-dioxygenase activity; P:oxidation-reduction process; F:2 iron, 2 sulfur cluster binding; P:transport |
| Efet.01.655933.g899.t1 | tripartite tricarboxylate transporter substrate binding | 684 | 6.83E-119 | 79.55 | C:outer membrane-bounded periplasmic space |
| Efet.01.655933.g900.t1 | 4,5-dihydroxyphthalate decarboxylase | 840 | 0 | 77.3 | F:4,5-dihydroxyphthalate decarboxylase activity |
| Efet.01.655933.g901.t1 | family transcriptional regulator | 1098 | 0 | 87.35 | F:iron ion binding; F:phthalate 4,5-dioxygenase activity; P:oxidation-reduction process; F:2 iron, 2 sulfur cluster binding |
| Efet.01.655964.g905.t1 | NADH-quinone oxidoreductase subunit G | 696 | 4.11E-114 | 87.65 | F:metal ion binding; F:electron carrier activity; C:membrane; F:NADH dehydrogenase (ubiquinone) activity; F:4 iron, 4 sulfur cluster binding; P:ATP synthesis coupled electron transport |
| Efet.01.655964.g906.t1 | NADH-quinone oxidoreductase subunit H | 1083 | 0 | 94.15 | F:quinone binding; C:integral component of membrane; P:oxidation-reduction process; C:plasma membrane; F:oxidoreductase activity, acting on NAD(P)H, quinone or similar compound as acceptor |
| Efet.01.655966.g907.t1 | acetyl propionyl methylcrotonyl- carboxylase subunit alpha | 1731 | 0 | 92.05 | F:ATP binding; F:metal ion binding; F:biotin carboxylase activity |
| Efet.01.655966.g908.t1 | dihydroxy-acid dehydratase | 1161 | 0 | 95.45 | F:dihydroxy-acid dehydratase activity; P:metabolic process |
| Efet.01.655974.g909.t1 | chorismate mutase | 615 | 1.72E-134 | 96.05 | F:chorismate mutase activity; P:L-phenylalanine biosynthetic process; C:cytoplasm; F:amino acid binding; P:chorismate metabolic process; F:prephenate dehydratase activity |
| Efet.01.655974.g910.t1 | 3-phosphoserine phosphohydroxythreonine transaminase | 1035 | 0 | 93.85 | F:pyridoxal phosphate binding; P:pyridoxine biosynthetic process; C:cytoplasm; F:O-phospho-L-serine:2-oxoglutarate aminotransferase activity; P:L-serine biosynthetic process |
| Efet.01.655974.g911.t1 | DNA gyrase subunit A | 2712 | 0 | 96.55 | F:DNA binding; F:ATP binding; C:cytoplasm; P:DNA-dependent DNA replication; C:chromosome; F:DNA topoisomerase type II (ATP-hydrolyzing) activity; P:DNA topological change |
| Efet.01.655974.g912.t1 | family | 708 | 8.45E-106 | 83.2 | C:integral component of membrane; C:cell outer membrane |
| Efet.01.655974.g913.t1 | bifunctional 2-polyprenyl-6-hydroxyphenol methylase 3-demethylubiquinol 3-O-methyltransferase | 711 | 4.38E-174 | 89.15 | F:3-demethylubiquinone-n 3-O-methyltransferase activity; P:ubiquinone biosynthetic process; F:2-polyprenyl-6-methoxy-1,4-benzoquinone methyltransferase activity; P:methylation |
| Efet.01.655978.g914.t1 | rootletin-like isoform X10 | 672 | 2.87E-28 | 56.5 |  |
| Efet.01.655982.g915.t1 | fumarylacetoacetate hydrolase | 861 | 9.63E-149 | 59.65 | F:catalytic activity; P:metabolic process; F:hydrolase activity |
| Efet.01.655982.g916.t1 | Macrolide export ATP-binding permease | 1311 | 1.73E-114 | 64.25 | C:membrane |
| Efet.01.655987.g917.t1 | ABC transporter permease | 801 | 3.61E-47 | 91.9 | F:ATPase activity; F:ATP binding; C:integral component of membrane; P:peptide transport; C:plasma membrane |
| Efet.01.655987.g918.t1 | ABC transporter permease | 918 | 0 | 93.4 | C:integral component of membrane; P:transport; C:plasma membrane |
| Efet.01.655987.g919.t1 | ABC transporter substrate-binding | 1530 | 0 | 89.85 | P:transmembrane transport; C:ATP-binding cassette (ABC) transporter complex |
| Efet.01.655987.g920.t1 | family transcriptional regulator | 918 | 0 | 88.15 | F:DNA binding; F:transcription factor activity, sequence-specific DNA binding; P:transcription, DNA-templated; P:regulation of transcription, DNA-templated |
| Efet.01.655987.g921.t1 | aspartate racemase | 513 | 2.64E-96 | 95.05 | F:racemase activity, acting on amino acids and derivatives; P:cellular amino acid metabolic process |
| Efet.01.655988.g922.t1 | biotin--[acetyl- -carboxylase] ligase | 708 | 2.46E-154 | 79.85 | P:cellular protein modification process; F:biotin-[acetyl-CoA-carboxylase] ligase activity |
| Efet.01.655988.g923.t1 | sporulation | 324 | 6.11E-69 | 85.4 | F:peptidoglycan binding |
| Efet.01.655988.g924.t1 | thymidylate synthase | 897 | 0 | 91.9 | F:thymidylate synthase activity; C:cytoplasm; P:dTMP biosynthetic process; P:dTTP biosynthetic process; P:methylation |
| Efet.01.655994.g925.t1 | sex peptide receptor-like | 387 | 4.60E-61 | 80.75 | F:G-protein coupled peptide receptor activity; P:G-protein coupled receptor signaling pathway; C:integral component of membrane |
| Efet.01.655999.g926.t1 | carboxyvinyl-carboxyphosphonate phosphorylmutase | 549 | 1.14E-130 | 93.7 | P:metabolic process; F:2,3-dimethylmalate lyase activity |
| Efet.01.655999.g927.t1 | dihydrolipoyl dehydrogenase | 1695 | 0 | 90.25 | C:cell; F:flavin adenine dinucleotide binding; P:cell redox homeostasis; P:oxidation-reduction process; F:dihydrolipoyl dehydrogenase activity |
| Efet.01.655999.g928.t1 | dihydrolipoyllysine-residue acetyltransferase | 786 | 1.81E-163 | 95.3 | C:pyruvate dehydrogenase complex; P:glycolytic process; F:dihydrolipoyllysine-residue acetyltransferase activity |
| Efet.01.656025.g929.t1 | ribosomal RNA-processing 8 | 363 | 1.65E-22 | 73.85 | F:methyltransferase activity; C:nucleolus; P:methylation |
| Efet.01.656032.g931.t1 | cholecystokinin receptor type A-like | 378 | 6.78E-36 | 67.15 | F:G-protein coupled receptor activity; P:signal transduction; C:membrane |
| Efet.01.656054.g932.t1 | uncharacterized protein LOC111107117 | 609 | 2.88E-34 | 56.65 |  |
| Efet.01.656076.g935.t1 | phosphohistidine phosphatase | 489 | 1.06E-104 | 60.4 |  |
| Efet.01.656090.g936.t1 | glycine oxidase | 828 | 1.20E-151 | 85.95 | F:oxidoreductase activity; F:flavin adenine dinucleotide binding; P:oxidation-reduction process |
| Efet.01.656090.g937.t1 | bifunctional hydroxymethylpyrimidine kinase phosphomethylpyrimidine kinase | 894 | 0 | 83.55 | F:ATP binding; F:phosphomethylpyrimidine kinase activity; P:phosphorylation; P:thiamine biosynthetic process |
| Efet.01.656090.g938.t1 | NAD(P) FAD-dependent oxidoreductase | 1317 | 0 | 85.45 | F:NADP binding; F:flavin adenine dinucleotide binding; P:oxidation-reduction process; F:ferredoxin-NADP+ reductase activity |
| Efet.01.656104.g940.t1 | inositol 2-dehydrogenase | 498 | 4.23E-92 | 72.85 | F:oxidoreductase activity; P:metabolic process |
| Efet.01.656107.g941.t1 | zinc finger BED domain-containing 1-like | 1887 | 1.00E-112 | 49.9 | F:binding; C:intracellular part; C:membrane-bounded organelle |
| Efet.01.656123.g942.t1 | Neuronal calcium sensor 2 | 486 | 2.11E-38 | 65.25 | F:calcium ion binding |
| Efet.01.656145.g943.t1 | non-ribosomal peptide synthetase | 2298 | 0 | 87.4 | F:catalytic activity; P:metabolic process; F:phosphopantetheine binding |
| Efet.01.656171.g946.t1 | hypothetical protein | 543 | 1.78E-123 | 63.65 |  |
| Efet.01.656171.g947.t1 | family transcriptional regulator | 663 | 2.65E-105 | 69.75 |  |
| Efet.01.656171.g948.t1 | hypothetical protein | 348 | 7.40E-76 | 81.3 |  |
| Efet.01.656171.g949.t1 | DUF4123 domain-containing | 408 | 3.58E-73 | 77.1 |  |
| Efet.01.656171.g950.t1 | PAAR domain-containing | 324 | 1.73E-54 | 80.35 |  |
| Efet.01.656171.g951.t1 | PAAR motif | 690 | 1.65E-143 | 74.55 | F:DNA binding; F:sequence-specific DNA binding |
| Efet.01.656171.g952.t1 | IS30 family transposase | 1020 | 6.07E-165 | 79.2 | F:nucleic acid binding; P:transposition; P:DNA recombination |
| Efet.01.656171.g953.t1 | resolvase | 681 | 1.67E-107 | 87.4 | F:DNA binding; F:recombinase activity; P:DNA recombination |
| Efet.01.656171.g954.t1 | hypothetical protein | 510 | 4.23E-99 | 88 |  |
| Efet.01.656171.g955.t1 | hypothetical protein | 336 | 8.05E-24 | 80.45 | C:integral component of membrane |
| Efet.01.656171.g956.t1 | 3 -5 exonuclease | 474 | 3.19E-99 | 87.9 | F:nucleic acid binding; P:nucleic acid phosphodiester bond hydrolysis; F:exonuclease activity |
| Efet.01.656171.g957.t1 | Hypothetical protein (plasmid) | 447 | 6.48E-72 | 63.35 |  |
| Efet.01.656171.g958.t1 | HAD family hydrolase | 582 | 1.12E-103 | 76.95 | P:metabolic process; F:hydrolase activity |
| Efet.01.656171.g959.t1 | hypothetical protein | 561 | 1.34E-118 | 70.7 |  |
| Efet.01.656171.g960.t1 | partition | 948 | 6.94E-145 | 86.85 |  |
| Efet.01.656171.g961.t1 | replication | 708 | 4.78E-164 | 85.8 |  |
| Efet.01.656174.g962.t1 | M23 family peptidase | 837 | 3.45E-151 | 75.5 | C:membrane |
| Efet.01.656190.g963.t1 | glycoside hydrolase | 966 | 1.34E-79 | 61.35 | F:hydrolase activity |
| Efet.01.656206.g964.t1 | carbohydrate ABC transporter permease | 474 | 1.35E-80 | 80.1 | C:integral component of membrane; P:carbohydrate transport; C:plasma membrane |
| Efet.01.656206.g965.t1 | transcriptional repressor | 483 | 4.21E-112 | 91.4 | F:DNA binding; F:ATP binding; F:zinc ion binding; P:negative regulation of transcription, DNA-templated; P:transcription, DNA-templated |
| Efet.01.656216.g966.t1 | hypothetical protein | 222 | 1.77E-40 | 93 |  |
| Efet.01.656216.g967.t1 | outer membrane | 915 | 2.91E-164 | 53.5 |  |
| Efet.01.656216.g968.t1 | tripartite tricarboxylate transporter substrate binding | 792 | 1.04E-139 | 72.2 | C:outer membrane-bounded periplasmic space |
| Efet.01.656216.g969.t1 | nitrate ABC transporter substrate-binding | 753 | 3.12E-179 | 79.25 |  |
| Efet.01.656238.g971.t1 | spermine synthase | 1011 | 3.72E-81 | 80.1 |  |
| Efet.01.656239.g972.t1 | hypothetical protein CAPTEDRAFT_202832 | 717 | 7.14E-17 | 75.33 | F:zinc ion binding |
| Efet.01.656246.g974.t1 | NAD-dependent dehydratase | 1308 | 2.80E-155 | 83 | F:coenzyme binding; F:transferase activity |
| Efet.01.65626.g956.t1 | dual oxidase 2-like isoform X1 | 279 | 1.32E-16 | 64.5 | F:catalytic activity |
| Efet.01.656277.g977.t1 | hypothetical protein CAPTEDRAFT_215105 | 1047 | 2.64E-09 | 60.5 |  |
| Efet.01.656278.g978.t1 | FH1 FH2 domain-containing 3-like isoform X9 | 390 | 1.61E-42 | 82.05 |  |
| Efet.01.656283.g980.t1 | type III restriction res subunit | 1953 | 0 | 77.05 | F:DNA binding; F:ATP binding; F:hydrolase activity |
| Efet.01.656283.g981.t1 | urease accessory | 558 | 1.62E-129 | 94.6 | F:GTP binding; C:cytoplasm; P:nitrogen compound metabolic process; F:GTPase activity; F:nickel cation binding |
| Efet.01.656283.g982.t1 | urease accessory | 630 | 5.73E-91 | 69.85 | C:cytoplasm; P:nitrogen compound metabolic process; F:nickel cation binding |
| Efet.01.656283.g983.t1 | urease accessory | 324 | 2.88E-52 | 91.9 | P:enzyme active site formation; P:urea metabolic process; C:cytoplasm; P:protein complex assembly; F:nickel cation binding; F:unfolded protein binding; P:protein folding |
| Efet.01.656283.g984.t1 | urease subunit alpha | 1773 | 0 | 89.15 | P:urea catabolic process; C:cytoplasm; F:nickel cation binding; F:urease activity |
| Efet.01.656283.g985.t1 | urease subunit beta | 306 | 1.92E-65 | 90 | P:urea catabolic process; C:cytoplasm; F:urease activity |
| Efet.01.656289.g987.t1 | polyribonucleotide nucleotidyltransferase | 1464 | 0 | 89.4 | F:magnesium ion binding; P:RNA phosphodiester bond hydrolysis, exonucleolytic; F:3'-5'-exoribonuclease activity; F:RNA binding; C:cytoplasm; P:RNA processing; P:mRNA catabolic process; F:polyribonucleotide nucleotidyltransferase activity |
| Efet.01.656303.g989.t1 | Serine threonine- kinase STY46 | 507 | 1.13E-10 | 63.25 | P:metabolic process; F:kinase activity |
| Efet.01.656306.g991.t1 | neuronal migration doublecortin-like isoform X3 | 747 | 1.05E-94 | 76 | F:nucleotide binding; F:protein kinase activity; P:phosphorylation |
| Efet.01.656307.g992.t1 | DNA-binding HEXBP-like | 210 | 2.38E-08 | 67.25 | F:binding |
| Efet.01.656312.g993.t1 | substance-K receptor-like | 591 | 1.08E-12 | 46.38 | F:G-protein coupled receptor activity; P:neuropeptide signaling pathway; P:G-protein coupled receptor signaling pathway; P:signal transduction; C:membrane; C:integral component of membrane; F:signal transducer activity; F:neuropeptide Y receptor activity |
| Efet.01.656315.g994.t1 | vang 2 | 1302 | 1.07E-117 | 72.95 | P:multicellular organism development; C:integral component of membrane |
| Efet.01.656319.g995.t1 | THAP domain-containing 2-like | 627 | 3.09E-12 | 54.95 | F:nucleic acid binding; P:phosphorylation; F:kinase activity |
| Efet.01.656321.g996.t1 | ATP synthase-coupling factor mitochondrial | 225 | 4.58E-15 | 71.75 | C:mitochondrial proton-transporting ATP synthase complex, coupling factor F(o); F:hydrogen ion transmembrane transporter activity; P:ATP synthesis coupled proton transport |
| Efet.01.656322.g997.t1 | matrix metallo ase-21-like | 462 | 1.03E-37 | 65.25 | F:metal ion binding; P:multicellular organism development; F:peptidase activity |
| Efet.01.656328.g998.t1 | nitrite reductase | 336 | 3.08E-19 | 63.3 | F:binding |
| Efet.01.656334.g1000.t1 | bifunctional 3-phosphoshikimate 1-carboxyvinyltransferase cytidylate kinase | 738 | 1.16E-153 | 90.45 | F:cytidylate kinase activity; F:ATP binding; P:nucleotide phosphorylation; P:aromatic amino acid family biosynthetic process; F:3-phosphoshikimate 1-carboxyvinyltransferase activity; C:cytoplasm; P:chorismate biosynthetic process |
| Efet.01.656334.g1001.t1 | cytidylate kinase | 234 | 6.28E-41 | 83.95 | F:cytidylate kinase activity; F:ATP binding; P:nucleotide phosphorylation; P:aromatic amino acid family biosynthetic process; F:3-phosphoshikimate 1-carboxyvinyltransferase activity; C:cytoplasm; P:chorismate biosynthetic process |
| Efet.01.656336.g1002.t1 | hydrolase | 669 | 3.71E-145 | 69.2 | F:catalytic activity |
| Efet.01.656340.g1003.t1 | dihydrodipicolinate synthase family | 441 | 2.02E-23 | 83.4 | F:catalytic activity |
| Efet.01.656340.g1004.t1 | dihydrodipicolinate synthase family | 825 | 1.17E-116 | 76.7 | F:catalytic activity |
| Efet.01.656340.g1005.t1 | 3-ketoacyl-ACP reductase | 288 | 2.48E-45 | 86.2 |  |
| Efet.01.656364.g1008.t1 | DC-STAMP domain-containing 2-like | 1953 | 9.84E-56 | 56.7 | F:zinc ion binding; C:membrane; C:integral component of membrane |
| Efet.01.656367.g1009.t1 | family | 510 | 6.04E-50 | 59.75 |  |
| Efet.01.65638.g957.t1 | dual specificity phosphatase 7-like | 300 | 3.78E-07 | 74 |  |
| Efet.01.656383.g1010.t1 | hypothetical protein CAPTEDRAFT_195684 | 561 | 8.24E-43 | 53.38 |  |
| Efet.01.656400.g1013.t1 | zinc finger OZF-like isoform X1 | 954 | 4.46E-13 | 52.95 | F:nucleic acid binding; F:metal ion binding |
| Efet.01.65641.g958.t1 | alpha-1A adrenergic receptor | 1056 | 1.22E-106 | 70.85 | F:adrenergic receptor activity; P:G-protein coupled receptor signaling pathway; P:positive regulation of multicellular organismal process; P:regulation of muscle contraction; C:integral component of membrane; P:regulation of heart contraction; P:regulation of blood vessel diameter; C:cell part; P:positive regulation of blood pressure by epinephrine-norepinephrine |
| Efet.01.656421.g1014.t1 | Mitochondrial dynamics MID51 | 633 | 1.16E-18 | 48.8 |  |
| Efet.01.656427.g1015.t1 | substance-K receptor | 684 | 4.66E-53 | 71.05 | P:neuropeptide signaling pathway; C:integral component of membrane; F:neuropeptide Y receptor activity |
| Efet.01.656433.g1018.t1 | hypothetical protein CAPTEDRAFT_227908 | 546 | 2.86E-11 | 72 |  |
| Efet.01.656454.g1019.t1 | Cell division | 417 | 9.94E-74 | 73.4 | C:membrane |
| Efet.01.656454.g1020.t1 | peptidase S8 | 237 | 2.99E-42 | 81.35 | P:proteolysis; C:integral component of membrane; F:serine-type endopeptidase activity |
| Efet.01.656457.g1021.t1 | arf6 guanine nucleotide exchange factor | 216 | 5.65E-16 | 70 | C:trans-Golgi network; P:vesicle-mediated transport; F:ARF guanyl-nucleotide exchange factor activity |
| Efet.01.656462.g1022.t1 | 14-3-3 beta alpha-A | 318 | 4.03E-53 | 92.3 | F:protein domain specific binding; P:phagocytosis, engulfment; C:cytoplasm |
| Efet.01.656470.g1025.t1 | tripartite tricarboxylate transporter substrate binding | 519 | 1.13E-102 | 79.55 | C:outer membrane-bounded periplasmic space |
| Efet.01.656470.g1026.t1 | family transcriptional regulator | 231 | 1.02E-30 | 78.27 | F:DNA binding; F:transcription factor activity, sequence-specific DNA binding; P:transcription, DNA-templated; P:regulation of transcription, DNA-templated |
| Efet.01.656470.g1027.t1 | tripartite tricarboxylate transporter substrate binding | 873 | 2.43E-171 | 92.8 | C:outer membrane-bounded periplasmic space |
| Efet.01.656470.g1028.t1 | DUF1254 domain-containing | 2478 | 0 | 91.15 | F:transferase activity |
| Efet.01.656470.g1029.t1 | family transcriptional regulator | 957 | 0 | 85.05 | F:DNA binding; F:transcription factor activity, sequence-specific DNA binding; P:transcription, DNA-templated; P:regulation of transcription, DNA-templated |
| Efet.01.656471.g1030.t1 | Carnitine O-acetyltransferase | 447 | 1.77E-17 | 73.45 | F:transferase activity, transferring acyl groups |
| Efet.01.656480.g1031.t1 | trimeric intracellular cation channel type B | 312 | 1.50E-18 | 76.6 | P:monovalent inorganic cation transport; F:cation channel activity; P:cation transmembrane transport; C:integral component of membrane |
| Efet.01.656495.g1033.t1 | NADH-quinone oxidoreductase subunit F | 249 | 1.25E-36 | 79.75 | F:FMN binding; F:NAD binding; F:NADH dehydrogenase (ubiquinone) activity; P:oxidation-reduction process; F:4 iron, 4 sulfur cluster binding |
| Efet.01.656506.g1034.t1 | sodium channel type 4 subunit alpha B-like isoform X1 | 423 | 5.36E-13 | 64.75 | F:ion channel activity; P:ion transport; P:transmembrane transport; C:membrane |
| Efet.01.656508.g1035.t1 | zinc finger BED domain-containing 4-like | 1047 | 1.03E-67 | 56.85 | F:binding; C:intracellular part |
| Efet.01.656513.g1036.t1 | allene oxide synthase-lipoxygenase -like | 420 | 2.27E-21 | 54.6 | F:oxidoreductase activity |
| Efet.01.656518.g1037.t1 | ABC transporter ATP-binding | 1455 | 0 | 88.2 | F:ATPase activity; F:ATP binding |
| Efet.01.656518.g1038.t1 | ABC transporter permease | 1113 | 2.03E-173 | 89.15 | C:integral component of membrane; P:transport; C:plasma membrane; F:transporter activity |
| Efet.01.656558.g1039.t1 | helicase | 2685 | 0 | 74.55 | F:ATP binding; F:helicase activity |
| Efet.01.656558.g1040.t1 | NAD(P) FAD-dependent oxidoreductase | 1449 | 0 | 87.2 | F:FAD binding; F:oxidoreductase activity; C:integral component of membrane; P:oxidation-reduction process |
| Efet.01.656558.g1041.t1 | ABC transporter permease | 921 | 0 | 95.8 | C:integral component of membrane; P:transport; C:plasma membrane |
| Efet.01.656558.g1042.t1 | ABC transporter permease | 936 | 0 | 93.35 | C:integral component of membrane; P:transport; C:plasma membrane |
| Efet.01.656558.g1043.t1 | ABC transporter ATP-binding | 459 | 3.40E-76 | 94.55 | F:ATPase activity; F:ATP binding; P:peptide transport |
| Efet.01.656558.g1044.t1 | ABC transporter ATP-binding | 258 | 5.52E-52 | 94.15 | F:ATPase activity; F:ATP binding; P:peptide transport |
| Efet.01.656558.g1045.t1 | peptide ABC transporter ATP-binding | 1050 | 0 | 90.15 | F:ATPase activity; F:ATP binding; P:peptide transport |
| Efet.01.656558.g1046.t1 | ABC transporter substrate-binding | 573 | 2.28E-96 | 96.1 | P:transmembrane transport; C:ATP-binding cassette (ABC) transporter complex |
| Efet.01.656558.g1047.t1 | ABC transporter substrate-binding | 984 | 0 | 94.15 | P:transmembrane transport; C:ATP-binding cassette (ABC) transporter complex |
| Efet.01.65656.g960.t1 | hypothetical protein HELRODRAFT_184904 | 372 | 1.34E-08 | 79.5 |  |
| Efet.01.656585.g1048.t1 | tripartite motif-containing 2-like isoform X5 | 633 | 5.40E-22 | 48.9 | F:zinc ion binding; F:metal ion binding; C:intracellular |
| Efet.01.656598.g1049.t1 | NAD(P)-dependent oxidoreductase | 372 | 2.15E-80 | 79.75 |  |
| Efet.01.656598.g1050.t1 | polysaccharide deacetylase | 501 | 4.78E-116 | 84.35 | F:hydrolase activity, acting on carbon-nitrogen (but not peptide) bonds; P:carbohydrate metabolic process |
| Efet.01.656598.g1051.t1 | polysaccharide deacetylase | 2169 | 0 | 89.5 | F:hydrolase activity, acting on carbon-nitrogen (but not peptide) bonds; P:xylan catabolic process; C:outer membrane-bounded periplasmic space; F:hydrolase activity, acting on glycosyl bonds |
| Efet.01.656639.g1055.t1 | atrial natriuretic peptide receptor 1-like | 690 | 4.43E-19 | 50.8 | P:cellular process |
| Efet.01.656644.g1056.t1 | mitogen-activated kinase kinase kinase 12 | 345 | 3.19E-11 | 60 | P:regulation of cellular process; P:single-organism cellular process; P:protein phosphorylation; F:kinase activity |
| Efet.01.656647.g1057.t1 | hypothetical protein HELRODRAFT_136819, partial | 216 | 2.08E-17 | 59.67 | F:calcium ion binding |
| Efet.01.656649.g1058.t1 | cyclin-dependent kinase inhibitor 1 | 312 | 2.04E-09 | 61.4 | P:regulation of cellular process; P:cellular process |
| Efet.01.656651.g1060.t1 | 4-hydroxyphenylpyruvate dioxygenase | 339 | 1.15E-40 | 86.25 | P:aromatic amino acid family metabolic process; F:4-hydroxyphenylpyruvate dioxygenase activity; F:metal ion binding; P:oxidation-reduction process; F:lactoylglutathione lyase activity |
| Efet.01.656672.g1061.t1 | hypothetical protein CAPTEDRAFT_21433 | 525 | 5.32E-21 | 52.25 | C:membrane; C:integral component of membrane |
| Efet.01.656695.g1063.t1 | hypothetical protein HELRODRAFT_160282 | 426 | 7.47E-27 | 65.65 | C:integral component of membrane; F:carbohydrate binding |
| Efet.01.656704.g1064.t1 | sugar ABC transporter permease | 972 | 4.38E-175 | 66.25 | C:membrane |
| Efet.01.656704.g1065.t1 | carbohydrate ABC transporter permease | 636 | 3.00E-134 | 77.3 | C:integral component of membrane; P:transport; C:plasma membrane |
| Efet.01.656705.g1066.t1 | Fido domain-containing | 459 | 4.20E-25 | 55.15 |  |
| Efet.01.656716.g1067.t1 | diaminopimelate epimerase | 531 | 7.63E-85 | 87.9 | F:diaminopimelate epimerase activity; C:cytoplasm; P:lysine biosynthetic process via diaminopimelate |
| Efet.01.656716.g1068.t1 | DNA mismatch repair | 609 | 2.70E-87 | 98 | F:ATP binding; P:mismatch repair; F:mismatched DNA binding; F:damaged DNA binding |
| Efet.01.656717.g1069.t1 | aldehyde dehydrogenase | 1233 | 0 | 95.65 | F:aldehyde dehydrogenase (NAD) activity; P:oxidation-reduction process |
| Efet.01.656717.g1070.t1 | DUF779 domain-containing | 387 | 1.14E-90 | 90.6 |  |
| Efet.01.656717.g1071.t1 | cytochrome c biogenesis | 372 | 9.35E-11 | 73.25 | F:antioxidant activity; F:oxidoreductase activity; C:integral component of membrane; P:cellular oxidant detoxification; P:oxidation-reduction process; P:cytochrome complex assembly; C:plasma membrane |
| Efet.01.656717.g1072.t1 | (Fe-S)-binding | 456 | 4.07E-68 | 71.85 | F:hydrolase activity |
| Efet.01.656717.g1073.t1 | Gamma-glutamyl AIG2-like | 390 | 2.90E-54 | 78.35 | F:ligase activity |
| Efet.01.656717.g1074.t1 | aldehyde dehydrogenase family | 1434 | 0 | 89.4 | F:aldehyde dehydrogenase (NAD) activity; P:oxidation-reduction process |
| Efet.01.656717.g1076.t1 | nitrate ABC transporter substrate-binding | 510 | 4.50E-56 | 88.85 | F:phosphoglycolate phosphatase activity; P:dephosphorylation; P:thiamine biosynthetic process |
| Efet.01.656717.g1077.t1 | nucleotidyltransferase domain-containing | 1131 | 1.20E-173 | 76.71 | C:integral component of membrane |
| Efet.01.656717.g1078.t1 | arabinose efflux MFS family | 900 | 8.42E-139 | 68.8 | C:integral component of membrane |
| Efet.01.656742.g1082.t1 | aspartate carbamoyltransferase | 1989 | 0 | 95.85 | F:aspartate carbamoyltransferase activity; P:'de novo' pyrimidine nucleobase biosynthetic process; P:cellular amino acid metabolic process; F:amino acid binding; P:'de novo' UMP biosynthetic process |
| Efet.01.656742.g1083.t1 | family transcriptional regulator | 954 | 0 | 66.45 | F:transcription factor activity, sequence-specific DNA binding; P:transcription, DNA-templated; P:regulation of transcription, DNA-templated; F:sequence-specific DNA binding |
| Efet.01.656742.g1084.t1 | hydantoinase | 3594 | 0 | 83.9 | F:5-oxoprolinase (ATP-hydrolyzing) activity; F:N-methylhydantoinase (ATP-hydrolyzing) activity |
| Efet.01.656742.g1085.t1 | NAD NADP octopine nopaline dehydrogenase | 822 | 0 | 78.9 | F:coenzyme binding; F:opine dehydrogenase activity; P:oxidation-reduction process; F:2-dehydropantoate 2-reductase activity |
| Efet.01.656742.g1086.t1 | tripartite tricarboxylate transporter substrate binding | 1419 | 2.49E-162 | 73.6 | C:outer membrane-bounded periplasmic space |
| Efet.01.656742.g1088.t1 | phosphomethylpyrimidine synthase | 1896 | 0 | 89.95 | F:zinc ion binding; F:lyase activity; F:4 iron, 4 sulfur cluster binding; P:thiamine diphosphate biosynthetic process; P:thiamine biosynthetic process |
| Efet.01.656757.g1089.t1 | family transcriptional regulator | 1437 | 4.55E-143 | 78.5 | F:DNA binding; P:transmembrane transport; P:transcription, DNA-templated; C:integral component of membrane; P:regulation of transcription, DNA-templated |
| Efet.01.656757.g1090.t1 | tripartite tricarboxylate transporter substrate binding | 903 | 0 | 75.6 | C:outer membrane-bounded periplasmic space |
| Efet.01.656757.g1091.t1 | arylsulfatase | 1329 | 0 | 89.35 | F:sulfuric ester hydrolase activity; P:metabolic process |
| Efet.01.656757.g1092.t1 | family transcriptional regulator | 921 | 0 | 67.4 | F:DNA binding; F:transcription factor activity, sequence-specific DNA binding; P:transcription, DNA-templated; P:regulation of transcription, DNA-templated |
| Efet.01.656757.g1093.t1 | ADP-ribose pyrophosphatase | 570 | 2.59E-137 | 91.85 | F:hydrolase activity |
| Efet.01.656757.g1094.t1 | cysteine synthase | 744 | 2.41E-163 | 88.3 | F:cysteine synthase activity; F:transferase activity; P:cysteine biosynthetic process from serine |
| Efet.01.65677.g961.t1 | CLK4-associating serine arginine rich | 342 | 4.86E-35 | 60.75 |  |
| Efet.01.656795.g1099.t1 | branched-chain amino acid ABC transporter permease | 459 | 2.87E-57 | 94.4 | C:integral component of membrane; P:transport; C:plasma membrane; F:transporter activity |
| Efet.01.656795.g1100.t1 | ABC transporter substrate-binding | 1926 | 0 | 96.45 | P:amino acid transport |
| Efet.01.656795.g1101.t1 | ornithine carbamoyltransferase | 921 | 0 | 92.55 | C:cytoplasm; F:amino acid binding; P:arginine deiminase pathway; F:ornithine carbamoyltransferase activity; P:arginine catabolic process to ornithine |
| Efet.01.656795.g1102.t1 | aspartate aminotransferase family | 1203 | 0 | 91.9 | F:pyridoxal phosphate binding; P:arginine biosynthetic process; C:cytoplasm; F:N2-acetyl-L-ornithine:2-oxoglutarate 5-aminotransferase activity |
| Efet.01.656795.g1103.t1 | thioesterase family | 843 | 4.51E-168 | 88.65 |  |
| Efet.01.656795.g1104.t1 | enoyl- hydratase | 777 | 4.52E-129 | 79.7 | P:metabolic process; F:enoyl-CoA hydratase activity |
| Efet.01.656795.g1105.t1 | family transcriptional regulator | 546 | 3.45E-99 | 85.9 | F:DNA binding; P:transcription, DNA-templated; P:regulation of transcription, DNA-templated; C:chromatin |
| Efet.01.656796.g1106.t1 | PRELI domain-containing mitochondrial-like | 321 | 1.57E-33 | 52.45 | C:mitochondrial intermembrane space; P:phospholipid transport; F:phosphatidic acid transporter activity |
| Efet.01.656797.g1107.t1 | conserved repeat domain-containing | 954 | 2.56E-123 | 61.4 | C:membrane; C:integral component of membrane |
| Efet.01.656799.g1108.t1 | ATPase WRNIP1-like | 1416 | 3.42E-74 | 70.55 | F:organic cyclic compound binding; F:heterocyclic compound binding; F:helicase activity; P:DNA metabolic process |
| Efet.01.656804.g1109.t1 | choline mitochondrial | 1485 | 0 | 70.3 | F:oxidoreductase activity, acting on CH-OH group of donors; P:metabolic process |
| Efet.01.656813.g1110.t1 | tripartite tricarboxylate transporter substrate binding | 804 | 2.81E-180 | 84.9 | C:outer membrane-bounded periplasmic space |
| Efet.01.656813.g1111.t1 | altronate dehydratase | 1554 | 0 | 88.35 | F:galactarate dehydratase activity; F:altronate dehydratase activity; F:hydrolase activity |
| Efet.01.656813.g1112.t1 | metalloprotease | 1434 | 0 | 92.7 | P:proteolysis; F:metallopeptidase activity |
| Efet.01.656813.g1113.t1 | molybdopterin adenylyltransferase | 1197 | 3.41E-152 | 91.25 | F:nucleotidyltransferase activity; P:Mo-molybdopterin cofactor biosynthetic process |
| Efet.01.656816.g1114.t1 | ABC transporter ATP-binding | 1089 | 0 | 70.5 | F:ATPase activity; F:ATP binding; P:peptide transport |
| Efet.01.656816.g1115.t1 | peptide ABC transporter substrate-binding | 1527 | 0 | 86.15 | P:transmembrane transport; C:ATP-binding cassette (ABC) transporter complex |
| Efet.01.656816.g1116.t1 | peptidase C45 | 999 | 0 | 85.8 | F:isopenicillin-N N-acyltransferase activity |
| Efet.01.656816.g1117.t1 | amidohydrolase | 1182 | 0 | 90.7 | F:carboxypeptidase activity; F:hippurate hydrolase activity; P:proteolysis; F:aminoacylase activity |
| Efet.01.656816.g1118.t1 | M20 peptidase family dipeptidase | 894 | 0 | 90.8 | P:metabolic process; F:hydrolase activity |
| Efet.01.656822.g1119.t1 | sugar ABC transporter substrate-binding | 1785 | 0 | 81.4 | P:carbohydrate transport |
| Efet.01.656822.g1120.t1 | D-threitol dehydrogenase | 765 | 5.67E-159 | 76.95 | F:oxidoreductase activity; P:oxidation-reduction process |
| Efet.01.656822.g1121.t1 | ribose 5-phosphate isomerase B | 429 | 2.03E-96 | 83.55 | P:carbohydrate metabolic process; P:pentose-phosphate shunt; F:galactose-6-phosphate isomerase activity; F:ribose-5-phosphate isomerase activity |
| Efet.01.656822.g1122.t1 | DNA-binding | 960 | 0 | 85.55 | F:DNA binding; F:carbohydrate binding |
| Efet.01.656822.g1123.t1 | PIN domain-containing | 420 | 4.59E-96 | 91.8 | F:magnesium ion binding; P:RNA phosphodiester bond hydrolysis; F:ribonuclease activity |
| Efet.01.656828.g1124.t1 | UDP-glucuronosyltransferase 2C1-like | 765 | 7.07E-73 | 64.45 | F:transferase activity, transferring hexosyl groups; P:flavonoid metabolic process |
| Efet.01.656836.g1126.t1 | serine threonine- kinase TAO1 | 483 | 9.41E-12 | 59.1 | F:nucleotide binding; F:ATP binding; F:protein kinase activity; P:phosphorylation; P:protein phosphorylation; F:kinase activity |
| Efet.01.656856.g1128.t1 | mitochondrial ATP synthase subunit beta precursor | 603 | 1.68E-91 | 95.3 | C:proton-transporting ATP synthase complex, catalytic core F(1); F:ATP binding; P:ATP hydrolysis coupled proton transport; C:mitochondrial inner membrane; P:ATP synthesis coupled proton transport; F:proton-transporting ATP synthase activity, rotational mechanism |
| Efet.01.656866.g1130.t1 | hypothetical protein CAPTEDRAFT_223681 | 672 | 3.79E-10 | 53 | C:membrane; C:integral component of membrane |
| Efet.01.656870.g1131.t1 | frizzled 9 10 | 951 | 1.58E-97 | 73.5 | F:transmembrane signaling receptor activity; C:membrane; P:cell surface receptor signaling pathway |
| Efet.01.65688.g963.t1 | eukaryotic translation initiation factor Y-chromosomal-like | 234 | 2.06E-28 | 90.45 | F:translation initiation factor activity; P:translational initiation |
| Efet.01.656883.g1133.t1 | carbamoyl-phosphate synthase large subunit | 1311 | 0 | 76.7 | F:ATP binding; F:metal ion binding; F:biotin carboxylase activity |
| Efet.01.656889.g1134.t1 | phosphoadenosine phosphosulfate sulfotransferase | 1239 | 0 | 56.55 | F:catalytic activity; P:metabolic process; F:transferase activity |
| Efet.01.656889.g1135.t1 | hypothetical protein | 639 | 1.58E-82 | 72.05 |  |
| Efet.01.656889.g1136.t1 | RES domain-containing | 414 | 2.27E-73 | 73.55 |  |
| Efet.01.656891.g1137.t1 | isoleucine--tRNA ligase | 3363 | 0 | 92.3 | P:isoleucyl-tRNA aminoacylation; F:ATP binding; F:zinc ion binding; F:isoleucine-tRNA ligase activity; C:cytoplasm; P:regulation of translational fidelity; F:aminoacyl-tRNA editing activity |
| Efet.01.656919.g1140.t1 | 1-deoxy-D-xylulose-5-phosphate reductoisomerase | 354 | 4.28E-55 | 91.35 | F:1-deoxy-D-xylulose-5-phosphate reductoisomerase activity; F:metal ion binding; F:NADPH binding; P:isopentenyl diphosphate biosynthetic process, methylerythritol 4-phosphate pathway; P:oxidation-reduction process; P:terpenoid biosynthetic process; F:isomerase activity |
| Efet.01.656919.g1141.t1 | di-trans,poly-cis-decaprenylcistransferase | 690 | 1.81E-134 | 93.1 | F:magnesium ion binding; F:prenyltransferase activity |
| Efet.01.656919.g1142.t1 | ribosome recycling factor | 561 | 2.11E-131 | 97.4 | P:translational termination; C:cytoplasm |
| Efet.01.656919.g1143.t1 | UMP kinase | 723 | 2.31E-161 | 92.05 | F:ATP binding; P:nucleotide phosphorylation; C:cytoplasm; P:'de novo' CTP biosynthetic process; F:UMP kinase activity |
| Efet.01.656929.g1144.t1 | U7 snRNA-associated Sm LSm10 | 456 | 1.08E-27 | 69.2 | C:nucleus |
| Efet.01.656932.g1145.t1 | D-glycerate dehydrogenase | 1179 | 9.11E-151 | 88.95 | F:oxidoreductase activity, acting on the CH-OH group of donors, NAD or NADP as acceptor; F:NAD binding; P:oxidation-reduction process |
| Efet.01.656939.g1146.t1 | polycystic kidney disease 1-like 2 | 702 | 2.27E-29 | 47.8 | P:detection of mechanical stimulus; F:calcium channel activity; F:calcium ion binding; C:membrane; C:integral component of membrane; P:calcium ion transmembrane transport |
| Efet.01.656952.g1147.t1 | 3-oxoacyl-ACP synthase | 786 | 2.52E-162 | 94.05 | F:3-oxoacyl-[acyl-carrier-protein] synthase activity; C:cytoplasm; F:beta-ketoacyl-acyl-carrier-protein synthase III activity; P:fatty acid biosynthetic process |
| Efet.01.656952.g1148.t1 | phosphate acyltransferase | 1071 | 0 | 91.4 | F:transferase activity, transferring acyl groups other than amino-acyl groups; F:oxidoreductase activity, acting on the CH-OH group of donors, NAD or NADP as acceptor; C:cytoplasm; P:phospholipid biosynthetic process; P:oxidation-reduction process; P:fatty acid biosynthetic process |
| Efet.01.656971.g1149.t1 | biopolymer transporter | 339 | 8.93E-65 | 92.15 | P:protein transport; C:integral component of membrane; C:plasma membrane; F:transporter activity |
| Efet.01.656971.g1150.t1 | biopolymer transporter | 720 | 5.92E-172 | 93.15 | P:protein transport; C:integral component of membrane; F:protein transporter activity |
| Efet.01.656971.g1151.t1 | energy transducer | 210 | 1.45E-42 | 97.4 | F:siderophore transmembrane transporter activity; P:siderophore transmembrane transport; P:protein transport; C:integral component of membrane; F:energy transducer activity; C:outer membrane-bounded periplasmic space; C:plasma membrane |
| Efet.01.656991.g1153.t1 | lytic transglycosylase F | 345 | 6.59E-64 | 65.65 | F:carbon-oxygen lyase activity, acting on polysaccharides; P:peptidoglycan metabolic process; C:membrane; F:lytic transglycosylase activity |
| Efet.01.657010.g1154.t1 | Inositol phosphoceramide mannosyltransferase 3 | 537 | 3.13E-33 | 58.9 | F:oxidoreductase activity; P:metabolic process; C:membrane; C:integral component of membrane; P:oxidation-reduction process |
| Efet.01.657025.g1155.t1 | murein biosynthesis integral membrane | 1002 | 7.62E-163 | 74.05 | C:membrane |
| Efet.01.657036.g1156.t1 | low-density lipo receptor-related 2-like | 678 | 1.61E-74 | 62.05 | P:peptidyl-tyrosine phosphorylation; F:transmembrane receptor protein tyrosine kinase activity; F:calcium ion binding; C:membrane; C:integral component of membrane; F:transferase activity; F:hydrolase activity |
| Efet.01.657048.g1157.t1 | octopamine receptor 1-like | 714 | 4.26E-44 | 55.15 | P:signal transduction; C:membrane; F:signal transducer activity |
| Efet.01.657066.g1158.t1 | chromodomain-helicase-DNA-binding 8-like isoform X3 | 1050 | 1.03E-88 | 76.05 | C:nucleus; F:DNA binding; F:ATP binding; F:helicase activity |
| Efet.01.657077.g1159.t1 | aldehyde dehydrogenase | 1398 | 0 | 78.9 | P:oxidation-reduction process; F:oxidoreductase activity, acting on the aldehyde or oxo group of donors, NAD or NADP as acceptor |
| Efet.01.657077.g1160.t1 | transcriptional regulator | 2505 | 0 | 73.5 | F:ATP binding; F:helicase activity |
| Efet.01.657077.g1161.t1 | hypothetical protein | 459 | 1.59E-84 | 84.15 |  |
| Efet.01.657078.g1162.t1 | 50S ribosomal L10 | 918 | 5.40E-65 | 93.15 | F:structural constituent of ribosome; P:ribosome biogenesis; C:ribosome; F:large ribosomal subunit rRNA binding; P:translation |
| Efet.01.657078.g1163.t1 | gfo Idh family oxidoreductase | 1146 | 0 | 84.4 | F:oxidoreductase activity; P:oxidation-reduction process |
| Efet.01.657094.g1165.t1 | mannosyl phosphorylinositol ceramide synthase CSH1-like | 360 | 2.41E-24 | 54.64 | C:membrane; C:integral component of membrane |
| Efet.01.657098.g1166.t1 | probable G- coupled receptor 83 | 633 | 4.72E-40 | 69.7 | F:G-protein coupled receptor activity; P:G-protein coupled receptor signaling pathway; C:membrane |
| Efet.01.657101.g1167.t1 | non-POU domain-containing octamer-binding -like | 549 | 2.84E-20 | 77.7 | F:nucleotide binding; C:nucleus; F:transcription regulatory region sequence-specific DNA binding; P:regulation of transcription, DNA-templated; P:mRNA splicing, via spliceosome |
| Efet.01.657110.g1168.t1 | carbohydrate ABC transporter permease | 477 | 5.65E-67 | 84.4 | C:integral component of membrane; P:transport; C:plasma membrane |
| Efet.01.657110.g1169.t1 | sugar ABC transporter permease | 552 | 1.03E-116 | 83.35 | C:integral component of membrane; P:transport; C:plasma membrane |
| Efet.01.657110.g1170.t1 | ABC transporter substrate-binding | 2241 | 0 | 87.6 | F:ATPase activity; F:ATP binding; P:transmembrane transport; C:ATP-binding cassette (ABC) transporter complex; F:hydrolase activity, acting on acid anhydrides, catalyzing transmembrane movement of substances |
| Efet.01.657110.g1171.t1 | xylulokinase | 765 | 1.42E-157 | 86.95 | P:xylulose metabolic process; F:xylulokinase activity; P:carbohydrate phosphorylation |
| Efet.01.657112.g1172.t1 | toll-like receptor 3 | 2181 | 1.61E-66 | 46.3 | C:extracellular exosome; P:G-protein coupled receptor signaling pathway; C:membrane; C:integral component of membrane; C:vesicle; P:positive regulation of Wnt signaling pathway; F:insulin-like growth factor binding; C:insulin-like growth factor ternary complex; F:protein-hormone receptor activity; P:positive regulation of cell migration; C:nucleoplasm |
| Efet.01.657123.g1174.t1 | acyl- dehydrogenase | 924 | 0 | 94.35 | F:acyl-CoA dehydrogenase activity; F:flavin adenine dinucleotide binding; P:oxidation-reduction process |
| Efet.01.657123.g1175.t1 | transferase | 1182 | 0 | 87.6 | F:formyl-CoA transferase activity |
| Efet.01.657123.g1176.t1 | tripartite tricarboxylate transporter substrate binding | 729 | 1.76E-145 | 93.55 | C:outer membrane-bounded periplasmic space |
| Efet.01.657129.g1178.t1 | tyramine receptor 1 | 1314 | 2.66E-100 | 54.3 | F:G-protein coupled receptor activity; P:G-protein coupled receptor signaling pathway; F:octopamine receptor activity; P:signal transduction; C:membrane; C:integral component of membrane; F:signal transducer activity |
| Efet.01.657130.g1179.t1 | PLP-dependent aminotransferase family | 1500 | 0 | 67.45 | F:DNA binding; F:transcription factor activity, sequence-specific DNA binding; F:pyridoxal phosphate binding; F:catalytic activity; P:transcription, DNA-templated; P:regulation of transcription, DNA-templated |
| Efet.01.657130.g1180.t1 | Sugar binding of ABC transporter | 984 | 0 | 60.45 | C:membrane; C:integral component of membrane |
| Efet.01.657149.g1182.t1 | extended synaptotagmin-2-like isoform X1 | 354 | 1.60E-08 | 67.69 | C:membrane |
| Efet.01.657150.g1183.t1 | restriction endonuclease subunit S | 297 | 4.86E-44 | 70.9 | F:DNA binding; F:nucleotidyltransferase activity; P:DNA modification |
| Efet.01.657150.g1184.t1 | restriction endonuclease subunit S | 975 | 0 | 71.05 | F:DNA binding; F:nucleotidyltransferase activity; P:DNA modification |
| Efet.01.657150.g1185.t1 | cell filamentation Fic | 1086 | 5.45E-115 | 84.75 | F:DNA binding |
| Efet.01.657152.g1186.t1 | hypothetical protein CAPTEDRAFT_222005 | 996 | 3.27E-10 | 53 | F:ATP binding |
| Efet.01.657158.g1189.t1 | type II secretion system F | 603 | 7.51E-141 | 92.55 | C:integral component of membrane |
| Efet.01.657158.g1190.t1 | type IV-A pilus assembly ATPase | 1509 | 0 | 84.85 | F:ATP binding; P:pilus assembly; P:protein transport; F:protein transporter activity |
| Efet.01.657158.g1191.t1 | site-specific integrase | 1059 | 0 | 90.05 | F:DNA binding; P:DNA recombination; P:DNA integration |
| Efet.01.657158.g1192.t1 | DNA-binding | 660 | 1.28E-94 | 62.8 | F:DNA binding |
| Efet.01.657183.g1193.t1 | DNA repair | 1233 | 0 | 69.5 | F:nucleotide binding; P:macromolecule metabolic process; F:DNA binding; F:peptidase activity, acting on L-amino acid peptides; F:ion binding; P:cellular response to DNA damage stimulus; P:primary metabolic process; F:ATPase activity, coupled |
| Efet.01.657185.g1194.t1 | BTB POZ domain-containing kctd15-like | 612 | 1.19E-76 | 71.75 | P:protein homooligomerization; F:3',5'-cyclic-nucleotide phosphodiesterase activity; F:hydrolase activity |
| Efet.01.657189.g1195.t1 | uracil phosphoribosyltransferase | 336 | 9.09E-72 | 92.45 | F:magnesium ion binding; F:GTP binding; F:uracil phosphoribosyltransferase activity; P:uracil salvage; P:UMP salvage |
| Efet.01.657189.g1196.t1 | nucleoside deaminase | 507 | 5.53E-89 | 81.5 | F:zinc ion binding; F:tRNA-specific adenosine-34 deaminase activity; P:tRNA wobble adenosine to inosine editing |
| Efet.01.657189.g1197.t1 | cation transporter | 801 | 2.50E-149 | 83.45 | P:cation transmembrane transport; C:integral component of membrane; F:cation transmembrane transporter activity |
| Efet.01.657206.g1198.t1 | MFS transporter | 618 | 2.86E-109 | 84.75 | P:transmembrane transport; C:integral component of membrane |
| Efet.01.657206.g1199.t1 | dihydrodipicolinate synthase family | 822 | 8.46E-167 | 88.15 | P:metabolic process; F:lyase activity |
| Efet.01.657206.g1200.t1 | dihydroxy-acid dehydratase | 372 | 8.08E-65 | 87.75 | F:dihydroxy-acid dehydratase activity; P:metabolic process |
| Efet.01.657207.g1201.t1 | basement membrane-specific heparan sulfate proteoglycan core -like | 399 | 5.23E-33 | 61.6 | F:calcium ion binding |
| Efet.01.657208.g1202.t1 | kelch 31 | 549 | 1.25E-37 | 59.25 | P:skeletal muscle tissue development; P:regulation of transcription, DNA-templated |
| Efet.01.657217.g1203.t1 | dihydroxy-acid dehydratase | 1557 | 0 | 74.3 | F:dihydroxy-acid dehydratase activity; P:metabolic process |
| Efet.01.657217.g1204.t1 | family transcriptional regulator | 708 | 4.72E-57 | 62.95 | F:DNA binding; F:transcription factor activity, sequence-specific DNA binding; P:transcription, DNA-templated; P:regulation of transcription, DNA-templated |
| Efet.01.657237.g1206.t1 | potassium voltage-gated channel subfamily H member 7-like isoform X3 | 591 | 7.77E-66 | 83.1 | F:voltage-gated potassium channel activity; P:potassium ion transmembrane transport; C:integral component of membrane |
| Efet.01.657239.g1207.t1 | ABC transporter ATP-binding | 1533 | 0 | 82.4 | F:ATP binding; F:ATPase activity, coupled to transmembrane movement of substances; P:transmembrane transport; C:integral component of membrane |
| Efet.01.657243.g1209.t1 | hypothetical protein CAPTEDRAFT_193384 | 486 | 2.00E-25 | 55.6 | P:proteolysis; F:serine-type peptidase activity |
| Efet.01.657248.g1210.t1 | oxidoreductase | 357 | 5.19E-75 | 89.15 | F:dioxygenase activity; F:metal ion binding; F:electron carrier activity; F:methyltransferase activity; P:oxidation-reduction process; F:2 iron, 2 sulfur cluster binding; P:transport; P:methylation |
| Efet.01.657248.g1211.t1 | family | 465 | 1.50E-106 | 93 |  |
| Efet.01.657248.g1212.t1 | amidohydrolase | 786 | 5.76E-133 | 85 | F:cytosine deaminase activity |
| Efet.01.657248.g1213.t1 | NAD-dependent epimerase | 714 | 7.16E-146 | 92.05 | F:coenzyme binding; F:ADP-glyceromanno-heptose 6-epimerase activity |
| Efet.01.657257.g1214.t1 | acyl :acetate 3-ketoacid transferase | 894 | 1.97E-161 | 90.4 | F:acetate CoA-transferase activity; P:ketone body catabolic process |
| Efet.01.657257.g1215.t1 | gfo Idh family oxidoreductase | 951 | 0 | 75.65 | F:oxidoreductase activity; P:oxidation-reduction process |
| Efet.01.657257.g1216.t1 | family transcriptional regulator | 1020 | 0 | 73.6 | F:DNA binding; F:transcription factor activity, sequence-specific DNA binding; P:transcription, DNA-templated; P:regulation of transcription, DNA-templated |
| Efet.01.657257.g1217.t1 | family transcriptional regulator | 738 | 3.03E-165 | 83.5 | F:DNA binding; F:transcription factor activity, sequence-specific DNA binding; P:transcription, DNA-templated; P:regulation of transcription, DNA-templated |
| Efet.01.657257.g1218.t1 | ABC transporter substrate-binding | 945 | 4.24E-175 | 91.9 | C:integral component of membrane |
| Efet.01.65726.g964.t1 | neuron navigator 3-like isoform X2 | 255 | 7.29E-47 | 92.1 | F:ATP binding |
| Efet.01.657266.g1219.t1 | peptidase | 975 | 2.23E-110 | 83.65 | C:integral component of membrane |
| Efet.01.657268.g1220.t1 | transient receptor potential cation channel subfamily M member 2-like | 249 | 9.00E-08 | 61 |  |
| Efet.01.657269.g1221.t1 | zinc finger 665-like | 378 | 3.15E-38 | 64.75 | F:metal ion binding |
| Efet.01.657286.g1223.t1 | Heparan sulfate glucosamine 3-O-sulfotransferase | 633 | 6.97E-82 | 71.7 | C:integral component of plasma membrane; P:heparan sulfate proteoglycan biosynthetic process, enzymatic modification; F:[heparan sulfate]-glucosamine 3-sulfotransferase 1 activity; P:glycosaminoglycan biosynthetic process; C:Golgi membrane |
| Efet.01.657293.g1224.t1 | pyruvate dehydrogenase complex dihydrolipoamide acetyltransferase | 651 | 3.65E-112 | 87.05 | C:pyruvate dehydrogenase complex; P:pyruvate metabolic process; F:dihydrolipoyllysine-residue acetyltransferase activity |
| Efet.01.657302.g1225.t1 | DUF480 domain-containing | 723 | 3.73E-129 | 77.6 |  |
| Efet.01.657302.g1226.t1 | phosphoribosylglycinamide formyltransferase | 474 | 3.42E-92 | 91.95 | F:phosphoribosylglycinamide formyltransferase activity; P:'de novo' IMP biosynthetic process |
| Efet.01.657302.g1227.t1 | SAM-dependent methyltransferase | 1356 | 0 | 90 | F:RNA binding; C:cytoplasm; F:methyltransferase activity; P:methylation |
| Efet.01.657302.g1228.t1 | lytic murein transglycosylase B | 1125 | 0 | 86.45 |  |
| Efet.01.657308.g1229.t1 | META domain-containing | 756 | 7.61E-147 | 75.9 |  |
| Efet.01.657308.g1230.t1 | thioredoxin | 909 | 2.06E-176 | 89.45 | C:cell; F:protein disulfide oxidoreductase activity; P:glycerol ether metabolic process; P:cell redox homeostasis; P:oxidation-reduction process |
| Efet.01.657308.g1231.t1 | 5-(carboxyamino)imidazole ribonucleotide mutase | 495 | 1.82E-69 | 95.9 | F:phosphoribosylaminoimidazole carboxylase activity; F:5-(carboxyamino)imidazole ribonucleotide mutase activity; C:integral component of membrane; P:'de novo' IMP biosynthetic process |
| Efet.01.657308.g1232.t1 | 5-(carboxyamino)imidazole ribonucleotide synthase | 888 | 3.83E-82 | 92.5 | F:ATP binding; F:phosphoribosylaminoimidazole carboxylase activity; F:metal ion binding; F:5-(carboxyamino)imidazole ribonucleotide synthase activity; P:'de novo' IMP biosynthetic process |
| Efet.01.657308.g1233.t1 | threonylcarbamoyl-AMP synthase | 1047 | 0 | 79.95 | F:ATP binding; C:cytoplasm; F:double-stranded RNA binding; P:tRNA processing; F:L-threonylcarbamoyladenylate synthase |
| Efet.01.657310.g1235.t1 | piccolo isoform X1 | 1638 | 5.88E-16 | 58.05 | F:zinc ion binding; F:metal ion binding; P:intracellular protein transport; F:Rab GTPase binding; C:intracellular |
| Efet.01.657312.g1236.t1 | aristaless-related homeobox -like | 471 | 2.27E-07 | 63 | F:DNA binding; C:nucleus; P:multicellular organism development; P:regulation of transcription, DNA-templated; F:sequence-specific DNA binding |
| Efet.01.657314.g1237.t1 | hypothetical protein CAPTEDRAFT_228538 | 603 | 1.03E-14 | 52 |  |
| Efet.01.657315.g1238.t1 | tRNA (adenosine(37)-N6)-threonylcarbamoyltransferase complex ATPase subunit type 1 | 678 | 1.17E-88 | 81.4 | P:tRNA threonylcarbamoyladenosine modification |
| Efet.01.657315.g1239.t1 | tRNA epoxyqueuosine(34) reductase | 849 | 0 | 91.3 | P:queuosine biosynthetic process; C:cytoplasm; F:metal ion binding; P:tRNA processing; F:epoxyqueuosine reductase activity; P:oxidation-reduction process; F:4 iron, 4 sulfur cluster binding |
| Efet.01.657315.g1240.t1 | transporter | 906 | 1.07E-148 | 90.85 | P:transmembrane transport; C:integral component of membrane |
| Efet.01.657315.g1241.t1 | ABC transporter substrate-binding | 963 | 0 | 93.55 | C:outer membrane-bounded periplasmic space |
| Efet.01.657315.g1242.t1 | site-specific tyrosine recombinase | 912 | 0 | 89.55 | P:cell division; F:DNA binding; C:cytoplasm; P:DNA integration; P:chromosome segregation; F:tyrosine-based site-specific recombinase activity; P:cell cycle; P:transposition, DNA-mediated |
| Efet.01.657315.g1243.t1 | ABC transporter substrate-binding | 984 | 0 | 85.2 | C:outer membrane-bounded periplasmic space |
| Efet.01.657315.g1244.t1 | diguanylate phosphodiesterase | 1347 | 0 | 91.25 |  |
| Efet.01.657315.g1245.t1 | gamma carbonic anhydrase family | 594 | 1.12E-104 | 92.35 | F:transferase activity |
| Efet.01.657315.g1246.t1 | tRNA (N6-isopentenyl adenosine(37)-C2)-methylthiotransferase | 1353 | 0 | 91.8 | C:cytoplasm; F:iron ion binding; F:4 iron, 4 sulfur cluster binding; F:transferase activity; P:tRNA modification |
| Efet.01.657315.g1247.t1 | hypothetical protein | 705 | 1.37E-63 | 83.9 | C:integral component of membrane |
| Efet.01.657315.g1248.t1 | aquaporin family | 672 | 2.26E-87 | 80.75 | C:integral component of membrane; P:transport; F:transporter activity |
| Efet.01.657315.g1249.t1 | DEAD DEAH box helicase | 2841 | 0 | 86.45 | F:ATP binding; F:helicase activity |
| Efet.01.657315.g1250.t1 | DNA damage-inducible D | 510 | 3.10E-07 | 88.44 | P:biological_process; F:molecular_function |
| Efet.01.657315.g1251.t1 | DUF4868 domain-containing | 660 | 1.85E-84 | 76.7 | P:biological_process; C:cellular_component; F:molecular_function |
| Efet.01.657315.g1252.t1 | Fic family | 1347 | 0 | 69 | F:transferase activity |
| Efet.01.657315.g1253.t1 | site-specific DNA-methyltransferase | 1686 | 0 | 81.45 | F:DNA binding; F:N-methyltransferase activity; P:DNA methylation |
| Efet.01.657318.g1254.t1 | integrase | 1134 | 2.58E-109 | 86.15 | F:DNA binding; P:DNA recombination; P:DNA integration |
| Efet.01.657318.g1255.t1 | cell filamentation Fic | 609 | 1.73E-133 | 87.55 | F:nucleotidyltransferase activity |
| Efet.01.657321.g1256.t1 | E3 ubiquitin- ligase TRIM71-like | 879 | 4.15E-17 | 47.95 | F:zinc ion binding; F:metal ion binding; C:intracellular |
| Efet.01.657322.g1257.t1 | adenosylhomocysteinase 3 isoform X2 | 252 | 1.23E-24 | 88.9 | P:S-adenosylmethionine cycle; C:cytosol; F:adenosylhomocysteinase activity; P:one-carbon metabolic process |
| Efet.01.657325.g1258.t1 | leucine-rich repeat 1-like | 255 | 3.30E-09 | 61.33 |  |
| Efet.01.657327.g1259.t1 | galactitol-1-phosphate 5-dehydrogenase | 552 | 3.97E-98 | 61.05 | F:zinc ion binding; F:metal ion binding; F:oxidoreductase activity; P:oxidation-reduction process |
| Efet.01.657327.g1260.t1 | family transcriptional regulator | 849 | 2.06E-176 | 61.95 | F:DNA binding; P:transcription, DNA-templated; P:regulation of transcription, DNA-templated |
| Efet.01.657334.g1261.t1 | family transcriptional regulator | 885 | 2.03E-165 | 64.45 | F:DNA binding; P:carbohydrate metabolic process; F:transcription factor activity, sequence-specific DNA binding; P:regulation of transcription, DNA-templated; F:carbohydrate binding |
| Efet.01.657334.g1262.t1 | isoaspartyl peptidase L-asparaginase | 648 | 5.41E-146 | 73.4 | F:hydrolase activity |
| Efet.01.657336.g1263.t1 | fumarate hydratase | 2331 | 0 | 92.35 | P:generation of precursor metabolites and energy; F:fumarate hydratase activity |
| Efet.01.657336.g1264.t1 | class II fumarate hydratase | 1512 | 0 | 93.45 | P:tricarboxylic acid cycle; F:fumarate hydratase activity; P:fumarate metabolic process; C:tricarboxylic acid cycle enzyme complex |
| Efet.01.657336.g1265.t1 | phosphoesterase | 2688 | 0 | 73.8 | F:catalytic activity |
| Efet.01.657336.g1266.t1 | ATP-dependent DNA helicase | 1182 | 0 | 73.15 | F:DNA binding; F:ATP binding; P:DNA repair; P:DNA recombination; F:helicase activity |
| Efet.01.657336.g1267.t1 | TIGR00645 family | 549 | 1.16E-126 | 96.45 | C:integral component of membrane; C:plasma membrane |
| Efet.01.657336.g1268.t1 | acetate-- ligase | 1995 | 0 | 92.85 | F:ATP binding; F:acetate-CoA ligase activity; F:AMP binding; F:metal ion binding; P:acetyl-CoA biosynthetic process from acetate |
| Efet.01.657336.g1269.t1 | Signal transduction histidine kinase | 312 | 6.51E-48 | 89.55 | C:integral component of membrane; P:phosphorylation; F:kinase activity |
| Efet.01.657339.g1270.t1 | hypothetical protein HELRODRAFT_160496 | 408 | 3.38E-23 | 66 | P:signal transduction |
| Efet.01.657343.g1271.t1 | glyco -N-acetylgalactosamine 3-beta-galactosyltransferase 1-like isoform X1 | 990 | 2.70E-107 | 74.8 | C:membrane; F:glycoprotein-N-acetylgalactosamine 3-beta-galactosyltransferase activity; P:O-glycan processing |
| Efet.01.65736.g966.t1 | hypothetical protein HELRODRAFT_161459 | 234 | 1.71E-12 | 55 | C:membrane; P:proteolysis; C:integral component of membrane; F:serine-type peptidase activity |
| Efet.01.657376.g1272.t1 | bark beetle-like isoform X1 | 849 | 2.11E-95 | 54.8 | C:membrane |
| Efet.01.657392.g1273.t1 | hypothetical protein HELRODRAFT_191437 | 858 | 4.62E-11 | 49 |  |
| Efet.01.65740.g967.t1 | hypothetical protein HELRODRAFT_172434 | 825 | 1.57E-08 | 72.67 | F:DNA binding |
| Efet.01.657430.g1277.t1 | amino acid ABC transporter substrate-binding | 1116 | 0 | 83.75 | P:amino acid transport |
| Efet.01.657430.g1278.t1 | family transcriptional regulator | 687 | 4.62E-150 | 91.5 | F:DNA binding; P:transcription, DNA-templated; P:regulation of transcription, DNA-templated |
| Efet.01.657430.g1279.t1 | AMP-dependent synthetase | 1608 | 0 | 88.6 | P:metabolic process; F:ligase activity |
| Efet.01.657433.g1280.t1 | cupin domain-containing | 564 | 1.76E-101 | 78.35 |  |
| Efet.01.657433.g1281.t1 | alpha beta hydrolase | 378 | 5.68E-50 | 58.25 | F:hydrolase activity |
| Efet.01.657433.g1282.t1 | TRAP-type C4-dicarboxylate transport substrate-binding | 972 | 1.11E-176 | 63.15 | C:outer membrane-bounded periplasmic space; P:transport |
| Efet.01.657445.g1284.t1 | ubiquitin carboxyl-terminal hydrolase 7-like | 816 | 1.44E-25 | 52.75 | P:protein deubiquitination; C:membrane; C:integral component of membrane; F:thiol-dependent ubiquitinyl hydrolase activity; P:ubiquitin-dependent protein catabolic process; F:hydrolase activity |
| Efet.01.657446.g1285.t1 | cadherin EGF LAG seven-pass G-type receptor 3-like | 960 | 1.37E-52 | 55.55 | P:G-protein coupled receptor signaling pathway |
| Efet.01.657452.g1287.t1 | probable G- coupled receptor 158 isoform X1 | 795 | 5.69E-25 | 45.95 | F:G-protein coupled receptor activity; P:G-protein coupled receptor signaling pathway; C:membrane; C:integral component of membrane |
| Efet.01.657453.g1288.t1 | hypothetical protein | 600 | 3.66E-76 | 72 |  |
| Efet.01.657453.g1289.t1 | site-specific integrase | 546 | 3.82E-106 | 71.55 | F:DNA binding; P:DNA integration; P:DNA recombination |
| Efet.01.657453.g1290.t1 | peptide chain release factor 3 | 231 | 3.59E-46 | 93.7 | F:GTP binding; P:translational termination; P:regulation of translational termination; C:cytoplasm; F:GTPase activity; F:translation release factor activity, codon specific |
| Efet.01.657457.g1291.t1 | ATP-dependent DNA helicase | 837 | 0 | 96.5 | F:nucleic acid binding; F:ATP binding; P:DNA repair; P:DNA duplex unwinding; P:DNA recombination; F:ATP-dependent DNA helicase activity |
| Efet.01.657457.g1292.t1 | family transcriptional regulator | 1161 | 0 | 91.85 | F:DNA binding; F:transcription factor activity, sequence-specific DNA binding; P:transcription, DNA-templated; P:regulation of transcription, DNA-templated |
| Efet.01.657457.g1293.t1 | DNA starvation stationary phase protection | 501 | 1.64E-100 | 90 | F:oxidoreductase activity, oxidizing metal ions; P:cellular iron ion homeostasis; C:cell; F:DNA binding; F:ferric iron binding; P:oxidation-reduction process; P:response to stress |
| Efet.01.657473.g1296.t1 | indole-3-glycerol-phosphate synthase | 804 | 8.70E-155 | 90.25 | F:indole-3-glycerol-phosphate synthase activity; P:tryptophan biosynthetic process |
| Efet.01.657473.g1297.t1 | transporter | 645 | 5.98E-111 | 91.9 | C:integral component of plasma membrane; P:lactate transmembrane transport; F:lactate transmembrane transporter activity |
| Efet.01.657473.g1298.t1 | peptidase M20 | 1512 | 0 | 93.25 | P:metabolic process; F:hydrolase activity |
| Efet.01.657473.g1299.t1 | outer membrane assembly factor | 2055 | 0 | 88.85 | C:cytoplasm; P:lysine biosynthetic process via diaminopimelate; F:4-hydroxy-tetrahydrodipicolinate synthase; P:diaminopimelate biosynthetic process |
| Efet.01.657484.g1300.t1 | winged helix family transcriptional regulator | 225 | 7.83E-45 | 90.25 | F:DNA binding; P:phosphorelay signal transduction system; P:regulation of transcription, DNA-templated; C:intracellular |
| Efet.01.657484.g1301.t1 | glycerophosphodiester phosphodiesterase | 885 | 0 | 65.2 | P:lipid metabolic process; F:glycerophosphodiester phosphodiesterase activity; F:phosphoric diester hydrolase activity |
| Efet.01.657484.g1302.t1 | 30S ribosomal S6 | 459 | 1.61E-76 | 91.25 | F:structural constituent of ribosome; C:ribosome; F:rRNA binding; P:translation |
| Efet.01.657484.g1303.t1 | 50S ribosomal L9 | 453 | 5.57E-100 | 85.35 | F:structural constituent of ribosome; C:ribosome; F:rRNA binding; P:translation |
| Efet.01.657492.g1304.t1 | ABC transporter ATP-binding | 1572 | 0 | 75.55 | F:ATPase activity; F:ATP binding; P:transmembrane transport; P:single-organism transport; C:ATP-binding cassette (ABC) transporter complex; F:hydrolase activity, acting on acid anhydrides, catalyzing transmembrane movement of substances; P:organic substance transport |
| Efet.01.657492.g1305.t1 | GMC family oxidoreductase | 1287 | 0 | 82.15 | F:oxidoreductase activity, acting on CH-OH group of donors; F:flavin adenine dinucleotide binding; P:oxidation-reduction process |
| Efet.01.657492.g1306.t1 | 3-ketoacyl-ACP reductase | 753 | 3.53E-116 | 73.4 | F:3-oxoacyl-[acyl-carrier-protein] reductase (NADPH) activity; F:oxidoreductase activity; P:oxidation-reduction process |
| Efet.01.657492.g1307.t1 | alanine-phosphoribitol ligase | 924 | 0 | 84.35 | F:oxidoreductase activity, acting on CH-OH group of donors; F:flavin adenine dinucleotide binding; P:oxidation-reduction process; F:ligase activity |
| Efet.01.657496.g1308.t1 | family transcriptional regulator | 579 | 6.36E-126 | 77.05 | F:DNA binding; F:transcription factor activity, sequence-specific DNA binding; P:transcription, DNA-templated; P:regulation of transcription, DNA-templated |
| Efet.01.657496.g1309.t1 | aminoacyl-tRNA hydrolase | 609 | 1.46E-133 | 93.8 | C:cytoplasm; F:aminoacyl-tRNA hydrolase activity; P:translation |
| Efet.01.657496.g1310.t1 | 50S ribosomal L25 general stress Ctc | 723 | 1.14E-129 | 95.2 | F:structural constituent of ribosome; F:5S rRNA binding; C:ribosome; P:translation |
| Efet.01.657496.g1311.t1 | ribose-phosphate pyrophosphokinase | 975 | 0 | 97.85 | F:magnesium ion binding; F:ATP binding; P:ribonucleoside monophosphate biosynthetic process; C:cytoplasm; P:nucleotide biosynthetic process; F:ribose phosphate diphosphokinase activity; P:phosphorylation; F:kinase activity; P:5-phosphoribose 1-diphosphate biosynthetic process |
| Efet.01.657496.g1312.t1 | 4-(cytidine 5 -diphospho)-2-C-methyl-D-erythritol kinase | 903 | 0 | 81.5 | F:ATP binding; P:isopentenyl diphosphate biosynthetic process, methylerythritol 4-phosphate pathway; F:4-(cytidine 5'-diphospho)-2-C-methyl-D-erythritol kinase activity; P:phosphorylation; P:terpenoid biosynthetic process |
| Efet.01.657496.g1313.t1 | outer membrane lipo | 327 | 7.90E-59 | 85.35 | P:protein transport; C:cell outer membrane |
| Efet.01.657496.g1314.t1 | TPR repeat-containing | 1725 | 0 | 85.9 |  |
| Efet.01.657496.g1315.t1 | bifunctional DNA-formamidopyrimidine glycosylase DNA-(apurinic or apyrimidinic site) lyase | 822 | 2.27E-168 | 88.75 | F:zinc ion binding; P:base-excision repair; P:nucleotide-excision repair; F:damaged DNA binding; F:oxidized purine nucleobase lesion DNA N-glycosylase activity |
| Efet.01.657496.g1316.t1 | 3-oxoacyl-ACP reductase | 747 | 1.34E-146 | 74.1 | F:oxidoreductase activity; P:oxidation-reduction process |
| Efet.01.657515.g1317.t1 | ADP-ribose pyrophosphatase NUDIX family | 675 | 6.22E-164 | 87.75 | F:nucleotidyltransferase activity; F:hydrolase activity |
| Efet.01.657516.g1318.t1 | spermidine putrescine ABC transporter ATP-binding | 1143 | 0 | 72.3 | F:ATPase activity; F:ATP binding; P:transmembrane transport; C:ATP-binding cassette (ABC) transporter complex; F:hydrolase activity, acting on acid anhydrides, catalyzing transmembrane movement of substances |
| Efet.01.657516.g1319.t1 | ABC transporter substrate-binding | 1041 | 0 | 76 |  |
| Efet.01.657516.g1320.t1 | ABC transporter permease | 1074 | 0 | 82.45 | C:integral component of membrane; P:monosaccharide transport; F:monosaccharide-transporting ATPase activity; C:plasma membrane |
| Efet.01.657528.g1321.t1 | rab GTPase-activating 1-like isoform X1 | 336 | 2.86E-40 | 70.65 | F:G-protein coupled receptor activity; P:G-protein coupled receptor signaling pathway; F:GTPase activator activity; C:integral component of membrane; P:cell cycle; P:positive regulation of GTPase activity |
| Efet.01.657532.g1322.t1 | collagen alpha-1(XII) chain-like isoform X1 | 1806 | 3.67E-57 | 56.5 | C:collagen trimer |
| Efet.01.657535.g1323.t1 | gastrula zinc finger -like | 2079 | 7.18E-67 | 44 | F:binding |
| Efet.01.657544.g1326.t1 | hypothetical protein CAPTEDRAFT_224319 | 207 | 6.20E-14 | 73 | C:membrane |
| Efet.01.657548.g1327.t1 | succinate dehydrogenase [ubiquinone] iron-sulfur mitochondrial | 330 | 1.24E-66 | 90.1 | C:extracellular exosome; C:mitochondrial respiratory chain complex II, succinate dehydrogenase complex (ubiquinone); F:succinate dehydrogenase (ubiquinone) activity; F:metal ion binding; F:electron carrier activity; F:3 iron, 4 sulfur cluster binding; P:tricarboxylic acid cycle; F:4 iron, 4 sulfur cluster binding; F:2 iron, 2 sulfur cluster binding; C:plasma membrane; C:nucleoplasm |
| Efet.01.657551.g1328.t1 | glutamate receptor 8 | 846 | 3.10E-116 | 66.1 | C:postsynaptic membrane; P:ion transmembrane transport; C:integral component of membrane; C:cell junction; P:ionotropic glutamate receptor signaling pathway; F:ionotropic glutamate receptor activity; F:extracellular-glutamate-gated ion channel activity |
| Efet.01.657559.g1329.t1 | ROK family transcriptional regulator | 660 | 1.49E-107 | 66.25 | F:DNA binding; P:regulation of transcription, DNA-templated |
| Efet.01.657559.g1330.t1 | sugar ABC transporter substrate-binding | 774 | 2.28E-158 | 79.8 | P:transport; F:transporter activity |
| Efet.01.657592.g1332.t1 | cytochrome P450 10-like | 399 | 6.46E-18 | 55.45 | F:iron ion binding; F:oxidoreductase activity, acting on paired donors, with incorporation or reduction of molecular oxygen; F:oxidoreductase activity; F:metal ion binding; C:membrane; C:integral component of membrane; P:oxidation-reduction process; F:monooxygenase activity; F:heme binding |
| Efet.01.657593.g1333.t1 | phosphoribosylamine--glycine ligase | 1407 | 0 | 75.8 | F:nucleotide binding; F:metal ion binding; P:purine nucleotide biosynthetic process; F:ligase activity |
| Efet.01.657604.g1334.t1 | dachsous | 1365 | 3.49E-40 | 58.05 | F:calcium ion binding; C:membrane; C:integral component of membrane; P:cell adhesion; P:homophilic cell adhesion via plasma membrane adhesion molecules; C:plasma membrane |
| Efet.01.657607.g1335.t1 | helicase | 1305 | 0 | 88.1 | F:ATP binding; P:DNA repair; F:DNA helicase activity; P:DNA duplex unwinding; P:telomere maintenance |
| Efet.01.657607.g1336.t1 | transposase | 267 | 1.48E-23 | 62.85 | F:DNA binding; F:transposase activity; P:transposition, DNA-mediated |
| Efet.01.657609.g1337.t1 | T family of potassium channels 18-like | 480 | 1.62E-56 | 62.05 | P:ion transport; C:membrane |
| Efet.01.657610.g1338.t1 | amidohydrolase | 960 | 0 | 74.05 | F:hydrolase activity |
| Efet.01.657610.g1339.t1 | Lrp family transcriptional regulator | 930 | 0 | 82.3 | F:transcription factor activity, sequence-specific DNA binding; P:transcription, DNA-templated; P:regulation of transcription, DNA-templated; F:sequence-specific DNA binding; C:intracellular |
| Efet.01.657617.g1340.t1 | E3 ubiquitin- ligase TRIM71-like | 738 | 2.03E-06 | 50 |  |
| Efet.01.657621.g1341.t1 | PREDICTED: uncharacterized protein LOC106168600 | 810 | 8.25E-11 | 43.5 | P:proteolysis; F:peptidase activity |
| Efet.01.657624.g1342.t1 | multidrug transporter subunit | 321 | 1.55E-42 | 90.95 | C:integral component of membrane; P:transport; F:transporter activity |
| Efet.01.657624.g1343.t1 | multidrug transporter subunit | 2634 | 0 | 85.85 | C:integral component of membrane; P:transport; F:transporter activity |
| Efet.01.657624.g1344.t1 | efflux RND transporter periplasmic adaptor subunit | 807 | 9.94E-159 | 81.9 | P:transmembrane transport; C:integral component of membrane |
| Efet.01.657624.g1345.t1 | PIN domain-containing | 528 | 1.13E-87 | 67.95 | F:hydrolase activity |
| Efet.01.657624.g1346.t1 | 2TM domain-containing | 300 | 3.71E-42 | 88.5 | C:integral component of membrane |
| Efet.01.657626.g1347.t1 | biotin transporter | 516 | 1.02E-59 | 69.65 | F:oxidoreductase activity; F:metal ion binding |
| Efet.01.657626.g1348.t1 | IS4 family | 1320 | 0 | 83.3 | F:DNA binding; F:transposase activity; P:transposition, DNA-mediated |
| Efet.01.657626.g1349.t1 | family transcriptional regulator | 450 | 2.18E-79 | 78.85 | F:DNA binding; F:transcription factor activity, sequence-specific DNA binding; P:transcription, DNA-templated; P:regulation of transcription, DNA-templated |
| Efet.01.657626.g1350.t1 | hypothetical protein | 723 | 3.54E-62 | 58.1 |  |
| Efet.01.657626.g1351.t1 | 2-methylcitrate dehydratase | 1167 | 0 | 86 | F:lyase activity |
| Efet.01.657626.g1352.t1 | tripartite tricarboxylate transporter substrate binding | 840 | 0 | 80.1 | C:outer membrane-bounded periplasmic space |
| Efet.01.657626.g1353.t1 | alpha-E domain-containing | 927 | 0 | 90.5 |  |
| Efet.01.657626.g1354.t1 | circularly permuted type 2 ATP-grasp | 1458 | 0 | 94.7 |  |
| Efet.01.657627.g1355.t1 | hypothetical protein CAPTEDRAFT_227990 | 672 | 2.46E-33 | 47.5 |  |
| Efet.01.657652.g1357.t1 | polypeptide N-acetylgalactosaminyltransferase 1-like | 717 | 8.92E-110 | 64.7 | C:membrane; C:cytoplasmic part; F:polypeptide N-acetylgalactosaminyltransferase activity |
| Efet.01.657660.g1358.t1 | mitogen-activated kinase 14-like | 210 | 5.53E-13 | 69.3 | F:ATP binding; P:MAPK cascade; F:MAP kinase activity; C:intracellular |
| Efet.01.657669.g1360.t1 | insecticidal toxin complex | 291 | 6.57E-18 | 55 | F:nucleic acid binding; P:self proteolysis; F:metal ion binding |
| Efet.01.657674.g1363.t1 | neuronal acetylcholine receptor subunit alpha-10-like | 552 | 1.28E-98 | 73.85 | P:synaptic transmission, cholinergic; F:acetylcholine receptor activity; P:neuromuscular synaptic transmission; C:postsynaptic membrane; P:cation transmembrane transport; C:cell junction; F:extracellular ligand-gated ion channel activity; F:acetylcholine binding; C:acetylcholine-gated channel complex; P:response to nicotine |
| Efet.01.657677.g1364.t1 | octaprenyl diphosphate synthase | 930 | 0 | 92.1 | F:dimethylallyltranstransferase activity; P:isoprenoid biosynthetic process |
| Efet.01.657677.g1365.t1 | hypothetical protein | 330 | 3.72E-69 | 83.3 |  |
| Efet.01.657694.g1368.t1 | family DNA-binding transcriptional regulator | 714 | 6.10E-140 | 80.75 | F:DNA binding; C:cytoplasm; P:transcription, DNA-templated; P:regulation of transcription, DNA-templated |
| Efet.01.657694.g1369.t1 | PQQ-dependent sugar dehydrogenase | 936 | 8.14E-165 | 78.4 | F:catalytic activity; P:metabolic process |
| Efet.01.657700.g1370.t1 | RNA polymerase subunit sigma | 480 | 2.84E-101 | 76.35 | F:DNA binding; F:sigma factor activity; F:transcription factor activity, sequence-specific DNA binding; P:regulation of transcription, DNA-templated; P:DNA-templated transcription, initiation |
| Efet.01.657700.g1371.t1 | propionate catabolism operon regulatory | 1368 | 0 | 85.15 | F:ATP binding; P:propionate catabolic process, 2-methylcitrate cycle; C:cytoplasm; P:transcription, DNA-templated; P:phosphorelay signal transduction system; P:regulation of transcription, DNA-templated; F:sequence-specific DNA binding; F:transcription factor binding; F:phosphorelay response regulator activity |
| Efet.01.657700.g1372.t1 | CoA transferase | 1161 | 0 | 92.9 | F:transferase activity |
| Efet.01.657700.g1373.t1 | hydroxymethylglutaryl- lyase | 279 | 4.73E-52 | 88.45 | F:hydroxymethylglutaryl-CoA lyase activity; F:transferase activity |
| Efet.01.657703.g1374.t1 | dTMP kinase | 651 | 2.32E-119 | 86.7 | F:thymidylate kinase activity; F:ATP binding; P:nucleotide phosphorylation; P:dTDP biosynthetic process; P:dTTP biosynthetic process |
| Efet.01.657703.g1375.t1 | aminodeoxychorismate lyase | 567 | 4.15E-132 | 96.25 | F:nucleic acid binding; F:lyase activity |
| Efet.01.657703.g1376.t1 | folate-binding | 849 | 3.63E-147 | 77.35 | F:transferase activity |
| Efet.01.657703.g1377.t1 | signal peptide prediction | 1239 | 0 | 76.55 |  |
| Efet.01.657703.g1378.t1 | GTP-binding | 1014 | 0 | 70.1 |  |
| Efet.01.657703.g1379.t1 | polysaccharide deacetylase | 600 | 2.88E-145 | 89.7 | F:hydrolase activity, acting on carbon-nitrogen (but not peptide) bonds; P:carbohydrate metabolic process |
| Efet.01.657703.g1380.t1 | ABC transporter ATP-binding | 969 | 0 | 70.75 | F:ATPase activity; F:ATP binding; P:transmembrane transport; C:ATP-binding cassette (ABC) transporter complex; F:hydrolase activity, acting on acid anhydrides, catalyzing transmembrane movement of substances |
| Efet.01.657703.g1381.t1 | ABC transporter substrate-binding | 1221 | 0 | 88.2 |  |
| Efet.01.657703.g1382.t1 | NAD(P)-dependent oxidoreductase | 744 | 4.78E-167 | 81.35 | F:oxidoreductase activity; P:oxidation-reduction process |
| Efet.01.657703.g1383.t1 | ABC transporter permease | 501 | 1.76E-95 | 89.6 | C:integral component of membrane; P:transport; C:plasma membrane |
| Efet.01.657703.g1384.t1 | ABC transporter permease | 798 | 1.52E-154 | 90.9 | C:integral component of membrane; P:transport; C:plasma membrane |
| Efet.01.657703.g1385.t1 | ABC transporter ATP-binding | 1314 | 0 | 67.15 | F:ATPase activity; F:ATP binding |
| Efet.01.657703.g1386.t1 | family transcriptional regulator | 351 | 2.57E-47 | 67.65 | F:DNA binding; F:transcription factor activity, sequence-specific DNA binding; P:transcription, DNA-templated; P:regulation of transcription, DNA-templated |
| Efet.01.657703.g1387.t1 | FAA hydrolase family | 1104 | 0 | 94.25 | P:metabolic process; F:hydrolase activity |
| Efet.01.657719.g1389.t1 | oxidative damage protection | 273 | 1.87E-60 | 94.8 | F:iron ion binding |
| Efet.01.657719.g1390.t1 | sulfate transporter subunit | 1077 | 0 | 94.45 | F:secondary active sulfate transmembrane transporter activity; P:sulfate transmembrane transport; F:ATPase-coupled sulfate transmembrane transporter activity; C:outer membrane-bounded periplasmic space |
| Efet.01.657719.g1391.t1 | acyl- dehydrogenase | 1767 | 0 | 87.15 | F:acyl-CoA dehydrogenase activity; F:flavin adenine dinucleotide binding; P:oxidation-reduction process |
| Efet.01.657719.g1392.t1 | family transcriptional regulator | 744 | 7.86E-161 | 91.25 | F:DNA binding; P:transcription, DNA-templated; P:regulation of transcription, DNA-templated |
| Efet.01.657719.g1393.t1 | acetylglutamate kinase | 891 | 0 | 97.4 | F:ATP binding; P:arginine biosynthetic process; C:cytoplasm; P:proline biosynthetic process; P:phosphorylation; F:acetylglutamate kinase activity |
| Efet.01.657719.g1394.t1 | glycosyl transferase | 939 | 0 | 92.45 | C:integral component of membrane; F:phospho-N-acetylmuramoyl-pentapeptide-transferase activity |
| Efet.01.657719.g1395.t1 | glycosyl transferase | 1191 | 6.82E-115 | 77.4 | F:transferase activity |
| Efet.01.657719.g1396.t1 | 6-carboxytetrahydropterin synthase | 387 | 8.13E-76 | 77.2 | P:queuosine biosynthetic process; F:metal ion binding; F:lyase activity |
| Efet.01.657719.g1397.t1 | FMN-dependent NADH-azoreductase | 738 | 7.90E-152 | 74.85 | F:FMN binding; F:electron carrier activity; F:FMN reductase activity; F:oxidoreductase activity, acting on other nitrogenous compounds as donors; P:oxidation-reduction process; F:oxidoreductase activity, acting on NAD(P)H, NAD(P) as acceptor |
| Efet.01.657719.g1398.t1 | 5-dehydro-4-deoxyglucarate dehydratase | 756 | 5.70E-106 | 67.3 | F:catalytic activity; P:carbohydrate catabolic process |
| Efet.01.657719.g1399.t1 | NAD-dependent dehydratase | 921 | 5.53E-139 | 71.7 | F:coenzyme binding; F:catalytic activity |
| Efet.01.657719.g1400.t1 | carbohydrate ABC transporter ATP-binding CUT1 family | 954 | 0 | 86.85 | F:ATP binding; P:glycerol-3-phosphate transport; P:transmembrane transport; F:glycerol-3-phosphate-transporting ATPase activity; P:carbohydrate transport; C:ATP-binding cassette (ABC) transporter complex |
| Efet.01.657719.g1401.t1 | carbohydrate ABC transporter permease | 756 | 1.59E-143 | 95.6 | C:integral component of membrane; P:carbohydrate transport; C:plasma membrane |
| Efet.01.657719.g1402.t1 | carbohydrate ABC transporter membrane CUT1 family | 873 | 7.59E-168 | 96.9 | C:integral component of membrane; P:carbohydrate transport; C:plasma membrane |
| Efet.01.657719.g1403.t1 | carbohydrate ABC transporter substrate-binding | 2034 | 0 | 91.45 | P:carbohydrate transport |
| Efet.01.657719.g1404.t1 | N-6 DNA methylase | 1746 | 0 | 87.7 | F:DNA binding; F:N-methyltransferase activity; P:DNA methylation |
| Efet.01.657730.g1406.t1 | dynamin-binding -like | 588 | 4.90E-16 | 65.5 | P:regulation of cellular process; F:guanyl-nucleotide exchange factor activity |
| Efet.01.657744.g1408.t1 | cyclic nucleotide-gated olfactory channel-like | 996 | 1.54E-130 | 70.85 | F:voltage-gated potassium channel activity; P:potassium ion transmembrane transport; C:integral component of membrane |
| Efet.01.657756.g1410.t1 | ABC transporter ATP-binding | 615 | 2.04E-141 | 74.1 | F:ATPase activity; F:ATP binding; C:membrane |
| Efet.01.657756.g1411.t1 | branched-chain amino acid ABC transporter permease | 756 | 6.27E-114 | 68.15 | C:integral component of membrane; P:transport; C:plasma membrane; F:transporter activity |
| Efet.01.657759.g1412.t1 | TonB-dependent hemoglobin/transferrin/lactoferrin family receptor | 585 | 1.67E-125 | 70.25 | C:cell outer membrane; F:receptor activity; P:transport; F:transporter activity |
| Efet.01.657759.g1413.t1 | TonB-dependent hemoglobin/transferrin/lactoferrin family receptor | 1371 | 0 | 72.75 | C:cell outer membrane; F:receptor activity; P:transport; F:transporter activity |
| Efet.01.657759.g1414.t1 | alpha-hydroxy-acid oxidizing | 903 | 8.56E-168 | 85.9 | F:FMN binding; F:oxidoreductase activity; P:oxidation-reduction process |
| Efet.01.657759.g1415.t1 | Fe2+-dependent dioxygenase | 510 | 5.65E-123 | 91.6 | F:L-ascorbic acid binding; F:iron ion binding; F:oxidoreductase activity, acting on paired donors, with incorporation or reduction of molecular oxygen, 2-oxoglutarate as one donor, and incorporation of one atom each of oxygen into both donors; P:oxidation-reduction process |
| Efet.01.657759.g1416.t1 | TonB-dependent hemoglobin/transferrin/lactoferrin family receptor | 2148 | 0 | 89.45 | F:iron ion binding; P:siderophore transport; C:integral component of membrane; C:cell outer membrane; F:receptor activity |
| Efet.01.657759.g1417.t1 | DNA topoisomerase III | 228 | 5.45E-47 | 99 | F:isomerase activity |
| Efet.01.657761.g1418.t1 | WbqC-like protein family protein | 429 | 2.06E-102 | 60.6 |  |
| Efet.01.657761.g1419.t1 | hypothetical protein | 453 | 1.46E-07 | 50.83 |  |
| Efet.01.657766.g1421.t1 | sulfite oxidase | 846 | 0 | 89.15 | F:sulfite dehydrogenase activity; F:molybdenum ion binding; P:oxidation-reduction process; P:nitrate assimilation |
| Efet.01.657766.g1422.t1 | cytochrome c family | 378 | 2.32E-76 | 77.95 | F:sulfite dehydrogenase activity; F:electron carrier activity; P:oxidation-reduction process; F:heme binding |
| Efet.01.657766.g1423.t1 | amino acid ABC transporter substrate-binding | 864 | 1.99E-165 | 93.75 | P:transport; F:transporter activity |
| Efet.01.657766.g1424.t1 | amino acid ABC transporter ATP-binding | 1362 | 5.29E-149 | 92.75 | F:ATP binding; P:organic phosphonate transport; P:organophosphate ester transport; F:ATPase-coupled organic phosphonate transmembrane transporter activity; P:amino acid transmembrane transport; F:amino acid-transporting ATPase activity |
| Efet.01.657766.g1425.t1 | porin | 1302 | 0 | 72.9 | F:porin activity; P:transmembrane transport; C:integral component of membrane |
| Efet.01.657766.g1426.t1 | family transporter | 894 | 9.12E-144 | 87.45 | C:integral component of membrane |
| Efet.01.657766.g1427.t1 | penicillin-binding 2 | 1953 | 0 | 94.25 | C:integral component of membrane; F:penicillin binding; F:peptidoglycan glycosyltransferase activity |
| Efet.01.657766.g1428.t1 | rod shape-determining | 519 | 2.02E-76 | 79.15 | P:regulation of cell shape; C:integral component of membrane |
| Efet.01.657766.g1429.t1 | rod shape-determining | 834 | 2.27E-161 | 82.35 | P:regulation of cell shape |
| Efet.01.657766.g1430.t1 | rod shape-determining | 558 | 4.24E-107 | 97.25 | P:cell morphogenesis |
| Efet.01.657766.g1431.t1 | Asp-tRNA(Asn) Glu-tRNA(Gln) amidotransferase subunit | 1512 | 0 | 82.2 | F:ATP binding; F:transferase activity; F:glutaminyl-tRNA synthase (glutamine-hydrolyzing) activity; P:translation |
| Efet.01.657766.g1432.t1 | Asp-tRNA(Asn) Glu-tRNA(Gln) amidotransferase subunit | 1542 | 0 | 90.3 | F:ATP binding; F:transferase activity; F:glutaminyl-tRNA synthase (glutamine-hydrolyzing) activity; P:translation |
| Efet.01.657766.g1433.t1 | DUF4124 domain-containing | 372 | 2.36E-71 | 76.65 |  |
| Efet.01.657766.g1434.t1 | orotate phosphoribosyltransferase | 474 | 3.36E-107 | 88.65 | F:magnesium ion binding; P:'de novo' UMP biosynthetic process; F:orotate phosphoribosyltransferase activity |
| Efet.01.657766.g1435.t1 | secretion system E | 2595 | 0 | 78.75 | F:ATP binding; P:transport |
| Efet.01.657766.g1436.t1 | water dikinase | 1131 | 0 | 83.8 | F:magnesium ion binding; F:ATP binding; F:selenide, water dikinase activity; P:selenocysteine biosynthetic process; P:phosphorylation |
| Efet.01.657766.g1437.t1 | tRNA 2-selenouridine(34) synthase | 978 | 0 | 81.45 | F:tRNA 2-selenouridine synthase activity; P:tRNA seleno-modification |
| Efet.01.657766.g1438.t1 | tryptophan--tRNA ligase | 1203 | 0 | 86.8 | F:ATP binding; C:cytoplasm; P:tryptophanyl-tRNA aminoacylation; F:tryptophan-tRNA ligase activity |
| Efet.01.657766.g1439.t1 | threonylcarbamoyl-AMP synthase | 1257 | 5.66E-110 | 92.35 | F:double-stranded RNA binding; C:integral component of membrane |
| Efet.01.657766.g1440.t1 | PHP domain-containing | 723 | 7.16E-173 | 91.55 | F:DNA binding; F:DNA-directed DNA polymerase activity; P:DNA replication; P:DNA biosynthetic process; F:hydrolase activity |
| Efet.01.657766.g1441.t1 | universal stress | 423 | 2.83E-85 | 88.3 | P:response to stress |
| Efet.01.657766.g1442.t1 | hypothetical protein | 444 | 1.22E-27 | 77 | C:membrane |
| Efet.01.657766.g1443.t1 | EF-hand domain-containing | 438 | 6.54E-72 | 81.25 | F:calcium ion binding |
| Efet.01.657766.g1444.t1 | type II secretion system | 2025 | 0 | 92.3 | C:integral component of membrane; F:protein transporter activity; P:protein secretion by the type II secretion system; C:type II protein secretion system complex |
| Efet.01.657766.g1445.t1 | septum site-determining | 756 | 2.67E-139 | 81.05 | P:cell division; P:regulation of cell division; P:cell morphogenesis; P:regulation of cell cycle; P:cell cycle |
| Efet.01.657766.g1446.t1 | septum site-determining | 1041 | 5.89E-166 | 87.8 | F:ATPase activity; P:cell division; F:ATP binding |
| Efet.01.657766.g1447.t1 | malate synthase G | 2601 | 0 | 90.65 | P:glyoxylate cycle; C:cytoplasm; F:metal ion binding; F:malate synthase activity; P:tricarboxylic acid cycle |
| Efet.01.657766.g1448.t1 | 4-oxalocrotonate tautomerase | 294 | 1.65E-37 | 98.4 | P:cellular aromatic compound metabolic process; F:isomerase activity |
| Efet.01.657766.g1449.t1 | ferredoxin | 492 | 8.19E-45 | 69.95 | F:iron-sulfur cluster binding |
| Efet.01.657766.g1450.t1 | channel | 1290 | 0 | 73.6 | P:transport; C:outer membrane; F:transporter activity |
| Efet.01.657766.g1451.t1 | family type I secretion periplasmic adaptor subunit | 1047 | 2.52E-146 | 84.65 | C:integral component of membrane; F:protein transporter activity; P:protein secretion |
| Efet.01.657766.g1452.t1 | type I secretion system permease ATPase | 1740 | 0 | 76.1 | F:ATP binding; F:ATPase activity, coupled to transmembrane movement of substances; C:integral component of membrane; P:protein secretion by the type I secretion system; F:protein transporter activity; C:type I protein secretion system complex |
| Efet.01.657766.g1453.t1 | type I secretion system permease ATPase | 1749 | 0 | 76.55 | F:ATP binding; F:ATPase activity, coupled to transmembrane movement of substances; C:integral component of membrane; P:protein secretion by the type I secretion system; F:protein transporter activity; C:type I protein secretion system complex |
| Efet.01.657766.g1454.t1 | NADP-dependent malic enzyme | 2268 | 0 | 90.7 | F:oxaloacetate decarboxylase activity; F:phosphate acetyltransferase activity; P:malate metabolic process; F:NAD binding; C:cytosol; F:malate dehydrogenase (decarboxylating) (NAD+) activity; F:manganese ion binding; P:oxidation-reduction process; F:malate dehydrogenase (decarboxylating) (NADP+) activity |
| Efet.01.657766.g1455.t1 | DNA polymerase IV | 1239 | 0 | 89.1 | F:magnesium ion binding; P:DNA repair; F:DNA-directed DNA polymerase activity; C:cytoplasm; P:DNA-dependent DNA replication; P:DNA biosynthetic process; F:damaged DNA binding |
| Efet.01.657766.g1456.t1 | DUF3225 domain-containing | 375 | 2.75E-84 | 84.65 |  |
| Efet.01.657767.g1457.t1 | sperm-associated antigen 17-like isoform X2 | 273 | 4.10E-14 | 63.95 |  |
| Efet.01.657770.g1458.t1 | motor neuron and pancreas homeobox 1 | 699 | 1.02E-09 | 88.45 | C:nucleus; P:regulation of transcription, DNA-templated; F:sequence-specific DNA binding |
| Efet.01.657788.g1460.t1 | beta-1,3-galactosyltransferase 1-like | 1038 | 2.89E-122 | 59 | F:transferase activity, transferring glycosyl groups; C:membrane; C:Golgi apparatus |
| Efet.01.657795.g1463.t1 | argininosuccinate lyase | 534 | 2.65E-121 | 79.6 | P:arginine biosynthetic process via ornithine; F:argininosuccinate lyase activity |
| Efet.01.657795.g1464.t1 | hypothetical protein | 873 | 2.39E-128 | 99 |  |
| Efet.01.657795.g1465.t1 | homoserine dehydrogenase | 2304 | 0 | 83.7 | F:NADP binding; F:oxidoreductase activity; P:oxidation-reduction process |
| Efet.01.657795.g1466.t1 | pyruvate dehydrogenase (acetyl-transferring) E1 component subunit alpha | 957 | 0 | 80.3 | P:acetyl-CoA biosynthetic process from pyruvate; C:intracellular membrane-bounded organelle; F:pyruvate dehydrogenase (acetyl-transferring) activity; P:oxidation-reduction process |
| Efet.01.657795.g1467.t1 | alpha-ketoacid dehydrogenase subunit beta | 942 | 0 | 87.95 | F:oxidoreductase activity; P:oxidation-reduction process |
| Efet.01.657795.g1468.t1 | acetoin dehydrogenase dihydrolipoyllysine-residue acetyltransferase subunit | 750 | 1.24E-128 | 56.9 | F:transferase activity, transferring acyl groups; P:metabolic process; F:hydrolase activity |
| Efet.01.657803.g1469.t1 | enoyl- hydratase | 765 | 6.31E-166 | 78 | P:metabolic process; F:enoyl-CoA hydratase activity |
| Efet.01.657803.g1470.t1 | SAM-dependent methyltransferase | 579 | 2.19E-132 | 78.25 | F:methyltransferase activity; P:methylation |
| Efet.01.657803.g1471.t1 | MFS transporter | 552 | 2.11E-121 | 93.05 | P:transmembrane transport; C:integral component of membrane |
| Efet.01.657803.g1472.t1 | MFS transporter | 255 | 6.35E-50 | 94.1 | P:transmembrane transport; C:integral component of membrane |
| Efet.01.657803.g1473.t1 | type IIA DNA topoisomerase subunit B | 1986 | 0 | 94.6 | F:magnesium ion binding; F:DNA binding; F:ATP binding; P:chromosome segregation; C:chromosome; F:DNA topoisomerase type II (ATP-hydrolyzing) activity; P:DNA topological change |
| Efet.01.657803.g1474.t1 | lytic transglycosylase domain-containing | 603 | 1.90E-127 | 86.15 | P:peptidoglycan metabolic process; C:membrane; F:lytic transglycosylase activity |
| Efet.01.657803.g1475.t1 | cation transporter | 858 | 6.77E-96 | 83.75 | P:cation transmembrane transport; C:integral component of membrane; F:cation transmembrane transporter activity |
| Efet.01.657803.g1476.t1 | blue (type 1) copper domain | 495 | 2.27E-117 | 78.35 | F:copper ion binding; F:electron carrier activity |
| Efet.01.657803.g1477.t1 | twin-arginine translocation pathway signal | 420 | 1.65E-98 | 85.55 |  |
| Efet.01.657803.g1478.t1 | peptidase S58 | 786 | 4.47E-146 | 90.35 |  |
| Efet.01.657805.g1479.t1 | hypothetical protein HELRODRAFT_184146 | 765 | 9.07E-07 | 75.67 | F:binding |
| Efet.01.657807.g1480.t1 | relaxase primase-like fusion (plasmid) | 405 | 4.49E-49 | 78.15 |  |
| Efet.01.657807.g1481.t1 | mobilization | 321 | 2.07E-66 | 81.55 | P:regulation of transcription, DNA-templated |
| Efet.01.657807.g1482.t1 | mobC | 444 | 9.65E-64 | 75.85 |  |
| Efet.01.657807.g1483.t1 | mobD | 687 | 2.93E-162 | 82.1 |  |
| Efet.01.657807.g1484.t1 | type IV secretion system | 2895 | 0 | 73.15 | C:membrane; C:integral component of membrane |
| Efet.01.657807.g1485.t1 | hypothetical protein | 270 | 1.35E-50 | 82.63 |  |
| Efet.01.657807.g1486.t1 | hypothetical protein | 420 | 2.80E-81 | 91.73 |  |
| Efet.01.657807.g1487.t1 | hypothetical protein | 633 | 4.87E-126 | 80.05 |  |
| Efet.01.657807.g1488.t1 | M23 family peptidase | 1056 | 0 | 81.25 |  |
| Efet.01.657807.g1489.t1 | hypothetical protein | 759 | 8.39E-138 | 73.55 | C:integral component of membrane |
| Efet.01.657807.g1490.t1 | hypothetical protein | 354 | 5.65E-60 | 89.45 |  |
| Efet.01.657820.g1491.t1 | hypothetical protein | 234 | 7.87E-27 | 75.75 |  |
| Efet.01.657820.g1492.t1 | hypothetical protein [Diaphorobacter polyhydroxybutyrativorans] | 450 | 8.87E-43 | 80 |  |
| Efet.01.657820.g1493.t1 | hypothetical protein [Diaphorobacter polyhydroxybutyrativorans] | 468 | 8.12E-78 | 69.2 | F:NAD+ ADP-ribosyltransferase activity |
| Efet.01.657820.g1494.t1 | hypothetical protein | 396 | 7.92E-27 | 79.75 |  |
| Efet.01.657820.g1495.t1 | chromosome partitioning | 828 | 2.32E-156 | 86.15 | F:DNA binding |
| Efet.01.657838.g1496.t1 | ABC transporter ATP-binding | 633 | 1.74E-101 | 78.85 | F:ATPase activity; F:ATP binding |
| Efet.01.657838.g1497.t1 | ABC transporter ATP-binding | 678 | 1.14E-100 | 77.6 | F:hydrolase activity |
| Efet.01.657838.g1498.t1 | branched-chain amino acid ABC transporter permease | 1527 | 6.26E-108 | 78.45 | C:integral component of membrane; P:transport; C:plasma membrane; F:transporter activity |
| Efet.01.657838.g1499.t1 | ABC transporter substrate-binding | 1080 | 0 | 84 |  |
| Efet.01.657838.g1500.t1 | aminocarboxymuconate-semialdehyde decarboxylase | 879 | 6.03E-179 | 84.7 | P:regulation of quinolinate biosynthetic process; P:tryptophan metabolic process; F:aminocarboxymuconate-semialdehyde decarboxylase activity; F:hydrolase activity |
| Efet.01.657838.g1501.t1 | 3-hydroxyanthranilate 3,4-dioxygenase | 843 | 2.66E-95 | 85.25 | P:tryptophan catabolic process; P:quinolinate biosynthetic process; P:'de novo' NAD biosynthetic process from tryptophan; F:ferrous iron binding; P:anthranilate metabolic process; P:oxidation-reduction process; F:3-hydroxyanthranilate 3,4-dioxygenase activity |
| Efet.01.657838.g1502.t1 | 4-oxalocrotonate decarboxylase | 786 | 3.46E-133 | 80.35 | F:catalytic activity; P:metabolic process |
| Efet.01.657838.g1503.t1 | 2-hydroxymuconic semialdehyde dehydrogenase | 1173 | 8.21E-96 | 85.95 | F:betaine-aldehyde dehydrogenase activity; P:oxidation-reduction process |
| Efet.01.657847.g1504.t1 | doublesex and mab-3 related transcription factor truncated-like | 1044 | 1.47E-27 | 67.35 | C:nucleus; F:transcription factor activity, sequence-specific DNA binding; P:regulation of transcription, DNA-templated; F:sequence-specific DNA binding |
| Efet.01.657855.g1506.t1 | spermidine putrescine ABC transporter substrate-binding | 1110 | 0 | 63.4 | F:polyamine binding; C:periplasmic space; P:polyamine transport |
| Efet.01.657855.g1507.t1 | mandelate racemase muconate lactonizing enzyme family | 891 | 0 | 83.95 | P:cellular amino acid catabolic process; F:catalytic activity; F:metal ion binding |
| Efet.01.657855.g1508.t1 | aldehyde dehydrogenase | 1491 | 0 | 77.85 | F:aldehyde dehydrogenase (quinone) activity; P:oxidation-reduction process; F:oxidoreductase activity, acting on the aldehyde or oxo group of donors, NAD or NADP as acceptor |
| Efet.01.657868.g1509.t1 | tripartite tricarboxylate transporter substrate binding | 1083 | 0 | 95.85 | C:outer membrane-bounded periplasmic space |
| Efet.01.657868.g1510.t1 | cystathionine beta-lyase | 1203 | 0 | 88.1 | F:pyridoxal phosphate binding; C:cytoplasm; P:cellular amino acid metabolic process; F:cystathionine beta-lyase activity |
| Efet.01.657876.g1512.t1 | enoyl- hydratase isomerase family | 696 | 4.87E-124 | 81.95 | F:3-hydroxybutyryl-CoA dehydratase activity; P:metabolic process; F:enoyl-CoA hydratase activity; F:isomerase activity |
| Efet.01.657876.g1513.t1 | sorbosone dehydrogenase | 1521 | 0 | 78.3 | F:betaine-aldehyde dehydrogenase activity; P:oxidation-reduction process |
| Efet.01.657876.g1514.t1 | carbohydrate ABC transporter substrate-binding | 1245 | 0 | 88.05 | P:carbohydrate transport; F:transporter activity |
| Efet.01.657876.g1515.t1 | sugar ABC transporter permease | 867 | 0 | 88.15 | C:integral component of membrane; P:carbohydrate transport; C:plasma membrane |
| Efet.01.657882.g1516.t1 | 60 kDa SS-A Ro ribonucleo -like | 549 | 5.33E-37 | 57.55 | F:RNA binding; C:intracellular ribonucleoprotein complex; P:single-organism process; P:cellular process |
| Efet.01.657888.g1518.t1 | potassium sodium hyperpolarization-activated cyclic nucleotide-gated channel 3-like isoform X2 | 1077 | 3.27E-37 | 62.15 | P:ion transport; P:transmembrane transport |
| Efet.01.657897.g1520.t1 | zinc finger SNAI2 | 573 | 9.70E-66 | 74.65 | P:neural crest cell fate specification; P:negative regulation of response to DNA damage stimulus; P:negative regulation of cell adhesion; P:negative regulation of cell proliferation; P:endocardial cushion formation; P:response to radiation; P:cell surface receptor signaling pathway; P:negative regulation of intracellular signal transduction; F:RNA polymerase II transcription factor activity, sequence-specific DNA binding; P:negative regulation of transcription from RNA polymerase II promoter; C:nucleus; F:DNA binding; P:regulation of localization; P:neural crest cell migration; P:regulation of cell differentiation; P:positive regulation of cellular process; F:metal ion binding; P:negative regulation of apoptotic signaling pathway; P:regulation of cellular component organization; P:regulation of multicellular organismal development |
| Efet.01.657899.g1521.t1 | oxidoreductase | 789 | 5.90E-163 | 81.9 | F:3-oxoacyl-[acyl-carrier-protein] reductase (NADPH) activity; P:oxidation-reduction process |
| Efet.01.657899.g1522.t1 | long-chain fatty acid-- ligase | 1509 | 0 | 72.95 | P:primary metabolic process; P:cellular metabolic process; P:organic substance metabolic process; F:ligase activity |
| Efet.01.657899.g1523.t1 | thiolase family | 1152 | 0 | 79.05 | F:transferase activity, transferring acyl groups other than amino-acyl groups; P:metabolic process |
| Efet.01.657900.g1524.t1 | Pyridoxal-dependent decarboxylase | 879 | 0 | 57.7 | F:pyridoxal phosphate binding; P:carboxylic acid metabolic process; F:carboxy-lyase activity |
| Efet.01.657900.g1525.t1 | choline dehydrogenase | 1338 | 0 | 74.8 | F:oxidoreductase activity, acting on CH-OH group of donors; F:flavin adenine dinucleotide binding; P:oxidation-reduction process |
| Efet.01.657900.g1526.t1 | carbohydrate ABC transporter permease | 741 | 8.35E-140 | 67.15 | C:integral component of membrane; P:transport; C:plasma membrane |
| Efet.01.657900.g1527.t1 | sugar ABC transporter permease | 456 | 3.80E-75 | 68.95 | C:integral component of membrane; P:transport; C:plasma membrane |
| Efet.01.657900.g1528.t1 | sugar ABC transporter substrate-binding | 1302 | 0 | 52.05 | P:transport; F:transporter activity |
| Efet.01.657900.g1529.t1 | family transcriptional regulator | 855 | 0 | 66.1 | F:DNA binding; F:transcription factor activity, sequence-specific DNA binding; P:transcription, DNA-templated; P:regulation of transcription, DNA-templated |
| Efet.01.657910.g1530.t1 | APOBEC1 complementation factor isoform X3 | 804 | 1.53E-109 | 84.3 | F:nucleotide binding; F:nucleic acid binding |
| Efet.01.657912.g1531.t1 | IS481 family transposase | 237 | 3.34E-14 | 73.4 | F:nucleic acid binding |
| Efet.01.657912.g1532.t1 | site-specific integrase | 870 | 0 | 69 | F:DNA binding; P:DNA integration; P:DNA recombination |
| Efet.01.657920.g1533.t1 | neurexin-3 isoform X3 | 786 | 3.98E-50 | 53.7 | F:metal ion binding; C:membrane; C:integral component of membrane; P:chemical synaptic transmission |
| Efet.01.657923.g1534.t1 | neuronal acetylcholine receptor subunit alpha-10-like isoform X1 | 534 | 1.72E-10 | 73 | F:ion channel activity; P:ion transmembrane transport; C:synapse; C:membrane part; C:plasma membrane |
| Efet.01.657925.g1535.t1 | hypothetical protein CAPTEDRAFT_199669 | 351 | 2.52E-09 | 54 | F:iron ion binding; F:oxidoreductase activity; F:oxidoreductase activity, acting on paired donors, with incorporation or reduction of molecular oxygen; F:metal ion binding; P:oxidation-reduction process; F:monooxygenase activity; F:heme binding |
| Efet.01.657934.g1537.t1 | growth arrest-specific 1-like | 1008 | 5.70E-21 | 45.6 | C:membrane; C:integral component of membrane |
| Efet.01.657942.g1539.t1 | small proline-rich 3 | 705 | 1.24E-14 | 52.65 | P:keratinocyte differentiation; C:cornified envelope; P:wound healing; F:protein binding; C:extracellular exosome; C:cytoplasm; P:epidermis development; F:structural molecule activity; P:peptide cross-linking; P:keratinization |
| Efet.01.657953.g1541.t1 | somatostatin receptor type 2-like | 1152 | 8.70E-72 | 52.1 | F:G-protein coupled receptor activity; P:G-protein coupled receptor signaling pathway |
| Efet.01.657961.g1543.t1 | Mt-collagen alpha I type XVIII | 297 | 3.20E-06 | 69 | C:collagen trimer; F:structural molecule activity; P:cell adhesion; C:extracellular matrix; C:proteinaceous extracellular matrix |
| Efet.01.657967.g1545.t1 | Chromosome transmission fidelity | 606 | 3.82E-63 | 71 | F:nucleotide binding |
| Efet.01.657976.g1546.t1 | 2-oxo-hepta-3-ene-1,7-dioic acid hydratase | 711 | 1.18E-172 | 91.4 | P:aromatic compound catabolic process; F:4-oxalocrotonate decarboxylase activity; F:2-oxopent-4-enoate hydratase activity |
| Efet.01.657976.g1547.t1 | CoA-binding protein | 522 | 2.60E-78 | 72.45 | F:catalytic activity; F:cofactor binding; F:ion binding |
| Efet.01.657982.g1548.t1 | Ras family | 984 | 1.00E-40 | 57.35 |  |
| Efet.01.657985.g1550.t1 | E3 ubiquitin- ligase TRIM71-like | 570 | 5.48E-11 | 50 |  |
| Efet.01.658002.g1551.t1 | phenol hydroxylase | 348 | 2.60E-50 | 88.95 | F:metal ion binding; F:electron carrier activity; F:phenol 2-monooxygenase activity; P:oxidation-reduction process; F:2 iron, 2 sulfur cluster binding |
| Efet.01.658002.g1552.t1 | phenol 2-monooxygenase | 1773 | 0 | 94.9 | P:cellular aromatic compound metabolic process; F:phenol 2-monooxygenase activity; P:oxidation-reduction process |
| Efet.01.658008.g1553.t1 | hypothetical protein BRAFLDRAFT_76362 | 2670 | 6.94E-163 | 53.05 |  |
| Efet.01.658011.g1554.t1 | sugar ABC transporter ATP-binding | 1086 | 0 | 78.85 | F:ATP binding; P:monosaccharide transport; F:monosaccharide-transporting ATPase activity |
| Efet.01.658012.g1555.t1 | NAD(P)-dependent alcohol dehydrogenase | 252 | 2.70E-52 | 94.5 | F:zinc ion binding; F:oxidoreductase activity; P:oxidation-reduction process |
| Efet.01.658012.g1556.t1 | NAD(P)-dependent alcohol dehydrogenase | 522 | 1.38E-107 | 95.1 | F:zinc ion binding; F:oxidoreductase activity; P:oxidation-reduction process |
| Efet.01.658012.g1557.t1 | methane monooxygenase | 768 | 0 | 98.85 | F:methane monooxygenase activity; P:cellular aromatic compound metabolic process; P:oxidation-reduction process |
| Efet.01.658014.g1558.t1 | fasciculation and elongation zeta-2-like isoform X1 | 246 | 8.79E-06 | 90 |  |
| Efet.01.658017.g1560.t1 | Coiled-coil domain-containing 102A | 780 | 9.35E-22 | 73.9 | C:myosin complex; F:motor activity |
| Efet.01.658019.g1561.t1 | helicase with zinc finger domain 2-like | 471 | 9.64E-30 | 65.7 | F:binding |
| Efet.01.658023.g1564.t1 | hypothetical protein CAPTEDRAFT_216764 | 2073 | 7.21E-91 | 49.2 |  |
| Efet.01.658025.g1565.t1 | TPA: zinc finger | 756 | 3.67E-39 | 45.75 | F:nucleic acid binding; F:zinc ion binding; F:aspartic-type endopeptidase activity; P:proteolysis; P:DNA integration |
| Efet.01.658028.g1566.t1 | dmX 1 isoform X11 | 642 | 1.56E-56 | 64.25 |  |
| Efet.01.658030.g1567.t1 | gastrula zinc finger -like | 1185 | 2.28E-38 | 44.75 | F:metal ion binding |
| Efet.01.658034.g1568.t1 | frizzled 1 2 7 | 540 | 8.04E-71 | 85.3 | F:G-protein coupled receptor activity; F:Wnt-activated receptor activity; P:G-protein coupled receptor signaling pathway; P:multicellular organism development; C:integral component of membrane; P:canonical Wnt signaling pathway; F:Wnt-protein binding; P:non-canonical Wnt signaling pathway |
| Efet.01.658034.g1569.t1 | Frizzled- | 939 | 6.27E-165 | 81.75 | P:hard palate development; F:Wnt-activated receptor activity; P:planar cell polarity pathway involved in neural tube closure; P:muscular septum morphogenesis; P:outflow tract morphogenesis; P:positive regulation of sequence-specific DNA binding transcription factor activity; P:canonical Wnt signaling pathway involved in osteoblast differentiation; C:cell surface; P:canonical Wnt signaling pathway involved in mesenchymal stem cell differentiation; P:negative regulation of BMP signaling pathway; P:epithelial cell differentiation; P:negative regulation of canonical Wnt signaling pathway; P:membranous septum morphogenesis; F:frizzled binding; C:focal adhesion; P:negative regulation of transcription, DNA-templated; P:positive regulation of transcription, DNA-templated; P:response to drug; P:positive regulation of protein phosphorylation; C:integral component of membrane; F:PDZ domain binding; F:Wnt-protein binding; P:negative regulation of catenin import into nucleus; P:autocrine signaling |
| Efet.01.658037.g1570.t1 | Leucine-rich repeat-containing 8A | 480 | 2.15E-68 | 77.3 |  |
| Efet.01.658040.g1571.t1 | transmembrane cytochrome oxidase | 570 | 2.51E-132 | 85.95 | C:integral component of membrane |
| Efet.01.658040.g1572.t1 | cytochrome oxidase assembly Sco1 family | 585 | 1.18E-102 | 86.7 | C:integral component of membrane |
| Efet.01.658040.g1573.t1 | heme A synthase | 1173 | 0 | 82.85 | F:oxidoreductase activity, acting on the CH-CH group of donors; C:integral component of membrane; P:heme a biosynthetic process; P:oxidation-reduction process |
| Efet.01.658040.g1574.t1 | protoheme IX farnesyltransferase | 792 | 2.09E-165 | 94.35 | P:heme O biosynthetic process; F:protoheme IX farnesyltransferase activity; C:integral component of membrane; C:plasma membrane |
| Efet.01.658040.g1575.t1 | SCO family | 354 | 3.68E-79 | 88.6 |  |
| Efet.01.658040.g1576.t1 | urease accessory | 579 | 1.28E-121 | 84.15 | C:cytoplasm; P:nitrogen compound metabolic process; F:nickel cation binding |
| Efet.01.658040.g1577.t1 | family transcriptional regulator | 1050 | 0 | 77.35 | F:DNA binding; F:transcription factor activity, sequence-specific DNA binding; P:transcription, DNA-templated; P:regulation of transcription, DNA-templated |
| Efet.01.658040.g1578.t1 | carbohydrate ABC transporter substrate-binding | 1386 | 0 | 83.65 | P:carbohydrate transport; F:transporter activity |
| Efet.01.658040.g1579.t1 | glucosamine-6-phosphate deaminase | 759 | 1.34E-156 | 76.95 | P:carbohydrate metabolic process; F:glucosamine-6-phosphate deaminase activity; P:N-acetylglucosamine metabolic process |
| Efet.01.658040.g1580.t1 | N-acetylglucosamine-6-phosphate deacetylase | 813 | 0 | 71.2 | P:carbohydrate metabolic process; P:N-acetylglucosamine metabolic process; F:N-acetylglucosamine-6-phosphate deacetylase activity |
| Efet.01.658040.g1581.t1 | gfo Idh family oxidoreductase | 990 | 0 | 62.5 | F:oxidoreductase activity; P:oxidation-reduction process |
| Efet.01.658040.g1582.t1 | penicillin acylase family | 927 | 0 | 73 | P:antibiotic biosynthetic process; F:penicillin amidase activity |
| Efet.01.658041.g1583.t1 | 3-hydroxybutyrate dehydrogenase | 807 | 6.08E-125 | 92.2 | F:3-hydroxybutyrate dehydrogenase activity; P:oxidation-reduction process |
| Efet.01.658041.g1584.t1 | two-component system response regulator | 732 | 7.29E-174 | 97 | F:DNA binding; P:transcription, DNA-templated; P:phosphorelay signal transduction system; P:regulation of transcription, DNA-templated; C:intracellular |
| Efet.01.658041.g1585.t1 | two-component sensor histidine kinase | 1233 | 0 | 89.2 | F:phosphorelay sensor kinase activity; F:ATP binding; P:phosphorelay signal transduction system; C:integral component of membrane; P:signal transduction by protein phosphorylation; C:intracellular |
| Efet.01.658041.g1586.t1 | 2-C-methyl-D-erythritol 4-phosphate cytidylyltransferase | 627 | 6.85E-117 | 88.4 | F:2-C-methyl-D-erythritol 4-phosphate cytidylyltransferase activity; P:isopentenyl diphosphate biosynthetic process, methylerythritol 4-phosphate pathway; P:terpenoid biosynthetic process |
| Efet.01.658041.g1587.t1 | transcription-repair coupling factor | 2694 | 0 | 92.65 | F:ATP binding; C:cytoplasm; P:transcription-coupled nucleotide-excision repair, DNA damage recognition; P:regulation of transcription, DNA-templated; F:helicase activity; F:damaged DNA binding |
| Efet.01.658044.g1588.t1 | ABC transporter ATP-binding | 366 | 2.43E-76 | 84.85 | F:ATPase activity; F:ATP binding |
| Efet.01.658044.g1589.t1 | ABC transporter ATP-binding | 591 | 1.21E-109 | 71.85 | F:ATPase activity; F:ATP binding |
| Efet.01.658044.g1590.t1 | branched-chain amino acid ABC transporter permease | 957 | 0 | 83.7 | C:integral component of membrane; P:transport; C:plasma membrane; F:transporter activity |
| Efet.01.658044.g1591.t1 | branched-chain amino acid ABC transporter permease | 834 | 1.66E-161 | 81.9 | C:integral component of membrane; P:transport; C:plasma membrane; F:transporter activity |
| Efet.01.658044.g1592.t1 | ABC transporter substrate-binding | 1266 | 0 | 80.55 | P:amino acid transport |
| Efet.01.658044.g1593.t1 | family transcriptional regulator | 825 | 1.66E-169 | 85.9 | F:DNA binding; F:transcription factor activity, sequence-specific DNA binding; P:transcription, DNA-templated; P:regulation of transcription, DNA-templated |
| Efet.01.658044.g1594.t1 | flagellar biosynthesis | 282 | 1.26E-48 | 89.4 | F:sulfolactate sulfo-lyase activity; F:hydrolase activity |
| Efet.01.658044.g1595.t1 | D-galactarate dehydratase | 1227 | 0 | 96.05 | F:sulfolactate sulfo-lyase activity; F:altronate dehydratase activity; F:hydrolase activity |
| Efet.01.658044.g1596.t1 | 3-phosphoglycerate dehydrogenase | 927 | 1.22E-177 | 76.55 | F:oxidoreductase activity, acting on the CH-OH group of donors, NAD or NADP as acceptor; F:NAD binding; P:oxidation-reduction process |
| Efet.01.658044.g1597.t1 | tripartite tricarboxylate transporter substrate binding | 1944 | 0 | 88.35 | C:outer membrane-bounded periplasmic space; F:malate dehydrogenase (NADP+) activity; P:oxidation-reduction process |
| Efet.01.658044.g1598.t1 | ABC transporter permease | 801 | 1.32E-133 | 92.7 | C:integral component of membrane; P:monosaccharide transport; F:monosaccharide-transporting ATPase activity; C:plasma membrane |
| Efet.01.658044.g1599.t1 | sugar ABC transporter ATP-binding | 453 | 3.78E-100 | 93.25 | F:ATPase activity; F:ATP binding; P:carbohydrate transport |
| Efet.01.658045.g1600.t1 | trissin receptor-like | 444 | 1.03E-09 | 63.35 | P:signal transduction; F:signal transducer activity |
| Efet.01.658045.g1601.t1 | alpha-2C adrenergic receptor isoform X3 | 453 | 3.66E-17 | 56.4 | F:G-protein coupled receptor activity; P:neuropeptide signaling pathway; P:G-protein coupled receptor signaling pathway; P:signal transduction; C:membrane; C:integral component of membrane; F:signal transducer activity; F:neuropeptide Y receptor activity |
| Efet.01.658052.g1602.t1 | ABC transporter ATP-binding | 1116 | 0 | 81.85 | F:ATP binding; P:glycine betaine transport; P:ferric iron transport; C:membrane; F:ferric-transporting ATPase activity |
| Efet.01.658052.g1603.t1 | ABC transporter permease | 648 | 1.22E-146 | 89.2 | C:integral component of membrane; P:transport; C:plasma membrane |
| Efet.01.658052.g1604.t1 | glycine betaine ABC transporter substrate-binding | 708 | 3.14E-168 | 85.05 | P:transport; F:transporter activity |
| Efet.01.658052.g1605.t1 | choline dehydrogenase | 1434 | 0 | 81.85 | F:flavin adenine dinucleotide binding; P:oxidation-reduction process; F:choline dehydrogenase activity |
| Efet.01.658052.g1606.t1 | mandelate racemase muconate lactonizing enzyme family | 954 | 0 | 89.1 | F:gluconate dehydratase activity; F:metal ion binding; P:metabolic process; F:isomerase activity |
| Efet.01.658052.g1607.t1 | MFS transporter | 489 | 4.20E-88 | 75.55 | P:transmembrane transport; C:integral component of membrane |
| Efet.01.658052.g1608.t1 | 5-oxoprolinase | 981 | 0 | 77.2 | F:5-oxoprolinase (ATP-hydrolyzing) activity |
| Efet.01.658052.g1609.t1 | 5-oxoprolinase | 1500 | 0 | 77.55 | F:5-oxoprolinase (ATP-hydrolyzing) activity |
| Efet.01.658057.g1610.t1 | DUF3696 domain-containing | 1137 | 0 | 84.95 |  |
| Efet.01.658057.g1611.t1 | restriction endonuclease | 444 | 2.75E-52 | 61.75 | F:catalytic activity; P:metabolic process |
| Efet.01.658062.g1614.t1 | hypothetical protein CAPTEDRAFT_226427 | 1254 | 8.34E-19 | 49 | F:calcium ion binding; C:membrane; C:integral component of membrane |
| Efet.01.658069.g1616.t1 | hypothetical protein CAPTEDRAFT_186654 | 990 | 1.51E-27 | 52.5 | F:G-protein coupled receptor activity; P:G-protein coupled receptor signaling pathway; C:membrane; C:integral component of membrane |
| Efet.01.65807.g969.t1 | neurexin-4 isoform X1 | 252 | 6.35E-20 | 50.7 | C:membrane; C:integral component of membrane |
| Efet.01.65808.g970.t1 | glycine-rich domain-containing 2-like | 1485 | 9.14E-112 | 47.7 |  |
| Efet.01.658081.g1618.t1 | GMP reductase | 969 | 0 | 92.85 | C:GMP reductase complex; F:GMP reductase activity; P:purine nucleotide metabolic process; P:oxidation-reduction process |
| Efet.01.658081.g1619.t1 | DNA helicase II | 2142 | 0 | 89.05 | F:DNA binding; F:ATP binding; P:DNA duplex unwinding; F:ATP-dependent DNA helicase activity |
| Efet.01.658081.g1620.t1 | polyamine ABC transporter substrate-binding | 1122 | 0 | 84.95 | F:polyamine binding; C:periplasmic space; P:polyamine transport |
| Efet.01.658084.g1621.t1 | efflux RND transporter periplasmic adaptor subunit | 1122 | 0 | 92.45 | P:transmembrane transport; C:membrane |
| Efet.01.658084.g1622.t1 | multidrug efflux RND transporter permease subunit | 3033 | 0 | 90.6 | C:integral component of membrane; P:transport; F:transporter activity |
| Efet.01.658084.g1623.t1 | multidrug transporter | 1374 | 0 | 87.2 | C:integral component of membrane; F:lipid binding; P:transport; C:plasma membrane; F:transporter activity |
| Efet.01.658084.g1624.t1 | family transcriptional regulator | 939 | 0 | 87.65 | F:transcription factor activity, sequence-specific DNA binding; P:transcription, DNA-templated; P:regulation of transcription, DNA-templated; F:sequence-specific DNA binding |
| Efet.01.658084.g1625.t1 | 2-methylcitrate dehydratase | 1287 | 0 | 74.95 | F:lyase activity |
| Efet.01.658084.g1626.t1 | AMP-dependent synthetase | 1572 | 0 | 85.5 | P:metabolic process; F:ligase activity |
| Efet.01.658109.g1627.t1 | sugar ABC transporter substrate-binding | 1041 | 0 | 83.3 | P:carbohydrate transport |
| Efet.01.658109.g1628.t1 | galactitol-1-phosphate 5-dehydrogenase | 783 | 2.34E-97 | 85.8 | F:zinc ion binding; F:oxidoreductase activity; P:oxidation-reduction process |
| Efet.01.658109.g1629.t1 | sugar kinase | 1422 | 0 | 70.25 | P:carbohydrate metabolic process; P:phosphorylation; F:phosphotransferase activity, alcohol group as acceptor; F:kinase activity |
| Efet.01.658109.g1630.t1 | family transcriptional regulator | 918 | 0 | 75.7 | F:carbohydrate binding |
| Efet.01.658109.g1631.t1 | sugar ABC transporter ATP-binding | 1146 | 0 | 75.9 | F:ATPase activity; F:ATP binding |
| Efet.01.658110.g1632.t1 | ATP GTP-binding | 219 | 3.03E-43 | 76.95 | F:helicase activity |
| Efet.01.658110.g1633.t1 | MFS transporter | 612 | 1.82E-125 | 80.15 | P:transmembrane transport; C:integral component of membrane |
| Efet.01.658110.g1634.t1 | MFS transporter | 645 | 1.33E-110 | 78.9 | P:transmembrane transport; C:integral component of membrane; F:transporter activity |
| Efet.01.658110.g1635.t1 | galactitol-1-phosphate 5-dehydrogenase | 1002 | 0 | 81.5 | F:zinc ion binding; P:oxidation-reduction process; F:L-threonine 3-dehydrogenase activity |
| Efet.01.658110.g1636.t1 | gluconate 5-dehydrogenase | 690 | 1.31E-116 | 88.15 | F:3-oxoacyl-[acyl-carrier-protein] reductase (NADPH) activity; P:oxidation-reduction process |
| Efet.01.658110.g1637.t1 | histidinol dehydrogenase | 1314 | 0 | 89.55 | F:zinc ion binding; F:NAD binding; P:oxidation-reduction process; P:histidine biosynthetic process; F:histidinol dehydrogenase activity |
| Efet.01.658110.g1638.t1 | nitrate ABC transporter substrate-binding | 846 | 6.44E-180 | 82.05 |  |
| Efet.01.658116.g1639.t1 | family transcriptional regulator | 822 | 0 | 68.4 | F:DNA binding; P:transcription, DNA-templated; P:regulation of transcription, DNA-templated |
| Efet.01.658116.g1640.t1 | FAD-dependent oxidoreductase | 1674 | 0 | 74 | F:FAD binding; P:oxidation-reduction process; F:monooxygenase activity |
| Efet.01.658116.g1641.t1 | tripartite tricarboxylate transporter substrate binding | 1017 | 0 | 87.7 | F:lyase activity; C:outer membrane-bounded periplasmic space |
| Efet.01.658116.g1642.t1 | VOC family | 450 | 2.28E-80 | 82.3 | F:dioxygenase activity; P:aromatic compound catabolic process; F:ferrous iron binding; F:lyase activity; P:oxidation-reduction process |
| Efet.01.658116.g1643.t1 | 2-keto-4-pentenoate hydratase | 894 | 0 | 80.05 | F:ureidoglycolate lyase activity; P:metabolic process; F:hydrolase activity |
| Efet.01.658116.g1644.t1 | family transcriptional regulator | 357 | 3.86E-81 | 64.6 | F:DNA binding; F:transcription factor activity, sequence-specific DNA binding; P:transcription, DNA-templated; P:regulation of transcription, DNA-templated; F:sequence-specific DNA binding |
| Efet.01.658116.g1645.t1 | family transcriptional regulator | 357 | 2.09E-33 | 58.95 | P:regulation of anion transmembrane transport; P:ion transmembrane transport; C:integral component of membrane; P:chloride transport; F:voltage-gated chloride channel activity |
| Efet.01.658116.g1646.t1 | short chain fatty acid transporter | 1002 | 0 | 96.85 | C:integral component of membrane |
| Efet.01.658116.g1647.t1 | ferredoxin:oxidoreductase FAD NAD(P)-binding | 3081 | 0 | 81.7 | F:oxidoreductase activity; F:electron carrier activity; F:molybdenum ion binding; P:oxidation-reduction process; F:2 iron, 2 sulfur cluster binding |
| Efet.01.658116.g1648.t1 | ornithine cyclodeaminase | 789 | 3.68E-167 | 79.05 | F:ornithine cyclodeaminase activity |
| Efet.01.658116.g1649.t1 | family transcriptional regulator | 852 | 0 | 92.9 | F:DNA binding; F:transcription factor activity, sequence-specific DNA binding; P:transcription, DNA-templated; P:regulation of transcription, DNA-templated |
| Efet.01.658116.g1650.t1 | tripartite tricarboxylate transporter substrate binding | 960 | 0 | 97.05 | C:outer membrane-bounded periplasmic space |
| Efet.01.658116.g1651.t1 | gamma-glutamyltransferase | 1617 | 0 | 96.45 | P:glutathione metabolic process; F:gamma-glutamyltransferase activity |
| Efet.01.658116.g1652.t1 | acetyl- acetyltransferase | 987 | 0 | 76.45 | F:acetyl-CoA C-acetyltransferase activity; P:metabolic process |
| Efet.01.658116.g1653.t1 | transferase | 882 | 0 | 77.95 | F:formyl-CoA transferase activity; F:isomerase activity |
| Efet.01.658121.g1654.t1 | biotin transporter | 663 | 1.16E-130 | 81.4 | F:electron carrier activity; F:molybdenum ion binding; P:oxidation-reduction process; F:trimethylamine-N-oxide reductase (cytochrome c) activity |
| Efet.01.658122.g1655.t1 | 3-methylcrotonyl- carboxylase | 1641 | 0 | 74.5 | F:ATP binding; F:metal ion binding; F:biotin carboxylase activity |
| Efet.01.658122.g1656.t1 | MBL fold metallo-hydrolase | 1116 | 0 | 84.35 | F:hydrolase activity |
| Efet.01.658133.g1658.t1 | family transcriptional regulator (plasmid) | 339 | 4.31E-74 | 80.15 | F:DNA binding; F:transcription factor activity, sequence-specific DNA binding; P:transcription, DNA-templated; P:regulation of transcription, DNA-templated; F:alanine racemase activity |
| Efet.01.658133.g1659.t1 | DUF2961 domain-containing | 1254 | 0 | 76.65 |  |
| Efet.01.658133.g1660.t1 | ABC transporter permease | 990 | 0 | 74.9 | C:integral component of membrane; P:transport; C:plasma membrane |
| Efet.01.658133.g1661.t1 | ABC transporter ATP-binding | 1590 | 0 | 64.55 | F:ATPase activity; F:ATP binding; C:integral component of membrane; P:peptide transport; C:plasma membrane |
| Efet.01.658133.g1662.t1 | ABC transporter ATP-binding | 789 | 1.03E-148 | 63.6 | F:ATPase activity; F:ATP binding |
| Efet.01.658133.g1663.t1 | peptide ABC transporter substrate-binding | 1881 | 0 | 71.85 | P:transmembrane transport; C:ATP-binding cassette (ABC) transporter complex |
| Efet.01.658133.g1664.t1 | SAM-dependent methyltransferase | 924 | 0 | 87.85 | F:methyltransferase activity; P:methylation |
| Efet.01.658133.g1665.t1 | ABC transporter permease | 711 | 3.17E-124 | 90.4 | C:integral component of membrane; C:plasma membrane |
| Efet.01.658135.g1666.t1 | angel homolog 2 isoform X3 | 894 | 8.38E-55 | 54.35 | P:3'-UTR-mediated mRNA stabilization; C:cytoplasm; C:Cajal body; P:negative regulation of mitotic cell cycle; F:mRNA 3'-UTR binding |
| Efet.01.658138.g1667.t1 | XRE family transcriptional regulator | 450 | 2.08E-78 | 89.3 | F:sequence-specific DNA binding |
| Efet.01.658138.g1668.t1 | molecular chaperone | 429 | 3.79E-97 | 84.7 |  |
| Efet.01.658138.g1669.t1 | aminotransferase | 1215 | 0 | 92.35 | F:transaminase activity; P:lipid metabolic process; C:integral component of membrane |
| Efet.01.658139.g1670.t1 | Transmembrane emp24 domain-containing 7 | 588 | 4.96E-73 | 65.5 | C:endoplasmic reticulum membrane; C:integral component of membrane; P:transport |
| Efet.01.658142.g1671.t1 | copper-translocating P-type ATPase | 1062 | 0 | 82.8 | F:nucleotide binding; F:cation-transporting ATPase activity; F:metal ion binding; P:cation transmembrane transport; C:integral component of membrane; P:metal ion transport; C:plasma membrane |
| Efet.01.658142.g1672.t1 | N-acetyl-gamma-glutamyl-phosphate reductase | 888 | 0 | 67.8 | F:N-acetyl-gamma-glutamyl-phosphate reductase activity; P:arginine biosynthetic process; C:cytoplasm; F:NAD binding; P:oxidation-reduction process |
| Efet.01.658142.g1673.t1 | N-acetyltransferase | 459 | 2.65E-56 | 57.5 | F:N-acetyltransferase activity |
| Efet.01.658142.g1674.t1 | NADPH-dependent FMN reductase | 687 | 9.50E-112 | 79.25 | F:oxidoreductase activity; P:oxidation-reduction process |
| Efet.01.658142.g1675.t1 | tRNA uridine-5-carboxymethylaminomethyl(34) synthesis enzyme | 1977 | 0 | 93.4 | C:cytoplasm; F:oxidoreductase activity; F:flavin adenine dinucleotide binding; P:oxidation-reduction process; P:tRNA wobble uridine modification |
| Efet.01.658142.g1676.t1 | 16S rRNA (guanine(527)-N(7))-methyltransferase | 549 | 8.09E-109 | 82.2 | C:cytoplasm; F:rRNA (guanine-N7-)-methyltransferase activity; P:rRNA (guanine-N7)-methylation |
| Efet.01.658142.g1677.t1 | lysine transporter | 615 | 1.76E-137 | 89.95 | C:integral component of membrane; P:amino acid transport; C:plasma membrane |
| Efet.01.658142.g1678.t1 | chromosome partitioning | 675 | 2.82E-152 | 92.5 |  |
| Efet.01.658142.g1679.t1 | alpha beta hydrolase | 534 | 5.92E-117 | 89.55 | F:hydrolase activity |
| Efet.01.658142.g1680.t1 | chromosome partitioning | 912 | 1.96E-152 | 92.8 | F:DNA binding |
| Efet.01.658150.g1681.t1 | sodium:proton antiporter | 1038 | 5.84E-134 | 90.35 | C:integral component of membrane |
| Efet.01.658150.g1682.t1 | nicotinate phosphoribosyltransferase | 1446 | 0 | 94.55 | F:nicotinate-nucleotide diphosphorylase (carboxylating) activity; F:nicotinate phosphoribosyltransferase activity; P:NAD biosynthetic process; P:nicotinate nucleotide biosynthetic process; F:ligase activity |
| Efet.01.658150.g1683.t1 | thioesterase | 531 | 5.59E-77 | 91.8 |  |
| Efet.01.658150.g1684.t1 | ABC transporter substrate-binding | 1545 | 0 | 95.55 | P:transmembrane transport; C:ATP-binding cassette (ABC) transporter complex |
| Efet.01.658150.g1685.t1 | ABC transporter permease | 954 | 0 | 94.15 | C:integral component of membrane; P:transport; C:plasma membrane |
| Efet.01.658150.g1686.t1 | ABC transporter permease | 903 | 2.99E-178 | 95.1 | C:integral component of membrane; P:transport; C:plasma membrane |
| Efet.01.658150.g1687.t1 | ABC transporter ATP-binding | 801 | 2.92E-153 | 81.5 | F:ATPase activity; F:ATP binding; P:peptide transport |
| Efet.01.658150.g1688.t1 | ABC transporter ATP-binding | 600 | 1.61E-113 | 93.85 | F:ATPase activity; F:ATP binding; P:peptide transport |
| Efet.01.658151.g1689.t1 | MFS transporter | 1197 | 0 | 76.1 | P:transmembrane transport; C:integral component of membrane |
| Efet.01.658151.g1690.t1 | ABC transporter permease | 873 | 0 | 87.75 | C:integral component of membrane; P:transport; C:plasma membrane |
| Efet.01.658169.g1692.t1 | rho GTPase-activating 100F-like isoform X2 | 1059 | 7.56E-91 | 62.95 | P:signal transduction |
| Efet.01.658170.g1693.t1 | 30S ribosomal S1 | 1566 | 0 | 98.5 | F:RNA binding; F:structural constituent of ribosome; C:ribosome; P:translation |
| Efet.01.658170.g1694.t1 | aspartate aminotransferase family | 1371 | 0 | 79.8 | F:pyridoxal phosphate binding; F:transaminase activity |
| Efet.01.658170.g1695.t1 | amidase | 1452 | 0 | 69.95 | F:amidase activity; F:carbon-nitrogen ligase activity, with glutamine as amido-N-donor |
| Efet.01.658171.g1696.t1 | transmembrane 26-like | 852 | 1.24E-19 | 67.05 | C:integral component of membrane |
| Efet.01.658176.g1698.t1 | HHIP 2 | 1179 | 1.16E-77 | 54.95 | P:carbohydrate metabolic process; F:oxidoreductase activity, acting on the CH-OH group of donors, quinone or similar compound as acceptor; F:quinone binding; F:catalytic activity; P:oxidation-reduction process |
| Efet.01.658177.g1699.t1 | phenol hydroxylase | 1191 | 0 | 88.1 | P:cellular aromatic compound metabolic process; F:phenol 2-monooxygenase activity; P:oxidation-reduction process |
| Efet.01.658177.g1700.t1 | sigma-54-dependent Fis family transcriptional regulator | 2271 | 0 | 82.35 | F:ATP binding; P:transcription, DNA-templated; P:phosphorelay signal transduction system; P:regulation of transcription, DNA-templated; F:sequence-specific DNA binding; F:transcription factor binding; C:intracellular |
| Efet.01.65818.g971.t1 | monocarboxylate transporter 12-like | 645 | 2.12E-36 | 70.35 | C:integral component of membrane; P:transport; F:monocarboxylic acid transmembrane transporter activity |
| Efet.01.658190.g1703.t1 | Bacterial membrane flanked domain | 1266 | 0 | 51.45 | C:membrane; C:integral component of membrane |
| Efet.01.658190.g1704.t1 | ABC transporter | 588 | 1.08E-117 | 65.75 | F:ATPase activity; F:ATP binding |
| Efet.01.658192.g1705.t1 | redox-regulated ATPase | 1095 | 0 | 95.15 | F:GTP binding; F:ATPase activity; F:ATP binding; F:ribosome binding; F:ribosomal large subunit binding |
| Efet.01.658192.g1706.t1 | addiction module toxin | 327 | 1.69E-37 | 92.8 |  |
| Efet.01.658192.g1707.t1 | DNA-binding helix-turn-helix | 285 | 1.07E-61 | 93.5 | F:sequence-specific DNA binding |
| Efet.01.658192.g1708.t1 | lipoyl synthase | 720 | 3.83E-174 | 95.15 | C:cytoplasm; F:metal ion binding; F:lipoate synthase activity; F:4 iron, 4 sulfur cluster binding; P:lipoate biosynthetic process; P:protein lipoylation |
| Efet.01.658193.g1709.t1 | excinuclease ABC subunit A | 870 | 0 | 94.55 | F:ATPase activity; F:DNA binding; F:ATP binding; P:nucleic acid phosphodiester bond hydrolysis; F:excinuclease ABC activity; C:excinuclease repair complex; C:cytoplasm; F:metal ion binding; P:nucleotide-excision repair |
| Efet.01.658193.g1710.t1 | C4-dicarboxylate transporter | 1458 | 0 | 95.1 | P:transmembrane transport; C:integral component of membrane; F:symporter activity |
| Efet.01.658193.g1711.t1 | NAD(P)-dependent oxidoreductase | 498 | 7.84E-104 | 76.95 | F:2-(R)-hydroxypropyl-CoM dehydrogenase activity; P:oxidation-reduction process |
| Efet.01.658198.g1712.t1 | mitogen-activated kinase kinase kinase 9-like | 600 | 7.22E-28 | 56 | P:phosphorylation; F:kinase activity |
| Efet.01.658207.g1713.t1 | protocadherin gamma-B5-like | 972 | 1.11E-57 | 53.75 | F:calcium ion binding; C:membrane; C:integral component of membrane; P:cell adhesion; P:homophilic cell adhesion via plasma membrane adhesion molecules; C:plasma membrane |
| Efet.01.658209.g1714.t1 | hypothetical protein S7711_09530 | 558 | 1.59E-06 | 52.67 | C:membrane; C:integral component of membrane |
| Efet.01.658211.g1715.t1 | ABC transporter substrate-binding | 525 | 2.95E-118 | 87 | P:carbohydrate transport |
| Efet.01.658211.g1716.t1 | altronate dehydratase | 1206 | 0 | 76.35 | F:lyase activity |
| Efet.01.658211.g1717.t1 | TRAP transporter large permease | 1401 | 0 | 93.95 | C:integral component of membrane |
| Efet.01.658211.g1718.t1 | TRAP transporter small permease | 561 | 7.42E-118 | 87.6 | C:integral component of membrane |
| Efet.01.658214.g1719.t1 | potassium voltage-gated channel Shaw-like | 1113 | 9.48E-50 | 63.05 | F:ion channel activity; P:ion transport; C:membrane |
| Efet.01.658217.g1720.t1 | tubulin alpha-1A | 1194 | 0 | 98.6 | F:GTP binding; C:cytoplasm; F:GTPase activity; F:structural constituent of cytoskeleton; C:microtubule; P:microtubule-based process |
| Efet.01.658218.g1721.t1 | indolepyruvate ferredoxin oxidoreductase | 3300 | 0 | 90.7 | F:thiamine pyrophosphate binding; F:indolepyruvate ferredoxin oxidoreductase activity; P:oxidation-reduction process |
| Efet.01.658218.g1722.t1 | arabinose ABC transporter permease | 1728 | 0 | 88.75 | C:integral component of membrane |
| Efet.01.658230.g1723.t1 | branched-chain amino acid ABC transporter permease | 1017 | 8.51E-153 | 77.5 | C:integral component of membrane; P:transport; C:plasma membrane; F:transporter activity |
| Efet.01.658230.g1724.t1 | branched-chain amino acid ABC transporter substrate-binding | 1047 | 0 | 85.65 |  |
| Efet.01.658230.g1725.t1 | family transcriptional regulator | 708 | 3.53E-145 | 85.6 | F:DNA binding; P:transcription, DNA-templated; P:regulation of transcription, DNA-templated |
| Efet.01.658230.g1726.t1 | phenylacetate- oxygenase reductase subunit | 1086 | 0 | 84.05 | F:metal ion binding; F:electron carrier activity; P:oxidation-reduction process; F:2 iron, 2 sulfur cluster binding; P:transport; F:nitric oxide dioxygenase activity; P:phenylacetate catabolic process |
| Efet.01.658235.g1729.t1 | transcriptional regulator | 909 | 0 | 81 | F:DNA binding; F:transcription factor activity, sequence-specific DNA binding; P:transcription, DNA-templated; P:regulation of transcription, DNA-templated |
| Efet.01.658235.g1730.t1 | formate dehydrogenase | 891 | 0 | 96.1 | F:oxidoreductase activity, acting on the CH-OH group of donors, NAD or NADP as acceptor; F:NAD binding; F:formate dehydrogenase (NAD+) activity; F:formate dehydrogenase (cytochrome) activity; P:oxidation-reduction process |
| Efet.01.658236.g1731.t1 | methylaspartate mutase | 663 | 3.75E-52 | 54.9 | F:intramolecular transferase activity; F:methylaspartate mutase activity; P:anaerobic glutamate catabolic process; F:catalytic activity; F:cobalamin binding; P:metabolic process |
| Efet.01.658236.g1732.t1 | ABC transporter ATP-binding | 885 | 0 | 55.9 | F:ATPase activity; F:ATP binding |
| Efet.01.658236.g1733.t1 | ABC transporter permease | 663 | 2.47E-112 | 58.65 | C:integral component of membrane; P:transport; C:plasma membrane |
| Efet.01.658236.g1734.t1 | acyl carrier | 243 | 7.27E-31 | 71.75 |  |
| Efet.01.658236.g1735.t1 | family transcriptional regulator | 882 | 0 | 86.3 | F:DNA binding; F:transcription factor activity, sequence-specific DNA binding; P:transcription, DNA-templated; P:regulation of transcription, DNA-templated |
| Efet.01.658236.g1736.t1 | ester lyase | 867 | 3.13E-162 | 73.75 | F:citryl-CoA lyase activity; F:metal ion binding |
| Efet.01.658236.g1737.t1 | tripartite tricarboxylate transporter substrate binding | 969 | 0 | 89.65 | C:outer membrane-bounded periplasmic space |
| Efet.01.658236.g1738.t1 | formyl- transferase | 1152 | 0 | 83.5 | F:cinnamoyl-CoA:phenyllactate CoA-transferase activity |
| Efet.01.658236.g1739.t1 | mechanosensitive ion channel | 486 | 2.33E-70 | 88.35 | P:transmembrane transport; C:integral component of membrane |
| Efet.01.658236.g1740.t1 | lysine--tRNA ligase | 1548 | 0 | 92.85 | F:magnesium ion binding; F:nucleic acid binding; F:ATP binding; C:cytoplasm; F:lysine-tRNA ligase activity; P:lysyl-tRNA aminoacylation |
| Efet.01.658237.g1741.t1 | sex peptide receptor-like | 1119 | 1.99E-89 | 63 | F:G-protein coupled receptor activity; F:G-protein coupled peptide receptor activity; P:G-protein coupled receptor signaling pathway; C:membrane; C:integral component of membrane |
| Efet.01.658238.g1742.t1 | catenin beta-like | 729 | 6.73E-135 | 85.55 | C:cytoskeleton; C:cytoplasm; P:signal transduction; C:integral component of membrane; P:cell adhesion; P:adherens junction assembly; F:signal transducer activity |
| Efet.01.658245.g1744.t1 | dihydropyrimidine dehydrogenase | 621 | 2.29E-133 | 87.8 | P:oxidation-reduction process; F:glutamate synthase (NADPH) activity; F:iron-sulfur cluster binding |
| Efet.01.658245.g1745.t1 | NAD-dependent dihydropyrimidine dehydrogenase subunit | 1119 | 0 | 90.5 | C:cytoplasm; P:oxidation-reduction process; F:dihydropyrimidine dehydrogenase (NADP+) activity; F:dihydrouracil dehydrogenase (NAD+) activity |
| Efet.01.658245.g1746.t1 | nitrate reductase | 1335 | 0 | 94.8 | P:transmembrane transport; C:integral component of membrane; F:transporter activity |
| Efet.01.658245.g1747.t1 | dihydropyrimidinase | 1257 | 0 | 91.05 | C:cytoplasm; F:dihydropyrimidinase activity |
| Efet.01.658245.g1748.t1 | Zn-dependent hydrolase | 507 | 1.43E-111 | 92.6 | F:hydrolase activity, acting on carbon-nitrogen (but not peptide) bonds, in linear amidines; P:metabolic process; F:N-carbamoyl-L-amino-acid hydrolase activity |
| Efet.01.658246.g1750.t1 | Integrase core domain-containing | 1197 | 1.38E-101 | 47.25 | F:nucleic acid binding; P:DNA integration |
| Efet.01.658246.g1751.t1 | ATPase | 450 | 5.37E-40 | 64.5 | F:helicase activity |
| Efet.01.658250.g1752.t1 | multiple PDZ domain -like isoform X1 | 441 | 4.05E-42 | 78.15 |  |
| Efet.01.658255.g1754.t1 | family transcriptional regulator | 630 | 9.67E-135 | 74.65 | F:DNA binding; F:transcription factor activity, sequence-specific DNA binding; P:transcription, DNA-templated; P:regulation of transcription, DNA-templated |
| Efet.01.658255.g1755.t1 | family transcriptional regulator | 1233 | 0 | 79.45 | F:transcription factor activity, sequence-specific DNA binding; P:transcription, DNA-templated; P:regulation of transcription, DNA-templated; F:sequence-specific DNA binding |
| Efet.01.658255.g1756.t1 | choline ABC transporter permease subunit | 576 | 9.25E-120 | 93.05 | C:integral component of membrane; P:transport; C:plasma membrane |
| Efet.01.658255.g1757.t1 | glycine betaine L-proline ABC transporter ATP-binding | 1107 | 0 | 83.75 | F:ATPase activity; F:ATP binding; P:glycine betaine transport; C:membrane |
| Efet.01.658257.g1759.t1 | periplasmic solute-binding family | 915 | 0 | 72.25 | F:metal ion binding; P:cell adhesion; P:metal ion transport |
| Efet.01.658257.g1760.t1 | ABC 3 transport family | 879 | 8.85E-148 | 69.95 | F:ATP binding; F:ATPase activity, coupled to transmembrane movement of substances; P:transmembrane transport; C:integral component of membrane; C:plasma membrane |
| Efet.01.658257.g1761.t1 | ABC transporter ATP-binding | 798 | 9.44E-161 | 69.4 | F:ATPase activity; F:ATP binding |
| Efet.01.658257.g1762.t1 | transcriptional repressor | 438 | 4.85E-90 | 82 | F:DNA binding; F:transcription factor activity, sequence-specific DNA binding; P:regulation of transcription, DNA-templated |
| Efet.01.658257.g1763.t1 | 3-hydroxyisobutyrate dehydrogenase | 258 | 2.42E-38 | 98.1 | F:phosphogluconate dehydrogenase (decarboxylating) activity; F:NAD binding; F:3-hydroxybutyrate dehydrogenase activity; P:oxidation-reduction process; F:3-hydroxyisobutyrate dehydrogenase activity |
| Efet.01.658261.g1765.t1 | ABC transporter substrate-binding | 750 | 1.58E-179 | 79.9 | P:transport; F:transporter activity |
| Efet.01.658261.g1766.t1 | amino acid ABC transporter ATP-binding | 486 | 4.76E-107 | 89.65 | F:ATP binding; P:amino acid transmembrane transport; F:amino acid-transporting ATPase activity |
| Efet.01.658261.g1767.t1 | argininosuccinate lyase | 2274 | 0 | 86.9 | C:cytoplasm; P:arginine biosynthetic process via ornithine; F:argininosuccinate lyase activity |
| Efet.01.658261.g1768.t1 | family transcriptional regulator | 915 | 0 | 76.45 | F:DNA binding; F:transcription factor activity, sequence-specific DNA binding; P:transcription, DNA-templated; P:regulation of transcription, DNA-templated |
| Efet.01.658261.g1769.t1 | ABC transporter substrate-binding | 1146 | 0 | 77.35 |  |
| Efet.01.658261.g1770.t1 | ABC transporter ATP-binding | 711 | 4.28E-169 | 84.6 | F:ATPase activity; F:ATP binding |
| Efet.01.658261.g1771.t1 | ABC transporter ATP-binding | 369 | 1.93E-45 | 77.45 | F:ATPase activity; F:ATP binding |
| Efet.01.658261.g1772.t1 | branched-chain amino acid ABC transporter permease | 909 | 7.11E-138 | 80.85 | C:integral component of membrane; P:transport; C:plasma membrane; F:transporter activity |
| Efet.01.658261.g1773.t1 | branched-chain amino acid ABC transporter permease | 921 | 2.18E-153 | 81.6 | C:integral component of membrane; P:transport; C:plasma membrane; F:transporter activity |
| Efet.01.658267.g1774.t1 | LD-carboxypeptidase | 993 | 1.11E-178 | 88 | F:carboxypeptidase activity; P:proteolysis |
| Efet.01.658267.g1775.t1 | tRNA adenosine(34) deaminase | 1347 | 0 | 72 | F:zinc ion binding; F:tRNA-specific adenosine deaminase activity; P:tRNA wobble adenosine to inosine editing |
| Efet.01.658267.g1776.t1 | hypothetical protein Veis_2621 | 312 | 2.24E-27 | 97 | C:integral component of membrane |
| Efet.01.658267.g1777.t1 | ribonuclease | 1488 | 0 | 95.45 | F:RNA binding; P:RNA phosphodiester bond hydrolysis; F:ribonuclease activity; P:RNA processing |
| Efet.01.658275.g1779.t1 | hypothetical protein | 969 | 0 | 93.7 |  |
| Efet.01.658275.g1780.t1 | nucleotidyl PF08843 domain | 1749 | 0 | 91.75 |  |
| Efet.01.658277.g1782.t1 | kelch domain-containing 9-like | 993 | 1.63E-94 | 59.25 |  |
| Efet.01.658279.g1783.t1 | family transcriptional regulator | 531 | 1.02E-90 | 69.55 | F:DNA binding; F:transcription factor activity, sequence-specific DNA binding; P:transcription, DNA-templated; P:regulation of transcription, DNA-templated |
| Efet.01.658279.g1784.t1 | ABC transporter substrate-binding | 1332 | 0 | 70.25 | P:transmembrane transport; C:ATP-binding cassette (ABC) transporter complex |
| Efet.01.658279.g1785.t1 | ABC transporter permease | 1017 | 1.33E-154 | 66.5 | C:integral component of membrane; P:transport; C:plasma membrane |
| Efet.01.658279.g1786.t1 | ABC transporter permease | 789 | 5.94E-157 | 86.75 | C:integral component of membrane; P:transport; C:plasma membrane |
| Efet.01.658279.g1787.t1 | dipeptide ABC transporter ATP-binding | 1722 | 0 | 76.15 | F:ATPase activity; F:ATP binding; P:peptide transport |
| Efet.01.658279.g1788.t1 | HNH endonuclease | 579 | 5.25E-21 | 70.46 | F:nucleic acid binding; P:nucleic acid phosphodiester bond hydrolysis; F:endonuclease activity |
| Efet.01.658279.g1789.t1 | spermidine putrescine ABC transporter substrate-binding | 567 | 5.82E-136 | 74.15 | F:polyamine binding; C:periplasmic space; P:polyamine transport |
| Efet.01.658281.g1790.t1 | alkaline phosphatase | 1446 | 0 | 89.7 | P:nucleic acid phosphodiester bond hydrolysis; F:phosphodiesterase I activity |
| Efet.01.658281.g1791.t1 | dehydratase | 453 | 1.80E-106 | 90.6 |  |
| Efet.01.658281.g1792.t1 | tripartite tricarboxylate transporter substrate binding | 951 | 0 | 84.3 | C:outer membrane-bounded periplasmic space |
| Efet.01.658282.g1793.t1 | phosphorylase | 894 | 0 | 86 | P:nucleoside metabolic process; F:methylthioadenosine nucleosidase activity |
| Efet.01.658282.g1794.t1 | BMP family ABC transporter substrate-binding | 1083 | 0 | 96.3 | C:plasma membrane |
| Efet.01.658282.g1795.t1 | chemotaxis | 501 | 3.60E-97 | 85.9 | F:phosphoprotein phosphatase activity; P:chemotaxis; C:bacterial-type flagellum; P:archaeal or bacterial-type flagellum-dependent cell motility; C:cytoplasm; P:protein dephosphorylation; P:regulation of chemotaxis |
| Efet.01.658282.g1796.t1 | two-component system response regulator | 342 | 5.27E-66 | 94.1 | P:phosphorelay signal transduction system; C:intracellular |
| Efet.01.658282.g1797.t1 | flagellar motor | 984 | 0 | 82 | C:integral component of membrane |
| Efet.01.658283.g1798.t1 | GTP 3 ,8-cyclase | 1146 | 0 | 88.5 | F:GTP binding; F:metal ion binding; F:lyase activity; F:4 iron, 4 sulfur cluster binding; P:Mo-molybdopterin cofactor biosynthetic process; C:molybdopterin synthase complex; F:hydrolase activity |
| Efet.01.658283.g1799.t1 | molybdenum cofactor guanylyltransferase | 627 | 2.46E-93 | 67 | F:GTP binding; C:cytoplasm; F:metal ion binding; F:molybdenum cofactor guanylyltransferase activity; P:Mo-molybdopterin cofactor biosynthetic process |
| Efet.01.658283.g1800.t1 | molybdopterin molybdenumtransferase | 1362 | 0 | 75.7 | C:integral component of membrane; P:Mo-molybdopterin cofactor biosynthetic process; P:molybdopterin cofactor biosynthetic process |
| Efet.01.658291.g1802.t1 | DNA-binding response regulator | 762 | 1.46E-93 | 90.15 | F:DNA binding; P:transcription, DNA-templated; P:phosphorelay signal transduction system; P:regulation of transcription, DNA-templated; C:intracellular |
| Efet.01.658291.g1803.t1 | 2-C-methyl-D-erythritol 2,4-cyclodiphosphate synthase | 1248 | 0 | 68.9 | P:isoprenoid biosynthetic process; F:transferase activity |
| Efet.01.658291.g1804.t1 | cysteine--tRNA ligase | 381 | 6.19E-83 | 83 | F:ATP binding; F:zinc ion binding; C:cytoplasm; F:cysteine-tRNA ligase activity; P:cysteinyl-tRNA aminoacylation |
| Efet.01.658292.g1805.t1 | 30S ribosomal S12 | 309 | 2.51E-67 | 98.85 | F:structural constituent of ribosome; C:small ribosomal subunit; F:tRNA binding; F:rRNA binding; P:translation |
| Efet.01.658292.g1806.t1 | 30S ribosomal S7 | 474 | 1.21E-108 | 98.35 | F:structural constituent of ribosome; C:small ribosomal subunit; F:tRNA binding; F:rRNA binding; P:translation |
| Efet.01.658292.g1807.t1 | elongation factor G | 2205 | 0 | 91.5 | F:GTP binding; F:translation elongation factor activity; C:cytoplasm; F:GTPase activity; P:translational elongation |
| Efet.01.658294.g1808.t1 | slit homolog 1 | 1317 | 1.64E-67 | 47.65 | P:Roundabout signaling pathway; F:Roundabout binding; C:extracellular space; P:regulation of axonogenesis; P:embryo development ending in birth or egg hatching; F:heparin binding; C:proteinaceous extracellular matrix; P:axon extension involved in axon guidance |
| Efet.01.658297.g1811.t1 | FAA hydrolase family | 1275 | 0 | 76.85 | F:5-carboxymethyl-2-hydroxymuconate delta-isomerase activity; P:metabolic process |
| Efet.01.658297.g1812.t1 | acyl- synthetase | 1449 | 0 | 77.35 | P:metabolic process; F:ligase activity |
| Efet.01.658297.g1813.t1 | AMP-dependent synthetase | 1707 | 0 | 69.6 | P:metabolic process; F:ligase activity |
| Efet.01.658297.g1814.t1 | ABC transporter permease | 864 | 2.37E-152 | 89.4 | C:integral component of membrane; P:transport; C:plasma membrane |
| Efet.01.658297.g1815.t1 | xylose isomerase | 912 | 0 | 83.75 | F:isomerase activity |
| Efet.01.658298.g1816.t1 | branched-chain amino acid ABC transporter substrate-binding | 942 | 0 | 87.95 | P:amino acid transport |
| Efet.01.658298.g1817.t1 | hydroxyproline-2-epimerase | 864 | 0 | 88.1 |  |
| Efet.01.658298.g1818.t1 | histidine kinase | 867 | 0 | 86.45 | P:phosphorylation; F:kinase activity |
| Efet.01.658298.g1819.t1 | RNA-binding transcriptional accessory | 2400 | 0 | 88.9 | F:nucleic acid binding; P:nucleobase-containing compound metabolic process |
| Efet.01.658298.g1820.t1 | phosphoenolpyruvate carboxylase | 342 | 2.50E-68 | 93.7 | F:magnesium ion binding; P:oxaloacetate metabolic process; P:tricarboxylic acid cycle; P:carbon fixation; F:phosphoenolpyruvate carboxylase activity |
| Efet.01.658303.g1821.t1 | hypothetical protein HELRODRAFT_171537 | 1338 | 4.00E-69 | 51.75 |  |
| Efet.01.658304.g1822.t1 | leukocyte elastase inhibitor-like | 1320 | 0 | 51.4 | F:serine-type endopeptidase inhibitor activity; C:extracellular space; P:regulation of blood coagulation, intrinsic pathway; P:negative regulation of endopeptidase activity; F:heparin binding |
| Efet.01.658306.g1824.t1 | DC-STAMP domain-containing 2-like | 867 | 7.19E-42 | 47.15 | C:membrane; C:integral component of membrane |
| Efet.01.658307.g1825.t1 | family transcriptional regulator | 930 | 0 | 78.3 | F:DNA binding; F:transcription factor activity, sequence-specific DNA binding; P:transcription, DNA-templated; P:regulation of transcription, DNA-templated |
| Efet.01.658307.g1826.t1 | MBL fold metallo-hydrolase | 933 | 0 | 87 | P:proteolysis; F:metallopeptidase activity |
| Efet.01.658307.g1827.t1 | RNA polymerase sigma factor | 2316 | 0 | 93.45 | P:transcription initiation from bacterial-type RNA polymerase promoter; F:DNA binding; F:sigma factor activity; F:transcription factor activity, sequence-specific DNA binding; C:cytoplasm; P:regulation of transcription, DNA-templated |
| Efet.01.658307.g1828.t1 | transferase | 1458 | 0 | 91.95 | F:transferase activity |
| Efet.01.658307.g1829.t1 | transcriptional regulator | 318 | 3.28E-72 | 94.2 |  |
| Efet.01.658307.g1830.t1 | hypothetical protein | 579 | 4.45E-83 | 73.45 |  |
| Efet.01.658307.g1831.t1 | cation acetate symporter | 741 | 8.29E-115 | 89.45 | P:transmembrane transport; C:integral component of membrane; F:transporter activity |
| Efet.01.65831.g972.t1 | Golgi apparatus 1- | 657 | 9.98E-62 | 64.65 | C:membrane |
| Efet.01.658310.g1832.t1 | gamma carbonic anhydrase family | 582 | 1.41E-107 | 76.9 | F:transferase activity |
| Efet.01.658310.g1833.t1 | family transcriptional regulator | 504 | 1.89E-93 | 92.65 |  |
| Efet.01.658310.g1834.t1 | GNAT family N-acetyltransferase | 495 | 3.76E-105 | 93.15 | F:N-acetyltransferase activity |
| Efet.01.658310.g1835.t1 | MULTISPECIES: DNA-binding | 378 | 1.24E-57 | 70.6 |  |
| Efet.01.658310.g1836.t1 | GNAT family N-acetyltransferase | 312 | 1.19E-19 | 75.35 | F:transferase activity, transferring acyl groups |
| Efet.01.658310.g1837.t1 | alpha beta hydrolase | 1029 | 7.02E-105 | 65.45 | F:hydrolase activity |
| Efet.01.658310.g1838.t1 | outer membrane lipo -sorting | 1746 | 2.40E-168 | 67.2 |  |
| Efet.01.658310.g1839.t1 | ABC transporter permease | 1137 | 0 | 63.6 | C:integral component of membrane; C:plasma membrane |
| Efet.01.658310.g1840.t1 | ABC transporter ATP-binding | 687 | 1.10E-148 | 75.05 | F:ATPase activity; F:ATP binding |
| Efet.01.658311.g1841.t1 | sex peptide receptor-like | 1170 | 2.24E-79 | 59.3 | F:G-protein coupled receptor activity; F:G-protein coupled peptide receptor activity; P:G-protein coupled receptor signaling pathway; C:membrane; C:integral component of membrane |
| Efet.01.658312.g1842.t1 | phospholipase | 405 | 1.25E-74 | 88.35 | F:hydrolase activity |
| Efet.01.658312.g1843.t1 | aminoacyl-tRNA hydrolase | 423 | 3.66E-94 | 90.45 | P:translational termination; F:translation release factor activity |
| Efet.01.658312.g1844.t1 | type II 3-dehydroquinate dehydratase | 480 | 9.07E-113 | 93.35 | P:aromatic amino acid family biosynthetic process; F:3-dehydroquinate dehydratase activity; P:chorismate biosynthetic process |
| Efet.01.658312.g1845.t1 | 50S ribosomal L17 | 333 | 6.81E-74 | 90.1 | F:structural constituent of ribosome; C:ribosome; P:translation |
| Efet.01.658312.g1846.t1 | DNA-directed RNA polymerase subunit alpha | 852 | 0 | 98.2 | F:DNA binding; F:DNA-directed 5'-3' RNA polymerase activity; P:transcription, DNA-templated; F:protein dimerization activity |
| Efet.01.658312.g1847.t1 | 30S ribosomal S4 | 432 | 1.00E-99 | 95.1 | F:structural constituent of ribosome; C:small ribosomal subunit; F:rRNA binding; P:translation |
| Efet.01.658312.g1848.t1 | 30S ribosomal S11 | 903 | 2.20E-88 | 99 | F:structural constituent of ribosome; C:ribosome; F:rRNA binding; P:translation |
| Efet.01.658312.g1849.t1 | pre translocase subunit | 687 | 1.04E-131 | 97.95 | P:protein targeting; P:intracellular protein transmembrane transport; C:integral component of membrane; P:protein transport by the Sec complex; C:intracellular; C:plasma membrane |
| Efet.01.658319.g1852.t1 | netrin-1-like isoform X3 | 339 | 4.87E-25 | 61.45 | C:extracellular region |
| Efet.01.658322.g1854.t1 | intraflagellar transport 88 homolog isoform X1 | 1740 | 7.14E-122 | 60.9 |  |
| Efet.01.658325.g1855.t1 | low-density lipo receptor-related 6- | 372 | 1.40E-44 | 74.5 | F:Wnt-activated receptor activity; P:negative regulation of smooth muscle cell apoptotic process; C:Golgi apparatus; C:neuronal cell body; C:synapse; P:positive regulation of sequence-specific DNA binding transcription factor activity; P:thalamus development; P:face morphogenesis; C:caveola; F:frizzled binding; F:coreceptor activity involved in canonical Wnt signaling pathway; F:Wnt-protein binding; F:apolipoprotein binding; P:embryonic pattern specification; F:low-density lipoprotein receptor activity; P:positive regulation of canonical Wnt signaling pathway; P:chemical synaptic transmission; C:receptor complex; P:cerebral cortex development; P:pericardium morphogenesis; P:cerebellum morphogenesis; C:early endosome membrane; C:Wnt-Frizzled-LRP5/6 complex; P:neural crest formation; P:protein localization to plasma membrane; C:Wnt signalosome; P:receptor-mediated endocytosis involved in cholesterol transport; P:external genitalia morphogenesis; P:palate development; P:Wnt signaling pathway involved in dorsal/ventral axis specification; P:positive regulation of cell cycle; P:odontogenesis of dentin-containing tooth; P:canonical Wnt signaling pathway involved in neural crest cell differentiation; P:Wnt signaling pathway involved in midbrain dopaminergic neuron differentiation; C:extracellular region; P:positive regulation of transcription from RNA polymerase II promoter; P:convergent extension; P:neural tube closure; P:canonical Wnt signaling pathway involved in regulation of cell proliferation; P:cellular response to cholesterol; P:positive regulation of Wnt signaling pathway involved in dorsal/ventral axis specification; F:protein homodimerization activity; P:axis elongation involved in somitogenesis; P:Wnt signaling pathway involved in somitogenesis; P:midbrain-hindbrain boundary development; P:beta-catenin destruction complex disassembly; P:embryonic retina morphogenesis in camera-type eye; C:cell surface; P:trachea cartilage morphogenesis; P:negative regulation of canonical Wnt signaling pathway; F:kinase inhibitor activity; C:integral component of membrane; P:negative regulation of protein serine/threonine kinase activity; F:toxin transporter activity; P:primitive streak formation |
| Efet.01.658331.g1856.t1 | D-galactarate dehydratase | 342 | 9.40E-75 | 77.95 | F:galactarate dehydratase activity |
| Efet.01.658331.g1857.t1 | hypothetical membrane associated | 438 | 4.76E-94 | 73.1 |  |
| Efet.01.658331.g1858.t1 | hypothetical protein | 408 | 3.82E-50 | 74.4 |  |
| Efet.01.658331.g1859.t1 | acryloyl- reductase | 807 | 1.04E-177 | 85.45 | F:zinc ion binding; F:acryloyl-CoA reductase (NADP+) activity; P:oxidation-reduction process; F:NADPH:quinone reductase activity |
| Efet.01.658331.g1860.t1 | family dioxygenase | 384 | 1.00E-85 | 87.8 | F:taurine dioxygenase activity; P:oxidation-reduction process |
| Efet.01.658334.g1861.t1 | methane monooxygenase | 471 | 3.98E-113 | 96.25 | F:methane monooxygenase activity; P:cellular aromatic compound metabolic process; P:oxidation-reduction process |
| Efet.01.658334.g1862.t1 | CDP-6-deoxy-delta-3,4-glucoseen reductase | 1095 | 0 | 93.35 | F:metal ion binding; F:electron carrier activity; P:oxidation-reduction process; F:ferredoxin-NAD+ reductase activity; F:monooxygenase activity; F:2 iron, 2 sulfur cluster binding |
| Efet.01.658334.g1863.t1 | toluene hydroxylase | 1098 | 0 | 90.6 | P:cellular aromatic compound metabolic process; F:oxidoreductase activity, acting on paired donors, with incorporation or reduction of molecular oxygen, NAD(P)H as one donor, and incorporation of one atom of oxygen; P:oxidation-reduction process |
| Efet.01.658334.g1864.t1 | monooxygenase component | 354 | 2.22E-81 | 94.1 | P:cellular aromatic compound metabolic process; F:phenol 2-monooxygenase activity; P:oxidation-reduction process |
| Efet.01.658334.g1865.t1 | amidohydrolase | 1053 | 0 | 87.95 | F:hydrolase activity |
| Efet.01.658334.g1866.t1 | DUF59 domain-containing | 630 | 1.46E-108 | 78.7 |  |
| Efet.01.658334.g1867.t1 | molecular chaperone | 1602 | 0 | 94 | F:ATP binding; C:cytoplasm; P:protein refolding |
| Efet.01.658334.g1868.t1 | divalent metal cation transporter | 873 | 6.29E-153 | 79.8 | P:transmembrane transport; F:metal ion binding; C:integral component of membrane; F:symporter activity; P:metal ion transport; C:plasma membrane |
| Efet.01.658338.g1869.t1 | serine threonine- phosphatase 6 regulatory ankyrin repeat subunit B-like | 1584 | 1.11E-49 | 48.95 |  |
| Efet.01.658339.g1870.t1 | site-specific DNA-methyltransferase | 3651 | 0 | 77.8 | F:DNA binding; F:N-methyltransferase activity; P:DNA methylation |
| Efet.01.658339.g1871.t1 | cytoplasmic | 552 | 2.97E-124 | 81.2 |  |
| Efet.01.658339.g1872.t1 | chromosome segregation SMC | 1200 | 0 | 93.85 | F:ATP binding |
| Efet.01.658339.g1873.t1 | restriction endonuclease | 3063 | 0 | 87.8 | F:DNA binding; F:ATP binding; P:nucleic acid phosphodiester bond hydrolysis; F:deoxyribonuclease activity; F:endonuclease activity; P:DNA metabolic process |
| Efet.01.658339.g1874.t1 | family transcriptional regulator | 1056 | 0 | 92.6 | F:nucleotidyltransferase activity |
| Efet.01.658339.g1875.t1 | hypothetical protein | 528 | 7.28E-108 | 80.14 | C:integral component of membrane |
| Efet.01.658342.g1877.t1 | transcription initiation factor TFIID subunit 1-like isoform X1 | 1647 | 2.21E-103 | 64.65 | P:regulation of signal transduction by p53 class mediator; P:protein autophosphorylation; F:protein serine/threonine kinase activity; P:cellular response to DNA damage stimulus; P:positive regulation of transcription from RNA polymerase II promoter; P:transcription initiation from RNA polymerase II promoter; P:transcription elongation from RNA polymerase II promoter; F:DNA binding; F:histone acetyltransferase activity; F:p53 binding; C:transcription factor TFIID complex; F:transcription coactivator activity; F:lysine-acetylated histone binding; P:cellular component organization; F:TBP-class protein binding; P:peptidyl-serine phosphorylation; P:positive regulation of proteasomal ubiquitin-dependent protein catabolic process; P:peptidyl-threonine phosphorylation; C:MLL1 complex |
| Efet.01.658343.g1878.t1 | Rho GTPase-activating 100F | 1845 | 6.01E-29 | 70.85 | P:signal transduction |
| Efet.01.65835.g974.t1 | tripartite motif-containing 2-like isoform X5 | 1485 | 2.72E-24 | 43.4 | F:zinc ion binding; F:metal ion binding; C:intracellular |
| Efet.01.658350.g1880.t1 | serine O-acetyltransferase | 282 | 4.09E-55 | 95.3 | C:cytoplasm; F:serine O-acetyltransferase activity; P:cysteine biosynthetic process from serine |
| Efet.01.658350.g1881.t1 | amidohydrolase | 1236 | 0 | 69.65 | F:hippurate hydrolase activity; P:metabolic process |
| Efet.01.658350.g1882.t1 | family transcriptional regulator | 651 | 1.53E-151 | 65.95 | F:DNA binding; P:transcription, DNA-templated; P:regulation of transcription, DNA-templated |
| Efet.01.658350.g1883.t1 | peptide ABC transporter substrate-binding | 1584 | 0 | 78.15 | P:transmembrane transport; C:ATP-binding cassette (ABC) transporter complex; F:hydrolase activity |
| Efet.01.658354.g1885.t1 | tripartite motif-containing 2-like | 648 | 7.63E-17 | 49.33 |  |
| Efet.01.658355.g1886.t1 | amidohydrolase | 1329 | 0 | 93.1 | F:hydrolase activity, acting on carbon-nitrogen (but not peptide) bonds |
| Efet.01.658355.g1887.t1 | G3E family | 345 | 6.78E-62 | 91.85 |  |
| Efet.01.658358.g1890.t1 | FLYWCH zinc finger domain | 222 | 3.69E-10 | 62.05 | F:ATP binding; P:response to antibiotic |
| Efet.01.658359.g1891.t1 | transcription factor Sp5 | 1275 | 2.34E-59 | 66.9 | F:nucleic acid binding; F:metal ion binding; P:mesoderm morphogenesis |
| Efet.01.658362.g1892.t1 | titin isoform X1 | 816 | 1.25E-10 | 60.85 | F:transferase activity |
| Efet.01.658363.g1893.t1 | FMRFamide-activated amiloride-sensitive sodium channel-like isoform X1 | 1905 | 0 | 49.05 | P:sodium ion transport; C:membrane |
| Efet.01.658364.g1894.t1 | amidinotransferase | 705 | 3.92E-170 | 79.8 | F:dimethylargininase activity; F:transferase activity |
| Efet.01.658364.g1895.t1 | family transcriptional regulator | 1125 | 0 | 55.8 | F:transcription factor activity, sequence-specific DNA binding; P:transcription, DNA-templated; P:regulation of transcription, DNA-templated; F:sequence-specific DNA binding |
| Efet.01.658364.g1896.t1 | aminotransferase | 1377 | 0 | 75.7 | F:pyridoxal phosphate binding; F:transaminase activity |
| Efet.01.658364.g1897.t1 | NAD-dependent succinate-semialdehyde dehydrogenase | 885 | 5.80E-173 | 82.35 | F:succinate-semialdehyde dehydrogenase [NAD(P)+] activity; P:oxidation-reduction process |
| Efet.01.658368.g1898.t1 | outer membrane lipo -sorting | 858 | 3.81E-173 | 76.65 |  |
| Efet.01.658368.g1899.t1 | short-chain dehydrogenase | 480 | 2.20E-106 | 72.65 | F:oxidoreductase activity; P:oxidation-reduction process |
| Efet.01.658368.g1900.t1 | ABC transporter permease | 1773 | 0 | 73.8 | C:integral component of membrane; C:plasma membrane |
| Efet.01.658368.g1901.t1 | ABC transporter ATP-binding | 1089 | 1.51E-164 | 79.1 | F:ATPase activity; F:ATP binding |
| Efet.01.658371.g1903.t1 | zinc finger 710-like isoform X2 | 1593 | 3.94E-32 | 39.95 | F:nucleic acid binding; F:metal ion binding |
| Efet.01.658371.g1904.t1 | gastrula zinc finger -like | 2079 | 2.35E-44 | 52.55 | F:nucleic acid binding; F:metal ion binding; F:methyltransferase activity; F:transferase activity; P:methylation |
| Efet.01.658372.g1905.t1 | DUF4212 domain-containing | 315 | 6.39E-44 | 81.3 | C:integral component of membrane |
| Efet.01.658372.g1906.t1 | phosphoenolpyruvate synthase | 2166 | 0 | 88.2 | F:ATP binding; P:gluconeogenesis; F:metal ion binding; P:phosphorylation; P:pyruvate metabolic process; F:pyruvate, water dikinase activity |
| Efet.01.658372.g1907.t1 | kinase pyrophosphorylase | 822 | 0 | 94.05 | F:ATP binding; P:protein dephosphorylation; F:ADP binding; F:protein serine/threonine kinase activity; P:protein phosphorylation; F:phosphotransferase activity, phosphate group as acceptor |
| Efet.01.658372.g1908.t1 | HAD family phosphatase | 717 | 7.99E-160 | 67.5 | P:metabolic process; F:hydrolase activity |
| Efet.01.658372.g1909.t1 | dihydroxyacetone kinase subunit L | 642 | 1.28E-94 | 74.3 | P:glycerol metabolic process; F:glycerone kinase activity; P:phosphorylation |
| Efet.01.658373.g1910.t1 | hypothetical protein CAPTEDRAFT_220792 | 606 | 9.04E-24 | 56.2 |  |
| Efet.01.658374.g1911.t1 | WD repeat-containing 25 | 936 | 3.89E-29 | 56.95 | F:second spliceosomal transesterification activity; P:generation of catalytic spliceosome for second transesterification step; C:catalytic step 2 spliceosome |
| Efet.01.658380.g1912.t1 | CoA-binding protein | 675 | 1.02E-136 | 77.2 | F:ATP binding; F:metal ion binding; F:cofactor binding; F:ligase activity |
| Efet.01.658380.g1913.t1 | 50S ribosomal L19 | 654 | 3.66E-78 | 97.95 | F:structural constituent of ribosome; C:ribosome; P:translation |
| Efet.01.658380.g1914.t1 | FAD-dependent oxidoreductase | 1320 | 0 | 79.05 | F:oxidoreductase activity; P:oxidation-reduction process |
| Efet.01.658380.g1915.t1 | glutamine synthetase | 1371 | 0 | 95.15 | P:glutamine biosynthetic process; F:glutamate-ammonia ligase activity |
| Efet.01.658380.g1916.t1 | aspartate aminotransferase family | 1398 | 0 | 88.85 | F:pyridoxal phosphate binding; F:adenosylmethionine-8-amino-7-oxononanoate transaminase activity |
| Efet.01.658380.g1917.t1 | polyamine ABC transporter ATP-binding | 1134 | 0 | 77.3 | F:ATP binding; P:polyamine transmembrane transport; F:polyamine-transporting ATPase activity; C:ATP-binding cassette (ABC) transporter complex |
| Efet.01.658380.g1918.t1 | putrescine ABC transporter permease | 771 | 0 | 85.65 | C:integral component of membrane; P:transport; C:plasma membrane |
| Efet.01.658380.g1919.t1 | putrescine ABC transporter permease | 789 | 5.53E-144 | 83.15 | C:integral component of membrane; P:transport; C:plasma membrane |
| Efet.01.658380.g1920.t1 | alkylhydroperoxidase | 603 | 5.15E-138 | 91.65 | F:peroxidase activity; P:cellular oxidant detoxification; P:oxidation-reduction process; F:peroxiredoxin activity |
| Efet.01.658380.g1921.t1 | CMD domain | 525 | 1.23E-93 | 89.05 | F:palmitoyl-CoA hydrolase activity |
| Efet.01.658380.g1922.t1 | palmitoyl- hydrolase | 375 | 4.19E-83 | 86.5 | P:acyl-CoA metabolic process; F:palmitoyl-CoA hydrolase activity; F:transferase activity |
| Efet.01.658383.g1923.t1 | gfo Idh family oxidoreductase | 1164 | 0 | 94.5 | F:oxidoreductase activity; P:oxidation-reduction process |
| Efet.01.658383.g1924.t1 | Enolase C-terminal domain-like | 1251 | 0 | 92.45 |  |
| Efet.01.658383.g1925.t1 | ABC transporter ATP-binding | 780 | 0 | 96.6 | F:ATPase activity; F:ATP binding |
| Efet.01.658383.g1926.t1 | nitrate ABC transporter substrate-binding | 1017 | 0 | 95.55 | P:thiamine biosynthetic process |
| Efet.01.658383.g1927.t1 | ABC transporter permease | 765 | 1.71E-131 | 97.85 | C:integral component of membrane; P:transport; C:plasma membrane |
| Efet.01.658383.g1928.t1 | tripartite tricarboxylate transporter substrate binding | 999 | 0 | 94.9 | C:outer membrane-bounded periplasmic space |
| Efet.01.658383.g1929.t1 | family transcriptional regulator | 807 | 2.48E-143 | 90.25 | F:DNA binding; P:transcription, DNA-templated; P:regulation of transcription, DNA-templated |
| Efet.01.658384.g1930.t1 | ABC transporter substrate-binding | 1101 | 0 | 87.65 | P:transmembrane transport; C:ATP-binding cassette (ABC) transporter complex |
| Efet.01.658384.g1931.t1 | enoyl-[acyl-carrier- ] reductase | 801 | 5.56E-179 | 93.8 | F:enoyl-[acyl-carrier-protein] reductase (NADH) activity; P:oxidation-reduction process; P:fatty acid biosynthetic process |
| Efet.01.658384.g1932.t1 | family radical SAM | 2310 | 0 | 87.1 | F:catalytic activity; F:iron ion binding; F:4 iron, 4 sulfur cluster binding |
| Efet.01.658384.g1933.t1 | DUF1949 domain-containing | 321 | 1.69E-67 | 88.45 |  |
| Efet.01.658385.g1934.t1 | sarcosine oxidase subunit alpha family | 627 | 1.13E-138 | 83.1 | F:sarcosine oxidase activity; P:tetrahydrofolate metabolic process; P:oxidation-reduction process; F:aminomethyltransferase activity; P:methylation |
| Efet.01.658385.g1935.t1 | sarcosine oxidase subunit gamma | 447 | 2.44E-99 | 71.75 |  |
| Efet.01.658385.g1936.t1 | membrane dipeptidase | 972 | 0 | 88.35 | F:dipeptidase activity; P:proteolysis |
| Efet.01.658385.g1937.t1 | glycine betaine ABC transporter substrate-binding | 654 | 1.36E-156 | 81.2 | P:transport; F:transporter activity |
| Efet.01.658385.g1938.t1 | N-methylproline demethylase | 2562 | 0 | 88.55 | F:FMN binding; F:oxidoreductase activity; F:methyltransferase activity; P:oxidation-reduction process; P:methylation |
| Efet.01.658387.g1939.t1 | CTP synthase | 2409 | 0 | 86.9 | F:ATP binding; P:'de novo' CTP biosynthetic process; P:glutamine metabolic process; F:CTP synthase activity |
| Efet.01.658387.g1940.t1 | 3-deoxy-8-phosphooctulonate synthase | 771 | 1.24E-141 | 91.95 | P:keto-3-deoxy-D-manno-octulosonic acid biosynthetic process; C:cytoplasm; F:3-deoxy-8-phosphooctulonate synthase activity |
| Efet.01.658387.g1941.t1 | phosphopyruvate hydratase | 1287 | 0 | 95.95 | F:magnesium ion binding; F:phosphopyruvate hydratase activity; P:glycolytic process; C:extracellular region; C:phosphopyruvate hydratase complex; C:cell surface |
| Efet.01.658389.g1942.t1 | gastrula zinc finger -like | 2223 | 1.24E-23 | 56.65 | F:nucleic acid binding; F:metal ion binding |
| Efet.01.658391.g1943.t1 | bis(5 -nucleosyl)-tetraphosphatase (symmetrical) | 948 | 0 | 85.45 | F:bis(5'-nucleosyl)-tetraphosphatase (symmetrical) activity |
| Efet.01.658391.g1944.t1 | histone | 312 | 3.67E-69 | 86.2 | F:DNA binding; P:regulation of transcription, DNA-templated; C:intracellular |
| Efet.01.658391.g1945.t1 | aconitate hydratase | 2658 | 0 | 80.05 | P:metabolic process; F:4 iron, 4 sulfur cluster binding; F:aconitate hydratase activity |
| Efet.01.658392.g1946.t1 | asparagine synthase (glutamine-hydrolyzing) | 390 | 9.75E-87 | 64.5 | P:asparagine biosynthetic process; F:asparagine synthase (glutamine-hydrolyzing) activity |
| Efet.01.658392.g1947.t1 | UDP-Glycosyltransferase glycogen phosphorylase | 1194 | 0 | 48.75 | C:membrane; C:integral component of membrane; F:transferase activity; F:isomerase activity |
| Efet.01.658392.g1948.t1 | N-acetyl sugar amidotransferase | 939 | 0 | 81.3 | F:transferase activity |
| Efet.01.658392.g1949.t1 | class I SAM-dependent methyltransferase | 534 | 1.55E-94 | 66.17 | F:methyltransferase activity; P:methylation |
| Efet.01.658392.g1950.t1 | short-chain dehydrogenase | 642 | 2.74E-143 | 68.3 |  |
| Efet.01.658392.g1951.t1 | acylneuraminate cytidylyltransferase family | 1455 | 0 | 71.25 | F:nucleotidyltransferase activity |
| Efet.01.658392.g1952.t1 | N-acetylneuraminate synthase | 627 | 4.36E-134 | 69.1 | P:carbohydrate biosynthetic process; F:N-acetylneuraminate synthase activity |
| Efet.01.658392.g1953.t1 | UDP-N-acetylglucosamine 2-epimerase (hydrolyzing) | 2586 | 0 | 67.7 | P:UDP-N-acetylglucosamine metabolic process; F:methyltransferase activity; F:hydrolase activity, hydrolyzing O-glycosyl compounds; P:methylation |
| Efet.01.658392.g1954.t1 | Flavin containing amine oxidoreductase | 1422 | 0 | 50.64 |  |
| Efet.01.658392.g1955.t1 | alcohol dehydrogenase | 1032 | 0 | 66.4 | F:nucleotidyltransferase activity; P:biosynthetic process |
| Efet.01.658392.g1956.t1 | aminotransferase | 966 | 0 | 72.6 | F:transaminase activity |
| Efet.01.658392.g1957.t1 | NAD-dependent dehydratase | 1185 | 0 | 76.35 |  |
| Efet.01.658392.g1958.t1 | imidazole glycerol phosphate synthase subunit | 609 | 2.99E-128 | 78 | F:catalytic activity; P:histidine biosynthetic process |
| Efet.01.658392.g1959.t1 | polysaccharide biosynthesis | 1803 | 0 | 76.75 | C:integral component of membrane; F:isomerase activity |
| Efet.01.658392.g1960.t1 | AAA family ATPase | 1050 | 1.35E-170 | 81.9 |  |
| Efet.01.658392.g1961.t1 | peptidase S16 | 561 | 6.21E-112 | 86.2 | P:proteolysis; C:integral component of membrane; F:ATP-dependent peptidase activity |
| Efet.01.658392.g1962.t1 | family | 627 | 1.49E-64 | 82.25 | C:integral component of membrane |
| Efet.01.658392.g1963.t1 | acetyl- carboxylase subunit beta | 1509 | 0 | 97.5 | F:ATP binding; P:malonyl-CoA biosynthetic process; F:zinc ion binding; F:acetyl-CoA carboxylase activity; F:transferase activity; P:fatty acid biosynthetic process; C:acetyl-CoA carboxylase complex |
| Efet.01.658392.g1964.t1 | tryptophan synthase subunit beta | 1371 | 0 | 94.4 | F:tryptophan synthase activity; P:tryptophan biosynthetic process |
| Efet.01.658392.g1965.t1 | tRNA pseudouridine(38-40) synthase | 819 | 2.17E-178 | 87.45 | F:RNA binding; P:tRNA pseudouridine synthesis; F:pseudouridine synthase activity |
| Efet.01.658392.g1966.t1 | pilus assembly | 1176 | 9.01E-80 | 93.25 | C:integral component of membrane |
| Efet.01.658393.g1967.t1 | uncharacterized protein LOC109918957 | 1101 | 1.03E-10 | 45.5 |  |
| Efet.01.658394.g1968.t1 | Coiled-coil domain-containing 15 | 2406 | 7.04E-18 | 50.65 | C:viral envelope; P:viral life cycle |
| Efet.01.658395.g1969.t1 | type I glutamate--ammonia ligase | 1416 | 0 | 98.75 | F:ATP binding; C:cytoplasm; P:glutamine biosynthetic process; P:nitrogen fixation; F:glutamate-ammonia ligase activity |
| Efet.01.658395.g1970.t1 | competence damage-inducible A | 633 | 6.48E-150 | 87.6 |  |
| Efet.01.658395.g1971.t1 | etoposide-induced 24 (EI24) | 669 | 3.19E-88 | 89.65 | C:integral component of membrane |
| Efet.01.658395.g1972.t1 | fatty acid hydroxylase | 996 | 0 | 89.85 | F:iron ion binding; F:oxidoreductase activity; C:integral component of membrane; P:oxidation-reduction process; P:fatty acid biosynthetic process |
| Efet.01.658395.g1973.t1 | peptidase A8 | 444 | 1.24E-86 | 92.5 | F:hydrolase activity, acting on carbon-nitrogen (but not peptide) bonds; P:carbohydrate metabolic process |
| Efet.01.658395.g1974.t1 | YVTN family beta-propeller | 1038 | 0 | 92.15 |  |
| Efet.01.658397.g1975.t1 | transcriptional regulator | 228 | 4.90E-47 | 93.95 | F:sequence-specific DNA binding |
| Efet.01.658397.g1976.t1 | type II toxin-antitoxin system family toxin | 333 | 3.53E-45 | 90.3 |  |
| Efet.01.658397.g1977.t1 | recombinase family | 693 | 3.90E-155 | 94.9 | F:DNA binding; F:recombinase activity; P:DNA recombination |
| Efet.01.658397.g1978.t1 | tape measure domain-containing | 2622 | 0 | 83.8 |  |
| Efet.01.658397.g1979.t1 | prevent-host-death | 246 | 3.73E-52 | 85.45 |  |
| Efet.01.658398.g1980.t1 | thiolase family | 1107 | 0 | 83.65 | F:propanoyl-CoA C-acyltransferase activity; P:metabolic process |
| Efet.01.658398.g1981.t1 | Uncharacterized OB-fold contains Zn-ribbon domain | 258 | 8.01E-52 | 66.18 |  |
| Efet.01.658398.g1982.t1 | tripartite tricarboxylate transporter substrate binding | 975 | 0 | 76.15 | C:outer membrane-bounded periplasmic space |
| Efet.01.658398.g1983.t1 | family transcriptional regulator | 552 | 2.59E-106 | 67.05 | F:DNA binding; P:transcription, DNA-templated; P:regulation of transcription, DNA-templated |
| Efet.01.658398.g1984.t1 | acyl- dehydrogenase | 330 | 3.42E-56 | 86 | F:acyl-CoA dehydrogenase activity; F:flavin adenine dinucleotide binding; F:butyryl-CoA dehydrogenase activity; P:oxidation-reduction process |
| Efet.01.658398.g1985.t1 | transferase | 771 | 7.65E-101 | 80.3 | F:formyl-CoA transferase activity |
| Efet.01.658398.g1986.t1 | family transcriptional regulator | 852 | 1.32E-167 | 67.4 | F:DNA binding; P:transcription, DNA-templated; P:regulation of transcription, DNA-templated |
| Efet.01.658399.g1987.t1 | transcriptional regulator | 1821 | 0 | 80.9 | F:ATP binding; F:helicase activity |
| Efet.01.658399.g1988.t1 | transferase | 573 | 3.96E-134 | 66.3 | F:catalytic activity |
| Efet.01.658399.g1989.t1 | PQQ-dependent methanol ethanol family | 2049 | 0 | 95.95 | F:oxidoreductase activity, acting on CH-OH group of donors; F:calcium ion binding; F:electron carrier activity; C:membrane; C:outer membrane-bounded periplasmic space; P:oxidation-reduction process; F:heme binding |
| Efet.01.658399.g1990.t1 | Fis family transcriptional regulator | 1227 | 0 | 86.1 | F:sequence-specific DNA binding |
| Efet.01.658401.g1991.t1 | amino acid ABC transporter permease | 657 | 3.74E-152 | 61.6 | C:integral component of membrane; P:transport; C:plasma membrane; F:transporter activity |
| Efet.01.658401.g1992.t1 | family transcriptional regulator | 2610 | 0 | 70.2 | F:DNA binding; P:transcription, DNA-templated; P:regulation of transcription, DNA-templated |
| Efet.01.658401.g1993.t1 | IS3 family transposase | 267 | 7.47E-17 | 83.6 | F:nucleic acid binding; P:DNA integration |
| Efet.01.658401.g1994.t1 | glyoxalase bleomycin resistance dioxygenase | 624 | 1.06E-97 | 68.8 | F:dioxygenase activity; P:oxidation-reduction process |
| Efet.01.658403.g1995.t1 | gastrula zinc finger -like | 1929 | 4.68E-36 | 45.7 | F:nucleic acid binding; F:transcription factor activity, sequence-specific DNA binding; F:metal ion binding |
| Efet.01.658404.g1996.t1 | E3 ubiquitin- ligase PDZRN3-like | 1347 | 4.83E-69 | 55 |  |
| Efet.01.658405.g1997.t1 | helicase | 1971 | 0 | 94.7 | F:helicase activity |
| Efet.01.658405.g1998.t1 | hypothetical protein | 753 | 5.54E-147 | 93.5 |  |
| Efet.01.658405.g1999.t1 | hypothetical protein | 1428 | 0 | 92.75 |  |
| Efet.01.658405.g2000.t1 | glutamine-hydrolyzing GMP synthase | 1620 | 0 | 93.55 | F:ATP binding; F:GMP synthase (glutamine-hydrolyzing) activity; P:glutamine metabolic process; F:pyrophosphatase activity; P:GMP biosynthetic process |
| Efet.01.658405.g2001.t1 | IMP dehydrogenase | 1476 | 0 | 95.6 | F:nucleotide binding; F:metal ion binding; F:IMP dehydrogenase activity; P:oxidation-reduction process; P:GMP biosynthetic process |
| Efet.01.658405.g2002.t1 | Zn-dependent hydrolase | 1074 | 0 | 66.5 | F:hydrolase activity, acting on carbon-nitrogen (but not peptide) bonds, in linear amidines; P:metabolic process; F:N-carbamoyl-L-amino-acid hydrolase activity |
| Efet.01.658405.g2003.t1 | mandelate racemase | 972 | 0 | 75.6 | F:metal ion binding; P:metabolic process; F:lyase activity; F:isomerase activity |
| Efet.01.658405.g2004.t1 | aspartate aminotransferase family | 1026 | 0 | 67.65 | F:pyridoxal phosphate binding; F:transaminase activity |
| Efet.01.658408.g2006.t1 | glutamine--tRNA ligase | 2736 | 0 | 89.85 | F:ATP binding; C:cytoplasm; F:glutamine-tRNA ligase activity; P:glutamyl-tRNA aminoacylation; P:glutaminyl-tRNA aminoacylation |
| Efet.01.658408.g2007.t1 | uracil phosphoribosyltransferase | 630 | 6.79E-150 | 94.15 | F:magnesium ion binding; F:GTP binding; F:uracil phosphoribosyltransferase activity; P:uracil salvage; P:UMP salvage |
| Efet.01.658408.g2008.t1 | family DNA-binding transcriptional regulator | 636 | 6.28E-114 | 92.2 | F:DNA binding; C:cytoplasm; P:transcription, DNA-templated; P:regulation of transcription, DNA-templated |
| Efet.01.658408.g2009.t1 | phosphoribosylamine--glycine ligase | 1281 | 0 | 92.6 | F:magnesium ion binding; F:ATP binding; F:phosphoribosylamine-glycine ligase activity; P:'de novo' IMP biosynthetic process; F:manganese ion binding; P:purine nucleobase biosynthetic process |
| Efet.01.658408.g2010.t1 | oxygen-dependent coproporphyrinogen oxidase | 654 | 1.32E-159 | 89.75 | F:protein homodimerization activity; C:cytoplasm; F:coproporphyrinogen oxidase activity; F:metal ion binding; P:oxidation-reduction process; P:protoporphyrinogen IX biosynthetic process |
| Efet.01.658408.g2011.t1 | DUF4347 domain-containing | 1599 | 0 | 55.85 | F:hydrolase activity |
| Efet.01.658409.g2012.t1 | hypothetical protein | 555 | 4.66E-130 | 87.45 |  |
| Efet.01.658409.g2013.t1 | family transcriptional regulator | 921 | 0 | 65.7 | F:DNA binding; F:transcription factor activity, sequence-specific DNA binding; P:transcription, DNA-templated; P:regulation of transcription, DNA-templated |
| Efet.01.658409.g2014.t1 | family transcriptional regulator | 705 | 7.94E-138 | 67.65 | F:hydrolase activity |
| Efet.01.658409.g2015.t1 | acyl- dehydrogenase | 1002 | 0 | 68.85 | F:acyl-CoA dehydrogenase activity; F:flavin adenine dinucleotide binding; P:oxidation-reduction process |
| Efet.01.658409.g2016.t1 | AMP-dependent synthetase and ligase | 861 | 0 | 66.9 | P:metabolic process; F:ligase activity |
| Efet.01.658409.g2017.t1 | tripartite tricarboxylate transporter substrate binding | 933 | 0 | 67.25 | C:outer membrane-bounded periplasmic space |
| Efet.01.658409.g2018.t1 | NAD(P)-dependent oxidoreductase | 699 | 4.57E-124 | 72.1 | F:phosphogluconate dehydrogenase (decarboxylating) activity; P:oxidation-reduction process |
| Efet.01.658410.g2019.t1 | GCN5 family acetyltransferase | 618 | 6.68E-147 | 88 | C:ribosome; F:N-acetyltransferase activity |
| Efet.01.658410.g2020.t1 | 4-hydroxyphenylpyruvate dioxygenase | 1134 | 0 | 91.95 | P:aromatic amino acid family metabolic process; F:4-hydroxyphenylpyruvate dioxygenase activity; F:metal ion binding; P:oxidation-reduction process |
| Efet.01.658410.g2021.t1 | phenylalanine 4-monooxygenase | 828 | 0 | 87.95 | P:L-phenylalanine catabolic process; F:phenylalanine 4-monooxygenase activity; F:iron ion binding; P:oxidation-reduction process |
| Efet.01.658410.g2022.t1 | family transcriptional regulator | 963 | 0 | 89.45 | P:carbohydrate transport |
| Efet.01.658410.g2023.t1 | sugar ABC transporter ATP-binding | 1500 | 0 | 83.2 | F:ATPase activity; F:ATP binding; P:carbohydrate transport |
| Efet.01.658410.g2024.t1 | ABC transporter permease | 903 | 1.32E-161 | 95.4 | C:integral component of membrane; P:transport; C:plasma membrane; F:transporter activity |
| Efet.01.658411.g2025.t1 | cytochrome C | 552 | 3.15E-113 | 85.8 | F:iron ion binding; F:electron carrier activity; C:integral component of membrane; F:heme binding |
| Efet.01.658411.g2026.t1 | DUF2946 domain-containing | 540 | 8.15E-128 | 83.55 |  |
| Efet.01.658411.g2027.t1 | ketosteroid isomerase | 450 | 6.65E-105 | 70.6 | F:isomerase activity |
| Efet.01.658411.g2028.t1 | 7-cyano-7-deazaguanine synthase | 714 | 6.67E-173 | 91.9 | F:ATP binding; F:zinc ion binding; P:queuosine biosynthetic process; F:ligase activity, forming carbon-nitrogen bonds |
| Efet.01.658412.g2029.t1 | taurine ABC transporter substrate-binding | 1908 | 0 | 92.9 | C:periplasmic space; P:taurine transport |
| Efet.01.658412.g2030.t1 | taurine ABC transporter permease | 825 | 1.48E-162 | 89.2 | C:integral component of membrane; P:transport; C:plasma membrane |
| Efet.01.658413.g2031.t1 | type I restriction-modification system subunit M | 1581 | 0 | 91.05 | F:DNA binding; P:nucleic acid phosphodiester bond hydrolysis; F:N-methyltransferase activity; F:endonuclease activity; P:DNA restriction-modification system; P:DNA methylation on adenine; F:site-specific DNA-methyltransferase (adenine-specific) activity |
| Efet.01.658413.g2032.t1 | DUF262 domain-containing | 354 | 3.19E-80 | 85.05 |  |
| Efet.01.658413.g2033.t1 | WYL domain-containing | 900 | 0 | 92.65 |  |
| Efet.01.658414.g2034.t1 | alpha beta hydrolase | 447 | 5.51E-84 | 85.1 | P:metabolic process; F:hydrolase activity |
| Efet.01.658414.g2035.t1 | autotransporter domain-containing esterase | 993 | 0 | 48.2 | F:hydrolase activity, acting on ester bonds |
| Efet.01.658414.g2036.t1 | MFS transporter | 1083 | 0 | 80.1 | P:transmembrane transport; C:integral component of membrane; F:transporter activity |
| Efet.01.658414.g2037.t1 | transcriptional regulator | 309 | 9.21E-35 | 82.95 | F:DNA binding |
| Efet.01.658414.g2038.t1 | glycine dehydrogenase (aminomethyl-transferring) | 2913 | 0 | 91.05 | P:glycine decarboxylation via glycine cleavage system; F:glycine dehydrogenase (decarboxylating) activity; P:oxidation-reduction process |
| Efet.01.658414.g2039.t1 | glycine cleavage system H | 375 | 9.23E-65 | 93.65 | P:glycine decarboxylation via glycine cleavage system; C:glycine cleavage complex |
| Efet.01.658414.g2040.t1 | glycine cleavage system T | 1038 | 0 | 91.7 | F:transaminase activity; F:aminomethyltransferase activity; P:glycine catabolic process; P:methylation |
| Efet.01.658414.g2041.t1 | C4-dicarboxylate ABC transporter permease | 1266 | 0 | 96.65 | C:integral component of membrane |
| Efet.01.658415.g2042.t1 | FAD-linked oxidase | 3195 | 0 | 90.55 | F:oxidoreductase activity, acting on CH-OH group of donors; F:flavin adenine dinucleotide binding; P:oxidation-reduction process; F:iron-sulfur cluster binding |
| Efet.01.658415.g2043.t1 | ABC transporter substrate-binding | 912 | 7.96E-161 | 81.9 |  |
| Efet.01.658415.g2044.t1 | amino acid ABC transporter permease ATP-binding | 1539 | 0 | 80.9 | F:ATPase activity; F:ATP binding; C:integral component of membrane; P:transport; C:plasma membrane; F:transporter activity |
| Efet.01.658415.g2045.t1 | DUF1989 domain-containing | 1464 | 0 | 93.15 |  |
| Efet.01.658415.g2046.t1 | DUF971 domain-containing | 480 | 9.49E-97 | 90.7 |  |
| Efet.01.658415.g2047.t1 | 1-acyl-sn-glycerol-3-phosphate acyltransferase | 600 | 2.17E-80 | 75.75 | F:transferase activity, transferring acyl groups; P:metabolic process; C:integral component of membrane |
| Efet.01.658415.g2048.t1 | acyl carrier | 360 | 3.45E-18 | 84.8 | C:cytoplasm; P:fatty acid biosynthetic process; F:ACP phosphopantetheine attachment site binding involved in fatty acid biosynthetic process |
| Efet.01.658415.g2049.t1 | membrane | 444 | 8.94E-50 | 75.85 | C:integral component of membrane |
| Efet.01.658415.g2050.t1 | AMP-binding | 1560 | 0 | 59.3 | F:catalytic activity; P:metabolic process; F:ligase activity |
| Efet.01.658415.g2051.t1 | glycosyltransferase family 2 | 684 | 1.97E-103 | 76.95 | F:transferase activity, transferring acyl groups |
| Efet.01.658415.g2052.t1 | acyltransferase | 912 | 1.11E-113 | 69.05 | C:membrane; F:transferase activity |
| Efet.01.658415.g2053.t1 | acyl- thioesterase | 366 | 2.08E-66 | 83.9 |  |
| Efet.01.658415.g2054.t1 | lipo transmembrane | 1269 | 1.17E-88 | 61.45 | C:membrane; C:integral component of membrane |
| Efet.01.658416.g2055.t1 | 2-amino-4-hydroxy-6-hydroxymethyldihydropteridine diphosphokinase | 432 | 1.79E-66 | 83.45 | P:folic acid-containing compound biosynthetic process; F:2-amino-4-hydroxy-6-hydroxymethyldihydropteridine diphosphokinase activity; P:phosphorylation; F:kinase activity |
| Efet.01.658416.g2056.t1 | poly(A) polymerase | 1554 | 0 | 95.35 | F:ATP binding; F:RNA binding; P:transcription, DNA-templated; F:polynucleotide adenylyltransferase activity; P:mRNA polyadenylation |
| Efet.01.658416.g2057.t1 | phosphoserine phosphatase | 705 | 1.67E-141 | 80.6 | F:phosphatase activity; P:dephosphorylation |
| Efet.01.658416.g2058.t1 | regulatory inactivator Hda | 546 | 7.05E-99 | 82.7 | P:negative regulation of DNA-dependent DNA replication initiation |
| Efet.01.658416.g2059.t1 | phosphoribosylformylglycinamidine cyclo-ligase | 1062 | 0 | 89.05 | F:ATP binding; C:cytoplasm; P:'de novo' IMP biosynthetic process; F:phosphoribosylformylglycinamidine cyclo-ligase activity |
| Efet.01.658416.g2060.t1 | permease | 1032 | 1.64E-150 | 87.35 | C:integral component of membrane |
| Efet.01.658416.g2061.t1 | polyphosphate kinase 2 | 930 | 0 | 94.8 | P:phosphorylation; F:polyphosphate kinase activity |
| Efet.01.658416.g2062.t1 | transglutaminase | 852 | 0 | 90.45 |  |
| Efet.01.658416.g2063.t1 | murein biosynthesis integral membrane | 1179 | 2.46E-152 | 91.2 | P:regulation of cell shape; P:peptidoglycan biosynthetic process; C:integral component of membrane; P:cell wall organization; P:transport; C:plasma membrane |
| Efet.01.658417.g2064.t1 | transketolase | 510 | 1.48E-100 | 75.05 | F:transketolase activity |
| Efet.01.658417.g2065.t1 | transketolase | 963 | 0 | 72.85 | F:transketolase activity; P:metabolic process |
| Efet.01.658417.g2066.t1 | glycerol dehydrogenase | 1092 | 0 | 66.85 | F:oxidoreductase activity; F:metal ion binding; P:oxidation-reduction process |
| Efet.01.658417.g2067.t1 | short-chain dehydrogenase | 327 | 1.75E-59 | 76.2 | F:oxidoreductase activity; P:oxidation-reduction process |
| Efet.01.658417.g2068.t1 | family transcriptional regulator | 612 | 5.43E-130 | 68.15 | F:DNA binding; P:transcription, DNA-templated; P:regulation of transcription, DNA-templated |
| Efet.01.658417.g2069.t1 | family transcriptional regulator | 339 | 6.58E-63 | 76.5 | F:DNA binding; P:transcription, DNA-templated; P:regulation of transcription, DNA-templated |
| Efet.01.658417.g2070.t1 | tripartite tricarboxylate transporter substrate binding | 369 | 2.59E-67 | 79.25 | C:outer membrane-bounded periplasmic space |
| Efet.01.658417.g2071.t1 | Appr-1-p processing | 1071 | 0 | 89.05 |  |
| Efet.01.658417.g2072.t1 | choline dehydrogenase | 1671 | 0 | 93.1 | F:oxidoreductase activity, acting on CH-OH group of donors; F:flavin adenine dinucleotide binding; P:oxidation-reduction process |
| Efet.01.658417.g2073.t1 | aldose epimerase | 1353 | 0 | 75.85 | P:carbohydrate metabolic process; F:carbohydrate binding; F:isomerase activity |
| Efet.01.658417.g2074.t1 | ABC transporter ATP-binding | 1557 | 1.16E-172 | 94.1 | F:ATP binding; C:integral component of membrane; P:monosaccharide transport; F:monosaccharide-transporting ATPase activity; C:plasma membrane |
| Efet.01.658417.g2075.t1 | ABC transporter substrate-binding | 981 | 0 | 85.65 | P:carbohydrate transport |
| Efet.01.658417.g2076.t1 | dehydratase | 957 | 0 | 90 | F:catalytic activity; F:metal ion binding; P:metabolic process |
| Efet.01.658417.g2077.t1 | family transcriptional regulator | 720 | 1.03E-146 | 82.4 | F:DNA binding; F:transcription factor activity, sequence-specific DNA binding; P:transcription, DNA-templated; P:regulation of transcription, DNA-templated |
| Efet.01.658417.g2078.t1 | gluconolaconase | 930 | 0 | 82.3 | F:gluconolactonase activity |
| Efet.01.658417.g2079.t1 | ABC transporter permease | 1125 | 0 | 88.35 | P:transport; C:ATP-binding cassette (ABC) transporter complex |
| Efet.01.658417.g2080.t1 | ABC transporter ATP-binding | 831 | 4.47E-179 | 93.9 | F:ATPase activity; F:ATP binding |
| Efet.01.658417.g2081.t1 | mammalian cell entry | 909 | 1.64E-123 | 81.75 | C:integral component of membrane |
| Efet.01.658417.g2082.t1 | cholesterol transport system auxiliary component | 615 | 4.91E-108 | 80.3 |  |
| Efet.01.658418.g2083.t1 | sulfatase | 1347 | 0 | 79.6 | F:sulfuric ester hydrolase activity; P:metabolic process |
| Efet.01.658418.g2084.t1 | hypothetical protein Veis_2243 | 486 | 5.64E-27 | 90 |  |
| Efet.01.658418.g2085.t1 | ABC transporter permease | 642 | 1.08E-125 | 93 | C:integral component of membrane; P:transport; C:plasma membrane |
| Efet.01.658418.g2086.t1 | ABC transporter ATP-binding | 1782 | 3.43E-162 | 87.25 | F:ATPase activity; F:ATP binding; P:glycine betaine transport; C:membrane |
| Efet.01.658418.g2087.t1 | glycine betaine ABC transporter substrate-binding | 906 | 0 | 88.9 | P:transport; F:transporter activity |
| Efet.01.658418.g2088.t1 | nitrilase | 663 | 5.29E-160 | 84.85 | F:hydrolase activity, acting on carbon-nitrogen (but not peptide) bonds; F:transferase activity, transferring acyl groups; P:glutamine metabolic process |
| Efet.01.658418.g2089.t1 | acyl- dehydrogenase | 702 | 3.87E-149 | 87.1 | F:acyl-CoA dehydrogenase activity; F:flavin adenine dinucleotide binding; P:oxidation-reduction process |
| Efet.01.658419.g2090.t1 | Endoribonuclease L-PSP | 804 | 7.86E-104 | 81.6 | F:iron ion binding; F:electron carrier activity; F:heme binding |
| Efet.01.658419.g2091.t1 | flavin monoamine oxidase family | 1533 | 0 | 83.25 | F:oxidoreductase activity; C:integral component of membrane; P:oxidation-reduction process |
| Efet.01.658419.g2092.t1 | NADP-dependent malic enzyme | 2337 | 0 | 93.35 | F:oxaloacetate decarboxylase activity; F:phosphate acetyltransferase activity; P:malate metabolic process; F:NAD binding; C:cytosol; F:malate dehydrogenase (decarboxylating) (NAD+) activity; F:manganese ion binding; P:oxidation-reduction process; F:malate dehydrogenase (decarboxylating) (NADP+) activity |
| Efet.01.658419.g2093.t1 | Uncharacterized conserved contains Zn finger domain | 1329 | 0 | 73.9 | F:zinc ion binding |
| Efet.01.658419.g2094.t1 | transcriptional regulator | 1095 | 0 | 80.95 | F:DNA binding; F:transcription factor activity, sequence-specific DNA binding; P:transcription, DNA-templated; P:regulation of transcription, DNA-templated |
| Efet.01.658419.g2095.t1 | sugar ABC transporter substrate-binding | 1929 | 0 | 84.7 | P:transport; F:transporter activity |
| Efet.01.658419.g2096.t1 | sugar ABC transporter permease | 798 | 7.37E-165 | 80.4 | C:integral component of membrane; P:transport; C:plasma membrane |
| Efet.01.658420.g2097.t1 | receptor-type tyrosine- phosphatase eta-like isoform X1 | 2364 | 1.93E-92 | 49.4 | F:phosphoprotein phosphatase activity; F:protein tyrosine phosphatase activity; C:membrane; P:protein dephosphorylation; C:integral component of membrane; F:phosphatase activity; P:dephosphorylation; P:peptidyl-tyrosine dephosphorylation; F:hydrolase activity |
| Efet.01.658421.g2098.t1 | FMRFamide receptor-like | 1425 | 3.63E-87 | 60.95 | F:G-protein coupled receptor activity; F:G-protein coupled peptide receptor activity; P:G-protein coupled receptor signaling pathway; C:membrane; C:integral component of membrane |
| Efet.01.658422.g2099.t1 | transcriptional regulator | 342 | 3.53E-76 | 89.35 | F:sequence-specific DNA binding |
| Efet.01.658422.g2100.t1 | family metallo-endopeptidase | 852 | 0 | 87.8 |  |
| Efet.01.658422.g2101.t1 | DUF262 domain-containing | 1833 | 0 | 94.2 |  |
| Efet.01.658422.g2102.t1 | OST-HTH LOTUS domain | 405 | 1.91E-41 | 86.9 |  |
| Efet.01.658423.g2103.t1 | nuclear transport factor 2 family | 426 | 2.05E-97 | 70.1 |  |
| Efet.01.658423.g2104.t1 | alpha beta hydrolase | 621 | 3.23E-143 | 77.95 | F:3-oxoadipate enol-lactonase activity |
| Efet.01.658423.g2105.t1 | ABC transporter ATP-binding | 636 | 4.52E-131 | 82.2 | F:ATPase activity; F:ATP binding |
| Efet.01.658423.g2106.t1 | ABC transporter ATP-binding | 567 | 7.82E-102 | 87.55 | F:ATP binding; P:sulfate transmembrane transport; F:ATPase-coupled sulfate transmembrane transporter activity |
| Efet.01.658423.g2107.t1 | branched-chain amino acid ABC transporter permease | 876 | 1.50E-125 | 84.5 | C:integral component of membrane; P:transport; C:plasma membrane; F:transporter activity |
| Efet.01.658423.g2108.t1 | branched-chain amino acid ABC transporter permease | 933 | 1.39E-177 | 87.75 | C:integral component of membrane; P:transport; C:plasma membrane; F:transporter activity |
| Efet.01.658423.g2109.t1 | ABC transporter substrate-binding | 1131 | 0 | 81 | P:amino acid transport |
| Efet.01.658423.g2110.t1 | family transcriptional regulator | 885 | 1.65E-157 | 75.05 | F:DNA binding; F:transcription factor activity, sequence-specific DNA binding; P:transcription, DNA-templated; P:regulation of transcription, DNA-templated |
| Efet.01.658423.g2111.t1 | oxidoreductase | 1023 | 0 | 70.45 | F:metal ion binding; F:electron carrier activity; P:oxidation-reduction process; F:monooxygenase activity; F:2 iron, 2 sulfur cluster binding; P:transport |
| Efet.01.658423.g2112.t1 | aromatic ring-hydroxylating dioxygenase subunit alpha | 801 | 0 | 83.9 | F:metal ion binding; P:oxidation-reduction process; P:demethylation; F:vanillate monooxygenase activity; F:2 iron, 2 sulfur cluster binding |
| Efet.01.658423.g2113.t1 | protocatechuate 3,4-dioxygenase | 258 | 2.07E-54 | 78.9 | F:dioxygenase activity; P:oxidation-reduction process |
| Efet.01.658423.g2114.t1 | aromatic ring-hydroxylating dioxygenase subunit alpha | 903 | 0 | 82.95 | F:metal ion binding; P:oxidation-reduction process; P:demethylation; F:vanillate monooxygenase activity; F:2 iron, 2 sulfur cluster binding |
| Efet.01.658423.g2115.t1 | oxidoreductase | 1038 | 0 | 70.1 | F:oxidoreductase activity; F:metal ion binding; F:electron carrier activity; P:oxidation-reduction process; F:2 iron, 2 sulfur cluster binding; P:transport |
| Efet.01.658423.g2116.t1 | family transcriptional regulator | 615 | 5.37E-131 | 74.7 | F:DNA binding; F:transcription factor activity, sequence-specific DNA binding; P:transcription, DNA-templated; P:regulation of transcription, DNA-templated |
| Efet.01.658424.g2117.t1 | family transcriptional regulator | 978 | 0 | 55.9 | F:DNA binding; F:transcription factor activity, sequence-specific DNA binding; P:transcription, DNA-templated; P:regulation of transcription, DNA-templated |
| Efet.01.658424.g2118.t1 | glycosyltransferase | 705 | 3.74E-152 | 90.65 | F:transferase activity |
| Efet.01.658424.g2119.t1 | RNA-binding | 285 | 3.30E-59 | 93.25 | F:RNA binding |
| Efet.01.658424.g2120.t1 | rRNA methyltransferase | 729 | 1.87E-175 | 87.15 | F:rRNA (uridine-2'-O-)-methyltransferase activity; C:cytoplasm; P:rRNA methylation |
| Efet.01.658424.g2121.t1 | ATP-dependent zinc metalloprotease | 726 | 1.50E-111 | 93.7 | F:ATPase activity; P:cell division; F:ATP binding; F:zinc ion binding; P:protein catabolic process; P:proteolysis; C:integral component of membrane; F:metalloendopeptidase activity; C:plasma membrane |
| Efet.01.658424.g2122.t1 | cell division | 1188 | 0 | 97.3 | F:ATPase activity; P:cell division; F:ATP binding; F:zinc ion binding; P:protein catabolic process; P:proteolysis; C:integral component of membrane; F:metalloendopeptidase activity; C:plasma membrane |
| Efet.01.658424.g2123.t1 | dihydropteroate synthase | 780 | 3.82E-162 | 88.4 | F:metal ion binding; P:folic acid biosynthetic process; P:tetrahydrofolate biosynthetic process; F:dihydropteroate synthase activity |
| Efet.01.658424.g2124.t1 | phosphoglucosamine mutase | 1383 | 0 | 91.9 | F:magnesium ion binding; P:carbohydrate metabolic process; F:phosphoglucosamine mutase activity |
| Efet.01.658424.g2125.t1 | histidinol dehydrogenase | 1347 | 0 | 75.85 | F:zinc ion binding; F:NAD binding; P:oxidation-reduction process; P:histidine biosynthetic process; F:histidinol dehydrogenase activity |
| Efet.01.658425.g2126.t1 | ABC transporter substrate-binding | 1125 | 0 | 78.3 | C:outer membrane-bounded periplasmic space |
| Efet.01.658425.g2127.t1 | transferase | 798 | 9.58E-90 | 69.7 | F:catalytic activity |
| Efet.01.658425.g2128.t1 | tRNA (uridine(34) cytosine(34) 5-carboxymethylaminomethyluridine(34)-2 -O)-methyltransferase | 507 | 1.24E-110 | 84.9 | F:RNA binding; C:cytoplasm; F:tRNA methyltransferase activity; F:S-adenosylmethionine-dependent methyltransferase activity; P:tRNA methylation |
| Efet.01.658425.g2129.t1 | dehydratase | 465 | 1.25E-110 | 95.7 |  |
| Efet.01.658426.g2130.t1 | xanthine dehydrogenase accessory | 582 | 4.47E-120 | 85.95 |  |
| Efet.01.658426.g2131.t1 | family transcriptional regulator | 747 | 4.38E-155 | 82.3 | F:DNA binding; F:transcription factor activity, sequence-specific DNA binding; P:transcription, DNA-templated; P:regulation of transcription, DNA-templated |
| Efet.01.658426.g2132.t1 | DEAD DEAH box helicase | 945 | 0 | 85.15 | F:DNA binding; F:ATP binding; F:helicase activity |
| Efet.01.658426.g2133.t1 | transcriptional regulator | 462 | 7.38E-103 | 92.4 | F:sequence-specific DNA binding |
| Efet.01.658426.g2134.t1 | cell filamentation Fic | 903 | 2.38E-44 | 85.8 | P:cell division |
| Efet.01.658426.g2135.t1 | DNA topoisomerase (ATP-hydrolyzing) subunit B | 2607 | 0 | 93 | F:magnesium ion binding; F:DNA binding; F:ATP binding; C:cytoplasm; P:DNA-dependent DNA replication; C:chromosome; F:DNA topoisomerase type II (ATP-hydrolyzing) activity; P:DNA topological change |
| Efet.01.658426.g2136.t1 | DNA polymerase III subunit beta | 1164 | 0 | 93.05 | C:DNA polymerase III complex; F:DNA binding; F:3'-5' exonuclease activity; P:nucleic acid phosphodiester bond hydrolysis; F:DNA-directed DNA polymerase activity; C:cytoplasm; P:DNA replication; P:DNA biosynthetic process |
| Efet.01.658426.g2137.t1 | chromosomal replication initiator | 1449 | 0 | 89.15 | F:DNA replication origin binding; F:ATP binding; P:DNA replication initiation; C:cytoplasm; P:regulation of DNA replication |
| Efet.01.658426.g2138.t1 | ribonuclease P component | 384 | 1.13E-81 | 75 | P:RNA phosphodiester bond hydrolysis, endonucleolytic; F:ribonuclease P activity; P:tRNA processing; F:tRNA binding |
| Efet.01.658427.g2139.t1 | Uncharacterized OB-fold contains Zn-ribbon domain | 288 | 2.78E-59 | 70.95 |  |
| Efet.01.658427.g2140.t1 | thiolase family | 1095 | 0 | 78.65 | F:transferase activity, transferring acyl groups other than amino-acyl groups; P:metabolic process |
| Efet.01.658427.g2141.t1 | crotonase | 807 | 2.14E-179 | 89.85 | P:metabolic process; F:enoyl-CoA hydratase activity |
| Efet.01.658427.g2142.t1 | short-chain dehydrogenase | 816 | 0 | 89.6 | F:oxidoreductase activity; P:oxidation-reduction process |
| Efet.01.658427.g2143.t1 | ATP-binding [Paraburkholderia graminis] | 1788 | 0 | 59.14 |  |
| Efet.01.658427.g2144.t1 | AAA+ ATPase superfamily | 4152 | 0 | 60 |  |
| Efet.01.658427.g2145.t1 | hypothetical protein | 906 | 0 | 61.45 |  |
| Efet.01.658427.g2146.t1 | SRPBCC family | 504 | 4.11E-98 | 64.9 | P:biological_process; F:molecular_function |
| Efet.01.658428.g2147.t1 | carboxymuconolactone decarboxylase | 828 | 0 | 72.75 | P:cellular oxidant detoxification; P:oxidation-reduction process; F:peroxiredoxin activity |
| Efet.01.658428.g2148.t1 | family transcriptional regulator | 1002 | 0 | 67.2 | F:DNA binding; F:transcription factor activity, sequence-specific DNA binding; P:transcription, DNA-templated; P:regulation of transcription, DNA-templated |
| Efet.01.658428.g2149.t1 | integrase | 1281 | 0 | 78.1 | F:DNA binding; P:DNA recombination; P:DNA integration |
| Efet.01.658428.g2150.t1 | ABC transporter permease | 1260 | 3.24E-172 | 80.95 | C:integral component of membrane; P:transport; C:plasma membrane; F:transporter activity |
| Efet.01.658428.g2151.t1 | sugar ABC transporter ATP-binding | 744 | 5.35E-174 | 93.6 | F:ATPase activity; F:ATP binding; P:carbohydrate transport |
| Efet.01.658428.g2152.t1 | sugar ABC transporter substrate-binding | 1071 | 0 | 89.55 | P:carbohydrate transport |
| Efet.01.658428.g2153.t1 | inositol 2-dehydrogenase | 1674 | 0 | 82.45 | F:inositol 2-dehydrogenase activity; P:oxidation-reduction process |
| Efet.01.658428.g2154.t1 | gfo Idh family oxidoreductase | 1086 | 0 | 84.55 | F:oxidoreductase activity; P:oxidation-reduction process |
| Efet.01.658428.g2155.t1 | maleylacetate reductase | 798 | 1.67E-168 | 86.35 | F:oxidoreductase activity; F:metal ion binding; P:oxidation-reduction process |
| Efet.01.658428.g2156.t1 | stress responsive | 309 | 5.95E-59 | 82.55 |  |
| Efet.01.658429.g2157.t1 | PF06634 family | 1647 | 0 | 92.35 | F:nucleic acid binding; F:methyltransferase activity; P:methylation |
| Efet.01.658429.g2158.t1 | DUF853 domain-containing | 1656 | 0 | 92.95 |  |
| Efet.01.658429.g2159.t1 | SIR2-like domain | 963 | 0 | 78 |  |
| Efet.01.658429.g2160.t1 | Uncharacterised [Mycobacterium abscessus abscessus] | 996 | 0 | 90.55 |  |
| Efet.01.658429.g2161.t1 | DUF499 domain-containing | 2109 | 0 | 90.1 | P:biological_process; C:cellular_component; F:molecular_function |
| Efet.01.658429.g2162.t1 | DNA-binding | 264 | 1.07E-57 | 80.2 | F:DNA binding |
| Efet.01.658429.g2163.t1 | DNA primase | 1971 | 0 | 84.5 | F:magnesium ion binding; F:DNA primase activity; F:DNA binding; F:zinc ion binding; C:primosome complex; P:DNA replication, synthesis of RNA primer |
| Efet.01.658429.g2164.t1 | alkaline phosphatase | 234 | 1.85E-21 | 84.75 |  |
| Efet.01.658429.g2165.t1 | NADPH:quinone oxidoreductase | 1026 | 0 | 93.55 | F:zinc ion binding; F:oxidoreductase activity; P:oxidation-reduction process |
| Efet.01.658429.g2166.t1 | 5 3 -nucleotidase | 384 | 5.74E-82 | 98.45 | F:nucleotide binding; C:cytoplasm; F:5'-nucleotidase activity; F:metal ion binding; P:dephosphorylation |
| Efet.01.658430.g2167.t1 | ferredoxin | 321 | 1.41E-52 | 64.25 | F:metal ion binding; F:electron carrier activity; F:2 iron, 2 sulfur cluster binding |
| Efet.01.658430.g2168.t1 | pyridine nucleotide-disulfide oxidoreductase | 1152 | 0 | 66.65 | C:cell; F:oxidoreductase activity; F:flavin adenine dinucleotide binding; P:cell redox homeostasis; P:oxidation-reduction process |
| Efet.01.658430.g2169.t1 | anthranilate 1,2-dioxygenase | 312 | 3.21E-69 | 66.1 | F:dioxygenase activity; P:cellular aromatic compound metabolic process; P:oxidation-reduction process |
| Efet.01.658430.g2170.t1 | Rieske (2Fe-2S) domain | 1320 | 0 | 73.35 | P:aromatic compound catabolic process; F:iron ion binding; F:oxidoreductase activity, acting on paired donors, with incorporation or reduction of molecular oxygen, NAD(P)H as one donor, and incorporation of two atoms of oxygen into one donor; P:oxidation-reduction process; F:2 iron, 2 sulfur cluster binding |
| Efet.01.658430.g2171.t1 | ABC transporter ATP-binding | 588 | 2.21E-135 | 83.8 | F:ATPase activity; F:ATP binding |
| Efet.01.658430.g2172.t1 | ABC transporter ATP-binding | 2517 | 5.46E-161 | 77.45 | F:ATPase activity; F:ATP binding; C:integral component of membrane; P:transport; C:plasma membrane; F:transporter activity |
| Efet.01.658430.g2173.t1 | ABC transporter substrate-binding | 1170 | 0 | 74.5 | P:amino acid transport |
| Efet.01.658430.g2174.t1 | maleylacetate reductase | 1062 | 0 | 77.5 | F:oxidoreductase activity; F:metal ion binding; P:oxidation-reduction process |
| Efet.01.658430.g2175.t1 | hydroxyquinol 1,2-dioxygenase | 873 | 0 | 81.4 | F:ferric iron binding; F:hydroxyquinol 1,2-dioxygenase activity; F:catechol 1,2-dioxygenase activity; P:oxidation-reduction process; F:protocatechuate 3,4-dioxygenase activity; P:catechol-containing compound metabolic process |
| Efet.01.658430.g2176.t1 | family transcriptional regulator | 1017 | 0 | 76.85 | F:DNA binding; F:transcription factor activity, sequence-specific DNA binding; P:transcription, DNA-templated; P:regulation of transcription, DNA-templated |
| Efet.01.658430.g2177.t1 | transferase | 2025 | 0 | 87.1 | F:DNA binding; F:transcription factor activity, sequence-specific DNA binding; P:transcription, DNA-templated; F:formyl-CoA transferase activity; P:regulation of transcription, DNA-templated |
| Efet.01.658430.g2178.t1 | enoyl- hydratase isomerase family | 795 | 0 | 85.15 | P:metabolic process; F:enoyl-CoA hydratase activity; F:isomerase activity |
| Efet.01.658430.g2179.t1 | acyl- dehydrogenase | 348 | 5.16E-74 | 77.9 | F:oxidoreductase activity, acting on the CH-CH group of donors; P:oxidation-reduction process |
| Efet.01.65862.g977.t1 | hypothetical protein HELRODRAFT_183289 | 1347 | 3.25E-10 | 41 | C:membrane; C:integral component of membrane |
| Efet.01.65892.g978.t1 | titin-like isoform X1 | 711 | 2.16E-23 | 62.7 | F:nucleotide binding; F:ATP binding; F:protein kinase activity; P:phosphorylation; F:transferase activity; P:protein phosphorylation; F:kinase activity |
| Efet.01.65897.g979.t1 | phosphatidylinositol 4-kinase beta-like | 627 | 6.66E-54 | 73.5 | P:phosphorylation; F:transferase activity; P:single-organism process |
| Efet.01.659.g64.t1 | Collagen alpha-5(VI) chain | 255 | 9.14E-09 | 56.14 |  |
| Efet.01.65909.g980.t1 | sex peptide receptor-like | 1089 | 5.60E-81 | 59.05 | F:G-protein coupled receptor activity; F:G-protein coupled peptide receptor activity; P:G-protein coupled receptor signaling pathway; C:membrane; C:integral component of membrane |
| Efet.01.65931.g982.t1 | Signal peptidase complex catalytic subunit SEC11A | 219 | 8.12E-28 | 86.75 | C:endoplasmic reticulum membrane; C:integral component of membrane; F:serine-type peptidase activity; P:signal peptide processing |
| Efet.01.66020.g984.t1 | neurofibromatosis type 1 | 654 | 5.69E-119 | 75.6 | P:phosphatidylinositol 3-kinase signaling; P:regulation of angiogenesis; P:visual learning; P:regulation of bone resorption; P:positive regulation of adenylate cyclase activity; P:positive regulation of GTPase activity; P:negative regulation of oligodendrocyte differentiation; P:spinal cord development; P:sympathetic nervous system development; C:dendrite; P:myelination in peripheral nervous system; P:wound healing; P:response to hypoxia; P:negative regulation of neuroblast proliferation; P:negative regulation of Ras protein signal transduction; P:pigmentation; P:regulation of synaptic plasticity; C:cytosol; C:axon; C:intrinsic component of the cytoplasmic side of the plasma membrane; P:artery morphogenesis; P:Ras protein signal transduction; P:cerebral cortex development; P:neurotransmitter secretion; C:nucleus; F:phosphatidylethanolamine binding; F:protein binding; P:regulation of cell-matrix adhesion; P:negative regulation of cell migration; P:neuron-neuron synaptic transmission; P:negative regulation of MAP kinase activity; P:acid secretion; F:GTPase activator activity; P:forebrain astrocyte development; P:negative regulation of transcription factor import into nucleus; P:forebrain morphogenesis; P:amino acid transport; P:positive regulation of neuron apoptotic process; P:MAPK cascade; P:heart development; P:negative regulation of fibroblast proliferation; P:liver development; P:osteoblast differentiation; F:phosphatidylcholine binding; P:adrenal gland development; P:smooth muscle tissue development; P:regulation of blood vessel endothelial cell migration; P:camera-type eye morphogenesis; P:metanephros development; P:negative regulation of endothelial cell proliferation; P:positive regulation of extrinsic apoptotic signaling pathway; P:collagen fibril organization; P:actin cytoskeleton organization |
| Efet.01.66022.g985.t1 | calsyntenin-1 | 366 | 2.70E-18 | 53.55 | F:calcium ion binding; C:membrane; C:integral component of membrane; P:homophilic cell adhesion via plasma membrane adhesion molecules |
| Efet.01.66033.g986.t1 | hypothetical protein CAPTEDRAFT_222036 | 597 | 6.69E-31 | 50.94 | C:membrane; C:integral component of membrane |
| Efet.01.66049.g987.t1 | hypothetical protein | 525 | 9.24E-08 | 53.25 | F:catalytic activity; C:integral component of membrane; F:sulfotransferase activity |
| Efet.01.6605.g516.t1 | dual specificity tyrosine-phosphorylation-regulated kinase 2 isoform X1 | 1518 | 0 | 79.55 | P:peptidyl-tyrosine phosphorylation; P:regulation of signal transduction by p53 class mediator; P:smoothened signaling pathway; P:intrinsic apoptotic signaling pathway in response to DNA damage by p53 class mediator; F:protein serine/threonine kinase activity; P:positive regulation of glycogen biosynthetic process; C:nucleoplasm; F:magnesium ion binding; F:ATP binding; F:protein tyrosine kinase activity; F:protein serine/threonine/tyrosine kinase activity; C:ubiquitin ligase complex; C:intracellular ribonucleoprotein complex; F:ubiquitin binding; C:cytoplasm; F:manganese ion binding; P:negative regulation of NFAT protein import into nucleus |
| Efet.01.66063.g988.t1 | cilia- and flagella-associated 44-like isoform X2 | 288 | 3.70E-11 | 61.5 |  |
| Efet.01.66074.g990.t1 | very long-chain specific acyl- mitochondrial | 399 | 3.09E-34 | 65.9 | F:acyl-CoA dehydrogenase activity; P:fatty acid catabolic process; F:coenzyme binding; P:oxidation-reduction process; F:anion binding |
| Efet.01.66092.g991.t1 | cytospin-A-like isoform X2 | 744 | 3.87E-11 | 52 |  |
| Efet.01.66092.g992.t1 | cytospin-A-like isoform X2 | 1008 | 5.43E-25 | 61.3 |  |
| Efet.01.66099.g993.t1 | sialin-like | 627 | 2.51E-18 | 63.4 | P:transmembrane transport; C:membrane; C:integral component of membrane |
| Efet.01.66143.g996.t1 | zinc finger Eos-like isoform X1 | 558 | 2.22E-14 | 75.3 | C:nucleus; P:regulation of lymphocyte differentiation; P:regulation of development, heterochronic; F:metal ion binding; P:transcription, DNA-templated; F:sequence-specific DNA binding; F:protein dimerization activity; F:RNA polymerase II transcription factor activity, sequence-specific DNA binding; P:regulation of transcription from RNA polymerase II promoter; P:regulation of B cell activation |
| Efet.01.66160.g997.t1 | cytosolic 5 -nucleotidase 3 isoform X2 | 204 | 6.39E-22 | 85.05 |  |
| Efet.01.6617.g517.t1 | transcription factor IIIB 50 kDa subunit | 1059 | 2.23E-32 | 47.85 | F:zinc ion binding; P:regulation of transcription, DNA-templated; P:DNA-templated transcription, initiation |
| Efet.01.66296.g1001.t1 | hormone-sensitive lipase-like | 1020 | 6.05E-27 | 67.65 | P:lipid metabolic process; F:hydrolase activity |
| Efet.01.66298.g1002.t1 | RING finger 141-like | 474 | 6.62E-37 | 53.5 | F:zinc ion binding |
| Efet.01.66307.g1003.t1 | oocyte zinc finger -like | 1104 | 6.74E-06 | 46 |  |
| Efet.01.66322.g1004.t1 | tyrosine- kinase SYK-like | 597 | 4.12E-54 | 60.95 | F:ion binding; F:protein kinase activity; P:phosphorylation |
| Efet.01.66351.g1005.t1 | HAUS augmin-like complex subunit 5 | 606 | 6.12E-27 | 52.89 | P:spindle assembly; C:HAUS complex |
| Efet.01.66352.g1006.t1 | Kyphoscoliosis peptidase | 276 | 1.17E-07 | 64.4 |  |
| Efet.01.6637.g518.t1 | titin-like isoform X4 | 2457 | 3.80E-128 | 53.35 | F:ATP binding; F:protein kinase activity; P:protein phosphorylation |
| Efet.01.66388.g1008.t1 | cell number regulator 10-like | 378 | 1.83E-30 | 66.55 | C:membrane |
| Efet.01.66407.g1009.t1 | PREDICTED: uncharacterized protein C7orf57 homolog | 252 | 9.89E-07 | 52 |  |
| Efet.01.66482.g1012.t1 | hypothetical protein CAPTEDRAFT_216691 | 2982 | 0 | 60 | C:membrane |
| Efet.01.66491.g1015.t1 | mushroom body large-type Kenyon cell-specific 1 isoform X4 | 1890 | 3.31E-51 | 63.2 | F:DNA binding |
| Efet.01.66506.g1016.t1 | voltage-dependent T-type calcium channel subunit alpha-1G-like isoform X11 | 231 | 1.82E-34 | 83.45 | P:calcium ion import; C:voltage-gated calcium channel complex; P:regulation of ion transmembrane transport; P:calcium ion transmembrane transport; F:low voltage-gated calcium channel activity |
| Efet.01.66507.g1017.t1 | pleckstrin homology domain-containing family H member 1 isoform X1 | 240 | 2.72E-15 | 84.4 | C:cytoskeleton |
| Efet.01.6662.g519.t1 | uncharacterized family 31 glucosidase KIAA1161-like | 1857 | 0 | 65.05 | P:metabolic process; F:hydrolase activity, acting on glycosyl bonds |
| Efet.01.66624.g1023.t1 | serine threonine- phosphatase 1 regulatory subunit 10 | 1380 | 1.31E-31 | 67.35 | C:nucleus; F:DNA binding; F:metal ion binding; P:transcription, DNA-templated |
| Efet.01.66657.g1025.t1 | A-kinase anchor 9-like isoform X14 | 3024 | 8.99E-56 | 55.4 |  |
| Efet.01.66676.g1027.t1 | ATP-binding cassette sub-family A member 3 | 267 | 1.52E-23 | 66.7 | F:ATP binding; P:lipid transport; F:ATPase activity, coupled to transmembrane movement of substances; P:transmembrane transport; C:intracellular membrane-bounded organelle; C:integral component of membrane; C:organelle part; C:alveolar lamellar body; C:plasma membrane |
| Efet.01.6675.g520.t1 | SZT2-like isoform X2 | 429 | 7.26E-38 | 64.1 | C:peroxisome |
| Efet.01.66761.g1031.t1 | leucine-rich repeat neuronal 1-like | 762 | 1.21E-26 | 50.8 | C:membrane; C:integral component of membrane |
| Efet.01.66808.g1032.t1 | FK506-binding 59-like isoform X1 | 645 | 7.26E-38 | 52.6 | P:protein folding; F:isomerase activity |
| Efet.01.66846.g1034.t1 | CREB-binding isoform X4 | 1485 | 1.62E-127 | 75.9 | F:histone acetyltransferase activity; F:zinc ion binding; F:transcription coactivator activity; P:regulation of transcription, DNA-templated; P:histone acetylation; C:histone acetyltransferase complex |
| Efet.01.66854.g1035.t1 | excitatory amino acid transporter 1-like | 570 | 3.53E-40 | 75.35 | P:transmembrane transport; P:nervous system development; P:response to chemical; P:cellular amino acid biosynthetic process; C:cell projection; P:response to stress; F:L-glutamate transmembrane transporter activity; P:glutamate metabolic process; P:response to abiotic stimulus; P:response to external stimulus; P:positive regulation of cellular process; C:neuron part; F:sodium:dicarboxylate symporter activity; C:integral component of membrane; P:L-glutamate transport; F:amino acid binding; P:neurological system process; C:cell periphery; P:amino acid import |
| Efet.01.66879.g1036.t1 | ADP-ribosylation factor 8A | 417 | 5.98E-87 | 93.95 | F:GTP binding; P:cell division; C:spindle midzone; C:endosome; C:extracellular exosome; P:small GTPase mediated signal transduction; P:chromosome segregation; P:mitotic nuclear division; C:midbody; C:lysosomal membrane |
| Efet.01.66895.g1038.t1 | ankyrin repeat domain | 1248 | 6.68E-103 | 45.35 | F:ubiquitin-protein transferase activity; F:actin binding; C:membrane; C:integral component of membrane; P:regulation of apoptotic process; F:ADP binding; P:protein ubiquitination |
| Efet.01.669.g65.t1 | phosphatase inhibitor 2 | 465 | 2.53E-22 | 62.9 | P:biological regulation |
| Efet.01.66923.g1039.t1 | Neurofilament heavy | 2181 | 1.54E-08 | 61.5 |  |
| Efet.01.66928.g1040.t1 | homolog isoform X1 | 576 | 8.44E-62 | 55.9 | P:cytoskeleton organization; C:cell part |
| Efet.01.66958.g1041.t1 | hypothetical protein CAPTEDRAFT_222265 | 807 | 1.72E-34 | 52.5 | F:calcium-activated potassium channel activity; P:potassium ion transport; P:potassium ion transmembrane transport; C:membrane; C:integral component of membrane |
| Efet.01.66965.g1042.t1 | G- coupled receptor 54-like | 276 | 6.85E-13 | 69.7 | F:G-protein coupled receptor activity; P:G-protein coupled receptor signaling pathway; C:integral component of membrane |
| Efet.01.6697.g522.t1 | coatomer subunit beta | 936 | 1.75E-124 | 57.2 | C:membrane coat; P:vesicle-mediated transport; F:structural molecule activity; P:intracellular protein transport |
| Efet.01.66985.g1043.t1 | one cut domain family member 2-like isoform X1 | 318 | 3.23E-13 | 88.55 | C:nucleus; F:DNA binding; P:transcription, DNA-templated; P:regulation of transcription, DNA-templated |
| Efet.01.67001.g1044.t1 | dual oxidase maturation factor 1-like | 276 | 5.61E-16 | 66.4 | C:membrane part |
| Efet.01.67025.g1045.t1 | U3 small nucleolar RNA-associated 4 homolog | 471 | 1.13E-28 | 50.3 |  |
| Efet.01.67030.g1046.t1 | polyubiquitin 8-like | 651 | 4.33E-44 | 54.35 | F:ubiquitin-protein transferase activity; F:ubiquitin protein ligase activity; F:ubiquitin conjugating enzyme binding; C:ubiquitin ligase complex; C:cytoplasm; P:protein polyubiquitination; P:positive regulation of proteasomal ubiquitin-dependent protein catabolic process; P:protein ubiquitination involved in ubiquitin-dependent protein catabolic process; P:protein ubiquitination |
| Efet.01.67064.g1048.t1 | nuclear pore complex Nup133-like | 312 | 8.43E-17 | 62.65 | P:nucleocytoplasmic transport; P:chromosome organization; C:protein complex; C:intracellular organelle part; P:cellular protein localization; P:organic substance transport |
| Efet.01.6707.g523.t1 | Mastermind 1 | 2406 | 6.76E-11 | 43.8 |  |
| Efet.01.67085.g1049.t1 | Inhibitor of growth 4 | 987 | 1.25E-13 | 50.9 | P:proteolysis; F:cysteine-type peptidase activity |
| Efet.01.67091.g1050.t1 | neuropeptide Y receptor-like | 513 | 1.36E-35 | 79.55 | F:rhodopsin kinase activity; P:neuropeptide signaling pathway; C:integral component of membrane; P:protein phosphorylation; P:tachykinin receptor signaling pathway; C:plasma membrane; F:tachykinin receptor activity; F:neuropeptide Y receptor activity |
| Efet.01.67129.g1051.t1 | E3 ubiquitin- ligase TRIM71-like | 1221 | 1.90E-32 | 50.55 |  |
| Efet.01.67186.g1056.t1 | Inositol phosphoceramide mannosyltransferase 3 | 726 | 1.17E-16 | 61.7 |  |
| Efet.01.67197.g1060.t1 | CD109 antigen | 699 | 9.38E-109 | 55.25 | C:extracellular space; F:endopeptidase inhibitor activity; P:negative regulation of endopeptidase activity |
| Efet.01.67214.g1061.t1 | AF282882_1plasminogen activator kringle | 231 | 2.40E-15 | 56.05 | P:embryo development; P:regulation of response to stimulus; P:regulation of biological quality; P:cell differentiation; P:cell surface receptor signaling pathway; P:anatomical structure morphogenesis; P:animal organ development; F:hydrolase activity |
| Efet.01.67252.g1063.t1 | Ubiquitin conjugation factor E4 A | 438 | 6.19E-44 | 73.05 | F:ubiquitin-protein transferase activity; P:cellular protein metabolic process |
| Efet.01.67327.g1065.t1 | methyltransferase DDB_G0268948 | 534 | 1.58E-41 | 64.7 | P:metabolic process; F:methyltransferase activity; P:methylation |
| Efet.01.67341.g1067.t1 | asparagine--tRNA cytoplasmic | 543 | 4.13E-86 | 66.7 | F:organic cyclic compound binding; F:heterocyclic compound binding; F:ligase activity; P:translation |
| Efet.01.67347.g1068.t1 | 60 kDa neurofilament -like | 516 | 9.32E-53 | 79 | F:structural molecule activity; C:intermediate filament |
| Efet.01.67351.g1069.t1 | POU class transcription factor 4-like | 1530 | 1.16E-46 | 85.5 | C:nucleus; F:transcription factor activity, sequence-specific DNA binding; P:transcription, DNA-templated; F:sequence-specific DNA binding; P:brain development; P:regulation of transcription from RNA polymerase II promoter |
| Efet.01.67354.g1070.t1 | serine threonine- kinase endoribonuclease IRE1-like | 273 | 2.18E-20 | 56.8 | P:cellular metabolic process |
| Efet.01.67373.g1071.t1 | Dual specificity | 552 | 5.51E-59 | 59.95 | F:phosphoprotein phosphatase activity; P:protein dephosphorylation |
| Efet.01.67405.g1073.t1 | 2-hydroxyacylsphingosine 1-beta-galactosyltransferase | 1197 | 4.01E-59 | 49.55 | F:transferase activity; P:cellular process |
| Efet.01.67419.g1074.t1 | PREDICTED: uncharacterized protein LOC105438107 | 714 | 3.66E-09 | 44 |  |
| Efet.01.6742.g524.t1 | polycystic kidney disease 1-like 2 | 435 | 2.54E-13 | 60.8 | P:kidney development; F:calcium ion binding; C:membrane; C:integral component of membrane; F:carbohydrate binding |
| Efet.01.6742.g525.t1 | polycystic kidney disease 1-like 2 | 270 | 3.60E-12 | 67.25 | P:detection of mechanical stimulus; F:calcium channel activity; C:membrane |
| Efet.01.67423.g1075.t1 | dmX 2 isoform X2 | 1431 | 6.02E-111 | 60.3 |  |
| Efet.01.67430.g1076.t1 | FMRFamide receptor-like | 1125 | 1.15E-70 | 53.05 | F:signal transducer activity |
| Efet.01.67444.g1077.t1 | arginine N-methyltransferase 1 isoform X4 | 720 | 2.38E-133 | 87.15 | F:protein-arginine omega-N asymmetric methyltransferase activity; P:histone arginine methylation; F:protein homodimerization activity; F:protein heterodimerization activity; P:peptidyl-arginine methylation, to asymmetrical-dimethyl arginine; F:protein-arginine omega-N monomethyltransferase activity; C:plasma membrane; F:histone-arginine N-methyltransferase activity |
| Efet.01.67458.g1078.t1 | NF-kappa-B inhibitor alpha-like | 1041 | 1.65E-26 | 50 |  |
| Efet.01.67467.g1079.t1 | discoidin domain-containing receptor 2-like isoform X1 | 525 | 3.95E-67 | 73.25 | F:transmembrane receptor protein tyrosine kinase activity; C:integral component of membrane; P:transmembrane receptor protein tyrosine kinase signaling pathway; P:protein phosphorylation |
| Efet.01.67551.g1082.t1 | glutamate receptor NMDA 3A-like | 564 | 6.51E-35 | 60.7 | C:postsynaptic membrane; P:ion transmembrane transport; C:integral component of membrane; C:cell junction; P:ionotropic glutamate receptor signaling pathway; F:ionotropic glutamate receptor activity; F:extracellular-glutamate-gated ion channel activity |
| Efet.01.67570.g1083.t1 | hypothetical protein OCBIM_22017953mg | 858 | 1.66E-68 | 65 | P:signal transduction |
| Efet.01.67602.g1084.t1 | tRNA pseudouridine synthase 2 | 276 | 9.23E-12 | 68.65 | P:RNA metabolic process |
| Efet.01.67665.g1086.t1 | RNA-binding 12 | 2913 | 5.31E-34 | 85.65 | F:nucleotide binding; F:nucleic acid binding |
| Efet.01.67677.g1087.t1 | glutathione S-transferase theta-1-like | 309 | 1.23E-15 | 62.4 | F:transferase activity |
| Efet.01.67685.g1088.t1 | TCDD-inducible poly [ADP-ribose] polymerase-like | 549 | 1.31E-34 | 63.55 | F:transferase activity, transferring glycosyl groups; F:metal ion binding; F:transferase activity; F:NAD+ ADP-ribosyltransferase activity |
| Efet.01.67688.g1089.t1 | solute carrier family 12 | 399 | 2.04E-64 | 80.9 | F:potassium:chloride symporter activity; P:chloride transmembrane transport; C:integral component of membrane |
| Efet.01.6772.g528.t1 | Mitogen-activated kinase kinase kinase | 432 | 1.07E-22 | 55.3 | P:regulation of macromolecule metabolic process; F:protein binding; P:positive regulation of cellular process; P:protein autophosphorylation; P:single-organism cellular process; F:protein serine/threonine kinase activity; P:regulation of primary metabolic process; P:regulation of cellular metabolic process |
| Efet.01.67734.g1091.t1 | serine threonine- kinase 32A-like | 294 | 7.06E-33 | 83.45 | F:ATP binding; F:protein serine/threonine kinase activity; P:peptidyl-serine phosphorylation; C:intracellular; P:intracellular signal transduction |
| Efet.01.67756.g1092.t1 | UPF0505 C16orf62- | 438 | 1.49E-60 | 82.75 | C:early endosome; F:protein binding; C:retromer complex; P:protein transport; C:integral component of membrane; P:Golgi to plasma membrane transport; F:protein transporter activity; P:retrograde transport, endosome to Golgi |
| Efet.01.67756.g1093.t1 | UPF0505 C16orf62 homolog | 474 | 8.23E-56 | 81.05 | F:ATP binding; C:membrane; P:Golgi to plasma membrane transport |
| Efet.01.67778.g1095.t1 | 39S ribosomal mitochondrial-like | 468 | 7.14E-33 | 61.6 | C:mitochondrion; C:ribosome |
| Efet.01.67780.g1097.t1 | microsomal glutathione S-transferase 3 | 312 | 5.32E-15 | 69.9 | C:membrane |
| Efet.01.67792.g1098.t1 | titin-like isoform X1 | 261 | 3.35E-20 | 56.1 |  |
| Efet.01.67810.g1100.t1 | degenerin deg-1-like | 528 | 3.45E-23 | 46.05 | P:sodium ion transport; P:ion transport; F:sodium channel activity; C:membrane; C:integral component of membrane; P:sodium ion transmembrane transport; P:transport |
| Efet.01.67832.g1102.t1 | mannan-binding lectin serine protease 1 isoform X2 | 264 | 1.34E-07 | 60.8 | F:hydrolase activity |
| Efet.01.67862.g1103.t1 | sodium channel alpha-subunit SCAP1 | 549 | 6.28E-38 | 68.7 | P:regulation of postsynaptic membrane potential; P:regulation of ion transmembrane transport; C:voltage-gated sodium channel complex; P:sodium ion transmembrane transport; F:voltage-gated sodium channel activity |
| Efet.01.67865.g1104.t1 | MDS1 and EVI1 complex locus EVI1-A | 1947 | 5.27E-47 | 90.7 | F:nucleic acid binding; P:proximal/distal pattern formation involved in pronephric nephron development; P:multi-ciliated epithelial cell differentiation; F:metal ion binding; C:integral component of membrane; P:embryonic neurocranium morphogenesis; P:regulation of transcription, DNA-templated; P:pronephric proximal tubule development; P:pronephric distal tubule development; P:embryonic viscerocranium morphogenesis |
| Efet.01.67866.g1106.t1 | MDS1 and EVI1 complex locus EVI1-A | 1671 | 9.05E-48 | 87.9 | F:nucleic acid binding; F:metal ion binding; C:integral component of membrane; P:regulation of transcription, DNA-templated |
| Efet.01.67880.g1108.t1 | Wnt-7b-like isoform X3 | 309 | 3.96E-28 | 68.8 | C:extracellular region; P:single-organism process |
| Efet.01.67889.g1109.t1 | WD repeat-containing | 1353 | 1.15E-113 | 57.2 |  |
| Efet.01.67906.g1110.t1 | leucine-rich repeat and fibronectin type III domain-containing 1 | 1374 | 4.19E-148 | 57.75 | C:membrane |
| Efet.01.67920.g1112.t1 | zinc finger 239-like | 1131 | 3.16E-63 | 59.55 | F:nucleic acid binding; F:metal ion binding |
| Efet.01.67924.g1113.t1 | cyclin-G2-like | 765 | 8.26E-36 | 55.2 | C:membrane |
| Efet.01.67970.g1115.t1 | WD repeat-containing 48 | 288 | 2.88E-35 | 77.8 |  |
| Efet.01.68027.g1116.t1 | Transcriptional activator Pur-alpha | 363 | 1.03E-18 | 63.4 |  |
| Efet.01.6810.g529.t1 | helicase with zinc finger domain 2 | 1662 | 2.06E-18 | 45.75 | F:metal ion binding; F:ligand-dependent nuclear receptor transcription coactivator activity; F:helicase activity; P:regulation of nucleic acid-templated transcription |
| Efet.01.68132.g1117.t1 | exportin-6 isoform X2 | 813 | 1.91E-41 | 64.3 | C:intracellular part |
| Efet.01.68133.g1118.t1 | homeobox orthopedia-like | 444 | 1.80E-10 | 64 | F:DNA binding; C:nucleus; P:regulation of transcription, DNA-templated; F:sequence-specific DNA binding |
| Efet.01.68170.g1119.t1 | hypothetical protein CAPTEDRAFT_208848 | 1101 | 9.47E-118 | 67.2 | F:catalytic activity |
| Efet.01.68176.g1120.t1 | 26S proteasome non-ATPase regulatory subunit 6-like | 804 | 3.59E-149 | 79.6 | C:proteasome complex |
| Efet.01.68188.g1122.t1 | hypothetical protein CAPTEDRAFT_224542 | 294 | 5.42E-17 | 56.94 | C:membrane; C:integral component of membrane |
| Efet.01.68203.g1123.t1 | disks large homolog 5 isoform X1 | 2901 | 3.64E-59 | 52.6 |  |
| Efet.01.68257.g1124.t1 | eukaryotic translation initiation factor 3 subunit B | 378 | 1.22E-55 | 67.9 | F:nucleotide binding; F:translation initiation factor activity; C:extracellular exosome; C:eukaryotic translation initiation factor 3 complex, eIF3m; C:translation preinitiation complex; F:translation initiation factor binding; P:translational initiation; P:regulation of translational initiation; C:nucleoplasm |
| Efet.01.68319.g1125.t1 | Hau-cytoplasmic intermediate filament | 294 | 7.02E-26 | 87.15 | F:structural molecule activity; C:intermediate filament |
| Efet.01.68336.g1126.t1 | twist-related 2 | 774 | 1.85E-44 | 74.5 | F:protein dimerization activity |
| Efet.01.68360.g1127.t1 | centromere kinetochore zw10 homolog | 1434 | 5.26E-60 | 46.55 | P:cellular component organization; C:intracellular organelle part |
| Efet.01.6840.g530.t1 | otopetrin-2-like isoform X1 | 282 | 9.45E-13 | 71.75 | C:integral component of membrane |
| Efet.01.68406.g1130.t1 | ATP-binding cassette sub-family F member 2-like | 606 | 2.59E-105 | 82.65 | F:ATPase activity; F:ATP binding |
| Efet.01.68409.g1131.t1 | type-1 angiotensin II receptor-like isoform X1 | 489 | 1.68E-27 | 57.6 | P:signal transduction; F:signal transducer activity |
| Efet.01.68412.g1132.t1 | neurogenic locus notch | 585 | 1.63E-41 | 58.3 | C:membrane; C:integral component of membrane |
| Efet.01.68476.g1134.t1 | receptor tyrosine- kinase erbB-2 | 417 | 2.10E-39 | 82.5 | P:positive regulation of MAP kinase activity; P:peptidyl-tyrosine phosphorylation; F:growth factor binding; C:myelin sheath; P:positive regulation of translation; P:positive regulation of protein targeting to membrane; P:motor neuron axon guidance; F:protein C-terminus binding; P:phosphatidylinositol 3-kinase signaling; P:negative regulation of immature T cell proliferation in thymus; P:protein autophosphorylation; P:myelination; P:positive regulation of cell adhesion; P:signal transduction by protein phosphorylation; P:positive regulation of transcription from RNA polymerase III promoter; P:positive regulation of GTPase activity; P:positive regulation of transcription from RNA polymerase I promoter; P:neuromuscular junction development; F:ATP binding; P:heart development; P:positive regulation of cell growth; P:wound healing; F:transmembrane receptor protein tyrosine kinase activity; F:protein heterodimerization activity; F:signal transducer, downstream of receptor, with protein tyrosine kinase activity; P:positive regulation of epithelial cell proliferation; P:regulation of microtubule-based process; P:oligodendrocyte differentiation; F:identical protein binding; F:protein phosphatase binding; P:transmembrane receptor protein tyrosine kinase signaling pathway; P:peripheral nervous system development; C:receptor complex; C:nucleus; P:regulation of ERK1 and ERK2 cascade; F:RNA polymerase I core binding; P:cellular response to growth factor stimulus; C:integral component of membrane; C:endosome membrane; C:basolateral plasma membrane; C:apical plasma membrane |
| Efet.01.68482.g1135.t1 | zinc finger BED domain-containing 1-like | 2139 | 6.32E-54 | 45.25 | F:nucleic acid binding; F:protein dimerization activity |
| Efet.01.68528.g1136.t1 | transcription factor SOX-14 | 336 | 1.77E-28 | 80.4 | F:DNA binding; P:entrainment of circadian clock; F:protein heterodimerization activity; P:transcription, DNA-templated; P:regulation of transcription, DNA-templated; F:chromatin binding; P:visual perception; C:nuclear transcription factor complex; P:regulation of neuron migration; F:RNA polymerase II transcription factor activity, sequence-specific DNA binding |
| Efet.01.68551.g1139.t1 | hypothetical protein CAPTEDRAFT_113983 | 315 | 3.44E-06 | 80 | P:transmembrane transport; C:membrane; C:integral component of membrane |
| Efet.01.68600.g1140.t1 | chromodomain-helicase-DNA-binding 8-like isoform X1 | 606 | 1.48E-62 | 74.3 | C:nucleus; F:ATP binding; F:hydrolase activity, acting on acid anhydrides |
| Efet.01.68600.g1141.t1 | chromodomain-helicase-DNA-binding 8-like isoform X3 | 705 | 4.97E-51 | 58.25 | F:ATP binding; C:nucleus; F:hydrolase activity, acting on acid anhydrides |
| Efet.01.68617.g1143.t1 | G- coupled receptor Mth2 | 2496 | 3.37E-46 | 40.55 | F:G-protein coupled receptor activity; F:transmembrane signaling receptor activity; P:G-protein coupled receptor signaling pathway; C:membrane; C:integral component of membrane; P:cell surface receptor signaling pathway |
| Efet.01.68655.g1145.t1 | histone-binding N1 N2-like isoform X1 | 888 | 1.81E-21 | 80.95 |  |
| Efet.01.68676.g1146.t1 | E3 ubiquitin- ligase TRAF7 isoform X2 | 324 | 3.69E-14 | 78.75 | F:ubiquitin-protein transferase activity; F:zinc ion binding; P:positive regulation of MAPK cascade; C:intracellular part; P:protein ubiquitination |
| Efet.01.68699.g1147.t1 | hypothetical protein HELRODRAFT_184008 | 1530 | 8.56E-07 | 75 | P:signal transduction |
| Efet.01.68699.g1148.t1 | afadin-like isoform X2 | 1242 | 6.75E-06 | 56 |  |
| Efet.01.68720.g1150.t1 | sodium channel type 4 subunit alpha B-like isoform X1 | 1092 | 5.62E-158 | 77.2 | P:regulation of postsynaptic membrane potential; F:calcium ion binding; P:regulation of ion transmembrane transport; C:voltage-gated sodium channel complex; P:sodium ion transmembrane transport; F:voltage-gated sodium channel activity |
| Efet.01.68739.g1151.t1 | myotubularin-related 13-like | 303 | 1.58E-24 | 70.75 | P:biological regulation |
| Efet.01.68748.g1152.t1 | FMRFamide receptor-like | 1218 | 4.36E-109 | 56.15 | F:signal transducer activity |
| Efet.01.6878.g533.t1 | hypothetical protein CAPTEDRAFT_216159 | 834 | 9.62E-71 | 53.4 | C:membrane |
| Efet.01.68807.g1157.t1 | hypothetical protein LOTGIDRAFT_154105 | 1302 | 3.42E-11 | 48.67 |  |
| Efet.01.68810.g1159.t1 | sal 3 | 2889 | 1.98E-27 | 80.8 | F:nucleic acid binding; F:metal ion binding |
| Efet.01.68854.g1160.t1 | uncharacterized protein LOC110044736 | 276 | 1.92E-06 | 55 |  |
| Efet.01.68866.g1161.t1 | muscarinic acetylcholine receptor M1-like | 1305 | 1.54E-107 | 73.3 | C:integral component of plasma membrane; F:G-protein coupled acetylcholine receptor activity; P:G-protein coupled acetylcholine receptor signaling pathway |
| Efet.01.68866.g1162.t1 | muscarinic acetylcholine receptor M1-like | 1074 | 8.42E-44 | 78.55 | C:integral component of plasma membrane; F:G-protein coupled acetylcholine receptor activity; P:G-protein coupled acetylcholine receptor signaling pathway |
| Efet.01.68869.g1163.t1 | selenocysteine-specific elongation factor selB homologue | 282 | 8.52E-15 | 52.6 |  |
| Efet.01.68872.g1164.t1 | hypothetical protein CAPTEDRAFT_227405 | 1338 | 9.90E-109 | 57.05 | C:membrane; C:integral component of membrane |
| Efet.01.68875.g1165.t1 | bestrophin-4-like isoform X1 | 231 | 2.74E-14 | 81.3 | C:integral component of membrane |
| Efet.01.6891.g534.t1 | hypothetical protein CAPTEDRAFT_201913 | 714 | 1.28E-14 | 55.75 | F:G-protein coupled receptor activity; F:G-protein coupled peptide receptor activity; P:G-protein coupled receptor signaling pathway; C:membrane; C:integral component of membrane |
| Efet.01.68919.g1166.t1 | hypothetical protein BRAFLDRAFT_99629 | 339 | 9.72E-06 | 50 | F:histamine N-methyltransferase activity; P:methylation |
| Efet.01.68938.g1169.t1 | crumbs homolog 2 | 240 | 3.30E-15 | 62.75 | P:establishment or maintenance of epithelial cell apical/basal polarity; P:retina morphogenesis in camera-type eye; F:calcium ion binding; C:membrane; C:integral component of membrane; P:mesoderm formation |
| Efet.01.68955.g1170.t1 | Dopamine D2-like receptor | 465 | 3.31E-22 | 78.85 | C:integral component of plasma membrane; F:dopamine neurotransmitter receptor activity; P:dopamine receptor signaling pathway |
| Efet.01.68974.g1172.t1 | hypothetical protein HELRODRAFT_190915 | 1098 | 9.57E-06 | 77 | C:cytoskeleton |
| Efet.01.68974.g1173.t1 | plectin-like isoform X4 | 612 | 3.20E-73 | 65.8 | C:cytoskeleton |
| Efet.01.69.g7.t1 | poly [ADP-ribose] polymerase 1 | 318 | 7.63E-28 | 79.2 | C:nucleus; F:DNA binding; F:zinc ion binding; P:protein ADP-ribosylation; F:NAD binding; F:NAD+ ADP-ribosyltransferase activity |
| Efet.01.69046.g1174.t1 | tripartite motif-containing 3-like isoform X1 | 1479 | 1.51E-49 | 48.3 | F:zinc ion binding; C:intracellular |
| Efet.01.6905.g535.t1 | cysteine serine-rich nuclear 3 isoform X6 | 1227 | 3.27E-11 | 60.5 |  |
| Efet.01.69061.g1176.t1 | dynein heavy chain axonemal | 222 | 1.05E-30 | 94.7 | F:ATPase activity; F:ATP binding; F:microtubule motor activity; C:dynein complex; P:microtubule-based movement |
| Efet.01.69155.g1180.t1 | acid trehalase 1 | 435 | 2.98E-24 | 57.4 | P:carbohydrate metabolic process; F:catalytic activity |
| Efet.01.69186.g1181.t1 | prominin-1-A-like isoform X3 | 2430 | 7.41E-56 | 43.8 | C:membrane; C:integral component of membrane |
| Efet.01.69204.g1183.t1 | signal transducer and activator of transcription 5B-like | 753 | 6.49E-30 | 47.85 | F:DNA binding; C:nucleus; F:transcription factor activity, sequence-specific DNA binding; C:cytoplasm; P:transcription, DNA-templated; P:signal transduction; P:regulation of transcription, DNA-templated; F:signal transducer activity |
| Efet.01.69212.g1184.t1 | UPF0676 -like | 738 | 2.08E-60 | 61.65 | F:dioxygenase activity; F:iron ion binding; F:metal ion binding; F:oxidoreductase activity; P:oxidation-reduction process |
| Efet.01.69243.g1185.t1 | WD repeat and FYVE domain-containing 3 isoform X2 | 549 | 2.66E-44 | 74.8 | P:carbohydrate metabolic process; F:poly(ADP-ribose) glycohydrolase activity; F:metal ion binding |
| Efet.01.69249.g1186.t1 | paladin-like isoform X1 | 426 | 2.01E-24 | 59.45 | C:nucleus; F:protein tyrosine phosphatase activity; C:cytoplasm; F:phosphatase activity; P:dephosphorylation; P:peptidyl-tyrosine dephosphorylation |
| Efet.01.69250.g1187.t1 | RNA pseudouridylate synthase domain-containing 2 isoform X2 | 381 | 9.91E-08 | 72.5 | P:pseudouridine synthesis; F:RNA binding; F:pseudouridine synthase activity |
| Efet.01.69315.g1190.t1 | Polypeptide N-acetylgalactosaminyltransferase 1 | 1869 | 2.51E-174 | 62.95 | F:transferase activity, transferring glycosyl groups; C:membrane; C:integral component of membrane; P:protein glycosylation; C:Golgi apparatus; C:Golgi membrane; F:transferase activity; F:carbohydrate binding |
| Efet.01.69325.g1191.t1 | BTB POZ domain-containing 6 isoform X1 | 762 | 6.81E-28 | 47.2 |  |
| Efet.01.69344.g1196.t1 | galactocerebrosidase isoform X3 | 276 | 1.51E-18 | 64.55 | F:galactosylceramidase activity; P:carbohydrate metabolic process; C:mitochondrion; P:galactosylceramide catabolic process |
| Efet.01.69383.g1198.t1 | G- coupled receptor 98 | 1227 | 2.44E-32 | 46.4 | P:G-protein coupled receptor signaling pathway; P:cell communication; P:cell surface receptor signaling pathway; C:receptor complex; C:cell surface; P:maintenance of animal organ identity; P:sensory perception of sound; F:G-protein coupled receptor activity; F:transmembrane signaling receptor activity; C:extracellular exosome; C:cytoplasm; P:photoreceptor cell maintenance; F:calcium ion binding; C:membrane; C:integral component of membrane; P:sensory perception of light stimulus; P:neurological system process |
| Efet.01.69420.g1200.t1 | muscle M-line assembly unc-89-like | 420 | 3.26E-47 | 62.45 | F:ATP binding; F:protein kinase activity; P:protein phosphorylation |
| Efet.01.69455.g1202.t1 | 60 kDa neurofilament -like | 594 | 4.16E-39 | 68.9 | F:structural molecule activity; C:intermediate filament |
| Efet.01.69506.g1206.t1 | cyclic nucleotide-gated cation channel alpha-3-like | 1302 | 3.99E-107 | 62.95 | P:ion transport; P:transmembrane transport; C:membrane |
| Efet.01.69522.g1207.t1 | dynein regulatory complex 1-like | 435 | 4.30E-13 | 61.7 | P:heart development; P:cilium or flagellum-dependent cell motility; C:cell part; P:determination of left/right symmetry |
| Efet.01.69534.g1208.t1 | Ubiquitin carboxyl-terminal hydrolase 32 | 939 | 1.49E-12 | 62.35 | F:thiol-dependent ubiquitin-specific protease activity; P:protein deubiquitination; F:calcium ion binding; P:ubiquitin-dependent protein catabolic process |
| Efet.01.69568.g1210.t1 | hypothetical protein LOTGIDRAFT_232226, partial | 393 | 2.59E-06 | 62.5 |  |
| Efet.01.69586.g1211.t1 | zinc finger AEBP2-like isoform X2 | 1143 | 6.16E-36 | 58.95 | F:nucleic acid binding; F:metal ion binding |
| Efet.01.69600.g1213.t1 | rolling stone | 561 | 1.36E-47 | 59.75 | C:membrane; C:integral component of membrane |
| Efet.01.6961.g537.t1 | ETS isoform P1 | 1239 | 4.42E-36 | 59.4 | C:membrane |
| Efet.01.69614.g1214.t1 | forkhead box O-like | 591 | 6.85E-45 | 63.7 | F:DNA binding; C:nucleus; F:transcription factor activity, sequence-specific DNA binding; P:transcription, DNA-templated; P:regulation of transcription, DNA-templated; F:sequence-specific DNA binding |
| Efet.01.69624.g1215.t1 | neuronal acetylcholine receptor subunit alpha-7-like isoform X1 | 477 | 1.56E-30 | 67.95 | P:ion transport; C:membrane |
| Efet.01.69641.g1217.t1 | plasma membrane calcium-transporting ATPase 2 isoform X1 | 1185 | 0 | 85.7 | F:ATP binding; F:metal ion binding; C:integral component of membrane; P:calcium ion transmembrane transport; F:calcium-transporting ATPase activity |
| Efet.01.6967.g538.t1 | rootletin-like isoform X7 | 555 | 2.33E-35 | 76.65 |  |
| Efet.01.69675.g1219.t1 | homeodomain transcription factor 2 isoform X1 | 1671 | 8.56E-27 | 63.4 | F:DNA binding; C:membrane; C:integral component of membrane |
| Efet.01.69870.g1223.t1 | neuronal acetylcholine receptor subunit alpha-10-like isoform X1 | 312 | 1.29E-31 | 61.15 | F:ion channel activity; F:transmembrane receptor activity; P:ion transmembrane transport; C:integral component of membrane; P:chemical synaptic transmission; C:synapse; P:response to stimulus; C:plasma membrane part |
| Efet.01.69878.g1224.t1 | baculoviral IAP repeat-containing 7 | 543 | 1.72E-06 | 46 |  |
| Efet.01.69879.g1225.t1 | dynein beta flagellar outer arm-like | 399 | 2.88E-26 | 66.4 | F:microtubule motor activity; C:dynein complex; P:microtubule-based movement |
| Efet.01.69882.g1226.t1 | hypothetical protein HELRODRAFT_163326 | 345 | 3.75E-06 | 57 |  |
| Efet.01.69893.g1227.t1 | arginine--tRNA cytoplasmic | 792 | 3.49E-101 | 65.95 | F:nucleotide binding; P:tRNA aminoacylation for protein translation; P:ncRNA processing; F:aminoacyl-tRNA ligase activity |
| Efet.01.69896.g1229.t1 | microsomal triglyceride transfer large subunit-like | 1020 | 3.29E-59 | 49.95 | P:lipid transport; F:lipid transporter activity; C:membrane; C:integral component of membrane; P:response to stress |
| Efet.01.70021.g1233.t1 | 5-hydroxytryptamine receptor 1A-like | 513 | 1.89E-17 | 68.3 | P:neuropeptide signaling pathway; C:integral component of membrane; P:octopamine or tyramine signaling pathway; F:neuropeptide Y receptor activity |
| Efet.01.70021.g1234.t1 | 5-hydroxytryptamine receptor 1A-like | 1197 | 4.63E-26 | 75.95 | P:regulation of biological quality; P:neuropeptide signaling pathway; C:integral component of membrane; C:plasma membrane; F:neuropeptide Y receptor activity |
| Efet.01.70053.g1236.t1 | hypothetical protein CAPTEDRAFT_155089 | 201 | 4.98E-07 | 64 | F:corticotropin-releasing hormone binding |
| Efet.01.70054.g1238.t1 | hypothetical protein CAPTEDRAFT_219305 | 1437 | 1.45E-14 | 52.75 | C:membrane; C:integral component of membrane |
| Efet.01.70055.g1239.t1 | amiloride-sensitive sodium channel subunit gamma-like isoform X2 | 1353 | 1.47E-79 | 43.65 | P:transport |
| Efet.01.70094.g1240.t1 | thyrotropin-releasing hormone receptor-like | 1185 | 2.97E-80 | 57.5 | P:signal transduction |
| Efet.01.7015.g540.t1 | roundabout homolog 2-like | 636 | 2.99E-52 | 62.85 | P:peptidyl-tyrosine phosphorylation; F:transmembrane receptor protein tyrosine kinase activity; C:membrane; C:integral component of membrane; P:transmembrane receptor protein tyrosine kinase signaling pathway |
| Efet.01.70168.g1241.t1 | neuroglian-like isoform X3 | 345 | 2.00E-10 | 54.55 | C:membrane; C:integral component of membrane |
| Efet.01.70274.g1245.t1 | potassium voltage-gated channel Shab isoform X1 | 498 | 2.86E-54 | 86.25 | F:voltage-gated potassium channel activity; P:potassium ion transmembrane transport; C:voltage-gated potassium channel complex; P:regulation of ion transmembrane transport; P:protein homooligomerization |
| Efet.01.70274.g1246.t1 | potassium voltage-gated channel subfamily B member 2-like isoform X5 | 537 | 6.36E-38 | 83.45 | F:voltage-gated potassium channel activity; P:potassium ion transmembrane transport; C:voltage-gated potassium channel complex; P:regulation of ion transmembrane transport; P:protein homooligomerization |
| Efet.01.7028.g541.t1 | MPDZ_RAT ame: Full=Multiple PDZ domain ame: Full=Multi-PDZ domain 1 | 354 | 3.86E-06 | 64 | C:apicolateral plasma membrane; C:postsynaptic density; F:protein C-terminus binding; P:cell adhesion; P:myelination; C:synapse; C:neuron projection; C:cell projection; C:plasma membrane; C:dendrite; C:bicellular tight junction; F:protein binding; C:cytoplasm; C:postsynaptic membrane; C:Schmidt-Lanterman incisure; C:membrane; C:cytoplasmic vesicle; C:endomembrane system; C:cell junction; C:apical plasma membrane |
| Efet.01.70314.g1248.t1 | eukaryotic translation initiation factor 4 gamma 1-like isoform X1 | 606 | 4.90E-14 | 77.95 | F:RNA binding |
| Efet.01.70319.g1249.t1 | OTU domain-containing 7B-like | 549 | 1.69E-49 | 69.9 | F:thiol-dependent ubiquitin-specific protease activity; C:nucleus; P:protein K11-linked deubiquitination; P:protein deubiquitination involved in ubiquitin-dependent protein catabolic process; P:immune response; C:cytoplasm; F:K63-linked polyubiquitin binding; P:negative regulation of cellular process; P:protein K63-linked deubiquitination; P:protein K48-linked deubiquitination |
| Efet.01.70335.g1251.t1 | cilia- and flagella-associated 61-like | 495 | 3.60E-41 | 82.55 | F:oxidoreductase activity; C:integral component of membrane; P:oxidation-reduction process |
| Efet.01.7034.g542.t1 | hypothetical protein CAPTEDRAFT_222036 | 393 | 9.21E-14 | 52.5 | C:membrane; C:integral component of membrane |
| Efet.01.70346.g1252.t1 | ribosome-releasing factor mitochondrial | 1116 | 3.67E-109 | 67.4 | F:organic cyclic compound binding; F:heterocyclic compound binding; C:intracellular; P:cellular process |
| Efet.01.70360.g1253.t1 | Visual system homeobox | 279 | 4.43E-12 | 86.65 | C:nucleus; P:retinal bipolar neuron differentiation; P:transcription, DNA-templated; P:regulation of transcription, DNA-templated; F:sequence-specific DNA binding |
| Efet.01.70364.g1254.t1 | hypothetical protein HELRODRAFT_184150 | 4329 | 3.08E-13 | 41 |  |
| Efet.01.70373.g1256.t1 | ras-related Rab- | 459 | 1.35E-27 | 61.4 | F:GTP binding; P:small GTPase mediated signal transduction; C:intracellular |
| Efet.01.70431.g1258.t1 | transport Sec24A-like | 504 | 9.10E-63 | 72.6 | F:zinc ion binding; P:ER to Golgi vesicle-mediated transport; P:intracellular protein transport; C:COPII vesicle coat |
| Efet.01.70461.g1260.t1 | Acid trehalase 1 | 390 | 1.49E-14 | 59.9 | F:catalytic activity |
| Efet.01.70563.g1266.t1 | FMRFamide receptor-like | 1053 | 2.00E-46 | 53.45 | F:G-protein coupled receptor activity; F:G-protein coupled peptide receptor activity; P:G-protein coupled receptor signaling pathway; P:signal transduction; C:membrane; C:integral component of membrane; F:signal transducer activity |
| Efet.01.70586.g1267.t1 | disintegrin and metallo ase domain-containing 19 | 657 | 1.21E-63 | 74.9 | P:proteolysis; C:integral component of membrane; F:metalloendopeptidase activity |
| Efet.01.70627.g1271.t1 | ceramide kinase | 210 | 2.39E-08 | 56.5 | P:phosphorylation; F:kinase activity |
| Efet.01.70681.g1274.t1 | Ribonuclease kappa | 372 | 2.58E-22 | 61.15 | P:RNA metabolic process; C:membrane |
| Efet.01.7070.g543.t1 | active breakpoint cluster region-related -like isoform X2 | 231 | 6.83E-21 | 65.35 | F:molecular function regulator; P:regulation of cellular process |
| Efet.01.70752.g1277.t1 | major egg antigen-like isoform X3 | 1140 | 4.80E-113 | 53.2 | F:nucleic acid binding |
| Efet.01.70776.g1281.t1 | heart- and neural crest derivatives-expressed protein 2-like | 780 | 4.08E-34 | 79.8 | P:coronary artery morphogenesis; P:visceral serous pericardium development; P:palate development; F:RNA polymerase II regulatory region sequence-specific DNA binding; P:odontogenesis of dentin-containing tooth; P:cardiac neural crest cell migration involved in outflow tract morphogenesis; P:tongue development; P:apoptotic process involved in heart morphogenesis; P:peripheral nervous system neuron development; P:adult heart development; P:embryonic digit morphogenesis; P:negative regulation of osteoblast differentiation; P:thymus development; P:sympathetic nervous system development; C:transcription factor complex; F:transcriptional activator activity, RNA polymerase II core promoter proximal region sequence-specific binding; P:negative regulation of DNA binding; P:positive regulation of transcription regulatory region DNA binding; F:protein heterodimerization activity; P:angiogenesis; P:in utero embryonic development; P:suckling behavior; P:heart looping; P:positive regulation of cardiac muscle hypertrophy; P:regulation of tissue remodeling; F:activating transcription factor binding; F:E-box binding; F:protein homodimerization activity; P:transcription, DNA-templated; P:cartilage morphogenesis; F:AT DNA binding; P:negative regulation of sequence-specific DNA binding transcription factor activity; P:regulation of secondary heart field cardioblast proliferation; P:cell proliferation involved in outflow tract morphogenesis; P:positive regulation of p38MAPK cascade; P:negative regulation of gene expression; C:nuclear chromatin; F:transcription coactivator activity; P:cardiac right ventricle formation; P:positive regulation of ERK1 and ERK2 cascade; P:positive regulation of transcription from RNA polymerase II promoter involved in norepinephrine biosynthetic process; P:positive regulation of semaphorin-plexin signaling pathway involved in outflow tract morphogenesis; P:negative regulation of cardiac muscle cell apoptotic process; P:mesenchymal cell proliferation |
| Efet.01.70778.g1282.t1 | protocadherin-9 isoform X1 | 1809 | 4.01E-61 | 51.2 | F:calcium ion binding; C:membrane; C:integral component of membrane; P:cell adhesion; P:homophilic cell adhesion via plasma membrane adhesion molecules; C:plasma membrane |
| Efet.01.70789.g1283.t1 | outer dense fiber 3 | 363 | 3.48E-12 | 57.35 | C:outer dense fiber |
| Efet.01.70881.g1285.t1 | myomodulin neuropeptides-like | 630 | 6.36E-07 | 51.75 |  |
| Efet.01.70895.g1286.t1 | hypothetical protein CAPTEDRAFT_212399 | 1260 | 2.54E-122 | 52.65 | C:membrane; C:integral component of membrane |
| Efet.01.70917.g1288.t1 | zinc finger ZIC 4-like | 870 | 8.85E-08 | 81.12 | F:nucleic acid binding; F:metal ion binding |
| Efet.01.70921.g1290.t1 | inositol 1,4,5-trisphosphate receptor type 1-like | 339 | 2.53E-23 | 89.1 | P:inositol phosphate-mediated signaling; C:integral component of membrane; F:inositol 1,4,5-trisphosphate-sensitive calcium-release channel activity; P:calcium ion transmembrane transport; C:endoplasmic reticulum |
| Efet.01.70928.g1291.t1 | zinc finger 474-like | 693 | 1.56E-40 | 74.3 |  |
| Efet.01.70960.g1293.t1 | spectrin beta chain-like | 426 | 1.54E-61 | 70.05 | F:binding |
| Efet.01.70976.g1294.t1 | 5-hydroxytryptamine receptor 1A-like | 507 | 4.62E-32 | 72.5 | F:adrenergic receptor activity; C:integral component of membrane; F:neurotransmitter receptor activity; P:adrenergic receptor signaling pathway; C:plasma membrane |
| Efet.01.70976.g1295.t1 | 5-hydroxytryptamine receptor 1A-like | 1008 | 1.02E-84 | 59.85 | F:G-protein coupled receptor activity; P:G-protein coupled receptor signaling pathway; C:membrane |
| Efet.01.71012.g1297.t1 | acid-sensing ion channel 1-like | 459 | 2.24E-23 | 56.15 | P:transport |
| Efet.01.71048.g1298.t1 | lysine-specific demethylase 5B | 657 | 2.96E-20 | 58.5 | F:catalytic activity; P:metabolic process; P:single-organism process |
| Efet.01.71128.g1301.t1 | mitochondrial ATP synthase subunit alpha precursor | 225 | 3.60E-34 | 89.25 | C:proton-transporting ATP synthase complex, catalytic core F(1); F:ATP binding; P:ATP hydrolysis coupled proton transport; F:proton-transporting ATPase activity, rotational mechanism; P:ATP synthesis coupled proton transport; F:proton-transporting ATP synthase activity, rotational mechanism |
| Efet.01.71139.g1302.t1 | zinc finger ZFPM2 isoform X1 | 1863 | 3.45E-31 | 42.85 | F:nucleic acid binding; F:metal ion binding |
| Efet.01.71139.g1303.t1 | zinc finger ZFPM2 isoform X1 | 957 | 2.49E-23 | 61.85 | F:binding |
| Efet.01.71147.g1304.t1 | zinc finger 239-like | 1485 | 4.33E-62 | 58.8 | F:nucleic acid binding; F:metal ion binding |
| Efet.01.71147.g1305.t1 | zinc finger 271-like | 1266 | 9.23E-117 | 57.5 | F:nucleotide binding; F:nucleic acid binding; F:metal ion binding |
| Efet.01.71147.g1306.t1 | gastrula zinc finger - | 666 | 1.04E-64 | 60.3 | F:binding |
| Efet.01.71147.g1307.t1 | oocyte zinc finger 6-like | 1257 | 3.15E-111 | 57.4 | F:histone methyltransferase activity (H3-K4 specific); C:nucleus; P:histone lysine methylation; P:positive regulation of reciprocal meiotic recombination; P:spermatogenesis; F:recombination hotspot binding; F:sequence-specific DNA binding; P:positive regulation of transcription from RNA polymerase II promoter |
| Efet.01.71192.g1308.t1 | Melanin-concentrating hormone receptor 1 | 744 | 4.41E-25 | 53.88 | F:G-protein coupled receptor activity; F:G-protein coupled peptide receptor activity; P:G-protein coupled receptor signaling pathway; C:membrane; C:integral component of membrane |
| Efet.01.71192.g1309.t1 | CX3C chemokine receptor 1-like | 528 | 1.49E-25 | 56.05 | F:signal transducer activity |
| Efet.01.71236.g1311.t1 | FMRFamide receptor-like | 477 | 3.17E-34 | 62.25 | F:G-protein coupled receptor activity; F:G-protein coupled peptide receptor activity; P:G-protein coupled receptor signaling pathway; C:membrane; C:integral component of membrane |
| Efet.01.71244.g1312.t1 | phosphatidylinositol 3-kinase regulatory subunit alpha isoform X4 | 294 | 1.50E-20 | 77.7 | P:regulation of phosphatidylinositol 3-kinase signaling; F:protein C-terminus binding; P:phosphatidylinositol 3-kinase signaling; P:epidermal growth factor receptor signaling pathway; P:negative regulation of cell-cell adhesion; P:phosphatidylinositol-3-phosphate biosynthetic process; P:phosphatidylinositol phosphorylation; P:regulation of stress fiber assembly; F:insulin receptor binding; F:phosphatidylinositol-4,5-bisphosphate 3-kinase activity; P:glucose metabolic process; P:extrinsic apoptotic signaling pathway via death domain receptors; F:1-phosphatidylinositol-3-kinase regulator activity; F:insulin-like growth factor receptor binding; C:phosphatidylinositol 3-kinase complex, class IA; F:protein heterodimerization activity; C:cytosol; P:negative regulation of heart rate; P:positive regulation of tumor necrosis factor production; P:negative regulation of muscle cell apoptotic process; P:growth hormone receptor signaling pathway; F:insulin binding; F:protein domain specific binding; F:protein phosphatase binding; F:platelet-derived growth factor receptor binding; P:B cell differentiation; P:insulin-like growth factor receptor signaling pathway; P:regulation of phosphatidylinositol 3-kinase activity; P:negative regulation of blood pressure; P:intrinsic apoptotic signaling pathway in response to DNA damage; C:protein complex; P:positive regulation of endoplasmic reticulum unfolded protein response; P:regulation of insulin receptor signaling pathway; F:neurotrophin TRKA receptor binding; C:plasma membrane; C:nucleus; F:calmodulin binding; P:vascular endothelial growth factor receptor signaling pathway; C:cell-cell junction; F:ubiquitin protein ligase binding; P:NFAT protein import into nucleus; P:cellular glucose homeostasis; P:negative regulation of cell-matrix adhesion; C:perinuclear endoplasmic reticulum membrane; F:metal ion binding; F:insulin receptor substrate binding; F:phosphoprotein binding; F:estrogen receptor binding; P:response to endoplasmic reticulum stress; P:positive regulation of cell migration; F:phosphatidylinositol 3-kinase regulatory subunit binding; P:positive regulation of glucose import in response to insulin stimulus; P:insulin receptor signaling pathway; P:positive regulation of RNA splicing; P:Fc-epsilon receptor signaling pathway; P:response to cAMP; F:ErbB-3 class receptor binding; P:positive regulation of myoblast differentiation; P:negative regulation of proteolysis; P:Fc-gamma receptor signaling pathway involved in phagocytosis; P:positive regulation of transcription factor import into nucleus; P:protein phosphorylation; P:positive regulation of transcription from RNA polymerase II promoter; C:neuron projection; C:cis-Golgi network; P:negative regulation of osteoclast differentiation; P:negative regulation of smooth muscle cell proliferation; P:response to glucocorticoid; P:viral process; P:cellular response to UV; P:platelet activation; P:ERBB2 signaling pathway; P:T cell receptor signaling pathway; P:T cell costimulation; P:positive regulation of establishment of protein localization to plasma membrane; F:transmembrane receptor protein tyrosine kinase adaptor activity; F:transcription factor binding; P:positive regulation of protein phosphorylation; P:leukocyte migration; P:protein stabilization; F:1-phosphatidylinositol-3-kinase activity; F:ATPase binding |
| Efet.01.71248.g1313.t1 | beta-1,4-glucuronyltransferase 1 | 729 | 2.99E-83 | 65.8 | F:transferase activity, transferring glycosyl groups; P:single-organism cellular process; P:carbohydrate derivative biosynthetic process; P:primary metabolic process; P:single-organism metabolic process; P:cellular macromolecule biosynthetic process |
| Efet.01.71264.g1315.t1 | pyroglutamylated RFamide peptide receptor-like | 855 | 1.36E-45 | 50.6 | F:G-protein coupled receptor activity; P:neuropeptide signaling pathway; P:G-protein coupled receptor signaling pathway; P:signal transduction; C:membrane; C:integral component of membrane; F:signal transducer activity; F:neuropeptide Y receptor activity |
| Efet.01.71350.g1322.t1 | A-kinase anchor mitochondrial-like | 618 | 4.08E-59 | 60.35 | F:binding |
| Efet.01.71374.g1323.t1 | CWF19 2 isoform X1 | 282 | 1.62E-14 | 84.55 | F:catalytic activity |
| Efet.01.71383.g1325.t1 | ADP-ribosylation factor 13B | 270 | 2.34E-28 | 73.8 | F:GTP binding; P:embryo development; P:small GTPase mediated signal transduction; C:ciliary part; P:tube development; P:anatomical structure morphogenesis; P:brain development; P:epithelium development; P:regionalization; P:cell migration; C:intracellular; P:determination of left/right symmetry |
| Efet.01.71390.g1326.t1 | ER membrane complex subunit 1-like | 393 | 6.02E-30 | 77.7 | C:ER membrane protein complex |
| Efet.01.71449.g1329.t1 | histone-lysine N-methyltransferase 2D | 285 | 4.37E-25 | 79 | F:zinc ion binding; P:response to estrogen; C:histone methyltransferase complex; P:positive regulation of cell proliferation; F:transcription regulatory region DNA binding; P:positive regulation of intracellular estrogen receptor signaling pathway; F:methyltransferase activity; P:positive regulation of transcription from RNA polymerase II promoter; P:methylation |
| Efet.01.715.g67.t1 | thimet oligopeptidase-like | 996 | 2.00E-76 | 71.95 | C:mitochondrial intermembrane space; P:peptide metabolic process; F:metalloendopeptidase activity |
| Efet.01.71502.g1332.t1 | high affinity cationic amino acid transporter 1 | 1215 | 4.21E-29 | 68.5 | C:integral component of plasma membrane; P:L-ornithine transmembrane transport; F:L-ornithine transmembrane transporter activity; F:L-lysine transmembrane transporter activity; P:arginine transport; F:antiporter activity; F:arginine transmembrane transporter activity |
| Efet.01.71503.g1333.t1 | hypothetical protein CAPTEDRAFT_186105 | 636 | 1.13E-08 | 54 | C:membrane; C:integral component of membrane; P:smoothened signaling pathway |
| Efet.01.71527.g1334.t1 | glomulin | 387 | 7.64E-17 | 61.85 | F:nucleotide binding; F:ATP binding; P:ubiquinone biosynthetic process |
| Efet.01.71530.g1335.t1 | ubiquitin carboxyl-terminal hydrolase CYLD-like | 726 | 6.26E-31 | 48.05 | F:thiol-dependent ubiquitin-specific protease activity; P:protein deubiquitination; P:apoptotic process; C:cytosol; C:intracellular; P:protein K63-linked deubiquitination; F:thiol-dependent ubiquitinyl hydrolase activity; F:hydrolase activity |
| Efet.01.71538.g1336.t1 | Endoplasmic reticulum mannosyl-oligosaccharide 1,2-alpha-mannosidase | 390 | 2.70E-31 | 55.45 |  |
| Efet.01.71550.g1337.t1 | hypothetical protein CAPTEDRAFT_99765 | 441 | 1.45E-13 | 49 | F:ion channel activity; P:ion transport; C:postsynaptic membrane; P:ion transmembrane transport; P:cation transmembrane transport; C:membrane; C:integral component of membrane; F:extracellular ligand-gated ion channel activity; C:cell junction; C:synapse; P:transport; C:plasma membrane |
| Efet.01.71577.g1338.t1 | prohibitin-2 isoform X2 | 420 | 9.89E-53 | 61.8 | P:positive regulation of cellular process; C:mitochondrion; C:membrane; P:regulation of transcription, DNA-templated; P:negative regulation of cellular process; F:binding; C:intracellular organelle part; P:single-organism process |
| Efet.01.7158.g546.t1 | singed-like | 393 | 2.54E-23 | 62.75 | F:protein binding |
| Efet.01.71592.g1339.t1 | DNA damage-inducible transcript 4 | 489 | 2.82E-23 | 50.9 | P:negative regulation of signal transduction; C:cytoplasm |
| Efet.01.71608.g1340.t1 | WD repeat-containing 90 | 210 | 7.82E-27 | 84.9 |  |
| Efet.01.7161.g547.t1 | glutamate receptor 1-like | 255 | 8.21E-07 | 61 | C:membrane |
| Efet.01.71619.g1342.t1 | hypothetical protein CAPTEDRAFT_220857 | 258 | 4.70E-13 | 56.55 | C:membrane; C:integral component of membrane |
| Efet.01.7162.g548.t1 | Gypsy retrotransposon integrase 1 | 453 | 3.67E-16 | 51.75 | F:nucleic acid binding; P:DNA integration; F:N-acetyltransferase activity |
| Efet.01.71682.g1344.t1 | hypothetical protein HELRODRAFT_180534 | 462 | 2.84E-18 | 64 |  |
| Efet.01.71699.g1346.t1 | sorting nexin-14- | 609 | 1.09E-51 | 63.45 | P:autophagosome maturation; C:membrane; C:integral component of membrane; C:late endosome; F:phosphatidylinositol binding; F:phosphatidylinositol-3,5-bisphosphate binding; C:lysosome |
| Efet.01.71713.g1347.t1 | cyclic nucleotide-gated olfactory channel-like | 549 | 1.50E-53 | 66.8 | P:potassium ion transport; F:ion channel activity; P:ion transmembrane transport; C:membrane |
| Efet.01.71838.g1352.t1 | ANKUB1-like | 1875 | 4.40E-122 | 60.85 |  |
| Efet.01.71880.g1355.t1 | sodium channel type 4 subunit alpha B-like isoform X1 | 642 | 1.23E-40 | 72.4 | P:regulation of postsynaptic membrane potential; P:regulation of ion transmembrane transport; C:voltage-gated sodium channel complex; P:sodium ion transmembrane transport; F:voltage-gated sodium channel activity |
| Efet.01.71880.g1356.t1 | sodium channel para isoform X46 | 567 | 2.17E-52 | 77.9 | P:regulation of postsynaptic membrane potential; P:regulation of ion transmembrane transport; C:voltage-gated sodium channel complex; P:sodium ion transmembrane transport; F:voltage-gated sodium channel activity |
| Efet.01.72032.g1361.t1 | regulation of nuclear pre-mRNA domain-containing 1B isoform X1 | 753 | 5.93E-93 | 68.45 |  |
| Efet.01.72107.g1363.t1 | tellurium resistance - | 456 | 1.90E-27 | 61.75 | P:response to stress |
| Efet.01.72172.g1364.t1 | cilia- and flagella-associated 54-like isoform X2 | 1071 | 1.44E-58 | 58.85 |  |
| Efet.01.72175.g1366.t1 | Heat shock 70 kDa 12B | 312 | 8.19E-46 | 75.1 | C:membrane; C:integral component of membrane |
| Efet.01.72210.g1368.t1 | neurabin-1-like isoform X2 | 216 | 2.04E-18 | 79.6 |  |
| Efet.01.7224.g551.t1 | A-kinase anchor mitochondrial | 414 | 1.78E-28 | 66.85 | F:RNA binding; C:integral component of membrane |
| Efet.01.72330.g1374.t1 | paladin isoform X2 | 288 | 9.16E-25 | 59.95 | P:angiogenesis |
| Efet.01.72405.g1377.t1 | lysine and serine-rich -like isoform X1 | 270 | 1.92E-15 | 77.8 | F:nucleic acid binding; C:nucleus; F:zinc ion binding |
| Efet.01.72408.g1378.t1 | hypothetical protein CAPTEDRAFT_219620 | 726 | 4.36E-07 | 52 | P:plasma membrane organization; F:actin binding |
| Efet.01.72427.g1379.t1 | lactate | 225 | 1.76E-34 | 86 | P:carbohydrate metabolic process; F:L-lactate dehydrogenase activity; C:cytoplasm; P:oxidation-reduction process; P:carboxylic acid metabolic process |
| Efet.01.72460.g1381.t1 | FMRFamide receptor-like | 885 | 1.08E-29 | 59.05 | F:G-protein coupled receptor activity; F:G-protein coupled peptide receptor activity; P:G-protein coupled receptor signaling pathway; C:membrane; C:integral component of membrane |
| Efet.01.72500.g1382.t1 | Nuclear respiratory factor 1 | 1185 | 1.47E-07 | 51.33 |  |
| Efet.01.72525.g1387.t1 | myosin-IA isoform X2 | 612 | 5.07E-54 | 57.8 | F:nucleotide binding; C:myosin complex; F:ATP binding; F:actin binding; F:motor activity |
| Efet.01.72544.g1388.t1 | monocarboxylate transporter 14-like | 348 | 2.82E-25 | 66.95 | C:membrane |
| Efet.01.72547.g1389.t1 | casein kinase II subunit alpha-like | 387 | 2.84E-47 | 90.7 | F:ATP binding; F:protein serine/threonine kinase activity; P:protein phosphorylation |
| Efet.01.72569.g1392.t1 | collagen alpha-3(VI) chain-like isoform X2 | 3276 | 0 | 51.05 | F:calcium ion binding |
| Efet.01.72587.g1393.t1 | microtubule-actin cross-linking factor isoforms 1 2 3 5-like | 618 | 3.89E-44 | 56.8 | C:cytoskeleton |
| Efet.01.72592.g1394.t1 | Retrovirus-related Pol poly from transposon 412 | 291 | 2.43E-10 | 61 | F:binding |
| Efet.01.72593.g1396.t1 | mitotic spindle assembly checkpoint MAD1-like | 480 | 5.48E-19 | 63.9 | C:nuclear envelope; P:regulation of chromosome segregation; P:regulation of mitotic cell cycle phase transition; C:cytoplasm; P:negative regulation of T cell proliferation; C:spindle pole; P:mitotic nuclear division; C:kinetochore; P:thymus development |
| Efet.01.72610.g1397.t1 | insulin-like growth factor 1 receptor | 489 | 3.39E-67 | 68.65 | F:protein kinase activity; P:phosphorylation; P:primary metabolic process; P:organic substance metabolic process |
| Efet.01.72626.g1398.t1 | neuronal acetylcholine receptor subunit alpha-9-like isoform X1 | 567 | 2.73E-93 | 73.65 | C:postsynaptic membrane; P:cation transmembrane transport; C:integral component of membrane; C:cell junction; F:extracellular ligand-gated ion channel activity; P:chemical synaptic transmission; P:response to stimulus |
| Efet.01.72631.g1399.t1 | transmembrane 6 superfamily member 1 isoform X2 | 225 | 7.22E-22 | 79.35 | F:protein binding; C:integral component of membrane; C:lysosomal membrane |
| Efet.01.72648.g1400.t1 | beta-(1,2)-xylosyltransferase-like | 1365 | 4.74E-107 | 56.65 | F:transferase activity, transferring glycosyl groups; C:membrane; C:integral component of membrane; F:transferase activity |
| Efet.01.72661.g1401.t1 | Kyphoscoliosis peptidase | 264 | 1.86E-20 | 62.75 |  |
| Efet.01.72679.g1403.t1 | Hau-cytoplasmic intermediate filament | 330 | 1.37E-28 | 77.67 | F:structural molecule activity; C:intermediate filament |
| Efet.01.72696.g1404.t1 | Exocyst complex component 2 | 1302 | 1.72E-33 | 68.75 | P:Golgi to plasma membrane transport; C:exocyst |
| Efet.01.72738.g1407.t1 | piggyBac transposable element-derived 4-like | 894 | 4.10E-38 | 58.2 | C:membrane |
| Efet.01.72812.g1408.t1 | methyltransferase 24 | 273 | 4.62E-13 | 55.2 |  |
| Efet.01.72844.g1409.t1 | glutamyl aminopeptidase | 297 | 3.82E-38 | 55.9 | F:peptidase activity, acting on L-amino acid peptides; C:cell part; C:membrane part |
| Efet.01.72870.g1411.t1 | hypothetical protein HELRODRAFT_81234, partial | 606 | 1.31E-43 | 61.7 |  |
| Efet.01.72941.g1414.t1 | probable G- coupled receptor 52 | 735 | 4.73E-89 | 63.8 | F:G-protein coupled receptor activity; P:G-protein coupled receptor signaling pathway; C:integral component of membrane |
| Efet.01.72961.g1415.t1 | hypothetical protein HELRODRAFT_160205 | 222 | 1.38E-06 | 65 | C:membrane; C:integral component of membrane |
| Efet.01.72973.g1416.t1 | retinol retinaldehyde reductase | 621 | 1.57E-48 | 68.45 | F:oxidoreductase activity; C:membrane; C:integral component of membrane; P:oxidation-reduction process |
| Efet.01.72989.g1418.t1 | cysteine and glycine-rich 2 | 1002 | 5.15E-79 | 55.6 | F:metal ion binding |
| Efet.01.73041.g1420.t1 | PC-esterase domain-containing 1A-like | 420 | 9.58E-31 | 69.8 | F:hydrolase activity, acting on ester bonds |
| Efet.01.73083.g1423.t1 | spectrin beta non-erythrocytic 1 isoform X1 | 432 | 9.17E-32 | 64.8 | F:actin binding; F:phospholipid binding |
| Efet.01.73094.g1425.t1 | dual specificity phosphatase 2 | 1095 | 1.19E-67 | 63.55 | F:protein tyrosine phosphatase activity; P:peptidyl-tyrosine dephosphorylation; F:protein tyrosine/serine/threonine phosphatase activity |
| Efet.01.73094.g1427.t1 | 3 -5 exoribonuclease 1 | 462 | 2.93E-33 | 68.1 | F:nucleic acid binding |
| Efet.01.73138.g1429.t1 | coiled-coil domain-containing 113-like | 468 | 3.04E-30 | 65.05 | C:motile cilium |
| Efet.01.73139.g1430.t1 | G- coupled receptor Mth2-like | 888 | 8.78E-30 | 50.6 | F:G-protein coupled receptor activity; F:transmembrane signaling receptor activity; P:G-protein coupled receptor signaling pathway; C:membrane; C:integral component of membrane; P:cell surface receptor signaling pathway |
| Efet.01.73139.g1431.t1 | class B secretin-like G- coupled receptor | 1341 | 6.41E-51 | 41.9 | F:G-protein coupled receptor activity; F:zinc ion binding; F:transmembrane signaling receptor activity; P:immune response; P:G-protein coupled receptor signaling pathway; C:membrane; C:integral component of membrane; F:scavenger receptor activity; P:cell surface receptor signaling pathway; P:receptor-mediated endocytosis; F:polysaccharide binding; F:SUMO transferase activity |
| Efet.01.73184.g1432.t1 | NEDD8-conjugating enzyme Ubc12 | 372 | 2.21E-76 | 96.1 | F:ATP binding; F:ubiquitin protein ligase activity; C:extracellular exosome; F:ubiquitin protein ligase binding; P:transforming growth factor beta receptor signaling pathway; C:cytosol; P:NIK/NF-kappaB signaling; P:protein neddylation; F:ribosomal S6-glutamic acid ligase activity; F:NEDD8 transferase activity; P:positive regulation of neuron apoptotic process; P:protein ubiquitination |
| Efet.01.73198.g1433.t1 | cGMP-dependent kinase 1-like | 222 | 7.91E-12 | 71.35 | C:mediator complex; P:phosphorylation; F:kinase activity; F:RNA polymerase II transcription cofactor activity; P:regulation of transcription from RNA polymerase II promoter |
| Efet.01.73207.g1434.t1 | zinc transporter ZIP10-like | 969 | 2.40E-10 | 44.33 | P:transmembrane transport; C:membrane; C:integral component of membrane; F:metal ion transmembrane transporter activity; P:metal ion transport |
| Efet.01.73235.g1435.t1 | zinc finger | 939 | 1.21E-48 | 83.75 | C:nucleus; F:DNA binding; F:metal ion binding; P:transcription, DNA-templated; P:regulation of transcription, DNA-templated |
| Efet.01.73320.g1439.t1 | zinc finger FYVE domain-containing 16-like isoform X1 | 549 | 1.16E-58 | 78.1 | F:metal ion binding |
| Efet.01.7333.g554.t1 | potassium sodium hyperpolarization-activated cyclic nucleotide-gated channel 2 isoform X5 | 327 | 1.49E-13 | 85.4 | F:ion channel activity; P:ion transmembrane transport; C:integral component of membrane |
| Efet.01.73350.g1440.t1 | glypican-5 isoform X3 | 831 | 2.33E-56 | 56.7 | F:heparan sulfate proteoglycan binding; C:membrane; C:proteinaceous extracellular matrix; C:plasma membrane; C:anchored component of membrane |
| Efet.01.73369.g1442.t1 | PREDICTED: uncharacterized protein LOC108914561 | 405 | 4.92E-06 | 59 |  |
| Efet.01.73374.g1443.t1 | alpha-1,6-mannosyl-glyco 2-beta-N-acetylglucosaminyltransferase-like isoform X1 | 576 | 1.70E-26 | 72.65 | C:integral component of membrane; F:alpha-1,6-mannosylglycoprotein 2-beta-N-acetylglucosaminyltransferase activity; C:Golgi membrane; P:oligosaccharide biosynthetic process; P:protein N-linked glycosylation via asparagine; C:Golgi stack |
| Efet.01.73419.g1444.t1 | importin subunit alpha-7 | 315 | 1.38E-44 | 82.65 | P:NLS-bearing protein import into nucleus; C:cytosol; F:protein transporter activity; F:nuclear localization sequence binding; C:nuclear pore; C:nucleoplasm |
| Efet.01.73494.g1448.t1 | tubulin--tyrosine ligase 12 | 720 | 2.81E-66 | 63.8 | P:cellular protein modification process; F:ligase activity |
| Efet.01.73627.g1452.t1 | Solute carrier family 12 member 6 | 378 | 7.90E-17 | 83.15 | F:potassium:chloride symporter activity; P:chloride transmembrane transport; C:integral component of membrane |
| Efet.01.73632.g1453.t1 | spectrin alpha chain-like | 1221 | 3.28E-168 | 77.45 | F:calcium ion binding |
| Efet.01.73649.g1455.t1 | metal transporter CNNM4-like | 1131 | 4.40E-102 | 64.3 | C:membrane |
| Efet.01.73674.g1456.t1 | regulatory-associated of mTOR-like isoform X1 | 204 | 1.26E-08 | 65.6 | P:TOR signaling; C:TORC1 complex |
| Efet.01.73701.g1458.t1 | hypothetical protein Smp_164070 | 237 | 2.39E-12 | 72 |  |
| Efet.01.73716.g1460.t1 | dystrobrevin beta- | 492 | 1.12E-24 | 76.1 | F:metal ion binding |
| Efet.01.73780.g1461.t1 | histamine H1 receptor-like | 327 | 2.56E-38 | 84.35 | C:integral component of plasma membrane; P:inflammatory response; F:G-protein coupled acetylcholine receptor activity; P:G-protein coupled acetylcholine receptor signaling pathway; P:modulation of synaptic transmission; P:phospholipase C-activating G-protein coupled receptor signaling pathway; P:regulation of vasoconstriction |
| Efet.01.73780.g1462.t1 | histamine H1 receptor-like | 840 | 8.65E-26 | 59.05 | P:signal transduction; C:membrane; F:signal transducer activity |
| Efet.01.73780.g1463.t1 | Histamine H1 receptor | 993 | 6.14E-34 | 73.6 | C:integral component of plasma membrane; P:regulation of biological quality; P:system process; F:G-protein coupled acetylcholine receptor activity; P:G-protein coupled acetylcholine receptor signaling pathway |
| Efet.01.73790.g1464.t1 | sedoheptulokinase isoform X2 | 390 | 1.52E-46 | 71.95 | F:nucleotide binding; F:sedoheptulokinase activity; P:carbohydrate metabolic process; P:pentose-phosphate shunt, non-oxidative branch; P:cellular response to lipopolysaccharide; C:cytoplasm; P:regulation of macrophage activation; P:cellular response to interleukin-4; P:regulation of inflammatory response; P:phosphorylation; P:cellular response to interleukin-13 |
| Efet.01.73791.g1465.t1 | ras-responsive element-binding 1-like isoform X1 | 1179 | 3.03E-107 | 63.55 | C:nucleus; F:transcription factor activity, sequence-specific DNA binding; P:signal transduction; P:multicellular organism development; F:transcription regulatory region DNA binding; P:regulation of transcription, DNA-templated; P:transcription from RNA polymerase II promoter; F:sequence-specific DNA binding |
| Efet.01.73807.g1466.t1 | digestive cysteine ase 1-like | 300 | 4.88E-26 | 86.45 | C:extracellular space; C:integral component of membrane; F:cysteine-type endopeptidase activity; P:proteolysis involved in cellular protein catabolic process; C:lysosome |
| Efet.01.73812.g1467.t1 | 14 kDa phosphohistidine phosphatase | 552 | 6.60E-32 | 62.9 | P:regulation of cellular process; C:cytoplasm; P:protein dephosphorylation; F:phosphatase activity |
| Efet.01.73865.g1469.t1 | major egg antigen-like | 1110 | 3.95E-40 | 52.8 |  |
| Efet.01.73868.g1470.t1 | anaphase-promoting complex subunit 2 | 486 | 1.47E-33 | 62.55 | F:ubiquitin protein ligase binding; P:ubiquitin-dependent protein catabolic process |
| Efet.01.73868.g1471.t1 | anaphase-promoting complex subunit 2-like | 957 | 1.67E-144 | 78.1 | F:ubiquitin protein ligase binding; P:ubiquitin-dependent protein catabolic process |
| Efet.01.73869.g1472.t1 | allene oxide synthase-lipoxygenase -like | 1833 | 0 | 57.8 | F:oxidoreductase activity, acting on single donors with incorporation of molecular oxygen, incorporation of two atoms of oxygen; F:metal ion binding; F:catalase activity; P:lipid metabolic process; P:cellular oxidant detoxification; P:oxidation-reduction process; F:heme binding |
| Efet.01.7395.g558.t1 | pecanex 3 | 1926 | 4.43E-20 | 73.05 | C:membrane |
| Efet.01.73968.g1477.t1 | unconventional myosin-Va isoform X1 | 630 | 1.28E-57 | 62.4 | F:binding |
| Efet.01.73993.g1479.t1 | hypothetical protein HELRODRAFT_173396 | 342 | 1.07E-08 | 58 | F:G-protein coupled receptor activity; P:G-protein coupled receptor signaling pathway; C:membrane; C:integral component of membrane |
| Efet.01.73993.g1480.t1 | FMRFamide receptor-like | 951 | 6.31E-69 | 57.1 | F:G-protein coupled receptor activity; P:signal transduction; C:membrane |
| Efet.01.74069.g1485.t1 | BTB POZ domain-containing KCTD12 | 393 | 9.88E-66 | 85.55 | C:integral component of membrane; P:protein homooligomerization |
| Efet.01.74070.g1486.t1 | casein kinase I isoform delta-B-like isoform X4 | 957 | 2.45E-112 | 94.5 | F:ATP binding; F:protein serine/threonine kinase activity; P:protein phosphorylation |
| Efet.01.74074.g1487.t1 | E3 ubiquitin- ligase PDZRN3-like | 429 | 8.18E-44 | 71.2 |  |
| Efet.01.74074.g1488.t1 | E3 ubiquitin- ligase PDZRN3-like | 1008 | 3.97E-17 | 45.55 |  |
| Efet.01.74090.g1489.t1 | ATP-binding cassette sub-family B member mitochondrial-like | 426 | 7.23E-58 | 77.65 | F:efflux transmembrane transporter activity; C:endoplasmic reticulum; C:Golgi apparatus; P:brain development; F:xenobiotic-transporting ATPase activity; P:skin development; C:mitochondrial inner membrane; F:heme binding; C:integral component of mitochondrial outer membrane; P:cellular iron ion homeostasis; F:ATP binding; P:drug transmembrane transport; C:endosome; C:extracellular exosome; P:porphyrin-containing compound biosynthetic process; P:xenobiotic transport; F:heme-transporting ATPase activity; C:ATP-binding cassette (ABC) transporter complex; P:heme transport |
| Efet.01.74098.g1490.t1 | virilizer homolog | 648 | 1.87E-08 | 81.5 |  |
| Efet.01.74123.g1491.t1 | carbohydrate sulfotransferase 11 isoform X2 | 669 | 5.49E-32 | 52.8 | P:single-organism process |
| Efet.01.74132.g1492.t1 | hypothetical protein HELRODRAFT_190994 | 288 | 4.03E-12 | 64 | P:metabolic process; P:lipid metabolic process |
| Efet.01.74133.g1493.t1 | hypothetical protein CAPTEDRAFT_227505 | 714 | 3.90E-09 | 55 | C:membrane; C:integral component of membrane |
| Efet.01.74154.g1494.t1 | sodium-potassium ATPase alpha- | 318 | 1.28E-32 | 72.2 | F:nucleotide binding; P:transmembrane transport; P:monovalent inorganic cation transport; C:membrane; F:ion binding; P:metal ion transport; F:hydrolase activity |
| Efet.01.74169.g1495.t1 | transmembrane 45B-like | 222 | 5.22E-20 | 65.67 | C:membrane |
| Efet.01.74171.g1496.t1 | SCBP2-like protein, partial | 339 | 1.10E-07 | 62 | F:calcium ion binding |
| Efet.01.74244.g1501.t1 | Eukaryotic initiation factor 4A-III | 384 | 2.04E-51 | 96.2 | F:translation initiation factor activity; F:ATP binding; P:RNA splicing; C:catalytic step 2 spliceosome; P:regulation of gene expression; P:RNA secondary structure unwinding; P:translational initiation; F:ATP-dependent RNA helicase activity |
| Efet.01.74263.g1503.t1 | 5-hydroxytryptamine receptor 2C | 351 | 5.92E-25 | 70.5 | C:integral component of plasma membrane; F:dopamine neurotransmitter receptor activity; P:dopamine receptor signaling pathway |
| Efet.01.74277.g1504.t1 | alpha- sarcomeric-like isoform X2 | 495 | 1.59E-52 | 55.25 | F:actin filament binding; P:actin crosslink formation; F:actin binding; F:calcium ion binding; P:actin filament bundle assembly |
| Efet.01.74289.g1505.t1 | cartilage matrix -like isoform X2 | 654 | 8.46E-34 | 53.75 | F:calcium ion binding; C:collagen trimer; C:membrane; C:integral component of membrane; C:proteinaceous extracellular matrix |
| Efet.01.74293.g1506.t1 | myosin heavy chain cardiac muscle specific isoform | 1419 | 1.56E-107 | 69.3 | F:nucleotide binding; F:anion binding |
| Efet.01.74325.g1508.t1 | nose resistant to fluoxetine 6-like | 240 | 2.49E-13 | 61.95 | C:membrane |
| Efet.01.74339.g1509.t1 | helicase SRCAP | 249 | 1.79E-12 | 68.65 | F:ATP binding |
| Efet.01.74339.g1510.t1 | helicase domino-like isoform X1 | 2202 | 0 | 63.4 | F:ATP binding |
| Efet.01.74343.g1511.t1 | enhancer of mRNA-decapping | 342 | 4.80E-31 | 67.75 | P:cellular process |
| Efet.01.74343.g1512.t1 | enhancer of mRNA-decapping 3 | 762 | 4.25E-28 | 48.15 |  |
| Efet.01.74410.g1513.t1 | germinal-center associated nuclear -like isoform X1 | 768 | 7.29E-08 | 47.5 | F:nucleotide binding; F:nucleic acid binding |
| Efet.01.74415.g1514.t1 | collagen alpha-1(XIV) chain- | 612 | 1.08E-32 | 58.3 | F:calcium ion binding |
| Efet.01.74488.g1515.t1 | probable beta-D-xylosidase 7 | 378 | 3.33E-27 | 66 | P:carbohydrate metabolic process; F:hydrolase activity, hydrolyzing O-glycosyl compounds |
| Efet.01.7451.g559.t1 | hypothetical protein HELRODRAFT_167866 | 339 | 1.62E-06 | 69 | C:membrane; C:integral component of membrane |
| Efet.01.7455.g560.t1 | glycogen-binding subunit 76A-like | 1473 | 2.03E-44 | 55.8 |  |
| Efet.01.74556.g1519.t1 | formin-2 isoform X1 | 210 | 7.79E-13 | 74.4 | P:actin nucleation; C:membrane; C:integral component of membrane; C:actin filament; F:microtubule binding |
| Efet.01.74614.g1521.t1 | Angiotensin-converting enzyme | 216 | 6.70E-17 | 75.45 | F:carboxypeptidase activity; F:metal ion binding; F:peptidyl-dipeptidase activity; C:membrane; P:proteolysis; F:metallopeptidase activity |
| Efet.01.74633.g1523.t1 | delta-like 4 (Drosophila) | 915 | 7.40E-19 | 60.75 | P:negative regulation of blood vessel endothelial cell migration; P:regulation of neural retina development; F:Notch binding; P:negative regulation of Notch signaling pathway; P:ventricular trabecula myocardium morphogenesis; P:positive regulation of Notch signaling pathway; P:Notch signaling involved in heart development; P:pericardium morphogenesis; P:negative regulation of transcription from RNA polymerase II promoter; P:T cell differentiation; P:blood vessel remodeling; P:blood vessel lumenization; P:branching involved in blood vessel morphogenesis; P:cardiac atrium morphogenesis; C:membrane; P:positive regulation of neural precursor cell proliferation; P:dorsal aorta morphogenesis; P:regulation of neurogenesis |
| Efet.01.74649.g1524.t1 | PREDICTED: uncharacterized protein LOC106156013 | 435 | 7.14E-25 | 50.65 | C:membrane; C:integral component of membrane |
| Efet.01.74673.g1526.t1 | hypothetical protein CAPTEDRAFT_223646 | 231 | 6.06E-07 | 58 |  |
| Efet.01.74750.g1528.t1 | MAP kinase-activated kinase 2-like | 222 | 2.66E-26 | 80.95 | F:ATP binding; F:protein serine/threonine kinase activity; P:protein phosphorylation |
| Efet.01.74765.g1529.t1 | E3 SUMO- ligase 2-like | 702 | 3.23E-56 | 67.1 | P:regulation of glucose metabolic process; C:intracellular membrane-bounded organelle; P:intracellular transport; C:protein complex; F:binding; C:intracellular organelle part; P:single-organism process; P:cellular process |
| Efet.01.74817.g1531.t1 | trapped in endoderm-1-like | 1266 | 7.87E-99 | 50.5 | F:G-protein coupled receptor activity; P:G-protein coupled receptor signaling pathway; P:signal transduction; C:membrane; C:integral component of membrane; F:signal transducer activity |
| Efet.01.74844.g1532.t1 | leucine-rich repeat and guanylate kinase domain-containing | 558 | 6.29E-26 | 63.3 | C:intracellular organelle |
| Efet.01.74876.g1533.t1 | cardiolipin synthase | 780 | 1.55E-155 | 80.55 | F:cardiolipin synthase activity; C:integral component of membrane; C:plasma membrane; P:cardiolipin biosynthetic process |
| Efet.01.74876.g1534.t1 | cardiolipin synthase | 729 | 1.19E-112 | 75.65 | C:membrane; F:phosphotransferase activity, for other substituted phosphate groups; P:phospholipid biosynthetic process |
| Efet.01.74876.g1535.t1 | lysine--tRNA ligase | 675 | 6.33E-147 | 90.2 | F:magnesium ion binding; F:nucleic acid binding; F:ATP binding; C:cytoplasm; F:lysine-tRNA ligase activity; P:lysyl-tRNA aminoacylation |
| Efet.01.74904.g1536.t1 | hypothetical protein CAPTEDRAFT_218172 | 1338 | 1.93E-89 | 54.1 | C:membrane; C:integral component of membrane |
| Efet.01.7491.g561.t1 | Small conductance calcium-activated potassium channel | 504 | 1.23E-68 | 85.3 | F:calmodulin binding; P:potassium ion transmembrane transport; C:integral component of membrane; F:small conductance calcium-activated potassium channel activity |
| Efet.01.74957.g1539.t1 | aryl hydrocarbon receptor | 1494 | 7.66E-67 | 55.95 | C:intracellular part |
| Efet.01.74978.g1540.t1 | tyrosine- phosphatase Lar isoform X1 | 873 | 1.55E-51 | 49.45 | F:phosphoprotein phosphatase activity; P:protein dephosphorylation |
| Efet.01.75019.g1.t1 | protocadherin-9-like isoform X1 | 951 | 2.03E-14 | 51.4 | F:calcium ion binding; C:membrane; C:integral component of membrane; P:cell adhesion; P:homophilic cell adhesion via plasma membrane adhesion molecules; C:plasma membrane |
| Efet.01.75028.g3.t1 | monocarboxylate transporter 13- | 237 | 1.19E-18 | 81.25 | P:transmembrane transport; C:integral component of membrane |
| Efet.01.75067.g8.t1 | glutamate receptor NMDA 3A-like | 1134 | 2.87E-89 | 62.9 | C:postsynaptic membrane; P:ion transmembrane transport; C:integral component of membrane; C:cell junction; P:ionotropic glutamate receptor signaling pathway; F:ionotropic glutamate receptor activity; F:extracellular-glutamate-gated ion channel activity |
| Efet.01.7507.g562.t1 | transmembrane 246-like | 1179 | 1.94E-65 | 49.8 | C:membrane; C:integral component of membrane |
| Efet.01.75095.g9.t1 | myosin heavy chain 95F isoform X1 | 294 | 1.60E-42 | 78.9 | C:myosin complex; F:ATP binding; F:actin binding; F:motor activity |
| Efet.01.75118.g11.t1 | hypothetical protein HELRODRAFT_89022, partial | 255 | 6.68E-14 | 82.67 | C:postsynaptic membrane; P:cation transmembrane transport; C:integral component of membrane; C:cell junction; F:extracellular ligand-gated ion channel activity |
| Efet.01.75207.g15.t1 | neuromedin-U receptor 2-like isoform X1 | 540 | 1.77E-11 | 63.05 | F:G-protein coupled receptor activity; P:G-protein coupled receptor signaling pathway; C:membrane |
| Efet.01.75217.g17.t1 | forkhead box O-like | 477 | 3.46E-21 | 74.9 | C:nucleus; F:transcription factor activity, sequence-specific DNA binding; P:transcription, DNA-templated; P:regulation of transcription, DNA-templated; F:sequence-specific DNA binding |
| Efet.01.75385.g19.t1 | zinc finger SWIM domain-containing 1-like | 1107 | 1.94E-69 | 51.5 | F:zinc ion binding |
| Efet.01.75411.g20.t1 | POU class transcription factor 3 | 840 | 4.46E-96 | 85.2 | P:retinal ganglion cell axon guidance; F:RNA polymerase II core promoter proximal region sequence-specific DNA binding; P:transcription from RNA polymerase II promoter; P:visual perception; P:vestibulocochlear nerve development; P:positive regulation of transcription from RNA polymerase II promoter; C:nucleoplasm; P:sensory perception of sound; F:transcriptional activator activity, RNA polymerase II core promoter proximal region sequence-specific binding; P:auditory receptor cell differentiation; P:axon extension; P:inner ear morphogenesis; P:neuromuscular process controlling balance; P:neuron apoptotic process |
| Efet.01.75418.g21.t1 | regulator of G- signaling 3- | 216 | 7.89E-17 | 73.9 | P:biological regulation |
| Efet.01.75425.g22.t1 | Lysine-specific demethylase 3B | 1770 | 1.67E-69 | 51.15 | F:methyltransferase activity; P:methylation |
| Efet.01.75429.g23.t1 | proline-rich transmembrane 1-like | 321 | 1.92E-14 | 66.95 | C:membrane |
| Efet.01.75439.g24.t1 | gastrula zinc finger -like isoform X2 | 1437 | 2.22E-56 | 56.45 | F:nucleic acid binding; F:metal ion binding |
| Efet.01.75457.g25.t1 | arginine N-methyltransferase 5 isoform X2 | 321 | 2.83E-26 | 75.05 | C:nucleus; C:cytoplasmic part; F:binding; P:organelle organization; P:peptidyl-arginine N-methylation; F:histone-arginine N-methyltransferase activity |
| Efet.01.7548.g563.t1 | transcription factor SOX- | 624 | 3.32E-34 | 64.1 | F:DNA binding; P:positive regulation of macromolecule metabolic process; P:regulation of transcription, DNA-templated; P:animal organ development; P:single-organism cellular process; P:negative regulation of cellular process; P:positive regulation of cellular metabolic process; C:intracellular part |
| Efet.01.75563.g32.t1 | titin-like isoform X1 | 1665 | 7.07E-17 | 65.75 | F:nucleotide binding; P:regulation of Rho protein signal transduction; F:ATP binding; C:membrane; C:integral component of membrane; F:protein kinase activity; F:Rho guanyl-nucleotide exchange factor activity; P:phosphorylation; F:transferase activity; P:protein phosphorylation; F:kinase activity; P:positive regulation of GTPase activity |
| Efet.01.7557.g564.t1 | sarcoplasmic calcium-binding -like | 402 | 2.00E-23 | 52.5 | F:calcium ion binding |
| Efet.01.75570.g33.t1 | netrin precursor | 1002 | 1.03E-114 | 73.9 | C:membrane; C:integral component of membrane; C:extracellular region |
| Efet.01.75582.g34.t1 | junction-mediating and -regulatory -like | 1086 | 4.16E-06 | 52 |  |
| Efet.01.75607.g37.t1 | Phosphatidylinositol transfer alpha isoform | 267 | 3.23E-23 | 66.8 | C:membrane |
| Efet.01.75649.g38.t1 | hypothetical protein HELRODRAFT_189710 | 1212 | 8.78E-36 | 49.8 |  |
| Efet.01.75681.g39.t1 | ras-related Rab-18A | 399 | 3.56E-66 | 72.45 | F:GTP binding; P:small GTPase mediated signal transduction; P:lipid particle organization; C:intracellular; C:membrane-bounded organelle |
| Efet.01.75687.g40.t1 | homeobox H55 | 276 | 5.17E-32 | 87 | C:nucleus; F:transcription factor activity, sequence-specific DNA binding; P:transcription, DNA-templated; P:multicellular organism development; P:regulation of transcription, DNA-templated; F:sequence-specific DNA binding |
| Efet.01.75731.g41.t1 | pyroglutamyl-peptidase 1-like | 486 | 2.66E-58 | 70.35 | F:peptidase activity |
| Efet.01.75737.g42.t1 | dynein beta ciliary-like | 255 | 5.13E-36 | 85.7 | F:ATPase activity; F:ATP binding; F:microtubule motor activity; C:dynein complex; P:microtubule-based movement |
| Efet.01.75798.g45.t1 | vacuolar-sorting SNF8-like | 510 | 5.61E-54 | 79.4 |  |
| Efet.01.7581.g565.t1 | tRNA (guanine-N(7)-)-methyltransferase | 270 | 7.42E-31 | 60.85 | C:tRNA methyltransferase complex; P:RNA (guanine-N7)-methylation; F:protein binding; F:tRNA (guanine-N7-)-methyltransferase activity; C:nucleolus; F:tRNA binding; P:tRNA methylation; C:nucleoplasm |
| Efet.01.75843.g46.t1 | Wiskott-Aldrich syndrome family member | 372 | 1.13E-08 | 61 |  |
| Efet.01.75863.g49.t1 | uncharacterized protein PFB0145c-like | 507 | 1.31E-11 | 63.95 | F:chitin binding; C:membrane; P:chitin metabolic process; C:integral component of membrane; C:extracellular region |
| Efet.01.75866.g50.t1 | wingless-type MMTV integration site member 9 | 693 | 6.23E-49 | 64 | P:chordate embryonic development; P:skeletal system development; P:animal organ development |
| Efet.01.75877.g52.t1 | PHD and RING finger domain-containing 1 | 2220 | 5.15E-16 | 70.75 | F:zinc ion binding |
| Efet.01.75886.g53.t1 | centrosomal of 131 kDa-like | 1884 | 1.39E-62 | 51 | C:cilium; P:intraciliary transport involved in cilium assembly |
| Efet.01.75887.g54.t1 | transmembrane 35A-like | 432 | 5.64E-38 | 68.75 | C:membrane |
| Efet.01.75903.g57.t1 | cysteine dioxygenase | 270 | 7.88E-15 | 75.55 | F:oxidoreductase activity, acting on single donors with incorporation of molecular oxygen, incorporation of two atoms of oxygen; F:iron ion binding; P:oxidation-reduction process |
| Efet.01.75975.g58.t1 | neuroligin- X-linked-like isoform X1 | 633 | 3.87E-53 | 63.4 | C:membrane; F:neurexin family protein binding; C:integral component of membrane |
| Efet.01.75999.g59.t1 | forkhead box J3-like | 429 | 9.21E-23 | 56.05 | F:DNA binding; F:transcription factor activity, sequence-specific DNA binding; P:regulation of transcription, DNA-templated; F:sequence-specific DNA binding |
| Efet.01.76009.g60.t1 | dual specificity phosphatase 1B isoform X2 | 615 | 7.75E-52 | 58.9 | F:phosphoprotein phosphatase activity; P:protein dephosphorylation |
| Efet.01.76022.g61.t1 | twitchin-like isoform X1 | 423 | 5.43E-13 | 56.95 |  |
| Efet.01.76034.g62.t1 | SRSF kinase 3 isoform X1 | 540 | 2.74E-49 | 71.5 | F:nucleotide binding; F:protein kinase activity; P:phosphorylation |
| Efet.01.76039.g63.t1 | adenylate kinase 7-like | 288 | 1.19E-15 | 80.65 | F:ATP binding; P:phosphorylation; F:nucleobase-containing compound kinase activity; P:nucleobase-containing compound metabolic process |
| Efet.01.76043.g64.t1 | cartilage matrix - | 654 | 6.16E-22 | 53.25 | F:calcium ion binding |
| Efet.01.76048.g65.t1 | 29-kDa galactose-binding lectin | 330 | 1.81E-12 | 53.81 | F:carbohydrate binding |
| Efet.01.76067.g66.t1 | dynein beta ciliary-like | 693 | 4.33E-44 | 65.7 | F:nucleotide binding; F:ATPase activity; F:ATP binding; F:microtubule motor activity; C:dynein complex; P:microtubule-based movement |
| Efet.01.76098.g68.t1 | retinol dehydrogenase 2-like | 600 | 2.08E-63 | 69.85 | F:oxidoreductase activity; C:membrane; C:integral component of membrane; P:oxidation-reduction process |
| Efet.01.76155.g72.t1 | trans-1,2-dihydrobenzene-1,2-diol dehydrogenase-like | 1185 | 2.01E-45 | 71.45 | P:oxidation-reduction process; F:D-xylose 1-dehydrogenase (NADP+) activity; P:D-xylose catabolic process |
| Efet.01.76171.g74.t1 | IQ motif and SEC7 domain-containing 1-like | 309 | 5.46E-40 | 75.7 | P:biological regulation |
| Efet.01.76175.g75.t1 | hypothetical protein HELRODRAFT_192545 | 252 | 3.46E-19 | 70.33 | F:metal ion binding |
| Efet.01.76198.g76.t1 | chromodomain-helicase-DNA-binding 7 isoform X1 | 1617 | 0 | 68.7 | F:organic cyclic compound binding; F:heterocyclic compound binding; P:embryonic morphogenesis; P:nervous system development; P:circulatory system development; P:sensory organ development; P:animal organ morphogenesis |
| Efet.01.76207.g77.t1 | beta-ketoacyl-[acyl-carrier- ] synthase II | 399 | 1.96E-88 | 87 | F:beta-ketoacyl-acyl-carrier-protein synthase II activity; P:fatty acid biosynthetic process |
| Efet.01.76227.g78.t1 | hillarin isoform X1 | 1179 | 7.05E-151 | 54.7 |  |
| Efet.01.7627.g567.t1 | THO complex subunit 2 | 3516 | 0 | 69.5 | P:mRNA export from nucleus; C:nuclear part; C:protein complex |
| Efet.01.76292.g81.t1 | protocadherin-9-like isoform X1 | 2586 | 4.03E-118 | 52.3 | F:calcium ion binding; C:membrane; C:integral component of membrane; P:cell adhesion; P:homophilic cell adhesion via plasma membrane adhesion molecules; C:plasma membrane |
| Efet.01.76316.g83.t1 | alan shepard | 246 | 3.94E-31 | 80.6 | F:nucleotide binding; F:RNA binding |
| Efet.01.76397.g84.t1 | dystonin isoform X1 | 360 | 6.64E-21 | 68.95 | F:binding |
| Efet.01.76427.g85.t1 | predicted protein | 261 | 2.57E-08 | 56.5 | C:membrane; C:integral component of membrane |
| Efet.01.76483.g87.t1 | amyloid beta A4 -like isoform X1 | 876 | 2.74E-41 | 62.05 | F:transition metal ion binding; C:membrane; C:integral component of membrane; F:heparin binding |
| Efet.01.76514.g88.t1 | pleckstrin homology domain-containing family G member 4B-like isoform X5 | 837 | 6.74E-56 | 64.4 | P:regulation of Rho protein signal transduction; F:Rho guanyl-nucleotide exchange factor activity; P:positive regulation of GTPase activity |
| Efet.01.76544.g90.t1 | guanylate cyclase 32E | 285 | 1.21E-24 | 81.05 | F:ATP binding; P:cGMP biosynthetic process; P:peptidyl-tyrosine phosphorylation; F:protein tyrosine kinase activity; C:integral component of membrane; F:guanylate cyclase activity; C:intracellular; P:intracellular signal transduction |
| Efet.01.76552.g91.t1 | paired box Pax- | 495 | 1.33E-71 | 91.1 | F:HMG box domain binding; P:nervous system development; P:transcription from RNA polymerase II promoter; F:sequence-specific DNA binding; F:adenosine deaminase activity; P:positive regulation of transcription from RNA polymerase II promoter; C:nucleoplasm; P:animal organ morphogenesis; P:muscle organ development; P:sensory perception of sound; F:transcription factor activity, sequence-specific DNA binding; P:apoptotic process; P:neural crest formation |
| Efet.01.766.g68.t1 | SCY1 2 | 330 | 1.97E-16 | 60.5 | F:ATP binding; C:membrane; C:integral component of membrane; F:protein kinase activity; P:protein phosphorylation |
| Efet.01.76614.g95.t1 | nanos homolog 1-like | 567 | 1.11E-50 | 74 | F:zinc ion binding; F:RNA binding |
| Efet.01.76616.g96.t1 | filamin-A isoform X1 | 630 | 5.63E-36 | 51.6 | F:actin binding |
| Efet.01.76686.g98.t1 | sodium bicarbonate cotransporter 3 isoform X1 | 636 | 1.94E-33 | 50.7 | P:anion transport |
| Efet.01.76696.g99.t1 | myosin-VIIa | 852 | 8.46E-133 | 74.8 | F:nucleotide binding; C:cytoskeleton |
| Efet.01.76813.g101.t1 | gamma-glutamyltranspeptidase 1-like isoform X2 | 333 | 1.90E-29 | 65.05 | P:glutathione metabolic process; C:membrane; C:integral component of membrane; F:gamma-glutamyltransferase activity |
| Efet.01.76825.g102.t1 | INO80 complex subunit D | 1626 | 7.71E-32 | 82.75 | C:nucleus; P:DNA repair; P:transcription, DNA-templated; P:DNA recombination; P:regulation of transcription, DNA-templated |
| Efet.01.7686.g570.t1 | Membrane-associated guanylate WW and PDZ domain-containing | 1281 | 6.63E-58 | 69.55 | P:phosphorylation; F:kinase activity |
| Efet.01.76887.g104.t1 | ATP-binding cassette sub-family D member 2-like | 621 | 4.96E-105 | 85.15 | F:ATP binding; F:ATPase activity, coupled to transmembrane movement of substances; P:fatty acid beta-oxidation; C:integral component of peroxisomal membrane; P:fatty acid transmembrane transport |
| Efet.01.76890.g105.t1 | dynein heavy chain axonemal-like | 414 | 6.05E-47 | 74.25 | F:nucleotide binding; C:cell part; F:nucleoside-triphosphatase activity; C:organelle part |
| Efet.01.76905.g106.t1 | dynein heavy chain axonemal-like isoform X1 | 540 | 2.46E-81 | 80.7 | F:ATPase activity; F:ATP binding; F:microtubule motor activity; C:axonemal dynein complex; C:integral component of membrane; P:cilium or flagellum-dependent cell motility; P:microtubule-based movement |
| Efet.01.76957.g108.t1 | extracellular hemoglobin linker L3 subunit precursor | 429 | 4.12E-23 | 54.83 |  |
| Efet.01.76977.g109.t1 | serologically defined colon cancer antigen 8 homolog | 828 | 1.19E-18 | 55.89 | C:centrosome; P:centrosome organization |
| Efet.01.7698.g572.t1 | hypothetical protein CAPTEDRAFT_204696 | 486 | 2.27E-30 | 47.3 | C:membrane; C:integral component of membrane; F:carbohydrate binding |
| Efet.01.77006.g110.t1 | hypothetical protein HELRODRAFT_183927 | 2997 | 4.08E-25 | 61.5 |  |
| Efet.01.77055.g111.t1 | transmembrane 68-like | 369 | 4.39E-29 | 71.85 | C:membrane |
| Efet.01.77055.g112.t1 | transmembrane 68 isoform X2 | 297 | 1.83E-35 | 75.4 | C:membrane |
| Efet.01.77071.g115.t1 | hypothetical protein CAPTEDRAFT_227162 | 372 | 3.51E-23 | 59.1 | F:zinc ion binding; F:metal ion binding |
| Efet.01.77086.g117.t1 | FERM domain-containing | 1155 | 8.85E-47 | 60.5 | C:intracellular part |
| Efet.01.77132.g119.t1 | neurexin-3 isoform X8 | 2364 | 2.25E-77 | 43.15 |  |
| Efet.01.77155.g121.t1 | octopamine receptor beta-2R-like | 1050 | 3.68E-115 | 64.3 | P:G-protein coupled receptor signaling pathway; C:membrane; F:G-protein coupled amine receptor activity |
| Efet.01.77164.g122.t1 | xylose isomerase-like | 330 | 3.65E-36 | 93.4 | C:cytoplasm; F:xylose isomerase activity; F:metal ion binding; P:pentose-phosphate shunt; C:membrane; P:D-xylose metabolic process |
| Efet.01.77165.g123.t1 | titin-like isoform X4 | 999 | 7.84E-80 | 55.7 |  |
| Efet.01.77174.g124.t1 | muscle calcium channel subunit alpha-1 isoform X6 | 222 | 5.28E-29 | 87.35 | C:voltage-gated calcium channel complex; P:regulation of ion transmembrane transport; P:calcium ion transmembrane transport; F:voltage-gated calcium channel activity |
| Efet.01.7719.g573.t1 | delta 2 | 363 | 8.18E-22 | 54.78 | F:calcium ion binding; C:membrane; C:integral component of membrane |
| Efet.01.77194.g126.t1 | dynein heavy chain axonemal | 681 | 3.37E-107 | 70.4 | F:ATP binding; F:microtubule motor activity; C:dynein complex; P:microtubule-based movement |
| Efet.01.77265.g127.t1 | tudor domain-containing 6-like isoform X1 | 1032 | 8.00E-20 | 48.3 | F:metal ion binding |
| Efet.01.77265.g128.t1 | tudor domain-containing 1 isoform X1 | 1227 | 5.11E-13 | 59.25 |  |
| Efet.01.77269.g129.t1 | probable isocitrate dehydrogenase [NAD] subunit mitochondrial | 204 | 4.57E-25 | 87.85 | F:magnesium ion binding; F:isocitrate dehydrogenase (NAD+) activity; F:NAD binding; C:mitochondrion; P:tricarboxylic acid cycle |
| Efet.01.77290.g130.t1 | pikachurin isoform X2 | 516 | 7.10E-20 | 52 | F:calcium ion binding |
| Efet.01.77301.g131.t1 | regulator of microtubule dynamics 1-like | 312 | 1.73E-17 | 60.15 | C:membrane; C:integral component of membrane |
| Efet.01.77324.g132.t1 | radial spoke head 1 homolog | 699 | 2.95E-68 | 80.6 | C:outer dense fiber; P:meiotic cell cycle; F:protein binding; P:spermatid development; C:cytosol; P:axoneme assembly; C:meiotic spindle; C:condensed nuclear chromosome |
| Efet.01.77328.g134.t1 | calsyntenin-1-like isoform X1 | 348 | 9.23E-27 | 64.95 | F:calcium ion binding; C:membrane; C:integral component of membrane; P:homophilic cell adhesion via plasma membrane adhesion molecules |
| Efet.01.77346.g136.t1 | syntaxin-binding 1-like isoform X3 | 477 | 6.81E-52 | 60.55 | P:vesicle-mediated transport; P:vesicle docking involved in exocytosis |
| Efet.01.77392.g137.t1 | REST_DANRE ame: Full=RE1-silencing transcription factor ame: Full=Neural-restrictive silencer factor | 2226 | 3.89E-06 | 42 | P:transcription, DNA-templated; P:facial nerve morphogenesis; P:regulation of transcription, DNA-templated; P:neurogenesis; P:negative regulation of smoothened signaling pathway; P:smoothened signaling pathway involved in dorsal/ventral neural tube patterning; F:RNA polymerase II transcription factor activity, sequence-specific DNA binding; P:branchiomotor neuron axon guidance; F:nucleic acid binding; P:negative regulation of gene expression; C:nucleus; P:neuron migration; P:locomotory behavior; F:metal ion binding |
| Efet.01.77431.g139.t1 | prolactin-releasing peptide receptor-like | 789 | 1.64E-32 | 66.8 | P:neuropeptide signaling pathway; C:integral component of membrane; F:neuropeptide Y receptor activity |
| Efet.01.77453.g140.t1 | C-Jun-amino-terminal kinase-interacting 4-like | 456 | 2.01E-43 | 81 |  |
| Efet.01.77457.g141.t1 | prepilin-type cleavage methylation domain-containing | 525 | 7.11E-59 | 70.85 | C:integral component of membrane; P:type IV pilus biogenesis |
| Efet.01.77457.g142.t1 | type IV pilus modification | 816 | 6.18E-126 | 70.8 | C:integral component of membrane |
| Efet.01.77457.g143.t1 | bifunctional diaminohydroxyphosphoribosylaminopyrimidine deaminase 5-amino-6-(5-phosphoribosylamino)uracil reductase | 948 | 0 | 85.1 | F:zinc ion binding; F:NADP binding; F:5-amino-6-(5-phosphoribosylamino)uracil reductase activity; F:diaminohydroxyphosphoribosylaminopyrimidine deaminase activity; P:riboflavin biosynthetic process; P:oxidation-reduction process |
| Efet.01.77457.g144.t1 | pyridoxal phosphate-dependent aminotransferase | 960 | 0 | 87.35 | F:pyridoxal phosphate binding; F:L-phenylalanine:2-oxoglutarate aminotransferase activity; P:biosynthetic process; F:L-aspartate:2-oxoglutarate aminotransferase activity |
| Efet.01.77465.g145.t1 | calmodulin 3 | 270 | 8.51E-18 | 68.1 | F:calcium ion binding; C:membrane; C:integral component of membrane |
| Efet.01.77571.g147.t1 | zinc finger and BTB domain-containing 6 | 462 | 8.23E-18 | 55 | F:nucleic acid binding; F:metal ion binding |
| Efet.01.77578.g148.t1 | FAM220A isoform X1 | 1407 | 4.26E-106 | 62.7 |  |
| Efet.01.77585.g149.t1 | UPF0568 C14orf166 homolog | 507 | 2.53E-19 | 57.1 | F:DNA binding |
| Efet.01.776.g69.t1 | PRRC2C-like isoform X3 | 870 | 1.70E-19 | 56.2 |  |
| Efet.01.77614.g150.t1 | paired amphipathic helix Sin3a | 1137 | 7.87E-40 | 48.05 | P:regulation of transcription, DNA-templated; F:binding; P:negative regulation of biological process; C:intracellular part; P:cellular process |
| Efet.01.77639.g152.t1 | Glycine receptor subunit alpha- | 501 | 2.93E-33 | 88.4 | F:transmitter-gated ion channel activity; P:chloride transmembrane transport; C:external side of plasma membrane; P:protein heterooligomerization; P:regulation of respiratory gaseous exchange by neurological system process; C:perikaryon; C:chloride channel complex; C:integral component of plasma membrane; C:dendrite; P:adult walking behavior; P:cellular response to amino acid stimulus; P:cellular response to zinc ion; P:startle response; C:postsynaptic membrane; P:muscle contraction; F:glycine binding; P:action potential; C:inhibitory synapse; P:synaptic transmission, glycinergic; F:zinc ion binding; P:righting reflex; F:extracellular-glycine-gated chloride channel activity; P:acrosome reaction; P:protein homooligomerization; C:endoplasmic reticulum; P:visual perception; P:cellular response to ethanol; P:neuromuscular process controlling posture; P:neuropeptide signaling pathway; P:negative regulation of transmission of nerve impulse; F:protein binding; F:taurine binding; P:inhibitory postsynaptic potential; P:positive regulation of acrosome reaction; C:cell junction |
| Efet.01.77643.g153.t1 | phosphatase mitochondrial | 468 | 2.17E-45 | 78.5 | F:protein serine/threonine phosphatase activity; F:metal ion binding; P:protein dephosphorylation |
| Efet.01.77651.g154.t1 | PREDICTED: otopetrin-1-like | 468 | 2.62E-08 | 46 |  |
| Efet.01.77663.g155.t1 | hypothetical protein CAPTEDRAFT_223299 | 954 | 7.07E-15 | 66 |  |
| Efet.01.77689.g156.t1 | mitogen-activated kinase-binding 1 isoform X2 | 579 | 6.38E-45 | 59.55 | P:phosphorylation; F:kinase activity |
| Efet.01.77706.g157.t1 | cadherin-related tumor suppressor | 354 | 3.21E-16 | 62.15 | C:membrane |
| Efet.01.77724.g159.t1 | lateral signaling target 2 homolog | 1215 | 1.43E-27 | 78.05 | F:1-phosphatidylinositol-3-phosphate 5-kinase activity; F:metal ion binding; P:phosphatidylinositol phosphorylation |
| Efet.01.77746.g161.t1 | stanniocalcin family | 765 | 6.55E-46 | 49.5 | F:phospholipase A2 activity; P:cell wall macromolecule catabolic process; F:calcium ion binding; P:peptidoglycan catabolic process; P:lipid catabolic process; C:extracellular region; F:hormone activity; F:lysozyme activity |
| Efet.01.77764.g162.t1 | hypothetical protein CAPTEDRAFT_205875 | 261 | 6.73E-19 | 61.5 | C:membrane |
| Efet.01.77777.g163.t1 | Glutathione S-Transferase | 348 | 2.14E-16 | 75.1 | F:glutathione transferase activity; F:binding |
| Efet.01.77786.g164.t1 | large proline-rich BAG6-like isoform X1 | 534 | 2.39E-34 | 57 |  |
| Efet.01.7781.g575.t1 | BTB POZ domain-containing 6-B-like | 960 | 2.14E-114 | 49.3 | P:regulation of neurogenesis |
| Efet.01.7781.g576.t1 | polycystic kidney disease 1-like 2 | 1038 | 1.78E-14 | 46.8 | P:detection of mechanical stimulus; F:zinc ion binding; F:metal ion binding; F:calcium channel activity; F:calcium ion binding; C:membrane; C:integral component of membrane; P:calcium ion transmembrane transport; F:carbohydrate binding |
| Efet.01.77842.g165.t1 | transmembrane 183-like | 546 | 1.08E-43 | 54.55 | C:membrane; C:integral component of membrane |
| Efet.01.77852.g166.t1 | ATP-dependent DNA helicase Q4 | 519 | 1.22E-68 | 77.8 | F:nucleic acid binding; F:ATP binding; F:metal ion binding; F:helicase activity |
| Efet.01.77864.g167.t1 | intraflagellar transport 80 homolog | 762 | 6.53E-23 | 57.85 |  |
| Efet.01.77877.g168.t1 | hypothetical protein HELRODRAFT_168860 | 345 | 7.24E-07 | 73 |  |
| Efet.01.77964.g172.t1 | round spermatid basic 1 | 708 | 9.48E-67 | 84.2 | C:nucleus |
| Efet.01.77982.g173.t1 | thioester containing | 489 | 3.26E-46 | 64.6 | C:extracellular region |
| Efet.01.78015.g174.t1 | Homeobox prospero | 2004 | 5.22E-18 | 96.5 | F:DNA binding; C:integral component of membrane |
| Efet.01.78019.g175.t1 | elongation of very long chain fatty acids 6-like | 831 | 1.37E-74 | 65.55 | P:lipid metabolic process |
| Efet.01.78022.g176.t1 | copper-transporting ATPase 1-like | 450 | 2.97E-08 | 74.75 | F:nucleotide binding; F:copper ion binding; F:cation-transporting ATPase activity; P:cation transmembrane transport; C:integral component of membrane; P:metal ion transport |
| Efet.01.78037.g177.t1 | malectin-B-like isoform X1 | 435 | 6.48E-48 | 71.45 | C:integral component of membrane |
| Efet.01.7805.g577.t1 | neurocalcin homolog | 408 | 8.07E-66 | 91.2 | F:calcium ion binding |
| Efet.01.78091.g181.t1 | solute carrier family 45 member 3-like | 288 | 1.25E-17 | 72.75 | C:membrane |
| Efet.01.78155.g183.t1 | thymidine phosphorylase | 225 | 6.01E-23 | 76.3 | F:phosphorylase activity; P:pyrimidine nucleobase metabolic process; F:thymidine phosphorylase activity; C:cytosol; F:pyrimidine-nucleoside phosphorylase activity; P:pyrimidine nucleotide metabolic process; P:pyrimidine nucleoside metabolic process |
| Efet.01.78157.g184.t1 | Mitogen-activated kinase 7 | 321 | 3.48E-09 | 57.3 | P:regulation of macromolecule metabolic process; P:negative regulation of signal transduction; P:negative regulation of apoptotic process; P:cellular response to stimulus; P:phosphorylation; C:intracellular part; F:kinase activity; P:regulation of primary metabolic process; P:regulation of cellular metabolic process |
| Efet.01.78179.g186.t1 | gastrula zinc finger -like | 342 | 2.02E-20 | 62.5 | F:nucleic acid binding; F:metal ion binding |
| Efet.01.78194.g187.t1 | T-complex 1 subunit epsilon | 474 | 6.21E-72 | 81.55 | C:myelin sheath; P:positive regulation of telomerase RNA localization to Cajal body; F:G-protein beta-subunit binding; P:positive regulation of establishment of protein localization to telomere; P:chaperone-mediated protein folding; P:positive regulation of protein localization to Cajal body; F:unfolded protein binding; C:nucleolus; P:response to virus; P:toxin transport; C:zona pellucida receptor complex; P:'de novo' protein folding; C:microtubule; F:nucleic acid binding; C:chaperonin-containing T-complex; F:ATP binding; C:centrosome; P:binding of sperm to zona pellucida; C:extracellular exosome; F:protein binding involved in protein folding; F:beta-tubulin binding; C:cell body; P:positive regulation of telomere maintenance via telomerase; P:protein stabilization |
| Efet.01.78208.g188.t1 | intraflagellar transport 57 homolog | 432 | 1.50E-41 | 71.6 | C:photoreceptor connecting cilium; C:ciliary basal body; P:smoothened signaling pathway; P:non-motile cilium assembly; C:Golgi apparatus; P:motile cilium assembly; P:left/right pattern formation; C:axoneme; C:dendrite terminus; C:centrosome; C:ciliary tip; F:protein binding; P:neural tube closure; P:intraciliary transport; P:apoptotic process; P:negative regulation of epithelial cell proliferation; P:heart looping; C:intraciliary transport particle B; P:activation of cysteine-type endopeptidase activity involved in apoptotic process |
| Efet.01.78215.g189.t1 | CREB ATF bZIP transcription factor isoform X2 | 567 | 8.20E-25 | 61.75 | F:transcription factor activity, sequence-specific DNA binding; P:regulation of transcription, DNA-templated; F:sequence-specific DNA binding |
| Efet.01.78232.g192.t1 | mitogen-activated kinase kinase kinase 9 | 1218 | 3.56E-106 | 67.55 | P:MAPK cascade; P:regulation of MAP kinase activity; P:cell death; P:activation of protein kinase activity; F:MAP kinase kinase kinase activity; P:positive regulation of MAPK cascade; C:intracellular |
| Efet.01.78244.g193.t1 | ROP isoform X2 | 552 | 7.97E-71 | 68.7 | C:intracellular organelle; P:vesicle docking involved in exocytosis; C:vesicle; C:cytoplasmic part |
| Efet.01.78255.g194.t1 | RNA-binding 48 | 381 | 4.53E-41 | 81.2 | F:nucleotide binding; F:RNA binding; C:nucleoplasm |
| Efet.01.78263.g195.t1 | Dual specificity phosphatase | 522 | 6.84E-31 | 59.3 | F:phosphoprotein phosphatase activity; P:dephosphorylation |
| Efet.01.78277.g197.t1 | splicing factor 3B subunit 1 isoform X2 | 996 | 2.84E-89 | 57.85 | P:neural crest cell development; P:anterior/posterior pattern specification; P:myeloid cell development; C:spliceosomal complex; P:hematopoietic stem cell differentiation; P:regulation of apoptotic process; F:chromatin binding; C:chromatin; P:mRNA splicing, via spliceosome; P:definitive hemopoiesis; P:melanocyte differentiation; F:protein binding; P:blastocyst formation; C:nuclear matrix; C:small nuclear ribonucleoprotein complex |
| Efet.01.78316.g200.t1 | hypothetical protein HELRODRAFT_170946 | 555 | 4.71E-13 | 51.67 | C:membrane; C:integral component of membrane; F:carbohydrate binding |
| Efet.01.78363.g201.t1 | serine threonine- phosphatase 6 regulatory ankyrin repeat subunit C-like isoform X4 | 738 | 3.34E-63 | 57.9 | P:intracellular signal transduction; C:intracellular |
| Efet.01.78378.g202.t1 | tribbles homolog 2-like | 816 | 1.05E-101 | 71.55 | C:nucleus; P:regulation of MAP kinase activity; P:negative regulation of cellular process; P:protein phosphorylation; P:positive regulation of proteasomal ubiquitin-dependent protein catabolic process; P:regulation of myeloid leukocyte differentiation; P:negative regulation of molecular function; F:mitogen-activated protein kinase kinase binding |
| Efet.01.78381.g203.t1 | titin isoform X1 | 459 | 1.12E-13 | 64.4 |  |
| Efet.01.78381.g204.t1 | hypothetical protein CAPTEDRAFT_196991 | 480 | 2.55E-09 | 51.67 |  |
| Efet.01.78443.g206.t1 | VPS9 domain-containing 1-like isoform X1 | 426 | 2.13E-06 | 57 |  |
| Efet.01.78487.g208.t1 | T-complex-associated testis-expressed protein 1-like | 1293 | 1.94E-86 | 59.8 |  |
| Efet.01.78498.g209.t1 | hypoxia-inducible factor 1-alpha-like | 375 | 7.77E-38 | 75.2 | P:response to glucose; P:response to hypoxia; P:neural crest cell migration; P:motor neuron axon guidance; P:negative regulation of transcription, DNA-templated; F:chromatin binding; P:positive regulation of transcription from RNA polymerase II promoter; C:intracellular part; P:neuron apoptotic process; F:RNA polymerase II transcription factor activity, sequence-specific DNA binding |
| Efet.01.78557.g212.t1 | PREDICTED: uncharacterized protein LOC106160744 | 264 | 4.37E-06 | 61 |  |
| Efet.01.78559.g213.t1 | alsin isoform X1 | 666 | 8.19E-68 | 58.25 |  |
| Efet.01.78559.g214.t1 | alsin-like isoform X1 | 1104 | 1.24E-99 | 56.65 |  |
| Efet.01.78577.g215.t1 | PREDICTED: uncharacterized protein LOC101846802 | 717 | 2.22E-17 | 43 |  |
| Efet.01.7865.g578.t1 | 2-oxo acid dehydrogenase subunit E2 | 489 | 6.11E-96 | 94.2 | P:metabolic process; F:dihydrolipoyllysine-residue acetyltransferase activity |
| Efet.01.78661.g218.t1 | hypothetical protein A3Q56_04706, partial | 438 | 4.39E-08 | 50 |  |
| Efet.01.78671.g220.t1 | fez family zinc finger 2-like | 1464 | 2.75E-108 | 79.75 | F:nucleic acid binding; F:metal ion binding |
| Efet.01.78699.g221.t1 | rap guanine nucleotide exchange factor 4 isoform X2 | 291 | 6.73E-27 | 82.8 | P:insulin secretion; P:small GTPase mediated signal transduction; F:guanyl-nucleotide exchange factor activity; F:cAMP binding; P:regulation of exocytosis; C:intracellular; P:cAMP-mediated signaling; P:positive regulation of GTPase activity |
| Efet.01.78712.g222.t1 | hypothetical protein HELRODRAFT_176249 | 1488 | 1.56E-06 | 49 |  |
| Efet.01.78755.g224.t1 | nuclear factor interleukin-3-regulated -like | 1044 | 1.27E-26 | 76.9 | P:negative regulation of transcription from RNA polymerase II promoter; C:nucleus; F:transcriptional repressor activity, RNA polymerase II core promoter proximal region sequence-specific binding; P:circadian regulation of gene expression; F:sequence-specific DNA binding |
| Efet.01.78798.g227.t1 | atrial natriuretic peptide receptor 1-like isoform X1 | 510 | 5.42E-45 | 61.5 | F:nucleotide binding; F:phosphorus-oxygen lyase activity; C:membrane; P:cyclic nucleotide biosynthetic process |
| Efet.01.7880.g580.t1 | EF-hand calcium-binding domain-containing 6-like | 486 | 1.55E-26 | 52.3 | F:calcium ion binding |
| Efet.01.78804.g228.t1 | hypothetical protein HELRODRAFT_183289 | 1095 | 4.79E-10 | 43 | C:membrane; C:integral component of membrane |
| Efet.01.78807.g229.t1 | hypothetical protein HELRODRAFT_175147 | 291 | 1.13E-32 | 64.75 | C:membrane; F:carbohydrate binding |
| Efet.01.78842.g230.t1 | potassium voltage-gated channel subfamily H member 7-like isoform X3 | 552 | 8.90E-50 | 75.15 | F:voltage-gated potassium channel activity; P:potassium ion transmembrane transport; C:integral component of membrane |
| Efet.01.78857.g231.t1 | neuroblast differentiation-associated AHNAK | 2853 | 2.67E-44 | 40.3 | C:cell part |
| Efet.01.78870.g232.t1 | FMRFamide receptor-like | 465 | 2.20E-15 | 61.65 | F:G-protein coupled receptor activity; P:signal transduction; C:membrane |
| Efet.01.78872.g233.t1 | Cephalotocin receptor 1 | 954 | 0 | 66.65 | P:G-protein coupled receptor signaling pathway; C:integral component of membrane; F:vasopressin receptor activity; P:oviposition; C:plasma membrane; F:protein-hormone receptor activity |
| Efet.01.78877.g234.t1 | neuronal acetylcholine receptor subunit alpha-10-like isoform X1 | 672 | 9.02E-46 | 51.35 | F:ion channel activity; P:ion transport; C:postsynaptic membrane; P:ion transmembrane transport; P:cation transmembrane transport; C:membrane; C:integral component of membrane; C:cell junction; F:extracellular ligand-gated ion channel activity; C:synapse; P:transport; C:plasma membrane |
| Efet.01.7890.g581.t1 | diphthine methyl ester synthase | 330 | 2.58E-38 | 81.15 | P:peptidyl-diphthamide biosynthetic process from peptidyl-histidine; F:diphthine synthase activity; P:methylation |
| Efet.01.78936.g236.t1 | 2-oxoglutarate mitochondrial-like isoform X2 | 1158 | 0 | 88.8 | F:thiamine pyrophosphate binding; C:oxoglutarate dehydrogenase complex; F:oxoglutarate dehydrogenase (succinyl-transferring) activity; C:mitochondrion; C:cytosol; P:tricarboxylic acid cycle |
| Efet.01.78937.g237.t1 | hypothetical protein HELRODRAFT_193524 | 327 | 1.79E-30 | 70.55 | C:integral component of membrane; F:carbohydrate binding |
| Efet.01.78945.g238.t1 | laminin subunit beta-1-like isoform X2 | 468 | 5.74E-42 | 64.3 | F:catalytic activity |
| Efet.01.78948.g239.t1 | serine protease mitochondrial-like | 678 | 4.23E-71 | 58.9 | P:proteolysis; F:serine-type endopeptidase activity; F:peptidase activity |
| Efet.01.78970.g242.t1 | one cut domain family member 2-like isoform X4 | 1146 | 1.53E-43 | 81.85 | C:nucleus; F:DNA binding |
| Efet.01.78976.g243.t1 | roundabout homolog 1-like isoform X1 | 225 | 1.65E-13 | 60.2 | C:membrane; C:integral component of membrane |
| Efet.01.7898.g582.t1 | ATP-dependent zinc metalloprotease YME1 homolog isoform X3 | 393 | 9.94E-40 | 85.4 | P:cell division; F:ATP binding; P:proteolysis; C:integral component of membrane; F:metalloendopeptidase activity |
| Efet.01.78983.g245.t1 | DC-STAMP domain-containing 2-like | 504 | 9.99E-06 | 60 |  |
| Efet.01.78992.g247.t1 | solute carrier organic anion transporter family member 5A1-like | 390 | 4.95E-11 | 62 | C:membrane |
| Efet.01.7902.g583.t1 | protocadherin-11 X-linked | 2742 | 3.90E-116 | 50.1 | F:calcium ion binding; C:membrane; C:integral component of membrane; P:cell adhesion; P:homophilic cell adhesion via plasma membrane adhesion molecules; C:plasma membrane |
| Efet.01.79028.g250.t1 | dimethylglycine mitochondrial-like | 300 | 9.57E-34 | 82.8 | F:oxidoreductase activity; P:oxidation-reduction process |
| Efet.01.79072.g252.t1 | 1-phosphatidylinositol 4,5-bisphosphate phosphodiesterase epsilon-1-like | 234 | 1.77E-14 | 79.8 | F:phosphatidylinositol phospholipase C activity; P:small GTPase mediated signal transduction; F:guanyl-nucleotide exchange factor activity; P:lipid catabolic process; F:signal transducer activity; C:intracellular; P:positive regulation of GTPase activity |
| Efet.01.79091.g253.t1 | kinesin KIF17 | 552 | 5.93E-90 | 94 | F:ATP binding; P:cell projection organization; C:kinesin complex; P:neurogenesis; F:ATP-dependent microtubule motor activity, plus-end-directed; P:microtubule-based movement; C:microtubule; F:microtubule binding |
| Efet.01.7910.g584.t1 | C3 and PZP-like alpha-2-macroglobulin domain-containing 8 | 876 | 8.85E-32 | 47.75 | F:catalytic activity; C:membrane; C:integral component of membrane; P:nucleoside metabolic process |
| Efet.01.79104.g254.t1 | calcium calmodulin-dependent kinase type II subunit gamma isoform X1 | 573 | 3.64E-20 | 78.25 | F:ATP binding; F:calmodulin binding; F:protein homodimerization activity; C:membrane; P:G1/S transition of mitotic cell cycle; P:calcium ion transport; P:protein autophosphorylation; F:calmodulin-dependent protein kinase activity; P:regulation of relaxation of cardiac muscle; P:protein oligomerization |
| Efet.01.79107.g255.t1 | dynein heavy chain axonemal-like | 267 | 2.52E-20 | 64.55 | F:nucleotide binding; F:ATPase activity; F:ATP binding; F:microtubule motor activity; C:dynein complex; P:microtubule-based movement |
| Efet.01.79107.g256.t1 | dynein heavy chain axonemal-like | 285 | 4.83E-33 | 82.3 | F:microtubule motor activity; P:microtubule-based movement |
| Efet.01.7915.g585.t1 | phosphatidylinositol 3-kinase catalytic subunit type 3 | 288 | 8.53E-31 | 69.75 | F:ATP binding; P:phosphatidylinositol-mediated signaling; P:phosphatidylinositol-3-phosphate biosynthetic process; P:phosphatidylinositol phosphorylation; C:intracellular; F:1-phosphatidylinositol-3-kinase activity |
| Efet.01.79190.g261.t1 | hypothetical protein HELRODRAFT_170772 | 1080 | 1.61E-09 | 53.67 | C:membrane; C:integral component of membrane |
| Efet.01.79215.g264.t1 | neurobeachin isoform X4 | 531 | 2.14E-65 | 69.8 |  |
| Efet.01.79229.g265.t1 | palmitoyltransferase ZDHHC23-like isoform X1 | 342 | 4.77E-28 | 74 | F:transferase activity, transferring acyl groups; F:metal ion binding; C:membrane |
| Efet.01.79238.g266.t1 | TBC1 domain family member 4-like | 576 | 3.87E-26 | 60.25 | P:proteolysis; F:metallopeptidase activity |
| Efet.01.79249.g267.t1 | hypothetical protein CAPTEDRAFT_220592 | 255 | 9.25E-21 | 75 | P:regulation of apoptotic process |
| Efet.01.79283.g268.t1 | vascular endothelial growth factor receptor 1-like isoform X3 | 537 | 2.15E-57 | 71.1 | F:nucleotide binding; C:membrane; F:ion binding; F:protein kinase activity; P:protein phosphorylation |
| Efet.01.79292.g270.t1 | sarcosine mitochondrial-like | 513 | 3.80E-63 | 78.65 | P:choline catabolic process; F:sarcosine dehydrogenase activity; C:mitochondrial matrix; F:flavin adenine dinucleotide binding; F:electron carrier activity; P:oxidation-reduction process |
| Efet.01.79303.g272.t1 | kinesin heavy chain | 975 | 2.34E-123 | 83.75 | F:ATP binding; F:microtubule motor activity; P:microtubule-based movement; C:microtubule; F:microtubule binding |
| Efet.01.79309.g273.t1 | hypothetical protein HELRODRAFT_181350 | 342 | 6.74E-13 | 69 | C:membrane; C:integral component of membrane |
| Efet.01.79311.g274.t1 | golgin subfamily A member 6 22 | 1200 | 6.41E-13 | 54.65 |  |
| Efet.01.79330.g275.t1 | B-cell CLL lymphoma 9 -like isoform X1 | 2877 | 1.16E-12 | 64.4 |  |
| Efet.01.79357.g277.t1 | glycogen-binding subunit 76A | 684 | 3.66E-46 | 46.95 |  |
| Efet.01.79360.g278.t1 | hypothetical protein HELRODRAFT_177171 | 219 | 8.90E-11 | 71 |  |
| Efet.01.79400.g280.t1 | B-box type zinc finger ncl-1-like | 1392 | 5.07E-49 | 49.85 | F:zinc ion binding; F:metal ion binding; C:intracellular |
| Efet.01.79400.g281.t1 | Brain tumor | 1017 | 5.12E-137 | 80.6 | F:zinc ion binding; C:intracellular |
| Efet.01.79415.g284.t1 | low-density lipo receptor | 975 | 3.99E-32 | 57.75 | P:biological regulation; P:lipid transport; C:endosome; P:transcytosis; C:membrane part; F:binding; P:primary metabolic process; P:organic substance metabolic process |
| Efet.01.79415.g285.t1 | prolow-density lipo receptor-related 1-like | 321 | 6.74E-15 | 75.95 | C:membrane |
| Efet.01.79446.g287.t1 | hypothetical protein CAPTEDRAFT_168270 | 930 | 4.19E-125 | 56.45 |  |
| Efet.01.79463.g288.t1 | dynein heavy chain axonemal-like | 636 | 1.19E-91 | 82.5 | F:ATPase activity; F:ATP binding; F:microtubule motor activity; C:axonemal dynein complex; P:cilium movement |
| Efet.01.79480.g289.t1 | general transcription factor II-I repeat domain-containing 2-like | 1359 | 2.81E-88 | 57 |  |
| Efet.01.79533.g292.t1 | mitochondrial 2-oxoglutarate malate carrier | 351 | 5.38E-59 | 81.6 | C:nucleus; F:structural constituent of ribosome; P:mitochondrial transport; C:integral component of membrane; C:mitochondrial inner membrane; F:RNA binding; P:translation |
| Efet.01.79536.g293.t1 | centrosomal of 83 kDa | 219 | 5.89E-13 | 75.85 | P:establishment of localization |
| Efet.01.79591.g295.t1 | E3 ubiquitin- ligase RNF213 | 3621 | 1.89E-20 | 43.86 | F:nucleotide binding; F:ATP binding |
| Efet.01.79591.g296.t1 | E3 ubiquitin- ligase RNF213 | 2283 | 0 | 63.35 | F:nucleotide binding; F:ATPase activity; F:ATP binding |
| Efet.01.79597.g297.t1 | hypothetical protein HELRODRAFT_191437 | 588 | 1.65E-21 | 52 |  |
| Efet.01.79605.g298.t1 | hypothetical protein CAPTEDRAFT_192914 | 492 | 2.59E-08 | 66 | F:G-protein coupled receptor activity; P:G-protein coupled receptor signaling pathway; P:signal transduction; C:membrane; C:integral component of membrane; F:signal transducer activity |
| Efet.01.79666.g300.t1 | sodium channel type 4 subunit alpha B-like isoform X1 | 900 | 4.31E-90 | 61.75 | P:sodium ion transport; P:ion transmembrane transport; F:sodium channel activity; C:integral component of membrane; F:voltage-gated ion channel activity; P:action potential; C:plasma membrane |
| Efet.01.79666.g301.t1 | voltage-gated sodium | 489 | 3.43E-58 | 84.05 | F:voltage-gated potassium channel activity; P:regulation of postsynaptic membrane potential; P:potassium ion transmembrane transport; C:voltage-gated potassium channel complex; F:calcium ion binding; P:regulation of ion transmembrane transport; C:voltage-gated sodium channel complex; P:sodium ion transmembrane transport; F:voltage-gated sodium channel activity |
| Efet.01.79666.g302.t1 | sodium channel para-like isoform X2 | 462 | 4.22E-35 | 77.1 | P:sodium ion transport; P:ion transmembrane transport; F:sodium channel activity; C:integral component of membrane; P:regulation of ion transmembrane transport; F:voltage-gated ion channel activity |
| Efet.01.79678.g303.t1 | probable phospholipid-transporting ATPase IM isoform X3 | 798 | 7.84E-33 | 68.6 | F:magnesium ion binding; F:ATP binding; P:phospholipid translocation; C:integral component of membrane; C:cell part; F:phospholipid-translocating ATPase activity |
| Efet.01.79702.g304.t1 | xylosyltransferase oxt | 237 | 7.34E-10 | 61.75 | F:transferase activity, transferring glycosyl groups; P:sulfur compound biosynthetic process; C:intracellular membrane-bounded organelle; C:membrane; P:glycosaminoglycan biosynthetic process; C:endomembrane system; C:cytoplasmic part; P:proteoglycan biosynthetic process; P:single-organism biosynthetic process; P:oxoacid metabolic process |
| Efet.01.79717.g305.t1 | ubiquitin carboxyl-terminal hydrolase 22-like | 249 | 1.20E-32 | 80 | P:embryo development; P:histone H4 acetylation; F:zinc ion binding; P:transcription, DNA-templated; C:SAGA complex; P:cell cycle; P:positive regulation of mitotic cell cycle; P:ubiquitin-dependent protein catabolic process; F:thiol-dependent ubiquitin-specific protease activity; P:positive regulation of transcription, DNA-templated; F:ligand-dependent nuclear receptor transcription coactivator activity; P:histone ubiquitination; F:H4 histone acetyltransferase activity; F:enzyme binding; P:histone deubiquitination |
| Efet.01.79725.g306.t1 | Hsc70-interacting | 591 | 3.20E-15 | 77.1 |  |
| Efet.01.7973.g587.t1 | follistatin-related 5-like | 756 | 1.45E-51 | 55.4 | F:calcium ion binding |
| Efet.01.79782.g308.t1 | dynein heavy chain axonemal-like isoform X4 | 2187 | 0 | 72.05 | F:nucleotide binding; F:ion binding |
| Efet.01.7979.g588.t1 | repulsive guidance molecule A-like | 690 | 3.05E-51 | 60.85 | C:membrane |
| Efet.01.798.g71.t1 | Heat shock 70 kDa | 2595 | 7.38E-44 | 83.05 | F:nucleotide binding; P:biological_process; F:ATP binding; C:extracellular exosome; F:molecular_function |
| Efet.01.79809.g310.t1 | neuronal acetylcholine receptor subunit alpha-9-like | 621 | 3.16E-25 | 43.4 | C:membrane; P:single-organism process |
| Efet.01.79833.g311.t1 | probable RNA-binding 18 | 699 | 1.50E-28 | 68 | F:organic cyclic compound binding; F:heterocyclic compound binding |
| Efet.01.79836.g312.t1 | 2-hydroxyacylsphingosine 1-beta-galactosyltransferase-like | 819 | 1.59E-60 | 60.3 | F:transferase activity, transferring glycosyl groups |
| Efet.01.79845.g313.t1 | E3 ubiquitin- ligase HECTD1-like isoform X1 | 414 | 2.32E-26 | 81.25 | F:ubiquitin-protein transferase activity; F:metal ion binding; F:ligase activity; P:protein ubiquitination |
| Efet.01.79885.g314.t1 | integrator complex subunit 11-like | 288 | 1.47E-30 | 91.85 | P:snRNA processing; C:cytoplasm; P:regulation of RNA splicing; C:blood microparticle; C:integrator complex; F:hydrolase activity |
| Efet.01.79897.g315.t1 | potassium voltage-gated channel Shaker isoform X10 | 1083 | 0 | 74.35 | F:voltage-gated potassium channel activity; P:potassium ion transmembrane transport; C:voltage-gated potassium channel complex; P:regulation of ion transmembrane transport; P:protein homooligomerization |
| Efet.01.7995.g589.t1 | arrestin domain-containing 3-like | 537 | 4.67E-23 | 56.75 |  |
| Efet.01.79955.g317.t1 | hypothetical protein CAPTEDRAFT_224006 | 726 | 2.56E-10 | 59.5 | F:G-protein coupled receptor activity; P:G-protein coupled receptor signaling pathway; P:signal transduction; C:membrane; C:integral component of membrane; F:signal transducer activity |
| Efet.01.79984.g320.t1 | acyl- dehydrogenase family member mitochondrial-like | 315 | 2.72E-28 | 69.7 | F:acyl-CoA dehydrogenase activity; P:fatty acid beta-oxidation using acyl-CoA dehydrogenase; F:fatty-acyl-CoA binding; F:flavin adenine dinucleotide binding; F:electron carrier activity; F:oxidoreductase activity, acting on the CH-CH group of donors, with a flavin as acceptor; P:lipid homeostasis; C:mitochondrion |
| Efet.01.79997.g321.t1 | isoform A | 318 | 1.97E-27 | 64.4 | C:integral component of membrane |
| Efet.01.80000.g322.t1 | prolactin-releasing peptide receptor-like | 1149 | 2.76E-155 | 74.65 | C:integral component of plasma membrane; P:neuropeptide signaling pathway; P:cell surface receptor signaling pathway; F:neuropeptide Y receptor activity |
| Efet.01.80003.g323.t1 | yippee-like 5 | 393 | 2.54E-25 | 75.25 |  |
| Efet.01.80038.g325.t1 | ABC transporter permease | 1458 | 0 | 55.42 | C:membrane; C:integral component of membrane; C:plasma membrane |
| Efet.01.80038.g326.t1 | cold shock domain-containing | 381 | 1.07E-83 | 93.3 | F:DNA binding; C:cytoplasm; P:regulation of transcription, DNA-templated |
| Efet.01.8004.g590.t1 | zinc finger FYVE domain-containing 1-like isoform X1 | 486 | 1.94E-54 | 70.75 | F:metal ion binding |
| Efet.01.80043.g327.t1 | Paramyosin | 276 | 2.08E-21 | 75.6 | C:myosin complex; F:motor activity |
| Efet.01.80065.g328.t1 | extracellular serine threonine kinase FAM20C-like | 468 | 1.32E-50 | 72 | C:membrane; P:phosphorylation; F:kinase activity |
| Efet.01.80073.g329.t1 | TNF receptor-associated factor 4 | 642 | 2.62E-65 | 68.5 | F:metal ion binding; P:signal transduction; P:positive regulation of protein phosphorylation; P:positive regulation of intracellular signal transduction |
| Efet.01.80079.g330.t1 | Small conductance calcium-activated potassium channel | 417 | 2.47E-60 | 83 | F:calmodulin binding; P:potassium ion transmembrane transport; C:integral component of membrane; F:small conductance calcium-activated potassium channel activity |
| Efet.01.80099.g332.t1 | 40S ribosomal S8 | 576 | 1.10E-121 | 89.15 | P:chordate embryonic development; F:structural constituent of ribosome; P:regulation of cell cycle; C:ribosome; P:translation |
| Efet.01.80115.g336.t1 | uncharacterized protein LOC111122316 isoform X2 | 3447 | 1.92E-06 | 55.93 |  |
| Efet.01.80143.g340.t1 | hypothetical protein CAPTEDRAFT_207080 | 372 | 5.54E-18 | 58.9 | C:membrane; C:integral component of membrane |
| Efet.01.80155.g342.t1 | Ankyrin repeat and SOCS box 2 | 1140 | 3.89E-94 | 55.2 | C:membrane |
| Efet.01.80155.g343.t1 | hypothetical protein HELRODRAFT_180676 | 858 | 7.74E-45 | 51.5 | C:membrane; C:integral component of membrane |
| Efet.01.80157.g344.t1 | DNA primase | 360 | 9.30E-16 | 57.55 | C:membrane; C:integral component of membrane |
| Efet.01.80174.g345.t1 | Proto-oncogene tyrosine- kinase | 270 | 7.39E-20 | 65.4 | F:nucleotide binding; F:protein tyrosine kinase activity; P:protein phosphorylation |
| Efet.01.80193.g346.t1 | chromodomain-helicase-DNA-binding 2 | 276 | 1.73E-17 | 73.95 | F:core promoter sequence-specific DNA binding; C:nucleus; F:ATP binding; P:regulation of transcription, DNA-templated |
| Efet.01.80205.g348.t1 | hypothetical protein OCBIM_22008057mg | 423 | 5.75E-06 | 68 | C:cytoskeleton |
| Efet.01.80219.g349.t1 | G- coupled receptor 54-like | 321 | 3.96E-11 | 81.55 | P:neuropeptide signaling pathway; C:integral component of membrane; F:neuropeptide Y receptor activity |
| Efet.01.80255.g350.t1 | dynein regulatory complex subunit 7 | 462 | 5.87E-10 | 55.6 | C:cytoplasm |
| Efet.01.80256.g351.t1 | hypothetical protein HELRODRAFT_172085 | 351 | 1.69E-23 | 71.33 | P:signal transduction |
| Efet.01.80316.g353.t1 | transcription factor SOX-14 | 1326 | 2.03E-25 | 77.8 |  |
| Efet.01.8032.g593.t1 | hypothetical protein CAPTEDRAFT_228921 | 264 | 1.62E-10 | 58 | C:membrane; C:integral component of membrane; F:carbohydrate binding |
| Efet.01.80345.g355.t1 | polyadenylate-binding 1 | 513 | 5.53E-45 | 78.1 | C:cytoplasmic stress granule; P:positive regulation of translation; F:protein C-terminus binding; F:poly(A) binding; F:translation activator activity; F:poly(U) RNA binding; P:translational initiation; P:positive regulation of nuclear-transcribed mRNA poly(A) tail shortening; P:nuclear-transcribed mRNA poly(A) tail shortening; P:mRNA polyadenylation; F:RNA binding; P:mRNA splicing, via spliceosome; F:nucleotide binding; C:extracellular exosome; C:focal adhesion; C:catalytic step 2 spliceosome; P:nuclear-transcribed mRNA catabolic process, nonsense-mediated decay; C:membrane; P:mRNA stabilization; C:cytosol; P:gene silencing by RNA; P:negative regulation of nuclear-transcribed mRNA catabolic process, nonsense-mediated decay; P:positive regulation of viral genome replication |
| Efet.01.80398.g357.t1 | histidine ammonia-lyase-like | 288 | 2.28E-07 | 50.75 | F:lyase activity |
| Efet.01.80399.g358.t1 | turtle-like isoform X1 | 291 | 2.81E-14 | 39.65 | C:membrane; C:integral component of membrane |
| Efet.01.8040.g594.t1 | Neuropeptide FF receptor 2 | 885 | 1.66E-55 | 59.65 | P:signal transduction; F:signal transducer activity |
| Efet.01.80408.g359.t1 | WD repeat-containing 24 | 522 | 3.46E-15 | 43.55 |  |
| Efet.01.80415.g360.t1 | phospholipid-transporting ATPase ID-like isoform X1 | 594 | 3.53E-25 | 62.05 | F:binding |
| Efet.01.80426.g361.t1 | melanotransferrin-like isoform X1 | 264 | 9.90E-23 | 60.45 | P:protein deubiquitination; P:iron ion homeostasis; F:protein disulfide oxidoreductase activity; F:electron carrier activity; P:cell redox homeostasis; C:extracellular region; P:transport; C:cell; C:extracellular space; P:ion transport; F:metal ion binding; P:oxidation-reduction process; F:thiol-dependent ubiquitinyl hydrolase activity |
| Efet.01.80440.g362.t1 | Enhancer of polycomb | 216 | 5.24E-22 | 88.4 | P:transcription, DNA-templated; C:Piccolo NuA4 histone acetyltransferase complex; P:regulation of transcription from RNA polymerase II promoter |
| Efet.01.8047.g595.t1 | MTO1 mitochondrial | 420 | 2.33E-58 | 85.6 | F:oxidoreductase activity; F:flavin adenine dinucleotide binding; P:oxidation-reduction process; P:tRNA wobble uridine modification |
| Efet.01.80474.g365.t1 | lisH domain-containing -like | 1881 | 3.71E-07 | 56 |  |
| Efet.01.80476.g366.t1 | hypothetical protein CRN43_19745, partial | 405 | 2.75E-14 | 57 |  |
| Efet.01.80477.g367.t1 | Forkhead box F1 | 1185 | 2.31E-50 | 91.35 | C:nucleus; F:transcription factor activity, sequence-specific DNA binding; P:transcription, DNA-templated; P:regulation of transcription, DNA-templated; F:sequence-specific DNA binding |
| Efet.01.80479.g368.t1 | barH-like 2 homeobox | 381 | 1.29E-35 | 68.2 | F:DNA binding |
| Efet.01.8049.g596.t1 | hypothetical protein HELRODRAFT_191437 | 1026 | 5.07E-24 | 46 |  |
| Efet.01.8052.g597.t1 | rootletin isoform X2 | 726 | 6.79E-14 | 67.5 |  |
| Efet.01.80523.g370.t1 | serine threonine- kinase unc-51 | 1113 | 7.11E-15 | 42.25 | F:nucleotide binding; F:ATP binding; F:protein kinase activity; P:phosphorylation; F:protein serine/threonine kinase activity; P:protein phosphorylation; F:kinase activity |
| Efet.01.80523.g371.t1 | Serine threonine- kinase unc- | 339 | 2.29E-11 | 65.05 | F:kinase activity |
| Efet.01.80533.g372.t1 | ain family | 288 | 1.04E-12 | 55.9 | F:peptidase activity |
| Efet.01.80557.g374.t1 | hyccin-like | 330 | 4.78E-16 | 55.5 | F:metal ion binding |
| Efet.01.80561.g375.t1 | AP-3 complex subunit delta-1-like isoform X2 | 417 | 1.42E-53 | 78.3 | P:vesicle-mediated transport; F:protein transporter activity; C:Golgi apparatus; C:AP-3 adaptor complex; P:intracellular protein transport |
| Efet.01.80571.g376.t1 | CCAAT enhancer-binding gamma-like | 396 | 4.29E-11 | 66.8 | F:transcription factor activity, sequence-specific DNA binding; P:transcription, DNA-templated; P:regulation of transcription, DNA-templated; F:sequence-specific DNA binding |
| Efet.01.80572.g377.t1 | gastrula zinc finger isoform X2 | 2013 | 2.75E-21 | 44.25 | F:binding |
| Efet.01.80612.g379.t1 | zinc finger CCCH domain-containing 13-like isoform X7 | 990 | 9.74E-25 | 63.55 |  |
| Efet.01.80642.g380.t1 | serine threonine- kinase par-1-like isoform X3 | 417 | 6.12E-33 | 71.65 | F:ATP binding; F:protein kinase activity; P:protein phosphorylation |
| Efet.01.80663.g381.t1 | urocanate hydratase-like | 216 | 6.29E-27 | 80.2 | F:urocanate hydratase activity |
| Efet.01.80668.g382.t1 | protocadherin Fat 1 isoform X5 | 453 | 1.23E-46 | 62.1 | C:membrane; P:cell adhesion |
| Efet.01.80670.g383.t1 | parkin coregulated gene homolog | 234 | 7.82E-41 | 91.4 | P:spermatid development; P:negative regulation of cell death; F:alpha-tubulin binding; C:neuron projection; C:nucleus; F:G-protein coupled receptor binding; F:ubiquitin protein ligase binding; C:sperm midpiece; F:actin binding; F:beta-tubulin binding; C:mitochondrion; C:cell body; F:chaperone binding; C:cytosol; C:vesicle; F:Hsp90 protein binding; P:cellular response to unfolded protein; F:Hsp70 protein binding |
| Efet.01.80674.g384.t1 | integral membrane 2B-like | 444 | 1.69E-18 | 44.33 | C:membrane; C:integral component of membrane |
| Efet.01.80722.g386.t1 | Phosphoenolpyruvate carboxykinase [GTP] | 693 | 6.32E-101 | 63.05 | F:catalytic activity |
| Efet.01.80735.g387.t1 | tripartite motif-containing 2-like | 843 | 1.54E-16 | 43.7 | F:zinc ion binding; F:metal ion binding; C:intracellular |
| Efet.01.80739.g388.t1 | KIAA0556 isoform X1 | 318 | 9.67E-40 | 79.7 | C:extracellular space; C:plasma membrane; C:nucleoplasm |
| Efet.01.80794.g389.t1 | cGMP-dependent kinase 1- | 291 | 6.30E-52 | 90.7 | F:ATP binding; F:cGMP-dependent protein kinase activity; P:protein phosphorylation; F:cGMP binding |
| Efet.01.80858.g390.t1 | neuronal acetylcholine receptor subunit alpha-5-like isoform X1 | 483 | 3.98E-65 | 82 | C:postsynaptic membrane; P:cation transmembrane transport; C:integral component of membrane; C:cell junction; F:extracellular ligand-gated ion channel activity |
| Efet.01.80872.g391.t1 | allatostatin-A receptor-like | 1161 | 4.01E-98 | 66.85 | P:signal transduction; C:membrane; F:signal transducer activity |
| Efet.01.80891.g393.t1 | Wnt-11b-like isoform X1 [Calidris pugnax] | 333 | 3.25E-09 | 68.25 | C:extracellular region; P:single-organism process |
| Efet.01.80904.g394.t1 | hypothetical protein HELRODRAFT_162187 | 420 | 1.80E-07 | 45 | F:metal ion binding |
| Efet.01.80905.g395.t1 | dopamine receptor 2-like | 1095 | 1.15E-139 | 69.1 | F:dopamine neurotransmitter receptor activity, coupled via Gs; P:adenylate cyclase-activating dopamine receptor signaling pathway; P:synaptic transmission, dopaminergic; F:dopamine binding; C:membrane; P:learning; P:phospholipase C-activating dopamine receptor signaling pathway |
| Efet.01.80921.g397.t1 | tyrosine- phosphatase Lar-like isoform X1 | 396 | 2.65E-31 | 72 | F:phosphoprotein phosphatase activity; P:protein dephosphorylation |
| Efet.01.80957.g398.t1 | fibronectin type III domain | 762 | 7.09E-53 | 55.35 | F:nucleotide binding; F:ATP binding; F:metal ion binding; F:protein kinase activity; P:phosphorylation; F:transferase activity; P:protein phosphorylation; F:kinase activity |
| Efet.01.8099.g598.t1 | guanylate cyclase 32E-like | 474 | 1.32E-28 | 43.6 | F:phosphorus-oxygen lyase activity; C:membrane; C:cell part; P:primary metabolic process; P:single-organism process; P:organic substance metabolic process; P:phosphate-containing compound metabolic process |
| Efet.01.81014.g401.t1 | interferon regulatory factor 2 isoform X2 | 1005 | 5.34E-36 | 69 | F:DNA binding |
| Efet.01.8102.g599.t1 | TELO2-interacting 1 homolog | 1011 | 6.33E-62 | 57.25 |  |
| Efet.01.81027.g402.t1 | hypothetical protein CAPTEDRAFT_205572 | 411 | 3.92E-27 | 83 | C:integral component of membrane; P:cell adhesion; P:tissue regeneration |
| Efet.01.81034.g403.t1 | helix-loop-helix 2 | 543 | 8.40E-27 | 96.1 | C:nucleus; F:RNA polymerase II regulatory region sequence-specific DNA binding; F:protein dimerization activity; F:RNA polymerase II transcription factor activity, sequence-specific DNA binding; P:regulation of transcription from RNA polymerase II promoter |
| Efet.01.81054.g405.t1 | coronin-6 | 312 | 1.13E-19 | 80.15 |  |
| Efet.01.81062.g406.t1 | 20 kDa calcium-binding (Antigen SM20) | 342 | 3.41E-54 | 76.45 | F:calcium ion binding; C:membrane |
| Efet.01.81070.g407.t1 | polyubiquitin 8-like | 642 | 2.99E-34 | 47.45 | F:ubiquitin-protein transferase activity; F:ubiquitin protein ligase activity; F:ubiquitin conjugating enzyme binding; C:ubiquitin ligase complex; C:cytoplasm; P:protein polyubiquitination; P:positive regulation of proteasomal ubiquitin-dependent protein catabolic process; P:protein ubiquitination involved in ubiquitin-dependent protein catabolic process; P:protein ubiquitination |
| Efet.01.81085.g408.t1 | 5-hydroxytryptamine receptor 1A-like | 333 | 1.11E-47 | 81.6 | C:integral component of plasma membrane; F:dopamine neurotransmitter receptor activity; F:adrenergic receptor activity; P:dopamine receptor signaling pathway; P:adrenergic receptor signaling pathway; F:G-protein coupled acetylcholine receptor activity; P:G-protein coupled acetylcholine receptor signaling pathway |
| Efet.01.811.g72.t1 | hemicentin-1 isoform X2 | 387 | 2.06E-17 | 67 | P:fin development |
| Efet.01.81109.g409.t1 | haloacid dehalogenase-like hydrolase domain-containing 3 | 621 | 4.22E-52 | 60.55 | P:metabolic process; C:mitochondrion; F:hydrolase activity |
| Efet.01.81121.g411.t1 | D-beta-hydroxybutyrate mitochondrial-like | 360 | 9.15E-32 | 59.4 | C:membrane |
| Efet.01.81169.g413.t1 | ATP-dependent DNA helicase PIF1 | 570 | 2.90E-45 | 60.9 |  |
| Efet.01.81169.g414.t1 | ATP-dependent DNA helicase PIF1 | 744 | 1.66E-80 | 60.45 | P:nucleic acid phosphodiester bond hydrolysis; F:endonuclease activity; F:helicase activity |
| Efet.01.81169.g415.t1 | helentron 5 helitron transposon replicase helicase endonuclease | 348 | 4.66E-10 | 62.5 |  |
| Efet.01.81169.g416.t1 | ATP-dependent DNA helicase pif1-like | 519 | 3.11E-36 | 56.75 |  |
| Efet.01.81216.g419.t1 | Myosin regulatory light polypeptide | 201 | 7.25E-30 | 92.85 | F:calcium ion binding |
| Efet.01.81222.g420.t1 | transglutaminase domain | 2589 | 0 | 59.75 | C:membrane; P:proteolysis; C:integral component of membrane; F:peptidase activity |
| Efet.01.81246.g422.t1 | ABC transporter permease | 588 | 4.39E-112 | 77.35 | C:integral component of membrane; P:transport; C:plasma membrane |
| Efet.01.81263.g423.t1 | C-1-tetrahydrofolate cytoplasmic | 798 | 4.58E-117 | 72.5 | P:purine nucleotide biosynthetic process; P:somite development; P:folic acid metabolic process; P:10-formyltetrahydrofolate biosynthetic process; F:methylenetetrahydrofolate dehydrogenase (NAD+) activity; F:methylenetetrahydrofolate dehydrogenase [NAD(P)+] activity; F:methylenetetrahydrofolate dehydrogenase (NADP+) activity; F:ATP binding; P:heart development; C:extracellular exosome; P:neural tube closure; C:cytosol; P:tetrahydrofolate interconversion; P:transsulfuration; P:serine family amino acid biosynthetic process; P:methionine biosynthetic process; P:embryonic neurocranium morphogenesis; P:histidine biosynthetic process; P:neutrophil homeostasis; F:methenyltetrahydrofolate cyclohydrolase activity; P:embryonic viscerocranium morphogenesis; F:protein binding; F:formate-tetrahydrofolate ligase activity; C:mitochondrion; C:membrane; P:oxidation-reduction process |
| Efet.01.8128.g601.t1 | nesprin-1-like isoform X1 | 693 | 2.07E-14 | 51.57 | C:nuclear envelope; F:actin filament binding; F:actin binding; P:cytoskeletal anchoring at nuclear membrane; C:LINC complex |
| Efet.01.81308.g424.t1 | nucleo TPR-like | 492 | 1.26E-14 | 60.75 |  |
| Efet.01.81318.g425.t1 | Zinc finger | 972 | 3.10E-44 | 54.6 | F:binding |
| Efet.01.81318.g426.t1 | hypothetical protein HELRODRAFT_168783 | 411 | 1.92E-19 | 75 | F:binding |
| Efet.01.81350.g428.t1 | 1-phosphatidylinositol 4,5-bisphosphate phosphodiesterase beta-1- | 483 | 8.42E-75 | 83.45 | F:phosphatidylinositol phospholipase C activity; P:lipid catabolic process; F:signal transducer activity; C:intracellular; P:intracellular signal transduction |
| Efet.01.81386.g429.t1 | Paramyosin | 1410 | 6.11E-151 | 74.85 | C:myosin complex; C:cytoplasm; F:motor activity |
| Efet.01.81412.g430.t1 | potassium voltage-gated channel subfamily H member 8-like | 744 | 1.65E-67 | 63.9 | P:ion transport; P:transmembrane transport; P:single-organism process; P:cellular process |
| Efet.01.81419.g432.t1 | electroneutral sodium bicarbonate exchanger 1-like isoform X1 | 351 | 1.49E-18 | 63.6 | C:membrane; P:anion transport; F:transporter activity |
| Efet.01.8142.g602.t1 | neuropeptide FF receptor 2-like | 261 | 4.03E-24 | 73.1 | F:nucleotide binding; P:neuropeptide signaling pathway; P:metabolic process; C:integral component of membrane; F:transferase activity; F:neuropeptide Y receptor activity |
| Efet.01.81423.g433.t1 | 3-oxoacyl-[acyl-carrier- ] reductase -like | 804 | 6.16E-76 | 60.6 | F:oxidoreductase activity; P:oxidation-reduction process |
| Efet.01.81463.g434.t1 | hypothetical protein CAPTEDRAFT_222884 | 384 | 3.19E-28 | 62.1 | C:membrane |
| Efet.01.8147.g603.t1 | rho guanine nucleotide exchange factor 11-like isoform X4 | 1035 | 6.07E-76 | 57.9 | P:regulation of Rho protein signal transduction; C:cytoplasm; F:metal ion binding; F:Rho guanyl-nucleotide exchange factor activity; P:intracellular signal transduction; P:positive regulation of GTPase activity |
| Efet.01.81497.g435.t1 | YIPF6 | 342 | 2.97E-18 | 75.8 | C:integral component of membrane |
| Efet.01.81524.g436.t1 | WASH complex subunit 7-like | 327 | 8.14E-21 | 77.95 | C:endosome; P:endosome organization; P:endosomal transport; C:WASH complex |
| Efet.01.81557.g437.t1 | orphan G- coupled receptor 49 | 609 | 1.10E-52 | 57.05 | F:G-protein coupled receptor activity; P:signal transduction; C:membrane |
| Efet.01.81580.g440.t1 | zinc finger 3 homolog | 1428 | 2.04E-23 | 43.35 | F:nucleic acid binding; C:nucleus; F:metal ion binding |
| Efet.01.81581.g441.t1 | pentraxin-related PTX3 | 372 | 2.35E-23 | 56.6 |  |
| Efet.01.81613.g442.t1 | hypothetical protein HELRODRAFT_171296 | 222 | 4.93E-11 | 67 |  |
| Efet.01.81659.g446.t1 | von Willebrand factor D and EGF domain-containing | 2244 | 0 | 52.2 |  |
| Efet.01.81674.g447.t1 | IQ and AAA domain-containing 1-like | 225 | 7.93E-30 | 90.4 | F:ATP binding |
| Efet.01.81693.g448.t1 | signal transducer and activator of transcription 5B | 507 | 2.77E-38 | 53.25 | P:regulation of cellular process; C:intracellular part; P:cellular process |
| Efet.01.81744.g450.t1 | hypothetical protein CAPTEDRAFT_215767 | 624 | 6.28E-98 | 66 |  |
| Efet.01.81750.g451.t1 | glutamate synthase [NADPH] | 1350 | 4.35E-180 | 76.5 | P:glutamate biosynthetic process; F:FMN binding; C:cytoplasm; F:iron ion binding; F:glutamate synthase (NADH) activity; F:flavin adenine dinucleotide binding; P:ammonia assimilation cycle; P:oxidation-reduction process; F:iron-sulfur cluster binding |
| Efet.01.81753.g452.t1 | synaptotagmin-5-like isoform X2 | 660 | 1.89E-13 | 55.05 | F:binding |
| Efet.01.81765.g453.t1 | SH3 and multiple ankyrin repeat domains 3-like isoform X3 | 1866 | 9.82E-10 | 56 |  |
| Efet.01.8178.g605.t1 | sodium potassium ATPase alpha | 348 | 4.94E-45 | 72.8 | F:nucleotide binding; C:extracellular region; P:single-organism process |
| Efet.01.8179.g606.t1 | Golgi-specific brefeldin A-resistance guanine nucleotide exchange factor 1-like isoform X2 | 663 | 1.34E-49 | 59.4 | F:ATP binding; F:ARF guanyl-nucleotide exchange factor activity; P:regulation of ARF protein signal transduction; F:protein kinase activity; P:protein phosphorylation; P:positive regulation of GTPase activity |
| Efet.01.81810.g455.t1 | lysoplasmalogenase TMEM86A | 540 | 9.10E-67 | 73.7 | C:membrane |
| Efet.01.81814.g456.t1 | slit homolog 2 isoform X3 | 906 | 1.99E-84 | 49 | P:Roundabout signaling pathway; F:Roundabout binding; C:extracellular space; P:regulation of axonogenesis; F:calcium ion binding; C:membrane; C:integral component of membrane; F:heparin binding; C:extracellular region; C:proteinaceous extracellular matrix; P:axon extension involved in axon guidance |
| Efet.01.81832.g458.t1 | ankyrin repeat and zinc finger domain-containing 1-like | 582 | 1.10E-06 | 44.5 |  |
| Efet.01.81837.g459.t1 | phosphoenolpyruvate carboxykinase | 282 | 3.70E-38 | 87.5 | F:GTP binding; P:gluconeogenesis; F:phosphoenolpyruvate carboxykinase (GTP) activity; P:phosphorylation; F:kinase activity |
| Efet.01.8184.g609.t1 | discoidin domain-containing receptor 2-like isoform X1 | 819 | 7.88E-110 | 70.8 | F:transmembrane receptor protein tyrosine kinase activity; C:integral component of membrane; P:transmembrane receptor protein tyrosine kinase signaling pathway; P:protein phosphorylation |
| Efet.01.81847.g461.t1 | coiled-coil domain-containing 77-like | 312 | 7.30E-40 | 79.1 |  |
| Efet.01.81873.g462.t1 | probable methyltransferase TARBP1 | 1284 | 1.39E-17 | 52.35 | P:metabolic process; F:methyltransferase activity |
| Efet.01.81876.g463.t1 | phospholipase membrane-associated-like | 336 | 6.48E-22 | 51.1 | F:hydrolase activity, acting on ester bonds |
| Efet.01.81891.g464.t1 | dynein heavy chain axonemal- | 621 | 1.75E-123 | 90.6 | F:ATPase activity; F:ATP binding; F:microtubule motor activity; C:integral component of membrane; C:dynein complex; P:microtubule-based movement |
| Efet.01.81894.g465.t1 | PDZ domain containing | 681 | 2.55E-08 | 66.5 |  |
| Efet.01.81910.g467.t1 | Retrovirus-related Pol poly from transposon | 372 | 3.50E-17 | 60.95 | P:macromolecule metabolic process; F:binding; P:primary metabolic process |
| Efet.01.81939.g468.t1 | receptor-type guanylate cyclase gcy-4-like isoform X2 | 720 | 4.15E-09 | 58.69 | F:catalytic activity; P:primary metabolic process; P:organic substance metabolic process; P:phosphate-containing compound metabolic process |
| Efet.01.82003.g470.t1 | hypothetical protein HELRODRAFT_161987 | 1068 | 1.54E-09 | 73.92 | F:metal ion binding |
| Efet.01.82056.g472.t1 | disulfide-isomerase isoform X2 | 201 | 1.90E-08 | 71.65 | P:regulation of oxidative stress-induced intrinsic apoptotic signaling pathway; F:protein disulfide isomerase activity; C:endoplasmic reticulum; P:cell redox homeostasis; F:peptidyl-proline 4-dioxygenase activity; P:protein folding; P:response to endoplasmic reticulum stress |
| Efet.01.82061.g473.t1 | active breakpoint cluster region-related | 282 | 2.63E-12 | 66.43 | F:molecular function regulator; P:regulation of cellular process |
| Efet.01.82142.g475.t1 | hypothetical protein HELRODRAFT_161790 | 1566 | 1.40E-67 | 46.35 | P:sodium ion transport; P:ion transport; F:sodium channel activity; C:membrane; C:integral component of membrane; P:sodium ion transmembrane transport; P:transport |
| Efet.01.82167.g477.t1 | Selenocysteine-specific elongation | 837 | 2.30E-94 | 73.45 | F:nucleotide binding; F:RNA binding; F:GTPase activity; C:intracellular part; P:translational elongation |
| Efet.01.82172.g478.t1 | orphan G- coupled receptor 49 | 906 | 2.38E-37 | 48.05 | F:signal transducer activity |
| Efet.01.82213.g480.t1 | monocarboxylate transporter 3 isoform X3 | 567 | 7.15E-20 | 59.7 | P:transmembrane transport; C:membrane; C:integral component of membrane |
| Efet.01.82251.g481.t1 | NAD(P)H dehydrogenase [quinone] 1 isoform X2 | 234 | 3.82E-16 | 66.7 | P:synaptic transmission, cholinergic; F:identical protein binding; P:response to organic substance; P:response to toxic substance; P:response to oxygen-containing compound; P:regulation of cellular amino acid metabolic process; F:RNA binding; F:cytochrome-b5 reductase activity, acting on NAD(P)H; C:extracellular exosome; F:NAD(P)H dehydrogenase (quinone) activity; P:xenobiotic metabolic process; C:cytosol; P:nitric oxide biosynthetic process |
| Efet.01.82304.g484.t1 | AF-9- | 261 | 7.88E-24 | 70.6 | P:regulation of cellular process |
| Efet.01.82318.g485.t1 | Kinesin KIF22 | 612 | 5.27E-14 | 65.55 | F:ATP binding; F:microtubule motor activity; P:multicellular organism development; P:cellular component organization; P:nuclear chromosome segregation; P:microtubule-based movement; C:microtubule; F:microtubule binding |
| Efet.01.82347.g486.t1 | transmembrane and TPR repeat-containing 1 | 483 | 2.99E-16 | 49.1 | C:mitochondrion; C:membrane; C:integral component of membrane |
| Efet.01.82356.g487.t1 | muscle M-line assembly unc-89-like | 804 | 2.37E-30 | 62.08 | F:ATP binding; F:protein kinase activity; P:protein phosphorylation |
| Efet.01.82358.g488.t1 | WD repeat-containing 11-like | 993 | 2.36E-133 | 73.65 |  |
| Efet.01.82427.g490.t1 | serine threonine- kinase H1 homolog | 954 | 1.18E-124 | 81.2 | F:ATP binding; P:heart development; F:protein serine/threonine kinase activity; P:protein phosphorylation; P:determination of left/right symmetry |
| Efet.01.82459.g493.t1 | serine threonine- kinase MARK1-like | 255 | 1.47E-47 | 94.85 | C:microtubule cytoskeleton; P:cytoskeleton organization; F:protein serine/threonine kinase activity; P:protein phosphorylation; C:plasma membrane; F:magnesium ion binding; F:tau-protein kinase activity; F:ATP binding; F:phosphatidylinositol-4,5-bisphosphate binding; F:phosphatidylserine binding; P:neuron migration; C:cytoplasm; P:Wnt signaling pathway; F:phosphatidic acid binding; P:intracellular signal transduction |
| Efet.01.82472.g494.t1 | leucine-rich repeats and immunoglobulin-like domains 1 | 375 | 1.14E-21 | 60.3 | C:membrane; C:integral component of membrane |
| Efet.01.8252.g610.t1 | ras-related Rab-32-like | 213 | 4.52E-18 | 67.95 | F:GTP binding; P:small GTPase mediated signal transduction; C:intracellular |
| Efet.01.8255.g611.t1 | neuronal acetylcholine receptor subunit alpha-10-like | 279 | 4.84E-16 | 69 | F:ion channel activity; P:synaptic transmission, cholinergic; F:acetylcholine receptor activity; P:neuromuscular synaptic transmission; P:signal transduction; P:cation transmembrane transport; F:acetylcholine binding; C:acetylcholine-gated channel complex; C:synapse; P:response to nicotine |
| Efet.01.8257.g612.t1 | lamin Dm0-like isoform X1 | 462 | 3.16E-12 | 70.2 | F:structural molecule activity; C:intermediate filament |
| Efet.01.82570.g500.t1 | hypothetical protein HELRODRAFT_166421 | 2811 | 1.61E-06 | 64 |  |
| Efet.01.826.g74.t1 | zinc finger SWIM domain-containing 8-like | 243 | 5.05E-09 | 85.75 | F:zinc ion binding |
| Efet.01.82624.g502.t1 | CDK5 regulatory subunit-associated 1 | 576 | 8.02E-17 | 84.7 | F:methylthiotransferase activity; P:regulation of protein kinase activity; C:cytoplasm; F:4 iron, 4 sulfur cluster binding; F:protein kinase regulator activity; P:tRNA methylthiolation |
| Efet.01.8269.g614.t1 | dynein heavy chain axonemal | 510 | 6.38E-73 | 89.35 | F:ATPase activity; F:ATP binding; F:microtubule motor activity; C:dynein complex; P:microtubule-based movement |
| Efet.01.82718.g506.t1 | sterile alpha motif domain-containing 9-like | 4191 | 1.05E-106 | 51.4 |  |
| Efet.01.82778.g508.t1 | gastrula zinc finger -like | 543 | 2.54E-13 | 49.2 | F:nucleic acid binding; F:metal ion binding |
| Efet.01.82781.g509.t1 | probable Bax inhibitor 1 | 354 | 3.75E-29 | 74.6 | C:integral component of membrane |
| Efet.01.82790.g511.t1 | helix-loop-helix 1 | 522 | 1.55E-31 | 94.25 | C:nucleus; F:RNA polymerase II regulatory region sequence-specific DNA binding; F:protein dimerization activity; F:RNA polymerase II transcription factor activity, sequence-specific DNA binding; P:regulation of transcription from RNA polymerase II promoter |
| Efet.01.82796.g513.t1 | golgin subfamily B member 1-like isoform X1 | 870 | 2.70E-15 | 72.6 |  |
| Efet.01.82813.g514.t1 | hypothetical protein HELRODRAFT_156550 | 237 | 2.14E-08 | 80.5 |  |
| Efet.01.82813.g515.t1 | N-acetylgalactosaminyltransferase 7 | 705 | 3.84E-34 | 72.2 | C:membrane; C:Golgi apparatus; F:transferase activity |
| Efet.01.8285.g615.t1 | probable G- coupled receptor 63 | 699 | 1.86E-25 | 60.85 | F:G-protein coupled receptor activity; P:G-protein coupled receptor signaling pathway; C:membrane; C:integral component of membrane |
| Efet.01.82920.g516.t1 | hypothetical protein CAPTEDRAFT_144547 | 1578 | 1.97E-89 | 57.5 | F:calcium ion binding; C:membrane; C:integral component of membrane; P:cell adhesion; P:homophilic cell adhesion via plasma membrane adhesion molecules; C:plasma membrane |
| Efet.01.82921.g517.t1 | gamma-tubulin complex component 3 homolog | 354 | 1.60E-26 | 66.35 | C:centrosome; F:microtubule minus-end binding; C:gamma-tubulin small complex; P:centrosome duplication; C:equatorial microtubule organizing center; C:spindle pole body; F:structural constituent of cytoskeleton; F:gamma-tubulin binding; P:cytoplasmic microtubule organization; P:interphase microtubule nucleation by interphase microtubule organizing center; P:meiotic nuclear division; P:mitotic spindle assembly |
| Efet.01.82953.g520.t1 | SWI SNF complex subunit SMARCC1- | 327 | 3.38E-39 | 80.7 | C:SWI/SNF complex; F:DNA binding; P:ATP-dependent chromatin remodeling; P:regulation of transcription from RNA polymerase II promoter |
| Efet.01.82959.g521.t1 | ABC transporter substrate-binding | 276 | 4.33E-57 | 85.45 | P:transmembrane transport; C:ATP-binding cassette (ABC) transporter complex |
| Efet.01.82970.g522.t1 | copper radical oxidase | 579 | 7.48E-16 | 59.8 |  |
| Efet.01.82999.g524.t1 | probable ATP-dependent RNA helicase DDX4 isoform X1 | 957 | 4.95E-168 | 82.25 | F:nucleic acid binding; F:ATP binding; F:zinc ion binding; C:perinuclear region of cytoplasm; C:P granule; P:RNA secondary structure unwinding; F:ATP-dependent RNA helicase activity |
| Efet.01.83016.g525.t1 | thyrotropin-releasing hormone receptor | 762 | 1.81E-66 | 62.8 | F:G-protein coupled receptor activity; P:G-protein coupled receptor signaling pathway; C:integral component of membrane |
| Efet.01.83019.g526.t1 | sidekick-2 isoform X1 | 1602 | 3.71E-76 | 53.9 | P:synapse assembly; C:membrane; C:integral component of membrane; P:retina layer formation; P:camera-type eye photoreceptor cell differentiation; P:homophilic cell adhesion via plasma membrane adhesion molecules; C:synapse |
| Efet.01.83040.g528.t1 | muscarinic acetylcholine receptor M1-like | 393 | 7.43E-45 | 77.2 | C:integral component of plasma membrane; F:G-protein coupled acetylcholine receptor activity; P:G-protein coupled acetylcholine receptor signaling pathway |
| Efet.01.83040.g530.t1 | muscarinic acetylcholine receptor M1-like | 1062 | 9.01E-100 | 76.15 | C:integral component of plasma membrane; F:G-protein coupled acetylcholine receptor activity; P:G-protein coupled acetylcholine receptor signaling pathway |
| Efet.01.83082.g531.t1 | potassium channel | 1563 | 1.20E-147 | 67.4 | F:ion channel activity; P:ion transport; C:membrane |
| Efet.01.83085.g532.t1 | epidermal growth factor receptor kinase substrate 8 isoform X1 | 390 | 3.36E-24 | 67 |  |
| Efet.01.83187.g535.t1 | c-binding -like | 1398 | 3.52E-21 | 44.3 |  |
| Efet.01.8319.g616.t1 | ryanodine receptor 44F-like | 489 | 5.54E-24 | 69.7 | F:ryanodine-sensitive calcium-release channel activity; C:membrane; P:release of sequestered calcium ion into cytosol; C:intracellular |
| Efet.01.83195.g536.t1 | Spec 1a | 285 | 3.41E-27 | 70.29 | F:calcium ion binding |
| Efet.01.83209.g537.t1 | Zn-dependent alcohol dehydrogenase | 1071 | 0 | 68.95 | F:zinc ion binding; F:metal ion binding; F:oxidoreductase activity; P:oxidation-reduction process |
| Efet.01.83209.g538.t1 | xylose isomerase | 747 | 0 | 74.15 | F:isomerase activity |
| Efet.01.83214.g539.t1 | leucine-rich repeat neuronal 3-like | 1035 | 8.07E-39 | 47.4 | F:phosphoprotein phosphatase activity; C:extracellular space; P:axonogenesis; C:membrane; P:protein dephosphorylation; C:integral component of membrane; C:proteinaceous extracellular matrix; F:hydrolase activity |
| Efet.01.83242.g541.t1 | Wnt-4 isoform X1 | 309 | 7.85E-49 | 70.15 | C:extracellular space; F:frizzled binding; P:neuron differentiation; P:cell fate commitment; P:Wnt signaling pathway; C:proteinaceous extracellular matrix |
| Efet.01.83284.g545.t1 | hypothetical protein HELRODRAFT_167174 | 477 | 3.38E-14 | 45.5 |  |
| Efet.01.83312.g546.t1 | sex peptide receptor-like | 861 | 1.63E-53 | 50.35 | F:G-protein coupled receptor activity; F:G-protein coupled peptide receptor activity; P:G-protein coupled receptor signaling pathway; C:membrane; C:integral component of membrane |
| Efet.01.83380.g548.t1 | microtubule-associated futsch-like isoform X2 | 603 | 6.92E-07 | 62.5 |  |
| Efet.01.83394.g550.t1 | cytochrome P450 10-like | 342 | 2.32E-41 | 66.75 | F:oxidoreductase activity; F:metal ion binding |
| Efet.01.83435.g551.t1 | large conductance calcium-activated potassium (BK) channel alpha subunit | 243 | 1.55E-11 | 64.6 | F:calcium-activated potassium channel activity; P:biological regulation; P:ion transport; F:protein binding; C:integral component of membrane; P:response to auditory stimulus; C:plasma membrane region; P:single-organism cellular process |
| Efet.01.83481.g552.t1 | cholinesterase 1- | 417 | 8.91E-06 | 51 |  |
| Efet.01.83559.g557.t1 | hypothetical protein CAPTEDRAFT_222036 | 237 | 5.57E-13 | 63.5 | C:membrane; C:integral component of membrane |
| Efet.01.83612.g558.t1 | WD repeat-containing 20-like | 1161 | 6.64E-123 | 76.2 |  |
| Efet.01.83613.g559.t1 | basement membrane-specific heparan sulfate proteoglycan core -like | 717 | 2.89E-07 | 60.8 |  |
| Efet.01.83657.g560.t1 | diacylglycerol kinase delta | 369 | 1.12E-15 | 56.05 | C:cell part; F:binding; F:transferase activity; P:cellular process; P:response to stimulus |
| Efet.01.83681.g561.t1 | breast cancer anti-estrogen resistance 3-like isoform X5 | 2313 | 8.85E-107 | 53.65 | P:small GTPase mediated signal transduction; F:guanyl-nucleotide exchange factor activity; P:positive regulation of GTPase activity; C:intracellular |
| Efet.01.83686.g562.t1 | inactive tyrosine- kinase 7 | 564 | 1.42E-50 | 64.35 | P:lung-associated mesenchyme development; P:planar cell polarity pathway involved in neural tube closure; P:cochlea morphogenesis; P:ventricular septum development; P:coronary vasculature development; P:protein phosphorylation; P:convergent extension; C:plasma membrane; P:axis elongation; F:ATP binding; P:wound healing; C:cell-cell junction; F:transmembrane receptor protein tyrosine kinase activity; P:establishment of planar polarity; P:establishment of epithelial cell apical/basal polarity |
| Efet.01.83692.g563.t1 | sodium-dependent serotonin transporter | 333 | 3.87E-31 | 76.45 | P:serotonin uptake; F:neurotransmitter:sodium symporter activity; F:serotonin transmembrane transporter activity; P:transmembrane transport; F:cation channel activity; C:integral component of membrane; P:phagocytosis; C:plasma membrane; C:axon; C:axon terminus |
| Efet.01.83702.g564.t1 | nesprin-1-like isoform X1 | 2436 | 1.37E-35 | 82.75 | F:actin filament binding; P:cytoskeletal anchoring at nuclear membrane; C:LINC complex |
| Efet.01.83710.g565.t1 | fat-like cadherin-related tumor suppressor homolog isoform X1 | 933 | 4.04E-85 | 57.65 | C:membrane |
| Efet.01.83816.g566.t1 | NADH-ubiquinone reductase 75 kDa subunit | 246 | 2.11E-39 | 70.3 | P:oxidation-reduction process; F:oxidoreductase activity, acting on NAD(P)H |
| Efet.01.8386.g618.t1 | D(1A) dopamine receptor | 1137 | 4.40E-38 | 46.2 | P:G-protein coupled receptor signaling pathway |
| Efet.01.83865.g568.t1 | Hau-cytoplasmic intermediate filament | 261 | 5.44E-25 | 79.45 | F:structural molecule activity; C:intermediate filament |
| Efet.01.83965.g570.t1 | major vault -like | 324 | 2.07E-40 | 92.8 | P:eye development; P:neuron projection regeneration; P:brain development |
| Efet.01.84024.g575.t1 | guanine nucleotide exchange factor DBS- | 339 | 2.06E-31 | 62.9 | P:regulation of Rho protein signal transduction; F:Rho guanyl-nucleotide exchange factor activity; P:positive regulation of GTPase activity |
| Efet.01.84032.g576.t1 | hypothetical protein CAPTEDRAFT_105822 | 429 | 7.99E-07 | 46 |  |
| Efet.01.8404.g619.t1 | G2 mitotic-specific cyclin-B-like | 414 | 2.44E-32 | 67 | C:nucleus |
| Efet.01.84076.g577.t1 | LOW QUALITY PROTEIN: conserved hypothetical protein | 1887 | 1.08E-12 | 45 |  |
| Efet.01.8411.g620.t1 | extracellular tyrosine- kinase PKDCC-like | 294 | 1.08E-15 | 72.6 | F:ATP binding; C:membrane; F:protein kinase activity; P:protein phosphorylation |
| Efet.01.84118.g578.t1 | Inter-alpha-trypsin inhibitor heavy chain H3 | 711 | 2.38E-53 | 56.4 | P:response to cytokine; F:serine-type endopeptidase inhibitor activity; C:extracellular exosome; C:cytoplasm; C:blood microparticle; C:membrane; C:integral component of membrane; P:negative regulation of endopeptidase activity; P:hyaluronan metabolic process; P:acute-phase response; C:plasma membrane |
| Efet.01.8420.g621.t1 | glycine receptor subunit alpha-4-like isoform X1 | 579 | 7.17E-64 | 73.85 | P:ion transport; P:signal transduction; C:integral component of membrane; F:extracellular ligand-gated ion channel activity; P:chemical synaptic transmission; C:synapse; C:plasma membrane |
| Efet.01.8421.g622.t1 | TRPC channel, partial | 399 | 5.63E-15 | 63 | F:ion channel activity; P:ion transport; P:transmembrane transport; F:calcium channel activity; C:membrane; C:integral component of membrane; P:calcium ion transmembrane transport; P:transport |
| Efet.01.84215.g581.t1 | zinc finger | 1620 | 1.98E-19 | 60.65 | F:nucleic acid binding; F:metal ion binding |
| Efet.01.84223.g582.t1 | regulator of G- signaling 9-binding -like | 477 | 6.33E-10 | 60.5 | C:membrane; C:integral component of membrane |
| Efet.01.84247.g583.t1 | junctophilin-1 isoform X1 | 657 | 6.73E-23 | 62.6 | P:pseudouridine synthesis; F:RNA binding; P:RNA modification; F:pseudouridine synthase activity |
| Efet.01.84253.g584.t1 | collagen alpha-1(XII) chain-like | 570 | 1.85E-25 | 52.95 | F:calcium ion binding |
| Efet.01.8426.g623.t1 | FMRFamide receptor-like | 1206 | 1.43E-121 | 64.4 | F:G-protein coupled peptide receptor activity; P:G-protein coupled receptor signaling pathway; C:integral component of membrane |
| Efet.01.84283.g585.t1 | hypothetical protein CAPTEDRAFT_226428 | 279 | 2.90E-08 | 71 | C:membrane |
| Efet.01.84293.g586.t1 | formin CG32138 | 465 | 1.09E-17 | 60.45 | F:actin binding; P:cellular component organization; F:Rho GTPase binding; P:actin cytoskeleton organization |
| Efet.01.84363.g587.t1 | meiotic nuclear division 1 homolog | 333 | 1.12E-44 | 78.55 |  |
| Efet.01.84390.g588.t1 | leucine-rich repeat serine threonine- kinase 1 | 696 | 9.23E-30 | 53.7 | F:GTP binding; F:ATP binding; P:small GTPase mediated signal transduction; F:protein kinase activity; P:phosphorylation; P:protein phosphorylation; F:kinase activity; C:intracellular |
| Efet.01.84401.g589.t1 | titin-like isoform X1 | 846 | 3.63E-22 | 56.9 |  |
| Efet.01.8442.g627.t1 | hypothetical protein CAPTEDRAFT_222213 | 228 | 1.17E-11 | 68 | C:membrane; C:integral component of membrane; F:sulfotransferase activity |
| Efet.01.84441.g591.t1 | kelch 24 | 708 | 8.02E-45 | 56.3 | F:ubiquitin-protein transferase activity; C:Cul3-RING ubiquitin ligase complex; P:regulation of kainate selective glutamate receptor activity; P:protein ubiquitination |
| Efet.01.84481.g593.t1 | Dynein heavy chain axonemal | 276 | 1.98E-18 | 80.65 | F:microtubule motor activity; C:dynein complex; P:microtubule-based movement |
| Efet.01.8450.g628.t1 | carbohydrate sulfotransferase 1-like | 1161 | 6.69E-49 | 44.45 | C:membrane; C:integral component of membrane; F:sulfotransferase activity |
| Efet.01.84526.g594.t1 | F-box only | 1416 | 1.88E-55 | 42.76 |  |
| Efet.01.84572.g595.t1 | udp-n-acetylglucosamine--peptide n-acetylglucosaminyltransferase | 750 | 9.66E-80 | 56.15 | F:transferase activity, transferring glycosyl groups; C:membrane; C:integral component of membrane; F:transferase activity |
| Efet.01.84585.g596.t1 | sphingosine kinase 2 isoform X4 | 441 | 4.59E-16 | 48.06 | P:phosphorylation; F:kinase activity |
| Efet.01.84597.g598.t1 | Upstream activation factor subunit spp27 | 207 | 4.31E-26 | 78.35 | F:DNA binding; P:nucleic acid phosphodiester bond hydrolysis; F:calcium ion binding; F:nuclease activity; P:DNA metabolic process |
| Efet.01.84597.g599.t1 | seleno O-like | 1035 | 9.34E-123 | 68.75 |  |
| Efet.01.84597.g600.t1 | seleno O-like | 834 | 8.80E-64 | 71.3 |  |
| Efet.01.84624.g601.t1 | ankyrin repeat | 1257 | 3.10E-39 | 50.9 |  |
| Efet.01.84624.g602.t1 | ankyrin repeat | 1884 | 3.16E-56 | 47.5 | F:ubiquitin-protein transferase activity; P:protein ubiquitination |
| Efet.01.84646.g603.t1 | potassium sodium hyperpolarization-activated cyclic nucleotide-gated channel 3 isoform X2 | 513 | 2.83E-17 | 80.25 | F:voltage-gated potassium channel activity; P:potassium ion transmembrane transport; C:integral component of membrane |
| Efet.01.84684.g606.t1 | cyclin-dependent kinase-like 1 isoform X3 | 294 | 2.19E-48 | 89.35 | F:ATP binding; C:extracellular exosome; F:protein serine/threonine kinase activity; P:protein phosphorylation |
| Efet.01.84712.g607.t1 | histone acetyltransferase p300 | 1695 | 5.43E-49 | 88.65 | F:histone acetyltransferase activity; F:zinc ion binding; F:transcription coactivator activity; P:regulation of transcription, DNA-templated; P:histone acetylation; C:histone acetyltransferase complex |
| Efet.01.84755.g608.t1 | laminin subunit alpha | 360 | 4.35E-32 | 47.8 |  |
| Efet.01.84780.g609.t1 | photoreceptor-specific nuclear receptor | 276 | 2.35E-23 | 78.2 | F:zinc ion binding; P:mushroom body development; P:transcription, DNA-templated; P:imaginal disc-derived wing expansion; P:axon guidance; F:sequence-specific DNA binding; P:positive regulation of axon extension involved in axon guidance; P:steroid hormone mediated signaling pathway; F:heme binding; P:regulation of transcription from RNA polymerase II promoter; C:nucleus; F:RNA polymerase II transcription factor activity, ligand-activated sequence-specific DNA binding; P:locomotor rhythm; F:steroid hormone receptor activity; P:neuron remodeling; P:axon regeneration; P:intracellular receptor signaling pathway |
| Efet.01.848.g76.t1 | neuronal acetylcholine receptor subunit alpha-10-like isoform X1 | 594 | 3.87E-26 | 66.25 | C:postsynaptic membrane; P:cation transmembrane transport; C:integral component of membrane; C:cell junction; F:extracellular ligand-gated ion channel activity |
| Efet.01.84848.g617.t1 | baculoviral IAP repeat-containing 3-like | 426 | 1.27E-23 | 76.45 | P:positive regulation of I-kappaB kinase/NF-kappaB signaling; P:regulation of nucleic acid-templated transcription; F:cysteine-type endopeptidase inhibitor activity involved in apoptotic process; C:nucleoplasm; P:negative regulation of necroptotic process; P:inhibition of cysteine-type endopeptidase activity involved in apoptotic process; P:regulation of cell differentiation; P:positive regulation of protein ubiquitination; C:cytosol; P:regulation of nucleotide-binding oligomerization domain containing signaling pathway; P:regulation of cell cycle; F:zinc ion binding; P:regulation of RIG-I signaling pathway; P:protein polyubiquitination; P:regulation of toll-like receptor signaling pathway; P:NIK/NF-kappaB signaling; P:I-kappaB kinase/NF-kappaB signaling; C:spindle microtubule; F:ubiquitin-protein transferase activity; P:tumor necrosis factor-mediated signaling pathway; F:protein binding; F:transcription coactivator activity; P:regulation of tumor necrosis factor-mediated signaling pathway; P:regulation of inflammatory response; P:TRIF-dependent toll-like receptor signaling pathway; P:mitotic spindle assembly |
| Efet.01.84875.g619.t1 | titin-like isoform X1 | 804 | 1.18E-69 | 58.05 |  |
| Efet.01.84885.g620.t1 | Neuron navigator 2 | 285 | 6.66E-10 | 45.8 | F:nucleotide binding; F:ATP binding; P:ubiquinone biosynthetic process |
| Efet.01.84888.g622.t1 | paraplegin | 348 | 2.34E-30 | 56.2 | F:binding |
| Efet.01.84945.g625.t1 | focal adhesion kinase 1-like isoform X1 | 342 | 1.94E-20 | 77.85 | F:ATP binding; P:signal complex assembly; P:peptidyl-tyrosine phosphorylation; F:protein tyrosine kinase activity; C:cytoskeleton; C:focal adhesion; P:signal transduction; F:signal transducer activity |
| Efet.01.84951.g626.t1 | Pyruvate dehydrogenase X mitochondrial | 264 | 7.38E-24 | 72.8 | F:octanoyltransferase activity; F:S-acetyltransferase activity; F:succinyltransferase activity; F:C-acyltransferase activity; F:protein-cysteine S-acyltransferase activity; F:malonyltransferase activity; F:sterol O-acyltransferase activity; F:N-acetyltransferase activity; F:sinapoyltransferase activity; F:N-palmitoyltransferase activity; F:dihydrolipoamide S-acyltransferase activity; F:myristoyltransferase activity |
| Efet.01.84966.g627.t1 | menin-like isoform X1 | 1083 | 4.03E-35 | 76.1 | C:nucleus |
| Efet.01.85036.g629.t1 | coiled-coil and C2 domain-containing 2A-like isoform X1 | 480 | 3.38E-43 | 62.75 |  |
| Efet.01.85062.g630.t1 | HHIP 1 isoform X4 | 963 | 1.44E-91 | 64.15 | P:carbohydrate metabolic process; F:oxidoreductase activity, acting on the CH-OH group of donors, quinone or similar compound as acceptor; F:quinone binding; F:catalytic activity; P:oxidation-reduction process |
| Efet.01.85077.g631.t1 | melatonin receptor type 1A-like | 1338 | 2.57E-14 | 45.75 | C:integral component of plasma membrane; F:G-protein coupled receptor activity; P:G-protein coupled receptor signaling pathway; P:signal transduction; C:membrane; C:integral component of membrane; P:cell surface receptor signaling pathway; F:signal transducer activity; F:melatonin receptor activity |
| Efet.01.85095.g632.t1 | novel [Xenopus tropicalis] | 1182 | 8.39E-116 | 53.2 |  |
| Efet.01.85131.g633.t1 | membrane progestin receptor beta-like | 927 | 1.70E-114 | 61.5 | C:membrane; P:response to stimulus |
| Efet.01.8515.g630.t1 | alcohol dehydrogenase-like | 1056 | 2.98E-71 | 48.8 | F:zinc ion binding; F:oxidoreductase activity; F:metal ion binding; P:oxidation-reduction process |
| Efet.01.85236.g637.t1 | matrix metallopeptidase-21-like | 930 | 1.07E-07 | 74 | F:peptidase activity |
| Efet.01.85248.g638.t1 | hypothetical protein CAPTEDRAFT_216764 | 1380 | 2.76E-34 | 54.2 |  |
| Efet.01.85264.g639.t1 | serine threonine- kinase PAK 1-like isoform X1 | 513 | 7.23E-17 | 59.05 | F:kinase activity |
| Efet.01.85286.g641.t1 | hypothetical protein CAPTEDRAFT_226946 | 1071 | 7.16E-19 | 49 |  |
| Efet.01.85300.g642.t1 | AP-4 complex subunit beta-1 | 1503 | 2.71E-83 | 57.9 | C:membrane; P:transport |
| Efet.01.85308.g643.t1 | clavesin-2 | 336 | 9.37E-25 | 60.3 | P:transport; F:transporter activity; C:intracellular |
| Efet.01.85327.g644.t1 | hypothetical protein HELRODRAFT_180676 | 2163 | 4.28E-147 | 48.75 | F:nucleotide binding; F:nucleic acid binding; F:RNA binding; C:membrane; C:integral component of membrane; P:RNA processing |
| Efet.01.85329.g645.t1 | PREDICTED: uncharacterized protein LOC101856271 | 786 | 3.83E-23 | 53.65 |  |
| Efet.01.85359.g646.t1 | follistatin-related 5-like | 627 | 6.74E-44 | 51.3 | F:calcium ion binding; C:membrane; P:protein glycosylation; F:galactosyltransferase activity |
| Efet.01.85372.g647.t1 | disintegrin and metallo ase domain-containing 12-like isoform X2 | 387 | 4.40E-33 | 68.1 | F:metallopeptidase activity |
| Efet.01.85380.g648.t1 | low-density lipo receptor-related 2-like | 270 | 3.13E-16 | 61.9 | F:calcium ion binding; C:membrane; C:integral component of membrane |
| Efet.01.85450.g651.t1 | hypothetical protein HELRODRAFT_193953 | 2292 | 1.56E-07 | 84 | C:integral component of membrane; P:transport; F:transporter activity |
| Efet.01.85484.g654.t1 | sterile alpha motif domain-containing 9-like | 267 | 1.63E-07 | 64.8 |  |
| Efet.01.85490.g655.t1 | 60 kDa neurofilament -like | 525 | 4.52E-23 | 68.5 | F:structural molecule activity; C:intermediate filament |
| Efet.01.85522.g657.t1 | GAS2 pickled | 663 | 1.55E-25 | 81.65 | P:cell cycle arrest |
| Efet.01.85527.g658.t1 | adenylate cyclase type 9-like | 1302 | 4.74E-142 | 66.2 | F:phosphorus-oxygen lyase activity; C:membrane; P:cyclic nucleotide biosynthetic process |
| Efet.01.85540.g659.t1 | hypothetical protein CAPTEDRAFT_73789, partial | 204 | 2.13E-06 | 73 | C:integral component of plasma membrane; P:riboflavin transport; C:membrane; C:integral component of membrane; F:riboflavin transporter activity |
| Efet.01.85563.g660.t1 | myosin-IIIb-like isoform X1 | 285 | 3.70E-31 | 85.45 | C:myosin complex; F:ATP binding; F:galactosylceramide sulfotransferase activity; F:actin binding; C:integral component of membrane; P:glycolipid biosynthetic process; C:Golgi apparatus; F:protein kinase activity; P:protein phosphorylation; F:motor activity |
| Efet.01.85576.g661.t1 | serine arginine-rich splicing factor 7-like | 231 | 3.55E-27 | 80.1 | F:nucleotide binding; F:nucleic acid binding; F:zinc ion binding |
| Efet.01.85609.g663.t1 | neurexin-3-beta-like isoform X1 | 555 | 7.84E-52 | 58.6 | P:social behavior; F:protein binding; F:metal ion binding; C:integral component of membrane; P:nervous system development; P:cellular component organization; P:chemical synaptic transmission; P:cell adhesion; P:learning; P:adult behavior; C:plasma membrane part; P:vocalization behavior |
| Efet.01.85612.g664.t1 | Wnt11 | 213 | 1.11E-07 | 74.5 | C:extracellular region; P:single-organism process |
| Efet.01.85630.g665.t1 | beta-soluble NSF attachment -like isoform X2 | 321 | 3.63E-23 | 59.5 | C:intracellular |
| Efet.01.85640.g667.t1 | EGF-like domain-containing | 624 | 6.62E-64 | 48.85 | F:calcium ion binding; C:membrane; C:integral component of membrane |
| Efet.01.85650.g669.t1 | sodium-dependent noradrenaline transporter-like | 474 | 4.80E-34 | 91 | C:integral component of plasma membrane; C:presynapse; P:dopamine uptake involved in synaptic transmission; P:ammonium transmembrane transport; F:calcium ion binding; F:dopamine:sodium symporter activity; C:neuron projection |
| Efet.01.85693.g672.t1 | allatostatin-A receptor-like | 729 | 4.28E-42 | 59.2 | F:G-protein coupled receptor activity; P:G-protein coupled receptor signaling pathway; C:integral component of membrane |
| Efet.01.85704.g673.t1 | neurogenic locus Notch | 237 | 1.77E-13 | 79.5 | P:regulation of developmental process; P:Notch signaling pathway; P:multicellular organism development; F:calcium ion binding; C:integral component of membrane; P:cell differentiation |
| Efet.01.85705.g674.t1 | ubiquitin thioesterase OTUB1-like | 225 | 1.85E-31 | 80 | F:thiol-dependent ubiquitin-specific protease activity; C:nucleus; F:ubiquitin binding; F:NEDD8-specific protease activity; P:protein K48-linked deubiquitination |
| Efet.01.85725.g676.t1 | hypothetical protein LOTGIDRAFT_108839 | 672 | 5.66E-06 | 51 | P:ion transport; P:potassium ion transmembrane transport; C:membrane; C:integral component of membrane; F:potassium channel activity; P:transport |
| Efet.01.85725.g677.t1 | T family of potassium channels 18 | 729 | 7.33E-55 | 58.9 | P:transport |
| Efet.01.85725.g678.t1 | Neurogenic locus delta | 897 | 2.07E-24 | 51.75 | P:Notch signaling pathway; P:multicellular organism development; P:cell communication; F:calcium ion binding; C:membrane; C:integral component of membrane |
| Efet.01.85730.g680.t1 | hepatocyte nuclear factor 4-gamma isoform X1 | 369 | 1.71E-18 | 85.95 | F:zinc ion binding; F:RNA polymerase II transcription factor activity, ligand-activated sequence-specific DNA binding; F:steroid hormone receptor activity; F:sequence-specific DNA binding; P:steroid hormone mediated signaling pathway; P:transcription initiation from RNA polymerase II promoter; C:nucleoplasm; P:regulation of transcription from RNA polymerase II promoter; P:intracellular receptor signaling pathway |
| Efet.01.85743.g681.t1 | Zinc transporter ZIP3 | 1137 | 1.14E-45 | 50 | P:ion transport |
| Efet.01.85794.g685.t1 | transient receptor potential cation channel subfamily M member 1-like | 258 | 1.67E-14 | 63.13 | P:ion transport; P:transmembrane transport |
| Efet.01.85824.g687.t1 | 78 kDa glucose-regulated | 273 | 4.28E-26 | 94.75 | F:ATPase activity; C:myelin sheath; C:smooth endoplasmic reticulum; C:endoplasmic reticulum-Golgi intermediate compartment; P:positive regulation of embryonic development; P:ER overload response; C:endoplasmic reticulum lumen; F:ATP binding; P:cerebellar Purkinje cell layer development; C:extracellular exosome; C:focal adhesion; P:cellular response to interleukin-4; P:positive regulation of protein ubiquitination; P:maintenance of protein localization in endoplasmic reticulum; F:glycoprotein binding; P:positive regulation of neuron projection development; C:extracellular matrix; C:endoplasmic reticulum chaperone complex; C:COP9 signalosome; P:proteolysis involved in cellular protein catabolic process; F:misfolded protein binding; F:protein domain specific binding; P:cerebellum structural organization; F:unfolded protein binding; P:toxin transport; C:plasma membrane; P:substantia nigra development; P:cellular response to glucose starvation; C:cell surface; F:cadherin binding involved in cell-cell adhesion; P:activation of signaling protein activity involved in unfolded protein response; F:ubiquitin protein ligase binding; C:cell-cell adherens junction; C:melanosome; F:ribosome binding; P:negative regulation of apoptotic process; C:mitochondrion; P:cellular response to manganese ion; C:integral component of endoplasmic reticulum membrane; C:midbody; P:cell-cell adhesion; P:negative regulation of transforming growth factor beta receptor signaling pathway; P:positive regulation of cell migration |
| Efet.01.85915.g689.t1 | Programmed cell death 6-interacting | 654 | 1.64E-36 | 83.55 |  |
| Efet.01.85922.g690.t1 | polyubiquitin 8-like | 657 | 5.06E-46 | 51.3 | F:ubiquitin-protein transferase activity; F:ubiquitin protein ligase activity; F:ubiquitin conjugating enzyme binding; C:ubiquitin ligase complex; C:cytoplasm; F:metal ion binding; P:protein polyubiquitination; P:positive regulation of proteasomal ubiquitin-dependent protein catabolic process; P:protein ubiquitination involved in ubiquitin-dependent protein catabolic process; P:protein ubiquitination |
| Efet.01.85940.g691.t1 | NACHT and WD repeat domain-containing 2-like | 3558 | 0 | 71.95 | F:GDP-dissociation inhibitor activity; C:Rab-protein geranylgeranyltransferase complex; P:small GTPase mediated signal transduction; F:oxidoreductase activity; P:regulation of catalytic activity; P:oxidation-reduction process; P:protein geranylgeranylation; P:intracellular protein transport |
| Efet.01.85957.g693.t1 | ATP-dependent RNA helicase me31b | 348 | 5.16E-62 | 94.8 | F:nucleic acid binding; P:regulation of translation; F:ATP binding; C:cytoplasmic stress granule; P:RNA secondary structure unwinding; P:cytoplasmic mRNA processing body assembly; C:P-body; F:ATP-dependent RNA helicase activity |
| Efet.01.86075.g694.t1 | autophagy-related 2 homolog B-like | 231 | 7.95E-16 | 61.8 | P:autophagy |
| Efet.01.86113.g695.t1 | lethal(2)essential for life | 372 | 4.34E-18 | 62.9 |  |
| Efet.01.86113.g696.t1 | heat shock beta-6 | 702 | 3.09E-14 | 58.4 | C:nucleus; C:cytoplasm; F:structural constituent of eye lens |
| Efet.01.86115.g697.t1 | Myotubularin-related 13 | 276 | 4.02E-14 | 75.9 | F:metal ion binding; C:intracellular; P:intracellular signal transduction |
| Efet.01.86135.g698.t1 | hypothetical protein HELRODRAFT_163113 | 285 | 4.47E-14 | 57.4 | C:membrane; C:integral component of membrane; F:carbohydrate binding |
| Efet.01.86138.g699.t1 | kinesin KIF24 | 1143 | 3.18E-14 | 60.45 | C:microtubule cytoskeleton; C:cytoskeletal part; F:binding |
| Efet.01.86154.g700.t1 | PREDICTED: uncharacterized protein LOC106011355 | 306 | 2.18E-07 | 62.5 |  |
| Efet.01.86191.g704.t1 | engulfment and cell motility 1 | 1395 | 2.14E-66 | 56.8 | P:single-organism process; P:cellular process |
| Efet.01.86205.g705.t1 | hypothetical protein CRN43_19050, partial | 327 | 3.63E-12 | 61.67 |  |
| Efet.01.8622.g633.t1 | hypothetical protein HELRODRAFT_164759 | 483 | 3.75E-17 | 50 | C:membrane; C:integral component of membrane |
| Efet.01.86258.g707.t1 | microtubule-associated RP EB family member 3-like | 261 | 5.31E-25 | 68.65 | P:biological_process; F:microtubule binding |
| Efet.01.86281.g709.t1 | ankyrin repeat and SOCS box 13 | 561 | 1.16E-41 | 65.65 | P:intracellular signal transduction; C:intracellular; P:protein ubiquitination |
| Efet.01.863.g78.t1 | regulator of G signaling domain | 315 | 8.82E-09 | 67.8 | P:intracellular signal transduction; C:intracellular |
| Efet.01.86316.g713.t1 | cationic amino acid transporter 4 | 1740 | 3.09E-56 | 56.95 | C:membrane; C:integral component of membrane; F:amino acid transmembrane transporter activity; P:amino acid transmembrane transport |
| Efet.01.86351.g716.t1 | coatomer subunit epsilon-like | 264 | 1.22E-38 | 84.2 | C:endoplasmic reticulum membrane; P:ER to Golgi vesicle-mediated transport; F:protein binding; P:retrograde vesicle-mediated transport, Golgi to ER; P:protein transport; F:structural molecule activity; C:cytosol; C:Golgi membrane; P:intra-Golgi vesicle-mediated transport; C:transport vesicle; C:nucleoplasm; C:COPI vesicle coat |
| Efet.01.8636.g635.t1 | metal tolerance 9-like | 288 | 1.95E-33 | 80.3 | P:response to zinc ion; P:regulation of sequestering of zinc ion; P:cation transmembrane transport; C:integral component of membrane; F:zinc ion transmembrane transporter activity |
| Efet.01.8644.g636.t1 | Ribonucleases P MRP subunit POP1 | 1596 | 6.57E-50 | 44.05 | P:RNA phosphodiester bond hydrolysis, endonucleolytic; F:ribonuclease P activity; P:RNA phosphodiester bond hydrolysis; P:RNA processing; P:tRNA 5'-leader removal |
| Efet.01.86457.g719.t1 | dynein heavy chain axonemal-like isoform X1 | 438 | 8.46E-64 | 89.95 | F:ATPase activity; F:ATP binding; F:microtubule motor activity; C:axonemal dynein complex; P:cilium movement |
| Efet.01.86475.g721.t1 | neurogenic locus Notch -like | 1011 | 1.41E-108 | 68 | P:single-organism process |
| Efet.01.86487.g722.t1 | RNA-binding MEX3B | 1437 | 4.49E-66 | 75.1 | F:zinc ion binding; F:RNA binding |
| Efet.01.86491.g723.t1 | NF-kappa-B-repressing factor | 1278 | 1.82E-66 | 54.85 | F:nucleic acid binding |
| Efet.01.86498.g724.t1 | CLIP-associating 1-A- | 588 | 1.32E-51 | 75.3 |  |
| Efet.01.86546.g725.t1 | adenylate cyclase type 9-like | 1350 | 2.81E-160 | 71.45 | F:phosphorus-oxygen lyase activity; C:integral component of membrane; C:intracellular; P:cyclic nucleotide biosynthetic process; P:intracellular signal transduction |
| Efet.01.86574.g727.t1 | gastrin cholecystokinin type B receptor-like | 579 | 1.10E-21 | 59.25 | P:signal transduction; F:signal transducer activity |
| Efet.01.86595.g728.t1 | ETS isoform P1 | 963 | 2.42E-28 | 55.14 | C:membrane; C:integral component of membrane |
| Efet.01.866.g79.t1 | probable E3 ubiquitin- ligase RNF144A-A | 780 | 7.20E-60 | 57.55 | F:binding; C:intracellular; P:protein ubiquitination |
| Efet.01.86621.g729.t1 | BDNF NT-3 growth factors receptor-like | 288 | 2.00E-14 | 82.75 | F:ATP binding; P:peptidyl-tyrosine phosphorylation; F:transmembrane receptor protein tyrosine kinase activity; C:integral component of membrane; P:transmembrane receptor protein tyrosine kinase signaling pathway |
| Efet.01.86672.g730.t1 | onco -induced transcript 3 | 543 | 3.49E-18 | 52.65 | C:nuclear envelope; C:nucleus; F:calcium ion binding; C:membrane; C:integral component of membrane; P:urate homeostasis |
| Efet.01.86682.g731.t1 | cyclic-nucleotide-gated cation | 417 | 6.61E-48 | 89.5 | C:integral component of plasma membrane; F:voltage-gated potassium channel activity; P:regulation of membrane potential; P:potassium ion transmembrane transport; F:cGMP-dependent protein kinase activity; P:protein phosphorylation |
| Efet.01.8669.g638.t1 | tyrosine- phosphatase Lar-like isoform X1 | 663 | 3.10E-90 | 73.6 | F:phosphoprotein phosphatase activity; C:membrane; P:protein dephosphorylation |
| Efet.01.86692.g733.t1 | superkiller viralicidic activity 2-like | 426 | 2.19E-47 | 92.6 | F:ATP binding; P:maturation of 5.8S rRNA; C:catalytic step 2 spliceosome; C:exosome (RNase complex); F:ATP-dependent RNA helicase activity; F:RNA binding; P:RNA catabolic process |
| Efet.01.86733.g735.t1 | hypothetical protein CAPTEDRAFT_185637 | 2604 | 7.60E-135 | 46.45 | C:membrane |
| Efet.01.86761.g736.t1 | zinc finger E-box-binding homeobox 2-like isoform X1 | 1209 | 4.78E-07 | 54.75 | F:nucleic acid binding; F:DNA binding; C:nucleus; F:metal ion binding |
| Efet.01.86776.g737.t1 | MAP kinase-interacting serine threonine- kinase 1-like | 537 | 4.37E-34 | 69.25 | F:nucleotide binding; F:protein kinase activity; P:phosphorylation |
| Efet.01.86786.g738.t1 | titin-like isoform X4 | 2289 | 2.50E-15 | 58.35 | F:transferase activity |
| Efet.01.8681.g639.t1 | upstream-binding 1 isoform X3 | 645 | 2.96E-65 | 77.45 | C:nucleus; F:transcription factor activity, sequence-specific DNA binding; F:sequence-specific DNA binding; P:regulation of transcription from RNA polymerase II promoter |
| Efet.01.86933.g743.t1 | atrial natriuretic peptide receptor 3-like | 603 | 7.18E-42 | 62.45 | C:membrane; C:integral component of membrane |
| Efet.01.86952.g744.t1 | hypothetical protein CAPTEDRAFT_25162, partial | 525 | 7.09E-16 | 64 | F:G-protein coupled receptor activity; P:G-protein coupled receptor signaling pathway; C:membrane; C:integral component of membrane |
| Efet.01.86952.g745.t1 | FMRFamide receptor-like | 531 | 3.45E-37 | 56.9 | F:signal transducer activity |
| Efet.01.86998.g747.t1 | histone acetyltransferase KAT2B-like | 324 | 2.62E-47 | 86.1 | P:cell cycle arrest; P:positive regulation of chromatin binding; P:positive regulation of vasodilation; P:negative regulation of cell proliferation; C:Ada2/Gcn5/Ada3 transcription activator complex; F:RNA polymerase II regulatory region sequence-specific DNA binding; C:kinetochore; F:cyclin-dependent protein serine/threonine kinase inhibitor activity; P:memory; P:transcription initiation from RNA polymerase II promoter; P:cellular response to insulin stimulus; P:positive regulation of histone H3-K9 acetylation; P:chromatin remodeling; P:regulation of protein ADP-ribosylation; P:cellular response to parathyroid hormone stimulus; P:N-terminal peptidyl-lysine acetylation; P:viral process; C:cytosol; C:I band; P:positive regulation of neuron projection development; C:A band; F:protein complex binding; F:protein kinase binding; F:histone acetyltransferase binding; P:positive regulation of gene expression, epigenetic; F:histone deacetylase binding; P:histone H3-K9 acetylation; C:actomyosin; F:chromatin binding; F:transcription factor binding; C:PCAF complex; P:negative regulation of cyclin-dependent protein serine/threonine kinase activity; F:histone acetyltransferase activity; F:lysine N-acetyltransferase activity, acting on acetyl phosphate as donor; C:nuclear chromatin; P:Notch signaling pathway; F:transcription coactivator activity; P:rhythmic process; P:positive regulation of histone H3-K14 acetylation; P:positive regulation of gluconeogenesis by positive regulation of transcription from RNA polymerase II promoter; P:cellular response to oxidative stress |
| Efet.01.87075.g749.t1 | RAS-like GTP-binding | 558 | 6.25E-64 | 77.3 | P:wound healing, spreading of cells; P:skeletal muscle satellite cell migration |
| Efet.01.87158.g752.t1 | hypothetical protein CAPTEDRAFT_222571 | 735 | 1.80E-20 | 50.14 | C:membrane; C:integral component of membrane |
| Efet.01.87188.g753.t1 | neuroglian-like isoform X1 | 1050 | 1.89E-48 | 41.35 | C:membrane; C:integral component of membrane |
| Efet.01.87204.g754.t1 | probable G- coupled receptor 158 | 384 | 1.29E-33 | 68 | F:G-protein coupled receptor activity; F:G-protein coupled GABA receptor activity; P:G-protein coupled receptor signaling pathway; C:membrane; C:integral component of membrane |
| Efet.01.8721.g643.t1 | microtubule-actin cross-linking factor isoforms 1 2 3 5-like | 285 | 1.86E-22 | 77.85 | P:cell cycle arrest; F:calcium ion binding |
| Efet.01.87214.g755.t1 | hypothetical protein HELRODRAFT_194828 | 390 | 1.08E-09 | 87 | F:nucleic acid binding; F:ATP binding; F:helicase activity |
| Efet.01.87233.g756.t1 | hypothetical protein HELRODRAFT_136819, partial | 273 | 2.53E-11 | 51 |  |
| Efet.01.87263.g761.t1 | serine threonine- kinase pim-2 | 600 | 5.94E-62 | 73.7 | P:positive regulation of macroautophagy; P:negative regulation of cell proliferation; P:positive regulation of I-kappaB kinase/NF-kappaB signaling; P:protein autophosphorylation; F:protein serine/threonine kinase activity; P:negative regulation of protein binding; P:regulation of mitotic cell cycle; F:nucleotide binding; F:protein binding; P:apoptotic mitochondrial changes; C:cytoplasm; P:positive regulation of transcription, DNA-templated; P:negative regulation of apoptotic process; P:G1/S transition of mitotic cell cycle; P:protein stabilization |
| Efet.01.87334.g763.t1 | Low-density lipo receptor-related | 531 | 8.63E-52 | 73.75 | C:integral component of membrane |
| Efet.01.87348.g764.t1 | secreted frizzled-related 5-like | 681 | 1.97E-08 | 60.33 | P:multicellular organism development; P:Wnt signaling pathway; C:extracellular region |
| Efet.01.87354.g765.t1 | cytoplasmic dynein 1 heavy chain 1-like | 354 | 3.77E-44 | 79.15 | F:ATPase activity; F:ATP binding; F:microtubule motor activity; C:dynein complex; P:microtubule-based movement |
| Efet.01.87357.g766.t1 | sprouty- EVH1 domain-containing 2-like | 216 | 7.71E-27 | 77 | P:multicellular organism development; C:membrane; P:regulation of signal transduction |
| Efet.01.87369.g767.t1 | T family of potassium channels 18-like | 402 | 8.84E-50 | 79.5 | P:potassium ion transmembrane transport; C:integral component of membrane; F:potassium channel activity |
| Efet.01.87466.g769.t1 | insulin receptor substrate- | 327 | 2.18E-35 | 71.15 | F:phosphatidylinositol 3-kinase binding; P:insulin receptor signaling pathway; C:cytosol; F:insulin receptor binding; P:regulation of lipid metabolic process; F:signal transducer activity; C:plasma membrane |
| Efet.01.87511.g770.t1 | ras GTPase-activating -binding 2 isoform X4 | 828 | 2.12E-42 | 56.2 | F:nucleotide binding; F:nucleic acid binding; C:cytoplasm; P:transport; C:intracellular; F:RNA binding |
| Efet.01.87543.g772.t1 | Pleckstrin homology domain-containing family A member | 1974 | 6.53E-06 | 49.14 |  |
| Efet.01.87593.g773.t1 | transmembrane 9 superfamily member 1-like | 531 | 1.26E-27 | 75.85 | C:integral component of membrane |
| Efet.01.87617.g776.t1 | acetolactate synthase | 336 | 4.71E-36 | 80.05 | F:magnesium ion binding; F:thiamine pyrophosphate binding; F:catalytic activity; C:integral component of membrane; P:embryonic cranial skeleton morphogenesis |
| Efet.01.87620.g777.t1 | hypothetical protein CAPTEDRAFT_218728 | 585 | 1.37E-27 | 51.55 | F:pyridoxal phosphate binding; P:tryptophan catabolic process; C:cytoplasm; F:kynureninase activity; F:catalytic activity; C:membrane; C:integral component of membrane; P:NAD biosynthetic process; F:carbohydrate binding |
| Efet.01.87633.g778.t1 | RNA polymerase II-associated 3-like | 681 | 4.04E-09 | 62.56 |  |
| Efet.01.87647.g779.t1 | synaptojanin-1 isoform X1 | 318 | 1.60E-24 | 70.35 | F:phosphoric ester hydrolase activity |
| Efet.01.87685.g781.t1 | hypothetical protein HELRODRAFT_175687 | 1866 | 1.29E-12 | 58 | C:nucleus; F:histone acetyltransferase activity; F:transferase activity, transferring acyl groups other than amino-acyl groups; P:regulation of transcription, DNA-templated; P:histone acetylation |
| Efet.01.87703.g783.t1 | protocadherin Fat 1-like isoform X1 | 705 | 4.11E-10 | 48.85 | F:calcium ion binding; C:membrane; C:integral component of membrane; P:cell adhesion; P:homophilic cell adhesion via plasma membrane adhesion molecules; C:plasma membrane |
| Efet.01.87721.g784.t1 | laminin subunit alpha | 750 | 8.60E-40 | 56.4 | P:regulation of biological process |
| Efet.01.87758.g786.t1 | cilia- and flagella-associated 57-like | 879 | 2.50E-69 | 75.4 | C:membrane |
| Efet.01.87855.g788.t1 | unc-119 homolog B | 423 | 6.76E-48 | 84.2 | P:cilium assembly; P:Kupffer's vesicle development; P:nervous system development; F:3',5'-cyclic-nucleotide phosphodiesterase activity |
| Efet.01.87870.g789.t1 | phosphatase 1 regulatory subunit 35-like | 993 | 1.19E-16 | 52.65 |  |
| Efet.01.87903.g792.t1 | nesprin-1-like isoform X1 | 852 | 1.08E-20 | 49.75 | C:nuclear envelope; F:actin filament binding; F:actin binding; P:cytoskeletal anchoring at nuclear membrane; C:LINC complex |
| Efet.01.87942.g793.t1 | 5 -AMP-activated kinase catalytic subunit alpha-2 isoform X1 | 237 | 5.57E-18 | 84.05 | C:nucleus; F:ATP binding; F:AMP-activated protein kinase activity; C:cytoplasm; F:histone serine kinase activity; P:histone-serine phosphorylation; F:calmodulin-dependent protein kinase activity; P:negative regulation of TOR signaling; P:intracellular signal transduction; P:cellular response to glucose starvation; C:nucleotide-activated protein kinase complex |
| Efet.01.87944.g794.t1 | tripartite motif-containing 45-like isoform X6 | 426 | 7.08E-33 | 77 | F:metal ion binding |
| Efet.01.87959.g795.t1 | ankyrin-3 isoform X1 | 2127 | 0 | 47.15 | C:intracellular; P:intracellular signal transduction |
| Efet.01.880.g82.t1 | Ankyrin repeat | 1473 | 8.24E-63 | 47.9 |  |
| Efet.01.880.g83.t1 | Ankyrin repeat | 894 | 6.47E-47 | 52.75 | F:ion channel activity; P:ion transport; P:transmembrane transport; P:ion transmembrane transport; C:membrane; C:integral component of membrane; P:transport |
| Efet.01.880.g84.t1 | transient receptor potential cation channel subfamily V member 3 | 705 | 2.07E-20 | 61.75 | P:ion transport; P:transmembrane transport; C:membrane |
| Efet.01.88009.g798.t1 | hypothetical protein HELRODRAFT_193047 | 441 | 5.68E-53 | 59.85 | F:calcium ion binding |
| Efet.01.88010.g799.t1 | calsyntenin-2 | 324 | 3.21E-19 | 62.8 | C:membrane |
| Efet.01.88126.g801.t1 | filamin-A-like isoform X1 | 951 | 1.28E-22 | 76.25 | F:actin filament binding |
| Efet.01.8816.g644.t1 | neuropilin and tolloid 2 | 375 | 3.09E-27 | 56.95 | C:membrane; C:integral component of membrane; F:hydrolase activity |
| Efet.01.88255.g805.t1 | Structural maintenance of chromosomes flexible hinge domain-containing 1 | 399 | 6.03E-17 | 59.5 | F:ATP binding; P:chromosome organization; C:chromosome |
| Efet.01.8827.g645.t1 | tigger transposable element-derived 6-like | 1275 | 2.01E-138 | 64.75 | F:nucleic acid binding |
| Efet.01.88290.g807.t1 | oligosaccharyltransferase complex subunit OSTC | 450 | 1.73E-79 | 88.8 | C:integral component of membrane; F:transferase activity |
| Efet.01.88291.g808.t1 | NEDD4-binding 2-like | 2148 | 6.46E-08 | 76.4 | P:negative regulation of transcription from RNA polymerase II promoter; C:nucleus; P:in utero embryonic development; P:blastocyst development; F:RNA polymerase II transcription corepressor activity |
| Efet.01.88326.g810.t1 | transmembrane GTPase Marf isoform X1 | 486 | 4.65E-31 | 60.85 | F:GTP binding; F:GTPase activity; P:mitochondrial fusion; P:negative regulation of smooth muscle cell proliferation; C:mitochondrion; C:membrane; C:integral component of membrane; C:mitochondrial outer membrane |
| Efet.01.8833.g646.t1 | la-related 1 | 1020 | 1.48E-53 | 56.3 | C:nucleus; F:RNA binding; C:intracellular ribonucleoprotein complex; P:RNA processing |
| Efet.01.8840.g647.t1 | kinesin KIF23 | 504 | 1.64E-54 | 77.7 | F:ATPase activity; F:ATP binding; F:microtubule motor activity; P:plus-end-directed vesicle transport along microtubule; P:mitotic spindle midzone assembly; P:actomyosin contractile ring assembly; C:kinesin complex; C:microtubule; F:microtubule binding |
| Efet.01.88433.g811.t1 | inactive tyrosine- kinase 7-like | 465 | 5.47E-18 | 68.5 | F:ATP binding; P:peptidyl-tyrosine phosphorylation; F:protein tyrosine kinase activity; C:integral component of membrane |
| Efet.01.88438.g812.t1 | DNA polymerase theta | 360 | 3.46E-30 | 50.1 | F:nucleic acid binding; F:DNA binding; F:ATP binding; F:DNA-directed DNA polymerase activity; P:DNA replication; P:DNA-dependent DNA replication; P:DNA biosynthetic process |
| Efet.01.88465.g814.t1 | sodium potassium-transporting ATPase subunit alpha-1 isoform X3 | 825 | 1.80E-156 | 94.1 | C:postsynaptic density; F:phosphatidylinositol 3-kinase binding; P:sodium ion export from cell; P:regulation of cardiac muscle cell membrane potential; F:drug binding; P:ATP hydrolysis coupled proton transport; F:sodium:potassium-exchanging ATPase activity involved in regulation of cardiac muscle cell membrane potential; P:cellular sodium ion homeostasis; C:Golgi apparatus; P:adult locomotory behavior; P:visual learning; P:regulation of respiratory gaseous exchange by neurological system process; P:cardiac muscle contraction; P:memory; C:caveola; F:ankyrin binding; P:neurotransmitter uptake; F:heparan sulfate proteoglycan binding; F:chaperone binding; C:cytosol; P:ionotropic glutamate receptor signaling pathway; C:axon; F:protein kinase binding; F:protein domain specific binding; P:regulation of cardiac muscle cell contraction; C:protein complex; P:membrane repolarization; C:dendritic spine neck; P:response to glycoside; C:dendritic spine head; P:potassium ion import across plasma membrane; C:nucleus; F:D1 dopamine receptor binding; P:negative regulation of glucocorticoid biosynthetic process; P:ATP metabolic process; C:intercalated disc; P:membrane hyperpolarization; C:basolateral plasma membrane; C:apical plasma membrane; C:myelin sheath; P:locomotion; C:sodium:potassium-exchanging ATPase complex; P:positive regulation of heart contraction; P:negative regulation of striated muscle contraction; P:positive regulation of striated muscle contraction; P:regulation of smooth muscle contraction; P:negative regulation of heart contraction; F:ATP binding; P:cellular potassium ion homeostasis; C:endosome; C:extracellular exosome; F:phosphatase activity; P:regulation of vasoconstriction; P:negative regulation of cytosolic calcium ion concentration; C:endoplasmic reticulum; F:ADP binding; P:regulation of blood pressure; P:regulation of the force of heart contraction; P:cellular response to steroid hormone stimulus; C:T-tubule; P:regulation of sodium ion transport; P:cellular response to mechanical stimulus; F:potassium ion binding; P:response to drug; F:steroid hormone binding; P:establishment or maintenance of transmembrane electrochemical gradient; F:sodium ion binding |
| Efet.01.88473.g815.t1 | TRPM8 channel-associated factor homolog | 750 | 1.25E-32 | 50.25 |  |
| Efet.01.8860.g648.t1 | zinc finger GLI4-like | 921 | 5.03E-28 | 53.08 | F:nucleic acid binding; F:metal ion binding; P:smoothened signaling pathway; P:regulation of transcription, DNA-templated; C:Hedgehog signaling complex |
| Efet.01.88640.g821.t1 | 60S ribosomal L9 | 294 | 2.67E-30 | 81.95 | F:structural constituent of ribosome; C:ribosome; F:rRNA binding; P:translation |
| Efet.01.88694.g822.t1 | hypothetical protein CAPTEDRAFT_171703 | 1038 | 3.44E-96 | 61.05 | F:catalytic activity; F:sulfuric ester hydrolase activity; P:metabolic process |
| Efet.01.88705.g823.t1 | hypothetical protein HELRODRAFT_194477 | 423 | 4.56E-18 | 72.6 | P:response to stress |
| Efet.01.88740.g825.t1 | hypothetical protein CAPTEDRAFT_222512 | 1887 | 7.70E-13 | 74 | F:DNA binding; P:regulation of cellular process; F:metal ion binding; P:cellular process |
| Efet.01.88761.g826.t1 | nesprin-1 isoform X1 | 1161 | 7.05E-50 | 57.1 | C:nuclear envelope; F:actin filament binding; F:actin binding; C:membrane; C:integral component of membrane; P:cytoskeletal anchoring at nuclear membrane; C:LINC complex |
| Efet.01.88768.g827.t1 | hypothetical protein CAPTEDRAFT_221239, partial | 744 | 7.57E-20 | 61.05 |  |
| Efet.01.88861.g829.t1 | cullin-associated NEDD8-dissociated 1-like | 342 | 9.59E-38 | 56.8 | C:extracellular exosome; F:protein binding; P:negative regulation of catalytic activity; C:cullin-RING ubiquitin ligase complex; C:membrane; P:cell differentiation; C:Golgi apparatus; P:SCF complex assembly; C:nucleoplasm; P:protein ubiquitination |
| Efet.01.88883.g830.t1 | src substrate cortactin-like | 300 | 1.09E-37 | 80.45 | F:protein kinase binding; P:positive regulation of phosphatidylinositol 3-kinase signaling; P:positive regulation of cell proliferation; P:actin filament polymerization; P:positive regulation of tyrosine phosphorylation of STAT protein; P:positive regulation of transcription factor import into nucleus; P:response to hormone; P:positive regulation of transcription from RNA polymerase II promoter; P:negative regulation of transcription from RNA polymerase II promoter; P:regulation of actin filament polymerization; C:nucleus; C:transcription factor complex; P:positive regulation of granulocyte differentiation; P:erythrocyte differentiation; P:negative regulation of leukocyte apoptotic process; F:actin binding; C:cytoplasm; F:RNA polymerase II transcription factor binding; P:positive regulation of macrophage differentiation; P:cellular response to cytokine stimulus; F:SH3 domain binding; F:protein complex binding; P:positive regulation of protein kinase B signaling; P:positive regulation of peptidyl-serine phosphorylation |
| Efet.01.88956.g831.t1 | neurofibromin-like isoform X2 | 252 | 1.88E-29 | 78.6 | P:signal transduction; F:GTPase activator activity; P:positive regulation of GTPase activity |
| Efet.01.88958.g832.t1 | prolactin-releasing peptide receptor-like | 1242 | 6.93E-83 | 59.4 | P:signal transduction; F:signal transducer activity |
| Efet.01.88963.g833.t1 | ubiquitin carboxyl-terminal hydrolase 16-like | 732 | 4.14E-63 | 62.5 | F:hydrolase activity |
| Efet.01.8897.g649.t1 | TYRAmine receptor | 1182 | 4.06E-46 | 48.1 | F:G-protein coupled receptor activity; P:G-protein coupled receptor signaling pathway; C:integral component of membrane |
| Efet.01.88971.g835.t1 | transcription factor Sox-11-like | 912 | 8.40E-30 | 60.5 | F:catalytic activity |
| Efet.01.89.g10.t1 | hypothetical protein CAPTEDRAFT_222036 | 228 | 1.30E-18 | 63.69 | C:membrane; C:integral component of membrane |
| Efet.01.89025.g836.t1 | ankyrin repeat | 1389 | 9.83E-77 | 47.3 | P:intracellular signal transduction; C:intracellular |
| Efet.01.89035.g837.t1 | zinc finger 678-like | 861 | 8.11E-56 | 53.85 | F:nucleic acid binding; F:metal ion binding |
| Efet.01.89110.g839.t1 | Growth differentiation factor 11 | 405 | 8.84E-55 | 75.3 | F:growth factor activity; P:regulation of MAPK cascade; F:transforming growth factor beta receptor binding; P:SMAD protein signal transduction; P:anterior/posterior pattern specification; P:regulation of apoptotic process; P:cell development; P:negative regulation of cellular process; F:cytokine activity; P:animal organ morphogenesis; C:extracellular space; P:kidney development; C:intracellular membrane-bounded organelle; P:positive regulation of pathway-restricted SMAD protein phosphorylation |
| Efet.01.89113.g840.t1 | potassium voltage-gated channel subfamily B member 1 | 387 | 4.34E-38 | 67.7 | P:potassium ion transport; F:voltage-gated potassium channel activity; P:regulation of transport; P:ion transmembrane transport; C:integral component of membrane; C:plasma membrane; P:cellular process |
| Efet.01.89152.g841.t1 | epidermal growth factor receptor-like isoform X1 | 294 | 6.07E-11 | 58.05 | F:kinase activity; P:cellular process |
| Efet.01.89157.g842.t1 | LIN 24 Like family member | 777 | 1.98E-98 | 62.75 |  |
| Efet.01.89166.g844.t1 | partitioning defective 3 homolog isoform X3 | 720 | 6.00E-12 | 49.2 |  |
| Efet.01.8918.g652.t1 | tubulin polyglutamylase | 411 | 6.86E-60 | 81.9 | C:dendrite; F:ATP binding; P:protein polyglutamylation; C:cytoplasm; F:beta-tubulin binding; C:cilium; P:nervous system development; P:cell differentiation; F:tubulin-glutamic acid ligase activity; F:alpha-tubulin binding; C:perikaryon; C:microtubule |
| Efet.01.89243.g848.t1 | transcription factor SOX-2 | 717 | 4.15E-20 | 44.7 | F:DNA binding; C:nucleus; P:nervous system development; P:regulation of transcription, DNA-templated |
| Efet.01.8928.g655.t1 | teneurin-m-like isoform X8 | 2571 | 0 | 55.65 | P:self proteolysis |
| Efet.01.89295.g851.t1 | epidermal growth factor receptor isoform X1 | 828 | 2.15E-41 | 51.25 | F:transferase activity |
| Efet.01.8931.g656.t1 | hypothetical protein CAPTEDRAFT_225483 | 219 | 1.25E-25 | 68.8 | C:integral component of membrane; F:carbohydrate binding |
| Efet.01.89345.g852.t1 | Niemann-Pick C1 | 867 | 9.32E-40 | 54.2 | P:lipid transport; C:intracellular membrane-bounded organelle; C:endomembrane system; C:cytoplasmic part; C:membrane part; P:cellular process |
| Efet.01.89373.g854.t1 | insulin-like growth factor 2 mRNA-binding 1 isoform X2 | 936 | 1.81E-88 | 53.3 | F:nucleotide binding; F:nucleic acid binding; F:RNA binding |
| Efet.01.89438.g855.t1 | ribonuclease 3-like | 537 | 1.37E-12 | 74.33 | P:RNA phosphodiester bond hydrolysis, endonucleolytic; F:RNA binding; F:ribonuclease III activity; P:rRNA catabolic process; P:RNA processing |
| Efet.01.89446.g856.t1 | WNT1-inducible-signaling pathway 3 isoform X2 | 390 | 2.35E-10 | 72.35 | P:regulation of cell growth; P:signal transduction; P:cell-cell signaling; P:negative regulation of cell death; F:integrin binding; F:heparin binding; P:cell adhesion; F:insulin-like growth factor binding; C:proteinaceous extracellular matrix |
| Efet.01.89450.g858.t1 | SMOC2_HUMAN ame: Full=SPARC-related modular calcium-binding 2 ame: Full=Secreted modular calcium-binding 2 Short=SMOC-2 ame: Full=Smooth muscle-associated 2 Short=SMAP-2 Flags: Precursor | 318 | 8.22E-06 | 78 | C:basement membrane; C:interstitial matrix; F:glycosaminoglycan binding; P:signal transduction; F:metal ion binding; F:calcium ion binding; P:extracellular matrix organization; P:positive regulation of cell-substrate adhesion; F:heparin binding; C:extracellular region; C:proteinaceous extracellular matrix |
| Efet.01.89479.g859.t1 | tRNA-dihydrouridine(47) synthase [NAD(P)(+)]-like | 942 | 3.35E-33 | 64.5 | F:oxidoreductase activity; P:metabolic process; F:RNA binding |
| Efet.01.89518.g861.t1 | CAD -like | 621 | 2.55E-56 | 84 | F:ATP binding; F:aspartate carbamoyltransferase activity; P:'de novo' pyrimidine nucleobase biosynthetic process; F:metal ion binding; F:dihydroorotase activity; F:carbamoyl-phosphate synthase (ammonia) activity; P:glutamine metabolic process; F:carbamoyl-phosphate synthase (glutamine-hydrolyzing) activity; F:amino acid binding |
| Efet.01.89523.g862.t1 | E3 ubiquitin- ligase PDZRN3-like | 849 | 2.20E-39 | 51.95 |  |
| Efet.01.89567.g863.t1 | transcriptional adapter 1 | 585 | 2.23E-48 | 62.55 | C:SAGA-type complex; P:regulation of nucleic acid-templated transcription |
| Efet.01.89587.g864.t1 | hypothetical protein CAPTEDRAFT_211244 | 534 | 4.74E-29 | 62 |  |
| Efet.01.89615.g866.t1 | PREDICTED: uncharacterized protein LOC106156781 | 279 | 3.20E-06 | 63 |  |
| Efet.01.89619.g867.t1 | adenosine receptor A2b-like | 408 | 1.08E-12 | 73.3 | F:G-protein coupled receptor activity; P:G-protein coupled receptor signaling pathway; P:regulation of hematopoietic stem cell differentiation; C:membrane; P:endothelial to hematopoietic transition |
| Efet.01.89626.g868.t1 | probable E3 ubiquitin- ligase RNF217 | 1584 | 1.49E-67 | 66.3 | F:ubiquitin-protein transferase activity; F:binding; C:intracellular part; P:protein ubiquitination |
| Efet.01.89634.g869.t1 | ELAV 2 | 411 | 1.51E-49 | 85.15 | F:nucleotide binding; F:RNA binding |
| Efet.01.89639.g870.t1 | orphan G- coupled receptor 7 | 1290 | 1.51E-73 | 50.7 | F:G-protein coupled receptor activity; F:G-protein coupled peptide receptor activity; P:G-protein coupled receptor signaling pathway; P:signal transduction; C:membrane; C:integral component of membrane; F:signal transducer activity |
| Efet.01.89695.g871.t1 | ATPase inhibitor mai- mitochondrial-like | 285 | 1.40E-24 | 76.55 | P:negative regulation of ATPase activity; F:ATPase inhibitor activity; C:mitochondrion |
| Efet.01.89695.g872.t1 | actin-related 2 3 complex subunit 4 | 300 | 2.43E-33 | 96.7 | F:actin filament binding; C:extracellular exosome; C:cytoplasm; P:actin filament polymerization; P:Arp2/3 complex-mediated actin nucleation; F:structural constituent of cytoskeleton; F:protein binding, bridging; C:Arp2/3 protein complex; F:enzyme binding |
| Efet.01.89700.g873.t1 | ubiquitin carboxyl-terminal hydrolase BAP1-like isoform X4 | 1131 | 6.83E-09 | 88.64 | F:thiol-dependent ubiquitin-specific protease activity; C:intracellular; P:ubiquitin-dependent protein catabolic process |
| Efet.01.89731.g875.t1 | hypothetical protein CAPTEDRAFT_217013 | 753 | 1.04E-17 | 45 |  |
| Efet.01.89741.g876.t1 | sacsin-like | 252 | 5.63E-30 | 58.85 |  |
| Efet.01.89750.g877.t1 | RBPJ-interacting and tubulin-associated 1 | 1023 | 3.93E-14 | 46.35 | F:tubulin binding; C:nucleus; C:centrosome; P:Notch signaling pathway; P:nuclear export; C:cytoplasm; P:negative regulation of Notch signaling pathway; C:intracellular |
| Efet.01.89766.g878.t1 | retina and anterior neural fold homeobox 2 | 519 | 2.03E-26 | 76.5 | C:nucleus; F:transcription factor activity, sequence-specific DNA binding; P:camera-type eye development; P:regulation of transcription, DNA-templated; P:transcription from RNA polymerase II promoter; F:sequence-specific DNA binding; P:visual perception |
| Efet.01.89786.g879.t1 | glyco -N-acetylgalactosamine 3-beta-galactosyltransferase 1-like | 564 | 1.75E-40 | 56.45 | F:transferase activity |
| Efet.01.8979.g657.t1 | apoptosis-stimulating of p53 1-like | 879 | 2.67E-21 | 49.9 | P:signal transduction |
| Efet.01.89814.g880.t1 | hypothetical protein CAPTEDRAFT_197284 | 333 | 4.81E-09 | 71 | F:G-protein coupled receptor activity; C:membrane |
| Efet.01.89815.g881.t1 | serine threonine- kinase WNK3-like isoform X3 | 1272 | 1.33E-16 | 86.6 | F:ATP binding; P:regulation of ion homeostasis; C:cytosol; F:protein serine/threonine kinase activity; P:protein phosphorylation; P:positive regulation of ion transmembrane transporter activity; P:intracellular signal transduction |
| Efet.01.89835.g883.t1 | neuronal acetylcholine receptor subunit non-alpha-2-like isoform X2 | 1347 | 7.18E-122 | 62.05 | P:ion transmembrane transport; C:integral component of membrane; F:extracellular ligand-gated ion channel activity |
| Efet.01.89871.g884.t1 | STAM-binding -like | 777 | 6.26E-32 | 71.55 |  |
| Efet.01.89880.g885.t1 | torsin-1B-like isoform X2 | 312 | 3.87E-24 | 63.9 | C:cytoplasm; C:intracellular membrane-bounded organelle; P:cellular component organization; C:endomembrane system |
| Efet.01.89901.g886.t1 | hypothetical protein CAPTEDRAFT_220800 | 459 | 5.87E-06 | 59 | P:regulation of apoptotic process |
| Efet.01.89929.g889.t1 | mitochondrial H+-transporting ATP synthase F1 complex alpha subunit | 465 | 1.59E-97 | 94.85 | P:embryo development; C:myelin sheath; P:ATP hydrolysis coupled proton transport; F:MHC class I protein binding; F:proton-transporting ATPase activity, rotational mechanism; F:ADP binding; C:plasma membrane; F:RNA binding; F:proton-transporting ATP synthase activity, rotational mechanism; C:mitochondrial proton-transporting ATP synthase complex, catalytic core F(1); F:ATP binding; C:extracellular exosome; P:lipid metabolic process; P:negative regulation of endothelial cell proliferation; C:extracellular matrix; C:COP9 signalosome; P:ATP synthesis coupled proton transport |
| Efet.01.89937.g891.t1 | discoidin domain-containing receptor 2-like isoform X1 | 306 | 1.13E-28 | 76.05 | F:ATP binding; P:peptidyl-tyrosine phosphorylation; F:transmembrane receptor protein tyrosine kinase activity; C:integral component of membrane; P:transmembrane receptor protein tyrosine kinase signaling pathway |
| Efet.01.89954.g893.t1 | carbonic anhydrase 4-like | 573 | 6.79E-19 | 58.6 | F:zinc ion binding; F:metal ion binding; C:membrane; C:integral component of membrane; F:carbonate dehydratase activity; P:one-carbon metabolic process |
| Efet.01.89985.g895.t1 | multiple epidermal growth factor-like domains 6 | 624 | 2.30E-14 | 57.7 | F:metal ion binding; F:calcium ion binding |
| Efet.01.90021.g896.t1 | carboxypeptidase N subunit 2 | 1686 | 2.68E-51 | 50.3 | P:regulation of cellular process; P:cellular process |
| Efet.01.90086.g900.t1 | DNA-directed RNA polymerase III subunit RPC9-like | 399 | 2.53E-27 | 63.55 | F:DNA-directed 5'-3' RNA polymerase activity; P:transcription, DNA-templated |
| Efet.01.901.g87.t1 | IQ and AAA domain-containing 1-like | 306 | 2.42E-46 | 89 | F:ATP binding |
| Efet.01.90130.g901.t1 | annexin A6 | 651 | 7.31E-84 | 64.9 | F:calcium ion binding; C:cytoplasmic part; C:membrane-bounded organelle; F:calcium-dependent phospholipid binding |
| Efet.01.90144.g902.t1 | dynein heavy chain axonemal-like isoform X3 | 516 | 3.72E-43 | 63.55 | F:microtubule motor activity; P:microtubule-based movement |
| Efet.01.90150.g903.t1 | steroid 17-alpha-hydroxylase 17,20 lyase-like isoform X2 | 903 | 8.45E-43 | 52.8 | F:iron ion binding; F:oxidoreductase activity; F:oxidoreductase activity, acting on paired donors, with incorporation or reduction of molecular oxygen; F:metal ion binding; C:membrane; C:integral component of membrane; P:oxidation-reduction process; F:monooxygenase activity; F:heme binding |
| Efet.01.90184.g905.t1 | adenosine deaminase CECR1-like | 1536 | 1.06E-156 | 58.5 | C:extracellular space; F:deaminase activity |
| Efet.01.9019.g661.t1 | glutamate synthase subunit beta | 291 | 6.63E-22 | 94.85 | P:glutamate biosynthetic process; P:oxidation-reduction process; F:glutamate synthase (NADPH) activity; F:iron-sulfur cluster binding |
| Efet.01.90206.g908.t1 | cytochrome c oxidase assembly factor 7 | 681 | 7.72E-71 | 62.4 | C:mitochondrial intermembrane space; C:mitochondrion |
| Efet.01.9021.g662.t1 | Zinc finger and SCAN domain-containing 22 | 1482 | 4.53E-48 | 71.35 | F:nucleic acid binding; F:metal ion binding |
| Efet.01.90223.g909.t1 | membralin-like isoform X1 | 891 | 7.53E-37 | 69 | C:membrane |
| Efet.01.90237.g910.t1 | hypothetical protein CAPTEDRAFT_21078 | 417 | 8.29E-16 | 57.33 | C:membrane; C:integral component of membrane; P:transport; F:transporter activity |
| Efet.01.9024.g663.t1 | calcium-binding CML24 | 360 | 3.85E-14 | 66.17 | F:calcium ion binding |
| Efet.01.90276.g915.t1 | WD repeat-containing 47-like isoform X1 | 1833 | 4.42E-166 | 76.8 |  |
| Efet.01.90297.g916.t1 | ATP-dependent RNA helicase DHX36 | 891 | 1.73E-53 | 50.15 | F:nucleic acid binding |
| Efet.01.90300.g917.t1 | DNA helicase INO80-like isoform X1 | 231 | 7.12E-29 | 94.65 | F:ATPase activity; F:DNA binding; F:ATP binding; P:DNA repair; P:chromatin remodeling; P:transcription, DNA-templated; C:Ino80 complex; F:helicase activity |
| Efet.01.90307.g918.t1 | C1q-related factor-like isoform X1 | 423 | 2.05E-31 | 72.2 | C:membrane |
| Efet.01.90316.g919.t1 | inactive tyrosine- kinase 7-like | 339 | 6.92E-28 | 69.9 | P:cellular macromolecule metabolic process; F:organic cyclic compound binding; F:heterocyclic compound binding; C:membrane; F:transferase activity, transferring phosphorus-containing groups; P:primary metabolic process |
| Efet.01.9033.g665.t1 | Calnexin | 429 | 1.34E-11 | 59 | F:metal ion binding; F:calcium ion binding; C:membrane; C:integral component of membrane; C:endoplasmic reticulum; F:unfolded protein binding; P:protein folding |
| Efet.01.90335.g920.t1 | myopalladin isoform X1 | 546 | 4.98E-29 | 57.45 | C:membrane; C:integral component of membrane |
| Efet.01.90542.g924.t1 | CCR4-NOT transcription complex subunit 1-like | 969 | 7.17E-106 | 63.15 | C:macromolecular complex; C:intracellular part |
| Efet.01.90661.g927.t1 | neogenin-like isoform X2 | 564 | 1.05E-66 | 67.15 | C:membrane |
| Efet.01.90712.g929.t1 | SPARC-related modular calcium-binding 1-like isoform X8 | 411 | 3.89E-18 | 70.1 |  |
| Efet.01.90720.g931.t1 | FMRFamide-activated amiloride-sensitive sodium channel-like isoform X2 | 1863 | 1.04E-162 | 48.3 | P:ion transport |
| Efet.01.90784.g932.t1 | PREDICTED: uncharacterized protein LOC101858907 | 1116 | 6.38E-45 | 44.35 |  |
| Efet.01.9081.g666.t1 | trifunctional enzyme subunit mitochondrial | 846 | 5.38E-115 | 68.8 | F:acetyl-CoA C-acetyltransferase activity; P:fatty acid beta-oxidation; F:protein binding; F:coenzyme binding; F:3-hydroxyacyl-CoA dehydrogenase activity; P:cardiolipin acyl-chain remodeling; F:enoyl-CoA hydratase activity; C:mitochondrial inner membrane; C:mitochondrial nucleoid |
| Efet.01.90841.g935.t1 | SCO-spondin-like isoform X2 | 450 | 8.24E-10 | 53.25 | F:calcium ion binding; C:membrane; F:scavenger receptor activity; P:receptor-mediated endocytosis |
| Efet.01.90868.g937.t1 | tctex1 domain-containing 1-B-like | 381 | 6.10E-22 | 66.2 |  |
| Efet.01.90895.g938.t1 | RAB6A-GEF complex partner 1-like | 279 | 9.35E-15 | 71.05 |  |
| Efet.01.90951.g940.t1 | SRC_DANRE ame: Full=Proto-oncogene tyrosine- kinase Src ame: Full=Proto-oncogene c-Src ame: Full=pp60c-src Short=p60-Src | 321 | 5.88E-07 | 52.67 | C:intracellular organelle; F:protein tyrosine kinase activity; P:intracellular steroid hormone receptor signaling pathway; P:response to yeast; P:transmembrane receptor protein tyrosine kinase signaling pathway; P:regulation of vascular permeability; P:posterior lateral line neuromast primordium migration; P:protein phosphorylation; F:receptor binding; C:plasma membrane; P:immune system process; P:negative regulation of apoptotic signaling pathway |
| Efet.01.90993.g943.t1 | hypothetical protein HELRODRAFT_190657 | 303 | 2.41E-12 | 69 | F:protein dimerization activity |
| Efet.01.91031.g945.t1 | chromodomain-helicase-DNA-binding 4-like isoform X1 | 285 | 2.88E-53 | 88.1 | F:DNA binding; F:ATP binding; P:spindle organization; F:zinc ion binding; P:centrosome organization; C:NuRD complex; F:ATP-dependent helicase activity; P:regulation of transcription, DNA-templated; P:chromatin organization |
| Efet.01.91171.g951.t1 | hypothetical protein HELRODRAFT_109123 | 381 | 1.70E-09 | 65 | F:metal ion binding |
| Efet.01.91235.g954.t1 | FMRFamide receptor-like | 1209 | 1.61E-78 | 52.25 | F:signal transducer activity |
| Efet.01.91237.g955.t1 | microtubule-actin cross-linking factor isoforms 1 2 3 5-like | 1614 | 2.05E-143 | 62.9 | P:cell cycle arrest; C:cytoskeleton; F:calcium ion binding |
| Efet.01.91308.g957.t1 | sarcoplasmic calcium-binding -like | 228 | 8.89E-09 | 63.33 | F:calcium ion binding |
| Efet.01.91358.g958.t1 | filamin-A isoform X4 | 786 | 1.47E-58 | 50.05 | P:response to external stimulus; P:anatomical structure morphogenesis; P:single-organism developmental process |
| Efet.01.91379.g959.t1 | transcription factor SOX-14 | 345 | 1.91E-11 | 76.15 | P:positive regulation of cellular process |
| Efet.01.91405.g961.t1 | double-stranded RNA-specific adenosine deaminase-like | 2445 | 3.02E-07 | 74.14 | F:RNA binding; F:double-stranded RNA adenosine deaminase activity; P:RNA processing; F:adenosine deaminase activity |
| Efet.01.9142.g668.t1 | transmembrane 151B-like | 1893 | 5.52E-54 | 62.85 | C:membrane; C:integral component of membrane |
| Efet.01.91432.g962.t1 | ankyrin repeat domain-containing ENSP00000383069 | 702 | 1.53E-06 | 68.1 | F:fatty-acyl-CoA binding |
| Efet.01.91437.g963.t1 | interference hedgehog-like isoform X1 | 213 | 2.93E-09 | 63.1 | C:membrane; C:integral component of membrane |
| Efet.01.91443.g964.t1 | rab GTPase-activating 1-like isoform X1 | 357 | 6.30E-21 | 72.1 | F:GTPase activator activity; P:cell cycle; P:positive regulation of GTPase activity |
| Efet.01.91454.g965.t1 | hypothetical protein CAPTEDRAFT_209261 | 381 | 9.75E-36 | 73.67 | C:integral component of membrane |
| Efet.01.91462.g966.t1 | acetylcholinesterase-like isoform X5 | 753 | 5.59E-109 | 61.6 | F:hydrolase activity |
| Efet.01.91500.g968.t1 | mitochondrial ribonuclease P 1 homolog | 828 | 5.47E-53 | 57.2 | C:mitochondrion; F:methyltransferase activity; F:transferase activity; P:methylation |
| Efet.01.91504.g969.t1 | eukaryotic translation initiation factor 4 gamma 1-like | 345 | 1.81E-07 | 51 | F:translation initiation factor activity; F:RNA binding; P:translational initiation |
| Efet.01.91525.g970.t1 | lipase maturation factor 1-like | 345 | 7.42E-20 | 82.8 | C:endoplasmic reticulum membrane; C:integral component of membrane |
| Efet.01.91538.g971.t1 | DNA ligase 4-like | 627 | 2.33E-64 | 77.55 | P:DNA ligation involved in DNA repair; F:DNA binding; F:ATP binding; F:DNA ligase (ATP) activity; C:DNA-dependent protein kinase-DNA ligase 4 complex; C:DNA ligase IV complex; P:lagging strand elongation; C:cytoplasm; P:DNA recombination; P:DNA biosynthetic process; P:nucleotide-excision repair, DNA gap filling; P:double-strand break repair via nonhomologous end joining |
| Efet.01.91605.g974.t1 | prosaposin-like isoform X2 | 729 | 9.21E-26 | 42.1 | F:G-protein coupled receptor binding; C:cytoplasm; P:positive regulation of catalytic activity; P:lipid metabolic process; F:enzyme activator activity; C:lysosome; P:sphingolipid metabolic process |
| Efet.01.91619.g975.t1 | retinoic acid receptor RXR-beta-A-like | 204 | 3.05E-20 | 77.55 | C:nucleus; F:transcription factor activity, sequence-specific DNA binding; F:zinc ion binding; P:transcription, DNA-templated; F:steroid hormone receptor activity; P:regulation of transcription, DNA-templated; F:sequence-specific DNA binding; P:steroid hormone mediated signaling pathway |
| Efet.01.91620.g976.t1 | misshapen-like kinase 1 | 618 | 2.07E-67 | 64.6 | F:ATP binding; P:regulation of response to stimulus; F:signal transducer, downstream of receptor, with serine/threonine kinase activity; C:cytoplasm; P:single-organism cellular process; P:negative regulation of cellular process; P:protein phosphorylation; P:response to stress; P:intracellular signal transduction |
| Efet.01.91669.g979.t1 | mitogen-activated kinase kinase kinase 13-like isoform X1 | 2133 | 0 | 84.8 | F:ATP binding; P:activation of MAPKK activity; F:protein homodimerization activity; P:protein autophosphorylation; P:JNK cascade; F:MAP kinase kinase kinase activity; P:positive regulation of NF-kappaB transcription factor activity; C:intracellular |
| Efet.01.91681.g981.t1 | poly [ADP-ribose] polymerase 1-like | 603 | 7.21E-57 | 63.2 | F:binding; F:transferase activity |
| Efet.01.91701.g983.t1 | calpain family cysteine protease | 351 | 4.17E-18 | 71.85 | F:metal ion binding; C:acrosomal vesicle; P:proteolysis; F:calcium-dependent cysteine-type endopeptidase activity |
| Efet.01.91716.g984.t1 | Dedicator of cytokinesis | 621 | 2.45E-21 | 67.7 | P:biological regulation; P:single-organism process; P:cellular process |
| Efet.01.91724.g985.t1 | cholinesterase 2-like isoform X5 | 231 | 9.98E-09 | 76.71 |  |
| Efet.01.9177.g670.t1 | GRIP and coiled-coil domain-containing 1-like | 327 | 1.36E-07 | 68 |  |
| Efet.01.91790.g986.t1 | type II transmembrane | 1278 | 7.79E-87 | 55.05 | P:self proteolysis; F:catalytic activity; C:membrane; C:integral component of membrane |
| Efet.01.918.g88.t1 | riboflavin-binding -like | 1005 | 8.99E-60 | 68 | C:membrane |
| Efet.01.91857.g990.t1 | solute carrier family | 219 | 4.86E-28 | 80.25 | P:inorganic anion transport; P:anion transmembrane transport; C:integral component of membrane; F:inorganic anion exchanger activity |
| Efet.01.91891.g992.t1 | kinase D-interacting substrate of 220 kDa-like | 1128 | 3.48E-73 | 65.8 | C:membrane; C:integral component of membrane; P:phosphorylation; F:kinase activity |
| Efet.01.91904.g993.t1 | unconventional myosin-XV isoform X1 | 609 | 2.21E-09 | 66.95 | C:myosin complex; F:ATP binding; C:cytoskeleton; F:motor activity |
| Efet.01.91963.g996.t1 | acidic mammalian chitinase-like | 333 | 4.86E-43 | 77.85 | F:chitinase activity; P:carbohydrate metabolic process; F:chitin binding; C:cytoplasm; P:chitin catabolic process; C:extracellular region |
| Efet.01.91985.g997.t1 | RNA-dependent RNA polymerase 1 | 5520 | 3.67E-170 | 57.15 | F:RNA-directed 5'-3' RNA polymerase activity; P:transcription, RNA-templated |
| Efet.01.91998.g998.t1 | regulator of G- signaling 12-like | 1425 | 8.87E-21 | 49.45 |  |
| Efet.01.9200.g671.t1 | pumilio homolog 2- | 222 | 7.84E-32 | 90.95 | F:RNA binding |
| Efet.01.92087.g1001.t1 | SET and MYND domain-containing 4-like | 2724 | 5.24E-52 | 42.55 |  |
| Efet.01.92098.g1002.t1 | ubiquitin-like modifier-activating enzyme 1 | 393 | 2.11E-62 | 83.35 | P:cellular response to DNA damage stimulus; C:lysosomal membrane; F:RNA binding; P:ubiquitin-dependent protein catabolic process; C:heterochromatin; C:nucleus; F:ubiquitin-protein transferase activity; F:ATP binding; C:rough endoplasmic reticulum membrane; C:extracellular exosome; F:protein binding; P:axonogenesis; C:mitochondrion; C:cytosol; F:ubiquitin activating enzyme activity; C:endosome membrane; C:desmosome; P:protein ubiquitination |
| Efet.01.92100.g1003.t1 | Collagen alpha-3(VI) chain | 1353 | 6.93E-68 | 48.65 | F:calcium ion binding; C:collagen trimer; C:membrane; C:integral component of membrane; C:proteinaceous extracellular matrix |
| Efet.01.92119.g1004.t1 | regulating synaptic membrane exocytosis 2-like | 1353 | 2.49E-33 | 60.95 | C:membrane |
| Efet.01.92221.g1006.t1 | MAGUK p55 subfamily member 5-like isoform X3 | 699 | 5.34E-21 | 54.3 | P:bicellular tight junction assembly; P:establishment of epithelial cell polarity |
| Efet.01.92243.g1007.t1 | hypothetical protein CAPTEDRAFT_228538 | 546 | 4.93E-24 | 59 |  |
| Efet.01.92258.g1008.t1 | hypothetical protein CAPTEDRAFT_220753 | 270 | 9.38E-10 | 70 | C:membrane |
| Efet.01.92325.g1009.t1 | dynein intermediate chain axonemal | 294 | 4.00E-30 | 86.75 | C:outer dynein arm; F:microtubule motor activity; P:outer dynein arm assembly; C:cytoplasm; C:external side of plasma membrane; P:cilium movement; C:sperm flagellum; P:determination of left/right symmetry; C:microtubule |
| Efet.01.9235.g673.t1 | APCDD1 isoform X1 | 417 | 2.72E-31 | 60.85 |  |
| Efet.01.92377.g1010.t1 | YEATS domain-containing 4-like | 285 | 2.48E-46 | 90 | F:translation initiation factor activity; P:regulation of transcription, DNA-templated; P:translational initiation |
| Efet.01.9240.g674.t1 | transcription factor grauzone | 2064 | 4.88E-15 | 45.2 | F:metal ion binding |
| Efet.01.92407.g1011.t1 | membralin-like isoform X1 | 765 | 1.27E-30 | 71.85 | C:integral component of membrane |
| Efet.01.92458.g1012.t1 | plasma membrane calcium-transporting ATPase | 234 | 1.01E-10 | 86.2 | F:ATP binding; F:metal ion binding; C:integral component of membrane; P:calcium ion transmembrane transport; F:calcium-transporting ATPase activity |
| Efet.01.92460.g1013.t1 | chondroitin sulfate proteoglycan 4-like | 1698 | 2.58E-64 | 51.1 | C:membrane; C:integral component of membrane |
| Efet.01.92463.g1014.t1 | PREDICTED: uncharacterized protein LOC106181940 | 339 | 3.97E-12 | 48.6 |  |
| Efet.01.92476.g1015.t1 | split ends-like | 498 | 9.52E-15 | 70.55 | F:organic cyclic compound binding; F:heterocyclic compound binding |
| Efet.01.92483.g1017.t1 | collagen alpha-1(XIV) chain | 360 | 1.19E-10 | 62.6 | F:calcium ion binding; C:membrane; C:integral component of membrane; C:proteinaceous extracellular matrix |
| Efet.01.92486.g1019.t1 | kyphoscoliosis peptidase | 1200 | 2.46E-151 | 54.85 |  |
| Efet.01.92486.g1020.t1 | kyphoscoliosis peptidase | 432 | 1.74E-43 | 56.35 |  |
| Efet.01.9249.g677.t1 | histone acetyltransferase KAT7-like | 1026 | 0 | 71.8 | C:nucleus; F:histone acetyltransferase activity; F:transcription factor activity, sequence-specific DNA binding; F:zinc ion binding; P:regulation of transcription, DNA-templated; P:histone acetylation |
| Efet.01.92493.g1021.t1 | zinc finger 711 isoform X1 | 303 | 3.00E-11 | 52.35 | F:nucleic acid binding |
| Efet.01.92493.g1022.t1 | zinc finger 366-like | 504 | 7.20E-12 | 43.9 | F:nucleic acid binding; F:metal ion binding |
| Efet.01.925.g89.t1 | mab-21-like 2 | 1116 | 0 | 86.9 |  |
| Efet.01.92513.g1025.t1 | hypothetical protein CAPTEDRAFT_227150, partial | 2190 | 0 | 71.95 | C:membrane |
| Efet.01.92543.g1026.t1 | Zinc finger and BTB domain-containing 40 | 3315 | 4.41E-79 | 50.6 | F:metal ion binding |
| Efet.01.92585.g1027.t1 | ceramide-1-phosphate transfer -like | 570 | 1.23E-47 | 63.55 | F:glycolipid binding; C:cytoplasm; F:glycolipid transporter activity; P:glycolipid transport |
| Efet.01.92642.g1030.t1 | RNA-directed DNA methylation 3-like | 771 | 1.04E-19 | 50.5 | P:neuropeptide signaling pathway |
| Efet.01.9268.g679.t1 | cholinesterase isoform X2 | 1377 | 1.46E-77 | 62.5 | C:extracellular space; F:carboxylic ester hydrolase activity |
| Efet.01.9268.g680.t1 | acetylcholinesterase-like isoform X1 | 426 | 1.16E-27 | 61.55 | C:membrane; C:integral component of membrane; F:cholinesterase activity; F:hydrolase activity |
| Efet.01.92699.g1032.t1 | disintegrin and metallo ase domain-containing 10-like | 609 | 2.64E-26 | 49.5 | F:zinc ion binding; P:integrin-mediated signaling pathway; P:Notch signaling pathway; P:membrane protein ectodomain proteolysis; P:proteolysis; C:membrane; C:integral component of membrane; F:metalloendopeptidase activity; C:intracellular; F:metallopeptidase activity |
| Efet.01.92735.g1034.t1 | glyceraldehyde-3-phosphate dehydrogenase | 690 | 3.27E-125 | 89.75 | F:NADP binding; F:NAD binding; P:glycolytic process; P:oxidation-reduction process; P:glucose metabolic process; F:glyceraldehyde-3-phosphate dehydrogenase (NAD+) (phosphorylating) activity |
| Efet.01.9278.g681.t1 | neuronal acetylcholine receptor subunit alpha-10-like | 486 | 3.06E-98 | 88.9 | P:chemical synaptic transmission, postsynaptic; P:synaptic transmission, cholinergic; F:acetylcholine receptor activity; P:neuromuscular synaptic transmission; C:postsynaptic membrane; P:cation transmembrane transport; C:cell junction; F:extracellular ligand-gated ion channel activity; F:acetylcholine binding; C:acetylcholine-gated channel complex; P:response to nicotine |
| Efet.01.92785.g1036.t1 | DNA repair XRCC1 | 339 | 4.27E-20 | 53.45 | P:DNA repair; P:hippocampus development |
| Efet.01.92788.g1038.t1 | FMRFamide receptor-like | 1281 | 1.40E-116 | 56.25 | F:G-protein coupled receptor activity; C:membrane |
| Efet.01.92849.g1042.t1 | atrial natriuretic peptide receptor 1-like isoform X1 | 549 | 2.05E-13 | 91.55 | F:ATP binding; P:cGMP biosynthetic process; C:integral component of membrane; F:guanylate cyclase activity; F:protein kinase activity; P:protein phosphorylation; C:intracellular; P:intracellular signal transduction |
| Efet.01.92862.g1043.t1 | polypyrimidine tract-binding 1 isoform X11 | 228 | 7.43E-19 | 83.35 | F:nucleotide binding; C:nucleus; F:RNA binding; P:regulation of cell differentiation; P:mRNA processing |
| Efet.01.92948.g1046.t1 | hypothetical protein HELRODRAFT_133959, partial | 807 | 3.70E-35 | 61.6 | P:transmembrane transport; C:integral component of membrane; F:metal ion transmembrane transporter activity; P:metal ion transport |
| Efet.01.92948.g1047.t1 | zinc transporter ZIP3-like isoform X1 | 222 | 2.02E-22 | 66.13 | P:transmembrane transport; C:integral component of membrane; F:metal ion transmembrane transporter activity; P:metal ion transport |
| Efet.01.92957.g1049.t1 | hypothetical protein HELRODRAFT_160583 | 369 | 1.10E-07 | 81 |  |
| Efet.01.92962.g1050.t1 | alpha- sarcomeric-like isoform X2 | 441 | 2.49E-69 | 95 | F:actin filament binding; P:actin crosslink formation; F:protein tyrosine phosphatase activity; F:2-alkenal reductase [NAD(P)] activity; F:calcium ion binding; P:oxidation-reduction process; P:peptidyl-tyrosine dephosphorylation; P:actin filament bundle assembly |
| Efet.01.92984.g1051.t1 | Wobble nucleotide-excising tRNase | 1062 | 3.94E-86 | 77.85 |  |
| Efet.01.92984.g1052.t1 | molecular chaperone Tir | 519 | 2.32E-103 | 75.65 |  |
| Efet.01.92984.g1053.t1 | hypothetical protein, partial | 339 | 1.48E-32 | 74.23 | C:integral component of membrane |
| Efet.01.93035.g1055.t1 | uncharacterized protein LOC110443823 | 642 | 1.11E-35 | 54.75 |  |
| Efet.01.93046.g1056.t1 | cyclin-dependent kinase inhibitor 1B | 621 | 1.24E-08 | 57.4 | P:regulation of protein metabolic process; P:positive regulation of cellular process; P:regulation of catalytic activity; P:negative regulation of cell cycle; P:cellular response to stimulus; P:single-organism cellular process; C:intracellular part; P:regulation of cell proliferation; P:negative regulation of phosphorylation |
| Efet.01.93083.g1058.t1 | tyrosine- kinase PR2 isoform X2 | 375 | 1.03E-19 | 73.7 | F:nucleotide binding; F:protein tyrosine kinase activity; P:protein phosphorylation |
| Efet.01.93086.g1059.t1 | transient receptor potential cation channel subfamily V member 6-like | 2001 | 2.73E-108 | 56.5 | C:integral component of membrane; P:transport |
| Efet.01.931.g90.t1 | sentrin-specific protease 6 isoform X1 | 726 | 3.05E-13 | 81.8 | P:proteolysis; F:cysteine-type peptidase activity |
| Efet.01.93118.g1060.t1 | Golgi phospho 3-like | 303 | 5.68E-50 | 85.8 | F:phosphatidylinositol-4-phosphate binding; C:intracellular; P:Golgi vesicle transport |
| Efet.01.93138.g1061.t1 | nicotinate-nucleotide pyrophosphorylase [carboxylating]-like | 951 | 1.16E-145 | 78.75 | F:nicotinate-nucleotide diphosphorylase (carboxylating) activity; C:cytoplasm; P:NAD biosynthetic process; P:quinolinate catabolic process |
| Efet.01.932.g91.t1 | carnitine O-palmitoyltransferase muscle isoform isoform X1 | 441 | 2.39E-47 | 51.8 | F:transferase activity, transferring acyl groups; C:membrane; C:integral component of membrane; F:transferase activity |
| Efet.01.93216.g1063.t1 | spectrin alpha chain-like isoform X4 | 1176 | 0 | 84.4 | F:calcium ion binding |
| Efet.01.93379.g1067.t1 | armadillo repeat-containing 2-like isoform X2 | 357 | 3.37E-06 | 74.8 | F:oxidoreductase activity, acting on CH-OH group of donors; F:oxidoreductase activity; P:oxidation-reduction process |
| Efet.01.93391.g1068.t1 | transcription initiation factor TFIID subunit 1-like isoform X1 | 303 | 9.16E-29 | 91.6 | F:translation initiation factor activity; P:translational initiation |
| Efet.01.9340.g684.t1 | hypothetical protein HELRODRAFT_109965 | 315 | 2.93E-07 | 76 | P:biological regulation; P:sodium ion transport; P:ion transmembrane transport; F:sodium channel activity; C:integral component of membrane; F:voltage-gated ion channel activity |
| Efet.01.9340.g685.t1 | sodium channel type 4 subunit alpha B-like isoform X1 | 303 | 4.63E-08 | 71.7 | P:biological regulation; P:sodium ion transport; P:ion transmembrane transport; F:sodium channel activity; C:integral component of membrane; F:voltage-gated ion channel activity |
| Efet.01.9340.g686.t1 | Sodium channel type 9 subunit alpha | 900 | 9.21E-51 | 67.95 | P:biological regulation; P:sodium ion transport; P:ion transmembrane transport; F:sodium channel activity; C:integral component of membrane; F:voltage-gated ion channel activity |
| Efet.01.9340.g687.t1 | sodium channel para- | 1326 | 7.00E-106 | 74.15 | P:regulation of postsynaptic membrane potential; F:calcium ion binding; P:regulation of ion transmembrane transport; C:voltage-gated sodium channel complex; P:sodium ion transmembrane transport; F:voltage-gated sodium channel activity |
| Efet.01.93459.g1071.t1 | 5-hydroxytryptamine receptor 2C isoform X2 | 417 | 2.90E-18 | 76.05 | P:positive regulation of phosphate metabolic process; P:dopamine receptor signaling pathway; P:serotonin receptor signaling pathway; P:phospholipase C-activating G-protein coupled receptor signaling pathway; F:G-protein coupled serotonin receptor activity; P:behavior; P:positive regulation of cytosolic calcium ion concentration; C:integral component of plasma membrane; F:dopamine neurotransmitter receptor activity; P:regulation of localization; F:organic cyclic compound binding; P:regulation of signaling; F:amine binding; P:regulation of cell communication; P:regulation of primary metabolic process |
| Efet.01.93483.g1072.t1 | hypothetical protein CAPTEDRAFT_222792 | 288 | 1.82E-07 | 60.5 | P:signal transduction |
| Efet.01.93508.g1073.t1 | sialate O-acetylesterase-like | 639 | 3.35E-35 | 52.25 | P:carbohydrate metabolic process; C:extracellular space; C:extracellular exosome; C:membrane; C:integral component of membrane; F:sialate O-acetylesterase activity; P:regulation of immune system process |
| Efet.01.93532.g1075.t1 | dynein heavy chain axonemal-like | 222 | 1.92E-12 | 86.05 | F:ATP binding; F:microtubule motor activity; F:calcium ion binding; C:dynein complex; P:microtubule-based movement |
| Efet.01.93535.g1076.t1 | serine threonine- kinase Tao-like | 369 | 1.96E-25 | 64 | F:nucleotide binding; P:cellular protein modification process; P:phosphorylation; F:kinase activity |
| Efet.01.9354.g688.t1 | dynein heavy chain axonemal-like isoform X1 | 897 | 2.99E-139 | 75.6 | F:nucleotide binding; C:dynein complex; F:nucleoside-triphosphatase activity; P:microtubule-based movement |
| Efet.01.93570.g1077.t1 | adenosine receptor A2b-like | 1056 | 9.25E-29 | 43.3 | F:G-protein coupled receptor activity; P:G-protein coupled receptor signaling pathway; C:membrane; C:integral component of membrane |
| Efet.01.93580.g1078.t1 | neurotrophin isoform 2 | 369 | 2.66E-33 | 56.05 | P:regulation of cellular process; P:nervous system development; P:cell differentiation; F:receptor binding |
| Efet.01.936.g93.t1 | RNA-binding 5-like | 843 | 1.28E-18 | 83.35 | F:nucleotide binding; F:zinc ion binding; F:identical protein binding; P:RNA splicing; P:regulation of alternative mRNA splicing, via spliceosome; P:regulation of apoptotic process; P:mRNA processing; F:protein complex binding; C:nucleoplasm; F:RNA binding |
| Efet.01.93618.g1080.t1 | beta-galactosidase | 267 | 6.41E-29 | 76.75 |  |
| Efet.01.93625.g1081.t1 | catenin delta-2-like isoform X1 | 339 | 3.66E-12 | 60.2 | P:single organismal cell-cell adhesion; P:regulation of actin cytoskeleton organization; C:cell-cell adherens junction; F:cadherin binding; P:embryonic body morphogenesis |
| Efet.01.93636.g1082.t1 | polypeptide N-acetylgalactosaminyltransferase 5 | 246 | 8.55E-33 | 84 | C:integral component of membrane; P:protein O-linked glycosylation via threonine; C:Golgi membrane; F:carbohydrate binding; F:polypeptide N-acetylgalactosaminyltransferase activity |
| Efet.01.93668.g1083.t1 | hypothetical protein HELRODRAFT_189003 | 285 | 9.86E-09 | 49 |  |
| Efet.01.93733.g1086.t1 | DNA (cytosine-5)-methyltransferase 3A-like | 321 | 1.31E-20 | 74.55 | F:DNA (cytosine-5-)-methyltransferase activity; P:C-5 methylation of cytosine; P:regulation of gene expression |
| Efet.01.93761.g1088.t1 | growth factor receptor-bound 2 | 492 | 4.03E-40 | 68.1 | F:organic cyclic compound binding; F:heterocyclic compound binding; F:transferase activity; P:cellular metabolic process |
| Efet.01.93804.g1090.t1 | substance-K receptor-like | 213 | 1.41E-24 | 66.95 | F:G-protein coupled receptor activity; P:G-protein coupled receptor signaling pathway; C:membrane |
| Efet.01.93828.g1092.t1 | spermine oxidase-like | 909 | 2.05E-119 | 62.6 | F:oxidoreductase activity; P:oxidation-reduction process |
| Efet.01.93883.g1096.t1 | Thrombospondin type-1 domain-containing | 1122 | 1.23E-47 | 47.95 | C:membrane; C:integral component of membrane |
| Efet.01.93957.g1098.t1 | potassium voltage-gated channel Shaw-like isoform X1 | 351 | 1.26E-50 | 59.2 | F:ion channel activity; P:transport |
| Efet.01.93975.g1099.t1 | vam6 Vps39 | 285 | 1.54E-26 | 72.55 | P:vesicle-mediated transport; C:lysosomal membrane |
| Efet.01.93984.g1100.t1 | sacsin-like | 9495 | 0 | 53.15 |  |
| Efet.01.9399.g695.t1 | deleted in lung and esophageal cancer 1-like | 477 | 9.14E-41 | 60.5 | P:negative regulation of cell proliferation |
| Efet.01.940.g94.t1 | 14-3-3 epsilon isoform X3 | 306 | 4.13E-44 | 89.9 | P:mitotic G2 DNA damage checkpoint; P:positive regulation of growth; F:protein domain specific binding; P:pole cell migration; P:regulation of mitotic nuclear division; P:germarium-derived oocyte fate determination; P:determination of adult lifespan; P:axon guidance; P:oocyte microtubule cytoskeleton polarization; P:positive regulation of Ras protein signal transduction; P:response to UV; C:chromosome; C:microtubule associated complex; C:plasma membrane; C:nucleoplasm; C:centrosome; C:germline ring canal; C:cytoplasm; F:protein heterodimerization activity; P:positive regulation of ERK1 and ERK2 cascade; F:phosphoserine binding; P:wing disc dorsal/ventral pattern formation |
| Efet.01.94028.g1103.t1 | hypothetical protein HELRODRAFT_165590 | 258 | 5.53E-10 | 71 | F:binding |
| Efet.01.94031.g1104.t1 | ubiquitin-like modifier-activating enzyme 1 | 585 | 9.11E-50 | 74.9 | F:nucleotide binding; F:ligase activity; P:cellular process |
| Efet.01.94034.g1105.t1 | cleft lip and palate transmembrane 1 homolog | 279 | 4.83E-11 | 76.85 | C:integral component of membrane |
| Efet.01.94051.g1106.t1 | innexin 1 | 1101 | 0 | 68.3 | C:gap junction; P:ion transport; C:integral component of membrane; P:cell communication by electrical coupling; C:synapse; F:gap junction channel activity |
| Efet.01.94070.g1107.t1 | hypothetical protein LOTGIDRAFT_188880 | 438 | 6.18E-09 | 51.5 | F:actin filament binding; P:synapse assembly; P:ruffle assembly; F:actin binding; P:neuron projection morphogenesis; P:receptor-mediated endocytosis; C:intracellular |
| Efet.01.94120.g1110.t1 | NADP-dependent malic enzyme-like | 714 | 2.16E-81 | 69.2 | C:intracellular membrane-bounded organelle; F:malic enzyme activity; F:binding; P:single-organism metabolic process |
| Efet.01.94133.g1111.t1 | PREDICTED: uncharacterized protein LOC106172818 | 741 | 1.94E-43 | 54.15 | C:membrane; C:integral component of membrane |
| Efet.01.94160.g1112.t1 | reticulon-1-A-like isoform X3 | 225 | 1.44E-09 | 91 | C:endoplasmic reticulum membrane; C:integral component of membrane |
| Efet.01.9417.g696.t1 | bridging integrator 3-like | 408 | 1.96E-44 | 67.15 | C:cytoplasm |
| Efet.01.94193.g1113.t1 | Stabilin-2 | 564 | 3.38E-16 | 60.4 | C:external side of plasma membrane; F:hyaluronic acid binding; P:endocytosis |
| Efet.01.94205.g1114.t1 | CRAL-TRIO domain-containing | 216 | 2.54E-34 | 81.15 |  |
| Efet.01.94211.g1115.t1 | rootletin-like isoform X8 | 1533 | 3.91E-147 | 69.65 |  |
| Efet.01.94242.g1116.t1 | protocadherin gamma subfamily 11 | 1368 | 8.14E-77 | 54.85 | F:calcium ion binding; C:membrane; C:integral component of membrane; P:cell adhesion; P:homophilic cell adhesion via plasma membrane adhesion molecules; C:plasma membrane |
| Efet.01.94242.g1117.t1 | protocadherin beta-6 | 342 | 1.03E-15 | 60.15 | F:calcium ion binding; C:membrane; C:integral component of membrane; P:cell adhesion; P:homophilic cell adhesion via plasma membrane adhesion molecules; C:plasma membrane |
| Efet.01.94261.g1119.t1 | lysoplasmalogenase TMEM86A | 702 | 1.07E-34 | 55.45 | C:membrane; C:integral component of membrane |
| Efet.01.94267.g1120.t1 | nuclear hormone receptor HR96-like isoform X8 | 840 | 1.61E-97 | 67.6 | P:regulation of cellular process; F:binding; P:cellular process |
| Efet.01.94299.g1121.t1 | OTU domain-containing 7B-like isoform X1 | 1266 | 1.28E-09 | 52.55 | F:DNA binding; F:zinc ion binding |
| Efet.01.943.g95.t1 | torsin-1A-interacting 2-like | 885 | 4.22E-39 | 49 | P:protein localization to nuclear envelope; P:positive regulation of ATPase activity; C:membrane; C:integral component of membrane; C:endoplasmic reticulum; P:endoplasmic reticulum organization; F:ATPase activator activity; F:ATPase binding |
| Efet.01.94359.g1125.t1 | sex peptide receptor-like | 1086 | 4.91E-97 | 64.1 | F:G-protein coupled receptor activity; F:G-protein coupled peptide receptor activity; P:G-protein coupled receptor signaling pathway; C:membrane; C:integral component of membrane |
| Efet.01.94396.g1130.t1 | E3 ubiquitin- ligase TRIM56-like isoform X2 | 534 | 1.94E-09 | 52.8 | F:zinc ion binding; F:metal ion binding; C:intracellular |
| Efet.01.94407.g1131.t1 | Ras-related Rab-5B | 243 | 9.07E-14 | 65.6 | F:GTP binding; F:DNA primase activity; P:small GTPase mediated signal transduction; C:membrane; C:integral component of membrane; C:intracellular; P:DNA replication, synthesis of RNA primer |
| Efet.01.94419.g1132.t1 | retinol dehydrogenase 7-like isoform X2 | 381 | 5.14E-23 | 60.1 | F:oxidoreductase activity; C:membrane; C:integral component of membrane; P:oxidation-reduction process |
| Efet.01.94444.g1133.t1 | FACT complex subunit Ssrp1-like | 774 | 1.86E-07 | 79.1 | F:DNA binding; C:nucleus |
| Efet.01.94459.g1134.t1 | cilia- and flagella-associated 44-like isoform X1 | 423 | 1.06E-61 | 87.6 |  |
| Efet.01.94459.g1135.t1 | cilia- and flagella-associated 44-like isoform X3 | 381 | 1.34E-43 | 83.4 |  |
| Efet.01.94492.g1137.t1 | Down syndrome cell adhesion molecule | 576 | 7.38E-20 | 56.86 | C:integral component of plasma membrane; C:membrane; C:integral component of membrane; P:nervous system development; P:cell adhesion |
| Efet.01.94513.g1138.t1 | probable G- coupled receptor | 1383 | 6.93E-39 | 43.9 | F:G-protein coupled receptor activity; F:G-protein coupled peptide receptor activity; P:G-protein coupled receptor signaling pathway; C:membrane; C:integral component of membrane |
| Efet.01.94548.g1139.t1 | hypothetical protein CAPTEDRAFT_224867 | 249 | 9.70E-14 | 70 | C:membrane; C:integral component of membrane |
| Efet.01.94555.g1140.t1 | sex peptide receptor-like | 744 | 1.47E-64 | 60.4 | F:G-protein coupled receptor activity; F:G-protein coupled peptide receptor activity; P:G-protein coupled receptor signaling pathway; C:membrane; C:integral component of membrane |
| Efet.01.9467.g699.t1 | gibberellin-regulated 14-like | 2169 | 1.60E-33 | 46.2 | C:membrane; C:integral component of membrane |
| Efet.01.94691.g1143.t1 | cyclic GMP-AMP synthase-like | 387 | 1.64E-15 | 52.6 | C:membrane; C:integral component of membrane |
| Efet.01.94709.g1145.t1 | solute carrier family 25 member 35 | 324 | 4.29E-33 | 75.2 | F:structural constituent of ribosome; C:membrane; P:translation |
| Efet.01.94758.g1146.t1 | rho GTPase-activating 44-like isoform X5 | 273 | 1.00E-17 | 68.35 | C:cytoplasm; P:signal transduction |
| Efet.01.94769.g1147.t1 | probable G- coupled receptor 158 | 381 | 1.85E-30 | 63.6 | C:membrane |
| Efet.01.9480.g700.t1 | glutamate receptor kainate 2-like | 936 | 1.40E-12 | 44.8 | P:ion transmembrane transport; C:synapse; F:receptor activity; F:extracellular-glutamate-gated ion channel activity; F:ionotropic glutamate receptor activity; P:transport; C:plasma membrane; F:ion channel activity; P:ion transport; C:postsynaptic membrane; C:membrane; C:integral component of membrane; C:cell junction; P:ionotropic glutamate receptor signaling pathway |
| Efet.01.94802.g1148.t1 | calpain-A isoform X3 | 234 | 2.14E-10 | 67.1 | P:proteolysis; F:calcium-dependent cysteine-type endopeptidase activity; C:intracellular |
| Efet.01.94858.g1150.t1 | inactive phospholipase C 2 | 738 | 9.14E-132 | 82.35 | F:phosphatidylinositol phospholipase C activity; F:calcium ion binding; P:lipid catabolic process; F:signal transducer activity; C:intracellular; P:intracellular signal transduction |
| Efet.01.94926.g1152.t1 | hypothetical protein CAPTEDRAFT_211921 | 234 | 6.15E-17 | 64.85 | P:cellular aromatic compound metabolic process; P:small molecule metabolic process; P:heterocycle metabolic process; P:organic cyclic compound metabolic process; F:catalytic activity; P:organonitrogen compound metabolic process; P:cellular nitrogen compound metabolic process; P:single-organism cellular process; F:binding; P:primary metabolic process |
| Efet.01.94956.g1154.t1 | pyruvate mitochondrial-like isoform X1 | 444 | 3.04E-70 | 89.25 | F:DNA binding; F:ATP binding; P:gluconeogenesis; F:pyruvate carboxylase activity; F:metal ion binding; F:biotin binding; F:biotin carboxylase activity; P:pyruvate metabolic process |
| Efet.01.94957.g1155.t1 | MTG16_MOUSE ame: Full= CBFA2T3 ame: Full=Eight twenty one 2 ame: Full=MTG8-related 2 ame: Full= ETO-2 | 231 | 2.18E-06 | 74 | P:negative regulation of cell proliferation; P:negative regulation of glycolytic process; C:Golgi apparatus; P:fat cell differentiation; C:nucleolus; C:nucleoplasm; P:negative regulation of transcription from RNA polymerase II promoter; F:transcription factor activity, sequence-specific DNA binding; P:regulation of DNA binding; P:response to hypoxia; P:negative regulation of transcription, DNA-templated; F:protein homodimerization activity; P:transcription, DNA-templated; P:negative regulation of fat cell differentiation; P:regulation of transcription, DNA-templated; F:chromatin binding; P:regulation of aerobic respiration; C:intracellular; F:DNA binding; C:nucleus; F:protein binding; F:metal ion binding; C:cytoplasm; F:transcription corepressor activity; C:membrane; C:mitochondrion; P:cell differentiation; C:nuclear matrix; P:granulocyte differentiation; P:positive regulation of proteasomal ubiquitin-dependent protein catabolic process |
| Efet.01.9503.g703.t1 | alpha-catulin isoform X1 | 297 | 2.02E-36 | 84.35 | F:actin filament binding; F:cadherin binding; C:integral component of membrane; P:cell adhesion; P:Rho protein signal transduction; C:intracellular |
| Efet.01.95041.g1160.t1 | fibrillin-2-like | 852 | 9.45E-84 | 62.95 | C:extracellular region |
| Efet.01.95043.g1161.t1 | otoferlin-like isoform X5 | 522 | 2.85E-88 | 86.55 | C:integral component of membrane |
| Efet.01.95052.g1162.t1 | hypothetical protein HELRODRAFT_162326 | 492 | 7.54E-12 | 67.5 | P:potassium ion transport; C:membrane |
| Efet.01.95067.g1163.t1 | zinc finger CCCH domain-containing 10 | 654 | 2.32E-12 | 59.2 | C:cytoplasm; F:metal ion binding; F:RNA binding |
| Efet.01.95083.g1164.t1 | PREDICTED: uncharacterized protein LOC106174227 isoform X1 | 444 | 2.28E-39 | 60.2 | C:membrane; C:integral component of membrane |
| Efet.01.95089.g1165.t1 | Peptidyl-prolyl cis-trans isomerase NIMA-interacting 1 | 252 | 3.11E-28 | 79.75 | F:peptidyl-prolyl cis-trans isomerase activity; P:protein peptidyl-prolyl isomerization |
| Efet.01.95090.g1166.t1 | estrogen-related receptor gamma isoform X3 | 408 | 2.65E-17 | 68.35 | F:DNA binding; P:regulation of cellular process; F:metal ion binding; C:membrane; P:cellular process |
| Efet.01.95093.g1167.t1 | zinc finger 66 | 624 | 1.27E-65 | 74.1 | F:binding; F:transferase activity |
| Efet.01.95161.g1170.t1 | UBX domain-containing 6-like | 1182 | 2.81E-116 | 68.45 |  |
| Efet.01.9520.g704.t1 | UPF0739 C1orf74 homolog | 651 | 5.74E-16 | 46.1 |  |
| Efet.01.95229.g1173.t1 | dynein heavy cytoplasmic | 624 | 1.62E-108 | 91.05 | F:ATPase activity; F:ATP binding; F:microtubule motor activity; C:dynein complex; P:microtubule-based movement |
| Efet.01.95233.g1174.t1 | golgin subfamily A member 4-like isoform X2 | 942 | 3.04E-34 | 64.25 | C:membrane; C:integral component of membrane |
| Efet.01.95249.g1175.t1 | atrial natriuretic peptide receptor 3-like | 591 | 2.91E-44 | 57.2 | F:nucleotide binding; F:phosphorus-oxygen lyase activity; C:membrane; P:cyclic nucleotide biosynthetic process |
| Efet.01.95340.g1177.t1 | serine threonine- kinase pim-1-like | 552 | 5.21E-61 | 74.8 | F:nucleotide binding; P:single-multicellular organism process; F:protein kinase activity |
| Efet.01.95344.g1178.t1 | major egg antigen-like | 1020 | 3.49E-88 | 56.8 |  |
| Efet.01.95375.g1179.t1 | guanylate cyclase soluble subunit beta-1-like | 705 | 7.48E-27 | 71.15 | C:guanylate cyclase complex, soluble; F:nucleotide binding; P:cGMP biosynthetic process; F:guanylate cyclase activity; C:plasma membrane; F:adenylate cyclase activity; P:intracellular signal transduction; F:heme binding |
| Efet.01.954.g96.t1 | hypothetical protein CAPTEDRAFT_146207 | 927 | 4.66E-08 | 59 |  |
| Efet.01.95410.g1181.t1 | arginine methyltransferase | 996 | 2.76E-69 | 60.65 | F:protein-arginine N-methyltransferase activity; P:regulation of transcription, DNA-templated; P:peptidyl-arginine methylation |
| Efet.01.95414.g1182.t1 | cholesterol 7-desaturase-like | 486 | 2.88E-35 | 66.05 | C:membrane; F:iron-sulfur cluster binding |
| Efet.01.95426.g1183.t1 | plexin-A2-like isoform X1 | 711 | 9.80E-16 | 47.7 | F:ATP binding; C:membrane; C:integral component of membrane; F:semaphorin receptor activity; F:protein kinase activity; P:protein phosphorylation; P:semaphorin-plexin signaling pathway |
| Efet.01.95451.g1188.t1 | hypothetical protein CAPTEDRAFT_169570 | 576 | 5.46E-21 | 77 | P:regulation of cellular process; F:guanyl-nucleotide exchange factor activity |
| Efet.01.95476.g1189.t1 | hypothetical protein CAPTEDRAFT_107262 | 1233 | 2.79E-23 | 50.55 | P:cellular response to stress; C:mitochondrial outer membrane |
| Efet.01.95496.g1191.t1 | hypothetical protein CAPTEDRAFT_204443 | 1164 | 2.88E-13 | 52 |  |
| Efet.01.95564.g1192.t1 | hypothetical protein CAPTEDRAFT_221462 | 390 | 5.22E-18 | 71.55 | P:signal transduction |
| Efet.01.95607.g1193.t1 | dicer 1 | 1161 | 4.48E-46 | 53.05 | F:binding |
| Efet.01.95625.g1195.t1 | discoidin domain-containing receptor 2-like isoform X1 | 348 | 1.54E-51 | 82.7 | C:integral component of plasma membrane; F:ATP binding; P:peptidyl-tyrosine phosphorylation; F:protein tyrosine kinase collagen receptor activity; P:collagen-activated tyrosine kinase receptor signaling pathway |
| Efet.01.95670.g1196.t1 | mitochondrial F-type ATP synthase subunit gamma | 459 | 1.34E-57 | 85.45 | C:proton-transporting ATP synthase complex, catalytic core F(1); F:proton-transporting ATPase activity, rotational mechanism; P:ATP synthesis coupled proton transport; F:proton-transporting ATP synthase activity, rotational mechanism |
| Efet.01.95672.g1197.t1 | nucleosome-remodeling factor subunit NURF301-like isoform X1 | 2040 | 2.19E-84 | 61.5 | F:binding |
| Efet.01.9572.g707.t1 | Transient receptor potential-gamma | 486 | 1.79E-49 | 65.6 | F:calcium channel activity; C:integral component of membrane; P:calcium ion transmembrane transport |
| Efet.01.95720.g1199.t1 | thyrotropin-releasing hormone receptor-like | 474 | 5.07E-41 | 66.95 | F:G-protein coupled receptor activity; P:signal transduction; C:membrane |
| Efet.01.95720.g1200.t1 | thyrotropin-releasing hormone receptor-like | 582 | 2.33E-46 | 69.95 | F:thyrotropin-releasing hormone receptor activity; P:G-protein coupled receptor signaling pathway; C:integral component of membrane; F:transferase activity |
| Efet.01.95726.g1201.t1 | Na(+) H(+) exchange regulatory cofactor NHE-RF1 | 321 | 2.47E-23 | 68.8 | F:protein complex scaffold; C:membrane; C:endomembrane system |
| Efet.01.95783.g1206.t1 | mitogen-activated kinase kinase kinase 11-like | 1260 | 1.57E-23 | 52.1 | F:ATP binding; C:membrane; C:integral component of membrane; F:protein kinase activity; P:protein phosphorylation |
| Efet.01.95798.g1207.t1 | sprouty homolog 4 | 804 | 4.38E-35 | 53.9 | P:respiratory system development; P:negative regulation of signal transduction; P:regulation of protein phosphorylation; P:regulation of intracellular signal transduction; P:tube development; P:animal organ development; P:positive regulation of biological process |
| Efet.01.95803.g1208.t1 | BAI1-associated 3-like isoform X1 | 375 | 1.88E-15 | 62.1 |  |
| Efet.01.95823.g1210.t1 | histone-lysine N-methyltransferase NSD2 isoform X3 | 993 | 3.49E-13 | 58.5 | C:nucleus; F:zinc ion binding; P:histone lysine methylation; F:metal ion binding; F:histone-lysine N-methyltransferase activity; F:methyltransferase activity; F:transferase activity; P:methylation |
| Efet.01.95824.g1211.t1 | nuclear hormone receptor HR3 | 1779 | 2.58E-11 | 69 | F:DNA binding; F:transcription factor activity, sequence-specific DNA binding; F:metal ion binding; P:signal transduction; F:signaling receptor activity |
| Efet.01.95896.g1214.t1 | vacuolar sorting-associated 41 homolog | 369 | 8.47E-34 | 76.75 | F:zinc ion binding; P:vesicle-mediated transport; P:regulation of SNARE complex assembly; C:CORVET complex; F:GTPase binding; C:late endosome; P:vacuole fusion, non-autophagic; P:protein targeting to vacuole; C:HOPS complex |
| Efet.01.95932.g1216.t1 | regulator of nonsense transcripts 3A isoform X1 | 231 | 5.08E-09 | 77.9 | F:nucleotide binding |
| Efet.01.96062.g1222.t1 | N-6 DNA methylase | 819 | 4.32E-115 | 74.15 | F:nucleic acid binding; P:DNA methylation; F:methyltransferase activity |
| Efet.01.96087.g1224.t1 | hypothetical protein HELRODRAFT_170285 | 837 | 1.38E-14 | 78 |  |
| Efet.01.96096.g1225.t1 | hypothetical protein HELRODRAFT_193970 | 747 | 6.74E-102 | 61.44 |  |
| Efet.01.96096.g1226.t1 | hypothetical protein HELRODRAFT_193970 | 453 | 1.87E-32 | 71 |  |
| Efet.01.96100.g1227.t1 | serine arginine-rich splicing factor 2 | 309 | 1.92E-43 | 89.35 | F:nucleotide binding; F:RNA binding |
| Efet.01.96273.g1232.t1 | CYR61-like | 279 | 5.59E-12 | 55.8 | P:regulation of cell growth; C:extracellular region; F:insulin-like growth factor binding |
| Efet.01.96286.g1233.t1 | thioredoxin domain-containing 9-like | 654 | 4.61E-101 | 81.25 | P:queuosine biosynthetic process; C:cytoplasm; P:cell redox homeostasis; F:queuine tRNA-ribosyltransferase activity |
| Efet.01.96358.g1234.t1 | solute carrier family 23 member 1 | 255 | 8.45E-20 | 78.25 | P:L-ascorbic acid transport; P:carboxylic acid transmembrane transport; F:sodium-dependent L-ascorbate transmembrane transporter activity; P:vitamin transmembrane transport; C:integral component of membrane |
| Efet.01.96362.g1235.t1 | spectrin alpha non-erythrocytic 1-like isoform X1 | 819 | 3.43E-102 | 75.65 | F:actin binding; F:phospholipid binding |
| Efet.01.96385.g1236.t1 | hypothetical protein CAPTEDRAFT_186947 | 480 | 3.62E-06 | 69 | C:membrane; C:integral component of membrane |
| Efet.01.96391.g1237.t1 | Protocadherin Fat 4 | 2787 | 0 | 47.7 | F:calcium ion binding; C:membrane; C:integral component of membrane; P:cell adhesion; P:homophilic cell adhesion via plasma membrane adhesion molecules; C:plasma membrane |
| Efet.01.96391.g1238.t1 | hypothetical protein CAPTEDRAFT_220753 | 270 | 1.13E-10 | 58 | F:calcium ion binding; C:membrane; C:integral component of membrane; P:cell adhesion; P:homophilic cell adhesion via plasma membrane adhesion molecules; C:plasma membrane |
| Efet.01.96420.g1239.t1 | low-density lipo receptor-related 6-like | 2133 | 2.18E-91 | 48.15 | P:biological regulation; P:multicellular organism development |
| Efet.01.96423.g1240.t1 | lethal(3)malignant brain tumor 1 isoform X1 | 747 | 1.42E-73 | 68.95 | C:nucleus; F:transcription factor activity, sequence-specific DNA binding; F:zinc ion binding; P:regulation of transcription, DNA-templated |
| Efet.01.96439.g1243.t1 | deleted in lung and esophageal cancer 1-like | 408 | 4.19E-22 | 78.65 | P:negative regulation of cell proliferation |
| Efet.01.96454.g1245.t1 | E3 ubiquitin- ligase TRIM71-like | 978 | 1.02E-19 | 44.95 | F:zinc ion binding; P:signal transduction; P:ion transmembrane transport; F:GABA-A receptor activity; F:extracellular ligand-gated ion channel activity; C:GABA-A receptor complex; C:synapse; P:transport; C:intracellular; C:plasma membrane; P:ion transport; F:metal ion binding; C:membrane; C:integral component of membrane; C:cell junction |
| Efet.01.96460.g1246.t1 | enhancer of mRNA-decapping 4 isoform X1 | 939 | 3.52E-42 | 59.15 | F:protein binding; C:intracellular membrane-bounded organelle |
| Efet.01.96503.g1248.t1 | hypothetical protein HELRODRAFT_188674 | 1392 | 4.87E-74 | 47.65 | C:membrane; C:integral component of membrane |
| Efet.01.96548.g1249.t1 | guanylate cyclase 32E-like | 366 | 1.46E-54 | 87.8 | C:guanylate cyclase complex, soluble; F:ATP binding; P:cGMP biosynthetic process; C:integral component of membrane; F:guanylate cyclase activity; F:protein kinase activity; P:protein phosphorylation; C:plasma membrane; F:adenylate cyclase activity; P:intracellular signal transduction |
| Efet.01.96552.g1251.t1 | hypothetical protein CAPTEDRAFT_216764 | 2022 | 1.06E-80 | 49.5 |  |
| Efet.01.96589.g1255.t1 | zinc metallo ase dpy-31-like | 636 | 5.71E-14 | 51.4 | P:single-multicellular organism process |
| Efet.01.96625.g1257.t1 | E3 ubiquitin- ligase MIB1-like isoform X2 | 441 | 1.08E-37 | 74.15 | F:ubiquitin-protein transferase activity; F:zinc ion binding; F:ligase activity; P:protein ubiquitination |
| Efet.01.96631.g1259.t1 | hypothetical protein HELRODRAFT_189841 | 1104 | 1.39E-16 | 48.67 | C:membrane; C:integral component of membrane |
| Efet.01.96649.g1260.t1 | Titin | 1467 | 3.07E-28 | 42.6 | F:ATP binding; F:protein kinase activity; P:protein phosphorylation |
| Efet.01.96649.g1261.t1 | hypothetical protein HELRODRAFT_78542 | 357 | 1.65E-10 | 69 |  |
| Efet.01.96653.g1262.t1 | dolichyl-diphosphooligosaccharide-- glycosyltransferase subunit DAD1-like | 261 | 5.14E-32 | 89.55 | C:oligosaccharyltransferase complex; F:dolichyl-diphosphooligosaccharide-protein glycotransferase activity; C:integral component of membrane; P:protein glycosylation |
| Efet.01.96750.g1264.t1 | kelch 35 | 783 | 1.36E-20 | 48.5 |  |
| Efet.01.96753.g1265.t1 | Lysine-specific demethylase 8 | 891 | 9.25E-117 | 69.35 | P:macromolecule metabolic process; C:nucleus; P:G2/M transition of mitotic cell cycle; P:positive regulation of transcription, DNA-templated; C:membrane; F:chromatin binding; F:histone demethylase activity (H3-K36 specific) |
| Efet.01.96762.g1267.t1 | glycogen-binding subunit 76A-like | 828 | 1.14E-33 | 60.05 |  |
| Efet.01.96793.g1268.t1 | hypothetical protein CAPTEDRAFT_204443 | 981 | 2.93E-09 | 47 |  |
| Efet.01.96819.g1269.t1 | FRAS1-related extracellular matrix 1-like | 2037 | 1.90E-129 | 50.05 | P:cell-matrix adhesion; C:basement membrane; P:cell communication; F:calcium ion binding; C:membrane; C:integral component of membrane; P:craniofacial suture morphogenesis; P:homophilic cell adhesion via plasma membrane adhesion molecules |
| Efet.01.96826.g1270.t1 | hypothetical protein HELRODRAFT_173331 | 264 | 7.32E-20 | 68.33 |  |
| Efet.01.96837.g1271.t1 | semaphorin-5A-like isoform X1 | 315 | 9.63E-22 | 75.4 | C:extracellular space; P:negative chemotaxis; P:neural crest cell migration; C:membrane; P:negative regulation of axon extension involved in axon guidance; F:chemorepellent activity; F:semaphorin receptor binding; P:positive regulation of cell migration; P:semaphorin-plexin signaling pathway |
| Efet.01.96842.g1272.t1 | carbohydrate sulfotransferase 1-like | 1056 | 7.74E-83 | 45.6 | C:membrane; C:integral component of membrane; F:sulfotransferase activity |
| Efet.01.96845.g1273.t1 | inositol 1,4,5-trisphosphate receptor type 3-like isoform X13 | 306 | 2.96E-48 | 87.5 | P:inositol phosphate-mediated signaling; C:integral component of membrane; F:inositol 1,4,5-trisphosphate-sensitive calcium-release channel activity; P:calcium ion transmembrane transport; C:endoplasmic reticulum |
| Efet.01.9693.g712.t1 | cytoglobin-2-like isoform X1 | 348 | 1.26E-41 | 57.6 | F:binding; P:transport |
| Efet.01.96937.g1276.t1 | hypothetical protein HELRODRAFT_194111 | 384 | 1.35E-09 | 49.7 | C:membrane; C:integral component of membrane; F:carbohydrate binding |
| Efet.01.96946.g1277.t1 | clathrin heavy chain | 228 | 2.48E-38 | 93.7 | F:protein kinase binding; C:myelin sheath; P:vesicle-mediated transport; C:clathrin coat of trans-Golgi network vesicle; C:mitochondrion; C:clathrin coat of coated pit; F:structural molecule activity; P:intracellular protein transport |
| Efet.01.96946.g1278.t1 | clathrin heavy chain 1 isoform X2 | 597 | 3.10E-109 | 97.2 | F:clathrin light chain binding; C:myelin sheath; F:protein C-terminus binding; C:clathrin coat of trans-Golgi network vesicle; P:Golgi organization; F:structural molecule activity; F:peptide binding; P:intracellular protein transport; P:cell division; C:clathrin-coated endocytic vesicle membrane; C:extracellular exosome; C:focal adhesion; C:terminal bouton; F:double-stranded RNA binding; F:ankyrin binding; P:autophagy; P:osteoblast differentiation; C:cytosol; P:Wnt signaling pathway, planar cell polarity pathway; C:extracellular matrix; C:clathrin complex; P:antigen processing and presentation of exogenous peptide antigen via MHC class II; C:trans-Golgi network membrane; F:protein kinase binding; F:heat shock protein binding; P:negative regulation of protein localization to plasma membrane; C:endolysosome membrane; P:transferrin transport; P:negative regulation of hyaluronan biosynthetic process; P:retrograde transport, endosome to Golgi; P:regulation of mitotic spindle organization; F:RNA binding; C:T-tubule; C:mitotic spindle microtubule; C:melanosome; C:mitochondrion; C:clathrin coat of coated pit; C:Myb complex; P:receptor internalization; P:mitotic spindle assembly |
| Efet.01.96961.g1281.t1 | E3 ubiquitin- ligase DTX3L-like | 1803 | 6.99E-70 | 55.05 |  |
| Efet.01.96965.g1283.t1 | invertebrate-type lysozyme | 363 | 2.28E-27 | 69.3 | P:oxidation-reduction process; F:glyoxylate dehydrogenase (acylating) activity; F:lysozyme activity |
| Efet.01.97049.g1284.t1 | protocadherin-9-like isoform X1 | 3108 | 5.58E-86 | 53.1 | F:calcium ion binding; C:membrane; C:integral component of membrane; P:cell adhesion; P:homophilic cell adhesion via plasma membrane adhesion molecules; C:plasma membrane |
| Efet.01.97060.g1288.t1 | cytospin-A-like isoform X2 | 696 | 2.71E-25 | 62.81 |  |
| Efet.01.97060.g1289.t1 | cytospin-A-like isoform X2 | 1965 | 1.06E-51 | 66.1 |  |
| Efet.01.97130.g1293.t1 | cyclic GMP-AMP synthase-like | 945 | 1.52E-30 | 51.85 |  |
| Efet.01.97183.g1294.t1 | FMRFamide receptor-like | 1146 | 6.86E-41 | 53.2 | F:G-protein coupled receptor activity; F:G-protein coupled peptide receptor activity; P:G-protein coupled receptor signaling pathway; C:membrane; C:integral component of membrane |
| Efet.01.97189.g1295.t1 | dopamine receptor 1-like | 1185 | 7.62E-110 | 61.35 | F:G-protein coupled receptor activity; P:signal transduction; C:membrane |
| Efet.01.97213.g1296.t1 | homeobox SIX6 | 528 | 3.60E-99 | 90 | C:nucleus; F:DNA binding; P:eye development; P:transcription from RNA polymerase II promoter; F:transcriptional activator activity, RNA polymerase II distal enhancer sequence-specific binding; P:visual perception; P:positive regulation of transcription from RNA polymerase II promoter; P:animal organ morphogenesis |
| Efet.01.97250.g1297.t1 | ATP-binding cassette sub-family A member 3-like | 1107 | 6.89E-42 | 53 | C:membrane; P:transport |
| Efet.01.9727.g713.t1 | hyperpolarizaion-activated cyclic nucleotide-gated cation channel | 543 | 3.62E-23 | 85.3 | F:voltage-gated potassium channel activity; P:potassium ion transmembrane transport; C:integral component of membrane |
| Efet.01.97308.g1300.t1 | dynein heavy chain axonemal-like isoform X1 | 408 | 2.10E-64 | 87.3 | F:ATPase activity; F:ATP binding; F:microtubule motor activity; C:dynein complex; P:microtubule-based movement |
| Efet.01.97309.g1301.t1 | calponin-2 isoform X3 | 591 | 5.03E-83 | 84.15 | F:calmodulin binding; F:actin binding; P:actomyosin structure organization |
| Efet.01.97316.g1302.t1 | adenosine receptor A2b isoform X1 | 201 | 1.01E-07 | 73.75 | F:G-protein coupled receptor activity; P:G-protein coupled receptor signaling pathway; C:membrane |
| Efet.01.97384.g1303.t1 | hypothetical protein CAPTEDRAFT_193207 | 684 | 6.01E-10 | 64.67 | C:membrane |
| Efet.01.97408.g1305.t1 | hypothetical protein HELRODRAFT_189125 | 429 | 3.41E-13 | 61 | F:G-protein coupled receptor activity; P:G-protein coupled receptor signaling pathway; C:membrane; C:integral component of membrane |
| Efet.01.97409.g1306.t1 | mitochondrial ATP synthase subunit alpha precursor | 696 | 6.97E-144 | 97.25 | C:myelin sheath; P:ATP hydrolysis coupled proton transport; F:MHC class I protein binding; F:proton-transporting ATPase activity, rotational mechanism; C:plasma membrane; C:mitochondrial proton-transporting ATP synthase complex; F:RNA binding; F:proton-transporting ATP synthase activity, rotational mechanism; C:proton-transporting ATP synthase complex, catalytic core F(1); F:ATP binding; C:extracellular exosome; P:lipid metabolic process; P:negative regulation of endothelial cell proliferation; C:COP9 signalosome; P:ATP synthesis coupled proton transport |
| Efet.01.97482.g1308.t1 | allatostatin-C neuropeptide precursor | 207 | 2.11E-18 | 85 | P:neuropeptide signaling pathway |
| Efet.01.97503.g1309.t1 | CAP-Gly domain-containing linker 1-like isoform X3 | 837 | 1.06E-07 | 74 |  |
| Efet.01.97534.g1311.t1 | discoidin domain-containing receptor 2-like isoform X1 | 312 | 4.65E-40 | 75.7 | F:ATP binding; P:peptidyl-tyrosine phosphorylation; F:protein tyrosine kinase activity; C:integral component of membrane |
| Efet.01.97537.g1312.t1 | neuroblastoma-amplified sequence | 405 | 1.36E-12 | 65.9 | C:intracellular |
| Efet.01.9754.g714.t1 | dynein heavy chain axonemal-like isoform X5 | 399 | 3.52E-25 | 83.8 | F:ATP binding; F:microtubule motor activity; C:dynein complex; P:microtubule-based movement |
| Efet.01.97595.g1317.t1 | sclerostin domain-containing 1-like | 399 | 3.59E-18 | 69.75 | C:extracellular space |
| Efet.01.97618.g1318.t1 | Ubiquitin carboxyl-terminal hydrolase 34 | 453 | 3.86E-57 | 78.2 | C:membrane; P:regulation of transcription, DNA-templated; C:SAGA complex; F:thiol-dependent ubiquitinyl hydrolase activity; P:ubiquitin-dependent protein catabolic process; P:histone deubiquitination |
| Efet.01.97666.g1320.t1 | Protocadherin-11 X-linked | 2130 | 9.03E-116 | 52.45 | F:calcium ion binding; C:membrane; C:integral component of membrane; P:cell adhesion; P:homophilic cell adhesion via plasma membrane adhesion molecules; C:plasma membrane |
| Efet.01.97666.g1321.t1 | protocadherin-23 isoform X3 | 771 | 3.53E-13 | 54.6 | F:calcium ion binding; C:membrane; C:integral component of membrane; P:cell adhesion; P:homophilic cell adhesion via plasma membrane adhesion molecules; C:plasma membrane |
| Efet.01.97684.g1322.t1 | calcium-activated potassium channel slowpoke | 693 | 4.83E-75 | 67.8 | P:ion transport; C:membrane; F:potassium channel activity |
| Efet.01.97720.g1324.t1 | RRNAD1-like isoform X1 | 312 | 3.03E-16 | 63.95 |  |
| Efet.01.9774.g716.t1 | Inositol phosphoceramide mannosyltransferase 3 | 348 | 5.53E-12 | 57.85 | F:oxidoreductase activity; P:metabolic process; C:membrane; C:integral component of membrane; P:oxidation-reduction process |
| Efet.01.97750.g1326.t1 | receptor-type tyrosine- phosphatase | 423 | 1.44E-72 | 87.4 | P:establishment of endothelial intestinal barrier; P:cerebellum development; P:neuron differentiation; F:cell adhesion molecule binding; P:heterophilic cell-cell adhesion via plasma membrane cell adhesion molecules; P:hippocampus development; P:cerebral cortex development; F:receptor binding; P:negative regulation of JAK-STAT cascade; P:spinal cord development; P:corpus callosum development; F:protein tyrosine phosphatase activity; P:presynaptic membrane assembly; C:integral component of membrane; P:peptidyl-tyrosine dephosphorylation; P:positive regulation of dendrite morphogenesis; P:regulation of immune response |
| Efet.01.97764.g1327.t1 | F-box At1g78280-like isoform X1 | 393 | 1.33E-18 | 65.45 | C:membrane |
| Efet.01.97789.g1328.t1 | tetratricopeptide repeat 25-like | 321 | 2.08E-21 | 68.05 |  |
| Efet.01.97790.g1329.t1 | transmembrane 94 isoform X2 | 441 | 8.15E-37 | 64.85 | F:nucleotide binding; C:membrane; C:integral component of membrane |
| Efet.01.97817.g1330.t1 | octopamine receptor beta-2R-like | 1446 | 5.01E-120 | 61.75 | F:G-protein coupled receptor activity; P:signal transduction; C:membrane |
| Efet.01.97818.g1331.t1 | mucin-4 isoform X2 | 1083 | 2.03E-37 | 43.85 | P:cell-matrix adhesion; F:transmembrane signaling receptor activity; P:signal transduction; F:calcium ion binding; C:membrane; C:integral component of membrane; F:extracellular matrix structural constituent; C:proteinaceous extracellular matrix |
| Efet.01.97861.g1332.t1 | hypothetical protein CRN43_11665, partial | 1569 | 3.11E-28 | 66 |  |
| Efet.01.97875.g1333.t1 | DNA replication licensing factor mcm7 | 228 | 7.27E-25 | 85.3 | C:nucleus; F:DNA binding; F:ATP binding; P:DNA replication initiation; F:DNA helicase activity; P:DNA duplex unwinding; C:MCM complex |
| Efet.01.97894.g1334.t1 | C-Jun-amino-terminal kinase-interacting 4-like isoform X3 | 480 | 6.53E-33 | 71.35 |  |
| Efet.01.97952.g1335.t1 | methylmalonic aciduria type A mitochondrial-like | 639 | 1.79E-20 | 69 | F:GTP binding; F:GTPase activity |
| Efet.01.97960.g1336.t1 | synaptotagmin 1 | 462 | 7.06E-77 | 91.6 | F:calcium ion binding; C:cytosol; C:cell junction; C:integral component of synaptic vesicle membrane; F:SNARE binding; P:synaptic vesicle exocytosis; P:negative regulation of calcium ion-dependent exocytosis |
| Efet.01.97972.g1337.t1 | cytochrome P450 1B1-like | 789 | 2.04E-25 | 48.05 | F:oxidoreductase activity, acting on paired donors, with incorporation or reduction of molecular oxygen, reduced flavin or flavoprotein as one donor, and incorporation of one atom of oxygen; F:oxidoreductase activity, acting on paired donors, with incorporation or reduction of molecular oxygen; P:retinol metabolic process; P:steroid metabolic process; C:endoplasmic reticulum; F:monooxygenase activity; F:heme binding; C:endoplasmic reticulum membrane; F:iron ion binding; F:oxidoreductase activity; F:metal ion binding; C:intracellular membrane-bounded organelle; P:xenobiotic metabolic process; F:aromatase activity; C:membrane; C:integral component of membrane; P:oxidation-reduction process |
| Efet.01.98035.g1338.t1 | von Willebrand factor A domain-containing 3B-like | 339 | 3.01E-06 | 58 |  |
| Efet.01.98057.g1339.t1 | hypothetical protein CAPTEDRAFT_160399, partial | 1029 | 5.87E-80 | 56.55 | C:extracellular space; P:multicellular organism reproduction; F:catalytic activity; P:metabolic process; C:membrane; C:integral component of membrane |
| Efet.01.98058.g1340.t1 | piezo-type mechanosensitive ion channel component isoform X4 | 210 | 1.75E-16 | 76.7 | P:mechanosensory behavior; F:mechanically-gated ion channel activity; P:cellular response to mechanical stimulus; P:ion transmembrane transport; C:integral component of membrane; P:detection of temperature stimulus involved in sensory perception of pain; C:plasma membrane; P:positive regulation of dendrite morphogenesis |
| Efet.01.98076.g1341.t1 | hypothetical protein HELRODRAFT_172268 | 1689 | 2.24E-19 | 61 | F:DNA binding; C:nucleus; P:transcription, DNA-templated; P:regulation of transcription, DNA-templated; F:steroid hormone receptor activity; P:steroid hormone mediated signaling pathway |
| Efet.01.98106.g1342.t1 | hemicentin-1 isoform X2 | 567 | 5.91E-33 | 55.45 | C:basement membrane; C:extracellular exosome; C:cell cortex; F:calcium ion binding; C:cell junction; C:extracellular region |
| Efet.01.98120.g1343.t1 | FMRFamide receptor-like | 1245 | 3.11E-125 | 55.25 | F:G-protein coupled receptor activity; F:G-protein coupled peptide receptor activity; P:G-protein coupled receptor signaling pathway; C:membrane; C:integral component of membrane |
| Efet.01.98149.g1344.t1 | dynein heavy chain axonemal-like | 543 | 7.20E-56 | 70.65 | F:nucleotide binding; F:ATP binding; F:microtubule motor activity; C:dynein complex; P:microtubule-based movement |
| Efet.01.98160.g1345.t1 | transcription initiation factor TFIID subunit 6-like | 1908 | 1.58E-177 | 68.7 | P:cellular nitrogen compound biosynthetic process; P:gene expression; F:binding; P:primary metabolic process; P:cellular macromolecule biosynthetic process |
| Efet.01.9821.g717.t1 | leucine-rich repeat-containing 15-like | 1290 | 1.77E-42 | 52.6 | P:peptidyl-tyrosine phosphorylation; F:ATP binding; F:protein tyrosine kinase activity; C:membrane; C:integral component of membrane; F:protein kinase activity; P:phosphorylation; P:protein phosphorylation; F:kinase activity |
| Efet.01.98266.g1346.t1 | E3 ubiquitin- ligase TRIM56-like | 684 | 2.70E-07 | 47 | F:zinc ion binding; F:metal ion binding; C:intracellular |
| Efet.01.98276.g1347.t1 | neuropeptide F receptor-like | 1242 | 5.36E-131 | 66.15 | F:G-protein coupled receptor activity; P:G-protein coupled receptor signaling pathway; C:membrane |
| Efet.01.98331.g1348.t1 | fasciculation and elongation zeta-2-like | 444 | 5.91E-29 | 63.75 |  |
| Efet.01.98332.g1350.t1 | hypothetical protein CAPTEDRAFT_218723 | 1653 | 5.09E-86 | 44 | C:membrane; C:integral component of membrane |
| Efet.01.98400.g1351.t1 | serine threonine- kinase D3-like isoform X1 | 264 | 1.48E-36 | 99.4 | F:ATP binding; F:metal ion binding; P:protein phosphorylation; C:intracellular; P:intracellular signal transduction; F:protein kinase C activity |
| Efet.01.98411.g1352.t1 | PREDICTED: uncharacterized protein LOC105844725 | 666 | 1.11E-69 | 61.55 | F:nucleic acid binding; F:metal ion binding |
| Efet.01.98475.g1355.t1 | discoidin domain-containing receptor 2-like isoform X1 | 405 | 4.31E-39 | 68.9 | C:membrane; F:protein kinase activity; P:protein phosphorylation |
| Efet.01.98481.g1357.t1 | atrial natriuretic peptide receptor 3- | 423 | 1.26E-18 | 60.25 | F:G-protein coupled receptor activity; P:G-protein coupled receptor signaling pathway; C:membrane; C:integral component of membrane |
| Efet.01.985.g97.t1 | potassium voltage-gated channel Shab-like | 798 | 8.79E-66 | 73.2 | P:potassium ion transport; P:transmembrane transport; C:integral component of membrane; F:voltage-gated ion channel activity; F:potassium channel activity; P:cellular process |
| Efet.01.9855.g719.t1 | leucine-rich repeats and immunoglobulin-like domains 1 | 600 | 5.75E-33 | 58.45 | C:membrane; C:integral component of membrane |
| Efet.01.98562.g1360.t1 | rootletin isoform X9 | 375 | 2.23E-15 | 72.9 | C:ciliary rootlet; P:centrosome organization |
| Efet.01.98588.g1361.t1 | atrial natriuretic peptide receptor 3-like | 384 | 8.02E-41 | 61.5 | F:nucleotide binding; F:phosphorus-oxygen lyase activity; C:membrane; P:cyclic nucleotide biosynthetic process |
| Efet.01.98627.g1363.t1 | glutamate receptor kainate 1-like isoform X1 | 330 | 1.16E-35 | 76.85 | F:transmembrane signaling receptor activity; F:extracellular ligand-gated ion channel activity; C:synapse; C:membrane part; P:transport; C:plasma membrane |
| Efet.01.98659.g1365.t1 | 5-hydroxytryptamine receptor 1A-like | 1704 | 1.46E-123 | 61.1 | F:G-protein coupled receptor activity; P:G-protein coupled receptor signaling pathway; C:membrane |
| Efet.01.98668.g1367.t1 | Transaldolase | 870 | 1.49E-109 | 87.2 | C:nucleus; P:carbohydrate metabolic process; F:sedoheptulose-7-phosphate:D-glyceraldehyde-3-phosphate glyceronetransferase activity; C:extracellular exosome; C:cytoplasm; P:pentose-phosphate shunt |
| Efet.01.98716.g1369.t1 | hypothetical protein CAPTEDRAFT_223229 | 393 | 4.30E-55 | 74.7 | P:RNA phosphodiester bond hydrolysis, endonucleolytic; P:rRNA transcription; F:endoribonuclease activity |
| Efet.01.9872.g720.t1 | serrate RNA effector molecule homolog isoform X1 | 261 | 3.43E-22 | 74.05 | F:nucleotide binding; C:integral component of membrane |
| Efet.01.98738.g1370.t1 | GTP-binding GEM-like | 357 | 1.39E-10 | 49.45 | F:nucleotide binding; P:signal transduction |
| Efet.01.98751.g1371.t1 | kintoun isoform X1 | 1083 | 1.36E-24 | 59.65 |  |
| Efet.01.98762.g1372.t1 | G -activated inward rectifier potassium channel 1 | 375 | 1.96E-21 | 65.6 | P:ion transport; F:voltage-gated ion channel activity |
| Efet.01.98774.g1373.t1 | zinc finger and BTB domain-containing 40 isoform X2 | 384 | 1.28E-09 | 59 | F:nucleic acid binding; F:metal ion binding |
| Efet.01.98805.g1374.t1 | meiosis-specific coiled-coil domain-containing MEIOC-like isoform X1 | 777 | 3.45E-14 | 55.9 |  |
| Efet.01.98821.g1375.t1 | 60S ribosomal L11 | 384 | 4.20E-66 | 93.75 | P:protein targeting; C:extracellular exosome; F:structural constituent of ribosome; C:membrane; C:cytosolic large ribosomal subunit; P:rRNA processing; C:nucleolus; P:ribosomal large subunit assembly; F:RNA binding; P:translation |
| Efet.01.98871.g1378.t1 | centrosomal of 112 kDa-like isoform X1 | 363 | 4.29E-06 | 60 |  |
| Efet.01.98873.g1379.t1 | protocadherin gamma-B1 | 2775 | 0 | 55.9 | C:membrane |
| Efet.01.9893.g721.t1 | jmjC domain-containing histone demethylation 1-like | 318 | 1.03E-16 | 79.45 | C:nucleus; F:DNA binding; F:zinc ion binding |
| Efet.01.98947.g1381.t1 | allatostatin-A receptor-like isoform X1 | 1137 | 9.90E-90 | 61.5 | F:G-protein coupled receptor activity; P:G-protein coupled receptor signaling pathway; C:membrane |
| Efet.01.9895.g722.t1 | 1-phosphatidylinositol 4,5-bisphosphate phosphodiesterase epsilon-1 | 1677 | 1.03E-34 | 60.08 |  |
| Efet.01.98955.g1382.t1 | phosphatidylinositol 4-kinase beta isoform X3 | 576 | 1.67E-102 | 85.75 | P:phosphatidylinositol phosphorylation; C:Golgi membrane; F:1-phosphatidylinositol 4-kinase activity; P:phosphatidylinositol biosynthetic process; C:mitochondrial outer membrane; P:receptor-mediated endocytosis; C:plasma membrane; F:ATP binding; C:endosome; C:rough endoplasmic reticulum membrane; C:perinuclear region of cytoplasm; P:phosphatidylinositol-mediated signaling; C:cytosol; F:14-3-3 protein binding |
| Efet.01.98961.g1385.t1 | homeobox aristaless-like | 384 | 4.77E-15 | 96.85 | F:HMG box domain binding; P:post-embryonic development; P:palate development; F:RNA polymerase II regulatory region sequence-specific DNA binding; P:anterior/posterior pattern specification; P:transcription from RNA polymerase II promoter; F:transcriptional activator activity, RNA polymerase II transcription regulatory region sequence-specific binding; P:regulation of apoptotic process; P:positive regulation of transcription from RNA polymerase II promoter; P:embryonic skeletal system morphogenesis; P:embryonic digit morphogenesis; P:muscle organ development; C:nucleus; C:transcription factor complex; P:hair follicle development; F:protein heterodimerization activity; P:digestive tract development; P:embryonic forelimb morphogenesis; P:embryonic hindlimb morphogenesis |
| Efet.01.99020.g1387.t1 | coiled-coil domain-containing 113-like | 348 | 5.20E-25 | 77.3 |  |
| Efet.01.99038.g1389.t1 | phosphatidylinositol glycan anchor biosynthesis class U | 1323 | 4.17E-164 | 66.4 | C:membrane |
| Efet.01.99041.g1390.t1 | F-BAR and double SH3 domains 2 isoform X1 | 381 | 4.28E-28 | 71.35 |  |
| Efet.01.99053.g1392.t1 | importin subunit alpha-3 | 513 | 9.76E-85 | 87.45 | C:extracellular exosome; F:protein binding; P:intracellular transport of virus; P:modulation by virus of host process; P:NLS-bearing protein import into nucleus; C:cytosol; F:protein transporter activity; P:response to hydrogen peroxide; F:nuclear localization sequence binding; C:nuclear pore; C:nucleoplasm |
| Efet.01.99066.g1393.t1 | DNA polymerase delta subunit 2 | 1095 | 5.56E-102 | 63.4 | F:DNA-directed DNA polymerase activity; P:DNA damage response, detection of DNA damage; F:protein binding; P:nucleotide-excision repair, DNA incision, 5'-to lesion; P:DNA strand elongation involved in DNA replication; P:transcription-coupled nucleotide-excision repair; P:telomere maintenance via recombination; P:nucleotide-excision repair, DNA gap filling; P:mismatch repair; P:translesion synthesis; C:nucleoplasm |
| Efet.01.9907.g723.t1 | Major egg antigen (p40) | 939 | 2.34E-13 | 49.94 | F:DNA binding; P:DNA replication; C:nuclear origin of replication recognition complex |
| Efet.01.99098.g1395.t1 | Regucalcin | 354 | 6.57E-51 | 73 | C:nucleus; F:zinc ion binding; C:cytoplasm; P:positive regulation of ATPase activity; F:calcium ion binding; P:regulation of calcium-mediated signaling; F:gluconolactonase activity; C:extracellular region; F:enzyme regulator activity; P:L-ascorbic acid biosynthetic process; P:cellular calcium ion homeostasis |
| Efet.01.99136.g1396.t1 | cell division cycle and apoptosis regulator 1-like | 525 | 1.46E-75 | 77.5 | F:nucleic acid binding; P:cell division; F:transcription cofactor activity; P:positive regulation of cellular process; P:regulation of transcription, DNA-templated |
| Efet.01.99161.g1397.t1 | probable G- coupled receptor 139 isoform X1 | 297 | 3.53E-16 | 70.35 | F:G-protein coupled receptor activity; P:G-protein coupled receptor signaling pathway; C:integral component of membrane |
| Efet.01.99175.g1398.t1 | prostacyclin synthase-like | 972 | 9.61E-100 | 60.27 | F:oxidoreductase activity; F:metal ion binding; C:endoplasmic reticulum; C:membrane part |
| Efet.01.99228.g1400.t1 | eukaryotic translation initiation factor 3 subunit G | 486 | 1.86E-68 | 72.6 | F:nucleotide binding; F:translation initiation factor activity; P:formation of translation preinitiation complex; C:eukaryotic 43S preinitiation complex; C:membrane; C:eukaryotic 48S preinitiation complex; P:regulation of translational initiation; C:eukaryotic translation initiation factor 3 complex |
| Efet.01.99237.g1401.t1 | low-density lipo receptor-related 1-like | 447 | 1.13E-51 | 59.55 | C:membrane |
| Efet.01.99237.g1402.t1 | low-density lipo receptor-related 2 | 213 | 1.41E-27 | 79.3 | P:cell proliferation; P:ventricular septum development; P:coronary vasculature development; C:endoplasmic reticulum; C:Golgi apparatus; C:receptor complex; P:vitamin metabolic process; P:receptor-mediated endocytosis; C:lysosomal membrane; C:endocytic vesicle; C:endosome; C:brush border membrane; C:extracellular exosome; P:forebrain development; P:aorta development; C:clathrin-coated pit; F:calcium ion binding; C:integral component of membrane; C:apical plasma membrane |
| Efet.01.99238.g1403.t1 | chondroitin sulfate proteoglycan 4 | 3444 | 3.67E-149 | 47.95 | C:membrane; C:integral component of membrane |
| Efet.01.99238.g1404.t1 | chondroitin sulfate proteoglycan 4-like | 573 | 1.02E-39 | 61.35 | C:membrane; C:integral component of membrane |
| Efet.01.99273.g1405.t1 | FAST kinase domain-containing 3-like | 513 | 4.81E-42 | 66.67 | F:protein kinase activity; P:protein phosphorylation |
| Efet.01.99274.g1406.t1 | alpha-mannosidase 2-like | 537 | 5.93E-48 | 71.6 | F:alpha-mannosidase activity; F:zinc ion binding; C:membrane; P:mannose metabolic process; F:carbohydrate binding |
| Efet.01.99316.g1408.t1 | dnaJ 60 | 231 | 5.17E-10 | 63.5 | C:membrane; C:integral component of membrane |
| Efet.01.99321.g1409.t1 | tripartite motif-containing 2-like | 2025 | 1.44E-19 | 37.6 | F:zinc ion binding; F:metal ion binding; C:intracellular |
| Efet.01.99354.g1410.t1 | transmembrane 45B-like | 258 | 3.08E-13 | 66.6 | C:membrane; C:integral component of membrane |
| Efet.01.99355.g1411.t1 | rho GTPase-activating 190-like isoform X1 | 1098 | 4.01E-65 | 71 | P:signal transduction |
| Efet.01.9939.g725.t1 | beta-1,3-glucosyltransferase | 261 | 5.45E-17 | 72.5 | F:transferase activity |
| Efet.01.9941.g726.t1 | Caseinolytic peptidase B | 279 | 5.86E-10 | 70.65 | F:nucleotide binding |
| Efet.01.99411.g1414.t1 | Muscle M-line assembly unc-89 | 480 | 2.11E-69 | 66.45 | F:ATP binding; P:regulation of Rho protein signal transduction; C:integral component of membrane; F:protein kinase activity; F:Rho guanyl-nucleotide exchange factor activity; P:protein phosphorylation; P:positive regulation of GTPase activity |
| Efet.01.99420.g1415.t1 | low-density lipo receptor-related 1B | 609 | 1.77E-34 | 59.45 | C:membrane; P:regulation of vasculature development; F:binding |
| Efet.01.99455.g1416.t1 | T-complex 1 subunit delta-like | 318 | 1.40E-55 | 93.1 | P:scaRNA localization to Cajal body; P:positive regulation of telomerase RNA localization to Cajal body; P:positive regulation of establishment of protein localization to telomere; P:positive regulation of protein localization to Cajal body; F:unfolded protein binding; P:protein folding; P:toxin transport; C:zona pellucida receptor complex; C:cell projection; C:nucleoplasm; C:microtubule; F:RNA binding; C:chaperonin-containing T-complex; F:ATP binding; P:positive regulation of telomerase activity; C:centrosome; P:binding of sperm to zona pellucida; C:extracellular exosome; C:melanosome; C:cell body; P:positive regulation of telomere maintenance via telomerase; P:protein stabilization |
| Efet.01.99517.g1421.t1 | polyphosphoinositide phosphatase-like isoform X1 | 801 | 9.06E-31 | 69.45 | P:nervous system development; P:cellular component organization; F:phosphoric ester hydrolase activity; P:cellular developmental process |
| Efet.01.99569.g1423.t1 | dachshund homolog 1-like isoform X2 | 402 | 1.37E-57 | 95.25 | C:nucleus; C:transcription factor complex; P:RNA polymerase II transcriptional preinitiation complex assembly; F:transcription factor activity, RNA polymerase II core promoter sequence-specific binding involved in preinitiation complex assembly; P:multicellular organism development; P:regulation of transcription, DNA-templated |
| Efet.01.99654.g1425.t1 | probable E3 ubiquitin- ligase HECTD2 | 321 | 1.31E-43 | 60 | F:ubiquitin-protein transferase activity; P:protein ubiquitination |
| Efet.01.99657.g1426.t1 | alpha-1A adrenergic receptor-like | 1224 | 1.24E-95 | 71.9 | F:adrenergic receptor activity; C:integral component of membrane; P:adrenergic receptor signaling pathway; C:plasma membrane |
| Efet.01.99663.g1427.t1 | ubiquitin-like modifier-activating enzyme 1 | 396 | 1.71E-37 | 71.05 | F:nucleotide binding; C:nucleus; F:ubiquitin-protein transferase activity; P:cellular protein modification process; C:cytosol; F:ubiquitin activating enzyme activity; P:modification-dependent protein catabolic process |
| Efet.01.9968.g728.t1 | hypothetical protein HELRODRAFT_159084 | 561 | 4.17E-07 | 96 | C:integral component of membrane |
| Efet.01.9970.g729.t1 | dystrophin-like isoform X1 | 1137 | 1.08E-38 | 45.1 | F:zinc ion binding; F:actin binding; F:metal ion binding; F:dystroglycan binding; F:structural constituent of cytoskeleton; P:muscle attachment |
| Efet.01.99705.g1432.t1 | heat shock 70 kDa 12B | 222 | 1.06E-31 | 83.35 | P:sprouting angiogenesis |
| Efet.01.99705.g1433.t1 | heat shock | 600 | 1.25E-31 | 74.15 |  |
| Efet.01.99733.g1434.t1 | reelin isoform X1 | 270 | 1.65E-18 | 71.35 | P:regulation of biological quality; P:positive regulation of synaptic transmission; P:protein localization; C:cell part; P:anatomical structure morphogenesis; P:cerebral cortex development; P:cell development; P:positive regulation of intracellular signal transduction; P:learning or memory; P:positive regulation of molecular function; P:regulation of localization; P:cellular localization; P:positive regulation of cellular component organization; P:positive regulation of protein phosphorylation; P:cellular component organization; P:positive regulation of neurogenesis; P:cell migration; P:response to stimulus |
| Efet.01.99792.g1438.t1 | low-density lipo receptor-related 2-like | 519 | 8.45E-28 | 57.6 | P:peptidyl-tyrosine phosphorylation; F:transmembrane receptor protein tyrosine kinase activity; F:calcium ion binding; C:membrane; C:integral component of membrane; F:transferase activity; F:hydrolase activity |
| Efet.01.9983.g730.t1 | kinetochore-associated 1-like | 738 | 5.49E-31 | 53 |  |
| Efet.01.99838.g1440.t1 | zinc finger 532 isoform X3 | 1797 | 7.69E-43 | 44.05 | F:metal ion binding |
| Efet.01.99896.g1443.t1 | mucin-17-like isoform X6 | 270 | 2.43E-18 | 72.4 |  |
| Efet.01.99900.g1444.t1 | midasin isoform X1 | 699 | 1.56E-27 | 67.4 | F:nucleotide binding |
| Efet.01.9995.g731.t1 | serrate RNA effector molecule homolog | 510 | 5.55E-33 | 74.35 | P:cell proliferation; P:primary miRNA processing; C:cytoplasm; C:nucleoplasm |
| Efet.01.99959.g1445.t1 | calcium-activated potassium channel subunit alpha-1-like | 273 | 1.93E-11 | 56.5 | P:ion transport; F:potassium channel activity |
