# Supplementary material for: Data on genome annotation and analysis of earthworm Eisenia fetida
Source: Data Brief. 2018 Aug 29;20:525–34. doi: 10.1016/j.dib.2018.08.067 (PMC6126081; doi:10.1016/j.dib.2018.08.067)
Supplement: Supplementary file 3 — Supplementary material [file mmc3.docx]

Table S2: List of *Eisenia fetida* genes showed sequence homology to bacterial genomic sequence

| **Sequence Name** | **Sequence Description**   \|  \| \| --- \| | **Alignment Length** | **e-Value** | **similarity** |
| --- | --- | --- | --- | --- | --- |
| Efet.01.618025.g797.t1 | Verminephrobacter eiseniae EF01-2, complete genome | 321 | 5.60E-162 | 100.00% |
| Efet.01.658339.g1875.t1 | Verminephrobacter eiseniae EF01-2, complete genome | 528 | 0.00E+00 | 100.00% |
| Efet.01.1646673.g353.t1 | Verminephrobacter eiseniae EF01-2, complete genome | 228 | 7.00E-112 | 100.00% |
| Efet.01.32340.g516.t1 | Verminephrobacter eiseniae EF01-2, complete genome | 378 | 0.00E+00 | 99.00% |
| Efet.01.151234.g66.t1 | Verminephrobacter eiseniae EF01-2, complete genome | 363 | 1.60E-180 | 99.00% |
| Efet.01.194571.g2072.t1 | Verminephrobacter eiseniae EF01-2, complete genome | 398 | 0.00E+00 | 99.00% |
| Efet.01.269359.g716.t1 | Verminephrobacter eiseniae EF01-2, complete genome | 300 | 3.40E-147 | 99.00% |
| Efet.01.316780.g530.t1 | Verminephrobacter eiseniae EF01-2, complete genome | 1080 | 0.00E+00 | 99.00% |
| Efet.01.316780.g531.t1 | Verminephrobacter eiseniae EF01-2, complete genome | 1195 | 0.00E+00 | 99.00% |
| Efet.01.440538.g970.t1 | Verminephrobacter eiseniae EF01-2, complete genome | 1014 | 0.00E+00 | 99.00% |
| Efet.01.496128.g1088.t1 | Verminephrobacter eiseniae EF01-2, complete genome | 435 | 0.00E+00 | 99.00% |
| Efet.01.609276.g383.t1 | Verminephrobacter eiseniae EF01-2, complete genome | 711 | 0.00E+00 | 99.00% |
| Efet.01.622611.g996.t1 | Verminephrobacter eiseniae EF01-2, complete genome | 624 | 0.00E+00 | 99.00% |
| Efet.01.625145.g1099.t1 | Verminephrobacter eiseniae EF01-2, complete genome | 136 | 8.50E-60 | 99.00% |
| Efet.01.642117.g659.t1 | Verminephrobacter eiseniae EF01-2, complete genome | 331 | 4.20E-163 | 99.00% |
| Efet.01.642485.g690.t1 | Verminephrobacter eiseniae EF01-2, complete genome | 576 | 0.00E+00 | 99.00% |
| Efet.01.642485.g691.t1 | Verminephrobacter eiseniae EF01-2, complete genome | 873 | 0.00E+00 | 99.00% |
| Efet.01.643898.g839.t1 | Verminephrobacter eiseniae EF01-2, complete genome | 417 | 0.00E+00 | 99.00% |
| Efet.01.650311.g21.t1 | Verminephrobacter eiseniae EF01-2, complete genome | 345 | 3.90E-172 | 99.00% |
| Efet.01.650357.g46.t1 | Verminephrobacter eiseniae EF01-2, complete genome | 201 | 5.90E-94 | 99.00% |
| Efet.01.652215.g266.t1 | Verminephrobacter eiseniae EF01-2, complete genome | 597 | 0.00E+00 | 99.00% |
| Efet.01.652442.g302.t1 | Verminephrobacter eiseniae EF01-2, complete genome | 201 | 8.90E-95 | 99.00% |
| Efet.01.653992.g511.t1 | Verminephrobacter eiseniae EF01-2, complete genome | 414 | 0.00E+00 | 99.00% |
| Efet.01.653992.g512.t1 | Verminephrobacter eiseniae EF01-2, complete genome | 660 | 0.00E+00 | 99.00% |
| Efet.01.654521.g623.t1 | Verminephrobacter eiseniae EF01-2, complete genome | 963 | 0.00E+00 | 99.00% |
| Efet.01.656717.g1077.t1 | Verminephrobacter eiseniae EF01-2, complete genome | 1131 | 0.00E+00 | 99.00% |
| Efet.01.656717.g1078.t1 | Verminephrobacter eiseniae EF01-2, complete genome | 834 | 0.00E+00 | 99.00% |
| Efet.01.657130.g1180.t1 | Verminephrobacter eiseniae EF01-2, complete genome | 982 | 0.00E+00 | 99.00% |
| Efet.01.657795.g1464.t1 | Verminephrobacter eiseniae EF01-2, complete genome | 872 | 0.00E+00 | 99.00% |
| Efet.01.658116.g1644.t1 | Verminephrobacter eiseniae EF01-2, complete genome | 357 | 5.90E-177 | 99.00% |
| Efet.01.658236.g1731.t1 | Verminephrobacter eiseniae EF01-2, complete genome | 235 | 2.70E-112 | 99.00% |
| Efet.01.658310.g1838.t1 | Verminephrobacter eiseniae EF01-2, complete genome | 764 | 0.00E+00 | 99.00% |
| Efet.01.658392.g1946.t1 | Verminephrobacter eiseniae EF01-2, complete genome | 390 | 0.00E+00 | 99.00% |
| Efet.01.658392.g1949.t1 | Verminephrobacter eiseniae EF01-2, complete genome | 521 | 0.00E+00 | 99.00% |
| Efet.01.658414.g2035.t1 | Verminephrobacter eiseniae EF01-2, complete genome | 993 | 0.00E+00 | 99.00% |
| Efet.01.658423.g2103.t1 | Verminephrobacter eiseniae EF01-2, complete genome | 426 | 0.00E+00 | 99.00% |
| Efet.01.658427.g2143.t1 | Verminephrobacter eiseniae EF01-2, complete genome | 1751 | 0.00E+00 | 99.00% |
| Efet.01.1610020.g22.t1 | Verminephrobacter eiseniae EF01-2, complete genome | 228 | 4.60E-110 | 99.00% |
| Efet.01.1659316.g1546.t1 | Verminephrobacter eiseniae EF01-2, complete genome | 432 | 0.00E+00 | 99.00% |
| Efet.01.1659339.g1568.t1 | Verminephrobacter eiseniae EF01-2, complete genome | 456 | 0.00E+00 | 99.00% |
| Efet.01.1659425.g1687.t1 | Verminephrobacter eiseniae EF01-2, complete genome | 1131 | 0.00E+00 | 99.00% |
| Efet.01.1659425.g1689.t1 | Verminephrobacter eiseniae EF01-2, complete genome | 563 | 0.00E+00 | 99.00% |
| Efet.01.1659456.g1758.t1 | Verminephrobacter eiseniae EF01-2, complete genome | 447 | 0.00E+00 | 99.00% |
| Efet.01.1659510.g1926.t1 | Verminephrobacter eiseniae EF01-2, complete genome | 465 | 0.00E+00 | 99.00% |
| Efet.01.1659518.g1972.t1 | Verminephrobacter eiseniae EF01-2, complete genome | 462 | 0.00E+00 | 99.00% |
| Efet.01.151234.g69.t1 | Verminephrobacter eiseniae EF01-2, complete genome | 1119 | 0.00E+00 | 98.00% |
| Efet.01.151234.g70.t1 | Verminephrobacter eiseniae EF01-2, complete genome | 495 | 0.00E+00 | 98.00% |
| Efet.01.305235.g172.t1 | Verminephrobacter eiseniae EF01-2, complete genome | 237 | 1.30E-110 | 98.00% |
| Efet.01.439340.g928.t1 | Xanthomonas oryzae pv. oryzicola strain CFBP2286, complete genome | 231 | 1.30E-107 | 98.00% |
| Efet.01.439340.g929.t1 | Verminephrobacter eiseniae EF01-2, complete genome | 685 | 0.00E+00 | 98.00% |
| Efet.01.440538.g971.t1 | Verminephrobacter eiseniae EF01-2, complete genome | 927 | 0.00E+00 | 98.00% |
| Efet.01.474932.g648.t1 | Verminephrobacter eiseniae EF01-2, complete genome | 378 | 0.00E+00 | 98.00% |
| Efet.01.504526.g116.t1 | Verminephrobacter eiseniae EF01-2, complete genome | 918 | 0.00E+00 | 98.00% |
| Efet.01.533383.g124.t1 | Verminephrobacter eiseniae EF01-2, complete genome | 825 | 0.00E+00 | 98.00% |
| Efet.01.603005.g120.t1 | Verminephrobacter eiseniae EF01-2, complete genome | 134 | 2.20E-57 | 98.00% |
| Efet.01.605364.g205.t1 | Verminephrobacter eiseniae EF01-2, complete genome | 252 | 6.10E-117 | 98.00% |
| Efet.01.614947.g675.t1 | Verminephrobacter eiseniae EF01-2, complete genome | 975 | 0.00E+00 | 98.00% |
| Efet.01.630858.g73.t1 | Verminephrobacter eiseniae EF01-2, complete genome | 387 | 0.00E+00 | 98.00% |
| Efet.01.642485.g688.t1 | Verminephrobacter eiseniae EF01-2, complete genome | 222 | 4.50E-103 | 98.00% |
| Efet.01.643464.g791.t1 | Verminephrobacter eiseniae EF01-2, complete genome | 876 | 0.00E+00 | 98.00% |
| Efet.01.644010.g857.t1 | Verminephrobacter eiseniae EF01-2, complete genome | 483 | 0.00E+00 | 98.00% |
| Efet.01.649821.g1358.t1 | Verminephrobacter eiseniae EF01-2, complete genome | 639 | 0.00E+00 | 98.00% |
| Efet.01.649916.g1363.t1 | Verminephrobacter eiseniae EF01-2, complete genome | 834 | 0.00E+00 | 98.00% |
| Efet.01.655756.g866.t1 | Verminephrobacter eiseniae EF01-2, complete genome | 298 | 4.10E-144 | 98.00% |
| Efet.01.656216.g966.t1 | Verminephrobacter eiseniae EF01-2, complete genome | 222 | 9.90E-103 | 98.00% |
| Efet.01.657624.g1345.t1 | Verminephrobacter eiseniae EF01-2, complete genome | 528 | 0.00E+00 | 98.00% |
| Efet.01.658040.g1581.t1 | Verminephrobacter eiseniae EF01-2, complete genome | 990 | 0.00E+00 | 98.00% |
| Efet.01.658142.g1673.t1 | Verminephrobacter eiseniae EF01-2, complete genome | 264 | 2.20E-125 | 98.00% |
| Efet.01.658236.g1733.t1 | Verminephrobacter eiseniae EF01-2, complete genome | 663 | 0.00E+00 | 98.00% |
| Efet.01.658398.g1981.t1 | Verminephrobacter eiseniae EF01-2, complete genome | 258 | 5.50E-123 | 98.00% |
| Efet.01.658401.g1991.t1 | Verminephrobacter eiseniae EF01-2, complete genome | 657 | 0.00E+00 | 98.00% |
| Efet.01.1655956.g807.t1 | Verminephrobacter eiseniae EF01-2, complete genome | 438 | 0.00E+00 | 98.00% |
| Efet.01.1659395.g1647.t1 | Verminephrobacter eiseniae EF01-2, complete genome | 297 | 2.50E-141 | 98.00% |
| Efet.01.1659518.g1968.t1 | Verminephrobacter eiseniae EF01-2, complete genome | 762 | 0.00E+00 | 98.00% |
| Efet.01.284847.g1261.t1 | Verminephrobacter eiseniae EF01-2, complete genome | 303 | 1.30E-147 | 97.20% |
| Efet.01.153212.g170.t1 | Verminephrobacter eiseniae EF01-2, complete genome | 269 | 4.70E-124 | 97.00% |
| Efet.01.187450.g1754.t1 | Verminephrobacter eiseniae EF01-2, complete genome | 529 | 0.00E+00 | 97.00% |
| Efet.01.559401.g735.t1 | Verminephrobacter eiseniae EF01-2, complete genome | 363 | 1.20E-173 | 97.00% |
| Efet.01.613419.g611.t1 | Verminephrobacter eiseniae EF01-2, complete genome | 309 | 8.10E-145 | 97.00% |
| Efet.01.630546.g44.t1 | Verminephrobacter eiseniae EF01-2, complete genome | 564 | 0.00E+00 | 97.00% |
| Efet.01.636598.g339.t1 | Verminephrobacter eiseniae EF01-2, complete genome | 288 | 2.40E-135 | 97.00% |
| Efet.01.644010.g858.t1 | Verminephrobacter eiseniae EF01-2, complete genome | 960 | 0.00E+00 | 97.00% |
| Efet.01.650311.g22.t1 | Verminephrobacter eiseniae EF01-2, complete genome | 1629 | 0.00E+00 | 97.00% |
| Efet.01.656598.g1049.t1 | Verminephrobacter eiseniae EF01-2, complete genome | 372 | 3.50E-179 | 97.00% |
| Efet.01.1651061.g497.t1 | Verminephrobacter eiseniae EF01-2, complete genome | 322 | 3.80E-148 | 97.00% |
| Efet.01.1659496.g1873.t1 | Verminephrobacter eiseniae EF01-2, complete genome | 375 | 6.90E-179 | 97.00% |
| Efet.01.1659518.g1969.t1 | Verminephrobacter eiseniae EF01-2, complete genome | 539 | 0.00E+00 | 97.00% |
| Efet.01.55841.g378.t1 | Verminephrobacter eiseniae EF01-2, complete genome | 230 | 3.40E-108 | 96.67% |
| Efet.01.427593.g657.t1 | Verminephrobacter eiseniae EF01-2, complete genome | 233 | 2.10E-103 | 96.00% |
| Efet.01.532996.g98.t1 | Verminephrobacter eiseniae EF01-2, complete genome | 550 | 0.00E+00 | 96.00% |
| Efet.01.598313.g1249.t1 | Verminephrobacter eiseniae EF01-2, complete genome | 349 | 7.90E-160 | 96.00% |
| Efet.01.605884.g248.t1 | Verminephrobacter eiseniae EF01-2, complete genome | 378 | 9.00E-172 | 96.00% |
| Efet.01.638425.g439.t1 | Verminephrobacter eiseniae EF01-2, complete genome | 246 | 1.90E-108 | 96.00% |
| Efet.01.646642.g1113.t1 | Verminephrobacter eiseniae EF01-2, complete genome | 196 | 7.10E-84 | 96.00% |
| Efet.01.654027.g521.t1 | Verminephrobacter eiseniae EF01-2, complete genome | 1269 | 0.00E+00 | 96.00% |
| Efet.01.656717.g1075.t1 | Verminephrobacter eiseniae EF01-2, complete genome | 310 | 6.30E-138 | 96.00% |
| Efet.01.657703.g1386.t1 | Verminephrobacter eiseniae EF01-2, complete genome | 300 | 1.80E-133 | 96.00% |
| Efet.01.1659518.g1971.t1 | Verminephrobacter eiseniae EF01-2, complete genome | 577 | 0.00E+00 | 96.00% |
| Efet.01.1659519.g1976.t1 | Verminephrobacter eiseniae EF01-2, complete genome | 295 | 5.40E-131 | 96.00% |
| Efet.01.151234.g73.t1 | Verminephrobacter eiseniae EF01-2, complete genome | 717 | 0.00E+00 | 95.40% |
| Efet.01.284847.g1260.t1 | Verminephrobacter eiseniae EF01-2, complete genome | 267 | 1.10E-129 | 95.40% |
| Efet.01.636814.g353.t1 | Verminephrobacter eiseniae EF01-2, complete genome | 228 | 4.40E-94 | 95.00% |
| Efet.01.1658878.g1308.t1 | Verminephrobacter eiseniae EF01-2, complete genome | 312 | 2.40E-139 | 95.00% |
| Efet.01.284847.g1262.t1 | Verminephrobacter eiseniae EF01-2, complete genome | 396 | 0.00E+00 | 94.50% |
| Efet.01.1659522.g1998.t1 | Verminephrobacter eiseniae EF01-2, complete genome | 312 | 1.60E-156 | 94.50% |
| Efet.01.654391.g583.t1 | Verminephrobacter eiseniae EF01-2, complete genome | 660 | 0.00E+00 | 94.20% |
| Efet.01.558699.g712.t1 | Massilia putida strain 6NM-7T, complete genome | 39 | 6.80E-06 | 94.00% |
| Efet.01.622654.g998.t1 | Verminephrobacter eiseniae EF01-2, complete genome | 942 | 0.00E+00 | 94.00% |
| Efet.01.658116.g1645.t1 | Verminephrobacter eiseniae EF01-2, complete genome | 227 | 1.60E-95 | 94.00% |
| Efet.01.1651662.g515.t1 | Verminephrobacter eiseniae EF01-2, complete genome | 135 | 2.00E-50 | 94.00% |
| Efet.01.1657234.g959.t1 | Verminephrobacter eiseniae EF01-2, complete genome | 688 | 0.00E+00 | 94.00% |
| Efet.01.1658350.g1161.t1 | Verminephrobacter eiseniae EF01-2, complete genome | 516 | 0.00E+00 | 94.00% |
| Efet.01.1658500.g1198.t1 | Verminephrobacter eiseniae EF01-2, complete genome | 996 | 0.00E+00 | 94.00% |
| Efet.01.565049.g165.t1 | Verminephrobacter eiseniae EF01-2, complete genome | 1150 | 0.00E+00 | 93.90% |
| Efet.01.284847.g1263.t1 | Verminephrobacter eiseniae EF01-2, complete genome | 422 | 0.00E+00 | 93.60% |
| Efet.01.267648.g652.t1 | Verminephrobacter eiseniae EF01-2, complete genome | 339 | 4.40E-171 | 93.50% |
| Efet.01.657318.g1254.t1 | Acidovorax sp. NA3, complete genome | 521 | 0.00E+00 | 93.20% |
| Efet.01.22048.g1619.t1 | Verminephrobacter eiseniae EF01-2, complete genome | 557 | 0.00E+00 | 93.00% |
| Efet.01.25255.g23.t1 | Verminephrobacter eiseniae EF01-2, complete genome | 1137 | 0.00E+00 | 93.00% |
| Efet.01.486596.g888.t1 | Verminephrobacter eiseniae EF01-2, complete genome | 339 | 9.60E-169 | 93.00% |
| Efet.01.1659131.g1417.t1 | Variovorax paradoxus B4 chromosome 2, complete sequence | 159 | 9.50E-59 | 93.00% |
| Efet.01.1659486.g1839.t1 | Verminephrobacter eiseniae EF01-2, complete genome | 697 | 0.00E+00 | 93.00% |
| Efet.01.1659524.g2010.t1 | Verminephrobacter eiseniae EF01-2, complete genome | 234 | 1.30E-94 | 93.00% |
| Efet.01.456900.g172.t1 | Verminephrobacter eiseniae EF01-2, complete genome | 213 | 2.10E-103 | 92.90% |
| Efet.01.628685.g1260.t1 | Verminephrobacter eiseniae EF01-2, complete genome | 735 | 0.00E+00 | 92.70% |
| Efet.01.651141.g129.t1 | Verminephrobacter eiseniae EF01-2, complete genome | 360 | 0.00E+00 | 92.70% |
| Efet.01.634415.g245.t1 | Verminephrobacter eiseniae EF01-2, complete genome | 538 | 0.00E+00 | 92.60% |
| Efet.01.605365.g216.t1 | Variovorax paradoxus B4 chromosome 1, complete sequence | 44 | 2.30E-08 | 92.50% |
| Efet.01.658292.g1805.t1 | Verminephrobacter eiseniae EF01-2, complete genome | 309 | 2.10E-153 | 92.50% |
| Efet.01.655573.g835.t1 | Verminephrobacter eiseniae EF01-2, complete genome | 413 | 0.00E+00 | 92.30% |
| Efet.01.648797.g1265.t1 | Verminephrobacter eiseniae EF01-2, complete genome | 312 | 5.70E-157 | 92.10% |
| Efet.01.651136.g125.t1 | Verminephrobacter eiseniae EF01-2, complete genome | 234 | 1.50E-112 | 92.10% |
| Efet.01.350955.g29.t1 | Bordetella bronchiseptica strain F709 chromosome, complete genome | 816 | 0.00E+00 | 92.00% |
| Efet.01.393322.g1083.t1 | Verminephrobacter eiseniae EF01-2, complete genome | 270 | 1.80E-105 | 92.00% |
| Efet.01.658292.g1806.t1 | Verminephrobacter eiseniae EF01-2, complete genome | 474 | 0.00E+00 | 91.90% |
| Efet.01.573518.g416.t1 | Verminephrobacter eiseniae EF01-2, complete genome | 2080 | 0.00E+00 | 91.70% |
| Efet.01.628685.g1262.t1 | Verminephrobacter eiseniae EF01-2, complete genome | 1154 | 0.00E+00 | 91.60% |
| Efet.01.267648.g651.t1 | Verminephrobacter eiseniae EF01-2, complete genome | 1510 | 0.00E+00 | 91.50% |
| Efet.01.1657977.g1074.t1 | Verminephrobacter eiseniae EF01-2, complete genome | 516 | 0.00E+00 | 91.50% |
| Efet.01.515650.g384.t1 | Verminephrobacter eiseniae EF01-2, complete genome | 279 | 3.20E-136 | 91.40% |
| Efet.01.652898.g378.t1 | Verminephrobacter eiseniae EF01-2, complete genome | 769 | 0.00E+00 | 91.40% |
| Efet.01.658170.g1693.t1 | Verminephrobacter eiseniae EF01-2, complete genome | 1232 | 0.00E+00 | 91.40% |
| Efet.01.1659522.g1996.t1 | Verminephrobacter eiseniae EF01-2, complete genome | 348 | 2.00E-174 | 91.20% |
| Efet.01.651136.g123.t1 | Verminephrobacter eiseniae EF01-2, complete genome | 226 | 7.70E-110 | 91.10% |
| Efet.01.657766.g1446.t1 | Verminephrobacter eiseniae EF01-2, complete genome | 473 | 0.00E+00 | 91.10% |
| Efet.01.140059.g775.t1 | Thalassospira sp. CSC3H3 chromosome, complete genome | 44 | 2.30E-06 | 91.00% |
| Efet.01.565049.g162.t1 | Verminephrobacter eiseniae EF01-2, complete genome | 1416 | 0.00E+00 | 91.00% |
| Efet.01.654316.g572.t1 | Verminephrobacter eiseniae EF01-2, complete genome | 1237 | 0.00E+00 | 91.00% |
| Efet.01.1658008.g1080.t1 | Verminephrobacter eiseniae EF01-2, complete genome | 429 | 0.00E+00 | 91.00% |
| Efet.01.31180.g429.t1 | Verminephrobacter eiseniae EF01-2, complete genome | 540 | 0.00E+00 | 90.80% |
| Efet.01.1651153.g500.t1 | Burkholderia thailandensis strain FDAARGOS_238 chromosome 2, complete sequence | 39 | 1.90E-06 | 90.80% |
| Efet.01.1659397.g1653.t1 | Verminephrobacter eiseniae EF01-2, complete genome | 573 | 0.00E+00 | 90.80% |
| Efet.01.1659466.g1780.t1 | Verminephrobacter eiseniae EF01-2, complete genome | 396 | 0.00E+00 | 90.70% |
| Efet.01.296519.g1635.t1 | Verminephrobacter eiseniae EF01-2, complete genome | 477 | 0.00E+00 | 90.60% |
| Efet.01.650480.g55.t1 | Verminephrobacter eiseniae EF01-2, complete genome | 612 | 0.00E+00 | 90.60% |
| Efet.01.655756.g864.t1 | Verminephrobacter eiseniae EF01-2, complete genome | 558 | 0.00E+00 | 90.60% |
| Efet.01.1643236.g274.t1 | Verminephrobacter eiseniae EF01-2, complete genome | 363 | 0.00E+00 | 90.60% |
| Efet.01.1659438.g1713.t1 | Verminephrobacter eiseniae EF01-2, complete genome | 678 | 0.00E+00 | 90.60% |
| Efet.01.468732.g503.t1 | Agromyces aureus strain AR33, complete genome | 201 | 5.90E-73 | 90.40% |
| Efet.01.622256.g979.t1 | Verminephrobacter eiseniae EF01-2, complete genome | 515 | 0.00E+00 | 90.40% |
| Efet.01.638026.g411.t1 | Verminephrobacter eiseniae EF01-2, complete genome | 1017 | 0.00E+00 | 90.30% |
| Efet.01.625145.g1102.t1 | Cupriavidus sp. USMAA2-4 plasmid unnamed1, complete sequence | 383 | 8.50E-155 | 90.20% |
| Efet.01.648372.g1234.t1 | Verminephrobacter eiseniae EF01-2, complete genome | 774 | 0.00E+00 | 90.20% |
| Efet.01.657766.g1430.t1 | Verminephrobacter eiseniae EF01-2, complete genome | 558 | 0.00E+00 | 90.20% |
| Efet.01.1658542.g1212.t1 | Verminephrobacter eiseniae EF01-2, complete genome | 255 | 3.20E-126 | 90.20% |
| Efet.01.1659466.g1782.t1 | Verminephrobacter eiseniae EF01-2, complete genome | 304 | 8.70E-153 | 90.20% |
| Efet.01.1659522.g1995.t1 | Verminephrobacter eiseniae EF01-2, complete genome | 825 | 0.00E+00 | 90.20% |
| Efet.01.143861.g970.t1 | Verminephrobacter eiseniae EF01-2, complete genome | 357 | 4.90E-180 | 90.10% |
| Efet.01.434985.g839.t1 | Agromyces sp. 30A chromosome, complete genome | 309 | 8.30E-123 | 90.10% |
| Efet.01.621705.g952.t1 | Variovorax boronicumulans strain J1 chromosome, complete genome | 792 | 0.00E+00 | 90.10% |
| Efet.01.648797.g1269.t1 | Verminephrobacter eiseniae EF01-2, complete genome | 216 | 7.50E-105 | 90.10% |
| Efet.01.653325.g419.t1 | Verminephrobacter eiseniae EF01-2, complete genome | 1791 | 0.00E+00 | 90.10% |
| Efet.01.2563.g235.t1 | Gloeobacter kilaueensis JS1, complete genome | 44 | 4.20E-06 | 90.00% |
| Efet.01.91308.g957.t1 | Actinoalloteichus sp. AHMU CJ021 chromosome, complete genome | 43 | 5.00E-06 | 90.00% |
| Efet.01.93618.g1080.t1 | Prevotella ruminicola 23, complete genome | 44 | 5.10E-06 | 90.00% |
| Efet.01.117723.g916.t1 | Mycobacterium paraseoulense strain DSM 45000 elongation factor Tu (tuf) gene cds | 44 | 4.00E-06 | 90.00% |
| Efet.01.203477.g160.t1 | Pseudomonas viridiflava strain CFBP 1590 genome assembly, chromosome: I | 50 | 6.00E-08 | 90.00% |
| Efet.01.549718.g486.t1 | Aeromicrobium sp. 592 chromosome, complete genome | 52 | 3.20E-09 | 90.00% |
| Efet.01.565049.g163.t1 | Verminephrobacter eiseniae EF01-2, complete genome | 879 | 0.00E+00 | 90.00% |
| Efet.01.595396.g1149.t1 | Rhodococcus opacus B4 DNA, complete genome | 44 | 5.10E-06 | 90.00% |
| Efet.01.613416.g598.t1 | Verminephrobacter eiseniae EF01-2, complete genome | 256 | 6.70E-127 | 90.00% |
| Efet.01.646487.g1088.t1 | Verminephrobacter eiseniae EF01-2, complete genome | 253 | 5.90E-95 | 90.00% |
| Efet.01.657807.g1486.t1 | Pseudomonas syringae pv. tomato strain B13-200 plasmid pB13-200A, complete sequence | 420 | 3.10E-161 | 90.00% |
| Efet.01.1658626.g1234.t1 | Verminephrobacter eiseniae EF01-2, complete genome | 918 | 0.00E+00 | 90.00% |
| Efet.01.622583.g989.t1 | Pseudomonas aeruginosa strain PA_D21, complete genome | 605 | 0.00E+00 | 89.90% |
| Efet.01.658257.g1763.t1 | Verminephrobacter eiseniae EF01-2, complete genome | 253 | 8.80E-121 | 89.90% |
| Efet.01.1653579.g626.t1 | Verminephrobacter eiseniae EF01-2, complete genome | 225 | 4.30E-100 | 89.90% |
| Efet.01.1658858.g1296.t1 | Verminephrobacter eiseniae EF01-2, complete genome | 387 | 0.00E+00 | 89.90% |
| Efet.01.275134.g928.t1 | Verminephrobacter eiseniae EF01-2, complete genome | 1198 | 0.00E+00 | 89.80% |
| Efet.01.544715.g353.t1 | Variovorax boronicumulans strain J1 chromosome, complete genome | 660 | 0.00E+00 | 89.80% |
| Efet.01.658350.g1880.t1 | Verminephrobacter eiseniae EF01-2, complete genome | 282 | 4.40E-141 | 89.80% |
| Efet.01.427593.g668.t1 | Verminephrobacter eiseniae EF01-2, complete genome | 467 | 0.00E+00 | 89.70% |
| Efet.01.520113.g478.t1 | Verminephrobacter eiseniae EF01-2, complete genome | 282 | 7.90E-141 | 89.70% |
| Efet.01.651810.g216.t1 | Verminephrobacter eiseniae EF01-2, complete genome | 361 | 1.50E-180 | 89.70% |
| Efet.01.1647192.g372.t1 | Verminephrobacter eiseniae EF01-2, complete genome | 276 | 3.20E-135 | 89.70% |
| Efet.01.114635.g765.t1 | Bacteroides vulgatus strain mpk genome | 47 | 1.60E-07 | 89.67% |
| Efet.01.612091.g521.t1 | Verminephrobacter eiseniae EF01-2, complete genome | 627 | 0.00E+00 | 89.60% |
| Efet.01.625494.g1120.t1 | Verminephrobacter eiseniae EF01-2, complete genome | 252 | 1.10E-121 | 89.60% |
| Efet.01.634752.g259.t1 | Verminephrobacter eiseniae EF01-2, complete genome | 385 | 0.00E+00 | 89.60% |
| Efet.01.645971.g1018.t1 | Verminephrobacter eiseniae EF01-2, complete genome | 783 | 0.00E+00 | 89.60% |
| Efet.01.655098.g752.t1 | Verminephrobacter eiseniae EF01-2, complete genome | 813 | 0.00E+00 | 89.60% |
| Efet.01.657719.g1389.t1 | Verminephrobacter eiseniae EF01-2, complete genome | 273 | 3.30E-130 | 89.60% |
| Efet.01.1657170.g948.t1 | Verminephrobacter eiseniae EF01-2, complete genome | 267 | 1.80E-132 | 89.60% |
| Efet.01.645199.g955.t1 | Verminephrobacter eiseniae EF01-2, complete genome | 1047 | 0.00E+00 | 89.50% |
| Efet.01.646642.g1115.t1 | Verminephrobacter eiseniae EF01-2, complete genome | 318 | 8.60E-159 | 89.50% |
| Efet.01.651141.g126.t1 | Verminephrobacter eiseniae EF01-2, complete genome | 1269 | 0.00E+00 | 89.50% |
| Efet.01.654418.g601.t1 | Verminephrobacter eiseniae EF01-2, complete genome | 156 | 9.90E-71 | 89.50% |
| Efet.01.654656.g652.t1 | Verminephrobacter eiseniae EF01-2, complete genome | 2758 | 0.00E+00 | 89.50% |
| Efet.01.658422.g2101.t1 | Hydrogenophaga sp. PBC, complete genome | 1836 | 0.00E+00 | 89.50% |
| Efet.01.1656769.g911.t1 | Verminephrobacter eiseniae EF01-2, complete genome | 210 | 1.70E-100 | 89.50% |
| Efet.01.655128.g761.t1 | Verminephrobacter eiseniae EF01-2, complete genome | 352 | 4.70E-177 | 89.40% |
| Efet.01.658116.g1651.t1 | Verminephrobacter eiseniae EF01-2, complete genome | 1617 | 0.00E+00 | 89.40% |
| Efet.01.1659117.g1412.t1 | Verminephrobacter eiseniae EF01-2, complete genome | 336 | 4.90E-170 | 89.40% |
| Efet.01.1659325.g1553.t1 | Verminephrobacter eiseniae EF01-2, complete genome | 288 | 3.50E-142 | 89.40% |
| Efet.01.1659486.g1838.t1 | Verminephrobacter eiseniae EF01-2, complete genome | 921 | 0.00E+00 | 89.40% |
| Efet.01.405260.g127.t1 | Uncultured Planctomycetales bacterium HF0500_02G17 genomic sequence | 47 | 1.20E-07 | 89.33% |
| Efet.01.578168.g629.t1 | Variovorax boronicumulans strain J1 chromosome, complete genome | 570 | 0.00E+00 | 89.30% |
| Efet.01.621705.g951.t1 | Variovorax boronicumulans strain J1 chromosome, complete genome | 621 | 0.00E+00 | 89.30% |
| Efet.01.638026.g412.t1 | Verminephrobacter eiseniae EF01-2, complete genome | 492 | 0.00E+00 | 89.30% |
| Efet.01.653479.g434.t1 | Verminephrobacter eiseniae EF01-2, complete genome | 303 | 1.20E-149 | 89.30% |
| Efet.01.654418.g594.t1 | Verminephrobacter eiseniae EF01-2, complete genome | 3105 | 0.00E+00 | 89.30% |
| Efet.01.658397.g1977.t1 | Thauera sp. K11 chromosome, complete genome | 692 | 0.00E+00 | 89.30% |
| Efet.01.658424.g2122.t1 | Verminephrobacter eiseniae EF01-2, complete genome | 1188 | 0.00E+00 | 89.30% |
| Efet.01.1659365.g1607.t1 | Verminephrobacter eiseniae EF01-2, complete genome | 741 | 0.00E+00 | 89.30% |
| Efet.01.300799.g29.t1 | Pseudomonas aeruginosa strain T38079, complete genome | 2841 | 0.00E+00 | 89.20% |
| Efet.01.515650.g381.t1 | Verminephrobacter eiseniae EF01-2, complete genome | 1146 | 0.00E+00 | 89.20% |
| Efet.01.573518.g420.t1 | Verminephrobacter eiseniae EF01-2, complete genome | 432 | 0.00E+00 | 89.20% |
| Efet.01.630390.g21.t1 | Verminephrobacter eiseniae EF01-2, complete genome | 908 | 0.00E+00 | 89.20% |
| Efet.01.654391.g582.t1 | Verminephrobacter eiseniae EF01-2, complete genome | 3867 | 0.00E+00 | 89.20% |
| Efet.01.656090.g938.t1 | Verminephrobacter eiseniae EF01-2, complete genome | 1010 | 0.00E+00 | 89.20% |
| Efet.01.658307.g1827.t1 | Verminephrobacter eiseniae EF01-2, complete genome | 1157 | 0.00E+00 | 89.20% |
| Efet.01.658387.g1941.t1 | Verminephrobacter eiseniae EF01-2, complete genome | 1287 | 0.00E+00 | 89.20% |
| Efet.01.494042.g1049.t1 | Verminephrobacter eiseniae EF01-2, complete genome | 480 | 0.00E+00 | 89.10% |
| Efet.01.528741.g656.t1 | Verminephrobacter eiseniae EF01-2, complete genome | 1662 | 0.00E+00 | 89.10% |
| Efet.01.658282.g1796.t1 | Verminephrobacter eiseniae EF01-2, complete genome | 342 | 1.00E-170 | 89.10% |
| Efet.01.658395.g1969.t1 | Verminephrobacter eiseniae EF01-2, complete genome | 1416 | 0.00E+00 | 89.10% |
| Efet.01.1659454.g1754.t1 | Verminephrobacter eiseniae EF01-2, complete genome | 1233 | 0.00E+00 | 89.10% |
| Efet.01.328568.g877.t1 | Emticicia oligotrophica DSM 17448, complete genome | 46 | 6.70E-06 | 89.00% |
| Efet.01.628685.g1259.t1 | Verminephrobacter eiseniae EF01-2, complete genome | 897 | 0.00E+00 | 89.00% |
| Efet.01.643099.g760.t1 | Verminephrobacter eiseniae EF01-2, complete genome | 220 | 1.10E-72 | 89.00% |
| Efet.01.653401.g424.t1 | Planctomyces sp. SH-PL62, complete genome | 49 | 3.80E-06 | 89.00% |
| Efet.01.656742.g1082.t1 | Verminephrobacter eiseniae EF01-2, complete genome | 974 | 0.00E+00 | 89.00% |
| Efet.01.657759.g1417.t1 | Verminephrobacter eiseniae EF01-2, complete genome | 228 | 2.00E-111 | 89.00% |
| Efet.01.658002.g1552.t1 | Verminephrobacter eiseniae EF01-2, complete genome | 1338 | 0.00E+00 | 89.00% |
| Efet.01.658380.g1913.t1 | Verminephrobacter eiseniae EF01-2, complete genome | 659 | 0.00E+00 | 89.00% |
| Efet.01.1658248.g1138.t1 | Hydrogenophaga sp. PBC, complete genome | 47 | 2.30E-06 | 89.00% |
| Efet.01.1659522.g1992.t1 | Verminephrobacter eiseniae EF01-2, complete genome | 270 | 4.40E-132 | 89.00% |
| Efet.01.25255.g19.t1 | Verminephrobacter eiseniae EF01-2, complete genome | 1387 | 0.00E+00 | 88.90% |
| Efet.01.565049.g168.t1 | Verminephrobacter eiseniae EF01-2, complete genome | 270 | 3.70E-134 | 88.90% |
| Efet.01.652898.g376.t1 | Verminephrobacter eiseniae EF01-2, complete genome | 681 | 0.00E+00 | 88.90% |
| Efet.01.653613.g459.t1 | Verminephrobacter eiseniae EF01-2, complete genome | 203 | 2.00E-96 | 88.90% |
| Efet.01.658041.g1584.t1 | Verminephrobacter eiseniae EF01-2, complete genome | 732 | 0.00E+00 | 88.90% |
| Efet.01.1659522.g1993.t1 | Verminephrobacter eiseniae EF01-2, complete genome | 742 | 0.00E+00 | 88.90% |
| Efet.01.1659522.g1994.t1 | Verminephrobacter eiseniae EF01-2, complete genome | 333 | 2.80E-166 | 88.90% |
| Efet.01.607412.g312.t1 | Verminephrobacter eiseniae EF01-2, complete genome | 1002 | 0.00E+00 | 88.80% |
| Efet.01.628433.g1244.t1 | Verminephrobacter eiseniae EF01-2, complete genome | 612 | 0.00E+00 | 88.80% |
| Efet.01.656971.g1151.t1 | Verminephrobacter eiseniae EF01-2, complete genome | 210 | 3.10E-99 | 88.80% |
| Efet.01.1659489.g1850.t1 | Verminephrobacter eiseniae EF01-2, complete genome | 483 | 0.00E+00 | 88.80% |
| Efet.01.427593.g666.t1 | Verminephrobacter eiseniae EF01-2, complete genome | 930 | 0.00E+00 | 88.70% |
| Efet.01.429122.g715.t1 | Verminephrobacter eiseniae EF01-2, complete genome | 402 | 0.00E+00 | 88.70% |
| Efet.01.471393.g560.t1 | Verminephrobacter eiseniae EF01-2, complete genome | 634 | 0.00E+00 | 88.70% |
| Efet.01.578168.g628.t1 | Paraburkholderia xenovorans LB400 chromosome 2, complete sequence | 480 | 0.00E+00 | 88.70% |
| Efet.01.631737.g131.t1 | Verminephrobacter eiseniae EF01-2, complete genome | 406 | 0.00E+00 | 88.70% |
| Efet.01.651229.g152.t1 | Verminephrobacter eiseniae EF01-2, complete genome | 201 | 2.90E-96 | 88.70% |
| Efet.01.654965.g725.t1 | Verminephrobacter eiseniae EF01-2, complete genome | 966 | 0.00E+00 | 88.70% |
| Efet.01.657496.g1311.t1 | Verminephrobacter eiseniae EF01-2, complete genome | 975 | 0.00E+00 | 88.70% |
| Efet.01.1656388.g859.t1 | Verminephrobacter eiseniae EF01-2, complete genome | 303 | 6.70E-150 | 88.70% |
| Efet.01.1659349.g1585.t1 | Verminephrobacter eiseniae EF01-2, complete genome | 1350 | 0.00E+00 | 88.70% |
| Efet.01.655669.g848.t1 | Verminephrobacter eiseniae EF01-2, complete genome | 519 | 0.00E+00 | 88.67% |
| Efet.01.656742.g1088.t1 | Verminephrobacter eiseniae EF01-2, complete genome | 1494 | 0.00E+00 | 88.60% |
| Efet.01.658392.g1963.t1 | Verminephrobacter eiseniae EF01-2, complete genome | 836 | 0.00E+00 | 88.60% |
| Efet.01.1659452.g1748.t1 | Verminephrobacter eiseniae EF01-2, complete genome | 582 | 0.00E+00 | 88.60% |
| Efet.01.151234.g68.t1 | Verminephrobacter eiseniae EF01-2, complete genome | 1185 | 0.00E+00 | 88.50% |
| Efet.01.204628.g201.t1 | Uncultured bacterium clone contig80424 genomic sequence | 206 | 1.20E-16 | 88.50% |
| Efet.01.563201.g81.t1 | Ralstonia solanacearum genome assembly 9 genomes, chromosome : I | 180 | 1.90E-55 | 88.50% |
| Efet.01.605357.g201.t1 | Verminephrobacter eiseniae EF01-2, complete genome | 435 | 0.00E+00 | 88.50% |
| Efet.01.625145.g1101.t1 | Acidovorax sp. P4, complete genome | 282 | 6.50E-112 | 88.50% |
| Efet.01.638674.g446.t1 | Verminephrobacter eiseniae EF01-2, complete genome | 858 | 0.00E+00 | 88.50% |
| Efet.01.639260.g496.t1 | Verminephrobacter eiseniae EF01-2, complete genome | 952 | 0.00E+00 | 88.50% |
| Efet.01.646487.g1092.t1 | Verminephrobacter eiseniae EF01-2, complete genome | 228 | 1.60E-108 | 88.50% |
| Efet.01.657807.g1490.t1 | Pseudomonas syringae pv. tomato strain B13-200 plasmid pB13-200A, complete sequence | 354 | 1.00E-135 | 88.50% |
| Efet.01.658312.g1848.t1 | Verminephrobacter eiseniae EF01-2, complete genome | 903 | 0.00E+00 | 88.50% |
| Efet.01.1659341.g1571.t1 | Verminephrobacter eiseniae EF01-2, complete genome | 569 | 0.00E+00 | 88.50% |
| Efet.01.1659522.g1997.t1 | Verminephrobacter eiseniae EF01-2, complete genome | 675 | 0.00E+00 | 88.50% |
| Efet.01.1659526.g2025.t1 | Verminephrobacter eiseniae EF01-2, complete genome | 2319 | 0.00E+00 | 88.50% |
| Efet.01.427593.g669.t1 | Verminephrobacter eiseniae EF01-2, complete genome | 654 | 0.00E+00 | 88.40% |
| Efet.01.553408.g590.t1 | Verminephrobacter eiseniae EF01-2, complete genome | 705 | 0.00E+00 | 88.40% |
| Efet.01.577373.g582.t1 | Verminephrobacter eiseniae EF01-2, complete genome | 1017 | 0.00E+00 | 88.40% |
| Efet.01.605365.g211.t1 | Verminephrobacter eiseniae EF01-2, complete genome | 738 | 0.00E+00 | 88.40% |
| Efet.01.638026.g406.t1 | Verminephrobacter eiseniae EF01-2, complete genome | 1389 | 0.00E+00 | 88.40% |
| Efet.01.643899.g843.t1 | Verminephrobacter eiseniae EF01-2, complete genome | 975 | 0.00E+00 | 88.40% |
| Efet.01.658397.g1978.t1 | Acidovorax ebreus TPSY, complete genome | 1383 | 0.00E+00 | 88.40% |
| Efet.01.1659377.g1626.t1 | Verminephrobacter eiseniae EF01-2, complete genome | 667 | 0.00E+00 | 88.40% |
| Efet.01.515650.g382.t1 | Verminephrobacter eiseniae EF01-2, complete genome | 1962 | 0.00E+00 | 88.30% |
| Efet.01.597630.g1216.t1 | Verminephrobacter eiseniae EF01-2, complete genome | 341 | 7.40E-173 | 88.30% |
| Efet.01.651141.g128.t1 | Verminephrobacter eiseniae EF01-2, complete genome | 540 | 0.00E+00 | 88.30% |
| Efet.01.654310.g562.t1 | Verminephrobacter eiseniae EF01-2, complete genome | 989 | 0.00E+00 | 88.30% |
| Efet.01.657766.g1424.t1 | Verminephrobacter eiseniae EF01-2, complete genome | 686 | 0.00E+00 | 88.30% |
| Efet.01.658211.g1716.t1 | Verminephrobacter eiseniae EF01-2, complete genome | 679 | 0.00E+00 | 88.30% |
| Efet.01.1645908.g335.t1 | Verminephrobacter eiseniae EF01-2, complete genome | 495 | 0.00E+00 | 88.30% |
| Efet.01.17103.g1258.t1 | Verminephrobacter eiseniae EF01-2, complete genome | 507 | 0.00E+00 | 88.20% |
| Efet.01.150930.g53.t1 | Verminephrobacter eiseniae EF01-2, complete genome | 372 | 0.00E+00 | 88.20% |
| Efet.01.365325.g417.t1 | Verminephrobacter eiseniae EF01-2, complete genome | 624 | 0.00E+00 | 88.20% |
| Efet.01.494042.g1051.t1 | Verminephrobacter eiseniae EF01-2, complete genome | 1254 | 0.00E+00 | 88.20% |
| Efet.01.494042.g1053.t1 | Verminephrobacter eiseniae EF01-2, complete genome | 669 | 0.00E+00 | 88.20% |
| Efet.01.565049.g164.t1 | Verminephrobacter eiseniae EF01-2, complete genome | 270 | 8.20E-130 | 88.20% |
| Efet.01.630546.g37.t1 | Verminephrobacter eiseniae EF01-2, complete genome | 849 | 0.00E+00 | 88.20% |
| Efet.01.634752.g257.t1 | Verminephrobacter eiseniae EF01-2, complete genome | 437 | 0.00E+00 | 88.20% |
| Efet.01.645138.g939.t1 | Verminephrobacter eiseniae EF01-2, complete genome | 237 | 4.00E-116 | 88.20% |
| Efet.01.651136.g113.t1 | Verminephrobacter eiseniae EF01-2, complete genome | 702 | 0.00E+00 | 88.20% |
| Efet.01.655399.g815.t1 | Verminephrobacter eiseniae EF01-2, complete genome | 705 | 0.00E+00 | 88.20% |
| Efet.01.658426.g2135.t1 | Verminephrobacter eiseniae EF01-2, complete genome | 2607 | 0.00E+00 | 88.20% |
| Efet.01.1659442.g1724.t1 | Verminephrobacter eiseniae EF01-2, complete genome | 222 | 9.30E-108 | 88.20% |
| Efet.01.440538.g967.t1 | Verminephrobacter eiseniae EF01-2, complete genome | 1347 | 0.00E+00 | 88.10% |
| Efet.01.471393.g562.t1 | Verminephrobacter eiseniae EF01-2, complete genome | 499 | 0.00E+00 | 88.10% |
| Efet.01.630768.g62.t1 | Verminephrobacter eiseniae EF01-2, complete genome | 812 | 0.00E+00 | 88.10% |
| Efet.01.654418.g587.t1 | Verminephrobacter eiseniae EF01-2, complete genome | 1542 | 0.00E+00 | 88.10% |
| Efet.01.655281.g788.t1 | Verminephrobacter eiseniae EF01-2, complete genome | 532 | 0.00E+00 | 88.10% |
| Efet.01.655974.g909.t1 | Verminephrobacter eiseniae EF01-2, complete genome | 615 | 0.00E+00 | 88.10% |
| Efet.01.657719.g1393.t1 | Verminephrobacter eiseniae EF01-2, complete genome | 891 | 0.00E+00 | 88.10% |
| Efet.01.658383.g1927.t1 | Verminephrobacter eiseniae EF01-2, complete genome | 765 | 0.00E+00 | 88.10% |
| Efet.01.1657388.g980.t1 | Verminephrobacter eiseniae EF01-2, complete genome | 317 | 2.80E-155 | 88.10% |
| Efet.01.79897.g315.t1 | Desulfobacterium autotrophicum HRM2, complete genome | 50 | 1.20E-06 | 88.00% |
| Efet.01.180113.g1405.t1 | Clostridium beijerinckii isolate WB53 chromosome, complete genome | 51 | 6.50E-08 | 88.00% |
| Efet.01.180300.g1409.t1 | Shewanella halifaxensis HAW-EB4, complete genome | 50 | 2.80E-06 | 88.00% |
| Efet.01.278935.g1070.t1 | Verminephrobacter eiseniae EF01-2, complete genome | 243 | 1.70E-113 | 88.00% |
| Efet.01.284106.g1241.t1 | Peptoclostridium acidaminophilum DSM 3953 chromosome, complete genome | 50 | 1.90E-06 | 88.00% |
| Efet.01.532148.g63.t1 | Verminephrobacter eiseniae EF01-2, complete genome | 135 | 3.50E-61 | 88.00% |
| Efet.01.573518.g417.t1 | Verminephrobacter eiseniae EF01-2, complete genome | 305 | 1.30E-143 | 88.00% |
| Efet.01.653613.g460.t1 | Verminephrobacter eiseniae EF01-2, complete genome | 639 | 0.00E+00 | 88.00% |
| Efet.01.656919.g1142.t1 | Verminephrobacter eiseniae EF01-2, complete genome | 561 | 0.00E+00 | 88.00% |
| Efet.01.1630309.g103.t1 | Verminephrobacter eiseniae EF01-2, complete genome | 252 | 1.90E-120 | 88.00% |
| Efet.01.1659085.g1395.t1 | Verminephrobacter eiseniae EF01-2, complete genome | 1107 | 0.00E+00 | 88.00% |
| Efet.01.1659466.g1781.t1 | Verminephrobacter eiseniae EF01-2, complete genome | 465 | 0.00E+00 | 88.00% |
| Efet.01.1659525.g2016.t1 | Verminephrobacter eiseniae EF01-2, complete genome | 1287 | 0.00E+00 | 88.00% |
| Efet.01.440935.g994.t1 | Verminephrobacter eiseniae EF01-2, complete genome | 474 | 0.00E+00 | 87.90% |
| Efet.01.611807.g499.t1 | Variovorax paradoxus EPS, complete genome | 616 | 0.00E+00 | 87.90% |
| Efet.01.613416.g609.t1 | Verminephrobacter eiseniae EF01-2, complete genome | 1365 | 0.00E+00 | 87.90% |
| Efet.01.619389.g865.t1 | Verminephrobacter eiseniae EF01-2, complete genome | 567 | 0.00E+00 | 87.90% |
| Efet.01.641218.g616.t1 | Verminephrobacter eiseniae EF01-2, complete genome | 1485 | 0.00E+00 | 87.90% |
| Efet.01.643497.g807.t1 | Verminephrobacter eiseniae EF01-2, complete genome | 1707 | 0.00E+00 | 87.90% |
| Efet.01.656289.g987.t1 | Verminephrobacter eiseniae EF01-2, complete genome | 766 | 0.00E+00 | 87.90% |
| Efet.01.656971.g1150.t1 | Verminephrobacter eiseniae EF01-2, complete genome | 720 | 0.00E+00 | 87.90% |
| Efet.01.658312.g1845.t1 | Verminephrobacter eiseniae EF01-2, complete genome | 333 | 3.70E-164 | 87.90% |
| Efet.01.1659117.g1413.t1 | Verminephrobacter eiseniae EF01-2, complete genome | 711 | 0.00E+00 | 87.90% |
| Efet.01.1659348.g1583.t1 | Verminephrobacter eiseniae EF01-2, complete genome | 696 | 0.00E+00 | 87.90% |
| Efet.01.119502.g1009.t1 | Verminephrobacter eiseniae EF01-2, complete genome | 1380 | 0.00E+00 | 87.80% |
| Efet.01.300799.g28.t1 | Verminephrobacter eiseniae EF01-2, complete genome | 2267 | 0.00E+00 | 87.80% |
| Efet.01.515650.g385.t1 | Verminephrobacter eiseniae EF01-2, complete genome | 430 | 0.00E+00 | 87.80% |
| Efet.01.566725.g223.t1 | Verminephrobacter eiseniae EF01-2, complete genome | 387 | 0.00E+00 | 87.80% |
| Efet.01.636372.g330.t1 | Verminephrobacter eiseniae EF01-2, complete genome | 567 | 0.00E+00 | 87.80% |
| Efet.01.646487.g1102.t1 | Verminephrobacter eiseniae EF01-2, complete genome | 462 | 0.00E+00 | 87.80% |
| Efet.01.656283.g981.t1 | Verminephrobacter eiseniae EF01-2, complete genome | 558 | 0.00E+00 | 87.80% |
| Efet.01.658292.g1807.t1 | Verminephrobacter eiseniae EF01-2, complete genome | 1826 | 0.00E+00 | 87.80% |
| Efet.01.1658864.g1302.t1 | Verminephrobacter eiseniae EF01-2, complete genome | 747 | 0.00E+00 | 87.80% |
| Efet.01.296519.g1636.t1 | Verminephrobacter eiseniae EF01-2, complete genome | 1909 | 0.00E+00 | 87.70% |
| Efet.01.300799.g34.t1 | Verminephrobacter eiseniae EF01-2, complete genome | 1461 | 0.00E+00 | 87.70% |
| Efet.01.427593.g671.t1 | Verminephrobacter eiseniae EF01-2, complete genome | 1080 | 0.00E+00 | 87.70% |
| Efet.01.607412.g307.t1 | Verminephrobacter eiseniae EF01-2, complete genome | 441 | 0.00E+00 | 87.70% |
| Efet.01.620750.g906.t1 | Verminephrobacter eiseniae EF01-2, complete genome | 1608 | 0.00E+00 | 87.70% |
| Efet.01.658298.g1820.t1 | Verminephrobacter eiseniae EF01-2, complete genome | 342 | 1.30E-168 | 87.70% |
| Efet.01.658408.g2007.t1 | Verminephrobacter eiseniae EF01-2, complete genome | 630 | 0.00E+00 | 87.70% |
| Efet.01.1659028.g1368.t1 | Agromyces sp. 30A chromosome, complete genome | 61 | 2.90E-08 | 87.67% |
| Efet.01.64436.g887.t1 | Verminephrobacter eiseniae EF01-2, complete genome | 342 | 8.30E-170 | 87.60% |
| Efet.01.445097.g1116.t1 | Verminephrobacter eiseniae EF01-2, complete genome | 1092 | 0.00E+00 | 87.60% |
| Efet.01.456900.g171.t1 | Verminephrobacter eiseniae EF01-2, complete genome | 576 | 0.00E+00 | 87.60% |
| Efet.01.610801.g453.t1 | Verminephrobacter eiseniae EF01-2, complete genome | 761 | 0.00E+00 | 87.60% |
| Efet.01.626245.g1158.t1 | Verminephrobacter eiseniae EF01-2, complete genome | 702 | 0.00E+00 | 87.60% |
| Efet.01.641291.g620.t1 | Verminephrobacter eiseniae EF01-2, complete genome | 697 | 0.00E+00 | 87.60% |
| Efet.01.642094.g657.t1 | Verminephrobacter eiseniae EF01-2, complete genome | 1818 | 0.00E+00 | 87.60% |
| Efet.01.658192.g1705.t1 | Verminephrobacter eiseniae EF01-2, complete genome | 1095 | 0.00E+00 | 87.60% |
| Efet.01.658312.g1847.t1 | Verminephrobacter eiseniae EF01-2, complete genome | 432 | 0.00E+00 | 87.60% |
| Efet.01.1659453.g1752.t1 | Verminephrobacter eiseniae EF01-2, complete genome | 462 | 0.00E+00 | 87.60% |
| Efet.01.515650.g388.t1 | Verminephrobacter eiseniae EF01-2, complete genome | 1302 | 0.00E+00 | 87.50% |
| Efet.01.572488.g395.t1 | Verminephrobacter eiseniae EF01-2, complete genome | 214 | 1.30E-99 | 87.50% |
| Efet.01.578612.g646.t1 | Verminephrobacter eiseniae EF01-2, complete genome | 399 | 1.70E-165 | 87.50% |
| Efet.01.597508.g1207.t1 | Verminephrobacter eiseniae EF01-2, complete genome | 372 | 0.00E+00 | 87.50% |
| Efet.01.628482.g1247.t1 | Verminephrobacter eiseniae EF01-2, complete genome | 915 | 0.00E+00 | 87.50% |
| Efet.01.633356.g190.t1 | Microterricola viridarii strain DSM 21772 genome assembly, chromosome: I | 249 | 1.80E-87 | 87.50% |
| Efet.01.633502.g194.t1 | Verminephrobacter eiseniae EF01-2, complete genome | 258 | 5.10E-126 | 87.50% |
| Efet.01.638023.g399.t1 | Verminephrobacter eiseniae EF01-2, complete genome | 327 | 5.00E-163 | 87.50% |
| Efet.01.652858.g355.t1 | Verminephrobacter eiseniae EF01-2, complete genome | 1158 | 0.00E+00 | 87.50% |
| Efet.01.654418.g590.t1 | Verminephrobacter eiseniae EF01-2, complete genome | 1146 | 0.00E+00 | 87.50% |
| Efet.01.655281.g786.t1 | Verminephrobacter eiseniae EF01-2, complete genome | 234 | 4.40E-112 | 87.50% |
| Efet.01.657766.g1431.t1 | Verminephrobacter eiseniae EF01-2, complete genome | 892 | 0.00E+00 | 87.50% |
| Efet.01.657803.g1473.t1 | Verminephrobacter eiseniae EF01-2, complete genome | 1986 | 0.00E+00 | 87.50% |
| Efet.01.658414.g2041.t1 | Verminephrobacter eiseniae EF01-2, complete genome | 1266 | 0.00E+00 | 87.50% |
| Efet.01.1653365.g610.t1 | Verminephrobacter eiseniae EF01-2, complete genome | 342 | 7.30E-167 | 87.50% |
| Efet.01.1659352.g1590.t1 | Verminephrobacter eiseniae EF01-2, complete genome | 906 | 0.00E+00 | 87.50% |
| Efet.01.1659395.g1648.t1 | Verminephrobacter eiseniae EF01-2, complete genome | 1533 | 0.00E+00 | 87.50% |
| Efet.01.318747.g591.t1 | Verminephrobacter eiseniae EF01-2, complete genome | 1587 | 0.00E+00 | 87.40% |
| Efet.01.378778.g769.t1 | Verminephrobacter eiseniae EF01-2, complete genome | 680 | 0.00E+00 | 87.40% |
| Efet.01.463370.g363.t1 | Verminephrobacter eiseniae EF01-2, complete genome | 993 | 0.00E+00 | 87.40% |
| Efet.01.513298.g318.t1 | Verminephrobacter eiseniae EF01-2, complete genome | 740 | 0.00E+00 | 87.40% |
| Efet.01.585463.g842.t1 | Verminephrobacter eiseniae EF01-2, complete genome | 1029 | 0.00E+00 | 87.40% |
| Efet.01.622256.g978.t1 | Verminephrobacter eiseniae EF01-2, complete genome | 390 | 0.00E+00 | 87.40% |
| Efet.01.657318.g1255.t1 | Verminephrobacter eiseniae EF01-2, complete genome | 609 | 0.00E+00 | 87.40% |
| Efet.01.658245.g1748.t1 | Verminephrobacter eiseniae EF01-2, complete genome | 486 | 0.00E+00 | 87.40% |
| Efet.01.658312.g1846.t1 | Verminephrobacter eiseniae EF01-2, complete genome | 852 | 0.00E+00 | 87.40% |
| Efet.01.1659154.g1434.t1 | Verminephrobacter eiseniae EF01-2, complete genome | 1281 | 0.00E+00 | 87.40% |
| Efet.01.1659328.g1556.t1 | Variovorax boronicumulans strain J1 chromosome, complete genome | 1131 | 0.00E+00 | 87.40% |
| Efet.01.597702.g1226.t1 | Verminephrobacter eiseniae EF01-2, complete genome | 1310 | 0.00E+00 | 87.33% |
| Efet.01.514247.g342.t1 | Verminephrobacter eiseniae EF01-2, complete genome | 676 | 0.00E+00 | 87.30% |
| Efet.01.573518.g418.t1 | Verminephrobacter eiseniae EF01-2, complete genome | 414 | 0.00E+00 | 87.30% |
| Efet.01.577664.g608.t1 | Verminephrobacter eiseniae EF01-2, complete genome | 510 | 0.00E+00 | 87.30% |
| Efet.01.594275.g1115.t1 | Verminephrobacter eiseniae EF01-2, complete genome | 540 | 0.00E+00 | 87.30% |
| Efet.01.601193.g57.t1 | Verminephrobacter eiseniae EF01-2, complete genome | 894 | 0.00E+00 | 87.30% |
| Efet.01.608047.g336.t1 | Verminephrobacter eiseniae EF01-2, complete genome | 801 | 0.00E+00 | 87.30% |
| Efet.01.623950.g1039.t1 | Verminephrobacter eiseniae EF01-2, complete genome | 542 | 0.00E+00 | 87.30% |
| Efet.01.643024.g746.t1 | Verminephrobacter eiseniae EF01-2, complete genome | 366 | 0.00E+00 | 87.30% |
| Efet.01.650749.g85.t1 | Verminephrobacter eiseniae EF01-2, complete genome | 567 | 0.00E+00 | 87.30% |
| Efet.01.651515.g181.t1 | Verminephrobacter eiseniae EF01-2, complete genome | 1275 | 0.00E+00 | 87.30% |
| Efet.01.655399.g814.t1 | Verminephrobacter eiseniae EF01-2, complete genome | 1806 | 0.00E+00 | 87.30% |
| Efet.01.655573.g831.t1 | Verminephrobacter eiseniae EF01-2, complete genome | 1281 | 0.00E+00 | 87.30% |
| Efet.01.655974.g911.t1 | Verminephrobacter eiseniae EF01-2, complete genome | 2712 | 0.00E+00 | 87.30% |
| Efet.01.658193.g1710.t1 | Verminephrobacter eiseniae EF01-2, complete genome | 1325 | 0.00E+00 | 87.30% |
| Efet.01.658267.g1777.t1 | Verminephrobacter eiseniae EF01-2, complete genome | 1488 | 0.00E+00 | 87.30% |
| Efet.01.658399.g1989.t1 | Verminephrobacter eiseniae EF01-2, complete genome | 2049 | 0.00E+00 | 87.30% |
| Efet.01.1659031.g1370.t1 | Verminephrobacter eiseniae EF01-2, complete genome | 741 | 0.00E+00 | 87.30% |
| Efet.01.1659370.g1611.t1 | Verminephrobacter eiseniae EF01-2, complete genome | 1272 | 0.00E+00 | 87.30% |
| Efet.01.58055.g502.t1 | Microbulbifer sp. CCB-MM1, complete genome | 75 | 2.00E-08 | 87.20% |
| Efet.01.515650.g379.t1 | Verminephrobacter eiseniae EF01-2, complete genome | 477 | 0.00E+00 | 87.20% |
| Efet.01.592294.g1071.t1 | Verminephrobacter eiseniae EF01-2, complete genome | 1608 | 0.00E+00 | 87.20% |
| Efet.01.602021.g86.t1 | Verminephrobacter eiseniae EF01-2, complete genome | 143 | 2.40E-63 | 87.20% |
| Efet.01.614463.g646.t1 | Verminephrobacter eiseniae EF01-2, complete genome | 607 | 0.00E+00 | 87.20% |
| Efet.01.625010.g1090.t1 | Verminephrobacter eiseniae EF01-2, complete genome | 773 | 0.00E+00 | 87.20% |
| Efet.01.635927.g304.t1 | Rhodoplanes sp. Z2-YC6860, complete genome | 114 | 2.30E-11 | 87.20% |
| Efet.01.657457.g1292.t1 | Verminephrobacter eiseniae EF01-2, complete genome | 1161 | 0.00E+00 | 87.20% |
| Efet.01.658218.g1722.t1 | Verminephrobacter eiseniae EF01-2, complete genome | 881 | 0.00E+00 | 87.20% |
| Efet.01.658372.g1906.t1 | Verminephrobacter eiseniae EF01-2, complete genome | 1345 | 0.00E+00 | 87.20% |
| Efet.01.1657814.g1042.t1 | Verminephrobacter eiseniae EF01-2 plasmid pVEIS01, complete sequence | 399 | 0.00E+00 | 87.17% |
| Efet.01.537684.g222.t1 | Verminephrobacter eiseniae EF01-2, complete genome | 246 | 1.40E-119 | 87.10% |
| Efet.01.604212.g155.t1 | Verminephrobacter eiseniae EF01-2, complete genome | 504 | 0.00E+00 | 87.10% |
| Efet.01.605483.g225.t1 | Verminephrobacter eiseniae EF01-2, complete genome | 861 | 0.00E+00 | 87.10% |
| Efet.01.608389.g350.t1 | Verminephrobacter eiseniae EF01-2, complete genome | 1809 | 0.00E+00 | 87.10% |
| Efet.01.623992.g1047.t1 | Verminephrobacter eiseniae EF01-2, complete genome | 549 | 0.00E+00 | 87.10% |
| Efet.01.640475.g574.t1 | Verminephrobacter eiseniae EF01-2, complete genome | 1134 | 0.00E+00 | 87.10% |
| Efet.01.642550.g704.t1 | Verminephrobacter eiseniae EF01-2, complete genome | 507 | 0.00E+00 | 87.10% |
| Efet.01.643394.g778.t1 | Verminephrobacter eiseniae EF01-2, complete genome | 637 | 0.00E+00 | 87.10% |
| Efet.01.654656.g654.t1 | Verminephrobacter eiseniae EF01-2, complete genome | 666 | 0.00E+00 | 87.10% |
| Efet.01.658012.g1557.t1 | Verminephrobacter eiseniae EF01-2, complete genome | 757 | 0.00E+00 | 87.10% |
| Efet.01.1659514.g1945.t1 | Verminephrobacter eiseniae EF01-2, complete genome | 2238 | 0.00E+00 | 87.10% |
| Efet.01.44841.g1293.t1 | Uncultured bacterium clone GYQASSE01BN2SJ genomic sequence | 54 | 3.60E-07 | 87.00% |
| Efet.01.61804.g731.t1 | Verminephrobacter eiseniae EF01-2, complete genome | 405 | 0.00E+00 | 87.00% |
| Efet.01.70486.g1261.t1 | Chlorobium chlorochromatii CaD3, complete genome | 66 | 2.60E-07 | 87.00% |
| Efet.01.274225.g886.t1 | Scytonema sp. NIES-4073 DNA, nearly complete genome | 44 | 8.30E-06 | 87.00% |
| Efet.01.320499.g646.t1 | Chlorobium luteolum DSM 273, complete genome | 49 | 6.90E-06 | 87.00% |
| Efet.01.390740.g1020.t1 | Lactococcus lactis subsp. lactis strain G50 chromosome, complete genome | 54 | 2.80E-07 | 87.00% |
| Efet.01.515650.g380.t1 | Verminephrobacter eiseniae EF01-2, complete genome | 681 | 0.00E+00 | 87.00% |
| Efet.01.534125.g144.t1 | Plantibacter flavus strain 251 genome | 48 | 8.20E-06 | 87.00% |
| Efet.01.546787.g409.t1 | Verminephrobacter eiseniae EF01-2, complete genome | 1023 | 0.00E+00 | 87.00% |
| Efet.01.560830.g19.t1 | Verminephrobacter eiseniae EF01-2, complete genome | 834 | 0.00E+00 | 87.00% |
| Efet.01.577373.g570.t1 | Verminephrobacter eiseniae EF01-2, complete genome | 1182 | 0.00E+00 | 87.00% |
| Efet.01.578494.g639.t1 | Cystobacter fuscus strain DSM 52655 chromosome, complete genome | 54 | 6.00E-07 | 87.00% |
| Efet.01.613026.g578.t1 | Verminephrobacter eiseniae EF01-2, complete genome | 1386 | 0.00E+00 | 87.00% |
| Efet.01.613416.g604.t1 | Verminephrobacter eiseniae EF01-2, complete genome | 1199 | 0.00E+00 | 87.00% |
| Efet.01.626245.g1149.t1 | Verminephrobacter eiseniae EF01-2, complete genome | 744 | 0.00E+00 | 87.00% |
| Efet.01.630860.g92.t1 | Verminephrobacter eiseniae EF01-2, complete genome | 531 | 0.00E+00 | 87.00% |
| Efet.01.638026.g413.t1 | Verminephrobacter eiseniae EF01-2, complete genome | 1875 | 0.00E+00 | 87.00% |
| Efet.01.642485.g687.t1 | Verminephrobacter eiseniae EF01-2, complete genome | 301 | 8.70E-106 | 87.00% |
| Efet.01.642485.g689.t1 | Verminephrobacter eiseniae EF01-2, complete genome | 1089 | 0.00E+00 | 87.00% |
| Efet.01.646274.g1066.t1 | Verminephrobacter eiseniae EF01-2, complete genome | 255 | 1.50E-121 | 87.00% |
| Efet.01.651148.g141.t1 | Verminephrobacter eiseniae EF01-2, complete genome | 983 | 0.00E+00 | 87.00% |
| Efet.01.654316.g569.t1 | Verminephrobacter eiseniae EF01-2, complete genome | 621 | 0.00E+00 | 87.00% |
| Efet.01.654965.g727.t1 | Verminephrobacter eiseniae EF01-2, complete genome | 1137 | 0.00E+00 | 87.00% |
| Efet.01.657308.g1231.t1 | Verminephrobacter eiseniae EF01-2, complete genome | 495 | 0.00E+00 | 87.00% |
| Efet.01.658383.g1926.t1 | Verminephrobacter eiseniae EF01-2, complete genome | 933 | 0.00E+00 | 87.00% |
| Efet.01.1656872.g920.t1 | Verminephrobacter eiseniae EF01-2, complete genome | 753 | 0.00E+00 | 87.00% |
| Efet.01.1659476.g1803.t1 | Verminephrobacter eiseniae EF01-2, complete genome | 204 | 1.20E-87 | 87.00% |
| Efet.01.318747.g602.t1 | Verminephrobacter eiseniae EF01-2, complete genome | 576 | 0.00E+00 | 86.90% |
| Efet.01.494042.g1052.t1 | Verminephrobacter eiseniae EF01-2, complete genome | 1271 | 0.00E+00 | 86.90% |
| Efet.01.585463.g844.t1 | Verminephrobacter eiseniae EF01-2, complete genome | 1119 | 0.00E+00 | 86.90% |
| Efet.01.607412.g308.t1 | Verminephrobacter eiseniae EF01-2, complete genome | 887 | 0.00E+00 | 86.90% |
| Efet.01.638026.g405.t1 | Verminephrobacter eiseniae EF01-2, complete genome | 495 | 0.00E+00 | 86.90% |
| Efet.01.650480.g57.t1 | Verminephrobacter eiseniae EF01-2, complete genome | 1308 | 0.00E+00 | 86.90% |
| Efet.01.650749.g84.t1 | Verminephrobacter eiseniae EF01-2, complete genome | 1095 | 0.00E+00 | 86.90% |
| Efet.01.651940.g247.t1 | Verminephrobacter eiseniae EF01-2, complete genome | 1077 | 0.00E+00 | 86.90% |
| Efet.01.652858.g353.t1 | Verminephrobacter eiseniae EF01-2, complete genome | 651 | 0.00E+00 | 86.90% |
| Efet.01.653312.g415.t1 | Verminephrobacter eiseniae EF01-2, complete genome | 804 | 0.00E+00 | 86.90% |
| Efet.01.654769.g695.t1 | Verminephrobacter eiseniae EF01-2, complete genome | 540 | 0.00E+00 | 86.90% |
| Efet.01.655821.g880.t1 | Verminephrobacter eiseniae EF01-2, complete genome | 587 | 0.00E+00 | 86.90% |
| Efet.01.657123.g1176.t1 | Verminephrobacter eiseniae EF01-2, complete genome | 736 | 0.00E+00 | 86.90% |
| Efet.01.657336.g1263.t1 | Verminephrobacter eiseniae EF01-2, complete genome | 1437 | 0.00E+00 | 86.90% |
| Efet.01.657766.g1448.t1 | Verminephrobacter eiseniae EF01-2, complete genome | 198 | 3.80E-95 | 86.90% |
| Efet.01.658192.g1708.t1 | Verminephrobacter eiseniae EF01-2, complete genome | 720 | 0.00E+00 | 86.90% |
| Efet.01.658426.g2136.t1 | Verminephrobacter eiseniae EF01-2, complete genome | 1164 | 0.00E+00 | 86.90% |
| Efet.01.1654783.g711.t1 | Verminephrobacter eiseniae EF01-2, complete genome | 609 | 0.00E+00 | 86.90% |
| Efet.01.1658553.g1215.t1 | Verminephrobacter eiseniae EF01-2, complete genome | 413 | 0.00E+00 | 86.90% |
| Efet.01.1659205.g1460.t1 | Verminephrobacter eiseniae EF01-2, complete genome | 1533 | 0.00E+00 | 86.90% |
| Efet.01.1659388.g1634.t1 | Verminephrobacter eiseniae EF01-2, complete genome | 1155 | 0.00E+00 | 86.90% |
| Efet.01.585033.g830.t1 | Verminephrobacter eiseniae EF01-2, complete genome | 891 | 0.00E+00 | 86.80% |
| Efet.01.585463.g841.t1 | Verminephrobacter eiseniae EF01-2, complete genome | 381 | 0.00E+00 | 86.80% |
| Efet.01.592294.g1070.t1 | Verminephrobacter eiseniae EF01-2, complete genome | 898 | 0.00E+00 | 86.80% |
| Efet.01.595595.g1162.t1 | Verminephrobacter eiseniae EF01-2, complete genome | 897 | 0.00E+00 | 86.80% |
| Efet.01.597630.g1215.t1 | Verminephrobacter eiseniae EF01-2, complete genome | 1013 | 0.00E+00 | 86.80% |
| Efet.01.613416.g599.t1 | Verminephrobacter eiseniae EF01-2, complete genome | 1263 | 0.00E+00 | 86.80% |
| Efet.01.623992.g1049.t1 | Verminephrobacter eiseniae EF01-2, complete genome | 605 | 0.00E+00 | 86.80% |
| Efet.01.626245.g1153.t1 | Verminephrobacter eiseniae EF01-2, complete genome | 1225 | 0.00E+00 | 86.80% |
| Efet.01.654656.g673.t1 | Verminephrobacter eiseniae EF01-2, complete genome | 2160 | 0.00E+00 | 86.80% |
| Efet.01.654851.g710.t1 | Verminephrobacter eiseniae EF01-2, complete genome | 936 | 0.00E+00 | 86.80% |
| Efet.01.658312.g1849.t1 | Verminephrobacter eiseniae EF01-2, complete genome | 687 | 0.00E+00 | 86.80% |
| Efet.01.1657970.g1072.t1 | Verminephrobacter eiseniae EF01-2, complete genome | 360 | 1.30E-180 | 86.80% |
| Efet.01.1659411.g1672.t1 | Verminephrobacter eiseniae EF01-2, complete genome | 1755 | 0.00E+00 | 86.80% |
| Efet.01.300799.g35.t1 | Verminephrobacter eiseniae EF01-2, complete genome | 564 | 0.00E+00 | 86.70% |
| Efet.01.515650.g386.t1 | Verminephrobacter eiseniae EF01-2, complete genome | 1431 | 0.00E+00 | 86.70% |
| Efet.01.530574.g22.t1 | Verminephrobacter eiseniae EF01-2, complete genome | 354 | 8.50E-175 | 86.70% |
| Efet.01.557665.g697.t1 | Verminephrobacter eiseniae EF01-2, complete genome | 444 | 0.00E+00 | 86.70% |
| Efet.01.622588.g991.t1 | Variovorax sp. HW608 genome assembly, chromosome: I | 1056 | 0.00E+00 | 86.70% |
| Efet.01.642395.g682.t1 | Verminephrobacter eiseniae EF01-2, complete genome | 796 | 0.00E+00 | 86.70% |
| Efet.01.643674.g822.t1 | Verminephrobacter eiseniae EF01-2, complete genome | 237 | 5.30E-116 | 86.70% |
| Efet.01.650480.g52.t1 | Verminephrobacter eiseniae EF01-2, complete genome | 1439 | 0.00E+00 | 86.70% |
| Efet.01.653479.g433.t1 | Verminephrobacter eiseniae EF01-2, complete genome | 1154 | 0.00E+00 | 86.70% |
| Efet.01.653626.g464.t1 | Verminephrobacter eiseniae EF01-2, complete genome | 987 | 0.00E+00 | 86.70% |
| Efet.01.654851.g708.t1 | Verminephrobacter eiseniae EF01-2, complete genome | 1440 | 0.00E+00 | 86.70% |
| Efet.01.656283.g985.t1 | Verminephrobacter eiseniae EF01-2, complete genome | 306 | 2.50E-148 | 86.70% |
| Efet.01.657473.g1299.t1 | Verminephrobacter eiseniae EF01-2, complete genome | 1123 | 0.00E+00 | 86.70% |
| Efet.01.658040.g1574.t1 | Verminephrobacter eiseniae EF01-2, complete genome | 792 | 0.00E+00 | 86.70% |
| Efet.01.658193.g1709.t1 | Verminephrobacter eiseniae EF01-2, complete genome | 870 | 0.00E+00 | 86.70% |
| Efet.01.1652652.g573.t1 | Verminephrobacter eiseniae EF01-2, complete genome | 336 | 8.90E-169 | 86.70% |
| Efet.01.1659408.g1666.t1 | Verminephrobacter eiseniae EF01-2, complete genome | 1347 | 0.00E+00 | 86.70% |
| Efet.01.1659411.g1673.t1 | Verminephrobacter eiseniae EF01-2, complete genome | 504 | 0.00E+00 | 86.70% |
| Efet.01.1659455.g1756.t1 | Verminephrobacter eiseniae EF01-2, complete genome | 1245 | 0.00E+00 | 86.70% |
| Efet.01.270790.g773.t1 | Verminephrobacter eiseniae EF01-2, complete genome | 938 | 0.00E+00 | 86.60% |
| Efet.01.394810.g1110.t1 | Verminephrobacter eiseniae EF01-2, complete genome | 1187 | 0.00E+00 | 86.60% |
| Efet.01.515650.g383.t1 | Verminephrobacter eiseniae EF01-2, complete genome | 546 | 0.00E+00 | 86.60% |
| Efet.01.544715.g354.t1 | Variovorax boronicumulans strain J1 chromosome, complete genome | 1749 | 0.00E+00 | 86.60% |
| Efet.01.570134.g331.t1 | Paeniclostridium sordellii strain AM370 chromosome, complete genome | 48 | 2.40E-08 | 86.60% |
| Efet.01.605884.g246.t1 | Verminephrobacter eiseniae EF01-2, complete genome | 250 | 9.00E-115 | 86.60% |
| Efet.01.622583.g988.t1 | Acidovorax ebreus TPSY, complete genome | 411 | 5.90E-154 | 86.60% |
| Efet.01.630390.g19.t1 | Verminephrobacter eiseniae EF01-2, complete genome | 1356 | 0.00E+00 | 86.60% |
| Efet.01.630860.g93.t1 | Verminephrobacter eiseniae EF01-2, complete genome | 1138 | 0.00E+00 | 86.60% |
| Efet.01.633916.g225.t1 | Verminephrobacter eiseniae EF01-2, complete genome | 2694 | 0.00E+00 | 86.60% |
| Efet.01.633916.g228.t1 | Verminephrobacter eiseniae EF01-2, complete genome | 561 | 0.00E+00 | 86.60% |
| Efet.01.638674.g445.t1 | Verminephrobacter eiseniae EF01-2, complete genome | 930 | 0.00E+00 | 86.60% |
| Efet.01.639761.g538.t1 | Variovorax boronicumulans strain J1 chromosome, complete genome | 1190 | 0.00E+00 | 86.60% |
| Efet.01.646149.g1043.t1 | Verminephrobacter eiseniae EF01-2, complete genome | 425 | 0.00E+00 | 86.60% |
| Efet.01.653992.g507.t1 | Verminephrobacter eiseniae EF01-2, complete genome | 1677 | 0.00E+00 | 86.60% |
| Efet.01.654656.g674.t1 | Verminephrobacter eiseniae EF01-2, complete genome | 1019 | 0.00E+00 | 86.60% |
| Efet.01.658372.g1907.t1 | Verminephrobacter eiseniae EF01-2, complete genome | 822 | 0.00E+00 | 86.60% |
| Efet.01.658383.g1923.t1 | Verminephrobacter eiseniae EF01-2, complete genome | 1164 | 0.00E+00 | 86.60% |
| Efet.01.658405.g2001.t1 | Verminephrobacter eiseniae EF01-2, complete genome | 1476 | 0.00E+00 | 86.60% |
| Efet.01.658410.g2020.t1 | Verminephrobacter eiseniae EF01-2, complete genome | 1134 | 0.00E+00 | 86.60% |
| Efet.01.1658864.g1301.t1 | Verminephrobacter eiseniae EF01-2, complete genome | 375 | 0.00E+00 | 86.60% |
| Efet.01.1659114.g1409.t1 | Acidovorax ebreus TPSY, complete genome | 359 | 9.40E-135 | 86.60% |
| Efet.01.1659248.g1488.t1 | Verminephrobacter eiseniae EF01-2, complete genome | 1239 | 0.00E+00 | 86.60% |
| Efet.01.1659428.g1693.t1 | Verminephrobacter eiseniae EF01-2, complete genome | 1302 | 0.00E+00 | 86.60% |
| Efet.01.1659466.g1779.t1 | Verminephrobacter eiseniae EF01-2, complete genome | 534 | 0.00E+00 | 86.60% |
| Efet.01.1659471.g1793.t1 | Verminephrobacter eiseniae EF01-2, complete genome | 258 | 4.20E-125 | 86.60% |
| Efet.01.17103.g1259.t1 | Verminephrobacter eiseniae EF01-2, complete genome | 500 | 0.00E+00 | 86.50% |
| Efet.01.61804.g730.t1 | Verminephrobacter eiseniae EF01-2, complete genome | 861 | 0.00E+00 | 86.50% |
| Efet.01.143861.g969.t1 | Verminephrobacter eiseniae EF01-2, complete genome | 1233 | 0.00E+00 | 86.50% |
| Efet.01.545302.g372.t1 | Verminephrobacter eiseniae EF01-2, complete genome | 891 | 0.00E+00 | 86.50% |
| Efet.01.565049.g167.t1 | Verminephrobacter eiseniae EF01-2, complete genome | 408 | 0.00E+00 | 86.50% |
| Efet.01.569748.g313.t1 | Agromyces aureus strain AR33, complete genome | 163 | 7.40E-53 | 86.50% |
| Efet.01.573348.g411.t1 | Verminephrobacter eiseniae EF01-2, complete genome | 70 | 3.60E-24 | 86.50% |
| Efet.01.622654.g1000.t1 | Verminephrobacter eiseniae EF01-2, complete genome | 199 | 1.60E-91 | 86.50% |
| Efet.01.626208.g1145.t1 | Verminephrobacter eiseniae EF01-2, complete genome | 1230 | 0.00E+00 | 86.50% |
| Efet.01.638894.g479.t1 | Verminephrobacter eiseniae EF01-2, complete genome | 459 | 0.00E+00 | 86.50% |
| Efet.01.639260.g495.t1 | Verminephrobacter eiseniae EF01-2, complete genome | 502 | 0.00E+00 | 86.50% |
| Efet.01.651940.g246.t1 | Verminephrobacter eiseniae EF01-2, complete genome | 807 | 0.00E+00 | 86.50% |
| Efet.01.652309.g277.t1 | Verminephrobacter eiseniae EF01-2, complete genome | 222 | 8.30E-108 | 86.50% |
| Efet.01.652858.g354.t1 | Verminephrobacter eiseniae EF01-2, complete genome | 1074 | 0.00E+00 | 86.50% |
| Efet.01.655750.g855.t1 | Verminephrobacter eiseniae EF01-2, complete genome | 393 | 0.00E+00 | 86.50% |
| Efet.01.655974.g912.t1 | Verminephrobacter eiseniae EF01-2, complete genome | 409 | 0.00E+00 | 86.50% |
| Efet.01.658116.g1650.t1 | Verminephrobacter eiseniae EF01-2, complete genome | 960 | 0.00E+00 | 86.50% |
| Efet.01.658384.g1931.t1 | Verminephrobacter eiseniae EF01-2, complete genome | 801 | 0.00E+00 | 86.50% |
| Efet.01.658405.g2000.t1 | Verminephrobacter eiseniae EF01-2, complete genome | 1620 | 0.00E+00 | 86.50% |
| Efet.01.1659376.g1623.t1 | Verminephrobacter eiseniae EF01-2, complete genome | 423 | 0.00E+00 | 86.50% |
| Efet.01.61804.g732.t1 | Verminephrobacter eiseniae EF01-2, complete genome | 324 | 2.50E-163 | 86.40% |
| Efet.01.327033.g834.t1 | Thioalkalivibrio sulfidiphilus HL-EbGr7, complete genome | 129 | 1.30E-08 | 86.40% |
| Efet.01.514247.g340.t1 | Verminephrobacter eiseniae EF01-2, complete genome | 395 | 0.00E+00 | 86.40% |
| Efet.01.560830.g18.t1 | Verminephrobacter eiseniae EF01-2, complete genome | 330 | 1.90E-158 | 86.40% |
| Efet.01.585463.g840.t1 | Verminephrobacter eiseniae EF01-2, complete genome | 303 | 1.90E-152 | 86.40% |
| Efet.01.646092.g1032.t1 | Verminephrobacter eiseniae EF01-2, complete genome | 2082 | 0.00E+00 | 86.40% |
| Efet.01.647470.g1182.t1 | Verminephrobacter eiseniae EF01-2, complete genome | 332 | 2.20E-166 | 86.40% |
| Efet.01.651141.g130.t1 | Verminephrobacter eiseniae EF01-2, complete genome | 1053 | 0.00E+00 | 86.40% |
| Efet.01.654656.g650.t1 | Verminephrobacter eiseniae EF01-2, complete genome | 896 | 0.00E+00 | 86.40% |
| Efet.01.654851.g706.t1 | Verminephrobacter eiseniae EF01-2, complete genome | 1044 | 0.00E+00 | 86.40% |
| Efet.01.654851.g707.t1 | Verminephrobacter eiseniae EF01-2, complete genome | 608 | 0.00E+00 | 86.40% |
| Efet.01.657150.g1184.t1 | Verminephrobacter eiseniae EF01-2, complete genome | 881 | 0.00E+00 | 86.40% |
| Efet.01.658142.g1678.t1 | Verminephrobacter eiseniae EF01-2, complete genome | 675 | 0.00E+00 | 86.40% |
| Efet.01.1635737.g164.t1 | Verminephrobacter eiseniae EF01-2, complete genome | 270 | 6.30E-133 | 86.40% |
| Efet.01.1658253.g1140.t1 | Verminephrobacter eiseniae EF01-2, complete genome | 831 | 0.00E+00 | 86.40% |
| Efet.01.110726.g542.t1 | Vibrio coralliilyticus strain SNUTY-1 plasmid pSNUTY1, complete sequence | 52 | 6.50E-09 | 86.30% |
| Efet.01.292188.g1516.t1 | Verminephrobacter eiseniae EF01-2, complete genome | 498 | 0.00E+00 | 86.30% |
| Efet.01.350955.g30.t1 | Paraburkholderia xenovorans LB400 chromosome 1, complete sequence | 493 | 1.90E-175 | 86.30% |
| Efet.01.440538.g964.t1 | Verminephrobacter eiseniae EF01-2, complete genome | 324 | 6.80E-162 | 86.30% |
| Efet.01.589035.g962.t1 | Micropruina glycogenica isolate 1 genome assembly, chromosome: 1 | 93 | 2.40E-13 | 86.30% |
| Efet.01.645947.g1014.t1 | Verminephrobacter eiseniae EF01-2, complete genome | 699 | 0.00E+00 | 86.30% |
| Efet.01.651816.g224.t1 | Verminephrobacter eiseniae EF01-2, complete genome | 270 | 8.20E-127 | 86.30% |
| Efet.01.654418.g592.t1 | Verminephrobacter eiseniae EF01-2, complete genome | 1008 | 0.00E+00 | 86.30% |
| Efet.01.654656.g671.t1 | Verminephrobacter eiseniae EF01-2, complete genome | 324 | 5.00E-159 | 86.30% |
| Efet.01.658383.g1925.t1 | Verminephrobacter eiseniae EF01-2, complete genome | 780 | 0.00E+00 | 86.30% |
| Efet.01.1659506.g1912.t1 | Verminephrobacter eiseniae EF01-2, complete genome | 967 | 0.00E+00 | 86.30% |
| Efet.01.20312.g1499.t1 | Micromonospora zamorensis strain DSM 45600 genome assembly, chromosome: I | 133 | 2.70E-14 | 86.20% |
| Efet.01.287526.g1369.t1 | Verminephrobacter eiseniae EF01-2, complete genome | 1398 | 0.00E+00 | 86.20% |
| Efet.01.318747.g590.t1 | Verminephrobacter eiseniae EF01-2, complete genome | 663 | 0.00E+00 | 86.20% |
| Efet.01.465744.g417.t1 | Verminephrobacter eiseniae EF01-2, complete genome | 1644 | 0.00E+00 | 86.20% |
| Efet.01.537099.g205.t1 | Verminephrobacter eiseniae EF01-2, complete genome | 498 | 0.00E+00 | 86.20% |
| Efet.01.565049.g161.t1 | Verminephrobacter eiseniae EF01-2, complete genome | 420 | 0.00E+00 | 86.20% |
| Efet.01.605357.g202.t1 | Verminephrobacter eiseniae EF01-2, complete genome | 278 | 1.90E-104 | 86.20% |
| Efet.01.644747.g896.t1 | Verminephrobacter eiseniae EF01-2, complete genome | 768 | 0.00E+00 | 86.20% |
| Efet.01.650749.g83.t1 | Verminephrobacter eiseniae EF01-2, complete genome | 318 | 3.50E-160 | 86.20% |
| Efet.01.653541.g449.t1 | Verminephrobacter eiseniae EF01-2, complete genome | 376 | 1.70E-179 | 86.20% |
| Efet.01.654316.g568.t1 | Verminephrobacter eiseniae EF01-2, complete genome | 330 | 2.20E-161 | 86.20% |
| Efet.01.654656.g672.t1 | Verminephrobacter eiseniae EF01-2, complete genome | 788 | 0.00E+00 | 86.20% |
| Efet.01.656795.g1100.t1 | Verminephrobacter eiseniae EF01-2, complete genome | 1178 | 0.00E+00 | 86.20% |
| Efet.01.656795.g1101.t1 | Verminephrobacter eiseniae EF01-2, complete genome | 921 | 0.00E+00 | 86.20% |
| Efet.01.657453.g1290.t1 | Verminephrobacter eiseniae EF01-2, complete genome | 231 | 3.70E-111 | 86.20% |
| Efet.01.658397.g1975.t1 | Verminephrobacter eiseniae EF01-2, complete genome | 228 | 2.10E-109 | 86.20% |
| Efet.01.1651340.g506.t1 | Verminephrobacter eiseniae EF01-2, complete genome | 338 | 7.30E-170 | 86.20% |
| Efet.01.1657921.g1062.t1 | Verminephrobacter eiseniae EF01-2, complete genome | 559 | 0.00E+00 | 86.20% |
| Efet.01.81246.g422.t1 | Verminephrobacter eiseniae EF01-2, complete genome | 328 | 5.20E-164 | 86.10% |
| Efet.01.581510.g717.t1 | Verminephrobacter eiseniae EF01-2, complete genome | 795 | 0.00E+00 | 86.10% |
| Efet.01.624347.g1071.t1 | Verminephrobacter eiseniae EF01-2, complete genome | 1434 | 0.00E+00 | 86.10% |
| Efet.01.626245.g1155.t1 | Verminephrobacter eiseniae EF01-2, complete genome | 1764 | 0.00E+00 | 86.10% |
| Efet.01.638023.g394.t1 | Verminephrobacter eiseniae EF01-2, complete genome | 188 | 1.40E-87 | 86.10% |
| Efet.01.638026.g401.t1 | Verminephrobacter eiseniae EF01-2, complete genome | 903 | 0.00E+00 | 86.10% |
| Efet.01.638026.g404.t1 | Verminephrobacter eiseniae EF01-2, complete genome | 945 | 0.00E+00 | 86.10% |
| Efet.01.642550.g699.t1 | Verminephrobacter eiseniae EF01-2, complete genome | 1455 | 0.00E+00 | 86.10% |
| Efet.01.655128.g760.t1 | Verminephrobacter eiseniae EF01-2, complete genome | 1326 | 0.00E+00 | 86.10% |
| Efet.01.656717.g1069.t1 | Verminephrobacter eiseniae EF01-2, complete genome | 1233 | 0.00E+00 | 86.10% |
| Efet.01.657457.g1291.t1 | Verminephrobacter eiseniae EF01-2, complete genome | 837 | 0.00E+00 | 86.10% |
| Efet.01.658429.g2166.t1 | Verminephrobacter eiseniae EF01-2, complete genome | 369 | 0.00E+00 | 86.10% |
| Efet.01.1659166.g1440.t1 | Verminephrobacter eiseniae EF01-2, complete genome | 638 | 0.00E+00 | 86.10% |
| Efet.01.1659506.g1911.t1 | Verminephrobacter eiseniae EF01-2, complete genome | 1422 | 0.00E+00 | 86.10% |
| Efet.01.310657.g340.t1 | Idiomarina sp. OT37-5b chromosome, complete genome | 53 | 1.20E-06 | 86.00% |
| Efet.01.314457.g454.t1 | Verminephrobacter eiseniae EF01-2, complete genome | 699 | 0.00E+00 | 86.00% |
| Efet.01.357077.g200.t1 | Xanthomonas campestris pv. campestris str. CN16 chromosome, complete genome | 79 | 3.10E-15 | 86.00% |
| Efet.01.573518.g419.t1 | Verminephrobacter eiseniae EF01-2, complete genome | 666 | 0.00E+00 | 86.00% |
| Efet.01.576819.g539.t1 | Verminephrobacter eiseniae EF01-2, complete genome | 327 | 9.90E-163 | 86.00% |
| Efet.01.585463.g845.t1 | Verminephrobacter eiseniae EF01-2, complete genome | 424 | 6.20E-146 | 86.00% |
| Efet.01.591923.g1052.t1 | Verminephrobacter eiseniae EF01-2, complete genome | 250 | 1.00E-123 | 86.00% |
| Efet.01.643898.g836.t1 | Acidovorax avenae subsp. avenae strain INV chromosome | 1074 | 0.00E+00 | 86.00% |
| Efet.01.645947.g1012.t1 | Verminephrobacter eiseniae EF01-2, complete genome | 669 | 0.00E+00 | 86.00% |
| Efet.01.649481.g1317.t1 | Hymenobacter sedentarius strain DG5B chromosome, complete genome | 53 | 1.40E-06 | 86.00% |
| Efet.01.654391.g584.t1 | Verminephrobacter eiseniae EF01-2, complete genome | 987 | 0.00E+00 | 86.00% |
| Efet.01.655399.g810.t1 | Verminephrobacter eiseniae EF01-2, complete genome | 987 | 0.00E+00 | 86.00% |
| Efet.01.656795.g1099.t1 | Verminephrobacter eiseniae EF01-2, complete genome | 459 | 0.00E+00 | 86.00% |
| Efet.01.658150.g1682.t1 | Verminephrobacter eiseniae EF01-2, complete genome | 1282 | 0.00E+00 | 86.00% |
| Efet.01.658392.g1964.t1 | Verminephrobacter eiseniae EF01-2, complete genome | 1371 | 0.00E+00 | 86.00% |
| Efet.01.1654997.g725.t1 | Amycolatopsis mediterranei S699, complete genome | 59 | 6.70E-09 | 86.00% |
| Efet.01.1656733.g908.t1 | Cellulosimicrobium cellulans strain PSBB019, complete genome | 152 | 7.10E-42 | 86.00% |
| Efet.01.1657186.g953.t1 | Verminephrobacter eiseniae EF01-2, complete genome | 434 | 0.00E+00 | 86.00% |
| Efet.01.1657283.g966.t1 | Verminephrobacter eiseniae EF01-2, complete genome | 549 | 0.00E+00 | 86.00% |
| Efet.01.1659484.g1829.t1 | Verminephrobacter eiseniae EF01-2, complete genome | 846 | 0.00E+00 | 86.00% |
| Efet.01.1659496.g1875.t1 | Verminephrobacter eiseniae EF01-2, complete genome | 1356 | 0.00E+00 | 86.00% |
| Efet.01.1659509.g1924.t1 | Verminephrobacter eiseniae EF01-2, complete genome | 405 | 0.00E+00 | 86.00% |
| Efet.01.118288.g943.t1 | Prosthecochloris aestuarii DSM 271, complete genome | 115 | 4.90E-24 | 85.90% |
| Efet.01.150930.g54.t1 | Verminephrobacter eiseniae EF01-2, complete genome | 1025 | 0.00E+00 | 85.90% |
| Efet.01.566725.g221.t1 | Verminephrobacter eiseniae EF01-2, complete genome | 912 | 0.00E+00 | 85.90% |
| Efet.01.577373.g584.t1 | Verminephrobacter eiseniae EF01-2, complete genome | 423 | 0.00E+00 | 85.90% |
| Efet.01.605365.g213.t1 | Verminephrobacter eiseniae EF01-2, complete genome | 659 | 0.00E+00 | 85.90% |
| Efet.01.641462.g627.t1 | Verminephrobacter eiseniae EF01-2, complete genome | 670 | 0.00E+00 | 85.90% |
| Efet.01.643097.g757.t1 | Verminephrobacter eiseniae EF01-2, complete genome | 653 | 0.00E+00 | 85.90% |
| Efet.01.645947.g1015.t1 | Verminephrobacter eiseniae EF01-2, complete genome | 261 | 3.70E-129 | 85.90% |
| Efet.01.651816.g225.t1 | Verminephrobacter eiseniae EF01-2, complete genome | 1779 | 0.00E+00 | 85.90% |
| Efet.01.652309.g279.t1 | Verminephrobacter eiseniae EF01-2, complete genome | 804 | 0.00E+00 | 85.90% |
| Efet.01.654656.g655.t1 | Verminephrobacter eiseniae EF01-2, complete genome | 883 | 0.00E+00 | 85.90% |
| Efet.01.655966.g908.t1 | Verminephrobacter eiseniae EF01-2, complete genome | 1114 | 0.00E+00 | 85.90% |
| Efet.01.1646951.g365.t1 | Verminephrobacter eiseniae EF01-2, complete genome | 287 | 4.10E-140 | 85.90% |
| Efet.01.1659277.g1505.t1 | Aminobacter sp. MSH1 chromosome, complete genome | 891 | 0.00E+00 | 85.90% |
| Efet.01.1659334.g1561.t1 | Verminephrobacter eiseniae EF01-2, complete genome | 450 | 0.00E+00 | 85.90% |
| Efet.01.1659418.g1682.t1 | Verminephrobacter eiseniae EF01-2, complete genome | 708 | 0.00E+00 | 85.90% |
| Efet.01.1659501.g1895.t1 | Verminephrobacter eiseniae EF01-2, complete genome | 684 | 0.00E+00 | 85.90% |
| Efet.01.1659525.g2018.t1 | Verminephrobacter eiseniae EF01-2, complete genome | 1761 | 0.00E+00 | 85.90% |
| Efet.01.215157.g633.t1 | Verminephrobacter eiseniae EF01-2, complete genome | 1467 | 0.00E+00 | 85.80% |
| Efet.01.275883.g960.t1 | Verminephrobacter eiseniae EF01-2, complete genome | 999 | 0.00E+00 | 85.80% |
| Efet.01.440935.g1001.t1 | Verminephrobacter eiseniae EF01-2, complete genome | 1320 | 0.00E+00 | 85.80% |
| Efet.01.464530.g383.t1 | Verminephrobacter eiseniae EF01-2, complete genome | 658 | 0.00E+00 | 85.80% |
| Efet.01.477677.g712.t1 | Verminephrobacter eiseniae EF01-2, complete genome | 1157 | 0.00E+00 | 85.80% |
| Efet.01.514247.g338.t1 | Verminephrobacter eiseniae EF01-2, complete genome | 894 | 0.00E+00 | 85.80% |
| Efet.01.586228.g870.t1 | Verminephrobacter eiseniae EF01-2, complete genome | 1242 | 0.00E+00 | 85.80% |
| Efet.01.605365.g207.t1 | Verminephrobacter eiseniae EF01-2, complete genome | 972 | 0.00E+00 | 85.80% |
| Efet.01.610308.g435.t1 | Verminephrobacter eiseniae EF01-2, complete genome | 1162 | 0.00E+00 | 85.80% |
| Efet.01.621705.g950.t1 | Variovorax boronicumulans strain J1 chromosome, complete genome | 724 | 0.00E+00 | 85.80% |
| Efet.01.626208.g1146.t1 | Verminephrobacter eiseniae EF01-2, complete genome | 754 | 0.00E+00 | 85.80% |
| Efet.01.627060.g1195.t1 | Verminephrobacter eiseniae EF01-2, complete genome | 1059 | 0.00E+00 | 85.80% |
| Efet.01.650654.g73.t1 | Verminephrobacter eiseniae EF01-2, complete genome | 486 | 0.00E+00 | 85.80% |
| Efet.01.651515.g182.t1 | Verminephrobacter eiseniae EF01-2, complete genome | 949 | 0.00E+00 | 85.80% |
| Efet.01.652108.g261.t1 | Verminephrobacter eiseniae EF01-2, complete genome | 630 | 0.00E+00 | 85.80% |
| Efet.01.653215.g408.t1 | Verminephrobacter eiseniae EF01-2, complete genome | 672 | 0.00E+00 | 85.80% |
| Efet.01.653526.g445.t1 | Verminephrobacter eiseniae EF01-2, complete genome | 400 | 0.00E+00 | 85.80% |
| Efet.01.654034.g529.t1 | Acidovorax ebreus TPSY, complete genome | 1005 | 0.00E+00 | 85.80% |
| Efet.01.655573.g834.t1 | Verminephrobacter eiseniae EF01-2, complete genome | 247 | 7.40E-122 | 85.80% |
| Efet.01.657473.g1298.t1 | Verminephrobacter eiseniae EF01-2, complete genome | 1512 | 0.00E+00 | 85.80% |
| Efet.01.657703.g1387.t1 | Verminephrobacter eiseniae EF01-2, complete genome | 1104 | 0.00E+00 | 85.80% |
| Efet.01.658044.g1595.t1 | Verminephrobacter eiseniae EF01-2, complete genome | 1227 | 0.00E+00 | 85.80% |
| Efet.01.658410.g2024.t1 | Verminephrobacter eiseniae EF01-2, complete genome | 903 | 0.00E+00 | 85.80% |
| Efet.01.658416.g2056.t1 | Verminephrobacter eiseniae EF01-2, complete genome | 1569 | 0.00E+00 | 85.80% |
| Efet.01.1656440.g868.t1 | Agromyces sp. 30A chromosome, complete genome | 136 | 1.10E-45 | 85.80% |
| Efet.01.1657492.g994.t1 | Paraburkholderia xenovorans LB400 chromosome 1, complete sequence | 60 | 1.60E-06 | 85.80% |
| Efet.01.1658315.g1154.t1 | Verminephrobacter eiseniae EF01-2, complete genome | 756 | 0.00E+00 | 85.80% |
| Efet.01.1659455.g1757.t1 | Verminephrobacter eiseniae EF01-2, complete genome | 717 | 0.00E+00 | 85.80% |
| Efet.01.1659478.g1810.t1 | Verminephrobacter eiseniae EF01-2, complete genome | 810 | 0.00E+00 | 85.80% |
| Efet.01.1659486.g1837.t1 | Verminephrobacter eiseniae EF01-2, complete genome | 1641 | 0.00E+00 | 85.80% |
| Efet.01.292188.g1515.t1 | Verminephrobacter eiseniae EF01-2, complete genome | 1128 | 0.00E+00 | 85.70% |
| Efet.01.592090.g1061.t1 | Verminephrobacter eiseniae EF01-2, complete genome | 794 | 0.00E+00 | 85.70% |
| Efet.01.629329.g1282.t1 | Verminephrobacter eiseniae EF01-2, complete genome | 479 | 0.00E+00 | 85.70% |
| Efet.01.630860.g94.t1 | Verminephrobacter eiseniae EF01-2, complete genome | 948 | 0.00E+00 | 85.70% |
| Efet.01.634939.g265.t1 | Verminephrobacter eiseniae EF01-2, complete genome | 798 | 0.00E+00 | 85.70% |
| Efet.01.638894.g478.t1 | Verminephrobacter eiseniae EF01-2, complete genome | 261 | 4.40E-129 | 85.70% |
| Efet.01.648797.g1268.t1 | Verminephrobacter eiseniae EF01-2, complete genome | 831 | 0.00E+00 | 85.70% |
| Efet.01.651148.g138.t1 | Verminephrobacter eiseniae EF01-2, complete genome | 972 | 0.00E+00 | 85.70% |
| Efet.01.651148.g140.t1 | Verminephrobacter eiseniae EF01-2, complete genome | 849 | 0.00E+00 | 85.70% |
| Efet.01.652283.g272.t1 | Verminephrobacter eiseniae EF01-2, complete genome | 996 | 0.00E+00 | 85.70% |
| Efet.01.653445.g428.t1 | Verminephrobacter eiseniae EF01-2, complete genome | 1547 | 0.00E+00 | 85.70% |
| Efet.01.655128.g759.t1 | Verminephrobacter eiseniae EF01-2, complete genome | 438 | 0.00E+00 | 85.70% |
| Efet.01.655224.g774.t1 | Verminephrobacter eiseniae EF01-2, complete genome | 444 | 0.00E+00 | 85.70% |
| Efet.01.655821.g883.t1 | Verminephrobacter eiseniae EF01-2, complete genome | 951 | 0.00E+00 | 85.70% |
| Efet.01.658416.g2061.t1 | Verminephrobacter eiseniae EF01-2, complete genome | 930 | 0.00E+00 | 85.70% |
| Efet.01.1658449.g1185.t1 | Verminephrobacter eiseniae EF01-2, complete genome | 685 | 0.00E+00 | 85.70% |
| Efet.01.1658883.g1313.t1 | Verminephrobacter eiseniae EF01-2, complete genome | 1089 | 0.00E+00 | 85.70% |
| Efet.01.1659300.g1531.t1 | Verminephrobacter eiseniae EF01-2, complete genome | 603 | 0.00E+00 | 85.70% |
| Efet.01.1659440.g1720.t1 | Verminephrobacter eiseniae EF01-2, complete genome | 618 | 0.00E+00 | 85.70% |
| Efet.01.314858.g469.t1 | Verminephrobacter eiseniae EF01-2, complete genome | 966 | 0.00E+00 | 85.60% |
| Efet.01.353298.g94.t1 | Verminephrobacter eiseniae EF01-2, complete genome | 773 | 0.00E+00 | 85.60% |
| Efet.01.421789.g519.t1 | Verminephrobacter eiseniae EF01-2, complete genome | 413 | 0.00E+00 | 85.60% |
| Efet.01.440538.g972.t1 | Verminephrobacter eiseniae EF01-2, complete genome | 1480 | 0.00E+00 | 85.60% |
| Efet.01.440935.g1003.t1 | Verminephrobacter eiseniae EF01-2, complete genome | 898 | 0.00E+00 | 85.60% |
| Efet.01.454467.g105.t1 | Verminephrobacter eiseniae EF01-2, complete genome | 372 | 0.00E+00 | 85.60% |
| Efet.01.455382.g133.t1 | Verminephrobacter eiseniae EF01-2, complete genome | 948 | 0.00E+00 | 85.60% |
| Efet.01.503541.g83.t1 | Verminephrobacter eiseniae EF01-2, complete genome | 2451 | 0.00E+00 | 85.60% |
| Efet.01.530574.g23.t1 | Verminephrobacter eiseniae EF01-2, complete genome | 1710 | 0.00E+00 | 85.60% |
| Efet.01.566222.g210.t1 | Verminephrobacter eiseniae EF01-2, complete genome | 251 | 4.00E-121 | 85.60% |
| Efet.01.577373.g594.t1 | Verminephrobacter eiseniae EF01-2, complete genome | 600 | 0.00E+00 | 85.60% |
| Efet.01.581510.g718.t1 | Verminephrobacter eiseniae EF01-2, complete genome | 1215 | 0.00E+00 | 85.60% |
| Efet.01.592563.g1076.t1 | Verminephrobacter eiseniae EF01-2, complete genome | 1347 | 0.00E+00 | 85.60% |
| Efet.01.597508.g1208.t1 | Verminephrobacter eiseniae EF01-2, complete genome | 2156 | 0.00E+00 | 85.60% |
| Efet.01.605365.g208.t1 | Verminephrobacter eiseniae EF01-2, complete genome | 351 | 5.00E-175 | 85.60% |
| Efet.01.613416.g606.t1 | Verminephrobacter eiseniae EF01-2, complete genome | 1543 | 0.00E+00 | 85.60% |
| Efet.01.628482.g1249.t1 | Verminephrobacter eiseniae EF01-2, complete genome | 1185 | 0.00E+00 | 85.60% |
| Efet.01.630521.g28.t1 | Verminephrobacter eiseniae EF01-2, complete genome | 1089 | 0.00E+00 | 85.60% |
| Efet.01.636372.g327.t1 | Verminephrobacter eiseniae EF01-2, complete genome | 333 | 1.60E-165 | 85.60% |
| Efet.01.646963.g1140.t1 | Verminephrobacter eiseniae EF01-2, complete genome | 1185 | 0.00E+00 | 85.60% |
| Efet.01.649588.g1336.t1 | Verminephrobacter eiseniae EF01-2, complete genome | 783 | 0.00E+00 | 85.60% |
| Efet.01.654418.g595.t1 | Verminephrobacter eiseniae EF01-2, complete genome | 477 | 0.00E+00 | 85.60% |
| Efet.01.654936.g716.t1 | Verminephrobacter eiseniae EF01-2, complete genome | 752 | 0.00E+00 | 85.60% |
| Efet.01.655999.g928.t1 | Verminephrobacter eiseniae EF01-2, complete genome | 786 | 0.00E+00 | 85.60% |
| Efet.01.656518.g1037.t1 | Verminephrobacter eiseniae EF01-2, complete genome | 775 | 0.00E+00 | 85.60% |
| Efet.01.658192.g1706.t1 | Verminephrobacter eiseniae EF01-2, complete genome | 197 | 2.10E-87 | 85.60% |
| Efet.01.658355.g1886.t1 | Verminephrobacter eiseniae EF01-2, complete genome | 1329 | 0.00E+00 | 85.60% |
| Efet.01.1656279.g847.t1 | Verminephrobacter eiseniae EF01-2, complete genome | 696 | 0.00E+00 | 85.60% |
| Efet.01.1659476.g1804.t1 | Verminephrobacter eiseniae EF01-2, complete genome | 701 | 0.00E+00 | 85.60% |
| Efet.01.1657814.g1041.t1 | Xylella fastidiosa MUL0034 plasmid unnamed2, complete sequence | 253 | 7.10E-68 | 85.57% |
| Efet.01.151661.g99.t1 | Verminephrobacter eiseniae EF01-2, complete genome | 1158 | 0.00E+00 | 85.50% |
| Efet.01.220990.g852.t1 | Verminephrobacter eiseniae EF01-2, complete genome | 714 | 0.00E+00 | 85.50% |
| Efet.01.365501.g422.t1 | Verminephrobacter eiseniae EF01-2, complete genome | 698 | 0.00E+00 | 85.50% |
| Efet.01.492121.g998.t1 | Verminephrobacter eiseniae EF01-2, complete genome | 633 | 0.00E+00 | 85.50% |
| Efet.01.503541.g86.t1 | Vibrio rumoiensis FERM P-14531 plasmid 1 DNA, complete genome | 281 | 1.80E-89 | 85.50% |
| Efet.01.524250.g564.t1 | Verminephrobacter eiseniae EF01-2, complete genome | 978 | 0.00E+00 | 85.50% |
| Efet.01.550874.g522.t1 | Bacteriovorax stolpii strain DSM 12778 chromosome, complete genome | 54 | 9.70E-06 | 85.50% |
| Efet.01.553292.g582.t1 | Verminephrobacter eiseniae EF01-2, complete genome | 806 | 0.00E+00 | 85.50% |
| Efet.01.591301.g1035.t1 | Verminephrobacter eiseniae EF01-2, complete genome | 1731 | 0.00E+00 | 85.50% |
| Efet.01.609092.g379.t1 | Verminephrobacter eiseniae EF01-2, complete genome | 403 | 0.00E+00 | 85.50% |
| Efet.01.623992.g1052.t1 | Verminephrobacter eiseniae EF01-2, complete genome | 396 | 0.00E+00 | 85.50% |
| Efet.01.625433.g1115.t1 | Verminephrobacter eiseniae EF01-2, complete genome | 2409 | 0.00E+00 | 85.50% |
| Efet.01.636598.g338.t1 | Verminephrobacter eiseniae EF01-2, complete genome | 948 | 0.00E+00 | 85.50% |
| Efet.01.646963.g1137.t1 | Verminephrobacter eiseniae EF01-2, complete genome | 1524 | 0.00E+00 | 85.50% |
| Efet.01.651441.g175.t1 | Verminephrobacter eiseniae EF01-2, complete genome | 744 | 0.00E+00 | 85.50% |
| Efet.01.656558.g1046.t1 | Verminephrobacter eiseniae EF01-2, complete genome | 471 | 0.00E+00 | 85.50% |
| Efet.01.657703.g1375.t1 | Verminephrobacter eiseniae EF01-2, complete genome | 567 | 0.00E+00 | 85.50% |
| Efet.01.658041.g1587.t1 | Verminephrobacter eiseniae EF01-2, complete genome | 2688 | 0.00E+00 | 85.50% |
| Efet.01.658138.g1667.t1 | Verminephrobacter eiseniae EF01-2, complete genome | 450 | 0.00E+00 | 85.50% |
| Efet.01.658417.g2072.t1 | Verminephrobacter eiseniae EF01-2, complete genome | 1671 | 0.00E+00 | 85.50% |
| Efet.01.1659311.g1539.t1 | Verminephrobacter eiseniae EF01-2, complete genome | 699 | 0.00E+00 | 85.50% |
| Efet.01.412509.g279.t1 | Verminephrobacter eiseniae EF01-2, complete genome | 234 | 3.80E-109 | 85.40% |
| Efet.01.429497.g727.t1 | Verminephrobacter eiseniae EF01-2, complete genome | 965 | 0.00E+00 | 85.40% |
| Efet.01.440935.g997.t1 | Verminephrobacter eiseniae EF01-2, complete genome | 777 | 0.00E+00 | 85.40% |
| Efet.01.533383.g128.t1 | Verminephrobacter eiseniae EF01-2, complete genome | 589 | 0.00E+00 | 85.40% |
| Efet.01.537684.g223.t1 | Verminephrobacter eiseniae EF01-2, complete genome | 232 | 5.50E-112 | 85.40% |
| Efet.01.550271.g509.t1 | Verminephrobacter eiseniae EF01-2, complete genome | 921 | 0.00E+00 | 85.40% |
| Efet.01.585033.g829.t1 | Verminephrobacter eiseniae EF01-2, complete genome | 903 | 0.00E+00 | 85.40% |
| Efet.01.597702.g1234.t1 | Verminephrobacter eiseniae EF01-2, complete genome | 285 | 6.50E-85 | 85.40% |
| Efet.01.617238.g757.t1 | Verminephrobacter eiseniae EF01-2, complete genome | 483 | 0.00E+00 | 85.40% |
| Efet.01.638894.g476.t1 | Verminephrobacter eiseniae EF01-2, complete genome | 645 | 0.00E+00 | 85.40% |
| Efet.01.644812.g901.t1 | Verminephrobacter eiseniae EF01-2, complete genome | 441 | 0.00E+00 | 85.40% |
| Efet.01.651088.g109.t1 | Verminephrobacter eiseniae EF01-2, complete genome | 795 | 0.00E+00 | 85.40% |
| Efet.01.653992.g508.t1 | Verminephrobacter eiseniae EF01-2, complete genome | 992 | 0.00E+00 | 85.40% |
| Efet.01.654851.g703.t1 | Verminephrobacter eiseniae EF01-2, complete genome | 794 | 0.00E+00 | 85.40% |
| Efet.01.657457.g1293.t1 | Verminephrobacter eiseniae EF01-2, complete genome | 448 | 0.00E+00 | 85.40% |
| Efet.01.657496.g1310.t1 | Verminephrobacter eiseniae EF01-2, complete genome | 660 | 0.00E+00 | 85.40% |
| Efet.01.658081.g1618.t1 | Verminephrobacter eiseniae EF01-2, complete genome | 969 | 0.00E+00 | 85.40% |
| Efet.01.1655217.g743.t1 | Verminephrobacter eiseniae EF01-2, complete genome | 381 | 0.00E+00 | 85.40% |
| Efet.01.1659432.g1701.t1 | Verminephrobacter eiseniae EF01-2, complete genome | 1959 | 0.00E+00 | 85.40% |
| Efet.01.1659433.g1703.t1 | Verminephrobacter eiseniae EF01-2, complete genome | 861 | 0.00E+00 | 85.40% |
| Efet.01.1659524.g2012.t1 | Verminephrobacter eiseniae EF01-2, complete genome | 1913 | 0.00E+00 | 85.40% |
| Efet.01.3425.g297.t1 | Streptomyces sp. 452 chromosome, complete genome | 50 | 1.70E-06 | 85.33% |
| Efet.01.658279.g1783.t1 | Verminephrobacter eiseniae EF01-2, complete genome | 531 | 0.00E+00 | 85.33% |
| Efet.01.32422.g525.t1 | Verminephrobacter eiseniae EF01-2, complete genome | 414 | 0.00E+00 | 85.30% |
| Efet.01.61804.g733.t1 | Verminephrobacter eiseniae EF01-2, complete genome | 717 | 0.00E+00 | 85.30% |
| Efet.01.220990.g850.t1 | Verminephrobacter eiseniae EF01-2, complete genome | 258 | 4.00E-125 | 85.30% |
| Efet.01.270790.g772.t1 | Verminephrobacter eiseniae EF01-2, complete genome | 1131 | 0.00E+00 | 85.30% |
| Efet.01.445097.g1117.t1 | Verminephrobacter eiseniae EF01-2, complete genome | 478 | 0.00E+00 | 85.30% |
| Efet.01.533383.g125.t1 | Verminephrobacter eiseniae EF01-2, complete genome | 471 | 0.00E+00 | 85.30% |
| Efet.01.569918.g322.t1 | Verminephrobacter eiseniae EF01-2, complete genome | 558 | 0.00E+00 | 85.30% |
| Efet.01.619389.g860.t1 | Verminephrobacter eiseniae EF01-2, complete genome | 1233 | 0.00E+00 | 85.30% |
| Efet.01.633502.g196.t1 | Verminephrobacter eiseniae EF01-2, complete genome | 357 | 0.00E+00 | 85.30% |
| Efet.01.638678.g449.t1 | Verminephrobacter eiseniae EF01-2, complete genome | 2358 | 0.00E+00 | 85.30% |
| Efet.01.645133.g934.t1 | Verminephrobacter eiseniae EF01-2, complete genome | 702 | 0.00E+00 | 85.30% |
| Efet.01.654851.g709.t1 | Verminephrobacter eiseniae EF01-2, complete genome | 573 | 0.00E+00 | 85.30% |
| Efet.01.656932.g1145.t1 | Verminephrobacter eiseniae EF01-2, complete genome | 709 | 0.00E+00 | 85.30% |
| Efet.01.657336.g1268.t1 | Verminephrobacter eiseniae EF01-2, complete genome | 1312 | 0.00E+00 | 85.30% |
| Efet.01.657719.g1402.t1 | Verminephrobacter eiseniae EF01-2, complete genome | 873 | 0.00E+00 | 85.30% |
| Efet.01.658150.g1684.t1 | Verminephrobacter eiseniae EF01-2, complete genome | 1545 | 0.00E+00 | 85.30% |
| Efet.01.658383.g1928.t1 | Verminephrobacter eiseniae EF01-2, complete genome | 999 | 0.00E+00 | 85.30% |
| Efet.01.1659389.g1637.t1 | Verminephrobacter eiseniae EF01-2, complete genome | 622 | 0.00E+00 | 85.30% |
| Efet.01.1659455.g1755.t1 | Verminephrobacter eiseniae EF01-2, complete genome | 1188 | 0.00E+00 | 85.30% |
| Efet.01.28403.g230.t1 | Verminephrobacter eiseniae EF01-2, complete genome | 618 | 0.00E+00 | 85.20% |
| Efet.01.41969.g1111.t1 | Alistipes shahii WAL 8301 draft genome | 73 | 1.70E-10 | 85.20% |
| Efet.01.528741.g654.t1 | Verminephrobacter eiseniae EF01-2, complete genome | 1009 | 0.00E+00 | 85.20% |
| Efet.01.561085.g21.t1 | Verminephrobacter eiseniae EF01-2, complete genome | 273 | 6.30E-133 | 85.20% |
| Efet.01.577373.g573.t1 | Verminephrobacter eiseniae EF01-2, complete genome | 333 | 6.20E-166 | 85.20% |
| Efet.01.592294.g1069.t1 | Verminephrobacter eiseniae EF01-2, complete genome | 450 | 0.00E+00 | 85.20% |
| Efet.01.609276.g382.t1 | Verminephrobacter eiseniae EF01-2, complete genome | 549 | 0.00E+00 | 85.20% |
| Efet.01.655750.g857.t1 | Verminephrobacter eiseniae EF01-2, complete genome | 789 | 0.00E+00 | 85.20% |
| Efet.01.656757.g1093.t1 | Verminephrobacter eiseniae EF01-2, complete genome | 570 | 0.00E+00 | 85.20% |
| Efet.01.657766.g1438.t1 | Verminephrobacter eiseniae EF01-2, complete genome | 1149 | 0.00E+00 | 85.20% |
| Efet.01.658408.g2008.t1 | Verminephrobacter eiseniae EF01-2, complete genome | 636 | 0.00E+00 | 85.20% |
| Efet.01.658414.g2039.t1 | Verminephrobacter eiseniae EF01-2, complete genome | 375 | 0.00E+00 | 85.20% |
| Efet.01.658426.g2137.t1 | Verminephrobacter eiseniae EF01-2, complete genome | 1452 | 0.00E+00 | 85.20% |
| Efet.01.1659352.g1589.t1 | Verminephrobacter eiseniae EF01-2, complete genome | 294 | 1.10E-144 | 85.20% |
| Efet.01.1659374.g1619.t1 | Verminephrobacter eiseniae EF01-2, complete genome | 936 | 0.00E+00 | 85.20% |
| Efet.01.1659389.g1636.t1 | Verminephrobacter eiseniae EF01-2, complete genome | 294 | 2.00E-142 | 85.20% |
| Efet.01.1659401.g1660.t1 | Verminephrobacter eiseniae EF01-2, complete genome | 756 | 0.00E+00 | 85.20% |
| Efet.01.1659467.g1784.t1 | Verminephrobacter eiseniae EF01-2, complete genome | 972 | 0.00E+00 | 85.20% |
| Efet.01.119502.g1008.t1 | Verminephrobacter eiseniae EF01-2, complete genome | 559 | 0.00E+00 | 85.10% |
| Efet.01.471393.g561.t1 | Verminephrobacter eiseniae EF01-2, complete genome | 735 | 0.00E+00 | 85.10% |
| Efet.01.540063.g271.t1 | Frondihabitans sp. PAMC28766, complete genome | 163 | 5.20E-49 | 85.10% |
| Efet.01.545291.g368.t1 | Verminephrobacter eiseniae EF01-2, complete genome | 493 | 0.00E+00 | 85.10% |
| Efet.01.581588.g723.t1 | Verminephrobacter eiseniae EF01-2, complete genome | 1172 | 0.00E+00 | 85.10% |
| Efet.01.590200.g997.t1 | Verminephrobacter eiseniae EF01-2, complete genome | 807 | 0.00E+00 | 85.10% |
| Efet.01.611343.g475.t1 | Verminephrobacter eiseniae EF01-2, complete genome | 968 | 0.00E+00 | 85.10% |
| Efet.01.618025.g801.t1 | Verminephrobacter eiseniae EF01-2, complete genome | 2619 | 0.00E+00 | 85.10% |
| Efet.01.651136.g121.t1 | Verminephrobacter eiseniae EF01-2, complete genome | 1416 | 0.00E+00 | 85.10% |
| Efet.01.652587.g317.t1 | Verminephrobacter eiseniae EF01-2, complete genome | 567 | 0.00E+00 | 85.10% |
| Efet.01.657302.g1226.t1 | Verminephrobacter eiseniae EF01-2, complete genome | 462 | 0.00E+00 | 85.10% |
| Efet.01.658116.g1649.t1 | Verminephrobacter eiseniae EF01-2, complete genome | 852 | 0.00E+00 | 85.10% |
| Efet.01.658307.g1829.t1 | Verminephrobacter eiseniae EF01-2, complete genome | 318 | 3.30E-157 | 85.10% |
| Efet.01.658387.g1939.t1 | Verminephrobacter eiseniae EF01-2, complete genome | 2053 | 0.00E+00 | 85.10% |
| Efet.01.658408.g2009.t1 | Verminephrobacter eiseniae EF01-2, complete genome | 1281 | 0.00E+00 | 85.10% |
| Efet.01.1659318.g1550.t1 | Verminephrobacter eiseniae EF01-2, complete genome | 810 | 0.00E+00 | 85.10% |
| Efet.01.1659452.g1747.t1 | Verminephrobacter eiseniae EF01-2, complete genome | 270 | 1.00E-132 | 85.10% |
| Efet.01.12283.g918.t1 | Natranaerobius thermophilus JW/NM-WN-LF, complete genome | 64 | 3.80E-10 | 85.00% |
| Efet.01.32422.g527.t1 | Verminephrobacter eiseniae EF01-2, complete genome | 537 | 0.00E+00 | 85.00% |
| Efet.01.111477.g574.t1 | Listeria monocytogenes isolate LMNC088 complete genome genome assembly, chromosome: 1 | 77 | 2.80E-14 | 85.00% |
| Efet.01.143861.g968.t1 | Verminephrobacter eiseniae EF01-2, complete genome | 627 | 0.00E+00 | 85.00% |
| Efet.01.147445.g1114.t1 | Verminephrobacter eiseniae EF01-2, complete genome | 449 | 0.00E+00 | 85.00% |
| Efet.01.152930.g157.t1 | Zhihengliuella sp. ISTPL4 chromosome | 560 | 0.00E+00 | 85.00% |
| Efet.01.154996.g257.t1 | Brachybacterium sp. VM2412 chromosome, complete genome | 56 | 4.40E-07 | 85.00% |
| Efet.01.169387.g949.t1 | Ruminococcus sp. SR1/5 draft genome | 61 | 8.20E-08 | 85.00% |
| Efet.01.234637.g1370.t1 | Cyanobium gracile PCC 6307, complete genome | 55 | 3.20E-06 | 85.00% |
| Efet.01.246753.g1770.t1 | Wenzhouxiangella marina strain KCTC 42284, complete genome | 54 | 2.40E-06 | 85.00% |
| Efet.01.275134.g933.t1 | Verminephrobacter eiseniae EF01-2, complete genome | 841 | 0.00E+00 | 85.00% |
| Efet.01.417053.g408.t1 | Verminephrobacter eiseniae EF01-2, complete genome | 985 | 0.00E+00 | 85.00% |
| Efet.01.427307.g653.t1 | Verminephrobacter eiseniae EF01-2, complete genome | 781 | 0.00E+00 | 85.00% |
| Efet.01.492504.g1008.t1 | Siansivirga zeaxanthinifaciens CC-SAMT-1, complete genome | 54 | 8.20E-06 | 85.00% |
| Efet.01.545302.g374.t1 | Verminephrobacter eiseniae EF01-2, complete genome | 757 | 0.00E+00 | 85.00% |
| Efet.01.564055.g129.t1 | Microbacterium aurum strain KACC 15219, complete genome | 134 | 2.90E-34 | 85.00% |
| Efet.01.577373.g571.t1 | Verminephrobacter eiseniae EF01-2, complete genome | 420 | 0.00E+00 | 85.00% |
| Efet.01.612007.g511.t1 | Verminephrobacter eiseniae EF01-2, complete genome | 661 | 0.00E+00 | 85.00% |
| Efet.01.624347.g1070.t1 | Verminephrobacter eiseniae EF01-2, complete genome | 762 | 0.00E+00 | 85.00% |
| Efet.01.631741.g132.t1 | Verminephrobacter eiseniae EF01-2, complete genome | 789 | 0.00E+00 | 85.00% |
| Efet.01.638426.g440.t1 | Streptococcus agalactiae strain C001, complete genome | 56 | 6.20E-07 | 85.00% |
| Efet.01.639343.g500.t1 | Azospirillum sp. CFH 70021 chromosome 3, complete sequence | 57 | 3.40E-10 | 85.00% |
| Efet.01.643464.g794.t1 | Verminephrobacter eiseniae EF01-2, complete genome | 597 | 0.00E+00 | 85.00% |
| Efet.01.643674.g823.t1 | Verminephrobacter eiseniae EF01-2, complete genome | 474 | 0.00E+00 | 85.00% |
| Efet.01.646099.g1033.t1 | Spirochaeta africana DSM 8902, complete genome | 56 | 5.20E-07 | 85.00% |
| Efet.01.646487.g1086.t1 | Verminephrobacter eiseniae EF01-2, complete genome | 498 | 0.00E+00 | 85.00% |
| Efet.01.649588.g1344.t1 | Verminephrobacter eiseniae EF01-2, complete genome | 1348 | 0.00E+00 | 85.00% |
| Efet.01.652863.g357.t1 | Achromobacter xylosoxidans strain FDAARGOS_162 chromosome, complete genome | 1619 | 0.00E+00 | 85.00% |
| Efet.01.653800.g476.t1 | Verminephrobacter eiseniae EF01-2, complete genome | 324 | 3.00E-159 | 85.00% |
| Efet.01.654310.g564.t1 | Verminephrobacter eiseniae EF01-2, complete genome | 1281 | 0.00E+00 | 85.00% |
| Efet.01.654851.g702.t1 | Verminephrobacter eiseniae EF01-2, complete genome | 720 | 0.00E+00 | 85.00% |
| Efet.01.655756.g860.t1 | Verminephrobacter eiseniae EF01-2, complete genome | 366 | 0.00E+00 | 85.00% |
| Efet.01.657496.g1313.t1 | Verminephrobacter eiseniae EF01-2, complete genome | 327 | 3.40E-162 | 85.00% |
| Efet.01.657719.g1401.t1 | Verminephrobacter eiseniae EF01-2, complete genome | 756 | 0.00E+00 | 85.00% |
| Efet.01.1659019.g1364.t1 | Verminephrobacter eiseniae EF01-2, complete genome | 219 | 8.30E-104 | 85.00% |
| Efet.01.1659518.g1970.t1 | Verminephrobacter eiseniae EF01-2, complete genome | 372 | 0.00E+00 | 85.00% |
| Efet.01.89113.g840.t1 | Chlorobium luteolum DSM 273, complete genome | 46 | 1.80E-08 | 84.90% |
| Efet.01.557665.g696.t1 | Variovorax sp. HW608 genome assembly, chromosome: I | 678 | 0.00E+00 | 84.90% |
| Efet.01.566222.g208.t1 | Verminephrobacter eiseniae EF01-2, complete genome | 1551 | 0.00E+00 | 84.90% |
| Efet.01.594275.g1112.t1 | Verminephrobacter eiseniae EF01-2, complete genome | 717 | 0.00E+00 | 84.90% |
| Efet.01.604212.g156.t1 | Verminephrobacter eiseniae EF01-2, complete genome | 630 | 0.00E+00 | 84.90% |
| Efet.01.616615.g732.t1 | Verminephrobacter eiseniae EF01-2, complete genome | 1486 | 0.00E+00 | 84.90% |
| Efet.01.623992.g1050.t1 | Verminephrobacter eiseniae EF01-2, complete genome | 1737 | 0.00E+00 | 84.90% |
| Efet.01.636598.g340.t1 | Verminephrobacter eiseniae EF01-2, complete genome | 642 | 0.00E+00 | 84.90% |
| Efet.01.640917.g602.t1 | Verminephrobacter eiseniae EF01-2, complete genome | 951 | 0.00E+00 | 84.90% |
| Efet.01.645971.g1017.t1 | Verminephrobacter eiseniae EF01-2, complete genome | 1020 | 0.00E+00 | 84.90% |
| Efet.01.651148.g133.t1 | Verminephrobacter eiseniae EF01-2, complete genome | 1230 | 0.00E+00 | 84.90% |
| Efet.01.652898.g375.t1 | Verminephrobacter eiseniae EF01-2, complete genome | 1395 | 0.00E+00 | 84.90% |
| Efet.01.654597.g640.t1 | Verminephrobacter eiseniae EF01-2, complete genome | 450 | 0.00E+00 | 84.90% |
| Efet.01.655224.g775.t1 | Verminephrobacter eiseniae EF01-2, complete genome | 882 | 0.00E+00 | 84.90% |
| Efet.01.657123.g1174.t1 | Verminephrobacter eiseniae EF01-2, complete genome | 924 | 0.00E+00 | 84.90% |
| Efet.01.657308.g1232.t1 | Verminephrobacter eiseniae EF01-2, complete genome | 474 | 0.00E+00 | 84.90% |
| Efet.01.657336.g1267.t1 | Verminephrobacter eiseniae EF01-2, complete genome | 549 | 0.00E+00 | 84.90% |
| Efet.01.657868.g1509.t1 | Verminephrobacter eiseniae EF01-2, complete genome | 1083 | 0.00E+00 | 84.90% |
| Efet.01.658417.g2080.t1 | Verminephrobacter eiseniae EF01-2, complete genome | 831 | 0.00E+00 | 84.90% |
| Efet.01.658419.g2096.t1 | Verminephrobacter eiseniae EF01-2, complete genome | 590 | 0.00E+00 | 84.90% |
| Efet.01.1659338.g1565.t1 | Verminephrobacter eiseniae EF01-2, complete genome | 543 | 0.00E+00 | 84.90% |
| Efet.01.1659448.g1737.t1 | Verminephrobacter eiseniae EF01-2, complete genome | 1038 | 0.00E+00 | 84.90% |
| Efet.01.1659491.g1855.t1 | Verminephrobacter eiseniae EF01-2, complete genome | 213 | 3.90E-103 | 84.90% |
| Efet.01.1659512.g1936.t1 | Verminephrobacter eiseniae EF01-2, complete genome | 663 | 0.00E+00 | 84.90% |
| Efet.01.5650.g440.t1 | Pseudomonas sabulinigri strain JCM 14963 genome assembly, chromosome: I | 56 | 4.20E-07 | 84.80% |
| Efet.01.275134.g931.t1 | Verminephrobacter eiseniae EF01-2, complete genome | 1020 | 0.00E+00 | 84.80% |
| Efet.01.392427.g1063.t1 | Verminephrobacter eiseniae EF01-2, complete genome | 222 | 4.20E-100 | 84.80% |
| Efet.01.512410.g292.t1 | Verminephrobacter eiseniae EF01-2, complete genome | 462 | 0.00E+00 | 84.80% |
| Efet.01.560830.g17.t1 | Verminephrobacter eiseniae EF01-2, complete genome | 501 | 0.00E+00 | 84.80% |
| Efet.01.569918.g323.t1 | Verminephrobacter eiseniae EF01-2, complete genome | 867 | 0.00E+00 | 84.80% |
| Efet.01.581684.g728.t1 | Verminephrobacter eiseniae EF01-2, complete genome | 683 | 0.00E+00 | 84.80% |
| Efet.01.586228.g869.t1 | Verminephrobacter eiseniae EF01-2, complete genome | 1259 | 0.00E+00 | 84.80% |
| Efet.01.627540.g1212.t1 | Microbacterium sp. BH-3-3-3, complete genome | 310 | 1.20E-97 | 84.80% |
| Efet.01.629376.g1291.t1 | Verminephrobacter eiseniae EF01-2, complete genome | 1104 | 0.00E+00 | 84.80% |
| Efet.01.639260.g497.t1 | Verminephrobacter eiseniae EF01-2, complete genome | 336 | 4.40E-167 | 84.80% |
| Efet.01.649588.g1341.t1 | Verminephrobacter eiseniae EF01-2, complete genome | 405 | 0.00E+00 | 84.80% |
| Efet.01.651148.g137.t1 | Verminephrobacter eiseniae EF01-2, complete genome | 954 | 0.00E+00 | 84.80% |
| Efet.01.654656.g648.t1 | Verminephrobacter eiseniae EF01-2, complete genome | 720 | 0.00E+00 | 84.80% |
| Efet.01.654946.g719.t1 | Verminephrobacter eiseniae EF01-2, complete genome | 423 | 0.00E+00 | 84.80% |
| Efet.01.655821.g879.t1 | Verminephrobacter eiseniae EF01-2, complete genome | 528 | 0.00E+00 | 84.80% |
| Efet.01.655964.g906.t1 | Verminephrobacter eiseniae EF01-2, complete genome | 1083 | 0.00E+00 | 84.80% |
| Efet.01.656795.g1104.t1 | Verminephrobacter eiseniae EF01-2, complete genome | 509 | 0.00E+00 | 84.80% |
| Efet.01.657315.g1239.t1 | Verminephrobacter eiseniae EF01-2, complete genome | 849 | 0.00E+00 | 84.80% |
| Efet.01.658150.g1686.t1 | Verminephrobacter eiseniae EF01-2, complete genome | 903 | 0.00E+00 | 84.80% |
| Efet.01.658236.g1738.t1 | Verminephrobacter eiseniae EF01-2, complete genome | 579 | 0.00E+00 | 84.80% |
| Efet.01.658307.g1828.t1 | Verminephrobacter eiseniae EF01-2, complete genome | 1287 | 0.00E+00 | 84.80% |
| Efet.01.1656219.g839.t1 | Verminephrobacter eiseniae EF01-2, complete genome | 387 | 0.00E+00 | 84.80% |
| Efet.01.1659237.g1480.t1 | Verminephrobacter eiseniae EF01-2, complete genome | 483 | 0.00E+00 | 84.80% |
| Efet.01.1659443.g1728.t1 | Verminephrobacter eiseniae EF01-2, complete genome | 450 | 0.00E+00 | 84.80% |
| Efet.01.20103.g1488.t1 | Verminephrobacter eiseniae EF01-2, complete genome | 399 | 0.00E+00 | 84.75% |
| Efet.01.47711.g1453.t1 | Verminephrobacter eiseniae EF01-2, complete genome | 130 | 5.80E-57 | 84.75% |
| Efet.01.609593.g410.t1 | Alteromonas mediterranea strain U10, complete genome | 56 | 4.30E-08 | 84.75% |
| Efet.01.658424.g2117.t1 | Verminephrobacter eiseniae EF01-2, complete genome | 978 | 0.00E+00 | 84.75% |
| Efet.01.533149.g111.t1 | Verminephrobacter eiseniae EF01-2, complete genome | 240 | 1.80E-115 | 84.70% |
| Efet.01.546787.g407.t1 | Verminephrobacter eiseniae EF01-2, complete genome | 600 | 0.00E+00 | 84.70% |
| Efet.01.598882.g1270.t1 | Verminephrobacter eiseniae EF01-2, complete genome | 1237 | 0.00E+00 | 84.70% |
| Efet.01.638026.g407.t1 | Verminephrobacter eiseniae EF01-2, complete genome | 837 | 0.00E+00 | 84.70% |
| Efet.01.649588.g1338.t1 | Verminephrobacter eiseniae EF01-2, complete genome | 311 | 5.80E-151 | 84.70% |
| Efet.01.652283.g271.t1 | Verminephrobacter eiseniae EF01-2, complete genome | 763 | 0.00E+00 | 84.70% |
| Efet.01.652898.g372.t1 | Verminephrobacter eiseniae EF01-2, complete genome | 478 | 0.00E+00 | 84.70% |
| Efet.01.653479.g435.t1 | Verminephrobacter eiseniae EF01-2, complete genome | 510 | 0.00E+00 | 84.70% |
| Efet.01.656919.g1141.t1 | Verminephrobacter eiseniae EF01-2, complete genome | 690 | 0.00E+00 | 84.70% |
| Efet.01.658211.g1717.t1 | Verminephrobacter eiseniae EF01-2, complete genome | 1401 | 0.00E+00 | 84.70% |
| Efet.01.658218.g1721.t1 | Verminephrobacter eiseniae EF01-2, complete genome | 1834 | 0.00E+00 | 84.70% |
| Efet.01.658245.g1747.t1 | Verminephrobacter eiseniae EF01-2, complete genome | 1257 | 0.00E+00 | 84.70% |
| Efet.01.658334.g1861.t1 | Verminephrobacter eiseniae EF01-2, complete genome | 471 | 0.00E+00 | 84.70% |
| Efet.01.658384.g1930.t1 | Verminephrobacter eiseniae EF01-2, complete genome | 1079 | 0.00E+00 | 84.70% |
| Efet.01.658387.g1940.t1 | Verminephrobacter eiseniae EF01-2, complete genome | 771 | 0.00E+00 | 84.70% |
| Efet.01.658408.g2010.t1 | Verminephrobacter eiseniae EF01-2, complete genome | 654 | 0.00E+00 | 84.70% |
| Efet.01.658419.g2092.t1 | Verminephrobacter eiseniae EF01-2, complete genome | 2337 | 0.00E+00 | 84.70% |
| Efet.01.1659429.g1695.t1 | Verminephrobacter eiseniae EF01-2, complete genome | 1203 | 0.00E+00 | 84.70% |
| Efet.01.1659477.g1806.t1 | Verminephrobacter eiseniae EF01-2, complete genome | 1404 | 0.00E+00 | 84.70% |
| Efet.01.405067.g120.t1 | Agromyces sp. 30A chromosome, complete genome | 61 | 8.40E-10 | 84.67% |
| Efet.01.577062.g559.t1 | Verminephrobacter eiseniae EF01-2, complete genome | 765 | 0.00E+00 | 84.67% |
| Efet.01.185951.g1684.t1 | Verminephrobacter eiseniae EF01-2, complete genome | 774 | 0.00E+00 | 84.60% |
| Efet.01.229456.g1177.t1 | Verminephrobacter eiseniae EF01-2, complete genome | 846 | 0.00E+00 | 84.60% |
| Efet.01.412509.g278.t1 | Verminephrobacter eiseniae EF01-2, complete genome | 1053 | 0.00E+00 | 84.60% |
| Efet.01.427593.g670.t1 | Verminephrobacter eiseniae EF01-2, complete genome | 648 | 0.00E+00 | 84.60% |
| Efet.01.548599.g458.t1 | Verminephrobacter eiseniae EF01-2, complete genome | 3102 | 0.00E+00 | 84.60% |
| Efet.01.598882.g1269.t1 | Verminephrobacter eiseniae EF01-2, complete genome | 534 | 0.00E+00 | 84.60% |
| Efet.01.605357.g203.t1 | Verminephrobacter eiseniae EF01-2, complete genome | 564 | 0.00E+00 | 84.60% |
| Efet.01.614177.g631.t1 | Verminephrobacter eiseniae EF01-2, complete genome | 452 | 0.00E+00 | 84.60% |
| Efet.01.626245.g1150.t1 | Verminephrobacter eiseniae EF01-2, complete genome | 741 | 0.00E+00 | 84.60% |
| Efet.01.630546.g39.t1 | Verminephrobacter eiseniae EF01-2, complete genome | 1173 | 0.00E+00 | 84.60% |
| Efet.01.630860.g82.t1 | Verminephrobacter eiseniae EF01-2, complete genome | 1116 | 0.00E+00 | 84.60% |
| Efet.01.638023.g397.t1 | Verminephrobacter eiseniae EF01-2, complete genome | 1210 | 0.00E+00 | 84.60% |
| Efet.01.646179.g1051.t1 | Verminephrobacter eiseniae EF01-2, complete genome | 924 | 0.00E+00 | 84.60% |
| Efet.01.652309.g278.t1 | Verminephrobacter eiseniae EF01-2, complete genome | 1002 | 0.00E+00 | 84.60% |
| Efet.01.653719.g470.t1 | Verminephrobacter eiseniae EF01-2, complete genome | 502 | 0.00E+00 | 84.60% |
| Efet.01.655128.g757.t1 | Verminephrobacter eiseniae EF01-2, complete genome | 588 | 0.00E+00 | 84.60% |
| Efet.01.656283.g983.t1 | Verminephrobacter eiseniae EF01-2, complete genome | 324 | 8.10E-161 | 84.60% |
| Efet.01.658429.g2157.t1 | Verminephrobacter eiseniae EF01-2, complete genome | 1614 | 0.00E+00 | 84.60% |
| Efet.01.1659513.g1938.t1 | Verminephrobacter eiseniae EF01-2, complete genome | 402 | 0.00E+00 | 84.60% |
| Efet.01.412509.g281.t1 | Verminephrobacter eiseniae EF01-2, complete genome | 1896 | 0.00E+00 | 84.50% |
| Efet.01.550127.g499.t1 | Verminephrobacter eiseniae EF01-2, complete genome | 738 | 0.00E+00 | 84.50% |
| Efet.01.574744.g470.t1 | Verminephrobacter eiseniae EF01-2, complete genome | 369 | 0.00E+00 | 84.50% |
| Efet.01.591301.g1032.t1 | Verminephrobacter eiseniae EF01-2, complete genome | 696 | 0.00E+00 | 84.50% |
| Efet.01.605365.g210.t1 | Verminephrobacter eiseniae EF01-2, complete genome | 745 | 0.00E+00 | 84.50% |
| Efet.01.608047.g337.t1 | Thauera aromatica K172 chromosome, complete genome | 306 | 2.50E-130 | 84.50% |
| Efet.01.613416.g605.t1 | Verminephrobacter eiseniae EF01-2, complete genome | 1035 | 0.00E+00 | 84.50% |
| Efet.01.622654.g999.t1 | Verminephrobacter eiseniae EF01-2, complete genome | 759 | 0.00E+00 | 84.50% |
| Efet.01.629329.g1286.t1 | Verminephrobacter eiseniae EF01-2, complete genome | 674 | 0.00E+00 | 84.50% |
| Efet.01.639369.g507.t1 | Verminephrobacter eiseniae EF01-2, complete genome | 267 | 3.00E-130 | 84.50% |
| Efet.01.640475.g575.t1 | Verminephrobacter eiseniae EF01-2, complete genome | 1092 | 0.00E+00 | 84.50% |
| Efet.01.646274.g1062.t1 | Verminephrobacter eiseniae EF01-2, complete genome | 1317 | 0.00E+00 | 84.50% |
| Efet.01.651940.g242.t1 | Verminephrobacter eiseniae EF01-2, complete genome | 870 | 0.00E+00 | 84.50% |
| Efet.01.651940.g243.t1 | Verminephrobacter eiseniae EF01-2, complete genome | 573 | 0.00E+00 | 84.50% |
| Efet.01.654969.g730.t1 | Verminephrobacter eiseniae EF01-2, complete genome | 1101 | 0.00E+00 | 84.50% |
| Efet.01.655999.g926.t1 | Verminephrobacter eiseniae EF01-2, complete genome | 549 | 0.00E+00 | 84.50% |
| Efet.01.656813.g1112.t1 | Verminephrobacter eiseniae EF01-2, complete genome | 1446 | 0.00E+00 | 84.50% |
| Efet.01.656822.g1123.t1 | Verminephrobacter eiseniae EF01-2, complete genome | 420 | 0.00E+00 | 84.50% |
| Efet.01.657766.g1439.t1 | Verminephrobacter eiseniae EF01-2, complete genome | 648 | 0.00E+00 | 84.50% |
| Efet.01.1657920.g1061.t1 | Agromyces sp. 30A chromosome, complete genome | 395 | 4.70E-129 | 84.50% |
| Efet.01.1659228.g1473.t1 | Verminephrobacter eiseniae EF01-2, complete genome | 1053 | 0.00E+00 | 84.50% |
| Efet.01.1659345.g1575.t1 | Verminephrobacter eiseniae EF01-2, complete genome | 1131 | 0.00E+00 | 84.50% |
| Efet.01.1659413.g1674.t1 | Verminephrobacter eiseniae EF01-2, complete genome | 1040 | 0.00E+00 | 84.50% |
| Efet.01.1659494.g1865.t1 | Verminephrobacter eiseniae EF01-2, complete genome | 405 | 0.00E+00 | 84.50% |
| Efet.01.213491.g572.t1 | Bdellovibrio bacteriovorus complete genome, strain HD100; segment 2/11 | 152 | 3.00E-32 | 84.40% |
| Efet.01.427593.g665.t1 | Verminephrobacter eiseniae EF01-2, complete genome | 981 | 0.00E+00 | 84.40% |
| Efet.01.587924.g927.t1 | Verminephrobacter eiseniae EF01-2, complete genome | 363 | 0.00E+00 | 84.40% |
| Efet.01.590200.g1003.t1 | Verminephrobacter eiseniae EF01-2, complete genome | 978 | 0.00E+00 | 84.40% |
| Efet.01.623992.g1051.t1 | Verminephrobacter eiseniae EF01-2, complete genome | 987 | 0.00E+00 | 84.40% |
| Efet.01.642550.g705.t1 | Verminephrobacter eiseniae EF01-2, complete genome | 549 | 0.00E+00 | 84.40% |
| Efet.01.649471.g1314.t1 | Verminephrobacter eiseniae EF01-2, complete genome | 336 | 5.40E-170 | 84.40% |
| Efet.01.651429.g169.t1 | Variovorax sp. PMC12 chromosome 1, complete sequence | 299 | 8.30E-97 | 84.40% |
| Efet.01.652977.g386.t1 | Verminephrobacter eiseniae EF01-2, complete genome | 1707 | 0.00E+00 | 84.40% |
| Efet.01.657496.g1309.t1 | Verminephrobacter eiseniae EF01-2, complete genome | 609 | 0.00E+00 | 84.40% |
| Efet.01.1659236.g1479.t1 | Verminephrobacter eiseniae EF01-2, complete genome | 1326 | 0.00E+00 | 84.40% |
| Efet.01.1659478.g1809.t1 | Verminephrobacter eiseniae EF01-2, complete genome | 465 | 0.00E+00 | 84.40% |
| Efet.01.1659491.g1857.t1 | Verminephrobacter eiseniae EF01-2, complete genome | 561 | 0.00E+00 | 84.40% |
| Efet.01.1659524.g2011.t1 | Verminephrobacter eiseniae EF01-2, complete genome | 1209 | 0.00E+00 | 84.40% |
| Efet.01.1658077.g1095.t1 | Variovorax boronicumulans strain J1 chromosome, complete genome | 530 | 0.00E+00 | 84.38% |
| Efet.01.597702.g1232.t1 | Verminephrobacter eiseniae EF01-2, complete genome | 867 | 0.00E+00 | 84.33% |
| Efet.01.28403.g231.t1 | Verminephrobacter eiseniae EF01-2, complete genome | 240 | 8.30E-117 | 84.30% |
| Efet.01.353298.g98.t1 | Verminephrobacter eiseniae EF01-2, complete genome | 450 | 0.00E+00 | 84.30% |
| Efet.01.639260.g498.t1 | Verminephrobacter eiseniae EF01-2, complete genome | 1349 | 0.00E+00 | 84.30% |
| Efet.01.640112.g558.t1 | Agromyces flavus strain CPCC 202695 genome assembly, chromosome: I | 382 | 4.10E-122 | 84.30% |
| Efet.01.643394.g779.t1 | Verminephrobacter eiseniae EF01-2, complete genome | 786 | 0.00E+00 | 84.30% |
| Efet.01.646642.g1114.t1 | Verminephrobacter eiseniae EF01-2, complete genome | 939 | 0.00E+00 | 84.30% |
| Efet.01.651441.g172.t1 | Verminephrobacter eiseniae EF01-2, complete genome | 1206 | 0.00E+00 | 84.30% |
| Efet.01.651810.g218.t1 | Verminephrobacter eiseniae EF01-2, complete genome | 1122 | 0.00E+00 | 84.30% |
| Efet.01.656757.g1094.t1 | Verminephrobacter eiseniae EF01-2, complete genome | 609 | 0.00E+00 | 84.30% |
| Efet.01.656813.g1111.t1 | Verminephrobacter eiseniae EF01-2, complete genome | 1554 | 0.00E+00 | 84.30% |
| Efet.01.657626.g1354.t1 | Verminephrobacter eiseniae EF01-2, complete genome | 1458 | 0.00E+00 | 84.30% |
| Efet.01.657719.g1390.t1 | Verminephrobacter eiseniae EF01-2, complete genome | 1077 | 0.00E+00 | 84.30% |
| Efet.01.658275.g1779.t1 | Xanthomonas citri pv. anacardii CFBP 2913 chromosome | 969 | 0.00E+00 | 84.30% |
| Efet.01.658411.g2028.t1 | Verminephrobacter eiseniae EF01-2, complete genome | 714 | 0.00E+00 | 84.30% |
| Efet.01.1658007.g1079.t1 | Verminephrobacter eiseniae EF01-2, complete genome | 831 | 0.00E+00 | 84.30% |
| Efet.01.1659376.g1622.t1 | Verminephrobacter eiseniae EF01-2, complete genome | 351 | 2.80E-172 | 84.30% |
| Efet.01.1659418.g1681.t1 | Verminephrobacter eiseniae EF01-2, complete genome | 1347 | 0.00E+00 | 84.30% |
| Efet.01.1659433.g1702.t1 | Verminephrobacter eiseniae EF01-2, complete genome | 408 | 0.00E+00 | 84.30% |
| Efet.01.1659497.g1881.t1 | Verminephrobacter eiseniae EF01-2, complete genome | 288 | 1.60E-142 | 84.30% |
| Efet.01.1659523.g2004.t1 | Verminephrobacter eiseniae EF01-2, complete genome | 1318 | 0.00E+00 | 84.30% |
| Efet.01.650357.g44.t1 | Pseudomonas aeruginosa strain Pa1207, complete genome | 712 | 0.00E+00 | 84.25% |
| Efet.01.173363.g1111.t1 | Verminephrobacter eiseniae EF01-2, complete genome | 958 | 0.00E+00 | 84.20% |
| Efet.01.226191.g1047.t1 | Martelella endophytica strain YC6887, complete genome | 65 | 2.50E-09 | 84.20% |
| Efet.01.282031.g1159.t1 | Verminephrobacter eiseniae EF01-2, complete genome | 954 | 0.00E+00 | 84.20% |
| Efet.01.427593.g664.t1 | Verminephrobacter eiseniae EF01-2, complete genome | 178 | 4.70E-80 | 84.20% |
| Efet.01.454467.g106.t1 | Verminephrobacter eiseniae EF01-2, complete genome | 873 | 0.00E+00 | 84.20% |
| Efet.01.524250.g565.t1 | Verminephrobacter eiseniae EF01-2, complete genome | 489 | 0.00E+00 | 84.20% |
| Efet.01.556379.g670.t1 | Verminephrobacter eiseniae EF01-2, complete genome | 1051 | 0.00E+00 | 84.20% |
| Efet.01.577373.g581.t1 | Verminephrobacter eiseniae EF01-2, complete genome | 1263 | 0.00E+00 | 84.20% |
| Efet.01.594275.g1114.t1 | Verminephrobacter eiseniae EF01-2, complete genome | 717 | 0.00E+00 | 84.20% |
| Efet.01.610801.g452.t1 | Verminephrobacter eiseniae EF01-2, complete genome | 1116 | 0.00E+00 | 84.20% |
| Efet.01.629329.g1283.t1 | Verminephrobacter eiseniae EF01-2, complete genome | 1061 | 0.00E+00 | 84.20% |
| Efet.01.630860.g78.t1 | Verminephrobacter eiseniae EF01-2, complete genome | 1118 | 0.00E+00 | 84.20% |
| Efet.01.646149.g1042.t1 | Verminephrobacter eiseniae EF01-2, complete genome | 1095 | 0.00E+00 | 84.20% |
| Efet.01.646487.g1091.t1 | Verminephrobacter eiseniae EF01-2, complete genome | 633 | 0.00E+00 | 84.20% |
| Efet.01.646963.g1141.t1 | Verminephrobacter eiseniae EF01-2, complete genome | 171 | 1.30E-80 | 84.20% |
| Efet.01.656283.g982.t1 | Verminephrobacter eiseniae EF01-2, complete genome | 630 | 0.00E+00 | 84.20% |
| Efet.01.657315.g1245.t1 | Verminephrobacter eiseniae EF01-2, complete genome | 594 | 0.00E+00 | 84.20% |
| Efet.01.657336.g1264.t1 | Verminephrobacter eiseniae EF01-2, complete genome | 1512 | 0.00E+00 | 84.20% |
| Efet.01.657624.g1342.t1 | Verminephrobacter eiseniae EF01-2, complete genome | 262 | 1.60E-128 | 84.20% |
| Efet.01.657700.g1372.t1 | Variovorax paradoxus S110 chromosome 1, complete sequence | 1161 | 0.00E+00 | 84.20% |
| Efet.01.657766.g1447.t1 | Verminephrobacter eiseniae EF01-2, complete genome | 2285 | 0.00E+00 | 84.20% |
| Efet.01.1657992.g1076.t1 | Verminephrobacter eiseniae EF01-2, complete genome | 444 | 0.00E+00 | 84.20% |
| Efet.01.1659277.g1506.t1 | Verminephrobacter eiseniae EF01-2, complete genome | 363 | 3.50E-180 | 84.20% |
| Efet.01.1659388.g1635.t1 | Verminephrobacter eiseniae EF01-2, complete genome | 939 | 0.00E+00 | 84.20% |
| Efet.01.1659414.g1676.t1 | Verminephrobacter eiseniae EF01-2, complete genome | 954 | 0.00E+00 | 84.20% |
| Efet.01.1659507.g1915.t1 | Verminephrobacter eiseniae EF01-2, complete genome | 2088 | 0.00E+00 | 84.20% |
| Efet.01.25255.g30.t1 | Verminephrobacter eiseniae EF01-2, complete genome | 1536 | 0.00E+00 | 84.10% |
| Efet.01.275134.g932.t1 | Verminephrobacter eiseniae EF01-2, complete genome | 372 | 0.00E+00 | 84.10% |
| Efet.01.300799.g26.t1 | Verminephrobacter eiseniae EF01-2, complete genome | 1065 | 0.00E+00 | 84.10% |
| Efet.01.319179.g615.t1 | Agromyces aureus strain AR33, complete genome | 1291 | 0.00E+00 | 84.10% |
| Efet.01.350955.g31.t1 | Thauera aromatica K172 chromosome, complete genome | 306 | 2.00E-86 | 84.10% |
| Efet.01.363215.g354.t1 | Acidihalobacter prosperus strain F5, complete genome | 270 | 3.00E-97 | 84.10% |
| Efet.01.365501.g421.t1 | Verminephrobacter eiseniae EF01-2, complete genome | 969 | 0.00E+00 | 84.10% |
| Efet.01.378778.g768.t1 | Verminephrobacter eiseniae EF01-2, complete genome | 420 | 0.00E+00 | 84.10% |
| Efet.01.412509.g277.t1 | Verminephrobacter eiseniae EF01-2, complete genome | 909 | 0.00E+00 | 84.10% |
| Efet.01.555240.g637.t1 | Agromyces flavus strain CPCC 202695 genome assembly, chromosome: I | 974 | 0.00E+00 | 84.10% |
| Efet.01.595633.g1166.t1 | Verminephrobacter eiseniae EF01-2, complete genome | 714 | 0.00E+00 | 84.10% |
| Efet.01.600711.g30.t1 | Verminephrobacter eiseniae EF01-2, complete genome | 1040 | 0.00E+00 | 84.10% |
| Efet.01.624933.g1086.t1 | Agromyces sp. 30A chromosome, complete genome | 489 | 2.40E-155 | 84.10% |
| Efet.01.638674.g447.t1 | Verminephrobacter eiseniae EF01-2, complete genome | 216 | 3.20E-80 | 84.10% |
| Efet.01.642123.g663.t1 | Verminephrobacter eiseniae EF01-2, complete genome | 1656 | 0.00E+00 | 84.10% |
| Efet.01.643394.g780.t1 | Verminephrobacter eiseniae EF01-2, complete genome | 294 | 4.50E-141 | 84.10% |
| Efet.01.651939.g238.t1 | Verminephrobacter eiseniae EF01-2, complete genome | 228 | 4.50E-111 | 84.10% |
| Efet.01.655966.g907.t1 | Verminephrobacter eiseniae EF01-2, complete genome | 1731 | 0.00E+00 | 84.10% |
| Efet.01.657257.g1218.t1 | Verminephrobacter eiseniae EF01-2, complete genome | 696 | 0.00E+00 | 84.10% |
| Efet.01.658150.g1687.t1 | Verminephrobacter eiseniae EF01-2, complete genome | 579 | 0.00E+00 | 84.10% |
| Efet.01.658413.g2032.t1 | Verminephrobacter eiseniae EF01-2, complete genome | 354 | 3.10E-172 | 84.10% |
| Efet.01.658425.g2129.t1 | Verminephrobacter eiseniae EF01-2, complete genome | 465 | 0.00E+00 | 84.10% |
| Efet.01.1659019.g1365.t1 | Verminephrobacter eiseniae EF01-2, complete genome | 432 | 0.00E+00 | 84.10% |
| Efet.01.1659188.g1450.t1 | Verminephrobacter eiseniae EF01-2, complete genome | 871 | 0.00E+00 | 84.10% |
| Efet.01.1659311.g1538.t1 | Verminephrobacter eiseniae EF01-2, complete genome | 777 | 0.00E+00 | 84.10% |
| Efet.01.1659348.g1584.t1 | Verminephrobacter eiseniae EF01-2, complete genome | 408 | 0.00E+00 | 84.10% |
| Efet.01.1659435.g1708.t1 | Verminephrobacter eiseniae EF01-2, complete genome | 645 | 0.00E+00 | 84.10% |
| Efet.01.1659440.g1719.t1 | Verminephrobacter eiseniae EF01-2, complete genome | 1191 | 0.00E+00 | 84.10% |
| Efet.01.1659471.g1789.t1 | Verminephrobacter eiseniae EF01-2, complete genome | 555 | 0.00E+00 | 84.10% |
| Efet.01.1659513.g1939.t1 | Verminephrobacter eiseniae EF01-2, complete genome | 369 | 0.00E+00 | 84.10% |
| Efet.01.1659514.g1946.t1 | Verminephrobacter eiseniae EF01-2, complete genome | 1773 | 0.00E+00 | 84.10% |
| Efet.01.1659527.g2038.t1 | Verminephrobacter eiseniae EF01-2, complete genome | 1458 | 0.00E+00 | 84.10% |
| Efet.01.11232.g823.t1 | Flavobacteriaceae bacterium UJ101, complete genome | 67 | 3.90E-08 | 84.00% |
| Efet.01.108910.g462.t1 | Pseudomonas fluorescens strain L111, complete genome | 76 | 8.30E-14 | 84.00% |
| Efet.01.169097.g940.t1 | Pseudomonas sabulinigri strain JCM 14963 genome assembly, chromosome: I | 58 | 3.50E-06 | 84.00% |
| Efet.01.188280.g1801.t1 | Thermosulfidibacter takaii ABI70S6 DNA, complete genome | 63 | 2.50E-08 | 84.00% |
| Efet.01.267648.g650.t1 | Verminephrobacter eiseniae EF01-2, complete genome | 1762 | 0.00E+00 | 84.00% |
| Efet.01.440935.g996.t1 | Verminephrobacter eiseniae EF01-2, complete genome | 258 | 7.50E-117 | 84.00% |
| Efet.01.492220.g1002.t1 | Elizabethkingia anophelis strain FDAARGOS_198 chromosome, complete genome | 57 | 8.20E-06 | 84.00% |
| Efet.01.494042.g1050.t1 | Verminephrobacter eiseniae EF01-2, complete genome | 474 | 0.00E+00 | 84.00% |
| Efet.01.497711.g1130.t1 | Verminephrobacter eiseniae EF01-2, complete genome | 408 | 0.00E+00 | 84.00% |
| Efet.01.548599.g457.t1 | Verminephrobacter eiseniae EF01-2, complete genome | 729 | 0.00E+00 | 84.00% |
| Efet.01.557838.g701.t1 | Verminephrobacter eiseniae EF01-2, complete genome | 1744 | 0.00E+00 | 84.00% |
| Efet.01.607412.g309.t1 | Verminephrobacter eiseniae EF01-2, complete genome | 1401 | 0.00E+00 | 84.00% |
| Efet.01.630546.g41.t1 | Verminephrobacter eiseniae EF01-2, complete genome | 438 | 0.00E+00 | 84.00% |
| Efet.01.631607.g128.t1 | Verminephrobacter eiseniae EF01-2, complete genome | 276 | 2.00E-137 | 84.00% |
| Efet.01.632992.g178.t1 | Verminephrobacter eiseniae EF01-2, complete genome | 360 | 1.10E-178 | 84.00% |
| Efet.01.646280.g1068.t1 | Micromonospora siamensis strain DSM 45097 genome assembly, chromosome: I | 129 | 2.30E-30 | 84.00% |
| Efet.01.650275.g19.t1 | Verminephrobacter eiseniae EF01-2, complete genome | 627 | 0.00E+00 | 84.00% |
| Efet.01.651136.g124.t1 | Verminephrobacter eiseniae EF01-2, complete genome | 672 | 0.00E+00 | 84.00% |
| Efet.01.652977.g387.t1 | Verminephrobacter eiseniae EF01-2, complete genome | 1399 | 0.00E+00 | 84.00% |
| Efet.01.655128.g764.t1 | Verminephrobacter eiseniae EF01-2, complete genome | 1170 | 0.00E+00 | 84.00% |
| Efet.01.655399.g808.t1 | Verminephrobacter eiseniae EF01-2, complete genome | 889 | 0.00E+00 | 84.00% |
| Efet.01.655987.g917.t1 | Verminephrobacter eiseniae EF01-2, complete genome | 439 | 0.00E+00 | 84.00% |
| Efet.01.656558.g1044.t1 | Verminephrobacter eiseniae EF01-2, complete genome | 258 | 8.30E-124 | 84.00% |
| Efet.01.657700.g1373.t1 | Variovorax paradoxus S110 chromosome 1, complete sequence | 276 | 2.90E-118 | 84.00% |
| Efet.01.657766.g1432.t1 | Verminephrobacter eiseniae EF01-2, complete genome | 1502 | 0.00E+00 | 84.00% |
| Efet.01.657807.g1485.t1 | Vitreoscilla filiformis strain ATCC 15551 plasmid pVF2, complete sequence | 270 | 1.60E-82 | 84.00% |
| Efet.01.658427.g2139.t1 | Verminephrobacter eiseniae EF01-2, complete genome | 288 | 2.60E-141 | 84.00% |
| Efet.01.1651663.g516.t1 | Variovorax sp. PAMC 28711, complete genome | 59 | 1.50E-07 | 84.00% |
| Efet.01.1654998.g726.t1 | Verminephrobacter eiseniae EF01-2, complete genome | 486 | 0.00E+00 | 84.00% |
| Efet.01.1659425.g1688.t1 | Verminephrobacter eiseniae EF01-2, complete genome | 386 | 0.00E+00 | 84.00% |
| Efet.01.1659427.g1691.t1 | Verminephrobacter eiseniae EF01-2, complete genome | 1479 | 0.00E+00 | 84.00% |
| Efet.01.1659438.g1715.t1 | Verminephrobacter eiseniae EF01-2, complete genome | 530 | 0.00E+00 | 84.00% |
| Efet.01.25255.g31.t1 | Verminephrobacter eiseniae EF01-2, complete genome | 951 | 0.00E+00 | 83.90% |
| Efet.01.494042.g1048.t1 | Verminephrobacter eiseniae EF01-2, complete genome | 411 | 0.00E+00 | 83.90% |
| Efet.01.577753.g613.t1 | Verminephrobacter eiseniae EF01-2, complete genome | 661 | 0.00E+00 | 83.90% |
| Efet.01.608047.g338.t1 | Comamonadaceae bacterium A1 DNA, complete genome | 303 | 5.60E-126 | 83.90% |
| Efet.01.610928.g461.t1 | Agromyces flavus strain CPCC 202695 genome assembly, chromosome: I | 368 | 5.90E-114 | 83.90% |
| Efet.01.626245.g1156.t1 | Verminephrobacter eiseniae EF01-2, complete genome | 738 | 0.00E+00 | 83.90% |
| Efet.01.638026.g403.t1 | Verminephrobacter eiseniae EF01-2, complete genome | 2388 | 0.00E+00 | 83.90% |
| Efet.01.651148.g139.t1 | Verminephrobacter eiseniae EF01-2, complete genome | 1581 | 0.00E+00 | 83.90% |
| Efet.01.657719.g1403.t1 | Verminephrobacter eiseniae EF01-2, complete genome | 2034 | 0.00E+00 | 83.90% |
| Efet.01.657803.g1471.t1 | Verminephrobacter eiseniae EF01-2, complete genome | 552 | 0.00E+00 | 83.90% |
| Efet.01.658002.g1551.t1 | Verminephrobacter eiseniae EF01-2, complete genome | 316 | 2.20E-157 | 83.90% |
| Efet.01.658236.g1740.t1 | Verminephrobacter eiseniae EF01-2, complete genome | 1548 | 0.00E+00 | 83.90% |
| Efet.01.658283.g1798.t1 | Verminephrobacter eiseniae EF01-2, complete genome | 1155 | 0.00E+00 | 83.90% |
| Efet.01.658401.g1993.t1 | Verminephrobacter eiseniae EF01-2, complete genome | 268 | 2.90E-120 | 83.90% |
| Efet.01.658415.g2046.t1 | Verminephrobacter eiseniae EF01-2, complete genome | 480 | 0.00E+00 | 83.90% |
| Efet.01.1658858.g1297.t1 | Verminephrobacter eiseniae EF01-2, complete genome | 620 | 0.00E+00 | 83.90% |
| Efet.01.1659399.g1656.t1 | Verminephrobacter eiseniae EF01-2, complete genome | 1473 | 0.00E+00 | 83.90% |
| Efet.01.1659408.g1665.t1 | Verminephrobacter eiseniae EF01-2, complete genome | 802 | 0.00E+00 | 83.90% |
| Efet.01.1659491.g1856.t1 | Verminephrobacter eiseniae EF01-2, complete genome | 2724 | 0.00E+00 | 83.90% |
| Efet.01.25255.g29.t1 | Verminephrobacter eiseniae EF01-2, complete genome | 330 | 9.60E-165 | 83.80% |
| Efet.01.284847.g1259.t1 | Verminephrobacter eiseniae EF01-2, complete genome | 743 | 0.00E+00 | 83.80% |
| Efet.01.300440.g17.t1 | Verminephrobacter eiseniae EF01-2, complete genome | 378 | 0.00E+00 | 83.80% |
| Efet.01.318747.g597.t1 | Verminephrobacter eiseniae EF01-2, complete genome | 954 | 0.00E+00 | 83.80% |
| Efet.01.369900.g542.t1 | Verminephrobacter eiseniae EF01-2, complete genome | 2535 | 0.00E+00 | 83.80% |
| Efet.01.369901.g548.t1 | Verminephrobacter eiseniae EF01-2, complete genome | 2535 | 0.00E+00 | 83.80% |
| Efet.01.419625.g458.t1 | Verminephrobacter eiseniae EF01-2, complete genome | 618 | 0.00E+00 | 83.80% |
| Efet.01.434359.g827.t1 | Verminephrobacter eiseniae EF01-2, complete genome | 1125 | 0.00E+00 | 83.80% |
| Efet.01.530573.g16.t1 | Verminephrobacter eiseniae EF01-2, complete genome | 1271 | 0.00E+00 | 83.80% |
| Efet.01.574381.g456.t1 | Verminephrobacter eiseniae EF01-2, complete genome | 681 | 0.00E+00 | 83.80% |
| Efet.01.577373.g579.t1 | Verminephrobacter eiseniae EF01-2, complete genome | 567 | 0.00E+00 | 83.80% |
| Efet.01.609514.g405.t1 | Verminephrobacter eiseniae EF01-2, complete genome | 879 | 0.00E+00 | 83.80% |
| Efet.01.609514.g406.t1 | Verminephrobacter eiseniae EF01-2, complete genome | 288 | 3.50E-142 | 83.80% |
| Efet.01.614947.g676.t1 | Verminephrobacter eiseniae EF01-2, complete genome | 740 | 0.00E+00 | 83.80% |
| Efet.01.623527.g1027.t1 | Verminephrobacter eiseniae EF01-2, complete genome | 2496 | 0.00E+00 | 83.80% |
| Efet.01.627824.g1223.t1 | Cryobacterium sp. LW097 plasmid unnamed1 sequence | 543 | 0.00E+00 | 83.80% |
| Efet.01.628433.g1243.t1 | Verminephrobacter eiseniae EF01-2, complete genome | 752 | 0.00E+00 | 83.80% |
| Efet.01.628482.g1248.t1 | Verminephrobacter eiseniae EF01-2, complete genome | 885 | 0.00E+00 | 83.80% |
| Efet.01.630860.g80.t1 | Burkholderia ambifaria AMMD chromosome 3, complete sequence | 237 | 1.60E-116 | 83.80% |
| Efet.01.631607.g127.t1 | Verminephrobacter eiseniae EF01-2, complete genome | 1911 | 0.00E+00 | 83.80% |
| Efet.01.651810.g217.t1 | Verminephrobacter eiseniae EF01-2, complete genome | 402 | 0.00E+00 | 83.80% |
| Efet.01.651940.g245.t1 | Verminephrobacter eiseniae EF01-2, complete genome | 363 | 0.00E+00 | 83.80% |
| Efet.01.652659.g331.t1 | Verminephrobacter eiseniae EF01-2, complete genome | 642 | 0.00E+00 | 83.80% |
| Efet.01.652898.g369.t1 | Verminephrobacter eiseniae EF01-2, complete genome | 700 | 0.00E+00 | 83.80% |
| Efet.01.653526.g444.t1 | Verminephrobacter eiseniae EF01-2, complete genome | 636 | 0.00E+00 | 83.80% |
| Efet.01.654310.g563.t1 | Verminephrobacter eiseniae EF01-2, complete genome | 1383 | 0.00E+00 | 83.80% |
| Efet.01.654769.g693.t1 | Verminephrobacter eiseniae EF01-2, complete genome | 551 | 0.00E+00 | 83.80% |
| Efet.01.655399.g813.t1 | Verminephrobacter eiseniae EF01-2, complete genome | 369 | 0.00E+00 | 83.80% |
| Efet.01.655988.g924.t1 | Verminephrobacter eiseniae EF01-2, complete genome | 897 | 0.00E+00 | 83.80% |
| Efet.01.656795.g1102.t1 | Verminephrobacter eiseniae EF01-2, complete genome | 1201 | 0.00E+00 | 83.80% |
| Efet.01.657315.g1241.t1 | Verminephrobacter eiseniae EF01-2, complete genome | 963 | 0.00E+00 | 83.80% |
| Efet.01.658116.g1646.t1 | Verminephrobacter eiseniae EF01-2, complete genome | 1002 | 0.00E+00 | 83.80% |
| Efet.01.658230.g1723.t1 | Verminephrobacter eiseniae EF01-2, complete genome | 1017 | 0.00E+00 | 83.80% |
| Efet.01.1658963.g1337.t1 | Agromyces aureus strain AR33, complete genome | 289 | 7.80E-95 | 83.80% |
| Efet.01.1659286.g1513.t1 | Verminephrobacter eiseniae EF01-2, complete genome | 1059 | 0.00E+00 | 83.80% |
| Efet.01.1659365.g1606.t1 | Verminephrobacter eiseniae EF01-2, complete genome | 704 | 0.00E+00 | 83.80% |
| Efet.01.1659451.g1744.t1 | Verminephrobacter eiseniae EF01-2, complete genome | 528 | 0.00E+00 | 83.80% |
| Efet.01.1659471.g1790.t1 | Verminephrobacter eiseniae EF01-2, complete genome | 612 | 0.00E+00 | 83.80% |
| Efet.01.1659471.g1791.t1 | Verminephrobacter eiseniae EF01-2, complete genome | 618 | 0.00E+00 | 83.80% |
| Efet.01.378313.g756.t1 | Microbulbifer sp. CCB-MM1, complete genome | 56 | 3.50E-07 | 83.75% |
| Efet.01.104091.g223.t1 | Magnetospira sp. QH-2 chromosome, complete genome | 70 | 3.50E-12 | 83.70% |
| Efet.01.104942.g270.t1 | Verminephrobacter eiseniae EF01-2, complete genome | 1148 | 0.00E+00 | 83.70% |
| Efet.01.325607.g792.t1 | Verminephrobacter eiseniae EF01-2, complete genome | 1290 | 0.00E+00 | 83.70% |
| Efet.01.417053.g407.t1 | Verminephrobacter eiseniae EF01-2, complete genome | 927 | 0.00E+00 | 83.70% |
| Efet.01.565049.g158.t1 | Verminephrobacter eiseniae EF01-2, complete genome | 1578 | 0.00E+00 | 83.70% |
| Efet.01.565049.g166.t1 | Verminephrobacter eiseniae EF01-2, complete genome | 321 | 4.00E-159 | 83.70% |
| Efet.01.577373.g592.t1 | Verminephrobacter eiseniae EF01-2, complete genome | 1091 | 0.00E+00 | 83.70% |
| Efet.01.585463.g843.t1 | Verminephrobacter eiseniae EF01-2, complete genome | 927 | 0.00E+00 | 83.70% |
| Efet.01.597547.g1211.t1 | Verminephrobacter eiseniae EF01-2, complete genome | 621 | 0.00E+00 | 83.70% |
| Efet.01.619389.g859.t1 | Verminephrobacter eiseniae EF01-2, complete genome | 867 | 0.00E+00 | 83.70% |
| Efet.01.630200.g12.t1 | Verminephrobacter eiseniae EF01-2, complete genome | 1053 | 0.00E+00 | 83.70% |
| Efet.01.638425.g430.t1 | Verminephrobacter eiseniae EF01-2, complete genome | 996 | 0.00E+00 | 83.70% |
| Efet.01.638678.g450.t1 | Verminephrobacter eiseniae EF01-2, complete genome | 1701 | 0.00E+00 | 83.70% |
| Efet.01.641218.g613.t1 | Verminephrobacter eiseniae EF01-2, complete genome | 309 | 4.50E-150 | 83.70% |
| Efet.01.643497.g803.t1 | Verminephrobacter eiseniae EF01-2, complete genome | 1044 | 0.00E+00 | 83.70% |
| Efet.01.645138.g942.t1 | Verminephrobacter eiseniae EF01-2, complete genome | 1157 | 0.00E+00 | 83.70% |
| Efet.01.645971.g1019.t1 | Verminephrobacter eiseniae EF01-2, complete genome | 657 | 0.00E+00 | 83.70% |
| Efet.01.650480.g56.t1 | Verminephrobacter eiseniae EF01-2, complete genome | 579 | 0.00E+00 | 83.70% |
| Efet.01.654418.g585.t1 | Verminephrobacter eiseniae EF01-2, complete genome | 444 | 0.00E+00 | 83.70% |
| Efet.01.654946.g720.t1 | Verminephrobacter eiseniae EF01-2, complete genome | 975 | 0.00E+00 | 83.70% |
| Efet.01.655750.g856.t1 | Verminephrobacter eiseniae EF01-2, complete genome | 1557 | 0.00E+00 | 83.70% |
| Efet.01.655999.g927.t1 | Verminephrobacter eiseniae EF01-2, complete genome | 1695 | 0.00E+00 | 83.70% |
| Efet.01.656558.g1041.t1 | Verminephrobacter eiseniae EF01-2, complete genome | 921 | 0.00E+00 | 83.70% |
| Efet.01.656558.g1047.t1 | Verminephrobacter eiseniae EF01-2, complete genome | 984 | 0.00E+00 | 83.70% |
| Efet.01.657248.g1211.t1 | Verminephrobacter eiseniae EF01-2, complete genome | 465 | 0.00E+00 | 83.70% |
| Efet.01.657766.g1454.t1 | Verminephrobacter eiseniae EF01-2, complete genome | 1821 | 0.00E+00 | 83.70% |
| Efet.01.1647571.g381.t1 | Verminephrobacter eiseniae EF01-2, complete genome | 303 | 5.70E-151 | 83.70% |
| Efet.01.1658055.g1090.t1 | Variovorax boronicumulans strain J1 chromosome, complete genome | 315 | 2.40E-145 | 83.70% |
| Efet.01.658392.g1953.t1 | Verminephrobacter eiseniae EF01-2, complete genome | 1104 | 0.00E+00 | 83.67% |
| Efet.01.300799.g32.t1 | Verminephrobacter eiseniae EF01-2, complete genome | 444 | 0.00E+00 | 83.60% |
| Efet.01.439764.g941.t1 | Verminephrobacter eiseniae EF01-2, complete genome | 804 | 0.00E+00 | 83.60% |
| Efet.01.526468.g616.t1 | Verminephrobacter eiseniae EF01-2, complete genome | 726 | 0.00E+00 | 83.60% |
| Efet.01.530573.g15.t1 | Verminephrobacter eiseniae EF01-2, complete genome | 495 | 0.00E+00 | 83.60% |
| Efet.01.533383.g127.t1 | Verminephrobacter eiseniae EF01-2, complete genome | 1229 | 0.00E+00 | 83.60% |
| Efet.01.553639.g603.t1 | Verminephrobacter eiseniae EF01-2, complete genome | 256 | 3.00E-126 | 83.60% |
| Efet.01.577373.g576.t1 | Verminephrobacter eiseniae EF01-2, complete genome | 1224 | 0.00E+00 | 83.60% |
| Efet.01.634939.g266.t1 | Verminephrobacter eiseniae EF01-2, complete genome | 580 | 0.00E+00 | 83.60% |
| Efet.01.637843.g386.t1 | Verminephrobacter eiseniae EF01-2, complete genome | 228 | 1.40E-106 | 83.60% |
| Efet.01.643394.g785.t1 | Verminephrobacter eiseniae EF01-2, complete genome | 1005 | 0.00E+00 | 83.60% |
| Efet.01.646274.g1060.t1 | Verminephrobacter eiseniae EF01-2, complete genome | 838 | 0.00E+00 | 83.60% |
| Efet.01.654538.g625.t1 | Verminephrobacter eiseniae EF01-2, complete genome | 396 | 0.00E+00 | 83.60% |
| Efet.01.654591.g635.t1 | Verminephrobacter eiseniae EF01-2, complete genome | 1002 | 0.00E+00 | 83.60% |
| Efet.01.654946.g721.t1 | Verminephrobacter eiseniae EF01-2, complete genome | 288 | 6.00E-141 | 83.60% |
| Efet.01.655128.g758.t1 | Verminephrobacter eiseniae EF01-2, complete genome | 801 | 0.00E+00 | 83.60% |
| Efet.01.655821.g881.t1 | Verminephrobacter eiseniae EF01-2, complete genome | 948 | 0.00E+00 | 83.60% |
| Efet.01.657803.g1472.t1 | Verminephrobacter eiseniae EF01-2, complete genome | 255 | 6.10E-126 | 83.60% |
| Efet.01.658116.g1647.t1 | Verminephrobacter eiseniae EF01-2, complete genome | 1197 | 0.00E+00 | 83.60% |
| Efet.01.658142.g1680.t1 | Verminephrobacter eiseniae EF01-2, complete genome | 912 | 0.00E+00 | 83.60% |
| Efet.01.658380.g1915.t1 | Verminephrobacter eiseniae EF01-2, complete genome | 1371 | 0.00E+00 | 83.60% |
| Efet.01.1659348.g1582.t1 | Verminephrobacter eiseniae EF01-2, complete genome | 402 | 0.00E+00 | 83.60% |
| Efet.01.160494.g550.t1 | Streptomyces laurentii DNA, complete genome, strain: ATCC 31255 | 55 | 6.50E-07 | 83.50% |
| Efet.01.300799.g36.t1 | Verminephrobacter eiseniae EF01-2, complete genome | 915 | 0.00E+00 | 83.50% |
| Efet.01.318747.g593.t1 | Verminephrobacter eiseniae EF01-2, complete genome | 1053 | 0.00E+00 | 83.50% |
| Efet.01.430820.g753.t1 | Verminephrobacter eiseniae EF01-2, complete genome | 557 | 0.00E+00 | 83.50% |
| Efet.01.440538.g974.t1 | Verminephrobacter eiseniae EF01-2, complete genome | 1684 | 0.00E+00 | 83.50% |
| Efet.01.440935.g993.t1 | Verminephrobacter eiseniae EF01-2, complete genome | 1413 | 0.00E+00 | 83.50% |
| Efet.01.583614.g784.t1 | Verminephrobacter eiseniae EF01-2, complete genome | 1026 | 0.00E+00 | 83.50% |
| Efet.01.592653.g1083.t1 | Verminephrobacter eiseniae EF01-2, complete genome | 762 | 0.00E+00 | 83.50% |
| Efet.01.617251.g762.t1 | Verminephrobacter eiseniae EF01-2, complete genome | 546 | 0.00E+00 | 83.50% |
| Efet.01.617251.g764.t1 | Verminephrobacter eiseniae EF01-2, complete genome | 167 | 6.00E-73 | 83.50% |
| Efet.01.625433.g1114.t1 | Verminephrobacter eiseniae EF01-2, complete genome | 656 | 0.00E+00 | 83.50% |
| Efet.01.626983.g1190.t1 | Verminephrobacter eiseniae EF01-2, complete genome | 839 | 0.00E+00 | 83.50% |
| Efet.01.634939.g264.t1 | Verminephrobacter eiseniae EF01-2, complete genome | 900 | 0.00E+00 | 83.50% |
| Efet.01.638425.g428.t1 | Verminephrobacter eiseniae EF01-2, complete genome | 694 | 0.00E+00 | 83.50% |
| Efet.01.638425.g436.t1 | Verminephrobacter eiseniae EF01-2, complete genome | 438 | 0.00E+00 | 83.50% |
| Efet.01.638713.g461.t1 | Agromyces flavus strain CPCC 202695 genome assembly, chromosome: I | 248 | 1.40E-78 | 83.50% |
| Efet.01.648797.g1267.t1 | Verminephrobacter eiseniae EF01-2, complete genome | 1143 | 0.00E+00 | 83.50% |
| Efet.01.649364.g1300.t1 | Verminephrobacter eiseniae EF01-2, complete genome | 597 | 0.00E+00 | 83.50% |
| Efet.01.650091.g5.t1 | Verminephrobacter eiseniae EF01-2, complete genome | 216 | 3.50E-103 | 83.50% |
| Efet.01.651136.g112.t1 | Verminephrobacter eiseniae EF01-2, complete genome | 468 | 0.00E+00 | 83.50% |
| Efet.01.652898.g377.t1 | Verminephrobacter eiseniae EF01-2, complete genome | 423 | 0.00E+00 | 83.50% |
| Efet.01.654257.g555.t1 | Verminephrobacter eiseniae EF01-2, complete genome | 822 | 0.00E+00 | 83.50% |
| Efet.01.655974.g910.t1 | Verminephrobacter eiseniae EF01-2, complete genome | 1035 | 0.00E+00 | 83.50% |
| Efet.01.656470.g1026.t1 | Verminephrobacter eiseniae EF01-2, complete genome | 229 | 5.60E-107 | 83.50% |
| Efet.01.656813.g1113.t1 | Verminephrobacter eiseniae EF01-2, complete genome | 616 | 0.00E+00 | 83.50% |
| Efet.01.657766.g1455.t1 | Verminephrobacter eiseniae EF01-2, complete genome | 1239 | 0.00E+00 | 83.50% |
| Efet.01.657868.g1510.t1 | Verminephrobacter eiseniae EF01-2, complete genome | 985 | 0.00E+00 | 83.50% |
| Efet.01.658138.g1669.t1 | Verminephrobacter eiseniae EF01-2, complete genome | 1215 | 0.00E+00 | 83.50% |
| Efet.01.658142.g1675.t1 | Verminephrobacter eiseniae EF01-2, complete genome | 1977 | 0.00E+00 | 83.50% |
| Efet.01.658398.g1983.t1 | Amycolatopsis mediterranei S699, complete genome | 511 | 0.00E+00 | 83.50% |
| Efet.01.658413.g2033.t1 | Verminephrobacter eiseniae EF01-2, complete genome | 900 | 0.00E+00 | 83.50% |
| Efet.01.658415.g2045.t1 | Verminephrobacter eiseniae EF01-2, complete genome | 807 | 0.00E+00 | 83.50% |
| Efet.01.658422.g2099.t1 | Hydrogenophaga sp. PBC, complete genome | 342 | 5.50E-166 | 83.50% |
| Efet.01.1658305.g1152.t1 | Verminephrobacter eiseniae EF01-2, complete genome | 435 | 0.00E+00 | 83.50% |
| Efet.01.1659497.g1878.t1 | Verminephrobacter eiseniae EF01-2, complete genome | 1551 | 0.00E+00 | 83.50% |
| Efet.01.1659497.g1882.t1 | Verminephrobacter eiseniae EF01-2, complete genome | 878 | 0.00E+00 | 83.50% |
| Efet.01.1659527.g2040.t1 | Verminephrobacter eiseniae EF01-2, complete genome | 786 | 0.00E+00 | 83.50% |
| Efet.01.279930.g1099.t1 | Verminephrobacter eiseniae EF01-2, complete genome | 445 | 0.00E+00 | 83.40% |
| Efet.01.287526.g1361.t1 | Verminephrobacter eiseniae EF01-2, complete genome | 1082 | 0.00E+00 | 83.40% |
| Efet.01.419552.g455.t1 | Verminephrobacter eiseniae EF01-2, complete genome | 897 | 0.00E+00 | 83.40% |
| Efet.01.497711.g1132.t1 | Verminephrobacter eiseniae EF01-2, complete genome | 987 | 0.00E+00 | 83.40% |
| Efet.01.578167.g626.t1 | Verminephrobacter eiseniae EF01-2, complete genome | 478 | 0.00E+00 | 83.40% |
| Efet.01.578612.g645.t1 | Verminephrobacter eiseniae EF01-2, complete genome | 1167 | 0.00E+00 | 83.40% |
| Efet.01.605884.g247.t1 | Verminephrobacter eiseniae EF01-2, complete genome | 327 | 1.70E-136 | 83.40% |
| Efet.01.611807.g500.t1 | Variovorax paradoxus EPS, complete genome | 795 | 0.00E+00 | 83.40% |
| Efet.01.629376.g1297.t1 | Verminephrobacter eiseniae EF01-2, complete genome | 354 | 9.60E-177 | 83.40% |
| Efet.01.633502.g192.t1 | Verminephrobacter eiseniae EF01-2, complete genome | 317 | 4.70E-158 | 83.40% |
| Efet.01.636814.g349.t1 | Verminephrobacter eiseniae EF01-2, complete genome | 1059 | 0.00E+00 | 83.40% |
| Efet.01.642395.g681.t1 | Verminephrobacter eiseniae EF01-2, complete genome | 822 | 0.00E+00 | 83.40% |
| Efet.01.645138.g940.t1 | Verminephrobacter eiseniae EF01-2, complete genome | 525 | 0.00E+00 | 83.40% |
| Efet.01.645500.g990.t1 | Verminephrobacter eiseniae EF01-2, complete genome | 1564 | 0.00E+00 | 83.40% |
| Efet.01.651229.g150.t1 | Verminephrobacter eiseniae EF01-2, complete genome | 306 | 1.20E-151 | 83.40% |
| Efet.01.653541.g447.t1 | Verminephrobacter eiseniae EF01-2, complete genome | 600 | 0.00E+00 | 83.40% |
| Efet.01.654418.g593.t1 | Verminephrobacter eiseniae EF01-2, complete genome | 1218 | 0.00E+00 | 83.40% |
| Efet.01.654656.g651.t1 | Verminephrobacter eiseniae EF01-2, complete genome | 375 | 0.00E+00 | 83.40% |
| Efet.01.656717.g1070.t1 | Verminephrobacter eiseniae EF01-2, complete genome | 387 | 0.00E+00 | 83.40% |
| Efet.01.657719.g1391.t1 | Verminephrobacter eiseniae EF01-2, complete genome | 1767 | 0.00E+00 | 83.40% |
| Efet.01.658052.g1602.t1 | Verminephrobacter eiseniae EF01-2, complete genome | 1116 | 0.00E+00 | 83.40% |
| Efet.01.658081.g1619.t1 | Verminephrobacter eiseniae EF01-2, complete genome | 2142 | 0.00E+00 | 83.40% |
| Efet.01.658150.g1685.t1 | Verminephrobacter eiseniae EF01-2, complete genome | 954 | 0.00E+00 | 83.40% |
| Efet.01.658312.g1844.t1 | Verminephrobacter eiseniae EF01-2, complete genome | 480 | 0.00E+00 | 83.40% |
| Efet.01.658410.g2022.t1 | Verminephrobacter eiseniae EF01-2, complete genome | 963 | 0.00E+00 | 83.40% |
| Efet.01.1659013.g1359.t1 | Verminephrobacter eiseniae EF01-2, complete genome | 453 | 0.00E+00 | 83.40% |
| Efet.01.654027.g520.t1 | Verminephrobacter eiseniae EF01-2, complete genome | 228 | 2.20E-101 | 83.33% |
| Efet.01.25255.g16.t1 | Verminephrobacter eiseniae EF01-2, complete genome | 1461 | 0.00E+00 | 83.30% |
| Efet.01.275134.g929.t1 | Verminephrobacter eiseniae EF01-2, complete genome | 1008 | 0.00E+00 | 83.30% |
| Efet.01.378778.g767.t1 | Verminephrobacter eiseniae EF01-2, complete genome | 737 | 0.00E+00 | 83.30% |
| Efet.01.393322.g1080.t1 | Verminephrobacter eiseniae EF01-2, complete genome | 534 | 0.00E+00 | 83.30% |
| Efet.01.463370.g361.t1 | Verminephrobacter eiseniae EF01-2, complete genome | 576 | 0.00E+00 | 83.30% |
| Efet.01.467295.g462.t1 | Verminephrobacter eiseniae EF01-2, complete genome | 520 | 0.00E+00 | 83.30% |
| Efet.01.608389.g349.t1 | Verminephrobacter eiseniae EF01-2, complete genome | 747 | 0.00E+00 | 83.30% |
| Efet.01.614731.g666.t1 | Verminephrobacter eiseniae EF01-2, complete genome | 594 | 0.00E+00 | 83.30% |
| Efet.01.615846.g705.t1 | Methylobacterium sp. 4-46, complete genome | 852 | 0.00E+00 | 83.30% |
| Efet.01.629153.g1275.t1 | Verminephrobacter eiseniae EF01-2, complete genome | 501 | 0.00E+00 | 83.30% |
| Efet.01.637843.g387.t1 | Paraburkholderia xenovorans LB400 chromosome 2, complete sequence | 448 | 0.00E+00 | 83.30% |
| Efet.01.650480.g61.t1 | Verminephrobacter eiseniae EF01-2, complete genome | 546 | 0.00E+00 | 83.30% |
| Efet.01.651810.g219.t1 | Verminephrobacter eiseniae EF01-2, complete genome | 542 | 0.00E+00 | 83.30% |
| Efet.01.652442.g299.t1 | Verminephrobacter eiseniae EF01-2, complete genome | 660 | 0.00E+00 | 83.30% |
| Efet.01.653312.g416.t1 | Verminephrobacter eiseniae EF01-2, complete genome | 690 | 0.00E+00 | 83.30% |
| Efet.01.655573.g833.t1 | Verminephrobacter eiseniae EF01-2, complete genome | 842 | 0.00E+00 | 83.30% |
| Efet.01.656518.g1038.t1 | Verminephrobacter eiseniae EF01-2, complete genome | 1113 | 0.00E+00 | 83.30% |
| Efet.01.658410.g2023.t1 | Verminephrobacter eiseniae EF01-2, complete genome | 792 | 0.00E+00 | 83.30% |
| Efet.01.658429.g2165.t1 | Verminephrobacter eiseniae EF01-2, complete genome | 1002 | 0.00E+00 | 83.30% |
| Efet.01.1640504.g221.t1 | Aeromonas sp. ASNIH3 chromosome, complete genome | 201 | 3.30E-29 | 83.30% |
| Efet.01.1658628.g1236.t1 | Leifsonia sp. 98AMF genome assembly, chromosome: I | 705 | 0.00E+00 | 83.30% |
| Efet.01.1659300.g1530.t1 | Verminephrobacter eiseniae EF01-2, complete genome | 798 | 0.00E+00 | 83.30% |
| Efet.01.1659427.g1690.t1 | Verminephrobacter eiseniae EF01-2, complete genome | 318 | 1.10E-154 | 83.30% |
| Efet.01.1659448.g1736.t1 | Verminephrobacter eiseniae EF01-2, complete genome | 918 | 0.00E+00 | 83.30% |
| Efet.01.1659514.g1947.t1 | Verminephrobacter eiseniae EF01-2, complete genome | 309 | 1.30E-152 | 83.30% |
| Efet.01.31180.g430.t1 | Verminephrobacter eiseniae EF01-2, complete genome | 579 | 0.00E+00 | 83.20% |
| Efet.01.185951.g1683.t1 | Verminephrobacter eiseniae EF01-2, complete genome | 637 | 0.00E+00 | 83.20% |
| Efet.01.185951.g1686.t1 | Verminephrobacter eiseniae EF01-2, complete genome | 1056 | 0.00E+00 | 83.20% |
| Efet.01.282031.g1160.t1 | Verminephrobacter eiseniae EF01-2, complete genome | 1290 | 0.00E+00 | 83.20% |
| Efet.01.440538.g973.t1 | Verminephrobacter eiseniae EF01-2, complete genome | 1885 | 0.00E+00 | 83.20% |
| Efet.01.445097.g1111.t1 | Verminephrobacter eiseniae EF01-2, complete genome | 1281 | 0.00E+00 | 83.20% |
| Efet.01.575546.g500.t1 | Verminephrobacter eiseniae EF01-2, complete genome | 408 | 0.00E+00 | 83.20% |
| Efet.01.576557.g532.t1 | Verminephrobacter eiseniae EF01-2, complete genome | 211 | 1.00E-100 | 83.20% |
| Efet.01.610928.g462.t1 | Microbacterium paludicola strain CC3, complete genome | 214 | 1.70E-57 | 83.20% |
| Efet.01.630860.g88.t1 | Verminephrobacter eiseniae EF01-2, complete genome | 540 | 0.00E+00 | 83.20% |
| Efet.01.636372.g328.t1 | Verminephrobacter eiseniae EF01-2, complete genome | 2182 | 0.00E+00 | 83.20% |
| Efet.01.648372.g1236.t1 | Verminephrobacter eiseniae EF01-2, complete genome | 522 | 0.00E+00 | 83.20% |
| Efet.01.650480.g58.t1 | Verminephrobacter eiseniae EF01-2, complete genome | 600 | 0.00E+00 | 83.20% |
| Efet.01.653626.g465.t1 | Verminephrobacter eiseniae EF01-2, complete genome | 1023 | 0.00E+00 | 83.20% |
| Efet.01.654303.g559.t1 | Verminephrobacter eiseniae EF01-2, complete genome | 714 | 0.00E+00 | 83.20% |
| Efet.01.654851.g705.t1 | Verminephrobacter eiseniae EF01-2, complete genome | 765 | 0.00E+00 | 83.20% |
| Efet.01.657766.g1441.t1 | Verminephrobacter eiseniae EF01-2, complete genome | 423 | 0.00E+00 | 83.20% |
| Efet.01.658255.g1756.t1 | Verminephrobacter eiseniae EF01-2, complete genome | 576 | 0.00E+00 | 83.20% |
| Efet.01.658275.g1780.t1 | Paraburkholderia xenovorans LB400 chromosome 1, complete sequence | 878 | 0.00E+00 | 83.20% |
| Efet.01.658416.g2059.t1 | Verminephrobacter eiseniae EF01-2, complete genome | 1062 | 0.00E+00 | 83.20% |
| Efet.01.1649523.g442.t1 | Verminephrobacter eiseniae EF01-2, complete genome | 264 | 8.50E-130 | 83.20% |
| Efet.01.1659334.g1560.t1 | Verminephrobacter eiseniae EF01-2, complete genome | 981 | 0.00E+00 | 83.20% |
| Efet.01.1659416.g1679.t1 | Verminephrobacter eiseniae EF01-2, complete genome | 659 | 0.00E+00 | 83.20% |
| Efet.01.1659360.g1599.t1 | Verminephrobacter eiseniae EF01-2, complete genome | 420 | 0.00E+00 | 83.17% |
| Efet.01.419552.g454.t1 | Verminephrobacter eiseniae EF01-2, complete genome | 780 | 0.00E+00 | 83.10% |
| Efet.01.575700.g505.t1 | Verminephrobacter eiseniae EF01-2, complete genome | 912 | 0.00E+00 | 83.10% |
| Efet.01.578612.g642.t1 | Verminephrobacter eiseniae EF01-2, complete genome | 803 | 0.00E+00 | 83.10% |
| Efet.01.594594.g1128.t1 | Verminephrobacter eiseniae EF01-2, complete genome | 441 | 0.00E+00 | 83.10% |
| Efet.01.601193.g55.t1 | Verminephrobacter eiseniae EF01-2, complete genome | 1786 | 0.00E+00 | 83.10% |
| Efet.01.601193.g56.t1 | Verminephrobacter eiseniae EF01-2, complete genome | 975 | 0.00E+00 | 83.10% |
| Efet.01.605365.g212.t1 | Verminephrobacter eiseniae EF01-2, complete genome | 669 | 0.00E+00 | 83.10% |
| Efet.01.613416.g608.t1 | Verminephrobacter eiseniae EF01-2, complete genome | 696 | 0.00E+00 | 83.10% |
| Efet.01.616615.g731.t1 | Verminephrobacter eiseniae EF01-2, complete genome | 483 | 0.00E+00 | 83.10% |
| Efet.01.621349.g930.t1 | Verminephrobacter eiseniae EF01-2, complete genome | 930 | 0.00E+00 | 83.10% |
| Efet.01.641218.g615.t1 | Verminephrobacter eiseniae EF01-2, complete genome | 1467 | 0.00E+00 | 83.10% |
| Efet.01.642094.g658.t1 | Verminephrobacter eiseniae EF01-2, complete genome | 915 | 0.00E+00 | 83.10% |
| Efet.01.644010.g854.t1 | Verminephrobacter eiseniae EF01-2, complete genome | 1177 | 0.00E+00 | 83.10% |
| Efet.01.646437.g1083.t1 | Agromyces sp. 30A chromosome, complete genome | 203 | 4.80E-77 | 83.10% |
| Efet.01.648654.g1251.t1 | Agromyces flavus strain CPCC 202695 genome assembly, chromosome: I | 485 | 8.00E-149 | 83.10% |
| Efet.01.655573.g832.t1 | Verminephrobacter eiseniae EF01-2, complete genome | 903 | 0.00E+00 | 83.10% |
| Efet.01.656919.g1143.t1 | Verminephrobacter eiseniae EF01-2, complete genome | 723 | 0.00E+00 | 83.10% |
| Efet.01.658041.g1583.t1 | Verminephrobacter eiseniae EF01-2, complete genome | 546 | 0.00E+00 | 83.10% |
| Efet.01.658245.g1745.t1 | Verminephrobacter eiseniae EF01-2, complete genome | 1119 | 0.00E+00 | 83.10% |
| Efet.01.658334.g1864.t1 | Verminephrobacter eiseniae EF01-2, complete genome | 354 | 3.50E-177 | 83.10% |
| Efet.01.1638827.g200.t1 | Verminephrobacter eiseniae EF01-2, complete genome | 255 | 5.90E-121 | 83.10% |
| Efet.01.1659409.g1668.t1 | Verminephrobacter eiseniae EF01-2, complete genome | 1167 | 0.00E+00 | 83.10% |
| Efet.01.1659484.g1830.t1 | Verminephrobacter eiseniae EF01-2, complete genome | 951 | 0.00E+00 | 83.10% |
| Efet.01.1659499.g1887.t1 | Verminephrobacter eiseniae EF01-2, complete genome | 330 | 3.60E-157 | 83.10% |
| Efet.01.1659522.g1991.t1 | Verminephrobacter eiseniae EF01-2, complete genome | 507 | 0.00E+00 | 83.10% |
| Efet.01.1659523.g2005.t1 | Verminephrobacter eiseniae EF01-2, complete genome | 1050 | 0.00E+00 | 83.10% |
| Efet.01.2417.g220.t1 | Streptomyces sp. CNQ-509, complete genome | 68 | 2.80E-08 | 83.00% |
| Efet.01.39673.g973.t1 | Spongiibacter sp. IMCC21906, complete genome | 59 | 3.30E-07 | 83.00% |
| Efet.01.137032.g606.t1 | Psychromonas ingrahamii 37, complete genome | 71 | 2.00E-07 | 83.00% |
| Efet.01.169028.g935.t1 | Methylobacterium extorquens strain PSBB040, complete genome | 60 | 3.50E-06 | 83.00% |
| Efet.01.200253.g18.t1 | Francisella halioticida strain DSM 23729, complete genome | 159 | 5.60E-09 | 83.00% |
| Efet.01.270738.g769.t1 | Verminephrobacter eiseniae EF01-2, complete genome | 618 | 0.00E+00 | 83.00% |
| Efet.01.394560.g1107.t1 | Neisseria sp. KEM232, complete genome | 59 | 4.10E-06 | 83.00% |
| Efet.01.454467.g107.t1 | Verminephrobacter eiseniae EF01-2, complete genome | 936 | 0.00E+00 | 83.00% |
| Efet.01.471393.g566.t1 | Verminephrobacter eiseniae EF01-2, complete genome | 564 | 0.00E+00 | 83.00% |
| Efet.01.503541.g85.t1 | Acidovorax sp. RAC01, complete genome | 1413 | 0.00E+00 | 83.00% |
| Efet.01.511938.g280.t1 | Bacillus thuringiensis strain M199 vegetative insecticidal protein gene, complete cds | 60 | 5.60E-06 | 83.00% |
| Efet.01.550127.g500.t1 | Verminephrobacter eiseniae EF01-2, complete genome | 477 | 0.00E+00 | 83.00% |
| Efet.01.577373.g593.t1 | Verminephrobacter eiseniae EF01-2, complete genome | 1464 | 0.00E+00 | 83.00% |
| Efet.01.577753.g612.t1 | Verminephrobacter eiseniae EF01-2, complete genome | 546 | 0.00E+00 | 83.00% |
| Efet.01.578167.g623.t1 | Verminephrobacter eiseniae EF01-2, complete genome | 402 | 0.00E+00 | 83.00% |
| Efet.01.581651.g726.t1 | Verminephrobacter eiseniae EF01-2, complete genome | 62 | 1.50E-18 | 83.00% |
| Efet.01.592582.g1077.t1 | Verminephrobacter eiseniae EF01-2, complete genome | 240 | 2.70E-118 | 83.00% |
| Efet.01.628009.g1227.t1 | Verminephrobacter eiseniae EF01-2, complete genome | 843 | 0.00E+00 | 83.00% |
| Efet.01.642859.g735.t1 | Diaphorobacter polyhydroxybutyrativorans strain SL-205, complete genome | 177 | 2.30E-43 | 83.00% |
| Efet.01.650542.g65.t1 | Verminephrobacter eiseniae EF01-2, complete genome | 342 | 4.50E-165 | 83.00% |
| Efet.01.651136.g119.t1 | Variovorax sp. HW608 genome assembly, chromosome: I | 2177 | 0.00E+00 | 83.00% |
| Efet.01.652858.g352.t1 | Verminephrobacter eiseniae EF01-2, complete genome | 1299 | 0.00E+00 | 83.00% |
| Efet.01.653325.g418.t1 | Verminephrobacter eiseniae EF01-2, complete genome | 360 | 0.00E+00 | 83.00% |
| Efet.01.655777.g873.t1 | Mesorhizobium ciceri biovar biserrulae WSM1271, complete genome | 67 | 7.40E-08 | 83.00% |
| Efet.01.657315.g1246.t1 | Verminephrobacter eiseniae EF01-2, complete genome | 1353 | 0.00E+00 | 83.00% |
| Efet.01.658133.g1663.t1 | Verminephrobacter eiseniae EF01-2, complete genome | 1881 | 0.00E+00 | 83.00% |
| Efet.01.658214.g1719.t1 | Mycobacterium fortuitum subsp. fortuitum DSM 46621 = ATCC 6841 genome | 61 | 4.70E-07 | 83.00% |
| Efet.01.658279.g1788.t1 | Pusillimonas sp. T7-7, complete genome | 74 | 1.10E-11 | 83.00% |
| Efet.01.1587334.g8.t1 | Microbacterium sp. TPU 3598 DNA, complete genome | 210 | 1.70E-58 | 83.00% |
| Efet.01.1648222.g401.t1 | Pontibacter actiniarum DSM 19842, complete genome | 72 | 6.60E-11 | 83.00% |
| Efet.01.1648930.g421.t1 | Haliangium ochraceum DSM 14365, complete genome | 56 | 5.10E-06 | 83.00% |
| Efet.01.1648991.g423.t1 | Bordetella genomosp. 9 strain AU14267, complete genome | 61 | 9.80E-09 | 83.00% |
| Efet.01.1655934.g804.t1 | Dyadobacter fermentans DSM 18053, complete genome | 67 | 6.70E-09 | 83.00% |
| Efet.01.1658956.g1333.t1 | Verminephrobacter eiseniae EF01-2, complete genome | 363 | 0.00E+00 | 83.00% |
| Efet.01.1659293.g1523.t1 | Verminephrobacter eiseniae EF01-2, complete genome | 297 | 4.00E-135 | 83.00% |
| Efet.01.1659438.g1714.t1 | Verminephrobacter eiseniae EF01-2, complete genome | 1179 | 0.00E+00 | 83.00% |
| Efet.01.287526.g1364.t1 | Verminephrobacter eiseniae EF01-2, complete genome | 267 | 5.20E-131 | 82.90% |
| Efet.01.287526.g1368.t1 | Verminephrobacter eiseniae EF01-2, complete genome | 1311 | 0.00E+00 | 82.90% |
| Efet.01.350740.g21.t1 | Verminephrobacter eiseniae EF01-2, complete genome | 266 | 9.10E-129 | 82.90% |
| Efet.01.428348.g691.t1 | Leifsonia sp. 98AMF genome assembly, chromosome: I | 417 | 4.30E-131 | 82.90% |
| Efet.01.439764.g942.t1 | Verminephrobacter eiseniae EF01-2, complete genome | 297 | 8.30E-142 | 82.90% |
| Efet.01.463370.g360.t1 | Verminephrobacter eiseniae EF01-2, complete genome | 843 | 0.00E+00 | 82.90% |
| Efet.01.502067.g50.t1 | Verminephrobacter eiseniae EF01-2, complete genome | 710 | 0.00E+00 | 82.90% |
| Efet.01.514247.g341.t1 | Verminephrobacter eiseniae EF01-2, complete genome | 384 | 0.00E+00 | 82.90% |
| Efet.01.561085.g22.t1 | Verminephrobacter eiseniae EF01-2, complete genome | 835 | 0.00E+00 | 82.90% |
| Efet.01.563201.g80.t1 | Paucibacter sp. KCTC 42545, complete genome | 623 | 0.00E+00 | 82.90% |
| Efet.01.612091.g518.t1 | Verminephrobacter eiseniae EF01-2, complete genome | 456 | 0.00E+00 | 82.90% |
| Efet.01.638026.g409.t1 | Verminephrobacter eiseniae EF01-2, complete genome | 1066 | 0.00E+00 | 82.90% |
| Efet.01.640251.g566.t1 | Agromyces flavus strain CPCC 202695 genome assembly, chromosome: I | 408 | 1.90E-126 | 82.90% |
| Efet.01.642550.g702.t1 | Verminephrobacter eiseniae EF01-2, complete genome | 326 | 4.20E-154 | 82.90% |
| Efet.01.645040.g922.t1 | Verminephrobacter eiseniae EF01-2, complete genome | 519 | 0.00E+00 | 82.90% |
| Efet.01.650749.g87.t1 | Verminephrobacter eiseniae EF01-2, complete genome | 1050 | 0.00E+00 | 82.90% |
| Efet.01.654965.g728.t1 | Verminephrobacter eiseniae EF01-2, complete genome | 843 | 0.00E+00 | 82.90% |
| Efet.01.654987.g732.t1 | Verminephrobacter eiseniae EF01-2, complete genome | 1827 | 0.00E+00 | 82.90% |
| Efet.01.655281.g787.t1 | Verminephrobacter eiseniae EF01-2, complete genome | 1605 | 0.00E+00 | 82.90% |
| Efet.01.657766.g1427.t1 | Verminephrobacter eiseniae EF01-2, complete genome | 1953 | 0.00E+00 | 82.90% |
| Efet.01.658235.g1730.t1 | Verminephrobacter eiseniae EF01-2, complete genome | 891 | 0.00E+00 | 82.90% |
| Efet.01.658391.g1945.t1 | Verminephrobacter eiseniae EF01-2, complete genome | 1960 | 0.00E+00 | 82.90% |
| Efet.01.658414.g2040.t1 | Verminephrobacter eiseniae EF01-2, complete genome | 1038 | 0.00E+00 | 82.90% |
| Efet.01.1658479.g1190.t1 | Verminephrobacter eiseniae EF01-2, complete genome | 291 | 2.00E-141 | 82.90% |
| Efet.01.1659131.g1418.t1 | Verminephrobacter eiseniae EF01-2, complete genome | 628 | 0.00E+00 | 82.90% |
| Efet.01.1659497.g1880.t1 | Verminephrobacter eiseniae EF01-2, complete genome | 798 | 0.00E+00 | 82.90% |
| Efet.01.1656247.g842.t1 | Candidatus Aquiluna sp. UB-MaderosW2red genome assembly, chromosome: I | 506 | 4.80E-63 | 82.83% |
| Efet.01.207785.g354.t1 | Verminephrobacter eiseniae EF01-2, complete genome | 269 | 5.90E-132 | 82.80% |
| Efet.01.275134.g937.t1 | Verminephrobacter eiseniae EF01-2, complete genome | 3213 | 0.00E+00 | 82.80% |
| Efet.01.487512.g905.t1 | Agromyces flavus strain CPCC 202695 genome assembly, chromosome: I | 211 | 7.30E-60 | 82.80% |
| Efet.01.616615.g730.t1 | Verminephrobacter eiseniae EF01-2, complete genome | 885 | 0.00E+00 | 82.80% |
| Efet.01.630860.g89.t1 | Verminephrobacter eiseniae EF01-2, complete genome | 825 | 0.00E+00 | 82.80% |
| Efet.01.639207.g490.t1 | Verminephrobacter eiseniae EF01-2, complete genome | 1593 | 0.00E+00 | 82.80% |
| Efet.01.648999.g1279.t1 | Verminephrobacter eiseniae EF01-2, complete genome | 252 | 2.10E-124 | 82.80% |
| Efet.01.654418.g586.t1 | Verminephrobacter eiseniae EF01-2, complete genome | 1350 | 0.00E+00 | 82.80% |
| Efet.01.655128.g763.t1 | Verminephrobacter eiseniae EF01-2, complete genome | 954 | 0.00E+00 | 82.80% |
| Efet.01.655987.g921.t1 | Verminephrobacter eiseniae EF01-2, complete genome | 513 | 0.00E+00 | 82.80% |
| Efet.01.656334.g1000.t1 | Verminephrobacter eiseniae EF01-2, complete genome | 731 | 0.00E+00 | 82.80% |
| Efet.01.656716.g1068.t1 | Verminephrobacter eiseniae EF01-2, complete genome | 609 | 0.00E+00 | 82.80% |
| Efet.01.657158.g1190.t1 | Verminephrobacter eiseniae EF01-2, complete genome | 883 | 0.00E+00 | 82.80% |
| Efet.01.657315.g1242.t1 | Verminephrobacter eiseniae EF01-2, complete genome | 912 | 0.00E+00 | 82.80% |
| Efet.01.657336.g1269.t1 | Verminephrobacter eiseniae EF01-2, complete genome | 312 | 4.80E-154 | 82.80% |
| Efet.01.657766.g1436.t1 | Verminephrobacter eiseniae EF01-2, complete genome | 574 | 0.00E+00 | 82.80% |
| Efet.01.658142.g1677.t1 | Verminephrobacter eiseniae EF01-2, complete genome | 615 | 0.00E+00 | 82.80% |
| Efet.01.658412.g2030.t1 | Verminephrobacter eiseniae EF01-2, complete genome | 825 | 0.00E+00 | 82.80% |
| Efet.01.1657907.g1058.t1 | Variovorax boronicumulans strain J1 chromosome, complete genome | 462 | 0.00E+00 | 82.80% |
| Efet.01.1657963.g1070.t1 | Verminephrobacter eiseniae EF01-2, complete genome | 369 | 1.90E-178 | 82.80% |
| Efet.01.1658216.g1133.t1 | Verminephrobacter eiseniae EF01-2, complete genome | 233 | 2.10E-114 | 82.80% |
| Efet.01.1659377.g1625.t1 | Verminephrobacter eiseniae EF01-2, complete genome | 1086 | 0.00E+00 | 82.80% |
| Efet.01.1659431.g1699.t1 | Verminephrobacter eiseniae EF01-2, complete genome | 1179 | 0.00E+00 | 82.80% |
| Efet.01.1659440.g1718.t1 | Verminephrobacter eiseniae EF01-2, complete genome | 450 | 0.00E+00 | 82.80% |
| Efet.01.1659525.g2023.t1 | Verminephrobacter eiseniae EF01-2, complete genome | 1116 | 0.00E+00 | 82.80% |
| Efet.01.444700.g1097.t1 | Flavobacteriaceae bacterium UJ101, complete genome | 68 | 2.80E-08 | 82.75% |
| Efet.01.1659273.g1500.t1 | Verminephrobacter eiseniae EF01-2, complete genome | 651 | 0.00E+00 | 82.75% |
| Efet.01.64436.g888.t1 | Verminephrobacter eiseniae EF01-2, complete genome | 787 | 0.00E+00 | 82.70% |
| Efet.01.70346.g1252.t1 | Campylobacter gracilis strain ATCC 33236, complete genome | 85 | 4.80E-09 | 82.70% |
| Efet.01.270738.g766.t1 | Verminephrobacter eiseniae EF01-2, complete genome | 852 | 0.00E+00 | 82.70% |
| Efet.01.318747.g589.t1 | Verminephrobacter eiseniae EF01-2, complete genome | 207 | 5.70E-100 | 82.70% |
| Efet.01.440935.g1002.t1 | Verminephrobacter eiseniae EF01-2, complete genome | 2030 | 0.00E+00 | 82.70% |
| Efet.01.550271.g510.t1 | Verminephrobacter eiseniae EF01-2, complete genome | 846 | 0.00E+00 | 82.70% |
| Efet.01.563201.g77.t1 | Verminephrobacter eiseniae EF01-2, complete genome | 1140 | 0.00E+00 | 82.70% |
| Efet.01.578651.g649.t1 | Verminephrobacter eiseniae EF01-2, complete genome | 1272 | 0.00E+00 | 82.70% |
| Efet.01.588418.g945.t1 | Agromyces sp. 30A chromosome, complete genome | 785 | 0.00E+00 | 82.70% |
| Efet.01.625145.g1103.t1 | Verminephrobacter eiseniae EF01-2, complete genome | 292 | 1.50E-132 | 82.70% |
| Efet.01.626208.g1147.t1 | Verminephrobacter eiseniae EF01-2, complete genome | 1428 | 0.00E+00 | 82.70% |
| Efet.01.630200.g7.t1 | Verminephrobacter eiseniae EF01-2, complete genome | 396 | 0.00E+00 | 82.70% |
| Efet.01.642375.g677.t1 | Bacillus cereus E33L, complete genome | 61 | 3.70E-09 | 82.70% |
| Efet.01.651141.g127.t1 | Verminephrobacter eiseniae EF01-2, complete genome | 1953 | 0.00E+00 | 82.70% |
| Efet.01.655622.g840.t1 | Verminephrobacter eiseniae EF01-2, complete genome | 696 | 0.00E+00 | 82.70% |
| Efet.01.656822.g1121.t1 | Verminephrobacter eiseniae EF01-2, complete genome | 429 | 0.00E+00 | 82.70% |
| Efet.01.656891.g1137.t1 | Verminephrobacter eiseniae EF01-2, complete genome | 3363 | 0.00E+00 | 82.70% |
| Efet.01.657766.g1444.t1 | Verminephrobacter eiseniae EF01-2, complete genome | 1173 | 0.00E+00 | 82.70% |
| Efet.01.657900.g1526.t1 | Verminephrobacter eiseniae EF01-2, complete genome | 741 | 0.00E+00 | 82.70% |
| Efet.01.658230.g1724.t1 | Verminephrobacter eiseniae EF01-2, complete genome | 747 | 0.00E+00 | 82.70% |
| Efet.01.658291.g1802.t1 | Microterricola viridarii strain DSM 21772 genome assembly, chromosome: I | 342 | 5.90E-104 | 82.70% |
| Efet.01.658384.g1932.t1 | Verminephrobacter eiseniae EF01-2, complete genome | 2283 | 0.00E+00 | 82.70% |
| Efet.01.658415.g2042.t1 | Verminephrobacter eiseniae EF01-2, complete genome | 3195 | 0.00E+00 | 82.70% |
| Efet.01.1652696.g575.t1 | Verminephrobacter eiseniae EF01-2, complete genome | 528 | 0.00E+00 | 82.70% |
| Efet.01.1655073.g736.t1 | Verminephrobacter eiseniae EF01-2, complete genome | 309 | 9.30E-153 | 82.70% |
| Efet.01.1655581.g769.t1 | Verminephrobacter eiseniae EF01-2, complete genome | 342 | 9.30E-172 | 82.70% |
| Efet.01.1659346.g1577.t1 | Verminephrobacter eiseniae EF01-2, complete genome | 435 | 0.00E+00 | 82.70% |
| Efet.01.1659454.g1753.t1 | Verminephrobacter eiseniae EF01-2, complete genome | 1230 | 0.00E+00 | 82.70% |
| Efet.01.1659465.g1778.t1 | Verminephrobacter eiseniae EF01-2, complete genome | 1149 | 0.00E+00 | 82.70% |
| Efet.01.139123.g728.t1 | Vibrio campbellii strain 151112C chromosome 1, complete sequence | 61 | 1.60E-08 | 82.67% |
| Efet.01.64436.g886.t1 | Verminephrobacter eiseniae EF01-2, complete genome | 981 | 0.00E+00 | 82.60% |
| Efet.01.314858.g470.t1 | Verminephrobacter eiseniae EF01-2, complete genome | 426 | 0.00E+00 | 82.60% |
| Efet.01.318747.g592.t1 | Verminephrobacter eiseniae EF01-2, complete genome | 639 | 0.00E+00 | 82.60% |
| Efet.01.378778.g770.t1 | Verminephrobacter eiseniae EF01-2, complete genome | 1701 | 0.00E+00 | 82.60% |
| Efet.01.471393.g563.t1 | Verminephrobacter eiseniae EF01-2, complete genome | 579 | 0.00E+00 | 82.60% |
| Efet.01.565869.g195.t1 | Verminephrobacter eiseniae EF01-2, complete genome | 381 | 0.00E+00 | 82.60% |
| Efet.01.623992.g1043.t1 | Verminephrobacter eiseniae EF01-2, complete genome | 1116 | 0.00E+00 | 82.60% |
| Efet.01.630200.g13.t1 | Verminephrobacter eiseniae EF01-2, complete genome | 980 | 0.00E+00 | 82.60% |
| Efet.01.646009.g1025.t1 | Verminephrobacter eiseniae EF01-2, complete genome | 420 | 0.00E+00 | 82.60% |
| Efet.01.646642.g1112.t1 | Verminephrobacter eiseniae EF01-2, complete genome | 557 | 0.00E+00 | 82.60% |
| Efet.01.654656.g649.t1 | Verminephrobacter eiseniae EF01-2, complete genome | 834 | 0.00E+00 | 82.60% |
| Efet.01.655224.g773.t1 | Verminephrobacter eiseniae EF01-2, complete genome | 363 | 1.70E-180 | 82.60% |
| Efet.01.656283.g984.t1 | Verminephrobacter eiseniae EF01-2, complete genome | 1773 | 0.00E+00 | 82.60% |
| Efet.01.656558.g1042.t1 | Verminephrobacter eiseniae EF01-2, complete genome | 936 | 0.00E+00 | 82.60% |
| Efet.01.656971.g1149.t1 | Verminephrobacter eiseniae EF01-2, complete genome | 339 | 1.50E-166 | 82.60% |
| Efet.01.657430.g1277.t1 | Verminephrobacter eiseniae EF01-2, complete genome | 909 | 0.00E+00 | 82.60% |
| Efet.01.657700.g1371.t1 | Variovorax paradoxus S110 chromosome 1, complete sequence | 843 | 0.00E+00 | 82.60% |
| Efet.01.657719.g1392.t1 | Verminephrobacter eiseniae EF01-2, complete genome | 750 | 0.00E+00 | 82.60% |
| Efet.01.658177.g1699.t1 | Verminephrobacter eiseniae EF01-2, complete genome | 948 | 0.00E+00 | 82.60% |
| Efet.01.658414.g2038.t1 | Verminephrobacter eiseniae EF01-2, complete genome | 2913 | 0.00E+00 | 82.60% |
| Efet.01.658424.g2121.t1 | Verminephrobacter eiseniae EF01-2, complete genome | 743 | 0.00E+00 | 82.60% |
| Efet.01.1659012.g1358.t1 | Verminephrobacter eiseniae EF01-2, complete genome | 862 | 0.00E+00 | 82.60% |
| Efet.01.1659229.g1474.t1 | Verminephrobacter eiseniae EF01-2, complete genome | 639 | 0.00E+00 | 82.60% |
| Efet.01.1659292.g1520.t1 | Corynebacterium mycetoides strain DSM 20632 genome assembly, chromosome: I | 795 | 0.00E+00 | 82.60% |
| Efet.01.1659489.g1848.t1 | Verminephrobacter eiseniae EF01-2, complete genome | 690 | 0.00E+00 | 82.60% |
| Efet.01.151234.g75.t1 | Verminephrobacter eiseniae EF01-2, complete genome | 267 | 5.80E-123 | 82.50% |
| Efet.01.163163.g671.t1 | Verminephrobacter eiseniae EF01-2, complete genome | 256 | 3.30E-121 | 82.50% |
| Efet.01.308074.g242.t1 | Verminephrobacter eiseniae EF01-2, complete genome | 142 | 3.60E-51 | 82.50% |
| Efet.01.427307.g652.t1 | Verminephrobacter eiseniae EF01-2, complete genome | 582 | 0.00E+00 | 82.50% |
| Efet.01.512410.g290.t1 | Verminephrobacter eiseniae EF01-2, complete genome | 582 | 0.00E+00 | 82.50% |
| Efet.01.591858.g1050.t1 | Acidovorax sp. KKS102, complete genome | 1460 | 0.00E+00 | 82.50% |
| Efet.01.592294.g1068.t1 | Verminephrobacter eiseniae EF01-2, complete genome | 921 | 0.00E+00 | 82.50% |
| Efet.01.602021.g87.t1 | Verminephrobacter eiseniae EF01-2, complete genome | 393 | 0.00E+00 | 82.50% |
| Efet.01.602573.g103.t1 | Verminephrobacter eiseniae EF01-2, complete genome | 684 | 0.00E+00 | 82.50% |
| Efet.01.604212.g154.t1 | Verminephrobacter eiseniae EF01-2, complete genome | 729 | 0.00E+00 | 82.50% |
| Efet.01.623992.g1048.t1 | Verminephrobacter eiseniae EF01-2, complete genome | 1062 | 0.00E+00 | 82.50% |
| Efet.01.632756.g171.t1 | Verminephrobacter eiseniae EF01-2, complete genome | 733 | 0.00E+00 | 82.50% |
| Efet.01.638678.g453.t1 | Verminephrobacter eiseniae EF01-2, complete genome | 924 | 0.00E+00 | 82.50% |
| Efet.01.644010.g856.t1 | Verminephrobacter eiseniae EF01-2, complete genome | 1911 | 0.00E+00 | 82.50% |
| Efet.01.650311.g24.t1 | Verminephrobacter eiseniae EF01-2, complete genome | 1269 | 0.00E+00 | 82.50% |
| Efet.01.653325.g417.t1 | Verminephrobacter eiseniae EF01-2, complete genome | 2718 | 0.00E+00 | 82.50% |
| Efet.01.653526.g443.t1 | Verminephrobacter eiseniae EF01-2, complete genome | 597 | 0.00E+00 | 82.50% |
| Efet.01.657473.g1296.t1 | Verminephrobacter eiseniae EF01-2, complete genome | 804 | 0.00E+00 | 82.50% |
| Efet.01.658142.g1679.t1 | Verminephrobacter eiseniae EF01-2, complete genome | 534 | 0.00E+00 | 82.50% |
| Efet.01.658298.g1819.t1 | Verminephrobacter eiseniae EF01-2, complete genome | 2400 | 0.00E+00 | 82.50% |
| Efet.01.658310.g1834.t1 | Thiomonas sp. CB2 genome assembly ThiCB2, scaffold THICB2_Contig_48 | 495 | 0.00E+00 | 82.50% |
| Efet.01.658427.g2145.t1 | Verminephrobacter eiseniae EF01-2, complete genome | 906 | 0.00E+00 | 82.50% |
| Efet.01.1657672.g1024.t1 | Verminephrobacter eiseniae EF01-2, complete genome | 708 | 0.00E+00 | 82.50% |
| Efet.01.1659242.g1485.t1 | Verminephrobacter eiseniae EF01-2, complete genome | 1234 | 0.00E+00 | 82.50% |
| Efet.01.1659477.g1807.t1 | Verminephrobacter eiseniae EF01-2, complete genome | 110 | 5.70E-43 | 82.50% |
| Efet.01.1658020.g1082.t1 | Verminephrobacter eiseniae EF01-2 plasmid pVEIS01, complete sequence | 189 | 3.60E-86 | 82.43% |
| Efet.01.258956.g337.t1 | Phreatobacter sp. S-12 chromosome, complete genome | 100 | 1.50E-12 | 82.40% |
| Efet.01.500837.g20.t1 | Comamonadaceae bacterium A1 DNA, complete genome | 288 | 7.70E-106 | 82.40% |
| Efet.01.513298.g317.t1 | Verminephrobacter eiseniae EF01-2, complete genome | 1320 | 0.00E+00 | 82.40% |
| Efet.01.546674.g404.t1 | Verminephrobacter eiseniae EF01-2, complete genome | 900 | 0.00E+00 | 82.40% |
| Efet.01.550271.g512.t1 | Verminephrobacter eiseniae EF01-2, complete genome | 993 | 0.00E+00 | 82.40% |
| Efet.01.565049.g170.t1 | Verminephrobacter eiseniae EF01-2, complete genome | 324 | 1.80E-163 | 82.40% |
| Efet.01.581510.g720.t1 | Verminephrobacter eiseniae EF01-2, complete genome | 2067 | 0.00E+00 | 82.40% |
| Efet.01.581759.g731.t1 | Verminephrobacter eiseniae EF01-2, complete genome | 910 | 0.00E+00 | 82.40% |
| Efet.01.616615.g733.t1 | Verminephrobacter eiseniae EF01-2, complete genome | 1161 | 0.00E+00 | 82.40% |
| Efet.01.617238.g758.t1 | Verminephrobacter eiseniae EF01-2, complete genome | 832 | 0.00E+00 | 82.40% |
| Efet.01.617606.g786.t1 | Variovorax boronicumulans strain J1 chromosome, complete genome | 924 | 0.00E+00 | 82.40% |
| Efet.01.626245.g1160.t1 | Verminephrobacter eiseniae EF01-2, complete genome | 1023 | 0.00E+00 | 82.40% |
| Efet.01.630860.g87.t1 | Verminephrobacter eiseniae EF01-2, complete genome | 669 | 0.00E+00 | 82.40% |
| Efet.01.631607.g126.t1 | Verminephrobacter eiseniae EF01-2, complete genome | 930 | 0.00E+00 | 82.40% |
| Efet.01.632798.g172.t1 | Verminephrobacter eiseniae EF01-2, complete genome | 1023 | 0.00E+00 | 82.40% |
| Efet.01.638425.g432.t1 | Verminephrobacter eiseniae EF01-2, complete genome | 253 | 4.10E-123 | 82.40% |
| Efet.01.652956.g383.t1 | Streptomyces lydicus strain A02, complete genome | 127 | 3.10E-14 | 82.40% |
| Efet.01.657248.g1213.t1 | Verminephrobacter eiseniae EF01-2, complete genome | 714 | 0.00E+00 | 82.40% |
| Efet.01.658150.g1688.t1 | Verminephrobacter eiseniae EF01-2, complete genome | 583 | 0.00E+00 | 82.40% |
| Efet.01.658236.g1739.t1 | Verminephrobacter eiseniae EF01-2, complete genome | 486 | 0.00E+00 | 82.40% |
| Efet.01.658281.g1791.t1 | Verminephrobacter eiseniae EF01-2, complete genome | 453 | 0.00E+00 | 82.40% |
| Efet.01.658364.g1897.t1 | Verminephrobacter eiseniae EF01-2, complete genome | 665 | 0.00E+00 | 82.40% |
| Efet.01.658429.g2163.t1 | Verminephrobacter eiseniae EF01-2, complete genome | 1971 | 0.00E+00 | 82.40% |
| Efet.01.1658456.g1187.t1 | Verminephrobacter eiseniae EF01-2, complete genome | 438 | 0.00E+00 | 82.40% |
| Efet.01.1658668.g1244.t1 | Verminephrobacter eiseniae EF01-2, complete genome | 1026 | 0.00E+00 | 82.40% |
| Efet.01.1659087.g1397.t1 | Agromyces flavus strain CPCC 202695 genome assembly, chromosome: I | 424 | 6.80E-124 | 82.40% |
| Efet.01.1659519.g1975.t1 | Verminephrobacter eiseniae EF01-2, complete genome | 924 | 0.00E+00 | 82.40% |
| Efet.01.1659527.g2037.t1 | Verminephrobacter eiseniae EF01-2, complete genome | 1681 | 0.00E+00 | 82.40% |
| Efet.01.658405.g1998.t1 | Pseudomonas aeruginosa strain T63266, complete genome | 653 | 0.00E+00 | 82.33% |
| Efet.01.1647343.g375.t1 | Polaribacter sejongensis strain KCTC 23670 chromosome | 65 | 4.20E-07 | 82.33% |
| Efet.01.1658401.g1172.t1 | Streptomyces peucetius subsp. caesius ATCC 27952 chromosome, complete genome | 68 | 1.70E-09 | 82.33% |
| Efet.01.114669.g767.t1 | Polaribacter sp. ALD11 chromosome, complete genome | 113 | 3.90E-09 | 82.30% |
| Efet.01.318747.g596.t1 | Verminephrobacter eiseniae EF01-2, complete genome | 942 | 0.00E+00 | 82.30% |
| Efet.01.392427.g1064.t1 | Verminephrobacter eiseniae EF01-2, complete genome | 496 | 0.00E+00 | 82.30% |
| Efet.01.427593.g659.t1 | Verminephrobacter eiseniae EF01-2, complete genome | 825 | 0.00E+00 | 82.30% |
| Efet.01.440538.g976.t1 | Verminephrobacter eiseniae EF01-2, complete genome | 453 | 0.00E+00 | 82.30% |
| Efet.01.440935.g998.t1 | Verminephrobacter eiseniae EF01-2, complete genome | 1154 | 0.00E+00 | 82.30% |
| Efet.01.504526.g115.t1 | Verminephrobacter eiseniae EF01-2, complete genome | 922 | 0.00E+00 | 82.30% |
| Efet.01.523923.g555.t1 | Verminephrobacter eiseniae EF01-2, complete genome | 1221 | 0.00E+00 | 82.30% |
| Efet.01.524250.g567.t1 | Verminephrobacter eiseniae EF01-2, complete genome | 568 | 0.00E+00 | 82.30% |
| Efet.01.528741.g657.t1 | Verminephrobacter eiseniae EF01-2, complete genome | 576 | 0.00E+00 | 82.30% |
| Efet.01.530574.g24.t1 | Verminephrobacter eiseniae EF01-2, complete genome | 2245 | 0.00E+00 | 82.30% |
| Efet.01.563201.g92.t1 | Verminephrobacter eiseniae EF01-2, complete genome | 1233 | 0.00E+00 | 82.30% |
| Efet.01.587005.g892.t1 | Verminephrobacter eiseniae EF01-2, complete genome | 381 | 0.00E+00 | 82.30% |
| Efet.01.595491.g1159.t1 | Verminephrobacter eiseniae EF01-2, complete genome | 1669 | 0.00E+00 | 82.30% |
| Efet.01.603056.g123.t1 | Verminephrobacter eiseniae EF01-2, complete genome | 1638 | 0.00E+00 | 82.30% |
| Efet.01.611343.g473.t1 | Verminephrobacter eiseniae EF01-2, complete genome | 453 | 0.00E+00 | 82.30% |
| Efet.01.626245.g1154.t1 | Verminephrobacter eiseniae EF01-2, complete genome | 726 | 0.00E+00 | 82.30% |
| Efet.01.629329.g1284.t1 | Verminephrobacter eiseniae EF01-2, complete genome | 914 | 0.00E+00 | 82.30% |
| Efet.01.638026.g414.t1 | Verminephrobacter eiseniae EF01-2, complete genome | 453 | 0.00E+00 | 82.30% |
| Efet.01.646179.g1050.t1 | Verminephrobacter eiseniae EF01-2, complete genome | 912 | 0.00E+00 | 82.30% |
| Efet.01.648372.g1235.t1 | Verminephrobacter eiseniae EF01-2, complete genome | 672 | 0.00E+00 | 82.30% |
| Efet.01.648797.g1266.t1 | Verminephrobacter eiseniae EF01-2, complete genome | 1092 | 0.00E+00 | 82.30% |
| Efet.01.651136.g122.t1 | Verminephrobacter eiseniae EF01-2, complete genome | 2022 | 0.00E+00 | 82.30% |
| Efet.01.652442.g300.t1 | Verminephrobacter eiseniae EF01-2, complete genome | 693 | 0.00E+00 | 82.30% |
| Efet.01.652587.g316.t1 | Verminephrobacter eiseniae EF01-2, complete genome | 651 | 0.00E+00 | 82.30% |
| Efet.01.654656.g668.t1 | Verminephrobacter eiseniae EF01-2, complete genome | 714 | 0.00E+00 | 82.30% |
| Efet.01.656813.g1110.t1 | Verminephrobacter eiseniae EF01-2, complete genome | 825 | 0.00E+00 | 82.30% |
| Efet.01.657308.g1230.t1 | Verminephrobacter eiseniae EF01-2, complete genome | 909 | 0.00E+00 | 82.30% |
| Efet.01.657766.g1445.t1 | Verminephrobacter eiseniae EF01-2, complete genome | 756 | 0.00E+00 | 82.30% |
| Efet.01.657803.g1478.t1 | Verminephrobacter eiseniae EF01-2, complete genome | 786 | 0.00E+00 | 82.30% |
| Efet.01.1659526.g2031.t1 | Verminephrobacter eiseniae EF01-2, complete genome | 1419 | 0.00E+00 | 82.30% |
| Efet.01.22048.g1617.t1 | Verminephrobacter eiseniae EF01-2, complete genome | 706 | 0.00E+00 | 82.20% |
| Efet.01.185951.g1685.t1 | Verminephrobacter eiseniae EF01-2, complete genome | 454 | 0.00E+00 | 82.20% |
| Efet.01.269025.g704.t1 | Verminephrobacter eiseniae EF01-2, complete genome | 681 | 0.00E+00 | 82.20% |
| Efet.01.300440.g18.t1 | Verminephrobacter eiseniae EF01-2, complete genome | 1377 | 0.00E+00 | 82.20% |
| Efet.01.455545.g144.t1 | Tessaracoccus sp. NSG39, complete genome | 173 | 9.10E-45 | 82.20% |
| Efet.01.544969.g362.t1 | Verminephrobacter eiseniae EF01-2, complete genome | 885 | 0.00E+00 | 82.20% |
| Efet.01.565049.g157.t1 | Verminephrobacter eiseniae EF01-2, complete genome | 603 | 0.00E+00 | 82.20% |
| Efet.01.630725.g57.t1 | Verminephrobacter eiseniae EF01-2, complete genome | 604 | 0.00E+00 | 82.20% |
| Efet.01.638023.g398.t1 | Verminephrobacter eiseniae EF01-2, complete genome | 1131 | 0.00E+00 | 82.20% |
| Efet.01.639369.g505.t1 | Verminephrobacter eiseniae EF01-2, complete genome | 792 | 0.00E+00 | 82.20% |
| Efet.01.641218.g614.t1 | Verminephrobacter eiseniae EF01-2, complete genome | 984 | 0.00E+00 | 82.20% |
| Efet.01.644010.g855.t1 | Verminephrobacter eiseniae EF01-2, complete genome | 565 | 0.00E+00 | 82.20% |
| Efet.01.646487.g1097.t1 | Verminephrobacter eiseniae EF01-2, complete genome | 708 | 0.00E+00 | 82.20% |
| Efet.01.646642.g1106.t1 | Verminephrobacter eiseniae EF01-2, complete genome | 1278 | 0.00E+00 | 82.20% |
| Efet.01.650480.g59.t1 | Verminephrobacter eiseniae EF01-2, complete genome | 682 | 0.00E+00 | 82.20% |
| Efet.01.654418.g591.t1 | Verminephrobacter eiseniae EF01-2, complete genome | 2742 | 0.00E+00 | 82.20% |
| Efet.01.655755.g859.t1 | Verminephrobacter eiseniae EF01-2, complete genome | 656 | 0.00E+00 | 82.20% |
| Efet.01.658012.g1555.t1 | Verminephrobacter eiseniae EF01-2, complete genome | 252 | 7.50E-119 | 82.20% |
| Efet.01.658044.g1594.t1 | Verminephrobacter eiseniae EF01-2, complete genome | 282 | 3.90E-135 | 82.20% |
| Efet.01.658331.g1860.t1 | Verminephrobacter eiseniae EF01-2, complete genome | 384 | 0.00E+00 | 82.20% |
| Efet.01.658339.g1874.t1 | Verminephrobacter eiseniae EF01-2, complete genome | 1056 | 0.00E+00 | 82.20% |
| Efet.01.1654205.g670.t1 | Frondihabitans sp. PAMC28766, complete genome | 249 | 2.90E-64 | 82.20% |
| Efet.01.1655278.g748.t1 | Verminephrobacter eiseniae EF01-2, complete genome | 347 | 9.30E-172 | 82.20% |
| Efet.01.1655883.g796.t1 | Verminephrobacter eiseniae EF01-2, complete genome | 226 | 2.10E-110 | 82.20% |
| Efet.01.1659346.g1576.t1 | Verminephrobacter eiseniae EF01-2, complete genome | 849 | 0.00E+00 | 82.20% |
| Efet.01.318747.g594.t1 | Verminephrobacter eiseniae EF01-2, complete genome | 1281 | 0.00E+00 | 82.10% |
| Efet.01.373468.g653.t1 | Verminephrobacter eiseniae EF01-2, complete genome | 1281 | 0.00E+00 | 82.10% |
| Efet.01.578167.g624.t1 | Verminephrobacter eiseniae EF01-2, complete genome | 1352 | 0.00E+00 | 82.10% |
| Efet.01.591301.g1031.t1 | Verminephrobacter eiseniae EF01-2, complete genome | 552 | 0.00E+00 | 82.10% |
| Efet.01.606486.g280.t1 | Verminephrobacter eiseniae EF01-2, complete genome | 1185 | 0.00E+00 | 82.10% |
| Efet.01.612091.g520.t1 | Verminephrobacter eiseniae EF01-2, complete genome | 400 | 0.00E+00 | 82.10% |
| Efet.01.613026.g577.t1 | Verminephrobacter eiseniae EF01-2, complete genome | 765 | 0.00E+00 | 82.10% |
| Efet.01.617238.g759.t1 | Verminephrobacter eiseniae EF01-2, complete genome | 975 | 0.00E+00 | 82.10% |
| Efet.01.625145.g1100.t1 | Acidovorax sp. T1, complete genome | 540 | 7.00E-156 | 82.10% |
| Efet.01.626208.g1148.t1 | Verminephrobacter eiseniae EF01-2, complete genome | 573 | 0.00E+00 | 82.10% |
| Efet.01.636814.g350.t1 | Verminephrobacter eiseniae EF01-2, complete genome | 744 | 0.00E+00 | 82.10% |
| Efet.01.637131.g365.t1 | Agromyces flavus strain CPCC 202695 genome assembly, chromosome: I | 528 | 4.70E-62 | 82.10% |
| Efet.01.638678.g451.t1 | Verminephrobacter eiseniae EF01-2, complete genome | 1432 | 0.00E+00 | 82.10% |
| Efet.01.642550.g700.t1 | Verminephrobacter eiseniae EF01-2, complete genome | 2531 | 0.00E+00 | 82.10% |
| Efet.01.644746.g893.t1 | Verminephrobacter eiseniae EF01-2, complete genome | 266 | 9.60E-132 | 82.10% |
| Efet.01.647987.g1209.t1 | Verminephrobacter eiseniae EF01-2, complete genome | 1509 | 0.00E+00 | 82.10% |
| Efet.01.651148.g136.t1 | Verminephrobacter eiseniae EF01-2, complete genome | 1581 | 0.00E+00 | 82.10% |
| Efet.01.654769.g694.t1 | Verminephrobacter eiseniae EF01-2, complete genome | 585 | 0.00E+00 | 82.10% |
| Efet.01.656558.g1043.t1 | Verminephrobacter eiseniae EF01-2, complete genome | 358 | 4.30E-178 | 82.10% |
| Efet.01.657766.g1443.t1 | Verminephrobacter eiseniae EF01-2, complete genome | 438 | 0.00E+00 | 82.10% |
| Efet.01.658310.g1833.t1 | Pandoraea pnomenusa strain RB-44, complete genome | 440 | 2.40E-146 | 82.10% |
| Efet.01.1632518.g123.t1 | Verminephrobacter eiseniae EF01-2, complete genome | 273 | 9.40E-131 | 82.10% |
| Efet.01.1658727.g1260.t1 | Verminephrobacter eiseniae EF01-2, complete genome | 705 | 0.00E+00 | 82.10% |
| Efet.01.1659445.g1730.t1 | Verminephrobacter eiseniae EF01-2, complete genome | 3182 | 0.00E+00 | 82.10% |
| Efet.01.1659523.g2006.t1 | Verminephrobacter eiseniae EF01-2, complete genome | 546 | 0.00E+00 | 82.10% |
| Efet.01.1659525.g2015.t1 | Verminephrobacter eiseniae EF01-2, complete genome | 1293 | 0.00E+00 | 82.10% |
| Efet.01.60696.g660.t1 | Flavobacteriaceae bacterium UJ101, complete genome | 66 | 3.70E-10 | 82.00% |
| Efet.01.67136.g1053.t1 | Amycolatopsis methanolica 239, complete genome | 67 | 3.30E-07 | 82.00% |
| Efet.01.104145.g228.t1 | Verminephrobacter eiseniae EF01-2, complete genome | 930 | 0.00E+00 | 82.00% |
| Efet.01.120927.g1085.t1 | [Pseudomonas] mesoacidophila strain ATCC 31433 chromosome 2, complete sequence | 67 | 7.60E-07 | 82.00% |
| Efet.01.147347.g1111.t1 | Uncultured bacterium clone GYQASSE01D3J1Z genomic sequence | 161 | 8.30E-38 | 82.00% |
| Efet.01.151234.g65.t1 | Verminephrobacter eiseniae EF01-2, complete genome | 432 | 0.00E+00 | 82.00% |
| Efet.01.180054.g1400.t1 | Streptomyces xinghaiensis S187 chromosome, complete genome | 78 | 9.60E-06 | 82.00% |
| Efet.01.271446.g803.t1 | Niabella ginsenosidivorans strain BS26, complete genome | 67 | 2.00E-07 | 82.00% |
| Efet.01.275134.g930.t1 | Verminephrobacter eiseniae EF01-2, complete genome | 552 | 0.00E+00 | 82.00% |
| Efet.01.325607.g791.t1 | Verminephrobacter eiseniae EF01-2, complete genome | 813 | 0.00E+00 | 82.00% |
| Efet.01.415394.g357.t1 | Bacillus infantis NRRL B-14911, complete genome | 83 | 3.00E-09 | 82.00% |
| Efet.01.419625.g457.t1 | Verminephrobacter eiseniae EF01-2, complete genome | 969 | 0.00E+00 | 82.00% |
| Efet.01.494042.g1047.t1 | Verminephrobacter eiseniae EF01-2, complete genome | 762 | 0.00E+00 | 82.00% |
| Efet.01.505702.g143.t1 | Actinoalloteichus sp. GBA129-24, complete genome | 69 | 2.00E-08 | 82.00% |
| Efet.01.530573.g17.t1 | Verminephrobacter eiseniae EF01-2, complete genome | 1722 | 0.00E+00 | 82.00% |
| Efet.01.532148.g66.t1 | Verminephrobacter eiseniae EF01-2, complete genome | 182 | 1.50E-78 | 82.00% |
| Efet.01.533383.g126.t1 | Verminephrobacter eiseniae EF01-2, complete genome | 351 | 8.20E-177 | 82.00% |
| Efet.01.549455.g480.t1 | Aeromonas hydrophila strain GYK1, complete genome | 73 | 9.10E-10 | 82.00% |
| Efet.01.561853.g33.t1 | Streptomyces peucetius subsp. caesius ATCC 27952 chromosome, complete genome | 77 | 1.60E-07 | 82.00% |
| Efet.01.594275.g1117.t1 | Verminephrobacter eiseniae EF01-2, complete genome | 423 | 0.00E+00 | 82.00% |
| Efet.01.628685.g1261.t1 | Verminephrobacter eiseniae EF01-2, complete genome | 297 | 1.40E-147 | 82.00% |
| Efet.01.633502.g195.t1 | Verminephrobacter eiseniae EF01-2, complete genome | 225 | 4.20E-108 | 82.00% |
| Efet.01.634939.g268.t1 | Verminephrobacter eiseniae EF01-2, complete genome | 585 | 0.00E+00 | 82.00% |
| Efet.01.638425.g433.t1 | Verminephrobacter eiseniae EF01-2, complete genome | 246 | 6.10E-121 | 82.00% |
| Efet.01.639706.g534.t1 | Agromyces flavus strain CPCC 202695 genome assembly, chromosome: I | 232 | 1.00E-53 | 82.00% |
| Efet.01.642837.g724.t1 | Verminephrobacter eiseniae EF01-2, complete genome | 285 | 1.30E-141 | 82.00% |
| Efet.01.651000.g104.t1 | Pontibacter actiniarum DSM 19842, complete genome | 70 | 7.30E-09 | 82.00% |
| Efet.01.654191.g542.t1 | Rathayibacter tritici strain NCPPB 1953, complete genome | 63 | 1.10E-06 | 82.00% |
| Efet.01.654418.g598.t1 | Verminephrobacter eiseniae EF01-2, complete genome | 843 | 0.00E+00 | 82.00% |
| Efet.01.654656.g663.t1 | Verminephrobacter eiseniae EF01-2, complete genome | 834 | 0.00E+00 | 82.00% |
| Efet.01.656216.g967.t1 | Verminephrobacter eiseniae EF01-2, complete genome | 785 | 0.00E+00 | 82.00% |
| Efet.01.657624.g1343.t1 | Verminephrobacter eiseniae EF01-2, complete genome | 2226 | 0.00E+00 | 82.00% |
| Efet.01.657626.g1353.t1 | Verminephrobacter eiseniae EF01-2, complete genome | 927 | 0.00E+00 | 82.00% |
| Efet.01.657766.g1422.t1 | Verminephrobacter eiseniae EF01-2, complete genome | 378 | 0.00E+00 | 82.00% |
| Efet.01.657766.g1426.t1 | Verminephrobacter eiseniae EF01-2, complete genome | 894 | 0.00E+00 | 82.00% |
| Efet.01.657820.g1492.t1 | Diaphorobacter polyhydroxybutyrativorans strain SL-205, complete genome | 384 | 9.00E-105 | 82.00% |
| Efet.01.657900.g1528.t1 | Verminephrobacter eiseniae EF01-2, complete genome | 1302 | 0.00E+00 | 82.00% |
| Efet.01.658084.g1622.t1 | Verminephrobacter eiseniae EF01-2, complete genome | 1750 | 0.00E+00 | 82.00% |
| Efet.01.658417.g2073.t1 | Verminephrobacter eiseniae EF01-2, complete genome | 845 | 0.00E+00 | 82.00% |
| Efet.01.1633463.g132.t1 | Zobellella denitrificans strain F13-1 chromosome, complete genome | 58 | 6.80E-06 | 82.00% |
| Efet.01.1646855.g359.t1 | Verminephrobacter eiseniae EF01-2, complete genome | 219 | 1.40E-101 | 82.00% |
| Efet.01.1653575.g625.t1 | Agromyces sp. 30A chromosome, complete genome | 324 | 4.90E-93 | 82.00% |
| Efet.01.1654284.g679.t1 | Brevibacterium linens strain SMQ-1335, complete genome | 68 | 3.60E-06 | 82.00% |
| Efet.01.1658685.g1250.t1 | Verminephrobacter eiseniae EF01-2, complete genome | 561 | 0.00E+00 | 82.00% |
| Efet.01.1658804.g1279.t1 | Verminephrobacter eiseniae EF01-2, complete genome | 417 | 0.00E+00 | 82.00% |
| Efet.01.1659314.g1543.t1 | Verminephrobacter eiseniae EF01-2, complete genome | 765 | 0.00E+00 | 82.00% |
| Efet.01.1659447.g1734.t1 | Verminephrobacter eiseniae EF01-2, complete genome | 1063 | 0.00E+00 | 82.00% |
| Efet.01.1659449.g1739.t1 | Verminephrobacter eiseniae EF01-2, complete genome | 393 | 0.00E+00 | 82.00% |
| Efet.01.279930.g1100.t1 | Verminephrobacter eiseniae EF01-2, complete genome | 1872 | 0.00E+00 | 81.90% |
| Efet.01.282031.g1162.t1 | Verminephrobacter eiseniae EF01-2, complete genome | 1731 | 0.00E+00 | 81.90% |
| Efet.01.320812.g657.t1 | Massilia sp. WG5, complete sequence | 95 | 1.40E-14 | 81.90% |
| Efet.01.325607.g793.t1 | Verminephrobacter eiseniae EF01-2, complete genome | 1056 | 0.00E+00 | 81.90% |
| Efet.01.569918.g321.t1 | Verminephrobacter eiseniae EF01-2, complete genome | 1695 | 0.00E+00 | 81.90% |
| Efet.01.591235.g1030.t1 | Verminephrobacter eiseniae EF01-2, complete genome | 957 | 0.00E+00 | 81.90% |
| Efet.01.606486.g281.t1 | Verminephrobacter eiseniae EF01-2, complete genome | 759 | 0.00E+00 | 81.90% |
| Efet.01.625433.g1116.t1 | Verminephrobacter eiseniae EF01-2, complete genome | 687 | 0.00E+00 | 81.90% |
| Efet.01.638425.g437.t1 | Verminephrobacter eiseniae EF01-2, complete genome | 705 | 0.00E+00 | 81.90% |
| Efet.01.642630.g707.t1 | Ralstonia pickettii DTP0602 chromosome 1, complete sequence | 191 | 7.10E-50 | 81.90% |
| Efet.01.645268.g968.t1 | Verminephrobacter eiseniae EF01-2, complete genome | 309 | 3.80E-148 | 81.90% |
| Efet.01.645500.g989.t1 | Verminephrobacter eiseniae EF01-2, complete genome | 828 | 0.00E+00 | 81.90% |
| Efet.01.650931.g98.t1 | Verminephrobacter eiseniae EF01-2, complete genome | 288 | 1.70E-143 | 81.90% |
| Efet.01.652858.g351.t1 | Verminephrobacter eiseniae EF01-2, complete genome | 870 | 0.00E+00 | 81.90% |
| Efet.01.653735.g473.t1 | Burkholderia cepacia ATCC 25416 chromosome 2, complete sequence | 814 | 0.00E+00 | 81.90% |
| Efet.01.656716.g1067.t1 | Verminephrobacter eiseniae EF01-2, complete genome | 501 | 0.00E+00 | 81.90% |
| Efet.01.656952.g1147.t1 | Verminephrobacter eiseniae EF01-2, complete genome | 751 | 0.00E+00 | 81.90% |
| Efet.01.657315.g1244.t1 | Verminephrobacter eiseniae EF01-2, complete genome | 1347 | 0.00E+00 | 81.90% |
| Efet.01.657766.g1423.t1 | Verminephrobacter eiseniae EF01-2, complete genome | 789 | 0.00E+00 | 81.90% |
| Efet.01.657766.g1440.t1 | Verminephrobacter eiseniae EF01-2, complete genome | 723 | 0.00E+00 | 81.90% |
| Efet.01.658040.g1576.t1 | Verminephrobacter eiseniae EF01-2, complete genome | 579 | 0.00E+00 | 81.90% |
| Efet.01.658041.g1585.t1 | Verminephrobacter eiseniae EF01-2, complete genome | 1233 | 0.00E+00 | 81.90% |
| Efet.01.658416.g2057.t1 | Verminephrobacter eiseniae EF01-2, complete genome | 647 | 0.00E+00 | 81.90% |
| Efet.01.658416.g2062.t1 | Verminephrobacter eiseniae EF01-2, complete genome | 852 | 0.00E+00 | 81.90% |
| Efet.01.1659421.g1684.t1 | Verminephrobacter eiseniae EF01-2, complete genome | 1215 | 0.00E+00 | 81.90% |
| Efet.01.1659496.g1876.t1 | Verminephrobacter eiseniae EF01-2, complete genome | 990 | 0.00E+00 | 81.90% |
| Efet.01.59384.g592.t1 | Verminephrobacter eiseniae EF01-2, complete genome | 212 | 5.40E-103 | 81.80% |
| Efet.01.377408.g740.t1 | Verminephrobacter eiseniae EF01-2, complete genome | 411 | 0.00E+00 | 81.80% |
| Efet.01.520113.g480.t1 | Verminephrobacter eiseniae EF01-2, complete genome | 1392 | 0.00E+00 | 81.80% |
| Efet.01.546787.g410.t1 | Verminephrobacter eiseniae EF01-2, complete genome | 530 | 0.00E+00 | 81.80% |
| Efet.01.592582.g1080.t1 | Verminephrobacter eiseniae EF01-2, complete genome | 335 | 7.20E-170 | 81.80% |
| Efet.01.611807.g505.t1 | Verminephrobacter eiseniae EF01-2, complete genome | 891 | 0.00E+00 | 81.80% |
| Efet.01.612091.g514.t1 | Verminephrobacter eiseniae EF01-2, complete genome | 1170 | 0.00E+00 | 81.80% |
| Efet.01.614331.g641.t1 | Agromyces sp. 30A chromosome, complete genome | 571 | 3.90E-174 | 81.80% |
| Efet.01.618401.g827.t1 | Verminephrobacter eiseniae EF01-2, complete genome | 891 | 0.00E+00 | 81.80% |
| Efet.01.629376.g1293.t1 | Verminephrobacter eiseniae EF01-2, complete genome | 594 | 0.00E+00 | 81.80% |
| Efet.01.636598.g341.t1 | Verminephrobacter eiseniae EF01-2, complete genome | 1092 | 0.00E+00 | 81.80% |
| Efet.01.643099.g758.t1 | Verminephrobacter eiseniae EF01-2, complete genome | 404 | 0.00E+00 | 81.80% |
| Efet.01.643898.g835.t1 | Variovorax sp. HW608 genome assembly, chromosome: I | 468 | 2.40E-139 | 81.80% |
| Efet.01.645268.g963.t1 | Verminephrobacter eiseniae EF01-2, complete genome | 948 | 0.00E+00 | 81.80% |
| Efet.01.646642.g1110.t1 | Verminephrobacter eiseniae EF01-2, complete genome | 606 | 0.00E+00 | 81.80% |
| Efet.01.654656.g647.t1 | Verminephrobacter eiseniae EF01-2, complete genome | 471 | 0.00E+00 | 81.80% |
| Efet.01.654656.g658.t1 | Verminephrobacter eiseniae EF01-2, complete genome | 840 | 0.00E+00 | 81.80% |
| Efet.01.655622.g842.t1 | Verminephrobacter eiseniae EF01-2, complete genome | 513 | 0.00E+00 | 81.80% |
| Efet.01.657766.g1421.t1 | Verminephrobacter eiseniae EF01-2, complete genome | 846 | 0.00E+00 | 81.80% |
| Efet.01.658282.g1794.t1 | Verminephrobacter eiseniae EF01-2, complete genome | 1083 | 0.00E+00 | 81.80% |
| Efet.01.658417.g2074.t1 | Verminephrobacter eiseniae EF01-2, complete genome | 832 | 0.00E+00 | 81.80% |
| Efet.01.1658215.g1131.t1 | Verminephrobacter eiseniae EF01-2, complete genome | 499 | 0.00E+00 | 81.80% |
| Efet.01.1658720.g1257.t1 | Verminephrobacter eiseniae EF01-2, complete genome | 471 | 0.00E+00 | 81.80% |
| Efet.01.1659356.g1597.t1 | Verminephrobacter eiseniae EF01-2, complete genome | 903 | 0.00E+00 | 81.80% |
| Efet.01.1659399.g1655.t1 | Verminephrobacter eiseniae EF01-2, complete genome | 477 | 0.00E+00 | 81.80% |
| Efet.01.655982.g916.t1 | Microbacterium sp. XT11, complete genome | 112 | 4.20E-06 | 81.75% |
| Efet.01.292188.g1514.t1 | Verminephrobacter eiseniae EF01-2, complete genome | 522 | 0.00E+00 | 81.70% |
| Efet.01.430820.g755.t1 | Verminephrobacter eiseniae EF01-2, complete genome | 534 | 0.00E+00 | 81.70% |
| Efet.01.471393.g565.t1 | Verminephrobacter eiseniae EF01-2, complete genome | 945 | 0.00E+00 | 81.70% |
| Efet.01.574381.g454.t1 | Verminephrobacter eiseniae EF01-2, complete genome | 1164 | 0.00E+00 | 81.70% |
| Efet.01.581759.g732.t1 | Verminephrobacter eiseniae EF01-2, complete genome | 644 | 0.00E+00 | 81.70% |
| Efet.01.587924.g924.t1 | Verminephrobacter eiseniae EF01-2, complete genome | 609 | 0.00E+00 | 81.70% |
| Efet.01.594275.g1113.t1 | Verminephrobacter eiseniae EF01-2, complete genome | 1023 | 0.00E+00 | 81.70% |
| Efet.01.607412.g311.t1 | Verminephrobacter eiseniae EF01-2, complete genome | 1983 | 0.00E+00 | 81.70% |
| Efet.01.612230.g532.t1 | Agromyces sp. 30A chromosome, complete genome | 270 | 1.10E-53 | 81.70% |
| Efet.01.617251.g761.t1 | Verminephrobacter eiseniae EF01-2, complete genome | 360 | 0.00E+00 | 81.70% |
| Efet.01.629376.g1290.t1 | Verminephrobacter eiseniae EF01-2, complete genome | 899 | 0.00E+00 | 81.70% |
| Efet.01.642149.g669.t1 | Agromyces aureus strain AR33, complete genome | 200 | 6.70E-53 | 81.70% |
| Efet.01.646009.g1024.t1 | Verminephrobacter eiseniae EF01-2, complete genome | 415 | 0.00E+00 | 81.70% |
| Efet.01.651940.g248.t1 | Verminephrobacter eiseniae EF01-2, complete genome | 298 | 1.90E-147 | 81.70% |
| Efet.01.652103.g258.t1 | Agromyces sp. 30A chromosome, complete genome | 242 | 8.20E-70 | 81.70% |
| Efet.01.658410.g2021.t1 | Verminephrobacter eiseniae EF01-2, complete genome | 828 | 0.00E+00 | 81.70% |
| Efet.01.658425.g2128.t1 | Verminephrobacter eiseniae EF01-2, complete genome | 507 | 0.00E+00 | 81.70% |
| Efet.01.1654225.g672.t1 | Paraburkholderia sprentiae WSM5005 chromosome 1, complete sequence | 101 | 1.50E-23 | 81.70% |
| Efet.01.1658810.g1282.t1 | Magnetospirillum sp. ME-1, complete genome | 89 | 4.70E-09 | 81.70% |
| Efet.01.1659180.g1446.t1 | Verminephrobacter eiseniae EF01-2, complete genome | 1218 | 0.00E+00 | 81.70% |
| Efet.01.1659338.g1566.t1 | Verminephrobacter eiseniae EF01-2, complete genome | 1374 | 0.00E+00 | 81.70% |
| Efet.01.1659396.g1650.t1 | Verminephrobacter eiseniae EF01-2, complete genome | 594 | 0.00E+00 | 81.70% |
| Efet.01.1659496.g1877.t1 | Verminephrobacter eiseniae EF01-2, complete genome | 375 | 0.00E+00 | 81.70% |
| Efet.01.647154.g1159.t1 | Verminephrobacter eiseniae EF01-2, complete genome | 318 | 1.10E-157 | 81.67% |
| Efet.01.658236.g1732.t1 | Verminephrobacter eiseniae EF01-2, complete genome | 885 | 0.00E+00 | 81.67% |
| Efet.01.658430.g2169.t1 | Verminephrobacter eiseniae EF01-2, complete genome | 312 | 1.40E-154 | 81.67% |
| Efet.01.270738.g768.t1 | Verminephrobacter eiseniae EF01-2, complete genome | 410 | 0.00E+00 | 81.60% |
| Efet.01.287526.g1372.t1 | Verminephrobacter eiseniae EF01-2, complete genome | 1254 | 0.00E+00 | 81.60% |
| Efet.01.464530.g384.t1 | Verminephrobacter eiseniae EF01-2, complete genome | 762 | 0.00E+00 | 81.60% |
| Efet.01.492121.g997.t1 | Verminephrobacter eiseniae EF01-2, complete genome | 457 | 0.00E+00 | 81.60% |
| Efet.01.494042.g1046.t1 | Verminephrobacter eiseniae EF01-2, complete genome | 503 | 0.00E+00 | 81.60% |
| Efet.01.546943.g418.t1 | Verminephrobacter eiseniae EF01-2, complete genome | 1593 | 0.00E+00 | 81.60% |
| Efet.01.563201.g79.t1 | Verminephrobacter eiseniae EF01-2, complete genome | 1536 | 0.00E+00 | 81.60% |
| Efet.01.577373.g569.t1 | Verminephrobacter eiseniae EF01-2, complete genome | 366 | 1.60E-179 | 81.60% |
| Efet.01.578167.g627.t1 | Verminephrobacter eiseniae EF01-2, complete genome | 571 | 0.00E+00 | 81.60% |
| Efet.01.578612.g647.t1 | Verminephrobacter eiseniae EF01-2, complete genome | 1230 | 0.00E+00 | 81.60% |
| Efet.01.624347.g1069.t1 | Verminephrobacter eiseniae EF01-2, complete genome | 1563 | 0.00E+00 | 81.60% |
| Efet.01.629720.g1309.t1 | Leifsonia sp. 98AMF genome assembly, chromosome: I | 381 | 5.70E-110 | 81.60% |
| Efet.01.642087.g655.t1 | Verminephrobacter eiseniae EF01-2, complete genome | 480 | 0.00E+00 | 81.60% |
| Efet.01.643024.g747.t1 | Verminephrobacter eiseniae EF01-2, complete genome | 2304 | 0.00E+00 | 81.60% |
| Efet.01.645138.g941.t1 | Verminephrobacter eiseniae EF01-2, complete genome | 843 | 0.00E+00 | 81.60% |
| Efet.01.645268.g967.t1 | Verminephrobacter eiseniae EF01-2, complete genome | 693 | 0.00E+00 | 81.60% |
| Efet.01.646487.g1093.t1 | Verminephrobacter eiseniae EF01-2, complete genome | 1419 | 0.00E+00 | 81.60% |
| Efet.01.651136.g120.t1 | Verminephrobacter eiseniae EF01-2, complete genome | 1470 | 0.00E+00 | 81.60% |
| Efet.01.651141.g131.t1 | Verminephrobacter eiseniae EF01-2, complete genome | 1465 | 0.00E+00 | 81.60% |
| Efet.01.651229.g151.t1 | Verminephrobacter eiseniae EF01-2, complete genome | 372 | 2.20E-148 | 81.60% |
| Efet.01.651441.g171.t1 | Verminephrobacter eiseniae EF01-2, complete genome | 336 | 4.00E-164 | 81.60% |
| Efet.01.656795.g1103.t1 | Verminephrobacter eiseniae EF01-2, complete genome | 843 | 0.00E+00 | 81.60% |
| Efet.01.656816.g1118.t1 | Verminephrobacter eiseniae EF01-2, complete genome | 872 | 0.00E+00 | 81.60% |
| Efet.01.658142.g1674.t1 | Verminephrobacter eiseniae EF01-2, complete genome | 687 | 0.00E+00 | 81.60% |
| Efet.01.658267.g1776.t1 | Verminephrobacter eiseniae EF01-2, complete genome | 312 | 2.20E-147 | 81.60% |
| Efet.01.658405.g1999.t1 | Achromobacter xylosoxidans strain FDAARGOS_162 chromosome, complete genome | 1429 | 0.00E+00 | 81.60% |
| Efet.01.1656182.g833.t1 | Verminephrobacter eiseniae EF01-2, complete genome | 570 | 0.00E+00 | 81.60% |
| Efet.01.1659505.g1904.t1 | Verminephrobacter eiseniae EF01-2, complete genome | 1140 | 0.00E+00 | 81.60% |
| Efet.01.1659506.g1909.t1 | Verminephrobacter eiseniae EF01-2, complete genome | 618 | 0.00E+00 | 81.60% |
| Efet.01.174699.g1161.t1 | Mycobacterium aurum isolate liquid genome assembly, chromosome: I | 79 | 5.80E-14 | 81.50% |
| Efet.01.305235.g170.t1 | Verminephrobacter eiseniae EF01-2, complete genome | 758 | 0.00E+00 | 81.50% |
| Efet.01.321963.g683.t1 | Verminephrobacter eiseniae EF01-2, complete genome | 249 | 5.70E-121 | 81.50% |
| Efet.01.419552.g453.t1 | Verminephrobacter eiseniae EF01-2, complete genome | 366 | 4.50E-140 | 81.50% |
| Efet.01.551303.g529.t1 | Agromyces aureus strain AR33, complete genome | 571 | 2.40E-165 | 81.50% |
| Efet.01.553408.g589.t1 | Verminephrobacter eiseniae EF01-2, complete genome | 837 | 0.00E+00 | 81.50% |
| Efet.01.565865.g194.t1 | Agromyces flavus strain CPCC 202695 genome assembly, chromosome: I | 574 | 4.00E-159 | 81.50% |
| Efet.01.590200.g999.t1 | Verminephrobacter eiseniae EF01-2, complete genome | 1040 | 0.00E+00 | 81.50% |
| Efet.01.594275.g1116.t1 | Verminephrobacter eiseniae EF01-2, complete genome | 639 | 0.00E+00 | 81.50% |
| Efet.01.594594.g1127.t1 | Verminephrobacter eiseniae EF01-2, complete genome | 291 | 6.60E-144 | 81.50% |
| Efet.01.598881.g1267.t1 | Microbacterium sp. BH-3-3-3, complete genome | 297 | 2.10E-78 | 81.50% |
| Efet.01.605365.g206.t1 | Verminephrobacter eiseniae EF01-2, complete genome | 575 | 0.00E+00 | 81.50% |
| Efet.01.611795.g489.t1 | Verminephrobacter eiseniae EF01-2, complete genome | 984 | 0.00E+00 | 81.50% |
| Efet.01.615273.g686.t1 | Verminephrobacter eiseniae EF01-2, complete genome | 756 | 0.00E+00 | 81.50% |
| Efet.01.624068.g1058.t1 | Hymenobacter sp. APR13, complete genome | 51 | 2.70E-06 | 81.50% |
| Efet.01.636372.g329.t1 | Verminephrobacter eiseniae EF01-2, complete genome | 540 | 0.00E+00 | 81.50% |
| Efet.01.645947.g1013.t1 | Verminephrobacter eiseniae EF01-2, complete genome | 1403 | 0.00E+00 | 81.50% |
| Efet.01.646149.g1041.t1 | Verminephrobacter eiseniae EF01-2, complete genome | 1167 | 0.00E+00 | 81.50% |
| Efet.01.653626.g463.t1 | Verminephrobacter eiseniae EF01-2, complete genome | 1362 | 0.00E+00 | 81.50% |
| Efet.01.655285.g790.t1 | Agromyces aureus strain AR33, complete genome | 616 | 2.10E-179 | 81.50% |
| Efet.01.655821.g882.t1 | Verminephrobacter eiseniae EF01-2, complete genome | 1461 | 0.00E+00 | 81.50% |
| Efet.01.655987.g918.t1 | Verminephrobacter eiseniae EF01-2, complete genome | 918 | 0.00E+00 | 81.50% |
| Efet.01.656883.g1133.t1 | Verminephrobacter eiseniae EF01-2, complete genome | 740 | 0.00E+00 | 81.50% |
| Efet.01.658380.g1920.t1 | Verminephrobacter eiseniae EF01-2, complete genome | 603 | 0.00E+00 | 81.50% |
| Efet.01.658397.g1976.t1 | Verminephrobacter eiseniae EF01-2, complete genome | 333 | 1.30E-162 | 81.50% |
| Efet.01.658398.g1984.t1 | Verminephrobacter eiseniae EF01-2, complete genome | 280 | 7.70E-134 | 81.50% |
| Efet.01.1650560.g478.t1 | Verminephrobacter eiseniae EF01-2, complete genome | 387 | 0.00E+00 | 81.50% |
| Efet.01.1659290.g1518.t1 | Verminephrobacter eiseniae EF01-2, complete genome | 473 | 0.00E+00 | 81.50% |
| Efet.01.630768.g67.t1 | Verminephrobacter eiseniae EF01-2, complete genome | 786 | 0.00E+00 | 81.44% |
| Efet.01.282031.g1161.t1 | Verminephrobacter eiseniae EF01-2, complete genome | 567 | 0.00E+00 | 81.40% |
| Efet.01.412509.g280.t1 | Verminephrobacter eiseniae EF01-2, complete genome | 425 | 0.00E+00 | 81.40% |
| Efet.01.529529.g687.t1 | Agromyces aureus strain AR33, complete genome | 475 | 8.60E-137 | 81.40% |
| Efet.01.557109.g682.t1 | Agromyces sp. 30A chromosome, complete genome | 564 | 1.50E-172 | 81.40% |
| Efet.01.598882.g1271.t1 | Verminephrobacter eiseniae EF01-2, complete genome | 627 | 0.00E+00 | 81.40% |
| Efet.01.634241.g242.t1 | Verminephrobacter eiseniae EF01-2, complete genome | 597 | 0.00E+00 | 81.40% |
| Efet.01.642823.g720.t1 | Verminephrobacter eiseniae EF01-2, complete genome | 1351 | 0.00E+00 | 81.40% |
| Efet.01.649588.g1334.t1 | Verminephrobacter eiseniae EF01-2, complete genome | 1158 | 0.00E+00 | 81.40% |
| Efet.01.653992.g506.t1 | Verminephrobacter eiseniae EF01-2, complete genome | 696 | 0.00E+00 | 81.40% |
| Efet.01.657976.g1546.t1 | Verminephrobacter eiseniae EF01-2, complete genome | 711 | 0.00E+00 | 81.40% |
| Efet.01.658150.g1681.t1 | Verminephrobacter eiseniae EF01-2, complete genome | 926 | 0.00E+00 | 81.40% |
| Efet.01.658412.g2029.t1 | Verminephrobacter eiseniae EF01-2, complete genome | 1174 | 0.00E+00 | 81.40% |
| Efet.01.658413.g2031.t1 | Verminephrobacter eiseniae EF01-2, complete genome | 1581 | 0.00E+00 | 81.40% |
| Efet.01.658422.g2100.t1 | Hydrogenophaga sp. PBC, complete genome | 852 | 0.00E+00 | 81.40% |
| Efet.01.1656606.g890.t1 | Verminephrobacter eiseniae EF01-2, complete genome | 763 | 0.00E+00 | 81.40% |
| Efet.01.1658917.g1324.t1 | Verminephrobacter eiseniae EF01-2, complete genome | 1057 | 0.00E+00 | 81.40% |
| Efet.01.1658958.g1334.t1 | Verminephrobacter eiseniae EF01-2, complete genome | 756 | 0.00E+00 | 81.40% |
| Efet.01.1659102.g1405.t1 | Verminephrobacter eiseniae EF01-2, complete genome | 1332 | 0.00E+00 | 81.40% |
| Efet.01.1659512.g1937.t1 | Verminephrobacter eiseniae EF01-2, complete genome | 2427 | 0.00E+00 | 81.40% |
| Efet.01.1659519.g1977.t1 | Verminephrobacter eiseniae EF01-2, complete genome | 1329 | 0.00E+00 | 81.40% |
| Efet.01.1659522.g1987.t1 | Verminephrobacter eiseniae EF01-2, complete genome | 597 | 0.00E+00 | 81.40% |
| Efet.01.151661.g98.t1 | Verminephrobacter eiseniae EF01-2, complete genome | 819 | 0.00E+00 | 81.30% |
| Efet.01.206518.g284.t1 | Sphingobium hydrophobicum strain C1 chromosome I, complete sequence | 143 | 1.30E-32 | 81.30% |
| Efet.01.215157.g632.t1 | Verminephrobacter eiseniae EF01-2, complete genome | 1155 | 0.00E+00 | 81.30% |
| Efet.01.445097.g1113.t1 | Verminephrobacter eiseniae EF01-2, complete genome | 531 | 0.00E+00 | 81.30% |
| Efet.01.594835.g1131.t1 | Agromyces flavus strain CPCC 202695 genome assembly, chromosome: I | 230 | 1.50E-63 | 81.30% |
| Efet.01.613026.g579.t1 | Verminephrobacter eiseniae EF01-2, complete genome | 801 | 0.00E+00 | 81.30% |
| Efet.01.646487.g1085.t1 | Verminephrobacter eiseniae EF01-2, complete genome | 1349 | 0.00E+00 | 81.30% |
| Efet.01.658044.g1599.t1 | Verminephrobacter eiseniae EF01-2, complete genome | 453 | 0.00E+00 | 81.30% |
| Efet.01.658122.g1655.t1 | Verminephrobacter eiseniae EF01-2, complete genome | 1653 | 0.00E+00 | 81.30% |
| Efet.01.658384.g1933.t1 | Verminephrobacter eiseniae EF01-2, complete genome | 321 | 4.90E-159 | 81.30% |
| Efet.01.1650606.g481.t1 | Verminephrobacter eiseniae EF01-2, complete genome | 480 | 0.00E+00 | 81.30% |
| Efet.01.1656205.g837.t1 | Verminephrobacter eiseniae EF01-2, complete genome | 451 | 0.00E+00 | 81.30% |
| Efet.01.1658150.g1113.t1 | Verminephrobacter eiseniae EF01-2, complete genome | 444 | 0.00E+00 | 81.30% |
| Efet.01.1658480.g1192.t1 | Verminephrobacter eiseniae EF01-2, complete genome | 777 | 0.00E+00 | 81.30% |
| Efet.01.1658542.g1213.t1 | Verminephrobacter eiseniae EF01-2, complete genome | 312 | 5.10E-149 | 81.30% |
| Efet.01.1659401.g1659.t1 | Verminephrobacter eiseniae EF01-2, complete genome | 1353 | 0.00E+00 | 81.30% |
| Efet.01.1659471.g1792.t1 | Verminephrobacter eiseniae EF01-2, complete genome | 957 | 0.00E+00 | 81.30% |
| Efet.01.1659499.g1891.t1 | Verminephrobacter eiseniae EF01-2, complete genome | 894 | 0.00E+00 | 81.30% |
| Efet.01.512343.g288.t1 | Verminephrobacter eiseniae EF01-2, complete genome | 774 | 0.00E+00 | 81.25% |
| Efet.01.206297.g272.t1 | Moraxella ovis strain 199/55, complete genome | 76 | 8.30E-10 | 81.22% |
| Efet.01.369901.g546.t1 | Verminephrobacter eiseniae EF01-2, complete genome | 216 | 2.70E-103 | 81.20% |
| Efet.01.479869.g754.t1 | Curtobacterium pusillum strain AA3, complete genome | 206 | 9.80E-54 | 81.20% |
| Efet.01.529220.g676.t1 | Verminephrobacter eiseniae EF01-2, complete genome | 498 | 0.00E+00 | 81.20% |
| Efet.01.545291.g369.t1 | Verminephrobacter eiseniae EF01-2, complete genome | 347 | 8.50E-175 | 81.20% |
| Efet.01.588731.g952.t1 | Comamonadaceae bacterium B1 DNA, complete genome | 346 | 5.50E-110 | 81.20% |
| Efet.01.595633.g1165.t1 | Verminephrobacter eiseniae EF01-2, complete genome | 2364 | 0.00E+00 | 81.20% |
| Efet.01.618025.g800.t1 | Verminephrobacter eiseniae EF01-2, complete genome | 1050 | 0.00E+00 | 81.20% |
| Efet.01.628537.g1254.t1 | Verminephrobacter eiseniae EF01-2, complete genome | 1777 | 0.00E+00 | 81.20% |
| Efet.01.630860.g96.t1 | Verminephrobacter eiseniae EF01-2, complete genome | 621 | 0.00E+00 | 81.20% |
| Efet.01.631607.g125.t1 | Verminephrobacter eiseniae EF01-2, complete genome | 2295 | 0.00E+00 | 81.20% |
| Efet.01.639369.g506.t1 | Verminephrobacter eiseniae EF01-2, complete genome | 330 | 2.10E-158 | 81.20% |
| Efet.01.643394.g782.t1 | Verminephrobacter eiseniae EF01-2, complete genome | 1017 | 0.00E+00 | 81.20% |
| Efet.01.645133.g935.t1 | Verminephrobacter eiseniae EF01-2, complete genome | 258 | 1.60E-127 | 81.20% |
| Efet.01.650749.g86.t1 | Verminephrobacter eiseniae EF01-2, complete genome | 1193 | 0.00E+00 | 81.20% |
| Efet.01.654418.g600.t1 | Verminephrobacter eiseniae EF01-2, complete genome | 1572 | 0.00E+00 | 81.20% |
| Efet.01.654591.g637.t1 | Verminephrobacter eiseniae EF01-2, complete genome | 520 | 0.00E+00 | 81.20% |
| Efet.01.656145.g943.t1 | Verminephrobacter eiseniae EF01-2, complete genome | 1956 | 0.00E+00 | 81.20% |
| Efet.01.657078.g1162.t1 | Agromyces sp. 30A chromosome, complete genome | 319 | 1.80E-89 | 81.20% |
| Efet.01.657302.g1227.t1 | Verminephrobacter eiseniae EF01-2, complete genome | 1356 | 0.00E+00 | 81.20% |
| Efet.01.658282.g1795.t1 | Verminephrobacter eiseniae EF01-2, complete genome | 501 | 0.00E+00 | 81.20% |
| Efet.01.658307.g1826.t1 | Verminephrobacter eiseniae EF01-2, complete genome | 933 | 0.00E+00 | 81.20% |
| Efet.01.658372.g1905.t1 | Verminephrobacter eiseniae EF01-2, complete genome | 315 | 6.10E-152 | 81.20% |
| Efet.01.658424.g2119.t1 | Verminephrobacter eiseniae EF01-2, complete genome | 285 | 2.20E-142 | 81.20% |
| Efet.01.1656193.g836.t1 | Verminephrobacter eiseniae EF01-2, complete genome | 638 | 0.00E+00 | 81.20% |
| Efet.01.1656473.g873.t1 | Microbacterium testaceum StLB037 DNA, complete genome | 204 | 4.20E-51 | 81.20% |
| Efet.01.1659217.g1468.t1 | Verminephrobacter eiseniae EF01-2, complete genome | 801 | 0.00E+00 | 81.20% |
| Efet.01.1659507.g1913.t1 | Verminephrobacter eiseniae EF01-2, complete genome | 618 | 0.00E+00 | 81.20% |
| Efet.01.653968.g502.t1 | Verminephrobacter eiseniae EF01-2, complete genome | 483 | 0.00E+00 | 81.17% |
| Efet.01.300799.g31.t1 | Verminephrobacter eiseniae EF01-2, complete genome | 1078 | 0.00E+00 | 81.10% |
| Efet.01.300799.g37.t1 | Verminephrobacter eiseniae EF01-2, complete genome | 1500 | 0.00E+00 | 81.10% |
| Efet.01.306217.g206.t1 | Verminephrobacter eiseniae EF01-2, complete genome | 582 | 0.00E+00 | 81.10% |
| Efet.01.401337.g29.t1 | Verminephrobacter eiseniae EF01-2, complete genome | 333 | 8.90E-154 | 81.10% |
| Efet.01.434985.g838.t1 | Agromyces sp. 30A chromosome, complete genome | 607 | 6.40E-175 | 81.10% |
| Efet.01.512343.g289.t1 | Verminephrobacter eiseniae EF01-2, complete genome | 368 | 1.30E-178 | 81.10% |
| Efet.01.575546.g501.t1 | Verminephrobacter eiseniae EF01-2, complete genome | 210 | 3.60E-100 | 81.10% |
| Efet.01.581510.g719.t1 | Verminephrobacter eiseniae EF01-2, complete genome | 1152 | 0.00E+00 | 81.10% |
| Efet.01.585033.g828.t1 | Verminephrobacter eiseniae EF01-2, complete genome | 801 | 0.00E+00 | 81.10% |
| Efet.01.633916.g226.t1 | Verminephrobacter eiseniae EF01-2, complete genome | 151 | 1.90E-69 | 81.10% |
| Efet.01.638026.g400.t1 | Verminephrobacter eiseniae EF01-2, complete genome | 276 | 2.40E-135 | 81.10% |
| Efet.01.639207.g489.t1 | Verminephrobacter eiseniae EF01-2, complete genome | 294 | 1.20E-142 | 81.10% |
| Efet.01.642390.g678.t1 | Verminephrobacter eiseniae EF01-2, complete genome | 2340 | 0.00E+00 | 81.10% |
| Efet.01.646274.g1061.t1 | Verminephrobacter eiseniae EF01-2, complete genome | 795 | 0.00E+00 | 81.10% |
| Efet.01.650297.g20.t1 | Verminephrobacter eiseniae EF01-2, complete genome | 564 | 0.00E+00 | 81.10% |
| Efet.01.651136.g118.t1 | Verminephrobacter eiseniae EF01-2, complete genome | 373 | 0.00E+00 | 81.10% |
| Efet.01.654418.g597.t1 | Verminephrobacter eiseniae EF01-2, complete genome | 966 | 0.00E+00 | 81.10% |
| Efet.01.655573.g829.t1 | Verminephrobacter eiseniae EF01-2, complete genome | 678 | 0.00E+00 | 81.10% |
| Efet.01.657315.g1250.t1 | Methylococcus capsulatus str. Bath, complete genome | 142 | 7.10E-19 | 81.10% |
| Efet.01.657803.g1477.t1 | Verminephrobacter eiseniae EF01-2, complete genome | 420 | 0.00E+00 | 81.10% |
| Efet.01.1657080.g939.t1 | Verminephrobacter eiseniae EF01-2, complete genome | 539 | 0.00E+00 | 81.10% |
| Efet.01.1659437.g1712.t1 | Verminephrobacter eiseniae EF01-2, complete genome | 807 | 0.00E+00 | 81.10% |
| Efet.01.14779.g1084.t1 | Fuerstia marisgermanicae strain NH11 chromosome, complete genome | 100 | 1.20E-11 | 81.00% |
| Efet.01.32340.g518.t1 | Verminephrobacter eiseniae EF01-2, complete genome | 1155 | 0.00E+00 | 81.00% |
| Efet.01.54008.g266.t1 | Paraburkholderia phymatum STM815 chromosome 2, complete sequence | 69 | 2.40E-07 | 81.00% |
| Efet.01.135728.g537.t1 | Streptococcus mitis strain SVGS_061, complete genome | 64 | 3.60E-06 | 81.00% |
| Efet.01.142769.g916.t1 | Chitinophaga sp. T22 chromosome | 67 | 6.00E-08 | 81.00% |
| Efet.01.176534.g1234.t1 | Gloeobacter violaceus PCC 7421 DNA, complete genome | 109 | 3.70E-10 | 81.00% |
| Efet.01.209985.g444.t1 | Actinoalloteichus sp. AHMU CJ021 chromosome, complete genome | 64 | 1.40E-06 | 81.00% |
| Efet.01.294677.g1589.t1 | Tenacibaculum sp. LPB0136, complete genome | 62 | 1.20E-07 | 81.00% |
| Efet.01.318747.g601.t1 | Verminephrobacter eiseniae EF01-2, complete genome | 1608 | 0.00E+00 | 81.00% |
| Efet.01.323858.g739.t1 | Azotobacter vinelandii CA6, complete genome | 69 | 5.70E-07 | 81.00% |
| Efet.01.330621.g936.t1 | Mycobacterium sp. QIA-37, complete genome | 65 | 5.50E-07 | 81.00% |
| Efet.01.394810.g1112.t1 | Verminephrobacter eiseniae EF01-2, complete genome | 1287 | 0.00E+00 | 81.00% |
| Efet.01.398399.g1201.t1 | Mycobacterium chimaera strain ZUERICH-1 plasmid unnamed 2, complete sequence | 82 | 3.60E-13 | 81.00% |
| Efet.01.532148.g65.t1 | Verminephrobacter eiseniae EF01-2, complete genome | 63 | 4.60E-09 | 81.00% |
| Efet.01.558703.g713.t1 | Parvularcula bermudensis HTCC2503 strain HTCC2503, complete genome | 64 | 6.80E-06 | 81.00% |
| Efet.01.565049.g169.t1 | Verminephrobacter eiseniae EF01-2, complete genome | 879 | 0.00E+00 | 81.00% |
| Efet.01.578167.g625.t1 | Verminephrobacter eiseniae EF01-2, complete genome | 531 | 0.00E+00 | 81.00% |
| Efet.01.578612.g644.t1 | Verminephrobacter eiseniae EF01-2, complete genome | 1083 | 0.00E+00 | 81.00% |
| Efet.01.595474.g1156.t1 | Sorangium cellulosum So0157-2, complete genome | 65 | 1.50E-06 | 81.00% |
| Efet.01.612787.g564.t1 | Verminephrobacter eiseniae EF01-2, complete genome | 406 | 9.90E-163 | 81.00% |
| Efet.01.616429.g723.t1 | Microbacterium foliorum strain 122 genome | 105 | 5.20E-23 | 81.00% |
| Efet.01.626245.g1159.t1 | Verminephrobacter eiseniae EF01-2, complete genome | 243 | 8.50E-117 | 81.00% |
| Efet.01.645138.g944.t1 | Verminephrobacter eiseniae EF01-2, complete genome | 609 | 0.00E+00 | 81.00% |
| Efet.01.646487.g1094.t1 | Verminephrobacter eiseniae EF01-2, complete genome | 864 | 0.00E+00 | 81.00% |
| Efet.01.647987.g1210.t1 | Verminephrobacter eiseniae EF01-2, complete genome | 918 | 0.00E+00 | 81.00% |
| Efet.01.647987.g1211.t1 | Verminephrobacter eiseniae EF01-2, complete genome | 411 | 0.00E+00 | 81.00% |
| Efet.01.648013.g1219.t1 | Verminephrobacter eiseniae EF01-2, complete genome | 875 | 0.00E+00 | 81.00% |
| Efet.01.651088.g110.t1 | Verminephrobacter eiseniae EF01-2, complete genome | 285 | 3.10E-140 | 81.00% |
| Efet.01.652863.g362.t1 | Verminephrobacter eiseniae EF01-2, complete genome | 459 | 0.00E+00 | 81.00% |
| Efet.01.653346.g420.t1 | Verminephrobacter eiseniae EF01-2, complete genome | 1081 | 0.00E+00 | 81.00% |
| Efet.01.654418.g606.t1 | Verminephrobacter eiseniae EF01-2, complete genome | 1101 | 0.00E+00 | 81.00% |
| Efet.01.654965.g724.t1 | Verminephrobacter eiseniae EF01-2, complete genome | 345 | 1.50E-167 | 81.00% |
| Efet.01.656171.g957.t1 | Pseudomonas aeruginosa strain HS87 plasmid pHS87a, complete sequence | 447 | 9.90E-141 | 81.00% |
| Efet.01.658297.g1814.t1 | Verminephrobacter eiseniae EF01-2, complete genome | 864 | 0.00E+00 | 81.00% |
| Efet.01.658350.g1882.t1 | Verminephrobacter eiseniae EF01-2, complete genome | 651 | 0.00E+00 | 81.00% |
| Efet.01.658385.g1936.t1 | Verminephrobacter eiseniae EF01-2, complete genome | 972 | 0.00E+00 | 81.00% |
| Efet.01.658417.g2076.t1 | Verminephrobacter eiseniae EF01-2, complete genome | 957 | 0.00E+00 | 81.00% |
| Efet.01.658417.g2077.t1 | Verminephrobacter eiseniae EF01-2, complete genome | 720 | 0.00E+00 | 81.00% |
| Efet.01.658429.g2162.t1 | Verminephrobacter eiseniae EF01-2, complete genome | 264 | 2.80E-128 | 81.00% |
| Efet.01.1654846.g717.t1 | Verminephrobacter eiseniae EF01-2, complete genome | 486 | 0.00E+00 | 81.00% |
| Efet.01.1656111.g828.t1 | Verminephrobacter eiseniae EF01-2, complete genome | 260 | 6.20E-125 | 81.00% |
| Efet.01.1658364.g1163.t1 | Verminephrobacter eiseniae EF01-2, complete genome | 879 | 0.00E+00 | 81.00% |
| Efet.01.1659138.g1425.t1 | Verminephrobacter eiseniae EF01-2, complete genome | 783 | 0.00E+00 | 81.00% |
| Efet.01.1659373.g1616.t1 | Verminephrobacter eiseniae EF01-2, complete genome | 849 | 0.00E+00 | 81.00% |
| Efet.01.1659392.g1641.t1 | Verminephrobacter eiseniae EF01-2, complete genome | 547 | 0.00E+00 | 81.00% |
| Efet.01.1659428.g1692.t1 | Verminephrobacter eiseniae EF01-2, complete genome | 894 | 0.00E+00 | 81.00% |
| Efet.01.1659525.g2019.t1 | Verminephrobacter eiseniae EF01-2, complete genome | 660 | 0.00E+00 | 81.00% |
| Efet.01.257895.g292.t1 | Aeromonas caviae strain 8LM chromosome, complete genome | 138 | 1.10E-26 | 80.90% |
| Efet.01.442655.g1053.t1 | Verminephrobacter eiseniae EF01-2, complete genome | 744 | 0.00E+00 | 80.90% |
| Efet.01.545223.g367.t1 | Agromyces flavus strain CPCC 202695 genome assembly, chromosome: I | 350 | 1.30E-102 | 80.90% |
| Efet.01.582665.g767.t1 | Clavibacter michiganensis subsp. nebraskensis NCPPB 2581 complete genome | 98 | 1.90E-19 | 80.90% |
| Efet.01.588731.g951.t1 | Comamonadaceae bacterium B1 DNA, complete genome | 303 | 1.90E-90 | 80.90% |
| Efet.01.606285.g268.t1 | Paraburkholderia xenovorans LB400 chromosome 2, complete sequence | 1044 | 0.00E+00 | 80.90% |
| Efet.01.626245.g1152.t1 | Verminephrobacter eiseniae EF01-2, complete genome | 498 | 0.00E+00 | 80.90% |
| Efet.01.629049.g1273.t1 | Verminephrobacter eiseniae EF01-2, complete genome | 704 | 0.00E+00 | 80.90% |
| Efet.01.630860.g95.t1 | Verminephrobacter eiseniae EF01-2, complete genome | 1929 | 0.00E+00 | 80.90% |
| Efet.01.631607.g122.t1 | Verminephrobacter eiseniae EF01-2, complete genome | 303 | 2.30E-149 | 80.90% |
| Efet.01.649588.g1339.t1 | Verminephrobacter eiseniae EF01-2, complete genome | 365 | 0.00E+00 | 80.90% |
| Efet.01.651939.g240.t1 | Verminephrobacter eiseniae EF01-2, complete genome | 537 | 0.00E+00 | 80.90% |
| Efet.01.652440.g297.t1 | Verminephrobacter eiseniae EF01-2, complete genome | 336 | 2.90E-164 | 80.90% |
| Efet.01.653479.g436.t1 | Verminephrobacter eiseniae EF01-2, complete genome | 1812 | 0.00E+00 | 80.90% |
| Efet.01.654591.g634.t1 | Verminephrobacter eiseniae EF01-2, complete genome | 957 | 0.00E+00 | 80.90% |
| Efet.01.654591.g638.t1 | Verminephrobacter eiseniae EF01-2, complete genome | 1191 | 0.00E+00 | 80.90% |
| Efet.01.654756.g692.t1 | Verminephrobacter eiseniae EF01-2, complete genome | 435 | 0.00E+00 | 80.90% |
| Efet.01.656757.g1091.t1 | Verminephrobacter eiseniae EF01-2, complete genome | 1329 | 0.00E+00 | 80.90% |
| Efet.01.657123.g1175.t1 | Verminephrobacter eiseniae EF01-2, complete genome | 1182 | 0.00E+00 | 80.90% |
| Efet.01.657719.g1400.t1 | Verminephrobacter eiseniae EF01-2, complete genome | 954 | 0.00E+00 | 80.90% |
| Efet.01.658150.g1683.t1 | Verminephrobacter eiseniae EF01-2, complete genome | 531 | 0.00E+00 | 80.90% |
| Efet.01.658408.g2006.t1 | Verminephrobacter eiseniae EF01-2, complete genome | 1448 | 0.00E+00 | 80.90% |
| Efet.01.658417.g2071.t1 | Verminephrobacter eiseniae EF01-2, complete genome | 1071 | 0.00E+00 | 80.90% |
| Efet.01.658424.g2118.t1 | Verminephrobacter eiseniae EF01-2, complete genome | 705 | 0.00E+00 | 80.90% |
| Efet.01.658429.g2160.t1 | Bordetella bronchiseptica strain D755 chromosome, complete genome | 999 | 0.00E+00 | 80.90% |
| Efet.01.1643086.g270.t1 | Verminephrobacter eiseniae EF01-2, complete genome | 366 | 0.00E+00 | 80.90% |
| Efet.01.1647925.g389.t1 | Synechococcus sp. KORDI-52, complete genome | 73 | 8.00E-10 | 80.90% |
| Efet.01.1657797.g1038.t1 | Verminephrobacter eiseniae EF01-2, complete genome | 517 | 0.00E+00 | 80.90% |
| Efet.01.1658865.g1303.t1 | Verminephrobacter eiseniae EF01-2, complete genome | 783 | 0.00E+00 | 80.90% |
| Efet.01.1659107.g1406.t1 | Verminephrobacter eiseniae EF01-2, complete genome | 663 | 0.00E+00 | 80.90% |
| Efet.01.1659289.g1516.t1 | Verminephrobacter eiseniae EF01-2, complete genome | 654 | 0.00E+00 | 80.90% |
| Efet.01.1659456.g1760.t1 | Verminephrobacter eiseniae EF01-2, complete genome | 861 | 0.00E+00 | 80.90% |
| Efet.01.1659494.g1866.t1 | Verminephrobacter eiseniae EF01-2, complete genome | 574 | 0.00E+00 | 80.90% |
| Efet.01.577578.g605.t1 | Verminephrobacter eiseniae EF01-2, complete genome | 237 | 5.30E-71 | 80.88% |
| Efet.01.151234.g72.t1 | Verminephrobacter eiseniae EF01-2, complete genome | 1305 | 0.00E+00 | 80.80% |
| Efet.01.164554.g740.t1 | Planctomyces sp. SH-PL14, complete genome | 77 | 1.50E-11 | 80.80% |
| Efet.01.207785.g357.t1 | Verminephrobacter eiseniae EF01-2, complete genome | 287 | 5.20E-139 | 80.80% |
| Efet.01.275134.g935.t1 | Verminephrobacter eiseniae EF01-2, complete genome | 336 | 1.10E-162 | 80.80% |
| Efet.01.295261.g1601.t1 | Shewanella putrefaciens strain WS13 chromosome, complete genome | 82 | 1.20E-12 | 80.80% |
| Efet.01.321826.g680.t1 | Agromyces sp. 30A chromosome, complete genome | 652 | 1.30E-179 | 80.80% |
| Efet.01.454467.g104.t1 | Verminephrobacter eiseniae EF01-2, complete genome | 615 | 0.00E+00 | 80.80% |
| Efet.01.476494.g676.t1 | Verminephrobacter eiseniae EF01-2, complete genome | 1254 | 0.00E+00 | 80.80% |
| Efet.01.532996.g96.t1 | Verminephrobacter eiseniae EF01-2, complete genome | 321 | 1.90E-159 | 80.80% |
| Efet.01.533149.g112.t1 | Verminephrobacter eiseniae EF01-2, complete genome | 85 | 4.90E-34 | 80.80% |
| Efet.01.544969.g360.t1 | Verminephrobacter eiseniae EF01-2, complete genome | 786 | 0.00E+00 | 80.80% |
| Efet.01.598882.g1268.t1 | Verminephrobacter eiseniae EF01-2, complete genome | 951 | 0.00E+00 | 80.80% |
| Efet.01.621349.g927.t1 | Verminephrobacter eiseniae EF01-2, complete genome | 379 | 0.00E+00 | 80.80% |
| Efet.01.625602.g1126.t1 | Verminephrobacter eiseniae EF01-2, complete genome | 682 | 0.00E+00 | 80.80% |
| Efet.01.626983.g1189.t1 | Verminephrobacter eiseniae EF01-2, complete genome | 612 | 0.00E+00 | 80.80% |
| Efet.01.640024.g551.t1 | Leifsonia xyli subsp. xyli str. CTCB07, complete genome | 171 | 3.90E-40 | 80.80% |
| Efet.01.644812.g900.t1 | Verminephrobacter eiseniae EF01-2, complete genome | 336 | 1.30E-161 | 80.80% |
| Efet.01.646274.g1064.t1 | Verminephrobacter eiseniae EF01-2, complete genome | 879 | 0.00E+00 | 80.80% |
| Efet.01.652442.g301.t1 | Verminephrobacter eiseniae EF01-2, complete genome | 1149 | 0.00E+00 | 80.80% |
| Efet.01.657315.g1240.t1 | Verminephrobacter eiseniae EF01-2, complete genome | 906 | 0.00E+00 | 80.80% |
| Efet.01.1658616.g1231.t1 | Verminephrobacter eiseniae EF01-2, complete genome | 1049 | 0.00E+00 | 80.80% |
| Efet.01.1659488.g1843.t1 | Uncultured bacterium clone fosmid H220-238 genomic sequence | 641 | 0.00E+00 | 80.80% |
| Efet.01.1659522.g1990.t1 | Verminephrobacter eiseniae EF01-2, complete genome | 444 | 0.00E+00 | 80.80% |
| Efet.01.1659527.g2033.t1 | Verminephrobacter eiseniae EF01-2, complete genome | 1266 | 0.00E+00 | 80.80% |
| Efet.01.575546.g494.t1 | Verminephrobacter eiseniae EF01-2, complete genome | 327 | 1.10E-163 | 80.75% |
| Efet.01.625602.g1125.t1 | Verminephrobacter eiseniae EF01-2, complete genome | 378 | 2.60E-180 | 80.75% |
| Efet.01.335919.g1070.t1 | Verminephrobacter eiseniae EF01-2, complete genome | 345 | 1.20E-173 | 80.70% |
| Efet.01.370035.g554.t1 | Verminephrobacter eiseniae EF01-2, complete genome | 780 | 0.00E+00 | 80.70% |
| Efet.01.399855.g1242.t1 | Aeromonas hydrophila strain AL06-06, complete genome | 157 | 8.40E-34 | 80.70% |
| Efet.01.440935.g999.t1 | Verminephrobacter eiseniae EF01-2, complete genome | 915 | 0.00E+00 | 80.70% |
| Efet.01.546787.g408.t1 | Verminephrobacter eiseniae EF01-2, complete genome | 840 | 0.00E+00 | 80.70% |
| Efet.01.550127.g501.t1 | Verminephrobacter eiseniae EF01-2, complete genome | 1098 | 0.00E+00 | 80.70% |
| Efet.01.566725.g222.t1 | Verminephrobacter eiseniae EF01-2, complete genome | 664 | 0.00E+00 | 80.70% |
| Efet.01.573518.g421.t1 | Verminephrobacter eiseniae EF01-2, complete genome | 755 | 0.00E+00 | 80.70% |
| Efet.01.587924.g926.t1 | Verminephrobacter eiseniae EF01-2, complete genome | 447 | 0.00E+00 | 80.70% |
| Efet.01.605483.g224.t1 | Verminephrobacter eiseniae EF01-2, complete genome | 1001 | 0.00E+00 | 80.70% |
| Efet.01.626245.g1151.t1 | Verminephrobacter eiseniae EF01-2, complete genome | 654 | 0.00E+00 | 80.70% |
| Efet.01.640917.g601.t1 | Verminephrobacter eiseniae EF01-2, complete genome | 558 | 0.00E+00 | 80.70% |
| Efet.01.643497.g806.t1 | Verminephrobacter eiseniae EF01-2, complete genome | 834 | 0.00E+00 | 80.70% |
| Efet.01.656470.g1029.t1 | Verminephrobacter eiseniae EF01-2, complete genome | 957 | 0.00E+00 | 80.70% |
| Efet.01.656952.g1148.t1 | Verminephrobacter eiseniae EF01-2, complete genome | 1071 | 0.00E+00 | 80.70% |
| Efet.01.658133.g1665.t1 | Verminephrobacter eiseniae EF01-2, complete genome | 711 | 0.00E+00 | 80.70% |
| Efet.01.658192.g1707.t1 | Verminephrobacter eiseniae EF01-2, complete genome | 285 | 1.30E-140 | 80.70% |
| Efet.01.658416.g2063.t1 | Verminephrobacter eiseniae EF01-2, complete genome | 1179 | 0.00E+00 | 80.70% |
| Efet.01.658429.g2161.t1 | Verminephrobacter eiseniae EF01-2, complete genome | 2028 | 0.00E+00 | 80.70% |
| Efet.01.1633599.g137.t1 | Zobellella denitrificans strain F13-1 chromosome, complete genome | 231 | 2.20E-56 | 80.70% |
| Efet.01.1657733.g1030.t1 | Verminephrobacter eiseniae EF01-2, complete genome | 738 | 0.00E+00 | 80.70% |
| Efet.01.1657891.g1054.t1 | Verminephrobacter eiseniae EF01-2, complete genome | 306 | 2.60E-151 | 80.70% |
| Efet.01.1659493.g1864.t1 | Verminephrobacter eiseniae EF01-2, complete genome | 540 | 0.00E+00 | 80.70% |
| Efet.01.1659526.g2024.t1 | Verminephrobacter eiseniae EF01-2, complete genome | 279 | 3.00E-134 | 80.70% |
| Efet.01.234348.g1354.t1 | Verminephrobacter eiseniae EF01-2, complete genome | 1876 | 0.00E+00 | 80.67% |
| Efet.01.1425.g134.t1 | Agromyces flavus strain CPCC 202695 genome assembly, chromosome: I | 466 | 2.20E-111 | 80.60% |
| Efet.01.61804.g735.t1 | Verminephrobacter eiseniae EF01-2, complete genome | 412 | 0.00E+00 | 80.60% |
| Efet.01.207785.g355.t1 | Verminephrobacter eiseniae EF01-2, complete genome | 1034 | 0.00E+00 | 80.60% |
| Efet.01.293477.g1552.t1 | Agromyces sp. 30A chromosome, complete genome | 305 | 1.40E-76 | 80.60% |
| Efet.01.365325.g416.t1 | Verminephrobacter eiseniae EF01-2, complete genome | 303 | 1.90E-150 | 80.60% |
| Efet.01.401337.g31.t1 | Verminephrobacter eiseniae EF01-2, complete genome | 603 | 0.00E+00 | 80.60% |
| Efet.01.454467.g102.t1 | Verminephrobacter eiseniae EF01-2, complete genome | 450 | 0.00E+00 | 80.60% |
| Efet.01.497719.g1133.t1 | Cryobacterium arcticum strain PAMC 27867 chromosome 1, complete sequence | 157 | 8.30E-36 | 80.60% |
| Efet.01.563201.g82.t1 | Ralstonia solanacearum Po82, complete genome | 120 | 3.10E-21 | 80.60% |
| Efet.01.565049.g159.t1 | Verminephrobacter eiseniae EF01-2, complete genome | 933 | 0.00E+00 | 80.60% |
| Efet.01.613416.g594.t1 | Verminephrobacter eiseniae EF01-2, complete genome | 1032 | 0.00E+00 | 80.60% |
| Efet.01.630768.g63.t1 | Verminephrobacter eiseniae EF01-2, complete genome | 1208 | 0.00E+00 | 80.60% |
| Efet.01.643024.g749.t1 | Verminephrobacter eiseniae EF01-2, complete genome | 518 | 0.00E+00 | 80.60% |
| Efet.01.646149.g1039.t1 | Verminephrobacter eiseniae EF01-2, complete genome | 1513 | 0.00E+00 | 80.60% |
| Efet.01.649364.g1301.t1 | Verminephrobacter eiseniae EF01-2, complete genome | 489 | 0.00E+00 | 80.60% |
| Efet.01.653844.g482.t1 | Verminephrobacter eiseniae EF01-2, complete genome | 1345 | 0.00E+00 | 80.60% |
| Efet.01.655756.g861.t1 | Verminephrobacter eiseniae EF01-2, complete genome | 348 | 4.70E-175 | 80.60% |
| Efet.01.657248.g1210.t1 | Verminephrobacter eiseniae EF01-2, complete genome | 357 | 2.00E-177 | 80.60% |
| Efet.01.658312.g1843.t1 | Verminephrobacter eiseniae EF01-2, complete genome | 423 | 0.00E+00 | 80.60% |
| Efet.01.1658479.g1191.t1 | Verminephrobacter eiseniae EF01-2, complete genome | 465 | 0.00E+00 | 80.60% |
| Efet.01.1658956.g1332.t1 | Verminephrobacter eiseniae EF01-2, complete genome | 609 | 0.00E+00 | 80.60% |
| Efet.01.1659433.g1704.t1 | Verminephrobacter eiseniae EF01-2, complete genome | 582 | 0.00E+00 | 80.60% |
| Efet.01.1659453.g1751.t1 | Verminephrobacter eiseniae EF01-2, complete genome | 1041 | 0.00E+00 | 80.60% |
| Efet.01.656171.g955.t1 | Pseudomonas aeruginosa strain CCBH4851 genome | 269 | 3.50E-64 | 80.57% |
| Efet.01.147445.g1116.t1 | Verminephrobacter eiseniae EF01-2, complete genome | 882 | 0.00E+00 | 80.50% |
| Efet.01.155144.g265.t1 | Verminephrobacter eiseniae EF01-2, complete genome | 599 | 0.00E+00 | 80.50% |
| Efet.01.287526.g1362.t1 | Verminephrobacter eiseniae EF01-2, complete genome | 621 | 0.00E+00 | 80.50% |
| Efet.01.287526.g1367.t1 | Verminephrobacter eiseniae EF01-2, complete genome | 384 | 1.30E-176 | 80.50% |
| Efet.01.305383.g181.t1 | Variovorax paradoxus S110 chromosome 1, complete sequence | 928 | 0.00E+00 | 80.50% |
| Efet.01.318747.g587.t1 | Verminephrobacter eiseniae EF01-2, complete genome | 783 | 0.00E+00 | 80.50% |
| Efet.01.413810.g323.t1 | Verminephrobacter eiseniae EF01-2, complete genome | 1290 | 0.00E+00 | 80.50% |
| Efet.01.469369.g517.t1 | Flavobacterium sp. HYN0056 chromosome, complete genome | 101 | 1.60E-10 | 80.50% |
| Efet.01.528516.g647.t1 | Cellulosimicrobium sp. TH-20, complete genome | 71 | 1.40E-07 | 80.50% |
| Efet.01.588731.g953.t1 | Pseudomonas savastanoi pv. phaseolicola 1448A plasmid small, complete sequence | 204 | 2.90E-50 | 80.50% |
| Efet.01.590200.g1002.t1 | Verminephrobacter eiseniae EF01-2, complete genome | 368 | 6.30E-179 | 80.50% |
| Efet.01.605884.g249.t1 | Verminephrobacter eiseniae EF01-2, complete genome | 273 | 4.70E-131 | 80.50% |
| Efet.01.611795.g491.t1 | Verminephrobacter eiseniae EF01-2, complete genome | 295 | 1.40E-115 | 80.50% |
| Efet.01.638026.g408.t1 | Verminephrobacter eiseniae EF01-2, complete genome | 3181 | 0.00E+00 | 80.50% |
| Efet.01.643060.g751.t1 | Burkholderia ubonensis strain MSMB1189WGS chromosome 2, complete sequence | 73 | 1.90E-06 | 80.50% |
| Efet.01.644581.g887.t1 | Stenotrophomonas rhizophila strain DSM14405 genome | 96 | 1.30E-09 | 80.50% |
| Efet.01.648013.g1214.t1 | Verminephrobacter eiseniae EF01-2, complete genome | 276 | 1.80E-129 | 80.50% |
| Efet.01.651515.g183.t1 | Verminephrobacter eiseniae EF01-2, complete genome | 720 | 0.00E+00 | 80.50% |
| Efet.01.656283.g980.t1 | Comamonadaceae bacterium A1 DNA, complete genome | 855 | 0.00E+00 | 80.50% |
| Efet.01.658133.g1664.t1 | Verminephrobacter eiseniae EF01-2, complete genome | 924 | 0.00E+00 | 80.50% |
| Efet.01.658414.g2034.t1 | Verminephrobacter eiseniae EF01-2, complete genome | 447 | 0.00E+00 | 80.50% |
| Efet.01.658418.g2085.t1 | Verminephrobacter eiseniae EF01-2, complete genome | 642 | 0.00E+00 | 80.50% |
| Efet.01.1573918.g5.t1 | Alcanivorax xenomutans strain P40, complete genome | 181 | 4.90E-40 | 80.50% |
| Efet.01.1597529.g22.t1 | Bacteroides heparinolyticus strain F0111 chromosome, complete genome | 153 | 2.20E-25 | 80.50% |
| Efet.01.1657574.g1010.t1 | Rhodococcus opacus strain R7 sequence | 142 | 3.90E-33 | 80.50% |
| Efet.01.1659046.g1380.t1 | Verminephrobacter eiseniae EF01-2, complete genome | 1209 | 0.00E+00 | 80.50% |
| Efet.01.1659513.g1943.t1 | Verminephrobacter eiseniae EF01-2, complete genome | 348 | 4.40E-165 | 80.50% |
| Efet.01.640737.g586.t1 | Verminephrobacter eiseniae EF01-2, complete genome | 316 | 1.10E-157 | 80.43% |
| Efet.01.104942.g269.t1 | Verminephrobacter eiseniae EF01-2, complete genome | 1305 | 0.00E+00 | 80.40% |
| Efet.01.278686.g1054.t1 | Bacteroides caccae strain ATCC 43185, complete genome | 77 | 1.30E-13 | 80.40% |
| Efet.01.287526.g1371.t1 | Verminephrobacter eiseniae EF01-2, complete genome | 681 | 0.00E+00 | 80.40% |
| Efet.01.318747.g588.t1 | Verminephrobacter eiseniae EF01-2, complete genome | 855 | 0.00E+00 | 80.40% |
| Efet.01.354578.g134.t1 | Verminephrobacter eiseniae EF01-2, complete genome | 392 | 0.00E+00 | 80.40% |
| Efet.01.393322.g1084.t1 | Verminephrobacter eiseniae EF01-2, complete genome | 696 | 0.00E+00 | 80.40% |
| Efet.01.411637.g260.t1 | Microbacterium sp. XT11, complete genome | 1955 | 0.00E+00 | 80.40% |
| Efet.01.445097.g1112.t1 | Verminephrobacter eiseniae EF01-2, complete genome | 586 | 0.00E+00 | 80.40% |
| Efet.01.497711.g1131.t1 | Verminephrobacter eiseniae EF01-2, complete genome | 912 | 0.00E+00 | 80.40% |
| Efet.01.500837.g21.t1 | Verminephrobacter eiseniae EF01-2, complete genome | 264 | 1.80E-126 | 80.40% |
| Efet.01.533383.g129.t1 | Verminephrobacter eiseniae EF01-2, complete genome | 1041 | 0.00E+00 | 80.40% |
| Efet.01.545291.g370.t1 | Verminephrobacter eiseniae EF01-2, complete genome | 387 | 0.00E+00 | 80.40% |
| Efet.01.562244.g43.t1 | Achromobacter spanius strain MYb73 chromosome, complete genome | 343 | 5.60E-102 | 80.40% |
| Efet.01.563201.g90.t1 | Verminephrobacter eiseniae EF01-2, complete genome | 369 | 9.30E-180 | 80.40% |
| Efet.01.600657.g22.t1 | Verminephrobacter eiseniae EF01-2, complete genome | 366 | 1.00E-156 | 80.40% |
| Efet.01.628009.g1226.t1 | Verminephrobacter eiseniae EF01-2, complete genome | 207 | 2.40E-100 | 80.40% |
| Efet.01.629329.g1285.t1 | Verminephrobacter eiseniae EF01-2, complete genome | 411 | 2.40E-176 | 80.40% |
| Efet.01.630860.g85.t1 | Verminephrobacter eiseniae EF01-2, complete genome | 1284 | 0.00E+00 | 80.40% |
| Efet.01.634752.g258.t1 | Verminephrobacter eiseniae EF01-2, complete genome | 610 | 0.00E+00 | 80.40% |
| Efet.01.636379.g332.t1 | Microbacterium aurum strain KACC 15219, complete genome | 904 | 0.00E+00 | 80.40% |
| Efet.01.638026.g410.t1 | Verminephrobacter eiseniae EF01-2, complete genome | 2775 | 0.00E+00 | 80.40% |
| Efet.01.638872.g472.t1 | Verminephrobacter eiseniae EF01-2, complete genome | 717 | 0.00E+00 | 80.40% |
| Efet.01.643099.g763.t1 | Verminephrobacter eiseniae EF01-2, complete genome | 888 | 0.00E+00 | 80.40% |
| Efet.01.643394.g777.t1 | Verminephrobacter eiseniae EF01-2, complete genome | 1306 | 0.00E+00 | 80.40% |
| Efet.01.643394.g781.t1 | Verminephrobacter eiseniae EF01-2, complete genome | 294 | 8.60E-144 | 80.40% |
| Efet.01.649511.g1326.t1 | Verminephrobacter eiseniae EF01-2, complete genome | 1083 | 0.00E+00 | 80.40% |
| Efet.01.649588.g1337.t1 | Verminephrobacter eiseniae EF01-2, complete genome | 717 | 0.00E+00 | 80.40% |
| Efet.01.651810.g220.t1 | Verminephrobacter eiseniae EF01-2, complete genome | 783 | 0.00E+00 | 80.40% |
| Efet.01.652898.g368.t1 | Verminephrobacter eiseniae EF01-2, complete genome | 858 | 0.00E+00 | 80.40% |
| Efet.01.652898.g371.t1 | Verminephrobacter eiseniae EF01-2, complete genome | 675 | 0.00E+00 | 80.40% |
| Efet.01.657150.g1183.t1 | Azoarcus sp. SY39 chromosome, complete genome | 247 | 2.60E-30 | 80.40% |
| Efet.01.657703.g1374.t1 | Verminephrobacter eiseniae EF01-2, complete genome | 644 | 0.00E+00 | 80.40% |
| Efet.01.658040.g1575.t1 | Verminephrobacter eiseniae EF01-2, complete genome | 354 | 6.20E-177 | 80.40% |
| Efet.01.658385.g1938.t1 | Verminephrobacter eiseniae EF01-2, complete genome | 2019 | 0.00E+00 | 80.40% |
| Efet.01.658405.g1997.t1 | Pseudomonas aeruginosa strain T63266, complete genome | 1970 | 0.00E+00 | 80.40% |
| Efet.01.1659429.g1694.t1 | Verminephrobacter eiseniae EF01-2, complete genome | 798 | 0.00E+00 | 80.40% |
| Efet.01.1659449.g1740.t1 | Verminephrobacter eiseniae EF01-2, complete genome | 629 | 0.00E+00 | 80.40% |
| Efet.01.1659498.g1884.t1 | Verminephrobacter eiseniae EF01-2, complete genome | 316 | 3.40E-151 | 80.40% |
| Efet.01.207474.g334.t1 | Bradyrhizobium sp. ORS 285 strain ORS285 genome assembly, chromosome: BRAD285 | 72 | 1.00E-08 | 80.33% |
| Efet.01.416122.g385.t1 | Xanthomonas citri pv. phaseoli var. fuscans strain CFBP6991 plasmid pF, complete sequence | 323 | 9.80E-98 | 80.33% |
| Efet.01.559401.g740.t1 | Verminephrobacter eiseniae EF01-2, complete genome | 569 | 0.00E+00 | 80.33% |
| Efet.01.1659523.g1999.t1 | Herminiimonas arsenicoxydans chromosome, complete sequence | 307 | 1.80E-29 | 80.33% |
| Efet.01.191906.g1955.t1 | Rubrobacter xylanophilus DSM 9941, complete genome | 103 | 3.70E-11 | 80.30% |
| Efet.01.237415.g1459.t1 | Verminephrobacter eiseniae EF01-2, complete genome | 279 | 1.10E-138 | 80.30% |
| Efet.01.270738.g770.t1 | Verminephrobacter eiseniae EF01-2, complete genome | 1338 | 0.00E+00 | 80.30% |
| Efet.01.574744.g471.t1 | Verminephrobacter eiseniae EF01-2, complete genome | 513 | 0.00E+00 | 80.30% |
| Efet.01.607412.g303.t1 | Verminephrobacter eiseniae EF01-2, complete genome | 727 | 0.00E+00 | 80.30% |
| Efet.01.615846.g704.t1 | Ralstonia mannitolilytica strain SN83A39 chromosome 1, complete sequence | 248 | 1.20E-81 | 80.30% |
| Efet.01.619389.g861.t1 | Verminephrobacter eiseniae EF01-2, complete genome | 1389 | 0.00E+00 | 80.30% |
| Efet.01.628433.g1240.t1 | Verminephrobacter eiseniae EF01-2, complete genome | 1584 | 0.00E+00 | 80.30% |
| Efet.01.630546.g36.t1 | Verminephrobacter eiseniae EF01-2, complete genome | 270 | 2.30E-127 | 80.30% |
| Efet.01.637843.g388.t1 | Verminephrobacter eiseniae EF01-2, complete genome | 712 | 0.00E+00 | 80.30% |
| Efet.01.641462.g629.t1 | Verminephrobacter eiseniae EF01-2, complete genome | 978 | 0.00E+00 | 80.30% |
| Efet.01.643464.g797.t1 | Verminephrobacter eiseniae EF01-2, complete genome | 675 | 0.00E+00 | 80.30% |
| Efet.01.645199.g950.t1 | Verminephrobacter eiseniae EF01-2, complete genome | 876 | 0.00E+00 | 80.30% |
| Efet.01.650480.g54.t1 | Verminephrobacter eiseniae EF01-2, complete genome | 531 | 0.00E+00 | 80.30% |
| Efet.01.650749.g79.t1 | Comamonadaceae bacterium A1 DNA, complete genome | 517 | 9.90E-161 | 80.30% |
| Efet.01.653635.g466.t1 | Amycolatopsis mediterranei S699, complete genome | 124 | 2.00E-20 | 80.30% |
| Efet.01.653915.g494.t1 | Verminephrobacter eiseniae EF01-2, complete genome | 333 | 4.00E-164 | 80.30% |
| Efet.01.654310.g567.t1 | Verminephrobacter eiseniae EF01-2, complete genome | 483 | 0.00E+00 | 80.30% |
| Efet.01.655933.g901.t1 | Verminephrobacter eiseniae EF01-2, complete genome | 1002 | 0.00E+00 | 80.30% |
| Efet.01.656598.g1051.t1 | Verminephrobacter eiseniae EF01-2, complete genome | 1040 | 0.00E+00 | 80.30% |
| Efet.01.657766.g1434.t1 | Verminephrobacter eiseniae EF01-2, complete genome | 474 | 0.00E+00 | 80.30% |
| Efet.01.657795.g1468.t1 | Verminephrobacter eiseniae EF01-2, complete genome | 734 | 0.00E+00 | 80.30% |
| Efet.01.658211.g1718.t1 | Verminephrobacter eiseniae EF01-2, complete genome | 561 | 0.00E+00 | 80.30% |
| Efet.01.658312.g1842.t1 | Verminephrobacter eiseniae EF01-2, complete genome | 404 | 0.00E+00 | 80.30% |
| Efet.01.658424.g2124.t1 | Verminephrobacter eiseniae EF01-2, complete genome | 1383 | 0.00E+00 | 80.30% |
| Efet.01.1648752.g415.t1 | Agromyces sp. 30A chromosome, complete genome | 212 | 6.60E-49 | 80.30% |
| Efet.01.1657947.g1066.t1 | Verminephrobacter eiseniae EF01-2, complete genome | 384 | 0.00E+00 | 80.30% |
| Efet.01.1658150.g1114.t1 | Verminephrobacter eiseniae EF01-2, complete genome | 423 | 0.00E+00 | 80.30% |
| Efet.01.1659161.g1438.t1 | Verminephrobacter eiseniae EF01-2, complete genome | 1023 | 0.00E+00 | 80.30% |
| Efet.01.1659373.g1617.t1 | Verminephrobacter eiseniae EF01-2, complete genome | 975 | 0.00E+00 | 80.30% |
| Efet.01.1659478.g1811.t1 | Verminephrobacter eiseniae EF01-2, complete genome | 990 | 0.00E+00 | 80.30% |
| Efet.01.1659506.g1910.t1 | Verminephrobacter eiseniae EF01-2, complete genome | 491 | 0.00E+00 | 80.30% |
| Efet.01.658110.g1632.t1 | Verminephrobacter eiseniae EF01-2, complete genome | 219 | 1.40E-96 | 80.22% |
| Efet.01.9019.g661.t1 | Agromyces aureus strain AR33, complete genome | 288 | 1.10E-85 | 80.20% |
| Efet.01.126065.g58.t1 | Bradyrhizobium sp. ORS 285 strain ORS285 genome assembly, chromosome: BRAD285 | 92 | 3.30E-14 | 80.20% |
| Efet.01.365325.g415.t1 | Verminephrobacter eiseniae EF01-2, complete genome | 779 | 0.00E+00 | 80.20% |
| Efet.01.440538.g977.t1 | Verminephrobacter eiseniae EF01-2, complete genome | 393 | 0.00E+00 | 80.20% |
| Efet.01.530573.g19.t1 | Verminephrobacter eiseniae EF01-2, complete genome | 1553 | 0.00E+00 | 80.20% |
| Efet.01.545302.g373.t1 | Verminephrobacter eiseniae EF01-2, complete genome | 2919 | 0.00E+00 | 80.20% |
| Efet.01.563201.g91.t1 | Verminephrobacter eiseniae EF01-2, complete genome | 831 | 0.00E+00 | 80.20% |
| Efet.01.597702.g1233.t1 | Verminephrobacter eiseniae EF01-2, complete genome | 1393 | 0.00E+00 | 80.20% |
| Efet.01.608389.g351.t1 | Verminephrobacter eiseniae EF01-2, complete genome | 1239 | 0.00E+00 | 80.20% |
| Efet.01.625494.g1119.t1 | Verminephrobacter eiseniae EF01-2, complete genome | 579 | 0.00E+00 | 80.20% |
| Efet.01.640048.g554.t1 | Verminephrobacter eiseniae EF01-2, complete genome | 359 | 0.00E+00 | 80.20% |
| Efet.01.647897.g1205.t1 | Variovorax sp. PMC12 chromosome 1, complete sequence | 746 | 0.00E+00 | 80.20% |
| Efet.01.657703.g1379.t1 | Verminephrobacter eiseniae EF01-2, complete genome | 600 | 0.00E+00 | 80.20% |
| Efet.01.657795.g1463.t1 | Verminephrobacter eiseniae EF01-2, complete genome | 534 | 0.00E+00 | 80.20% |
| Efet.01.658012.g1556.t1 | Verminephrobacter eiseniae EF01-2, complete genome | 477 | 0.00E+00 | 80.20% |
| Efet.01.658142.g1676.t1 | Verminephrobacter eiseniae EF01-2, complete genome | 549 | 0.00E+00 | 80.20% |
| Efet.01.658245.g1744.t1 | Verminephrobacter eiseniae EF01-2, complete genome | 621 | 0.00E+00 | 80.20% |
| Efet.01.658298.g1817.t1 | Verminephrobacter eiseniae EF01-2, complete genome | 864 | 0.00E+00 | 80.20% |
| Efet.01.658401.g1994.t1 | Verminephrobacter eiseniae EF01-2, complete genome | 527 | 0.00E+00 | 80.20% |
| Efet.01.658410.g2019.t1 | Verminephrobacter eiseniae EF01-2, complete genome | 618 | 0.00E+00 | 80.20% |
| Efet.01.1658765.g1268.t1 | Verminephrobacter eiseniae EF01-2, complete genome | 635 | 0.00E+00 | 80.20% |
| Efet.01.1658958.g1335.t1 | Verminephrobacter eiseniae EF01-2, complete genome | 405 | 0.00E+00 | 80.20% |
| Efet.01.1659290.g1519.t1 | Verminephrobacter eiseniae EF01-2, complete genome | 996 | 0.00E+00 | 80.20% |
| Efet.01.1659389.g1638.t1 | Verminephrobacter eiseniae EF01-2, complete genome | 483 | 0.00E+00 | 80.20% |
| Efet.01.1659462.g1771.t1 | Verminephrobacter eiseniae EF01-2, complete genome | 696 | 0.00E+00 | 80.20% |
| Efet.01.1659515.g1950.t1 | Verminephrobacter eiseniae EF01-2, complete genome | 452 | 0.00E+00 | 80.20% |
| Efet.01.633916.g224.t1 | Verminephrobacter eiseniae EF01-2, complete genome | 1085 | 0.00E+00 | 80.17% |
| Efet.01.559401.g738.t1 | Verminephrobacter eiseniae EF01-2, complete genome | 222 | 8.30E-100 | 80.14% |
| Efet.01.654569.g630.t1 | Verminephrobacter eiseniae EF01-2, complete genome | 384 | 0.00E+00 | 80.14% |
| Efet.01.649821.g1353.t1 | Verminephrobacter eiseniae EF01-2, complete genome | 204 | 5.90E-94 | 80.11% |
| Efet.01.28403.g228.t1 | Verminephrobacter eiseniae EF01-2, complete genome | 207 | 3.50E-96 | 80.10% |
| Efet.01.29780.g329.t1 | Allokutzneria albata strain DSM 44149 genome assembly, chromosome: I | 151 | 6.00E-11 | 80.10% |
| Efet.01.275134.g936.t1 | Verminephrobacter eiseniae EF01-2, complete genome | 714 | 0.00E+00 | 80.10% |
| Efet.01.445097.g1114.t1 | Verminephrobacter eiseniae EF01-2, complete genome | 450 | 0.00E+00 | 80.10% |
| Efet.01.474932.g647.t1 | Verminephrobacter eiseniae EF01-2, complete genome | 1621 | 0.00E+00 | 80.10% |
| Efet.01.530573.g18.t1 | Verminephrobacter eiseniae EF01-2, complete genome | 920 | 0.00E+00 | 80.10% |
| Efet.01.563201.g75.t1 | Verminephrobacter eiseniae EF01-2, complete genome | 1023 | 0.00E+00 | 80.10% |
| Efet.01.563201.g76.t1 | Verminephrobacter eiseniae EF01-2, complete genome | 342 | 6.70E-131 | 80.10% |
| Efet.01.592891.g1093.t1 | Leifsonia xyli subsp. cynodontis DSM 46306, complete genome | 150 | 1.10E-14 | 80.10% |
| Efet.01.613504.g616.t1 | Agromyces sp. 30A chromosome, complete genome | 212 | 1.90E-51 | 80.10% |
| Efet.01.643899.g844.t1 | Verminephrobacter eiseniae EF01-2, complete genome | 315 | 1.40E-148 | 80.10% |
| Efet.01.645268.g961.t1 | Verminephrobacter eiseniae EF01-2, complete genome | 580 | 0.00E+00 | 80.10% |
| Efet.01.646487.g1087.t1 | Verminephrobacter eiseniae EF01-2, complete genome | 990 | 0.00E+00 | 80.10% |
| Efet.01.646487.g1095.t1 | Verminephrobacter eiseniae EF01-2, complete genome | 897 | 0.00E+00 | 80.10% |
| Efet.01.654656.g669.t1 | Verminephrobacter eiseniae EF01-2, complete genome | 1869 | 0.00E+00 | 80.10% |
| Efet.01.654656.g678.t1 | Verminephrobacter eiseniae EF01-2, complete genome | 1113 | 0.00E+00 | 80.10% |
| Efet.01.657626.g1351.t1 | Verminephrobacter eiseniae EF01-2, complete genome | 1167 | 0.00E+00 | 80.10% |
| Efet.01.658395.g1973.t1 | Verminephrobacter eiseniae EF01-2, complete genome | 444 | 0.00E+00 | 80.10% |
| Efet.01.1659007.g1356.t1 | Verminephrobacter eiseniae EF01-2, complete genome | 858 | 0.00E+00 | 80.10% |
| Efet.01.1659452.g1749.t1 | Verminephrobacter eiseniae EF01-2, complete genome | 1140 | 0.00E+00 | 80.10% |
| Efet.01.1659482.g1825.t1 | Pseudomonas aeruginosa PA38182, complete genome | 428 | 4.10E-112 | 80.10% |
| Efet.01.1659483.g1827.t1 | Verminephrobacter eiseniae EF01-2, complete genome | 1375 | 0.00E+00 | 80.10% |
| Efet.01.21481.g1579.t1 | Turneriella parva DSM 21527, complete genome | 76 | 2.80E-06 | 80.00% |
| Efet.01.22077.g1621.t1 | Chondromyces crocatus strain Cm c5, complete genome | 67 | 2.80E-06 | 80.00% |
| Efet.01.117435.g904.t1 | Uncultured bacterium clone contig27074 genomic sequence | 87 | 1.50E-11 | 80.00% |
| Efet.01.120585.g1072.t1 | Candidatus Fluviicola riflensis chromosome, complete genome | 187 | 3.10E-13 | 80.00% |
| Efet.01.138016.g673.t1 | Phyllobacterium zundukense strain Tri-48 chromosome, complete genome | 67 | 2.30E-06 | 80.00% |
| Efet.01.196477.g2150.t1 | Cylindrospermum sp. NIES-4074 DNA, nearly complete genome | 72 | 1.30E-07 | 80.00% |
| Efet.01.201471.g72.t1 | Myxococcus fulvus 124B02, complete genome | 68 | 8.30E-07 | 80.00% |
| Efet.01.300799.g30.t1 | Verminephrobacter eiseniae EF01-2, complete genome | 363 | 1.40E-173 | 80.00% |
| Efet.01.341935.g1207.t1 | Frankia casuarinae strain CcI3, complete genome | 72 | 4.80E-08 | 80.00% |
| Efet.01.429114.g713.t1 | Agromyces flavus strain CPCC 202695 genome assembly, chromosome: I | 522 | 3.80E-138 | 80.00% |
| Efet.01.434985.g837.t1 | Agromyces flavus strain CPCC 202695 genome assembly, chromosome: I | 386 | 3.70E-72 | 80.00% |
| Efet.01.451660.g39.t1 | Cystobacter fuscus strain DSM 52655 chromosome, complete genome | 70 | 3.30E-06 | 80.00% |
| Efet.01.479337.g747.t1 | Agromyces sp. 30A chromosome, complete genome | 301 | 1.60E-76 | 80.00% |
| Efet.01.529220.g677.t1 | Verminephrobacter eiseniae EF01-2, complete genome | 857 | 0.00E+00 | 80.00% |
| Efet.01.546084.g391.t1 | Cellulosimicrobium cellulans strain PSBB019, complete genome | 93 | 3.40E-15 | 80.00% |
| Efet.01.576621.g534.t1 | Microbacterium pygmaeum strain DSM 23142 genome assembly, chromosome: I | 152 | 2.10E-33 | 80.00% |
| Efet.01.595218.g1141.t1 | Microterricola viridarii strain ERGS5:02, complete genome | 415 | 1.60E-31 | 80.00% |
| Efet.01.597264.g1202.t1 | Microterricola viridarii strain DSM 21772 genome assembly, chromosome: I | 181 | 1.20E-43 | 80.00% |
| Efet.01.599924.g1308.t1 | Isoptericola variabilis 225, complete genome | 169 | 4.70E-36 | 80.00% |
| Efet.01.611807.g507.t1 | Verminephrobacter eiseniae EF01-2, complete genome | 936 | 0.00E+00 | 80.00% |
| Efet.01.614731.g667.t1 | Verminephrobacter eiseniae EF01-2, complete genome | 768 | 0.00E+00 | 80.00% |
| Efet.01.618401.g825.t1 | Verminephrobacter eiseniae EF01-2, complete genome | 936 | 0.00E+00 | 80.00% |
| Efet.01.618401.g826.t1 | Verminephrobacter eiseniae EF01-2, complete genome | 2397 | 0.00E+00 | 80.00% |
| Efet.01.620235.g886.t1 | Sanguibacter keddieii DSM 10542, complete genome | 167 | 6.50E-08 | 80.00% |
| Efet.01.622187.g974.t1 | Agromyces aureus strain AR33, complete genome | 243 | 9.40E-63 | 80.00% |
| Efet.01.626474.g1166.t1 | Curtobacterium sp. MR_MD2014, complete genome | 141 | 1.60E-26 | 80.00% |
| Efet.01.629153.g1276.t1 | Verminephrobacter eiseniae EF01-2, complete genome | 856 | 0.00E+00 | 80.00% |
| Efet.01.629376.g1296.t1 | Verminephrobacter eiseniae EF01-2, complete genome | 618 | 0.00E+00 | 80.00% |
| Efet.01.631289.g110.t1 | Verminephrobacter eiseniae EF01-2, complete genome | 1443 | 0.00E+00 | 80.00% |
| Efet.01.633916.g227.t1 | Verminephrobacter eiseniae EF01-2, complete genome | 823 | 0.00E+00 | 80.00% |
| Efet.01.639535.g520.t1 | Diaphorobacter polyhydroxybutyrativorans strain SL-205, complete genome | 266 | 4.70E-58 | 80.00% |
| Efet.01.641291.g619.t1 | Verminephrobacter eiseniae EF01-2, complete genome | 1281 | 0.00E+00 | 80.00% |
| Efet.01.642550.g698.t1 | Verminephrobacter eiseniae EF01-2, complete genome | 1548 | 0.00E+00 | 80.00% |
| Efet.01.644100.g865.t1 | Thermus brockianus strain GE-1, complete genome | 68 | 2.40E-06 | 80.00% |
| Efet.01.645199.g951.t1 | Verminephrobacter eiseniae EF01-2, complete genome | 276 | 2.70E-137 | 80.00% |
| Efet.01.649916.g1362.t1 | Verminephrobacter eiseniae EF01-2, complete genome | 688 | 0.00E+00 | 80.00% |
| Efet.01.650262.g17.t1 | Microbacterium pygmaeum strain DSM 23142 genome assembly, chromosome: I | 104 | 6.70E-11 | 80.00% |
| Efet.01.656171.g958.t1 | Pseudomonas syringae pv. tomato strain B13-200 plasmid pB13-200A, complete sequence | 548 | 1.70E-150 | 80.00% |
| Efet.01.656816.g1117.t1 | Verminephrobacter eiseniae EF01-2, complete genome | 1182 | 0.00E+00 | 80.00% |
| Efet.01.658417.g2075.t1 | Verminephrobacter eiseniae EF01-2, complete genome | 981 | 0.00E+00 | 80.00% |
| Efet.01.658427.g2146.t1 | Verminephrobacter eiseniae EF01-2, complete genome | 503 | 0.00E+00 | 80.00% |
| Efet.01.658430.g2179.t1 | Verminephrobacter eiseniae EF01-2, complete genome | 348 | 3.30E-175 | 80.00% |
| Efet.01.1564905.g1.t1 | Salinibacterium sp. CGMCC 1.16371 chromosome, complete genome | 88 | 2.40E-12 | 80.00% |
| Efet.01.1658659.g1242.t1 | Verminephrobacter eiseniae EF01-2, complete genome | 653 | 0.00E+00 | 80.00% |
| Efet.01.1659128.g1416.t1 | Verminephrobacter eiseniae EF01-2, complete genome | 1386 | 0.00E+00 | 80.00% |
| Efet.01.1659397.g1654.t1 | Verminephrobacter eiseniae EF01-2, complete genome | 1092 | 0.00E+00 | 80.00% |
| Efet.01.1659442.g1725.t1 | Verminephrobacter eiseniae EF01-2, complete genome | 2535 | 0.00E+00 | 80.00% |
| Efet.01.25255.g28.t1 | Verminephrobacter eiseniae EF01-2, complete genome | 1825 | 0.00E+00 | 79.90% |
| Efet.01.471393.g567.t1 | Verminephrobacter eiseniae EF01-2, complete genome | 732 | 0.00E+00 | 79.90% |
| Efet.01.563201.g89.t1 | Verminephrobacter eiseniae EF01-2, complete genome | 613 | 0.00E+00 | 79.90% |
| Efet.01.566222.g209.t1 | Verminephrobacter eiseniae EF01-2, complete genome | 881 | 0.00E+00 | 79.90% |
| Efet.01.569313.g296.t1 | Verminephrobacter eiseniae EF01-2, complete genome | 354 | 2.90E-178 | 79.90% |
| Efet.01.597547.g1213.t1 | Verminephrobacter eiseniae EF01-2, complete genome | 819 | 0.00E+00 | 79.90% |
| Efet.01.605365.g214.t1 | Janthinobacterium agaricidamnosum NBRC 102515 = DSM 9628, complete genome | 727 | 7.90E-65 | 79.90% |
| Efet.01.614463.g648.t1 | Verminephrobacter eiseniae EF01-2, complete genome | 1091 | 0.00E+00 | 79.90% |
| Efet.01.619389.g866.t1 | Verminephrobacter eiseniae EF01-2, complete genome | 669 | 0.00E+00 | 79.90% |
| Efet.01.620076.g881.t1 | Verminephrobacter eiseniae EF01-2, complete genome | 1288 | 0.00E+00 | 79.90% |
| Efet.01.626245.g1157.t1 | Verminephrobacter eiseniae EF01-2, complete genome | 1431 | 0.00E+00 | 79.90% |
| Efet.01.637025.g361.t1 | Microterricola viridarii strain DSM 21772 genome assembly, chromosome: I | 331 | 2.90E-77 | 79.90% |
| Efet.01.639931.g544.t1 | Verminephrobacter eiseniae EF01-2, complete genome | 633 | 0.00E+00 | 79.90% |
| Efet.01.642837.g730.t1 | Verminephrobacter eiseniae EF01-2, complete genome | 1197 | 0.00E+00 | 79.90% |
| Efet.01.643394.g783.t1 | Verminephrobacter eiseniae EF01-2, complete genome | 1038 | 0.00E+00 | 79.90% |
| Efet.01.648999.g1280.t1 | Verminephrobacter eiseniae EF01-2, complete genome | 456 | 0.00E+00 | 79.90% |
| Efet.01.652659.g332.t1 | Verminephrobacter eiseniae EF01-2, complete genome | 753 | 0.00E+00 | 79.90% |
| Efet.01.653184.g406.t1 | Agromyces sp. 30A chromosome, complete genome | 468 | 1.40E-125 | 79.90% |
| Efet.01.653904.g490.t1 | Paraburkholderia xenovorans LB400 chromosome 2, complete sequence | 1827 | 0.00E+00 | 79.90% |
| Efet.01.654965.g726.t1 | Verminephrobacter eiseniae EF01-2, complete genome | 663 | 0.00E+00 | 79.90% |
| Efet.01.655933.g900.t1 | Verminephrobacter eiseniae EF01-2, complete genome | 422 | 0.00E+00 | 79.90% |
| Efet.01.656171.g953.t1 | Achromobacter xylosoxidans strain FDAARGOS_147 plasmid, complete sequence | 596 | 7.60E-155 | 79.90% |
| Efet.01.658041.g1586.t1 | Verminephrobacter eiseniae EF01-2, complete genome | 557 | 0.00E+00 | 79.90% |
| Efet.01.658211.g1715.t1 | Verminephrobacter eiseniae EF01-2, complete genome | 508 | 0.00E+00 | 79.90% |
| Efet.01.658261.g1766.t1 | Burkholderia ambifaria AMMD chromosome 3, complete sequence | 486 | 0.00E+00 | 79.90% |
| Efet.01.658339.g1872.t1 | Verminephrobacter eiseniae EF01-2, complete genome | 1200 | 0.00E+00 | 79.90% |
| Efet.01.1650343.g469.t1 | Agromyces aureus strain AR33, complete genome | 233 | 4.40E-46 | 79.90% |
| Efet.01.1653800.g639.t1 | Agromyces sp. 30A chromosome, complete genome | 299 | 1.80E-79 | 79.90% |
| Efet.01.1659507.g1914.t1 | Verminephrobacter eiseniae EF01-2, complete genome | 441 | 0.00E+00 | 79.90% |
| Efet.01.657900.g1527.t1 | Verminephrobacter eiseniae EF01-2, complete genome | 456 | 0.00E+00 | 79.88% |
| Efet.01.546787.g411.t1 | Verminephrobacter eiseniae EF01-2, complete genome | 658 | 0.00E+00 | 79.83% |
| Efet.01.77457.g144.t1 | Verminephrobacter eiseniae EF01-2, complete genome | 955 | 0.00E+00 | 79.80% |
| Efet.01.318747.g600.t1 | Verminephrobacter eiseniae EF01-2, complete genome | 762 | 0.00E+00 | 79.80% |
| Efet.01.343951.g1250.t1 | Microterricola viridarii strain ERGS5:02, complete genome | 94 | 1.50E-14 | 79.80% |
| Efet.01.393322.g1086.t1 | Verminephrobacter eiseniae EF01-2, complete genome | 468 | 0.00E+00 | 79.80% |
| Efet.01.440538.g966.t1 | Verminephrobacter eiseniae EF01-2, complete genome | 534 | 0.00E+00 | 79.80% |
| Efet.01.440538.g975.t1 | Verminephrobacter eiseniae EF01-2, complete genome | 841 | 0.00E+00 | 79.80% |
| Efet.01.477289.g694.t1 | Alpha proteobacterium HIMB59, complete genome | 70 | 7.50E-11 | 79.80% |
| Efet.01.514559.g351.t1 | Verminephrobacter eiseniae EF01-2, complete genome | 3588 | 0.00E+00 | 79.80% |
| Efet.01.563201.g83.t1 | Simplicispira sp. SC1-8 chromosome, complete genome | 215 | 1.00E-52 | 79.80% |
| Efet.01.585033.g825.t1 | Paraburkholderia xenovorans LB400 chromosome 2, complete sequence | 327 | 7.50E-164 | 79.80% |
| Efet.01.591858.g1049.t1 | Verminephrobacter eiseniae EF01-2, complete genome | 678 | 0.00E+00 | 79.80% |
| Efet.01.598882.g1272.t1 | Verminephrobacter eiseniae EF01-2, complete genome | 603 | 0.00E+00 | 79.80% |
| Efet.01.607412.g310.t1 | Verminephrobacter eiseniae EF01-2, complete genome | 735 | 0.00E+00 | 79.80% |
| Efet.01.612091.g519.t1 | Verminephrobacter eiseniae EF01-2, complete genome | 1213 | 0.00E+00 | 79.80% |
| Efet.01.626208.g1144.t1 | Verminephrobacter eiseniae EF01-2, complete genome | 366 | 0.00E+00 | 79.80% |
| Efet.01.635927.g305.t1 | Alicycliphilus denitrificans K601, complete genome | 834 | 0.00E+00 | 79.80% |
| Efet.01.640475.g576.t1 | Verminephrobacter eiseniae EF01-2, complete genome | 225 | 3.60E-110 | 79.80% |
| Efet.01.642485.g685.t1 | Verminephrobacter eiseniae EF01-2, complete genome | 1187 | 0.00E+00 | 79.80% |
| Efet.01.652588.g318.t1 | Diaphorobacter polyhydroxybutyrativorans strain SL-205, complete genome | 109 | 6.90E-33 | 79.80% |
| Efet.01.653904.g493.t1 | Verminephrobacter eiseniae EF01-2, complete genome | 219 | 7.20E-104 | 79.80% |
| Efet.01.654310.g565.t1 | Verminephrobacter eiseniae EF01-2, complete genome | 1183 | 0.00E+00 | 79.80% |
| Efet.01.654656.g677.t1 | Verminephrobacter eiseniae EF01-2, complete genome | 1602 | 0.00E+00 | 79.80% |
| Efet.01.654656.g679.t1 | Verminephrobacter eiseniae EF01-2, complete genome | 831 | 0.00E+00 | 79.80% |
| Efet.01.657158.g1189.t1 | Verminephrobacter eiseniae EF01-2, complete genome | 600 | 0.00E+00 | 79.80% |
| Efet.01.658116.g1648.t1 | Verminephrobacter eiseniae EF01-2, complete genome | 721 | 0.00E+00 | 79.80% |
| Efet.01.658355.g1887.t1 | Verminephrobacter eiseniae EF01-2, complete genome | 331 | 6.50E-158 | 79.80% |
| Efet.01.1658679.g1249.t1 | Verminephrobacter eiseniae EF01-2, complete genome | 962 | 0.00E+00 | 79.80% |
| Efet.01.1659142.g1427.t1 | Verminephrobacter eiseniae EF01-2, complete genome | 693 | 0.00E+00 | 79.80% |
| Efet.01.1659519.g1979.t1 | Verminephrobacter eiseniae EF01-2, complete genome | 1263 | 0.00E+00 | 79.80% |
| Efet.01.630858.g72.t1 | Verminephrobacter eiseniae EF01-2, complete genome | 318 | 1.50E-129 | 79.71% |
| Efet.01.8178.g605.t1 | Mycobacterium sp. YC-RL4, complete genome | 126 | 1.00E-12 | 79.70% |
| Efet.01.25255.g27.t1 | Verminephrobacter eiseniae EF01-2, complete genome | 1071 | 0.00E+00 | 79.70% |
| Efet.01.165713.g780.t1 | Paenibacillus sp. FSL R5-0345, complete genome | 75 | 2.30E-09 | 79.70% |
| Efet.01.176760.g1241.t1 | Verminephrobacter eiseniae EF01-2, complete genome | 740 | 0.00E+00 | 79.70% |
| Efet.01.279930.g1101.t1 | Verminephrobacter eiseniae EF01-2, complete genome | 336 | 1.20E-159 | 79.70% |
| Efet.01.369900.g544.t1 | Verminephrobacter eiseniae EF01-2, complete genome | 297 | 2.80E-147 | 79.70% |
| Efet.01.440935.g1004.t1 | Verminephrobacter eiseniae EF01-2, complete genome | 1030 | 0.00E+00 | 79.70% |
| Efet.01.465744.g415.t1 | Verminephrobacter eiseniae EF01-2, complete genome | 1071 | 0.00E+00 | 79.70% |
| Efet.01.514247.g333.t1 | Verminephrobacter eiseniae EF01-2, complete genome | 788 | 0.00E+00 | 79.70% |
| Efet.01.514247.g337.t1 | Verminephrobacter eiseniae EF01-2, complete genome | 813 | 0.00E+00 | 79.70% |
| Efet.01.514315.g345.t1 | Verminephrobacter eiseniae EF01-2, complete genome | 759 | 0.00E+00 | 79.70% |
| Efet.01.545291.g371.t1 | Verminephrobacter eiseniae EF01-2, complete genome | 676 | 0.00E+00 | 79.70% |
| Efet.01.546674.g403.t1 | Verminephrobacter eiseniae EF01-2, complete genome | 524 | 0.00E+00 | 79.70% |
| Efet.01.554757.g626.t1 | Pseudomonas syringae pv. tomato strain B13-200 plasmid pB13-200A, complete sequence | 87 | 1.60E-15 | 79.70% |
| Efet.01.559401.g732.t1 | Verminephrobacter eiseniae EF01-2, complete genome | 1026 | 0.00E+00 | 79.70% |
| Efet.01.578612.g643.t1 | Verminephrobacter eiseniae EF01-2, complete genome | 921 | 0.00E+00 | 79.70% |
| Efet.01.622882.g1005.t1 | Carnobacterium sp. CP1, complete genome | 59 | 2.20E-07 | 79.70% |
| Efet.01.630390.g22.t1 | Verminephrobacter eiseniae EF01-2, complete genome | 838 | 0.00E+00 | 79.70% |
| Efet.01.643464.g798.t1 | Verminephrobacter eiseniae EF01-2, complete genome | 2202 | 0.00E+00 | 79.70% |
| Efet.01.647059.g1157.t1 | Verminephrobacter eiseniae EF01-2, complete genome | 343 | 4.50E-156 | 79.70% |
| Efet.01.655933.g897.t1 | Verminephrobacter eiseniae EF01-2, complete genome | 297 | 3.70E-146 | 79.70% |
| Efet.01.657206.g1200.t1 | Verminephrobacter eiseniae EF01-2, complete genome | 372 | 0.00E+00 | 79.70% |
| Efet.01.657257.g1214.t1 | Verminephrobacter eiseniae EF01-2, complete genome | 895 | 0.00E+00 | 79.70% |
| Efet.01.657315.g1252.t1 | Pedobacter cryoconitis strain PAMC 27485, complete genome | 568 | 1.70E-58 | 79.70% |
| Efet.01.657719.g1394.t1 | Verminephrobacter eiseniae EF01-2, complete genome | 939 | 0.00E+00 | 79.70% |
| Efet.01.658230.g1725.t1 | Verminephrobacter eiseniae EF01-2, complete genome | 708 | 0.00E+00 | 79.70% |
| Efet.01.1636673.g179.t1 | Agromyces flavus strain CPCC 202695 genome assembly, chromosome: I | 230 | 6.40E-57 | 79.70% |
| Efet.01.1644773.g312.t1 | Thauera sp. MZ1T, complete genome | 201 | 6.40E-51 | 79.70% |
| Efet.01.1658179.g1120.t1 | Verminephrobacter eiseniae EF01-2, complete genome | 831 | 0.00E+00 | 79.70% |
| Efet.01.1659057.g1386.t1 | Verminephrobacter eiseniae EF01-2, complete genome | 279 | 1.10E-133 | 79.70% |
| Efet.01.1659462.g1773.t1 | Verminephrobacter eiseniae EF01-2, complete genome | 699 | 0.00E+00 | 79.70% |
| Efet.01.650336.g39.t1 | Microbacterium chocolatum strain SIT 101, complete genome | 318 | 1.80E-100 | 79.67% |
| Efet.01.652442.g303.t1 | Verminephrobacter eiseniae EF01-2, complete genome | 387 | 0.00E+00 | 79.67% |
| Efet.01.68406.g1130.t1 | Paenibacillus borealis strain DSM 13188, complete genome | 51 | 5.40E-08 | 79.60% |
| Efet.01.440538.g983.t1 | Verminephrobacter eiseniae EF01-2, complete genome | 399 | 0.00E+00 | 79.60% |
| Efet.01.557665.g694.t1 | Verminephrobacter eiseniae EF01-2, complete genome | 1185 | 0.00E+00 | 79.60% |
| Efet.01.563201.g78.t1 | Verminephrobacter eiseniae EF01-2, complete genome | 717 | 0.00E+00 | 79.60% |
| Efet.01.564563.g135.t1 | Aeromonas salmonicida subsp. masoucida strain RFAS1 chromosome, complete genome | 267 | 3.70E-58 | 79.60% |
| Efet.01.577373.g580.t1 | Verminephrobacter eiseniae EF01-2, complete genome | 485 | 0.00E+00 | 79.60% |
| Efet.01.583627.g785.t1 | Microterricola viridarii strain DSM 21772 genome assembly, chromosome: I | 257 | 1.30E-59 | 79.60% |
| Efet.01.587924.g922.t1 | Verminephrobacter eiseniae EF01-2, complete genome | 687 | 0.00E+00 | 79.60% |
| Efet.01.605365.g209.t1 | Verminephrobacter eiseniae EF01-2, complete genome | 609 | 0.00E+00 | 79.60% |
| Efet.01.611343.g474.t1 | Verminephrobacter eiseniae EF01-2, complete genome | 975 | 0.00E+00 | 79.60% |
| Efet.01.613416.g602.t1 | Verminephrobacter eiseniae EF01-2, complete genome | 897 | 0.00E+00 | 79.60% |
| Efet.01.613416.g607.t1 | Verminephrobacter eiseniae EF01-2, complete genome | 1576 | 0.00E+00 | 79.60% |
| Efet.01.629703.g1306.t1 | Zhihengliuella sp. ISTPL4 chromosome | 358 | 2.00E-90 | 79.60% |
| Efet.01.643464.g787.t1 | Verminephrobacter eiseniae EF01-2, complete genome | 785 | 0.00E+00 | 79.60% |
| Efet.01.645971.g1016.t1 | Verminephrobacter eiseniae EF01-2, complete genome | 1259 | 0.00E+00 | 79.60% |
| Efet.01.649821.g1355.t1 | Verminephrobacter eiseniae EF01-2, complete genome | 1530 | 0.00E+00 | 79.60% |
| Efet.01.652858.g349.t1 | Verminephrobacter eiseniae EF01-2, complete genome | 837 | 0.00E+00 | 79.60% |
| Efet.01.653063.g393.t1 | Verminephrobacter eiseniae EF01-2, complete genome | 545 | 0.00E+00 | 79.60% |
| Efet.01.653719.g471.t1 | Verminephrobacter eiseniae EF01-2, complete genome | 252 | 4.50E-119 | 79.60% |
| Efet.01.658281.g1790.t1 | Verminephrobacter eiseniae EF01-2, complete genome | 1446 | 0.00E+00 | 79.60% |
| Efet.01.658307.g1831.t1 | Verminephrobacter eiseniae EF01-2, complete genome | 741 | 0.00E+00 | 79.60% |
| Efet.01.1659349.g1586.t1 | Verminephrobacter eiseniae EF01-2, complete genome | 382 | 0.00E+00 | 79.60% |
| Efet.01.1659473.g1798.t1 | Paraburkholderia xenovorans LB400 chromosome 1, complete sequence | 237 | 8.70E-115 | 79.60% |
| Efet.01.1659479.g1814.t1 | Variovorax sp. PMC12 chromosome 1, complete sequence | 840 | 0.00E+00 | 79.60% |
| Efet.01.1659481.g1820.t1 | Diaphorobacter polyhydroxybutyrativorans strain SL-205, complete genome | 258 | 2.60E-104 | 79.60% |
| Efet.01.1659525.g2021.t1 | Verminephrobacter eiseniae EF01-2, complete genome | 771 | 0.00E+00 | 79.60% |
| Efet.01.59384.g593.t1 | Verminephrobacter eiseniae EF01-2, complete genome | 246 | 9.80E-119 | 79.50% |
| Efet.01.333865.g1009.t1 | Bacillus mycoides strain Gnyt1, complete genome | 70 | 5.50E-07 | 79.50% |
| Efet.01.471393.g564.t1 | Verminephrobacter eiseniae EF01-2, complete genome | 1231 | 0.00E+00 | 79.50% |
| Efet.01.506828.g172.t1 | Verminephrobacter eiseniae EF01-2, complete genome | 1013 | 0.00E+00 | 79.50% |
| Efet.01.512410.g293.t1 | Verminephrobacter eiseniae EF01-2, complete genome | 1281 | 0.00E+00 | 79.50% |
| Efet.01.605802.g240.t1 | Verminephrobacter eiseniae EF01-2, complete genome | 780 | 0.00E+00 | 79.50% |
| Efet.01.616228.g716.t1 | Verminephrobacter eiseniae EF01-2, complete genome | 1063 | 0.00E+00 | 79.50% |
| Efet.01.625010.g1091.t1 | Verminephrobacter eiseniae EF01-2, complete genome | 1078 | 0.00E+00 | 79.50% |
| Efet.01.629376.g1294.t1 | Verminephrobacter eiseniae EF01-2, complete genome | 645 | 0.00E+00 | 79.50% |
| Efet.01.630390.g24.t1 | Verminephrobacter eiseniae EF01-2, complete genome | 264 | 1.90E-128 | 79.50% |
| Efet.01.630768.g65.t1 | Verminephrobacter eiseniae EF01-2, complete genome | 1032 | 0.00E+00 | 79.50% |
| Efet.01.630860.g79.t1 | Verminephrobacter eiseniae EF01-2, complete genome | 1479 | 0.00E+00 | 79.50% |
| Efet.01.640619.g581.t1 | Plantibacter flavus strain 251 genome | 312 | 9.10E-80 | 79.50% |
| Efet.01.642094.g656.t1 | Verminephrobacter eiseniae EF01-2, complete genome | 303 | 1.60E-149 | 79.50% |
| Efet.01.651441.g174.t1 | Verminephrobacter eiseniae EF01-2, complete genome | 639 | 0.00E+00 | 79.50% |
| Efet.01.651567.g189.t1 | Verminephrobacter eiseniae EF01-2, complete genome | 680 | 0.00E+00 | 79.50% |
| Efet.01.652693.g334.t1 | Clavibacter insidiosus strain ATCC 10253 chromosome, complete genome | 75 | 1.00E-07 | 79.50% |
| Efet.01.657302.g1228.t1 | Verminephrobacter eiseniae EF01-2, complete genome | 1137 | 0.00E+00 | 79.50% |
| Efet.01.657807.g1482.t1 | Vitreoscilla filiformis strain ATCC 15551 plasmid pVF2, complete sequence | 442 | 1.40E-165 | 79.50% |
| Efet.01.657899.g1522.t1 | Verminephrobacter eiseniae EF01-2, complete genome | 1492 | 0.00E+00 | 79.50% |
| Efet.01.658283.g1800.t1 | Verminephrobacter eiseniae EF01-2, complete genome | 735 | 0.00E+00 | 79.50% |
| Efet.01.658298.g1816.t1 | Verminephrobacter eiseniae EF01-2, complete genome | 942 | 0.00E+00 | 79.50% |
| Efet.01.1646835.g358.t1 | Verminephrobacter eiseniae EF01-2, complete genome | 435 | 0.00E+00 | 79.50% |
| Efet.01.1656766.g910.t1 | Microbacterium testaceum StLB037 DNA, complete genome | 81 | 9.30E-09 | 79.50% |
| Efet.01.1658209.g1129.t1 | Verminephrobacter eiseniae EF01-2, complete genome | 585 | 0.00E+00 | 79.50% |
| Efet.01.1659476.g1801.t1 | Verminephrobacter eiseniae EF01-2, complete genome | 204 | 7.10E-97 | 79.50% |
| Efet.01.220148.g818.t1 | Arthrobacter agilis strain UMCV2 chromosome | 70 | 2.10E-08 | 79.43% |
| Efet.01.25255.g22.t1 | Verminephrobacter eiseniae EF01-2, complete genome | 430 | 0.00E+00 | 79.40% |
| Efet.01.278716.g1055.t1 | Providencia stuartii strain FDAARGOS_291 chromosome, complete genome | 89 | 2.90E-09 | 79.40% |
| Efet.01.307993.g236.t1 | Verminephrobacter eiseniae EF01-2, complete genome | 652 | 0.00E+00 | 79.40% |
| Efet.01.440538.g978.t1 | Verminephrobacter eiseniae EF01-2, complete genome | 300 | 4.40E-145 | 79.40% |
| Efet.01.500652.g15.t1 | Agromyces aureus strain AR33, complete genome | 347 | 2.20E-87 | 79.40% |
| Efet.01.512410.g291.t1 | Verminephrobacter eiseniae EF01-2, complete genome | 219 | 7.30E-106 | 79.40% |
| Efet.01.530573.g14.t1 | Verminephrobacter eiseniae EF01-2, complete genome | 604 | 0.00E+00 | 79.40% |
| Efet.01.623992.g1046.t1 | Verminephrobacter eiseniae EF01-2, complete genome | 300 | 2.30E-146 | 79.40% |
| Efet.01.632423.g157.t1 | Verminephrobacter eiseniae EF01-2, complete genome | 1615 | 0.00E+00 | 79.40% |
| Efet.01.636184.g319.t1 | Verminephrobacter eiseniae EF01-2, complete genome | 279 | 1.70E-133 | 79.40% |
| Efet.01.636814.g351.t1 | Verminephrobacter eiseniae EF01-2, complete genome | 756 | 0.00E+00 | 79.40% |
| Efet.01.638894.g475.t1 | Verminephrobacter eiseniae EF01-2, complete genome | 298 | 3.20E-118 | 79.40% |
| Efet.01.642149.g668.t1 | Agromyces aureus strain AR33, complete genome | 348 | 3.50E-88 | 79.40% |
| Efet.01.645947.g1010.t1 | Verminephrobacter eiseniae EF01-2, complete genome | 930 | 0.00E+00 | 79.40% |
| Efet.01.653445.g429.t1 | Verminephrobacter eiseniae EF01-2, complete genome | 561 | 0.00E+00 | 79.40% |
| Efet.01.653479.g432.t1 | Verminephrobacter eiseniae EF01-2, complete genome | 676 | 0.00E+00 | 79.40% |
| Efet.01.654333.g577.t1 | Verminephrobacter eiseniae EF01-2, complete genome | 474 | 0.00E+00 | 79.40% |
| Efet.01.655791.g875.t1 | Agromyces sp. 30A chromosome, complete genome | 254 | 6.10E-66 | 79.40% |
| Efet.01.1634867.g156.t1 | Cryobacterium arcticum strain PAMC 27867 plasmid pP27867_2, complete sequence | 273 | 2.90E-80 | 79.40% |
| Efet.01.1659077.g1391.t1 | Verminephrobacter eiseniae EF01-2, complete genome | 1216 | 0.00E+00 | 79.40% |
| Efet.01.1659462.g1770.t1 | Verminephrobacter eiseniae EF01-2, complete genome | 969 | 0.00E+00 | 79.40% |
| Efet.01.1659465.g1777.t1 | Verminephrobacter eiseniae EF01-2, complete genome | 1266 | 0.00E+00 | 79.40% |
| Efet.01.1659523.g2002.t1 | Verminephrobacter eiseniae EF01-2, complete genome | 1273 | 0.00E+00 | 79.40% |
| Efet.01.1659526.g2026.t1 | Verminephrobacter eiseniae EF01-2, complete genome | 1014 | 0.00E+00 | 79.40% |
| Efet.01.32340.g515.t1 | Verminephrobacter eiseniae EF01-2, complete genome | 342 | 8.90E-171 | 79.33% |
| Efet.01.132372.g369.t1 | Myxococcus macrosporus DSM 14697 chromosome, complete genome | 136 | 1.20E-12 | 79.33% |
| Efet.01.624203.g1064.t1 | Prosthecochloris sp. CIB 2401, complete genome | 63 | 1.80E-08 | 79.33% |
| Efet.01.10219.g747.t1 | Verminephrobacter eiseniae EF01-2, complete genome | 771 | 0.00E+00 | 79.30% |
| Efet.01.312578.g395.t1 | Bradyrhizobium erythrophlei strain GAS242 genome assembly, chromosome: I | 80 | 4.10E-13 | 79.30% |
| Efet.01.318747.g603.t1 | Verminephrobacter eiseniae EF01-2, complete genome | 814 | 0.00E+00 | 79.30% |
| Efet.01.393322.g1082.t1 | Verminephrobacter eiseniae EF01-2, complete genome | 831 | 0.00E+00 | 79.30% |
| Efet.01.410874.g244.t1 | Microbacterium sp. CGR1, complete genome | 284 | 2.00E-68 | 79.30% |
| Efet.01.424172.g576.t1 | Verminephrobacter eiseniae EF01-2, complete genome | 1019 | 0.00E+00 | 79.30% |
| Efet.01.440538.g968.t1 | Verminephrobacter eiseniae EF01-2, complete genome | 840 | 0.00E+00 | 79.30% |
| Efet.01.562840.g62.t1 | Agromyces sp. 30A chromosome, complete genome | 495 | 1.60E-26 | 79.30% |
| Efet.01.605483.g226.t1 | Verminephrobacter eiseniae EF01-2, complete genome | 696 | 0.00E+00 | 79.30% |
| Efet.01.608349.g346.t1 | Verminephrobacter eiseniae EF01-2, complete genome | 930 | 0.00E+00 | 79.30% |
| Efet.01.635927.g298.t1 | Aminobacter sp. MSH1 plasmid pBAM1, complete sequence | 488 | 1.70E-133 | 79.30% |
| Efet.01.642550.g703.t1 | Verminephrobacter eiseniae EF01-2, complete genome | 801 | 0.00E+00 | 79.30% |
| Efet.01.642837.g727.t1 | Verminephrobacter eiseniae EF01-2, complete genome | 1587 | 0.00E+00 | 79.30% |
| Efet.01.643922.g846.t1 | Verminephrobacter eiseniae EF01-2, complete genome | 383 | 0.00E+00 | 79.30% |
| Efet.01.646178.g1046.t1 | Verminephrobacter eiseniae EF01-2, complete genome | 219 | 9.90E-105 | 79.30% |
| Efet.01.646487.g1101.t1 | Verminephrobacter eiseniae EF01-2, complete genome | 822 | 0.00E+00 | 79.30% |
| Efet.01.648372.g1233.t1 | Verminephrobacter eiseniae EF01-2, complete genome | 974 | 0.00E+00 | 79.30% |
| Efet.01.649511.g1328.t1 | Verminephrobacter eiseniae EF01-2, complete genome | 231 | 5.40E-113 | 79.30% |
| Efet.01.650480.g60.t1 | Verminephrobacter eiseniae EF01-2, complete genome | 360 | 0.00E+00 | 79.30% |
| Efet.01.652659.g329.t1 | Verminephrobacter eiseniae EF01-2, complete genome | 376 | 2.10E-172 | 79.30% |
| Efet.01.653992.g509.t1 | Verminephrobacter eiseniae EF01-2, complete genome | 711 | 0.00E+00 | 79.30% |
| Efet.01.654569.g631.t1 | Verminephrobacter eiseniae EF01-2, complete genome | 678 | 0.00E+00 | 79.30% |
| Efet.01.657766.g1456.t1 | Verminephrobacter eiseniae EF01-2, complete genome | 375 | 0.00E+00 | 79.30% |
| Efet.01.657803.g1474.t1 | Verminephrobacter eiseniae EF01-2, complete genome | 603 | 0.00E+00 | 79.30% |
| Efet.01.658052.g1606.t1 | Verminephrobacter eiseniae EF01-2, complete genome | 954 | 0.00E+00 | 79.30% |
| Efet.01.658151.g1690.t1 | Verminephrobacter eiseniae EF01-2, complete genome | 873 | 0.00E+00 | 79.30% |
| Efet.01.658380.g1916.t1 | Verminephrobacter eiseniae EF01-2, complete genome | 1398 | 0.00E+00 | 79.30% |
| Efet.01.658416.g2060.t1 | Verminephrobacter eiseniae EF01-2, complete genome | 1032 | 0.00E+00 | 79.30% |
| Efet.01.1659264.g1492.t1 | Verminephrobacter eiseniae EF01-2, complete genome | 330 | 2.90E-162 | 79.30% |
| Efet.01.1659515.g1952.t1 | Verminephrobacter eiseniae EF01-2, complete genome | 276 | 4.70E-133 | 79.30% |
| Efet.01.524813.g582.t1 | Agromyces sp. 30A chromosome, complete genome | 129 | 5.90E-13 | 79.22% |
| Efet.01.456900.g173.t1 | Verminephrobacter eiseniae EF01-2, complete genome | 288 | 5.00E-105 | 79.20% |
| Efet.01.492854.g1014.t1 | Verminephrobacter eiseniae EF01-2, complete genome | 468 | 0.00E+00 | 79.20% |
| Efet.01.523999.g559.t1 | Verminephrobacter eiseniae EF01-2, complete genome | 1194 | 0.00E+00 | 79.20% |
| Efet.01.554586.g619.t1 | Verminephrobacter eiseniae EF01-2, complete genome | 702 | 0.00E+00 | 79.20% |
| Efet.01.594126.g1110.t1 | Variovorax boronicumulans strain J1 chromosome, complete genome | 550 | 0.00E+00 | 79.20% |
| Efet.01.612196.g531.t1 | Agromyces sp. 30A chromosome, complete genome | 456 | 6.30E-120 | 79.20% |
| Efet.01.613038.g581.t1 | Gammaproteobacteria bacterium DM2 chromosome, complete genome | 188 | 1.70E-39 | 79.20% |
| Efet.01.613416.g595.t1 | Verminephrobacter eiseniae EF01-2, complete genome | 1237 | 0.00E+00 | 79.20% |
| Efet.01.619389.g863.t1 | Verminephrobacter eiseniae EF01-2, complete genome | 399 | 0.00E+00 | 79.20% |
| Efet.01.621349.g928.t1 | Paraburkholderia xenovorans LB400 chromosome 2, complete sequence | 1254 | 0.00E+00 | 79.20% |
| Efet.01.646487.g1099.t1 | Verminephrobacter eiseniae EF01-2, complete genome | 1014 | 0.00E+00 | 79.20% |
| Efet.01.657315.g1253.t1 | Pseudomonas aeruginosa strain ATCC 27853, complete genome | 668 | 0.00E+00 | 79.20% |
| Efet.01.657430.g1279.t1 | Verminephrobacter eiseniae EF01-2, complete genome | 1608 | 0.00E+00 | 79.20% |
| Efet.01.658334.g1865.t1 | Verminephrobacter eiseniae EF01-2, complete genome | 705 | 0.00E+00 | 79.20% |
| Efet.01.658426.g2130.t1 | Verminephrobacter eiseniae EF01-2, complete genome | 582 | 0.00E+00 | 79.20% |
| Efet.01.1659325.g1552.t1 | Verminephrobacter eiseniae EF01-2, complete genome | 1407 | 0.00E+00 | 79.20% |
| Efet.01.1659494.g1868.t1 | Verminephrobacter eiseniae EF01-2, complete genome | 1158 | 0.00E+00 | 79.20% |
| Efet.01.1659505.g1907.t1 | Verminephrobacter eiseniae EF01-2, complete genome | 942 | 0.00E+00 | 79.20% |
| Efet.01.1659516.g1958.t1 | Verminephrobacter eiseniae EF01-2, complete genome | 873 | 0.00E+00 | 79.20% |
| Efet.01.83816.g566.t1 | Tistrella mobilis KA081020-065, complete genome | 148 | 1.70E-12 | 79.10% |
| Efet.01.284847.g1264.t1 | Verminephrobacter eiseniae EF01-2, complete genome | 504 | 0.00E+00 | 79.10% |
| Efet.01.287526.g1365.t1 | Verminephrobacter eiseniae EF01-2, complete genome | 606 | 0.00E+00 | 79.10% |
| Efet.01.318747.g598.t1 | Verminephrobacter eiseniae EF01-2, complete genome | 1368 | 0.00E+00 | 79.10% |
| Efet.01.318747.g604.t1 | Verminephrobacter eiseniae EF01-2, complete genome | 1025 | 0.00E+00 | 79.10% |
| Efet.01.394810.g1111.t1 | Verminephrobacter eiseniae EF01-2, complete genome | 564 | 0.00E+00 | 79.10% |
| Efet.01.514247.g336.t1 | Verminephrobacter eiseniae EF01-2, complete genome | 1058 | 0.00E+00 | 79.10% |
| Efet.01.514247.g339.t1 | Verminephrobacter eiseniae EF01-2, complete genome | 327 | 3.90E-154 | 79.10% |
| Efet.01.532996.g102.t1 | Verminephrobacter eiseniae EF01-2, complete genome | 1047 | 0.00E+00 | 79.10% |
| Efet.01.553292.g581.t1 | Verminephrobacter eiseniae EF01-2, complete genome | 852 | 0.00E+00 | 79.10% |
| Efet.01.577664.g610.t1 | Verminephrobacter eiseniae EF01-2, complete genome | 606 | 0.00E+00 | 79.10% |
| Efet.01.624010.g1055.t1 | Verminephrobacter eiseniae EF01-2, complete genome | 730 | 0.00E+00 | 79.10% |
| Efet.01.630993.g103.t1 | Leifsonia sp. 21MFCrub1.1 genome assembly, chromosome: I | 220 | 2.20E-48 | 79.10% |
| Efet.01.639369.g504.t1 | Verminephrobacter eiseniae EF01-2, complete genome | 2221 | 0.00E+00 | 79.10% |
| Efet.01.643024.g748.t1 | Verminephrobacter eiseniae EF01-2, complete genome | 1131 | 0.00E+00 | 79.10% |
| Efet.01.651816.g223.t1 | Verminephrobacter eiseniae EF01-2, complete genome | 576 | 0.00E+00 | 79.10% |
| Efet.01.652843.g347.t1 | Verminephrobacter eiseniae EF01-2, complete genome | 914 | 0.00E+00 | 79.10% |
| Efet.01.654418.g588.t1 | Verminephrobacter eiseniae EF01-2, complete genome | 979 | 0.00E+00 | 79.10% |
| Efet.01.655399.g809.t1 | Verminephrobacter eiseniae EF01-2, complete genome | 369 | 2.40E-172 | 79.10% |
| Efet.01.655821.g886.t1 | Verminephrobacter eiseniae EF01-2, complete genome | 237 | 1.00E-113 | 79.10% |
| Efet.01.655964.g905.t1 | Verminephrobacter eiseniae EF01-2, complete genome | 651 | 0.00E+00 | 79.10% |
| Efet.01.657803.g1476.t1 | Verminephrobacter eiseniae EF01-2, complete genome | 495 | 0.00E+00 | 79.10% |
| Efet.01.1654548.g698.t1 | Verminephrobacter eiseniae EF01-2, complete genome | 343 | 2.70E-172 | 79.10% |
| Efet.01.1659356.g1596.t1 | Verminephrobacter eiseniae EF01-2, complete genome | 759 | 0.00E+00 | 79.10% |
| Efet.01.1659457.g1763.t1 | Rhodoferax saidenbachensis strain DSM 22694, complete genome | 582 | 1.70E-142 | 79.10% |
| Efet.01.1659520.g1981.t1 | Burkholderia lata strain FL-7-5-30-S1-D0 chromosome 2, complete sequence | 710 | 3.20E-178 | 79.10% |
| Efet.01.7898.g582.t1 | Acidithiobacillus ferrivorans isolate PRJEB5721 genome assembly, chromosome: AFERRI | 73 | 9.70E-07 | 79.00% |
| Efet.01.14845.g1091.t1 | Agromyces aureus strain AR33, complete genome | 227 | 1.50E-54 | 79.00% |
| Efet.01.34490.g652.t1 | Opitutus sp. GAS368 genome assembly, chromosome: I | 84 | 4.60E-09 | 79.00% |
| Efet.01.38489.g905.t1 | Streptomyces sp. DUT11 chromosome | 101 | 1.30E-16 | 79.00% |
| Efet.01.59384.g591.t1 | Verminephrobacter eiseniae EF01-2, complete genome | 1287 | 0.00E+00 | 79.00% |
| Efet.01.143134.g927.t1 | Xanthomonadales bacterium D13 chromosome, complete genome | 47 | 6.00E-08 | 79.00% |
| Efet.01.172818.g1086.t1 | Deinococcus radiodurans R1 chromosome 1, complete sequence | 68 | 8.30E-06 | 79.00% |
| Efet.01.187552.g1763.t1 | Streptomyces alfalfae strain ACCC40021 chromosome, complete genome | 64 | 3.00E-07 | 79.00% |
| Efet.01.187613.g1764.t1 | Coxiella-like endosymbiont strain CRt, complete genome | 77 | 8.70E-08 | 79.00% |
| Efet.01.188187.g1795.t1 | gamma proteobacterium HdN1 complete genome | 88 | 1.70E-10 | 79.00% |
| Efet.01.190792.g1901.t1 | Leifsonia xyli subsp. xyli str. CTCB07, complete genome | 330 | 5.10E-79 | 79.00% |
| Efet.01.235339.g1391.t1 | Bacillus cereus strain MBGJa3 chromosome | 79 | 3.20E-06 | 79.00% |
| Efet.01.248603.g1838.t1 | Stackebrandtia nassauensis DSM 44728, complete genome | 73 | 1.20E-06 | 79.00% |
| Efet.01.281370.g1138.t1 | Planococcus rifietoensis strain M8, complete genome | 79 | 1.50E-07 | 79.00% |
| Efet.01.285725.g1292.t1 | Halomonas elongata DSM 2581, complete genome | 69 | 3.80E-06 | 79.00% |
| Efet.01.353298.g95.t1 | Verminephrobacter eiseniae EF01-2, complete genome | 672 | 0.00E+00 | 79.00% |
| Efet.01.441173.g1009.t1 | Flavobacterium gilvum strain EM1308, complete genome | 69 | 4.20E-06 | 79.00% |
| Efet.01.530744.g27.t1 | Microbacterium sp. 1.5R, complete genome | 162 | 1.20E-33 | 79.00% |
| Efet.01.544967.g359.t1 | Clostridium estertheticum subsp. estertheticum strain DSM 8809, complete genome | 68 | 5.80E-06 | 79.00% |
| Efet.01.556379.g669.t1 | Verminephrobacter eiseniae EF01-2, complete genome | 425 | 0.00E+00 | 79.00% |
| Efet.01.559401.g734.t1 | Aminobacter sp. MSH1 plasmid pBAM1, complete sequence | 272 | 5.20E-58 | 79.00% |
| Efet.01.571102.g355.t1 | Nonlabens marinus S1-08 DNA, nearly complete genome | 99 | 7.00E-09 | 79.00% |
| Efet.01.597547.g1212.t1 | Verminephrobacter eiseniae EF01-2, complete genome | 783 | 0.00E+00 | 79.00% |
| Efet.01.606285.g261.t1 | Verminephrobacter eiseniae EF01-2, complete genome | 588 | 0.00E+00 | 79.00% |
| Efet.01.606285.g266.t1 | Verminephrobacter eiseniae EF01-2, complete genome | 444 | 0.00E+00 | 79.00% |
| Efet.01.611250.g470.t1 | Fibrella aestuarina BUZ 2 drat genome | 76 | 4.30E-08 | 79.00% |
| Efet.01.612521.g549.t1 | Microcella alkaliphila DNA, complete genome, strain: JAM AC0309 | 264 | 1.70E-57 | 79.00% |
| Efet.01.615846.g703.t1 | Variovorax sp. HW608 genome assembly, chromosome: I | 239 | 5.60E-48 | 79.00% |
| Efet.01.623992.g1042.t1 | Verminephrobacter eiseniae EF01-2, complete genome | 1640 | 0.00E+00 | 79.00% |
| Efet.01.630860.g84.t1 | Verminephrobacter eiseniae EF01-2, complete genome | 867 | 0.00E+00 | 79.00% |
| Efet.01.635927.g297.t1 | Aminobacter sp. MSH1 plasmid pBAM1, complete sequence | 301 | 2.30E-68 | 79.00% |
| Efet.01.645197.g948.t1 | Agromyces sp. 30A chromosome, complete genome | 326 | 2.70E-80 | 79.00% |
| Efet.01.652898.g370.t1 | Verminephrobacter eiseniae EF01-2, complete genome | 759 | 0.00E+00 | 79.00% |
| Efet.01.655687.g850.t1 | Agromyces sp. 30A chromosome, complete genome | 684 | 6.90E-173 | 79.00% |
| Efet.01.657266.g1219.t1 | Verminephrobacter eiseniae EF01-2, complete genome | 565 | 0.00E+00 | 79.00% |
| Efet.01.657308.g1233.t1 | Verminephrobacter eiseniae EF01-2, complete genome | 977 | 0.00E+00 | 79.00% |
| Efet.01.658395.g1974.t1 | Verminephrobacter eiseniae EF01-2, complete genome | 1038 | 0.00E+00 | 79.00% |
| Efet.01.1656111.g827.t1 | Verminephrobacter eiseniae EF01-2, complete genome | 222 | 1.10E-102 | 79.00% |
| Efet.01.1658036.g1086.t1 | Alicycliphilus denitrificans K601, complete genome | 552 | 2.30E-139 | 79.00% |
| Efet.01.1659352.g1591.t1 | Verminephrobacter eiseniae EF01-2, complete genome | 363 | 0.00E+00 | 79.00% |
| Efet.01.1659394.g1645.t1 | Deinococcus radiodurans R1 chromosome 2, complete sequence | 74 | 3.70E-06 | 79.00% |
| Efet.01.25255.g25.t1 | Verminephrobacter eiseniae EF01-2, complete genome | 537 | 0.00E+00 | 78.90% |
| Efet.01.111819.g594.t1 | Sulfobacillus acidophilus DSM 10332, complete genome | 82 | 1.80E-10 | 78.90% |
| Efet.01.150930.g52.t1 | Verminephrobacter eiseniae EF01-2, complete genome | 1761 | 0.00E+00 | 78.90% |
| Efet.01.176760.g1250.t1 | Verminephrobacter eiseniae EF01-2, complete genome | 438 | 0.00E+00 | 78.90% |
| Efet.01.300799.g33.t1 | Verminephrobacter eiseniae EF01-2, complete genome | 1505 | 0.00E+00 | 78.90% |
| Efet.01.334417.g1026.t1 | Haematospirillum jordaniae strain H5569, complete genome | 131 | 1.60E-26 | 78.90% |
| Efet.01.445097.g1110.t1 | Verminephrobacter eiseniae EF01-2, complete genome | 884 | 0.00E+00 | 78.90% |
| Efet.01.573904.g432.t1 | Pseudomonas salegens strain CECT 8338 genome assembly, chromosome: I | 173 | 2.00E-46 | 78.90% |
| Efet.01.574381.g453.t1 | Verminephrobacter eiseniae EF01-2, complete genome | 1395 | 0.00E+00 | 78.90% |
| Efet.01.600657.g24.t1 | Verminephrobacter eiseniae EF01-2, complete genome | 879 | 0.00E+00 | 78.90% |
| Efet.01.611807.g508.t1 | Verminephrobacter eiseniae EF01-2, complete genome | 870 | 0.00E+00 | 78.90% |
| Efet.01.618401.g824.t1 | Verminephrobacter eiseniae EF01-2, complete genome | 870 | 0.00E+00 | 78.90% |
| Efet.01.626729.g1172.t1 | Agromyces sp. 30A chromosome, complete genome | 1276 | 0.00E+00 | 78.90% |
| Efet.01.631766.g135.t1 | Verminephrobacter eiseniae EF01-2, complete genome | 278 | 7.00E-137 | 78.90% |
| Efet.01.641462.g630.t1 | Verminephrobacter eiseniae EF01-2, complete genome | 558 | 0.00E+00 | 78.90% |
| Efet.01.642550.g701.t1 | Verminephrobacter eiseniae EF01-2, complete genome | 1355 | 0.00E+00 | 78.90% |
| Efet.01.645589.g994.t1 | Agromyces flavus strain CPCC 202695 genome assembly, chromosome: I | 479 | 2.00E-126 | 78.90% |
| Efet.01.646274.g1065.t1 | Verminephrobacter eiseniae EF01-2, complete genome | 312 | 2.30E-144 | 78.90% |
| Efet.01.646642.g1109.t1 | Morganella morganii strain FDAARGOS_63 chromosome, complete genome | 747 | 0.00E+00 | 78.90% |
| Efet.01.646715.g1120.t1 | Salinibacterium sp. CGMCC 1.16371 chromosome, complete genome | 382 | 2.50E-102 | 78.90% |
| Efet.01.646963.g1136.t1 | Verminephrobacter eiseniae EF01-2, complete genome | 1452 | 0.00E+00 | 78.90% |
| Efet.01.654418.g604.t1 | Verminephrobacter eiseniae EF01-2, complete genome | 1239 | 0.00E+00 | 78.90% |
| Efet.01.655750.g858.t1 | Verminephrobacter eiseniae EF01-2, complete genome | 552 | 0.00E+00 | 78.90% |
| Efet.01.655756.g863.t1 | Verminephrobacter eiseniae EF01-2, complete genome | 750 | 0.00E+00 | 78.90% |
| Efet.01.656090.g937.t1 | Verminephrobacter eiseniae EF01-2, complete genome | 894 | 0.00E+00 | 78.90% |
| Efet.01.657677.g1364.t1 | Verminephrobacter eiseniae EF01-2, complete genome | 930 | 0.00E+00 | 78.90% |
| Efet.01.658399.g1990.t1 | Verminephrobacter eiseniae EF01-2, complete genome | 1227 | 0.00E+00 | 78.90% |
| Efet.01.658417.g2081.t1 | Verminephrobacter eiseniae EF01-2, complete genome | 608 | 0.00E+00 | 78.90% |
| Efet.01.658426.g2134.t1 | Verminephrobacter eiseniae EF01-2, complete genome | 903 | 0.00E+00 | 78.90% |
| Efet.01.658430.g2177.t1 | Verminephrobacter eiseniae EF01-2, complete genome | 898 | 0.00E+00 | 78.90% |
| Efet.01.1657891.g1053.t1 | Verminephrobacter eiseniae EF01-2, complete genome | 396 | 0.00E+00 | 78.90% |
| Efet.01.1659439.g1716.t1 | Verminephrobacter eiseniae EF01-2, complete genome | 519 | 0.00E+00 | 78.90% |
| Efet.01.1658583.g1220.t1 | Verminephrobacter eiseniae EF01-2, complete genome | 801 | 0.00E+00 | 78.89% |
| Efet.01.377804.g745.t1 | Kocuria turfanensis strain HO-9042 genome | 94 | 2.90E-08 | 78.88% |
| Efet.01.1656988.g925.t1 | Alloactinosynnema sp. L-07 genome assembly Alloactinosynnema sp. L-07, chromosome : I | 89 | 2.20E-10 | 78.83% |
| Efet.01.282030.g1158.t1 | Verminephrobacter eiseniae EF01-2, complete genome | 311 | 7.40E-151 | 78.80% |
| Efet.01.350771.g22.t1 | Rhodococcus rhodochrous strain NCTC10210 genome assembly, chromosome: 1 | 125 | 1.90E-23 | 78.80% |
| Efet.01.413810.g320.t1 | Verminephrobacter eiseniae EF01-2, complete genome | 699 | 0.00E+00 | 78.80% |
| Efet.01.440538.g979.t1 | Verminephrobacter eiseniae EF01-2, complete genome | 1248 | 0.00E+00 | 78.80% |
| Efet.01.524250.g566.t1 | Verminephrobacter eiseniae EF01-2, complete genome | 891 | 0.00E+00 | 78.80% |
| Efet.01.577373.g572.t1 | Verminephrobacter eiseniae EF01-2, complete genome | 1281 | 0.00E+00 | 78.80% |
| Efet.01.577664.g609.t1 | Verminephrobacter eiseniae EF01-2, complete genome | 720 | 0.00E+00 | 78.80% |
| Efet.01.600693.g27.t1 | Capnocytophaga sp. ChDC OS43, complete genome | 113 | 6.40E-20 | 78.80% |
| Efet.01.613416.g596.t1 | Verminephrobacter eiseniae EF01-2, complete genome | 597 | 0.00E+00 | 78.80% |
| Efet.01.623992.g1044.t1 | Verminephrobacter eiseniae EF01-2, complete genome | 1206 | 0.00E+00 | 78.80% |
| Efet.01.639356.g503.t1 | Verminephrobacter eiseniae EF01-2, complete genome | 2345 | 0.00E+00 | 78.80% |
| Efet.01.643099.g759.t1 | Verminephrobacter eiseniae EF01-2, complete genome | 1108 | 0.00E+00 | 78.80% |
| Efet.01.644746.g895.t1 | Verminephrobacter eiseniae EF01-2, complete genome | 1083 | 0.00E+00 | 78.80% |
| Efet.01.645199.g954.t1 | Verminephrobacter eiseniae EF01-2, complete genome | 1743 | 0.00E+00 | 78.80% |
| Efet.01.647241.g1162.t1 | Verminephrobacter eiseniae EF01-2, complete genome | 255 | 3.20E-126 | 78.80% |
| Efet.01.647444.g1179.t1 | Verminephrobacter eiseniae EF01-2, complete genome | 1416 | 0.00E+00 | 78.80% |
| Efet.01.650480.g53.t1 | Verminephrobacter eiseniae EF01-2, complete genome | 1101 | 0.00E+00 | 78.80% |
| Efet.01.653800.g478.t1 | Verminephrobacter eiseniae EF01-2, complete genome | 625 | 0.00E+00 | 78.80% |
| Efet.01.656558.g1045.t1 | Verminephrobacter eiseniae EF01-2, complete genome | 1049 | 0.00E+00 | 78.80% |
| Efet.01.657110.g1168.t1 | Verminephrobacter eiseniae EF01-2, complete genome | 377 | 0.00E+00 | 78.80% |
| Efet.01.657766.g1449.t1 | Verminephrobacter eiseniae EF01-2, complete genome | 502 | 0.00E+00 | 78.80% |
| Efet.01.658395.g1971.t1 | Verminephrobacter eiseniae EF01-2, complete genome | 669 | 0.00E+00 | 78.80% |
| Efet.01.1649327.g433.t1 | Brevibacterium linens strain SMQ-1335, complete genome | 139 | 7.50E-42 | 78.80% |
| Efet.01.1657590.g1013.t1 | Agromyces sp. 30A chromosome, complete genome | 486 | 5.10E-133 | 78.80% |
| Efet.01.1658886.g1314.t1 | Rathayibacter tritici strain NCPPB 1953, complete genome | 886 | 0.00E+00 | 78.80% |
| Efet.01.1659194.g1456.t1 | Verminephrobacter eiseniae EF01-2, complete genome | 387 | 1.40E-170 | 78.80% |
| Efet.01.1659527.g2035.t1 | Verminephrobacter eiseniae EF01-2, complete genome | 666 | 0.00E+00 | 78.80% |
| Efet.01.653968.g497.t1 | Verminephrobacter eiseniae EF01-2, complete genome | 297 | 3.70E-139 | 78.75% |
| Efet.01.1658216.g1132.t1 | Verminephrobacter eiseniae EF01-2, complete genome | 486 | 0.00E+00 | 78.75% |
| Efet.01.32672.g536.t1 | Kushneria sp. X49, complete genome | 129 | 5.30E-09 | 78.71% |
| Efet.01.1659376.g1624.t1 | Variovorax paradoxus B4 chromosome 1, complete sequence | 178 | 1.20E-56 | 78.71% |
| Efet.01.25255.g18.t1 | Verminephrobacter eiseniae EF01-2, complete genome | 495 | 0.00E+00 | 78.70% |
| Efet.01.91462.g966.t1 | Mycobacterium sp. EPa45, complete genome | 86 | 4.40E-13 | 78.70% |
| Efet.01.183662.g1580.t1 | Verminephrobacter eiseniae EF01-2, complete genome | 526 | 0.00E+00 | 78.70% |
| Efet.01.395805.g1135.t1 | Rhodococcus pyridinivorans strain GF3, complete genome | 94 | 1.80E-10 | 78.70% |
| Efet.01.514559.g352.t1 | Verminephrobacter eiseniae EF01-2, complete genome | 822 | 0.00E+00 | 78.70% |
| Efet.01.591854.g1047.t1 | Microterricola viridarii strain ERGS5:02, complete genome | 120 | 5.20E-26 | 78.70% |
| Efet.01.595491.g1158.t1 | Verminephrobacter eiseniae EF01-2, complete genome | 1017 | 0.00E+00 | 78.70% |
| Efet.01.607412.g304.t1 | Verminephrobacter eiseniae EF01-2, complete genome | 974 | 0.00E+00 | 78.70% |
| Efet.01.611343.g472.t1 | Verminephrobacter eiseniae EF01-2, complete genome | 1158 | 0.00E+00 | 78.70% |
| Efet.01.613088.g584.t1 | Verminephrobacter eiseniae EF01-2, complete genome | 441 | 0.00E+00 | 78.70% |
| Efet.01.625371.g1113.t1 | Nonlabens sp. Hel1_33_55 genome assembly, chromosome: I | 158 | 5.00E-12 | 78.70% |
| Efet.01.635927.g308.t1 | Pandoraea oxalativorans strain DSM 23570 plasmid pPO70-4, complete sequence | 725 | 3.20E-174 | 78.70% |
| Efet.01.642123.g660.t1 | Verminephrobacter eiseniae EF01-2, complete genome | 477 | 0.00E+00 | 78.70% |
| Efet.01.643898.g842.t1 | Verminephrobacter eiseniae EF01-2, complete genome | 822 | 0.00E+00 | 78.70% |
| Efet.01.654120.g536.t1 | Agromyces albus rpoB gene for RNA polymerase subunit B, type strain DSM 15934 | 855 | 0.00E+00 | 78.70% |
| Efet.01.656919.g1140.t1 | Verminephrobacter eiseniae EF01-2, complete genome | 354 | 2.50E-176 | 78.70% |
| Efet.01.657077.g1161.t1 | Verminephrobacter eiseniae EF01-2, complete genome | 459 | 0.00E+00 | 78.70% |
| Efet.01.658298.g1818.t1 | Verminephrobacter eiseniae EF01-2, complete genome | 867 | 0.00E+00 | 78.70% |
| Efet.01.658391.g1943.t1 | Verminephrobacter eiseniae EF01-2, complete genome | 928 | 0.00E+00 | 78.70% |
| Efet.01.658429.g2164.t1 | Verminephrobacter eiseniae EF01-2, complete genome | 235 | 2.20E-104 | 78.70% |
| Efet.01.1659335.g1562.t1 | Agromyces sp. 30A chromosome, complete genome | 1670 | 0.00E+00 | 78.70% |
| Efet.01.1659487.g1842.t1 | Verminephrobacter eiseniae EF01-2, complete genome | 811 | 0.00E+00 | 78.70% |
| Efet.01.1659526.g2030.t1 | Verminephrobacter eiseniae EF01-2, complete genome | 972 | 0.00E+00 | 78.70% |
| Efet.01.9268.g679.t1 | Mycobacterium dioxanotrophicus strain PH-06, complete genome | 93 | 8.90E-11 | 78.67% |
| Efet.01.617238.g756.t1 | Verminephrobacter eiseniae EF01-2, complete genome | 924 | 0.00E+00 | 78.67% |
| Efet.01.645199.g949.t1 | Verminephrobacter eiseniae EF01-2, complete genome | 1022 | 0.00E+00 | 78.67% |
| Efet.01.658417.g2068.t1 | Verminephrobacter eiseniae EF01-2, complete genome | 612 | 0.00E+00 | 78.67% |
| Efet.01.34783.g673.t1 | Corynebacterium doosanense CAU 212 = DSM 45436, complete genome | 59 | 4.60E-09 | 78.60% |
| Efet.01.39921.g984.t1 | [Clostridium] stercorarium subsp. stercorarium DSM 8532, complete genome | 104 | 7.70E-09 | 78.60% |
| Efet.01.318747.g595.t1 | Verminephrobacter eiseniae EF01-2, complete genome | 828 | 0.00E+00 | 78.60% |
| Efet.01.464530.g382.t1 | Verminephrobacter eiseniae EF01-2, complete genome | 1026 | 0.00E+00 | 78.60% |
| Efet.01.514247.g334.t1 | Verminephrobacter eiseniae EF01-2, complete genome | 992 | 0.00E+00 | 78.60% |
| Efet.01.523923.g554.t1 | Verminephrobacter eiseniae EF01-2, complete genome | 488 | 0.00E+00 | 78.60% |
| Efet.01.559401.g736.t1 | Verminephrobacter eiseniae EF01-2, complete genome | 264 | 1.10E-123 | 78.60% |
| Efet.01.586228.g873.t1 | Verminephrobacter eiseniae EF01-2, complete genome | 399 | 0.00E+00 | 78.60% |
| Efet.01.605872.g245.t1 | Microterricola viridarii strain DSM 21772 genome assembly, chromosome: I | 584 | 6.90E-148 | 78.60% |
| Efet.01.623992.g1041.t1 | Verminephrobacter eiseniae EF01-2, complete genome | 699 | 0.00E+00 | 78.60% |
| Efet.01.623992.g1045.t1 | Verminephrobacter eiseniae EF01-2, complete genome | 1158 | 0.00E+00 | 78.60% |
| Efet.01.633916.g219.t1 | Verminephrobacter eiseniae EF01-2, complete genome | 705 | 0.00E+00 | 78.60% |
| Efet.01.643464.g786.t1 | Verminephrobacter eiseniae EF01-2, complete genome | 715 | 0.00E+00 | 78.60% |
| Efet.01.643637.g816.t1 | Microbacterium sp. LKL04 genome assembly, chromosome: I | 126 | 3.60E-23 | 78.60% |
| Efet.01.654310.g566.t1 | Verminephrobacter eiseniae EF01-2, complete genome | 771 | 0.00E+00 | 78.60% |
| Efet.01.655988.g922.t1 | Verminephrobacter eiseniae EF01-2, complete genome | 711 | 0.00E+00 | 78.60% |
| Efet.01.656470.g1027.t1 | Verminephrobacter eiseniae EF01-2, complete genome | 873 | 0.00E+00 | 78.60% |
| Efet.01.658133.g1662.t1 | Verminephrobacter eiseniae EF01-2, complete genome | 550 | 0.00E+00 | 78.60% |
| Efet.01.658417.g2079.t1 | Verminephrobacter eiseniae EF01-2, complete genome | 1125 | 0.00E+00 | 78.60% |
| Efet.01.658430.g2178.t1 | Verminephrobacter eiseniae EF01-2, complete genome | 795 | 0.00E+00 | 78.60% |
| Efet.01.1657070.g938.t1 | Azoarcus aromaticum EbN1 complete genome | 541 | 1.10E-166 | 78.60% |
| Efet.01.1659346.g1578.t1 | Verminephrobacter eiseniae EF01-2, complete genome | 585 | 0.00E+00 | 78.60% |
| Efet.01.1659437.g1711.t1 | Verminephrobacter eiseniae EF01-2, complete genome | 1458 | 0.00E+00 | 78.60% |
| Efet.01.1659451.g1746.t1 | Verminephrobacter eiseniae EF01-2, complete genome | 552 | 0.00E+00 | 78.60% |
| Efet.01.1659495.g1872.t1 | Verminephrobacter eiseniae EF01-2, complete genome | 429 | 0.00E+00 | 78.60% |
| Efet.01.10434.g766.t1 | Hymenobacter sp. APR13, complete genome | 82 | 6.60E-08 | 78.50% |
| Efet.01.42798.g1158.t1 | Candidatus Kuenenia stuttgartiensis isolate kuenenia_mbr1_ru-nijmegen genome assembly, chromosome: Kuenenia_stuttgartiensis_MBR1 | 75 | 3.10E-07 | 78.50% |
| Efet.01.49324.g1550.t1 | Vibrio vulnificus strain VV2014DJH chromosome 1, complete sequence | 89 | 1.10E-08 | 78.50% |
| Efet.01.267648.g641.t1 | Verminephrobacter eiseniae EF01-2, complete genome | 424 | 6.20E-168 | 78.50% |
| Efet.01.353298.g93.t1 | Verminephrobacter eiseniae EF01-2, complete genome | 780 | 0.00E+00 | 78.50% |
| Efet.01.530573.g20.t1 | Verminephrobacter eiseniae EF01-2, complete genome | 555 | 0.00E+00 | 78.50% |
| Efet.01.577373.g575.t1 | Verminephrobacter eiseniae EF01-2, complete genome | 777 | 0.00E+00 | 78.50% |
| Efet.01.593811.g1105.t1 | Plantibacter flavus strain 251 genome | 298 | 1.90E-68 | 78.50% |
| Efet.01.599543.g1293.t1 | Streptomyces albulus strain CK-15 chromosome, complete genome | 120 | 1.10E-18 | 78.50% |
| Efet.01.606285.g259.t1 | Verminephrobacter eiseniae EF01-2, complete genome | 435 | 6.90E-167 | 78.50% |
| Efet.01.612091.g512.t1 | Verminephrobacter eiseniae EF01-2, complete genome | 450 | 0.00E+00 | 78.50% |
| Efet.01.626983.g1177.t1 | Verminephrobacter eiseniae EF01-2, complete genome | 1263 | 0.00E+00 | 78.50% |
| Efet.01.638425.g431.t1 | Verminephrobacter eiseniae EF01-2, complete genome | 1415 | 0.00E+00 | 78.50% |
| Efet.01.646179.g1049.t1 | Verminephrobacter eiseniae EF01-2, complete genome | 933 | 0.00E+00 | 78.50% |
| Efet.01.647059.g1155.t1 | Verminephrobacter eiseniae EF01-2, complete genome | 804 | 0.00E+00 | 78.50% |
| Efet.01.655987.g919.t1 | Verminephrobacter eiseniae EF01-2, complete genome | 1530 | 0.00E+00 | 78.50% |
| Efet.01.656171.g950.t1 | Diaphorobacter polyhydroxybutyrativorans strain SL-205, complete genome | 324 | 4.20E-101 | 78.50% |
| Efet.01.657453.g1288.t1 | Verminephrobacter eiseniae EF01-2, complete genome | 357 | 3.00E-169 | 78.50% |
| Efet.01.657703.g1376.t1 | Verminephrobacter eiseniae EF01-2, complete genome | 634 | 0.00E+00 | 78.50% |
| Efet.01.658122.g1656.t1 | Verminephrobacter eiseniae EF01-2, complete genome | 1116 | 0.00E+00 | 78.50% |
| Efet.01.658383.g1924.t1 | Verminephrobacter eiseniae EF01-2, complete genome | 1257 | 0.00E+00 | 78.50% |
| Efet.01.658392.g1966.t1 | Verminephrobacter eiseniae EF01-2, complete genome | 610 | 0.00E+00 | 78.50% |
| Efet.01.658421.g2098.t1 | Streptomyces spongiicola strain HNM0071 chromosome, complete genome | 64 | 6.30E-07 | 78.50% |
| Efet.01.658427.g2144.t1 | Verminephrobacter eiseniae EF01-2, complete genome | 4152 | 0.00E+00 | 78.50% |
| Efet.01.1629704.g97.t1 | Microbacterium aurum strain KACC 15219, complete genome | 167 | 8.80E-49 | 78.50% |
| Efet.01.1640270.g219.t1 | Microterricola viridarii strain ERGS5:02, complete genome | 188 | 7.20E-44 | 78.50% |
| Efet.01.1658918.g1325.t1 | Agromyces sp. 30A chromosome, complete genome | 1007 | 0.00E+00 | 78.50% |
| Efet.01.1659519.g1974.t1 | Verminephrobacter eiseniae EF01-2, complete genome | 666 | 0.00E+00 | 78.50% |
| Efet.01.1659519.g1978.t1 | Verminephrobacter eiseniae EF01-2, complete genome | 1038 | 0.00E+00 | 78.50% |
| Efet.01.658372.g1908.t1 | Verminephrobacter eiseniae EF01-2, complete genome | 717 | 0.00E+00 | 78.43% |
| Efet.01.1659484.g1832.t1 | Verminephrobacter eiseniae EF01-2, complete genome | 1125 | 0.00E+00 | 78.43% |
| Efet.01.220990.g851.t1 | Verminephrobacter eiseniae EF01-2, complete genome | 936 | 0.00E+00 | 78.40% |
| Efet.01.225483.g1017.t1 | Microcystis aeruginosa NIES-2481, complete genome | 103 | 5.80E-11 | 78.40% |
| Efet.01.424172.g573.t1 | Verminephrobacter eiseniae EF01-2, complete genome | 216 | 2.10E-103 | 78.40% |
| Efet.01.467295.g463.t1 | Verminephrobacter eiseniae EF01-2, complete genome | 402 | 4.70E-172 | 78.40% |
| Efet.01.577664.g607.t1 | Verminephrobacter eiseniae EF01-2, complete genome | 411 | 0.00E+00 | 78.40% |
| Efet.01.616615.g734.t1 | Verminephrobacter eiseniae EF01-2, complete genome | 888 | 0.00E+00 | 78.40% |
| Efet.01.618025.g799.t1 | Verminephrobacter eiseniae EF01-2, complete genome | 834 | 0.00E+00 | 78.40% |
| Efet.01.629153.g1274.t1 | Verminephrobacter eiseniae EF01-2, complete genome | 487 | 0.00E+00 | 78.40% |
| Efet.01.636379.g331.t1 | Leifsonia sp. 98AMF genome assembly, chromosome: I | 797 | 0.00E+00 | 78.40% |
| Efet.01.640048.g555.t1 | Verminephrobacter eiseniae EF01-2, complete genome | 1188 | 0.00E+00 | 78.40% |
| Efet.01.651136.g114.t1 | Verminephrobacter eiseniae EF01-2, complete genome | 1035 | 0.00E+00 | 78.40% |
| Efet.01.651939.g239.t1 | Verminephrobacter eiseniae EF01-2, complete genome | 912 | 0.00E+00 | 78.40% |
| Efet.01.653460.g431.t1 | Paludibacter propionicigenes WB4, complete genome | 210 | 1.50E-15 | 78.40% |
| Efet.01.654656.g645.t1 | Verminephrobacter eiseniae EF01-2, complete genome | 525 | 0.00E+00 | 78.40% |
| Efet.01.654851.g701.t1 | Verminephrobacter eiseniae EF01-2, complete genome | 427 | 0.00E+00 | 78.40% |
| Efet.01.657315.g1238.t1 | Verminephrobacter eiseniae EF01-2, complete genome | 678 | 0.00E+00 | 78.40% |
| Efet.01.657430.g1278.t1 | Verminephrobacter eiseniae EF01-2, complete genome | 687 | 0.00E+00 | 78.40% |
| Efet.01.657766.g1425.t1 | Verminephrobacter eiseniae EF01-2, complete genome | 739 | 0.00E+00 | 78.40% |
| Efet.01.658236.g1737.t1 | Verminephrobacter eiseniae EF01-2, complete genome | 993 | 0.00E+00 | 78.40% |
| Efet.01.658423.g2115.t1 | Verminephrobacter eiseniae EF01-2, complete genome | 947 | 0.00E+00 | 78.40% |
| Efet.01.1659347.g1581.t1 | Verminephrobacter eiseniae EF01-2, complete genome | 444 | 0.00E+00 | 78.40% |
| Efet.01.1659525.g2014.t1 | Burkholderia cepacia ATCC 25416 chromosome 2, complete sequence | 291 | 1.10E-139 | 78.40% |
| Efet.01.1659526.g2032.t1 | Verminephrobacter eiseniae EF01-2, complete genome | 756 | 0.00E+00 | 78.40% |
| Efet.01.257008.g263.t1 | Gemmata obscuriglobus strain DSM 5831 chromosome, complete genome | 82 | 3.90E-07 | 78.33% |
| Efet.01.645275.g970.t1 | Verminephrobacter eiseniae EF01-2, complete genome | 597 | 0.00E+00 | 78.33% |
| Efet.01.32422.g524.t1 | Verminephrobacter eiseniae EF01-2, complete genome | 645 | 0.00E+00 | 78.30% |
| Efet.01.370035.g556.t1 | Verminephrobacter eiseniae EF01-2, complete genome | 563 | 0.00E+00 | 78.30% |
| Efet.01.531412.g46.t1 | Miniimonas sp. S16 plasmid pS16-2, complete sequence | 240 | 3.90E-65 | 78.30% |
| Efet.01.553421.g592.t1 | Agromyces aureus strain AR33, complete genome | 420 | 4.40E-105 | 78.30% |
| Efet.01.564720.g140.t1 | Devosia sp. A16, complete genome | 197 | 1.90E-17 | 78.30% |
| Efet.01.584286.g800.t1 | Thiohalobacter thiocyanaticus DNA, complete genome, strain: FOKN1 | 197 | 1.10E-41 | 78.30% |
| Efet.01.592653.g1084.t1 | Verminephrobacter eiseniae EF01-2, complete genome | 1069 | 0.00E+00 | 78.30% |
| Efet.01.612521.g551.t1 | Agromyces sp. 30A chromosome, complete genome | 537 | 5.50E-146 | 78.30% |
| Efet.01.614177.g630.t1 | Verminephrobacter eiseniae EF01-2, complete genome | 342 | 4.20E-161 | 78.30% |
| Efet.01.616228.g718.t1 | Verminephrobacter eiseniae EF01-2, complete genome | 570 | 0.00E+00 | 78.30% |
| Efet.01.650749.g80.t1 | Verminephrobacter eiseniae EF01-2, complete genome | 612 | 0.00E+00 | 78.30% |
| Efet.01.651148.g142.t1 | Verminephrobacter eiseniae EF01-2, complete genome | 466 | 0.00E+00 | 78.30% |
| Efet.01.653968.g504.t1 | Verminephrobacter eiseniae EF01-2, complete genome | 769 | 0.00E+00 | 78.30% |
| Efet.01.654200.g546.t1 | Agromyces flavus strain CPCC 202695 genome assembly, chromosome: I | 698 | 0.00E+00 | 78.30% |
| Efet.01.655281.g789.t1 | Verminephrobacter eiseniae EF01-2, complete genome | 951 | 0.00E+00 | 78.30% |
| Efet.01.655399.g812.t1 | Verminephrobacter eiseniae EF01-2, complete genome | 366 | 0.00E+00 | 78.30% |
| Efet.01.656717.g1074.t1 | Verminephrobacter eiseniae EF01-2, complete genome | 1434 | 0.00E+00 | 78.30% |
| Efet.01.658044.g1598.t1 | Verminephrobacter eiseniae EF01-2, complete genome | 801 | 0.00E+00 | 78.30% |
| Efet.01.658052.g1603.t1 | Verminephrobacter eiseniae EF01-2, complete genome | 648 | 0.00E+00 | 78.30% |
| Efet.01.658385.g1934.t1 | Verminephrobacter eiseniae EF01-2, complete genome | 627 | 0.00E+00 | 78.30% |
| Efet.01.658392.g1965.t1 | Verminephrobacter eiseniae EF01-2, complete genome | 819 | 0.00E+00 | 78.30% |
| Efet.01.1603813.g4.t1 | Thauera sp. MZ1T, complete genome | 196 | 3.50E-48 | 78.30% |
| Efet.01.1654151.g664.t1 | Microbacterium aurum strain KACC 15219, complete genome | 448 | 5.60E-105 | 78.30% |
| Efet.01.1655572.g768.t1 | Verminephrobacter eiseniae EF01-2, complete genome | 246 | 1.20E-119 | 78.30% |
| Efet.01.1659289.g1517.t1 | Verminephrobacter eiseniae EF01-2, complete genome | 169 | 8.90E-74 | 78.30% |
| Efet.01.1659487.g1840.t1 | Verminephrobacter eiseniae EF01-2, complete genome | 1314 | 0.00E+00 | 78.30% |
| Efet.01.651136.g116.t1 | Variovorax sp. HW608 genome assembly, chromosome: I | 1047 | 0.00E+00 | 78.29% |
| Efet.01.657807.g1489.t1 | Pseudomonas syringae pv. tomato strain B13-200 plasmid pB13-200A, complete sequence | 759 | 0.00E+00 | 78.29% |
| Efet.01.487768.g913.t1 | Bacillus coagulans LA204, complete genome | 71 | 3.30E-07 | 78.25% |
| Efet.01.61804.g734.t1 | Verminephrobacter eiseniae EF01-2, complete genome | 462 | 0.00E+00 | 78.20% |
| Efet.01.267648.g647.t1 | Verminephrobacter eiseniae EF01-2, complete genome | 1179 | 0.00E+00 | 78.20% |
| Efet.01.300440.g20.t1 | Verminephrobacter eiseniae EF01-2, complete genome | 591 | 0.00E+00 | 78.20% |
| Efet.01.463370.g364.t1 | Verminephrobacter eiseniae EF01-2, complete genome | 306 | 6.90E-151 | 78.20% |
| Efet.01.504526.g112.t1 | Verminephrobacter eiseniae EF01-2, complete genome | 1090 | 0.00E+00 | 78.20% |
| Efet.01.553639.g598.t1 | Verminephrobacter eiseniae EF01-2, complete genome | 3375 | 0.00E+00 | 78.20% |
| Efet.01.565049.g171.t1 | Verminephrobacter eiseniae EF01-2, complete genome | 639 | 0.00E+00 | 78.20% |
| Efet.01.573202.g409.t1 | Agromyces flavus strain CPCC 202695 genome assembly, chromosome: I | 1517 | 0.00E+00 | 78.20% |
| Efet.01.598881.g1266.t1 | Agromyces flavus strain CPCC 202695 genome assembly, chromosome: I | 896 | 0.00E+00 | 78.20% |
| Efet.01.605483.g223.t1 | Verminephrobacter eiseniae EF01-2, complete genome | 364 | 1.90E-180 | 78.20% |
| Efet.01.613416.g600.t1 | Verminephrobacter eiseniae EF01-2, complete genome | 444 | 0.00E+00 | 78.20% |
| Efet.01.623996.g1053.t1 | Verminephrobacter eiseniae EF01-2, complete genome | 589 | 0.00E+00 | 78.20% |
| Efet.01.630860.g97.t1 | Verminephrobacter eiseniae EF01-2, complete genome | 718 | 0.00E+00 | 78.20% |
| Efet.01.642357.g676.t1 | Verminephrobacter eiseniae EF01-2, complete genome | 2603 | 0.00E+00 | 78.20% |
| Efet.01.645268.g964.t1 | Verminephrobacter eiseniae EF01-2, complete genome | 720 | 0.00E+00 | 78.20% |
| Efet.01.646129.g1036.t1 | Arthrobacter sp. QXT-31, complete genome | 213 | 5.20E-45 | 78.20% |
| Efet.01.646487.g1100.t1 | Verminephrobacter eiseniae EF01-2, complete genome | 1116 | 0.00E+00 | 78.20% |
| Efet.01.649998.g1367.t1 | Verminephrobacter eiseniae EF01-2, complete genome | 900 | 0.00E+00 | 78.20% |
| Efet.01.654418.g603.t1 | Verminephrobacter eiseniae EF01-2, complete genome | 895 | 0.00E+00 | 78.20% |
| Efet.01.654756.g691.t1 | Verminephrobacter eiseniae EF01-2, complete genome | 1194 | 0.00E+00 | 78.20% |
| Efet.01.654965.g723.t1 | Verminephrobacter eiseniae EF01-2, complete genome | 798 | 0.00E+00 | 78.20% |
| Efet.01.656856.g1128.t1 | Methylobacterium nodulans ORS 2060, complete genome | 406 | 5.40E-92 | 78.20% |
| Efet.01.657484.g1300.t1 | Agromyces aureus strain AR33, complete genome | 212 | 4.50E-34 | 78.20% |
| Efet.01.658142.g1671.t1 | Verminephrobacter eiseniae EF01-2, complete genome | 1062 | 0.00E+00 | 78.20% |
| Efet.01.1659392.g1642.t1 | Verminephrobacter eiseniae EF01-2, complete genome | 771 | 0.00E+00 | 78.20% |
| Efet.01.1659489.g1851.t1 | Verminephrobacter eiseniae EF01-2, complete genome | 940 | 0.00E+00 | 78.20% |
| Efet.01.1659501.g1896.t1 | Verminephrobacter eiseniae EF01-2, complete genome | 3006 | 0.00E+00 | 78.20% |
| Efet.01.654066.g533.t1 | Frondihabitans sp. PAMC28766, complete genome | 84 | 6.30E-10 | 78.17% |
| Efet.01.1659475.g1800.t1 | Achromobacter xylosoxidans strain FDAARGOS_162 chromosome, complete genome | 1342 | 0.00E+00 | 78.13% |
| Efet.01.59384.g590.t1 | Verminephrobacter eiseniae EF01-2, complete genome | 1374 | 0.00E+00 | 78.10% |
| Efet.01.182140.g1505.t1 | Streptomyces sp. TLI_053 genome assembly, chromosome: I | 119 | 1.10E-17 | 78.10% |
| Efet.01.240933.g1564.t1 | Agromyces sp. 30A chromosome, complete genome | 689 | 5.70E-170 | 78.10% |
| Efet.01.293477.g1553.t1 | Agromyces sp. 30A chromosome, complete genome | 515 | 2.60E-117 | 78.10% |
| Efet.01.393322.g1081.t1 | Verminephrobacter eiseniae EF01-2, complete genome | 1322 | 0.00E+00 | 78.10% |
| Efet.01.466628.g442.t1 | Rhodococcus sp. H-CA8f chromosome, complete genome | 100 | 5.40E-13 | 78.10% |
| Efet.01.471393.g573.t1 | Verminephrobacter eiseniae EF01-2, complete genome | 261 | 1.60E-124 | 78.10% |
| Efet.01.558742.g715.t1 | Verminephrobacter eiseniae EF01-2, complete genome | 1226 | 0.00E+00 | 78.10% |
| Efet.01.559401.g737.t1 | Verminephrobacter eiseniae EF01-2, complete genome | 216 | 2.40E-100 | 78.10% |
| Efet.01.573348.g412.t1 | Verminephrobacter eiseniae EF01-2, complete genome | 1215 | 0.00E+00 | 78.10% |
| Efet.01.585033.g822.t1 | Verminephrobacter eiseniae EF01-2, complete genome | 357 | 1.00E-174 | 78.10% |
| Efet.01.596935.g1198.t1 | Alistipes finegoldii DSM 17242, complete genome | 153 | 2.80E-28 | 78.10% |
| Efet.01.655428.g819.t1 | Clavibacter michiganensis subsp. nebraskensis NCPPB 2581 complete genome | 153 | 7.90E-33 | 78.10% |
| Efet.01.655987.g920.t1 | Verminephrobacter eiseniae EF01-2, complete genome | 918 | 0.00E+00 | 78.10% |
| Efet.01.656816.g1116.t1 | Verminephrobacter eiseniae EF01-2, complete genome | 958 | 0.00E+00 | 78.10% |
| Efet.01.656822.g1122.t1 | Verminephrobacter eiseniae EF01-2, complete genome | 960 | 0.00E+00 | 78.10% |
| Efet.01.1649509.g439.t1 | Alkalilimnicola ehrlichii MLHE-1, complete genome | 365 | 5.00E-82 | 78.10% |
| Efet.01.1659142.g1428.t1 | Verminephrobacter eiseniae EF01-2, complete genome | 630 | 0.00E+00 | 78.10% |
| Efet.01.1659209.g1461.t1 | Verminephrobacter eiseniae EF01-2, complete genome | 958 | 0.00E+00 | 78.10% |
| Efet.01.1659414.g1675.t1 | Verminephrobacter eiseniae EF01-2, complete genome | 1102 | 0.00E+00 | 78.10% |
| Efet.01.1659493.g1862.t1 | Verminephrobacter eiseniae EF01-2, complete genome | 1155 | 0.00E+00 | 78.10% |
| Efet.01.1659527.g2034.t1 | Verminephrobacter eiseniae EF01-2, complete genome | 549 | 0.00E+00 | 78.10% |
| Efet.01.34431.g649.t1 | Sorangium cellulosum strain So ce26 chromosome, complete genome | 88 | 3.80E-10 | 78.00% |
| Efet.01.43891.g1220.t1 | Dokdonia sp. Dokd-P16 chromosome, complete genome | 66 | 4.30E-07 | 78.00% |
| Efet.01.105872.g313.t1 | Streptomyces gilvosporeus strain F607, complete genome | 186 | 2.40E-32 | 78.00% |
| Efet.01.139973.g767.t1 | Desulfocapsa sulfexigens DSM 10523, complete genome | 71 | 8.10E-06 | 78.00% |
| Efet.01.150866.g42.t1 | Arthrobacter sp. Hiyo4 DNA, complete genome, strain: Hiyo4 | 79 | 3.00E-07 | 78.00% |
| Efet.01.152294.g132.t1 | Candidatus Pelagibacter sp. IMCC9063, complete genome | 106 | 2.00E-08 | 78.00% |
| Efet.01.175082.g1174.t1 | Beijerinckia indica subsp. indica ATCC 9039, complete genome | 86 | 6.60E-07 | 78.00% |
| Efet.01.176760.g1240.t1 | Verminephrobacter eiseniae EF01-2, complete genome | 1197 | 0.00E+00 | 78.00% |
| Efet.01.226555.g1061.t1 | Corynebacterium sp. ATCC 6931, complete genome | 139 | 6.20E-17 | 78.00% |
| Efet.01.278935.g1068.t1 | Verminephrobacter eiseniae EF01-2, complete genome | 840 | 0.00E+00 | 78.00% |
| Efet.01.296729.g1644.t1 | Mycobacteroides abscessus strain G153 chromosome, complete genome | 793 | 0.00E+00 | 78.00% |
| Efet.01.333614.g1006.t1 | Paenibacillus mucilaginosus K02, complete genome | 73 | 6.70E-06 | 78.00% |
| Efet.01.413810.g322.t1 | Verminephrobacter eiseniae EF01-2, complete genome | 270 | 1.40E-108 | 78.00% |
| Efet.01.423212.g548.t1 | Microbulbifer sp. CCB-MM1, complete genome | 80 | 7.40E-08 | 78.00% |
| Efet.01.551303.g530.t1 | Curtobacterium sp. MR_MD2014, complete genome | 76 | 8.00E-07 | 78.00% |
| Efet.01.574381.g455.t1 | Verminephrobacter eiseniae EF01-2, complete genome | 1434 | 0.00E+00 | 78.00% |
| Efet.01.601193.g63.t1 | Verminephrobacter eiseniae EF01-2, complete genome | 498 | 0.00E+00 | 78.00% |
| Efet.01.606285.g271.t1 | Verminephrobacter eiseniae EF01-2, complete genome | 1647 | 0.00E+00 | 78.00% |
| Efet.01.611807.g506.t1 | Verminephrobacter eiseniae EF01-2, complete genome | 2397 | 0.00E+00 | 78.00% |
| Efet.01.622583.g985.t1 | Ralstonia solanacearum strain CQPS-1, complete genome | 656 | 4.80E-155 | 78.00% |
| Efet.01.622583.g990.t1 | Verminephrobacter eiseniae EF01-2, complete genome | 269 | 8.20E-127 | 78.00% |
| Efet.01.625458.g1118.t1 | Akkermansia glycaniphila isolate APytT genome assembly, chromosome: I | 169 | 2.40E-41 | 78.00% |
| Efet.01.626983.g1191.t1 | Verminephrobacter eiseniae EF01-2, complete genome | 792 | 0.00E+00 | 78.00% |
| Efet.01.630546.g35.t1 | Verminephrobacter eiseniae EF01-2, complete genome | 771 | 0.00E+00 | 78.00% |
| Efet.01.630711.g55.t1 | Lysobacter enzymogenes strain OH11 led gene cluster, complete sequence | 170 | 2.40E-19 | 78.00% |
| Efet.01.630774.g68.t1 | Verminephrobacter eiseniae EF01-2, complete genome | 1044 | 0.00E+00 | 78.00% |
| Efet.01.630860.g86.t1 | Verminephrobacter eiseniae EF01-2, complete genome | 924 | 0.00E+00 | 78.00% |
| Efet.01.640829.g594.t1 | Sinorhizobium fredii USDA 257, complete genome | 76 | 2.70E-07 | 78.00% |
| Efet.01.643097.g756.t1 | Verminephrobacter eiseniae EF01-2, complete genome | 729 | 0.00E+00 | 78.00% |
| Efet.01.654656.g657.t1 | Verminephrobacter eiseniae EF01-2, complete genome | 768 | 0.00E+00 | 78.00% |
| Efet.01.655193.g770.t1 | Verminephrobacter eiseniae EF01-2, complete genome | 413 | 0.00E+00 | 78.00% |
| Efet.01.656799.g1108.t1 | [Clostridium] innocuum strain I46 genome | 115 | 2.50E-16 | 78.00% |
| Efet.01.656889.g1134.t1 | Diaphorobacter polyhydroxybutyrativorans strain SL-205, complete genome | 761 | 3.00E-171 | 78.00% |
| Efet.01.658279.g1785.t1 | Verminephrobacter eiseniae EF01-2, complete genome | 371 | 0.00E+00 | 78.00% |
| Efet.01.658409.g2012.t1 | Verminephrobacter eiseniae EF01-2, complete genome | 555 | 0.00E+00 | 78.00% |
| Efet.01.658428.g2151.t1 | Mycobacterium smegmatis str. MC2 155, complete genome | 744 | 0.00E+00 | 78.00% |
| Efet.01.1594716.g18.t1 | Uncultured bacterium BD_contig02123 genomic sequence | 73 | 7.70E-06 | 78.00% |
| Efet.01.1655942.g805.t1 | Microterricola viridarii strain DSM 21772 genome assembly, chromosome: I | 367 | 4.20E-81 | 78.00% |
| Efet.01.1659309.g1537.t1 | Micromonospora sp. WMMA2032 chromosome | 80 | 1.50E-07 | 78.00% |
| Efet.01.1659511.g1932.t1 | Delftia sp. Cs1-4, complete genome | 324 | 2.30E-67 | 78.00% |
| Efet.01.176795.g1255.t1 | Verminephrobacter eiseniae EF01-2, complete genome | 1236 | 0.00E+00 | 77.90% |
| Efet.01.300388.g14.t1 | Streptomyces sp. PAMC26508, complete genome | 196 | 7.30E-41 | 77.90% |
| Efet.01.325607.g790.t1 | Verminephrobacter eiseniae EF01-2, complete genome | 603 | 0.00E+00 | 77.90% |
| Efet.01.406381.g157.t1 | Flavobacterium sp. AJ004 chromosome, complete genome | 141 | 1.30E-13 | 77.90% |
| Efet.01.456900.g170.t1 | Verminephrobacter eiseniae EF01-2, complete genome | 237 | 1.40E-86 | 77.90% |
| Efet.01.548599.g461.t1 | Verminephrobacter eiseniae EF01-2, complete genome | 1395 | 0.00E+00 | 77.90% |
| Efet.01.592582.g1079.t1 | Verminephrobacter eiseniae EF01-2, complete genome | 1671 | 0.00E+00 | 77.90% |
| Efet.01.609276.g389.t1 | Paraburkholderia xenovorans LB400 chromosome 2, complete sequence | 1202 | 0.00E+00 | 77.90% |
| Efet.01.613416.g593.t1 | Verminephrobacter eiseniae EF01-2, complete genome | 693 | 0.00E+00 | 77.90% |
| Efet.01.619581.g872.t1 | Agromyces aureus strain AR33, complete genome | 502 | 3.40E-138 | 77.90% |
| Efet.01.647241.g1163.t1 | Verminephrobacter eiseniae EF01-2, complete genome | 468 | 0.00E+00 | 77.90% |
| Efet.01.649588.g1333.t1 | Verminephrobacter eiseniae EF01-2, complete genome | 302 | 4.10E-146 | 77.90% |
| Efet.01.653541.g450.t1 | Verminephrobacter eiseniae EF01-2, complete genome | 1020 | 0.00E+00 | 77.90% |
| Efet.01.654656.g670.t1 | Verminephrobacter eiseniae EF01-2, complete genome | 537 | 0.00E+00 | 77.90% |
| Efet.01.655756.g865.t1 | Verminephrobacter eiseniae EF01-2, complete genome | 858 | 0.00E+00 | 77.90% |
| Efet.01.655920.g893.t1 | Pedobacter ginsengisoli strain T01R-27 chromosome, complete genome | 78 | 2.30E-09 | 77.90% |
| Efet.01.657206.g1198.t1 | Verminephrobacter eiseniae EF01-2, complete genome | 575 | 0.00E+00 | 77.90% |
| Efet.01.657759.g1414.t1 | Verminephrobacter eiseniae EF01-2, complete genome | 903 | 0.00E+00 | 77.90% |
| Efet.01.658334.g1867.t1 | Verminephrobacter eiseniae EF01-2, complete genome | 1602 | 0.00E+00 | 77.90% |
| Efet.01.658380.g1921.t1 | Verminephrobacter eiseniae EF01-2, complete genome | 525 | 0.00E+00 | 77.90% |
| Efet.01.658411.g2026.t1 | Verminephrobacter eiseniae EF01-2, complete genome | 540 | 0.00E+00 | 77.90% |
| Efet.01.658415.g2043.t1 | Verminephrobacter eiseniae EF01-2, complete genome | 912 | 0.00E+00 | 77.90% |
| Efet.01.658416.g2055.t1 | Verminephrobacter eiseniae EF01-2, complete genome | 432 | 0.00E+00 | 77.90% |
| Efet.01.658424.g2123.t1 | Verminephrobacter eiseniae EF01-2, complete genome | 780 | 0.00E+00 | 77.90% |
| Efet.01.1655588.g770.t1 | Microbacterium sp. XT11, complete genome | 276 | 8.70E-71 | 77.90% |
| Efet.01.1659421.g1683.t1 | Verminephrobacter eiseniae EF01-2, complete genome | 303 | 2.30E-150 | 77.90% |
| Efet.01.1659450.g1742.t1 | Pseudomonas mediterranea strain DSM 16733 genome assembly, chromosome: I | 556 | 5.60E-79 | 77.90% |
| Efet.01.1659505.g1905.t1 | Verminephrobacter eiseniae EF01-2, complete genome | 375 | 0.00E+00 | 77.90% |
| Efet.01.643922.g847.t1 | Verminephrobacter eiseniae EF01-2, complete genome | 291 | 5.60E-141 | 77.86% |
| Efet.01.620235.g888.t1 | Microbacterium aurum strain KACC 15219, complete genome | 713 | 0.00E+00 | 77.83% |
| Efet.01.33473.g580.t1 | Candidatus Accumulibacter phosphatis clade IIA str. UW-1, complete genome | 97 | 1.70E-14 | 77.80% |
| Efet.01.176795.g1252.t1 | Verminephrobacter eiseniae EF01-2, complete genome | 591 | 0.00E+00 | 77.80% |
| Efet.01.275134.g934.t1 | Verminephrobacter eiseniae EF01-2, complete genome | 1341 | 0.00E+00 | 77.80% |
| Efet.01.287526.g1373.t1 | Verminephrobacter eiseniae EF01-2, complete genome | 408 | 0.00E+00 | 77.80% |
| Efet.01.353298.g96.t1 | Verminephrobacter eiseniae EF01-2, complete genome | 753 | 0.00E+00 | 77.80% |
| Efet.01.386431.g937.t1 | Verminephrobacter eiseniae EF01-2, complete genome | 367 | 1.70E-130 | 77.80% |
| Efet.01.413810.g310.t1 | Verminephrobacter eiseniae EF01-2, complete genome | 1113 | 0.00E+00 | 77.80% |
| Efet.01.430820.g754.t1 | Verminephrobacter eiseniae EF01-2, complete genome | 904 | 0.00E+00 | 77.80% |
| Efet.01.455382.g136.t1 | Verminephrobacter eiseniae EF01-2, complete genome | 746 | 0.00E+00 | 77.80% |
| Efet.01.487632.g908.t1 | Anaerococcus sp. Marseille-P2765 strain Marseille-P2765T genome assembly, chromosome: contig00001 | 89 | 1.50E-11 | 77.80% |
| Efet.01.587031.g896.t1 | Agromyces flavus strain CPCC 202695 genome assembly, chromosome: I | 939 | 0.00E+00 | 77.80% |
| Efet.01.600657.g26.t1 | Verminephrobacter eiseniae EF01-2, complete genome | 1673 | 0.00E+00 | 77.80% |
| Efet.01.609276.g393.t1 | Paraburkholderia xenovorans LB400 chromosome 2, complete sequence | 579 | 0.00E+00 | 77.80% |
| Efet.01.612122.g526.t1 | Verminephrobacter eiseniae EF01-2, complete genome | 486 | 0.00E+00 | 77.80% |
| Efet.01.616898.g744.t1 | Agromyces flavus strain CPCC 202695 genome assembly, chromosome: I | 228 | 1.40E-55 | 77.80% |
| Efet.01.623153.g1013.t1 | Agromyces aureus strain AR33, complete genome | 389 | 1.90E-90 | 77.80% |
| Efet.01.630546.g40.t1 | Verminephrobacter eiseniae EF01-2, complete genome | 867 | 0.00E+00 | 77.80% |
| Efet.01.633916.g229.t1 | Verminephrobacter eiseniae EF01-2, complete genome | 597 | 0.00E+00 | 77.80% |
| Efet.01.642390.g679.t1 | Verminephrobacter eiseniae EF01-2, complete genome | 444 | 1.80E-171 | 77.80% |
| Efet.01.647774.g1200.t1 | Agromyces sp. 30A chromosome, complete genome | 330 | 2.30E-96 | 77.80% |
| Efet.01.649494.g1322.t1 | Microterricola viridarii strain DSM 21772 genome assembly, chromosome: I | 216 | 2.00E-42 | 77.80% |
| Efet.01.651148.g134.t1 | Verminephrobacter eiseniae EF01-2, complete genome | 834 | 0.00E+00 | 77.80% |
| Efet.01.654569.g632.t1 | Verminephrobacter eiseniae EF01-2, complete genome | 1002 | 0.00E+00 | 77.80% |
| Efet.01.654656.g646.t1 | Verminephrobacter eiseniae EF01-2, complete genome | 627 | 0.00E+00 | 77.80% |
| Efet.01.654656.g676.t1 | Verminephrobacter eiseniae EF01-2, complete genome | 906 | 0.00E+00 | 77.80% |
| Efet.01.656470.g1028.t1 | Verminephrobacter eiseniae EF01-2, complete genome | 1372 | 0.00E+00 | 77.80% |
| Efet.01.657759.g1412.t1 | Verminephrobacter eiseniae EF01-2, complete genome | 585 | 0.00E+00 | 77.80% |
| Efet.01.657803.g1475.t1 | Verminephrobacter eiseniae EF01-2, complete genome | 858 | 0.00E+00 | 77.80% |
| Efet.01.657876.g1515.t1 | Verminephrobacter eiseniae EF01-2, complete genome | 867 | 0.00E+00 | 77.80% |
| Efet.01.658040.g1571.t1 | Verminephrobacter eiseniae EF01-2, complete genome | 570 | 0.00E+00 | 77.80% |
| Efet.01.658230.g1726.t1 | Verminephrobacter eiseniae EF01-2, complete genome | 1086 | 0.00E+00 | 77.80% |
| Efet.01.658282.g1797.t1 | Verminephrobacter eiseniae EF01-2, complete genome | 984 | 0.00E+00 | 77.80% |
| Efet.01.658392.g1962.t1 | Verminephrobacter eiseniae EF01-2, complete genome | 627 | 0.00E+00 | 77.80% |
| Efet.01.1655662.g777.t1 | Verminephrobacter eiseniae EF01-2, complete genome | 570 | 0.00E+00 | 77.80% |
| Efet.01.1658935.g1328.t1 | Verminephrobacter eiseniae EF01-2, complete genome | 363 | 1.30E-180 | 77.80% |
| Efet.01.1659371.g1614.t1 | Verminephrobacter eiseniae EF01-2, complete genome | 909 | 0.00E+00 | 77.80% |
| Efet.01.1659451.g1745.t1 | Verminephrobacter eiseniae EF01-2, complete genome | 1938 | 0.00E+00 | 77.80% |
| Efet.01.574744.g468.t1 | Verminephrobacter eiseniae EF01-2, complete genome | 432 | 2.30E-172 | 77.75% |
| Efet.01.577003.g555.t1 | Flavobacterium sp. AJ004 chromosome, complete genome | 62 | 5.20E-08 | 77.75% |
| Efet.01.654521.g622.t1 | Verminephrobacter eiseniae EF01-2, complete genome | 1116 | 0.00E+00 | 77.75% |
| Efet.01.658399.g1988.t1 | Verminephrobacter eiseniae EF01-2, complete genome | 573 | 0.00E+00 | 77.75% |
| Efet.01.16684.g1234.t1 | Winogradskyella sp. PG-2 DNA, complete genome | 105 | 2.20E-11 | 77.70% |
| Efet.01.25255.g21.t1 | Verminephrobacter eiseniae EF01-2, complete genome | 513 | 0.00E+00 | 77.70% |
| Efet.01.59384.g587.t1 | Verminephrobacter eiseniae EF01-2, complete genome | 1356 | 0.00E+00 | 77.70% |
| Efet.01.82624.g502.t1 | Flavobacterium johnsoniae strain GSE09, complete genome | 93 | 3.90E-15 | 77.70% |
| Efet.01.104145.g226.t1 | Verminephrobacter eiseniae EF01-2, complete genome | 1587 | 0.00E+00 | 77.70% |
| Efet.01.440538.g965.t1 | Verminephrobacter eiseniae EF01-2, complete genome | 1005 | 0.00E+00 | 77.70% |
| Efet.01.489798.g957.t1 | Verminephrobacter eiseniae EF01-2, complete genome | 331 | 1.00E-165 | 77.70% |
| Efet.01.566728.g224.t1 | Agromyces sp. 30A chromosome, complete genome | 305 | 1.50E-12 | 77.70% |
| Efet.01.575546.g498.t1 | Verminephrobacter eiseniae EF01-2, complete genome | 656 | 0.00E+00 | 77.70% |
| Efet.01.578168.g630.t1 | Variovorax boronicumulans strain J1 chromosome, complete genome | 930 | 0.00E+00 | 77.70% |
| Efet.01.586228.g872.t1 | Verminephrobacter eiseniae EF01-2, complete genome | 774 | 0.00E+00 | 77.70% |
| Efet.01.586314.g879.t1 | Arthrobacter sp. Rue61a, complete genome | 136 | 3.20E-29 | 77.70% |
| Efet.01.590200.g1001.t1 | Verminephrobacter eiseniae EF01-2, complete genome | 261 | 2.90E-126 | 77.70% |
| Efet.01.597702.g1224.t1 | Verminephrobacter eiseniae EF01-2, complete genome | 735 | 0.00E+00 | 77.70% |
| Efet.01.611343.g471.t1 | Verminephrobacter eiseniae EF01-2, complete genome | 1118 | 0.00E+00 | 77.70% |
| Efet.01.618083.g804.t1 | Brachybacterium ginsengisoli strain DCY80 chromosome, complete genome | 266 | 1.70E-54 | 77.70% |
| Efet.01.621685.g947.t1 | Cryobacterium arcticum strain PAMC 27867 chromosome 1, complete sequence | 189 | 2.40E-32 | 77.70% |
| Efet.01.644990.g919.t1 | Saccharomonospora viridis DSM 43017, complete genome | 101 | 3.80E-15 | 77.70% |
| Efet.01.648013.g1216.t1 | Verminephrobacter eiseniae EF01-2, complete genome | 1089 | 0.00E+00 | 77.70% |
| Efet.01.652587.g315.t1 | Verminephrobacter eiseniae EF01-2, complete genome | 696 | 0.00E+00 | 77.70% |
| Efet.01.653541.g448.t1 | Verminephrobacter eiseniae EF01-2, complete genome | 915 | 0.00E+00 | 77.70% |
| Efet.01.653968.g501.t1 | Verminephrobacter eiseniae EF01-2, complete genome | 841 | 0.00E+00 | 77.70% |
| Efet.01.654264.g557.t1 | Verminephrobacter eiseniae EF01-2, complete genome | 517 | 0.00E+00 | 77.70% |
| Efet.01.656334.g1001.t1 | Verminephrobacter eiseniae EF01-2, complete genome | 234 | 2.10E-112 | 77.70% |
| Efet.01.657703.g1381.t1 | Verminephrobacter eiseniae EF01-2, complete genome | 1221 | 0.00E+00 | 77.70% |
| Efet.01.658116.g1652.t1 | Verminephrobacter eiseniae EF01-2, complete genome | 987 | 0.00E+00 | 77.70% |
| Efet.01.658334.g1863.t1 | Verminephrobacter eiseniae EF01-2, complete genome | 1098 | 0.00E+00 | 77.70% |
| Efet.01.658395.g1972.t1 | Verminephrobacter eiseniae EF01-2, complete genome | 996 | 0.00E+00 | 77.70% |
| Efet.01.1636724.g180.t1 | Aurantimicrobium minutum DNA, complete genome, strain: KNC | 112 | 7.30E-12 | 77.70% |
| Efet.01.1659293.g1522.t1 | Verminephrobacter eiseniae EF01-2, complete genome | 1101 | 0.00E+00 | 77.70% |
| Efet.01.1659397.g1652.t1 | Verminephrobacter eiseniae EF01-2, complete genome | 253 | 7.60E-122 | 77.70% |
| Efet.01.1659479.g1812.t1 | Variovorax sp. PMC12 chromosome 1, complete sequence | 832 | 0.00E+00 | 77.70% |
| Efet.01.1659509.g1920.t1 | Verminephrobacter eiseniae EF01-2, complete genome | 408 | 0.00E+00 | 77.70% |
| Efet.01.1659522.g1988.t1 | Verminephrobacter eiseniae EF01-2, complete genome | 591 | 0.00E+00 | 77.70% |
| Efet.01.348061.g1359.t1 | Clavibacter insidiosus strain ATCC 10253 chromosome, complete genome | 95 | 7.10E-10 | 77.63% |
| Efet.01.296729.g1646.t1 | Agromyces sp. 30A chromosome, complete genome | 253 | 3.50E-51 | 77.60% |
| Efet.01.323909.g741.t1 | Bernardetia litoralis DSM 6794, complete genome | 340 | 1.70E-51 | 77.60% |
| Efet.01.413810.g316.t1 | Verminephrobacter eiseniae EF01-2, complete genome | 444 | 0.00E+00 | 77.60% |
| Efet.01.465744.g418.t1 | Verminephrobacter eiseniae EF01-2, complete genome | 696 | 0.00E+00 | 77.60% |
| Efet.01.529529.g688.t1 | Agromyces flavus strain CPCC 202695 genome assembly, chromosome: I | 878 | 0.00E+00 | 77.60% |
| Efet.01.548329.g450.t1 | Agromyces sp. 30A chromosome, complete genome | 198 | 4.10E-52 | 77.60% |
| Efet.01.556410.g671.t1 | Verminephrobacter eiseniae EF01-2, complete genome | 277 | 1.80E-133 | 77.60% |
| Efet.01.592090.g1062.t1 | Verminephrobacter eiseniae EF01-2, complete genome | 972 | 0.00E+00 | 77.60% |
| Efet.01.596181.g1185.t1 | Sphingobacteriaceae bacterium GW460-11-11-14-LB5, complete genome | 170 | 1.60E-12 | 77.60% |
| Efet.01.614463.g649.t1 | Verminephrobacter eiseniae EF01-2, complete genome | 213 | 6.20E-102 | 77.60% |
| Efet.01.621349.g929.t1 | Verminephrobacter eiseniae EF01-2, complete genome | 1353 | 0.00E+00 | 77.60% |
| Efet.01.630546.g46.t1 | Verminephrobacter eiseniae EF01-2, complete genome | 957 | 0.00E+00 | 77.60% |
| Efet.01.635568.g281.t1 | Verminephrobacter eiseniae EF01-2, complete genome | 834 | 0.00E+00 | 77.60% |
| Efet.01.638023.g395.t1 | Verminephrobacter eiseniae EF01-2, complete genome | 704 | 0.00E+00 | 77.60% |
| Efet.01.639914.g542.t1 | Agromyces sp. 30A chromosome, complete genome | 939 | 0.00E+00 | 77.60% |
| Efet.01.643394.g784.t1 | Verminephrobacter eiseniae EF01-2, complete genome | 1482 | 0.00E+00 | 77.60% |
| Efet.01.649588.g1332.t1 | Verminephrobacter eiseniae EF01-2, complete genome | 1140 | 0.00E+00 | 77.60% |
| Efet.01.650091.g7.t1 | Verminephrobacter eiseniae EF01-2, complete genome | 594 | 0.00E+00 | 77.60% |
| Efet.01.651441.g173.t1 | Verminephrobacter eiseniae EF01-2, complete genome | 2124 | 0.00E+00 | 77.60% |
| Efet.01.656717.g1076.t1 | Verminephrobacter eiseniae EF01-2, complete genome | 280 | 4.90E-133 | 77.60% |
| Efet.01.656816.g1114.t1 | Verminephrobacter eiseniae EF01-2, complete genome | 747 | 0.00E+00 | 77.60% |
| Efet.01.657302.g1225.t1 | Verminephrobacter eiseniae EF01-2, complete genome | 723 | 0.00E+00 | 77.60% |
| Efet.01.657484.g1302.t1 | Agromyces aureus strain AR33, complete genome | 350 | 4.80E-78 | 77.60% |
| Efet.01.657795.g1467.t1 | Verminephrobacter eiseniae EF01-2, complete genome | 942 | 0.00E+00 | 77.60% |
| Efet.01.658310.g1839.t1 | Verminephrobacter eiseniae EF01-2, complete genome | 1137 | 0.00E+00 | 77.60% |
| Efet.01.1643789.g285.t1 | Agromyces sp. 30A chromosome, complete genome | 201 | 1.40E-46 | 77.60% |
| Efet.01.1659504.g1901.t1 | Bordetella sp. N genome | 453 | 3.10E-107 | 77.60% |
| Efet.01.65442.g947.t1 | Verminephrobacter eiseniae EF01-2, complete genome | 1818 | 0.00E+00 | 77.50% |
| Efet.01.102678.g153.t1 | Haliangium ochraceum DSM 14365, complete genome | 95 | 1.00E-09 | 77.50% |
| Efet.01.150234.g9.t1 | Gordonia sp. QH-11, complete genome | 144 | 5.80E-10 | 77.50% |
| Efet.01.157336.g386.t1 | Bacteriovorax stolpii strain DSM 12778 chromosome, complete genome | 177 | 1.20E-14 | 77.50% |
| Efet.01.176760.g1244.t1 | Verminephrobacter eiseniae EF01-2, complete genome | 846 | 0.00E+00 | 77.50% |
| Efet.01.176760.g1248.t1 | Verminephrobacter eiseniae EF01-2, complete genome | 1714 | 0.00E+00 | 77.50% |
| Efet.01.185627.g1662.t1 | Clostridium botulinum strain Mfbjulcb6 chromosome, complete genome | 111 | 2.40E-10 | 77.50% |
| Efet.01.269359.g717.t1 | Verminephrobacter eiseniae EF01-2, complete genome | 421 | 0.00E+00 | 77.50% |
| Efet.01.305235.g169.t1 | Verminephrobacter eiseniae EF01-2, complete genome | 873 | 0.00E+00 | 77.50% |
| Efet.01.363886.g373.t1 | Spirosoma linguale DSM 74, complete genome | 73 | 3.20E-08 | 77.50% |
| Efet.01.430820.g752.t1 | Verminephrobacter eiseniae EF01-2, complete genome | 411 | 0.00E+00 | 77.50% |
| Efet.01.514247.g335.t1 | Verminephrobacter eiseniae EF01-2, complete genome | 990 | 0.00E+00 | 77.50% |
| Efet.01.532148.g64.t1 | Verminephrobacter eiseniae EF01-2, complete genome | 399 | 0.00E+00 | 77.50% |
| Efet.01.548599.g454.t1 | Rhizobacter gummiphilus strain NBRC 109400 chromosome, complete genome | 1218 | 0.00E+00 | 77.50% |
| Efet.01.565049.g160.t1 | Verminephrobacter eiseniae EF01-2, complete genome | 438 | 0.00E+00 | 77.50% |
| Efet.01.586228.g874.t1 | Verminephrobacter eiseniae EF01-2, complete genome | 213 | 3.70E-98 | 77.50% |
| Efet.01.616228.g717.t1 | Verminephrobacter eiseniae EF01-2, complete genome | 363 | 1.40E-169 | 77.50% |
| Efet.01.618695.g841.t1 | Agromyces aureus strain AR33, complete genome | 559 | 4.10E-137 | 77.50% |
| Efet.01.627410.g1207.t1 | Prevotella ruminicola 23, complete genome | 171 | 3.30E-16 | 77.50% |
| Efet.01.630200.g11.t1 | Verminephrobacter eiseniae EF01-2, complete genome | 927 | 0.00E+00 | 77.50% |
| Efet.01.631940.g142.t1 | Verminephrobacter eiseniae EF01-2, complete genome | 333 | 9.80E-167 | 77.50% |
| Efet.01.638425.g429.t1 | Verminephrobacter eiseniae EF01-2, complete genome | 477 | 0.00E+00 | 77.50% |
| Efet.01.640141.g559.t1 | Jiangella alkaliphila strain DSM 45079 genome assembly, chromosome: I | 478 | 3.00E-54 | 77.50% |
| Efet.01.646179.g1048.t1 | Verminephrobacter eiseniae EF01-2, complete genome | 510 | 0.00E+00 | 77.50% |
| Efet.01.646179.g1052.t1 | Verminephrobacter eiseniae EF01-2, complete genome | 267 | 1.10E-129 | 77.50% |
| Efet.01.652898.g374.t1 | Verminephrobacter eiseniae EF01-2, complete genome | 557 | 0.00E+00 | 77.50% |
| Efet.01.656742.g1084.t1 | Verminephrobacter eiseniae EF01-2, complete genome | 1949 | 0.00E+00 | 77.50% |
| Efet.01.656795.g1105.t1 | Verminephrobacter eiseniae EF01-2, complete genome | 546 | 0.00E+00 | 77.50% |
| Efet.01.658110.g1637.t1 | Verminephrobacter eiseniae EF01-2, complete genome | 1314 | 0.00E+00 | 77.50% |
| Efet.01.658261.g1767.t1 | Verminephrobacter eiseniae EF01-2, complete genome | 1324 | 0.00E+00 | 77.50% |
| Efet.01.658334.g1868.t1 | Verminephrobacter eiseniae EF01-2, complete genome | 705 | 0.00E+00 | 77.50% |
| Efet.01.1650866.g488.t1 | Microbacterium sp. BH-3-3-3, complete genome | 218 | 1.20E-46 | 77.50% |
| Efet.01.1654295.g680.t1 | Agromyces flavus strain CPCC 202695 genome assembly, chromosome: I | 315 | 9.60E-39 | 77.50% |
| Efet.01.1656231.g840.t1 | Agromyces flavus strain CPCC 202695 genome assembly, chromosome: I | 240 | 1.50E-50 | 77.50% |
| Efet.01.1658942.g1329.t1 | Verminephrobacter eiseniae EF01-2, complete genome | 891 | 0.00E+00 | 77.50% |
| Efet.01.1659193.g1455.t1 | Verminephrobacter eiseniae EF01-2, complete genome | 726 | 0.00E+00 | 77.50% |
| Efet.01.646274.g1063.t1 | Verminephrobacter eiseniae EF01-2, complete genome | 288 | 3.50E-142 | 77.44% |
| Efet.01.57096.g442.t1 | Azoarcus sp. SY39 chromosome, complete genome | 101 | 5.50E-21 | 77.40% |
| Efet.01.185951.g1682.t1 | Verminephrobacter eiseniae EF01-2, complete genome | 819 | 0.00E+00 | 77.40% |
| Efet.01.270738.g767.t1 | Verminephrobacter eiseniae EF01-2, complete genome | 1080 | 0.00E+00 | 77.40% |
| Efet.01.316410.g520.t1 | Cobetia marina strain JCM 21022, complete genome | 93 | 5.00E-12 | 77.40% |
| Efet.01.468278.g485.t1 | Sorangium cellulosum 'So ce 56' complete genome | 141 | 6.00E-16 | 77.40% |
| Efet.01.557665.g699.t1 | Verminephrobacter eiseniae EF01-2, complete genome | 629 | 0.00E+00 | 77.40% |
| Efet.01.576819.g540.t1 | Verminephrobacter eiseniae EF01-2, complete genome | 285 | 1.20E-142 | 77.40% |
| Efet.01.580499.g685.t1 | Rufibacter sp. DG31D, complete genome | 311 | 1.90E-36 | 77.40% |
| Efet.01.598670.g1260.t1 | Verminephrobacter eiseniae EF01-2, complete genome | 228 | 3.60E-107 | 77.40% |
| Efet.01.604766.g177.t1 | Microbacterium aurum strain KACC 15219, complete genome | 452 | 2.90E-108 | 77.40% |
| Efet.01.609276.g390.t1 | Verminephrobacter eiseniae EF01-2, complete genome | 909 | 0.00E+00 | 77.40% |
| Efet.01.623527.g1028.t1 | Verminephrobacter eiseniae EF01-2, complete genome | 1221 | 0.00E+00 | 77.40% |
| Efet.01.631813.g137.t1 | Variovorax sp. PMC12 chromosome 1, complete sequence | 882 | 0.00E+00 | 77.40% |
| Efet.01.642837.g725.t1 | Verminephrobacter eiseniae EF01-2, complete genome | 462 | 0.00E+00 | 77.40% |
| Efet.01.643674.g824.t1 | Verminephrobacter eiseniae EF01-2, complete genome | 417 | 0.00E+00 | 77.40% |
| Efet.01.649588.g1335.t1 | Verminephrobacter eiseniae EF01-2, complete genome | 480 | 0.00E+00 | 77.40% |
| Efet.01.650311.g26.t1 | Verminephrobacter eiseniae EF01-2, complete genome | 777 | 0.00E+00 | 77.40% |
| Efet.01.652898.g364.t1 | Verminephrobacter eiseniae EF01-2, complete genome | 256 | 6.80E-124 | 77.40% |
| Efet.01.654067.g534.t1 | Verminephrobacter eiseniae EF01-2, complete genome | 1314 | 0.00E+00 | 77.40% |
| Efet.01.657110.g1169.t1 | Verminephrobacter eiseniae EF01-2, complete genome | 571 | 0.00E+00 | 77.40% |
| Efet.01.657110.g1170.t1 | Verminephrobacter eiseniae EF01-2, complete genome | 1199 | 0.00E+00 | 77.40% |
| Efet.01.658040.g1573.t1 | Verminephrobacter eiseniae EF01-2, complete genome | 1173 | 0.00E+00 | 77.40% |
| Efet.01.1651621.g513.t1 | Cnuibacter physcomitrellae strain XA(T), complete genome | 290 | 7.40E-56 | 77.40% |
| Efet.01.1652570.g566.t1 | Vibrio rotiferianus B64D1 chromosome 1, complete sequence | 129 | 9.60E-23 | 77.40% |
| Efet.01.1656537.g880.t1 | Verminephrobacter eiseniae EF01-2, complete genome | 432 | 0.00E+00 | 77.40% |
| Efet.01.1658370.g1165.t1 | Verminephrobacter eiseniae EF01-2, complete genome | 489 | 0.00E+00 | 77.40% |
| Efet.01.1658720.g1256.t1 | Verminephrobacter eiseniae EF01-2, complete genome | 555 | 0.00E+00 | 77.40% |
| Efet.01.1659318.g1549.t1 | Verminephrobacter eiseniae EF01-2, complete genome | 348 | 3.10E-168 | 77.40% |
| Efet.01.1659435.g1707.t1 | Verminephrobacter eiseniae EF01-2, complete genome | 924 | 0.00E+00 | 77.40% |
| Efet.01.1659447.g1735.t1 | Verminephrobacter eiseniae EF01-2, complete genome | 1126 | 0.00E+00 | 77.40% |
| Efet.01.1659448.g1738.t1 | Verminephrobacter eiseniae EF01-2, complete genome | 693 | 0.00E+00 | 77.40% |
| Efet.01.1659460.g1769.t1 | Verminephrobacter eiseniae EF01-2, complete genome | 750 | 0.00E+00 | 77.40% |
| Efet.01.1659487.g1841.t1 | Verminephrobacter eiseniae EF01-2, complete genome | 936 | 0.00E+00 | 77.40% |
| Efet.01.1659523.g2000.t1 | Verminephrobacter eiseniae EF01-2, complete genome | 1332 | 0.00E+00 | 77.40% |
| Efet.01.267712.g654.t1 | Arthrobacter sp. U41, complete genome | 80 | 7.50E-10 | 77.33% |
| Efet.01.656171.g956.t1 | Thiomonas intermedia K12 plasmid pTINT01, complete sequence | 460 | 1.40E-151 | 77.33% |
| Efet.01.38447.g896.t1 | Solitalea canadensis DSM 3403, complete genome | 112 | 1.90E-14 | 77.30% |
| Efet.01.292188.g1517.t1 | Verminephrobacter eiseniae EF01-2, complete genome | 655 | 0.00E+00 | 77.30% |
| Efet.01.370637.g575.t1 | Verminephrobacter eiseniae EF01-2, complete genome | 987 | 0.00E+00 | 77.30% |
| Efet.01.430820.g756.t1 | Verminephrobacter eiseniae EF01-2, complete genome | 335 | 5.70E-117 | 77.30% |
| Efet.01.590200.g996.t1 | Verminephrobacter eiseniae EF01-2, complete genome | 446 | 0.00E+00 | 77.30% |
| Efet.01.592091.g1063.t1 | Leifsonia sp. 21MFCrub1.1 genome assembly, chromosome: I | 225 | 5.20E-45 | 77.30% |
| Efet.01.614731.g665.t1 | Verminephrobacter eiseniae EF01-2, complete genome | 879 | 0.00E+00 | 77.30% |
| Efet.01.617461.g778.t1 | Corynebacterium mycetoides strain DSM 20632 genome assembly, chromosome: I | 383 | 6.80E-104 | 77.30% |
| Efet.01.625202.g1106.t1 | Magnetospira sp. QH-2 chromosome, complete genome | 303 | 6.10E-68 | 77.30% |
| Efet.01.637997.g393.t1 | Frondihabitans sp. 762G35, complete genome | 326 | 6.50E-73 | 77.30% |
| Efet.01.652843.g345.t1 | Verminephrobacter eiseniae EF01-2, complete genome | 303 | 2.90E-141 | 77.30% |
| Efet.01.654656.g660.t1 | Verminephrobacter eiseniae EF01-2, complete genome | 1128 | 0.00E+00 | 77.30% |
| Efet.01.657795.g1466.t1 | Verminephrobacter eiseniae EF01-2, complete genome | 957 | 0.00E+00 | 77.30% |
| Efet.01.658236.g1735.t1 | Verminephrobacter eiseniae EF01-2, complete genome | 845 | 0.00E+00 | 77.30% |
| Efet.01.658267.g1774.t1 | Verminephrobacter eiseniae EF01-2, complete genome | 905 | 0.00E+00 | 77.30% |
| Efet.01.1653412.g614.t1 | Verminephrobacter eiseniae EF01-2, complete genome | 576 | 0.00E+00 | 77.30% |
| Efet.01.1654506.g696.t1 | Agromyces sp. 30A chromosome, complete genome | 274 | 2.90E-64 | 77.30% |
| Efet.01.1655262.g746.t1 | Microbacterium sp. TPU 3598 DNA, complete genome | 283 | 1.00E-63 | 77.30% |
| Efet.01.1658890.g1315.t1 | Verminephrobacter eiseniae EF01-2, complete genome | 927 | 0.00E+00 | 77.30% |
| Efet.01.1659237.g1481.t1 | Verminephrobacter eiseniae EF01-2, complete genome | 662 | 0.00E+00 | 77.30% |
| Efet.01.1659446.g1733.t1 | Verminephrobacter eiseniae EF01-2, complete genome | 813 | 0.00E+00 | 77.30% |
| Efet.01.1659515.g1949.t1 | Verminephrobacter eiseniae EF01-2, complete genome | 579 | 0.00E+00 | 77.30% |
| Efet.01.658418.g2084.t1 | Verminephrobacter eiseniae EF01-2, complete genome | 486 | 0.00E+00 | 77.29% |
| Efet.01.530627.g25.t1 | Actinoalloteichus hymeniacidonis strain HPA177(T) (=DSM 45092(T)), complete genome | 88 | 4.80E-07 | 77.25% |
| Efet.01.640857.g600.t1 | Bradyrhizobium erythrophlei strain GAS138 genome assembly, chromosome: I | 82 | 1.50E-10 | 77.25% |
| Efet.01.648204.g1228.t1 | Pediococcus claussenii strain TMW 2.54, complete genome | 83 | 3.20E-08 | 77.25% |
| Efet.01.1659518.g1973.t1 | Verminephrobacter eiseniae EF01-2, complete genome | 477 | 0.00E+00 | 77.25% |
| Efet.01.107793.g401.t1 | Devosia sp. I507 chromosome, complete genome | 110 | 4.90E-19 | 77.20% |
| Efet.01.287526.g1374.t1 | Verminephrobacter eiseniae EF01-2, complete genome | 645 | 0.00E+00 | 77.20% |
| Efet.01.300440.g19.t1 | Verminephrobacter eiseniae EF01-2, complete genome | 1149 | 0.00E+00 | 77.20% |
| Efet.01.316780.g532.t1 | Verminephrobacter eiseniae EF01-2, complete genome | 384 | 9.60E-148 | 77.20% |
| Efet.01.358765.g246.t1 | Streptomyces sp. SM18 chromosome, complete genome | 79 | 4.30E-07 | 77.20% |
| Efet.01.426161.g624.t1 | Cellulosimicrobium sp. TH-20, complete genome | 79 | 6.10E-09 | 77.20% |
| Efet.01.440039.g951.t1 | Agromyces sp. 30A chromosome, complete genome | 408 | 2.20E-41 | 77.20% |
| Efet.01.577373.g578.t1 | Verminephrobacter eiseniae EF01-2, complete genome | 406 | 0.00E+00 | 77.20% |
| Efet.01.581651.g727.t1 | Verminephrobacter eiseniae EF01-2, complete genome | 1068 | 0.00E+00 | 77.20% |
| Efet.01.587920.g919.t1 | Verminephrobacter eiseniae EF01-2, complete genome | 1825 | 0.00E+00 | 77.20% |
| Efet.01.604870.g183.t1 | Solitalea canadensis DSM 3403, complete genome | 121 | 8.00E-08 | 77.20% |
| Efet.01.622184.g973.t1 | Spirosoma aerolatum strain KACC 17939, complete genome | 106 | 8.20E-13 | 77.20% |
| Efet.01.624818.g1078.t1 | Verminephrobacter eiseniae EF01-2, complete genome | 258 | 1.30E-120 | 77.20% |
| Efet.01.628433.g1242.t1 | Verminephrobacter eiseniae EF01-2, complete genome | 962 | 0.00E+00 | 77.20% |
| Efet.01.630860.g81.t1 | Verminephrobacter eiseniae EF01-2, complete genome | 924 | 0.00E+00 | 77.20% |
| Efet.01.630860.g90.t1 | Verminephrobacter eiseniae EF01-2, complete genome | 983 | 0.00E+00 | 77.20% |
| Efet.01.643099.g761.t1 | Verminephrobacter eiseniae EF01-2, complete genome | 1077 | 0.00E+00 | 77.20% |
| Efet.01.647059.g1154.t1 | Verminephrobacter eiseniae EF01-2, complete genome | 885 | 0.00E+00 | 77.20% |
| Efet.01.648013.g1215.t1 | Verminephrobacter eiseniae EF01-2, complete genome | 1029 | 0.00E+00 | 77.20% |
| Efet.01.650931.g99.t1 | Verminephrobacter eiseniae EF01-2, complete genome | 1269 | 0.00E+00 | 77.20% |
| Efet.01.652659.g330.t1 | Verminephrobacter eiseniae EF01-2, complete genome | 606 | 0.00E+00 | 77.20% |
| Efet.01.653800.g477.t1 | Verminephrobacter eiseniae EF01-2, complete genome | 875 | 0.00E+00 | 77.20% |
| Efet.01.657719.g1398.t1 | Leptothrix cholodnii SP-6, complete genome | 441 | 1.60E-102 | 77.20% |
| Efet.01.658372.g1909.t1 | Verminephrobacter eiseniae EF01-2, complete genome | 642 | 0.00E+00 | 77.20% |
| Efet.01.1658273.g1147.t1 | Agromyces flavus strain CPCC 202695 genome assembly, chromosome: I | 288 | 1.50E-65 | 77.20% |
| Efet.01.1659504.g1900.t1 | Bordetella sp. N genome | 600 | 3.10E-126 | 77.20% |
| Efet.01.1659510.g1927.t1 | Verminephrobacter eiseniae EF01-2, complete genome | 611 | 0.00E+00 | 77.20% |
| Efet.01.1659516.g1954.t1 | Verminephrobacter eiseniae EF01-2, complete genome | 104 | 1.10E-39 | 77.20% |
| Efet.01.614177.g635.t1 | Verminephrobacter eiseniae EF01-2, complete genome | 1512 | 0.00E+00 | 77.17% |
| Efet.01.642837.g723.t1 | Verminephrobacter eiseniae EF01-2, complete genome | 267 | 6.20E-114 | 77.14% |
| Efet.01.52284.g155.t1 | Streptomyces noursei ATCC 11455, complete genome | 54 | 2.40E-07 | 77.10% |
| Efet.01.370035.g555.t1 | Verminephrobacter eiseniae EF01-2, complete genome | 1779 | 0.00E+00 | 77.10% |
| Efet.01.434359.g826.t1 | Verminephrobacter eiseniae EF01-2, complete genome | 414 | 2.60E-156 | 77.10% |
| Efet.01.492121.g999.t1 | Verminephrobacter eiseniae EF01-2, complete genome | 789 | 0.00E+00 | 77.10% |
| Efet.01.511346.g269.t1 | Microterricola viridarii strain ERGS5:02, complete genome | 243 | 1.00E-40 | 77.10% |
| Efet.01.516565.g413.t1 | Lacunisphaera limnophila strain IG16b chromosome, complete genome | 208 | 1.90E-12 | 77.10% |
| Efet.01.530573.g21.t1 | Verminephrobacter eiseniae EF01-2, complete genome | 372 | 0.00E+00 | 77.10% |
| Efet.01.585463.g847.t1 | Verminephrobacter eiseniae EF01-2, complete genome | 795 | 0.00E+00 | 77.10% |
| Efet.01.597702.g1220.t1 | Verminephrobacter eiseniae EF01-2, complete genome | 1503 | 0.00E+00 | 77.10% |
| Efet.01.612787.g565.t1 | Verminephrobacter eiseniae EF01-2, complete genome | 558 | 0.00E+00 | 77.10% |
| Efet.01.620408.g895.t1 | Agromyces aureus strain AR33, complete genome | 292 | 1.50E-66 | 77.10% |
| Efet.01.626983.g1188.t1 | Verminephrobacter eiseniae EF01-2, complete genome | 651 | 0.00E+00 | 77.10% |
| Efet.01.636323.g323.t1 | Cryobacterium arcticum strain PAMC 27867 chromosome 1, complete sequence | 445 | 1.80E-101 | 77.10% |
| Efet.01.638872.g471.t1 | Verminephrobacter eiseniae EF01-2, complete genome | 1033 | 0.00E+00 | 77.10% |
| Efet.01.640737.g588.t1 | Verminephrobacter eiseniae EF01-2, complete genome | 898 | 0.00E+00 | 77.10% |
| Efet.01.642722.g713.t1 | Azospirillum brasilense strain Sp7 plasmid ABSP7_p1, complete sequence | 809 | 0.00E+00 | 77.10% |
| Efet.01.646642.g1107.t1 | Sterolibacterium denitrificans strain Chol genome assembly, chromosome: SDENCHOL | 1249 | 0.00E+00 | 77.10% |
| Efet.01.647720.g1196.t1 | Mycobacterium sp. NRRL B-3805, complete genome | 309 | 5.80E-66 | 77.10% |
| Efet.01.651515.g180.t1 | Verminephrobacter eiseniae EF01-2, complete genome | 1260 | 0.00E+00 | 77.10% |
| Efet.01.651762.g211.t1 | Leifsonia sp. 98AMF genome assembly, chromosome: I | 767 | 0.00E+00 | 77.10% |
| Efet.01.652384.g284.t1 | Verminephrobacter eiseniae EF01-2, complete genome | 2019 | 0.00E+00 | 77.10% |
| Efet.01.656171.g947.t1 | Diaphorobacter polyhydroxybutyrativorans strain SL-205, complete genome | 258 | 1.10E-70 | 77.10% |
| Efet.01.657766.g1429.t1 | Verminephrobacter eiseniae EF01-2, complete genome | 834 | 0.00E+00 | 77.10% |
| Efet.01.658044.g1593.t1 | Verminephrobacter eiseniae EF01-2, complete genome | 825 | 0.00E+00 | 77.10% |
| Efet.01.658084.g1621.t1 | Verminephrobacter eiseniae EF01-2, complete genome | 1045 | 0.00E+00 | 77.10% |
| Efet.01.658282.g1793.t1 | Verminephrobacter eiseniae EF01-2, complete genome | 812 | 0.00E+00 | 77.10% |
| Efet.01.658383.g1929.t1 | Verminephrobacter eiseniae EF01-2, complete genome | 809 | 0.00E+00 | 77.10% |
| Efet.01.658411.g2025.t1 | Verminephrobacter eiseniae EF01-2, complete genome | 552 | 0.00E+00 | 77.10% |
| Efet.01.658418.g2087.t1 | Verminephrobacter eiseniae EF01-2, complete genome | 906 | 0.00E+00 | 77.10% |
| Efet.01.1656266.g845.t1 | Verminephrobacter eiseniae EF01-2, complete genome | 378 | 0.00E+00 | 77.10% |
| Efet.01.1657387.g979.t1 | Verminephrobacter eiseniae EF01-2, complete genome | 742 | 0.00E+00 | 77.10% |
| Efet.01.1659395.g1649.t1 | Verminephrobacter eiseniae EF01-2, complete genome | 222 | 1.10E-94 | 77.10% |
| Efet.01.1659433.g1705.t1 | Verminephrobacter eiseniae EF01-2, complete genome | 573 | 0.00E+00 | 77.10% |
| Efet.01.1659500.g1893.t1 | Paraburkholderia xenovorans LB400 chromosome 1, complete sequence | 963 | 0.00E+00 | 77.10% |
| Efet.01.1659525.g2022.t1 | Burkholderia ambifaria AMMD chromosome 3, complete sequence | 576 | 0.00E+00 | 77.10% |
| Efet.01.14494.g1065.t1 | Dietzia psychralcaliphila strain ILA-1 chromosome, complete genome | 81 | 9.20E-07 | 77.00% |
| Efet.01.65646.g959.t1 | Burkholderia pseudomallei strain 2013833057 chromosome 2, complete sequence | 83 | 5.90E-07 | 77.00% |
| Efet.01.75902.g56.t1 | Nostoc carneum NIES-2107 plasmid plasmid1 DNA, nearly complete genome | 77 | 3.30E-06 | 77.00% |
| Efet.01.77457.g141.t1 | Verminephrobacter eiseniae EF01-2, complete genome | 352 | 1.70E-174 | 77.00% |
| Efet.01.108412.g437.t1 | Arsenicicoccus sp. oral taxon 190, complete genome | 102 | 2.90E-13 | 77.00% |
| Efet.01.112926.g652.t1 | Planctomyces sp. SH-PL62, complete genome | 88 | 2.10E-08 | 77.00% |
| Efet.01.117335.g901.t1 | Actinomyces sp. Marseille-P2985 strain Marseille-P2985T genome assembly, chromosome: contig00001 | 101 | 2.40E-15 | 77.00% |
| Efet.01.147445.g1115.t1 | Verminephrobacter eiseniae EF01-2, complete genome | 462 | 0.00E+00 | 77.00% |
| Efet.01.176795.g1254.t1 | Verminephrobacter eiseniae EF01-2, complete genome | 984 | 0.00E+00 | 77.00% |
| Efet.01.267648.g644.t1 | Verminephrobacter eiseniae EF01-2, complete genome | 2265 | 0.00E+00 | 77.00% |
| Efet.01.267648.g649.t1 | Verminephrobacter eiseniae EF01-2, complete genome | 900 | 0.00E+00 | 77.00% |
| Efet.01.315538.g497.t1 | Parabacteroides sp. CT06, complete genome | 83 | 1.30E-06 | 77.00% |
| Efet.01.383782.g883.t1 | Paraburkholderia phytofirmans PsJN chromosome 2, complete sequence | 83 | 4.30E-06 | 77.00% |
| Efet.01.392427.g1062.t1 | Verminephrobacter eiseniae EF01-2, complete genome | 464 | 1.70E-130 | 77.00% |
| Efet.01.466400.g438.t1 | Curtobacterium pusillum strain AA3, complete genome | 81 | 5.00E-07 | 77.00% |
| Efet.01.504526.g114.t1 | Verminephrobacter eiseniae EF01-2, complete genome | 867 | 0.00E+00 | 77.00% |
| Efet.01.507373.g189.t1 | Mucilaginibacter gotjawali DNA, complete genome | 86 | 7.20E-08 | 77.00% |
| Efet.01.527451.g629.t1 | Paludibacter propionicigenes WB4, complete genome | 172 | 8.20E-24 | 77.00% |
| Efet.01.553292.g580.t1 | Verminephrobacter eiseniae EF01-2, complete genome | 1677 | 0.00E+00 | 77.00% |
| Efet.01.567190.g237.t1 | Stenotrophomonas maltophilia strain AB550 chromosome, complete genome | 78 | 1.00E-06 | 77.00% |
| Efet.01.569313.g291.t1 | Verminephrobacter eiseniae EF01-2, complete genome | 278 | 1.20E-132 | 77.00% |
| Efet.01.575546.g499.t1 | Verminephrobacter eiseniae EF01-2, complete genome | 807 | 0.00E+00 | 77.00% |
| Efet.01.595633.g1167.t1 | Verminephrobacter eiseniae EF01-2, complete genome | 633 | 0.00E+00 | 77.00% |
| Efet.01.601193.g62.t1 | Verminephrobacter eiseniae EF01-2, complete genome | 625 | 0.00E+00 | 77.00% |
| Efet.01.606285.g260.t1 | Verminephrobacter eiseniae EF01-2, complete genome | 1437 | 0.00E+00 | 77.00% |
| Efet.01.616135.g713.t1 | Marinifilaceae bacterium SPP2 DNA, complete genome | 97 | 2.00E-09 | 77.00% |
| Efet.01.617630.g787.t1 | Verminephrobacter eiseniae EF01-2, complete genome | 1614 | 0.00E+00 | 77.00% |
| Efet.01.619389.g862.t1 | Verminephrobacter eiseniae EF01-2, complete genome | 1110 | 0.00E+00 | 77.00% |
| Efet.01.638776.g465.t1 | Verminephrobacter eiseniae EF01-2, complete genome | 1631 | 0.00E+00 | 77.00% |
| Efet.01.645268.g965.t1 | Verminephrobacter eiseniae EF01-2, complete genome | 372 | 0.00E+00 | 77.00% |
| Efet.01.647059.g1152.t1 | Verminephrobacter eiseniae EF01-2, complete genome | 900 | 0.00E+00 | 77.00% |
| Efet.01.650091.g6.t1 | Verminephrobacter eiseniae EF01-2, complete genome | 462 | 0.00E+00 | 77.00% |
| Efet.01.651204.g148.t1 | Geobacter sulfurreducens strain AM-1 genome | 513 | 4.60E-138 | 77.00% |
| Efet.01.651438.g170.t1 | Microterricola viridarii strain DSM 21772 genome assembly, chromosome: I | 108 | 3.30E-13 | 77.00% |
| Efet.01.654486.g616.t1 | Agromyces sp. 30A chromosome, complete genome | 1109 | 0.00E+00 | 77.00% |
| Efet.01.655399.g811.t1 | Verminephrobacter eiseniae EF01-2, complete genome | 774 | 0.00E+00 | 77.00% |
| Efet.01.656558.g1040.t1 | Verminephrobacter eiseniae EF01-2, complete genome | 1447 | 0.00E+00 | 77.00% |
| Efet.01.1597384.g21.t1 | Rhizobium sp. NXC24 chromosome, complete genome | 95 | 1.80E-07 | 77.00% |
| Efet.01.1638395.g194.t1 | Agarivorans gilvus strain WH0801, complete genome | 88 | 2.10E-06 | 77.00% |
| Efet.01.1644488.g300.t1 | Geobacter uraniireducens Rf4, complete genome | 77 | 7.30E-06 | 77.00% |
| Efet.01.1647264.g374.t1 | Chlorobium chlorochromatii CaD3, complete genome | 147 | 6.60E-11 | 77.00% |
| Efet.01.1654050.g657.t1 | Agromyces sp. 30A chromosome, complete genome | 333 | 9.50E-77 | 77.00% |
| Efet.01.1654813.g715.t1 | Thiocystis violascens DSM 198, complete genome | 69 | 6.90E-09 | 77.00% |
| Efet.01.1657446.g986.t1 | Verminephrobacter eiseniae EF01-2, complete genome | 606 | 0.00E+00 | 77.00% |
| Efet.01.1657830.g1044.t1 | Diaphorobacter polyhydroxybutyrativorans strain SL-205, complete genome | 331 | 3.00E-68 | 77.00% |
| Efet.01.1658398.g1171.t1 | Cnuibacter physcomitrellae strain XA(T), complete genome | 676 | 5.10E-149 | 77.00% |
| Efet.01.1659374.g1618.t1 | Verminephrobacter eiseniae EF01-2, complete genome | 516 | 0.00E+00 | 77.00% |
| Efet.01.1659473.g1795.t1 | Burkholderia pseudomallei PB08298010 chromosome I, complete sequence | 693 | 0.00E+00 | 77.00% |
| Efet.01.1659482.g1824.t1 | Delftia acidovorans isolate ANG1, complete genome | 432 | 4.10E-93 | 77.00% |
| Efet.01.1659492.g1860.t1 | Verminephrobacter eiseniae EF01-2, complete genome | 858 | 0.00E+00 | 77.00% |
| Efet.01.1659510.g1928.t1 | Verminephrobacter eiseniae EF01-2, complete genome | 2220 | 0.00E+00 | 77.00% |
| Efet.01.1659520.g1980.t1 | Verminephrobacter eiseniae EF01-2, complete genome | 1281 | 0.00E+00 | 77.00% |
| Efet.01.10485.g769.t1 | Verminephrobacter eiseniae EF01-2, complete genome | 471 | 7.00E-128 | 76.90% |
| Efet.01.59384.g589.t1 | Verminephrobacter eiseniae EF01-2, complete genome | 843 | 0.00E+00 | 76.90% |
| Efet.01.239492.g1521.t1 | Fibrella sp. ES10-3-2-2, complete genome | 125 | 8.60E-20 | 76.90% |
| Efet.01.308074.g243.t1 | Ralstonia solanacearum strain 10319 genome | 936 | 0.00E+00 | 76.90% |
| Efet.01.449969.g1221.t1 | Agromyces sp. 30A chromosome, complete genome | 599 | 6.70E-148 | 76.90% |
| Efet.01.458277.g228.t1 | Thermotoga maritima MSB8, complete genome | 91 | 9.20E-07 | 76.90% |
| Efet.01.533149.g108.t1 | Verminephrobacter eiseniae EF01-2, complete genome | 843 | 0.00E+00 | 76.90% |
| Efet.01.563393.g103.t1 | Verminephrobacter eiseniae EF01-2, complete genome | 765 | 0.00E+00 | 76.90% |
| Efet.01.577373.g583.t1 | Verminephrobacter eiseniae EF01-2, complete genome | 586 | 0.00E+00 | 76.90% |
| Efet.01.600657.g21.t1 | Verminephrobacter eiseniae EF01-2, complete genome | 354 | 1.60E-160 | 76.90% |
| Efet.01.611807.g504.t1 | Verminephrobacter eiseniae EF01-2, complete genome | 1299 | 0.00E+00 | 76.90% |
| Efet.01.616477.g726.t1 | Fibrella aestuarina BUZ 2 drat genome | 146 | 4.60E-30 | 76.90% |
| Efet.01.618401.g828.t1 | Verminephrobacter eiseniae EF01-2, complete genome | 1299 | 0.00E+00 | 76.90% |
| Efet.01.624818.g1079.t1 | Verminephrobacter eiseniae EF01-2, complete genome | 1062 | 0.00E+00 | 76.90% |
| Efet.01.641462.g628.t1 | Verminephrobacter eiseniae EF01-2, complete genome | 975 | 0.00E+00 | 76.90% |
| Efet.01.650275.g18.t1 | Verminephrobacter eiseniae EF01-2, complete genome | 432 | 0.00E+00 | 76.90% |
| Efet.01.651263.g153.t1 | Agromyces flavus strain CPCC 202695 genome assembly, chromosome: I | 203 | 5.20E-36 | 76.90% |
| Efet.01.651429.g168.t1 | Ramlibacter tataouinensis TTB310, complete genome | 593 | 7.80E-91 | 76.90% |
| Efet.01.652384.g286.t1 | Verminephrobacter eiseniae EF01-2, complete genome | 1452 | 0.00E+00 | 76.90% |
| Efet.01.654034.g530.t1 | Acidovorax sp. KKS102, complete genome | 596 | 1.40E-154 | 76.90% |
| Efet.01.656090.g936.t1 | Verminephrobacter eiseniae EF01-2, complete genome | 843 | 0.00E+00 | 76.90% |
| Efet.01.658245.g1746.t1 | Verminephrobacter eiseniae EF01-2, complete genome | 1335 | 0.00E+00 | 76.90% |
| Efet.01.658415.g2044.t1 | Burkholderia ambifaria AMMD chromosome 2, complete sequence | 1539 | 0.00E+00 | 76.90% |
| Efet.01.658430.g2171.t1 | Verminephrobacter eiseniae EF01-2, complete genome | 588 | 0.00E+00 | 76.90% |
| Efet.01.1652885.g587.t1 | Microterricola viridarii strain DSM 21772 genome assembly, chromosome: I | 294 | 1.90E-63 | 76.90% |
| Efet.01.1656175.g831.t1 | Verminephrobacter eiseniae EF01-2, complete genome | 654 | 0.00E+00 | 76.90% |
| Efet.01.1659337.g1564.t1 | Micromonospora zamorensis strain DSM 45600 genome assembly, chromosome: I | 124 | 1.30E-14 | 76.90% |
| Efet.01.1659370.g1612.t1 | Verminephrobacter eiseniae EF01-2, complete genome | 405 | 0.00E+00 | 76.90% |
| Efet.01.1659494.g1867.t1 | Verminephrobacter eiseniae EF01-2, complete genome | 833 | 0.00E+00 | 76.90% |
| Efet.01.1659475.g1799.t1 | Burkholderia pseudomallei strain vgh16W chromosome 1, complete sequence | 1839 | 0.00E+00 | 76.88% |
| Efet.01.645138.g937.t1 | Verminephrobacter eiseniae EF01-2, complete genome | 351 | 1.40E-172 | 76.86% |
| Efet.01.454313.g97.t1 | Streptomyces chartreusis NRRL 3882 isolate NRRL3882 genome assembly, chromosome: I | 81 | 7.60E-08 | 76.83% |
| Efet.01.80038.g326.t1 | Agromyces sp. 30A chromosome, complete genome | 383 | 6.90E-92 | 76.80% |
| Efet.01.138845.g711.t1 | Salinibacterium sp. CGMCC 1.16371 chromosome, complete genome | 121 | 2.20E-19 | 76.80% |
| Efet.01.157857.g413.t1 | Cnuibacter physcomitrellae strain XA(T), complete genome | 81 | 7.60E-11 | 76.80% |
| Efet.01.381410.g819.t1 | Catenulispora acidiphila DSM 44928, complete genome | 115 | 4.60E-12 | 76.80% |
| Efet.01.436194.g857.t1 | Agromyces sp. 30A chromosome, complete genome | 644 | 6.80E-162 | 76.80% |
| Efet.01.544715.g355.t1 | Variovorax boronicumulans strain J1 chromosome, complete genome | 414 | 0.00E+00 | 76.80% |
| Efet.01.559401.g733.t1 | Verminephrobacter eiseniae EF01-2, complete genome | 387 | 0.00E+00 | 76.80% |
| Efet.01.581510.g715.t1 | Verminephrobacter eiseniae EF01-2, complete genome | 431 | 0.00E+00 | 76.80% |
| Efet.01.593574.g1103.t1 | Hymenobacter sp. DG25A, complete genome | 115 | 4.50E-13 | 76.80% |
| Efet.01.606285.g269.t1 | Verminephrobacter eiseniae EF01-2, complete genome | 1112 | 0.00E+00 | 76.80% |
| Efet.01.618287.g815.t1 | Verminephrobacter eiseniae EF01-2, complete genome | 551 | 0.00E+00 | 76.80% |
| Efet.01.630200.g10.t1 | Verminephrobacter eiseniae EF01-2, complete genome | 849 | 0.00E+00 | 76.80% |
| Efet.01.632850.g174.t1 | Agromyces sp. 30A chromosome, complete genome | 275 | 3.00E-59 | 76.80% |
| Efet.01.643898.g841.t1 | Streptomyces chartreusis NRRL 3882 isolate NRRL3882 genome assembly, chromosome: I | 1093 | 0.00E+00 | 76.80% |
| Efet.01.645275.g972.t1 | Verminephrobacter eiseniae EF01-2, complete genome | 1118 | 0.00E+00 | 76.80% |
| Efet.01.651575.g196.t1 | Paraburkholderia xenovorans LB400 chromosome 3, complete sequence | 299 | 6.70E-147 | 76.80% |
| Efet.01.654656.g664.t1 | Burkholderia thailandensis strain 2003015869 chromosome 1, complete sequence | 768 | 1.20E-141 | 76.80% |
| Efet.01.656816.g1115.t1 | Verminephrobacter eiseniae EF01-2, complete genome | 1527 | 0.00E+00 | 76.80% |
| Efet.01.657703.g1384.t1 | Verminephrobacter eiseniae EF01-2, complete genome | 798 | 0.00E+00 | 76.80% |
| Efet.01.657759.g1415.t1 | Verminephrobacter eiseniae EF01-2, complete genome | 510 | 0.00E+00 | 76.80% |
| Efet.01.657807.g1487.t1 | Pseudomonas syringae pv. tomato strain B13-200 plasmid pB13-200A, complete sequence | 645 | 0.00E+00 | 76.80% |
| Efet.01.658044.g1591.t1 | Verminephrobacter eiseniae EF01-2, complete genome | 834 | 0.00E+00 | 76.80% |
| Efet.01.658255.g1754.t1 | Verminephrobacter eiseniae EF01-2, complete genome | 630 | 0.00E+00 | 76.80% |
| Efet.01.658261.g1771.t1 | Verminephrobacter eiseniae EF01-2, complete genome | 369 | 8.70E-159 | 76.80% |
| Efet.01.658297.g1812.t1 | Verminephrobacter eiseniae EF01-2, complete genome | 1458 | 0.00E+00 | 76.80% |
| Efet.01.1659354.g1592.t1 | Verminephrobacter eiseniae EF01-2, complete genome | 525 | 0.00E+00 | 76.80% |
| Efet.01.1659497.g1879.t1 | Verminephrobacter eiseniae EF01-2, complete genome | 642 | 0.00E+00 | 76.80% |
| Efet.01.1659514.g1944.t1 | Verminephrobacter eiseniae EF01-2, complete genome | 942 | 0.00E+00 | 76.80% |
| Efet.01.1659516.g1959.t1 | Verminephrobacter eiseniae EF01-2, complete genome | 405 | 0.00E+00 | 76.80% |
| Efet.01.539243.g258.t1 | Verminephrobacter eiseniae EF01-2, complete genome | 723 | 0.00E+00 | 76.75% |
| Efet.01.658310.g1835.t1 | Comamonadaceae bacterium B1 DNA, complete genome | 378 | 1.70E-111 | 76.75% |
| Efet.01.658385.g1935.t1 | Verminephrobacter eiseniae EF01-2, complete genome | 447 | 0.00E+00 | 76.75% |
| Efet.01.1659402.g1662.t1 | Verminephrobacter eiseniae EF01-2, complete genome | 933 | 0.00E+00 | 76.75% |
| Efet.01.642649.g709.t1 | Zobellia galactanivorans strain DsiJT chromosome, complete genome | 134 | 3.70E-09 | 76.71% |
| Efet.01.1642880.g267.t1 | Bernardetia litoralis DSM 6794, complete genome | 107 | 2.90E-17 | 76.71% |
| Efet.01.136471.g573.t1 | Chlorobium phaeobacteroides BS1, complete genome | 94 | 2.60E-14 | 76.70% |
| Efet.01.151234.g78.t1 | Verminephrobacter eiseniae EF01-2, complete genome | 324 | 4.70E-162 | 76.70% |
| Efet.01.213964.g593.t1 | Anaeromyxobacter sp. Fw109-5, complete genome | 171 | 4.40E-11 | 76.70% |
| Efet.01.293816.g1570.t1 | Rufibacter sp. DG15C, complete genome | 393 | 2.50E-16 | 76.70% |
| Efet.01.447463.g1161.t1 | Verminephrobacter eiseniae EF01-2, complete genome | 1066 | 0.00E+00 | 76.70% |
| Efet.01.513298.g316.t1 | Verminephrobacter eiseniae EF01-2, complete genome | 429 | 0.00E+00 | 76.70% |
| Efet.01.553408.g591.t1 | Verminephrobacter eiseniae EF01-2, complete genome | 621 | 0.00E+00 | 76.70% |
| Efet.01.575546.g495.t1 | Verminephrobacter eiseniae EF01-2, complete genome | 1566 | 0.00E+00 | 76.70% |
| Efet.01.601193.g58.t1 | Burkholderia ambifaria AMMD chromosome 2, complete sequence | 354 | 6.80E-178 | 76.70% |
| Efet.01.605802.g241.t1 | Verminephrobacter eiseniae EF01-2, complete genome | 246 | 2.10E-116 | 76.70% |
| Efet.01.614463.g647.t1 | Verminephrobacter eiseniae EF01-2, complete genome | 972 | 0.00E+00 | 76.70% |
| Efet.01.628537.g1256.t1 | Verminephrobacter eiseniae EF01-2, complete genome | 1270 | 0.00E+00 | 76.70% |
| Efet.01.629376.g1292.t1 | Verminephrobacter eiseniae EF01-2, complete genome | 2564 | 0.00E+00 | 76.70% |
| Efet.01.634939.g267.t1 | Verminephrobacter eiseniae EF01-2, complete genome | 461 | 0.00E+00 | 76.70% |
| Efet.01.643394.g776.t1 | Verminephrobacter eiseniae EF01-2, complete genome | 1269 | 0.00E+00 | 76.70% |
| Efet.01.648705.g1258.t1 | Agromyces aureus strain AR33, complete genome | 303 | 2.60E-66 | 76.70% |
| Efet.01.654656.g662.t1 | Verminephrobacter eiseniae EF01-2, complete genome | 2763 | 0.00E+00 | 76.70% |
| Efet.01.654993.g735.t1 | Verminephrobacter eiseniae EF01-2, complete genome | 1266 | 0.00E+00 | 76.70% |
| Efet.01.657206.g1199.t1 | Aminobacter sp. MSH1 chromosome, complete genome | 701 | 0.00E+00 | 76.70% |
| Efet.01.657719.g1399.t1 | Verminephrobacter eiseniae EF01-2, complete genome | 632 | 0.00E+00 | 76.70% |
| Efet.01.657876.g1514.t1 | Verminephrobacter eiseniae EF01-2, complete genome | 1245 | 0.00E+00 | 76.70% |
| Efet.01.658193.g1711.t1 | Verminephrobacter eiseniae EF01-2, complete genome | 498 | 0.00E+00 | 76.70% |
| Efet.01.658261.g1765.t1 | Burkholderia ambifaria AMMD chromosome 3, complete sequence | 750 | 0.00E+00 | 76.70% |
| Efet.01.658310.g1840.t1 | Verminephrobacter eiseniae EF01-2, complete genome | 687 | 0.00E+00 | 76.70% |
| Efet.01.658395.g1970.t1 | Verminephrobacter eiseniae EF01-2, complete genome | 633 | 0.00E+00 | 76.70% |
| Efet.01.1659026.g1367.t1 | Verminephrobacter eiseniae EF01-2, complete genome | 1008 | 0.00E+00 | 76.70% |
| Efet.01.1659367.g1608.t1 | Verminephrobacter eiseniae EF01-2, complete genome | 989 | 0.00E+00 | 76.70% |
| Efet.01.1659431.g1698.t1 | Verminephrobacter eiseniae EF01-2, complete genome | 1446 | 0.00E+00 | 76.70% |
| Efet.01.1659480.g1818.t1 | Verminephrobacter eiseniae EF01-2, complete genome | 960 | 0.00E+00 | 76.70% |
| Efet.01.595921.g1178.t1 | Bacillus cytotoxicus strain CH_23 chromosome, complete genome | 121 | 1.50E-06 | 76.67% |
| Efet.01.627247.g1203.t1 | Leifsonia sp. 21MFCrub1.1 genome assembly, chromosome: I | 121 | 8.40E-24 | 76.67% |
| Efet.01.118788.g967.t1 | Melittangium boletus DSM 14713 chromosome, complete genome | 203 | 3.10E-13 | 76.60% |
| Efet.01.267355.g630.t1 | Achromobacter sp. MFA1 R4 genome assembly, chromosome: I | 257 | 3.70E-44 | 76.60% |
| Efet.01.376846.g723.t1 | Flammeovirgaceae bacterium 311, complete genome | 277 | 6.00E-36 | 76.60% |
| Efet.01.538715.g246.t1 | Kosakonia sacchari SP1 chromosome, complete genome | 104 | 1.20E-12 | 76.60% |
| Efet.01.546202.g397.t1 | Fibrella aestuarina BUZ 2 drat genome | 232 | 3.90E-46 | 76.60% |
| Efet.01.569313.g294.t1 | Verminephrobacter eiseniae EF01-2, complete genome | 441 | 0.00E+00 | 76.60% |
| Efet.01.600657.g23.t1 | Verminephrobacter eiseniae EF01-2, complete genome | 933 | 0.00E+00 | 76.60% |
| Efet.01.609276.g391.t1 | Paraburkholderia xenovorans LB400 chromosome 2, complete sequence | 445 | 0.00E+00 | 76.60% |
| Efet.01.610801.g451.t1 | Verminephrobacter eiseniae EF01-2, complete genome | 876 | 0.00E+00 | 76.60% |
| Efet.01.612104.g523.t1 | Verminephrobacter eiseniae EF01-2, complete genome | 498 | 0.00E+00 | 76.60% |
| Efet.01.614177.g633.t1 | Verminephrobacter eiseniae EF01-2, complete genome | 669 | 0.00E+00 | 76.60% |
| Efet.01.614640.g657.t1 | Microterricola viridarii strain ERGS5:02, complete genome | 289 | 1.10E-60 | 76.60% |
| Efet.01.614719.g664.t1 | Verminephrobacter eiseniae EF01-2, complete genome | 504 | 0.00E+00 | 76.60% |
| Efet.01.620076.g879.t1 | Verminephrobacter eiseniae EF01-2, complete genome | 911 | 0.00E+00 | 76.60% |
| Efet.01.630860.g75.t1 | Verminephrobacter eiseniae EF01-2, complete genome | 1227 | 0.00E+00 | 76.60% |
| Efet.01.642837.g726.t1 | Verminephrobacter eiseniae EF01-2, complete genome | 375 | 0.00E+00 | 76.60% |
| Efet.01.643536.g811.t1 | Agromyces sp. 30A chromosome, complete genome | 306 | 5.00E-34 | 76.60% |
| Efet.01.649821.g1352.t1 | Verminephrobacter eiseniae EF01-2, complete genome | 903 | 0.00E+00 | 76.60% |
| Efet.01.651148.g132.t1 | Verminephrobacter eiseniae EF01-2, complete genome | 2677 | 0.00E+00 | 76.60% |
| Efet.01.651940.g241.t1 | Verminephrobacter eiseniae EF01-2, complete genome | 1129 | 0.00E+00 | 76.60% |
| Efet.01.655974.g913.t1 | Verminephrobacter eiseniae EF01-2, complete genome | 711 | 0.00E+00 | 76.60% |
| Efet.01.657130.g1179.t1 | Verminephrobacter eiseniae EF01-2, complete genome | 1500 | 0.00E+00 | 76.60% |
| Efet.01.657315.g1243.t1 | Verminephrobacter eiseniae EF01-2, complete genome | 984 | 0.00E+00 | 76.60% |
| Efet.01.658040.g1572.t1 | Verminephrobacter eiseniae EF01-2, complete genome | 585 | 0.00E+00 | 76.60% |
| Efet.01.658044.g1597.t1 | Verminephrobacter eiseniae EF01-2, complete genome | 975 | 0.00E+00 | 76.60% |
| Efet.01.658052.g1604.t1 | Verminephrobacter eiseniae EF01-2, complete genome | 708 | 0.00E+00 | 76.60% |
| Efet.01.658084.g1623.t1 | Verminephrobacter eiseniae EF01-2, complete genome | 1374 | 0.00E+00 | 76.60% |
| Efet.01.658116.g1639.t1 | Verminephrobacter eiseniae EF01-2, complete genome | 822 | 0.00E+00 | 76.60% |
| Efet.01.658121.g1654.t1 | Verminephrobacter eiseniae EF01-2, complete genome | 640 | 0.00E+00 | 76.60% |
| Efet.01.658177.g1700.t1 | Verminephrobacter eiseniae EF01-2, complete genome | 1476 | 0.00E+00 | 76.60% |
| Efet.01.658283.g1799.t1 | Verminephrobacter eiseniae EF01-2, complete genome | 393 | 1.70E-161 | 76.60% |
| Efet.01.658368.g1901.t1 | Verminephrobacter eiseniae EF01-2, complete genome | 692 | 0.00E+00 | 76.60% |
| Efet.01.658380.g1918.t1 | Verminephrobacter eiseniae EF01-2, complete genome | 771 | 0.00E+00 | 76.60% |
| Efet.01.1642306.g252.t1 | Acidovorax sp. P4, complete genome | 377 | 1.40E-84 | 76.60% |
| Efet.01.1646950.g364.t1 | Verminephrobacter eiseniae EF01-2, complete genome | 315 | 4.10E-159 | 76.60% |
| Efet.01.1648211.g400.t1 | Streptomyces pactum strain ACT12, complete genome | 96 | 1.90E-11 | 76.60% |
| Efet.01.1657368.g977.t1 | Verminephrobacter eiseniae EF01-2, complete genome | 678 | 0.00E+00 | 76.60% |
| Efet.01.1659314.g1541.t1 | Verminephrobacter eiseniae EF01-2, complete genome | 255 | 7.80E-119 | 76.60% |
| Efet.01.638872.g473.t1 | Verminephrobacter eiseniae EF01-2, complete genome | 491 | 0.00E+00 | 76.57% |
| Efet.01.6605.g516.t1 | Sorangium cellulosum 'So ce 56' complete genome | 85 | 3.90E-07 | 76.50% |
| Efet.01.7865.g578.t1 | Agromyces sp. 30A chromosome, complete genome | 487 | 1.70E-117 | 76.50% |
| Efet.01.22048.g1618.t1 | Verminephrobacter eiseniae EF01-2, complete genome | 1704 | 0.00E+00 | 76.50% |
| Efet.01.296729.g1645.t1 | Agromyces flavus strain CPCC 202695 genome assembly, chromosome: I | 1192 | 0.00E+00 | 76.50% |
| Efet.01.324717.g760.t1 | Paraburkholderia xenovorans LB400 chromosome 2, complete sequence | 90 | 3.80E-09 | 76.50% |
| Efet.01.402143.g54.t1 | Laribacter hongkongensis strain HLGZ1 chromosome, complete genome | 91 | 4.40E-07 | 76.50% |
| Efet.01.508646.g226.t1 | Verminephrobacter eiseniae EF01-2, complete genome | 846 | 0.00E+00 | 76.50% |
| Efet.01.526064.g605.t1 | Agromyces aureus strain AR33, complete genome | 269 | 2.90E-61 | 76.50% |
| Efet.01.562385.g46.t1 | Gordonia sp. QH-11, complete genome | 172 | 1.20E-34 | 76.50% |
| Efet.01.563785.g118.t1 | Verminephrobacter eiseniae EF01-2, complete genome | 624 | 0.00E+00 | 76.50% |
| Efet.01.565724.g190.t1 | Verminephrobacter eiseniae EF01-2, complete genome | 667 | 0.00E+00 | 76.50% |
| Efet.01.576819.g544.t1 | Serratia sp. ATCC 39006 chromosome, complete genome | 765 | 0.00E+00 | 76.50% |
| Efet.01.587924.g921.t1 | Verminephrobacter eiseniae EF01-2, complete genome | 903 | 0.00E+00 | 76.50% |
| Efet.01.605364.g204.t1 | Verminephrobacter eiseniae EF01-2, complete genome | 186 | 1.90E-85 | 76.50% |
| Efet.01.606285.g265.t1 | Verminephrobacter eiseniae EF01-2, complete genome | 944 | 0.00E+00 | 76.50% |
| Efet.01.606486.g279.t1 | Verminephrobacter eiseniae EF01-2, complete genome | 1026 | 0.00E+00 | 76.50% |
| Efet.01.616864.g743.t1 | Agromyces aureus strain AR33, complete genome | 367 | 1.20E-94 | 76.50% |
| Efet.01.633916.g221.t1 | Verminephrobacter eiseniae EF01-2, complete genome | 831 | 0.00E+00 | 76.50% |
| Efet.01.638894.g477.t1 | Verminephrobacter eiseniae EF01-2, complete genome | 999 | 0.00E+00 | 76.50% |
| Efet.01.642722.g717.t1 | Variovorax paradoxus B4 chromosome 1, complete sequence | 410 | 3.00E-86 | 76.50% |
| Efet.01.643497.g801.t1 | Verminephrobacter eiseniae EF01-2, complete genome | 1187 | 0.00E+00 | 76.50% |
| Efet.01.643637.g817.t1 | Microbacterium sp. LKL04 genome assembly, chromosome: I | 525 | 2.10E-70 | 76.50% |
| Efet.01.649588.g1340.t1 | Verminephrobacter eiseniae EF01-2, complete genome | 711 | 0.00E+00 | 76.50% |
| Efet.01.652843.g346.t1 | Verminephrobacter eiseniae EF01-2, complete genome | 188 | 5.60E-87 | 76.50% |
| Efet.01.653992.g510.t1 | Verminephrobacter eiseniae EF01-2, complete genome | 1557 | 0.00E+00 | 76.50% |
| Efet.01.654597.g639.t1 | Verminephrobacter eiseniae EF01-2, complete genome | 1090 | 0.00E+00 | 76.50% |
| Efet.01.654851.g704.t1 | Verminephrobacter eiseniae EF01-2, complete genome | 534 | 0.00E+00 | 76.50% |
| Efet.01.655128.g762.t1 | Verminephrobacter eiseniae EF01-2, complete genome | 1072 | 0.00E+00 | 76.50% |
| Efet.01.657703.g1383.t1 | Verminephrobacter eiseniae EF01-2, complete genome | 501 | 0.00E+00 | 76.50% |
| Efet.01.657807.g1480.t1 | Vitreoscilla filiformis strain ATCC 15551 plasmid pVF2, complete sequence | 377 | 5.00E-89 | 76.50% |
| Efet.01.658261.g1770.t1 | Verminephrobacter eiseniae EF01-2, complete genome | 711 | 0.00E+00 | 76.50% |
| Efet.01.658368.g1898.t1 | Verminephrobacter eiseniae EF01-2, complete genome | 858 | 0.00E+00 | 76.50% |
| Efet.01.658409.g2016.t1 | Verminephrobacter eiseniae EF01-2, complete genome | 861 | 0.00E+00 | 76.50% |
| Efet.01.1643972.g288.t1 | Agromyces flavus strain CPCC 202695 genome assembly, chromosome: I | 232 | 4.00E-47 | 76.50% |
| Efet.01.1652040.g538.t1 | Verminephrobacter eiseniae EF01-2 plasmid pVEIS01, complete sequence | 519 | 2.10E-132 | 76.50% |
| Efet.01.1658859.g1298.t1 | Agromyces flavus strain CPCC 202695 genome assembly, chromosome: I | 914 | 0.00E+00 | 76.50% |
| Efet.01.1659033.g1372.t1 | Verminephrobacter eiseniae EF01-2, complete genome | 462 | 0.00E+00 | 76.50% |
| Efet.01.1659233.g1476.t1 | Ralstonia pickettii DTP0602 chromosome 3, complete sequence | 207 | 8.90E-36 | 76.50% |
| Efet.01.658364.g1895.t1 | Verminephrobacter eiseniae EF01-2, complete genome | 1125 | 0.00E+00 | 76.44% |
| Efet.01.40766.g1050.t1 | Agromyces sp. 30A chromosome, complete genome | 1080 | 0.00E+00 | 76.40% |
| Efet.01.68869.g1163.t1 | Burkholderia pseudomallei strain TSV202 chromosome 1, complete sequence | 155 | 4.70E-15 | 76.40% |
| Efet.01.74876.g1535.t1 | Agromyces sp. 30A chromosome, complete genome | 673 | 4.40E-153 | 76.40% |
| Efet.01.104145.g230.t1 | Verminephrobacter eiseniae EF01-2, complete genome | 792 | 0.00E+00 | 76.40% |
| Efet.01.104145.g231.t1 | Verminephrobacter eiseniae EF01-2, complete genome | 1146 | 0.00E+00 | 76.40% |
| Efet.01.241488.g1583.t1 | Microterricola viridarii strain DSM 21772 genome assembly, chromosome: I | 345 | 1.00E-71 | 76.40% |
| Efet.01.257112.g271.t1 | Pseudomonas moraviensis strain BS3668 genome assembly, chromosome: I | 115 | 6.70E-17 | 76.40% |
| Efet.01.440538.g981.t1 | Verminephrobacter eiseniae EF01-2, complete genome | 984 | 0.00E+00 | 76.40% |
| Efet.01.533149.g109.t1 | Verminephrobacter eiseniae EF01-2, complete genome | 542 | 0.00E+00 | 76.40% |
| Efet.01.534987.g160.t1 | Leifsonia xyli strain SE134, complete genome | 177 | 8.80E-31 | 76.40% |
| Efet.01.548599.g459.t1 | Verminephrobacter eiseniae EF01-2, complete genome | 816 | 0.00E+00 | 76.40% |
| Efet.01.550271.g511.t1 | Verminephrobacter eiseniae EF01-2, complete genome | 1056 | 0.00E+00 | 76.40% |
| Efet.01.587924.g925.t1 | Verminephrobacter eiseniae EF01-2, complete genome | 1053 | 0.00E+00 | 76.40% |
| Efet.01.588154.g932.t1 | Verminephrobacter eiseniae EF01-2, complete genome | 677 | 0.00E+00 | 76.40% |
| Efet.01.606690.g287.t1 | Spirosoma linguale DSM 74, complete genome | 163 | 1.20E-29 | 76.40% |
| Efet.01.609276.g387.t1 | Verminephrobacter eiseniae EF01-2, complete genome | 396 | 0.00E+00 | 76.40% |
| Efet.01.613416.g592.t1 | Verminephrobacter eiseniae EF01-2, complete genome | 609 | 0.00E+00 | 76.40% |
| Efet.01.621343.g926.t1 | Microterricola viridarii strain ERGS5:02, complete genome | 251 | 2.20E-45 | 76.40% |
| Efet.01.630546.g45.t1 | Verminephrobacter eiseniae EF01-2, complete genome | 248 | 1.80E-122 | 76.40% |
| Efet.01.643497.g802.t1 | Verminephrobacter eiseniae EF01-2, complete genome | 951 | 0.00E+00 | 76.40% |
| Efet.01.651575.g199.t1 | Verminephrobacter eiseniae EF01-2, complete genome | 1389 | 0.00E+00 | 76.40% |
| Efet.01.652384.g292.t1 | Verminephrobacter eiseniae EF01-2, complete genome | 491 | 0.00E+00 | 76.40% |
| Efet.01.654591.g633.t1 | Verminephrobacter eiseniae EF01-2, complete genome | 699 | 0.00E+00 | 76.40% |
| Efet.01.658392.g1961.t1 | Verminephrobacter eiseniae EF01-2, complete genome | 561 | 0.00E+00 | 76.40% |
| Efet.01.658405.g2004.t1 | Verminephrobacter eiseniae EF01-2, complete genome | 1026 | 0.00E+00 | 76.40% |
| Efet.01.658417.g2082.t1 | Verminephrobacter eiseniae EF01-2, complete genome | 615 | 0.00E+00 | 76.40% |
| Efet.01.1651974.g537.t1 | Leifsonia xyli subsp. cynodontis DSM 46306, complete genome | 157 | 9.00E-17 | 76.40% |
| Efet.01.1657781.g1036.t1 | Verminephrobacter eiseniae EF01-2, complete genome | 423 | 0.00E+00 | 76.40% |
| Efet.01.1658584.g1221.t1 | Verminephrobacter eiseniae EF01-2, complete genome | 312 | 3.60E-157 | 76.40% |
| Efet.01.1658963.g1338.t1 | Agromyces sp. 30A chromosome, complete genome | 305 | 5.70E-65 | 76.40% |
| Efet.01.1659182.g1447.t1 | Uncultured bacterium BD_contig00014 genomic sequence | 105 | 6.00E-17 | 76.40% |
| Efet.01.1659477.g1808.t1 | Bordetella bronchiseptica strain I943 chromosome, complete genome | 565 | 3.00E-116 | 76.40% |
| Efet.01.1659509.g1922.t1 | Verminephrobacter eiseniae EF01-2, complete genome | 878 | 0.00E+00 | 76.40% |
| Efet.01.59144.g576.t1 | Myroides sp. A21, complete genome | 101 | 3.00E-11 | 76.33% |
| Efet.01.277954.g1028.t1 | Verminephrobacter eiseniae EF01-2, complete genome | 485 | 3.50E-179 | 76.33% |
| Efet.01.639535.g519.t1 | Diaphorobacter polyhydroxybutyrativorans strain SL-205, complete genome | 173 | 6.10E-44 | 76.33% |
| Efet.01.658418.g2089.t1 | Verminephrobacter eiseniae EF01-2, complete genome | 624 | 0.00E+00 | 76.33% |
| Efet.01.177666.g1296.t1 | Agromyces sp. 30A chromosome, complete genome | 407 | 5.50E-103 | 76.30% |
| Efet.01.523999.g557.t1 | Verminephrobacter eiseniae EF01-2, complete genome | 729 | 0.00E+00 | 76.30% |
| Efet.01.600657.g20.t1 | Verminephrobacter eiseniae EF01-2, complete genome | 1441 | 0.00E+00 | 76.30% |
| Efet.01.617251.g763.t1 | Verminephrobacter eiseniae EF01-2, complete genome | 348 | 5.20E-175 | 76.30% |
| Efet.01.630390.g23.t1 | Verminephrobacter eiseniae EF01-2, complete genome | 993 | 0.00E+00 | 76.30% |
| Efet.01.630860.g76.t1 | Verminephrobacter eiseniae EF01-2, complete genome | 1098 | 0.00E+00 | 76.30% |
| Efet.01.644987.g912.t1 | Shinella sp. HZN7, complete genome | 714 | 3.10E-168 | 76.30% |
| Efet.01.650338.g41.t1 | Leifsonia sp. 98AMF genome assembly, chromosome: I | 541 | 7.60E-118 | 76.30% |
| Efet.01.652108.g259.t1 | Verminephrobacter eiseniae EF01-2, complete genome | 837 | 0.00E+00 | 76.30% |
| Efet.01.655098.g751.t1 | Aminobacter sp. MSH1 chromosome, complete genome | 1165 | 0.00E+00 | 76.30% |
| Efet.01.657257.g1217.t1 | Verminephrobacter eiseniae EF01-2, complete genome | 738 | 0.00E+00 | 76.30% |
| Efet.01.657766.g1435.t1 | Verminephrobacter eiseniae EF01-2, complete genome | 1859 | 0.00E+00 | 76.30% |
| Efet.01.658084.g1626.t1 | Verminephrobacter eiseniae EF01-2, complete genome | 1617 | 0.00E+00 | 76.30% |
| Efet.01.658138.g1668.t1 | Verminephrobacter eiseniae EF01-2, complete genome | 429 | 0.00E+00 | 76.30% |
| Efet.01.658236.g1736.t1 | Verminephrobacter eiseniae EF01-2, complete genome | 485 | 0.00E+00 | 76.30% |
| Efet.01.658255.g1757.t1 | Verminephrobacter eiseniae EF01-2, complete genome | 1107 | 0.00E+00 | 76.30% |
| Efet.01.658424.g2120.t1 | Verminephrobacter eiseniae EF01-2, complete genome | 729 | 0.00E+00 | 76.30% |
| Efet.01.1630802.g107.t1 | Agromyces sp. 30A chromosome, complete genome | 237 | 5.90E-51 | 76.30% |
| Efet.01.1638668.g196.t1 | Echinicola vietnamensis DSM 17526, complete genome | 221 | 7.20E-44 | 76.30% |
| Efet.01.1656772.g913.t1 | Agromyces aureus strain AR33, complete genome | 141 | 5.90E-24 | 76.30% |
| Efet.01.95089.g1165.t1 | Methyloceanibacter caenitepidi DNA, complete genome, strain: Gela4 | 128 | 4.70E-07 | 76.25% |
| Efet.01.562188.g41.t1 | Gemmatimonas phototrophica strain AP64, complete genome | 91 | 8.90E-11 | 76.25% |
| Efet.01.59384.g594.t1 | Verminephrobacter eiseniae EF01-2, complete genome | 1350 | 0.00E+00 | 76.20% |
| Efet.01.206769.g298.t1 | Psychroflexus torquis ATCC 700755, complete genome | 107 | 8.80E-16 | 76.20% |
| Efet.01.223676.g952.t1 | Arthrobacter sp. U41, complete genome | 113 | 3.60E-18 | 76.20% |
| Efet.01.258920.g335.t1 | Deinococcus radiodurans R1 chromosome 1, complete sequence | 104 | 4.20E-13 | 76.20% |
| Efet.01.369900.g545.t1 | Verminephrobacter eiseniae EF01-2, complete genome | 204 | 8.70E-97 | 76.20% |
| Efet.01.440538.g969.t1 | Verminephrobacter eiseniae EF01-2, complete genome | 1056 | 0.00E+00 | 76.20% |
| Efet.01.533149.g106.t1 | Verminephrobacter eiseniae EF01-2, complete genome | 183 | 8.20E-82 | 76.20% |
| Efet.01.538552.g244.t1 | Agromyces aureus strain AR33, complete genome | 239 | 1.70E-42 | 76.20% |
| Efet.01.601873.g79.t1 | Xanthomonas citri pv. mangiferaeindicae strain XC01, complete genome | 1316 | 0.00E+00 | 76.20% |
| Efet.01.602630.g106.t1 | Rufibacter sp. DG31D, complete genome | 182 | 7.30E-32 | 76.20% |
| Efet.01.614177.g634.t1 | Verminephrobacter eiseniae EF01-2, complete genome | 732 | 0.00E+00 | 76.20% |
| Efet.01.627489.g1209.t1 | Rhodococcus opacus B4 DNA, complete genome | 464 | 3.50E-98 | 76.20% |
| Efet.01.639260.g494.t1 | Verminephrobacter eiseniae EF01-2, complete genome | 1232 | 0.00E+00 | 76.20% |
| Efet.01.645133.g933.t1 | Verminephrobacter eiseniae EF01-2, complete genome | 297 | 4.60E-147 | 76.20% |
| Efet.01.645947.g1011.t1 | Verminephrobacter eiseniae EF01-2, complete genome | 1254 | 0.00E+00 | 76.20% |
| Efet.01.646487.g1096.t1 | Verminephrobacter eiseniae EF01-2, complete genome | 612 | 0.00E+00 | 76.20% |
| Efet.01.647324.g1169.t1 | Hymenobacter sp. DG25B, complete genome | 296 | 6.70E-59 | 76.20% |
| Efet.01.651575.g192.t1 | Verminephrobacter eiseniae EF01-2, complete genome | 927 | 0.00E+00 | 76.20% |
| Efet.01.651631.g203.t1 | Agromyces aureus strain AR33, complete genome | 420 | 7.20E-96 | 76.20% |
| Efet.01.654034.g527.t1 | Alicycliphilus denitrificans BC, complete genome | 1434 | 0.00E+00 | 76.20% |
| Efet.01.655193.g772.t1 | Verminephrobacter eiseniae EF01-2, complete genome | 690 | 0.00E+00 | 76.20% |
| Efet.01.657248.g1212.t1 | Verminephrobacter eiseniae EF01-2, complete genome | 690 | 0.00E+00 | 76.20% |
| Efet.01.658116.g1641.t1 | Verminephrobacter eiseniae EF01-2, complete genome | 1017 | 0.00E+00 | 76.20% |
| Efet.01.658116.g1642.t1 | Verminephrobacter eiseniae EF01-2, complete genome | 450 | 0.00E+00 | 76.20% |
| Efet.01.658334.g1862.t1 | Verminephrobacter eiseniae EF01-2, complete genome | 1095 | 0.00E+00 | 76.20% |
| Efet.01.658380.g1922.t1 | Verminephrobacter eiseniae EF01-2, complete genome | 375 | 1.00E-180 | 76.20% |
| Efet.01.658430.g2167.t1 | Verminephrobacter eiseniae EF01-2, complete genome | 321 | 3.50E-162 | 76.17% |
| Efet.01.1658872.g1306.t1 | Nocardiopsis dassonvillei strain NOCA502F, complete genome | 260 | 1.60E-08 | 76.14% |
| Efet.01.171446.g1033.t1 | Paenibacillus sp. FSL H7-0357, complete genome | 99 | 4.60E-09 | 76.13% |
| Efet.01.11025.g810.t1 | Synechococcus sp. KORDI-49, complete genome | 153 | 4.80E-26 | 76.10% |
| Efet.01.190792.g1902.t1 | Microbacterium sp. No. 7, complete genome | 314 | 2.50E-70 | 76.10% |
| Efet.01.195994.g2128.t1 | Cellvibrio sp. PSBB023, complete genome | 193 | 8.50E-10 | 76.10% |
| Efet.01.310180.g325.t1 | Chitinophaga pinensis DSM 2588, complete genome | 97 | 2.30E-09 | 76.10% |
| Efet.01.440538.g980.t1 | Verminephrobacter eiseniae EF01-2, complete genome | 1662 | 0.00E+00 | 76.10% |
| Efet.01.464584.g387.t1 | Pseudomonas syringae pv. cerasicola isolate CFBP6109 genome assembly, chromosome: 1 | 92 | 1.20E-08 | 76.10% |
| Efet.01.489798.g956.t1 | Verminephrobacter eiseniae EF01-2, complete genome | 434 | 0.00E+00 | 76.10% |
| Efet.01.531405.g45.t1 | Kitasatospora aureofaciens strain DM-1, complete genome | 222 | 1.60E-19 | 76.10% |
| Efet.01.560210.g3.t1 | Agromyces flavus strain CPCC 202695 genome assembly, chromosome: I | 386 | 3.30E-73 | 76.10% |
| Efet.01.566222.g207.t1 | Verminephrobacter eiseniae EF01-2, complete genome | 969 | 0.00E+00 | 76.10% |
| Efet.01.585033.g827.t1 | Verminephrobacter eiseniae EF01-2, complete genome | 879 | 0.00E+00 | 76.10% |
| Efet.01.590200.g998.t1 | Verminephrobacter eiseniae EF01-2, complete genome | 1385 | 0.00E+00 | 76.10% |
| Efet.01.597702.g1221.t1 | Verminephrobacter eiseniae EF01-2, complete genome | 810 | 0.00E+00 | 76.10% |
| Efet.01.607412.g302.t1 | Verminephrobacter eiseniae EF01-2, complete genome | 1440 | 0.00E+00 | 76.10% |
| Efet.01.611210.g467.t1 | Agromyces sp. 30A chromosome, complete genome | 225 | 7.70E-24 | 76.10% |
| Efet.01.628537.g1257.t1 | Verminephrobacter eiseniae EF01-2, complete genome | 877 | 0.00E+00 | 76.10% |
| Efet.01.632756.g166.t1 | Verminephrobacter eiseniae EF01-2, complete genome | 441 | 0.00E+00 | 76.10% |
| Efet.01.632756.g169.t1 | Verminephrobacter eiseniae EF01-2, complete genome | 1254 | 0.00E+00 | 76.10% |
| Efet.01.633502.g193.t1 | Verminephrobacter eiseniae EF01-2, complete genome | 465 | 0.00E+00 | 76.10% |
| Efet.01.638678.g452.t1 | Verminephrobacter eiseniae EF01-2, complete genome | 366 | 0.00E+00 | 76.10% |
| Efet.01.639963.g546.t1 | Verminephrobacter eiseniae EF01-2, complete genome | 1598 | 0.00E+00 | 76.10% |
| Efet.01.640737.g587.t1 | Verminephrobacter eiseniae EF01-2, complete genome | 294 | 9.90E-146 | 76.10% |
| Efet.01.643464.g799.t1 | Verminephrobacter eiseniae EF01-2, complete genome | 429 | 0.00E+00 | 76.10% |
| Efet.01.646487.g1098.t1 | Verminephrobacter eiseniae EF01-2, complete genome | 828 | 0.00E+00 | 76.10% |
| Efet.01.648953.g1277.t1 | Verminephrobacter eiseniae EF01-2, complete genome | 1624 | 0.00E+00 | 76.10% |
| Efet.01.649821.g1357.t1 | Verminephrobacter eiseniae EF01-2, complete genome | 555 | 0.00E+00 | 76.10% |
| Efet.01.650311.g32.t1 | Pseudomonas aeruginosa strain AR441 chromosome, complete genome | 209 | 4.50E-32 | 76.10% |
| Efet.01.651940.g244.t1 | Verminephrobacter eiseniae EF01-2, complete genome | 426 | 8.10E-165 | 76.10% |
| Efet.01.654656.g653.t1 | Verminephrobacter eiseniae EF01-2, complete genome | 696 | 0.00E+00 | 76.10% |
| Efet.01.655224.g776.t1 | Verminephrobacter eiseniae EF01-2, complete genome | 237 | 2.00E-116 | 76.10% |
| Efet.01.655756.g862.t1 | Verminephrobacter eiseniae EF01-2, complete genome | 612 | 0.00E+00 | 76.10% |
| Efet.01.656206.g965.t1 | Microbacterium hominis strain SJTG1 chromosome, complete genome | 437 | 1.60E-87 | 76.10% |
| Efet.01.658422.g2102.t1 | Hydrogenophaga sp. PBC, complete genome | 384 | 0.00E+00 | 76.10% |
| Efet.01.658430.g2172.t1 | Verminephrobacter eiseniae EF01-2, complete genome | 835 | 0.00E+00 | 76.10% |
| Efet.01.1652260.g550.t1 | Verminephrobacter eiseniae EF01-2, complete genome | 451 | 0.00E+00 | 76.10% |
| Efet.01.1658105.g1105.t1 | Agromyces sp. 30A chromosome, complete genome | 608 | 1.10E-130 | 76.10% |
| Efet.01.1659403.g1663.t1 | Verminephrobacter eiseniae EF01-2, complete genome | 1933 | 0.00E+00 | 76.10% |
| Efet.01.1659460.g1768.t1 | Verminephrobacter eiseniae EF01-2, complete genome | 939 | 0.00E+00 | 76.10% |
| Efet.01.1659515.g1948.t1 | Verminephrobacter eiseniae EF01-2, complete genome | 1497 | 0.00E+00 | 76.10% |
| Efet.01.1659516.g1957.t1 | Verminephrobacter eiseniae EF01-2, complete genome | 555 | 0.00E+00 | 76.10% |
| Efet.01.1659524.g2007.t1 | Verminephrobacter eiseniae EF01-2, complete genome | 969 | 0.00E+00 | 76.10% |
| Efet.01.1659525.g2017.t1 | Verminephrobacter eiseniae EF01-2, complete genome | 543 | 0.00E+00 | 76.10% |
| Efet.01.3361.g288.t1 | [Haemophilus] parasuis strain SC1401, complete genome | 93 | 1.70E-06 | 76.00% |
| Efet.01.4757.g380.t1 | Uncultured bacterium clone contig27074 genomic sequence | 121 | 3.00E-15 | 76.00% |
| Efet.01.43997.g1227.t1 | Virgibacillus sp. SK37, complete genome | 105 | 1.20E-07 | 76.00% |
| Efet.01.51025.g72.t1 | Geitlerinema sp. PCC 7407, complete genome | 109 | 1.90E-09 | 76.00% |
| Efet.01.57291.g453.t1 | Ferrimonas balearica DSM 9799, complete genome | 176 | 5.80E-08 | 76.00% |
| Efet.01.76155.g72.t1 | Paenibacillus sp. FSL P4-0081, complete genome | 84 | 3.30E-06 | 76.00% |
| Efet.01.79725.g306.t1 | Providencia rettgeri strain 06-1619 plasmid p06-1619-NDM, complete sequence | 150 | 3.20E-20 | 76.00% |
| Efet.01.85563.g660.t1 | Uncultured bacterium clone GN8LFNR02I2JQ7 genomic sequence | 97 | 1.30E-08 | 76.00% |
| Efet.01.110329.g526.t1 | Rhodoferax sp. DCY110, complete genome | 81 | 3.40E-06 | 76.00% |
| Efet.01.287526.g1370.t1 | Verminephrobacter eiseniae EF01-2, complete genome | 444 | 0.00E+00 | 76.00% |
| Efet.01.369119.g519.t1 | Uncultured bacterium BD_contig01554 genomic sequence | 161 | 1.50E-12 | 76.00% |
| Efet.01.395721.g1133.t1 | Streptomyces sp. TLI_053 genome assembly, chromosome: I | 82 | 4.10E-06 | 76.00% |
| Efet.01.459793.g272.t1 | Prochlorococcus marinus str. MIT 9301, complete genome | 99 | 7.80E-06 | 76.00% |
| Efet.01.464530.g385.t1 | Verminephrobacter eiseniae EF01-2, complete genome | 432 | 0.00E+00 | 76.00% |
| Efet.01.508646.g227.t1 | Verminephrobacter eiseniae EF01-2, complete genome | 726 | 0.00E+00 | 76.00% |
| Efet.01.530881.g30.t1 | Variovorax paradoxus S110 chromosome 1, complete sequence | 960 | 0.00E+00 | 76.00% |
| Efet.01.533383.g123.t1 | Verminephrobacter eiseniae EF01-2, complete genome | 560 | 0.00E+00 | 76.00% |
| Efet.01.562540.g52.t1 | Planctomyces sp. SH-PL62, complete genome | 88 | 2.00E-06 | 76.00% |
| Efet.01.568134.g263.t1 | Agromyces flavus strain CPCC 202695 genome assembly, chromosome: I | 947 | 0.00E+00 | 76.00% |
| Efet.01.574381.g452.t1 | Verminephrobacter eiseniae EF01-2, complete genome | 599 | 0.00E+00 | 76.00% |
| Efet.01.606493.g282.t1 | Tenacibaculum jejuense strain KCTC 22618(T) genome assembly, chromosome: TJEJU | 88 | 2.60E-06 | 76.00% |
| Efet.01.609092.g377.t1 | Verminephrobacter eiseniae EF01-2, complete genome | 786 | 0.00E+00 | 76.00% |
| Efet.01.609830.g417.t1 | Leifsonia sp. 98AMF genome assembly, chromosome: I | 404 | 2.50E-74 | 76.00% |
| Efet.01.619389.g864.t1 | Verminephrobacter eiseniae EF01-2, complete genome | 591 | 0.00E+00 | 76.00% |
| Efet.01.622611.g995.t1 | Verminephrobacter eiseniae EF01-2, complete genome | 1083 | 0.00E+00 | 76.00% |
| Efet.01.630521.g31.t1 | Verminephrobacter eiseniae EF01-2, complete genome | 942 | 0.00E+00 | 76.00% |
| Efet.01.630546.g42.t1 | Verminephrobacter eiseniae EF01-2, complete genome | 1087 | 0.00E+00 | 76.00% |
| Efet.01.643464.g790.t1 | Verminephrobacter eiseniae EF01-2, complete genome | 735 | 0.00E+00 | 76.00% |
| Efet.01.643497.g808.t1 | Verminephrobacter eiseniae EF01-2, complete genome | 249 | 8.40E-120 | 76.00% |
| Efet.01.644987.g915.t1 | Verminephrobacter eiseniae EF01-2, complete genome | 997 | 0.00E+00 | 76.00% |
| Efet.01.645268.g966.t1 | Verminephrobacter eiseniae EF01-2, complete genome | 1104 | 0.00E+00 | 76.00% |
| Efet.01.651978.g250.t1 | Agromyces aureus strain AR33, complete genome | 309 | 7.70E-64 | 76.00% |
| Efet.01.652215.g265.t1 | Verminephrobacter eiseniae EF01-2, complete genome | 1750 | 0.00E+00 | 76.00% |
| Efet.01.652384.g288.t1 | Verminephrobacter eiseniae EF01-2, complete genome | 813 | 0.00E+00 | 76.00% |
| Efet.01.652863.g358.t1 | Variovorax sp. HW608 genome assembly, chromosome: I | 103 | 5.60E-11 | 76.00% |
| Efet.01.653063.g396.t1 | Verminephrobacter eiseniae EF01-2, complete genome | 1058 | 0.00E+00 | 76.00% |
| Efet.01.654418.g599.t1 | Verminephrobacter eiseniae EF01-2, complete genome | 985 | 0.00E+00 | 76.00% |
| Efet.01.656495.g1033.t1 | Bacteroidetes bacterium UKL13-3, complete genome | 195 | 6.00E-18 | 76.00% |
| Efet.01.656889.g1136.t1 | gamma proteobacterium HdN1 complete genome | 356 | 1.80E-66 | 76.00% |
| Efet.01.657077.g1159.t1 | Verminephrobacter eiseniae EF01-2, complete genome | 735 | 0.00E+00 | 76.00% |
| Efet.01.657719.g1397.t1 | Verminephrobacter eiseniae EF01-2, complete genome | 641 | 0.00E+00 | 76.00% |
| Efet.01.658044.g1588.t1 | Verminephrobacter eiseniae EF01-2, complete genome | 366 | 4.20E-179 | 76.00% |
| Efet.01.658084.g1625.t1 | Verminephrobacter eiseniae EF01-2, complete genome | 1019 | 0.00E+00 | 76.00% |
| Efet.01.658236.g1734.t1 | Verminephrobacter eiseniae EF01-2, complete genome | 111 | 3.10E-10 | 76.00% |
| Efet.01.658426.g2133.t1 | Verminephrobacter eiseniae EF01-2, complete genome | 462 | 0.00E+00 | 76.00% |
| Efet.01.1651673.g518.t1 | Agromyces sp. 30A chromosome, complete genome | 359 | 2.70E-80 | 76.00% |
| Efet.01.1657936.g1064.t1 | Agromyces aureus strain AR33, complete genome | 111 | 3.00E-11 | 76.00% |
| Efet.01.1659255.g1490.t1 | Pseudomonas aeruginosa strain AR439 chromosome, complete genome | 237 | 1.70E-38 | 76.00% |
| Efet.01.1659351.g1587.t1 | Ensifer adhaerens strain Casida A, complete genome | 96 | 2.00E-08 | 76.00% |
| Efet.01.1659473.g1796.t1 | Burkholderia pseudomallei PB08298010 chromosome I, complete sequence | 431 | 3.20E-120 | 76.00% |
| Efet.01.1659479.g1815.t1 | Variovorax sp. PMC12 chromosome 1, complete sequence | 678 | 0.00E+00 | 76.00% |
| Efet.01.1659523.g2001.t1 | Verminephrobacter eiseniae EF01-2, complete genome | 627 | 0.00E+00 | 76.00% |
| Efet.01.1659523.g2003.t1 | Verminephrobacter eiseniae EF01-2, complete genome | 846 | 0.00E+00 | 76.00% |
| Efet.01.151234.g76.t1 | Verminephrobacter eiseniae EF01-2, complete genome | 1311 | 0.00E+00 | 75.90% |
| Efet.01.275134.g927.t1 | Verminephrobacter eiseniae EF01-2, complete genome | 1597 | 0.00E+00 | 75.90% |
| Efet.01.444678.g1095.t1 | Agromyces flavus strain CPCC 202695 genome assembly, chromosome: I | 376 | 9.10E-78 | 75.90% |
| Efet.01.471393.g574.t1 | Verminephrobacter eiseniae EF01-2, complete genome | 267 | 5.50E-124 | 75.90% |
| Efet.01.553257.g578.t1 | Rufibacter sp. DG31D, complete genome | 343 | 5.40E-66 | 75.90% |
| Efet.01.570164.g334.t1 | Leifsonia xyli strain SE134, complete genome | 501 | 7.40E-91 | 75.90% |
| Efet.01.618287.g813.t1 | Verminephrobacter eiseniae EF01-2, complete genome | 404 | 0.00E+00 | 75.90% |
| Efet.01.629510.g1298.t1 | Agromyces sp. 30A chromosome, complete genome | 215 | 7.40E-39 | 75.90% |
| Efet.01.630200.g8.t1 | Paraburkholderia xenovorans LB400 chromosome 3, complete sequence | 393 | 0.00E+00 | 75.90% |
| Efet.01.632992.g179.t1 | Verminephrobacter eiseniae EF01-2, complete genome | 645 | 0.00E+00 | 75.90% |
| Efet.01.638678.g448.t1 | Verminephrobacter eiseniae EF01-2, complete genome | 1404 | 0.00E+00 | 75.90% |
| Efet.01.643099.g762.t1 | Verminephrobacter eiseniae EF01-2, complete genome | 1353 | 0.00E+00 | 75.90% |
| Efet.01.649588.g1343.t1 | Verminephrobacter eiseniae EF01-2, complete genome | 999 | 0.00E+00 | 75.90% |
| Efet.01.652898.g373.t1 | Verminephrobacter eiseniae EF01-2, complete genome | 540 | 0.00E+00 | 75.90% |
| Efet.01.657624.g1346.t1 | Verminephrobacter eiseniae EF01-2, complete genome | 300 | 2.40E-145 | 75.90% |
| Efet.01.658350.g1881.t1 | Verminephrobacter eiseniae EF01-2, complete genome | 1236 | 0.00E+00 | 75.90% |
| Efet.01.658392.g1951.t1 | Verminephrobacter eiseniae EF01-2, complete genome | 781 | 0.00E+00 | 75.90% |
| Efet.01.658427.g2140.t1 | Verminephrobacter eiseniae EF01-2, complete genome | 1095 | 0.00E+00 | 75.90% |
| Efet.01.1637086.g184.t1 | Aeromonas schubertii strain WL1483, complete genome | 271 | 1.70E-51 | 75.90% |
| Efet.01.1654190.g668.t1 | Leifsonia sp. 98AMF genome assembly, chromosome: I | 260 | 7.30E-53 | 75.90% |
| Efet.01.1657281.g965.t1 | Variovorax paradoxus S110 chromosome 1, complete sequence | 183 | 3.00E-47 | 75.90% |
| Efet.01.1659396.g1651.t1 | Verminephrobacter eiseniae EF01-2, complete genome | 1698 | 0.00E+00 | 75.90% |
| Efet.01.1659466.g1783.t1 | Verminephrobacter eiseniae EF01-2, complete genome | 219 | 8.70E-96 | 75.90% |
| Efet.01.1659467.g1785.t1 | Verminephrobacter eiseniae EF01-2, complete genome | 1155 | 0.00E+00 | 75.90% |
| Efet.01.635754.g291.t1 | Mycobacterium aurum isolate liquid genome assembly, chromosome: I | 108 | 2.30E-11 | 75.89% |
| Efet.01.650262.g16.t1 | Mycobacterium thermoresistibile strain NCTC10409 genome assembly, chromosome: 1 | 89 | 8.20E-10 | 75.89% |
| Efet.01.633916.g218.t1 | Verminephrobacter eiseniae EF01-2, complete genome | 945 | 0.00E+00 | 75.86% |
| Efet.01.590200.g1000.t1 | Verminephrobacter eiseniae EF01-2, complete genome | 354 | 5.50E-167 | 75.83% |
| Efet.01.658398.g1986.t1 | Amycolatopsis mediterranei S699, complete genome | 699 | 0.00E+00 | 75.83% |
| Efet.01.32368.g521.t1 | Azospirillum humicireducens strain SgZ-5, complete genome | 186 | 8.40E-32 | 75.80% |
| Efet.01.58768.g545.t1 | Leifsonia xyli subsp. xyli str. CTCB07, complete genome | 627 | 0.00E+00 | 75.80% |
| Efet.01.59384.g588.t1 | Verminephrobacter eiseniae EF01-2, complete genome | 653 | 0.00E+00 | 75.80% |
| Efet.01.258476.g315.t1 | Verminephrobacter eiseniae EF01-2, complete genome | 1875 | 0.00E+00 | 75.80% |
| Efet.01.398399.g1202.t1 | Agromyces sp. 30A chromosome, complete genome | 465 | 1.80E-105 | 75.80% |
| Efet.01.440538.g982.t1 | Verminephrobacter eiseniae EF01-2, complete genome | 948 | 0.00E+00 | 75.80% |
| Efet.01.464530.g380.t1 | Verminephrobacter eiseniae EF01-2, complete genome | 360 | 4.70E-172 | 75.80% |
| Efet.01.465744.g416.t1 | Verminephrobacter eiseniae EF01-2, complete genome | 997 | 0.00E+00 | 75.80% |
| Efet.01.520113.g479.t1 | Verminephrobacter eiseniae EF01-2, complete genome | 711 | 0.00E+00 | 75.80% |
| Efet.01.560128.g1.t1 | Hymenobacter sp. PAMC26628, complete genome | 356 | 7.30E-69 | 75.80% |
| Efet.01.577373.g574.t1 | Verminephrobacter eiseniae EF01-2, complete genome | 650 | 0.00E+00 | 75.80% |
| Efet.01.587858.g916.t1 | Agromyces sp. 30A chromosome, complete genome | 1140 | 0.00E+00 | 75.80% |
| Efet.01.593134.g1096.t1 | Fibrella sp. ES10-3-2-2, complete genome | 150 | 1.70E-18 | 75.80% |
| Efet.01.597702.g1219.t1 | Verminephrobacter eiseniae EF01-2, complete genome | 255 | 7.90E-122 | 75.80% |
| Efet.01.606285.g270.t1 | Verminephrobacter eiseniae EF01-2, complete genome | 399 | 0.00E+00 | 75.80% |
| Efet.01.607412.g305.t1 | Verminephrobacter eiseniae EF01-2, complete genome | 1038 | 0.00E+00 | 75.80% |
| Efet.01.618821.g847.t1 | Chryseobacterium gallinarum strain DSM 27622, complete genome | 212 | 1.40E-41 | 75.80% |
| Efet.01.621328.g923.t1 | Agromyces sp. 30A chromosome, complete genome | 706 | 0.00E+00 | 75.80% |
| Efet.01.626983.g1184.t1 | Verminephrobacter eiseniae EF01-2, complete genome | 1389 | 0.00E+00 | 75.80% |
| Efet.01.629376.g1295.t1 | Verminephrobacter eiseniae EF01-2, complete genome | 948 | 0.00E+00 | 75.80% |
| Efet.01.630768.g66.t1 | Verminephrobacter eiseniae EF01-2, complete genome | 441 | 0.00E+00 | 75.80% |
| Efet.01.638425.g434.t1 | Verminephrobacter eiseniae EF01-2, complete genome | 441 | 0.00E+00 | 75.80% |
| Efet.01.642123.g667.t1 | Verminephrobacter eiseniae EF01-2, complete genome | 972 | 0.00E+00 | 75.80% |
| Efet.01.643497.g805.t1 | Verminephrobacter eiseniae EF01-2, complete genome | 1359 | 0.00E+00 | 75.80% |
| Efet.01.644507.g882.t1 | Verminephrobacter eiseniae EF01-2, complete genome | 1101 | 0.00E+00 | 75.80% |
| Efet.01.645138.g943.t1 | Verminephrobacter eiseniae EF01-2, complete genome | 621 | 0.00E+00 | 75.80% |
| Efet.01.650749.g82.t1 | Verminephrobacter eiseniae EF01-2, complete genome | 1509 | 0.00E+00 | 75.80% |
| Efet.01.654034.g522.t1 | Diaphorobacter polyhydroxybutyrativorans strain SL-205, complete genome | 117 | 6.60E-13 | 75.80% |
| Efet.01.654310.g561.t1 | Verminephrobacter eiseniae EF01-2, complete genome | 933 | 0.00E+00 | 75.80% |
| Efet.01.655622.g841.t1 | Verminephrobacter eiseniae EF01-2, complete genome | 726 | 0.00E+00 | 75.80% |
| Efet.01.655933.g898.t1 | Verminephrobacter eiseniae EF01-2, complete genome | 939 | 0.00E+00 | 75.80% |
| Efet.01.656558.g1039.t1 | Verminephrobacter eiseniae EF01-2, complete genome | 1922 | 0.00E+00 | 75.80% |
| Efet.01.657077.g1160.t1 | Verminephrobacter eiseniae EF01-2, complete genome | 1633 | 0.00E+00 | 75.80% |
| Efet.01.657189.g1195.t1 | Agromyces sp. 30A chromosome, complete genome | 328 | 2.10E-69 | 75.80% |
| Efet.01.657624.g1344.t1 | Verminephrobacter eiseniae EF01-2, complete genome | 807 | 0.00E+00 | 75.80% |
| Efet.01.658081.g1620.t1 | Verminephrobacter eiseniae EF01-2, complete genome | 1122 | 0.00E+00 | 75.80% |
| Efet.01.658423.g2108.t1 | Verminephrobacter eiseniae EF01-2, complete genome | 857 | 0.00E+00 | 75.80% |
| Efet.01.1655038.g733.t1 | Streptomyces sp. TLI_053 genome assembly, chromosome: I | 305 | 1.70E-35 | 75.80% |
| Efet.01.1658086.g1100.t1 | Verminephrobacter eiseniae EF01-2, complete genome | 873 | 0.00E+00 | 75.80% |
| Efet.01.1659446.g1731.t1 | Verminephrobacter eiseniae EF01-2, complete genome | 1527 | 0.00E+00 | 75.80% |
| Efet.01.1659477.g1805.t1 | Verminephrobacter eiseniae EF01-2, complete genome | 657 | 0.00E+00 | 75.80% |
| Efet.01.1659515.g1951.t1 | Verminephrobacter eiseniae EF01-2, complete genome | 1584 | 0.00E+00 | 75.80% |
| Efet.01.1659527.g2039.t1 | Verminephrobacter eiseniae EF01-2, complete genome | 2259 | 0.00E+00 | 75.80% |
| Efet.01.182758.g1538.t1 | Streptomyces peucetius subsp. caesius ATCC 27952 chromosome, complete genome | 111 | 8.40E-13 | 75.75% |
| Efet.01.1658501.g1199.t1 | Verminephrobacter eiseniae EF01-2, complete genome | 690 | 0.00E+00 | 75.75% |
| Efet.01.124508.g1264.t1 | [Bacillus] selenitireducens MLS10, complete genome | 114 | 1.70E-11 | 75.70% |
| Efet.01.488568.g930.t1 | Verminephrobacter eiseniae EF01-2, complete genome | 1248 | 0.00E+00 | 75.70% |
| Efet.01.519674.g471.t1 | Elizabethkingia genomosp. 3 strain G0146, complete genome | 152 | 5.70E-16 | 75.70% |
| Efet.01.533610.g133.t1 | Agromyces flavus strain CPCC 202695 genome assembly, chromosome: I | 126 | 1.00E-23 | 75.70% |
| Efet.01.563201.g74.t1 | Verminephrobacter eiseniae EF01-2, complete genome | 666 | 0.00E+00 | 75.70% |
| Efet.01.578243.g632.t1 | Agromyces flavus strain CPCC 202695 genome assembly, chromosome: I | 245 | 2.60E-43 | 75.70% |
| Efet.01.585033.g821.t1 | Verminephrobacter eiseniae EF01-2, complete genome | 378 | 0.00E+00 | 75.70% |
| Efet.01.585463.g846.t1 | Verminephrobacter eiseniae EF01-2, complete genome | 1476 | 0.00E+00 | 75.70% |
| Efet.01.587924.g920.t1 | Verminephrobacter eiseniae EF01-2, complete genome | 414 | 0.00E+00 | 75.70% |
| Efet.01.591301.g1033.t1 | Verminephrobacter eiseniae EF01-2, complete genome | 765 | 0.00E+00 | 75.70% |
| Efet.01.606285.g264.t1 | Verminephrobacter eiseniae EF01-2, complete genome | 1298 | 0.00E+00 | 75.70% |
| Efet.01.613322.g590.t1 | Agromyces sp. 30A chromosome, complete genome | 548 | 2.30E-107 | 75.70% |
| Efet.01.614719.g662.t1 | Verminephrobacter eiseniae EF01-2, complete genome | 271 | 1.10E-130 | 75.70% |
| Efet.01.618287.g814.t1 | Verminephrobacter eiseniae EF01-2, complete genome | 1356 | 0.00E+00 | 75.70% |
| Efet.01.627540.g1211.t1 | Microterricola viridarii strain ERGS5:02, complete genome | 580 | 3.10E-143 | 75.70% |
| Efet.01.640054.g556.t1 | Cnuibacter physcomitrellae strain XA(T), complete genome | 506 | 5.00E-102 | 75.70% |
| Efet.01.642071.g652.t1 | Devosia sp. A16, complete genome | 1081 | 0.00E+00 | 75.70% |
| Efet.01.643674.g821.t1 | Verminephrobacter eiseniae EF01-2, complete genome | 404 | 0.00E+00 | 75.70% |
| Efet.01.649588.g1342.t1 | Verminephrobacter eiseniae EF01-2, complete genome | 426 | 0.00E+00 | 75.70% |
| Efet.01.650357.g45.t1 | Verminephrobacter eiseniae EF01-2, complete genome | 296 | 1.70E-132 | 75.70% |
| Efet.01.651136.g115.t1 | Burkholderia vietnamiensis strain FL-2-3-30-S1-D0 chromosome 1, complete sequence | 686 | 6.40E-130 | 75.70% |
| Efet.01.651575.g195.t1 | Verminephrobacter eiseniae EF01-2, complete genome | 705 | 0.00E+00 | 75.70% |
| Efet.01.655650.g844.t1 | Verminephrobacter eiseniae EF01-2, complete genome | 506 | 0.00E+00 | 75.70% |
| Efet.01.655650.g845.t1 | Verminephrobacter eiseniae EF01-2, complete genome | 636 | 0.00E+00 | 75.70% |
| Efet.01.655988.g923.t1 | Verminephrobacter eiseniae EF01-2, complete genome | 324 | 1.80E-156 | 75.70% |
| Efet.01.657492.g1307.t1 | Verminephrobacter eiseniae EF01-2, complete genome | 891 | 0.00E+00 | 75.70% |
| Efet.01.657876.g1513.t1 | Verminephrobacter eiseniae EF01-2, complete genome | 1219 | 0.00E+00 | 75.70% |
| Efet.01.1657332.g971.t1 | Agromyces aureus strain AR33, complete genome | 578 | 9.20E-130 | 75.70% |
| Efet.01.1659354.g1594.t1 | Verminephrobacter eiseniae EF01-2, complete genome | 438 | 0.00E+00 | 75.70% |
| Efet.01.1659443.g1726.t1 | Verminephrobacter eiseniae EF01-2, complete genome | 753 | 0.00E+00 | 75.70% |
| Efet.01.1659504.g1902.t1 | Variovorax paradoxus S110 chromosome 2, complete sequence | 1156 | 0.00E+00 | 75.70% |
| Efet.01.1659517.g1962.t1 | Verminephrobacter eiseniae EF01-2, complete genome | 417 | 0.00E+00 | 75.70% |
| Efet.01.82959.g521.t1 | Microbacterium sp. str. 'China' chromosome, complete genome | 259 | 1.30E-46 | 75.67% |
| Efet.01.151234.g71.t1 | Verminephrobacter eiseniae EF01-2, complete genome | 971 | 0.00E+00 | 75.67% |
| Efet.01.401337.g30.t1 | Verminephrobacter eiseniae EF01-2, complete genome | 486 | 0.00E+00 | 75.67% |
| Efet.01.574820.g473.t1 | Algoriphagus sp. M8-2, complete genome | 154 | 4.70E-10 | 75.67% |
| Efet.01.590828.g1018.t1 | Paucibacter sp. KCTC 42545, complete genome | 96 | 2.90E-10 | 75.67% |
| Efet.01.642392.g680.t1 | Persicobacter sp. JZB09, complete genome | 104 | 1.40E-14 | 75.67% |
| Efet.01.1658878.g1307.t1 | Verminephrobacter eiseniae EF01-2, complete genome | 741 | 0.00E+00 | 75.67% |
| Efet.01.575925.g514.t1 | Lacunisphaera limnophila strain IG16b chromosome, complete genome | 117 | 2.60E-13 | 75.63% |
| Efet.01.622077.g966.t1 | Streptomyces niveus strain SCSIO 3406, complete genome | 173 | 1.80E-08 | 75.63% |
| Efet.01.31176.g428.t1 | Cryobacterium sp. LW097, complete genome | 301 | 1.20E-61 | 75.60% |
| Efet.01.62716.g791.t1 | Methylocaldum marinum DNA, complete genome, strain: S8 | 273 | 7.10E-31 | 75.60% |
| Efet.01.176923.g1265.t1 | Bacteroidales bacterium CF, complete genome | 187 | 1.10E-16 | 75.60% |
| Efet.01.323272.g722.t1 | Bradyrhizobium sp. 3 85S1MB chromosome, complete genome | 332 | 1.90E-57 | 75.60% |
| Efet.01.472695.g619.t1 | Thalassococcus sp. SH-1 chromosome, complete genome | 152 | 9.80E-16 | 75.60% |
| Efet.01.550127.g498.t1 | Verminephrobacter eiseniae EF01-2, complete genome | 915 | 0.00E+00 | 75.60% |
| Efet.01.565977.g196.t1 | Verminephrobacter eiseniae EF01-2, complete genome | 335 | 1.40E-139 | 75.60% |
| Efet.01.592077.g1060.t1 | Verminephrobacter eiseniae EF01-2, complete genome | 825 | 0.00E+00 | 75.60% |
| Efet.01.606285.g262.t1 | Verminephrobacter eiseniae EF01-2, complete genome | 1680 | 0.00E+00 | 75.60% |
| Efet.01.612521.g550.t1 | Microcella alkaliphila DNA, complete genome, strain: JAM AC0309 | 436 | 1.90E-88 | 75.60% |
| Efet.01.615846.g706.t1 | Pseudomonas orientalis strain BS2775 genome assembly, chromosome: I | 127 | 5.60E-10 | 75.60% |
[truncated: 262,592 more chars]
