# Supplementary material for: Data on genome annotation and analysis of earthworm Eisenia fetida
Source: Data Brief. 2018 Aug 29;20:525–34. doi: 10.1016/j.dib.2018.08.067 (PMC6126081; doi:10.1016/j.dib.2018.08.067)
Supplement: Supplementary file 4 — Supplementary material [file mmc4.docx]

Table S3: Summary of KEGG pathway annotation

| **Pathways** | Number of Sequences |
| --- | --- |
| **Metabolism** | |
| Purine metabolism | 96 |
| Oxidative phosphorylation | 67 |
| Pyrimidine metabolism | 67 |
| Pyruvate metabolism | 45 |
| Glycine, serine and threonine metabolism | 42 |
| Glyoxylate and dicarboxylate metabolism | 39 |
| Amino sugar and nucleotide sugar metabolism | 38 |
| Glycolysis / Gluconeogenesis | 37 |
| Propanoate metabolism | 35 |
| Cysteine and methionine metabolism | 34 |
| Arginine and proline metabolism | 33 |
| Butanoate metabolism | 32 |
| Glycerophospholipid metabolism | 32 |
| Valine, leucine and isoleucine degradation | 32 |
| Alanine, aspartate and glutamate metabolism | 31 |
| Lysine degradation | 30 |
| Pentose phosphate pathway | 30 |
| Citrate cycle (TCA cycle) | 28 |
| Methane metabolism | 28 |
| Phenylalanine metabolism | 28 |
| Fructose and mannose metabolism | 27 |
| Carbon fixation pathways in prokaryotes | 26 |
| Fatty acid degradation | 25 |
| Histidine metabolism | 23 |
| Tryptophan metabolism | 23 |
| Nicotinate and nicotinamide metabolism | 22 |
| Porphyrin and chlorophyll metabolism | 22 |
| Arginine biosynthesis | 21 |
| Inositol phosphate metabolism | 21 |
| Phenylalanine, tyrosine and tryptophan biosynthesis | 21 |
| Starch and sucrose metabolism | 21 |
| beta-Alanine metabolism | 20 |
| Pentose and glucuronate interconversions | 20 |
| Folate biosynthesis | 19 |
| Glutathione metabolism | 19 |
| Glycerolipid metabolism | 19 |
| Terpenoid backbone biosynthesis | 19 |
| Carbon fixation in photosynthetic organisms | 18 |
| Drug metabolism - other enzymes | 17 |
| Pantothenate and CoA biosynthesis | 17 |
| Sulfur metabolism | 17 |
| Tyrosine metabolism | 17 |
| Fatty acid biosynthesis | 16 |
| Galactose metabolism | 16 |
| Lysine biosynthesis | 16 |
| Sphingolipid metabolism | 16 |
| Various types of N-glycan biosynthesis | 16 |
| N-Glycan biosynthesis | 15 |
| Peptidoglycan biosynthesis | 15 |
| Lipopolysaccharide biosynthesis | 14 |
| Ascorbate and aldarate metabolism | 12 |
| Metabolism of xenobiotics by cytochrome P450 | 12 |
| Nitrogen metabolism | 12 |
| Retinol metabolism | 12 |
| Selenocompound metabolism | 12 |
| Valine, leucine and isoleucine biosynthesis | 12 |
| Glycosaminoglycan biosynthesis - heparan sulfate / heparin | 11 |
| One carbon pool by folate | 11 |
| Thiamine metabolism | 11 |
| Ubiquinone and other terpenoid-quinone biosynthesis | 11 |
| Benzoate degradation | 10 |
| Biotin metabolism | 10 |
| Biosynthesis of unsaturated fatty acids | 9 |
| Drug metabolism - cytochrome P450 | 9 |
| Glycosylphosphatidylinositol (GPI)-anchor biosynthesis | 9 |
| Photosynthesis | 9 |
| Riboflavin metabolism | 9 |
| Glycosaminoglycan biosynthesis - chondroitin sulfate / dermatan sulfate | 8 |
| Other types of O-glycan biosynthesis | 8 |
| Steroid biosynthesis | 8 |
| Streptomycin biosynthesis | 8 |
| Arachidonic acid metabolism | 7 |
| C5-Branched dibasic acid metabolism | 7 |
| Caprolactam degradation | 7 |
| Chloroalkane and chloroalkene degradation | 7 |
| Ether lipid metabolism | 7 |
| Taurine and hypotaurine metabolism | 7 |
| Aminobenzoate degradation | 6 |
| Cyanoamino acid metabolism | 6 |
| Glycosaminoglycan biosynthesis - keratan sulfate | 6 |
| Glycosaminoglycan degradation | 6 |
| Isoquinoline alkaloid biosynthesis | 6 |
| Mannose type O-glycan biosyntheis | 6 |
| Monobactam biosynthesis | 6 |
| Vitamin B6 metabolism | 6 |
| Fatty acid elongation | 5 |
| Geraniol degradation | 5 |
| Glycosphingolipid biosynthesis - globo and isoglobo series | 5 |
| Glycosphingolipid biosynthesis - lacto and neolacto series | 5 |
| Mucin type O-glycan biosynthesis | 5 |
| Phosphonate and phosphinate metabolism | 5 |
| Steroid hormone biosynthesis | 5 |
| Synthesis and degradation of ketone bodies | 5 |
| Atrazine degradation | 4 |
| D-Glutamine and D-glutamate metabolism | 4 |
| Naphthalene degradation | 4 |
| Primary bile acid biosynthesis | 4 |
| Tropane, piperidine and pyridine alkaloid biosynthesis | 4 |
| alpha-Linolenic acid metabolism | 3 |
| Betalain biosynthesis | 3 |
| Dioxin degradation | 3 |
| Glucosinolate biosynthesis | 3 |
| Limonene and pinene degradation | 3 |
| Lipoic acid metabolism | 3 |
| Novobiocin biosynthesis | 3 |
| Other glycan degradation | 3 |
| Phenylpropanoid biosynthesis | 3 |
| Polyketide sugar unit biosynthesis | 3 |
| Prodigiosin biosyntheses | 3 |
| Steroid degradation | 3 |
| Styrene degradation | 3 |
| Xylene degradation | 3 |
| Acarbose and validamycin biosynthesis | 2 |
| Biosynthesis of siderophore group nonribosomal peptides | 2 |
| Caffeine metabolism | 2 |
| Carbapenem biosynthesis | 2 |
| Carotenoid biosynthesis | 2 |
| Chlorocyclohexane and chlorobenzene degradation | 2 |
| D-Alanine metabolism | 2 |
| Glycosphingolipid biosynthesis - ganglio series | 2 |
| Insect hormone biosynthesis | 2 |
| Phenazine biosynthesis | 2 |
| Aflatoxin biosynthesis | 1 |
| Biosynthesis of ansamycins | 1 |
| Biosynthesis of vancomycin group antibiotics | 1 |
| Cutin, suberine and wax biosynthesis | 1 |
| Flavone and flavonol biosynthesis | 1 |
| Fluorobenzoate degradation | 1 |
| Indole alkaloid biosynthesis | 1 |
| Linoleic acid metabolism | 1 |
| Neomycin, kanamycin and gentamicin biosynthesis | 1 |
| Nonribosomal peptide structures | 1 |
| Penicillin and cephalosporin biosynthesis | 1 |
| Secondary bile acid biosynthesis | 1 |
| Sesquiterpenoid and triterpenoid biosynthesis | 1 |
| Toluene degradation | 1 |
| Zeatin biosynthesis | 1 |
|  |  |
| **Genetic Information Processing** | |
| Ribosome | 74 |
| Ubiquitin mediated proteolysis | 50 |
| RNA transport | 44 |
| Spliceosome | 44 |
| Protein processing in endoplasmic reticulum | 43 |
| RNA degradation | 29 |
| Aminoacyl-tRNA biosynthesis | 28 |
| Homologous recombination | 28 |
| Ribosome biogenesis in eukaryotes | 28 |
| DNA replication | 24 |
| Nucleotide excision repair | 23 |
| mRNA surveillance pathway | 22 |
| Base excision repair | 21 |
| Mismatch repair | 20 |
| Proteasome | 17 |
| Protein export | 17 |
| RNA polymerase | 14 |
| Basal transcription factors | 11 |
| Fanconi anemia pathway | 10 |
| Sulfur relay system | 7 |
| SNARE interactions in vesicular transport | 5 |
| Non-homologous end-joining | 2 |
|  |  |
| **Environmental Information Processing** | |
| ABC transporters | 125 |
| Two-component system | 70 |
| PI3K-Akt signaling pathway | 52 |
| Neuroactive ligand-receptor interaction | 46 |
| mTOR signaling pathway | 45 |
| Hippo signaling pathway | 43 |
| MAPK signaling pathway | 43 |
| Calcium signaling pathway | 41 |
| Wnt signaling pathway | 40 |
| cAMP signaling pathway | 39 |
| AMPK signaling pathway | 34 |
| Ras signaling pathway | 34 |
| cGMP - PKG signaling pathway | 33 |
| FoxO signaling pathway | 31 |
| Rap1 signaling pathway | 30 |
| MAPK signaling pathway - fly | 28 |
| Apelin signaling pathway | 27 |
| Phospholipase D signaling pathway | 27 |
| Sphingolipid signaling pathway | 25 |
| Phosphatidylinositol signaling system | 23 |
| HIF-1 signaling pathway | 22 |
| Hippo signaling pathway -fly | 22 |
| Jak-STAT signaling pathway | 18 |
| NF-kappa B signaling pathway | 18 |
| TGF-beta signaling pathway | 18 |
| Bacterial secretion system | 17 |
| TNF signaling pathway | 16 |
| ErbB signaling pathway | 15 |
| ECM-receptor interaction | 13 |
| MAPK signaling pathway - yeast | 12 |
| Notch signaling pathway | 12 |
| Cell adhesion molecules (CAMs) | 11 |
| Hedgehog signaling pathway | 11 |
| Hedgehog signaling pathway - fly | 11 |
| Hippo signaling pathway - multiple species | 8 |
| VEGF signaling pathway | 8 |
| Phosphotransferase system (PTS) | 5 |
| Cytokine-cytokine receptor interaction | 4 |
| MAPK signaling pathway - plant | 3 |
| Plant hormone signal transduction | 2 |
|  |  |
| **Cellular Processes** | |
| Endocytosis | 60 |
| Quorum sensing | 44 |
| Tight junction | 38 |
| Lysosome | 36 |
| Signaling pathways regulating pluripotency of stem cells | 35 |
| Focal adhesion | 34 |
| Peroxisome | 34 |
| Regulation of actin cytoskeleton | 34 |
| Autophagy - animal | 30 |
| Cell cycle | 30 |
| Autophagy - yeast | 28 |
| Apoptosis | 27 |
| Necroptosis | 27 |
| Cell cycle - yeast | 26 |
| Cellular senescence | 26 |
| Flagellar assembly | 26 |
| Apoptosis - fly | 24 |
| Oocyte meiosis | 22 |
| Adherens junction | 19 |
| Phagosome | 18 |
| Gap junction | 17 |
| Meiosis - yeast | 15 |
| p53 signaling pathway | 15 |
| Bacterial chemotaxis | 14 |
| Biofilm formation - Escherichia coli | 13 |
| Cell cycle - Caulobacter | 11 |
| Biofilm formation - Vibrio cholerae | 10 |
| Autophagy - other eukaryotes | 9 |
| Mitophagy - animal | 9 |
| Mitophagy - yeast | 9 |
| Apoptosis - multiple species | 8 |
| Biofilm formation - Pseudomonas aeruginosa | 8 |
| Ferroptosis | 5 |
|  |  |
| **Organismal Systems** | |
| Thermogenesis | 61 |
| Axon guidance | 35 |
| Insulin signaling pathway | 35 |
| Thyroid hormone signaling pathway | 33 |
| NOD-like receptor signaling pathway | 29 |
| Oxytocin signaling pathway | 29 |
| Glucagon signaling pathway | 25 |
| Adrenergic signaling in cardiomyocytes | 24 |
| Dopaminergic synapse | 24 |
| Glutamatergic synapse | 24 |
| Retrograde endocannabinoid signaling | 24 |
| Cholinergic synapse | 23 |
| Longevity regulating pathway - mammal | 23 |
| Vascular smooth muscle contraction | 23 |
| Chemokine signaling pathway | 22 |
| Neurotrophin signaling pathway | 22 |
| Platelet activation | 22 |
| Serotonergic synapse | 21 |
| Circadian entrainment | 20 |
| GABAergic synapse | 20 |
| Longevity regulating pathway - worm | 20 |
| Salivary secretion | 20 |
| Synaptic vesicle cycle | 20 |
| Estrogen signaling pathway | 19 |
| Insulin secretion | 19 |
| Melanogenesis | 19 |
| Bile secretion | 18 |
| Cardiac muscle contraction | 18 |
| Osteoclast differentiation | 18 |
| PPAR signaling pathway | 18 |
| Fc gamma R-mediated phagocytosis | 17 |
| GnRH signaling pathway | 17 |
| Longevity regulating pathway - multiple species | 17 |
| Aldosterone synthesis and secretion | 16 |
| IL-17 signaling pathway | 16 |
| Leukocyte transendothelial migration | 16 |
| Relaxin signaling pathway | 16 |
| Inflammatory mediator regulation of TRP channels | 15 |
| Pancreatic secretion | 15 |
| Progesterone-mediated oocyte maturation | 15 |
| Renin secretion | 15 |
| Endocrine and other factor-regulated calcium reabsorption | 14 |
| Protein digestion and absorption | 14 |
| Adipocytokine signaling pathway | 13 |
| Gastric acid secretion | 13 |
| Toll-like receptor signaling pathway | 13 |
| Cortisol synthesis and secretion | 12 |
| Prolactin signaling pathway | 12 |
| Regulation of lipolysis in adipocyte | 11 |
| T cell receptor signaling pathway | 11 |
| B cell receptor signaling pathway | 9 |
| Carbohydrate digestion and absorption | 9 |
| Fc epsilon RI signaling pathway | 9 |
| Long-term depression | 9 |
| Long-term potentiation | 9 |
| Olfactory transduction | 9 |
| Proximal tubule bicarbonate reclamation | 9 |
| RIG-I-like receptor signaling pathway | 9 |
| Taste transduction | 9 |
| Th17 cell differentiation | 9 |
| Thyroid hormone synthesis | 9 |
| Vasopressin-regulated water reabsorption | 9 |
| Aldosterone-regulated sodium reabsorption | 8 |
| Cholesterol metabolism | 8 |
| Natural killer cell mediated cytotoxicity | 8 |
| Plant-pathogen interaction | 8 |
| Th1 and Th2 cell differentiation | 8 |
| Toll and Imd signaling pathway | 8 |
| Antigen processing and presentation | 7 |
| Cytosolic DNA-sensing pathway | 7 |
| Ovarian Steroidogenesis | 7 |
| Phototransduction - fly | 7 |
| Dorso-ventral axis formation | 6 |
| Circadian rhythm | 5 |
| Mineral absorption | 5 |
| Vitamin digestion and absorption | 5 |
| Collecting duct acid secretion | 4 |
| Fat digestion and absorption | 4 |
| Renin-angiotensin system | 4 |
| Circadian rhythm - fly | 2 |
| Complement and coagulation cascades | 2 |
| Phototransduction | 2 |
| Circadian rhythm - plant | 1 |
| Hematopoietic cell lineage | 1 |
|  |  |
| **Human Diseases** | |
| Pathways in cancer | 95 |
| Human papillomavirus infection | 72 |
| Huntington's disease | 56 |
| Epstein-Barr virus infection | 54 |
| HTLV-I infection | 49 |
| Proteoglycans in cancer | 46 |
| Viral carcinogenesis | 46 |
| Alzheimer's disease | 43 |
| Hepatocellular carcinoma | 42 |
| MicroRNAs in cancer | 41 |
| Non-alcoholic fatty liver disease (NAFLD | 38 |
| Transcriptional misregulation in cancers | 36 |
| Parkinson's disease | 36 |
| Cushing's syndrome | 34 |
| Gastric cancer | 31 |
| Herpes simplex infection | 31 |
| Breast cancer | 29 |
| Fluid shear stress and atherosclerosis | 27 |
| Insulin resistance | 26 |
| Kaposi's sarcoma-associated herpesvirus infection | 25 |
| Tuberculosis | 24 |
| Hepatitis B | 23 |
| Hepatitis C | 22 |
| Small cell lung cancer | 21 |
| Measles | 21 |
| Influenza A | 20 |
| Choline metabolism in cancer | 19 |
| Colorectal cancer | 19 |
| Morphine addiction | 19 |
| Basal cell carcinoma | 18 |
| Central carbon metabolism in cancer | 17 |
| Alcoholism | 17 |
| Bacterial invasion of epithelial cells | 17 |
| Prostate cancer | 16 |
| AGE-RAGE signaling pathway in diabetic complications | 16 |
| Toxoplasmosis | 16 |
| Endocrine resistance | 16 |
| Platinum drug resistance | 16 |
| Chronic myeloid leukemia | 15 |
| Renal cell carcinoma | 15 |
| Salmonella infection | 15 |
| EGFR tyrosine kinase inhibitor resistance | 15 |
| Non-small cell lung cancer | 14 |
| Arrhythmogenic right ventricular cardiomyopathy (ARVC) | 14 |
| Type II diabetes mellitus | 14 |
| Epithelial cell signaling in Helicobacter pylori infection | 14 |
| Amoebiasis | 14 |
| Acute myeloid leukemia | 13 |
| Endometrial cancer | 13 |
| Glioma | 13 |
| Dilated cardiomyopathy (DCM | 13 |
| Hypertrophic cardiomyopathy (HCM | 13 |
| Pathogenic Escherichia coli infection | 13 |
| Chagas disease (American trypanosomiasis | 13 |
| beta-Lactam resistance | 13 |
| Amphetamine addiction | 12 |
| Vibrio cholerae infection | 12 |
| Legionellosis | 11 |
| Shigellosis | 11 |
| Pancreatic cancer | 10 |
| Amyotrophic lateral sclerosis (ALS) | 10 |
| Pertussis | 10 |
| Antifolate resistance | 10 |
| Cocaine addiction | 9 |
| Maturity onset diabetes of the young | 9 |
| Cationic antimicrobial peptide (CAMP) resistance | 9 |
| Chemical carcinogenesis | 8 |
| Bladder cancer | 8 |
| Melanoma | 8 |
| Rheumatoid arthritis | 8 |
| Viral myocarditis | 8 |
| Vancomycin resistance | 7 |
| Thyroid cancer | 6 |
| Nicotine addiction | 6 |
| Leishmaniasis | 5 |
| Primary immunodeficiency | 4 |
| Systemic lupus erythematosus | 4 |
| Prion diseases | 4 |
| Inflammatory bowel disease (IBD) | 3 |
| African trypanosomiasis | 3 |
| Malaria | 3 |
| Type I diabetes mellitus | 2 |
| Staphylococcus aureus infection | 1 |
