# Supplementary material for: Data on genome annotation and analysis of earthworm Eisenia fetida
Source: Data Brief. 2018 Aug 29;20:525–34. doi: 10.1016/j.dib.2018.08.067 (PMC6126081; doi:10.1016/j.dib.2018.08.067)
Supplement: Supplementary file 5 — Supplementary material [file mmc5.docx]

Table S4: List of Riboflavin metabolism related enzymes showed BLAST sequence homology to bacterial sequences

| **SeqName** | **Description** | **Species** | **Length** | **e-Value** | **sim mean** |
| --- | --- | --- | --- | --- | --- |
| Efet.01.635643.g284.t1 | bifunctional 3,4-dihydroxy-2-butanone-4-phosphate synthase GTP cyclohydrolase II | Microbacteriaceae bacterium EaCA-K | 822 | 0 | 74.57 |
| Efet.01.77457.g143.t1 | bifunctional diaminohydroxyphosphoribosylaminopyrimidine deaminase 5-amino-6-(5-phosphoribosylamino)uracil reductase | Verminephrobacter eiseniae | 948 | 0 | 85.56 |
| Efet.01.651810.g218.t1 | 3,4-dihydroxy-2-butanone 4-phosphate synthase | Verminephrobacter eiseniae | 1122 | 0 | 91.49 |
| Efet.01.651567.g189.t1 | riboflavin synthase | Verminephrobacter eiseniae | 702 | 3.81E-158 | 85.22 |
| Efet.01.189547.g1856.t1 | acid phosphatase | Cytophagales bacterium TFI 002 | 789 | 1.25E-76 | 52.04 |
| Efet.01.1659341.g1571.t1 | restriction endonuclease | Verminephrobacter eiseniae | 840 | 4.35E-131 | 95.81 |
| Efet.01.591301.g1031.t1 | NUDIX hydrolase | Verminephrobacter eiseniae | 552 | 1.93E-133 | 93.88 |
