# Supplementary material for: Data on genome annotation and analysis of earthworm Eisenia fetida
Source: Data Brief. 2018 Aug 29;20:525–34. doi: 10.1016/j.dib.2018.08.067 (PMC6126081; doi:10.1016/j.dib.2018.08.067)
Supplement: Supplementary file 6 — Supplementary material [file mmc6.docx]

Table S5: List of stem cell and regeneration associated genes identified in the genome dataset of earthworm *Eisenia fetida*

| **SeqName** | **Description** | **Length** | **e-Value** | **Mean similarity (Percentage)** |
| --- | --- | --- | --- | --- |
| Efet.01.49266.g1547.t1 | Hepatocyte nuclear factor 6 | 684 | 1.48E-49 | 100 |
| Efet.01.606599.g285.t1 | SWI/SNF-related matrix-associated actin-dependent regulator of chromatin subfamily A member 5 | 213 | 2.75E-27 | 100 |
| Efet.01.283238.g1210.t1 | Tubulin alpha-1 chain | 453 | 1.35E-95 | 100 |
| Efet.01.283238.g1209.t1 | Tubulin alpha-3 chain | 225 | 1.84E-36 | 100 |
| Efet.01.165905.g792.t1 | Tubulin alpha-1C chain | 747 | 2.07E-178 | 99 |
| Efet.01.548251.g447.t1 | Histone deacetylase 2 | 552 | 1.31E-110 | 98 |
| Efet.01.658217.g1720.t1 | Tubulin alpha-1 chain | 1194 | 0 | 98 |
| Efet.01.149454.g1204.t1 | Tubulin alpha-1C chain | 420 | 4.82E-50 | 98 |
| Efet.01.95090.g1166.t1 | Steroid hormone receptor ERR2 | 408 | 2.92E-17 | 97 |
| Efet.01.126264.g74.t1 | Hepatocyte nuclear factor 6 | 975 | 5.91E-46 | 97 |
| Efet.01.477434.g699.t1 | Eukaryotic initiation factor 4A-I | 399 | 2.52E-86 | 97 |
| Efet.01.164462.g733.t1 | Homeobox protein Nkx-2.2 | 1194 | 5.65E-37 | 97 |
| Efet.01.214912.g625.t1 | PR domain zinc finger protein 16 | 1464 | 1.18E-47 | 97 |
| Efet.01.484317.g840.t1 | Sodium-dependent serotonin transporter | 420 | 4.65E-21 | 97 |
| Efet.01.288169.g1387.t1 | Tubulin alpha-1 chain | 294 | 7.12E-56 | 97 |
| Efet.01.33146.g561.t1 | Heat shock cognate 71 kDa protein | 1947 | 0 | 96 |
| Efet.01.167334.g857.t1 | Mitogen-activated protein kinase 3 | 267 | 1.91E-31 | 96 |
| Efet.01.506033.g151.t1 | Mitogen-activated protein kinase 8 | 261 | 3.67E-30 | 96 |
| Efet.01.313009.g414.t1 | Polyadenylate-binding protein 2 | 276 | 1.60E-50 | 96 |
| Efet.01.576541.g531.t1 | Semaphorin-2A | 201 | 1.57E-12 | 96 |
| Efet.01.89243.g848.t1 | Transcription factor Sox-2 | 717 | 3.01E-07 | 96 |
| Efet.01.247142.g1782.t1 | Translin | 204 | 2.08E-24 | 96 |
| Efet.01.229658.g1182.t1 | Hepatocyte nuclear factor 6 | 1167 | 1.06E-35 | 95 |
| Efet.01.75687.g40.t1 | Homeobox protein Hox-B4a | 276 | 1.60E-33 | 95 |
| Efet.01.370593.g571.t1 | Homeobox protein Hox-B4a | 276 | 1.60E-33 | 95 |
| Efet.01.109714.g504.t1 | Myocyte-specific enhancer factor 2C | 255 | 2.92E-34 | 95 |
| Efet.01.133754.g426.t1 | Myocyte-specific enhancer factor 2D | 324 | 1.51E-53 | 95 |
| Efet.01.425351.g603.t1 | Peroxiredoxin-1 | 222 | 2.98E-33 | 95 |
| Efet.01.129732.g252.t1 | Rho-associated protein kinase 1 | 471 | 1.32E-87 | 95 |
| Efet.01.216175.g670.t1 | Caspase-3 | 222 | 2.30E-12 | 94 |
| Efet.01.204119.g182.t1 | CCR4-NOT transcription complex subunit 1 | 414 | 2.94E-82 | 94 |
| Efet.01.61418.g708.t1 | ELAV-like protein 4 | 1083 | 7.50E-38 | 94 |
| Efet.01.431861.g775.t1 | Forkhead box protein D3 | 1113 | 7.51E-45 | 94 |
| Efet.01.638685.g459.t1 | Paired box protein Pax-3 | 813 | 5.96E-12 | 94 |
| Efet.01.67865.g1104.t1 | PR domain zinc finger protein 16 | 1947 | 7.51E-45 | 94 |
| Efet.01.67866.g1106.t1 | PR domain zinc finger protein 16 | 1671 | 2.40E-45 | 94 |
| Efet.01.89766.g878.t1 | Retinal homeobox protein Rx | 519 | 3.67E-27 | 94 |
| Efet.01.463449.g366.t1 | SWI/SNF-related matrix-associated actin-dependent regulator of chromatin subfamily A member 5 | 390 | 1.75E-77 | 94 |
| Efet.01.149454.g1205.t1 | Tubulin alpha-1C chain | 447 | 4.34E-87 | 94 |
| Efet.01.17573.g1294.t1 | Transcriptional enhancer factor TEF-4 | 960 | 1.41E-16 | 94 |
| Efet.01.498215.g1144.t1 | Transcriptional enhancer factor TEF-4 | 216 | 1.08E-21 | 94 |
| Efet.01.481492.g780.t1 | Glutamate receptor 3 | 210 | 7.57E-13 | 93 |
| Efet.01.419168.g443.t1 | Nuclear hormone receptor HR96 | 282 | 6.08E-14 | 93 |
| Efet.01.1656424.g864.t1 | ATP-dependent RNA helicase eIF4A | 456 | 6.03E-43 | 93 |
| Efet.01.98400.g1351.t1 | Serine/threonine-protein kinase D1 | 264 | 1.04E-34 | 93 |
| Efet.01.225823.g1029.t1 | Pancreas/duodenum homeobox protein 1 | 696 | 2.70E-32 | 93 |
| Efet.01.279682.g1088.t1 | Transforming protein RhoA | 579 | 1.50E-125 | 93 |
| Efet.01.622883.g1006.t1 | SWI/SNF-related matrix-associated actin-dependent regulator of chromatin subfamily A member 5 | 219 | 3.09E-41 | 93 |
| Efet.01.545636.g384.t1 | Zinc finger protein ZIC 5 | 459 | 2.77E-31 | 93 |
| Efet.01.67444.g1077.t1 | Protein arginine N-methyltransferase 1 | 720 | 5.95E-69 | 92 |
| Efet.01.440292.g955.t1 | Serine/threonine-protein kinase ATR | 222 | 2.81E-27 | 92 |
| Efet.01.37305.g814.t1 | Beta-enolase | 381 | 5.92E-71 | 92 |
| Efet.01.69614.g1214.t1 | Forkhead box protein O3 | 591 | 1.61E-30 | 92 |
| Efet.01.112926.g652.t1 | Glutamine--fructose-6-phosphate aminotransferase [isomerizing] 1 | 447 | 5.23E-44 | 92 |
| Efet.01.13456.g1006.t1 | Homeobox protein Hox-B3a | 429 | 6.35E-10 | 92 |
| Efet.01.86692.g733.t1 | Exosome RNA helicase MTR4 | 426 | 9.13E-48 | 92 |
| Efet.01.33291.g569.t1 | Receptor-type tyrosine-protein phosphatase S | 936 | 1.38E-82 | 92 |
| Efet.01.28183.g210.t1 | Transforming protein RhoA | 336 | 3.17E-60 | 92 |
| Efet.01.386779.g943.t1 | Transcriptional repressor protein YY1 | 321 | 3.89E-66 | 92 |
| Efet.01.1629507.g96.t1 | RAC serine/threonine-protein kinase | 213 | 1.80E-21 | 91 |
| Efet.01.279225.g1076.t1 | Forkhead box protein K1 | 768 | 3.58E-58 | 91 |
| Efet.01.200208.g10.t1 | Forkhead box protein O3 | 411 | 1.25E-28 | 91 |
| Efet.01.66985.g1043.t1 | Hepatocyte nuclear factor 6 | 318 | 5.77E-11 | 91 |
| Efet.01.378958.g777.t1 | Homeobox protein Hox-B3 | 276 | 3.28E-13 | 91 |
| Efet.01.296517.g1634.t1 | Stress-activated protein kinase jnk-1 | 252 | 4.52E-16 | 91 |
| Efet.01.191906.g1955.t1 | Potassium voltage-gated channel subfamily B member 1 | 381 | 8.08E-33 | 91 |
| Efet.01.621517.g938.t1 | Myogenin | 867 | 8.11E-15 | 91 |
| Efet.01.140993.g826.t1 | Homeobox protein Nkx-2.2 | 1128 | 2.99E-25 | 91 |
| Efet.01.43469.g1200.t1 | PR domain zinc finger protein 16 | 660 | 4.35E-51 | 91 |
| Efet.01.182350.g1514.t1 | DNA topoisomerase 1 | 426 | 2.94E-73 | 91 |
| Efet.01.122424.g1169.t1 | DNA topoisomerase 2-alpha | 636 | 2.68E-111 | 91 |
| Efet.01.15111.g1108.t1 | Y-box-binding protein 3 | 345 | 1.43E-20 | 91 |
| Efet.01.188016.g1788.t1 | RAC-alpha serine/threonine-protein kinase | 258 | 1.32E-16 | 90 |
| Efet.01.8099.g598.t1 | Atrial natriuretic peptide receptor 1 | 474 | 1.72E-13 | 90 |
| Efet.01.81263.g423.t1 | C-1-tetrahydrofolate synthase, cytoplasmic | 798 | 4.23E-69 | 90 |
| Efet.01.1636384.g176.t1 | Forkhead box protein O3 | 408 | 7.48E-28 | 90 |
| Efet.01.251256.g45.t1 | Hepatocyte nuclear factor 6 | 408 | 4.50E-26 | 90 |
| Efet.01.407960.g194.t1 | Insulin-like receptor | 225 | 3.63E-41 | 90 |
| Efet.01.156672.g349.t1 | Insulin gene enhancer protein isl-1 | 405 | 3.55E-39 | 90 |
| Efet.01.19309.g1429.t1 | Inactive histone-lysine N-methyltransferase 2E | 411 | 4.25E-08 | 90 |
| Efet.01.388165.g973.t1 | E3 ubiquitin-protein ligase MYCBP2 | 246 | 2.90E-20 | 90 |
| Efet.01.76552.g91.t1 | Paired box protein Pax-3 | 495 | 9.16E-72 | 90 |
| Efet.01.4658.g372.t1 | Receptor-type tyrosine-protein phosphatase S | 468 | 6.43E-07 | 90 |
| Efet.01.1616067.g35.t1 | Receptor-type tyrosine-protein phosphatase S | 231 | 8.50E-39 | 90 |
| Efet.01.640207.g562.t1 | Protein SOX-15 | 1032 | 1.54E-20 | 90 |
| Efet.01.143193.g935.t1 | Spectrin beta chain, non-erythrocytic 1 | 417 | 1.48E-64 | 90 |
| Efet.01.36410.g759.t1 | Cohesin subunit SA-1 | 243 | 3.05E-35 | 90 |
| Efet.01.122416.g1168.t1 | CREB-binding protein | 477 | 9.85E-28 | 89 |
| Efet.01.277700.g1022.t1 | CREB-binding protein | 1605 | 9.48E-09 | 89 |
| Efet.01.73235.g1435.t1 | Early growth response protein 1 | 939 | 7.88E-51 | 89 |
| Efet.01.331361.g947.t1 | Transforming protein p54/c-ets-1 | 423 | 2.97E-61 | 89 |
| Efet.01.488594.g932.t1 | Forkhead box protein D3 | 1176 | 1.58E-48 | 89 |
| Efet.01.151662.g100.t1 | Potassium voltage-gated channel subfamily B member 1 | 558 | 1.20E-22 | 89 |
| Efet.01.285725.g1292.t1 | Potassium voltage-gated channel subfamily B member 1 | 543 | 5.78E-64 | 89 |
| Efet.01.284368.g1248.t1 | Krueppel-like factor 2 | 813 | 2.33E-47 | 89 |
| Efet.01.376846.g723.t1 | S-adenosylmethionine synthase isoform type-1 | 588 | 2.65E-80 | 89 |
| Efet.01.191246.g1926.t1 | S-adenosylmethionine synthase isoform type-1 | 423 | 7.82E-66 | 89 |
| Efet.01.199090.g2262.t1 | Proliferating cell nuclear antigen | 537 | 2.11E-74 | 89 |
| Efet.01.38090.g867.t1 | Receptor-type tyrosine-protein phosphatase S | 351 | 3.47E-45 | 89 |
| Efet.01.192821.g1995.t1 | Receptor-type tyrosine-protein phosphatase S | 384 | 2.77E-53 | 89 |
| Efet.01.492122.g1000.t1 | Dexamethasone-induced Ras-related protein 1 | 378 | 9.41E-38 | 89 |
| Efet.01.585143.g834.t1 | Dexamethasone-induced Ras-related protein 1 | 429 | 8.57E-39 | 89 |
| Efet.01.12365.g921.t1 | Sodium channel protein type 9 subunit alpha | 714 | 1.24E-10 | 89 |
| Efet.01.18451.g1368.t1 | Structural maintenance of chromosomes protein 1A | 636 | 7.14E-103 | 89 |
| Efet.01.307677.g230.t1 | Zinc finger protein SNAI1 | 285 | 2.16E-51 | 89 |
| Efet.01.144207.g984.t1 | Transcription factor Sp7 | 1383 | 2.14E-61 | 89 |
| Efet.01.609804.g415.t1 | Serum response factor | 747 | 1.70E-66 | 89 |
| Efet.01.649489.g1319.t1 | Zinc finger homeobox protein 3 | 4503 | 3.25E-29 | 89 |
| Efet.01.22003.g1616.t1 | CREB-binding protein | 291 | 3.06E-20 | 88 |
| Efet.01.164554.g740.t1 | Cyclin-dependent kinase 9 | 930 | 5.82E-146 | 88 |
| Efet.01.285802.g1294.t1 | Alpha-(1,3)-fucosyltransferase 4 | 264 | 1.11E-07 | 88 |
| Efet.01.85730.g680.t1 | Hepatocyte nuclear factor 4-alpha | 369 | 2.22E-20 | 88 |
| Efet.01.647165.g1160.t1 | Heat shock 70 kDa protein cognate 1 | 459 | 2.73E-44 | 88 |
| Efet.01.109476.g496.t1 | Eukaryotic initiation factor 4A-I | 342 | 1.28E-13 | 88 |
| Efet.01.50347.g29.t1 | Insulin gene enhancer protein ISL-1 | 210 | 3.13E-28 | 88 |
| Efet.01.332814.g985.t1 | Kinesin-like protein KIF3C | 270 | 5.10E-19 | 88 |
| Efet.01.15446.g1135.t1 | Krueppel-like factor 2 | 480 | 4.31E-45 | 88 |
| Efet.01.49096.g1531.t1 | Octopamine receptor beta-3R | 504 | 5.61E-22 | 88 |
| Efet.01.9200.g671.t1 | Pumilio homolog 2 | 222 | 2.25E-31 | 88 |
| Efet.01.35339.g697.t1 | SMARCA4 isoform 2 | 213 | 1.32E-19 | 88 |
| Efet.01.68810.g1159.t1 | Sal-like protein 1 | 2889 | 9.85E-27 | 88 |
| Efet.01.590974.g1022.t1 | SWI/SNF-related matrix-associated actin-dependent regulator of chromatin subfamily B member 1 | 411 | 3.11E-69 | 88 |
| Efet.01.528100.g640.t1 | Transcription factor Sox-11 | 1236 | 8.07E-15 | 88 |
| Efet.01.517829.g437.t1 | Protein SOX-15 | 978 | 1.27E-20 | 88 |
| Efet.01.645117.g932.t1 | Transcription factor Sox-2 | 555 | 2.13E-08 | 88 |
| Efet.01.349344.g1391.t1 | Spectrin beta chain, non-erythrocytic 1 | 534 | 1.34E-82 | 88 |
| Efet.01.307809.g233.t1 | Thymidylate synthase | 759 | 2.66E-152 | 88 |
| Efet.01.139553.g749.t1 | Exportin-7 | 201 | 1.16E-17 | 88 |
| Efet.01.194757.g2084.t1 | Chromodomain-helicase-DNA-binding protein 1 | 489 | 4.20E-54 | 87 |
| Efet.01.599318.g1286.t1 | Catenin alpha-1 | 273 | 2.53E-33 | 87 |
| Efet.01.560299.g7.t1 | Early growth response protein 1 | 1008 | 4.93E-43 | 87 |
| Efet.01.47558.g1448.t1 | ELAV-like protein 4 | 399 | 4.90E-44 | 87 |
| Efet.01.421099.g505.t1 | Transforming protein p54/c-ets-1 | 405 | 6.16E-11 | 87 |
| Efet.01.108854.g460.t1 | Forkhead box protein C2 | 834 | 4.00E-34 | 87 |
| Efet.01.318200.g571.t1 | Forkhead box protein C2 | 396 | 2.70E-15 | 87 |
| Efet.01.436356.g862.t1 | Forkhead box protein C2 | 831 | 1.31E-37 | 87 |
| Efet.01.284106.g1241.t1 | ATP-dependent RNA helicase eIF4A | 225 | 2.28E-31 | 87 |
| Efet.01.323858.g739.t1 | Potassium voltage-gated channel subfamily A member 5 | 852 | 2.12E-48 | 87 |
| Efet.01.41250.g1070.t1 | Krueppel-like factor 2 | 1365 | 1.78E-44 | 87 |
| Efet.01.124124.g1245.t1 | Krueppel-like factor 2 | 1470 | 3.99E-44 | 87 |
| Efet.01.13773.g1025.t1 | Methionine aminopeptidase 2 | 1005 | 0 | 87 |
| Efet.01.119260.g997.t1 | Mediator of RNA polymerase II transcription subunit 12 | 318 | 5.39E-13 | 87 |
| Efet.01.648808.g1271.t1 | Paired box protein Pax-6 | 447 | 4.68E-58 | 87 |
| Efet.01.115691.g814.t1 | Ribose-phosphate pyrophosphokinase 1 | 249 | 7.40E-07 | 87 |
| Efet.01.369822.g540.t1 | CAD protein | 567 | 2.05E-79 | 87 |
| Efet.01.30014.g340.t1 | CAD protein | 300 | 4.75E-45 | 87 |
| Efet.01.144382.g989.t1 | Dexamethasone-induced Ras-related protein 1 | 297 | 3.28E-41 | 87 |
| Efet.01.94160.g1112.t1 | Reticulon-4 | 225 | 9.51E-11 | 87 |
| Efet.01.286082.g1305.t1 | Transcription factor Sox-2 | 216 | 1.52E-16 | 87 |
| Efet.01.274369.g890.t1 | DNA topoisomerase 2-alpha | 246 | 6.10E-15 | 87 |
| Efet.01.281756.g1147.t1 | DNA topoisomerase 2-alpha | 330 | 2.01E-50 | 87 |
| Efet.01.271034.g785.t1 | Protein atonal homolog 1 | 675 | 9.82E-15 | 86 |
| Efet.01.271035.g786.t1 | Protein atonal homolog 1 | 279 | 6.64E-15 | 86 |
| Efet.01.284289.g1246.t1 | Cyclin-dependent kinase 2 | 330 | 3.00E-17 | 86 |
| Efet.01.16540.g1221.t1 | Early growth response protein 1 | 729 | 2.79E-56 | 86 |
| Efet.01.68476.g1134.t1 | Receptor tyrosine-protein kinase erbB-4 | 417 | 2.19E-24 | 86 |
| Efet.01.10434.g766.t1 | Glutamine--fructose-6-phosphate aminotransferase [isomerizing] 1 | 735 | 1.41E-124 | 86 |
| Efet.01.532551.g76.t1 | Glycine N-methyltransferase | 210 | 4.18E-32 | 86 |
| Efet.01.201782.g89.t1 | Hepatocyte nuclear factor 6 | 849 | 3.81E-33 | 86 |
| Efet.01.203005.g143.t1 | Histone-lysine N-methyltransferase 2A | 453 | 1.89E-79 | 86 |
| Efet.01.91669.g979.t1 | Mitogen-activated protein kinase kinase kinase 12 | 2133 | 3.17E-162 | 86 |
| Efet.01.168137.g896.t1 | Paired box protein Pax-2a | 1527 | 7.18E-09 | 86 |
| Efet.01.216349.g678.t1 | Pumilio homolog 2 | 219 | 2.86E-32 | 86 |
| Efet.01.37663.g833.t1 | Kruppel-like factor 5 | 270 | 1.15E-21 | 86 |
| Efet.01.171047.g1014.t1 | Kruppel-like factor 5 | 444 | 2.08E-27 | 86 |
| Efet.01.173336.g1109.t1 | SWI/SNF-related matrix-associated actin-dependent regulator of chromatin subfamily A containing DEAD/H box 1 | 291 | 8.20E-42 | 86 |
| Efet.01.658359.g1891.t1 | Transcription factor Sp1 | 1275 | 6.52E-48 | 86 |
| Efet.01.204094.g181.t1 | Spectrin beta chain, non-erythrocytic 1 | 879 | 1.83E-127 | 86 |
| Efet.01.38168.g875.t1 | Spectrin alpha chain, non-erythrocytic 1 | 438 | 1.27E-73 | 86 |
| Efet.01.36288.g750.t1 | Sterol regulatory element-binding protein 1 | 1203 | 4.95E-09 | 86 |
| Efet.01.28205.g212.t1 | TGF-beta receptor type-1 | 525 | 1.99E-39 | 86 |
| Efet.01.625531.g1123.t1 | APOBEC1 complementation factor | 696 | 1.64E-111 | 85 |
| Efet.01.657910.g1530.t1 | APOBEC1 complementation factor | 804 | 4.00E-109 | 85 |
| Efet.01.26825.g124.t1 | Atrial natriuretic peptide receptor 1 | 633 | 2.53E-75 | 85 |
| Efet.01.540423.g280.t1 | Collagen alpha-1(II) chain | 264 | 5.02E-07 | 85 |
| Efet.01.121668.g1127.t1 | Collagen alpha-1(II) chain | 294 | 2.15E-15 | 85 |
| Efet.01.333614.g1006.t1 | Citron Rho-interacting kinase | 234 | 2.80E-21 | 85 |
| Efet.01.316410.g520.t1 | Dihydropyrimidinase-related protein 2 | 264 | 3.09E-21 | 85 |
| Efet.01.10983.g806.t1 | Dual specificity protein phosphatase 1 | 501 | 2.52E-07 | 85 |
| Efet.01.169384.g948.t1 | Early growth response protein 1 | 1467 | 1.94E-54 | 85 |
| Efet.01.419320.g447.t1 | Histone acetyltransferase p300 | 315 | 8.13E-31 | 85 |
| Efet.01.5919.g472.t1 | Glial fibrillary acidic protein | 444 | 2.43E-06 | 85 |
| Efet.01.1647785.g386.t1 | Nuclear hormone receptor HR96 | 243 | 1.16E-14 | 85 |
| Efet.01.1652197.g548.t1 | Homeobox protein Hox-B3a | 357 | 9.28E-22 | 85 |
| Efet.01.86998.g747.t1 | Histone acetyltransferase KAT2B | 324 | 5.08E-43 | 85 |
| Efet.01.496426.g1092.t1 | Homeobox protein Nkx-2.2 | 507 | 2.52E-41 | 85 |
| Efet.01.11025.g810.t1 | Peroxiredoxin 1 | 255 | 1.63E-43 | 85 |
| Efet.01.97750.g1326.t1 | Receptor-type tyrosine-protein phosphatase S | 423 | 1.34E-67 | 85 |
| Efet.01.178385.g1327.t1 | Dexamethasone-induced Ras-related protein 1 | 300 | 1.88E-30 | 85 |
| Efet.01.391709.g1042.t1 | Repulsive guidance molecule A | 207 | 5.70E-17 | 85 |
| Efet.01.18734.g1391.t1 | Sodium channel protein type 9 subunit alpha | 207 | 6.26E-12 | 85 |
| Efet.01.40511.g1023.t1 | Protein SET | 747 | 1.73E-111 | 85 |
| Efet.01.1638252.g192.t1 | Protein SET | 318 | 3.44E-52 | 85 |
| Efet.01.597763.g1235.t1 | SWI/SNF-related matrix-associated actin-dependent regulator of chromatin subfamily A member 5 | 468 | 2.11E-62 | 85 |
| Efet.01.3356.g287.t1 | Spectrin beta chain, non-erythrocytic 1 | 210 | 1.15E-11 | 85 |
| Efet.01.135314.g509.t1 | Zinc finger homeobox protein 3 | 5415 | 3.51E-30 | 85 |
| Efet.01.403416.g89.t1 | 5-hydroxytryptamine receptor 2C | 465 | 3.50E-09 | 84 |
| Efet.01.467801.g475.t1 | 5-hydroxytryptamine receptor 2C | 465 | 3.50E-09 | 84 |
| Efet.01.482711.g802.t1 | 5-hydroxytryptamine receptor 2C | 273 | 1.07E-11 | 84 |
| Efet.01.10868.g796.t1 | Histone acetyltransferase p300 | 2145 | 1.11E-126 | 84 |
| Efet.01.609219.g381.t1 | Histone acetyltransferase p300 | 654 | 1.57E-99 | 84 |
| Efet.01.541011.g291.t1 | Receptor tyrosine-protein kinase erbB-4 | 213 | 1.03E-12 | 84 |
| Efet.01.52888.g200.t1 | Forkhead box protein D3 | 762 | 1.18E-44 | 84 |
| Efet.01.539567.g261.t1 | Forkhead box protein N1 | 597 | 1.45E-24 | 84 |
| Efet.01.74244.g1501.t1 | ATP-dependent RNA helicase eIF4A | 384 | 1.42E-38 | 84 |
| Efet.01.1603858.g5.t1 | Histone acetyltransferase KAT6A | 243 | 3.81E-36 | 84 |
| Efet.01.2417.g220.t1 | Potassium voltage-gated channel subfamily B member 1 | 597 | 1.51E-34 | 84 |
| Efet.01.636970.g358.t1 | Lysine-specific demethylase 4A | 465 | 6.03E-68 | 84 |
| Efet.01.425067.g594.t1 | Mitogen-activated protein kinase 8 | 378 | 2.25E-41 | 84 |
| Efet.01.143720.g959.t1 | Protein CBFA2T3 | 393 | 1.22E-16 | 84 |
| Efet.01.37915.g860.t1 | Exosome RNA helicase MTR4 | 291 | 7.05E-30 | 84 |
| Efet.01.257560.g281.t1 | Receptor-type tyrosine-protein phosphatase S | 525 | 4.97E-84 | 84 |
| Efet.01.193627.g2027.t1 | Double-strand-break repair protein rad21 homolog | 378 | 3.45E-61 | 84 |
| Efet.01.98961.g1385.t1 | Retinal homeobox protein Rx3 | 384 | 5.87E-12 | 84 |
| Efet.01.339857.g1170.t1 | Retinoic acid receptor RXR-alpha | 402 | 1.36E-63 | 84 |
| Efet.01.3010.g266.t1 | Sodium channel protein type 1 subunit alpha | 234 | 2.25E-15 | 84 |
| Efet.01.204442.g193.t1 | Protein SET | 645 | 1.76E-110 | 84 |
| Efet.01.143193.g934.t1 | Spectrin beta chain, non-erythrocytic 1 | 375 | 2.75E-43 | 84 |
| Efet.01.1611785.g24.t1 | Protein unc-112 | 264 | 8.51E-39 | 84 |
| Efet.01.1648656.g414.t1 | Vasopressin V1a receptor | 264 | 2.06E-09 | 84 |
| Efet.01.65277.g928.t1 | Zinc finger protein ZIC 5 | 756 | 4.46E-34 | 84 |
| Efet.01.443588.g1075.t1 | Angiotensin-converting enzyme | 294 | 1.57E-35 | 83 |
| Efet.01.382296.g849.t1 | Acidic leucine-rich nuclear phosphoprotein 32 family member A | 774 | 1.80E-29 | 83 |
| Efet.01.96548.g1249.t1 | Atrial natriuretic peptide receptor 1 | 366 | 7.66E-51 | 83 |
| Efet.01.658238.g1742.t1 | Armadillo segment polarity protein | 729 | 7.07E-117 | 83 |
| Efet.01.1655655.g776.t1 | Cholecystokinin receptor type A | 366 | 1.95E-09 | 83 |
| Efet.01.552187.g556.t1 | Catenin beta-1 | 1011 | 3.98E-158 | 83 |
| Efet.01.575832.g511.t1 | ELAV-like protein 4 | 351 | 6.67E-44 | 83 |
| Efet.01.142614.g913.t1 | Protein C-ets-1 | 240 | 1.80E-36 | 83 |
| Efet.01.14047.g1038.t1 | Gamma-aminobutyric acid receptor subunit beta-3 | 264 | 4.53E-10 | 83 |
| Efet.01.43558.g1203.t1 | PDZ domain-containing protein GIPC1 | 321 | 5.12E-32 | 83 |
| Efet.01.70274.g1245.t1 | Potassium voltage-gated channel subfamily B member 1 | 498 | 6.31E-54 | 83 |
| Efet.01.653239.g411.t1 | Plasma kallikrein | 231 | 3.93E-29 | 83 |
| Efet.01.1632263.g119.t1 | Serine/threonine-protein kinase D1 | 270 | 3.22E-21 | 83 |
| Efet.01.458423.g233.t1 | Homeobox protein Nkx-2.2 | 1074 | 1.05E-38 | 83 |
| Efet.01.71244.g1312.t1 | Phosphatidylinositol 3-kinase regulatory subunit alpha | 294 | 8.05E-20 | 83 |
| Efet.01.145589.g1041.t1 | RNA polymerase II-associated factor 1 homolog | 351 | 1.86E-56 | 83 |
| Efet.01.160557.g552.t1 | Paxillin | 228 | 8.21E-13 | 83 |
| Efet.01.164747.g750.t1 | PR domain zinc finger protein 14 | 348 | 1.16E-34 | 83 |
| Efet.01.504193.g105.t1 | Tyrosine-protein phosphatase non-receptor type 1 | 222 | 5.35E-16 | 83 |
| Efet.01.179781.g1389.t1 | SWI/SNF-related matrix-associated actin-dependent regulator of chromatin subfamily A member 5 | 243 | 2.39E-12 | 83 |
| Efet.01.555586.g647.t1 | Transcription factor SOX-17 | 684 | 1.17E-15 | 83 |
| Efet.01.341935.g1207.t1 | Extracellular sulfatase Sulf-2 | 339 | 3.26E-43 | 83 |
| Efet.01.336665.g1083.t1 | Vacuolar protein sorting-associated protein 35 | 354 | 5.57E-54 | 83 |
| Efet.01.365305.g414.t1 | Protein Wnt-4 | 453 | 1.45E-76 | 83 |
| Efet.01.294664.g1586.t1 | GTP-binding protein ypt1 | 312 | 1.45E-29 | 83 |
| Efet.01.157687.g407.t1 | Beta-2 adrenergic receptor | 270 | 7.81E-06 | 82 |
| Efet.01.84712.g607.t1 | CREB-binding protein | 1695 | 8.73E-47 | 82 |
| Efet.01.393823.g1094.t1 | CREB-binding protein | 219 | 1.54E-30 | 82 |
| Efet.01.413150.g296.t1 | Dihydrofolate reductase | 282 | 8.44E-08 | 82 |
| Efet.01.210912.g482.t1 | Early growth response protein 1 | 1503 | 4.56E-48 | 82 |
| Efet.01.209460.g427.t1 | Forkhead box protein C2 | 987 | 1.66E-32 | 82 |
| Efet.01.106547.g344.t1 | Protein furry homolog | 447 | 8.84E-61 | 82 |
| Efet.01.152059.g121.t1 | Hepatocyte nuclear factor 4-alpha | 978 | 2.05E-58 | 82 |
| Efet.01.357018.g197.t1 | DNA-binding protein inhibitor ID-2 | 435 | 1.14E-15 | 82 |
| Efet.01.1642338.g254.t1 | Potassium voltage-gated channel subfamily B member 1 | 213 | 1.13E-13 | 82 |
| Efet.01.154297.g215.t1 | Inactive histone-lysine N-methyltransferase 2E | 822 | 8.86E-29 | 82 |
| Efet.01.15175.g1113.t1 | Protein kinase C theta type | 246 | 6.01E-19 | 82 |
| Efet.01.67351.g1069.t1 | POU domain protein | 1530 | 1.11E-17 | 82 |
| Efet.01.485993.g877.t1 | Hepatocyte growth factor receptor | 303 | 1.31E-33 | 82 |
| Efet.01.252382.g84.t1 | Nascent polypeptide-associated complex subunit alpha | 417 | 1.70E-54 | 82 |
| Efet.01.76614.g95.t1 | Nanos homolog 1 | 567 | 1.54E-22 | 82 |
| Efet.01.57438.g464.t1 | Neurogenic differentiation factor 1 | 1200 | 4.65E-42 | 82 |
| Efet.01.324510.g755.t1 | Octopamine receptor beta-2R | 372 | 1.68E-12 | 82 |
| Efet.01.89518.g861.t1 | CAD protein | 621 | 3.51E-43 | 82 |
| Efet.01.318739.g586.t1 | SMARCA4 isoform 2 | 1080 | 1.36E-40 | 82 |
| Efet.01.1632648.g125.t1 | Double-strand-break repair protein rad21 homolog | 243 | 1.59E-16 | 82 |
| Efet.01.119082.g989.t1 | RNA polymerase-associated protein RTF1 homolog | 684 | 7.66E-23 | 82 |
| Efet.01.546654.g401.t1 | RNA polymerase-associated protein RTF1 homolog | 315 | 5.47E-15 | 82 |
| Efet.01.53253.g219.t1 | Sonic hedgehog protein | 204 | 1.35E-27 | 82 |
| Efet.01.209105.g413.t1 | SWI/SNF complex subunit SMARCC1 | 651 | 7.54E-54 | 82 |
| Efet.01.168675.g921.t1 | Transcription factor Sox-11 | 1152 | 1.22E-36 | 82 |
| Efet.01.64637.g892.t1 | Transcription factor Sox-2 | 792 | 9.83E-34 | 82 |
| Efet.01.3977.g334.t1 | Transcription factor Sp7 | 1290 | 4.25E-55 | 82 |
| Efet.01.536272.g190.t1 | Spectrin beta chain, non-erythrocytic 1 | 249 | 1.82E-15 | 82 |
| Efet.01.271724.g814.t1 | Protein Wnt-2 | 249 | 6.75E-26 | 82 |
| Efet.01.62256.g768.t1 | Aldehyde dehydrogenase, dimeric NADP-preferring | 369 | 3.58E-26 | 81 |
| Efet.01.132302.g364.t1 | Fructose-bisphosphate aldolase 1 | 426 | 1.48E-59 | 81 |
| Efet.01.1649227.g430.t1 | Fructose-bisphosphate aldolase 1 | 297 | 7.88E-41 | 81 |
| Efet.01.563274.g97.t1 | Metal response element binding transcription factor 2, isoform CRA_c | 498 | 2.00E-14 | 81 |
| Efet.01.194970.g2089.t1 | Chromobox protein homolog 1 | 363 | 7.14E-14 | 81 |
| Efet.01.158093.g428.t1 | Chromodomain-helicase-DNA-binding protein 7 | 300 | 2.23E-24 | 81 |
| Efet.01.303057.g94.t1 | Histone acetyltransferase p300 | 435 | 3.90E-62 | 81 |
| Efet.01.409839.g220.t1 | Forkhead box protein C2 | 1578 | 1.93E-35 | 81 |
| Efet.01.153418.g184.t1 | Forkhead box protein C2 | 534 | 2.84E-25 | 81 |
| Efet.01.465638.g410.t1 | Gamma-aminobutyric acid receptor subunit beta-3 | 405 | 1.96E-14 | 81 |
| Efet.01.310939.g346.t1 | Glutamate receptor 3 | 354 | 1.27E-48 | 81 |
| Efet.01.292570.g1528.t1 | Hepatocyte nuclear factor 6 | 264 | 3.07E-29 | 81 |
| Efet.01.545625.g382.t1 | Hepatocyte nuclear factor 6 | 1092 | 4.13E-48 | 81 |
| Efet.01.119304.g1000.t1 | Heterogeneous nuclear ribonucleoprotein K | 213 | 1.40E-21 | 81 |
| Efet.01.465072.g403.t1 | Potassium voltage-gated channel subfamily B member 1 | 753 | 1.75E-21 | 81 |
| Efet.01.611250.g470.t1 | Potassium voltage-gated channel subfamily B member 1 | 1077 | 1.53E-72 | 81 |
| Efet.01.137525.g641.t1 | Lysine-specific histone demethylase 1A | 525 | 1.03E-33 | 81 |
| Efet.01.237261.g1454.t1 | Lysine-specific demethylase 6B | 726 | 1.10E-104 | 81 |
| Efet.01.215456.g643.t1 | Histone-lysine N-methyltransferase 2C | 258 | 3.04E-39 | 81 |
| Efet.01.231723.g1267.t1 | DNA replication licensing factor MCM2 | 297 | 3.12E-35 | 81 |
| Efet.01.243407.g1650.t1 | Microspherule protein 1 | 207 | 3.50E-14 | 81 |
| Efet.01.168338.g902.t1 | Canalicular multispecific organic anion transporter 2 | 501 | 7.50E-69 | 81 |
| Efet.01.457023.g178.t1 | Homeobox protein Nkx-2.2 | 678 | 2.02E-31 | 81 |
| Efet.01.42547.g1143.t1 | Pancreas/duodenum homeobox protein 1 | 351 | 5.84E-13 | 81 |
| Efet.01.71577.g1338.t1 | Prohibitin-1 | 420 | 1.35E-17 | 81 |
| Efet.01.166830.g831.t1 | Receptor-type tyrosine-protein phosphatase S | 1416 | 6.16E-125 | 81 |
| Efet.01.564043.g128.t1 | Receptor-type tyrosine-protein phosphatase S | 252 | 3.17E-32 | 81 |
| Efet.01.85650.g669.t1 | Sodium-dependent serotonin transporter | 474 | 3.75E-08 | 81 |
| Efet.01.79666.g301.t1 | Sodium channel protein type 9 subunit alpha | 489 | 1.13E-45 | 81 |
| Efet.01.225198.g1010.t1 | Splicing factor 3A subunit 3 | 324 | 2.15E-46 | 81 |
| Efet.01.106921.g356.t1 | Zinc finger protein SNAI1 | 903 | 2.56E-53 | 81 |
| Efet.01.91379.g959.t1 | Transcription factor Sox-9 | 345 | 2.39E-11 | 81 |
| Efet.01.55694.g370.t1 | Transcription factor Sp7 | 942 | 3.31E-55 | 81 |
| Efet.01.93216.g1063.t1 | Spectrin alpha chain, non-erythrocytic 1 | 1176 | 1.36E-166 | 81 |
| Efet.01.2135.g188.t1 | Protein Wnt-5b | 330 | 3.27E-46 | 81 |
| Efet.01.231215.g1235.t1 | GTP-binding protein YPT1 | 336 | 2.26E-21 | 81 |
| Efet.01.452829.g63.t1 | C-1-tetrahydrofolate synthase, cytoplasmic | 324 | 7.30E-31 | 80 |
| Efet.01.11855.g878.t1 | Cholecystokinin receptor type A | 375 | 2.87E-20 | 80 |
| Efet.01.159951.g520.t1 | Cyclin-dependent kinase 1 | 492 | 7.45E-21 | 80 |
| Efet.01.114928.g781.t1 | Early growth response protein 1 | 1182 | 2.19E-55 | 80 |
| Efet.01.609920.g423.t1 | Ephrin type-A receptor 4 | 441 | 3.43E-59 | 80 |
| Efet.01.44786.g1289.t1 | Forkhead box protein C2 | 987 | 1.26E-32 | 80 |
| Efet.01.80477.g367.t1 | Forkhead box protein C2 | 1185 | 3.34E-29 | 80 |
| Efet.01.75217.g17.t1 | Forkhead box protein O3 | 477 | 1.13E-21 | 80 |
| Efet.01.658034.g1569.t1 | Frizzled-7 | 939 | 1.41E-152 | 80 |
| Efet.01.77639.g152.t1 | Gamma-aminobutyric acid receptor subunit beta-3 | 501 | 5.36E-23 | 80 |
| Efet.01.288723.g1404.t1 | Glutamine--fructose-6-phosphate aminotransferase [isomerizing] 1 | 318 | 2.43E-39 | 80 |
| Efet.01.645478.g988.t1 | Glutamate receptor 3 | 321 | 2.29E-49 | 80 |
| Efet.01.648009.g1213.t1 | Glutamate receptor 3 | 378 | 5.16E-43 | 80 |
| Efet.01.1583077.g6.t1 | Homeobox protein GHOX-7 | 210 | 3.83E-19 | 80 |
| Efet.01.107849.g403.t1 | Heterogeneous nuclear ribonucleoprotein K | 411 | 4.60E-16 | 80 |
| Efet.01.452987.g67.t1 | Heat shock 70 kDa protein | 204 | 1.08E-23 | 80 |
| Efet.01.1658296.g1151.t1 | Eukaryotic initiation factor 4A-I | 225 | 1.78E-18 | 80 |
| Efet.01.554796.g628.t1 | Potassium voltage-gated channel subfamily B member 1 | 219 | 4.99E-11 | 80 |
| Efet.01.124318.g1254.t1 | Potassium channel subfamily K member 3 | 645 | 3.47E-34 | 80 |
| Efet.01.155565.g283.t1 | Lysine-specific demethylase 6B | 612 | 1.80E-33 | 80 |
| Efet.01.458128.g221.t1 | Muscleblind-like protein 3 | 237 | 4.95E-19 | 80 |
| Efet.01.152924.g156.t1 | Neuroendocrine convertase 1 | 354 | 8.64E-08 | 80 |
| Efet.01.227313.g1092.t1 | Nuclear factor 1 C-type | 474 | 2.61E-70 | 80 |
| Efet.01.143799.g964.t1 | Homeobox protein Nkx-2.2 | 1050 | 1.08E-36 | 80 |
| Efet.01.303724.g124.t1 | Tyrosine-protein phosphatase non-receptor type 11 | 273 | 1.58E-16 | 80 |
| Efet.01.227532.g1100.t1 | 2-iminobutanoate/2-iminopropanoate deaminase | 354 | 2.08E-40 | 80 |
| Efet.01.164424.g732.t1 | Sodium channel protein type 9 subunit alpha | 528 | 1.85E-06 | 80 |
| Efet.01.602849.g116.t1 | STE20-like serine/threonine-protein kinase | 276 | 1.12E-38 | 80 |
| Efet.01.82953.g520.t1 | SWI/SNF complex subunit SMARCC1 | 327 | 8.44E-36 | 80 |
| Efet.01.255662.g208.t1 | Transcription factor Sox-11 | 612 | 2.71E-26 | 80 |
| Efet.01.68528.g1136.t1 | Transcription factor Sox-2 | 336 | 1.97E-30 | 80 |
| Efet.01.23732.g1723.t1 | Spectrin alpha chain, non-erythrocytic 1 | 291 | 1.19E-27 | 80 |
| Efet.01.88883.g830.t1 | Src substrate cortactin | 300 | 1.20E-34 | 80 |
| Efet.01.30272.g356.t1 | TGF-beta receptor type-1 | 591 | 1.03E-93 | 80 |
| Efet.01.219841.g801.t1 | Transcription intermediary factor 1-alpha | 588 | 3.06E-21 | 80 |
| Efet.01.182660.g1532.t1 | TNF receptor-associated factor 6 | 330 | 7.96E-15 | 80 |
| Efet.01.262367.g466.t1 | Short transient receptor potential channel 4 | 222 | 9.41E-09 | 80 |
| Efet.01.441490.g1019.t1 | Protein Wnt-7b | 426 | 4.42E-23 | 80 |
| Efet.01.109096.g474.t1 | Serine/threonine-protein kinase YPK2/YKR2 | 210 | 1.45E-16 | 80 |
| Efet.01.1654352.g684.t1 | 5-hydroxytryptamine receptor 2C | 393 | 1.17E-17 | 79 |
| Efet.01.237901.g1476.t1 | RAC-beta serine/threonine-protein kinase | 288 | 8.14E-30 | 79 |
| Efet.01.297493.g1672.t1 | Achaete-scute homolog 1 | 615 | 2.39E-08 | 79 |
| Efet.01.1653352.g609.t1 | Chromodomain-helicase-DNA-binding protein 7 | 282 | 8.32E-33 | 79 |
| Efet.01.228341.g1145.t1 | Catenin alpha-1 | 222 | 1.42E-17 | 79 |
| Efet.01.186217.g1697.t1 | Early growth response protein 1 | 435 | 5.10E-23 | 79 |
| Efet.01.1635692.g163.t1 | Receptor tyrosine-protein kinase erbB-4 | 270 | 5.40E-19 | 79 |
| Efet.01.15180.g1114.t1 | Protein C-ets-1 | 510 | 1.36E-15 | 79 |
| Efet.01.454345.g99.t1 | Forkhead box protein C2 | 837 | 6.25E-23 | 79 |
| Efet.01.499170.g1157.t1 | Forkhead box protein C2 | 480 | 1.52E-28 | 79 |
| Efet.01.645075.g925.t1 | Tyrosine-protein kinase Fyn | 405 | 5.95E-51 | 79 |
| Efet.01.406286.g153.t1 | Glutamine synthetase | 321 | 1.68E-24 | 79 |
| Efet.01.368523.g498.t1 | Glutamate receptor 3 | 390 | 2.62E-58 | 79 |
| Efet.01.343830.g1247.t1 | Glutamate receptor 3 | 315 | 3.61E-22 | 79 |
| Efet.01.78970.g242.t1 | Hepatocyte nuclear factor 6 | 1146 | 1.03E-40 | 79 |
| Efet.01.14155.g1048.t1 | Nuclear hormone receptor HR96 | 327 | 2.06E-31 | 79 |
| Efet.01.405260.g127.t1 | Potassium voltage-gated channel subfamily A member 5 | 300 | 1.43E-28 | 79 |
| Efet.01.258956.g337.t1 | Potassium voltage-gated channel subfamily A member 5 | 552 | 2.99E-50 | 79 |
| Efet.01.651897.g233.t1 | Potassium voltage-gated channel subfamily A member 5 | 603 | 2.51E-42 | 79 |
| Efet.01.214501.g614.t1 | Mitogen-activated protein kinase 7 | 540 | 1.72E-70 | 79 |
| Efet.01.1635333.g160.t1 | Serine/threonine-protein kinase mTOR | 279 | 2.47E-14 | 79 |
| Efet.01.439588.g938.t1 | Exosome RNA helicase MTR4 | 414 | 8.41E-57 | 79 |
| Efet.01.104975.g273.t1 | Myogenic factor 6 | 1179 | 3.82E-23 | 79 |
| Efet.01.1658488.g1194.t1 | Netrin-1 | 927 | 1.17E-102 | 79 |
| Efet.01.1633285.g131.t1 | BDNF/NT-3 growth factors receptor | 216 | 2.64E-22 | 79 |
| Efet.01.561911.g37.t1 | One cut domain family member 2 | 234 | 1.80E-19 | 79 |
| Efet.01.575763.g507.t1 | Paired box protein Pax-3 | 438 | 3.10E-18 | 79 |
| Efet.01.53633.g240.t1 | Serine/threonine-protein kinase pim-1 | 651 | 1.87E-77 | 79 |
| Efet.01.394995.g1117.t1 | Ras-related protein Rab-1A | 204 | 9.48E-13 | 79 |
| Efet.01.23372.g1693.t1 | Sodium-dependent serotonin transporter | 234 | 1.95E-08 | 79 |
| Efet.01.332080.g965.t1 | Septin-4 | 393 | 2.97E-48 | 79 |
| Efet.01.115608.g806.t1 | Proto-oncogene tyrosine-protein kinase Src | 879 | 1.20E-134 | 79 |
| Efet.01.314519.g458.t1 | GTP-binding protein YPT1 | 381 | 3.83E-21 | 79 |
| Efet.01.652346.g281.t1 | Apoptosis-inducing factor 1, mitochondrial | 393 | 6.97E-46 | 78 |
| Efet.01.11294.g827.t1 | Atrial natriuretic peptide receptor 1 | 1962 | 5.66E-57 | 78 |
| Efet.01.76544.g90.t1 | Atrial natriuretic peptide receptor 1 | 285 | 6.61E-25 | 78 |
| Efet.01.92849.g1042.t1 | Atrial natriuretic peptide receptor 1 | 549 | 5.77E-11 | 78 |
| Efet.01.597232.g1201.t1 | Achaete-scute homolog 1 | 975 | 1.45E-11 | 78 |
| Efet.01.560587.g15.t1 | G1/S-specific cyclin-D1 | 279 | 3.97E-19 | 78 |
| Efet.01.1658305.g1153.t1 | Cystic fibrosis transmembrane conductance regulator | 324 | 5.87E-06 | 78 |
| Efet.01.73716.g1460.t1 | Dystrobrevin alpha | 492 | 3.11E-20 | 78 |
| Efet.01.18228.g1350.t1 | Epidermal growth factor receptor | 222 | 2.26E-21 | 78 |
| Efet.01.76316.g83.t1 | ELAV-like protein 4 | 246 | 2.46E-17 | 78 |
| Efet.01.11926.g884.t1 | Histone acetyltransferase p300 | 618 | 1.29E-63 | 78 |
| Efet.01.277850.g1026.t1 | Four and a half LIM domains protein 2 | 570 | 1.10E-73 | 78 |
| Efet.01.553238.g577.t1 | Forkhead box protein J3 | 321 | 4.73E-16 | 78 |
| Efet.01.291697.g1501.t1 | Protein furry homolog | 498 | 1.25E-14 | 78 |
| Efet.01.562974.g70.t1 | Gamma-aminobutyric acid receptor subunit beta-3 | 327 | 8.40E-32 | 78 |
| Efet.01.38735.g918.t1 | Guanine nucleotide-binding protein G(s) subunit alpha isoforms XLas | 276 | 3.40E-15 | 78 |
| Efet.01.1654027.g655.t1 | Glutamate receptor 3 | 621 | 9.88E-77 | 78 |
| Efet.01.14025.g1036.t1 | Glutamate receptor 3 | 222 | 4.39E-30 | 78 |
| Efet.01.276854.g989.t1 | Hypoxia-inducible factor 1-alpha | 267 | 2.27E-16 | 78 |
| Efet.01.58581.g526.t1 | Homeobox protein Hox-B4 | 426 | 1.53E-09 | 78 |
| Efet.01.247722.g1814.t1 | Potassium voltage-gated channel subfamily A member 5 | 441 | 5.02E-17 | 78 |
| Efet.01.54970.g333.t1 | Potassium voltage-gated channel subfamily B member 1 | 894 | 1.44E-74 | 78 |
| Efet.01.51926.g127.t1 | Inactive histone-lysine N-methyltransferase 2E | 804 | 1.94E-32 | 78 |
| Efet.01.510023.g246.t1 | Protein kinase C theta type | 288 | 2.39E-19 | 78 |
| Efet.01.402937.g78.t1 | Pyruvate kinase PKM | 738 | 7.01E-100 | 78 |
| Efet.01.444232.g1089.t1 | Homeobox protein Nkx-2.2 | 933 | 4.02E-31 | 78 |
| Efet.01.139887.g762.t1 | Neurogenic locus notch homolog protein 2 | 1206 | 2.84E-21 | 78 |
| Efet.01.136977.g601.t1 | Octopamine receptor beta-2R | 450 | 5.00E-12 | 78 |
| Efet.01.224599.g982.t1 | Octopamine receptor beta-2R | 837 | 1.46E-31 | 78 |
| Efet.01.532190.g70.t1 | Cellular tumor antigen p53 | 348 | 4.08E-17 | 78 |
| Efet.01.3425.g297.t1 | Serine/threonine-protein kinase pim-1 | 330 | 1.67E-39 | 78 |
| Efet.01.383830.g885.t1 | PR domain zinc finger protein 16 | 2088 | 6.42E-51 | 78 |
| Efet.01.229837.g1192.t1 | MYC associated factor X | 225 | 1.46E-08 | 78 |
| Efet.01.201860.g95.t1 | Ras-related protein Rab-30 | 216 | 2.11E-18 | 78 |
| Efet.01.494785.g1066.t1 | Ras-related C3 botulinum toxin substrate 1 | 264 | 6.16E-07 | 78 |
| Efet.01.348061.g1359.t1 | Regucalcin | 201 | 5.55E-07 | 78 |
| Efet.01.120981.g1089.t1 | Reticulon-4 | 201 | 2.91E-16 | 78 |
| Efet.01.47442.g1443.t1 | Retinoic acid receptor RXR-alpha | 417 | 1.18E-30 | 78 |
| Efet.01.337302.g1098.t1 | Structural maintenance of chromosomes protein 1A | 411 | 8.46E-33 | 78 |
| Efet.01.177689.g1297.t1 | Spectrin beta chain, non-erythrocytic 1 | 516 | 7.55E-53 | 78 |
| Efet.01.214447.g612.t1 | Tubulin alpha-1C chain | 1206 | 0 | 78 |
| Efet.01.312693.g400.t1 | FAD-linked sulfhydryl oxidase ALR | 462 | 1.59E-19 | 77 |
| Efet.01.493038.g1020.t1 | Beta-1,4-galactosyltransferase 1 | 237 | 7.36E-24 | 77 |
| Efet.01.189875.g1865.t1 | Cholecystokinin receptor type A | 432 | 2.40E-12 | 77 |
| Efet.01.117982.g924.t1 | Chromodomain-helicase-DNA-binding protein 1 | 357 | 1.52E-57 | 77 |
| Efet.01.64708.g898.t1 | DNA damage-binding protein 1 | 810 | 4.79E-106 | 77 |
| Efet.01.89937.g891.t1 | Epithelial discoidin domain-containing receptor 1 | 306 | 1.64E-25 | 77 |
| Efet.01.40211.g1001.t1 | 6-phosphofructo-2-kinase/fructose-2,6-bisphosphatase 1 | 297 | 1.76E-29 | 77 |
| Efet.01.39498.g964.t1 | Gamma-aminobutyric acid receptor subunit beta-3 | 321 | 5.83E-17 | 77 |
| Efet.01.235102.g1385.t1 | Gamma-aminobutyric acid receptor subunit beta-3 | 372 | 1.29E-10 | 77 |
| Efet.01.289275.g1427.t1 | Gelsolin | 243 | 5.86E-13 | 77 |
| Efet.01.635054.g271.t1 | Glutamate receptor 3 | 501 | 4.14E-66 | 77 |
| Efet.01.73780.g1461.t1 | Histamine H1 receptor | 327 | 8.81E-27 | 77 |
| Efet.01.70976.g1294.t1 | Histamine H2 receptor | 507 | 1.09E-16 | 77 |
| Efet.01.636379.g332.t1 | Heat shock cognate 71 kDa protein | 888 | 4.55E-71 | 77 |
| Efet.01.251356.g50.t1 | Heat shock cognate 71 kDa protein | 585 | 1.47E-54 | 77 |
| Efet.01.165193.g763.t1 | ATP-dependent RNA helicase eIF4A | 339 | 7.08E-35 | 77 |
| Efet.01.63299.g817.t1 | Inhibitor of growth protein 5 | 480 | 1.09E-20 | 77 |
| Efet.01.71449.g1329.t1 | Histone-lysine N-methyltransferase 2D | 285 | 2.42E-23 | 77 |
| Efet.01.86115.g697.t1 | Serine/threonine-protein kinase D1 | 276 | 5.28E-07 | 77 |
| Efet.01.38849.g923.t1 | Protein kinase C theta type | 318 | 4.99E-08 | 77 |
| Efet.01.163117.g668.t1 | Raf homolog serine/threonine-protein kinase phl | 654 | 3.25E-09 | 77 |
| Efet.01.260625.g395.t1 | Laminin subunit beta-2 | 204 | 5.98E-24 | 77 |
| Efet.01.116376.g854.t1 | [F-actin]-monooxygenase MICAL2 | 3777 | 5.45E-12 | 77 |
| Efet.01.584882.g815.t1 | Canalicular multispecific organic anion transporter 1 | 279 | 5.52E-23 | 77 |
| Efet.01.206375.g279.t1 | Musculin | 726 | 3.44E-09 | 77 |
| Efet.01.85563.g660.t1 | Unconventional myosin-Va | 285 | 1.19E-22 | 77 |
| Efet.01.152908.g155.t1 | Neurogenic differentiation factor 1 | 831 | 1.18E-14 | 77 |
| Efet.01.30813.g401.t1 | Nuclear factor interleukin-3-regulated protein | 540 | 2.29E-21 | 77 |
| Efet.01.60291.g640.t1 | Neurogenic locus notch homolog protein 2 | 768 | 2.75E-07 | 77 |
| Efet.01.540295.g276.t1 | Polyadenylate-binding protein 2 | 261 | 1.72E-06 | 77 |
| Efet.01.26374.g104.t1 | Paired box protein Pax-2 | 1038 | 3.96E-61 | 77 |
| Efet.01.523793.g548.t1 | Paired box protein Pax-2 | 948 | 3.54E-47 | 77 |
| Efet.01.544334.g345.t1 | Serine/threonine-protein kinase pim-1 | 345 | 3.97E-29 | 77 |
| Efet.01.518849.g454.t1 | Kruppel-like factor 5 | 1164 | 1.63E-35 | 77 |
| Efet.01.654000.g516.t1 | Kruppel-like factor 5 | 1356 | 8.12E-37 | 77 |
| Efet.01.644427.g876.t1 | Ribonucleoside-diphosphate reductase large subunit | 654 | 9.56E-89 | 77 |
| Efet.01.596412.g1189.t1 | Ribonucleoside-diphosphate reductase large subunit | 588 | 1.59E-81 | 77 |
| Efet.01.150032.g1.t1 | Runt-related transcription factor 1 | 378 | 3.35E-39 | 77 |
| Efet.01.543615.g333.t1 | Sodium channel protein type 9 subunit alpha | 288 | 1.38E-25 | 77 |
| Efet.01.528379.g646.t1 | Transient receptor potential-gamma protein | 399 | 1.05E-39 | 77 |
| Efet.01.121746.g1131.t1 | 5-hydroxytryptamine receptor 2A | 1296 | 1.85E-52 | 76 |
| Efet.01.128888.g218.t1 | APOBEC1 complementation factor | 423 | 2.06E-18 | 76 |
| Efet.01.628471.g1246.t1 | RAC-alpha serine/threonine-protein kinase | 387 | 9.87E-34 | 76 |
| Efet.01.20280.g1498.t1 | RAC-beta serine/threonine-protein kinase | 687 | 1.22E-18 | 76 |
| Efet.01.267259.g627.t1 | Fructose-bisphosphate aldolase 1 | 477 | 3.38E-57 | 76 |
| Efet.01.151815.g108.t1 | Atrial natriuretic peptide receptor 1 | 228 | 1.11E-22 | 76 |
| Efet.01.384324.g894.t1 | Atrial natriuretic peptide receptor 1 | 294 | 6.48E-32 | 76 |
| Efet.01.573678.g425.t1 | Atrial natriuretic peptide receptor 1 | 207 | 4.51E-26 | 76 |
| Efet.01.257694.g285.t1 | Adenomatous polyposis coli protein | 2379 | 3.47E-68 | 76 |
| Efet.01.575796.g509.t1 | Set1/Ash2 histone methyltransferase complex subunit ASH2 | 255 | 5.93E-26 | 76 |
| Efet.01.75418.g21.t1 | Axin-1 | 216 | 5.42E-07 | 76 |
| Efet.01.10287.g751.t1 | Calpain-3 | 225 | 7.56E-12 | 76 |
| Efet.01.142275.g891.t1 | Catenin alpha-1 | 771 | 4.57E-92 | 76 |
| Efet.01.156546.g342.t1 | 6-phosphofructo-2-kinase/fructose-2,6-bisphosphatase 1 | 1248 | 0 | 76 |
| Efet.01.18489.g1370.t1 | Protein flightless-1 homolog | 231 | 2.28E-18 | 76 |
| Efet.01.529093.g668.t1 | Gamma-aminobutyric acid receptor subunit beta-3 | 252 | 3.07E-24 | 76 |
| Efet.01.383036.g866.t1 | High mobility group protein B2 | 351 | 1.44E-34 | 76 |
| Efet.01.305666.g196.t1 | Histamine H2 receptor | 474 | 1.46E-26 | 76 |
| Efet.01.514423.g348.t1 | Histamine H2 receptor | 399 | 1.62E-19 | 76 |
| Efet.01.640793.g591.t1 | Heat shock 70 kDa protein A | 705 | 2.99E-82 | 76 |
| Efet.01.169097.g940.t1 | Potassium voltage-gated channel subfamily A member 5 | 1407 | 3.91E-143 | 76 |
| Efet.01.291609.g1499.t1 | Mast/stem cell growth factor receptor kita | 291 | 2.07E-11 | 76 |
| Efet.01.22077.g1621.t1 | Protein kinase C theta type | 255 | 9.34E-23 | 76 |
| Efet.01.24261.g1762.t1 | Pyruvate kinase PKM | 1206 | 1.37E-159 | 76 |
| Efet.01.32294.g509.t1 | LARGE xylosyl- and glucuronyltransferase 1 | 948 | 2.98E-120 | 76 |
| Efet.01.256676.g249.t1 | Mitogen-activated protein kinase 14A | 216 | 1.66E-11 | 76 |
| Efet.01.85704.g673.t1 | Neurogenic locus notch homolog protein 2 | 237 | 3.44E-14 | 76 |
| Efet.01.185812.g1672.t1 | Neurogenic locus Notch protein | 288 | 1.27E-20 | 76 |
| Efet.01.69.g7.t1 | Poly [ADP-ribose] polymerase 1 | 318 | 5.62E-28 | 76 |
| Efet.01.288672.g1402.t1 | E3 ubiquitin-protein ligase pellino homolog 1 | 906 | 1.52E-71 | 76 |
| Efet.01.154996.g257.t1 | Serine/threonine-protein kinase pim-1 | 657 | 1.57E-91 | 76 |
| Efet.01.21931.g1608.t1 | 1-phosphatidylinositol 4,5-bisphosphate phosphodiesterase gamma-1 | 399 | 6.07E-25 | 76 |
| Efet.01.366886.g456.t1 | Peroxidasin homolog | 1137 | 2.04E-122 | 76 |
| Efet.01.523430.g540.t1 | Kruppel-like factor 5 | 1392 | 6.99E-35 | 76 |
| Efet.01.87075.g749.t1 | Rho-related GTP-binding protein RhoA-B | 558 | 1.08E-65 | 76 |
| Efet.01.339069.g1139.t1 | Runt-related transcription factor 1 | 369 | 2.02E-30 | 76 |
| Efet.01.288799.g1408.t1 | Sodium channel protein type 1 subunit alpha | 297 | 2.46E-23 | 76 |
| Efet.01.499285.g1158.t1 | Sodium channel protein type 9 subunit alpha | 291 | 1.23E-06 | 76 |
| Efet.01.584181.g796.t1 | Sodium channel protein type 9 subunit alpha | 384 | 4.51E-33 | 76 |
| Efet.01.438635.g914.t1 | Septin-4 | 201 | 8.68E-14 | 76 |
| Efet.01.204865.g209.t1 | Protein SOX-15 | 1053 | 1.08E-29 | 76 |
| Efet.01.185967.g1688.t1 | Transcription factor 21 | 726 | 6.99E-15 | 76 |
| Efet.01.213325.g563.t1 | TGF-beta receptor type-1 | 846 | 5.37E-110 | 76 |
| Efet.01.423054.g543.t1 | DNA topoisomerase 2-alpha | 366 | 1.14E-23 | 76 |
| Efet.01.184536.g1613.t1 | Vang-like protein 2 | 483 | 2.01E-41 | 76 |
| Efet.01.70021.g1234.t1 | 5-hydroxytryptamine receptor 1A | 1197 | 8.49E-20 | 75 |
| Efet.01.93459.g1071.t1 | 5-hydroxytryptamine receptor 2C | 417 | 5.02E-14 | 75 |
| Efet.01.276696.g984.t1 | APOBEC1 complementation factor | 330 | 9.43E-24 | 75 |
| Efet.01.605483.g226.t1 | Probable ATP-dependent permease | 1665 | 1.76E-06 | 75 |
| Efet.01.426161.g624.t1 | Serine/threonine-protein kinase akt-2 | 315 | 4.68E-24 | 75 |
| Efet.01.617314.g770.t1 | MGA protein | 303 | 3.88E-20 | 75 |
| Efet.01.165203.g764.t1 | Cadherin-99C | 261 | 3.37E-06 | 75 |
| Efet.01.62034.g752.t1 | Chromodomain-helicase-DNA-binding protein 7 | 537 | 1.41E-42 | 75 |
| Efet.01.505473.g138.t1 | Chromodomain-helicase-DNA-binding protein 7 | 207 | 9.66E-25 | 75 |
| Efet.01.178337.g1326.t1 | CCR4-NOT transcription complex subunit 1 | 513 | 1.40E-38 | 75 |
| Efet.01.303502.g112.t1 | Dystrophin | 342 | 8.21E-26 | 75 |
| Efet.01.228315.g1144.t1 | ELAV-like protein 4 | 381 | 2.25E-43 | 75 |
| Efet.01.366129.g435.t1 | Fibroblast growth factor receptor homolog 2 | 462 | 6.52E-47 | 75 |
| Efet.01.12578.g939.t1 | Flotillin-2a | 306 | 7.83E-26 | 75 |
| Efet.01.45649.g1333.t1 | Forkhead box protein C2 | 696 | 1.73E-61 | 75 |
| Efet.01.310554.g334.t1 | Forkhead box protein O3 | 1716 | 5.11E-10 | 75 |
| Efet.01.38448.g897.t1 | Tyrosine-protein kinase Fyn | 297 | 1.11E-33 | 75 |
| Efet.01.68319.g1125.t1 | Glial fibrillary acidic protein | 294 | 5.93E-07 | 75 |
| Efet.01.257315.g276.t1 | PDZ domain-containing protein GIPC1 | 345 | 2.09E-13 | 75 |
| Efet.01.78498.g209.t1 | Hypoxia-inducible factor 1-alpha | 375 | 2.00E-36 | 75 |
| Efet.01.94444.g1133.t1 | High mobility group protein B2 | 774 | 7.12E-07 | 75 |
| Efet.01.199281.g2270.t1 | Hepatocyte nuclear factor 4-alpha | 693 | 1.33E-43 | 75 |
| Efet.01.31806.g476.t1 | Hepatocyte nuclear factor 6 | 1017 | 3.05E-68 | 75 |
| Efet.01.596027.g1180.t1 | Heat shock protein 60, mitochondrial | 939 | 4.36E-104 | 75 |
| Efet.01.85824.g687.t1 | Probable heat shock protein ssa2 | 273 | 6.64E-15 | 75 |
| Efet.01.384045.g889.t1 | Heat shock 70 kDa protein 4 | 339 | 1.60E-09 | 75 |
| Efet.01.59126.g574.t1 | Homeobox protein Hox-B4 | 645 | 4.26E-31 | 75 |
| Efet.01.183541.g1574.t1 | Homeobox protein Hox-B4 | 612 | 2.93E-08 | 75 |
| Efet.01.11981.g889.t1 | DNA-binding protein inhibitor ID-2 | 345 | 2.97E-09 | 75 |
| Efet.01.512803.g301.t1 | Transcription factor jun-D | 1062 | 2.74E-17 | 75 |
| Efet.01.402143.g54.t1 | Potassium voltage-gated channel subfamily A member 5 | 747 | 5.88E-59 | 75 |
| Efet.01.216873.g696.t1 | Potassium voltage-gated channel subfamily B member 1 | 567 | 8.74E-63 | 75 |
| Efet.01.303844.g131.t1 | Potassium voltage-gated channel subfamily B member 1 | 876 | 9.56E-64 | 75 |
| Efet.01.206322.g275.t1 | Potassium channel subfamily K member 3 | 489 | 1.66E-33 | 75 |
| Efet.01.331228.g945.t1 | Potassium channel subfamily K member 3 | 351 | 3.35E-35 | 75 |
| Efet.01.147229.g1108.t1 | Laminin subunit beta-2 | 630 | 2.82E-74 | 75 |
| Efet.01.99237.g1402.t1 | Low-density lipoprotein receptor-related protein 2 | 213 | 1.34E-26 | 75 |
| Efet.01.157126.g374.t1 | Neurogenic differentiation factor 1 | 672 | 1.79E-13 | 75 |
| Efet.01.349899.g1403.t1 | Nuclear factor 1 C-type | 939 | 6.97E-33 | 75 |
| Efet.01.78755.g224.t1 | Nuclear factor interleukin-3-regulated protein | 1044 | 8.03E-24 | 75 |
| Efet.01.129913.g261.t1 | Neurogenic locus notch homolog protein 1 | 234 | 2.15E-24 | 75 |
| Efet.01.404361.g108.t1 | Octopamine receptor beta-2R | 402 | 3.94E-25 | 75 |
| Efet.01.492264.g1003.t1 | Paired box protein Pax-7 | 249 | 1.64E-14 | 75 |
| Efet.01.262752.g484.t1 | Retinal homeobox protein Rx | 348 | 2.01E-11 | 75 |
| Efet.01.525220.g588.t1 | Retinoic acid receptor RXR-alpha | 573 | 1.05E-29 | 75 |
| Efet.01.461650.g328.t1 | Sodium channel protein type 1 subunit alpha | 279 | 3.38E-33 | 75 |
| Efet.01.148480.g1160.t1 | SWI/SNF complex subunit SMARCC1 | 285 | 2.64E-22 | 75 |
| Efet.01.32174.g499.t1 | SWI/SNF-related matrix-associated actin-dependent regulator of chromatin subfamily D member 1 | 777 | 2.33E-138 | 75 |
| Efet.01.64936.g905.t1 | Speedy protein A | 357 | 3.68E-10 | 75 |
| Efet.01.328571.g878.t1 | Spectrin beta chain, non-erythrocytic 1 | 444 | 4.81E-47 | 75 |
| Efet.01.492942.g1018.t1 | Proto-oncogene tyrosine-protein kinase Src | 213 | 7.79E-20 | 75 |
| Efet.01.192196.g1969.t1 | Tyrosine protein-kinase src-1 | 216 | 6.68E-19 | 75 |
| Efet.01.9872.g720.t1 | Serrate RNA effector molecule homolog | 261 | 1.05E-13 | 75 |
| Efet.01.299564.g1732.t1 | Transient receptor potential-gamma protein | 414 | 1.99E-35 | 75 |
| Efet.01.221949.g893.t1 | Protein Wnt-5b | 564 | 7.33E-73 | 75 |
| Efet.01.258575.g319.t1 | X-box-binding protein 1 | 1545 | 1.24E-15 | 75 |
| Efet.01.289040.g1419.t1 | GTP-binding protein YPT1 | 237 | 1.21E-18 | 75 |
| Efet.01.70917.g1288.t1 | Zinc finger protein ZIC 3 | 870 | 2.87E-06 | 75 |
| Efet.01.637720.g383.t1 | 5-hydroxytryptamine receptor 2C | 417 | 2.80E-13 | 74 |
| Efet.01.116098.g837.t1 | RAC-alpha serine/threonine-protein kinase | 489 | 1.03E-52 | 74 |
| Efet.01.539084.g254.t1 | Atrial natriuretic peptide receptor 1 | 279 | 1.57E-18 | 74 |
| Efet.01.439950.g948.t1 | Calpain-3 | 432 | 2.38E-46 | 74 |
| Efet.01.297736.g1681.t1 | Chromodomain Y-like protein | 231 | 1.16E-08 | 74 |
| Efet.01.246246.g1749.t1 | CCAAT/enhancer-binding protein beta | 1221 | 4.69E-14 | 74 |
| Efet.01.647387.g1174.t1 | Carbohydrate sulfotransferase 3 | 204 | 5.76E-06 | 74 |
| Efet.01.386635.g940.t1 | Serine/threonine-protein kinase cst-1 | 246 | 1.04E-08 | 74 |
| Efet.01.151411.g87.t1 | Protein decapentaplegic | 1155 | 2.39E-24 | 74 |
| Efet.01.443028.g1066.t1 | ETS-related transcription factor Elf-4 | 1197 | 1.01E-30 | 74 |
| Efet.01.289879.g1443.t1 | Histone acetyltransferase p300 | 903 | 4.47E-40 | 74 |
| Efet.01.637884.g389.t1 | Four and a half LIM domains protein 2 | 207 | 1.52E-17 | 74 |
| Efet.01.369719.g537.t1 | Forkhead box protein K1 | 396 | 7.23E-29 | 74 |
| Efet.01.160017.g521.t1 | PDZ domain-containing protein GIPC1 | 705 | 2.85E-81 | 74 |
| Efet.01.308044.g238.t1 | Histamine H1 receptor | 408 | 4.67E-26 | 74 |
| Efet.01.275054.g924.t1 | Histamine H2 receptor | 345 | 1.65E-06 | 74 |
| Efet.01.276480.g979.t1 | Insulinoma-associated protein 1 | 759 | 6.38E-24 | 74 |
| Efet.01.260885.g408.t1 | Potassium voltage-gated channel subfamily A member 5 | 342 | 5.27E-25 | 74 |
| Efet.01.70274.g1246.t1 | Potassium voltage-gated channel subfamily B member 1 | 537 | 4.19E-30 | 74 |
| Efet.01.282511.g1177.t1 | Potassium voltage-gated channel subfamily B member 1 | 327 | 1.79E-31 | 74 |
| Efet.01.10032.g735.t1 | Krueppel-like factor 9 | 750 | 5.39E-33 | 74 |
| Efet.01.194197.g2057.t1 | Pyruvate kinase PKM | 231 | 2.66E-17 | 74 |
| Efet.01.94957.g1155.t1 | Protein CBFA2T3 | 231 | 2.69E-08 | 74 |
| Efet.01.500598.g12.t1 | Nascent polypeptide-associated complex subunit alpha | 318 | 1.94E-20 | 74 |
| Efet.01.567190.g237.t1 | Nicotinamide phosphoribosyltransferase | 273 | 8.27E-29 | 74 |
| Efet.01.262148.g456.t1 | Neurogenic differentiation factor 1 | 987 | 2.22E-09 | 74 |
| Efet.01.168408.g909.t1 | Neuroendocrine convertase 1 | 306 | 4.81E-19 | 74 |
| Efet.01.370469.g569.t1 | BDNF/NT-3 growth factors receptor | 663 | 5.02E-29 | 74 |
| Efet.01.407760.g182.t1 | Paired box protein Pax-7 | 486 | 1.65E-35 | 74 |
| Efet.01.95340.g1177.t1 | Serine/threonine-protein kinase pim-1 | 552 | 1.48E-63 | 74 |
| Efet.01.208690.g396.t1 | Pumilio homolog 1 | 276 | 3.70E-33 | 74 |
| Efet.01.100870.g59.t1 | Ras-related protein Rab-1A | 441 | 8.73E-64 | 74 |
| Efet.01.255795.g211.t1 | Telomere-associated protein RIF1 | 2235 | 6.59E-18 | 74 |
| Efet.01.91619.g975.t1 | Retinoic acid receptor RXR-alpha | 204 | 7.70E-22 | 74 |
| Efet.01.625818.g1135.t1 | Sal-like protein 1 | 1116 | 2.15E-45 | 74 |
| Efet.01.264832.g557.t1 | Sonic hedgehog protein | 273 | 8.19E-14 | 74 |
| Efet.01.129827.g258.t1 | Zinc finger protein SNAI1 | 696 | 1.14E-44 | 74 |
| Efet.01.366483.g447.t1 | Zinc finger protein SNAI1 | 354 | 1.15E-37 | 74 |
| Efet.01.657897.g1520.t1 | Zinc finger protein SNAI1 | 573 | 3.65E-58 | 74 |
| Efet.01.628051.g1229.t1 | Spectrin beta chain, non-erythrocytic 1 | 450 | 1.31E-38 | 74 |
| Efet.01.180342.g1414.t1 | Spectrin beta chain, non-erythrocytic 1 | 420 | 1.11E-34 | 74 |
| Efet.01.1652856.g586.t1 | 5-hydroxytryptamine receptor 1A | 441 | 1.85E-20 | 73 |
| Efet.01.74614.g1521.t1 | Angiotensin-converting enzyme | 216 | 5.63E-16 | 73 |
| Efet.01.318395.g576.t1 | Cadherin-4 | 282 | 2.26E-11 | 73 |
| Efet.01.184871.g1629.t1 | Calumenin | 363 | 3.39E-26 | 73 |
| Efet.01.66846.g1034.t1 | CREB-binding protein | 1485 | 1.81E-117 | 73 |
| Efet.01.180113.g1405.t1 | Chromodomain-helicase-DNA-binding protein 1 | 846 | 2.26E-113 | 73 |
| Efet.01.13838.g1027.t1 | Chromodomain-helicase-DNA-binding protein 7 | 549 | 1.02E-40 | 73 |
| Efet.01.1635971.g170.t1 | Chromodomain-helicase-DNA-binding protein 7 | 306 | 3.26E-19 | 73 |
| Efet.01.95625.g1195.t1 | Epithelial discoidin domain-containing receptor 1 | 348 | 2.16E-33 | 73 |
| Efet.01.89634.g869.t1 | ELAV-like protein 4 | 411 | 8.29E-44 | 73 |
| Efet.01.232893.g1303.t1 | Ephrin type-A receptor 4 | 546 | 2.13E-85 | 73 |
| Efet.01.169305.g947.t1 | Receptor tyrosine-protein kinase erbB-4 | 222 | 7.70E-20 | 73 |
| Efet.01.399580.g1235.t1 | Forkhead box protein J3 | 423 | 2.92E-24 | 73 |
| Efet.01.277975.g1029.t1 | Tyrosine-protein kinase fynb | 339 | 3.74E-28 | 73 |
| Efet.01.387336.g954.t1 | Gamma-aminobutyric acid receptor subunit beta-3 | 390 | 2.56E-08 | 73 |
| Efet.01.470455.g540.t1 | Gamma-aminobutyric acid receptor subunit beta-3 | 498 | 5.04E-09 | 73 |
| Efet.01.190176.g1878.t1 | Homeobox protein GBX-2 | 915 | 1.14E-13 | 73 |
| Efet.01.130643.g304.t1 | Germinal center kinase 1 | 285 | 1.35E-14 | 73 |
| Efet.01.89110.g839.t1 | Growth/differentiation factor 8 | 405 | 4.62E-55 | 73 |
| Efet.01.146716.g1089.t1 | Growth/differentiation factor 8 | 324 | 3.07E-38 | 73 |
| Efet.01.488935.g942.t1 | Nuclear hormone receptor HR96 | 435 | 1.84E-33 | 73 |
| Efet.01.516705.g419.t1 | Heat shock cognate 71 kDa protein | 564 | 3.38E-32 | 73 |
| Efet.01.59035.g566.t1 | ATP-dependent RNA helicase eIF4A | 339 | 6.24E-14 | 73 |
| Efet.01.513780.g327.t1 | Potassium channel subfamily K member 3 | 402 | 7.26E-10 | 73 |
| Efet.01.219066.g769.t1 | Lysine-specific demethylase 3A | 483 | 7.10E-41 | 73 |
| Efet.01.445197.g1119.t1 | Lysine-specific demethylase 5C | 516 | 1.86E-27 | 73 |
| Efet.01.128333.g190.t1 | Kinesin-like protein KIF3C | 210 | 2.57E-15 | 73 |
| Efet.01.613959.g627.t1 | Protein kinase C theta type | 264 | 7.65E-13 | 73 |
| Efet.01.310784.g343.t1 | Protein kinase C theta type | 216 | 2.35E-11 | 73 |
| Efet.01.402937.g79.t1 | Pyruvate kinase PKM | 987 | 1.28E-98 | 73 |
| Efet.01.365656.g428.t1 | Canalicular multispecific organic anion transporter 1 | 414 | 2.23E-50 | 73 |
| Efet.01.490393.g964.t1 | Canalicular multispecific organic anion transporter 1 | 360 | 1.20E-29 | 73 |
| Efet.01.221778.g890.t1 | Nuclear factor interleukin-3-regulated protein | 426 | 1.51E-10 | 73 |
| Efet.01.115606.g805.t1 | Homeobox protein Nkx-2.2 | 558 | 4.42E-29 | 73 |
| Efet.01.11571.g843.t1 | Neurogenic locus notch homolog protein 2 | 474 | 2.04E-22 | 73 |
| Efet.01.562218.g42.t1 | Octopamine receptor beta-3R | 780 | 1.07E-33 | 73 |
| Efet.01.2740.g250.t1 | Poly [ADP-ribose] polymerase 1 | 579 | 4.52E-92 | 73 |
| Efet.01.87263.g761.t1 | Serine/threonine-protein kinase pim-2 | 600 | 1.45E-62 | 73 |
| Efet.01.339111.g1140.t1 | Tyrosine-protein phosphatase non-receptor type 3 | 687 | 1.11E-77 | 73 |
| Efet.01.138459.g688.t1 | Receptor-type tyrosine-protein phosphatase S | 594 | 1.01E-06 | 73 |
| Efet.01.41609.g1095.t1 | Pumilio homolog 2 | 879 | 1.21E-121 | 73 |
| Efet.01.161592.g600.t1 | Ras-related protein Rab-1A | 291 | 1.26E-11 | 73 |
| Efet.01.11635.g851.t1 | Proto-oncogene tyrosine-protein kinase receptor Ret | 264 | 6.22E-06 | 73 |
| Efet.01.239605.g1522.t1 | Proto-oncogene tyrosine-protein kinase receptor Ret | 474 | 8.51E-43 | 73 |
| Efet.01.653926.g495.t1 | E3 ubiquitin-protein ligase RNF13 | 888 | 2.41E-12 | 73 |
| Efet.01.19562.g1449.t1 | Sodium channel protein type 1 subunit alpha | 375 | 2.71E-29 | 73 |
| Efet.01.71880.g1356.t1 | Sodium channel protein type 1 subunit alpha | 567 | 1.47E-39 | 73 |
| Efet.01.502615.g62.t1 | Sodium channel protein type 1 subunit alpha | 297 | 7.58E-27 | 73 |
| Efet.01.35947.g729.t1 | Serine/threonine-protein kinase Sgk1 | 342 | 3.91E-22 | 73 |
| Efet.01.473583.g630.t1 | Survival motor neuron protein | 240 | 2.09E-10 | 73 |
| Efet.01.548703.g463.t1 | Spectrin beta chain, non-erythrocytic 1 | 363 | 2.31E-40 | 73 |
| Efet.01.637419.g375.t1 | Spectrin beta chain, non-erythrocytic 1 | 450 | 3.74E-24 | 73 |
| Efet.01.73632.g1453.t1 | Spectrin alpha chain, non-erythrocytic 1 | 1221 | 1.51E-118 | 73 |
| Efet.01.103468.g185.t1 | Sterol regulatory element-binding protein 1 | 1887 | 7.34E-32 | 73 |
| Efet.01.526328.g615.t1 | Proto-oncogene tyrosine-protein kinase Src | 288 | 5.51E-27 | 73 |
| Efet.01.204838.g208.t1 | Short transient receptor potential channel 4 | 762 | 3.13E-42 | 73 |
| Efet.01.229079.g1165.t1 | Thrombospondin-2 | 288 | 5.65E-39 | 73 |
| Efet.01.269936.g742.t1 | Protein Wnt-5a | 420 | 3.35E-54 | 73 |
| Efet.01.139194.g730.t1 | 5-hydroxytryptamine receptor 1A | 1551 | 1.54E-72 | 72 |
| Efet.01.70021.g1233.t1 | 5-hydroxytryptamine receptor 2A | 513 | 1.22E-14 | 72 |
| Efet.01.81085.g408.t1 | 5-hydroxytryptamine receptor 2A | 333 | 3.51E-33 | 72 |
| Efet.01.528516.g647.t1 | Serine/threonine-protein kinase akt-2 | 255 | 1.53E-23 | 72 |
| Efet.01.301345.g48.t1 | Annexin A3 | 393 | 5.67E-38 | 72 |
| Efet.01.91701.g983.t1 | Calpain-3 | 351 | 4.34E-16 | 72 |
| Efet.01.195800.g2121.t1 | Cholecystokinin receptor type A | 309 | 7.90E-17 | 72 |
| Efet.01.177423.g1288.t1 | Cholecystokinin receptor type A | 498 | 3.23E-16 | 72 |
| Efet.01.46000.g1355.t1 | Dystrophin | 393 | 4.07E-22 | 72 |
| Efet.01.93733.g1086.t1 | DNA (cytosine-5)-methyltransferase 3B | 321 | 6.77E-20 | 72 |
| Efet.01.61281.g703.t1 | Egl nine homolog 1 | 1641 | 2.80E-80 | 72 |
| Efet.01.225184.g1009.t1 | Focal adhesion kinase 1 | 237 | 2.70E-19 | 72 |
| Efet.01.43142.g1178.t1 | Frizzled-7 | 861 | 9.04E-51 | 72 |
| Efet.01.537660.g220.t1 | Serine/threonine-protein kinase gad8 | 225 | 9.66E-11 | 72 |
| Efet.01.321720.g676.t1 | Guanine nucleotide-binding protein G(s) subunit alpha isoforms XLas | 315 | 1.03E-30 | 72 |
| Efet.01.5060.g392.t1 | Glutamate receptor 3 | 282 | 7.33E-30 | 72 |
| Efet.01.130056.g271.t1 | Glutamate receptor 3 | 597 | 1.07E-19 | 72 |
| Efet.01.412551.g282.t1 | Histamine H1 receptor | 438 | 7.58E-25 | 72 |
| Efet.01.218983.g765.t1 | Probable heat shock protein ssa2 | 207 | 2.98E-18 | 72 |
| Efet.01.11753.g867.t1 | Zinc finger and SCAN domain-containing protein 10 | 411 | 3.10E-14 | 72 |
| Efet.01.274331.g887.t1 | Eukaryotic translation initiation factor 5A-1 | 222 | 1.51E-06 | 72 |
| Efet.01.216183.g671.t1 | Transcription factor AP-1 | 474 | 1.07E-29 | 72 |
| Efet.01.320499.g647.t1 | Potassium voltage-gated channel subfamily A member 5 | 573 | 4.74E-07 | 72 |
| Efet.01.51631.g104.t1 | Potassium voltage-gated channel subfamily B member 1 | 648 | 1.28E-64 | 72 |
| Efet.01.54943.g331.t1 | Potassium voltage-gated channel subfamily B member 1 | 357 | 6.13E-10 | 72 |
| Efet.01.609516.g407.t1 | Potassium voltage-gated channel subfamily B member 1 | 813 | 5.08E-53 | 72 |
| Efet.01.257895.g292.t1 | Pyruvate kinase PKM | 1425 | 1.48E-135 | 72 |
| Efet.01.186255.g1699.t1 | Tyrosine-protein kinase Lyn | 285 | 2.24E-26 | 72 |
| Efet.01.1659186.g1449.t1 | POU domain protein | 432 | 2.04E-15 | 72 |
| Efet.01.125383.g22.t1 | Muscleblind-like protein 3 | 390 | 2.19E-24 | 72 |
| Efet.01.286720.g1333.t1 | Mediator of RNA polymerase II transcription subunit 13-like | 288 | 8.31E-23 | 72 |
| Efet.01.47515.g1447.t1 | Dual specificity mitogen-activated protein kinase kinase 4 | 609 | 9.62E-44 | 72 |
| Efet.01.351600.g46.t1 | Unconventional myosin-Va | 351 | 1.06E-21 | 72 |
| Efet.01.423212.g548.t1 | Nicotinamide phosphoribosyltransferase | 471 | 7.82E-48 | 72 |
| Efet.01.103327.g178.t1 | Neurogenic differentiation factor 1 | 555 | 2.67E-30 | 72 |
| Efet.01.75570.g33.t1 | Netrin-1 | 1002 | 9.34E-104 | 72 |
| Efet.01.5856.g465.t1 | Neurofilament heavy polypeptide | 315 | 8.61E-08 | 72 |
| Efet.01.1655808.g792.t1 | Neurogenic locus notch homolog protein 2 | 417 | 1.93E-47 | 72 |
| Efet.01.34490.g652.t1 | 3-phosphoinositide-dependent protein kinase 1 | 306 | 1.59E-17 | 72 |
| Efet.01.23041.g1679.t1 | PR domain zinc finger protein 14 | 321 | 5.25E-26 | 72 |
| Efet.01.37135.g802.t1 | Dexamethasone-induced Ras-related protein 1 | 441 | 3.13E-21 | 72 |
| Efet.01.449767.g1211.t1 | Reelin | 279 | 1.03E-28 | 72 |
| Efet.01.369469.g528.t1 | Proto-oncogene tyrosine-protein kinase receptor Ret | 336 | 1.53E-21 | 72 |
| Efet.01.178394.g1328.t1 | Roundabout homolog 2 | 414 | 3.04E-36 | 72 |
| Efet.01.601050.g46.t1 | RNA polymerase-associated protein RTF1 homolog | 264 | 1.59E-19 | 72 |
| Efet.01.570764.g343.t1 | Retinal homeobox protein Rx | 231 | 4.58E-15 | 72 |
| Efet.01.115845.g824.t1 | Retinoic acid receptor RXR-alpha | 369 | 1.44E-21 | 72 |
| Efet.01.17339.g1278.t1 | Sodium channel protein type 1 subunit alpha | 951 | 1.22E-18 | 72 |
| Efet.01.118867.g976.t1 | Sodium channel protein type 9 subunit alpha | 369 | 1.89E-22 | 72 |
| Efet.01.101473.g95.t1 | Alpha-1-syntrophin | 285 | 1.26E-08 | 72 |
| Efet.01.80316.g353.t1 | Transcription factor SOX-9 | 1326 | 1.67E-25 | 72 |
| Efet.01.19055.g1414.t1 | Transcription factor Sp1 | 462 | 7.07E-52 | 72 |
| Efet.01.198386.g2229.t1 | Transcription factor 21 | 417 | 4.58E-29 | 72 |
| Efet.01.81034.g403.t1 | Transcription factor 21 | 543 | 1.47E-11 | 72 |
| Efet.01.1612062.g25.t1 | Protein Wnt-4 | 243 | 3.56E-27 | 72 |
| Efet.01.197167.g2184.t1 | Protein Wnt-7b | 417 | 2.36E-19 | 72 |
| Efet.01.173182.g1105.t1 | 5-hydroxytryptamine receptor 1A | 1704 | 1.75E-34 | 71 |
| Efet.01.446475.g1150.t1 | 5-hydroxytryptamine receptor 1A | 774 | 1.47E-21 | 71 |
| Efet.01.21275.g1571.t1 | Angiotensin-converting enzyme | 405 | 3.92E-42 | 71 |
| Efet.01.589679.g980.t1 | Disintegrin and metalloproteinase domain-containing protein 10 | 351 | 4.37E-14 | 71 |
| Efet.01.205455.g235.t1 | RAC-beta serine/threonine-protein kinase | 324 | 4.12E-18 | 71 |
| Efet.01.282064.g1163.t1 | Atrial natriuretic peptide receptor 1 | 1455 | 2.42E-15 | 71 |
| Efet.01.3848.g327.t1 | AT-rich interactive domain-containing protein 1B | 2097 | 3.05E-27 | 71 |
| Efet.01.13376.g1001.t1 | CREB-binding protein | 1464 | 2.25E-73 | 71 |
| Efet.01.49916.g1583.t1 | Cholecystokinin receptor type A | 576 | 2.17E-14 | 71 |
| Efet.01.257008.g263.t1 | Probable cyclin-dependent kinase 9 | 219 | 7.33E-15 | 71 |
| Efet.01.655451.g822.t1 | Cystic fibrosis transmembrane conductance regulator | 435 | 5.94E-07 | 71 |
| Efet.01.267648.g651.t1 | 60 kDa heat shock protein, mitochondrial | 1545 | 7.50E-144 | 71 |
| Efet.01.89446.g856.t1 | Connective tissue growth factor | 390 | 1.83E-10 | 71 |
| Efet.01.67467.g1079.t1 | Epithelial discoidin domain-containing receptor 1 | 525 | 4.56E-55 | 71 |
| Efet.01.122724.g1183.t1 | Transforming protein p54/c-ets-1 | 501 | 8.57E-12 | 71 |
| Efet.01.46837.g1404.t1 | Frizzled-7 | 1509 | 0 | 71 |
| Efet.01.191556.g1939.t1 | Gamma-aminobutyric acid receptor subunit beta-3 | 372 | 6.36E-16 | 71 |
| Efet.01.48095.g1473.t1 | Histone acetyltransferase gcn5 | 678 | 2.55E-25 | 71 |
| Efet.01.165344.g767.t1 | Guanine nucleotide-binding protein G(s) subunit alpha | 240 | 1.50E-25 | 71 |
| Efet.01.20441.g1505.t1 | Guanine nucleotide-binding protein G(s) subunit alpha isoforms XLas | 606 | 4.26E-38 | 71 |
| Efet.01.537432.g212.t1 | Histamine H1 receptor | 786 | 4.34E-22 | 71 |
| Efet.01.604040.g151.t1 | Histamine H2 receptor | 471 | 5.13E-19 | 71 |
| Efet.01.319179.g615.t1 | Heat shock protein 60, mitochondrial | 1485 | 1.50E-105 | 71 |
| Efet.01.578494.g639.t1 | Potassium voltage-gated channel subfamily A member 5 | 528 | 5.15E-44 | 71 |
| Efet.01.41442.g1082.t1 | Potassium voltage-gated channel subfamily A member 5 | 1650 | 1.42E-161 | 71 |
| Efet.01.573711.g427.t1 | Potassium channel subfamily K member 3 | 642 | 7.46E-45 | 71 |
| Efet.01.336823.g1087.t1 | Pyruvate kinase PKM | 1731 | 0 | 71 |
| Efet.01.248603.g1838.t1 | Raf homolog serine/threonine-protein kinase | 561 | 1.93E-07 | 71 |
| Efet.01.168130.g895.t1 | Low-density lipoprotein receptor-related protein 1 | 609 | 4.99E-76 | 71 |
| Efet.01.258373.g306.t1 | Prolow-density lipoprotein receptor-related protein 1 | 840 | 1.23E-95 | 71 |
| Efet.01.5487.g426.t1 | MAX gene-associated protein | 504 | 2.66E-36 | 71 |
| Efet.01.121428.g1112.t1 | Mitogen-activated protein kinase 1 | 342 | 3.93E-35 | 71 |
| Efet.01.47104.g1420.t1 | Canalicular multispecific organic anion transporter 1 | 312 | 9.21E-23 | 71 |
| Efet.01.636758.g347.t1 | Canalicular multispecific organic anion transporter 1 | 237 | 2.00E-21 | 71 |
| Efet.01.459793.g272.t1 | ATP-dependent RNA helicase DOB1 | 564 | 8.32E-53 | 71 |
| Efet.01.28650.g249.t1 | Musculin | 696 | 4.25E-12 | 71 |
| Efet.01.55101.g343.t1 | E3 ubiquitin-protein ligase MYCBP2 | 1551 | 2.92E-160 | 71 |
| Efet.01.503659.g92.t1 | Unconventional myosin-Va | 279 | 8.15E-21 | 71 |
| Efet.01.229002.g1162.t1 | Netrin-1 | 570 | 1.67E-55 | 71 |
| Efet.01.115317.g795.t1 | Homeobox protein Nkx-2.2 | 867 | 1.39E-27 | 71 |
| Efet.01.185297.g1646.t1 | Neurogenic locus Notch protein | 306 | 5.52E-13 | 71 |
| Efet.01.423964.g569.t1 | BDNF/NT-3 growth factors receptor | 324 | 4.08E-31 | 71 |
| Efet.01.294922.g1594.t1 | Octopamine receptor beta-3R | 540 | 1.47E-25 | 71 |
| Efet.01.446475.g1151.t1 | Octopamine receptor beta-3R | 1110 | 4.83E-19 | 71 |
| Efet.01.8669.g638.t1 | Receptor-type tyrosine-protein phosphatase S | 663 | 3.79E-64 | 71 |
| Efet.01.33719.g600.t1 | E3 ubiquitin-protein ligase RNF13 | 207 | 1.47E-15 | 71 |
| Efet.01.529096.g669.t1 | Rho-associated protein kinase let-502 | 1632 | 8.59E-16 | 71 |
| Efet.01.73627.g1452.t1 | Solute carrier family 12 member 2 | 378 | 1.40E-07 | 71 |
| Efet.01.168503.g912.t1 | Solute carrier family 12 member 2 | 237 | 5.76E-16 | 71 |
| Efet.01.379736.g788.t1 | Sodium channel protein type 1 subunit alpha | 330 | 1.21E-17 | 71 |
| Efet.01.420676.g491.t1 | Sodium channel protein type 9 subunit alpha | 219 | 7.17E-12 | 71 |
| Efet.01.278273.g1046.t1 | Sodium channel protein type 9 subunit alpha | 288 | 4.78E-22 | 71 |
| Efet.01.439498.g936.t1 | Structural maintenance of chromosomes protein 1A | 492 | 6.68E-23 | 71 |
| Efet.01.100624.g46.t1 | Transcription factor Sox-11 | 1134 | 1.88E-36 | 71 |
| Efet.01.27016.g133.t1 | Transcription factor Sp1 | 759 | 5.70E-48 | 71 |
| Efet.01.83702.g564.t1 | Spectrin beta chain, non-erythrocytic 1 | 2436 | 6.83E-24 | 71 |
| Efet.01.230377.g1209.t1 | Proto-oncogene tyrosine-protein kinase Src | 615 | 1.74E-61 | 71 |
| Efet.01.355154.g146.t1 | Tyrosine protein-kinase src-1 | 459 | 1.45E-50 | 71 |
| Efet.01.9995.g731.t1 | Serrate RNA effector molecule homolog | 510 | 1.46E-32 | 71 |
| Efet.01.595643.g1169.t1 | Transient receptor potential-gamma protein | 207 | 1.53E-11 | 71 |
| Efet.01.11564.g842.t1 | Protein Wnt-5a | 321 | 6.62E-40 | 71 |
| Efet.01.375186.g685.t1 | Protein Wnt-5b | 447 | 1.90E-52 | 71 |
| Efet.01.106665.g348.t1 | Protein Wnt-7b | 423 | 9.36E-43 | 71 |
| Efet.01.504560.g119.t1 | Zinc finger protein ZIC 3 | 690 | 2.57E-45 | 71 |
| Efet.01.438810.g921.t1 | NAD-dependent protein deacetylase sirtuin-2 | 282 | 3.48E-06 | 70 |
| Efet.01.203545.g162.t1 | APOBEC1 complementation factor | 1137 | 3.61E-21 | 70 |
| Efet.01.540563.g282.t1 | Alcohol dehydrogenase 1 | 396 | 2.75E-39 | 70 |
| Efet.01.552145.g554.t1 | Serine/threonine-protein kinase akt-1 | 441 | 1.41E-14 | 70 |
| Efet.01.16831.g1244.t1 | Aldose reductase | 210 | 3.74E-11 | 70 |
| Efet.01.415394.g357.t1 | Acidic leucine-rich nuclear phosphoprotein 32 family member A | 285 | 6.32E-09 | 70 |
| Efet.01.1650523.g477.t1 | Angiopoietin-2 | 312 | 1.42E-10 | 70 |
| Efet.01.169883.g972.t1 | Atrial natriuretic peptide receptor 1 | 231 | 1.68E-18 | 70 |
| Efet.01.541570.g299.t1 | Calretinin | 267 | 1.36E-12 | 70 |
| Efet.01.1658974.g1344.t1 | G1/S-specific cyclin-D2 | 414 | 1.12E-16 | 70 |
| Efet.01.305150.g168.t1 | Cyclin-dependent kinase 9 | 480 | 4.31E-39 | 70 |
| Efet.01.238691.g1501.t1 | Cyclin-dependent kinase inhibitor 1B | 417 | 1.22E-12 | 70 |
| Efet.01.1656108.g826.t1 | Cytochrome P450 26A1 | 690 | 1.38E-15 | 70 |
| Efet.01.509552.g241.t1 | Epithelial discoidin domain-containing receptor 1 | 291 | 4.59E-07 | 70 |
| Efet.01.552216.g557.t1 | Ecdysone receptor | 330 | 3.11E-33 | 70 |
| Efet.01.632329.g155.t1 | Early growth response protein 1 | 3147 | 5.00E-24 | 70 |
| Efet.01.24531.g1780.t1 | ELAV-like protein 4 | 699 | 2.12E-76 | 70 |
| Efet.01.373825.g663.t1 | Gamma-aminobutyric acid receptor subunit beta-3 | 285 | 5.81E-09 | 70 |
| Efet.01.343704.g1241.t1 | Guanine nucleotide-binding protein G(s) subunit alpha isoforms XLas | 222 | 7.87E-11 | 70 |
| Efet.01.635880.g294.t1 | Glutamate receptor 3 | 204 | 3.37E-22 | 70 |
| Efet.01.414.g50.t1 | High mobility group protein B2 | 492 | 5.83E-51 | 70 |
| Efet.01.347451.g1339.t1 | Homeobox protein GHOX-7 | 570 | 4.81E-09 | 70 |
| Efet.01.600986.g43.t1 | Tyrosine-protein kinase JAK2 | 720 | 1.68E-09 | 70 |
| Efet.01.258956.g338.t1 | Potassium voltage-gated channel subfamily A member 5 | 909 | 8.18E-75 | 70 |
| Efet.01.320812.g657.t1 | Potassium voltage-gated channel subfamily B member 1 | 753 | 1.12E-39 | 70 |
| Efet.01.362119.g325.t1 | Potassium channel subfamily K member 3 | 315 | 1.06E-06 | 70 |
| Efet.01.282018.g1156.t1 | Kinesin-like protein KIF3C | 654 | 9.65E-74 | 70 |
| Efet.01.154453.g228.t1 | Histone-lysine N-methyltransferase 2A | 348 | 1.91E-38 | 70 |
| Efet.01.307957.g235.t1 | E3 ubiquitin-protein ligase TRIM71 | 300 | 1.30E-06 | 70 |
| Efet.01.645508.g993.t1 | Phosphatidate phosphatase LPIN1 | 234 | 4.56E-14 | 70 |
| Efet.01.79415.g285.t1 | Prolow-density lipoprotein receptor-related protein 1 | 321 | 3.67E-11 | 70 |
| Efet.01.1646869.g361.t1 | Low-density lipoprotein receptor-related protein 2 | 216 | 2.22E-13 | 70 |
| Efet.01.243480.g1653.t1 | POU domain protein | 324 | 9.97E-14 | 70 |
| Efet.01.140856.g813.t1 | Mediator of RNA polymerase II transcription subunit 12 | 804 | 2.61E-79 | 70 |
| Efet.01.190959.g1914.t1 | Hepatocyte growth factor receptor | 528 | 1.97E-52 | 70 |
| Efet.01.1651700.g522.t1 | MAX gene-associated protein | 303 | 3.04E-26 | 70 |
| Efet.01.1655691.g780.t1 | Matrix metalloproteinase-9 | 333 | 9.72E-08 | 70 |
| Efet.01.367527.g473.t1 | Neuroendocrine convertase 1 | 342 | 2.84E-17 | 70 |
| Efet.01.154995.g256.t1 | Serine/threonine-protein kinase pim-1 | 480 | 2.31E-40 | 70 |
| Efet.01.224777.g994.t1 | 1-phosphatidylinositol 4,5-bisphosphate phosphodiesterase delta-4 | 327 | 4.95E-15 | 70 |
| Efet.01.1944.g174.t1 | Plastin-2 | 399 | 2.54E-40 | 70 |
| Efet.01.173893.g1130.t1 | Protein phosphatase 1D | 339 | 3.48E-16 | 70 |
| Efet.01.251242.g43.t1 | PR domain zinc finger protein 16 | 237 | 5.21E-08 | 70 |
| Efet.01.507395.g191.t1 | Tyrosine-protein phosphatase non-receptor type 3 | 336 | 1.24E-30 | 70 |
| Efet.01.386675.g941.t1 | Tyrosine-protein phosphatase non-receptor type 3 | 201 | 3.89E-14 | 70 |
| Efet.01.41609.g1096.t1 | Pumilio homolog 1 | 366 | 2.22E-06 | 70 |
| Efet.01.164265.g721.t1 | Peroxidasin homolog | 852 | 4.94E-89 | 70 |
| Efet.01.615102.g681.t1 | Putative hydrolase RBBP9 | 570 | 3.61E-68 | 70 |
| Efet.01.99098.g1395.t1 | Regucalcin | 354 | 4.49E-38 | 70 |
| Efet.01.446673.g1155.t1 | Roundabout homolog 2 | 318 | 1.49E-34 | 70 |
| Efet.01.344506.g1257.t1 | Retinal Mueller cells isomerohydrolase | 273 | 3.38E-27 | 70 |
| Efet.01.294086.g1576.t1 | Ras-related protein R-Ras | 282 | 1.41E-25 | 70 |
| Efet.01.122331.g1163.t1 | Sodium-dependent serotonin transporter | 360 | 7.96E-14 | 70 |
| Efet.01.45584.g1330.t1 | Septin-4 | 723 | 9.23E-60 | 70 |
| Efet.01.80794.g389.t1 | Serine/threonine-protein kinase sgk-1 | 291 | 9.56E-32 | 70 |
| Efet.01.323739.g732.t1 | SH2B adapter protein 3 | 444 | 1.31E-13 | 70 |
| Efet.01.125724.g44.t1 | Extracellular sulfatase Sulf-2 | 243 | 1.09E-17 | 70 |
| Efet.01.131207.g329.t1 | Transcription factor 7-like 2 | 351 | 6.81E-41 | 70 |
| Efet.01.328055.g863.t1 | Tropomyosin alpha-4 chain | 282 | 3.47E-15 | 70 |
| Efet.01.656315.g994.t1 | Vang-like protein 2 | 1302 | 3.67E-67 | 70 |
| Efet.01.34460.g650.t1 | Wee1-like protein kinase | 249 | 2.12E-20 | 70 |
| Efet.01.361028.g298.t1 | Zinc finger homeobox protein 3 | 2211 | 1.66E-68 | 70 |
| Efet.01.157687.g406.t1 | 5-hydroxytryptamine receptor 1A | 744 | 1.19E-27 | 69 |
| Efet.01.283023.g1193.t1 | 5-hydroxytryptamine receptor 1A | 1590 | 5.79E-49 | 69 |
| Efet.01.11637.g854.t1 | Disintegrin and metalloproteinase domain-containing protein 10 | 348 | 1.79E-20 | 69 |
| Efet.01.121050.g1094.t1 | Atrial natriuretic peptide receptor 1 | 288 | 2.32E-38 | 69 |
| Efet.01.258243.g304.t1 | Aquaporin-5 | 639 | 9.76E-26 | 69 |
| Efet.01.330561.g934.t1 | Beta-1,4-galactosyltransferase 1 | 237 | 4.92E-07 | 69 |
| Efet.01.94802.g1148.t1 | Calpain-3 | 234 | 3.61E-08 | 69 |
| Efet.01.572068.g383.t1 | Cholecystokinin receptor type A | 348 | 7.75E-07 | 69 |
| Efet.01.244693.g1687.t1 | CCAAT/enhancer-binding protein alpha | 501 | 2.82E-06 | 69 |
| Efet.01.68600.g1140.t1 | Chromodomain-helicase-DNA-binding protein 7 | 606 | 6.01E-52 | 69 |
| Efet.01.657066.g1158.t1 | Chromodomain-helicase-DNA-binding protein 7 | 1050 | 4.48E-57 | 69 |
| Efet.01.516480.g411.t1 | Epithelial discoidin domain-containing receptor 1 | 501 | 6.72E-42 | 69 |
| Efet.01.651287.g156.t1 | Epithelial discoidin domain-containing receptor 1 | 339 | 1.05E-28 | 69 |
| Efet.01.195713.g2117.t1 | Dysferlin | 480 | 6.68E-35 | 69 |
| Efet.01.84945.g625.t1 | Focal adhesion kinase 1 | 342 | 1.12E-16 | 69 |
| Efet.01.127766.g152.t1 | Four and a half LIM domains protein 2 | 588 | 3.20E-59 | 69 |
| Efet.01.58075.g503.t1 | Forkhead box protein C2 | 255 | 9.96E-12 | 69 |
| Efet.01.338451.g1123.t1 | Tyrosine-protein kinase fynb | 219 | 1.14E-18 | 69 |
| Efet.01.658034.g1568.t1 | Frizzled-7 | 540 | 1.64E-62 | 69 |
| Efet.01.181834.g1493.t1 | Gamma-aminobutyric acid receptor subunit beta-3 | 495 | 5.13E-28 | 69 |
| Efet.01.415758.g368.t1 | Gamma-aminobutyric acid receptor subunit beta-3 | 243 | 3.85E-16 | 69 |
| Efet.01.371428.g609.t1 | Glutamate receptor 1 | 261 | 1.66E-17 | 69 |
| Efet.01.200918.g46.t1 | Glutamate receptor 3 | 276 | 2.65E-24 | 69 |
| Efet.01.1658203.g1124.t1 | Glutamate receptor 3 | 276 | 1.34E-25 | 69 |
| Efet.01.515650.g382.t1 | Heat shock 70 kDa protein | 1962 | 0 | 69 |
| Efet.01.113356.g676.t1 | Insulin-like growth factor 1 receptor | 1164 | 6.30E-49 | 69 |
| Efet.01.180681.g1432.t1 | Inter-alpha-trypsin inhibitor heavy chain H4 | 270 | 1.69E-11 | 69 |
| Efet.01.445227.g1121.t1 | Potassium voltage-gated channel subfamily A member 5 | 1005 | 1.17E-98 | 69 |
| Efet.01.466010.g425.t1 | Potassium voltage-gated channel subfamily B member 1 | 702 | 3.28E-80 | 69 |
| Efet.01.52547.g177.t1 | Potassium channel subfamily K member 3 | 891 | 1.48E-51 | 69 |
| Efet.01.267321.g628.t1 | Kinesin-like protein KIF3C | 210 | 6.30E-15 | 69 |
| Efet.01.507997.g205.t1 | Low-density lipoprotein receptor-related protein 1 | 279 | 2.79E-22 | 69 |
| Efet.01.192282.g1974.t1 | POU domain protein | 1077 | 4.37E-19 | 69 |
| Efet.01.524439.g573.t1 | Metastasis suppressor protein 1 | 234 | 1.27E-17 | 69 |
| Efet.01.292670.g1532.t1 | Myogenic factor 5 | 1134 | 1.37E-15 | 69 |
| Efet.01.562590.g55.t1 | Nanos homolog 1 | 600 | 8.95E-17 | 69 |
| Efet.01.116755.g874.t1 | Neurogenic differentiation factor 1 | 828 | 8.66E-14 | 69 |
| Efet.01.352391.g68.t1 | Nuclear factor erythroid 2-related factor 2 | 915 | 3.40E-45 | 69 |
| Efet.01.654159.g538.t1 | Neuromedin-U receptor 1 | 294 | 4.56E-12 | 69 |
| Efet.01.657048.g1157.t1 | Octopamine receptor beta-2R | 714 | 3.91E-24 | 69 |
| Efet.01.20693.g1531.t1 | 1-phosphatidylinositol 4,5-bisphosphate phosphodiesterase delta-1 | 258 | 2.17E-15 | 69 |
| Efet.01.1626162.g80.t1 | Receptor-type tyrosine-protein phosphatase U | 234 | 6.78E-19 | 69 |
| Efet.01.279647.g1086.t1 | Peroxidasin homolog | 255 | 1.87E-25 | 69 |
| Efet.01.339583.g1161.t1 | CAD protein | 615 | 8.15E-76 | 69 |
| Efet.01.99733.g1434.t1 | Reelin | 270 | 2.16E-21 | 69 |
| Efet.01.364933.g402.t1 | Sodium channel protein type 1 subunit alpha | 387 | 2.94E-17 | 69 |
| Efet.01.304280.g148.t1 | Sodium channel protein type 1 subunit alpha | 264 | 1.51E-06 | 69 |
| Efet.01.182168.g1508.t1 | Sodium channel protein type 9 subunit alpha | 264 | 2.68E-16 | 69 |
| Efet.01.79666.g302.t1 | Sodium channel protein type 9 subunit alpha | 462 | 3.95E-30 | 69 |
| Efet.01.321180.g665.t1 | Serine/threonine-protein kinase Sgk1 | 342 | 8.67E-11 | 69 |
| Efet.01.542780.g321.t1 | STE20-like serine/threonine-protein kinase | 234 | 3.88E-11 | 69 |
| Efet.01.251453.g52.t1 | Transcription factor Sox-2 | 732 | 3.13E-48 | 69 |
| Efet.01.536272.g191.t1 | Spectrin beta chain, non-erythrocytic 1 | 444 | 3.54E-36 | 69 |
| Efet.01.23732.g1722.t1 | Spectrin alpha chain, non-erythrocytic 1 | 1380 | 1.07E-138 | 69 |
| Efet.01.32105.g494.t1 | Tropomyosin alpha-4 chain | 303 | 2.34E-06 | 69 |
| Efet.01.33715.g598.t1 | Transient receptor potential-gamma protein | 570 | 1.51E-41 | 69 |
| Efet.01.133136.g408.t1 | Hamartin | 1023 | 1.67E-11 | 69 |
| Efet.01.628006.g1225.t1 | Protein Wnt-7a | 468 | 1.68E-59 | 69 |
| Efet.01.175585.g1194.t1 | Exportin-7 | 537 | 6.84E-52 | 69 |
| Efet.01.489806.g958.t1 | Metal resistance protein YCF1 | 231 | 1.22E-16 | 69 |
| Efet.01.1659525.g2023.t1 | Alcohol dehydrogenase 1 | 1116 | 2.82E-111 | 68 |
| Efet.01.288887.g1415.t1 | Beta-2 adrenergic receptor | 528 | 1.51E-14 | 68 |
| Efet.01.4774.g381.t1 | Atrial natriuretic peptide receptor 1 | 291 | 3.81E-20 | 68 |
| Efet.01.20668.g1526.t1 | Aquaporin-5 | 582 | 1.03E-46 | 68 |
| Efet.01.51183.g81.t1 | Aquaporin-5 | 279 | 4.88E-10 | 68 |
| Efet.01.26141.g86.t1 | Serine/threonine-protein kinase ATR | 570 | 2.12E-26 | 68 |
| Efet.01.323400.g725.t1 | Calpain-6 | 204 | 1.29E-13 | 68 |
| Efet.01.497208.g1117.t1 | Carbonyl reductase [NADPH] 1 | 216 | 9.86E-10 | 68 |
| Efet.01.124388.g1258.t1 | Cholecystokinin receptor type A | 303 | 8.29E-12 | 68 |
| Efet.01.323044.g713.t1 | Chromodomain-helicase-DNA-binding protein 1 | 738 | 5.01E-07 | 68 |
| Efet.01.213338.g564.t1 | Collagen alpha-1(XVIII) chain | 435 | 6.64E-30 | 68 |
| Efet.01.290621.g1477.t1 | Catenin alpha-1 | 528 | 2.37E-29 | 68 |
| Efet.01.541560.g298.t1 | Epithelial discoidin domain-containing receptor 1 | 231 | 6.92E-16 | 68 |
| Efet.01.44746.g1283.t1 | Corticosteroid 11-beta-dehydrogenase isozyme 1 | 486 | 3.12E-06 | 68 |
| Efet.01.646005.g1022.t1 | Dedicator of cytokinesis protein 3 | 207 | 1.16E-11 | 68 |
| Efet.01.114530.g756.t1 | Early growth response protein 1 | 759 | 1.02E-44 | 68 |
| Efet.01.217103.g706.t1 | ELAV-like protein 4 | 546 | 2.86E-22 | 68 |
| Efet.01.658387.g1941.t1 | Beta-enolase | 1287 | 4.55E-132 | 68 |
| Efet.01.441139.g1007.t1 | Homeobox protein OTX2 | 522 | 5.25E-18 | 68 |
| Efet.01.483133.g811.t1 | Four and a half LIM domains protein 2 | 222 | 4.55E-14 | 68 |
| Efet.01.534932.g158.t1 | Four and a half LIM domains protein 2 | 270 | 7.90E-13 | 68 |
| Efet.01.477781.g715.t1 | Forkhead box protein C2 | 1533 | 9.10E-39 | 68 |
| Efet.01.265657.g576.t1 | Tyrosine-protein kinase fynb | 414 | 1.85E-21 | 68 |
| Efet.01.521270.g501.t1 | Homeobox protein GBX-2 | 402 | 4.66E-41 | 68 |
| Efet.01.310959.g349.t1 | Growth/differentiation factor 8 | 291 | 1.83E-34 | 68 |
| Efet.01.205990.g259.t1 | Glial fibrillary acidic protein | 381 | 2.10E-15 | 68 |
| Efet.01.1829.g162.t1 | Guanine nucleotide-binding protein G(s) subunit alpha | 468 | 5.91E-06 | 68 |
| Efet.01.466476.g439.t1 | Potassium voltage-gated channel subfamily A member 5 | 1311 | 5.89E-111 | 68 |
| Efet.01.79091.g253.t1 | Kinesin-like protein KIF3C | 552 | 2.67E-50 | 68 |
| Efet.01.140326.g789.t1 | Histone-lysine N-methyltransferase 2A | 903 | 5.00E-68 | 68 |
| Efet.01.643291.g769.t1 | Pyruvate kinase 1 | 558 | 6.85E-16 | 68 |
| Efet.01.267558.g635.t1 | Neural cell adhesion molecule L1 | 351 | 2.74E-07 | 68 |
| Efet.01.27830.g186.t1 | Neural cell adhesion molecule L1 | 204 | 3.37E-07 | 68 |
| Efet.01.14921.g1096.t1 | Prolow-density lipoprotein receptor-related protein 1 | 357 | 2.79E-33 | 68 |
| Efet.01.114629.g761.t1 | Prolow-density lipoprotein receptor-related protein 1 | 288 | 2.70E-09 | 68 |
| Efet.01.649669.g1347.t1 | Prolow-density lipoprotein receptor-related protein 1 | 390 | 2.57E-28 | 68 |
| Efet.01.195111.g2097.t1 | Low-density lipoprotein receptor-related protein 2 | 312 | 5.60E-19 | 68 |
| Efet.01.164472.g735.t1 | Canalicular multispecific organic anion transporter 1 | 435 | 2.88E-18 | 68 |
| Efet.01.6534.g511.t1 | RNA-binding protein Musashi homolog 1 | 576 | 3.86E-14 | 68 |
| Efet.01.400310.g7.t1 | Neurogenic differentiation factor 6 | 1032 | 3.15E-07 | 68 |
| Efet.01.219977.g807.t1 | Nuclear factor interleukin-3-regulated protein | 714 | 2.78E-10 | 68 |
| Efet.01.38025.g865.t1 | Nuclear factor interleukin-3-regulated protein | 318 | 4.64E-12 | 68 |
| Efet.01.129786.g254.t1 | Neurogenic locus notch homolog protein 1 | 1812 | 4.79E-11 | 68 |
| Efet.01.376308.g710.t1 | Neurogenic locus notch homolog protein 2 | 453 | 4.65E-20 | 68 |
| Efet.01.265233.g564.t1 | Paired box protein Pax-3 | 408 | 3.54E-18 | 68 |
| Efet.01.1328.g122.t1 | Serine/threonine-protein kinase pim-1 | 861 | 1.77E-102 | 68 |
| Efet.01.496527.g1100.t1 | 1-phosphatidylinositol 4,5-bisphosphate phosphodiesterase gamma plc-3 | 234 | 3.04E-07 | 68 |
| Efet.01.13630.g1016.t1 | Plastin-2 | 249 | 2.59E-16 | 68 |
| Efet.01.272790.g849.t1 | PR domain zinc finger protein 16 | 225 | 1.30E-06 | 68 |
| Efet.01.657496.g1311.t1 | Ribose-phosphate pyrophosphokinase 2 | 975 | 1.44E-103 | 68 |
| Efet.01.108059.g419.t1 | Receptor-type tyrosine-protein phosphatase zeta | 348 | 1.52E-26 | 68 |
| Efet.01.380739.g808.t1 | Tyrosine-protein phosphatase non-receptor type 11 | 216 | 4.50E-15 | 68 |
| Efet.01.350874.g25.t1 | Tyrosine-protein phosphatase non-receptor type 3 | 414 | 6.43E-06 | 68 |
| Efet.01.6357.g503.t1 | Receptor-type tyrosine-protein phosphatase S | 465 | 4.23E-39 | 68 |
| Efet.01.50244.g20.t1 | Rho-related GTP-binding protein RhoA-B | 540 | 6.33E-73 | 68 |
| Efet.01.158179.g431.t1 | Rho-related GTP-binding protein RhoA-B | 720 | 1.56E-75 | 68 |
| Efet.01.128293.g186.t1 | GTP-binding protein Rit1 | 540 | 4.95E-44 | 68 |
| Efet.01.324653.g758.t1 | Rho-associated protein kinase 2 | 576 | 2.74E-26 | 68 |
| Efet.01.67862.g1103.t1 | Sodium channel protein type 1 subunit alpha | 549 | 8.27E-22 | 68 |
| Efet.01.48186.g1479.t1 | Secreted frizzled-related protein 2 | 423 | 1.91E-40 | 68 |
| Efet.01.509666.g243.t1 | SWI/SNF complex subunit SMARCC1 | 348 | 2.33E-32 | 68 |
| Efet.01.644765.g897.t1 | Solute carrier organic anion transporter family member 1A5 | 354 | 5.45E-11 | 68 |
| Efet.01.70960.g1293.t1 | Spectrin beta chain, non-erythrocytic 1 | 426 | 3.17E-58 | 68 |
| Efet.01.236756.g1428.t1 | Spectrin beta chain, non-erythrocytic 1 | 288 | 9.61E-18 | 68 |
| Efet.01.401518.g34.t1 | Spectrin beta chain, non-erythrocytic 1 | 567 | 6.74E-45 | 68 |
| Efet.01.624119.g1062.t1 | Spectrin alpha chain, non-erythrocytic 1 | 393 | 2.58E-50 | 68 |
| Efet.01.193330.g2009.t1 | Homeobox protein TGIF1 | 522 | 2.16E-12 | 68 |
| Efet.01.1649921.g456.t1 | Protein Wnt-4 | 489 | 1.56E-54 | 68 |
| Efet.01.42283.g1130.t1 | 5-hydroxytryptamine receptor 2A | 840 | 7.22E-09 | 67 |
| Efet.01.219516.g790.t1 | 5-hydroxytryptamine receptor 2B | 1707 | 1.15E-19 | 67 |
| Efet.01.643474.g800.t1 | 5-hydroxytryptamine receptor 2B | 477 | 4.07E-13 | 67 |
| Efet.01.213034.g552.t1 | 5-hydroxytryptamine receptor 2C | 3039 | 3.39E-20 | 67 |
| Efet.01.483470.g822.t1 | 5-hydroxytryptamine receptor 2C | 408 | 1.10E-11 | 67 |
| Efet.01.75457.g25.t1 | Protein arginine N-methyltransferase 5 | 321 | 1.36E-26 | 67 |
| Efet.01.151563.g92.t1 | Aquaporin-1 | 417 | 4.11E-09 | 67 |
| Efet.01.300.g34.t1 | Protein atonal homolog 1 | 645 | 3.41E-11 | 67 |
| Efet.01.8142.g602.t1 | Cholecystokinin receptor type A | 261 | 6.26E-11 | 67 |
| Efet.01.1625073.g73.t1 | Cholecystokinin receptor type A | 246 | 3.68E-08 | 67 |
| Efet.01.67091.g1050.t1 | Cholecystokinin receptor type A | 513 | 1.97E-18 | 67 |
| Efet.01.234586.g1366.t1 | G1/S-specific cyclin-D2 | 222 | 3.05E-16 | 67 |
| Efet.01.84684.g606.t1 | Cyclin-dependent kinase 1 | 294 | 2.07E-20 | 67 |
| Efet.01.236322.g1416.t1 | Epithelial discoidin domain-containing receptor 1 | 432 | 2.18E-39 | 67 |
| Efet.01.251124.g33.t1 | Epithelial discoidin domain-containing receptor 1 | 234 | 1.86E-06 | 67 |
| Efet.01.122920.g1189.t1 | Endoribonuclease Dicer | 324 | 4.26E-28 | 67 |
| Efet.01.91716.g984.t1 | Dedicator of cytokinesis protein 3 | 621 | 1.13E-19 | 67 |
| Efet.01.604801.g179.t1 | Dual specificity protein phosphatase 1 | 429 | 4.24E-12 | 67 |
| Efet.01.1655957.g808.t1 | Beta-enolase | 558 | 1.21E-56 | 67 |
| Efet.01.12857.g969.t1 | Ephrin type-B receptor 3 | 642 | 8.68E-86 | 67 |
| Efet.01.162174.g630.t1 | Receptor tyrosine-protein kinase erbB-2 | 402 | 7.63E-12 | 67 |
| Efet.01.124196.g1248.t1 | Exostosin-like 2 | 255 | 1.63E-09 | 67 |
| Efet.01.391121.g1030.t1 | Coagulation factor VII | 369 | 3.91E-06 | 67 |
| Efet.01.377118.g729.t1 | Fibrinogen alpha chain | 471 | 3.90E-07 | 67 |
| Efet.01.5036.g391.t1 | Forkhead box protein C2 | 1203 | 1.18E-34 | 67 |
| Efet.01.176942.g1266.t1 | Forkhead box protein C2 | 870 | 1.66E-25 | 67 |
| Efet.01.627326.g1205.t1 | Protein furry homolog | 204 | 1.71E-10 | 67 |
| Efet.01.648525.g1245.t1 | Gamma-aminobutyric acid receptor subunit beta-3 | 258 | 8.91E-11 | 67 |
| Efet.01.59716.g614.t1 | Glial fibrillary acidic protein | 432 | 1.24E-06 | 67 |
| Efet.01.182447.g1519.t1 | Glutamate receptor 1 | 969 | 7.80E-20 | 67 |
| Efet.01.302220.g74.t1 | Homeobox protein GHOX-7 | 951 | 8.44E-19 | 67 |
| Efet.01.26888.g129.t1 | Heterogeneous nuclear ribonucleoprotein K | 447 | 5.67E-07 | 67 |
| Efet.01.168402.g908.t1 | Histamine H1 receptor | 771 | 2.34E-08 | 67 |
| Efet.01.634561.g250.t1 | Histamine H1 receptor | 882 | 9.67E-22 | 67 |
| Efet.01.284823.g1258.t1 | Homeobox protein Hox-B4a | 216 | 1.87E-06 | 67 |
| Efet.01.32672.g536.t1 | ATP-dependent RNA helicase eIF4A | 384 | 1.15E-11 | 67 |
| Efet.01.224739.g990.t1 | Insulin-like growth factor 1 receptor | 345 | 3.99E-22 | 67 |
| Efet.01.1658050.g1089.t1 | Potassium voltage-gated channel subfamily A member 5 | 258 | 5.84E-08 | 67 |
| Efet.01.985.g97.t1 | Potassium voltage-gated channel subfamily B member 1 | 798 | 1.47E-56 | 67 |
| Efet.01.89113.g840.t1 | Potassium voltage-gated channel subfamily B member 1 | 387 | 2.80E-38 | 67 |
| Efet.01.555069.g632.t1 | Potassium channel subfamily K member 3 | 405 | 2.42E-11 | 67 |
| Efet.01.553365.g585.t1 | Histone-lysine N-methyltransferase 2A | 207 | 1.94E-06 | 67 |
| Efet.01.128245.g180.t1 | Laminin subunit beta-2 | 1155 | 2.88E-33 | 67 |
| Efet.01.658325.g1855.t1 | Prolow-density lipoprotein receptor-related protein 1 | 372 | 4.32E-37 | 67 |
| Efet.01.286635.g1326.t1 | Prolow-density lipoprotein receptor-related protein 1 | 648 | 1.33E-65 | 67 |
| Efet.01.486631.g890.t1 | Low-density lipoprotein receptor-related protein 2 | 726 | 1.82E-44 | 67 |
| Efet.01.247864.g1818.t1 | Mitogen-activated protein kinase kinase kinase 12 | 207 | 3.04E-11 | 67 |
| Efet.01.648372.g1234.t1 | S-adenosylmethionine synthase 2 | 1146 | 1.10E-122 | 67 |
| Efet.01.162123.g629.t1 | MAX gene-associated protein | 378 | 1.06E-17 | 67 |
| Efet.01.313716.g433.t1 | Myelin transcription factor 1 | 1092 | 3.62E-14 | 67 |
| Efet.01.21882.g1602.t1 | Neurogenic differentiation factor 6 | 426 | 3.61E-08 | 67 |
| Efet.01.425716.g613.t1 | Bifunctional heparan sulfate N-deacetylase/N-sulfotransferase 1 | 1194 | 8.41E-81 | 67 |
| Efet.01.544428.g347.t1 | Neuroendocrine convertase 1 | 267 | 1.38E-14 | 67 |
| Efet.01.282230.g1168.t1 | Neurogenin-1 | 441 | 4.19E-16 | 67 |
| Efet.01.39361.g956.t1 | Neurogenic locus Notch protein | 375 | 1.27E-35 | 67 |
| Efet.01.313025.g415.t1 | Neurogenic locus Notch protein | 324 | 2.35E-24 | 67 |
| Efet.01.381855.g837.t1 | Paired box protein Pax-2 | 327 | 2.79E-16 | 67 |
| Efet.01.650005.g1.t1 | 1-phosphatidylinositol 4,5-bisphosphate phosphodiesterase delta-4 | 360 | 1.32E-07 | 67 |
| Efet.01.187884.g1777.t1 | Ras-related protein Rab-1A | 210 | 3.63E-15 | 67 |
| Efet.01.542240.g309.t1 | Transforming protein RhoA | 429 | 1.29E-50 | 67 |
| Efet.01.112740.g644.t1 | Retinoic acid receptor RXR-alpha | 345 | 6.91E-20 | 67 |
| Efet.01.83692.g563.t1 | Sodium-dependent serotonin transporter | 333 | 4.84E-23 | 67 |
| Efet.01.217124.g707.t1 | Sodium channel protein type 1 subunit alpha | 327 | 2.00E-19 | 67 |
| Efet.01.9340.g687.t1 | Sodium channel protein type 9 subunit alpha | 1326 | 3.67E-76 | 67 |
| Efet.01.14766.g1081.t1 | Sodium channel protein type 9 subunit alpha | 792 | 2.84E-68 | 67 |
| Efet.01.9340.g686.t1 | Sodium channel protein type 9 subunit alpha | 900 | 2.03E-41 | 67 |
| Efet.01.19816.g1469.t1 | Slit homolog 1 protein | 285 | 3.61E-17 | 67 |
| Efet.01.118523.g954.t1 | SWI/SNF-related matrix-associated actin-dependent regulator of chromatin subfamily A containing DEAD/H box 1 | 312 | 5.85E-23 | 67 |
| Efet.01.82790.g511.t1 | Transcription factor 21 | 522 | 2.51E-11 | 67 |
| Efet.01.469334.g516.t1 | Protein Wnt-5b | 426 | 8.27E-48 | 67 |
| Efet.01.67880.g1108.t1 | Protein Wnt-7a | 309 | 2.58E-26 | 67 |
| Efet.01.1644061.g290.t1 | GTP-binding protein ypt1 | 231 | 1.33E-20 | 67 |
| Efet.01.535313.g170.t1 | GTP-binding protein YPT1 | 291 | 1.33E-09 | 67 |
| Efet.01.187462.g1755.t1 | Palmitoyltransferase ZDHHC23 | 450 | 3.13E-07 | 67 |
| Efet.01.537432.g211.t1 | 5-hydroxytryptamine receptor 1A | 897 | 1.21E-16 | 66 |
| Efet.01.456769.g166.t1 | 5-hydroxytryptamine receptor 1A | 270 | 1.36E-19 | 66 |
| Efet.01.164833.g754.t1 | 5-hydroxytryptamine receptor 1A | 969 | 1.28E-51 | 66 |
| Efet.01.371278.g597.t1 | 5-hydroxytryptamine receptor 2A | 1629 | 2.50E-20 | 66 |
| Efet.01.19637.g1456.t1 | 5-hydroxytryptamine receptor 2C | 2169 | 5.94E-22 | 66 |
| Efet.01.254119.g149.t1 | 5-hydroxytryptamine receptor 2C | 345 | 1.84E-13 | 66 |
| Efet.01.211660.g509.t1 | Beta-2 adrenergic receptor | 1062 | 3.57E-32 | 66 |
| Efet.01.90130.g901.t1 | Annexin A1 | 651 | 1.79E-32 | 66 |
| Efet.01.463340.g359.t1 | Protein atonal homolog 1 | 570 | 9.88E-10 | 66 |
| Efet.01.375927.g702.t1 | Cholecystokinin receptor type A | 423 | 6.21E-18 | 66 |
| Efet.01.193637.g2029.t1 | Cholecystokinin receptor type A | 627 | 1.55E-15 | 66 |
| Efet.01.396287.g1151.t1 | Cholecystokinin receptor type A | 270 | 3.82E-17 | 66 |
| Efet.01.142769.g916.t1 | Beta-enolase | 375 | 3.45E-27 | 66 |
| Efet.01.114632.g762.t1 | Receptor tyrosine-protein kinase erbB-3 | 549 | 5.21E-37 | 66 |
| Efet.01.118004.g927.t1 | Forkhead box protein K1 | 519 | 3.75E-25 | 66 |
| Efet.01.186608.g1707.t1 | Frizzled-7 | 1521 | 3.57E-56 | 66 |
| Efet.01.656870.g1131.t1 | Frizzled-7 | 951 | 3.65E-71 | 66 |
| Efet.01.232471.g1288.t1 | Frizzled-7 | 1068 | 9.58E-77 | 66 |
| Efet.01.371294.g598.t1 | Gamma-aminobutyric acid receptor subunit beta-3 | 390 | 1.21E-12 | 66 |
| Efet.01.1643636.g283.t1 | Gamma-aminobutyric acid receptor subunit beta-3 | 348 | 4.54E-15 | 66 |
| Efet.01.210847.g480.t1 | Glial fibrillary acidic protein | 258 | 3.00E-11 | 66 |
| Efet.01.166417.g814.t1 | Glutamate receptor 1 | 207 | 2.03E-15 | 66 |
| Efet.01.103302.g177.t1 | Glypican-1 | 351 | 3.14E-32 | 66 |
| Efet.01.49245.g1546.t1 | Hepatocyte nuclear factor 6 | 339 | 2.08E-12 | 66 |
| Efet.01.606399.g275.t1 | Hepatocyte nuclear factor 6 | 492 | 7.03E-14 | 66 |
| Efet.01.32239.g506.t1 | Heat shock protein SSA1 | 396 | 4.22E-29 | 66 |
| Efet.01.628190.g1233.t1 | Homeobox protein Hox-B3 | 252 | 2.46E-12 | 66 |
| Efet.01.395045.g1118.t1 | Insulin-like growth factor 2 mRNA-binding protein 1 | 342 | 1.28E-10 | 66 |
| Efet.01.624933.g1086.t1 | ATP-dependent RNA helicase eIF4A | 876 | 1.31E-20 | 66 |
| Efet.01.85957.g693.t1 | ATP-dependent RNA helicase eIF4A | 348 | 1.36E-25 | 66 |
| Efet.01.79897.g315.t1 | Potassium voltage-gated channel subfamily A member 5 | 1083 | 4.46E-118 | 66 |
| Efet.01.1634772.g155.t1 | Kinesin-like protein KIF3C | 234 | 4.75E-11 | 66 |
| Efet.01.21004.g1552.t1 | Kinesin-like protein KIF3C | 3768 | 2.18E-127 | 66 |
| Efet.01.61675.g726.t1 | Mast/stem cell growth factor receptor kita | 627 | 1.26E-08 | 66 |
| Efet.01.407970.g196.t1 | Inactive histone-lysine N-methyltransferase 2E | 810 | 1.79E-17 | 66 |
| Efet.01.1652593.g569.t1 | Low-density lipoprotein receptor-related protein 1 | 204 | 1.04E-16 | 66 |
| Efet.01.87334.g763.t1 | Prolow-density lipoprotein receptor-related protein 1 | 531 | 3.93E-34 | 66 |
| Efet.01.97875.g1333.t1 | DNA replication licensing factor MCM2 | 228 | 7.80E-07 | 66 |
| Efet.01.187552.g1763.t1 | Mitogen-activated protein kinase 7 | 954 | 6.96E-115 | 66 |
| Efet.01.343607.g1240.t1 | 72 kDa type IV collagenase | 222 | 4.78E-12 | 66 |
| Efet.01.135095.g491.t1 | Homeobox protein NANOG | 273 | 2.40E-07 | 66 |
| Efet.01.333751.g1007.t1 | Netrin-1 | 990 | 5.22E-109 | 66 |
| Efet.01.464992.g401.t1 | Neurogenin-1 | 816 | 1.21E-27 | 66 |
| Efet.01.184491.g1611.t1 | Neurogenin-1 | 234 | 3.07E-08 | 66 |
| Efet.01.294922.g1593.t1 | Octopamine receptor beta-3R | 576 | 1.19E-36 | 66 |
| Efet.01.119208.g992.t1 | Melanopsin | 537 | 1.36E-09 | 66 |
| Efet.01.185603.g1658.t1 | Plasminogen activator inhibitor 1 | 210 | 1.93E-10 | 66 |
| Efet.01.583627.g785.t1 | Protein ura1 | 261 | 8.74E-13 | 66 |
| Efet.01.312622.g397.t1 | MYC associated factor X | 2265 | 7.06E-07 | 66 |
| Efet.01.508634.g225.t1 | Ras-related protein Rab-30 | 546 | 1.31E-11 | 66 |
| Efet.01.119353.g1001.t1 | RE1-silencing transcription factor | 273 | 8.25E-09 | 66 |
| Efet.01.421050.g502.t1 | RE1-silencing transcription factor | 2673 | 7.53E-06 | 66 |
| Efet.01.572470.g393.t1 | GTP-binding protein Rit1 | 384 | 6.14E-09 | 66 |
| Efet.01.68720.g1150.t1 | Sodium channel protein type 1 subunit alpha | 1092 | 4.14E-95 | 66 |
| Efet.01.71880.g1355.t1 | Sodium channel protein type 1 subunit alpha | 642 | 5.35E-29 | 66 |
| Efet.01.285707.g1291.t1 | Sodium channel protein type 9 subunit alpha | 303 | 2.16E-15 | 66 |
| Efet.01.96362.g1235.t1 | Spectrin alpha chain, non-erythrocytic 1 | 819 | 1.94E-60 | 66 |
| Efet.01.124546.g1266.t1 | Spectrin alpha chain, non-erythrocytic 1 | 2091 | 5.54E-07 | 66 |
| Efet.01.18459.g1369.t1 | Short transient receptor potential channel 4 | 732 | 3.70E-32 | 66 |
| Efet.01.1638728.g198.t1 | Thymidylate synthase | 237 | 2.51E-21 | 66 |
| Efet.01.470851.g549.t1 | Vascular endothelial growth factor receptor 1 | 405 | 4.21E-24 | 66 |
| Efet.01.83242.g541.t1 | Protein Wnt-4 | 309 | 5.71E-32 | 66 |
| Efet.01.21925.g1606.t1 | Serine/threonine-protein kinase YPK1 | 771 | 5.02E-15 | 66 |
| Efet.01.517765.g436.t1 | Zinc finger homeobox protein 3 | 1488 | 4.87E-09 | 66 |
| Efet.01.608984.g372.t1 | 5-hydroxytryptamine receptor 1A | 1959 | 4.80E-53 | 65 |
| Efet.01.74263.g1503.t1 | 5-hydroxytryptamine receptor 2C | 351 | 2.01E-17 | 65 |
| Efet.01.278227.g1044.t1 | 5-hydroxytryptamine receptor 2C | 465 | 6.61E-13 | 65 |
| Efet.01.70586.g1267.t1 | Disintegrin and metalloproteinase domain-containing protein 15 | 657 | 1.21E-36 | 65 |
| Efet.01.491721.g989.t1 | Homeobox protein AKR | 429 | 1.09E-08 | 65 |
| Efet.01.45444.g1324.t1 | Angiopoietin-1 | 933 | 1.79E-39 | 65 |
| Efet.01.289880.g1444.t1 | Atrial natriuretic peptide receptor 1 | 378 | 1.96E-31 | 65 |
| Efet.01.290323.g1468.t1 | Atrial natriuretic peptide receptor 1 | 1368 | 3.27E-18 | 65 |
| Efet.01.536439.g197.t1 | Atrial natriuretic peptide receptor 1 | 303 | 1.17E-18 | 65 |
| Efet.01.172932.g1092.t1 | Armadillo segment polarity protein | 690 | 3.48E-63 | 65 |
| Efet.01.613715.g619.t1 | Tyrosine-protein kinase Btk29A | 1578 | 1.55E-07 | 65 |
| Efet.01.306527.g213.t1 | Cholecystokinin receptor type A | 402 | 1.16E-21 | 65 |
| Efet.01.420546.g488.t1 | Cholecystokinin receptor type A | 381 | 2.32E-27 | 65 |
| Efet.01.583549.g783.t1 | Cholecystokinin receptor type A | 408 | 1.41E-26 | 65 |
| Efet.01.181045.g1450.t1 | Cyclin-dependent kinase 9 | 342 | 4.14E-12 | 65 |
| Efet.01.601193.g62.t1 | Cystic fibrosis transmembrane conductance regulator | 1428 | 1.27E-07 | 65 |
| Efet.01.329563.g902.t1 | Chromodomain-helicase-DNA-binding protein 7 | 303 | 6.42E-28 | 65 |
| Efet.01.252322.g81.t1 | C-X-C chemokine receptor type 2 | 303 | 3.34E-06 | 65 |
| Efet.01.97534.g1311.t1 | Epithelial discoidin domain-containing receptor 1 | 312 | 1.66E-24 | 65 |
| Efet.01.472391.g605.t1 | Steroid hormone receptor ERR2 | 483 | 5.88E-16 | 65 |
| Efet.01.322458.g702.t1 | Protein furry homolog | 264 | 1.61E-07 | 65 |
| Efet.01.137261.g615.t1 | Gelsolin | 612 | 3.35E-06 | 65 |
| Efet.01.653635.g466.t1 | Glutamine--fructose-6-phosphate aminotransferase [isomerizing] 1 | 285 | 2.15E-07 | 65 |
| Efet.01.53353.g226.t1 | Glucagon-like peptide 1 receptor | 462 | 2.22E-09 | 65 |
| Efet.01.45284.g1316.t1 | Glutamate receptor 1 | 276 | 1.49E-13 | 65 |
| Efet.01.121615.g1126.t1 | Glutamate receptor 1 | 219 | 5.05E-14 | 65 |
| Efet.01.1604645.g9.t1 | Glutamate receptor 1 | 207 | 3.32E-14 | 65 |
| Efet.01.19789.g1467.t1 | Glypican-1 | 432 | 5.63E-25 | 65 |
| Efet.01.47357.g1438.t1 | Histamine H1 receptor | 612 | 1.18E-17 | 65 |
| Efet.01.73780.g1463.t1 | Histamine H1 receptor | 993 | 1.33E-25 | 65 |
| Efet.01.198217.g2224.t1 | Heat shock protein beta-1 | 1134 | 9.27E-12 | 65 |
| Efet.01.253758.g139.t1 | Insulin-like growth factor 1 receptor | 636 | 2.27E-42 | 65 |
| Efet.01.585951.g858.t1 | Potassium channel subfamily K member 3 | 531 | 2.41E-08 | 65 |
| Efet.01.14494.g1065.t1 | Lysine-specific histone demethylase 1A | 1539 | 1.18E-10 | 65 |
| Efet.01.208546.g390.t1 | Laminin subunit beta-2 | 921 | 7.05E-93 | 65 |
| Efet.01.450196.g5.t1 | S-adenosylmethionine synthase isoform type-2 | 321 | 8.83E-28 | 65 |
| Efet.01.475707.g669.t1 | Collagenase 3 | 210 | 3.20E-18 | 65 |
| Efet.01.410874.g244.t1 | Canalicular multispecific organic anion transporter 1 | 384 | 3.50E-15 | 65 |
| Efet.01.255064.g182.t1 | Unconventional myosin-Va | 600 | 2.34E-49 | 65 |
| Efet.01.370047.g558.t1 | Unconventional myosin-Va | 216 | 1.10E-07 | 65 |
| Efet.01.573373.g413.t1 | Unconventional myosin-Va | 276 | 1.72E-12 | 65 |
| Efet.01.337317.g1099.t1 | Netrin-1 | 1218 | 1.14E-107 | 65 |
| Efet.01.278892.g1064.t1 | Nuclear factor interleukin-3-regulated protein | 1209 | 1.22E-10 | 65 |
| Efet.01.70776.g1281.t1 | Neurogenin-1 | 780 | 6.65E-15 | 65 |
| Efet.01.352234.g65.t1 | Bile acid receptor | 324 | 4.52E-13 | 65 |
| Efet.01.288887.g1414.t1 | Octopamine receptor beta-2R | 1209 | 1.52E-22 | 65 |
| Efet.01.298804.g1710.t1 | Phosphoglycerate kinase | 258 | 5.12E-18 | 65 |
| Efet.01.164747.g749.t1 | PR domain zinc finger protein 14 | 222 | 2.12E-15 | 65 |
| Efet.01.251024.g29.t1 | PR domain zinc finger protein 5 | 576 | 7.25E-31 | 65 |
| Efet.01.74978.g1540.t1 | Receptor-type tyrosine-protein phosphatase S | 873 | 1.67E-23 | 65 |
| Efet.01.225708.g1023.t1 | SMARCA4 isoform 2 | 669 | 6.01E-44 | 65 |
| Efet.01.172060.g1058.t1 | Double-strand-break repair protein rad21 homolog | 207 | 2.17E-16 | 65 |
| Efet.01.382875.g863.t1 | Runt-related transcription factor 1 | 363 | 1.24E-24 | 65 |
| Efet.01.58214.g511.t1 | Sodium channel protein type 1 subunit alpha | 861 | 4.03E-67 | 65 |
| Efet.01.152440.g138.t1 | Histone-lysine N-methyltransferase SETDB1 | 702 | 1.15E-42 | 65 |
| Efet.01.38006.g862.t1 | Splicing factor 3A subunit 1 | 420 | 2.93E-06 | 65 |
| Efet.01.7548.g563.t1 | Transcription factor Sox-2 | 624 | 1.69E-35 | 65 |
| Efet.01.367334.g467.t1 | Spectrin beta chain, non-erythrocytic 1 | 243 | 1.67E-07 | 65 |
| Efet.01.581030.g700.t1 | Spectrin beta chain, non-erythrocytic 1 | 318 | 3.89E-08 | 65 |
| Efet.01.650606.g67.t1 | Spectrin alpha chain, non-erythrocytic 1 | 546 | 7.13E-39 | 65 |
| Efet.01.297755.g1682.t1 | Src substrate cortactin | 1599 | 2.12E-08 | 65 |
| Efet.01.605308.g199.t1 | Tumor necrosis factor alpha-induced protein 3 | 450 | 3.44E-09 | 65 |
| Efet.01.144794.g1006.t1 | Short transient receptor potential channel 4 | 429 | 3.54E-30 | 65 |
| Efet.01.188256.g1800.t1 | Transient receptor potential-gamma protein | 822 | 6.96E-37 | 65 |
| Efet.01.56401.g400.t1 | Thrombospondin-2 | 765 | 2.14E-12 | 65 |
| Efet.01.179721.g1384.t1 | Tyrosine-protein kinase receptor UFO | 216 | 7.19E-12 | 65 |
| Efet.01.79283.g268.t1 | Vascular endothelial growth factor receptor 1 | 537 | 4.04E-47 | 65 |
| Efet.01.653787.g475.t1 | von Willebrand factor | 213 | 1.28E-08 | 65 |
| Efet.01.361028.g297.t1 | Zinc finger homeobox protein 3 | 762 | 6.76E-48 | 65 |
| Efet.01.603758.g141.t1 | 5-hydroxytryptamine receptor 1A | 1116 | 1.25E-09 | 64 |
| Efet.01.649562.g1329.t1 | 5-hydroxytryptamine receptor 2A | 285 | 2.18E-16 | 64 |
| Efet.01.276957.g997.t1 | Disintegrin and metalloproteinase domain-containing protein 10 | 906 | 6.47E-30 | 64 |
| Efet.01.543655.g336.t1 | Atrial natriuretic peptide receptor 1 | 213 | 3.41E-14 | 64 |
| Efet.01.1658158.g1117.t1 | Protein atonal homolog 1 | 537 | 1.49E-12 | 64 |
| Efet.01.259307.g356.t1 | Beta-1,4-galactosyltransferase 5 | 273 | 1.15E-16 | 64 |
| Efet.01.58850.g552.t1 | Calumenin | 657 | 3.88E-37 | 64 |
| Efet.01.270586.g761.t1 | Calpain-3 | 372 | 3.39E-26 | 64 |
| Efet.01.144556.g998.t1 | G2/mitotic-specific cyclin-B1 | 375 | 4.17E-26 | 64 |
| Efet.01.199879.g2295.t1 | G2/mitotic-specific cyclin-B1 | 939 | 5.94E-89 | 64 |
| Efet.01.80193.g346.t1 | Chromodomain-helicase-DNA-binding protein 1 | 276 | 5.36E-15 | 64 |
| Efet.01.449958.g1219.t1 | Collagen alpha-1(II) chain | 1533 | 7.72E-06 | 64 |
| Efet.01.541621.g301.t1 | Collagen alpha-1(X) chain | 261 | 7.47E-07 | 64 |
| Efet.01.199447.g2281.t1 | C-X-C chemokine receptor type 4 | 1167 | 8.01E-11 | 64 |
| Efet.01.520921.g494.t1 | Corticosteroid 11-beta-dehydrogenase isozyme 1 | 420 | 8.35E-09 | 64 |
| Efet.01.610445.g439.t1 | Dixin | 519 | 3.33E-19 | 64 |
| Efet.01.65153.g922.t1 | Epidermal growth factor receptor | 906 | 1.26E-89 | 64 |
| Efet.01.243235.g1646.t1 | Early growth response protein 1 | 231 | 1.86E-16 | 64 |
| Efet.01.565819.g192.t1 | ELAV-like protein 4 | 1170 | 3.71E-54 | 64 |
| Efet.01.168410.g910.t1 | Coagulation factor VII | 453 | 4.41E-09 | 64 |
| Efet.01.3114.g271.t1 | Fibroblast growth factor receptor 1 | 1362 | 1.86E-84 | 64 |
| Efet.01.8420.g621.t1 | Gamma-aminobutyric acid receptor subunit beta-3 | 579 | 1.95E-42 | 64 |
| Efet.01.15460.g1138.t1 | Gamma-aminobutyric acid receptor subunit beta-3 | 321 | 3.31E-28 | 64 |
| Efet.01.515187.g366.t1 | Gamma-aminobutyric acid receptor subunit beta-3 | 309 | 2.84E-23 | 64 |
| Efet.01.618826.g848.t1 | Histone acetyltransferase GCN5 | 345 | 7.62E-23 | 64 |
| Efet.01.638173.g419.t1 | Histone acetyltransferase GCN5 | 210 | 1.53E-09 | 64 |
| Efet.01.162760.g645.t1 | Glial fibrillary acidic protein | 507 | 7.13E-15 | 64 |
| Efet.01.434538.g831.t1 | Glial fibrillary acidic protein | 387 | 6.06E-17 | 64 |
| Efet.01.2557.g234.t1 | Solute carrier family 2, facilitated glucose transporter member 4 | 561 | 1.95E-24 | 64 |
| Efet.01.370733.g581.t1 | Hepatocyte nuclear factor 6 | 2925 | 9.99E-11 | 64 |
| Efet.01.1646325.g346.t1 | Homeobox protein Hox-B4a | 300 | 8.23E-24 | 64 |
| Efet.01.87466.g769.t1 | Insulin receptor substrate 2 | 327 | 7.03E-24 | 64 |
| Efet.01.521131.g498.t1 | Potassium voltage-gated channel subfamily A member 5 | 972 | 6.67E-77 | 64 |
| Efet.01.625146.g1104.t1 | Potassium voltage-gated channel subfamily A member 5 | 1632 | 1.38E-100 | 64 |
| Efet.01.648921.g1276.t1 | Potassium voltage-gated channel subfamily B member 1 | 1050 | 2.47E-55 | 64 |
| Efet.01.568360.g272.t1 | Potassium channel subfamily K member 3 | 534 | 4.45E-49 | 64 |
| Efet.01.183459.g1568.t1 | Potassium channel subfamily K member 3 | 822 | 1.05E-14 | 64 |
| Efet.01.358688.g243.t1 | Protein kinase C theta type | 240 | 7.45E-13 | 64 |
| Efet.01.42898.g1171.t1 | Ribosomal protein S6 kinase beta-1 | 417 | 2.38E-28 | 64 |
| Efet.01.466221.g435.t1 | Mediator of RNA polymerase II transcription subunit 13-like | 324 | 4.23E-09 | 64 |
| Efet.01.338669.g1128.t1 | Mediator of RNA polymerase II transcription subunit 13 | 1236 | 2.60E-33 | 64 |
| Efet.01.657660.g1358.t1 | Mitogen-activated protein kinase 14A | 210 | 1.75E-11 | 64 |
| Efet.01.597541.g1210.t1 | Neurogenic locus notch homolog protein 3 | 402 | 1.20E-32 | 64 |
| Efet.01.94858.g1150.t1 | 1-phosphatidylinositol 4,5-bisphosphate phosphodiesterase delta-4 | 738 | 3.47E-62 | 64 |
| Efet.01.218078.g738.t1 | 1-phosphatidylinositol 4,5-bisphosphate phosphodiesterase gamma plc-3 | 426 | 7.69E-26 | 64 |
| Efet.01.587031.g896.t1 | Ribose-phosphate pyrophosphokinase 1 | 918 | 2.23E-64 | 64 |
| Efet.01.426237.g628.t1 | Receptor-type tyrosine-protein phosphatase zeta | 489 | 7.35E-08 | 64 |
| Efet.01.522687.g530.t1 | Receptor-type tyrosine-protein phosphatase S | 465 | 2.77E-39 | 64 |
| Efet.01.379755.g790.t1 | Double-strand-break repair protein rad21 homolog | 705 | 8.29E-16 | 64 |
| Efet.01.174458.g1152.t1 | Retinoblastoma-like protein 2 | 255 | 5.88E-09 | 64 |
| Efet.01.458795.g251.t1 | GTP-binding protein Rit1 | 381 | 2.38E-12 | 64 |
| Efet.01.167398.g860.t1 | Sodium channel protein type 1 subunit alpha | 291 | 1.52E-16 | 64 |
| Efet.01.446102.g1141.t1 | Sodium channel protein type 9 subunit alpha | 300 | 3.24E-16 | 64 |
| Efet.01.408769.g209.t1 | STE20-like serine/threonine-protein kinase | 477 | 9.44E-33 | 64 |
| Efet.01.655882.g892.t1 | SWI/SNF-related matrix-associated actin-dependent regulator of chromatin subfamily A member 5 | 921 | 4.82E-09 | 64 |
| Efet.01.217538.g721.t1 | SWI/SNF-related matrix-associated actin-dependent regulator of chromatin subfamily A containing DEAD/H box 1 | 276 | 2.86E-11 | 64 |
| Efet.01.218309.g745.t1 | Alpha-1-syntrophin | 489 | 1.24E-38 | 64 |
| Efet.01.88971.g835.t1 | Transcription factor Sox-11 | 912 | 2.31E-21 | 64 |
| Efet.01.298396.g1694.t1 | Syntaxin-12 | 387 | 6.72E-11 | 64 |
| Efet.01.242597.g1626.t1 | Tubulin alpha-2 chain | 567 | 7.06E-45 | 64 |
| Efet.01.44229.g1240.t1 | Transcription intermediary factor 1-alpha | 447 | 1.88E-12 | 64 |
| Efet.01.501312.g30.t1 | Transient receptor potential-gamma protein | 633 | 6.53E-31 | 64 |
| Efet.01.362724.g342.t1 | Utrophin | 309 | 5.09E-07 | 64 |
| Efet.01.1657106.g943.t1 | Guanine nucleotide exchange factor VAV2 | 285 | 3.70E-10 | 64 |
| Efet.01.52636.g184.t1 | Vimentin | 453 | 1.19E-11 | 64 |
| Efet.01.646437.g1083.t1 | Y-box-binding protein 3 | 204 | 1.26E-14 | 64 |
| Efet.01.232781.g1300.t1 | Zinc finger protein 37A | 1350 | 5.09E-17 | 64 |
| Efet.01.619725.g874.t1 | Zinc finger protein 37A | 1212 | 4.69E-27 | 64 |
| Efet.01.16387.g1210.t1 | 5-hydroxytryptamine receptor 1A | 399 | 4.45E-07 | 63 |
| Efet.01.59365.g586.t1 | Beta-2 adrenergic receptor | 522 | 1.32E-09 | 63 |
| Efet.01.25057.g6.t1 | AT-rich interactive domain-containing protein 1B | 1716 | 1.33E-71 | 63 |
| Efet.01.1646664.g352.t1 | Armadillo segment polarity protein | 243 | 5.88E-16 | 63 |
| Efet.01.146103.g1058.t1 | Beta-1,4-galactosyltransferase 5 | 216 | 8.14E-14 | 63 |
| Efet.01.47013.g1417.t1 | Serine/threonine-protein kinase B-raf | 897 | 1.71E-33 | 63 |
| Efet.01.190560.g1896.t1 | Cadherin-4 | 468 | 2.03E-11 | 63 |
| Efet.01.39695.g976.t1 | Cholecystokinin receptor type A | 534 | 8.65E-14 | 63 |
| Efet.01.532534.g75.t1 | Cholecystokinin receptor type A | 675 | 7.51E-12 | 63 |
| Efet.01.159489.g495.t1 | Cholecystokinin receptor type A | 453 | 4.67E-16 | 63 |
| Efet.01.319861.g636.t1 | Cholecystokinin receptor type A | 345 | 8.53E-20 | 63 |
| Efet.01.657098.g1166.t1 | Cholecystokinin receptor type A | 633 | 3.39E-19 | 63 |
| Efet.01.325363.g777.t1 | CCAAT/enhancer-binding protein beta | 456 | 3.70E-20 | 63 |
| Efet.01.8404.g619.t1 | G2/mitotic-specific cyclin-3 | 414 | 6.05E-27 | 63 |
| Efet.01.76198.g76.t1 | Chromodomain-helicase-DNA-binding protein 7 | 1617 | 5.85E-177 | 63 |
| Efet.01.90542.g924.t1 | CCR4-NOT transcription complex subunit 1 | 969 | 6.56E-99 | 63 |
| Efet.01.391218.g1032.t1 | Cytochrome P450 3A2 | 450 | 6.14E-22 | 63 |
| Efet.01.401473.g33.t1 | Cytochrome P450 3A2 | 324 | 1.08E-19 | 63 |
| Efet.01.458510.g239.t1 | DAZ-associated protein 1 | 1029 | 4.20E-09 | 63 |
| Efet.01.8184.g609.t1 | Epithelial discoidin domain-containing receptor 1 | 819 | 1.90E-76 | 63 |
| Efet.01.58408.g519.t1 | Protein decapentaplegic | 387 | 5.51E-16 | 63 |
| Efet.01.354879.g139.t1 | Dysferlin | 342 | 9.69E-16 | 63 |
| Efet.01.43410.g1197.t1 | Epidermal growth factor receptor | 1473 | 5.21E-18 | 63 |
| Efet.01.141662.g860.t1 | Receptor tyrosine-protein kinase erbB-4 | 744 | 1.98E-54 | 63 |
| Efet.01.19706.g1460.t1 | Mitogen-activated protein kinase ERK-A | 507 | 2.73E-31 | 63 |
| Efet.01.339877.g1171.t1 | Frizzled-7 | 1866 | 1.80E-171 | 63 |
| Efet.01.30468.g371.t1 | Serine/threonine-protein kinase gad8 | 279 | 4.82E-06 | 63 |
| Efet.01.119547.g1013.t1 | Serine/threonine-protein kinase gad8 | 618 | 1.50E-56 | 63 |
| Efet.01.64906.g904.t1 | Trans-acting T-cell-specific transcription factor GATA-3 | 939 | 2.49E-06 | 63 |
| Efet.01.252654.g99.t1 | Germinal center kinase 1 | 618 | 5.14E-13 | 63 |
| Efet.01.320250.g643.t1 | Glutamate receptor 1 | 660 | 4.16E-51 | 63 |
| Efet.01.10996.g807.t1 | Solute carrier family 2, facilitated glucose transporter member 4 | 396 | 6.51E-20 | 63 |
| Efet.01.3101.g270.t1 | Hypoxia-inducible factor 1-alpha | 396 | 3.32E-34 | 63 |
| Efet.01.657838.g1501.t1 | Protein HMF1 | 843 | 4.70E-12 | 63 |
| Efet.01.277898.g1027.t1 | Homeobox protein Hox-A13 | 345 | 2.49E-13 | 63 |
| Efet.01.231715.g1266.t1 | Homeobox protein Hox-B3 | 312 | 6.37E-16 | 63 |
| Efet.01.444254.g1090.t1 | Homeobox protein Hox-B3 | 480 | 6.69E-13 | 63 |
| Efet.01.528741.g657.t1 | ATP-dependent RNA helicase eIF4A | 576 | 2.07E-16 | 63 |
| Efet.01.59935.g621.t1 | Eukaryotic initiation factor 4A-I | 519 | 7.26E-14 | 63 |
| Efet.01.506751.g171.t1 | Insulin-like growth factor 1 receptor | 498 | 8.67E-40 | 63 |
| Efet.01.422683.g533.t1 | Insulin-like growth factor 1 receptor | 249 | 1.22E-08 | 63 |
| Efet.01.234335.g1352.t1 | Potassium voltage-gated channel subfamily A member 5 | 1008 | 3.17E-60 | 63 |
| Efet.01.405260.g128.t1 | Potassium voltage-gated channel subfamily A member 5 | 807 | 5.41E-59 | 63 |
| Efet.01.640851.g598.t1 | Potassium voltage-gated channel subfamily A member 5 | 1233 | 7.72E-92 | 63 |
| Efet.01.281370.g1138.t1 | Potassium voltage-gated channel subfamily B member 1 | 909 | 8.60E-45 | 63 |
| Efet.01.186595.g1706.t1 | Lysine-specific histone demethylase 1A | 1014 | 4.89E-10 | 63 |
| Efet.01.231384.g1245.t1 | Krueppel-like factor 12 | 1380 | 6.68E-07 | 63 |
| Efet.01.505702.g143.t1 | Plasma kallikrein | 717 | 8.12E-42 | 63 |
| Efet.01.63758.g845.t1 | Protein kinase C theta type | 297 | 1.13E-21 | 63 |
| Efet.01.2155.g191.t1 | Laminin subunit beta-2 | 243 | 5.23E-15 | 63 |
| Efet.01.99420.g1415.t1 | Low-density lipoprotein receptor-related protein 1 | 609 | 1.20E-31 | 63 |
| Efet.01.52691.g189.t1 | Low-density lipoprotein receptor-related protein 2 | 306 | 1.37E-15 | 63 |
| Efet.01.620686.g903.t1 | POU domain protein | 1149 | 3.50E-17 | 63 |
| Efet.01.513435.g323.t1 | Hepatocyte growth factor receptor | 222 | 1.65E-10 | 63 |
| Efet.01.532993.g95.t1 | Matrix metalloproteinase-14 | 342 | 1.24E-12 | 63 |
| Efet.01.603056.g123.t1 | Canalicular multispecific organic anion transporter 1 | 1671 | 2.36E-12 | 63 |
| Efet.01.215215.g637.t1 | Canalicular multispecific organic anion transporter 2 | 300 | 2.22E-09 | 63 |
| Efet.01.1653250.g605.t1 | Myotrophin | 447 | 1.98E-12 | 63 |
| Efet.01.50215.g18.t1 | Neurogenic locus notch homolog protein 2 | 369 | 3.24E-21 | 63 |
| Efet.01.604040.g150.t1 | Octopamine receptor beta-3R | 1110 | 1.04E-17 | 63 |
| Efet.01.619569.g871.t1 | Palladin | 321 | 1.34E-21 | 63 |
| Efet.01.77643.g153.t1 | Protein phosphatase 1D | 468 | 1.93E-10 | 63 |
| Efet.01.44643.g1273.t1 | PR domain zinc finger protein 16 | 531 | 2.21E-13 | 63 |
| Efet.01.267720.g656.t1 | Receptor-type tyrosine-protein phosphatase U | 285 | 4.93E-15 | 63 |
| Efet.01.123153.g1200.t1 | SMARCA4 isoform 2 | 768 | 6.65E-68 | 63 |
| Efet.01.234304.g1350.t1 | Ras-related protein Rab-1A | 468 | 2.69E-32 | 63 |
| Efet.01.338654.g1126.t1 | Ras-related C3 botulinum toxin substrate 1 | 534 | 1.94E-33 | 63 |
| Efet.01.129444.g245.t1 | Ras-related protein Rac2 | 228 | 2.93E-21 | 63 |
| Efet.01.102785.g160.t1 | Regulator of nonsense transcripts 2 | 837 | 2.13E-64 | 63 |
| Efet.01.211491.g499.t1 | RE1-silencing transcription factor | 1464 | 5.89E-12 | 63 |
| Efet.01.3372.g290.t1 | Proto-oncogene tyrosine-protein kinase receptor Ret | 405 | 7.13E-27 | 63 |
| Efet.01.46903.g1406.t1 | Transforming protein RhoA | 297 | 4.39E-29 | 63 |
| Efet.01.638760.g464.t1 | Ras-related protein R-Ras | 237 | 4.19E-06 | 63 |
| Efet.01.212893.g549.t1 | Ras-related protein R-Ras | 537 | 9.29E-46 | 63 |
| Efet.01.1657789.g1037.t1 | Reticulon-4 receptor | 249 | 1.03E-06 | 63 |
| Efet.01.32134.g496.t1 | Solute carrier family 12 member 2 | 762 | 1.57E-34 | 63 |
| Efet.01.1659281.g1509.t1 | Sodium channel protein type 1 subunit alpha | 813 | 2.29E-65 | 63 |
| Efet.01.356415.g180.t1 | Sodium channel protein type 9 subunit alpha | 693 | 7.83E-39 | 63 |
| Efet.01.33073.g558.t1 | Mothers against decapentaplegic homolog 1 | 513 | 5.18E-43 | 63 |
| Efet.01.90300.g917.t1 | SWI/SNF-related matrix-associated actin-dependent regulator of chromatin subfamily A member 5 | 231 | 1.72E-09 | 63 |
| Efet.01.165204.g765.t1 | Spectrin alpha chain, non-erythrocytic 1 | 336 | 6.50E-18 | 63 |
| Efet.01.315484.g489.t1 | Spectrin alpha chain, non-erythrocytic 1 | 471 | 2.01E-28 | 63 |
| Efet.01.406957.g167.t1 | Spectrin alpha chain, non-erythrocytic 1 | 255 | 1.66E-07 | 63 |
| Efet.01.90951.g940.t1 | Proto-oncogene tyrosine-protein kinase Src | 321 | 7.26E-09 | 63 |
| Efet.01.433836.g818.t1 | Histone-lysine N-methyltransferase SUV39H2 | 690 | 2.05E-58 | 63 |
| Efet.01.370247.g564.t1 | Tubulin alpha-2 chain | 531 | 2.92E-41 | 63 |
| Efet.01.9572.g707.t1 | Transient receptor potential-gamma protein | 486 | 1.38E-20 | 63 |
| Efet.01.319767.g634.t1 | Transient receptor potential-gamma protein | 351 | 1.67E-26 | 63 |
| Efet.01.423962.g568.t1 | Vasopressin V1a receptor | 540 | 9.64E-26 | 63 |
| Efet.01.347085.g1323.t1 | Protein white | 288 | 5.91E-13 | 63 |
| Efet.01.273997.g884.t1 | Zinc finger protein 41 homolog | 294 | 7.05E-22 | 63 |
| Efet.01.205904.g256.t1 | 5-hydroxytryptamine receptor 1A | 972 | 1.27E-51 | 62 |
| Efet.01.224622.g983.t1 | 5-hydroxytryptamine receptor 1A | 2676 | 1.44E-30 | 62 |
| Efet.01.99657.g1426.t1 | 5-hydroxytryptamine receptor 1A | 1224 | 2.69E-47 | 62 |
| Efet.01.179495.g1367.t1 | 5-hydroxytryptamine receptor 2A | 285 | 1.79E-18 | 62 |
| Efet.01.224394.g975.t1 | 5-hydroxytryptamine receptor 2A | 1074 | 1.17E-16 | 62 |
| Efet.01.239919.g1534.t1 | 5-hydroxytryptamine receptor 2A | 705 | 2.40E-18 | 62 |
| Efet.01.536532.g200.t1 | 5-hydroxytryptamine receptor 2B | 1848 | 3.25E-22 | 62 |
| Efet.01.564720.g140.t1 | Fructose-bisphosphate aldolase C | 993 | 1.07E-51 | 62 |
| Efet.01.182112.g1504.t1 | AT-rich interactive domain-containing protein 1B | 1944 | 5.42E-45 | 62 |
| Efet.01.227748.g1112.t1 | MGA protein | 363 | 2.30E-21 | 62 |
| Efet.01.361892.g322.t1 | NF-kappa-B inhibitor cactus | 1926 | 1.11E-06 | 62 |
| Efet.01.656032.g931.t1 | Cholecystokinin receptor type A | 378 | 7.82E-24 | 62 |
| Efet.01.1145.g106.t1 | Cholecystokinin receptor type A | 219 | 2.15E-11 | 62 |
| Efet.01.126965.g117.t1 | Cholecystokinin receptor type A | 1026 | 6.79E-21 | 62 |
| Efet.01.268321.g675.t1 | C-C chemokine receptor type 2 | 1032 | 2.81E-16 | 62 |
| Efet.01.656649.g1058.t1 | Cyclin-dependent kinase inhibitor 1 | 312 | 8.26E-12 | 62 |
| Efet.01.658334.g1867.t1 | 60 kDa heat shock protein, mitochondrial | 1602 | 7.73E-101 | 62 |
| Efet.01.52967.g206.t1 | Cytochrome P450 3A12 | 318 | 1.61E-26 | 62 |
| Efet.01.322080.g686.t1 | C-X-C chemokine receptor type 1 | 984 | 1.49E-19 | 62 |
| Efet.01.658419.g2095.t1 | Corticosteroid 11-beta-dehydrogenase isozyme 1 | 1929 | 6.27E-06 | 62 |
| Efet.01.567719.g251.t1 | Endoribonuclease Dicer | 1527 | 2.28E-74 | 62 |
| Efet.01.20078.g1487.t1 | Dedicator of cytokinesis protein 3 | 528 | 2.18E-31 | 62 |
| Efet.01.541676.g305.t1 | Dual specificity protein phosphatase 1 | 516 | 5.65E-14 | 62 |
| Efet.01.357665.g214.t1 | Transcription factor E2F7 | 522 | 1.42E-14 | 62 |
| Efet.01.9021.g662.t1 | Early growth response protein 1 | 1482 | 4.75E-24 | 62 |
| Efet.01.467887.g476.t1 | ELAV-like protein 4 | 219 | 7.90E-07 | 62 |
| Efet.01.621374.g933.t1 | Ephrin type-A receptor 4 | 354 | 5.05E-20 | 62 |
| Efet.01.297866.g1684.t1 | Steroid hormone receptor ERR2 | 276 | 4.66E-14 | 62 |
| Efet.01.338659.g1127.t1 | Steroid hormone receptor ERR2 | 474 | 6.26E-23 | 62 |
| Efet.01.1654540.g697.t1 | Steroid hormone receptor ERR2 | 273 | 4.11E-13 | 62 |
| Efet.01.644201.g871.t1 | Alpha-(1,3)-fucosyltransferase 4 | 912 | 3.34E-06 | 62 |
| Efet.01.417019.g404.t1 | Tyrosine-protein kinase Fyn | 471 | 1.16E-08 | 62 |
| Efet.01.12346.g920.t1 | Gamma-aminobutyric acid receptor subunit beta-3 | 219 | 6.60E-13 | 62 |
| Efet.01.308265.g250.t1 | Germinal center kinase 1 | 495 | 3.71E-32 | 62 |
| Efet.01.248669.g1843.t1 | Glutamate receptor 2 | 1059 | 6.49E-19 | 62 |
| Efet.01.280891.g1127.t1 | Glypican-1 | 405 | 8.93E-29 | 62 |
| Efet.01.140506.g799.t1 | Nuclear hormone receptor HR96 | 780 | 4.29E-42 | 62 |
| Efet.01.349854.g1401.t1 | Nuclear hormone receptor HR96 | 747 | 7.21E-14 | 62 |
| Efet.01.197807.g2211.t1 | Histamine H1 receptor | 2004 | 9.07E-16 | 62 |
| Efet.01.47357.g1439.t1 | Histamine H1 receptor | 1533 | 6.98E-17 | 62 |
| Efet.01.552742.g571.t1 | Histamine H2 receptor | 534 | 5.36E-10 | 62 |
| Efet.01.215751.g655.t1 | Heat shock 70 kDa protein | 567 | 2.24E-36 | 62 |
| Efet.01.269109.g709.t1 | ATP-dependent RNA helicase eIF4A | 402 | 7.60E-15 | 62 |
| Efet.01.171183.g1024.t1 | Indian hedgehog protein | 1110 | 3.32E-92 | 62 |
| Efet.01.86761.g736.t1 | Insulin gene enhancer protein ISL-1 | 1209 | 1.05E-08 | 62 |
| Efet.01.413886.g324.t1 | Insulin gene enhancer protein ISL-1 | 228 | 1.45E-10 | 62 |
| Efet.01.402876.g75.t1 | Potassium voltage-gated channel subfamily A member 5 | 306 | 1.50E-09 | 62 |
| Efet.01.25653.g55.t1 | Potassium voltage-gated channel subfamily A member 5 | 1302 | 7.67E-76 | 62 |
| Efet.01.310657.g340.t1 | Potassium voltage-gated channel subfamily A member 5 | 999 | 2.21E-70 | 62 |
| Efet.01.501260.g28.t1 | Lysine-specific demethylase 3A | 288 | 1.25E-20 | 62 |
| Efet.01.553365.g587.t1 | Histone-lysine N-methyltransferase 2A | 1200 | 4.92E-07 | 62 |
| Efet.01.449198.g1199.t1 | Histone-lysine N-methyltransferase 2D | 483 | 4.74E-18 | 62 |
| Efet.01.101223.g81.t1 | Serine/threonine-protein kinase D1 | 1575 | 1.29E-09 | 62 |
| Efet.01.19609.g1453.t1 | Neural cell adhesion molecule L1 | 207 | 1.24E-08 | 62 |
| Efet.01.62805.g793.t1 | Low-density lipoprotein receptor-related protein 2 | 558 | 3.48E-35 | 62 |
| Efet.01.80174.g345.t1 | Tyrosine-protein kinase Lyn | 270 | 3.75E-15 | 62 |
| Efet.01.342115.g1210.t1 | POU domain protein | 888 | 1.10E-30 | 62 |
| Efet.01.628078.g1230.t1 | MAX gene-associated protein | 660 | 3.23E-31 | 62 |
| Efet.01.489283.g947.t1 | Mitogen-activated protein kinase 12 | 555 | 4.87E-27 | 62 |
| Efet.01.311559.g369.t1 | Neuroendocrine convertase 1 | 471 | 8.57E-26 | 62 |
| Efet.01.266383.g596.t1 | Homeobox protein Nkx-2.2 | 681 | 2.81E-18 | 62 |
| Efet.01.254952.g180.t1 | Neuromedin-U receptor 1 | 477 | 4.44E-06 | 62 |
| Efet.01.86475.g721.t1 | Neurogenic locus Notch protein | 1011 | 1.43E-87 | 62 |
| Efet.01.56912.g428.t1 | Octopamine receptor beta-2R | 1413 | 2.42E-104 | 62 |
| Efet.01.376288.g709.t1 | Tumor protein p73 | 285 | 6.47E-15 | 62 |
| Efet.01.1645469.g323.t1 | Palladin | 252 | 1.33E-19 | 62 |
| Efet.01.318823.g606.t1 | Protocadherin-15 | 447 | 1.00E-06 | 62 |
| Efet.01.128219.g178.t1 | PR domain zinc finger protein 16 | 1086 | 6.08E-52 | 62 |
| Efet.01.591317.g1036.t1 | PR domain zinc finger protein 5 | 1281 | 1.67E-06 | 62 |
| Efet.01.160746.g559.t1 | Receptor-type tyrosine-protein phosphatase S | 222 | 5.90E-09 | 62 |
| Efet.01.294470.g1582.t1 | E3 ubiquitin-protein ligase RNF13 | 2223 | 3.69E-06 | 62 |
| Efet.01.78699.g221.t1 | Rap guanine nucleotide exchange factor 3 | 291 | 6.04E-17 | 62 |
| Efet.01.476046.g673.t1 | Ras-related protein R-Ras | 390 | 2.17E-21 | 62 |
| Efet.01.319627.g628.t1 | Solute carrier family 12 member 2 | 240 | 4.83E-15 | 62 |
| Efet.01.261413.g432.t1 | Sal-like protein 1 | 2307 | 4.85E-48 | 62 |
| Efet.01.1655.g153.t1 | Sodium channel protein type 1 subunit alpha | 552 | 2.07E-31 | 62 |
| Efet.01.64587.g891.t1 | Sodium channel protein type 1 subunit alpha | 342 | 4.62E-13 | 62 |
| Efet.01.312.g36.t1 | Sodium channel protein type 9 subunit alpha | 402 | 1.76E-16 | 62 |
| Efet.01.135109.g494.t1 | Septin-4 | 252 | 1.93E-12 | 62 |
| Efet.01.547139.g423.t1 | Sonic hedgehog protein | 972 | 2.04E-89 | 62 |
| Efet.01.45882.g1350.t1 | S-phase kinase-associated protein 2 | 414 | 1.79E-07 | 62 |
| Efet.01.643605.g813.t1 | Transcription factor Sox-2 | 1407 | 1.12E-32 | 62 |
| Efet.01.644693.g891.t1 | Transcription factor Sox-2 | 1362 | 3.13E-25 | 62 |
| Efet.01.38223.g876.t1 | Spectrin beta chain, non-erythrocytic 1 | 1407 | 8.69E-120 | 62 |
| Efet.01.43739.g1216.t1 | Spectrin beta chain, non-erythrocytic 1 | 342 | 1.61E-13 | 62 |
| Efet.01.104751.g263.t1 | Spectrin beta chain, non-erythrocytic 1 | 516 | 9.99E-23 | 62 |
| Efet.01.368253.g491.t1 | Spectrin beta chain, non-erythrocytic 1 | 270 | 3.15E-08 | 62 |
| Efet.01.188078.g1791.t1 | Spectrin alpha chain, non-erythrocytic 1 | 717 | 2.06E-43 | 62 |
| Efet.01.215816.g658.t1 | Tyrosine-protein kinase Src64B | 393 | 1.68E-12 | 62 |
| Efet.01.347437.g1337.t1 | Serrate RNA effector molecule homolog | 426 | 4.12E-16 | 62 |
| Efet.01.241023.g1568.t1 | Serine/threonine-protein kinase 3 | 282 | 1.36E-12 | 62 |
| Efet.01.119982.g1031.t1 | Transcription factor 21 | 513 | 2.72E-13 | 62 |
| Efet.01.432187.g777.t1 | Tyrosine-protein kinase Tec | 870 | 1.96E-37 | 62 |
| Efet.01.607077.g295.t1 | Transcription factor p65 | 381 | 2.93E-17 | 62 |
| Efet.01.32366.g520.t1 | TGF-beta receptor type-1 | 1548 | 8.16E-141 | 62 |
| Efet.01.181701.g1489.t1 | TNF receptor-associated factor 6 | 414 | 1.31E-08 | 62 |
| Efet.01.178419.g1330.t1 | TNF receptor-associated factor 6 | 252 | 2.66E-09 | 62 |
| Efet.01.187700.g1768.t1 | Short transient receptor potential channel 4 | 879 | 9.67E-50 | 62 |
| Efet.01.13075.g987.t1 | Dual specificity protein kinase Ttk | 390 | 1.49E-23 | 62 |
| Efet.01.655988.g924.t1 | Thymidylate synthase | 897 | 4.74E-97 | 62 |
| Efet.01.154386.g220.t1 | Ubiquitin-conjugating enzyme E2 2 | 303 | 7.51E-24 | 62 |
| Efet.01.392427.g1063.t1 | RutC family protein UK114 | 222 | 8.40E-12 | 62 |
| Efet.01.216967.g700.t1 | Vasopressin V1a receptor | 609 | 3.80E-40 | 62 |
| Efet.01.241486.g1582.t1 | Vasopressin V1a receptor | 462 | 6.15E-12 | 62 |
| Efet.01.33301.g571.t1 | Vang-like protein 2 | 1689 | 9.29E-101 | 62 |
| Efet.01.183407.g1564.t1 | Vimentin | 474 | 3.22E-07 | 62 |
| Efet.01.138103.g677.t1 | Protein Wnt-4 | 447 | 2.36E-45 | 62 |
| Efet.01.234175.g1347.t1 | GTP-binding protein YPT1 | 405 | 3.66E-24 | 62 |
| Efet.01.193608.g2023.t1 | Zinc finger protein 37A | 501 | 2.48E-42 | 62 |
| Efet.01.1631066.g111.t1 | Zinc finger protein 37A | 369 | 4.81E-28 | 62 |
| Efet.01.38438.g895.t1 | 5-hydroxytryptamine receptor 1A | 1893 | 8.04E-41 | 61 |
| Efet.01.456769.g165.t1 | 5-hydroxytryptamine receptor 1A | 1098 | 5.48E-39 | 61 |
| Efet.01.15226.g1120.t1 | 5-hydroxytryptamine receptor 2A | 2493 | 1.22E-16 | 61 |
| Efet.01.232820.g1302.t1 | 5-hydroxytryptamine receptor 2A | 387 | 1.63E-18 | 61 |
| Efet.01.570091.g328.t1 | Atypical chemokine receptor 3 | 738 | 2.98E-25 | 61 |
| Efet.01.85372.g647.t1 | Disintegrin and metalloproteinase domain-containing protein 15 | 387 | 2.04E-16 | 61 |
| Efet.01.647720.g1196.t1 | Alcohol dehydrogenase 1C | 513 | 5.46E-20 | 61 |
| Efet.01.658012.g1555.t1 | Alcohol dehydrogenase 1C | 252 | 2.36E-12 | 61 |
| Efet.01.1656827.g917.t1 | Beta-2 adrenergic receptor | 495 | 1.01E-09 | 61 |
| Efet.01.193858.g2044.t1 | Atrial natriuretic peptide receptor 1 | 327 | 3.41E-09 | 61 |
| Efet.01.518799.g452.t1 | Atrial natriuretic peptide receptor 1 | 402 | 8.36E-11 | 61 |
| Efet.01.9417.g696.t1 | Bridging integrator 3 | 408 | 3.83E-25 | 61 |
| Efet.01.159707.g507.t1 | Cadherin-4 | 423 | 1.38E-09 | 61 |
| Efet.01.192031.g1964.t1 | Calcitonin receptor | 279 | 3.72E-06 | 61 |
| Efet.01.60523.g651.t1 | Carboxypeptidase B2 | 252 | 1.34E-09 | 61 |
| Efet.01.271589.g808.t1 | Cholecystokinin receptor type A | 411 | 1.15E-18 | 61 |
| Efet.01.374880.g680.t1 | Cholecystokinin receptor type A | 909 | 2.28E-13 | 61 |
| Efet.01.354456.g127.t1 | Cyclin-dependent kinase 2 | 282 | 1.01E-11 | 61 |
| Efet.01.449436.g1203.t1 | Cyclin-dependent kinase 9 | 591 | 2.28E-25 | 61 |
| Efet.01.525091.g586.t1 | Cytochrome P450 3A2 | 294 | 4.94E-20 | 61 |
| Efet.01.303006.g93.t1 | Cholesterol 7-alpha-monooxygenase | 429 | 4.21E-28 | 61 |
| Efet.01.12748.g954.t1 | Endoribonuclease Dcr-1 | 405 | 8.99E-35 | 61 |
| Efet.01.8816.g644.t1 | Deleted in malignant brain tumors 1 protein | 375 | 6.06E-10 | 61 |
| Efet.01.270288.g752.t1 | Dipeptidyl peptidase 4 | 216 | 7.67E-09 | 61 |
| Efet.01.5975.g475.t1 | Dysferlin | 375 | 1.62E-29 | 61 |
| Efet.01.277007.g998.t1 | Ephrin type-B receptor 3 | 240 | 7.99E-16 | 61 |
| Efet.01.638688.g460.t1 | Fibroblast growth factor receptor 1 | 273 | 1.73E-14 | 61 |
| Efet.01.399586.g1236.t1 | Fibrinogen-like protein 1 | 567 | 1.61E-46 | 61 |
| Efet.01.354564.g132.t1 | Four and a half LIM domains protein 2 | 1806 | 2.31E-17 | 61 |
| Efet.01.606524.g283.t1 | Four and a half LIM domains protein 2 | 1287 | 3.68E-19 | 61 |
| Efet.01.211986.g518.t1 | Forkhead box protein C2 | 1308 | 5.81E-32 | 61 |
| Efet.01.497535.g1126.t1 | Frizzled-7 | 1878 | 8.99E-140 | 61 |
| Efet.01.33507.g584.t1 | Gamma-aminobutyric acid receptor subunit beta-3 | 270 | 3.89E-13 | 61 |
| Efet.01.303542.g115.t1 | Gamma-aminobutyric acid receptor subunit beta-3 | 351 | 2.18E-14 | 61 |
| Efet.01.564919.g153.t1 | Gamma-aminobutyric acid receptor subunit beta-3 | 615 | 9.03E-22 | 61 |
| Efet.01.483637.g827.t1 | Histone acetyltransferase GCN5 | 459 | 6.13E-11 | 61 |
| Efet.01.85490.g655.t1 | Glial fibrillary acidic protein | 525 | 2.94E-07 | 61 |
| Efet.01.176130.g1211.t1 | Glutamate receptor 1 | 282 | 9.52E-07 | 61 |
| Efet.01.201280.g66.t1 | Histone deacetylase 4 | 576 | 3.82E-22 | 61 |
| Efet.01.654303.g559.t1 | Porphobilinogen deaminase | 942 | 7.92E-48 | 61 |
| Efet.01.95410.g1181.t1 | Protein arginine N-methyltransferase 1 | 996 | 2.67E-55 | 61 |
| Efet.01.636379.g331.t1 | Heat shock protein SSA1 | 828 | 1.25E-71 | 61 |
| Efet.01.25685.g58.t1 | Transcription factor AP-1 | 1077 | 1.45E-18 | 61 |
| Efet.01.258920.g335.t1 | Potassium voltage-gated channel subfamily A member 5 | 579 | 3.81E-33 | 61 |
| Efet.01.83082.g531.t1 | Potassium voltage-gated channel subfamily A member 5 | 1563 | 2.59E-114 | 61 |
| Efet.01.1658810.g1282.t1 | Potassium voltage-gated channel subfamily A member 5 | 1221 | 1.26E-114 | 61 |
| Efet.01.653210.g407.t1 | Laminin subunit alpha-5 | 408 | 2.11E-17 | 61 |
| Efet.01.579669.g664.t1 | Low-density lipoprotein receptor-related protein | 438 | 4.36E-35 | 61 |
| Efet.01.579669.g663.t1 | Low-density lipoprotein receptor-related protein 2 | 783 | 5.74E-50 | 61 |
| Efet.01.75411.g20.t1 | POU domain protein | 840 | 3.00E-26 | 61 |
| Efet.01.78232.g192.t1 | Mitogen-activated protein kinase kinase kinase 12 | 1218 | 5.84E-51 | 61 |
| Efet.01.655926.g895.t1 | Microtubule-associated protein 1B | 681 | 1.11E-14 | 61 |
| Efet.01.203125.g150.t1 | [F-actin]-monooxygenase MICAL3 | 279 | 5.32E-15 | 61 |
| Efet.01.366245.g437.t1 | [F-actin]-monooxygenase MICAL3 | 1887 | 9.02E-07 | 61 |
| Efet.01.607698.g321.t1 | Canalicular multispecific organic anion transporter 1 | 498 | 1.28E-09 | 61 |
| Efet.01.452136.g54.t1 | RNA-binding protein Musashi homolog 1 | 309 | 5.69E-13 | 61 |
| Efet.01.636391.g333.t1 | RNA-binding protein Musashi homolog 1 | 222 | 5.17E-12 | 61 |
| Efet.01.444919.g1104.t1 | Myotrophin | 870 | 8.20E-09 | 61 |
| Efet.01.240723.g1557.t1 | Nucleus accumbens-associated protein 1 | 498 | 2.09E-06 | 61 |
| Efet.01.293225.g1546.t1 | Neurogenic differentiation factor 1 | 594 | 2.28E-13 | 61 |
| Efet.01.129807.g256.t1 | Protein NDRG1 | 255 | 1.59E-06 | 61 |
| Efet.01.111804.g593.t1 | Nuclear factor of activated T-cells, cytoplasmic 3 | 441 | 1.79E-26 | 61 |
| Efet.01.467973.g478.t1 | Neurogenic locus Notch protein | 462 | 5.84E-27 | 61 |
| Efet.01.19353.g1433.t1 | Oxysterols receptor LXR-beta | 219 | 3.53E-13 | 61 |
| Efet.01.518908.g455.t1 | 3-phosphoinositide-dependent protein kinase 1 | 486 | 1.35E-08 | 61 |
| Efet.01.577665.g611.t1 | Phosphoglycerate kinase | 216 | 1.79E-07 | 61 |
| Efet.01.161840.g613.t1 | Phosphatidylinositol 4,5-bisphosphate 3-kinase catalytic subunit gamma isoform | 519 | 1.00E-18 | 61 |
| Efet.01.515990.g398.t1 | 1-phosphatidylinositol 4,5-bisphosphate phosphodiesterase gamma-1 | 444 | 2.44E-07 | 61 |
| Efet.01.42663.g1151.t1 | PR domain zinc finger protein 16 | 1905 | 2.02E-14 | 61 |
| Efet.01.451554.g36.t1 | PR domain zinc finger protein 5 | 780 | 1.47E-11 | 61 |
| Efet.01.610211.g433.t1 | Receptor-type tyrosine-protein phosphatase U | 402 | 1.22E-21 | 61 |
| Efet.01.512594.g299.t1 | Dexamethasone-induced Ras-related protein 1 | 726 | 1.33E-40 | 61 |
| Efet.01.613817.g622.t1 | Retinoblastoma-like protein 2 | 372 | 2.37E-12 | 61 |
| Efet.01.64697.g895.t1 | Reelin | 378 | 5.62E-07 | 61 |
| Efet.01.109181.g478.t1 | GTP-binding protein Rit1 | 408 | 5.10E-19 | 61 |
| Efet.01.563953.g127.t1 | E3 ubiquitin-protein ligase RNF13 | 510 | 4.70E-12 | 61 |
| Efet.01.7015.g540.t1 | Roundabout homolog 2 | 636 | 5.02E-47 | 61 |
| Efet.01.23001.g1676.t1 | Retinoic acid receptor RXR-alpha | 762 | 1.83E-49 | 61 |
| Efet.01.173457.g1116.t1 | Semaphorin-2A | 771 | 3.40E-52 | 61 |
| Efet.01.626070.g1138.t1 | Slit homolog 1 protein | 267 | 9.81E-19 | 61 |
| Efet.01.3649.g311.t1 | STE20-like serine/threonine-protein kinase | 456 | 1.71E-10 | 61 |
| Efet.01.171446.g1033.t1 | Superoxide dismutase [Mn], mitochondrial | 423 | 3.33E-22 | 61 |
| Efet.01.398399.g1202.t1 | Superoxide dismutase [Mn], mitochondrial | 558 | 1.06E-43 | 61 |
| Efet.01.1659093.g1402.t1 | Spastin | 744 | 3.05E-11 | 61 |
| Efet.01.284784.g1255.t1 | Spectrin alpha chain, non-erythrocytic 1 | 387 | 7.73E-21 | 61 |
| Efet.01.172277.g1063.t1 | Src substrate cortactin | 318 | 4.22E-11 | 61 |
| Efet.01.200243.g17.t1 | Serine/threonine-protein kinase 25 | 429 | 1.26E-33 | 61 |
| Efet.01.441947.g1031.t1 | Serine/threonine-protein kinase 3 | 273 | 4.00E-17 | 61 |
| Efet.01.439279.g927.t1 | Tubulin alpha-1 chain | 735 | 1.11E-47 | 61 |
| Efet.01.1649255.g431.t1 | Tenascin | 330 | 7.76E-30 | 61 |
| Efet.01.31498.g451.t1 | TNFAIP3-interacting protein 2 | 423 | 3.94E-08 | 61 |
| Efet.01.134765.g477.t1 | Transient receptor potential-gamma protein | 387 | 5.91E-14 | 61 |
| Efet.01.80523.g371.t1 | Serine/threonine-protein kinase ULK1 | 339 | 9.26E-12 | 61 |
| Efet.01.1945.g175.t1 | Utrophin | 291 | 2.09E-06 | 61 |
| Efet.01.643528.g810.t1 | Guanine nucleotide exchange factor VAV3 | 1008 | 5.41E-08 | 61 |
| Efet.01.57859.g492.t1 | Vitamin D3 receptor A | 399 | 3.10E-08 | 61 |
| Efet.01.290247.g1459.t1 | Vascular endothelial growth factor receptor 1 | 855 | 2.30E-19 | 61 |
| Efet.01.629351.g1288.t1 | Protein Wnt-2b | 447 | 7.07E-27 | 61 |
| Efet.01.649156.g1286.t1 | Zinc finger homeobox protein 3 | 1095 | 1.53E-10 | 61 |
| Efet.01.351182.g38.t1 | Zinc finger protein 37A | 1488 | 4.24E-56 | 61 |
| Efet.01.66965.g1042.t1 | 5-hydroxytryptamine receptor 1A | 276 | 1.42E-06 | 60 |
| Efet.01.23843.g1728.t1 | 5-hydroxytryptamine receptor 1A | 369 | 8.34E-10 | 60 |
| Efet.01.83040.g528.t1 | 5-hydroxytryptamine receptor 1A | 393 | 1.94E-18 | 60 |
| Efet.01.27710.g172.t1 | 5-hydroxytryptamine receptor 2C | 1341 | 1.15E-21 | 60 |
| Efet.01.47379.g1440.t1 | Serine/threonine-protein kinase akt-1 | 381 | 2.54E-26 | 60 |
| Efet.01.344802.g1264.t1 | Serine/threonine-protein kinase akt-1 | 255 | 1.43E-15 | 60 |
| Efet.01.16513.g1218.t1 | Androgen receptor | 339 | 1.21E-08 | 60 |
| Efet.01.95375.g1179.t1 | Atrial natriuretic peptide receptor 1 | 705 | 2.68E-15 | 60 |
| Efet.01.31463.g449.t1 | Aquaporin-1 | 825 | 9.07E-44 | 60 |
| Efet.01.6257.g493.t1 | Calretinin | 261 | 2.56E-07 | 60 |
| Efet.01.1628109.g85.t1 | Carboxypeptidase B2 | 240 | 4.63E-11 | 60 |
| Efet.01.19407.g1436.t1 | Cholecystokinin receptor type A | 483 | 1.09E-16 | 60 |
| Efet.01.27763.g179.t1 | Cholecystokinin receptor type A | 840 | 4.29E-18 | 60 |
| Efet.01.54581.g303.t1 | C-C chemokine receptor type 7 | 483 | 1.06E-06 | 60 |
| Efet.01.656795.g1104.t1 | Chromodomain Y-like protein | 777 | 6.89E-16 | 60 |
| Efet.01.160307.g542.t1 | Chromodomain-helicase-DNA-binding protein 1 | 318 | 4.69E-12 | 60 |
| Efet.01.294674.g1588.t1 | Chromodomain-helicase-DNA-binding protein 7 | 1122 | 4.54E-13 | 60 |
| Efet.01.372855.g643.t1 | Chromodomain-helicase-DNA-binding protein 7 | 330 | 1.19E-19 | 60 |
| Efet.01.3361.g288.t1 | Collagen alpha-1(II) chain | 822 | 9.39E-07 | 60 |
| Efet.01.162043.g624.t1 | Complement C3 | 255 | 3.41E-11 | 60 |
| Efet.01.140944.g820.t1 | Catenin beta-1 | 954 | 6.03E-76 | 60 |
| Efet.01.282810.g1188.t1 | C-X-C chemokine receptor type 1 | 441 | 3.96E-11 | 60 |
| Efet.01.321727.g677.t1 | Endoribonuclease dcr-1 | 429 | 5.66E-07 | 60 |
| Efet.01.537674.g221.t1 | Epithelial discoidin domain-containing receptor 1 | 306 | 9.19E-15 | 60 |
| Efet.01.242998.g1637.t1 | Deleted in malignant brain tumors 1 protein | 237 | 1.77E-07 | 60 |
| Efet.01.658245.g1747.t1 | Dihydropyrimidinase-related protein 2 | 1257 | 4.14E-108 | 60 |
| Efet.01.307.g35.t1 | Dysferlin | 531 | 1.94E-18 | 60 |
| Efet.01.279695.g1089.t1 | Dysferlin | 315 | 2.38E-17 | 60 |
| Efet.01.259657.g369.t1 | Ephrin type-B receptor 3 | 279 | 6.94E-20 | 60 |
| Efet.01.186881.g1718.t1 | Protein C-ets-1 | 327 | 9.41E-10 | 60 |
| Efet.01.1649902.g455.t1 | Fibrinogen-like protein 1 | 399 | 2.63E-15 | 60 |
| Efet.01.389262.g992.t1 | Four and a half LIM domains protein 2 | 225 | 2.73E-13 | 60 |
| Efet.01.419997.g474.t1 | Four and a half LIM domains protein 2 | 1680 | 3.65E-16 | 60 |
| Efet.01.496522.g1099.t1 | Fibrinogen alpha chain | 387 | 9.05E-08 | 60 |
| Efet.01.655549.g826.t1 | Fibrinogen alpha chain | 225 | 4.53E-18 | 60 |
| Efet.01.589955.g990.t1 | Forkhead box protein J3 | 642 | 2.09E-26 | 60 |
| Efet.01.323111.g715.t1 | Forkhead box protein M1 | 975 | 8.88E-13 | 60 |
| Efet.01.56158.g389.t1 | Gamma-aminobutyric acid receptor subunit beta-3 | 309 | 6.07E-12 | 60 |
| Efet.01.442147.g1038.t1 | Gamma-aminobutyric acid receptor subunit beta-3 | 234 | 1.04E-11 | 60 |
| Efet.01.658342.g1877.t1 | Histone acetyltransferase gcn5 | 1647 | 3.34E-17 | 60 |
| Efet.01.113921.g709.t1 | Gelsolin | 252 | 2.83E-16 | 60 |
| Efet.01.14748.g1077.t1 | Guanine nucleotide-binding protein-like 3 | 714 | 4.57E-39 | 60 |
| Efet.01.98627.g1363.t1 | Glutamate receptor 3 | 330 | 1.31E-23 | 60 |
| Efet.01.477481.g701.t1 | Hepatocyte nuclear factor 6 | 1092 | 4.90E-13 | 60 |
| Efet.01.429340.g722.t1 | Nuclear hormone receptor HR96 | 1185 | 2.34E-38 | 60 |
| Efet.01.360459.g281.t1 | Histamine H1 receptor | 1758 | 8.40E-16 | 60 |
| Efet.01.550874.g522.t1 | Probable heat shock protein ssa1 | 522 | 5.93E-23 | 60 |
| Efet.01.627560.g1213.t1 | Zinc finger and SCAN domain-containing protein 10 | 699 | 7.52E-41 | 60 |
| Efet.01.224880.g997.t1 | Integrin-linked protein kinase | 426 | 1.02E-09 | 60 |
| Efet.01.288824.g1412.t1 | Integrin-linked protein kinase homolog pat-4 | 294 | 1.34E-10 | 60 |
| Efet.01.455425.g141.t1 | Insulin gene enhancer protein isl-1 | 330 | 1.45E-13 | 60 |
| Efet.01.1304.g119.t1 | Integrin alpha-V | 423 | 4.41E-15 | 60 |
| Efet.01.25721.g60.t1 | Potassium voltage-gated channel subfamily A member 5 | 1359 | 2.39E-68 | 60 |
| Efet.01.163725.g691.t1 | Potassium voltage-gated channel subfamily A member 5 | 1056 | 1.18E-59 | 60 |
| Efet.01.262936.g495.t1 | Potassium voltage-gated channel subfamily A member 5 | 1320 | 2.81E-90 | 60 |
| Efet.01.128390.g192.t1 | Potassium voltage-gated channel subfamily B member 1 | 474 | 2.19E-09 | 60 |
| Efet.01.612737.g562.t1 | Lysine-specific demethylase 3A | 288 | 2.70E-11 | 60 |
| Efet.01.58869.g555.t1 | Kinesin-like protein KIF3C | 771 | 1.67E-36 | 60 |
| Efet.01.65243.g927.t1 | Kinesin-like protein KIF3C | 804 | 3.07E-33 | 60 |
| Efet.01.152944.g158.t1 | Laminin subunit alpha-1 | 267 | 1.04E-08 | 60 |
| Efet.01.354504.g128.t1 | Low-density lipoprotein receptor-related protein 1 | 477 | 6.94E-14 | 60 |
| Efet.01.5833.g459.t1 | Matrilin-2 | 951 | 1.66E-36 | 60 |
| Efet.01.229254.g1173.t1 | DNA replication licensing factor MCM2 | 477 | 2.63E-09 | 60 |
| Efet.01.59460.g598.t1 | [F-actin]-monooxygenase MICAL3 | 399 | 2.59E-12 | 60 |
| Efet.01.140871.g814.t1 | Protein CBFA2T3 | 399 | 2.53E-28 | 60 |
| Efet.01.156857.g360.t1 | Myogenic factor 6 | 1128 | 8.47E-09 | 60 |
| Efet.01.73968.g1477.t1 | Unconventional myosin-Va | 630 | 1.19E-46 | 60 |
| Efet.01.142242.g890.t1 | Nucleus accumbens-associated protein 1 | 2106 | 3.37E-17 | 60 |
| Efet.01.22865.g1667.t1 | Neural cell adhesion molecule 1 | 612 | 1.17E-09 | 60 |
| Efet.01.620247.g889.t1 | Netrin-1 | 300 | 9.26E-20 | 60 |
| Efet.01.113299.g674.t1 | Nuclear factor NF-kappa-B p105 subunit | 315 | 2.05E-15 | 60 |
| Efet.01.194314.g2063.t1 | Neuromedin-U receptor 1 | 375 | 8.12E-13 | 60 |
| Efet.01.16642.g1228.t1 | Neuromedin-U receptor 1 | 540 | 3.93E-21 | 60 |
| Efet.01.181257.g1465.t1 | Neuromedin-U receptor 1 | 882 | 2.40E-36 | 60 |
| Efet.01.658334.g1868.t1 | Natural resistance-associated macrophage protein 1 | 873 | 4.86E-34 | 60 |
| Efet.01.101628.g106.t1 | Octopamine receptor beta-2R | 594 | 2.02E-37 | 60 |
| Efet.01.392803.g1072.t1 | Octopamine receptor beta-3R | 894 | 1.41E-09 | 60 |
| Efet.01.19332.g1431.t1 | Palladin | 1068 | 9.67E-19 | 60 |
| Efet.01.67832.g1102.t1 | Inactive serine protease PAMR1 | 264 | 7.03E-07 | 60 |
| Efet.01.201192.g60.t1 | Protocadherin-15 | 705 | 8.64E-20 | 60 |
| Efet.01.81350.g428.t1 | 1-phosphatidylinositol 4,5-bisphosphate phosphodiesterase delta-1 | 483 | 1.17E-35 | 60 |
| Efet.01.205760.g250.t1 | 1-phosphatidylinositol 4,5-bisphosphate phosphodiesterase gamma-1 | 345 | 5.16E-20 | 60 |
| Efet.01.359684.g263.t1 | PR domain zinc finger protein 16 | 639 | 3.15E-09 | 60 |
| Efet.01.130402.g294.t1 | Tyrosine-protein phosphatase non-receptor type 3 | 459 | 2.09E-09 | 60 |
| Efet.01.118045.g929.t1 | Receptor-type tyrosine-protein phosphatase S | 654 | 3.69E-19 | 60 |
| Efet.01.80921.g397.t1 | Receptor-type tyrosine-protein phosphatase S | 396 | 2.20E-18 | 60 |
| Efet.01.184056.g1598.t1 | Peroxidasin | 348 | 1.31E-21 | 60 |
| Efet.01.82718.g506.t1 | Uncharacterized protein DKFZp686A1782 | 4191 | 2.36E-06 | 60 |
| Efet.01.416099.g378.t1 | Ras-related protein Rab-1A | 303 | 2.54E-15 | 60 |
| Efet.01.608299.g343.t1 | Reelin | 486 | 2.19E-25 | 60 |
| Efet.01.649586.g1331.t1 | Roundabout homolog 2 | 228 | 3.25E-12 | 60 |
| Efet.01.502484.g58.t1 | Retinoic acid receptor RXR-alpha | 360 | 4.24E-08 | 60 |
| Efet.01.40449.g1019.t1 | Solute carrier family 12 member 2 | 390 | 5.56E-08 | 60 |
| Efet.01.164062.g711.t1 | Sodium channel protein type 9 subunit alpha | 546 | 1.11E-19 | 60 |
| Efet.01.497455.g1122.t1 | STE20-like serine/threonine-protein kinase | 363 | 5.55E-17 | 60 |
| Efet.01.503482.g81.t1 | SWI/SNF-related matrix-associated actin-dependent regulator of chromatin subfamily A member 5 | 744 | 2.37E-27 | 60 |
| Efet.01.360655.g288.t1 | Transcription factor Sox-2 | 993 | 6.22E-40 | 60 |
| Efet.01.390740.g1020.t1 | Spastin | 441 | 5.14E-17 | 60 |
| Efet.01.303203.g98.t1 | Spastin | 501 | 7.24E-23 | 60 |
| Efet.01.51521.g99.t1 | Spectrin beta chain, non-erythrocytic 1 | 372 | 7.61E-14 | 60 |
| Efet.01.92962.g1050.t1 | Spectrin beta chain, non-erythrocytic 1 | 441 | 2.68E-27 | 60 |
| Efet.01.286820.g1336.t1 | Serine/threonine-protein kinase 3 | 729 | 6.61E-54 | 60 |
| Efet.01.30749.g396.t1 | Tubulin alpha-1C chain | 954 | 1.05E-65 | 60 |
| Efet.01.1216.g110.t1 | Tyrosine-protein kinase Tec | 642 | 7.28E-06 | 60 |
| Efet.01.326409.g813.t1 | TNF receptor-associated factor 6 | 426 | 1.17E-32 | 60 |
| Efet.01.1655969.g809.t1 | Short transient receptor potential channel 4 | 273 | 6.27E-07 | 60 |
| Efet.01.45600.g1331.t1 | Transient receptor potential-gamma protein | 456 | 4.22E-22 | 60 |
| Efet.01.226428.g1054.t1 | Transient receptor potential-gamma protein | 573 | 2.61E-41 | 60 |
| Efet.01.73184.g1432.t1 | Ubiquitin-conjugating enzyme E2 B | 372 | 1.18E-23 | 60 |
| Efet.01.656828.g1124.t1 | UDP-glucuronosyltransferase 1-2 | 765 | 2.79E-48 | 60 |
| Efet.01.228204.g1133.t1 | Vasopressin V1a receptor | 948 | 2.10E-47 | 60 |
| Efet.01.256420.g237.t1 | GTP-binding protein YPT1 | 600 | 6.72E-41 | 60 |
| Efet.01.125508.g30.t1 | Palmitoyltransferase ZDHHC23 | 1035 | 1.43E-08 | 60 |
| Efet.01.123569.g1224.t1 | Zinc finger protein 37A | 732 | 5.48E-56 | 60 |
| Efet.01.653068.g397.t1 | Zinc finger protein 37A | 1497 | 4.72E-76 | 60 |
| Efet.01.1635206.g159.t1 | Zinc finger protein 37A | 375 | 1.18E-20 | 60 |
| Efet.01.457868.g215.t1 | 5-hydroxytryptamine receptor 1A | 1260 | 4.38E-82 | 59 |
| Efet.01.68955.g1170.t1 | 5-hydroxytryptamine receptor 2A | 465 | 2.20E-12 | 59 |
| Efet.01.77431.g139.t1 | 5-hydroxytryptamine receptor 2B | 789 | 3.05E-08 | 59 |
| Efet.01.282770.g1184.t1 | 5-hydroxytryptamine receptor 2B | 261 | 3.36E-07 | 59 |
| Efet.01.133581.g418.t1 | Akirin-1 | 291 | 2.46E-08 | 59 |
| Efet.01.257809.g290.t1 | RAC-beta serine/threonine-protein kinase | 303 | 3.92E-17 | 59 |
| Efet.01.175771.g1201.t1 | Cadherin-1 | 441 | 1.99E-16 | 59 |
| Efet.01.411005.g248.t1 | Caspase-8 | 345 | 1.67E-12 | 59 |
| Efet.01.598882.g1269.t1 | Carbonyl reductase [NADPH] 1 | 594 | 5.47E-11 | 59 |
| Efet.01.232962.g1306.t1 | CD9 antigen | 246 | 4.32E-06 | 59 |
| Efet.01.651940.g243.t1 | Cystic fibrosis transmembrane conductance regulator | 573 | 2.96E-06 | 59 |
| Efet.01.189.g23.t1 | Chromodomain-helicase-DNA-binding protein 1 | 1263 | 4.15E-24 | 59 |
| Efet.01.194464.g2068.t1 | Chromodomain-helicase-DNA-binding protein 1 | 915 | 2.02E-113 | 59 |
| Efet.01.167274.g854.t1 | Cytochrome P450 3A2 | 366 | 3.29E-19 | 59 |
| Efet.01.96273.g1232.t1 | Connective tissue growth factor | 279 | 2.12E-12 | 59 |
| Efet.01.50534.g40.t1 | Citron Rho-interacting kinase | 1200 | 3.35E-49 | 59 |
| Efet.01.168346.g904.t1 | Dystroglycan | 921 | 8.23E-26 | 59 |
| Efet.01.1651955.g536.t1 | DAZ-associated protein 1 | 261 | 1.74E-12 | 59 |
| Efet.01.233779.g1327.t1 | Deleted in malignant brain tumors 1 protein | 378 | 9.16E-17 | 59 |
| Efet.01.211445.g496.t1 | Dihydropyrimidinase-related protein 2 | 258 | 5.31E-09 | 59 |
| Efet.01.145056.g1018.t1 | Dual specificity protein phosphatase 1 | 507 | 2.07E-07 | 59 |
| Efet.01.12984.g978.t1 | Ephrin type-B receptor 3 | 528 | 2.29E-26 | 59 |
| Efet.01.491629.g988.t1 | Fibroblast growth factor receptor 1 | 396 | 3.51E-21 | 59 |
| Efet.01.651187.g145.t1 | GRB2-associated-binding protein 1 | 249 | 7.14E-08 | 59 |
| Efet.01.140299.g787.t1 | Glycine amidinotransferase, mitochondrial | 336 | 3.11E-26 | 59 |
| Efet.01.657551.g1328.t1 | Glutamate receptor 1 | 846 | 1.30E-54 | 59 |
| Efet.01.471635.g581.t1 | Glutamate receptor 1 | 279 | 7.14E-12 | 59 |
| Efet.01.428715.g703.t1 | Glutamate receptor 3 | 486 | 4.01E-22 | 59 |
| Efet.01.280092.g1104.t1 | E3 ISG15--protein ligase HERC5 | 1137 | 2.18E-29 | 59 |
| Efet.01.377895.g748.t1 | E3 ISG15--protein ligase HERC5 | 666 | 1.15E-07 | 59 |
| Efet.01.58977.g563.t1 | Nuclear hormone receptor HR96 | 1302 | 5.69E-27 | 59 |
| Efet.01.336401.g1079.t1 | Nuclear hormone receptor HR96 | 762 | 2.10E-34 | 59 |
| Efet.01.44391.g1250.t1 | Insulin-like growth factor 2 mRNA-binding protein 1 | 216 | 5.24E-10 | 59 |
| Efet.01.64706.g897.t1 | Insulin-like growth factor 2 mRNA-binding protein 1 | 687 | 5.06E-18 | 59 |
| Efet.01.547070.g420.t1 | Eukaryotic initiation factor 4A-I | 528 | 3.07E-24 | 59 |
| Efet.01.312852.g408.t1 | Insulin-like growth factor 1 receptor | 510 | 5.17E-27 | 59 |
| Efet.01.155465.g277.t1 | Insulin gene enhancer protein ISL-1 | 390 | 6.48E-14 | 59 |
| Efet.01.5650.g440.t1 | Potassium voltage-gated channel subfamily A member 5 | 1107 | 2.12E-107 | 59 |
| Efet.01.150866.g42.t1 | Potassium voltage-gated channel subfamily A member 5 | 1467 | 6.01E-57 | 59 |
| Efet.01.320499.g646.t1 | Potassium voltage-gated channel subfamily A member 5 | 954 | 1.96E-80 | 59 |
| Efet.01.167362.g858.t1 | Lysine-specific demethylase 3A | 273 | 6.06E-06 | 59 |
| Efet.01.259865.g376.t1 | Lysine-specific demethylase 4A | 759 | 2.62E-69 | 59 |
| Efet.01.196095.g2131.t1 | Lysine-specific demethylase 4B | 798 | 4.96E-31 | 59 |
| Efet.01.79303.g272.t1 | Kinesin-like protein KIF3C | 975 | 1.46E-34 | 59 |
| Efet.01.34250.g640.t1 | Laminin subunit alpha | 504 | 2.69E-07 | 59 |
| Efet.01.87721.g784.t1 | Laminin subunit alpha | 750 | 2.52E-26 | 59 |
| Efet.01.486631.g889.t1 | Low-density lipoprotein receptor-related protein | 249 | 1.25E-12 | 59 |
| Efet.01.113110.g659.t1 | Prolow-density lipoprotein receptor-related protein 1 | 588 | 1.70E-35 | 59 |
| Efet.01.603047.g122.t1 | Latent-transforming growth factor beta-binding protein 4 | 405 | 3.37E-26 | 59 |
| Efet.01.554457.g614.t1 | Mitogen-activated protein kinase kinase kinase 12 | 363 | 3.41E-21 | 59 |
| Efet.01.591949.g1053.t1 | Mitogen-activated protein kinase kinase kinase 12 | 708 | 3.59E-22 | 59 |
| Efet.01.191963.g1959.t1 | Hepatocyte growth factor receptor | 690 | 1.69E-24 | 59 |
| Efet.01.551655.g542.t1 | RNA-binding protein Musashi homolog 1 | 429 | 5.25E-13 | 59 |
| Efet.01.194568.g2070.t1 | Myotrophin | 864 | 6.48E-10 | 59 |
| Efet.01.1658795.g1278.t1 | Unconventional myosin-Va | 402 | 6.11E-12 | 59 |
| Efet.01.75095.g9.t1 | Unconventional myosin-Va | 294 | 9.36E-11 | 59 |
| Efet.01.396818.g1164.t1 | Unconventional myosin-Va | 363 | 8.76E-17 | 59 |
| Efet.01.571127.g356.t1 | Neural cell adhesion molecule 1 | 327 | 8.11E-08 | 59 |
| Efet.01.658319.g1852.t1 | Netrin-1 | 339 | 5.40E-20 | 59 |
| Efet.01.227538.g1101.t1 | Nuclear factor interleukin-3-regulated protein | 366 | 7.14E-08 | 59 |
| Efet.01.226079.g1043.t1 | Homeobox protein Nkx-2.2 | 849 | 5.57E-21 | 59 |
| Efet.01.17140.g1264.t1 | Neuromedin-U receptor 1 | 375 | 1.31E-15 | 59 |
| Efet.01.658250.g1752.t1 | Nitric oxide synthase, brain | 441 | 2.24E-09 | 59 |
| Efet.01.144008.g976.t1 | Neuropilin-2 | 414 | 1.39E-12 | 59 |
| Efet.01.77155.g121.t1 | Octopamine receptor beta-2R | 1050 | 5.68E-72 | 59 |
| Efet.01.97817.g1330.t1 | Octopamine receptor beta-2R | 1446 | 1.17E-85 | 59 |
| Efet.01.194314.g2064.t1 | Melanopsin | 1251 | 5.20E-06 | 59 |
| Efet.01.52751.g192.t1 | Melanopsin | 876 | 6.57E-42 | 59 |
| Efet.01.435664.g846.t1 | Inactive serine protease PAMR1 | 399 | 2.04E-09 | 59 |
| Efet.01.91681.g981.t1 | Poly [ADP-ribose] polymerase 1 | 603 | 4.95E-37 | 59 |
| Efet.01.548789.g465.t1 | Paxillin | 252 | 4.55E-10 | 59 |
| Efet.01.579347.g660.t1 | Protocadherin-15 | 267 | 6.80E-08 | 59 |
| Efet.01.10961.g804.t1 | 1-phosphatidylinositol 4,5-bisphosphate phosphodiesterase delta-4 | 360 | 3.27E-21 | 59 |
| Efet.01.105471.g292.t1 | Plastin-2 | 216 | 2.60E-07 | 59 |
| Efet.01.402163.g56.t1 | PR domain zinc finger protein 16 | 1050 | 1.81E-17 | 59 |
| Efet.01.43904.g1221.t1 | Tyrosine-protein phosphatase non-receptor type 3 | 306 | 3.19E-17 | 59 |
| Efet.01.200.g27.t1 | Roundabout homolog 2 | 504 | 7.68E-39 | 59 |
| Efet.01.155842.g302.t1 | R-spondin-1 | 225 | 1.49E-06 | 59 |
| Efet.01.67688.g1089.t1 | Solute carrier family 12 member 2 | 399 | 2.05E-16 | 59 |
| Efet.01.77174.g124.t1 | Sodium channel protein type 1 subunit alpha | 222 | 3.77E-10 | 59 |
| Efet.01.431156.g764.t1 | Sodium channel protein type 9 subunit alpha | 210 | 6.46E-15 | 59 |
| Efet.01.656506.g1034.t1 | Sodium channel protein type 9 subunit alpha | 423 | 1.75E-08 | 59 |
| Efet.01.96837.g1271.t1 | Semaphorin-2A | 315 | 4.28E-16 | 59 |
| Efet.01.80572.g377.t1 | Zinc finger protein SNAI1 | 2013 | 5.74E-12 | 59 |
| Efet.01.478766.g731.t1 | Zinc finger protein SNAI1 | 387 | 1.41E-27 | 59 |
| Efet.01.625264.g1111.t1 | Superoxide dismutase [Mn], mitochondrial | 678 | 3.02E-48 | 59 |
| Efet.01.359293.g255.t1 | Spectrin beta chain, non-erythrocytic 1 | 4248 | 0 | 59 |
| Efet.01.428848.g705.t1 | Serine/threonine-protein kinase STK11 | 504 | 5.30E-17 | 59 |
| Efet.01.68336.g1126.t1 | Transcription factor 21 | 774 | 1.66E-13 | 59 |
| Efet.01.336929.g1089.t1 | Tenascin | 345 | 5.71E-07 | 59 |
| Efet.01.167584.g872.t1 | TNF receptor-associated factor 6 | 432 | 1.73E-18 | 59 |
| Efet.01.534802.g154.t1 | UDP-glucuronosyltransferase 1-1 | 429 | 1.63E-19 | 59 |
| Efet.01.78872.g233.t1 | Vasopressin V1a receptor | 954 | 1.68E-53 | 59 |
| Efet.01.1644771.g311.t1 | Protein white | 414 | 8.87E-10 | 59 |
| Efet.01.445097.g1114.t1 | ABC transporter ATP-binding protein/permease wht-1 | 450 | 3.83E-13 | 59 |
| Efet.01.57379.g461.t1 | GTP-binding protein YPT1 | 432 | 1.23E-21 | 59 |
| Efet.01.520061.g477.t1 | Zinc finger homeobox protein 3 | 3477 | 1.00E-108 | 59 |
| Efet.01.261138.g418.t1 | Zinc finger protein ZIC 2 | 900 | 7.75E-63 | 59 |
| Efet.01.46569.g1385.t1 | Zinc finger protein 37A | 648 | 1.18E-38 | 59 |
| Efet.01.95093.g1167.t1 | Zinc finger protein 37A | 624 | 2.44E-47 | 59 |
| Efet.01.186906.g1721.t1 | Zinc finger protein 37A | 825 | 1.03E-53 | 59 |
| Efet.01.249213.g1867.t1 | 5-hydroxytryptamine receptor 1A | 957 | 1.85E-06 | 58 |
| Efet.01.23590.g1709.t1 | 5-hydroxytryptamine receptor 2A | 312 | 6.72E-20 | 58 |
| Efet.01.556705.g673.t1 | 5-hydroxytryptamine receptor 2B | 1374 | 9.85E-22 | 58 |
| Efet.01.177893.g1307.t1 | 5-hydroxytryptamine receptor 2B | 612 | 2.42E-11 | 58 |
| Efet.01.531405.g45.t1 | ATP-binding cassette sub-family G member 2 | 384 | 3.00E-10 | 58 |
| Efet.01.328117.g865.t1 | Disintegrin and metalloproteinase domain-containing protein 15 | 279 | 6.09E-14 | 58 |
| Efet.01.152236.g130.t1 | Beta-2 adrenergic receptor | 1047 | 4.59E-61 | 58 |
| Efet.01.206847.g303.t1 | Atrial natriuretic peptide receptor 1 | 225 | 2.88E-07 | 58 |
| Efet.01.552276.g558.t1 | Atrial natriuretic peptide receptor 1 | 852 | 3.25E-14 | 58 |
| Efet.01.170284.g990.t1 | AT-rich interactive domain-containing protein 1B | 1806 | 1.51E-70 | 58 |
| Efet.01.288638.g1400.t1 | Aurora kinase A | 579 | 5.49E-32 | 58 |
| Efet.01.326123.g805.t1 | Aurora kinase A | 384 | 3.56E-13 | 58 |
| Efet.01.37955.g861.t1 | Axin-1 | 990 | 1.93E-33 | 58 |
| Efet.01.137584.g644.t1 | Axin-1 | 882 | 2.70E-42 | 58 |
| Efet.01.653635.g467.t1 | C-1-tetrahydrofolate synthase, cytoplasmic | 549 | 4.73E-31 | 58 |
| Efet.01.96625.g1257.t1 | NF-kappa-B inhibitor cactus | 441 | 8.52E-07 | 58 |
| Efet.01.298810.g1711.t1 | Cadherin-99C | 222 | 1.55E-08 | 58 |
| Efet.01.542241.g310.t1 | Calpain-3 | 255 | 3.11E-07 | 58 |
| Efet.01.651586.g200.t1 | CREB-binding protein | 408 | 3.74E-11 | 58 |
| Efet.01.576944.g553.t1 | Cholecystokinin receptor type A | 813 | 1.51E-14 | 58 |
| Efet.01.200726.g39.t1 | C-C chemokine receptor type 2 | 747 | 1.37E-15 | 58 |
| Efet.01.653365.g423.t1 | C-C chemokine receptor type 2 | 606 | 7.27E-17 | 58 |
| Efet.01.365522.g423.t1 | Cyclin-dependent kinase 9 | 489 | 3.51E-09 | 58 |
| Efet.01.398129.g1195.t1 | Probable cyclin-dependent kinase 9 | 435 | 3.66E-19 | 58 |
| Efet.01.583689.g790.t1 | Cyclin-dependent kinase inhibitor 1B | 351 | 6.28E-10 | 58 |
| Efet.01.38271.g882.t1 | Complement C5 | 663 | 8.14E-15 | 58 |
| Efet.01.552303.g559.t1 | Sterol 26-hydroxylase, mitochondrial | 231 | 1.38E-06 | 58 |
| Efet.01.24289.g1764.t1 | COP9 signalosome complex subunit 2 | 312 | 1.10E-07 | 58 |
| Efet.01.227482.g1096.t1 | Endoribonuclease Dcr-1 | 1020 | 1.54E-78 | 58 |
| Efet.01.49683.g1576.t1 | Epithelial discoidin domain-containing receptor 1 | 1257 | 1.06E-08 | 58 |
| Efet.01.1659192.g1454.t1 | Deleted in malignant brain tumors 1 protein | 378 | 2.77E-20 | 58 |
| Efet.01.652674.g333.t1 | Dysferlin | 453 | 1.10E-22 | 58 |
| Efet.01.195382.g2107.t1 | Dysferlin | 303 | 3.61E-06 | 58 |
| Efet.01.62007.g750.t1 | Dysferlin | 555 | 1.29E-10 | 58 |
| Efet.01.372580.g631.t1 | Egl nine homolog 1 | 1026 | 1.96E-07 | 58 |
| Efet.01.78179.g186.t1 | Early growth response protein 1 | 342 | 1.53E-16 | 58 |
| Efet.01.400102.g2.t1 | Receptor tyrosine-protein kinase erbB-4 | 597 | 5.35E-10 | 58 |
| Efet.01.46849.g1405.t1 | Germinal center kinase 1 | 306 | 3.34E-08 | 58 |
| Efet.01.634438.g246.t1 | Glutamine--fructose-6-phosphate aminotransferase [isomerizing] 1 | 1080 | 1.83E-64 | 58 |
| Efet.01.192165.g1967.t1 | Glucagon-like peptide 1 receptor | 357 | 4.23E-16 | 58 |
| Efet.01.93535.g1076.t1 | Serine/threonine-protein kinase hippo | 369 | 6.56E-12 | 58 |
| Efet.01.10588.g777.t1 | Histamine H1 receptor | 1371 | 1.39E-12 | 58 |
| Efet.01.143655.g955.t1 | Histamine H1 receptor | 2394 | 7.16E-32 | 58 |
| Efet.01.430569.g748.t1 | Histamine H1 receptor | 1914 | 4.76E-18 | 58 |
| Efet.01.68866.g1162.t1 | Histamine H1 receptor | 1074 | 2.62E-15 | 58 |
| Efet.01.60297.g641.t1 | Zinc finger and SCAN domain-containing protein 10 | 273 | 3.06E-12 | 58 |
| Efet.01.330278.g922.t1 | Zinc finger and SCAN domain-containing protein 10 | 1020 | 7.58E-48 | 58 |
| Efet.01.423413.g555.t1 | Zinc finger and SCAN domain-containing protein 10 | 825 | 6.55E-27 | 58 |
| Efet.01.1657637.g1020.t1 | Zinc finger and SCAN domain-containing protein 10 | 255 | 3.60E-12 | 58 |
| Efet.01.204628.g201.t1 | ATP-dependent RNA helicase eIF4A | 435 | 2.40E-16 | 58 |
| Efet.01.412154.g273.t1 | ATP-dependent RNA helicase eIF4A | 279 | 2.86E-26 | 58 |
| Efet.01.654418.g586.t1 | ATP-dependent RNA helicase eIF4A | 1350 | 1.23E-58 | 58 |
| Efet.01.481701.g784.t1 | Interferon-related developmental regulator 1 | 354 | 2.55E-12 | 58 |
| Efet.01.533832.g138.t1 | Integrin-linked protein kinase | 438 | 2.65E-10 | 58 |
| Efet.01.154210.g211.t1 | Potassium voltage-gated channel subfamily B member 1 | 561 | 1.79E-13 | 58 |
| Efet.01.506207.g159.t1 | Potassium channel subfamily K member 3 | 321 | 1.02E-06 | 58 |
| Efet.01.248063.g1824.t1 | Potassium channel subfamily K member 3 | 1611 | 1.71E-06 | 58 |
| Efet.01.615474.g691.t1 | Lysine-specific demethylase 4B | 885 | 2.60E-30 | 58 |
| Efet.01.614863.g674.t1 | Plasma kallikrein | 258 | 2.11E-10 | 58 |
| Efet.01.578127.g622.t1 | Ribosomal protein S6 kinase beta-1 | 276 | 1.43E-08 | 58 |
| Efet.01.506990.g181.t1 | Laminin subunit alpha-5 | 300 | 1.05E-12 | 58 |
| Efet.01.1645249.g319.t1 | Protein lin-41 | 354 | 7.05E-06 | 58 |
| Efet.01.506957.g178.t1 | Prolow-density lipoprotein receptor-related protein 1 | 324 | 1.71E-18 | 58 |
| Efet.01.177191.g1276.t1 | Lumican | 1026 | 4.37E-07 | 58 |
| Efet.01.582142.g749.t1 | POU domain protein | 582 | 3.04E-12 | 58 |
| Efet.01.53017.g209.t1 | Microtubule-associated protein 1B | 1644 | 7.72E-20 | 58 |
| Efet.01.143327.g946.t1 | Microspherule protein 1 | 231 | 1.14E-15 | 58 |
| Efet.01.37225.g806.t1 | Mediator of RNA polymerase II transcription subunit 14 | 849 | 2.78E-28 | 58 |
| Efet.01.74090.g1489.t1 | Canalicular multispecific organic anion transporter 2 | 426 | 1.33E-16 | 58 |
| Efet.01.128690.g209.t1 | Neurocan core protein | 327 | 1.90E-08 | 58 |
| Efet.01.264731.g555.t1 | Neurogenic differentiation factor 1 | 849 | 7.66E-09 | 58 |
| Efet.01.27761.g178.t1 | Necdin | 780 | 1.27E-34 | 58 |
| Efet.01.149786.g1217.t1 | Neuromedin-U receptor 1 | 1203 | 6.32E-09 | 58 |
| Efet.01.428255.g686.t1 | Neuromedin-U receptor 1 | 630 | 2.19E-12 | 58 |
| Efet.01.216373.g682.t1 | Nitric oxide synthase, brain | 315 | 3.68E-11 | 58 |
| Efet.01.324176.g746.t1 | Neurogenic locus notch homolog protein 1 | 564 | 4.94E-26 | 58 |
| Efet.01.107332.g380.t1 | Neurogenic locus Notch protein | 996 | 1.15E-72 | 58 |
| Efet.01.279731.g1090.t1 | Neuropilin-1 | 291 | 3.44E-13 | 58 |
| Efet.01.335490.g1062.t1 | Nematocin receptor 2 | 792 | 4.10E-06 | 58 |
| Efet.01.547849.g439.t1 | BDNF/NT-3 growth factors receptor | 486 | 1.46E-31 | 58 |
| Efet.01.139400.g740.t1 | BDNF/NT-3 growth factors receptor | 1197 | 5.42E-72 | 58 |
| Efet.01.397305.g1178.t1 | Octopamine receptor beta-2R | 720 | 3.31E-06 | 58 |
| Efet.01.25268.g34.t1 | Octopamine receptor beta-3R | 273 | 9.54E-20 | 58 |
| Efet.01.171920.g1053.t1 | Melanopsin | 639 | 6.45E-07 | 58 |
| Efet.01.65341.g933.t1 | Melanopsin | 459 | 2.41E-12 | 58 |
| Efet.01.553112.g576.t1 | Melanopsin | 705 | 7.49E-37 | 58 |
| Efet.01.419761.g464.t1 | Palladin | 1062 | 4.79E-15 | 58 |
| Efet.01.603066.g124.t1 | Inactive serine protease PAMR1 | 216 | 1.21E-06 | 58 |
| Efet.01.658189.g1702.t1 | Pappalysin-1 | 438 | 5.52E-06 | 58 |
| Efet.01.161247.g580.t1 | Poly [ADP-ribose] polymerase 1 | 489 | 8.88E-06 | 58 |
| Efet.01.113895.g707.t1 | Protocadherin-15 | 354 | 8.25E-06 | 58 |
| Efet.01.575084.g482.t1 | Phosphatidylinositol 4,5-bisphosphate 3-kinase catalytic subunit gamma isoform | 255 | 1.91E-13 | 58 |
| Efet.01.17744.g1309.t1 | 1-phosphatidylinositol 4,5-bisphosphate phosphodiesterase gamma plc-3 | 408 | 2.62E-16 | 58 |
| Efet.01.17494.g1290.t1 | PR domain zinc finger protein 16 | 1092 | 3.04E-13 | 58 |
| Efet.01.190689.g1900.t1 | PR domain zinc finger protein 16 | 2517 | 9.98E-13 | 58 |
| Efet.01.22804.g1661.t1 | Receptor-type tyrosine-protein phosphatase S | 402 | 1.41E-16 | 58 |
| Efet.01.618695.g841.t1 | Protein ura1 | 642 | 3.55E-19 | 58 |
| Efet.01.654418.g594.t1 | Protein URA2 | 3105 | 0 | 58 |
| Efet.01.502787.g69.t1 | Dexamethasone-induced Ras-related protein 1 | 732 | 1.29E-37 | 58 |
| Efet.01.613416.g606.t1 | Ribonucleoside-diphosphate reductase large subunit | 1680 | 3.36E-75 | 58 |
| Efet.01.654050.g532.t1 | Roundabout homolog 2 | 378 | 3.25E-22 | 58 |
| Efet.01.207156.g320.t1 | Rap guanine nucleotide exchange factor 3 | 1323 | 9.12E-07 | 58 |
| Efet.01.656025.g929.t1 | Ribosomal RNA-processing protein 8 | 363 | 3.58E-19 | 58 |
| Efet.01.279329.g1077.t1 | Sodium channel protein type 9 subunit alpha | 582 | 9.64E-12 | 58 |
| Efet.01.5268.g410.t1 | Serine/threonine-protein kinase Sgk1 | 396 | 4.18E-21 | 58 |
| Efet.01.104261.g240.t1 | Slit homolog 1 protein | 663 | 1.24E-09 | 58 |
| Efet.01.171232.g1025.t1 | Alpha-1-syntrophin | 375 | 7.64E-06 | 58 |
| Efet.01.617979.g792.t1 | Transcription factor Sox-2 | 882 | 3.15E-23 | 58 |
| Efet.01.84888.g622.t1 | Spastin | 348 | 1.81E-08 | 58 |
| Efet.01.181324.g1472.t1 | Spastin | 666 | 8.70E-06 | 58 |
| Efet.01.5848.g464.t1 | Spectrin beta chain, non-erythrocytic 1 | 489 | 2.30E-14 | 58 |
| Efet.01.73083.g1423.t1 | Spectrin alpha chain, non-erythrocytic 1 | 432 | 4.50E-25 | 58 |
| Efet.01.107118.g367.t1 | Serine/threonine-protein kinase 3 | 273 | 3.07E-15 | 58 |
| Efet.01.1659086.g1396.t1 | Tenascin | 1176 | 3.37E-10 | 58 |
| Efet.01.3630.g309.t1 | Angiopoietin-1 receptor | 273 | 2.15E-16 | 58 |
| Efet.01.70319.g1249.t1 | Tumor necrosis factor alpha-induced protein 3 | 549 | 1.34E-27 | 58 |
| Efet.01.1653071.g597.t1 | TNF receptor-associated factor 6 | 297 | 7.84E-11 | 58 |
| Efet.01.16198.g1197.t1 | TNF receptor-associated factor 6 | 957 | 6.82E-41 | 58 |
| Efet.01.415847.g370.t1 | Transient receptor potential-gamma protein | 288 | 5.12E-12 | 58 |
| Efet.01.318747.g602.t1 | Peroxiredoxin TSA1 | 576 | 1.01E-39 | 58 |
| Efet.01.1655997.g811.t1 | Serine/threonine-protein kinase ULK1 | 270 | 2.81E-07 | 58 |
| Efet.01.101039.g71.t1 | Vasopressin V1a receptor | 462 | 2.31E-22 | 58 |
| Efet.01.11418.g832.t1 | Vasopressin V1a receptor | 1368 | 7.29E-07 | 58 |
| Efet.01.83686.g562.t1 | Vascular endothelial growth factor receptor 1 | 564 | 4.81E-36 | 58 |
| Efet.01.51129.g77.t1 | von Willebrand factor | 1248 | 1.36E-19 | 58 |
| Efet.01.1659487.g1842.t1 | Protein white | 1365 | 1.42E-07 | 58 |
| Efet.01.162818.g649.t1 | Protein Wnt-2b | 225 | 9.46E-14 | 58 |
| Efet.01.586731.g887.t1 | Protein Wnt-7b | 231 | 7.48E-13 | 58 |
| Efet.01.11508.g839.t1 | GTP-binding protein ypt1 | 288 | 3.19E-06 | 58 |
| Efet.01.416263.g390.t1 | GTP-binding protein ypt1 | 645 | 2.69E-53 | 58 |
| Efet.01.483378.g817.t1 | Palmitoyltransferase ZDHHC23 | 954 | 8.38E-13 | 58 |
| Efet.01.562142.g40.t1 | Zinc finger protein 143 | 366 | 1.13E-27 | 58 |
| Efet.01.236595.g1426.t1 | Zinc finger protein 37A | 675 | 1.27E-48 | 58 |
| Efet.01.125379.g21.t1 | 5-hydroxytryptamine receptor 1A | 645 | 1.79E-08 | 57 |
| Efet.01.1600837.g2.t1 | 5-hydroxytryptamine receptor 1A | 207 | 7.50E-07 | 57 |
| Efet.01.58590.g529.t1 | 5-hydroxytryptamine receptor 1A | 1710 | 7.83E-33 | 57 |
| Efet.01.181544.g1481.t1 | 5-hydroxytryptamine receptor 1A | 1476 | 7.32E-49 | 57 |
| Efet.01.527677.g633.t1 | 5-hydroxytryptamine receptor 1A | 828 | 3.28E-11 | 57 |
| Efet.01.484071.g837.t1 | 5-hydroxytryptamine receptor 2A | 603 | 6.12E-18 | 57 |
| Efet.01.86546.g725.t1 | Atrial natriuretic peptide receptor 1 | 1350 | 1.32E-22 | 57 |
| Efet.01.462288.g338.t1 | Atrial natriuretic peptide receptor 1 | 378 | 1.55E-17 | 57 |
| Efet.01.33314.g573.t1 | Annexin A3 | 552 | 2.66E-31 | 57 |
| Efet.01.83394.g550.t1 | Probable cytochrome P450 49a1 | 342 | 2.62E-23 | 57 |
| Efet.01.265246.g565.t1 | NF-kappa-B inhibitor cactus | 285 | 8.98E-10 | 57 |
| Efet.01.543955.g340.t1 | Cadherin-1 | 609 | 1.20E-26 | 57 |
| Efet.01.341693.g1201.t1 | Calumenin | 297 | 2.45E-11 | 57 |
| Efet.01.129147.g232.t1 | Calpain-6 | 1752 | 5.39E-141 | 57 |
| Efet.01.62210.g765.t1 | Caspase-2 | 267 | 5.84E-06 | 57 |
| Efet.01.65039.g911.t1 | Cholecystokinin receptor type A | 462 | 8.15E-07 | 57 |
| Efet.01.572761.g400.t1 | Cholecystokinin receptor type A | 615 | 2.88E-11 | 57 |
| Efet.01.25007.g1.t1 | Cholecystokinin receptor type A | 786 | 1.48E-21 | 57 |
| Efet.01.93046.g1056.t1 | Cyclin-dependent kinase inhibitor 1B | 621 | 2.39E-10 | 57 |
| Efet.01.78755.g223.t1 | CCAAT/enhancer-binding protein delta | 1098 | 6.11E-06 | 57 |
| Efet.01.196234.g2144.t1 | Polyribonucleotide 5'-hydroxyl-kinase Clp1 | 258 | 1.51E-21 | 57 |
| Efet.01.29013.g279.t1 | Cytochrome P450 3A12 | 576 | 3.37E-10 | 57 |
| Efet.01.335091.g1053.t1 | Citron Rho-interacting kinase | 429 | 6.63E-16 | 57 |
| Efet.01.534512.g148.t1 | C-X-C chemokine receptor type 1 | 588 | 8.38E-07 | 57 |
| Efet.01.195070.g2094.t1 | Neurogenic locus protein delta | 765 | 3.31E-45 | 57 |
| Efet.01.18399.g1364.t1 | Dystrophin | 390 | 4.76E-09 | 57 |
| Efet.01.262124.g451.t1 | Dual specificity protein phosphatase 1 | 531 | 4.38E-27 | 57 |
| Efet.01.254372.g160.t1 | Dysferlin | 618 | 1.68E-08 | 57 |
| Efet.01.243079.g1638.t1 | Epidermal growth factor receptor | 636 | 4.45E-24 | 57 |
| Efet.01.46536.g1383.t1 | ELAV-like protein 4 | 1026 | 3.33E-80 | 57 |
| Efet.01.559401.g732.t1 | Beta-enolase | 1026 | 1.08E-57 | 57 |
| Efet.01.514275.g343.t1 | Histone-lysine N-methyltransferase EZH2 | 216 | 5.55E-12 | 57 |
| Efet.01.49800.g1581.t1 | Four and a half LIM domains protein 2 | 864 | 3.59E-47 | 57 |
| Efet.01.164792.g752.t1 | Alpha-(1,3)-fucosyltransferase 4 | 366 | 3.98E-14 | 57 |
| Efet.01.228168.g1130.t1 | Growth arrest-specific protein 6 | 396 | 2.16E-20 | 57 |
| Efet.01.202977.g140.t1 | Gamma-aminobutyric acid receptor subunit beta-3 | 432 | 3.44E-24 | 57 |
| Efet.01.204214.g184.t1 | Gamma-aminobutyric acid receptor subunit beta-3 | 474 | 2.71E-28 | 57 |
| Efet.01.237760.g1473.t1 | Gamma-aminobutyric acid receptor subunit beta-3 | 369 | 9.01E-07 | 57 |
| Efet.01.649393.g1305.t1 | Gamma-aminobutyric acid receptor subunit beta-3 | 216 | 9.62E-10 | 57 |
| Efet.01.345963.g1297.t1 | Glial fibrillary acidic protein | 492 | 1.28E-15 | 57 |
| Efet.01.130388.g293.t1 | Glial fibrillary acidic protein | 579 | 6.82E-18 | 57 |
| Efet.01.52250.g151.t1 | Glutamate receptor 1 | 240 | 1.11E-06 | 57 |
| Efet.01.66322.g1004.t1 | Growth factor receptor-bound protein 7 | 597 | 1.44E-09 | 57 |
| Efet.01.565356.g181.t1 | Solute carrier family 2, facilitated glucose transporter member 4 | 795 | 3.19E-43 | 57 |
| Efet.01.125671.g39.t1 | E3 ISG15--protein ligase HERC5 | 864 | 4.43E-47 | 57 |
| Efet.01.654656.g669.t1 | Probable heat shock protein ssa2 | 1869 | 3.18E-124 | 57 |
| Efet.01.73865.g1469.t1 | Heat shock protein beta-1 | 1110 | 5.48E-06 | 57 |
| Efet.01.89373.g854.t1 | Insulin-like growth factor 2 mRNA-binding protein 1 | 936 | 9.22E-46 | 57 |
| Efet.01.239691.g1527.t1 | Insulin-like growth factor 2 mRNA-binding protein 1 | 339 | 3.81E-06 | 57 |
| Efet.01.82999.g524.t1 | ATP-dependent RNA helicase eIF4A | 957 | 5.38E-48 | 57 |
| Efet.01.155178.g267.t1 | Eukaryotic initiation factor 4A-I | 453 | 1.13E-20 | 57 |
| Efet.01.84624.g601.t1 | Integrin-linked protein kinase | 1257 | 2.79E-16 | 57 |
| Efet.01.190823.g1904.t1 | Insulin gene enhancer protein isl-1 | 474 | 4.83E-12 | 57 |
| Efet.01.260885.g409.t1 | Potassium voltage-gated channel subfamily A member 5 | 1047 | 3.37E-54 | 57 |
| Efet.01.263321.g502.t1 | Potassium voltage-gated channel subfamily B member 1 | 609 | 1.78E-40 | 57 |
| Efet.01.87369.g767.t1 | Potassium channel subfamily K member 3 | 402 | 8.80E-10 | 57 |
| Efet.01.555692.g648.t1 | Potassium channel subfamily K member 3 | 915 | 2.54E-09 | 57 |
| Efet.01.334920.g1047.t1 | Lysine-specific demethylase 4A | 522 | 5.01E-26 | 57 |
| Efet.01.129079.g226.t1 | Plasma kallikrein | 399 | 3.08E-11 | 57 |
| Efet.01.146892.g1097.t1 | Histone-lysine N-methyltransferase 2C | 1578 | 1.03E-127 | 57 |
| Efet.01.547414.g425.t1 | Laminin subunit alpha-1 | 432 | 8.79E-08 | 57 |
| Efet.01.245206.g1702.t1 | E3 ubiquitin-protein ligase TRIM71 | 354 | 1.96E-09 | 57 |
| Efet.01.452252.g56.t1 | Prolow-density lipoprotein receptor-related protein 1 | 948 | 1.78E-30 | 57 |
| Efet.01.99792.g1438.t1 | Low-density lipoprotein receptor-related protein 2 | 519 | 6.66E-26 | 57 |
| Efet.01.323764.g734.t1 | Matrilin-2 | 336 | 6.09E-12 | 57 |
| Efet.01.33261.g565.t1 | E3 ubiquitin-protein ligase Mdm2 | 648 | 5.06E-07 | 57 |
| Efet.01.624000.g1054.t1 | Mediator of RNA polymerase II transcription subunit 17 | 576 | 9.55E-34 | 57 |
| Efet.01.433171.g804.t1 | Cation-independent mannose-6-phosphate receptor | 747 | 5.26E-21 | 57 |
| Efet.01.63891.g856.t1 | Myotrophin | 1347 | 1.92E-11 | 57 |
| Efet.01.146303.g1069.t1 | Neural cell adhesion molecule L1-like protein | 450 | 2.49E-22 | 57 |
| Efet.01.339772.g1168.t1 | Neural cell adhesion molecule L1-like protein | 261 | 2.42E-06 | 57 |
| Efet.01.1641038.g228.t1 | Nuclear factor NF-kappa-B p105 subunit | 243 | 2.15E-07 | 57 |
| Efet.01.240113.g1539.t1 | Homeobox protein Nkx-2.2 | 753 | 5.85E-22 | 57 |
| Efet.01.80668.g382.t1 | Neurogenic locus notch homolog protein 2 | 453 | 2.45E-20 | 57 |
| Efet.01.193366.g2011.t1 | Neurogenic locus notch homolog protein 2 | 519 | 3.85E-08 | 57 |
| Efet.01.128015.g169.t1 | BDNF/NT-3 growth factors receptor | 1410 | 5.24E-66 | 57 |
| Efet.01.254056.g146.t1 | Palladin | 1080 | 1.05E-13 | 57 |
| Efet.01.514711.g356.t1 | Inactive serine protease PAMR1 | 453 | 1.00E-07 | 57 |
| Efet.01.519211.g459.t1 | Poly [ADP-ribose] polymerase 1 | 330 | 1.11E-10 | 57 |
| Efet.01.231126.g1230.t1 | Tyrosine-protein phosphatase non-receptor type 11 | 447 | 6.03E-26 | 57 |
| Efet.01.66928.g1040.t1 | Tyrosine-protein phosphatase non-receptor type 3 | 576 | 3.08E-19 | 57 |
| Efet.01.77086.g117.t1 | Tyrosine-protein phosphatase non-receptor type 3 | 1155 | 7.79E-17 | 57 |
| Efet.01.472791.g620.t1 | Receptor-type tyrosine-protein phosphatase S | 1407 | 8.43E-11 | 57 |
| Efet.01.56425.g401.t1 | Ras-related protein Rab-1A | 417 | 1.17E-18 | 57 |
| Efet.01.187047.g1734.t1 | Histone-binding protein RBBP4 | 207 | 4.67E-08 | 57 |
| Efet.01.613416.g605.t1 | Ribonucleoside-diphosphate reductase large subunit | 1035 | 7.73E-65 | 57 |
| Efet.01.553529.g596.t1 | Roundabout homolog 2 | 321 | 7.45E-13 | 57 |
| Efet.01.493463.g1033.t1 | Sal-like protein 4 | 273 | 7.32E-11 | 57 |
| Efet.01.42558.g1145.t1 | Sodium channel protein type 1 subunit alpha | 252 | 3.76E-18 | 57 |
| Efet.01.143912.g972.t1 | Sodium channel protein type 1 subunit alpha | 1092 | 7.89E-77 | 57 |
| Efet.01.204928.g212.t1 | Sodium channel protein type 9 subunit alpha | 456 | 1.65E-08 | 57 |
| Efet.01.48455.g1492.t1 | Serine/threonine-protein kinase sgk-1 | 300 | 7.03E-12 | 57 |
| Efet.01.10938.g801.t1 | Serine/threonine-protein kinase Sgk1 | 405 | 1.56E-19 | 57 |
| Efet.01.657544.g1326.t1 | Slit homolog 1 protein | 207 | 1.25E-07 | 57 |
| Efet.01.43363.g1193.t1 | STE20-like serine/threonine-protein kinase | 666 | 3.52E-11 | 57 |
| Efet.01.246146.g1739.t1 | Alpha-1-syntrophin | 276 | 8.49E-10 | 57 |
| Efet.01.135513.g518.t1 | Proto-oncogene tyrosine-protein kinase Src | 336 | 2.52E-07 | 57 |
| Efet.01.11031.g811.t1 | Src substrate cortactin | 465 | 1.63E-06 | 57 |
| Efet.01.50643.g46.t1 | Serine/threonine-protein kinase STK11 | 483 | 3.07E-39 | 57 |
| Efet.01.222808.g921.t1 | Serine/threonine-protein kinase 3 | 264 | 3.71E-15 | 57 |
| Efet.01.515782.g391.t1 | Tenascin | 216 | 8.05E-16 | 57 |
| Efet.01.367531.g474.t1 | TNFAIP3-interacting protein 2 | 360 | 9.99E-08 | 57 |
| Efet.01.201410.g68.t1 | TNF receptor-associated factor 6 | 576 | 5.35E-06 | 57 |
| Efet.01.559264.g729.t1 | Transcriptional repressor protein YY1 | 498 | 9.93E-16 | 57 |
| Efet.01.407831.g188.t1 | Serine/threonine-protein kinase ULK1 | 489 | 6.67E-25 | 57 |
| Efet.01.31892.g481.t1 | Vasopressin V1a receptor | 711 | 6.21E-25 | 57 |
| Efet.01.652644.g326.t1 | Vasodilator-stimulated phosphoprotein | 360 | 1.57E-20 | 57 |
| Efet.01.35659.g710.t1 | WD repeat-containing protein 5 | 447 | 2.40E-17 | 57 |
| Efet.01.647059.g1154.t1 | Protein white | 885 | 8.33E-10 | 57 |
| Efet.01.1653796.g638.t1 | Protein Wnt-4 | 387 | 1.06E-30 | 57 |
| Efet.01.173855.g1128.t1 | Protein Wnt-5b | 354 | 1.35E-08 | 57 |
| Efet.01.268818.g696.t1 | Protein Wnt-7b | 282 | 9.00E-14 | 57 |
| Efet.01.1659497.g1878.t1 | Metal resistance protein YCF1 | 1551 | 3.62E-32 | 57 |
| Efet.01.117435.g904.t1 | GTP-binding protein ypt1 | 258 | 2.11E-14 | 57 |
| Efet.01.631414.g115.t1 | Zinc finger X-chromosomal protein | 3087 | 5.44E-07 | 57 |
| Efet.01.46589.g1388.t1 | Zinc finger protein 37A | 1455 | 6.24E-76 | 57 |
| Efet.01.71147.g1306.t1 | Zinc finger protein 37A | 666 | 2.26E-54 | 57 |
| Efet.01.78671.g220.t1 | Zinc finger protein 37A | 1464 | 7.71E-37 | 57 |
| Efet.01.269859.g737.t1 | Zinc finger protein 37A | 996 | 3.55E-69 | 57 |
| Efet.01.3779.g323.t1 | 5-hydroxytryptamine receptor 1A | 1467 | 5.41E-91 | 56 |
| Efet.01.534728.g150.t1 | 5-hydroxytryptamine receptor 1A | 1794 | 2.19E-39 | 56 |
| Efet.01.101430.g93.t1 | 5-hydroxytryptamine receptor 1A | 1416 | 2.18E-72 | 56 |
| Efet.01.332901.g987.t1 | 5-hydroxytryptamine receptor 1A | 1365 | 1.15E-95 | 56 |
| Efet.01.1654936.g723.t1 | 5-hydroxytryptamine receptor 1A | 237 | 4.69E-13 | 56 |
| Efet.01.221628.g884.t1 | 5-hydroxytryptamine receptor 1A | 1437 | 5.17E-102 | 56 |
| Efet.01.180448.g1423.t1 | 5-hydroxytryptamine receptor 2C | 573 | 2.05E-17 | 56 |
| Efet.01.107327.g379.t1 | Disintegrin and metalloproteinase domain-containing protein 10 | 330 | 1.89E-14 | 56 |
| Efet.01.60754.g669.t1 | Disintegrin and metalloproteinase domain-containing protein 15 | 267 | 4.55E-07 | 56 |
| Efet.01.118295.g945.t1 | Calretinin | 297 | 8.89E-15 | 56 |
| Efet.01.638084.g418.t1 | Calretinin | 381 | 8.55E-10 | 56 |
| Efet.01.51744.g112.t1 | CREB-binding protein | 825 | 5.06E-74 | 56 |
| Efet.01.37851.g855.t1 | Cholecystokinin receptor type A | 891 | 8.55E-09 | 56 |
| Efet.01.115985.g833.t1 | Cholecystokinin receptor type A | 540 | 2.66E-16 | 56 |
| Efet.01.187525.g1761.t1 | Cholecystokinin receptor type A | 939 | 2.00E-31 | 56 |
| Efet.01.206695.g295.t1 | Cholecystokinin receptor type A | 726 | 7.93E-24 | 56 |
| Efet.01.471393.g573.t1 | Cystic fibrosis transmembrane conductance regulator | 261 | 8.78E-08 | 56 |
| Efet.01.203719.g168.t1 | Collagen alpha-1(II) chain | 705 | 5.05E-08 | 56 |
| Efet.01.77982.g173.t1 | Complement C3 | 489 | 1.46E-13 | 56 |
| Efet.01.391903.g1047.t1 | Cytochrome P450 26A1 | 351 | 9.91E-14 | 56 |
| Efet.01.116602.g864.t1 | Cytochrome P450 3A2 | 1695 | 1.78E-21 | 56 |
| Efet.01.4040.g342.t1 | Delta-like protein 1 | 1053 | 3.00E-32 | 56 |
| Efet.01.496456.g1093.t1 | Dual specificity protein phosphatase 1 | 696 | 9.22E-19 | 56 |
| Efet.01.118494.g953.t1 | Dysferlin | 648 | 3.34E-34 | 56 |
| Efet.01.339664.g1163.t1 | Translation initiation factor eIF-2B subunit alpha | 405 | 9.33E-12 | 56 |
| Efet.01.85576.g661.t1 | ELAV-like protein 4 | 231 | 5.82E-08 | 56 |
| Efet.01.174549.g1155.t1 | Ephrin type-A receptor 4 | 282 | 2.16E-06 | 56 |
| Efet.01.644595.g888.t1 | Receptor tyrosine-protein kinase erbB-3 | 582 | 2.45E-15 | 56 |
| Efet.01.221275.g868.t1 | Receptor tyrosine-protein kinase erbB-4 | 492 | 5.94E-18 | 56 |
| Efet.01.654956.g722.t1 | Fibrinogen-like protein 1 | 387 | 2.59E-16 | 56 |
| Efet.01.72870.g1411.t1 | Fibrinogen-like protein 1 | 606 | 1.62E-27 | 56 |
| Efet.01.171406.g1031.t1 | Fibrinogen alpha chain | 462 | 1.82E-17 | 56 |
| Efet.01.582422.g760.t1 | Forkhead box protein J3 | 447 | 6.81E-16 | 56 |
| Efet.01.13566.g1012.t1 | Gamma-aminobutyric acid receptor subunit beta-3 | 207 | 1.11E-09 | 56 |
| Efet.01.126049.g57.t1 | Gamma-aminobutyric acid receptor subunit beta-3 | 309 | 2.32E-09 | 56 |
| Efet.01.1645250.g320.t1 | Gamma-aminobutyric acid receptor subunit beta-3 | 228 | 9.87E-13 | 56 |
| Efet.01.241906.g1595.t1 | Growth/differentiation factor 3 | 1068 | 1.19E-18 | 56 |
| Efet.01.536087.g185.t1 | Growth/differentiation factor 8 | 225 | 9.85E-06 | 56 |
| Efet.01.366320.g441.t1 | Glial fibrillary acidic protein | 822 | 7.78E-14 | 56 |
| Efet.01.1658883.g1313.t1 | Glutamine--fructose-6-phosphate aminotransferase [isomerizing] 1 | 1089 | 5.37E-68 | 56 |
| Efet.01.351649.g49.t1 | Glypican-1 | 567 | 2.76E-34 | 56 |
| Efet.01.533219.g116.t1 | Glutamate receptor 3 | 495 | 1.68E-17 | 56 |
| Efet.01.2563.g235.t1 | Histone deacetylase 4 | 612 | 8.43E-43 | 56 |
| Efet.01.102473.g142.t1 | Mitogen-activated protein kinase sty1 | 705 | 2.60E-28 | 56 |
| Efet.01.61636.g723.t1 | Nuclear hormone receptor HR96 | 402 | 1.32E-13 | 56 |
| Efet.01.100925.g66.t1 | Nuclear hormone receptor HR96 | 387 | 2.96E-18 | 56 |
| Efet.01.130066.g272.t1 | Histamine H1 receptor | 1746 | 1.07E-35 | 56 |
| Efet.01.42283.g1129.t1 | Histamine H1 receptor | 1209 | 1.14E-38 | 56 |
| Efet.01.215751.g654.t1 | Heat shock 70 kDa protein 1-like | 261 | 2.54E-09 | 56 |
| Efet.01.155767.g296.t1 | Heat shock protein beta-1 | 1122 | 6.42E-15 | 56 |
| Efet.01.86113.g696.t1 | Heat shock protein beta-1 | 702 | 9.33E-07 | 56 |
| Efet.01.216626.g688.t1 | Integrin-linked protein kinase homolog pat-4 | 846 | 3.39E-10 | 56 |
| Efet.01.209323.g419.t1 | Potassium voltage-gated channel subfamily A member 5 | 1038 | 7.39E-63 | 56 |
| Efet.01.405236.g126.t1 | Potassium channel subfamily K member 3 | 609 | 1.70E-08 | 56 |
| Efet.01.181839.g1495.t1 | Lysine-specific demethylase 3A | 1737 | 3.82E-55 | 56 |
| Efet.01.103840.g210.t1 | Lysine-specific demethylase 4B | 885 | 1.97E-28 | 56 |
| Efet.01.569311.g290.t1 | Kinesin-like protein KIF3C | 528 | 1.28E-14 | 56 |
| Efet.01.482748.g803.t1 | Plasma kallikrein | 339 | 1.04E-12 | 56 |
| Efet.01.134179.g450.t1 | Plasma kallikrein | 576 | 7.34E-28 | 56 |
| Efet.01.109887.g509.t1 | Serine/threonine-protein kinase D1 | 315 | 2.07E-08 | 56 |
| Efet.01.178010.g1313.t1 | Protein kinase C theta type | 414 | 2.26E-10 | 56 |
| Efet.01.236483.g1421.t1 | Neural cell adhesion molecule L1 | 300 | 3.31E-09 | 56 |
| Efet.01.79415.g284.t1 | Low-density lipoprotein receptor | 975 | 3.29E-32 | 56 |
| Efet.01.218844.g763.t1 | Low-density lipoprotein receptor-related protein 2 | 468 | 2.02E-16 | 56 |
| Efet.01.371355.g602.t1 | Low-density lipoprotein receptor-related protein 2 | 441 | 8.95E-23 | 56 |
| Efet.01.85380.g648.t1 | Low-density lipoprotein receptor-related protein 2 | 270 | 1.43E-16 | 56 |
| Efet.01.657036.g1156.t1 | Low-density lipoprotein receptor-related protein 2 | 678 | 3.65E-44 | 56 |
| Efet.01.153308.g179.t1 | Mediator of RNA polymerase II transcription subunit 13 | 1380 | 5.10E-91 | 56 |
| Efet.01.1076.g100.t1 | Myotrophin | 744 | 2.51E-13 | 56 |
| Efet.01.604656.g173.t1 | Myotrophin | 1098 | 1.41E-08 | 56 |
| Efet.01.420107.g477.t1 | E3 ubiquitin-protein ligase MYCBP2 | 288 | 4.14E-22 | 56 |
| Efet.01.462929.g353.t1 | Unconventional myosin-Va | 204 | 8.24E-09 | 56 |
| Efet.01.463735.g368.t1 | Unconventional myosin-Va | 375 | 7.54E-17 | 56 |
| Efet.01.125339.g19.t1 | Netrin-1 | 468 | 5.12E-17 | 56 |
| Efet.01.201905.g97.t1 | Neuromedin-U receptor 1 | 1002 | 1.36E-42 | 56 |
| Efet.01.486994.g897.t1 | Neuromedin-U receptor 1 | 1230 | 6.66E-08 | 56 |
| Efet.01.85725.g678.t1 | Neurogenic locus notch homolog protein 1 | 897 | 2.12E-23 | 56 |
| Efet.01.222386.g905.t1 | Nuclear receptor subfamily 1 group I member 2 | 291 | 5.57E-06 | 56 |
| Efet.01.562842.g66.t1 | Neuropilin-1 | 270 | 2.62E-10 | 56 |
| Efet.01.525686.g600.t1 | Octopamine receptor beta-2R | 1356 | 1.33E-59 | 56 |
| Efet.01.288863.g1413.t1 | Octopamine receptor beta-3R | 1041 | 8.02E-19 | 56 |
| Efet.01.164135.g713.t1 | Phosphatidylinositol 3-kinase regulatory subunit alpha | 1527 | 2.25E-77 | 56 |
| Efet.01.133068.g403.t1 | Inactive serine protease PAMR1 | 261 | 7.40E-06 | 56 |
| Efet.01.322947.g711.t1 | Paxillin | 465 | 3.68E-07 | 56 |
| Efet.01.124113.g1244.t1 | Protocadherin-15 | 738 | 2.18E-21 | 56 |
| Efet.01.365169.g407.t1 | Tyrosine-protein phosphatase non-receptor type 3 | 2529 | 3.85E-48 | 56 |
| Efet.01.4277.g357.t1 | Receptor-type tyrosine-protein phosphatase S | 570 | 1.48E-38 | 56 |
| Efet.01.29465.g307.t1 | Peroxidasin | 261 | 1.11E-12 | 56 |
| Efet.01.74339.g1510.t1 | SMARCA4 isoform 2 | 2202 | 5.40E-56 | 56 |
| Efet.01.186014.g1689.t1 | Reelin | 429 | 9.20E-27 | 56 |
| Efet.01.38482.g904.t1 | Regulator of nonsense transcripts 2 | 1659 | 8.64E-58 | 56 |
| Efet.01.1658267.g1144.t1 | Retinoic acid receptor RXR-alpha | 339 | 5.93E-14 | 56 |
| Efet.01.175657.g1197.t1 | Sodium channel protein type 1 subunit alpha | 321 | 1.62E-13 | 56 |
| Efet.01.53310.g223.t1 | Sodium channel protein type 9 subunit alpha | 1101 | 1.24E-06 | 56 |
| Efet.01.103473.g186.t1 | Pulmonary surfactant-associated protein D | 333 | 3.70E-06 | 56 |
| Efet.01.369069.g516.t1 | Serine/threonine-protein kinase Sgk1 | 1308 | 1.21E-46 | 56 |
| Efet.01.375887.g700.t1 | SWI/SNF-related matrix-associated actin-dependent regulator of chromatin subfamily A member 5 | 696 | 1.13E-38 | 56 |
| Efet.01.251185.g39.t1 | Suppressor of cytokine signaling 3 | 879 | 9.31E-32 | 56 |
| Efet.01.641291.g620.t1 | Spastin | 1269 | 2.65E-07 | 56 |
| Efet.01.658424.g2122.t1 | Spastin | 1188 | 2.07E-15 | 56 |
| Efet.01.643930.g850.t1 | Sterol regulatory element-binding protein 1 | 597 | 2.76E-21 | 56 |
| Efet.01.244152.g1670.t1 | Proto-oncogene tyrosine-protein kinase Src | 450 | 7.25E-20 | 56 |
| Efet.01.371952.g617.t1 | Serine/threonine-protein kinase STK11 | 477 | 6.14E-11 | 56 |
| Efet.01.401970.g52.t1 | Tubulin alpha-2 chain | 828 | 1.36E-33 | 56 |
| Efet.01.530307.g8.t1 | Tenascin | 219 | 7.67E-06 | 56 |
| Efet.01.100458.g28.t1 | TNF receptor-associated factor 6 | 573 | 4.10E-20 | 56 |
| Efet.01.64704.g896.t1 | Short transient receptor potential channel 4 | 1053 | 2.09E-29 | 56 |
| Efet.01.555350.g639.t1 | Thrombospondin-1 | 771 | 7.98E-11 | 56 |
| Efet.01.185157.g1639.t1 | Ubiquitin-conjugating enzyme E2 B | 267 | 7.52E-16 | 56 |
| Efet.01.222847.g924.t1 | Vasopressin V1a receptor | 669 | 3.84E-21 | 56 |
| Efet.01.210718.g472.t1 | von Willebrand factor | 558 | 2.20E-06 | 56 |
| Efet.01.646487.g1096.t1 | Protein white | 612 | 8.39E-09 | 56 |
| Efet.01.129961.g264.t1 | Protein Wnt-3a | 354 | 1.43E-22 | 56 |
| Efet.01.453629.g90.t1 | GTP-binding protein ypt1 | 315 | 2.24E-18 | 56 |
| Efet.01.20643.g1522.t1 | Zinc finger X-chromosomal protein | 330 | 6.97E-08 | 56 |
| Efet.01.24177.g1757.t1 | Zinc finger protein 37A | 1011 | 1.37E-48 | 56 |
| Efet.01.138347.g684.t1 | Zinc finger protein 37A | 1968 | 3.14E-37 | 56 |
| Efet.01.144561.g999.t1 | Zinc finger protein 37A | 2106 | 1.62E-24 | 56 |
| Efet.01.209372.g421.t1 | Zinc finger protein 37A | 894 | 2.09E-57 | 56 |
| Efet.01.347874.g1353.t1 | Zinc finger protein 37A | 849 | 1.12E-53 | 56 |
| Efet.01.407630.g177.t1 | Zinc finger protein 37A | 819 | 1.10E-61 | 56 |
| Efet.01.1658606.g1227.t1 | Zinc finger protein 37A | 852 | 8.79E-60 | 56 |
| Efet.01.54171.g277.t1 | 5-hydroxytryptamine receptor 1A | 981 | 3.75E-07 | 55 |
| Efet.01.302008.g67.t1 | 5-hydroxytryptamine receptor 1A | 2205 | 3.68E-34 | 55 |
| Efet.01.507448.g192.t1 | 5-hydroxytryptamine receptor 1A | 1188 | 1.29E-74 | 55 |
| Efet.01.119388.g1003.t1 | 5-hydroxytryptamine receptor 1A | 1614 | 3.11E-86 | 55 |
| Efet.01.26568.g109.t1 | 5-hydroxytryptamine receptor 2C | 525 | 3.48E-13 | 55 |
| Efet.01.263931.g523.t1 | APOBEC1 complementation factor | 480 | 1.01E-38 | 55 |
| Efet.01.658150.g1687.t1 | ATP-binding cassette transporter abc2 | 801 | 1.83E-06 | 55 |
| Efet.01.370192.g561.t1 | ATP-binding cassette sub-family G member 2 | 603 | 5.96E-34 | 55 |
| Efet.01.476829.g686.t1 | Aurora/IPL1-related protein kinase 2 | 534 | 3.94E-19 | 55 |
| Efet.01.307993.g236.t1 | Aldehyde dehydrogenase, dimeric NADP-preferring | 663 | 1.15E-06 | 55 |
| Efet.01.1659287.g1514.t1 | Aldose reductase | 837 | 3.70E-54 | 55 |
| Efet.01.213312.g562.t1 | Atrial natriuretic peptide receptor 1 | 627 | 2.58E-52 | 55 |
| Efet.01.407773.g185.t1 | Atrial natriuretic peptide receptor 1 | 345 | 1.08E-09 | 55 |
| Efet.01.271475.g806.t1 | Aquaporin-5 | 807 | 3.11E-22 | 55 |
| Efet.01.354513.g129.t1 | Protein atonal homolog 1 | 432 | 5.97E-11 | 55 |
| Efet.01.625368.g1112.t1 | Beta-1,4-galactosyltransferase 1 | 609 | 2.33E-16 | 55 |
| Efet.01.139722.g756.t1 | Bile acid-CoA:amino acid N-acyltransferase | 225 | 4.14E-09 | 55 |
| Efet.01.338161.g1115.t1 | C5a anaphylatoxin chemotactic receptor 1 | 1338 | 6.71E-07 | 55 |
| Efet.01.146794.g1093.t1 | Cadherin-99C | 2058 | 7.49E-17 | 55 |
| Efet.01.27707.g171.t1 | Calpain-3 | 225 | 6.27E-09 | 55 |
| Efet.01.656427.g1015.t1 | Cholecystokinin receptor type A | 684 | 4.16E-17 | 55 |
| Efet.01.302504.g81.t1 | Cholecystokinin receptor type A | 870 | 1.10E-29 | 55 |
| Efet.01.324841.g765.t1 | C-C chemokine receptor type 2 | 810 | 1.71E-31 | 55 |
| Efet.01.211785.g513.t1 | C-C chemokine receptor type 7 | 669 | 4.39E-08 | 55 |
| Efet.01.648013.g1216.t1 | Cystic fibrosis transmembrane conductance regulator | 1089 | 3.63E-07 | 55 |
| Efet.01.21625.g1590.t1 | Cytochrome P450 3A2 | 249 | 7.70E-07 | 55 |
| Efet.01.271704.g812.t1 | Cytochrome P450 3A12 | 813 | 2.42E-17 | 55 |
| Efet.01.74633.g1523.t1 | Versican core protein | 915 | 3.59E-16 | 55 |
| Efet.01.9503.g703.t1 | Catenin alpha-1 | 297 | 2.50E-10 | 55 |
| Efet.01.537981.g232.t1 | C-X-C chemokine receptor type 1 | 1044 | 7.19E-24 | 55 |
| Efet.01.86621.g729.t1 | Epithelial discoidin domain-containing receptor 1 | 288 | 3.99E-08 | 55 |
| Efet.01.1656127.g830.t1 | Corticosteroid 11-beta-dehydrogenase isozyme 1 | 297 | 3.13E-09 | 55 |
| Efet.01.11121.g816.t1 | Deleted in malignant brain tumors 1 protein | 1377 | 9.92E-15 | 55 |
| Efet.01.121829.g1136.t1 | Dual specificity protein phosphatase 1 | 624 | 3.01E-19 | 55 |
| Efet.01.73094.g1425.t1 | Dual specificity protein phosphatase 1 | 1095 | 1.30E-20 | 55 |
| Efet.01.307104.g222.t1 | Epidermal growth factor receptor | 435 | 2.23E-21 | 55 |
| Efet.01.314463.g455.t1 | Epidermal growth factor receptor | 372 | 8.07E-16 | 55 |
| Efet.01.86281.g709.t1 | Histone-lysine N-methyltransferase EHMT2 | 561 | 1.15E-12 | 55 |
| Efet.01.143406.g947.t1 | Histone-lysine N-methyltransferase EHMT2 | 669 | 4.20E-26 | 55 |
| Efet.01.165229.g766.t1 | Steroid hormone receptor ERR2 | 594 | 3.55E-15 | 55 |
| Efet.01.63903.g858.t1 | Protein C-ets-1 | 240 | 2.70E-06 | 55 |
| Efet.01.498867.g1153.t1 | Transforming protein p54/c-ets-1 | 570 | 1.12E-08 | 55 |
| Efet.01.44055.g1231.t1 | Histone-lysine N-methyltransferase EZH1 | 279 | 5.58E-16 | 55 |
| Efet.01.125602.g37.t1 | Four and a half LIM domains protein 2 | 363 | 5.60E-45 | 55 |
| Efet.01.576304.g523.t1 | Protein flightless-1 | 798 | 3.37E-16 | 55 |
| Efet.01.630355.g16.t1 | Protein flightless-1 | 3561 | 2.95E-06 | 55 |
| Efet.01.120233.g1049.t1 | Frizzled-7 | 426 | 4.34E-20 | 55 |
| Efet.01.89985.g895.t1 | Growth arrest-specific protein 6 | 624 | 7.60E-16 | 55 |
| Efet.01.451716.g41.t1 | Growth/differentiation factor 8 | 369 | 9.82E-11 | 55 |
| Efet.01.614627.g655.t1 | Glutamine--fructose-6-phosphate aminotransferase [isomerizing] 1 | 1311 | 7.03E-90 | 55 |
| Efet.01.10485.g768.t1 | Bifunctional UDP-N-acetylglucosamine 2-epimerase/N-acetylmannosamine kinase | 579 | 1.52E-16 | 55 |
| Efet.01.117617.g913.t1 | Guanine nucleotide-binding protein G(s) subunit alpha | 507 | 1.85E-26 | 55 |
| Efet.01.563018.g72.t1 | Guanine nucleotide-binding protein G(s) subunit alpha | 528 | 1.17E-23 | 55 |
| Efet.01.6243.g491.t1 | Guanine nucleotide-binding protein G(s) subunit alpha isoforms XLas | 717 | 7.25E-19 | 55 |
| Efet.01.411192.g254.t1 | Glypican-3 | 678 | 3.94E-36 | 55 |
| Efet.01.118442.g950.t1 | Glutamate receptor 3 | 777 | 2.10E-26 | 55 |
| Efet.01.608766.g363.t1 | Glutamate receptor 3 | 285 | 3.17E-10 | 55 |
| Efet.01.655821.g879.t1 | Histone deacetylase 6 | 528 | 1.92E-30 | 55 |
| Efet.01.439592.g939.t1 | Histone deacetylase 8 | 222 | 5.41E-08 | 55 |
| Efet.01.29999.g339.t1 | Protein hedgehog | 573 | 2.60E-30 | 55 |
| Efet.01.191976.g1960.t1 | Nuclear hormone receptor HR96 | 570 | 6.91E-24 | 55 |
| Efet.01.111627.g583.t1 | Histamine H1 receptor | 1902 | 1.49E-31 | 55 |
| Efet.01.396152.g1143.t1 | Histamine H1 receptor | 651 | 5.46E-07 | 55 |
| Efet.01.156618.g345.t1 | ATP-dependent RNA helicase eIF4A | 2325 | 1.56E-56 | 55 |
| Efet.01.205875.g254.t1 | Insulin-like growth factor 1 receptor | 255 | 4.80E-10 | 55 |
| Efet.01.613800.g620.t1 | Insulinoma-associated protein 1 | 1563 | 3.91E-30 | 55 |
| Efet.01.9249.g677.t1 | Histone acetyltransferase KAT6A | 1026 | 1.54E-76 | 55 |
| Efet.01.416574.g396.t1 | Potassium voltage-gated channel subfamily A member 5 | 1038 | 5.06E-59 | 55 |
| Efet.01.345403.g1280.t1 | Potassium voltage-gated channel subfamily B member 1 | 381 | 1.14E-19 | 55 |
| Efet.01.571578.g371.t1 | Potassium channel subfamily K member 3 | 813 | 2.81E-07 | 55 |
| Efet.01.56510.g405.t1 | Kinesin-like protein KIF3C | 369 | 7.67E-15 | 55 |
| Efet.01.5845.g461.t1 | Histone-lysine N-methyltransferase 2A | 1740 | 4.05E-07 | 55 |
| Efet.01.162228.g631.t1 | Serine/threonine-protein kinase D1 | 462 | 7.98E-23 | 55 |
| Efet.01.175928.g1208.t1 | Serine/threonine-protein kinase D1 | 537 | 1.51E-26 | 55 |
| Efet.01.131026.g320.t1 | Raf homolog serine/threonine-protein kinase | 531 | 4.42E-16 | 55 |
| Efet.01.649449.g1309.t1 | Laminin subunit alpha | 723 | 5.58E-06 | 55 |
| Efet.01.302542.g83.t1 | Leucine-rich repeat-containing G-protein coupled receptor 5 | 1416 | 9.18E-09 | 55 |
| Efet.01.214524.g616.t1 | Protein lin-41 | 1641 | 7.77E-06 | 55 |
| Efet.01.655474.g825.t1 | Low-density lipoprotein receptor-related protein 1 | 420 | 3.97E-09 | 55 |
| Efet.01.206239.g270.t1 | Latent-transforming growth factor beta-binding protein 4 | 369 | 1.27E-22 | 55 |
| Efet.01.1655895.g800.t1 | Tyrosine-protein kinase Lyn | 417 | 3.06E-18 | 55 |
| Efet.01.34989.g678.t1 | POU domain protein | 1029 | 1.05E-12 | 55 |
| Efet.01.645029.g921.t1 | Mitogen-activated protein kinase kinase kinase 12 | 651 | 8.57E-21 | 55 |
| Efet.01.12151.g904.t1 | DNA replication licensing factor MCM2 | 612 | 5.12E-09 | 55 |
| Efet.01.271982.g827.t1 | Canalicular multispecific organic anion transporter 1 | 918 | 5.38E-33 | 55 |
| Efet.01.14070.g1039.t1 | Myotrophin | 1053 | 3.09E-07 | 55 |
| Efet.01.226864.g1072.t1 | Nucleus accumbens-associated protein 1 | 348 | 3.34E-11 | 55 |
| Efet.01.144972.g1013.t1 | Neural cell adhesion molecule 1 | 453 | 4.64E-08 | 55 |
| Efet.01.374937.g682.t1 | Neurogenic differentiation factor 1 | 1008 | 5.34E-23 | 55 |
| Efet.01.477544.g707.t1 | Nuclear factor NF-kappa-B p105 subunit | 1707 | 2.04E-27 | 55 |
| Efet.01.43974.g1225.t1 | Neurofilament light polypeptide | 327 | 2.74E-09 | 55 |
| Efet.01.1655718.g785.t1 | Neuromedin-U receptor 1 | 528 | 1.41E-23 | 55 |
| Efet.01.226191.g1047.t1 | Nitric oxide synthase, inducible | 852 | 1.45E-33 | 55 |
| Efet.01.43341.g1190.t1 | Neurogenic locus notch homolog protein 1 | 567 | 7.71E-25 | 55 |
| Efet.01.39720.g978.t1 | Neurogenic locus Notch protein | 1368 | 9.67E-17 | 55 |
| Efet.01.505346.g135.t1 | Melanopsin | 2589 | 2.55E-12 | 55 |
| Efet.01.606340.g272.t1 | Melanopsin | 546 | 2.02E-07 | 55 |
| Efet.01.607618.g320.t1 | Melanopsin | 447 | 2.00E-09 | 55 |
| Efet.01.34534.g655.t1 | Plasminogen activator inhibitor 1 | 978 | 2.04E-45 | 55 |
| Efet.01.1654736.g707.t1 | Plasminogen activator inhibitor 1 | 558 | 3.81E-26 | 55 |
| Efet.01.344.g41.t1 | Palladin | 1071 | 5.44E-38 | 55 |
| Efet.01.658362.g1892.t1 | Palladin | 816 | 2.03E-07 | 55 |
| Efet.01.67734.g1091.t1 | 3-phosphoinositide-dependent protein kinase 1 | 294 | 2.96E-11 | 55 |
| Efet.01.67214.g1061.t1 | Plasminogen | 231 | 3.43E-12 | 55 |
| Efet.01.54188.g278.t1 | Plastin-2 | 252 | 5.42E-07 | 55 |
| Efet.01.105872.g313.t1 | Plastin-2 | 234 | 7.90E-07 | 55 |
| Efet.01.190657.g1898.t1 | Protein O-linked-mannose beta-1,2-N-acetylglucosaminyltransferase 1 | 315 | 1.13E-08 | 55 |
| Efet.01.266089.g588.t1 | PR domain zinc finger protein 14 | 243 | 1.12E-09 | 55 |
| Efet.01.73791.g1465.t1 | PR domain zinc finger protein 16 | 1179 | 1.31E-13 | 55 |
| Efet.01.627199.g1201.t1 | PR domain zinc finger protein 16 | 2349 | 1.12E-13 | 55 |
| Efet.01.64671.g894.t1 | Paired mesoderm homeobox protein 1 | 477 | 2.93E-08 | 55 |
| Efet.01.179564.g1376.t1 | Receptor-type tyrosine-protein phosphatase S | 375 | 6.19E-18 | 55 |
| Efet.01.1658966.g1339.t1 | SMARCA4 isoform 2 | 993 | 9.80E-13 | 55 |
| Efet.01.20621.g1517.t1 | RE1-silencing transcription factor | 5742 | 1.18E-14 | 55 |
| Efet.01.320949.g662.t1 | Repulsive guidance molecule A | 438 | 2.32E-26 | 55 |
| Efet.01.70373.g1256.t1 | GTP-binding protein RHO1 | 459 | 3.49E-13 | 55 |
| Efet.01.559164.g725.t1 | E3 ubiquitin-protein ligase RNF13 | 1410 | 3.90E-07 | 55 |
| Efet.01.193439.g2014.t1 | Roundabout homolog 2 | 474 | 2.46E-10 | 55 |
| Efet.01.649448.g1308.t1 | Roundabout homolog 2 | 258 | 1.38E-07 | 55 |
| Efet.01.510009.g245.t1 | Sal-like protein 1 | 1623 | 2.41E-08 | 55 |
| Efet.01.126454.g85.t1 | Sal-like protein 4 | 1284 | 2.77E-06 | 55 |
| Efet.01.353784.g109.t1 | Sodium channel protein type 1 subunit alpha | 309 | 2.87E-11 | 55 |
| Efet.01.82427.g490.t1 | Serine/threonine-protein kinase Sgk1 | 954 | 1.30E-43 | 55 |
| Efet.01.83681.g561.t1 | SHC-transforming protein 1 | 2313 | 2.15E-13 | 55 |
| Efet.01.230086.g1200.t1 | S-phase kinase-associated protein 2 | 942 | 5.17E-18 | 55 |
| Efet.01.607469.g314.t1 | Slit homolog 1 protein | 258 | 8.62E-08 | 55 |
| Efet.01.1659452.g1748.t1 | Superoxide dismutase [Mn], mitochondrial | 582 | 3.06E-29 | 55 |
| Efet.01.197751.g2207.t1 | Spectrin beta chain, non-erythrocytic 1 | 285 | 1.64E-12 | 55 |
| Efet.01.286207.g1310.t1 | Spectrin beta chain, non-erythrocytic 1 | 651 | 7.73E-26 | 55 |
| Efet.01.1324.g121.t1 | Src substrate cortactin | 843 | 2.12E-09 | 55 |
| Efet.01.218753.g759.t1 | Src substrate cortactin | 438 | 8.34E-06 | 55 |
| Efet.01.347255.g1329.t1 | Tubulin alpha chain | 927 | 8.00E-52 | 55 |
| Efet.01.237679.g1470.t1 | Tubulin alpha-1C chain | 828 | 2.16E-69 | 55 |
| Efet.01.429511.g728.t1 | Tenascin | 1296 | 3.24E-09 | 55 |
| Efet.01.171370.g1028.t1 | Transcription intermediary factor 1-alpha | 2385 | 5.29E-09 | 55 |
| Efet.01.162340.g634.t1 | DNA topoisomerase 3 | 1437 | 2.56E-41 | 55 |
| Efet.01.305731.g198.t1 | TNF receptor-associated factor 6 | 723 | 1.43E-19 | 55 |
| Efet.01.658387.g1939.t1 | Probable thymidylate synthase | 2409 | 5.23E-20 | 55 |
| Efet.01.170740.g1005.t1 | UDP-glucuronosyltransferase 1-2 | 435 | 7.48E-10 | 55 |
| Efet.01.102678.g153.t1 | Serine/threonine-protein kinase ULK1 | 903 | 6.65E-34 | 55 |
| Efet.01.172552.g1074.t1 | Vasopressin V1a receptor | 552 | 1.52E-24 | 55 |
| Efet.01.285241.g1280.t1 | Protein vav-1 | 264 | 2.75E-06 | 55 |
| Efet.01.231434.g1247.t1 | Vascular endothelial growth factor receptor 2 | 525 | 3.76E-11 | 55 |
| Efet.01.304054.g141.t1 | Vimentin | 318 | 2.89E-07 | 55 |
| Efet.01.14885.g1092.t1 | WD repeat-containing protein 5 | 1578 | 2.11E-13 | 55 |
| Efet.01.266312.g594.t1 | WD repeat-containing protein 5 | 282 | 2.84E-13 | 55 |
| Efet.01.271010.g782.t1 | WD repeat-containing protein 5 | 597 | 2.14E-29 | 55 |
| Efet.01.585463.g840.t1 | Protein white | 303 | 3.40E-10 | 55 |
| Efet.01.658044.g1599.t1 | Protein white | 453 | 2.83E-08 | 55 |
| Efet.01.658257.g1761.t1 | Protein white | 798 | 7.99E-09 | 55 |
| Efet.01.61428.g710.t1 | Protein wingless | 234 | 4.65E-13 | 55 |
| Efet.01.350607.g18.t1 | Serine/threonine-protein kinase YPK1 | 357 | 3.92E-17 | 55 |
| Efet.01.626647.g1168.t1 | Serine/threonine-protein kinase YPK1 | 480 | 8.75E-10 | 55 |
| Efet.01.323242.g721.t1 | Zinc finger homeobox protein 3 | 312 | 9.18E-07 | 55 |
| Efet.01.15688.g1154.t1 | Zinc finger protein 37A | 1071 | 3.43E-47 | 55 |
| Efet.01.60114.g630.t1 | Zinc finger protein 37A | 1077 | 1.24E-68 | 55 |
| Efet.01.71147.g1304.t1 | Zinc finger protein 37A | 1485 | 2.30E-47 | 55 |
| Efet.01.146998.g1100.t1 | Zinc finger protein 37A | 1311 | 2.97E-76 | 55 |
| Efet.01.238231.g1486.t1 | Zinc finger protein 37A | 783 | 1.12E-36 | 55 |
| Efet.01.282394.g1172.t1 | Zinc finger protein 37A | 1413 | 3.56E-54 | 55 |
| Efet.01.356320.g177.t1 | Zinc finger protein 37A | 741 | 6.32E-51 | 55 |
| Efet.01.408488.g203.t1 | Zinc finger protein 37A | 990 | 1.47E-64 | 55 |
| Efet.01.585955.g860.t1 | Zinc finger protein 37A | 2319 | 9.86E-36 | 55 |
| Efet.01.657269.g1221.t1 | Zinc finger protein 37A | 378 | 3.71E-20 | 55 |
| Efet.01.153611.g193.t1 | Protein Z-dependent protease inhibitor | 243 | 2.14E-09 | 55 |
| Efet.01.626280.g1161.t1 | Zinc finger and SCAN domain-containing protein 10 | 792 | 1.28E-38 | 55 |
| Efet.01.608093.g342.t1 | 5-hydroxytryptamine receptor 1A | 1884 | 8.21E-47 | 54 |
| Efet.01.70976.g1295.t1 | 5-hydroxytryptamine receptor 1A | 1008 | 3.93E-50 | 54 |
| Efet.01.390862.g1024.t1 | 5-hydroxytryptamine receptor 1A | 912 | 3.59E-21 | 54 |
| Efet.01.65641.g958.t1 | 5-hydroxytryptamine receptor 1A | 1056 | 2.26E-51 | 54 |
| Efet.01.155968.g309.t1 | 5-hydroxytryptamine receptor 1A | 1713 | 1.30E-29 | 54 |
| Efet.01.227917.g1123.t1 | 5-hydroxytryptamine receptor 2A | 1722 | 1.40E-08 | 54 |
| Efet.01.458583.g242.t1 | Probable ATP-dependent permease | 624 | 2.75E-09 | 54 |
| Efet.01.109444.g494.t1 | Angiopoietin-2 | 1251 | 1.54E-28 | 54 |
| Efet.01.432204.g779.t1 | Protein arginine N-methyltransferase 1 | 474 | 1.17E-11 | 54 |
| Efet.01.186761.g1711.t1 | Atrial natriuretic peptide receptor 1 | 1656 | 8.20E-12 | 54 |
| Efet.01.1655550.g767.t1 | Atrial natriuretic peptide receptor 1 | 315 | 4.80E-12 | 54 |
| Efet.01.580788.g692.t1 | Aurora kinase A | 636 | 2.71E-07 | 54 |
| Efet.01.41772.g1103.t1 | Activin receptor type-2B | 1014 | 1.74E-59 | 54 |
| Efet.01.360588.g284.t1 | Apoptosis regulator Bcl-2 | 501 | 2.01E-23 | 54 |
| Efet.01.487337.g900.t1 | Serine/threonine-protein kinase B-raf | 981 | 3.83E-21 | 54 |
| Efet.01.605603.g231.t1 | Tyrosine-protein kinase Btk29A | 3231 | 5.43E-62 | 54 |
| Efet.01.256078.g221.t1 | Calumenin | 444 | 5.61E-18 | 54 |
| Efet.01.107634.g393.t1 | Cholecystokinin receptor type A | 621 | 2.05E-10 | 54 |
| Efet.01.226540.g1059.t1 | Cholecystokinin receptor type A | 564 | 6.56E-18 | 54 |
| Efet.01.210782.g476.t1 | Cholecystokinin receptor type A | 1074 | 4.66E-21 | 54 |
| Efet.01.354363.g124.t1 | C-C chemokine receptor type 2 | 1254 | 2.33E-13 | 54 |
| Efet.01.558703.g713.t1 | Cystic fibrosis transmembrane conductance regulator | 600 | 6.35E-06 | 54 |
| Efet.01.1639369.g206.t1 | Contactin-1 | 270 | 2.50E-06 | 54 |
| Efet.01.55702.g372.t1 | Cytochrome P450 3A12 | 894 | 6.80E-22 | 54 |
| Efet.01.213261.g558.t1 | Cytochrome P450 3A12 | 678 | 5.99E-09 | 54 |
| Efet.01.93975.g1099.t1 | Citron Rho-interacting kinase | 285 | 9.79E-06 | 54 |
| Efet.01.167044.g842.t1 | C-X-C chemokine receptor type 1 | 759 | 7.01E-10 | 54 |
| Efet.01.655322.g797.t1 | Epithelial discoidin domain-containing receptor 1 | 558 | 2.21E-23 | 54 |
| Efet.01.143711.g957.t1 | Neurogenic locus protein delta | 261 | 3.40E-12 | 54 |
| Efet.01.1659012.g1358.t1 | Dihydropyrimidinase-related protein 2 | 984 | 5.79E-48 | 54 |
| Efet.01.95043.g1161.t1 | Dysferlin | 522 | 1.88E-30 | 54 |
| Efet.01.213709.g582.t1 | Histone-lysine N-methyltransferase EHMT2 | 423 | 4.95E-12 | 54 |
| Efet.01.40205.g999.t1 | ELAV-like protein 4 | 273 | 4.49E-16 | 54 |
| Efet.01.288803.g1410.t1 | Ephrin type-B receptor 3 | 336 | 5.27E-10 | 54 |
| Efet.01.260813.g406.t1 | Coagulation factor VII | 297 | 1.65E-09 | 54 |
| Efet.01.1656600.g889.t1 | FAS-associated death domain protein | 258 | 7.32E-07 | 54 |
| Efet.01.197703.g2205.t1 | Fibrinogen-like protein 1 | 420 | 2.62E-16 | 54 |
| Efet.01.287526.g1372.t1 | Folylpolyglutamate synthase, mitochondrial | 1254 | 4.47E-32 | 54 |
| Efet.01.75999.g59.t1 | Forkhead box protein J3 | 429 | 1.56E-21 | 54 |
| Efet.01.655099.g753.t1 | Follistatin | 243 | 2.84E-06 | 54 |
| Efet.01.458432.g234.t1 | Alpha-(1,3)-fucosyltransferase 4 | 288 | 2.41E-09 | 54 |
| Efet.01.67347.g1068.t1 | Glial fibrillary acidic protein | 516 | 2.19E-19 | 54 |
| Efet.01.121040.g1092.t1 | Glypican-3 | 753 | 5.22E-37 | 54 |
| Efet.01.383813.g884.t1 | Glutamate receptor 3 | 777 | 1.16E-24 | 54 |
| Efet.01.99654.g1425.t1 | E3 ISG15--protein ligase HERC5 | 321 | 3.41E-07 | 54 |
| Efet.01.178125.g1315.t1 | Transcription factor HES-1 | 909 | 1.49E-11 | 54 |
| Efet.01.95344.g1178.t1 | Heat shock protein beta-1 | 1020 | 1.89E-14 | 54 |
| Efet.01.643899.g843.t1 | ATP-dependent RNA helicase eIF4A | 1152 | 2.24E-49 | 54 |
| Efet.01.21880.g1600.t1 | Integrin-linked protein kinase | 1068 | 8.76E-12 | 54 |
| Efet.01.52756.g193.t1 | Integrin-linked protein kinase | 1107 | 5.75E-08 | 54 |
| Efet.01.567084.g233.t1 | Integrin-linked protein kinase homolog pat-4 | 384 | 5.84E-09 | 54 |
| Efet.01.84118.g578.t1 | Inter-alpha-trypsin inhibitor heavy chain H4 | 711 | 1.56E-31 | 54 |
| Efet.01.566481.g216.t1 | Potassium voltage-gated channel subfamily B member 1 | 993 | 2.03E-56 | 54 |
| Efet.01.518526.g447.t1 | Potassium channel subfamily K member 3 | 918 | 5.73E-07 | 54 |
| Efet.01.562940.g69.t1 | Potassium channel subfamily K member 3 | 537 | 5.57E-07 | 54 |
| Efet.01.146232.g1063.t1 | Histone-lysine N-methyltransferase 2A | 561 | 6.43E-15 | 54 |
| Efet.01.363782.g369.t1 | Serine/threonine-protein kinase D1 | 441 | 1.76E-11 | 54 |
| Efet.01.157022.g367.t1 | Neural cell adhesion molecule L1 | 498 | 3.30E-09 | 54 |
| Efet.01.559694.g744.t1 | Low-density lipoprotein receptor-related protein 1 | 573 | 2.79E-07 | 54 |
| Efet.01.1655191.g742.t1 | Prolow-density lipoprotein receptor-related protein 1 | 591 | 4.11E-27 | 54 |
| Efet.01.99237.g1401.t1 | Low-density lipoprotein receptor-related protein 2 | 447 | 9.54E-48 | 54 |
| Efet.01.108219.g426.t1 | Mitogen-activated protein kinase kinase kinase 12 | 429 | 3.18E-14 | 54 |
| Efet.01.285894.g1297.t1 | Mitogen-activated protein kinase kinase kinase 12 | 825 | 3.80E-20 | 54 |
| Efet.01.573477.g415.t1 | Mitogen-activated protein kinase kinase kinase 12 | 816 | 3.76E-23 | 54 |
| Efet.01.53797.g253.t1 | Mitogen-activated protein kinase kinase kinase 12 | 831 | 1.80E-19 | 54 |
| Efet.01.320638.g651.t1 | Matrilin-2 | 627 | 3.33E-27 | 54 |
| Efet.01.657532.g1322.t1 | Matrilin-2 | 1806 | 4.29E-19 | 54 |
| Efet.01.229254.g1172.t1 | DNA replication licensing factor MCM2 | 987 | 4.39E-58 | 54 |
| Efet.01.287459.g1356.t1 | Mediator of RNA polymerase II transcription subunit 14 | 468 | 8.77E-21 | 54 |
| Efet.01.12164.g906.t1 | Hepatocyte growth factor receptor | 603 | 1.87E-20 | 54 |
| Efet.01.31396.g445.t1 | 72 kDa type IV collagenase | 312 | 1.16E-10 | 54 |
| Efet.01.139023.g723.t1 | Myotrophin | 1875 | 5.87E-13 | 54 |
| Efet.01.1659497.g1879.t1 | Myotrophin | 642 | 1.05E-14 | 54 |
| Efet.01.76696.g99.t1 | Myosin-2 | 852 | 2.04E-59 | 54 |
| Efet.01.1372.g128.t1 | Unconventional myosin-Va | 450 | 1.37E-28 | 54 |
| Efet.01.179556.g1375.t1 | Neuromedin-U receptor 1 | 480 | 3.96E-14 | 54 |
| Efet.01.654669.g681.t1 | Neuromedin-U receptor 1 | 1209 | 8.59E-15 | 54 |
| Efet.01.446106.g1142.t1 | Neuropilin-1 | 234 | 1.94E-08 | 54 |
| Efet.01.1619149.g53.t1 | Neuropilin-2 | 201 | 2.36E-06 | 54 |
| Efet.01.387979.g969.t1 | BDNF/NT-3 growth factors receptor | 360 | 4.17E-09 | 54 |
| Efet.01.526612.g619.t1 | BDNF/NT-3 growth factors receptor | 597 | 1.23E-24 | 54 |
| Efet.01.212656.g545.t1 | Octopamine receptor beta-3R | 453 | 1.82E-13 | 54 |
| Efet.01.137754.g654.t1 | Phosphatidylinositol 3-kinase regulatory subunit alpha | 480 | 3.80E-06 | 54 |
| Efet.01.196489.g2151.t1 | Polyadenylate-binding protein 2 | 309 | 9.82E-10 | 54 |
| Efet.01.1474.g140.t1 | Plasminogen activator inhibitor 1 | 459 | 2.81E-10 | 54 |
| Efet.01.98955.g1382.t1 | Phosphatidylinositol 4,5-bisphosphate 3-kinase catalytic subunit gamma isoform | 576 | 1.50E-23 | 54 |
| Efet.01.274540.g896.t1 | Phosphatidylinositol 4,5-bisphosphate 3-kinase catalytic subunit gamma isoform | 348 | 9.64E-10 | 54 |
| Efet.01.203477.g160.t1 | Plasminogen | 669 | 4.34E-33 | 54 |
| Efet.01.277984.g1031.t1 | Plastin-2 | 384 | 1.80E-18 | 54 |
| Efet.01.148771.g1169.t1 | Receptor-type tyrosine-protein phosphatase S | 549 | 3.92E-15 | 54 |
| Efet.01.373741.g660.t1 | Receptor-type tyrosine-protein phosphatase S | 378 | 1.08E-10 | 54 |
| Efet.01.9855.g719.t1 | Receptor-type tyrosine-protein phosphatase S | 600 | 3.06E-06 | 54 |
| Efet.01.303499.g111.t1 | Receptor-type tyrosine-protein phosphatase S | 327 | 3.19E-13 | 54 |
| Efet.01.176388.g1229.t1 | Peroxidasin | 696 | 3.71E-35 | 54 |
| Efet.01.472493.g610.t1 | SMARCA4 isoform 2 | 333 | 1.24E-08 | 54 |
| Efet.01.257033.g267.t1 | Retinoic acid receptor beta | 891 | 2.59E-27 | 54 |
| Efet.01.7979.g588.t1 | Repulsive guidance molecule A | 690 | 2.21E-32 | 54 |
| Efet.01.258504.g316.t1 | Repulsive guidance molecule A | 480 | 7.69E-30 | 54 |
| Efet.01.1658029.g1083.t1 | Repulsive guidance molecule A | 729 | 1.85E-21 | 54 |
| Efet.01.78976.g243.t1 | Roundabout homolog 2 | 225 | 9.98E-07 | 54 |
| Efet.01.224592.g981.t1 | Retinoid isomerohydrolase | 567 | 2.15E-27 | 54 |
| Efet.01.274930.g916.t1 | Solute carrier family 12 member 2 | 276 | 1.75E-12 | 54 |
| Efet.01.322707.g706.t1 | Solute carrier family 12 member 2 | 321 | 9.44E-18 | 54 |
| Efet.01.53441.g229.t1 | Sal-like protein 1 | 924 | 1.28E-08 | 54 |
| Efet.01.458517.g240.t1 | Sal-like protein 1 | 2562 | 8.01E-55 | 54 |
| Efet.01.265556.g569.t1 | Sodium channel protein type 9 subunit alpha | 207 | 3.52E-09 | 54 |
| Efet.01.6158.g487.t1 | Semaphorin-3A | 270 | 8.22E-09 | 54 |
| Efet.01.230071.g1199.t1 | Slit homolog 1 protein | 465 | 1.86E-08 | 54 |
| Efet.01.10407.g763.t1 | Suppressor of cytokine signaling 3 | 1044 | 1.05E-29 | 54 |
| Efet.01.167554.g870.t1 | Suppressor of cytokine signaling 3 | 576 | 2.30E-29 | 54 |
| Efet.01.347007.g1320.t1 | Suppressor of cytokine signaling 3 | 1287 | 8.87E-23 | 54 |
| Efet.01.117865.g919.t1 | Spectrin alpha chain, non-erythrocytic 1 | 297 | 1.40E-07 | 54 |
| Efet.01.199402.g2278.t1 | Sterol regulatory element-binding protein 1 | 327 | 8.55E-15 | 54 |
| Efet.01.217335.g716.t1 | Tenascin | 417 | 5.05E-13 | 54 |
| Efet.01.141269.g842.t1 | Fructose-2,6-bisphosphatase TIGAR | 1011 | 5.41E-06 | 54 |
| Efet.01.37705.g838.t1 | Toll-like receptor Tollo | 798 | 3.03E-15 | 54 |
| Efet.01.295656.g1606.t1 | E3 ubiquitin-protein ligase TRIM32 | 1518 | 2.99E-06 | 54 |
| Efet.01.12399.g924.t1 | Transient receptor potential-gamma protein | 696 | 5.41E-31 | 54 |
| Efet.01.185932.g1678.t1 | Transcriptional repressor protein YY1 | 1392 | 2.56E-12 | 54 |
| Efet.01.477273.g693.t1 | Tyrosine-protein kinase receptor UFO | 666 | 5.57E-08 | 54 |
| Efet.01.28951.g274.t1 | Serine/threonine-protein kinase ULK1 | 1011 | 3.31E-17 | 54 |
| Efet.01.208970.g405.t1 | Vasopressin V1a receptor | 639 | 1.07E-10 | 54 |
| Efet.01.412692.g288.t1 | Vasopressin V1a receptor | 585 | 2.27E-14 | 54 |
| Efet.01.500525.g11.t1 | Vasopressin V1a receptor | 417 | 9.66E-16 | 54 |
| Efet.01.58092.g504.t1 | WD repeat-containing protein 5 | 357 | 2.10E-10 | 54 |
| Efet.01.406600.g163.t1 | WD repeat-containing protein 5 | 906 | 3.68E-16 | 54 |
| Efet.01.456900.g171.t1 | Protein white | 576 | 4.16E-09 | 54 |
| Efet.01.275883.g958.t1 | Protein white | 687 | 3.26E-07 | 54 |
| Efet.01.381535.g823.t1 | Protein white | 603 | 2.28E-29 | 54 |
| Efet.01.577373.g594.t1 | Protein white | 600 | 2.76E-10 | 54 |
| Efet.01.658261.g1766.t1 | ABC transporter ATP-binding protein/permease wht-1 | 486 | 3.61E-11 | 54 |
| Efet.01.310488.g331.t1 | Protein Wnt-5 | 402 | 1.02E-21 | 54 |
| Efet.01.295634.g1605.t1 | Zinc finger protein 281 | 573 | 1.50E-07 | 54 |
| Efet.01.227117.g1087.t1 | Zinc finger protein 37A | 861 | 3.74E-31 | 54 |
| Efet.01.305761.g200.t1 | Zinc finger protein 37A | 903 | 1.79E-45 | 54 |
| Efet.01.59101.g572.t1 | 5-hydroxytryptamine receptor 1A | 1134 | 3.77E-18 | 53 |
| Efet.01.103538.g191.t1 | 5-hydroxytryptamine receptor 1A | 1719 | 1.75E-79 | 53 |
| Efet.01.401737.g46.t1 | 5-hydroxytryptamine receptor 1A | 1275 | 1.70E-67 | 53 |
| Efet.01.68866.g1161.t1 | 5-hydroxytryptamine receptor 2A | 1305 | 4.28E-28 | 53 |
| Efet.01.399157.g1220.t1 | 5-hydroxytryptamine receptor 2A | 354 | 4.54E-09 | 53 |
| Efet.01.529496.g684.t1 | 5-hydroxytryptamine receptor 2B | 282 | 5.11E-06 | 53 |
| Efet.01.107779.g399.t1 | 5-hydroxytryptamine receptor 2C | 981 | 5.31E-12 | 53 |
| Efet.01.654257.g555.t1 | ATP-binding cassette sub-family G member 2 | 1080 | 4.34E-14 | 53 |
| Efet.01.458583.g243.t1 | ATP-binding cassette sub-family G member 2 | 735 | 1.14E-10 | 53 |
| Efet.01.401777.g48.t1 | Atypical chemokine receptor 3 | 570 | 1.51E-06 | 53 |
| Efet.01.472687.g617.t1 | Atypical chemokine receptor 3 | 1215 | 2.78E-07 | 53 |
| Efet.01.333555.g1004.t1 | ADAM 17-like protease | 297 | 1.37E-13 | 53 |
| Efet.01.1654813.g715.t1 | Probable ATP-dependent permease | 390 | 4.99E-06 | 53 |
| Efet.01.86776.g737.t1 | Aurora/IPL1-related protein kinase 2 | 537 | 4.22E-06 | 53 |
| Efet.01.86933.g743.t1 | Atrial natriuretic peptide receptor 1 | 603 | 2.02E-17 | 53 |
| Efet.01.354576.g133.t1 | Atrial natriuretic peptide receptor 1 | 393 | 6.70E-10 | 53 |
| Efet.01.421681.g518.t1 | Atrial natriuretic peptide receptor 1 | 387 | 3.91E-15 | 53 |
| Efet.01.522129.g520.t1 | Aurora kinase A | 255 | 5.13E-07 | 53 |
| Efet.01.215127.g629.t1 | Bone morphogenetic protein 4 | 1440 | 1.96E-79 | 53 |
| Efet.01.147479.g1118.t1 | Cadherin-99C | 2055 | 3.04E-17 | 53 |
| Efet.01.654468.g610.t1 | Cadherin-1 | 306 | 9.95E-12 | 53 |
| Efet.01.1643610.g282.t1 | Cadherin-4 | 312 | 3.60E-06 | 53 |
| Efet.01.62261.g769.t1 | Caspase-3 | 405 | 1.12E-25 | 53 |
| Efet.01.208232.g376.t1 | Cholecystokinin receptor type A | 831 | 1.25E-23 | 53 |
| Efet.01.138652.g698.t1 | G1/S-specific cyclin-D2 | 405 | 1.95E-09 | 53 |
| Efet.01.572225.g385.t1 | C-C chemokine receptor type 2 | 738 | 6.00E-27 | 53 |
| Efet.01.657719.g1400.t1 | Cystic fibrosis transmembrane conductance regulator | 954 | 1.40E-10 | 53 |
| Efet.01.453507.g77.t1 | Chromodomain-helicase-DNA-binding protein 1 | 684 | 4.91E-40 | 53 |
| Efet.01.68600.g1141.t1 | Chromodomain-helicase-DNA-binding protein 7 | 705 | 2.98E-26 | 53 |
| Efet.01.495120.g1072.t1 | Chromodomain-helicase-DNA-binding protein 7 | 216 | 1.10E-06 | 53 |
| Efet.01.97972.g1337.t1 | 25-hydroxyvitamin D-1 alpha hydroxylase, mitochondrial | 789 | 3.40E-08 | 53 |
| Efet.01.568048.g259.t1 | 25-hydroxyvitamin D-1 alpha hydroxylase, mitochondrial | 516 | 2.65E-06 | 53 |
| Efet.01.100686.g52.t1 | Cytochrome P450 3A2 | 363 | 9.32E-11 | 53 |
| Efet.01.126447.g84.t1 | Cytochrome P450 3A12 | 762 | 1.46E-12 | 53 |
| Efet.01.99238.g1404.t1 | Chondroitin sulfate proteoglycan 4 | 573 | 1.01E-29 | 53 |
| Efet.01.52633.g183.t1 | C-X-C chemokine receptor type 1 | 720 | 1.90E-06 | 53 |
| Efet.01.95607.g1193.t1 | Protein Dicer | 1161 | 5.02E-17 | 53 |
| Efet.01.449761.g1210.t1 | Endoribonuclease Dicer | 990 | 2.20E-19 | 53 |
| Efet.01.280613.g1119.t1 | Dual specificity protein phosphatase 1 | 879 | 1.57E-20 | 53 |
| Efet.01.50680.g48.t1 | Dual specificity protein phosphatase 1 | 522 | 5.88E-26 | 53 |
| Efet.01.10686.g787.t1 | Histone-lysine N-methyltransferase EHMT2 | 687 | 4.50E-15 | 53 |
| Efet.01.35526.g700.t1 | Receptor tyrosine-protein kinase erbB-4 | 435 | 7.88E-19 | 53 |
| Efet.01.2314.g206.t1 | Fibroblast growth factor receptor homolog 2 | 621 | 6.73E-19 | 53 |
| Efet.01.187083.g1736.t1 | Fibroblast growth factor receptor 4 | 684 | 1.13E-14 | 53 |
| Efet.01.161682.g606.t1 | Protein flightless-1 | 888 | 1.71E-17 | 53 |
| Efet.01.44655.g1275.t1 | Alpha-(1,3)-fucosyltransferase 4 | 576 | 7.90E-17 | 53 |
| Efet.01.80858.g390.t1 | Gamma-aminobutyric acid receptor subunit beta-3 | 483 | 3.85E-10 | 53 |
| Efet.01.310452.g330.t1 | Gamma-aminobutyric acid receptor subunit beta-3 | 426 | 3.41E-13 | 53 |
| Efet.01.643099.g761.t1 | Bifunctional UDP-N-acetylglucosamine 2-epimerase/N-acetylmannosamine kinase | 1077 | 3.47E-14 | 53 |
| Efet.01.274959.g920.t1 | Guanine nucleotide-binding protein G(s) subunit alpha | 1080 | 1.28E-68 | 53 |
| Efet.01.43074.g1175.t1 | G protein alpha s subunit | 342 | 7.53E-16 | 53 |
| Efet.01.108200.g425.t1 | Solute carrier family 2, facilitated glucose transporter member 4 | 840 | 4.53E-44 | 53 |
| Efet.01.39151.g946.t1 | Nuclear hormone receptor HR96 | 1029 | 2.49E-22 | 53 |
| Efet.01.282098.g1165.t1 | Histamine H2 receptor | 1734 | 6.88E-28 | 53 |
| Efet.01.429104.g712.t1 | Histamine H2 receptor | 861 | 1.73E-06 | 53 |
| Efet.01.19994.g1480.t1 | ATP-dependent RNA helicase eIF4A | 603 | 1.70E-27 | 53 |
| Efet.01.5443.g422.t1 | Integrin-linked protein kinase | 2073 | 5.14E-12 | 53 |
| Efet.01.32323.g513.t1 | Integrin-linked protein kinase | 816 | 2.34E-09 | 53 |
| Efet.01.214770.g620.t1 | Integrin-linked protein kinase | 480 | 1.68E-11 | 53 |
| Efet.01.330277.g921.t1 | Integrin-linked protein kinase | 279 | 9.09E-06 | 53 |
| Efet.01.167644.g875.t1 | Integrin-linked protein kinase | 219 | 5.87E-08 | 53 |
| Efet.01.185271.g1642.t1 | Transcription factor AP-1 | 948 | 2.26E-46 | 53 |
| Efet.01.311866.g376.t1 | Potassium voltage-gated channel subfamily A member 5 | 681 | 2.87E-27 | 53 |
| Efet.01.658214.g1719.t1 | Potassium voltage-gated channel subfamily A member 5 | 1113 | 7.51E-32 | 53 |
| Efet.01.126884.g111.t1 | Potassium voltage-gated channel subfamily B member 1 | 1032 | 2.13E-33 | 53 |
| Efet.01.262822.g486.t1 | Kinesin-like protein KIF3C | 339 | 7.82E-09 | 53 |
| Efet.01.61049.g689.t1 | Raf homolog serine/threonine-protein kinase phl | 378 | 4.84E-13 | 53 |
| Efet.01.78945.g238.t1 | Laminin subunit beta-2 | 468 | 5.66E-22 | 53 |
| Efet.01.348247.g1363.t1 | Low-density lipoprotein receptor | 363 | 4.00E-08 | 53 |
| Efet.01.108959.g465.t1 | Lymphoid enhancer-binding factor 1 | 603 | 1.26E-22 | 53 |
| Efet.01.27357.g151.t1 | E3 ubiquitin-protein ligase TRIM71 | 210 | 2.55E-07 | 53 |
| Efet.01.112787.g646.t1 | Low-density lipoprotein receptor-related protein 1 | 459 | 1.14E-11 | 53 |
| Efet.01.125155.g7.t1 | Matrilin-2 | 1098 | 4.56E-19 | 53 |
| Efet.01.231624.g1260.t1 | Matrilin-2 | 621 | 1.05E-20 | 53 |
| Efet.01.323828.g738.t1 | Matrilin-2 | 1026 | 1.76E-23 | 53 |
| Efet.01.1657811.g1040.t1 | Induced myeloid leukemia cell differentiation protein Mcl-1 homolog | 495 | 2.12E-14 | 53 |
| Efet.01.586576.g885.t1 | Hepatocyte growth factor receptor | 909 | 8.71E-20 | 53 |
| Efet.01.271245.g795.t1 | MAX gene-associated protein | 678 | 1.62E-09 | 53 |
| Efet.01.538409.g239.t1 | Mitogen-activated protein kinase 14A | 408 | 1.10E-15 | 53 |
| Efet.01.34225.g639.t1 | Mitogen-activated protein kinase 14B | 1125 | 3.05E-36 | 53 |
| Efet.01.214444.g611.t1 | Matrix metalloproteinase-14 | 579 | 1.74E-18 | 53 |
| Efet.01.2598.g238.t1 | Canalicular multispecific organic anion transporter 1 | 981 | 2.75E-60 | 53 |
| Efet.01.645424.g985.t1 | Canalicular multispecific organic anion transporter 1 | 612 | 2.71E-10 | 53 |
| Efet.01.656518.g1037.t1 | Canalicular multispecific organic anion transporter 1 | 1455 | 2.22E-10 | 53 |
| Efet.01.532173.g69.t1 | Canalicular multispecific organic anion transporter 2 | 501 | 5.18E-14 | 53 |
| Efet.01.1638137.g190.t1 | Myosin-2 | 219 | 1.38E-08 | 53 |
| Efet.01.561311.g25.t1 | Nucleus accumbens-associated protein 1 | 1620 | 1.14E-06 | 53 |
| Efet.01.469285.g515.t1 | Bifunctional heparan sulfate N-deacetylase/N-sulfotransferase 1 | 525 | 5.27E-15 | 53 |
| Efet.01.254136.g152.t1 | Neuromedin-U receptor 1 | 1041 | 8.72E-16 | 53 |
| Efet.01.330621.g936.t1 | Nitric oxide synthase, inducible | 762 | 2.08E-38 | 53 |
| Efet.01.5872.g467.t1 | Neurogenic locus Notch protein | 459 | 1.60E-14 | 53 |
| Efet.01.269911.g738.t1 | Neurogenic locus Notch protein | 1095 | 1.34E-08 | 53 |
| Efet.01.227631.g1108.t1 | Neuropilin-1 | 483 | 5.84E-20 | 53 |
| Efet.01.33299.g570.t1 | Octopamine receptor beta-3R | 978 | 1.70E-07 | 53 |
| Efet.01.335772.g1068.t1 | Melanopsin | 513 | 8.96E-09 | 53 |
| Efet.01.583853.g793.t1 | Phosphatidylinositol 3-kinase regulatory subunit alpha | 1026 | 4.49E-12 | 53 |
| Efet.01.138762.g708.t1 | Palladin | 663 | 2.33E-10 | 53 |
| Efet.01.142415.g901.t1 | Palladin | 5718 | 3.30E-10 | 53 |
| Efet.01.314672.g462.t1 | Protocadherin-15 | 1206 | 1.62E-25 | 53 |
| Efet.01.532983.g93.t1 | Protocadherin-15 | 687 | 3.96E-13 | 53 |
| Efet.01.525310.g590.t1 | Protocadherin-15 | 468 | 7.62E-14 | 53 |
| Efet.01.324563.g756.t1 | 3-phosphoinositide-dependent protein kinase 1 | 273 | 6.30E-08 | 53 |
| Efet.01.141017.g828.t1 | 1-phosphatidylinositol 4,5-bisphosphate phosphodiesterase gamma plc-3 | 543 | 6.17E-25 | 53 |
| Efet.01.106619.g347.t1 | Plasminogen | 270 | 8.24E-09 | 53 |
| Efet.01.148529.g1161.t1 | PR domain zinc finger protein 16 | 264 | 6.01E-08 | 53 |
| Efet.01.308049.g240.t1 | PR domain zinc finger protein 16 | 1035 | 7.50E-12 | 53 |
| Efet.01.287526.g1369.t1 | Amidophosphoribosyltransferase | 1398 | 1.56E-73 | 53 |
| Efet.01.654418.g593.t1 | CAD protein | 1218 | 8.99E-70 | 53 |
| Efet.01.656742.g1082.t1 | CAD protein | 1989 | 5.30E-41 | 53 |
| Efet.01.239492.g1521.t1 | CAD protein | 648 | 1.42E-25 | 53 |
| Efet.01.177769.g1300.t1 | Protein phosphatase 1 regulatory subunit | 534 | 1.24E-10 | 53 |
| Efet.01.105150.g277.t1 | Reticulon-4 receptor-like 2 | 1182 | 6.79E-11 | 53 |
| Efet.01.127471.g139.t1 | Ras-related protein Rab-1A | 543 | 2.48E-27 | 53 |
| Efet.01.204317.g188.t1 | Ras-related protein Rab-1A | 393 | 1.48E-17 | 53 |
| Efet.01.238089.g1481.t1 | Reelin | 627 | 1.61E-20 | 53 |
| Efet.01.576188.g518.t1 | Receptor-interacting serine/threonine-protein kinase 2 | 408 | 3.12E-08 | 53 |
| Efet.01.27500.g158.t1 | Roundabout homolog 2 | 258 | 1.45E-09 | 53 |
| Efet.01.225780.g1026.t1 | Rho-associated protein kinase 2 | 360 | 1.12E-20 | 53 |
| Efet.01.113788.g699.t1 | Reticulon-4 | 567 | 4.20E-12 | 53 |
| Efet.01.389997.g1006.t1 | Retinoic acid receptor RXR-alpha | 567 | 7.03E-08 | 53 |
| Efet.01.289710.g1442.t1 | Slit homolog 1 protein | 1665 | 5.75E-10 | 53 |
| Efet.01.64243.g878.t1 | Structural maintenance of chromosomes protein 1A | 1026 | 7.63E-14 | 53 |
| Efet.01.156547.g343.t1 | Zinc finger protein SNAI1 | 246 | 4.85E-07 | 53 |
| Efet.01.10601.g779.t1 | Solute carrier organic anion transporter family member 1A5 | 621 | 1.97E-20 | 53 |
| Efet.01.116704.g872.t1 | Spectrin alpha chain, non-erythrocytic 1 | 444 | 1.24E-07 | 53 |
| Efet.01.187044.g1733.t1 | Src substrate protein p85 | 1248 | 7.14E-07 | 53 |
| Efet.01.361526.g308.t1 | Tenascin | 297 | 6.78E-07 | 53 |
| Efet.01.441784.g1027.t1 | Tenascin | 555 | 2.62E-24 | 53 |
| Efet.01.259315.g357.t1 | Transcription factor 7-like 2 | 225 | 3.18E-16 | 53 |
| Efet.01.564507.g134.t1 | Transcription intermediary factor 1-alpha | 1200 | 1.82E-16 | 53 |
| Efet.01.423283.g550.t1 | Thrombospondin-2 | 930 | 9.93E-08 | 53 |
| Efet.01.636780.g348.t1 | Ubiquitin-conjugating enzyme E2 2 | 264 | 7.62E-08 | 53 |
| Efet.01.237744.g1472.t1 | Mitochondrial uncoupling protein 2 | 312 | 1.28E-09 | 53 |
| Efet.01.79836.g312.t1 | UDP-glucuronosyltransferase 1-8 | 819 | 4.21E-45 | 53 |
| Efet.01.4227.g352.t1 | von Willebrand factor | 411 | 1.31E-09 | 53 |
| Efet.01.612643.g556.t1 | Protein wech | 1176 | 1.96E-06 | 53 |
| Efet.01.626983.g1184.t1 | Protein white | 1356 | 1.57E-06 | 53 |
| Efet.01.44757.g1286.t1 | Metal resistance protein YCF1 | 975 | 1.04E-26 | 53 |
| Efet.01.533790.g136.t1 | Palmitoyltransferase ZDHHC23 | 633 | 2.11E-09 | 53 |
| Efet.01.303664.g120.t1 | Zinc finger homeobox protein 3 | 345 | 3.05E-07 | 53 |
| Efet.01.173358.g1110.t1 | Zinc finger X-chromosomal protein | 432 | 7.22E-10 | 53 |
| Efet.01.39004.g936.t1 | Zinc finger protein 37A | 684 | 7.81E-17 | 53 |
| Efet.01.57472.g467.t1 | Zinc finger protein 37A | 1209 | 4.77E-78 | 53 |
| Efet.01.447897.g1172.t1 | Zinc finger and SCAN domain-containing protein 10 | 624 | 7.77E-38 | 53 |
| Efet.01.559102.g723.t1 | 5-hydroxytryptamine receptor 1A | 375 | 7.09E-08 | 52 |
| Efet.01.98659.g1365.t1 | 5-hydroxytryptamine receptor 1A | 1704 | 8.97E-62 | 52 |
| Efet.01.131221.g330.t1 | 5-hydroxytryptamine receptor 1A | 1443 | 9.41E-24 | 52 |
| Efet.01.657838.g1496.t1 | ATP-binding cassette transporter abc2 | 633 | 8.79E-13 | 52 |
| Efet.01.658310.g1840.t1 | ATP-binding cassette sub-family G member 2 | 687 | 1.49E-11 | 52 |
| Efet.01.597702.g1221.t1 | Alcohol dehydrogenase 1C | 810 | 5.34E-25 | 52 |
| Efet.01.287526.g1362.t1 | Probable ATP-dependent permease | 642 | 1.09E-16 | 52 |
| Efet.01.215501.g647.t1 | Aurora/IPL1-related protein kinase 2 | 1182 | 9.10E-31 | 52 |
| Efet.01.633916.g227.t1 | Aldehyde dehydrogenase, dimeric NADP-preferring | 882 | 7.50E-48 | 52 |
| Efet.01.55780.g375.t1 | Atrial natriuretic peptide receptor 1 | 741 | 6.97E-26 | 52 |
| Efet.01.172280.g1064.t1 | Atrial natriuretic peptide receptor 1 | 474 | 3.26E-18 | 52 |
| Efet.01.122286.g1159.t1 | Apolipoprotein(a) | 294 | 6.69E-07 | 52 |
| Efet.01.572648.g396.t1 | Aquaporin-1 | 549 | 4.52E-26 | 52 |
| Efet.01.648013.g1219.t1 | Arginase-1 | 1035 | 1.40E-40 | 52 |
| Efet.01.179434.g1361.t1 | Axin-1 | 1008 | 1.50E-39 | 52 |
| Efet.01.338861.g1137.t1 | Bone morphogenetic protein 2 | 1095 | 9.13E-66 | 52 |
| Efet.01.16311.g1205.t1 | Tyrosine-protein kinase Btk29A | 3684 | 3.10E-50 | 52 |
| Efet.01.11849.g876.t1 | C5a anaphylatoxin chemotactic receptor 1 | 1227 | 1.94E-08 | 52 |
| Efet.01.34868.g676.t1 | Calpain-3 | 237 | 5.72E-07 | 52 |
| Efet.01.80533.g372.t1 | Calpain-3 | 288 | 6.39E-08 | 52 |
| Efet.01.332717.g981.t1 | Carboxypeptidase B2 | 336 | 6.28E-08 | 52 |
| Efet.01.601035.g44.t1 | Cholecystokinin receptor type A | 603 | 1.19E-18 | 52 |
| Efet.01.86574.g727.t1 | Cholecystokinin receptor type A | 579 | 2.57E-07 | 52 |
| Efet.01.85693.g672.t1 | Cholecystokinin receptor type A | 729 | 1.47E-06 | 52 |
| Efet.01.267253.g626.t1 | Cholecystokinin receptor type A | 726 | 1.09E-07 | 52 |
| Efet.01.341256.g1192.t1 | G2/mitotic-specific cyclin-B1 | 921 | 4.49E-18 | 52 |
| Efet.01.40170.g996.t1 | C-C chemokine receptor type 2 | 501 | 1.09E-18 | 52 |
| Efet.01.302773.g88.t1 | Cyclin-dependent kinase 1 | 444 | 3.46E-18 | 52 |
| Efet.01.651136.g119.t1 | Chromodomain-helicase-DNA-binding protein 1 | 2961 | 3.54E-09 | 52 |
| Efet.01.360473.g283.t1 | Carbohydrate sulfotransferase 3 | 279 | 3.49E-06 | 52 |
| Efet.01.246116.g1736.t1 | C-X-C chemokine receptor type 1 | 675 | 1.77E-23 | 52 |
| Efet.01.177474.g1291.t1 | C-X-C chemokine receptor type 4 | 1185 | 7.49E-11 | 52 |
| Efet.01.38405.g891.t1 | Dystroglycan | 2574 | 8.66E-37 | 52 |
| Efet.01.87511.g770.t1 | DAZ-associated protein 1 | 828 | 6.21E-08 | 52 |
| Efet.01.96100.g1227.t1 | DAZ-associated protein 1 | 309 | 2.36E-10 | 52 |
| Efet.01.138483.g690.t1 | Epithelial discoidin domain-containing receptor 1 | 513 | 9.77E-21 | 52 |
| Efet.01.234414.g1359.t1 | Epithelial discoidin domain-containing receptor 1 | 504 | 1.96E-32 | 52 |
| Efet.01.188519.g1811.t1 | Dystrophin | 309 | 3.77E-07 | 52 |
| Efet.01.36827.g781.t1 | Dystrophin | 561 | 4.75E-10 | 52 |
| Efet.01.126610.g99.t1 | Ephrin type-B receptor 3 | 765 | 2.94E-48 | 52 |
| Efet.01.5934.g474.t1 | Protein eyes shut homolog | 570 | 2.08E-16 | 52 |
| Efet.01.161059.g576.t1 | FAS-associated death domain protein | 1365 | 3.37E-06 | 52 |
| Efet.01.490897.g973.t1 | Fibroblast growth factor receptor homolog 2 | 276 | 4.81E-07 | 52 |
| Efet.01.256.g32.t1 | Fibroblast growth factor receptor 2 | 672 | 8.41E-07 | 52 |
| Efet.01.13472.g1007.t1 | Fibroblast growth factor receptor 4 | 450 | 7.35E-11 | 52 |
| Efet.01.199268.g2269.t1 | Fibrinogen-like protein 1 | 693 | 1.83E-39 | 52 |
| Efet.01.135897.g544.t1 | Four and a half LIM domains protein 2 | 321 | 1.16E-10 | 52 |
| Efet.01.180341.g1413.t1 | Four and a half LIM domains protein 2 | 1590 | 4.60E-08 | 52 |
| Efet.01.181455.g1475.t1 | Protein flightless-1 homolog | 1041 | 6.36E-14 | 52 |
| Efet.01.524324.g570.t1 | Protein flightless-1 homolog | 798 | 4.80E-14 | 52 |
| Efet.01.1643975.g289.t1 | Alpha-(1,3)-fucosyltransferase 4 | 372 | 8.91E-11 | 52 |
| Efet.01.239185.g1511.t1 | GRB2-associated-binding protein 1 | 360 | 2.38E-10 | 52 |
| Efet.01.1652285.g551.t1 | Growth/differentiation factor 8 | 369 | 4.15E-14 | 52 |
| Efet.01.1630939.g109.t1 | Glial fibrillary acidic protein | 288 | 2.57E-09 | 52 |
| Efet.01.540949.g290.t1 | Glial fibrillary acidic protein | 630 | 7.49E-15 | 52 |
| Efet.01.230803.g1219.t1 | Protein hedgehog | 567 | 7.33E-24 | 52 |
| Efet.01.177294.g1284.t1 | Histamine H1 receptor | 2199 | 2.88E-24 | 52 |
| Efet.01.491091.g978.t1 | Histamine H1 receptor | 1920 | 2.44E-30 | 52 |
| Efet.01.73780.g1462.t1 | Histamine H1 receptor | 840 | 1.34E-14 | 52 |
| Efet.01.30637.g381.t1 | Histamine H2 receptor | 2019 | 2.72E-36 | 52 |
| Efet.01.608807.g366.t1 | Histamine H2 receptor | 282 | 8.11E-08 | 52 |
| Efet.01.175082.g1174.t1 | ATP-dependent RNA helicase eIF4A | 1284 | 3.03E-42 | 52 |
| Efet.01.639914.g542.t1 | ATP-dependent RNA helicase eIF4A | 1038 | 6.78E-42 | 52 |
| Efet.01.143134.g927.t1 | ATP-dependent RNA helicase eIF4A | 600 | 6.75E-22 | 52 |
| Efet.01.634415.g245.t1 | ATP-dependent RNA helicase eIF4A | 603 | 8.54E-25 | 52 |
| Efet.01.303580.g116.t1 | Transcription factor AP-1 | 1014 | 4.50E-31 | 52 |
| Efet.01.181193.g1464.t1 | Potassium voltage-gated channel subfamily A member 5 | 1188 | 1.36E-58 | 52 |
| Efet.01.27301.g148.t1 | Potassium voltage-gated channel subfamily B member 1 | 1161 | 1.48E-62 | 52 |
| Efet.01.120224.g1048.t1 | Potassium channel subfamily K member 3 | 390 | 1.39E-06 | 52 |
| Efet.01.107154.g370.t1 | Lysine-specific demethylase 3A | 1458 | 4.13E-96 | 52 |
| Efet.01.11807.g872.t1 | Plasma kallikrein | 258 | 7.38E-09 | 52 |
| Efet.01.104527.g253.t1 | Serine/threonine-protein kinase D1 | 462 | 2.66E-14 | 52 |
| Efet.01.607412.g308.t1 | Pyruvate kinase PKM | 969 | 1.05E-35 | 52 |
| Efet.01.317832.g562.t1 | Laminin subunit alpha | 285 | 1.61E-08 | 52 |
| Efet.01.371545.g611.t1 | Laminin subunit alpha-1 | 417 | 3.83E-06 | 52 |
| Efet.01.71248.g1313.t1 | LARGE xylosyl- and glucuronyltransferase 1 | 729 | 5.61E-29 | 52 |
| Efet.01.49153.g1534.t1 | Low-density lipoprotein receptor | 789 | 3.35E-33 | 52 |
| Efet.01.630701.g54.t1 | E3 ubiquitin-protein ligase TRIM71 | 642 | 1.88E-08 | 52 |
| Efet.01.101392.g90.t1 | Matrilin-2 | 3411 | 9.13E-17 | 52 |
| Efet.01.123452.g1216.t1 | E3 ubiquitin-protein ligase Mdm2 | 699 | 7.72E-06 | 52 |
| Efet.01.598556.g1255.t1 | Hepatocyte growth factor receptor | 840 | 2.36E-15 | 52 |
| Efet.01.318747.g598.t1 | Canalicular multispecific organic anion transporter 1 | 1416 | 2.05E-07 | 52 |
| Efet.01.576455.g528.t1 | Canalicular multispecific organic anion transporter 2 | 945 | 6.66E-31 | 52 |
| Efet.01.658133.g1662.t1 | Canalicular multispecific organic anion transporter 2 | 789 | 1.34E-06 | 52 |
| Efet.01.38692.g913.t1 | Myotrophin | 561 | 1.35E-07 | 52 |
| Efet.01.44551.g1265.t1 | Unconventional myosin-Va | 462 | 1.37E-14 | 52 |
| Efet.01.118863.g975.t1 | Nucleus accumbens-associated protein 1 | 1434 | 4.39E-07 | 52 |
| Efet.01.214221.g601.t1 | Nucleus accumbens-associated protein 1 | 1803 | 1.13E-08 | 52 |
| Efet.01.220466.g830.t1 | Neural cell adhesion molecule 1 | 330 | 2.08E-07 | 52 |
| Efet.01.537256.g209.t1 | Neurocan core protein | 288 | 2.87E-12 | 52 |
| Efet.01.489168.g946.t1 | Neural cell adhesion molecule L1-like protein | 426 | 1.12E-08 | 52 |
| Efet.01.173460.g1117.t1 | Neuromedin-U receptor 1 | 288 | 8.21E-09 | 52 |
| Efet.01.10432.g765.t1 | Neuromedin-U receptor 1 | 1200 | 6.40E-10 | 52 |
| Efet.01.151299.g84.t1 | Neuromedin-U receptor 1 | 1200 | 9.44E-11 | 52 |
| Efet.01.247538.g1805.t1 | Neurogenic locus Notch protein | 495 | 9.66E-10 | 52 |
| Efet.01.5596.g433.t1 | Bile acid receptor | 222 | 9.61E-09 | 52 |
| Efet.01.18596.g1378.t1 | Neuropilin-1 | 495 | 2.58E-13 | 52 |
| Efet.01.38674.g910.t1 | Phosphatidylinositol 3-kinase regulatory subunit alpha | 1440 | 1.03E-10 | 52 |
| Efet.01.27686.g168.t1 | Plasminogen activator inhibitor 1 | 480 | 3.32E-10 | 52 |
| Efet.01.257790.g289.t1 | Protocadherin-15 | 363 | 3.65E-08 | 52 |
| Efet.01.203950.g174.t1 | 1-phosphatidylinositol 4,5-bisphosphate phosphodiesterase gamma-1 | 894 | 1.03E-40 | 52 |
| Efet.01.124056.g1242.t1 | PR domain zinc finger protein 14 | 330 | 1.07E-12 | 52 |
| Efet.01.655281.g787.t1 | Bifunctional purine biosynthesis protein PURH | 1605 | 3.19E-80 | 52 |
| Efet.01.490573.g967.t1 | Peroxidasin homolog | 2052 | 1.95E-08 | 52 |
| Efet.01.7973.g587.t1 | Peroxidasin | 756 | 2.16E-07 | 52 |
| Efet.01.79357.g277.t1 | Protein phosphatase 1 regulatory subunit | 684 | 3.05E-12 | 52 |
| Efet.01.96762.g1267.t1 | Protein phosphatase 1 regulatory subunit | 828 | 9.59E-17 | 52 |
| Efet.01.515.g56.t1 | Ras-related protein Rab-30 | 789 | 1.37E-28 | 52 |
| Efet.01.346491.g1309.t1 | Dexamethasone-induced Ras-related protein 1 | 435 | 4.87E-18 | 52 |
| Efet.01.51975.g132.t1 | Reelin | 831 | 4.09E-42 | 52 |
| Efet.01.21198.g1566.t1 | Repulsive guidance molecule A | 480 | 7.84E-09 | 52 |
| Efet.01.574744.g467.t1 | 2-iminobutanoate/2-iminopropanoate deaminase | 405 | 2.61E-06 | 52 |
| Efet.01.264153.g530.t1 | Retinoic acid receptor RXR-alpha | 708 | 1.53E-06 | 52 |
| Efet.01.20108.g1489.t1 | Sal-like protein 1 | 354 | 1.45E-10 | 52 |
| Efet.01.79666.g300.t1 | Sodium channel protein type 1 subunit alpha | 900 | 2.73E-57 | 52 |
| Efet.01.419529.g452.t1 | Slit homolog 3 protein | 1068 | 5.52E-10 | 52 |
| Efet.01.241097.g1570.t1 | Mothers against decapentaplegic homolog 2 | 429 | 1.49E-18 | 52 |
| Efet.01.617285.g767.t1 | Suppressor of cytokine signaling 3 | 384 | 1.05E-14 | 52 |
| Efet.01.131246.g332.t1 | Spastin | 1053 | 7.43E-43 | 52 |
| Efet.01.1638608.g195.t1 | Spectrin alpha chain, non-erythrocytic 1 | 312 | 4.41E-06 | 52 |
| Efet.01.260742.g400.t1 | Spectrin alpha chain, non-erythrocytic 1 | 207 | 3.85E-12 | 52 |
| Efet.01.72610.g1397.t1 | Tyrosine protein-kinase src-1 | 489 | 1.30E-38 | 52 |
| Efet.01.284857.g1266.t1 | Transcription factor 21 | 420 | 8.32E-15 | 52 |
| Efet.01.235778.g1400.t1 | Transcription intermediary factor 1-beta | 903 | 7.19E-10 | 52 |
| Efet.01.607560.g316.t1 | Tumor necrosis factor alpha-induced protein 3 | 1224 | 1.11E-24 | 52 |
| Efet.01.115844.g823.t1 | Serotransferrin | 477 | 2.97E-09 | 52 |
| Efet.01.658198.g1712.t1 | Serine/threonine-protein kinase ULK1 | 600 | 2.04E-18 | 52 |
| Efet.01.452012.g52.t1 | Vasopressin V1a receptor | 1167 | 6.80E-52 | 52 |
| Efet.01.280752.g1124.t1 | Vasopressin V1a receptor | 1065 | 1.29E-58 | 52 |
| Efet.01.57705.g481.t1 | Vascular endothelial growth factor receptor 1 | 369 | 7.84E-14 | 52 |
| Efet.01.549763.g488.t1 | Vascular endothelial growth factor receptor 1 | 357 | 3.35E-07 | 52 |
| Efet.01.6697.g522.t1 | WD repeat-containing protein 5 | 936 | 4.09E-11 | 52 |
| Efet.01.652475.g308.t1 | WD repeat-containing protein 5 | 453 | 8.52E-23 | 52 |
| Efet.01.282031.g1161.t1 | Protein white | 567 | 1.00E-11 | 52 |
| Efet.01.656816.g1114.t1 | Protein white | 1089 | 9.64E-07 | 52 |
| Efet.01.75866.g50.t1 | Protein Wnt-7b | 693 | 1.12E-27 | 52 |
| Efet.01.488482.g925.t1 | GTP-binding protein YPT1 | 465 | 4.15E-10 | 52 |
| Efet.01.347406.g1335.t1 | Zinc finger homeobox protein 3 | 4599 | 5.01E-36 | 52 |
| Efet.01.11656.g856.t1 | Zinc finger protein 37A | 1608 | 2.73E-55 | 52 |
| Efet.01.120059.g1036.t1 | Zinc finger protein 37A | 2235 | 1.89E-10 | 52 |
| Efet.01.255258.g194.t1 | Zinc finger protein 37A | 762 | 3.96E-12 | 52 |
| Efet.01.312463.g391.t1 | Zinc finger protein 37A | 969 | 6.85E-45 | 52 |
| Efet.01.496822.g1108.t1 | Zinc finger protein 37A | 1422 | 3.60E-47 | 52 |
| Efet.01.1657429.g984.t1 | Zinc finger protein 37A | 432 | 4.22E-23 | 52 |
| Efet.01.37398.g822.t1 | 5-hydroxytryptamine receptor 1A | 1338 | 9.19E-71 | 51 |
| Efet.01.53101.g212.t1 | 5-hydroxytryptamine receptor 1A | 840 | 4.59E-35 | 51 |
| Efet.01.578471.g638.t1 | 5-hydroxytryptamine receptor 2A | 1338 | 1.95E-18 | 51 |
| Efet.01.658255.g1757.t1 | ATP-binding cassette transporter abc2 | 1107 | 6.46E-14 | 51 |
| Efet.01.646487.g1097.t1 | ATP-binding cassette transporter abc3 | 708 | 1.84E-15 | 51 |
| Efet.01.1657368.g977.t1 | ATP-binding cassette transporter abc3 | 678 | 7.03E-07 | 51 |
| Efet.01.652384.g288.t1 | ATP-binding cassette sub-family G member 2 | 813 | 1.51E-09 | 51 |
| Efet.01.10385.g760.t1 | ATP-binding cassette sub-family G member 2 | 846 | 3.65E-16 | 51 |
| Efet.01.658368.g1901.t1 | Probable ATP-dependent permease | 1089 | 1.93E-14 | 51 |
| Efet.01.1654661.g701.t1 | Aldehyde dehydrogenase, dimeric NADP-preferring | 606 | 1.57E-17 | 51 |
| Efet.01.98588.g1361.t1 | Atrial natriuretic peptide receptor 1 | 384 | 1.94E-11 | 51 |
| Efet.01.245886.g1726.t1 | Atrial natriuretic peptide receptor 1 | 237 | 4.74E-07 | 51 |
| Efet.01.334791.g1041.t1 | Atrial natriuretic peptide receptor 1 | 906 | 2.72E-26 | 51 |
| Efet.01.274389.g892.t1 | Aquaporin-1 | 603 | 3.50E-23 | 51 |
| Efet.01.22823.g1666.t1 | MGA protein | 306 | 1.29E-07 | 51 |
| Efet.01.612091.g520.t1 | C-1-tetrahydrofolate synthase, cytoplasmic | 762 | 5.95E-45 | 51 |
| Efet.01.100270.g15.t1 | Cadherin-99C | 537 | 2.47E-07 | 51 |
| Efet.01.94242.g1117.t1 | Cadherin-2 | 342 | 3.81E-06 | 51 |
| Efet.01.553639.g599.t1 | Carbonyl reductase [NADPH] 1 | 756 | 9.54E-07 | 51 |
| Efet.01.45076.g1308.t1 | Cholecystokinin receptor type A | 582 | 1.89E-12 | 51 |
| Efet.01.655193.g770.t1 | Cystic fibrosis transmembrane conductance regulator | 450 | 1.02E-15 | 51 |
| Efet.01.466628.g442.t1 | Cytochrome P450 26A1 | 354 | 5.71E-14 | 51 |
| Efet.01.657592.g1332.t1 | Sterol 26-hydroxylase, mitochondrial | 399 | 8.12E-08 | 51 |
| Efet.01.51907.g126.t1 | 25-hydroxyvitamin D-1 alpha hydroxylase, mitochondrial | 1521 | 3.39E-22 | 51 |
| Efet.01.419729.g461.t1 | Versican core protein | 219 | 9.38E-08 | 51 |
| Efet.01.541637.g302.t1 | Versican core protein | 474 | 2.12E-06 | 51 |
| Efet.01.27958.g194.t1 | Chondroitin sulfate proteoglycan 4 | 1503 | 5.38E-19 | 51 |
| Efet.01.376672.g721.t1 | C-X-C chemokine receptor type 1 | 228 | 6.71E-09 | 51 |
| Efet.01.28159.g206.t1 | C-X-C chemokine receptor type 4 | 861 | 9.44E-35 | 51 |
| Efet.01.150286.g14.t1 | Dystroglycan | 786 | 2.80E-31 | 51 |
| Efet.01.168346.g905.t1 | Dystroglycan | 1353 | 7.22E-43 | 51 |
| Efet.01.132446.g373.t1 | Epithelial discoidin domain-containing receptor 1 | 1182 | 9.94E-18 | 51 |
| Efet.01.369489.g529.t1 | Epithelial discoidin domain-containing receptor 1 | 558 | 1.22E-22 | 51 |
| Efet.01.16560.g1223.t1 | Endoribonuclease Dicer | 2079 | 1.38E-16 | 51 |
| Efet.01.652565.g314.t1 | Dystrophin | 1317 | 2.26E-09 | 51 |
| Efet.01.645424.g983.t1 | Dihydropyrimidinase-related protein 2 | 1464 | 8.97E-66 | 51 |
| Efet.01.268360.g678.t1 | Dual specificity protein phosphatase 1 | 1656 | 1.48E-14 | 51 |
| Efet.01.102779.g159.t1 | Early growth response protein 1 | 1608 | 1.32E-15 | 51 |
| Efet.01.4639.g371.t1 | Histone-lysine N-methyltransferase EHMT2 | 1275 | 2.26E-19 | 51 |
| Efet.01.20745.g1534.t1 | Histone-lysine N-methyltransferase EHMT2 | 1803 | 6.15E-07 | 51 |
| Efet.01.89152.g841.t1 | Receptor tyrosine-protein kinase erbB-4 | 294 | 3.04E-10 | 51 |
| Efet.01.279341.g1078.t1 | Protein eyes shut homolog | 258 | 4.54E-06 | 51 |
| Efet.01.19413.g1437.t1 | Fibroblast growth factor receptor 4 | 450 | 1.14E-22 | 51 |
| Efet.01.420782.g496.t1 | Protein flightless-1 | 3723 | 6.43E-10 | 51 |
| Efet.01.553917.g605.t1 | Leucine-rich repeat transmembrane protein FLRT3 | 834 | 9.30E-08 | 51 |
| Efet.01.149684.g1212.t1 | Alpha-(1,3)-fucosyltransferase 4 | 996 | 2.52E-18 | 51 |
| Efet.01.240301.g1542.t1 | Frizzled-7 | 2022 | 1.77E-76 | 51 |
| Efet.01.126895.g112.t1 | Zinc finger protein GLI1 | 3444 | 2.26E-12 | 51 |
| Efet.01.75067.g8.t1 | Glutamate receptor 2 | 1134 | 2.68E-18 | 51 |
| Efet.01.556379.g669.t1 | Glutamate receptor 2 | 612 | 1.40E-06 | 51 |
| Efet.01.60829.g676.t1 | Guanine nucleotide-binding protein G(s) subunit alpha | 210 | 5.18E-07 | 51 |
| Efet.01.187432.g1751.t1 | Heme oxygenase 1 | 726 | 9.19E-28 | 51 |
| Efet.01.81580.g440.t1 | Zinc finger and SCAN domain-containing protein 10 | 1428 | 2.00E-20 | 51 |
| Efet.01.134140.g449.t1 | Zinc finger and SCAN domain-containing protein 10 | 1029 | 7.24E-22 | 51 |
| Efet.01.150884.g44.t1 | Immunoglobulin superfamily member 10 | 1137 | 1.92E-21 | 51 |
| Efet.01.605603.g232.t1 | Integrin-linked protein kinase homolog pat-4 | 285 | 5.69E-06 | 51 |
| Efet.01.28728.g260.t1 | Transcription factor AP-1 | 891 | 1.74E-44 | 51 |
| Efet.01.136375.g567.t1 | Transcription factor AP-1 | 972 | 1.66E-31 | 51 |
| Efet.01.63036.g803.t1 | Potassium voltage-gated channel subfamily B member 1 | 1161 | 8.98E-58 | 51 |
| Efet.01.160140.g531.t1 | Potassium channel subfamily K member 3 | 402 | 1.28E-09 | 51 |
| Efet.01.626144.g1142.t1 | Plasma kallikrein | 720 | 1.75E-20 | 51 |
| Efet.01.351623.g48.t1 | Protein kinase C theta type | 621 | 1.02E-31 | 51 |
| Efet.01.314685.g463.t1 | Neural cell adhesion molecule L1 | 276 | 3.80E-06 | 51 |
| Efet.01.392261.g1057.t1 | Leucine-rich repeat-containing G-protein coupled receptor 5 | 390 | 1.47E-06 | 51 |
| Efet.01.327016.g832.t1 | Prolow-density lipoprotein receptor-related protein 1 | 444 | 2.21E-14 | 51 |
| Efet.01.303916.g136.t1 | Latent-transforming growth factor beta-binding protein 4 | 666 | 8.30E-42 | 51 |
| Efet.01.603047.g121.t1 | Latent-transforming growth factor beta-binding protein 4 | 366 | 1.17E-20 | 51 |
| Efet.01.38448.g898.t1 | Tyrosine-protein kinase Lyn | 348 | 3.76E-31 | 51 |
| Efet.01.149279.g1196.t1 | Matrilin-2 | 918 | 1.45E-10 | 51 |
| Efet.01.607412.g304.t1 | Canalicular multispecific organic anion transporter 2 | 1851 | 3.69E-10 | 51 |
| Efet.01.642087.g655.t1 | Canalicular multispecific organic anion transporter 2 | 480 | 1.22E-06 | 51 |
| Efet.01.550127.g501.t1 | Canalicular multispecific organic anion transporter 2 | 1098 | 1.19E-13 | 51 |
| Efet.01.658383.g1925.t1 | Canalicular multispecific organic anion transporter 2 | 780 | 3.27E-13 | 51 |
| Efet.01.66895.g1038.t1 | Myotrophin | 1248 | 7.09E-12 | 51 |
| Efet.01.113369.g680.t1 | Myotrophin | 939 | 3.18E-09 | 51 |
| Efet.01.215417.g640.t1 | Nuclear factor NF-kappa-B p105 subunit | 909 | 2.89E-08 | 51 |
| Efet.01.65099.g917.t1 | Neuromedin-U receptor 1 | 1008 | 5.07E-06 | 51 |
| Efet.01.204323.g189.t1 | Neuromedin-U receptor 1 | 1242 | 5.67E-07 | 51 |
| Efet.01.362212.g328.t1 | Neuromedin-U receptor 1 | 297 | 1.12E-06 | 51 |
| Efet.01.494448.g1058.t1 | Neuromedin-U receptor 1 | 1011 | 1.94E-08 | 51 |
| Efet.01.49027.g1525.t1 | Nitric oxide synthase, brain | 1125 | 9.16E-06 | 51 |
| Efet.01.261097.g415.t1 | Neurogenic locus notch homolog protein 1 | 342 | 3.81E-07 | 51 |
| Efet.01.101982.g121.t1 | Neurogenic locus notch homolog protein 2 | 801 | 6.01E-07 | 51 |
| Efet.01.285731.g1293.t1 | P2Y purinoceptor 1 | 849 | 3.30E-12 | 51 |
| Efet.01.29692.g319.t1 | Polyadenylate-binding protein 2 | 660 | 5.58E-06 | 51 |
| Efet.01.595474.g1156.t1 | Plasminogen activator inhibitor 1 | 564 | 1.40E-20 | 51 |
| Efet.01.119970.g1029.t1 | Protocadherin-15 | 513 | 1.80E-12 | 51 |
| Efet.01.327140.g836.t1 | Protocadherin-15 | 906 | 1.04E-18 | 51 |
| Efet.01.379381.g784.t1 | Protocadherin-15 | 540 | 6.96E-11 | 51 |
| Efet.01.81318.g425.t1 | PR domain zinc finger protein 14 | 972 | 3.56E-09 | 51 |
| Efet.01.167467.g868.t1 | PR domain zinc finger protein 16 | 1350 | 2.25E-16 | 51 |
| Efet.01.369241.g521.t1 | PR domain zinc finger protein 5 | 1323 | 1.14E-42 | 51 |
| Efet.01.425207.g600.t1 | Parvalbumin, thymic CPV3 | 246 | 1.67E-08 | 51 |
| Efet.01.602628.g105.t1 | Receptor-type tyrosine-protein phosphatase S | 600 | 1.24E-11 | 51 |
| Efet.01.326202.g808.t1 | Peroxidasin | 660 | 8.12E-11 | 51 |
| Efet.01.657207.g1201.t1 | Peroxidasin homolog | 399 | 1.94E-17 | 51 |
| Efet.01.172503.g1070.t1 | Peroxidasin | 1638 | 4.51E-27 | 51 |
| Efet.01.305137.g165.t1 | Peroxidasin | 306 | 3.40E-07 | 51 |
| Efet.01.94956.g1154.t1 | CAD protein | 444 | 4.51E-09 | 51 |
| Efet.01.25272.g37.t1 | Protein phosphatase 1 regulatory subunit | 1074 | 1.61E-27 | 51 |
| Efet.01.381944.g839.t1 | Dexamethasone-induced Ras-related protein 1 | 420 | 4.25E-18 | 51 |
| Efet.01.109319.g485.t1 | Roundabout homolog 2 | 633 | 1.26E-06 | 51 |
| Efet.01.135161.g500.t1 | Roundabout homolog 2 | 849 | 9.26E-39 | 51 |
| Efet.01.124209.g1250.t1 | R-spondin-1 | 594 | 7.51E-08 | 51 |
| Efet.01.47595.g1449.t1 | Retinoic acid receptor RXR-alpha | 480 | 1.37E-19 | 51 |
| Efet.01.638470.g442.t1 | Sodium channel protein type 1 subunit alpha | 303 | 1.60E-13 | 51 |
| Efet.01.286362.g1316.t1 | Ski-like protein | 477 | 1.37E-06 | 51 |
| Efet.01.1659032.g1371.t1 | Slit homolog 1 protein | 825 | 6.37E-15 | 51 |
| Efet.01.37704.g837.t1 | Slit homolog 1 protein | 579 | 6.64E-10 | 51 |
| Efet.01.20643.g1523.t1 | Zinc finger protein SNAI1 | 261 | 9.74E-11 | 51 |
| Efet.01.568886.g285.t1 | Spastin | 450 | 1.11E-11 | 51 |
| Efet.01.167816.g882.t1 | Spastin | 411 | 4.24E-07 | 51 |
| Efet.01.93761.g1088.t1 | Proto-oncogene tyrosine-protein kinase Src | 492 | 1.36E-15 | 51 |
| Efet.01.486245.g882.t1 | Serine/threonine-protein kinase STK11 | 411 | 1.81E-19 | 51 |
| Efet.01.272519.g837.t1 | Serine/threonine-protein kinase 3 | 501 | 3.34E-07 | 51 |
| Efet.01.600388.g10.t1 | Serine/threonine-protein kinase 3 | 513 | 2.11E-10 | 51 |
| Efet.01.193784.g2036.t1 | Tenascin | 588 | 1.75E-17 | 51 |
| Efet.01.403912.g100.t1 | Transducin-like enhancer protein 1 | 345 | 8.08E-07 | 51 |
| Efet.01.248972.g1858.t1 | Toll-like receptor Tollo | 2880 | 9.91E-13 | 51 |
| Efet.01.1649392.g435.t1 | TNF receptor-associated factor 6 | 309 | 2.91E-12 | 51 |
| Efet.01.94709.g1145.t1 | Mitochondrial uncoupling protein 2 | 324 | 9.45E-08 | 51 |
| Efet.01.532989.g94.t1 | UDP-glucuronosyltransferase 1-1 | 1416 | 2.22E-45 | 51 |
| Efet.01.212268.g531.t1 | Serine/threonine-protein kinase ULK1 | 450 | 5.87E-06 | 51 |
| Efet.01.245278.g1704.t1 | Netrin unc-6 | 366 | 9.65E-06 | 51 |
| Efet.01.289987.g1447.t1 | Vasopressin V1a receptor | 1254 | 1.82E-07 | 51 |
| Efet.01.2954.g263.t1 | Vitamin D3 receptor | 1245 | 3.23E-46 | 51 |
| Efet.01.187355.g1747.t1 | Vascular endothelial growth factor receptor 2 | 690 | 5.80E-11 | 51 |
| Efet.01.53863.g254.t1 | WD repeat-containing protein 5 | 561 | 3.48E-11 | 51 |
| Efet.01.131945.g353.t1 | WD repeat-containing protein 5 | 972 | 1.31E-09 | 51 |
| Efet.01.464530.g383.t1 | ABC transporter ATP-binding protein/permease wht-1 | 1272 | 6.70E-17 | 51 |
| Efet.01.600908.g36.t1 | Protein Wnt-7b | 258 | 1.35E-11 | 51 |
| Efet.01.614177.g633.t1 | Metal resistance protein YCF1 | 669 | 1.65E-13 | 51 |
| Efet.01.66879.g1036.t1 | GTP-binding protein ypt1 | 417 | 1.23E-07 | 51 |
| Efet.01.92493.g1021.t1 | Zinc finger X-chromosomal protein | 303 | 6.66E-12 | 51 |
| Efet.01.67920.g1112.t1 | Zinc finger protein 37A | 1131 | 3.98E-40 | 51 |
| Efet.01.170251.g987.t1 | Zinc finger protein 37A | 1248 | 9.32E-53 | 51 |
| Efet.01.80905.g395.t1 | 5-hydroxytryptamine receptor 1A | 1095 | 7.32E-57 | 50 |
| Efet.01.83040.g530.t1 | 5-hydroxytryptamine receptor 1A | 1062 | 8.73E-32 | 50 |
| Efet.01.366620.g449.t1 | 5-hydroxytryptamine receptor 2B | 879 | 3.83E-15 | 50 |
| Efet.01.585463.g841.t1 | ATP-binding cassette transporter abc3 | 633 | 1.19E-16 | 50 |
| Efet.01.1659460.g1769.t1 | ATP-binding cassette transporter abc3 | 750 | 5.92E-14 | 50 |
| Efet.01.1658864.g1301.t1 | ATP-binding cassette sub-family G member 2 | 375 | 5.09E-10 | 50 |
| Efet.01.1659450.g1742.t1 | Probable ATP-dependent permease | 717 | 9.22E-20 | 50 |
| Efet.01.97189.g1295.t1 | Beta-2 adrenergic receptor | 1185 | 3.14E-55 | 50 |
| Efet.01.331133.g944.t1 | Beta-2 adrenergic receptor | 906 | 6.89E-55 | 50 |
| Efet.01.434.g52.t1 | RAC-beta serine/threonine-protein kinase | 1095 | 1.23E-20 | 50 |
| Efet.01.1659505.g1904.t1 | Aldehyde dehydrogenase, dimeric NADP-preferring | 1140 | 9.63E-42 | 50 |
| Efet.01.238309.g1487.t1 | Protein arginine N-methyltransferase 6 | 597 | 8.80E-47 | 50 |
| Efet.01.78798.g227.t1 | Atrial natriuretic peptide receptor 1 | 510 | 2.28E-23 | 50 |
| Efet.01.85527.g658.t1 | Atrial natriuretic peptide receptor 1 | 1302 | 6.47E-19 | 50 |
| Efet.01.196477.g2150.t1 | Atrial natriuretic peptide receptor 1 | 1230 | 1.90E-21 | 50 |
| Efet.01.45427.g1322.t1 | Axin-1 | 276 | 1.76E-06 | 50 |
| Efet.01.549171.g474.t1 | Apoptosis regulator Bcl-2 | 1128 | 4.22E-06 | 50 |
| Efet.01.635430.g277.t1 | Baculoviral IAP repeat-containing protein 5 | 825 | 3.61E-08 | 50 |
| Efet.01.43870.g1219.t1 | Tyrosine-protein kinase Btk29A | 2847 | 9.66E-54 | 50 |
| Efet.01.67458.g1078.t1 | NF-kappa-B inhibitor cactus | 1041 | 3.42E-24 | 50 |
| Efet.01.226824.g1070.t1 | Cadherin-15 | 303 | 1.73E-06 | 50 |
| Efet.01.19107.g1422.t1 | Cadherin-99C | 2628 | 1.48E-20 | 50 |
| Efet.01.658041.g1583.t1 | Carbonyl reductase [NADPH] 1 | 807 | 1.03E-08 | 50 |
| Efet.01.118190.g941.t1 | Cholecystokinin receptor type A | 447 | 1.01E-10 | 50 |
| Efet.01.216065.g665.t1 | Cholecystokinin receptor type A | 1455 | 2.22E-07 | 50 |
| Efet.01.95720.g1200.t1 | C-C chemokine receptor type 2 | 582 | 1.98E-06 | 50 |
| Efet.01.3992.g336.t1 | C-C chemokine receptor type 2 | 1269 | 8.04E-22 | 50 |
| Efet.01.12775.g960.t1 | Cyclin-dependent kinase 4 | 1524 | 1.91E-34 | 50 |
| Efet.01.633829.g212.t1 | Cystic fibrosis transmembrane conductance regulator | 2313 | 1.75E-18 | 50 |
| Efet.01.209427.g425.t1 | Contactin-1 | 225 | 2.59E-06 | 50 |
| Efet.01.574275.g443.t1 | Sterol 26-hydroxylase, mitochondrial | 390 | 5.23E-11 | 50 |
| Efet.01.372426.g628.t1 | C-X-C chemokine receptor type 2 | 1119 | 4.21E-39 | 50 |
| Efet.01.466101.g432.t1 | C-X-C chemokine receptor type 4 | 879 | 8.73E-26 | 50 |
| Efet.01.528348.g645.t1 | Cytoglobin | 543 | 9.96E-14 | 50 |
| Efet.01.98475.g1355.t1 | Epithelial discoidin domain-containing receptor 1 | 405 | 6.10E-09 | 50 |
| Efet.01.103884.g215.t1 | Epithelial discoidin domain-containing receptor 1 | 948 | 2.78E-55 | 50 |
| Efet.01.505417.g136.t1 | Epithelial discoidin domain-containing receptor 1 | 609 | 4.40E-14 | 50 |
| Efet.01.62690.g789.t1 | Endoribonuclease Dicer | 1500 | 1.29E-16 | 50 |
| Efet.01.27820.g185.t1 | Deleted in malignant brain tumors 1 protein | 465 | 5.74E-11 | 50 |
| Efet.01.67373.g1071.t1 | Dual specificity protein phosphatase 1 | 552 | 4.75E-24 | 50 |
| Efet.01.12644.g947.t1 | Histone-lysine N-methyltransferase EHMT2 | 1224 | 3.11E-23 | 50 |
| Efet.01.113369.g679.t1 | Histone-lysine N-methyltransferase EHMT2 | 2175 | 6.69E-21 | 50 |
| Efet.01.208269.g379.t1 | Histone-lysine N-methyltransferase EHMT2 | 3315 | 1.23E-20 | 50 |
| Efet.01.167098.g844.t1 | Fermitin family homolog 2 | 384 | 6.80E-31 | 50 |
| Efet.01.50213.g17.t1 | Fibroblast growth factor receptor 4 | 594 | 7.79E-28 | 50 |
| Efet.01.543473.g331.t1 | Fibrinogen-like protein 1 | 420 | 3.55E-17 | 50 |
| Efet.01.1658843.g1295.t1 | Fibrinogen-like protein 1 | 630 | 1.76E-22 | 50 |
| Efet.01.364419.g391.t1 | Fibrinogen alpha chain | 429 | 2.27E-10 | 50 |
| Efet.01.252987.g113.t1 | Protein flightless-1 | 387 | 3.65E-07 | 50 |
| Efet.01.448506.g1186.t1 | Protein flightless-1 | 2805 | 2.08E-08 | 50 |
| Efet.01.296245.g1622.t1 | Tyrosine-protein kinase Fyn | 543 | 7.49E-15 | 50 |
| Efet.01.57017.g437.t1 | Homeobox protein GBX-2 | 858 | 2.34E-10 | 50 |
| Efet.01.69455.g1202.t1 | Glial fibrillary acidic protein | 594 | 2.04E-08 | 50 |
| Efet.01.595647.g1170.t1 | Bifunctional UDP-N-acetylglucosamine 2-epimerase/N-acetylmannosamine kinase | 687 | 2.92E-10 | 50 |
| Efet.01.591014.g1024.t1 | Bifunctional UDP-N-acetylglucosamine 2-epimerase/N-acetylmannosamine kinase | 636 | 3.39E-11 | 50 |
| Efet.01.213854.g589.t1 | Solute carrier family 2, facilitated glucose transporter member 4 | 1449 | 1.68E-33 | 50 |
| Efet.01.315502.g492.t1 | Mitogen-activated protein kinase hog-1 | 1089 | 4.96E-36 | 50 |
| Efet.01.94267.g1120.t1 | Nuclear hormone receptor HR96 | 840 | 6.80E-24 | 50 |
| Efet.01.5446.g423.t1 | Histamine H2 receptor | 1251 | 8.97E-62 | 50 |
| Efet.01.389950.g1005.t1 | Histamine H2 receptor | 1680 | 1.61E-63 | 50 |
| Efet.01.1632146.g118.t1 | Histamine H2 receptor | 303 | 1.32E-09 | 50 |
| Efet.01.150928.g49.t1 | Zinc finger and SCAN domain-containing protein 10 | 948 | 1.51E-17 | 50 |
| Efet.01.272705.g845.t1 | Zinc finger and SCAN domain-containing protein 10 | 1191 | 1.56E-06 | 50 |
| Efet.01.567964.g255.t1 | ATP-dependent RNA helicase eIF4A | 591 | 7.57E-12 | 50 |
| Efet.01.89025.g836.t1 | Integrin-linked protein kinase | 1389 | 6.72E-10 | 50 |
| Efet.01.506340.g163.t1 | Integrin-linked protein kinase | 855 | 3.42E-08 | 50 |
| Efet.01.123185.g1203.t1 | Integrin-linked protein kinase homolog pat-4 | 393 | 4.51E-08 | 50 |
| Efet.01.312306.g387.t1 | Insulin-like receptor | 408 | 7.34E-07 | 50 |
| Efet.01.537781.g226.t1 | Insulin gene enhancer protein ISL-1 | 312 | 2.33E-06 | 50 |
| Efet.01.621650.g944.t1 | Integrin beta-4 | 726 | 8.07E-12 | 50 |
| Efet.01.146631.g1086.t1 | Histone acetyltransferase KAT6A | 816 | 1.23E-16 | 50 |
| Efet.01.140629.g803.t1 | Potassium voltage-gated channel subfamily B member 1 | 249 | 7.02E-13 | 50 |
| Efet.01.84215.g581.t1 | Krueppel-like factor 9 | 1620 | 1.11E-08 | 50 |
| Efet.01.163558.g687.t1 | Protein kinase C theta type | 291 | 1.92E-20 | 50 |
| Efet.01.60885.g679.t1 | Laminin subunit alpha-1 | 528 | 2.98E-20 | 50 |
| Efet.01.16840.g1245.t1 | Laminin subunit alpha-1 | 630 | 5.74E-12 | 50 |
| Efet.01.85609.g663.t1 | Laminin subunit alpha-1 | 555 | 2.75E-11 | 50 |
| Efet.01.428617.g697.t1 | Laminin subunit alpha-3 | 246 | 1.56E-06 | 50 |
| Efet.01.152439.g137.t1 | LARGE xylosyl- and glucuronyltransferase 1 | 3159 | 9.42E-27 | 50 |
| Efet.01.203834.g172.t1 | LARGE xylosyl- and glucuronyltransferase 1 | 621 | 8.37E-22 | 50 |
| Efet.01.615480.g692.t1 | Low-density lipoprotein receptor | 366 | 3.93E-09 | 50 |
| Efet.01.1655294.g750.t1 | Protein lin-41 | 441 | 4.58E-14 | 50 |
| Efet.01.657985.g1550.t1 | E3 ubiquitin-protein ligase TRIM71 | 570 | 7.05E-08 | 50 |
| Efet.01.119857.g1024.t1 | E3 ubiquitin-protein ligase TRIM71 | 954 | 3.30E-23 | 50 |
| Efet.01.41773.g1105.t1 | Low-density lipoprotein receptor-related protein 1 | 888 | 1.92E-57 | 50 |
| Efet.01.1659417.g1680.t1 | Tyrosine-protein kinase Lyn | 489 | 2.93E-14 | 50 |
| Efet.01.308972.g283.t1 | Induced myeloid leukemia cell differentiation protein Mcl-1 homolog | 582 | 1.05E-14 | 50 |
| Efet.01.171839.g1051.t1 | Hepatocyte growth factor receptor | 969 | 4.65E-12 | 50 |
| Efet.01.56518.g406.t1 | MAX gene-associated protein | 354 | 1.61E-29 | 50 |
| Efet.01.457479.g200.t1 | [F-actin]-monooxygenase MICAL1 | 504 | 1.28E-08 | 50 |
| Efet.01.3792.g324.t1 | Mitogen-activated protein kinase 14A | 972 | 1.50E-11 | 50 |
| Efet.01.652446.g304.t1 | Myotrophin | 1938 | 7.02E-12 | 50 |
| Efet.01.31175.g427.t1 | Myosin-2 | 420 | 9.63E-18 | 50 |
| Efet.01.11125.g817.t1 | Nucleus accumbens-associated protein 1 | 1437 | 1.24E-06 | 50 |
| Efet.01.576662.g536.t1 | Neural cell adhesion molecule 1 | 285 | 1.24E-07 | 50 |
| Efet.01.194053.g2051.t1 | Bifunctional heparan sulfate N-deacetylase/N-sulfotransferase 1 | 390 | 2.16E-09 | 50 |
| Efet.01.38692.g914.t1 | Nuclear factor NF-kappa-B p105 subunit | 1167 | 7.25E-12 | 50 |
| Efet.01.632909.g176.t1 | Neuronal-glial cell adhesion molecule | 684 | 5.04E-14 | 50 |
| Efet.01.331755.g958.t1 | Neuromedin-U receptor 1 | 363 | 2.60E-08 | 50 |
| Efet.01.104908.g267.t1 | Neuromedin-U receptor 1 | 483 | 6.75E-08 | 50 |
| Efet.01.240487.g1553.t1 | Neuromedin-U receptor 1 | 1047 | 3.65E-06 | 50 |
| Efet.01.396930.g1168.t1 | Neuromedin-U receptor 1 | 483 | 6.75E-08 | 50 |
| Efet.01.580237.g679.t1 | Nitric oxide synthase, inducible | 3948 | 7.84E-09 | 50 |
| Efet.01.265565.g571.t1 | Neurogenic locus notch homolog protein 3 | 939 | 5.57E-18 | 50 |
| Efet.01.1627232.g83.t1 | Neurogenic locus notch homolog protein 3 | 258 | 1.14E-11 | 50 |
| Efet.01.49451.g1558.t1 | Neurogenic locus Notch protein | 4587 | 2.23E-06 | 50 |
| Efet.01.445529.g1131.t1 | Neurogenic locus Notch protein | 1404 | 4.62E-08 | 50 |
| Efet.01.378411.g757.t1 | BDNF/NT-3 growth factors receptor | 429 | 2.71E-32 | 50 |
| Efet.01.148148.g1148.t1 | Melanopsin | 378 | 3.12E-13 | 50 |
| Efet.01.344.g42.t1 | Palladin | 765 | 2.48E-14 | 50 |
| Efet.01.65892.g978.t1 | Palladin | 711 | 2.80E-07 | 50 |
| Efet.01.55530.g362.t1 | Protocadherin-15 | 768 | 6.42E-07 | 50 |
| Efet.01.22489.g1635.t1 | Protocadherin-15 | 1614 | 5.25E-14 | 50 |
| Efet.01.651921.g234.t1 | Protocadherin-15 | 339 | 1.03E-10 | 50 |
| Efet.01.652440.g296.t1 | Phosphoglycerate kinase | 528 | 1.18E-18 | 50 |
| Efet.01.155474.g279.t1 | Decorin | 1050 | 3.90E-19 | 50 |
| Efet.01.642313.g673.t1 | PR domain zinc finger protein 14 | 411 | 5.64E-16 | 50 |
| Efet.01.77571.g147.t1 | PR domain zinc finger protein 5 | 462 | 1.54E-10 | 50 |
| Efet.01.158801.g462.t1 | Receptor-type tyrosine-protein phosphatase S | 1899 | 3.99E-11 | 50 |
| Efet.01.123223.g1206.t1 | Receptor-type tyrosine-protein phosphatase S | 897 | 5.85E-18 | 50 |
| Efet.01.89954.g893.t1 | Receptor-type tyrosine-protein phosphatase zeta | 573 | 5.75E-08 | 50 |
| Efet.01.132988.g397.t1 | Peroxidasin homolog | 276 | 2.80E-09 | 50 |
| Efet.01.48009.g1466.t1 | Peroxidasin homolog | 432 | 1.19E-11 | 50 |
| Efet.01.133987.g441.t1 | Peroxidasin homolog | 291 | 6.34E-08 | 50 |
| Efet.01.509178.g236.t1 | Peroxidasin homolog | 1338 | 3.43E-16 | 50 |
| Efet.01.448981.g1192.t1 | X-linked zinc finger protein | 588 | 1.50E-14 | 50 |
| Efet.01.57826.g489.t1 | Eukaryotic translation initiation factor 2B, subunit 3 gamma | 960 | 1.69E-15 | 50 |
| Efet.01.169.g19.t1 | Roundabout homolog 2 | 1725 | 7.91E-14 | 50 |
| Efet.01.76022.g61.t1 | Roundabout homolog 2 | 423 | 6.54E-10 | 50 |
| Efet.01.80957.g398.t1 | Roundabout homolog 2 | 762 | 1.43E-10 | 50 |
| Efet.01.126469.g90.t1 | Roundabout homolog 2 | 648 | 1.35E-10 | 50 |
| Efet.01.58477.g522.t1 | Reticulon-4 receptor | 2661 | 7.80E-20 | 50 |
| Efet.01.322098.g688.t1 | Sodium channel protein type 1 subunit alpha | 405 | 5.82E-15 | 50 |
| Efet.01.351277.g40.t1 | Sodium channel protein type 1 subunit alpha | 342 | 6.42E-12 | 50 |
| Efet.01.48825.g1511.t1 | Sodium channel protein type 9 subunit alpha | 723 | 4.39E-13 | 50 |
| Efet.01.83214.g539.t1 | Slit homolog 3 protein | 1035 | 5.30E-13 | 50 |
| Efet.01.656799.g1108.t1 | Probable spastin homolog spas-1 | 1416 | 7.58E-09 | 50 |
| Efet.01.164922.g757.t1 | Spastin | 489 | 1.05E-12 | 50 |
| Efet.01.369119.g519.t1 | Spastin | 783 | 5.78E-19 | 50 |
| Efet.01.232319.g1284.t1 | Spectrin alpha chain, non-erythrocytic 1 | 975 | 6.20E-30 | 50 |
| Efet.01.286600.g1324.t1 | Spectrin alpha chain, non-erythrocytic 1 | 429 | 2.29E-10 | 50 |
| Efet.01.99041.g1390.t1 | Src substrate cortactin | 381 | 1.11E-08 | 50 |
| Efet.01.38392.g890.t1 | Serine/threonine-protein kinase STK11 | 321 | 2.46E-08 | 50 |
| Efet.01.658418.g2083.t1 | Extracellular sulfatase Sulf-2 | 1347 | 4.35E-11 | 50 |
| Efet.01.59298.g583.t1 | Tenascin | 552 | 1.14E-26 | 50 |
| Efet.01.272611.g839.t1 | Transcription intermediary factor 1-alpha | 1110 | 8.92E-06 | 50 |
| Efet.01.263882.g522.t1 | Toll-like receptor Tollo | 903 | 9.67E-24 | 50 |
| Efet.01.1655707.g782.t1 | E3 ubiquitin-protein ligase TRIM32 | 561 | 3.70E-08 | 50 |
| Efet.01.1659015.g1361.t1 | Thrombospondin-1 | 1083 | 4.96E-07 | 50 |
| Efet.01.298689.g1704.t1 | Transcriptional repressor protein YY1 | 480 | 5.47E-09 | 50 |
| Efet.01.312980.g413.t1 | Mitochondrial uncoupling protein 2 | 438 | 1.29E-08 | 50 |
| Efet.01.63094.g804.t1 | UDP-glucuronosyltransferase 1-2 | 1011 | 8.09E-35 | 50 |
| Efet.01.6772.g528.t1 | Serine/threonine-protein kinase ULK1 | 432 | 2.92E-16 | 50 |
| Efet.01.31049.g418.t1 | Urokinase-type plasminogen activator | 378 | 8.48E-09 | 50 |
| Efet.01.508540.g220.t1 | Urokinase-type plasminogen activator | 252 | 2.50E-10 | 50 |
| Efet.01.380192.g799.t1 | Vasopressin V1a receptor | 1233 | 1.87E-11 | 50 |
| Efet.01.58473.g521.t1 | Vasopressin V1a receptor | 861 | 3.65E-07 | 50 |
| Efet.01.127823.g155.t1 | Vasopressin V1a receptor | 1404 | 1.34E-44 | 50 |
| Efet.01.628200.g1235.t1 | von Willebrand factor | 489 | 1.18E-15 | 50 |
| Efet.01.83612.g558.t1 | WD repeat-containing protein 5 | 1161 | 2.97E-08 | 50 |
| Efet.01.288284.g1392.t1 | WD repeat-containing protein 5 | 939 | 4.26E-13 | 50 |
| Efet.01.120161.g1043.t1 | Protein wech | 2526 | 9.32E-20 | 50 |
| Efet.01.440935.g1003.t1 | Protein white | 1623 | 8.55E-17 | 50 |
| Efet.01.654418.g597.t1 | Protein white | 966 | 2.22E-19 | 50 |
| Efet.01.654756.g692.t1 | Protein white | 435 | 6.72E-07 | 50 |
| Efet.01.657766.g1424.t1 | ABC transporter ATP-binding protein/permease wht-1 | 1362 | 2.39E-14 | 50 |
| Efet.01.193521.g2020.t1 | Palmitoyltransferase ZDHHC23 | 837 | 1.24E-07 | 50 |
| Efet.01.351135.g37.t1 | Zinc finger X-chromosomal protein | 927 | 5.03E-11 | 50 |
| Efet.01.102725.g154.t1 | Zinc finger protein 37A | 1317 | 2.66E-48 | 50 |
| Efet.01.325602.g787.t1 | Zinc finger protein 37A | 819 | 3.74E-51 | 50 |
| Efet.01.380433.g802.t1 | Zinc finger protein 37A | 771 | 9.51E-41 | 50 |
| Efet.01.406366.g156.t1 | Zinc finger protein 37A | 1500 | 1.99E-49 | 50 |
| Efet.01.408562.g205.t1 | Zinc finger protein 37A | 354 | 5.90E-21 | 50 |
| Efet.01.606065.g252.t1 | Zinc finger protein 37A | 1611 | 2.97E-39 | 50 |
| Efet.01.72941.g1414.t1 | 5-hydroxytryptamine receptor 2C | 735 | 9.94E-14 | 49 |
| Efet.01.639343.g500.t1 | ATP-binding cassette transporter abc2 | 1542 | 1.15E-09 | 49 |
| Efet.01.1659481.g1823.t1 | ATP-binding cassette transporter abc2 | 588 | 3.58E-10 | 49 |
| Efet.01.611807.g506.t1 | ATP-binding cassette transporter abc3 | 2397 | 2.65E-10 | 49 |
| Efet.01.618401.g826.t1 | ATP-binding cassette transporter abc3 | 2397 | 2.65E-10 | 49 |
| Efet.01.656558.g1045.t1 | ATP-binding cassette transporter abc3 | 1050 | 9.35E-11 | 49 |
| Efet.01.635754.g291.t1 | ATP-binding cassette sub-family G member 2 | 684 | 6.02E-12 | 49 |
| Efet.01.638425.g437.t1 | ATP-binding cassette sub-family G member 2 | 705 | 9.77E-16 | 49 |
| Efet.01.252450.g89.t1 | Atypical chemokine receptor 3 | 738 | 2.01E-23 | 49 |
| Efet.01.380171.g798.t1 | Atypical chemokine receptor 3 | 636 | 8.65E-21 | 49 |
| Efet.01.270738.g770.t1 | Probable ATP-dependent permease | 1359 | 9.95E-10 | 49 |
| Efet.01.655821.g885.t1 | Aldehyde dehydrogenase, dimeric NADP-preferring | 1458 | 1.87E-29 | 49 |
| Efet.01.390398.g1013.t1 | Atrial natriuretic peptide receptor 1 | 822 | 3.47E-13 | 49 |
| Efet.01.1659499.g1891.t1 | Arginase | 894 | 1.66E-18 | 49 |
| Efet.01.387540.g959.t1 | Axin-1 | 1221 | 8.96E-15 | 49 |
| Efet.01.1656574.g885.t1 | Bone morphogenetic protein 4 | 420 | 5.58E-17 | 49 |
| Efet.01.1657589.g1012.t1 | Cystic fibrosis transmembrane conductance regulator | 810 | 2.18E-10 | 49 |
| Efet.01.651518.g185.t1 | Cystic fibrosis transmembrane conductance regulator | 1296 | 4.08E-17 | 49 |
| Efet.01.654851.g705.t1 | Cystic fibrosis transmembrane conductance regulator | 765 | 2.90E-09 | 49 |
| Efet.01.132501.g377.t1 | Collagen alpha-1(II) chain | 651 | 1.19E-11 | 49 |
| Efet.01.202517.g119.t1 | Collagen alpha-3(V) chain | 885 | 5.24E-31 | 49 |
| Efet.01.39963.g985.t1 | Collagen alpha-1(VI) chain | 705 | 1.58E-07 | 49 |
| Efet.01.321268.g668.t1 | C-X-C chemokine receptor type 1 | 417 | 5.89E-06 | 49 |
| Efet.01.657953.g1541.t1 | C-X-C chemokine receptor type 1 | 1152 | 4.67E-33 | 49 |
| Efet.01.297332.g1667.t1 | C-X-C chemokine receptor type 1 | 1173 | 9.65E-28 | 49 |
| Efet.01.98947.g1381.t1 | C-X-C chemokine receptor type 4 | 1137 | 7.95E-14 | 49 |
| Efet.01.242647.g1628.t1 | Epithelial discoidin domain-containing receptor 1 | 534 | 2.23E-15 | 49 |
| Efet.01.314930.g473.t1 | Epithelial discoidin domain-containing receptor 1 | 630 | 5.67E-20 | 49 |
| Efet.01.156944.g364.t1 | Dual specificity protein phosphatase 1 | 849 | 7.50E-47 | 49 |
| Efet.01.368485.g497.t1 | Dual specificity protein phosphatase 1 | 405 | 2.99E-14 | 49 |
| Efet.01.76009.g60.t1 | Dual specificity protein phosphatase 1 | 615 | 2.55E-16 | 49 |
| Efet.01.658374.g1911.t1 | Polycomb protein EED | 936 | 1.36E-06 | 49 |
| Efet.01.84624.g602.t1 | Histone-lysine N-methyltransferase EHMT2 | 1884 | 3.35E-16 | 49 |
| Efet.01.159641.g504.t1 | Histone-lysine N-methyltransferase EHMT2 | 2331 | 4.21E-15 | 49 |
| Efet.01.568152.g268.t1 | Histone-lysine N-methyltransferase EHMT2 | 1524 | 6.07E-20 | 49 |
| Efet.01.649470.g1312.t1 | Fibrinogen alpha chain | 366 | 2.58E-13 | 49 |
| Efet.01.19840.g1470.t1 | Frizzled-7 | 2031 | 5.29E-70 | 49 |
| Efet.01.363635.g364.t1 | GRB2-associated-binding protein 1 | 342 | 4.78E-09 | 49 |
| Efet.01.32170.g498.t1 | Gelsolin | 735 | 3.02E-19 | 49 |
| Efet.01.549223.g477.t1 | Bifunctional UDP-N-acetylglucosamine 2-epimerase/N-acetylmannosamine kinase | 783 | 3.46E-11 | 49 |
| Efet.01.658392.g1953.t1 | Bifunctional UDP-N-acetylglucosamine 2-epimerase/N-acetylmannosamine kinase | 2586 | 1.56E-18 | 49 |
| Efet.01.14170.g1049.t1 | Transcriptional activator GLI3 | 651 | 2.20E-09 | 49 |
| Efet.01.73350.g1440.t1 | Glypican-3 | 831 | 2.90E-34 | 49 |
| Efet.01.317845.g563.t1 | G-protein-signaling modulator 1 | 1542 | 3.76E-30 | 49 |
| Efet.01.271797.g817.t1 | Glutamate receptor 3 | 1212 | 1.25E-43 | 49 |
| Efet.01.565356.g182.t1 | Solute carrier family 2, facilitated glucose transporter member 4 | 801 | 1.20E-28 | 49 |
| Efet.01.638466.g441.t1 | Solute carrier family 2, facilitated glucose transporter member 4 | 1119 | 2.86E-31 | 49 |
| Efet.01.252050.g76.t1 | E3 ISG15--protein ligase HERC5 | 1404 | 1.11E-19 | 49 |
| Efet.01.34431.g649.t1 | Mitogen-activated protein kinase hog-1 | 1629 | 4.12E-39 | 49 |
| Efet.01.214184.g599.t1 | Histamine H1 receptor | 2382 | 3.14E-33 | 49 |
| Efet.01.237262.g1455.t1 | Histamine H2 receptor | 660 | 8.40E-07 | 49 |
| Efet.01.31198.g431.t1 | Zinc finger and SCAN domain-containing protein 10 | 1494 | 6.03E-29 | 49 |
| Efet.01.474362.g640.t1 | Zinc finger and SCAN domain-containing protein 10 | 768 | 1.49E-34 | 49 |
| Efet.01.489389.g948.t1 | Zinc finger and SCAN domain-containing protein 10 | 423 | 6.15E-18 | 49 |
| Efet.01.530517.g13.t1 | Inter-alpha-trypsin inhibitor heavy chain H4 | 471 | 4.11E-06 | 49 |
| Efet.01.75425.g22.t1 | Lysine-specific demethylase 3A | 1770 | 1.30E-31 | 49 |
| Efet.01.257585.g282.t1 | Kinesin-like protein KIF3C | 576 | 8.88E-15 | 49 |
| Efet.01.195876.g2123.t1 | Neural cell adhesion molecule L1 | 435 | 2.12E-09 | 49 |
| Efet.01.381835.g836.t1 | Laminin subunit alpha-1 | 870 | 1.28E-08 | 49 |
| Efet.01.79400.g281.t1 | E3 ubiquitin-protein ligase TRIM71 | 1017 | 1.03E-27 | 49 |
| Efet.01.154433.g222.t1 | E3 ubiquitin-protein ligase TRIM71 | 1503 | 5.86E-34 | 49 |
| Efet.01.122559.g1176.t1 | E3 ubiquitin-protein ligase TRIM71 | 2718 | 4.78E-22 | 49 |
| Efet.01.632298.g153.t1 | Low-density lipoprotein receptor-related protein | 462 | 1.68E-07 | 49 |
| Efet.01.348247.g1362.t1 | Low-density lipoprotein receptor-related protein 1 | 411 | 6.80E-18 | 49 |
| Efet.01.95041.g1160.t1 | Latent-transforming growth factor beta-binding protein 4 | 852 | 5.56E-32 | 49 |
| Efet.01.47225.g1428.t1 | Microtubule-associated protein 1B | 5265 | 1.02E-60 | 49 |
| Efet.01.1643329.g276.t1 | Induced myeloid leukemia cell differentiation protein Mcl-1 | 309 | 2.22E-15 | 49 |
| Efet.01.244195.g1675.t1 | Cation-independent mannose-6-phosphate receptor | 375 | 2.85E-14 | 49 |
| Efet.01.657110.g1170.t1 | Canalicular multispecific organic anion transporter 2 | 2241 | 2.50E-12 | 49 |
| Efet.01.657239.g1207.t1 | Canalicular multispecific organic anion transporter 2 | 1533 | 4.77E-42 | 49 |
| Efet.01.553639.g603.t1 | Canalicular multispecific organic anion transporter 2 | 402 | 3.09E-07 | 49 |
| Efet.01.357317.g207.t1 | RNA-binding protein Musashi homolog 1 | 735 | 1.47E-13 | 49 |
| Efet.01.219431.g785.t1 | Myotrophin | 1056 | 5.11E-14 | 49 |
| Efet.01.271014.g783.t1 | Neuromedin-U receptor 1 | 1047 | 9.01E-06 | 49 |
| Efet.01.271853.g821.t1 | Neuromedin-U receptor 1 | 1104 | 4.53E-08 | 49 |
| Efet.01.611100.g465.t1 | Neurogenic locus notch homolog protein 1 | 984 | 2.45E-28 | 49 |
| Efet.01.1076.g99.t1 | Neurogenic locus notch homolog protein 2 | 1848 | 5.20E-13 | 49 |
| Efet.01.401055.g20.t1 | Neurogenic locus Notch protein | 2181 | 1.48E-69 | 49 |
| Efet.01.548630.g462.t1 | Neurogenic locus Notch protein | 846 | 6.79E-35 | 49 |
| Efet.01.446132.g1143.t1 | Neuropilin-1 | 684 | 3.54E-06 | 49 |
| Efet.01.425662.g611.t1 | Nematocin receptor 1 | 636 | 2.88E-06 | 49 |
| Efet.01.243146.g1642.t1 | BDNF/NT-3 growth factors receptor | 1596 | 1.20E-43 | 49 |
| Efet.01.221225.g865.t1 | Protein numb homolog | 546 | 1.57E-10 | 49 |
| Efet.01.404361.g109.t1 | Octopamine receptor beta-2R | 882 | 3.49E-46 | 49 |
| Efet.01.120660.g1077.t1 | Melanopsin | 684 | 1.76E-13 | 49 |
| Efet.01.38734.g917.t1 | Cellular tumor antigen p53 | 249 | 8.08E-07 | 49 |
| Efet.01.421566.g516.t1 | Polyadenylate-binding protein 2 | 1749 | 1.25E-06 | 49 |
| Efet.01.27686.g169.t1 | Plasminogen activator inhibitor 1 | 936 | 1.23E-22 | 49 |
| Efet.01.172588.g1076.t1 | Palladin | 1071 | 8.81E-15 | 49 |
| Efet.01.269856.g736.t1 | Palladin | 324 | 1.69E-08 | 49 |
| Efet.01.460118.g285.t1 | Protocadherin-15 | 1188 | 7.67E-10 | 49 |
| Efet.01.133708.g423.t1 | Protocadherin-15 | 888 | 2.94E-11 | 49 |
| Efet.01.189006.g1836.t1 | Protocadherin-15 | 888 | 2.94E-11 | 49 |
| Efet.01.156196.g318.t1 | Decorin | 1470 | 9.60E-12 | 49 |
| Efet.01.223452.g946.t1 | Decorin | 2514 | 1.34E-18 | 49 |
| Efet.01.44302.g1246.t1 | PR domain zinc finger protein 16 | 1788 | 1.44E-06 | 49 |
| Efet.01.479453.g749.t1 | PR domain zinc finger protein 16 | 2100 | 1.29E-06 | 49 |
| Efet.01.18916.g1404.t1 | PR domain zinc finger protein 5 | 900 | 1.07E-15 | 49 |
| Efet.01.1655266.g747.t1 | Prominin-1-A | 348 | 4.92E-06 | 49 |
| Efet.01.403601.g93.t1 | Tyrosine-protein phosphatase non-receptor type 3 | 444 | 1.91E-15 | 49 |
| Efet.01.638713.g462.t1 | Amidophosphoribosyltransferase | 1011 | 9.95E-50 | 49 |
| Efet.01.7455.g560.t1 | Protein phosphatase 1 regulatory subunit | 1473 | 6.14E-25 | 49 |
| Efet.01.602679.g111.t1 | Protein phosphatase 1 regulatory subunit | 1188 | 3.70E-23 | 49 |
| Efet.01.389533.g996.t1 | SMARCA4 isoform 2 | 570 | 9.29E-13 | 49 |
| Efet.01.606668.g286.t1 | Dexamethasone-induced Ras-related protein 1 | 654 | 1.87E-23 | 49 |
| Efet.01.34405.g647.t1 | Dexamethasone-induced Ras-related protein 1 | 687 | 4.93E-22 | 49 |
| Efet.01.625096.g1095.t1 | Histone-binding protein RBBP4 | 1272 | 2.78E-06 | 49 |
| Efet.01.60946.g683.t1 | RE1-silencing transcription factor | 1248 | 2.19E-07 | 49 |
| Efet.01.499920.g1170.t1 | Repulsive guidance molecule A | 735 | 3.93E-22 | 49 |
| Efet.01.370163.g560.t1 | Rho-related GTP-binding protein RhoA-B | 576 | 1.03E-06 | 49 |
| Efet.01.371070.g592.t1 | Ribonuclease inhibitor | 2067 | 4.70E-14 | 49 |
| Efet.01.1652532.g564.t1 | Roundabout homolog 2 | 420 | 2.83E-09 | 49 |
| Efet.01.1658037.g1087.t1 | Roundabout homolog 2 | 375 | 4.43E-12 | 49 |
| Efet.01.141182.g837.t1 | Ski-like protein | 1185 | 4.68E-28 | 49 |
| Efet.01.1918.g169.t1 | Slit homolog 1 protein | 858 | 2.22E-16 | 49 |
| Efet.01.460797.g306.t1 | Slit homolog 2 protein | 729 | 3.08E-10 | 49 |
| Efet.01.1659342.g1572.t1 | Structural maintenance of chromosomes protein 1A | 1095 | 4.55E-09 | 49 |
| Efet.01.656558.g1039.t1 | SWI/SNF-related matrix-associated actin-dependent regulator of chromatin subfamily A member 5 | 2685 | 6.70E-53 | 49 |
| Efet.01.22955.g1673.t1 | Suppressor of cytokine signaling 3 | 1254 | 1.50E-13 | 49 |
| Efet.01.139002.g720.t1 | Suppressor of cytokine signaling 3 | 1488 | 9.82E-16 | 49 |
| Efet.01.90712.g929.t1 | SPARC | 411 | 2.28E-06 | 49 |
| Efet.01.656303.g989.t1 | Serine/threonine-protein kinase STK11 | 507 | 8.96E-10 | 49 |
| Efet.01.656644.g1056.t1 | Tyrosine-protein kinase Tec | 345 | 1.68E-10 | 49 |
| Efet.01.356039.g166.t1 | Telomerase reverse transcriptase | 3486 | 8.62E-63 | 49 |
| Efet.01.214849.g624.t1 | Transducin-like enhancer protein 1 | 1197 | 3.28E-06 | 49 |
| Efet.01.654486.g616.t1 | DNA topoisomerase 2 top-2 | 1194 | 2.37E-08 | 49 |
| Efet.01.116149.g841.t1 | Transient receptor potential-gamma protein | 810 | 3.36E-11 | 49 |
| Efet.01.5096.g394.t1 | Vasopressin V1a receptor | 1320 | 2.07E-27 | 49 |
| Efet.01.151865.g112.t1 | Vasopressin V1a receptor | 1203 | 2.50E-09 | 49 |
| Efet.01.361627.g310.t1 | Vasopressin V1a receptor | 1098 | 9.98E-10 | 49 |
| Efet.01.428050.g682.t1 | Vasopressin V1a receptor | 1149 | 2.92E-51 | 49 |
| Efet.01.288652.g1401.t1 | Vascular endothelial growth factor receptor 1 | 513 | 9.50E-09 | 49 |
| Efet.01.16951.g1250.t1 | WD repeat-containing protein 5 | 366 | 7.81E-12 | 49 |
| Efet.01.601188.g53.t1 | WD repeat-containing protein 5 | 624 | 3.49E-08 | 49 |
| Efet.01.1659500.g1892.t1 | Protein white | 1530 | 8.23E-09 | 49 |
| Efet.01.565724.g190.t1 | Protein white | 840 | 5.77E-17 | 49 |
| Efet.01.35746.g717.t1 | Transcriptional coactivator YAP1 | 423 | 4.46E-07 | 49 |
| Efet.01.387053.g946.t1 | Zinc finger X-chromosomal protein | 984 | 2.21E-07 | 49 |
| Efet.01.1135.g104.t1 | Zinc finger protein 37A | 468 | 1.52E-18 | 49 |
| Efet.01.56656.g414.t1 | Zinc finger protein 37A | 1158 | 1.30E-62 | 49 |
| Efet.01.176741.g1238.t1 | Zinc finger protein 37A | 1791 | 6.10E-73 | 49 |
| Efet.01.424735.g588.t1 | Zinc finger protein 37A | 828 | 7.30E-16 | 49 |
| Efet.01.445984.g1137.t1 | Zinc finger protein 37A | 1602 | 1.91E-48 | 49 |
| Efet.01.532831.g88.t1 | Zinc finger protein 37A | 1674 | 3.89E-69 | 49 |
| Efet.01.261972.g446.t1 | Zinc finger and SCAN domain-containing protein 10 | 3924 | 6.52E-82 | 49 |
| Efet.01.180648.g1429.t1 | 3 beta-hydroxysteroid dehydrogenase type 7 | 816 | 2.90E-32 | 48 |
| Efet.01.296467.g1631.t1 | 5-hydroxytryptamine receptor 1A | 924 | 6.88E-08 | 48 |
| Efet.01.618480.g837.t1 | 5-hydroxytryptamine receptor 1A | 1152 | 1.11E-49 | 48 |
| Efet.01.189318.g1847.t1 | 5-hydroxytryptamine receptor 2C | 948 | 6.50E-37 | 48 |
| Efet.01.25268.g35.t1 | 5-hydroxytryptamine receptor 2C | 426 | 8.50E-15 | 48 |
| Efet.01.658430.g2171.t1 | ATP-binding cassette transporter abc2 | 588 | 3.58E-11 | 48 |
| Efet.01.588154.g932.t1 | ATP-binding cassette sub-family G member 2 | 699 | 9.78E-13 | 48 |
| Efet.01.658417.g2080.t1 | Probable ATP-dependent permease | 831 | 5.38E-18 | 48 |
| Efet.01.1658958.g1334.t1 | Probable ATP-dependent permease | 756 | 1.18E-13 | 48 |
| Efet.01.1659316.g1547.t1 | Aldehyde dehydrogenase, dimeric NADP-preferring | 1470 | 6.47E-33 | 48 |
| Efet.01.1658901.g1320.t1 | Aldehyde dehydrogenase, dimeric NADP-preferring | 834 | 1.32E-10 | 48 |
| Efet.01.657876.g1513.t1 | Aldehyde dehydrogenase, dimeric NADP-preferring | 1521 | 3.50E-41 | 48 |
| Efet.01.644987.g916.t1 | Aldose reductase | 1899 | 7.08E-28 | 48 |
| Efet.01.575925.g514.t1 | Atrial natriuretic peptide receptor 1 | 816 | 3.30E-21 | 48 |
| Efet.01.232025.g1275.t1 | Aurora kinase A | 423 | 1.92E-13 | 48 |
| Efet.01.231687.g1263.t1 | Bone morphogenetic protein 7 | 594 | 5.28E-22 | 48 |
| Efet.01.245601.g1718.t1 | Cadherin-99C | 813 | 3.03E-06 | 48 |
| Efet.01.140014.g769.t1 | Cadherin-2 | 951 | 1.09E-14 | 48 |
| Efet.01.237184.g1450.t1 | Cadherin-3 | 945 | 2.05E-13 | 48 |
| Efet.01.470219.g535.t1 | Cadherin-3 | 1539 | 4.90E-09 | 48 |
| Efet.01.609438.g402.t1 | Cadherin-4 | 1113 | 1.24E-11 | 48 |
| Efet.01.98276.g1347.t1 | Cholecystokinin receptor type A | 1242 | 2.02E-28 | 48 |
| Efet.01.300204.g8.t1 | Cholecystokinin receptor type A | 546 | 5.66E-11 | 48 |
| Efet.01.657766.g1452.t1 | Cystic fibrosis transmembrane conductance regulator | 1740 | 1.19E-14 | 48 |
| Efet.01.56698.g416.t1 | Collagen alpha-1(XVIII) chain | 735 | 1.20E-17 | 48 |
| Efet.01.623094.g1010.t1 | C-X-C chemokine receptor type 4 | 1053 | 7.85E-18 | 48 |
| Efet.01.218578.g751.t1 | Dicer-like protein 1 | 1263 | 4.92E-13 | 48 |
| Efet.01.290544.g1473.t1 | Epithelial discoidin domain-containing receptor 1 | 705 | 4.12E-12 | 48 |
| Efet.01.363551.g361.t1 | Epithelial discoidin domain-containing receptor 1 | 1476 | 2.42E-16 | 48 |
| Efet.01.606395.g274.t1 | Neurogenic locus protein delta | 1053 | 4.57E-37 | 48 |
| Efet.01.334564.g1034.t1 | Deleted in malignant brain tumors 1 protein | 231 | 4.12E-08 | 48 |
| Efet.01.196576.g2153.t1 | Early growth response protein 1 | 336 | 2.37E-12 | 48 |
| Efet.01.13025.g981.t1 | Histone-lysine N-methyltransferase EHMT2 | 1308 | 7.38E-08 | 48 |
| Efet.01.194568.g2071.t1 | Histone-lysine N-methyltransferase EHMT2 | 381 | 2.12E-09 | 48 |
| Efet.01.214833.g622.t1 | Histone-lysine N-methyltransferase EHMT2 | 2982 | 3.00E-23 | 48 |
| Efet.01.308920.g278.t1 | Histone-lysine N-methyltransferase EHMT2 | 1323 | 3.22E-11 | 48 |
| Efet.01.424604.g585.t1 | Histone-lysine N-methyltransferase EHMT2 | 1014 | 1.03E-17 | 48 |
| Efet.01.2633.g240.t1 | ELAV-like protein 4 | 726 | 1.38E-34 | 48 |
| Efet.01.135665.g528.t1 | Histone acetyltransferase p300 | 855 | 4.67E-30 | 48 |
| Efet.01.621374.g934.t1 | Ephrin type-B receptor 3 | 345 | 7.40E-14 | 48 |
| Efet.01.195918.g2124.t1 | Protein eyes shut homolog | 435 | 4.29E-12 | 48 |
| Efet.01.69420.g1200.t1 | Fibroblast growth factor receptor 4 | 420 | 1.01E-08 | 48 |
| Efet.01.2186.g196.t1 | Protein flightless-1 | 2532 | 2.81E-27 | 48 |
| Efet.01.172324.g1065.t1 | Protein flightless-1 | 2745 | 4.54E-21 | 48 |
| Efet.01.657982.g1548.t1 | Protein flightless-1 | 984 | 4.25E-14 | 48 |
| Efet.01.169940.g973.t1 | Follistatin | 378 | 3.88E-13 | 48 |
| Efet.01.69586.g1211.t1 | Zinc finger protein GLI1 | 1143 | 3.37E-10 | 48 |
| Efet.01.155910.g307.t1 | E3 ubiquitin-protein ligase highwire | 1065 | 3.03E-06 | 48 |
| Efet.01.49085.g1530.t1 | Histamine H1 receptor | 531 | 9.77E-09 | 48 |
| Efet.01.591473.g1037.t1 | Zinc finger and SCAN domain-containing protein 10 | 801 | 2.38E-21 | 48 |
| Efet.01.406317.g154.t1 | ATP-dependent RNA helicase eIF4A | 504 | 2.16E-11 | 48 |
| Efet.01.67906.g1110.t1 | Immunoglobulin superfamily member 10 | 1374 | 8.59E-23 | 48 |
| Efet.01.880.g83.t1 | NF-kappa-B inhibitor alpha | 894 | 1.67E-08 | 48 |
| Efet.01.20745.g1533.t1 | Integrin-linked protein kinase homolog pat-4 | 780 | 1.48E-08 | 48 |
| Efet.01.334119.g1019.t1 | Integrin-linked protein kinase homolog pat-4 | 1158 | 1.02E-06 | 48 |
| Efet.01.59965.g624.t1 | Insulin gene enhancer protein ISL-1 | 327 | 3.20E-09 | 48 |
| Efet.01.25460.g46.t1 | Kazal-type serine protease inhibitor domain-containing protein 1 | 264 | 7.49E-08 | 48 |
| Efet.01.179606.g1378.t1 | Potassium voltage-gated channel subfamily B member 1 | 906 | 2.95E-31 | 48 |
| Efet.01.205468.g239.t1 | Potassium voltage-gated channel subfamily B member 1 | 1200 | 1.56E-52 | 48 |
| Efet.01.484831.g853.t1 | Potassium voltage-gated channel subfamily B member 1 | 315 | 3.19E-08 | 48 |
| Efet.01.291261.g1488.t1 | Potassium channel subfamily K member 3 | 846 | 4.95E-09 | 48 |
| Efet.01.634208.g240.t1 | Neural cell adhesion molecule L1 | 465 | 4.27E-13 | 48 |
| Efet.01.347287.g1330.t1 | Laminin subunit alpha | 888 | 1.16E-08 | 48 |
| Efet.01.40704.g1046.t1 | Laminin subunit alpha-1 | 1593 | 3.91E-22 | 48 |
| Efet.01.293547.g1554.t1 | Laminin subunit alpha-1 | 615 | 7.90E-11 | 48 |
| Efet.01.235632.g1396.t1 | E3 ubiquitin-protein ligase TRIM71 | 876 | 2.30E-09 | 48 |
| Efet.01.140020.g772.t1 | E3 ubiquitin-protein ligase TRIM71 | 2409 | 4.63E-19 | 48 |
| Efet.01.100157.g8.t1 | Low-density lipoprotein receptor-related protein | 714 | 2.96E-17 | 48 |
| Efet.01.72569.g1392.t1 | Matrilin-2 | 3276 | 4.77E-22 | 48 |
| Efet.01.200062.g5.t1 | Hepatocyte growth factor receptor | 294 | 1.91E-10 | 48 |
| Efet.01.548599.g461.t1 | Canalicular multispecific organic anion transporter 1 | 2115 | 4.27E-10 | 48 |
| Efet.01.655285.g790.t1 | Canalicular multispecific organic anion transporter 1 | 648 | 3.37E-13 | 48 |
| Efet.01.657766.g1453.t1 | Canalicular multispecific organic anion transporter 2 | 1749 | 2.59E-22 | 48 |
| Efet.01.626536.g1167.t1 | Myotrophin | 306 | 3.73E-12 | 48 |
| Efet.01.27021.g134.t1 | Nucleus accumbens-associated protein 1 | 963 | 3.20E-07 | 48 |
| Efet.01.254126.g150.t1 | Nucleus accumbens-associated protein 1 | 1806 | 1.91E-11 | 48 |
| Efet.01.312728.g402.t1 | Nucleus accumbens-associated protein 1 | 1776 | 2.16E-07 | 48 |
| Efet.01.504602.g120.t1 | Bifunctional heparan sulfate N-deacetylase/N-sulfotransferase 1 | 645 | 4.93E-21 | 48 |
| Efet.01.612968.g574.t1 | Nuclear factor NF-kappa-B p105 subunit | 1398 | 1.60E-08 | 48 |
| Efet.01.58211.g510.t1 | Homeobox protein Nkx-2.2 | 717 | 9.36E-15 | 48 |
| Efet.01.550416.g517.t1 | Neuromedin-U receptor 1 | 627 | 2.55E-06 | 48 |
| Efet.01.97183.g1294.t1 | Neuromedin-U receptor 1 | 1146 | 2.53E-06 | 48 |
| Efet.01.447789.g1170.t1 | Neuromedin-U receptor 1 | 1251 | 1.83E-06 | 48 |
| Efet.01.19462.g1441.t1 | Neurogenic locus notch homolog protein 2 | 624 | 4.36E-11 | 48 |
| Efet.01.487066.g898.t1 | Neuropilin-1 | 633 | 3.22E-21 | 48 |
| Efet.01.93570.g1077.t1 | Octopamine receptor beta-3R | 1056 | 3.64E-06 | 48 |
| Efet.01.191806.g1950.t1 | Octopamine receptor beta-3R | 342 | 6.96E-13 | 48 |
| Efet.01.325095.g770.t1 | Palladin | 744 | 2.96E-14 | 48 |
| Efet.01.232238.g1280.t1 | Inactive serine protease PAMR1 | 438 | 1.78E-11 | 48 |
| Efet.01.82920.g516.t1 | Protocadherin-15 | 1578 | 1.47E-35 | 48 |
| Efet.01.150634.g35.t1 | Protocadherin-15 | 1248 | 3.74E-37 | 48 |
| Efet.01.22489.g1634.t1 | Protocadherin-15 | 1050 | 4.17E-23 | 48 |
| Efet.01.193753.g2032.t1 | Protocadherin-15 | 2217 | 7.19E-22 | 48 |
| Efet.01.210405.g461.t1 | Protocadherin-15 | 1311 | 1.24E-31 | 48 |
| Efet.01.657604.g1334.t1 | Protocadherin-15 | 1365 | 3.74E-08 | 48 |
| Efet.01.220561.g834.t1 | Protocadherin-15 | 612 | 5.69E-10 | 48 |
| Efet.01.643183.g766.t1 | Protocadherin-15 | 1194 | 2.84E-23 | 48 |
| Efet.01.318970.g609.t1 | Serine/threonine-protein kinase pim-2 | 339 | 9.73E-09 | 48 |
| Efet.01.350900.g27.t1 | Serine/threonine-protein kinase pim-2 | 984 | 6.31E-11 | 48 |
| Efet.01.102725.g155.t1 | PR domain zinc finger protein 5 | 816 | 2.76E-43 | 48 |
| Efet.01.180649.g1430.t1 | Receptor-type tyrosine-protein phosphatase S | 288 | 4.10E-09 | 48 |
| Efet.01.639728.g536.t1 | Receptor-type tyrosine-protein phosphatase S | 2709 | 7.19E-84 | 48 |
| Efet.01.173036.g1099.t1 | Peroxidasin homolog | 795 | 2.31E-06 | 48 |
| Efet.01.98106.g1342.t1 | Peroxidasin homolog | 567 | 3.28E-22 | 48 |
| Efet.01.234116.g1345.t1 | Peroxidasin homolog | 294 | 7.86E-10 | 48 |
| Efet.01.312955.g412.t1 | Peroxidasin homolog | 2523 | 8.46E-17 | 48 |
| Efet.01.369812.g539.t1 | Peroxidasin homolog | 570 | 2.42E-21 | 48 |
| Efet.01.190962.g1916.t1 | Peroxidasin | 1089 | 6.87E-24 | 48 |
| Efet.01.272756.g848.t1 | Peroxidasin | 909 | 5.31E-08 | 48 |
| Efet.01.343203.g1225.t1 | Peroxidasin | 660 | 1.12E-17 | 48 |
| Efet.01.272336.g833.t1 | Histone-binding protein RBBP7 | 591 | 1.63E-08 | 48 |
| Efet.01.255509.g201.t1 | Ribonuclease inhibitor | 2619 | 4.84E-22 | 48 |
| Efet.01.226070.g1040.t1 | GTP-binding protein Rit1 | 585 | 2.45E-12 | 48 |
| Efet.01.49383.g1553.t1 | Roundabout homolog 2 | 795 | 4.04E-56 | 48 |
| Efet.01.497157.g1116.t1 | Roundabout homolog 2 | 351 | 5.82E-11 | 48 |
| Efet.01.13857.g1028.t1 | Sal-like protein 4 | 684 | 5.91E-10 | 48 |
| Efet.01.30910.g409.t1 | Sodium channel protein type 1 subunit alpha | 438 | 2.61E-12 | 48 |
| Efet.01.58675.g539.t1 | Slit homolog 1 protein | 612 | 4.14E-10 | 48 |
| Efet.01.166008.g797.t1 | Structural maintenance of chromosomes protein 1A | 498 | 3.63E-12 | 48 |
| Efet.01.239850.g1530.t1 | Suppressor of cytokine signaling 3 | 456 | 2.22E-11 | 48 |
| Efet.01.224947.g1001.t1 | Transcription factor Sp1 | 306 | 2.37E-13 | 48 |
| Efet.01.253281.g127.t1 | Mitogen-activated protein kinase spk1 | 606 | 3.72E-17 | 48 |
| Efet.01.78378.g202.t1 | Serine/threonine-protein kinase STK11 | 816 | 1.21E-25 | 48 |
| Efet.01.213727.g585.t1 | Histone-lysine N-methyltransferase SUV39H2 | 2355 | 2.10E-09 | 48 |
| Efet.01.146252.g1067.t1 | Transcription intermediary factor 1-alpha | 1857 | 5.36E-08 | 48 |
| Efet.01.40656.g1037.t1 | Transcription intermediary factor 1-beta | 1020 | 6.94E-14 | 48 |
| Efet.01.638678.g449.t1 | DNA topoisomerase 2 top-2 | 2358 | 1.30E-12 | 48 |
| Efet.01.636598.g340.t1 | Peroxiredoxin tpx1 | 642 | 5.54E-26 | 48 |
| Efet.01.80073.g329.t1 | TNF receptor-associated factor 6 | 642 | 1.36E-22 | 48 |
| Efet.01.2207.g200.t1 | E3 ubiquitin-protein ligase TRIM32 | 381 | 6.67E-06 | 48 |
| Efet.01.170165.g981.t1 | Transient receptor potential-gamma protein | 879 | 1.92E-06 | 48 |
| Efet.01.114292.g739.t1 | Ubiquitin-conjugating enzyme E2 2 | 297 | 4.21E-08 | 48 |
| Efet.01.474209.g638.t1 | UDP-glucuronosyltransferase 1-1 | 1668 | 2.08E-54 | 48 |
| Efet.01.534802.g153.t1 | UDP-glucuronosyltransferase 1-2 | 966 | 1.52E-14 | 48 |
| Efet.01.212011.g519.t1 | Protein wech | 909 | 1.70E-16 | 48 |
| Efet.01.585033.g830.t1 | Protein white | 891 | 1.94E-10 | 48 |
| Efet.01.654656.g649.t1 | Protein white | 834 | 1.06E-08 | 48 |
| Efet.01.1659380.g1628.t1 | Protein white | 2013 | 4.28E-11 | 48 |
| Efet.01.658415.g2044.t1 | Protein white | 1539 | 7.10E-16 | 48 |
| Efet.01.658428.g2151.t1 | ABC transporter ATP-binding protein/permease wht-1 | 744 | 1.86E-14 | 48 |
| Efet.01.1659501.g1895.t1 | ABC transporter ATP-binding protein/permease wht-1 | 1191 | 1.11E-15 | 48 |
| Efet.01.545657.g385.t1 | Transcriptional coactivator YAP1 | 453 | 5.83E-14 | 48 |
| Efet.01.17597.g1297.t1 | Zinc finger and SCAN domain-containing protein 10 | 1281 | 1.58E-56 | 48 |
| Efet.01.544463.g348.t1 | Zinc finger and SCAN domain-containing protein 10 | 1539 | 1.26E-79 | 48 |
| Efet.01.528754.g658.t1 | 5-hydroxytryptamine receptor 1A | 1497 | 7.12E-63 | 47 |
| Efet.01.43615.g1207.t1 | 5-hydroxytryptamine receptor 1A | 1542 | 5.21E-49 | 47 |
| Efet.01.267648.g644.t1 | ATP-binding cassette transporter abc2 | 2265 | 2.01E-44 | 47 |
| Efet.01.533383.g128.t1 | ATP-binding cassette transporter abc2 | 987 | 6.88E-12 | 47 |
| Efet.01.658261.g1770.t1 | ATP-binding cassette transporter abc2 | 711 | 3.72E-13 | 47 |
| Efet.01.1658449.g1185.t1 | ATP-binding cassette transporter abc2 | 885 | 1.46E-12 | 47 |
| Efet.01.580583.g687.t1 | ATP-binding cassette sub-family G member 2 | 606 | 2.05E-14 | 47 |
| Efet.01.621705.g952.t1 | ATP-binding cassette sub-family G member 2 | 792 | 3.20E-13 | 47 |
| Efet.01.629376.g1293.t1 | ATP-binding cassette sub-family G member 2 | 594 | 1.67E-07 | 47 |
| Efet.01.645268.g962.t1 | ATP-binding cassette sub-family G member 2 | 1563 | 2.39E-09 | 47 |
| Efet.01.654656.g678.t1 | ATP-binding cassette sub-family G member 2 | 1113 | 1.82E-15 | 47 |
| Efet.01.658430.g2172.t1 | ATP-binding cassette sub-family G member 2 | 2517 | 9.37E-09 | 47 |
| Efet.01.657703.g1385.t1 | ATP-binding cassette sub-family G member 2 | 1314 | 7.66E-11 | 47 |
| Efet.01.282031.g1160.t1 | ATP-binding cassette sub-family G member 2 | 2010 | 3.12E-13 | 47 |
| Efet.01.155169.g266.t1 | Atypical chemokine receptor 3 | 1200 | 1.29E-23 | 47 |
| Efet.01.658109.g1628.t1 | Alcohol dehydrogenase 1 | 783 | 5.63E-07 | 47 |
| Efet.01.386139.g933.t1 | Adiponectin | 582 | 8.30E-19 | 47 |
| Efet.01.558794.g717.t1 | Beta-2 adrenergic receptor | 1314 | 4.51E-25 | 47 |
| Efet.01.203301.g156.t1 | Aurora/IPL1-related protein kinase 2 | 570 | 3.05E-19 | 47 |
| Efet.01.322944.g710.t1 | Aurora/IPL1-related protein kinase 2 | 834 | 5.21E-09 | 47 |
| Efet.01.1659526.g2031.t1 | Aurora/IPL1-related protein kinase 2 | 1419 | 3.21E-16 | 47 |
| Efet.01.1659026.g1367.t1 | Aldehyde dehydrogenase, dimeric NADP-preferring | 1008 | 2.49E-30 | 47 |
| Efet.01.278716.g1055.t1 | Aldehyde dehydrogenase, dimeric NADP-preferring | 468 | 1.68E-13 | 47 |
| Efet.01.624347.g1071.t1 | Aldehyde dehydrogenase, dimeric NADP-preferring | 1434 | 4.18E-35 | 47 |
| Efet.01.115447.g800.t1 | Beta-2-glycoprotein 1 | 2586 | 9.41E-28 | 47 |
| Efet.01.653365.g422.t1 | C5a anaphylatoxin chemotactic receptor 1 | 519 | 1.14E-06 | 47 |
| Efet.01.263392.g505.t1 | Cadherin-15 | 741 | 1.67E-11 | 47 |
| Efet.01.315709.g504.t1 | Cadherin-15 | 348 | 2.83E-06 | 47 |
| Efet.01.432422.g784.t1 | Cadherin-99C | 525 | 2.88E-10 | 47 |
| Efet.01.443364.g1070.t1 | Cadherin-99C | 1128 | 5.72E-17 | 47 |
| Efet.01.322182.g693.t1 | Cadherin-1 | 804 | 1.79E-17 | 47 |
| Efet.01.140057.g774.t1 | Cadherin-4 | 1518 | 2.58E-16 | 47 |
| Efet.01.83710.g565.t1 | Cadherin-4 | 933 | 1.74E-24 | 47 |
| Efet.01.545525.g381.t1 | Cadherin-4 | 789 | 4.44E-08 | 47 |
| Efet.01.301942.g66.t1 | Calpain-3 | 501 | 7.73E-06 | 47 |
| Efet.01.472242.g600.t1 | Carbonyl reductase [NADPH] 1 | 771 | 8.34E-10 | 47 |
| Efet.01.81557.g437.t1 | Cholecystokinin receptor type A | 609 | 3.60E-09 | 47 |
| Efet.01.658380.g1917.t1 | Cystic fibrosis transmembrane conductance regulator | 1134 | 3.69E-06 | 47 |
| Efet.01.571102.g355.t1 | Cystic fibrosis transmembrane conductance regulator | 885 | 4.07E-12 | 47 |
| Efet.01.60321.g642.t1 | Chondroitin sulfate proteoglycan 4 | 2907 | 4.68E-59 | 47 |
| Efet.01.72989.g1418.t1 | Cysteine and glycine-rich protein 1 | 1002 | 6.40E-31 | 47 |
| Efet.01.158239.g433.t1 | C-X-C chemokine receptor type 1 | 1098 | 7.01E-18 | 47 |
| Efet.01.575886.g512.t1 | C-X-C chemokine receptor type 1 | 1218 | 4.84E-16 | 47 |
| Efet.01.54566.g302.t1 | Dystrophin | 330 | 2.09E-17 | 47 |
| Efet.01.412424.g275.t1 | Dipeptidyl peptidase 4 | 1548 | 6.91E-52 | 47 |
| Efet.01.113002.g655.t1 | Dual specificity protein phosphatase 1 | 999 | 7.75E-41 | 47 |
| Efet.01.54995.g337.t1 | Histone-lysine N-methyltransferase EHMT2 | 681 | 6.20E-06 | 47 |
| Efet.01.516348.g407.t1 | ELAV-like protein 4 | 777 | 3.90E-07 | 47 |
| Efet.01.112053.g610.t1 | Fasciclin-2 | 417 | 5.94E-17 | 47 |
| Efet.01.527516.g630.t1 | Fasciclin-2 | 513 | 1.49E-09 | 47 |
| Efet.01.256481.g241.t1 | Protein flightless-1 homolog | 2454 | 1.01E-27 | 47 |
| Efet.01.4098.g345.t1 | Follistatin-A | 711 | 9.97E-41 | 47 |
| Efet.01.569805.g314.t1 | Glutamate receptor 1 | 552 | 9.21E-11 | 47 |
| Efet.01.246753.g1770.t1 | Glycogen synthase kinase-3 beta | 990 | 1.64E-09 | 47 |
| Efet.01.266525.g603.t1 | Hepatocyte nuclear factor 4-alpha | 987 | 5.99E-13 | 47 |
| Efet.01.634043.g235.t1 | Histamine H2 receptor | 738 | 1.77E-06 | 47 |
| Efet.01.260157.g382.t1 | Zinc finger and SCAN domain-containing protein 10 | 1266 | 1.43E-09 | 47 |
| Efet.01.656400.g1013.t1 | Zinc finger and SCAN domain-containing protein 10 | 954 | 1.82E-10 | 47 |
| Efet.01.1648839.g418.t1 | Immunoglobulin superfamily member 10 | 288 | 8.15E-08 | 47 |
| Efet.01.582094.g747.t1 | Integrin-linked protein kinase | 597 | 3.48E-14 | 47 |
| Efet.01.20147.g1491.t1 | Potassium voltage-gated channel subfamily B member 1 | 549 | 5.72E-08 | 47 |
| Efet.01.83019.g526.t1 | Neural cell adhesion molecule L1 | 1602 | 9.14E-34 | 47 |
| Efet.01.135278.g506.t1 | Laminin subunit alpha | 336 | 6.70E-27 | 47 |
| Efet.01.428610.g696.t1 | Laminin subunit alpha-1 | 525 | 9.69E-08 | 47 |
| Efet.01.84755.g608.t1 | Laminin subunit alpha-3 | 360 | 3.45E-07 | 47 |
| Efet.01.332623.g977.t1 | Laminin subunit beta-2 | 585 | 1.96E-15 | 47 |
| Efet.01.492983.g1019.t1 | Leucine-rich repeat-containing G-protein coupled receptor 5 | 777 | 1.25E-20 | 47 |
| Efet.01.1658141.g1111.t1 | E3 ubiquitin-protein ligase TRIM71 | 501 | 2.28E-07 | 47 |
| Efet.01.457074.g182.t1 | E3 ubiquitin-protein ligase TRIM71 | 531 | 1.59E-11 | 47 |
| Efet.01.581638.g725.t1 | E3 ubiquitin-protein ligase TRIM71 | 867 | 2.96E-06 | 47 |
| Efet.01.26381.g105.t1 | Latent-transforming growth factor beta-binding protein 4 | 609 | 1.13E-23 | 47 |
| Efet.01.110486.g533.t1 | Mitogen-activated protein kinase kinase kinase 12 | 2355 | 1.35E-07 | 47 |
| Efet.01.151196.g63.t1 | Microtubule-associated protein 1B | 2823 | 5.64E-63 | 47 |
| Efet.01.92100.g1003.t1 | Matrilin-2 | 1353 | 1.57E-15 | 47 |
| Efet.01.35780.g719.t1 | Canalicular multispecific organic anion transporter 1 | 771 | 1.39E-15 | 47 |
| Efet.01.630774.g68.t1 | Canalicular multispecific organic anion transporter 1 | 1044 | 3.32E-16 | 47 |
| Efet.01.635927.g301.t1 | Canalicular multispecific organic anion transporter 2 | 1038 | 2.25E-14 | 47 |
| Efet.01.268411.g681.t1 | Unconventional myosin-Va | 837 | 1.08E-28 | 47 |
| Efet.01.119477.g1007.t1 | Neural cell adhesion molecule L1-like protein | 627 | 2.46E-18 | 47 |
| Efet.01.229555.g1179.t1 | Neural cell adhesion molecule L1-like protein | 1116 | 2.01E-41 | 47 |
| Efet.01.516197.g403.t1 | Bifunctional heparan sulfate N-deacetylase/N-sulfotransferase 1 | 1047 | 2.08E-17 | 47 |
| Efet.01.40595.g1031.t1 | Bifunctional heparan sulfate N-deacetylase/N-sulfotransferase 1 | 678 | 2.30E-20 | 47 |
| Efet.01.161270.g581.t1 | Neuronal-glial cell adhesion molecule | 426 | 3.71E-16 | 47 |
| Efet.01.612380.g540.t1 | Neuronal-glial cell adhesion molecule | 414 | 7.16E-10 | 47 |
| Efet.01.130094.g274.t1 | Neuromedin-U receptor 1 | 1032 | 4.15E-26 | 47 |
| Efet.01.549512.g481.t1 | Neurogenic locus notch homolog protein 2 | 483 | 6.14E-09 | 47 |
| Efet.01.215811.g657.t1 | Neurogenic locus notch homolog protein 3 | 639 | 9.12E-27 | 47 |
| Efet.01.258765.g328.t1 | Neurogenic locus notch homolog protein 3 | 4038 | 3.91E-12 | 47 |
| Efet.01.177775.g1302.t1 | Neurogenic locus Notch protein | 744 | 7.64E-11 | 47 |
| Efet.01.93580.g1078.t1 | Neurotrophin-3 | 369 | 4.97E-08 | 47 |
| Efet.01.168063.g891.t1 | Octopamine receptor beta-2R | 771 | 2.96E-11 | 47 |
| Efet.01.338850.g1136.t1 | Octopamine receptor beta-3R | 408 | 3.87E-07 | 47 |
| Efet.01.597855.g1239.t1 | Melanopsin | 1059 | 5.20E-14 | 47 |
| Efet.01.85077.g631.t1 | Melanopsin | 1338 | 5.09E-14 | 47 |
| Efet.01.82172.g478.t1 | P2Y purinoceptor 1 | 906 | 8.15E-09 | 47 |
| Efet.01.1496.g143.t1 | Protocadherin-15 | 564 | 4.71E-07 | 47 |
| Efet.01.100889.g62.t1 | Protocadherin-15 | 2925 | 7.04E-46 | 47 |
| Efet.01.142313.g892.t1 | Protocadherin-15 | 1320 | 9.35E-21 | 47 |
| Efet.01.248016.g1823.t1 | Protocadherin-15 | 3201 | 3.69E-37 | 47 |
| Efet.01.275935.g964.t1 | Protocadherin-15 | 1980 | 1.01E-30 | 47 |
| Efet.01.321798.g679.t1 | Protocadherin-15 | 951 | 1.37E-11 | 47 |
| Efet.01.147809.g1135.t1 | Protocadherin-15 | 948 | 5.16E-16 | 47 |
| Efet.01.410278.g226.t1 | Protocadherin-15 | 2232 | 7.97E-27 | 47 |
| Efet.01.616830.g741.t1 | Protocadherin-15 | 2259 | 4.84E-23 | 47 |
| Efet.01.658207.g1713.t1 | Protocadherin-15 | 972 | 1.50E-25 | 47 |
| Efet.01.658389.g1942.t1 | PR domain zinc finger protein 14 | 2223 | 1.96E-14 | 47 |
| Efet.01.647603.g1190.t1 | PR domain zinc finger protein 16 | 1560 | 2.37E-06 | 47 |
| Efet.01.53691.g247.t1 | PR domain zinc finger protein 5 | 2157 | 3.41E-09 | 47 |
| Efet.01.340441.g1179.t1 | PR domain zinc finger protein 5 | 801 | 5.57E-17 | 47 |
| Efet.01.652554.g313.t1 | Receptor-type tyrosine-protein phosphatase S | 318 | 4.58E-07 | 47 |
| Efet.01.206171.g267.t1 | Peroxidasin | 288 | 3.19E-09 | 47 |
| Efet.01.17477.g1287.t1 | Peroxidasin homolog | 1233 | 1.55E-12 | 47 |
| Efet.01.363563.g362.t1 | Peroxidasin homolog | 1011 | 2.04E-16 | 47 |
| Efet.01.405153.g124.t1 | SMC1A protein | 558 | 4.28E-07 | 47 |
| Efet.01.257263.g274.t1 | Protein phosphatase 1 regulatory subunit | 2952 | 8.53E-27 | 47 |
| Efet.01.75681.g39.t1 | Ras-related protein Rab-30 | 399 | 1.03E-20 | 47 |
| Efet.01.169.g18.t1 | Roundabout homolog 2 | 318 | 1.47E-07 | 47 |
| Efet.01.113833.g703.t1 | Roundabout homolog 2 | 888 | 7.61E-19 | 47 |
| Efet.01.255862.g214.t1 | Reticulon-4 receptor | 1041 | 1.29E-16 | 47 |
| Efet.01.494798.g1067.t1 | Reticulon-4 receptor | 1128 | 4.82E-26 | 47 |
| Efet.01.617194.g753.t1 | Reticulon-4 receptor | 969 | 1.92E-21 | 47 |
| Efet.01.84780.g609.t1 | Retinoic acid receptor RXR-alpha | 276 | 8.77E-11 | 47 |
| Efet.01.10562.g775.t1 | Sphingosine 1-phosphate receptor 2 | 1650 | 5.60E-07 | 47 |
| Efet.01.138522.g694.t1 | Phosphatidylinositol 3,4,5-trisphosphate 5-phosphatase 1 | 522 | 5.12E-12 | 47 |
| Efet.01.286375.g1317.t1 | Slit homolog 1 protein | 1032 | 9.69E-17 | 47 |
| Efet.01.117912.g920.t1 | Slit homolog 2 protein | 1824 | 1.44E-31 | 47 |
| Efet.01.1657906.g1057.t1 | Structural maintenance of chromosomes protein 1A | 888 | 5.18E-14 | 47 |
| Efet.01.514566.g355.t1 | Alpha-1-syntrophin | 624 | 6.28E-34 | 47 |
| Efet.01.332031.g961.t1 | Suppressor of cytokine signaling 3 | 549 | 5.83E-21 | 47 |
| Efet.01.1658952.g1330.t1 | Serine/threonine-protein kinase 25 | 936 | 9.87E-26 | 47 |
| Efet.01.110471.g532.t1 | Transcription intermediary factor 1-beta | 1695 | 1.54E-09 | 47 |
| Efet.01.214273.g603.t1 | Transcription intermediary factor 1-beta | 2082 | 1.13E-09 | 47 |
| Efet.01.577234.g565.t1 | Transcription intermediary factor 1-beta | 417 | 3.12E-12 | 47 |
| Efet.01.9821.g717.t1 | Toll-like receptor Tollo | 1290 | 2.28E-13 | 47 |
| Efet.01.205270.g228.t1 | Toll-like receptor Tollo | 882 | 5.72E-19 | 47 |
| Efet.01.605501.g228.t1 | DNA topoisomerase 2 top-2 | 489 | 4.47E-08 | 47 |
| Efet.01.302544.g84.t1 | Mitochondrial uncoupling protein 2 | 537 | 2.10E-10 | 47 |
| Efet.01.137915.g667.t1 | UDP-glucuronosyltransferase 1-1 | 1608 | 1.59E-45 | 47 |
| Efet.01.129692.g250.t1 | UDP-glucuronosyltransferase 1-2 | 762 | 7.16E-17 | 47 |
| Efet.01.355548.g155.t1 | UDP-glucuronosyltransferase 1-2 | 1467 | 2.26E-47 | 47 |
| Efet.01.255191.g189.t1 | Serine/threonine-protein kinase ULK1 | 372 | 8.26E-10 | 47 |
| Efet.01.286960.g1341.t1 | Vasopressin V1a receptor | 804 | 2.52E-21 | 47 |
| Efet.01.162667.g643.t1 | Vascular endothelial growth factor receptor 2 | 318 | 2.41E-07 | 47 |
| Efet.01.453351.g73.t1 | von Willebrand factor | 480 | 2.76E-07 | 47 |
| Efet.01.575546.g498.t1 | Protein white | 795 | 6.53E-07 | 47 |
| Efet.01.1659471.g1791.t1 | Protein white | 618 | 3.02E-12 | 47 |
| Efet.01.126673.g103.t1 | Zinc finger X-chromosomal protein | 555 | 7.63E-09 | 47 |
| Efet.01.24566.g1781.t1 | Zinc finger protein 281 | 333 | 2.99E-08 | 47 |
| Efet.01.61208.g698.t1 | Zinc finger protein 281 | 210 | 1.61E-06 | 47 |
| Efet.01.37251.g810.t1 | Zinc finger protein 37A | 1401 | 5.77E-41 | 47 |
| Efet.01.222600.g910.t1 | Zinc finger protein 37A | 1287 | 5.00E-52 | 47 |
| Efet.01.71147.g1305.t1 | Zinc finger and SCAN domain-containing protein 10 | 1266 | 9.60E-74 | 47 |
| Efet.01.71147.g1307.t1 | Zinc finger and SCAN domain-containing protein 10 | 1257 | 6.42E-74 | 47 |
| Efet.01.329399.g900.t1 | Zinc finger and SCAN domain-containing protein 10 | 1221 | 2.33E-40 | 47 |
| Efet.01.658371.g1904.t1 | Zinc finger and SCAN domain-containing protein 10 | 2079 | 2.79E-17 | 47 |
| Efet.01.657129.g1178.t1 | 5-hydroxytryptamine receptor 1A | 1314 | 9.04E-49 | 46 |
| Efet.01.101276.g83.t1 | 5-hydroxytryptamine receptor 2C | 1017 | 1.55E-10 | 46 |
| Efet.01.630860.g89.t1 | ATP-binding cassette transporter abc3 | 825 | 2.41E-15 | 46 |
| Efet.01.658418.g2086.t1 | ATP-binding cassette transporter abc3 | 1782 | 1.92E-09 | 46 |
| Efet.01.581684.g728.t1 | ATP-binding cassette sub-family G member 2 | 2346 | 1.82E-12 | 46 |
| Efet.01.299005.g1716.t1 | Atypical chemokine receptor 3 | 1029 | 7.46E-27 | 46 |
| Efet.01.531211.g41.t1 | Atypical chemokine receptor 3 | 630 | 2.42E-15 | 46 |
| Efet.01.609514.g405.t1 | Probable ATP-dependent permease | 870 | 6.45E-11 | 46 |
| Efet.01.628433.g1244.t1 | Probable ATP-dependent permease | 612 | 2.40E-07 | 46 |
| Efet.01.651148.g141.t1 | Probable ATP-dependent permease | 1989 | 1.41E-11 | 46 |
| Efet.01.657838.g1503.t1 | Aldehyde dehydrogenase, dimeric NADP-preferring | 1173 | 4.22E-10 | 46 |
| Efet.01.427593.g671.t1 | Aldehyde dehydrogenase, dimeric NADP-preferring | 1080 | 9.57E-37 | 46 |
| Efet.01.616615.g732.t1 | Aldehyde dehydrogenase, dimeric NADP-preferring | 2982 | 2.78E-30 | 46 |
| Efet.01.644987.g918.t1 | Aldehyde dehydrogenase, dimeric NADP-preferring | 540 | 1.31E-10 | 46 |
| Efet.01.656717.g1074.t1 | Aldehyde dehydrogenase, dimeric NADP-preferring | 1434 | 3.32E-36 | 46 |
| Efet.01.1657652.g1023.t1 | Aldehyde dehydrogenase, dimeric NADP-preferring | 678 | 9.34E-11 | 46 |
| Efet.01.606285.g260.t1 | Aldehyde dehydrogenase, dimeric NADP-preferring | 1437 | 2.30E-35 | 46 |
| Efet.01.654034.g527.t1 | Aldehyde dehydrogenase, dimeric NADP-preferring | 1434 | 3.73E-28 | 46 |
| Efet.01.1659480.g1819.t1 | Aldehyde dehydrogenase, dimeric NADP-preferring | 1782 | 1.15E-35 | 46 |
| Efet.01.65177.g924.t1 | Beta-2-glycoprotein 1 | 252 | 7.04E-08 | 46 |
| Efet.01.1265.g113.t1 | Aurora kinase A | 849 | 9.49E-10 | 46 |
| Efet.01.1636455.g177.t1 | MGA protein | 204 | 2.32E-07 | 46 |
| Efet.01.201747.g86.t1 | Cadherin-99C | 777 | 9.67E-18 | 46 |
| Efet.01.608804.g365.t1 | Cadherin-99C | 1221 | 3.31E-18 | 46 |
| Efet.01.21624.g1588.t1 | Cadherin-3 | 639 | 5.46E-10 | 46 |
| Efet.01.553408.g590.t1 | Carbonyl reductase [NADPH] 1 | 705 | 7.00E-06 | 46 |
| Efet.01.80000.g322.t1 | Cholecystokinin receptor type A | 1149 | 6.27E-35 | 46 |
| Efet.01.1659088.g1398.t1 | Cholecystokinin receptor type A | 1338 | 4.29E-31 | 46 |
| Efet.01.98120.g1343.t1 | Cholecystokinin receptor type A | 1245 | 7.57E-06 | 46 |
| Efet.01.131135.g327.t1 | Cholecystokinin receptor type A | 1101 | 5.67E-27 | 46 |
| Efet.01.80872.g391.t1 | C-C chemokine receptor type 2 | 1161 | 6.27E-25 | 46 |
| Efet.01.151043.g59.t1 | C-C chemokine receptor type 2 | 1377 | 3.21E-25 | 46 |
| Efet.01.168046.g890.t1 | C-C chemokine receptor type 2 | 750 | 4.73E-06 | 46 |
| Efet.01.532166.g67.t1 | C-C chemokine receptor type 2 | 1428 | 1.22E-30 | 46 |
| Efet.01.6605.g516.t1 | Cyclin-dependent kinase 4 | 1518 | 2.24E-38 | 46 |
| Efet.01.633916.g219.t1 | Chromodomain Y-like protein | 705 | 2.09E-10 | 46 |
| Efet.01.617238.g758.t1 | Cystic fibrosis transmembrane conductance regulator | 837 | 1.75E-12 | 46 |
| Efet.01.658052.g1602.t1 | Cystic fibrosis transmembrane conductance regulator | 1116 | 1.17E-15 | 46 |
| Efet.01.642722.g712.t1 | Cystic fibrosis transmembrane conductance regulator | 672 | 3.65E-09 | 46 |
| Efet.01.657315.g1249.t1 | Chromodomain-helicase-DNA-binding protein 7 | 2841 | 8.70E-17 | 46 |
| Efet.01.291648.g1500.t1 | Collagen alpha-3(V) chain | 705 | 1.46E-07 | 46 |
| Efet.01.90150.g903.t1 | Sterol 26-hydroxylase, mitochondrial | 903 | 1.02E-17 | 46 |
| Efet.01.30684.g388.t1 | Cholesterol 7-alpha-monooxygenase | 1392 | 1.84E-44 | 46 |
| Efet.01.34535.g656.t1 | C-X-C chemokine receptor type 1 | 783 | 4.27E-16 | 46 |
| Efet.01.375277.g687.t1 | C-X-C chemokine receptor type 1 | 1107 | 1.68E-30 | 46 |
| Efet.01.245650.g1721.t1 | C-X-C chemokine receptor type 2 | 1152 | 7.98E-23 | 46 |
| Efet.01.11624.g850.t1 | C-X-C chemokine receptor type 3 | 855 | 6.72E-16 | 46 |
| Efet.01.16795.g1242.t1 | Corticosteroid 11-beta-dehydrogenase isozyme 1 | 693 | 1.97E-12 | 46 |
| Efet.01.539695.g263.t1 | Corticosteroid 11-beta-dehydrogenase isozyme 1 | 582 | 4.25E-09 | 46 |
| Efet.01.158399.g440.t1 | Deleted in malignant brain tumors 1 protein | 1629 | 7.65E-12 | 46 |
| Efet.01.78263.g195.t1 | Dual specificity protein phosphatase 1 | 522 | 2.14E-11 | 46 |
| Efet.01.194380.g2066.t1 | Histone-lysine N-methyltransferase EHMT2 | 1668 | 3.65E-14 | 46 |
| Efet.01.551547.g538.t1 | Histone-lysine N-methyltransferase EHMT2 | 5421 | 2.67E-22 | 46 |
| Efet.01.574697.g463.t1 | Histone-lysine N-methyltransferase EHMT2 | 2763 | 8.45E-13 | 46 |
| Efet.01.126489.g92.t1 | Ephrin type-A receptor 4 | 744 | 1.39E-08 | 46 |
| Efet.01.131917.g351.t1 | Protein eyes shut homolog | 702 | 7.39E-23 | 46 |
| Efet.01.117203.g895.t1 | Fasciclin-2 | 468 | 2.73E-06 | 46 |
| Efet.01.127169.g125.t1 | Protein flightless-1 homolog | 1464 | 8.59E-14 | 46 |
| Efet.01.148442.g1158.t1 | Protein flightless-1 | 2679 | 9.11E-14 | 46 |
| Efet.01.35528.g702.t1 | Alpha-(1,3)-fucosyltransferase 4 | 732 | 4.18E-17 | 46 |
| Efet.01.654309.g560.t1 | Alpha-(1,3)-fucosyltransferase 4 | 1062 | 5.84E-11 | 46 |
| Efet.01.44655.g1274.t1 | Alpha-(1,3)-fucosyltransferase 9 | 684 | 3.60E-07 | 46 |
| Efet.01.2677.g245.t1 | Gamma-aminobutyric acid receptor subunit beta-3 | 408 | 1.41E-09 | 46 |
| Efet.01.194411.g2067.t1 | Glial fibrillary acidic protein | 609 | 3.81E-12 | 46 |
| Efet.01.627107.g1199.t1 | Zinc finger protein GLI1 | 231 | 9.94E-06 | 46 |
| Efet.01.533365.g122.t1 | Hepatocyte growth factor | 243 | 4.44E-06 | 46 |
| Efet.01.622209.g975.t1 | Hepatocyte growth factor | 243 | 3.34E-06 | 46 |
| Efet.01.10811.g795.t1 | Histamine H2 receptor | 1026 | 5.92E-14 | 46 |
| Efet.01.181348.g1474.t1 | Histamine H2 receptor | 957 | 6.45E-15 | 46 |
| Efet.01.631644.g129.t1 | Histamine H2 receptor | 930 | 2.23E-11 | 46 |
| Efet.01.148280.g1153.t1 | Zinc finger and SCAN domain-containing protein 10 | 1701 | 4.80E-51 | 46 |
| Efet.01.177133.g1274.t1 | Potassium channel subfamily K member 3 | 660 | 7.31E-07 | 46 |
| Efet.01.356392.g179.t1 | Neural cell adhesion molecule L1 | 309 | 6.17E-06 | 46 |
| Efet.01.33512.g586.t1 | Laminin subunit alpha-1 | 669 | 4.24E-11 | 46 |
| Efet.01.107380.g382.t1 | Leucine-rich repeat-containing G-protein coupled receptor 5 | 1302 | 4.15E-09 | 46 |
| Efet.01.353188.g86.t1 | E3 ubiquitin-protein ligase TRIM71 | 1950 | 1.32E-14 | 46 |
| Efet.01.283461.g1218.t1 | E3 ubiquitin-protein ligase TRIM71 | 2220 | 6.08E-22 | 46 |
| Efet.01.210718.g473.t1 | Matrilin-2 | 567 | 5.15E-12 | 46 |
| Efet.01.496743.g1105.t1 | Matrilin-2 | 1596 | 1.34E-18 | 46 |
| Efet.01.2227.g201.t1 | Hepatocyte growth factor receptor | 1350 | 1.74E-21 | 46 |
| Efet.01.61556.g717.t1 | Hepatocyte growth factor receptor | 420 | 3.20E-09 | 46 |
| Efet.01.207785.g356.t1 | Canalicular multispecific organic anion transporter 1 | 1023 | 5.82E-13 | 46 |
| Efet.01.1659447.g1735.t1 | Canalicular multispecific organic anion transporter 1 | 1695 | 6.02E-11 | 46 |
| Efet.01.611807.g494.t1 | Canalicular multispecific organic anion transporter 2 | 1131 | 5.71E-06 | 46 |
| Efet.01.618287.g815.t1 | Canalicular multispecific organic anion transporter 2 | 597 | 7.60E-14 | 46 |
| Efet.01.366296.g439.t1 | RNA-binding protein Musashi homolog 1 | 264 | 1.20E-07 | 46 |
| Efet.01.7781.g575.t1 | E3 ubiquitin-protein ligase MYCBP2 | 960 | 5.34E-12 | 46 |
| Efet.01.96423.g1240.t1 | Myelin transcription factor 1 | 747 | 7.30E-18 | 46 |
| Efet.01.333335.g1000.t1 | Neural cell adhesion molecule 1 | 465 | 1.21E-07 | 46 |
| Efet.01.58685.g540.t1 | Neural cell adhesion molecule L1-like protein | 1479 | 3.22E-16 | 46 |
| Efet.01.285433.g1286.t1 | Neural cell adhesion molecule L1-like protein | 519 | 3.97E-11 | 46 |
| Efet.01.657286.g1223.t1 | Bifunctional heparan sulfate N-deacetylase/N-sulfotransferase 1 | 633 | 9.87E-19 | 46 |
| Efet.01.286501.g1321.t1 | Neuromedin-U receptor 1 | 1242 | 4.59E-07 | 46 |
| Efet.01.410526.g236.t1 | Neuromedin-U receptor 1 | 945 | 7.86E-11 | 46 |
| Efet.01.530573.g15.t1 | Nitric oxide synthase, inducible | 546 | 4.05E-08 | 46 |
| Efet.01.28254.g217.t1 | Neurogenic locus notch homolog protein 2 | 1521 | 6.25E-11 | 46 |
| Efet.01.299359.g1728.t1 | Neurogenic locus notch homolog protein 3 | 1632 | 6.91E-29 | 46 |
| Efet.01.101832.g110.t1 | Nuclear receptor subfamily 0 group B member 2 | 1464 | 3.14E-09 | 46 |
| Efet.01.111788.g592.t1 | Neuropilin-2 | 726 | 3.82E-07 | 46 |
| Efet.01.15726.g1158.t1 | P2Y purinoceptor 1 | 474 | 5.37E-07 | 46 |
| Efet.01.5326.g418.t1 | Protocadherin-15 | 2325 | 1.62E-39 | 46 |
| Efet.01.21624.g1589.t1 | Protocadherin-15 | 1461 | 1.13E-24 | 46 |
| Efet.01.102846.g164.t1 | Protocadherin-15 | 1800 | 1.06E-23 | 46 |
| Efet.01.194729.g2082.t1 | Protocadherin-15 | 3327 | 2.40E-62 | 46 |
| Efet.01.381785.g835.t1 | Protocadherin-15 | 2859 | 2.53E-33 | 46 |
| Efet.01.77706.g157.t1 | Protocadherin-15 | 354 | 6.01E-07 | 46 |
| Efet.01.114158.g720.t1 | Protocadherin-15 | 2046 | 9.33E-31 | 46 |
| Efet.01.296469.g1632.t1 | Protocadherin-15 | 3513 | 1.27E-60 | 46 |
| Efet.01.646337.g1075.t1 | Protocadherin-15 | 1491 | 2.62E-23 | 46 |
| Efet.01.2734.g249.t1 | Protocadherin-15 | 2334 | 9.12E-32 | 46 |
| Efet.01.114562.g758.t1 | Protocadherin-15 | 2817 | 5.36E-52 | 46 |
| Efet.01.379243.g781.t1 | Protein phosphatase 1D | 885 | 4.44E-21 | 46 |
| Efet.01.298057.g1686.t1 | PR domain zinc finger protein 16 | 285 | 2.14E-12 | 46 |
| Efet.01.112585.g637.t1 | PR domain zinc finger protein 5 | 1149 | 2.62E-26 | 46 |
| Efet.01.195183.g2099.t1 | PR domain zinc finger protein 5 | 1011 | 4.09E-41 | 46 |
| Efet.01.220926.g847.t1 | PR domain zinc finger protein 5 | 522 | 1.79E-19 | 46 |
| Efet.01.23226.g1686.t1 | Phosphatidylinositol 3,4,5-trisphosphate 3-phosphatase and dual-specificity protein phosphatase PTEN | 408 | 5.01E-08 | 46 |
| Efet.01.535585.g177.t1 | Peroxidasin | 327 | 2.17E-10 | 46 |
| Efet.01.141290.g844.t1 | Peroxidasin homolog | 1311 | 3.37E-09 | 46 |
| Efet.01.458460.g236.t1 | Peroxidasin homolog | 264 | 2.50E-06 | 46 |
| Efet.01.63900.g857.t1 | RE1-silencing transcription factor | 792 | 1.00E-11 | 46 |
| Efet.01.658417.g2078.t1 | Regucalcin | 930 | 3.02E-35 | 46 |
| Efet.01.54977.g335.t1 | E3 ubiquitin-protein ligase RNF13 | 2247 | 4.09E-08 | 46 |
| Efet.01.189358.g1848.t1 | Reticulon-4 receptor | 1692 | 4.58E-21 | 46 |
| Efet.01.537957.g231.t1 | Reticulon-4 receptor | 1335 | 4.31E-18 | 46 |
| Efet.01.90021.g896.t1 | Reticulon-4 receptor | 1686 | 9.76E-22 | 46 |
| Efet.01.81814.g456.t1 | Slit homolog 2 protein | 906 | 5.17E-61 | 46 |
| Efet.01.188605.g1815.t1 | Slit homolog 3 protein | 528 | 1.39E-07 | 46 |
| Efet.01.19488.g1443.t1 | Suppressor of cytokine signaling 3 | 1194 | 1.02E-13 | 46 |
| Efet.01.425604.g609.t1 | Transcription factor Sox-2 | 762 | 4.28E-33 | 46 |
| Efet.01.91620.g976.t1 | Serine/threonine-protein kinase 3 | 618 | 6.75E-13 | 46 |
| Efet.01.21091.g1558.t1 | Tubulin alpha-3 chain | 585 | 4.13E-10 | 46 |
| Efet.01.113702.g694.t1 | Transcription intermediary factor 1-alpha | 444 | 5.22E-13 | 46 |
| Efet.01.96454.g1245.t1 | Transcription intermediary factor 1-beta | 978 | 4.07E-10 | 46 |
| Efet.01.126148.g63.t1 | Tumor necrosis factor alpha-induced protein 3 | 1950 | 9.10E-23 | 46 |
| Efet.01.153765.g199.t1 | Toll-like receptor Tollo | 2829 | 4.23E-28 | 46 |
| Efet.01.378160.g753.t1 | Utrophin | 1026 | 2.16E-06 | 46 |
| Efet.01.428470.g692.t1 | Vasopressin V1a receptor | 831 | 3.43E-19 | 46 |
| Efet.01.500434.g7.t1 | Vasopressin V1a receptor | 1344 | 3.41E-07 | 46 |
| Efet.01.624397.g1072.t1 | von Willebrand factor | 738 | 5.22E-09 | 46 |
| Efet.01.124977.g1291.t1 | WD repeat-containing protein 5 | 564 | 3.64E-18 | 46 |
| Efet.01.262309.g463.t1 | WD repeat-containing protein 5 | 741 | 2.99E-21 | 46 |
| Efet.01.1659451.g1745.t1 | Protein white | 1920 | 3.53E-07 | 46 |
| Efet.01.59384.g594.t1 | Protein white | 1350 | 1.25E-07 | 46 |
| Efet.01.649916.g1360.t1 | Protein white | 1197 | 3.82E-12 | 46 |
| Efet.01.642395.g681.t1 | ABC transporter ATP-binding protein/permease wht-1 | 822 | 3.20E-09 | 46 |
| Efet.01.652384.g287.t1 | ABC transporter ATP-binding protein/permease wht-1 | 939 | 9.77E-11 | 46 |
| Efet.01.502741.g67.t1 | Zinc finger X-chromosomal protein | 357 | 6.49E-09 | 46 |
| Efet.01.14110.g1041.t1 | Zinc finger protein 37A | 1524 | 1.09E-43 | 46 |
| Efet.01.109107.g475.t1 | Zinc finger protein 37A | 1182 | 4.50E-51 | 46 |
| Efet.01.618726.g843.t1 | Zinc finger and SCAN domain-containing protein 10 | 2463 | 5.52E-33 | 46 |
| Efet.01.507761.g201.t1 | 5-hydroxytryptamine receptor 1A | 1020 | 6.60E-40 | 45 |
| Efet.01.315581.g500.t1 | 5-hydroxytryptamine receptor 2C | 1119 | 1.05E-06 | 45 |
| Efet.01.621705.g951.t1 | ATP-binding cassette sub-family G member 2 | 621 | 1.29E-07 | 45 |
| Efet.01.590200.g999.t1 | ATP-binding cassette sub-family G member 2 | 1089 | 2.65E-06 | 45 |
| Efet.01.658279.g1787.t1 | Probable ATP-dependent permease | 1722 | 3.11E-13 | 45 |
| Efet.01.1659157.g1436.t1 | Probable ATP-dependent permease | 999 | 3.81E-12 | 45 |
| Efet.01.25091.g10.t1 | Beta-2 adrenergic receptor | 1107 | 2.62E-22 | 45 |
| Efet.01.138016.g673.t1 | Aldehyde dehydrogenase, dimeric NADP-preferring | 351 | 7.55E-06 | 45 |
| Efet.01.206818.g300.t1 | Atrial natriuretic peptide receptor 1 | 2250 | 1.05E-21 | 45 |
| Efet.01.633584.g200.t1 | Aurora kinase A | 438 | 1.82E-14 | 45 |
| Efet.01.109981.g511.t1 | Axin-1 | 1068 | 1.15E-17 | 45 |
| Efet.01.137589.g645.t1 | Cadherin-1 | 1821 | 4.95E-17 | 45 |
| Efet.01.226912.g1074.t1 | Cadherin-1 | 2109 | 1.62E-28 | 45 |
| Efet.01.257013.g264.t1 | Cadherin-2 | 654 | 5.30E-10 | 45 |
| Efet.01.607739.g323.t1 | Cholecystokinin receptor type A | 1236 | 6.15E-28 | 45 |
| Efet.01.121917.g1141.t1 | Cholecystokinin receptor type A | 1215 | 3.20E-08 | 45 |
| Efet.01.242277.g1616.t1 | Chromodomain Y-like protein | 489 | 1.80E-12 | 45 |
| Efet.01.642630.g708.t1 | Chromodomain Y-like protein | 435 | 4.95E-07 | 45 |
| Efet.01.657803.g1469.t1 | Chromodomain Y-like protein | 765 | 1.05E-07 | 45 |
| Efet.01.569313.g293.t1 | Cystic fibrosis transmembrane conductance regulator | 1974 | 7.71E-11 | 45 |
| Efet.01.628537.g1256.t1 | Cystic fibrosis transmembrane conductance regulator | 1542 | 2.90E-13 | 45 |
| Efet.01.658133.g1661.t1 | Cystic fibrosis transmembrane conductance regulator | 1590 | 8.42E-11 | 45 |
| Efet.01.658417.g2074.t1 | Cystic fibrosis transmembrane conductance regulator | 1557 | 1.42E-11 | 45 |
| Efet.01.96842.g1272.t1 | Carbohydrate sulfotransferase 3 | 1056 | 6.97E-10 | 45 |
| Efet.01.152511.g139.t1 | Contactin-1 | 399 | 2.11E-12 | 45 |
| Efet.01.103900.g217.t1 | Complement C3 | 504 | 8.36E-08 | 45 |
| Efet.01.129786.g253.t1 | Collagen alpha-1(VI) chain | 1254 | 2.11E-16 | 45 |
| Efet.01.475948.g671.t1 | Collagen alpha-1(VI) chain | 468 | 4.70E-07 | 45 |
| Efet.01.381141.g816.t1 | 25-hydroxyvitamin D-1 alpha hydroxylase, mitochondrial | 1563 | 7.48E-18 | 45 |
| Efet.01.96819.g1269.t1 | Chondroitin sulfate proteoglycan 4 | 2037 | 4.37E-07 | 45 |
| Efet.01.99238.g1403.t1 | Chondroitin sulfate proteoglycan 4 | 3444 | 9.99E-50 | 45 |
| Efet.01.159207.g480.t1 | Chondroitin sulfate proteoglycan 4 | 1716 | 1.56E-22 | 45 |
| Efet.01.88958.g832.t1 | C-X-C chemokine receptor type 3 | 1242 | 3.21E-23 | 45 |
| Efet.01.76098.g68.t1 | Corticosteroid 11-beta-dehydrogenase isozyme 1 | 600 | 1.35E-14 | 45 |
| Efet.01.544969.g360.t1 | Corticosteroid 11-beta-dehydrogenase isozyme 1 | 786 | 2.35E-07 | 45 |
| Efet.01.633916.g221.t1 | Corticosteroid 11-beta-dehydrogenase isozyme 1 | 1416 | 6.28E-10 | 45 |
| Efet.01.100653.g50.t1 | Dixin | 753 | 6.83E-16 | 45 |
| Efet.01.210314.g456.t1 | Deleted in malignant brain tumors 1 protein | 729 | 2.67E-18 | 45 |
| Efet.01.9970.g729.t1 | Dystrophin | 1137 | 1.70E-06 | 45 |
| Efet.01.414475.g338.t1 | Histone-lysine N-methyltransferase EHMT2 | 5916 | 8.23E-14 | 45 |
| Efet.01.653099.g398.t1 | Histone-lysine N-methyltransferase EHMT2 | 1113 | 1.05E-11 | 45 |
| Efet.01.658338.g1869.t1 | Histone-lysine N-methyltransferase EHMT2 | 1584 | 1.27E-18 | 45 |
| Efet.01.77290.g130.t1 | Protein eyes shut homolog | 516 | 8.66E-08 | 45 |
| Efet.01.204481.g194.t1 | Protein eyes shut homolog | 636 | 1.84E-14 | 45 |
| Efet.01.223295.g940.t1 | Protein flightless-1 homolog | 2361 | 1.69E-14 | 45 |
| Efet.01.561295.g24.t1 | Leucine-rich repeat transmembrane protein FLRT3 | 1518 | 3.36E-24 | 45 |
| Efet.01.475314.g659.t1 | Growth/differentiation factor 8 | 861 | 5.44E-24 | 45 |
| Efet.01.652659.g332.t1 | Glutamine--fructose-6-phosphate aminotransferase [isomerizing] 1 | 711 | 2.23E-32 | 45 |
| Efet.01.338426.g1122.t1 | Glutamate receptor 1 | 561 | 3.49E-09 | 45 |
| Efet.01.276381.g975.t1 | Glutamate receptor 2 | 957 | 5.61E-17 | 45 |
| Efet.01.107149.g369.t1 | Mitogen-activated protein kinase hog-1 | 609 | 3.54E-17 | 45 |
| Efet.01.300366.g11.t1 | Histamine H1 receptor | 1461 | 5.66E-47 | 45 |
| Efet.01.28233.g215.t1 | Heat shock 70 kDa protein 4 | 861 | 4.93E-09 | 45 |
| Efet.01.301100.g43.t1 | Integrin beta-4 | 492 | 3.48E-06 | 45 |
| Efet.01.327761.g853.t1 | Histone acetyltransferase KAT6A | 636 | 2.01E-07 | 45 |
| Efet.01.576160.g517.t1 | Potassium voltage-gated channel subfamily B member 1 | 480 | 4.93E-13 | 45 |
| Efet.01.171729.g1046.t1 | Potassium voltage-gated channel subfamily B member 1 | 444 | 6.54E-15 | 45 |
| Efet.01.93828.g1092.t1 | Lysine-specific histone demethylase 1A | 909 | 3.82E-07 | 45 |
| Efet.01.209094.g411.t1 | Protein kinase C theta type | 951 | 6.69E-11 | 45 |
| Efet.01.1657335.g972.t1 | Pyruvate kinase PKM | 498 | 2.73E-12 | 45 |
| Efet.01.657920.g1533.t1 | Laminin subunit alpha-1 | 786 | 9.97E-07 | 45 |
| Efet.01.562032.g38.t1 | Leucine-rich repeat-containing G-protein coupled receptor 5 | 2124 | 5.54E-22 | 45 |
| Efet.01.102244.g129.t1 | E3 ubiquitin-protein ligase TRIM71 | 2235 | 6.10E-20 | 45 |
| Efet.01.128237.g179.t1 | E3 ubiquitin-protein ligase TRIM71 | 2262 | 1.70E-68 | 45 |
| Efet.01.141154.g834.t1 | Microtubule-associated protein 1B | 654 | 1.81E-38 | 45 |
| Efet.01.325515.g784.t1 | Hepatocyte growth factor receptor | 729 | 3.61E-09 | 45 |
| Efet.01.359651.g262.t1 | [F-actin]-monooxygenase MICAL3 | 492 | 6.49E-09 | 45 |
| Efet.01.1659154.g1434.t1 | Canalicular multispecific organic anion transporter 1 | 1299 | 5.46E-21 | 45 |
| Efet.01.23383.g1694.t1 | Myosin-2 | 399 | 1.84E-07 | 45 |
| Efet.01.334473.g1029.t1 | Nuclear factor NF-kappa-B p105 subunit | 1371 | 1.21E-09 | 45 |
| Efet.01.48664.g1499.t1 | Neuromedin-U receptor 1 | 1356 | 8.84E-06 | 45 |
| Efet.01.179995.g1395.t1 | Neuromedin-U receptor 1 | 1014 | 2.30E-15 | 45 |
| Efet.01.542867.g322.t1 | Neuromedin-U receptor 1 | 1134 | 1.73E-24 | 45 |
| Efet.01.625114.g1096.t1 | Neuromedin-U receptor 1 | 1074 | 2.31E-19 | 45 |
| Efet.01.143622.g953.t1 | BDNF/NT-3 growth factors receptor | 411 | 3.85E-07 | 45 |
| Efet.01.432939.g798.t1 | Octopamine receptor beta-2R | 1230 | 3.24E-27 | 45 |
| Efet.01.44468.g1255.t1 | Protocadherin-15 | 1056 | 2.63E-18 | 45 |
| Efet.01.139446.g743.t1 | Protocadherin-15 | 2430 | 5.03E-32 | 45 |
| Efet.01.325968.g799.t1 | Protocadherin-15 | 2715 | 2.97E-35 | 45 |
| Efet.01.547603.g434.t1 | Protocadherin-15 | 1299 | 2.46E-18 | 45 |
| Efet.01.25068.g9.t1 | Protocadherin-15 | 2514 | 2.77E-47 | 45 |
| Efet.01.97049.g1284.t1 | Protocadherin-15 | 3108 | 4.98E-30 | 45 |
| Efet.01.190369.g1886.t1 | Protocadherin-15 | 771 | 1.41E-10 | 45 |
| Efet.01.438803.g920.t1 | Protocadherin-15 | 2622 | 1.78E-47 | 45 |
| Efet.01.460118.g284.t1 | Protocadherin-15 | 1491 | 5.80E-27 | 45 |
| Efet.01.205050.g217.t1 | Protocadherin-15 | 2787 | 2.86E-47 | 45 |
| Efet.01.399093.g1219.t1 | Protocadherin-15 | 2757 | 1.93E-44 | 45 |
| Efet.01.25304.g39.t1 | Protein phosphatase 1D | 903 | 1.44E-19 | 45 |
| Efet.01.236525.g1423.t1 | PR domain zinc finger protein 5 | 741 | 3.00E-09 | 45 |
| Efet.01.618860.g851.t1 | Receptor-type tyrosine-protein phosphatase S | 510 | 2.54E-09 | 45 |
| Efet.01.53795.g252.t1 | Receptor-type tyrosine-protein phosphatase U | 297 | 6.90E-07 | 45 |
| Efet.01.599555.g1297.t1 | Peroxidasin | 363 | 4.91E-10 | 45 |
| Efet.01.168697.g923.t1 | Peroxidasin | 1527 | 9.36E-19 | 45 |
| Efet.01.210609.g469.t1 | Peroxidasin | 1587 | 2.26E-26 | 45 |
| Efet.01.656795.g1101.t1 | CAD protein | 921 | 1.12E-26 | 45 |
| Efet.01.590200.g1003.t1 | CAD protein | 978 | 1.74E-09 | 45 |
| Efet.01.654987.g732.t1 | CAD protein | 1827 | 7.99E-07 | 45 |
| Efet.01.174585.g1156.t1 | Dexamethasone-induced Ras-related protein 1 | 684 | 5.64E-22 | 45 |
| Efet.01.35531.g703.t1 | RE1-silencing transcription factor | 1137 | 1.38E-12 | 45 |
| Efet.01.190280.g1881.t1 | Sodium channel protein type 1 subunit alpha | 354 | 1.45E-10 | 45 |
| Efet.01.288799.g1409.t1 | Sodium channel protein type 1 subunit alpha | 534 | 1.19E-25 | 45 |
| Efet.01.493319.g1030.t1 | Mothers against decapentaplegic homolog 2 | 621 | 7.90E-16 | 45 |
| Efet.01.1658253.g1140.t1 | Structural maintenance of chromosomes protein 1A | 831 | 1.20E-15 | 45 |
| Efet.01.657942.g1539.t1 | Cornifin-A | 705 | 9.74E-06 | 45 |
| Efet.01.100503.g37.t1 | Proto-oncogene tyrosine-protein kinase Src | 366 | 2.22E-09 | 45 |
| Efet.01.373950.g664.t1 | Serine/threonine-protein kinase STK11 | 483 | 7.67E-20 | 45 |
| Efet.01.641084.g607.t1 | Toll-like receptor Tollo | 1755 | 1.66E-24 | 45 |
| Efet.01.655974.g911.t1 | DNA topoisomerase 2-alpha | 2712 | 1.95E-17 | 45 |
| Efet.01.657669.g1360.t1 | Transcriptional repressor protein YY1 | 291 | 2.89E-07 | 45 |
| Efet.01.377309.g736.t1 | Serine/threonine-protein kinase ULK1 | 366 | 3.10E-07 | 45 |
| Efet.01.114400.g751.t1 | Vasopressin V1a receptor | 1431 | 4.50E-07 | 45 |
| Efet.01.135694.g531.t1 | Vasopressin V1a receptor | 936 | 3.13E-06 | 45 |
| Efet.01.249521.g1874.t1 | Vasopressin V1a receptor | 726 | 3.26E-09 | 45 |
| Efet.01.52249.g150.t1 | Vasopressin V1a receptor | 1251 | 7.74E-24 | 45 |
| Efet.01.46526.g1382.t1 | WD repeat-containing protein 5 | 675 | 9.80E-19 | 45 |
| Efet.01.411322.g257.t1 | WD repeat-containing protein 5 | 870 | 4.14E-19 | 45 |
| Efet.01.48933.g1519.t1 | Protein wech | 3255 | 2.36E-16 | 45 |
| Efet.01.493760.g1042.t1 | Zinc finger protein 143 | 1104 | 2.04E-09 | 45 |
| Efet.01.207039.g312.t1 | Zinc finger protein 219 | 2340 | 3.36E-10 | 45 |
| Efet.01.89035.g837.t1 | Zinc finger protein 37A | 861 | 2.19E-41 | 45 |
| Efet.01.197569.g2200.t1 | Zinc finger protein 37A | 1377 | 1.19E-23 | 45 |
| Efet.01.396319.g1152.t1 | Zinc finger protein 37A | 3084 | 1.15E-31 | 45 |
| Efet.01.587803.g914.t1 | Zinc finger protein 37A | 336 | 6.59E-09 | 45 |
| Efet.01.658304.g1822.t1 | Protein Z-dependent protease inhibitor | 1320 | 1.38E-21 | 45 |
| Efet.01.153611.g192.t1 | Protein Z-dependent protease inhibitor | 432 | 2.64E-06 | 45 |
| Efet.01.212159.g521.t1 | Zinc finger and SCAN domain-containing protein 10 | 1458 | 1.55E-51 | 45 |
| Efet.01.476997.g689.t1 | Zinc finger and SCAN domain-containing protein 10 | 5253 | 1.08E-64 | 45 |
| Efet.01.207325.g325.t1 | 5-hydroxytryptamine receptor 1A | 852 | 4.48E-26 | 44 |
| Efet.01.643394.g779.t1 | ATP-binding cassette transporter abc2 | 786 | 1.40E-09 | 44 |
| Efet.01.592582.g1079.t1 | ATP-binding cassette transporter abc3 | 3213 | 2.34E-07 | 44 |
| Efet.01.550084.g495.t1 | ATP-binding cassette sub-family G member 2 | 1317 | 4.79E-11 | 44 |
| Efet.01.657492.g1304.t1 | ATP-binding cassette sub-family G member 2 | 1572 | 1.15E-13 | 44 |
| Efet.01.658423.g2105.t1 | ATP-binding cassette sub-family G member 2 | 636 | 7.91E-10 | 44 |
| Efet.01.235118.g1387.t1 | Atypical chemokine receptor 3 | 804 | 5.80E-12 | 44 |
| Efet.01.92699.g1032.t1 | Disintegrin and metalloproteinase domain-containing protein 10 | 609 | 4.87E-17 | 44 |
| Efet.01.643497.g803.t1 | Alcohol dehydrogenase 1C | 1044 | 3.25E-09 | 44 |
| Efet.01.655193.g771.t1 | Probable ATP-dependent permease | 1095 | 5.06E-07 | 44 |
| Efet.01.241488.g1583.t1 | Aurora/IPL1-related protein kinase 2 | 471 | 1.01E-18 | 44 |
| Efet.01.656717.g1069.t1 | Aldehyde dehydrogenase, dimeric NADP-preferring | 1233 | 3.91E-32 | 44 |
| Efet.01.657855.g1508.t1 | Aldehyde dehydrogenase, dimeric NADP-preferring | 1491 | 7.77E-27 | 44 |
| Efet.01.658364.g1897.t1 | Aldehyde dehydrogenase, dimeric NADP-preferring | 885 | 4.01E-13 | 44 |
| Efet.01.95249.g1175.t1 | Atrial natriuretic peptide receptor 1 | 591 | 4.21E-16 | 44 |
| Efet.01.199457.g2282.t1 | Atrial natriuretic peptide receptor 1 | 639 | 2.53E-09 | 44 |
| Efet.01.115447.g799.t1 | Beta-2-glycoprotein 1 | 1989 | 7.53E-23 | 44 |
| Efet.01.502147.g51.t1 | Cadherin-1 | 486 | 2.12E-08 | 44 |
| Efet.01.522670.g529.t1 | Cadherin-2 | 1356 | 9.27E-14 | 44 |
| Efet.01.61623.g721.t1 | Cadherin-4 | 642 | 7.80E-13 | 44 |
| Efet.01.1658861.g1299.t1 | Carbonyl reductase [NADPH] 1 | 744 | 2.42E-13 | 44 |
| Efet.01.1658617.g1232.t1 | C-C chemokine receptor type 2 | 1089 | 1.92E-20 | 44 |
| Efet.01.115311.g794.t1 | C-C chemokine receptor type 2 | 1161 | 1.19E-08 | 44 |
| Efet.01.625120.g1098.t1 | C-C chemokine receptor type 2 | 1215 | 3.75E-17 | 44 |
| Efet.01.244226.g1676.t1 | Cyclin-dependent kinase 1 | 285 | 2.81E-11 | 44 |
| Efet.01.654418.g607.t1 | Chromodomain Y-like protein | 810 | 7.42E-07 | 44 |
| Efet.01.1659485.g1836.t1 | Chromodomain Y-like protein | 699 | 7.26E-14 | 44 |
| Efet.01.1659526.g2029.t1 | Chromodomain Y-like protein | 738 | 7.21E-11 | 44 |
| Efet.01.650311.g32.t1 | Cystic fibrosis transmembrane conductance regulator | 807 | 3.67E-13 | 44 |
| Efet.01.597702.g1220.t1 | Cystic fibrosis transmembrane conductance regulator | 1503 | 1.02E-07 | 44 |
| Efet.01.376597.g718.t1 | Carbohydrate sulfotransferase 3 | 1092 | 1.47E-14 | 44 |
| Efet.01.92460.g1013.t1 | Chondroitin sulfate proteoglycan 4 | 1698 | 4.63E-31 | 44 |
| Efet.01.353129.g82.t1 | Chondroitin sulfate proteoglycan 4 | 1902 | 1.54E-37 | 44 |
| Efet.01.50358.g30.t1 | C-X-C chemokine receptor type 2 | 954 | 4.63E-06 | 44 |
| Efet.01.157121.g373.t1 | C-X-C chemokine receptor type 2 | 840 | 2.69E-09 | 44 |
| Efet.01.188981.g1834.t1 | DNA damage-binding protein 1 | 1848 | 1.19E-13 | 44 |
| Efet.01.611807.g507.t1 | Corticosteroid 11-beta-dehydrogenase isozyme 1 | 1632 | 4.21E-12 | 44 |
| Efet.01.618401.g825.t1 | Corticosteroid 11-beta-dehydrogenase isozyme 1 | 1632 | 4.21E-12 | 44 |
| Efet.01.209668.g433.t1 | Neurogenic locus protein delta | 843 | 1.10E-20 | 44 |
| Efet.01.39045.g939.t1 | Dysferlin | 2361 | 8.03E-07 | 44 |
| Efet.01.37717.g839.t1 | Pro-epidermal growth factor | 1002 | 2.68E-06 | 44 |
| Efet.01.54879.g328.t1 | Glutamate receptor 1 | 495 | 8.06E-09 | 44 |
| Efet.01.652659.g331.t1 | Guanine nucleotide-binding protein-like 3 | 642 | 1.15E-08 | 44 |
| Efet.01.142449.g903.t1 | Glypican-1 | 828 | 5.70E-13 | 44 |
| Efet.01.120884.g1084.t1 | Histamine H2 receptor | 1053 | 4.03E-09 | 44 |
| Efet.01.586480.g883.t1 | Histamine H2 receptor | 603 | 8.00E-09 | 44 |
| Efet.01.75439.g24.t1 | Zinc finger and SCAN domain-containing protein 10 | 1437 | 1.65E-17 | 44 |
| Efet.01.153003.g165.t1 | NF-kappa-B inhibitor alpha | 1119 | 9.74E-06 | 44 |
| Efet.01.312578.g395.t1 | Potassium voltage-gated channel subfamily B member 1 | 1230 | 3.08E-35 | 44 |
| Efet.01.77132.g119.t1 | Laminin subunit alpha | 2364 | 3.57E-07 | 44 |
| Efet.01.278918.g1066.t1 | Leucine-rich repeat-containing G-protein coupled receptor 5 | 2349 | 8.28E-22 | 44 |
| Efet.01.49163.g1536.t1 | Protein lin-41 | 771 | 3.09E-06 | 44 |
| Efet.01.572116.g384.t1 | Protein lin-41 | 504 | 3.04E-06 | 44 |
| Efet.01.65437.g945.t1 | E3 ubiquitin-protein ligase TRIM71 | 912 | 7.85E-07 | 44 |
| Efet.01.467324.g465.t1 | E3 ubiquitin-protein ligase TRIM71 | 2040 | 3.22E-26 | 44 |
| Efet.01.96420.g1239.t1 | Prolow-density lipoprotein receptor-related protein 1 | 2133 | 6.07E-45 | 44 |
| Efet.01.6271.g494.t1 | Hepatocyte growth factor receptor | 972 | 2.10E-11 | 44 |
| Efet.01.333984.g1015.t1 | Hepatocyte growth factor receptor | 1080 | 3.32E-15 | 44 |
| Efet.01.308311.g253.t1 | Neural cell adhesion molecule 1 | 1395 | 2.46E-32 | 44 |
| Efet.01.129420.g244.t1 | Neurogenic locus notch homolog protein 1 | 969 | 2.33E-18 | 44 |
| Efet.01.145886.g1049.t1 | Neuropilin-2 | 594 | 2.15E-18 | 44 |
| Efet.01.620294.g890.t1 | P2Y purinoceptor 1 | 717 | 5.06E-08 | 44 |
| Efet.01.582019.g745.t1 | P2Y purinoceptor 2 | 1251 | 7.71E-13 | 44 |
| Efet.01.110423.g531.t1 | Palladin | 903 | 7.87E-15 | 44 |
| Efet.01.27587.g163.t1 | Protocadherin-15 | 2841 | 5.12E-46 | 44 |
| Efet.01.98873.g1379.t1 | Protocadherin-15 | 2775 | 5.42E-37 | 44 |
| Efet.01.100812.g57.t1 | Protocadherin-15 | 2376 | 7.23E-42 | 44 |
| Efet.01.236229.g1413.t1 | Protocadherin-15 | 2763 | 4.18E-45 | 44 |
| Efet.01.291362.g1491.t1 | Protocadherin-15 | 2781 | 2.72E-29 | 44 |
| Efet.01.225954.g1034.t1 | Protocadherin-15 | 2655 | 2.20E-40 | 44 |
| Efet.01.250796.g23.t1 | Protocadherin-15 | 2385 | 1.43E-44 | 44 |
| Efet.01.258659.g323.t1 | Protocadherin-15 | 2934 | 9.05E-42 | 44 |
| Efet.01.380911.g810.t1 | Protocadherin-15 | 2826 | 1.21E-41 | 44 |
| Efet.01.382878.g864.t1 | Protocadherin-15 | 1119 | 5.48E-10 | 44 |
| Efet.01.601319.g66.t1 | Protocadherin-15 | 2319 | 2.75E-44 | 44 |
| Efet.01.657446.g1285.t1 | Protocadherin-15 | 960 | 1.32E-14 | 44 |
| Efet.01.1653592.g628.t1 | Protein phosphatase 1D | 579 | 9.64E-17 | 44 |
| Efet.01.18206.g1346.t1 | PR domain zinc finger protein 14 | 1614 | 1.24E-07 | 44 |
| Efet.01.243796.g1662.t1 | PR domain zinc finger protein 5 | 1872 | 1.89E-23 | 44 |
| Efet.01.268108.g669.t1 | PR domain zinc finger protein 5 | 1227 | 1.21E-22 | 44 |
| Efet.01.350991.g33.t1 | PR domain zinc finger protein 5 | 1482 | 3.54E-16 | 44 |
| Efet.01.396570.g1158.t1 | PR domain zinc finger protein 5 | 1056 | 2.06E-26 | 44 |
| Efet.01.658420.g2097.t1 | Receptor-type tyrosine-protein phosphatase S | 2364 | 4.38E-54 | 44 |
| Efet.01.148859.g1176.t1 | Peroxidasin homolog | 606 | 2.33E-13 | 44 |
| Efet.01.151826.g109.t1 | Peroxidasin homolog | 555 | 3.03E-13 | 44 |
| Efet.01.220462.g826.t1 | Peroxidasin homolog | 1875 | 1.04E-16 | 44 |
| Efet.01.28462.g236.t1 | Peroxidasin | 1725 | 3.89E-26 | 44 |
| Efet.01.187627.g1767.t1 | Peroxidasin | 312 | 2.38E-06 | 44 |
| Efet.01.2840.g256.t1 | Protein phosphatase 1 regulatory subunit | 1053 | 1.13E-15 | 44 |
| Efet.01.123351.g1211.t1 | X-linked zinc finger protein | 1989 | 1.08E-09 | 44 |
| Efet.01.570920.g348.t1 | Reticulon-4 receptor-like 1 | 1305 | 7.08E-22 | 44 |
| Efet.01.19626.g1455.t1 | Ribonuclease inhibitor | 2103 | 2.49E-16 | 44 |
| Efet.01.403654.g95.t1 | Retinal homeobox protein Rx3 | 627 | 1.89E-21 | 44 |
| Efet.01.201591.g77.t1 | Retinoic acid receptor RXR-alpha | 528 | 1.28E-14 | 44 |
| Efet.01.300516.g21.t1 | Slit homolog 1 protein | 1833 | 1.43E-16 | 44 |
| Efet.01.65218.g925.t1 | Slit homolog 3 protein | 1494 | 5.95E-20 | 44 |
| Efet.01.117731.g917.t1 | Slit homolog 3 protein | 1755 | 7.49E-23 | 44 |
| Efet.01.643064.g754.t1 | DNA topoisomerase 2-alpha | 1623 | 5.73E-13 | 44 |
| Efet.01.362172.g326.t1 | Dual specificity protein kinase Ttk | 483 | 1.65E-10 | 44 |
| Efet.01.70563.g1266.t1 | Vasopressin V1a receptor | 1053 | 1.43E-06 | 44 |
| Efet.01.50040.g4.t1 | Vasopressin V1a receptor | 882 | 4.50E-11 | 44 |
| Efet.01.99411.g1414.t1 | Vascular endothelial growth factor receptor 1 | 480 | 7.78E-13 | 44 |
| Efet.01.209929.g443.t1 | von Willebrand factor | 687 | 1.55E-06 | 44 |
| Efet.01.580979.g699.t1 | WD repeat-containing protein 5 | 945 | 8.34E-13 | 44 |
| Efet.01.275883.g957.t1 | Protein white | 2466 | 7.40E-08 | 44 |
| Efet.01.1658657.g1240.t1 | Protein white | 822 | 1.75E-06 | 44 |
| Efet.01.432304.g781.t1 | Transcriptional coactivator YAP1 | 471 | 1.33E-07 | 44 |
| Efet.01.35531.g704.t1 | Zinc finger X-chromosomal protein | 1062 | 2.57E-10 | 44 |
| Efet.01.82778.g508.t1 | Zinc finger protein 37A | 543 | 2.79E-09 | 44 |
| Efet.01.247061.g1778.t1 | Zinc finger protein 37A | 1110 | 1.99E-29 | 44 |
| Efet.01.516989.g421.t1 | Zinc finger protein 37A | 1548 | 8.74E-46 | 44 |
| Efet.01.46938.g1410.t1 | 5-hydroxytryptamine receptor 1A | 1401 | 7.07E-24 | 43 |
| Efet.01.316608.g524.t1 | 5-hydroxytryptamine receptor 2C | 726 | 4.72E-15 | 43 |
| Efet.01.595491.g1158.t1 | ATP-binding cassette transporter abc3 | 957 | 2.18E-08 | 43 |
| Efet.01.1659515.g1948.t1 | ATP-binding cassette transporter abc3 | 1497 | 4.54E-16 | 43 |
| Efet.01.40709.g1047.t1 | ATP-binding cassette sub-family G member 2 | 1713 | 7.28E-08 | 43 |
| Efet.01.83209.g537.t1 | Alcohol dehydrogenase 1C | 1071 | 6.31E-25 | 43 |
| Efet.01.156506.g340.t1 | Beta-2 adrenergic receptor | 939 | 2.31E-07 | 43 |
| Efet.01.610340.g437.t1 | Aldehyde dehydrogenase, dimeric NADP-preferring | 1029 | 9.11E-14 | 43 |
| Efet.01.646963.g1137.t1 | Aldehyde dehydrogenase, dimeric NADP-preferring | 1524 | 2.15E-20 | 43 |
| Efet.01.657077.g1159.t1 | Aldehyde dehydrogenase, dimeric NADP-preferring | 1398 | 1.01E-29 | 43 |
| Efet.01.3484.g302.t1 | Apoptotic protease-activating factor 1 | 720 | 4.93E-07 | 43 |
| Efet.01.147059.g1102.t1 | Bone morphogenetic protein 7 | 873 | 3.92E-22 | 43 |
| Efet.01.341071.g1189.t1 | Cadherin-1 | 792 | 1.64E-06 | 43 |
| Efet.01.70778.g1282.t1 | Cadherin-2 | 1809 | 1.77E-19 | 43 |
| Efet.01.430165.g738.t1 | Cadherin-3 | 687 | 1.64E-11 | 43 |
| Efet.01.5699.g445.t1 | Carbohydrate sulfotransferase 3 | 957 | 2.43E-08 | 43 |
| Efet.01.363997.g379.t1 | Cytochrome P450 3A2 | 1584 | 3.50E-14 | 43 |
| Efet.01.312008.g380.t1 | Cholesterol 7-alpha-monooxygenase | 828 | 4.34E-11 | 43 |
| Efet.01.359090.g251.t1 | C-X-C chemokine receptor type 1 | 795 | 1.13E-12 | 43 |
| Efet.01.416118.g380.t1 | C-X-C chemokine receptor type 2 | 1089 | 2.58E-27 | 43 |
| Efet.01.531003.g33.t1 | C-X-C chemokine receptor type 2 | 2871 | 3.34E-15 | 43 |
| Efet.01.159648.g505.t1 | Dystrophin | 3519 | 6.67E-16 | 43 |
| Efet.01.91237.g955.t1 | Dystrophin | 1614 | 2.28E-07 | 43 |
| Efet.01.880.g82.t1 | Histone-lysine N-methyltransferase EHMT2 | 1473 | 5.89E-15 | 43 |
| Efet.01.109200.g480.t1 | Histone-lysine N-methyltransferase EHMT2 | 1128 | 7.42E-17 | 43 |
| Efet.01.218940.g764.t1 | Histone-lysine N-methyltransferase EHMT2 | 1131 | 2.96E-17 | 43 |
| Efet.01.225893.g1032.t1 | Gamma-aminobutyric acid receptor subunit beta-3 | 594 | 3.72E-09 | 43 |
| Efet.01.471393.g562.t1 | Eukaryotic initiation factor 4A-I | 1203 | 1.41E-23 | 43 |
| Efet.01.104466.g249.t1 | Immunoglobulin superfamily member 10 | 1236 | 3.64E-20 | 43 |
| Efet.01.289119.g1423.t1 | Insulin gene enhancer protein ISL-1 | 702 | 3.44E-07 | 43 |
| Efet.01.85725.g677.t1 | Potassium channel subfamily K member 3 | 729 | 8.03E-11 | 43 |
| Efet.01.590049.g991.t1 | Low-density lipoprotein receptor | 690 | 1.54E-07 | 43 |
| Efet.01.348325.g1367.t1 | Leucine-rich repeat-containing G-protein coupled receptor 5 | 633 | 7.05E-06 | 43 |
| Efet.01.80735.g387.t1 | E3 ubiquitin-protein ligase TRIM71 | 843 | 1.10E-06 | 43 |
| Efet.01.238548.g1495.t1 | E3 ubiquitin-protein ligase TRIM71 | 879 | 5.95E-06 | 43 |
| Efet.01.219590.g795.t1 | E3 ubiquitin-protein ligase TRIM71 | 1428 | 2.22E-25 | 43 |
| Efet.01.559811.g748.t1 | Hepatocyte growth factor receptor | 1110 | 1.91E-12 | 43 |
| Efet.01.536169.g187.t1 | Stromelysin-1 | 531 | 2.23E-20 | 43 |
| Efet.01.618025.g795.t1 | Canalicular multispecific organic anion transporter 1 | 2514 | 1.00E-06 | 43 |
| Efet.01.658236.g1732.t1 | Canalicular multispecific organic anion transporter 1 | 885 | 4.43E-10 | 43 |
| Efet.01.566222.g208.t1 | Canalicular multispecific organic anion transporter 2 | 1551 | 2.42E-11 | 43 |
| Efet.01.224347.g974.t1 | mRNA transport homolog 4 | 1446 | 3.17E-16 | 43 |
| Efet.01.1335.g123.t1 | Neural cell adhesion molecule 1 | 504 | 4.46E-07 | 43 |
| Efet.01.361767.g315.t1 | Bifunctional heparan sulfate N-deacetylase/N-sulfotransferase 1 | 606 | 8.74E-15 | 43 |
| Efet.01.231113.g1229.t1 | Neuromedin-U receptor 1 | 1293 | 9.44E-11 | 43 |
| Efet.01.10360.g758.t1 | Palladin | 591 | 8.44E-14 | 43 |
| Efet.01.28472.g238.t1 | Protocadherin-15 | 2676 | 9.27E-45 | 43 |
| Efet.01.108486.g445.t1 | Protocadherin-15 | 2499 | 4.47E-26 | 43 |
| Efet.01.125233.g13.t1 | Protocadherin-15 | 2313 | 7.97E-31 | 43 |
| Efet.01.128550.g203.t1 | Protocadherin-15 | 2661 | 8.44E-47 | 43 |
| Efet.01.118189.g940.t1 | Protocadherin-15 | 2454 | 9.15E-38 | 43 |
| Efet.01.622543.g982.t1 | Protocadherin-15 | 2853 | 3.94E-32 | 43 |
| Efet.01.44468.g1254.t1 | Protocadherin-15 | 1476 | 3.33E-19 | 43 |
| Efet.01.52387.g164.t1 | Protocadherin-15 | 1359 | 1.17E-07 | 43 |
| Efet.01.215469.g645.t1 | Protocadherin-15 | 1803 | 3.14E-41 | 43 |
| Efet.01.379695.g787.t1 | Protocadherin-15 | 3024 | 4.29E-38 | 43 |
| Efet.01.630441.g25.t1 | Protocadherin-15 | 768 | 1.30E-11 | 43 |
| Efet.01.424158.g572.t1 | PR domain zinc finger protein 5 | 2373 | 5.75E-24 | 43 |
| Efet.01.252963.g110.t1 | Receptor-type tyrosine-protein phosphatase S | 468 | 1.01E-10 | 43 |
| Efet.01.129282.g238.t1 | Peroxidasin homolog | 2220 | 1.11E-16 | 43 |
| Efet.01.544502.g350.t1 | Peroxidasin | 2103 | 8.55E-19 | 43 |
| Efet.01.605426.g218.t1 | Peroxidasin | 1383 | 2.38E-28 | 43 |
| Efet.01.654851.g709.t1 | CAD protein | 573 | 2.24E-14 | 43 |
| Efet.01.267648.g649.t1 | Regucalcin | 900 | 8.83E-35 | 43 |
| Efet.01.296729.g1645.t1 | Ribonucleoside-diphosphate reductase large chain | 2175 | 1.28E-38 | 43 |
| Efet.01.295038.g1597.t1 | Roundabout homolog 2 | 486 | 2.37E-13 | 43 |
| Efet.01.36804.g778.t1 | Slit homolog 2 protein | 2247 | 4.69E-08 | 43 |
| Efet.01.36323.g756.t1 | Slit homolog 3 protein | 4065 | 5.13E-16 | 43 |
| Efet.01.40026.g992.t1 | Slit homolog 3 protein | 1263 | 1.89E-11 | 43 |
| Efet.01.311103.g353.t1 | Slit homolog 3 protein | 948 | 1.27E-26 | 43 |
| Efet.01.281521.g1142.t1 | Solute carrier organic anion transporter family member 1A2 | 600 | 3.57E-15 | 43 |
| Efet.01.187902.g1779.t1 | Solute carrier organic anion transporter family member 1A5 | 837 | 5.73E-08 | 43 |
| Efet.01.65835.g974.t1 | Transcription intermediary factor 1-beta | 1485 | 1.25E-09 | 43 |
| Efet.01.324488.g754.t1 | Toll-like receptor 4 | 2010 | 3.35E-25 | 43 |
| Efet.01.191636.g1942.t1 | Toll-like receptor Tollo | 2418 | 1.02E-23 | 43 |
| Efet.01.642062.g651.t1 | DNA topoisomerase 2-alpha | 1017 | 5.72E-10 | 43 |
| Efet.01.610558.g443.t1 | E3 ubiquitin-protein ligase TRIM32 | 801 | 7.12E-08 | 43 |
| Efet.01.333148.g993.t1 | Vasopressin V1a receptor | 1020 | 1.57E-06 | 43 |
| Efet.01.233410.g1316.t1 | Vascular endothelial growth factor receptor 1 | 510 | 1.38E-10 | 43 |
| Efet.01.246000.g1732.t1 | von Willebrand factor | 450 | 4.51E-12 | 43 |
| Efet.01.633916.g222.t1 | Protein white | 1467 | 1.61E-08 | 43 |
| Efet.01.1658249.g1139.t1 | Protein white | 633 | 7.60E-06 | 43 |
| Efet.01.658412.g2029.t1 | Protein white | 1908 | 4.25E-11 | 43 |
| Efet.01.575546.g499.t1 | ABC transporter ATP-binding protein/permease wht-1 | 807 | 6.85E-09 | 43 |
| Efet.01.643099.g762.t1 | ABC transporter ATP-binding protein/permease wht-1 | 1353 | 3.07E-06 | 43 |
| Efet.01.265946.g584.t1 | Zinc finger protein 37A | 1857 | 3.92E-30 | 43 |
| Efet.01.658403.g1995.t1 | Zinc finger and SCAN domain-containing protein 10 | 1929 | 8.37E-28 | 43 |
| Efet.01.133008.g400.t1 | APOBEC1 complementation factor | 738 | 3.30E-09 | 42 |
| Efet.01.578540.g640.t1 | ATP-binding cassette sub-family G member 2 | 927 | 1.91E-07 | 42 |
| Efet.01.601193.g63.t1 | ATP-binding cassette sub-family G member 2 | 498 | 1.27E-11 | 42 |
| Efet.01.57472.g466.t1 | Disintegrin and metalloproteinase domain-containing protein 10 | 828 | 1.72E-08 | 42 |
| Efet.01.13349.g998.t1 | Beta-2 adrenergic receptor | 831 | 2.62E-07 | 42 |
| Efet.01.563201.g79.t1 | Aldehyde dehydrogenase, dimeric NADP-preferring | 1536 | 1.35E-18 | 42 |
| Efet.01.630860.g79.t1 | Aldehyde dehydrogenase, dimeric NADP-preferring | 1479 | 9.77E-26 | 42 |
| Efet.01.515933.g395.t1 | Aldehyde dehydrogenase, dimeric NADP-preferring | 915 | 2.43E-26 | 42 |
| Efet.01.211042.g487.t1 | Atrial natriuretic peptide receptor 1 | 453 | 7.34E-07 | 42 |
| Efet.01.108288.g432.t1 | NF-kappa-B inhibitor cactus | 828 | 4.81E-08 | 42 |
| Efet.01.552029.g552.t1 | Carboxypeptidase B2 | 516 | 9.07E-09 | 42 |
| Efet.01.406639.g165.t1 | Cholecystokinin receptor type A | 639 | 1.85E-06 | 42 |
| Efet.01.412888.g293.t1 | C-C chemokine receptor type 2 | 1032 | 9.10E-07 | 42 |
| Efet.01.646963.g1136.t1 | Cystic fibrosis transmembrane conductance regulator | 1452 | 6.51E-09 | 42 |
| Efet.01.100279.g16.t1 | C-X-C chemokine receptor type 1 | 702 | 2.77E-08 | 42 |
| Efet.01.179398.g1358.t1 | Dystroglycan | 1854 | 9.08E-27 | 42 |
| Efet.01.274962.g921.t1 | Epithelial discoidin domain-containing receptor 1 | 1284 | 3.70E-09 | 42 |
| Efet.01.23708.g1721.t1 | Fibrinogen alpha chain | 450 | 6.01E-10 | 42 |
| Efet.01.589191.g966.t1 | Protein flightless-1 homolog | 1821 | 5.01E-18 | 42 |
| Efet.01.546787.g409.t1 | Germinal center kinase 1 | 1023 | 8.18E-16 | 42 |
| Efet.01.425111.g596.t1 | Bifunctional UDP-N-acetylglucosamine 2-epimerase/N-acetylmannosamine kinase | 630 | 1.28E-11 | 42 |
| Efet.01.9480.g700.t1 | Glutamate receptor 3 | 936 | 2.55E-12 | 42 |
| Efet.01.296052.g1614.t1 | Zinc finger and SCAN domain-containing protein 10 | 1704 | 1.44E-21 | 42 |
| Efet.01.1658990.g1350.t1 | Zinc finger and SCAN domain-containing protein 10 | 1203 | 9.55E-10 | 42 |
| Efet.01.450560.g19.t1 | Potassium voltage-gated channel subfamily B member 1 | 591 | 1.34E-12 | 42 |
| Efet.01.20556.g1513.t1 | Protein kinase C theta type | 618 | 1.30E-13 | 42 |
| Efet.01.27795.g181.t1 | Neural cell adhesion molecule L1 | 1842 | 8.07E-19 | 42 |
| Efet.01.34812.g674.t1 | Neural cell adhesion molecule L1 | 1362 | 4.47E-09 | 42 |
| Efet.01.83613.g559.t1 | Laminin subunit alpha-1 | 717 | 2.05E-06 | 42 |
| Efet.01.658294.g1808.t1 | Leucine-rich repeat-containing G-protein coupled receptor 5 | 1317 | 1.07E-10 | 42 |
| Efet.01.158998.g470.t1 | E3 ubiquitin-protein ligase TRIM71 | 630 | 9.00E-06 | 42 |
| Efet.01.549594.g483.t1 | Hepatocyte growth factor receptor | 978 | 7.25E-18 | 42 |
| Efet.01.591235.g1030.t1 | Canalicular multispecific organic anion transporter 1 | 957 | 3.30E-09 | 42 |
| Efet.01.236986.g1440.t1 | Neuromedin-U receptor 1 | 1209 | 1.45E-11 | 42 |
| Efet.01.305267.g178.t1 | Neuromedin-U receptor 1 | 1170 | 1.42E-07 | 42 |
| Efet.01.38692.g915.t1 | Neurogenic locus notch homolog protein 2 | 1209 | 5.28E-11 | 42 |
| Efet.01.126150.g64.t1 | Rhodopsin | 1272 | 7.68E-07 | 42 |
| Efet.01.24505.g1778.t1 | Palladin | 7593 | 1.13E-22 | 42 |
| Efet.01.56668.g415.t1 | Palladin | 1059 | 1.98E-25 | 42 |
| Efet.01.90335.g920.t1 | Palladin | 546 | 3.05E-29 | 42 |
| Efet.01.7902.g583.t1 | Protocadherin-15 | 2742 | 4.08E-39 | 42 |
| Efet.01.20638.g1521.t1 | Protocadherin-15 | 2664 | 2.93E-40 | 42 |
| Efet.01.42470.g1140.t1 | Protocadherin-15 | 2637 | 3.39E-35 | 42 |
| Efet.01.44676.g1278.t1 | Protocadherin-15 | 2436 | 8.63E-31 | 42 |
| Efet.01.290988.g1485.t1 | Protocadherin-15 | 2826 | 1.13E-41 | 42 |
| Efet.01.76292.g81.t1 | Protocadherin-15 | 2586 | 2.29E-37 | 42 |
| Efet.01.94242.g1116.t1 | Protocadherin-15 | 1368 | 3.43E-27 | 42 |
| Efet.01.97666.g1320.t1 | Protocadherin-15 | 2130 | 2.08E-25 | 42 |
| Efet.01.142004.g881.t1 | Protocadherin-15 | 2124 | 3.24E-28 | 42 |
| Efet.01.654678.g682.t1 | Protocadherin-15 | 1332 | 4.97E-17 | 42 |
| Efet.01.508265.g216.t1 | Protocadherin-15 | 930 | 3.57E-12 | 42 |
| Efet.01.420818.g497.t1 | PR domain zinc finger protein 16 | 333 | 5.43E-06 | 42 |
| Efet.01.140965.g822.t1 | PR domain zinc finger protein 5 | 1749 | 1.23E-38 | 42 |
| Efet.01.209218.g416.t1 | PR domain zinc finger protein 5 | 1386 | 1.07E-18 | 42 |
| Efet.01.248735.g1846.t1 | PR domain zinc finger protein 5 | 1815 | 3.09E-11 | 42 |
| Efet.01.176278.g1222.t1 | Receptor-type tyrosine-protein phosphatase S | 1401 | 3.93E-10 | 42 |
| Efet.01.120355.g1059.t1 | Peroxidasin homolog | 543 | 1.63E-22 | 42 |
| Efet.01.154796.g249.t1 | Peroxidasin homolog | 1716 | 1.76E-17 | 42 |
| Efet.01.355783.g163.t1 | Peroxidasin | 1935 | 2.39E-14 | 42 |
| Efet.01.17048.g1257.t1 | X-linked zinc finger protein | 621 | 1.13E-07 | 42 |
| Efet.01.22442.g1631.t1 | Reticulon-4 receptor-like 1 | 1362 | 8.27E-26 | 42 |
| Efet.01.57085.g440.t1 | RE1-silencing transcription factor | 1854 | 3.51E-07 | 42 |
| Efet.01.230142.g1203.t1 | RE1-silencing transcription factor | 1227 | 1.08E-06 | 42 |
| Efet.01.642390.g678.t1 | Ribonucleoside-diphosphate reductase large subunit | 2328 | 2.89E-29 | 42 |
| Efet.01.28426.g235.t1 | Retinoid isomerohydrolase | 1464 | 3.04E-29 | 42 |
| Efet.01.18433.g1367.t1 | Slit homolog 2 protein | 2436 | 2.52E-26 | 42 |
| Efet.01.74277.g1504.t1 | Spectrin beta chain, non-erythrocytic 1 | 495 | 9.39E-22 | 42 |
| Efet.01.274975.g922.t1 | Tyrosine protein-kinase src-1 | 2925 | 6.23E-16 | 42 |
| Efet.01.262461.g472.t1 | Transcription intermediary factor 1-alpha | 984 | 2.89E-11 | 42 |
| Efet.01.399789.g1240.t1 | Transcription intermediary factor 1-alpha | 1182 | 1.21E-11 | 42 |
| Efet.01.503172.g77.t1 | Transcription intermediary factor 1-alpha | 1143 | 6.75E-16 | 42 |
| Efet.01.206399.g280.t1 | Transcription intermediary factor 1-beta | 1296 | 7.90E-15 | 42 |
| Efet.01.656585.g1048.t1 | Transcription intermediary factor 1-beta | 633 | 3.00E-07 | 42 |
| Efet.01.657803.g1473.t1 | DNA topoisomerase 2-alpha | 1986 | 1.92E-28 | 42 |
| Efet.01.69046.g1174.t1 | E3 ubiquitin-protein ligase TRIM32 | 1479 | 2.31E-08 | 42 |
| Efet.01.67405.g1073.t1 | UDP-glucuronosyltransferase 1-8 | 1197 | 6.90E-16 | 42 |
| Efet.01.381231.g818.t1 | Vasopressin V1a receptor | 1047 | 1.68E-13 | 42 |
| Efet.01.18112.g1338.t1 | Protein vav-1 | 393 | 1.51E-07 | 42 |
| Efet.01.377271.g735.t1 | von Willebrand factor | 777 | 5.84E-11 | 42 |
| Efet.01.506406.g165.t1 | Protein wech | 1083 | 1.01E-07 | 42 |
| Efet.01.131003.g318.t1 | Zinc finger protein 281 | 1269 | 7.47E-06 | 42 |
| Efet.01.291324.g1489.t1 | Zinc finger protein 37A | 858 | 4.98E-21 | 42 |
| Efet.01.206470.g282.t1 | Zinc finger and SCAN domain-containing protein 10 | 2022 | 7.14E-52 | 42 |
| Efet.01.130073.g273.t1 | 5-hydroxytryptamine receptor 1A | 1290 | 7.97E-27 | 41 |
| Efet.01.614177.g632.t1 | ATP-binding cassette transporter abc3 | 909 | 8.65E-10 | 41 |
| Efet.01.633190.g184.t1 | Aldehyde dehydrogenase, dimeric NADP-preferring | 768 | 6.73E-09 | 41 |
| Efet.01.48712.g1501.t1 | Apoptotic protease-activating factor 1 | 978 | 2.12E-07 | 41 |
| Efet.01.322182.g692.t1 | Cadherin-4 | 1386 | 2.03E-14 | 41 |
| Efet.01.562540.g52.t1 | Carboxypeptidase Q | 1440 | 2.49E-07 | 41 |
| Efet.01.275134.g933.t1 | Carbonyl reductase [NADPH] 1 | 843 | 4.70E-08 | 41 |
| Efet.01.378778.g769.t1 | Carbonyl reductase [NADPH] 1 | 801 | 2.62E-08 | 41 |
| Efet.01.8040.g594.t1 | Cholecystokinin receptor type A | 885 | 2.88E-12 | 41 |
| Efet.01.71264.g1315.t1 | Cholecystokinin receptor type A | 855 | 7.07E-12 | 41 |
| Efet.01.84875.g619.t1 | Contactin-1 | 804 | 1.18E-12 | 41 |
| Efet.01.12240.g914.t1 | Chondroitin sulfate proteoglycan 4 | 2616 | 6.87E-29 | 41 |
| Efet.01.15840.g1168.t1 | Chondroitin sulfate proteoglycan 4 | 1557 | 1.08E-15 | 41 |
| Efet.01.402538.g66.t1 | Chondroitin sulfate proteoglycan 4 | 1911 | 5.44E-29 | 41 |
| Efet.01.640707.g584.t1 | Deleted in malignant brain tumors 1 protein | 1218 | 1.58E-11 | 41 |
| Efet.01.152705.g146.t1 | Dysferlin | 822 | 7.95E-24 | 41 |
| Efet.01.161827.g612.t1 | Histone-lysine N-methyltransferase EHMT2 | 1734 | 1.70E-15 | 41 |
| Efet.01.102935.g167.t1 | Fibroblast growth factor receptor 2 | 273 | 2.40E-12 | 41 |
| Efet.01.205237.g225.t1 | E3 ISG15--protein ligase HERC5 | 1548 | 3.87E-14 | 41 |
| Efet.01.460071.g281.t1 | Histamine H1 receptor | 1017 | 3.92E-20 | 41 |
| Efet.01.305275.g179.t1 | Histamine H2 receptor | 1401 | 2.77E-08 | 41 |
| Efet.01.1659248.g1488.t1 | ATP-dependent RNA helicase eIF4A | 1254 | 3.66E-06 | 41 |
| Efet.01.1658788.g1276.t1 | Integrin-linked protein kinase homolog pat-4 | 621 | 6.29E-09 | 41 |
| Efet.01.29207.g290.t1 | Tyrosine-protein kinase JAK2 | 822 | 2.13E-16 | 41 |
| Efet.01.93957.g1098.t1 | Potassium voltage-gated channel subfamily B member 1 | 351 | 2.89E-10 | 41 |
| Efet.01.284049.g1237.t1 | Potassium voltage-gated channel subfamily B member 1 | 336 | 3.24E-06 | 41 |
| Efet.01.531039.g36.t1 | Potassium channel subfamily K member 3 | 936 | 3.49E-07 | 41 |
| Efet.01.17644.g1300.t1 | Potassium channel subfamily K member 3 | 744 | 3.48E-13 | 41 |
| Efet.01.454754.g113.t1 | Leucine-rich repeat-containing G-protein coupled receptor 5 | 654 | 5.10E-06 | 41 |
| Efet.01.657112.g1172.t1 | Leucine-rich repeat-containing G-protein coupled receptor 5 | 2181 | 7.11E-12 | 41 |
| Efet.01.483441.g820.t1 | Leucine-rich repeat-containing G-protein coupled receptor 5 | 2571 | 7.44E-16 | 41 |
| Efet.01.262143.g455.t1 | E3 ubiquitin-protein ligase TRIM71 | 1473 | 1.47E-18 | 41 |
| Efet.01.366963.g459.t1 | E3 ubiquitin-protein ligase TRIM71 | 843 | 2.76E-07 | 41 |
| Efet.01.415242.g350.t1 | E3 ubiquitin-protein ligase TRIM71 | 1350 | 1.26E-07 | 41 |
| Efet.01.575699.g504.t1 | E3 ubiquitin-protein ligase TRIM71 | 2358 | 1.15E-37 | 41 |
| Efet.01.18764.g1392.t1 | E3 ubiquitin-protein ligase Mdm2 | 885 | 2.16E-23 | 41 |
| Efet.01.604243.g163.t1 | Canalicular multispecific organic anion transporter 2 | 798 | 4.42E-10 | 41 |
| Efet.01.500190.g5.t1 | Neurogenic locus notch homolog protein 3 | 1152 | 9.92E-15 | 41 |
| Efet.01.285095.g1272.t1 | Rhodopsin | 981 | 1.94E-10 | 41 |
| Efet.01.77165.g123.t1 | Palladin | 999 | 1.49E-27 | 41 |
| Efet.01.96391.g1237.t1 | Protocadherin-15 | 2787 | 1.19E-17 | 41 |
| Efet.01.315253.g478.t1 | Protocadherin-15 | 1551 | 4.24E-11 | 41 |
| Efet.01.364737.g397.t1 | Protocadherin-15 | 2640 | 8.04E-39 | 41 |
| Efet.01.448174.g1178.t1 | Protocadherin-15 | 1296 | 2.78E-18 | 41 |
| Efet.01.508554.g221.t1 | Protocadherin-15 | 2691 | 7.32E-26 | 41 |
| Efet.01.170966.g1011.t1 | Protocadherin-15 | 2787 | 3.03E-44 | 41 |
| Efet.01.140014.g770.t1 | Protocadherin-15 | 1686 | 1.56E-18 | 41 |
| Efet.01.100938.g68.t1 | Mitogen-activated protein kinase pmk-1 | 438 | 6.47E-09 | 41 |
| Efet.01.20598.g1515.t1 | PR domain zinc finger protein 16 | 1698 | 1.99E-06 | 41 |
| Efet.01.325542.g785.t1 | PR domain zinc finger protein 16 | 1023 | 4.37E-10 | 41 |
| Efet.01.23579.g1708.t1 | PR domain zinc finger protein 5 | 1242 | 2.85E-24 | 41 |
| Efet.01.476997.g688.t1 | PR domain zinc finger protein 5 | 1059 | 1.83E-40 | 41 |
| Efet.01.69186.g1181.t1 | Prominin-1-A | 2430 | 7.70E-23 | 41 |
| Efet.01.428633.g698.t1 | Receptor-type tyrosine-protein phosphatase S | 576 | 2.28E-11 | 41 |
| Efet.01.658030.g1567.t1 | X-linked zinc finger protein | 1185 | 1.37E-06 | 41 |
| Efet.01.374889.g681.t1 | Reticulon-4 receptor | 1161 | 1.46E-15 | 41 |
| Efet.01.91605.g974.t1 | Prosaposin | 729 | 1.70E-11 | 41 |
| Efet.01.118933.g982.t1 | S-phase kinase-associated protein 2 | 1527 | 3.99E-14 | 41 |
| Efet.01.127962.g166.t1 | Slit homolog 1 protein | 1665 | 7.25E-13 | 41 |
| Efet.01.211583.g506.t1 | Slit homolog 2 protein | 1470 | 3.50E-14 | 41 |
| Efet.01.184432.g1608.t1 | Slit homolog 2 protein | 807 | 2.44E-09 | 41 |
| Efet.01.18361.g1360.t1 | Slit homolog 3 protein | 1068 | 8.85E-16 | 41 |
| Efet.01.110869.g550.t1 | Slit homolog 3 protein | 1788 | 1.75E-19 | 41 |
| Efet.01.356095.g168.t1 | Solute carrier organic anion transporter family member 1A2 | 408 | 2.84E-09 | 41 |
| Efet.01.448496.g1183.t1 | Suppressor of cytokine signaling 1 | 1485 | 9.76E-12 | 41 |
| Efet.01.160082.g525.t1 | Telomeric repeat-binding factor 1 | 1287 | 6.04E-14 | 41 |
| Efet.01.180873.g1440.t1 | Transcription intermediary factor 1-beta | 1137 | 7.03E-12 | 41 |
| Efet.01.357075.g199.t1 | Transcription intermediary factor 1-beta | 1131 | 3.81E-10 | 41 |
| Efet.01.658426.g2135.t1 | DNA topoisomerase 2-alpha | 2607 | 1.82E-23 | 41 |
| Efet.01.193113.g2001.t1 | Tissue-type plasminogen activator | 462 | 1.30E-06 | 41 |
| Efet.01.351507.g44.t1 | UDP-glucuronosyltransferase 1-2 | 1017 | 2.54E-13 | 41 |
| Efet.01.101219.g80.t1 | Utrophin | 624 | 3.64E-06 | 41 |
| Efet.01.401292.g28.t1 | Vascular endothelial growth factor receptor 1 | 462 | 6.73E-06 | 41 |
| Efet.01.4022.g337.t1 | Vimentin | 828 | 1.01E-06 | 41 |
| Efet.01.29951.g336.t1 | von Willebrand factor | 1932 | 8.60E-16 | 41 |
| Efet.01.169949.g974.t1 | Palmitoyltransferase ZDHHC23 | 762 | 5.18E-09 | 41 |
| Efet.01.213291.g559.t1 | Zinc finger protein 37A | 1080 | 1.41E-14 | 41 |
| Efet.01.219186.g773.t1 | Zinc finger protein 37A | 1659 | 5.58E-69 | 41 |
| Efet.01.325919.g797.t1 | Zinc finger protein 37A | 660 | 1.85E-15 | 41 |
| Efet.01.44302.g1245.t1 | Zinc finger and SCAN domain-containing protein 10 | 1632 | 2.15E-16 | 41 |
| Efet.01.644990.g919.t1 | ATP-binding cassette sub-family G member 2 | 966 | 1.67E-07 | 40 |
| Efet.01.655750.g856.t1 | Aldehyde dehydrogenase, dimeric NADP-preferring | 1557 | 3.89E-16 | 40 |
| Efet.01.588154.g937.t1 | Aldehyde dehydrogenase, dimeric NADP-preferring | 1317 | 1.33E-10 | 40 |
| Efet.01.446.g53.t1 | Contactin-1 | 2211 | 3.38E-12 | 40 |
| Efet.01.456290.g158.t1 | Cytochrome P450 3A12 | 1434 | 1.18E-21 | 40 |
| Efet.01.135715.g533.t1 | C-X-C chemokine receptor type 2 | 1176 | 1.41E-14 | 40 |
| Efet.01.591855.g1048.t1 | Fibronectin | 1245 | 2.23E-19 | 40 |
| Efet.01.178091.g1314.t1 | Gamma-aminobutyric acid receptor subunit beta-3 | 345 | 7.20E-11 | 40 |
| Efet.01.573469.g414.t1 | E3 ISG15--protein ligase HERC5 | 1236 | 2.36E-12 | 40 |
| Efet.01.295933.g1612.t1 | Histamine H2 receptor | 1233 | 9.05E-07 | 40 |
| Efet.01.8386.g618.t1 | Histamine H2 receptor | 1137 | 2.74E-06 | 40 |
| Efet.01.38135.g871.t1 | Histamine H2 receptor | 1044 | 2.07E-09 | 40 |
| Efet.01.603337.g130.t1 | Zinc finger and SCAN domain-containing protein 10 | 3072 | 7.59E-18 | 40 |
| Efet.01.158739.g458.t1 | Protein lin-41 | 1452 | 8.85E-07 | 40 |
| Efet.01.158046.g426.t1 | E3 ubiquitin-protein ligase TRIM71 | 1692 | 1.68E-30 | 40 |
| Efet.01.639465.g512.t1 | E3 ubiquitin-protein ligase TRIM71 | 1914 | 1.00E-11 | 40 |
| Efet.01.140240.g784.t1 | Neurogenic locus Notch protein | 1611 | 3.94E-11 | 40 |
| Efet.01.352960.g80.t1 | Neuropilin-2 | 696 | 4.52E-08 | 40 |
| Efet.01.22543.g1639.t1 | Opsin Rh1 | 1446 | 1.76E-06 | 40 |
| Efet.01.24505.g1777.t1 | Palladin | 1065 | 2.50E-15 | 40 |
| Efet.01.322538.g703.t1 | Protocadherin-15 | 1824 | 3.23E-23 | 40 |
| Efet.01.245444.g1710.t1 | PR domain zinc finger protein 5 | 1788 | 5.44E-14 | 40 |
| Efet.01.126460.g87.t1 | Receptor-type tyrosine-protein phosphatase zeta | 603 | 1.89E-18 | 40 |
| Efet.01.6637.g518.t1 | Receptor-type tyrosine-protein phosphatase S | 2457 | 7.90E-18 | 40 |
| Efet.01.47121.g1422.t1 | Peroxidasin homolog | 2013 | 3.57E-13 | 40 |
| Efet.01.306437.g211.t1 | Peroxidasin homolog | 429 | 4.86E-08 | 40 |
| Efet.01.390489.g1015.t1 | X-linked zinc finger protein | 681 | 4.24E-13 | 40 |
| Efet.01.14139.g1046.t1 | Roundabout homolog 2 | 1233 | 9.85E-25 | 40 |
| Efet.01.521297.g504.t1 | Reticulon-4 receptor | 1053 | 7.65E-13 | 40 |
| Efet.01.517044.g422.t1 | Slit homolog 2 protein | 1326 | 2.18E-19 | 40 |
| Efet.01.231136.g1231.t1 | Slit homolog 3 protein | 1521 | 8.12E-13 | 40 |
| Efet.01.368337.g494.t1 | Slit homolog 3 protein | 3240 | 4.99E-14 | 40 |
| Efet.01.67129.g1051.t1 | Transcription intermediary factor 1-alpha | 1221 | 1.21E-17 | 40 |
| Efet.01.79400.g280.t1 | Transcription intermediary factor 1-alpha | 1392 | 6.33E-12 | 40 |
| Efet.01.359740.g267.t1 | Transcription intermediary factor 1-alpha | 927 | 7.08E-08 | 40 |
| Efet.01.551910.g549.t1 | Transcription intermediary factor 1-alpha | 2019 | 6.05E-21 | 40 |
| Efet.01.408352.g200.t1 | Transcription intermediary factor 1-beta | 945 | 2.64E-08 | 40 |
| Efet.01.521053.g496.t1 | Transcription intermediary factor 1-beta | 1011 | 6.12E-14 | 40 |
| Efet.01.32090.g491.t1 | Toll-like receptor 3 | 1815 | 1.83E-14 | 40 |
| Efet.01.230071.g1198.t1 | Toll-like receptor 4 | 1473 | 7.87E-08 | 40 |
| Efet.01.448052.g1175.t1 | Protein wech | 831 | 2.90E-06 | 40 |
| Efet.01.647897.g1205.t1 | Protein white | 1410 | 4.33E-09 | 40 |
| Efet.01.652309.g279.t1 | Protein white | 804 | 3.35E-12 | 40 |
| Efet.01.11637.g853.t1 | Disintegrin and metalloproteinase domain-containing protein 10 | 759 | 2.42E-10 | 39 |
| Efet.01.438619.g913.t1 | Aldehyde dehydrogenase, dimeric NADP-preferring | 918 | 1.52E-09 | 39 |
| Efet.01.226824.g1071.t1 | Cadherin-15 | 1068 | 1.88E-08 | 39 |
| Efet.01.128998.g224.t1 | Cadherin-99C | 1335 | 5.06E-11 | 39 |
| Efet.01.207713.g349.t1 | Chromodomain-helicase-DNA-binding protein 7 | 201 | 6.12E-12 | 39 |
| Efet.01.1659523.g2002.t1 | Glutamine synthetase 1, mitochondrial | 1293 | 1.73E-09 | 39 |
| Efet.01.8897.g649.t1 | Histamine H1 receptor | 1182 | 2.19E-18 | 39 |
| Efet.01.102833.g163.t1 | Histamine H2 receptor | 1743 | 3.09E-13 | 39 |
| Efet.01.99321.g1409.t1 | E3 ubiquitin-protein ligase TRIM71 | 2025 | 3.01E-13 | 39 |
| Efet.01.156737.g352.t1 | Stromelysin-1 | 942 | 5.64E-09 | 39 |
| Efet.01.131739.g347.t1 | Exosome RNA helicase MTR4 | 1857 | 9.32E-14 | 39 |
| Efet.01.38231.g879.t1 | PR domain zinc finger protein 14 | 1719 | 6.79E-09 | 39 |
| Efet.01.316830.g535.t1 | Receptor-type tyrosine-protein phosphatase S | 1737 | 3.42E-25 | 39 |
| Efet.01.252916.g107.t1 | von Willebrand factor | 1404 | 6.22E-20 | 39 |
| Efet.01.9240.g674.t1 | Zinc finger X-chromosomal protein | 2064 | 9.05E-07 | 39 |
| Efet.01.194666.g2077.t1 | Zinc finger and SCAN domain-containing protein 10 | 834 | 5.73E-22 | 39 |
| Efet.01.649506.g1325.t1 | Zinc finger and SCAN domain-containing protein 10 | 1674 | 4.40E-23 | 39 |
| Efet.01.291216.g1487.t1 | Chondroitin sulfate proteoglycan 4 | 816 | 1.88E-06 | 38 |
| Efet.01.656322.g997.t1 | Matrix metalloproteinase-14 | 462 | 2.16E-13 | 38 |
| Efet.01.138649.g697.t1 | Neuromedin-U receptor 1 | 1116 | 7.51E-07 | 38 |
| Efet.01.96649.g1260.t1 | Palladin | 1467 | 4.28E-14 | 38 |
| Efet.01.350436.g12.t1 | 1-phosphatidylinositol 4,5-bisphosphate phosphodiesterase gamma-1 | 495 | 5.45E-08 | 38 |
| Efet.01.250406.g12.t1 | PR domain zinc finger protein 5 | 1728 | 8.19E-07 | 38 |
| Efet.01.423677.g560.t1 | PR domain zinc finger protein 5 | 669 | 4.78E-06 | 38 |
| Efet.01.13936.g1032.t1 | RE1-silencing transcription factor | 3087 | 1.07E-14 | 38 |
| Efet.01.68170.g1119.t1 | Extracellular sulfatase Sulf-2 | 1101 | 9.91E-07 | 38 |
| Efet.01.54808.g325.t1 | Transcription intermediary factor 1-alpha | 2259 | 3.21E-14 | 38 |
| Efet.01.125413.g24.t1 | Transcription intermediary factor 1-beta | 2013 | 9.33E-13 | 38 |
| Efet.01.92493.g1022.t1 | Zinc finger protein 37A | 504 | 1.27E-08 | 38 |
| Efet.01.99838.g1440.t1 | Zinc finger protein 37A | 1797 | 5.90E-08 | 38 |
| Efet.01.658371.g1903.t1 | Zinc finger protein 37A | 1593 | 1.18E-09 | 38 |
| Efet.01.318420.g577.t1 | E3 ubiquitin-protein ligase TRIM71 | 633 | 4.03E-08 | 37 |
| Efet.01.206117.g265.t1 | RE1-silencing transcription factor | 6186 | 4.02E-09 | 37 |
| Efet.01.30465.g370.t1 | Roundabout homolog 2 | 1149 | 3.92E-11 | 37 |
| Efet.01.136213.g559.t1 | Roundabout homolog 2 | 1248 | 3.48E-18 | 37 |
| Efet.01.518735.g450.t1 | Transcription intermediary factor 1-beta | 1203 | 8.44E-08 | 37 |
| Efet.01.597702.g1227.t1 | Aldehyde dehydrogenase, dimeric NADP-preferring | 1071 | 1.92E-14 | 36 |
| Efet.01.657535.g1323.t1 | PR domain zinc finger protein 5 | 2079 | 2.64E-13 | 36 |
| Efet.01.209765.g437.t1 | Reticulon-4 receptor | 2037 | 8.04E-14 | 36 |
| Efet.01.217012.g703.t1 | Zinc finger and SCAN domain-containing protein 10 | 846 | 3.67E-10 | 35 |
| Efet.01.240865.g1563.t1 | PR domain zinc finger protein 5 | 1779 | 8.11E-14 | 35 |
| Efet.01.181099.g1456.t1 | PR domain zinc finger protein 5 | 1164 | 3.53E-10 | 34 |
| Efet.01.89295.g851.t1 | Receptor tyrosine-protein kinase erbB-3 | 828 | 6.55E-06 | 33 |
