# Supplementary material for: Data on genome annotation and analysis of earthworm Eisenia fetida
Source: Data Brief. 2018 Aug 29;20:525–34. doi: 10.1016/j.dib.2018.08.067 (PMC6126081; doi:10.1016/j.dib.2018.08.067)
Supplement: Supplementary file 7 — Supplementary material [file mmc7.docx]

Table S6: List of immune-related genes identified in the genome dataset of earthworm *Eisenia fetida*

| **SeqName** | **Description** | **Length** | **e-Value** | **Mean similarity (Percentage)** |
| --- | --- | --- | --- | --- |
| Efet.01.650655.g74.t1 | Calmodulin | 240 | 1.79E-44 | 100 |
| Efet.01.110119.g517.t1 | Calcineurin subunit B type 1 | 306 | 3.18E-33 | 100 |
| Efet.01.321720.g676.t1 | Guanine nucleotide-binding protein G(q) subunit alpha | 315 | 7.29E-64 | 100 |
| Efet.01.343704.g1241.t1 | Guanine nucleotide-binding protein G(q) subunit alpha | 222 | 2.24E-29 | 100 |
| Efet.01.49266.g1547.t1 | Hepatocyte nuclear factor 6 | 684 | 2.61E-49 | 100 |
| Efet.01.384679.g902.t1 | LIM/homeobox protein Lhx4 | 378 | 1.31E-09 | 100 |
| Efet.01.254930.g179.t1 | Misshapen-like kinase 1 | 225 | 1.79E-15 | 100 |
| Efet.01.167388.g859.t1 | Serine/threonine-protein phosphatase 2A catalytic subunit alpha isoform | 348 | 1.45E-35 | 100 |
| Efet.01.242597.g1626.t1 | Tubulin beta chain | 567 | 1.19E-114 | 100 |
| Efet.01.135109.g494.t1 | Septin-7 | 252 | 1.21E-13 | 100 |
| Efet.01.370247.g564.t1 | Tubulin beta-4B chain | 531 | 3.16E-111 | 100 |
| Efet.01.110194.g522.t1 | Testicular tissue protein Li 149 | 204 | 5.35E-32 | 98 |
| Efet.01.96946.g1278.t1 | Clathrin heavy chain 1 | 597 | 4.07E-104 | 98 |
| Efet.01.271454.g804.t1 | COUP transcription factor 2 | 306 | 8.33E-37 | 98 |
| Efet.01.548251.g447.t1 | Histone deacetylase 2 | 552 | 2.14E-110 | 98 |
| Efet.01.628471.g1246.t1 | Serine/threonine-protein kinase N2 | 387 | 7.75E-60 | 98 |
| Efet.01.416099.g378.t1 | Ras-related protein Rab-11A | 303 | 6.68E-42 | 98 |
| Efet.01.98961.g1385.t1 | Homeobox protein aristaless-like 4 | 384 | 2.60E-17 | 97 |
| Efet.01.97409.g1306.t1 | ATP synthase subunit alpha, mitochondrial | 696 | 3.47E-140 | 97 |
| Efet.01.301942.g66.t1 | Calmodulin | 501 | 2.17E-91 | 97 |
| Efet.01.377814.g746.t1 | Calcium-dependent secretion activator 1 | 624 | 3.00E-20 | 97 |
| Efet.01.33771.g606.t1 | Excitatory amino acid transporter 2 | 366 | 6.13E-09 | 97 |
| Efet.01.126264.g74.t1 | Hepatocyte nuclear factor 6 | 975 | 1.25E-45 | 97 |
| Efet.01.100540.g40.t1 | Inositol 1,4,5-trisphosphate receptor type 1 | 453 | 3.89E-47 | 97 |
| Efet.01.303664.g120.t1 | LIM/homeobox protein Lhx4 | 345 | 8.72E-17 | 97 |
| Efet.01.246146.g1739.t1 | Protein lin-7 homolog B | 276 | 6.66E-47 | 97 |
| Efet.01.164462.g733.t1 | Homeobox protein Nkx-2.2 | 1194 | 9.03E-37 | 97 |
| Efet.01.59935.g621.t1 | DDX39B (HCG2005638, isoform CRA_a) | 519 | 1.30E-51 | 96 |
| Efet.01.364911.g401.t1 | AP-1 complex subunit beta-1 | 267 | 5.91E-37 | 96 |
| Efet.01.20280.g1498.t1 | Beta-adrenergic receptor kinase 1 | 687 | 5.35E-45 | 96 |
| Efet.01.237901.g1476.t1 | Beta-adrenergic receptor kinase 1 | 288 | 2.70E-56 | 96 |
| Efet.01.625202.g1106.t1 | ATP synthase subunit beta, mitochondrial | 309 | 5.14E-63 | 96 |
| Efet.01.1658267.g1144.t1 | COUP transcription factor 2 | 339 | 2.89E-54 | 96 |
| Efet.01.648861.g1273.t1 | Cullin-4A | 387 | 1.04E-06 | 96 |
| Efet.01.506033.g151.t1 | Mitogen-activated protein kinase | 261 | 2.63E-32 | 96 |
| Efet.01.5657.g441.t1 | Dynamin-2 | 270 | 2.01E-10 | 96 |
| Efet.01.165344.g767.t1 | Guanine nucleotide-binding protein G(q) subunit alpha | 240 | 1.56E-47 | 96 |
| Efet.01.653548.g452.t1 | Vesicle-fusing ATPase | 300 | 1.89E-54 | 96 |
| Efet.01.181324.g1472.t1 | Proteasome (Prosome, macropain) 26S subunit, ATPase, 1 | 666 | 6.85E-132 | 96 |
| Efet.01.390740.g1020.t1 | Proteasome (Prosome, macropain) 26S subunit, ATPase, 1 | 441 | 4.34E-88 | 96 |
| Efet.01.294664.g1586.t1 | Ras-related protein Rab-3A | 312 | 9.26E-46 | 96 |
| Efet.01.89243.g848.t1 | Transcription factor SOX-2 | 717 | 6.10E-07 | 96 |
| Efet.01.33146.g561.t1 | Epididymis luminal protein 33 | 1947 | 0 | 96 |
| Efet.01.3356.g287.t1 | Alpha-actinin-4 | 210 | 7.04E-18 | 95 |
| Efet.01.656856.g1128.t1 | ATP synthase subunit beta, mitochondrial | 603 | 7.59E-87 | 95 |
| Efet.01.85824.g687.t1 | Endoplasmic reticulum chaperone BiP | 273 | 3.28E-27 | 95 |
| Efet.01.594190.g1111.t1 | Calcineurin subunit B type 1 | 273 | 4.14E-44 | 95 |
| Efet.01.452987.g67.t1 | Stress-70 protein, mitochondrial | 204 | 6.91E-37 | 95 |
| Efet.01.229658.g1182.t1 | Hepatocyte nuclear factor 6 | 1167 | 1.77E-35 | 95 |
| Efet.01.109096.g474.t1 | cAMP-dependent protein kinase catalytic subunit gamma | 210 | 1.33E-23 | 95 |
| Efet.01.128333.g190.t1 | Kinesin-like protein KIF13B | 210 | 2.33E-29 | 95 |
| Efet.01.109714.g504.t1 | Myocyte-specific enhancer factor 2C | 255 | 6.53E-34 | 95 |
| Efet.01.133754.g426.t1 | Myocyte-specific enhancer factor 2D | 324 | 3.07E-53 | 95 |
| Efet.01.67351.g1069.t1 | POU domain, class 3, transcription factor 3 | 1530 | 3.05E-44 | 95 |
| Efet.01.279682.g1088.t1 | Transforming protein RhoA | 579 | 2.92E-125 | 95 |
| Efet.01.129732.g252.t1 | Rho-associated protein kinase 1 | 471 | 2.11E-87 | 95 |
| Efet.01.112046.g609.t1 | 40S ribosomal protein S6 | 543 | 6.95E-106 | 95 |
| Efet.01.89815.g881.t1 | Serine/threonine-protein kinase WNK3 | 1272 | 2.15E-16 | 95 |
| Efet.01.89929.g889.t1 | ATP synthase subunit alpha, mitochondrial | 465 | 1.73E-96 | 94 |
| Efet.01.647165.g1160.t1 | Endoplasmic reticulum chaperone BiP | 459 | 4.63E-56 | 94 |
| Efet.01.23001.g1676.t1 | COUP transcription factor 2 | 762 | 1.73E-159 | 94 |
| Efet.01.236273.g1414.t1 | Epididymis luminal protein 4 | 447 | 1.40E-36 | 94 |
| Efet.01.421099.g505.t1 | Friend leukemia integration 1 transcription factor | 405 | 6.73E-14 | 94 |
| Efet.01.431861.g775.t1 | Forkhead box protein D3 | 1113 | 1.18E-44 | 94 |
| Efet.01.70921.g1290.t1 | Inositol 1,4,5-trisphosphate receptor type 1 | 339 | 3.34E-24 | 94 |
| Efet.01.613959.g627.t1 | Protein kinase C alpha type | 264 | 9.52E-19 | 94 |
| Efet.01.98400.g1351.t1 | Serine/threonine-protein kinase D3 | 264 | 5.27E-35 | 94 |
| Efet.01.410808.g241.t1 | Polyadenylate-binding protein 4 | 261 | 1.83E-30 | 94 |
| Efet.01.638685.g459.t1 | Paired box protein Pax-3 | 813 | 1.10E-11 | 94 |
| Efet.01.1616067.g35.t1 | Receptor-type tyrosine-protein phosphatase F | 231 | 2.88E-42 | 94 |
| Efet.01.179480.g1364.t1 | Peptidylprolyl isomerase | 282 | 2.71E-49 | 94 |
| Efet.01.167334.g857.t1 | MAPK3 protein | 267 | 3.27E-33 | 94 |
| Efet.01.98821.g1375.t1 | 60S ribosomal protein L11 | 384 | 1.65E-65 | 94 |
| Efet.01.245408.g1708.t1 | 40S ribosomal protein S27-like | 249 | 2.97E-41 | 94 |
| Efet.01.640207.g562.t1 | Transcription factor SOX-2 | 1032 | 5.92E-20 | 94 |
| Efet.01.1651700.g522.t1 | T-box transcription factor TBX3 | 303 | 8.08E-56 | 94 |
| Efet.01.131246.g332.t1 | Transitional endoplasmic reticulum ATPase | 1053 | 0 | 94 |
| Efet.01.231238.g1236.t1 | Transitional endoplasmic reticulum ATPase | 210 | 1.64E-15 | 94 |
| Efet.01.274369.g890.t1 | DNA topoisomerase 2-beta | 246 | 2.44E-15 | 94 |
| Efet.01.547070.g420.t1 | DDX39B (HCG2005638, isoform CRA_a) | 528 | 1.53E-76 | 93 |
| Efet.01.655301.g794.t1 | Proteasome (Prosome, macropain) 26S subunit, non-ATPase, 12, isoform CRA_a | 276 | 3.42E-40 | 93 |
| Efet.01.167816.g882.t1 | Testis secretory sperm-binding protein Li 197a | 411 | 1.14E-45 | 93 |
| Efet.01.42663.g1151.t1 | B-cell lymphoma/leukemia 11B | 1905 | 3.67E-41 | 93 |
| Efet.01.21882.g1602.t1 | Class E basic helix-loop-helix protein 23 | 426 | 4.99E-30 | 93 |
| Efet.01.103667.g199.t1 | Brefeldin A-inhibited guanine nucleotide-exchange protein 2 | 411 | 1.98E-79 | 93 |
| Efet.01.640793.g591.t1 | Endoplasmic reticulum chaperone BiP | 705 | 8.67E-116 | 93 |
| Efet.01.207750.g350.t1 | Cullin-3 | 360 | 6.50E-59 | 93 |
| Efet.01.488594.g932.t1 | Forkhead box protein D1 | 1176 | 1.71E-48 | 93 |
| Efet.01.363890.g374.t1 | Guanine nucleotide-binding protein G(i) subunit alpha-2 | 213 | 1.01E-34 | 93 |
| Efet.01.32239.g506.t1 | Stress-70 protein, mitochondrial | 396 | 2.32E-67 | 93 |
| Efet.01.17966.g1325.t1 | GTP-binding protein 1 | 279 | 1.80E-41 | 93 |
| Efet.01.78970.g242.t1 | Hepatocyte nuclear factor 6 | 1146 | 2.14E-40 | 93 |
| Efet.01.225823.g1029.t1 | Homeobox protein Hox-A3 | 696 | 8.07E-27 | 93 |
| Efet.01.300383.g13.t1 | Serine/threonine-protein kinase MARK1 | 273 | 1.51E-50 | 93 |
| Efet.01.425351.g603.t1 | Peroxiredoxin-1 | 222 | 9.32E-33 | 93 |
| Efet.01.458277.g228.t1 | tRNA-splicing ligase RtcB homolog | 1053 | 0 | 93 |
| Efet.01.332080.g965.t1 | Septin-7 | 393 | 3.83E-68 | 93 |
| Efet.01.401970.g52.t1 | Tubulin beta-2A chain | 828 | 1.46E-104 | 93 |
| Efet.01.5487.g426.t1 | T-box transcription factor TBX3 | 504 | 4.09E-69 | 93 |
| Efet.01.298402.g1695.t1 | Protein unc-13 homolog B | 222 | 2.20E-32 | 93 |
| Efet.01.41602.g1094.t1 | NEDD4-like E3 ubiquitin-protein ligase WWP1 | 453 | 1.92E-91 | 93 |
| Efet.01.269109.g709.t1 | DDX39B (HCG2005638, isoform CRA_a) | 402 | 1.49E-74 | 92 |
| Efet.01.488482.g925.t1 | ADP-ribosylation factor 6 | 465 | 6.81E-88 | 92 |
| Efet.01.12049.g898.t1 | Sarcoplasmic/endoplasmic reticulum calcium ATPase 1 | 315 | 7.24E-35 | 92 |
| Efet.01.440292.g955.t1 | Serine/threonine-protein kinase ATR | 222 | 4.49E-27 | 92 |
| Efet.01.96946.g1277.t1 | Clathrin heavy chain 1 | 228 | 2.86E-40 | 92 |
| Efet.01.115845.g824.t1 | COUP transcription factor 2 | 369 | 2.70E-54 | 92 |
| Efet.01.377322.g737.t1 | Peripheral plasma membrane protein CASK | 246 | 2.13E-34 | 92 |
| Efet.01.635582.g282.t1 | Epididymis luminal protein 4 | 318 | 1.33E-54 | 92 |
| Efet.01.69614.g1214.t1 | Forkhead box protein O3 | 591 | 2.58E-30 | 92 |
| Efet.01.257809.g290.t1 | G protein-coupled receptor kinase 5 | 303 | 6.73E-45 | 92 |
| Efet.01.218983.g765.t1 | Stress-70 protein, mitochondrial | 207 | 1.49E-36 | 92 |
| Efet.01.13456.g1006.t1 | Homeobox protein Hox-A3 | 429 | 1.40E-09 | 92 |
| Efet.01.82459.g493.t1 | Serine/threonine-protein kinase MARK1 | 255 | 8.07E-47 | 92 |
| Efet.01.573373.g413.t1 | Myosin-9 | 276 | 1.37E-26 | 92 |
| Efet.01.198432.g2232.t1 | 26S proteasome non-ATPase regulatory subunit 7 | 414 | 1.98E-43 | 92 |
| Efet.01.33291.g569.t1 | Receptor-type tyrosine-protein phosphatase S | 936 | 2.21E-82 | 92 |
| Efet.01.30749.g396.t1 | Tubulin beta chain | 954 | 5.61E-172 | 92 |
| Efet.01.439279.g927.t1 | Tubulin beta chain | 735 | 1.00E-134 | 92 |
| Efet.01.235801.g1401.t1 | PBX3 protein | 279 | 8.60E-21 | 92 |
| Efet.01.231215.g1235.t1 | Ras-related protein Rab-3D | 336 | 8.88E-39 | 92 |
| Efet.01.28183.g210.t1 | Transforming protein RhoA | 336 | 5.07E-60 | 92 |
| Efet.01.46116.g1363.t1 | E3 ubiquitin-protein ligase SIAH1 | 279 | 1.23E-25 | 92 |
| Efet.01.288638.g1400.t1 | SNF-related serine/threonine-protein kinase | 579 | 4.94E-111 | 92 |
| Efet.01.517829.g437.t1 | Transcription factor SOX-2 | 978 | 4.40E-18 | 92 |
| Efet.01.266312.g594.t1 | F-box-like/WD repeat-containing protein TBL1Y | 282 | 9.58E-51 | 92 |
| Efet.01.122718.g1182.t1 | Serine/threonine-protein kinase WNK3 | 1284 | 3.77E-17 | 92 |
| Efet.01.53441.g229.t1 | Zinc finger protein 40 | 924 | 1.63E-43 | 92 |
| Efet.01.609097.g380.t1 | Serine/threonine-protein phosphatase 2A 55 kDa regulatory subunit B beta isoform | 492 | 4.86E-82 | 91 |
| Efet.01.197487.g2198.t1 | AP-2 complex subunit alpha-2 | 630 | 1.14E-94 | 91 |
| Efet.01.18115.g1339.t1 | Sarcoplasmic/endoplasmic reticulum calcium ATPase 2 | 219 | 9.55E-19 | 91 |
| Efet.01.218386.g748.t1 | Proteasome activator complex subunit 3 | 282 | 1.40E-43 | 91 |
| Efet.01.251356.g50.t1 | Endoplasmic reticulum chaperone BiP | 585 | 5.21E-77 | 91 |
| Efet.01.312.g36.t1 | Voltage-dependent T-type calcium channel subunit alpha-1G | 402 | 1.03E-38 | 91 |
| Efet.01.42139.g1121.t1 | Peripheral plasma membrane protein CASK | 384 | 2.78E-52 | 91 |
| Efet.01.599318.g1286.t1 | Catenin alpha-2 | 273 | 3.68E-34 | 91 |
| Efet.01.656462.g1022.t1 | Epididymis luminal protein 4 | 318 | 2.29E-54 | 91 |
| Efet.01.622640.g997.t1 | Diacylglycerol kinase eta | 321 | 1.16E-54 | 91 |
| Efet.01.500982.g23.t1 | Dynamin-3 | 270 | 2.08E-36 | 91 |
| Efet.01.43904.g1221.t1 | Ezrin | 306 | 2.36E-46 | 91 |
| Efet.01.200208.g10.t1 | Forkhead box protein O3 | 411 | 1.99E-28 | 91 |
| Efet.01.234778.g1373.t1 | Huntingtin | 408 | 3.63E-21 | 91 |
| Efet.01.66985.g1043.t1 | Hepatocyte nuclear factor 6 | 318 | 9.04E-11 | 91 |
| Efet.01.378958.g777.t1 | Homeobox protein Hox-A3 | 276 | 1.78E-13 | 91 |
| Efet.01.75687.g40.t1 | Homeobox protein Hox-A5 | 276 | 1.82E-34 | 91 |
| Efet.01.370593.g571.t1 | Homeobox protein Hox-A5 | 276 | 1.82E-34 | 91 |
| Efet.01.267321.g628.t1 | Kinesin-like protein KIF1B | 210 | 1.46E-27 | 91 |
| Efet.01.386675.g941.t1 | Moesin | 201 | 2.60E-28 | 91 |
| Efet.01.43469.g1200.t1 | PR domain zinc finger protein 16 | 660 | 6.95E-51 | 91 |
| Efet.01.936.g93.t1 | RNA-binding protein 5 | 843 | 7.94E-13 | 91 |
| Efet.01.284287.g1245.t1 | Serine/threonine-protein kinase SIK1 | 381 | 1.95E-11 | 91 |
| Efet.01.208415.g386.t1 | Synaptotagmin-1 | 306 | 2.49E-40 | 91 |
| Efet.01.162123.g629.t1 | T-box transcription factor TBX1 | 378 | 3.15E-38 | 91 |
| Efet.01.182350.g1514.t1 | DNA topoisomerase 1 | 426 | 4.70E-73 | 91 |
| Efet.01.65051.g912.t1 | Tubby protein homolog | 642 | 5.68E-45 | 91 |
| Efet.01.15111.g1108.t1 | Nuclease-sensitive element-binding protein 1 | 345 | 3.64E-21 | 91 |
| Efet.01.34168.g632.t1 | Serine/threonine-protein phosphatase 2A 65 kDa regulatory subunit A alpha isoform | 531 | 3.48E-92 | 90 |
| Efet.01.22162.g1624.t1 | Serine/threonine-protein phosphatase 2A 55 kDa regulatory subunit B beta isoform | 399 | 7.22E-74 | 90 |
| Efet.01.234900.g1380.t1 | HCG2039588 (PBX2) | 381 | 5.97E-57 | 90 |
| Efet.01.317552.g554.t1 | Tyrosine--tRNA ligase | 387 | 1.55E-64 | 90 |
| Efet.01.331361.g947.t1 | cDNA FLJ75737, highly similar to Homo sapiens v-ets erythroblastosis virus E26 oncogene homolog 1 | 423 | 2.78E-61 | 90 |
| Efet.01.205455.g235.t1 | 5'-AMP-activated protein kinase catalytic subunit alpha-1 | 324 | 2.37E-44 | 90 |
| Efet.01.188016.g1788.t1 | RAC-beta serine/threonine-protein kinase | 258 | 3.56E-16 | 90 |
| Efet.01.30244.g354.t1 | Beta-adrenergic receptor kinase 1 | 216 | 8.88E-18 | 90 |
| Efet.01.499285.g1158.t1 | Voltage-dependent T-type calcium channel subunit alpha-1G | 291 | 5.33E-20 | 90 |
| Efet.01.326961.g829.t1 | Voltage-dependent L-type calcium channel subunit beta-4 | 1149 | 3.57E-11 | 90 |
| Efet.01.488005.g917.t1 | Calcium-dependent secretion activator 1 | 375 | 4.04E-61 | 90 |
| Efet.01.525220.g588.t1 | COUP transcription factor 2 | 573 | 1.36E-42 | 90 |
| Efet.01.172246.g1062.t1 | Cullin-5 | 351 | 4.92E-52 | 90 |
| Efet.01.227685.g1109.t1 | H/ACA ribonucleoprotein complex subunit 4 | 549 | 3.82E-92 | 90 |
| Efet.01.175628.g1196.t1 | Epsin-2 | 639 | 3.60E-89 | 90 |
| Efet.01.78671.g220.t1 | Fez family zinc finger protein 2 | 1464 | 2.64E-103 | 90 |
| Efet.01.1636384.g176.t1 | Forkhead box protein O3 | 408 | 1.19E-27 | 90 |
| Efet.01.30832.g402.t1 | GDP-mannose 4,6 dehydratase | 399 | 2.00E-75 | 90 |
| Efet.01.251256.g45.t1 | Hepatocyte nuclear factor 6 | 408 | 1.65E-25 | 90 |
| Efet.01.24059.g1749.t1 | Hypoxanthine-guanine phosphoribosyltransferase | 207 | 1.97E-18 | 90 |
| Efet.01.124954.g1289.t1 | Kalirin | 285 | 2.35E-35 | 90 |
| Efet.01.124124.g1245.t1 | Krueppel-like factor 1 | 1470 | 7.20E-46 | 90 |
| Efet.01.19309.g1429.t1 | Inactive histone-lysine N-methyltransferase 2E | 411 | 6.78E-08 | 90 |
| Efet.01.370047.g558.t1 | Myosin-9 | 216 | 1.23E-20 | 90 |
| Efet.01.26374.g104.t1 | Paired box protein Pax-1 | 1038 | 1.44E-74 | 90 |
| Efet.01.76552.g91.t1 | Paired box protein Pax-3 | 495 | 1.46E-71 | 90 |
| Efet.01.359526.g258.t1 | POU domain, class 3, transcription factor 3 | 612 | 6.14E-32 | 90 |
| Efet.01.73235.g1435.t1 | Early growth response protein | 939 | 7.16E-50 | 90 |
| Efet.01.391709.g1042.t1 | RGM domain family member B | 207 | 7.49E-17 | 90 |
| Efet.01.58055.g502.t1 | Solute carrier family 13 member 2 | 417 | 2.01E-11 | 90 |
| Efet.01.576541.g531.t1 | Semaphorin-5A | 201 | 1.08E-09 | 90 |
| Efet.01.289119.g1423.t1 | Homeobox protein SIX1 | 702 | 3.24E-115 | 90 |
| Efet.01.462758.g349.t1 | Recombining binding protein suppressor of hairless | 420 | 7.45E-50 | 90 |
| Efet.01.122424.g1169.t1 | DNA topoisomerase 2-beta | 636 | 1.83E-110 | 90 |
| Efet.01.1633572.g136.t1 | Protein unc-13 homolog A | 318 | 4.04E-18 | 90 |
| Efet.01.1658204.g1125.t1 | Serine/threonine-protein kinase WNK4 | 705 | 3.36E-17 | 90 |
| Efet.01.336099.g1074.t1 | NEDD4-like E3 ubiquitin-protein ligase WWP1 | 252 | 3.19E-38 | 90 |
| Efet.01.623940.g1038.t1 | 14-3-3 protein epsilon | 453 | 6.32E-79 | 89 |
| Efet.01.242568.g1625.t1 | Alpha-actinin-2 | 204 | 3.53E-17 | 89 |
| Efet.01.236756.g1428.t1 | Alpha-actinin-3 | 288 | 2.54E-37 | 89 |
| Efet.01.196353.g2147.t1 | AP-2 complex subunit sigma | 315 | 1.62E-37 | 89 |
| Efet.01.105872.g313.t1 | Calmodulin | 234 | 1.30E-38 | 89 |
| Efet.01.122416.g1168.t1 | CREB-binding protein | 477 | 1.57E-27 | 89 |
| Efet.01.277700.g1022.t1 | CREB-binding protein | 1605 | 1.52E-08 | 89 |
| Efet.01.130536.g298.t1 | Disks large homolog 1 | 276 | 1.07E-19 | 89 |
| Efet.01.396105.g1140.t1 | Dynein light chain Tctex-type 1 | 375 | 3.15E-58 | 89 |
| Efet.01.585955.g860.t1 | Fez family zinc finger protein 2 | 2319 | 1.09E-100 | 89 |
| Efet.01.284368.g1248.t1 | Krueppel-like factor 2 | 813 | 3.73E-47 | 89 |
| Efet.01.310784.g343.t1 | Protein kinase C alpha type | 216 | 2.49E-17 | 89 |
| Efet.01.110329.g526.t1 | Polyadenylate-binding protein 4 | 489 | 1.51E-77 | 89 |
| Efet.01.523793.g548.t1 | Paired box protein Pax-1 | 948 | 1.42E-57 | 89 |
| Efet.01.192282.g1974.t1 | POU domain, class 3, transcription factor 3 | 1077 | 3.27E-46 | 89 |
| Efet.01.582142.g749.t1 | POU domain, class 4, transcription factor 3 | 582 | 1.54E-68 | 89 |
| Efet.01.192821.g1995.t1 | Receptor-type tyrosine-protein phosphatase F | 384 | 3.15E-51 | 89 |
| Efet.01.187884.g1777.t1 | Ras-related protein Rab-5B | 210 | 4.32E-28 | 89 |
| Efet.01.307677.g230.t1 | Zinc finger protein SNAI1 | 285 | 3.46E-51 | 89 |
| Efet.01.609804.g415.t1 | Serum response factor | 747 | 2.72E-66 | 89 |
| Efet.01.237679.g1470.t1 | Tubulin beta-4B chain | 828 | 0 | 89 |
| Efet.01.92962.g1050.t1 | Alpha-actinin-2 | 441 | 1.11E-54 | 88 |
| Efet.01.254660.g170.t1 | AP-2 complex subunit alpha-1 | 249 | 7.20E-35 | 88 |
| Efet.01.517765.g436.t1 | cDNA FLJ61244, highly similar to Transcription factor 8 | 1488 | 7.91E-14 | 88 |
| Efet.01.112228.g621.t1 | BTB/POZ domain-containing protein 9 | 675 | 8.74E-80 | 88 |
| Efet.01.472269.g601.t1 | Calmodulin-A | 213 | 6.09E-24 | 88 |
| Efet.01.39405.g958.t1 | Calcineurin subunit B type 2 | 417 | 2.09E-25 | 88 |
| Efet.01.22003.g1616.t1 | CREB-binding protein | 291 | 4.88E-20 | 88 |
| Efet.01.284917.g1267.t1 | Epididymis luminal protein 4 | 381 | 7.22E-50 | 88 |
| Efet.01.101884.g114.t1 | Dynamin-1-like protein | 204 | 5.15E-32 | 88 |
| Efet.01.142616.g914.t1 | Dedicator of cytokinesis protein 11 | 270 | 1.05E-24 | 88 |
| Efet.01.34431.g649.t1 | Dual specificity tyrosine-phosphorylation-regulated kinase 2 | 1629 | 0 | 88 |
| Efet.01.151599.g94.t1 | EH domain-containing protein 1 | 231 | 9.86E-35 | 88 |
| Efet.01.151599.g95.t1 | EH domain-containing protein 1 | 621 | 1.11E-103 | 88 |
| Efet.01.336837.g1088.t1 | EH domain-containing protein 1 | 1008 | 0 | 88 |
| Efet.01.443028.g1066.t1 | ETS-related transcription factor Elf-1 | 1197 | 1.32E-30 | 88 |
| Efet.01.85730.g680.t1 | Hepatocyte nuclear factor 4 4 alpha variant 1 | 369 | 2.85E-20 | 88 |
| Efet.01.409839.g220.t1 | Forkhead box protein L2 | 1578 | 2.89E-49 | 88 |
| Efet.01.43074.g1175.t1 | Guanine nucleotide-binding protein G(o) subunit alpha | 342 | 1.78E-62 | 88 |
| Efet.01.35947.g729.t1 | cAMP-dependent protein kinase catalytic subunit alpha | 342 | 8.86E-37 | 88 |
| Efet.01.15446.g1135.t1 | Krueppel-like factor 1 | 480 | 6.52E-46 | 88 |
| Efet.01.333614.g1006.t1 | Serine/threonine-protein kinase LATS2 | 234 | 2.36E-42 | 88 |
| Efet.01.289531.g1436.t1 | E3 ubiquitin-protein ligase MARCH8 | 381 | 3.59E-51 | 88 |
| Efet.01.140993.g826.t1 | Homeobox protein Nkx-2.1 | 1128 | 6.99E-27 | 88 |
| Efet.01.35953.g730.t1 | 26S proteasome non-ATPase regulatory subunit 1 | 600 | 1.98E-109 | 88 |
| Efet.01.314519.g458.t1 | Ras-related protein Rab-3C | 381 | 3.12E-39 | 88 |
| Efet.01.134364.g456.t1 | 60S ribosomal protein L11 | 213 | 1.45E-25 | 88 |
| Efet.01.594585.g1126.t1 | 40S ribosomal protein S3a | 456 | 4.96E-68 | 88 |
| Efet.01.645117.g932.t1 | Transcription factor SOX-2 | 555 | 5.18E-08 | 88 |
| Efet.01.143193.g935.t1 | Spectrin beta chain, erythrocytic | 417 | 9.38E-63 | 88 |
| Efet.01.229079.g1165.t1 | Thrombospondin-4 | 288 | 3.24E-49 | 88 |
| Efet.01.227313.g1092.t1 | Nuclear factor 1 | 474 | 2.47E-69 | 88 |
| Efet.01.620822.g909.t1 | Calreticulin, isoform CRA_b | 798 | 1.78E-103 | 88 |
| Efet.01.614411.g644.t1 | Epididymis luminal protein 35 | 363 | 7.12E-48 | 88 |
| Efet.01.315508.g493.t1 | 14-3-3 protein epsilon | 375 | 4.39E-65 | 87 |
| Efet.01.347085.g1323.t1 | ATP-binding cassette sub-family G member 1 | 288 | 1.68E-32 | 87 |
| Efet.01.129195.g234.t1 | Aryl hydrocarbon receptor nuclear translocator 2 | 378 | 3.81E-15 | 87 |
| Efet.01.216175.g670.t1 | cDNA FLJ52786, highly similar to Caspase-7 | 222 | 1.57E-10 | 87 |
| Efet.01.627199.g1201.t1 | B-cell lymphoma/leukemia 11B | 2349 | 5.95E-38 | 87 |
| Efet.01.148332.g1154.t1 | E3 ubiquitin-protein ligase CBL | 495 | 8.17E-69 | 87 |
| Efet.01.500045.g2.t1 | Glutamate decarboxylase 1 | 243 | 9.60E-26 | 87 |
| Efet.01.365522.g423.t1 | Dual specificity tyrosine-phosphorylation-regulated kinase 2 | 489 | 7.00E-90 | 87 |
| Efet.01.436356.g862.t1 | Forkhead box protein C1 | 831 | 5.50E-37 | 87 |
| Efet.01.108854.g460.t1 | Forkhead box protein C2 | 834 | 9.09E-34 | 87 |
| Efet.01.407960.g194.t1 | Insulin receptor | 225 | 9.75E-40 | 87 |
| Efet.01.191461.g1935.t1 | Calcium-activated potassium channel subunit alpha-1 | 357 | 9.17E-29 | 87 |
| Efet.01.41250.g1070.t1 | Krueppel-like factor 1 | 1365 | 1.11E-45 | 87 |
| Efet.01.38849.g923.t1 | Protein kinase C beta type | 318 | 5.55E-11 | 87 |
| Efet.01.1617647.g41.t1 | DNA mismatch repair protein Mlh1 | 210 | 3.42E-09 | 87 |
| Efet.01.146547.g1082.t1 | E3 ubiquitin-protein ligase NEDD4-like | 201 | 5.73E-33 | 87 |
| Efet.01.240113.g1539.t1 | Homeobox protein Nkx-2.3 | 753 | 5.05E-25 | 87 |
| Efet.01.80345.g355.t1 | Polyadenylate-binding protein 4 | 513 | 4.09E-40 | 87 |
| Efet.01.645413.g982.t1 | PRKCA-binding protein | 204 | 1.09E-08 | 87 |
| Efet.01.43607.g1206.t1 | Plexin-A3 | 507 | 1.12E-63 | 87 |
| Efet.01.38090.g867.t1 | Receptor-type tyrosine-protein phosphatase S | 351 | 1.48E-43 | 87 |
| Efet.01.560299.g7.t1 | Early growth response protein | 1008 | 2.25E-42 | 87 |
| Efet.01.392378.g1060.t1 | ELKS/Rab6-interacting/CAST family member 1 | 417 | 4.22E-16 | 87 |
| Efet.01.94160.g1112.t1 | Reticulon-4 | 225 | 1.52E-10 | 87 |
| Efet.01.111501.g577.t1 | Ryanodine receptor 2 | 447 | 3.92E-66 | 87 |
| Efet.01.12365.g921.t1 | Sodium channel protein type 2 subunit alpha | 714 | 2.64E-10 | 87 |
| Efet.01.251212.g41.t1 | Homeobox protein SIX4 | 612 | 8.22E-90 | 87 |
| Efet.01.37915.g860.t1 | Helicase SKI2W | 291 | 7.00E-35 | 87 |
| Efet.01.141182.g837.t1 | SKI family transcriptional corepressor 2 | 1185 | 6.24E-108 | 87 |
| Efet.01.64637.g892.t1 | Transcription factor SOX-2 | 792 | 3.29E-32 | 87 |
| Efet.01.97960.g1336.t1 | Synaptotagmin-1 | 462 | 2.11E-72 | 87 |
| Efet.01.200253.g18.t1 | Epididymis luminal protein 35 | 291 | 8.81E-42 | 87 |
| Efet.01.19034.g1410.t1 | V-type proton ATPase subunit H | 453 | 9.01E-64 | 87 |
| Efet.01.22955.g1673.t1 | Suppressor of cytokine signaling 6, isoform CRA_a | 1254 | 9.55E-82 | 86 |
| Efet.01.204442.g193.t1 | SET translocation (Myeloid leukemia-associated), isoform CRA_b | 645 | 9.07E-111 | 86 |
| Efet.01.234126.g1346.t1 | Alpha-1B adrenergic receptor | 294 | 2.28E-06 | 86 |
| Efet.01.271034.g785.t1 | Protein atonal homolog 1 | 675 | 1.57E-14 | 86 |
| Efet.01.271035.g786.t1 | Protein atonal homolog 1 | 279 | 1.67E-14 | 86 |
| Efet.01.152930.g157.t1 | ATP synthase subunit beta, mitochondrial | 570 | 9.49E-98 | 86 |
| Efet.01.15175.g1113.t1 | Protein kinase C | 246 | 1.26E-19 | 86 |
| Efet.01.440903.g992.t1 | Calcium-dependent secretion activator 1 | 579 | 1.94E-87 | 86 |
| Efet.01.284289.g1246.t1 | Cyclin-dependent-like kinase 5 | 330 | 3.54E-18 | 86 |
| Efet.01.1656422.g863.t1 | Calcineurin B homologous protein 1 | 243 | 1.61E-18 | 86 |
| Efet.01.360769.g292.t1 | Beta-catenin-like protein 1 | 456 | 5.84E-46 | 86 |
| Efet.01.190689.g1900.t1 | B-cell CLL/lymphoma 11A (Zinc finger protein), isoform CRA_b | 2517 | 2.02E-34 | 86 |
| Efet.01.190449.g1889.t1 | Dedicator of cytokinesis protein 7 | 252 | 2.61E-19 | 86 |
| Efet.01.324510.g755.t1 | D(2) dopamine receptor | 372 | 2.93E-13 | 86 |
| Efet.01.50957.g65.t1 | Epsin-2 | 795 | 6.87E-81 | 86 |
| Efet.01.1655753.g788.t1 | Epsin-2 | 582 | 2.56E-73 | 86 |
| Efet.01.68476.g1134.t1 | Receptor tyrosine-protein kinase erbB-4 | 417 | 3.36E-24 | 86 |
| Efet.01.323044.g713.t1 | DNA excision repair protein ERCC-6 | 738 | 1.01E-43 | 86 |
| Efet.01.499170.g1157.t1 | Hepatocyte nuclear factor 3-beta | 480 | 3.32E-34 | 86 |
| Efet.01.38735.g918.t1 | Guanine nucleotide-binding protein G(i) subunit alpha-2 | 276 | 6.07E-28 | 86 |
| Efet.01.4709.g376.t1 | Inositol 1,4,5-trisphosphate receptor type 1 | 471 | 1.94E-41 | 86 |
| Efet.01.647709.g1195.t1 | Potassium voltage-gated channel subfamily H member 8 | 1395 | 7.24E-06 | 86 |
| Efet.01.37663.g833.t1 | Krueppel-like factor 6 | 270 | 8.32E-21 | 86 |
| Efet.01.171047.g1014.t1 | Krueppel-like factor 6 | 444 | 6.15E-29 | 86 |
| Efet.01.203005.g143.t1 | Histone-lysine N-methyltransferase 2A | 453 | 3.01E-79 | 86 |
| Efet.01.91669.g979.t1 | Mitogen-activated protein kinase kinase kinase 12 | 2133 | 5.06E-162 | 86 |
| Efet.01.522832.g532.t1 | MAP/microtubule affinity-regulating kinase 3 | 276 | 4.77E-46 | 86 |
| Efet.01.38316.g886.t1 | N6-adenosine-methyltransferase non-catalytic subunit | 411 | 6.67E-50 | 86 |
| Efet.01.255064.g182.t1 | Myosin-10 | 600 | 5.46E-100 | 86 |
| Efet.01.351600.g46.t1 | Unconventional myosin-Ie | 351 | 2.92E-48 | 86 |
| Efet.01.653584.g455.t1 | Nucleoside diphosphate kinase homolog 5 | 345 | 5.70E-31 | 86 |
| Efet.01.168137.g896.t1 | Paired box protein Pax-5 | 1527 | 1.13E-08 | 86 |
| Efet.01.98955.g1382.t1 | Phosphatidylinositol 4-kinase beta | 576 | 8.06E-102 | 86 |
| Efet.01.272700.g844.t1 | Proteasome subunit beta type-5 | 420 | 4.73E-38 | 86 |
| Efet.01.491040.g976.t1 | 26S proteasome non-ATPase regulatory subunit 1 | 597 | 1.99E-52 | 86 |
| Efet.01.4658.g372.t1 | Receptor-type tyrosine-protein phosphatase F | 468 | 4.79E-07 | 86 |
| Efet.01.296517.g1634.t1 | MAPK10 protein | 252 | 2.24E-15 | 86 |
| Efet.01.16540.g1221.t1 | Early growth response protein | 729 | 6.01E-56 | 86 |
| Efet.01.234304.g1350.t1 | Ras-related protein Rab-27A | 468 | 1.73E-86 | 86 |
| Efet.01.378313.g756.t1 | Solute carrier family 13 member 2 | 423 | 8.20E-17 | 86 |
| Efet.01.528100.g640.t1 | Transcription factor SOX-4 | 1236 | 1.94E-13 | 86 |
| Efet.01.23444.g1698.t1 | E3 ubiquitin-protein ligase synoviolin | 471 | 4.51E-33 | 86 |
| Efet.01.281756.g1147.t1 | DNA topoisomerase 2-alpha | 330 | 8.05E-49 | 86 |
| Efet.01.416653.g397.t1 | Zinc finger CCCH domain-containing protein 15 | 318 | 1.53E-26 | 86 |
| Efet.01.940.g94.t1 | 14-3-3 protein epsilon | 306 | 3.47E-38 | 85 |
| Efet.01.34868.g676.t1 | HCG1985580, isoform CRA_c | 237 | 2.28E-35 | 85 |
| Efet.01.131503.g339.t1 | Suppressor of cytokine signaling 6, isoform CRA_a | 858 | 2.44E-56 | 85 |
| Efet.01.1638252.g192.t1 | SET translocation (Myeloid leukemia-associated), isoform CRA_b | 318 | 4.11E-52 | 85 |
| Efet.01.1659186.g1449.t1 | POU domain protein | 432 | 3.44E-26 | 85 |
| Efet.01.444576.g1093.t1 | Proteasome subunit alpha type | 207 | 1.55E-29 | 85 |
| Efet.01.625531.g1123.t1 | APOBEC1 complementation factor | 696 | 2.63E-111 | 85 |
| Efet.01.657910.g1530.t1 | APOBEC1 complementation factor | 804 | 6.39E-109 | 85 |
| Efet.01.142614.g913.t1 | cDNA FLJ75737, highly similar to Homo sapiens v-ets erythroblastosis virus E26 oncogene homolog 1 | 240 | 1.41E-36 | 85 |
| Efet.01.165204.g765.t1 | Alpha-actinin-4 | 336 | 1.77E-51 | 85 |
| Efet.01.436562.g869.t1 | AP-3 complex subunit beta-2 | 1200 | 9.92E-87 | 85 |
| Efet.01.309444.g297.t1 | Putative Polycomb group protein ASXL2 | 888 | 2.59E-12 | 85 |
| Efet.01.71128.g1301.t1 | ATP synthase subunit alpha, mitochondrial | 225 | 3.74E-28 | 85 |
| Efet.01.328976.g892.t1 | Voltage-dependent L-type calcium channel subunit beta-4 | 204 | 2.01E-15 | 85 |
| Efet.01.425207.g600.t1 | Calmodulin-A | 246 | 1.32E-28 | 85 |
| Efet.01.540423.g280.t1 | Collagen alpha-1(II) chain | 264 | 2.16E-06 | 85 |
| Efet.01.96653.g1262.t1 | Dolichyl-diphosphooligosaccharide--protein glycosyltransferase subunit DAD1 | 261 | 9.32E-30 | 85 |
| Efet.01.143134.g927.t1 | Probable ATP-dependent RNA helicase DDX41 | 600 | 1.67E-85 | 85 |
| Efet.01.202263.g110.t1 | Delta(24)-sterol reductase | 267 | 5.50E-36 | 85 |
| Efet.01.10983.g806.t1 | Dual specificity protein phosphatase 1 | 501 | 4.03E-07 | 85 |
| Efet.01.121829.g1136.t1 | Dual specificity protein phosphatase 6 | 624 | 3.24E-31 | 85 |
| Efet.01.117723.g916.t1 | EH domain-containing protein 1 | 948 | 1.97E-157 | 85 |
| Efet.01.211986.g518.t1 | Forkhead box protein Q1 | 1308 | 5.44E-50 | 85 |
| Efet.01.198828.g2247.t1 | GTPase-activating protein and VPS9 domain-containing protein 1 | 225 | 8.59E-34 | 85 |
| Efet.01.658021.g1563.t1 | Growth arrest-specific protein 2 | 381 | 7.99E-07 | 85 |
| Efet.01.1632263.g119.t1 | Serine/threonine-protein kinase D3 | 270 | 1.27E-24 | 85 |
| Efet.01.537660.g220.t1 | Ribosomal protein S6 kinase alpha-1 | 225 | 6.10E-16 | 85 |
| Efet.01.282912.g1189.t1 | Lipopolysaccharide-responsive and beige-like anchor protein | 225 | 2.90E-06 | 85 |
| Efet.01.649669.g1347.t1 | Low-density lipoprotein receptor-related protein 5 | 390 | 2.73E-47 | 85 |
| Efet.01.130643.g304.t1 | Misshapen-like kinase 1 | 285 | 7.53E-31 | 85 |
| Efet.01.200243.g17.t1 | Serine/threonine-protein kinase PAK 1 | 429 | 3.54E-72 | 85 |
| Efet.01.308265.g250.t1 | Serine/threonine-protein kinase PAK 1 | 495 | 1.52E-66 | 85 |
| Efet.01.213501.g573.t1 | Peptidyl-prolyl cis-trans isomerase F, mitochondrial | 216 | 7.89E-14 | 85 |
| Efet.01.78015.g174.t1 | Prospero homeobox protein 1 | 2004 | 3.42E-14 | 85 |
| Efet.01.591126.g1028.t1 | Prospero homeobox protein 1 | 213 | 7.29E-21 | 85 |
| Efet.01.26975.g130.t1 | 26S proteasome non-ATPase regulatory subunit 4 | 327 | 2.78E-42 | 85 |
| Efet.01.18913.g1403.t1 | Presenilin-2 | 708 | 7.77E-14 | 85 |
| Efet.01.97750.g1326.t1 | Receptor-type tyrosine-protein phosphatase F | 423 | 2.24E-66 | 85 |
| Efet.01.208334.g381.t1 | Poly(U)-binding-splicing factor PUF60 | 429 | 1.70E-45 | 85 |
| Efet.01.169384.g948.t1 | Early growth response protein | 1467 | 1.43E-53 | 85 |
| Efet.01.40511.g1023.t1 | SET (SET translocation (Myeloid leukemia-associated), isoform CRA_a) | 747 | 2.76E-111 | 85 |
| Efet.01.339857.g1170.t1 | Retinoic acid receptor RXR-alpha | 402 | 2.11E-63 | 85 |
| Efet.01.18734.g1391.t1 | Sodium channel protein type 2 subunit alpha | 207 | 8.69E-11 | 85 |
| Efet.01.303971.g138.t1 | SRSF protein kinase 1 | 231 | 1.19E-32 | 85 |
| Efet.01.298396.g1694.t1 | Syntaxin-1A | 387 | 1.66E-47 | 85 |
| Efet.01.403070.g82.t1 | Tryptophan--tRNA ligase, cytoplasmic | 1227 | 0 | 85 |
| Efet.01.320524.g648.t1 | E3 ubiquitin-protein ligase TRIP12 | 237 | 9.16E-28 | 85 |
| Efet.01.37888.g857.t1 | Ubiquitin-protein ligase E3C | 456 | 1.71E-65 | 85 |
| Efet.01.497567.g1127.t1 | Protein unc-13 homolog B | 417 | 1.03E-66 | 85 |
| Efet.01.402762.g71.t1 | NEDD4-like E3 ubiquitin-protein ligase WWP1 | 228 | 8.17E-15 | 85 |
| Efet.01.26141.g86.t1 | Ataxia telangiectasia mutated (Includes complementation groups A, C and D) | 570 | 1.40E-50 | 84 |
| Efet.01.233319.g1315.t1 | 60S ribosomal protein L7 | 258 | 3.48E-23 | 84 |
| Efet.01.608883.g369.t1 | Neuronal acetylcholine receptor subunit alpha-7 | 219 | 6.46E-25 | 84 |
| Efet.01.334180.g1022.t1 | Actin, alpha cardiac muscle 1 | 552 | 3.93E-97 | 84 |
| Efet.01.540563.g282.t1 | Alcohol dehydrogenase class-3 | 396 | 6.46E-65 | 84 |
| Efet.01.111819.g594.t1 | Sarcoplasmic/endoplasmic reticulum calcium ATPase 1 | 213 | 6.24E-32 | 84 |
| Efet.01.377804.g745.t1 | Calcium-transporting ATPase type 2C member 1 | 246 | 1.41E-35 | 84 |
| Efet.01.18708.g1386.t1 | Interleukin enhancer-binding factor 2 | 789 | 1.28E-135 | 84 |
| Efet.01.262148.g456.t1 | Class E basic helix-loop-helix protein 23 | 987 | 1.45E-17 | 84 |
| Efet.01.58408.g519.t1 | Bone morphogenetic protein 10 | 387 | 3.69E-29 | 84 |
| Efet.01.30272.g356.t1 | Bone morphogenetic protein receptor type-1B | 591 | 3.87E-102 | 84 |
| Efet.01.42557.g1144.t1 | Voltage-dependent T-type calcium channel subunit alpha-1G | 624 | 6.10E-13 | 84 |
| Efet.01.49765.g1577.t1 | Voltage-dependent T-type calcium channel subunit alpha-1G | 495 | 4.67E-61 | 84 |
| Efet.01.562681.g59.t1 | Voltage-dependent T-type calcium channel subunit alpha-1G | 231 | 3.65E-25 | 84 |
| Efet.01.152042.g120.t1 | Calmodulin-A | 219 | 2.69E-26 | 84 |
| Efet.01.1644717.g308.t1 | Hsp90 co-chaperone Cdc37 | 255 | 2.51E-18 | 84 |
| Efet.01.56739.g419.t1 | Cullin-2 | 234 | 1.45E-24 | 84 |
| Efet.01.1594087.g17.t1 | Cytoplasmic FMR1-interacting protein 2 | 222 | 4.40E-28 | 84 |
| Efet.01.191884.g1953.t1 | Epididymis luminal protein 4 | 435 | 4.01E-73 | 84 |
| Efet.01.315502.g492.t1 | Dual specificity tyrosine-phosphorylation-regulated kinase 2 | 1089 | 1.47E-165 | 84 |
| Efet.01.10868.g796.t1 | Histone acetyltransferase p300 | 2145 | 3.22E-126 | 84 |
| Efet.01.541011.g291.t1 | Receptor tyrosine-protein kinase erbB-4 | 213 | 1.86E-12 | 84 |
| Efet.01.651825.g229.t1 | Protein farnesyltransferase/geranylgeranyltransferase type-1 subunit alpha | 390 | 1.85E-41 | 84 |
| Efet.01.52888.g200.t1 | Forkhead box protein D3 | 762 | 1.89E-44 | 84 |
| Efet.01.58075.g503.t1 | Forkhead box protein P1 | 255 | 1.54E-28 | 84 |
| Efet.01.60829.g676.t1 | Guanine nucleotide-binding protein G(q) subunit alpha | 210 | 3.96E-30 | 84 |
| Efet.01.1654027.g655.t1 | Glutamate receptor ionotropic, kainate 2 | 621 | 6.69E-89 | 84 |
| Efet.01.201782.g89.t1 | Hepatocyte nuclear factor 6 | 849 | 4.97E-33 | 84 |
| Efet.01.224929.g1000.t1 | Inositol 1,4,5-trisphosphate receptor type 1 | 261 | 4.44E-22 | 84 |
| Efet.01.34490.g652.t1 | cAMP-dependent protein kinase catalytic subunit alpha | 306 | 3.72E-19 | 84 |
| Efet.01.1603858.g5.t1 | Histone acetyltransferase KAT6A | 243 | 6.09E-36 | 84 |
| Efet.01.118795.g970.t1 | Calcium-activated potassium channel subunit alpha-1 | 375 | 7.92E-27 | 84 |
| Efet.01.601081.g47.t1 | Potassium voltage-gated channel subfamily H member 8 | 288 | 1.21E-27 | 84 |
| Efet.01.47379.g1440.t1 | Protein kinase C iota type | 381 | 2.26E-65 | 84 |
| Efet.01.425067.g594.t1 | Mitogen-activated protein kinase 8 | 378 | 6.03E-41 | 84 |
| Efet.01.143720.g959.t1 | Protein CBFA2T3 | 393 | 1.99E-16 | 84 |
| Efet.01.2204.g199.t1 | Unconventional myosin-VI | 546 | 3.03E-43 | 84 |
| Efet.01.639970.g548.t1 | Nipped-B-like protein | 246 | 3.28E-17 | 84 |
| Efet.01.475053.g654.t1 | Phosphofurin acidic cluster sorting protein 1 | 204 | 1.29E-11 | 84 |
| Efet.01.33473.g580.t1 | ATP-dependent 6-phosphofructokinase, platelet type | 345 | 1.14E-47 | 84 |
| Efet.01.26741.g120.t1 | PH-interacting protein | 345 | 2.50E-27 | 84 |
| Efet.01.75411.g20.t1 | POU domain, class 4, transcription factor 3 | 840 | 2.19E-91 | 84 |
| Efet.01.300388.g14.t1 | Peptidyl-prolyl cis-trans isomerase F, mitochondrial | 327 | 5.66E-47 | 84 |
| Efet.01.596555.g1192.t1 | Peroxiredoxin-6 | 327 | 3.06E-49 | 84 |
| Efet.01.16466.g1215.t1 | Prospero homeobox protein 1 | 417 | 1.09E-17 | 84 |
| Efet.01.257560.g281.t1 | Receptor-type tyrosine-protein phosphatase F | 525 | 1.46E-82 | 84 |
| Efet.01.289040.g1419.t1 | Ras-related protein Rab-3D | 237 | 2.46E-32 | 84 |
| Efet.01.193627.g2027.t1 | Double-strand-break repair protein rad21 homolog | 378 | 5.51E-61 | 84 |
| Efet.01.287139.g1342.t1 | Transcription factor RFX3 | 423 | 1.08E-58 | 84 |
| Efet.01.206089.g263.t1 | 40S ribosomal protein S3a | 240 | 2.42E-18 | 84 |
| Efet.01.105929.g314.t1 | Radial spoke head protein 6 homolog A | 222 | 3.00E-19 | 84 |
| Efet.01.3010.g266.t1 | Sodium channel protein type 2 subunit alpha | 234 | 4.34E-13 | 84 |
| Efet.01.599013.g1277.t1 | Protein scribble homolog | 255 | 3.16E-06 | 84 |
| Efet.01.286082.g1305.t1 | Transcription factor SOX-2 | 216 | 2.42E-15 | 84 |
| Efet.01.555586.g647.t1 | Transcription factor SOX-7 | 684 | 1.26E-14 | 84 |
| Efet.01.24599.g1785.t1 | Epididymis luminal protein 35 | 801 | 4.58E-131 | 84 |
| Efet.01.4030.g340.t1 | YTH domain-containing family protein 2 | 1359 | 2.45E-94 | 84 |
| Efet.01.100458.g28.t1 | TNF receptor-associated factor 3 | 573 | 7.84E-90 | 83 |
| Efet.01.305731.g198.t1 | TNF receptor-associated factor 3 | 723 | 7.88E-82 | 83 |
| Efet.01.552187.g556.t1 | Catenin beta-1 | 1011 | 1.44E-157 | 83 |
| Efet.01.504193.g105.t1 | Tyrosine-protein phosphatase non-receptor type | 222 | 1.28E-15 | 83 |
| Efet.01.62636.g783.t1 | Voltage-dependent T-type calcium channel subunit alpha-1G | 525 | 1.04E-16 | 83 |
| Efet.01.598816.g1262.t1 | Voltage-dependent T-type calcium channel subunit alpha-1G | 2004 | 5.22E-18 | 83 |
| Efet.01.1655655.g776.t1 | Cholecystokinin receptor type A | 366 | 4.48E-09 | 83 |
| Efet.01.1658367.g1164.t1 | Collagen alpha-6(IV) chain | 441 | 9.10E-41 | 83 |
| Efet.01.236887.g1435.t1 | Cytochrome c | 333 | 8.21E-53 | 83 |
| Efet.01.32743.g538.t1 | Dynamin-1-like protein | 417 | 3.77E-46 | 83 |
| Efet.01.368485.g497.t1 | Dual specificity protein phosphatase 6 | 405 | 1.13E-30 | 83 |
| Efet.01.509455.g240.t1 | Dynamin-2 | 207 | 7.10E-30 | 83 |
| Efet.01.136471.g573.t1 | Excitatory amino acid transporter 2 | 519 | 2.36E-58 | 83 |
| Efet.01.419320.g447.t1 | Histone acetyltransferase p300 | 315 | 4.17E-30 | 83 |
| Efet.01.253320.g131.t1 | Ezrin | 306 | 6.65E-11 | 83 |
| Efet.01.15180.g1114.t1 | Friend leukemia integration 1 transcription factor | 510 | 5.82E-21 | 83 |
| Efet.01.153418.g184.t1 | Hepatocyte nuclear factor 3-beta | 534 | 1.89E-39 | 83 |
| Efet.01.45649.g1333.t1 | Forkhead box protein C1 | 696 | 7.61E-60 | 83 |
| Efet.01.318200.g571.t1 | Forkhead box protein C1 | 396 | 6.93E-16 | 83 |
| Efet.01.192196.g1969.t1 | Tyrosine-protein kinase FRK | 216 | 4.55E-20 | 83 |
| Efet.01.519525.g466.t1 | Glucose-6-phosphate 1-dehydrogenase | 492 | 8.49E-67 | 83 |
| Efet.01.200918.g46.t1 | Glutamate receptor ionotropic, kainate 2 | 276 | 1.67E-31 | 83 |
| Efet.01.70776.g1281.t1 | Heart- and neural crest derivatives-expressed protein 2 | 780 | 6.41E-31 | 83 |
| Efet.01.253281.g127.t1 | Homeodomain-interacting protein kinase 3 | 606 | 5.35E-101 | 83 |
| Efet.01.383036.g866.t1 | High mobility group protein B2 | 351 | 5.71E-34 | 83 |
| Efet.01.59126.g574.t1 | Homeobox protein Hox-A5 | 645 | 4.77E-32 | 83 |
| Efet.01.267612.g639.t1 | KN motif and ankyrin repeat domain-containing protein 1 | 2988 | 2.00E-11 | 83 |
| Efet.01.194660.g2076.t1 | cAMP-dependent protein kinase catalytic subunit gamma | 312 | 2.24E-14 | 83 |
| Efet.01.653239.g411.t1 | Plasma kallikrein | 231 | 6.28E-29 | 83 |
| Efet.01.1652593.g569.t1 | Low-density lipoprotein receptor-related protein 5 | 204 | 3.03E-29 | 83 |
| Efet.01.476829.g686.t1 | Myosin light chain kinase, smooth muscle | 534 | 1.57E-61 | 83 |
| Efet.01.458423.g233.t1 | Homeobox protein Nkx-2.2 | 1074 | 1.68E-38 | 83 |
| Efet.01.213526.g576.t1 | NPC intracellular cholesterol transporter 1 | 708 | 3.33E-65 | 83 |
| Efet.01.228195.g1132.t1 | Nuclear receptor-binding protein 2 | 336 | 6.23E-19 | 83 |
| Efet.01.283980.g1235.t1 | Vesicle-fusing ATPase | 369 | 5.34E-47 | 83 |
| Efet.01.160557.g552.t1 | Paxillin | 228 | 1.63E-12 | 83 |
| Efet.01.114928.g781.t1 | Early growth response protein | 1182 | 1.57E-54 | 83 |
| Efet.01.299613.g1735.t1 | DNA-binding protein RFX2 | 255 | 2.27E-38 | 83 |
| Efet.01.78699.g221.t1 | Rap guanine nucleotide exchange factor 4 | 291 | 9.92E-30 | 83 |
| Efet.01.265923.g583.t1 | Radial spoke head protein 3 homolog | 693 | 2.00E-27 | 83 |
| Efet.01.120938.g1086.t1 | Solute carrier family 13 member 2 | 228 | 1.35E-19 | 83 |
| Efet.01.459793.g272.t1 | Helicase SKI2W | 564 | 3.10E-78 | 83 |
| Efet.01.448496.g1183.t1 | Suppressor of cytokine signaling 5 | 1485 | 9.65E-84 | 83 |
| Efet.01.56518.g406.t1 | T-box transcription factor TBX3 | 354 | 9.33E-48 | 83 |
| Efet.01.154553.g231.t1 | Twist-related protein 2 | 393 | 7.05E-11 | 83 |
| Efet.01.271724.g814.t1 | Protein Wnt-2 | 249 | 2.65E-25 | 83 |
| Efet.01.365305.g414.t1 | Protein Wnt-4 | 453 | 2.32E-76 | 83 |
| Efet.01.312576.g394.t1 | 14-3-3 protein epsilon | 660 | 9.76E-115 | 82 |
| Efet.01.213127.g554.t1 | Proteasome subunit alpha type | 495 | 3.31E-53 | 82 |
| Efet.01.76887.g104.t1 | ATP-binding cassette sub-family D member 2 | 621 | 2.14E-90 | 82 |
| Efet.01.443588.g1075.t1 | Angiotensin-converting enzyme | 294 | 7.30E-35 | 82 |
| Efet.01.353008.g81.t1 | Neuronal acetylcholine receptor subunit beta-2 | 243 | 1.64E-08 | 82 |
| Efet.01.640020.g549.t1 | Actin, alpha cardiac muscle 1 | 480 | 3.63E-73 | 82 |
| Efet.01.1656677.g901.t1 | Actin, alpha cardiac muscle 1 | 567 | 5.13E-93 | 82 |
| Efet.01.28205.g212.t1 | Activin receptor type-1 | 525 | 1.48E-41 | 82 |
| Efet.01.403654.g95.t1 | Homeobox protein aristaless-like 3 | 627 | 6.25E-19 | 82 |
| Efet.01.38489.g905.t1 | Aminopeptidase N | 279 | 8.11E-31 | 82 |
| Efet.01.382296.g849.t1 | Acidic leucine-rich nuclear phosphoprotein 32 family member B | 774 | 4.11E-27 | 82 |
| Efet.01.97894.g1334.t1 | MAPK8IP3 protein | 480 | 8.17E-24 | 82 |
| Efet.01.2485.g224.t1 | Brefeldin A-inhibited guanine nucleotide-exchange protein 1 | 291 | 2.08E-36 | 82 |
| Efet.01.66506.g1016.t1 | Voltage-dependent T-type calcium channel subunit alpha-1G | 231 | 1.18E-26 | 82 |
| Efet.01.379736.g788.t1 | Voltage-dependent T-type calcium channel subunit alpha-1G | 330 | 7.78E-28 | 82 |
| Efet.01.50666.g47.t1 | Calmodulin-alpha | 222 | 8.75E-17 | 82 |
| Efet.01.1656547.g881.t1 | E3 ubiquitin-protein ligase CBL | 513 | 4.81E-61 | 82 |
| Efet.01.84712.g607.t1 | CREB-binding protein | 1695 | 1.40E-46 | 82 |
| Efet.01.393823.g1094.t1 | CREB-binding protein | 219 | 2.45E-30 | 82 |
| Efet.01.609219.g381.t1 | CREB-binding protein | 654 | 6.86E-99 | 82 |
| Efet.01.532811.g86.t1 | Cyclin-dependent kinase 5 activator 1 | 1131 | 7.27E-73 | 82 |
| Efet.01.1653352.g609.t1 | Chromodomain-helicase-DNA-binding protein 8 | 282 | 2.14E-34 | 82 |
| Efet.01.480730.g771.t1 | Collagen alpha-1(IV) chain | 405 | 1.86E-39 | 82 |
| Efet.01.50257.g22.t1 | Cullin-3 | 666 | 2.89E-76 | 82 |
| Efet.01.441530.g1021.t1 | ATP-dependent RNA helicase DDX42 | 357 | 8.12E-16 | 82 |
| Efet.01.383830.g885.t1 | MDS1 and EVI1 complex locus protein EVI1 | 2088 | 1.56E-51 | 82 |
| Efet.01.152059.g121.t1 | Hepatocyte nuclear factor 4 4 alpha variant 4 | 978 | 2.45E-56 | 82 |
| Efet.01.209460.g427.t1 | Forkhead box protein C2 | 987 | 4.26E-32 | 82 |
| Efet.01.121322.g1108.t1 | Alpha-(1,6)-fucosyltransferase | 393 | 7.10E-54 | 82 |
| Efet.01.1625073.g73.t1 | Gastrin/cholecystokinin type B receptor | 246 | 2.96E-07 | 82 |
| Efet.01.43558.g1203.t1 | PDZ domain-containing protein GIPC1 | 321 | 2.83E-30 | 82 |
| Efet.01.34225.g639.t1 | Homeodomain-interacting protein kinase 2 | 1125 | 2.99E-174 | 82 |
| Efet.01.4907.g385.t1 | cAMP-dependent protein kinase type I-alpha regulatory subunit | 333 | 5.11E-43 | 82 |
| Efet.01.552145.g554.t1 | cAMP-dependent protein kinase catalytic subunit alpha | 441 | 5.07E-24 | 82 |
| Efet.01.35862.g723.t1 | Calcium-activated potassium channel subunit alpha-1 | 261 | 5.50E-27 | 82 |
| Efet.01.154297.g215.t1 | Inactive histone-lysine N-methyltransferase 2E | 822 | 1.42E-28 | 82 |
| Efet.01.510023.g246.t1 | Protein kinase C epsilon type | 288 | 2.68E-21 | 82 |
| Efet.01.57817.g487.t1 | Protein kinase C gamma type | 330 | 6.51E-07 | 82 |
| Efet.01.637471.g378.t1 | Lipoyl synthase, mitochondrial | 249 | 3.68E-16 | 82 |
| Efet.01.140300.g788.t1 | Multidrug resistance protein 1 | 297 | 9.39E-26 | 82 |
| Efet.01.596611.g1193.t1 | Myosin-10 | 369 | 1.12E-47 | 82 |
| Efet.01.57438.g464.t1 | Neurogenic differentiation factor 1 | 1200 | 8.41E-42 | 82 |
| Efet.01.121428.g1112.t1 | Serine/threonine-protein kinase NLK | 342 | 3.51E-48 | 82 |
| Efet.01.497455.g1122.t1 | Serine/threonine-protein kinase PAK 1 | 363 | 1.45E-46 | 82 |
| Efet.01.620549.g898.t1 | Phospholipase D1 | 1074 | 2.30E-07 | 82 |
| Efet.01.11025.g810.t1 | Thioredoxin-dependent peroxide reductase, mitochondrial | 255 | 9.18E-41 | 82 |
| Efet.01.239850.g1530.t1 | Full-length cDNA clone CS0DD006YF02 of Neuroblastoma of Homo sapiens (human) | 456 | 2.56E-69 | 82 |
| Efet.01.394995.g1117.t1 | Ras-related protein Rab-8A | 204 | 3.61E-26 | 82 |
| Efet.01.1632648.g125.t1 | Double-strand-break repair protein rad21 homolog | 243 | 2.54E-16 | 82 |
| Efet.01.520.g57.t1 | Regulating synaptic membrane exocytosis protein 2 | 327 | 7.37E-11 | 82 |
| Efet.01.649786.g1350.t1 | Ribonucleoside-diphosphate reductase subunit M2 B | 573 | 5.23E-66 | 82 |
| Efet.01.199526.g2284.t1 | DNA-directed RNA polymerase III subunit RPC1 | 387 | 1.12E-44 | 82 |
| Efet.01.95090.g1166.t1 | Retinoic acid receptor RXR-beta | 408 | 5.42E-13 | 82 |
| Efet.01.53253.g219.t1 | Sonic hedgehog protein | 204 | 2.26E-26 | 82 |
| Efet.01.47816.g1458.t1 | Homeobox protein SIX1 | 393 | 2.56E-57 | 82 |
| Efet.01.204094.g181.t1 | Spectrin beta chain, non-erythrocytic 2 | 879 | 1.59E-116 | 82 |
| Efet.01.310168.g323.t1 | Syntaxin-binding protein 1 | 267 | 2.83E-35 | 82 |
| Efet.01.164807.g753.t1 | Synaptotagmin-7 | 390 | 2.16E-37 | 82 |
| Efet.01.617314.g770.t1 | T-box transcription factor TBX3 | 303 | 1.57E-23 | 82 |
| Efet.01.104428.g248.t1 | Terminal uridylyltransferase 4 | 327 | 2.31E-27 | 82 |
| Efet.01.125671.g39.t1 | Ubiquitin-protein ligase E3A | 864 | 2.63E-144 | 82 |
| Efet.01.134061.g445.t1 | Serine/threonine-protein phosphatase 2A 65 kDa regulatory subunit A alpha isoform | 342 | 5.61E-34 | 81 |
| Efet.01.17470.g1286.t1 | Interleukin enhancer binding factor 3, 90kDa | 297 | 2.59E-08 | 81 |
| Efet.01.26010.g74.t1 | Minor histocompatibility antigen H13 | 249 | 1.49E-14 | 81 |
| Efet.01.565049.g162.t1 | ATP synthase subunit beta, mitochondrial | 1416 | 0 | 81 |
| Efet.01.315456.g487.t1 | ATP-binding cassette, sub-family A (ABC1), member 1 | 264 | 1.35E-20 | 81 |
| Efet.01.60297.g641.t1 | cDNA FLJ61244, highly similar to Transcription factor 8 | 273 | 6.51E-30 | 81 |
| Efet.01.135095.g491.t1 | BarH-like 1 homeobox protein | 273 | 2.19E-12 | 81 |
| Efet.01.158093.g428.t1 | Chromodomain-helicase-DNA-binding protein 7 | 300 | 3.57E-24 | 81 |
| Efet.01.369276.g523.t1 | Coronin-1A | 228 | 6.03E-33 | 81 |
| Efet.01.34363.g643.t1 | Beta-catenin-like protein 1 | 360 | 6.30E-48 | 81 |
| Efet.01.406317.g154.t1 | ATP-dependent RNA helicase DDX42 | 504 | 1.20E-36 | 81 |
| Efet.01.648808.g1271.t1 | Paired box protein Pax-5 | 447 | 3.57E-50 | 81 |
| Efet.01.38447.g896.t1 | Endothelin-converting enzyme 1 | 465 | 2.99E-53 | 81 |
| Efet.01.303057.g94.t1 | Histone acetyltransferase p300 | 435 | 6.23E-62 | 81 |
| Efet.01.1647925.g389.t1 | DNA excision repair protein ERCC-6-like | 222 | 8.96E-06 | 81 |
| Efet.01.99041.g1390.t1 | F-BAR and double SH3 domains protein 2 | 381 | 6.53E-21 | 81 |
| Efet.01.457146.g185.t1 | Dimethylaniline monooxygenase [N-oxide-forming] 1 | 231 | 8.91E-21 | 81 |
| Efet.01.117617.g913.t1 | Guanine nucleotide-binding protein G(q) subunit alpha | 507 | 2.23E-84 | 81 |
| Efet.01.516705.g419.t1 | Stress-70 protein, mitochondrial | 564 | 1.21E-37 | 81 |
| Efet.01.206375.g279.t1 | Heart- and neural crest derivatives-expressed protein 2 | 726 | 9.19E-23 | 81 |
| Efet.01.545625.g382.t1 | Hepatocyte nuclear factor 6 | 1092 | 7.31E-48 | 81 |
| Efet.01.1652197.g548.t1 | Homeobox protein Hox-A3 | 357 | 1.48E-19 | 81 |
| Efet.01.61532.g715.t1 | Intraflagellar transport protein 88 homolog | 453 | 9.64E-44 | 81 |
| Efet.01.121926.g1142.t1 | Calcium-activated potassium channel subunit alpha-1 | 321 | 2.77E-38 | 81 |
| Efet.01.646144.g1037.t1 | Calcium-activated potassium channel subunit alpha-1 | 318 | 5.31E-26 | 81 |
| Efet.01.391562.g1038.t1 | Potassium voltage-gated channel subfamily H member 8 | 684 | 5.88E-58 | 81 |
| Efet.01.215456.g643.t1 | Histone-lysine N-methyltransferase 2C | 258 | 4.86E-39 | 81 |
| Efet.01.260625.g395.t1 | Laminin subunit beta-1 | 204 | 2.59E-24 | 81 |
| Efet.01.113110.g659.t1 | Low-density lipoprotein receptor-related protein 6 | 588 | 1.93E-81 | 81 |
| Efet.01.506957.g178.t1 | Low-density lipoprotein receptor-related protein 6 | 324 | 1.67E-39 | 81 |
| Efet.01.326123.g805.t1 | Myosin light chain kinase, smooth muscle | 384 | 3.17E-36 | 81 |
| Efet.01.457023.g178.t1 | Homeobox protein Nkx-2.2 | 678 | 3.23E-31 | 81 |
| Efet.01.266383.g596.t1 | Homeobox protein Nkx-3.2 | 681 | 1.67E-19 | 81 |
| Efet.01.32095.g493.t1 | Phosphatidylinositol 4,5-bisphosphate 3-kinase catalytic subunit alpha isoform | 363 | 2.05E-46 | 81 |
| Efet.01.166830.g831.t1 | Receptor-type tyrosine-protein phosphatase F | 1416 | 1.09E-127 | 81 |
| Efet.01.513258.g314.t1 | MRE11A protein | 1068 | 3.37E-140 | 81 |
| Efet.01.210912.g482.t1 | Early growth response protein | 1503 | 2.58E-46 | 81 |
| Efet.01.485141.g859.t1 | Ras-specific guanine nucleotide-releasing factor 1 | 240 | 9.42E-33 | 81 |
| Efet.01.399471.g1229.t1 | Radial spoke head protein 4 homolog A | 237 | 2.87E-20 | 81 |
| Efet.01.48186.g1479.t1 | Secreted frizzled-related protein 5 | 423 | 3.11E-52 | 81 |
| Efet.01.106921.g356.t1 | Zinc finger protein SNAI1 | 903 | 4.23E-53 | 81 |
| Efet.01.91379.g959.t1 | Transcription factor SOX-9 | 345 | 6.30E-11 | 81 |
| Efet.01.282971.g1191.t1 | Synaptotagmin-7 | 336 | 7.84E-40 | 81 |
| Efet.01.284857.g1266.t1 | Twist-related protein 2 | 420 | 8.69E-50 | 81 |
| Efet.01.1658998.g1353.t1 | Calreticulin, isoform CRA_b | 351 | 6.52E-44 | 81 |
| Efet.01.2135.g188.t1 | Protein Wnt-5b | 330 | 5.23E-46 | 81 |
| Efet.01.604633.g170.t1 | Serine/threonine-protein phosphatase 2A 56 kDa regulatory subunit gamma isoform | 336 | 2.57E-44 | 80 |
| Efet.01.169089.g939.t1 | Diacylglycerol kinase | 243 | 1.83E-11 | 80 |
| Efet.01.91538.g971.t1 | DNA ligase | 627 | 4.61E-48 | 80 |
| Efet.01.177285.g1283.t1 | Neuronal acetylcholine receptor subunit alpha-7 | 210 | 5.96E-26 | 80 |
| Efet.01.429353.g723.t1 | Neuronal acetylcholine receptor subunit alpha-7 | 201 | 7.05E-25 | 80 |
| Efet.01.368253.g491.t1 | Alpha-actinin-1 | 270 | 3.16E-24 | 80 |
| Efet.01.1659525.g2023.t1 | Alcohol dehydrogenase class-3 | 1116 | 6.32E-149 | 80 |
| Efet.01.552317.g560.t1 | Band 3 anion transport protein | 330 | 3.95E-14 | 80 |
| Efet.01.129224.g236.t1 | BTB/POZ domain-containing adapter for CUL3-mediated RhoA degradation protein 2 | 453 | 7.11E-54 | 80 |
| Efet.01.578120.g621.t1 | Bone morphogenetic protein 3 | 447 | 4.55E-07 | 80 |
| Efet.01.408157.g199.t1 | BTB/POZ domain-containing protein 9 | 234 | 2.44E-21 | 80 |
| Efet.01.1649875.g453.t1 | Calmodulin-A | 207 | 1.02E-17 | 80 |
| Efet.01.11855.g878.t1 | Cholecystokinin receptor type A | 375 | 6.38E-20 | 80 |
| Efet.01.306527.g213.t1 | Cholecystokinin receptor type A | 402 | 2.28E-21 | 80 |
| Efet.01.94034.g1105.t1 | Cleft lip and palate transmembrane protein 1 | 279 | 4.16E-11 | 80 |
| Efet.01.121668.g1127.t1 | Collagen alpha-1(III) chain | 294 | 3.85E-14 | 80 |
| Efet.01.112740.g644.t1 | COUP transcription factor 2 | 345 | 6.68E-35 | 80 |
| Efet.01.168087.g893.t1 | Cullin-5 | 396 | 8.49E-34 | 80 |
| Efet.01.131588.g343.t1 | Dual specificity protein phosphatase 6 | 354 | 7.23E-12 | 80 |
| Efet.01.188880.g1831.t1 | Dual specificity tyrosine-phosphorylation-regulated kinase 2 | 660 | 3.96E-90 | 80 |
| Efet.01.183691.g1581.t1 | EH domain-containing protein 1 | 648 | 1.18E-93 | 80 |
| Efet.01.116692.g871.t1 | Fasciculation and elongation protein zeta-1 | 222 | 1.40E-11 | 80 |
| Efet.01.27357.g151.t1 | Filamin-A | 210 | 3.53E-21 | 80 |
| Efet.01.44786.g1289.t1 | Forkhead box protein C2 | 987 | 3.23E-32 | 80 |
| Efet.01.80477.g367.t1 | Forkhead box protein C2 | 1185 | 6.71E-29 | 80 |
| Efet.01.75217.g17.t1 | Forkhead box protein O3 | 477 | 1.81E-21 | 80 |
| Efet.01.515650.g382.t1 | Stress-70 protein, mitochondrial | 1962 | 0 | 80 |
| Efet.01.505473.g138.t1 | Lymphoid-specific helicase | 207 | 2.06E-31 | 80 |
| Efet.01.276854.g989.t1 | Hypoxia-inducible factor 1-alpha | 267 | 9.15E-16 | 80 |
| Efet.01.357018.g197.t1 | DNA-binding protein inhibitor ID-2 | 435 | 8.58E-15 | 80 |
| Efet.01.1550.g148.t1 | Intraflagellar transport protein 46 homolog | 447 | 3.96E-51 | 80 |
| Efet.01.426161.g624.t1 | cAMP-dependent protein kinase catalytic subunit alpha | 315 | 9.03E-29 | 80 |
| Efet.01.321180.g665.t1 | cAMP-dependent protein kinase catalytic subunit beta | 342 | 8.90E-17 | 80 |
| Efet.01.86998.g747.t1 | Histone acetyltransferase KAT2A | 324 | 1.45E-38 | 80 |
| Efet.01.597775.g1237.t1 | Potassium voltage-gated channel subfamily H member 8 | 369 | 2.62E-13 | 80 |
| Efet.01.1629507.g96.t1 | Protein kinase C zeta type | 213 | 3.28E-21 | 80 |
| Efet.01.5268.g410.t1 | Ribosomal protein S6 kinase alpha-5 | 396 | 4.60E-47 | 80 |
| Efet.01.115880.g827.t1 | Lethal(2) giant larvae protein homolog 1 | 252 | 4.69E-14 | 80 |
| Efet.01.59965.g624.t1 | LIM/homeobox protein Lhx4 | 327 | 2.71E-08 | 80 |
| Efet.01.256670.g248.t1 | Lipopolysaccharide-responsive and beige-like anchor protein | 1623 | 1.98E-92 | 80 |
| Efet.01.19816.g1469.t1 | Leucine-rich repeats and immunoglobulin-like domains protein 3 | 285 | 4.00E-25 | 80 |
| Efet.01.507997.g205.t1 | Low-density lipoprotein receptor-related protein 6 | 279 | 5.32E-34 | 80 |
| Efet.01.258991.g343.t1 | Mitogen-activated protein kinase kinase kinase 1 | 369 | 1.53E-18 | 80 |
| Efet.01.28395.g226.t1 | Myosin-10 | 375 | 1.58E-43 | 80 |
| Efet.01.163808.g695.t1 | Negative elongation factor C/D | 246 | 5.35E-22 | 80 |
| Efet.01.143799.g964.t1 | Homeobox protein Nkx-2.6 | 1050 | 1.77E-41 | 80 |
| Efet.01.292570.g1528.t1 | One cut domain family member 2 | 264 | 2.62E-28 | 80 |
| Efet.01.286820.g1336.t1 | Serine/threonine-protein kinase PAK 1 | 729 | 1.58E-111 | 80 |
| Efet.01.62056.g753.t1 | Proline dehydrogenase 1, mitochondrial | 345 | 1.94E-18 | 80 |
| Efet.01.24289.g1764.t1 | 26S proteasome non-ATPase regulatory subunit 11 | 312 | 4.17E-28 | 80 |
| Efet.01.470851.g549.t1 | Inactive tyrosine-protein kinase 7 | 405 | 6.07E-35 | 80 |
| Efet.01.564043.g128.t1 | Receptor-type tyrosine-protein phosphatase F | 252 | 3.20E-34 | 80 |
| Efet.01.463340.g359.t1 | TAL-1 | 570 | 3.07E-33 | 80 |
| Efet.01.114530.g756.t1 | Early growth response protein | 759 | 5.96E-44 | 80 |
| Efet.01.254293.g158.t1 | Double-stranded RNA-specific editase 1 | 315 | 6.61E-12 | 80 |
| Efet.01.79666.g301.t1 | Sodium channel protein type 2 subunit alpha | 489 | 3.91E-45 | 80 |
| Efet.01.164424.g732.t1 | Sodium channel protein type 2 subunit alpha | 528 | 1.46E-07 | 80 |
| Efet.01.323739.g732.t1 | SH2B adapter protein 2 | 444 | 7.06E-20 | 80 |
| Efet.01.264832.g557.t1 | Sonic hedgehog protein | 273 | 4.71E-13 | 80 |
| Efet.01.602849.g116.t1 | STE20-like serine/threonine-protein kinase | 276 | 5.61E-38 | 80 |
| Efet.01.251453.g52.t1 | Transcription factor SOX-2 | 732 | 4.89E-47 | 80 |
| Efet.01.143193.g934.t1 | Spectrin beta chain, non-erythrocytic 2 | 375 | 1.10E-42 | 80 |
| Efet.01.244152.g1670.t1 | Proto-oncogene tyrosine-protein kinase Src | 450 | 2.31E-19 | 80 |
| Efet.01.113474.g684.t1 | Serine/threonine-protein kinase 17A | 795 | 2.57E-12 | 80 |
| Efet.01.1642265.g251.t1 | Synaptotagmin-7 | 276 | 1.65E-20 | 80 |
| Efet.01.114077.g716.t1 | YTH domain-containing family protein 2 | 1368 | 1.49E-94 | 80 |
| Efet.01.43976.g1226.t1 | Zinc finger FYVE domain-containing protein 9 | 858 | 1.42E-23 | 80 |
| Efet.01.19314.g1430.t1 | Proteasome subunit alpha type | 552 | 1.35E-77 | 79 |
| Efet.01.381535.g823.t1 | ATP-binding cassette sub-family G member 1 | 603 | 9.44E-86 | 79 |
| Efet.01.297493.g1672.t1 | Achaete-scute homolog 1 | 615 | 4.46E-08 | 79 |
| Efet.01.555240.g637.t1 | ATP synthase subunit alpha, mitochondrial | 1179 | 4.28E-145 | 79 |
| Efet.01.73419.g1444.t1 | cDNA FLJ46417 fis, clone THYMU3012402, highly similar to Importin alpha-1 subunit (cDNA FLJ55353, highly similar to Importin alpha-1 subunit) | 315 | 3.14E-41 | 79 |
| Efet.01.658238.g1742.t1 | Catenin beta-1 | 729 | 3.41E-103 | 79 |
| Efet.01.485993.g877.t1 | cDNA FLJ50323, highly similar to Macrophage-stimulating protein receptor | 303 | 1.20E-34 | 79 |
| Efet.01.84848.g617.t1 | Baculoviral IAP repeat-containing protein 3 | 426 | 3.93E-24 | 79 |
| Efet.01.633301.g189.t1 | Bone morphogenetic protein 10 | 564 | 3.44E-06 | 79 |
| Efet.01.415860.g371.t1 | Calmodulin-regulated spectrin-associated protein 3 | 2811 | 1.33E-06 | 79 |
| Efet.01.162043.g624.t1 | CD109 antigen | 255 | 2.69E-36 | 79 |
| Efet.01.460181.g288.t1 | Collagen alpha-6(IV) chain | 267 | 1.89E-38 | 79 |
| Efet.01.228341.g1145.t1 | Catenin alpha-1 | 222 | 2.27E-17 | 79 |
| Efet.01.6605.g516.t1 | Dual specificity tyrosine-phosphorylation-regulated kinase 2 | 1518 | 0 | 79 |
| Efet.01.350874.g25.t1 | Band 4.1-like protein 5 | 414 | 4.73E-34 | 79 |
| Efet.01.1611785.g24.t1 | Fermitin family homolog 2 | 264 | 1.54E-36 | 79 |
| Efet.01.43739.g1216.t1 | Filamin-A | 342 | 9.37E-36 | 79 |
| Efet.01.446074.g1139.t1 | Filamin-A | 444 | 4.85E-29 | 79 |
| Efet.01.645075.g925.t1 | Tyrosine-protein kinase Fyn | 405 | 1.26E-50 | 79 |
| Efet.01.6243.g491.t1 | Guanine nucleotide-binding protein subunit alpha-12 | 717 | 8.33E-61 | 79 |
| Efet.01.519408.g464.t1 | Inosine-5'-monophosphate dehydrogenase 2 | 435 | 7.31E-22 | 79 |
| Efet.01.12946.g971.t1 | Intersectin-1 | 207 | 2.11E-20 | 79 |
| Efet.01.135232.g505.t1 | Adenylate kinase isoenzyme 6 | 567 | 1.31E-74 | 79 |
| Efet.01.528516.g647.t1 | cAMP-dependent protein kinase catalytic subunit alpha | 255 | 1.57E-27 | 79 |
| Efet.01.403178.g86.t1 | Kinesin-like protein KIF1B | 285 | 2.07E-28 | 79 |
| Efet.01.523430.g540.t1 | Krueppel-like factor 1 | 1392 | 3.48E-33 | 79 |
| Efet.01.654000.g516.t1 | Krueppel-like factor 2 | 1356 | 1.16E-34 | 79 |
| Efet.01.63758.g845.t1 | Protein kinase C beta type | 297 | 3.33E-42 | 79 |
| Efet.01.147229.g1108.t1 | Laminin subunit beta-1 | 630 | 2.34E-77 | 79 |
| Efet.01.284677.g1254.t1 | Membrane-associated guanylate kinase, WW and PDZ domain-containing protein 1 | 267 | 7.40E-19 | 79 |
| Efet.01.126151.g65.t1 | Inner nuclear membrane protein Man1 | 354 | 3.41E-42 | 79 |
| Efet.01.214501.g614.t1 | Mitogen-activated protein kinase 7 | 540 | 4.30E-70 | 79 |
| Efet.01.438619.g913.t1 | Methylmalonate-semialdehyde dehydrogenase [acylating], mitochondrial | 918 | 1.26E-132 | 79 |
| Efet.01.1635333.g160.t1 | Serine/threonine-protein kinase mTOR | 279 | 3.94E-14 | 79 |
| Efet.01.104975.g273.t1 | Myoblast determination protein 1 | 1179 | 4.84E-21 | 79 |
| Efet.01.104578.g255.t1 | Neuronal calcium sensor 1 | 396 | 1.41E-57 | 79 |
| Efet.01.464992.g401.t1 | Neurogenic differentiation factor 1 | 816 | 8.74E-17 | 79 |
| Efet.01.1658488.g1194.t1 | Netrin-1 | 927 | 2.26E-99 | 79 |
| Efet.01.44481.g1257.t1 | Neuroligin-2 | 309 | 2.07E-31 | 79 |
| Efet.01.561911.g37.t1 | One cut domain family member 2 | 234 | 2.88E-19 | 79 |
| Efet.01.133008.g400.t1 | Polyadenylate-binding protein 4 | 738 | 8.92E-118 | 79 |
| Efet.01.408769.g209.t1 | Serine/threonine-protein kinase PAK 1 | 477 | 8.06E-61 | 79 |
| Efet.01.575763.g507.t1 | Paired box protein Pax-3 | 438 | 6.13E-18 | 79 |
| Efet.01.53633.g240.t1 | Serine/threonine-protein kinase pim-1 | 651 | 2.53E-76 | 79 |
| Efet.01.95089.g1165.t1 | Peptidyl-prolyl cis-trans isomerase NIMA-interacting 1 | 252 | 1.45E-25 | 79 |
| Efet.01.161840.g613.t1 | Phosphatidylinositol 4,5-bisphosphate 3-kinase catalytic subunit alpha isoform | 519 | 1.24E-33 | 79 |
| Efet.01.243480.g1653.t1 | POU domain, class 4, transcription factor 3 | 324 | 1.64E-32 | 79 |
| Efet.01.1404.g132.t1 | Poly(U)-binding-splicing factor PUF60 | 291 | 6.43E-35 | 79 |
| Efet.01.68406.g1130.t1 | ABC50 protein (ATP-binding cassette, sub-family F (GCN20), member 1) | 606 | 1.36E-20 | 79 |
| Efet.01.560587.g15.t1 | CCND1 protein (Cyclin D1 (PRAD1: parathyroid adenomatosis 1)) (Cyclin D1, isoform CRA_c) | 279 | 8.06E-18 | 79 |
| Efet.01.535313.g170.t1 | Ras-related protein Rab-3C | 291 | 1.81E-23 | 79 |
| Efet.01.14825.g1090.t1 | Serine/threonine-protein kinase SIK2 | 1446 | 4.46E-10 | 79 |
| Efet.01.159466.g492.t1 | Homeobox protein SIX1 | 645 | 8.51E-79 | 79 |
| Efet.01.10938.g801.t1 | SNF-related serine/threonine-protein kinase | 405 | 4.99E-48 | 79 |
| Efet.01.68528.g1136.t1 | Transcription factor SOX-2 | 336 | 1.56E-29 | 79 |
| Efet.01.144207.g984.t1 | Transcription factor Sp3 | 1383 | 5.49E-57 | 79 |
| Efet.01.349344.g1391.t1 | Spectrin beta chain, erythrocytic | 534 | 4.95E-71 | 79 |
| Efet.01.341935.g1207.t1 | Extracellular sulfatase Sulf-1 | 339 | 8.63E-42 | 79 |
| Efet.01.118916.g980.t1 | Synaptotagmin-7 | 669 | 3.27E-28 | 79 |
| Efet.01.245427.g1709.t1 | Synaptotagmin-7 | 594 | 3.59E-52 | 79 |
| Efet.01.246409.g1756.t1 | Synaptotagmin-7 | 285 | 1.48E-21 | 79 |
| Efet.01.1649392.g435.t1 | TNF receptor-associated factor 1 | 309 | 3.52E-45 | 79 |
| Efet.01.349899.g1403.t1 | Nuclear factor 1 | 939 | 1.81E-33 | 79 |
| Efet.01.187337.g1745.t1 | Ubiquitin carboxyl-terminal hydrolase 28 | 231 | 8.55E-26 | 79 |
| Efet.01.4757.g380.t1 | 14-3-3 protein epsilon | 717 | 3.61E-121 | 78 |
| Efet.01.403416.g89.t1 | 5-hydroxytryptamine receptor 2B | 465 | 1.00E-08 | 78 |
| Efet.01.467801.g475.t1 | 5-hydroxytryptamine receptor 2B | 465 | 1.00E-08 | 78 |
| Efet.01.30813.g401.t1 | Nuclear factor interleukin-3-regulated protein | 540 | 1.80E-20 | 78 |
| Efet.01.315484.g489.t1 | Alpha-actinin-1 | 471 | 1.51E-65 | 78 |
| Efet.01.652346.g281.t1 | Apoptosis-inducing factor 1, mitochondrial | 393 | 5.01E-46 | 78 |
| Efet.01.637136.g366.t1 | A-kinase anchor protein 17A | 810 | 9.36E-113 | 78 |
| Efet.01.278716.g1055.t1 | Aldehyde dehydrogenase, mitochondrial | 468 | 9.08E-60 | 78 |
| Efet.01.301345.g48.t1 | Annexin A4 | 393 | 5.24E-47 | 78 |
| Efet.01.597232.g1201.t1 | Achaete-scute homolog 1 | 975 | 2.35E-11 | 78 |
| Efet.01.400774.g11.t1 | Sarcoplasmic/endoplasmic reticulum calcium ATPase 1 | 324 | 3.17E-09 | 78 |
| Efet.01.165507.g774.t1 | Sarcoplasmic/endoplasmic reticulum calcium ATPase 2 | 1002 | 1.71E-80 | 78 |
| Efet.01.468581.g493.t1 | Sarcoplasmic/endoplasmic reticulum calcium ATPase 2 | 339 | 7.81E-41 | 78 |
| Efet.01.24328.g1769.t1 | Calcium-transporting ATPase type 2C member 1 | 246 | 2.16E-14 | 78 |
| Efet.01.439123.g924.t1 | Band 3 anion transport protein | 477 | 9.89E-15 | 78 |
| Efet.01.1657637.g1020.t1 | cDNA FLJ61244, highly similar to Transcription factor 8 | 255 | 2.05E-24 | 78 |
| Efet.01.101363.g88.t1 | BRCA1-associated RING domain protein 1 | 792 | 2.90E-07 | 78 |
| Efet.01.251242.g43.t1 | B-cell lymphoma/leukemia 11B | 237 | 1.84E-16 | 78 |
| Efet.01.82781.g509.t1 | Bax inhibitor 1 | 354 | 2.66E-23 | 78 |
| Efet.01.189150.g1842.t1 | Coatomer subunit gamma-2 | 345 | 6.98E-42 | 78 |
| Efet.01.206659.g292.t1 | Delta(24)-sterol reductase | 363 | 8.37E-52 | 78 |
| Efet.01.142360.g898.t1 | Disks large homolog 1 | 336 | 5.64E-37 | 78 |
| Efet.01.278263.g1045.t1 | C-Jun-amino-terminal kinase-interacting protein 3 | 756 | 3.65E-08 | 78 |
| Efet.01.643324.g771.t1 | EH domain-containing protein 1 | 465 | 3.11E-58 | 78 |
| Efet.01.11926.g884.t1 | Histone acetyltransferase p300 | 618 | 2.59E-63 | 78 |
| Efet.01.472493.g610.t1 | DNA excision repair protein ERCC-6-like | 333 | 1.10E-28 | 78 |
| Efet.01.51521.g99.t1 | Filamin-A | 372 | 3.92E-34 | 78 |
| Efet.01.115608.g806.t1 | Tyrosine-protein kinase FRK | 879 | 9.12E-139 | 78 |
| Efet.01.186608.g1707.t1 | Frizzled-4 | 1521 | 2.62E-80 | 78 |
| Efet.01.20441.g1505.t1 | Guanine nucleotide-binding protein subunit alpha-12 | 606 | 6.13E-64 | 78 |
| Efet.01.38025.g865.t1 | Hepatic leukemia factor | 318 | 3.42E-26 | 78 |
| Efet.01.58581.g526.t1 | Homeobox protein Hox-B4 | 426 | 6.33E-09 | 78 |
| Efet.01.271657.g811.t1 | Interferon regulatory factor 2-binding protein 2 | 804 | 3.11E-35 | 78 |
| Efet.01.136375.g567.t1 | Transcription factor AP-1 | 972 | 1.48E-18 | 78 |
| Efet.01.16539.g1220.t1 | KN motif and ankyrin repeat domain-containing protein 1 | 1260 | 2.12E-06 | 78 |
| Efet.01.127944.g164.t1 | Histone acetyltransferase KAT8 | 216 | 4.10E-26 | 78 |
| Efet.01.51926.g127.t1 | Inactive histone-lysine N-methyltransferase 2E | 804 | 3.10E-32 | 78 |
| Efet.01.402937.g78.t1 | Pyruvate kinase PKM | 738 | 1.12E-99 | 78 |
| Efet.01.529096.g669.t1 | Serine/threonine-protein kinase LATS2 | 1632 | 5.03E-59 | 78 |
| Efet.01.100069.g2.t1 | Lipopolysaccharide-responsive and beige-like anchor protein | 1320 | 1.05E-76 | 78 |
| Efet.01.48455.g1492.t1 | Microtubule-associated serine/threonine-protein kinase 2 | 300 | 7.11E-24 | 78 |
| Efet.01.515990.g398.t1 | Cytoplasmic protein NCK2 | 444 | 1.68E-15 | 78 |
| Efet.01.7805.g577.t1 | Neuronal calcium sensor 1 | 408 | 1.70E-50 | 78 |
| Efet.01.157126.g374.t1 | Neurogenic differentiation factor 1 | 672 | 7.40E-13 | 78 |
| Efet.01.444232.g1089.t1 | Homeobox protein Nkx-2.2 | 933 | 7.90E-31 | 78 |
| Efet.01.139887.g762.t1 | Neurogenic locus notch homolog protein 2 | 1206 | 4.54E-21 | 78 |
| Efet.01.532190.g70.t1 | Cellular tumor antigen p53 | 348 | 1.94E-16 | 78 |
| Efet.01.71244.g1312.t1 | Phosphatidylinositol 3-kinase regulatory subunit beta | 294 | 7.98E-15 | 78 |
| Efet.01.282729.g1182.t1 | cGMP-inhibited 3',5'-cyclic phosphodiesterase B | 369 | 3.13E-44 | 78 |
| Efet.01.215264.g639.t1 | Phosphatidylinositol 4-kinase beta | 453 | 3.05E-25 | 78 |
| Efet.01.3425.g297.t1 | Serine/threonine-protein kinase pim-1 | 330 | 2.67E-39 | 78 |
| Efet.01.169395.g953.t1 | Serine/threonine-protein phosphatase 2A activator | 411 | 1.61E-23 | 78 |
| Efet.01.347255.g1329.t1 | Tubulin beta chain | 927 | 1.76E-153 | 78 |
| Efet.01.494785.g1066.t1 | Ras-related C3 botulinum toxin substrate 1 | 264 | 9.84E-07 | 78 |
| Efet.01.318728.g584.t1 | Ras-related protein Ral-B | 252 | 2.32E-06 | 78 |
| Efet.01.77324.g132.t1 | Radial spoke head 1 homolog | 699 | 1.97E-63 | 78 |
| Efet.01.47442.g1443.t1 | Retinoic acid receptor RXR-alpha | 417 | 2.38E-30 | 78 |
| Efet.01.96837.g1271.t1 | Semaphorin-5A | 315 | 9.23E-19 | 78 |
| Efet.01.308953.g280.t1 | Homeobox protein SIX1 | 642 | 1.85E-69 | 78 |
| Efet.01.439588.g938.t1 | Helicase SKI2W | 414 | 7.21E-35 | 78 |
| Efet.01.89450.g858.t1 | SPARC-related modular calcium-binding protein 2 | 318 | 1.62E-07 | 78 |
| Efet.01.168675.g921.t1 | Transcription factor SOX-4 | 1152 | 9.40E-35 | 78 |
| Efet.01.255662.g208.t1 | Transcription factor SOX-4 | 612 | 2.46E-25 | 78 |
| Efet.01.401587.g39.t1 | SRSF protein kinase 2 | 1239 | 1.05E-77 | 78 |
| Efet.01.260967.g413.t1 | Syntaxin-binding protein 1 | 1140 | 3.70E-174 | 78 |
| Efet.01.81034.g403.t1 | T-cell acute lymphocytic leukemia protein 2 | 543 | 4.10E-13 | 78 |
| Efet.01.250343.g11.t1 | E3 ubiquitin-protein ligase Topors | 429 | 9.82E-15 | 78 |
| Efet.01.18112.g1338.t1 | Triple functional domain protein | 393 | 3.26E-45 | 78 |
| Efet.01.28650.g249.t1 | Twist-related protein 1 | 696 | 6.28E-15 | 78 |
| Efet.01.202250.g109.t1 | Vinexin | 951 | 6.30E-19 | 78 |
| Efet.01.645063.g924.t1 | Serine/threonine-protein kinase WNK2 | 216 | 2.92E-21 | 78 |
| Efet.01.441490.g1019.t1 | Protein Wnt-7a | 426 | 9.59E-23 | 78 |
| Efet.01.258575.g319.t1 | X-box-binding protein 1 | 1545 | 2.63E-14 | 78 |
| Efet.01.655798.g876.t1 | Serine/threonine-protein phosphatase 2A 55 kDa regulatory subunit B beta isoform | 405 | 3.94E-49 | 77 |
| Efet.01.89937.g891.t1 | Epithelial discoidin domain-containing receptor 1 | 306 | 2.60E-25 | 77 |
| Efet.01.369119.g519.t1 | Proteasome (Prosome, macropain) 26S subunit, ATPase, 4, isoform CRA_b | 783 | 3.08E-109 | 77 |
| Efet.01.47515.g1447.t1 | Mitogen-activated protein kinase kinase 6 | 609 | 3.64E-70 | 77 |
| Efet.01.349746.g1398.t1 | Neuronal acetylcholine receptor subunit alpha-4 | 1164 | 2.93E-42 | 77 |
| Efet.01.598493.g1252.t1 | Neuronal acetylcholine receptor subunit alpha-4 | 231 | 1.33E-08 | 77 |
| Efet.01.213325.g563.t1 | Activin receptor type-1 | 846 | 1.16E-118 | 77 |
| Efet.01.275054.g924.t1 | Alpha-2A adrenergic receptor | 345 | 9.41E-08 | 77 |
| Efet.01.209679.g434.t1 | Gamma-adducin | 387 | 2.60E-35 | 77 |
| Efet.01.492264.g1003.t1 | Homeobox protein aristaless-like 4 | 249 | 2.26E-15 | 77 |
| Efet.01.167644.g875.t1 | Ankyrin-1 | 219 | 3.47E-22 | 77 |
| Efet.01.65318.g931.t1 | AP-2 complex subunit alpha-1 | 225 | 3.86E-09 | 77 |
| Efet.01.190094.g1876.t1 | Rho guanine nucleotide exchange factor 12 | 315 | 5.42E-21 | 77 |
| Efet.01.135513.g518.t1 | Apoptosis-stimulating of p53 protein 1 | 336 | 3.69E-43 | 77 |
| Efet.01.1583077.g6.t1 | BarH-like 1 homeobox protein | 210 | 6.49E-21 | 77 |
| Efet.01.151411.g87.t1 | Bone morphogenetic protein 2 | 1155 | 1.50E-22 | 77 |
| Efet.01.105471.g292.t1 | Calmodulin-alpha | 216 | 3.88E-17 | 77 |
| Efet.01.99136.g1396.t1 | Cell division cycle and apoptosis regulator protein 1 | 525 | 4.89E-71 | 77 |
| Efet.01.189875.g1865.t1 | Cholecystokinin receptor type A | 432 | 3.84E-12 | 77 |
| Efet.01.64177.g875.t1 | Cytochrome c | 396 | 3.64E-11 | 77 |
| Efet.01.283573.g1222.t1 | DDB1- and CUL4-associated factor 1 | 462 | 4.82E-47 | 77 |
| Efet.01.398517.g1206.t1 | DDB1- and CUL4-associated factor 1 | 234 | 3.23E-12 | 77 |
| Efet.01.170352.g991.t1 | Glutamate decarboxylase 1 | 285 | 2.28E-21 | 77 |
| Efet.01.1634715.g153.t1 | Dynamin-1 | 231 | 1.53E-20 | 77 |
| Efet.01.12775.g960.t1 | Dual specificity tyrosine-phosphorylation-regulated kinase 2 | 1524 | 0 | 77 |
| Efet.01.18228.g1350.t1 | Epidermal growth factor receptor | 222 | 9.57E-21 | 77 |
| Efet.01.214912.g625.t1 | MDS1 and EVI1 complex locus protein EVI1 | 1464 | 4.22E-49 | 77 |
| Efet.01.17273.g1274.t1 | Fas apoptotic inhibitory molecule 1 | 282 | 1.14E-24 | 77 |
| Efet.01.208210.g374.t1 | Filamin-A | 594 | 6.05E-70 | 77 |
| Efet.01.477781.g715.t1 | Hepatocyte nuclear factor 3-beta | 1533 | 3.21E-60 | 77 |
| Efet.01.265657.g576.t1 | Tyrosine-protein kinase Fyn | 414 | 8.08E-21 | 77 |
| Efet.01.172827.g1087.t1 | 1-phosphatidylinositol 3-phosphate 5-kinase | 465 | 4.32E-24 | 77 |
| Efet.01.274959.g920.t1 | Guanine nucleotide-binding protein subunit alpha-12 | 1080 | 8.04E-149 | 77 |
| Efet.01.426224.g626.t1 | Hemicentin-1 | 450 | 4.57E-21 | 77 |
| Efet.01.73780.g1461.t1 | Histamine H1 receptor | 327 | 2.20E-26 | 77 |
| Efet.01.579690.g666.t1 | Heparan sulfate glucosamine 3-O-sulfotransferase 5 | 453 | 4.71E-17 | 77 |
| Efet.01.63299.g817.t1 | Inhibitor of growth protein 4 | 480 | 1.43E-21 | 77 |
| Efet.01.13736.g1022.t1 | Inositol 1,4,5-trisphosphate receptor type 1 | 690 | 6.60E-70 | 77 |
| Efet.01.119547.g1013.t1 | cAMP-dependent protein kinase catalytic subunit alpha | 618 | 8.92E-87 | 77 |
| Efet.01.371309.g599.t1 | Potassium voltage-gated channel subfamily H member 8 | 372 | 2.02E-13 | 77 |
| Efet.01.518849.g454.t1 | Krueppel-like factor 2 | 1164 | 8.58E-34 | 77 |
| Efet.01.71449.g1329.t1 | Histone-lysine N-methyltransferase 2D | 285 | 3.86E-23 | 77 |
| Efet.01.86115.g697.t1 | Serine/threonine-protein kinase D1 | 276 | 1.27E-06 | 77 |
| Efet.01.246645.g1767.t1 | KICSTOR complex protein kaptin | 288 | 8.28E-12 | 77 |
| Efet.01.190823.g1904.t1 | LIM/homeobox protein Lhx4 | 474 | 7.04E-41 | 77 |
| Efet.01.14921.g1096.t1 | Low-density lipoprotein receptor-related protein 6 | 357 | 1.28E-43 | 77 |
| Efet.01.571572.g369.t1 | Mitogen-activated protein kinase kinase kinase 4 | 243 | 3.83E-24 | 77 |
| Efet.01.74750.g1528.t1 | MAP kinase-activated protein kinase 2 | 222 | 3.78E-20 | 77 |
| Efet.01.21925.g1606.t1 | Microtubule-associated serine/threonine-protein kinase 2 | 771 | 5.64E-16 | 77 |
| Efet.01.490393.g964.t1 | Multidrug resistance-associated protein 4 | 360 | 2.81E-32 | 77 |
| Efet.01.1372.g128.t1 | Myosin-10 | 450 | 1.58E-56 | 77 |
| Efet.01.652915.g381.t1 | Neuronal calcium sensor 1 | 378 | 1.12E-50 | 77 |
| Efet.01.226079.g1043.t1 | Homeobox protein Nkx-3.2 | 849 | 1.75E-31 | 77 |
| Efet.01.19706.g1460.t1 | Serine/threonine-protein kinase NLK | 507 | 1.46E-55 | 77 |
| Efet.01.129913.g261.t1 | Neurogenic locus notch homolog protein 1 | 234 | 6.41E-24 | 77 |
| Efet.01.60291.g640.t1 | Neurogenic locus notch homolog protein 2 | 768 | 4.39E-07 | 77 |
| Efet.01.107118.g367.t1 | Serine/threonine-protein kinase PAK 2 | 273 | 4.02E-34 | 77 |
| Efet.01.241023.g1568.t1 | Serine/threonine-protein kinase PAK 2 | 282 | 7.80E-24 | 77 |
| Efet.01.544334.g345.t1 | Serine/threonine-protein kinase pim-3 | 345 | 4.59E-30 | 77 |
| Efet.01.338309.g1121.t1 | Plexin-A3 | 297 | 4.87E-35 | 77 |
| Efet.01.182140.g1505.t1 | Peptidyl-prolyl cis-trans isomerase B | 411 | 3.43E-37 | 77 |
| Efet.01.122617.g1179.t1 | 26S proteasome non-ATPase regulatory subunit 2 | 222 | 8.38E-15 | 77 |
| Efet.01.68176.g1120.t1 | 26S proteasome non-ATPase regulatory subunit 6 | 804 | 1.83E-122 | 77 |
| Efet.01.654545.g627.t1 | Tyrosine-protein phosphatase non-receptor type 9 | 213 | 1.06E-12 | 77 |
| Efet.01.38006.g862.t1 | SFRS8 protein | 420 | 5.84E-17 | 77 |
| Efet.01.201860.g95.t1 | Ras-related protein Rab-13 | 216 | 1.73E-17 | 77 |
| Efet.01.354377.g125.t1 | Rho GTPase-activating protein 7 | 225 | 8.36E-22 | 77 |
| Efet.01.52506.g174.t1 | Regulating synaptic membrane exocytosis protein 1 | 645 | 2.87E-43 | 77 |
| Efet.01.150032.g1.t1 | Runt-related transcription factor 1 | 378 | 5.41E-39 | 77 |
| Efet.01.617179.g752.t1 | Ryanodine receptor 2 | 363 | 4.34E-14 | 77 |
| Efet.01.97213.g1296.t1 | Homeobox protein SIX1 | 528 | 8.57E-73 | 77 |
| Efet.01.267193.g625.t1 | Sodium/hydrogen exchanger 3 | 330 | 2.63E-22 | 77 |
| Efet.01.613649.g617.t1 | Sorting nexin-6 | 297 | 3.68E-23 | 77 |
| Efet.01.100624.g46.t1 | Transcription factor SOX-4 | 1134 | 3.35E-36 | 77 |
| Efet.01.3977.g334.t1 | Transcription factor Sp3 | 1290 | 1.00E-50 | 77 |
| Efet.01.19055.g1414.t1 | Transcription factor Sp3 | 462 | 1.62E-57 | 77 |
| Efet.01.227748.g1112.t1 | T-box transcription factor TBX1 | 363 | 1.30E-37 | 77 |
| Efet.01.570134.g331.t1 | Thioredoxin, mitochondrial | 426 | 7.15E-45 | 77 |
| Efet.01.78155.g183.t1 | Thymidine phosphorylase | 225 | 4.56E-23 | 77 |
| Efet.01.185157.g1639.t1 | Ubiquitin-conjugating enzyme E2 D3 | 267 | 4.12E-32 | 77 |
| Efet.01.4486.g361.t1 | Calreticulin, isoform CRA_b | 594 | 2.17E-58 | 77 |
| Efet.01.600388.g10.t1 | Serine/threonine-protein kinase WNK3 | 513 | 3.38E-63 | 77 |
| Efet.01.111457.g570.t1 | Zinc finger FYVE domain-containing protein 9 | 201 | 5.38E-11 | 77 |
| Efet.01.1654352.g684.t1 | 5-hydroxytryptamine receptor 2B | 393 | 1.14E-13 | 76 |
| Efet.01.22077.g1621.t1 | Protein kinase C delta type | 255 | 1.40E-22 | 76 |
| Efet.01.1651186.g501.t1 | ATP-binding cassette sub-family G member 1 | 279 | 6.47E-24 | 76 |
| Efet.01.537568.g217.t1 | Neuronal acetylcholine receptor subunit alpha-4 | 351 | 4.47E-19 | 76 |
| Efet.01.166936.g839.t1 | Neuronal acetylcholine receptor subunit alpha-7 | 249 | 5.29E-14 | 76 |
| Efet.01.182737.g1537.t1 | Alpha-actinin-1 | 261 | 2.33E-07 | 76 |
| Efet.01.51463.g94.t1 | Proteasomal ubiquitin receptor ADRM1 | 417 | 1.91E-28 | 76 |
| Efet.01.257694.g285.t1 | Adenomatous polyposis coli protein | 2379 | 5.54E-68 | 76 |
| Efet.01.163999.g708.t1 | Apoptosis-stimulating of p53 protein 2 | 2085 | 2.02E-27 | 76 |
| Efet.01.124508.g1264.t1 | Sarcoplasmic/endoplasmic reticulum calcium ATPase 1 | 627 | 7.84E-79 | 76 |
| Efet.01.487632.g908.t1 | Sarcoplasmic/endoplasmic reticulum calcium ATPase 2 | 489 | 3.95E-13 | 76 |
| Efet.01.291609.g1499.t1 | Tyrosine-protein kinase | 291 | 4.30E-11 | 76 |
| Efet.01.330623.g937.t1 | Elongator complex protein 1 | 378 | 5.72E-43 | 76 |
| Efet.01.77174.g124.t1 | Voltage-dependent P/Q-type calcium channel subunit alpha-1A | 222 | 6.48E-21 | 76 |
| Efet.01.16428.g1214.t1 | Calmodulin | 297 | 6.62E-16 | 76 |
| Efet.01.179440.g1362.t1 | Calmodulin-A | 225 | 2.99E-08 | 76 |
| Efet.01.177423.g1288.t1 | Cholecystokinin receptor type A | 498 | 9.55E-16 | 76 |
| Efet.01.647387.g1174.t1 | Carbohydrate sulfotransferase 4 | 204 | 5.52E-06 | 76 |
| Efet.01.189228.g1844.t1 | Cullin-5 | 258 | 7.05E-22 | 76 |
| Efet.01.611895.g509.t1 | Protein diaphanous homolog 2 | 234 | 4.61E-08 | 76 |
| Efet.01.1650143.g465.t1 | EH domain-containing protein 1 | 456 | 6.23E-56 | 76 |
| Efet.01.609920.g423.t1 | Ephrin type-A receptor 7 | 441 | 2.03E-54 | 76 |
| Efet.01.67865.g1104.t1 | MDS1 and EVI1 complex locus protein EVI1 | 1947 | 4.58E-46 | 76 |
| Efet.01.67866.g1106.t1 | MDS1 and EVI1 complex locus protein EVI1 | 1671 | 1.52E-46 | 76 |
| Efet.01.14155.g1048.t1 | Vitamin D (1,25-dihydroxyvitamin D3) receptor, isoform CRA_c | 327 | 1.64E-25 | 76 |
| Efet.01.40211.g1001.t1 | 6-phosphofructo-2-kinase/fructose-2,6-bisphosphatase 1 | 297 | 3.37E-28 | 76 |
| Efet.01.156546.g342.t1 | 6-phosphofructo-2-kinase/fructose-2,6-bisphosphatase 1 | 1248 | 0 | 76 |
| Efet.01.563308.g99.t1 | FYVE, RhoGEF and PH domain-containing protein 2 | 840 | 1.02E-43 | 76 |
| Efet.01.5848.g464.t1 | Filamin-A | 489 | 1.43E-35 | 76 |
| Efet.01.286207.g1310.t1 | Filamin-A | 651 | 7.15E-62 | 76 |
| Efet.01.12578.g939.t1 | Flotillin-2 | 306 | 1.50E-24 | 76 |
| Efet.01.68810.g1159.t1 | Zinc finger protein ZFPM1 | 2889 | 3.14E-11 | 76 |
| Efet.01.5036.g391.t1 | Hepatocyte nuclear factor 3-beta | 1203 | 2.04E-46 | 76 |
| Efet.01.16172.g1195.t1 | Germinal-center associated nuclear protein | 414 | 1.19E-51 | 76 |
| Efet.01.635880.g294.t1 | Glutamate receptor ionotropic, kainate 2 | 204 | 9.02E-24 | 76 |
| Efet.01.345072.g1270.t1 | Huntingtin | 603 | 2.64E-37 | 76 |
| Efet.01.495120.g1072.t1 | Lymphoid-specific helicase | 216 | 6.65E-16 | 76 |
| Efet.01.17707.g1305.t1 | Interferon regulatory factor 2-binding protein 2 | 2001 | 4.85E-38 | 76 |
| Efet.01.80794.g389.t1 | cAMP-dependent protein kinase catalytic subunit alpha | 291 | 2.67E-37 | 76 |
| Efet.01.544010.g341.t1 | Potassium voltage-gated channel subfamily H member 8 | 261 | 4.14E-20 | 76 |
| Efet.01.573127.g406.t1 | Potassium voltage-gated channel subfamily H member 8 | 363 | 7.11E-31 | 76 |
| Efet.01.332814.g985.t1 | Kinesin-like protein KIFC3 | 270 | 7.85E-10 | 76 |
| Efet.01.24261.g1762.t1 | Pyruvate kinase PKM | 1206 | 2.20E-159 | 76 |
| Efet.01.24295.g1766.t1 | Ribosomal protein S6 kinase alpha-1 | 297 | 1.58E-23 | 76 |
| Efet.01.350607.g18.t1 | Ribosomal protein S6 kinase alpha-5 | 357 | 5.88E-30 | 76 |
| Efet.01.2155.g191.t1 | Laminin subunit beta-1 | 243 | 6.99E-16 | 76 |
| Efet.01.53797.g253.t1 | Mitogen-activated protein kinase kinase kinase 11 | 831 | 2.22E-81 | 76 |
| Efet.01.573477.g415.t1 | Mitogen-activated protein kinase kinase kinase 11 | 816 | 5.67E-88 | 76 |
| Efet.01.532993.g95.t1 | Stromelysin-1 | 342 | 5.71E-08 | 76 |
| Efet.01.646963.g1137.t1 | Methylmalonate-semialdehyde dehydrogenase [acylating], mitochondrial | 1524 | 0 | 76 |
| Efet.01.152924.g156.t1 | Neuroendocrine convertase 1 | 354 | 3.82E-07 | 76 |
| Efet.01.85704.g673.t1 | Neurogenic locus notch homolog protein 2 | 237 | 5.50E-14 | 76 |
| Efet.01.441947.g1031.t1 | Serine/threonine-protein kinase PAK 2 | 273 | 2.46E-33 | 76 |
| Efet.01.69.g7.t1 | Poly [ADP-ribose] polymerase 1 | 318 | 8.98E-28 | 76 |
| Efet.01.160883.g569.t1 | High affinity cAMP-specific 3',5'-cyclic phosphodiesterase 7A | 201 | 8.06E-11 | 76 |
| Efet.01.288672.g1402.t1 | E3 ubiquitin-protein ligase pellino homolog 1 | 906 | 2.43E-71 | 76 |
| Efet.01.154996.g257.t1 | Serine/threonine-protein kinase pim-1 | 657 | 4.42E-89 | 76 |
| Efet.01.1658489.g1195.t1 | Tartrate-resistant acid phosphatase type 5 | 300 | 9.24E-27 | 76 |
| Efet.01.426237.g628.t1 | Tyrosine-protein phosphatase non-receptor type 9 | 489 | 5.70E-14 | 76 |
| Efet.01.138459.g688.t1 | Receptor-type tyrosine-protein phosphatase epsilon | 594 | 1.58E-06 | 76 |
| Efet.01.366886.g456.t1 | Peroxidasin homolog | 1137 | 3.26E-122 | 76 |
| Efet.01.208563.g393.t1 | Ras-related protein Rab-18 | 237 | 2.40E-27 | 76 |
| Efet.01.133179.g410.t1 | DNA-binding protein RFX2 | 426 | 1.30E-12 | 76 |
| Efet.01.87075.g749.t1 | Transforming protein RhoA | 558 | 1.97E-64 | 76 |
| Efet.01.120981.g1089.t1 | Reticulon-4 | 201 | 2.28E-15 | 76 |
| Efet.01.339069.g1139.t1 | Runt-related transcription factor 1 | 369 | 3.24E-30 | 76 |
| Efet.01.288799.g1408.t1 | Sodium channel protein type 2 subunit alpha | 297 | 3.18E-23 | 76 |
| Efet.01.543615.g333.t1 | Sodium channel protein type 2 subunit alpha | 288 | 3.88E-25 | 76 |
| Efet.01.114171.g722.t1 | Septin-7 | 468 | 2.59E-27 | 76 |
| Efet.01.204865.g209.t1 | Transcription factor SOX-2 | 1053 | 3.20E-29 | 76 |
| Efet.01.27016.g133.t1 | Transcription factor Sp3 | 759 | 8.65E-53 | 76 |
| Efet.01.145017.g1014.t1 | T-lymphoma invasion and metastasis-inducing protein 2 | 1701 | 1.91E-11 | 76 |
| Efet.01.373950.g664.t1 | Tribbles homolog 2 | 483 | 2.24E-48 | 76 |
| Efet.01.486245.g882.t1 | Tribbles homolog 2 | 411 | 1.01E-49 | 76 |
| Efet.01.202003.g100.t1 | mRNA decay activator protein ZFP36 | 786 | 1.10E-27 | 76 |
| Efet.01.8681.g639.t1 | Upstream-binding protein 1 | 645 | 2.18E-66 | 76 |
| Efet.01.12564.g936.t1 | V-type proton ATPase subunit H | 528 | 3.09E-39 | 76 |
| Efet.01.231146.g1232.t1 | Vesicle transport through interaction with t-SNAREs homolog 1B | 249 | 2.49E-16 | 76 |
| Efet.01.141662.g860.t1 | Tyrosine-protein kinase ZAP-70 | 744 | 1.30E-84 | 76 |
| Efet.01.162174.g630.t1 | Tyrosine-protein kinase ZAP-70 | 402 | 2.15E-20 | 76 |
| Efet.01.603784.g142.t1 | Serine/threonine-protein phosphatase 2A 56 kDa regulatory subunit gamma isoform | 348 | 1.62E-32 | 75 |
| Efet.01.177893.g1307.t1 | 5-hydroxytryptamine receptor 2B | 612 | 6.08E-10 | 75 |
| Efet.01.78755.g224.t1 | Nuclear factor interleukin-3-regulated protein | 1044 | 1.29E-23 | 75 |
| Efet.01.139002.g720.t1 | Suppressor of cytokine signaling 6, isoform CRA_a | 1488 | 1.40E-68 | 75 |
| Efet.01.201410.g68.t1 | TNF receptor-associated factor 2, isoform CRA_a | 576 | 1.37E-31 | 75 |
| Efet.01.74957.g1539.t1 | Aryl hydrocarbon receptor, isoform CRA_a | 1494 | 1.88E-09 | 75 |
| Efet.01.424716.g587.t1 | Myeloid/lymphoid or mixed-lineage leukemia translocated to 3 isoform 1 | 1584 | 4.29E-11 | 75 |
| Efet.01.14281.g1055.t1 | Testicular tissue protein Li 153 | 357 | 1.90E-40 | 75 |
| Efet.01.276696.g984.t1 | APOBEC1 complementation factor | 330 | 2.25E-23 | 75 |
| Efet.01.140823.g810.t1 | 5'-AMP-activated protein kinase subunit gamma-1 | 261 | 2.59E-25 | 75 |
| Efet.01.138414.g685.t1 | Neuronal acetylcholine receptor subunit alpha-4 | 324 | 2.15E-17 | 75 |
| Efet.01.1551.g149.t1 | Neuronal acetylcholine receptor subunit alpha-7 | 558 | 7.17E-35 | 75 |
| Efet.01.102426.g139.t1 | Neuronal acetylcholine receptor subunit alpha-7 | 657 | 1.66E-61 | 75 |
| Efet.01.143655.g955.t1 | Muscarinic acetylcholine receptor M1 | 2394 | 9.50E-65 | 75 |
| Efet.01.491091.g978.t1 | Muscarinic acetylcholine receptor M1 | 1920 | 8.69E-65 | 75 |
| Efet.01.577929.g618.t1 | tRNA-specific adenosine deaminase 2 | 414 | 7.64E-55 | 75 |
| Efet.01.138016.g673.t1 | Aldehyde dehydrogenase, mitochondrial | 351 | 6.29E-32 | 75 |
| Efet.01.235810.g1402.t1 | Aminopeptidase N | 204 | 2.37E-10 | 75 |
| Efet.01.621744.g953.t1 | Ankyrin-1 | 261 | 4.49E-13 | 75 |
| Efet.01.650329.g37.t1 | Rho guanine nucleotide exchange factor 4 | 282 | 8.85E-31 | 75 |
| Efet.01.558740.g714.t1 | ADP-ribosylation factor-like protein 4A | 528 | 3.81E-67 | 75 |
| Efet.01.174699.g1161.t1 | Sarcoplasmic/endoplasmic reticulum calcium ATPase 2 | 288 | 1.79E-13 | 75 |
| Efet.01.546084.g391.t1 | Sarcoplasmic/endoplasmic reticulum calcium ATPase 2 | 201 | 2.47E-13 | 75 |
| Efet.01.565049.g165.t1 | ATP synthase subunit alpha, mitochondrial | 1215 | 9.82E-158 | 75 |
| Efet.01.2320.g208.t1 | ATP-binding cassette, sub-family A (ABC1), member 1 | 537 | 3.01E-20 | 75 |
| Efet.01.76686.g98.t1 | Band 3 anion transport protein | 636 | 4.81E-08 | 75 |
| Efet.01.493038.g1020.t1 | Beta-1,4-galactosyltransferase 1 | 237 | 9.46E-22 | 75 |
| Efet.01.62034.g752.t1 | Chromodomain-helicase-DNA-binding protein 7 | 537 | 2.25E-42 | 75 |
| Efet.01.172645.g1080.t1 | Centriolin | 531 | 3.66E-30 | 75 |
| Efet.01.166891.g834.t1 | C-terminal-binding protein 1 | 660 | 5.76E-36 | 75 |
| Efet.01.212603.g543.t1 | Glutamate decarboxylase 1 | 303 | 6.66E-22 | 75 |
| Efet.01.272147.g830.t1 | Glutamate decarboxylase 1 | 318 | 2.24E-32 | 75 |
| Efet.01.60699.g661.t1 | Diacylglycerol kinase zeta | 288 | 4.15E-09 | 75 |
| Efet.01.221319.g869.t1 | Protein diaphanous homolog 2 | 288 | 7.30E-13 | 75 |
| Efet.01.630034.g1.t1 | Disks large homolog 1 | 207 | 2.95E-14 | 75 |
| Efet.01.298810.g1711.t1 | Protocadherin Fat 1 | 222 | 3.35E-16 | 75 |
| Efet.01.594332.g1121.t1 | F-BAR domain only protein 2 | 471 | 5.01E-26 | 75 |
| Efet.01.429708.g729.t1 | Filamin-A | 207 | 2.84E-17 | 75 |
| Efet.01.71139.g1303.t1 | Zinc finger protein ZFPM1 | 957 | 3.79E-10 | 75 |
| Efet.01.310554.g334.t1 | Forkhead box protein O3 | 1716 | 9.89E-10 | 75 |
| Efet.01.38448.g897.t1 | Tyrosine-protein kinase FRK | 297 | 2.79E-34 | 75 |
| Efet.01.89110.g839.t1 | Growth/differentiation factor 11 | 405 | 1.54E-55 | 75 |
| Efet.01.289275.g1427.t1 | Gelsolin | 243 | 2.64E-12 | 75 |
| Efet.01.155922.g308.t1 | Guanine nucleotide-binding protein G(i) subunit alpha-2 | 261 | 9.25E-19 | 75 |
| Efet.01.310939.g346.t1 | Glutamate receptor ionotropic, kainate 2 | 354 | 2.95E-36 | 75 |
| Efet.01.368523.g498.t1 | Glutamate receptor ionotropic, kainate 2 | 390 | 1.36E-42 | 75 |
| Efet.01.645478.g988.t1 | Glutamate receptor ionotropic, kainate 2 | 321 | 1.29E-35 | 75 |
| Efet.01.185967.g1688.t1 | Heart- and neural crest derivatives-expressed protein 2 | 726 | 6.36E-15 | 75 |
| Efet.01.225708.g1023.t1 | Lymphoid-specific helicase | 669 | 1.36E-63 | 75 |
| Efet.01.463449.g366.t1 | Lymphoid-specific helicase | 390 | 7.49E-46 | 75 |
| Efet.01.655882.g892.t1 | Lymphoid-specific helicase | 921 | 2.75E-15 | 75 |
| Efet.01.78498.g209.t1 | Hypoxia-inducible factor 1-alpha | 375 | 3.19E-36 | 75 |
| Efet.01.31806.g476.t1 | Hepatocyte nuclear factor 6 | 1017 | 8.22E-68 | 75 |
| Efet.01.151663.g101.t1 | Heparan sulfate glucosamine 3-O-sulfotransferase 5 | 609 | 4.54E-14 | 75 |
| Efet.01.42547.g1143.t1 | Homeobox protein Hox-A3 | 351 | 1.51E-12 | 75 |
| Efet.01.628190.g1233.t1 | Homeobox protein Hox-A5 | 252 | 4.20E-13 | 75 |
| Efet.01.107723.g396.t1 | Intraflagellar transport protein 88 homolog | 399 | 2.62E-26 | 75 |
| Efet.01.512803.g301.t1 | Transcription factor jun-D | 1062 | 2.01E-16 | 75 |
| Efet.01.152379.g135.t1 | Potassium voltage-gated channel subfamily H member 8 | 264 | 4.76E-18 | 75 |
| Efet.01.100504.g38.t1 | ATP-sensitive inward rectifier potassium channel 8 | 642 | 9.36E-31 | 75 |
| Efet.01.10032.g735.t1 | Krueppel-like factor 6 | 750 | 5.84E-31 | 75 |
| Efet.01.218220.g742.t1 | LIM/homeobox protein Lhx4 | 459 | 3.94E-18 | 75 |
| Efet.01.137568.g642.t1 | Protein LMBR1L | 396 | 5.73E-41 | 75 |
| Efet.01.148956.g1182.t1 | Lipopolysaccharide-responsive and beige-like anchor protein | 270 | 1.49E-07 | 75 |
| Efet.01.99237.g1402.t1 | Low-density lipoprotein receptor-related protein 2 | 213 | 2.13E-26 | 75 |
| Efet.01.168338.g902.t1 | Canalicular multispecific organic anion transporter 1 | 501 | 2.27E-62 | 75 |
| Efet.01.584882.g815.t1 | Canalicular multispecific organic anion transporter 1 | 279 | 3.76E-22 | 75 |
| Efet.01.636758.g347.t1 | Canalicular multispecific organic anion transporter 1 | 237 | 1.43E-20 | 75 |
| Efet.01.66020.g984.t1 | Neurofibromin | 654 | 5.24E-89 | 75 |
| Efet.01.111804.g593.t1 | Nuclear factor of activated T-cells 5 | 441 | 1.14E-44 | 75 |
| Efet.01.151176.g61.t1 | Neuroligin-1 | 279 | 1.26E-15 | 75 |
| Efet.01.16168.g1194.t1 | Neuroligin-2 | 207 | 3.06E-17 | 75 |
| Efet.01.127939.g163.t1 | Protein kinase C and casein kinase substrate in neurons protein 1 | 240 | 3.82E-19 | 75 |
| Efet.01.601896.g83.t1 | Calcium/calmodulin-dependent 3',5'-cyclic nucleotide phosphodiesterase 1B | 363 | 3.09E-11 | 75 |
| Efet.01.19056.g1415.t1 | Serine/threonine-protein kinase N2 | 516 | 2.92E-14 | 75 |
| Efet.01.109087.g473.t1 | Protein PRRC2C | 2283 | 2.96E-07 | 75 |
| Efet.01.296738.g1648.t1 | TSC22 domain family protein 3 | 273 | 6.93E-10 | 75 |
| Efet.01.161592.g600.t1 | Ras-related protein Rab-8A | 291 | 3.30E-11 | 75 |
| Efet.01.342019.g1208.t1 | Rab GTPase-binding effector protein 1 | 303 | 6.23E-09 | 75 |
| Efet.01.461650.g328.t1 | Sodium channel protein type 2 subunit alpha | 279 | 1.47E-33 | 75 |
| Efet.01.357349.g209.t1 | Serine/threonine-protein kinase SIK2 | 279 | 4.55E-06 | 75 |
| Efet.01.143468.g949.t1 | Homeobox protein SIX1 | 687 | 4.58E-68 | 75 |
| Efet.01.245982.g1731.t1 | Sorting nexin-3 | 207 | 3.89E-15 | 75 |
| Efet.01.338451.g1123.t1 | Proto-oncogene tyrosine-protein kinase Src | 219 | 4.31E-17 | 75 |
| Efet.01.511938.g280.t1 | SRSF protein kinase 1 | 696 | 3.79E-15 | 75 |
| Efet.01.40401.g1015.t1 | Synaptotagmin-7 | 291 | 3.71E-27 | 75 |
| Efet.01.150464.g24.t1 | Synaptotagmin-7 | 501 | 5.87E-27 | 75 |
| Efet.01.284823.g1258.t1 | T-cell leukemia homeobox protein 3 | 216 | 1.96E-22 | 75 |
| Efet.01.143099.g926.t1 | Triple functional domain protein | 417 | 9.84E-37 | 75 |
| Efet.01.212161.g523.t1 | mRNA decay activator protein ZFP36 | 1722 | 2.43E-21 | 75 |
| Efet.01.418601.g435.t1 | mRNA decay activator protein ZFP36 | 1110 | 4.49E-27 | 75 |
| Efet.01.569658.g310.t1 | Thymidine phosphorylase | 279 | 8.44E-23 | 75 |
| Efet.01.215576.g648.t1 | Epididymis secretory protein Li 31 | 690 | 3.44E-94 | 75 |
| Efet.01.73320.g1439.t1 | Zinc finger FYVE domain-containing protein 9 | 549 | 5.34E-53 | 75 |
| Efet.01.74263.g1503.t1 | 5-hydroxytryptamine receptor 2B | 351 | 1.75E-12 | 74 |
| Efet.01.128888.g218.t1 | APOBEC1 complementation factor | 423 | 5.83E-18 | 74 |
| Efet.01.9278.g681.t1 | Neuronal acetylcholine receptor subunit alpha-7 | 486 | 1.55E-58 | 74 |
| Efet.01.111627.g583.t1 | Muscarinic acetylcholine receptor M1 | 1902 | 5.49E-65 | 74 |
| Efet.01.130066.g272.t1 | Muscarinic acetylcholine receptor M1 | 1746 | 1.22E-69 | 74 |
| Efet.01.430569.g748.t1 | Muscarinic acetylcholine receptor M1 | 1914 | 1.80E-18 | 74 |
| Efet.01.1649227.g430.t1 | Fructose-bisphosphate aldolase C | 297 | 1.10E-35 | 74 |
| Efet.01.570764.g343.t1 | Homeobox protein aristaless-like 4 | 231 | 2.86E-15 | 74 |
| Efet.01.5872.g467.t1 | Ankyrin-1 | 459 | 8.57E-38 | 74 |
| Efet.01.478316.g724.t1 | Anillin | 282 | 9.19E-35 | 74 |
| Efet.01.90130.g901.t1 | Annexin A4 | 651 | 2.57E-38 | 74 |
| Efet.01.41213.g1064.t1 | AP-1 complex subunit gamma-1 | 738 | 3.42E-64 | 74 |
| Efet.01.94957.g1155.t1 | cDNA, FLJ92968, highly similar to Homo sapiens runt-related transcription factor 1; translocated to, 1 | 231 | 1.07E-07 | 74 |
| Efet.01.68676.g1146.t1 | cDNA FLJ33305 fis, clone BNGH42003529, highly similar to E3 ubiquitin protein ligase TRAF7 | 324 | 7.08E-16 | 74 |
| Efet.01.77453.g140.t1 | MAPK8IP3 protein | 456 | 1.69E-36 | 74 |
| Efet.01.485619.g874.t1 | Calmodulin-A | 507 | 1.03E-15 | 74 |
| Efet.01.246246.g1749.t1 | CCAAT/enhancer-binding protein beta | 1221 | 9.02E-14 | 74 |
| Efet.01.91963.g996.t1 | Acidic mammalian chitinase | 333 | 2.51E-41 | 74 |
| Efet.01.162919.g657.t1 | COUP transcription factor 2 | 249 | 6.44E-38 | 74 |
| Efet.01.142275.g891.t1 | Catenin alpha-2 | 771 | 8.38E-95 | 74 |
| Efet.01.290621.g1477.t1 | Catenin alpha-2 | 528 | 2.98E-40 | 74 |
| Efet.01.9503.g703.t1 | Alpha-catulin | 297 | 3.39E-27 | 74 |
| Efet.01.169719.g965.t1 | Histidine decarboxylase | 261 | 3.13E-24 | 74 |
| Efet.01.559562.g742.t1 | DnaJ homolog subfamily A member 3, mitochondrial | 693 | 1.80E-17 | 74 |
| Efet.01.289879.g1443.t1 | Histone acetyltransferase p300 | 903 | 8.49E-40 | 74 |
| Efet.01.403601.g93.t1 | Ezrin | 444 | 2.71E-51 | 74 |
| Efet.01.199281.g2270.t1 | Hepatocyte nuclear factor 4 4 alpha variant 1 | 693 | 8.29E-42 | 74 |
| Efet.01.539676.g262.t1 | Filamin-A | 402 | 2.04E-33 | 74 |
| Efet.01.160017.g521.t1 | PDZ domain-containing protein GIPC1 | 705 | 1.26E-80 | 74 |
| Efet.01.257315.g276.t1 | PDZ domain-containing protein GIPC1 | 345 | 2.10E-10 | 74 |
| Efet.01.121615.g1126.t1 | Glutamate receptor ionotropic, kainate 2 | 219 | 5.78E-21 | 74 |
| Efet.01.635054.g271.t1 | Glutamate receptor ionotropic, kainate 2 | 501 | 1.45E-46 | 74 |
| Efet.01.308044.g238.t1 | Histamine H1 receptor | 408 | 8.17E-26 | 74 |
| Efet.01.516197.g403.t1 | Heparan sulfate glucosamine 3-O-sulfotransferase 5 | 1047 | 1.20E-81 | 74 |
| Efet.01.309640.g304.t1 | Intraflagellar transport protein 80 homolog | 318 | 3.37E-33 | 74 |
| Efet.01.87466.g769.t1 | Insulin receptor substrate 1 | 327 | 1.88E-25 | 74 |
| Efet.01.344802.g1264.t1 | Calcium/calmodulin-dependent protein kinase type 1G | 255 | 1.48E-27 | 74 |
| Efet.01.79104.g254.t1 | Calcium/calmodulin-dependent protein kinase type II subunit delta | 573 | 1.13E-20 | 74 |
| Efet.01.180028.g1398.t1 | Kinesin-like protein KIF1B | 474 | 7.11E-31 | 74 |
| Efet.01.285894.g1297.t1 | Mitogen-activated protein kinase kinase kinase 11 | 825 | 2.92E-87 | 74 |
| Efet.01.272519.g837.t1 | Mitogen-activated protein kinase kinase kinase 2 | 501 | 4.76E-25 | 74 |
| Efet.01.81216.g419.t1 | Myosin regulatory light chain 2, skeletal muscle isoform | 201 | 4.24E-18 | 74 |
| Efet.01.656716.g1068.t1 | DNA mismatch repair protein Msh6 | 609 | 1.35E-42 | 74 |
| Efet.01.640805.g592.t1 | Unconventional myosin-XVIIIa | 210 | 2.26E-13 | 74 |
| Efet.01.292670.g1532.t1 | Myoblast determination protein 1 | 1134 | 6.03E-12 | 74 |
| Efet.01.621517.g938.t1 | Myoblast determination protein 1 | 867 | 1.31E-13 | 74 |
| Efet.01.168408.g909.t1 | Neuroendocrine convertase 1 | 306 | 7.69E-19 | 74 |
| Efet.01.152768.g151.t1 | Vesicle-fusing ATPase | 525 | 9.18E-63 | 74 |
| Efet.01.407760.g182.t1 | Paired box protein Pax-7 | 486 | 2.64E-35 | 74 |
| Efet.01.483637.g827.t1 | PH-interacting protein | 459 | 4.08E-36 | 74 |
| Efet.01.1639631.g210.t1 | PH-interacting protein | 222 | 2.35E-10 | 74 |
| Efet.01.95340.g1177.t1 | Serine/threonine-protein kinase pim-1 | 552 | 4.13E-62 | 74 |
| Efet.01.21931.g1608.t1 | 1-phosphatidylinositol 4,5-bisphosphate phosphodiesterase gamma-1 | 399 | 7.14E-24 | 74 |
| Efet.01.252956.g109.t1 | Prospero homeobox protein 1 | 312 | 3.01E-34 | 74 |
| Efet.01.225184.g1009.t1 | PTK2 protein tyrosine kinase 2 isoform b variant | 237 | 4.34E-18 | 74 |
| Efet.01.25039.g3.t1 | GTP cyclohydrolase I type IV | 381 | 1.79E-10 | 74 |
| Efet.01.194731.g2083.t1 | Regulator of G-protein signaling 1 | 438 | 1.69E-06 | 74 |
| Efet.01.430627.g749.t1 | DNA-directed RNA polymerase III subunit RPC1 | 426 | 1.89E-39 | 74 |
| Efet.01.91619.g975.t1 | Retinoic acid receptor RXR-alpha | 204 | 1.29E-21 | 74 |
| Efet.01.460097.g282.t1 | Solute carrier family 17 member 9 | 372 | 5.15E-07 | 74 |
| Efet.01.404906.g119.t1 | Stearoyl-CoA desaturase 5 | 402 | 7.38E-54 | 74 |
| Efet.01.19562.g1449.t1 | Sodium channel protein type 2 subunit alpha | 375 | 2.63E-30 | 74 |
| Efet.01.204928.g212.t1 | Sodium channel protein type 2 subunit alpha | 456 | 4.96E-07 | 74 |
| Efet.01.502615.g62.t1 | Sodium channel protein type 2 subunit alpha | 297 | 1.87E-27 | 74 |
| Efet.01.129827.g258.t1 | Zinc finger protein SNAI1 | 696 | 1.82E-44 | 74 |
| Efet.01.366483.g447.t1 | Zinc finger protein SNAI1 | 354 | 1.85E-37 | 74 |
| Efet.01.657897.g1520.t1 | Zinc finger protein SNAI1 | 573 | 5.83E-58 | 74 |
| Efet.01.36487.g764.t1 | Synaptotagmin-7 | 315 | 3.19E-29 | 74 |
| Efet.01.310684.g341.t1 | Mitochondrial import inner membrane translocase subunit Tim8 A | 306 | 1.10E-28 | 74 |
| Efet.01.423054.g543.t1 | DNA topoisomerase 2-beta | 366 | 3.75E-25 | 74 |
| Efet.01.475266.g656.t1 | Tribbles homolog 1 | 600 | 3.86E-33 | 74 |
| Efet.01.169624.g958.t1 | Protein unc-13 homolog A | 204 | 5.61E-20 | 74 |
| Efet.01.1648656.g414.t1 | Vasopressin V1b receptor | 264 | 3.89E-09 | 74 |
| Efet.01.190020.g1872.t1 | Wiskott-Aldrich syndrome protein family member 3 | 342 | 4.12E-29 | 74 |
| Efet.01.221949.g893.t1 | Protein Wnt-5a | 564 | 1.74E-72 | 74 |
| Efet.01.221778.g890.t1 | Nuclear factor interleukin-3-regulated protein | 426 | 4.13E-10 | 73 |
| Efet.01.229837.g1192.t1 | MYC associated factor X, isoform CRA_d | 225 | 3.13E-10 | 73 |
| Efet.01.95625.g1195.t1 | Epithelial discoidin domain-containing receptor 1 | 348 | 3.27E-33 | 73 |
| Efet.01.82304.g484.t1 | Myeloid/lymphoid or mixed-lineage leukemia translocated to 3 isoform 1 | 261 | 7.72E-24 | 73 |
| Efet.01.123299.g1210.t1 | Neuronal acetylcholine receptor subunit alpha-7 | 318 | 2.58E-17 | 73 |
| Efet.01.579135.g656.t1 | Neuronal acetylcholine receptor subunit alpha-7 | 321 | 8.81E-36 | 73 |
| Efet.01.249208.g1866.t1 | Neuronal acetylcholine receptor subunit beta-2 | 366 | 3.11E-18 | 73 |
| Efet.01.42283.g1129.t1 | Muscarinic acetylcholine receptor M1 | 1209 | 7.90E-78 | 73 |
| Efet.01.212656.g545.t1 | Muscarinic acetylcholine receptor M1 | 453 | 1.23E-42 | 73 |
| Efet.01.618480.g837.t1 | Alpha-1A adrenergic receptor | 1152 | 2.22E-69 | 73 |
| Efet.01.657048.g1157.t1 | Alpha-1A adrenergic receptor | 714 | 2.82E-24 | 73 |
| Efet.01.514423.g348.t1 | Alpha-1B adrenergic receptor | 399 | 2.61E-22 | 73 |
| Efet.01.305666.g196.t1 | Alpha-2A adrenergic receptor | 474 | 4.12E-25 | 73 |
| Efet.01.267259.g627.t1 | Fructose-bisphosphate aldolase C | 477 | 2.99E-47 | 73 |
| Efet.01.48268.g1484.t1 | Angiomotin-like protein 1 | 243 | 2.98E-19 | 73 |
| Efet.01.389586.g999.t1 | AP-1 complex subunit beta-1 | 528 | 7.83E-74 | 73 |
| Efet.01.370163.g560.t1 | ADP-ribosylation factor-like protein 4A | 576 | 9.37E-66 | 73 |
| Efet.01.531474.g48.t1 | ADP-ribosylation factor-like protein 4A | 534 | 1.20E-64 | 73 |
| Efet.01.646005.g1022.t1 | Dedicator of cytokinesis 1 | 207 | 4.19E-17 | 73 |
| Efet.01.224947.g1001.t1 | cDNA FLJ61244, highly similar to Transcription factor 8 | 306 | 3.75E-29 | 73 |
| Efet.01.256676.g249.t1 | Mitogen-activated protein kinase | 216 | 1.73E-10 | 73 |
| Efet.01.279329.g1077.t1 | Voltage-dependent P/Q-type calcium channel subunit alpha-1A | 582 | 4.82E-63 | 73 |
| Efet.01.318395.g576.t1 | Cadherin-4 | 282 | 6.19E-11 | 73 |
| Efet.01.66846.g1034.t1 | CREB-binding protein | 1485 | 2.88E-117 | 73 |
| Efet.01.49176.g1538.t1 | Cyclin-dependent kinase 5 activator 1 | 756 | 4.36E-57 | 73 |
| Efet.01.13838.g1027.t1 | Chromodomain-helicase-DNA-binding protein 7 | 549 | 1.63E-40 | 73 |
| Efet.01.1635971.g170.t1 | Chromodomain-helicase-DNA-binding protein 7 | 306 | 5.22E-19 | 73 |
| Efet.01.211440.g495.t1 | Cholinephosphotransferase 1 | 237 | 6.83E-14 | 73 |
| Efet.01.414910.g343.t1 | Clathrin light chain A | 213 | 5.44E-08 | 73 |
| Efet.01.602643.g107.t1 | Calsenilin | 219 | 5.92E-23 | 73 |
| Efet.01.29499.g309.t1 | Cytoplasmic FMR1-interacting protein 2 | 393 | 9.25E-35 | 73 |
| Efet.01.261191.g421.t1 | Cytoplasmic FMR1-interacting protein 2 | 330 | 1.20E-34 | 73 |
| Efet.01.207916.g363.t1 | DDB1- and CUL4-associated factor 1 | 312 | 4.08E-23 | 73 |
| Efet.01.5901.g471.t1 | Death-inducer obliterator 1 | 732 | 4.22E-07 | 73 |
| Efet.01.280756.g1125.t1 | DnaJ homolog subfamily B member 6 | 687 | 1.05E-07 | 73 |
| Efet.01.338972.g1138.t1 | Double C2-like domain-containing protein alpha | 318 | 3.90E-35 | 73 |
| Efet.01.136977.g601.t1 | D(2) dopamine receptor | 450 | 1.69E-12 | 73 |
| Efet.01.294922.g1594.t1 | D(4) dopamine receptor | 540 | 8.50E-23 | 73 |
| Efet.01.66854.g1035.t1 | Excitatory amino acid transporter 4 | 570 | 1.09E-29 | 73 |
| Efet.01.169305.g947.t1 | Epidermal growth factor receptor | 222 | 1.63E-19 | 73 |
| Efet.01.173336.g1109.t1 | DNA excision repair protein ERCC-6 | 291 | 3.88E-29 | 73 |
| Efet.01.113415.g681.t1 | Splicing factor ESS-2 homolog | 294 | 5.19E-22 | 73 |
| Efet.01.188519.g1811.t1 | Filamin-A | 309 | 1.38E-22 | 73 |
| Efet.01.454345.g99.t1 | Forkhead box protein L2 | 837 | 5.17E-23 | 73 |
| Efet.01.277975.g1029.t1 | Tyrosine-protein kinase Fyn | 339 | 1.50E-27 | 73 |
| Efet.01.173443.g1115.t1 | Polypeptide N-acetylgalactosaminyltransferase 2 | 426 | 5.46E-20 | 73 |
| Efet.01.460320.g296.t1 | Polypeptide N-acetylgalactosaminyltransferase 2 | 201 | 8.18E-23 | 73 |
| Efet.01.146716.g1089.t1 | Growth/differentiation factor 8 | 324 | 5.00E-38 | 73 |
| Efet.01.545636.g384.t1 | Zinc finger protein GLI2 | 459 | 1.63E-18 | 73 |
| Efet.01.1829.g162.t1 | Guanine nucleotide-binding protein subunit alpha-12 | 468 | 3.82E-23 | 73 |
| Efet.01.98627.g1363.t1 | Glutamate receptor ionotropic, kainate 2 | 330 | 1.58E-32 | 73 |
| Efet.01.94444.g1133.t1 | High mobility group protein B2 | 774 | 2.28E-06 | 73 |
| Efet.01.369469.g528.t1 | Insulin receptor | 336 | 6.98E-22 | 73 |
| Efet.01.2690.g246.t1 | C-Jun-amino-terminal kinase-interacting protein 3 | 330 | 2.75E-23 | 73 |
| Efet.01.425430.g604.t1 | Calcium-activated potassium channel subunit alpha-1 | 492 | 1.08E-52 | 73 |
| Efet.01.345196.g1274.t1 | Protein kinase C alpha type | 297 | 8.27E-27 | 73 |
| Efet.01.363390.g356.t1 | Protein kinase C epsilon type | 330 | 6.04E-12 | 73 |
| Efet.01.410874.g244.t1 | Multidrug resistance protein 1 | 384 | 1.26E-26 | 73 |
| Efet.01.365656.g428.t1 | Canalicular multispecific organic anion transporter 1 | 414 | 3.56E-50 | 73 |
| Efet.01.387933.g966.t1 | Mitochondrial fission process protein 1 | 255 | 2.98E-26 | 73 |
| Efet.01.75095.g9.t1 | Unconventional myosin-VI | 294 | 2.06E-29 | 73 |
| Efet.01.567190.g237.t1 | Nicotinamide phosphoribosyltransferase | 273 | 3.07E-28 | 73 |
| Efet.01.59329.g584.t1 | Nipped-B-like protein | 1131 | 2.81E-90 | 73 |
| Efet.01.622077.g966.t1 | Neuroligin-1 | 312 | 1.30E-35 | 73 |
| Efet.01.489283.g947.t1 | Serine/threonine-protein kinase NLK | 555 | 9.67E-65 | 73 |
| Efet.01.185297.g1646.t1 | Neurogenic locus notch homolog protein 1 | 306 | 4.02E-12 | 73 |
| Efet.01.180017.g1397.t1 | Phosphofurin acidic cluster sorting protein 1 | 564 | 1.07E-38 | 73 |
| Efet.01.2740.g250.t1 | Poly [ADP-ribose] polymerase 1 | 579 | 5.48E-91 | 73 |
| Efet.01.225885.g1031.t1 | PDZ domain-containing protein 2 | 591 | 5.99E-09 | 73 |
| Efet.01.65897.g979.t1 | Phosphatidylinositol 4-kinase beta | 627 | 7.67E-34 | 73 |
| Efet.01.87263.g761.t1 | Serine/threonine-protein kinase pim-2 | 600 | 2.31E-62 | 73 |
| Efet.01.450135.g2.t1 | Serine/threonine-protein kinase PLK3 | 276 | 2.01E-06 | 73 |
| Efet.01.8669.g638.t1 | Receptor-type tyrosine-protein phosphatase F | 663 | 1.50E-66 | 73 |
| Efet.01.1644061.g290.t1 | Ras-related protein Rab-35 | 231 | 6.62E-22 | 73 |
| Efet.01.54939.g330.t1 | Rab5 GDP/GTP exchange factor | 921 | 5.71E-65 | 73 |
| Efet.01.17339.g1278.t1 | Sodium channel protein type 2 subunit alpha | 951 | 6.77E-19 | 73 |
| Efet.01.71880.g1356.t1 | Sodium channel protein type 2 subunit alpha | 567 | 1.58E-39 | 73 |
| Efet.01.438635.g914.t1 | Septin-7 | 201 | 5.46E-15 | 73 |
| Efet.01.483652.g829.t1 | Homeobox protein SIX1 | 684 | 1.05E-80 | 73 |
| Efet.01.55694.g370.t1 | Transcription factor Sp3 | 942 | 4.66E-50 | 73 |
| Efet.01.177689.g1297.t1 | Spectrin beta chain, erythrocytic | 516 | 1.34E-43 | 73 |
| Efet.01.536272.g190.t1 | Spectrin beta chain, erythrocytic | 249 | 8.23E-13 | 73 |
| Efet.01.45686.g1337.t1 | Tumor protein p53-inducible nuclear protein 1 | 1347 | 8.73E-11 | 73 |
| Efet.01.628078.g1230.t1 | T-box transcription factor TBX1 | 660 | 1.40E-51 | 73 |
| Efet.01.175532.g1192.t1 | Transcription factor AP-4 | 723 | 1.78E-20 | 73 |
| Efet.01.39587.g969.t1 | Thimet oligopeptidase | 264 | 1.09E-10 | 73 |
| Efet.01.347451.g1339.t1 | T-cell leukemia homeobox protein 2 | 570 | 1.38E-10 | 73 |
| Efet.01.182660.g1532.t1 | TNF receptor-associated factor 6 | 330 | 5.83E-14 | 73 |
| Efet.01.282230.g1168.t1 | Twist-related protein 1 | 441 | 1.05E-13 | 73 |
| Efet.01.68336.g1126.t1 | Twist-related protein 2 | 774 | 1.85E-46 | 73 |
| Efet.01.250016.g1.t1 | Vacuolar protein sorting-associated protein 33A | 249 | 2.05E-17 | 73 |
| Efet.01.269936.g742.t1 | Protein Wnt-5a | 420 | 5.35E-54 | 73 |
| Efet.01.476525.g679.t1 | AN1-type zinc finger protein 5 | 234 | 1.92E-09 | 73 |
| Efet.01.111457.g571.t1 | Zinc finger FYVE domain-containing protein 9 | 738 | 2.84E-34 | 73 |
| Efet.01.1650088.g463.t1 | Zinc finger FYVE domain-containing protein 9 | 321 | 2.92E-25 | 73 |
| Efet.01.619725.g874.t1 | Zinc finger protein 675 | 1212 | 3.77E-28 | 73 |
| Efet.01.93459.g1071.t1 | 5-hydroxytryptamine receptor 2B | 417 | 1.18E-11 | 72 |
| Efet.01.87942.g793.t1 | 5'-AMP-activated protein kinase catalytic subunit alpha-2 | 237 | 1.46E-09 | 72 |
| Efet.01.336817.g1086.t1 | Active breakpoint cluster region-related protein | 222 | 2.27E-13 | 72 |
| Efet.01.470636.g546.t1 | Neuronal acetylcholine receptor subunit alpha-4 | 1059 | 5.06E-66 | 72 |
| Efet.01.242282.g1617.t1 | Neuronal acetylcholine receptor subunit alpha-7 | 222 | 7.60E-13 | 72 |
| Efet.01.42283.g1130.t1 | Muscarinic acetylcholine receptor M1 | 840 | 2.80E-13 | 72 |
| Efet.01.70976.g1294.t1 | Alpha-1B adrenergic receptor | 507 | 1.73E-17 | 72 |
| Efet.01.446475.g1151.t1 | Alpha-2A adrenergic receptor | 1110 | 3.45E-21 | 72 |
| Efet.01.137401.g629.t1 | Aldehyde dehydrogenase family 1 member A3 | 273 | 6.52E-09 | 72 |
| Efet.01.274389.g892.t1 | Aquaporin-4 | 603 | 2.44E-26 | 72 |
| Efet.01.117940.g923.t1 | Sarcoplasmic/endoplasmic reticulum calcium ATPase 2 | 435 | 1.16E-48 | 72 |
| Efet.01.506845.g177.t1 | ATP-binding cassette, sub-family A (ABC1), member 1 | 507 | 4.36E-41 | 72 |
| Efet.01.38317.g887.t1 | Band 3 anion transport protein | 306 | 1.70E-08 | 72 |
| Efet.01.412555.g285.t1 | Band 3 anion transport protein | 312 | 1.16E-11 | 72 |
| Efet.01.179511.g1370.t1 | Interleukin enhancer-binding factor 2 | 327 | 4.87E-27 | 72 |
| Efet.01.304280.g148.t1 | Voltage-dependent T-type calcium channel subunit alpha-1G | 264 | 3.94E-11 | 72 |
| Efet.01.195800.g2121.t1 | Cholecystokinin receptor type A | 309 | 1.73E-16 | 72 |
| Efet.01.325363.g777.t1 | CCAAT/enhancer-binding protein beta | 456 | 1.70E-18 | 72 |
| Efet.01.157331.g383.t1 | Cysteine/serine-rich nuclear protein 3 | 777 | 3.75E-64 | 72 |
| Efet.01.181114.g1457.t1 | B-cell CLL/lymphoma 11A (Zinc finger protein) isoform 2 | 342 | 1.35E-06 | 72 |
| Efet.01.209427.g425.t1 | Netrin receptor DCC | 225 | 1.70E-21 | 72 |
| Efet.01.251124.g33.t1 | Discoidin domain-containing receptor 2 | 234 | 2.13E-08 | 72 |
| Efet.01.516480.g411.t1 | Discoidin domain-containing receptor 2 | 501 | 8.67E-47 | 72 |
| Efet.01.45822.g1347.t1 | ATP-dependent RNA helicase DDX42 | 210 | 5.05E-09 | 72 |
| Efet.01.204628.g201.t1 | ATP-dependent RNA helicase DDX42 | 435 | 3.20E-30 | 72 |
| Efet.01.483470.g822.t1 | D(2) dopamine receptor | 408 | 7.82E-17 | 72 |
| Efet.01.643474.g800.t1 | D(2) dopamine receptor | 477 | 4.61E-15 | 72 |
| Efet.01.54171.g277.t1 | D(3) dopamine receptor | 981 | 1.44E-07 | 72 |
| Efet.01.138915.g716.t1 | Dual specificity protein phosphatase 6 | 627 | 3.57E-08 | 72 |
| Efet.01.47403.g1441.t1 | Excitatory amino acid transporter 2 | 234 | 1.78E-09 | 72 |
| Efet.01.1635692.g163.t1 | Epidermal growth factor receptor | 270 | 9.86E-19 | 72 |
| Efet.01.232893.g1303.t1 | Ephrin type-A receptor 3 | 546 | 2.97E-83 | 72 |
| Efet.01.517413.g428.t1 | Eyes absent homolog 2 | 351 | 1.24E-32 | 72 |
| Efet.01.1650523.g477.t1 | Fibroleukin | 312 | 6.32E-11 | 72 |
| Efet.01.326574.g820.t1 | Protein farnesyltransferase/geranylgeranyltransferase type-1 subunit alpha | 624 | 1.39E-80 | 72 |
| Efet.01.25937.g72.t1 | Alpha-(1,6)-fucosyltransferase | 276 | 1.72E-31 | 72 |
| Efet.01.339877.g1171.t1 | Frizzled-5 | 1866 | 0 | 72 |
| Efet.01.421392.g514.t1 | Glutamate--cysteine ligase regulatory subunit | 486 | 1.42E-51 | 72 |
| Efet.01.653283.g412.t1 | Aquaporin-9 | 279 | 2.12E-24 | 72 |
| Efet.01.1648211.g400.t1 | Aquaporin-9 | 210 | 1.03E-19 | 72 |
| Efet.01.412551.g282.t1 | Histamine H1 receptor | 438 | 1.21E-24 | 72 |
| Efet.01.274331.g887.t1 | Eukaryotic translation initiation factor 5A-1 | 222 | 2.41E-06 | 72 |
| Efet.01.78208.g188.t1 | Intraflagellar transport protein 57 homolog | 432 | 1.04E-40 | 72 |
| Efet.01.113356.g676.t1 | Insulin receptor | 1164 | 1.31E-50 | 72 |
| Efet.01.639184.g488.t1 | Afadin | 207 | 2.22E-14 | 72 |
| Efet.01.468407.g488.t1 | Protein Jade-1 | 2529 | 2.65E-30 | 72 |
| Efet.01.216183.g671.t1 | Transcription factor AP-1 | 474 | 2.33E-29 | 72 |
| Efet.01.51500.g98.t1 | Potassium voltage-gated channel subfamily H member 8 | 960 | 6.09E-60 | 72 |
| Efet.01.153445.g185.t1 | Kelch-like protein 20 | 381 | 4.74E-47 | 72 |
| Efet.01.257895.g292.t1 | Pyruvate kinase PKM | 1425 | 2.36E-135 | 72 |
| Efet.01.402937.g79.t1 | Pyruvate kinase PKM | 987 | 2.28E-98 | 72 |
| Efet.01.579669.g664.t1 | Low-density lipoprotein receptor-related protein 2 | 438 | 7.05E-34 | 72 |
| Efet.01.658325.g1855.t1 | Low-density lipoprotein receptor-related protein 6 | 372 | 3.33E-43 | 72 |
| Efet.01.135066.g489.t1 | Protein LSM14 homolog A | 216 | 1.80E-12 | 72 |
| Efet.01.42747.g1153.t1 | Transcription factor MafB | 918 | 9.48E-17 | 72 |
| Efet.01.205764.g251.t1 | Transcription factor MafB | 471 | 3.07E-17 | 72 |
| Efet.01.84966.g627.t1 | Menin | 1083 | 1.38E-23 | 72 |
| Efet.01.513435.g323.t1 | Tyrosine-protein kinase Mer | 222 | 1.53E-12 | 72 |
| Efet.01.211936.g515.t1 | Unconventional myosin-VI | 249 | 8.62E-19 | 72 |
| Efet.01.274856.g913.t1 | Unconventional myosin-VI | 435 | 3.83E-34 | 72 |
| Efet.01.423212.g548.t1 | Nicotinamide phosphoribosyltransferase | 471 | 3.25E-47 | 72 |
| Efet.01.103327.g178.t1 | Neurogenic differentiation factor 1 | 555 | 1.63E-27 | 72 |
| Efet.01.75570.g33.t1 | Netrin-1 | 1002 | 2.61E-102 | 72 |
| Efet.01.88956.g831.t1 | Neurofibromin | 252 | 1.77E-21 | 72 |
| Efet.01.396287.g1151.t1 | Substance-P receptor | 270 | 4.04E-23 | 72 |
| Efet.01.496426.g1092.t1 | Homeobox protein Nkx-2.6 | 507 | 1.34E-49 | 72 |
| Efet.01.1655808.g792.t1 | Neurogenic locus notch homolog protein 2 | 417 | 1.54E-46 | 72 |
| Efet.01.1647785.g386.t1 | Oxysterols receptor LXR-alpha | 243 | 4.50E-12 | 72 |
| Efet.01.131113.g324.t1 | Serine/threonine-protein kinase N1 | 363 | 2.06E-10 | 72 |
| Efet.01.620686.g903.t1 | POU domain, class 4, transcription factor 3 | 1149 | 2.79E-72 | 72 |
| Efet.01.84945.g625.t1 | PTK2 protein tyrosine kinase 2 isoform b variant | 342 | 1.47E-15 | 72 |
| Efet.01.25685.g58.t1 | Jun B proto-oncogene | 1077 | 1.79E-20 | 72 |
| Efet.01.107675.g394.t1 | UV excision repair protein RAD23 homolog B | 312 | 6.78E-29 | 72 |
| Efet.01.239605.g1522.t1 | Proto-oncogene tyrosine-protein kinase receptor Ret | 474 | 1.96E-42 | 72 |
| Efet.01.248345.g1832.t1 | E3 ubiquitin-protein ligase rififylin | 255 | 6.80E-07 | 72 |
| Efet.01.54406.g289.t1 | Ras-specific guanine nucleotide-releasing factor 1 | 345 | 1.16E-09 | 72 |
| Efet.01.653926.g495.t1 | E3 ubiquitin-protein ligase RNF128 | 888 | 8.40E-13 | 72 |
| Efet.01.178394.g1328.t1 | Roundabout homolog 2 | 414 | 4.86E-36 | 72 |
| Efet.01.113675.g691.t1 | RUN and FYVE domain-containing protein 1 | 387 | 2.22E-11 | 72 |
| Efet.01.219531.g792.t1 | Sodium/glucose cotransporter 4 | 657 | 3.84E-46 | 72 |
| Efet.01.182168.g1508.t1 | Sodium channel protein type 2 subunit alpha | 264 | 3.74E-10 | 72 |
| Efet.01.278273.g1046.t1 | Sodium channel protein type 2 subunit alpha | 288 | 9.55E-22 | 72 |
| Efet.01.364933.g402.t1 | Sodium channel protein type 2 subunit alpha | 387 | 1.69E-18 | 72 |
| Efet.01.584181.g796.t1 | Sodium channel protein type 2 subunit alpha | 384 | 6.53E-30 | 72 |
| Efet.01.438810.g921.t1 | NAD-dependent protein deacetylase sirtuin-1 | 282 | 7.51E-06 | 72 |
| Efet.01.176.g20.t1 | SNF-related serine/threonine-protein kinase | 372 | 1.17E-25 | 72 |
| Efet.01.549162.g473.t1 | Sorting nexin-6 | 459 | 4.10E-54 | 72 |
| Efet.01.7548.g563.t1 | Transcription factor SOX-2 | 624 | 2.31E-34 | 72 |
| Efet.01.80316.g353.t1 | Transcription factor SOX-9 | 1326 | 2.67E-25 | 72 |
| Efet.01.83702.g564.t1 | Spectrin beta chain, non-erythrocytic 2 | 2436 | 8.58E-22 | 72 |
| Efet.01.328571.g878.t1 | Spectrin beta chain, non-erythrocytic 2 | 444 | 2.07E-45 | 72 |
| Efet.01.12769.g957.t1 | Synaptotagmin-7 | 288 | 1.01E-23 | 72 |
| Efet.01.172896.g1090.t1 | Synaptotagmin-7 | 558 | 2.07E-68 | 72 |
| Efet.01.82790.g511.t1 | T-cell acute lymphocytic leukemia protein 2 | 522 | 7.36E-15 | 72 |
| Efet.01.400310.g7.t1 | T-cell acute lymphocytic leukemia protein 2 | 1032 | 3.95E-07 | 72 |
| Efet.01.198386.g2229.t1 | Transcription factor 21 | 417 | 7.89E-29 | 72 |
| Efet.01.4048.g344.t1 | Protein Tob1 | 1086 | 4.21E-47 | 72 |
| Efet.01.18545.g1374.t1 | E3 ubiquitin-protein ligase Topors | 2772 | 8.18E-14 | 72 |
| Efet.01.35201.g689.t1 | mRNA decay activator protein ZFP36 | 870 | 3.21E-26 | 72 |
| Efet.01.416518.g395.t1 | Tubby protein homolog | 1584 | 8.91E-07 | 72 |
| Efet.01.520470.g486.t1 | Probable ubiquitin carboxyl-terminal hydrolase FAF-X | 1395 | 2.42E-96 | 72 |
| Efet.01.127336.g135.t1 | Wiskott-Aldrich syndrome protein family member 3 | 291 | 4.14E-22 | 72 |
| Efet.01.1612062.g25.t1 | Protein Wnt-4 | 243 | 5.69E-27 | 72 |
| Efet.01.197167.g2184.t1 | Protein Wnt-7a | 417 | 1.45E-18 | 72 |
| Efet.01.241051.g1569.t1 | NEDD4-like E3 ubiquitin-protein ligase WWP2 | 669 | 4.68E-91 | 72 |
| Efet.01.596027.g1180.t1 | Heat shock 60kDa protein 1 (Chaperonin) | 939 | 1.36E-93 | 71 |
| Efet.01.589679.g980.t1 | ADAM metallopeptidase domain 10, isoform CRA_b | 351 | 2.83E-13 | 71 |
| Efet.01.67467.g1079.t1 | Epithelial discoidin domain-containing receptor 1 | 525 | 4.41E-55 | 71 |
| Efet.01.86621.g729.t1 | Epithelial discoidin domain-containing receptor 1 | 288 | 1.46E-07 | 71 |
| Efet.01.186255.g1699.t1 | Tyrosine-protein kinase | 285 | 2.48E-26 | 71 |
| Efet.01.341567.g1198.t1 | ATP-binding cassette sub-family G member 1 | 534 | 2.49E-21 | 71 |
| Efet.01.21275.g1571.t1 | Angiotensin-converting enzyme | 405 | 6.26E-42 | 71 |
| Efet.01.19105.g1420.t1 | Neuronal acetylcholine receptor subunit alpha-4 | 1098 | 2.37E-41 | 71 |
| Efet.01.225893.g1032.t1 | Neuronal acetylcholine receptor subunit alpha-7 | 594 | 6.49E-62 | 71 |
| Efet.01.372232.g624.t1 | Neuronal acetylcholine receptor subunit alpha-7 | 363 | 2.61E-34 | 71 |
| Efet.01.197751.g2207.t1 | Alpha-actinin-4 | 285 | 2.51E-25 | 71 |
| Efet.01.294922.g1593.t1 | Alpha-1B adrenergic receptor | 576 | 4.96E-37 | 71 |
| Efet.01.371278.g597.t1 | Alpha-2A adrenergic receptor | 1629 | 8.32E-21 | 71 |
| Efet.01.142966.g923.t1 | Gamma-adducin | 549 | 1.48E-09 | 71 |
| Efet.01.150063.g3.t1 | Allograft inflammatory factor 1-like | 381 | 5.85E-06 | 71 |
| Efet.01.116098.g837.t1 | RAC-alpha serine/threonine-protein kinase | 489 | 1.91E-49 | 71 |
| Efet.01.132302.g364.t1 | Fructose-bisphosphate aldolase C | 426 | 3.75E-47 | 71 |
| Efet.01.160923.g572.t1 | Ankyrin-1 | 330 | 3.11E-17 | 71 |
| Efet.01.185603.g1658.t1 | Antithrombin-III | 210 | 3.69E-16 | 71 |
| Efet.01.435187.g842.t1 | ADP-ribosylation factor 6 | 432 | 5.02E-46 | 71 |
| Efet.01.99053.g1392.t1 | cDNA FLJ46417 fis, clone THYMU3012402, highly similar to Importin alpha-1 subunit (cDNA FLJ55353, highly similar to Importin alpha-1 subunit) | 513 | 9.13E-50 | 71 |
| Efet.01.93138.g1061.t1 | cDNA FLJ58446, highly similar to Nicotinate-nucleotide pyrophosphorylase (carboxylating) | 951 | 2.86E-28 | 71 |
| Efet.01.498867.g1153.t1 | cDNA FLJ59231, highly similar to C-ets-1 protein | 570 | 1.14E-08 | 71 |
| Efet.01.397083.g1175.t1 | Baculoviral IAP repeat-containing protein 6 | 297 | 1.81E-28 | 71 |
| Efet.01.585438.g838.t1 | Baculoviral IAP repeat-containing protein 6 | 756 | 1.08E-38 | 71 |
| Efet.01.648535.g1246.t1 | Baculoviral IAP repeat-containing protein 6 | 297 | 1.81E-28 | 71 |
| Efet.01.624241.g1065.t1 | BCL2/adenovirus E1B 19 kDa protein-interacting protein 2 | 450 | 7.82E-12 | 71 |
| Efet.01.210030.g448.t1 | Calmodulin-A | 297 | 4.09E-21 | 71 |
| Efet.01.13376.g1001.t1 | CREB-binding protein | 1464 | 3.59E-73 | 71 |
| Efet.01.194970.g2089.t1 | E3 SUMO-protein ligase CBX4 | 363 | 1.55E-07 | 71 |
| Efet.01.49916.g1583.t1 | Cholecystokinin receptor type A | 576 | 3.47E-14 | 71 |
| Efet.01.84684.g606.t1 | Cyclin-dependent kinase 1 | 294 | 7.89E-20 | 71 |
| Efet.01.80571.g376.t1 | CCAAT/enhancer-binding protein gamma | 396 | 6.78E-09 | 71 |
| Efet.01.307049.g220.t1 | Cadherin EGF LAG seven-pass G-type receptor 1 | 285 | 4.21E-25 | 71 |
| Efet.01.213338.g564.t1 | Collagen alpha-1(XV) chain | 435 | 1.78E-33 | 71 |
| Efet.01.502484.g58.t1 | COUP transcription factor 2 | 360 | 1.72E-12 | 71 |
| Efet.01.89518.g861.t1 | Carbamoyl-phosphate synthase [ammonia], mitochondrial | 621 | 2.47E-32 | 71 |
| Efet.01.369822.g540.t1 | Carbamoyl-phosphate synthase [ammonia], mitochondrial | 567 | 5.36E-51 | 71 |
| Efet.01.327033.g834.t1 | Probable ATP-dependent RNA helicase DDX41 | 234 | 2.00E-14 | 71 |
| Efet.01.482711.g802.t1 | D(2) dopamine receptor | 273 | 4.12E-15 | 71 |
| Efet.01.115066.g784.t1 | Eukaryotic translation initiation factor 2-alpha kinase 3 | 237 | 2.95E-18 | 71 |
| Efet.01.184744.g1625.t1 | Filamin-A | 276 | 4.84E-25 | 71 |
| Efet.01.211491.g499.t1 | Zinc finger protein ZFPM1 | 1464 | 4.26E-11 | 71 |
| Efet.01.526328.g615.t1 | Tyrosine-protein kinase FRK | 288 | 3.31E-28 | 71 |
| Efet.01.200931.g49.t1 | Afadin | 372 | 5.11E-30 | 71 |
| Efet.01.124388.g1258.t1 | Gastrin/cholecystokinin type B receptor | 303 | 9.53E-12 | 71 |
| Efet.01.163521.g685.t1 | Guanine nucleotide-binding protein G(I)/G(S)/G(O) subunit gamma-12 | 213 | 2.04E-13 | 71 |
| Efet.01.1648952.g422.t1 | E3 ubiquitin-protein ligase RNF130 | 267 | 6.21E-18 | 71 |
| Efet.01.407332.g173.t1 | Gremlin-2 | 570 | 3.75E-38 | 71 |
| Efet.01.170965.g1010.t1 | Glutamate receptor ionotropic, kainate 2 | 240 | 3.25E-15 | 71 |
| Efet.01.343830.g1247.t1 | Glutamate receptor ionotropic, kainate 2 | 315 | 3.33E-14 | 71 |
| Efet.01.1604645.g9.t1 | Glutamate receptor ionotropic, kainate 2 | 207 | 4.75E-16 | 71 |
| Efet.01.1646325.g346.t1 | Homeobox protein Hox-A7 | 300 | 2.74E-23 | 71 |
| Efet.01.66143.g996.t1 | Zinc finger protein Aiolos | 558 | 1.50E-15 | 71 |
| Efet.01.449769.g1212.t1 | Intersectin-2 | 216 | 4.07E-06 | 71 |
| Efet.01.170695.g1004.t1 | KN motif and ankyrin repeat domain-containing protein 1 | 276 | 8.69E-18 | 71 |
| Efet.01.18637.g1383.t1 | Calcium-activated potassium channel subunit alpha-1 | 261 | 7.32E-14 | 71 |
| Efet.01.528809.g660.t1 | Potassium voltage-gated channel subfamily H member 8 | 243 | 3.35E-18 | 71 |
| Efet.01.572354.g390.t1 | Potassium voltage-gated channel subfamily H member 8 | 327 | 7.82E-16 | 71 |
| Efet.01.79091.g253.t1 | Kinesin-like protein KIF13B | 552 | 5.59E-43 | 71 |
| Efet.01.1634772.g155.t1 | Kinesin-like protein KIF13B | 234 | 1.76E-10 | 71 |
| Efet.01.336823.g1087.t1 | Pyruvate kinase PKM | 1731 | 0 | 71 |
| Efet.01.642837.g725.t1 | Lactoylglutathione lyase | 462 | 1.58E-60 | 71 |
| Efet.01.168130.g895.t1 | Prolow-density lipoprotein receptor-related protein 1 | 609 | 6.44E-75 | 71 |
| Efet.01.407607.g176.t1 | MKL/myocardin-like protein 1 | 1980 | 6.70E-07 | 71 |
| Efet.01.47104.g1420.t1 | Canalicular multispecific organic anion transporter 1 | 312 | 1.47E-22 | 71 |
| Efet.01.381769.g834.t1 | Unconventional myosin-XVIIIa | 1122 | 1.88E-18 | 71 |
| Efet.01.19597.g1452.t1 | Myosin-binding protein H | 201 | 1.01E-06 | 71 |
| Efet.01.371952.g617.t1 | Myosin light chain kinase, smooth muscle | 477 | 7.90E-27 | 71 |
| Efet.01.116755.g874.t1 | Neurogenic differentiation factor 1 | 828 | 1.45E-12 | 71 |
| Efet.01.83816.g566.t1 | NADH-ubiquinone oxidoreductase 75 kDa subunit, mitochondrial | 246 | 5.23E-35 | 71 |
| Efet.01.229002.g1162.t1 | Netrin-1 | 570 | 2.67E-55 | 71 |
| Efet.01.115317.g795.t1 | Homeobox protein Nkx-2.2 | 867 | 2.27E-27 | 71 |
| Efet.01.376215.g707.t1 | Neuroligin-4, X-linked | 333 | 7.35E-20 | 71 |
| Efet.01.50643.g46.t1 | Serine/threonine-protein kinase NLK | 483 | 2.62E-11 | 71 |
| Efet.01.185812.g1672.t1 | Neurogenic locus notch homolog protein 2 | 288 | 1.65E-19 | 71 |
| Efet.01.552216.g557.t1 | Oxysterols receptor LXR-alpha | 330 | 1.24E-28 | 71 |
| Efet.01.57776.g484.t1 | Neurexin-3-beta | 315 | 1.13E-11 | 71 |
| Efet.01.422630.g531.t1 | Phosphofurin acidic cluster sorting protein 1 | 441 | 1.90E-22 | 71 |
| Efet.01.222808.g921.t1 | Serine/threonine-protein kinase PAK 2 | 264 | 1.06E-23 | 71 |
| Efet.01.544428.g347.t1 | Proprotein convertase subtilisin/kexin type 5 | 267 | 4.32E-17 | 71 |
| Efet.01.1328.g122.t1 | Serine/threonine-protein kinase pim-1 | 861 | 4.60E-100 | 71 |
| Efet.01.271606.g810.t1 | Pinin | 315 | 2.82E-17 | 71 |
| Efet.01.288284.g1392.t1 | Phospholipase A-2-activating protein | 939 | 9.44E-64 | 71 |
| Efet.01.257008.g263.t1 | Serine/threonine-protein kinase PLK3 | 219 | 9.96E-21 | 71 |
| Efet.01.1944.g174.t1 | Plastin-2 | 399 | 1.07E-35 | 71 |
| Efet.01.320901.g660.t1 | 26S proteasome non-ATPase regulatory subunit 4 | 339 | 7.16E-20 | 71 |
| Efet.01.1626162.g80.t1 | Receptor-type tyrosine-protein phosphatase T | 234 | 5.23E-20 | 71 |
| Efet.01.421611.g517.t1 | Transcriptional activator protein Pur-beta | 246 | 3.81E-11 | 71 |
| Efet.01.366129.g435.t1 | Receptor protein-tyrosine kinase | 462 | 2.52E-48 | 71 |
| Efet.01.258373.g306.t1 | Low density lipoprotein-related protein 1 variant | 840 | 7.07E-101 | 71 |
| Efet.01.116175.g842.t1 | cDNA FLJ45819 fis, clone NT2RP8001363, highly similar to Mus musculus signal peptide, CUB domain, EGF-like 1 | 426 | 3.39E-45 | 71 |
| Efet.01.294086.g1576.t1 | GTPase HRas | 282 | 1.51E-24 | 71 |
| Efet.01.212893.g549.t1 | GTPase KRas | 537 | 7.89E-46 | 71 |
| Efet.01.449767.g1211.t1 | Reelin | 279 | 2.41E-26 | 71 |
| Efet.01.130010.g268.t1 | Sodium/myo-inositol cotransporter 2 | 255 | 4.18E-14 | 71 |
| Efet.01.646046.g1029.t1 | Sorting nexin-3 | 240 | 4.06E-17 | 71 |
| Efet.01.658359.g1891.t1 | Transcription factor Sp3 | 1275 | 8.27E-51 | 71 |
| Efet.01.628051.g1229.t1 | Spectrin beta chain, non-erythrocytic 2 | 450 | 6.95E-34 | 71 |
| Efet.01.201157.g57.t1 | Synaptotagmin-7 | 330 | 2.88E-33 | 71 |
| Efet.01.273345.g864.t1 | Synaptotagmin-7 | 501 | 8.50E-32 | 71 |
| Efet.01.120962.g1087.t1 | TGF-beta-activated kinase 1 and MAP3K7-binding protein 1 | 339 | 5.21E-32 | 71 |
| Efet.01.420410.g484.t1 | Ubiquitin carboxyl-terminal hydrolase 28 | 444 | 8.38E-35 | 71 |
| Efet.01.11564.g842.t1 | Protein Wnt-5a | 321 | 1.06E-39 | 71 |
| Efet.01.375186.g685.t1 | Protein Wnt-5b | 447 | 3.04E-52 | 71 |
| Efet.01.1633285.g131.t1 | Tyrosine-protein kinase receptor | 216 | 3.14E-18 | 71 |
| Efet.01.1651695.g521.t1 | Protein kinase C delta type | 300 | 7.04E-13 | 70 |
| Efet.01.267648.g651.t1 | Heat shock 60kDa protein 1 (Chaperonin) | 1545 | 3.34E-141 | 70 |
| Efet.01.319179.g615.t1 | Heat shock 60kDa protein 1 (Chaperonin) | 1485 | 2.66E-100 | 70 |
| Efet.01.208353.g382.t1 | Collagen, type IV, alpha 3 (Goodpasture antigen) binding protein | 441 | 5.59E-41 | 70 |
| Efet.01.50843.g57.t1 | Macrophage-stimulating protein receptor, MSP receptor | 228 | 5.02E-06 | 70 |
| Efet.01.203545.g162.t1 | APOBEC1 complementation factor | 1137 | 1.23E-20 | 70 |
| Efet.01.56431.g402.t1 | CAP-GLY domain containing linker protein 2 | 207 | 1.05E-09 | 70 |
| Efet.01.255502.g200.t1 | Neuronal acetylcholine receptor subunit alpha-4 | 216 | 4.51E-14 | 70 |
| Efet.01.588565.g948.t1 | Neuronal acetylcholine receptor subunit alpha-4 | 351 | 4.68E-11 | 70 |
| Efet.01.1625906.g78.t1 | Neuronal acetylcholine receptor subunit alpha-4 | 213 | 5.93E-13 | 70 |
| Efet.01.12961.g974.t1 | Neuronal acetylcholine receptor subunit alpha-7 | 984 | 1.49E-09 | 70 |
| Efet.01.143758.g961.t1 | Neuronal acetylcholine receptor subunit alpha-7 | 315 | 5.41E-23 | 70 |
| Efet.01.183939.g1595.t1 | Neuronal acetylcholine receptor subunit alpha-7 | 339 | 4.60E-37 | 70 |
| Efet.01.34869.g677.t1 | Peroxisomal acyl-coenzyme A oxidase 1 | 513 | 1.21E-37 | 70 |
| Efet.01.99657.g1426.t1 | Alpha-1A adrenergic receptor | 1224 | 1.20E-71 | 70 |
| Efet.01.81085.g408.t1 | Alpha-1B adrenergic receptor | 333 | 4.63E-30 | 70 |
| Efet.01.173182.g1105.t1 | Alpha-1B adrenergic receptor | 1704 | 2.36E-34 | 70 |
| Efet.01.224599.g982.t1 | Alpha-1B adrenergic receptor | 837 | 5.06E-22 | 70 |
| Efet.01.288887.g1415.t1 | Alpha-1B adrenergic receptor | 528 | 1.63E-16 | 70 |
| Efet.01.634561.g250.t1 | Alpha-1B adrenergic receptor | 882 | 3.57E-22 | 70 |
| Efet.01.415394.g357.t1 | Acidic leucine-rich nuclear phosphoprotein 32 family member B | 285 | 1.65E-07 | 70 |
| Efet.01.515095.g364.t1 | UDP-GlcNAc:betaGal beta-1,3-N-acetylglucosaminyltransferase-like protein 1 | 1077 | 2.93E-129 | 70 |
| Efet.01.27883.g191.t1 | ATP-binding cassette transporter A1 | 462 | 5.40E-40 | 70 |
| Efet.01.494920.g1069.t1 | BAG family molecular chaperone regulator 3 | 1137 | 1.18E-15 | 70 |
| Efet.01.164062.g711.t1 | Voltage-dependent P/Q-type calcium channel subunit alpha-1A | 546 | 7.93E-56 | 70 |
| Efet.01.238691.g1501.t1 | Cyclin-dependent kinase inhibitor 1B | 417 | 1.95E-12 | 70 |
| Efet.01.68600.g1140.t1 | Chromodomain-helicase-DNA-binding protein 8 | 606 | 1.16E-54 | 70 |
| Efet.01.580112.g674.t1 | CAP-Gly domain-containing linker protein 1 | 2406 | 3.62E-21 | 70 |
| Efet.01.113358.g677.t1 | Coronin-1C | 333 | 3.42E-35 | 70 |
| Efet.01.1656108.g826.t1 | Cytochrome P450 26A1 | 690 | 2.20E-15 | 70 |
| Efet.01.1297.g117.t1 | DnaJ homolog subfamily B member 6 | 729 | 8.64E-08 | 70 |
| Efet.01.33299.g570.t1 | D(4) dopamine receptor | 978 | 1.92E-14 | 70 |
| Efet.01.634840.g262.t1 | Nuclear receptor coactivator 6 | 2124 | 2.98E-16 | 70 |
| Efet.01.377118.g729.t1 | Fibrinogen alpha chain | 471 | 1.08E-06 | 70 |
| Efet.01.88126.g801.t1 | Filamin-A | 951 | 1.19E-09 | 70 |
| Efet.01.220412.g824.t1 | Filamin-A | 474 | 3.62E-48 | 70 |
| Efet.01.279225.g1076.t1 | Forkhead box protein K2 | 768 | 7.49E-63 | 70 |
| Efet.01.176942.g1266.t1 | Forkhead box protein L2 | 870 | 2.16E-25 | 70 |
| Efet.01.553238.g577.t1 | Forkhead box protein N2 | 321 | 6.06E-14 | 70 |
| Efet.01.355154.g146.t1 | Tyrosine-protein kinase FRK | 459 | 3.12E-49 | 70 |
| Efet.01.656870.g1131.t1 | Frizzled-4 | 951 | 2.82E-86 | 70 |
| Efet.01.528131.g642.t1 | Gastrin/cholecystokinin type B receptor | 315 | 9.92E-06 | 70 |
| Efet.01.646642.g1115.t1 | Glutaredoxin-3 | 318 | 9.51E-30 | 70 |
| Efet.01.203066.g148.t1 | N-acetyllactosaminide beta-1,6-N-acetylglucosaminyl-transferase | 666 | 8.47E-44 | 70 |
| Efet.01.371428.g609.t1 | Glutamate receptor ionotropic, kainate 2 | 261 | 1.24E-17 | 70 |
| Efet.01.414.g50.t1 | High mobility group protein B2 | 492 | 2.22E-49 | 70 |
| Efet.01.40595.g1031.t1 | Heparan sulfate glucosamine 3-O-sulfotransferase 5 | 678 | 9.22E-87 | 70 |
| Efet.01.32487.g531.t1 | HIV Tat-specific factor 1 | 444 | 1.21E-08 | 70 |
| Efet.01.231632.g1261.t1 | Intraflagellar transport protein 122 homolog | 555 | 3.26E-49 | 70 |
| Efet.01.266670.g609.t1 | Intraflagellar transport protein 81 homolog | 705 | 4.37E-51 | 70 |
| Efet.01.256116.g223.t1 | Inhibitor of nuclear factor kappa-B kinase subunit beta | 228 | 1.46E-20 | 70 |
| Efet.01.142155.g885.t1 | Inositol-trisphosphate 3-kinase B | 261 | 4.73E-18 | 70 |
| Efet.01.44845.g1294.t1 | Afadin | 465 | 2.55E-30 | 70 |
| Efet.01.21481.g1579.t1 | Potassium voltage-gated channel subfamily H member 8 | 267 | 2.75E-10 | 70 |
| Efet.01.65243.g927.t1 | Kinesin-like protein KIF1B | 804 | 3.80E-122 | 70 |
| Efet.01.154453.g228.t1 | Histone-lysine N-methyltransferase 2A | 348 | 3.06E-38 | 70 |
| Efet.01.194197.g2057.t1 | Pyruvate kinase PKM | 231 | 9.92E-15 | 70 |
| Efet.01.31730.g473.t1 | Lethal(2) giant larvae protein homolog 1 | 237 | 8.27E-07 | 70 |
| Efet.01.442011.g1033.t1 | Serine/threonine-protein kinase LATS2 | 1062 | 8.86E-18 | 70 |
| Efet.01.79415.g285.t1 | Prolow-density lipoprotein receptor-related protein 1 | 321 | 5.87E-11 | 70 |
| Efet.01.87334.g763.t1 | Low-density lipoprotein receptor-related protein 6 | 531 | 1.28E-50 | 70 |
| Efet.01.137298.g619.t1 | Serine/threonine-protein kinase MARK1 | 420 | 8.48E-53 | 70 |
| Efet.01.311269.g358.t1 | DNA mismatch repair protein Msh6 | 384 | 4.70E-32 | 70 |
| Efet.01.23113.g1683.t1 | Myosin-10 | 222 | 1.44E-10 | 70 |
| Efet.01.494042.g1053.t1 | NADH-ubiquinone oxidoreductase 75 kDa subunit, mitochondrial | 678 | 3.95E-83 | 70 |
| Efet.01.367527.g473.t1 | Neuroendocrine convertase 1 | 342 | 7.02E-17 | 70 |
| Efet.01.13938.g1033.t1 | Neogenin | 330 | 5.95E-27 | 70 |
| Efet.01.5856.g465.t1 | Neurofilament light polypeptide | 315 | 5.87E-07 | 70 |
| Efet.01.481364.g778.t1 | High affinity cAMP-specific 3',5'-cyclic phosphodiesterase 7A | 507 | 1.57E-23 | 70 |
| Efet.01.154995.g256.t1 | Serine/threonine-protein kinase pim-1 | 480 | 2.22E-39 | 70 |
| Efet.01.20693.g1531.t1 | 1-phosphatidylinositol 4,5-bisphosphate phosphodiesterase delta-4 | 258 | 2.58E-14 | 70 |
| Efet.01.650005.g1.t1 | 1-phosphatidylinositol 4,5-bisphosphate phosphodiesterase epsilon-1 | 360 | 4.70E-09 | 70 |
| Efet.01.609353.g399.t1 | Phospholipase D1 | 561 | 1.11E-61 | 70 |
| Efet.01.164265.g721.t1 | Peroxidasin homolog | 852 | 3.10E-88 | 70 |
| Efet.01.225027.g1004.t1 | Atlastin variant | 477 | 1.94E-30 | 70 |
| Efet.01.632329.g155.t1 | Early growth response protein | 3147 | 1.24E-23 | 70 |
| Efet.01.495764.g1081.t1 | Rab5 GDP/GTP exchange factor | 222 | 4.43E-19 | 70 |
| Efet.01.128293.g186.t1 | GTPase KRas | 540 | 1.25E-46 | 70 |
| Efet.01.371075.g593.t1 | Putative RNA-binding protein 15 | 1689 | 1.34E-45 | 70 |
| Efet.01.75418.g21.t1 | Regulator of G-protein signaling 1 | 216 | 2.15E-17 | 70 |
| Efet.01.591691.g1040.t1 | Rho GTPase-activating protein 5 | 432 | 2.18E-49 | 70 |
| Efet.01.446673.g1155.t1 | Roundabout homolog 1 | 318 | 4.12E-35 | 70 |
| Efet.01.8319.g616.t1 | Ryanodine receptor 2 | 489 | 7.63E-15 | 70 |
| Efet.01.53310.g223.t1 | Sodium channel protein type 2 subunit alpha | 1101 | 3.56E-06 | 70 |
| Efet.01.79666.g302.t1 | Sodium channel protein type 2 subunit alpha | 462 | 5.73E-30 | 70 |
| Efet.01.118867.g976.t1 | Sodium channel protein type 2 subunit alpha | 369 | 3.33E-21 | 70 |
| Efet.01.45584.g1330.t1 | Septin-4 | 723 | 1.48E-59 | 70 |
| Efet.01.32174.g499.t1 | SWI/SNF-related matrix-associated actin-dependent regulator of chromatin subfamily D member 2 | 777 | 1.55E-112 | 70 |
| Efet.01.51664.g106.t1 | Sorting nexin-6 | 351 | 4.17E-26 | 70 |
| Efet.01.643605.g813.t1 | Transcription factor SOX-2 | 1407 | 3.10E-31 | 70 |
| Efet.01.548703.g463.t1 | Spectrin beta chain, non-erythrocytic 2 | 363 | 9.32E-41 | 70 |
| Efet.01.386635.g940.t1 | Serine/threonine-protein kinase 26 | 246 | 5.85E-08 | 70 |
| Efet.01.125724.g44.t1 | Extracellular sulfatase Sulf-1 | 243 | 9.50E-16 | 70 |
| Efet.01.1646823.g357.t1 | Synaptotagmin-7 | 333 | 3.46E-26 | 70 |
| Efet.01.93535.g1076.t1 | Serine/threonine-protein kinase TAO2 | 369 | 1.45E-24 | 70 |
| Efet.01.229264.g1174.t1 | Heat shock protein 75 kDa, mitochondrial | 489 | 2.44E-42 | 70 |
| Efet.01.318970.g609.t1 | Tribbles homolog 2 | 339 | 2.55E-28 | 70 |
| Efet.01.284585.g1251.t1 | DNA repair protein XRCC3 | 597 | 2.34E-12 | 70 |
| Efet.01.302232.g75.t1 | E3 ubiquitin-protein ligase ZSWIM2 | 336 | 1.09E-15 | 70 |
| Efet.01.11637.g854.t1 | ADAM metallopeptidase domain 10, isoform CRA_b | 348 | 2.98E-20 | 69 |
| Efet.01.651287.g156.t1 | Discoidin domain receptor family, member 1, isoform CRA_a | 339 | 1.91E-28 | 69 |
| Efet.01.230377.g1209.t1 | Tyrosine-protein kinase | 615 | 7.62E-62 | 69 |
| Efet.01.89619.g867.t1 | Adenosine receptor A2b | 408 | 1.88E-10 | 69 |
| Efet.01.11750.g866.t1 | Neuronal acetylcholine receptor subunit alpha-4 | 1134 | 6.05E-74 | 69 |
| Efet.01.80858.g390.t1 | Neuronal acetylcholine receptor subunit alpha-7 | 483 | 5.02E-36 | 69 |
| Efet.01.100931.g67.t1 | Neuronal acetylcholine receptor subunit alpha-7 | 333 | 1.01E-20 | 69 |
| Efet.01.211483.g498.t1 | Neuronal acetylcholine receptor subunit alpha-7 | 357 | 1.67E-28 | 69 |
| Efet.01.334603.g1036.t1 | Neuronal acetylcholine receptor subunit alpha-7 | 552 | 6.92E-55 | 69 |
| Efet.01.426977.g646.t1 | Long-chain-fatty-acid--CoA ligase 4 | 1032 | 2.44E-127 | 69 |
| Efet.01.512876.g303.t1 | Alpha-actinin-1 | 246 | 1.05E-14 | 69 |
| Efet.01.65641.g958.t1 | Alpha-1A adrenergic receptor | 1056 | 6.63E-81 | 69 |
| Efet.01.127082.g122.t1 | Ankyrin-1 | 336 | 1.62E-33 | 69 |
| Efet.01.1631129.g112.t1 | AT-rich interactive domain-containing protein 5B | 282 | 8.85E-14 | 69 |
| Efet.01.190959.g1914.t1 | cDNA FLJ50323, highly similar to Macrophage-stimulating protein receptor | 528 | 1.00E-50 | 69 |
| Efet.01.330561.g934.t1 | Beta-1,4-galactosyltransferase 1 | 237 | 3.23E-06 | 69 |
| Efet.01.10385.g760.t1 | ATP-binding cassette transporter A1 | 846 | 1.48E-83 | 69 |
| Efet.01.156258.g323.t1 | Baculoviral IAP repeat-containing protein 6 | 300 | 1.17E-15 | 69 |
| Efet.01.166489.g817.t1 | Bloom syndrome protein | 267 | 2.60E-25 | 69 |
| Efet.01.358041.g224.t1 | Bone morphogenetic protein 10 | 831 | 1.12E-22 | 69 |
| Efet.01.5717.g447.t1 | Scavenger receptor cysteine-rich type 1 protein M160 | 264 | 3.72E-09 | 69 |
| Efet.01.131207.g329.t1 | Transcription factor 7-like 2 | 351 | 2.44E-41 | 69 |
| Efet.01.476973.g687.t1 | Calmodulin-A | 558 | 7.34E-22 | 69 |
| Efet.01.572068.g383.t1 | Cholecystokinin receptor type A | 348 | 1.61E-06 | 69 |
| Efet.01.583549.g783.t1 | Cholecystokinin receptor type A | 408 | 1.41E-25 | 69 |
| Efet.01.338654.g1126.t1 | Cell division control protein 42 homolog | 534 | 4.42E-37 | 69 |
| Efet.01.657066.g1158.t1 | Chromodomain-helicase-DNA-binding protein 7 | 1050 | 7.16E-57 | 69 |
| Efet.01.156415.g336.t1 | Calsyntenin-1 | 324 | 1.79E-11 | 69 |
| Efet.01.110841.g548.t1 | Death-associated protein kinase 3 | 522 | 2.16E-07 | 69 |
| Efet.01.423964.g569.t1 | Discoidin domain-containing receptor 2 | 324 | 1.56E-28 | 69 |
| Efet.01.624933.g1086.t1 | Probable ATP-dependent RNA helicase DDX41 | 876 | 7.46E-18 | 69 |
| Efet.01.32199.g501.t1 | Dystonin | 621 | 1.76E-45 | 69 |
| Efet.01.198577.g2237.t1 | C-Jun-amino-terminal kinase-interacting protein 3 | 453 | 5.07E-39 | 69 |
| Efet.01.127714.g149.t1 | Excitatory amino acid transporter 2 | 228 | 2.54E-16 | 69 |
| Efet.01.12857.g969.t1 | Ephrin type-A receptor 7 | 642 | 3.35E-86 | 69 |
| Efet.01.372855.g643.t1 | DNA excision repair protein ERCC-6 | 330 | 2.71E-21 | 69 |
| Efet.01.67354.g1070.t1 | Serine/threonine-protein kinase/endoribonuclease IRE2 | 273 | 1.14E-07 | 69 |
| Efet.01.652446.g304.t1 | Protein fem-1 homolog B | 1938 | 0 | 69 |
| Efet.01.285074.g1271.t1 | Dimethylaniline monooxygenase [N-oxide-forming] 1 | 324 | 9.64E-19 | 69 |
| Efet.01.399580.g1235.t1 | Forkhead box protein D4 | 423 | 9.41E-25 | 69 |
| Efet.01.658034.g1568.t1 | Frizzled-5 | 540 | 3.04E-41 | 69 |
| Efet.01.658034.g1569.t1 | Frizzled-5 | 939 | 6.75E-109 | 69 |
| Efet.01.506990.g181.t1 | Laminin subunit beta-1 | 300 | 1.58E-13 | 69 |
| Efet.01.565302.g180.t1 | Glucose-6-phosphate 1-dehydrogenase | 378 | 7.87E-22 | 69 |
| Efet.01.375689.g693.t1 | Polypeptide N-acetylgalactosaminyltransferase 2 | 414 | 4.92E-20 | 69 |
| Efet.01.85522.g657.t1 | Growth arrest-specific protein 2 | 663 | 5.46E-15 | 69 |
| Efet.01.562218.g42.t1 | Histamine H2 receptor | 780 | 2.97E-22 | 69 |
| Efet.01.25416.g44.t1 | Heparan sulfate glucosamine 3-O-sulfotransferase 5 | 714 | 4.94E-07 | 69 |
| Efet.01.338200.g1118.t1 | Heat shock protein HSP 90-beta | 1029 | 1.96E-33 | 69 |
| Efet.01.81014.g401.t1 | Interferon regulatory factor 2 | 1005 | 2.11E-36 | 69 |
| Efet.01.180681.g1432.t1 | Inter-alpha-trypsin inhibitor heavy chain H4 | 270 | 2.70E-11 | 69 |
| Efet.01.15124.g1110.t1 | Calcium/calmodulin-dependent protein kinase type II subunit delta | 276 | 6.53E-17 | 69 |
| Efet.01.125339.g19.t1 | Laminin subunit gamma-1 | 468 | 1.09E-30 | 69 |
| Efet.01.113833.g703.t1 | Leucine-rich repeats and immunoglobulin-like domains protein 2 | 888 | 5.57E-61 | 69 |
| Efet.01.238085.g1480.t1 | MAP kinase-activating death domain protein | 582 | 3.87E-65 | 69 |
| Efet.01.14618.g1071.t1 | TRAF3-interacting protein 1 | 2493 | 1.21E-06 | 69 |
| Efet.01.524439.g573.t1 | Metastasis suppressor protein 1 | 234 | 2.02E-17 | 69 |
| Efet.01.578127.g622.t1 | Myosin light chain kinase, smooth muscle | 276 | 4.58E-22 | 69 |
| Efet.01.85563.g660.t1 | Unconventional myosin-VI | 285 | 3.41E-18 | 69 |
| Efet.01.8023.g592.t1 | Nuclear receptor coactivator 6 | 2685 | 2.63E-06 | 69 |
| Efet.01.184674.g1622.t1 | Homeobox protein Nkx-3.2 | 363 | 2.58E-06 | 69 |
| Efet.01.302220.g74.t1 | Homeobox protein Nkx-3.2 | 951 | 2.21E-21 | 69 |
| Efet.01.297755.g1682.t1 | Nostrin | 1599 | 1.89E-11 | 69 |
| Efet.01.1653216.g601.t1 | Nephrocystin-4 | 471 | 9.55E-51 | 69 |
| Efet.01.526563.g618.t1 | Calcium/calmodulin-dependent 3',5'-cyclic nucleotide phosphodiesterase 1B | 273 | 6.78E-09 | 69 |
| Efet.01.83613.g559.t1 | Basement membrane-specific heparan sulfate proteoglycan core protein | 717 | 5.14E-09 | 69 |
| Efet.01.165477.g773.t1 | Homeobox protein PKNOX1 | 525 | 2.29E-24 | 69 |
| Efet.01.342115.g1210.t1 | POU domain, class 3, transcription factor 2 | 888 | 9.38E-43 | 69 |
| Efet.01.383063.g868.t1 | Protein phosphatase 1F | 240 | 9.91E-14 | 69 |
| Efet.01.54995.g337.t1 | 26S proteasome non-ATPase regulatory subunit 10 | 681 | 7.00E-06 | 69 |
| Efet.01.506751.g171.t1 | Inactive tyrosine-protein kinase 7 | 498 | 7.31E-51 | 69 |
| Efet.01.508634.g225.t1 | Ras-related protein Rab-13 | 546 | 3.45E-10 | 69 |
| Efet.01.271981.g826.t1 | RNA-binding protein 5 | 270 | 2.89E-06 | 69 |
| Efet.01.371981.g618.t1 | Double-stranded RNA-specific editase 1 | 1077 | 6.86E-122 | 69 |
| Efet.01.121950.g1143.t1 | Rho GTPase-activating protein 4 | 312 | 5.52E-32 | 69 |
| Efet.01.99355.g1411.t1 | Rho GTPase-activating protein 5 | 1098 | 1.87E-47 | 69 |
| Efet.01.137784.g656.t1 | Secretory carrier-associated membrane protein 3 | 315 | 1.96E-14 | 69 |
| Efet.01.217124.g707.t1 | Sodium channel protein type 2 subunit alpha | 327 | 1.19E-17 | 69 |
| Efet.01.172277.g1063.t1 | SH3 domain-containing kinase-binding protein 1 | 318 | 3.90E-22 | 69 |
| Efet.01.542780.g321.t1 | STE20-like serine/threonine-protein kinase | 234 | 6.21E-11 | 69 |
| Efet.01.360655.g288.t1 | Transcription factor SOX-2 | 993 | 1.86E-38 | 69 |
| Efet.01.115245.g792.t1 | Syntaxin-binding protein 5 | 1395 | 1.29E-12 | 69 |
| Efet.01.467973.g478.t1 | Sushi, von Willebrand factor type A, EGF and pentraxin domain-containing protein 1 | 462 | 1.44E-26 | 69 |
| Efet.01.87647.g779.t1 | Synaptojanin-1 | 318 | 5.20E-23 | 69 |
| Efet.01.27904.g192.t1 | Synaptotagmin-7 | 498 | 2.52E-29 | 69 |
| Efet.01.548169.g442.t1 | Tumor necrosis factor alpha-induced protein 8 | 498 | 8.19E-46 | 69 |
| Efet.01.26159.g89.t1 | Tropomyosin alpha-1 chain | 354 | 9.28E-06 | 69 |
| Efet.01.167584.g872.t1 | TNF receptor-associated factor 5 | 432 | 3.28E-39 | 69 |
| Efet.01.78378.g202.t1 | Tribbles homolog 1 | 816 | 6.10E-96 | 69 |
| Efet.01.44913.g1296.t1 | Transient receptor potential cation channel subfamily M member 7 | 303 | 6.38E-20 | 69 |
| Efet.01.91620.g976.t1 | Epididymis secretory protein Li 31 | 618 | 3.55E-45 | 69 |
| Efet.01.37227.g807.t1 | Serine/threonine-protein kinase WNK3 | 282 | 4.67E-14 | 69 |
| Efet.01.1649921.g456.t1 | Protein Wnt-4 | 489 | 3.92E-52 | 69 |
| Efet.01.628006.g1225.t1 | Protein Wnt-7a | 468 | 2.57E-59 | 69 |
| Efet.01.370469.g569.t1 | Tyrosine-protein kinase receptor | 663 | 5.57E-26 | 69 |
| Efet.01.219977.g807.t1 | Nuclear factor interleukin-3-regulated protein | 714 | 5.06E-10 | 68 |
| Efet.01.178419.g1330.t1 | TNF receptor-associated factor 2, isoform CRA_a | 252 | 1.67E-12 | 68 |
| Efet.01.608021.g332.t1 | Vitamin K epoxide reductase complex subunit 1 isoform 2 | 219 | 1.52E-07 | 68 |
| Efet.01.310452.g330.t1 | Neuronal acetylcholine receptor subunit alpha-7 | 426 | 8.10E-43 | 68 |
| Efet.01.649562.g1329.t1 | Muscarinic acetylcholine receptor M1 | 285 | 8.53E-21 | 68 |
| Efet.01.1652577.g567.t1 | Actin, alpha cardiac muscle 1 | 234 | 9.78E-16 | 68 |
| Efet.01.117865.g919.t1 | Alpha-actinin-1 | 297 | 1.68E-21 | 68 |
| Efet.01.537432.g211.t1 | Alpha-1A adrenergic receptor | 897 | 1.48E-18 | 68 |
| Efet.01.604040.g150.t1 | Alpha-2A adrenergic receptor | 1110 | 9.20E-20 | 68 |
| Efet.01.403289.g87.t1 | ADP/ATP translocase 3 | 435 | 4.12E-44 | 68 |
| Efet.01.353333.g99.t1 | AP-3 complex subunit beta-2 | 240 | 3.29E-19 | 68 |
| Efet.01.256488.g242.t1 | Beta-adrenergic receptor kinase 1 | 525 | 7.84E-58 | 68 |
| Efet.01.432961.g799.t1 | Band 3 anion transport protein | 579 | 1.19E-07 | 68 |
| Efet.01.1474.g139.t1 | cDNA FLJ53503, highly similar to Plasma serine protease inhibitor | 453 | 1.43E-18 | 68 |
| Efet.01.24566.g1781.t1 | cDNA FLJ61244, highly similar to Transcription factor 8 | 333 | 4.09E-15 | 68 |
| Efet.01.600986.g43.t1 | Tyrosine-protein kinase | 720 | 5.86E-09 | 68 |
| Efet.01.220119.g816.t1 | Homeobox protein BarH-like 1 | 375 | 7.82E-09 | 68 |
| Efet.01.247437.g1793.t1 | Protein bicaudal D homolog 2 | 234 | 5.25E-09 | 68 |
| Efet.01.32366.g520.t1 | Bone morphogenetic protein receptor type-1B | 1548 | 1.52E-169 | 68 |
| Efet.01.1638608.g195.t1 | Calmodulin | 312 | 6.39E-11 | 68 |
| Efet.01.77465.g145.t1 | Calmodulin-alpha | 270 | 6.10E-15 | 68 |
| Efet.01.191678.g1945.t1 | Cathepsin B | 282 | 6.41E-31 | 68 |
| Efet.01.341256.g1192.t1 | G2/mitotic-specific cyclin-B2 | 921 | 4.72E-15 | 68 |
| Efet.01.38271.g882.t1 | CD109 antigen | 663 | 1.79E-26 | 68 |
| Efet.01.173958.g1134.t1 | Acidic mammalian chitinase | 783 | 3.76E-53 | 68 |
| Efet.01.360473.g283.t1 | Carbohydrate sulfotransferase 4 | 279 | 7.61E-06 | 68 |
| Efet.01.277980.g1030.t1 | Cullin-4A | 300 | 8.20E-46 | 68 |
| Efet.01.395303.g1124.t1 | DCN1-like protein 3 | 861 | 1.31E-70 | 68 |
| Efet.01.509552.g241.t1 | Discoidin domain-containing receptor 2 | 291 | 2.31E-19 | 68 |
| Efet.01.155881.g305.t1 | Diacylglycerol kinase gamma | 246 | 3.33E-21 | 68 |
| Efet.01.268657.g687.t1 | Docking protein 1 | 1173 | 1.39E-11 | 68 |
| Efet.01.239919.g1534.t1 | D(3) dopamine receptor | 705 | 1.20E-21 | 68 |
| Efet.01.181985.g1500.t1 | Dual specificity protein phosphatase 3 | 237 | 5.36E-17 | 68 |
| Efet.01.100938.g68.t1 | Dual specificity tyrosine-phosphorylation-regulated kinase 2 | 438 | 1.06E-34 | 68 |
| Efet.01.107149.g369.t1 | Dual specificity tyrosine-phosphorylation-regulated kinase 2 | 609 | 5.27E-72 | 68 |
| Efet.01.8721.g643.t1 | Dystonin | 285 | 3.47E-16 | 68 |
| Efet.01.24733.g1792.t1 | 55 kDa erythrocyte membrane protein | 204 | 1.90E-19 | 68 |
| Efet.01.168410.g910.t1 | Protocadherin Fat 4 | 453 | 8.31E-11 | 68 |
| Efet.01.532098.g60.t1 | Filamin-A | 534 | 8.87E-49 | 68 |
| Efet.01.354314.g123.t1 | Dimethylaniline monooxygenase [N-oxide-forming] 1 | 573 | 8.39E-20 | 68 |
| Efet.01.75999.g59.t1 | Forkhead box protein D2 | 429 | 4.38E-12 | 68 |
| Efet.01.323111.g715.t1 | Forkhead box protein S1 | 975 | 1.16E-08 | 68 |
| Efet.01.521270.g501.t1 | Homeobox protein GBX-2 | 402 | 7.21E-41 | 68 |
| Efet.01.310959.g349.t1 | Growth/differentiation factor 8 | 291 | 4.75E-34 | 68 |
| Efet.01.252336.g82.t1 | GRAM domain-containing protein 4 | 255 | 2.20E-08 | 68 |
| Efet.01.5060.g392.t1 | Glutamate receptor ionotropic, kainate 2 | 282 | 6.09E-18 | 68 |
| Efet.01.636379.g332.t1 | Stress-70 protein, mitochondrial | 888 | 1.11E-76 | 68 |
| Efet.01.227538.g1101.t1 | Hepatic leukemia factor | 366 | 2.43E-10 | 68 |
| Efet.01.444919.g1104.t1 | Inhibitor of Bruton tyrosine kinase | 870 | 3.12E-61 | 68 |
| Efet.01.395045.g1118.t1 | Insulin-like growth factor 2 mRNA-binding protein 2 | 342 | 5.81E-12 | 68 |
| Efet.01.253758.g139.t1 | Insulin receptor | 636 | 5.45E-45 | 68 |
| Efet.01.334920.g1047.t1 | Protein Jade-1 | 522 | 2.53E-46 | 68 |
| Efet.01.58382.g516.t1 | Calcium-activated potassium channel subunit alpha-1 | 210 | 7.23E-10 | 68 |
| Efet.01.311866.g376.t1 | Calcium-activated potassium channel subunit alpha-1 | 681 | 6.27E-06 | 68 |
| Efet.01.642328.g674.t1 | Potassium voltage-gated channel subfamily H member 8 | 240 | 1.19E-17 | 68 |
| Efet.01.449263.g1201.t1 | ATP-sensitive inward rectifier potassium channel 8 | 867 | 2.77E-44 | 68 |
| Efet.01.231384.g1245.t1 | Krueppel-like factor 10 | 1380 | 5.74E-06 | 68 |
| Efet.01.140326.g789.t1 | Histone-lysine N-methyltransferase 2A | 903 | 7.99E-68 | 68 |
| Efet.01.42898.g1171.t1 | Ribosomal protein S6 kinase alpha-1 | 417 | 2.34E-32 | 68 |
| Efet.01.27830.g186.t1 | Neural cell adhesion molecule L1 | 204 | 5.44E-07 | 68 |
| Efet.01.267558.g635.t1 | Neural cell adhesion molecule L1 | 351 | 4.38E-07 | 68 |
| Efet.01.328646.g881.t1 | Lethal(2) giant larvae protein homolog 2 | 567 | 5.58E-50 | 68 |
| Efet.01.173512.g1118.t1 | Liprin-beta-1 | 297 | 3.78E-23 | 68 |
| Efet.01.195111.g2097.t1 | Low-density lipoprotein receptor-related protein 2 | 312 | 8.95E-19 | 68 |
| Efet.01.628746.g1267.t1 | Low-density lipoprotein receptor-related protein 6 | 222 | 5.96E-06 | 68 |
| Efet.01.591949.g1053.t1 | Mitogen-activated protein kinase kinase kinase 11 | 708 | 4.95E-66 | 68 |
| Efet.01.62369.g777.t1 | Multidrug resistance protein 1 | 432 | 2.64E-19 | 68 |
| Efet.01.451660.g39.t1 | Multidrug resistance protein 1 | 594 | 2.32E-56 | 68 |
| Efet.01.1635206.g159.t1 | Myeloid zinc finger 1 | 375 | 4.93E-25 | 68 |
| Efet.01.494042.g1050.t1 | NADH dehydrogenase [ubiquinone] iron-sulfur protein 3, mitochondrial | 474 | 3.08E-42 | 68 |
| Efet.01.134538.g469.t1 | Nischarin | 288 | 6.77E-10 | 68 |
| Efet.01.573548.g422.t1 | Protein kinase C and casein kinase substrate in neurons protein 2 | 216 | 5.44E-09 | 68 |
| Efet.01.445279.g1123.t1 | TCDD-inducible poly [ADP-ribose] polymerase | 630 | 1.81E-14 | 68 |
| Efet.01.265233.g564.t1 | Paired box protein Pax-3 | 408 | 5.77E-18 | 68 |
| Efet.01.423326.g551.t1 | PDZ domain-containing protein 2 | 714 | 2.22E-08 | 68 |
| Efet.01.301613.g57.t1 | Phosphoglucomutase-like protein 5 | 345 | 5.78E-22 | 68 |
| Efet.01.26741.g122.t1 | PH-interacting protein | 375 | 5.36E-18 | 68 |
| Efet.01.138671.g699.t1 | Pleckstrin homology domain-containing family G member 5 | 285 | 2.03E-11 | 68 |
| Efet.01.13630.g1016.t1 | Plastin-2 | 249 | 7.07E-16 | 68 |
| Efet.01.475796.g670.t1 | Plexin-A3 | 339 | 1.32E-25 | 68 |
| Efet.01.611353.g476.t1 | Plexin-A3 | 540 | 1.08E-39 | 68 |
| Efet.01.1642204.g249.t1 | Plexin-A3 | 276 | 5.18E-10 | 68 |
| Efet.01.469150.g511.t1 | Tartrate-resistant acid phosphatase type 5 | 402 | 5.42E-30 | 68 |
| Efet.01.212268.g531.t1 | TP53-regulating kinase | 450 | 1.82E-47 | 68 |
| Efet.01.189949.g1869.t1 | 26S proteasome non-ATPase regulatory subunit 10 | 366 | 1.98E-07 | 68 |
| Efet.01.610211.g433.t1 | Receptor-type tyrosine-protein phosphatase kappa | 402 | 2.61E-25 | 68 |
| Efet.01.82427.g490.t1 | Calcium/calmodulin-dependent protein kinase ID, isoform CRA_b | 954 | 1.06E-66 | 68 |
| Efet.01.369069.g516.t1 | Calcium/calmodulin-dependent protein kinase ID, isoform CRA_b | 1308 | 3.97E-91 | 68 |
| Efet.01.467253.g461.t1 | Jun B proto-oncogene | 903 | 2.12E-14 | 68 |
| Efet.01.360349.g279.t1 | 5'-nucleotidase, ecto (CD73) | 402 | 4.41E-32 | 68 |
| Efet.01.193608.g2023.t1 | Zinc finger protein 3 | 501 | 8.22E-48 | 68 |
| Efet.01.208546.g390.t1 | LAMB1 protein | 921 | 3.69E-89 | 68 |
| Efet.01.13036.g982.t1 | ELKS/Rab6-interacting/CAST family member 1 | 528 | 5.26E-17 | 68 |
| Efet.01.232163.g1278.t1 | Protein Red | 945 | 3.39E-41 | 68 |
| Efet.01.607077.g295.t1 | Proto-oncogene c-Rel | 381 | 8.72E-23 | 68 |
| Efet.01.250457.g14.t1 | Rho GTPase-activating protein 17 | 357 | 1.15E-19 | 68 |
| Efet.01.42558.g1145.t1 | Sodium channel protein type 2 subunit alpha | 252 | 4.40E-18 | 68 |
| Efet.01.58214.g511.t1 | Sodium channel protein type 2 subunit alpha | 861 | 2.34E-64 | 68 |
| Efet.01.389533.g996.t1 | SWI/SNF-related matrix-associated actin-dependent regulator of chromatin subfamily A-like protein 1 | 570 | 1.06E-33 | 68 |
| Efet.01.76034.g62.t1 | SRSF protein kinase 1 | 540 | 1.90E-45 | 68 |
| Efet.01.89871.g884.t1 | STAM-binding protein | 777 | 1.42E-21 | 68 |
| Efet.01.122927.g1190.t1 | Synaptotagmin-1 | 345 | 2.19E-14 | 68 |
| Efet.01.149454.g1204.t1 | Tubulin beta-2A chain | 420 | 1.48E-17 | 68 |
| Efet.01.569638.g309.t1 | Alpha-taxilin | 393 | 2.60E-30 | 68 |
| Efet.01.309809.g310.t1 | Ubiquitin conjugation factor E4 B | 291 | 1.56E-20 | 68 |
| Efet.01.356392.g179.t1 | Netrin receptor UNC5D | 309 | 2.66E-07 | 68 |
| Efet.01.33232.g562.t1 | Voltage-dependent anion-selective channel protein 1 | 282 | 6.97E-20 | 68 |
| Efet.01.231351.g1242.t1 | V-type proton ATPase 116 kDa subunit a isoform 2 | 246 | 1.46E-19 | 68 |
| Efet.01.106665.g348.t1 | Protein Wnt-7a | 423 | 1.95E-39 | 68 |
| Efet.01.11753.g867.t1 | Zinc finger protein 675 | 411 | 3.70E-15 | 68 |
| Efet.01.219516.g790.t1 | 5-hydroxytryptamine receptor 2B | 1707 | 1.99E-18 | 67 |
| Efet.01.254119.g149.t1 | 5-hydroxytryptamine receptor 2B | 345 | 8.07E-13 | 67 |
| Efet.01.536532.g200.t1 | 5-hydroxytryptamine receptor 2B | 1848 | 6.13E-22 | 67 |
| Efet.01.204180.g183.t1 | TNF receptor-associated factor 2, isoform CRA_a | 585 | 2.40E-24 | 67 |
| Efet.01.1216.g110.t1 | Proline-serine-threonine phosphatase interacting protein 1 isoform 1 | 642 | 3.61E-10 | 67 |
| Efet.01.629376.g1292.t1 | ATP-binding cassette sub-family A member 5 | 2850 | 4.70E-06 | 67 |
| Efet.01.79809.g310.t1 | Neuronal acetylcholine receptor subunit alpha-4 | 621 | 7.41E-06 | 67 |
| Efet.01.386404.g936.t1 | Neuronal acetylcholine receptor subunit alpha-7 | 213 | 6.05E-06 | 67 |
| Efet.01.1630598.g106.t1 | Neuronal acetylcholine receptor subunit alpha-7 | 234 | 1.80E-11 | 67 |
| Efet.01.47357.g1439.t1 | Muscarinic acetylcholine receptor M1 | 1533 | 1.44E-18 | 67 |
| Efet.01.197807.g2211.t1 | Muscarinic acetylcholine receptor M1 | 2004 | 5.46E-18 | 67 |
| Efet.01.205904.g256.t1 | Alpha-1A adrenergic receptor | 972 | 6.79E-48 | 67 |
| Efet.01.651609.g202.t1 | ADP/ATP translocase 3 | 564 | 7.92E-57 | 67 |
| Efet.01.1657574.g1010.t1 | Aldehyde dehydrogenase family 1 member A3 | 204 | 2.16E-11 | 67 |
| Efet.01.14494.g1065.t1 | Amine oxidase [flavin-containing] B | 1539 | 4.86E-173 | 67 |
| Efet.01.151563.g92.t1 | Aquaporin-4 | 417 | 4.49E-10 | 67 |
| Efet.01.118824.g972.t1 | Rho guanine nucleotide exchange factor 12 | 540 | 1.61E-06 | 67 |
| Efet.01.223676.g952.t1 | Sarcoplasmic/endoplasmic reticulum calcium ATPase 1 | 513 | 3.19E-13 | 67 |
| Efet.01.423585.g559.t1 | Sarcoplasmic/endoplasmic reticulum calcium ATPase 1 | 483 | 1.60E-07 | 67 |
| Efet.01.225483.g1017.t1 | Calcium-transporting ATPase type 2C member 1 | 306 | 3.09E-16 | 67 |
| Efet.01.241936.g1598.t1 | Cyclic AMP-dependent transcription factor ATF-5 | 987 | 1.27E-15 | 67 |
| Efet.01.300.g34.t1 | Protein atonal homolog 1 | 645 | 6.32E-11 | 67 |
| Efet.01.28238.g216.t1 | ATP-binding cassette, sub-family A (ABC1), member 1 | 330 | 4.14E-15 | 67 |
| Efet.01.373055.g646.t1 | ATP-binding cassette, sub-family A (ABC1), member 1 | 420 | 7.51E-29 | 67 |
| Efet.01.1654813.g715.t1 | ATP-binding cassette, sub-family A (ABC1), member 1 | 390 | 1.46E-33 | 67 |
| Efet.01.40709.g1047.t1 | ATP-binding cassette transporter A1 | 1713 | 4.57E-65 | 67 |
| Efet.01.152987.g163.t1 | Protein bicaudal D homolog 2 | 279 | 2.44E-19 | 67 |
| Efet.01.356924.g194.t1 | Calmodulin | 354 | 6.78E-18 | 67 |
| Efet.01.73807.g1466.t1 | Cathepsin K | 300 | 9.56E-15 | 67 |
| Efet.01.518738.g451.t1 | Cathepsin S | 267 | 1.59E-09 | 67 |
| Efet.01.8142.g602.t1 | Cholecystokinin receptor type A | 261 | 1.00E-10 | 67 |
| Efet.01.67091.g1050.t1 | Cholecystokinin receptor type A | 513 | 4.22E-18 | 67 |
| Efet.01.199879.g2295.t1 | G2/mitotic-specific cyclin-B2 | 939 | 3.04E-91 | 67 |
| Efet.01.334916.g1046.t1 | Cyclin-dependent kinase 5 activator 1 | 432 | 7.99E-14 | 67 |
| Efet.01.84780.g609.t1 | COUP transcription factor 2 | 276 | 1.71E-12 | 67 |
| Efet.01.236322.g1416.t1 | Discoidin domain-containing receptor 2 | 432 | 3.09E-40 | 67 |
| Efet.01.541560.g298.t1 | Discoidin domain-containing receptor 2 | 231 | 6.75E-19 | 67 |
| Efet.01.59035.g566.t1 | Probable ATP-dependent RNA helicase DDX41 | 339 | 1.37E-25 | 67 |
| Efet.01.471393.g562.t1 | Probable ATP-dependent RNA helicase DDX41 | 1203 | 5.23E-24 | 67 |
| Efet.01.23590.g1709.t1 | D(2) dopamine receptor | 312 | 8.20E-21 | 67 |
| Efet.01.604801.g179.t1 | Dual specificity protein phosphatase 6 | 429 | 8.11E-34 | 67 |
| Efet.01.655549.g826.t1 | Ficolin-1 | 225 | 5.02E-24 | 67 |
| Efet.01.496900.g1111.t1 | Filamin-A | 564 | 1.07E-42 | 67 |
| Efet.01.589955.g990.t1 | Forkhead box protein N2 | 642 | 5.31E-42 | 67 |
| Efet.01.572761.g400.t1 | Gastrin/cholecystokinin type B receptor | 615 | 1.40E-11 | 67 |
| Efet.01.1657544.g1004.t1 | Guanylate-binding protein 1 | 348 | 1.85E-09 | 67 |
| Efet.01.65277.g928.t1 | Zinc finger protein GLI2 | 756 | 2.98E-17 | 67 |
| Efet.01.31309.g441.t1 | Glutaredoxin-2, mitochondrial | 336 | 1.29E-24 | 67 |
| Efet.01.19058.g1416.t1 | Glutaredoxin-3 | 444 | 2.21E-25 | 67 |
| Efet.01.93636.g1082.t1 | Polypeptide N-acetylgalactosaminyltransferase 16 | 246 | 5.44E-20 | 67 |
| Efet.01.563018.g72.t1 | Guanine nucleotide-binding protein subunit alpha-13 | 528 | 5.34E-42 | 67 |
| Efet.01.14025.g1036.t1 | Glutamate receptor ionotropic, kainate 2 | 222 | 4.07E-16 | 67 |
| Efet.01.130056.g271.t1 | Glutamate receptor ionotropic, kainate 2 | 597 | 1.70E-13 | 67 |
| Efet.01.166417.g814.t1 | Glutamate receptor ionotropic, kainate 2 | 207 | 5.19E-15 | 67 |
| Efet.01.11981.g889.t1 | DNA-binding protein inhibitor ID-1 | 345 | 6.26E-09 | 67 |
| Efet.01.2753.g252.t1 | Zinc finger protein Aiolos | 1077 | 1.66E-06 | 67 |
| Efet.01.224739.g990.t1 | Insulin receptor | 345 | 9.40E-22 | 67 |
| Efet.01.213709.g582.t1 | KN motif and ankyrin repeat domain-containing protein 1 | 423 | 1.86E-27 | 67 |
| Efet.01.261097.g415.t1 | KN motif and ankyrin repeat domain-containing protein 1 | 342 | 1.56E-22 | 67 |
| Efet.01.248572.g1837.t1 | cAMP-dependent protein kinase type II-beta regulatory subunit | 462 | 6.29E-34 | 67 |
| Efet.01.275664.g954.t1 | ATP-sensitive inward rectifier potassium channel 8 | 1515 | 1.53E-87 | 67 |
| Efet.01.282018.g1156.t1 | Kinesin-like protein KIF13B | 654 | 7.49E-49 | 67 |
| Efet.01.259108.g350.t1 | Kelch-like protein 20 | 249 | 5.53E-16 | 67 |
| Efet.01.553365.g585.t1 | Histone-lysine N-methyltransferase 2A | 207 | 3.11E-06 | 67 |
| Efet.01.421207.g509.t1 | Protein kinase C epsilon type | 360 | 4.13E-31 | 67 |
| Efet.01.486631.g890.t1 | Low-density lipoprotein receptor-related protein 2 | 726 | 2.90E-44 | 67 |
| Efet.01.78232.g192.t1 | Mitogen-activated protein kinase kinase kinase 11 | 1218 | 7.97E-96 | 67 |
| Efet.01.626647.g1168.t1 | Microtubule-associated serine/threonine-protein kinase 2 | 480 | 1.36E-36 | 67 |
| Efet.01.1659497.g1878.t1 | Multidrug resistance protein 1 | 1551 | 3.00E-56 | 67 |
| Efet.01.165880.g789.t1 | Menin | 207 | 9.69E-08 | 67 |
| Efet.01.110569.g537.t1 | Hippocampus abundant transcript-like protein 1 | 309 | 2.98E-10 | 67 |
| Efet.01.44757.g1286.t1 | Multidrug resistance-associated protein 5 | 975 | 3.33E-64 | 67 |
| Efet.01.1656827.g917.t1 | Substance-P receptor | 495 | 6.18E-18 | 67 |
| Efet.01.11571.g843.t1 | Neurogenic locus notch homolog protein 1 | 474 | 3.10E-21 | 67 |
| Efet.01.39361.g956.t1 | Neurogenic locus notch homolog protein 2 | 375 | 2.94E-35 | 67 |
| Efet.01.82251.g481.t1 | NAD(P)H dehydrogenase [quinone] 1 | 234 | 4.33E-16 | 67 |
| Efet.01.419168.g443.t1 | Bile acid receptor | 282 | 2.34E-10 | 67 |
| Efet.01.17140.g1264.t1 | Kappa-type opioid receptor | 375 | 8.55E-09 | 67 |
| Efet.01.381855.g837.t1 | Paired box protein Pax-5 | 327 | 2.13E-15 | 67 |
| Efet.01.85915.g689.t1 | Programmed cell death 6-interacting protein | 654 | 1.31E-22 | 67 |
| Efet.01.1643010.g269.t1 | Protein-glucosylgalactosylhydroxylysine glucosidase | 270 | 2.46E-16 | 67 |
| Efet.01.468161.g480.t1 | PH domain leucine-rich repeat-containing protein phosphatase 1 | 528 | 6.01E-12 | 67 |
| Efet.01.575084.g482.t1 | Phosphatidylinositol 4,5-bisphosphate 3-kinase catalytic subunit beta isoform | 255 | 1.86E-22 | 67 |
| Efet.01.162431.g635.t1 | Phospholipase A-2-activating protein | 597 | 4.78E-45 | 67 |
| Efet.01.547849.g439.t1 | Inactive tyrosine-protein kinase 7 | 486 | 1.18E-46 | 67 |
| Efet.01.303724.g124.t1 | Tyrosine-protein phosphatase non-receptor type | 273 | 5.51E-16 | 67 |
| Efet.01.286635.g1326.t1 | Low density lipoprotein-related protein 1 variant | 648 | 7.89E-65 | 67 |
| Efet.01.89446.g856.t1 | Connective tissue growth factor | 390 | 6.84E-11 | 67 |
| Efet.01.381944.g839.t1 | Ras-related protein Rab-3A | 420 | 3.85E-08 | 67 |
| Efet.01.127471.g139.t1 | Ras-related protein Rab-7a | 543 | 7.36E-53 | 67 |
| Efet.01.416263.g390.t1 | Ras-related protein Rab-11A | 645 | 8.82E-54 | 67 |
| Efet.01.99733.g1434.t1 | Reelin | 270 | 4.13E-20 | 67 |
| Efet.01.50244.g20.t1 | Transforming protein RhoA | 540 | 1.15E-71 | 67 |
| Efet.01.158179.g431.t1 | Transforming protein RhoA | 720 | 2.10E-74 | 67 |
| Efet.01.542240.g309.t1 | Transforming protein RhoA | 429 | 4.06E-50 | 67 |
| Efet.01.651547.g187.t1 | Sodium-coupled monocarboxylate transporter 1 | 393 | 9.72E-19 | 67 |
| Efet.01.9340.g687.t1 | Sodium channel protein type 2 subunit alpha | 1326 | 4.07E-74 | 67 |
| Efet.01.14766.g1081.t1 | Sodium channel protein type 2 subunit alpha | 792 | 1.43E-67 | 67 |
| Efet.01.71880.g1355.t1 | Sodium channel protein type 2 subunit alpha | 642 | 6.03E-27 | 67 |
| Efet.01.247441.g1795.t1 | Secernin-1 | 207 | 4.71E-13 | 67 |
| Efet.01.312671.g399.t1 | Homeobox protein SIX4 | 915 | 4.00E-48 | 67 |
| Efet.01.204620.g200.t1 | Sushi, von Willebrand factor type A, EGF and pentraxin domain-containing protein 1 | 630 | 2.88E-21 | 67 |
| Efet.01.254372.g160.t1 | Synaptotagmin-9 | 618 | 2.75E-39 | 67 |
| Efet.01.528798.g659.t1 | Mitochondrial import inner membrane translocase subunit Tim8 A | 249 | 3.51E-24 | 67 |
| Efet.01.194286.g2060.t1 | Triple functional domain protein | 597 | 1.91E-49 | 67 |
| Efet.01.12683.g951.t1 | Ubiquitin conjugation factor E4 B | 693 | 1.56E-87 | 67 |
| Efet.01.194817.g2086.t1 | Ubiquitin carboxyl-terminal hydrolase 4 | 204 | 1.47E-14 | 67 |
| Efet.01.415059.g346.t1 | Wiskott-Aldrich syndrome protein family member 3 | 1482 | 3.39E-18 | 67 |
| Efet.01.469334.g516.t1 | Protein Wnt-5b | 426 | 1.32E-47 | 67 |
| Efet.01.67880.g1108.t1 | Protein Wnt-7a | 309 | 4.29E-26 | 67 |
| Efet.01.168063.g891.t1 | 5-hydroxytryptamine receptor 6 | 771 | 5.66E-12 | 66 |
| Efet.01.80073.g329.t1 | TNF receptor-associated factor 4, isoform CRA_a | 642 | 2.93E-51 | 66 |
| Efet.01.130466.g297.t1 | DAXX (Death-associated protein 6, isoform CRA_a) | 324 | 2.49E-21 | 66 |
| Efet.01.256941.g262.t1 | Transcription factor 7-like 2 | 396 | 8.24E-06 | 66 |
| Efet.01.259579.g366.t1 | cDNA FLJ77467, highly similar to Homo sapiens dipeptidylpeptidase 8 | 204 | 1.89E-08 | 66 |
| Efet.01.558703.g713.t1 | ATP-binding cassette sub-family F member 1 | 600 | 3.43E-46 | 66 |
| Efet.01.41283.g1074.t1 | Active breakpoint cluster region-related protein | 240 | 1.96E-09 | 66 |
| Efet.01.505751.g144.t1 | Active breakpoint cluster region-related protein | 288 | 3.33E-15 | 66 |
| Efet.01.1658806.g1280.t1 | Neuronal acetylcholine receptor subunit alpha-4 | 1020 | 1.61E-48 | 66 |
| Efet.01.514565.g354.t1 | Neuronal acetylcholine receptor subunit alpha-7 | 525 | 1.61E-36 | 66 |
| Efet.01.630132.g6.t1 | Neuronal acetylcholine receptor subunit alpha-7 | 336 | 1.30E-27 | 66 |
| Efet.01.286600.g1324.t1 | Alpha-actinin-2 | 429 | 6.11E-31 | 66 |
| Efet.01.10588.g777.t1 | Alpha-1A adrenergic receptor | 1371 | 4.26E-18 | 66 |
| Efet.01.288887.g1414.t1 | Alpha-1A adrenergic receptor | 1209 | 4.34E-23 | 66 |
| Efet.01.446475.g1150.t1 | Alpha-2A adrenergic receptor | 774 | 4.74E-21 | 66 |
| Efet.01.174695.g1160.t1 | Adenylate cyclase type 10 | 1638 | 4.07E-40 | 66 |
| Efet.01.632855.g175.t1 | Cytosol aminopeptidase | 651 | 6.35E-26 | 66 |
| Efet.01.39202.g948.t1 | Aminopeptidase N | 438 | 6.93E-09 | 66 |
| Efet.01.399586.g1236.t1 | Angiopoietin-related protein 1 | 567 | 7.99E-54 | 66 |
| Efet.01.330277.g921.t1 | Ankyrin-1 | 279 | 7.79E-09 | 66 |
| Efet.01.595454.g1155.t1 | Rho guanine nucleotide exchange factor 4 | 327 | 8.35E-09 | 66 |
| Efet.01.259307.g356.t1 | Beta-1,4-galactosyltransferase 1 | 273 | 6.95E-12 | 66 |
| Efet.01.138845.g711.t1 | BLM protein | 2943 | 8.00E-176 | 66 |
| Efet.01.6376.g505.t1 | BAG family molecular chaperone regulator 4 | 243 | 4.09E-16 | 66 |
| Efet.01.455498.g143.t1 | Calmodulin-A | 366 | 5.35E-33 | 66 |
| Efet.01.191763.g1946.t1 | Dipeptidyl peptidase 1 | 312 | 7.36E-20 | 66 |
| Efet.01.1628109.g85.t1 | Mast cell carboxypeptidase A | 240 | 2.12E-14 | 66 |
| Efet.01.375927.g702.t1 | Cholecystokinin receptor type A | 423 | 1.12E-17 | 66 |
| Efet.01.420546.g488.t1 | Cholecystokinin receptor type A | 381 | 1.14E-24 | 66 |
| Efet.01.144556.g998.t1 | G2/mitotic-specific cyclin-B2 | 375 | 4.94E-26 | 66 |
| Efet.01.1658974.g1344.t1 | G1/S-specific cyclin-D3 | 414 | 1.10E-12 | 66 |
| Efet.01.552742.g571.t1 | C-C chemokine receptor type 6 | 534 | 1.78E-11 | 66 |
| Efet.01.46903.g1406.t1 | Cell division control protein 42 homolog | 297 | 5.56E-29 | 66 |
| Efet.01.194757.g2084.t1 | Chromodomain-helicase-DNA-binding protein 8 | 489 | 9.81E-31 | 66 |
| Efet.01.606599.g285.t1 | Chromodomain-helicase-DNA-binding protein 8 | 213 | 5.04E-08 | 66 |
| Efet.01.391218.g1032.t1 | Cytochrome P450 4F8 | 450 | 3.57E-24 | 66 |
| Efet.01.198217.g2224.t1 | Alpha-crystallin B chain | 1134 | 4.96E-10 | 66 |
| Efet.01.376308.g710.t1 | Cubilin | 453 | 2.87E-22 | 66 |
| Efet.01.252393.g86.t1 | Disks large homolog 1 | 537 | 1.31E-25 | 66 |
| Efet.01.603758.g141.t1 | D(4) dopamine receptor | 1116 | 7.57E-11 | 66 |
| Efet.01.581030.g700.t1 | Dystonin | 318 | 1.04E-09 | 66 |
| Efet.01.293791.g1567.t1 | Amyloid-beta A4 protein | 270 | 4.07E-15 | 66 |
| Efet.01.61281.g703.t1 | Egl nine homolog 3 | 1641 | 1.15E-70 | 66 |
| Efet.01.350988.g32.t1 | EH domain-containing protein 1 | 771 | 1.32E-87 | 66 |
| Efet.01.21118.g1560.t1 | Protein ENL | 630 | 9.23E-38 | 66 |
| Efet.01.114632.g762.t1 | Receptor tyrosine-protein kinase erbB-3 | 549 | 8.33E-37 | 66 |
| Efet.01.58977.g563.t1 | Vitamin D (1,25-dihydroxyvitamin D3) receptor, isoform CRA_c | 1302 | 4.34E-19 | 66 |
| Efet.01.187083.g1736.t1 | Fibroblast growth factor receptor 3 | 684 | 5.78E-15 | 66 |
| Efet.01.30764.g398.t1 | Filamin-A | 288 | 3.05E-26 | 66 |
| Efet.01.135733.g538.t1 | Guanine nucleotide-binding protein G(I)/G(S)/G(O) subunit gamma-12 | 201 | 2.13E-13 | 66 |
| Efet.01.113921.g709.t1 | Gelsolin | 252 | 5.07E-14 | 66 |
| Efet.01.278771.g1057.t1 | Growth hormone secretagogue receptor type 1 | 750 | 6.31E-07 | 66 |
| Efet.01.296407.g1630.t1 | Polypeptide N-acetylgalactosaminyltransferase 16 | 735 | 2.02E-20 | 66 |
| Efet.01.453348.g72.t1 | Glutamate receptor ionotropic, kainate 2 | 255 | 1.18E-06 | 66 |
| Efet.01.49245.g1546.t1 | Hepatocyte nuclear factor 6 | 339 | 3.48E-12 | 66 |
| Efet.01.606399.g275.t1 | Hepatocyte nuclear factor 6 | 492 | 8.34E-14 | 66 |
| Efet.01.77777.g163.t1 | Hematopoietic prostaglandin D synthase | 348 | 8.70E-12 | 66 |
| Efet.01.183541.g1574.t1 | Homeobox protein Hox-A5 | 612 | 8.83E-08 | 66 |
| Efet.01.602169.g92.t1 | Zinc finger protein Pegasus | 1599 | 4.84E-13 | 66 |
| Efet.01.256148.g225.t1 | Interferon regulatory factor 2 | 660 | 1.29E-31 | 66 |
| Efet.01.240304.g1543.t1 | Kalirin | 258 | 8.85E-07 | 66 |
| Efet.01.367455.g468.t1 | Potassium voltage-gated channel subfamily H member 8 | 924 | 6.16E-73 | 66 |
| Efet.01.385949.g930.t1 | Potassium voltage-gated channel subfamily H member 8 | 492 | 1.94E-29 | 66 |
| Efet.01.569509.g301.t1 | Potassium voltage-gated channel subfamily H member 8 | 315 | 4.71E-10 | 66 |
| Efet.01.491516.g983.t1 | ATP-sensitive inward rectifier potassium channel 8 | 729 | 3.64E-35 | 66 |
| Efet.01.1920.g170.t1 | Kelch-like protein 20 | 321 | 5.68E-45 | 66 |
| Efet.01.454759.g115.t1 | Kelch-like protein 20 | 264 | 1.96E-18 | 66 |
| Efet.01.407970.g196.t1 | Inactive histone-lysine N-methyltransferase 2E | 810 | 2.87E-17 | 66 |
| Efet.01.467188.g460.t1 | Protein kinase C epsilon type | 363 | 5.00E-28 | 66 |
| Efet.01.86761.g736.t1 | LIM/homeobox protein Lhx4 | 1209 | 9.80E-07 | 66 |
| Efet.01.156672.g349.t1 | LIM/homeobox protein Lhx4 | 405 | 3.49E-11 | 66 |
| Efet.01.114629.g761.t1 | Low-density lipoprotein receptor-related protein 1B | 288 | 2.84E-10 | 66 |
| Efet.01.163811.g696.t1 | Membrane-associated guanylate kinase, WW and PDZ domain-containing protein 3 | 285 | 3.93E-14 | 66 |
| Efet.01.531405.g45.t1 | Multidrug resistance protein 1 | 384 | 1.68E-09 | 66 |
| Efet.01.187552.g1763.t1 | Mitogen-activated protein kinase 7 | 954 | 1.62E-114 | 66 |
| Efet.01.177805.g1304.t1 | Myosin regulatory light chain 2, skeletal muscle isoform | 384 | 1.13E-33 | 66 |
| Efet.01.532173.g69.t1 | Multidrug resistance-associated protein 4 | 501 | 3.45E-12 | 66 |
| Efet.01.44551.g1265.t1 | Unconventional myosin-Ie | 462 | 1.29E-28 | 66 |
| Efet.01.164184.g716.t1 | Neuronal calcium sensor 1 | 357 | 6.74E-24 | 66 |
| Efet.01.333751.g1007.t1 | Netrin-1 | 990 | 8.48E-107 | 66 |
| Efet.01.307480.g226.t1 | Substance-P receptor | 405 | 1.25E-07 | 66 |
| Efet.01.313025.g415.t1 | Neurogenic locus notch homolog protein 1 | 324 | 2.26E-23 | 66 |
| Efet.01.20905.g1548.t1 | NAD(P)H dehydrogenase [quinone] 1 | 435 | 1.87E-09 | 66 |
| Efet.01.226824.g1070.t1 | Protocadherin-23 | 303 | 3.96E-12 | 66 |
| Efet.01.130636.g302.t1 | Pericentriolar material 1 protein | 561 | 2.52E-41 | 66 |
| Efet.01.171232.g1025.t1 | PDZ domain-containing protein 2 | 375 | 7.82E-17 | 66 |
| Efet.01.436194.g857.t1 | Phosphoglycerate mutase 1 | 705 | 7.03E-64 | 66 |
| Efet.01.653312.g416.t1 | Phosphoglycerate mutase 1 | 738 | 2.59E-85 | 66 |
| Efet.01.1657366.g976.t1 | GPI transamidase component PIG-T | 333 | 2.36E-24 | 66 |
| Efet.01.32095.g492.t1 | Phosphatidylinositol 4,5-bisphosphate 3-kinase catalytic subunit alpha isoform | 477 | 8.66E-35 | 66 |
| Efet.01.66624.g1023.t1 | Serine/threonine-protein phosphatase 1 regulatory subunit 10 | 1380 | 4.59E-28 | 66 |
| Efet.01.503313.g79.t1 | Prospero homeobox protein 1 | 264 | 3.01E-09 | 66 |
| Efet.01.240853.g1562.t1 | Prostacyclin synthase | 1011 | 2.13E-11 | 66 |
| Efet.01.655767.g870.t1 | ABC50 protein (ATP-binding cassette, sub-family F (GCN20), member 1) | 786 | 3.68E-15 | 66 |
| Efet.01.312622.g397.t1 | MYC associated factor X | 2265 | 1.13E-06 | 66 |
| Efet.01.657660.g1358.t1 | MAPK3 protein | 210 | 5.81E-10 | 66 |
| Efet.01.75681.g39.t1 | Ras-related protein Rab-18 | 399 | 1.03E-60 | 66 |
| Efet.01.256420.g237.t1 | Ras-related protein Rab-7a | 600 | 2.66E-66 | 66 |
| Efet.01.3871.g329.t1 | GTP-binding protein RAD | 402 | 1.49E-08 | 66 |
| Efet.01.492122.g1000.t1 | Ras-related protein Ral-B | 378 | 1.47E-10 | 66 |
| Efet.01.453629.g90.t1 | Ras-related protein Rab-11A | 315 | 1.97E-10 | 66 |
| Efet.01.654391.g584.t1 | DNA-directed RNA polymerase III subunit RPC2 | 987 | 8.11E-11 | 66 |
| Efet.01.10662.g784.t1 | Radial spoke head protein 3 homolog | 402 | 4.99E-15 | 66 |
| Efet.01.446498.g1153.t1 | Sacsin | 480 | 6.87E-14 | 66 |
| Efet.01.9340.g686.t1 | Sodium channel protein type 2 subunit alpha | 900 | 2.10E-37 | 66 |
| Efet.01.68720.g1150.t1 | Sodium channel protein type 2 subunit alpha | 1092 | 2.16E-92 | 66 |
| Efet.01.6158.g487.t1 | Semaphorin-5A | 270 | 4.93E-17 | 66 |
| Efet.01.178681.g1339.t1 | Sodium/hydrogen exchanger 3 | 396 | 2.71E-06 | 66 |
| Efet.01.565253.g179.t1 | SWI/SNF-related matrix-associated actin-dependent regulator of chromatin subfamily A-like protein 1 | 273 | 3.36E-06 | 66 |
| Efet.01.415307.g354.t1 | Sphingosine kinase 2 | 789 | 7.31E-29 | 66 |
| Efet.01.180342.g1414.t1 | Spectrin beta chain, erythrocytic | 420 | 4.80E-29 | 66 |
| Efet.01.293106.g1543.t1 | Synaptotagmin-9 | 870 | 4.03E-28 | 66 |
| Efet.01.658424.g2122.t1 | Transitional endoplasmic reticulum ATPase | 1188 | 2.71E-32 | 66 |
| Efet.01.715.g67.t1 | Thimet oligopeptidase | 996 | 1.99E-52 | 66 |
| Efet.01.243125.g1641.t1 | Tubulointerstitial nephritis antigen | 378 | 6.77E-25 | 66 |
| Efet.01.107350.g381.t1 | mRNA decay activator protein ZFP36 | 1185 | 1.32E-26 | 66 |
| Efet.01.241121.g1573.t1 | mRNA decay activator protein ZFP36 | 759 | 1.51E-28 | 66 |
| Efet.01.147804.g1134.t1 | Voltage-dependent anion-selective channel protein 1 | 216 | 6.00E-13 | 66 |
| Efet.01.83242.g541.t1 | Protein Wnt-4 | 309 | 9.22E-32 | 66 |
| Efet.01.91171.g951.t1 | Zinc finger protein 443 | 381 | 2.40E-06 | 66 |
| Efet.01.207101.g316.t1 | Restin (Reed-Steinberg cell-expressed intermediate filament-associated protein) | 375 | 5.15E-06 | 65 |
| Efet.01.165193.g763.t1 | DDX39B (HCG2005638, isoform CRA_a) | 339 | 8.87E-25 | 65 |
| Efet.01.97534.g1311.t1 | Epithelial discoidin domain-containing receptor 1 | 312 | 2.47E-24 | 65 |
| Efet.01.614331.g641.t1 | Testis secretory sperm-binding protein Li 197a | 765 | 4.41E-27 | 65 |
| Efet.01.80174.g345.t1 | Tyrosine-protein kinase | 270 | 5.07E-15 | 65 |
| Efet.01.74614.g1521.t1 | Angiotensin-converting enzyme | 216 | 3.53E-15 | 65 |
| Efet.01.72626.g1398.t1 | Neuronal acetylcholine receptor subunit alpha-7 | 567 | 9.19E-42 | 65 |
| Efet.01.328763.g884.t1 | Neuronal acetylcholine receptor subunit alpha-7 | 609 | 1.14E-38 | 65 |
| Efet.01.657674.g1363.t1 | Neuronal acetylcholine receptor subunit alpha-7 | 552 | 2.81E-41 | 65 |
| Efet.01.136066.g552.t1 | Neuronal acetylcholine receptor subunit beta-2 | 546 | 2.28E-25 | 65 |
| Efet.01.157687.g406.t1 | Alpha-1B adrenergic receptor | 744 | 1.44E-17 | 65 |
| Efet.01.164833.g754.t1 | Alpha-2A adrenergic receptor | 969 | 1.99E-51 | 65 |
| Efet.01.456769.g166.t1 | Alpha-2A adrenergic receptor | 270 | 8.81E-21 | 65 |
| Efet.01.45444.g1324.t1 | Angiopoietin-1 | 933 | 2.86E-39 | 65 |
| Efet.01.20668.g1526.t1 | Aquaporin-4 | 582 | 1.07E-49 | 65 |
| Efet.01.327891.g858.t1 | Calcium-transporting ATPase type 2C member 1 | 207 | 9.20E-07 | 65 |
| Efet.01.268580.g685.t1 | Band 3 anion transport protein | 342 | 1.09E-11 | 65 |
| Efet.01.172932.g1092.t1 | Catenin beta-1 | 690 | 4.70E-57 | 65 |
| Efet.01.61675.g726.t1 | Fibroblast growth factor receptor | 627 | 1.11E-07 | 65 |
| Efet.01.19688.g1458.t1 | BTB/POZ domain-containing adapter for CUL3-mediated RhoA degradation protein 2 | 1527 | 7.54E-19 | 65 |
| Efet.01.396858.g1165.t1 | BTB/POZ domain-containing adapter for CUL3-mediated RhoA degradation protein 2 | 1671 | 1.36E-18 | 65 |
| Efet.01.103667.g200.t1 | Brefeldin A-inhibited guanine nucleotide-exchange protein 1 | 573 | 1.84E-75 | 65 |
| Efet.01.516205.g404.t1 | Protein BTG1 | 483 | 1.03E-43 | 65 |
| Efet.01.578197.g631.t1 | Voltage-dependent P/Q-type calcium channel subunit alpha-1A | 204 | 3.69E-18 | 65 |
| Efet.01.1652388.g555.t1 | Calcineurin subunit B type 1 | 225 | 2.60E-08 | 65 |
| Efet.01.251816.g68.t1 | Coiled-coil domain-containing protein 85B | 681 | 4.00E-27 | 65 |
| Efet.01.8404.g619.t1 | G2/mitotic-specific cyclin-B2 | 414 | 2.62E-26 | 65 |
| Efet.01.194729.g2081.t1 | Cadherin EGF LAG seven-pass G-type receptor 1 | 396 | 1.54E-26 | 65 |
| Efet.01.329563.g902.t1 | Chromodomain-helicase-DNA-binding protein 7 | 303 | 1.03E-27 | 65 |
| Efet.01.617285.g767.t1 | Cytokine-inducible SH2-containing protein | 384 | 9.29E-14 | 65 |
| Efet.01.81054.g405.t1 | Coronin-1A | 312 | 8.25E-14 | 65 |
| Efet.01.208617.g395.t1 | Coronin-1C | 438 | 2.54E-20 | 65 |
| Efet.01.99896.g1443.t1 | Centriolar coiled-coil protein of 110 kDa | 270 | 2.85E-17 | 65 |
| Efet.01.21625.g1590.t1 | Cytochrome P450 1A1 | 249 | 1.18E-13 | 65 |
| Efet.01.525091.g586.t1 | Cytochrome P450 4F8 | 294 | 6.49E-26 | 65 |
| Efet.01.51052.g74.t1 | Calsyntenin-1 | 204 | 6.09E-07 | 65 |
| Efet.01.51052.g75.t1 | Calsyntenin-1 | 474 | 1.86E-10 | 65 |
| Efet.01.93625.g1081.t1 | Catenin delta-2 | 339 | 1.39E-12 | 65 |
| Efet.01.2082.g183.t1 | Cytohesin-interacting protein | 1476 | 1.89E-08 | 65 |
| Efet.01.358765.g246.t1 | DnaJ homolog subfamily C member 3 | 498 | 1.17E-10 | 65 |
| Efet.01.91716.g984.t1 | Dedicator of cytokinesis protein 4 | 621 | 9.39E-21 | 65 |
| Efet.01.637720.g383.t1 | D(2) dopamine receptor | 417 | 4.99E-09 | 65 |
| Efet.01.292914.g1538.t1 | Dual specificity protein phosphatase 6 | 453 | 5.08E-10 | 65 |
| Efet.01.580788.g692.t1 | Eukaryotic translation initiation factor 2-alpha kinase 3 | 636 | 3.58E-14 | 65 |
| Efet.01.182563.g1527.t1 | Filamin-A | 327 | 2.78E-19 | 65 |
| Efet.01.210398.g460.t1 | Filamin-A | 345 | 1.10E-22 | 65 |
| Efet.01.497535.g1126.t1 | Frizzled-5 | 1878 | 0 | 65 |
| Efet.01.252322.g81.t1 | Mu opioid receptor hMOR-1a | 303 | 4.78E-07 | 65 |
| Efet.01.570091.g328.t1 | Mu opioid receptor hMOR-1a | 738 | 1.22E-27 | 65 |
| Efet.01.640044.g553.t1 | Guanylate-binding protein 1 | 375 | 6.61E-19 | 65 |
| Efet.01.294470.g1582.t1 | E3 ubiquitin-protein ligase RNF130 | 2223 | 4.99E-06 | 65 |
| Efet.01.529496.g684.t1 | G-protein coupled receptor 84 | 282 | 3.13E-16 | 65 |
| Efet.01.496820.g1106.t1 | Glutamate receptor ionotropic, delta-2 | 210 | 6.95E-13 | 65 |
| Efet.01.636379.g331.t1 | Stress-70 protein, mitochondrial | 828 | 3.24E-89 | 65 |
| Efet.01.174672.g1159.t1 | GTP-binding protein 1 | 594 | 2.28E-41 | 65 |
| Efet.01.1655890.g798.t1 | GTP-binding protein 1 | 390 | 5.55E-20 | 65 |
| Efet.01.560453.g13.t1 | Aquaporin-9 | 414 | 6.61E-39 | 65 |
| Efet.01.811.g72.t1 | Hemicentin-1 | 387 | 1.86E-15 | 65 |
| Efet.01.73780.g1463.t1 | Histamine H1 receptor | 993 | 3.67E-25 | 65 |
| Efet.01.194053.g2051.t1 | Heparan sulfate glucosamine 3-O-sulfotransferase 5 | 390 | 3.70E-31 | 65 |
| Efet.01.95344.g1178.t1 | Heat shock protein beta-1 | 1020 | 1.24E-12 | 65 |
| Efet.01.107973.g415.t1 | Inosine-5'-monophosphate dehydrogenase 2 | 531 | 1.46E-50 | 65 |
| Efet.01.603747.g140.t1 | Interferon regulatory factor 2 | 393 | 1.87E-14 | 65 |
| Efet.01.203997.g176.t1 | Janus kinase and microtubule-interacting protein 3 | 663 | 1.16E-19 | 65 |
| Efet.01.160824.g565.t1 | cAMP-dependent protein kinase type II-alpha regulatory subunit | 1110 | 1.93E-116 | 65 |
| Efet.01.567995.g256.t1 | ATP-sensitive inward rectifier potassium channel 8 | 1203 | 6.39E-56 | 65 |
| Efet.01.79303.g272.t1 | Kinesin-like protein KIF13B | 975 | 2.56E-37 | 65 |
| Efet.01.137515.g640.t1 | Kinesin-associated protein 3 | 633 | 2.39E-74 | 65 |
| Efet.01.358688.g243.t1 | Protein kinase C epsilon type | 240 | 3.12E-21 | 65 |
| Efet.01.128245.g180.t1 | Laminin subunit beta-1 | 1155 | 4.32E-37 | 65 |
| Efet.01.36239.g746.t1 | Laminin subunit gamma-1 | 339 | 1.66E-26 | 65 |
| Efet.01.50347.g29.t1 | LIM/homeobox protein Lhx4 | 210 | 5.56E-12 | 65 |
| Efet.01.155465.g277.t1 | LIM/homeobox protein Lhx4 | 390 | 2.23E-13 | 65 |
| Efet.01.313716.g433.t1 | Lethal(3)malignant brain tumor-like protein 1 | 1092 | 6.78E-09 | 65 |
| Efet.01.1646869.g361.t1 | Low-density lipoprotein receptor-related protein 8 | 216 | 2.97E-16 | 65 |
| Efet.01.353192.g87.t1 | Mitogen-activated protein kinase kinase kinase 10 | 387 | 1.76E-17 | 65 |
| Efet.01.247864.g1818.t1 | Mitogen-activated protein kinase kinase kinase 11 | 207 | 1.87E-12 | 65 |
| Efet.01.162109.g628.t1 | Guanine nucleotide exchange factor DBS | 369 | 5.40E-20 | 65 |
| Efet.01.246214.g1747.t1 | Multidrug resistance protein 1 | 444 | 1.05E-31 | 65 |
| Efet.01.116376.g854.t1 | MICAL-like protein 2 | 3777 | 2.98E-07 | 65 |
| Efet.01.86776.g737.t1 | MAP kinase-interacting serine/threonine-protein kinase 1 | 537 | 1.89E-25 | 65 |
| Efet.01.5518.g429.t1 | NADH dehydrogenase [ubiquinone] iron-sulfur protein 3, mitochondrial | 753 | 2.63E-80 | 65 |
| Efet.01.195876.g2123.t1 | Neogenin | 435 | 1.70E-37 | 65 |
| Efet.01.337317.g1099.t1 | Netrin-1 | 1218 | 1.59E-106 | 65 |
| Efet.01.19407.g1436.t1 | Substance-P receptor | 483 | 2.27E-23 | 65 |
| Efet.01.597541.g1210.t1 | Neurogenic locus notch homolog protein 2 | 402 | 1.57E-31 | 65 |
| Efet.01.46849.g1405.t1 | Serine/threonine-protein kinase OSR1 | 306 | 1.05E-27 | 65 |
| Efet.01.188445.g1809.t1 | Protein kinase C and casein kinase substrate in neurons protein 1 | 426 | 1.39E-35 | 65 |
| Efet.01.199459.g2283.t1 | TCDD-inducible poly [ADP-ribose] polymerase | 690 | 1.16E-15 | 65 |
| Efet.01.579347.g660.t1 | Protocadherin gamma-B6 | 267 | 1.44E-16 | 65 |
| Efet.01.215916.g659.t1 | Calcium/calmodulin-dependent 3',5'-cyclic nucleotide phosphodiesterase 1B | 561 | 1.42E-20 | 65 |
| Efet.01.503634.g90.t1 | cGMP-specific 3',5'-cyclic phosphodiesterase | 213 | 6.32E-08 | 65 |
| Efet.01.600657.g24.t1 | Peroxisomal trans-2-enoyl-CoA reductase | 879 | 4.18E-60 | 65 |
| Efet.01.27554.g160.t1 | Protein-glucosylgalactosylhydroxylysine glucosidase | 270 | 6.71E-07 | 65 |
| Efet.01.145380.g1034.t1 | Peptidoglycan recognition protein 1 | 375 | 4.57E-32 | 65 |
| Efet.01.224777.g994.t1 | 1-phosphatidylinositol 4,5-bisphosphate phosphodiesterase delta-4 | 327 | 1.13E-11 | 65 |
| Efet.01.636598.g340.t1 | Peroxiredoxin-6 | 642 | 5.30E-70 | 65 |
| Efet.01.83686.g562.t1 | Inactive tyrosine-protein kinase 7 | 564 | 3.33E-50 | 65 |
| Efet.01.6357.g503.t1 | Receptor-type tyrosine-protein phosphatase F | 465 | 2.00E-42 | 65 |
| Efet.01.522687.g530.t1 | Receptor-type tyrosine-protein phosphatase F | 465 | 1.21E-43 | 65 |
| Efet.01.100870.g59.t1 | Ras-related protein Rab-13 | 441 | 1.61E-36 | 65 |
| Efet.01.172060.g1058.t1 | Double-strand-break repair protein rad21 homolog | 207 | 3.47E-16 | 65 |
| Efet.01.144382.g989.t1 | GTPase HRas | 297 | 1.03E-10 | 65 |
| Efet.01.178385.g1327.t1 | GTPase HRas | 300 | 1.94E-10 | 65 |
| Efet.01.304956.g163.t1 | GTPase KRas | 249 | 2.18E-07 | 65 |
| Efet.01.204317.g188.t1 | Ras-related protein Rab-11A | 393 | 7.80E-21 | 65 |
| Efet.01.599369.g1289.t1 | Ras-specific guanine nucleotide-releasing factor 1 | 279 | 5.24E-23 | 65 |
| Efet.01.181838.g1494.t1 | Regulator of G-protein signaling 1 | 204 | 4.36E-15 | 65 |
| Efet.01.45427.g1322.t1 | Regulator of G-protein signaling 2 | 276 | 3.52E-22 | 65 |
| Efet.01.183058.g1550.t1 | Rho GTPase-activating protein 7 | 411 | 4.07E-29 | 65 |
| Efet.01.92119.g1004.t1 | Regulating synaptic membrane exocytosis protein 2 | 1353 | 1.79E-18 | 65 |
| Efet.01.478397.g725.t1 | 28S ribosomal protein S29, mitochondrial | 909 | 7.52E-88 | 65 |
| Efet.01.55629.g365.t1 | Sodium-coupled monocarboxylate transporter 1 | 501 | 8.21E-19 | 65 |
| Efet.01.20754.g1537.t1 | Sodium/glucose cotransporter 4 | 387 | 3.61E-13 | 65 |
| Efet.01.1659281.g1509.t1 | Sodium channel protein type 2 subunit alpha | 813 | 3.03E-62 | 65 |
| Efet.01.100503.g37.t1 | Endophilin-A1 | 366 | 6.98E-34 | 65 |
| Efet.01.58900.g557.t1 | DNA-binding protein SMUBP-2 | 339 | 7.12E-16 | 65 |
| Efet.01.255222.g191.t1 | E3 ubiquitin-protein ligase SMURF1 | 1317 | 3.09E-39 | 65 |
| Efet.01.644765.g897.t1 | Solute carrier organic anion transporter family member 1A2 | 354 | 3.64E-09 | 65 |
| Efet.01.70960.g1293.t1 | Spectrin beta chain, non-erythrocytic 2 | 426 | 1.92E-53 | 65 |
| Efet.01.637419.g375.t1 | Spectrin beta chain, non-erythrocytic 2 | 450 | 5.26E-20 | 65 |
| Efet.01.288169.g1387.t1 | Tubulin beta-4A chain | 294 | 2.49E-23 | 65 |
| Efet.01.1659093.g1402.t1 | Transitional endoplasmic reticulum ATPase | 744 | 3.34E-15 | 65 |
| Efet.01.605308.g199.t1 | Tumor necrosis factor alpha-induced protein 3 | 450 | 5.50E-09 | 65 |
| Efet.01.548286.g448.t1 | Triple functional domain protein | 1125 | 7.59E-15 | 65 |
| Efet.01.212207.g528.t1 | E3 ubiquitin-protein ligase TRIP12 | 663 | 1.45E-56 | 65 |
| Efet.01.212473.g539.t1 | Thrombospondin-type laminin G domain and EAR repeat-containing protein | 393 | 7.36E-16 | 65 |
| Efet.01.436441.g864.t1 | Protein turtle homolog A | 276 | 2.23E-19 | 65 |
| Efet.01.279676.g1087.t1 | Protein turtle homolog B | 516 | 9.32E-19 | 65 |
| Efet.01.432187.g777.t1 | Tyrosine-protein kinase TXK | 870 | 1.23E-37 | 65 |
| Efet.01.250649.g16.t1 | Epididymis secretory protein Li 303 | 312 | 1.18E-17 | 65 |
| Efet.01.129452.g246.t1 | Serine/threonine-protein kinase WNK2 | 327 | 7.72E-07 | 65 |
| Efet.01.251024.g29.t1 | Zinc finger protein 3 | 576 | 3.71E-34 | 65 |
| Efet.01.200688.g35.t1 | HCG1985580, isoform CRA_c | 285 | 6.57E-16 | 64 |
| Efet.01.595746.g1174.t1 | HCG1985580, isoform CRA_c | 306 | 9.33E-11 | 64 |
| Efet.01.13729.g1021.t1 | Suppressor of cytokine signaling 6, isoform CRA_a | 348 | 6.14E-13 | 64 |
| Efet.01.276957.g997.t1 | ADAM metallopeptidase domain 10, isoform CRA_b | 906 | 1.03E-29 | 64 |
| Efet.01.1654936.g723.t1 | Adenosine receptor A1 | 237 | 3.01E-11 | 64 |
| Efet.01.13005.g979.t1 | Neuronal acetylcholine receptor subunit alpha-7 | 396 | 3.60E-27 | 64 |
| Efet.01.38168.g875.t1 | Alpha-actinin-1 | 438 | 6.63E-30 | 64 |
| Efet.01.34054.g624.t1 | Adenosine deaminase 2 | 381 | 1.02E-14 | 64 |
| Efet.01.401737.g46.t1 | Alpha-2A adrenergic receptor | 1275 | 1.68E-46 | 64 |
| Efet.01.262752.g484.t1 | Homeobox protein aristaless-like 4 | 348 | 1.21E-10 | 64 |
| Efet.01.288824.g1412.t1 | Ankyrin-1 | 294 | 2.74E-15 | 64 |
| Efet.01.6408.g508.t1 | Aquaporin-3 | 210 | 6.20E-17 | 64 |
| Efet.01.71383.g1325.t1 | ADP-ribosylation factor 6 | 270 | 3.07E-15 | 64 |
| Efet.01.587022.g893.t1 | AT-rich interactive domain-containing protein 5B | 282 | 7.16E-06 | 64 |
| Efet.01.6037.g478.t1 | Sarcoplasmic/endoplasmic reticulum calcium ATPase 1 | 306 | 7.80E-20 | 64 |
| Efet.01.130911.g315.t1 | Calcium-transporting ATPase type 2C member 1 | 471 | 1.79E-26 | 64 |
| Efet.01.113046.g657.t1 | Cyclic AMP-dependent transcription factor ATF-5 | 660 | 2.18E-10 | 64 |
| Efet.01.1658158.g1117.t1 | Protein atonal homolog 1 | 537 | 2.39E-12 | 64 |
| Efet.01.195919.g2125.t1 | Band 3 anion transport protein | 771 | 4.10E-11 | 64 |
| Efet.01.546558.g399.t1 | ATP-binding cassette transporter A1 | 243 | 1.22E-06 | 64 |
| Efet.01.646715.g1120.t1 | cDNA FLJ53627, highly similar to Antigen peptide transporter 1 | 1170 | 2.31E-09 | 64 |
| Efet.01.247181.g1785.t1 | Bone morphogenetic protein 1 | 204 | 4.98E-12 | 64 |
| Efet.01.81062.g406.t1 | Calmodulin | 342 | 6.93E-20 | 64 |
| Efet.01.59142.g575.t1 | Calmodulin-A | 312 | 1.86E-20 | 64 |
| Efet.01.138697.g703.t1 | Calmodulin-alpha | 249 | 1.23E-13 | 64 |
| Efet.01.345236.g1276.t1 | Carbohydrate sulfotransferase 11 | 567 | 1.93E-47 | 64 |
| Efet.01.200726.g39.t1 | Cysteinyl leukotriene receptor 2 | 747 | 8.90E-19 | 64 |
| Efet.01.541621.g301.t1 | Collagen alpha-1(IV) chain | 261 | 6.32E-06 | 64 |
| Efet.01.114237.g727.t1 | Collagen alpha-1(XII) chain | 303 | 5.98E-08 | 64 |
| Efet.01.167274.g854.t1 | Cytochrome P450 4F8 | 366 | 2.28E-23 | 64 |
| Efet.01.401473.g33.t1 | Cytochrome P450 4F8 | 324 | 4.69E-22 | 64 |
| Efet.01.592296.g1072.t1 | Calsyntenin-1 | 318 | 4.76E-16 | 64 |
| Efet.01.144957.g1012.t1 | Catenin delta-2 | 714 | 1.48E-50 | 64 |
| Efet.01.284106.g1241.t1 | Probable ATP-dependent RNA helicase DDX20 | 225 | 2.90E-10 | 64 |
| Efet.01.155178.g267.t1 | Probable ATP-dependent RNA helicase DDX41 | 453 | 4.39E-39 | 64 |
| Efet.01.175082.g1174.t1 | ATP-dependent RNA helicase DDX42 | 1284 | 3.69E-104 | 64 |
| Efet.01.534728.g150.t1 | D(2) dopamine receptor | 1794 | 6.66E-44 | 64 |
| Efet.01.249213.g1867.t1 | D(4) dopamine receptor | 957 | 7.61E-09 | 64 |
| Efet.01.541676.g305.t1 | Dual specificity protein phosphatase 1 | 516 | 1.35E-12 | 64 |
| Efet.01.3436.g298.t1 | Dual specificity tyrosine-phosphorylation-regulated kinase 2 | 339 | 2.82E-06 | 64 |
| Efet.01.65153.g922.t1 | Epidermal growth factor receptor | 906 | 4.14E-89 | 64 |
| Efet.01.33854.g612.t1 | EH domain-containing protein 1 | 669 | 3.80E-64 | 64 |
| Efet.01.212390.g536.t1 | Endoplasmic reticulum aminopeptidase 2 | 480 | 8.64E-09 | 64 |
| Efet.01.601188.g53.t1 | DNA excision repair protein ERCC-8 | 624 | 1.46E-45 | 64 |
| Efet.01.72696.g1404.t1 | Exocyst complex component 2 | 1302 | 2.75E-16 | 64 |
| Efet.01.134257.g451.t1 | Ezrin | 780 | 1.58E-17 | 64 |
| Efet.01.62210.g765.t1 | Death domain-containing protein CRADD | 267 | 2.09E-13 | 64 |
| Efet.01.485390.g869.t1 | Fasciculation and elongation protein zeta-1 | 408 | 3.47E-07 | 64 |
| Efet.01.248270.g1831.t1 | Peptidyl-prolyl cis-trans isomerase FKBP3 | 225 | 6.46E-12 | 64 |
| Efet.01.369719.g537.t1 | Forkhead box protein K2 | 396 | 1.57E-27 | 64 |
| Efet.01.422683.g533.t1 | Tyrosine-protein kinase FRK | 249 | 7.68E-09 | 64 |
| Efet.01.492942.g1018.t1 | Tyrosine-protein kinase FRK | 213 | 1.47E-19 | 64 |
| Efet.01.43142.g1178.t1 | Frizzled-5 | 861 | 3.35E-35 | 64 |
| Efet.01.335772.g1068.t1 | Mu opioid receptor hMOR-1a | 513 | 9.13E-10 | 64 |
| Efet.01.123197.g1204.t1 | GTPase-activating protein and VPS9 domain-containing protein 1 | 378 | 5.64E-08 | 64 |
| Efet.01.359746.g268.t1 | Polypeptide N-acetylgalactosaminyltransferase 16 | 351 | 3.41E-17 | 64 |
| Efet.01.2557.g234.t1 | Solute carrier family 2, facilitated glucose transporter member 4 | 561 | 2.26E-22 | 64 |
| Efet.01.178125.g1315.t1 | Transcription factor HES-1 | 909 | 1.78E-10 | 64 |
| Efet.01.370733.g581.t1 | Hepatocyte nuclear factor 6 | 2925 | 1.19E-10 | 64 |
| Efet.01.432939.g798.t1 | Histamine H3 receptor | 1230 | 1.49E-37 | 64 |
| Efet.01.102138.g127.t1 | Heparan sulfate glucosamine 3-O-sulfotransferase 5 | 471 | 8.69E-28 | 64 |
| Efet.01.30037.g342.t1 | Zinc finger protein Pegasus | 831 | 6.00E-11 | 64 |
| Efet.01.591615.g1039.t1 | cAMP-dependent protein kinase type II-alpha regulatory subunit | 744 | 3.09E-72 | 64 |
| Efet.01.83435.g551.t1 | Calcium-activated potassium channel subunit alpha-1 | 243 | 2.68E-12 | 64 |
| Efet.01.178576.g1335.t1 | Potassium voltage-gated channel subfamily H member 8 | 1062 | 2.08E-32 | 64 |
| Efet.01.100504.g39.t1 | ATP-sensitive inward rectifier potassium channel 8 | 768 | 2.06E-21 | 64 |
| Efet.01.32675.g537.t1 | Kelch-like protein 20 | 210 | 4.74E-12 | 64 |
| Efet.01.201271.g65.t1 | Kelch-like protein 20 | 546 | 4.85E-51 | 64 |
| Efet.01.565394.g185.t1 | Kelch-like protein 20 | 249 | 5.47E-15 | 64 |
| Efet.01.45731.g1342.t1 | Protein kinase C epsilon type | 417 | 1.53E-25 | 64 |
| Efet.01.497753.g1134.t1 | Leukocyte receptor cluster member 8 | 1557 | 5.27E-07 | 64 |
| Efet.01.401571.g37.t1 | Protein ERGIC-53 | 252 | 1.43E-13 | 64 |
| Efet.01.1655191.g742.t1 | Low-density lipoprotein receptor-related protein 6 | 591 | 1.64E-45 | 64 |
| Efet.01.100429.g27.t1 | Lysosomal-trafficking regulator | 531 | 3.15E-14 | 64 |
| Efet.01.363782.g369.t1 | Serine/threonine-protein kinase MARK1 | 441 | 7.78E-23 | 64 |
| Efet.01.428848.g705.t1 | MAP/microtubule affinity-regulating kinase 4 | 504 | 1.10E-25 | 64 |
| Efet.01.5179.g403.t1 | Multidrug resistance protein 1 | 546 | 2.60E-26 | 64 |
| Efet.01.271982.g827.t1 | Multidrug resistance protein 1 | 918 | 1.13E-59 | 64 |
| Efet.01.3630.g309.t1 | Multiple epidermal growth factor-like domains protein 10 | 273 | 3.41E-19 | 64 |
| Efet.01.465183.g404.t1 | Menin | 1257 | 9.09E-50 | 64 |
| Efet.01.62256.g768.t1 | Methylmalonate-semialdehyde dehydrogenase [acylating], mitochondrial | 369 | 1.07E-07 | 64 |
| Efet.01.23383.g1694.t1 | Myosin-10 | 399 | 4.67E-22 | 64 |
| Efet.01.165896.g791.t1 | Nucleolar GTP-binding protein 1 | 522 | 2.76E-53 | 64 |
| Efet.01.58303.g513.t1 | Neurexin-1 | 477 | 5.81E-27 | 64 |
| Efet.01.1645757.g330.t1 | Calcium/calmodulin-dependent 3',5'-cyclic nucleotide phosphodiesterase 1B | 249 | 6.74E-11 | 64 |
| Efet.01.46950.g1414.t1 | cGMP-specific 3',5'-cyclic phosphodiesterase | 651 | 2.01E-17 | 64 |
| Efet.01.1593073.g16.t1 | Basement membrane-specific heparan sulfate proteoglycan core protein | 222 | 9.41E-12 | 64 |
| Efet.01.94858.g1150.t1 | 1-phosphatidylinositol 4,5-bisphosphate phosphodiesterase delta-4 | 738 | 5.54E-62 | 64 |
| Efet.01.311516.g368.t1 | Peptidyl-prolyl cis-trans isomerase B | 267 | 6.20E-18 | 64 |
| Efet.01.365252.g409.t1 | Protein PTHB1 | 696 | 2.86E-16 | 64 |
| Efet.01.108059.g419.t1 | Receptor-type tyrosine-protein phosphatase mu | 348 | 1.28E-24 | 64 |
| Efet.01.507395.g191.t1 | Receptor-type tyrosine-protein phosphatase S | 336 | 1.08E-19 | 64 |
| Efet.01.279647.g1086.t1 | Peroxidasin homolog | 255 | 5.98E-18 | 64 |
| Efet.01.532667.g84.t1 | SARM1 protein | 1494 | 8.09E-56 | 64 |
| Efet.01.655687.g850.t1 | ABC50 protein (ATP-binding cassette, sub-family F (GCN20), member 1) | 1329 | 1.08E-11 | 64 |
| Efet.01.9021.g662.t1 | Early growth response protein | 1482 | 4.30E-23 | 64 |
| Efet.01.243235.g1646.t1 | Early growth response protein | 231 | 2.99E-16 | 64 |
| Efet.01.60114.g630.t1 | Zinc finger protein 3 | 1077 | 3.32E-73 | 64 |
| Efet.01.626245.g1158.t1 | GTP cyclohydrolase I type IV | 702 | 3.37E-30 | 64 |
| Efet.01.13936.g1032.t1 | DNA-binding protein Ikaros | 3087 | 1.07E-12 | 64 |
| Efet.01.379755.g790.t1 | Double-strand-break repair protein rad21 homolog | 705 | 1.32E-15 | 64 |
| Efet.01.304010.g139.t1 | Rap guanine nucleotide exchange factor 4 | 468 | 2.73E-10 | 64 |
| Efet.01.312379.g389.t1 | Ryanodine receptor 2 | 246 | 2.88E-08 | 64 |
| Efet.01.600467.g13.t1 | Ryanodine receptor 2 | 534 | 1.12E-39 | 64 |
| Efet.01.49668.g1575.t1 | Protein strawberry notch homolog 2 | 1467 | 1.20E-114 | 64 |
| Efet.01.1655.g153.t1 | Sodium channel protein type 2 subunit alpha | 552 | 6.31E-31 | 64 |
| Efet.01.67862.g1103.t1 | Sodium channel protein type 2 subunit alpha | 549 | 1.06E-19 | 64 |
| Efet.01.167398.g860.t1 | Sodium channel protein type 2 subunit alpha | 291 | 3.38E-16 | 64 |
| Efet.01.356415.g180.t1 | Sodium channel protein type 2 subunit alpha | 693 | 3.66E-40 | 64 |
| Efet.01.420676.g491.t1 | Sodium channel protein type 2 subunit alpha | 219 | 5.87E-11 | 64 |
| Efet.01.446102.g1141.t1 | Sodium channel protein type 2 subunit alpha | 300 | 5.44E-16 | 64 |
| Efet.01.81753.g452.t1 | Synaptotagmin-1 | 660 | 4.25E-06 | 64 |
| Efet.01.17138.g1263.t1 | Synaptotagmin-7 | 582 | 5.24E-49 | 64 |
| Efet.01.283238.g1209.t1 | Tubulin beta-4B chain | 225 | 9.44E-08 | 64 |
| Efet.01.1649255.g431.t1 | Tenascin-R | 330 | 5.34E-34 | 64 |
| Efet.01.7898.g582.t1 | Transitional endoplasmic reticulum ATPase | 393 | 3.24E-09 | 64 |
| Efet.01.585749.g854.t1 | Protein turtle homolog A | 279 | 8.86E-22 | 64 |
| Efet.01.14452.g1063.t1 | Ubiquitin carboxyl-terminal hydrolase isozyme L5 | 303 | 4.93E-08 | 64 |
| Efet.01.78872.g233.t1 | Vasopressin V1b receptor | 954 | 1.15E-55 | 64 |
| Efet.01.228204.g1133.t1 | Vasopressin V1b receptor | 948 | 4.46E-47 | 64 |
| Efet.01.61974.g746.t1 | Vinexin | 318 | 6.18E-11 | 64 |
| Efet.01.131026.g320.t1 | Serine/threonine-protein kinase WNK3 | 531 | 2.52E-68 | 64 |
| Efet.01.34064.g628.t1 | XIAP-associated factor 1 | 480 | 1.04E-24 | 64 |
| Efet.01.646437.g1083.t1 | Y-box-binding protein 3 | 204 | 2.01E-14 | 64 |
| Efet.01.187462.g1755.t1 | Palmitoyltransferase ZDHHC16 | 450 | 6.60E-06 | 64 |
| Efet.01.273997.g884.t1 | Zinc finger protein 675 | 294 | 4.95E-20 | 64 |
| Efet.01.129444.g245.t1 | Ras-related C3 botulinum toxin substrate 2 (Rho family, small GTP binding protein Rac2) | 228 | 5.67E-21 | 63 |
| Efet.01.532868.g90.t1 | Atlastin GTPase 1 isoform 2 | 399 | 2.36E-27 | 63 |
| Efet.01.282685.g1180.t1 | Adenosine receptor A1 | 282 | 4.81E-06 | 63 |
| Efet.01.468278.g485.t1 | ATP-binding cassette sub-family A member 5 | 1656 | 2.16E-06 | 63 |
| Efet.01.2314.g206.t1 | Abelson tyrosine-protein kinase 2 | 621 | 3.41E-19 | 63 |
| Efet.01.199312.g2271.t1 | Active breakpoint cluster region-related protein | 426 | 2.53E-12 | 63 |
| Efet.01.60429.g645.t1 | Neuronal acetylcholine receptor subunit alpha-7 | 594 | 1.74E-33 | 63 |
| Efet.01.112709.g640.t1 | Neuronal acetylcholine receptor subunit alpha-7 | 450 | 3.82E-32 | 63 |
| Efet.01.187507.g1758.t1 | Neuronal acetylcholine receptor subunit beta-2 | 306 | 8.94E-11 | 63 |
| Efet.01.85372.g647.t1 | Disintegrin and metalloproteinase domain-containing protein 12 | 387 | 3.49E-19 | 63 |
| Efet.01.70586.g1267.t1 | Disintegrin and metalloproteinase domain-containing protein 19 | 657 | 8.10E-38 | 63 |
| Efet.01.224622.g983.t1 | Alpha-1B adrenergic receptor | 2676 | 1.81E-31 | 63 |
| Efet.01.404361.g108.t1 | Alpha-1B adrenergic receptor | 402 | 7.32E-14 | 63 |
| Efet.01.283023.g1193.t1 | Alpha-2A adrenergic receptor | 1590 | 1.68E-48 | 63 |
| Efet.01.1659320.g1551.t1 | Gamma-adducin | 405 | 1.41E-29 | 63 |
| Efet.01.61208.g698.t1 | cDNA, FLJ95535, highly similar to Homo sapiens transcription factor 8 | 210 | 2.21E-14 | 63 |
| Efet.01.1620234.g57.t1 | ATP-binding cassette, sub-family A (ABC1), member 1 | 270 | 7.85E-19 | 63 |
| Efet.01.146103.g1058.t1 | Beta-1,4-galactosyltransferase 1 | 216 | 4.04E-12 | 63 |
| Efet.01.657185.g1194.t1 | BTB/POZ domain-containing adapter for CUL3-mediated RhoA degradation protein 2 | 612 | 2.32E-06 | 63 |
| Efet.01.146249.g1065.t1 | BarH-like 1 homeobox protein | 381 | 4.45E-14 | 63 |
| Efet.01.123375.g1213.t1 | Bax inhibitor 1 | 354 | 6.26E-18 | 63 |
| Efet.01.47013.g1417.t1 | Serine/threonine-protein kinase B-raf | 897 | 2.74E-33 | 63 |
| Efet.01.459990.g275.t1 | Protein BTG1 | 489 | 7.61E-43 | 63 |
| Efet.01.259315.g357.t1 | Transcription factor 7-like 2 | 225 | 1.39E-16 | 63 |
| Efet.01.124511.g1265.t1 | Calmodulin-alpha | 480 | 2.12E-31 | 63 |
| Efet.01.91701.g983.t1 | Calpain-10 | 351 | 1.10E-09 | 63 |
| Efet.01.39695.g976.t1 | Cholecystokinin receptor type A | 534 | 1.38E-13 | 63 |
| Efet.01.532534.g75.t1 | Cholecystokinin receptor type A | 675 | 1.20E-11 | 63 |
| Efet.01.305150.g168.t1 | Cyclin-dependent kinase 1 | 480 | 2.40E-29 | 63 |
| Efet.01.52193.g148.t1 | Glutathione-specific gamma-glutamylcyclotransferase 1 | 555 | 4.68E-10 | 63 |
| Efet.01.76198.g76.t1 | Chromodomain-helicase-DNA-binding protein 7 | 1617 | 9.35E-177 | 63 |
| Efet.01.1622408.g65.t1 | Collagen alpha-3(VI) chain | 249 | 6.18E-12 | 63 |
| Efet.01.236996.g1441.t1 | COUP transcription factor 2 | 240 | 1.34E-12 | 63 |
| Efet.01.6905.g535.t1 | Cysteine/serine-rich nuclear protein 3 | 1227 | 4.94E-14 | 63 |
| Efet.01.144008.g976.t1 | Cubilin | 414 | 8.54E-15 | 63 |
| Efet.01.450481.g14.t1 | Cytoplasmic FMR1-interacting protein 2 | 246 | 5.35E-32 | 63 |
| Efet.01.144034.g977.t1 | DCN1-like protein 3 | 966 | 1.53E-72 | 63 |
| Efet.01.246354.g1753.t1 | DCN1-like protein 3 | 639 | 2.27E-19 | 63 |
| Efet.01.16736.g1238.t1 | Diacylglycerol kinase delta | 519 | 1.22E-16 | 63 |
| Efet.01.278227.g1044.t1 | D(2) dopamine receptor | 465 | 2.27E-18 | 63 |
| Efet.01.224394.g975.t1 | D(3) dopamine receptor | 1074 | 2.00E-16 | 63 |
| Efet.01.50559.g42.t1 | Dystonin | 669 | 7.52E-21 | 63 |
| Efet.01.76397.g84.t1 | Dystonin | 360 | 7.49E-15 | 63 |
| Efet.01.363465.g358.t1 | Dystonin | 630 | 1.57E-40 | 63 |
| Efet.01.140797.g809.t1 | Excitatory amino acid transporter 2 | 702 | 7.18E-37 | 63 |
| Efet.01.43410.g1197.t1 | Epidermal growth factor receptor | 1473 | 8.33E-18 | 63 |
| Efet.01.472391.g605.t1 | Estrogen receptor beta | 483 | 1.39E-12 | 63 |
| Efet.01.44643.g1273.t1 | MDS1 and EVI1 complex locus protein EVI1 | 531 | 2.31E-13 | 63 |
| Efet.01.391121.g1030.t1 | Coagulation factor IX | 369 | 6.10E-06 | 63 |
| Efet.01.308434.g260.t1 | Peptidyl-prolyl cis-trans isomerase FKBP4 | 1248 | 5.53E-117 | 63 |
| Efet.01.539567.g261.t1 | Forkhead box protein N2 | 597 | 9.83E-34 | 63 |
| Efet.01.169129.g942.t1 | Fascin-2 | 819 | 4.99E-76 | 63 |
| Efet.01.311559.g369.t1 | Furin | 471 | 2.51E-26 | 63 |
| Efet.01.46837.g1404.t1 | Frizzled-5 | 1509 | 1.72E-149 | 63 |
| Efet.01.347335.g1333.t1 | Polypeptide N-acetylgalactosaminyltransferase 2 | 648 | 3.49E-14 | 63 |
| Efet.01.64906.g904.t1 | Trans-acting T-cell-specific transcription factor GATA-3 | 939 | 5.89E-06 | 63 |
| Efet.01.82813.g515.t1 | Polypeptide N-acetylgalactosaminyltransferase 16 | 705 | 2.45E-14 | 63 |
| Efet.01.320250.g643.t1 | Glutamate receptor ionotropic, kainate 2 | 660 | 3.96E-55 | 63 |
| Efet.01.482748.g803.t1 | Hyaluronan-binding protein 2 | 339 | 5.42E-13 | 63 |
| Efet.01.3101.g270.t1 | Hypoxia-inducible factor 1-alpha | 396 | 5.51E-34 | 63 |
| Efet.01.633502.g196.t1 | Histidine triad nucleotide-binding protein 1 | 357 | 5.22E-28 | 63 |
| Efet.01.244693.g1687.t1 | Hepatic leukemia factor | 501 | 5.77E-08 | 63 |
| Efet.01.278892.g1064.t1 | Hepatic leukemia factor | 1209 | 1.63E-13 | 63 |
| Efet.01.361767.g315.t1 | Heparan sulfate glucosamine 3-O-sulfotransferase 5 | 606 | 6.91E-58 | 63 |
| Efet.01.657286.g1223.t1 | Heparan sulfate glucosamine 3-O-sulfotransferase 5 | 633 | 2.13E-58 | 63 |
| Efet.01.277898.g1027.t1 | Homeobox protein Hox-A13 | 345 | 2.20E-13 | 63 |
| Efet.01.444254.g1090.t1 | Homeobox protein Hox-A3 | 480 | 2.00E-13 | 63 |
| Efet.01.567719.g251.t1 | Interferon-induced helicase C domain-containing protein 1 | 1527 | 5.12E-13 | 63 |
| Efet.01.47983.g1462.t1 | Bifunctional arginine demethylase and lysyl-hydroxylase JMJD6 | 204 | 6.55E-10 | 63 |
| Efet.01.237034.g1443.t1 | ATP-sensitive inward rectifier potassium channel 8 | 1659 | 2.62E-91 | 63 |
| Efet.01.386744.g942.t1 | ATP-sensitive inward rectifier potassium channel 8 | 1275 | 2.09E-52 | 63 |
| Efet.01.56510.g405.t1 | Kinesin-like protein KIF1B | 369 | 2.23E-31 | 63 |
| Efet.01.505702.g143.t1 | Plasma kallikrein | 717 | 1.30E-41 | 63 |
| Efet.01.178010.g1313.t1 | Protein kinase C epsilon type | 414 | 1.75E-10 | 63 |
| Efet.01.67734.g1091.t1 | Ribosomal protein S6 kinase alpha-6 | 294 | 1.51E-10 | 63 |
| Efet.01.135314.g509.t1 | LIM/homeobox protein Lhx4 | 5415 | 6.50E-09 | 63 |
| Efet.01.413886.g324.t1 | LIM/homeobox protein Lhx4 | 228 | 2.16E-10 | 63 |
| Efet.01.537781.g226.t1 | LIM/homeobox protein Lhx4 | 312 | 9.25E-09 | 63 |
| Efet.01.79215.g264.t1 | Lipopolysaccharide-responsive and beige-like anchor protein | 531 | 2.09E-47 | 63 |
| Efet.01.35276.g694.t1 | Low-density lipoprotein receptor-related protein 12 | 246 | 4.32E-06 | 63 |
| Efet.01.554457.g614.t1 | Mitogen-activated protein kinase kinase kinase 11 | 363 | 2.72E-27 | 63 |
| Efet.01.128861.g217.t1 | Membrane-associated guanylate kinase, WW and PDZ domain-containing protein 3 | 723 | 3.01E-06 | 63 |
| Efet.01.101223.g81.t1 | Serine/threonine-protein kinase MARK1 | 1575 | 8.30E-15 | 63 |
| Efet.01.610340.g437.t1 | Methylmalonate-semialdehyde dehydrogenase [acylating], mitochondrial | 1029 | 8.56E-91 | 63 |
| Efet.01.496985.g1113.t1 | Putative helicase MOV-10 | 1734 | 1.52E-163 | 63 |
| Efet.01.652439.g295.t1 | Myosin-10 | 810 | 7.24E-28 | 63 |
| Efet.01.462929.g353.t1 | Myosin-9 | 204 | 1.15E-12 | 63 |
| Efet.01.1638137.g190.t1 | Unconventional myosin-Ie | 219 | 2.45E-12 | 63 |
| Efet.01.1659453.g1752.t1 | Nucleoside diphosphate kinase 3 | 462 | 4.97E-36 | 63 |
| Efet.01.419081.g442.t1 | Neogenin | 378 | 8.07E-22 | 63 |
| Efet.01.352391.g68.t1 | Endoplasmic reticulum membrane sensor NFE2L1 | 915 | 1.63E-49 | 63 |
| Efet.01.59365.g586.t1 | Substance-P receptor | 522 | 1.51E-18 | 63 |
| Efet.01.297112.g1659.t1 | Substance-P receptor | 366 | 7.45E-13 | 63 |
| Efet.01.137045.g607.t1 | Nucleolar complex protein 3 homolog | 330 | 3.03E-23 | 63 |
| Efet.01.285241.g1280.t1 | Nostrin | 264 | 1.07E-10 | 63 |
| Efet.01.50215.g18.t1 | Neurogenic locus notch homolog protein 2 | 369 | 6.86E-21 | 63 |
| Efet.01.319861.g636.t1 | Neuropeptide Y receptor type 5 | 345 | 2.56E-19 | 63 |
| Efet.01.352234.g65.t1 | Bile acid receptor | 324 | 1.84E-12 | 63 |
| Efet.01.210557.g465.t1 | Neurexin-1 | 522 | 9.34E-24 | 63 |
| Efet.01.302791.g89.t1 | Protein kinase C and casein kinase substrate in neurons protein 1 | 450 | 1.35E-29 | 63 |
| Efet.01.142004.g880.t1 | Protocadherin-19 | 513 | 2.14E-22 | 63 |
| Efet.01.651921.g234.t1 | Protocadherin-8 | 339 | 9.66E-18 | 63 |
| Efet.01.515599.g378.t1 | PDZ domain-containing protein 2 | 735 | 9.01E-09 | 63 |
| Efet.01.3750.g319.t1 | PH domain leucine-rich repeat-containing protein phosphatase 1 | 318 | 4.49E-18 | 63 |
| Efet.01.1632087.g116.t1 | Polycystin-1 | 324 | 6.17E-13 | 63 |
| Efet.01.306163.g204.t1 | Peripherin-2 | 321 | 6.08E-27 | 63 |
| Efet.01.267720.g656.t1 | Receptor-type tyrosine-protein phosphatase U | 285 | 7.87E-15 | 63 |
| Efet.01.184056.g1598.t1 | Peroxidasin homolog | 348 | 1.50E-20 | 63 |
| Efet.01.172588.g1076.t1 | MYLK protein | 1071 | 3.27E-19 | 63 |
| Efet.01.66808.g1032.t1 | Peptidylprolyl isomerase | 645 | 2.00E-19 | 63 |
| Efet.01.880.g83.t1 | Uncharacterized protein | 894 | 1.77E-08 | 63 |
| Efet.01.150601.g34.t1 | GTP-binding protein RAD | 219 | 4.07E-07 | 63 |
| Efet.01.534125.g144.t1 | Retinol dehydrogenase 10 | 360 | 2.63E-12 | 63 |
| Efet.01.3372.g290.t1 | Proto-oncogene tyrosine-protein kinase receptor Ret | 405 | 1.14E-26 | 63 |
| Efet.01.382875.g863.t1 | Runt-related transcription factor 1 | 363 | 1.61E-23 | 63 |
| Efet.01.151664.g102.t1 | Sacsin | 519 | 8.85E-23 | 63 |
| Efet.01.33073.g558.t1 | Mothers against decapentaplegic homolog 5 | 513 | 5.39E-43 | 63 |
| Efet.01.228094.g1129.t1 | DNA-binding protein SMUBP-2 | 606 | 2.32E-13 | 63 |
| Efet.01.536272.g191.t1 | Spectrin beta chain, erythrocytic | 444 | 2.78E-32 | 63 |
| Efet.01.78244.g193.t1 | Syntaxin-binding protein 1 | 552 | 1.42E-53 | 63 |
| Efet.01.606395.g274.t1 | Sushi, von Willebrand factor type A, EGF and pentraxin domain-containing protein 1 | 1053 | 3.66E-32 | 63 |
| Efet.01.11049.g814.t1 | Synaptotagmin-7 | 837 | 8.57E-25 | 63 |
| Efet.01.22823.g1666.t1 | T-box transcription factor TBX1 | 306 | 2.84E-20 | 63 |
| Efet.01.420374.g483.t1 | E3 ubiquitin-protein ligase TRIP12 | 357 | 2.12E-08 | 63 |
| Efet.01.27761.g178.t1 | Trophinin | 780 | 2.17E-46 | 63 |
| Efet.01.357335.g208.t1 | Tax1-binding protein 3 | 516 | 4.39E-08 | 63 |
| Efet.01.154386.g220.t1 | Ubiquitin-conjugating enzyme E2 B | 303 | 2.36E-23 | 63 |
| Efet.01.193790.g2037.t1 | Ubiquitin-protein ligase E3A | 405 | 7.11E-15 | 63 |
| Efet.01.231419.g1246.t1 | E3 ubiquitin-protein ligase UBR1 | 201 | 1.65E-10 | 63 |
| Efet.01.248603.g1838.t1 | Serine/threonine-protein kinase WNK1 | 561 | 1.47E-25 | 63 |
| Efet.01.155734.g294.t1 | Serine/threonine-protein kinase WNK2 | 4377 | 9.98E-11 | 63 |
| Efet.01.638688.g460.t1 | Receptor protein-tyrosine kinase | 273 | 2.00E-13 | 63 |
| Efet.01.563953.g127.t1 | E3 ubiquitin-protein ligase ZSWIM2 | 510 | 3.18E-11 | 63 |
| Efet.01.19637.g1456.t1 | 5-hydroxytryptamine receptor 2B | 2169 | 7.32E-20 | 62 |
| Efet.01.122310.g1160.t1 | Ataxia telangiectasia mutated (Includes complementation groups A, C and D) | 696 | 2.04E-09 | 62 |
| Efet.01.459294.g262.t1 | Amyloid-beta A4 protein | 474 | 1.18E-31 | 62 |
| Efet.01.179721.g1384.t1 | Tyrosine-protein kinase | 216 | 1.68E-14 | 62 |
| Efet.01.605974.g251.t1 | DNA ligase | 297 | 5.70E-09 | 62 |
| Efet.01.633829.g210.t1 | ATP-binding cassette sub-family A member 5 | 408 | 9.29E-06 | 62 |
| Efet.01.39673.g973.t1 | ATP-binding cassette sub-family A member 7 | 369 | 1.28E-18 | 62 |
| Efet.01.7070.g543.t1 | Active breakpoint cluster region-related protein | 231 | 1.68E-12 | 62 |
| Efet.01.339351.g1151.t1 | Neuronal acetylcholine receptor subunit beta-2 | 420 | 1.22E-11 | 62 |
| Efet.01.83040.g528.t1 | Muscarinic acetylcholine receptor M1 | 393 | 9.01E-19 | 62 |
| Efet.01.194991.g2090.t1 | Actin, alpha cardiac muscle 1 | 441 | 1.52E-35 | 62 |
| Efet.01.60754.g669.t1 | Disintegrin and metalloproteinase domain-containing protein 12 | 267 | 9.74E-08 | 62 |
| Efet.01.1633975.g141.t1 | Disintegrin and metalloproteinase domain-containing protein 12 | 234 | 1.69E-07 | 62 |
| Efet.01.537432.g212.t1 | Alpha-1A adrenergic receptor | 786 | 1.77E-25 | 62 |
| Efet.01.70021.g1233.t1 | Alpha-1B adrenergic receptor | 513 | 1.04E-10 | 62 |
| Efet.01.77431.g139.t1 | Alpha-1B adrenergic receptor | 789 | 1.28E-08 | 62 |
| Efet.01.139194.g730.t1 | Alpha-1B adrenergic receptor | 1551 | 1.39E-41 | 62 |
| Efet.01.608984.g372.t1 | Alpha-2A adrenergic receptor | 1959 | 3.21E-51 | 62 |
| Efet.01.655750.g857.t1 | Gamma-adducin | 789 | 6.42E-43 | 62 |
| Efet.01.1659479.g1815.t1 | Gamma-adducin | 675 | 3.56E-31 | 62 |
| Efet.01.657098.g1166.t1 | Type-1 angiotensin II receptor | 633 | 1.04E-19 | 62 |
| Efet.01.1658901.g1320.t1 | Aldehyde dehydrogenase, mitochondrial | 834 | 9.15E-28 | 62 |
| Efet.01.564720.g140.t1 | Fructose-bisphosphate aldolase C | 993 | 5.27E-51 | 62 |
| Efet.01.19846.g1473.t1 | Amyloid-like protein 1 | 480 | 2.22E-25 | 62 |
| Efet.01.1658772.g1269.t1 | ADP-ribosylation factor 6 | 546 | 9.98E-39 | 62 |
| Efet.01.30935.g411.t1 | Rho guanine nucleotide exchange factor 9 | 525 | 7.03E-09 | 62 |
| Efet.01.32281.g508.t1 | ADAMTS-like protein 4 | 231 | 4.40E-09 | 62 |
| Efet.01.2406.g219.t1 | A disintegrin and metalloproteinase with thrombospondin motifs 13 | 375 | 6.05E-07 | 62 |
| Efet.01.137584.g644.t1 | Axin-1 | 882 | 4.42E-41 | 62 |
| Efet.01.176840.g1259.t1 | cDNA FLJ58014, highly similar to Homo sapiens programmed cell death 4, transcript variant 1, mRNA | 408 | 8.44E-10 | 62 |
| Efet.01.420673.g490.t1 | Voltage-dependent P/Q-type calcium channel subunit alpha-1A | 360 | 6.35E-14 | 62 |
| Efet.01.296194.g1618.t1 | Cell cycle and apoptosis regulator protein 2 | 570 | 3.40E-07 | 62 |
| Efet.01.126965.g117.t1 | Cholecystokinin receptor type A | 1026 | 5.07E-20 | 62 |
| Efet.01.103900.g217.t1 | CD109 antigen | 504 | 9.30E-36 | 62 |
| Efet.01.296469.g1632.t1 | Cadherin EGF LAG seven-pass G-type receptor 3 | 3513 | 0 | 62 |
| Efet.01.438019.g901.t1 | Carbohydrate sulfotransferase 11 | 663 | 1.14E-63 | 62 |
| Efet.01.652534.g312.t1 | Protein cornichon homolog 1 | 228 | 1.87E-10 | 62 |
| Efet.01.52967.g206.t1 | Cytochrome P450 4F8 | 318 | 5.54E-30 | 62 |
| Efet.01.566223.g211.t1 | Cytochrome P450 4F8 | 288 | 3.02E-14 | 62 |
| Efet.01.8184.g609.t1 | Discoidin domain-containing receptor 2 | 819 | 5.51E-81 | 62 |
| Efet.01.138483.g690.t1 | Discoidin domain-containing receptor 2 | 513 | 2.87E-35 | 62 |
| Efet.01.74244.g1501.t1 | Probable ATP-dependent RNA helicase DDX20 | 384 | 1.34E-16 | 62 |
| Efet.01.270845.g776.t1 | DENN domain-containing protein 1A | 216 | 1.58E-11 | 62 |
| Efet.01.83657.g560.t1 | Diacylglycerol kinase eta | 369 | 1.41E-10 | 62 |
| Efet.01.2899.g261.t1 | DnaJ homolog subfamily B member 13 | 810 | 2.05E-52 | 62 |
| Efet.01.536832.g203.t1 | Disks large homolog 5 | 363 | 5.84E-20 | 62 |
| Efet.01.20078.g1487.t1 | Dedicator of cytokinesis protein 4 | 528 | 9.21E-33 | 62 |
| Efet.01.13700.g1020.t1 | Docking protein 1 | 858 | 6.44E-10 | 62 |
| Efet.01.1652856.g586.t1 | D(1A) dopamine receptor | 441 | 2.66E-16 | 62 |
| Efet.01.38438.g895.t1 | D(2) dopamine receptor | 1893 | 2.83E-39 | 62 |
| Efet.01.58590.g529.t1 | D(2) dopamine receptor | 1710 | 8.46E-34 | 62 |
| Efet.01.213034.g552.t1 | D(3) dopamine receptor | 3039 | 8.75E-18 | 62 |
| Efet.01.131221.g330.t1 | D(4) dopamine receptor | 1443 | 4.21E-25 | 62 |
| Efet.01.234295.g1349.t1 | Dual specificity protein phosphatase 22 | 816 | 2.24E-15 | 62 |
| Efet.01.102473.g142.t1 | Dual specificity tyrosine-phosphorylation-regulated kinase 2 | 705 | 6.52E-57 | 62 |
| Efet.01.646875.g1131.t1 | Dual specificity tyrosine-phosphorylation-regulated kinase 2 | 231 | 5.77E-06 | 62 |
| Efet.01.1655997.g811.t1 | Dual specificity tyrosine-phosphorylation-regulated kinase 2 | 270 | 2.16E-20 | 62 |
| Efet.01.363465.g359.t1 | Dystonin | 330 | 3.83E-06 | 62 |
| Efet.01.548217.g446.t1 | Ectonucleoside triphosphate diphosphohydrolase 1 | 366 | 1.64E-29 | 62 |
| Efet.01.621374.g934.t1 | Ephrin type-A receptor 3 | 345 | 1.38E-12 | 62 |
| Efet.01.582422.g760.t1 | Forkhead box protein P1 | 447 | 2.64E-12 | 62 |
| Efet.01.104529.g254.t1 | Fascin | 930 | 5.00E-71 | 62 |
| Efet.01.644201.g871.t1 | Alpha-(1,3)-fucosyltransferase 4 | 912 | 5.34E-06 | 62 |
| Efet.01.417019.g404.t1 | Tyrosine-protein kinase Fyn | 471 | 1.86E-08 | 62 |
| Efet.01.149786.g1217.t1 | Growth hormone secretagogue receptor type 1 | 1203 | 8.30E-08 | 62 |
| Efet.01.16800.g1243.t1 | N-acetyllactosaminide beta-1,6-N-acetylglucosaminyl-transferase | 342 | 2.67E-13 | 62 |
| Efet.01.379162.g780.t1 | Gremlin-1 | 420 | 4.39E-34 | 62 |
| Efet.01.648009.g1213.t1 | Glutamate receptor ionotropic, kainate 2 | 378 | 7.86E-26 | 62 |
| Efet.01.1658203.g1124.t1 | Glutamate receptor ionotropic, kainate 2 | 276 | 9.09E-16 | 62 |
| Efet.01.10996.g807.t1 | Solute carrier family 2, facilitated glucose transporter member 4 | 396 | 1.14E-19 | 62 |
| Efet.01.469285.g515.t1 | Heparan sulfate glucosamine 3-O-sulfotransferase 5 | 525 | 8.91E-50 | 62 |
| Efet.01.171183.g1024.t1 | Indian hedgehog protein | 1110 | 2.55E-83 | 62 |
| Efet.01.77643.g153.t1 | Integrin-linked kinase-associated serine/threonine phosphatase 2C | 468 | 4.78E-15 | 62 |
| Efet.01.78842.g230.t1 | Potassium voltage-gated channel subfamily H member 8 | 552 | 3.54E-32 | 62 |
| Efet.01.180403.g1417.t1 | Potassium voltage-gated channel subfamily H member 8 | 582 | 1.78E-24 | 62 |
| Efet.01.31679.g469.t1 | ATP-sensitive inward rectifier potassium channel 8 | 1641 | 4.83E-90 | 62 |
| Efet.01.131669.g345.t1 | ATP-sensitive inward rectifier potassium channel 8 | 1101 | 7.29E-41 | 62 |
| Efet.01.313196.g420.t1 | ATP-sensitive inward rectifier potassium channel 8 | 1491 | 6.95E-88 | 62 |
| Efet.01.1658091.g1102.t1 | ATP-sensitive inward rectifier potassium channel 8 | 666 | 2.01E-27 | 62 |
| Efet.01.58869.g555.t1 | Kinesin-like protein KIF1B | 771 | 9.78E-63 | 62 |
| Efet.01.240723.g1557.t1 | Kelch-like protein 20 | 498 | 3.70E-31 | 62 |
| Efet.01.330998.g940.t1 | Kelch-like protein 20 | 771 | 3.44E-43 | 62 |
| Efet.01.650832.g91.t1 | Kelch-like protein 20 | 279 | 7.08E-07 | 62 |
| Efet.01.553365.g587.t1 | Histone-lysine N-methyltransferase 2A | 1200 | 7.86E-07 | 62 |
| Efet.01.449198.g1199.t1 | Histone-lysine N-methyltransferase 2D | 483 | 7.58E-18 | 62 |
| Efet.01.643291.g769.t1 | Pyruvate kinase PKM | 558 | 3.43E-14 | 62 |
| Efet.01.19609.g1453.t1 | Neural cell adhesion molecule L1 | 207 | 1.98E-08 | 62 |
| Efet.01.4486.g362.t1 | Leukocyte receptor cluster member 1 | 372 | 5.06E-11 | 62 |
| Efet.01.658192.g1708.t1 | Lipoyl synthase, mitochondrial | 720 | 2.95E-63 | 62 |
| Efet.01.18764.g1392.t1 | Protein Mdm4 | 885 | 8.92E-10 | 62 |
| Efet.01.658150.g1687.t1 | Multidrug resistance protein 1 | 801 | 6.48E-12 | 62 |
| Efet.01.1597384.g21.t1 | Multidrug resistance protein 1 | 207 | 3.97E-10 | 62 |
| Efet.01.445217.g1120.t1 | Methylated-DNA--protein-cysteine methyltransferase | 513 | 2.08E-21 | 62 |
| Efet.01.354456.g127.t1 | Mitogen-activated protein kinase 7 | 282 | 3.20E-11 | 62 |
| Efet.01.300117.g5.t1 | Myosin regulatory light chain 2, skeletal muscle isoform | 258 | 6.48E-13 | 62 |
| Efet.01.164472.g735.t1 | Canalicular multispecific organic anion transporter 1 | 435 | 6.51E-17 | 62 |
| Efet.01.489806.g958.t1 | Canalicular multispecific organic anion transporter 1 | 231 | 2.17E-09 | 62 |
| Efet.01.191998.g1962.t1 | Myosin-10 | 390 | 2.36E-16 | 62 |
| Efet.01.269997.g744.t1 | Myosin-10 | 468 | 2.06E-23 | 62 |
| Efet.01.396818.g1164.t1 | Myosin-9 | 363 | 3.30E-20 | 62 |
| Efet.01.141383.g849.t1 | Myosin-binding protein C, fast-type | 507 | 7.04E-24 | 62 |
| Efet.01.1657106.g943.t1 | Neutrophil cytosol factor 4 | 285 | 1.77E-14 | 62 |
| Efet.01.207527.g340.t1 | Neuronal calcium sensor 1 | 387 | 4.12E-22 | 62 |
| Efet.01.1640710.g224.t1 | Neuronal calcium sensor 1 | 387 | 1.39E-30 | 62 |
| Efet.01.162760.g645.t1 | Neurofilament light polypeptide | 507 | 9.45E-11 | 62 |
| Efet.01.194411.g2067.t1 | Neurofilament light polypeptide | 609 | 7.97E-06 | 62 |
| Efet.01.115606.g805.t1 | Homeobox protein Nkx-2.6 | 558 | 3.91E-34 | 62 |
| Efet.01.341377.g1194.t1 | Neurexin-2 | 315 | 4.00E-18 | 62 |
| Efet.01.376288.g709.t1 | Tumor protein p73 | 285 | 2.48E-14 | 62 |
| Efet.01.188445.g1808.t1 | Protein kinase C and casein kinase substrate in neurons protein 3 | 306 | 1.65E-10 | 62 |
| Efet.01.142179.g887.t1 | Serine/threonine-protein kinase PAK 1 | 501 | 7.48E-15 | 62 |
| Efet.01.67685.g1088.t1 | TCDD-inducible poly [ADP-ribose] polymerase | 549 | 8.95E-29 | 62 |
| Efet.01.576941.g551.t1 | Programmed cell death protein 5 | 300 | 2.65E-16 | 62 |
| Efet.01.372774.g641.t1 | High affinity cAMP-specific 3',5'-cyclic phosphodiesterase 7A | 600 | 2.35E-22 | 62 |
| Efet.01.5934.g474.t1 | Basement membrane-specific heparan sulfate proteoglycan core protein | 570 | 5.58E-28 | 62 |
| Efet.01.650749.g82.t1 | Peptidyl-prolyl cis-trans isomerase NIMA-interacting 1 | 1650 | 1.54E-11 | 62 |
| Efet.01.193330.g2009.t1 | Homeobox protein PKNOX1 | 522 | 2.17E-15 | 62 |
| Efet.01.491721.g989.t1 | Homeobox protein PKNOX1 | 429 | 2.85E-11 | 62 |
| Efet.01.187760.g1773.t1 | Serine/threonine-protein phosphatase 2A catalytic subunit beta isoform | 309 | 2.50E-25 | 62 |
| Efet.01.128219.g178.t1 | PR domain zinc finger protein 16 | 1086 | 9.72E-52 | 62 |
| Efet.01.495296.g1074.t1 | DNA primase large subunit | 408 | 2.01E-18 | 62 |
| Efet.01.3114.g271.t1 | Receptor protein-tyrosine kinase | 1362 | 5.96E-86 | 62 |
| Efet.01.206847.g303.t1 | Receptor protein-tyrosine kinase | 225 | 4.70E-06 | 62 |
| Efet.01.24177.g1757.t1 | Zinc finger protein 3 | 1011 | 5.85E-50 | 62 |
| Efet.01.95093.g1167.t1 | Zinc finger protein 3 | 624 | 3.38E-54 | 62 |
| Efet.01.493760.g1042.t1 | DNA-binding protein Ikaros | 1104 | 2.01E-10 | 62 |
| Efet.01.610319.g436.t1 | Double-stranded RNA-specific editase 1 | 348 | 2.57E-18 | 62 |
| Efet.01.387540.g959.t1 | Regulator of G-protein signaling 1 | 1221 | 8.10E-34 | 62 |
| Efet.01.153578.g191.t1 | Rho GTPase-activating protein 5 | 300 | 4.59E-13 | 62 |
| Efet.01.137274.g617.t1 | E3 ubiquitin-protein ligase RNF34 | 345 | 1.91E-23 | 62 |
| Efet.01.338659.g1127.t1 | Retinoic acid receptor RXR-alpha | 474 | 1.24E-21 | 62 |
| Efet.01.64587.g891.t1 | Sodium channel protein type 2 subunit alpha | 342 | 3.14E-13 | 62 |
| Efet.01.1657493.g995.t1 | Serine incorporator 3 | 342 | 4.79E-21 | 62 |
| Efet.01.547139.g423.t1 | Sonic hedgehog protein | 972 | 1.09E-83 | 62 |
| Efet.01.493319.g1030.t1 | Mothers against decapentaplegic homolog 6 | 621 | 1.33E-51 | 62 |
| Efet.01.122992.g1194.t1 | Sorting nexin-2 | 408 | 1.61E-37 | 62 |
| Efet.01.617979.g792.t1 | Transcription factor SOX-2 | 882 | 4.44E-22 | 62 |
| Efet.01.323702.g730.t1 | Synaptotagmin-1 | 717 | 1.35E-12 | 62 |
| Efet.01.577981.g620.t1 | Synaptotagmin-7 | 369 | 6.91E-23 | 62 |
| Efet.01.24354.g1770.t1 | Tumor protein p53-inducible nuclear protein 1 | 915 | 8.01E-08 | 62 |
| Efet.01.140902.g817.t1 | Tenascin-X | 1119 | 2.79E-06 | 62 |
| Efet.01.219841.g801.t1 | E3 ubiquitin-protein ligase TRIM33 | 588 | 2.55E-20 | 62 |
| Efet.01.170165.g981.t1 | Transient receptor potential cation channel subfamily M member 7 | 879 | 3.84E-24 | 62 |
| Efet.01.53144.g213.t1 | Protein turtle homolog A | 483 | 5.12E-22 | 62 |
| Efet.01.1634377.g146.t1 | Protein turtle homolog A | 306 | 2.58E-24 | 62 |
| Efet.01.184491.g1611.t1 | Twist-related protein 1 | 234 | 2.80E-09 | 62 |
| Efet.01.353382.g100.t1 | Ubiquitin carboxyl-terminal hydrolase 4 | 966 | 4.22E-30 | 62 |
| Efet.01.262435.g470.t1 | Netrin receptor UNC5C | 255 | 6.52E-15 | 62 |
| Efet.01.507549.g197.t1 | Netrin receptor UNC5D | 267 | 2.32E-08 | 62 |
| Efet.01.241486.g1582.t1 | Vasopressin V1b receptor | 462 | 1.49E-11 | 62 |
| Efet.01.173855.g1128.t1 | Protein Wnt-11 | 354 | 2.98E-15 | 62 |
| Efet.01.423962.g568.t1 | Vasopressin V1a receptor | 540 | 7.14E-25 | 62 |
| Efet.01.139400.g740.t1 | Tyrosine-protein kinase receptor | 1197 | 3.76E-71 | 62 |
| Efet.01.259173.g353.t1 | XK-related protein 6 | 906 | 2.01E-23 | 62 |
| Efet.01.458077.g220.t1 | Zinc finger FYVE domain-containing protein 9 | 213 | 2.51E-14 | 62 |
| Efet.01.330278.g922.t1 | Zinc finger protein 3 | 1020 | 1.17E-54 | 62 |
| Efet.01.423413.g555.t1 | Zinc finger protein 3 | 825 | 7.95E-28 | 62 |
| Efet.01.346290.g1307.t1 | HCG1985580, isoform CRA_c | 282 | 2.64E-09 | 61 |
| Efet.01.153611.g193.t1 | Serpin peptidase inhibitor, clade B (Ovalbumin), member 9 | 243 | 2.90E-14 | 61 |
| Efet.01.658334.g1867.t1 | Heat shock 60kDa protein 1 (Chaperonin) | 1602 | 2.28E-93 | 61 |
| Efet.01.65177.g924.t1 | HCG40889, isoform CRA_b | 252 | 2.29E-11 | 61 |
| Efet.01.658044.g1588.t1 | ATP-binding cassette sub-family A member 5 | 366 | 1.14E-06 | 61 |
| Efet.01.1659389.g1636.t1 | ATP-binding cassette sub-family B member 9 | 294 | 3.69E-13 | 61 |
| Efet.01.9268.g679.t1 | Acetylcholinesterase | 1377 | 1.78E-73 | 61 |
| Efet.01.29780.g329.t1 | Acetylcholinesterase | 1503 | 8.68E-127 | 61 |
| Efet.01.91724.g985.t1 | Acetylcholinesterase | 231 | 2.74E-07 | 61 |
| Efet.01.1659337.g1564.t1 | Acetylcholinesterase | 1578 | 2.43E-146 | 61 |
| Efet.01.106459.g339.t1 | Neuronal acetylcholine receptor subunit alpha-7 | 516 | 3.23E-29 | 61 |
| Efet.01.133698.g422.t1 | Neuronal acetylcholine receptor subunit alpha-7 | 531 | 4.43E-34 | 61 |
| Efet.01.307000.g218.t1 | Neuronal acetylcholine receptor subunit alpha-7 | 594 | 2.66E-36 | 61 |
| Efet.01.478212.g723.t1 | Neuronal acetylcholine receptor subunit alpha-7 | 399 | 1.11E-26 | 61 |
| Efet.01.177294.g1284.t1 | Muscarinic acetylcholine receptor M1 | 2199 | 3.02E-41 | 61 |
| Efet.01.104751.g263.t1 | Alpha-actinin-2 | 516 | 3.79E-27 | 61 |
| Efet.01.456769.g165.t1 | Alpha-1A adrenergic receptor | 1098 | 5.31E-38 | 61 |
| Efet.01.578471.g638.t1 | Alpha-1A adrenergic receptor | 1338 | 8.83E-26 | 61 |
| Efet.01.70021.g1234.t1 | Alpha-1B adrenergic receptor | 1197 | 1.24E-13 | 61 |
| Efet.01.8897.g649.t1 | Alpha-2A adrenergic receptor | 1182 | 1.87E-18 | 61 |
| Efet.01.124138.g1246.t1 | Gamma-adducin | 570 | 1.16E-37 | 61 |
| Efet.01.647720.g1196.t1 | Alcohol dehydrogenase class-3 | 513 | 9.41E-19 | 61 |
| Efet.01.656717.g1069.t1 | Aldehyde dehydrogenase, mitochondrial | 1233 | 6.23E-117 | 61 |
| Efet.01.630616.g51.t1 | Alstrom syndrome protein 1 | 453 | 1.50E-10 | 61 |
| Efet.01.214770.g620.t1 | Ankyrin-1 | 480 | 1.45E-46 | 61 |
| Efet.01.1653899.g642.t1 | Ankyrin-1 | 396 | 6.16E-11 | 61 |
| Efet.01.258243.g304.t1 | Aquaporin-4 | 639 | 2.66E-25 | 61 |
| Efet.01.572295.g388.t1 | Rho guanine nucleotide exchange factor 11 | 405 | 3.69E-07 | 61 |
| Efet.01.224880.g997.t1 | Apoptosis-stimulating of p53 protein 2 | 426 | 1.13E-10 | 61 |
| Efet.01.127962.g166.t1 | TLR7 (Toll-like receptor 7) | 1665 | 3.45E-08 | 61 |
| Efet.01.477544.g707.t1 | cDNA FLJ51484, highly similar to B-cell lymphoma 3-encoded protein | 1707 | 1.86E-44 | 61 |
| Efet.01.133173.g409.t1 | Breast cancer anti-estrogen resistance protein 1 | 1848 | 5.74E-26 | 61 |
| Efet.01.501312.g30.t1 | B-cell lymphoma 3 protein | 633 | 1.97E-06 | 61 |
| Efet.01.9417.g696.t1 | Bridging integrator 3 | 408 | 6.12E-25 | 61 |
| Efet.01.215751.g655.t1 | Endoplasmic reticulum chaperone BiP | 567 | 1.90E-35 | 61 |
| Efet.01.652956.g384.t1 | Bloom syndrome protein | 279 | 2.44E-14 | 61 |
| Efet.01.514711.g356.t1 | Bone morphogenetic protein 1 | 453 | 1.62E-07 | 61 |
| Efet.01.275103.g926.t1 | Brother of CDO | 267 | 1.05E-09 | 61 |
| Efet.01.1659192.g1454.t1 | Scavenger receptor cysteine-rich type 1 protein M160 | 378 | 2.32E-23 | 61 |
| Efet.01.124003.g1240.t1 | Voltage-dependent T-type calcium channel subunit alpha-1G | 1221 | 1.97E-28 | 61 |
| Efet.01.253306.g129.t1 | Voltage-dependent T-type calcium channel subunit alpha-1G | 309 | 3.02E-15 | 61 |
| Efet.01.119208.g992.t1 | Cholecystokinin receptor type A | 537 | 8.87E-09 | 61 |
| Efet.01.271589.g808.t1 | Cholecystokinin receptor type A | 411 | 1.83E-18 | 61 |
| Efet.01.37135.g802.t1 | Cell division control protein 42 homolog | 441 | 3.48E-09 | 61 |
| Efet.01.91437.g963.t1 | Cell adhesion molecule-related/down-regulated by oncogenes | 213 | 2.45E-10 | 61 |
| Efet.01.651871.g231.t1 | Cadherin EGF LAG seven-pass G-type receptor 1 | 357 | 1.26E-21 | 61 |
| Efet.01.5833.g459.t1 | Collagen alpha-1(XII) chain | 951 | 4.24E-34 | 61 |
| Efet.01.268287.g674.t1 | Cartilage oligomeric matrix protein | 381 | 2.75E-10 | 61 |
| Efet.01.618695.g841.t1 | Carbamoyl-phosphate synthase [ammonia], mitochondrial | 642 | 5.82E-26 | 61 |
| Efet.01.552029.g552.t1 | Probable carboxypeptidase X1 | 516 | 5.95E-17 | 61 |
| Efet.01.646112.g1035.t1 | Inactive carboxypeptidase-like protein X2 | 270 | 3.43E-07 | 61 |
| Efet.01.549120.g471.t1 | Cytohesin-3 | 1359 | 8.62E-09 | 61 |
| Efet.01.605603.g232.t1 | Death-associated protein kinase 1 | 285 | 6.14E-08 | 61 |
| Efet.01.270288.g752.t1 | Dipeptidyl peptidase 4 | 216 | 1.23E-08 | 61 |
| Efet.01.16387.g1210.t1 | D(4) dopamine receptor | 399 | 4.32E-10 | 61 |
| Efet.01.65638.g957.t1 | Dual specificity protein phosphatase 6 | 300 | 1.23E-06 | 61 |
| Efet.01.302034.g68.t1 | Proteasome subunit beta type | 351 | 7.46E-17 | 61 |
| Efet.01.465852.g423.t1 | Endothelin-converting enzyme 1 | 516 | 1.56E-45 | 61 |
| Efet.01.259657.g369.t1 | Ephrin type-A receptor 7 | 279 | 5.52E-19 | 61 |
| Efet.01.1652994.g594.t1 | Chimeric ERCC6-PGBD3 protein | 570 | 6.02E-25 | 61 |
| Efet.01.63903.g858.t1 | Transcription factor ETV6 | 240 | 1.25E-11 | 61 |
| Efet.01.402163.g56.t1 | MDS1 and EVI1 complex locus protein EVI1 | 1050 | 1.69E-17 | 61 |
| Efet.01.331421.g949.t1 | Exocyst complex component 2 | 243 | 3.53E-11 | 61 |
| Efet.01.565205.g176.t1 | Exocyst complex component 7 | 222 | 4.87E-14 | 61 |
| Efet.01.209668.g433.t1 | Protocadherin Fat 4 | 843 | 8.43E-21 | 61 |
| Efet.01.599964.g1312.t1 | Fez family zinc finger protein 2 | 1056 | 9.58E-06 | 61 |
| Efet.01.289089.g1422.t1 | Dimethylaniline monooxygenase [N-oxide-forming] 1 | 393 | 3.91E-20 | 61 |
| Efet.01.240301.g1542.t1 | Frizzled-4 | 2022 | 1.39E-128 | 61 |
| Efet.01.651187.g145.t1 | GRB2-associated-binding protein 2 | 249 | 1.12E-07 | 61 |
| Efet.01.159489.g495.t1 | Gastrin/cholecystokinin type B receptor | 453 | 1.89E-18 | 61 |
| Efet.01.601035.g44.t1 | Gastrin/cholecystokinin type B receptor | 603 | 1.13E-18 | 61 |
| Efet.01.656032.g931.t1 | Gastrin/cholecystokinin type B receptor | 378 | 1.05E-23 | 61 |
| Efet.01.261138.g418.t1 | Zinc finger protein GLI2 | 900 | 2.24E-25 | 61 |
| Efet.01.504560.g119.t1 | Transcriptional activator GLI3 | 690 | 1.22E-21 | 61 |
| Efet.01.42893.g1170.t1 | N-acetyllactosaminide beta-1,6-N-acetylglucosaminyl-transferase | 843 | 1.41E-39 | 61 |
| Efet.01.397522.g1182.t1 | G-protein coupled receptor 98 | 1293 | 8.79E-39 | 61 |
| Efet.01.182447.g1519.t1 | Glutamate receptor ionotropic, kainate 2 | 969 | 1.30E-15 | 61 |
| Efet.01.303950.g137.t1 | Huntingtin | 906 | 1.10E-11 | 61 |
| Efet.01.187322.g1744.t1 | Hepatic leukemia factor | 291 | 6.34E-06 | 61 |
| Efet.01.527516.g630.t1 | Hemicentin-1 | 513 | 2.02E-18 | 61 |
| Efet.01.155767.g296.t1 | Heat shock protein beta-1 | 1122 | 1.11E-12 | 61 |
| Efet.01.239691.g1527.t1 | Insulin-like growth factor 2 mRNA-binding protein 2 | 339 | 8.88E-07 | 61 |
| Efet.01.240865.g1563.t1 | DNA-binding protein Ikaros | 1779 | 3.43E-13 | 61 |
| Efet.01.107111.g366.t1 | Intersectin-2 | 981 | 1.56E-11 | 61 |
| Efet.01.32862.g546.t1 | Kalirin | 249 | 4.59E-10 | 61 |
| Efet.01.150164.g8.t1 | Potassium voltage-gated channel subfamily H member 8 | 420 | 4.44E-27 | 61 |
| Efet.01.657237.g1206.t1 | Potassium voltage-gated channel subfamily H member 8 | 591 | 3.81E-26 | 61 |
| Efet.01.98762.g1372.t1 | ATP-sensitive inward rectifier potassium channel 8 | 375 | 7.28E-16 | 61 |
| Efet.01.52418.g166.t1 | Kelch-like protein 20 | 342 | 4.61E-23 | 61 |
| Efet.01.131945.g353.t1 | Platelet-activating factor acetylhydrolase IB subunit alpha | 972 | 3.51E-10 | 61 |
| Efet.01.99420.g1415.t1 | Prolow-density lipoprotein receptor-related protein 1 | 609 | 9.92E-31 | 61 |
| Efet.01.102678.g153.t1 | MAP/microtubule affinity-regulating kinase 3 | 903 | 3.88E-49 | 61 |
| Efet.01.576455.g528.t1 | Multidrug resistance protein 1 | 945 | 5.32E-51 | 61 |
| Efet.01.343607.g1240.t1 | Stromelysin-1 | 222 | 1.21E-08 | 61 |
| Efet.01.1657652.g1023.t1 | Methylmalonate-semialdehyde dehydrogenase [acylating], mitochondrial | 678 | 5.79E-53 | 61 |
| Efet.01.114007.g714.t1 | DNA mismatch repair protein Msh6 | 1368 | 2.87E-11 | 61 |
| Efet.01.80043.g327.t1 | Myosin-10 | 276 | 7.38E-08 | 61 |
| Efet.01.643528.g810.t1 | Cytoplasmic protein NCK2 | 1008 | 1.50E-73 | 61 |
| Efet.01.293225.g1546.t1 | Neurogenic differentiation factor 1 | 594 | 3.65E-13 | 61 |
| Efet.01.35723.g715.t1 | NPC intracellular cholesterol transporter 1 | 294 | 6.19E-17 | 61 |
| Efet.01.19353.g1433.t1 | Oxysterols receptor LXR-beta | 219 | 5.64E-13 | 61 |
| Efet.01.238417.g1492.t1 | Neurexin-3 | 603 | 3.59E-43 | 61 |
| Efet.01.50974.g66.t1 | TCDD-inducible poly [ADP-ribose] polymerase | 1347 | 2.56E-11 | 61 |
| Efet.01.75019.g1.t1 | Protocadherin-17 | 951 | 3.46E-12 | 61 |
| Efet.01.14215.g1051.t1 | Calcium/calmodulin-dependent 3',5'-cyclic nucleotide phosphodiesterase 1B | 531 | 1.12E-07 | 61 |
| Efet.01.228296.g1143.t1 | Polycystic kidney disease protein 1-like 1 | 312 | 1.15E-12 | 61 |
| Efet.01.218078.g738.t1 | 1-phosphatidylinositol 4,5-bisphosphate phosphodiesterase gamma-1 | 426 | 2.46E-20 | 61 |
| Efet.01.347406.g1335.t1 | Homeobox protein prophet of Pit-1 | 4599 | 4.59E-08 | 61 |
| Efet.01.168152.g898.t1 | Peripherin-2 | 654 | 7.32E-27 | 61 |
| Efet.01.380739.g808.t1 | Tyrosine-protein phosphatase non-receptor type 6 | 216 | 3.07E-11 | 61 |
| Efet.01.179564.g1376.t1 | Receptor-type tyrosine-protein phosphatase F | 375 | 4.60E-21 | 61 |
| Efet.01.652554.g313.t1 | Receptor-type tyrosine-protein phosphatase F | 318 | 3.34E-13 | 61 |
| Efet.01.40799.g1053.t1 | Poly(U)-binding-splicing factor PUF60 | 507 | 4.52E-07 | 61 |
| Efet.01.646145.g1038.t1 | Poly(U)-binding-splicing factor PUF60 | 273 | 1.38E-06 | 61 |
| Efet.01.68027.g1116.t1 | Transcriptional activator protein Pur-beta | 363 | 1.26E-13 | 61 |
| Efet.01.619569.g871.t1 | MYLK protein | 321 | 2.34E-20 | 61 |
| Efet.01.157456.g393.t1 | Macrophage receptor with collagenous structure | 270 | 3.47E-06 | 61 |
| Efet.01.658217.g1720.t1 | Tubulin beta chain | 1194 | 1.25E-99 | 61 |
| Efet.01.458795.g251.t1 | Ras-related protein Ral-A | 381 | 3.46E-11 | 61 |
| Efet.01.585143.g834.t1 | GTPase HRas | 429 | 8.32E-10 | 61 |
| Efet.01.64697.g895.t1 | Reelin | 378 | 8.98E-07 | 61 |
| Efet.01.145642.g1043.t1 | Ras-specific guanine nucleotide-releasing factor 2 | 450 | 8.04E-39 | 61 |
| Efet.01.144789.g1005.t1 | Regulating synaptic membrane exocytosis protein 2 | 825 | 7.11E-06 | 61 |
| Efet.01.7015.g540.t1 | Roundabout homolog 2 | 636 | 8.02E-47 | 61 |
| Efet.01.324653.g758.t1 | Rho-associated protein kinase 1 | 576 | 3.18E-26 | 61 |
| Efet.01.257033.g267.t1 | Nuclear receptor ROR-alpha | 891 | 2.42E-30 | 61 |
| Efet.01.312306.g387.t1 | Tyrosine-protein kinase RYK | 408 | 1.64E-13 | 61 |
| Efet.01.523046.g537.t1 | Ryanodine receptor 2 | 540 | 1.94E-07 | 61 |
| Efet.01.656506.g1034.t1 | Sodium channel protein type 2 subunit alpha | 423 | 5.33E-08 | 61 |
| Efet.01.415242.g350.t1 | E3 ubiquitin-protein ligase SH3RF1 | 1350 | 9.68E-10 | 61 |
| Efet.01.1607092.g14.t1 | Helicase SKI2W | 240 | 2.57E-12 | 61 |
| Efet.01.88971.g835.t1 | Transcription factor SOX-4 | 912 | 1.59E-20 | 61 |
| Efet.01.58573.g525.t1 | Sphingosine kinase 2 | 294 | 7.33E-15 | 61 |
| Efet.01.401518.g34.t1 | Spectrin beta chain, non-erythrocytic 2 | 567 | 1.81E-36 | 61 |
| Efet.01.653735.g473.t1 | Serine racemase | 900 | 7.52E-50 | 61 |
| Efet.01.94193.g1113.t1 | Stabilin-2 | 564 | 6.89E-12 | 61 |
| Efet.01.193204.g2005.t1 | Sushi, von Willebrand factor type A, EGF and pentraxin domain-containing protein 1 | 282 | 1.88E-13 | 61 |
| Efet.01.222614.g911.t1 | Synaptotagmin-7 | 921 | 2.19E-22 | 61 |
| Efet.01.227552.g1103.t1 | Tissue factor pathway inhibitor | 801 | 8.87E-06 | 61 |
| Efet.01.488935.g942.t1 | Thyroid hormone receptor alpha | 435 | 3.41E-24 | 61 |
| Efet.01.458510.g239.t1 | Nucleolysin TIA-1 isoform p40 | 1029 | 2.00E-11 | 61 |
| Efet.01.31498.g451.t1 | TNFAIP3-interacting protein 2 | 423 | 6.30E-08 | 61 |
| Efet.01.181701.g1489.t1 | TNF receptor-associated factor 6 | 414 | 7.76E-08 | 61 |
| Efet.01.326409.g813.t1 | TNF receptor-associated factor 6 | 426 | 5.76E-32 | 61 |
| Efet.01.56401.g400.t1 | Thrombospondin-1 | 765 | 1.50E-11 | 61 |
| Efet.01.652812.g342.t1 | Protein turtle homolog A | 390 | 2.32E-24 | 61 |
| Efet.01.269856.g736.t1 | Protein turtle homolog B | 324 | 3.25E-21 | 61 |
| Efet.01.640198.g561.t1 | Protein turtle homolog B | 357 | 3.81E-25 | 61 |
| Efet.01.300577.g22.t1 | Ubiquitin-conjugating enzyme E2 D3 | 282 | 6.49E-09 | 61 |
| Efet.01.79717.g305.t1 | Ubiquitin carboxyl-terminal hydrolase 14 | 249 | 4.23E-07 | 61 |
| Efet.01.1483.g142.t1 | Ubiquitin carboxyl-terminal hydrolase 4 | 393 | 1.12E-07 | 61 |
| Efet.01.534802.g154.t1 | UDP-glucuronosyltransferase 1-10 | 429 | 2.73E-19 | 61 |
| Efet.01.158006.g424.t1 | Netrin receptor UNC5B | 576 | 1.55E-26 | 61 |
| Efet.01.24163.g1756.t1 | Probable ubiquitin carboxyl-terminal hydrolase FAF-X | 627 | 6.45E-23 | 61 |
| Efet.01.216967.g700.t1 | Vasopressin V1b receptor | 609 | 5.29E-43 | 61 |
| Efet.01.80891.g393.t1 | Protein Wnt-11 | 333 | 5.17E-07 | 61 |
| Efet.01.129961.g264.t1 | Protein Wnt-4 | 354 | 5.25E-22 | 61 |
| Efet.01.497208.g1117.t1 | WW domain-containing oxidoreductase | 216 | 9.99E-12 | 61 |
| Efet.01.261972.g446.t1 | Zinc finger protein 675 | 3924 | 2.19E-96 | 61 |
| Efet.01.654940.g717.t1 | 4F2 cell-surface antigen heavy chain | 459 | 5.36E-08 | 60 |
| Efet.01.1646664.g352.t1 | Catenin (Cadherin-associated protein), beta 1, 88kDa, isoform CRA_a | 243 | 6.21E-08 | 60 |
| Efet.01.537674.g221.t1 | Discoidin domain receptor family, member 1, isoform CRA_b | 306 | 1.99E-14 | 60 |
| Efet.01.477434.g699.t1 | DDX39B (HCG2005638, isoform CRA_a) | 399 | 1.85E-22 | 60 |
| Efet.01.79283.g268.t1 | Kinase insert domain receptor (A type III receptor tyrosine kinase), isoform CRA_a | 537 | 6.53E-38 | 60 |
| Efet.01.113299.g674.t1 | Nuclear factor of kappa light polypeptide gene enhancer in B-cells 1 (P105), isoform CRA_b | 315 | 9.29E-14 | 60 |
| Efet.01.22865.g1667.t1 | Neural cell adhesion molecule 1 | 612 | 2.50E-09 | 60 |
| Efet.01.92377.g1010.t1 | Myeloid/lymphoid or mixed-lineage leukemia translocated to 3 isoform 1 | 285 | 5.46E-07 | 60 |
| Efet.01.54581.g303.t1 | Chemokine (C-C motif) receptor 7 | 483 | 1.69E-06 | 60 |
| Efet.01.561737.g31.t1 | 5'-AMP-activated protein kinase subunit gamma-2 | 291 | 6.89E-11 | 60 |
| Efet.01.585463.g840.t1 | ATP-binding cassette sub-family A member 5 | 303 | 9.97E-10 | 60 |
| Efet.01.69624.g1215.t1 | Neuronal acetylcholine receptor subunit alpha-7 | 477 | 8.96E-09 | 60 |
| Efet.01.271851.g820.t1 | Neuronal acetylcholine receptor subunit alpha-7 | 582 | 3.63E-44 | 60 |
| Efet.01.655360.g800.t1 | Neuronal acetylcholine receptor subunit alpha-7 | 309 | 1.86E-20 | 60 |
| Efet.01.259156.g352.t1 | Neuronal acetylcholine receptor subunit beta-2 | 666 | 1.92E-40 | 60 |
| Efet.01.343852.g1249.t1 | Neuronal acetylcholine receptor subunit beta-2 | 717 | 3.28E-31 | 60 |
| Efet.01.47357.g1438.t1 | Muscarinic acetylcholine receptor M1 | 612 | 3.72E-22 | 60 |
| Efet.01.214184.g599.t1 | Muscarinic acetylcholine receptor M1 | 2382 | 6.04E-46 | 60 |
| Efet.01.282098.g1165.t1 | Muscarinic acetylcholine receptor M1 | 1734 | 1.56E-42 | 60 |
| Efet.01.633829.g213.t1 | Acyl-CoA synthetase family member 2, mitochondrial | 1311 | 4.45E-113 | 60 |
| Efet.01.328117.g865.t1 | Disintegrin and metalloproteinase domain-containing protein 12 | 279 | 8.99E-18 | 60 |
| Efet.01.249155.g1865.t1 | Alpha-1B adrenergic receptor | 630 | 2.37E-06 | 60 |
| Efet.01.164386.g728.t1 | Adenosine deaminase 2 | 357 | 3.43E-17 | 60 |
| Efet.01.604040.g151.t1 | Alpha-2A adrenergic receptor | 471 | 1.36E-24 | 60 |
| Efet.01.16831.g1244.t1 | Aldo-keto reductase family 1 member C2 | 210 | 1.60E-08 | 60 |
| Efet.01.171406.g1031.t1 | Angiopoietin-related protein 1 | 462 | 5.11E-19 | 60 |
| Efet.01.129420.g244.t1 | Ankyrin-1 | 969 | 1.93E-64 | 60 |
| Efet.01.11593.g846.t1 | Rho guanine nucleotide exchange factor 17 | 543 | 4.31E-13 | 60 |
| Efet.01.567084.g233.t1 | Apoptosis-stimulating of p53 protein 2 | 384 | 8.70E-14 | 60 |
| Efet.01.53984.g263.t1 | Atlastin-2 | 237 | 1.28E-09 | 60 |
| Efet.01.477289.g694.t1 | ATP synthase subunit beta, mitochondrial | 399 | 4.92E-27 | 60 |
| Efet.01.140871.g814.t1 | cDNA, FLJ92968, highly similar to Homo sapiens runt-related transcription factor 1; translocated to, 1 | 399 | 3.50E-29 | 60 |
| Efet.01.635430.g277.t1 | cDNA, FLJ94517, highly similar to Homo sapiens baculoviral IAP repeat-containing 4 (BIRC4), mRNA | 825 | 3.97E-17 | 60 |
| Efet.01.433992.g820.t1 | Band 3 anion transport protein | 753 | 3.01E-12 | 60 |
| Efet.01.599360.g1288.t1 | Lactosylceramide 1,3-N-acetyl-beta-D-glucosaminyltransferase | 1047 | 3.05E-26 | 60 |
| Efet.01.140944.g820.t1 | Catenin beta-1 | 954 | 9.08E-76 | 60 |
| Efet.01.193473.g2017.t1 | BTB/POZ domain-containing adapter for CUL3-mediated RhoA degradation protein 2 | 648 | 4.67E-07 | 60 |
| Efet.01.80479.g368.t1 | BarH-like 1 homeobox protein | 381 | 1.77E-15 | 60 |
| Efet.01.152987.g162.t1 | Protein bicaudal D homolog 2 | 939 | 3.34E-12 | 60 |
| Efet.01.334564.g1034.t1 | Scavenger receptor cysteine-rich type 1 protein M130 | 231 | 5.24E-11 | 60 |
| Efet.01.203719.g168.t1 | Complement C1q and tumor necrosis factor-related protein 9A | 705 | 2.00E-10 | 60 |
| Efet.01.471289.g558.t1 | Calmodulin | 246 | 2.70E-08 | 60 |
| Efet.01.134348.g455.t1 | Calmodulin-A | 315 | 6.49E-15 | 60 |
| Efet.01.46657.g1394.t1 | CD109 antigen | 465 | 2.55E-11 | 60 |
| Efet.01.118853.g974.t1 | Cadherin EGF LAG seven-pass G-type receptor 2 | 252 | 9.00E-10 | 60 |
| Efet.01.9024.g663.t1 | Centrin-2 | 360 | 2.14E-07 | 60 |
| Efet.01.267648.g652.t1 | 10 kDa heat shock protein, mitochondrial | 339 | 1.04E-11 | 60 |
| Efet.01.117982.g924.t1 | Chromodomain-helicase-DNA-binding protein 7 | 357 | 1.01E-36 | 60 |
| Efet.01.294674.g1588.t1 | Chromodomain-helicase-DNA-binding protein 7 | 1122 | 7.26E-13 | 60 |
| Efet.01.622883.g1006.t1 | Chromodomain-helicase-DNA-binding protein 8 | 219 | 1.93E-14 | 60 |
| Efet.01.57000.g434.t1 | Acidic mammalian chitinase | 246 | 7.73E-13 | 60 |
| Efet.01.29309.g300.t1 | Collagen alpha-3(VI) chain | 264 | 7.68E-15 | 60 |
| Efet.01.605763.g237.t1 | Collagen alpha-6(VI) chain | 333 | 9.46E-12 | 60 |
| Efet.01.339583.g1161.t1 | Carbamoyl-phosphate synthase [ammonia], mitochondrial | 615 | 1.15E-53 | 60 |
| Efet.01.73865.g1469.t1 | Alpha-crystallin B chain | 1110 | 3.16E-11 | 60 |
| Efet.01.27820.g185.t1 | Cubilin | 465 | 2.76E-21 | 60 |
| Efet.01.229790.g1189.t1 | Cubilin | 285 | 7.82E-06 | 60 |
| Efet.01.562842.g66.t1 | Cubilin | 270 | 1.06E-14 | 60 |
| Efet.01.1438.g137.t1 | Cullin-4A | 246 | 5.19E-24 | 60 |
| Efet.01.657766.g1422.t1 | Cytochrome c | 378 | 1.76E-25 | 60 |
| Efet.01.548172.g443.t1 | Disabled homolog 1 | 744 | 5.57E-23 | 60 |
| Efet.01.314930.g473.t1 | Discoidin domain-containing receptor 2 | 630 | 2.73E-34 | 60 |
| Efet.01.363551.g361.t1 | Discoidin domain-containing receptor 2 | 1476 | 5.50E-32 | 60 |
| Efet.01.32672.g536.t1 | Probable ATP-dependent RNA helicase DDX41 | 384 | 1.06E-09 | 60 |
| Efet.01.208865.g401.t1 | Protein diaphanous homolog 2 | 327 | 2.53E-09 | 60 |
| Efet.01.95726.g1201.t1 | Disks large homolog 5 | 321 | 1.13E-06 | 60 |
| Efet.01.242998.g1637.t1 | Deleted in malignant brain tumors 1 protein | 237 | 2.83E-07 | 60 |
| Efet.01.372691.g636.t1 | DnaJ homolog subfamily A member 3, mitochondrial | 741 | 6.51E-13 | 60 |
| Efet.01.623265.g1017.t1 | Dedicator of cytokinesis protein 7 | 291 | 4.63E-11 | 60 |
| Efet.01.146303.g1069.t1 | Down syndrome cell adhesion molecule | 450 | 6.58E-29 | 60 |
| Efet.01.159648.g505.t1 | Dystonin | 3519 | 0 | 60 |
| Efet.01.206695.g294.t1 | Endothelin-1 receptor | 393 | 1.06E-08 | 60 |
| Efet.01.621374.g933.t1 | Ephrin type-A receptor 3 | 354 | 2.84E-17 | 60 |
| Efet.01.31664.g468.t1 | DNA repair protein complementing XP-G cells | 387 | 1.13E-23 | 60 |
| Efet.01.16513.g1218.t1 | Estrogen receptor beta | 339 | 8.88E-08 | 60 |
| Efet.01.272790.g849.t1 | MDS1 and EVI1 complex locus protein EVI1 | 225 | 1.03E-06 | 60 |
| Efet.01.359684.g263.t1 | MDS1 and EVI1 complex locus protein EVI1 | 639 | 3.32E-09 | 60 |
| Efet.01.10949.g803.t1 | Exocyst complex component 4 | 531 | 3.98E-24 | 60 |
| Efet.01.52345.g159.t1 | Fatty acid-binding protein, adipocyte | 315 | 8.46E-14 | 60 |
| Efet.01.362790.g344.t1 | Protocadherin Fat 3 | 342 | 4.80E-06 | 60 |
| Efet.01.325919.g797.t1 | Fez family zinc finger protein 2 | 660 | 2.99E-18 | 60 |
| Efet.01.466200.g434.t1 | Peptidyl-prolyl cis-trans isomerase FKBP4 | 369 | 1.64E-18 | 60 |
| Efet.01.272635.g840.t1 | Filamin-A | 267 | 2.28E-17 | 60 |
| Efet.01.151237.g79.t1 | FRAS1-related extracellular matrix protein 1 | 627 | 1.92E-38 | 60 |
| Efet.01.109181.g478.t1 | GTP-binding protein GEM | 408 | 8.04E-17 | 60 |
| Efet.01.638760.g464.t1 | GTP-binding protein GEM | 237 | 1.60E-06 | 60 |
| Efet.01.71527.g1334.t1 | Glomulin | 387 | 1.18E-17 | 60 |
| Efet.01.53353.g226.t1 | Glucagon receptor | 462 | 1.67E-07 | 60 |
| Efet.01.654656.g669.t1 | Stress-70 protein, mitochondrial | 1869 | 1.03E-131 | 60 |
| Efet.01.201280.g66.t1 | Histone deacetylase 4 | 576 | 2.89E-21 | 60 |
| Efet.01.477481.g701.t1 | Hepatocyte nuclear factor 6 | 1092 | 7.22E-13 | 60 |
| Efet.01.231715.g1266.t1 | Homeobox protein Hox-A3 | 312 | 8.66E-17 | 60 |
| Efet.01.276421.g976.t1 | Intraflagellar transport protein 172 homolog | 366 | 3.47E-36 | 60 |
| Efet.01.481701.g784.t1 | Interferon-related developmental regulator 1 | 354 | 4.82E-13 | 60 |
| Efet.01.618860.g851.t1 | Immunoglobulin superfamily member 22 | 510 | 3.49E-18 | 60 |
| Efet.01.627540.g1211.t1 | Inosine-5'-monophosphate dehydrogenase 2 | 609 | 1.48E-32 | 60 |
| Efet.01.351434.g42.t1 | Interferon regulatory factor 2 | 954 | 1.45E-14 | 60 |
| Efet.01.557284.g686.t1 | KN motif and ankyrin repeat domain-containing protein 1 | 342 | 1.94E-11 | 60 |
| Efet.01.37320.g815.t1 | cAMP-dependent protein kinase type II-alpha regulatory subunit | 927 | 2.42E-76 | 60 |
| Efet.01.9249.g677.t1 | Histone acetyltransferase KAT8 | 1026 | 1.51E-79 | 60 |
| Efet.01.18637.g1384.t1 | Calcium-activated potassium channel subunit alpha-1 | 681 | 1.42E-72 | 60 |
| Efet.01.97684.g1322.t1 | Calcium-activated potassium channel subunit alpha-1 | 693 | 2.48E-57 | 60 |
| Efet.01.276451.g977.t1 | Calcium-activated potassium channel subunit alpha-1 | 459 | 1.28E-20 | 60 |
| Efet.01.347523.g1343.t1 | Potassium voltage-gated channel subfamily H member 8 | 480 | 4.72E-24 | 60 |
| Efet.01.232969.g1307.t1 | Kelch-like protein 13 | 978 | 1.03E-47 | 60 |
| Efet.01.52885.g199.t1 | Kelch-like protein 20 | 396 | 1.50E-06 | 60 |
| Efet.01.193777.g2035.t1 | Kelch-like protein 20 | 411 | 6.22E-27 | 60 |
| Efet.01.251665.g61.t1 | Kelch-like protein 20 | 459 | 1.56E-23 | 60 |
| Efet.01.330998.g941.t1 | Kelch-like protein 20 | 483 | 9.33E-18 | 60 |
| Efet.01.175928.g1208.t1 | Serine/threonine-protein kinase D3 | 537 | 3.64E-34 | 60 |
| Efet.01.5268.g411.t1 | Ribosomal protein S6 kinase alpha-5 | 1212 | 1.33E-27 | 60 |
| Efet.01.455425.g141.t1 | LIM/homeobox protein Lhx4 | 330 | 9.23E-36 | 60 |
| Efet.01.115982.g832.t1 | Leucine-rich repeats and immunoglobulin-like domains protein 3 | 339 | 1.56E-09 | 60 |
| Efet.01.43596.g1205.t1 | Mitogen-activated protein kinase kinase kinase 10 | 396 | 1.86E-09 | 60 |
| Efet.01.40530.g1025.t1 | Methyltransferase-like protein 13 | 492 | 2.49E-22 | 60 |
| Efet.01.203125.g150.t1 | MICAL-like protein 2 | 279 | 2.88E-16 | 60 |
| Efet.01.103468.g185.t1 | Microphthalmia-associated transcription factor | 1887 | 3.06E-07 | 60 |
| Efet.01.655750.g856.t1 | Methylmalonate-semialdehyde dehydrogenase [acylating], mitochondrial | 1557 | 2.53E-123 | 60 |
| Efet.01.103454.g183.t1 | Myosin-9 | 288 | 8.50E-07 | 60 |
| Efet.01.25013.g2.t1 | Neuronal calcium sensor 1 | 468 | 3.54E-21 | 60 |
| Efet.01.248938.g1857.t1 | Neuronal calcium sensor 1 | 213 | 4.76E-10 | 60 |
| Efet.01.620247.g889.t1 | Netrin-1 | 300 | 1.48E-19 | 60 |
| Efet.01.128524.g202.t1 | Neuroligin-3 | 1002 | 4.88E-08 | 60 |
| Efet.01.483397.g818.t1 | Neuroligin-4, Y-linked | 525 | 1.55E-22 | 60 |
| Efet.01.526119.g606.t1 | Neurexin-3 | 348 | 5.11E-11 | 60 |
| Efet.01.547414.g425.t1 | Neurexin-3 | 432 | 4.36E-24 | 60 |
| Efet.01.2882.g259.t1 | N-terminal Xaa-Pro-Lys N-methyltransferase 1 | 645 | 6.74E-41 | 60 |
| Efet.01.1651955.g536.t1 | Polyadenylate-binding protein 4 | 261 | 1.66E-11 | 60 |
| Efet.01.218753.g759.t1 | Protein kinase C and casein kinase substrate in neurons protein 2 | 438 | 1.71E-19 | 60 |
| Efet.01.595474.g1156.t1 | Plasminogen activator inhibitor 2 | 564 | 6.38E-32 | 60 |
| Efet.01.226961.g1079.t1 | Partitioning defective 3 homolog | 618 | 1.84E-13 | 60 |
| Efet.01.525310.g590.t1 | Protocadherin-23 | 468 | 5.41E-26 | 60 |
| Efet.01.464304.g377.t1 | High affinity cAMP-specific 3',5'-cyclic phosphodiesterase 7A | 924 | 1.01E-06 | 60 |
| Efet.01.264332.g536.t1 | Peptidyl-prolyl cis-trans isomerase NIMA-interacting 1 | 246 | 1.37E-06 | 60 |
| Efet.01.112717.g641.t1 | Phosphatidylinositol 4,5-bisphosphate 3-kinase catalytic subunit beta isoform | 315 | 3.39E-12 | 60 |
| Efet.01.102773.g158.t1 | POC1 centriolar protein homolog A | 351 | 6.24E-08 | 60 |
| Efet.01.151322.g85.t1 | Peptidyl-prolyl cis-trans isomerase-like 3 | 1359 | 1.02E-38 | 60 |
| Efet.01.118045.g929.t1 | Receptor-type tyrosine-protein phosphatase S | 654 | 5.90E-19 | 60 |
| Efet.01.644746.g895.t1 | Dihydroorotate dehydrogenase (quinone), mitochondrial | 1083 | 1.07E-78 | 60 |
| Efet.01.657961.g1543.t1 | Macrophage receptor with collagenous structure | 297 | 1.47E-06 | 60 |
| Efet.01.653365.g423.t1 | Chemokine (C-C motif) receptor 1 | 606 | 4.54E-17 | 60 |
| Efet.01.186906.g1721.t1 | Zinc finger protein 3 | 825 | 1.40E-54 | 60 |
| Efet.01.209372.g421.t1 | Zinc finger protein 3 | 894 | 2.99E-61 | 60 |
| Efet.01.236595.g1426.t1 | Zinc finger protein 3 | 675 | 2.68E-51 | 60 |
| Efet.01.627560.g1213.t1 | Zinc finger protein 3 | 699 | 1.98E-43 | 60 |
| Efet.01.471393.g573.t1 | Transporter 2 isoform (Transporter 2, ATP-binding cassette, sub-family B (MDR/TAP), isoform CRA_a) | 261 | 9.71E-09 | 60 |
| Efet.01.158986.g469.t1 | Ras-related protein Rab-11A | 270 | 6.49E-06 | 60 |
| Efet.01.181964.g1499.t1 | Double-stranded RNA-specific editase 1 | 1554 | 3.67E-11 | 60 |
| Efet.01.608299.g343.t1 | Reelin | 486 | 3.50E-25 | 60 |
| Efet.01.48741.g1502.t1 | Ras-specific guanine nucleotide-releasing factor 1 | 357 | 8.15E-11 | 60 |
| Efet.01.166453.g816.t1 | Ras-specific guanine nucleotide-releasing factor 1 | 267 | 2.07E-07 | 60 |
| Efet.01.124546.g1266.t1 | Rho GTPase-activating protein 4 | 2091 | 4.64E-52 | 60 |
| Efet.01.137754.g654.t1 | Rho GTPase-activating protein 17 | 480 | 2.77E-27 | 60 |
| Efet.01.200.g27.t1 | Roundabout homolog 1 | 504 | 3.50E-39 | 60 |
| Efet.01.649586.g1331.t1 | Roundabout homolog 2 | 228 | 5.19E-12 | 60 |
| Efet.01.592788.g1090.t1 | Protein RRP5 homolog | 282 | 1.76E-14 | 60 |
| Efet.01.319872.g637.t1 | Radial spoke head protein 9 homolog | 822 | 2.16E-60 | 60 |
| Efet.01.3265.g278.t1 | RUN and FYVE domain-containing protein 1 | 354 | 1.93E-17 | 60 |
| Efet.01.61556.g717.t1 | Tyrosine-protein kinase RYK | 420 | 4.04E-25 | 60 |
| Efet.01.149819.g1218.t1 | Sodium-coupled monocarboxylate transporter 1 | 396 | 1.25E-13 | 60 |
| Efet.01.408894.g210.t1 | Sodium/hydrogen exchanger 3 | 213 | 3.21E-09 | 60 |
| Efet.01.171446.g1033.t1 | Superoxide dismutase [Mn], mitochondrial | 423 | 4.34E-21 | 60 |
| Efet.01.398399.g1202.t1 | Superoxide dismutase [Mn], mitochondrial | 558 | 9.25E-41 | 60 |
| Efet.01.486631.g889.t1 | Sortilin-related receptor | 249 | 1.11E-10 | 60 |
| Efet.01.303203.g98.t1 | Spastin | 501 | 2.66E-22 | 60 |
| Efet.01.388854.g986.t1 | Spermatogenesis-associated protein 4 | 771 | 7.74E-32 | 60 |
| Efet.01.90951.g940.t1 | Proto-oncogene tyrosine-protein kinase Src | 321 | 2.29E-07 | 60 |
| Efet.01.1657876.g1049.t1 | Serine racemase | 969 | 1.10E-67 | 60 |
| Efet.01.246000.g1732.t1 | SCO-spondin | 450 | 3.25E-27 | 60 |
| Efet.01.515782.g391.t1 | Tenascin-R | 216 | 1.72E-18 | 60 |
| Efet.01.230423.g1210.t1 | Tensin-4 | 393 | 1.41E-27 | 60 |
| Efet.01.333597.g1005.t1 | THAP domain-containing protein 2 | 900 | 5.62E-08 | 60 |
| Efet.01.657308.g1230.t1 | Thioredoxin, mitochondrial | 909 | 3.49E-20 | 60 |
| Efet.01.458360.g230.t1 | Troponin C, skeletal muscle | 225 | 1.13E-07 | 60 |
| Efet.01.430892.g759.t1 | E3 ubiquitin-protein ligase TRIP12 | 801 | 1.28E-49 | 60 |
| Efet.01.268958.g701.t1 | Protein turtle homolog B | 399 | 1.67E-20 | 60 |
| Efet.01.73184.g1432.t1 | Ubiquitin-conjugating enzyme E2 D3 | 372 | 6.91E-24 | 60 |
| Efet.01.99654.g1425.t1 | Ubiquitin-protein ligase E3A | 321 | 9.03E-13 | 60 |
| Efet.01.16762.g1239.t1 | Ubiquitin carboxyl-terminal hydrolase 14 | 366 | 6.67E-06 | 60 |
| Efet.01.139856.g759.t1 | E3 ubiquitin-protein ligase UBR1 | 372 | 6.02E-09 | 60 |
| Efet.01.656828.g1124.t1 | UDP-glucuronosyltransferase 1-1 | 765 | 8.50E-48 | 60 |
| Efet.01.464979.g400.t1 | Vacuolar protein sorting-associated protein 4A | 246 | 3.32E-06 | 60 |
| Efet.01.138103.g677.t1 | Protein Wnt-4 | 447 | 5.48E-45 | 60 |
| Efet.01.121585.g1125.t1 | Transcription factor HIVEP2 | 744 | 4.04E-08 | 60 |
| Efet.01.46569.g1385.t1 | Zinc finger protein 675 | 648 | 2.18E-35 | 60 |
| Efet.01.408488.g203.t1 | Zinc finger protein 675 | 990 | 3.24E-72 | 60 |
| Efet.01.658334.g1868.t1 | Solute carrier family 11 (Proton-coupled divalent metal ion transporters), member 1 | 873 | 4.99E-32 | 59 |
| Efet.01.525779.g602.t1 | V-type proton ATPase subunit a | 258 | 3.59E-19 | 59 |
| Efet.01.232962.g1306.t1 | Tetraspanin | 246 | 1.60E-06 | 59 |
| Efet.01.76483.g87.t1 | Testicular tissue protein Li 2 | 876 | 5.24E-19 | 59 |
| Efet.01.196864.g2165.t1 | Testicular tissue protein Li 14 | 1302 | 3.99E-82 | 59 |
| Efet.01.1658864.g1301.t1 | ATP-binding cassette sub-family B member 9 | 375 | 5.50E-10 | 59 |
| Efet.01.395805.g1135.t1 | Acetylcholinesterase | 1650 | 5.71E-120 | 59 |
| Efet.01.20626.g1519.t1 | Neuronal acetylcholine receptor subunit alpha-4 | 1203 | 1.38E-47 | 59 |
| Efet.01.83040.g530.t1 | Muscarinic acetylcholine receptor M1 | 1062 | 6.68E-46 | 59 |
| Efet.01.460071.g281.t1 | Muscarinic acetylcholine receptor M1 | 1017 | 3.19E-30 | 59 |
| Efet.01.656145.g943.t1 | Long-chain-fatty-acid--CoA ligase 4 | 2298 | 2.60E-12 | 59 |
| Efet.01.19974.g1479.t1 | Actin, alpha cardiac muscle 1 | 540 | 3.02E-43 | 59 |
| Efet.01.155968.g309.t1 | Alpha-1A adrenergic receptor | 1713 | 4.56E-31 | 59 |
| Efet.01.507761.g201.t1 | Alpha-1A adrenergic receptor | 1020 | 3.83E-69 | 59 |
| Efet.01.608093.g342.t1 | Alpha-1A adrenergic receptor | 1884 | 2.04E-49 | 59 |
| Efet.01.654956.g722.t1 | Angiopoietin-related protein 1 | 387 | 2.79E-16 | 59 |
| Efet.01.45821.g1346.t1 | Ankyrin-1 | 1359 | 1.10E-33 | 59 |
| Efet.01.141582.g859.t1 | Attractin | 402 | 2.95E-23 | 59 |
| Efet.01.655451.g822.t1 | ATP-binding cassette, sub-family A (ABC1), member 1 | 435 | 3.70E-09 | 59 |
| Efet.01.178670.g1338.t1 | cDNA FLJ60590, highly similar to Interferon-inducible double strandedRNA-dependent protein kinase activator A | 246 | 1.67E-15 | 59 |
| Efet.01.67214.g1061.t1 | Tissue-type plasminogen activator | 231 | 1.50E-10 | 59 |
| Efet.01.186881.g1718.t1 | cDNA FLJ59231, highly similar to C-ets-1 protein | 327 | 8.21E-09 | 59 |
| Efet.01.493463.g1033.t1 | Zinc finger transcription factor BCL6S | 273 | 4.02E-10 | 59 |
| Efet.01.97250.g1297.t1 | ATP-binding cassette transporter A1 | 1107 | 3.15E-18 | 59 |
| Efet.01.533832.g138.t1 | BRCA1-associated RING domain protein 1 | 438 | 7.93E-15 | 59 |
| Efet.01.386779.g943.t1 | B-cell lymphoma 6 protein | 321 | 1.98E-21 | 59 |
| Efet.01.148789.g1170.t1 | Brefeldin A-inhibited guanine nucleotide-exchange protein 1 | 1653 | 3.46E-07 | 59 |
| Efet.01.9033.g665.t1 | Calnexin | 429 | 8.80E-12 | 59 |
| Efet.01.439950.g948.t1 | Calpain-10 | 432 | 4.41E-21 | 59 |
| Efet.01.86574.g727.t1 | Cholecystokinin receptor type A | 579 | 1.69E-06 | 59 |
| Efet.01.226540.g1059.t1 | Cholecystokinin receptor type A | 564 | 1.87E-15 | 59 |
| Efet.01.449436.g1203.t1 | Cyclin-dependent-like kinase 5 | 591 | 5.25E-19 | 59 |
| Efet.01.93046.g1056.t1 | Cyclin-dependent kinase inhibitor 1C | 621 | 2.45E-10 | 59 |
| Efet.01.309519.g301.t1 | Ceramide kinase | 348 | 3.30E-21 | 59 |
| Efet.01.627540.g1212.t1 | 10 kDa heat shock protein, mitochondrial | 324 | 7.02E-12 | 59 |
| Efet.01.13356.g999.t1 | Glutathione-specific gamma-glutamylcyclotransferase 1 | 354 | 1.64E-19 | 59 |
| Efet.01.654418.g594.t1 | Carbamoyl-phosphate synthase [ammonia], mitochondrial | 3105 | 0 | 59 |
| Efet.01.88010.g799.t1 | Calsyntenin-2 | 324 | 1.04E-12 | 59 |
| Efet.01.96273.g1232.t1 | Connective tissue growth factor | 279 | 1.17E-11 | 59 |
| Efet.01.132446.g373.t1 | Discoidin domain-containing receptor 2 | 1182 | 2.12E-26 | 59 |
| Efet.01.634415.g245.t1 | Probable ATP-dependent RNA helicase DDX41 | 603 | 4.65E-36 | 59 |
| Efet.01.317115.g545.t1 | Dehydrogenase/reductase SDR family member 2, mitochondrial | 309 | 1.47E-11 | 59 |
| Efet.01.593134.g1096.t1 | Dehydrogenase/reductase SDR family member 2, mitochondrial | 399 | 4.40E-07 | 59 |
| Efet.01.101567.g103.t1 | Docking protein 1 | 564 | 1.40E-11 | 59 |
| Efet.01.25268.g34.t1 | D(2) dopamine receptor | 273 | 1.97E-16 | 59 |
| Efet.01.68955.g1170.t1 | D(2) dopamine receptor | 465 | 2.58E-15 | 59 |
| Efet.01.288863.g1413.t1 | D(3) dopamine receptor | 1041 | 1.76E-16 | 59 |
| Efet.01.90307.g918.t1 | EMILIN-1 | 423 | 1.73E-08 | 59 |
| Efet.01.116915.g881.t1 | Epidermal growth factor receptor substrate 15-like 1 | 2157 | 1.01E-108 | 59 |
| Efet.01.129079.g226.t1 | Coagulation factor XI | 399 | 1.83E-11 | 59 |
| Efet.01.614863.g674.t1 | Coagulation factor XI | 258 | 3.26E-10 | 59 |
| Efet.01.315709.g504.t1 | Protocadherin Fat 1 | 348 | 4.19E-11 | 59 |
| Efet.01.451768.g44.t1 | Protocadherin Fat 1 | 276 | 6.03E-07 | 59 |
| Efet.01.175771.g1201.t1 | Protocadherin Fat 4 | 441 | 2.63E-23 | 59 |
| Efet.01.139023.g723.t1 | Protein fem-1 homolog B | 1875 | 2.10E-142 | 59 |
| Efet.01.7158.g546.t1 | Fascin | 393 | 2.79E-16 | 59 |
| Efet.01.554376.g613.t1 | Fascin | 675 | 9.60E-46 | 59 |
| Efet.01.19840.g1470.t1 | Frizzled-4 | 2031 | 8.77E-119 | 59 |
| Efet.01.69315.g1190.t1 | Polypeptide N-acetylgalactosaminyltransferase 2 | 1869 | 3.80E-127 | 59 |
| Efet.01.118190.g941.t1 | Gastrin/cholecystokinin type B receptor | 447 | 6.51E-13 | 59 |
| Efet.01.608039.g333.t1 | N-acetyllactosaminide beta-1,6-N-acetylglucosaminyl-transferase | 207 | 1.96E-07 | 59 |
| Efet.01.52250.g151.t1 | Glutamate receptor ionotropic, kainate 2 | 240 | 4.18E-10 | 59 |
| Efet.01.657551.g1328.t1 | Glutamate receptor ionotropic, kainate 2 | 846 | 2.85E-56 | 59 |
| Efet.01.565356.g181.t1 | Solute carrier family 2, facilitated glucose transporter member 4 | 795 | 1.24E-39 | 59 |
| Efet.01.1639369.g206.t1 | Hemicentin-1 | 270 | 2.05E-08 | 59 |
| Efet.01.599423.g1291.t1 | Heparan sulfate glucosamine 3-O-sulfotransferase 5 | 726 | 8.42E-17 | 59 |
| Efet.01.44391.g1250.t1 | Insulin-like growth factor 2 mRNA-binding protein 1 | 216 | 8.38E-10 | 59 |
| Efet.01.64706.g897.t1 | Insulin-like growth factor 2 mRNA-binding protein 1 | 687 | 8.09E-18 | 59 |
| Efet.01.312852.g408.t1 | Insulin-like growth factor 1 receptor | 510 | 9.19E-27 | 59 |
| Efet.01.497157.g1116.t1 | Immunoglobulin-like and fibronectin type III domain-containing protein 1 | 351 | 2.21E-19 | 59 |
| Efet.01.205237.g225.t1 | E3 ubiquitin-protein ligase Itchy homolog | 1548 | 1.67E-57 | 59 |
| Efet.01.336929.g1089.t1 | Tenascin | 345 | 1.15E-06 | 59 |
| Efet.01.542389.g311.t1 | Signal transducer and activator of transcription | 216 | 1.25E-09 | 59 |
| Efet.01.232250.g1282.t1 | Anosmin-1 | 468 | 9.39E-29 | 59 |
| Efet.01.38692.g913.t1 | KN motif and ankyrin repeat domain-containing protein 1 | 561 | 1.93E-10 | 59 |
| Efet.01.81412.g430.t1 | Potassium voltage-gated channel subfamily H member 8 | 744 | 9.77E-47 | 59 |
| Efet.01.528809.g661.t1 | Potassium voltage-gated channel subfamily H member 8 | 474 | 6.06E-08 | 59 |
| Efet.01.634762.g260.t1 | Potassium voltage-gated channel subfamily H member 8 | 549 | 1.00E-32 | 59 |
| Efet.01.234090.g1340.t1 | ATP-sensitive inward rectifier potassium channel 8 | 1473 | 3.67E-79 | 59 |
| Efet.01.449263.g1200.t1 | ATP-sensitive inward rectifier potassium channel 8 | 252 | 1.03E-10 | 59 |
| Efet.01.21004.g1552.t1 | Kinesin-like protein KIF1B | 3768 | 3.18E-80 | 59 |
| Efet.01.27021.g135.t1 | Kelch-like protein 20 | 225 | 6.42E-11 | 59 |
| Efet.01.29663.g317.t1 | Lethal(2) giant larvae protein homolog 2 | 855 | 3.23E-50 | 59 |
| Efet.01.186953.g1724.t1 | Lethal(2) giant larvae protein homolog 2 | 528 | 4.62E-21 | 59 |
| Efet.01.32294.g509.t1 | LARGE xylosyl- and glucuronyltransferase 2 | 948 | 1.48E-101 | 59 |
| Efet.01.1658718.g1255.t1 | Lipoyl synthase, mitochondrial | 963 | 1.09E-69 | 59 |
| Efet.01.1619149.g53.t1 | Low-density lipoprotein receptor-related protein 12 | 201 | 1.08E-06 | 59 |
| Efet.01.579669.g663.t1 | Low-density lipoprotein receptor-related protein 2 | 783 | 1.53E-49 | 59 |
| Efet.01.104527.g253.t1 | Leucine-rich repeat serine/threonine-protein kinase 2 | 462 | 1.49E-23 | 59 |
| Efet.01.603047.g122.t1 | Latent-transforming growth factor beta-binding protein 4 | 405 | 5.39E-26 | 59 |
| Efet.01.21591.g1586.t1 | Multidrug resistance protein 1 | 345 | 5.89E-24 | 59 |
| Efet.01.74090.g1489.t1 | Multidrug resistance protein 1 | 426 | 8.65E-21 | 59 |
| Efet.01.656322.g997.t1 | Stromelysin-1 | 462 | 4.10E-08 | 59 |
| Efet.01.653787.g475.t1 | Mucin-5B | 213 | 7.27E-09 | 59 |
| Efet.01.72525.g1387.t1 | Unconventional myosin-Ie | 612 | 6.89E-10 | 59 |
| Efet.01.173358.g1110.t1 | Myeloid zinc finger 1 | 432 | 1.18E-10 | 59 |
| Efet.01.90661.g927.t1 | Neogenin | 564 | 2.48E-46 | 59 |
| Efet.01.486960.g895.t1 | Neprilysin | 912 | 7.95E-14 | 59 |
| Efet.01.205990.g259.t1 | Neurofilament light polypeptide | 381 | 4.04E-10 | 59 |
| Efet.01.345963.g1297.t1 | Neurofilament light polypeptide | 492 | 5.59E-14 | 59 |
| Efet.01.434538.g831.t1 | Neurofilament light polypeptide | 387 | 1.32E-10 | 59 |
| Efet.01.30203.g351.t1 | Nipped-B-like protein | 375 | 9.18E-16 | 59 |
| Efet.01.65341.g933.t1 | Substance-P receptor | 459 | 1.47E-12 | 59 |
| Efet.01.321268.g668.t1 | Substance-P receptor | 417 | 2.13E-09 | 59 |
| Efet.01.190176.g1878.t1 | Homeobox protein Nkx-2.6 | 915 | 6.53E-16 | 59 |
| Efet.01.1145.g106.t1 | Neuropeptide Y receptor type 5 | 219 | 1.44E-11 | 59 |
| Efet.01.160487.g549.t1 | Neuropeptide Y receptor type 5 | 477 | 1.02E-09 | 59 |
| Efet.01.428610.g696.t1 | Neurexin-2 | 525 | 4.13E-19 | 59 |
| Efet.01.381835.g836.t1 | Neurexin-3 | 870 | 2.47E-29 | 59 |
| Efet.01.114445.g753.t1 | Ileal sodium/bile acid cotransporter | 801 | 6.29E-18 | 59 |
| Efet.01.428255.g686.t1 | Neurotensin receptor type 1 | 630 | 2.43E-13 | 59 |
| Efet.01.617383.g775.t1 | Endonuclease G, mitochondrial | 1074 | 4.93E-54 | 59 |
| Efet.01.591317.g1036.t1 | Transcription factor Ovo-like 2 | 1281 | 2.35E-09 | 59 |
| Efet.01.61418.g708.t1 | Polyadenylate-binding protein 4 | 1083 | 1.08E-09 | 59 |
| Efet.01.296318.g1626.t1 | Partitioning defective 3 homolog | 837 | 6.25E-22 | 59 |
| Efet.01.91681.g981.t1 | Poly [ADP-ribose] polymerase 1 | 603 | 7.91E-37 | 59 |
| Efet.01.201192.g60.t1 | Protocadherin-19 | 705 | 1.02E-31 | 59 |
| Efet.01.257790.g289.t1 | Protocadherin gamma-A11 | 363 | 2.02E-17 | 59 |
| Efet.01.548789.g465.t1 | PDZ and LIM domain protein 7 | 252 | 2.84E-12 | 59 |
| Efet.01.205760.g250.t1 | 1-phosphatidylinositol 4,5-bisphosphate phosphodiesterase gamma-1 | 345 | 7.05E-19 | 59 |
| Efet.01.1659226.g1471.t1 | Proline dehydrogenase 1, mitochondrial | 690 | 7.40E-47 | 59 |
| Efet.01.172508.g1071.t1 | 26S proteasome non-ATPase regulatory subunit 10 | 843 | 8.87E-07 | 59 |
| Efet.01.165905.g792.t1 | Tubulin beta chain | 747 | 3.30E-63 | 59 |
| Efet.01.241537.g1584.t1 | Radial spoke head 10 homolog B2 | 897 | 6.09E-07 | 59 |
| Efet.01.11508.g839.t1 | Ras-related protein Rab-4A | 288 | 5.76E-11 | 59 |
| Efet.01.119901.g1027.t1 | Putative RNA-binding protein 15 | 1137 | 8.35E-32 | 59 |
| Efet.01.128805.g214.t1 | Rho GTPase-activating protein 27 | 375 | 4.90E-21 | 59 |
| Efet.01.649448.g1308.t1 | Roundabout homolog 1 | 258 | 1.09E-08 | 59 |
| Efet.01.500602.g13.t1 | DNA-directed RNA polymerase III subunit RPC1 | 1653 | 4.00E-13 | 59 |
| Efet.01.59586.g606.t1 | 40S ribosomal protein SA | 369 | 5.27E-20 | 59 |
| Efet.01.348325.g1367.t1 | Relaxin receptor 2 | 633 | 1.75E-31 | 59 |
| Efet.01.250659.g17.t1 | Pendrin | 1593 | 7.06E-57 | 59 |
| Efet.01.285707.g1291.t1 | Sodium channel protein type 2 subunit alpha | 303 | 2.58E-13 | 59 |
| Efet.01.599013.g1276.t1 | Protein scribble homolog | 207 | 3.03E-12 | 59 |
| Efet.01.100110.g5.t1 | SH2B adapter protein 2 | 681 | 6.71E-07 | 59 |
| Efet.01.626070.g1138.t1 | Slit homolog 1 protein | 267 | 3.12E-17 | 59 |
| Efet.01.90712.g929.t1 | SPARC-related modular calcium-binding protein 1 | 411 | 3.73E-11 | 59 |
| Efet.01.80572.g377.t1 | Zinc finger protein SNAI1 | 2013 | 9.17E-12 | 59 |
| Efet.01.625264.g1111.t1 | Superoxide dismutase [Mn], mitochondrial | 678 | 4.82E-48 | 59 |
| Efet.01.4040.g342.t1 | Sushi, von Willebrand factor type A, EGF and pentraxin domain-containing protein 1 | 1053 | 1.59E-31 | 59 |
| Efet.01.89634.g869.t1 | Nucleolysin TIAR | 411 | 4.43E-13 | 59 |
| Efet.01.346087.g1301.t1 | Tropomyosin alpha-1 chain | 291 | 5.93E-06 | 59 |
| Efet.01.123153.g1200.t1 | E3 ubiquitin-protein ligase TRIM33 | 768 | 3.49E-06 | 59 |
| Efet.01.592151.g1064.t1 | Ubiquitin carboxyl-terminal hydrolase 4 | 924 | 8.43E-28 | 59 |
| Efet.01.71147.g1306.t1 | Zinc finger protein 675 | 666 | 5.74E-59 | 59 |
| Efet.01.146998.g1100.t1 | Zinc finger protein 675 | 1311 | 3.65E-83 | 59 |
| Efet.01.544463.g348.t1 | Zinc finger protein 675 | 1539 | 1.20E-94 | 59 |
| Efet.01.556705.g673.t1 | 5-hydroxytryptamine receptor 2B | 1374 | 1.57E-21 | 58 |
| Efet.01.450404.g11.t1 | Interleukin enhancer binding factor 3, 90kDa | 1341 | 5.31E-09 | 58 |
| Efet.01.348645.g1374.t1 | Tetraspanin | 276 | 1.63E-06 | 58 |
| Efet.01.1656424.g864.t1 | DDX39B (HCG2005638, isoform CRA_a) | 456 | 8.85E-17 | 58 |
| Efet.01.34783.g673.t1 | Acetylcholinesterase | 1587 | 1.02E-132 | 58 |
| Efet.01.516565.g413.t1 | Acetylcholinesterase | 1530 | 5.97E-125 | 58 |
| Efet.01.541120.g294.t1 | Neuronal acetylcholine receptor subunit alpha-7 | 570 | 2.87E-23 | 58 |
| Efet.01.612104.g522.t1 | Gamma-adducin | 852 | 3.40E-34 | 58 |
| Efet.01.85693.g672.t1 | Type-1 angiotensin II receptor | 729 | 9.93E-10 | 58 |
| Efet.01.655821.g885.t1 | Retinal dehydrogenase 2 | 1458 | 1.74E-109 | 58 |
| Efet.01.1659316.g1547.t1 | Aldehyde dehydrogenase family 1 member A3 | 1470 | 3.42E-99 | 58 |
| Efet.01.644987.g918.t1 | Aldehyde dehydrogenase, mitochondrial | 540 | 5.42E-35 | 58 |
| Efet.01.657312.g1236.t1 | Homeobox protein aristaless-like 4 | 471 | 1.86E-07 | 58 |
| Efet.01.634241.g242.t1 | Cytosol aminopeptidase | 576 | 3.55E-35 | 58 |
| Efet.01.1644605.g304.t1 | Ankyrin-1 | 252 | 9.25E-06 | 58 |
| Efet.01.1653250.g605.t1 | Ankyrin-1 | 447 | 2.73E-19 | 58 |
| Efet.01.31463.g449.t1 | Aquaporin-4 | 825 | 5.59E-41 | 58 |
| Efet.01.112073.g611.t1 | Neuroepithelial cell-transforming gene 1 protein | 387 | 8.17E-27 | 58 |
| Efet.01.319321.g620.t1 | Rho guanine nucleotide exchange factor 17 | 717 | 1.87E-54 | 58 |
| Efet.01.62716.g791.t1 | Sarcoplasmic/endoplasmic reticulum calcium ATPase 1 | 1314 | 5.79E-50 | 58 |
| Efet.01.364437.g392.t1 | A disintegrin and metalloproteinase with thrombospondin motifs 20 | 444 | 5.42E-20 | 58 |
| Efet.01.91857.g990.t1 | Band 3 anion transport protein | 219 | 4.74E-10 | 58 |
| Efet.01.551491.g534.t1 | Band 3 anion transport protein | 276 | 1.48E-12 | 58 |
| Efet.01.20312.g1499.t1 | BLM protein | 699 | 2.29E-35 | 58 |
| Efet.01.552138.g553.t1 | BTB/POZ domain-containing adapter for CUL3-mediated RhoA degradation protein 2 | 234 | 3.74E-12 | 58 |
| Efet.01.44674.g1276.t1 | Protein bicaudal D homolog 2 | 1965 | 7.50E-123 | 58 |
| Efet.01.33261.g565.t1 | Baculoviral IAP repeat-containing protein 2 | 648 | 1.96E-07 | 58 |
| Efet.01.651586.g200.t1 | CREB-binding protein | 408 | 5.98E-11 | 58 |
| Efet.01.398129.g1195.t1 | Cyclin-dependent kinase 11A | 435 | 3.65E-18 | 58 |
| Efet.01.164554.g740.t1 | Cyclin-dependent kinase 11B | 930 | 6.65E-46 | 58 |
| Efet.01.583689.g790.t1 | Cyclin-dependent kinase inhibitor 1B | 351 | 1.00E-09 | 58 |
| Efet.01.194729.g2082.t1 | Cadherin EGF LAG seven-pass G-type receptor 2 | 3327 | 0 | 58 |
| Efet.01.152511.g139.t1 | Contactin-5 | 399 | 9.96E-18 | 58 |
| Efet.01.25127.g13.t1 | Centriolin | 705 | 1.34E-07 | 58 |
| Efet.01.152661.g145.t1 | Collagen alpha-6(VI) chain | 246 | 7.70E-12 | 58 |
| Efet.01.618629.g839.t1 | Collagen alpha-1(VII) chain | 831 | 3.34E-08 | 58 |
| Efet.01.227331.g1094.t1 | Collagen alpha-1(IX) chain | 246 | 2.73E-09 | 58 |
| Efet.01.323764.g734.t1 | Collagen alpha-1(XII) chain | 336 | 1.85E-13 | 58 |
| Efet.01.116192.g843.t1 | Collagen alpha-1(XIV) chain | 327 | 3.63E-15 | 58 |
| Efet.01.271704.g812.t1 | Cytochrome P450 1A1 | 813 | 1.62E-29 | 58 |
| Efet.01.453264.g71.t1 | Calsyntenin-1 | 480 | 4.48E-31 | 58 |
| Efet.01.446106.g1142.t1 | Cubilin | 234 | 4.58E-10 | 58 |
| Efet.01.515957.g397.t1 | Cullin-1 | 432 | 8.09E-38 | 58 |
| Efet.01.168346.g904.t1 | Dystroglycan | 921 | 5.21E-25 | 58 |
| Efet.01.85957.g693.t1 | Probable ATP-dependent RNA helicase DDX20 | 348 | 5.10E-16 | 58 |
| Efet.01.82999.g524.t1 | Probable ATP-dependent RNA helicase DDX41 | 957 | 4.07E-71 | 58 |
| Efet.01.156618.g345.t1 | ATP-dependent RNA helicase DDX42 | 2325 | 2.14E-91 | 58 |
| Efet.01.62833.g795.t1 | Diacylglycerol kinase beta | 372 | 6.84E-18 | 58 |
| Efet.01.658250.g1752.t1 | Disks large homolog 1 | 441 | 4.09E-14 | 58 |
| Efet.01.268360.g678.t1 | Dual specificity protein phosphatase 3 | 1656 | 1.20E-21 | 58 |
| Efet.01.30539.g374.t1 | Dual specificity protein phosphatase 6 | 3570 | 6.12E-08 | 58 |
| Efet.01.198283.g2226.t1 | Dual specificity protein phosphatase 6 | 453 | 2.81E-09 | 58 |
| Efet.01.101219.g80.t1 | Dystonin | 624 | 4.80E-33 | 58 |
| Efet.01.34460.g650.t1 | Eukaryotic translation initiation factor 2-alpha kinase 3 | 249 | 5.78E-06 | 58 |
| Efet.01.77086.g117.t1 | Band 4.1-like protein 5 | 1155 | 1.19E-19 | 58 |
| Efet.01.243079.g1638.t1 | Receptor tyrosine-protein kinase erbB-4 | 636 | 9.15E-22 | 58 |
| Efet.01.217538.g721.t1 | DNA excision repair protein ERCC-6-like | 276 | 1.95E-08 | 58 |
| Efet.01.642149.g669.t1 | Eukaryotic peptide chain release factor GTP-binding subunit ERF3A | 201 | 3.89E-09 | 58 |
| Efet.01.17494.g1290.t1 | MDS1 and EVI1 complex locus protein EVI1 | 1092 | 1.95E-13 | 58 |
| Efet.01.120627.g1075.t1 | Fanconi anemia group I protein | 1176 | 4.94E-24 | 58 |
| Efet.01.201747.g86.t1 | Protocadherin Fat 1 | 777 | 2.32E-51 | 58 |
| Efet.01.39720.g978.t1 | Protocadherin Fat 4 | 1368 | 3.02E-16 | 58 |
| Efet.01.491629.g988.t1 | Fibroblast growth factor receptor 3 | 396 | 4.84E-21 | 58 |
| Efet.01.28842.g265.t1 | Filamin-A | 1365 | 6.42E-08 | 58 |
| Efet.01.52277.g153.t1 | Filamin-A | 1362 | 1.47E-91 | 58 |
| Efet.01.245206.g1702.t1 | Filamin-A | 354 | 1.90E-18 | 58 |
| Efet.01.642550.g698.t1 | Glucose-6-phosphate isomerase | 2835 | 1.70E-129 | 58 |
| Efet.01.200062.g5.t1 | RON variant E2E3 | 294 | 4.02E-08 | 58 |
| Efet.01.562142.g40.t1 | Zinc finger protein GLI2 | 366 | 2.49E-21 | 58 |
| Efet.01.20071.g1485.t1 | N-acetyllactosaminide beta-1,6-N-acetylglucosaminyl-transferase | 309 | 9.35E-09 | 58 |
| Efet.01.242364.g1618.t1 | N-acetyllactosaminide beta-1,6-N-acetylglucosaminyl-transferase | 690 | 3.14E-41 | 58 |
| Efet.01.33719.g600.t1 | E3 ubiquitin-protein ligase RNF130 | 207 | 1.65E-10 | 58 |
| Efet.01.11807.g872.t1 | Hyaluronan-binding protein 2 | 258 | 9.38E-11 | 58 |
| Efet.01.27500.g158.t1 | Hemicentin-1 | 258 | 7.02E-10 | 58 |
| Efet.01.160746.g559.t1 | Hemicentin-1 | 222 | 7.75E-12 | 58 |
| Efet.01.369812.g539.t1 | Hemicentin-1 | 570 | 4.12E-33 | 58 |
| Efet.01.504602.g120.t1 | Heparan sulfate glucosamine 3-O-sulfotransferase 5 | 645 | 3.27E-56 | 58 |
| Efet.01.427672.g673.t1 | Intraflagellar transport protein 81 homolog | 345 | 6.65E-25 | 58 |
| Efet.01.658322.g1854.t1 | Intraflagellar transport protein 88 homolog | 1740 | 1.17E-104 | 58 |
| Efet.01.213966.g595.t1 | Interferon regulatory factor 2 | 678 | 8.85E-09 | 58 |
| Efet.01.1304.g119.t1 | Integrin alpha-V | 423 | 9.12E-14 | 58 |
| Efet.01.80523.g371.t1 | cAMP-dependent protein kinase catalytic subunit gamma | 339 | 7.96E-06 | 58 |
| Efet.01.41703.g1101.t1 | Calcium-activated potassium channel subunit alpha-1 | 465 | 1.53E-19 | 58 |
| Efet.01.544550.g351.t1 | Potassium voltage-gated channel subfamily H member 8 | 390 | 1.52E-12 | 58 |
| Efet.01.1657052.g936.t1 | Potassium voltage-gated channel subfamily H member 8 | 447 | 1.29E-22 | 58 |
| Efet.01.25348.g41.t1 | Kinesin-like protein KIF1B | 294 | 4.68E-13 | 58 |
| Efet.01.63028.g802.t1 | Kinesin-like protein KIF1B | 243 | 6.41E-10 | 58 |
| Efet.01.226864.g1072.t1 | Kelch-like protein 21 | 348 | 1.97E-21 | 58 |
| Efet.01.87721.g784.t1 | Laminin subunit alpha-5 | 750 | 1.32E-22 | 58 |
| Efet.01.160917.g570.t1 | Protein lifeguard 2 | 297 | 2.35E-11 | 58 |
| Efet.01.62805.g793.t1 | Low-density lipoprotein receptor-related protein 2 | 558 | 1.40E-30 | 58 |
| Efet.01.553639.g603.t1 | Multidrug resistance protein 1 | 402 | 1.69E-10 | 58 |
| Efet.01.475707.g669.t1 | Stromelysin-1 | 210 | 1.48E-16 | 58 |
| Efet.01.460565.g302.t1 | Myosin-9 | 522 | 5.01E-11 | 58 |
| Efet.01.463735.g368.t1 | Myosin-9 | 375 | 1.18E-17 | 58 |
| Efet.01.655057.g743.t1 | Myosin light chain 1/3, skeletal muscle isoform | 201 | 1.50E-06 | 58 |
| Efet.01.451554.g36.t1 | Myeloid zinc finger 1 | 780 | 3.53E-10 | 58 |
| Efet.01.128690.g209.t1 | Neurocan core protein | 327 | 3.04E-08 | 58 |
| Efet.01.357087.g201.t1 | Neuronal calcium sensor 1 | 249 | 1.72E-11 | 58 |
| Efet.01.61400.g707.t1 | Neprilysin | 240 | 8.99E-10 | 58 |
| Efet.01.658319.g1852.t1 | Netrin-1 | 339 | 2.69E-18 | 58 |
| Efet.01.1655711.g783.t1 | Substance-P receptor | 438 | 7.46E-17 | 58 |
| Efet.01.54879.g328.t1 | Glutamate receptor ionotropic, NMDA 2A | 495 | 2.53E-26 | 58 |
| Efet.01.216373.g682.t1 | Nitric oxide synthase, brain | 315 | 5.87E-11 | 58 |
| Efet.01.550416.g517.t1 | Neurotensin receptor type 1 | 627 | 7.50E-08 | 58 |
| Efet.01.1645469.g323.t1 | Obscurin | 252 | 4.25E-12 | 58 |
| Efet.01.28159.g206.t1 | Kappa-type opioid receptor | 861 | 5.69E-43 | 58 |
| Efet.01.322182.g693.t1 | Protocadherin alpha-13 | 804 | 3.46E-40 | 58 |
| Efet.01.586228.g872.t1 | Peroxisomal trans-2-enoyl-CoA reductase | 774 | 2.92E-09 | 58 |
| Efet.01.152944.g158.t1 | Basement membrane-specific heparan sulfate proteoglycan core protein | 267 | 4.82E-12 | 58 |
| Efet.01.274540.g896.t1 | Phosphatidylinositol 4-kinase beta | 348 | 9.57E-15 | 58 |
| Efet.01.361764.g313.t1 | Phospholipase A-2-activating protein | 333 | 9.66E-10 | 58 |
| Efet.01.81350.g428.t1 | 1-phosphatidylinositol 4,5-bisphosphate phosphodiesterase delta-4 | 483 | 3.93E-31 | 58 |
| Efet.01.203301.g156.t1 | Serine/threonine-protein kinase PLK3 | 570 | 2.26E-36 | 58 |
| Efet.01.606117.g253.t1 | Serine/threonine-protein kinase PLK3 | 300 | 2.29E-08 | 58 |
| Efet.01.403912.g100.t1 | POC1 centriolar protein homolog A | 345 | 5.03E-08 | 58 |
| Efet.01.516348.g407.t1 | Peptidyl-prolyl cis-trans isomerase-like 4 | 777 | 6.03E-10 | 58 |
| Efet.01.318747.g602.t1 | Thioredoxin-dependent peroxide reductase, mitochondrial | 576 | 1.65E-40 | 58 |
| Efet.01.1659522.g1991.t1 | Peroxiredoxin-5, mitochondrial | 507 | 4.28E-37 | 58 |
| Efet.01.183596.g1578.t1 | Inactive tyrosine-protein kinase 7 | 315 | 4.52E-13 | 58 |
| Efet.01.22804.g1661.t1 | Receptor-type tyrosine-protein phosphatase S | 402 | 3.13E-16 | 58 |
| Efet.01.339111.g1140.t1 | Receptor-type tyrosine-protein phosphatase S | 687 | 3.20E-48 | 58 |
| Efet.01.78179.g186.t1 | Early growth response protein | 342 | 2.47E-16 | 58 |
| Efet.01.324841.g765.t1 | Chemokine (C-C motif) receptor 1 | 810 | 1.69E-34 | 58 |
| Efet.01.279731.g1090.t1 | Neuropilin | 291 | 6.73E-12 | 58 |
| Efet.01.15688.g1154.t1 | Zinc finger protein 3 | 1071 | 1.98E-53 | 58 |
| Efet.01.265246.g565.t1 | NFKBIE protein | 285 | 3.43E-09 | 58 |
| Efet.01.120447.g1064.t1 | Pro-interleukin-16 | 213 | 1.89E-07 | 58 |
| Efet.01.512594.g299.t1 | GTPase HRas | 726 | 6.61E-26 | 58 |
| Efet.01.222309.g902.t1 | Rho GTPase-activating protein 5 | 723 | 1.27E-38 | 58 |
| Efet.01.647991.g1212.t1 | E3 ubiquitin-protein ligase RNF34 | 252 | 1.35E-07 | 58 |
| Efet.01.266681.g610.t1 | Nuclear receptor ROR-alpha | 408 | 9.36E-12 | 58 |
| Efet.01.454754.g114.t1 | Relaxin receptor 2 | 330 | 3.22E-13 | 58 |
| Efet.01.653970.g505.t1 | Sodium-coupled monocarboxylate transporter 1 | 306 | 5.38E-11 | 58 |
| Efet.01.9340.g685.t1 | Sodium channel protein type 2 subunit alpha | 303 | 4.26E-06 | 58 |
| Efet.01.175657.g1197.t1 | Sodium channel protein type 2 subunit alpha | 321 | 1.82E-14 | 58 |
| Efet.01.86692.g733.t1 | Helicase SKI2W | 426 | 3.76E-13 | 58 |
| Efet.01.555721.g650.t1 | Transmembrane protein 161A | 912 | 1.70E-47 | 58 |
| Efet.01.1649902.g455.t1 | Tenascin-X | 399 | 1.13E-17 | 58 |
| Efet.01.649471.g1314.t1 | Thioredoxin, mitochondrial | 336 | 6.26E-18 | 58 |
| Efet.01.228315.g1144.t1 | Nucleolysin TIAR | 381 | 4.81E-12 | 58 |
| Efet.01.70319.g1249.t1 | Tumor necrosis factor alpha-induced protein 3 | 549 | 2.15E-27 | 58 |
| Efet.01.187085.g1737.t1 | Protein turtle homolog B | 348 | 1.96E-12 | 58 |
| Efet.01.229279.g1175.t1 | Ubiquitin-protein ligase E3A | 1392 | 3.77E-83 | 58 |
| Efet.01.120660.g1077.t1 | Vasopressin V1b receptor | 684 | 1.65E-16 | 58 |
| Efet.01.1324.g121.t1 | Vinexin | 843 | 1.09E-09 | 58 |
| Efet.01.162818.g649.t1 | Protein Wnt-2 | 225 | 1.52E-12 | 58 |
| Efet.01.1653796.g638.t1 | Protein Wnt-4 | 387 | 3.41E-30 | 58 |
| Efet.01.629351.g1288.t1 | Protein Wnt-7a | 447 | 9.53E-26 | 58 |
| Efet.01.79702.g304.t1 | WSC domain-containing protein 2 | 237 | 9.43E-07 | 58 |
| Efet.01.31892.g481.t1 | Vasopressin V1a receptor | 711 | 2.29E-25 | 58 |
| Efet.01.101039.g71.t1 | Vasopressin V1a receptor | 462 | 8.52E-23 | 58 |
| Efet.01.1659476.g1804.t1 | Xanthine dehydrogenase/oxidase | 1401 | 4.71E-112 | 58 |
| Efet.01.249907.g1884.t1 | YTH domain-containing family protein 2 | 1383 | 1.65E-99 | 58 |
| Efet.01.295634.g1605.t1 | Transcription factor HIVEP3 | 573 | 2.29E-09 | 58 |
| Efet.01.46589.g1388.t1 | Zinc finger protein 675 | 1455 | 7.30E-90 | 58 |
| Efet.01.269859.g737.t1 | Zinc finger protein 675 | 996 | 4.08E-79 | 58 |
| Efet.01.407630.g177.t1 | Zinc finger protein 675 | 819 | 6.17E-65 | 58 |
| Efet.01.657269.g1221.t1 | Zinc finger protein 675 | 378 | 9.81E-23 | 58 |
| Efet.01.1656600.g889.t1 | Receptor (TNFRSF)-interacting serine-threonine kinase 1, isoform CRA_a | 258 | 2.64E-11 | 57 |
| Efet.01.352643.g74.t1 | Serine/threonine-protein phosphatase | 282 | 6.68E-10 | 57 |
| Efet.01.85576.g661.t1 | APOBEC1 complementation factor | 231 | 1.07E-06 | 57 |
| Efet.01.1657589.g1012.t1 | ATP-binding cassette sub-family A member 5 | 810 | 8.39E-15 | 57 |
| Efet.01.655767.g871.t1 | ATP-binding cassette sub-family F member 1 | 501 | 5.01E-15 | 57 |
| Efet.01.20247.g1495.t1 | Neuronal acetylcholine receptor subunit alpha-4 | 222 | 3.81E-12 | 57 |
| Efet.01.196959.g2169.t1 | Neuronal acetylcholine receptor subunit alpha-4 | 474 | 2.68E-23 | 57 |
| Efet.01.360459.g281.t1 | Muscarinic acetylcholine receptor M1 | 1758 | 2.72E-21 | 57 |
| Efet.01.59460.g598.t1 | Alpha-actinin-3 | 399 | 1.83E-15 | 57 |
| Efet.01.307993.g236.t1 | Retinal dehydrogenase 2 | 663 | 2.40E-30 | 57 |
| Efet.01.654034.g527.t1 | Retinal dehydrogenase 2 | 1434 | 6.72E-93 | 57 |
| Efet.01.1659026.g1367.t1 | Retinal dehydrogenase 2 | 1008 | 1.23E-72 | 57 |
| Efet.01.1659480.g1819.t1 | Retinal dehydrogenase 2 | 1782 | 5.97E-99 | 57 |
| Efet.01.657876.g1513.t1 | Aldehyde dehydrogenase family 1 member A3 | 1521 | 8.07E-118 | 57 |
| Efet.01.89766.g878.t1 | Homeobox protein aristaless-like 4 | 519 | 3.76E-17 | 57 |
| Efet.01.174166.g1138.t1 | Aminopeptidase N | 354 | 8.85E-18 | 57 |
| Efet.01.1658843.g1295.t1 | Angiopoietin-related protein 1 | 630 | 3.19E-24 | 57 |
| Efet.01.52756.g193.t1 | Ankyrin-1 | 1107 | 5.22E-09 | 57 |
| Efet.01.124679.g1279.t1 | Ankyrin-1 | 339 | 4.87E-07 | 57 |
| Efet.01.315491.g490.t1 | AP-1 complex subunit gamma-1 | 456 | 4.45E-17 | 57 |
| Efet.01.51183.g81.t1 | Aquaporin-4 | 279 | 3.57E-12 | 57 |
| Efet.01.62079.g755.t1 | Rho guanine nucleotide exchange factor 4 | 357 | 5.48E-13 | 57 |
| Efet.01.636111.g317.t1 | Rho guanine nucleotide exchange factor 11 | 477 | 6.31E-06 | 57 |
| Efet.01.88465.g814.t1 | Sarcoplasmic/endoplasmic reticulum calcium ATPase 1 | 825 | 3.69E-46 | 57 |
| Efet.01.462860.g351.t1 | Lactosylceramide 1,3-N-acetyl-beta-D-glucosaminyltransferase | 1101 | 4.28E-42 | 57 |
| Efet.01.149454.g1205.t1 | Tubulin beta chain | 447 | 9.63E-21 | 57 |
| Efet.01.479453.g749.t1 | Zinc finger transcription factor BCL6S | 2100 | 6.57E-08 | 57 |
| Efet.01.378919.g776.t1 | BLM protein | 1185 | 2.95E-85 | 57 |
| Efet.01.229219.g1168.t1 | Bardet-Biedl syndrome 7 protein | 1731 | 1.42E-124 | 57 |
| Efet.01.123452.g1216.t1 | Baculoviral IAP repeat-containing protein 2 | 699 | 1.73E-08 | 57 |
| Efet.01.346237.g1305.t1 | Bcl-2-related ovarian killer protein | 522 | 3.13E-27 | 57 |
| Efet.01.310032.g316.t1 | Protein BTG1 | 504 | 6.84E-35 | 57 |
| Efet.01.605603.g231.t1 | Tyrosine-protein kinase BTK | 3231 | 1.58E-68 | 57 |
| Efet.01.543955.g340.t1 | Cadherin-11 | 609 | 1.76E-34 | 57 |
| Efet.01.318823.g606.t1 | Cadherin-7 | 447 | 1.21E-23 | 57 |
| Efet.01.126086.g59.t1 | Calmodulin-regulated spectrin-associated protein 3 | 1398 | 4.95E-06 | 57 |
| Efet.01.65039.g911.t1 | Cholecystokinin receptor type A | 462 | 1.30E-06 | 57 |
| Efet.01.138652.g698.t1 | G1/S-specific cyclin-D3 | 405 | 1.24E-09 | 57 |
| Efet.01.77982.g173.t1 | CD109 antigen | 489 | 1.78E-24 | 57 |
| Efet.01.14070.g1039.t1 | Cyclin-dependent kinase inhibitor 2A | 1053 | 6.32E-06 | 57 |
| Efet.01.257013.g264.t1 | Cadherin EGF LAG seven-pass G-type receptor 3 | 654 | 4.04E-24 | 57 |
| Efet.01.340783.g1183.t1 | CAP-Gly domain-containing linker protein 1 | 1911 | 1.16E-10 | 57 |
| Efet.01.74415.g1514.t1 | Collagen alpha-6(VI) chain | 612 | 1.24E-07 | 57 |
| Efet.01.477628.g711.t1 | Collagen alpha-6(VI) chain | 360 | 7.57E-06 | 57 |
| Efet.01.59281.g580.t1 | Collagen alpha-2(XI) chain | 498 | 4.06E-12 | 57 |
| Efet.01.588431.g946.t1 | Collagen alpha-1(XIV) chain | 510 | 4.39E-16 | 57 |
| Efet.01.233781.g1328.t1 | Cytochrome P450 1A1 | 516 | 3.69E-17 | 57 |
| Efet.01.239492.g1521.t1 | Carbamoyl-phosphate synthase [ammonia], mitochondrial | 648 | 7.40E-25 | 57 |
| Efet.01.233779.g1327.t1 | Cubilin | 378 | 1.02E-22 | 57 |
| Efet.01.216626.g688.t1 | Death-associated protein kinase 1 | 846 | 8.06E-12 | 57 |
| Efet.01.38230.g878.t1 | DNA damage-induced apoptosis suppressor protein | 285 | 2.02E-12 | 57 |
| Efet.01.1655466.g761.t1 | Diacylglycerol kinase epsilon | 684 | 5.99E-43 | 57 |
| Efet.01.653346.g420.t1 | Dehydrogenase/reductase SDR family member 2, mitochondrial | 1143 | 1.65E-06 | 57 |
| Efet.01.232820.g1302.t1 | D(4) dopamine receptor | 387 | 2.51E-20 | 57 |
| Efet.01.145056.g1018.t1 | Dual specificity protein phosphatase 1 | 507 | 7.47E-07 | 57 |
| Efet.01.262124.g451.t1 | Dual specificity protein phosphatase 1 | 531 | 6.00E-26 | 57 |
| Efet.01.186854.g1716.t1 | Dual specificity protein phosphatase 3 | 1908 | 4.23E-20 | 57 |
| Efet.01.624178.g1063.t1 | Dystonin | 606 | 6.64E-23 | 57 |
| Efet.01.214397.g610.t1 | C-Jun-amino-terminal kinase-interacting protein 3 | 627 | 4.94E-25 | 57 |
| Efet.01.400102.g2.t1 | Receptor tyrosine-protein kinase erbB-4 | 597 | 2.36E-09 | 57 |
| Efet.01.132814.g390.t1 | Protocadherin Fat 3 | 456 | 2.76E-10 | 57 |
| Efet.01.1643610.g282.t1 | Protocadherin Fat 3 | 312 | 8.35E-13 | 57 |
| Efet.01.464105.g375.t1 | Filamin-A | 360 | 9.72E-17 | 57 |
| Efet.01.289089.g1421.t1 | Dimethylaniline monooxygenase [N-oxide-forming] 1 | 618 | 3.89E-40 | 57 |
| Efet.01.164792.g752.t1 | Alpha-(1,3)-fucosyltransferase 4 | 366 | 6.37E-14 | 57 |
| Efet.01.16642.g1228.t1 | Growth hormone secretagogue receptor type 1 | 540 | 4.86E-15 | 57 |
| Efet.01.1632146.g118.t1 | Growth hormone secretagogue receptor type 1 | 303 | 1.61E-09 | 57 |
| Efet.01.439592.g939.t1 | Histone deacetylase 7 | 222 | 8.54E-10 | 57 |
| Efet.01.449160.g1196.t1 | Heat shock factor protein 2 | 528 | 2.34E-06 | 57 |
| Efet.01.550874.g522.t1 | Putative heat shock 70 kDa protein 7 | 522 | 2.92E-24 | 57 |
| Efet.01.428633.g698.t1 | Immunoglobulin superfamily member 22 | 576 | 1.08E-32 | 57 |
| Efet.01.51746.g113.t1 | NF-kappa-B inhibitor zeta | 225 | 1.92E-06 | 57 |
| Efet.01.52636.g184.t1 | Keratin, type I cytoskeletal 18 | 453 | 8.73E-07 | 57 |
| Efet.01.48095.g1473.t1 | Histone acetyltransferase KAT2A | 678 | 2.34E-22 | 57 |
| Efet.01.618826.g848.t1 | Histone acetyltransferase KAT2A | 345 | 2.68E-19 | 57 |
| Efet.01.136013.g549.t1 | Calcium-activated potassium channel subunit alpha-1 | 441 | 1.38E-19 | 57 |
| Efet.01.130371.g291.t1 | ATP-sensitive inward rectifier potassium channel 8 | 660 | 2.19E-41 | 57 |
| Efet.01.569311.g290.t1 | Kinesin-like protein KIF13B | 528 | 3.12E-12 | 57 |
| Efet.01.84441.g591.t1 | Kelch-like protein 20 | 708 | 3.52E-29 | 57 |
| Efet.01.213727.g585.t1 | Histone-lysine N-methyltransferase 2A | 2355 | 6.20E-09 | 57 |
| Efet.01.146892.g1097.t1 | Histone-lysine N-methyltransferase 2C | 1578 | 1.64E-127 | 57 |
| Efet.01.299058.g1718.t1 | Lethal(3)malignant brain tumor-like protein 1 | 312 | 2.82E-12 | 57 |
| Efet.01.47719.g1455.t1 | LIM domain only protein 7 | 366 | 4.09E-10 | 57 |
| Efet.01.452252.g56.t1 | Prolow-density lipoprotein receptor-related protein 1 | 948 | 2.84E-30 | 57 |
| Efet.01.657036.g1156.t1 | Low-density lipoprotein receptor-related protein 2 | 678 | 5.96E-43 | 57 |
| Efet.01.219618.g797.t1 | Low-density lipoprotein receptor-related protein 6 | 531 | 1.27E-11 | 57 |
| Efet.01.576662.g536.t1 | Limbic system-associated membrane protein | 285 | 1.53E-08 | 57 |
| Efet.01.108219.g426.t1 | Mitogen-activated protein kinase kinase kinase 10 | 429 | 3.73E-19 | 57 |
| Efet.01.156916.g361.t1 | Serine/threonine-protein kinase MARK1 | 288 | 3.35E-07 | 57 |
| Efet.01.215501.g647.t1 | MAP/microtubule affinity-regulating kinase 3 | 1182 | 1.18E-42 | 57 |
| Efet.01.657766.g1453.t1 | Multidrug resistance protein 1 | 1749 | 7.62E-35 | 57 |
| Efet.01.433171.g804.t1 | Cation-independent mannose-6-phosphate receptor | 747 | 8.41E-21 | 57 |
| Efet.01.1372.g125.t1 | Myosin-10 | 438 | 1.18E-08 | 57 |
| Efet.01.14695.g1074.t1 | Myosin-binding protein C, cardiac-type | 360 | 1.40E-13 | 57 |
| Efet.01.656123.g942.t1 | Neuronal calcium sensor 1 | 486 | 1.28E-20 | 57 |
| Efet.01.28907.g271.t1 | Neogenin | 465 | 9.29E-13 | 57 |
| Efet.01.134877.g485.t1 | Neogenin | 291 | 4.87E-08 | 57 |
| Efet.01.67347.g1068.t1 | Neurofilament light polypeptide | 516 | 1.39E-09 | 57 |
| Efet.01.130388.g293.t1 | Neurofilament light polypeptide | 579 | 3.96E-12 | 57 |
| Efet.01.369905.g550.t1 | Neuroligin-4, Y-linked | 738 | 1.82E-23 | 57 |
| Efet.01.324176.g746.t1 | Neurogenic locus notch homolog protein 1 | 564 | 9.22E-26 | 57 |
| Efet.01.86475.g721.t1 | Neurogenic locus notch homolog protein 2 | 1011 | 1.68E-69 | 57 |
| Efet.01.27763.g179.t1 | Neuropeptide Y receptor type 5 | 840 | 1.90E-25 | 57 |
| Efet.01.85609.g663.t1 | Neurexin-3 | 555 | 2.11E-34 | 57 |
| Efet.01.210782.g476.t1 | Kappa-type opioid receptor | 1074 | 1.84E-23 | 57 |
| Efet.01.1649513.g440.t1 | Mu-type opioid receptor | 519 | 7.49E-06 | 57 |
| Efet.01.457697.g207.t1 | TCDD-inducible poly [ADP-ribose] polymerase | 408 | 5.06E-22 | 57 |
| Efet.01.441139.g1007.t1 | Paired box protein Pax-3 | 522 | 1.21E-16 | 57 |
| Efet.01.1496.g143.t1 | Protocadherin-11 X-linked | 564 | 3.79E-10 | 57 |
| Efet.01.390288.g1011.t1 | Protocadherin-10 | 1581 | 1.68E-18 | 57 |
| Efet.01.314672.g462.t1 | Protocadherin-16 | 1206 | 3.51E-44 | 57 |
| Efet.01.327140.g836.t1 | Protocadherin-19 | 906 | 3.54E-43 | 57 |
| Efet.01.94242.g1117.t1 | Protocadherin beta-4 | 342 | 2.05E-17 | 57 |
| Efet.01.430165.g738.t1 | Protocadherin gamma-B7 | 687 | 9.99E-40 | 57 |
| Efet.01.413810.g313.t1 | Peroxisomal trans-2-enoyl-CoA reductase | 780 | 2.18E-36 | 57 |
| Efet.01.569313.g296.t1 | Peroxisomal trans-2-enoyl-CoA reductase | 354 | 2.61E-13 | 57 |
| Efet.01.117335.g901.t1 | ATP-dependent 6-phosphofructokinase, platelet type | 570 | 1.19E-59 | 57 |
| Efet.01.554660.g622.t1 | ATP-dependent 6-phosphofructokinase, platelet type | 477 | 6.49E-36 | 57 |
| Efet.01.126454.g85.t1 | Zinc finger protein PLAGL1 | 1284 | 1.58E-06 | 57 |
| Efet.01.14779.g1084.t1 | Peptidyl-prolyl cis-trans isomerase B | 531 | 3.95E-45 | 57 |
| Efet.01.355886.g165.t1 | Protein phosphatase 1 regulatory subunit 15A | 249 | 4.39E-06 | 57 |
| Efet.01.387979.g969.t1 | Inactive tyrosine-protein kinase 7 | 360 | 3.15E-12 | 57 |
| Efet.01.345177.g1273.t1 | Tyrosine-protein phosphatase non-receptor type 13 | 1131 | 2.29E-08 | 57 |
| Efet.01.231126.g1230.t1 | Tyrosine-protein phosphatase non-receptor type 6 | 447 | 3.36E-20 | 57 |
| Efet.01.4277.g357.t1 | Receptor-type tyrosine-protein phosphatase F | 570 | 2.52E-39 | 57 |
| Efet.01.148771.g1169.t1 | Receptor-type tyrosine-protein phosphatase F | 549 | 2.20E-17 | 57 |
| Efet.01.142415.g901.t1 | MYLK protein | 5718 | 1.79E-13 | 57 |
| Efet.01.283238.g1210.t1 | Tubulin beta chain | 453 | 4.56E-28 | 57 |
| Efet.01.537981.g232.t1 | Chemokine (C-C motif) receptor 1 | 1044 | 8.84E-25 | 57 |
| Efet.01.186217.g1697.t1 | Zinc finger protein 3 | 435 | 7.01E-23 | 57 |
| Efet.01.347874.g1353.t1 | Zinc finger protein 3 | 849 | 1.17E-56 | 57 |
| Efet.01.356320.g177.t1 | Zinc finger protein 3 | 741 | 1.76E-51 | 57 |
| Efet.01.1631066.g111.t1 | Zinc finger protein 3 | 369 | 1.92E-28 | 57 |
| Efet.01.476046.g673.t1 | Ras-related protein Ral-B | 390 | 3.91E-20 | 57 |
| Efet.01.502787.g69.t1 | Ras-related protein Ral-B | 732 | 8.34E-26 | 57 |
| Efet.01.1594716.g18.t1 | DNA-directed RNA polymerase III subunit RPC2 | 228 | 4.35E-15 | 57 |
| Efet.01.62310.g772.t1 | Sacsin | 357 | 1.38E-11 | 57 |
| Efet.01.316166.g517.t1 | Spermidine/spermine N(1)-acetyltransferase-like protein 1 | 258 | 5.35E-10 | 57 |
| Efet.01.79666.g300.t1 | Sodium channel protein type 2 subunit alpha | 900 | 1.08E-54 | 57 |
| Efet.01.265556.g569.t1 | Sodium channel protein type 2 subunit alpha | 207 | 8.67E-08 | 57 |
| Efet.01.448506.g1186.t1 | Protein scribble homolog | 2805 | 4.89E-12 | 57 |
| Efet.01.630355.g16.t1 | Protein scribble homolog | 3561 | 4.97E-16 | 57 |
| Efet.01.242070.g1610.t1 | Secernin-1 | 513 | 1.80E-29 | 57 |
| Efet.01.316830.g535.t1 | Protein sidekick-2 | 1737 | 2.05E-57 | 57 |
| Efet.01.162228.g631.t1 | Serine/threonine-protein kinase SIK1 | 462 | 3.43E-35 | 57 |
| Efet.01.27432.g154.t1 | Paired amphipathic helix protein Sin3a | 519 | 4.29E-18 | 57 |
| Efet.01.445086.g1109.t1 | Paired amphipathic helix protein Sin3a | 1110 | 6.86E-07 | 57 |
| Efet.01.241097.g1570.t1 | Mothers against decapentaplegic homolog 6 | 429 | 1.23E-34 | 57 |
| Efet.01.478766.g731.t1 | Zinc finger protein SNAI1 | 387 | 2.27E-26 | 57 |
| Efet.01.649414.g1306.t1 | Sequestosome-1 | 336 | 2.59E-17 | 57 |
| Efet.01.377309.g736.t1 | Serine/threonine-protein kinase TBK1 | 366 | 1.37E-30 | 57 |
| Efet.01.199268.g2269.t1 | Tenascin-R | 693 | 1.79E-44 | 57 |
| Efet.01.575832.g511.t1 | Nucleolysin TIAR | 351 | 1.28E-10 | 57 |
| Efet.01.305137.g165.t1 | Protein turtle homolog B | 306 | 1.27E-13 | 57 |
| Efet.01.405644.g137.t1 | Ubiquitin carboxyl-terminal hydrolase 33 | 2769 | 0 | 57 |
| Efet.01.61428.g710.t1 | Proto-oncogene Wnt-1 | 234 | 2.77E-10 | 57 |
| Efet.01.222847.g924.t1 | Vasopressin V1a receptor | 669 | 6.68E-22 | 57 |
| Efet.01.526612.g619.t1 | Tyrosine-protein kinase receptor | 597 | 3.08E-24 | 57 |
| Efet.01.532816.g87.t1 | DNA repair protein XRCC2 | 1119 | 8.32E-27 | 57 |
| Efet.01.210718.g472.t1 | Zonadhesin | 558 | 4.78E-15 | 57 |
| Efet.01.228010.g1126.t1 | Zinc finger protein 40 | 405 | 5.96E-09 | 57 |
| Efet.01.1658606.g1227.t1 | Zinc finger protein 443 | 852 | 2.23E-61 | 57 |
| Efet.01.71147.g1304.t1 | Zinc finger protein 675 | 1485 | 9.73E-58 | 57 |
| Efet.01.123569.g1224.t1 | Zinc finger protein 675 | 732 | 8.33E-61 | 57 |
| Efet.01.138347.g684.t1 | Zinc finger protein 675 | 1968 | 2.13E-39 | 57 |
| Efet.01.227117.g1087.t1 | Zinc finger protein 3 | 861 | 3.91E-34 | 57 |
| Efet.01.298328.g1692.t1 | Serine/threonine-protein phosphatase 2A 55 kDa regulatory subunit B beta isoform | 900 | 4.05E-73 | 56 |
| Efet.01.27710.g172.t1 | 5-hydroxytryptamine receptor 2B | 1341 | 1.27E-15 | 56 |
| Efet.01.34534.g655.t1 | Serpin peptidase inhibitor, clade B (Ovalbumin), member 9 | 978 | 2.86E-60 | 56 |
| Efet.01.107327.g379.t1 | ADAM metallopeptidase domain 10, isoform CRA_b | 330 | 3.18E-13 | 56 |
| Efet.01.215816.g658.t1 | Tyrosine-protein kinase | 393 | 1.11E-11 | 56 |
| Efet.01.45767.g1344.t1 | Restin (Reed-Steinberg cell-expressed intermediate filament-associated protein) | 822 | 9.76E-55 | 56 |
| Efet.01.1659455.g1757.t1 | Acetoacetyl-CoA synthetase | 717 | 7.94E-46 | 56 |
| Efet.01.1658305.g1153.t1 | ATP-binding cassette sub-family A member 5 | 324 | 7.05E-08 | 56 |
| Efet.01.597508.g1207.t1 | ATP-binding cassette sub-family A member 7 | 372 | 3.44E-07 | 56 |
| Efet.01.658410.g2023.t1 | ATP-binding cassette sub-family A member 7 | 1500 | 1.01E-09 | 56 |
| Efet.01.657239.g1207.t1 | ATP-binding cassette sub-family B member 9 | 1533 | 5.74E-81 | 56 |
| Efet.01.24419.g1772.t1 | Alkaline ceramidase 2 | 642 | 4.67E-43 | 56 |
| Efet.01.508523.g219.t1 | Acetylcholinesterase | 1752 | 4.94E-125 | 56 |
| Efet.01.68866.g1162.t1 | Muscarinic acetylcholine receptor M1 | 1074 | 1.01E-18 | 56 |
| Efet.01.302008.g67.t1 | Alpha-1B adrenergic receptor | 2205 | 1.66E-37 | 56 |
| Efet.01.559102.g723.t1 | Alpha-1B adrenergic receptor | 375 | 2.29E-09 | 56 |
| Efet.01.607618.g320.t1 | Alpha-1B adrenergic receptor | 447 | 2.16E-08 | 56 |
| Efet.01.525686.g600.t1 | Beta-2 adrenergic receptor | 1356 | 7.50E-57 | 56 |
| Efet.01.11624.g850.t1 | Type-1 angiotensin II receptor | 855 | 5.37E-20 | 56 |
| Efet.01.427593.g671.t1 | Retinal dehydrogenase 2 | 1080 | 3.52E-83 | 56 |
| Efet.01.624347.g1071.t1 | Retinal dehydrogenase 2 | 1434 | 3.17E-99 | 56 |
| Efet.01.1654661.g701.t1 | Aldehyde dehydrogenase family 1 member A3 | 606 | 1.40E-31 | 56 |
| Efet.01.305117.g164.t1 | Ankyrin-1 | 1041 | 4.07E-09 | 56 |
| Efet.01.33314.g573.t1 | Annexin A4 | 552 | 3.50E-35 | 56 |
| Efet.01.370612.g572.t1 | Rho guanine nucleotide exchange factor 12 | 285 | 1.39E-11 | 56 |
| Efet.01.518124.g443.t1 | Rho guanine nucleotide exchange factor 16 | 213 | 8.11E-10 | 56 |
| Efet.01.171582.g1037.t1 | Sarcoplasmic/endoplasmic reticulum calcium ATPase 2 | 1194 | 6.24E-42 | 56 |
| Efet.01.364850.g400.t1 | ATP-binding cassette, sub-family A (ABC1), member 1 | 822 | 6.29E-22 | 56 |
| Efet.01.589173.g965.t1 | ATP-binding cassette, sub-family A (ABC1), member 1 | 744 | 1.76E-13 | 56 |
| Efet.01.658044.g1599.t1 | ATP-binding cassette, sub-family A (ABC1), member 1 | 453 | 3.24E-10 | 56 |
| Efet.01.191806.g1950.t1 | Adenosine receptor A2 | 342 | 4.95E-19 | 56 |
| Efet.01.1659248.g1488.t1 | BLM protein | 1254 | 4.43E-86 | 56 |
| Efet.01.34187.g634.t1 | Bone morphogenetic protein 1 | 228 | 2.71E-06 | 56 |
| Efet.01.372247.g626.t1 | Voltage-dependent P/Q-type calcium channel subunit alpha-1A | 495 | 3.89E-22 | 56 |
| Efet.01.434114.g824.t1 | Calmodulin-A | 372 | 3.64E-10 | 56 |
| Efet.01.377327.g738.t1 | Caspase recruitment domain-containing protein 9 | 609 | 3.72E-09 | 56 |
| Efet.01.51744.g112.t1 | CREB-binding protein | 825 | 8.08E-74 | 56 |
| Efet.01.115985.g833.t1 | Cholecystokinin receptor type A | 540 | 4.25E-16 | 56 |
| Efet.01.148148.g1148.t1 | Cholecystokinin receptor type A | 378 | 5.01E-12 | 56 |
| Efet.01.187525.g1761.t1 | Cholecystokinin receptor type A | 939 | 3.20E-31 | 56 |
| Efet.01.16592.g1225.t1 | Contactin-associated protein-like 3 | 390 | 2.31E-08 | 56 |
| Efet.01.629309.g1280.t1 | Collagen alpha-1(IX) chain | 414 | 3.82E-18 | 56 |
| Efet.01.37890.g858.t1 | Calsyntenin-1 | 252 | 1.68E-15 | 56 |
| Efet.01.366337.g442.t1 | Doublecortin domain-containing protein 2 | 504 | 3.45E-12 | 56 |
| Efet.01.49683.g1576.t1 | Discoidin domain-containing receptor 2 | 1257 | 1.70E-17 | 56 |
| Efet.01.369489.g529.t1 | Discoidin domain-containing receptor 2 | 558 | 9.34E-31 | 56 |
| Efet.01.218309.g745.t1 | Disks large homolog 1 | 489 | 3.69E-14 | 56 |
| Efet.01.30637.g381.t1 | D(1A) dopamine receptor | 2019 | 1.62E-40 | 56 |
| Efet.01.23843.g1728.t1 | D(2) dopamine receptor | 369 | 7.47E-09 | 56 |
| Efet.01.179495.g1367.t1 | D(4) dopamine receptor | 285 | 2.11E-21 | 56 |
| Efet.01.1649379.g434.t1 | Dystonin | 300 | 3.07E-06 | 56 |
| Efet.01.396285.g1150.t1 | C-Jun-amino-terminal kinase-interacting protein 3 | 369 | 1.34E-08 | 56 |
| Efet.01.114845.g776.t1 | Endothelin-converting enzyme 1 | 798 | 4.47E-17 | 56 |
| Efet.01.19245.g1428.t1 | Ectonucleoside triphosphate diphosphohydrolase 1 | 396 | 3.82E-09 | 56 |
| Efet.01.171370.g1028.t1 | Histone acetyltransferase p300 | 2385 | 1.73E-06 | 56 |
| Efet.01.174549.g1155.t1 | Ephrin type-A receptor 2 | 282 | 6.02E-06 | 56 |
| Efet.01.221275.g868.t1 | Receptor tyrosine-protein kinase erbB-4 | 492 | 1.55E-17 | 56 |
| Efet.01.73791.g1465.t1 | MDS1 and EVI1 complex locus protein EVI1 | 1179 | 1.83E-13 | 56 |
| Efet.01.636391.g333.t1 | Nucleolysin TIA-1 isoform p40 | 222 | 6.55E-11 | 56 |
| Efet.01.237184.g1450.t1 | Protocadherin Fat 4 | 945 | 6.95E-24 | 56 |
| Efet.01.157252.g382.t1 | Tyrosine-protein kinase Fer | 600 | 5.83E-25 | 56 |
| Efet.01.139358.g737.t1 | Filamin-A | 507 | 8.48E-53 | 56 |
| Efet.01.293437.g1550.t1 | Filamin-A | 336 | 2.31E-16 | 56 |
| Efet.01.336965.g1091.t1 | Dimethylaniline monooxygenase [N-oxide-forming] 1 | 1179 | 1.14E-52 | 56 |
| Efet.01.501813.g41.t1 | Fascin | 972 | 3.74E-46 | 56 |
| Efet.01.374880.g680.t1 | Gastrin/cholecystokinin type B receptor | 909 | 4.10E-15 | 56 |
| Efet.01.241906.g1595.t1 | Growth/differentiation factor 3 | 1068 | 1.90E-18 | 56 |
| Efet.01.95720.g1200.t1 | Growth hormone secretagogue receptor type 1 | 582 | 2.06E-09 | 56 |
| Efet.01.148158.g1149.t1 | N-acetyllactosaminide beta-1,6-N-acetylglucosaminyl-transferase | 1053 | 1.39E-44 | 56 |
| Efet.01.505346.g135.t1 | G-protein coupled receptor 84 | 2589 | 6.30E-12 | 56 |
| Efet.01.2563.g235.t1 | Histone deacetylase 4 | 612 | 1.35E-42 | 56 |
| Efet.01.90300.g917.t1 | Lymphoid-specific helicase | 231 | 1.01E-06 | 56 |
| Efet.01.64393.g884.t1 | Hemicentin-1 | 270 | 9.57E-09 | 56 |
| Efet.01.180448.g1423.t1 | Histamine H3 receptor | 573 | 6.61E-18 | 56 |
| Efet.01.558794.g717.t1 | Histamine H3 receptor | 1314 | 1.16E-32 | 56 |
| Efet.01.252907.g106.t1 | Heparan sulfate glucosamine 3-O-sulfotransferase 5 | 576 | 1.65E-38 | 56 |
| Efet.01.215751.g654.t1 | Heat shock 70 kDa protein 1-like | 261 | 4.06E-09 | 56 |
| Efet.01.86113.g696.t1 | Heat shock protein beta-1 | 702 | 1.49E-06 | 56 |
| Efet.01.606486.g280.t1 | Serine protease HTRA1 | 1185 | 3.41E-34 | 56 |
| Efet.01.78948.g239.t1 | Serine protease HTRA2, mitochondrial | 678 | 5.97E-57 | 56 |
| Efet.01.169.g18.t1 | Immunoglobulin superfamily member 22 | 318 | 5.85E-18 | 56 |
| Efet.01.144972.g1013.t1 | Immunoglobulin superfamily member 22 | 453 | 2.88E-21 | 56 |
| Efet.01.157022.g367.t1 | Immunoglobulin superfamily member 22 | 498 | 1.81E-15 | 56 |
| Efet.01.658405.g2001.t1 | Inosine-5'-monophosphate dehydrogenase 2 | 1476 | 5.38E-83 | 56 |
| Efet.01.196095.g2131.t1 | Protein Jade-1 | 798 | 2.80E-42 | 56 |
| Efet.01.658342.g1877.t1 | Histone acetyltransferase KAT2A | 1647 | 7.99E-13 | 56 |
| Efet.01.201672.g82.t1 | Kelch-like protein 20 | 963 | 4.10E-53 | 56 |
| Efet.01.109887.g509.t1 | Serine/threonine-protein kinase D1 | 315 | 3.30E-08 | 56 |
| Efet.01.79415.g284.t1 | Low-density lipoprotein receptor | 975 | 5.16E-32 | 56 |
| Efet.01.649156.g1286.t1 | LIM/homeobox protein Lhx4 | 1095 | 9.21E-10 | 56 |
| Efet.01.649489.g1319.t1 | LIM/homeobox protein Lhx4 | 4503 | 8.43E-10 | 56 |
| Efet.01.121904.g1140.t1 | Protein lin-7 homolog A | 750 | 6.06E-07 | 56 |
| Efet.01.99792.g1438.t1 | Low-density lipoprotein receptor-related protein 2 | 519 | 5.67E-24 | 56 |
| Efet.01.35015.g681.t1 | Leucine-rich repeat serine/threonine-protein kinase 2 | 498 | 2.64E-21 | 56 |
| Efet.01.155474.g279.t1 | Leucine-rich repeat neuronal protein 2 | 1050 | 9.52E-37 | 56 |
| Efet.01.387159.g947.t1 | E3 ubiquitin-protein ligase listerin | 1179 | 2.59E-75 | 56 |
| Efet.01.597811.g1238.t1 | Lysosomal-trafficking regulator | 438 | 2.13E-21 | 56 |
| Efet.01.6772.g528.t1 | Mitogen-activated protein kinase kinase kinase 10 | 432 | 1.27E-21 | 56 |
| Efet.01.42467.g1139.t1 | E3 ubiquitin-protein ligase MARCH3 | 747 | 4.59E-06 | 56 |
| Efet.01.173963.g1136.t1 | Multidrug resistance protein 1 | 201 | 2.81E-09 | 56 |
| Efet.01.458583.g243.t1 | Multidrug resistance protein 1 | 735 | 5.08E-10 | 56 |
| Efet.01.607412.g304.t1 | Multidrug resistance protein 1 | 1851 | 8.11E-24 | 56 |
| Efet.01.618287.g815.t1 | Multidrug resistance protein 1 | 597 | 5.62E-20 | 56 |
| Efet.01.638425.g437.t1 | Multidrug resistance protein 1 | 705 | 1.92E-25 | 56 |
| Efet.01.657703.g1385.t1 | Multidrug resistance protein 1 | 1314 | 3.71E-16 | 56 |
| Efet.01.536169.g187.t1 | Stromelysin-1 | 531 | 1.85E-17 | 56 |
| Efet.01.31175.g427.t1 | Myosin-9 | 420 | 1.31E-21 | 56 |
| Efet.01.38392.g890.t1 | Myosin light chain kinase, smooth muscle | 321 | 1.05E-14 | 56 |
| Efet.01.655964.g905.t1 | NADH-ubiquinone oxidoreductase 75 kDa subunit, mitochondrial | 696 | 6.28E-08 | 56 |
| Efet.01.343203.g1225.t1 | Neogenin | 660 | 5.16E-33 | 56 |
| Efet.01.505184.g132.t1 | Neogenin | 453 | 7.24E-21 | 56 |
| Efet.01.43974.g1225.t1 | Neurofilament light polypeptide | 327 | 9.17E-09 | 56 |
| Efet.01.533528.g131.t1 | Neuroligin-4, Y-linked | 546 | 1.68E-21 | 56 |
| Efet.01.85725.g678.t1 | Neurogenic locus notch homolog protein 1 | 897 | 3.39E-23 | 56 |
| Efet.01.193637.g2029.t1 | Neuropeptide Y receptor type 5 | 627 | 8.49E-20 | 56 |
| Efet.01.293547.g1554.t1 | Neurexin-3 | 615 | 1.23E-35 | 56 |
| Efet.01.294182.g1578.t1 | Peroxisomal coenzyme A diphosphatase NUDT7 | 897 | 1.64E-31 | 56 |
| Efet.01.285731.g1293.t1 | Kappa-type opioid receptor | 849 | 4.89E-16 | 56 |
| Efet.01.372426.g628.t1 | Kappa-type opioid receptor | 1119 | 7.59E-48 | 56 |
| Efet.01.196489.g2151.t1 | Polyadenylate-binding protein 4 | 309 | 2.21E-08 | 56 |
| Efet.01.1654736.g707.t1 | Plasminogen activator inhibitor 2 | 558 | 1.49E-32 | 56 |
| Efet.01.358609.g238.t1 | Partitioning defective 3 homolog | 1572 | 1.14E-08 | 56 |
| Efet.01.24190.g1758.t1 | TCDD-inducible poly [ADP-ribose] polymerase | 507 | 1.93E-23 | 56 |
| Efet.01.238211.g1485.t1 | TCDD-inducible poly [ADP-ribose] polymerase | 348 | 1.06E-15 | 56 |
| Efet.01.322947.g711.t1 | Paxillin | 465 | 5.87E-07 | 56 |
| Efet.01.547603.g434.t1 | Protocadherin-18 | 1299 | 1.55E-71 | 56 |
| Efet.01.245601.g1719.t1 | Protocadherin alpha-5 | 429 | 8.00E-18 | 56 |
| Efet.01.124113.g1244.t1 | Protocadherin alpha-9 | 738 | 4.55E-35 | 56 |
| Efet.01.535023.g163.t1 | Protein piccolo | 1359 | 1.29E-35 | 56 |
| Efet.01.153118.g169.t1 | Calcium/calmodulin-dependent 3',5'-cyclic nucleotide phosphodiesterase 1B | 519 | 8.91E-13 | 56 |
| Efet.01.235721.g1398.t1 | Homeobox protein PKNOX1 | 519 | 4.01E-20 | 56 |
| Efet.01.10961.g804.t1 | 1-phosphatidylinositol 4,5-bisphosphate phosphodiesterase delta-4 | 360 | 7.48E-19 | 56 |
| Efet.01.141017.g828.t1 | 1-phosphatidylinositol 4,5-bisphosphate phosphodiesterase gamma-1 | 543 | 2.12E-22 | 56 |
| Efet.01.361705.g312.t1 | Protein phosphatase 1F | 1098 | 2.84E-10 | 56 |
| Efet.01.20585.g1514.t1 | Pentatricopeptide repeat-containing protein 2, mitochondrial | 318 | 2.48E-12 | 56 |
| Efet.01.133987.g441.t1 | Inactive tyrosine-protein kinase 7 | 291 | 4.62E-14 | 56 |
| Efet.01.258463.g314.t1 | Inactive tyrosine-protein kinase 7 | 225 | 1.40E-12 | 56 |
| Efet.01.49027.g1525.t1 | Tyrosine-protein phosphatase non-receptor type 13 | 1125 | 9.24E-13 | 56 |
| Efet.01.365169.g407.t1 | Tyrosine-protein phosphatase non-receptor type 13 | 2529 | 2.30E-50 | 56 |
| Efet.01.180649.g1430.t1 | Receptor-type tyrosine-protein phosphatase F | 288 | 7.69E-12 | 56 |
| Efet.01.80921.g397.t1 | Receptor-type tyrosine-protein phosphatase S | 396 | 6.94E-18 | 56 |
| Efet.01.117247.g896.t1 | SARM1 protein | 1923 | 6.27E-90 | 56 |
| Efet.01.125379.g21.t1 | Interleukin 8 receptor, beta | 645 | 5.07E-07 | 56 |
| Efet.01.215417.g640.t1 | Uncharacterized protein | 909 | 2.84E-07 | 56 |
| Efet.01.81557.g437.t1 | Chemokine (C-C motif) receptor 1 | 609 | 8.53E-09 | 56 |
| Efet.01.199447.g2281.t1 | Chemokine (C-C motif) receptor 1 | 1167 | 2.96E-11 | 56 |
| Efet.01.305761.g200.t1 | Zinc finger protein 3 | 903 | 1.77E-49 | 56 |
| Efet.01.559264.g729.t1 | Zinc finger protein 3 | 498 | 9.05E-18 | 56 |
| Efet.01.286397.g1318.t1 | Hexosyltransferase | 1059 | 9.26E-19 | 56 |
| Efet.01.234175.g1347.t1 | Ras-related protein Rab-3C | 405 | 2.41E-13 | 56 |
| Efet.01.70373.g1256.t1 | Ras-related protein Rab-4A | 459 | 1.27E-16 | 56 |
| Efet.01.186014.g1689.t1 | Reelin | 429 | 1.47E-26 | 56 |
| Efet.01.159902.g519.t1 | E3 ubiquitin-protein ligase rififylin | 567 | 4.36E-10 | 56 |
| Efet.01.7979.g588.t1 | RGM domain family member B | 690 | 4.57E-33 | 56 |
| Efet.01.610928.g460.t1 | 60S ribosomal protein L11 | 525 | 6.06E-07 | 56 |
| Efet.01.252963.g110.t1 | Roundabout homolog 1 | 468 | 7.90E-17 | 56 |
| Efet.01.553529.g596.t1 | Roundabout homolog 1 | 321 | 3.73E-17 | 56 |
| Efet.01.654050.g532.t1 | Roundabout homolog 1 | 378 | 1.57E-22 | 56 |
| Efet.01.198882.g2249.t1 | Radial spoke head protein 4 homolog A | 573 | 6.90E-27 | 56 |
| Efet.01.30223.g352.t1 | Sodium-coupled monocarboxylate transporter 1 | 267 | 7.50E-12 | 56 |
| Efet.01.431156.g764.t1 | Sodium channel protein type 2 subunit alpha | 210 | 4.96E-12 | 56 |
| Efet.01.107380.g382.t1 | Protein scribble homolog | 1302 | 3.41E-14 | 56 |
| Efet.01.159430.g488.t1 | Protein scribble homolog | 819 | 4.10E-08 | 56 |
| Efet.01.236483.g1421.t1 | Protein sidekick-2 | 300 | 1.40E-11 | 56 |
| Efet.01.40583.g1029.t1 | Serine/threonine-protein kinase SIK1 | 234 | 2.75E-11 | 56 |
| Efet.01.43363.g1193.t1 | STE20-like serine/threonine-protein kinase | 666 | 8.41E-11 | 56 |
| Efet.01.230524.g1211.t1 | Solute carrier organic anion transporter family member 2A1 | 261 | 1.58E-06 | 56 |
| Efet.01.395351.g1125.t1 | Sortilin | 351 | 4.18E-08 | 56 |
| Efet.01.284784.g1255.t1 | Spectrin beta chain, erythrocytic | 387 | 4.44E-17 | 56 |
| Efet.01.38223.g876.t1 | Spectrin beta chain, non-erythrocytic 2 | 1407 | 2.06E-96 | 56 |
| Efet.01.77346.g136.t1 | Syntaxin-binding protein 1 | 477 | 6.30E-45 | 56 |
| Efet.01.250799.g24.t1 | Synaptotagmin-1 | 339 | 7.54E-11 | 56 |
| Efet.01.54977.g335.t1 | E3 ubiquitin-protein ligase synoviolin | 2247 | 6.85E-12 | 56 |
| Efet.01.27178.g140.t1 | Serine/threonine-protein kinase TBK1 | 411 | 1.81E-10 | 56 |
| Efet.01.361526.g308.t1 | Tenascin-R | 297 | 5.69E-08 | 56 |
| Efet.01.167939.g888.t1 | THO complex subunit 1 | 645 | 3.29E-23 | 56 |
| Efet.01.335070.g1051.t1 | E3 ubiquitin-protein ligase Topors | 1332 | 2.07E-06 | 56 |
| Efet.01.198926.g2254.t1 | Targeting protein for Xklp2 | 408 | 3.54E-16 | 56 |
| Efet.01.16198.g1197.t1 | TNF receptor-associated factor 6 | 957 | 1.97E-39 | 56 |
| Efet.01.160657.g554.t1 | E3 ubiquitin-protein ligase TRAIP | 300 | 7.62E-07 | 56 |
| Efet.01.564507.g134.t1 | E3 ubiquitin-protein ligase TRIM33 | 1200 | 9.16E-19 | 56 |
| Efet.01.601084.g48.t1 | E3 ubiquitin-protein ligase TRIP12 | 414 | 1.19E-28 | 56 |
| Efet.01.7581.g565.t1 | tRNA (guanine-N(7)-)-methyltransferase | 270 | 1.25E-28 | 56 |
| Efet.01.93086.g1059.t1 | Transient receptor potential cation channel subfamily V member 6 | 2001 | 9.82E-13 | 56 |
| Efet.01.8816.g644.t1 | Tumor necrosis factor-inducible gene 6 protein | 375 | 6.92E-11 | 56 |
| Efet.01.535585.g177.t1 | Protein turtle homolog A | 327 | 1.50E-20 | 56 |
| Efet.01.40313.g1009.t1 | Protein turtle homolog B | 309 | 3.26E-16 | 56 |
| Efet.01.114533.g757.t1 | V-type proton ATPase 116 kDa subunit a isoform 2 | 306 | 1.32E-18 | 56 |
| Efet.01.128015.g169.t1 | Tyrosine-protein kinase receptor | 1410 | 3.87E-61 | 56 |
| Efet.01.631414.g115.t1 | Transcription factor HIVEP2 | 3087 | 1.02E-06 | 56 |
| Efet.01.124056.g1242.t1 | Zinc finger protein 175 | 330 | 1.51E-13 | 56 |
| Efet.01.653068.g397.t1 | Zinc finger protein 443 | 1497 | 6.09E-93 | 56 |
| Efet.01.351182.g38.t1 | Zinc finger protein 675 | 1488 | 2.22E-59 | 56 |
| Efet.01.626280.g1161.t1 | Zinc finger protein 675 | 792 | 2.19E-41 | 56 |
| Efet.01.153611.g192.t1 | Serpin peptidase inhibitor, clade B (Ovalbumin), member 9 | 432 | 1.55E-16 | 55 |
| Efet.01.212596.g542.t1 | ArfGAP with FG repeats 1 isoform 4 | 336 | 3.67E-12 | 55 |
| Efet.01.576188.g518.t1 | Receptor-interacting serine-threonine kinase 2 isoform 1 | 408 | 8.63E-08 | 55 |
| Efet.01.242062.g1608.t1 | Atlastin GTPase 1 isoform 2 | 483 | 2.40E-28 | 55 |
| Efet.01.193784.g2036.t1 | Testicular tissue protein Li 70 | 588 | 2.64E-21 | 55 |
| Efet.01.211785.g513.t1 | Chemokine (C-C motif) receptor 7 | 669 | 7.01E-08 | 55 |
| Efet.01.263931.g523.t1 | APOBEC1 complementation factor | 480 | 1.61E-38 | 55 |
| Efet.01.252987.g113.t1 | Leucine-rich alpha-2-glycoprotein | 387 | 5.59E-09 | 55 |
| Efet.01.605357.g203.t1 | Acetoacetyl-CoA synthetase | 597 | 2.62E-26 | 55 |
| Efet.01.441504.g1020.t1 | 5'-AMP-activated protein kinase subunit gamma-2 | 357 | 2.53E-25 | 55 |
| Efet.01.569918.g322.t1 | ATP-binding cassette sub-family A member 5 | 558 | 3.95E-09 | 55 |
| Efet.01.654257.g555.t1 | ATP-binding cassette sub-family A member 5 | 1080 | 4.29E-25 | 55 |
| Efet.01.658423.g2105.t1 | ATP-binding cassette sub-family A member 5 | 636 | 1.12E-14 | 55 |
| Efet.01.279930.g1099.t1 | ATP-binding cassette sub-family A member 7 | 846 | 8.66E-10 | 55 |
| Efet.01.440935.g1003.t1 | ATP-binding cassette sub-family A member 7 | 1623 | 3.82E-36 | 55 |
| Efet.01.647059.g1155.t1 | ATP-binding cassette sub-family B member 9 | 957 | 1.21E-06 | 55 |
| Efet.01.458583.g242.t1 | ATP-binding cassette sub-family G member 1 | 624 | 3.60E-09 | 55 |
| Efet.01.275960.g965.t1 | Acetylcholinesterase | 1215 | 5.39E-92 | 55 |
| Efet.01.41390.g1080.t1 | Neuronal acetylcholine receptor subunit alpha-7 | 513 | 3.88E-17 | 55 |
| Efet.01.21119.g1561.t1 | Neuronal acetylcholine receptor subunit beta-2 | 1260 | 6.38E-74 | 55 |
| Efet.01.277426.g1011.t1 | Peroxisomal acyl-coenzyme A oxidase 1 | 1170 | 6.89E-27 | 55 |
| Efet.01.56912.g428.t1 | Beta-2 adrenergic receptor | 1413 | 7.75E-68 | 55 |
| Efet.01.121917.g1141.t1 | Type-1 angiotensin II receptor | 1215 | 4.64E-08 | 55 |
| Efet.01.657855.g1508.t1 | Retinal dehydrogenase 2 | 1491 | 9.56E-76 | 55 |
| Efet.01.657838.g1503.t1 | Aldehyde dehydrogenase family 1 member A3 | 1173 | 5.30E-29 | 55 |
| Efet.01.5235.g408.t1 | Ankyrin-1 | 1269 | 1.96E-12 | 55 |
| Efet.01.20745.g1533.t1 | Ankyrin-1 | 780 | 2.22E-14 | 55 |
| Efet.01.582094.g747.t1 | Ankyrin-1 | 597 | 2.23E-24 | 55 |
| Efet.01.625433.g1114.t1 | Aldehyde oxidase | 654 | 4.67E-27 | 55 |
| Efet.01.58092.g504.t1 | Apoptotic protease-activating factor 1 | 357 | 3.90E-09 | 55 |
| Efet.01.66879.g1036.t1 | ADP-ribosylation factor 6 | 417 | 4.52E-28 | 55 |
| Efet.01.8147.g603.t1 | Rho guanine nucleotide exchange factor 12 | 1035 | 1.89E-55 | 55 |
| Efet.01.222759.g915.t1 | Rho guanine nucleotide exchange factor 12 | 2067 | 4.93E-06 | 55 |
| Efet.01.3848.g327.t1 | AT-rich interactive domain-containing protein 5B | 2097 | 2.49E-08 | 55 |
| Efet.01.174699.g1162.t1 | Sarcoplasmic/endoplasmic reticulum calcium ATPase 2 | 1341 | 3.59E-52 | 55 |
| Efet.01.354513.g129.t1 | Protein atonal homolog 1 | 432 | 9.54E-11 | 55 |
| Efet.01.37705.g838.t1 | TLR7 (Toll-like receptor 7) | 798 | 1.10E-12 | 55 |
| Efet.01.333555.g1004.t1 | ADAM metallopeptidase domain 17 (ADAM metallopeptidase domain 17 | 297 | 4.43E-07 | 55 |
| Efet.01.594275.g1115.t1 | ATP-binding cassette, sub-family A (ABC1), member 1 | 540 | 8.83E-17 | 55 |
| Efet.01.33257.g563.t1 | Lactosylceramide 1,3-N-acetyl-beta-D-glucosaminyltransferase | 702 | 1.29E-26 | 55 |
| Efet.01.104959.g272.t1 | Acetylgalactosaminyl-O-glycosyl-glycoprotein beta-1,3-N-acetylglucosaminyltransferase | 921 | 3.83E-11 | 55 |
| Efet.01.255258.g194.t1 | cDNA FLJ34665 fis, clone LIVER2000626, highly similar to Zinc finger protein 38 | 762 | 1.33E-13 | 55 |
| Efet.01.351623.g48.t1 | Protein kinase C | 621 | 1.22E-37 | 55 |
| Efet.01.1648606.g412.t1 | Zinc finger transcription factor BCL6S | 357 | 1.40E-06 | 55 |
| Efet.01.247437.g1794.t1 | Protein bicaudal D homolog 2 | 2040 | 2.14E-94 | 55 |
| Efet.01.3908.g331.t1 | Baculoviral IAP repeat-containing protein 6 | 483 | 1.64E-10 | 55 |
| Efet.01.205875.g254.t1 | Tyrosine-protein kinase receptor | 255 | 7.52E-10 | 55 |
| Efet.01.316952.g541.t1 | Calmodulin-A | 222 | 1.54E-09 | 55 |
| Efet.01.502147.g51.t1 | Cadherin EGF LAG seven-pass G-type receptor 2 | 486 | 2.22E-16 | 55 |
| Efet.01.68600.g1141.t1 | Chromodomain-helicase-DNA-binding protein 8 | 705 | 2.29E-29 | 55 |
| Efet.01.607027.g294.t1 | Cleft lip and palate transmembrane protein 1-like protein | 390 | 1.27E-07 | 55 |
| Efet.01.3361.g288.t1 | Collagen alpha-1(I) chain | 822 | 7.75E-07 | 55 |
| Efet.01.320638.g651.t1 | Collagen alpha-1(XII) chain | 627 | 6.85E-28 | 55 |
| Efet.01.101392.g90.t1 | Collagen alpha-1(XXI) chain | 3411 | 1.53E-21 | 55 |
| Efet.01.391903.g1047.t1 | Cytochrome P450 26A1 | 351 | 3.69E-13 | 55 |
| Efet.01.74633.g1523.t1 | Versican core protein | 915 | 2.98E-15 | 55 |
| Efet.01.641462.g630.t1 | C-terminal-binding protein 1 | 543 | 4.41E-28 | 55 |
| Efet.01.487066.g898.t1 | Cubilin | 633 | 2.02E-24 | 55 |
| Efet.01.242647.g1628.t1 | Discoidin domain-containing receptor 2 | 534 | 1.14E-20 | 55 |
| Efet.01.643899.g843.t1 | Probable ATP-dependent RNA helicase DDX41 | 1152 | 3.25E-59 | 55 |
| Efet.01.16560.g1223.t1 | Probable ATP-dependent RNA helicase DDX58 | 2079 | 8.98E-37 | 55 |
| Efet.01.608807.g366.t1 | D(2) dopamine receptor | 282 | 2.15E-09 | 55 |
| Efet.01.496456.g1093.t1 | Dual specificity protein phosphatase 1 | 696 | 2.27E-18 | 55 |
| Efet.01.73094.g1425.t1 | Dual specificity protein phosphatase 3 | 1095 | 1.79E-21 | 55 |
| Efet.01.59060.g568.t1 | Dual specificity tyrosine-phosphorylation-regulated kinase 2 | 378 | 3.96E-06 | 55 |
| Efet.01.563393.g103.t1 | Uracil-DNA glycosylase | 765 | 3.33E-39 | 55 |
| Efet.01.273837.g881.t1 | Endothelin-converting enzyme 1 | 285 | 2.32E-07 | 55 |
| Efet.01.278686.g1054.t1 | Endothelin-converting enzyme 1 | 399 | 1.44E-42 | 55 |
| Efet.01.18596.g1378.t1 | EGF-like repeat and discoidin I-like domain-containing protein 3 | 495 | 5.96E-21 | 55 |
| Efet.01.401777.g48.t1 | Endothelin receptor type B | 570 | 1.31E-11 | 55 |
| Efet.01.307104.g222.t1 | Epidermal growth factor receptor | 435 | 3.56E-21 | 55 |
| Efet.01.39616.g970.t1 | 55 kDa erythrocyte membrane protein | 351 | 6.54E-17 | 55 |
| Efet.01.196922.g2168.t1 | Endoplasmic reticulum aminopeptidase 2 | 729 | 1.37E-27 | 55 |
| Efet.01.644595.g888.t1 | Receptor tyrosine-protein kinase erbB-3 | 582 | 1.50E-14 | 55 |
| Efet.01.314463.g455.t1 | Receptor tyrosine-protein kinase erbB-4 | 372 | 2.43E-15 | 55 |
| Efet.01.181455.g1475.t1 | Erbin | 1041 | 2.02E-21 | 55 |
| Efet.01.576304.g523.t1 | Erbin | 798 | 4.07E-22 | 55 |
| Efet.01.503482.g81.t1 | DNA excision repair protein ERCC-6 | 744 | 1.70E-27 | 55 |
| Efet.01.47558.g1448.t1 | Nucleolysin TIA-1 isoform p40 | 399 | 8.60E-12 | 55 |
| Efet.01.43341.g1190.t1 | Protocadherin Fat 1 | 567 | 3.98E-27 | 55 |
| Efet.01.1627232.g83.t1 | Protocadherin Fat 1 | 258 | 4.33E-15 | 55 |
| Efet.01.22489.g1634.t1 | Protocadherin Fat 4 | 1050 | 1.33E-37 | 55 |
| Efet.01.288803.g1410.t1 | Tyrosine-protein kinase Fer | 336 | 4.69E-12 | 55 |
| Efet.01.340441.g1179.t1 | Fez family zinc finger protein 2 | 801 | 4.31E-19 | 55 |
| Efet.01.155056.g259.t1 | Feline leukemia virus subgroup C receptor-related protein 1 | 519 | 8.37E-06 | 55 |
| Efet.01.63716.g842.t1 | Fibronectin type III domain-containing protein 3B | 285 | 1.07E-09 | 55 |
| Efet.01.118004.g927.t1 | Forkhead box protein K2 | 519 | 2.12E-22 | 55 |
| Efet.01.151237.g80.t1 | FRAS1-related extracellular matrix protein 1 | 873 | 1.22E-31 | 55 |
| Efet.01.169940.g973.t1 | Follistatin-related protein 1 | 378 | 9.42E-14 | 55 |
| Efet.01.232471.g1288.t1 | Frizzled-5 | 1068 | 6.87E-55 | 55 |
| Efet.01.642550.g699.t1 | Glucose-6-phosphate 1-dehydrogenase | 1455 | 3.18E-93 | 55 |
| Efet.01.173550.g1119.t1 | Polypeptide N-acetylgalactosaminyltransferase 2 | 1860 | 2.41E-73 | 55 |
| Efet.01.189944.g1868.t1 | Polypeptide N-acetylgalactosaminyltransferase 2 | 456 | 1.60E-12 | 55 |
| Efet.01.632184.g152.t1 | Polypeptide N-acetylgalactosaminyltransferase 2 | 213 | 2.32E-06 | 55 |
| Efet.01.576944.g553.t1 | Gastrin/cholecystokinin type B receptor | 813 | 6.31E-22 | 55 |
| Efet.01.231687.g1263.t1 | Growth/differentiation factor 3 | 594 | 4.03E-21 | 55 |
| Efet.01.584674.g811.t1 | GTP-binding protein GEM | 324 | 2.44E-06 | 55 |
| Efet.01.10485.g768.t1 | Bifunctional UDP-N-acetylglucosamine 2-epimerase/N-acetylmannosamine kinase | 579 | 2.42E-16 | 55 |
| Efet.01.14748.g1077.t1 | Guanine nucleotide-binding protein-like 1 | 714 | 2.60E-12 | 55 |
| Efet.01.137871.g664.t1 | N-acetyllactosaminide beta-1,6-N-acetylglucosaminyl-transferase | 1293 | 5.26E-49 | 55 |
| Efet.01.188710.g1824.t1 | N-acetyllactosaminide beta-1,6-N-acetylglucosaminyl-transferase | 921 | 7.02E-57 | 55 |
| Efet.01.578902.g652.t1 | N-acetyllactosaminide beta-1,6-N-acetylglucosaminyl-transferase | 528 | 2.11E-30 | 55 |
| Efet.01.411192.g254.t1 | Glypican-3 | 678 | 6.29E-36 | 55 |
| Efet.01.395065.g1119.t1 | Hemicentin-1 | 216 | 3.32E-09 | 55 |
| Efet.01.300366.g11.t1 | Histamine H1 receptor | 1461 | 5.61E-26 | 55 |
| Efet.01.107849.g403.t1 | Insulin-like growth factor 2 mRNA-binding protein 2 | 411 | 4.66E-06 | 55 |
| Efet.01.89373.g854.t1 | Insulin-like growth factor 2 mRNA-binding protein 3 | 936 | 4.14E-49 | 55 |
| Efet.01.11031.g811.t1 | Intersectin-1 | 465 | 8.27E-09 | 55 |
| Efet.01.103840.g210.t1 | Protein Jade-1 | 885 | 3.73E-48 | 55 |
| Efet.01.334278.g1024.t1 | Junction-mediating and -regulatory protein | 705 | 1.96E-33 | 55 |
| Efet.01.227961.g1125.t1 | Kalirin | 651 | 2.00E-14 | 55 |
| Efet.01.134086.g447.t1 | Kelch-like protein 20 | 375 | 2.21E-06 | 55 |
| Efet.01.5845.g461.t1 | Histone-lysine N-methyltransferase 2A | 1740 | 6.46E-07 | 55 |
| Efet.01.324563.g756.t1 | Ribosomal protein S6 kinase alpha-4 | 273 | 1.42E-07 | 55 |
| Efet.01.35659.g710.t1 | Platelet-activating factor acetylhydrolase IB subunit alpha | 447 | 1.48E-12 | 55 |
| Efet.01.200548.g29.t1 | LIM domain only protein 7 | 615 | 8.89E-06 | 55 |
| Efet.01.577820.g614.t1 | Volume-regulated anion channel subunit LRRC8A | 564 | 1.99E-09 | 55 |
| Efet.01.371355.g602.t1 | Prolow-density lipoprotein receptor-related protein 1 | 441 | 6.28E-22 | 55 |
| Efet.01.655474.g825.t1 | Low-density lipoprotein receptor-related protein 1B | 420 | 2.64E-11 | 55 |
| Efet.01.85380.g648.t1 | Low-density lipoprotein receptor-related protein 2 | 270 | 5.44E-15 | 55 |
| Efet.01.206239.g270.t1 | Latent-transforming growth factor beta-binding protein 4 | 369 | 2.04E-22 | 55 |
| Efet.01.73869.g1472.t1 | Arachidonate 12-lipoxygenase, 12R-type | 1833 | 3.19E-93 | 55 |
| Efet.01.645029.g921.t1 | Mitogen-activated protein kinase kinase kinase 12 | 651 | 3.42E-19 | 55 |
| Efet.01.655926.g895.t1 | Microtubule-associated protein 1S | 681 | 2.33E-09 | 55 |
| Efet.01.67832.g1102.t1 | Mannan-binding lectin serine protease 1 | 264 | 1.32E-09 | 55 |
| Efet.01.275883.g958.t1 | Multidrug resistance protein 1 | 687 | 4.70E-10 | 55 |
| Efet.01.611807.g494.t1 | Multidrug resistance protein 1 | 1131 | 2.87E-13 | 55 |
| Efet.01.1644771.g311.t1 | Multidrug resistance protein 1 | 414 | 9.34E-09 | 55 |
| Efet.01.1658958.g1334.t1 | Multidrug resistance protein 1 | 756 | 3.90E-25 | 55 |
| Efet.01.1655691.g780.t1 | Stromelysin-1 | 333 | 1.77E-07 | 55 |
| Efet.01.60578.g653.t1 | Unconventional myosin-XVIIIa | 1176 | 8.73E-21 | 55 |
| Efet.01.1372.g126.t1 | Myosin-10 | 2133 | 3.63E-30 | 55 |
| Efet.01.109319.g485.t1 | Myosin-binding protein C, slow-type | 633 | 1.19E-13 | 55 |
| Efet.01.71147.g1305.t1 | Myeloid zinc finger 1 | 1266 | 7.82E-89 | 55 |
| Efet.01.189783.g1863.t1 | Neuronal calcium sensor 1 | 453 | 3.94E-24 | 55 |
| Efet.01.374937.g682.t1 | Neurogenic differentiation factor 1 | 1008 | 8.53E-23 | 55 |
| Efet.01.366320.g441.t1 | Neurofilament light polypeptide | 822 | 3.10E-09 | 55 |
| Efet.01.508993.g234.t1 | Neuroligin-4, X-linked | 513 | 9.12E-19 | 55 |
| Efet.01.274741.g907.t1 | Neuroligin-4, Y-linked | 891 | 3.26E-46 | 55 |
| Efet.01.172665.g1082.t1 | NLR family CARD domain-containing protein 3 | 804 | 2.28E-19 | 55 |
| Efet.01.368540.g499.t1 | Nephrin | 324 | 6.70E-09 | 55 |
| Efet.01.302504.g81.t1 | Neuropeptide Y receptor type 5 | 870 | 2.29E-31 | 55 |
| Efet.01.191976.g1960.t1 | Oxysterols receptor LXR-alpha | 570 | 1.73E-11 | 55 |
| Efet.01.70168.g1241.t1 | Neuronal cell adhesion molecule | 345 | 1.19E-07 | 55 |
| Efet.01.314685.g463.t1 | Neuronal cell adhesion molecule | 276 | 6.69E-07 | 55 |
| Efet.01.184432.g1608.t1 | Neurexin-1 | 807 | 1.51E-43 | 55 |
| Efet.01.33512.g586.t1 | Neurexin-2 | 669 | 1.04E-30 | 55 |
| Efet.01.179556.g1375.t1 | Neurotensin receptor type 1 | 480 | 3.98E-12 | 55 |
| Efet.01.84401.g589.t1 | Obscurin | 846 | 3.39E-09 | 55 |
| Efet.01.531211.g41.t1 | Mu-type opioid receptor | 630 | 3.75E-22 | 55 |
| Efet.01.58473.g521.t1 | Oxytocin receptor | 861 | 2.07E-07 | 55 |
| Efet.01.164135.g713.t1 | Phosphatidylinositol 3-kinase regulatory subunit beta | 1527 | 2.84E-73 | 55 |
| Efet.01.76316.g83.t1 | Polyadenylate-binding protein 4 | 246 | 1.55E-10 | 55 |
| Efet.01.443364.g1070.t1 | Protocadherin-16 | 1128 | 2.16E-33 | 55 |
| Efet.01.525310.g591.t1 | Protocadherin-16 | 225 | 1.55E-08 | 55 |
| Efet.01.460118.g284.t1 | Protocadherin gamma-A9 | 1491 | 1.80E-73 | 55 |
| Efet.01.272052.g828.t1 | Protocadherin gamma-B7 | 426 | 1.57E-07 | 55 |
| Efet.01.505477.g139.t1 | cGMP-specific 3',5'-cyclic phosphodiesterase | 885 | 1.80E-54 | 55 |
| Efet.01.301530.g52.t1 | Protein disulfide-isomerase A2 | 285 | 1.28E-07 | 55 |
| Efet.01.658417.g2067.t1 | Peroxisomal trans-2-enoyl-CoA reductase | 327 | 1.27E-07 | 55 |
| Efet.01.52691.g189.t1 | Basement membrane-specific heparan sulfate proteoglycan core protein | 306 | 1.46E-17 | 55 |
| Efet.01.131917.g351.t1 | Basement membrane-specific heparan sulfate proteoglycan core protein | 702 | 1.62E-37 | 55 |
| Efet.01.1658861.g1299.t1 | 15-hydroxyprostaglandin dehydrogenase [NAD(+)] | 744 | 7.38E-34 | 55 |
| Efet.01.51876.g122.t1 | Serine/threonine-protein kinase N2 | 573 | 7.87E-06 | 55 |
| Efet.01.203950.g174.t1 | 1-phosphatidylinositol 4,5-bisphosphate phosphodiesterase gamma-1 | 894 | 3.06E-40 | 55 |
| Efet.01.203477.g160.t1 | Plasminogen | 669 | 2.70E-32 | 55 |
| Efet.01.54188.g278.t1 | Plastin-2 | 252 | 8.66E-07 | 55 |
| Efet.01.27735.g174.t1 | Plexin-B1 | 1035 | 5.14E-48 | 55 |
| Efet.01.125089.g5.t1 | Blood vessel epicardial substance | 519 | 8.60E-30 | 55 |
| Efet.01.633150.g181.t1 | Galectin | 267 | 5.22E-06 | 55 |
| Efet.01.115477.g801.t1 | L-Fucosyltransferase | 816 | 2.66E-27 | 55 |
| Efet.01.1475.g141.t1 | E3 ubiquitin-protein ligase rififylin | 312 | 2.02E-09 | 55 |
| Efet.01.320949.g662.t1 | RGM domain family member B | 438 | 6.46E-26 | 55 |
| Efet.01.193439.g2014.t1 | Roundabout homolog 2 | 474 | 3.94E-10 | 55 |
| Efet.01.253833.g141.t1 | DNA-directed RNA polymerase III subunit RPC1 | 537 | 1.78E-12 | 55 |
| Efet.01.454754.g113.t1 | Relaxin receptor 2 | 654 | 5.79E-33 | 55 |
| Efet.01.28977.g276.t1 | Sodium/glucose cotransporter 4 | 687 | 1.07E-09 | 55 |
| Efet.01.353784.g109.t1 | Sodium channel protein type 2 subunit alpha | 309 | 2.31E-10 | 55 |
| Efet.01.123223.g1206.t1 | Protein sidekick-2 | 897 | 4.61E-47 | 55 |
| Efet.01.230071.g1199.t1 | Slit homolog 2 protein | 465 | 1.24E-06 | 55 |
| Efet.01.1659452.g1748.t1 | Superoxide dismutase [Mn], mitochondrial | 582 | 6.87E-28 | 55 |
| Efet.01.644693.g891.t1 | Transcription factor SOX-4 | 1362 | 4.70E-26 | 55 |
| Efet.01.450516.g16.t1 | Nuclear autoantigen Sp-100 | 687 | 2.19E-09 | 55 |
| Efet.01.359293.g255.t1 | Spectrin beta chain, non-erythrocytic 2 | 4248 | 0 | 55 |
| Efet.01.52053.g137.t1 | Syntaxin-binding protein 5 | 423 | 1.98E-24 | 55 |
| Efet.01.266546.g604.t1 | Synaptotagmin-7 | 468 | 1.70E-11 | 55 |
| Efet.01.452136.g54.t1 | Nucleolysin TIAR | 309 | 1.74E-11 | 55 |
| Efet.01.553257.g578.t1 | Transketolase-like protein 1 | 444 | 6.34E-20 | 55 |
| Efet.01.1653071.g597.t1 | TNF receptor-associated factor 6 | 297 | 2.36E-10 | 55 |
| Efet.01.115844.g823.t1 | Melanotransferrin | 477 | 6.67E-22 | 55 |
| Efet.01.102475.g143.t1 | Ubiquitin carboxyl-terminal hydrolase 17-like protein 5 | 4278 | 2.13E-46 | 55 |
| Efet.01.256385.g234.t1 | Protein unc-13 homolog D | 384 | 5.90E-07 | 55 |
| Efet.01.183867.g1590.t1 | WW domain-containing oxidoreductase | 858 | 2.62E-45 | 55 |
| Efet.01.412692.g288.t1 | Vasopressin V1a receptor | 585 | 8.06E-15 | 55 |
| Efet.01.227631.g1108.t1 | Retinoschisin | 483 | 1.18E-29 | 55 |
| Efet.01.100486.g32.t1 | AN1-type zinc finger protein 5 | 252 | 4.79E-06 | 55 |
| Efet.01.185932.g1678.t1 | Zinc finger protein 148 | 1392 | 5.90E-12 | 55 |
| Efet.01.134140.g449.t1 | Zinc finger protein 175 | 1029 | 3.15E-21 | 55 |
| Efet.01.17597.g1297.t1 | Zinc finger protein 675 | 1281 | 3.38E-74 | 55 |
| Efet.01.176741.g1238.t1 | Zinc finger protein 675 | 1791 | 8.38E-87 | 55 |
| Efet.01.66322.g1004.t1 | Tyrosine-protein kinase | 597 | 1.93E-34 | 54 |
| Efet.01.78487.g208.t1 | Caspase recruitment domain family, member 4 | 1293 | 8.40E-11 | 54 |
| Efet.01.347007.g1320.t1 | Suppressor of cytokine signaling 2 | 1287 | 5.18E-23 | 54 |
| Efet.01.285802.g1294.t1 | Fucosyltransferase | 264 | 5.44E-08 | 54 |
| Efet.01.134179.g450.t1 | cDNA FLJ77744, highly similar to Homo sapiens kallikrein B, plasma | 576 | 2.14E-27 | 54 |
| Efet.01.234082.g1339.t1 | 5'-AMP-activated protein kinase subunit gamma-2 | 483 | 3.24E-35 | 54 |
| Efet.01.588154.g932.t1 | ATP-binding cassette sub-family A member 7 | 699 | 2.30E-21 | 54 |
| Efet.01.267648.g644.t1 | ATP-binding cassette sub-family B member 9 | 2265 | 3.28E-82 | 54 |
| Efet.01.565724.g190.t1 | ATP-binding cassette sub-family G member 1 | 840 | 1.08E-26 | 54 |
| Efet.01.38674.g910.t1 | Active breakpoint cluster region-related protein | 1440 | 3.25E-20 | 54 |
| Efet.01.82061.g473.t1 | Active breakpoint cluster region-related protein | 282 | 1.84E-07 | 54 |
| Efet.01.91462.g966.t1 | Acetylcholinesterase | 753 | 1.26E-52 | 54 |
| Efet.01.69870.g1223.t1 | Neuronal acetylcholine receptor subunit beta-2 | 312 | 2.31E-09 | 54 |
| Efet.01.68866.g1161.t1 | Muscarinic acetylcholine receptor M1 | 1305 | 1.65E-43 | 54 |
| Efet.01.606404.g277.t1 | Peroxisomal acyl-coenzyme A oxidase 1 | 1176 | 2.70E-42 | 54 |
| Efet.01.639963.g546.t1 | Acyl-CoA synthetase family member 3, mitochondrial | 2928 | 1.84E-13 | 54 |
| Efet.01.658012.g1555.t1 | Alcohol dehydrogenase class-3 | 252 | 8.19E-08 | 54 |
| Efet.01.515933.g395.t1 | Retinal dehydrogenase 2 | 915 | 4.21E-59 | 54 |
| Efet.01.656717.g1074.t1 | Aldehyde dehydrogenase family 1 member A3 | 1434 | 3.82E-91 | 54 |
| Efet.01.1659505.g1904.t1 | Aldehyde dehydrogenase family 1 member A3 | 1140 | 4.32E-68 | 54 |
| Efet.01.517044.g422.t1 | Insulin-like growth factor-binding protein complex acid labile subunit | 1326 | 3.93E-29 | 54 |
| Efet.01.86281.g709.t1 | Ankyrin-1 | 561 | 5.38E-14 | 54 |
| Efet.01.271475.g806.t1 | Aquaporin-2 | 807 | 1.64E-24 | 54 |
| Efet.01.572648.g396.t1 | Aquaporin-4 | 549 | 6.10E-26 | 54 |
| Efet.01.445197.g1119.t1 | AT-rich interactive domain-containing protein 5B | 516 | 1.03E-06 | 54 |
| Efet.01.433154.g803.t1 | Calcium-transporting ATPase type 2C member 1 | 876 | 3.68E-23 | 54 |
| Efet.01.419529.g452.t1 | TLR7 (Toll-like receptor 7) | 1068 | 2.64E-11 | 54 |
| Efet.01.1659462.g1771.t1 | ATP-binding cassette, sub-family A (ABC1), member 1 | 696 | 1.05E-11 | 54 |
| Efet.01.12448.g926.t1 | Lactosylceramide 1,3-N-acetyl-beta-D-glucosaminyltransferase | 1161 | 9.50E-41 | 54 |
| Efet.01.305147.g167.t1 | Lactosylceramide 1,3-N-acetyl-beta-D-glucosaminyltransferase | 1476 | 4.53E-38 | 54 |
| Efet.01.1659098.g1404.t1 | Lactosylceramide 1,3-N-acetyl-beta-D-glucosaminyltransferase | 1188 | 7.99E-28 | 54 |
| Efet.01.654418.g597.t1 | cDNA FLJ40025 fis, clone STOMA2008050, highly similar to Homo sapiens ATP-binding cassette, sub-family A | 966 | 7.98E-39 | 54 |
| Efet.01.1657368.g977.t1 | cDNA FLJ40025 fis, clone STOMA2008050, highly similar to Homo sapiens ATP-binding cassette, sub-family A | 678 | 1.07E-06 | 54 |
| Efet.01.508540.g220.t1 | cDNA FLJ58124, highly similar to Complement factor I | 252 | 2.14E-10 | 54 |
| Efet.01.290247.g1459.t1 | cDNA FLJ50323, highly similar to Macrophage-stimulating protein receptor | 855 | 2.60E-22 | 54 |
| Efet.01.625368.g1112.t1 | Beta-1,4-galactosyltransferase 1 | 609 | 1.79E-14 | 54 |
| Efet.01.489389.g948.t1 | Zinc finger transcription factor BCL6S | 423 | 8.28E-20 | 54 |
| Efet.01.187527.g1762.t1 | BTB/POZ domain-containing adapter for CUL3-mediated RhoA degradation protein 2 | 645 | 9.75E-09 | 54 |
| Efet.01.158399.g440.t1 | Bone morphogenetic protein 1 | 1629 | 1.08E-13 | 54 |
| Efet.01.487337.g900.t1 | Serine/threonine-protein kinase B-raf | 981 | 6.12E-21 | 54 |
| Efet.01.108232.g427.t1 | BTB/POZ domain-containing protein 9 | 687 | 6.16E-06 | 54 |
| Efet.01.108959.g465.t1 | TCF7L2 isoform pFC8A | 603 | 1.17E-25 | 54 |
| Efet.01.228888.g1158.t1 | Calmodulin-A | 351 | 8.71E-06 | 54 |
| Efet.01.107634.g393.t1 | Cholecystokinin receptor type A | 621 | 4.07E-09 | 54 |
| Efet.01.656649.g1058.t1 | Cyclin-dependent kinase inhibitor 1C | 312 | 1.89E-08 | 54 |
| Efet.01.119970.g1029.t1 | Cadherin EGF LAG seven-pass G-type receptor 2 | 513 | 1.98E-22 | 54 |
| Efet.01.287664.g1376.t1 | CAP-Gly domain-containing linker protein 2 | 435 | 4.75E-07 | 54 |
| Efet.01.152984.g161.t1 | Contactin-2 | 522 | 1.22E-13 | 54 |
| Efet.01.32808.g541.t1 | Contactin-associated protein-like 4 | 630 | 7.65E-31 | 54 |
| Efet.01.603471.g135.t1 | Coronin-1C | 384 | 4.81E-14 | 54 |
| Efet.01.657592.g1332.t1 | Cholesterol side-chain cleavage enzyme, mitochondrial | 399 | 6.58E-08 | 54 |
| Efet.01.83394.g550.t1 | 25-hydroxyvitamin D-1 alpha hydroxylase, mitochondrial | 342 | 4.25E-17 | 54 |
| Efet.01.167044.g842.t1 | C-X-C chemokine receptor type 1 | 759 | 1.31E-09 | 54 |
| Efet.01.505417.g136.t1 | Discoidin domain-containing receptor 2 | 609 | 2.91E-26 | 54 |
| Efet.01.655322.g797.t1 | Discoidin domain-containing receptor 2 | 558 | 3.88E-33 | 54 |
| Efet.01.528741.g657.t1 | Probable ATP-dependent RNA helicase DDX41 | 576 | 9.61E-11 | 54 |
| Efet.01.449761.g1210.t1 | Probable ATP-dependent RNA helicase DDX58 | 990 | 2.80E-25 | 54 |
| Efet.01.1659457.g1763.t1 | Dehydrogenase/reductase SDR family member 2, mitochondrial | 588 | 1.44E-11 | 54 |
| Efet.01.562555.g53.t1 | Disks large homolog 1 | 789 | 8.16E-12 | 54 |
| Efet.01.571127.g356.t1 | Down syndrome cell adhesion molecule-like protein 1 | 327 | 5.47E-10 | 54 |
| Efet.01.50680.g48.t1 | Dual specificity protein phosphatase 1 | 522 | 7.52E-25 | 54 |
| Efet.01.206695.g295.t1 | Endothelin receptor type B | 726 | 3.68E-28 | 54 |
| Efet.01.86191.g704.t1 | Engulfment and cell motility protein 2 | 1395 | 4.15E-38 | 54 |
| Efet.01.12984.g978.t1 | Ephrin type-A receptor 7 | 528 | 3.02E-18 | 54 |
| Efet.01.277007.g998.t1 | Ephrin type-A receptor 7 | 240 | 2.17E-09 | 54 |
| Efet.01.429497.g727.t1 | Eukaryotic peptide chain release factor GTP-binding subunit ERF3A | 2496 | 2.41E-07 | 54 |
| Efet.01.594862.g1132.t1 | Exocyst complex component 2 | 1698 | 7.61E-11 | 54 |
| Efet.01.39151.g946.t1 | Vitamin D (1,25-dihydroxyvitamin D3) receptor, isoform CRA_c | 1029 | 3.67E-20 | 54 |
| Efet.01.140506.g799.t1 | Vitamin D (1,25-dihydroxyvitamin D3) receptor, isoform CRA_c | 780 | 3.21E-18 | 54 |
| Efet.01.80668.g382.t1 | Protocadherin Fat 1 | 453 | 1.23E-31 | 54 |
| Efet.01.83710.g565.t1 | Protocadherin Fat 1 | 933 | 2.66E-46 | 54 |
| Efet.01.77706.g157.t1 | Protocadherin Fat 2 | 354 | 2.10E-15 | 54 |
| Efet.01.654468.g610.t1 | Protocadherin Fat 3 | 306 | 1.55E-09 | 54 |
| Efet.01.89985.g895.t1 | Fibulin-5 | 624 | 4.66E-12 | 54 |
| Efet.01.83681.g561.t1 | Tyrosine-protein kinase Fer | 2313 | 9.20E-16 | 54 |
| Efet.01.197703.g2205.t1 | Fibrinogen-like protein 1 | 420 | 4.18E-16 | 54 |
| Efet.01.643389.g773.t1 | Filamin-A | 249 | 7.09E-07 | 54 |
| Efet.01.650664.g75.t1 | Mu opioid receptor hMOR-1a | 351 | 8.55E-06 | 54 |
| Efet.01.363635.g364.t1 | GRB2-associated-binding protein 2 | 342 | 7.56E-07 | 54 |
| Efet.01.11849.g876.t1 | Gastrin/cholecystokinin type B receptor | 1227 | 1.44E-09 | 54 |
| Efet.01.338161.g1115.t1 | Gastrin/cholecystokinin type B receptor | 1338 | 1.87E-09 | 54 |
| Efet.01.451716.g41.t1 | Growth/differentiation factor 8 | 369 | 2.20E-10 | 54 |
| Efet.01.331755.g958.t1 | Growth hormone secretagogue receptor type 1 | 363 | 1.01E-07 | 54 |
| Efet.01.121040.g1092.t1 | Glypican-3 | 753 | 8.33E-37 | 54 |
| Efet.01.74339.g1510.t1 | Lymphoid-specific helicase | 2202 | 3.70E-44 | 54 |
| Efet.01.375887.g700.t1 | Lymphoid-specific helicase | 696 | 2.31E-38 | 54 |
| Efet.01.159951.g520.t1 | Serine/threonine-protein kinase ICK | 492 | 5.44E-47 | 54 |
| Efet.01.95607.g1193.t1 | Interferon-induced helicase C domain-containing protein 1 | 1161 | 6.62E-12 | 54 |
| Efet.01.218578.g751.t1 | Interferon-induced helicase C domain-containing protein 1 | 1263 | 3.49E-21 | 54 |
| Efet.01.29999.g339.t1 | Indian hedgehog protein | 573 | 2.43E-27 | 54 |
| Efet.01.298689.g1704.t1 | Zinc finger protein Eos | 480 | 1.04E-09 | 54 |
| Efet.01.72610.g1397.t1 | Insulin receptor | 489 | 2.17E-38 | 54 |
| Efet.01.121075.g1098.t1 | Inositol hexakisphosphate kinase 2 | 396 | 9.44E-18 | 54 |
| Efet.01.84118.g578.t1 | Inter-alpha-trypsin inhibitor heavy chain H4 | 711 | 2.50E-31 | 54 |
| Efet.01.186787.g1712.t1 | Kalirin | 648 | 6.11E-20 | 54 |
| Efet.01.99959.g1445.t1 | Calcium-activated potassium channel subunit alpha-1 | 273 | 5.35E-08 | 54 |
| Efet.01.254268.g156.t1 | Kelch-like protein 20 | 858 | 4.78E-41 | 54 |
| Efet.01.318554.g580.t1 | Kelch-like protein 20 | 1008 | 9.98E-51 | 54 |
| Efet.01.127209.g129.t1 | Kelch-like protein 9 | 1677 | 2.22E-112 | 54 |
| Efet.01.44055.g1231.t1 | Histone-lysine N-methyltransferase 2A | 279 | 6.83E-13 | 54 |
| Efet.01.146232.g1063.t1 | Histone-lysine N-methyltransferase 2A | 561 | 1.03E-14 | 54 |
| Efet.01.19230.g1427.t1 | Laminin subunit alpha-2 | 615 | 6.59E-06 | 54 |
| Efet.01.52786.g196.t1 | Protein lin-7 homolog A | 912 | 3.52E-06 | 54 |
| Efet.01.96423.g1240.t1 | Lethal(3)malignant brain tumor-like protein 1 | 747 | 2.01E-34 | 54 |
| Efet.01.99237.g1401.t1 | Low-density lipoprotein receptor-related protein 2 | 447 | 1.52E-47 | 54 |
| Efet.01.226348.g1050.t1 | RNA helicase Mov10l1 | 645 | 1.50E-08 | 54 |
| Efet.01.658198.g1712.t1 | Mitogen-activated protein kinase kinase kinase 10 | 600 | 8.40E-28 | 54 |
| Efet.01.1655895.g800.t1 | Mitogen-activated protein kinase kinase kinase 10 | 417 | 8.84E-19 | 54 |
| Efet.01.53017.g209.t1 | Microtubule-associated protein 1S | 1644 | 7.77E-14 | 54 |
| Efet.01.254703.g171.t1 | Myc-associated zinc finger protein | 498 | 1.53E-06 | 54 |
| Efet.01.533383.g128.t1 | Multidrug resistance protein 1 | 987 | 5.55E-25 | 54 |
| Efet.01.550127.g501.t1 | Multidrug resistance protein 1 | 1098 | 2.49E-23 | 54 |
| Efet.01.635927.g301.t1 | Multidrug resistance protein 1 | 1038 | 1.74E-25 | 54 |
| Efet.01.96625.g1257.t1 | E3 ubiquitin-protein ligase MIB1 | 441 | 2.78E-29 | 54 |
| Efet.01.642550.g700.t1 | DNA mismatch repair protein Mlh1 | 2514 | 3.59E-48 | 54 |
| Efet.01.51022.g70.t1 | Putative helicase MOV-10 | 648 | 6.58E-08 | 54 |
| Efet.01.49571.g1565.t1 | Interferon-induced GTP-binding protein Mx1 | 624 | 6.81E-11 | 54 |
| Efet.01.419761.g464.t1 | Myosin light chain kinase, smooth muscle | 1062 | 8.35E-15 | 54 |
| Efet.01.1630939.g109.t1 | Neurofilament light polypeptide | 288 | 5.21E-08 | 54 |
| Efet.01.239686.g1526.t1 | Neuroligin-1 | 834 | 1.66E-48 | 54 |
| Efet.01.110051.g513.t1 | Neuroligin-2 | 543 | 2.71E-30 | 54 |
| Efet.01.347811.g1349.t1 | Neuroligin-2 | 558 | 1.89E-29 | 54 |
| Efet.01.286669.g1331.t1 | Neuroligin-3 | 285 | 5.00E-09 | 54 |
| Efet.01.135203.g503.t1 | Neuroligin-4, X-linked | 486 | 3.63E-23 | 54 |
| Efet.01.156308.g326.t1 | NLR family CARD domain-containing protein 3 | 849 | 5.34E-15 | 54 |
| Efet.01.5596.g433.t1 | Bile acid receptor | 222 | 1.54E-08 | 54 |
| Efet.01.34250.g640.t1 | Neurexin-1 | 504 | 9.09E-25 | 54 |
| Efet.01.204481.g194.t1 | Neurexin-1 | 636 | 1.87E-30 | 54 |
| Efet.01.16840.g1245.t1 | Neurexin-3 | 630 | 3.55E-24 | 54 |
| Efet.01.461078.g314.t1 | Ileal sodium/bile acid cotransporter | 378 | 3.08E-07 | 54 |
| Efet.01.221225.g865.t1 | Numb-like protein | 546 | 1.09E-07 | 54 |
| Efet.01.173460.g1117.t1 | Kappa-type opioid receptor | 288 | 1.36E-08 | 54 |
| Efet.01.282810.g1188.t1 | Kappa-type opioid receptor | 441 | 1.36E-12 | 54 |
| Efet.01.380171.g798.t1 | Mu-type opioid receptor | 636 | 4.95E-27 | 54 |
| Efet.01.466101.g432.t1 | Mu-type opioid receptor | 879 | 8.62E-38 | 54 |
| Efet.01.77571.g147.t1 | Transcription factor Ovo-like 2 | 462 | 2.73E-13 | 54 |
| Efet.01.2633.g240.t1 | Polyadenylate-binding protein 4 | 726 | 2.80E-09 | 54 |
| Efet.01.467887.g476.t1 | Polyadenylate-binding protein 4 | 219 | 3.80E-07 | 54 |
| Efet.01.85264.g639.t1 | Serine/threonine-protein kinase PAK 1 | 513 | 3.29E-15 | 54 |
| Efet.01.188605.g1816.t1 | Protocadherin-18 | 714 | 4.51E-06 | 54 |
| Efet.01.44468.g1255.t1 | Protocadherin-19 | 1056 | 8.15E-45 | 54 |
| Efet.01.646337.g1075.t1 | Protocadherin-19 | 1491 | 5.11E-70 | 54 |
| Efet.01.146794.g1093.t1 | Protocadherin-23 | 2058 | 6.58E-29 | 54 |
| Efet.01.147809.g1135.t1 | Protocadherin gamma-A8 | 948 | 1.24E-34 | 54 |
| Efet.01.533093.g104.t1 | Protocadherin gamma-A10 | 879 | 1.10E-13 | 54 |
| Efet.01.518467.g446.t1 | Calcium/calmodulin-dependent 3',5'-cyclic nucleotide phosphodiesterase 1B | 294 | 9.76E-13 | 54 |
| Efet.01.302866.g90.t1 | cGMP-specific 3',5'-cyclic phosphodiesterase | 915 | 1.30E-33 | 54 |
| Efet.01.441227.g1011.t1 | cGMP-specific 3',5'-cyclic phosphodiesterase | 1272 | 3.28E-09 | 54 |
| Efet.01.658419.g2095.t1 | Peroxisomal trans-2-enoyl-CoA reductase | 1929 | 3.32E-23 | 54 |
| Efet.01.159620.g502.t1 | Basement membrane-specific heparan sulfate proteoglycan core protein | 279 | 2.54E-09 | 54 |
| Efet.01.277984.g1031.t1 | Plastin-2 | 384 | 2.87E-18 | 54 |
| Efet.01.239761.g1529.t1 | Protein phosphatase 1F | 558 | 1.52E-18 | 54 |
| Efet.01.1657001.g929.t1 | Protein phosphatase 1F | 423 | 2.13E-14 | 54 |
| Efet.01.43637.g1210.t1 | Protein regulator of cytokinesis 1 | 678 | 8.74E-24 | 54 |
| Efet.01.228168.g1130.t1 | Vitamin K-dependent protein S | 396 | 5.85E-17 | 54 |
| Efet.01.641291.g620.t1 | 26S proteasome regulatory subunit 10B | 1269 | 3.12E-06 | 54 |
| Efet.01.10686.g787.t1 | 26S proteasome non-ATPase regulatory subunit 10 | 687 | 1.74E-18 | 54 |
| Efet.01.373741.g660.t1 | Receptor-type tyrosine-protein phosphatase S | 378 | 1.73E-10 | 54 |
| Efet.01.325095.g770.t1 | MYLK protein | 744 | 7.05E-13 | 54 |
| Efet.01.25307.g40.t1 | Uncharacterized protein | 885 | 9.48E-06 | 54 |
| Efet.01.315097.g475.t1 | NF-kappa-B inhibitor-like protein 1 | 264 | 2.99E-12 | 54 |
| Efet.01.434985.g838.t1 | Ribosomal protein L3 | 606 | 4.71E-06 | 54 |
| Efet.01.117435.g904.t1 | Ras-related protein Rab-13 | 258 | 1.74E-14 | 54 |
| Efet.01.138477.g689.t1 | Putative RNA-binding protein 15 | 2070 | 3.97E-62 | 54 |
| Efet.01.258504.g316.t1 | RGM domain family member B | 480 | 3.27E-26 | 54 |
| Efet.01.78976.g243.t1 | Roundabout homolog 2 | 225 | 1.59E-06 | 54 |
| Efet.01.654391.g583.t1 | DNA-directed RNA polymerase III subunit RPC2 | 660 | 1.23E-17 | 54 |
| Efet.01.161682.g606.t1 | Protein scribble homolog | 888 | 6.61E-25 | 54 |
| Efet.01.56193.g393.t1 | Signal peptide, CUB and EGF-like domain-containing protein 1 | 993 | 2.38E-06 | 54 |
| Efet.01.1644859.g315.t1 | Protein sidekick-2 | 231 | 1.04E-10 | 54 |
| Efet.01.173457.g1116.t1 | Semaphorin-5A | 771 | 5.71E-52 | 54 |
| Efet.01.340129.g1175.t1 | Sphingosine-1-phosphate lyase 1 | 318 | 1.30E-12 | 54 |
| Efet.01.337386.g1100.t1 | E3 ubiquitin-protein ligase SH3RF1 | 342 | 8.37E-06 | 54 |
| Efet.01.189666.g1861.t1 | Serine/threonine-protein kinase SIK2 | 258 | 1.58E-06 | 54 |
| Efet.01.284135.g1242.t1 | Helicase SKI2W | 408 | 2.88E-15 | 54 |
| Efet.01.92493.g1021.t1 | Zinc finger protein SNAI1 | 303 | 1.02E-09 | 54 |
| Efet.01.281521.g1142.t1 | Solute carrier organic anion transporter family member 2A1 | 600 | 6.44E-17 | 54 |
| Efet.01.650606.g67.t1 | Spectrin beta chain, erythrocytic | 546 | 1.23E-22 | 54 |
| Efet.01.28160.g208.t1 | Sushi, von Willebrand factor type A, EGF and pentraxin domain-containing protein 1 | 252 | 2.35E-06 | 54 |
| Efet.01.384680.g903.t1 | Synaptotagmin-7 | 537 | 6.35E-16 | 54 |
| Efet.01.145838.g1048.t1 | Transmembrane protein 229A | 555 | 1.68E-21 | 54 |
| Efet.01.328721.g883.t1 | Serine/threonine-protein kinase TAO2 | 621 | 8.87E-16 | 54 |
| Efet.01.81873.g462.t1 | Probable methyltransferase TARBP1 | 1284 | 3.17E-12 | 54 |
| Efet.01.200486.g23.t1 | Transcription factor AP-4 | 828 | 9.72E-14 | 54 |
| Efet.01.295656.g1606.t1 | E3 ubiquitin-protein ligase TRIM32 | 1518 | 4.77E-06 | 54 |
| Efet.01.42473.g1141.t1 | Transient receptor potential cation channel subfamily M member 7 | 564 | 4.39E-18 | 54 |
| Efet.01.477273.g693.t1 | Tyrosine-protein kinase receptor UFO | 666 | 8.90E-08 | 54 |
| Efet.01.280752.g1124.t1 | Vasopressin V1b receptor | 1065 | 1.88E-60 | 54 |
| Efet.01.21200.g1567.t1 | Protein disulfide-isomerase | 567 | 1.05E-45 | 54 |
| Efet.01.49153.g1534.t1 | Very low-density lipoprotein receptor | 789 | 2.97E-34 | 54 |
| Efet.01.268818.g696.t1 | Protein Wnt-2 | 282 | 3.45E-12 | 54 |
| Efet.01.170251.g987.t1 | Zinc finger protein 175 | 1248 | 6.88E-58 | 54 |
| Efet.01.238231.g1486.t1 | Zinc finger protein 175 | 783 | 2.16E-39 | 54 |
| Efet.01.282394.g1172.t1 | Zinc finger protein 675 | 1413 | 5.83E-55 | 54 |
| Efet.01.67920.g1112.t1 | Zinc finger protein 3 | 1131 | 8.90E-47 | 54 |
| Efet.01.424735.g588.t1 | Zinc finger protein 3 | 828 | 1.86E-14 | 54 |
| Efet.01.658389.g1942.t1 | Zinc finger protein 3 | 2223 | 1.25E-14 | 54 |
| Efet.01.31382.g444.t1 | DDX39B (HCG2005638, isoform CRA_a) | 444 | 1.73E-06 | 53 |
| Efet.01.472687.g617.t1 | Epstein-Barr virus induced gene 2 (Lymphocyte-specific G protein-coupled receptor), isoform CRA_a | 1215 | 5.81E-12 | 53 |
| Efet.01.654656.g678.t1 | ATP-binding cassette sub-family A member 1 | 1113 | 1.01E-23 | 53 |
| Efet.01.658368.g1901.t1 | ATP-binding cassette sub-family A member 5 | 1089 | 5.28E-19 | 53 |
| Efet.01.658383.g1925.t1 | ATP-binding cassette sub-family A member 5 | 780 | 2.65E-23 | 53 |
| Efet.01.658310.g1840.t1 | ATP-binding cassette sub-family G member 1 | 687 | 2.58E-14 | 53 |
| Efet.01.9268.g680.t1 | Acetylcholinesterase | 426 | 4.69E-15 | 53 |
| Efet.01.503314.g80.t1 | Neuronal acetylcholine receptor subunit alpha-7 | 357 | 1.83E-10 | 53 |
| Efet.01.190934.g1912.t1 | Neuronal acetylcholine receptor subunit beta-2 | 975 | 5.89E-45 | 53 |
| Efet.01.610445.g439.t1 | Alpha-actinin-1 | 519 | 8.19E-07 | 53 |
| Efet.01.18399.g1364.t1 | Alpha-actinin-3 | 390 | 6.39E-10 | 53 |
| Efet.01.80905.g395.t1 | Alpha-1A adrenergic receptor | 1095 | 1.19E-51 | 53 |
| Efet.01.227590.g1105.t1 | Adenosine deaminase 2 | 1257 | 1.00E-42 | 53 |
| Efet.01.423076.g544.t1 | Adenosine deaminase 2 | 480 | 3.71E-08 | 53 |
| Efet.01.152236.g130.t1 | Beta-2 adrenergic receptor | 1047 | 3.30E-60 | 53 |
| Efet.01.289987.g1447.t1 | Type-1 angiotensin II receptor | 1254 | 8.89E-07 | 53 |
| Efet.01.194314.g2063.t1 | Type-2 angiotensin II receptor | 375 | 1.96E-10 | 53 |
| Efet.01.1659287.g1514.t1 | Aldo-keto reductase family 1 member C2 | 837 | 4.53E-56 | 53 |
| Efet.01.1076.g100.t1 | Ankyrin-1 | 744 | 7.83E-19 | 53 |
| Efet.01.247538.g1805.t1 | Ankyrin-1 | 495 | 1.95E-16 | 53 |
| Efet.01.586718.g886.t1 | Ankyrin-1 | 1077 | 5.29E-07 | 53 |
| Efet.01.85300.g642.t1 | AP-1 complex subunit beta-1 | 1503 | 3.67E-35 | 53 |
| Efet.01.281396.g1139.t1 | Ankyrin repeat and SOCS box protein 1 | 555 | 4.24E-12 | 53 |
| Efet.01.658142.g1671.t1 | Calcium-transporting ATPase type 2C member 1 | 1062 | 1.01E-12 | 53 |
| Efet.01.264731.g555.t1 | Protein atonal homolog 1 | 849 | 4.32E-08 | 53 |
| Efet.01.601193.g63.t1 | ATP-binding cassette, sub-family A (ABC1), member 1 | 498 | 1.58E-10 | 53 |
| Efet.01.646963.g1136.t1 | ATP-binding cassette, sub-family A (ABC1), member 1 | 1452 | 1.70E-17 | 53 |
| Efet.01.657110.g1170.t1 | ATP-binding cassette, sub-family A (ABC1), member 1 | 2241 | 3.29E-18 | 53 |
| Efet.01.658052.g1602.t1 | ATP-binding cassette, sub-family A (ABC1), member 1 | 1116 | 3.64E-27 | 53 |
| Efet.01.181767.g1491.t1 | Lactosylceramide 1,3-N-acetyl-beta-D-glucosaminyltransferase | 1176 | 8.96E-26 | 53 |
| Efet.01.445097.g1114.t1 | cDNA FLJ40025 fis, clone STOMA2008050, highly similar to Homo sapiens ATP-binding cassette, sub-family A | 450 | 5.26E-15 | 53 |
| Efet.01.658190.g1704.t1 | cDNA FLJ40025 fis, clone STOMA2008050, highly similar to Homo sapiens ATP-binding cassette, sub-family A | 588 | 6.42E-11 | 53 |
| Efet.01.626536.g1167.t1 | cDNA FLJ60536, highly similar to Death-associated protein kinase 1 | 306 | 5.31E-14 | 53 |
| Efet.01.1659380.g1628.t1 | cDNA FLJ53627, highly similar to Antigen peptide transporter 1 | 2013 | 3.45E-15 | 53 |
| Efet.01.2515.g227.t1 | Beta-glucuronidase | 414 | 2.29E-43 | 53 |
| Efet.01.215127.g629.t1 | Bone morphogenetic protein 4 | 1440 | 3.13E-79 | 53 |
| Efet.01.338861.g1137.t1 | Bone morphogenetic protein 4 | 1095 | 5.86E-64 | 53 |
| Efet.01.261110.g416.t1 | Bactericidal permeability-increasing protein | 870 | 1.11E-37 | 53 |
| Efet.01.208232.g376.t1 | Cholecystokinin receptor type A | 831 | 2.00E-23 | 53 |
| Efet.01.532983.g93.t1 | Cadherin EGF LAG seven-pass G-type receptor 3 | 687 | 3.39E-20 | 53 |
| Efet.01.29465.g307.t1 | Contactin-5 | 261 | 1.44E-12 | 53 |
| Efet.01.99411.g1414.t1 | Contactin-5 | 480 | 1.70E-15 | 53 |
| Efet.01.132501.g377.t1 | Collagen alpha-1(XXIV) chain | 651 | 2.81E-12 | 53 |
| Efet.01.51907.g126.t1 | Cytochrome P450 1A1 | 1521 | 3.75E-69 | 53 |
| Efet.01.381141.g816.t1 | Cytochrome P450 1A1 | 1563 | 1.19E-91 | 53 |
| Efet.01.643669.g820.t1 | C-terminal-binding protein 1 | 354 | 6.73E-16 | 53 |
| Efet.01.653479.g432.t1 | C-terminal-binding protein 1 | 1011 | 1.06E-06 | 53 |
| Efet.01.376672.g721.t1 | C-X-C chemokine receptor type 1 | 228 | 1.63E-08 | 53 |
| Efet.01.76171.g74.t1 | Cytohesin-3 | 309 | 7.24E-14 | 53 |
| Efet.01.156547.g343.t1 | B-cell CLL/lymphoma 11A (Zinc finger protein), isoform CRA_b | 246 | 2.40E-07 | 53 |
| Efet.01.170002.g977.t1 | Discoidin domain-containing receptor 2 | 1146 | 8.86E-17 | 53 |
| Efet.01.567964.g255.t1 | ATP-dependent RNA helicase DDX42 | 591 | 2.98E-20 | 53 |
| Efet.01.33837.g611.t1 | Docking protein 1 | 303 | 5.02E-08 | 53 |
| Efet.01.41308.g1077.t1 | D(3) dopamine receptor | 900 | 1.77E-07 | 53 |
| Efet.01.280613.g1119.t1 | Dual specificity protein phosphatase 1 | 879 | 3.11E-20 | 53 |
| Efet.01.168567.g916.t1 | Dual specificity protein phosphatase 3 | 552 | 2.75E-17 | 53 |
| Efet.01.78263.g195.t1 | Dual specificity protein phosphatase 6 | 522 | 4.26E-25 | 53 |
| Efet.01.122724.g1183.t1 | ETS domain-containing protein Elk-1 | 501 | 9.15E-13 | 53 |
| Efet.01.35526.g700.t1 | Receptor tyrosine-protein kinase erbB-4 | 435 | 2.08E-18 | 53 |
| Efet.01.589191.g966.t1 | Erbin | 1821 | 3.15E-35 | 53 |
| Efet.01.1658966.g1339.t1 | DNA excision repair protein ERCC-6-like | 993 | 1.06E-15 | 53 |
| Efet.01.469171.g512.t1 | Chimeric ERCC6-PGBD3 protein | 399 | 4.06E-28 | 53 |
| Efet.01.550298.g513.t1 | Exocyst complex component 4 | 900 | 3.37E-20 | 53 |
| Efet.01.336401.g1079.t1 | Vitamin D (1,25-dihydroxyvitamin D3) receptor, isoform CRA_c | 762 | 7.08E-18 | 53 |
| Efet.01.2390.g214.t1 | Protocadherin Fat 1 | 522 | 8.53E-13 | 53 |
| Efet.01.448174.g1178.t1 | Protocadherin Fat 1 | 1296 | 4.81E-79 | 53 |
| Efet.01.419729.g461.t1 | Protocadherin Fat 3 | 219 | 1.79E-08 | 53 |
| Efet.01.140014.g771.t1 | Protocadherin Fat 4 | 762 | 1.87E-19 | 53 |
| Efet.01.59298.g583.t1 | Fibroleukin | 552 | 4.67E-29 | 53 |
| Efet.01.22670.g1647.t1 | Filamin-A | 879 | 3.72E-39 | 53 |
| Efet.01.56486.g403.t1 | Dimethylaniline monooxygenase [N-oxide-forming] 1 | 687 | 4.64E-41 | 53 |
| Efet.01.44655.g1275.t1 | Alpha-(1,3)-fucosyltransferase 4 | 576 | 1.26E-16 | 53 |
| Efet.01.239185.g1511.t1 | GRB2-associated-binding protein 4 | 360 | 6.46E-12 | 53 |
| Efet.01.430435.g744.t1 | Guanylate-binding protein 1 | 1056 | 4.14E-31 | 53 |
| Efet.01.1652285.g551.t1 | Growth/differentiation factor 11 | 369 | 5.28E-15 | 53 |
| Efet.01.10432.g765.t1 | Growth hormone secretagogue receptor type 1 | 1200 | 4.60E-10 | 53 |
| Efet.01.181257.g1465.t1 | Growth hormone secretagogue receptor type 1 | 882 | 4.70E-24 | 53 |
| Efet.01.201905.g97.t1 | Growth hormone secretagogue receptor type 1 | 1002 | 3.73E-31 | 53 |
| Efet.01.643099.g761.t1 | Bifunctional UDP-N-acetylglucosamine 2-epimerase/N-acetylmannosamine kinase | 1077 | 5.54E-14 | 53 |
| Efet.01.192165.g1967.t1 | Glucagon receptor | 357 | 7.94E-17 | 53 |
| Efet.01.1658522.g1206.t1 | Gremlin-1 | 714 | 9.33E-23 | 53 |
| Efet.01.108200.g425.t1 | Solute carrier family 2, facilitated glucose transporter member 4 | 840 | 7.24E-44 | 53 |
| Efet.01.216065.g665.t1 | Hydroxycarboxylic acid receptor 2 | 1455 | 2.80E-07 | 53 |
| Efet.01.98106.g1342.t1 | Hemicentin-1 | 567 | 1.18E-30 | 53 |
| Efet.01.73780.g1462.t1 | Histamine H1 receptor | 840 | 1.01E-13 | 53 |
| Efet.01.599555.g1297.t1 | Immunoglobulin superfamily DCC subclass member 3 | 363 | 2.18E-15 | 53 |
| Efet.01.1657789.g1037.t1 | Immunoglobulin superfamily member 10 | 249 | 2.56E-06 | 53 |
| Efet.01.649466.g1311.t1 | Inosine-5'-monophosphate dehydrogenase 2 | 1410 | 2.38E-25 | 53 |
| Efet.01.543313.g328.t1 | Tyrosine-protein kinase ITK/TSK | 405 | 1.36E-07 | 53 |
| Efet.01.25058.g7.t1 | KN motif and ankyrin repeat domain-containing protein 1 | 2331 | 2.60E-08 | 53 |
| Efet.01.336941.g1090.t1 | ATP-sensitive inward rectifier potassium channel 8 | 1854 | 8.26E-62 | 53 |
| Efet.01.232754.g1298.t1 | Kelch-like protein 20 | 300 | 3.19E-07 | 53 |
| Efet.01.326363.g811.t1 | Kelch-like protein 20 | 291 | 2.33E-08 | 53 |
| Efet.01.657208.g1202.t1 | Kelch-like protein 9 | 549 | 2.53E-14 | 53 |
| Efet.01.514275.g343.t1 | Histone-lysine N-methyltransferase 2A | 216 | 4.69E-10 | 53 |
| Efet.01.354504.g128.t1 | Low-density lipoprotein receptor-related protein 1B | 477 | 9.89E-14 | 53 |
| Efet.01.656513.g1036.t1 | Arachidonate 12-lipoxygenase, 12R-type | 420 | 2.19E-10 | 53 |
| Efet.01.421681.g518.t1 | Mitogen-activated protein kinase kinase kinase 10 | 387 | 4.66E-10 | 53 |
| Efet.01.287526.g1362.t1 | Multidrug resistance protein 1 | 642 | 5.13E-22 | 53 |
| Efet.01.645424.g985.t1 | Multidrug resistance protein 1 | 612 | 2.75E-17 | 53 |
| Efet.01.648013.g1216.t1 | Multidrug resistance protein 1 | 1089 | 2.24E-10 | 53 |
| Efet.01.658150.g1688.t1 | Multidrug resistance protein 1 | 600 | 3.51E-12 | 53 |
| Efet.01.311777.g375.t1 | E3 ubiquitin-protein ligase MIB1 | 1626 | 8.75E-08 | 53 |
| Efet.01.1658788.g1276.t1 | E3 ubiquitin-protein ligase MIB1 | 621 | 1.01E-19 | 53 |
| Efet.01.29274.g295.t1 | Carbohydrate-responsive element-binding protein | 768 | 6.46E-24 | 53 |
| Efet.01.2598.g238.t1 | Canalicular multispecific organic anion transporter 1 | 981 | 4.39E-60 | 53 |
| Efet.01.340194.g1176.t1 | DNA mismatch repair protein Msh6 | 2007 | 7.21E-08 | 53 |
| Efet.01.76696.g99.t1 | Myosin-9 | 852 | 1.43E-59 | 53 |
| Efet.01.252426.g88.t1 | Myosin-9 | 936 | 9.69E-10 | 53 |
| Efet.01.256.g32.t1 | Myosin light chain kinase, smooth muscle | 672 | 4.47E-22 | 53 |
| Efet.01.19332.g1431.t1 | Myosin light chain kinase, smooth muscle | 1068 | 5.48E-17 | 53 |
| Efet.01.120059.g1036.t1 | Myeloid zinc finger 1 | 2235 | 1.94E-10 | 53 |
| Efet.01.1657429.g984.t1 | Myeloid zinc finger 1 | 432 | 1.16E-24 | 53 |
| Efet.01.10360.g758.t1 | Neogenin | 591 | 3.09E-15 | 53 |
| Efet.01.57853.g491.t1 | Nicastrin | 684 | 3.45E-38 | 53 |
| Efet.01.226191.g1047.t1 | Nitric oxide synthase, brain | 852 | 1.39E-30 | 53 |
| Efet.01.107332.g380.t1 | Neurogenic locus notch homolog protein 1 | 996 | 9.45E-70 | 53 |
| Efet.01.548630.g462.t1 | Neurogenic locus notch homolog protein 2 | 846 | 2.58E-33 | 53 |
| Efet.01.161270.g581.t1 | Neuronal cell adhesion molecule | 426 | 6.18E-18 | 53 |
| Efet.01.620132.g882.t1 | Ileal sodium/bile acid cotransporter | 1005 | 4.15E-33 | 53 |
| Efet.01.471393.g567.t1 | Peroxisomal coenzyme A diphosphatase NUDT7 | 732 | 3.14E-27 | 53 |
| Efet.01.333335.g1000.t1 | Opioid-binding protein/cell adhesion molecule | 465 | 9.63E-09 | 53 |
| Efet.01.80872.g391.t1 | Kappa-type opioid receptor | 1161 | 8.21E-32 | 53 |
| Efet.01.254136.g152.t1 | Kappa-type opioid receptor | 1041 | 1.79E-15 | 53 |
| Efet.01.322080.g686.t1 | Kappa-type opioid receptor | 984 | 3.55E-18 | 53 |
| Efet.01.187247.g1740.t1 | Partitioning defective 3 homolog | 1311 | 8.85E-08 | 53 |
| Efet.01.262709.g481.t1 | TCDD-inducible poly [ADP-ribose] polymerase | 399 | 4.04E-18 | 53 |
| Efet.01.657446.g1285.t1 | Protocadherin-11 Y-linked | 960 | 1.98E-37 | 53 |
| Efet.01.21624.g1588.t1 | Protocadherin-16 | 639 | 1.91E-22 | 53 |
| Efet.01.190560.g1896.t1 | Protocadherin-16 | 468 | 8.54E-14 | 53 |
| Efet.01.657604.g1334.t1 | Protocadherin-16 | 1365 | 1.62E-29 | 53 |
| Efet.01.1655754.g789.t1 | Protocadherin-18 | 315 | 9.19E-14 | 53 |
| Efet.01.100889.g62.t1 | Protocadherin-19 | 2925 | 4.42E-101 | 53 |
| Efet.01.275935.g964.t1 | Protocadherin-19 | 1980 | 5.97E-82 | 53 |
| Efet.01.522670.g529.t1 | Protocadherin-19 | 1356 | 3.01E-45 | 53 |
| Efet.01.379381.g784.t1 | Protocadherin alpha-7 | 540 | 8.23E-25 | 53 |
| Efet.01.94242.g1116.t1 | Protocadherin gamma-A9 | 1368 | 8.94E-71 | 53 |
| Efet.01.220561.g834.t1 | Protocadherin-7 | 612 | 1.34E-20 | 53 |
| Efet.01.55530.g362.t1 | Protocadherin-9 | 768 | 1.10E-28 | 53 |
| Efet.01.100270.g15.t1 | Protocadherin-9 | 537 | 1.65E-16 | 53 |
| Efet.01.622543.g982.t1 | Protocadherin-9 | 2853 | 3.56E-113 | 53 |
| Efet.01.626983.g1188.t1 | Peroxisomal trans-2-enoyl-CoA reductase | 651 | 5.27E-32 | 53 |
| Efet.01.278002.g1032.t1 | PH domain leucine-rich repeat-containing protein phosphatase 1 | 1905 | 6.75E-37 | 53 |
| Efet.01.36229.g745.t1 | Pleckstrin homology domain-containing family G member 5 | 312 | 1.09E-20 | 53 |
| Efet.01.407831.g188.t1 | Serine/threonine-protein kinase PLK4 | 489 | 2.05E-26 | 53 |
| Efet.01.2325.g209.t1 | Plexin-A3 | 681 | 1.51E-46 | 53 |
| Efet.01.563706.g112.t1 | Plexin-A3 | 327 | 1.34E-11 | 53 |
| Efet.01.149916.g1225.t1 | Tartrate-resistant acid phosphatase type 5 | 261 | 1.10E-09 | 53 |
| Efet.01.551655.g542.t1 | Peptidyl-prolyl cis-trans isomerase E | 429 | 3.70E-06 | 53 |
| Efet.01.148529.g1161.t1 | PR domain zinc finger protein 16 | 264 | 9.60E-08 | 53 |
| Efet.01.63891.g856.t1 | 26S proteasome non-ATPase regulatory subunit 10 | 1347 | 8.53E-12 | 53 |
| Efet.01.303006.g93.t1 | Prostacyclin synthase | 429 | 7.34E-17 | 53 |
| Efet.01.275134.g931.t1 | Prostaglandin reductase 1 | 1020 | 8.92E-46 | 53 |
| Efet.01.22382.g1628.t1 | Inactive tyrosine-protein kinase 7 | 333 | 6.27E-16 | 53 |
| Efet.01.441784.g1027.t1 | TNC variant protein | 555 | 3.42E-24 | 53 |
| Efet.01.84888.g622.t1 | Proteasome (Prosome, macropain) 26S subunit, ATPase, 1 | 348 | 2.53E-12 | 53 |
| Efet.01.40170.g996.t1 | Chemokine (C-C motif) receptor 1 | 501 | 5.45E-19 | 53 |
| Efet.01.251185.g39.t1 | SOCS3 protein (Suppressor of cytokine signaling 3, isoform CRA_a) | 879 | 2.13E-30 | 53 |
| Efet.01.447897.g1172.t1 | Zinc finger protein 3 | 624 | 1.54E-39 | 53 |
| Efet.01.606065.g252.t1 | Zinc finger protein 3 | 1611 | 5.25E-40 | 53 |
| Efet.01.113449.g682.t1 | Uncharacterized protein | 300 | 2.01E-12 | 53 |
| Efet.01.238089.g1481.t1 | Reelin | 627 | 2.57E-20 | 53 |
| Efet.01.1658029.g1083.t1 | RGM domain family member B | 729 | 1.96E-21 | 53 |
| Efet.01.225780.g1026.t1 | Rho-associated protein kinase 2 | 360 | 3.32E-20 | 53 |
| Efet.01.310446.g329.t1 | DNA-directed RNA polymerase III subunit RPC1 | 477 | 7.00E-18 | 53 |
| Efet.01.143912.g972.t1 | Sodium channel protein type 2 subunit alpha | 1092 | 3.22E-75 | 53 |
| Efet.01.322098.g688.t1 | Sodium channel protein type 2 subunit alpha | 405 | 8.77E-16 | 53 |
| Efet.01.118537.g956.t1 | Protein scribble homolog | 480 | 2.07E-08 | 53 |
| Efet.01.310948.g348.t1 | Sortilin-related receptor | 390 | 7.65E-16 | 53 |
| Efet.01.367334.g467.t1 | Spectrin beta chain, erythrocytic | 243 | 1.95E-07 | 53 |
| Efet.01.628200.g1235.t1 | SCO-spondin | 489 | 2.70E-24 | 53 |
| Efet.01.101055.g73.t1 | Synaptotagmin-9 | 375 | 1.15E-11 | 53 |
| Efet.01.21316.g1572.t1 | F-box-like/WD repeat-containing protein TBL1Y | 345 | 1.15E-07 | 53 |
| Efet.01.1636455.g177.t1 | T-box transcription factor TBX3 | 204 | 9.58E-11 | 53 |
| Efet.01.562540.g52.t1 | Transferrin receptor protein 2 | 1440 | 8.24E-44 | 53 |
| Efet.01.367531.g474.t1 | TNFAIP3-interacting protein 2 | 360 | 2.35E-07 | 53 |
| Efet.01.297209.g1662.t1 | Troponin C, skeletal muscle | 240 | 2.97E-06 | 53 |
| Efet.01.366963.g459.t1 | E3 ubiquitin-protein ligase TRIM39 | 843 | 1.75E-07 | 53 |
| Efet.01.121156.g1100.t1 | Protein turtle homolog B | 789 | 5.51E-06 | 53 |
| Efet.01.16311.g1205.t1 | Tyrosine-protein kinase TXK | 3684 | 2.40E-52 | 53 |
| Efet.01.79836.g312.t1 | UDP-glucuronosyltransferase 1-10 | 819 | 6.80E-45 | 53 |
| Efet.01.452012.g52.t1 | Vasopressin V1b receptor | 1167 | 4.75E-49 | 53 |
| Efet.01.218844.g763.t1 | Very low-density lipoprotein receptor | 468 | 9.89E-17 | 53 |
| Efet.01.348247.g1363.t1 | Very low-density lipoprotein receptor | 363 | 3.22E-07 | 53 |
| Efet.01.75866.g50.t1 | Protein Wnt-4 | 693 | 9.33E-26 | 53 |
| Efet.01.586731.g887.t1 | Protein Wnt-7a | 231 | 3.86E-12 | 53 |
| Efet.01.172552.g1074.t1 | Vasopressin V1a receptor | 552 | 2.08E-23 | 53 |
| Efet.01.500525.g11.t1 | Vasopressin V1a receptor | 417 | 9.75E-13 | 53 |
| Efet.01.404132.g105.t1 | DNA repair protein XRCC2 | 285 | 6.62E-06 | 53 |
| Efet.01.4227.g352.t1 | Zonadhesin | 411 | 8.77E-18 | 53 |
| Efet.01.71147.g1307.t1 | Zinc finger protein 675 | 1257 | 2.86E-87 | 53 |
| Efet.01.496822.g1108.t1 | Zinc finger protein 675 | 1422 | 3.74E-52 | 53 |
| Efet.01.366620.g449.t1 | 5-hydroxytryptamine receptor 2B | 879 | 1.03E-14 | 52 |
| Efet.01.234414.g1359.t1 | DDR1 (Discoidin domain receptor family, member 1, isoform CRA_d) | 504 | 3.12E-32 | 52 |
| Efet.01.489168.g946.t1 | Neural cell adhesion molecule L1-like protein | 426 | 1.73E-08 | 52 |
| Efet.01.411005.g248.t1 | Caspase 10 apoptosis-related cysteine peptidase isoform 3 | 345 | 2.26E-08 | 52 |
| Efet.01.194061.g2052.t1 | Acetoacetyl-CoA synthetase | 567 | 5.00E-13 | 52 |
| Efet.01.571102.g355.t1 | ATP-binding cassette sub-family A member 5 | 885 | 3.34E-17 | 52 |
| Efet.01.655285.g790.t1 | ATP-binding cassette sub-family A member 5 | 648 | 6.59E-14 | 52 |
| Efet.01.39673.g974.t1 | ATP-binding cassette sub-family A member 7 | 957 | 1.15E-25 | 52 |
| Efet.01.597702.g1230.t1 | ATP-binding cassette sub-family B member 9 | 681 | 2.50E-15 | 52 |
| Efet.01.657766.g1452.t1 | ATP-binding cassette sub-family B member 9 | 1740 | 1.68E-31 | 52 |
| Efet.01.646487.g1096.t1 | ATP-binding cassette sub-family G member 1 | 612 | 2.25E-10 | 52 |
| Efet.01.658428.g2151.t1 | ATP-binding cassette sub-family G member 1 | 744 | 3.85E-18 | 52 |
| Efet.01.1659450.g1742.t1 | ATP-binding cassette sub-family G member 1 | 717 | 3.62E-19 | 52 |
| Efet.01.608919.g371.t1 | Acyl-CoA synthetase family member 2, mitochondrial | 378 | 3.93E-17 | 52 |
| Efet.01.74277.g1504.t1 | Alpha-actinin-3 | 495 | 2.33E-42 | 52 |
| Efet.01.97189.g1295.t1 | Alpha-1A adrenergic receptor | 1185 | 4.10E-56 | 52 |
| Efet.01.119388.g1003.t1 | Alpha-1A adrenergic receptor | 1614 | 5.28E-66 | 52 |
| Efet.01.338850.g1136.t1 | Alpha-1A adrenergic receptor | 408 | 2.31E-07 | 52 |
| Efet.01.507448.g192.t1 | Alpha-1A adrenergic receptor | 1188 | 1.36E-61 | 52 |
| Efet.01.296467.g1631.t1 | Alpha-1B adrenergic receptor | 924 | 1.80E-09 | 52 |
| Efet.01.30262.g355.t1 | Adenosine deaminase 2 | 1098 | 2.26E-40 | 52 |
| Efet.01.597855.g1239.t1 | Beta-1 adrenergic receptor | 1059 | 2.48E-18 | 52 |
| Efet.01.77155.g121.t1 | Beta-2 adrenergic receptor | 1050 | 3.69E-47 | 52 |
| Efet.01.630860.g79.t1 | Aldehyde dehydrogenase family 1 member A3 | 1479 | 3.64E-76 | 52 |
| Efet.01.5443.g422.t1 | Ankyrin-1 | 2073 | 2.64E-18 | 52 |
| Efet.01.161827.g612.t1 | Ankyrin-1 | 1734 | 1.12E-77 | 52 |
| Efet.01.214833.g622.t1 | Ankyrin-1 | 2982 | 1.05E-111 | 52 |
| Efet.01.402068.g53.t1 | Ankyrin-1 | 822 | 7.04E-21 | 52 |
| Efet.01.500190.g5.t1 | Ankyrin-1 | 1152 | 7.13E-50 | 52 |
| Efet.01.53900.g258.t1 | Aquaporin-4 | 471 | 2.20E-09 | 52 |
| Efet.01.105150.g277.t1 | Asporin | 1182 | 8.58E-19 | 52 |
| Efet.01.267712.g654.t1 | Sarcoplasmic/endoplasmic reticulum calcium ATPase 2 | 525 | 1.18E-21 | 52 |
| Efet.01.360588.g284.t1 | Bcl-2-like protein 2 | 501 | 5.05E-26 | 52 |
| Efet.01.1659487.g1842.t1 | cDNA FLJ40025 fis, clone STOMA2008050, highly similar to Homo sapiens ATP-binding cassette, sub-family A | 1365 | 2.24E-07 | 52 |
| Efet.01.387053.g946.t1 | Zinc finger transcription factor BCL6S | 984 | 3.78E-07 | 52 |
| Efet.01.597702.g1220.t1 | ATP-binding cassette transporter A1 | 1503 | 2.56E-17 | 52 |
| Efet.01.77852.g166.t1 | BLM protein | 519 | 5.80E-22 | 52 |
| Efet.01.296052.g1614.t1 | B-cell lymphoma 6 protein | 1704 | 1.38E-22 | 52 |
| Efet.01.408562.g205.t1 | B-cell lymphoma 6 protein | 354 | 1.05E-22 | 52 |
| Efet.01.384014.g887.t1 | Protein bicaudal D homolog 2 | 2088 | 4.83E-89 | 52 |
| Efet.01.232238.g1280.t1 | Bone morphogenetic protein 1 | 438 | 5.02E-18 | 52 |
| Efet.01.307770.g232.t1 | Calmodulin | 1374 | 9.87E-11 | 52 |
| Efet.01.67924.g1113.t1 | Cyclin-G1 | 765 | 7.20E-17 | 52 |
| Efet.01.87188.g753.t1 | Cell adhesion molecule-related/down-regulated by oncogenes | 1050 | 3.28E-07 | 52 |
| Efet.01.169044.g937.t1 | Cadherin EGF LAG seven-pass G-type receptor 2 | 495 | 1.07E-10 | 52 |
| Efet.01.180113.g1405.t1 | Chromodomain-helicase-DNA-binding protein 8 | 846 | 1.22E-33 | 52 |
| Efet.01.168410.g911.t1 | Collagen alpha-3(VI) chain | 348 | 4.10E-14 | 52 |
| Efet.01.125155.g7.t1 | Collagen alpha-6(VI) chain | 1098 | 1.40E-34 | 52 |
| Efet.01.475948.g671.t1 | Collagen alpha-1(XIV) chain | 468 | 9.60E-18 | 52 |
| Efet.01.630903.g99.t1 | Collagen alpha-1(XIV) chain | 498 | 2.60E-16 | 52 |
| Efet.01.101539.g100.t1 | Collagen alpha-1(XXI) chain | 492 | 1.40E-11 | 52 |
| Efet.01.77328.g134.t1 | Calsyntenin-2 | 348 | 1.50E-15 | 52 |
| Efet.01.651148.g133.t1 | C-terminal-binding protein 2 | 1230 | 1.57E-28 | 52 |
| Efet.01.653968.g501.t1 | C-terminal-binding protein 2 | 954 | 3.42E-28 | 52 |
| Efet.01.111788.g592.t1 | Cubilin | 726 | 2.60E-08 | 52 |
| Efet.01.468658.g498.t1 | Cubilin | 390 | 5.12E-06 | 52 |
| Efet.01.38405.g891.t1 | Dystroglycan | 2574 | 4.28E-34 | 52 |
| Efet.01.143711.g957.t1 | Delta-like protein 1 | 261 | 3.10E-10 | 52 |
| Efet.01.195070.g2094.t1 | Delta-like protein 1 | 765 | 8.23E-41 | 52 |
| Efet.01.515650.g381.t1 | DnaJ homolog subfamily A member 3, mitochondrial | 1146 | 4.64E-63 | 52 |
| Efet.01.646833.g1128.t1 | Dual specificity protein phosphatase 22 | 402 | 3.37E-21 | 52 |
| Efet.01.130094.g274.t1 | Endothelin receptor type B | 1032 | 2.64E-39 | 52 |
| Efet.01.317876.g567.t1 | Endoplasmic reticulum aminopeptidase 1 | 825 | 2.32E-66 | 52 |
| Efet.01.297866.g1684.t1 | Estrogen receptor beta | 276 | 4.31E-10 | 52 |
| Efet.01.308049.g240.t1 | MDS1 and EVI1 complex locus protein EVI1 | 1035 | 2.00E-12 | 52 |
| Efet.01.124196.g1248.t1 | Exostosin-like 1 | 255 | 4.03E-06 | 52 |
| Efet.01.122286.g1159.t1 | Coagulation factor X | 294 | 4.88E-06 | 52 |
| Efet.01.143159.g929.t1 | Protocadherin Fat 1 | 345 | 1.73E-09 | 52 |
| Efet.01.545525.g381.t1 | Protocadherin Fat 1 | 789 | 1.80E-20 | 52 |
| Efet.01.159707.g507.t1 | Protocadherin Fat 3 | 423 | 2.24E-14 | 52 |
| Efet.01.490897.g973.t1 | Fibroblast growth factor receptor 3 | 276 | 6.68E-08 | 52 |
| Efet.01.13472.g1007.t1 | Fibroblast growth factor receptor 4 | 450 | 1.17E-10 | 52 |
| Efet.01.109444.g494.t1 | Fibroleukin | 1251 | 7.26E-31 | 52 |
| Efet.01.165146.g762.t1 | Peptidyl-prolyl cis-trans isomerase FKBP4 | 807 | 3.60E-17 | 52 |
| Efet.01.125754.g45.t1 | FRAS1-related extracellular matrix protein 1 | 813 | 1.10E-21 | 52 |
| Efet.01.264096.g527.t1 | FRAS1-related extracellular matrix protein 1 | 435 | 7.37E-11 | 52 |
| Efet.01.113717.g697.t1 | Follistatin-related protein 1 | 414 | 4.49E-07 | 52 |
| Efet.01.1643975.g289.t1 | Alpha-(1,3)-fucosyltransferase 4 | 372 | 1.42E-10 | 52 |
| Efet.01.153261.g174.t1 | Polypeptide N-acetylgalactosaminyltransferase 2 | 1983 | 3.81E-84 | 52 |
| Efet.01.657652.g1357.t1 | Polypeptide N-acetylgalactosaminyltransferase 2 | 717 | 8.39E-32 | 52 |
| Efet.01.168046.g890.t1 | Growth hormone secretagogue receptor type 1 | 750 | 1.63E-09 | 52 |
| Efet.01.18203.g1345.t1 | Glomulin | 729 | 6.09E-17 | 52 |
| Efet.01.638894.g478.t1 | Glutaredoxin-2, mitochondrial | 261 | 6.14E-10 | 52 |
| Efet.01.485734.g876.t1 | N-acetyllactosaminide beta-1,6-N-acetylglucosaminyl-transferase | 744 | 3.01E-20 | 52 |
| Efet.01.1648008.g390.t1 | N-acetyllactosaminide beta-1,6-N-acetylglucosaminyl-transferase | 387 | 1.32E-09 | 52 |
| Efet.01.357773.g217.t1 | E3 ubiquitin-protein ligase RNF130 | 555 | 1.49E-11 | 52 |
| Efet.01.39258.g950.t1 | G-protein coupled receptor 98 | 1404 | 5.28E-27 | 52 |
| Efet.01.655821.g879.t1 | Histone deacetylase 6 | 528 | 3.50E-26 | 52 |
| Efet.01.162667.g643.t1 | Hemicentin-1 | 318 | 3.17E-07 | 52 |
| Efet.01.187432.g1751.t1 | Heme oxygenase 1 | 726 | 1.87E-27 | 52 |
| Efet.01.1600837.g2.t1 | Histamine H1 receptor | 207 | 2.20E-06 | 52 |
| Efet.01.608389.g351.t1 | Serine protease HTRA1 | 1515 | 8.98E-39 | 52 |
| Efet.01.86786.g738.t1 | Immunoglobulin-like and fibronectin type III domain-containing protein 1 | 2289 | 3.39E-08 | 52 |
| Efet.01.132988.g397.t1 | Immunoglobulin superfamily member 22 | 276 | 9.72E-11 | 52 |
| Efet.01.185271.g1642.t1 | Transcription factor AP-1 | 948 | 1.43E-40 | 52 |
| Efet.01.54157.g274.t1 | Calcium-activated potassium channel subunit alpha-1 | 312 | 1.44E-19 | 52 |
| Efet.01.54158.g275.t1 | Calcium-activated potassium channel subunit alpha-1 | 312 | 1.44E-19 | 52 |
| Efet.01.161841.g614.t1 | Kelch-like protein 20 | 936 | 7.08E-20 | 52 |
| Efet.01.607412.g308.t1 | Pyruvate kinase PKM | 969 | 5.36E-35 | 52 |
| Efet.01.491732.g990.t1 | Leucine-rich repeat protein 1 | 471 | 1.84E-27 | 52 |
| Efet.01.326202.g808.t1 | Leucine-rich repeats and immunoglobulin-like domains protein 3 | 660 | 1.27E-08 | 52 |
| Efet.01.41773.g1105.t1 | Low-density lipoprotein receptor-related protein 1B | 888 | 3.83E-60 | 52 |
| Efet.01.615480.g692.t1 | Low-density lipoprotein receptor-related protein 1B | 366 | 9.12E-09 | 52 |
| Efet.01.1659417.g1680.t1 | Mitogen-activated protein kinase kinase kinase 10 | 489 | 2.36E-21 | 52 |
| Efet.01.76514.g88.t1 | Guanine nucleotide exchange factor DBS | 837 | 2.40E-24 | 52 |
| Efet.01.604243.g163.t1 | Multidrug resistance protein 1 | 798 | 8.46E-22 | 52 |
| Efet.01.647059.g1154.t1 | Multidrug resistance protein 1 | 885 | 3.20E-16 | 52 |
| Efet.01.1659501.g1895.t1 | Multidrug resistance protein 1 | 1191 | 1.02E-24 | 52 |
| Efet.01.1372.g127.t1 | Myosin-10 | 642 | 7.51E-10 | 52 |
| Efet.01.74293.g1506.t1 | Myosin-9 | 1419 | 7.36E-22 | 52 |
| Efet.01.144561.g999.t1 | Myeloid zinc finger 1 | 2106 | 6.09E-24 | 52 |
| Efet.01.118863.g975.t1 | Nucleus accumbens-associated protein 1 | 1434 | 7.02E-07 | 52 |
| Efet.01.537256.g209.t1 | Neurocan core protein | 288 | 6.27E-12 | 52 |
| Efet.01.210718.g473.t1 | Nidogen-1 | 567 | 3.60E-16 | 52 |
| Efet.01.224874.g996.t1 | Neuroligin-4, X-linked | 507 | 9.54E-19 | 52 |
| Efet.01.283523.g1219.t1 | Neuroligin-4, X-linked | 435 | 4.91E-20 | 52 |
| Efet.01.168210.g901.t1 | Nucleolar GTP-binding protein 1 | 549 | 1.32E-31 | 52 |
| Efet.01.35723.g716.t1 | NPC intracellular cholesterol transporter 1 | 828 | 2.54E-37 | 52 |
| Efet.01.89345.g852.t1 | NPC intracellular cholesterol transporter 1 | 867 | 4.81E-24 | 52 |
| Efet.01.25007.g1.t1 | Neuropeptide Y receptor type 5 | 786 | 1.00E-24 | 52 |
| Efet.01.120884.g1084.t1 | Neuropeptide Y receptor type 5 | 1053 | 2.78E-12 | 52 |
| Efet.01.656427.g1015.t1 | Neuropeptide Y receptor type 5 | 684 | 4.25E-17 | 52 |
| Efet.01.339772.g1168.t1 | Neuronal cell adhesion molecule | 261 | 7.37E-09 | 52 |
| Efet.01.347287.g1330.t1 | Neurexin-1 | 888 | 1.19E-25 | 52 |
| Efet.01.371545.g611.t1 | Neurexin-1 | 417 | 4.25E-18 | 52 |
| Efet.01.401292.g28.t1 | Obscurin | 462 | 2.18E-15 | 52 |
| Efet.01.513005.g305.t1 | Kappa-type opioid receptor | 1164 | 5.42E-06 | 52 |
| Efet.01.532166.g67.t1 | Kappa-type opioid receptor | 1428 | 1.16E-45 | 52 |
| Efet.01.572225.g385.t1 | Kappa-type opioid receptor | 738 | 2.07E-35 | 52 |
| Efet.01.606340.g272.t1 | Kappa-type opioid receptor | 546 | 1.14E-07 | 52 |
| Efet.01.34535.g656.t1 | Mu-type opioid receptor | 783 | 1.29E-27 | 52 |
| Efet.01.653365.g422.t1 | Mu-type opioid receptor | 519 | 1.79E-08 | 52 |
| Efet.01.565819.g192.t1 | Polyadenylate-binding protein 4 | 1170 | 2.26E-20 | 52 |
| Efet.01.519211.g459.t1 | Poly [ADP-ribose] polymerase 1 | 330 | 3.05E-09 | 52 |
| Efet.01.290988.g1485.t1 | Protocadherin-11 X-linked | 2826 | 2.66E-119 | 52 |
| Efet.01.139446.g743.t1 | Protocadherin-11 Y-linked | 2430 | 6.97E-78 | 52 |
| Efet.01.142313.g892.t1 | Protocadherin-10 | 1320 | 1.87E-66 | 52 |
| Efet.01.22489.g1635.t1 | Protocadherin-18 | 1614 | 6.47E-55 | 52 |
| Efet.01.341071.g1189.t1 | Protocadherin-18 | 792 | 1.78E-27 | 52 |
| Efet.01.601319.g66.t1 | Protocadherin-19 | 2319 | 1.65E-109 | 52 |
| Efet.01.19107.g1422.t1 | Protocadherin gamma-A5 | 2628 | 2.98E-61 | 52 |
| Efet.01.21624.g1589.t1 | Protocadherin gamma-A5 | 1461 | 5.42E-69 | 52 |
| Efet.01.100812.g57.t1 | Protocadherin gamma-A5 | 2376 | 1.72E-97 | 52 |
| Efet.01.643183.g766.t1 | Protocadherin gamma-C5 | 1194 | 1.50E-39 | 52 |
| Efet.01.128550.g203.t1 | Protocadherin-1 | 2661 | 3.68E-125 | 52 |
| Efet.01.379695.g787.t1 | Protocadherin-1 | 3024 | 3.62E-97 | 52 |
| Efet.01.225954.g1034.t1 | Protocadherin-9 | 2655 | 1.80E-97 | 52 |
| Efet.01.250796.g23.t1 | Protocadherin-9 | 2385 | 1.24E-105 | 52 |
| Efet.01.492745.g1011.t1 | cGMP-specific 3',5'-cyclic phosphodiesterase | 1578 | 1.09E-35 | 52 |
| Efet.01.145963.g1051.t1 | Piwi-like protein 2 | 447 | 1.32E-13 | 52 |
| Efet.01.18939.g1406.t1 | Serine/threonine-protein kinase PLK3 | 741 | 1.42E-27 | 52 |
| Efet.01.106619.g347.t1 | Plasminogen | 270 | 4.51E-08 | 52 |
| Efet.01.14885.g1092.t1 | POC1 centriolar protein homolog B | 1578 | 1.71E-11 | 52 |
| Efet.01.472549.g611.t1 | Peptidyl-prolyl cis-trans isomerase C | 369 | 6.28E-12 | 52 |
| Efet.01.96100.g1227.t1 | Peptidyl-prolyl cis-trans isomerase E | 309 | 1.86E-11 | 52 |
| Efet.01.193366.g2011.t1 | 26S proteasome non-ATPase regulatory subunit 10 | 519 | 5.94E-08 | 52 |
| Efet.01.90316.g919.t1 | Inactive tyrosine-protein kinase 7 | 339 | 2.68E-07 | 52 |
| Efet.01.303499.g111.t1 | Receptor-type tyrosine-protein phosphatase F | 327 | 3.45E-13 | 52 |
| Efet.01.176388.g1229.t1 | Peroxidasin homolog | 696 | 3.80E-34 | 52 |
| Efet.01.302773.g88.t1 | Mitogen-activated protein kinase | 444 | 2.63E-15 | 52 |
| Efet.01.494042.g1046.t1 | Quinone oxidoreductase PIG3 | 669 | 5.92E-24 | 52 |
| Efet.01.866.g79.t1 | E3 ubiquitin-protein ligase RNF144B | 780 | 4.60E-42 | 52 |
| Efet.01.190460.g1890.t1 | Ras-related protein Rab-18 | 240 | 1.69E-08 | 52 |
| Efet.01.34405.g647.t1 | GTPase HRas | 687 | 6.57E-15 | 52 |
| Efet.01.11662.g857.t1 | Rho GTPase-activating protein 5 | 318 | 8.47E-08 | 52 |
| Efet.01.649450.g1310.t1 | Rho GTPase-activating protein 17 | 393 | 1.57E-08 | 52 |
| Efet.01.113788.g699.t1 | Reticulon-4 | 567 | 2.07E-11 | 52 |
| Efet.01.264153.g530.t1 | Retinoic acid receptor RXR-alpha | 708 | 2.51E-06 | 52 |
| Efet.01.389997.g1006.t1 | Retinoic acid receptor RXR-alpha | 567 | 1.25E-07 | 52 |
| Efet.01.57160.g447.t1 | Sacsin | 1590 | 1.46E-84 | 52 |
| Efet.01.260531.g390.t1 | Deoxynucleoside triphosphate triphosphohydrolase SAMHD1 | 624 | 7.96E-10 | 52 |
| Efet.01.106987.g360.t1 | Sodium/glucose cotransporter 4 | 567 | 3.17E-17 | 52 |
| Efet.01.638470.g442.t1 | Sodium channel protein type 2 subunit alpha | 303 | 1.43E-14 | 52 |
| Efet.01.524324.g570.t1 | Protein scribble homolog | 798 | 1.04E-21 | 52 |
| Efet.01.518908.g455.t1 | Serine/threonine-protein kinase Sgk2 | 486 | 2.67E-08 | 52 |
| Efet.01.96362.g1235.t1 | Spectrin beta chain, non-erythrocytic 2 | 819 | 2.24E-32 | 52 |
| Efet.01.138522.g694.t1 | Synaptojanin-1 | 522 | 1.91E-24 | 52 |
| Efet.01.3137.g273.t1 | Synaptotagmin-7 | 837 | 2.71E-09 | 52 |
| Efet.01.72870.g1411.t1 | Tenascin-N | 606 | 1.74E-27 | 52 |
| Efet.01.607560.g316.t1 | Tumor necrosis factor alpha-induced protein 3 | 1224 | 1.77E-24 | 52 |
| Efet.01.605501.g228.t1 | DNA topoisomerase 2-beta | 489 | 6.71E-08 | 52 |
| Efet.01.652475.g308.t1 | E3 ubiquitin-protein ligase TRAF7 | 453 | 1.52E-20 | 52 |
| Efet.01.262143.g455.t1 | Tripartite motif-containing protein 6 | 1473 | 3.37E-06 | 52 |
| Efet.01.206171.g267.t1 | Protein turtle homolog A | 288 | 1.33E-17 | 52 |
| Efet.01.231434.g1247.t1 | Protein turtle homolog B | 525 | 5.32E-13 | 52 |
| Efet.01.432193.g778.t1 | Protein turtle homolog B | 288 | 7.72E-09 | 52 |
| Efet.01.150508.g27.t1 | Uveal autoantigen with coiled-coil domains and ankyrin repeats | 285 | 3.96E-08 | 52 |
| Efet.01.226463.g1057.t1 | Protein disulfide-isomerase | 285 | 1.39E-08 | 52 |
| Efet.01.82970.g522.t1 | WSC domain-containing protein 2 | 579 | 4.37E-08 | 52 |
| Efet.01.127823.g155.t1 | Vasopressin V1a receptor | 1404 | 6.61E-41 | 52 |
| Efet.01.532831.g88.t1 | Zinc finger protein 175 | 1674 | 1.16E-75 | 52 |
| Efet.01.476997.g689.t1 | Zinc finger protein 443 | 5253 | 2.93E-93 | 52 |
| Efet.01.56656.g414.t1 | Zinc finger protein 675 | 1158 | 7.48E-66 | 52 |
| Efet.01.102725.g155.t1 | Zinc finger protein 675 | 816 | 8.75E-47 | 52 |
| Efet.01.148280.g1153.t1 | Zinc finger protein 675 | 1701 | 1.21E-65 | 52 |
| Efet.01.474362.g640.t1 | Zinc finger protein 675 | 768 | 6.29E-41 | 52 |
| Efet.01.37251.g810.t1 | Zinc finger protein 3 | 1401 | 7.46E-43 | 52 |
| Efet.01.27686.g169.t1 | Serpin peptidase inhibitor, clade B (Ovalbumin), member 9 | 936 | 4.14E-30 | 51 |
| Efet.01.348252.g1365.t1 | Tyrosine-protein kinase | 585 | 3.89E-24 | 51 |
| Efet.01.655193.g770.t1 | Cystic fibrosis transmembrane conductance regulator | 450 | 1.13E-13 | 51 |
| Efet.01.386139.g933.t1 | Complement C1q tumor necrosis factor-related protein 1 | 582 | 7.30E-26 | 51 |
| Efet.01.362212.g328.t1 | Adenosine receptor A2 | 297 | 1.80E-09 | 51 |
| Efet.01.658261.g1770.t1 | ATP-binding cassette sub-family A member 5 | 711 | 2.26E-24 | 51 |
| Efet.01.654656.g649.t1 | ATP-binding cassette sub-family A member 7 | 834 | 2.00E-19 | 51 |
| Efet.01.643394.g779.t1 | ATP-binding cassette sub-family B member 9 | 786 | 1.33E-14 | 51 |
| Efet.01.658255.g1757.t1 | ATP-binding cassette sub-family B member 9 | 1107 | 1.87E-21 | 51 |
| Efet.01.654756.g692.t1 | ATP-binding cassette sub-family G member 1 | 435 | 2.46E-07 | 51 |
| Efet.01.658257.g1761.t1 | ATP-binding cassette sub-family G member 1 | 798 | 1.25E-09 | 51 |
| Efet.01.179549.g1373.t1 | Neuronal acetylcholine receptor subunit alpha-7 | 1161 | 7.77E-54 | 51 |
| Efet.01.352855.g78.t1 | Neuronal acetylcholine receptor subunit beta-2 | 1320 | 2.89E-26 | 51 |
| Efet.01.1659189.g1451.t1 | Acyl-CoA synthetase family member 2, mitochondrial | 1224 | 5.16E-47 | 51 |
| Efet.01.224166.g970.t1 | Acyl-CoA synthetase family member 3, mitochondrial | 762 | 4.42E-36 | 51 |
| Efet.01.658297.g1813.t1 | Acyl-CoA synthetase family member 3, mitochondrial | 1707 | 3.13E-07 | 51 |
| Efet.01.2663.g243.t1 | Alpha-1B adrenergic receptor | 384 | 2.73E-07 | 51 |
| Efet.01.98659.g1365.t1 | Alpha-1B adrenergic receptor | 1704 | 3.86E-46 | 51 |
| Efet.01.237744.g1472.t1 | ADP/ATP translocase 3 | 312 | 9.14E-09 | 51 |
| Efet.01.616615.g732.t1 | Retinal dehydrogenase 2 | 2982 | 5.00E-75 | 51 |
| Efet.01.606285.g260.t1 | Aldehyde dehydrogenase family 1 member A3 | 1437 | 1.01E-80 | 51 |
| Efet.01.632798.g172.t1 | Aldehyde dehydrogenase family 1 member A3 | 1155 | 6.50E-39 | 51 |
| Efet.01.90021.g896.t1 | Insulin-like growth factor-binding protein complex acid labile subunit | 1686 | 9.54E-22 | 51 |
| Efet.01.208269.g379.t1 | Ankyrin-1 | 3315 | 1.59E-114 | 51 |
| Efet.01.218940.g764.t1 | Ankyrin-1 | 1131 | 1.73E-29 | 51 |
| Efet.01.574697.g463.t1 | Ankyrin-1 | 2763 | 9.90E-37 | 51 |
| Efet.01.6134.g485.t1 | Anillin | 1059 | 2.19E-45 | 51 |
| Efet.01.123185.g1203.t1 | Ankyrin repeat domain-containing protein 54 | 393 | 7.78E-09 | 51 |
| Efet.01.457823.g214.t1 | Ankyrin repeat and SOCS box protein 1 | 810 | 4.65E-06 | 51 |
| Efet.01.8178.g605.t1 | Sarcoplasmic/endoplasmic reticulum calcium ATPase 2 | 348 | 2.48E-11 | 51 |
| Efet.01.69641.g1217.t1 | Sarcoplasmic/endoplasmic reticulum calcium ATPase 2 | 1185 | 3.94E-53 | 51 |
| Efet.01.258767.g329.t1 | Sarcoplasmic/endoplasmic reticulum calcium ATPase 2 | 690 | 1.15E-08 | 51 |
| Efet.01.463936.g372.t1 | Sarcoplasmic/endoplasmic reticulum calcium ATPase 2 | 558 | 1.32E-16 | 51 |
| Efet.01.276655.g983.t1 | Calcium-transporting ATPase type 2C member 1 | 516 | 1.83E-17 | 51 |
| Efet.01.555350.g639.t1 | A disintegrin and metalloproteinase with thrombospondin motifs 1 | 771 | 1.51E-12 | 51 |
| Efet.01.434.g52.t1 | Aurora kinase C | 1095 | 1.37E-24 | 51 |
| Efet.01.481519.g782.t1 | Bcl-2-like protein 2 | 420 | 4.14E-20 | 51 |
| Efet.01.633829.g212.t1 | cDNA FLJ53627, highly similar to Antigen peptide transporter 1 | 2313 | 1.40E-15 | 51 |
| Efet.01.351135.g37.t1 | B-cell lymphoma/leukemia 11B | 927 | 3.21E-10 | 51 |
| Efet.01.161735.g609.t1 | Baculoviral IAP repeat-containing protein 6 | 423 | 2.38E-17 | 51 |
| Efet.01.276178.g969.t1 | Baculoviral IAP repeat-containing protein 6 | 945 | 1.92E-31 | 51 |
| Efet.01.289926.g1445.t1 | Bone morphogenetic protein 1 | 618 | 8.30E-09 | 51 |
| Efet.01.120524.g1069.t1 | BTB/POZ domain-containing protein 9 | 774 | 2.54E-07 | 51 |
| Efet.01.43870.g1219.t1 | Tyrosine-protein kinase BTK | 2847 | 9.01E-58 | 51 |
| Efet.01.114272.g735.t1 | Voltage-dependent T-type calcium channel subunit alpha-1G | 549 | 5.79E-16 | 51 |
| Efet.01.27707.g171.t1 | Calpain-10 | 225 | 7.41E-07 | 51 |
| Efet.01.60523.g651.t1 | Mast cell carboxypeptidase A | 252 | 2.18E-07 | 51 |
| Efet.01.210405.g461.t1 | Cadherin EGF LAG seven-pass G-type receptor 2 | 1311 | 8.98E-45 | 51 |
| Efet.01.74123.g1491.t1 | Carbohydrate sulfotransferase 11 | 669 | 1.01E-24 | 51 |
| Efet.01.39963.g985.t1 | Collagen alpha-1(XII) chain | 705 | 1.12E-19 | 51 |
| Efet.01.41847.g1107.t1 | Collagen alpha-1(XII) chain | 576 | 1.56E-23 | 51 |
| Efet.01.441429.g1018.t1 | Collagen alpha-1(XXI) chain | 630 | 5.43E-08 | 51 |
| Efet.01.614702.g660.t1 | Catechol O-methyltransferase | 642 | 4.10E-11 | 51 |
| Efet.01.126447.g84.t1 | Cytochrome P450 1A1 | 762 | 1.83E-29 | 51 |
| Efet.01.456290.g158.t1 | Cytochrome P450 1A1 | 1434 | 6.95E-70 | 51 |
| Efet.01.29013.g279.t1 | Cytochrome P450 4F8 | 576 | 1.38E-06 | 51 |
| Efet.01.654418.g593.t1 | Carbamoyl-phosphate synthase [ammonia], mitochondrial | 1218 | 1.21E-54 | 51 |
| Efet.01.70752.g1277.t1 | Alpha-crystallin B chain | 1140 | 1.68E-06 | 51 |
| Efet.01.150286.g14.t1 | Dystroglycan | 786 | 4.84E-31 | 51 |
| Efet.01.880.g82.t1 | Death-associated protein kinase 1 | 1473 | 1.65E-21 | 51 |
| Efet.01.98475.g1355.t1 | Discoidin domain-containing receptor 2 | 405 | 4.74E-15 | 51 |
| Efet.01.62690.g789.t1 | Probable ATP-dependent RNA helicase DDX58 | 1500 | 9.72E-51 | 51 |
| Efet.01.565869.g195.t1 | Dehydrogenase/reductase SDR family member 2, mitochondrial | 381 | 2.81E-14 | 51 |
| Efet.01.633916.g221.t1 | Dehydrogenase/reductase SDR family member 2, mitochondrial | 1416 | 8.59E-28 | 51 |
| Efet.01.656278.g978.t1 | Protein diaphanous homolog 2 | 390 | 2.89E-08 | 51 |
| Efet.01.221628.g884.t1 | D(2) dopamine receptor | 1437 | 7.87E-64 | 51 |
| Efet.01.528754.g658.t1 | D(2) dopamine receptor | 1497 | 9.57E-59 | 51 |
| Efet.01.537525.g216.t1 | D(4) dopamine receptor | 315 | 2.51E-06 | 51 |
| Efet.01.251277.g46.t1 | Ectonucleotide pyrophosphatase/phosphodiesterase family member 1 | 906 | 1.68E-40 | 51 |
| Efet.01.126610.g99.t1 | Ephrin type-A receptor 3 | 765 | 5.95E-38 | 51 |
| Efet.01.339172.g1142.t1 | Exostosin-like 1 | 2025 | 6.66E-10 | 51 |
| Efet.01.503764.g95.t1 | Nuclear receptor coactivator 6 | 1665 | 8.78E-13 | 51 |
| Efet.01.61623.g721.t1 | Protocadherin Fat 4 | 642 | 4.62E-17 | 51 |
| Efet.01.68938.g1169.t1 | Protocadherin Fat 4 | 240 | 1.62E-07 | 51 |
| Efet.01.140014.g769.t1 | Protocadherin Fat 4 | 951 | 2.63E-41 | 51 |
| Efet.01.140014.g770.t1 | Protocadherin Fat 4 | 1686 | 1.22E-57 | 51 |
| Efet.01.299359.g1728.t1 | Fibulin-5 | 1632 | 2.38E-31 | 51 |
| Efet.01.120233.g1049.t1 | Frizzled-5 | 426 | 8.26E-17 | 51 |
| Efet.01.68409.g1131.t1 | Gastrin/cholecystokinin type B receptor | 489 | 2.69E-06 | 51 |
| Efet.01.283670.g1227.t1 | Growth hormone secretagogue receptor type 1 | 1209 | 1.53E-06 | 51 |
| Efet.01.653425.g427.t1 | Growth hormone secretagogue receptor type 1 | 723 | 1.50E-06 | 51 |
| Efet.01.591014.g1024.t1 | Bifunctional UDP-N-acetylglucosamine 2-epimerase/N-acetylmannosamine kinase | 636 | 6.92E-11 | 51 |
| Efet.01.126895.g112.t1 | Zinc finger protein GLI2 | 3444 | 4.12E-12 | 51 |
| Efet.01.383813.g884.t1 | Glutamate receptor ionotropic, delta-2 | 777 | 1.60E-22 | 51 |
| Efet.01.119982.g1031.t1 | Heart- and neural crest derivatives-expressed protein 2 | 513 | 4.89E-14 | 51 |
| Efet.01.5446.g423.t1 | Histamine H2 receptor | 1251 | 1.52E-60 | 51 |
| Efet.01.65892.g978.t1 | Immunoglobulin-like and fibronectin type III domain-containing protein 1 | 711 | 1.52E-09 | 51 |
| Efet.01.150884.g44.t1 | Immunoglobulin superfamily member 10 | 1137 | 3.21E-19 | 51 |
| Efet.01.80957.g398.t1 | Immunoglobulin superfamily member 22 | 762 | 3.49E-24 | 51 |
| Efet.01.126469.g90.t1 | Immunoglobulin superfamily member 22 | 648 | 6.05E-31 | 51 |
| Efet.01.1652532.g564.t1 | Immunoglobulin superfamily member 22 | 420 | 4.28E-17 | 51 |
| Efet.01.656303.g989.t1 | Inhibitor of nuclear factor kappa-B kinase subunit alpha | 507 | 5.08E-08 | 51 |
| Efet.01.229812.g1191.t1 | Inositol-trisphosphate 3-kinase B | 489 | 1.06E-33 | 51 |
| Efet.01.615474.g691.t1 | Protein Jade-1 | 885 | 5.14E-50 | 51 |
| Efet.01.28728.g260.t1 | Transcription factor AP-1 | 891 | 1.93E-37 | 51 |
| Efet.01.1658789.g1277.t1 | Kinesin-like protein KIF1B | 255 | 3.32E-08 | 51 |
| Efet.01.62320.g773.t1 | Kelch-like protein 20 | 990 | 3.18E-50 | 51 |
| Efet.01.140965.g823.t1 | Kelch-like protein 20 | 522 | 8.06E-06 | 51 |
| Efet.01.167162.g849.t1 | Kelch-like protein 20 | 1281 | 2.01E-57 | 51 |
| Efet.01.142242.g890.t1 | Kelch-like protein 21 | 2106 | 4.51E-25 | 51 |
| Efet.01.71248.g1313.t1 | LARGE xylosyl- and glucuronyltransferase 2 | 729 | 2.31E-25 | 51 |
| Efet.01.45234.g1311.t1 | Platelet-activating factor acetylhydrolase IB subunit alpha | 357 | 1.28E-06 | 51 |
| Efet.01.46526.g1382.t1 | Platelet-activating factor acetylhydrolase IB subunit alpha | 675 | 7.37E-18 | 51 |
| Efet.01.104261.g240.t1 | Leucine-rich repeats and immunoglobulin-like domains protein 2 | 663 | 1.69E-10 | 51 |
| Efet.01.327016.g832.t1 | Low-density lipoprotein receptor-related protein 1B | 444 | 5.22E-15 | 51 |
| Efet.01.26381.g105.t1 | Latent-transforming growth factor beta-binding protein 2 | 609 | 1.00E-23 | 51 |
| Efet.01.303916.g136.t1 | Latent-transforming growth factor beta-binding protein 4 | 666 | 1.33E-41 | 51 |
| Efet.01.603047.g121.t1 | Latent-transforming growth factor beta-binding protein 4 | 366 | 1.86E-20 | 51 |
| Efet.01.656644.g1056.t1 | Mitogen-activated protein kinase kinase kinase 10 | 345 | 2.04E-10 | 51 |
| Efet.01.3649.g311.t1 | Mitogen-activated protein kinase kinase kinase 20 | 456 | 2.87E-12 | 51 |
| Efet.01.614779.g670.t1 | Inner nuclear membrane protein Man1 | 1617 | 3.19E-34 | 51 |
| Efet.01.651148.g141.t1 | Multidrug resistance protein 1 | 1989 | 1.56E-21 | 51 |
| Efet.01.652309.g279.t1 | Multidrug resistance protein 1 | 804 | 1.85E-14 | 51 |
| Efet.01.658412.g2029.t1 | Multidrug resistance protein 1 | 1908 | 1.76E-14 | 51 |
| Efet.01.642087.g655.t1 | Multidrug resistance-associated protein 5 | 480 | 8.37E-10 | 51 |
| Efet.01.81386.g429.t1 | Myosin-10 | 1410 | 2.08E-12 | 51 |
| Efet.01.16184.g1196.t1 | Myosin-9 | 1623 | 2.56E-11 | 51 |
| Efet.01.112376.g627.t1 | Myosin light chain kinase, smooth muscle | 786 | 4.08E-06 | 51 |
| Efet.01.126673.g103.t1 | Myeloid zinc finger 1 | 555 | 8.23E-09 | 51 |
| Efet.01.156857.g360.t1 | Neurogenic differentiation factor 1 | 1128 | 1.83E-07 | 51 |
| Efet.01.129807.g256.t1 | Protein NDRG1 | 255 | 3.76E-06 | 51 |
| Efet.01.202644.g124.t1 | Neogenin | 270 | 3.05E-06 | 51 |
| Efet.01.285433.g1286.t1 | Neurofascin | 519 | 1.76E-14 | 51 |
| Efet.01.207762.g352.t1 | Nischarin | 2733 | 1.76E-31 | 51 |
| Efet.01.371070.g592.t1 | NLR family CARD domain-containing protein 3 | 2067 | 9.44E-23 | 51 |
| Efet.01.545951.g389.t1 | Noggin | 579 | 1.25E-37 | 51 |
| Efet.01.101982.g121.t1 | Neurogenic locus notch homolog protein 2 | 801 | 9.61E-07 | 51 |
| Efet.01.37851.g855.t1 | Neuropeptide Y receptor type 5 | 891 | 3.60E-13 | 51 |
| Efet.01.27958.g194.t1 | Neurexin-3 | 1503 | 6.91E-16 | 51 |
| Efet.01.100279.g16.t1 | Neurotensin receptor type 1 | 702 | 7.01E-07 | 51 |
| Efet.01.151043.g59.t1 | Mu-type opioid receptor | 1377 | 6.28E-36 | 51 |
| Efet.01.155169.g266.t1 | Mu-type opioid receptor | 1200 | 1.82E-38 | 51 |
| Efet.01.177474.g1291.t1 | Mu-type opioid receptor | 1185 | 1.93E-14 | 51 |
| Efet.01.354363.g124.t1 | Mu-type opioid receptor | 1254 | 4.77E-21 | 51 |
| Efet.01.620294.g890.t1 | Mu-type opioid receptor | 717 | 4.94E-24 | 51 |
| Efet.01.46536.g1383.t1 | Polyadenylate-binding protein 4 | 1026 | 4.09E-23 | 51 |
| Efet.01.114562.g758.t1 | Protocadherin-11 X-linked | 2817 | 1.65E-109 | 51 |
| Efet.01.27587.g163.t1 | Protocadherin-11 Y-linked | 2841 | 2.22E-113 | 51 |
| Efet.01.48605.g1496.t1 | Protocadherin-11 Y-linked | 711 | 1.35E-12 | 51 |
| Efet.01.381785.g835.t1 | Protocadherin-11 Y-linked | 2859 | 2.58E-90 | 51 |
| Efet.01.399093.g1219.t1 | Protocadherin-11 Y-linked | 2757 | 5.09E-111 | 51 |
| Efet.01.150634.g35.t1 | Protocadherin-16 | 1248 | 3.33E-65 | 51 |
| Efet.01.215469.g645.t1 | Protocadherin-19 | 1803 | 3.26E-91 | 51 |
| Efet.01.147479.g1117.t1 | Protocadherin gamma-B4 | 636 | 2.26E-21 | 51 |
| Efet.01.5326.g418.t1 | Protocadherin-9 | 2325 | 1.13E-102 | 51 |
| Efet.01.98873.g1379.t1 | Protocadherin-9 | 2775 | 1.08E-110 | 51 |
| Efet.01.133708.g423.t1 | Protocadherin-9 | 888 | 1.97E-28 | 51 |
| Efet.01.189006.g1836.t1 | Protocadherin-9 | 888 | 1.97E-28 | 51 |
| Efet.01.380911.g810.t1 | Protocadherin-9 | 2826 | 2.34E-120 | 51 |
| Efet.01.89152.g841.t1 | Proprotein convertase subtilisin/kexin type 5 | 294 | 2.02E-09 | 51 |
| Efet.01.522934.g535.t1 | Programmed cell death 6-interacting protein | 630 | 1.11E-24 | 51 |
| Efet.01.49068.g1529.t1 | Calcium/calmodulin-dependent 3',5'-cyclic nucleotide phosphodiesterase 1B | 693 | 7.50E-29 | 51 |
| Efet.01.413810.g309.t1 | Peroxisomal trans-2-enoyl-CoA reductase | 759 | 2.71E-27 | 51 |
| Efet.01.472242.g600.t1 | Peroxisomal trans-2-enoyl-CoA reductase | 771 | 1.76E-38 | 51 |
| Efet.01.533149.g108.t1 | Peroxisomal trans-2-enoyl-CoA reductase | 843 | 3.42E-16 | 51 |
| Efet.01.569313.g298.t1 | Peroxisomal trans-2-enoyl-CoA reductase | 588 | 9.75E-22 | 51 |
| Efet.01.643011.g742.t1 | Peroxisomal trans-2-enoyl-CoA reductase | 714 | 5.58E-11 | 51 |
| Efet.01.657703.g1382.t1 | Peroxisomal trans-2-enoyl-CoA reductase | 744 | 3.72E-30 | 51 |
| Efet.01.1659033.g1372.t1 | Peroxisomal trans-2-enoyl-CoA reductase | 462 | 9.74E-14 | 51 |
| Efet.01.345219.g1275.t1 | PiggyBac transposable element-derived protein 3 | 1284 | 1.14E-37 | 51 |
| Efet.01.657207.g1201.t1 | Basement membrane-specific heparan sulfate proteoglycan core protein | 399 | 2.14E-23 | 51 |
| Efet.01.289710.g1442.t1 | p53-induced death domain-containing protein 1 | 1665 | 6.57E-10 | 51 |
| Efet.01.509585.g242.t1 | Polycystic kidney disease protein 1-like 1 | 549 | 4.23E-07 | 51 |
| Efet.01.656939.g1146.t1 | Polycystin-1 | 702 | 1.15E-07 | 51 |
| Efet.01.17744.g1309.t1 | 1-phosphatidylinositol 4,5-bisphosphate phosphodiesterase delta-4 | 408 | 1.82E-10 | 51 |
| Efet.01.12164.g906.t1 | Plexin-A3 | 603 | 5.22E-23 | 51 |
| Efet.01.325515.g784.t1 | Plexin-A3 | 729 | 1.66E-16 | 51 |
| Efet.01.598556.g1255.t1 | Plexin-A3 | 840 | 4.07E-17 | 51 |
| Efet.01.189547.g1856.t1 | Tartrate-resistant acid phosphatase type 5 | 789 | 1.03E-24 | 51 |
| Efet.01.642550.g705.t1 | Peptidyl-prolyl cis-trans isomerase-like 3 | 549 | 2.82E-20 | 51 |
| Efet.01.639728.g536.t1 | Receptor-type tyrosine-protein phosphatase mu | 2709 | 2.83E-97 | 51 |
| Efet.01.528741.g656.t1 | ABC50 protein (ATP-binding cassette, sub-family F (GCN20), member 1) | 1662 | 1.20E-29 | 51 |
| Efet.01.57379.g461.t1 | Ras-related protein Rab-13 | 432 | 7.48E-15 | 51 |
| Efet.01.213691.g581.t1 | Ras-specific guanine nucleotide-releasing factor 1 | 621 | 1.23E-12 | 51 |
| Efet.01.492369.g1006.t1 | Ras-specific guanine nucleotide-releasing factor 1 | 351 | 7.83E-10 | 51 |
| Efet.01.135161.g500.t1 | Roundabout homolog 2 | 849 | 1.48E-38 | 51 |
| Efet.01.47595.g1449.t1 | Retinoic acid receptor RXR-alpha | 480 | 2.13E-19 | 51 |
| Efet.01.297726.g1677.t1 | Sacsin | 10029 | 0 | 51 |
| Efet.01.351277.g40.t1 | Sodium channel protein type 2 subunit alpha | 342 | 1.75E-13 | 51 |
| Efet.01.658343.g1878.t1 | Protein scribble homolog | 1845 | 4.73E-06 | 51 |
| Efet.01.1646040.g339.t1 | Protein sidekick-1 | 330 | 3.28E-06 | 51 |
| Efet.01.83019.g526.t1 | Protein sidekick-2 | 1602 | 1.66E-47 | 51 |
| Efet.01.1657769.g1035.t1 | Sphingosine-1-phosphate phosphatase 1 | 504 | 2.87E-19 | 51 |
| Efet.01.182493.g1522.t1 | E3 ubiquitin-protein ligase SH3RF1 | 1350 | 7.05E-07 | 51 |
| Efet.01.14911.g1095.t1 | SH2 domain-containing adapter protein B | 309 | 4.48E-21 | 51 |
| Efet.01.286362.g1316.t1 | Ski-like protein | 477 | 2.90E-06 | 51 |
| Efet.01.1918.g169.t1 | Slit homolog 3 protein | 858 | 4.33E-15 | 51 |
| Efet.01.10601.g779.t1 | Solute carrier organic anion transporter family member 2A1 | 621 | 4.56E-20 | 51 |
| Efet.01.1756.g157.t1 | Sterol O-acyltransferase 1 | 555 | 9.13E-31 | 51 |
| Efet.01.93761.g1088.t1 | Proto-oncogene tyrosine-protein kinase Src | 492 | 2.54E-15 | 51 |
| Efet.01.530574.g23.t1 | Serine racemase | 1710 | 8.53E-38 | 51 |
| Efet.01.630860.g84.t1 | Serine racemase | 867 | 3.17E-35 | 51 |
| Efet.01.1659086.g1396.t1 | Sushi, von Willebrand factor type A, EGF and pentraxin domain-containing protein 1 | 1176 | 7.99E-11 | 51 |
| Efet.01.128111.g173.t1 | Synaptotagmin-1 | 285 | 3.53E-08 | 51 |
| Efet.01.543473.g331.t1 | Tenascin-X | 420 | 1.67E-15 | 51 |
| Efet.01.442193.g1041.t1 | E3 ubiquitin-protein ligase TRIP12 | 984 | 1.15E-26 | 51 |
| Efet.01.458460.g236.t1 | Protein turtle homolog A | 264 | 1.04E-10 | 51 |
| Efet.01.218715.g757.t1 | Protein turtle homolog B | 573 | 4.15E-18 | 51 |
| Efet.01.1654414.g688.t1 | Protein turtle homolog B | 390 | 1.01E-12 | 51 |
| Efet.01.636780.g348.t1 | Ubiquitin-conjugating enzyme E2 B | 264 | 3.18E-06 | 51 |
| Efet.01.657445.g1284.t1 | Ubiquitin carboxyl-terminal hydrolase 4 | 816 | 5.66E-11 | 51 |
| Efet.01.312980.g413.t1 | Mitochondrial brown fat uncoupling protein 1 | 438 | 1.10E-08 | 51 |
| Efet.01.532989.g94.t1 | UDP-glucuronosyltransferase 1-1 | 1416 | 3.55E-45 | 51 |
| Efet.01.600908.g36.t1 | Protein Wnt-7a | 258 | 1.23E-08 | 51 |
| Efet.01.151865.g112.t1 | Vasopressin V1a receptor | 1203 | 9.43E-09 | 51 |
| Efet.01.208970.g405.t1 | Vasopressin V1a receptor | 639 | 1.39E-09 | 51 |
| Efet.01.550400.g516.t1 | Xylosyltransferase 1 | 867 | 2.54E-46 | 51 |
| Efet.01.11656.g856.t1 | Zinc finger protein 175 | 1608 | 5.27E-62 | 51 |
| Efet.01.196576.g2153.t1 | Zinc finger protein 175 | 336 | 5.11E-13 | 51 |
| Efet.01.20643.g1523.t1 | Zinc finger protein 443 | 261 | 8.93E-11 | 51 |
| Efet.01.102725.g154.t1 | Zinc finger protein 675 | 1317 | 1.32E-53 | 51 |
| Efet.01.380433.g802.t1 | Zinc finger protein 675 | 771 | 1.94E-40 | 51 |
| Efet.01.406366.g156.t1 | Zinc finger protein 675 | 1500 | 1.05E-55 | 51 |
| Efet.01.549171.g474.t1 | B-cell CLL/lymphoma 2, isoform CRA_b | 1128 | 2.75E-07 | 50 |
| Efet.01.538409.g239.t1 | Mitogen-activated protein kinase | 408 | 2.97E-14 | 50 |
| Efet.01.361627.g310.t1 | Epstein-Barr virus induced gene 2 (Lymphocyte-specific G protein-coupled receptor), isoform CRA_a | 1098 | 1.31E-10 | 50 |
| Efet.01.19413.g1437.t1 | Receptor protein-tyrosine kinase | 450 | 6.18E-22 | 50 |
| Efet.01.313865.g439.t1 | Testicular tissue protein Li 14 | 1134 | 3.82E-41 | 50 |
| Efet.01.389950.g1005.t1 | Histamine H2 receptor | 1680 | 1.86E-63 | 50 |
| Efet.01.289367.g1429.t1 | ATP-binding cassette sub-family A member 1 | 1164 | 8.22E-60 | 50 |
| Efet.01.628537.g1256.t1 | ATP-binding cassette sub-family A member 5 | 1542 | 3.37E-16 | 50 |
| Efet.01.629376.g1293.t1 | ATP-binding cassette sub-family A member 5 | 594 | 1.46E-13 | 50 |
| Efet.01.658417.g2080.t1 | ATP-binding cassette sub-family A member 5 | 831 | 9.46E-21 | 50 |
| Efet.01.621705.g951.t1 | ATP-binding cassette sub-family A member 7 | 621 | 1.69E-20 | 50 |
| Efet.01.639343.g500.t1 | ATP-binding cassette sub-family A member 7 | 1542 | 7.96E-18 | 50 |
| Efet.01.657516.g1318.t1 | ATP-binding cassette sub-family A member 7 | 1143 | 6.15E-09 | 50 |
| Efet.01.654851.g705.t1 | ATP-binding cassette sub-family G member 1 | 765 | 1.14E-24 | 50 |
| Efet.01.33053.g557.t1 | Neuronal acetylcholine receptor subunit alpha-4 | 357 | 3.86E-07 | 50 |
| Efet.01.168402.g908.t1 | Muscarinic acetylcholine receptor M1 | 771 | 6.56E-12 | 50 |
| Efet.01.427593.g661.t1 | Acyl-CoA synthetase family member 2, mitochondrial | 1422 | 1.11E-46 | 50 |
| Efet.01.390862.g1024.t1 | Alpha-1A adrenergic receptor | 912 | 1.37E-23 | 50 |
| Efet.01.70976.g1295.t1 | Alpha-1B adrenergic receptor | 1008 | 7.55E-34 | 50 |
| Efet.01.597702.g1221.t1 | Alcohol dehydrogenase class-3 | 810 | 1.51E-24 | 50 |
| Efet.01.97817.g1330.t1 | Beta-2 adrenergic receptor | 1446 | 4.90E-57 | 50 |
| Efet.01.331133.g944.t1 | Beta-2 adrenergic receptor | 906 | 2.99E-54 | 50 |
| Efet.01.563201.g79.t1 | Aldehyde dehydrogenase family 1 member A3 | 1536 | 2.43E-45 | 50 |
| Efet.01.588154.g937.t1 | Aldehyde dehydrogenase family 1 member A3 | 1317 | 3.89E-40 | 50 |
| Efet.01.599761.g1303.t1 | Aldehyde dehydrogenase family 1 member A3 | 501 | 1.80E-10 | 50 |
| Efet.01.658364.g1897.t1 | Aldehyde dehydrogenase family 1 member A3 | 885 | 1.67E-36 | 50 |
| Efet.01.12644.g947.t1 | Ankyrin-1 | 1224 | 8.10E-42 | 50 |
| Efet.01.67570.g1083.t1 | Ankyrin-1 | 858 | 1.16E-33 | 50 |
| Efet.01.27686.g168.t1 | Antithrombin-III | 480 | 2.26E-18 | 50 |
| Efet.01.191763.g1947.t1 | ADP-ribosylation factor 6 | 459 | 9.31E-14 | 50 |
| Efet.01.310941.g347.t1 | Calcium-transporting ATPase type 2C member 1 | 456 | 4.71E-06 | 50 |
| Efet.01.127396.g136.t1 | ADAMTS-like protein 4 | 513 | 1.68E-25 | 50 |
| Efet.01.483853.g834.t1 | ATP synthase subunit beta, mitochondrial | 489 | 4.65E-10 | 50 |
| Efet.01.1658952.g1330.t1 | Aurora kinase C | 936 | 3.50E-27 | 50 |
| Efet.01.179434.g1361.t1 | Axin-1 | 1008 | 5.08E-38 | 50 |
| Efet.01.203040.g146.t1 | cDNA, FLJ93261, highly similar to Homo sapiens chemokine (C-C motif) receptor 9 | 456 | 4.91E-09 | 50 |
| Efet.01.318747.g598.t1 | ATP-binding cassette, sub-family A (ABC1), member 1 | 1416 | 4.44E-13 | 50 |
| Efet.01.592653.g1083.t1 | ATP-binding cassette, sub-family A (ABC1), member 1 | 1620 | 1.85E-14 | 50 |
| Efet.01.650311.g31.t1 | ATP-binding cassette, sub-family A (ABC1), member 1 | 759 | 6.84E-15 | 50 |
| Efet.01.658418.g2086.t1 | ATP-binding cassette, sub-family A (ABC1), member 1 | 1782 | 1.47E-18 | 50 |
| Efet.01.1658449.g1185.t1 | ATP-binding cassette, sub-family A (ABC1), member 1 | 885 | 7.69E-18 | 50 |
| Efet.01.514247.g340.t1 | cDNA FLJ40025 fis, clone STOMA2008050, highly similar to Homo sapiens ATP-binding cassette, sub-family A | 477 | 5.12E-07 | 50 |
| Efet.01.621705.g952.t1 | cDNA FLJ40025 fis, clone STOMA2008050, highly similar to Homo sapiens ATP-binding cassette, sub-family A | 792 | 3.95E-27 | 50 |
| Efet.01.67458.g1078.t1 | cDNA FLJ51484, highly similar to B-cell lymphoma 3-encoded protein | 1041 | 1.54E-21 | 50 |
| Efet.01.510009.g245.t1 | B-cell CLL/lymphoma 6 member B protein | 1623 | 2.43E-07 | 50 |
| Efet.01.161735.g610.t1 | Baculoviral IAP repeat-containing protein 6 | 489 | 2.31E-20 | 50 |
| Efet.01.57315.g455.t1 | Bone morphogenetic protein 1 | 591 | 6.56E-15 | 50 |
| Efet.01.105280.g284.t1 | Bone morphogenetic protein 1 | 912 | 7.62E-27 | 50 |
| Efet.01.410669.g239.t1 | Bone morphogenetic protein 1 | 354 | 2.14E-07 | 50 |
| Efet.01.553364.g584.t1 | Dipeptidyl peptidase 1 | 225 | 4.68E-07 | 50 |
| Efet.01.51527.g100.t1 | Cathepsin D | 453 | 1.66E-34 | 50 |
| Efet.01.20750.g1536.t1 | Uncharacterized protein C3orf38 | 780 | 2.59E-33 | 50 |
| Efet.01.658207.g1713.t1 | Cadherin EGF LAG seven-pass G-type receptor 3 | 972 | 5.11E-40 | 50 |
| Efet.01.453507.g77.t1 | Chromodomain-helicase-DNA-binding protein 8 | 684 | 1.47E-21 | 50 |
| Efet.01.294804.g1591.t1 | H(+)/Cl(-) exchange transporter 5 | 570 | 2.73E-19 | 50 |
| Efet.01.47302.g1433.t1 | Contactin-5 | 360 | 2.39E-09 | 50 |
| Efet.01.140413.g791.t1 | Collagen alpha-3(VI) chain | 456 | 2.94E-11 | 50 |
| Efet.01.149279.g1196.t1 | Collagen alpha-6(VI) chain | 918 | 1.41E-33 | 50 |
| Efet.01.56698.g416.t1 | Collagen alpha-1(XVIII) chain | 735 | 8.99E-14 | 50 |
| Efet.01.201591.g77.t1 | COUP transcription factor 2 | 528 | 1.16E-18 | 50 |
| Efet.01.153003.g165.t1 | Death-associated protein kinase 1 | 1119 | 1.48E-10 | 50 |
| Efet.01.290544.g1473.t1 | Discoidin domain-containing receptor 2 | 705 | 7.85E-23 | 50 |
| Efet.01.19994.g1480.t1 | Probable ATP-dependent RNA helicase DDX41 | 603 | 1.73E-29 | 50 |
| Efet.01.639914.g542.t1 | ATP-dependent RNA helicase DDX42 | 1038 | 2.33E-45 | 50 |
| Efet.01.654656.g673.t1 | ATP-dependent RNA helicase DDX42 | 2151 | 5.18E-06 | 50 |
| Efet.01.471393.g575.t1 | Dehydrogenase/reductase SDR family member 2, mitochondrial | 978 | 4.25E-20 | 50 |
| Efet.01.656598.g1049.t1 | Dehydrogenase/reductase SDR family member 2, mitochondrial | 372 | 7.23E-11 | 50 |
| Efet.01.68203.g1123.t1 | Disks large homolog 5 | 2901 | 4.40E-47 | 50 |
| Efet.01.5975.g475.t1 | Double C2-like domain-containing protein alpha | 375 | 1.52E-10 | 50 |
| Efet.01.101628.g106.t1 | D(1A) dopamine receptor | 594 | 1.10E-22 | 50 |
| Efet.01.103538.g191.t1 | D(3) dopamine receptor | 1719 | 9.85E-57 | 50 |
| Efet.01.67373.g1071.t1 | Dual specificity protein phosphatase 1 | 552 | 8.08E-24 | 50 |
| Efet.01.436318.g861.t1 | Ectonucleoside triphosphate diphosphohydrolase 1 | 204 | 5.13E-06 | 50 |
| Efet.01.127169.g125.t1 | Erbin | 1464 | 2.79E-19 | 50 |
| Efet.01.184366.g1605.t1 | Exostosin-like 1 | 306 | 2.63E-12 | 50 |
| Efet.01.446132.g1143.t1 | Mannan-binding lectin serine protease 1 | 684 | 9.03E-06 | 50 |
| Efet.01.609438.g402.t1 | Protocadherin Fat 2 | 1113 | 1.22E-32 | 50 |
| Efet.01.167098.g844.t1 | Fermitin family homolog 2 | 384 | 6.52E-30 | 50 |
| Efet.01.20108.g1489.t1 | Fez family zinc finger protein 2 | 354 | 1.44E-06 | 50 |
| Efet.01.652468.g307.t1 | Furin | 543 | 1.27E-06 | 50 |
| Efet.01.296245.g1622.t1 | Tyrosine-protein kinase Fyn | 543 | 1.23E-14 | 50 |
| Efet.01.50040.g4.t1 | Mu opioid receptor hMOR-1a | 882 | 3.45E-20 | 50 |
| Efet.01.1645605.g327.t1 | Polypeptide N-acetylgalactosaminyltransferase 2 | 444 | 3.55E-19 | 50 |
| Efet.01.273656.g870.t1 | Growth arrest-specific protein 2 | 792 | 2.55E-27 | 50 |
| Efet.01.15726.g1158.t1 | Gastrin/cholecystokinin type B receptor | 474 | 1.94E-08 | 50 |
| Efet.01.549827.g490.t1 | Gastrin/cholecystokinin type B receptor | 1119 | 7.03E-10 | 50 |
| Efet.01.57017.g437.t1 | Homeobox protein GBX-2 | 858 | 3.78E-10 | 50 |
| Efet.01.399406.g1227.t1 | Growth hormone secretagogue receptor type 1 | 1152 | 3.21E-09 | 50 |
| Efet.01.494448.g1058.t1 | Growth hormone secretagogue receptor type 1 | 1011 | 6.75E-10 | 50 |
| Efet.01.1645788.g333.t1 | Growth hormone secretagogue receptor type 1 | 246 | 7.73E-06 | 50 |
| Efet.01.595647.g1170.t1 | Bifunctional UDP-N-acetylglucosamine 2-epimerase/N-acetylmannosamine kinase | 687 | 8.00E-10 | 50 |
| Efet.01.657669.g1360.t1 | Transcriptional activator GLI3 | 291 | 1.99E-06 | 50 |
| Efet.01.103302.g177.t1 | Glypican-3 | 351 | 2.83E-12 | 50 |
| Efet.01.343304.g1229.t1 | Glutamate receptor ionotropic, kainate 2 | 633 | 9.92E-17 | 50 |
| Efet.01.213854.g589.t1 | Solute carrier family 2, facilitated glucose transporter member 4 | 1449 | 4.47E-33 | 50 |
| Efet.01.533365.g122.t1 | Hepatocyte growth factor | 243 | 8.79E-06 | 50 |
| Efet.01.43616.g1208.t1 | Serpin-like protein HMSD | 285 | 5.47E-10 | 50 |
| Efet.01.484071.g837.t1 | Histamine H3 receptor | 603 | 7.68E-19 | 50 |
| Efet.01.565049.g158.t1 | Heat shock protein HSP 90-beta | 1935 | 1.89E-113 | 50 |
| Efet.01.206117.g265.t1 | Zinc finger protein Pegasus | 6186 | 2.79E-06 | 50 |
| Efet.01.364584.g394.t1 | Importin subunit alpha-5 | 300 | 2.06E-13 | 50 |
| Efet.01.75563.g32.t1 | Kalirin | 1665 | 2.38E-07 | 50 |
| Efet.01.388037.g971.t1 | cAMP-dependent protein kinase type II-alpha regulatory subunit | 474 | 8.56E-17 | 50 |
| Efet.01.146631.g1086.t1 | Histone acetyltransferase KAT6A | 816 | 1.97E-16 | 50 |
| Efet.01.232025.g1275.t1 | Ribosomal protein S6 kinase alpha-2 | 423 | 4.39E-12 | 50 |
| Efet.01.623094.g1010.t1 | Micro opioid receptor isoform hMOR-1A2 | 1053 | 8.19E-25 | 50 |
| Efet.01.60885.g679.t1 | Laminin subunit alpha-1 | 528 | 4.76E-20 | 50 |
| Efet.01.428617.g697.t1 | Laminin subunit alpha-3 | 246 | 2.49E-06 | 50 |
| Efet.01.343951.g1250.t1 | Large neutral amino acids transporter small subunit 1 | 1638 | 8.72E-06 | 50 |
| Efet.01.31878.g479.t1 | LIM domain only protein 7 | 291 | 1.93E-06 | 50 |
| Efet.01.112787.g646.t1 | Low-density lipoprotein receptor-related protein 1B | 459 | 1.20E-10 | 50 |
| Efet.01.151815.g108.t1 | Mitogen-activated protein kinase kinase kinase 10 | 228 | 3.64E-06 | 50 |
| Efet.01.523305.g539.t1 | Microtubule-associated serine/threonine-protein kinase 2 | 1746 | 3.92E-13 | 50 |
| Efet.01.270738.g770.t1 | Multidrug resistance protein 1 | 1359 | 8.56E-11 | 50 |
| Efet.01.575546.g499.t1 | Multidrug resistance protein 1 | 807 | 1.65E-11 | 50 |
| Efet.01.655098.g751.t1 | Multidrug resistance protein 1 | 1098 | 1.02E-07 | 50 |
| Efet.01.657766.g1424.t1 | Multidrug resistance protein 1 | 1362 | 2.42E-20 | 50 |
| Efet.01.658261.g1766.t1 | Multidrug resistance protein 1 | 486 | 3.40E-16 | 50 |
| Efet.01.571841.g380.t1 | Methylated-DNA--protein-cysteine methyltransferase | 495 | 7.53E-22 | 50 |
| Efet.01.402896.g77.t1 | rRNA methyltransferase 3, mitochondrial | 423 | 6.49E-14 | 50 |
| Efet.01.267864.g659.t1 | Mitochondrial ubiquitin ligase activator of NFKB 1 | 456 | 1.47E-12 | 50 |
| Efet.01.141290.g844.t1 | Myosin light chain kinase, smooth muscle | 1311 | 1.92E-09 | 50 |
| Efet.01.234116.g1345.t1 | Myosin light chain kinase, smooth muscle | 294 | 4.29E-10 | 50 |
| Efet.01.268411.g681.t1 | Unconventional myosin-Ie | 837 | 6.39E-35 | 50 |
| Efet.01.344.g42.t1 | Myosin-binding protein C, slow-type | 765 | 3.19E-09 | 50 |
| Efet.01.158801.g461.t1 | Myosin-binding protein C, cardiac-type | 351 | 1.28E-15 | 50 |
| Efet.01.618726.g843.t1 | Myeloid zinc finger 1 | 2463 | 1.24E-37 | 50 |
| Efet.01.168.g17.t1 | Nucleoside diphosphate kinase homolog 5 | 741 | 2.50E-08 | 50 |
| Efet.01.301100.g43.t1 | Neogenin | 492 | 5.88E-11 | 50 |
| Efet.01.63396.g823.t1 | Substance-P receptor | 846 | 2.45E-12 | 50 |
| Efet.01.581598.g724.t1 | Neuroligin-1 | 840 | 1.98E-40 | 50 |
| Efet.01.75975.g58.t1 | Neuroligin-4, Y-linked | 633 | 1.40E-29 | 50 |
| Efet.01.255509.g201.t1 | NLR family CARD domain-containing protein 3 | 2619 | 1.69E-19 | 50 |
| Efet.01.330621.g936.t1 | Nitric oxide synthase, endothelial | 762 | 1.56E-35 | 50 |
| Efet.01.607739.g323.t1 | Neuropeptide Y receptor type 5 | 1236 | 1.37E-28 | 50 |
| Efet.01.2954.g263.t1 | Oxysterols receptor LXR-alpha | 1245 | 1.07E-24 | 50 |
| Efet.01.216008.g661.t1 | Neurexin-1 | 1707 | 7.51E-08 | 50 |
| Efet.01.329708.g908.t1 | Neurexin-1 | 699 | 1.28E-12 | 50 |
| Efet.01.45076.g1308.t1 | Neurotensin receptor type 1 | 582 | 1.43E-12 | 50 |
| Efet.01.104908.g267.t1 | Neurotensin receptor type 1 | 483 | 2.79E-10 | 50 |
| Efet.01.396930.g1168.t1 | Neurotensin receptor type 1 | 483 | 2.79E-10 | 50 |
| Efet.01.138762.g708.t1 | Obscurin | 663 | 2.06E-11 | 50 |
| Efet.01.425662.g611.t1 | Kappa-type opioid receptor | 636 | 6.49E-08 | 50 |
| Efet.01.3992.g336.t1 | Mu-type opioid receptor | 1269 | 2.02E-29 | 50 |
| Efet.01.135694.g531.t1 | Mu-type opioid receptor | 936 | 7.30E-08 | 50 |
| Efet.01.135715.g533.t1 | Mu-type opioid receptor | 1176 | 6.45E-27 | 50 |
| Efet.01.268321.g675.t1 | Mu-type opioid receptor | 1032 | 3.36E-17 | 50 |
| Efet.01.575886.g512.t1 | Mu-type opioid receptor | 1218 | 1.14E-20 | 50 |
| Efet.01.232364.g1285.t1 | Polyadenylate-binding protein 4 | 552 | 6.84E-07 | 50 |
| Efet.01.25068.g9.t1 | Protocadherin-11 Y-linked | 2514 | 2.16E-108 | 50 |
| Efet.01.114158.g720.t1 | Protocadherin-11 Y-linked | 2046 | 8.47E-71 | 50 |
| Efet.01.1639240.g204.t1 | Lysophosphatidylcholine acyltransferase 2 | 300 | 4.57E-07 | 50 |
| Efet.01.102846.g164.t1 | Protocadherin-16 | 1800 | 2.26E-62 | 50 |
| Efet.01.278788.g1062.t1 | Protocadherin-18 | 471 | 2.55E-19 | 50 |
| Efet.01.315253.g478.t1 | Protocadherin-19 | 1551 | 6.45E-52 | 50 |
| Efet.01.44676.g1278.t1 | Protocadherin gamma-B2 | 2436 | 1.05E-79 | 50 |
| Efet.01.108486.g445.t1 | Protocadherin gamma-B4 | 2499 | 5.47E-78 | 50 |
| Efet.01.608804.g365.t1 | Protocadherin gamma-C4 | 1221 | 2.04E-51 | 50 |
| Efet.01.245601.g1718.t1 | Protocadherin-1 | 813 | 6.53E-13 | 50 |
| Efet.01.322182.g692.t1 | Protocadherin-1 | 1386 | 2.41E-37 | 50 |
| Efet.01.20638.g1521.t1 | Protocadherin-9 | 2664 | 2.85E-110 | 50 |
| Efet.01.28472.g238.t1 | Protocadherin-9 | 2676 | 4.21E-100 | 50 |
| Efet.01.118189.g940.t1 | Protocadherin-9 | 2454 | 1.08E-84 | 50 |
| Efet.01.364737.g397.t1 | Protocadherin-9 | 2640 | 1.69E-88 | 50 |
| Efet.01.318747.g599.t1 | Peroxisomal trans-2-enoyl-CoA reductase | 753 | 2.97E-14 | 50 |
| Efet.01.506188.g156.t1 | Peroxisomal trans-2-enoyl-CoA reductase | 765 | 5.96E-12 | 50 |
| Efet.01.646487.g1098.t1 | Peroxisomal trans-2-enoyl-CoA reductase | 828 | 9.64E-25 | 50 |
| Efet.01.220923.g846.t1 | Basement membrane-specific heparan sulfate proteoglycan core protein | 417 | 9.17E-07 | 50 |
| Efet.01.176795.g1257.t1 | 15-hydroxyprostaglandin dehydrogenase [NAD(+)] | 636 | 3.07E-15 | 50 |
| Efet.01.212760.g546.t1 | 15-hydroxyprostaglandin dehydrogenase [NAD(+)] | 483 | 2.98E-11 | 50 |
| Efet.01.611807.g493.t1 | 15-hydroxyprostaglandin dehydrogenase [NAD(+)] | 600 | 1.83E-06 | 50 |
| Efet.01.645971.g1019.t1 | 15-hydroxyprostaglandin dehydrogenase [NAD(+)] | 657 | 2.95E-16 | 50 |
| Efet.01.12032.g897.t1 | Peptidoglycan recognition protein 1 | 1281 | 3.11E-11 | 50 |
| Efet.01.520989.g495.t1 | Pituitary homeobox 1 | 453 | 6.63E-09 | 50 |
| Efet.01.108288.g430.t1 | Polycystin-1 | 876 | 7.19E-18 | 50 |
| Efet.01.288920.g1417.t1 | Polycystin-1 | 465 | 8.17E-17 | 50 |
| Efet.01.553917.g605.t1 | Polycystin-1 | 834 | 9.33E-08 | 50 |
| Efet.01.191963.g1959.t1 | Plexin-A3 | 690 | 2.26E-28 | 50 |
| Efet.01.598556.g1254.t1 | Plexin-B1 | 489 | 3.30E-07 | 50 |
| Efet.01.173893.g1130.t1 | Protein phosphatase 1F | 339 | 9.74E-07 | 50 |
| Efet.01.72295.g1372.t1 | Platelet-activating factor receptor | 225 | 1.27E-06 | 50 |
| Efet.01.120308.g1054.t1 | Receptor-type tyrosine-protein phosphatase eta | 324 | 8.48E-10 | 50 |
| Efet.01.120355.g1059.t1 | Peroxidasin homolog | 543 | 7.36E-22 | 50 |
| Efet.01.509178.g236.t1 | Peroxidasin homolog | 1338 | 2.35E-15 | 50 |
| Efet.01.197051.g2179.t1 | Thyroid peroxidase | 708 | 4.10E-07 | 50 |
| Efet.01.348247.g1362.t1 | Low density lipoprotein-related protein 1 variant | 411 | 1.50E-17 | 50 |
| Efet.01.214447.g612.t1 | Tubulin beta chain | 1206 | 1.03E-57 | 50 |
| Efet.01.245650.g1721.t1 | Chemokine (C-C motif) receptor 1 | 1152 | 2.22E-25 | 50 |
| Efet.01.10407.g763.t1 | SOCS3 protein (Suppressor of cytokine signaling 3, isoform CRA_a) | 1044 | 2.06E-27 | 50 |
[truncated: 121,917 more chars]
