# Supplementary material for: Data on genome annotation and analysis of earthworm Eisenia fetida
Source: Data Brief. 2018 Aug 29;20:525–34. doi: 10.1016/j.dib.2018.08.067 (PMC6126081; doi:10.1016/j.dib.2018.08.067)
Supplement: Supplementary file 8 — Supplementary material [file mmc8.docx]

Table S7: List of functionally enriched GO terms associated with the stem cell and regeneration specific genes

| **GO ID** | **GO Category** | **GO Name** | **FDR** | **P-Value** |
| --- | --- | --- | --- | --- |
| GO:0050789 | BIOLOGICAL_PROCESS | regulation of biological process | 8.19E-207 | 7.23E-211 |
| GO:0065007 | BIOLOGICAL_PROCESS | biological regulation | 1.18E-198 | 2.08E-202 |
| GO:0007165 | BIOLOGICAL_PROCESS | signal transduction | 1.09E-187 | 2.88E-191 |
| GO:0044700 | BIOLOGICAL_PROCESS | single organism signaling | 3.94E-187 | 1.74E-190 |
| GO:0023052 | BIOLOGICAL_PROCESS | signaling | 3.94E-187 | 1.74E-190 |
| GO:0007154 | BIOLOGICAL_PROCESS | cell communication | 1.00E-179 | 5.31E-183 |
| GO:0050794 | BIOLOGICAL_PROCESS | regulation of cellular process | 3.49E-174 | 2.16E-177 |
| GO:0051716 | BIOLOGICAL_PROCESS | cellular response to stimulus | 3.97E-171 | 2.81E-174 |
| GO:0050896 | BIOLOGICAL_PROCESS | response to stimulus | 8.36E-167 | 6.64E-170 |
| GO:0004871 | MOLECULAR_FUNCTION | signal transducer activity | 4.19E-148 | 3.70E-151 |
| GO:0004888 | MOLECULAR_FUNCTION | transmembrane signaling receptor activity | 1.11E-123 | 1.08E-126 |
| GO:0038023 | MOLECULAR_FUNCTION | signaling receptor activity | 5.44E-121 | 5.76E-124 |
| GO:0099600 | MOLECULAR_FUNCTION | transmembrane receptor activity | 5.67E-101 | 6.50E-104 |
| GO:0004872 | MOLECULAR_FUNCTION | receptor activity | 2.30E-95 | 2.84E-98 |
| GO:0060089 | MOLECULAR_FUNCTION | molecular transducer activity | 3.87E-95 | 5.13E-98 |
| GO:0007186 | BIOLOGICAL_PROCESS | G-protein coupled receptor signaling pathway | 4.65E-94 | 6.57E-97 |
| GO:0032501 | BIOLOGICAL_PROCESS | multicellular organismal process | 8.62E-94 | 1.29E-96 |
| GO:0004930 | MOLECULAR_FUNCTION | G-protein coupled receptor activity | 9.43E-94 | 1.50E-96 |
| GO:0044707 | BIOLOGICAL_PROCESS | single-multicellular organism process | 2.61E-91 | 4.39E-94 |
| GO:0007275 | BIOLOGICAL_PROCESS | multicellular organism development | 6.27E-83 | 1.11E-85 |
| GO:0044767 | BIOLOGICAL_PROCESS | single-organism developmental process | 1.60E-79 | 2.97E-82 |
| GO:0032502 | BIOLOGICAL_PROCESS | developmental process | 9.79E-79 | 1.90E-81 |
| GO:0048856 | BIOLOGICAL_PROCESS | anatomical structure development | 1.70E-76 | 3.45E-79 |
| GO:0048731 | BIOLOGICAL_PROCESS | system development | 2.02E-71 | 4.29E-74 |
| GO:0006468 | BIOLOGICAL_PROCESS | protein phosphorylation | 2.51E-71 | 5.53E-74 |
| GO:0030154 | BIOLOGICAL_PROCESS | cell differentiation | 4.61E-65 | 1.06E-67 |
| GO:0007166 | BIOLOGICAL_PROCESS | cell surface receptor signaling pathway | 4.46E-64 | 1.06E-66 |
| GO:0004672 | MOLECULAR_FUNCTION | protein kinase activity | 5.94E-64 | 1.47E-66 |
| GO:0009987 | BIOLOGICAL_PROCESS | cellular process | 1.26E-62 | 3.23E-65 |
| GO:0005634 | CELLULAR_COMPONENT | nucleus | 3.83E-59 | 1.01E-61 |
| GO:0048513 | BIOLOGICAL_PROCESS | animal organ development | 2.56E-58 | 7.02E-61 |
| GO:0048869 | BIOLOGICAL_PROCESS | cellular developmental process | 6.35E-58 | 1.79E-60 |
| GO:0044699 | BIOLOGICAL_PROCESS | single-organism process | 1.03E-55 | 3.00E-58 |
| GO:0070887 | BIOLOGICAL_PROCESS | cellular response to chemical stimulus | 1.87E-55 | 5.63E-58 |
| GO:0010033 | BIOLOGICAL_PROCESS | response to organic substance | 1.90E-55 | 5.86E-58 |
| GO:0005887 | CELLULAR_COMPONENT | integral component of plasma membrane | 2.69E-54 | 8.54E-57 |
| GO:0016773 | MOLECULAR_FUNCTION | phosphotransferase activity, alcohol group as acceptor | 3.39E-53 | 1.11E-55 |
| GO:0031226 | CELLULAR_COMPONENT | intrinsic component of plasma membrane | 1.76E-52 | 5.90E-55 |
| GO:0006464 | BIOLOGICAL_PROCESS | cellular protein modification process | 4.86E-51 | 1.72E-53 |
| GO:0036211 | BIOLOGICAL_PROCESS | protein modification process | 4.86E-51 | 1.72E-53 |
| GO:0042221 | BIOLOGICAL_PROCESS | response to chemical | 2.61E-50 | 9.46E-53 |
| GO:0048518 | BIOLOGICAL_PROCESS | positive regulation of biological process | 6.60E-50 | 2.45E-52 |
| GO:0016310 | BIOLOGICAL_PROCESS | phosphorylation | 9.15E-49 | 3.47E-51 |
| GO:0048522 | BIOLOGICAL_PROCESS | positive regulation of cellular process | 3.42E-46 | 1.33E-48 |
| GO:0044459 | CELLULAR_COMPONENT | plasma membrane part | 1.40E-45 | 5.57E-48 |
| GO:0004713 | MOLECULAR_FUNCTION | protein tyrosine kinase activity | 3.29E-45 | 1.34E-47 |
| GO:0009653 | BIOLOGICAL_PROCESS | anatomical structure morphogenesis | 8.45E-45 | 3.51E-47 |
| GO:0006357 | BIOLOGICAL_PROCESS | regulation of transcription from RNA polymerase II promoter | 1.12E-44 | 4.74E-47 |
| GO:0007399 | BIOLOGICAL_PROCESS | nervous system development | 4.47E-44 | 1.93E-46 |
| GO:0006366 | BIOLOGICAL_PROCESS | transcription from RNA polymerase II promoter | 5.12E-44 | 2.26E-46 |
| GO:0048519 | BIOLOGICAL_PROCESS | negative regulation of biological process | 2.55E-43 | 1.15E-45 |
| GO:0009888 | BIOLOGICAL_PROCESS | tissue development | 9.47E-43 | 4.35E-45 |
| GO:0016301 | MOLECULAR_FUNCTION | kinase activity | 1.79E-42 | 8.37E-45 |
| GO:0010604 | BIOLOGICAL_PROCESS | positive regulation of macromolecule metabolic process | 2.26E-42 | 1.08E-44 |
| GO:0071310 | BIOLOGICAL_PROCESS | cellular response to organic substance | 7.36E-42 | 3.57E-44 |
| GO:0031325 | BIOLOGICAL_PROCESS | positive regulation of cellular metabolic process | 4.39E-41 | 2.17E-43 |
| GO:0060255 | BIOLOGICAL_PROCESS | regulation of macromolecule metabolic process | 4.70E-41 | 2.37E-43 |
| GO:0009893 | BIOLOGICAL_PROCESS | positive regulation of metabolic process | 6.92E-41 | 3.54E-43 |
| GO:0043565 | MOLECULAR_FUNCTION | sequence-specific DNA binding | 3.81E-40 | 1.99E-42 |
| GO:0008227 | MOLECULAR_FUNCTION | G-protein coupled amine receptor activity | 4.01E-40 | 2.13E-42 |
| GO:0043412 | BIOLOGICAL_PROCESS | macromolecule modification | 1.60E-39 | 8.63E-42 |
| GO:0019222 | BIOLOGICAL_PROCESS | regulation of metabolic process | 7.73E-39 | 4.23E-41 |
| GO:0048523 | BIOLOGICAL_PROCESS | negative regulation of cellular process | 2.01E-38 | 1.12E-40 |
| GO:0022008 | BIOLOGICAL_PROCESS | neurogenesis | 8.35E-38 | 4.72E-40 |
| GO:0034703 | CELLULAR_COMPONENT | cation channel complex | 2.53E-37 | 1.45E-39 |
| GO:0031323 | BIOLOGICAL_PROCESS | regulation of cellular metabolic process | 3.31E-37 | 1.93E-39 |
| GO:0006796 | BIOLOGICAL_PROCESS | phosphate-containing compound metabolic process | 9.35E-37 | 5.53E-39 |
| GO:0080090 | BIOLOGICAL_PROCESS | regulation of primary metabolic process | 1.46E-36 | 8.78E-39 |
| GO:0071495 | BIOLOGICAL_PROCESS | cellular response to endogenous stimulus | 1.83E-36 | 1.12E-38 |
| GO:0005515 | MOLECULAR_FUNCTION | protein binding | 3.22E-36 | 1.99E-38 |
| GO:0051252 | BIOLOGICAL_PROCESS | regulation of RNA metabolic process | 3.58E-36 | 2.24E-38 |
| GO:1902680 | BIOLOGICAL_PROCESS | positive regulation of RNA biosynthetic process | 4.07E-36 | 2.59E-38 |
| GO:0032879 | BIOLOGICAL_PROCESS | regulation of localization | 5.48E-36 | 3.53E-38 |
| GO:2001141 | BIOLOGICAL_PROCESS | regulation of RNA biosynthetic process | 5.73E-36 | 3.75E-38 |
| GO:1903506 | BIOLOGICAL_PROCESS | regulation of nucleic acid-templated transcription | 7.92E-36 | 5.24E-38 |
| GO:0010557 | BIOLOGICAL_PROCESS | positive regulation of macromolecule biosynthetic process | 1.05E-35 | 7.06E-38 |
| GO:0006355 | BIOLOGICAL_PROCESS | regulation of transcription, DNA-templated | 1.17E-35 | 7.97E-38 |
| GO:0010628 | BIOLOGICAL_PROCESS | positive regulation of gene expression | 1.34E-35 | 9.33E-38 |
| GO:0019219 | BIOLOGICAL_PROCESS | regulation of nucleobase-containing compound metabolic process | 1.34E-35 | 9.25E-38 |
| GO:0051254 | BIOLOGICAL_PROCESS | positive regulation of RNA metabolic process | 1.80E-35 | 1.27E-37 |
| GO:0009891 | BIOLOGICAL_PROCESS | positive regulation of biosynthetic process | 2.00E-35 | 1.43E-37 |
| GO:0048699 | BIOLOGICAL_PROCESS | generation of neurons | 2.31E-35 | 1.68E-37 |
| GO:0051239 | BIOLOGICAL_PROCESS | regulation of multicellular organismal process | 3.30E-35 | 2.41E-37 |
| GO:0010468 | BIOLOGICAL_PROCESS | regulation of gene expression | 7.26E-35 | 5.38E-37 |
| GO:0034702 | CELLULAR_COMPONENT | ion channel complex | 9.55E-35 | 7.17E-37 |
| GO:0030594 | MOLECULAR_FUNCTION | neurotransmitter receptor activity | 1.60E-34 | 1.21E-36 |
| GO:1903508 | BIOLOGICAL_PROCESS | positive regulation of nucleic acid-templated transcription | 3.33E-34 | 2.59E-36 |
| GO:0045893 | BIOLOGICAL_PROCESS | positive regulation of transcription, DNA-templated | 3.33E-34 | 2.59E-36 |
| GO:0006793 | BIOLOGICAL_PROCESS | phosphorus metabolic process | 3.45E-34 | 2.74E-36 |
| GO:0045935 | BIOLOGICAL_PROCESS | positive regulation of nucleobase-containing compound metabolic process | 3.45E-34 | 2.72E-36 |
| GO:0031328 | BIOLOGICAL_PROCESS | positive regulation of cellular biosynthetic process | 4.07E-34 | 3.27E-36 |
| GO:0000981 | MOLECULAR_FUNCTION | RNA polymerase II transcription factor activity, sequence-specific DNA binding | 4.46E-34 | 3.63E-36 |
| GO:0018108 | BIOLOGICAL_PROCESS | peptidyl-tyrosine phosphorylation | 7.94E-34 | 6.52E-36 |
| GO:2000112 | BIOLOGICAL_PROCESS | regulation of cellular macromolecule biosynthetic process | 9.22E-34 | 7.65E-36 |
| GO:0005488 | MOLECULAR_FUNCTION | binding | 1.23E-33 | 1.04E-35 |
| GO:0010556 | BIOLOGICAL_PROCESS | regulation of macromolecule biosynthetic process | 1.75E-33 | 1.49E-35 |
| GO:0050793 | BIOLOGICAL_PROCESS | regulation of developmental process | 2.89E-33 | 2.48E-35 |
| GO:0030182 | BIOLOGICAL_PROCESS | neuron differentiation | 3.16E-33 | 2.74E-35 |
| GO:0010646 | BIOLOGICAL_PROCESS | regulation of cell communication | 6.52E-33 | 5.70E-35 |
| GO:0009719 | BIOLOGICAL_PROCESS | response to endogenous stimulus | 8.27E-33 | 7.31E-35 |
| GO:0009889 | BIOLOGICAL_PROCESS | regulation of biosynthetic process | 9.03E-33 | 8.05E-35 |
| GO:0001883 | MOLECULAR_FUNCTION | purine nucleoside binding | 1.53E-32 | 1.39E-34 |
| GO:0032550 | MOLECULAR_FUNCTION | purine ribonucleoside binding | 1.53E-32 | 1.39E-34 |
| GO:0048468 | BIOLOGICAL_PROCESS | cell development | 1.67E-32 | 1.53E-34 |
| GO:0031326 | BIOLOGICAL_PROCESS | regulation of cellular biosynthetic process | 1.74E-32 | 1.61E-34 |
| GO:0097659 | BIOLOGICAL_PROCESS | nucleic acid-templated transcription | 1.84E-32 | 1.72E-34 |
| GO:0035639 | MOLECULAR_FUNCTION | purine ribonucleoside triphosphate binding | 2.17E-32 | 2.05E-34 |
| GO:0048583 | BIOLOGICAL_PROCESS | regulation of response to stimulus | 2.42E-32 | 2.31E-34 |
| GO:0001882 | MOLECULAR_FUNCTION | nucleoside binding | 2.73E-32 | 2.63E-34 |
| GO:0023051 | BIOLOGICAL_PROCESS | regulation of signaling | 2.76E-32 | 2.68E-34 |
| GO:2000026 | BIOLOGICAL_PROCESS | regulation of multicellular organismal development | 3.24E-32 | 3.18E-34 |
| GO:0051173 | BIOLOGICAL_PROCESS | positive regulation of nitrogen compound metabolic process | 3.77E-32 | 3.73E-34 |
| GO:0006351 | BIOLOGICAL_PROCESS | transcription, DNA-templated | 4.78E-32 | 4.76E-34 |
| GO:0032549 | MOLECULAR_FUNCTION | ribonucleoside binding | 7.04E-32 | 7.08E-34 |
| GO:0043231 | CELLULAR_COMPONENT | intracellular membrane-bounded organelle | 1.04E-31 | 1.05E-33 |
| GO:0032555 | MOLECULAR_FUNCTION | purine ribonucleotide binding | 1.15E-31 | 1.18E-33 |
| GO:0017076 | MOLECULAR_FUNCTION | purine nucleotide binding | 1.28E-31 | 1.32E-33 |
| GO:0045944 | BIOLOGICAL_PROCESS | positive regulation of transcription from RNA polymerase II promoter | 2.08E-31 | 2.17E-33 |
| GO:1901701 | BIOLOGICAL_PROCESS | cellular response to oxygen-containing compound | 2.12E-31 | 2.23E-33 |
| GO:0032774 | BIOLOGICAL_PROCESS | RNA biosynthetic process | 3.28E-31 | 3.47E-33 |
| GO:0051049 | BIOLOGICAL_PROCESS | regulation of transport | 4.76E-31 | 5.09E-33 |
| GO:0043227 | CELLULAR_COMPONENT | membrane-bounded organelle | 4.92E-31 | 5.30E-33 |
| GO:0051171 | BIOLOGICAL_PROCESS | regulation of nitrogen compound metabolic process | 5.59E-31 | 6.08E-33 |
| GO:0097367 | MOLECULAR_FUNCTION | carbohydrate derivative binding | 8.55E-31 | 9.36E-33 |
| GO:0045595 | BIOLOGICAL_PROCESS | regulation of cell differentiation | 1.06E-30 | 1.16E-32 |
| GO:0032553 | MOLECULAR_FUNCTION | ribonucleotide binding | 2.32E-30 | 2.58E-32 |
| GO:0006915 | BIOLOGICAL_PROCESS | apoptotic process | 5.64E-30 | 6.37E-32 |
| GO:0009887 | BIOLOGICAL_PROCESS | animal organ morphogenesis | 5.64E-30 | 6.37E-32 |
| GO:0008219 | BIOLOGICAL_PROCESS | cell death | 8.12E-30 | 9.25E-32 |
| GO:1901700 | BIOLOGICAL_PROCESS | response to oxygen-containing compound | 8.69E-30 | 9.97E-32 |
| GO:0018212 | BIOLOGICAL_PROCESS | peptidyl-tyrosine modification | 1.67E-29 | 1.93E-31 |
| GO:0051094 | BIOLOGICAL_PROCESS | positive regulation of developmental process | 2.14E-29 | 2.50E-31 |
| GO:1901699 | BIOLOGICAL_PROCESS | cellular response to nitrogen compound | 5.45E-29 | 6.44E-31 |
| GO:0018193 | BIOLOGICAL_PROCESS | peptidyl-amino acid modification | 5.45E-29 | 6.45E-31 |
| GO:0051240 | BIOLOGICAL_PROCESS | positive regulation of multicellular organismal process | 5.53E-29 | 6.60E-31 |
| GO:0004674 | MOLECULAR_FUNCTION | protein serine/threonine kinase activity | 6.06E-29 | 7.27E-31 |
| GO:0034705 | CELLULAR_COMPONENT | potassium channel complex | 6.95E-29 | 8.41E-31 |
| GO:0012501 | BIOLOGICAL_PROCESS | programmed cell death | 8.73E-29 | 1.07E-30 |
| GO:0034654 | BIOLOGICAL_PROCESS | nucleobase-containing compound biosynthetic process | 8.73E-29 | 1.07E-30 |
| GO:0009966 | BIOLOGICAL_PROCESS | regulation of signal transduction | 1.98E-28 | 2.45E-30 |
| GO:0060429 | BIOLOGICAL_PROCESS | epithelium development | 2.67E-28 | 3.33E-30 |
| GO:0009725 | BIOLOGICAL_PROCESS | response to hormone | 6.80E-28 | 8.53E-30 |
| GO:0016043 | BIOLOGICAL_PROCESS | cellular component organization | 2.46E-27 | 3.11E-29 |
| GO:0007167 | BIOLOGICAL_PROCESS | enzyme linked receptor protein signaling pathway | 3.60E-27 | 4.58E-29 |
| GO:0032870 | BIOLOGICAL_PROCESS | cellular response to hormone stimulus | 6.31E-27 | 8.08E-29 |
| GO:0005524 | MOLECULAR_FUNCTION | ATP binding | 1.46E-26 | 1.88E-28 |
| GO:0032559 | MOLECULAR_FUNCTION | adenyl ribonucleotide binding | 1.99E-26 | 2.59E-28 |
| GO:0000975 | MOLECULAR_FUNCTION | regulatory region DNA binding | 2.17E-26 | 2.88E-28 |
| GO:0001067 | MOLECULAR_FUNCTION | regulatory region nucleic acid binding | 2.17E-26 | 2.88E-28 |
| GO:0044212 | MOLECULAR_FUNCTION | transcription regulatory region DNA binding | 2.17E-26 | 2.88E-28 |
| GO:0030554 | MOLECULAR_FUNCTION | adenyl nucleotide binding | 2.85E-26 | 3.80E-28 |
| GO:0008076 | CELLULAR_COMPONENT | voltage-gated potassium channel complex | 3.27E-26 | 4.39E-28 |
| GO:0072359 | BIOLOGICAL_PROCESS | circulatory system development | 3.70E-26 | 5.00E-28 |
| GO:1902495 | CELLULAR_COMPONENT | transmembrane transporter complex | 2.63E-25 | 3.60E-27 |
| GO:1990351 | CELLULAR_COMPONENT | transporter complex | 2.63E-25 | 3.60E-27 |
| GO:0016772 | MOLECULAR_FUNCTION | transferase activity, transferring phosphorus-containing groups | 3.34E-25 | 4.60E-27 |
| GO:0001653 | MOLECULAR_FUNCTION | peptide receptor activity | 7.25E-25 | 1.00E-26 |
| GO:0008188 | MOLECULAR_FUNCTION | neuropeptide receptor activity | 7.80E-25 | 1.09E-26 |
| GO:0008283 | BIOLOGICAL_PROCESS | cell proliferation | 1.34E-24 | 1.89E-26 |
| GO:0007267 | BIOLOGICAL_PROCESS | cell-cell signaling | 1.55E-24 | 2.19E-26 |
| GO:0051260 | BIOLOGICAL_PROCESS | protein homooligomerization | 1.70E-24 | 2.41E-26 |
| GO:0008528 | MOLECULAR_FUNCTION | G-protein coupled peptide receptor activity | 2.09E-24 | 2.99E-26 |
| GO:0034765 | BIOLOGICAL_PROCESS | regulation of ion transmembrane transport | 2.18E-24 | 3.16E-26 |
| GO:0034762 | BIOLOGICAL_PROCESS | regulation of transmembrane transport | 2.18E-24 | 3.16E-26 |
| GO:0048729 | BIOLOGICAL_PROCESS | tissue morphogenesis | 3.55E-24 | 5.17E-26 |
| GO:0051259 | BIOLOGICAL_PROCESS | protein oligomerization | 6.15E-24 | 9.01E-26 |
| GO:0009790 | BIOLOGICAL_PROCESS | embryo development | 7.76E-24 | 1.14E-25 |
| GO:0097159 | MOLECULAR_FUNCTION | organic cyclic compound binding | 9.51E-24 | 1.41E-25 |
| GO:0071840 | BIOLOGICAL_PROCESS | cellular component organization or biogenesis | 1.47E-23 | 2.20E-25 |
| GO:0071407 | BIOLOGICAL_PROCESS | cellular response to organic cyclic compound | 2.09E-23 | 3.14E-25 |
| GO:0071944 | CELLULAR_COMPONENT | cell periphery | 2.53E-23 | 3.82E-25 |
| GO:0010941 | BIOLOGICAL_PROCESS | regulation of cell death | 3.24E-23 | 4.92E-25 |
| GO:0043167 | MOLECULAR_FUNCTION | ion binding | 4.44E-23 | 6.79E-25 |
| GO:0007218 | BIOLOGICAL_PROCESS | neuropeptide signaling pathway | 5.10E-23 | 7.83E-25 |
| GO:0044267 | BIOLOGICAL_PROCESS | cellular protein metabolic process | 8.43E-23 | 1.30E-24 |
| GO:1901698 | BIOLOGICAL_PROCESS | response to nitrogen compound | 8.54E-23 | 1.33E-24 |
| GO:1901363 | MOLECULAR_FUNCTION | heterocyclic compound binding | 1.09E-22 | 1.71E-24 |
| GO:0042127 | BIOLOGICAL_PROCESS | regulation of cell proliferation | 1.34E-22 | 2.11E-24 |
| GO:0043269 | BIOLOGICAL_PROCESS | regulation of ion transport | 2.35E-22 | 3.72E-24 |
| GO:1901265 | MOLECULAR_FUNCTION | nucleoside phosphate binding | 2.38E-22 | 3.81E-24 |
| GO:0000166 | MOLECULAR_FUNCTION | nucleotide binding | 2.38E-22 | 3.81E-24 |
| GO:0019438 | BIOLOGICAL_PROCESS | aromatic compound biosynthetic process | 2.76E-22 | 4.44E-24 |
| GO:0022838 | MOLECULAR_FUNCTION | substrate-specific channel activity | 3.02E-22 | 4.88E-24 |
| GO:0042981 | BIOLOGICAL_PROCESS | regulation of apoptotic process | 3.44E-22 | 5.59E-24 |
| GO:0005886 | CELLULAR_COMPONENT | plasma membrane | 3.52E-22 | 5.74E-24 |
| GO:0035295 | BIOLOGICAL_PROCESS | tube development | 4.02E-22 | 6.61E-24 |
| GO:0005216 | MOLECULAR_FUNCTION | ion channel activity | 4.07E-22 | 6.72E-24 |
| GO:0033993 | BIOLOGICAL_PROCESS | response to lipid | 5.13E-22 | 8.51E-24 |
| GO:0043067 | BIOLOGICAL_PROCESS | regulation of programmed cell death | 5.77E-22 | 9.63E-24 |
| GO:0048598 | BIOLOGICAL_PROCESS | embryonic morphogenesis | 1.15E-21 | 1.92E-23 |
| GO:0005261 | MOLECULAR_FUNCTION | cation channel activity | 1.57E-21 | 2.65E-23 |
| GO:0045597 | BIOLOGICAL_PROCESS | positive regulation of cell differentiation | 1.98E-21 | 3.36E-23 |
| GO:0018130 | BIOLOGICAL_PROCESS | heterocycle biosynthetic process | 2.12E-21 | 3.62E-23 |
| GO:0010647 | BIOLOGICAL_PROCESS | positive regulation of cell communication | 2.48E-21 | 4.24E-23 |
| GO:0035556 | BIOLOGICAL_PROCESS | intracellular signal transduction | 3.06E-21 | 5.27E-23 |
| GO:0048584 | BIOLOGICAL_PROCESS | positive regulation of response to stimulus | 3.14E-21 | 5.44E-23 |
| GO:0042325 | BIOLOGICAL_PROCESS | regulation of phosphorylation | 3.75E-21 | 6.52E-23 |
| GO:0002376 | BIOLOGICAL_PROCESS | immune system process | 3.97E-21 | 6.94E-23 |
| GO:0010605 | BIOLOGICAL_PROCESS | negative regulation of macromolecule metabolic process | 8.49E-21 | 1.49E-22 |
| GO:1905114 | BIOLOGICAL_PROCESS | cell surface receptor signaling pathway involved in cell-cell signaling | 9.09E-21 | 1.60E-22 |
| GO:0023056 | BIOLOGICAL_PROCESS | positive regulation of signaling | 1.09E-20 | 1.94E-22 |
| GO:0005102 | MOLECULAR_FUNCTION | receptor binding | 2.79E-20 | 4.97E-22 |
| GO:1901362 | BIOLOGICAL_PROCESS | organic cyclic compound biosynthetic process | 4.08E-20 | 7.32E-22 |
| GO:0071396 | BIOLOGICAL_PROCESS | cellular response to lipid | 5.58E-20 | 1.00E-21 |
| GO:0007389 | BIOLOGICAL_PROCESS | pattern specification process | 5.95E-20 | 1.08E-21 |
| GO:0048646 | BIOLOGICAL_PROCESS | anatomical structure formation involved in morphogenesis | 5.97E-20 | 1.08E-21 |
| GO:0048666 | BIOLOGICAL_PROCESS | neuron development | 6.34E-20 | 1.16E-21 |
| GO:0036094 | MOLECULAR_FUNCTION | small molecule binding | 6.34E-20 | 1.16E-21 |
| GO:0009892 | BIOLOGICAL_PROCESS | negative regulation of metabolic process | 7.02E-20 | 1.30E-21 |
| GO:0060359 | BIOLOGICAL_PROCESS | response to ammonium ion | 7.89E-20 | 1.46E-21 |
| GO:0071242 | BIOLOGICAL_PROCESS | cellular response to ammonium ion | 8.46E-20 | 1.58E-21 |
| GO:0045934 | BIOLOGICAL_PROCESS | negative regulation of nucleobase-containing compound metabolic process | 1.34E-19 | 2.51E-21 |
| GO:0002009 | BIOLOGICAL_PROCESS | morphogenesis of an epithelium | 2.41E-19 | 4.54E-21 |
| GO:0015267 | MOLECULAR_FUNCTION | channel activity | 2.45E-19 | 4.65E-21 |
| GO:0022803 | MOLECULAR_FUNCTION | passive transmembrane transporter activity | 2.45E-19 | 4.65E-21 |
| GO:1902679 | BIOLOGICAL_PROCESS | negative regulation of RNA biosynthetic process | 2.54E-19 | 4.84E-21 |
| GO:0016055 | BIOLOGICAL_PROCESS | Wnt signaling pathway | 2.74E-19 | 5.27E-21 |
| GO:0198738 | BIOLOGICAL_PROCESS | cell-cell signaling by wnt | 2.74E-19 | 5.27E-21 |
| GO:0001932 | BIOLOGICAL_PROCESS | regulation of protein phosphorylation | 3.04E-19 | 5.89E-21 |
| GO:0006325 | BIOLOGICAL_PROCESS | chromatin organization | 3.21E-19 | 6.23E-21 |
| GO:0032270 | BIOLOGICAL_PROCESS | positive regulation of cellular protein metabolic process | 4.64E-19 | 9.06E-21 |
| GO:0007417 | BIOLOGICAL_PROCESS | central nervous system development | 5.24E-19 | 1.03E-20 |
| GO:0019538 | BIOLOGICAL_PROCESS | protein metabolic process | 5.50E-19 | 1.08E-20 |
| GO:0051247 | BIOLOGICAL_PROCESS | positive regulation of protein metabolic process | 8.72E-19 | 1.73E-20 |
| GO:0044260 | BIOLOGICAL_PROCESS | cellular macromolecule metabolic process | 8.72E-19 | 1.73E-20 |
| GO:0051253 | BIOLOGICAL_PROCESS | negative regulation of RNA metabolic process | 8.96E-19 | 1.79E-20 |
| GO:0009755 | BIOLOGICAL_PROCESS | hormone-mediated signaling pathway | 9.93E-19 | 1.99E-20 |
| GO:0060548 | BIOLOGICAL_PROCESS | negative regulation of cell death | 1.03E-18 | 2.08E-20 |
| GO:1903831 | BIOLOGICAL_PROCESS | signal transduction involved in cellular response to ammonium ion | 1.26E-18 | 2.59E-20 |
| GO:1905145 | BIOLOGICAL_PROCESS | cellular response to acetylcholine | 1.26E-18 | 2.59E-20 |
| GO:1905144 | BIOLOGICAL_PROCESS | response to acetylcholine | 1.26E-18 | 2.59E-20 |
| GO:0007213 | BIOLOGICAL_PROCESS | G-protein coupled acetylcholine receptor signaling pathway | 1.26E-18 | 2.59E-20 |
| GO:0095500 | BIOLOGICAL_PROCESS | acetylcholine receptor signaling pathway | 1.26E-18 | 2.59E-20 |
| GO:0000976 | MOLECULAR_FUNCTION | transcription regulatory region sequence-specific DNA binding | 1.57E-18 | 3.24E-20 |
| GO:0035239 | BIOLOGICAL_PROCESS | tube morphogenesis | 1.95E-18 | 4.05E-20 |
| GO:0031324 | BIOLOGICAL_PROCESS | negative regulation of cellular metabolic process | 2.14E-18 | 4.45E-20 |
| GO:0004983 | MOLECULAR_FUNCTION | neuropeptide Y receptor activity | 2.83E-18 | 5.93E-20 |
| GO:0010629 | BIOLOGICAL_PROCESS | negative regulation of gene expression | 3.48E-18 | 7.31E-20 |
| GO:0009890 | BIOLOGICAL_PROCESS | negative regulation of biosynthetic process | 3.61E-18 | 7.61E-20 |
| GO:0014070 | BIOLOGICAL_PROCESS | response to organic cyclic compound | 4.42E-18 | 9.37E-20 |
| GO:0016477 | BIOLOGICAL_PROCESS | cell migration | 4.49E-18 | 9.56E-20 |
| GO:0005272 | MOLECULAR_FUNCTION | sodium channel activity | 4.68E-18 | 9.99E-20 |
| GO:0031327 | BIOLOGICAL_PROCESS | negative regulation of cellular biosynthetic process | 4.82E-18 | 1.03E-19 |
| GO:0010558 | BIOLOGICAL_PROCESS | negative regulation of macromolecule biosynthetic process | 4.89E-18 | 1.05E-19 |
| GO:0009967 | BIOLOGICAL_PROCESS | positive regulation of signal transduction | 5.68E-18 | 1.23E-19 |
| GO:0007507 | BIOLOGICAL_PROCESS | heart development | 5.89E-18 | 1.28E-19 |
| GO:0000977 | MOLECULAR_FUNCTION | RNA polymerase II regulatory region sequence-specific DNA binding | 6.02E-18 | 1.32E-19 |
| GO:0001012 | MOLECULAR_FUNCTION | RNA polymerase II regulatory region DNA binding | 6.02E-18 | 1.32E-19 |
| GO:0003008 | BIOLOGICAL_PROCESS | system process | 6.27E-18 | 1.38E-19 |
| GO:0043229 | CELLULAR_COMPONENT | intracellular organelle | 7.47E-18 | 1.65E-19 |
| GO:0043401 | BIOLOGICAL_PROCESS | steroid hormone mediated signaling pathway | 8.59E-18 | 1.90E-19 |
| GO:0040007 | BIOLOGICAL_PROCESS | growth | 1.05E-17 | 2.34E-19 |
| GO:1903507 | BIOLOGICAL_PROCESS | negative regulation of nucleic acid-templated transcription | 1.06E-17 | 2.38E-19 |
| GO:0060284 | BIOLOGICAL_PROCESS | regulation of cell development | 1.08E-17 | 2.43E-19 |
| GO:0016907 | MOLECULAR_FUNCTION | G-protein coupled acetylcholine receptor activity | 1.29E-17 | 2.91E-19 |
| GO:0099528 | MOLECULAR_FUNCTION | G-protein coupled neurotransmitter receptor activity | 1.29E-17 | 2.91E-19 |
| GO:0071383 | BIOLOGICAL_PROCESS | cellular response to steroid hormone stimulus | 1.29E-17 | 2.93E-19 |
| GO:0022610 | BIOLOGICAL_PROCESS | biological adhesion | 1.36E-17 | 3.10E-19 |
| GO:0007155 | BIOLOGICAL_PROCESS | cell adhesion | 1.36E-17 | 3.10E-19 |
| GO:0005244 | MOLECULAR_FUNCTION | voltage-gated ion channel activity | 1.56E-17 | 3.60E-19 |
| GO:0022832 | MOLECULAR_FUNCTION | voltage-gated channel activity | 1.56E-17 | 3.60E-19 |
| GO:0048545 | BIOLOGICAL_PROCESS | response to steroid hormone | 1.63E-17 | 3.76E-19 |
| GO:0031399 | BIOLOGICAL_PROCESS | regulation of protein modification process | 1.68E-17 | 3.89E-19 |
| GO:0045892 | BIOLOGICAL_PROCESS | negative regulation of transcription, DNA-templated | 1.73E-17 | 4.04E-19 |
| GO:0051172 | BIOLOGICAL_PROCESS | negative regulation of nitrogen compound metabolic process | 2.07E-17 | 4.88E-19 |
| GO:0051174 | BIOLOGICAL_PROCESS | regulation of phosphorus metabolic process | 2.07E-17 | 4.88E-19 |
| GO:0019220 | BIOLOGICAL_PROCESS | regulation of phosphate metabolic process | 2.07E-17 | 4.88E-19 |
| GO:0022836 | MOLECULAR_FUNCTION | gated channel activity | 2.61E-17 | 6.18E-19 |
| GO:0045165 | BIOLOGICAL_PROCESS | cell fate commitment | 2.82E-17 | 6.71E-19 |
| GO:0051241 | BIOLOGICAL_PROCESS | negative regulation of multicellular organismal process | 3.48E-17 | 8.30E-19 |
| GO:0000122 | BIOLOGICAL_PROCESS | negative regulation of transcription from RNA polymerase II promoter | 4.33E-17 | 1.04E-18 |
| GO:1990837 | MOLECULAR_FUNCTION | sequence-specific double-stranded DNA binding | 6.71E-17 | 1.62E-18 |
| GO:0031175 | BIOLOGICAL_PROCESS | neuron projection development | 6.71E-17 | 1.62E-18 |
| GO:0034706 | CELLULAR_COMPONENT | sodium channel complex | 7.26E-17 | 1.76E-18 |
| GO:0043069 | BIOLOGICAL_PROCESS | negative regulation of programmed cell death | 7.78E-17 | 1.89E-18 |
| GO:0048534 | BIOLOGICAL_PROCESS | hematopoietic or lymphoid organ development | 8.93E-17 | 2.18E-18 |
| GO:0043066 | BIOLOGICAL_PROCESS | negative regulation of apoptotic process | 8.93E-17 | 2.18E-18 |
| GO:0043226 | CELLULAR_COMPONENT | organelle | 1.23E-16 | 3.03E-18 |
| GO:0000982 | MOLECULAR_FUNCTION | transcription factor activity, RNA polymerase II core promoter proximal region sequence-specific binding | 1.38E-16 | 3.39E-18 |
| GO:2000113 | BIOLOGICAL_PROCESS | negative regulation of cellular macromolecule biosynthetic process | 1.54E-16 | 3.80E-18 |
| GO:0060562 | BIOLOGICAL_PROCESS | epithelial tube morphogenesis | 1.79E-16 | 4.44E-18 |
| GO:0006461 | BIOLOGICAL_PROCESS | protein complex assembly | 1.94E-16 | 4.85E-18 |
| GO:0070271 | BIOLOGICAL_PROCESS | protein complex biogenesis | 1.94E-16 | 4.85E-18 |
| GO:0051960 | BIOLOGICAL_PROCESS | regulation of nervous system development | 2.22E-16 | 5.58E-18 |
| GO:0001934 | BIOLOGICAL_PROCESS | positive regulation of protein phosphorylation | 2.57E-16 | 6.50E-18 |
| GO:0003002 | BIOLOGICAL_PROCESS | regionalization | 2.57E-16 | 6.50E-18 |
| GO:0043168 | MOLECULAR_FUNCTION | anion binding | 4.16E-16 | 1.05E-17 |
| GO:0042327 | BIOLOGICAL_PROCESS | positive regulation of phosphorylation | 4.56E-16 | 1.16E-17 |
| GO:1902531 | BIOLOGICAL_PROCESS | regulation of intracellular signal transduction | 5.04E-16 | 1.29E-17 |
| GO:0051093 | BIOLOGICAL_PROCESS | negative regulation of developmental process | 5.37E-16 | 1.38E-17 |
| GO:0023057 | BIOLOGICAL_PROCESS | negative regulation of signaling | 6.67E-16 | 1.72E-17 |
| GO:0010648 | BIOLOGICAL_PROCESS | negative regulation of cell communication | 6.67E-16 | 1.72E-17 |
| GO:0000165 | BIOLOGICAL_PROCESS | MAPK cascade | 7.43E-16 | 1.92E-17 |
| GO:0004952 | MOLECULAR_FUNCTION | dopamine neurotransmitter receptor activity | 8.09E-16 | 2.10E-17 |
| GO:0019199 | MOLECULAR_FUNCTION | transmembrane receptor protein kinase activity | 8.35E-16 | 2.17E-17 |
| GO:0048585 | BIOLOGICAL_PROCESS | negative regulation of response to stimulus | 1.19E-15 | 3.12E-17 |
| GO:0071822 | BIOLOGICAL_PROCESS | protein complex subunit organization | 1.20E-15 | 3.15E-17 |
| GO:0001071 | MOLECULAR_FUNCTION | nucleic acid binding transcription factor activity | 1.94E-15 | 5.12E-17 |
| GO:0003700 | MOLECULAR_FUNCTION | transcription factor activity, sequence-specific DNA binding | 1.94E-15 | 5.12E-17 |
| GO:0050767 | BIOLOGICAL_PROCESS | regulation of neurogenesis | 3.31E-15 | 8.79E-17 |
| GO:0007169 | BIOLOGICAL_PROCESS | transmembrane receptor protein tyrosine kinase signaling pathway | 3.31E-15 | 8.79E-17 |
| GO:0071363 | BIOLOGICAL_PROCESS | cellular response to growth factor stimulus | 3.72E-15 | 9.95E-17 |
| GO:0070848 | BIOLOGICAL_PROCESS | response to growth factor | 3.72E-15 | 9.95E-17 |
| GO:0031401 | BIOLOGICAL_PROCESS | positive regulation of protein modification process | 4.58E-15 | 1.23E-16 |
| GO:0010562 | BIOLOGICAL_PROCESS | positive regulation of phosphorus metabolic process | 4.67E-15 | 1.26E-16 |
| GO:0045937 | BIOLOGICAL_PROCESS | positive regulation of phosphate metabolic process | 4.67E-15 | 1.26E-16 |
| GO:0030855 | BIOLOGICAL_PROCESS | epithelial cell differentiation | 4.89E-15 | 1.33E-16 |
| GO:0048562 | BIOLOGICAL_PROCESS | embryonic organ morphogenesis | 5.18E-15 | 1.41E-16 |
| GO:0060485 | BIOLOGICAL_PROCESS | mesenchyme development | 5.42E-15 | 1.48E-16 |
| GO:0072358 | BIOLOGICAL_PROCESS | cardiovascular system development | 6.58E-15 | 1.81E-16 |
| GO:0002520 | BIOLOGICAL_PROCESS | immune system development | 6.58E-15 | 1.81E-16 |
| GO:0007517 | BIOLOGICAL_PROCESS | muscle organ development | 7.70E-15 | 2.12E-16 |
| GO:0001518 | CELLULAR_COMPONENT | voltage-gated sodium channel complex | 1.24E-14 | 3.43E-16 |
| GO:0005576 | CELLULAR_COMPONENT | extracellular region | 1.30E-14 | 3.60E-16 |
| GO:0000904 | BIOLOGICAL_PROCESS | cell morphogenesis involved in differentiation | 1.35E-14 | 3.76E-16 |
| GO:0048589 | BIOLOGICAL_PROCESS | developmental growth | 1.44E-14 | 4.01E-16 |
| GO:0048812 | BIOLOGICAL_PROCESS | neuron projection morphogenesis | 1.51E-14 | 4.21E-16 |
| GO:0007420 | BIOLOGICAL_PROCESS | brain development | 1.59E-14 | 4.48E-16 |
| GO:0007212 | BIOLOGICAL_PROCESS | dopamine receptor signaling pathway | 1.60E-14 | 4.52E-16 |
| GO:0061061 | BIOLOGICAL_PROCESS | muscle structure development | 1.70E-14 | 4.80E-16 |
| GO:0001944 | BIOLOGICAL_PROCESS | vasculature development | 1.78E-14 | 5.06E-16 |
| GO:0003690 | MOLECULAR_FUNCTION | double-stranded DNA binding | 1.92E-14 | 5.46E-16 |
| GO:0060322 | BIOLOGICAL_PROCESS | head development | 2.18E-14 | 6.22E-16 |
| GO:0051276 | BIOLOGICAL_PROCESS | chromosome organization | 2.19E-14 | 6.28E-16 |
| GO:0043170 | BIOLOGICAL_PROCESS | macromolecule metabolic process | 2.70E-14 | 7.74E-16 |
| GO:0048858 | BIOLOGICAL_PROCESS | cell projection morphogenesis | 2.95E-14 | 8.49E-16 |
| GO:0060078 | BIOLOGICAL_PROCESS | regulation of postsynaptic membrane potential | 4.08E-14 | 1.18E-15 |
| GO:0032990 | BIOLOGICAL_PROCESS | cell part morphogenesis | 5.81E-14 | 1.68E-15 |
| GO:0048667 | BIOLOGICAL_PROCESS | cell morphogenesis involved in neuron differentiation | 6.45E-14 | 1.87E-15 |
| GO:0005248 | MOLECULAR_FUNCTION | voltage-gated sodium channel activity | 6.66E-14 | 1.95E-15 |
| GO:1905030 | MOLECULAR_FUNCTION | voltage-gated ion channel activity involved in regulation of postsynaptic membrane potential | 6.66E-14 | 1.95E-15 |
| GO:0016070 | BIOLOGICAL_PROCESS | RNA metabolic process | 1.06E-13 | 3.11E-15 |
| GO:0032268 | BIOLOGICAL_PROCESS | regulation of cellular protein metabolic process | 1.46E-13 | 4.28E-15 |
| GO:0051246 | BIOLOGICAL_PROCESS | regulation of protein metabolic process | 1.56E-13 | 4.61E-15 |
| GO:0004714 | MOLECULAR_FUNCTION | transmembrane receptor protein tyrosine kinase activity | 1.70E-13 | 5.03E-15 |
| GO:0001077 | MOLECULAR_FUNCTION | transcriptional activator activity, RNA polymerase II core promoter proximal region sequence-specific binding | 2.12E-13 | 6.28E-15 |
| GO:0009968 | BIOLOGICAL_PROCESS | negative regulation of signal transduction | 2.23E-13 | 6.63E-15 |
| GO:0065003 | BIOLOGICAL_PROCESS | macromolecular complex assembly | 2.51E-13 | 7.49E-15 |
| GO:0061564 | BIOLOGICAL_PROCESS | axon development | 2.81E-13 | 8.41E-15 |
| GO:0048568 | BIOLOGICAL_PROCESS | embryonic organ development | 3.55E-13 | 1.07E-14 |
| GO:0030097 | BIOLOGICAL_PROCESS | hemopoiesis | 4.58E-13 | 1.38E-14 |
| GO:0007423 | BIOLOGICAL_PROCESS | sensory organ development | 4.87E-13 | 1.47E-14 |
| GO:0030522 | BIOLOGICAL_PROCESS | intracellular receptor signaling pathway | 5.25E-13 | 1.60E-14 |
| GO:0003007 | BIOLOGICAL_PROCESS | heart morphogenesis | 5.25E-13 | 1.60E-14 |
| GO:0008284 | BIOLOGICAL_PROCESS | positive regulation of cell proliferation | 5.25E-13 | 1.60E-14 |
| GO:0016569 | BIOLOGICAL_PROCESS | covalent chromatin modification | 5.53E-13 | 1.69E-14 |
| GO:0004935 | MOLECULAR_FUNCTION | adrenergic receptor activity | 8.52E-13 | 2.61E-14 |
| GO:0001568 | BIOLOGICAL_PROCESS | blood vessel development | 1.20E-12 | 3.69E-14 |
| GO:0043933 | BIOLOGICAL_PROCESS | macromolecular complex subunit organization | 1.36E-12 | 4.19E-14 |
| GO:0001501 | BIOLOGICAL_PROCESS | skeletal system development | 1.55E-12 | 4.78E-14 |
| GO:0007409 | BIOLOGICAL_PROCESS | axonogenesis | 1.98E-12 | 6.14E-14 |
| GO:0046873 | MOLECULAR_FUNCTION | metal ion transmembrane transporter activity | 2.33E-12 | 7.25E-14 |
| GO:0003682 | MOLECULAR_FUNCTION | chromatin binding | 2.52E-12 | 7.87E-14 |
| GO:0043408 | BIOLOGICAL_PROCESS | regulation of MAPK cascade | 2.59E-12 | 8.09E-14 |
| GO:0005231 | MOLECULAR_FUNCTION | excitatory extracellular ligand-gated ion channel activity | 2.87E-12 | 9.01E-14 |
| GO:0043009 | BIOLOGICAL_PROCESS | chordate embryonic development | 3.24E-12 | 1.02E-13 |
| GO:0051128 | BIOLOGICAL_PROCESS | regulation of cellular component organization | 3.41E-12 | 1.08E-13 |
| GO:0048732 | BIOLOGICAL_PROCESS | gland development | 3.43E-12 | 1.08E-13 |
| GO:0048511 | BIOLOGICAL_PROCESS | rhythmic process | 3.66E-12 | 1.16E-13 |
| GO:0009792 | BIOLOGICAL_PROCESS | embryo development ending in birth or egg hatching | 4.26E-12 | 1.35E-13 |
| GO:0000978 | MOLECULAR_FUNCTION | RNA polymerase II core promoter proximal region sequence-specific DNA binding | 4.54E-12 | 1.45E-13 |
| GO:0048762 | BIOLOGICAL_PROCESS | mesenchymal cell differentiation | 4.54E-12 | 1.45E-13 |
| GO:0044464 | CELLULAR_COMPONENT | cell part | 4.70E-12 | 1.50E-13 |
| GO:0022835 | MOLECULAR_FUNCTION | transmitter-gated channel activity | 4.79E-12 | 1.55E-13 |
| GO:0022824 | MOLECULAR_FUNCTION | transmitter-gated ion channel activity | 4.79E-12 | 1.55E-13 |
| GO:0045596 | BIOLOGICAL_PROCESS | negative regulation of cell differentiation | 4.79E-12 | 1.55E-13 |
| GO:0001228 | MOLECULAR_FUNCTION | transcriptional activator activity, RNA polymerase II transcription regulatory region sequence-specific binding | 4.79E-12 | 1.55E-13 |
| GO:0030900 | BIOLOGICAL_PROCESS | forebrain development | 5.10E-12 | 1.66E-13 |
| GO:0022607 | BIOLOGICAL_PROCESS | cellular component assembly | 5.82E-12 | 1.90E-13 |
| GO:0016570 | BIOLOGICAL_PROCESS | histone modification | 6.03E-12 | 1.97E-13 |
| GO:0005200 | MOLECULAR_FUNCTION | structural constituent of cytoskeleton | 7.03E-12 | 2.30E-13 |
| GO:0065008 | BIOLOGICAL_PROCESS | regulation of biological quality | 7.31E-12 | 2.40E-13 |
| GO:1902533 | BIOLOGICAL_PROCESS | positive regulation of intracellular signal transduction | 9.53E-12 | 3.14E-13 |
| GO:0003707 | MOLECULAR_FUNCTION | steroid hormone receptor activity | 9.68E-12 | 3.20E-13 |
| GO:0046983 | MOLECULAR_FUNCTION | protein dimerization activity | 9.68E-12 | 3.20E-13 |
| GO:0005623 | CELLULAR_COMPONENT | cell | 1.16E-11 | 3.84E-13 |
| GO:0007178 | BIOLOGICAL_PROCESS | transmembrane receptor protein serine/threonine kinase signaling pathway | 1.26E-11 | 4.19E-13 |
| GO:0022603 | BIOLOGICAL_PROCESS | regulation of anatomical structure morphogenesis | 1.38E-11 | 4.61E-13 |
| GO:0005251 | MOLECULAR_FUNCTION | delayed rectifier potassium channel activity | 1.38E-11 | 4.62E-13 |
| GO:0014706 | BIOLOGICAL_PROCESS | striated muscle tissue development | 1.50E-11 | 5.03E-13 |
| GO:0032989 | BIOLOGICAL_PROCESS | cellular component morphogenesis | 1.72E-11 | 5.78E-13 |
| GO:0044428 | CELLULAR_COMPONENT | nuclear part | 1.72E-11 | 5.80E-13 |
| GO:0040011 | BIOLOGICAL_PROCESS | locomotion | 1.77E-11 | 6.00E-13 |
| GO:0000989 | MOLECULAR_FUNCTION | transcription factor activity, transcription factor binding | 2.15E-11 | 7.28E-13 |
| GO:0060537 | BIOLOGICAL_PROCESS | muscle tissue development | 3.74E-11 | 1.27E-12 |
| GO:0005057 | MOLECULAR_FUNCTION | signal transducer activity, downstream of receptor | 4.09E-11 | 1.39E-12 |
| GO:0000902 | BIOLOGICAL_PROCESS | cell morphogenesis | 4.15E-11 | 1.42E-12 |
| GO:0003713 | MOLECULAR_FUNCTION | transcription coactivator activity | 4.57E-11 | 1.56E-12 |
| GO:0001159 | MOLECULAR_FUNCTION | core promoter proximal region DNA binding | 5.01E-11 | 1.72E-12 |
| GO:0045664 | BIOLOGICAL_PROCESS | regulation of neuron differentiation | 6.93E-11 | 2.39E-12 |
| GO:0005267 | MOLECULAR_FUNCTION | potassium channel activity | 1.12E-10 | 3.87E-12 |
| GO:0004702 | MOLECULAR_FUNCTION | signal transducer, downstream of receptor, with serine/threonine kinase activity | 1.15E-10 | 3.98E-12 |
| GO:0007623 | BIOLOGICAL_PROCESS | circadian rhythm | 1.15E-10 | 3.98E-12 |
| GO:0051704 | BIOLOGICAL_PROCESS | multi-organism process | 1.40E-10 | 4.88E-12 |
| GO:2000027 | BIOLOGICAL_PROCESS | regulation of organ morphogenesis | 1.63E-10 | 5.67E-12 |
| GO:0008134 | MOLECULAR_FUNCTION | transcription factor binding | 1.90E-10 | 6.65E-12 |
| GO:0003712 | MOLECULAR_FUNCTION | transcription cofactor activity | 1.90E-10 | 6.65E-12 |
| GO:0006996 | BIOLOGICAL_PROCESS | organelle organization | 2.12E-10 | 7.46E-12 |
| GO:0022891 | MOLECULAR_FUNCTION | substrate-specific transmembrane transporter activity | 2.22E-10 | 7.83E-12 |
| GO:0009611 | BIOLOGICAL_PROCESS | response to wounding | 2.34E-10 | 8.27E-12 |
| GO:0018205 | BIOLOGICAL_PROCESS | peptidyl-lysine modification | 2.43E-10 | 8.61E-12 |
| GO:0080134 | BIOLOGICAL_PROCESS | regulation of response to stress | 2.53E-10 | 8.99E-12 |
| GO:0022857 | MOLECULAR_FUNCTION | transmembrane transporter activity | 2.57E-10 | 9.16E-12 |
| GO:0051130 | BIOLOGICAL_PROCESS | positive regulation of cellular component organization | 3.78E-10 | 1.35E-11 |
| GO:0048863 | BIOLOGICAL_PROCESS | stem cell differentiation | 3.89E-10 | 1.39E-11 |
| GO:0034645 | BIOLOGICAL_PROCESS | cellular macromolecule biosynthetic process | 4.19E-10 | 1.50E-11 |
| GO:0071875 | BIOLOGICAL_PROCESS | adrenergic receptor signaling pathway | 4.53E-10 | 1.63E-11 |
| GO:0071417 | BIOLOGICAL_PROCESS | cellular response to organonitrogen compound | 4.54E-10 | 1.63E-11 |
| GO:0046777 | BIOLOGICAL_PROCESS | protein autophosphorylation | 4.87E-10 | 1.76E-11 |
| GO:0030335 | BIOLOGICAL_PROCESS | positive regulation of cell migration | 5.08E-10 | 1.84E-11 |
| GO:0021953 | BIOLOGICAL_PROCESS | central nervous system neuron differentiation | 5.11E-10 | 1.85E-11 |
| GO:0015077 | MOLECULAR_FUNCTION | monovalent inorganic cation transmembrane transporter activity | 5.26E-10 | 1.91E-11 |
| GO:0051726 | BIOLOGICAL_PROCESS | regulation of cell cycle | 7.06E-10 | 2.58E-11 |
| GO:0003231 | BIOLOGICAL_PROCESS | cardiac ventricle development | 7.50E-10 | 2.74E-11 |
| GO:0030334 | BIOLOGICAL_PROCESS | regulation of cell migration | 7.96E-10 | 2.92E-11 |
| GO:0010720 | BIOLOGICAL_PROCESS | positive regulation of cell development | 9.21E-10 | 3.38E-11 |
| GO:0008285 | BIOLOGICAL_PROCESS | negative regulation of cell proliferation | 1.04E-09 | 3.82E-11 |
| GO:0009059 | BIOLOGICAL_PROCESS | macromolecule biosynthetic process | 1.05E-09 | 3.88E-11 |
| GO:0040017 | BIOLOGICAL_PROCESS | positive regulation of locomotion | 1.06E-09 | 3.93E-11 |
| GO:0040008 | BIOLOGICAL_PROCESS | regulation of growth | 1.33E-09 | 4.91E-11 |
| GO:0060070 | BIOLOGICAL_PROCESS | canonical Wnt signaling pathway | 1.35E-09 | 5.04E-11 |
| GO:0051272 | BIOLOGICAL_PROCESS | positive regulation of cellular component movement | 1.35E-09 | 5.04E-11 |
| GO:0007187 | BIOLOGICAL_PROCESS | G-protein coupled receptor signaling pathway, coupled to cyclic nucleotide second messenger | 1.59E-09 | 5.92E-11 |
| GO:1901652 | BIOLOGICAL_PROCESS | response to peptide | 1.60E-09 | 6.02E-11 |
| GO:0000987 | MOLECULAR_FUNCTION | core promoter proximal region sequence-specific DNA binding | 1.60E-09 | 6.02E-11 |
| GO:0004970 | MOLECULAR_FUNCTION | ionotropic glutamate receptor activity | 1.60E-09 | 6.02E-11 |
| GO:0007369 | BIOLOGICAL_PROCESS | gastrulation | 1.60E-09 | 6.02E-11 |
| GO:0001227 | MOLECULAR_FUNCTION | transcriptional repressor activity, RNA polymerase II transcription regulatory region sequence-specific binding | 1.64E-09 | 6.19E-11 |
| GO:0040012 | BIOLOGICAL_PROCESS | regulation of locomotion | 2.55E-09 | 9.65E-11 |
| GO:0031981 | CELLULAR_COMPONENT | nuclear lumen | 2.63E-09 | 1.00E-10 |
| GO:2000145 | BIOLOGICAL_PROCESS | regulation of cell motility | 2.67E-09 | 1.02E-10 |
| GO:0042391 | BIOLOGICAL_PROCESS | regulation of membrane potential | 2.73E-09 | 1.04E-10 |
| GO:0044421 | CELLULAR_COMPONENT | extracellular region part | 2.88E-09 | 1.10E-10 |
| GO:0051338 | BIOLOGICAL_PROCESS | regulation of transferase activity | 3.02E-09 | 1.16E-10 |
| GO:0016020 | CELLULAR_COMPONENT | membrane | 3.02E-09 | 1.16E-10 |
| GO:0015075 | MOLECULAR_FUNCTION | ion transmembrane transporter activity | 3.31E-09 | 1.27E-10 |
| GO:0050877 | BIOLOGICAL_PROCESS | neurological system process | 3.34E-09 | 1.29E-10 |
| GO:0001763 | BIOLOGICAL_PROCESS | morphogenesis of a branching structure | 3.54E-09 | 1.37E-10 |
| GO:0042063 | BIOLOGICAL_PROCESS | gliogenesis | 3.54E-09 | 1.37E-10 |
| GO:2000147 | BIOLOGICAL_PROCESS | positive regulation of cell motility | 3.96E-09 | 1.54E-10 |
| GO:0048514 | BIOLOGICAL_PROCESS | blood vessel morphogenesis | 4.06E-09 | 1.58E-10 |
| GO:0050769 | BIOLOGICAL_PROCESS | positive regulation of neurogenesis | 4.37E-09 | 1.71E-10 |
| GO:0060560 | BIOLOGICAL_PROCESS | developmental growth involved in morphogenesis | 4.37E-09 | 1.71E-10 |
| GO:0044877 | MOLECULAR_FUNCTION | macromolecular complex binding | 4.45E-09 | 1.74E-10 |
| GO:0016887 | MOLECULAR_FUNCTION | ATPase activity | 4.88E-09 | 1.92E-10 |
| GO:0006338 | BIOLOGICAL_PROCESS | chromatin remodeling | 4.94E-09 | 1.95E-10 |
| GO:0014033 | BIOLOGICAL_PROCESS | neural crest cell differentiation | 5.93E-09 | 2.34E-10 |
| GO:0015081 | MOLECULAR_FUNCTION | sodium ion transmembrane transporter activity | 6.03E-09 | 2.39E-10 |
| GO:0016849 | MOLECULAR_FUNCTION | phosphorus-oxygen lyase activity | 6.03E-09 | 2.39E-10 |
| GO:0051270 | BIOLOGICAL_PROCESS | regulation of cellular component movement | 6.05E-09 | 2.41E-10 |
| GO:0015464 | MOLECULAR_FUNCTION | acetylcholine receptor activity | 7.25E-09 | 2.89E-10 |
| GO:0051962 | BIOLOGICAL_PROCESS | positive regulation of nervous system development | 8.63E-09 | 3.44E-10 |
| GO:0043010 | BIOLOGICAL_PROCESS | camera-type eye development | 8.78E-09 | 3.51E-10 |
| GO:0044271 | BIOLOGICAL_PROCESS | cellular nitrogen compound biosynthetic process | 9.08E-09 | 3.64E-10 |
| GO:0009798 | BIOLOGICAL_PROCESS | axis specification | 9.10E-09 | 3.66E-10 |
| GO:0030001 | BIOLOGICAL_PROCESS | metal ion transport | 1.04E-08 | 4.19E-10 |
| GO:0009190 | BIOLOGICAL_PROCESS | cyclic nucleotide biosynthetic process | 1.09E-08 | 4.41E-10 |
| GO:0000278 | BIOLOGICAL_PROCESS | mitotic cell cycle | 1.17E-08 | 4.73E-10 |
| GO:0005234 | MOLECULAR_FUNCTION | extracellular-glutamate-gated ion channel activity | 1.19E-08 | 4.84E-10 |
| GO:0004715 | MOLECULAR_FUNCTION | non-membrane spanning protein tyrosine kinase activity | 1.19E-08 | 4.84E-10 |
| GO:0061138 | BIOLOGICAL_PROCESS | morphogenesis of a branching epithelium | 1.19E-08 | 4.84E-10 |
| GO:0022414 | BIOLOGICAL_PROCESS | reproductive process | 1.20E-08 | 4.90E-10 |
| GO:0008066 | MOLECULAR_FUNCTION | glutamate receptor activity | 1.29E-08 | 5.26E-10 |
| GO:0007215 | BIOLOGICAL_PROCESS | glutamate receptor signaling pathway | 1.29E-08 | 5.26E-10 |
| GO:0007610 | BIOLOGICAL_PROCESS | behavior | 1.34E-08 | 5.52E-10 |
| GO:0048870 | BIOLOGICAL_PROCESS | cell motility | 1.38E-08 | 5.69E-10 |
| GO:0051674 | BIOLOGICAL_PROCESS | localization of cell | 1.38E-08 | 5.69E-10 |
| GO:0003205 | BIOLOGICAL_PROCESS | cardiac chamber development | 1.40E-08 | 5.79E-10 |
| GO:0061448 | BIOLOGICAL_PROCESS | connective tissue development | 1.53E-08 | 6.34E-10 |
| GO:0000003 | BIOLOGICAL_PROCESS | reproduction | 1.76E-08 | 7.29E-10 |
| GO:0022890 | MOLECULAR_FUNCTION | inorganic cation transmembrane transporter activity | 1.89E-08 | 7.85E-10 |
| GO:0032403 | MOLECULAR_FUNCTION | protein complex binding | 2.17E-08 | 9.05E-10 |
| GO:0043549 | BIOLOGICAL_PROCESS | regulation of kinase activity | 2.27E-08 | 9.49E-10 |
| GO:0044703 | BIOLOGICAL_PROCESS | multi-organism reproductive process | 2.57E-08 | 1.08E-09 |
| GO:0000988 | MOLECULAR_FUNCTION | transcription factor activity, protein binding | 2.79E-08 | 1.17E-09 |
| GO:0098531 | MOLECULAR_FUNCTION | transcription factor activity, direct ligand regulated sequence-specific DNA binding | 3.14E-08 | 1.32E-09 |
| GO:0060538 | BIOLOGICAL_PROCESS | skeletal muscle organ development | 3.14E-08 | 1.32E-09 |
| GO:0004879 | MOLECULAR_FUNCTION | RNA polymerase II transcription factor activity, ligand-activated sequence-specific DNA binding | 3.14E-08 | 1.32E-09 |
| GO:0005912 | CELLULAR_COMPONENT | adherens junction | 3.42E-08 | 1.45E-09 |
| GO:0015079 | MOLECULAR_FUNCTION | potassium ion transmembrane transporter activity | 3.42E-08 | 1.45E-09 |
| GO:0031012 | CELLULAR_COMPONENT | extracellular matrix | 3.67E-08 | 1.56E-09 |
| GO:0043410 | BIOLOGICAL_PROCESS | positive regulation of MAPK cascade | 3.77E-08 | 1.60E-09 |
| GO:0003676 | MOLECULAR_FUNCTION | nucleic acid binding | 3.84E-08 | 1.64E-09 |
| GO:0009605 | BIOLOGICAL_PROCESS | response to external stimulus | 3.85E-08 | 1.64E-09 |
| GO:0030030 | BIOLOGICAL_PROCESS | cell projection organization | 3.95E-08 | 1.69E-09 |
| GO:0030278 | BIOLOGICAL_PROCESS | regulation of ossification | 4.09E-08 | 1.76E-09 |
| GO:0035257 | MOLECULAR_FUNCTION | nuclear hormone receptor binding | 4.09E-08 | 1.76E-09 |
| GO:0051098 | BIOLOGICAL_PROCESS | regulation of binding | 4.17E-08 | 1.80E-09 |
| GO:0005249 | MOLECULAR_FUNCTION | voltage-gated potassium channel activity | 4.25E-08 | 1.83E-09 |
| GO:0021510 | BIOLOGICAL_PROCESS | spinal cord development | 4.48E-08 | 1.94E-09 |
| GO:0002521 | BIOLOGICAL_PROCESS | leukocyte differentiation | 4.51E-08 | 1.95E-09 |
| GO:0098609 | BIOLOGICAL_PROCESS | cell-cell adhesion | 4.53E-08 | 1.97E-09 |
| GO:0097485 | BIOLOGICAL_PROCESS | neuron projection guidance | 4.64E-08 | 2.02E-09 |
| GO:0051216 | BIOLOGICAL_PROCESS | cartilage development | 4.71E-08 | 2.05E-09 |
| GO:0017147 | MOLECULAR_FUNCTION | Wnt-protein binding | 5.23E-08 | 2.30E-09 |
| GO:0042813 | MOLECULAR_FUNCTION | Wnt-activated receptor activity | 5.23E-08 | 2.30E-09 |
| GO:0007210 | BIOLOGICAL_PROCESS | serotonin receptor signaling pathway | 5.23E-08 | 2.30E-09 |
| GO:0004993 | MOLECULAR_FUNCTION | G-protein coupled serotonin receptor activity | 5.23E-08 | 2.30E-09 |
| GO:0099589 | MOLECULAR_FUNCTION | serotonin receptor activity | 5.23E-08 | 2.30E-09 |
| GO:0003677 | MOLECULAR_FUNCTION | DNA binding | 5.25E-08 | 2.32E-09 |
| GO:0006955 | BIOLOGICAL_PROCESS | immune response | 5.85E-08 | 2.59E-09 |
| GO:0002682 | BIOLOGICAL_PROCESS | regulation of immune system process | 5.85E-08 | 2.59E-09 |
| GO:0007411 | BIOLOGICAL_PROCESS | axon guidance | 5.86E-08 | 2.60E-09 |
| GO:0099512 | CELLULAR_COMPONENT | supramolecular fiber | 6.48E-08 | 2.88E-09 |
| GO:0009952 | BIOLOGICAL_PROCESS | anterior/posterior pattern specification | 6.50E-08 | 2.90E-09 |
| GO:0006952 | BIOLOGICAL_PROCESS | defense response | 6.78E-08 | 3.03E-09 |
| GO:0080135 | BIOLOGICAL_PROCESS | regulation of cellular response to stress | 7.00E-08 | 3.14E-09 |
| GO:0023014 | BIOLOGICAL_PROCESS | signal transduction by protein phosphorylation | 7.09E-08 | 3.18E-09 |
| GO:0004721 | MOLECULAR_FUNCTION | phosphoprotein phosphatase activity | 7.15E-08 | 3.22E-09 |
| GO:0022892 | MOLECULAR_FUNCTION | substrate-specific transporter activity | 7.18E-08 | 3.23E-09 |
| GO:0051427 | MOLECULAR_FUNCTION | hormone receptor binding | 7.30E-08 | 3.29E-09 |
| GO:0044702 | BIOLOGICAL_PROCESS | single organism reproductive process | 8.93E-08 | 4.04E-09 |
| GO:0099080 | CELLULAR_COMPONENT | supramolecular complex | 8.93E-08 | 4.05E-09 |
| GO:0099081 | CELLULAR_COMPONENT | supramolecular polymer | 8.93E-08 | 4.05E-09 |
| GO:0090092 | BIOLOGICAL_PROCESS | regulation of transmembrane receptor protein serine/threonine kinase signaling pathway | 9.13E-08 | 4.15E-09 |
| GO:0090596 | BIOLOGICAL_PROCESS | sensory organ morphogenesis | 9.30E-08 | 4.24E-09 |
| GO:0022843 | MOLECULAR_FUNCTION | voltage-gated cation channel activity | 9.41E-08 | 4.30E-09 |
| GO:0019901 | MOLECULAR_FUNCTION | protein kinase binding | 9.91E-08 | 4.53E-09 |
| GO:0006950 | BIOLOGICAL_PROCESS | response to stress | 1.03E-07 | 4.70E-09 |
| GO:0004402 | MOLECULAR_FUNCTION | histone acetyltransferase activity | 1.03E-07 | 4.76E-09 |
| GO:0061733 | MOLECULAR_FUNCTION | peptide-lysine-N-acetyltransferase activity | 1.03E-07 | 4.76E-09 |
| GO:0005654 | CELLULAR_COMPONENT | nucleoplasm | 1.06E-07 | 4.89E-09 |
| GO:0016049 | BIOLOGICAL_PROCESS | cell growth | 1.15E-07 | 5.35E-09 |
| GO:0097190 | BIOLOGICAL_PROCESS | apoptotic signaling pathway | 1.15E-07 | 5.35E-09 |
| GO:0099568 | CELLULAR_COMPONENT | cytoplasmic region | 1.15E-07 | 5.35E-09 |
| GO:0035235 | BIOLOGICAL_PROCESS | ionotropic glutamate receptor signaling pathway | 1.19E-07 | 5.52E-09 |
| GO:0045859 | BIOLOGICAL_PROCESS | regulation of protein kinase activity | 1.22E-07 | 5.69E-09 |
| GO:0048754 | BIOLOGICAL_PROCESS | branching morphogenesis of an epithelial tube | 1.26E-07 | 5.87E-09 |
| GO:0001503 | BIOLOGICAL_PROCESS | ossification | 1.31E-07 | 6.12E-09 |
| GO:0009187 | BIOLOGICAL_PROCESS | cyclic nucleotide metabolic process | 1.31E-07 | 6.13E-09 |
| GO:0030323 | BIOLOGICAL_PROCESS | respiratory tube development | 1.38E-07 | 6.55E-09 |
| GO:0032868 | BIOLOGICAL_PROCESS | response to insulin | 1.38E-07 | 6.52E-09 |
| GO:0043434 | BIOLOGICAL_PROCESS | response to peptide hormone | 1.38E-07 | 6.55E-09 |
| GO:1901653 | BIOLOGICAL_PROCESS | cellular response to peptide | 1.38E-07 | 6.52E-09 |
| GO:0071375 | BIOLOGICAL_PROCESS | cellular response to peptide hormone stimulus | 1.38E-07 | 6.52E-09 |
| GO:0007188 | BIOLOGICAL_PROCESS | adenylate cyclase-modulating G-protein coupled receptor signaling pathway | 1.38E-07 | 6.55E-09 |
| GO:0030902 | BIOLOGICAL_PROCESS | hindbrain development | 1.42E-07 | 6.72E-09 |
| GO:0007270 | BIOLOGICAL_PROCESS | neuron-neuron synaptic transmission | 1.42E-07 | 6.72E-09 |
| GO:0010243 | BIOLOGICAL_PROCESS | response to organonitrogen compound | 1.46E-07 | 6.99E-09 |
| GO:0070013 | CELLULAR_COMPONENT | intracellular organelle lumen | 1.46E-07 | 6.99E-09 |
| GO:0031974 | CELLULAR_COMPONENT | membrane-enclosed lumen | 1.46E-07 | 6.99E-09 |
| GO:0043233 | CELLULAR_COMPONENT | organelle lumen | 1.46E-07 | 6.99E-09 |
| GO:0017111 | MOLECULAR_FUNCTION | nucleoside-triphosphatase activity | 1.56E-07 | 7.47E-09 |
| GO:0033043 | BIOLOGICAL_PROCESS | regulation of organelle organization | 1.56E-07 | 7.50E-09 |
| GO:0001525 | BIOLOGICAL_PROCESS | angiogenesis | 1.72E-07 | 8.28E-09 |
| GO:0070161 | CELLULAR_COMPONENT | anchoring junction | 1.75E-07 | 8.44E-09 |
| GO:0010467 | BIOLOGICAL_PROCESS | gene expression | 1.97E-07 | 9.51E-09 |
| GO:0019226 | BIOLOGICAL_PROCESS | transmission of nerve impulse | 1.97E-07 | 9.55E-09 |
| GO:0019900 | MOLECULAR_FUNCTION | kinase binding | 1.98E-07 | 9.61E-09 |
| GO:0048638 | BIOLOGICAL_PROCESS | regulation of developmental growth | 2.08E-07 | 1.01E-08 |
| GO:0097458 | CELLULAR_COMPONENT | neuron part | 2.21E-07 | 1.08E-08 |
| GO:0071804 | BIOLOGICAL_PROCESS | cellular potassium ion transport | 2.21E-07 | 1.08E-08 |
| GO:0071805 | BIOLOGICAL_PROCESS | potassium ion transmembrane transport | 2.21E-07 | 1.08E-08 |
| GO:0051223 | BIOLOGICAL_PROCESS | regulation of protein transport | 2.43E-07 | 1.19E-08 |
| GO:0001704 | BIOLOGICAL_PROCESS | formation of primary germ layer | 2.49E-07 | 1.22E-08 |
| GO:0001508 | BIOLOGICAL_PROCESS | action potential | 2.49E-07 | 1.22E-08 |
| GO:0051091 | BIOLOGICAL_PROCESS | positive regulation of sequence-specific DNA binding transcription factor activity | 2.49E-07 | 1.22E-08 |
| GO:0043005 | CELLULAR_COMPONENT | neuron projection | 2.49E-07 | 1.22E-08 |
| GO:0016817 | MOLECULAR_FUNCTION | hydrolase activity, acting on acid anhydrides | 2.67E-07 | 1.32E-08 |
| GO:0042060 | BIOLOGICAL_PROCESS | wound healing | 2.98E-07 | 1.47E-08 |
| GO:0019953 | BIOLOGICAL_PROCESS | sexual reproduction | 3.16E-07 | 1.57E-08 |
| GO:0001655 | BIOLOGICAL_PROCESS | urogenital system development | 3.36E-07 | 1.68E-08 |
| GO:0002064 | BIOLOGICAL_PROCESS | epithelial cell development | 3.36E-07 | 1.68E-08 |
| GO:0045927 | BIOLOGICAL_PROCESS | positive regulation of growth | 3.36E-07 | 1.68E-08 |
| GO:0010001 | BIOLOGICAL_PROCESS | glial cell differentiation | 3.39E-07 | 1.70E-08 |
| GO:0007519 | BIOLOGICAL_PROCESS | skeletal muscle tissue development | 3.39E-07 | 1.70E-08 |
| GO:0070201 | BIOLOGICAL_PROCESS | regulation of establishment of protein localization | 4.00E-07 | 2.00E-08 |
| GO:0000790 | CELLULAR_COMPONENT | nuclear chromatin | 4.03E-07 | 2.02E-08 |
| GO:1903533 | BIOLOGICAL_PROCESS | regulation of protein targeting | 4.12E-07 | 2.07E-08 |
| GO:0072331 | BIOLOGICAL_PROCESS | signal transduction by p53 class mediator | 4.12E-07 | 2.07E-08 |
| GO:0032869 | BIOLOGICAL_PROCESS | cellular response to insulin stimulus | 4.29E-07 | 2.18E-08 |
| GO:0003206 | BIOLOGICAL_PROCESS | cardiac chamber morphogenesis | 4.29E-07 | 2.18E-08 |
| GO:0046068 | BIOLOGICAL_PROCESS | cGMP metabolic process | 4.29E-07 | 2.18E-08 |
| GO:0051090 | BIOLOGICAL_PROCESS | regulation of sequence-specific DNA binding transcription factor activity | 4.29E-07 | 2.18E-08 |
| GO:0006182 | BIOLOGICAL_PROCESS | cGMP biosynthetic process | 4.29E-07 | 2.18E-08 |
| GO:0021543 | BIOLOGICAL_PROCESS | pallium development | 4.29E-07 | 2.18E-08 |
| GO:0044085 | BIOLOGICAL_PROCESS | cellular component biogenesis | 4.63E-07 | 2.36E-08 |
| GO:0035258 | MOLECULAR_FUNCTION | steroid hormone receptor binding | 5.21E-07 | 2.68E-08 |
| GO:0003151 | BIOLOGICAL_PROCESS | outflow tract morphogenesis | 5.21E-07 | 2.68E-08 |
| GO:0042752 | BIOLOGICAL_PROCESS | regulation of circadian rhythm | 5.21E-07 | 2.68E-08 |
| GO:0021515 | BIOLOGICAL_PROCESS | cell differentiation in spinal cord | 5.21E-07 | 2.68E-08 |
| GO:0007498 | BIOLOGICAL_PROCESS | mesoderm development | 5.21E-07 | 2.68E-08 |
| GO:0001654 | BIOLOGICAL_PROCESS | eye development | 5.32E-07 | 2.74E-08 |
| GO:0021537 | BIOLOGICAL_PROCESS | telencephalon development | 5.61E-07 | 2.89E-08 |
| GO:0008324 | MOLECULAR_FUNCTION | cation transmembrane transporter activity | 5.63E-07 | 2.91E-08 |
| GO:0001775 | BIOLOGICAL_PROCESS | cell activation | 6.03E-07 | 3.12E-08 |
| GO:0098660 | BIOLOGICAL_PROCESS | inorganic ion transmembrane transport | 6.52E-07 | 3.38E-08 |
| GO:0007049 | BIOLOGICAL_PROCESS | cell cycle | 6.62E-07 | 3.44E-08 |
| GO:0032880 | BIOLOGICAL_PROCESS | regulation of protein localization | 6.77E-07 | 3.52E-08 |
| GO:0001817 | BIOLOGICAL_PROCESS | regulation of cytokine production | 6.78E-07 | 3.54E-08 |
| GO:0050839 | MOLECULAR_FUNCTION | cell adhesion molecule binding | 6.78E-07 | 3.54E-08 |
| GO:0042802 | MOLECULAR_FUNCTION | identical protein binding | 6.85E-07 | 3.58E-08 |
| GO:0016818 | MOLECULAR_FUNCTION | hydrolase activity, acting on acid anhydrides, in phosphorus-containing anhydrides | 7.47E-07 | 3.91E-08 |
| GO:0005109 | MOLECULAR_FUNCTION | frizzled binding | 7.54E-07 | 3.96E-08 |
| GO:0008091 | CELLULAR_COMPONENT | spectrin | 7.54E-07 | 3.96E-08 |
| GO:0005938 | CELLULAR_COMPONENT | cell cortex | 7.64E-07 | 4.02E-08 |
| GO:0016462 | MOLECULAR_FUNCTION | pyrophosphatase activity | 7.98E-07 | 4.21E-08 |
| GO:0090304 | BIOLOGICAL_PROCESS | nucleic acid metabolic process | 8.44E-07 | 4.46E-08 |
| GO:0048864 | BIOLOGICAL_PROCESS | stem cell development | 8.47E-07 | 4.53E-08 |
| GO:0016202 | BIOLOGICAL_PROCESS | regulation of striated muscle tissue development | 8.47E-07 | 4.53E-08 |
| GO:0048634 | BIOLOGICAL_PROCESS | regulation of muscle organ development | 8.47E-07 | 4.53E-08 |
| GO:0001078 | MOLECULAR_FUNCTION | transcriptional repressor activity, RNA polymerase II core promoter proximal region sequence-specific binding | 8.47E-07 | 4.53E-08 |
| GO:0014032 | BIOLOGICAL_PROCESS | neural crest cell development | 8.47E-07 | 4.53E-08 |
| GO:0014031 | BIOLOGICAL_PROCESS | mesenchymal cell development | 8.47E-07 | 4.53E-08 |
| GO:1901861 | BIOLOGICAL_PROCESS | regulation of muscle tissue development | 8.47E-07 | 4.53E-08 |
| GO:0015672 | BIOLOGICAL_PROCESS | monovalent inorganic cation transport | 8.51E-07 | 4.55E-08 |
| GO:0005694 | CELLULAR_COMPONENT | chromosome | 8.95E-07 | 4.80E-08 |
| GO:0009953 | BIOLOGICAL_PROCESS | dorsal/ventral pattern formation | 9.37E-07 | 5.03E-08 |
| GO:0051046 | BIOLOGICAL_PROCESS | regulation of secretion | 9.90E-07 | 5.32E-08 |
| GO:0044087 | BIOLOGICAL_PROCESS | regulation of cellular component biogenesis | 1.03E-06 | 5.53E-08 |
| GO:0007219 | BIOLOGICAL_PROCESS | Notch signaling pathway | 1.07E-06 | 5.80E-08 |
| GO:0030324 | BIOLOGICAL_PROCESS | lung development | 1.20E-06 | 6.49E-08 |
| GO:0046649 | BIOLOGICAL_PROCESS | lymphocyte activation | 1.20E-06 | 6.51E-08 |
| GO:0065009 | BIOLOGICAL_PROCESS | regulation of molecular function | 1.26E-06 | 6.81E-08 |
| GO:0034212 | MOLECULAR_FUNCTION | peptide N-acetyltransferase activity | 1.32E-06 | 7.18E-08 |
| GO:0051129 | BIOLOGICAL_PROCESS | negative regulation of cellular component organization | 1.33E-06 | 7.21E-08 |
| GO:0006813 | BIOLOGICAL_PROCESS | potassium ion transport | 1.47E-06 | 8.03E-08 |
| GO:0030111 | BIOLOGICAL_PROCESS | regulation of Wnt signaling pathway | 1.49E-06 | 8.18E-08 |
| GO:0060541 | BIOLOGICAL_PROCESS | respiratory system development | 1.49E-06 | 8.18E-08 |
| GO:0033157 | BIOLOGICAL_PROCESS | regulation of intracellular protein transport | 1.49E-06 | 8.18E-08 |
| GO:0031344 | BIOLOGICAL_PROCESS | regulation of cell projection organization | 1.78E-06 | 9.74E-08 |
| GO:0030054 | CELLULAR_COMPONENT | cell junction | 1.84E-06 | 1.01E-07 |
| GO:0060026 | BIOLOGICAL_PROCESS | convergent extension | 1.87E-06 | 1.04E-07 |
| GO:0048636 | BIOLOGICAL_PROCESS | positive regulation of muscle organ development | 1.87E-06 | 1.04E-07 |
| GO:0009743 | BIOLOGICAL_PROCESS | response to carbohydrate | 1.87E-06 | 1.04E-07 |
| GO:1901863 | BIOLOGICAL_PROCESS | positive regulation of muscle tissue development | 1.87E-06 | 1.04E-07 |
| GO:0045844 | BIOLOGICAL_PROCESS | positive regulation of striated muscle tissue development | 1.87E-06 | 1.04E-07 |
| GO:0009986 | CELLULAR_COMPONENT | cell surface | 1.95E-06 | 1.08E-07 |
| GO:0030099 | BIOLOGICAL_PROCESS | myeloid cell differentiation | 2.23E-06 | 1.24E-07 |
| GO:0001664 | MOLECULAR_FUNCTION | G-protein coupled receptor binding | 2.24E-06 | 1.25E-07 |
| GO:0000785 | CELLULAR_COMPONENT | chromatin | 2.24E-06 | 1.25E-07 |
| GO:0035725 | BIOLOGICAL_PROCESS | sodium ion transmembrane transport | 2.32E-06 | 1.29E-07 |
| GO:0030055 | CELLULAR_COMPONENT | cell-substrate junction | 2.34E-06 | 1.31E-07 |
| GO:0000228 | CELLULAR_COMPONENT | nuclear chromosome | 2.34E-06 | 1.31E-07 |
| GO:0009314 | BIOLOGICAL_PROCESS | response to radiation | 2.34E-06 | 1.31E-07 |
| GO:0005925 | CELLULAR_COMPONENT | focal adhesion | 2.34E-06 | 1.31E-07 |
| GO:0005924 | CELLULAR_COMPONENT | cell-substrate adherens junction | 2.34E-06 | 1.31E-07 |
| GO:0045202 | CELLULAR_COMPONENT | synapse | 2.37E-06 | 1.34E-07 |
| GO:0006475 | BIOLOGICAL_PROCESS | internal protein amino acid acetylation | 2.47E-06 | 1.40E-07 |
| GO:0018394 | BIOLOGICAL_PROCESS | peptidyl-lysine acetylation | 2.47E-06 | 1.40E-07 |
| GO:0018393 | BIOLOGICAL_PROCESS | internal peptidyl-lysine acetylation | 2.47E-06 | 1.40E-07 |
| GO:0016573 | BIOLOGICAL_PROCESS | histone acetylation | 2.47E-06 | 1.40E-07 |
| GO:0097193 | BIOLOGICAL_PROCESS | intrinsic apoptotic signaling pathway | 2.62E-06 | 1.49E-07 |
| GO:0048738 | BIOLOGICAL_PROCESS | cardiac muscle tissue development | 2.62E-06 | 1.49E-07 |
| GO:0044448 | CELLULAR_COMPONENT | cell cortex part | 2.62E-06 | 1.49E-07 |
| GO:0001755 | BIOLOGICAL_PROCESS | neural crest cell migration | 2.87E-06 | 1.64E-07 |
| GO:0045778 | BIOLOGICAL_PROCESS | positive regulation of ossification | 2.87E-06 | 1.64E-07 |
| GO:0008630 | BIOLOGICAL_PROCESS | intrinsic apoptotic signaling pathway in response to DNA damage | 2.87E-06 | 1.64E-07 |
| GO:0043235 | CELLULAR_COMPONENT | receptor complex | 2.89E-06 | 1.66E-07 |
| GO:0048565 | BIOLOGICAL_PROCESS | digestive tract development | 2.89E-06 | 1.66E-07 |
| GO:0001667 | BIOLOGICAL_PROCESS | ameboidal-type cell migration | 3.05E-06 | 1.75E-07 |
| GO:0001816 | BIOLOGICAL_PROCESS | cytokine production | 3.11E-06 | 1.79E-07 |
| GO:0090287 | BIOLOGICAL_PROCESS | regulation of cellular response to growth factor stimulus | 3.11E-06 | 1.79E-07 |
| GO:0045787 | BIOLOGICAL_PROCESS | positive regulation of cell cycle | 3.11E-06 | 1.79E-07 |
| GO:1903827 | BIOLOGICAL_PROCESS | regulation of cellular protein localization | 3.56E-06 | 2.06E-07 |
| GO:0097305 | BIOLOGICAL_PROCESS | response to alcohol | 3.62E-06 | 2.10E-07 |
| GO:0030518 | BIOLOGICAL_PROCESS | intracellular steroid hormone receptor signaling pathway | 3.62E-06 | 2.10E-07 |
| GO:0007338 | BIOLOGICAL_PROCESS | single fertilization | 3.62E-06 | 2.10E-07 |
| GO:0010975 | BIOLOGICAL_PROCESS | regulation of neuron projection development | 3.76E-06 | 2.19E-07 |
| GO:0004383 | MOLECULAR_FUNCTION | guanylate cyclase activity | 3.80E-06 | 2.22E-07 |
| GO:0071560 | BIOLOGICAL_PROCESS | cellular response to transforming growth factor beta stimulus | 3.80E-06 | 2.22E-07 |
| GO:0071559 | BIOLOGICAL_PROCESS | response to transforming growth factor beta | 3.80E-06 | 2.22E-07 |
| GO:0044057 | BIOLOGICAL_PROCESS | regulation of system process | 3.87E-06 | 2.27E-07 |
| GO:0004029 | MOLECULAR_FUNCTION | aldehyde dehydrogenase (NAD) activity | 3.95E-06 | 2.32E-07 |
| GO:0061371 | BIOLOGICAL_PROCESS | determination of heart left/right asymmetry | 3.95E-06 | 2.32E-07 |
| GO:0030863 | CELLULAR_COMPONENT | cortical cytoskeleton | 3.96E-06 | 2.34E-07 |
| GO:0048639 | BIOLOGICAL_PROCESS | positive regulation of developmental growth | 3.96E-06 | 2.34E-07 |
| GO:0022037 | BIOLOGICAL_PROCESS | metencephalon development | 3.96E-06 | 2.34E-07 |
| GO:0021987 | BIOLOGICAL_PROCESS | cerebral cortex development | 3.96E-06 | 2.34E-07 |
| GO:0021549 | BIOLOGICAL_PROCESS | cerebellum development | 3.96E-06 | 2.34E-07 |
| GO:0005230 | MOLECULAR_FUNCTION | extracellular ligand-gated ion channel activity | 4.14E-06 | 2.45E-07 |
| GO:0005615 | CELLULAR_COMPONENT | extracellular space | 4.20E-06 | 2.49E-07 |
| GO:0045321 | BIOLOGICAL_PROCESS | leukocyte activation | 4.82E-06 | 2.87E-07 |
| GO:0002684 | BIOLOGICAL_PROCESS | positive regulation of immune system process | 4.82E-06 | 2.87E-07 |
| GO:0098662 | BIOLOGICAL_PROCESS | inorganic cation transmembrane transport | 6.26E-06 | 3.73E-07 |
| GO:0061458 | BIOLOGICAL_PROCESS | reproductive system development | 6.27E-06 | 3.75E-07 |
| GO:0008092 | MOLECULAR_FUNCTION | cytoskeletal protein binding | 6.27E-06 | 3.74E-07 |
| GO:0048608 | BIOLOGICAL_PROCESS | reproductive structure development | 6.27E-06 | 3.75E-07 |
| GO:0060021 | BIOLOGICAL_PROCESS | palate development | 6.62E-06 | 4.00E-07 |
| GO:0097306 | BIOLOGICAL_PROCESS | cellular response to alcohol | 6.62E-06 | 4.00E-07 |
| GO:0042476 | BIOLOGICAL_PROCESS | odontogenesis | 6.62E-06 | 4.00E-07 |
| GO:0021517 | BIOLOGICAL_PROCESS | ventral spinal cord development | 6.62E-06 | 4.00E-07 |
| GO:0009948 | BIOLOGICAL_PROCESS | anterior/posterior axis specification | 6.62E-06 | 4.00E-07 |
| GO:0033613 | MOLECULAR_FUNCTION | activating transcription factor binding | 6.62E-06 | 4.00E-07 |
| GO:0045637 | BIOLOGICAL_PROCESS | regulation of myeloid cell differentiation | 6.62E-06 | 4.00E-07 |
| GO:0043025 | CELLULAR_COMPONENT | neuronal cell body | 7.03E-06 | 4.26E-07 |
| GO:0048588 | BIOLOGICAL_PROCESS | developmental cell growth | 7.17E-06 | 4.36E-07 |
| GO:0050673 | BIOLOGICAL_PROCESS | epithelial cell proliferation | 7.17E-06 | 4.36E-07 |
| GO:0051222 | BIOLOGICAL_PROCESS | positive regulation of protein transport | 7.17E-06 | 4.36E-07 |
| GO:0043900 | BIOLOGICAL_PROCESS | regulation of multi-organism process | 8.59E-06 | 5.23E-07 |
| GO:0048609 | BIOLOGICAL_PROCESS | multicellular organismal reproductive process | 8.74E-06 | 5.34E-07 |
| GO:0032504 | BIOLOGICAL_PROCESS | multicellular organism reproduction | 8.74E-06 | 5.34E-07 |
| GO:0009416 | BIOLOGICAL_PROCESS | response to light stimulus | 9.26E-06 | 5.67E-07 |
| GO:0044093 | BIOLOGICAL_PROCESS | positive regulation of molecular function | 9.30E-06 | 5.70E-07 |
| GO:1903530 | BIOLOGICAL_PROCESS | regulation of secretion by cell | 9.42E-06 | 5.78E-07 |
| GO:0018209 | BIOLOGICAL_PROCESS | peptidyl-serine modification | 9.65E-06 | 5.94E-07 |
| GO:0018105 | BIOLOGICAL_PROCESS | peptidyl-serine phosphorylation | 9.65E-06 | 5.94E-07 |
| GO:0061505 | MOLECULAR_FUNCTION | DNA topoisomerase II activity | 9.76E-06 | 6.04E-07 |
| GO:0007200 | BIOLOGICAL_PROCESS | phospholipase C-activating G-protein coupled receptor signaling pathway | 9.76E-06 | 6.04E-07 |
| GO:0003918 | MOLECULAR_FUNCTION | DNA topoisomerase type II (ATP-hydrolyzing) activity | 9.76E-06 | 6.04E-07 |
| GO:0007422 | BIOLOGICAL_PROCESS | peripheral nervous system development | 9.76E-06 | 6.04E-07 |
| GO:0001822 | BIOLOGICAL_PROCESS | kidney development | 9.85E-06 | 6.13E-07 |
| GO:1990138 | BIOLOGICAL_PROCESS | neuron projection extension | 9.85E-06 | 6.13E-07 |
| GO:0046822 | BIOLOGICAL_PROCESS | regulation of nucleocytoplasmic transport | 9.85E-06 | 6.13E-07 |
| GO:1904951 | BIOLOGICAL_PROCESS | positive regulation of establishment of protein localization | 1.01E-05 | 6.27E-07 |
| GO:0015833 | BIOLOGICAL_PROCESS | peptide transport | 1.05E-05 | 6.56E-07 |
| GO:0050731 | BIOLOGICAL_PROCESS | positive regulation of peptidyl-tyrosine phosphorylation | 1.09E-05 | 6.80E-07 |
| GO:0072332 | BIOLOGICAL_PROCESS | intrinsic apoptotic signaling pathway by p53 class mediator | 1.09E-05 | 6.80E-07 |
| GO:0004707 | MOLECULAR_FUNCTION | MAP kinase activity | 1.09E-05 | 6.80E-07 |
| GO:0003143 | BIOLOGICAL_PROCESS | embryonic heart tube morphogenesis | 1.10E-05 | 6.95E-07 |
| GO:0010721 | BIOLOGICAL_PROCESS | negative regulation of cell development | 1.10E-05 | 6.95E-07 |
| GO:1905330 | BIOLOGICAL_PROCESS | regulation of morphogenesis of an epithelium | 1.10E-05 | 6.95E-07 |
| GO:0070603 | CELLULAR_COMPONENT | SWI/SNF superfamily-type complex | 1.10E-05 | 6.95E-07 |
| GO:0070371 | BIOLOGICAL_PROCESS | ERK1 and ERK2 cascade | 1.15E-05 | 7.30E-07 |
| GO:0055123 | BIOLOGICAL_PROCESS | digestive system development | 1.15E-05 | 7.30E-07 |
| GO:0031016 | BIOLOGICAL_PROCESS | pancreas development | 1.15E-05 | 7.30E-07 |
| GO:0051050 | BIOLOGICAL_PROCESS | positive regulation of transport | 1.16E-05 | 7.37E-07 |
| GO:0042306 | BIOLOGICAL_PROCESS | regulation of protein import into nucleus | 1.19E-05 | 7.55E-07 |
| GO:0048675 | BIOLOGICAL_PROCESS | axon extension | 1.19E-05 | 7.55E-07 |
| GO:0010501 | BIOLOGICAL_PROCESS | RNA secondary structure unwinding | 1.19E-05 | 7.55E-07 |
| GO:1904589 | BIOLOGICAL_PROCESS | regulation of protein import | 1.19E-05 | 7.55E-07 |
| GO:0035270 | BIOLOGICAL_PROCESS | endocrine system development | 1.21E-05 | 7.74E-07 |
| GO:0030864 | CELLULAR_COMPONENT | cortical actin cytoskeleton | 1.21E-05 | 7.74E-07 |
| GO:0002011 | BIOLOGICAL_PROCESS | morphogenesis of an epithelial sheet | 1.21E-05 | 7.74E-07 |
| GO:0051402 | BIOLOGICAL_PROCESS | neuron apoptotic process | 1.21E-05 | 7.74E-07 |
| GO:0048839 | BIOLOGICAL_PROCESS | inner ear development | 1.25E-05 | 8.01E-07 |
| GO:0005667 | CELLULAR_COMPONENT | transcription factor complex | 1.25E-05 | 8.01E-07 |
| GO:0042692 | BIOLOGICAL_PROCESS | muscle cell differentiation | 1.25E-05 | 8.01E-07 |
| GO:0030036 | BIOLOGICAL_PROCESS | actin cytoskeleton organization | 1.41E-05 | 9.08E-07 |
| GO:1903047 | BIOLOGICAL_PROCESS | mitotic cell cycle process | 1.66E-05 | 1.07E-06 |
| GO:0006473 | BIOLOGICAL_PROCESS | protein acetylation | 1.67E-05 | 1.08E-06 |
| GO:0007264 | BIOLOGICAL_PROCESS | small GTPase mediated signal transduction | 1.78E-05 | 1.15E-06 |
| GO:0048705 | BIOLOGICAL_PROCESS | skeletal system morphogenesis | 1.94E-05 | 1.26E-06 |
| GO:0045666 | BIOLOGICAL_PROCESS | positive regulation of neuron differentiation | 1.94E-05 | 1.26E-06 |
| GO:0034220 | BIOLOGICAL_PROCESS | ion transmembrane transport | 2.01E-05 | 1.30E-06 |
| GO:0006139 | BIOLOGICAL_PROCESS | nucleobase-containing compound metabolic process | 2.18E-05 | 1.42E-06 |
| GO:0009855 | BIOLOGICAL_PROCESS | determination of bilateral symmetry | 2.24E-05 | 1.46E-06 |
| GO:0009799 | BIOLOGICAL_PROCESS | specification of symmetry | 2.24E-05 | 1.46E-06 |
| GO:0044708 | BIOLOGICAL_PROCESS | single-organism behavior | 2.28E-05 | 1.49E-06 |
| GO:0034284 | BIOLOGICAL_PROCESS | response to monosaccharide | 2.34E-05 | 1.53E-06 |
| GO:0009746 | BIOLOGICAL_PROCESS | response to hexose | 2.34E-05 | 1.53E-06 |
| GO:0009950 | BIOLOGICAL_PROCESS | dorsal/ventral axis specification | 2.34E-05 | 1.53E-06 |
| GO:0021575 | BIOLOGICAL_PROCESS | hindbrain morphogenesis | 2.34E-05 | 1.53E-06 |
| GO:0045111 | CELLULAR_COMPONENT | intermediate filament cytoskeleton | 2.34E-05 | 1.54E-06 |
| GO:0001101 | BIOLOGICAL_PROCESS | response to acid chemical | 2.34E-05 | 1.54E-06 |
| GO:0009628 | BIOLOGICAL_PROCESS | response to abiotic stimulus | 2.38E-05 | 1.57E-06 |
| GO:0016791 | MOLECULAR_FUNCTION | phosphatase activity | 2.41E-05 | 1.59E-06 |
| GO:0099513 | CELLULAR_COMPONENT | polymeric cytoskeletal fiber | 2.42E-05 | 1.60E-06 |
| GO:0043583 | BIOLOGICAL_PROCESS | ear development | 2.56E-05 | 1.70E-06 |
| GO:0005578 | CELLULAR_COMPONENT | proteinaceous extracellular matrix | 2.56E-05 | 1.70E-06 |
| GO:0005622 | CELLULAR_COMPONENT | intracellular | 2.56E-05 | 1.70E-06 |
| GO:0052652 | BIOLOGICAL_PROCESS | cyclic purine nucleotide metabolic process | 2.56E-05 | 1.70E-06 |
| GO:0006913 | BIOLOGICAL_PROCESS | nucleocytoplasmic transport | 2.62E-05 | 1.74E-06 |
| GO:0051169 | BIOLOGICAL_PROCESS | nuclear transport | 2.62E-05 | 1.74E-06 |
| GO:0036477 | CELLULAR_COMPONENT | somatodendritic compartment | 2.69E-05 | 1.80E-06 |
| GO:0051961 | BIOLOGICAL_PROCESS | negative regulation of nervous system development | 2.76E-05 | 1.84E-06 |
| GO:0005882 | CELLULAR_COMPONENT | intermediate filament | 2.76E-05 | 1.84E-06 |
| GO:0044403 | BIOLOGICAL_PROCESS | symbiosis, encompassing mutualism through parasitism | 2.83E-05 | 1.89E-06 |
| GO:0050776 | BIOLOGICAL_PROCESS | regulation of immune response | 2.89E-05 | 1.94E-06 |
| GO:0030155 | BIOLOGICAL_PROCESS | regulation of cell adhesion | 2.89E-05 | 1.94E-06 |
| GO:0030029 | BIOLOGICAL_PROCESS | actin filament-based process | 2.94E-05 | 1.97E-06 |
| GO:0016032 | BIOLOGICAL_PROCESS | viral process | 2.95E-05 | 1.99E-06 |
| GO:0098916 | BIOLOGICAL_PROCESS | anterograde trans-synaptic signaling | 3.10E-05 | 2.09E-06 |
| GO:0007268 | BIOLOGICAL_PROCESS | chemical synaptic transmission | 3.10E-05 | 2.09E-06 |
| GO:0099536 | BIOLOGICAL_PROCESS | synaptic signaling | 3.10E-05 | 2.09E-06 |
| GO:0099537 | BIOLOGICAL_PROCESS | trans-synaptic signaling | 3.10E-05 | 2.09E-06 |
| GO:0070997 | BIOLOGICAL_PROCESS | neuron death | 3.15E-05 | 2.14E-06 |
| GO:0090101 | BIOLOGICAL_PROCESS | negative regulation of transmembrane receptor protein serine/threonine kinase signaling pathway | 3.15E-05 | 2.14E-06 |
| GO:0007179 | BIOLOGICAL_PROCESS | transforming growth factor beta receptor signaling pathway | 3.15E-05 | 2.14E-06 |
| GO:0009411 | BIOLOGICAL_PROCESS | response to UV | 3.15E-05 | 2.14E-06 |
| GO:0031683 | MOLECULAR_FUNCTION | G-protein beta/gamma-subunit complex binding | 3.19E-05 | 2.18E-06 |
| GO:0001047 | MOLECULAR_FUNCTION | core promoter binding | 3.19E-05 | 2.18E-06 |
| GO:0051100 | BIOLOGICAL_PROCESS | negative regulation of binding | 3.19E-05 | 2.18E-06 |
| GO:0051099 | BIOLOGICAL_PROCESS | positive regulation of binding | 3.19E-05 | 2.18E-06 |
| GO:0048592 | BIOLOGICAL_PROCESS | eye morphogenesis | 3.21E-05 | 2.20E-06 |
| GO:0044297 | CELLULAR_COMPONENT | cell body | 3.44E-05 | 2.36E-06 |
| GO:1903706 | BIOLOGICAL_PROCESS | regulation of hemopoiesis | 3.49E-05 | 2.40E-06 |
| GO:0030098 | BIOLOGICAL_PROCESS | lymphocyte differentiation | 3.49E-05 | 2.40E-06 |
| GO:0050730 | BIOLOGICAL_PROCESS | regulation of peptidyl-tyrosine phosphorylation | 3.62E-05 | 2.50E-06 |
| GO:0072073 | BIOLOGICAL_PROCESS | kidney epithelium development | 3.62E-05 | 2.50E-06 |
| GO:0043523 | BIOLOGICAL_PROCESS | regulation of neuron apoptotic process | 3.62E-05 | 2.50E-06 |
| GO:0042886 | BIOLOGICAL_PROCESS | amide transport | 3.62E-05 | 2.49E-06 |
| GO:0035637 | BIOLOGICAL_PROCESS | multicellular organismal signaling | 3.62E-05 | 2.50E-06 |
| GO:0070372 | BIOLOGICAL_PROCESS | regulation of ERK1 and ERK2 cascade | 3.62E-05 | 2.50E-06 |
| GO:0035265 | BIOLOGICAL_PROCESS | organ growth | 3.67E-05 | 2.55E-06 |
| GO:0001947 | BIOLOGICAL_PROCESS | heart looping | 3.67E-05 | 2.55E-06 |
| GO:0045087 | BIOLOGICAL_PROCESS | innate immune response | 3.72E-05 | 2.58E-06 |
| GO:0050679 | BIOLOGICAL_PROCESS | positive regulation of epithelial cell proliferation | 4.01E-05 | 2.82E-06 |
| GO:0035050 | BIOLOGICAL_PROCESS | embryonic heart tube development | 4.01E-05 | 2.82E-06 |
| GO:0060039 | BIOLOGICAL_PROCESS | pericardium development | 4.01E-05 | 2.82E-06 |
| GO:0042771 | BIOLOGICAL_PROCESS | intrinsic apoptotic signaling pathway in response to DNA damage by p53 class mediator | 4.01E-05 | 2.82E-06 |
| GO:0003197 | BIOLOGICAL_PROCESS | endocardial cushion development | 4.01E-05 | 2.82E-06 |
| GO:0048641 | BIOLOGICAL_PROCESS | regulation of skeletal muscle tissue development | 4.01E-05 | 2.82E-06 |
| GO:0010660 | BIOLOGICAL_PROCESS | regulation of muscle cell apoptotic process | 4.01E-05 | 2.82E-06 |
| GO:0045926 | BIOLOGICAL_PROCESS | negative regulation of growth | 4.01E-05 | 2.82E-06 |
| GO:0019228 | BIOLOGICAL_PROCESS | neuronal action potential | 4.01E-05 | 2.82E-06 |
| GO:0051568 | BIOLOGICAL_PROCESS | histone H3-K4 methylation | 4.01E-05 | 2.82E-06 |
| GO:0006814 | BIOLOGICAL_PROCESS | sodium ion transport | 4.06E-05 | 2.86E-06 |
| GO:0003006 | BIOLOGICAL_PROCESS | developmental process involved in reproduction | 4.73E-05 | 3.34E-06 |
| GO:0071900 | BIOLOGICAL_PROCESS | regulation of protein serine/threonine kinase activity | 4.73E-05 | 3.34E-06 |
| GO:0031347 | BIOLOGICAL_PROCESS | regulation of defense response | 4.85E-05 | 3.43E-06 |
| GO:0043543 | BIOLOGICAL_PROCESS | protein acylation | 5.01E-05 | 3.54E-06 |
| GO:0072001 | BIOLOGICAL_PROCESS | renal system development | 5.04E-05 | 3.57E-06 |
| GO:0004725 | MOLECULAR_FUNCTION | protein tyrosine phosphatase activity | 5.13E-05 | 3.64E-06 |
| GO:0003724 | MOLECULAR_FUNCTION | RNA helicase activity | 5.13E-05 | 3.64E-06 |
| GO:0048593 | BIOLOGICAL_PROCESS | camera-type eye morphogenesis | 6.25E-05 | 4.46E-06 |
| GO:0050678 | BIOLOGICAL_PROCESS | regulation of epithelial cell proliferation | 6.25E-05 | 4.46E-06 |
| GO:0009566 | BIOLOGICAL_PROCESS | fertilization | 6.25E-05 | 4.46E-06 |
| GO:0032386 | BIOLOGICAL_PROCESS | regulation of intracellular transport | 6.25E-05 | 4.47E-06 |
| GO:0019001 | MOLECULAR_FUNCTION | guanyl nucleotide binding | 6.42E-05 | 4.59E-06 |
| GO:0016620 | MOLECULAR_FUNCTION | oxidoreductase activity, acting on the aldehyde or oxo group of donors, NAD or NADP as acceptor | 6.70E-05 | 4.80E-06 |
| GO:0044454 | CELLULAR_COMPONENT | nuclear chromosome part | 6.76E-05 | 4.85E-06 |
| GO:0043405 | BIOLOGICAL_PROCESS | regulation of MAP kinase activity | 6.84E-05 | 4.92E-06 |
| GO:0035335 | BIOLOGICAL_PROCESS | peptidyl-tyrosine dephosphorylation | 6.84E-05 | 4.92E-06 |
| GO:0045860 | BIOLOGICAL_PROCESS | positive regulation of protein kinase activity | 6.84E-05 | 4.92E-06 |
| GO:0060341 | BIOLOGICAL_PROCESS | regulation of cellular localization | 6.95E-05 | 5.01E-06 |
| GO:0045786 | BIOLOGICAL_PROCESS | negative regulation of cell cycle | 7.39E-05 | 5.33E-06 |
| GO:0005525 | MOLECULAR_FUNCTION | GTP binding | 7.80E-05 | 5.64E-06 |
| GO:0097435 | BIOLOGICAL_PROCESS | supramolecular fiber organization | 7.81E-05 | 5.65E-06 |
| GO:0001707 | BIOLOGICAL_PROCESS | mesoderm formation | 7.97E-05 | 5.84E-06 |
| GO:0098878 | CELLULAR_COMPONENT | neurotransmitter receptor complex | 7.97E-05 | 5.84E-06 |
| GO:0001837 | BIOLOGICAL_PROCESS | epithelial to mesenchymal transition | 7.97E-05 | 5.84E-06 |
| GO:0050803 | BIOLOGICAL_PROCESS | regulation of synapse structure or activity | 7.97E-05 | 5.84E-06 |
| GO:0048332 | BIOLOGICAL_PROCESS | mesoderm morphogenesis | 7.97E-05 | 5.84E-06 |
| GO:0003208 | BIOLOGICAL_PROCESS | cardiac ventricle morphogenesis | 7.97E-05 | 5.84E-06 |
| GO:0016500 | MOLECULAR_FUNCTION | protein-hormone receptor activity | 7.97E-05 | 5.84E-06 |
| GO:0008328 | CELLULAR_COMPONENT | ionotropic glutamate receptor complex | 7.97E-05 | 5.84E-06 |
| GO:0031214 | BIOLOGICAL_PROCESS | biomineral tissue development | 7.97E-05 | 5.84E-06 |
| GO:0021522 | BIOLOGICAL_PROCESS | spinal cord motor neuron differentiation | 7.97E-05 | 5.84E-06 |
| GO:0021587 | BIOLOGICAL_PROCESS | cerebellum morphogenesis | 7.97E-05 | 5.84E-06 |
| GO:0044843 | BIOLOGICAL_PROCESS | cell cycle G1/S phase transition | 8.09E-05 | 5.94E-06 |
| GO:0051047 | BIOLOGICAL_PROCESS | positive regulation of secretion | 8.09E-05 | 5.94E-06 |
| GO:0042326 | BIOLOGICAL_PROCESS | negative regulation of phosphorylation | 8.71E-05 | 6.42E-06 |
| GO:0090066 | BIOLOGICAL_PROCESS | regulation of anatomical structure size | 8.71E-05 | 6.42E-06 |
| GO:0050768 | BIOLOGICAL_PROCESS | negative regulation of neurogenesis | 8.78E-05 | 6.51E-06 |
| GO:0035107 | BIOLOGICAL_PROCESS | appendage morphogenesis | 8.78E-05 | 6.51E-06 |
| GO:0016331 | BIOLOGICAL_PROCESS | morphogenesis of embryonic epithelium | 8.78E-05 | 6.51E-06 |
| GO:0071772 | BIOLOGICAL_PROCESS | response to BMP | 8.78E-05 | 6.51E-06 |
| GO:0071773 | BIOLOGICAL_PROCESS | cellular response to BMP stimulus | 8.78E-05 | 6.51E-06 |
| GO:0007548 | BIOLOGICAL_PROCESS | sex differentiation | 8.78E-05 | 6.51E-06 |
| GO:0033674 | BIOLOGICAL_PROCESS | positive regulation of kinase activity | 8.91E-05 | 6.62E-06 |
| GO:0035148 | BIOLOGICAL_PROCESS | tube formation | 1.01E-04 | 7.51E-06 |
| GO:1901214 | BIOLOGICAL_PROCESS | regulation of neuron death | 1.01E-04 | 7.51E-06 |
| GO:0072175 | BIOLOGICAL_PROCESS | epithelial tube formation | 1.01E-04 | 7.51E-06 |
| GO:0043393 | BIOLOGICAL_PROCESS | regulation of protein binding | 1.01E-04 | 7.51E-06 |
| GO:0032561 | MOLECULAR_FUNCTION | guanyl ribonucleotide binding | 1.01E-04 | 7.50E-06 |
| GO:0006470 | BIOLOGICAL_PROCESS | protein dephosphorylation | 1.02E-04 | 7.60E-06 |
| GO:0098742 | BIOLOGICAL_PROCESS | cell-cell adhesion via plasma-membrane adhesion molecules | 1.02E-04 | 7.73E-06 |
| GO:0098727 | BIOLOGICAL_PROCESS | maintenance of cell number | 1.02E-04 | 7.77E-06 |
| GO:0072006 | BIOLOGICAL_PROCESS | nephron development | 1.02E-04 | 7.77E-06 |
| GO:0072009 | BIOLOGICAL_PROCESS | nephron epithelium development | 1.02E-04 | 7.77E-06 |
| GO:0019827 | BIOLOGICAL_PROCESS | stem cell population maintenance | 1.02E-04 | 7.77E-06 |
| GO:0072089 | BIOLOGICAL_PROCESS | stem cell proliferation | 1.02E-04 | 7.77E-06 |
| GO:1900180 | BIOLOGICAL_PROCESS | regulation of protein localization to nucleus | 1.02E-04 | 7.73E-06 |
| GO:0048709 | BIOLOGICAL_PROCESS | oligodendrocyte differentiation | 1.02E-04 | 7.77E-06 |
| GO:0046620 | BIOLOGICAL_PROCESS | regulation of organ growth | 1.02E-04 | 7.77E-06 |
| GO:0060976 | BIOLOGICAL_PROCESS | coronary vasculature development | 1.02E-04 | 7.77E-06 |
| GO:0009880 | BIOLOGICAL_PROCESS | embryonic pattern specification | 1.02E-04 | 7.77E-06 |
| GO:0033143 | BIOLOGICAL_PROCESS | regulation of intracellular steroid hormone receptor signaling pathway | 1.02E-04 | 7.77E-06 |
| GO:0015424 | MOLECULAR_FUNCTION | amino acid-transporting ATPase activity | 1.02E-04 | 7.77E-06 |
| GO:0021536 | BIOLOGICAL_PROCESS | diencephalon development | 1.02E-04 | 7.77E-06 |
| GO:0015758 | BIOLOGICAL_PROCESS | glucose transport | 1.02E-04 | 7.77E-06 |
| GO:0001708 | BIOLOGICAL_PROCESS | cell fate specification | 1.08E-04 | 8.27E-06 |
| GO:0042054 | MOLECULAR_FUNCTION | histone methyltransferase activity | 1.08E-04 | 8.27E-06 |
| GO:0071229 | BIOLOGICAL_PROCESS | cellular response to acid chemical | 1.08E-04 | 8.27E-06 |
| GO:0006954 | BIOLOGICAL_PROCESS | inflammatory response | 1.08E-04 | 8.27E-06 |
| GO:1902275 | BIOLOGICAL_PROCESS | regulation of chromatin organization | 1.10E-04 | 8.47E-06 |
| GO:0034097 | BIOLOGICAL_PROCESS | response to cytokine | 1.10E-04 | 8.46E-06 |
| GO:0001085 | MOLECULAR_FUNCTION | RNA polymerase II transcription factor binding | 1.10E-04 | 8.47E-06 |
| GO:0004004 | MOLECULAR_FUNCTION | ATP-dependent RNA helicase activity | 1.17E-04 | 9.02E-06 |
| GO:0044424 | CELLULAR_COMPONENT | intracellular part | 1.24E-04 | 9.50E-06 |
| GO:0003779 | MOLECULAR_FUNCTION | actin binding | 1.26E-04 | 9.67E-06 |
| GO:0019899 | MOLECULAR_FUNCTION | enzyme binding | 1.29E-04 | 9.91E-06 |
| GO:1903532 | BIOLOGICAL_PROCESS | positive regulation of secretion by cell | 1.29E-04 | 9.97E-06 |
| GO:0044419 | BIOLOGICAL_PROCESS | interspecies interaction between organisms | 1.33E-04 | 1.03E-05 |
| GO:0001607 | MOLECULAR_FUNCTION | neuromedin U receptor activity | 1.49E-04 | 1.17E-05 |
| GO:0003207 | BIOLOGICAL_PROCESS | cardiac chamber formation | 1.49E-04 | 1.17E-05 |
| GO:0060688 | BIOLOGICAL_PROCESS | regulation of morphogenesis of a branching structure | 1.49E-04 | 1.17E-05 |
| GO:0001963 | BIOLOGICAL_PROCESS | synaptic transmission, dopaminergic | 1.49E-04 | 1.17E-05 |
| GO:0043921 | BIOLOGICAL_PROCESS | modulation by host of viral transcription | 1.49E-04 | 1.17E-05 |
| GO:0004360 | MOLECULAR_FUNCTION | glutamine-fructose-6-phosphate transaminase (isomerizing) activity | 1.49E-04 | 1.17E-05 |
| GO:0048703 | BIOLOGICAL_PROCESS | embryonic viscerocranium morphogenesis | 1.49E-04 | 1.17E-05 |
| GO:1905477 | BIOLOGICAL_PROCESS | positive regulation of protein localization to membrane | 1.49E-04 | 1.17E-05 |
| GO:0052312 | BIOLOGICAL_PROCESS | modulation of transcription in other organism involved in symbiotic interaction | 1.49E-04 | 1.17E-05 |
| GO:0052472 | BIOLOGICAL_PROCESS | modulation by host of symbiont transcription | 1.49E-04 | 1.17E-05 |
| GO:0008802 | MOLECULAR_FUNCTION | betaine-aldehyde dehydrogenase activity | 1.49E-04 | 1.17E-05 |
| GO:0007346 | BIOLOGICAL_PROCESS | regulation of mitotic cell cycle | 1.49E-04 | 1.16E-05 |
| GO:0070548 | MOLECULAR_FUNCTION | L-glutamine aminotransferase activity | 1.49E-04 | 1.17E-05 |
| GO:0001558 | BIOLOGICAL_PROCESS | regulation of cell growth | 1.51E-04 | 1.19E-05 |
| GO:0010035 | BIOLOGICAL_PROCESS | response to inorganic substance | 1.57E-04 | 1.23E-05 |
| GO:0042330 | BIOLOGICAL_PROCESS | taxis | 1.61E-04 | 1.27E-05 |
| GO:0035051 | BIOLOGICAL_PROCESS | cardiocyte differentiation | 1.61E-04 | 1.27E-05 |
| GO:0019897 | CELLULAR_COMPONENT | extrinsic component of plasma membrane | 1.61E-04 | 1.27E-05 |
| GO:0003279 | BIOLOGICAL_PROCESS | cardiac septum development | 1.61E-04 | 1.27E-05 |
| GO:0006935 | BIOLOGICAL_PROCESS | chemotaxis | 1.61E-04 | 1.27E-05 |
| GO:0040029 | BIOLOGICAL_PROCESS | regulation of gene expression, epigenetic | 1.61E-04 | 1.27E-05 |
| GO:0000082 | BIOLOGICAL_PROCESS | G1/S transition of mitotic cell cycle | 1.99E-04 | 1.58E-05 |
| GO:0021915 | BIOLOGICAL_PROCESS | neural tube development | 1.99E-04 | 1.58E-05 |
| GO:0009975 | MOLECULAR_FUNCTION | cyclase activity | 1.99E-04 | 1.58E-05 |
| GO:0001819 | BIOLOGICAL_PROCESS | positive regulation of cytokine production | 2.00E-04 | 1.59E-05 |
| GO:0090288 | BIOLOGICAL_PROCESS | negative regulation of cellular response to growth factor stimulus | 2.00E-04 | 1.59E-05 |
| GO:0044092 | BIOLOGICAL_PROCESS | negative regulation of molecular function | 2.40E-04 | 1.91E-05 |
| GO:0050792 | BIOLOGICAL_PROCESS | regulation of viral process | 2.42E-04 | 1.94E-05 |
| GO:0042110 | BIOLOGICAL_PROCESS | T cell activation | 2.42E-04 | 1.94E-05 |
| GO:0035567 | BIOLOGICAL_PROCESS | non-canonical Wnt signaling pathway | 2.42E-04 | 1.94E-05 |
| GO:0033002 | BIOLOGICAL_PROCESS | muscle cell proliferation | 2.42E-04 | 1.94E-05 |
| GO:0030509 | BIOLOGICAL_PROCESS | BMP signaling pathway | 2.42E-04 | 1.94E-05 |
| GO:0043903 | BIOLOGICAL_PROCESS | regulation of symbiosis, encompassing mutualism through parasitism | 2.42E-04 | 1.94E-05 |
| GO:1903900 | BIOLOGICAL_PROCESS | regulation of viral life cycle | 2.42E-04 | 1.94E-05 |
| GO:0007368 | BIOLOGICAL_PROCESS | determination of left/right symmetry | 2.47E-04 | 1.98E-05 |
| GO:2001233 | BIOLOGICAL_PROCESS | regulation of apoptotic signaling pathway | 2.58E-04 | 2.08E-05 |
| GO:0048736 | BIOLOGICAL_PROCESS | appendage development | 2.58E-04 | 2.08E-05 |
| GO:0060828 | BIOLOGICAL_PROCESS | regulation of canonical Wnt signaling pathway | 2.58E-04 | 2.08E-05 |
| GO:0031672 | CELLULAR_COMPONENT | A band | 2.69E-04 | 2.20E-05 |
| GO:0006479 | BIOLOGICAL_PROCESS | protein methylation | 2.69E-04 | 2.19E-05 |
| GO:0098602 | BIOLOGICAL_PROCESS | single organism cell adhesion | 2.69E-04 | 2.19E-05 |
| GO:0071322 | BIOLOGICAL_PROCESS | cellular response to carbohydrate stimulus | 2.69E-04 | 2.20E-05 |
| GO:0030520 | BIOLOGICAL_PROCESS | intracellular estrogen receptor signaling pathway | 2.69E-04 | 2.20E-05 |
| GO:0042475 | BIOLOGICAL_PROCESS | odontogenesis of dentin-containing tooth | 2.69E-04 | 2.20E-05 |
| GO:0048663 | BIOLOGICAL_PROCESS | neuron fate commitment | 2.69E-04 | 2.20E-05 |
| GO:0009749 | BIOLOGICAL_PROCESS | response to glucose | 2.69E-04 | 2.20E-05 |
| GO:0046622 | BIOLOGICAL_PROCESS | positive regulation of organ growth | 2.69E-04 | 2.20E-05 |
| GO:0051101 | BIOLOGICAL_PROCESS | regulation of DNA binding | 2.69E-04 | 2.20E-05 |
| GO:1905269 | BIOLOGICAL_PROCESS | positive regulation of chromatin organization | 2.69E-04 | 2.20E-05 |
| GO:0008213 | BIOLOGICAL_PROCESS | protein alkylation | 2.69E-04 | 2.19E-05 |
| GO:0003401 | BIOLOGICAL_PROCESS | axis elongation | 2.69E-04 | 2.20E-05 |
| GO:0007492 | BIOLOGICAL_PROCESS | endoderm development | 2.69E-04 | 2.20E-05 |
| GO:0001102 | MOLECULAR_FUNCTION | RNA polymerase II activating transcription factor binding | 2.69E-04 | 2.20E-05 |
| GO:0045667 | BIOLOGICAL_PROCESS | regulation of osteoblast differentiation | 2.69E-04 | 2.20E-05 |
| GO:0031018 | BIOLOGICAL_PROCESS | endocrine pancreas development | 2.69E-04 | 2.20E-05 |
| GO:0001838 | BIOLOGICAL_PROCESS | embryonic epithelial tube formation | 2.81E-04 | 2.31E-05 |
| GO:0045995 | BIOLOGICAL_PROCESS | regulation of embryonic development | 2.81E-04 | 2.31E-05 |
| GO:1903844 | BIOLOGICAL_PROCESS | regulation of cellular response to transforming growth factor beta stimulus | 3.19E-04 | 2.64E-05 |
| GO:0031333 | BIOLOGICAL_PROCESS | negative regulation of protein complex assembly | 3.19E-04 | 2.64E-05 |
| GO:0030901 | BIOLOGICAL_PROCESS | midbrain development | 3.19E-04 | 2.64E-05 |
| GO:0051147 | BIOLOGICAL_PROCESS | regulation of muscle cell differentiation | 3.19E-04 | 2.64E-05 |
| GO:0017015 | BIOLOGICAL_PROCESS | regulation of transforming growth factor beta receptor signaling pathway | 3.19E-04 | 2.64E-05 |
| GO:0050714 | BIOLOGICAL_PROCESS | positive regulation of protein secretion | 3.26E-04 | 2.74E-05 |
| GO:0050808 | BIOLOGICAL_PROCESS | synapse organization | 3.26E-04 | 2.73E-05 |
| GO:0043467 | BIOLOGICAL_PROCESS | regulation of generation of precursor metabolites and energy | 3.26E-04 | 2.74E-05 |
| GO:0016571 | BIOLOGICAL_PROCESS | histone methylation | 3.26E-04 | 2.73E-05 |
| GO:0050905 | BIOLOGICAL_PROCESS | neuromuscular process | 3.26E-04 | 2.74E-05 |
| GO:0048644 | BIOLOGICAL_PROCESS | muscle organ morphogenesis | 3.26E-04 | 2.74E-05 |
| GO:0060415 | BIOLOGICAL_PROCESS | muscle tissue morphogenesis | 3.26E-04 | 2.74E-05 |
| GO:0070374 | BIOLOGICAL_PROCESS | positive regulation of ERK1 and ERK2 cascade | 3.26E-04 | 2.74E-05 |
| GO:0034644 | BIOLOGICAL_PROCESS | cellular response to UV | 3.26E-04 | 2.74E-05 |
| GO:0001046 | MOLECULAR_FUNCTION | core promoter sequence-specific DNA binding | 3.26E-04 | 2.74E-05 |
| GO:0002065 | BIOLOGICAL_PROCESS | columnar/cuboidal epithelial cell differentiation | 3.26E-04 | 2.74E-05 |
| GO:0007586 | BIOLOGICAL_PROCESS | digestion | 3.26E-04 | 2.74E-05 |
| GO:0043044 | BIOLOGICAL_PROCESS | ATP-dependent chromatin remodeling | 3.26E-04 | 2.74E-05 |
| GO:0051347 | BIOLOGICAL_PROCESS | positive regulation of transferase activity | 3.28E-04 | 2.75E-05 |
| GO:0050806 | BIOLOGICAL_PROCESS | positive regulation of synaptic transmission | 3.34E-04 | 2.83E-05 |
| GO:0005275 | MOLECULAR_FUNCTION | amine transmembrane transporter activity | 3.34E-04 | 2.83E-05 |
| GO:0060348 | BIOLOGICAL_PROCESS | bone development | 3.34E-04 | 2.83E-05 |
| GO:0060411 | BIOLOGICAL_PROCESS | cardiac septum morphogenesis | 3.34E-04 | 2.83E-05 |
| GO:0031263 | MOLECULAR_FUNCTION | amine-transporting ATPase activity | 3.34E-04 | 2.83E-05 |
| GO:0031056 | BIOLOGICAL_PROCESS | regulation of histone modification | 3.34E-04 | 2.83E-05 |
| GO:0002573 | BIOLOGICAL_PROCESS | myeloid leukocyte differentiation | 3.34E-04 | 2.83E-05 |
| GO:0031099 | BIOLOGICAL_PROCESS | regeneration | 3.34E-04 | 2.83E-05 |
| GO:0030162 | BIOLOGICAL_PROCESS | regulation of proteolysis | 3.43E-04 | 2.90E-05 |
| GO:0016358 | BIOLOGICAL_PROCESS | dendrite development | 3.59E-04 | 3.05E-05 |
| GO:0051146 | BIOLOGICAL_PROCESS | striated muscle cell differentiation | 3.59E-04 | 3.05E-05 |
| GO:0044451 | CELLULAR_COMPONENT | nucleoplasm part | 3.73E-04 | 3.17E-05 |
| GO:0044763 | BIOLOGICAL_PROCESS | single-organism cellular process | 3.75E-04 | 3.19E-05 |
| GO:0022402 | BIOLOGICAL_PROCESS | cell cycle process | 3.77E-04 | 3.21E-05 |
| GO:0019904 | MOLECULAR_FUNCTION | protein domain specific binding | 3.79E-04 | 3.23E-05 |
| GO:1902532 | BIOLOGICAL_PROCESS | negative regulation of intracellular signal transduction | 4.04E-04 | 3.45E-05 |
| GO:0010038 | BIOLOGICAL_PROCESS | response to metal ion | 4.43E-04 | 3.79E-05 |
| GO:0045862 | BIOLOGICAL_PROCESS | positive regulation of proteolysis | 4.43E-04 | 3.79E-05 |
| GO:1903829 | BIOLOGICAL_PROCESS | positive regulation of cellular protein localization | 4.81E-04 | 4.12E-05 |
| GO:0016337 | BIOLOGICAL_PROCESS | single organismal cell-cell adhesion | 4.81E-04 | 4.12E-05 |
| GO:0010638 | BIOLOGICAL_PROCESS | positive regulation of organelle organization | 4.98E-04 | 4.27E-05 |
| GO:0090068 | BIOLOGICAL_PROCESS | positive regulation of cell cycle process | 5.25E-04 | 4.53E-05 |
| GO:0031349 | BIOLOGICAL_PROCESS | positive regulation of defense response | 5.25E-04 | 4.53E-05 |
| GO:1901796 | BIOLOGICAL_PROCESS | regulation of signal transduction by p53 class mediator | 5.25E-04 | 4.53E-05 |
| GO:0003916 | MOLECULAR_FUNCTION | DNA topoisomerase activity | 5.25E-04 | 4.53E-05 |
| GO:0006265 | BIOLOGICAL_PROCESS | DNA topological change | 5.25E-04 | 4.53E-05 |
| GO:0008186 | MOLECULAR_FUNCTION | RNA-dependent ATPase activity | 5.29E-04 | 4.57E-05 |
| GO:0003156 | BIOLOGICAL_PROCESS | regulation of animal organ formation | 5.50E-04 | 4.83E-05 |
| GO:0060536 | BIOLOGICAL_PROCESS | cartilage morphogenesis | 5.50E-04 | 4.83E-05 |
| GO:0003203 | BIOLOGICAL_PROCESS | endocardial cushion morphogenesis | 5.50E-04 | 4.83E-05 |
| GO:0008074 | CELLULAR_COMPONENT | guanylate cyclase complex, soluble | 5.50E-04 | 4.83E-05 |
| GO:0048643 | BIOLOGICAL_PROCESS | positive regulation of skeletal muscle tissue development | 5.50E-04 | 4.83E-05 |
| GO:0060425 | BIOLOGICAL_PROCESS | lung morphogenesis | 5.50E-04 | 4.83E-05 |
| GO:0010656 | BIOLOGICAL_PROCESS | negative regulation of muscle cell apoptotic process | 5.50E-04 | 4.83E-05 |
| GO:0004971 | MOLECULAR_FUNCTION | AMPA glutamate receptor activity | 5.50E-04 | 4.83E-05 |
| GO:0070167 | BIOLOGICAL_PROCESS | regulation of biomineral tissue development | 5.50E-04 | 4.83E-05 |
| GO:0003344 | BIOLOGICAL_PROCESS | pericardium morphogenesis | 5.50E-04 | 4.83E-05 |
| GO:0021879 | BIOLOGICAL_PROCESS | forebrain neuron differentiation | 5.50E-04 | 4.83E-05 |
| GO:0021872 | BIOLOGICAL_PROCESS | forebrain generation of neurons | 5.50E-04 | 4.83E-05 |
| GO:0032281 | CELLULAR_COMPONENT | AMPA glutamate receptor complex | 5.50E-04 | 4.83E-05 |
| GO:0044332 | BIOLOGICAL_PROCESS | Wnt signaling pathway involved in dorsal/ventral axis specification | 5.50E-04 | 4.83E-05 |
| GO:1904377 | BIOLOGICAL_PROCESS | positive regulation of protein localization to cell periphery | 5.50E-04 | 4.83E-05 |
| GO:1903078 | BIOLOGICAL_PROCESS | positive regulation of protein localization to plasma membrane | 5.50E-04 | 4.83E-05 |
| GO:0042578 | MOLECULAR_FUNCTION | phosphoric ester hydrolase activity | 5.50E-04 | 4.84E-05 |
| GO:0006606 | BIOLOGICAL_PROCESS | protein import into nucleus | 5.92E-04 | 5.23E-05 |
| GO:1902593 | BIOLOGICAL_PROCESS | single-organism nuclear import | 5.92E-04 | 5.23E-05 |
| GO:0050804 | BIOLOGICAL_PROCESS | modulation of synaptic transmission | 5.92E-04 | 5.23E-05 |
| GO:0044744 | BIOLOGICAL_PROCESS | protein targeting to nucleus | 5.92E-04 | 5.23E-05 |
| GO:0051170 | BIOLOGICAL_PROCESS | nuclear import | 5.92E-04 | 5.23E-05 |
| GO:0043209 | CELLULAR_COMPONENT | myelin sheath | 5.92E-04 | 5.23E-05 |
| GO:0003012 | BIOLOGICAL_PROCESS | muscle system process | 6.02E-04 | 5.33E-05 |
| GO:0045296 | MOLECULAR_FUNCTION | cadherin binding | 6.22E-04 | 5.50E-05 |
| GO:0030017 | CELLULAR_COMPONENT | sarcomere | 6.35E-04 | 5.64E-05 |
| GO:0010769 | BIOLOGICAL_PROCESS | regulation of cell morphogenesis involved in differentiation | 6.35E-04 | 5.64E-05 |
| GO:0001649 | BIOLOGICAL_PROCESS | osteoblast differentiation | 6.42E-04 | 5.70E-05 |
| GO:0032101 | BIOLOGICAL_PROCESS | regulation of response to external stimulus | 7.13E-04 | 6.34E-05 |
| GO:0051052 | BIOLOGICAL_PROCESS | regulation of DNA metabolic process | 7.13E-04 | 6.34E-05 |
| GO:0035282 | BIOLOGICAL_PROCESS | segmentation | 7.81E-04 | 6.99E-05 |
| GO:0001841 | BIOLOGICAL_PROCESS | neural tube formation | 7.81E-04 | 6.99E-05 |
| GO:0014020 | BIOLOGICAL_PROCESS | primary neural tube formation | 7.81E-04 | 6.99E-05 |
| GO:0004697 | MOLECULAR_FUNCTION | protein kinase C activity | 7.81E-04 | 6.99E-05 |
| GO:0008645 | BIOLOGICAL_PROCESS | hexose transport | 7.81E-04 | 6.99E-05 |
| GO:0046890 | BIOLOGICAL_PROCESS | regulation of lipid biosynthetic process | 7.81E-04 | 6.99E-05 |
| GO:0030016 | CELLULAR_COMPONENT | myofibril | 8.06E-04 | 7.23E-05 |
| GO:0060041 | BIOLOGICAL_PROCESS | retina development in camera-type eye | 8.06E-04 | 7.23E-05 |
| GO:0099572 | CELLULAR_COMPONENT | postsynaptic specialization | 8.16E-04 | 7.33E-05 |
| GO:0043620 | BIOLOGICAL_PROCESS | regulation of DNA-templated transcription in response to stress | 8.98E-04 | 8.25E-05 |
| GO:0072132 | BIOLOGICAL_PROCESS | mesenchyme morphogenesis | 8.98E-04 | 8.25E-05 |
| GO:0043618 | BIOLOGICAL_PROCESS | regulation of transcription from RNA polymerase II promoter in response to stress | 8.98E-04 | 8.25E-05 |
| GO:0030282 | BIOLOGICAL_PROCESS | bone mineralization | 8.98E-04 | 8.25E-05 |
| GO:0001823 | BIOLOGICAL_PROCESS | mesonephros development | 8.98E-04 | 8.25E-05 |
| GO:0014897 | BIOLOGICAL_PROCESS | striated muscle hypertrophy | 8.98E-04 | 8.25E-05 |
| GO:0014896 | BIOLOGICAL_PROCESS | muscle hypertrophy | 8.98E-04 | 8.25E-05 |
| GO:0030217 | BIOLOGICAL_PROCESS | T cell differentiation | 8.98E-04 | 8.25E-05 |
| GO:0003179 | BIOLOGICAL_PROCESS | heart valve morphogenesis | 8.98E-04 | 8.25E-05 |
| GO:0008045 | BIOLOGICAL_PROCESS | motor neuron axon guidance | 8.98E-04 | 8.25E-05 |
| GO:0090102 | BIOLOGICAL_PROCESS | cochlea development | 8.98E-04 | 8.25E-05 |
| GO:0004890 | MOLECULAR_FUNCTION | GABA-A receptor activity | 8.98E-04 | 8.25E-05 |
| GO:0004887 | MOLECULAR_FUNCTION | thyroid hormone receptor activity | 8.98E-04 | 8.25E-05 |
| GO:0004997 | MOLECULAR_FUNCTION | thyrotropin-releasing hormone receptor activity | 8.98E-04 | 8.25E-05 |
| GO:1901983 | BIOLOGICAL_PROCESS | regulation of protein acetylation | 8.98E-04 | 8.25E-05 |
| GO:0046782 | BIOLOGICAL_PROCESS | regulation of viral transcription | 8.98E-04 | 8.25E-05 |
| GO:0004995 | MOLECULAR_FUNCTION | tachykinin receptor activity | 8.98E-04 | 8.25E-05 |
| GO:0003300 | BIOLOGICAL_PROCESS | cardiac muscle hypertrophy | 8.98E-04 | 8.25E-05 |
| GO:0051153 | BIOLOGICAL_PROCESS | regulation of striated muscle cell differentiation | 8.98E-04 | 8.25E-05 |
| GO:0045913 | BIOLOGICAL_PROCESS | positive regulation of carbohydrate metabolic process | 8.98E-04 | 8.25E-05 |
| GO:0002761 | BIOLOGICAL_PROCESS | regulation of myeloid leukocyte differentiation | 8.98E-04 | 8.25E-05 |
| GO:2000826 | BIOLOGICAL_PROCESS | regulation of heart morphogenesis | 8.98E-04 | 8.25E-05 |
| GO:0031058 | BIOLOGICAL_PROCESS | positive regulation of histone modification | 8.98E-04 | 8.25E-05 |
| GO:1903845 | BIOLOGICAL_PROCESS | negative regulation of cellular response to transforming growth factor beta stimulus | 8.98E-04 | 8.27E-05 |
| GO:2001234 | BIOLOGICAL_PROCESS | negative regulation of apoptotic signaling pathway | 8.98E-04 | 8.27E-05 |
| GO:0030512 | BIOLOGICAL_PROCESS | negative regulation of transforming growth factor beta receptor signaling pathway | 8.98E-04 | 8.27E-05 |
| GO:0046872 | MOLECULAR_FUNCTION | metal ion binding | 9.16E-04 | 8.45E-05 |
| GO:1902578 | BIOLOGICAL_PROCESS | single-organism localization | 9.45E-04 | 8.72E-05 |
| GO:0042113 | BIOLOGICAL_PROCESS | B cell activation | 9.96E-04 | 9.27E-05 |
| GO:0071241 | BIOLOGICAL_PROCESS | cellular response to inorganic substance | 9.96E-04 | 9.27E-05 |
| GO:0042472 | BIOLOGICAL_PROCESS | inner ear morphogenesis | 9.96E-04 | 9.27E-05 |
| GO:0008406 | BIOLOGICAL_PROCESS | gonad development | 9.96E-04 | 9.27E-05 |
| GO:0022612 | BIOLOGICAL_PROCESS | gland morphogenesis | 9.96E-04 | 9.27E-05 |
| GO:0008544 | BIOLOGICAL_PROCESS | epidermis development | 9.96E-04 | 9.27E-05 |
| GO:0016917 | MOLECULAR_FUNCTION | GABA receptor activity | 9.96E-04 | 9.27E-05 |
| GO:0045137 | BIOLOGICAL_PROCESS | development of primary sexual characteristics | 9.96E-04 | 9.27E-05 |
| GO:1902105 | BIOLOGICAL_PROCESS | regulation of leukocyte differentiation | 9.96E-04 | 9.27E-05 |
| GO:0001702 | BIOLOGICAL_PROCESS | gastrulation with mouth forming second | 0.001008 | 9.50E-05 |
| GO:0051966 | BIOLOGICAL_PROCESS | regulation of synaptic transmission, glutamatergic | 0.001008 | 9.50E-05 |
| GO:0043500 | BIOLOGICAL_PROCESS | muscle adaptation | 0.001008 | 9.50E-05 |
| GO:0071248 | BIOLOGICAL_PROCESS | cellular response to metal ion | 0.001008 | 9.50E-05 |
| GO:0048844 | BIOLOGICAL_PROCESS | artery morphogenesis | 0.001008 | 9.50E-05 |
| GO:0010657 | BIOLOGICAL_PROCESS | muscle cell apoptotic process | 0.001008 | 9.50E-05 |
| GO:0032091 | BIOLOGICAL_PROCESS | negative regulation of protein binding | 0.001008 | 9.50E-05 |
| GO:0090544 | CELLULAR_COMPONENT | BAF-type complex | 0.001008 | 9.50E-05 |
| GO:0022600 | BIOLOGICAL_PROCESS | digestive system process | 0.001008 | 9.50E-05 |
| GO:0008286 | BIOLOGICAL_PROCESS | insulin receptor signaling pathway | 0.001008 | 9.50E-05 |
| GO:0004743 | MOLECULAR_FUNCTION | pyruvate kinase activity | 0.001008 | 9.50E-05 |
| GO:0021782 | BIOLOGICAL_PROCESS | glial cell development | 0.001008 | 9.50E-05 |
| GO:0043204 | CELLULAR_COMPONENT | perikaryon | 0.001008 | 9.50E-05 |
| GO:0031570 | BIOLOGICAL_PROCESS | DNA integrity checkpoint | 0.001023 | 9.69E-05 |
| GO:0042593 | BIOLOGICAL_PROCESS | glucose homeostasis | 0.001023 | 9.69E-05 |
| GO:0033500 | BIOLOGICAL_PROCESS | carbohydrate homeostasis | 0.001023 | 9.69E-05 |
| GO:0018022 | BIOLOGICAL_PROCESS | peptidyl-lysine methylation | 0.001023 | 9.69E-05 |
| GO:0034968 | BIOLOGICAL_PROCESS | histone lysine methylation | 0.001023 | 9.69E-05 |
| GO:0071705 | BIOLOGICAL_PROCESS | nitrogen compound transport | 0.001036 | 9.82E-05 |
| GO:0044425 | CELLULAR_COMPONENT | membrane part | 0.001047 | 9.93E-05 |
| GO:0044449 | CELLULAR_COMPONENT | contractile fiber part | 0.001051 | 9.98E-05 |
| GO:0000075 | BIOLOGICAL_PROCESS | cell cycle checkpoint | 0.001103 | 1.05E-04 |
| GO:0007010 | BIOLOGICAL_PROCESS | cytoskeleton organization | 0.00116 | 1.10E-04 |
| GO:0008270 | MOLECULAR_FUNCTION | zinc ion binding | 0.001215 | 1.16E-04 |
| GO:0019898 | CELLULAR_COMPONENT | extrinsic component of membrane | 0.001232 | 1.17E-04 |
| GO:0009894 | BIOLOGICAL_PROCESS | regulation of catabolic process | 0.001313 | 1.25E-04 |
| GO:0045088 | BIOLOGICAL_PROCESS | regulation of innate immune response | 0.001326 | 1.27E-04 |
| GO:1902806 | BIOLOGICAL_PROCESS | regulation of cell cycle G1/S phase transition | 0.001326 | 1.27E-04 |
| GO:0001933 | BIOLOGICAL_PROCESS | negative regulation of protein phosphorylation | 0.001358 | 1.30E-04 |
| GO:0010942 | BIOLOGICAL_PROCESS | positive regulation of cell death | 0.001358 | 1.30E-04 |
| GO:0016740 | MOLECULAR_FUNCTION | transferase activity | 0.001407 | 1.35E-04 |
| GO:0050778 | BIOLOGICAL_PROCESS | positive regulation of immune response | 0.001433 | 1.38E-04 |
| GO:0071214 | BIOLOGICAL_PROCESS | cellular response to abiotic stimulus | 0.001433 | 1.38E-04 |
| GO:0043225 | MOLECULAR_FUNCTION | ATPase-coupled anion transmembrane transporter activity | 0.001433 | 1.38E-04 |
| GO:0007156 | BIOLOGICAL_PROCESS | homophilic cell adhesion via plasma membrane adhesion molecules | 0.00148 | 1.42E-04 |
| GO:0071345 | BIOLOGICAL_PROCESS | cellular response to cytokine stimulus | 0.001495 | 1.44E-04 |
| GO:0043169 | MOLECULAR_FUNCTION | cation binding | 0.001553 | 1.50E-04 |
| GO:0044764 | BIOLOGICAL_PROCESS | multi-organism cellular process | 0.001562 | 1.51E-04 |
| GO:0007015 | BIOLOGICAL_PROCESS | actin filament organization | 0.001642 | 1.58E-04 |
| GO:0005509 | MOLECULAR_FUNCTION | calcium ion binding | 0.001674 | 1.62E-04 |
| GO:0031224 | CELLULAR_COMPONENT | intrinsic component of membrane | 0.001675 | 1.62E-04 |
| GO:0050773 | BIOLOGICAL_PROCESS | regulation of dendrite development | 0.001684 | 1.63E-04 |
| GO:0043902 | BIOLOGICAL_PROCESS | positive regulation of multi-organism process | 0.001684 | 1.63E-04 |
| GO:0071478 | BIOLOGICAL_PROCESS | cellular response to radiation | 0.001684 | 1.63E-04 |
| GO:0061053 | BIOLOGICAL_PROCESS | somite development | 0.001684 | 1.63E-04 |
| GO:0008015 | BIOLOGICAL_PROCESS | blood circulation | 0.001684 | 1.64E-04 |
| GO:0003013 | BIOLOGICAL_PROCESS | circulatory system process | 0.001684 | 1.64E-04 |
| GO:0010564 | BIOLOGICAL_PROCESS | regulation of cell cycle process | 0.001693 | 1.65E-04 |
| GO:0016021 | CELLULAR_COMPONENT | integral component of membrane | 0.00174 | 1.70E-04 |
| GO:0007600 | BIOLOGICAL_PROCESS | sensory perception | 0.00174 | 1.69E-04 |
| GO:0016311 | BIOLOGICAL_PROCESS | dephosphorylation | 0.00176 | 1.72E-04 |
| GO:0010563 | BIOLOGICAL_PROCESS | negative regulation of phosphorus metabolic process | 0.001768 | 1.73E-04 |
| GO:0045936 | BIOLOGICAL_PROCESS | negative regulation of phosphate metabolic process | 0.001768 | 1.73E-04 |
| GO:0033044 | BIOLOGICAL_PROCESS | regulation of chromosome organization | 0.001936 | 1.90E-04 |
| GO:0002253 | BIOLOGICAL_PROCESS | activation of immune response | 0.001936 | 1.90E-04 |
| GO:0002757 | BIOLOGICAL_PROCESS | immune response-activating signal transduction | 0.001936 | 1.90E-04 |
| GO:0031667 | BIOLOGICAL_PROCESS | response to nutrient levels | 0.001951 | 1.92E-04 |
| GO:0098984 | CELLULAR_COMPONENT | neuron to neuron synapse | 0.001951 | 1.92E-04 |
| GO:0014069 | CELLULAR_COMPONENT | postsynaptic density | 0.001951 | 1.92E-04 |
| GO:0045216 | BIOLOGICAL_PROCESS | cell-cell junction organization | 0.001951 | 1.92E-04 |
| GO:0010976 | BIOLOGICAL_PROCESS | positive regulation of neuron projection development | 0.001951 | 1.92E-04 |
| GO:0019216 | BIOLOGICAL_PROCESS | regulation of lipid metabolic process | 0.001951 | 1.92E-04 |
| GO:0032279 | CELLULAR_COMPONENT | asymmetric synapse | 0.001951 | 1.92E-04 |
| GO:2000179 | BIOLOGICAL_PROCESS | positive regulation of neural precursor cell proliferation | 0.00196 | 2.00E-04 |
| GO:0030308 | BIOLOGICAL_PROCESS | negative regulation of cell growth | 0.00196 | 2.00E-04 |
| GO:0030331 | MOLECULAR_FUNCTION | estrogen receptor binding | 0.00196 | 2.00E-04 |
| GO:0050707 | BIOLOGICAL_PROCESS | regulation of cytokine secretion | 0.00196 | 2.00E-04 |
| GO:0048536 | BIOLOGICAL_PROCESS | spleen development | 0.00196 | 2.00E-04 |
| GO:1903708 | BIOLOGICAL_PROCESS | positive regulation of hemopoiesis | 0.00196 | 2.00E-04 |
| GO:0035240 | MOLECULAR_FUNCTION | dopamine binding | 0.00196 | 2.00E-04 |
| GO:0001588 | MOLECULAR_FUNCTION | dopamine neurotransmitter receptor activity, coupled via Gs | 0.00196 | 2.00E-04 |
| GO:0050663 | BIOLOGICAL_PROCESS | cytokine secretion | 0.00196 | 2.00E-04 |
| GO:0001696 | BIOLOGICAL_PROCESS | gastric acid secretion | 0.00196 | 2.00E-04 |
| GO:0071236 | BIOLOGICAL_PROCESS | cellular response to antibiotic | 0.00196 | 2.00E-04 |
| GO:0003171 | BIOLOGICAL_PROCESS | atrioventricular valve development | 0.00196 | 2.00E-04 |
| GO:0048880 | BIOLOGICAL_PROCESS | sensory system development | 0.00196 | 2.00E-04 |
| GO:0071361 | BIOLOGICAL_PROCESS | cellular response to ethanol | 0.00196 | 2.00E-04 |
| GO:0003215 | BIOLOGICAL_PROCESS | cardiac right ventricle morphogenesis | 0.00196 | 2.00E-04 |
| GO:0042800 | MOLECULAR_FUNCTION | histone methyltransferase activity (H3-K4 specific) | 0.00196 | 2.00E-04 |
| GO:0015277 | MOLECULAR_FUNCTION | kainate selective glutamate receptor activity | 0.00196 | 2.00E-04 |
| GO:0030879 | BIOLOGICAL_PROCESS | mammary gland development | 0.00196 | 2.00E-04 |
| GO:0048935 | BIOLOGICAL_PROCESS | peripheral nervous system neuron development | 0.00196 | 2.00E-04 |
| GO:0048934 | BIOLOGICAL_PROCESS | peripheral nervous system neuron differentiation | 0.00196 | 2.00E-04 |
| GO:0048678 | BIOLOGICAL_PROCESS | response to axon injury | 0.00196 | 2.00E-04 |
| GO:0030500 | BIOLOGICAL_PROCESS | regulation of bone mineralization | 0.00196 | 2.00E-04 |
| GO:0090103 | BIOLOGICAL_PROCESS | cochlea morphogenesis | 0.00196 | 2.00E-04 |
| GO:0022038 | BIOLOGICAL_PROCESS | corpus callosum development | 0.00196 | 2.00E-04 |
| GO:1901338 | MOLECULAR_FUNCTION | catecholamine binding | 0.00196 | 2.00E-04 |
| GO:0021514 | BIOLOGICAL_PROCESS | ventral spinal cord interneuron differentiation | 0.00196 | 2.00E-04 |
| GO:0007207 | BIOLOGICAL_PROCESS | phospholipase C-activating G-protein coupled acetylcholine receptor signaling pathway | 0.00196 | 2.00E-04 |
| GO:1901985 | BIOLOGICAL_PROCESS | positive regulation of protein acetylation | 0.00196 | 2.00E-04 |
| GO:0007197 | BIOLOGICAL_PROCESS | adenylate cyclase-inhibiting G-protein coupled acetylcholine receptor signaling pathway | 0.00196 | 2.00E-04 |
| GO:0010613 | BIOLOGICAL_PROCESS | positive regulation of cardiac muscle hypertrophy | 0.00196 | 2.00E-04 |
| GO:0071542 | BIOLOGICAL_PROCESS | dopaminergic neuron differentiation | 0.00196 | 2.00E-04 |
| GO:0045815 | BIOLOGICAL_PROCESS | positive regulation of gene expression, epigenetic | 0.00196 | 2.00E-04 |
| GO:2000648 | BIOLOGICAL_PROCESS | positive regulation of stem cell proliferation | 0.00196 | 2.00E-04 |
| GO:2000677 | BIOLOGICAL_PROCESS | regulation of transcription regulatory region DNA binding | 0.00196 | 2.00E-04 |
| GO:0014742 | BIOLOGICAL_PROCESS | positive regulation of muscle hypertrophy | 0.00196 | 2.00E-04 |
| GO:0070888 | MOLECULAR_FUNCTION | E-box binding | 0.00196 | 2.00E-04 |
| GO:0051531 | BIOLOGICAL_PROCESS | NFAT protein import into nucleus | 0.00196 | 2.00E-04 |
| GO:0045669 | BIOLOGICAL_PROCESS | positive regulation of osteoblast differentiation | 0.00196 | 2.00E-04 |
| GO:0043292 | CELLULAR_COMPONENT | contractile fiber | 0.001964 | 2.01E-04 |
| GO:0046982 | MOLECULAR_FUNCTION | protein heterodimerization activity | 0.001964 | 2.01E-04 |
| GO:0030307 | BIOLOGICAL_PROCESS | positive regulation of cell growth | 0.00201 | 2.07E-04 |
| GO:0050878 | BIOLOGICAL_PROCESS | regulation of body fluid levels | 0.00201 | 2.07E-04 |
| GO:0001843 | BIOLOGICAL_PROCESS | neural tube closure | 0.00201 | 2.07E-04 |
| GO:0060606 | BIOLOGICAL_PROCESS | tube closure | 0.00201 | 2.07E-04 |
| GO:0060840 | BIOLOGICAL_PROCESS | artery development | 0.00201 | 2.07E-04 |
| GO:0021675 | BIOLOGICAL_PROCESS | nerve development | 0.00201 | 2.07E-04 |
| GO:0045665 | BIOLOGICAL_PROCESS | negative regulation of neuron differentiation | 0.00201 | 2.07E-04 |
| GO:0031329 | BIOLOGICAL_PROCESS | regulation of cellular catabolic process | 0.002038 | 2.10E-04 |
| GO:0031400 | BIOLOGICAL_PROCESS | negative regulation of protein modification process | 0.002138 | 2.20E-04 |
| GO:0031674 | CELLULAR_COMPONENT | I band | 0.002437 | 2.54E-04 |
| GO:0001756 | BIOLOGICAL_PROCESS | somitogenesis | 0.002437 | 2.54E-04 |
| GO:0006476 | BIOLOGICAL_PROCESS | protein deacetylation | 0.002437 | 2.54E-04 |
| GO:2000241 | BIOLOGICAL_PROCESS | regulation of reproductive process | 0.002437 | 2.54E-04 |
| GO:0016278 | MOLECULAR_FUNCTION | lysine N-methyltransferase activity | 0.002437 | 2.54E-04 |
| GO:0016279 | MOLECULAR_FUNCTION | protein-lysine N-methyltransferase activity | 0.002437 | 2.54E-04 |
| GO:0042471 | BIOLOGICAL_PROCESS | ear morphogenesis | 0.002437 | 2.54E-04 |
| GO:0010675 | BIOLOGICAL_PROCESS | regulation of cellular carbohydrate metabolic process | 0.002437 | 2.54E-04 |
| GO:0044033 | BIOLOGICAL_PROCESS | multi-organism metabolic process | 0.002437 | 2.54E-04 |
| GO:0007159 | BIOLOGICAL_PROCESS | leukocyte cell-cell adhesion | 0.002437 | 2.54E-04 |
| GO:0071482 | BIOLOGICAL_PROCESS | cellular response to light stimulus | 0.002437 | 2.54E-04 |
| GO:0015749 | BIOLOGICAL_PROCESS | monosaccharide transport | 0.002437 | 2.53E-04 |
| GO:0018024 | MOLECULAR_FUNCTION | histone-lysine N-methyltransferase activity | 0.002437 | 2.54E-04 |
| GO:0098590 | CELLULAR_COMPONENT | plasma membrane region | 0.002464 | 2.57E-04 |
| GO:0008361 | BIOLOGICAL_PROCESS | regulation of cell size | 0.002479 | 2.59E-04 |
| GO:0022407 | BIOLOGICAL_PROCESS | regulation of cell-cell adhesion | 0.002479 | 2.59E-04 |
| GO:0006323 | BIOLOGICAL_PROCESS | DNA packaging | 0.002479 | 2.59E-04 |
| GO:0001889 | BIOLOGICAL_PROCESS | liver development | 0.002491 | 2.61E-04 |
| GO:0051248 | BIOLOGICAL_PROCESS | negative regulation of protein metabolic process | 0.002491 | 2.61E-04 |
| GO:0061008 | BIOLOGICAL_PROCESS | hepaticobiliary system development | 0.002491 | 2.61E-04 |
| GO:0044427 | CELLULAR_COMPONENT | chromosomal part | 0.002491 | 2.61E-04 |
| GO:0031252 | CELLULAR_COMPONENT | cell leading edge | 0.002491 | 2.61E-04 |
| GO:0051051 | BIOLOGICAL_PROCESS | negative regulation of transport | 0.002668 | 2.80E-04 |
| GO:0044765 | BIOLOGICAL_PROCESS | single-organism transport | 0.002672 | 2.81E-04 |
| GO:0033554 | BIOLOGICAL_PROCESS | cellular response to stress | 0.002743 | 2.88E-04 |
| GO:0098552 | CELLULAR_COMPONENT | side of membrane | 0.002774 | 2.92E-04 |
| GO:0072148 | BIOLOGICAL_PROCESS | epithelial cell fate commitment | 0.002801 | 3.05E-04 |
| GO:0072164 | BIOLOGICAL_PROCESS | mesonephric tubule development | 0.002801 | 3.05E-04 |
| GO:0072163 | BIOLOGICAL_PROCESS | mesonephric epithelium development | 0.002801 | 3.05E-04 |
| GO:0035249 | BIOLOGICAL_PROCESS | synaptic transmission, glutamatergic | 0.002801 | 2.98E-04 |
| GO:0061245 | BIOLOGICAL_PROCESS | establishment or maintenance of bipolar cell polarity | 0.002801 | 3.05E-04 |
| GO:0043388 | BIOLOGICAL_PROCESS | positive regulation of DNA binding | 0.002801 | 3.05E-04 |
| GO:2000243 | BIOLOGICAL_PROCESS | positive regulation of reproductive process | 0.002801 | 3.05E-04 |
| GO:0035088 | BIOLOGICAL_PROCESS | establishment or maintenance of apical/basal cell polarity | 0.002801 | 3.05E-04 |
| GO:0035065 | BIOLOGICAL_PROCESS | regulation of histone acetylation | 0.002801 | 3.05E-04 |
| GO:0043525 | BIOLOGICAL_PROCESS | positive regulation of neuron apoptotic process | 0.002801 | 3.05E-04 |
| GO:0072080 | BIOLOGICAL_PROCESS | nephron tubule development | 0.002801 | 3.05E-04 |
| GO:1901654 | BIOLOGICAL_PROCESS | response to ketone | 0.002801 | 2.98E-04 |
| GO:0071331 | BIOLOGICAL_PROCESS | cellular response to hexose stimulus | 0.002801 | 3.05E-04 |
| GO:0071326 | BIOLOGICAL_PROCESS | cellular response to monosaccharide stimulus | 0.002801 | 3.05E-04 |
| GO:0048645 | BIOLOGICAL_PROCESS | animal organ formation | 0.002801 | 2.98E-04 |
| GO:0048665 | BIOLOGICAL_PROCESS | neuron fate specification | 0.002801 | 3.05E-04 |
| GO:0048708 | BIOLOGICAL_PROCESS | astrocyte differentiation | 0.002801 | 3.05E-04 |
| GO:0060420 | BIOLOGICAL_PROCESS | regulation of heart growth | 0.002801 | 3.05E-04 |
| GO:0060421 | BIOLOGICAL_PROCESS | positive regulation of heart growth | 0.002801 | 3.05E-04 |
| GO:0060419 | BIOLOGICAL_PROCESS | heart growth | 0.002801 | 2.98E-04 |
| GO:0010676 | BIOLOGICAL_PROCESS | positive regulation of cellular carbohydrate metabolic process | 0.002801 | 3.05E-04 |
| GO:0010827 | BIOLOGICAL_PROCESS | regulation of glucose transport | 0.002801 | 3.05E-04 |
| GO:0007173 | BIOLOGICAL_PROCESS | epidermal growth factor receptor signaling pathway | 0.002801 | 2.98E-04 |
| GO:0042990 | BIOLOGICAL_PROCESS | regulation of transcription factor import into nucleus | 0.002801 | 2.98E-04 |
| GO:0042991 | BIOLOGICAL_PROCESS | transcription factor import into nucleus | 0.002801 | 2.98E-04 |
| GO:0033146 | BIOLOGICAL_PROCESS | regulation of intracellular estrogen receptor signaling pathway | 0.002801 | 3.05E-04 |
| GO:0032526 | BIOLOGICAL_PROCESS | response to retinoic acid | 0.002801 | 3.05E-04 |
| GO:0021761 | BIOLOGICAL_PROCESS | limbic system development | 0.002801 | 2.98E-04 |
| GO:0007599 | BIOLOGICAL_PROCESS | hemostasis | 0.002801 | 2.98E-04 |
| GO:0051702 | BIOLOGICAL_PROCESS | interaction with symbiont | 0.002801 | 2.98E-04 |
| GO:0010907 | BIOLOGICAL_PROCESS | positive regulation of glucose metabolic process | 0.002801 | 3.05E-04 |
| GO:0046889 | BIOLOGICAL_PROCESS | positive regulation of lipid biosynthetic process | 0.002801 | 3.05E-04 |
| GO:0045598 | BIOLOGICAL_PROCESS | regulation of fat cell differentiation | 0.002801 | 3.05E-04 |
| GO:2000756 | BIOLOGICAL_PROCESS | regulation of peptidyl-lysine acetylation | 0.002801 | 3.05E-04 |
| GO:0045685 | BIOLOGICAL_PROCESS | regulation of glial cell differentiation | 0.002801 | 3.05E-04 |
| GO:0055008 | BIOLOGICAL_PROCESS | cardiac muscle tissue morphogenesis | 0.002801 | 3.05E-04 |
| GO:0055021 | BIOLOGICAL_PROCESS | regulation of cardiac muscle tissue growth | 0.002801 | 3.05E-04 |
| GO:0055023 | BIOLOGICAL_PROCESS | positive regulation of cardiac muscle tissue growth | 0.002801 | 3.05E-04 |
| GO:0055017 | BIOLOGICAL_PROCESS | cardiac muscle tissue growth | 0.002801 | 2.98E-04 |
| GO:0055025 | BIOLOGICAL_PROCESS | positive regulation of cardiac muscle tissue development | 0.002801 | 3.05E-04 |
| GO:0006757 | BIOLOGICAL_PROCESS | ATP generation from ADP | 0.002905 | 3.17E-04 |
| GO:0006096 | BIOLOGICAL_PROCESS | glycolytic process | 0.002905 | 3.17E-04 |
| GO:0015629 | CELLULAR_COMPONENT | actin cytoskeleton | 0.002929 | 3.19E-04 |
| GO:1901216 | BIOLOGICAL_PROCESS | positive regulation of neuron death | 0.002934 | 3.24E-04 |
| GO:0042308 | BIOLOGICAL_PROCESS | negative regulation of protein import into nucleus | 0.002934 | 3.24E-04 |
| GO:0001709 | BIOLOGICAL_PROCESS | cell fate determination | 0.002934 | 3.24E-04 |
| GO:0043502 | BIOLOGICAL_PROCESS | regulation of muscle adaptation | 0.002934 | 3.24E-04 |
| GO:0035097 | CELLULAR_COMPONENT | histone methyltransferase complex | 0.002934 | 3.24E-04 |
| GO:0004435 | MOLECULAR_FUNCTION | phosphatidylinositol phospholipase C activity | 0.002934 | 3.24E-04 |
| GO:0042733 | BIOLOGICAL_PROCESS | embryonic digit morphogenesis | 0.002934 | 3.24E-04 |
| GO:0003281 | BIOLOGICAL_PROCESS | ventricular septum development | 0.002934 | 3.24E-04 |
| GO:0008013 | MOLECULAR_FUNCTION | beta-catenin binding | 0.002934 | 3.24E-04 |
| GO:1900181 | BIOLOGICAL_PROCESS | negative regulation of protein localization to nucleus | 0.002934 | 3.24E-04 |
| GO:0007217 | BIOLOGICAL_PROCESS | tachykinin receptor signaling pathway | 0.002934 | 3.24E-04 |
| GO:0046717 | BIOLOGICAL_PROCESS | acid secretion | 0.002934 | 3.24E-04 |
| GO:0045471 | BIOLOGICAL_PROCESS | response to ethanol | 0.002934 | 3.24E-04 |
| GO:0014013 | BIOLOGICAL_PROCESS | regulation of gliogenesis | 0.002934 | 3.24E-04 |
| GO:1904590 | BIOLOGICAL_PROCESS | negative regulation of protein import | 0.002934 | 3.24E-04 |
| GO:0046823 | BIOLOGICAL_PROCESS | negative regulation of nucleocytoplasmic transport | 0.002934 | 3.24E-04 |
| GO:0002764 | BIOLOGICAL_PROCESS | immune response-regulating signaling pathway | 0.002972 | 3.29E-04 |
| GO:0001738 | BIOLOGICAL_PROCESS | morphogenesis of a polarized epithelium | 0.003118 | 3.46E-04 |
| GO:2000045 | BIOLOGICAL_PROCESS | regulation of G1/S transition of mitotic cell cycle | 0.003118 | 3.46E-04 |
| GO:0051054 | BIOLOGICAL_PROCESS | positive regulation of DNA metabolic process | 0.003118 | 3.46E-04 |
| GO:0019233 | BIOLOGICAL_PROCESS | sensory perception of pain | 0.003118 | 3.46E-04 |
| GO:0016604 | CELLULAR_COMPONENT | nuclear body | 0.003187 | 3.54E-04 |
| GO:0043068 | BIOLOGICAL_PROCESS | positive regulation of programmed cell death | 0.003187 | 3.54E-04 |
| GO:0050708 | BIOLOGICAL_PROCESS | regulation of protein secretion | 0.003229 | 3.59E-04 |
| GO:0034330 | BIOLOGICAL_PROCESS | cell junction organization | 0.003229 | 3.59E-04 |
| GO:0032269 | BIOLOGICAL_PROCESS | negative regulation of cellular protein metabolic process | 0.003265 | 3.63E-04 |
| GO:0051348 | BIOLOGICAL_PROCESS | negative regulation of transferase activity | 0.003435 | 3.83E-04 |
| GO:0032535 | BIOLOGICAL_PROCESS | regulation of cellular component size | 0.003435 | 3.83E-04 |
| GO:0032991 | CELLULAR_COMPONENT | macromolecular complex | 0.003865 | 4.31E-04 |
| GO:0030326 | BIOLOGICAL_PROCESS | embryonic limb morphogenesis | 0.004064 | 4.57E-04 |
| GO:0035113 | BIOLOGICAL_PROCESS | embryonic appendage morphogenesis | 0.004064 | 4.57E-04 |
| GO:0035108 | BIOLOGICAL_PROCESS | limb morphogenesis | 0.004064 | 4.57E-04 |
| GO:0042770 | BIOLOGICAL_PROCESS | signal transduction in response to DNA damage | 0.004064 | 4.57E-04 |
| GO:0002218 | BIOLOGICAL_PROCESS | activation of innate immune response | 0.004064 | 4.57E-04 |
| GO:0051224 | BIOLOGICAL_PROCESS | negative regulation of protein transport | 0.004064 | 4.57E-04 |
| GO:0010639 | BIOLOGICAL_PROCESS | negative regulation of organelle organization | 0.004064 | 4.57E-04 |
| GO:0045444 | BIOLOGICAL_PROCESS | fat cell differentiation | 0.004064 | 4.57E-04 |
| GO:0022411 | BIOLOGICAL_PROCESS | cellular component disassembly | 0.004064 | 4.55E-04 |
| GO:0002758 | BIOLOGICAL_PROCESS | innate immune response-activating signal transduction | 0.004064 | 4.57E-04 |
| GO:0042623 | MOLECULAR_FUNCTION | ATPase activity, coupled | 0.004286 | 4.83E-04 |
| GO:0021700 | BIOLOGICAL_PROCESS | developmental maturation | 0.004375 | 4.93E-04 |
| GO:0061136 | BIOLOGICAL_PROCESS | regulation of proteasomal protein catabolic process | 0.00485 | 5.47E-04 |
| GO:0034504 | BIOLOGICAL_PROCESS | protein localization to nucleus | 0.005026 | 5.67E-04 |
| GO:0098732 | BIOLOGICAL_PROCESS | macromolecule deacylation | 0.005243 | 5.96E-04 |
| GO:0030330 | BIOLOGICAL_PROCESS | DNA damage response, signal transduction by p53 class mediator | 0.005243 | 5.96E-04 |
| GO:0001894 | BIOLOGICAL_PROCESS | tissue homeostasis | 0.005243 | 5.96E-04 |
| GO:0031345 | BIOLOGICAL_PROCESS | negative regulation of cell projection organization | 0.005243 | 5.96E-04 |
| GO:0035601 | BIOLOGICAL_PROCESS | protein deacylation | 0.005243 | 5.96E-04 |
| GO:0097696 | BIOLOGICAL_PROCESS | STAT cascade | 0.005243 | 5.96E-04 |
| GO:0007193 | BIOLOGICAL_PROCESS | adenylate cyclase-inhibiting G-protein coupled receptor signaling pathway | 0.005243 | 5.96E-04 |
| GO:0030968 | BIOLOGICAL_PROCESS | endoplasmic reticulum unfolded protein response | 0.005243 | 5.96E-04 |
| GO:0007259 | BIOLOGICAL_PROCESS | JAK-STAT cascade | 0.005243 | 5.96E-04 |
| GO:0046031 | BIOLOGICAL_PROCESS | ADP metabolic process | 0.005488 | 6.26E-04 |
| GO:0019058 | BIOLOGICAL_PROCESS | viral life cycle | 0.005488 | 6.26E-04 |
| GO:0009607 | BIOLOGICAL_PROCESS | response to biotic stimulus | 0.005488 | 6.26E-04 |
| GO:0009898 | CELLULAR_COMPONENT | cytoplasmic side of plasma membrane | 0.005488 | 6.26E-04 |
| GO:1903050 | BIOLOGICAL_PROCESS | regulation of proteolysis involved in cellular protein catabolic process | 0.005488 | 6.26E-04 |
| GO:0031346 | BIOLOGICAL_PROCESS | positive regulation of cell projection organization | 0.005557 | 6.35E-04 |
| GO:0007163 | BIOLOGICAL_PROCESS | establishment or maintenance of cell polarity | 0.005557 | 6.35E-04 |
| GO:0008276 | MOLECULAR_FUNCTION | protein methyltransferase activity | 0.005557 | 6.35E-04 |
| GO:1903561 | CELLULAR_COMPONENT | extracellular vesicle | 0.005845 | 6.69E-04 |
| GO:0043230 | CELLULAR_COMPONENT | extracellular organelle | 0.005845 | 6.69E-04 |
| GO:0030178 | BIOLOGICAL_PROCESS | negative regulation of Wnt signaling pathway | 0.005866 | 6.74E-04 |
| GO:0034329 | BIOLOGICAL_PROCESS | cell junction assembly | 0.005866 | 6.74E-04 |
| GO:0048706 | BIOLOGICAL_PROCESS | embryonic skeletal system development | 0.005866 | 6.74E-04 |
| GO:0034599 | BIOLOGICAL_PROCESS | cellular response to oxidative stress | 0.005866 | 6.74E-04 |
| GO:0006109 | BIOLOGICAL_PROCESS | regulation of carbohydrate metabolic process | 0.005866 | 6.74E-04 |
| GO:1903362 | BIOLOGICAL_PROCESS | regulation of cellular protein catabolic process | 0.006499 | 7.48E-04 |
| GO:0043065 | BIOLOGICAL_PROCESS | positive regulation of apoptotic process | 0.006499 | 7.48E-04 |
| GO:0032872 | BIOLOGICAL_PROCESS | regulation of stress-activated MAPK cascade | 0.006562 | 7.60E-04 |
| GO:2001242 | BIOLOGICAL_PROCESS | regulation of intrinsic apoptotic signaling pathway | 0.006562 | 7.60E-04 |
| GO:0051261 | BIOLOGICAL_PROCESS | protein depolymerization | 0.006562 | 7.60E-04 |
| GO:0008366 | BIOLOGICAL_PROCESS | axon ensheathment | 0.006562 | 7.60E-04 |
| GO:0070302 | BIOLOGICAL_PROCESS | regulation of stress-activated protein kinase signaling cascade | 0.006562 | 7.60E-04 |
| GO:0007272 | BIOLOGICAL_PROCESS | ensheathment of neurons | 0.006562 | 7.60E-04 |
| GO:0003705 | MOLECULAR_FUNCTION | transcription factor activity, RNA polymerase II distal enhancer sequence-specific binding | 0.006562 | 7.60E-04 |
| GO:0016607 | CELLULAR_COMPONENT | nuclear speck | 0.006605 | 7.65E-04 |
| GO:0032922 | BIOLOGICAL_PROCESS | circadian regulation of gene expression | 0.006789 | 8.27E-04 |
| GO:0042249 | BIOLOGICAL_PROCESS | establishment of planar polarity of embryonic epithelium | 0.006789 | 8.27E-04 |
| GO:0035112 | BIOLOGICAL_PROCESS | genitalia morphogenesis | 0.006789 | 8.27E-04 |
| GO:0048532 | BIOLOGICAL_PROCESS | anatomical structure arrangement | 0.006789 | 8.27E-04 |
| GO:0090009 | BIOLOGICAL_PROCESS | primitive streak formation | 0.006789 | 8.27E-04 |
| GO:1904948 | BIOLOGICAL_PROCESS | midbrain dopaminergic neuron differentiation | 0.006789 | 8.27E-04 |
| GO:0031331 | BIOLOGICAL_PROCESS | positive regulation of cellular catabolic process | 0.006789 | 7.91E-04 |
| GO:0061339 | BIOLOGICAL_PROCESS | establishment or maintenance of monopolar cell polarity | 0.006789 | 8.27E-04 |
| GO:0035089 | BIOLOGICAL_PROCESS | establishment of apical/basal cell polarity | 0.006789 | 8.27E-04 |
| GO:0035066 | BIOLOGICAL_PROCESS | positive regulation of histone acetylation | 0.006789 | 8.27E-04 |
| GO:0060046 | BIOLOGICAL_PROCESS | regulation of acrosome reaction | 0.006789 | 8.27E-04 |
| GO:0043496 | BIOLOGICAL_PROCESS | regulation of protein homodimerization activity | 0.006789 | 8.27E-04 |
| GO:0060581 | BIOLOGICAL_PROCESS | cell fate commitment involved in pattern specification | 0.006789 | 8.27E-04 |
| GO:0010226 | BIOLOGICAL_PROCESS | response to lithium ion | 0.006789 | 8.27E-04 |
| GO:0033077 | BIOLOGICAL_PROCESS | T cell differentiation in thymus | 0.006789 | 8.27E-04 |
| GO:0030859 | BIOLOGICAL_PROCESS | polarized epithelial cell differentiation | 0.006789 | 8.27E-04 |
| GO:0003272 | BIOLOGICAL_PROCESS | endocardial cushion formation | 0.006789 | 8.27E-04 |
| GO:0048925 | BIOLOGICAL_PROCESS | lateral line system development | 0.006789 | 8.27E-04 |
| GO:0071300 | BIOLOGICAL_PROCESS | cellular response to retinoic acid | 0.006789 | 8.27E-04 |
| GO:0010092 | BIOLOGICAL_PROCESS | specification of animal organ identity | 0.006789 | 8.27E-04 |
| GO:0060324 | BIOLOGICAL_PROCESS | face development | 0.006789 | 8.27E-04 |
| GO:0060325 | BIOLOGICAL_PROCESS | face morphogenesis | 0.006789 | 8.27E-04 |
| GO:0030501 | BIOLOGICAL_PROCESS | positive regulation of bone mineralization | 0.006789 | 8.27E-04 |
| GO:1900101 | BIOLOGICAL_PROCESS | regulation of endoplasmic reticulum unfolded protein response | 0.006789 | 8.27E-04 |
| GO:0090179 | BIOLOGICAL_PROCESS | planar cell polarity pathway involved in neural tube closure | 0.006789 | 8.27E-04 |
| GO:0090177 | BIOLOGICAL_PROCESS | establishment of planar polarity involved in neural tube closure | 0.006789 | 8.27E-04 |
| GO:0090178 | BIOLOGICAL_PROCESS | regulation of establishment of planar polarity involved in neural tube closure | 0.006789 | 8.27E-04 |
| GO:0048640 | BIOLOGICAL_PROCESS | negative regulation of developmental growth | 0.006789 | 8.27E-04 |
| GO:0090183 | BIOLOGICAL_PROCESS | regulation of kidney development | 0.006789 | 8.27E-04 |
| GO:0060484 | BIOLOGICAL_PROCESS | lung-associated mesenchyme development | 0.006789 | 8.27E-04 |
| GO:0043923 | BIOLOGICAL_PROCESS | positive regulation by host of viral transcription | 0.006789 | 8.27E-04 |
| GO:0003680 | MOLECULAR_FUNCTION | AT DNA binding | 0.006789 | 8.27E-04 |
| GO:0004989 | MOLECULAR_FUNCTION | octopamine receptor activity | 0.006789 | 8.27E-04 |
| GO:0070169 | BIOLOGICAL_PROCESS | positive regulation of biomineral tissue development | 0.006789 | 8.27E-04 |
| GO:0005979 | BIOLOGICAL_PROCESS | regulation of glycogen biosynthetic process | 0.006789 | 8.27E-04 |
| GO:0060795 | BIOLOGICAL_PROCESS | cell fate commitment involved in formation of primary germ layer | 0.006789 | 8.27E-04 |
| GO:0014029 | BIOLOGICAL_PROCESS | neural crest formation | 0.006789 | 8.27E-04 |
| GO:0045198 | BIOLOGICAL_PROCESS | establishment of epithelial cell apical/basal polarity | 0.006789 | 8.27E-04 |
| GO:0002067 | BIOLOGICAL_PROCESS | glandular epithelial cell differentiation | 0.006789 | 8.27E-04 |
| GO:0090557 | BIOLOGICAL_PROCESS | establishment of endothelial intestinal barrier | 0.006789 | 8.27E-04 |
| GO:0010463 | BIOLOGICAL_PROCESS | mesenchymal cell proliferation | 0.006789 | 8.27E-04 |
| GO:0008301 | MOLECULAR_FUNCTION | DNA binding, bending | 0.006789 | 8.27E-04 |
| GO:0035914 | BIOLOGICAL_PROCESS | skeletal muscle cell differentiation | 0.006789 | 8.27E-04 |
| GO:1905276 | BIOLOGICAL_PROCESS | regulation of epithelial tube formation | 0.006789 | 8.27E-04 |
| GO:0038083 | BIOLOGICAL_PROCESS | peptidyl-tyrosine autophosphorylation | 0.006789 | 8.27E-04 |
| GO:0080154 | BIOLOGICAL_PROCESS | regulation of fertilization | 0.006789 | 8.27E-04 |
| GO:0031103 | BIOLOGICAL_PROCESS | axon regeneration | 0.006789 | 8.27E-04 |
| GO:0021781 | BIOLOGICAL_PROCESS | glial cell fate commitment | 0.006789 | 8.27E-04 |
| GO:0045725 | BIOLOGICAL_PROCESS | positive regulation of glycogen biosynthetic process | 0.006789 | 8.27E-04 |
| GO:0031128 | BIOLOGICAL_PROCESS | developmental induction | 0.006789 | 8.27E-04 |
| GO:2000679 | BIOLOGICAL_PROCESS | positive regulation of transcription regulatory region DNA binding | 0.006789 | 8.27E-04 |
| GO:0061162 | BIOLOGICAL_PROCESS | establishment of monopolar cell polarity | 0.006789 | 8.27E-04 |
| GO:0070875 | BIOLOGICAL_PROCESS | positive regulation of glycogen metabolic process | 0.006789 | 8.27E-04 |
| GO:0070873 | BIOLOGICAL_PROCESS | regulation of glycogen metabolic process | 0.006789 | 8.27E-04 |
| GO:0007340 | BIOLOGICAL_PROCESS | acrosome reaction | 0.006789 | 8.27E-04 |
| GO:0010962 | BIOLOGICAL_PROCESS | regulation of glucan biosynthetic process | 0.006789 | 8.27E-04 |
| GO:0071837 | MOLECULAR_FUNCTION | HMG box domain binding | 0.006789 | 8.27E-04 |
| GO:0033555 | BIOLOGICAL_PROCESS | multicellular organismal response to stress | 0.006789 | 8.27E-04 |
| GO:0071887 | BIOLOGICAL_PROCESS | leukocyte apoptotic process | 0.006789 | 8.27E-04 |
| GO:0051532 | BIOLOGICAL_PROCESS | regulation of NFAT protein import into nucleus | 0.006789 | 8.27E-04 |
| GO:0051534 | BIOLOGICAL_PROCESS | negative regulation of NFAT protein import into nucleus | 0.006789 | 8.27E-04 |
| GO:0051569 | BIOLOGICAL_PROCESS | regulation of histone H3-K4 methylation | 0.006789 | 8.27E-04 |
| GO:0050254 | MOLECULAR_FUNCTION | rhodopsin kinase activity | 0.006789 | 8.27E-04 |
| GO:0045600 | BIOLOGICAL_PROCESS | positive regulation of fat cell differentiation | 0.006789 | 8.27E-04 |
| GO:0018076 | BIOLOGICAL_PROCESS | N-terminal peptidyl-lysine acetylation | 0.006789 | 8.27E-04 |
| GO:0045639 | BIOLOGICAL_PROCESS | positive regulation of myeloid cell differentiation | 0.006789 | 8.27E-04 |
| GO:0031010 | CELLULAR_COMPONENT | ISWI-type complex | 0.006789 | 8.27E-04 |
| GO:2000758 | BIOLOGICAL_PROCESS | positive regulation of peptidyl-lysine acetylation | 0.006789 | 8.27E-04 |
| GO:0051493 | BIOLOGICAL_PROCESS | regulation of cytoskeleton organization | 0.006931 | 8.45E-04 |
| GO:0098794 | CELLULAR_COMPONENT | postsynapse | 0.006977 | 8.51E-04 |
| GO:0006811 | BIOLOGICAL_PROCESS | ion transport | 0.007115 | 8.69E-04 |
| GO:0003158 | BIOLOGICAL_PROCESS | endothelium development | 0.00722 | 8.84E-04 |
| GO:0031253 | CELLULAR_COMPONENT | cell projection membrane | 0.00722 | 8.84E-04 |
| GO:0018107 | BIOLOGICAL_PROCESS | peptidyl-threonine phosphorylation | 0.00722 | 8.84E-04 |
| GO:0060173 | BIOLOGICAL_PROCESS | limb development | 0.007455 | 9.19E-04 |
| GO:0001736 | BIOLOGICAL_PROCESS | establishment of planar polarity | 0.007455 | 9.19E-04 |
| GO:1904950 | BIOLOGICAL_PROCESS | negative regulation of establishment of protein localization | 0.007455 | 9.19E-04 |
| GO:0048813 | BIOLOGICAL_PROCESS | dendrite morphogenesis | 0.007455 | 9.19E-04 |
| GO:0048871 | BIOLOGICAL_PROCESS | multicellular organismal homeostasis | 0.007455 | 9.19E-04 |
| GO:0045089 | BIOLOGICAL_PROCESS | positive regulation of innate immune response | 0.007455 | 9.19E-04 |
| GO:0048704 | BIOLOGICAL_PROCESS | embryonic skeletal system morphogenesis | 0.007455 | 9.19E-04 |
| GO:0007164 | BIOLOGICAL_PROCESS | establishment of tissue polarity | 0.007455 | 9.19E-04 |
| GO:0051403 | BIOLOGICAL_PROCESS | stress-activated MAPK cascade | 0.007455 | 9.19E-04 |
| GO:0031098 | BIOLOGICAL_PROCESS | stress-activated protein kinase signaling cascade | 0.007455 | 9.19E-04 |
| GO:0031644 | BIOLOGICAL_PROCESS | regulation of neurological system process | 0.007494 | 9.35E-04 |
| GO:0051851 | BIOLOGICAL_PROCESS | modification by host of symbiont morphology or physiology | 0.007494 | 9.35E-04 |
| GO:0031420 | MOLECULAR_FUNCTION | alkali metal ion binding | 0.007494 | 9.35E-04 |
| GO:0030042 | BIOLOGICAL_PROCESS | actin filament depolymerization | 0.007494 | 9.35E-04 |
| GO:0090317 | BIOLOGICAL_PROCESS | negative regulation of intracellular protein transport | 0.007494 | 9.35E-04 |
| GO:0042826 | MOLECULAR_FUNCTION | histone deacetylase binding | 0.007494 | 9.35E-04 |
| GO:0030835 | BIOLOGICAL_PROCESS | negative regulation of actin filament depolymerization | 0.007494 | 9.35E-04 |
| GO:0030834 | BIOLOGICAL_PROCESS | regulation of actin filament depolymerization | 0.007494 | 9.35E-04 |
| GO:0090504 | BIOLOGICAL_PROCESS | epiboly | 0.007494 | 9.35E-04 |
| GO:0046332 | MOLECULAR_FUNCTION | SMAD binding | 0.007494 | 9.35E-04 |
| GO:0030955 | MOLECULAR_FUNCTION | potassium ion binding | 0.007494 | 9.35E-04 |
| GO:0002062 | BIOLOGICAL_PROCESS | chondrocyte differentiation | 0.007494 | 9.35E-04 |
| GO:0004629 | MOLECULAR_FUNCTION | phospholipase C activity | 0.007494 | 9.35E-04 |
| GO:0004712 | MOLECULAR_FUNCTION | protein serine/threonine/tyrosine kinase activity | 0.007494 | 9.35E-04 |
| GO:0051693 | BIOLOGICAL_PROCESS | actin filament capping | 0.007494 | 9.35E-04 |
| GO:0021766 | BIOLOGICAL_PROCESS | hippocampus development | 0.007494 | 9.35E-04 |
| GO:0021545 | BIOLOGICAL_PROCESS | cranial nerve development | 0.007494 | 9.35E-04 |
| GO:2000177 | BIOLOGICAL_PROCESS | regulation of neural precursor cell proliferation | 0.008503 | 0.001082643 |
| GO:1901215 | BIOLOGICAL_PROCESS | negative regulation of neuron death | 0.008503 | 0.001082643 |
| GO:0004016 | MOLECULAR_FUNCTION | adenylate cyclase activity | 0.008503 | 0.001082643 |
| GO:0030322 | BIOLOGICAL_PROCESS | stabilization of membrane potential | 0.008503 | 0.001109583 |
| GO:0001706 | BIOLOGICAL_PROCESS | endoderm formation | 0.008503 | 0.001109583 |
| GO:1902476 | BIOLOGICAL_PROCESS | chloride transmembrane transport | 0.008503 | 0.001071768 |
| GO:0004088 | MOLECULAR_FUNCTION | carbamoyl-phosphate synthase (glutamine-hydrolyzing) activity | 0.008503 | 0.001109583 |
| GO:0001818 | BIOLOGICAL_PROCESS | negative regulation of cytokine production | 0.008503 | 0.001082643 |
| GO:0048546 | BIOLOGICAL_PROCESS | digestive tract morphogenesis | 0.008503 | 0.001109583 |
| GO:0031430 | CELLULAR_COMPONENT | M band | 0.008503 | 0.001109583 |
| GO:0001569 | BIOLOGICAL_PROCESS | branching involved in blood vessel morphogenesis | 0.008503 | 0.001109583 |
| GO:0061326 | BIOLOGICAL_PROCESS | renal tubule development | 0.008503 | 0.001082643 |
| GO:0050681 | MOLECULAR_FUNCTION | androgen receptor binding | 0.008503 | 0.001109583 |
| GO:0019838 | MOLECULAR_FUNCTION | growth factor binding | 0.008503 | 0.001082643 |
| GO:0005237 | MOLECULAR_FUNCTION | inhibitory extracellular ligand-gated ion channel activity | 0.008503 | 0.001109583 |
| GO:0072091 | BIOLOGICAL_PROCESS | regulation of stem cell proliferation | 0.008503 | 0.001082643 |
| GO:0030183 | BIOLOGICAL_PROCESS | B cell differentiation | 0.008503 | 0.001109583 |
| GO:0001657 | BIOLOGICAL_PROCESS | ureteric bud development | 0.008503 | 0.001109583 |
| GO:0060561 | BIOLOGICAL_PROCESS | apoptotic process involved in morphogenesis | 0.008503 | 0.001109583 |
| GO:0003148 | BIOLOGICAL_PROCESS | outflow tract septum morphogenesis | 0.008503 | 0.001109583 |
| GO:0003209 | BIOLOGICAL_PROCESS | cardiac atrium morphogenesis | 0.008503 | 0.001082643 |
| GO:0016514 | CELLULAR_COMPONENT | SWI/SNF complex | 0.008503 | 0.001109583 |
| GO:0003230 | BIOLOGICAL_PROCESS | cardiac atrium development | 0.008503 | 0.001082643 |
| GO:0071353 | BIOLOGICAL_PROCESS | cellular response to interleukin-4 | 0.008503 | 0.001109583 |
| GO:0015276 | MOLECULAR_FUNCTION | ligand-gated ion channel activity | 0.008503 | 0.001101288 |
| GO:0016594 | MOLECULAR_FUNCTION | glycine binding | 0.008503 | 0.001109583 |
| GO:0003283 | BIOLOGICAL_PROCESS | atrial septum development | 0.008503 | 0.001109583 |
| GO:0046323 | BIOLOGICAL_PROCESS | glucose import | 0.008503 | 0.001082643 |
| GO:0046324 | BIOLOGICAL_PROCESS | regulation of glucose import | 0.008503 | 0.001109583 |
| GO:0060389 | BIOLOGICAL_PROCESS | pathway-restricted SMAD protein phosphorylation | 0.008503 | 0.001109583 |
| GO:0006828 | BIOLOGICAL_PROCESS | manganese ion transport | 0.008503 | 0.001082643 |
| GO:2001243 | BIOLOGICAL_PROCESS | negative regulation of intrinsic apoptotic signaling pathway | 0.008503 | 0.001109583 |
| GO:0060393 | BIOLOGICAL_PROCESS | regulation of pathway-restricted SMAD protein phosphorylation | 0.008503 | 0.001109583 |
| GO:1902742 | BIOLOGICAL_PROCESS | apoptotic process involved in development | 0.008503 | 0.001109583 |
| GO:0071156 | BIOLOGICAL_PROCESS | regulation of cell cycle arrest | 0.008503 | 0.001082643 |
| GO:1900076 | BIOLOGICAL_PROCESS | regulation of cellular response to insulin stimulus | 0.008503 | 0.001082643 |
| GO:0042641 | CELLULAR_COMPONENT | actomyosin | 0.008503 | 0.001109583 |
| GO:0060413 | BIOLOGICAL_PROCESS | atrial septum morphogenesis | 0.008503 | 0.001109583 |
| GO:0048713 | BIOLOGICAL_PROCESS | regulation of oligodendrocyte differentiation | 0.008503 | 0.001109583 |
| GO:0034707 | CELLULAR_COMPONENT | chloride channel complex | 0.008503 | 0.001109583 |
| GO:0019098 | BIOLOGICAL_PROCESS | reproductive behavior | 0.008503 | 0.001082643 |
| GO:0086010 | BIOLOGICAL_PROCESS | membrane depolarization during action potential | 0.008503 | 0.001109583 |
| GO:0021511 | BIOLOGICAL_PROCESS | spinal cord patterning | 0.008503 | 0.001109583 |
| GO:0022842 | MOLECULAR_FUNCTION | narrow pore channel activity | 0.008503 | 0.001109583 |
| GO:0022840 | MOLECULAR_FUNCTION | leak channel activity | 0.008503 | 0.001109583 |
| GO:0022841 | MOLECULAR_FUNCTION | potassium ion leak channel activity | 0.008503 | 0.001109583 |
| GO:0022834 | MOLECULAR_FUNCTION | ligand-gated channel activity | 0.008503 | 0.001101288 |
| GO:0016925 | BIOLOGICAL_PROCESS | protein sumoylation | 0.008503 | 0.001082643 |
| GO:0016903 | MOLECULAR_FUNCTION | oxidoreductase activity, acting on the aldehyde or oxo group of donors | 0.008503 | 0.001062213 |
| GO:0016933 | MOLECULAR_FUNCTION | extracellular-glycine-gated ion channel activity | 0.008503 | 0.001109583 |
| GO:0016934 | MOLECULAR_FUNCTION | extracellular-glycine-gated chloride channel activity | 0.008503 | 0.001109583 |
| GO:0030971 | MOLECULAR_FUNCTION | receptor tyrosine kinase binding | 0.008503 | 0.001109583 |
| GO:0045197 | BIOLOGICAL_PROCESS | establishment or maintenance of epithelial cell apical/basal polarity | 0.008503 | 0.001109583 |
| GO:1901800 | BIOLOGICAL_PROCESS | positive regulation of proteasomal protein catabolic process | 0.008503 | 0.001071768 |
| GO:0051155 | BIOLOGICAL_PROCESS | positive regulation of striated muscle cell differentiation | 0.008503 | 0.001109583 |
| GO:0010611 | BIOLOGICAL_PROCESS | regulation of cardiac muscle hypertrophy | 0.008503 | 0.001109583 |
| GO:0051055 | BIOLOGICAL_PROCESS | negative regulation of lipid biosynthetic process | 0.008503 | 0.001109583 |
| GO:0099095 | MOLECULAR_FUNCTION | ligand-gated anion channel activity | 0.008503 | 0.001082643 |
| GO:0045834 | BIOLOGICAL_PROCESS | positive regulation of lipid metabolic process | 0.008503 | 0.001082643 |
| GO:0045833 | BIOLOGICAL_PROCESS | negative regulation of lipid metabolic process | 0.008503 | 0.001082643 |
| GO:0032440 | MOLECULAR_FUNCTION | 2-alkenal reductase [NAD(P)] activity | 0.008503 | 0.001109583 |
| GO:0014743 | BIOLOGICAL_PROCESS | regulation of muscle hypertrophy | 0.008503 | 0.001109583 |
| GO:0009913 | BIOLOGICAL_PROCESS | epidermal cell differentiation | 0.008503 | 0.001082643 |
| GO:0070670 | BIOLOGICAL_PROCESS | response to interleukin-4 | 0.008503 | 0.001109583 |
| GO:0055024 | BIOLOGICAL_PROCESS | regulation of cardiac muscle tissue development | 0.008503 | 0.001082643 |
| GO:0046483 | BIOLOGICAL_PROCESS | heterocycle metabolic process | 0.008837 | 0.001153991 |
| GO:0006165 | BIOLOGICAL_PROCESS | nucleoside diphosphate phosphorylation | 0.008955 | 0.001170191 |
| GO:0043254 | BIOLOGICAL_PROCESS | regulation of protein complex assembly | 0.009079 | 0.001187183 |
| GO:0030425 | CELLULAR_COMPONENT | dendrite | 0.009097 | 0.001190285 |
| GO:0055085 | BIOLOGICAL_PROCESS | transmembrane transport | 0.009221 | 0.001207322 |
| GO:0070062 | CELLULAR_COMPONENT | extracellular exosome | 0.009264 | 0.00121386 |
| GO:0001764 | BIOLOGICAL_PROCESS | neuron migration | 0.009362 | 0.001228363 |
| GO:2001020 | BIOLOGICAL_PROCESS | regulation of response to DNA damage stimulus | 0.009362 | 0.001228363 |
| GO:0007611 | BIOLOGICAL_PROCESS | learning or memory | 0.00939 | 0.001232817 |
| GO:0043406 | BIOLOGICAL_PROCESS | positive regulation of MAP kinase activity | 0.009446 | 0.001244384 |
| GO:0030010 | BIOLOGICAL_PROCESS | establishment of cell polarity | 0.009446 | 0.001244384 |
| GO:2001252 | BIOLOGICAL_PROCESS | positive regulation of chromosome organization | 0.009446 | 0.001244384 |
| GO:0034620 | BIOLOGICAL_PROCESS | cellular response to unfolded protein | 0.009446 | 0.001244384 |
| GO:0035967 | BIOLOGICAL_PROCESS | cellular response to topologically incorrect protein | 0.009446 | 0.001244384 |
| GO:0006897 | BIOLOGICAL_PROCESS | endocytosis | 0.009634 | 0.001269923 |
| GO:0050790 | BIOLOGICAL_PROCESS | regulation of catalytic activity | 0.010108 | 0.001333387 |
| GO:0010506 | BIOLOGICAL_PROCESS | regulation of autophagy | 0.010339 | 0.001364671 |
| GO:0060249 | BIOLOGICAL_PROCESS | anatomical structure homeostasis | 0.011113 | 0.001467908 |
| GO:1902115 | BIOLOGICAL_PROCESS | regulation of organelle assembly | 0.011365 | 0.001503138 |
| GO:0018210 | BIOLOGICAL_PROCESS | peptidyl-threonine modification | 0.011365 | 0.001503138 |
| GO:0098562 | CELLULAR_COMPONENT | cytoplasmic side of membrane | 0.011662 | 0.001544536 |
| GO:0033673 | BIOLOGICAL_PROCESS | negative regulation of kinase activity | 0.011662 | 0.001544536 |
| GO:0044089 | BIOLOGICAL_PROCESS | positive regulation of cellular component biogenesis | 0.012052 | 0.001597267 |
| GO:0046903 | BIOLOGICAL_PROCESS | secretion | 0.012205 | 0.001618628 |
| GO:0017038 | BIOLOGICAL_PROCESS | protein import | 0.01226 | 0.001629061 |
| GO:0042803 | MOLECULAR_FUNCTION | protein homodimerization activity | 0.01226 | 0.001627386 |
| GO:0044389 | MOLECULAR_FUNCTION | ubiquitin-like protein ligase binding | 0.01226 | 0.001629061 |
| GO:0051015 | MOLECULAR_FUNCTION | actin filament binding | 0.012347 | 0.001642863 |
| GO:0009896 | BIOLOGICAL_PROCESS | positive regulation of catabolic process | 0.012347 | 0.001642863 |
| GO:1902904 | BIOLOGICAL_PROCESS | negative regulation of supramolecular fiber organization | 0.012509 | 0.001669979 |
| GO:0090100 | BIOLOGICAL_PROCESS | positive regulation of transmembrane receptor protein serine/threonine kinase signaling pathway | 0.012509 | 0.001669979 |
| GO:0050954 | BIOLOGICAL_PROCESS | sensory perception of mechanical stimulus | 0.012509 | 0.001669979 |
| GO:0010770 | BIOLOGICAL_PROCESS | positive regulation of cell morphogenesis involved in differentiation | 0.012509 | 0.001669979 |
| GO:0006275 | BIOLOGICAL_PROCESS | regulation of DNA replication | 0.012509 | 0.001669979 |
| GO:0031589 | BIOLOGICAL_PROCESS | cell-substrate adhesion | 0.012743 | 0.00170682 |
| GO:0048872 | BIOLOGICAL_PROCESS | homeostasis of number of cells | 0.012743 | 0.00170682 |
| GO:0001076 | MOLECULAR_FUNCTION | transcription factor activity, RNA polymerase II transcription factor binding | 0.012743 | 0.00170682 |
| GO:0005913 | CELLULAR_COMPONENT | cell-cell adherens junction | 0.012743 | 0.00170682 |
| GO:0031234 | CELLULAR_COMPONENT | extrinsic component of cytoplasmic side of plasma membrane | 0.012743 | 0.00170682 |
| GO:1903052 | BIOLOGICAL_PROCESS | positive regulation of proteolysis involved in cellular protein catabolic process | 0.012937 | 0.001733959 |
| GO:0006812 | BIOLOGICAL_PROCESS | cation transport | 0.015068 | 0.002020828 |
| GO:0001701 | BIOLOGICAL_PROCESS | in utero embryonic development | 0.015339 | 0.002059854 |
| GO:1903364 | BIOLOGICAL_PROCESS | positive regulation of cellular protein catabolic process | 0.015339 | 0.002059854 |
| GO:0009135 | BIOLOGICAL_PROCESS | purine nucleoside diphosphate metabolic process | 0.01547 | 0.002081661 |
| GO:0009179 | BIOLOGICAL_PROCESS | purine ribonucleoside diphosphate metabolic process | 0.01547 | 0.002081661 |
| GO:0009185 | BIOLOGICAL_PROCESS | ribonucleoside diphosphate metabolic process | 0.01547 | 0.002081661 |
| GO:0007162 | BIOLOGICAL_PROCESS | negative regulation of cell adhesion | 0.015667 | 0.002110904 |
| GO:0046883 | BIOLOGICAL_PROCESS | regulation of hormone secretion | 0.015667 | 0.002110904 |
| GO:0044770 | BIOLOGICAL_PROCESS | cell cycle phase transition | 0.01589 | 0.002142279 |
| GO:0006469 | BIOLOGICAL_PROCESS | negative regulation of protein kinase activity | 0.016018 | 0.002160977 |
| GO:0031669 | BIOLOGICAL_PROCESS | cellular response to nutrient levels | 0.016121 | 0.00220912 |
| GO:0050863 | BIOLOGICAL_PROCESS | regulation of T cell activation | 0.016121 | 0.00220912 |
| GO:0050817 | BIOLOGICAL_PROCESS | coagulation | 0.016121 | 0.00220912 |
| GO:0051899 | BIOLOGICAL_PROCESS | membrane depolarization | 0.016121 | 0.00220912 |
| GO:0030018 | CELLULAR_COMPONENT | Z disc | 0.016121 | 0.00220912 |
| GO:0014855 | BIOLOGICAL_PROCESS | striated muscle cell proliferation | 0.016121 | 0.00220912 |
| GO:0051817 | BIOLOGICAL_PROCESS | modification of morphology or physiology of other organism involved in symbiotic interaction | 0.016121 | 0.00220912 |
| GO:0061351 | BIOLOGICAL_PROCESS | neural precursor cell proliferation | 0.016121 | 0.00220912 |
| GO:1904892 | BIOLOGICAL_PROCESS | regulation of STAT cascade | 0.016121 | 0.00220912 |
| GO:0003170 | BIOLOGICAL_PROCESS | heart valve development | 0.016121 | 0.00220912 |
| GO:0030837 | BIOLOGICAL_PROCESS | negative regulation of actin filament polymerization | 0.016121 | 0.00220912 |
| GO:1903037 | BIOLOGICAL_PROCESS | regulation of leukocyte cell-cell adhesion | 0.016121 | 0.00220912 |
| GO:0034708 | CELLULAR_COMPONENT | methyltransferase complex | 0.016121 | 0.00220912 |
| GO:0019080 | BIOLOGICAL_PROCESS | viral gene expression | 0.016121 | 0.00220912 |
| GO:0019083 | BIOLOGICAL_PROCESS | viral transcription | 0.016121 | 0.00220912 |
| GO:1901879 | BIOLOGICAL_PROCESS | regulation of protein depolymerization | 0.016121 | 0.00220912 |
| GO:1901880 | BIOLOGICAL_PROCESS | negative regulation of protein depolymerization | 0.016121 | 0.00220912 |
| GO:0046425 | BIOLOGICAL_PROCESS | regulation of JAK-STAT cascade | 0.016121 | 0.00220912 |
| GO:0007605 | BIOLOGICAL_PROCESS | sensory perception of sound | 0.016121 | 0.00220912 |
| GO:0043242 | BIOLOGICAL_PROCESS | negative regulation of protein complex disassembly | 0.016121 | 0.00220912 |
| GO:0043200 | BIOLOGICAL_PROCESS | response to amino acid | 0.016121 | 0.00220912 |
| GO:0010906 | BIOLOGICAL_PROCESS | regulation of glucose metabolic process | 0.016121 | 0.00220912 |
| GO:0032272 | BIOLOGICAL_PROCESS | negative regulation of protein polymerization | 0.016121 | 0.00220912 |
| GO:0032387 | BIOLOGICAL_PROCESS | negative regulation of intracellular transport | 0.016121 | 0.00220912 |
| GO:0007276 | BIOLOGICAL_PROCESS | gamete generation | 0.016242 | 0.002227055 |
| GO:0042176 | BIOLOGICAL_PROCESS | regulation of protein catabolic process | 0.016295 | 0.002235808 |
| GO:0048471 | CELLULAR_COMPONENT | perinuclear region of cytoplasm | 0.016483 | 0.002263051 |
| GO:0006928 | BIOLOGICAL_PROCESS | movement of cell or subcellular component | 0.017187 | 0.00236114 |
| GO:1904888 | BIOLOGICAL_PROCESS | cranial skeletal system development | 0.017199 | 0.002368985 |
| GO:0090316 | BIOLOGICAL_PROCESS | positive regulation of intracellular protein transport | 0.017199 | 0.002368985 |
| GO:0048771 | BIOLOGICAL_PROCESS | tissue remodeling | 0.017199 | 0.002368985 |
| GO:0009791 | BIOLOGICAL_PROCESS | post-embryonic development | 0.017199 | 0.002368985 |
| GO:0036293 | BIOLOGICAL_PROCESS | response to decreased oxygen levels | 0.017693 | 0.002443242 |
| GO:0042493 | BIOLOGICAL_PROCESS | response to drug | 0.017693 | 0.002443242 |
| GO:0070482 | BIOLOGICAL_PROCESS | response to oxygen levels | 0.017693 | 0.002443242 |
| GO:0071902 | BIOLOGICAL_PROCESS | positive regulation of protein serine/threonine kinase activity | 0.017693 | 0.002443242 |
| GO:0071103 | BIOLOGICAL_PROCESS | DNA conformation change | 0.017955 | 0.002481017 |
| GO:0004386 | MOLECULAR_FUNCTION | helicase activity | 0.019048 | 0.002633686 |
| GO:0009914 | BIOLOGICAL_PROCESS | hormone transport | 0.019523 | 0.002702813 |
| GO:0034976 | BIOLOGICAL_PROCESS | response to endoplasmic reticulum stress | 0.019523 | 0.002702813 |
| GO:0019903 | MOLECULAR_FUNCTION | protein phosphatase binding | 0.020462 | 0.002852649 |
| GO:0035150 | BIOLOGICAL_PROCESS | regulation of tube size | 0.020462 | 0.002852649 |
| GO:0060038 | BIOLOGICAL_PROCESS | cardiac muscle cell proliferation | 0.020462 | 0.002852649 |
| GO:0031532 | BIOLOGICAL_PROCESS | actin cytoskeleton reorganization | 0.020462 | 0.002852649 |
| GO:0015279 | MOLECULAR_FUNCTION | store-operated calcium channel activity | 0.020462 | 0.002852649 |
| GO:0030516 | BIOLOGICAL_PROCESS | regulation of axon extension | 0.020462 | 0.002852649 |
| GO:0002221 | BIOLOGICAL_PROCESS | pattern recognition receptor signaling pathway | 0.020462 | 0.002852649 |
| GO:0034614 | BIOLOGICAL_PROCESS | cellular response to reactive oxygen species | 0.020462 | 0.002852649 |
| GO:0051149 | BIOLOGICAL_PROCESS | positive regulation of muscle cell differentiation | 0.020462 | 0.002852649 |
| GO:0007596 | BIOLOGICAL_PROCESS | blood coagulation | 0.020462 | 0.002852649 |
| GO:0001191 | MOLECULAR_FUNCTION | transcriptional repressor activity, RNA polymerase II transcription factor binding | 0.020462 | 0.002852649 |
| GO:0050890 | BIOLOGICAL_PROCESS | cognition | 0.020711 | 0.002894746 |
| GO:0098802 | CELLULAR_COMPONENT | plasma membrane receptor complex | 0.020711 | 0.002894746 |
| GO:0002252 | BIOLOGICAL_PROCESS | immune effector process | 0.020711 | 0.002894746 |
| GO:0000123 | CELLULAR_COMPONENT | histone acetyltransferase complex | 0.020711 | 0.002894746 |
| GO:0006725 | BIOLOGICAL_PROCESS | cellular aromatic compound metabolic process | 0.020884 | 0.002920782 |
| GO:0042393 | MOLECULAR_FUNCTION | histone binding | 0.021187 | 0.002968699 |
| GO:0045446 | BIOLOGICAL_PROCESS | endothelial cell differentiation | 0.021187 | 0.002968699 |
| GO:0055002 | BIOLOGICAL_PROCESS | striated muscle cell development | 0.021187 | 0.002968699 |
| GO:0006936 | BIOLOGICAL_PROCESS | muscle contraction | 0.021588 | 0.003026806 |
| GO:0032956 | BIOLOGICAL_PROCESS | regulation of actin cytoskeleton organization | 0.022135 | 0.00310749 |
| GO:1902903 | BIOLOGICAL_PROCESS | regulation of supramolecular fiber organization | 0.022135 | 0.00310749 |
| GO:0031625 | MOLECULAR_FUNCTION | ubiquitin protein ligase binding | 0.022404 | 0.003147097 |
| GO:1901222 | BIOLOGICAL_PROCESS | regulation of NIK/NF-kappaB signaling | 0.022655 | 0.003422406 |
| GO:1901224 | BIOLOGICAL_PROCESS | positive regulation of NIK/NF-kappaB signaling | 0.022655 | 0.003422406 |
| GO:1903897 | BIOLOGICAL_PROCESS | regulation of PERK-mediated unfolded protein response | 0.022655 | 0.003422406 |
| GO:0043627 | BIOLOGICAL_PROCESS | response to estrogen | 0.022655 | 0.003422406 |
| GO:0004070 | MOLECULAR_FUNCTION | aspartate carbamoyltransferase activity | 0.022655 | 0.003422406 |
| GO:0032983 | CELLULAR_COMPONENT | kainate selective glutamate receptor complex | 0.022655 | 0.003422406 |
| GO:0043586 | BIOLOGICAL_PROCESS | tongue development | 0.022655 | 0.003422406 |
| GO:0050715 | BIOLOGICAL_PROCESS | positive regulation of cytokine secretion | 0.022655 | 0.003422406 |
| GO:2000017 | BIOLOGICAL_PROCESS | positive regulation of determination of dorsal identity | 0.022655 | 0.003422406 |
| GO:2000015 | BIOLOGICAL_PROCESS | regulation of determination of dorsal identity | 0.022655 | 0.003422406 |
| GO:0098810 | BIOLOGICAL_PROCESS | neurotransmitter reuptake | 0.022655 | 0.003422406 |
| GO:0090074 | BIOLOGICAL_PROCESS | negative regulation of protein homodimerization activity | 0.022655 | 0.003422406 |
| GO:0004157 | MOLECULAR_FUNCTION | dihydropyrimidinase activity | 0.022655 | 0.003422406 |
| GO:0001885 | BIOLOGICAL_PROCESS | endothelial cell development | 0.022655 | 0.003281323 |
| GO:0090004 | BIOLOGICAL_PROCESS | positive regulation of establishment of protein localization to plasma membrane | 0.022655 | 0.003422406 |
| GO:0048263 | BIOLOGICAL_PROCESS | determination of dorsal identity | 0.022655 | 0.003422406 |
| GO:0048262 | BIOLOGICAL_PROCESS | determination of dorsal/ventral asymmetry | 0.022655 | 0.003422406 |
| GO:1990909 | CELLULAR_COMPONENT | Wnt signalosome | 0.022655 | 0.003422406 |
| GO:0043403 | BIOLOGICAL_PROCESS | skeletal muscle tissue regeneration | 0.022655 | 0.003422406 |
| GO:0031434 | MOLECULAR_FUNCTION | mitogen-activated protein kinase kinase binding | 0.022655 | 0.003422406 |
| GO:0030027 | CELLULAR_COMPONENT | lamellipodium | 0.022655 | 0.003281323 |
| GO:0070970 | BIOLOGICAL_PROCESS | interleukin-2 secretion | 0.022655 | 0.003422406 |
| GO:0045978 | BIOLOGICAL_PROCESS | negative regulation of nucleoside metabolic process | 0.022655 | 0.003422406 |
| GO:0036344 | BIOLOGICAL_PROCESS | platelet morphogenesis | 0.022655 | 0.003422406 |
| GO:1904886 | BIOLOGICAL_PROCESS | beta-catenin destruction complex disassembly | 0.022655 | 0.003422406 |
| GO:0051969 | BIOLOGICAL_PROCESS | regulation of transmission of nerve impulse | 0.022655 | 0.003422406 |
| GO:0051963 | BIOLOGICAL_PROCESS | regulation of synapse assembly | 0.022655 | 0.003422406 |
| GO:0051970 | BIOLOGICAL_PROCESS | negative regulation of transmission of nerve impulse | 0.022655 | 0.003422406 |
| GO:0061311 | BIOLOGICAL_PROCESS | cell surface receptor signaling pathway involved in heart development | 0.022655 | 0.003422406 |
| GO:0006556 | BIOLOGICAL_PROCESS | S-adenosylmethionine biosynthetic process | 0.022655 | 0.003422406 |
| GO:0030228 | MOLECULAR_FUNCTION | lipoprotein particle receptor activity | 0.022655 | 0.003422406 |
| GO:0030220 | BIOLOGICAL_PROCESS | platelet formation | 0.022655 | 0.003422406 |
| GO:1903579 | BIOLOGICAL_PROCESS | negative regulation of ATP metabolic process | 0.022655 | 0.003422406 |
| GO:0005250 | MOLECULAR_FUNCTION | A-type (transient outward) potassium channel activity | 0.022655 | 0.003422406 |
| GO:0031526 | CELLULAR_COMPONENT | brush border membrane | 0.022655 | 0.003422406 |
| GO:0001662 | BIOLOGICAL_PROCESS | behavioral fear response | 0.022655 | 0.003422406 |
| GO:0036314 | BIOLOGICAL_PROCESS | response to sterol | 0.022655 | 0.003422406 |
| GO:0036315 | BIOLOGICAL_PROCESS | cellular response to sterol | 0.022655 | 0.003422406 |
| GO:2000344 | BIOLOGICAL_PROCESS | positive regulation of acrosome reaction | 0.022655 | 0.003422406 |
| GO:0004478 | MOLECULAR_FUNCTION | methionine adenosyltransferase activity | 0.022655 | 0.003422406 |
| GO:0004468 | MOLECULAR_FUNCTION | lysine N-acetyltransferase activity, acting on acetyl phosphate as donor | 0.022655 | 0.003422406 |
| GO:0060579 | BIOLOGICAL_PROCESS | ventral spinal cord interneuron fate commitment | 0.022655 | 0.003422406 |
| GO:0071294 | BIOLOGICAL_PROCESS | cellular response to zinc ion | 0.022655 | 0.003422406 |
| GO:0048881 | BIOLOGICAL_PROCESS | mechanosensory lateral line system development | 0.022655 | 0.003422406 |
| GO:0048882 | BIOLOGICAL_PROCESS | lateral line development | 0.022655 | 0.003422406 |
| GO:0071384 | BIOLOGICAL_PROCESS | cellular response to corticosteroid stimulus | 0.022655 | 0.003422406 |
| GO:0003263 | BIOLOGICAL_PROCESS | cardioblast proliferation | 0.022655 | 0.003422406 |
| GO:0003264 | BIOLOGICAL_PROCESS | regulation of cardioblast proliferation | 0.022655 | 0.003422406 |
| GO:0003266 | BIOLOGICAL_PROCESS | regulation of secondary heart field cardioblast proliferation | 0.022655 | 0.003422406 |
| GO:0030850 | BIOLOGICAL_PROCESS | prostate gland development | 0.022655 | 0.003422406 |
| GO:0015271 | MOLECULAR_FUNCTION | outward rectifier potassium channel activity | 0.022655 | 0.003422406 |
| GO:0016589 | CELLULAR_COMPONENT | NURF complex | 0.022655 | 0.003422406 |
| GO:0048915 | BIOLOGICAL_PROCESS | posterior lateral line system development | 0.022655 | 0.003422406 |
| GO:1902766 | BIOLOGICAL_PROCESS | skeletal muscle satellite cell migration | 0.022655 | 0.003422406 |
| GO:0001938 | BIOLOGICAL_PROCESS | positive regulation of endothelial cell proliferation | 0.022655 | 0.003422406 |
| GO:0001964 | BIOLOGICAL_PROCESS | startle response | 0.022655 | 0.003422406 |
| GO:0090185 | BIOLOGICAL_PROCESS | negative regulation of kidney development | 0.022655 | 0.003422406 |
| GO:0048661 | BIOLOGICAL_PROCESS | positive regulation of smooth muscle cell proliferation | 0.022655 | 0.003422406 |
| GO:1901342 | BIOLOGICAL_PROCESS | regulation of vasculature development | 0.022655 | 0.003281323 |
| GO:1900040 | BIOLOGICAL_PROCESS | regulation of interleukin-2 secretion | 0.022655 | 0.003422406 |
| GO:0060441 | BIOLOGICAL_PROCESS | epithelial tube branching involved in lung morphogenesis | 0.022655 | 0.003422406 |
| GO:0060443 | BIOLOGICAL_PROCESS | mammary gland morphogenesis | 0.022655 | 0.003422406 |
| GO:0060438 | BIOLOGICAL_PROCESS | trachea development | 0.022655 | 0.003422406 |
| GO:0060439 | BIOLOGICAL_PROCESS | trachea morphogenesis | 0.022655 | 0.003422406 |
| GO:0006987 | BIOLOGICAL_PROCESS | activation of signaling protein activity involved in unfolded protein response | 0.022655 | 0.003422406 |
| GO:0015026 | MOLECULAR_FUNCTION | coreceptor activity | 0.022655 | 0.003422406 |
| GO:0015054 | MOLECULAR_FUNCTION | gastrin receptor activity | 0.022655 | 0.003422406 |
| GO:0042596 | BIOLOGICAL_PROCESS | fear response | 0.022655 | 0.003422406 |
| GO:0008347 | BIOLOGICAL_PROCESS | glial cell migration | 0.022655 | 0.003422406 |
| GO:0010662 | BIOLOGICAL_PROCESS | regulation of striated muscle cell apoptotic process | 0.022655 | 0.003422406 |
| GO:0002209 | BIOLOGICAL_PROCESS | behavioral defense response | 0.022655 | 0.003422406 |
| GO:0010829 | BIOLOGICAL_PROCESS | negative regulation of glucose transport | 0.022655 | 0.003422406 |
| GO:1905516 | BIOLOGICAL_PROCESS | positive regulation of fertilization | 0.022655 | 0.003422406 |
| GO:0010830 | BIOLOGICAL_PROCESS | regulation of myotube differentiation | 0.022655 | 0.003422406 |
| GO:0007183 | BIOLOGICAL_PROCESS | SMAD protein complex assembly | 0.022655 | 0.003422406 |
| GO:0007184 | BIOLOGICAL_PROCESS | SMAD protein import into nucleus | 0.022655 | 0.003422406 |
| GO:0030947 | BIOLOGICAL_PROCESS | regulation of vascular endothelial growth factor receptor signaling pathway | 0.022655 | 0.003422406 |
| GO:0004675 | MOLECULAR_FUNCTION | transmembrane receptor protein serine/threonine kinase activity | 0.022655 | 0.003422406 |
| GO:0003310 | BIOLOGICAL_PROCESS | pancreatic A cell differentiation | 0.022655 | 0.003422406 |
| GO:0004634 | MOLECULAR_FUNCTION | phosphopyruvate hydratase activity | 0.022655 | 0.003422406 |
| GO:0002052 | BIOLOGICAL_PROCESS | positive regulation of neuroblast proliferation | 0.022655 | 0.003422406 |
| GO:0030977 | MOLECULAR_FUNCTION | taurine binding | 0.022655 | 0.003422406 |
| GO:0042974 | MOLECULAR_FUNCTION | retinoic acid receptor binding | 0.022655 | 0.003422406 |
| GO:0038065 | BIOLOGICAL_PROCESS | collagen-activated signaling pathway | 0.022655 | 0.003422406 |
| GO:0038062 | MOLECULAR_FUNCTION | protein tyrosine kinase collagen receptor activity | 0.022655 | 0.003422406 |
| GO:0038064 | MOLECULAR_FUNCTION | collagen receptor activity | 0.022655 | 0.003422406 |
| GO:0038063 | BIOLOGICAL_PROCESS | collagen-activated tyrosine kinase receptor signaling pathway | 0.022655 | 0.003422406 |
| GO:0022404 | BIOLOGICAL_PROCESS | molting cycle process | 0.022655 | 0.003422406 |
| GO:1904019 | BIOLOGICAL_PROCESS | epithelial cell apoptotic process | 0.022655 | 0.003422406 |
| GO:0060765 | BIOLOGICAL_PROCESS | regulation of androgen receptor signaling pathway | 0.022655 | 0.003422406 |
| GO:0051145 | BIOLOGICAL_PROCESS | smooth muscle cell differentiation | 0.022655 | 0.003422406 |
| GO:0038127 | BIOLOGICAL_PROCESS | ERBB signaling pathway | 0.022655 | 0.003281323 |
| GO:0035909 | BIOLOGICAL_PROCESS | aorta morphogenesis | 0.022655 | 0.003422406 |
| GO:0035883 | BIOLOGICAL_PROCESS | enteroendocrine cell differentiation | 0.022655 | 0.003422406 |
| GO:0036003 | BIOLOGICAL_PROCESS | positive regulation of transcription from RNA polymerase II promoter in response to stress | 0.022655 | 0.003422406 |
| GO:1902107 | BIOLOGICAL_PROCESS | positive regulation of leukocyte differentiation | 0.022655 | 0.003422406 |
| GO:0007560 | BIOLOGICAL_PROCESS | imaginal disc morphogenesis | 0.022655 | 0.003422406 |
| GO:0021779 | BIOLOGICAL_PROCESS | oligodendrocyte cell fate commitment | 0.022655 | 0.003422406 |
| GO:0021778 | BIOLOGICAL_PROCESS | oligodendrocyte cell fate specification | 0.022655 | 0.003422406 |
| GO:0021780 | BIOLOGICAL_PROCESS | glial cell fate specification | 0.022655 | 0.003422406 |
| GO:0031143 | CELLULAR_COMPONENT | pseudopodium | 0.022655 | 0.003422406 |
| GO:0002683 | BIOLOGICAL_PROCESS | negative regulation of immune system process | 0.022655 | 0.003281323 |
| GO:0000015 | CELLULAR_COMPONENT | phosphopyruvate hydratase complex | 0.022655 | 0.003422406 |
| GO:0061180 | BIOLOGICAL_PROCESS | mammary gland epithelium development | 0.022655 | 0.003422406 |
| GO:0006333 | BIOLOGICAL_PROCESS | chromatin assembly or disassembly | 0.022655 | 0.003281323 |
| GO:0005041 | MOLECULAR_FUNCTION | low-density lipoprotein receptor activity | 0.022655 | 0.003422406 |
| GO:0043210 | MOLECULAR_FUNCTION | alkanesulfonate binding | 0.022655 | 0.003422406 |
| GO:0002763 | BIOLOGICAL_PROCESS | positive regulation of myeloid leukocyte differentiation | 0.022655 | 0.003422406 |
| GO:0010990 | BIOLOGICAL_PROCESS | regulation of SMAD protein complex assembly | 0.022655 | 0.003422406 |
| GO:0021524 | BIOLOGICAL_PROCESS | visceral motor neuron differentiation | 0.022655 | 0.003422406 |
| GO:0021589 | BIOLOGICAL_PROCESS | cerebellum structural organization | 0.022655 | 0.003422406 |
| GO:0021577 | BIOLOGICAL_PROCESS | hindbrain structural organization | 0.022655 | 0.003422406 |
| GO:0033558 | MOLECULAR_FUNCTION | protein deacetylase activity | 0.022655 | 0.003422406 |
| GO:0003725 | MOLECULAR_FUNCTION | double-stranded RNA binding | 0.022655 | 0.003281323 |
| GO:0007416 | BIOLOGICAL_PROCESS | synapse assembly | 0.022655 | 0.003281323 |
| GO:0071936 | MOLECULAR_FUNCTION | coreceptor activity involved in Wnt signaling pathway | 0.022655 | 0.003422406 |
| GO:0044340 | BIOLOGICAL_PROCESS | canonical Wnt signaling pathway involved in regulation of cell proliferation | 0.022655 | 0.003422406 |
| GO:0002526 | BIOLOGICAL_PROCESS | acute inflammatory response | 0.022655 | 0.003422406 |
| GO:0045687 | BIOLOGICAL_PROCESS | positive regulation of glial cell differentiation | 0.022655 | 0.003422406 |
| GO:0055007 | BIOLOGICAL_PROCESS | cardiac muscle cell differentiation | 0.022655 | 0.003281323 |
| GO:0005215 | MOLECULAR_FUNCTION | transporter activity | 0.022688 | 0.003429418 |
| GO:0035194 | BIOLOGICAL_PROCESS | posttranscriptional gene silencing by RNA | 0.022795 | 0.003524182 |
| GO:0048512 | BIOLOGICAL_PROCESS | circadian behavior | 0.022795 | 0.003524182 |
| GO:0050867 | BIOLOGICAL_PROCESS | positive regulation of cell activation | 0.022795 | 0.003524182 |
| GO:0098655 | BIOLOGICAL_PROCESS | cation transmembrane transport | 0.022795 | 0.003475862 |
| GO:1903578 | BIOLOGICAL_PROCESS | regulation of ATP metabolic process | 0.022795 | 0.003524182 |
| GO:0060037 | BIOLOGICAL_PROCESS | pharyngeal system development | 0.022795 | 0.003524182 |
| GO:0032874 | BIOLOGICAL_PROCESS | positive regulation of stress-activated MAPK cascade | 0.022795 | 0.003524182 |
| GO:1990782 | MOLECULAR_FUNCTION | protein tyrosine kinase binding | 0.022795 | 0.003524182 |
| GO:0001678 | BIOLOGICAL_PROCESS | cellular glucose homeostasis | 0.022795 | 0.003524182 |
| GO:0016441 | BIOLOGICAL_PROCESS | posttranscriptional gene silencing | 0.022795 | 0.003524182 |
| GO:0048806 | BIOLOGICAL_PROCESS | genitalia development | 0.022795 | 0.003524182 |
| GO:1901655 | BIOLOGICAL_PROCESS | cellular response to ketone | 0.022795 | 0.003524182 |
| GO:0016572 | BIOLOGICAL_PROCESS | histone phosphorylation | 0.022795 | 0.003524182 |
| GO:0090505 | BIOLOGICAL_PROCESS | epiboly involved in wound healing | 0.022795 | 0.003524182 |
| GO:0048659 | BIOLOGICAL_PROCESS | smooth muscle cell proliferation | 0.022795 | 0.003524182 |
| GO:0048660 | BIOLOGICAL_PROCESS | regulation of smooth muscle cell proliferation | 0.022795 | 0.003524182 |
| GO:1901360 | BIOLOGICAL_PROCESS | organic cyclic compound metabolic process | 0.022795 | 0.003457287 |
| GO:0071158 | BIOLOGICAL_PROCESS | positive regulation of cell cycle arrest | 0.022795 | 0.003524182 |
| GO:0006984 | BIOLOGICAL_PROCESS | ER-nucleus signaling pathway | 0.022795 | 0.003524182 |
| GO:0043966 | BIOLOGICAL_PROCESS | histone H3 acetylation | 0.022795 | 0.003524182 |
| GO:0009118 | BIOLOGICAL_PROCESS | regulation of nucleoside metabolic process | 0.022795 | 0.003524182 |
| GO:0061731 | MOLECULAR_FUNCTION | ribonucleoside-diphosphate reductase activity | 0.022795 | 0.003524182 |
| GO:0051251 | BIOLOGICAL_PROCESS | positive regulation of lymphocyte activation | 0.022795 | 0.003524182 |
| GO:0070304 | BIOLOGICAL_PROCESS | positive regulation of stress-activated protein kinase signaling cascade | 0.022795 | 0.003524182 |
| GO:0045445 | BIOLOGICAL_PROCESS | myoblast differentiation | 0.022795 | 0.003524182 |
| GO:0002039 | MOLECULAR_FUNCTION | p53 binding | 0.022795 | 0.003524182 |
| GO:0002066 | BIOLOGICAL_PROCESS | columnar/cuboidal epithelial cell development | 0.022795 | 0.003524182 |
| GO:0042992 | BIOLOGICAL_PROCESS | negative regulation of transcription factor import into nucleus | 0.022795 | 0.003524182 |
| GO:0016728 | MOLECULAR_FUNCTION | oxidoreductase activity, acting on CH or CH2 groups, disulfide as acceptor | 0.022795 | 0.003524182 |
| GO:0004748 | MOLECULAR_FUNCTION | ribonucleoside-diphosphate reductase activity, thioredoxin disulfide as acceptor | 0.022795 | 0.003524182 |
| GO:0048010 | BIOLOGICAL_PROCESS | vascular endothelial growth factor receptor signaling pathway | 0.022795 | 0.003524182 |
| GO:0061035 | BIOLOGICAL_PROCESS | regulation of cartilage development | 0.022795 | 0.003524182 |
| GO:0002696 | BIOLOGICAL_PROCESS | positive regulation of leukocyte activation | 0.022795 | 0.003524182 |
| GO:0007622 | BIOLOGICAL_PROCESS | rhythmic behavior | 0.022795 | 0.003524182 |
| GO:0021983 | BIOLOGICAL_PROCESS | pituitary gland development | 0.022795 | 0.003524182 |
| GO:0008593 | BIOLOGICAL_PROCESS | regulation of Notch signaling pathway | 0.022795 | 0.003524182 |
| GO:0046887 | BIOLOGICAL_PROCESS | positive regulation of hormone secretion | 0.022795 | 0.003524182 |
| GO:0044319 | BIOLOGICAL_PROCESS | wound healing, spreading of cells | 0.022795 | 0.003524182 |
| GO:0032388 | BIOLOGICAL_PROCESS | positive regulation of intracellular transport | 0.022795 | 0.003449861 |
| GO:0043086 | BIOLOGICAL_PROCESS | negative regulation of catalytic activity | 0.023763 | 0.003675821 |
| GO:0044772 | BIOLOGICAL_PROCESS | mitotic cell cycle phase transition | 0.024804 | 0.003839142 |
| GO:2000136 | BIOLOGICAL_PROCESS | regulation of cell proliferation involved in heart morphogenesis | 0.024846 | 0.003963961 |
| GO:0035195 | BIOLOGICAL_PROCESS | gene silencing by miRNA | 0.024846 | 0.003963961 |
| GO:0035173 | MOLECULAR_FUNCTION | histone kinase activity | 0.024846 | 0.003963961 |
| GO:0031571 | BIOLOGICAL_PROCESS | mitotic G1 DNA damage checkpoint | 0.024846 | 0.003963961 |
| GO:0001725 | CELLULAR_COMPONENT | stress fiber | 0.024846 | 0.003963961 |
| GO:0042246 | BIOLOGICAL_PROCESS | tissue regeneration | 0.024846 | 0.003963961 |
| GO:0097517 | CELLULAR_COMPONENT | contractile actin filament bundle | 0.024846 | 0.003963961 |
| GO:0048569 | BIOLOGICAL_PROCESS | post-embryonic animal organ development | 0.024846 | 0.003963961 |
| GO:1902400 | BIOLOGICAL_PROCESS | intracellular signal transduction involved in G1 DNA damage checkpoint | 0.024846 | 0.003963961 |
| GO:0000578 | BIOLOGICAL_PROCESS | embryonic axis specification | 0.024846 | 0.003963961 |
| GO:0050807 | BIOLOGICAL_PROCESS | regulation of synapse organization | 0.024846 | 0.003963961 |
| GO:0050810 | BIOLOGICAL_PROCESS | regulation of steroid biosynthetic process | 0.024846 | 0.003963961 |
| GO:0045980 | BIOLOGICAL_PROCESS | negative regulation of nucleotide metabolic process | 0.024846 | 0.003963961 |
| GO:0060004 | BIOLOGICAL_PROCESS | reflex | 0.024846 | 0.003963961 |
| GO:0061323 | BIOLOGICAL_PROCESS | cell proliferation involved in heart morphogenesis | 0.024846 | 0.003963961 |
| GO:1902284 | BIOLOGICAL_PROCESS | neuron projection extension involved in neuron projection guidance | 0.024846 | 0.003963961 |
| GO:0072088 | BIOLOGICAL_PROCESS | nephron epithelium morphogenesis | 0.024846 | 0.003963961 |
| GO:0060045 | BIOLOGICAL_PROCESS | positive regulation of cardiac muscle cell proliferation | 0.024846 | 0.003963961 |
| GO:0060043 | BIOLOGICAL_PROCESS | regulation of cardiac muscle cell proliferation | 0.024846 | 0.003963961 |
| GO:0098657 | BIOLOGICAL_PROCESS | import into cell | 0.024846 | 0.003963961 |
| GO:0072028 | BIOLOGICAL_PROCESS | nephron morphogenesis | 0.024846 | 0.003963961 |
| GO:0032881 | BIOLOGICAL_PROCESS | regulation of polysaccharide metabolic process | 0.024846 | 0.003963961 |
| GO:0032885 | BIOLOGICAL_PROCESS | regulation of polysaccharide biosynthetic process | 0.024846 | 0.003963961 |
| GO:0030219 | BIOLOGICAL_PROCESS | megakaryocyte differentiation | 0.024846 | 0.003963961 |
| GO:0044819 | BIOLOGICAL_PROCESS | mitotic G1/S transition checkpoint | 0.024846 | 0.003963961 |
| GO:0043470 | BIOLOGICAL_PROCESS | regulation of carbohydrate catabolic process | 0.024846 | 0.003963961 |
| GO:0014902 | BIOLOGICAL_PROCESS | myotube differentiation | 0.024846 | 0.003963961 |
| GO:0043425 | MOLECULAR_FUNCTION | bHLH transcription factor binding | 0.024846 | 0.003963961 |
| GO:0044783 | BIOLOGICAL_PROCESS | G1 DNA damage checkpoint | 0.024846 | 0.003963961 |
| GO:0004491 | MOLECULAR_FUNCTION | methylmalonate-semialdehyde dehydrogenase (acylating) activity | 0.024846 | 0.003963961 |
| GO:0048846 | BIOLOGICAL_PROCESS | axon extension involved in axon guidance | 0.024846 | 0.003963961 |
| GO:0070035 | MOLECULAR_FUNCTION | purine NTP-dependent helicase activity | 0.024846 | 0.003884276 |
| GO:0071333 | BIOLOGICAL_PROCESS | cellular response to glucose stimulus | 0.024846 | 0.003963961 |
| GO:0008026 | MOLECULAR_FUNCTION | ATP-dependent helicase activity | 0.024846 | 0.003884276 |
| GO:0060323 | BIOLOGICAL_PROCESS | head morphogenesis | 0.024846 | 0.003963961 |
| GO:0030521 | BIOLOGICAL_PROCESS | androgen receptor signaling pathway | 0.024846 | 0.003963961 |
| GO:0003016 | BIOLOGICAL_PROCESS | respiratory system process | 0.024846 | 0.003963961 |
| GO:0006977 | BIOLOGICAL_PROCESS | DNA damage response, signal transduction by p53 class mediator resulting in cell cycle arrest | 0.024846 | 0.003963961 |
| GO:0072431 | BIOLOGICAL_PROCESS | signal transduction involved in mitotic G1 DNA damage checkpoint | 0.024846 | 0.003963961 |
| GO:1900077 | BIOLOGICAL_PROCESS | negative regulation of cellular response to insulin stimulus | 0.024846 | 0.003963961 |
| GO:0035411 | BIOLOGICAL_PROCESS | catenin import into nucleus | 0.024846 | 0.003963961 |
| GO:0035412 | BIOLOGICAL_PROCESS | regulation of catenin import into nucleus | 0.024846 | 0.003963961 |
| GO:0060993 | BIOLOGICAL_PROCESS | kidney morphogenesis | 0.024846 | 0.003963961 |
| GO:0032075 | BIOLOGICAL_PROCESS | positive regulation of nuclease activity | 0.024846 | 0.003963961 |
| GO:0032069 | BIOLOGICAL_PROCESS | regulation of nuclease activity | 0.024846 | 0.003963961 |
| GO:0051384 | BIOLOGICAL_PROCESS | response to glucocorticoid | 0.024846 | 0.003963961 |
| GO:0021513 | BIOLOGICAL_PROCESS | spinal cord dorsal/ventral patterning | 0.024846 | 0.003963961 |
| GO:0010862 | BIOLOGICAL_PROCESS | positive regulation of pathway-restricted SMAD protein phosphorylation | 0.024846 | 0.003963961 |
| GO:1900543 | BIOLOGICAL_PROCESS | negative regulation of purine nucleotide metabolic process | 0.024846 | 0.003963961 |
| GO:0061005 | BIOLOGICAL_PROCESS | cell differentiation involved in kidney development | 0.024846 | 0.003963961 |
| GO:0032432 | CELLULAR_COMPONENT | actin filament bundle | 0.024846 | 0.003963961 |
| GO:0050434 | BIOLOGICAL_PROCESS | positive regulation of viral transcription | 0.024846 | 0.003963961 |
| GO:0007444 | BIOLOGICAL_PROCESS | imaginal disc development | 0.024846 | 0.003963961 |
| GO:0006110 | BIOLOGICAL_PROCESS | regulation of glycolytic process | 0.024846 | 0.003963961 |
| GO:0044456 | CELLULAR_COMPONENT | synapse part | 0.025976 | 0.004146642 |
| GO:0061572 | BIOLOGICAL_PROCESS | actin filament bundle organization | 0.026095 | 0.00418167 |
| GO:0090090 | BIOLOGICAL_PROCESS | negative regulation of canonical Wnt signaling pathway | 0.026095 | 0.00418167 |
| GO:0016458 | BIOLOGICAL_PROCESS | gene silencing | 0.026095 | 0.00418167 |
| GO:0051249 | BIOLOGICAL_PROCESS | regulation of lymphocyte activation | 0.026095 | 0.00418167 |
| GO:0051017 | BIOLOGICAL_PROCESS | actin filament bundle assembly | 0.026095 | 0.00418167 |
| GO:0000077 | BIOLOGICAL_PROCESS | DNA damage checkpoint | 0.026095 | 0.00418167 |
| GO:0099565 | BIOLOGICAL_PROCESS | chemical synaptic transmission, postsynaptic | 0.026095 | 0.00418167 |
| GO:0005768 | CELLULAR_COMPONENT | endosome | 0.02693 | 0.00431801 |
| GO:0006821 | BIOLOGICAL_PROCESS | chloride transport | 0.02705 | 0.004339562 |
| GO:0048524 | BIOLOGICAL_PROCESS | positive regulation of viral process | 0.028058 | 0.004528475 |
| GO:0097191 | BIOLOGICAL_PROCESS | extrinsic apoptotic signaling pathway | 0.028058 | 0.004528475 |
| GO:0060071 | BIOLOGICAL_PROCESS | Wnt signaling pathway, planar cell polarity pathway | 0.028058 | 0.004528475 |
| GO:0044774 | BIOLOGICAL_PROCESS | mitotic DNA integrity checkpoint | 0.028058 | 0.004528475 |
| GO:0090175 | BIOLOGICAL_PROCESS | regulation of establishment of planar polarity | 0.028058 | 0.004528475 |
| GO:1903902 | BIOLOGICAL_PROCESS | positive regulation of viral life cycle | 0.028058 | 0.004528475 |
| GO:0007043 | BIOLOGICAL_PROCESS | cell-cell junction assembly | 0.028058 | 0.004528475 |
| GO:0032102 | BIOLOGICAL_PROCESS | negative regulation of response to external stimulus | 0.028058 | 0.004528475 |
| GO:0045185 | BIOLOGICAL_PROCESS | maintenance of protein location | 0.028058 | 0.004528475 |
| GO:0061028 | BIOLOGICAL_PROCESS | establishment of endothelial barrier | 0.028058 | 0.004528475 |
| GO:0009895 | BIOLOGICAL_PROCESS | negative regulation of catabolic process | 0.028058 | 0.004528475 |
| GO:0050865 | BIOLOGICAL_PROCESS | regulation of cell activation | 0.030222 | 0.004883069 |
| GO:0055001 | BIOLOGICAL_PROCESS | muscle cell development | 0.030222 | 0.004883069 |
| GO:0001666 | BIOLOGICAL_PROCESS | response to hypoxia | 0.033382 | 0.005396583 |
| GO:0007059 | BIOLOGICAL_PROCESS | chromosome segregation | 0.034674 | 0.005608501 |
| GO:0044238 | BIOLOGICAL_PROCESS | primary metabolic process | 0.034873 | 0.005643899 |
| GO:0009165 | BIOLOGICAL_PROCESS | nucleotide biosynthetic process | 0.035443 | 0.005739288 |
| GO:0046879 | BIOLOGICAL_PROCESS | hormone secretion | 0.035451 | 0.005746846 |
| GO:0015850 | BIOLOGICAL_PROCESS | organic hydroxy compound transport | 0.035451 | 0.005746846 |
| GO:0005874 | CELLULAR_COMPONENT | microtubule | 0.035485 | 0.005755435 |
| GO:0000302 | BIOLOGICAL_PROCESS | response to reactive oxygen species | 0.036279 | 0.005900246 |
| GO:0001948 | MOLECULAR_FUNCTION | glycoprotein binding | 0.036279 | 0.005900246 |
| GO:0030496 | CELLULAR_COMPONENT | midbody | 0.036279 | 0.005900246 |
| GO:0048701 | BIOLOGICAL_PROCESS | embryonic cranial skeleton morphogenesis | 0.036279 | 0.005900246 |
| GO:0032147 | BIOLOGICAL_PROCESS | activation of protein kinase activity | 0.036279 | 0.005900246 |
| GO:0000819 | BIOLOGICAL_PROCESS | sister chromatid segregation | 0.036622 | 0.005961268 |
| GO:0016820 | MOLECULAR_FUNCTION | hydrolase activity, acting on acid anhydrides, catalyzing transmembrane movement of substances | 0.036622 | 0.005962512 |
| GO:0032984 | BIOLOGICAL_PROCESS | macromolecular complex disassembly | 0.037021 | 0.006030767 |
| GO:0098813 | BIOLOGICAL_PROCESS | nuclear chromosome segregation | 0.037135 | 0.006065612 |
| GO:1902493 | CELLULAR_COMPONENT | acetyltransferase complex | 0.037135 | 0.006065612 |
| GO:0015108 | MOLECULAR_FUNCTION | chloride transmembrane transporter activity | 0.037135 | 0.006065612 |
| GO:0005911 | CELLULAR_COMPONENT | cell-cell junction | 0.037135 | 0.006061798 |
| GO:0031248 | CELLULAR_COMPONENT | protein acetyltransferase complex | 0.037135 | 0.006065612 |
| GO:0060113 | BIOLOGICAL_PROCESS | inner ear receptor cell differentiation | 0.037725 | 0.006208627 |
| GO:0048469 | BIOLOGICAL_PROCESS | cell maturation | 0.037725 | 0.006208627 |
| GO:0032943 | BIOLOGICAL_PROCESS | mononuclear cell proliferation | 0.037725 | 0.006208627 |
| GO:0061387 | BIOLOGICAL_PROCESS | regulation of extent of cell growth | 0.037725 | 0.006208627 |
| GO:0030175 | CELLULAR_COMPONENT | filopodium | 0.037725 | 0.006208627 |
| GO:0031497 | BIOLOGICAL_PROCESS | chromatin assembly | 0.037725 | 0.006208627 |
| GO:0048814 | BIOLOGICAL_PROCESS | regulation of dendrite morphogenesis | 0.037725 | 0.006208627 |
| GO:0042552 | BIOLOGICAL_PROCESS | myelination | 0.037725 | 0.006208627 |
| GO:0042490 | BIOLOGICAL_PROCESS | mechanoreceptor differentiation | 0.037725 | 0.006208627 |
| GO:0046651 | BIOLOGICAL_PROCESS | lymphocyte proliferation | 0.037725 | 0.006208627 |
| GO:0010771 | BIOLOGICAL_PROCESS | negative regulation of cell morphogenesis involved in differentiation | 0.037725 | 0.006208627 |
| GO:1901989 | BIOLOGICAL_PROCESS | positive regulation of cell cycle phase transition | 0.037725 | 0.006208627 |
| GO:1901992 | BIOLOGICAL_PROCESS | positive regulation of mitotic cell cycle phase transition | 0.037725 | 0.006208627 |
| GO:0070661 | BIOLOGICAL_PROCESS | leukocyte proliferation | 0.037725 | 0.006208627 |
| GO:1901293 | BIOLOGICAL_PROCESS | nucleoside phosphate biosynthetic process | 0.039664 | 0.006531349 |
| GO:0022604 | BIOLOGICAL_PROCESS | regulation of cell morphogenesis | 0.039871 | 0.006568842 |
| GO:0043484 | BIOLOGICAL_PROCESS | regulation of RNA splicing | 0.041937 | 0.006931503 |
| GO:0072593 | BIOLOGICAL_PROCESS | reactive oxygen species metabolic process | 0.041937 | 0.006931503 |
| GO:0006986 | BIOLOGICAL_PROCESS | response to unfolded protein | 0.041937 | 0.006931503 |
| GO:0035966 | BIOLOGICAL_PROCESS | response to topologically incorrect protein | 0.041937 | 0.006931503 |
| GO:0002694 | BIOLOGICAL_PROCESS | regulation of leukocyte activation | 0.041937 | 0.006931503 |
| GO:0043062 | BIOLOGICAL_PROCESS | extracellular structure organization | 0.041937 | 0.006931503 |
| GO:0031982 | CELLULAR_COMPONENT | vesicle | 0.042237 | 0.006984733 |
| GO:1901576 | BIOLOGICAL_PROCESS | organic substance biosynthetic process | 0.04429 | 0.007328165 |
| GO:0071216 | BIOLOGICAL_PROCESS | cellular response to biotic stimulus | 0.046217 | 0.007655235 |
| GO:0032434 | BIOLOGICAL_PROCESS | regulation of proteasomal ubiquitin-dependent protein catabolic process | 0.046217 | 0.007655235 |
| GO:0006260 | BIOLOGICAL_PROCESS | DNA replication | 0.046974 | 0.007784816 |
| GO:0044237 | BIOLOGICAL_PROCESS | cellular metabolic process | 0.047257 | 0.007835864 |
| GO:0005262 | MOLECULAR_FUNCTION | calcium channel activity | 0.048274 | 0.008008773 |
| GO:0065004 | BIOLOGICAL_PROCESS | protein-DNA complex assembly | 0.048909 | 0.008122745 |
| GO:0007050 | BIOLOGICAL_PROCESS | cell cycle arrest | 0.048909 | 0.008122745 |
| GO:0033365 | BIOLOGICAL_PROCESS | protein localization to organelle | 0.049033 | 0.008151918 |
| GO:0003924 | MOLECULAR_FUNCTION | GTPase activity | 0.049033 | 0.008151918 |
| GO:0032970 | BIOLOGICAL_PROCESS | regulation of actin filament-based process | 0.049051 | 0.008163679 |
| GO:0030424 | CELLULAR_COMPONENT | axon | 0.049051 | 0.008163679 |
| GO:0044249 | BIOLOGICAL_PROCESS | cellular biosynthetic process | 0.049272 | 0.00820481 |
| GO:0043588 | BIOLOGICAL_PROCESS | skin development | 0.049776 | 0.008407261 |
| GO:0030261 | BIOLOGICAL_PROCESS | chromosome condensation | 0.049776 | 0.008407261 |
| GO:0098858 | CELLULAR_COMPONENT | actin-based cell projection | 0.049776 | 0.008339804 |
| GO:0098811 | MOLECULAR_FUNCTION | transcriptional repressor activity, RNA polymerase II activating transcription factor binding | 0.049776 | 0.008407261 |
| GO:0050880 | BIOLOGICAL_PROCESS | regulation of blood vessel size | 0.049776 | 0.008407261 |
| GO:0030165 | MOLECULAR_FUNCTION | PDZ domain binding | 0.049776 | 0.008407261 |
| GO:2001021 | BIOLOGICAL_PROCESS | negative regulation of response to DNA damage stimulus | 0.049776 | 0.008407261 |
| GO:1901618 | MOLECULAR_FUNCTION | organic hydroxy compound transmembrane transporter activity | 0.049776 | 0.008339804 |
| GO:1902808 | BIOLOGICAL_PROCESS | positive regulation of cell cycle G1/S phase transition | 0.049776 | 0.008407261 |
| GO:0050900 | BIOLOGICAL_PROCESS | leukocyte migration | 0.049776 | 0.008407261 |
| GO:0035326 | MOLECULAR_FUNCTION | enhancer binding | 0.049776 | 0.008407261 |
| GO:1900087 | BIOLOGICAL_PROCESS | positive regulation of G1/S transition of mitotic cell cycle | 0.049776 | 0.008407261 |
| GO:0000792 | CELLULAR_COMPONENT | heterochromatin | 0.049776 | 0.008407261 |
| GO:0046677 | BIOLOGICAL_PROCESS | response to antibiotic | 0.049776 | 0.008407261 |
| GO:0015605 | MOLECULAR_FUNCTION | organophosphate ester transmembrane transporter activity | 0.049776 | 0.008407261 |
| GO:0005903 | CELLULAR_COMPONENT | brush border | 0.049776 | 0.008407261 |
| GO:0035821 | BIOLOGICAL_PROCESS | modification of morphology or physiology of other organism | 0.049776 | 0.008339804 |
| GO:0006207 | BIOLOGICAL_PROCESS | 'de novo' pyrimidine nucleobase biosynthetic process | 0.049776 | 0.008407261 |
| GO:0045732 | BIOLOGICAL_PROCESS | positive regulation of protein catabolic process | 0.049776 | 0.00834905 |
| GO:0045930 | BIOLOGICAL_PROCESS | negative regulation of mitotic cell cycle | 0.049776 | 0.008385008 |
| GO:0031256 | CELLULAR_COMPONENT | leading edge membrane | 0.049776 | 0.008407261 |
| GO:0001158 | MOLECULAR_FUNCTION | enhancer sequence-specific DNA binding | 0.049776 | 0.008407261 |
| GO:0001190 | MOLECULAR_FUNCTION | transcriptional activator activity, RNA polymerase II transcription factor binding | 0.049776 | 0.008407261 |
| GO:0051494 | BIOLOGICAL_PROCESS | negative regulation of cytoskeleton organization | 0.049776 | 0.008339804 |
| GO:0070555 | BIOLOGICAL_PROCESS | response to interleukin-1 | 0.049776 | 0.008407261 |
| GO:0001105 | MOLECULAR_FUNCTION | RNA polymerase II transcription coactivator activity | 0.049776 | 0.008407261 |
| GO:0021695 | BIOLOGICAL_PROCESS | cerebellar cortex development | 0.049776 | 0.008407261 |
