# Supplementary material for: Data on genome annotation and analysis of earthworm Eisenia fetida
Source: Data Brief. 2018 Aug 29;20:525–34. doi: 10.1016/j.dib.2018.08.067 (PMC6126081; doi:10.1016/j.dib.2018.08.067)
Supplement: Supplementary file 9 — Supplementary material [file mmc9.docx]

Table S8: List of functionally enriched GO terms associated with the immune specific genes

| **GO ID** | **GO Category** | **GO Name** | **FDR** | **P-Value** |
| --- | --- | --- | --- | --- |
| GO:0044700 | BIOLOGICAL_PROCESS | single organism signaling | 1.34E-222 | 2.37E-226 |
| GO:0023052 | BIOLOGICAL_PROCESS | signaling | 1.34E-222 | 2.37E-226 |
| GO:0050789 | BIOLOGICAL_PROCESS | regulation of biological process | 1.66E-222 | 4.40E-226 |
| GO:0065007 | BIOLOGICAL_PROCESS | biological regulation | 2.47E-215 | 8.73E-219 |
| GO:0007154 | BIOLOGICAL_PROCESS | cell communication | 9.76E-212 | 4.31E-215 |
| GO:0050794 | BIOLOGICAL_PROCESS | regulation of cellular process | 5.03E-210 | 2.66E-213 |
| GO:0007165 | BIOLOGICAL_PROCESS | signal transduction | 1.05E-208 | 6.49E-212 |
| GO:0050896 | BIOLOGICAL_PROCESS | response to stimulus | 3.19E-204 | 2.25E-207 |
| GO:0051716 | BIOLOGICAL_PROCESS | cellular response to stimulus | 3.30E-195 | 2.62E-198 |
| GO:0032501 | BIOLOGICAL_PROCESS | multicellular organismal process | 4.41E-122 | 3.89E-125 |
| GO:0044707 | BIOLOGICAL_PROCESS | single-multicellular organism process | 7.21E-121 | 7.00E-124 |
| GO:0004871 | MOLECULAR_FUNCTION | signal transducer activity | 6.92E-111 | 7.33E-114 |
| GO:0007275 | BIOLOGICAL_PROCESS | multicellular organism development | 8.95E-106 | 1.03E-108 |
| GO:0032502 | BIOLOGICAL_PROCESS | developmental process | 6.64E-104 | 8.20E-107 |
| GO:0044767 | BIOLOGICAL_PROCESS | single-organism developmental process | 1.13E-103 | 1.50E-106 |
| GO:0048856 | BIOLOGICAL_PROCESS | anatomical structure development | 1.06E-100 | 1.49E-103 |
| GO:0048731 | BIOLOGICAL_PROCESS | system development | 2.47E-95 | 3.71E-98 |
| GO:0099600 | MOLECULAR_FUNCTION | transmembrane receptor activity | 9.97E-95 | 1.58E-97 |
| GO:0004872 | MOLECULAR_FUNCTION | receptor activity | 3.55E-93 | 5.96E-96 |
| GO:0060089 | MOLECULAR_FUNCTION | molecular transducer activity | 7.28E-93 | 1.29E-95 |
| GO:0006464 | BIOLOGICAL_PROCESS | cellular protein modification process | 3.29E-87 | 6.39E-90 |
| GO:0036211 | BIOLOGICAL_PROCESS | protein modification process | 3.29E-87 | 6.39E-90 |
| GO:0004888 | MOLECULAR_FUNCTION | transmembrane signaling receptor activity | 4.39E-86 | 8.92E-89 |
| GO:0038023 | MOLECULAR_FUNCTION | signaling receptor activity | 5.58E-86 | 1.18E-88 |
| GO:0006468 | BIOLOGICAL_PROCESS | protein phosphorylation | 3.14E-84 | 6.92E-87 |
| GO:0005515 | MOLECULAR_FUNCTION | protein binding | 3.91E-80 | 8.97E-83 |
| GO:0007166 | BIOLOGICAL_PROCESS | cell surface receptor signaling pathway | 1.29E-79 | 3.07E-82 |
| GO:0048518 | BIOLOGICAL_PROCESS | positive regulation of biological process | 9.79E-78 | 2.42E-80 |
| GO:0030154 | BIOLOGICAL_PROCESS | cell differentiation | 1.25E-77 | 3.21E-80 |
| GO:0007186 | BIOLOGICAL_PROCESS | G-protein coupled receptor signaling pathway | 2.80E-76 | 7.42E-79 |
| GO:0048519 | BIOLOGICAL_PROCESS | negative regulation of biological process | 4.92E-75 | 1.35E-77 |
| GO:0048522 | BIOLOGICAL_PROCESS | positive regulation of cellular process | 5.56E-74 | 1.57E-76 |
| GO:0048513 | BIOLOGICAL_PROCESS | animal organ development | 1.19E-72 | 3.47E-75 |
| GO:0004672 | MOLECULAR_FUNCTION | protein kinase activity | 1.57E-69 | 4.72E-72 |
| GO:0043412 | BIOLOGICAL_PROCESS | macromolecule modification | 3.43E-68 | 1.06E-70 |
| GO:0048523 | BIOLOGICAL_PROCESS | negative regulation of cellular process | 3.81E-68 | 1.21E-70 |
| GO:0043227 | CELLULAR_COMPONENT | membrane-bounded organelle | 1.92E-67 | 6.26E-70 |
| GO:0048869 | BIOLOGICAL_PROCESS | cellular developmental process | 7.21E-66 | 2.42E-68 |
| GO:0004930 | MOLECULAR_FUNCTION | G-protein coupled receptor activity | 3.14E-65 | 1.08E-67 |
| GO:0010033 | BIOLOGICAL_PROCESS | response to organic substance | 2.76E-63 | 9.75E-66 |
| GO:0048583 | BIOLOGICAL_PROCESS | regulation of response to stimulus | 4.45E-61 | 1.61E-63 |
| GO:0023051 | BIOLOGICAL_PROCESS | regulation of signaling | 1.63E-60 | 6.05E-63 |
| GO:0010646 | BIOLOGICAL_PROCESS | regulation of cell communication | 1.98E-59 | 7.52E-62 |
| GO:0042221 | BIOLOGICAL_PROCESS | response to chemical | 2.48E-59 | 9.63E-62 |
| GO:0043231 | CELLULAR_COMPONENT | intracellular membrane-bounded organelle | 1.08E-57 | 4.29E-60 |
| GO:0007399 | BIOLOGICAL_PROCESS | nervous system development | 9.25E-57 | 3.76E-59 |
| GO:0016773 | MOLECULAR_FUNCTION | phosphotransferase activity, alcohol group as acceptor | 1.84E-56 | 7.62E-59 |
| GO:0005634 | CELLULAR_COMPONENT | nucleus | 6.25E-56 | 2.65E-58 |
| GO:0009893 | BIOLOGICAL_PROCESS | positive regulation of metabolic process | 3.03E-55 | 1.31E-57 |
| GO:0016310 | BIOLOGICAL_PROCESS | phosphorylation | 4.70E-55 | 2.08E-57 |
| GO:0009966 | BIOLOGICAL_PROCESS | regulation of signal transduction | 5.48E-55 | 2.47E-57 |
| GO:0031325 | BIOLOGICAL_PROCESS | positive regulation of cellular metabolic process | 6.79E-54 | 3.12E-56 |
| GO:0070887 | BIOLOGICAL_PROCESS | cellular response to chemical stimulus | 2.72E-53 | 1.27E-55 |
| GO:0010604 | BIOLOGICAL_PROCESS | positive regulation of macromolecule metabolic process | 3.53E-53 | 1.68E-55 |
| GO:0009888 | BIOLOGICAL_PROCESS | tissue development | 1.39E-52 | 6.73E-55 |
| GO:0009987 | BIOLOGICAL_PROCESS | cellular process | 9.48E-52 | 4.69E-54 |
| GO:0044699 | BIOLOGICAL_PROCESS | single-organism process | 1.72E-51 | 8.68E-54 |
| GO:0009653 | BIOLOGICAL_PROCESS | anatomical structure morphogenesis | 8.62E-51 | 4.42E-53 |
| GO:0051239 | BIOLOGICAL_PROCESS | regulation of multicellular organismal process | 1.58E-48 | 8.23E-51 |
| GO:0044267 | BIOLOGICAL_PROCESS | cellular protein metabolic process | 3.28E-46 | 1.74E-48 |
| GO:0008219 | BIOLOGICAL_PROCESS | cell death | 1.02E-45 | 5.47E-48 |
| GO:0006915 | BIOLOGICAL_PROCESS | apoptotic process | 8.61E-44 | 4.72E-46 |
| GO:0004674 | MOLECULAR_FUNCTION | protein serine/threonine kinase activity | 1.47E-43 | 8.19E-46 |
| GO:0051174 | BIOLOGICAL_PROCESS | regulation of phosphorus metabolic process | 1.86E-43 | 1.07E-45 |
| GO:0019220 | BIOLOGICAL_PROCESS | regulation of phosphate metabolic process | 1.86E-43 | 1.07E-45 |
| GO:0016301 | MOLECULAR_FUNCTION | kinase activity | 4.04E-43 | 2.35E-45 |
| GO:0035556 | BIOLOGICAL_PROCESS | intracellular signal transduction | 6.17E-43 | 3.65E-45 |
| GO:0012501 | BIOLOGICAL_PROCESS | programmed cell death | 1.14E-42 | 6.86E-45 |
| GO:0048468 | BIOLOGICAL_PROCESS | cell development | 2.18E-42 | 1.33E-44 |
| GO:0031399 | BIOLOGICAL_PROCESS | regulation of protein modification process | 2.81E-42 | 1.74E-44 |
| GO:0022008 | BIOLOGICAL_PROCESS | neurogenesis | 3.78E-42 | 2.37E-44 |
| GO:0019538 | BIOLOGICAL_PROCESS | protein metabolic process | 4.70E-42 | 2.99E-44 |
| GO:0071310 | BIOLOGICAL_PROCESS | cellular response to organic substance | 5.97E-42 | 3.85E-44 |
| GO:0007267 | BIOLOGICAL_PROCESS | cell-cell signaling | 8.30E-42 | 5.42E-44 |
| GO:0042325 | BIOLOGICAL_PROCESS | regulation of phosphorylation | 2.63E-40 | 1.74E-42 |
| GO:0032879 | BIOLOGICAL_PROCESS | regulation of localization | 5.32E-40 | 3.57E-42 |
| GO:0048699 | BIOLOGICAL_PROCESS | generation of neurons | 7.32E-40 | 4.98E-42 |
| GO:0065009 | BIOLOGICAL_PROCESS | regulation of molecular function | 2.33E-39 | 1.61E-41 |
| GO:0005488 | MOLECULAR_FUNCTION | binding | 4.27E-39 | 2.98E-41 |
| GO:0016477 | BIOLOGICAL_PROCESS | cell migration | 5.48E-39 | 3.87E-41 |
| GO:0060429 | BIOLOGICAL_PROCESS | epithelium development | 9.80E-39 | 7.01E-41 |
| GO:0010941 | BIOLOGICAL_PROCESS | regulation of cell death | 2.25E-38 | 1.63E-40 |
| GO:2000026 | BIOLOGICAL_PROCESS | regulation of multicellular organismal development | 3.38E-38 | 2.48E-40 |
| GO:0044459 | CELLULAR_COMPONENT | plasma membrane part | 8.67E-38 | 6.43E-40 |
| GO:0006796 | BIOLOGICAL_PROCESS | phosphate-containing compound metabolic process | 4.70E-37 | 3.52E-39 |
| GO:0045595 | BIOLOGICAL_PROCESS | regulation of cell differentiation | 5.62E-37 | 4.27E-39 |
| GO:0009719 | BIOLOGICAL_PROCESS | response to endogenous stimulus | 7.53E-37 | 5.78E-39 |
| GO:0043226 | CELLULAR_COMPONENT | organelle | 1.09E-36 | 8.47E-39 |
| GO:0001932 | BIOLOGICAL_PROCESS | regulation of protein phosphorylation | 1.69E-36 | 1.33E-38 |
| GO:0050793 | BIOLOGICAL_PROCESS | regulation of developmental process | 3.08E-36 | 2.45E-38 |
| GO:0006357 | BIOLOGICAL_PROCESS | regulation of transcription from RNA polymerase II promoter | 8.19E-36 | 6.58E-38 |
| GO:0043229 | CELLULAR_COMPONENT | intracellular organelle | 1.06E-35 | 8.59E-38 |
| GO:1902531 | BIOLOGICAL_PROCESS | regulation of intracellular signal transduction | 1.28E-35 | 1.05E-37 |
| GO:0060255 | BIOLOGICAL_PROCESS | regulation of macromolecule metabolic process | 1.29E-35 | 1.07E-37 |
| GO:0019222 | BIOLOGICAL_PROCESS | regulation of metabolic process | 1.33E-35 | 1.11E-37 |
| GO:0043067 | BIOLOGICAL_PROCESS | regulation of programmed cell death | 1.78E-35 | 1.51E-37 |
| GO:0045935 | BIOLOGICAL_PROCESS | positive regulation of nucleobase-containing compound metabolic process | 2.07E-35 | 1.78E-37 |
| GO:0005576 | CELLULAR_COMPONENT | extracellular region | 5.83E-35 | 5.04E-37 |
| GO:0006366 | BIOLOGICAL_PROCESS | transcription from RNA polymerase II promoter | 1.60E-34 | 1.40E-36 |
| GO:0006793 | BIOLOGICAL_PROCESS | phosphorus metabolic process | 2.31E-34 | 2.04E-36 |
| GO:0007167 | BIOLOGICAL_PROCESS | enzyme linked receptor protein signaling pathway | 2.40E-34 | 2.14E-36 |
| GO:0042981 | BIOLOGICAL_PROCESS | regulation of apoptotic process | 2.79E-34 | 2.52E-36 |
| GO:0030182 | BIOLOGICAL_PROCESS | neuron differentiation | 5.86E-34 | 5.33E-36 |
| GO:0009891 | BIOLOGICAL_PROCESS | positive regulation of biosynthetic process | 5.99E-34 | 5.50E-36 |
| GO:0002376 | BIOLOGICAL_PROCESS | immune system process | 1.06E-33 | 9.80E-36 |
| GO:0010628 | BIOLOGICAL_PROCESS | positive regulation of gene expression | 5.44E-33 | 5.09E-35 |
| GO:0031328 | BIOLOGICAL_PROCESS | positive regulation of cellular biosynthetic process | 6.94E-33 | 6.55E-35 |
| GO:0009887 | BIOLOGICAL_PROCESS | animal organ morphogenesis | 8.68E-33 | 8.27E-35 |
| GO:0080090 | BIOLOGICAL_PROCESS | regulation of primary metabolic process | 8.77E-33 | 8.44E-35 |
| GO:0071495 | BIOLOGICAL_PROCESS | cellular response to endogenous stimulus | 9.70E-33 | 9.42E-35 |
| GO:0051173 | BIOLOGICAL_PROCESS | positive regulation of nitrogen compound metabolic process | 1.36E-32 | 1.33E-34 |
| GO:0005102 | MOLECULAR_FUNCTION | receptor binding | 1.85E-32 | 1.83E-34 |
| GO:0048584 | BIOLOGICAL_PROCESS | positive regulation of response to stimulus | 5.20E-32 | 5.19E-34 |
| GO:0031323 | BIOLOGICAL_PROCESS | regulation of cellular metabolic process | 6.50E-32 | 6.54E-34 |
| GO:0051254 | BIOLOGICAL_PROCESS | positive regulation of RNA metabolic process | 3.06E-31 | 3.10E-33 |
| GO:0008283 | BIOLOGICAL_PROCESS | cell proliferation | 3.11E-31 | 3.19E-33 |
| GO:0001882 | MOLECULAR_FUNCTION | nucleoside binding | 5.17E-31 | 5.34E-33 |
| GO:1902680 | BIOLOGICAL_PROCESS | positive regulation of RNA biosynthetic process | 5.96E-31 | 6.21E-33 |
| GO:0044421 | CELLULAR_COMPONENT | extracellular region part | 8.95E-31 | 9.41E-33 |
| GO:0051246 | BIOLOGICAL_PROCESS | regulation of protein metabolic process | 1.02E-30 | 1.08E-32 |
| GO:0032549 | MOLECULAR_FUNCTION | ribonucleoside binding | 1.08E-30 | 1.15E-32 |
| GO:0097367 | MOLECULAR_FUNCTION | carbohydrate derivative binding | 1.08E-30 | 1.17E-32 |
| GO:0010562 | BIOLOGICAL_PROCESS | positive regulation of phosphorus metabolic process | 1.37E-30 | 1.51E-32 |
| GO:0045937 | BIOLOGICAL_PROCESS | positive regulation of phosphate metabolic process | 1.37E-30 | 1.51E-32 |
| GO:0032555 | MOLECULAR_FUNCTION | purine ribonucleotide binding | 1.37E-30 | 1.49E-32 |
| GO:0010557 | BIOLOGICAL_PROCESS | positive regulation of macromolecule biosynthetic process | 1.44E-30 | 1.60E-32 |
| GO:0017076 | MOLECULAR_FUNCTION | purine nucleotide binding | 1.95E-30 | 2.18E-32 |
| GO:0001883 | MOLECULAR_FUNCTION | purine nucleoside binding | 3.15E-30 | 3.59E-32 |
| GO:0032550 | MOLECULAR_FUNCTION | purine ribonucleoside binding | 3.15E-30 | 3.59E-32 |
| GO:0035639 | MOLECULAR_FUNCTION | purine ribonucleoside triphosphate binding | 5.87E-30 | 6.74E-32 |
| GO:0051247 | BIOLOGICAL_PROCESS | positive regulation of protein metabolic process | 9.81E-30 | 1.14E-31 |
| GO:1903508 | BIOLOGICAL_PROCESS | positive regulation of nucleic acid-templated transcription | 1.15E-29 | 1.35E-31 |
| GO:0045893 | BIOLOGICAL_PROCESS | positive regulation of transcription, DNA-templated | 1.15E-29 | 1.35E-31 |
| GO:0044093 | BIOLOGICAL_PROCESS | positive regulation of molecular function | 1.23E-29 | 1.46E-31 |
| GO:0000981 | MOLECULAR_FUNCTION | RNA polymerase II transcription factor activity, sequence-specific DNA binding | 1.66E-29 | 1.98E-31 |
| GO:0072359 | BIOLOGICAL_PROCESS | circulatory system development | 1.87E-29 | 2.24E-31 |
| GO:0008227 | MOLECULAR_FUNCTION | G-protein coupled amine receptor activity | 2.15E-29 | 2.60E-31 |
| GO:0004713 | MOLECULAR_FUNCTION | protein tyrosine kinase activity | 2.58E-29 | 3.15E-31 |
| GO:0032270 | BIOLOGICAL_PROCESS | positive regulation of cellular protein metabolic process | 2.60E-29 | 3.19E-31 |
| GO:0022610 | BIOLOGICAL_PROCESS | biological adhesion | 6.28E-29 | 7.82E-31 |
| GO:0007155 | BIOLOGICAL_PROCESS | cell adhesion | 6.28E-29 | 7.82E-31 |
| GO:0050790 | BIOLOGICAL_PROCESS | regulation of catalytic activity | 9.56E-29 | 1.20E-30 |
| GO:0043565 | MOLECULAR_FUNCTION | sequence-specific DNA binding | 1.16E-28 | 1.46E-30 |
| GO:0032553 | MOLECULAR_FUNCTION | ribonucleotide binding | 1.18E-28 | 1.50E-30 |
| GO:0016772 | MOLECULAR_FUNCTION | transferase activity, transferring phosphorus-containing groups | 2.65E-28 | 3.39E-30 |
| GO:0051240 | BIOLOGICAL_PROCESS | positive regulation of multicellular organismal process | 3.75E-28 | 4.83E-30 |
| GO:0042127 | BIOLOGICAL_PROCESS | regulation of cell proliferation | 4.30E-28 | 5.59E-30 |
| GO:0043167 | MOLECULAR_FUNCTION | ion binding | 1.84E-27 | 2.40E-29 |
| GO:0048729 | BIOLOGICAL_PROCESS | tissue morphogenesis | 2.48E-27 | 3.26E-29 |
| GO:0009790 | BIOLOGICAL_PROCESS | embryo development | 2.53E-27 | 3.35E-29 |
| GO:0023056 | BIOLOGICAL_PROCESS | positive regulation of signaling | 2.55E-27 | 3.40E-29 |
| GO:0032403 | MOLECULAR_FUNCTION | protein complex binding | 3.17E-27 | 4.25E-29 |
| GO:0032268 | BIOLOGICAL_PROCESS | regulation of cellular protein metabolic process | 5.75E-27 | 7.77E-29 |
| GO:0051094 | BIOLOGICAL_PROCESS | positive regulation of developmental process | 6.02E-27 | 8.20E-29 |
| GO:0051049 | BIOLOGICAL_PROCESS | regulation of transport | 6.02E-27 | 8.23E-29 |
| GO:0010647 | BIOLOGICAL_PROCESS | positive regulation of cell communication | 1.41E-26 | 1.94E-28 |
| GO:0031401 | BIOLOGICAL_PROCESS | positive regulation of protein modification process | 2.63E-26 | 3.64E-28 |
| GO:0098590 | CELLULAR_COMPONENT | plasma membrane region | 4.81E-26 | 6.71E-28 |
| GO:0042327 | BIOLOGICAL_PROCESS | positive regulation of phosphorylation | 1.49E-25 | 2.09E-27 |
| GO:1901700 | BIOLOGICAL_PROCESS | response to oxygen-containing compound | 7.21E-25 | 1.02E-26 |
| GO:0001934 | BIOLOGICAL_PROCESS | positive regulation of protein phosphorylation | 9.85E-25 | 1.40E-26 |
| GO:0009967 | BIOLOGICAL_PROCESS | positive regulation of signal transduction | 1.18E-24 | 1.68E-26 |
| GO:0048585 | BIOLOGICAL_PROCESS | negative regulation of response to stimulus | 2.97E-24 | 4.27E-26 |
| GO:0009892 | BIOLOGICAL_PROCESS | negative regulation of metabolic process | 4.34E-24 | 6.28E-26 |
| GO:0003008 | BIOLOGICAL_PROCESS | system process | 5.18E-24 | 7.54E-26 |
| GO:0019899 | MOLECULAR_FUNCTION | enzyme binding | 9.03E-24 | 1.32E-25 |
| GO:0014070 | BIOLOGICAL_PROCESS | response to organic cyclic compound | 1.03E-23 | 1.52E-25 |
| GO:0010605 | BIOLOGICAL_PROCESS | negative regulation of macromolecule metabolic process | 1.44E-23 | 2.14E-25 |
| GO:0030594 | MOLECULAR_FUNCTION | neurotransmitter receptor activity | 1.56E-23 | 2.35E-25 |
| GO:0045944 | BIOLOGICAL_PROCESS | positive regulation of transcription from RNA polymerase II promoter | 1.56E-23 | 2.35E-25 |
| GO:1901698 | BIOLOGICAL_PROCESS | response to nitrogen compound | 2.11E-23 | 3.18E-25 |
| GO:0018193 | BIOLOGICAL_PROCESS | peptidyl-amino acid modification | 3.07E-23 | 4.67E-25 |
| GO:0000165 | BIOLOGICAL_PROCESS | MAPK cascade | 4.79E-23 | 7.31E-25 |
| GO:0031324 | BIOLOGICAL_PROCESS | negative regulation of cellular metabolic process | 5.75E-23 | 8.83E-25 |
| GO:0008188 | MOLECULAR_FUNCTION | neuropeptide receptor activity | 6.31E-23 | 9.75E-25 |
| GO:0032559 | MOLECULAR_FUNCTION | adenyl ribonucleotide binding | 6.42E-23 | 9.97E-25 |
| GO:0030554 | MOLECULAR_FUNCTION | adenyl nucleotide binding | 1.01E-22 | 1.58E-24 |
| GO:0016192 | BIOLOGICAL_PROCESS | vesicle-mediated transport | 1.06E-22 | 1.66E-24 |
| GO:0007417 | BIOLOGICAL_PROCESS | central nervous system development | 1.50E-22 | 2.37E-24 |
| GO:0060548 | BIOLOGICAL_PROCESS | negative regulation of cell death | 1.69E-22 | 2.69E-24 |
| GO:0008092 | MOLECULAR_FUNCTION | cytoskeletal protein binding | 1.94E-22 | 3.10E-24 |
| GO:0048666 | BIOLOGICAL_PROCESS | neuron development | 2.24E-22 | 3.60E-24 |
| GO:0016055 | BIOLOGICAL_PROCESS | Wnt signaling pathway | 2.34E-22 | 3.80E-24 |
| GO:0198738 | BIOLOGICAL_PROCESS | cell-cell signaling by wnt | 2.34E-22 | 3.80E-24 |
| GO:0030855 | BIOLOGICAL_PROCESS | epithelial cell differentiation | 4.56E-22 | 7.45E-24 |
| GO:0009725 | BIOLOGICAL_PROCESS | response to hormone | 4.89E-22 | 8.03E-24 |
| GO:0043549 | BIOLOGICAL_PROCESS | regulation of kinase activity | 5.26E-22 | 8.69E-24 |
| GO:0007264 | BIOLOGICAL_PROCESS | small GTPase mediated signal transduction | 8.12E-22 | 1.35E-23 |
| GO:0044456 | CELLULAR_COMPONENT | synapse part | 8.23E-22 | 1.37E-23 |
| GO:0005524 | MOLECULAR_FUNCTION | ATP binding | 1.18E-21 | 1.97E-23 |
| GO:0051270 | BIOLOGICAL_PROCESS | regulation of cellular component movement | 2.09E-21 | 3.53E-23 |
| GO:0051338 | BIOLOGICAL_PROCESS | regulation of transferase activity | 2.42E-21 | 4.12E-23 |
| GO:0018108 | BIOLOGICAL_PROCESS | peptidyl-tyrosine phosphorylation | 2.42E-21 | 4.12E-23 |
| GO:0060284 | BIOLOGICAL_PROCESS | regulation of cell development | 3.29E-21 | 5.67E-23 |
| GO:0043069 | BIOLOGICAL_PROCESS | negative regulation of programmed cell death | 3.29E-21 | 5.67E-23 |
| GO:0032870 | BIOLOGICAL_PROCESS | cellular response to hormone stimulus | 3.97E-21 | 6.91E-23 |
| GO:0002009 | BIOLOGICAL_PROCESS | morphogenesis of an epithelium | 3.97E-21 | 6.91E-23 |
| GO:0051241 | BIOLOGICAL_PROCESS | negative regulation of multicellular organismal process | 4.02E-21 | 7.04E-23 |
| GO:0045597 | BIOLOGICAL_PROCESS | positive regulation of cell differentiation | 4.73E-21 | 8.31E-23 |
| GO:0031982 | CELLULAR_COMPONENT | vesicle | 5.49E-21 | 9.70E-23 |
| GO:0019219 | BIOLOGICAL_PROCESS | regulation of nucleobase-containing compound metabolic process | 6.34E-21 | 1.13E-22 |
| GO:0035295 | BIOLOGICAL_PROCESS | tube development | 6.88E-21 | 1.23E-22 |
| GO:0005509 | MOLECULAR_FUNCTION | calcium ion binding | 8.14E-21 | 1.46E-22 |
| GO:1901701 | BIOLOGICAL_PROCESS | cellular response to oxygen-containing compound | 9.27E-21 | 1.67E-22 |
| GO:0006897 | BIOLOGICAL_PROCESS | endocytosis | 9.33E-21 | 1.69E-22 |
| GO:0040011 | BIOLOGICAL_PROCESS | locomotion | 1.00E-20 | 1.82E-22 |
| GO:1905114 | BIOLOGICAL_PROCESS | cell surface receptor signaling pathway involved in cell-cell signaling | 1.13E-20 | 2.07E-22 |
| GO:0023057 | BIOLOGICAL_PROCESS | negative regulation of signaling | 1.27E-20 | 2.35E-22 |
| GO:0010648 | BIOLOGICAL_PROCESS | negative regulation of cell communication | 1.27E-20 | 2.35E-22 |
| GO:0071363 | BIOLOGICAL_PROCESS | cellular response to growth factor stimulus | 1.37E-20 | 2.55E-22 |
| GO:0070848 | BIOLOGICAL_PROCESS | response to growth factor | 1.37E-20 | 2.55E-22 |
| GO:0048598 | BIOLOGICAL_PROCESS | embryonic morphogenesis | 1.51E-20 | 2.85E-22 |
| GO:0065008 | BIOLOGICAL_PROCESS | regulation of biological quality | 1.51E-20 | 2.85E-22 |
| GO:0043066 | BIOLOGICAL_PROCESS | negative regulation of apoptotic process | 1.94E-20 | 3.67E-22 |
| GO:0030334 | BIOLOGICAL_PROCESS | regulation of cell migration | 2.26E-20 | 4.30E-22 |
| GO:0045859 | BIOLOGICAL_PROCESS | regulation of protein kinase activity | 2.43E-20 | 4.64E-22 |
| GO:0009968 | BIOLOGICAL_PROCESS | negative regulation of signal transduction | 2.54E-20 | 4.87E-22 |
| GO:0044260 | BIOLOGICAL_PROCESS | cellular macromolecule metabolic process | 3.50E-20 | 6.74E-22 |
| GO:1901265 | MOLECULAR_FUNCTION | nucleoside phosphate binding | 5.12E-20 | 9.94E-22 |
| GO:0000166 | MOLECULAR_FUNCTION | nucleotide binding | 5.12E-20 | 9.94E-22 |
| GO:0001944 | BIOLOGICAL_PROCESS | vasculature development | 6.54E-20 | 1.28E-21 |
| GO:0048646 | BIOLOGICAL_PROCESS | anatomical structure formation involved in morphogenesis | 7.06E-20 | 1.38E-21 |
| GO:0007507 | BIOLOGICAL_PROCESS | heart development | 1.12E-19 | 2.21E-21 |
| GO:0097060 | CELLULAR_COMPONENT | synaptic membrane | 1.12E-19 | 2.21E-21 |
| GO:0098794 | CELLULAR_COMPONENT | postsynapse | 1.34E-19 | 2.67E-21 |
| GO:0051252 | BIOLOGICAL_PROCESS | regulation of RNA metabolic process | 1.45E-19 | 2.89E-21 |
| GO:0051093 | BIOLOGICAL_PROCESS | negative regulation of developmental process | 1.62E-19 | 3.25E-21 |
| GO:0051128 | BIOLOGICAL_PROCESS | regulation of cellular component organization | 2.04E-19 | 4.10E-21 |
| GO:0048534 | BIOLOGICAL_PROCESS | hematopoietic or lymphoid organ development | 2.19E-19 | 4.42E-21 |
| GO:0005622 | CELLULAR_COMPONENT | intracellular | 2.51E-19 | 5.10E-21 |
| GO:0072358 | BIOLOGICAL_PROCESS | cardiovascular system development | 2.67E-19 | 5.45E-21 |
| GO:0044877 | MOLECULAR_FUNCTION | macromolecular complex binding | 2.72E-19 | 5.57E-21 |
| GO:0000904 | BIOLOGICAL_PROCESS | cell morphogenesis involved in differentiation | 2.92E-19 | 6.01E-21 |
| GO:0016887 | MOLECULAR_FUNCTION | ATPase activity | 4.09E-19 | 8.44E-21 |
| GO:0006950 | BIOLOGICAL_PROCESS | response to stress | 4.14E-19 | 8.60E-21 |
| GO:0060322 | BIOLOGICAL_PROCESS | head development | 5.92E-19 | 1.23E-20 |
| GO:0051960 | BIOLOGICAL_PROCESS | regulation of nervous system development | 9.31E-19 | 1.95E-20 |
| GO:0007420 | BIOLOGICAL_PROCESS | brain development | 1.05E-18 | 2.21E-20 |
| GO:0043408 | BIOLOGICAL_PROCESS | regulation of MAPK cascade | 1.58E-18 | 3.33E-20 |
| GO:2001141 | BIOLOGICAL_PROCESS | regulation of RNA biosynthetic process | 1.59E-18 | 3.37E-20 |
| GO:0040012 | BIOLOGICAL_PROCESS | regulation of locomotion | 1.78E-18 | 3.79E-20 |
| GO:0010468 | BIOLOGICAL_PROCESS | regulation of gene expression | 2.15E-18 | 4.58E-20 |
| GO:0002520 | BIOLOGICAL_PROCESS | immune system development | 2.47E-18 | 5.31E-20 |
| GO:1903506 | BIOLOGICAL_PROCESS | regulation of nucleic acid-templated transcription | 3.35E-18 | 7.22E-20 |
| GO:0043085 | BIOLOGICAL_PROCESS | positive regulation of catalytic activity | 3.68E-18 | 7.95E-20 |
| GO:0048870 | BIOLOGICAL_PROCESS | cell motility | 3.68E-18 | 8.05E-20 |
| GO:0051050 | BIOLOGICAL_PROCESS | positive regulation of transport | 3.68E-18 | 8.02E-20 |
| GO:0051674 | BIOLOGICAL_PROCESS | localization of cell | 3.68E-18 | 8.05E-20 |
| GO:2000145 | BIOLOGICAL_PROCESS | regulation of cell motility | 3.88E-18 | 8.54E-20 |
| GO:0006355 | BIOLOGICAL_PROCESS | regulation of transcription, DNA-templated | 4.42E-18 | 9.75E-20 |
| GO:0050767 | BIOLOGICAL_PROCESS | regulation of neurogenesis | 5.73E-18 | 1.27E-19 |
| GO:1901699 | BIOLOGICAL_PROCESS | cellular response to nitrogen compound | 5.76E-18 | 1.29E-19 |
| GO:0071407 | BIOLOGICAL_PROCESS | cellular response to organic cyclic compound | 5.76E-18 | 1.29E-19 |
| GO:0097659 | BIOLOGICAL_PROCESS | nucleic acid-templated transcription | 8.05E-18 | 1.80E-19 |
| GO:0098916 | BIOLOGICAL_PROCESS | anterograde trans-synaptic signaling | 8.60E-18 | 1.97E-19 |
| GO:0043170 | BIOLOGICAL_PROCESS | macromolecule metabolic process | 8.60E-18 | 1.96E-19 |
| GO:0007268 | BIOLOGICAL_PROCESS | chemical synaptic transmission | 8.60E-18 | 1.97E-19 |
| GO:0099536 | BIOLOGICAL_PROCESS | synaptic signaling | 8.60E-18 | 1.97E-19 |
| GO:0099537 | BIOLOGICAL_PROCESS | trans-synaptic signaling | 8.60E-18 | 1.97E-19 |
| GO:0003779 | MOLECULAR_FUNCTION | actin binding | 8.82E-18 | 2.02E-19 |
| GO:0031175 | BIOLOGICAL_PROCESS | neuron projection development | 1.01E-17 | 2.34E-19 |
| GO:0002682 | BIOLOGICAL_PROCESS | regulation of immune system process | 1.03E-17 | 2.38E-19 |
| GO:0016820 | MOLECULAR_FUNCTION | hydrolase activity, acting on acid anhydrides, catalyzing transmembrane movement of substances | 1.13E-17 | 2.61E-19 |
| GO:0018212 | BIOLOGICAL_PROCESS | peptidyl-tyrosine modification | 1.24E-17 | 2.90E-19 |
| GO:0001568 | BIOLOGICAL_PROCESS | blood vessel development | 1.25E-17 | 2.91E-19 |
| GO:0030097 | BIOLOGICAL_PROCESS | hemopoiesis | 1.37E-17 | 3.21E-19 |
| GO:0006351 | BIOLOGICAL_PROCESS | transcription, DNA-templated | 1.74E-17 | 4.11E-19 |
| GO:0007389 | BIOLOGICAL_PROCESS | pattern specification process | 1.87E-17 | 4.42E-19 |
| GO:0007178 | BIOLOGICAL_PROCESS | transmembrane receptor protein serine/threonine kinase signaling pathway | 2.10E-17 | 4.98E-19 |
| GO:0045211 | CELLULAR_COMPONENT | postsynaptic membrane | 2.96E-17 | 7.06E-19 |
| GO:0030054 | CELLULAR_COMPONENT | cell junction | 3.53E-17 | 8.44E-19 |
| GO:0048858 | BIOLOGICAL_PROCESS | cell projection morphogenesis | 3.63E-17 | 8.71E-19 |
| GO:0008528 | MOLECULAR_FUNCTION | G-protein coupled peptide receptor activity | 3.87E-17 | 9.37E-19 |
| GO:0051726 | BIOLOGICAL_PROCESS | regulation of cell cycle | 3.87E-17 | 9.37E-19 |
| GO:0007169 | BIOLOGICAL_PROCESS | transmembrane receptor protein tyrosine kinase signaling pathway | 6.12E-17 | 1.49E-18 |
| GO:0051171 | BIOLOGICAL_PROCESS | regulation of nitrogen compound metabolic process | 6.51E-17 | 1.59E-18 |
| GO:0032880 | BIOLOGICAL_PROCESS | regulation of protein localization | 6.83E-17 | 1.67E-18 |
| GO:0048812 | BIOLOGICAL_PROCESS | neuron projection morphogenesis | 8.05E-17 | 1.98E-18 |
| GO:0001653 | MOLECULAR_FUNCTION | peptide receptor activity | 9.65E-17 | 2.38E-18 |
| GO:0032990 | BIOLOGICAL_PROCESS | cell part morphogenesis | 1.02E-16 | 2.53E-18 |
| GO:0033993 | BIOLOGICAL_PROCESS | response to lipid | 1.06E-16 | 2.63E-18 |
| GO:0040007 | BIOLOGICAL_PROCESS | growth | 1.28E-16 | 3.18E-18 |
| GO:0032774 | BIOLOGICAL_PROCESS | RNA biosynthetic process | 1.31E-16 | 3.27E-18 |
| GO:0098609 | BIOLOGICAL_PROCESS | cell-cell adhesion | 1.47E-16 | 3.70E-18 |
| GO:0036094 | MOLECULAR_FUNCTION | small molecule binding | 1.56E-16 | 3.92E-18 |
| GO:0061061 | BIOLOGICAL_PROCESS | muscle structure development | 1.69E-16 | 4.26E-18 |
| GO:0009611 | BIOLOGICAL_PROCESS | response to wounding | 1.69E-16 | 4.28E-18 |
| GO:1902533 | BIOLOGICAL_PROCESS | positive regulation of intracellular signal transduction | 2.02E-16 | 5.13E-18 |
| GO:0044424 | CELLULAR_COMPONENT | intracellular part | 2.12E-16 | 5.42E-18 |
| GO:0023014 | BIOLOGICAL_PROCESS | signal transduction by protein phosphorylation | 2.35E-16 | 6.03E-18 |
| GO:0043168 | MOLECULAR_FUNCTION | anion binding | 2.91E-16 | 7.46E-18 |
| GO:0030029 | BIOLOGICAL_PROCESS | actin filament-based process | 3.05E-16 | 7.87E-18 |
| GO:0009889 | BIOLOGICAL_PROCESS | regulation of biosynthetic process | 3.05E-16 | 7.88E-18 |
| GO:0007423 | BIOLOGICAL_PROCESS | sensory organ development | 3.89E-16 | 1.01E-17 |
| GO:0003002 | BIOLOGICAL_PROCESS | regionalization | 3.96E-16 | 1.03E-17 |
| GO:0042626 | MOLECULAR_FUNCTION | ATPase activity, coupled to transmembrane movement of substances | 5.13E-16 | 1.34E-17 |
| GO:0031326 | BIOLOGICAL_PROCESS | regulation of cellular biosynthetic process | 5.92E-16 | 1.55E-17 |
| GO:0000975 | MOLECULAR_FUNCTION | regulatory region DNA binding | 5.96E-16 | 1.58E-17 |
| GO:0001067 | MOLECULAR_FUNCTION | regulatory region nucleic acid binding | 5.96E-16 | 1.58E-17 |
| GO:0044212 | MOLECULAR_FUNCTION | transcription regulatory region DNA binding | 5.96E-16 | 1.58E-17 |
| GO:0097190 | BIOLOGICAL_PROCESS | apoptotic signaling pathway | 7.57E-16 | 2.01E-17 |
| GO:0035239 | BIOLOGICAL_PROCESS | tube morphogenesis | 8.52E-16 | 2.27E-17 |
| GO:0019900 | MOLECULAR_FUNCTION | kinase binding | 9.18E-16 | 2.46E-17 |
| GO:0017111 | MOLECULAR_FUNCTION | nucleoside-triphosphatase activity | 9.96E-16 | 2.67E-17 |
| GO:0071396 | BIOLOGICAL_PROCESS | cellular response to lipid | 1.12E-15 | 3.03E-17 |
| GO:0045596 | BIOLOGICAL_PROCESS | negative regulation of cell differentiation | 1.15E-15 | 3.11E-17 |
| GO:0044092 | BIOLOGICAL_PROCESS | negative regulation of molecular function | 1.57E-15 | 4.26E-17 |
| GO:0010556 | BIOLOGICAL_PROCESS | regulation of macromolecule biosynthetic process | 1.75E-15 | 4.75E-17 |
| GO:0012505 | CELLULAR_COMPONENT | endomembrane system | 2.17E-15 | 5.91E-17 |
| GO:0000278 | BIOLOGICAL_PROCESS | mitotic cell cycle | 2.33E-15 | 6.38E-17 |
| GO:0051336 | BIOLOGICAL_PROCESS | regulation of hydrolase activity | 2.36E-15 | 6.48E-17 |
| GO:1903561 | CELLULAR_COMPONENT | extracellular vesicle | 2.55E-15 | 7.04E-17 |
| GO:0043230 | CELLULAR_COMPONENT | extracellular organelle | 2.55E-15 | 7.04E-17 |
| GO:0001664 | MOLECULAR_FUNCTION | G-protein coupled receptor binding | 2.66E-15 | 7.39E-17 |
| GO:1903831 | BIOLOGICAL_PROCESS | signal transduction involved in cellular response to ammonium ion | 2.90E-15 | 8.18E-17 |
| GO:1905145 | BIOLOGICAL_PROCESS | cellular response to acetylcholine | 2.90E-15 | 8.18E-17 |
| GO:1905144 | BIOLOGICAL_PROCESS | response to acetylcholine | 2.90E-15 | 8.18E-17 |
| GO:0007213 | BIOLOGICAL_PROCESS | G-protein coupled acetylcholine receptor signaling pathway | 2.90E-15 | 8.18E-17 |
| GO:0095500 | BIOLOGICAL_PROCESS | acetylcholine receptor signaling pathway | 2.90E-15 | 8.18E-17 |
| GO:0048667 | BIOLOGICAL_PROCESS | cell morphogenesis involved in neuron differentiation | 4.02E-15 | 1.14E-16 |
| GO:0070062 | CELLULAR_COMPONENT | extracellular exosome | 4.76E-15 | 1.35E-16 |
| GO:0051046 | BIOLOGICAL_PROCESS | regulation of secretion | 5.06E-15 | 1.44E-16 |
| GO:0071900 | BIOLOGICAL_PROCESS | regulation of protein serine/threonine kinase activity | 5.06E-15 | 1.44E-16 |
| GO:0008289 | MOLECULAR_FUNCTION | lipid binding | 5.96E-15 | 1.71E-16 |
| GO:0015399 | MOLECULAR_FUNCTION | primary active transmembrane transporter activity | 6.28E-15 | 1.81E-16 |
| GO:0015405 | MOLECULAR_FUNCTION | P-P-bond-hydrolysis-driven transmembrane transporter activity | 6.28E-15 | 1.81E-16 |
| GO:0006955 | BIOLOGICAL_PROCESS | immune response | 6.89E-15 | 1.99E-16 |
| GO:0015464 | MOLECULAR_FUNCTION | acetylcholine receptor activity | 6.90E-15 | 2.00E-16 |
| GO:0045202 | CELLULAR_COMPONENT | synapse | 7.96E-15 | 2.31E-16 |
| GO:0005057 | MOLECULAR_FUNCTION | signal transducer activity, downstream of receptor | 8.11E-15 | 2.36E-16 |
| GO:0006470 | BIOLOGICAL_PROCESS | protein dephosphorylation | 8.29E-15 | 2.42E-16 |
| GO:0016817 | MOLECULAR_FUNCTION | hydrolase activity, acting on acid anhydrides | 1.01E-14 | 2.96E-16 |
| GO:2000112 | BIOLOGICAL_PROCESS | regulation of cellular macromolecule biosynthetic process | 1.51E-14 | 4.45E-16 |
| GO:0030036 | BIOLOGICAL_PROCESS | actin cytoskeleton organization | 1.54E-14 | 4.55E-16 |
| GO:0042326 | BIOLOGICAL_PROCESS | negative regulation of phosphorylation | 1.56E-14 | 4.61E-16 |
| GO:0060562 | BIOLOGICAL_PROCESS | epithelial tube morphogenesis | 1.66E-14 | 4.93E-16 |
| GO:0051130 | BIOLOGICAL_PROCESS | positive regulation of cellular component organization | 1.79E-14 | 5.33E-16 |
| GO:0005912 | CELLULAR_COMPONENT | adherens junction | 1.84E-14 | 5.48E-16 |
| GO:0051253 | BIOLOGICAL_PROCESS | negative regulation of RNA metabolic process | 1.87E-14 | 5.60E-16 |
| GO:0051015 | MOLECULAR_FUNCTION | actin filament binding | 1.91E-14 | 5.75E-16 |
| GO:0022603 | BIOLOGICAL_PROCESS | regulation of anatomical structure morphogenesis | 1.93E-14 | 5.82E-16 |
| GO:0010629 | BIOLOGICAL_PROCESS | negative regulation of gene expression | 2.25E-14 | 6.79E-16 |
| GO:0019901 | MOLECULAR_FUNCTION | protein kinase binding | 2.54E-14 | 7.69E-16 |
| GO:0016462 | MOLECULAR_FUNCTION | pyrophosphatase activity | 2.61E-14 | 7.91E-16 |
| GO:0008285 | BIOLOGICAL_PROCESS | negative regulation of cell proliferation | 2.72E-14 | 8.29E-16 |
| GO:0005543 | MOLECULAR_FUNCTION | phospholipid binding | 2.76E-14 | 8.47E-16 |
| GO:0070201 | BIOLOGICAL_PROCESS | regulation of establishment of protein localization | 2.76E-14 | 8.47E-16 |
| GO:0045664 | BIOLOGICAL_PROCESS | regulation of neuron differentiation | 3.03E-14 | 9.31E-16 |
| GO:0005887 | CELLULAR_COMPONENT | integral component of plasma membrane | 3.46E-14 | 1.06E-15 |
| GO:0061564 | BIOLOGICAL_PROCESS | axon development | 3.64E-14 | 1.12E-15 |
| GO:0060359 | BIOLOGICAL_PROCESS | response to ammonium ion | 3.74E-14 | 1.16E-15 |
| GO:0004983 | MOLECULAR_FUNCTION | neuropeptide Y receptor activity | 3.74E-14 | 1.16E-15 |
| GO:0016043 | BIOLOGICAL_PROCESS | cellular component organization | 3.93E-14 | 1.22E-15 |
| GO:0016818 | MOLECULAR_FUNCTION | hydrolase activity, acting on acid anhydrides, in phosphorus-containing anhydrides | 4.86E-14 | 1.52E-15 |
| GO:0010942 | BIOLOGICAL_PROCESS | positive regulation of cell death | 4.86E-14 | 1.52E-15 |
| GO:0042623 | MOLECULAR_FUNCTION | ATPase activity, coupled | 5.02E-14 | 1.58E-15 |
| GO:0060341 | BIOLOGICAL_PROCESS | regulation of cellular localization | 5.77E-14 | 1.82E-15 |
| GO:0009792 | BIOLOGICAL_PROCESS | embryo development ending in birth or egg hatching | 6.02E-14 | 1.90E-15 |
| GO:0051223 | BIOLOGICAL_PROCESS | regulation of protein transport | 6.38E-14 | 2.02E-15 |
| GO:0045934 | BIOLOGICAL_PROCESS | negative regulation of nucleobase-containing compound metabolic process | 8.11E-14 | 2.58E-15 |
| GO:0005623 | CELLULAR_COMPONENT | cell | 8.41E-14 | 2.68E-15 |
| GO:0080134 | BIOLOGICAL_PROCESS | regulation of response to stress | 8.58E-14 | 2.74E-15 |
| GO:0070161 | CELLULAR_COMPONENT | anchoring junction | 9.27E-14 | 2.97E-15 |
| GO:0048545 | BIOLOGICAL_PROCESS | response to steroid hormone | 9.35E-14 | 3.01E-15 |
| GO:1903530 | BIOLOGICAL_PROCESS | regulation of secretion by cell | 9.42E-14 | 3.04E-15 |
| GO:0045892 | BIOLOGICAL_PROCESS | negative regulation of transcription, DNA-templated | 9.89E-14 | 3.20E-15 |
| GO:0098772 | MOLECULAR_FUNCTION | molecular function regulator | 1.14E-13 | 3.71E-15 |
| GO:0043492 | MOLECULAR_FUNCTION | ATPase activity, coupled to movement of substances | 1.14E-13 | 3.71E-15 |
| GO:0032989 | BIOLOGICAL_PROCESS | cellular component morphogenesis | 1.22E-13 | 3.96E-15 |
| GO:1902679 | BIOLOGICAL_PROCESS | negative regulation of RNA biosynthetic process | 1.22E-13 | 3.97E-15 |
| GO:0031226 | CELLULAR_COMPONENT | intrinsic component of plasma membrane | 1.29E-13 | 4.21E-15 |
| GO:0046903 | BIOLOGICAL_PROCESS | secretion | 1.35E-13 | 4.43E-15 |
| GO:0004702 | MOLECULAR_FUNCTION | signal transducer, downstream of receptor, with serine/threonine kinase activity | 1.73E-13 | 5.69E-15 |
| GO:0007409 | BIOLOGICAL_PROCESS | axonogenesis | 1.75E-13 | 5.76E-15 |
| GO:0031400 | BIOLOGICAL_PROCESS | negative regulation of protein modification process | 1.79E-13 | 5.94E-15 |
| GO:0019904 | MOLECULAR_FUNCTION | protein domain specific binding | 2.11E-13 | 7.00E-15 |
| GO:0060537 | BIOLOGICAL_PROCESS | muscle tissue development | 2.31E-13 | 7.76E-15 |
| GO:0006887 | BIOLOGICAL_PROCESS | exocytosis | 2.31E-13 | 7.76E-15 |
| GO:0045165 | BIOLOGICAL_PROCESS | cell fate commitment | 2.31E-13 | 7.76E-15 |
| GO:0044464 | CELLULAR_COMPONENT | cell part | 2.31E-13 | 7.73E-15 |
| GO:0030155 | BIOLOGICAL_PROCESS | regulation of cell adhesion | 2.60E-13 | 8.75E-15 |
| GO:0022836 | MOLECULAR_FUNCTION | gated channel activity | 2.80E-13 | 9.45E-15 |
| GO:0014706 | BIOLOGICAL_PROCESS | striated muscle tissue development | 2.80E-13 | 9.48E-15 |
| GO:0046983 | MOLECULAR_FUNCTION | protein dimerization activity | 2.83E-13 | 9.58E-15 |
| GO:0007517 | BIOLOGICAL_PROCESS | muscle organ development | 3.10E-13 | 1.05E-14 |
| GO:0001775 | BIOLOGICAL_PROCESS | cell activation | 3.11E-13 | 1.06E-14 |
| GO:0044389 | MOLECULAR_FUNCTION | ubiquitin-like protein ligase binding | 3.11E-13 | 1.06E-14 |
| GO:0071383 | BIOLOGICAL_PROCESS | cellular response to steroid hormone stimulus | 3.13E-13 | 1.07E-14 |
| GO:1903507 | BIOLOGICAL_PROCESS | negative regulation of nucleic acid-templated transcription | 4.45E-13 | 1.53E-14 |
| GO:0016907 | MOLECULAR_FUNCTION | G-protein coupled acetylcholine receptor activity | 4.76E-13 | 1.64E-14 |
| GO:0099528 | MOLECULAR_FUNCTION | G-protein coupled neurotransmitter receptor activity | 4.76E-13 | 1.64E-14 |
| GO:0043009 | BIOLOGICAL_PROCESS | chordate embryonic development | 5.05E-13 | 1.75E-14 |
| GO:0097458 | CELLULAR_COMPONENT | neuron part | 5.08E-13 | 1.76E-14 |
| GO:0098602 | BIOLOGICAL_PROCESS | single organism cell adhesion | 6.88E-13 | 2.40E-14 |
| GO:0071242 | BIOLOGICAL_PROCESS | cellular response to ammonium ion | 6.88E-13 | 2.40E-14 |
| GO:0001933 | BIOLOGICAL_PROCESS | negative regulation of protein phosphorylation | 7.02E-13 | 2.45E-14 |
| GO:0051172 | BIOLOGICAL_PROCESS | negative regulation of nitrogen compound metabolic process | 7.12E-13 | 2.50E-14 |
| GO:1902580 | BIOLOGICAL_PROCESS | single-organism cellular localization | 7.22E-13 | 2.54E-14 |
| GO:0031012 | CELLULAR_COMPONENT | extracellular matrix | 7.54E-13 | 2.66E-14 |
| GO:0000902 | BIOLOGICAL_PROCESS | cell morphogenesis | 7.86E-13 | 2.78E-14 |
| GO:0031625 | MOLECULAR_FUNCTION | ubiquitin protein ligase binding | 7.87E-13 | 2.79E-14 |
| GO:0042060 | BIOLOGICAL_PROCESS | wound healing | 7.87E-13 | 2.79E-14 |
| GO:0002684 | BIOLOGICAL_PROCESS | positive regulation of immune system process | 7.93E-13 | 2.82E-14 |
| GO:0030055 | CELLULAR_COMPONENT | cell-substrate junction | 1.04E-12 | 3.75E-14 |
| GO:0005925 | CELLULAR_COMPONENT | focal adhesion | 1.04E-12 | 3.75E-14 |
| GO:0005924 | CELLULAR_COMPONENT | cell-substrate adherens junction | 1.04E-12 | 3.75E-14 |
| GO:0043086 | BIOLOGICAL_PROCESS | negative regulation of catalytic activity | 1.44E-12 | 5.18E-14 |
| GO:0000977 | MOLECULAR_FUNCTION | RNA polymerase II regulatory region sequence-specific DNA binding | 1.46E-12 | 5.28E-14 |
| GO:0001012 | MOLECULAR_FUNCTION | RNA polymerase II regulatory region DNA binding | 1.46E-12 | 5.28E-14 |
| GO:0009890 | BIOLOGICAL_PROCESS | negative regulation of biosynthetic process | 1.51E-12 | 5.48E-14 |
| GO:0043410 | BIOLOGICAL_PROCESS | positive regulation of MAPK cascade | 1.81E-12 | 6.56E-14 |
| GO:0051641 | BIOLOGICAL_PROCESS | cellular localization | 1.91E-12 | 6.95E-14 |
| GO:0004721 | MOLECULAR_FUNCTION | phosphoprotein phosphatase activity | 1.99E-12 | 7.27E-14 |
| GO:0097435 | BIOLOGICAL_PROCESS | supramolecular fiber organization | 2.51E-12 | 9.17E-14 |
| GO:0008284 | BIOLOGICAL_PROCESS | positive regulation of cell proliferation | 2.56E-12 | 9.36E-14 |
| GO:0009755 | BIOLOGICAL_PROCESS | hormone-mediated signaling pathway | 2.93E-12 | 1.07E-13 |
| GO:0010558 | BIOLOGICAL_PROCESS | negative regulation of macromolecule biosynthetic process | 3.03E-12 | 1.11E-13 |
| GO:0010720 | BIOLOGICAL_PROCESS | positive regulation of cell development | 3.45E-12 | 1.27E-13 |
| GO:0007212 | BIOLOGICAL_PROCESS | dopamine receptor signaling pathway | 3.70E-12 | 1.37E-13 |
| GO:0048589 | BIOLOGICAL_PROCESS | developmental growth | 3.90E-12 | 1.45E-13 |
| GO:0007049 | BIOLOGICAL_PROCESS | cell cycle | 4.34E-12 | 1.61E-13 |
| GO:0031327 | BIOLOGICAL_PROCESS | negative regulation of cellular biosynthetic process | 5.02E-12 | 1.87E-13 |
| GO:0007010 | BIOLOGICAL_PROCESS | cytoskeleton organization | 5.19E-12 | 1.94E-13 |
| GO:0050776 | BIOLOGICAL_PROCESS | regulation of immune response | 5.35E-12 | 2.02E-13 |
| GO:0030335 | BIOLOGICAL_PROCESS | positive regulation of cell migration | 5.35E-12 | 2.02E-13 |
| GO:0046649 | BIOLOGICAL_PROCESS | lymphocyte activation | 5.35E-12 | 2.02E-13 |
| GO:0033673 | BIOLOGICAL_PROCESS | negative regulation of kinase activity | 5.35E-12 | 2.02E-13 |
| GO:0015276 | MOLECULAR_FUNCTION | ligand-gated ion channel activity | 5.40E-12 | 2.04E-13 |
| GO:0022834 | MOLECULAR_FUNCTION | ligand-gated channel activity | 5.40E-12 | 2.04E-13 |
| GO:0010563 | BIOLOGICAL_PROCESS | negative regulation of phosphorus metabolic process | 5.47E-12 | 2.08E-13 |
| GO:0045936 | BIOLOGICAL_PROCESS | negative regulation of phosphate metabolic process | 5.47E-12 | 2.08E-13 |
| GO:2000113 | BIOLOGICAL_PROCESS | negative regulation of cellular macromolecule biosynthetic process | 6.04E-12 | 2.30E-13 |
| GO:0007610 | BIOLOGICAL_PROCESS | behavior | 6.54E-12 | 2.50E-13 |
| GO:0001501 | BIOLOGICAL_PROCESS | skeletal system development | 7.30E-12 | 2.80E-13 |
| GO:0018209 | BIOLOGICAL_PROCESS | peptidyl-serine modification | 8.34E-12 | 3.22E-13 |
| GO:0003713 | MOLECULAR_FUNCTION | transcription coactivator activity | 8.34E-12 | 3.22E-13 |
| GO:0018105 | BIOLOGICAL_PROCESS | peptidyl-serine phosphorylation | 8.34E-12 | 3.22E-13 |
| GO:0043401 | BIOLOGICAL_PROCESS | steroid hormone mediated signaling pathway | 1.02E-11 | 3.96E-13 |
| GO:0004952 | MOLECULAR_FUNCTION | dopamine neurotransmitter receptor activity | 1.02E-11 | 3.95E-13 |
| GO:0000976 | MOLECULAR_FUNCTION | transcription regulatory region sequence-specific DNA binding | 1.19E-11 | 4.62E-13 |
| GO:0007218 | BIOLOGICAL_PROCESS | neuropeptide signaling pathway | 1.23E-11 | 4.81E-13 |
| GO:0030900 | BIOLOGICAL_PROCESS | forebrain development | 1.23E-11 | 4.81E-13 |
| GO:0045321 | BIOLOGICAL_PROCESS | leukocyte activation | 1.28E-11 | 5.01E-13 |
| GO:0043068 | BIOLOGICAL_PROCESS | positive regulation of programmed cell death | 1.28E-11 | 5.01E-13 |
| GO:0001228 | MOLECULAR_FUNCTION | transcriptional activator activity, RNA polymerase II transcription regulatory region sequence-specific binding | 1.28E-11 | 5.01E-13 |
| GO:2000027 | BIOLOGICAL_PROCESS | regulation of organ morphogenesis | 1.36E-11 | 5.37E-13 |
| GO:0090092 | BIOLOGICAL_PROCESS | regulation of transmembrane receptor protein serine/threonine kinase signaling pathway | 1.36E-11 | 5.37E-13 |
| GO:0000982 | MOLECULAR_FUNCTION | transcription factor activity, RNA polymerase II core promoter proximal region sequence-specific binding | 1.36E-11 | 5.37E-13 |
| GO:0015081 | MOLECULAR_FUNCTION | sodium ion transmembrane transporter activity | 1.42E-11 | 5.63E-13 |
| GO:0051248 | BIOLOGICAL_PROCESS | negative regulation of protein metabolic process | 1.42E-11 | 5.63E-13 |
| GO:0051347 | BIOLOGICAL_PROCESS | positive regulation of transferase activity | 1.45E-11 | 5.79E-13 |
| GO:0005272 | MOLECULAR_FUNCTION | sodium channel activity | 1.46E-11 | 5.82E-13 |
| GO:0006996 | BIOLOGICAL_PROCESS | organelle organization | 1.47E-11 | 5.89E-13 |
| GO:0031344 | BIOLOGICAL_PROCESS | regulation of cell projection organization | 1.57E-11 | 6.29E-13 |
| GO:0016567 | BIOLOGICAL_PROCESS | protein ubiquitination | 2.31E-11 | 9.26E-13 |
| GO:0051129 | BIOLOGICAL_PROCESS | negative regulation of cellular component organization | 2.36E-11 | 9.49E-13 |
| GO:0030522 | BIOLOGICAL_PROCESS | intracellular receptor signaling pathway | 2.39E-11 | 9.66E-13 |
| GO:0003007 | BIOLOGICAL_PROCESS | heart morphogenesis | 2.39E-11 | 9.66E-13 |
| GO:1903827 | BIOLOGICAL_PROCESS | regulation of cellular protein localization | 2.73E-11 | 1.11E-12 |
| GO:0003690 | MOLECULAR_FUNCTION | double-stranded DNA binding | 3.07E-11 | 1.25E-12 |
| GO:0060485 | BIOLOGICAL_PROCESS | mesenchyme development | 3.20E-11 | 1.31E-12 |
| GO:0043065 | BIOLOGICAL_PROCESS | positive regulation of apoptotic process | 3.20E-11 | 1.31E-12 |
| GO:0016337 | BIOLOGICAL_PROCESS | single organismal cell-cell adhesion | 3.23E-11 | 1.33E-12 |
| GO:0019199 | MOLECULAR_FUNCTION | transmembrane receptor protein kinase activity | 3.23E-11 | 1.33E-12 |
| GO:0071944 | CELLULAR_COMPONENT | cell periphery | 3.23E-11 | 1.33E-12 |
| GO:0006469 | BIOLOGICAL_PROCESS | negative regulation of protein kinase activity | 3.47E-11 | 1.43E-12 |
| GO:0071840 | BIOLOGICAL_PROCESS | cellular component organization or biogenesis | 3.48E-11 | 1.43E-12 |
| GO:0032446 | BIOLOGICAL_PROCESS | protein modification by small protein conjugation | 4.24E-11 | 1.75E-12 |
| GO:0099568 | CELLULAR_COMPONENT | cytoplasmic region | 4.90E-11 | 2.03E-12 |
| GO:0043235 | CELLULAR_COMPONENT | receptor complex | 4.90E-11 | 2.03E-12 |
| GO:0010243 | BIOLOGICAL_PROCESS | response to organonitrogen compound | 5.99E-11 | 2.49E-12 |
| GO:0050673 | BIOLOGICAL_PROCESS | epithelial cell proliferation | 6.34E-11 | 2.64E-12 |
| GO:0008015 | BIOLOGICAL_PROCESS | blood circulation | 6.50E-11 | 2.73E-12 |
| GO:0003013 | BIOLOGICAL_PROCESS | circulatory system process | 6.50E-11 | 2.73E-12 |
| GO:0051272 | BIOLOGICAL_PROCESS | positive regulation of cellular component movement | 6.50E-11 | 2.73E-12 |
| GO:0044428 | CELLULAR_COMPONENT | nuclear part | 6.75E-11 | 2.84E-12 |
| GO:0032940 | BIOLOGICAL_PROCESS | secretion by cell | 8.23E-11 | 3.47E-12 |
| GO:0002064 | BIOLOGICAL_PROCESS | epithelial cell development | 8.52E-11 | 3.60E-12 |
| GO:0002521 | BIOLOGICAL_PROCESS | leukocyte differentiation | 8.52E-11 | 3.60E-12 |
| GO:0048514 | BIOLOGICAL_PROCESS | blood vessel morphogenesis | 8.57E-11 | 3.63E-12 |
| GO:0070647 | BIOLOGICAL_PROCESS | protein modification by small protein conjugation or removal | 8.87E-11 | 3.77E-12 |
| GO:0000122 | BIOLOGICAL_PROCESS | negative regulation of transcription from RNA polymerase II promoter | 9.43E-11 | 4.01E-12 |
| GO:0032269 | BIOLOGICAL_PROCESS | negative regulation of cellular protein metabolic process | 1.07E-10 | 4.55E-12 |
| GO:0043269 | BIOLOGICAL_PROCESS | regulation of ion transport | 1.13E-10 | 4.81E-12 |
| GO:0051348 | BIOLOGICAL_PROCESS | negative regulation of transferase activity | 1.19E-10 | 5.09E-12 |
| GO:0006814 | BIOLOGICAL_PROCESS | sodium ion transport | 1.32E-10 | 5.68E-12 |
| GO:0009605 | BIOLOGICAL_PROCESS | response to external stimulus | 1.34E-10 | 5.78E-12 |
| GO:2000147 | BIOLOGICAL_PROCESS | positive regulation of cell motility | 1.60E-10 | 6.93E-12 |
| GO:0034706 | CELLULAR_COMPONENT | sodium channel complex | 1.60E-10 | 6.93E-12 |
| GO:0033674 | BIOLOGICAL_PROCESS | positive regulation of kinase activity | 1.60E-10 | 6.93E-12 |
| GO:0030162 | BIOLOGICAL_PROCESS | regulation of proteolysis | 1.72E-10 | 7.45E-12 |
| GO:0080135 | BIOLOGICAL_PROCESS | regulation of cellular response to stress | 1.72E-10 | 7.46E-12 |
| GO:0044087 | BIOLOGICAL_PROCESS | regulation of cellular component biogenesis | 1.85E-10 | 8.04E-12 |
| GO:0005886 | CELLULAR_COMPONENT | plasma membrane | 1.87E-10 | 8.18E-12 |
| GO:0040017 | BIOLOGICAL_PROCESS | positive regulation of locomotion | 1.99E-10 | 8.68E-12 |
| GO:0001816 | BIOLOGICAL_PROCESS | cytokine production | 2.00E-10 | 8.77E-12 |
| GO:0090287 | BIOLOGICAL_PROCESS | regulation of cellular response to growth factor stimulus | 2.00E-10 | 8.77E-12 |
| GO:0045860 | BIOLOGICAL_PROCESS | positive regulation of protein kinase activity | 2.00E-10 | 8.77E-12 |
| GO:0051345 | BIOLOGICAL_PROCESS | positive regulation of hydrolase activity | 2.24E-10 | 9.88E-12 |
| GO:0001654 | BIOLOGICAL_PROCESS | eye development | 2.58E-10 | 1.14E-11 |
| GO:0009894 | BIOLOGICAL_PROCESS | regulation of catabolic process | 2.58E-10 | 1.14E-11 |
| GO:1903047 | BIOLOGICAL_PROCESS | mitotic cell cycle process | 2.72E-10 | 1.21E-11 |
| GO:0097159 | MOLECULAR_FUNCTION | organic cyclic compound binding | 2.80E-10 | 1.24E-11 |
| GO:0097485 | BIOLOGICAL_PROCESS | neuron projection guidance | 2.81E-10 | 1.25E-11 |
| GO:0005938 | CELLULAR_COMPONENT | cell cortex | 2.84E-10 | 1.27E-11 |
| GO:0050877 | BIOLOGICAL_PROCESS | neurological system process | 2.85E-10 | 1.27E-11 |
| GO:0030030 | BIOLOGICAL_PROCESS | cell projection organization | 3.01E-10 | 1.35E-11 |
| GO:0016311 | BIOLOGICAL_PROCESS | dephosphorylation | 3.17E-10 | 1.42E-11 |
| GO:1990837 | MOLECULAR_FUNCTION | sequence-specific double-stranded DNA binding | 3.40E-10 | 1.53E-11 |
| GO:0022804 | MOLECULAR_FUNCTION | active transmembrane transporter activity | 3.52E-10 | 1.58E-11 |
| GO:0042578 | MOLECULAR_FUNCTION | phosphoric ester hydrolase activity | 3.70E-10 | 1.67E-11 |
| GO:0040008 | BIOLOGICAL_PROCESS | regulation of growth | 3.70E-10 | 1.67E-11 |
| GO:0022402 | BIOLOGICAL_PROCESS | cell cycle process | 3.95E-10 | 1.79E-11 |
| GO:0031410 | CELLULAR_COMPONENT | cytoplasmic vesicle | 4.23E-10 | 1.92E-11 |
| GO:0097708 | CELLULAR_COMPONENT | intracellular vesicle | 4.23E-10 | 1.92E-11 |
| GO:0000989 | MOLECULAR_FUNCTION | transcription factor activity, transcription factor binding | 4.66E-10 | 2.12E-11 |
| GO:1902582 | BIOLOGICAL_PROCESS | single-organism intracellular transport | 4.95E-10 | 2.26E-11 |
| GO:0001518 | CELLULAR_COMPONENT | voltage-gated sodium channel complex | 4.95E-10 | 2.26E-11 |
| GO:0048732 | BIOLOGICAL_PROCESS | gland development | 5.04E-10 | 2.31E-11 |
| GO:0050778 | BIOLOGICAL_PROCESS | positive regulation of immune response | 5.71E-10 | 2.64E-11 |
| GO:0097193 | BIOLOGICAL_PROCESS | intrinsic apoptotic signaling pathway | 5.71E-10 | 2.64E-11 |
| GO:0048738 | BIOLOGICAL_PROCESS | cardiac muscle tissue development | 5.71E-10 | 2.64E-11 |
| GO:0007187 | BIOLOGICAL_PROCESS | G-protein coupled receptor signaling pathway, coupled to cyclic nucleotide second messenger | 5.71E-10 | 2.64E-11 |
| GO:0044448 | CELLULAR_COMPONENT | cell cortex part | 5.71E-10 | 2.64E-11 |
| GO:0003158 | BIOLOGICAL_PROCESS | endothelium development | 5.90E-10 | 2.74E-11 |
| GO:0071875 | BIOLOGICAL_PROCESS | adrenergic receptor signaling pathway | 5.90E-10 | 2.74E-11 |
| GO:0045786 | BIOLOGICAL_PROCESS | negative regulation of cell cycle | 6.41E-10 | 2.98E-11 |
| GO:0060070 | BIOLOGICAL_PROCESS | canonical Wnt signaling pathway | 6.75E-10 | 3.15E-11 |
| GO:0023061 | BIOLOGICAL_PROCESS | signal release | 8.56E-10 | 4.00E-11 |
| GO:0050769 | BIOLOGICAL_PROCESS | positive regulation of neurogenesis | 9.47E-10 | 4.43E-11 |
| GO:0007015 | BIOLOGICAL_PROCESS | actin filament organization | 1.12E-09 | 5.26E-11 |
| GO:1901363 | MOLECULAR_FUNCTION | heterocyclic compound binding | 1.15E-09 | 5.39E-11 |
| GO:0001817 | BIOLOGICAL_PROCESS | regulation of cytokine production | 1.23E-09 | 5.82E-11 |
| GO:0050839 | MOLECULAR_FUNCTION | cell adhesion molecule binding | 1.23E-09 | 5.82E-11 |
| GO:0007411 | BIOLOGICAL_PROCESS | axon guidance | 1.45E-09 | 6.83E-11 |
| GO:0004714 | MOLECULAR_FUNCTION | transmembrane receptor protein tyrosine kinase activity | 1.50E-09 | 7.12E-11 |
| GO:0002764 | BIOLOGICAL_PROCESS | immune response-regulating signaling pathway | 1.50E-09 | 7.12E-11 |
| GO:0070997 | BIOLOGICAL_PROCESS | neuron death | 1.66E-09 | 7.90E-11 |
| GO:0004935 | MOLECULAR_FUNCTION | adrenergic receptor activity | 1.66E-09 | 7.90E-11 |
| GO:0042886 | BIOLOGICAL_PROCESS | amide transport | 1.77E-09 | 8.47E-11 |
| GO:0003712 | MOLECULAR_FUNCTION | transcription cofactor activity | 1.77E-09 | 8.47E-11 |
| GO:0031981 | CELLULAR_COMPONENT | nuclear lumen | 2.27E-09 | 1.09E-10 |
| GO:0043405 | BIOLOGICAL_PROCESS | regulation of MAP kinase activity | 2.31E-09 | 1.11E-10 |
| GO:0005248 | MOLECULAR_FUNCTION | voltage-gated sodium channel activity | 2.31E-09 | 1.11E-10 |
| GO:1905030 | MOLECULAR_FUNCTION | voltage-gated ion channel activity involved in regulation of postsynaptic membrane potential | 2.31E-09 | 1.11E-10 |
| GO:0044257 | BIOLOGICAL_PROCESS | cellular protein catabolic process | 2.54E-09 | 1.23E-10 |
| GO:0010975 | BIOLOGICAL_PROCESS | regulation of neuron projection development | 2.60E-09 | 1.25E-10 |
| GO:0044444 | CELLULAR_COMPONENT | cytoplasmic part | 2.64E-09 | 1.28E-10 |
| GO:0048568 | BIOLOGICAL_PROCESS | embryonic organ development | 2.74E-09 | 1.33E-10 |
| GO:0030234 | MOLECULAR_FUNCTION | enzyme regulator activity | 3.15E-09 | 1.53E-10 |
| GO:0050678 | BIOLOGICAL_PROCESS | regulation of epithelial cell proliferation | 3.25E-09 | 1.58E-10 |
| GO:0043270 | BIOLOGICAL_PROCESS | positive regulation of ion transport | 3.25E-09 | 1.58E-10 |
| GO:0042802 | MOLECULAR_FUNCTION | identical protein binding | 3.58E-09 | 1.75E-10 |
| GO:0061448 | BIOLOGICAL_PROCESS | connective tissue development | 3.70E-09 | 1.82E-10 |
| GO:0001503 | BIOLOGICAL_PROCESS | ossification | 3.70E-09 | 1.82E-10 |
| GO:0005200 | MOLECULAR_FUNCTION | structural constituent of cytoskeleton | 3.70E-09 | 1.82E-10 |
| GO:0098589 | CELLULAR_COMPONENT | membrane region | 3.87E-09 | 1.91E-10 |
| GO:0001667 | BIOLOGICAL_PROCESS | ameboidal-type cell migration | 3.87E-09 | 1.91E-10 |
| GO:0002253 | BIOLOGICAL_PROCESS | activation of immune response | 3.87E-09 | 1.91E-10 |
| GO:0002757 | BIOLOGICAL_PROCESS | immune response-activating signal transduction | 3.87E-09 | 1.91E-10 |
| GO:0030163 | BIOLOGICAL_PROCESS | protein catabolic process | 4.04E-09 | 2.00E-10 |
| GO:0090596 | BIOLOGICAL_PROCESS | sensory organ morphogenesis | 5.42E-09 | 2.69E-10 |
| GO:0051649 | BIOLOGICAL_PROCESS | establishment of localization in cell | 6.12E-09 | 3.04E-10 |
| GO:0006511 | BIOLOGICAL_PROCESS | ubiquitin-dependent protein catabolic process | 6.35E-09 | 3.17E-10 |
| GO:0009628 | BIOLOGICAL_PROCESS | response to abiotic stimulus | 6.35E-09 | 3.17E-10 |
| GO:0005578 | CELLULAR_COMPONENT | proteinaceous extracellular matrix | 7.62E-09 | 3.81E-10 |
| GO:0003006 | BIOLOGICAL_PROCESS | developmental process involved in reproduction | 8.09E-09 | 4.06E-10 |
| GO:0007346 | BIOLOGICAL_PROCESS | regulation of mitotic cell cycle | 8.09E-09 | 4.06E-10 |
| GO:0005230 | MOLECULAR_FUNCTION | extracellular ligand-gated ion channel activity | 8.48E-09 | 4.26E-10 |
| GO:0034330 | BIOLOGICAL_PROCESS | cell junction organization | 8.69E-09 | 4.39E-10 |
| GO:0071560 | BIOLOGICAL_PROCESS | cellular response to transforming growth factor beta stimulus | 8.69E-09 | 4.39E-10 |
| GO:0071559 | BIOLOGICAL_PROCESS | response to transforming growth factor beta | 8.69E-09 | 4.39E-10 |
| GO:0051962 | BIOLOGICAL_PROCESS | positive regulation of nervous system development | 8.85E-09 | 4.48E-10 |
| GO:0005525 | MOLECULAR_FUNCTION | GTP binding | 9.19E-09 | 4.66E-10 |
| GO:0048863 | BIOLOGICAL_PROCESS | stem cell differentiation | 9.34E-09 | 4.74E-10 |
| GO:2001233 | BIOLOGICAL_PROCESS | regulation of apoptotic signaling pathway | 1.01E-08 | 5.14E-10 |
| GO:0007265 | BIOLOGICAL_PROCESS | Ras protein signal transduction | 1.18E-08 | 6.04E-10 |
| GO:0043010 | BIOLOGICAL_PROCESS | camera-type eye development | 1.18E-08 | 6.04E-10 |
| GO:0004842 | MOLECULAR_FUNCTION | ubiquitin-protein transferase activity | 1.25E-08 | 6.41E-10 |
| GO:0045446 | BIOLOGICAL_PROCESS | endothelial cell differentiation | 1.28E-08 | 6.56E-10 |
| GO:0005615 | CELLULAR_COMPONENT | extracellular space | 1.39E-08 | 7.14E-10 |
| GO:0001525 | BIOLOGICAL_PROCESS | angiogenesis | 1.41E-08 | 7.27E-10 |
| GO:0046777 | BIOLOGICAL_PROCESS | protein autophosphorylation | 1.41E-08 | 7.27E-10 |
| GO:1902532 | BIOLOGICAL_PROCESS | negative regulation of intracellular signal transduction | 1.44E-08 | 7.42E-10 |
| GO:0051603 | BIOLOGICAL_PROCESS | proteolysis involved in cellular protein catabolic process | 1.47E-08 | 7.61E-10 |
| GO:0015833 | BIOLOGICAL_PROCESS | peptide transport | 1.53E-08 | 7.90E-10 |
| GO:0033043 | BIOLOGICAL_PROCESS | regulation of organelle organization | 1.54E-08 | 8.00E-10 |
| GO:0005829 | CELLULAR_COMPONENT | cytosol | 1.78E-08 | 9.23E-10 |
| GO:0043632 | BIOLOGICAL_PROCESS | modification-dependent macromolecule catabolic process | 1.91E-08 | 9.95E-10 |
| GO:0043169 | MOLECULAR_FUNCTION | cation binding | 2.14E-08 | 1.11E-09 |
| GO:0021537 | BIOLOGICAL_PROCESS | telencephalon development | 2.19E-08 | 1.14E-09 |
| GO:0048562 | BIOLOGICAL_PROCESS | embryonic organ morphogenesis | 2.29E-08 | 1.20E-09 |
| GO:0030100 | BIOLOGICAL_PROCESS | regulation of endocytosis | 2.30E-08 | 1.21E-09 |
| GO:0010721 | BIOLOGICAL_PROCESS | negative regulation of cell development | 2.30E-08 | 1.21E-09 |
| GO:0001077 | MOLECULAR_FUNCTION | transcriptional activator activity, RNA polymerase II core promoter proximal region sequence-specific binding | 2.30E-08 | 1.21E-09 |
| GO:0045216 | BIOLOGICAL_PROCESS | cell-cell junction organization | 2.30E-08 | 1.21E-09 |
| GO:0043005 | CELLULAR_COMPONENT | neuron projection | 2.30E-08 | 1.21E-09 |
| GO:0048762 | BIOLOGICAL_PROCESS | mesenchymal cell differentiation | 2.33E-08 | 1.23E-09 |
| GO:0007188 | BIOLOGICAL_PROCESS | adenylate cyclase-modulating G-protein coupled receptor signaling pathway | 2.33E-08 | 1.23E-09 |
| GO:0016791 | MOLECULAR_FUNCTION | phosphatase activity | 2.58E-08 | 1.37E-09 |
| GO:0042063 | BIOLOGICAL_PROCESS | gliogenesis | 2.58E-08 | 1.37E-09 |
| GO:0021510 | BIOLOGICAL_PROCESS | spinal cord development | 2.58E-08 | 1.37E-09 |
| GO:0051090 | BIOLOGICAL_PROCESS | regulation of sequence-specific DNA binding transcription factor activity | 2.58E-08 | 1.37E-09 |
| GO:0019941 | BIOLOGICAL_PROCESS | modification-dependent protein catabolic process | 3.41E-08 | 1.82E-09 |
| GO:1901214 | BIOLOGICAL_PROCESS | regulation of neuron death | 3.53E-08 | 1.89E-09 |
| GO:0030864 | CELLULAR_COMPONENT | cortical actin cytoskeleton | 3.53E-08 | 1.89E-09 |
| GO:0051402 | BIOLOGICAL_PROCESS | neuron apoptotic process | 3.53E-08 | 1.89E-09 |
| GO:0042625 | MOLECULAR_FUNCTION | ATPase coupled ion transmembrane transporter activity | 4.16E-08 | 2.23E-09 |
| GO:0044057 | BIOLOGICAL_PROCESS | regulation of system process | 4.17E-08 | 2.24E-09 |
| GO:0006952 | BIOLOGICAL_PROCESS | defense response | 4.34E-08 | 2.34E-09 |
| GO:0034654 | BIOLOGICAL_PROCESS | nucleobase-containing compound biosynthetic process | 4.45E-08 | 2.40E-09 |
| GO:0009953 | BIOLOGICAL_PROCESS | dorsal/ventral pattern formation | 4.53E-08 | 2.45E-09 |
| GO:0009952 | BIOLOGICAL_PROCESS | anterior/posterior pattern specification | 4.58E-08 | 2.48E-09 |
| GO:0046872 | MOLECULAR_FUNCTION | metal ion binding | 4.70E-08 | 2.55E-09 |
| GO:0048511 | BIOLOGICAL_PROCESS | rhythmic process | 5.09E-08 | 2.77E-09 |
| GO:0045087 | BIOLOGICAL_PROCESS | innate immune response | 5.09E-08 | 2.77E-09 |
| GO:0021953 | BIOLOGICAL_PROCESS | central nervous system neuron differentiation | 5.09E-08 | 2.77E-09 |
| GO:0044702 | BIOLOGICAL_PROCESS | single organism reproductive process | 5.80E-08 | 3.17E-09 |
| GO:1903533 | BIOLOGICAL_PROCESS | regulation of protein targeting | 5.83E-08 | 3.20E-09 |
| GO:0045055 | BIOLOGICAL_PROCESS | regulated exocytosis | 5.83E-08 | 3.20E-09 |
| GO:0051216 | BIOLOGICAL_PROCESS | cartilage development | 5.83E-08 | 3.20E-09 |
| GO:0016032 | BIOLOGICAL_PROCESS | viral process | 6.09E-08 | 3.35E-09 |
| GO:0030863 | CELLULAR_COMPONENT | cortical cytoskeleton | 6.09E-08 | 3.35E-09 |
| GO:0007179 | BIOLOGICAL_PROCESS | transforming growth factor beta receptor signaling pathway | 6.09E-08 | 3.35E-09 |
| GO:0019001 | MOLECULAR_FUNCTION | guanyl nucleotide binding | 7.38E-08 | 4.07E-09 |
| GO:0061458 | BIOLOGICAL_PROCESS | reproductive system development | 7.45E-08 | 4.13E-09 |
| GO:0048608 | BIOLOGICAL_PROCESS | reproductive structure development | 7.45E-08 | 4.13E-09 |
| GO:0099094 | MOLECULAR_FUNCTION | ligand-gated cation channel activity | 7.45E-08 | 4.13E-09 |
| GO:0042592 | BIOLOGICAL_PROCESS | homeostatic process | 8.85E-08 | 4.91E-09 |
| GO:0015629 | CELLULAR_COMPONENT | actin cytoskeleton | 9.25E-08 | 5.14E-09 |
| GO:0044708 | BIOLOGICAL_PROCESS | single-organism behavior | 9.66E-08 | 5.39E-09 |
| GO:0010564 | BIOLOGICAL_PROCESS | regulation of cell cycle process | 9.66E-08 | 5.39E-09 |
| GO:0032386 | BIOLOGICAL_PROCESS | regulation of intracellular transport | 9.74E-08 | 5.44E-09 |
| GO:0060627 | BIOLOGICAL_PROCESS | regulation of vesicle-mediated transport | 1.07E-07 | 5.96E-09 |
| GO:0044843 | BIOLOGICAL_PROCESS | cell cycle G1/S phase transition | 1.10E-07 | 6.17E-09 |
| GO:0051047 | BIOLOGICAL_PROCESS | positive regulation of secretion | 1.10E-07 | 6.17E-09 |
| GO:0032561 | MOLECULAR_FUNCTION | guanyl ribonucleotide binding | 1.17E-07 | 6.56E-09 |
| GO:1901652 | BIOLOGICAL_PROCESS | response to peptide | 1.18E-07 | 6.70E-09 |
| GO:0003205 | BIOLOGICAL_PROCESS | cardiac chamber development | 1.18E-07 | 6.70E-09 |
| GO:0007369 | BIOLOGICAL_PROCESS | gastrulation | 1.18E-07 | 6.70E-09 |
| GO:0042176 | BIOLOGICAL_PROCESS | regulation of protein catabolic process | 1.38E-07 | 7.80E-09 |
| GO:0009314 | BIOLOGICAL_PROCESS | response to radiation | 1.38E-07 | 7.80E-09 |
| GO:0008134 | MOLECULAR_FUNCTION | transcription factor binding | 1.44E-07 | 8.18E-09 |
| GO:0048736 | BIOLOGICAL_PROCESS | appendage development | 1.45E-07 | 8.26E-09 |
| GO:0019787 | MOLECULAR_FUNCTION | ubiquitin-like protein transferase activity | 1.48E-07 | 8.41E-09 |
| GO:0016020 | CELLULAR_COMPONENT | membrane | 1.56E-07 | 8.88E-09 |
| GO:0090066 | BIOLOGICAL_PROCESS | regulation of anatomical structure size | 1.58E-07 | 9.01E-09 |
| GO:0050768 | BIOLOGICAL_PROCESS | negative regulation of neurogenesis | 1.61E-07 | 9.23E-09 |
| GO:0022407 | BIOLOGICAL_PROCESS | regulation of cell-cell adhesion | 1.61E-07 | 9.23E-09 |
| GO:0002768 | BIOLOGICAL_PROCESS | immune response-regulating cell surface receptor signaling pathway | 1.61E-07 | 9.23E-09 |
| GO:0050708 | BIOLOGICAL_PROCESS | regulation of protein secretion | 1.69E-07 | 9.74E-09 |
| GO:0051961 | BIOLOGICAL_PROCESS | negative regulation of nervous system development | 1.69E-07 | 9.74E-09 |
| GO:0035051 | BIOLOGICAL_PROCESS | cardiocyte differentiation | 1.69E-07 | 9.74E-09 |
| GO:0035335 | BIOLOGICAL_PROCESS | peptidyl-tyrosine dephosphorylation | 1.69E-07 | 9.76E-09 |
| GO:0045787 | BIOLOGICAL_PROCESS | positive regulation of cell cycle | 1.69E-07 | 9.76E-09 |
| GO:0060078 | BIOLOGICAL_PROCESS | regulation of postsynaptic membrane potential | 1.69E-07 | 9.81E-09 |
| GO:0051656 | BIOLOGICAL_PROCESS | establishment of organelle localization | 1.69E-07 | 9.81E-09 |
| GO:0005794 | CELLULAR_COMPONENT | Golgi apparatus | 1.73E-07 | 1.00E-08 |
| GO:0030099 | BIOLOGICAL_PROCESS | myeloid cell differentiation | 1.79E-07 | 1.05E-08 |
| GO:0045927 | BIOLOGICAL_PROCESS | positive regulation of growth | 1.79E-07 | 1.05E-08 |
| GO:0005654 | CELLULAR_COMPONENT | nucleoplasm | 2.02E-07 | 1.18E-08 |
| GO:0070013 | CELLULAR_COMPONENT | intracellular organelle lumen | 2.08E-07 | 1.22E-08 |
| GO:0031974 | CELLULAR_COMPONENT | membrane-enclosed lumen | 2.08E-07 | 1.22E-08 |
| GO:0043233 | CELLULAR_COMPONENT | organelle lumen | 2.08E-07 | 1.22E-08 |
| GO:0004725 | MOLECULAR_FUNCTION | protein tyrosine phosphatase activity | 2.28E-07 | 1.34E-08 |
| GO:0022414 | BIOLOGICAL_PROCESS | reproductive process | 2.43E-07 | 1.43E-08 |
| GO:0051056 | BIOLOGICAL_PROCESS | regulation of small GTPase mediated signal transduction | 2.45E-07 | 1.45E-08 |
| GO:0044403 | BIOLOGICAL_PROCESS | symbiosis, encompassing mutualism through parasitism | 2.45E-07 | 1.45E-08 |
| GO:0007219 | BIOLOGICAL_PROCESS | Notch signaling pathway | 2.62E-07 | 1.55E-08 |
| GO:0000082 | BIOLOGICAL_PROCESS | G1/S transition of mitotic cell cycle | 2.62E-07 | 1.55E-08 |
| GO:0030278 | BIOLOGICAL_PROCESS | regulation of ossification | 2.64E-07 | 1.57E-08 |
| GO:0048771 | BIOLOGICAL_PROCESS | tissue remodeling | 2.64E-07 | 1.57E-08 |
| GO:0070371 | BIOLOGICAL_PROCESS | ERK1 and ERK2 cascade | 2.64E-07 | 1.57E-08 |
| GO:0004697 | MOLECULAR_FUNCTION | protein kinase C activity | 2.64E-07 | 1.57E-08 |
| GO:0007009 | BIOLOGICAL_PROCESS | plasma membrane organization | 2.69E-07 | 1.61E-08 |
| GO:0051098 | BIOLOGICAL_PROCESS | regulation of binding | 2.69E-07 | 1.61E-08 |
| GO:0051051 | BIOLOGICAL_PROCESS | negative regulation of transport | 2.69E-07 | 1.61E-08 |
| GO:0042330 | BIOLOGICAL_PROCESS | taxis | 2.90E-07 | 1.74E-08 |
| GO:0006935 | BIOLOGICAL_PROCESS | chemotaxis | 2.90E-07 | 1.74E-08 |
| GO:0034220 | BIOLOGICAL_PROCESS | ion transmembrane transport | 3.35E-07 | 2.01E-08 |
| GO:1903532 | BIOLOGICAL_PROCESS | positive regulation of secretion by cell | 3.52E-07 | 2.12E-08 |
| GO:0021543 | BIOLOGICAL_PROCESS | pallium development | 3.52E-07 | 2.12E-08 |
| GO:1902578 | BIOLOGICAL_PROCESS | single-organism localization | 3.55E-07 | 2.14E-08 |
| GO:1902589 | BIOLOGICAL_PROCESS | single-organism organelle organization | 3.55E-07 | 2.14E-08 |
| GO:0034097 | BIOLOGICAL_PROCESS | response to cytokine | 3.64E-07 | 2.20E-08 |
| GO:0098805 | CELLULAR_COMPONENT | whole membrane | 3.74E-07 | 2.27E-08 |
| GO:0000003 | BIOLOGICAL_PROCESS | reproduction | 3.74E-07 | 2.26E-08 |
| GO:0050804 | BIOLOGICAL_PROCESS | modulation of synaptic transmission | 3.76E-07 | 2.29E-08 |
| GO:0033157 | BIOLOGICAL_PROCESS | regulation of intracellular protein transport | 3.76E-07 | 2.29E-08 |
| GO:0043209 | CELLULAR_COMPONENT | myelin sheath | 3.76E-07 | 2.29E-08 |
| GO:0070509 | BIOLOGICAL_PROCESS | calcium ion import | 3.76E-07 | 2.29E-08 |
| GO:0099080 | CELLULAR_COMPONENT | supramolecular complex | 3.80E-07 | 2.33E-08 |
| GO:0099081 | CELLULAR_COMPONENT | supramolecular polymer | 3.80E-07 | 2.33E-08 |
| GO:0048592 | BIOLOGICAL_PROCESS | eye morphogenesis | 4.09E-07 | 2.55E-08 |
| GO:0098802 | CELLULAR_COMPONENT | plasma membrane receptor complex | 4.09E-07 | 2.55E-08 |
| GO:1903706 | BIOLOGICAL_PROCESS | regulation of hemopoiesis | 4.09E-07 | 2.54E-08 |
| GO:0030098 | BIOLOGICAL_PROCESS | lymphocyte differentiation | 4.09E-07 | 2.54E-08 |
| GO:0060047 | BIOLOGICAL_PROCESS | heart contraction | 4.09E-07 | 2.54E-08 |
| GO:0042110 | BIOLOGICAL_PROCESS | T cell activation | 4.09E-07 | 2.54E-08 |
| GO:0034329 | BIOLOGICAL_PROCESS | cell junction assembly | 4.09E-07 | 2.54E-08 |
| GO:0010001 | BIOLOGICAL_PROCESS | glial cell differentiation | 4.09E-07 | 2.54E-08 |
| GO:0003015 | BIOLOGICAL_PROCESS | heart process | 4.09E-07 | 2.54E-08 |
| GO:0010769 | BIOLOGICAL_PROCESS | regulation of cell morphogenesis involved in differentiation | 4.09E-07 | 2.55E-08 |
| GO:0007623 | BIOLOGICAL_PROCESS | circadian rhythm | 4.09E-07 | 2.55E-08 |
| GO:0010959 | BIOLOGICAL_PROCESS | regulation of metal ion transport | 4.09E-07 | 2.55E-08 |
| GO:0022857 | MOLECULAR_FUNCTION | transmembrane transporter activity | 4.09E-07 | 2.53E-08 |
| GO:0002429 | BIOLOGICAL_PROCESS | immune response-activating cell surface receptor signaling pathway | 4.09E-07 | 2.54E-08 |
| GO:0017157 | BIOLOGICAL_PROCESS | regulation of exocytosis | 4.12E-07 | 2.58E-08 |
| GO:0009798 | BIOLOGICAL_PROCESS | axis specification | 4.12E-07 | 2.58E-08 |
| GO:0060560 | BIOLOGICAL_PROCESS | developmental growth involved in morphogenesis | 4.90E-07 | 3.09E-08 |
| GO:0048638 | BIOLOGICAL_PROCESS | regulation of developmental growth | 4.90E-07 | 3.09E-08 |
| GO:0051648 | BIOLOGICAL_PROCESS | vesicle localization | 4.90E-07 | 3.09E-08 |
| GO:0051650 | BIOLOGICAL_PROCESS | establishment of vesicle localization | 4.90E-07 | 3.09E-08 |
| GO:0045862 | BIOLOGICAL_PROCESS | positive regulation of proteolysis | 4.90E-07 | 3.09E-08 |
| GO:0051640 | BIOLOGICAL_PROCESS | organelle localization | 4.99E-07 | 3.15E-08 |
| GO:0022838 | MOLECULAR_FUNCTION | substrate-specific channel activity | 5.44E-07 | 3.44E-08 |
| GO:0006909 | BIOLOGICAL_PROCESS | phagocytosis | 6.04E-07 | 3.83E-08 |
| GO:0003707 | MOLECULAR_FUNCTION | steroid hormone receptor activity | 6.04E-07 | 3.83E-08 |
| GO:0099512 | CELLULAR_COMPONENT | supramolecular fiber | 6.04E-07 | 3.83E-08 |
| GO:1900180 | BIOLOGICAL_PROCESS | regulation of protein localization to nucleus | 6.06E-07 | 3.86E-08 |
| GO:0009914 | BIOLOGICAL_PROCESS | hormone transport | 6.06E-07 | 3.86E-08 |
| GO:0045666 | BIOLOGICAL_PROCESS | positive regulation of neuron differentiation | 6.06E-07 | 3.86E-08 |
| GO:0098588 | CELLULAR_COMPONENT | bounding membrane of organelle | 6.15E-07 | 3.92E-08 |
| GO:0033554 | BIOLOGICAL_PROCESS | cellular response to stress | 6.46E-07 | 4.13E-08 |
| GO:0043523 | BIOLOGICAL_PROCESS | regulation of neuron apoptotic process | 7.04E-07 | 4.53E-08 |
| GO:0035304 | BIOLOGICAL_PROCESS | regulation of protein dephosphorylation | 7.04E-07 | 4.53E-08 |
| GO:0070372 | BIOLOGICAL_PROCESS | regulation of ERK1 and ERK2 cascade | 7.04E-07 | 4.53E-08 |
| GO:0030901 | BIOLOGICAL_PROCESS | midbrain development | 7.04E-07 | 4.53E-08 |
| GO:0044770 | BIOLOGICAL_PROCESS | cell cycle phase transition | 8.20E-07 | 5.28E-08 |
| GO:0099643 | BIOLOGICAL_PROCESS | signal release from synapse | 8.30E-07 | 5.36E-08 |
| GO:0007269 | BIOLOGICAL_PROCESS | neurotransmitter secretion | 8.30E-07 | 5.36E-08 |
| GO:0050808 | BIOLOGICAL_PROCESS | synapse organization | 8.35E-07 | 5.43E-08 |
| GO:0007163 | BIOLOGICAL_PROCESS | establishment or maintenance of cell polarity | 8.35E-07 | 5.43E-08 |
| GO:0004715 | MOLECULAR_FUNCTION | non-membrane spanning protein tyrosine kinase activity | 8.35E-07 | 5.43E-08 |
| GO:0061138 | BIOLOGICAL_PROCESS | morphogenesis of a branching epithelium | 8.35E-07 | 5.43E-08 |
| GO:0071901 | BIOLOGICAL_PROCESS | negative regulation of protein serine/threonine kinase activity | 8.35E-07 | 5.43E-08 |
| GO:0051704 | BIOLOGICAL_PROCESS | multi-organism process | 8.45E-07 | 5.50E-08 |
| GO:0008047 | MOLECULAR_FUNCTION | enzyme activator activity | 8.58E-07 | 5.59E-08 |
| GO:0042692 | BIOLOGICAL_PROCESS | muscle cell differentiation | 8.97E-07 | 5.86E-08 |
| GO:0005216 | MOLECULAR_FUNCTION | ion channel activity | 9.45E-07 | 6.17E-08 |
| GO:0045177 | CELLULAR_COMPONENT | apical part of cell | 9.48E-07 | 6.20E-08 |
| GO:0001819 | BIOLOGICAL_PROCESS | positive regulation of cytokine production | 1.03E-06 | 6.80E-08 |
| GO:0090101 | BIOLOGICAL_PROCESS | negative regulation of transmembrane receptor protein serine/threonine kinase signaling pathway | 1.03E-06 | 6.80E-08 |
| GO:0090288 | BIOLOGICAL_PROCESS | negative regulation of cellular response to growth factor stimulus | 1.03E-06 | 6.80E-08 |
| GO:0007162 | BIOLOGICAL_PROCESS | negative regulation of cell adhesion | 1.03E-06 | 6.80E-08 |
| GO:0021987 | BIOLOGICAL_PROCESS | cerebral cortex development | 1.03E-06 | 6.80E-08 |
| GO:0046883 | BIOLOGICAL_PROCESS | regulation of hormone secretion | 1.03E-06 | 6.80E-08 |
| GO:0001227 | MOLECULAR_FUNCTION | transcriptional repressor activity, RNA polymerase II transcription regulatory region sequence-specific binding | 1.03E-06 | 6.80E-08 |
| GO:0031589 | BIOLOGICAL_PROCESS | cell-substrate adhesion | 1.05E-06 | 6.96E-08 |
| GO:0099531 | BIOLOGICAL_PROCESS | presynaptic process involved in chemical synaptic transmission | 1.07E-06 | 7.07E-08 |
| GO:0006812 | BIOLOGICAL_PROCESS | cation transport | 1.12E-06 | 7.43E-08 |
| GO:0034613 | BIOLOGICAL_PROCESS | cellular protein localization | 1.16E-06 | 7.72E-08 |
| GO:0070727 | BIOLOGICAL_PROCESS | cellular macromolecule localization | 1.16E-06 | 7.72E-08 |
| GO:0032844 | BIOLOGICAL_PROCESS | regulation of homeostatic process | 1.16E-06 | 7.73E-08 |
| GO:0044265 | BIOLOGICAL_PROCESS | cellular macromolecule catabolic process | 1.28E-06 | 8.54E-08 |
| GO:0031329 | BIOLOGICAL_PROCESS | regulation of cellular catabolic process | 1.29E-06 | 8.61E-08 |
| GO:0032101 | BIOLOGICAL_PROCESS | regulation of response to external stimulus | 1.33E-06 | 8.92E-08 |
| GO:0001655 | BIOLOGICAL_PROCESS | urogenital system development | 1.36E-06 | 9.09E-08 |
| GO:0048839 | BIOLOGICAL_PROCESS | inner ear development | 1.36E-06 | 9.09E-08 |
| GO:0048489 | BIOLOGICAL_PROCESS | synaptic vesicle transport | 1.42E-06 | 9.58E-08 |
| GO:0097480 | BIOLOGICAL_PROCESS | establishment of synaptic vesicle localization | 1.42E-06 | 9.58E-08 |
| GO:0097479 | BIOLOGICAL_PROCESS | synaptic vesicle localization | 1.42E-06 | 9.58E-08 |
| GO:0000502 | CELLULAR_COMPONENT | proteasome complex | 1.42E-06 | 9.58E-08 |
| GO:1905369 | CELLULAR_COMPONENT | endopeptidase complex | 1.42E-06 | 9.58E-08 |
| GO:0099003 | BIOLOGICAL_PROCESS | vesicle-mediated transport in synapse | 1.42E-06 | 9.58E-08 |
| GO:0046879 | BIOLOGICAL_PROCESS | hormone secretion | 1.42E-06 | 9.58E-08 |
| GO:0000988 | MOLECULAR_FUNCTION | transcription factor activity, protein binding | 1.52E-06 | 1.03E-07 |
| GO:0044772 | BIOLOGICAL_PROCESS | mitotic cell cycle phase transition | 1.58E-06 | 1.08E-07 |
| GO:0048609 | BIOLOGICAL_PROCESS | multicellular organismal reproductive process | 1.58E-06 | 1.08E-07 |
| GO:0035725 | BIOLOGICAL_PROCESS | sodium ion transmembrane transport | 1.58E-06 | 1.08E-07 |
| GO:0032504 | BIOLOGICAL_PROCESS | multicellular organism reproduction | 1.58E-06 | 1.08E-07 |
| GO:0009416 | BIOLOGICAL_PROCESS | response to light stimulus | 1.59E-06 | 1.08E-07 |
| GO:0009986 | CELLULAR_COMPONENT | cell surface | 1.59E-06 | 1.08E-07 |
| GO:0043161 | BIOLOGICAL_PROCESS | proteasome-mediated ubiquitin-dependent protein catabolic process | 1.65E-06 | 1.13E-07 |
| GO:0003682 | MOLECULAR_FUNCTION | chromatin binding | 1.71E-06 | 1.17E-07 |
| GO:0010498 | BIOLOGICAL_PROCESS | proteasomal protein catabolic process | 1.71E-06 | 1.17E-07 |
| GO:0044446 | CELLULAR_COMPONENT | intracellular organelle part | 1.72E-06 | 1.18E-07 |
| GO:0031347 | BIOLOGICAL_PROCESS | regulation of defense response | 1.80E-06 | 1.24E-07 |
| GO:0006898 | BIOLOGICAL_PROCESS | receptor-mediated endocytosis | 1.80E-06 | 1.24E-07 |
| GO:0010817 | BIOLOGICAL_PROCESS | regulation of hormone levels | 1.80E-06 | 1.24E-07 |
| GO:0019898 | CELLULAR_COMPONENT | extrinsic component of membrane | 1.85E-06 | 1.27E-07 |
| GO:0001885 | BIOLOGICAL_PROCESS | endothelial cell development | 1.88E-06 | 1.30E-07 |
| GO:0003151 | BIOLOGICAL_PROCESS | outflow tract morphogenesis | 1.88E-06 | 1.30E-07 |
| GO:2001234 | BIOLOGICAL_PROCESS | negative regulation of apoptotic signaling pathway | 1.88E-06 | 1.30E-07 |
| GO:0007416 | BIOLOGICAL_PROCESS | synapse assembly | 1.88E-06 | 1.30E-07 |
| GO:0003206 | BIOLOGICAL_PROCESS | cardiac chamber morphogenesis | 1.99E-06 | 1.38E-07 |
| GO:0035050 | BIOLOGICAL_PROCESS | embryonic heart tube development | 2.15E-06 | 1.50E-07 |
| GO:0000978 | MOLECULAR_FUNCTION | RNA polymerase II core promoter proximal region sequence-specific DNA binding | 2.15E-06 | 1.50E-07 |
| GO:0030111 | BIOLOGICAL_PROCESS | regulation of Wnt signaling pathway | 2.30E-06 | 1.61E-07 |
| GO:0046982 | MOLECULAR_FUNCTION | protein heterodimerization activity | 2.30E-06 | 1.61E-07 |
| GO:0009896 | BIOLOGICAL_PROCESS | positive regulation of catabolic process | 2.35E-06 | 1.64E-07 |
| GO:0034765 | BIOLOGICAL_PROCESS | regulation of ion transmembrane transport | 2.37E-06 | 1.66E-07 |
| GO:0034762 | BIOLOGICAL_PROCESS | regulation of transmembrane transport | 2.37E-06 | 1.66E-07 |
| GO:0042306 | BIOLOGICAL_PROCESS | regulation of protein import into nucleus | 2.52E-06 | 1.79E-07 |
| GO:0001764 | BIOLOGICAL_PROCESS | neuron migration | 2.52E-06 | 1.79E-07 |
| GO:0035107 | BIOLOGICAL_PROCESS | appendage morphogenesis | 2.52E-06 | 1.79E-07 |
| GO:0031349 | BIOLOGICAL_PROCESS | positive regulation of defense response | 2.52E-06 | 1.79E-07 |
| GO:1903522 | BIOLOGICAL_PROCESS | regulation of blood circulation | 2.52E-06 | 1.79E-07 |
| GO:0004402 | MOLECULAR_FUNCTION | histone acetyltransferase activity | 2.52E-06 | 1.79E-07 |
| GO:0061733 | MOLECULAR_FUNCTION | peptide-lysine-N-acetyltransferase activity | 2.52E-06 | 1.79E-07 |
| GO:0071772 | BIOLOGICAL_PROCESS | response to BMP | 2.52E-06 | 1.79E-07 |
| GO:0071773 | BIOLOGICAL_PROCESS | cellular response to BMP stimulus | 2.52E-06 | 1.79E-07 |
| GO:0014033 | BIOLOGICAL_PROCESS | neural crest cell differentiation | 2.52E-06 | 1.79E-07 |
| GO:1904589 | BIOLOGICAL_PROCESS | regulation of protein import | 2.52E-06 | 1.79E-07 |
| GO:0098857 | CELLULAR_COMPONENT | membrane microdomain | 2.66E-06 | 1.90E-07 |
| GO:0035265 | BIOLOGICAL_PROCESS | organ growth | 2.66E-06 | 1.90E-07 |
| GO:0045121 | CELLULAR_COMPONENT | membrane raft | 2.66E-06 | 1.90E-07 |
| GO:0051091 | BIOLOGICAL_PROCESS | positive regulation of sequence-specific DNA binding transcription factor activity | 2.66E-06 | 1.90E-07 |
| GO:1903305 | BIOLOGICAL_PROCESS | regulation of regulated secretory pathway | 2.66E-06 | 1.90E-07 |
| GO:0001071 | MOLECULAR_FUNCTION | nucleic acid binding transcription factor activity | 2.84E-06 | 2.03E-07 |
| GO:0003700 | MOLECULAR_FUNCTION | transcription factor activity, sequence-specific DNA binding | 2.84E-06 | 2.03E-07 |
| GO:0043087 | BIOLOGICAL_PROCESS | regulation of GTPase activity | 2.84E-06 | 2.04E-07 |
| GO:0050890 | BIOLOGICAL_PROCESS | cognition | 2.98E-06 | 2.14E-07 |
| GO:0019829 | MOLECULAR_FUNCTION | cation-transporting ATPase activity | 3.27E-06 | 2.35E-07 |
| GO:0001763 | BIOLOGICAL_PROCESS | morphogenesis of a branching structure | 3.28E-06 | 2.36E-07 |
| GO:0045296 | MOLECULAR_FUNCTION | cadherin binding | 3.28E-06 | 2.36E-07 |
| GO:0006457 | BIOLOGICAL_PROCESS | protein folding | 3.34E-06 | 2.41E-07 |
| GO:0008104 | BIOLOGICAL_PROCESS | protein localization | 3.67E-06 | 2.65E-07 |
| GO:0001505 | BIOLOGICAL_PROCESS | regulation of neurotransmitter levels | 3.81E-06 | 2.75E-07 |
| GO:0043583 | BIOLOGICAL_PROCESS | ear development | 3.95E-06 | 2.86E-07 |
| GO:0019058 | BIOLOGICAL_PROCESS | viral life cycle | 3.95E-06 | 2.86E-07 |
| GO:0016569 | BIOLOGICAL_PROCESS | covalent chromatin modification | 4.01E-06 | 2.91E-07 |
| GO:0033036 | BIOLOGICAL_PROCESS | macromolecule localization | 4.04E-06 | 2.94E-07 |
| GO:0022891 | MOLECULAR_FUNCTION | substrate-specific transmembrane transporter activity | 4.13E-06 | 3.00E-07 |
| GO:0034504 | BIOLOGICAL_PROCESS | protein localization to nucleus | 4.27E-06 | 3.11E-07 |
| GO:0030072 | BIOLOGICAL_PROCESS | peptide hormone secretion | 4.77E-06 | 3.50E-07 |
| GO:0003143 | BIOLOGICAL_PROCESS | embryonic heart tube morphogenesis | 4.77E-06 | 3.50E-07 |
| GO:0048754 | BIOLOGICAL_PROCESS | branching morphogenesis of an epithelial tube | 4.77E-06 | 3.50E-07 |
| GO:1905330 | BIOLOGICAL_PROCESS | regulation of morphogenesis of an epithelium | 4.77E-06 | 3.50E-07 |
| GO:1903320 | BIOLOGICAL_PROCESS | regulation of protein modification by small protein conjugation or removal | 4.77E-06 | 3.50E-07 |
| GO:0002790 | BIOLOGICAL_PROCESS | peptide secretion | 4.77E-06 | 3.50E-07 |
| GO:0034704 | CELLULAR_COMPONENT | calcium channel complex | 4.88E-06 | 3.59E-07 |
| GO:0030301 | BIOLOGICAL_PROCESS | cholesterol transport | 5.07E-06 | 3.76E-07 |
| GO:0031683 | MOLECULAR_FUNCTION | G-protein beta/gamma-subunit complex binding | 5.07E-06 | 3.76E-07 |
| GO:0017147 | MOLECULAR_FUNCTION | Wnt-protein binding | 5.07E-06 | 3.76E-07 |
| GO:0042813 | MOLECULAR_FUNCTION | Wnt-activated receptor activity | 5.07E-06 | 3.76E-07 |
| GO:0007210 | BIOLOGICAL_PROCESS | serotonin receptor signaling pathway | 5.07E-06 | 3.76E-07 |
| GO:0004993 | MOLECULAR_FUNCTION | G-protein coupled serotonin receptor activity | 5.07E-06 | 3.76E-07 |
| GO:0099589 | MOLECULAR_FUNCTION | serotonin receptor activity | 5.07E-06 | 3.76E-07 |
| GO:0015918 | BIOLOGICAL_PROCESS | sterol transport | 5.07E-06 | 3.76E-07 |
| GO:0048878 | BIOLOGICAL_PROCESS | chemical homeostasis | 5.29E-06 | 3.92E-07 |
| GO:0044765 | BIOLOGICAL_PROCESS | single-organism transport | 5.51E-06 | 4.09E-07 |
| GO:0006816 | BIOLOGICAL_PROCESS | calcium ion transport | 5.60E-06 | 4.17E-07 |
| GO:0036477 | CELLULAR_COMPONENT | somatodendritic compartment | 5.79E-06 | 4.31E-07 |
| GO:0044419 | BIOLOGICAL_PROCESS | interspecies interaction between organisms | 5.87E-06 | 4.38E-07 |
| GO:0090662 | BIOLOGICAL_PROCESS | ATP hydrolysis coupled transmembrane transport | 5.90E-06 | 4.41E-07 |
| GO:0004029 | MOLECULAR_FUNCTION | aldehyde dehydrogenase (NAD) activity | 6.20E-06 | 4.67E-07 |
| GO:0031396 | BIOLOGICAL_PROCESS | regulation of protein ubiquitination | 6.20E-06 | 4.67E-07 |
| GO:0061371 | BIOLOGICAL_PROCESS | determination of heart left/right asymmetry | 6.20E-06 | 4.67E-07 |
| GO:0030139 | CELLULAR_COMPONENT | endocytic vesicle | 6.20E-06 | 4.67E-07 |
| GO:0035567 | BIOLOGICAL_PROCESS | non-canonical Wnt signaling pathway | 6.20E-06 | 4.67E-07 |
| GO:0045088 | BIOLOGICAL_PROCESS | regulation of innate immune response | 6.20E-06 | 4.67E-07 |
| GO:0030509 | BIOLOGICAL_PROCESS | BMP signaling pathway | 6.20E-06 | 4.67E-07 |
| GO:0044422 | CELLULAR_COMPONENT | organelle part | 6.35E-06 | 4.79E-07 |
| GO:0051146 | BIOLOGICAL_PROCESS | striated muscle cell differentiation | 6.63E-06 | 5.00E-07 |
| GO:0008016 | BIOLOGICAL_PROCESS | regulation of heart contraction | 6.82E-06 | 5.18E-07 |
| GO:0016202 | BIOLOGICAL_PROCESS | regulation of striated muscle tissue development | 6.82E-06 | 5.18E-07 |
| GO:0048634 | BIOLOGICAL_PROCESS | regulation of muscle organ development | 6.82E-06 | 5.18E-07 |
| GO:1901861 | BIOLOGICAL_PROCESS | regulation of muscle tissue development | 6.82E-06 | 5.18E-07 |
| GO:0045931 | BIOLOGICAL_PROCESS | positive regulation of mitotic cell cycle | 6.82E-06 | 5.18E-07 |
| GO:0031016 | BIOLOGICAL_PROCESS | pancreas development | 6.82E-06 | 5.18E-07 |
| GO:0006508 | BIOLOGICAL_PROCESS | proteolysis | 7.00E-06 | 5.33E-07 |
| GO:0006325 | BIOLOGICAL_PROCESS | chromatin organization | 7.17E-06 | 5.46E-07 |
| GO:0046907 | BIOLOGICAL_PROCESS | intracellular transport | 7.47E-06 | 5.70E-07 |
| GO:0048593 | BIOLOGICAL_PROCESS | camera-type eye morphogenesis | 7.54E-06 | 5.78E-07 |
| GO:0003231 | BIOLOGICAL_PROCESS | cardiac ventricle development | 7.54E-06 | 5.78E-07 |
| GO:0042493 | BIOLOGICAL_PROCESS | response to drug | 7.54E-06 | 5.78E-07 |
| GO:0046822 | BIOLOGICAL_PROCESS | regulation of nucleocytoplasmic transport | 7.54E-06 | 5.78E-07 |
| GO:0071902 | BIOLOGICAL_PROCESS | positive regulation of protein serine/threonine kinase activity | 7.54E-06 | 5.78E-07 |
| GO:0015662 | MOLECULAR_FUNCTION | ATPase activity, coupled to transmembrane movement of ions, phosphorylative mechanism | 7.69E-06 | 5.91E-07 |
| GO:0009855 | BIOLOGICAL_PROCESS | determination of bilateral symmetry | 9.32E-06 | 7.18E-07 |
| GO:0009799 | BIOLOGICAL_PROCESS | specification of symmetry | 9.32E-06 | 7.18E-07 |
| GO:0098655 | BIOLOGICAL_PROCESS | cation transmembrane transport | 1.05E-05 | 8.09E-07 |
| GO:0060828 | BIOLOGICAL_PROCESS | regulation of canonical Wnt signaling pathway | 1.11E-05 | 8.54E-07 |
| GO:0099504 | BIOLOGICAL_PROCESS | synaptic vesicle cycle | 1.11E-05 | 8.54E-07 |
| GO:0042803 | MOLECULAR_FUNCTION | protein homodimerization activity | 1.13E-05 | 8.73E-07 |
| GO:0098531 | MOLECULAR_FUNCTION | transcription factor activity, direct ligand regulated sequence-specific DNA binding | 1.13E-05 | 8.80E-07 |
| GO:0060538 | BIOLOGICAL_PROCESS | skeletal muscle organ development | 1.13E-05 | 8.80E-07 |
| GO:0048639 | BIOLOGICAL_PROCESS | positive regulation of developmental growth | 1.13E-05 | 8.80E-07 |
| GO:0004879 | MOLECULAR_FUNCTION | RNA polymerase II transcription factor activity, ligand-activated sequence-specific DNA binding | 1.13E-05 | 8.80E-07 |
| GO:0007271 | BIOLOGICAL_PROCESS | synaptic transmission, cholinergic | 1.13E-05 | 8.80E-07 |
| GO:0006913 | BIOLOGICAL_PROCESS | nucleocytoplasmic transport | 1.15E-05 | 8.97E-07 |
| GO:0051169 | BIOLOGICAL_PROCESS | nuclear transport | 1.15E-05 | 8.97E-07 |
| GO:0016570 | BIOLOGICAL_PROCESS | histone modification | 1.33E-05 | 1.04E-06 |
| GO:0043666 | BIOLOGICAL_PROCESS | regulation of phosphoprotein phosphatase activity | 1.37E-05 | 1.08E-06 |
| GO:0097191 | BIOLOGICAL_PROCESS | extrinsic apoptotic signaling pathway | 1.37E-05 | 1.08E-06 |
| GO:0060021 | BIOLOGICAL_PROCESS | palate development | 1.37E-05 | 1.08E-06 |
| GO:0072089 | BIOLOGICAL_PROCESS | stem cell proliferation | 1.37E-05 | 1.08E-06 |
| GO:0042476 | BIOLOGICAL_PROCESS | odontogenesis | 1.37E-05 | 1.08E-06 |
| GO:0046620 | BIOLOGICAL_PROCESS | regulation of organ growth | 1.37E-05 | 1.08E-06 |
| GO:0007043 | BIOLOGICAL_PROCESS | cell-cell junction assembly | 1.37E-05 | 1.08E-06 |
| GO:0061028 | BIOLOGICAL_PROCESS | establishment of endothelial barrier | 1.37E-05 | 1.08E-06 |
| GO:0033613 | MOLECULAR_FUNCTION | activating transcription factor binding | 1.37E-05 | 1.08E-06 |
| GO:0071345 | BIOLOGICAL_PROCESS | cellular response to cytokine stimulus | 1.41E-05 | 1.12E-06 |
| GO:0001159 | MOLECULAR_FUNCTION | core promoter proximal region DNA binding | 1.41E-05 | 1.12E-06 |
| GO:0009057 | BIOLOGICAL_PROCESS | macromolecule catabolic process | 1.42E-05 | 1.13E-06 |
| GO:1990778 | BIOLOGICAL_PROCESS | protein localization to cell periphery | 1.46E-05 | 1.16E-06 |
| GO:0043434 | BIOLOGICAL_PROCESS | response to peptide hormone | 1.46E-05 | 1.16E-06 |
| GO:0007611 | BIOLOGICAL_PROCESS | learning or memory | 1.46E-05 | 1.16E-06 |
| GO:0022853 | MOLECULAR_FUNCTION | active ion transmembrane transporter activity | 1.48E-05 | 1.18E-06 |
| GO:0035270 | BIOLOGICAL_PROCESS | endocrine system development | 1.51E-05 | 1.22E-06 |
| GO:0090087 | BIOLOGICAL_PROCESS | regulation of peptide transport | 1.51E-05 | 1.22E-06 |
| GO:0043393 | BIOLOGICAL_PROCESS | regulation of protein binding | 1.51E-05 | 1.22E-06 |
| GO:0090276 | BIOLOGICAL_PROCESS | regulation of peptide hormone secretion | 1.51E-05 | 1.22E-06 |
| GO:0052548 | BIOLOGICAL_PROCESS | regulation of endopeptidase activity | 1.51E-05 | 1.22E-06 |
| GO:0002011 | BIOLOGICAL_PROCESS | morphogenesis of an epithelial sheet | 1.51E-05 | 1.22E-06 |
| GO:0006140 | BIOLOGICAL_PROCESS | regulation of nucleotide metabolic process | 1.51E-05 | 1.22E-06 |
| GO:0002791 | BIOLOGICAL_PROCESS | regulation of peptide secretion | 1.51E-05 | 1.22E-06 |
| GO:0051427 | MOLECULAR_FUNCTION | hormone receptor binding | 1.51E-05 | 1.22E-06 |
| GO:0030424 | CELLULAR_COMPONENT | axon | 1.56E-05 | 1.26E-06 |
| GO:0031331 | BIOLOGICAL_PROCESS | positive regulation of cellular catabolic process | 1.71E-05 | 1.38E-06 |
| GO:0006836 | BIOLOGICAL_PROCESS | neurotransmitter transport | 1.71E-05 | 1.38E-06 |
| GO:0071417 | BIOLOGICAL_PROCESS | cellular response to organonitrogen compound | 1.71E-05 | 1.38E-06 |
| GO:0051082 | MOLECULAR_FUNCTION | unfolded protein binding | 1.71E-05 | 1.38E-06 |
| GO:2000116 | BIOLOGICAL_PROCESS | regulation of cysteine-type endopeptidase activity | 1.72E-05 | 1.41E-06 |
| GO:1903844 | BIOLOGICAL_PROCESS | regulation of cellular response to transforming growth factor beta stimulus | 1.72E-05 | 1.41E-06 |
| GO:0030010 | BIOLOGICAL_PROCESS | establishment of cell polarity | 1.72E-05 | 1.41E-06 |
| GO:0031333 | BIOLOGICAL_PROCESS | negative regulation of protein complex assembly | 1.72E-05 | 1.41E-06 |
| GO:0050680 | BIOLOGICAL_PROCESS | negative regulation of epithelial cell proliferation | 1.72E-05 | 1.41E-06 |
| GO:0005891 | CELLULAR_COMPONENT | voltage-gated calcium channel complex | 1.72E-05 | 1.41E-06 |
| GO:0003279 | BIOLOGICAL_PROCESS | cardiac septum development | 1.72E-05 | 1.41E-06 |
| GO:0032147 | BIOLOGICAL_PROCESS | activation of protein kinase activity | 1.72E-05 | 1.41E-06 |
| GO:0017015 | BIOLOGICAL_PROCESS | regulation of transforming growth factor beta receptor signaling pathway | 1.72E-05 | 1.41E-06 |
| GO:0043281 | BIOLOGICAL_PROCESS | regulation of cysteine-type endopeptidase activity involved in apoptotic process | 1.72E-05 | 1.41E-06 |
| GO:0032409 | BIOLOGICAL_PROCESS | regulation of transporter activity | 1.72E-05 | 1.41E-06 |
| GO:0045665 | BIOLOGICAL_PROCESS | negative regulation of neuron differentiation | 1.72E-05 | 1.41E-06 |
| GO:0016049 | BIOLOGICAL_PROCESS | cell growth | 1.80E-05 | 1.48E-06 |
| GO:0048285 | BIOLOGICAL_PROCESS | organelle fission | 1.80E-05 | 1.48E-06 |
| GO:0003012 | BIOLOGICAL_PROCESS | muscle system process | 1.80E-05 | 1.48E-06 |
| GO:0006979 | BIOLOGICAL_PROCESS | response to oxidative stress | 1.80E-05 | 1.48E-06 |
| GO:1905368 | CELLULAR_COMPONENT | peptidase complex | 1.80E-05 | 1.48E-06 |
| GO:0099503 | CELLULAR_COMPONENT | secretory vesicle | 1.80E-05 | 1.48E-06 |
| GO:0005768 | CELLULAR_COMPONENT | endosome | 1.93E-05 | 1.59E-06 |
| GO:0030425 | CELLULAR_COMPONENT | dendrite | 2.09E-05 | 1.73E-06 |
| GO:0031090 | CELLULAR_COMPONENT | organelle membrane | 2.10E-05 | 1.73E-06 |
| GO:1903829 | BIOLOGICAL_PROCESS | positive regulation of cellular protein localization | 2.18E-05 | 1.80E-06 |
| GO:0035091 | MOLECULAR_FUNCTION | phosphatidylinositol binding | 2.18E-05 | 1.80E-06 |
| GO:0015075 | MOLECULAR_FUNCTION | ion transmembrane transporter activity | 2.21E-05 | 1.83E-06 |
| GO:0072659 | BIOLOGICAL_PROCESS | protein localization to plasma membrane | 2.43E-05 | 2.02E-06 |
| GO:0070405 | MOLECULAR_FUNCTION | ammonium ion binding | 2.43E-05 | 2.02E-06 |
| GO:0031252 | CELLULAR_COMPONENT | cell leading edge | 2.43E-05 | 2.02E-06 |
| GO:0016079 | BIOLOGICAL_PROCESS | synaptic vesicle exocytosis | 2.63E-05 | 2.20E-06 |
| GO:0050865 | BIOLOGICAL_PROCESS | regulation of cell activation | 2.63E-05 | 2.20E-06 |
| GO:0017156 | BIOLOGICAL_PROCESS | calcium ion regulated exocytosis | 2.63E-05 | 2.20E-06 |
| GO:0008361 | BIOLOGICAL_PROCESS | regulation of cell size | 2.63E-05 | 2.20E-06 |
| GO:0070588 | BIOLOGICAL_PROCESS | calcium ion transmembrane transport | 2.83E-05 | 2.36E-06 |
| GO:0015267 | MOLECULAR_FUNCTION | channel activity | 2.94E-05 | 2.46E-06 |
| GO:0022803 | MOLECULAR_FUNCTION | passive transmembrane transporter activity | 2.94E-05 | 2.46E-06 |
| GO:0006475 | BIOLOGICAL_PROCESS | internal protein amino acid acetylation | 3.14E-05 | 2.65E-06 |
| GO:0018394 | BIOLOGICAL_PROCESS | peptidyl-lysine acetylation | 3.14E-05 | 2.65E-06 |
| GO:0018393 | BIOLOGICAL_PROCESS | internal peptidyl-lysine acetylation | 3.14E-05 | 2.65E-06 |
| GO:0016573 | BIOLOGICAL_PROCESS | histone acetylation | 3.14E-05 | 2.65E-06 |
| GO:1903362 | BIOLOGICAL_PROCESS | regulation of cellular protein catabolic process | 3.14E-05 | 2.65E-06 |
| GO:0098742 | BIOLOGICAL_PROCESS | cell-cell adhesion via plasma-membrane adhesion molecules | 3.17E-05 | 2.67E-06 |
| GO:0019207 | MOLECULAR_FUNCTION | kinase regulator activity | 3.17E-05 | 2.67E-06 |
| GO:0000280 | BIOLOGICAL_PROCESS | nuclear division | 3.36E-05 | 2.84E-06 |
| GO:0046578 | BIOLOGICAL_PROCESS | regulation of Ras protein signal transduction | 3.39E-05 | 2.86E-06 |
| GO:0032535 | BIOLOGICAL_PROCESS | regulation of cellular component size | 3.60E-05 | 3.04E-06 |
| GO:0060173 | BIOLOGICAL_PROCESS | limb development | 3.65E-05 | 3.14E-06 |
| GO:0061572 | BIOLOGICAL_PROCESS | actin filament bundle organization | 3.65E-05 | 3.14E-06 |
| GO:1904950 | BIOLOGICAL_PROCESS | negative regulation of establishment of protein localization | 3.65E-05 | 3.14E-06 |
| GO:0005109 | MOLECULAR_FUNCTION | frizzled binding | 3.65E-05 | 3.12E-06 |
| GO:0043467 | BIOLOGICAL_PROCESS | regulation of generation of precursor metabolites and energy | 3.65E-05 | 3.12E-06 |
| GO:0045089 | BIOLOGICAL_PROCESS | positive regulation of innate immune response | 3.65E-05 | 3.14E-06 |
| GO:0008091 | CELLULAR_COMPONENT | spectrin | 3.65E-05 | 3.12E-06 |
| GO:0030518 | BIOLOGICAL_PROCESS | intracellular steroid hormone receptor signaling pathway | 3.65E-05 | 3.14E-06 |
| GO:0001947 | BIOLOGICAL_PROCESS | heart looping | 3.65E-05 | 3.14E-06 |
| GO:0048644 | BIOLOGICAL_PROCESS | muscle organ morphogenesis | 3.65E-05 | 3.12E-06 |
| GO:0060415 | BIOLOGICAL_PROCESS | muscle tissue morphogenesis | 3.65E-05 | 3.12E-06 |
| GO:0070374 | BIOLOGICAL_PROCESS | positive regulation of ERK1 and ERK2 cascade | 3.65E-05 | 3.12E-06 |
| GO:0051249 | BIOLOGICAL_PROCESS | regulation of lymphocyte activation | 3.65E-05 | 3.14E-06 |
| GO:0051017 | BIOLOGICAL_PROCESS | actin filament bundle assembly | 3.65E-05 | 3.14E-06 |
| GO:0043297 | BIOLOGICAL_PROCESS | apical junction assembly | 3.65E-05 | 3.12E-06 |
| GO:0009950 | BIOLOGICAL_PROCESS | dorsal/ventral axis specification | 3.65E-05 | 3.12E-06 |
| GO:0019221 | BIOLOGICAL_PROCESS | cytokine-mediated signaling pathway | 3.65E-05 | 3.14E-06 |
| GO:0034702 | CELLULAR_COMPONENT | ion channel complex | 3.78E-05 | 3.26E-06 |
| GO:0043254 | BIOLOGICAL_PROCESS | regulation of protein complex assembly | 3.88E-05 | 3.35E-06 |
| GO:0000228 | CELLULAR_COMPONENT | nuclear chromosome | 3.92E-05 | 3.38E-06 |
| GO:0034212 | MOLECULAR_FUNCTION | peptide N-acetyltransferase activity | 3.92E-05 | 3.40E-06 |
| GO:0052547 | BIOLOGICAL_PROCESS | regulation of peptidase activity | 3.92E-05 | 3.40E-06 |
| GO:0010976 | BIOLOGICAL_PROCESS | positive regulation of neuron projection development | 3.92E-05 | 3.40E-06 |
| GO:0019216 | BIOLOGICAL_PROCESS | regulation of lipid metabolic process | 3.92E-05 | 3.40E-06 |
| GO:1903845 | BIOLOGICAL_PROCESS | negative regulation of cellular response to transforming growth factor beta stimulus | 4.35E-05 | 3.81E-06 |
| GO:0031345 | BIOLOGICAL_PROCESS | negative regulation of cell projection organization | 4.35E-05 | 3.81E-06 |
| GO:0030512 | BIOLOGICAL_PROCESS | negative regulation of transforming growth factor beta receptor signaling pathway | 4.35E-05 | 3.81E-06 |
| GO:1901342 | BIOLOGICAL_PROCESS | regulation of vasculature development | 4.35E-05 | 3.81E-06 |
| GO:0004316 | MOLECULAR_FUNCTION | 3-oxoacyl-[acyl-carrier-protein] reductase (NADPH) activity | 4.35E-05 | 3.81E-06 |
| GO:0021515 | BIOLOGICAL_PROCESS | cell differentiation in spinal cord | 4.35E-05 | 3.81E-06 |
| GO:0007498 | BIOLOGICAL_PROCESS | mesoderm development | 4.35E-05 | 3.81E-06 |
| GO:0032411 | BIOLOGICAL_PROCESS | positive regulation of transporter activity | 4.35E-05 | 3.81E-06 |
| GO:0007422 | BIOLOGICAL_PROCESS | peripheral nervous system development | 4.35E-05 | 3.81E-06 |
| GO:0055007 | BIOLOGICAL_PROCESS | cardiac muscle cell differentiation | 4.35E-05 | 3.81E-06 |
| GO:0042277 | MOLECULAR_FUNCTION | peptide binding | 4.52E-05 | 3.96E-06 |
| GO:0044449 | CELLULAR_COMPONENT | contractile fiber part | 4.52E-05 | 3.96E-06 |
| GO:0006811 | BIOLOGICAL_PROCESS | ion transport | 4.78E-05 | 4.20E-06 |
| GO:0022892 | MOLECULAR_FUNCTION | substrate-specific transporter activity | 5.17E-05 | 4.54E-06 |
| GO:1904951 | BIOLOGICAL_PROCESS | positive regulation of establishment of protein localization | 5.17E-05 | 4.55E-06 |
| GO:0001822 | BIOLOGICAL_PROCESS | kidney development | 5.33E-05 | 4.72E-06 |
| GO:0031346 | BIOLOGICAL_PROCESS | positive regulation of cell projection organization | 5.33E-05 | 4.72E-06 |
| GO:0032868 | BIOLOGICAL_PROCESS | response to insulin | 5.33E-05 | 4.72E-06 |
| GO:1901653 | BIOLOGICAL_PROCESS | cellular response to peptide | 5.33E-05 | 4.72E-06 |
| GO:0071375 | BIOLOGICAL_PROCESS | cellular response to peptide hormone stimulus | 5.33E-05 | 4.72E-06 |
| GO:0035303 | BIOLOGICAL_PROCESS | regulation of dephosphorylation | 5.33E-05 | 4.72E-06 |
| GO:0007050 | BIOLOGICAL_PROCESS | cell cycle arrest | 5.33E-05 | 4.72E-06 |
| GO:0043547 | BIOLOGICAL_PROCESS | positive regulation of GTPase activity | 5.56E-05 | 4.93E-06 |
| GO:0006928 | BIOLOGICAL_PROCESS | movement of cell or subcellular component | 5.64E-05 | 5.01E-06 |
| GO:0006606 | BIOLOGICAL_PROCESS | protein import into nucleus | 5.69E-05 | 5.08E-06 |
| GO:1902593 | BIOLOGICAL_PROCESS | single-organism nuclear import | 5.69E-05 | 5.08E-06 |
| GO:0044744 | BIOLOGICAL_PROCESS | protein targeting to nucleus | 5.69E-05 | 5.08E-06 |
| GO:0051170 | BIOLOGICAL_PROCESS | nuclear import | 5.69E-05 | 5.08E-06 |
| GO:0043292 | CELLULAR_COMPONENT | contractile fiber | 5.69E-05 | 5.08E-06 |
| GO:0005244 | MOLECULAR_FUNCTION | voltage-gated ion channel activity | 5.83E-05 | 5.21E-06 |
| GO:0022832 | MOLECULAR_FUNCTION | voltage-gated channel activity | 5.83E-05 | 5.21E-06 |
| GO:0030073 | BIOLOGICAL_PROCESS | insulin secretion | 6.07E-05 | 5.45E-06 |
| GO:0030178 | BIOLOGICAL_PROCESS | negative regulation of Wnt signaling pathway | 6.07E-05 | 5.45E-06 |
| GO:0034599 | BIOLOGICAL_PROCESS | cellular response to oxidative stress | 6.07E-05 | 5.45E-06 |
| GO:0007519 | BIOLOGICAL_PROCESS | skeletal muscle tissue development | 6.07E-05 | 5.45E-06 |
| GO:0098562 | CELLULAR_COMPONENT | cytoplasmic side of membrane | 6.14E-05 | 5.52E-06 |
| GO:0042787 | BIOLOGICAL_PROCESS | protein ubiquitination involved in ubiquitin-dependent protein catabolic process | 6.14E-05 | 5.52E-06 |
| GO:0032970 | BIOLOGICAL_PROCESS | regulation of actin filament-based process | 6.54E-05 | 5.89E-06 |
| GO:0072001 | BIOLOGICAL_PROCESS | renal system development | 6.74E-05 | 6.09E-06 |
| GO:0010639 | BIOLOGICAL_PROCESS | negative regulation of organelle organization | 6.74E-05 | 6.09E-06 |
| GO:0030902 | BIOLOGICAL_PROCESS | hindbrain development | 6.74E-05 | 6.09E-06 |
| GO:0048471 | CELLULAR_COMPONENT | perinuclear region of cytoplasm | 6.82E-05 | 6.17E-06 |
| GO:0007368 | BIOLOGICAL_PROCESS | determination of left/right symmetry | 6.82E-05 | 6.17E-06 |
| GO:0044764 | BIOLOGICAL_PROCESS | multi-organism cellular process | 7.18E-05 | 6.51E-06 |
| GO:0051493 | BIOLOGICAL_PROCESS | regulation of cytoskeleton organization | 7.24E-05 | 6.57E-06 |
| GO:0033365 | BIOLOGICAL_PROCESS | protein localization to organelle | 7.90E-05 | 7.17E-06 |
| GO:0072511 | BIOLOGICAL_PROCESS | divalent inorganic cation transport | 7.95E-05 | 7.23E-06 |
| GO:0070838 | BIOLOGICAL_PROCESS | divalent metal ion transport | 7.95E-05 | 7.23E-06 |
| GO:0030323 | BIOLOGICAL_PROCESS | respiratory tube development | 8.13E-05 | 7.41E-06 |
| GO:0048588 | BIOLOGICAL_PROCESS | developmental cell growth | 8.13E-05 | 7.41E-06 |
| GO:0051222 | BIOLOGICAL_PROCESS | positive regulation of protein transport | 8.13E-05 | 7.41E-06 |
| GO:0016070 | BIOLOGICAL_PROCESS | RNA metabolic process | 8.44E-05 | 7.71E-06 |
| GO:0030326 | BIOLOGICAL_PROCESS | embryonic limb morphogenesis | 8.69E-05 | 8.08E-06 |
| GO:0001708 | BIOLOGICAL_PROCESS | cell fate specification | 8.69E-05 | 8.08E-06 |
| GO:0035113 | BIOLOGICAL_PROCESS | embryonic appendage morphogenesis | 8.69E-05 | 8.08E-06 |
| GO:0035108 | BIOLOGICAL_PROCESS | limb morphogenesis | 8.69E-05 | 8.08E-06 |
| GO:0035257 | MOLECULAR_FUNCTION | nuclear hormone receptor binding | 8.69E-05 | 8.08E-06 |
| GO:0030141 | CELLULAR_COMPONENT | secretory granule | 8.69E-05 | 8.08E-06 |
| GO:0048864 | BIOLOGICAL_PROCESS | stem cell development | 8.69E-05 | 8.08E-06 |
| GO:0005544 | MOLECULAR_FUNCTION | calcium-dependent phospholipid binding | 8.69E-05 | 8.08E-06 |
| GO:0006954 | BIOLOGICAL_PROCESS | inflammatory response | 8.69E-05 | 8.08E-06 |
| GO:0002218 | BIOLOGICAL_PROCESS | activation of innate immune response | 8.69E-05 | 8.08E-06 |
| GO:0051224 | BIOLOGICAL_PROCESS | negative regulation of protein transport | 8.69E-05 | 8.08E-06 |
| GO:0001078 | MOLECULAR_FUNCTION | transcriptional repressor activity, RNA polymerase II core promoter proximal region sequence-specific binding | 8.69E-05 | 8.08E-06 |
| GO:0045444 | BIOLOGICAL_PROCESS | fat cell differentiation | 8.69E-05 | 8.08E-06 |
| GO:0014032 | BIOLOGICAL_PROCESS | neural crest cell development | 8.69E-05 | 8.08E-06 |
| GO:0014031 | BIOLOGICAL_PROCESS | mesenchymal cell development | 8.69E-05 | 8.08E-06 |
| GO:1900542 | BIOLOGICAL_PROCESS | regulation of purine nucleotide metabolic process | 8.69E-05 | 8.08E-06 |
| GO:0043112 | BIOLOGICAL_PROCESS | receptor metabolic process | 8.69E-05 | 8.08E-06 |
| GO:0051783 | BIOLOGICAL_PROCESS | regulation of nuclear division | 8.69E-05 | 8.08E-06 |
| GO:0002758 | BIOLOGICAL_PROCESS | innate immune response-activating signal transduction | 8.69E-05 | 8.08E-06 |
| GO:0009411 | BIOLOGICAL_PROCESS | response to UV | 8.72E-05 | 8.13E-06 |
| GO:0032412 | BIOLOGICAL_PROCESS | regulation of ion transmembrane transporter activity | 8.72E-05 | 8.13E-06 |
| GO:0022898 | BIOLOGICAL_PROCESS | regulation of transmembrane transporter activity | 8.72E-05 | 8.13E-06 |
| GO:0030017 | CELLULAR_COMPONENT | sarcomere | 9.25E-05 | 8.64E-06 |
| GO:0002252 | BIOLOGICAL_PROCESS | immune effector process | 9.25E-05 | 8.64E-06 |
| GO:0050764 | BIOLOGICAL_PROCESS | regulation of phagocytosis | 9.48E-05 | 8.97E-06 |
| GO:0001702 | BIOLOGICAL_PROCESS | gastrulation with mouth forming second | 9.48E-05 | 8.97E-06 |
| GO:0001755 | BIOLOGICAL_PROCESS | neural crest cell migration | 9.48E-05 | 8.97E-06 |
| GO:0005242 | MOLECULAR_FUNCTION | inward rectifier potassium channel activity | 9.48E-05 | 8.97E-06 |
| GO:0003208 | BIOLOGICAL_PROCESS | cardiac ventricle morphogenesis | 9.48E-05 | 8.97E-06 |
| GO:0016500 | MOLECULAR_FUNCTION | protein-hormone receptor activity | 9.48E-05 | 8.97E-06 |
| GO:2001236 | BIOLOGICAL_PROCESS | regulation of extrinsic apoptotic signaling pathway | 9.48E-05 | 8.97E-06 |
| GO:0005546 | MOLECULAR_FUNCTION | phosphatidylinositol-4,5-bisphosphate binding | 9.48E-05 | 8.97E-06 |
| GO:0034103 | BIOLOGICAL_PROCESS | regulation of tissue remodeling | 9.48E-05 | 8.97E-06 |
| GO:1901989 | BIOLOGICAL_PROCESS | positive regulation of cell cycle phase transition | 9.48E-05 | 8.97E-06 |
| GO:1901992 | BIOLOGICAL_PROCESS | positive regulation of mitotic cell cycle phase transition | 9.48E-05 | 8.97E-06 |
| GO:0045778 | BIOLOGICAL_PROCESS | positive regulation of ossification | 9.48E-05 | 8.97E-06 |
| GO:0031214 | BIOLOGICAL_PROCESS | biomineral tissue development | 9.48E-05 | 8.97E-06 |
| GO:0009898 | CELLULAR_COMPONENT | cytoplasmic side of plasma membrane | 1.03E-04 | 9.73E-06 |
| GO:1903050 | BIOLOGICAL_PROCESS | regulation of proteolysis involved in cellular protein catabolic process | 1.03E-04 | 9.73E-06 |
| GO:0010638 | BIOLOGICAL_PROCESS | positive regulation of organelle organization | 1.04E-04 | 9.90E-06 |
| GO:0031674 | CELLULAR_COMPONENT | I band | 1.07E-04 | 1.03E-05 |
| GO:0050851 | BIOLOGICAL_PROCESS | antigen receptor-mediated signaling pathway | 1.07E-04 | 1.03E-05 |
| GO:0060026 | BIOLOGICAL_PROCESS | convergent extension | 1.07E-04 | 1.03E-05 |
| GO:0017158 | BIOLOGICAL_PROCESS | regulation of calcium ion-dependent exocytosis | 1.07E-04 | 1.03E-05 |
| GO:0005783 | CELLULAR_COMPONENT | endoplasmic reticulum | 1.07E-04 | 1.02E-05 |
| GO:0090100 | BIOLOGICAL_PROCESS | positive regulation of transmembrane receptor protein serine/threonine kinase signaling pathway | 1.07E-04 | 1.03E-05 |
| GO:0048636 | BIOLOGICAL_PROCESS | positive regulation of muscle organ development | 1.07E-04 | 1.03E-05 |
| GO:0048791 | BIOLOGICAL_PROCESS | calcium ion-regulated exocytosis of neurotransmitter | 1.07E-04 | 1.03E-05 |
| GO:0051271 | BIOLOGICAL_PROCESS | negative regulation of cellular component movement | 1.07E-04 | 1.03E-05 |
| GO:0007159 | BIOLOGICAL_PROCESS | leukocyte cell-cell adhesion | 1.07E-04 | 1.03E-05 |
| GO:1901863 | BIOLOGICAL_PROCESS | positive regulation of muscle tissue development | 1.07E-04 | 1.03E-05 |
| GO:0045844 | BIOLOGICAL_PROCESS | positive regulation of striated muscle tissue development | 1.07E-04 | 1.03E-05 |
| GO:0002573 | BIOLOGICAL_PROCESS | myeloid leukocyte differentiation | 1.07E-04 | 1.03E-05 |
| GO:0000987 | MOLECULAR_FUNCTION | core promoter proximal region sequence-specific DNA binding | 1.11E-04 | 1.07E-05 |
| GO:0032869 | BIOLOGICAL_PROCESS | cellular response to insulin stimulus | 1.14E-04 | 1.10E-05 |
| GO:0019897 | CELLULAR_COMPONENT | extrinsic component of plasma membrane | 1.14E-04 | 1.10E-05 |
| GO:0016740 | MOLECULAR_FUNCTION | transferase activity | 1.14E-04 | 1.09E-05 |
| GO:0018210 | BIOLOGICAL_PROCESS | peptidyl-threonine modification | 1.14E-04 | 1.10E-05 |
| GO:0099572 | CELLULAR_COMPONENT | postsynaptic specialization | 1.14E-04 | 1.10E-05 |
| GO:1902903 | BIOLOGICAL_PROCESS | regulation of supramolecular fiber organization | 1.18E-04 | 1.14E-05 |
| GO:0007215 | BIOLOGICAL_PROCESS | glutamate receptor signaling pathway | 1.18E-04 | 1.14E-05 |
| GO:0045732 | BIOLOGICAL_PROCESS | positive regulation of protein catabolic process | 1.18E-04 | 1.14E-05 |
| GO:0051235 | BIOLOGICAL_PROCESS | maintenance of location | 1.24E-04 | 1.20E-05 |
| GO:0044089 | BIOLOGICAL_PROCESS | positive regulation of cellular component biogenesis | 1.28E-04 | 1.24E-05 |
| GO:0001649 | BIOLOGICAL_PROCESS | osteoblast differentiation | 1.38E-04 | 1.34E-05 |
| GO:0016324 | CELLULAR_COMPONENT | apical plasma membrane | 1.38E-04 | 1.34E-05 |
| GO:0005913 | CELLULAR_COMPONENT | cell-cell adherens junction | 1.38E-04 | 1.34E-05 |
| GO:0002694 | BIOLOGICAL_PROCESS | regulation of leukocyte activation | 1.38E-04 | 1.34E-05 |
| GO:0031234 | CELLULAR_COMPONENT | extrinsic component of cytoplasmic side of plasma membrane | 1.38E-04 | 1.34E-05 |
| GO:0043062 | BIOLOGICAL_PROCESS | extracellular structure organization | 1.38E-04 | 1.34E-05 |
| GO:0051276 | BIOLOGICAL_PROCESS | chromosome organization | 1.44E-04 | 1.41E-05 |
| GO:0050801 | BIOLOGICAL_PROCESS | ion homeostasis | 1.45E-04 | 1.42E-05 |
| GO:0018205 | BIOLOGICAL_PROCESS | peptidyl-lysine modification | 1.52E-04 | 1.49E-05 |
| GO:2000021 | BIOLOGICAL_PROCESS | regulation of ion homeostasis | 1.90E-04 | 1.86E-05 |
| GO:0016358 | BIOLOGICAL_PROCESS | dendrite development | 1.90E-04 | 1.86E-05 |
| GO:0090257 | BIOLOGICAL_PROCESS | regulation of muscle system process | 1.90E-04 | 1.86E-05 |
| GO:0061136 | BIOLOGICAL_PROCESS | regulation of proteasomal protein catabolic process | 1.90E-04 | 1.86E-05 |
| GO:0090068 | BIOLOGICAL_PROCESS | positive regulation of cell cycle process | 1.96E-04 | 1.93E-05 |
| GO:0007548 | BIOLOGICAL_PROCESS | sex differentiation | 1.96E-04 | 1.93E-05 |
| GO:0043279 | BIOLOGICAL_PROCESS | response to alkaloid | 1.96E-04 | 1.93E-05 |
| GO:0050796 | BIOLOGICAL_PROCESS | regulation of insulin secretion | 2.08E-04 | 2.06E-05 |
| GO:0050730 | BIOLOGICAL_PROCESS | regulation of peptidyl-tyrosine phosphorylation | 2.08E-04 | 2.06E-05 |
| GO:0048565 | BIOLOGICAL_PROCESS | digestive tract development | 2.08E-04 | 2.06E-05 |
| GO:0050878 | BIOLOGICAL_PROCESS | regulation of body fluid levels | 2.08E-04 | 2.06E-05 |
| GO:0043406 | BIOLOGICAL_PROCESS | positive regulation of MAP kinase activity | 2.08E-04 | 2.06E-05 |
| GO:0072073 | BIOLOGICAL_PROCESS | kidney epithelium development | 2.08E-04 | 2.06E-05 |
| GO:0030496 | CELLULAR_COMPONENT | midbody | 2.08E-04 | 2.06E-05 |
| GO:0010506 | BIOLOGICAL_PROCESS | regulation of autophagy | 2.08E-04 | 2.06E-05 |
| GO:0060840 | BIOLOGICAL_PROCESS | artery development | 2.08E-04 | 2.06E-05 |
| GO:0044325 | MOLECULAR_FUNCTION | ion channel binding | 2.08E-04 | 2.06E-05 |
| GO:0030016 | CELLULAR_COMPONENT | myofibril | 2.23E-04 | 2.22E-05 |
| GO:0060041 | BIOLOGICAL_PROCESS | retina development in camera-type eye | 2.23E-04 | 2.22E-05 |
| GO:0030001 | BIOLOGICAL_PROCESS | metal ion transport | 2.34E-04 | 2.33E-05 |
| GO:0098552 | CELLULAR_COMPONENT | side of membrane | 2.41E-04 | 2.40E-05 |
| GO:1901987 | BIOLOGICAL_PROCESS | regulation of cell cycle phase transition | 2.43E-04 | 2.42E-05 |
| GO:0006473 | BIOLOGICAL_PROCESS | protein acetylation | 2.49E-04 | 2.49E-05 |
| GO:0005245 | MOLECULAR_FUNCTION | voltage-gated calcium channel activity | 2.51E-04 | 2.52E-05 |
| GO:0098984 | CELLULAR_COMPONENT | neuron to neuron synapse | 2.51E-04 | 2.52E-05 |
| GO:0014069 | CELLULAR_COMPONENT | postsynaptic density | 2.51E-04 | 2.52E-05 |
| GO:0031253 | CELLULAR_COMPONENT | cell projection membrane | 2.51E-04 | 2.52E-05 |
| GO:0032279 | CELLULAR_COMPONENT | asymmetric synapse | 2.51E-04 | 2.52E-05 |
| GO:0018107 | BIOLOGICAL_PROCESS | peptidyl-threonine phosphorylation | 2.51E-04 | 2.52E-05 |
| GO:1901216 | BIOLOGICAL_PROCESS | positive regulation of neuron death | 2.52E-04 | 2.58E-05 |
| GO:0001709 | BIOLOGICAL_PROCESS | cell fate determination | 2.52E-04 | 2.58E-05 |
| GO:0001786 | MOLECULAR_FUNCTION | phosphatidylserine binding | 2.52E-04 | 2.58E-05 |
| GO:0044853 | CELLULAR_COMPONENT | plasma membrane raft | 2.52E-04 | 2.58E-05 |
| GO:0042733 | BIOLOGICAL_PROCESS | embryonic digit morphogenesis | 2.52E-04 | 2.58E-05 |
| GO:0048857 | BIOLOGICAL_PROCESS | neural nucleus development | 2.52E-04 | 2.58E-05 |
| GO:0008013 | MOLECULAR_FUNCTION | beta-catenin binding | 2.52E-04 | 2.58E-05 |
| GO:0042475 | BIOLOGICAL_PROCESS | odontogenesis of dentin-containing tooth | 2.52E-04 | 2.58E-05 |
| GO:0048663 | BIOLOGICAL_PROCESS | neuron fate commitment | 2.52E-04 | 2.58E-05 |
| GO:0046622 | BIOLOGICAL_PROCESS | positive regulation of organ growth | 2.52E-04 | 2.58E-05 |
| GO:0007217 | BIOLOGICAL_PROCESS | tachykinin receptor signaling pathway | 2.52E-04 | 2.58E-05 |
| GO:0046717 | BIOLOGICAL_PROCESS | acid secretion | 2.52E-04 | 2.58E-05 |
| GO:0051101 | BIOLOGICAL_PROCESS | regulation of DNA binding | 2.52E-04 | 2.58E-05 |
| GO:0004707 | MOLECULAR_FUNCTION | MAP kinase activity | 2.52E-04 | 2.58E-05 |
| GO:0071526 | BIOLOGICAL_PROCESS | semaphorin-plexin signaling pathway | 2.52E-04 | 2.58E-05 |
| GO:0045785 | BIOLOGICAL_PROCESS | positive regulation of cell adhesion | 2.52E-04 | 2.58E-05 |
| GO:0070830 | BIOLOGICAL_PROCESS | bicellular tight junction assembly | 2.52E-04 | 2.58E-05 |
| GO:0001191 | MOLECULAR_FUNCTION | transcriptional repressor activity, RNA polymerase II transcription factor binding | 2.52E-04 | 2.58E-05 |
| GO:0019209 | MOLECULAR_FUNCTION | kinase activator activity | 2.52E-04 | 2.58E-05 |
| GO:0046849 | BIOLOGICAL_PROCESS | bone remodeling | 2.52E-04 | 2.58E-05 |
| GO:0001102 | MOLECULAR_FUNCTION | RNA polymerase II activating transcription factor binding | 2.52E-04 | 2.58E-05 |
| GO:0045667 | BIOLOGICAL_PROCESS | regulation of osteoblast differentiation | 2.52E-04 | 2.58E-05 |
| GO:0032366 | BIOLOGICAL_PROCESS | intracellular sterol transport | 2.52E-04 | 2.58E-05 |
| GO:0032367 | BIOLOGICAL_PROCESS | intracellular cholesterol transport | 2.52E-04 | 2.58E-05 |
| GO:0031018 | BIOLOGICAL_PROCESS | endocrine pancreas development | 2.52E-04 | 2.58E-05 |
| GO:0072006 | BIOLOGICAL_PROCESS | nephron development | 2.63E-04 | 2.75E-05 |
| GO:0072009 | BIOLOGICAL_PROCESS | nephron epithelium development | 2.63E-04 | 2.75E-05 |
| GO:0032872 | BIOLOGICAL_PROCESS | regulation of stress-activated MAPK cascade | 2.63E-04 | 2.75E-05 |
| GO:0042113 | BIOLOGICAL_PROCESS | B cell activation | 2.63E-04 | 2.75E-05 |
| GO:1902936 | MOLECULAR_FUNCTION | phosphatidylinositol bisphosphate binding | 2.63E-04 | 2.75E-05 |
| GO:0006941 | BIOLOGICAL_PROCESS | striated muscle contraction | 2.63E-04 | 2.75E-05 |
| GO:0048709 | BIOLOGICAL_PROCESS | oligodendrocyte differentiation | 2.63E-04 | 2.75E-05 |
| GO:0007088 | BIOLOGICAL_PROCESS | regulation of mitotic nuclear division | 2.63E-04 | 2.75E-05 |
| GO:0070302 | BIOLOGICAL_PROCESS | regulation of stress-activated protein kinase signaling cascade | 2.63E-04 | 2.75E-05 |
| GO:0060976 | BIOLOGICAL_PROCESS | coronary vasculature development | 2.63E-04 | 2.75E-05 |
| GO:0010810 | BIOLOGICAL_PROCESS | regulation of cell-substrate adhesion | 2.63E-04 | 2.75E-05 |
| GO:0021517 | BIOLOGICAL_PROCESS | ventral spinal cord development | 2.63E-04 | 2.75E-05 |
| GO:0008544 | BIOLOGICAL_PROCESS | epidermis development | 2.63E-04 | 2.75E-05 |
| GO:0034764 | BIOLOGICAL_PROCESS | positive regulation of transmembrane transport | 2.63E-04 | 2.75E-05 |
| GO:0034767 | BIOLOGICAL_PROCESS | positive regulation of ion transmembrane transport | 2.63E-04 | 2.75E-05 |
| GO:0022408 | BIOLOGICAL_PROCESS | negative regulation of cell-cell adhesion | 2.63E-04 | 2.75E-05 |
| GO:0015424 | MOLECULAR_FUNCTION | amino acid-transporting ATPase activity | 2.63E-04 | 2.75E-05 |
| GO:0008217 | BIOLOGICAL_PROCESS | regulation of blood pressure | 2.63E-04 | 2.75E-05 |
| GO:1902105 | BIOLOGICAL_PROCESS | regulation of leukocyte differentiation | 2.63E-04 | 2.75E-05 |
| GO:0045765 | BIOLOGICAL_PROCESS | regulation of angiogenesis | 2.63E-04 | 2.75E-05 |
| GO:0032414 | BIOLOGICAL_PROCESS | positive regulation of ion transmembrane transporter activity | 2.63E-04 | 2.75E-05 |
| GO:0009895 | BIOLOGICAL_PROCESS | negative regulation of catabolic process | 2.63E-04 | 2.75E-05 |
| GO:0003705 | MOLECULAR_FUNCTION | transcription factor activity, RNA polymerase II distal enhancer sequence-specific binding | 2.63E-04 | 2.75E-05 |
| GO:0015758 | BIOLOGICAL_PROCESS | glucose transport | 2.63E-04 | 2.75E-05 |
| GO:0045637 | BIOLOGICAL_PROCESS | regulation of myeloid cell differentiation | 2.63E-04 | 2.75E-05 |
| GO:0019438 | BIOLOGICAL_PROCESS | aromatic compound biosynthetic process | 2.66E-04 | 2.79E-05 |
| GO:0005234 | MOLECULAR_FUNCTION | extracellular-glutamate-gated ion channel activity | 2.92E-04 | 3.07E-05 |
| GO:1990138 | BIOLOGICAL_PROCESS | neuron projection extension | 2.92E-04 | 3.07E-05 |
| GO:0007156 | BIOLOGICAL_PROCESS | homophilic cell adhesion via plasma membrane adhesion molecules | 2.92E-04 | 3.07E-05 |
| GO:0034703 | CELLULAR_COMPONENT | cation channel complex | 3.01E-04 | 3.17E-05 |
| GO:0007600 | BIOLOGICAL_PROCESS | sensory perception | 3.04E-04 | 3.20E-05 |
| GO:0098862 | CELLULAR_COMPONENT | cluster of actin-based cell projections | 3.09E-04 | 3.28E-05 |
| GO:0090090 | BIOLOGICAL_PROCESS | negative regulation of canonical Wnt signaling pathway | 3.09E-04 | 3.28E-05 |
| GO:0090002 | BIOLOGICAL_PROCESS | establishment of protein localization to plasma membrane | 3.09E-04 | 3.28E-05 |
| GO:0030198 | BIOLOGICAL_PROCESS | extracellular matrix organization | 3.09E-04 | 3.28E-05 |
| GO:0048871 | BIOLOGICAL_PROCESS | multicellular organismal homeostasis | 3.09E-04 | 3.28E-05 |
| GO:0051403 | BIOLOGICAL_PROCESS | stress-activated MAPK cascade | 3.09E-04 | 3.28E-05 |
| GO:0031098 | BIOLOGICAL_PROCESS | stress-activated protein kinase signaling cascade | 3.09E-04 | 3.28E-05 |
| GO:0005911 | CELLULAR_COMPONENT | cell-cell junction | 3.16E-04 | 3.35E-05 |
| GO:0030324 | BIOLOGICAL_PROCESS | lung development | 3.36E-04 | 3.56E-05 |
| GO:0005085 | MOLECULAR_FUNCTION | guanyl-nucleotide exchange factor activity | 3.39E-04 | 3.60E-05 |
| GO:0004857 | MOLECULAR_FUNCTION | enzyme inhibitor activity | 3.74E-04 | 3.98E-05 |
| GO:0018130 | BIOLOGICAL_PROCESS | heterocycle biosynthetic process | 4.02E-04 | 4.27E-05 |
| GO:0001558 | BIOLOGICAL_PROCESS | regulation of cell growth | 4.04E-04 | 4.31E-05 |
| GO:0061630 | MOLECULAR_FUNCTION | ubiquitin protein ligase activity | 4.04E-04 | 4.31E-05 |
| GO:0051346 | BIOLOGICAL_PROCESS | negative regulation of hydrolase activity | 4.04E-04 | 4.31E-05 |
| GO:0010035 | BIOLOGICAL_PROCESS | response to inorganic substance | 4.10E-04 | 4.37E-05 |
| GO:0050792 | BIOLOGICAL_PROCESS | regulation of viral process | 4.23E-04 | 4.53E-05 |
| GO:0051924 | BIOLOGICAL_PROCESS | regulation of calcium ion transport | 4.23E-04 | 4.53E-05 |
| GO:0033002 | BIOLOGICAL_PROCESS | muscle cell proliferation | 4.23E-04 | 4.53E-05 |
| GO:1902806 | BIOLOGICAL_PROCESS | regulation of cell cycle G1/S phase transition | 4.23E-04 | 4.53E-05 |
| GO:0043903 | BIOLOGICAL_PROCESS | regulation of symbiosis, encompassing mutualism through parasitism | 4.23E-04 | 4.53E-05 |
| GO:1903900 | BIOLOGICAL_PROCESS | regulation of viral life cycle | 4.23E-04 | 4.53E-05 |
| GO:0035235 | BIOLOGICAL_PROCESS | ionotropic glutamate receptor signaling pathway | 4.24E-04 | 4.56E-05 |
| GO:1903364 | BIOLOGICAL_PROCESS | positive regulation of cellular protein catabolic process | 4.24E-04 | 4.56E-05 |
| GO:0016620 | MOLECULAR_FUNCTION | oxidoreductase activity, acting on the aldehyde or oxo group of donors, NAD or NADP as acceptor | 4.25E-04 | 4.57E-05 |
| GO:0098793 | CELLULAR_COMPONENT | presynapse | 4.33E-04 | 4.66E-05 |
| GO:0000151 | CELLULAR_COMPONENT | ubiquitin ligase complex | 4.33E-04 | 4.66E-05 |
| GO:0060249 | BIOLOGICAL_PROCESS | anatomical structure homeostasis | 4.38E-04 | 4.73E-05 |
| GO:0004970 | MOLECULAR_FUNCTION | ionotropic glutamate receptor activity | 4.38E-04 | 4.73E-05 |
| GO:0032956 | BIOLOGICAL_PROCESS | regulation of actin cytoskeleton organization | 4.47E-04 | 4.82E-05 |
| GO:1901990 | BIOLOGICAL_PROCESS | regulation of mitotic cell cycle phase transition | 4.47E-04 | 4.82E-05 |
| GO:0008270 | MOLECULAR_FUNCTION | zinc ion binding | 4.54E-04 | 4.91E-05 |
| GO:0030276 | MOLECULAR_FUNCTION | clathrin binding | 4.78E-04 | 5.22E-05 |
| GO:0035258 | MOLECULAR_FUNCTION | steroid hormone receptor binding | 4.78E-04 | 5.22E-05 |
| GO:0031398 | BIOLOGICAL_PROCESS | positive regulation of protein ubiquitination | 4.78E-04 | 5.22E-05 |
| GO:0042734 | CELLULAR_COMPONENT | presynaptic membrane | 4.78E-04 | 5.22E-05 |
| GO:0090132 | BIOLOGICAL_PROCESS | epithelium migration | 4.78E-04 | 5.22E-05 |
| GO:0090130 | BIOLOGICAL_PROCESS | tissue migration | 4.78E-04 | 5.22E-05 |
| GO:0010631 | BIOLOGICAL_PROCESS | epithelial cell migration | 4.78E-04 | 5.22E-05 |
| GO:0001085 | MOLECULAR_FUNCTION | RNA polymerase II transcription factor binding | 4.78E-04 | 5.22E-05 |
| GO:0007200 | BIOLOGICAL_PROCESS | phospholipase C-activating G-protein coupled receptor signaling pathway | 4.78E-04 | 5.22E-05 |
| GO:0007193 | BIOLOGICAL_PROCESS | adenylate cyclase-inhibiting G-protein coupled receptor signaling pathway | 4.78E-04 | 5.22E-05 |
| GO:0030968 | BIOLOGICAL_PROCESS | endoplasmic reticulum unfolded protein response | 4.78E-04 | 5.22E-05 |
| GO:0038127 | BIOLOGICAL_PROCESS | ERBB signaling pathway | 4.78E-04 | 5.22E-05 |
| GO:0002683 | BIOLOGICAL_PROCESS | negative regulation of immune system process | 4.78E-04 | 5.22E-05 |
| GO:0031570 | BIOLOGICAL_PROCESS | DNA integrity checkpoint | 5.23E-04 | 5.72E-05 |
| GO:0010921 | BIOLOGICAL_PROCESS | regulation of phosphatase activity | 5.23E-04 | 5.72E-05 |
| GO:0046873 | MOLECULAR_FUNCTION | metal ion transmembrane transporter activity | 5.55E-04 | 6.07E-05 |
| GO:0009607 | BIOLOGICAL_PROCESS | response to biotic stimulus | 5.90E-04 | 6.46E-05 |
| GO:0099513 | CELLULAR_COMPONENT | polymeric cytoskeletal fiber | 6.09E-04 | 6.67E-05 |
| GO:0071702 | BIOLOGICAL_PROCESS | organic substance transport | 6.34E-04 | 6.96E-05 |
| GO:2000177 | BIOLOGICAL_PROCESS | regulation of neural precursor cell proliferation | 6.47E-04 | 7.43E-05 |
| GO:1901215 | BIOLOGICAL_PROCESS | negative regulation of neuron death | 6.47E-04 | 7.43E-05 |
| GO:0072132 | BIOLOGICAL_PROCESS | mesenchyme morphogenesis | 6.47E-04 | 7.43E-05 |
| GO:0043588 | BIOLOGICAL_PROCESS | skin development | 6.47E-04 | 7.43E-05 |
| GO:0030295 | MOLECULAR_FUNCTION | protein kinase activator activity | 6.47E-04 | 7.43E-05 |
| GO:0030282 | BIOLOGICAL_PROCESS | bone mineralization | 6.47E-04 | 7.43E-05 |
| GO:0098811 | MOLECULAR_FUNCTION | transcriptional repressor activity, RNA polymerase II activating transcription factor binding | 6.47E-04 | 7.43E-05 |
| GO:0050852 | BIOLOGICAL_PROCESS | T cell receptor signaling pathway | 6.47E-04 | 7.34E-05 |
| GO:0050863 | BIOLOGICAL_PROCESS | regulation of T cell activation | 6.47E-04 | 7.34E-05 |
| GO:0050817 | BIOLOGICAL_PROCESS | coagulation | 6.47E-04 | 7.34E-05 |
| GO:0051899 | BIOLOGICAL_PROCESS | membrane depolarization | 6.47E-04 | 7.34E-05 |
| GO:0030018 | CELLULAR_COMPONENT | Z disc | 6.47E-04 | 7.34E-05 |
| GO:0045981 | BIOLOGICAL_PROCESS | positive regulation of nucleotide metabolic process | 6.47E-04 | 7.43E-05 |
| GO:0014897 | BIOLOGICAL_PROCESS | striated muscle hypertrophy | 6.47E-04 | 7.43E-05 |
| GO:0014896 | BIOLOGICAL_PROCESS | muscle hypertrophy | 6.47E-04 | 7.43E-05 |
| GO:0050679 | BIOLOGICAL_PROCESS | positive regulation of epithelial cell proliferation | 6.47E-04 | 7.43E-05 |
| GO:0061351 | BIOLOGICAL_PROCESS | neural precursor cell proliferation | 6.47E-04 | 7.34E-05 |
| GO:0072091 | BIOLOGICAL_PROCESS | regulation of stem cell proliferation | 6.47E-04 | 7.43E-05 |
| GO:0060039 | BIOLOGICAL_PROCESS | pericardium development | 6.47E-04 | 7.43E-05 |
| GO:0030217 | BIOLOGICAL_PROCESS | T cell differentiation | 6.47E-04 | 7.43E-05 |
| GO:0003179 | BIOLOGICAL_PROCESS | heart valve morphogenesis | 6.47E-04 | 7.43E-05 |
| GO:0003197 | BIOLOGICAL_PROCESS | endocardial cushion development | 6.47E-04 | 7.43E-05 |
| GO:0003209 | BIOLOGICAL_PROCESS | cardiac atrium morphogenesis | 6.47E-04 | 7.43E-05 |
| GO:0003230 | BIOLOGICAL_PROCESS | cardiac atrium development | 6.47E-04 | 7.43E-05 |
| GO:0030837 | BIOLOGICAL_PROCESS | negative regulation of actin filament polymerization | 6.47E-04 | 7.34E-05 |
| GO:1902808 | BIOLOGICAL_PROCESS | positive regulation of cell cycle G1/S phase transition | 6.47E-04 | 7.43E-05 |
| GO:0001935 | BIOLOGICAL_PROCESS | endothelial cell proliferation | 6.47E-04 | 7.43E-05 |
| GO:0050900 | BIOLOGICAL_PROCESS | leukocyte migration | 6.47E-04 | 7.43E-05 |
| GO:0048645 | BIOLOGICAL_PROCESS | animal organ formation | 6.47E-04 | 7.34E-05 |
| GO:0047485 | MOLECULAR_FUNCTION | protein N-terminus binding | 6.47E-04 | 7.34E-05 |
| GO:0030666 | CELLULAR_COMPONENT | endocytic vesicle membrane | 6.47E-04 | 7.34E-05 |
| GO:0040013 | BIOLOGICAL_PROCESS | negative regulation of locomotion | 6.47E-04 | 7.34E-05 |
| GO:1900087 | BIOLOGICAL_PROCESS | positive regulation of G1/S transition of mitotic cell cycle | 6.47E-04 | 7.43E-05 |
| GO:0060419 | BIOLOGICAL_PROCESS | heart growth | 6.47E-04 | 7.34E-05 |
| GO:1903037 | BIOLOGICAL_PROCESS | regulation of leukocyte cell-cell adhesion | 6.47E-04 | 7.34E-05 |
| GO:0008332 | MOLECULAR_FUNCTION | low voltage-gated calcium channel activity | 6.47E-04 | 7.34E-05 |
| GO:0007127 | BIOLOGICAL_PROCESS | meiosis I | 6.47E-04 | 7.43E-05 |
| GO:0007173 | BIOLOGICAL_PROCESS | epidermal growth factor receptor signaling pathway | 6.47E-04 | 7.34E-05 |
| GO:0007160 | BIOLOGICAL_PROCESS | cell-matrix adhesion | 6.47E-04 | 7.34E-05 |
| GO:1901983 | BIOLOGICAL_PROCESS | regulation of protein acetylation | 6.47E-04 | 7.43E-05 |
| GO:0004995 | MOLECULAR_FUNCTION | tachykinin receptor activity | 6.47E-04 | 7.43E-05 |
| GO:0003300 | BIOLOGICAL_PROCESS | cardiac muscle hypertrophy | 6.47E-04 | 7.43E-05 |
| GO:0045834 | BIOLOGICAL_PROCESS | positive regulation of lipid metabolic process | 6.47E-04 | 7.43E-05 |
| GO:0045833 | BIOLOGICAL_PROCESS | negative regulation of lipid metabolic process | 6.47E-04 | 7.43E-05 |
| GO:0021762 | BIOLOGICAL_PROCESS | substantia nigra development | 6.47E-04 | 7.43E-05 |
| GO:1903307 | BIOLOGICAL_PROCESS | positive regulation of regulated secretory pathway | 6.47E-04 | 7.43E-05 |
| GO:0007599 | BIOLOGICAL_PROCESS | hemostasis | 6.47E-04 | 7.34E-05 |
| GO:0001190 | MOLECULAR_FUNCTION | transcriptional activator activity, RNA polymerase II transcription factor binding | 6.47E-04 | 7.43E-05 |
| GO:0021575 | BIOLOGICAL_PROCESS | hindbrain morphogenesis | 6.47E-04 | 7.34E-05 |
| GO:0070555 | BIOLOGICAL_PROCESS | response to interleukin-1 | 6.47E-04 | 7.43E-05 |
| GO:0032272 | BIOLOGICAL_PROCESS | negative regulation of protein polymerization | 6.47E-04 | 7.34E-05 |
| GO:0001105 | MOLECULAR_FUNCTION | RNA polymerase II transcription coactivator activity | 6.47E-04 | 7.43E-05 |
| GO:0003823 | MOLECULAR_FUNCTION | antigen binding | 6.47E-04 | 7.43E-05 |
| GO:0032387 | BIOLOGICAL_PROCESS | negative regulation of intracellular transport | 6.47E-04 | 7.34E-05 |
| GO:0055006 | BIOLOGICAL_PROCESS | cardiac cell development | 6.47E-04 | 7.43E-05 |
| GO:0055013 | BIOLOGICAL_PROCESS | cardiac muscle cell development | 6.47E-04 | 7.43E-05 |
| GO:0055017 | BIOLOGICAL_PROCESS | cardiac muscle tissue growth | 6.47E-04 | 7.34E-05 |
| GO:0055024 | BIOLOGICAL_PROCESS | regulation of cardiac muscle tissue development | 6.47E-04 | 7.43E-05 |
| GO:0031647 | BIOLOGICAL_PROCESS | regulation of protein stability | 6.65E-04 | 7.68E-05 |
| GO:0099132 | BIOLOGICAL_PROCESS | ATP hydrolysis coupled cation transmembrane transport | 6.65E-04 | 7.68E-05 |
| GO:0099131 | BIOLOGICAL_PROCESS | ATP hydrolysis coupled ion transmembrane transport | 6.65E-04 | 7.68E-05 |
| GO:1901800 | BIOLOGICAL_PROCESS | positive regulation of proteasomal protein catabolic process | 6.65E-04 | 7.68E-05 |
| GO:0015991 | BIOLOGICAL_PROCESS | ATP hydrolysis coupled proton transport | 6.65E-04 | 7.68E-05 |
| GO:0015988 | BIOLOGICAL_PROCESS | energy coupled proton transmembrane transport, against electrochemical gradient | 6.65E-04 | 7.68E-05 |
| GO:0050770 | BIOLOGICAL_PROCESS | regulation of axonogenesis | 6.82E-04 | 7.91E-05 |
| GO:0098631 | MOLECULAR_FUNCTION | protein binding involved in cell adhesion | 6.82E-04 | 7.91E-05 |
| GO:0098632 | MOLECULAR_FUNCTION | protein binding involved in cell-cell adhesion | 6.82E-04 | 7.91E-05 |
| GO:0098641 | MOLECULAR_FUNCTION | cadherin binding involved in cell-cell adhesion | 6.82E-04 | 7.91E-05 |
| GO:0055123 | BIOLOGICAL_PROCESS | digestive system development | 6.82E-04 | 7.91E-05 |
| GO:1903322 | BIOLOGICAL_PROCESS | positive regulation of protein modification by small protein conjugation or removal | 6.82E-04 | 7.91E-05 |
| GO:0046890 | BIOLOGICAL_PROCESS | regulation of lipid biosynthetic process | 6.82E-04 | 7.91E-05 |
| GO:0007274 | BIOLOGICAL_PROCESS | neuromuscular synaptic transmission | 6.82E-04 | 7.91E-05 |
| GO:0033044 | BIOLOGICAL_PROCESS | regulation of chromosome organization | 7.21E-04 | 8.38E-05 |
| GO:0007266 | BIOLOGICAL_PROCESS | Rho protein signal transduction | 7.21E-04 | 8.38E-05 |
| GO:0006914 | BIOLOGICAL_PROCESS | autophagy | 7.25E-04 | 8.44E-05 |
| GO:0043543 | BIOLOGICAL_PROCESS | protein acylation | 7.53E-04 | 8.77E-05 |
| GO:0071705 | BIOLOGICAL_PROCESS | nitrogen compound transport | 7.59E-04 | 8.85E-05 |
| GO:0071214 | BIOLOGICAL_PROCESS | cellular response to abiotic stimulus | 7.63E-04 | 8.90E-05 |
| GO:0044454 | CELLULAR_COMPONENT | nuclear chromosome part | 7.76E-04 | 9.06E-05 |
| GO:0009101 | BIOLOGICAL_PROCESS | glycoprotein biosynthetic process | 7.89E-04 | 9.22E-05 |
| GO:0019932 | BIOLOGICAL_PROCESS | second-messenger-mediated signaling | 7.91E-04 | 9.25E-05 |
| GO:0061659 | MOLECULAR_FUNCTION | ubiquitin-like protein ligase activity | 7.91E-04 | 9.25E-05 |
| GO:0060541 | BIOLOGICAL_PROCESS | respiratory system development | 8.12E-04 | 9.50E-05 |
| GO:1901362 | BIOLOGICAL_PROCESS | organic cyclic compound biosynthetic process | 8.40E-04 | 9.85E-05 |
| GO:0001738 | BIOLOGICAL_PROCESS | morphogenesis of a polarized epithelium | 8.94E-04 | 1.05E-04 |
| GO:2000045 | BIOLOGICAL_PROCESS | regulation of G1/S transition of mitotic cell cycle | 8.94E-04 | 1.05E-04 |
| GO:0072593 | BIOLOGICAL_PROCESS | reactive oxygen species metabolic process | 8.94E-04 | 1.05E-04 |
| GO:0006986 | BIOLOGICAL_PROCESS | response to unfolded protein | 8.94E-04 | 1.05E-04 |
| GO:0035966 | BIOLOGICAL_PROCESS | response to topologically incorrect protein | 8.94E-04 | 1.05E-04 |
| GO:0051054 | BIOLOGICAL_PROCESS | positive regulation of DNA metabolic process | 8.94E-04 | 1.05E-04 |
| GO:0042391 | BIOLOGICAL_PROCESS | regulation of membrane potential | 8.99E-04 | 1.06E-04 |
| GO:0061024 | BIOLOGICAL_PROCESS | membrane organization | 9.34E-04 | 1.10E-04 |
| GO:0008022 | MOLECULAR_FUNCTION | protein C-terminus binding | 0.001088351 | 1.29E-04 |
| GO:0048675 | BIOLOGICAL_PROCESS | axon extension | 0.001088351 | 1.29E-04 |
| GO:0010501 | BIOLOGICAL_PROCESS | RNA secondary structure unwinding | 0.001088351 | 1.29E-04 |
| GO:0001726 | CELLULAR_COMPONENT | ruffle | 0.001098524 | 1.31E-04 |
| GO:0098858 | CELLULAR_COMPONENT | actin-based cell projection | 0.001098524 | 1.31E-04 |
| GO:0050806 | BIOLOGICAL_PROCESS | positive regulation of synaptic transmission | 0.001098524 | 1.31E-04 |
| GO:0005275 | MOLECULAR_FUNCTION | amine transmembrane transporter activity | 0.001098524 | 1.31E-04 |
| GO:1902904 | BIOLOGICAL_PROCESS | negative regulation of supramolecular fiber organization | 0.001098524 | 1.31E-04 |
| GO:0060348 | BIOLOGICAL_PROCESS | bone development | 0.001098524 | 1.31E-04 |
| GO:0060411 | BIOLOGICAL_PROCESS | cardiac septum morphogenesis | 0.001098524 | 1.31E-04 |
| GO:0009743 | BIOLOGICAL_PROCESS | response to carbohydrate | 0.001098524 | 1.31E-04 |
| GO:0010770 | BIOLOGICAL_PROCESS | positive regulation of cell morphogenesis involved in differentiation | 0.001098524 | 1.31E-04 |
| GO:0051100 | BIOLOGICAL_PROCESS | negative regulation of binding | 0.001098524 | 1.31E-04 |
| GO:0051099 | BIOLOGICAL_PROCESS | positive regulation of binding | 0.001098524 | 1.31E-04 |
| GO:0048167 | BIOLOGICAL_PROCESS | regulation of synaptic plasticity | 0.001098524 | 1.31E-04 |
| GO:0031263 | MOLECULAR_FUNCTION | amine-transporting ATPase activity | 0.001098524 | 1.31E-04 |
| GO:0022607 | BIOLOGICAL_PROCESS | cellular component assembly | 0.00111254 | 1.33E-04 |
| GO:0019887 | MOLECULAR_FUNCTION | protein kinase regulator activity | 0.001238539 | 1.48E-04 |
| GO:0022412 | BIOLOGICAL_PROCESS | cellular process involved in reproduction in multicellular organism | 0.001238539 | 1.48E-04 |
| GO:0043900 | BIOLOGICAL_PROCESS | regulation of multi-organism process | 0.001455057 | 1.74E-04 |
| GO:0000075 | BIOLOGICAL_PROCESS | cell cycle checkpoint | 0.001455057 | 1.74E-04 |
| GO:1903052 | BIOLOGICAL_PROCESS | positive regulation of proteolysis involved in cellular protein catabolic process | 0.001455057 | 1.74E-04 |
| GO:0015850 | BIOLOGICAL_PROCESS | organic hydroxy compound transport | 0.001455057 | 1.74E-04 |
| GO:0044432 | CELLULAR_COMPONENT | endoplasmic reticulum part | 0.001461552 | 1.75E-04 |
| GO:0007276 | BIOLOGICAL_PROCESS | gamete generation | 0.001468078 | 1.76E-04 |
| GO:0003724 | MOLECULAR_FUNCTION | RNA helicase activity | 0.001468078 | 1.76E-04 |
| GO:0008066 | MOLECULAR_FUNCTION | glutamate receptor activity | 0.001508966 | 1.81E-04 |
| GO:0021915 | BIOLOGICAL_PROCESS | neural tube development | 0.001510051 | 1.82E-04 |
| GO:0001701 | BIOLOGICAL_PROCESS | in utero embryonic development | 0.001548424 | 1.86E-04 |
| GO:0030307 | BIOLOGICAL_PROCESS | positive regulation of cell growth | 0.001565753 | 1.89E-04 |
| GO:0000302 | BIOLOGICAL_PROCESS | response to reactive oxygen species | 0.001565753 | 1.89E-04 |
| GO:0034620 | BIOLOGICAL_PROCESS | cellular response to unfolded protein | 0.001565753 | 1.89E-04 |
| GO:0035967 | BIOLOGICAL_PROCESS | cellular response to topologically incorrect protein | 0.001565753 | 1.89E-04 |
| GO:0036465 | BIOLOGICAL_PROCESS | synaptic vesicle recycling | 0.001569686 | 1.94E-04 |
| GO:0048469 | BIOLOGICAL_PROCESS | cell maturation | 0.001569686 | 1.94E-04 |
| GO:0031623 | BIOLOGICAL_PROCESS | receptor internalization | 0.001569686 | 1.94E-04 |
| GO:0032943 | BIOLOGICAL_PROCESS | mononuclear cell proliferation | 0.001569686 | 1.94E-04 |
| GO:0001707 | BIOLOGICAL_PROCESS | mesoderm formation | 0.001569686 | 1.94E-04 |
| GO:0001837 | BIOLOGICAL_PROCESS | epithelial to mesenchymal transition | 0.001569686 | 1.94E-04 |
| GO:0030042 | BIOLOGICAL_PROCESS | actin filament depolymerization | 0.001569686 | 1.94E-04 |
| GO:0048332 | BIOLOGICAL_PROCESS | mesoderm morphogenesis | 0.001569686 | 1.94E-04 |
| GO:0043500 | BIOLOGICAL_PROCESS | muscle adaptation | 0.001569686 | 1.94E-04 |
| GO:0060048 | BIOLOGICAL_PROCESS | cardiac muscle contraction | 0.001569686 | 1.94E-04 |
| GO:0030175 | CELLULAR_COMPONENT | filopodium | 0.001569686 | 1.94E-04 |
| GO:0090317 | BIOLOGICAL_PROCESS | negative regulation of intracellular protein transport | 0.001569686 | 1.94E-04 |
| GO:0048844 | BIOLOGICAL_PROCESS | artery morphogenesis | 0.001569686 | 1.94E-04 |
| GO:0042826 | MOLECULAR_FUNCTION | histone deacetylase binding | 0.001569686 | 1.94E-04 |
| GO:0030835 | BIOLOGICAL_PROCESS | negative regulation of actin filament depolymerization | 0.001569686 | 1.94E-04 |
| GO:0030834 | BIOLOGICAL_PROCESS | regulation of actin filament depolymerization | 0.001569686 | 1.94E-04 |
| GO:0090504 | BIOLOGICAL_PROCESS | epiboly | 0.001569686 | 1.94E-04 |
| GO:0030665 | CELLULAR_COMPONENT | clathrin-coated vesicle membrane | 0.001569686 | 1.94E-04 |
| GO:0015085 | MOLECULAR_FUNCTION | calcium ion transmembrane transporter activity | 0.001569686 | 1.91E-04 |
| GO:0010632 | BIOLOGICAL_PROCESS | regulation of epithelial cell migration | 0.001569686 | 1.94E-04 |
| GO:0046651 | BIOLOGICAL_PROCESS | lymphocyte proliferation | 0.001569686 | 1.94E-04 |
| GO:0010771 | BIOLOGICAL_PROCESS | negative regulation of cell morphogenesis involved in differentiation | 0.001569686 | 1.94E-04 |
| GO:0002062 | BIOLOGICAL_PROCESS | chondrocyte differentiation | 0.001569686 | 1.94E-04 |
| GO:0051693 | BIOLOGICAL_PROCESS | actin filament capping | 0.001569686 | 1.94E-04 |
| GO:0021782 | BIOLOGICAL_PROCESS | glial cell development | 0.001569686 | 1.94E-04 |
| GO:0043195 | CELLULAR_COMPONENT | terminal bouton | 0.001569686 | 1.94E-04 |
| GO:0007613 | BIOLOGICAL_PROCESS | memory | 0.001569686 | 1.94E-04 |
| GO:0045921 | BIOLOGICAL_PROCESS | positive regulation of exocytosis | 0.001569686 | 1.94E-04 |
| GO:0008630 | BIOLOGICAL_PROCESS | intrinsic apoptotic signaling pathway in response to DNA damage | 0.001569686 | 1.94E-04 |
| GO:0010876 | BIOLOGICAL_PROCESS | lipid localization | 0.001569686 | 1.91E-04 |
| GO:0021522 | BIOLOGICAL_PROCESS | spinal cord motor neuron differentiation | 0.001569686 | 1.94E-04 |
| GO:0021587 | BIOLOGICAL_PROCESS | cerebellum morphogenesis | 0.001569686 | 1.94E-04 |
| GO:0032365 | BIOLOGICAL_PROCESS | intracellular lipid transport | 0.001569686 | 1.94E-04 |
| GO:0070661 | BIOLOGICAL_PROCESS | leukocyte proliferation | 0.001569686 | 1.94E-04 |
| GO:0072148 | BIOLOGICAL_PROCESS | epithelial cell fate commitment | 0.001662198 | 2.14E-04 |
| GO:0043542 | BIOLOGICAL_PROCESS | endothelial cell migration | 0.001662198 | 2.14E-04 |
| GO:1902495 | CELLULAR_COMPONENT | transmembrane transporter complex | 0.001662198 | 2.12E-04 |
| GO:0042383 | CELLULAR_COMPONENT | sarcolemma | 0.001662198 | 2.14E-04 |
| GO:0050867 | BIOLOGICAL_PROCESS | positive regulation of cell activation | 0.001662198 | 2.14E-04 |
| GO:0005158 | MOLECULAR_FUNCTION | insulin receptor binding | 0.001662198 | 2.14E-04 |
| GO:0061245 | BIOLOGICAL_PROCESS | establishment or maintenance of bipolar cell polarity | 0.001662198 | 2.14E-04 |
| GO:0030048 | BIOLOGICAL_PROCESS | actin filament-based movement | 0.001662198 | 2.14E-04 |
| GO:0043388 | BIOLOGICAL_PROCESS | positive regulation of DNA binding | 0.001662198 | 2.14E-04 |
| GO:0035088 | BIOLOGICAL_PROCESS | establishment or maintenance of apical/basal cell polarity | 0.001662198 | 2.14E-04 |
| GO:0035065 | BIOLOGICAL_PROCESS | regulation of histone acetylation | 0.001662198 | 2.14E-04 |
| GO:0019882 | BIOLOGICAL_PROCESS | antigen processing and presentation | 0.001662198 | 2.14E-04 |
| GO:0043525 | BIOLOGICAL_PROCESS | positive regulation of neuron apoptotic process | 0.001662198 | 2.14E-04 |
| GO:1903578 | BIOLOGICAL_PROCESS | regulation of ATP metabolic process | 0.001662198 | 2.14E-04 |
| GO:0060037 | BIOLOGICAL_PROCESS | pharyngeal system development | 0.001662198 | 2.14E-04 |
| GO:0048806 | BIOLOGICAL_PROCESS | genitalia development | 0.001662198 | 2.14E-04 |
| GO:0003207 | BIOLOGICAL_PROCESS | cardiac chamber formation | 0.001662198 | 2.14E-04 |
| GO:0016572 | BIOLOGICAL_PROCESS | histone phosphorylation | 0.001662198 | 2.14E-04 |
| GO:0016247 | MOLECULAR_FUNCTION | channel regulator activity | 0.001662198 | 2.14E-04 |
| GO:0001936 | BIOLOGICAL_PROCESS | regulation of endothelial cell proliferation | 0.001662198 | 2.14E-04 |
| GO:0035306 | BIOLOGICAL_PROCESS | positive regulation of dephosphorylation | 0.001662198 | 2.14E-04 |
| GO:0048665 | BIOLOGICAL_PROCESS | neuron fate specification | 0.001662198 | 2.14E-04 |
| GO:0048708 | BIOLOGICAL_PROCESS | astrocyte differentiation | 0.001662198 | 2.14E-04 |
| GO:0009118 | BIOLOGICAL_PROCESS | regulation of nucleoside metabolic process | 0.001662198 | 2.14E-04 |
| GO:0060420 | BIOLOGICAL_PROCESS | regulation of heart growth | 0.001662198 | 2.14E-04 |
| GO:0060421 | BIOLOGICAL_PROCESS | positive regulation of heart growth | 0.001662198 | 2.14E-04 |
| GO:0051251 | BIOLOGICAL_PROCESS | positive regulation of lymphocyte activation | 0.001662198 | 2.14E-04 |
| GO:0099106 | MOLECULAR_FUNCTION | ion channel regulator activity | 0.001662198 | 2.14E-04 |
| GO:0010827 | BIOLOGICAL_PROCESS | regulation of glucose transport | 0.001662198 | 2.14E-04 |
| GO:0007131 | BIOLOGICAL_PROCESS | reciprocal meiotic recombination | 0.001662198 | 2.14E-04 |
| GO:1905477 | BIOLOGICAL_PROCESS | positive regulation of protein localization to membrane | 0.001662198 | 2.14E-04 |
| GO:0002039 | MOLECULAR_FUNCTION | p53 binding | 0.001662198 | 2.14E-04 |
| GO:0002028 | BIOLOGICAL_PROCESS | regulation of sodium ion transport | 0.001662198 | 2.14E-04 |
| GO:0005901 | CELLULAR_COMPONENT | caveola | 0.001662198 | 2.14E-04 |
| GO:0045132 | BIOLOGICAL_PROCESS | meiotic chromosome segregation | 0.001662198 | 2.14E-04 |
| GO:0035825 | BIOLOGICAL_PROCESS | reciprocal DNA recombination | 0.001662198 | 2.14E-04 |
| GO:0051048 | BIOLOGICAL_PROCESS | negative regulation of secretion | 0.001662198 | 2.14E-04 |
| GO:0010594 | BIOLOGICAL_PROCESS | regulation of endothelial cell migration | 0.001662198 | 2.14E-04 |
| GO:0048010 | BIOLOGICAL_PROCESS | vascular endothelial growth factor receptor signaling pathway | 0.001662198 | 2.14E-04 |
| GO:0008802 | MOLECULAR_FUNCTION | betaine-aldehyde dehydrogenase activity | 0.001662198 | 2.14E-04 |
| GO:0061035 | BIOLOGICAL_PROCESS | regulation of cartilage development | 0.001662198 | 2.14E-04 |
| GO:0044420 | CELLULAR_COMPONENT | extracellular matrix component | 0.001662198 | 2.14E-04 |
| GO:0002696 | BIOLOGICAL_PROCESS | positive regulation of leukocyte activation | 0.001662198 | 2.14E-04 |
| GO:0010952 | BIOLOGICAL_PROCESS | positive regulation of peptidase activity | 0.001662198 | 2.14E-04 |
| GO:0010950 | BIOLOGICAL_PROCESS | positive regulation of endopeptidase activity | 0.001662198 | 2.14E-04 |
| GO:0046889 | BIOLOGICAL_PROCESS | positive regulation of lipid biosynthetic process | 0.001662198 | 2.14E-04 |
| GO:0046850 | BIOLOGICAL_PROCESS | regulation of bone remodeling | 0.001662198 | 2.14E-04 |
| GO:0045598 | BIOLOGICAL_PROCESS | regulation of fat cell differentiation | 0.001662198 | 2.14E-04 |
| GO:1990351 | CELLULAR_COMPONENT | transporter complex | 0.001662198 | 2.12E-04 |
| GO:2000756 | BIOLOGICAL_PROCESS | regulation of peptidyl-lysine acetylation | 0.001662198 | 2.14E-04 |
| GO:0045685 | BIOLOGICAL_PROCESS | regulation of glial cell differentiation | 0.001662198 | 2.14E-04 |
| GO:0055008 | BIOLOGICAL_PROCESS | cardiac muscle tissue morphogenesis | 0.001662198 | 2.14E-04 |
| GO:0055021 | BIOLOGICAL_PROCESS | regulation of cardiac muscle tissue growth | 0.001662198 | 2.14E-04 |
| GO:0055023 | BIOLOGICAL_PROCESS | positive regulation of cardiac muscle tissue growth | 0.001662198 | 2.14E-04 |
| GO:0055025 | BIOLOGICAL_PROCESS | positive regulation of cardiac muscle tissue development | 0.001662198 | 2.14E-04 |
| GO:0055065 | BIOLOGICAL_PROCESS | metal ion homeostasis | 0.001856279 | 2.39E-04 |
| GO:0050773 | BIOLOGICAL_PROCESS | regulation of dendrite development | 0.001856855 | 2.41E-04 |
| GO:0005391 | MOLECULAR_FUNCTION | sodium:potassium-exchanging ATPase activity | 0.001856855 | 2.41E-04 |
| GO:0001704 | BIOLOGICAL_PROCESS | formation of primary germ layer | 0.001856855 | 2.41E-04 |
| GO:0001736 | BIOLOGICAL_PROCESS | establishment of planar polarity | 0.001856855 | 2.41E-04 |
| GO:1903828 | BIOLOGICAL_PROCESS | negative regulation of cellular protein localization | 0.001856855 | 2.41E-04 |
| GO:0045995 | BIOLOGICAL_PROCESS | regulation of embryonic development | 0.001856855 | 2.41E-04 |
| GO:0030118 | CELLULAR_COMPONENT | clathrin coat | 0.001856855 | 2.41E-04 |
| GO:0048813 | BIOLOGICAL_PROCESS | dendrite morphogenesis | 0.001856855 | 2.41E-04 |
| GO:0008556 | MOLECULAR_FUNCTION | potassium-transporting ATPase activity | 0.001856855 | 2.41E-04 |
| GO:0007164 | BIOLOGICAL_PROCESS | establishment of tissue polarity | 0.001856855 | 2.41E-04 |
| GO:0045184 | BIOLOGICAL_PROCESS | establishment of protein localization | 0.001856855 | 2.39E-04 |
| GO:0071478 | BIOLOGICAL_PROCESS | cellular response to radiation | 0.001856855 | 2.41E-04 |
| GO:1904062 | BIOLOGICAL_PROCESS | regulation of cation transmembrane transport | 0.001856855 | 2.41E-04 |
| GO:0045861 | BIOLOGICAL_PROCESS | negative regulation of proteolysis | 0.001856855 | 2.41E-04 |
| GO:0000149 | MOLECULAR_FUNCTION | SNARE binding | 0.001856855 | 2.41E-04 |
| GO:0022411 | BIOLOGICAL_PROCESS | cellular component disassembly | 0.001913833 | 2.49E-04 |
| GO:0044433 | CELLULAR_COMPONENT | cytoplasmic vesicle part | 0.001913833 | 2.49E-04 |
| GO:1990234 | CELLULAR_COMPONENT | transferase complex | 0.001975696 | 2.57E-04 |
| GO:0042175 | CELLULAR_COMPONENT | nuclear outer membrane-endoplasmic reticulum membrane network | 0.002007022 | 2.62E-04 |
| GO:0010256 | BIOLOGICAL_PROCESS | endomembrane system organization | 0.002082106 | 2.72E-04 |
| GO:0017038 | BIOLOGICAL_PROCESS | protein import | 0.002165578 | 2.83E-04 |
| GO:0060402 | BIOLOGICAL_PROCESS | calcium ion transport into cytosol | 0.002174199 | 2.85E-04 |
| GO:0070382 | CELLULAR_COMPONENT | exocytic vesicle | 0.002174199 | 2.85E-04 |
| GO:0008138 | MOLECULAR_FUNCTION | protein tyrosine/serine/threonine phosphatase activity | 0.002174199 | 2.85E-04 |
| GO:0033267 | CELLULAR_COMPONENT | axon part | 0.002174199 | 2.85E-04 |
| GO:0006109 | BIOLOGICAL_PROCESS | regulation of carbohydrate metabolic process | 0.002174199 | 2.85E-04 |
| GO:0055002 | BIOLOGICAL_PROCESS | striated muscle cell development | 0.002174199 | 2.85E-04 |
| GO:0044703 | BIOLOGICAL_PROCESS | multi-organism reproductive process | 0.002202074 | 2.89E-04 |
| GO:0016604 | CELLULAR_COMPONENT | nuclear body | 0.002403416 | 3.16E-04 |
| GO:0022037 | BIOLOGICAL_PROCESS | metencephalon development | 0.002421697 | 3.19E-04 |
| GO:0021549 | BIOLOGICAL_PROCESS | cerebellum development | 0.002421697 | 3.19E-04 |
| GO:0021700 | BIOLOGICAL_PROCESS | developmental maturation | 0.002421697 | 3.19E-04 |
| GO:0031032 | BIOLOGICAL_PROCESS | actomyosin structure organization | 0.002421697 | 3.19E-04 |
| GO:0019905 | MOLECULAR_FUNCTION | syntaxin binding | 0.002446309 | 3.25E-04 |
| GO:0060071 | BIOLOGICAL_PROCESS | Wnt signaling pathway, planar cell polarity pathway | 0.002446309 | 3.25E-04 |
| GO:0030136 | CELLULAR_COMPONENT | clathrin-coated vesicle | 0.002446309 | 3.25E-04 |
| GO:0043487 | BIOLOGICAL_PROCESS | regulation of RNA stability | 0.002446309 | 3.25E-04 |
| GO:2001242 | BIOLOGICAL_PROCESS | regulation of intrinsic apoptotic signaling pathway | 0.002446309 | 3.25E-04 |
| GO:0042472 | BIOLOGICAL_PROCESS | inner ear morphogenesis | 0.002446309 | 3.25E-04 |
| GO:0090175 | BIOLOGICAL_PROCESS | regulation of establishment of planar polarity | 0.002446309 | 3.25E-04 |
| GO:0051261 | BIOLOGICAL_PROCESS | protein depolymerization | 0.002446309 | 3.25E-04 |
| GO:0022612 | BIOLOGICAL_PROCESS | gland morphogenesis | 0.002446309 | 3.25E-04 |
| GO:0008366 | BIOLOGICAL_PROCESS | axon ensheathment | 0.002446309 | 3.25E-04 |
| GO:0032102 | BIOLOGICAL_PROCESS | negative regulation of response to external stimulus | 0.002446309 | 3.25E-04 |
| GO:0033143 | BIOLOGICAL_PROCESS | regulation of intracellular steroid hormone receptor signaling pathway | 0.002446309 | 3.25E-04 |
| GO:0009948 | BIOLOGICAL_PROCESS | anterior/posterior axis specification | 0.002446309 | 3.25E-04 |
| GO:0007254 | BIOLOGICAL_PROCESS | JNK cascade | 0.002446309 | 3.25E-04 |
| GO:0021536 | BIOLOGICAL_PROCESS | diencephalon development | 0.002446309 | 3.25E-04 |
| GO:0007272 | BIOLOGICAL_PROCESS | ensheathment of neurons | 0.002446309 | 3.25E-04 |
| GO:0004004 | MOLECULAR_FUNCTION | ATP-dependent RNA helicase activity | 0.002482967 | 3.31E-04 |
| GO:0022890 | MOLECULAR_FUNCTION | inorganic cation transmembrane transporter activity | 0.002486216 | 3.31E-04 |
| GO:0006936 | BIOLOGICAL_PROCESS | muscle contraction | 0.002664005 | 3.55E-04 |
| GO:0001889 | BIOLOGICAL_PROCESS | liver development | 0.00268122 | 3.58E-04 |
| GO:0000209 | BIOLOGICAL_PROCESS | protein polyubiquitination | 0.00268122 | 3.58E-04 |
| GO:0061008 | BIOLOGICAL_PROCESS | hepaticobiliary system development | 0.00268122 | 3.58E-04 |
| GO:0005667 | CELLULAR_COMPONENT | transcription factor complex | 0.00271689 | 3.63E-04 |
| GO:0072331 | BIOLOGICAL_PROCESS | signal transduction by p53 class mediator | 0.002731318 | 3.66E-04 |
| GO:0051321 | BIOLOGICAL_PROCESS | meiotic cell cycle | 0.002731318 | 3.66E-04 |
| GO:0034976 | BIOLOGICAL_PROCESS | response to endoplasmic reticulum stress | 0.002731318 | 3.66E-04 |
| GO:0005874 | CELLULAR_COMPONENT | microtubule | 0.002925702 | 3.92E-04 |
| GO:0055085 | BIOLOGICAL_PROCESS | transmembrane transport | 0.002928832 | 3.93E-04 |
| GO:0007067 | BIOLOGICAL_PROCESS | mitotic nuclear division | 0.003044669 | 4.09E-04 |
| GO:0005789 | CELLULAR_COMPONENT | endoplasmic reticulum membrane | 0.003109016 | 4.18E-04 |
| GO:0005773 | CELLULAR_COMPONENT | vacuole | 0.003278931 | 4.41E-04 |
| GO:1903729 | BIOLOGICAL_PROCESS | regulation of plasma membrane organization | 0.003282514 | 4.46E-04 |
| GO:0001894 | BIOLOGICAL_PROCESS | tissue homeostasis | 0.003282514 | 4.46E-04 |
| GO:0030027 | CELLULAR_COMPONENT | lamellipodium | 0.003282514 | 4.46E-04 |
| GO:0035094 | BIOLOGICAL_PROCESS | response to nicotine | 0.003282514 | 4.46E-04 |
| GO:0042165 | MOLECULAR_FUNCTION | neurotransmitter binding | 0.003282514 | 4.46E-04 |
| GO:0042166 | MOLECULAR_FUNCTION | acetylcholine binding | 0.003282514 | 4.46E-04 |
| GO:0042752 | BIOLOGICAL_PROCESS | regulation of circadian rhythm | 0.003282514 | 4.46E-04 |
| GO:0005892 | CELLULAR_COMPONENT | acetylcholine-gated channel complex | 0.003282514 | 4.46E-04 |
| GO:0005834 | CELLULAR_COMPONENT | heterotrimeric G-protein complex | 0.003282514 | 4.46E-04 |
| GO:0097696 | BIOLOGICAL_PROCESS | STAT cascade | 0.003282514 | 4.46E-04 |
| GO:0060491 | BIOLOGICAL_PROCESS | regulation of cell projection assembly | 0.003282514 | 4.46E-04 |
| GO:1905475 | BIOLOGICAL_PROCESS | regulation of protein localization to membrane | 0.003282514 | 4.46E-04 |
| GO:0010466 | BIOLOGICAL_PROCESS | negative regulation of peptidase activity | 0.003282514 | 4.46E-04 |
| GO:1905360 | CELLULAR_COMPONENT | GTPase complex | 0.003282514 | 4.46E-04 |
| GO:0003407 | BIOLOGICAL_PROCESS | neural retina development | 0.003282514 | 4.46E-04 |
| GO:0003924 | MOLECULAR_FUNCTION | GTPase activity | 0.003282514 | 4.43E-04 |
| GO:0061134 | MOLECULAR_FUNCTION | peptidase regulator activity | 0.003282514 | 4.46E-04 |
| GO:0007259 | BIOLOGICAL_PROCESS | JAK-STAT cascade | 0.003282514 | 4.46E-04 |
| GO:0016787 | MOLECULAR_FUNCTION | hydrolase activity | 0.003526112 | 4.80E-04 |
| GO:2000117 | BIOLOGICAL_PROCESS | negative regulation of cysteine-type endopeptidase activity | 0.003681871 | 5.11E-04 |
| GO:0019903 | MOLECULAR_FUNCTION | protein phosphatase binding | 0.003681871 | 5.11E-04 |
| GO:0035150 | BIOLOGICAL_PROCESS | regulation of tube size | 0.003681871 | 5.11E-04 |
| GO:0042308 | BIOLOGICAL_PROCESS | negative regulation of protein import into nucleus | 0.003681871 | 5.11E-04 |
| GO:0031672 | CELLULAR_COMPONENT | A band | 0.003681871 | 5.11E-04 |
| GO:0050731 | BIOLOGICAL_PROCESS | positive regulation of peptidyl-tyrosine phosphorylation | 0.003681871 | 5.11E-04 |
| GO:0043502 | BIOLOGICAL_PROCESS | regulation of muscle adaptation | 0.003681871 | 5.11E-04 |
| GO:0060038 | BIOLOGICAL_PROCESS | cardiac muscle cell proliferation | 0.003681871 | 5.11E-04 |
| GO:0031529 | BIOLOGICAL_PROCESS | ruffle organization | 0.003681871 | 5.11E-04 |
| GO:0051928 | BIOLOGICAL_PROCESS | positive regulation of calcium ion transport | 0.003681871 | 5.11E-04 |
| GO:0016529 | CELLULAR_COMPONENT | sarcoplasmic reticulum | 0.003681871 | 5.11E-04 |
| GO:0034332 | BIOLOGICAL_PROCESS | adherens junction organization | 0.003681871 | 5.11E-04 |
| GO:0046328 | BIOLOGICAL_PROCESS | regulation of JNK cascade | 0.003681871 | 5.11E-04 |
| GO:0072332 | BIOLOGICAL_PROCESS | intrinsic apoptotic signaling pathway by p53 class mediator | 0.003681871 | 5.11E-04 |
| GO:1900181 | BIOLOGICAL_PROCESS | negative regulation of protein localization to nucleus | 0.003681871 | 5.11E-04 |
| GO:0090162 | BIOLOGICAL_PROCESS | establishment of epithelial cell polarity | 0.003681871 | 5.11E-04 |
| GO:0003018 | BIOLOGICAL_PROCESS | vascular process in circulatory system | 0.003681871 | 5.11E-04 |
| GO:1903034 | BIOLOGICAL_PROCESS | regulation of response to wounding | 0.003681871 | 5.11E-04 |
| GO:0034614 | BIOLOGICAL_PROCESS | cellular response to reactive oxygen species | 0.003681871 | 5.11E-04 |
| GO:0014013 | BIOLOGICAL_PROCESS | regulation of gliogenesis | 0.003681871 | 5.11E-04 |
| GO:0022406 | BIOLOGICAL_PROCESS | membrane docking | 0.003681871 | 5.11E-04 |
| GO:0038093 | BIOLOGICAL_PROCESS | Fc receptor signaling pathway | 0.003681871 | 5.11E-04 |
| GO:0003401 | BIOLOGICAL_PROCESS | axis elongation | 0.003681871 | 5.11E-04 |
| GO:0032421 | CELLULAR_COMPONENT | stereocilium bundle | 0.003681871 | 5.11E-04 |
| GO:0043154 | BIOLOGICAL_PROCESS | negative regulation of cysteine-type endopeptidase activity involved in apoptotic process | 0.003681871 | 5.11E-04 |
| GO:0007596 | BIOLOGICAL_PROCESS | blood coagulation | 0.003681871 | 5.11E-04 |
| GO:1904590 | BIOLOGICAL_PROCESS | negative regulation of protein import | 0.003681871 | 5.11E-04 |
| GO:0046823 | BIOLOGICAL_PROCESS | negative regulation of nucleocytoplasmic transport | 0.003681871 | 5.11E-04 |
| GO:0032231 | BIOLOGICAL_PROCESS | regulation of actin filament bundle assembly | 0.003681871 | 5.11E-04 |
| GO:0043025 | CELLULAR_COMPONENT | neuronal cell body | 0.003681871 | 5.08E-04 |
| GO:0022604 | BIOLOGICAL_PROCESS | regulation of cell morphogenesis | 0.003857156 | 5.36E-04 |
| GO:0055080 | BIOLOGICAL_PROCESS | cation homeostasis | 0.003857156 | 5.36E-04 |
| GO:0035282 | BIOLOGICAL_PROCESS | segmentation | 0.003922734 | 5.47E-04 |
| GO:0050821 | BIOLOGICAL_PROCESS | protein stabilization | 0.003922734 | 5.47E-04 |
| GO:0043409 | BIOLOGICAL_PROCESS | negative regulation of MAPK cascade | 0.003922734 | 5.47E-04 |
| GO:0009791 | BIOLOGICAL_PROCESS | post-embryonic development | 0.003922734 | 5.47E-04 |
| GO:0008645 | BIOLOGICAL_PROCESS | hexose transport | 0.003922734 | 5.47E-04 |
| GO:0030659 | CELLULAR_COMPONENT | cytoplasmic vesicle membrane | 0.004140805 | 5.77E-04 |
| GO:0030235 | MOLECULAR_FUNCTION | nitric-oxide synthase regulator activity | 0.004230802 | 6.15E-04 |
| GO:0043548 | MOLECULAR_FUNCTION | phosphatidylinositol 3-kinase binding | 0.004230802 | 6.15E-04 |
| GO:0044706 | BIOLOGICAL_PROCESS | multi-multicellular organism process | 0.004230802 | 6.15E-04 |
| GO:0032663 | BIOLOGICAL_PROCESS | regulation of interleukin-2 production | 0.004230802 | 6.15E-04 |
| GO:0097178 | BIOLOGICAL_PROCESS | ruffle assembly | 0.004230802 | 6.15E-04 |
| GO:0001569 | BIOLOGICAL_PROCESS | branching involved in blood vessel morphogenesis | 0.004230802 | 6.15E-04 |
| GO:0050681 | MOLECULAR_FUNCTION | androgen receptor binding | 0.004230802 | 6.15E-04 |
| GO:0005201 | MOLECULAR_FUNCTION | extracellular matrix structural constituent | 0.004230802 | 6.15E-04 |
| GO:0043524 | BIOLOGICAL_PROCESS | negative regulation of neuron apoptotic process | 0.004230802 | 6.15E-04 |
| GO:0043534 | BIOLOGICAL_PROCESS | blood vessel endothelial cell migration | 0.004230802 | 6.15E-04 |
| GO:0043535 | BIOLOGICAL_PROCESS | regulation of blood vessel endothelial cell migration | 0.004230802 | 6.15E-04 |
| GO:1904893 | BIOLOGICAL_PROCESS | negative regulation of STAT cascade | 0.004230802 | 6.15E-04 |
| GO:0031463 | CELLULAR_COMPONENT | Cul3-RING ubiquitin ligase complex | 0.004230802 | 6.15E-04 |
| GO:0030183 | BIOLOGICAL_PROCESS | B cell differentiation | 0.004230802 | 6.15E-04 |
| GO:0000323 | CELLULAR_COMPONENT | lytic vacuole | 0.004230802 | 6.11E-04 |
| GO:0005764 | CELLULAR_COMPONENT | lysosome | 0.004230802 | 6.11E-04 |
| GO:0060561 | BIOLOGICAL_PROCESS | apoptotic process involved in morphogenesis | 0.004230802 | 6.15E-04 |
| GO:2001056 | BIOLOGICAL_PROCESS | positive regulation of cysteine-type endopeptidase activity | 0.004230802 | 6.15E-04 |
| GO:0003156 | BIOLOGICAL_PROCESS | regulation of animal organ formation | 0.004230802 | 6.15E-04 |
| GO:0003148 | BIOLOGICAL_PROCESS | outflow tract septum morphogenesis | 0.004230802 | 6.15E-04 |
| GO:0060536 | BIOLOGICAL_PROCESS | cartilage morphogenesis | 0.004230802 | 6.15E-04 |
| GO:0003203 | BIOLOGICAL_PROCESS | endocardial cushion morphogenesis | 0.004230802 | 6.15E-04 |
| GO:0071347 | BIOLOGICAL_PROCESS | cellular response to interleukin-1 | 0.004230802 | 6.15E-04 |
| GO:0003283 | BIOLOGICAL_PROCESS | atrial septum development | 0.004230802 | 6.15E-04 |
| GO:0033017 | CELLULAR_COMPONENT | sarcoplasmic reticulum membrane | 0.004230802 | 6.15E-04 |
| GO:0005838 | CELLULAR_COMPONENT | proteasome regulatory particle | 0.004230802 | 6.15E-04 |
| GO:0046324 | BIOLOGICAL_PROCESS | regulation of glucose import | 0.004230802 | 6.15E-04 |
| GO:0060389 | BIOLOGICAL_PROCESS | pathway-restricted SMAD protein phosphorylation | 0.004230802 | 6.15E-04 |
| GO:2001235 | BIOLOGICAL_PROCESS | positive regulation of apoptotic signaling pathway | 0.004230802 | 6.15E-04 |
| GO:2001243 | BIOLOGICAL_PROCESS | negative regulation of intrinsic apoptotic signaling pathway | 0.004230802 | 6.15E-04 |
| GO:0060393 | BIOLOGICAL_PROCESS | regulation of pathway-restricted SMAD protein phosphorylation | 0.004230802 | 6.15E-04 |
| GO:0030552 | MOLECULAR_FUNCTION | cAMP binding | 0.004230802 | 6.15E-04 |
| GO:0035307 | BIOLOGICAL_PROCESS | positive regulation of protein dephosphorylation | 0.004230802 | 6.15E-04 |
| GO:1902742 | BIOLOGICAL_PROCESS | apoptotic process involved in development | 0.004230802 | 6.15E-04 |
| GO:1900027 | BIOLOGICAL_PROCESS | regulation of ruffle assembly | 0.004230802 | 6.15E-04 |
| GO:0005604 | CELLULAR_COMPONENT | basement membrane | 0.004230802 | 6.15E-04 |
| GO:0042641 | CELLULAR_COMPONENT | actomyosin | 0.004230802 | 6.15E-04 |
| GO:0034109 | BIOLOGICAL_PROCESS | homotypic cell-cell adhesion | 0.004230802 | 6.15E-04 |
| GO:0060413 | BIOLOGICAL_PROCESS | atrial septum morphogenesis | 0.004230802 | 6.15E-04 |
| GO:0048713 | BIOLOGICAL_PROCESS | regulation of oligodendrocyte differentiation | 0.004230802 | 6.15E-04 |
| GO:0086010 | BIOLOGICAL_PROCESS | membrane depolarization during action potential | 0.004230802 | 6.15E-04 |
| GO:0021511 | BIOLOGICAL_PROCESS | spinal cord patterning | 0.004230802 | 6.15E-04 |
| GO:0032103 | BIOLOGICAL_PROCESS | positive regulation of response to external stimulus | 0.004230802 | 6.15E-04 |
| GO:0070167 | BIOLOGICAL_PROCESS | regulation of biomineral tissue development | 0.004230802 | 6.15E-04 |
| GO:0003344 | BIOLOGICAL_PROCESS | pericardium morphogenesis | 0.004230802 | 6.15E-04 |
| GO:0045197 | BIOLOGICAL_PROCESS | establishment or maintenance of epithelial cell apical/basal polarity | 0.004230802 | 6.15E-04 |
| GO:0002027 | BIOLOGICAL_PROCESS | regulation of heart rate | 0.004230802 | 6.15E-04 |
| GO:1900544 | BIOLOGICAL_PROCESS | positive regulation of purine nucleotide metabolic process | 0.004230802 | 6.15E-04 |
| GO:0016601 | BIOLOGICAL_PROCESS | Rac protein signal transduction | 0.004230802 | 6.15E-04 |
| GO:0046426 | BIOLOGICAL_PROCESS | negative regulation of JAK-STAT cascade | 0.004230802 | 6.15E-04 |
| GO:0010611 | BIOLOGICAL_PROCESS | regulation of cardiac muscle hypertrophy | 0.004230802 | 6.15E-04 |
| GO:0038094 | BIOLOGICAL_PROCESS | Fc-gamma receptor signaling pathway | 0.004230802 | 6.15E-04 |
| GO:0038096 | BIOLOGICAL_PROCESS | Fc-gamma receptor signaling pathway involved in phagocytosis | 0.004230802 | 6.15E-04 |
| GO:0051055 | BIOLOGICAL_PROCESS | negative regulation of lipid biosynthetic process | 0.004230802 | 6.15E-04 |
| GO:0048013 | BIOLOGICAL_PROCESS | ephrin receptor signaling pathway | 0.004230802 | 6.15E-04 |
| GO:0021879 | BIOLOGICAL_PROCESS | forebrain neuron differentiation | 0.004230802 | 6.15E-04 |
| GO:0021872 | BIOLOGICAL_PROCESS | forebrain generation of neurons | 0.004230802 | 6.15E-04 |
| GO:0032440 | MOLECULAR_FUNCTION | 2-alkenal reductase [NAD(P)] activity | 0.004230802 | 6.15E-04 |
| GO:0032623 | BIOLOGICAL_PROCESS | interleukin-2 production | 0.004230802 | 6.15E-04 |
| GO:0021885 | BIOLOGICAL_PROCESS | forebrain cell migration | 0.004230802 | 6.15E-04 |
| GO:0014743 | BIOLOGICAL_PROCESS | regulation of muscle hypertrophy | 0.004230802 | 6.15E-04 |
| GO:0043280 | BIOLOGICAL_PROCESS | positive regulation of cysteine-type endopeptidase activity involved in apoptotic process | 0.004230802 | 6.15E-04 |
| GO:0002433 | BIOLOGICAL_PROCESS | immune response-regulating cell surface receptor signaling pathway involved in phagocytosis | 0.004230802 | 6.15E-04 |
| GO:0002431 | BIOLOGICAL_PROCESS | Fc receptor mediated stimulatory signaling pathway | 0.004230802 | 6.15E-04 |
| GO:0007405 | BIOLOGICAL_PROCESS | neuroblast proliferation | 0.004230802 | 6.15E-04 |
| GO:0044332 | BIOLOGICAL_PROCESS | Wnt signaling pathway involved in dorsal/ventral axis specification | 0.004230802 | 6.15E-04 |
| GO:1904377 | BIOLOGICAL_PROCESS | positive regulation of protein localization to cell periphery | 0.004230802 | 6.15E-04 |
| GO:1903078 | BIOLOGICAL_PROCESS | positive regulation of protein localization to plasma membrane | 0.004230802 | 6.15E-04 |
| GO:0097553 | BIOLOGICAL_PROCESS | calcium ion transmembrane import into cytosol | 0.004265879 | 6.23E-04 |
| GO:1902656 | BIOLOGICAL_PROCESS | calcium ion import into cytosol | 0.004265879 | 6.23E-04 |
| GO:0051282 | BIOLOGICAL_PROCESS | regulation of sequestering of calcium ion | 0.004265879 | 6.23E-04 |
| GO:0051283 | BIOLOGICAL_PROCESS | negative regulation of sequestering of calcium ion | 0.004265879 | 6.23E-04 |
| GO:0051208 | BIOLOGICAL_PROCESS | sequestering of calcium ion | 0.004265879 | 6.23E-04 |
| GO:0051209 | BIOLOGICAL_PROCESS | release of sequestered calcium ion into cytosol | 0.004265879 | 6.23E-04 |
| GO:1901981 | MOLECULAR_FUNCTION | phosphatidylinositol phosphate binding | 0.004265879 | 6.23E-04 |
| GO:0060401 | BIOLOGICAL_PROCESS | cytosolic calcium ion transport | 0.004625522 | 6.76E-04 |
| GO:0055001 | BIOLOGICAL_PROCESS | muscle cell development | 0.004625522 | 6.76E-04 |
| GO:0030133 | CELLULAR_COMPONENT | transport vesicle | 0.004765272 | 6.97E-04 |
| GO:0007126 | BIOLOGICAL_PROCESS | meiotic nuclear division | 0.004840744 | 7.09E-04 |
| GO:0032388 | BIOLOGICAL_PROCESS | positive regulation of intracellular transport | 0.004840744 | 7.09E-04 |
| GO:0007204 | BIOLOGICAL_PROCESS | positive regulation of cytosolic calcium ion concentration | 0.004939571 | 7.24E-04 |
| GO:1903046 | BIOLOGICAL_PROCESS | meiotic cell cycle process | 0.004939571 | 7.24E-04 |
| GO:0031334 | BIOLOGICAL_PROCESS | positive regulation of protein complex assembly | 0.004941745 | 7.26E-04 |
| GO:0016482 | BIOLOGICAL_PROCESS | cytosolic transport | 0.004941745 | 7.26E-04 |
| GO:0019725 | BIOLOGICAL_PROCESS | cellular homeostasis | 0.005084087 | 7.47E-04 |
| GO:0031669 | BIOLOGICAL_PROCESS | cellular response to nutrient levels | 0.005358109 | 7.99E-04 |
| GO:0050714 | BIOLOGICAL_PROCESS | positive regulation of protein secretion | 0.005358109 | 7.99E-04 |
| GO:0005126 | MOLECULAR_FUNCTION | cytokine receptor binding | 0.005358109 | 7.99E-04 |
| GO:0017048 | MOLECULAR_FUNCTION | Rho GTPase binding | 0.005358109 | 7.99E-04 |
| GO:0014855 | BIOLOGICAL_PROCESS | striated muscle cell proliferation | 0.005358109 | 7.99E-04 |
| GO:1904892 | BIOLOGICAL_PROCESS | regulation of STAT cascade | 0.005358109 | 7.99E-04 |
| GO:0043488 | BIOLOGICAL_PROCESS | regulation of mRNA stability | 0.005358109 | 7.99E-04 |
| GO:0034284 | BIOLOGICAL_PROCESS | response to monosaccharide | 0.005358109 | 7.99E-04 |
| GO:0003170 | BIOLOGICAL_PROCESS | heart valve development | 0.005358109 | 7.99E-04 |
| GO:1901654 | BIOLOGICAL_PROCESS | response to ketone | 0.005358109 | 7.99E-04 |
| GO:0050920 | BIOLOGICAL_PROCESS | regulation of chemotaxis | 0.005358109 | 7.99E-04 |
| GO:0009746 | BIOLOGICAL_PROCESS | response to hexose | 0.005358109 | 7.99E-04 |
| GO:0034644 | BIOLOGICAL_PROCESS | cellular response to UV | 0.005358109 | 7.99E-04 |
| GO:0019079 | BIOLOGICAL_PROCESS | viral genome replication | 0.005358109 | 7.99E-04 |
| GO:0002065 | BIOLOGICAL_PROCESS | columnar/cuboidal epithelial cell differentiation | 0.005358109 | 7.99E-04 |
| GO:0042990 | BIOLOGICAL_PROCESS | regulation of transcription factor import into nucleus | 0.005358109 | 7.99E-04 |
| GO:0042991 | BIOLOGICAL_PROCESS | transcription factor import into nucleus | 0.005358109 | 7.99E-04 |
| GO:1901879 | BIOLOGICAL_PROCESS | regulation of protein depolymerization | 0.005358109 | 7.99E-04 |
| GO:1901880 | BIOLOGICAL_PROCESS | negative regulation of protein depolymerization | 0.005358109 | 7.99E-04 |
| GO:0046425 | BIOLOGICAL_PROCESS | regulation of JAK-STAT cascade | 0.005358109 | 7.99E-04 |
| GO:0007586 | BIOLOGICAL_PROCESS | digestion | 0.005358109 | 7.99E-04 |
| GO:0043197 | CELLULAR_COMPONENT | dendritic spine | 0.005358109 | 7.99E-04 |
| GO:0043242 | BIOLOGICAL_PROCESS | negative regulation of protein complex disassembly | 0.005358109 | 7.99E-04 |
| GO:0010951 | BIOLOGICAL_PROCESS | negative regulation of endopeptidase activity | 0.005358109 | 7.99E-04 |
| GO:0044309 | CELLULAR_COMPONENT | neuron spine | 0.005358109 | 7.99E-04 |
| GO:0006874 | BIOLOGICAL_PROCESS | cellular calcium ion homeostasis | 0.005581524 | 8.33E-04 |
| GO:0055074 | BIOLOGICAL_PROCESS | calcium ion homeostasis | 0.005581524 | 8.33E-04 |
| GO:0006869 | BIOLOGICAL_PROCESS | lipid transport | 0.005672604 | 8.47E-04 |
| GO:0005261 | MOLECULAR_FUNCTION | cation channel activity | 0.005781416 | 8.64E-04 |
| GO:0098771 | BIOLOGICAL_PROCESS | inorganic ion homeostasis | 0.006244414 | 9.34E-04 |
| GO:0012506 | CELLULAR_COMPONENT | vesicle membrane | 0.006473192 | 9.70E-04 |
| GO:0022835 | MOLECULAR_FUNCTION | transmitter-gated channel activity | 0.006473192 | 9.70E-04 |
| GO:0022824 | MOLECULAR_FUNCTION | transmitter-gated ion channel activity | 0.006473192 | 9.70E-04 |
| GO:0009100 | BIOLOGICAL_PROCESS | glycoprotein metabolic process | 0.006659012 | 9.98E-04 |
| GO:0070085 | BIOLOGICAL_PROCESS | glycosylation | 0.006744496 | 0.00101233 |
| GO:0044297 | CELLULAR_COMPONENT | cell body | 0.006744496 | 0.00101233 |
| GO:0004175 | MOLECULAR_FUNCTION | endopeptidase activity | 0.006849327 | 0.001028669 |
| GO:0001756 | BIOLOGICAL_PROCESS | somitogenesis | 0.006870534 | 0.001038527 |
| GO:0043407 | BIOLOGICAL_PROCESS | negative regulation of MAP kinase activity | 0.006870534 | 0.001038527 |
| GO:0072341 | MOLECULAR_FUNCTION | modified amino acid binding | 0.006870534 | 0.001038527 |
| GO:0042471 | BIOLOGICAL_PROCESS | ear morphogenesis | 0.006870534 | 0.001038527 |
| GO:0001047 | MOLECULAR_FUNCTION | core promoter binding | 0.006870534 | 0.001038527 |
| GO:0071482 | BIOLOGICAL_PROCESS | cellular response to light stimulus | 0.006870534 | 0.001038527 |
| GO:0007568 | BIOLOGICAL_PROCESS | aging | 0.006870534 | 0.001038527 |
| GO:0051494 | BIOLOGICAL_PROCESS | negative regulation of cytoskeleton organization | 0.006870534 | 0.001038527 |
| GO:0019210 | MOLECULAR_FUNCTION | kinase inhibitor activity | 0.006870534 | 0.001038527 |
| GO:1904375 | BIOLOGICAL_PROCESS | regulation of protein localization to cell periphery | 0.006870534 | 0.001038527 |
| GO:1903076 | BIOLOGICAL_PROCESS | regulation of protein localization to plasma membrane | 0.006870534 | 0.001038527 |
| GO:0008017 | MOLECULAR_FUNCTION | microtubule binding | 0.007331927 | 0.001108917 |
| GO:0005231 | MOLECULAR_FUNCTION | excitatory extracellular ligand-gated ion channel activity | 0.00782497 | 0.001184869 |
| GO:0044437 | CELLULAR_COMPONENT | vacuolar part | 0.00782497 | 0.001184869 |
| GO:0019902 | MOLECULAR_FUNCTION | phosphatase binding | 0.00802002 | 0.001217944 |
| GO:0010248 | BIOLOGICAL_PROCESS | establishment or maintenance of transmembrane electrochemical gradient | 0.00802002 | 0.001217944 |
| GO:0010469 | BIOLOGICAL_PROCESS | regulation of receptor activity | 0.00802002 | 0.001217944 |
| GO:0051147 | BIOLOGICAL_PROCESS | regulation of muscle cell differentiation | 0.00802002 | 0.001217944 |
| GO:0021675 | BIOLOGICAL_PROCESS | nerve development | 0.00802002 | 0.001217944 |
| GO:0019953 | BIOLOGICAL_PROCESS | sexual reproduction | 0.008074574 | 0.001227655 |
| GO:0005730 | CELLULAR_COMPONENT | nucleolus | 0.008074574 | 0.001227655 |
| GO:0005774 | CELLULAR_COMPONENT | vacuolar membrane | 0.008241747 | 0.001253799 |
| GO:2000146 | BIOLOGICAL_PROCESS | negative regulation of cell motility | 0.008558899 | 0.001330007 |
| GO:0043620 | BIOLOGICAL_PROCESS | regulation of DNA-templated transcription in response to stress | 0.008558899 | 0.001330007 |
| GO:0030336 | BIOLOGICAL_PROCESS | negative regulation of cell migration | 0.008558899 | 0.001330007 |
| GO:0043618 | BIOLOGICAL_PROCESS | regulation of transcription from RNA polymerase II promoter in response to stress | 0.008558899 | 0.001330007 |
| GO:0050880 | BIOLOGICAL_PROCESS | regulation of blood vessel size | 0.008558899 | 0.001330007 |
| GO:0001823 | BIOLOGICAL_PROCESS | mesonephros development | 0.008558899 | 0.001330007 |
| GO:0001818 | BIOLOGICAL_PROCESS | negative regulation of cytokine production | 0.008558899 | 0.001330007 |
| GO:2000377 | BIOLOGICAL_PROCESS | regulation of reactive oxygen species metabolic process | 0.008558899 | 0.001330007 |
| GO:0061326 | BIOLOGICAL_PROCESS | renal tubule development | 0.008558899 | 0.001330007 |
| GO:0019838 | MOLECULAR_FUNCTION | growth factor binding | 0.008558899 | 0.001330007 |
| GO:0072583 | BIOLOGICAL_PROCESS | clathrin-dependent endocytosis | 0.008558899 | 0.001330007 |
| GO:0042771 | BIOLOGICAL_PROCESS | intrinsic apoptotic signaling pathway in response to DNA damage by p53 class mediator | 0.008558899 | 0.001330007 |
| GO:0008045 | BIOLOGICAL_PROCESS | motor neuron axon guidance | 0.008558899 | 0.001330007 |
| GO:0046323 | BIOLOGICAL_PROCESS | glucose import | 0.008558899 | 0.001330007 |
| GO:0046037 | BIOLOGICAL_PROCESS | GMP metabolic process | 0.008558899 | 0.001330007 |
| GO:0090102 | BIOLOGICAL_PROCESS | cochlea development | 0.008558899 | 0.001330007 |
| GO:0001952 | BIOLOGICAL_PROCESS | regulation of cell-matrix adhesion | 0.008558899 | 0.001330007 |
| GO:0048641 | BIOLOGICAL_PROCESS | regulation of skeletal muscle tissue development | 0.008558899 | 0.001330007 |
| GO:0006942 | BIOLOGICAL_PROCESS | regulation of striated muscle contraction | 0.008558899 | 0.001330007 |
| GO:0030672 | CELLULAR_COMPONENT | synaptic vesicle membrane | 0.008558899 | 0.001330007 |
| GO:0002220 | BIOLOGICAL_PROCESS | innate immune response activating cell surface receptor signaling pathway | 0.008558899 | 0.001330007 |
| GO:0010660 | BIOLOGICAL_PROCESS | regulation of muscle cell apoptotic process | 0.008558899 | 0.001330007 |
| GO:0022624 | CELLULAR_COMPONENT | proteasome accessory complex | 0.008558899 | 0.001330007 |
| GO:0004997 | MOLECULAR_FUNCTION | thyrotropin-releasing hormone receptor activity | 0.008558899 | 0.001330007 |
| GO:0070160 | CELLULAR_COMPONENT | occluding junction | 0.008558899 | 0.001330007 |
| GO:0014074 | BIOLOGICAL_PROCESS | response to purine-containing compound | 0.008558899 | 0.001330007 |
| GO:0005905 | CELLULAR_COMPONENT | clathrin-coated pit | 0.008558899 | 0.001330007 |
| GO:0033135 | BIOLOGICAL_PROCESS | regulation of peptidyl-serine phosphorylation | 0.008558899 | 0.001330007 |
| GO:0045926 | BIOLOGICAL_PROCESS | negative regulation of growth | 0.008558899 | 0.001330007 |
| GO:0045913 | BIOLOGICAL_PROCESS | positive regulation of carbohydrate metabolic process | 0.008558899 | 0.001330007 |
| GO:0043225 | MOLECULAR_FUNCTION | ATPase-coupled anion transmembrane transporter activity | 0.008558899 | 0.001317658 |
| GO:0002761 | BIOLOGICAL_PROCESS | regulation of myeloid leukocyte differentiation | 0.008558899 | 0.001330007 |
| GO:0009913 | BIOLOGICAL_PROCESS | epidermal cell differentiation | 0.008558899 | 0.001330007 |
| GO:0010977 | BIOLOGICAL_PROCESS | negative regulation of neuron projection development | 0.008558899 | 0.001330007 |
| GO:2000826 | BIOLOGICAL_PROCESS | regulation of heart morphogenesis | 0.008558899 | 0.001330007 |
| GO:0099501 | CELLULAR_COMPONENT | exocytic vesicle membrane | 0.008558899 | 0.001330007 |
| GO:0051592 | BIOLOGICAL_PROCESS | response to calcium ion | 0.008558899 | 0.001330007 |
| GO:0015278 | MOLECULAR_FUNCTION | calcium-release channel activity | 0.008606288 | 0.001339651 |
| GO:0000077 | BIOLOGICAL_PROCESS | DNA damage checkpoint | 0.008606288 | 0.001339651 |
| GO:0099604 | MOLECULAR_FUNCTION | ligand-gated calcium channel activity | 0.008606288 | 0.001339651 |
| GO:0048705 | BIOLOGICAL_PROCESS | skeletal system morphogenesis | 0.008816668 | 0.001373177 |
| GO:0044802 | BIOLOGICAL_PROCESS | single-organism membrane organization | 0.008898646 | 0.001386731 |
| GO:0007281 | BIOLOGICAL_PROCESS | germ cell development | 0.009051876 | 0.001411409 |
| GO:0005388 | MOLECULAR_FUNCTION | calcium-transporting ATPase activity | 0.009214064 | 0.001442392 |
| GO:0032845 | BIOLOGICAL_PROCESS | negative regulation of homeostatic process | 0.009214064 | 0.001442392 |
| GO:0071216 | BIOLOGICAL_PROCESS | cellular response to biotic stimulus | 0.009214064 | 0.001442392 |
| GO:0000790 | CELLULAR_COMPONENT | nuclear chromatin | 0.009214064 | 0.001442392 |
| GO:0048017 | BIOLOGICAL_PROCESS | inositol lipid-mediated signaling | 0.009214064 | 0.001442392 |
| GO:1902115 | BIOLOGICAL_PROCESS | regulation of organelle assembly | 0.009214064 | 0.001441406 |
| GO:0032434 | BIOLOGICAL_PROCESS | regulation of proteasomal ubiquitin-dependent protein catabolic process | 0.009214064 | 0.001442392 |
| GO:0008081 | MOLECULAR_FUNCTION | phosphoric diester hydrolase activity | 0.009797479 | 0.001534587 |
| GO:0008186 | MOLECULAR_FUNCTION | RNA-dependent ATPase activity | 0.010037234 | 0.001573026 |
| GO:2000179 | BIOLOGICAL_PROCESS | positive regulation of neural precursor cell proliferation | 0.010623118 | 0.001769894 |
| GO:0035173 | MOLECULAR_FUNCTION | histone kinase activity | 0.010623118 | 0.001769894 |
| GO:0030260 | BIOLOGICAL_PROCESS | entry into host cell | 0.010623118 | 0.001769894 |
| GO:0001725 | CELLULAR_COMPONENT | stress fiber | 0.010623118 | 0.001769894 |
| GO:0001778 | BIOLOGICAL_PROCESS | plasma membrane repair | 0.010623118 | 0.001769894 |
| GO:0050707 | BIOLOGICAL_PROCESS | regulation of cytokine secretion | 0.010623118 | 0.001769894 |
| GO:0035254 | MOLECULAR_FUNCTION | glutamate receptor binding | 0.010623118 | 0.001769894 |
| GO:1903793 | BIOLOGICAL_PROCESS | positive regulation of anion transport | 0.010623118 | 0.001769894 |
| GO:0097517 | CELLULAR_COMPONENT | contractile actin filament bundle | 0.010623118 | 0.001769894 |
| GO:1990927 | BIOLOGICAL_PROCESS | calcium ion regulated lysosome exocytosis | 0.010623118 | 0.001769894 |
| GO:0048536 | BIOLOGICAL_PROCESS | spleen development | 0.010623118 | 0.001769894 |
| GO:0035240 | MOLECULAR_FUNCTION | dopamine binding | 0.010623118 | 0.001769894 |
| GO:0050818 | BIOLOGICAL_PROCESS | regulation of coagulation | 0.010623118 | 0.001769894 |
| GO:0050810 | BIOLOGICAL_PROCESS | regulation of steroid biosynthetic process | 0.010623118 | 0.001769894 |
| GO:0032743 | BIOLOGICAL_PROCESS | positive regulation of interleukin-2 production | 0.010623118 | 0.001769894 |
| GO:0042043 | MOLECULAR_FUNCTION | neurexin family protein binding | 0.010623118 | 0.001769894 |
| GO:0051806 | BIOLOGICAL_PROCESS | entry into cell of other organism involved in symbiotic interaction | 0.010623118 | 0.001769894 |
| GO:0051828 | BIOLOGICAL_PROCESS | entry into other organism involved in symbiotic interaction | 0.010623118 | 0.001769894 |
| GO:0001588 | MOLECULAR_FUNCTION | dopamine neurotransmitter receptor activity, coupled via Gs | 0.010623118 | 0.001769894 |
| GO:1903523 | BIOLOGICAL_PROCESS | negative regulation of blood circulation | 0.010623118 | 0.001769894 |
| GO:0048365 | MOLECULAR_FUNCTION | Rac GTPase binding | 0.010623118 | 0.001769894 |
| GO:1903531 | BIOLOGICAL_PROCESS | negative regulation of secretion by cell | 0.010623118 | 0.001769894 |
| GO:0050663 | BIOLOGICAL_PROCESS | cytokine secretion | 0.010623118 | 0.001769894 |
| GO:0060045 | BIOLOGICAL_PROCESS | positive regulation of cardiac muscle cell proliferation | 0.010623118 | 0.001769894 |
| GO:0060043 | BIOLOGICAL_PROCESS | regulation of cardiac muscle cell proliferation | 0.010623118 | 0.001769894 |
| GO:0032881 | BIOLOGICAL_PROCESS | regulation of polysaccharide metabolic process | 0.010623118 | 0.001769894 |
| GO:0032885 | BIOLOGICAL_PROCESS | regulation of polysaccharide biosynthetic process | 0.010623118 | 0.001769894 |
| GO:0030219 | BIOLOGICAL_PROCESS | megakaryocyte differentiation | 0.010623118 | 0.001769894 |
| GO:0043470 | BIOLOGICAL_PROCESS | regulation of carbohydrate catabolic process | 0.010623118 | 0.001769894 |
| GO:0030168 | BIOLOGICAL_PROCESS | platelet activation | 0.010623118 | 0.001769894 |
| GO:0001696 | BIOLOGICAL_PROCESS | gastric acid secretion | 0.010623118 | 0.001769894 |
| GO:0071236 | BIOLOGICAL_PROCESS | cellular response to antibiotic | 0.010623118 | 0.001769894 |
| GO:0003171 | BIOLOGICAL_PROCESS | atrioventricular valve development | 0.010623118 | 0.001769894 |
| GO:0048880 | BIOLOGICAL_PROCESS | sensory system development | 0.010623118 | 0.001769894 |
| GO:0090385 | BIOLOGICAL_PROCESS | phagosome-lysosome fusion | 0.010623118 | 0.001769894 |
| GO:0015203 | MOLECULAR_FUNCTION | polyamine transmembrane transporter activity | 0.010623118 | 0.001769894 |
| GO:0003215 | BIOLOGICAL_PROCESS | cardiac right ventricle morphogenesis | 0.010623118 | 0.001769894 |
| GO:0030801 | BIOLOGICAL_PROCESS | positive regulation of cyclic nucleotide metabolic process | 0.010623118 | 0.001769894 |
| GO:0042800 | MOLECULAR_FUNCTION | histone methyltransferase activity (H3-K4 specific) | 0.010623118 | 0.001769894 |
| GO:0030879 | BIOLOGICAL_PROCESS | mammary gland development | 0.010623118 | 0.001769894 |
| GO:0070092 | BIOLOGICAL_PROCESS | regulation of glucagon secretion | 0.010623118 | 0.001769894 |
| GO:0070091 | BIOLOGICAL_PROCESS | glucagon secretion | 0.010623118 | 0.001769894 |
| GO:0048935 | BIOLOGICAL_PROCESS | peripheral nervous system neuron development | 0.010623118 | 0.001769894 |
| GO:0048934 | BIOLOGICAL_PROCESS | peripheral nervous system neuron differentiation | 0.010623118 | 0.001769894 |
| GO:0008083 | MOLECULAR_FUNCTION | growth factor activity | 0.010623118 | 0.001769894 |
| GO:0033045 | BIOLOGICAL_PROCESS | regulation of sister chromatid segregation | 0.010623118 | 0.001769894 |
| GO:2001237 | BIOLOGICAL_PROCESS | negative regulation of extrinsic apoptotic signaling pathway | 0.010623118 | 0.001769894 |
| GO:0048678 | BIOLOGICAL_PROCESS | response to axon injury | 0.010623118 | 0.001769894 |
| GO:0060323 | BIOLOGICAL_PROCESS | head morphogenesis | 0.010623118 | 0.001769894 |
| GO:0060326 | BIOLOGICAL_PROCESS | cell chemotaxis | 0.010623118 | 0.001769894 |
| GO:0030506 | MOLECULAR_FUNCTION | ankyrin binding | 0.010623118 | 0.001769894 |
| GO:0030500 | BIOLOGICAL_PROCESS | regulation of bone mineralization | 0.010623118 | 0.001769894 |
| GO:0097610 | CELLULAR_COMPONENT | cell surface furrow | 0.010623118 | 0.001769894 |
| GO:0030521 | BIOLOGICAL_PROCESS | androgen receptor signaling pathway | 0.010623118 | 0.001769894 |
| GO:0090103 | BIOLOGICAL_PROCESS | cochlea morphogenesis | 0.010623118 | 0.001769894 |
| GO:0022038 | BIOLOGICAL_PROCESS | corpus callosum development | 0.010623118 | 0.001769894 |
| GO:0022029 | BIOLOGICAL_PROCESS | telencephalon cell migration | 0.010623118 | 0.001769894 |
| GO:0009059 | BIOLOGICAL_PROCESS | macromolecule biosynthetic process | 0.010623118 | 0.001667914 |
| GO:0090119 | BIOLOGICAL_PROCESS | vesicle-mediated cholesterol transport | 0.010623118 | 0.001769894 |
| GO:1902692 | BIOLOGICAL_PROCESS | regulation of neuroblast proliferation | 0.010623118 | 0.001769894 |
| GO:1901338 | MOLECULAR_FUNCTION | catecholamine binding | 0.010623118 | 0.001769894 |
| GO:0030669 | CELLULAR_COMPONENT | clathrin-coated endocytic vesicle membrane | 0.010623118 | 0.001769894 |
| GO:0031996 | MOLECULAR_FUNCTION | thioesterase binding | 0.010623118 | 0.001769894 |
| GO:0034110 | BIOLOGICAL_PROCESS | regulation of homotypic cell-cell adhesion | 0.010623118 | 0.001769894 |
| GO:0035411 | BIOLOGICAL_PROCESS | catenin import into nucleus | 0.010623118 | 0.001769894 |
| GO:0035412 | BIOLOGICAL_PROCESS | regulation of catenin import into nucleus | 0.010623118 | 0.001769894 |
| GO:0045309 | MOLECULAR_FUNCTION | protein phosphorylated amino acid binding | 0.010623118 | 0.001769894 |
| GO:0032009 | CELLULAR_COMPONENT | early phagosome | 0.010623118 | 0.001769894 |
| GO:0045335 | CELLULAR_COMPONENT | phagocytic vesicle | 0.010623118 | 0.001769894 |
| GO:0045334 | CELLULAR_COMPONENT | clathrin-coated endocytic vesicle | 0.010623118 | 0.001769894 |
| GO:0032075 | BIOLOGICAL_PROCESS | positive regulation of nuclease activity | 0.010623118 | 0.001769894 |
| GO:0032069 | BIOLOGICAL_PROCESS | regulation of nuclease activity | 0.010623118 | 0.001769894 |
| GO:0051306 | BIOLOGICAL_PROCESS | mitotic sister chromatid separation | 0.010623118 | 0.001769894 |
| GO:0051384 | BIOLOGICAL_PROCESS | response to glucocorticoid | 0.010623118 | 0.001769894 |
| GO:0008540 | CELLULAR_COMPONENT | proteasome regulatory particle, base subcomplex | 0.010623118 | 0.001769894 |
| GO:0021513 | BIOLOGICAL_PROCESS | spinal cord dorsal/ventral patterning | 0.010623118 | 0.001769894 |
| GO:0021514 | BIOLOGICAL_PROCESS | ventral spinal cord interneuron differentiation | 0.010623118 | 0.001769894 |
| GO:0007207 | BIOLOGICAL_PROCESS | phospholipase C-activating G-protein coupled acetylcholine receptor signaling pathway | 0.010623118 | 0.001769894 |
| GO:0010862 | BIOLOGICAL_PROCESS | positive regulation of pathway-restricted SMAD protein phosphorylation | 0.010623118 | 0.001769894 |
| GO:0046718 | BIOLOGICAL_PROCESS | viral entry into host cell | 0.010623118 | 0.001769894 |
| GO:1901985 | BIOLOGICAL_PROCESS | positive regulation of protein acetylation | 0.010623118 | 0.001769894 |
| GO:0007197 | BIOLOGICAL_PROCESS | adenylate cyclase-inhibiting G-protein coupled acetylcholine receptor signaling pathway | 0.010623118 | 0.001769894 |
| GO:0032154 | CELLULAR_COMPONENT | cleavage furrow | 0.010623118 | 0.001769894 |
| GO:0034446 | BIOLOGICAL_PROCESS | substrate adhesion-dependent cell spreading | 0.010623118 | 0.001769894 |
| GO:0015417 | MOLECULAR_FUNCTION | polyamine-transporting ATPase activity | 0.010623118 | 0.001769894 |
| GO:0070252 | BIOLOGICAL_PROCESS | actin-mediated cell contraction | 0.010623118 | 0.001769894 |
| GO:0010613 | BIOLOGICAL_PROCESS | positive regulation of cardiac muscle hypertrophy | 0.010623118 | 0.001769894 |
| GO:0051052 | BIOLOGICAL_PROCESS | regulation of DNA metabolic process | 0.010623118 | 0.001708202 |
| GO:0071542 | BIOLOGICAL_PROCESS | dopaminergic neuron differentiation | 0.010623118 | 0.001769894 |
| GO:0045815 | BIOLOGICAL_PROCESS | positive regulation of gene expression, epigenetic | 0.010623118 | 0.001769894 |
| GO:0045822 | BIOLOGICAL_PROCESS | negative regulation of heart contraction | 0.010623118 | 0.001769894 |
| GO:0032528 | BIOLOGICAL_PROCESS | microvillus organization | 0.010623118 | 0.001769894 |
| GO:0061005 | BIOLOGICAL_PROCESS | cell differentiation involved in kidney development | 0.010623118 | 0.001769894 |
| GO:0007569 | BIOLOGICAL_PROCESS | cell aging | 0.010623118 | 0.001769894 |
| GO:0021795 | BIOLOGICAL_PROCESS | cerebral cortex cell migration | 0.010623118 | 0.001769894 |
| GO:0045777 | BIOLOGICAL_PROCESS | positive regulation of blood pressure | 0.010623118 | 0.001769894 |
| GO:0032432 | CELLULAR_COMPONENT | actin filament bundle | 0.010623118 | 0.001769894 |
| GO:0044409 | BIOLOGICAL_PROCESS | entry into host | 0.010623118 | 0.001769894 |
| GO:2000648 | BIOLOGICAL_PROCESS | positive regulation of stem cell proliferation | 0.010623118 | 0.001769894 |
| GO:2000677 | BIOLOGICAL_PROCESS | regulation of transcription regulatory region DNA binding | 0.010623118 | 0.001769894 |
| GO:0051764 | BIOLOGICAL_PROCESS | actin crosslink formation | 0.010623118 | 0.001769894 |
| GO:0000159 | CELLULAR_COMPONENT | protein phosphatase type 2A complex | 0.010623118 | 0.001769894 |
| GO:0043207 | BIOLOGICAL_PROCESS | response to external biotic stimulus | 0.010623118 | 0.001690907 |
| GO:0014742 | BIOLOGICAL_PROCESS | positive regulation of muscle hypertrophy | 0.010623118 | 0.001769894 |
| GO:0051707 | BIOLOGICAL_PROCESS | response to other organism | 0.010623118 | 0.001690907 |
| GO:0070888 | MOLECULAR_FUNCTION | E-box binding | 0.010623118 | 0.001769894 |
| GO:0007229 | BIOLOGICAL_PROCESS | integrin-mediated signaling pathway | 0.010623118 | 0.001769894 |
| GO:0019208 | MOLECULAR_FUNCTION | phosphatase regulator activity | 0.010623118 | 0.001769894 |
| GO:0051531 | BIOLOGICAL_PROCESS | NFAT protein import into nucleus | 0.010623118 | 0.001769894 |
| GO:0007444 | BIOLOGICAL_PROCESS | imaginal disc development | 0.010623118 | 0.001769894 |
| GO:0006110 | BIOLOGICAL_PROCESS | regulation of glycolytic process | 0.010623118 | 0.001769894 |
| GO:0045669 | BIOLOGICAL_PROCESS | positive regulation of osteoblast differentiation | 0.010623118 | 0.001769894 |
| GO:0042995 | CELLULAR_COMPONENT | cell projection | 0.011033674 | 0.00183927 |
| GO:0060113 | BIOLOGICAL_PROCESS | inner ear receptor cell differentiation | 0.011465232 | 0.001934492 |
| GO:0006694 | BIOLOGICAL_PROCESS | steroid biosynthetic process | 0.011465232 | 0.001934492 |
| GO:0098878 | CELLULAR_COMPONENT | neurotransmitter receptor complex | 0.011465232 | 0.001934492 |
| GO:0031420 | MOLECULAR_FUNCTION | alkali metal ion binding | 0.011465232 | 0.001934492 |
| GO:0051966 | BIOLOGICAL_PROCESS | regulation of synaptic transmission, glutamatergic | 0.011465232 | 0.001934492 |
| GO:0061387 | BIOLOGICAL_PROCESS | regulation of extent of cell growth | 0.011465232 | 0.001934492 |
| GO:0030799 | BIOLOGICAL_PROCESS | regulation of cyclic nucleotide metabolic process | 0.011465232 | 0.001934492 |
| GO:0016528 | CELLULAR_COMPONENT | sarcoplasm | 0.011465232 | 0.001934492 |
| GO:0046332 | MOLECULAR_FUNCTION | SMAD binding | 0.011465232 | 0.001934492 |
| GO:0042552 | BIOLOGICAL_PROCESS | myelination | 0.011465232 | 0.001934492 |
| GO:0030510 | BIOLOGICAL_PROCESS | regulation of BMP signaling pathway | 0.011465232 | 0.001934492 |
| GO:0042490 | BIOLOGICAL_PROCESS | mechanoreceptor differentiation | 0.011465232 | 0.001934492 |
| GO:0002244 | BIOLOGICAL_PROCESS | hematopoietic progenitor cell differentiation | 0.011465232 | 0.001934492 |
| GO:0008328 | CELLULAR_COMPONENT | ionotropic glutamate receptor complex | 0.011465232 | 0.001934492 |
| GO:0032091 | BIOLOGICAL_PROCESS | negative regulation of protein binding | 0.011465232 | 0.001934492 |
| GO:0030955 | MOLECULAR_FUNCTION | potassium ion binding | 0.011465232 | 0.001934492 |
| GO:0022600 | BIOLOGICAL_PROCESS | digestive system process | 0.011465232 | 0.001934492 |
| GO:0010565 | BIOLOGICAL_PROCESS | regulation of cellular ketone metabolic process | 0.011465232 | 0.001934492 |
| GO:0008286 | BIOLOGICAL_PROCESS | insulin receptor signaling pathway | 0.011465232 | 0.001934492 |
| GO:0004743 | MOLECULAR_FUNCTION | pyruvate kinase activity | 0.011465232 | 0.001934492 |
| GO:0043296 | CELLULAR_COMPONENT | apical junction complex | 0.011465232 | 0.001934492 |
| GO:0007249 | BIOLOGICAL_PROCESS | I-kappaB kinase/NF-kappaB signaling | 0.011465232 | 0.001934492 |
| GO:0032368 | BIOLOGICAL_PROCESS | regulation of lipid transport | 0.011465232 | 0.001934492 |
| GO:0005694 | CELLULAR_COMPONENT | chromosome | 0.011660958 | 0.001968546 |
| GO:0005096 | MOLECULAR_FUNCTION | GTPase activator activity | 0.01182531 | 0.001997335 |
| GO:0000785 | CELLULAR_COMPONENT | chromatin | 0.012118342 | 0.002047899 |
| GO:0006875 | BIOLOGICAL_PROCESS | cellular metal ion homeostasis | 0.012774777 | 0.002159959 |
| GO:0034645 | BIOLOGICAL_PROCESS | cellular macromolecule biosynthetic process | 0.013408251 | 0.002268251 |
| GO:0006886 | BIOLOGICAL_PROCESS | intracellular protein transport | 0.013604692 | 0.002302684 |
| GO:0048524 | BIOLOGICAL_PROCESS | positive regulation of viral process | 0.013988246 | 0.002377483 |
| GO:0098662 | BIOLOGICAL_PROCESS | inorganic cation transmembrane transport | 0.013988246 | 0.00237371 |
| GO:1900182 | BIOLOGICAL_PROCESS | positive regulation of protein localization to nucleus | 0.013988246 | 0.002377483 |
| GO:1903902 | BIOLOGICAL_PROCESS | positive regulation of viral life cycle | 0.013988246 | 0.002377483 |
| GO:0008406 | BIOLOGICAL_PROCESS | gonad development | 0.013988246 | 0.002377483 |
| GO:0051304 | BIOLOGICAL_PROCESS | chromosome separation | 0.013988246 | 0.002377483 |
| GO:0009880 | BIOLOGICAL_PROCESS | embryonic pattern specification | 0.013988246 | 0.002377483 |
| GO:0045137 | BIOLOGICAL_PROCESS | development of primary sexual characteristics | 0.013988246 | 0.002377483 |
| GO:0055082 | BIOLOGICAL_PROCESS | cellular chemical homeostasis | 0.014016247 | 0.00238348 |
| GO:0032271 | BIOLOGICAL_PROCESS | regulation of protein polymerization | 0.014394957 | 0.002449151 |
| GO:0015749 | BIOLOGICAL_PROCESS | monosaccharide transport | 0.014509589 | 0.002469935 |
| GO:0015031 | BIOLOGICAL_PROCESS | protein transport | 0.01469101 | 0.002502115 |
| GO:0030041 | BIOLOGICAL_PROCESS | actin filament polymerization | 0.015152991 | 0.002590163 |
| GO:0031461 | CELLULAR_COMPONENT | cullin-RING ubiquitin ligase complex | 0.015152991 | 0.002590163 |
| GO:0030833 | BIOLOGICAL_PROCESS | regulation of actin filament polymerization | 0.015152991 | 0.002590163 |
| GO:0030832 | BIOLOGICAL_PROCESS | regulation of actin filament length | 0.015152991 | 0.002590163 |
| GO:0008064 | BIOLOGICAL_PROCESS | regulation of actin polymerization or depolymerization | 0.015152991 | 0.002590163 |
| GO:0008154 | BIOLOGICAL_PROCESS | actin polymerization or depolymerization | 0.015152991 | 0.002590163 |
| GO:0007270 | BIOLOGICAL_PROCESS | neuron-neuron synaptic transmission | 0.015152991 | 0.002590163 |
| GO:0072509 | MOLECULAR_FUNCTION | divalent inorganic cation transmembrane transporter activity | 0.015444503 | 0.002641356 |
| GO:0001890 | BIOLOGICAL_PROCESS | placenta development | 0.015546774 | 0.002667083 |
| GO:0061505 | MOLECULAR_FUNCTION | DNA topoisomerase II activity | 0.015546774 | 0.002667083 |
| GO:1902275 | BIOLOGICAL_PROCESS | regulation of chromatin organization | 0.015546774 | 0.002667083 |
| GO:0016323 | CELLULAR_COMPONENT | basolateral plasma membrane | 0.015546774 | 0.002667083 |
| GO:0090279 | BIOLOGICAL_PROCESS | regulation of calcium ion import | 0.015546774 | 0.002667083 |
| GO:0003918 | MOLECULAR_FUNCTION | DNA topoisomerase type II (ATP-hydrolyzing) activity | 0.015546774 | 0.002667083 |
| GO:0065004 | BIOLOGICAL_PROCESS | protein-DNA complex assembly | 0.015824188 | 0.002716071 |
| GO:0031667 | BIOLOGICAL_PROCESS | response to nutrient levels | 0.016389213 | 0.002817393 |
| GO:0035023 | BIOLOGICAL_PROCESS | regulation of Rho protein signal transduction | 0.016389213 | 0.002817393 |
| GO:0007224 | BIOLOGICAL_PROCESS | smoothened signaling pathway | 0.016389213 | 0.002817393 |
| GO:0090316 | BIOLOGICAL_PROCESS | positive regulation of intracellular protein transport | 0.016441711 | 0.002829321 |
| GO:0008021 | CELLULAR_COMPONENT | synaptic vesicle | 0.016441711 | 0.002829321 |
| GO:0006937 | BIOLOGICAL_PROCESS | regulation of muscle contraction | 0.016702718 | 0.00288161 |
| GO:0016331 | BIOLOGICAL_PROCESS | morphogenesis of embryonic epithelium | 0.016702718 | 0.00288161 |
| GO:0033178 | CELLULAR_COMPONENT | proton-transporting two-sector ATPase complex, catalytic domain | 0.016702718 | 0.00288161 |
| GO:1901796 | BIOLOGICAL_PROCESS | regulation of signal transduction by p53 class mediator | 0.016702718 | 0.00288161 |
| GO:0032436 | BIOLOGICAL_PROCESS | positive regulation of proteasomal ubiquitin-dependent protein catabolic process | 0.016702718 | 0.00288161 |
| GO:0035148 | BIOLOGICAL_PROCESS | tube formation | 0.01672281 | 0.002892459 |
| GO:0072175 | BIOLOGICAL_PROCESS | epithelial tube formation | 0.01672281 | 0.002892459 |
| GO:0050953 | BIOLOGICAL_PROCESS | sensory perception of light stimulus | 0.01672281 | 0.002892459 |
| GO:0030658 | CELLULAR_COMPONENT | transport vesicle membrane | 0.01672281 | 0.002892459 |
| GO:0007601 | BIOLOGICAL_PROCESS | visual perception | 0.01672281 | 0.002892459 |
| GO:0005737 | CELLULAR_COMPONENT | cytoplasm | 0.016908315 | 0.002926037 |
| GO:0010038 | BIOLOGICAL_PROCESS | response to metal ion | 0.017108135 | 0.002962127 |
| GO:0072657 | BIOLOGICAL_PROCESS | protein localization to membrane | 0.017120756 | 0.002965824 |
| GO:0036473 | BIOLOGICAL_PROCESS | cell death in response to oxidative stress | 0.019251757 | 0.003425065 |
| GO:0032944 | BIOLOGICAL_PROCESS | regulation of mononuclear cell proliferation | 0.019251757 | 0.003425065 |
| GO:0072164 | BIOLOGICAL_PROCESS | mesonephric tubule development | 0.019251757 | 0.003425065 |
| GO:0072163 | BIOLOGICAL_PROCESS | mesonephric epithelium development | 0.019251757 | 0.003425065 |
| GO:0050727 | BIOLOGICAL_PROCESS | regulation of inflammatory response | 0.019251757 | 0.003425065 |
| GO:2000058 | BIOLOGICAL_PROCESS | regulation of protein ubiquitination involved in ubiquitin-dependent protein catabolic process | 0.019251757 | 0.003425065 |
| GO:0048278 | BIOLOGICAL_PROCESS | vesicle docking | 0.019251757 | 0.003425065 |
| GO:0043413 | BIOLOGICAL_PROCESS | macromolecule glycosylation | 0.019251757 | 0.003406823 |
| GO:0006486 | BIOLOGICAL_PROCESS | protein glycosylation | 0.019251757 | 0.003406823 |
| GO:0050670 | BIOLOGICAL_PROCESS | regulation of lymphocyte proliferation | 0.019251757 | 0.003425065 |
| GO:0072080 | BIOLOGICAL_PROCESS | nephron tubule development | 0.019251757 | 0.003425065 |
| GO:0032874 | BIOLOGICAL_PROCESS | positive regulation of stress-activated MAPK cascade | 0.019251757 | 0.003425065 |
| GO:0042149 | BIOLOGICAL_PROCESS | cellular response to glucose starvation | 0.019251757 | 0.003425065 |
| GO:0030125 | CELLULAR_COMPONENT | clathrin vesicle coat | 0.019251757 | 0.003425065 |
| GO:0043433 | BIOLOGICAL_PROCESS | negative regulation of sequence-specific DNA binding transcription factor activity | 0.019251757 | 0.003425065 |
| GO:0001607 | MOLECULAR_FUNCTION | neuromedin U receptor activity | 0.019251757 | 0.003425065 |
| GO:0060688 | BIOLOGICAL_PROCESS | regulation of morphogenesis of a branching structure | 0.019251757 | 0.003425065 |
| GO:0090505 | BIOLOGICAL_PROCESS | epiboly involved in wound healing | 0.019251757 | 0.003425065 |
| GO:0042542 | BIOLOGICAL_PROCESS | response to hydrogen peroxide | 0.019251757 | 0.003425065 |
| GO:0001963 | BIOLOGICAL_PROCESS | synaptic transmission, dopaminergic | 0.019251757 | 0.003425065 |
| GO:0031901 | CELLULAR_COMPONENT | early endosome membrane | 0.019251757 | 0.003425065 |
| GO:0006984 | BIOLOGICAL_PROCESS | ER-nucleus signaling pathway | 0.019251757 | 0.003425065 |
| GO:0043966 | BIOLOGICAL_PROCESS | histone H3 acetylation | 0.019251757 | 0.003425065 |
| GO:0003073 | BIOLOGICAL_PROCESS | regulation of systemic arterial blood pressure | 0.019251757 | 0.003425065 |
| GO:0048703 | BIOLOGICAL_PROCESS | embryonic viscerocranium morphogenesis | 0.019251757 | 0.003425065 |
| GO:0000794 | CELLULAR_COMPONENT | condensed nuclear chromosome | 0.019251757 | 0.003425065 |
| GO:0002223 | BIOLOGICAL_PROCESS | stimulatory C-type lectin receptor signaling pathway | 0.019251757 | 0.003425065 |
| GO:0060914 | BIOLOGICAL_PROCESS | heart formation | 0.019251757 | 0.003425065 |
| GO:0010676 | BIOLOGICAL_PROCESS | positive regulation of cellular carbohydrate metabolic process | 0.019251757 | 0.003425065 |
| GO:0046661 | BIOLOGICAL_PROCESS | male sex differentiation | 0.019251757 | 0.003425065 |
| GO:0070304 | BIOLOGICAL_PROCESS | positive regulation of stress-activated protein kinase signaling cascade | 0.019251757 | 0.003425065 |
| GO:0007189 | BIOLOGICAL_PROCESS | adenylate cyclase-activating G-protein coupled receptor signaling pathway | 0.019251757 | 0.003425065 |
| GO:0045445 | BIOLOGICAL_PROCESS | myoblast differentiation | 0.019251757 | 0.003425065 |
| GO:0038061 | BIOLOGICAL_PROCESS | NIK/NF-kappaB signaling | 0.019251757 | 0.003425065 |
| GO:0042992 | BIOLOGICAL_PROCESS | negative regulation of transcription factor import into nucleus | 0.019251757 | 0.003425065 |
| GO:1904064 | BIOLOGICAL_PROCESS | positive regulation of cation transmembrane transport | 0.019251757 | 0.003425065 |
| GO:0033176 | CELLULAR_COMPONENT | proton-transporting V-type ATPase complex | 0.019251757 | 0.003425065 |
| GO:0005923 | CELLULAR_COMPONENT | bicellular tight junction | 0.019251757 | 0.003425065 |
| GO:1900407 | BIOLOGICAL_PROCESS | regulation of cellular response to oxidative stress | 0.019251757 | 0.003425065 |
| GO:0008287 | CELLULAR_COMPONENT | protein serine/threonine phosphatase complex | 0.019251757 | 0.003425065 |
| GO:0045807 | BIOLOGICAL_PROCESS | positive regulation of endocytosis | 0.019251757 | 0.003425065 |
| GO:0061041 | BIOLOGICAL_PROCESS | regulation of wound healing | 0.019251757 | 0.003425065 |
| GO:0032526 | BIOLOGICAL_PROCESS | response to retinoic acid | 0.019251757 | 0.003425065 |
| GO:0043198 | CELLULAR_COMPONENT | dendritic shaft | 0.019251757 | 0.003425065 |
| GO:0007632 | BIOLOGICAL_PROCESS | visual behavior | 0.019251757 | 0.003425065 |
| GO:0061135 | MOLECULAR_FUNCTION | endopeptidase regulator activity | 0.019251757 | 0.003425065 |
| GO:1903293 | CELLULAR_COMPONENT | phosphatase complex | 0.019251757 | 0.003425065 |
| GO:0010907 | BIOLOGICAL_PROCESS | positive regulation of glucose metabolic process | 0.019251757 | 0.003425065 |
| GO:0008593 | BIOLOGICAL_PROCESS | regulation of Notch signaling pathway | 0.019251757 | 0.003425065 |
| GO:0051591 | BIOLOGICAL_PROCESS | response to cAMP | 0.019251757 | 0.003425065 |
| GO:0044319 | BIOLOGICAL_PROCESS | wound healing, spreading of cells | 0.019251757 | 0.003425065 |
| GO:0032370 | BIOLOGICAL_PROCESS | positive regulation of lipid transport | 0.019251757 | 0.003425065 |
| GO:0070663 | BIOLOGICAL_PROCESS | regulation of leukocyte proliferation | 0.019251757 | 0.003425065 |
| GO:0016197 | BIOLOGICAL_PROCESS | endosomal transport | 0.019800861 | 0.003524504 |
| GO:0044425 | CELLULAR_COMPONENT | membrane part | 0.021367804 | 0.003805303 |
| GO:0045930 | BIOLOGICAL_PROCESS | negative regulation of mitotic cell cycle | 0.025093211 | 0.004470961 |
| GO:0072503 | BIOLOGICAL_PROCESS | cellular divalent inorganic cation homeostasis | 0.025346703 | 0.00452284 |
| GO:0072507 | BIOLOGICAL_PROCESS | divalent inorganic cation homeostasis | 0.025346703 | 0.00452284 |
| GO:0008514 | MOLECULAR_FUNCTION | organic anion transmembrane transporter activity | 0.025346703 | 0.00452284 |
| GO:0030239 | BIOLOGICAL_PROCESS | myofibril assembly | 0.025604237 | 0.004607225 |
| GO:0031532 | BIOLOGICAL_PROCESS | actin cytoskeleton reorganization | 0.025604237 | 0.004607225 |
| GO:0009267 | BIOLOGICAL_PROCESS | cellular response to starvation | 0.025604237 | 0.004607225 |
| GO:1902911 | CELLULAR_COMPONENT | protein kinase complex | 0.025604237 | 0.004607225 |
| GO:0005876 | CELLULAR_COMPONENT | spindle microtubule | 0.025604237 | 0.004607225 |
| GO:0071322 | BIOLOGICAL_PROCESS | cellular response to carbohydrate stimulus | 0.025604237 | 0.004607225 |
| GO:0003281 | BIOLOGICAL_PROCESS | ventricular septum development | 0.025604237 | 0.004607225 |
| GO:0030520 | BIOLOGICAL_PROCESS | intracellular estrogen receptor signaling pathway | 0.025604237 | 0.004607225 |
| GO:0030516 | BIOLOGICAL_PROCESS | regulation of axon extension | 0.025604237 | 0.004607225 |
| GO:0042470 | CELLULAR_COMPONENT | melanosome | 0.025604237 | 0.004607225 |
| GO:0048770 | CELLULAR_COMPONENT | pigment granule | 0.025604237 | 0.004607225 |
| GO:0010171 | BIOLOGICAL_PROCESS | body morphogenesis | 0.025604237 | 0.004607225 |
| GO:0002221 | BIOLOGICAL_PROCESS | pattern recognition receptor signaling pathway | 0.025604237 | 0.004607225 |
| GO:0009749 | BIOLOGICAL_PROCESS | response to glucose | 0.025604237 | 0.004607225 |
| GO:0051149 | BIOLOGICAL_PROCESS | positive regulation of muscle cell differentiation | 0.025604237 | 0.004607225 |
| GO:0043122 | BIOLOGICAL_PROCESS | regulation of I-kappaB kinase/NF-kappaB signaling | 0.025604237 | 0.004607225 |
| GO:0032456 | BIOLOGICAL_PROCESS | endocytic recycling | 0.025604237 | 0.004607225 |
| GO:0032991 | CELLULAR_COMPONENT | macromolecular complex | 0.025827728 | 0.004649721 |
| GO:0015631 | MOLECULAR_FUNCTION | tubulin binding | 0.026000342 | 0.004683092 |
| GO:0036499 | BIOLOGICAL_PROCESS | PERK-mediated unfolded protein response | 0.026376366 | 0.00509083 |
| GO:0032924 | BIOLOGICAL_PROCESS | activin receptor signaling pathway | 0.026376366 | 0.00509083 |
| GO:0032925 | BIOLOGICAL_PROCESS | regulation of activin receptor signaling pathway | 0.026376366 | 0.00509083 |
| GO:0032922 | BIOLOGICAL_PROCESS | circadian regulation of gene expression | 0.026376366 | 0.00509083 |
| GO:0050771 | BIOLOGICAL_PROCESS | negative regulation of axonogenesis | 0.026376366 | 0.00509083 |
| GO:0050772 | BIOLOGICAL_PROCESS | positive regulation of axonogenesis | 0.026376366 | 0.00509083 |
| GO:0050780 | MOLECULAR_FUNCTION | dopamine receptor binding | 0.026376366 | 0.00509083 |
| GO:0098751 | BIOLOGICAL_PROCESS | bone cell development | 0.026376366 | 0.00509083 |
| GO:0031648 | BIOLOGICAL_PROCESS | protein destabilization | 0.026376366 | 0.00509083 |
| GO:0030315 | CELLULAR_COMPONENT | T-tubule | 0.026376366 | 0.00509083 |
| GO:0031595 | CELLULAR_COMPONENT | nuclear proteasome complex | 0.026376366 | 0.00509083 |
| GO:0042249 | BIOLOGICAL_PROCESS | establishment of planar polarity of embryonic epithelium | 0.026376366 | 0.00509083 |
| GO:0030279 | BIOLOGICAL_PROCESS | negative regulation of ossification | 0.026376366 | 0.00509083 |
| GO:0035112 | BIOLOGICAL_PROCESS | genitalia morphogenesis | 0.026376366 | 0.00509083 |
| GO:0035256 | MOLECULAR_FUNCTION | G-protein coupled glutamate receptor binding | 0.026376366 | 0.00509083 |
| GO:0004104 | MOLECULAR_FUNCTION | cholinesterase activity | 0.026376366 | 0.00509083 |
| GO:0050870 | BIOLOGICAL_PROCESS | positive regulation of T cell activation | 0.026376366 | 0.00509083 |
| GO:1903707 | BIOLOGICAL_PROCESS | negative regulation of hemopoiesis | 0.026376366 | 0.00509083 |
| GO:0035220 | BIOLOGICAL_PROCESS | wing disc development | 0.026376366 | 0.00509083 |
| GO:0090009 | BIOLOGICAL_PROCESS | primitive streak formation | 0.026376366 | 0.00509083 |
| GO:1904948 | BIOLOGICAL_PROCESS | midbrain dopaminergic neuron differentiation | 0.026376366 | 0.00509083 |
| GO:0005160 | MOLECULAR_FUNCTION | transforming growth factor beta receptor binding | 0.026376366 | 0.00509083 |
| GO:0031330 | BIOLOGICAL_PROCESS | negative regulation of cellular catabolic process | 0.026376366 | 0.00509083 |
| GO:0030071 | BIOLOGICAL_PROCESS | regulation of mitotic metaphase/anaphase transition | 0.026376366 | 0.00509083 |
| GO:0070936 | BIOLOGICAL_PROCESS | protein K48-linked ubiquitination | 0.026376366 | 0.00509083 |
| GO:0014808 | BIOLOGICAL_PROCESS | release of sequestered calcium ion into cytosol by sarcoplasmic reticulum | 0.026376366 | 0.00509083 |
| GO:0030033 | BIOLOGICAL_PROCESS | microvillus assembly | 0.026376366 | 0.00509083 |
| GO:0001570 | BIOLOGICAL_PROCESS | vasculogenesis | 0.026376366 | 0.00509083 |
| GO:1903524 | BIOLOGICAL_PROCESS | positive regulation of blood circulation | 0.026376366 | 0.00509083 |
| GO:0061339 | BIOLOGICAL_PROCESS | establishment or maintenance of monopolar cell polarity | 0.026376366 | 0.00509083 |
| GO:0035089 | BIOLOGICAL_PROCESS | establishment of apical/basal cell polarity | 0.026376366 | 0.00509083 |
| GO:0035066 | BIOLOGICAL_PROCESS | positive regulation of histone acetylation | 0.026376366 | 0.00509083 |
| GO:1903514 | BIOLOGICAL_PROCESS | calcium ion transport from endoplasmic reticulum to cytosol | 0.026376366 | 0.00509083 |
| GO:0019888 | MOLECULAR_FUNCTION | protein phosphatase regulator activity | 0.026376366 | 0.00509083 |
| GO:0005178 | MOLECULAR_FUNCTION | integrin binding | 0.026376366 | 0.00509083 |
| GO:0043496 | BIOLOGICAL_PROCESS | regulation of protein homodimerization activity | 0.026376366 | 0.00509083 |
| GO:0044784 | BIOLOGICAL_PROCESS | metaphase/anaphase transition of cell cycle | 0.026376366 | 0.00509083 |
| GO:1900373 | BIOLOGICAL_PROCESS | positive regulation of purine nucleotide biosynthetic process | 0.026376366 | 0.00509083 |
| GO:0004467 | MOLECULAR_FUNCTION | long-chain fatty acid-CoA ligase activity | 0.026376366 | 0.00509083 |
| GO:0060581 | BIOLOGICAL_PROCESS | cell fate commitment involved in pattern specification | 0.026376366 | 0.00509083 |
| GO:0034260 | BIOLOGICAL_PROCESS | negative regulation of GTPase activity | 0.026376366 | 0.00509083 |
| GO:0030810 | BIOLOGICAL_PROCESS | positive regulation of nucleotide biosynthetic process | 0.026376366 | 0.00509083 |
| GO:0033077 | BIOLOGICAL_PROCESS | T cell differentiation in thymus | 0.026376366 | 0.00509083 |
| GO:0030856 | BIOLOGICAL_PROCESS | regulation of epithelial cell differentiation | 0.026376366 | 0.00509083 |
| GO:0030859 | BIOLOGICAL_PROCESS | polarized epithelial cell differentiation | 0.026376366 | 0.00509083 |
| GO:0071320 | BIOLOGICAL_PROCESS | cellular response to cAMP | 0.026376366 | 0.00509083 |
| GO:0070011 | MOLECULAR_FUNCTION | peptidase activity, acting on L-amino acid peptides | 0.026376366 | 0.004852456 |
| GO:0003272 | BIOLOGICAL_PROCESS | endocardial cushion formation | 0.026376366 | 0.00509083 |
| GO:0048925 | BIOLOGICAL_PROCESS | lateral line system development | 0.026376366 | 0.00509083 |
| GO:0071300 | BIOLOGICAL_PROCESS | cellular response to retinoic acid | 0.026376366 | 0.00509083 |
| GO:0033047 | BIOLOGICAL_PROCESS | regulation of mitotic sister chromatid segregation | 0.026376366 | 0.00509083 |
| GO:0035690 | BIOLOGICAL_PROCESS | cellular response to drug | 0.026376366 | 0.00509083 |
| GO:0072337 | BIOLOGICAL_PROCESS | modified amino acid transport | 0.026376366 | 0.00509083 |
| GO:0010092 | BIOLOGICAL_PROCESS | specification of animal organ identity | 0.026376366 | 0.00509083 |
| GO:2001238 | BIOLOGICAL_PROCESS | positive regulation of extrinsic apoptotic signaling pathway | 0.026376366 | 0.00509083 |
| GO:0060324 | BIOLOGICAL_PROCESS | face development | 0.026376366 | 0.00509083 |
| GO:0060325 | BIOLOGICAL_PROCESS | face morphogenesis | 0.026376366 | 0.00509083 |
| GO:0050998 | MOLECULAR_FUNCTION | nitric-oxide synthase binding | 0.026376366 | 0.00509083 |
| GO:0030501 | BIOLOGICAL_PROCESS | positive regulation of bone mineralization | 0.026376366 | 0.00509083 |
| GO:1900101 | BIOLOGICAL_PROCESS | regulation of endoplasmic reticulum unfolded protein response | 0.026376366 | 0.00509083 |
| GO:0090179 | BIOLOGICAL_PROCESS | planar cell polarity pathway involved in neural tube closure | 0.026376366 | 0.00509083 |
| GO:0090177 | BIOLOGICAL_PROCESS | establishment of planar polarity involved in neural tube closure | 0.026376366 | 0.00509083 |
| GO:0090178 | BIOLOGICAL_PROCESS | regulation of establishment of planar polarity involved in neural tube closure | 0.026376366 | 0.00509083 |
| GO:0048640 | BIOLOGICAL_PROCESS | negative regulation of developmental growth | 0.026376366 | 0.00509083 |
| GO:0090183 | BIOLOGICAL_PROCESS | regulation of kidney development | 0.026376366 | 0.00509083 |
| GO:0050921 | BIOLOGICAL_PROCESS | positive regulation of chemotaxis | 0.026376366 | 0.00509083 |
| GO:0060484 | BIOLOGICAL_PROCESS | lung-associated mesenchyme development | 0.026376366 | 0.00509083 |
| GO:0034185 | MOLECULAR_FUNCTION | apolipoprotein binding | 0.026376366 | 0.00509083 |
| GO:0006983 | BIOLOGICAL_PROCESS | ER overload response | 0.026376366 | 0.00509083 |
| GO:0004385 | MOLECULAR_FUNCTION | guanylate kinase activity | 0.026376366 | 0.00509083 |
| GO:0030670 | CELLULAR_COMPONENT | phagocytic vesicle membrane | 0.026376366 | 0.00509083 |
| GO:0030595 | BIOLOGICAL_PROCESS | leukocyte chemotaxis | 0.026376366 | 0.00509083 |
| GO:0034111 | BIOLOGICAL_PROCESS | negative regulation of homotypic cell-cell adhesion | 0.026376366 | 0.00509083 |
| GO:0090244 | BIOLOGICAL_PROCESS | Wnt signaling pathway involved in somitogenesis | 0.026376366 | 0.00509083 |
| GO:0002260 | BIOLOGICAL_PROCESS | lymphocyte homeostasis | 0.026376366 | 0.00509083 |
| GO:1903039 | BIOLOGICAL_PROCESS | positive regulation of leukocyte cell-cell adhesion | 0.026376366 | 0.00509083 |
| GO:0008356 | BIOLOGICAL_PROCESS | asymmetric cell division | 0.026376366 | 0.00509083 |
| GO:0034663 | CELLULAR_COMPONENT | endoplasmic reticulum chaperone complex | 0.026376366 | 0.00509083 |
| GO:0007091 | BIOLOGICAL_PROCESS | metaphase/anaphase transition of mitotic cell cycle | 0.026376366 | 0.00509083 |
| GO:0070300 | MOLECULAR_FUNCTION | phosphatidic acid binding | 0.026376366 | 0.00509083 |
| GO:0003680 | MOLECULAR_FUNCTION | AT DNA binding | 0.026376366 | 0.00509083 |
| GO:0010765 | BIOLOGICAL_PROCESS | positive regulation of sodium ion transport | 0.026376366 | 0.00509083 |
| GO:0004989 | MOLECULAR_FUNCTION | octopamine receptor activity | 0.026376366 | 0.00509083 |
| GO:0070169 | BIOLOGICAL_PROCESS | positive regulation of biomineral tissue development | 0.026376366 | 0.00509083 |
| GO:0005979 | BIOLOGICAL_PROCESS | regulation of glycogen biosynthetic process | 0.026376366 | 0.00509083 |
| GO:0014029 | BIOLOGICAL_PROCESS | neural crest formation | 0.026376366 | 0.00509083 |
| GO:0045198 | BIOLOGICAL_PROCESS | establishment of epithelial cell apical/basal polarity | 0.026376366 | 0.00509083 |
| GO:0014014 | BIOLOGICAL_PROCESS | negative regulation of gliogenesis | 0.026376366 | 0.00509083 |
| GO:0002067 | BIOLOGICAL_PROCESS | glandular epithelial cell differentiation | 0.026376366 | 0.00509083 |
| GO:0022409 | BIOLOGICAL_PROCESS | positive regulation of cell-cell adhesion | 0.026376366 | 0.00509083 |
| GO:0090557 | BIOLOGICAL_PROCESS | establishment of endothelial intestinal barrier | 0.026376366 | 0.00509083 |
| GO:1904018 | BIOLOGICAL_PROCESS | positive regulation of vasculature development | 0.026376366 | 0.00509083 |
| GO:0010463 | BIOLOGICAL_PROCESS | mesenchymal cell proliferation | 0.026376366 | 0.00509083 |
| GO:0033148 | BIOLOGICAL_PROCESS | positive regulation of intracellular estrogen receptor signaling pathway | 0.026376366 | 0.00509083 |
| GO:0033145 | BIOLOGICAL_PROCESS | positive regulation of intracellular steroid hormone receptor signaling pathway | 0.026376366 | 0.00509083 |
| GO:0004794 | MOLECULAR_FUNCTION | L-threonine ammonia-lyase activity | 0.026376366 | 0.00509083 |
| GO:0070296 | BIOLOGICAL_PROCESS | sarcoplasmic reticulum calcium ion transport | 0.026376366 | 0.00509083 |
| GO:0008301 | MOLECULAR_FUNCTION | DNA binding, bending | 0.026376366 | 0.00509083 |
| GO:0035914 | BIOLOGICAL_PROCESS | skeletal muscle cell differentiation | 0.026376366 | 0.00509083 |
| GO:0038179 | BIOLOGICAL_PROCESS | neurotrophin signaling pathway | 0.026376366 | 0.00509083 |
| GO:1905276 | BIOLOGICAL_PROCESS | regulation of epithelial tube formation | 0.026376366 | 0.00509083 |
| GO:0038083 | BIOLOGICAL_PROCESS | peptidyl-tyrosine autophosphorylation | 0.026376366 | 0.00509083 |
| GO:0035855 | BIOLOGICAL_PROCESS | megakaryocyte development | 0.026376366 | 0.00509083 |
| GO:0036010 | BIOLOGICAL_PROCESS | protein localization to endosome | 0.026376366 | 0.00509083 |
| GO:0048002 | BIOLOGICAL_PROCESS | antigen processing and presentation of peptide antigen | 0.026376366 | 0.00509083 |
| GO:0050321 | MOLECULAR_FUNCTION | tau-protein kinase activity | 0.026376366 | 0.00509083 |
| GO:1902106 | BIOLOGICAL_PROCESS | negative regulation of leukocyte differentiation | 0.026376366 | 0.00509083 |
| GO:0045839 | BIOLOGICAL_PROCESS | negative regulation of mitotic nuclear division | 0.026376366 | 0.00509083 |
| GO:0007565 | BIOLOGICAL_PROCESS | female pregnancy | 0.026376366 | 0.00509083 |
| GO:0045766 | BIOLOGICAL_PROCESS | positive regulation of angiogenesis | 0.026376366 | 0.00509083 |
| GO:0044451 | CELLULAR_COMPONENT | nucleoplasm part | 0.026376366 | 0.00506104 |
| GO:0031103 | BIOLOGICAL_PROCESS | axon regeneration | 0.026376366 | 0.00509083 |
| GO:0021781 | BIOLOGICAL_PROCESS | glial cell fate commitment | 0.026376366 | 0.00509083 |
| GO:0045725 | BIOLOGICAL_PROCESS | positive regulation of glycogen biosynthetic process | 0.026376366 | 0.00509083 |
| GO:1902099 | BIOLOGICAL_PROCESS | regulation of metaphase/anaphase transition of cell cycle | 0.026376366 | 0.00509083 |
| GO:0031128 | BIOLOGICAL_PROCESS | developmental induction | 0.026376366 | 0.00509083 |
| GO:2000679 | BIOLOGICAL_PROCESS | positive regulation of transcription regulatory region DNA binding | 0.026376366 | 0.00509083 |
| GO:0051784 | BIOLOGICAL_PROCESS | negative regulation of nuclear division | 0.026376366 | 0.00509083 |
| GO:0061162 | BIOLOGICAL_PROCESS | establishment of monopolar cell polarity | 0.026376366 | 0.00509083 |
| GO:1902047 | BIOLOGICAL_PROCESS | polyamine transmembrane transport | 0.026376366 | 0.00509083 |
| GO:0045956 | BIOLOGICAL_PROCESS | positive regulation of calcium ion-dependent exocytosis | 0.026376366 | 0.00509083 |
| GO:0097067 | BIOLOGICAL_PROCESS | cellular response to thyroid hormone stimulus | 0.026376366 | 0.00509083 |
| GO:0097066 | BIOLOGICAL_PROCESS | response to thyroid hormone | 0.026376366 | 0.00509083 |
| GO:0014704 | CELLULAR_COMPONENT | intercalated disc | 0.026376366 | 0.00509083 |
| GO:0051705 | BIOLOGICAL_PROCESS | multi-organism behavior | 0.026376366 | 0.00509083 |
| GO:0031295 | BIOLOGICAL_PROCESS | T cell costimulation | 0.026376366 | 0.00509083 |
| GO:0031294 | BIOLOGICAL_PROCESS | lymphocyte costimulation | 0.026376366 | 0.00509083 |
| GO:0043274 | MOLECULAR_FUNCTION | phospholipase binding | 0.026376366 | 0.00509083 |
| GO:0070875 | BIOLOGICAL_PROCESS | positive regulation of glycogen metabolic process | 0.026376366 | 0.00509083 |
| GO:0070873 | BIOLOGICAL_PROCESS | regulation of glycogen metabolic process | 0.026376366 | 0.00509083 |
| GO:1905818 | BIOLOGICAL_PROCESS | regulation of chromosome separation | 0.026376366 | 0.00509083 |
| GO:0010922 | BIOLOGICAL_PROCESS | positive regulation of phosphatase activity | 0.026376366 | 0.00509083 |
| GO:0010962 | BIOLOGICAL_PROCESS | regulation of glucan biosynthetic process | 0.026376366 | 0.00509083 |
| GO:0010965 | BIOLOGICAL_PROCESS | regulation of mitotic sister chromatid separation | 0.026376366 | 0.00509083 |
| GO:0071837 | MOLECULAR_FUNCTION | HMG box domain binding | 0.026376366 | 0.00509083 |
| GO:0010880 | BIOLOGICAL_PROCESS | regulation of release of sequestered calcium ion into cytosol by sarcoplasmic reticulum | 0.026376366 | 0.00509083 |
| GO:0033555 | BIOLOGICAL_PROCESS | multicellular organismal response to stress | 0.026376366 | 0.00509083 |
| GO:0044291 | CELLULAR_COMPONENT | cell-cell contact zone | 0.026376366 | 0.00509083 |
| GO:0051532 | BIOLOGICAL_PROCESS | regulation of NFAT protein import into nucleus | 0.026376366 | 0.00509083 |
| GO:0051534 | BIOLOGICAL_PROCESS | negative regulation of NFAT protein import into nucleus | 0.026376366 | 0.00509083 |
| GO:0055092 | BIOLOGICAL_PROCESS | sterol homeostasis | 0.026376366 | 0.00509083 |
| GO:0050254 | MOLECULAR_FUNCTION | rhodopsin kinase activity | 0.026376366 | 0.00509083 |
| GO:0045600 | BIOLOGICAL_PROCESS | positive regulation of fat cell differentiation | 0.026376366 | 0.00509083 |
| GO:0018076 | BIOLOGICAL_PROCESS | N-terminal peptidyl-lysine acetylation | 0.026376366 | 0.00509083 |
| GO:2000736 | BIOLOGICAL_PROCESS | regulation of stem cell differentiation | 0.026376366 | 0.00509083 |
| GO:2000758 | BIOLOGICAL_PROCESS | positive regulation of peptidyl-lysine acetylation | 0.026376366 | 0.00509083 |
| GO:0005088 | MOLECULAR_FUNCTION | Ras guanyl-nucleotide exchange factor activity | 0.026455147 | 0.005110706 |
| GO:0040029 | BIOLOGICAL_PROCESS | regulation of gene expression, epigenetic | 0.026455147 | 0.005110706 |
| GO:0044431 | CELLULAR_COMPONENT | Golgi apparatus part | 0.02709896 | 0.005237473 |
| GO:0048483 | BIOLOGICAL_PROCESS | autonomic nervous system development | 0.027545853 | 0.00534087 |
| GO:0035249 | BIOLOGICAL_PROCESS | synaptic transmission, glutamatergic | 0.027545853 | 0.00534087 |
| GO:0001046 | MOLECULAR_FUNCTION | core promoter sequence-specific DNA binding | 0.027545853 | 0.00534087 |
| GO:0048016 | BIOLOGICAL_PROCESS | inositol phosphate-mediated signaling | 0.027545853 | 0.00534087 |
| GO:0021761 | BIOLOGICAL_PROCESS | limbic system development | 0.027545853 | 0.00534087 |
| GO:0007612 | BIOLOGICAL_PROCESS | learning | 0.027545853 | 0.00534087 |
| GO:0007605 | BIOLOGICAL_PROCESS | sensory perception of sound | 0.027545853 | 0.00534087 |
| GO:0005089 | MOLECULAR_FUNCTION | Rho guanyl-nucleotide exchange factor activity | 0.027738376 | 0.00540024 |
| GO:1902905 | BIOLOGICAL_PROCESS | positive regulation of supramolecular fiber organization | 0.027738376 | 0.00540024 |
| GO:0004312 | MOLECULAR_FUNCTION | fatty acid synthase activity | 0.027738376 | 0.00540024 |
| GO:0042593 | BIOLOGICAL_PROCESS | glucose homeostasis | 0.027738376 | 0.00540024 |
| GO:0051238 | BIOLOGICAL_PROCESS | sequestering of metal ion | 0.027738376 | 0.00540024 |
| GO:0008146 | MOLECULAR_FUNCTION | sulfotransferase activity | 0.027738376 | 0.00540024 |
| GO:0006302 | BIOLOGICAL_PROCESS | double-strand break repair | 0.027738376 | 0.00540024 |
| GO:0051495 | BIOLOGICAL_PROCESS | positive regulation of cytoskeleton organization | 0.027738376 | 0.00540024 |
| GO:0033500 | BIOLOGICAL_PROCESS | carbohydrate homeostasis | 0.027738376 | 0.00540024 |
| GO:0010008 | CELLULAR_COMPONENT | endosome membrane | 0.028958945 | 0.00564298 |
| GO:0048015 | BIOLOGICAL_PROCESS | phosphatidylinositol-mediated signaling | 0.028958945 | 0.00564298 |
| GO:0030426 | CELLULAR_COMPONENT | growth cone | 0.02923351 | 0.005724874 |
| GO:0030433 | BIOLOGICAL_PROCESS | ER-associated ubiquitin-dependent protein catabolic process | 0.02923351 | 0.005724874 |
| GO:0006476 | BIOLOGICAL_PROCESS | protein deacetylation | 0.02923351 | 0.005724874 |
| GO:0060042 | BIOLOGICAL_PROCESS | retina morphogenesis in camera-type eye | 0.02923351 | 0.005724874 |
| GO:2001257 | BIOLOGICAL_PROCESS | regulation of cation channel activity | 0.02923351 | 0.005724874 |
| GO:0050954 | BIOLOGICAL_PROCESS | sensory perception of mechanical stimulus | 0.02923351 | 0.005724874 |
| GO:0042594 | BIOLOGICAL_PROCESS | response to starvation | 0.02923351 | 0.005724874 |
| GO:0010675 | BIOLOGICAL_PROCESS | regulation of cellular carbohydrate metabolic process | 0.02923351 | 0.005724874 |
| GO:0044070 | BIOLOGICAL_PROCESS | regulation of anion transport | 0.02923351 | 0.005724874 |
| GO:0031056 | BIOLOGICAL_PROCESS | regulation of histone modification | 0.02923351 | 0.005724874 |
| GO:0031099 | BIOLOGICAL_PROCESS | regeneration | 0.02923351 | 0.005724874 |
| GO:0001838 | BIOLOGICAL_PROCESS | embryonic epithelial tube formation | 0.029634645 | 0.005808663 |
| GO:0061053 | BIOLOGICAL_PROCESS | somite development | 0.029634645 | 0.005808663 |
| GO:0008234 | MOLECULAR_FUNCTION | cysteine-type peptidase activity | 0.02967089 | 0.005818387 |
| GO:0004114 | MOLECULAR_FUNCTION | 3',5'-cyclic-nucleotide phosphodiesterase activity | 0.029804886 | 0.005855189 |
| GO:0030427 | CELLULAR_COMPONENT | site of polarized growth | 0.029804886 | 0.005855189 |
| GO:0043679 | CELLULAR_COMPONENT | axon terminus | 0.029804886 | 0.005855189 |
| GO:0001948 | MOLECULAR_FUNCTION | glycoprotein binding | 0.029804886 | 0.005855189 |
| GO:0098813 | BIOLOGICAL_PROCESS | nuclear chromosome segregation | 0.031092085 | 0.006110805 |
| GO:0005262 | MOLECULAR_FUNCTION | calcium channel activity | 0.031176528 | 0.006130154 |
| GO:0006974 | BIOLOGICAL_PROCESS | cellular response to DNA damage stimulus | 0.033774248 | 0.006643919 |
| GO:0030003 | BIOLOGICAL_PROCESS | cellular cation homeostasis | 0.034948971 | 0.006878091 |
| GO:0033218 | MOLECULAR_FUNCTION | amide binding | 0.036734694 | 0.007232771 |
| GO:0098852 | CELLULAR_COMPONENT | lytic vacuole membrane | 0.037204825 | 0.007335191 |
| GO:0005765 | CELLULAR_COMPONENT | lysosomal membrane | 0.037204825 | 0.007335191 |
| GO:0071824 | BIOLOGICAL_PROCESS | protein-DNA complex subunit organization | 0.037204825 | 0.007335191 |
| GO:0044763 | BIOLOGICAL_PROCESS | single-organism cellular process | 0.038445589 | 0.007583211 |
| GO:0098796 | CELLULAR_COMPONENT | membrane protein complex | 0.038458326 | 0.007589119 |
| GO:0008233 | MOLECULAR_FUNCTION | peptidase activity | 0.041272896 | 0.008148172 |
| GO:0019933 | BIOLOGICAL_PROCESS | cAMP-mediated signaling | 0.042669403 | 0.008697861 |
| GO:0042307 | BIOLOGICAL_PROCESS | positive regulation of protein import into nucleus | 0.042669403 | 0.008697861 |
| GO:0004000 | MOLECULAR_FUNCTION | adenosine deaminase activity | 0.042669403 | 0.008697861 |
| GO:0001776 | BIOLOGICAL_PROCESS | leukocyte homeostasis | 0.042669403 | 0.008697861 |
| GO:0035296 | BIOLOGICAL_PROCESS | regulation of tube diameter | 0.042669403 | 0.008697861 |
| GO:1901184 | BIOLOGICAL_PROCESS | regulation of ERBB signaling pathway | 0.042669403 | 0.008697861 |
| GO:0001919 | BIOLOGICAL_PROCESS | regulation of receptor recycling | 0.042669403 | 0.008697861 |
| GO:0001881 | BIOLOGICAL_PROCESS | receptor recycling | 0.042669403 | 0.008697861 |
| GO:0001845 | BIOLOGICAL_PROCESS | phagolysosome assembly | 0.042669403 | 0.008697861 |
| GO:0048546 | BIOLOGICAL_PROCESS | digestive tract morphogenesis | 0.042669403 | 0.008697861 |
| GO:0050866 | BIOLOGICAL_PROCESS | negative regulation of cell activation | 0.042669403 | 0.008697861 |
| GO:0000281 | BIOLOGICAL_PROCESS | mitotic cytokinesis | 0.042669403 | 0.008697861 |
| GO:2000379 | BIOLOGICAL_PROCESS | positive regulation of reactive oxygen species metabolic process | 0.042669403 | 0.008697861 |
| GO:0036293 | BIOLOGICAL_PROCESS | response to decreased oxygen levels | 0.042669403 | 0.00870266 |
| GO:0031430 | CELLULAR_COMPONENT | M band | 0.042669403 | 0.008697861 |
| GO:0042026 | BIOLOGICAL_PROCESS | protein refolding | 0.042669403 | 0.008697861 |
| GO:0097194 | BIOLOGICAL_PROCESS | execution phase of apoptosis | 0.042669403 | 0.008697861 |
| GO:0030038 | BIOLOGICAL_PROCESS | contractile actin filament bundle assembly | 0.042669403 | 0.008697861 |
| GO:0042058 | BIOLOGICAL_PROCESS | regulation of epidermal growth factor receptor signaling pathway | 0.042669403 | 0.008697861 |
| GO:0051983 | BIOLOGICAL_PROCESS | regulation of chromosome segregation | 0.042669403 | 0.008697861 |
| GO:0043506 | BIOLOGICAL_PROCESS | regulation of JUN kinase activity | 0.042669403 | 0.008697861 |
| GO:0098687 | CELLULAR_COMPONENT | chromosomal region | 0.042669403 | 0.008468058 |
| GO:0001676 | BIOLOGICAL_PROCESS | long-chain fatty acid metabolic process | 0.042669403 | 0.008697861 |
| GO:0001657 | BIOLOGICAL_PROCESS | ureteric bud development | 0.042669403 | 0.008697861 |
| GO:1900371 | BIOLOGICAL_PROCESS | regulation of purine nucleotide biosynthetic process | 0.042669403 | 0.008697861 |
| GO:0005790 | CELLULAR_COMPONENT | smooth endoplasmic reticulum | 0.042669403 | 0.008697861 |
| GO:0005788 | CELLULAR_COMPONENT | endoplasmic reticulum lumen | 0.042669403 | 0.008697861 |
| GO:0090382 | BIOLOGICAL_PROCESS | phagosome maturation | 0.042669403 | 0.008697861 |
| GO:0030808 | BIOLOGICAL_PROCESS | regulation of nucleotide biosynthetic process | 0.042669403 | 0.008697861 |
| GO:0030866 | BIOLOGICAL_PROCESS | cortical actin cytoskeleton organization | 0.042669403 | 0.008697861 |
| GO:0030865 | BIOLOGICAL_PROCESS | cortical cytoskeleton organization | 0.042669403 | 0.008697861 |
| GO:0071353 | BIOLOGICAL_PROCESS | cellular response to interleukin-4 | 0.042669403 | 0.008697861 |
| GO:0006835 | BIOLOGICAL_PROCESS | dicarboxylic acid transport | 0.042669403 | 0.008697861 |
| GO:2001259 | BIOLOGICAL_PROCESS | positive regulation of cation channel activity | 0.042669403 | 0.008697861 |
| GO:0048643 | BIOLOGICAL_PROCESS | positive regulation of skeletal muscle tissue development | 0.042669403 | 0.008697861 |
| GO:0035315 | BIOLOGICAL_PROCESS | hair cell differentiation | 0.042669403 | 0.008697861 |
| GO:0006801 | BIOLOGICAL_PROCESS | superoxide metabolic process | 0.042669403 | 0.008697861 |
| GO:0006939 | BIOLOGICAL_PROCESS | smooth muscle contraction | 0.042669403 | 0.008697861 |
| GO:0097746 | BIOLOGICAL_PROCESS | regulation of blood vessel diameter | 0.042669403 | 0.008697861 |
| GO:0031960 | BIOLOGICAL_PROCESS | response to corticosteroid | 0.042669403 | 0.008697861 |
| GO:0060425 | BIOLOGICAL_PROCESS | lung morphogenesis | 0.042669403 | 0.008697861 |
| GO:0090280 | BIOLOGICAL_PROCESS | positive regulation of calcium ion import | 0.042669403 | 0.008697861 |
| GO:0002224 | BIOLOGICAL_PROCESS | toll-like receptor signaling pathway | 0.042669403 | 0.008697861 |
| GO:0010656 | BIOLOGICAL_PROCESS | negative regulation of muscle cell apoptotic process | 0.042669403 | 0.008697861 |
| GO:0051193 | BIOLOGICAL_PROCESS | regulation of cofactor metabolic process | 0.042669403 | 0.008697861 |
| GO:0051196 | BIOLOGICAL_PROCESS | regulation of coenzyme metabolic process | 0.042669403 | 0.008697861 |
| GO:0060996 | BIOLOGICAL_PROCESS | dendritic spine development | 0.042669403 | 0.008697861 |
| GO:0015645 | MOLECULAR_FUNCTION | fatty acid ligase activity | 0.042669403 | 0.008697861 |
| GO:0070482 | BIOLOGICAL_PROCESS | response to oxygen levels | 0.042669403 | 0.00870266 |
| GO:0030971 | MOLECULAR_FUNCTION | receptor tyrosine kinase binding | 0.042669403 | 0.008697861 |
| GO:0010467 | BIOLOGICAL_PROCESS | gene expression | 0.042669403 | 0.008583696 |
| GO:0005902 | CELLULAR_COMPONENT | microvillus | 0.042669403 | 0.008697861 |
| GO:0051155 | BIOLOGICAL_PROCESS | positive regulation of striated muscle cell differentiation | 0.042669403 | 0.008697861 |
| GO:0009617 | BIOLOGICAL_PROCESS | response to bacterium | 0.042669403 | 0.00870266 |
| GO:0038095 | BIOLOGICAL_PROCESS | Fc-epsilon receptor signaling pathway | 0.042669403 | 0.008697861 |
| GO:0008201 | MOLECULAR_FUNCTION | heparin binding | 0.042669403 | 0.008697861 |
| GO:0007528 | BIOLOGICAL_PROCESS | neuromuscular junction development | 0.042669403 | 0.008697861 |
| GO:0032420 | CELLULAR_COMPONENT | stereocilium | 0.042669403 | 0.008697861 |
| GO:0043138 | MOLECULAR_FUNCTION | 3'-5' DNA helicase activity | 0.042669403 | 0.008697861 |
| GO:0043140 | MOLECULAR_FUNCTION | ATP-dependent 3'-5' DNA helicase activity | 0.042669403 | 0.008697861 |
| GO:0055117 | BIOLOGICAL_PROCESS | regulation of cardiac muscle contraction | 0.042669403 | 0.008697861 |
| GO:0032479 | BIOLOGICAL_PROCESS | regulation of type I interferon production | 0.042669403 | 0.008697861 |
| GO:0043149 | BIOLOGICAL_PROCESS | stress fiber assembly | 0.042669403 | 0.008697861 |
| GO:0021903 | BIOLOGICAL_PROCESS | rostrocaudal neural tube patterning | 0.042669403 | 0.008697861 |
| GO:0032606 | BIOLOGICAL_PROCESS | type I interferon production | 0.042669403 | 0.008697861 |
| GO:0006312 | BIOLOGICAL_PROCESS | mitotic recombination | 0.042669403 | 0.008697861 |
| GO:0051438 | BIOLOGICAL_PROCESS | regulation of ubiquitin-protein transferase activity | 0.042669403 | 0.008697861 |
| GO:0008631 | BIOLOGICAL_PROCESS | intrinsic apoptotic signaling pathway in response to oxidative stress | 0.042669403 | 0.008697861 |
| GO:1904591 | BIOLOGICAL_PROCESS | positive regulation of protein import | 0.042669403 | 0.008697861 |
| GO:1903201 | BIOLOGICAL_PROCESS | regulation of oxidative stress-induced cell death | 0.042669403 | 0.008697861 |
| GO:0051480 | BIOLOGICAL_PROCESS | regulation of cytosolic calcium ion concentration | 0.042669403 | 0.008468058 |
| GO:0019218 | BIOLOGICAL_PROCESS | regulation of steroid metabolic process | 0.042669403 | 0.008697861 |
| GO:0070670 | BIOLOGICAL_PROCESS | response to interleukin-4 | 0.042669403 | 0.008697861 |
| GO:0001205 | MOLECULAR_FUNCTION | transcriptional activator activity, RNA polymerase II distal enhancer sequence-specific binding | 0.042669403 | 0.008697861 |
| GO:0072594 | BIOLOGICAL_PROCESS | establishment of protein localization to organelle | 0.044584391 | 0.009097168 |
| GO:0016459 | CELLULAR_COMPONENT | myosin complex | 0.044599492 | 0.009104187 |
| GO:0005516 | MOLECULAR_FUNCTION | calmodulin binding | 0.045964006 | 0.009386787 |
| GO:0044427 | CELLULAR_COMPONENT | chromosomal part | 0.046094496 | 0.009417505 |
| GO:2001020 | BIOLOGICAL_PROCESS | regulation of response to DNA damage stimulus | 0.049561434 | 0.010130207 |
| GO:0009132 | BIOLOGICAL_PROCESS | nucleoside diphosphate metabolic process | 0.049725327 | 0.010168096 |
