# Supplementary material for: Data on genome annotation and analysis of earthworm Eisenia fetida
Source: Data Brief. 2018 Aug 29;20:525–34. doi: 10.1016/j.dib.2018.08.067 (PMC6126081; doi:10.1016/j.dib.2018.08.067)
Supplement: Supplementary file 10 — Supplementary material [file mmc10.docx]

Table S9: Annotation details of noncoding RNA (ncRNAs) genes identified in the genome of *Eisenia fetida*

| **Sequence** | **Family ACC** | **Family ID** | **Biotype** | **Seq from** | **Seq to** | **Strand** |
| --- | --- | --- | --- | --- | --- | --- |
| Efet.01.1002931 | RF01959 | SSU_rRNA_archaea | Gene; rRNA | 1 | 61 | + |
| Efet.01.1002931 | RF00177 | SSU_rRNA_bacteria | Gene; rRNA | 1 | 63 | + |
| Efet.01.1011443 | RF00006 | Vault | Gene | 110 | 6 | - |
| Efet.01.101627 | RF00006 | Vault | Gene | 250 | 331 | + |
| Efet.01.101627 | RF00006 | Vault | Gene | 518 | 417 | - |
| Efet.01.1026331 | RF00222 | IRES_Bag1 | Cis-reg; IRES | 1 | 112 | + |
| Efet.01.1066129 | RF00004 | U2 | Gene; snRNA; splicing | 57 | 1 | - |
| Efet.01.1073632 | RF00657 | mir-184 | Gene; miRNA | 89 | 7 | - |
| Efet.01.1078903 | RF00114 | S15 | Cis-reg; leader | 96 | 1 | - |
| Efet.01.1081096 | RF00005 | tRNA | Gene; tRNA | 87 | 16 | - |
| Efet.01.1091993 | RF00177 | SSU_rRNA_bacteria | Gene; rRNA | 117 | 1 | - |
| Efet.01.1129693 | RF00005 | tRNA | Gene; tRNA | 1 | 61 | + |
| Efet.01.1140976 | RF00003 | U1 | Gene; snRNA; splicing | 1 | 120 | + |
| Efet.01.1144028 | RF00177 | SSU_rRNA_bacteria | Gene; rRNA | 120 | 1 | - |
| Efet.01.1202651 | RF00001 | 5S_rRNA | Gene; rRNA | 1 | 96 | + |
| Efet.01.1215051 | RF01959 | SSU_rRNA_archaea | Gene; rRNA | 1 | 126 | + |
| Efet.01.1215051 | RF00177 | SSU_rRNA_bacteria | Gene; rRNA | 1 | 126 | + |
| Efet.01.1215051 | RF01960 | SSU_rRNA_eukarya | Gene; rRNA | 1 | 126 | + |
| Efet.01.1272564 | RF01705 | Flavo-1 | Gene; sRNA | 84 | 131 | + |
| Efet.01.128389 | RF00485 | K_chan_RES | Cis-reg | 461 | 506 | + |
| Efet.01.128389 | RF00485 | K_chan_RES | Cis-reg | 1 | 100 | + |
| Efet.01.1285448 | RF00177 | SSU_rRNA_bacteria | Gene; rRNA | 133 | 1 | - |
| Efet.01.1294782 | RF00573 | SNORD67 | Gene; snRNA; snoRNA; CD-box | 131 | 10 | - |
| Efet.01.1299482 | RF00059 | TPP | Cis-reg; riboswitch | 135 | 64 | - |
| Efet.01.1299998 | RF02540 | LSU_rRNA_archaea | Gene; rRNA | 1 | 135 | + |
| Efet.01.1299998 | RF02541 | LSU_rRNA_bacteria | Gene; rRNA | 1 | 135 | + |
| Efet.01.1305377 | RF02540 | LSU_rRNA_archaea | Gene; rRNA | 135 | 1 | - |
| Efet.01.1305377 | RF02541 | LSU_rRNA_bacteria | Gene; rRNA | 135 | 1 | - |
| Efet.01.1306873 | RF01959 | SSU_rRNA_archaea | Gene; rRNA | 136 | 1 | - |
| Efet.01.1306873 | RF00177 | SSU_rRNA_bacteria | Gene; rRNA | 136 | 1 | - |
| Efet.01.1306873 | RF01960 | SSU_rRNA_eukarya | Gene; rRNA | 136 | 1 | - |
| Efet.01.1306873 | RF02542 | SSU_rRNA_microsporidia | Gene; rRNA | 136 | 1 | - |
| Efet.01.1313678 | RF00005 | tRNA | Gene; tRNA | 1 | 121 | + |
| Efet.01.131457 | RF01296 | snoU85 | Gene; snRNA; snoRNA; HACA-box | 32 | 77 | + |
| Efet.01.131457 | RF01296 | snoU85 | Gene; snRNA; snoRNA; HACA-box | 814 | 542 | - |
| Efet.01.1317638 | RF00032 | Histone3 | Cis-reg | 3 | 48 | + |
| Efet.01.1317701 | RF00005 | tRNA | Gene; tRNA | 101 | 30 | - |
| Efet.01.1328997 | RF00005 | tRNA | Gene; tRNA | 132 | 59 | - |
| Efet.01.1332592 | RF00005 | tRNA | Gene; tRNA | 1 | 66 | + |
| Efet.01.1334244 | RF00005 | tRNA | Gene; tRNA | 111 | 40 | - |
| Efet.01.1338388 | RF00281 | SNORD47 | Gene; snRNA; snoRNA; CD-box | 54 | 134 | + |
| Efet.01.1342136 | RF00013 | 6S | Gene | 141 | 1 | - |
| Efet.01.1350178 | RF01959 | SSU_rRNA_archaea | Gene; rRNA | 142 | 1 | - |
| Efet.01.1350178 | RF00177 | SSU_rRNA_bacteria | Gene; rRNA | 142 | 1 | - |
| Efet.01.1350178 | RF01960 | SSU_rRNA_eukarya | Gene; rRNA | 142 | 1 | - |
| Efet.01.1350178 | RF02542 | SSU_rRNA_microsporidia | Gene; rRNA | 142 | 1 | - |
| Efet.01.1351024 | RF00023 | tmRNA | Gene | 143 | 21 | - |
| Efet.01.1358748 | RF00003 | U1 | Gene; snRNA; splicing | 144 | 1 | - |
| Efet.01.1360008 | RF00104 | mir-10 | Gene; miRNA | 112 | 43 | - |
| Efet.01.13670 | RF02000 | MIR1846 | Gene; miRNA | 764 | 684 | - |
| Efet.01.13670 | RF02000 | MIR1846 | Gene; miRNA | 684 | 764 | + |
| Efet.01.1374872 | RF00005 | tRNA | Gene; tRNA | 19 | 91 | + |
| Efet.01.1380669 | RF00237 | mir-9 | Gene; miRNA | 124 | 64 | - |
| Efet.01.1390833 | RF00005 | tRNA | Gene; tRNA | 72 | 151 | + |
| Efet.01.1397416 | RF00177 | SSU_rRNA_bacteria | Gene; rRNA | 1 | 152 | + |
| Efet.01.140147 | RF00005 | tRNA | Gene; tRNA | 48 | 120 | + |
| Efet.01.140147 | RF00005 | tRNA | Gene; tRNA | 1396 | 1310 | - |
| Efet.01.140209 | RF00694 | mir-137 | Gene; miRNA | 1276 | 1163 | - |
| Efet.01.140209 | RF00694 | mir-137 | Gene; miRNA | 542 | 646 | + |
| Efet.01.1405713 | RF00005 | tRNA | Gene; tRNA | 52 | 124 | + |
| Efet.01.1427036 | RF00005 | tRNA | Gene; tRNA | 34 | 96 | + |
| Efet.01.1428625 | RF00005 | tRNA | Gene; tRNA | 1 | 71 | + |
| Efet.01.1450978 | RF01959 | SSU_rRNA_archaea | Gene; rRNA | 167 | 1 | - |
| Efet.01.1450978 | RF00177 | SSU_rRNA_bacteria | Gene; rRNA | 167 | 1 | - |
| Efet.01.1457839 | RF00032 | Histone3 | Cis-reg | 83 | 39 | - |
| Efet.01.1461246 | RF01959 | SSU_rRNA_archaea | Gene; rRNA | 1 | 171 | + |
| Efet.01.1461246 | RF00177 | SSU_rRNA_bacteria | Gene; rRNA | 1 | 171 | + |
| Efet.01.1464767 | RF00485 | K_chan_RES | Cis-reg | 67 | 172 | + |
| Efet.01.1468326 | RF00005 | tRNA | Gene; tRNA | 1 | 68 | + |
| Efet.01.1472817 | RF00005 | tRNA | Gene; tRNA | 118 | 46 | - |
| Efet.01.1478990 | RF00239 | mir-124 | Gene; miRNA | 81 | 160 | + |
| Efet.01.1479435 | RF00005 | tRNA | Gene; tRNA | 153 | 81 | - |
| Efet.01.1483728 | RF00005 | tRNA | Gene; tRNA | 35 | 106 | + |
| Efet.01.1483927 | RF00005 | tRNA | Gene; tRNA | 180 | 108 | - |
| Efet.01.1488561 | RF00005 | tRNA | Gene; tRNA | 161 | 89 | - |
| Efet.01.1488561 | RF00005 | tRNA | Gene; tRNA | 67 | 1 | - |
| Efet.01.1493952 | RF02540 | LSU_rRNA_archaea | Gene; rRNA | 1 | 185 | + |
| Efet.01.1493952 | RF02541 | LSU_rRNA_bacteria | Gene; rRNA | 1 | 185 | + |
| Efet.01.1493952 | RF02543 | LSU_rRNA_eukarya | Gene; rRNA | 49 | 185 | + |
| Efet.01.1495951 | RF00005 | tRNA | Gene; tRNA | 1 | 56 | + |
| Efet.01.1501429 | RF00174 | Cobalamin | Cis-reg; riboswitch | 155 | 1 | - |
| Efet.01.1505021 | RF00005 | tRNA | Gene; tRNA | 104 | 186 | + |
| Efet.01.1505820 | RF00032 | Histone3 | Cis-reg | 72 | 117 | + |
| Efet.01.1506705 | RF02540 | LSU_rRNA_archaea | Gene; rRNA | 96 | 192 | + |
| Efet.01.1506705 | RF02541 | LSU_rRNA_bacteria | Gene; rRNA | 96 | 192 | + |
| Efet.01.1518296 | RF00032 | Histone3 | Cis-reg | 72 | 117 | + |
| Efet.01.1521763 | RF01959 | SSU_rRNA_archaea | Gene; rRNA | 201 | 1 | - |
| Efet.01.1521763 | RF00177 | SSU_rRNA_bacteria | Gene; rRNA | 201 | 1 | - |
| Efet.01.1521763 | RF02542 | SSU_rRNA_microsporidia | Gene; rRNA | 201 | 1 | - |
| Efet.01.1521766 | RF01959 | SSU_rRNA_archaea | Gene; rRNA | 1 | 201 | + |
| Efet.01.1521766 | RF00177 | SSU_rRNA_bacteria | Gene; rRNA | 1 | 201 | + |
| Efet.01.1524586 | RF00005 | tRNA | Gene; tRNA | 1 | 59 | + |
| Efet.01.1536537 | RF00005 | tRNA | Gene; tRNA | 139 | 209 | + |
| Efet.01.1540227 | RF00005 | tRNA | Gene; tRNA | 131 | 214 | + |
| Efet.01.1545844 | RF00005 | tRNA | Gene; tRNA | 142 | 72 | - |
| Efet.01.1549932 | RF00008 | Hammerhead_3 | Gene; ribozyme | 123 | 177 | + |
| Efet.01.1551921 | RF00548 | U11 | Gene; snRNA; splicing | 93 | 1 | - |
| Efet.01.1553805 | RF00005 | tRNA | Gene; tRNA | 111 | 184 | + |
| Efet.01.1560786 | RF00485 | K_chan_RES | Cis-reg | 192 | 77 | - |
| Efet.01.156255 | RF00005 | tRNA | Gene; tRNA | 852 | 976 | + |
| Efet.01.156255 | RF00005 | tRNA | Gene; tRNA | 852 | 976 | + |
| Efet.01.1568729 | RF01959 | SSU_rRNA_archaea | Gene; rRNA | 1 | 244 | + |
| Efet.01.1568729 | RF00177 | SSU_rRNA_bacteria | Gene; rRNA | 1 | 244 | + |
| Efet.01.1568729 | RF01960 | SSU_rRNA_eukarya | Gene; rRNA | 1 | 244 | + |
| Efet.01.1568729 | RF02542 | SSU_rRNA_microsporidia | Gene; rRNA | 1 | 244 | + |
| Efet.01.1573962 | RF00005 | tRNA | Gene; tRNA | 66 | 1 | - |
| Efet.01.1577761 | RF01959 | SSU_rRNA_archaea | Gene; rRNA | 254 | 1 | - |
| Efet.01.1577761 | RF00177 | SSU_rRNA_bacteria | Gene; rRNA | 256 | 1 | - |
| Efet.01.1577761 | RF02542 | SSU_rRNA_microsporidia | Gene; rRNA | 213 | 1 | - |
| Efet.01.1585614 | RF00005 | tRNA | Gene; tRNA | 177 | 121 | - |
| Efet.01.1586741 | RF00485 | K_chan_RES | Cis-reg | 111 | 223 | + |
| Efet.01.1593019 | RF00005 | tRNA | Gene; tRNA | 138 | 66 | - |
| Efet.01.1593019 | RF00005 | tRNA | Gene; tRNA | 223 | 152 | - |
| Efet.01.1594470 | RF00630 | P26 | Gene | 190 | 135 | - |
| Efet.01.1596669 | RF00005 | tRNA | Gene; tRNA | 60 | 141 | + |
| Efet.01.1602090 | RF00005 | tRNA | Gene; tRNA | 300 | 239 | - |
| Efet.01.1603320 | RF00005 | tRNA | Gene; tRNA | 203 | 274 | + |
| Efet.01.1603605 | RF00005 | tRNA | Gene; tRNA | 232 | 159 | - |
| Efet.01.1613496 | RF00005 | tRNA | Gene; tRNA | 140 | 210 | + |
| Efet.01.1617117 | RF00005 | tRNA | Gene; tRNA | 1 | 56 | + |
| Efet.01.1618267 | RF00005 | tRNA | Gene; tRNA | 244 | 158 | - |
| Efet.01.1620837 | RF00005 | tRNA | Gene; tRNA | 206 | 278 | + |
| Efet.01.1621172 | RF00005 | tRNA | Gene; tRNA | 293 | 356 | + |
| Efet.01.1622033 | RF00032 | Histone3 | Cis-reg | 127 | 173 | + |
| Efet.01.1623397 | RF00005 | tRNA | Gene; tRNA | 114 | 41 | - |
| Efet.01.1624013 | RF00032 | Histone3 | Cis-reg | 345 | 299 | - |
| Efet.01.162450 | RF00485 | K_chan_RES | Cis-reg | 542 | 646 | + |
| Efet.01.162450 | RF00485 | K_chan_RES | Cis-reg | 1276 | 1163 | - |
| Efet.01.1631761 | RF00002 | 5_8S_rRNA | Gene; rRNA | 75 | 229 | + |
| Efet.01.1637200 | RF01705 | Flavo-1 | Gene; sRNA | 298 | 212 | - |
| Efet.01.1637593 | RF00600 | SNORA79 | Gene; snRNA; snoRNA; HACA-box | 32 | 157 | + |
| Efet.01.1637743 | RF00005 | tRNA | Gene; tRNA | 434 | 362 | - |
| Efet.01.1638491 | RF00005 | tRNA | Gene; tRNA | 262 | 189 | - |
| Efet.01.1640897 | RF02543 | LSU_rRNA_eukarya | Gene; rRNA | 1 | 411 | + |
| Efet.01.1642338 | RF00485 | K_chan_RES | Cis-reg | 380 | 267 | - |
| Efet.01.1642659 | RF00031 | SECIS_1 | Cis-reg | 169 | 104 | - |
| Efet.01.1648372 | RF01277 | snoU54 | Gene; snRNA; snoRNA; CD-box | 365 | 297 | - |
| Efet.01.1651600 | RF00005 | tRNA | Gene; tRNA | 260 | 197 | - |
| Efet.01.1651650 | RF00162 | SAM | Cis-reg; riboswitch | 535 | 434 | - |
| Efet.01.1652253 | RF00001 | 5S_rRNA | Gene; rRNA | 164 | 52 | - |
| Efet.01.1652253 | RF02541 | LSU_rRNA_bacteria | Gene; rRNA | 371 | 287 | - |
| Efet.01.1652723 | RF00005 | tRNA | Gene; tRNA | 72 | 1 | - |
| Efet.01.1652723 | RF00005 | tRNA | Gene; tRNA | 149 | 77 | - |
| Efet.01.1652723 | RF00005 | tRNA | Gene; tRNA | 262 | 180 | - |
| Efet.01.1656440 | RF00023 | tmRNA | Gene | 598 | 230 | - |
| Efet.01.1657123 | RF00005 | tRNA | Gene; tRNA | 1 | 70 | + |
| Efet.01.1657888 | RF00005 | tRNA | Gene; tRNA | 358 | 288 | - |
| Efet.01.1657888 | RF00005 | tRNA | Gene; tRNA | 779 | 852 | + |
| Efet.01.1658339 | RF00023 | tmRNA | Gene | 660 | 1046 | + |
| Efet.01.1658685 | RF00005 | tRNA | Gene; tRNA | 861 | 931 | + |
| Efet.01.1658819 | RF00005 | tRNA | Gene; tRNA | 1186 | 1113 | - |
| Efet.01.1658897 | RF00005 | tRNA | Gene; tRNA | 879 | 952 | + |
| Efet.01.1658980 | RF00005 | tRNA | Gene; tRNA | 1289 | 1361 | + |
| Efet.01.1658980 | RF00005 | tRNA | Gene; tRNA | 92 | 4 | - |
| Efet.01.1659 | RF02278 | Betaproteobacteria_toxic_sRNA | Gene; sRNA | 64 | 118 | - |
| Efet.01.1659 | RF02278 | Betaproteobacteria_toxic_sRNA | Gene; sRNA | 64 | 118 |  |
| Efet.01.1659285 | RF00005 | tRNA | Gene; tRNA | 1167 | 1095 | - |
| Efet.01.1659285 | RF00005 | tRNA | Gene; tRNA | 1040 | 970 | - |
| Efet.01.1659285 | RF00005 | tRNA | Gene; tRNA | 941 | 870 | - |
| Efet.01.1659285 | RF00005 | tRNA | Gene; tRNA | 1402 | 1474 | + |
| Efet.01.1659289 | RF00010 | RNaseP_bact_a | Gene; ribozyme | 878 | 1207 | + |
| Efet.01.1659376 | RF00005 | tRNA | Gene; tRNA | 1555 | 1628 | + |
| Efet.01.1659376 | RF00005 | tRNA | Gene; tRNA | 1555 | 1628 | + |
| Efet.01.1659462 | RF00442 | ykkC-yxkD | Cis-reg; riboswitch | 70 | 185 | + |
| Efet.01.1659462 | RF00442 | ykkC-yxkD | Cis-reg; riboswitch | 70 | 185 | + |
| Efet.01.1659477 | RF00005 | tRNA | Gene; tRNA | 2700 | 2772 | + |
| Efet.01.1659477 | RF00005 | tRNA | Gene; tRNA | 2700 | 2772 | + |
| Efet.01.1659506 | RF01057 | SAH_riboswitch | Cis-reg; riboswitch | 3434 | 3348 | - |
| Efet.01.1659506 | RF01057 | SAH_riboswitch | Cis-reg; riboswitch | 3434 | 3348 | - |
| Efet.01.1659527 | RF00005 | tRNA | Gene; tRNA | 4043 | 4115 | + |
| Efet.01.1659527 | RF00005 | tRNA | Gene; tRNA | 4043 | 4115 | + |
| Efet.01.167328 | RF00005 | tRNA | Gene; tRNA | 1396 | 1310 | - |
| Efet.01.167328 | RF00005 | tRNA | Gene; tRNA | 48 | 120 | + |
| Efet.01.16968 | RF00008 | Hammerhead_3 | Gene; ribozyme | 642 | 588 | - |
| Efet.01.201739 | RF00032 | Histone3 | Cis-reg | 814 | 542 | - |
| Efet.01.201739 | RF00032 | Histone3 | Cis-reg | 32 | 77 | + |
| Efet.01.216629 | RF00032 | Histone3 | Cis-reg | 1 | 100 | + |
| Efet.01.216629 | RF00032 | Histone3 | Cis-reg | 461 | 506 | + |
| Efet.01.219704 | RF00005 | tRNA | Gene; tRNA | 518 | 417 | - |
| Efet.01.219704 | RF00005 | tRNA | Gene; tRNA | 250 | 331 | + |
| Efet.01.220341 | RF00005 | tRNA | Gene; tRNA | 3612 | 3681 | + |
| Efet.01.221654 | RF00030 | RNase_MRP | Gene; ribozyme | 2327 | 2061 | - |
| Efet.01.227858 | RF00005 | tRNA | Gene; tRNA | 692 | 620 | - |
| Efet.01.232162 | RF00020 | U5 | Gene; snRNA; splicing | 504 | 389 | - |
| Efet.01.233473 | RF00005 | tRNA | Gene; tRNA | 4077 | 4148 | + |
| Efet.01.237800 | RF00005 | tRNA | Gene; tRNA | 244 | 315 | + |
| Efet.01.242765 | RF00006 | Vault | Gene | 242 | 347 | + |
| Efet.01.244333 | RF00005 | tRNA | Gene; tRNA | 322 | 251 | - |
| Efet.01.247722 | RF00485 | K_chan_RES | Cis-reg | 1501 | 1388 | - |
| Efet.01.253024 | RF01959 | SSU_rRNA_archaea | Gene; rRNA | 1 | 552 | + |
| Efet.01.253024 | RF00177 | SSU_rRNA_bacteria | Gene; rRNA | 1 | 552 | + |
| Efet.01.253024 | RF02542 | SSU_rRNA_microsporidia | Gene; rRNA | 1 | 552 | + |
| Efet.01.253032 | RF00032 | Histone3 | Cis-reg | 2241 | 2286 | + |
| Efet.01.257625 | RF00005 | tRNA | Gene; tRNA | 487 | 557 | + |
| Efet.01.258920 | RF00485 | K_chan_RES | Cis-reg | 1105 | 992 | - |
| Efet.01.258956 | RF00485 | K_chan_RES | Cis-reg | 1640 | 1527 | - |
| Efet.01.259336 | RF00007 | U12 | Gene; snRNA; splicing | 9204 | 9056 | - |
| Efet.01.262936 | RF00485 | K_chan_RES | Cis-reg | 2120 | 2233 | + |
| Efet.01.270468 | RF00005 | tRNA | Gene; tRNA | 1038 | 972 | - |
| Efet.01.271737 | RF00005 | tRNA | Gene; tRNA | 663 | 591 | - |
| Efet.01.271737 | RF00005 | tRNA | Gene; tRNA | 886 | 814 | - |
| Efet.01.273055 | RF00685 | mir-36 | Gene; miRNA | 1979 | 1891 | - |
| Efet.01.274905 | RF00012 | U3 | Gene; snRNA; snoRNA; CD-box | 760 | 501 | - |
| Efet.01.276165 | RF00005 | tRNA | Gene; tRNA | 211 | 279 | + |
| Efet.01.28060 | RF02540 | LSU_rRNA_archaea | Gene; rRNA | 705 | 1 | - |
| Efet.01.28060 | RF02540 | LSU_rRNA_archaea | Gene; rRNA | 705 | 1 | - |
| Efet.01.28060 | RF02541 | LSU_rRNA_bacteria | Gene; rRNA | 705 | 1 | - |
| Efet.01.28060 | RF02541 | LSU_rRNA_bacteria | Gene; rRNA | 705 | 1 | - |
| Efet.01.280853 | RF00005 | tRNA | Gene; tRNA | 1189 | 1251 | + |
| Efet.01.281370 | RF00485 | K_chan_RES | Cis-reg | 1020 | 1133 | + |
| Efet.01.281519 | RF00005 | tRNA | Gene; tRNA | 42 | 113 | + |
| Efet.01.284253 | RF00005 | tRNA | Gene; tRNA | 113 | 43 | - |
| Efet.01.287526 | RF00005 | tRNA | Gene; tRNA | 7820 | 7748 | - |
| Efet.01.287526 | RF00005 | tRNA | Gene; tRNA | 7693 | 7621 | - |
| Efet.01.293678 | RF00015 | U4 | Gene; snRNA; splicing | 818 | 950 | + |
| Efet.01.300440 | RF02540 | LSU_rRNA_archaea | Gene; rRNA | 2646 | 1 | - |
| Efet.01.300440 | RF02541 | LSU_rRNA_bacteria | Gene; rRNA | 2645 | 1 | - |
| Efet.01.300440 | RF02543 | LSU_rRNA_eukarya | Gene; rRNA | 2467 | 992 | - |
| Efet.01.300440 | RF01959 | SSU_rRNA_archaea | Gene; rRNA | 4852 | 3204 | - |
| Efet.01.300440 | RF00177 | SSU_rRNA_bacteria | Gene; rRNA | 4857 | 3202 | - |
| Efet.01.300440 | RF00005 | tRNA | Gene; tRNA | 3002 | 2930 | - |
| Efet.01.300440 | RF00005 | tRNA | Gene; tRNA | 3108 | 3050 | - |
| Efet.01.300577 | RF00032 | Histone3 | Cis-reg | 3126 | 3171 | + |
| Efet.01.304046 | RF00005 | tRNA | Gene; tRNA | 113 | 185 | + |
| Efet.01.305661 | RF00005 | tRNA | Gene; tRNA | 721 | 649 | - |
| Efet.01.30589 | RF02001 | group-II-D1D4-3 | Intron | 537 | 703 | + |
| Efet.01.30589 | RF02001 | group-II-D1D4-3 | Intron | 537 | 703 | + |
| Efet.01.308568 | RF00026 | U6 | Gene; snRNA; splicing | 847 | 942 | + |
| Efet.01.310145 | RF01959 | SSU_rRNA_archaea | Gene; rRNA | 2337 | 1697 | - |
| Efet.01.310145 | RF00177 | SSU_rRNA_bacteria | Gene; rRNA | 2368 | 518 | - |
| Efet.01.310145 | RF00005 | tRNA | Gene; tRNA | 413 | 340 | - |
| Efet.01.310145 | RF00005 | tRNA | Gene; tRNA | 248 | 175 | - |
| Efet.01.310657 | RF00485 | K_chan_RES | Cis-reg | 627 | 514 | - |
| Efet.01.311866 | RF00485 | K_chan_RES | Cis-reg | 3164 | 3277 | + |
| Efet.01.312578 | RF00485 | K_chan_RES | Cis-reg | 1126 | 1013 | - |
| Efet.01.319042 | RF00005 | tRNA | Gene; tRNA | 131 | 203 | + |
| Efet.01.320510 | RF00003 | U1 | Gene; snRNA; splicing | 1348 | 1497 | + |
| Efet.01.320812 | RF00485 | K_chan_RES | Cis-reg | 432 | 319 | - |
| Efet.01.321641 | RF00032 | Histone3 | Cis-reg | 699 | 743 | + |
| Efet.01.322442 | RF01959 | SSU_rRNA_archaea | Gene; rRNA | 1 | 323 | + |
| Efet.01.322442 | RF00177 | SSU_rRNA_bacteria | Gene; rRNA | 1 | 486 | + |
| Efet.01.323858 | RF00485 | K_chan_RES | Cis-reg | 1503 | 1616 | + |
| Efet.01.323909 | RF00005 | tRNA | Gene; tRNA | 3205 | 3132 | - |
| Efet.01.327212 | RF00032 | Histone3 | Cis-reg | 334 | 289 | - |
| Efet.01.327891 | RF00005 | tRNA | Gene; tRNA | 533 | 464 | - |
| Efet.01.331664 | RF00032 | Histone3 | Cis-reg | 2798 | 2754 | - |
| Efet.01.334737 | RF00005 | tRNA | Gene; tRNA | 2 | 83 | + |
| Efet.01.338819 | RF00005 | tRNA | Gene; tRNA | 804 | 736 | - |
| Efet.01.341562 | RF00005 | tRNA | Gene; tRNA | 513 | 440 | - |
| Efet.01.345494 | RF00005 | tRNA | Gene; tRNA | 488 | 335 | - |
| Efet.01.345921 | RF00032 | Histone3 | Cis-reg | 1811 | 1855 | + |
| Efet.01.347108 | RF00005 | tRNA | Gene; tRNA | 216 | 270 | + |
| Efet.01.353085 | RF00001 | 5S_rRNA | Gene; rRNA | 4582 | 4696 | + |
| Efet.01.353085 | RF02540 | LSU_rRNA_archaea | Gene; rRNA | 1123 | 4467 | + |
| Efet.01.353085 | RF02541 | LSU_rRNA_bacteria | Gene; rRNA | 1124 | 4468 | + |
| Efet.01.353085 | RF02543 | LSU_rRNA_eukarya | Gene; rRNA | 1279 | 3329 | + |
| Efet.01.353085 | RF01959 | SSU_rRNA_archaea | Gene; rRNA | 1 | 512 | + |
| Efet.01.353085 | RF00177 | SSU_rRNA_bacteria | Gene; rRNA | 1 | 514 | + |
| Efet.01.353085 | RF01960 | SSU_rRNA_eukarya | Gene; rRNA | 1 | 509 | + |
| Efet.01.353085 | RF00005 | tRNA | Gene; tRNA | 852 | 924 | + |
| Efet.01.353085 | RF00005 | tRNA | Gene; tRNA | 639 | 727 | + |
| Efet.01.356293 | RF00104 | mir-10 | Gene; miRNA | 1050 | 979 | - |
| Efet.01.363886 | RF00001 | 5S_rRNA | Gene; rRNA | 1258 | 1147 | - |
| Efet.01.363886 | RF02540 | LSU_rRNA_archaea | Gene; rRNA | 2131 | 1365 | - |
| Efet.01.363886 | RF02541 | LSU_rRNA_bacteria | Gene; rRNA | 2733 | 1350 | - |
| Efet.01.363886 | RF02543 | LSU_rRNA_eukarya | Gene; rRNA | 1606 | 1448 | - |
| Efet.01.365501 | RF00005 | tRNA | Gene; tRNA | 1341 | 1412 | + |
| Efet.01.370035 | RF00005 | tRNA | Gene; tRNA | 920 | 847 | - |
| Efet.01.371127 | RF00027 | let-7 | Gene; miRNA | 1237 | 1157 | - |
| Efet.01.373053 | RF00032 | Histone3 | Cis-reg | 727 | 773 | + |
| Efet.01.378774 | RF00005 | tRNA | Gene; tRNA | 578 | 497 | - |
| Efet.01.383831 | RF00005 | tRNA | Gene; tRNA | 1249 | 1332 | + |
| Efet.01.385563 | RF00005 | tRNA | Gene; tRNA | 44 | 127 | + |
| Efet.01.385798 | RF00694 | mir-137 | Gene; miRNA | 4299 | 4398 | + |
| Efet.01.386454 | RF02253 | IRE_II | Cis-reg | 100 | 70 | - |
| Efet.01.387750 | RF00032 | Histone3 | Cis-reg | 990 | 1034 | + |
| Efet.01.397118 | RF00005 | tRNA | Gene; tRNA | 60 | 130 | + |
| Efet.01.398701 | RF00005 | tRNA | Gene; tRNA | 120 | 47 | - |
| Efet.01.399335 | RF01746 | mraW | Cis-reg | 365 | 268 | - |
| Efet.01.405067 | RF00504 | Glycine | Cis-reg; riboswitch | 852 | 765 | - |
| Efet.01.405260 | RF00485 | K_chan_RES | Cis-reg | 613 | 500 | - |
| Efet.01.411886 | RF00005 | tRNA | Gene; tRNA | 761 | 833 | + |
| Efet.01.415711 | RF00005 | tRNA | Gene; tRNA | 1173 | 1101 | - |
| Efet.01.417053 | RF00005 | tRNA | Gene; tRNA | 2393 | 2320 | - |
| Efet.01.418416 | RF00003 | U1 | Gene; snRNA; splicing | 331 | 269 | - |
| Efet.01.424539 | RF00032 | Histone3 | Cis-reg | 23 | 69 | + |
| Efet.01.426361 | RF00005 | tRNA | Gene; tRNA | 756 | 709 | - |
| Efet.01.435182 | RF00005 | tRNA | Gene; tRNA | 333 | 407 | + |
| Efet.01.439340 | RF00005 | tRNA | Gene; tRNA | 1093 | 1021 | - |
| Efet.01.443583 | RF00005 | tRNA | Gene; tRNA | 2274 | 2208 | - |
| Efet.01.444482 | RF00485 | K_chan_RES | Cis-reg | 1440 | 1555 | + |
| Efet.01.445227 | RF00485 | K_chan_RES | Cis-reg | 1593 | 1706 | + |
| Efet.01.450560 | RF00485 | K_chan_RES | Cis-reg | 1186 | 1299 | + |
| Efet.01.452754 | RF00005 | tRNA | Gene; tRNA | 155 | 91 | - |
| Efet.01.452841 | RF01959 | SSU_rRNA_archaea | Gene; rRNA | 924 | 1 | - |
| Efet.01.452841 | RF00177 | SSU_rRNA_bacteria | Gene; rRNA | 929 | 1 | - |
| Efet.01.452841 | RF01960 | SSU_rRNA_eukarya | Gene; rRNA | 916 | 1 | - |
| Efet.01.452841 | RF02542 | SSU_rRNA_microsporidia | Gene; rRNA | 918 | 1 | - |
| Efet.01.453519 | RF00002 | 5_8S_rRNA | Gene; rRNA | 580 | 741 | + |
| Efet.01.453519 | RF00001 | 5S_rRNA | Gene; rRNA | 3856 | 3972 | + |
| Efet.01.453519 | RF02540 | LSU_rRNA_archaea | Gene; rRNA | 566 | 3686 | + |
| Efet.01.453519 | RF02541 | LSU_rRNA_bacteria | Gene; rRNA | 568 | 3686 | + |
| Efet.01.460527 | RF00005 | tRNA | Gene; tRNA | 1351 | 1419 | + |
| Efet.01.465744 | RF00005 | tRNA | Gene; tRNA | 3745 | 3664 | - |
| Efet.01.466010 | RF00485 | K_chan_RES | Cis-reg | 2161 | 2274 | + |
| Efet.01.466400 | RF00005 | tRNA | Gene; tRNA | 3475 | 3391 | - |
| Efet.01.466476 | RF00485 | K_chan_RES | Cis-reg | 2669 | 2782 | + |
| Efet.01.466869 | RF00657 | mir-184 | Gene; miRNA | 19 | 105 | + |
| Efet.01.473816 | RF00032 | Histone3 | Cis-reg | 347 | 391 | + |
| Efet.01.475027 | RF00005 | tRNA | Gene; tRNA | 1224 | 1078 | - |
| Efet.01.487143 | RF00005 | tRNA | Gene; tRNA | 34 | 107 | + |
| Efet.01.487186 | RF00005 | tRNA | Gene; tRNA | 46 | 118 | + |
| Efet.01.488260 | RF00032 | Histone3 | Cis-reg | 448 | 493 | + |
| Efet.01.491746 | RF00032 | Histone3 | Cis-reg | 120 | 74 | - |
| Efet.01.492589 | RF00005 | tRNA | Gene; tRNA | 1649 | 1718 | + |
| Efet.01.498517 | RF00266 | snoZ17 | Gene; snRNA; snoRNA; CD-box | 481 | 554 | + |
| Efet.01.501294 | RF00001 | 5S_rRNA | Gene; rRNA | 249 | 358 | + |
| Efet.01.503541 | RF00005 | tRNA | Gene; tRNA | 3040 | 3112 | + |
| Efet.01.504811 | RF00032 | Histone3 | Cis-reg | 1374 | 1419 | + |
| Efet.01.505344 | RF02540 | LSU_rRNA_archaea | Gene; rRNA | 1 | 1219 | + |
| Efet.01.505344 | RF02541 | LSU_rRNA_bacteria | Gene; rRNA | 1 | 1219 | + |
| Efet.01.505344 | RF02543 | LSU_rRNA_eukarya | Gene; rRNA | 1 | 1219 | + |
| Efet.01.508022 | RF00005 | tRNA | Gene; tRNA | 3795 | 3868 | + |
| Efet.01.511850 | RF01959 | SSU_rRNA_archaea | Gene; rRNA | 1 | 1030 | + |
| Efet.01.511850 | RF00177 | SSU_rRNA_bacteria | Gene; rRNA | 1 | 1032 | + |
| Efet.01.511850 | RF01960 | SSU_rRNA_eukarya | Gene; rRNA | 1 | 1017 | + |
| Efet.01.511850 | RF02542 | SSU_rRNA_microsporidia | Gene; rRNA | 1 | 1029 | + |
| Efet.01.513765 | RF00657 | mir-184 | Gene; miRNA | 495 | 415 | - |
| Efet.01.521131 | RF00485 | K_chan_RES | Cis-reg | 1648 | 1761 | + |
| Efet.01.521754 | RF00005 | tRNA | Gene; tRNA | 1868 | 1941 | + |
| Efet.01.521754 | RF00005 | tRNA | Gene; tRNA | 2002 | 2074 | + |
| Efet.01.521754 | RF00005 | tRNA | Gene; tRNA | 1746 | 1824 | + |
| Efet.01.522529 | RF00005 | tRNA | Gene; tRNA | 345 | 260 | - |
| Efet.01.522529 | RF00005 | tRNA | Gene; tRNA | 471 | 406 | - |
| Efet.01.526223 | RF00005 | tRNA | Gene; tRNA | 673 | 601 | - |
| Efet.01.530270 | RF00005 | tRNA | Gene; tRNA | 4499 | 4558 | + |
| Efet.01.538032 | RF00001 | 5S_rRNA | Gene; rRNA | 159 | 45 | - |
| Efet.01.538032 | RF02540 | LSU_rRNA_archaea | Gene; rRNA | 575 | 260 | - |
| Efet.01.538032 | RF02541 | LSU_rRNA_bacteria | Gene; rRNA | 575 | 259 | - |
| Efet.01.538032 | RF02543 | LSU_rRNA_eukarya | Gene; rRNA | 575 | 270 | - |
| Efet.01.539607 | RF00270 | SNORD61 | Gene; snRNA; snoRNA; CD-box | 1183 | 1112 | - |
| Efet.01.553743 | RF00005 | tRNA | Gene; tRNA | 28 | 96 | + |
| Efet.01.563718 | RF00005 | tRNA | Gene; tRNA | 20 | 90 | + |
| Efet.01.564055 | RF00005 | tRNA | Gene; tRNA | 1193 | 1123 | - |
| Efet.01.564242 | RF00005 | tRNA | Gene; tRNA | 68 | 150 | + |
| Efet.01.564242 | RF01852 | tRNA-Sec | Gene; tRNA | 66 | 151 | + |
| Efet.01.566902 | RF00005 | tRNA | Gene; tRNA | 384 | 457 | + |
| Efet.01.566902 | RF00005 | tRNA | Gene; tRNA | 298 | 370 | + |
| Efet.01.567042 | RF00032 | Histone3 | Cis-reg | 1753 | 1709 | - |
| Efet.01.571073 | RF00005 | tRNA | Gene; tRNA | 365 | 292 | - |
| Efet.01.571073 | RF00005 | tRNA | Gene; tRNA | 165 | 94 | - |
| Efet.01.571073 | RF00005 | tRNA | Gene; tRNA | 60 | 1 | - |
| Efet.01.572780 | RF00005 | tRNA | Gene; tRNA | 415 | 329 | - |
| Efet.01.573047 | RF00005 | tRNA | Gene; tRNA | 517 | 589 | + |
| Efet.01.573518 | RF00005 | tRNA | Gene; tRNA | 6252 | 6180 | - |
| Efet.01.574650 | RF00059 | TPP | Cis-reg; riboswitch | 957 | 860 | - |
| Efet.01.577785 | RF00140 | Alpha_RBS | Cis-reg | 497 | 459 | - |
| Efet.01.578494 | RF00485 | K_chan_RES | Cis-reg | 1961 | 2074 | + |
| Efet.01.578634 | RF00005 | tRNA | Gene; tRNA | 1056 | 1127 | + |
| Efet.01.583614 | RF01070 | sucA | Cis-reg | 1367 | 1286 | - |
| Efet.01.584719 | RF00005 | tRNA | Gene; tRNA | 857 | 785 | - |
| Efet.01.586614 | RF01959 | SSU_rRNA_archaea | Gene; rRNA | 482 | 812 | + |
| Efet.01.586614 | RF00177 | SSU_rRNA_bacteria | Gene; rRNA | 477 | 812 | + |
| Efet.01.586614 | RF01960 | SSU_rRNA_eukarya | Gene; rRNA | 482 | 812 | + |
| Efet.01.586614 | RF02542 | SSU_rRNA_microsporidia | Gene; rRNA | 482 | 812 | + |
| Efet.01.588117 | RF02540 | LSU_rRNA_archaea | Gene; rRNA | 858 | 1 | - |
| Efet.01.588117 | RF02541 | LSU_rRNA_bacteria | Gene; rRNA | 858 | 1 | - |
| Efet.01.588117 | RF02543 | LSU_rRNA_eukarya | Gene; rRNA | 679 | 1 | - |
| Efet.01.590987 | RF00694 | mir-137 | Gene; miRNA | 848 | 948 | + |
| Efet.01.592045 | RF00857 | mir-233 | Gene; miRNA | 152 | 55 | - |
| Efet.01.593259 | RF00059 | TPP | Cis-reg; riboswitch | 304 | 196 | - |
| Efet.01.594313 | RF02540 | LSU_rRNA_archaea | Gene; rRNA | 1 | 1469 | + |
| Efet.01.594313 | RF02541 | LSU_rRNA_bacteria | Gene; rRNA | 1 | 1469 | + |
| Efet.01.594313 | RF02543 | LSU_rRNA_eukarya | Gene; rRNA | 1 | 1469 | + |
| Efet.01.594981 | RF00005 | tRNA | Gene; tRNA | 138 | 209 | + |
| Efet.01.596592 | RF00005 | tRNA | Gene; tRNA | 76 | 160 | + |
| Efet.01.597087 | RF00005 | tRNA | Gene; tRNA | 439 | 511 | + |
| Efet.01.597489 | RF00023 | tmRNA | Gene | 669 | 1063 | + |
| Efet.01.597489 | RF00005 | tRNA | Gene; tRNA | 1080 | 1151 | + |
| Efet.01.597547 | RF00005 | tRNA | Gene; tRNA | 2642 | 2715 | + |
| Efet.01.598219 | RF00005 | tRNA | Gene; tRNA | 506 | 590 | + |
| Efet.01.599499 | RF00239 | mir-124 | Gene; miRNA | 590 | 672 | + |
| Efet.01.600573 | RF00241 | mir-8 | Gene; miRNA | 592 | 515 | - |
| Efet.01.601805 | RF00002 | 5_8S_rRNA | Gene; rRNA | 804 | 650 | - |
| Efet.01.601805 | RF02541 | LSU_rRNA_bacteria | Gene; rRNA | 816 | 1 | - |
| Efet.01.601805 | RF00005 | tRNA | Gene; tRNA | 1117 | 1058 | - |
| Efet.01.602510 | RF00005 | tRNA | Gene; tRNA | 649 | 579 | - |
| Efet.01.603284 | RF00188 | SNORD103 | Gene; snRNA; snoRNA; CD-box | 1014 | 954 | - |
| Efet.01.603871 | RF00005 | tRNA | Gene; tRNA | 59 | 140 | + |
| Efet.01.605365 | RF00005 | tRNA | Gene; tRNA | 8486 | 8416 | - |
| Efet.01.607053 | RF00162 | SAM | Cis-reg; riboswitch | 426 | 321 | - |
| Efet.01.608906 | RF00005 | tRNA | Gene; tRNA | 122 | 193 | + |
| Efet.01.611250 | RF00485 | K_chan_RES | Cis-reg | 482 | 595 | + |
| Efet.01.612077 | RF00005 | tRNA | Gene; tRNA | 545 | 642 | + |
| Efet.01.613088 | RF00174 | Cobalamin | Cis-reg; riboswitch | 2858 | 2757 | - |
| Efet.01.616258 | RF00005 | tRNA | Gene; tRNA | 182 | 94 | - |
| Efet.01.618467 | RF00005 | tRNA | Gene; tRNA | 57 | 1 | - |
| Efet.01.618821 | RF00174 | Cobalamin | Cis-reg; riboswitch | 1083 | 914 | - |
| Efet.01.619061 | RF00005 | tRNA | Gene; tRNA | 1118 | 1046 | - |
| Efet.01.621434 | RF00005 | tRNA | Gene; tRNA | 709 | 637 | - |
| Efet.01.621756 | RF00032 | Histone3 | Cis-reg | 249 | 205 | - |
| Efet.01.624189 | RF00667 | mir-33 | Gene; miRNA | 922 | 992 | + |
| Efet.01.625373 | RF00485 | K_chan_RES | Cis-reg | 252 | 140 | - |
| Efet.01.626403 | RF00694 | mir-137 | Gene; miRNA | 894 | 996 | + |
| Efet.01.627966 | RF00005 | tRNA | Gene; tRNA | 109 | 38 | - |
| Efet.01.628555 | RF00005 | tRNA | Gene; tRNA | 272 | 190 | - |
| Efet.01.628992 | RF00093 | SNORD18 | Gene; snRNA; snoRNA; CD-box | 932 | 861 | - |
| Efet.01.628992 | RF00093 | SNORD18 | Gene; snRNA; snoRNA; CD-box | 500 | 429 | - |
| Efet.01.629178 | RF01959 | SSU_rRNA_archaea | Gene; rRNA | 298 | 1 | - |
| Efet.01.629178 | RF00177 | SSU_rRNA_bacteria | Gene; rRNA | 296 | 1 | - |
| Efet.01.629178 | RF02542 | SSU_rRNA_microsporidia | Gene; rRNA | 289 | 1 | - |
| Efet.01.629773 | RF00005 | tRNA | Gene; tRNA | 164 | 232 | + |
| Efet.01.630637 | RF00005 | tRNA | Gene; tRNA | 1386 | 1458 | + |
| Efet.01.635465 | RF00485 | K_chan_RES | Cis-reg | 481 | 346 | - |
| Efet.01.637009 | RF00485 | K_chan_RES | Cis-reg | 714 | 826 | + |
| Efet.01.638488 | RF00005 | tRNA | Gene; tRNA | 498 | 592 | + |
| Efet.01.640077 | RF00169 | Bacteria_small_SRP | Gene | 144 | 240 | + |
| Efet.01.641291 | RF00005 | tRNA | Gene; tRNA | 288 | 371 | + |
| Efet.01.643419 | RF00485 | K_chan_RES | Cis-reg | 808 | 696 | - |
| Efet.01.644581 | RF00174 | Cobalamin | Cis-reg; riboswitch | 1970 | 1778 | - |
| Efet.01.645338 | RF02514 | 5_ureB_sRNA | Gene; sRNA | 888 | 1164 | + |
| Efet.01.645892 | RF00010 | RNaseP_bact_a | Gene; ribozyme | 647 | 347 | - |
| Efet.01.646274 | RF00005 | tRNA | Gene; tRNA | 5433 | 5519 | + |
| Efet.01.646642 | RF00005 | tRNA | Gene; tRNA | 1562 | 1634 | + |
| Efet.01.648231 | RF00005 | tRNA | Gene; tRNA | 324 | 397 | + |
| Efet.01.650086 | RF00237 | mir-9 | Gene; miRNA | 1539 | 1599 | + |
| Efet.01.650463 | RF00005 | tRNA | Gene; tRNA | 396 | 468 | + |
| Efet.01.650480 | RF00005 | tRNA | Gene; tRNA | 4474 | 4402 | - |
| Efet.01.650749 | RF00005 | tRNA | Gene; tRNA | 5083 | 5011 | - |
| Efet.01.650749 | RF00005 | tRNA | Gene; tRNA | 2850 | 2751 | - |
| Efet.01.652858 | RF00005 | tRNA | Gene; tRNA | 6430 | 6503 | + |
| Efet.01.653479 | RF00169 | Bacteria_small_SRP | Gene | 1732 | 1637 | - |
| Efet.01.654656 | RF00005 | tRNA | Gene; tRNA | 39859 | 39788 | - |
| Efet.01.656283 | RF02514 | 5_ureB_sRNA | Gene; sRNA | 6326 | 6615 | + |
| Efet.01.656283 | RF00005 | tRNA | Gene; tRNA | 2747 | 2661 | - |
| Efet.01.656742 | RF00059 | TPP | Cis-reg; riboswitch | 13149 | 13024 | - |
| Efet.01.656757 | RF00005 | tRNA | Gene; tRNA | 7369 | 7450 | + |
| Efet.01.657183 | RF00005 | tRNA | Gene; tRNA | 1707 | 1793 | + |
| Efet.01.657453 | RF00005 | tRNA | Gene; tRNA | 2323 | 2235 | - |
| Efet.01.657496 | RF00005 | tRNA | Gene; tRNA | 3801 | 3728 | - |
| Efet.01.657761 | RF00005 | tRNA | Gene; tRNA | 77 | 150 | + |
| Efet.01.658084 | RF00005 | tRNA | Gene; tRNA | 178 | 107 | - |
| Efet.01.658084 | RF00005 | tRNA | Gene; tRNA | 340 | 270 | - |
| Efet.01.658084 | RF00005 | tRNA | Gene; tRNA | 627 | 555 | - |
| Efet.01.658084 | RF00005 | tRNA | Gene; tRNA | 468 | 386 | - |
| Efet.01.658312 | RF00005 | tRNA | Gene; tRNA | 1772 | 1727 | - |
| Efet.01.658414 | RF00504 | Glycine | Cis-reg; riboswitch | 8338 | 8218 | - |
| Efet.01.658414 | RF00504 | Glycine | Cis-reg; riboswitch | 8451 | 8356 | - |
| Efet.01.658428 | RF00005 | tRNA | Gene; tRNA | 2864 | 2937 | + |
| Efet.01.666759 | RF00005 | tRNA | Gene; tRNA | 76 | 4 | - |
| Efet.01.667566 | RF00001 | 5S_rRNA | Gene; rRNA | 44 | 100 | + |
| Efet.01.669266 | RF02541 | LSU_rRNA_bacteria | Gene; rRNA | 100 | 1 | - |
| Efet.01.669521 | RF00005 | tRNA | Gene; tRNA | 55 | 1 | - |
| Efet.01.671092 | RF02541 | LSU_rRNA_bacteria | Gene; rRNA | 100 | 8 | - |
| Efet.01.673224 | RF02541 | LSU_rRNA_bacteria | Gene; rRNA | 100 | 1 | - |
| Efet.01.674206 | RF00005 | tRNA | Gene; tRNA | 33 | 100 | + |
| Efet.01.674469 | RF00005 | tRNA | Gene; tRNA | 38 | 100 | + |
| Efet.01.679633 | RF00005 | tRNA | Gene; tRNA | 80 | 8 | - |
| Efet.01.683448 | RF01055 | MOCO_RNA_motif | Cis-reg; riboswitch | 2 | 100 | + |
| Efet.01.684389 | RF00005 | tRNA | Gene; tRNA | 57 | 100 | + |
| Efet.01.686554 | RF00005 | tRNA | Gene; tRNA | 3 | 84 | + |
| Efet.01.688975 | RF00005 | tRNA | Gene; tRNA | 2 | 68 | + |
| Efet.01.689013 | RF00174 | Cobalamin | Cis-reg; riboswitch | 78 | 1 | - |
| Efet.01.689601 | RF00005 | tRNA | Gene; tRNA | 100 | 44 | - |
| Efet.01.693538 | RF01959 | SSU_rRNA_archaea | Gene; rRNA | 100 | 1 | - |
| Efet.01.693538 | RF00177 | SSU_rRNA_bacteria | Gene; rRNA | 100 | 1 | - |
| Efet.01.695111 | RF02541 | LSU_rRNA_bacteria | Gene; rRNA | 1 | 98 | + |
| Efet.01.697331 | RF02782 | CpoB_ybgF_thermometer | Cis-reg; thermoregulator | 99 | 1 | - |
| Efet.01.698921 | RF01051 | c-di-GMP-I | Cis-reg | 56 | 1 | - |
| Efet.01.699507 | RF00127 | t44 | Gene; sRNA | 97 | 10 | - |
| Efet.01.700733 | RF01705 | Flavo-1 | Gene; sRNA | 100 | 35 | - |
| Efet.01.700782 | RF02540 | LSU_rRNA_archaea | Gene; rRNA | 100 | 1 | - |
| Efet.01.700782 | RF02541 | LSU_rRNA_bacteria | Gene; rRNA | 100 | 1 | - |
| Efet.01.708253 | RF00003 | U1 | Gene; snRNA; splicing | 28 | 101 | + |
| Efet.01.714468 | RF02540 | LSU_rRNA_archaea | Gene; rRNA | 1 | 101 | + |
| Efet.01.714468 | RF02541 | LSU_rRNA_bacteria | Gene; rRNA | 1 | 101 | + |
| Efet.01.716334 | RF00005 | tRNA | Gene; tRNA | 16 | 87 | + |
| Efet.01.754877 | RF00032 | Histone3 | Cis-reg | 62 | 18 | - |
| Efet.01.793427 | RF00857 | mir-233 | Gene; miRNA | 101 | 4 | - |
| Efet.01.804982 | RF00005 | tRNA | Gene; tRNA | 41 | 104 | + |
| Efet.01.81094 | RF00485 | K_chan_RES | Cis-reg | 315 | 202 | - |
| Efet.01.81094 | RF00485 | K_chan_RES | Cis-reg | 315 | 202 | - |
| Efet.01.832220 | RF00005 | tRNA | Gene; tRNA | 28 | 99 | + |
| Efet.01.845712 | RF00005 | tRNA | Gene; tRNA | 54 | 1 | - |
| Efet.01.846025 | RF02541 | LSU_rRNA_bacteria | Gene; rRNA | 1 | 105 | + |
| Efet.01.88184 | RF00032 | Histone3 | Cis-reg | 76 | 32 | - |
| Efet.01.88184 | RF00032 | Histone3 | Cis-reg | 76 | 32 | - |
| Efet.01.898215 | RF00005 | tRNA | Gene; tRNA | 60 | 107 | + |
| Efet.01.914309 | RF00005 | tRNA | Gene; tRNA | 91 | 7 | - |
| Efet.01.928745 | RF00008 | Hammerhead_3 | Gene; ribozyme | 15 | 73 | + |
| Efet.01.934839 | RF01959 | SSU_rRNA_archaea | Gene; rRNA | 109 | 1 | - |
| Efet.01.934839 | RF00177 | SSU_rRNA_bacteria | Gene; rRNA | 109 | 1 | - |
| Efet.01.988863 | RF00005 | tRNA | Gene; tRNA | 107 | 35 | - |
